# Population and fertility by age and sex for 195 countries and territories, 1950–2017: a systematic analysis for the Global Burden of Disease Study 2017

**DOI:** 10.1016/S0140-6736(18)32278-5

**Published:** 2018-11-10

**Authors:** GBD 2017 Population and Fertility Collaborators, Christopher J L Murray, Charlton S K H Callender, Xie Rachel Kulikoff, Vinay Srinivasan, Degu Abate, Kalkidan Hassen Abate, Solomon M Abay, Nooshin Abbasi, Hedayat Abbastabar, Jemal Abdela, Ahmed Abdelalim, Omar Abdel-Rahman, Alireza Abdi, Nasrin Abdoli, Ibrahim Abdollahpour, Rizwan Suliankatchi Abdulkader, Haftom Temesgen Abebe, Molla Abebe, Zegeye Abebe, Teshome Abuka Abebo, Ayenew Negesse Abejie, Victor Aboyans, Haftom Niguse Abraha, Daisy Maria Xavier Abreu, Aklilu Roba Abrham, Laith Jamal Abu-Raddad, Niveen M E Abu-Rmeileh, Manfred Mario Kokou Accrombessi, Pawan Acharya, Abdu A Adamu, Oladimeji M Adebayo, Isaac Akinkunmi Adedeji, Victor Adekanmbi, Olatunji O Adetokunboh, Beyene Meressa Adhena, Tara Ballav Adhikari, Mina G Adib, Arsène Kouablan Adou, Jose C Adsuar, Mohsen Afarideh, Ashkan Afshin, Gina Agarwal, Kareha M Agesa, Sargis Aghasi Aghayan, Sutapa Agrawal, Alireza Ahmadi, Mehdi Ahmadi, Muktar Beshir Ahmed, Sayem Ahmed, Amani Nidhal Aichour, Ibtihel Aichour, Miloud Taki Eddine Aichour, Ali S Akanda, Mohammad Esmaeil Akbari, Mohammed Akibu, Rufus Olusola Akinyemi, Tomi Akinyemiju, Nadia Akseer, Fares Alahdab, Ziyad Al-Aly, Khurshid Alam, Animut Alebel, Alicia V Aleman, Kefyalew Addis Alene, Ayman Al-Eyadhy, Raghib Ali, Mehran Alijanzadeh, Reza Alizadeh-Navaei, Syed Mohamed Aljunid, Ala'a Alkerwi, François Alla, Peter Allebeck, Ali Almasi, Jordi Alonso, Rajaa M Al-Raddadi, Ubai Alsharif, Khalid Altirkawi, Nelson Alvis-Guzman, Azmeraw T Amare, Walid Ammar, Nahla Hamed Anber, Catalina Liliana Andrei, Sofia Androudi, Megbaru Debalkie Animut, Hossein Ansari, Mustafa Geleto Ansha, Carl Abelardo T Antonio, Seth Christopher Yaw Appiah, Olatunde Aremu, Habtamu Abera Areri, Nicholas Arian, Johan Ärnlöv, Al Artaman, Krishna K Aryal, Hamid Asayesh, Ephrem Tsegay Asfaw, Solomon Weldegebreal Asgedom, Reza Assadi, Tesfay Mehari Mehari Atey, Suleman Atique, Madhu Sudhan Atteraya, Marcel Ausloos, Euripide F G A Avokpaho, Ashish Awasthi, Beatriz Paulina Ayala Quintanilla, Yohanes Ayele, Rakesh Ayer, Tambe B Ayuk, Peter S Azzopardi, Tesleem Kayode Babalola, Arefeh Babazadeh, Hamid Badali, Alaa Badawi, Ayele Geleto Bali, Maciej Banach, Suzanne Lyn Barker-Collo, Till Winfried Bärnighausen, Lope H Barrero, Huda Basaleem, Quique Bassat, Arindam Basu, Bernhard T Baune, Habtamu Wondifraw Baynes, Ettore Beghi, Masoud Behzadifar, Meysam Behzadifar, Bayu Begashaw Bekele, Abate Bekele Belachew, Aregawi Gebreyesus Belay, Ezra Belay, Saba Abraham Belay, Yihalem Abebe Belay, Michelle L Bell, Aminu K Bello, Derrick A Bennett, Isabela M Bensenor, Gilles Bergeron, Adugnaw Berhane, Adam E Berman, Eduardo Bernabe, Robert S Bernstein, Gregory J Bertolacci, Mircea Beuran, Suraj Bhattarai, Soumyadeep Bhaumik, Zulfiqar A Bhutta, Belete Biadgo, Ali Bijani, Boris Bikbov, Nigus Bililign, Muhammad Shahdaat Bin Sayeed, Sait Mentes Birlik, Charles Birungi, Tuhin Biswas, Hailemichael Bizuneh, Archie Bleyer, Berrak Bora Basara, Cristina Bosetti, Soufiane Boufous, Oliver J Brady, Nicola Luigi Bragazzi, Michael Brainin, Alexandra Brazinova, Nicholas J K Breitborde, Hermann Brenner, Jerry D Brewer, Paul Svitil Briant, Gabrielle Britton, Roy Burstein, Reinhard Busse, Zahid A Butt, Lucero Cahuana-Hurtado, Ismael R Campos-Nonato, Julio Cesar Campuzano Rincon, Jorge Cano, Mate Car, Rosario Cárdenas, Juan J Carrero, Félix Carvalho, Carlos A Castañeda-Orjuela, Jacqueline Castillo Rivas, Franz Castro, Ferrán Catalá-López, Alanur Çavlin, Ester Cerin, Julian Chalek, Hsing-Yi Chang, Jung-Chen Chang, Aparajita Chattopadhyay, Pankaj Chaturvedi, Peggy Pei-Chia Chiang, Ken Lee Chin, Vesper Hichilombwe Chisumpa, Abdulaal Chitheer, Jee-Young J Choi, Rajiv Chowdhury, Devasahayam J Christopher, Flavia M Cicuttini, Liliana G Ciobanu, Massimo Cirillo, Rafael M Claro, Daniel Collado-Mateo, Maria-Magdalena Constantin, Sara Conti, Cyrus Cooper, Leslie Trumbull Cooper, Leslie Cornaby, Paolo Angelo Cortesi, Monica Cortinovis, Megan Costa, Elizabeth Cromwell, Christopher Stephen Crowe, Petra Cukelj, Matthew Cunningham, Alemneh Kabeta Daba, Berihun Assefa Dachew, Lalit Dandona, Rakhi Dandona, Paul I Dargan, Ahmad Daryani, Rajat Das Gupta, José Das Neves, Tamirat Tesfaye Dasa, Aditya Prasad Dash, Nicole Davis Weaver, Dragos Virgil Davitoiu, Kairat Davletov, Diego De Leo, Jan-Walter De Neve, Meaza Girma Degefa, Louisa Degenhardt, Tizta Tilahun Degfie, Selina Deiparine, Gebre Teklemariam Demoz, Balem Demtsu, Edgar Denova-Gutiérrez, Kebede Deribe, Nikolaos Dervenis, Don C Des Jarlais, Getenet Ayalew Dessie, Samath D Dharmaratne, Meghnath Dhimal, Daniel Dicker, Eric L Ding, Girmaye Deye Dinsa, Shirin Djalalinia, Huyen Phuc Do, Klara Dokova, David Teye Doku, Kate A Dolan, Kerrie E Doyle, Tim R Driscoll, Manisha Dubey, Eleonora Dubljanin, Eyasu Ejeta Duken, Andre R Duraes, Soheil Ebrahimpour, David Edvardsson, Charbel El Bcheraoui, Ziad El-Khatib, Iqbal Rf Elyazar, Ahmadali Enayati, Aman Yesuf Endries, Sergey Petrovich Ermakov, Babak Eshrati, Sharareh Eskandarieh, Reza Esmaeili, Alireza Esteghamati, Sadaf Esteghamati, Kara Estep, Hamed Fakhim, Tamer Farag, Mahbobeh Faramarzi, Mohammad Fareed, Carla Sofia E Sá Farinha, Andre Faro, Maryam S Farvid, Farshad Farzadfar, Mohammad Hosein Farzaei, Kairsten A Fay, Mir Sohail Fazeli, Valery L Feigin, Andrea B Feigl, Fariba Feizy, Ama P Fenny, Netsanet Fentahun, Seyed-Mohammad Fereshtehnejad, Eduarda Fernandes, Garumma Tolu Feyissa, Irina Filip, Samuel Finegold, Florian Fischer, Luisa Sorio Flor, Nataliya A Foigt, Kyle J Foreman, Carla Fornari, Thomas Fürst, Takeshi Fukumoto, John E Fuller, Nancy Fullman, Emmanuela Gakidou, Silvano Gallus, Amiran Gamkrelidze, Morsaleh Ganji, Fortune Gbetoho Gankpe, Gregory M Garcia, Miguel Á Garcia-Gordillo, Abadi Kahsu Gebre, Teshome Gebre, Gebremedhin Berhe Gebregergs, Tsegaye Tewelde Gebrehiwot, Amanuel Tesfay Gebremedhin, Tilayie Feto Gelano, Yalemzewod Assefa Gelaw, Johanna M Geleijnse, Ricard Genova-Maleras, Peter Gething, Kebede Embaye Gezae, Mohammad Rasoul Ghadami, Reza Ghadimi, Keyghobad Ghadiri, Khalil Ghasemi Falavarjani, Maryam Ghasemi-Kasman, Hesam Ghiasvand, Mamata Ghimire, Aloke Gopal Ghoshal, Paramjit Singh Gill, Tiffany K Gill, Giorgia Giussani, Elena V Gnedovskaya, Srinivas Goli, Ricardo Santiago Gomez, Hector Gómez-Dantés, Philimon N Gona, Amador Goodridge, Sameer Vali Gopalani, Alessandra C Goulart, Bárbara Niegia Garcia Goulart, Ayman Grada, Giuseppe Grosso, Harish Chander C Gugnani, Jingwen Guo, Yuming Guo, Prakash C Gupta, Rahul Gupta, Rajeev Gupta, Tanush Gupta, Juanita A Haagsma, Vladimir Hachinski, Nima Hafezi-Nejad, Tekleberhan B Hagos, Tewodros Tesfa Hailegiyorgis, Gessessew Bugssa Hailu, Arvin Haj-Mirzaian, Arya Haj-Mirzaian, Randah R Hamadeh, Samer Hamidi, Alexis J Handal, Graeme J Hankey, Yuantao Hao, Hilda L Harb, Hamidreza Haririan, Josep Maria Haro, Mehedi Hasan, Hadi Hassankhani, Hamid Yimam Hassen, Rasmus Havmoeller, Simon I Hay, Yihua He, Akbar Hedayatizadeh-Omran, Mohamed I Hegazy, Behzad Heibati, Behnam Heidari, Delia Hendrie, Andualem Henok, Nathaniel J Henry, Claudiu Herteliu, Fatemeh Heydarpour, Desalegn T Hibstu, Michael K Hole, Enayatollah Homaie Rad, Praveen Hoogar, H Dean Hosgood, Seyed Mostafa Hosseini, Meimanat M Hosseini Chavoshi, Mehdi Hosseinzadeh, Mihaela Hostiuc, Sorin Hostiuc, Mohamed Hsairi, Thomas Hsiao, Guoqing Hu, John J Huang, Kim Moesgaard Iburg, Ehimario U Igumbor, Chad Thomas Ikeda, Olayinka Stephen Ilesanmi, Usman Iqbal, Asnake Ararsa Irenso, Seyed Sina Naghibi Irvani, Oluwaseyi Oluwakemi Isehunwa, Sheikh Mohammed Shariful Islam, Leila Jahangiry, Nader Jahanmehr, Sudhir Kumar Jain, Mihajlo Jakovljevic, Moti Tolera Jalu, Spencer L James, Simerjot K Jassal, Mehdi Javanbakht, Achala Upendra Jayatilleke, Panniyammakal Jeemon, Ravi Prakash Jha, Vivekanand Jha, John S Ji, Jost B Jonas, Jacek Jerzy Jozwiak, Suresh Banayya Jungari, Mikk Jürisson, Zubair Kabir, Rajendra Kadel, Amaha Kahsay, Rizwan Kalani, Umesh Kapil, Manoochehr Karami, Behzad Karami Matin, André Karch, Corine Karema, Seyed M Karimi, Amir Kasaeian, Dessalegn H Kassa, Getachew Mullu Kassa, Tesfaye Dessale Kassa, Zemenu Yohannes Kassa, Nicholas J Kassebaum, Anshul Kastor, Srinivasa Vittal Katikireddi, Anil Kaul, Norito Kawakami, Ali Kazemi Karyani, Seifu Kebede, Peter Njenga Keiyoro, Grant Rodgers Kemp, Andre Pascal Kengne, Andre Keren, Maia Kereselidze, Yousef Saleh Khader, Morteza Abdullatif Khafaie, Alireza Khajavi, Nauman Khalid, Ibrahim A Khalil, Ejaz Ahmad Khan, Muhammad Shahzeb Khan, Young-Ho Khang, Tripti Khanna, Mona M Khater, Alireza Khatony, Zahra Khazaeipour, Habibolah Khazaie, Abdullah T Khoja, Ardeshir Khosravi, Mohammad Hossein Khosravi, Getiye D Kibret, Zelalem Teklemariam Kidanemariam, Daniel N Kiirithio, Paul Evan Kilgore, Daniel Kim, Jun Y Kim, Young-Eun Kim, Yun Jin Kim, Ruth W Kimokoti, Yohannes Kinfu, Sanjay Kinra, Adnan Kisa, Mika Kivimäki, Sonali Kochhar, Yoshihiro Kokubo, Tufa Kolola, Jacek A Kopec, Margaret N Kosek, Soewarta Kosen, Parvaiz A Koul, Ai Koyanagi, Kewal Krishan, Sanjay Krishnaswami, Kristopher J Krohn, Barthelemy Kuate Defo, Burcu Kucuk Bicer, G Anil Kumar, Manasi Kumar, Pushpendra Kumar, Fekede Asefa Kumsa, Michael J Kutz, Sheetal D Lad, Alessandra Lafranconi, Dharmesh Kumar Lal, Ratilal Lalloo, Hilton Lam, Faris Hasan Lami, Justin J Lang, Sonia Lanksy, Van C Lansingh, Dennis Odai Laryea, Zohra S Lassi, Arman Latifi, Avula Laxmaiah, Jeffrey V Lazarus, James B Lee, Paul H Lee, James Leigh, Cheru Tesema Leshargie, Samson Leta, Miriam Levi, Shanshan Li, Xiaohong Li, Yichong Li, Juan Liang, Xiaofeng Liang, Misgan Legesse Liben, Lee-Ling Lim, Miteku Andualem Limenih, Shai Linn, Shiwei Liu, Stefan Lorkowski, Paulo A Lotufo, Rafael Lozano, Raimundas Lunevicius, Crispin Mabika Mabika, Erlyn Rachelle King Macarayan, Mark T Mackay, Fabiana Madotto, Tarek Abd Elaziz Mahmood, Narayan Bahadur Mahotra, Marek Majdan, Reza Majdzadeh, Azeem Majeed, Reza Malekzadeh, Manzoor Ahmad Malik, Abdullah A Mamun, Wondimu Ayele Manamo, Ana-Laura Manda, Srikanth Mangalam, Mohammad Ali Mansournia, Lorenzo Giovanni Mantovani, Chabila Christopher Mapoma, Dadi Marami, Joemer C Maravilla, Wagner Marcenes, Shakhnazarova Marina, Francisco Rogerlândio Martins-Melo, Winfried März, Melvin B Marzan, Tivani Phosa Mashamba-Thompson, Felix Masiye, Amanda J Mason-Jones, Benjamin Ballard Massenburg, Manu Raj Mathur, Pallab K Maulik, Mohsen Mazidi, John J McGrath, Suresh Mehata, Sanjay Madhav Mehendale, Man Mohan Mehndiratta, Ravi Mehrotra, Saeed Mehrzadi, Kala M Mehta, Varshil Mehta, Tefera C Mekonnen, Hagazi Gebre Meles, Kidanu Gebremariam Meles, Addisu Melese, Mulugeta Melku, Peter T N Memiah, Ziad A Memish, Walter Mendoza, Melkamu Merid Mengesha, Desalegn Tadese Mengistu, Getnet Mengistu, George A Mensah, Seid Tiku Mereta, Atte Meretoja, Tuomo J Meretoja, Tomislav Mestrovic, Haftay Berhane Mezgebe, Yode Miangotar, Bartosz Miazgowski, Tomasz Miazgowski, Ted R Miller, Molly Katherine Miller-Petrie, G K Mini, Parvaneh Mirabi, Andreea Mirica, Erkin M Mirrakhimov, Awoke Temesgen Misganaw, Babak Moazen, Karzan Abdulmuhsin Mohammad, Moslem Mohammadi, Noushin Mohammadifard, Maryam Mohammadi-Khanaposhtani, Mohammed A Mohammed, Shafiu Mohammed, Ali H Mokdad, Glen Dl Mola, Mariam Molokhia, Lorenzo Monasta, Julio Cesar Montañez, Ghobad Moradi, Mahmoudreza Moradi, Maziar Moradi-Lakeh, Mehdi Moradinazar, Paula Moraga, Joana Morgado-Da-Costa, Rintaro Mori, Shane Douglas Morrison, Abbas Mosapour, Marilita M Moschos, Seyyed Meysam Mousavi, Achenef Asmamaw Muche, Kindie Fentahun Muchie, Ulrich Otto Mueller, Satinath Mukhopadhyay, Kate Muller, Tasha B Murphy, G V S Murthy, Jonah Musa, Kamarul Imran Musa, Ghulam Mustafa, Saravanan Muthupandian, Jean B Nachega, Gabriele Nagel, Mohsen Naghavi, Aliya Naheed, Azin Nahvijou, Gurudatta Naik, Paulami Naik, Farid Najafi, Luigi Naldi, Vinay Nangia, Jobert Richie Nansseu, Bruno Ramos Nascimento, Haseeb Nawaz, Busisiwe P Ncama, Nahid Neamati, Ionut Negoi, Ruxandra Irina Negoi, Subas Neupane, Charles Richard James Newton, Frida N Ngalesoni, Josephine W Ngunjiri, Grant Nguyen, Long Hoang Nguyen, Trang Huyen Nguyen, Dina Nur Anggraini Ningrum, Yirga Legesse Nirayo, Muhammad Imran Nisar, Molly R Nixon, Shuhei Nomura, Mehdi Noroozi, Jean Jacques Noubiap, Hamid Reza Nouri, Malihe Nourollahpour Shiadeh, Mohammad Reza Nowroozi, Alypio Nyandwi, Peter S Nyasulu, Christopher M Odell, Richard Ofori-Asenso, Okechukwu Samuel Ogah, Felix Akpojene Ogbo, In-Hwan Oh, Anselm Okoro, Olanrewaju Oladimeji, Andrew T Olagunju, Tinuke O Olagunju, Pedro R Olivares, Bolajoko Olubukunola Olusanya, Jacob Olusegun Olusanya, Sok King Ong, Alberto Ortiz, Aaron Osgood-Zimmerman, Erika Ota, Brenda Achieng Otieno, Stanislav S Otstavnov, Mayowa Ojo Owolabi, Abayomi Samuel Oyekale, Mahesh P A, Smita Pakhale, Abhijit P Pakhare, Adrian Pana, Basant Kumar Panda, Songhomitra Panda-Jonas, Achyut Raj Pandey, Eun-Kee Park, Hadi Parsian, Shanti Patel, Snehal T Patil, Ajay Patle, George C Patton, Vishnupriya Rao Paturi, Deepak Paudel, Marcel Moraes Pedroso, Emmanuel K Peprah, David M Pereira, Norberto Perico, Konrad Pesudovs, William A Petri, Max Petzold, Maxwell Pierce, David M Pigott, Julian David Pillay, Meghdad Pirsaheb, Guilherme V Polanczyk, Maarten J Postma, Farshad Pourmalek, Akram Pourshams, Hossein Poustchi, Swayam Prakash, Narayan Prasad, Caroline A Purcell, Manorama B Purwar, Mostafa Qorbani, Reginald Quansah, Amir Radfar, Anwar Rafay, Alireza Rafiei, Fakher Rahim, Afarin Rahimi-Movaghar, Vafa Rahimi-Movaghar, Mahfuzar Rahman, Md Shafiur Rahman, Mohammad Hifz Ur Rahman, Muhammad Aziz Rahman, Sajjad Ur Rahman, Rajesh Kumar Rai, Fatemeh Rajati, Sasa Rajsic, Usha Ram, Chhabi Lal Ranabhat, Prabhat Ranjan, David Laith Rawaf, Salman Rawaf, Sarah E Ray, Christian Razo-García, Robert C Reiner, Cesar Reis, Giuseppe Remuzzi, Andre M N Renzaho, Serge Resnikoff, Satar Rezaei, Shahab Rezaeian, Mohammad Sadegh Rezai, Seyed Mohammad Riahi, Maria Jesus Rios-Blancas, Kedir Teji Roba, Nicholas L S Roberts, Leonardo Roever, Luca Ronfani, Gholamreza Roshandel, Ali Rostami, Enrico Rubagotti, George Mugambage Ruhago, Yogesh Damodar Sabde, Perminder S Sachdev, Basema Saddik, Sahar Saeedi Moghaddam, Hosein Safari, Yahya Safari, Roya Safari-Faramani, Mahdi Safdarian, Sare Safi, Saeid Safiri, Rajesh Sagar, Amirhossein Sahebkar, Mohammad Ali Sahraian, Haniye Sadat Sajadi, Mohamadreza Salahshoor, Nasir Salam, Joseph S Salama, Payman Salamati, Raphael De Freitas Saldanha, Zikria Saleem, Yahya Salimi, Hamideh Salimzadeh, Joshua A Salomon, Sundeep Santosh Salvi, Inbal Salz, Evanson Zondani Sambala, Abdallah M Samy, Juan Sanabria, Maria Dolores Sanchez-Niño, Itamar S Santos, Milena M Santric Milicevic, Bruno Piassi Sao Jose, Mayank Sardana, Abdur Razzaque Sarker, Rodrigo Sarmiento-Suárez, Satish Saroshe, Nizal Sarrafzadegan, Benn Sartorius, Shahabeddin Sarvi, Brijesh Sathian, Maheswar Satpathy, Arundhati R Sawant, Monika Sawhney, Sonia Saxena, Elke Schaeffner, Kathryn Schelonka, Ione J C Schneider, David C Schwebel, Falk Schwendicke, Soraya Seedat, Mario Sekerija, Sadaf G Sepanlou, Edson Serván-Mori, Hosein Shabaninejad, Katya Anne Shackelford, Azadeh Shafieesabet, Amira A Shaheen, Masood Ali Shaikh, Raad A Shakir, Mehran Shams-Beyranvand, Mohammadbagher Shamsi, Morteza Shamsizadeh, Heidar Sharafi, Kiomars Sharafi, Mehdi Sharif, Mahdi Sharif-Alhoseini, Jayendra Sharma, Rajesh Sharma, Jun She, Aziz Sheikh, Peilin Shi, Kenji Shibuya, Mika Shigematsu, Rahman Shiri, Reza Shirkoohi, Ivy Shiue, Farhad Shokraneh, Sharvari Rahul Shukla, Si Si, Soraya Siabani, Abla Mehio Sibai, Tariq J Siddiqi, Inga Dora Sigfusdottir, Rannveig Sigurvinsdottir, Naris Silpakit, Diego Augusto Santos Silva, João Pedro Silva, Dayane Gabriele Alves Silveira, Narayana Sarma Venkata Singam, Jasvinder A Singh, Narinder Pal Singh, Virendra Singh, Dhirendra Narain Sinha, Karen Sliwa, Adauto Martins Soares Filho, Badr Hasan Sobaih, Soheila Sobhani, Moslem Soofi, Joan B Soriano, Ireneous N Soyiri, Chandrashekhar T Sreeramareddy, Vladimir I Starodubov, Caitlyn Steiner, Leo G Stewart, Mark A Stokes, Mark Strong, Michelle L Subart, Mu'awiyyah Babale Sufiyan, Gerhard Sulo, Bruno F Sunguya, Patrick John Sur, Ipsita Sutradhar, Bryan L Sykes, P N Sylaja, Dillon O Sylte, Cassandra E I Szoeke, Rafael Tabarés-Seisdedos, Karen M Tabb, Santosh Kumar Tadakamadla, Nikhil Tandon, Aberash Abay Tassew, Segen Gebremeskel Tassew, Nuno Taveira, Nega Yimer Tawye, Arash Tehrani-Banihashemi, Tigist Gashaw Tekalign, Merhawi Gebremedhin Tekle, Mohamad-Hani Temsah, Abdullah Sulieman Terkawi, Manaye Yihune Teshale, Belay Tessema, Mebrahtu Teweldemedhin, Jarnail Singh Thakur, Kavumpurathu Raman Thankappan, Sathish Thirunavukkarasu, Nihal Thomas, Alan J Thomson, Binyam Tilahun, Quyen G To, Marcello Tonelli, Roman Topor-Madry, Anna E Torre, Miguel Tortajada-Girbés, Marcos Roberto Tovani-Palone, Hideaki Toyoshima, Bach Xuan Tran, Khanh Bao Tran, Srikanth Prasad Tripathy, Thomas Clement Truelsen, Nu Thi Truong, Afewerki Gebremeskel Tsadik, Amanuel Tsegay, Nikolaos Tsilimparis, Lorainne Tudor Car, Kingsley N Ukwaja, Irfan Ullah, Muhammad Shariq Usman, Olalekan A Uthman, Selen Begüm Uzun, Muthiah Vaduganathan, Afsane Vaezi, Gaurang Vaidya, Pascual R Valdez, Elena Varavikova, Santosh Varughese, Tommi Juhani Vasankari, Ana Maria Nogales Vasconcelos, Narayanaswamy Venketasubramanian, Santos Villafaina, Francesco S Violante, Sergey Konstantinovitch Vladimirov, Vasily Vlassov, Stein Emil Vollset, Theo Vos, Kia Vosoughi, Isidora S Vujcic, Fasil Shiferaw Wagnew, Yasir Waheed, Judd L Walson, Yanping Wang, Yuan-Pang Wang, Elisabete Weiderpass, Robert G Weintraub, Kidu Gidey Weldegwergs, Andrea Werdecker, Ronny Westerman, Harvey Whiteford, Justyna Widecka, Katarzyna Widecka, Tissa Wijeratne, Andrea Sylvia Winkler, Charles Shey Wiysonge, Charles D A Wolfe, Shouling Wu, Grant M A Wyper, Gelin Xu, Tomohide Yamada, Yuichiro Yano, Mehdi Yaseri, Yasin Jemal Yasin, Pengpeng Ye, Gökalp Kadri Yentür, Alex Yeshaneh, Ebrahim M Yimer, Paul Yip, Engida Yisma, Naohiro Yonemoto, Seok-Jun Yoon, Marcel Yotebieng, Mustafa Z Younis, Mahmoud Yousefifard, Chuanhua Yu, Vesna Zadnik, Zoubida Zaidi, Sojib Bin Zaman, Mohammad Zamani, Zohreh Zare, Mulugeta Molla Zeleke, Zerihun Menlkalew Zenebe, Taddese Alemu Zerfu, Xueying Zhang, Xiu-Ju Zhao, Maigeng Zhou, Jun Zhu, Stephanie R M Zimsen, Sanjay Zodpey, Leo Zoeckler, Alan D Lopez, Stephen S Lim

## Abstract

**Background:**

Population estimates underpin demographic and epidemiological research and are used to track progress on numerous international indicators of health and development. To date, internationally available estimates of population and fertility, although useful, have not been produced with transparent and replicable methods and do not use standardised estimates of mortality. We present single-calendar year and single-year of age estimates of fertility and population by sex with standardised and replicable methods.

**Methods:**

We estimated population in 195 locations by single year of age and single calendar year from 1950 to 2017 with standardised and replicable methods. We based the estimates on the demographic balancing equation, with inputs of fertility, mortality, population, and migration data. Fertility data came from 7817 location-years of vital registration data, 429 surveys reporting complete birth histories, and 977 surveys and censuses reporting summary birth histories. We estimated age-specific fertility rates (ASFRs; the annual number of livebirths to women of a specified age group per 1000 women in that age group) by use of spatiotemporal Gaussian process regression and used the ASFRs to estimate total fertility rates (TFRs; the average number of children a woman would bear if she survived through the end of the reproductive age span [age 10–54 years] and experienced at each age a particular set of ASFRs observed in the year of interest). Because of sparse data, fertility at ages 10–14 years and 50–54 years was estimated from data on fertility in women aged 15–19 years and 45–49 years, through use of linear regression. Age-specific mortality data came from the Global Burden of Diseases, Injuries, and Risk Factors Study (GBD) 2017 estimates. Data on population came from 1257 censuses and 761 population registry location-years and were adjusted for underenumeration and age misreporting with standard demographic methods. Migration was estimated with the GBD Bayesian demographic balancing model, after incorporating information about refugee migration into the model prior. Final population estimates used the cohort-component method of population projection, with inputs of fertility, mortality, and migration data. Population uncertainty was estimated by use of out-of-sample predictive validity testing. With these data, we estimated the trends in population by age and sex and in fertility by age between 1950 and 2017 in 195 countries and territories.

**Findings:**

From 1950 to 2017, TFRs decreased by 49·4% (95% uncertainty interval [UI] 46·4–52·0). The TFR decreased from 4·7 livebirths (4·5–4·9) to 2·4 livebirths (2·2–2·5), and the ASFR of mothers aged 10–19 years decreased from 37 livebirths (34–40) to 22 livebirths (19–24) per 1000 women. Despite reductions in the TFR, the global population has been increasing by an average of 83·8 million people per year since 1985. The global population increased by 197·2% (193·3–200·8) since 1950, from 2·6 billion (2·5–2·6) to 7·6 billion (7·4–7·9) people in 2017; much of this increase was in the proportion of the global population in south Asia and sub-Saharan Africa. The global annual rate of population growth increased between 1950 and 1964, when it peaked at 2·0%; this rate then remained nearly constant until 1970 and then decreased to 1·1% in 2017. Population growth rates in the southeast Asia, east Asia, and Oceania GBD super-region decreased from 2·5% in 1963 to 0·7% in 2017, whereas in sub-Saharan Africa, population growth rates were almost at the highest reported levels ever in 2017, when they were at 2·7%. The global average age increased from 26·6 years in 1950 to 32·1 years in 2017, and the proportion of the population that is of working age (age 15–64 years) increased from 59·9% to 65·3%. At the national level, the TFR decreased in all countries and territories between 1950 and 2017; in 2017, TFRs ranged from a low of 1·0 livebirths (95% UI 0·9–1·2) in Cyprus to a high of 7·1 livebirths (6·8–7·4) in Niger. The TFR under age 25 years (TFU25; number of livebirths expected by age 25 years for a hypothetical woman who survived the age group and was exposed to current ASFRs) in 2017 ranged from 0·08 livebirths (0·07–0·09) in South Korea to 2·4 livebirths (2·2–2·6) in Niger, and the TFR over age 30 years (TFO30; number of livebirths expected for a hypothetical woman ageing from 30 to 54 years who survived the age group and was exposed to current ASFRs) ranged from a low of 0·3 livebirths (0·3–0·4) in Puerto Rico to a high of 3·1 livebirths (3·0–3·2) in Niger. TFO30 was higher than TFU25 in 145 countries and territories in 2017. 33 countries had a negative population growth rate from 2010 to 2017, most of which were located in central, eastern, and western Europe, whereas population growth rates of more than 2·0% were seen in 33 of 46 countries in sub-Saharan Africa. In 2017, less than 65% of the national population was of working age in 12 of 34 high-income countries, and less than 50% of the national population was of working age in Mali, Chad, and Niger.

**Interpretation:**

Population trends create demographic dividends and headwinds (ie, economic benefits and detriments) that affect national economies and determine national planning needs. Although TFRs are decreasing, the global population continues to grow as mortality declines, with diverse patterns at the national level and across age groups. To our knowledge, this is the first study to provide transparent and replicable estimates of population and fertility, which can be used to inform decision making and to monitor progress.

**Funding:**

Bill & Melinda Gates Foundation.

## Introduction

Age-sex-specific estimates of population are a bedrock of epidemiological and economic analyses, and they are integral to planning across several sectors of society. As the denominator for most indicators, such estimates permeate every aspect of our understanding of health and development. Errors in population estimates affect national and international target tracking and time-series and cross-country analyses of development outcomes. The importance of accurate population estimates for government planning cannot be overstated: population size, age, and composition dictate the national need for infrastructure, housing, education, employment, health care, care of older people, electoral representation, provision of public health and services, food supply, and security.^1^ Similarly, fertility rates, both by maternal age and overall, are key drivers of population growth and important social outcomes in their own right.

Many governments typically produce national population estimates by age and sex for planning purposes. Most international studies and comparative indicators, including the Millennium Development Goals and the Sustainable Development Goals, rely on the estimates generated by the UN Population Division at the Department of Economics and Social Affairs (UNPOP) for population denominators,^2^, ^3^ although it is not well documented how often these estimates are used by national governments. The UNPOP has produced population estimates since 1951, and it uses a decentralised approach to estimation.^4^ For example, the Latin American and Caribbean Demographic Centre produces estimates for Latin America, whereas estimates for all other groups of countries are developed by analysts in New York. Although the UNPOP describes a general approach of examining data on fertility, mortality, migration, and population and searching for consistency,^5^ replicable statistical methods are not used. Decisions on how to deal with inconsistency between the components of fertility, mortality, and migration within population counts are left to individual analysts, leading to considerable heterogeneity in approaches across countries. Accordingly, discrepancies between UNPOP and nationally produced estimates—for instance, in 2015, the population estimates for Mexico by UNPOP were 4·6 million more than those of Mexico's National Population Council (125·9 million by UNPOP *vs* 121·3 million by National Population Council)—cannot currently be resolved.^4^, ^6^

**Research in context**

**Evidence before this study**

Population estimates by age and sex are extensively used in all forms of epidemiological and demographic analysis. National estimates of population and fertility for age and sex groups have been produced by the UN Population Division since 1951. The US Census Bureau produces revised demographic estimates for 15 to 30 countries each year. Several national authorities produce their own population estimates, particularly those in high and middle Socio-demographic Index countries. These efforts are all based on the cohort-component method of population projection, namely that population in an age group at a given time *t* must equal the population in that cohort at the start of the time period (*t*–1) plus new entrants and minus people exiting the population because of migration and death. Although these estimates are based on the demographic balancing equation, estimates are not based on standardised, transparent, or replicable statistical methods.

**Added value of this study**

To our knowledge, this study presents the first estimates of population by location from 1950 to 2017 that are based on transparent data and replicable analytical code, applying a standardised approach to the estimation of population for each single year of age for each calendar year from 1950 to 2017 for 195 countries and territories and for the globe. This study provides improved population estimates that are internally consistent with the Global Burden of Diseases, Injuries, and Risk Factors Study's assessment of fertility and mortality, which are important inputs to other epidemiological research and government planning.

**Implications of all the available evidence**

Population counts by age and sex that are produced with a transparent and empirical approach will be useful for epidemiological and demographic analyses. The production of annual estimates will also facilitate timely tracking of progress on global indicators, including the Sustainable Development Goals. In the future, the methods applied here can be used to enhance population estimation at the subnational level.

The US Census Bureau's International Division periodically releases detailed population analyses for selected countries, with new revisions produced for 15 to 30 countries per year.^7^ Other organisations, such as the Population Reference Bureau,^8^ the World Bank,^9^ the Wittgenstein Centre,^10^ and Gapminder Foundation^11^ also release population estimates, but these are largely combinations of national estimates with selected UNPOP or US Census Bureau analyses. Many of the organisations who estimate or report on population also provide fertility estimates, which, in addition to affecting population trends, are used to monitor reproductive health service delivery in many locations. To our knowledge, global estimates of annual population by age and sex with underlying primary data and replicable computer code and statistical modelling details are not available from any source.

The Global Burden of Diseases, Injuries, and Risk Factors Study (GBD) is committed to the Guidelines on Accurate and Transparent Health Estimates Reporting (GATHER).^12^ Continued use of the UNPOP population estimates in GBD is not compatible with GATHER because the methods used for UNPOP estimation are not transparent and uncertainty intervals are not estimated for populations.^4^ Moreover, UNPOP population estimates, especially in years between or after a census, are inconsistent with GBD estimates because there is a marked difference between UNPOP and GBD estimates of age-specific mortality in many instances.^13^, ^14^ For this GBD 2017 paper, we sought to produce population estimates and associated fertility estimates for 195 countries and territories from 1950 to 2017 that were based on the available census or population registry data and survey and census data on age-specific fertility rates (ASFR; ie, the annual number of livebirths to women of a specified age group per 1000 women in that age group) by use of replicable methods, leveraging the previous GBD work that estimated age-sex-specific mortality rates.^15^ To achieve this goal, we aimed to conduct systematic analyses of available sources that could inform ASFR estimation and to systematically identify and extract census and population registry data.

## Methods

### Overview

As with all population estimation, the underlying equation used for GBD is based on the demographic balancing equation^16^

N(T)=N(0)+B(0,T)-D(0,T)+G(0,T)

where *N (T)* is the population at a given time, *N (0)* is the population at the start of the interval, *B (0,T)* is livebirths during the interval, *D (0,T)* is deaths during the interval, and *G (0,T)* is net migration during the interval.

The cohort-component method of population projection extends this demographic balancing equation to estimate internally consistent age-sex-specific populations. The method requires estimates of ASFRs, sex ratio at birth, age-sex-specific net migration, and age-sex-specific mortality rates that are consistent with observed population counts that have been corrected for underenumeration or overenumeration. GBD provides a consistent set of age-sex-specific mortality rates with standardised methods;^15^ in this analysis, we estimated the sex ratio at birth, ASFR, and age-sex-specific migration rates consistent with the available population data to create a full time series of population estimates by age and sex.

These estimates comply with GATHER (appendix 1 section 5). Analyses were done with R version 3.3.2, Python version 2.7.14, or Stata version 13.1. Data and statistical code for all analyses are publicly available online.

### Geographical units and time periods

We produced single calendar-year and single year-of-age population estimates for 195 countries and territories that were grouped into 21 regions and seven super-regions. The seven super-regions are central Europe, eastern Europe, and central Asia; high income; Latin America and the Caribbean; north Africa and the Middle East; south Asia; southeast Asia, east Asia, and Oceania; and sub-Saharan Africa. Each year, GBD includes subnational analyses for a few new countries and continues to provide subnational estimates for countries that were added in previous cycles. Subnational estimation in GBD 2017 includes five new countries (Ethiopia, Iran, New Zealand, Norway, Russia) and countries previously estimated at subnational levels (GBD 2013: China, Mexico, and the UK [regional level]; GBD 2015: Brazil, India, Japan, Kenya, South Africa, Sweden, and the USA; GBD 2016: Indonesia and the UK [local government authority level]). All analyses are at the first level of administrative organisation within each country except for New Zealand (by Māori ethnicity), Sweden (by Stockholm and non-Stockholm), and the UK (by local government authorities). All subnational estimates for these countries were incorporated into model development and evaluation as part of GBD 2017. To meet data use requirements, in this publication we present all subnational estimates excluding those pending publication (Brazil, India, Japan, Kenya, Mexico, Sweden, the UK, and the USA); given space constraints, these results are presented in appendix 2 instead of the main text. Subnational estimates for countries with populations of more than 200 million people (assessed by use of our most recent year of published estimates) that have not yet been published elsewhere are presented wherever estimates are illustrated with maps but are not included in tables. Estimates were produced for the years 1950–2017. 1950 was selected as the start year for the analysis because we were unable to locate sufficient data on ASFR, mortality, and population before 1950.

### Fertility

Fertility data are obtained from vital registration systems, complete birth histories, or summary birth histories. Complete birth histories include the date of birth and, if applicable, the dates of death of all children ever born alive to each woman that is interviewed, whereas summary birth histories include the total number of children ever born alive to each mother and the total number of those children born alive to each mother that have died. In countries with complete birth registration, vital registration systems typically provide tabulations of births by age of the mother. From 1890,^17^ some censuses asked about the number of children ever born to a woman, and this question has been widely asked in censuses and many household surveys in the past 70 years. From the 1970s, fertility information has also been collected through complete birth histories, beginning with the World Fertility Survey, then the Demographic and Health Surveys, and, in some countries, the Multiple Indicator Cluster Surveys, sponsored by the UN Children's Fund. We identified 977 censuses and household surveys that had summary birth history data, 429 household surveys that had complete birth history data, and 7817 country-years of birth registration systems through searches of national statistical sources and the Demographic Yearbooks produced by the UN Statistics Division from 1948 to present.^18^ The number and type of sources for each location are provided in appendix 1 (section 5). The Global Health Data Exchange provides the metadata for all these sources.

Given the hetergeneous nature of the data (vital registration, summary birth histories, complete birth histories), we used a two-stage approach to modelling the ASFR for the age groups 15–19 years, 20–24 years, 25–29 years, 30–34 years, 35–39 years, 40–44 years, and 45–49 years. The two-stage approach was designed to take advantage of the greater availability of some summary birth history data for the period 1950 to 1975 and to help to compensate for the lower availability of complete birth history data in some low-income countries. For the fertility rates in those aged 10–14 years and 50–54 years, which are much lower than in other age groups and for which only vital registration data were available, we used a separate, simpler approach, described later in this section.

In the first stage of our analysis, we used spatiotemporal Gaussian process regression to analyse vital registration and complete birth history data.^15^, ^19^ For spatiotemporal Gaussian process regression, the prior was estimated separately for women aged 20–24 years, with average years of schooling in women aged 20–24 years as the covariate. For all other age groups, the prior was estimated with a spline on the estimated ASFR for women aged 20–24 years and with the average years of schooling for the age group of interest. The prior for GBD locations in the high-income super-region did not include average years of schooling as a covariate. Spline knots were selected by inspection of the data to identify where there was a reversal in trend. The purpose of this approach was to capture an increase in fertility rates in women aged 30 years or older while the ASFR for women aged 20–24 years decreased below a specific threshold. Given that the point of inflection for the ASFR for women aged 30 years or older relative to the ASFR for women aged 20–24 years varied by super-region, we fit the models separately for some GBD super-regions (high income; sub-Saharan Africa; and central Europe, eastern Europe, and central Asia) and modelled the rest of the super-regions together. The first step of the model also included location-and-source-specific random effects to correct bias from non-sampling error in different source types, such as incomplete vital registration. Hyperparameters for the model were selected on the basis of a measure of data density. Further details on this process are provided in appendix 1 (section 2).

In the second stage of the analysis, we used the ASFR estimates from the first stage to process and incorporate several forms of aggregated data. First, we split cumulative cohort fertility data (ie, children ever born) from summary birth history into period ASFR data. For this split, we computed the ratio between reported children ever born alive from each 5-year cohort of women represented in a given data source and the total fertility for each of these cohorts that was implied by the first-stage estimates of ASFR by location and year. This ratio was applied as a scaling factor to our estimated cohort ASFR at 5-year intervals (when all members of the cohort all belong to a single 5-year GBD age group), to distribute experienced fertility (ie, from age 10 years until the date of the survey in women interviewed from the cohorts specified in the original data) back across age and time. Additionally, we used the estimated age proportion of livebirths from the first stage to distribute total reported livebirths by the age of the mother. Lastly, for historical location aggregates for which we had registry data (eg, the Soviet Union), we used the estimated proportions of age-specific livebirths in constituent locations from the first stage to allocate births back in time to their current GBD geographies. This new set of methods allowed us to supplement the model with a substantial amount of additional information about the overall fertility. We then re-estimated ASFR as described, with all vital registration, complete birth history, and split data to produce final fertility estimates for women aged 15–49 years.

In both the first and second stage, data were adjusted in the mixed-effects model on the basis of random effects values (appendix 1 section 2) by selecting a reference or benchmark source. In locations with complete child death registration (see previous GBD analyses),^15^, ^20^ vital registration was typically the benchmark or reference source. In other locations, Demographic and Health Survey complete birth history data were used as the reference source. If neither vital registration nor Demographic and Health Survey complete birth histories were available, other complete birth history sources were used as the reference. If no vital registration or complete birth history data were used, then the average of all remaining summary birth history sources were used as reference. Where sources were inconsistent or implausible time trends were identified, some reference source designations were modified; the final choice of reference sources for each location are provided in the appendix 1 (section 5).

Many household surveys on fertility excluded women in the age groups 10–14 years and 50–54 years, and these data were limited to 3947 country-years of vital registration data. To estimate fertility in girls aged 10–14 years, we used a linear regression of the log of the ratio of the ASFR of girls aged 10–14 years to the ASFR for girls aged 15–19 years as a function of the ASFR for girls aged 15–19 years. For women aged 50–54 years, we found no covariates that predicted variation in the ratio of ASFR for women aged 50–54 years to the ASFR for those aged 45–49 years. In this case, we assumed the ratio of ASFR for women aged 50–54 years to ASFR for women aged 45–49 years was constant across locations and over time.

Our analysis generated a full set of ASFRs for each location and year from 1950 to 2017; we used these ASFRs to compute the total fertility rate (TFR), which is the average number of children a woman would bear if she survived through the end of the reproductive age span (age 10–54 years) and experienced at each age a particular set of ASFRs observed in the year of interest. We also estimated the total fertility rate under age 25 years (TFU25; number of livebirths expected by age 25 years for a hypothetical woman who survived the age group and was exposed to current ASFRs) and the total fertility in women older than 30 years (TFO30; number of livebirths expected for a hypothetical woman ageing from 30 to 54 years who survived the age group and was exposed to current ASFRs). These age ranges were computed because nearly all locations show decreases in the TFU25 over time, with few or no reversals. In women aged 30 years or older, there is a clear U-shaped curve, with decreases followed by sustained increases; in women aged 25–29 years, the pattern is less consistent. The fertility rate in girls aged 10–19 years is a Sustainable Development Goal (SDG) indicator for goal 3, target 3.7: ensure universal access to sexual and reproductive health-care services, including for family planning, information and education, and the integration of reproductive health into national strategies and programmes.^21^

We estimated the sex ratio at birth with 4690 unique location-years of registered livebirths by sex, 1756 location-years of census and population registry counts that included children younger than 1 year and younger than 5 years by sex, and 2490 location-years of the proportion of live-born males from complete birth history. These data informed a spatiotemporal Gaussian process regression model of the proportion of live-born males, assuming a time-invariant prior for the mean because, in the absence of sex-selective abortion, we would not expect the sex ratio at birth to deviate significantly from its natural equilibrium. Hyperparameters for spatiotemporal smoothing and Gaussian process regression were chosen on the basis of data-density scores, taking into account both the quantity and quality of available data. Our analysis only produced national estimates of sex ratio at birth—including for Hong Kong and Macau—for all years from 1950 to 2017; thus, we assume that subnational sex ratio at birth equals the national sex ratio at birth. With additional data seeking and extraction, we will extend the analysis to all GBD locations in the next GBD study. Further details regarding sex ratio at birth estimation are shown in appendix 1 (section 2).

### Population

To determine national and subnational populations, we searched the Integrated Public Use Microdata Series questionnaires, the UN Demographic Yearbook, the UN census programme census dates, and the International Population Census Biography to identify all censuses conducted between 1950 and 2017 and available population registers.^22^, ^23^, ^24^, ^25^ We included 1233 censuses and 26 population registers that contained 730 location-years of census or population registry data. In some cases, the same census was reported by different sources in different years. We resolved these inconsistencies through a review of available documentation. A list of all confirmed censuses is shown in the appendix 1 (section 5). We obtained population counts that were age-sex-specific from 1171 censuses and only by sex from 62 censuses. We sought to identify whether the counts in each census were de facto (allocated to the place of enumeration) or de jure (allocated to the place of regular or legal residence). Our basis for population estimation is the de-facto population and, where both counts were available, we used de-facto counts. Where only de-jure counts were available—typically in lower Socio-demographic Index (SDI) countries—we assumed that de-jure and de-facto populations were similar. The main difference between the counts at the national level is the exclusion of some migrant workers in some de-jure counts; where migrant workers are known to be an important fraction of the population and de-facto counts were not available, we searched directly for data on documented migration.

In several cases, the UN does not recognise administrative splits in territories, including Kosovo and Serbia, Transnistria and Moldova, and the so-called Turkish Republic of Northern Cyprus and Cyprus.^26^ In these cases, we obtained census counts for the components and interpolated to generate census counts for the full territory. For east and west Germany before unification, as the input to the model, we used census counts for each component and interpolation to generate estimates of joint census counts in years closest to the censuses in both locations. We were able to obtain census counts for five of the six constituent components that made up Yugoslavia; for Serbia we split aggregate Yugoslavia census data with previous population estimates. For Singapore, we estimated the population for residents and non-resident workers combined (appendix 1 section 2). Of the 1963 location-years of census or population registry data, 72 location-years were identified as outliers that were inconsistent with adjacent data, model analysis, or excluded subpopulations.

Census counts are typically undercounts of the actual population, although there are known cases in which censuses have overcounted the population.^27^, ^28^, ^29^ Post-enumeration surveys (PESs) aim to identify instances of overcounts or undercounts by comparing data. Many, if not most, PESs are not published or are only reported in government releases, presentations, or online reports. PESs themselves are subject to considerable error, whether they use a direct or indirect method of estimating census completeness. We searched for all available PES results and supplemented these results with publications or presentations that provided summaries of other PESs.^30^, ^31^, ^32^, ^33^, ^34^ We identified 165 PESs, although it is likely that many more were done that did not publicly report their results. We analysed the 165 PESs to generate a general model of census completeness as a function of SDI. Because of variable quality of PESs, we assumed that, in aggregate, the 165 PESs provided an unbiased view of the association between enumeration completeness and SDI, so we adjusted census counts by the predictions from this model. We used nationally reported PES results to adjust census counts in high SDI countries and used the estimated census completeness to adjust data in other settings. To account for systematic age variation in census enumeration, we input age-sex-specific PES results into DisMod-MR 2.1, a Bayesian meta-regression tool, to estimate a global age pattern of enumeration. This age pattern was then used to adjust the overall predicted enumeration to vary by age (appendix 1 section 2).

**Figure 1:**
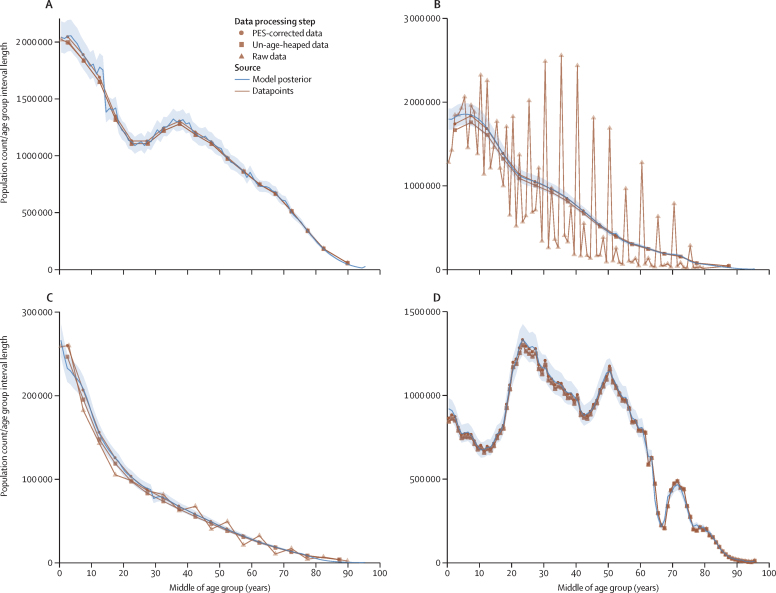
Census age patterns for females in 1970 in the USA (A), males in 2001 in Bangladesh (B), females in 1979 in Afghanistan (C), and males in 2010 in Russia (D) Lines show the model posterior and datapoints. Data processing steps are indicated by symbols. The 95% uncertainty interval is shown by light blue shading around the model posterior. PES=post-enumeration survey.

As has been extensively noted in the demographic literature, census counts have several common problems: undercounts (particularly of children younger than 5 years), a tendency to exaggerate age at older ages, and age heaping (reporting ages rounded to the nearest 5 or 10 years).^35^, ^36^, ^37^, ^38^ The population counts from four different censuses, illustrating the different types of age heaping and undercounts, are shown in figure 1. We evaluated the age structure and consistency of census data by calculating sex and age ratios for each census. These ratios were then used to calculate sex and age ratio scores, which were combined into a joint score. The joint score was used to determine whether to apply a correction to the census counts or not. For census counts available in 1-year age groups, we used the Feeney correction; for counts available in 5-year or 10-year age groups, we used either the Arriaga or Arriaga strong correction.^39^, ^40^ More details on the age-heaping corrections are shown in appendix 1 (section 2). For all censuses in low and middle SDI countries, we did not use the census count of children younger than 5 years in our model estimation. In other words, population estimates in these age groups were driven by fertility and mortality estimates and consistency with the later census counts for the same cohort. Systematic overestimation of age, particularly in some countries in sub-Saharan Africa and Latin America, was apparent in the data; for example, census counts could only be explained by large immigration of populations at older ages, which appears implausible. We were unable to correct the data for these issues and used the modelling strategy that is subsequently described to deal with these challenges.

Our approach requires an estimate of the population in 1950 in all locations for detailed age and sex groups; only 54 countries had a census count in 1950. For most other locations, we used backwards application of the cohort-component method of population projection by use of the oldest available census and the reverse application of estimated mortality rates and an assumption of zero net migration (appendix 1 section 2). As subsequently noted, in our GBD Bayesian demographic balancing modelling framework, the baseline population is assumed to be measured with substantial error, and the model produced posterior estimates that varied considerably from this initial baseline.

We used the estimates of population by location and year for each single year of age to generate other summary measures, including population growth rates that assumed logarithmic growth and the proportion of the population that was of working age, which is defined by the Organisation for Economic Co-operation and Development and the World Bank as those aged 15–64 years.^41^, ^42^

### Mortality

The GBD mortality process produced annual abridged life tables that comprised 24 age groups: younger than 1 year, 1–4 years, and then 5-year age groups up to age 110 years or older.^13^ To project populations forwards in time with the cohort-component method of population projection, we needed annual period life tables with single-year age groups up to 95 years or older. For ages 15–99 years, we interpolated abridged *l*_x_ values (the number of people still alive at age *x* for a hypothetical cohort in a period life table) by use of a monotone cubic spline with Hyman filtering.^43^, ^44^ For people younger than 15 years and older than 100 years, we applied regression coefficients to predict single-year age group probability of death values. The Human Mortality Database provided 4557 empirical full-period life tables for 48 locations. We excluded 1280 of the life tables because they were identified by the Human Mortality Database as problematic or occurred during time periods with extremely high mortality, such as World War 2 or the 1918 influenza pandemic. To predict probability of death *q*_x_ at age *x* for single-year age groups, we fit the following separate linear regression by single-year age group between ages zero and 110:

$${log(}_{1}q_{xf})=\beta_{0}+\beta_{1}{log(}_{5}q_{xa})+{ɛ}_{xf}$$

where _1_*q*_xf_ is the single-year age group *q*_x_ value from the full-period life table, β_0_ is the coefficient for the intercept, β_1_ is the coefficient for the slope, ɛ_xf_ is the error term, and _5_*q*_xa_ is the correponding abridged life-table age group's *q*_x_ value. These predicted _1_*q*_xf_ values were scaled to the GBD abridged life-table _5_*q*_x_ values for consistency.

For those aged 15–99 years, the non-parametric spline approach did not require rescaling to match the abridged _5_*q*_x_ values and, consequently, produced smooth steps in mortality across single-year ages and between 5-year age groups. The regression coefficients were applied to children younger than 15 years because of the unique patterns of single-year mortality younger than 15 years and to adults older than 100 years because of instability caused by low *l*_x_ values at older ages. To mitigate instability caused by spikes in mortality due to fatal discontinuities such as wars and natural disasters, full-period life tables were first generated based on abridged life tables without fatal discontinuities, and then fatal discontinuities were added to _n_*m*_x_ (the death rate in age group *x* to *x* + 1 for a hypothetical cohort in a period life table) assuming a constant death rate for fatal discontinuities within each age group. To produce full life tables with the complete set of single-year age group _1_*q*_x_ values, we assumed _1_*a*_x_ (the average number of years lived in age group *x* to *x* + 1 by people who died during the interval for a hypothetical cohort in a period life table) was 0·5 in all age groups except for those younger than 1 year and older than 110 years; these groups were assumed to be identical to the abridged life-table _1_*a*_x_ values.

### Migration

Real data on age-specific net migration are more difficult to obtain than data on fertility, population, and mortality. Net migration includes any change in the de-facto population that is not accounted for by births or deaths; this number would include refugees and temporary workers. For most country-years, documented net migration data are not reported and undocumented net migration is not estimated. For some high-SDI countries, net migration is tracked and reported,^45^ and the UN High Commission for Refugees (UNHCR) reports the stock of refugees (the count of people not born in the country that they currently live in) in each country by country of origin at the end of year. In more recent census rounds, census questions on the number of foreign-born individuals living in a country have been used, as have assumptions on differential survival to estimate when migration occurred;^46^ however, these approaches, especially for the period before 2000, have considerable uncertainty associated with them and are heavily dependent on fertility and mortality assumptions for migrants.

We developed and applied the GBD Bayesian demographic balancing model to estimate net migration by single year of age and single calendar year, consistent with our estimates of age-sex-specific mortality and ASFR and the observed population data. Our model was developed on the basis of the work of Wheldon and colleagues^47^, ^48^, ^49^ but includes important modifications, such as correlation of migration rates across ages and over time and single-year, single-age estimation. Details on our GBD Bayesian demographic balancing model, developed in Template Model Builder, an open-source statistical package for R,^50^ are shown in the appendix 1 (section 2).

In applying the model, we dealt with known issues of age misreporting by including larger input data variance for population counts at the youngest ages and input variance that steadily increases after age 45 years. The choice of data variance was based on testing of a range of variance assumptions; variance assumptions only change the point estimates of the results in settings where there is substantial inconsistency between adjacent census counts or between census counts (or both) and in the key inputs. To address age misreporting in the oldest ages, we ran several model versions for each location. For each model version, we excluded census counts above a given maximum age from the model fitting process (appendix 1 section 5). We then selected the best model version by prioritising versions that used the highest maximum age, predicted low absolute values of migration in the age groups older than 55 years, and had good in-sample fits. In high-income locations, the selection algorithm often chose the model version that did not exclude any of the census data for older ages but, in other regions, the population estimates at older ages were driven by the census counts for younger ages and the mortality estimates that aged those people forwards in time (appendix 1 section 2).

**Figure 2:**
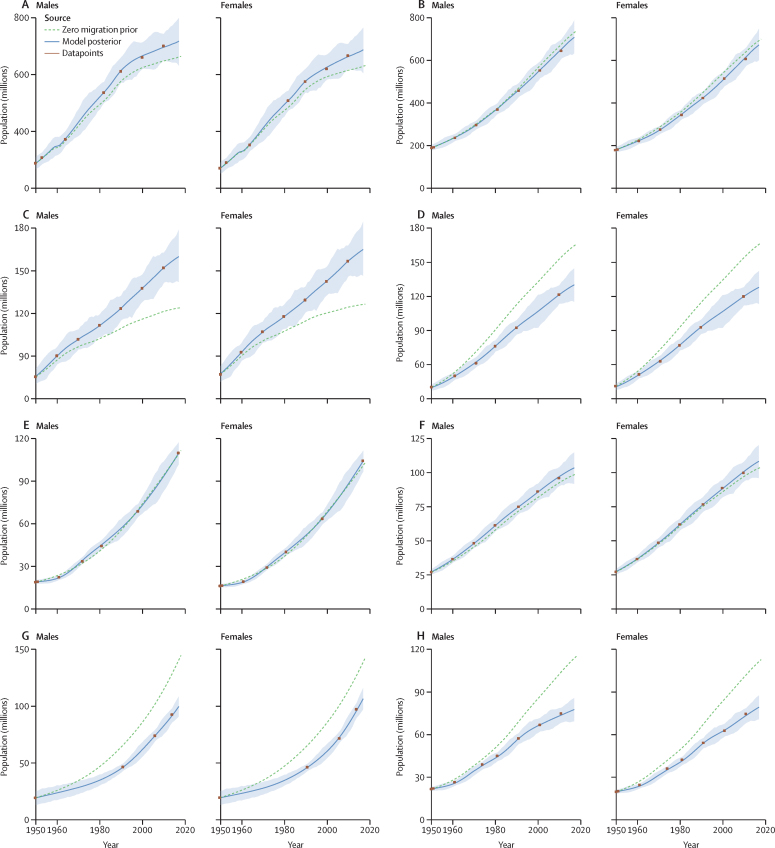
Fit of the GBD Bayesian demographic balancing model for the total population of males and females, from 1950 to 2017, in mainland China (A), India (B), the USA (C), Indonesia (D), Pakistan (E), Brazil (F), Nigeria (G), and Bangladesh (H) The 95% uncertainty interval is shown by light blue shading around the model posterior line. Mainland China excludes Hong Kong and Macao. GBD=Global Burden of Diseases, Injuries, and Risk Factors Study.

An example of the fit to the available population data for the eight largest populations in 2017 is shown in figure 2. Overall, the in-sample fit of the model for age-sex-specific population log space had an *R*^2^ value of 0·99. These fits show that the model closely tracks the available corrected census counts for all ages combined and by age. Code for the GBD Bayesian demographic balancing model is available at the Global Health Data Exchange. The population estimates and census and registry data for all 195 countries and territories are shown in appendix 2.

### The cohort-component method of population projection and uncertainty

We produced final population estimates by single year and by single-year age groups with the cohort-component method of population projection.^16^ The population in each single-year age group in each year was estimated on the basis of the estimated starting population and single-year, single-age rates of migration, fertility, and mortality. Uncertainty in population estimates comes from two fundamental sources: uncertainty about the completeness of a census count in a census year and uncertainty between censuses due to errors in estimates of migration, fertility, and mortality. Uncertainty in the counts was estimated by sampling the variance-covariance matrix of the model that predicted census completeness. We estimated the uncertainty between counts by use of out-of-sample predictive validity. We held out data and estimated the error in estimates as a function of the minimum of the number of years to the next or previous census. We combined these two sources of uncertainty and generated 1000 draws of percentage error in the population for each location-year. The 1000 draws of percentage error in the population and the population mean, generated by the GBD Bayesian demographic balancing model, were then combined to create 1000 draws of population by age, sex, location, and year. 95% uncertainty intervals (UIs) were calculated with the 2·5th and 97·5th percentiles. Details of this out-of-sample estimation of uncertainty are shown in appendix 1 (section 2). Out-of-sample estimates of uncertainty yielded larger uncertainty than in-sample methods because of the nearly perfect inverse correlation between migration and death rates, which was conditional on census counts with low error. A dot plot comparison of our total population counts by country for different age groups in 2017 with UNPOP estimates is shown in appendix 2.

### SDI

GBD 2015 developed the SDI as a composite measure of TFR in a population, lag-distributed income per capita, and average years of education in the population older than 15 years.^15^, ^20^ Each component was rescaled to a value between 0 and 1, and the SDI was derived from their geometric mean. The TFR was used in this overall measure of development as a proxy for the status of women in society; other plausible measures capturing the status of women are not available for all countries over a long time period. Our analysis of detailed ASFR revealed in many countries that, through the process of development the TFO30 generally decreased and then increased. For example, in the USA, the TFO30 has increased steadily from 1975. In exploratory analysis, we found that the TFU25 did not show this U-shaped pattern as countries develop. For GBD 2017, we have recalculated the SDI by use of the TFU25 as a better proxy for the status of women in society. The TFU25 not only does not show a U-shaped pattern with development but also remains highly correlated with under-5 mortality (Pearson correlation coefficient *r*=0·873) and other mortality measures. The revised method for computing SDI compared with the GBD 2016 method is correlated with the GBD 2017 method (*r*=0·992). Detailed comparisons of the GBD 2015 and GBD 2016 methods compared with the approach we used are shown in appendix 1 (section 3).

### Role of the funding source

The funder of the study had no role in study design, data collection, data analysis, data interpretation, or writing of the report. All authors had full access to all the data in the study and had final responsibility for the decision to submit for publication.

## Results

### Global

**Figure 3:**
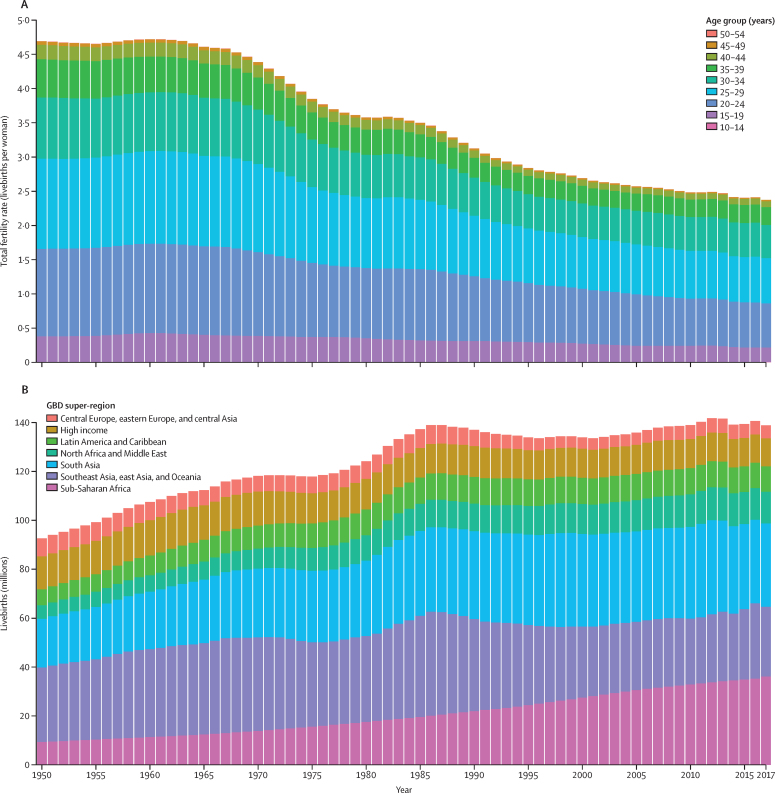
Global total fertility rate distributed by maternal age group (A) and number of livebirths by GBD super-region, for both sexes combined (B), 1950–2017 Total fertility rate is the number of births expected per woman in each age group if she were to survive through the reproductive years (10–54 years) under the age-specific fertility rates at that timepoint. GBD=Global Burden of Diseases, Injuries, and Risk Factors Study.

The global TFR by maternal age group from 1950 to 2017 is shown in figure 3. In 1950, the TFR was 4·7 livebirths (95% UI 4·5–4·9) and, by 2017, the TFR had decreased by 49·4% (46·4–52·0) to 2·4 livebirths (2·2–2·5). From 1950 to 1995, the TFR within all 5-year maternal age groups decreased: the greatest decrease in terms of contribution to TFR was in women aged 20–24 years (who showed a decrease of 0·42 livebirths), 25–29 years (0·52 livebirths), and 30–34 years (0·38 livebirths). Since 1995, decreases in the contribution to TFR from women aged 30–34 years, 35–39 years, and 40–44 years effectively plateaued at the global level, whereas decreases in women at younger ages continued. This slowing trend in reductions in the number of livebirths per woman in these age groups masks marked heterogeneity across countries, as we subsequently discuss. Of the total livebirths globally in 2017, 9·4% occurred in teenage mothers, which is a reduction from 9·9% of livebirths to teenage mothers in 1950. The age-specific fertility rate per 1000 women aged 10–19 years decreased from 37 livebirths (34–40) per 1000 women in 1950 to 22 livebirths (19–24) per 1000 women in 2017. The number of livebirths globally increased from 92·6 million livebirths (88·9–96·4 million) in 1950 to a peak of 141·7 million livebirths (135·8–147·3 million) in 2012. Over the past 35 years, the number of livebirths annually has varied within a relatively narrow range of 133·2 million (130·1–136·2) livebirths to 141·7 million (135·8–147·3) livebirths.

**Figure 4:**
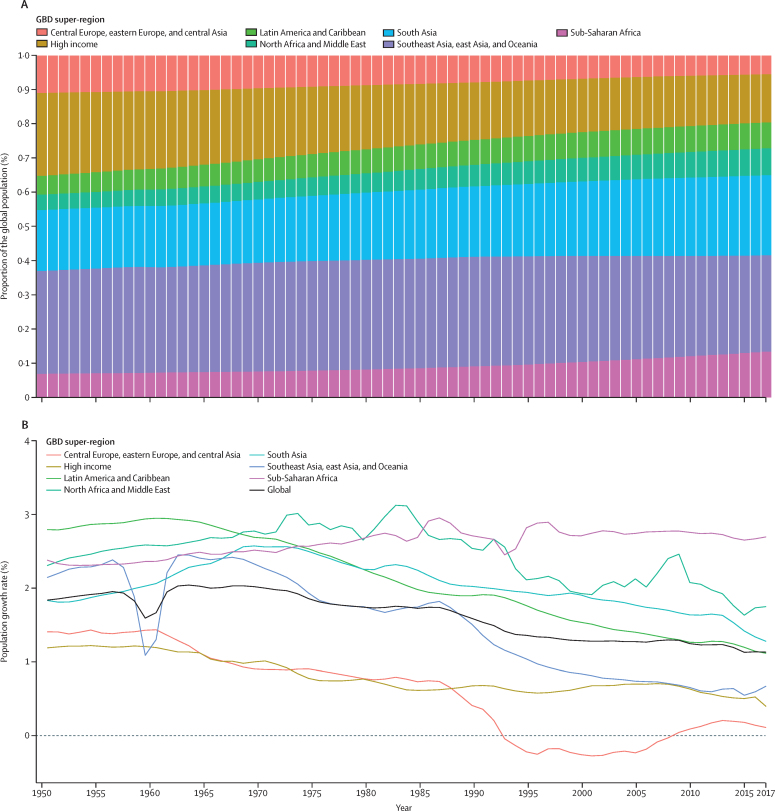
Proportion of the global population accounted for by the GBD super-regions (A) and the annual population growth rates, globally and for the super-regions (B) Data are shown for both sexes combined, from 1950 to 2017. GBD=Global Burden of Diseases, Injuries, and Risk Factors Study.

The trend in world population from 1950 to 2017 by GBD super-region is shown in figure 4. From 1950 to 1980, the global population increased exponentially at an annualised rate of 1·9% (95% UI 1·88–1·92). From 1981 to 2017, however, the pace of the global population increase has been largely linear, increasing by 83·6 million (79·8–87·5) people per year. Over the past 10 years (2007–17), the average annual increase in population has been by 87·2 million (80·8–93·2) people, compared with 81·5 million (79·0–84·5) people per year in the previous 10 years (1997–2007). The global population increased by 197·2% (95% UI 193·3–200·8), from 2·6 billion (2·5–2·6) people in 1950 to 7·6 billion (7·4–7·9) people in 2017. Over this period, the composition of the world's population changed substantially. In 1950, the high-income, central Europe, eastern Europe, and central Asia GBD super-regions accounted for 35·2% of the global population but, in 2017, the populations of these countries accounted for 19·5% of the global population. Large increases occurred in the proportion of the world's population living in south Asia, sub-Saharan Africa, Latin America and the Caribbean, and north Africa and the Middle East.

The annual population growth rate between 1950 and 2017, globally and for the GBD super-regions, is shown in figure 4. Growth of the global population increased in the 1950s and reached 2·0% per year in 1964, then slowly decreased to 1·1% in 2017. The slow shift in the global population growth rate is determined by markedly different trends by super-region. Growth of the population in north Africa and the Middle East increased until the 1970s, and it has remained quite high, at 1·7% in 2017. Population growth rates in sub-Saharan Africa increased from 1950 to 1985, decreased during 1985–1993, increased again until 1997, and then plateaued; at 2·7% in 2017, population growth rates were almost the highest rates ever recorded in this region. The most substantial changes to population growth rates were in the southeast Asia, east Asia, and Oceania super-region, where the population growth rate decreased from 2·5% in 1963 to 0·7% in 2017. The large reduction in the population growth rate for this super-region around 1960 was due to the Great Leap Forward in China. In central Europe, eastern Europe, and central Asia, the population growth rate dropped rapidly after 1987 and was negative from 1993 to 2008. Growth rates in the high-income super-region have changed the least, starting at 1·2% in 1950 and reaching 0·4% in 2017.

**Figure 5:**
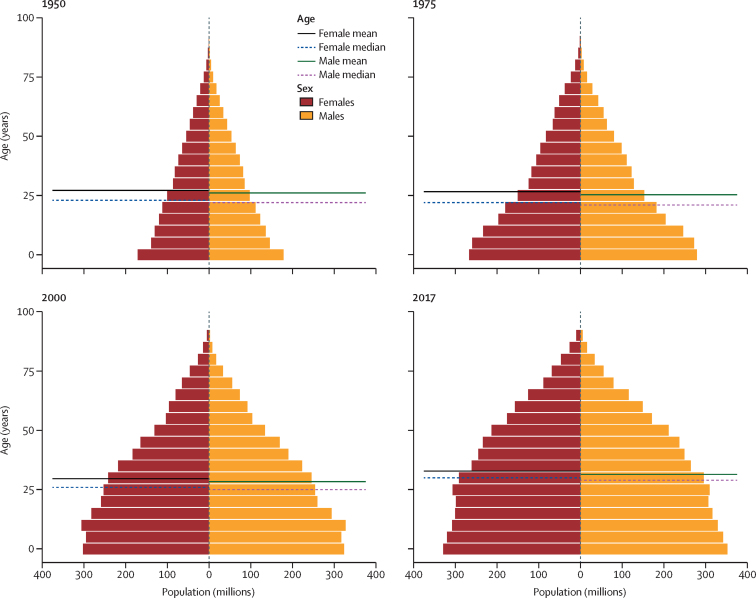
Global population pyramids for females and males by age, in 1950, 1975, 2000, and 2017

Global population pyramids in 1950, 1975, 2000, and 2017 are shown in figure 5. As the world's population has grown, not only has the distribution of the global population shifted toward sub-Saharan Africa and south Asia, but the age structure of the global population has also changed considerably. In 1950, the global mean age of a person was 26·6 years, decreasing to 26·0 years, in 1975, then increasing to 29·0 years in 2000 and 32·1 years in 2017. Demographic change has economic consequences, and the proportion of the population that is of working age (15–64 years) decreased from 59·9% in 1950 to 57·1% in 1975, then increased to 62·9% in 2000 and 65·3% in 2017. Another dimension of the global population is the proportion of the population that is female, which decreased from 50·1% to 49·8% over the 67-year period.

### National

Fertility rates vary substantially across countries and over time (table 1; appendix 2). In 1950, TFR ranged from a low of 1·7 livebirths (95% UI 1·4–2·0) in Andorra to a high of 8·9 livebirths (8·7–9·0) in Jordan. The TFR decreased in all 195 countries and territories between 1950 and 2017, and 102 countries and territories showed a decrease of more than 50%. By 2017, the TFR ranged from a low of 1·0 livebirths (0·9–1·2) in Cyprus to a high of 7·1 livebirths (6·8–7·4) in Niger. Although a useful summary, the TFR masks variation in trends in fertility at different ages in many countries. The global decrease in median ASFRs from 1950 to 2017 was 43·4% in women aged 15–19 years and 49·4% in women aged 20–24 years, which contrasts with the observed decreases in the median ASFR in older age groups of mothers of 59·4% in women aged 40–44 years, 65·6% in women aged 45–49 years, and 68·7% in women aged 50–54 years.

**Table 1:** Age-specific fertility rates, total fertility rate, total fertility up to a maternal age of 25 years and during ages 30–54 years; the number of livebirths; and net reproductive rate, globally and for the SDI groups, GBD regions, super-regions, countries, and territories, 2017

		**Age-specific fertility rate (livebirths per 1000 women annually)**									**Total fertility rate**	**Total fertility rate under age 25 years**	**Total fertility rate from ages 30 to 54 years**	**Number of livebirths**	**Net reproductive rate**
		10–14 years	15–19 years	20–24 years	25–29 years	30–34 years	35–39 years	40–44 years	45–49 years	50–54 years					
**Global**		**0·81 (0·35–1·69)**	**42·9 (38·6–48·0)**	**129·4 (117·8–142·7)**	**131·9 (125·9–138·7)**	**96·8 (91·4–102·9)**	**52·4 (47·7–57·5)**	**17·2 (15·4–19·2)**	**3·4 (3·1–3·8)**	**0·06 (0·06–0·06)**	**2·4 (2·2–2·5)**	**0·87 (0·78–0·96)**	**0·85 (0·79–0·92)**	**138 810 622 (129 960 385–149 058 367)**	**1·08 (1·02–1·16)**
Low SDI		1·4 (0·6–2·9)	71·9 (65·0–80·0)	202·6 (181·7–225·9)	188·4 (177·3–200·9)	147·0 (136·9–158·2)	93·4 (83·6–103·5)	44·6 (39·5–49·9)	15·1 (13·7–16·7)	0·29 (0·28–0·3)	3·8 (3·6–4·1)	1·4 (1·2–1·5)	1·5 (1·4–1·6)	37 891 965 (35 159 071–41 108 482)	1·68 (1·58–1·81)
Low-middle SDI		0·88 (0·39–1·85)	51·7 (45·5–59·3)	156·6 (141·0–174·3)	152·0 (143·4–161·4)	112·4 (104·4–121·8)	63·8 (57·0–72·0)	24·1 (21·0–27·7)	6·4 (5·5–7·5)	0·12 (0·11–0·12)	2·8 (2·6–3·1)	1·0 (0·9–1·2)	1·0 (0·9–1·1)	40 394 490 (37 088 216–44 296 344)	1·28 (1·18–1·4)
Middle SDI		0·61 (0·27–1·27)	33·5 (30·1–37·6)	112·4 (100·6–125·9)	120·1 (113·3–127·9)	79·2 (73·7–85·3)	39·0 (34·4–44·2)	11·3 (9·9–13·1)	1·4 (1·1–1·6)	0·03 (0·03–0·03)	2·0 (1·8–2·2)	0·73 (0·66–0·82)	0·65 (0·6–0·72)	26 502 966 (24 536 281–28 871 941)	0·83 (0·77–0·9)
High-middle SDI		0·42 (0·18–0·86)	19·8 (18·3–21·6)	84·2 (79·0–89·9)	107·0 (103·4–110·7)	69·4 (65·8–73·1)	32·1 (29·2–35·3)	8·0 (7·2–8·8)	0·63 (0·53–0·74)	0·01 (0·01–0·01)	1·6 (1·5–1·7)	0·52 (0·49–0·56)	0·55 (0·51–0·59)	22 028 156 (20 983 021–23 184 413)	0·86 (0·81–0·9)
High SDI		0·25 (0·11–0·5)	12·5 (11·3–14·0)	49·6 (44·4–55·7)	89·5 (84·3–95·2)	98·6 (91·2–106·8)	51·8 (45·2–59·4)	11·1 (9·4–13·2)	0·63 (0·55–0·72)	0·01 (0·01–0·01)	1·6 (1·4–1·7)	0·31 (0·28–0·35)	0·81 (0·73–0·9)	11 638 396 (10 631 265–12 780 564)	0·76 (0·69–0·83)
**Central Europe, eastern Europe, and central Asia**		**0·08 (0·03–0·15)**	**27·0 (23·5–31·2)**	**102·9 (89·3–118·2)**	**110·0 (102·9–117·7)**	**77·0 (70·1–84·8)**	**32·6 (27·8–38·2)**	**6·3 (5·3–7·5)**	**0·29 (0·24–0·35)**	**0·01 (0·01–0·01)**	**1·8 (1·6–2·0)**	**0·65 (0·56–0·75)**	**0·58 (0·52–0·66)**	**5 224 690 (4 687 984–5 805 610)**	**0·84 (0·76–0·94)**
Central Asia		0·05 (0·02–0·1)	35·8 (30·7–41·8)	172·3 (151·6–195·2)	145·6 (137·2–154·7)	91·2 (82·3–102·1)	39·3 (33·4–47·0)	9·6 (7·8–12·0)	0·53 (0·37–0·79)	0·01 (0·01–0·01)	2·5 (2·3–2·7)	1·0 (0·9–1·2)	0·7 (0·62–0·81)	1 910 928 (1 754 242–2 076 808)	1·15 (1·05–1·25)
	Armenia	0·04 (0·02–0·09)	24·8 (21·3–28·8)	113·7 (99·3–129·9)	103·4 (95·0–113·6)	50·3 (44·0–58·1)	20·5 (16·9–24·9)	3·7 (2·9–4·6)	0·2 (0·13–0·31)	0·0 (0·0–0·0)	1·6 (1·4–1·7)	0·69 (0·6–0·79)	0·37 (0·33–0·42)	38 128 (34 976–41 387)	0·73 (0·67–0·8)
	Azerbaijan	0·01 (0·0–0·02)	44·1 (37·4–52·0)	148·3 (128·8–169·9)	118·6 (108·9–129·2)	55·6 (48·9–63·2)	21·0 (17·5–25·2)	4·5 (3·6–5·5)	0·46 (0·31–0·72)	0·01 (0·01–0·01)	2·0 (1·7–2·2)	0·96 (0·83–1·11)	0·41 (0·35–0·47)	173 728 (153 488–196 430)	0·87 (0·77–0·99)
	Georgia	0·26 (0·11–0·54)	46·3 (39·3–54·6)	126·8 (109·2–146·5)	119·8 (109·9–131·5)	71·1 (62·7–81·3)	35·8 (29·6–43·9)	9·0 (7·3–11·3)	0·89 (0·67–1·21)	0·02 (0·02–0·02)	2·0 (1·9–2·2)	0·87 (0·74–1·01)	0·58 (0·5–0·69)	50 298 (45 798–55 247)	0·97 (0·88–1·07)
	Kazakhstan	0·05 (0·02–0·1)	30·1 (26·5–34·4)	140·1 (120·0–162·7)	149·2 (137·1–162·3)	93·7 (81·5–108·8)	52·5 (41·6–66·6)	12·4 (9·3–16·8)	0·5 (0·31–0·81)	0·01 (0·01–0·01)	2·4 (2·2–2·6)	0·85 (0·73–0·99)	0·8 (0·66–0·97)	347 980 (315 168–381 856)	1·13 (1·03–1·25)
	Kyrgyzstan	0·01 (0·01–0·03)	38·2 (32·6–44·7)	151·2 (132·1–172·4)	171·9 (159·8–186·0)	119·1 (107·2–131·6)	57·0 (48·0–66·9)	18·0 (14·6–22·0)	0·26 (0·16–0·39)	0·0 (0·0–0·01)	2·8 (2·6–3·0)	0·95 (0·82–1·09)	0·97 (0·85–1·1)	151 035 (141 013–161 162)	1·31 (1·22–1·4)
	Mongolia	0·21 (0·09–0·43)	27·2 (23·9–30·8)	147·3 (130·5–165·0)	159·7 (148·4–171·4)	114·5 (103·9–125·6)	69·6 (60·3–79·7)	20·1 (16·6–24·1)	1·5 (1·0–2·1)	0·03 (0·03–0·03)	2·7 (2·5–2·9)	0·87 (0·77–0·98)	1·0 (0·9–1·2)	75 835 (70 120–81 639)	1·27 (1·17–1·37)
	Tajikistan	0·06 (0·03–0·13)	55·7 (47·3–65·4)	226·8 (199·4–255·5)	208·3 (193·6–223·8)	128·9 (112·4–148·6)	68·3 (53·5–87·5)	19·2 (14·0–26·4)	2·1 (1·4–3·4)	0·04 (0·04–0·04)	3·5 (3·2–3·9)	1·4 (1·2–1·6)	1·1 (0·9–1·3)	285 161 (259 803–310 494)	1·62 (1·47–1·77)
	Turkmenistan	0·04 (0·02–0·07)	19·2 (16·4–22·5)	156·1 (135·1–181·5)	190·9 (176·5–207·5)	125·8 (113·3–140·8)	49·1 (41·1–59·4)	10·4 (8·3–13·2)	0·01 (0·01–0·01)	0·0 (0·0–0·0)	2·8 (2·5–3·1)	0·88 (0·77–1·0)	0·93 (0·81–1·07)	109 634 (98 243–123 307)	1·29 (1·15–1·45)
	Uzbekistan	0·03 (0·01–0·06)	32·3 (27·4–38·0)	194·3 (169·4–221·0)	127·8 (115·6–142·5)	85·2 (74·9–97·8)	25·4 (20·8–31·5)	5·1 (3·9–6·7)	0·2 (0·13–0·31)	0·0 (0·0–0·0)	2·4 (2·1–2·6)	1·1 (1·0–1·3)	0·58 (0·5–0·68)	679 125 (619 142–740 880)	1·09 (1·0–1·19)
Central Europe		0·19 (0·08–0·39)	19·5 (17·5–21·6)	57·2 (49·7–66·0)	93·1 (86·7–99·8)	78·0 (70·9–86·2)	31·9 (27·0–37·8)	5·5 (4·8–6·4)	0·24 (0·2–0·28)	0·0 (0·0–0·0)	1·4 (1·3–1·6)	0·38 (0·34–0·44)	0·58 (0·52–0·65)	1 066 904 (960 814–1 187 258)	0·69 (0·62–0·76)
	Albania	0·07 (0·03–0·13)	19·0 (15·9–22·7)	104·0 (86·8–123·8)	144·9 (132·5–158·3)	73·8 (63·5–85·5)	28·4 (21·9–36·5)	5·9 (4·2–8·1)	0·37 (0·26–0·52)	0·01 (0·01–0·01)	1·9 (1·6–2·2)	0·62 (0·51–0·73)	0·54 (0·45–0·65)	37 047 (32 029–42 830)	0·88 (0·76–1·02)
	Bosnia and Herzegovina	0·05 (0·02–0·11)	10·1 (8·6–11·7)	48·3 (42·7–54·6)	87·1 (81·7–92·8)	74·0 (67·7–80·7)	27·3 (23·2–31·9)	4·8 (3·9–5·9)	0·32 (0·23–0·45)	0·01 (0·01–0·01)	1·3 (1·2–1·4)	0·29 (0·26–0·33)	0·53 (0·49–0·58)	27 688 (25 627–29 913)	0·6 (0·56–0·65)
	Bulgaria	0·74 (0·33–1·54)	39·3 (34·2–45·4)	72·2 (61·9–84·0)	87·0 (79·8–94·9)	65·4 (57·6–74·1)	25·7 (20·8–31·7)	3·9 (3·0–4·9)	0·2 (0·14–0·27)	0·0 (0·0–0·0)	1·5 (1·3–1·7)	0·56 (0·48–0·65)	0·48 (0·41–0·55)	58 874 (51 873–66 693)	0·71 (0·62–0·8)
	Croatia	0·06 (0·03–0·12)	9·8 (8·5–11·6)	45·6 (40·5–51·3)	87·7 (82·7–93·0)	85·9 (79·4–92·6)	38·4 (33·5–43·8)	6·4 (5·2–7·7)	0·32 (0·23–0·44)	0·01 (0·01–0·01)	1·4 (1·3–1·4)	0·28 (0·25–0·31)	0·66 (0·59–0·72)	36 549 (34 544–38 688)	0·66 (0·63–0·7)
	Czech Republic	0·03 (0·01–0·06)	12·6 (10·7–14·8)	51·1 (44·9–58·2)	99·4 (93·5–105·9)	103·0 (95·5–111·2)	43·4 (37·6–50·0)	6·2 (4·7–7·9)	0·2 (0·12–0·32)	0·0 (0·0–0·0)	1·6 (1·4–1·7)	0·32 (0·28–0·37)	0·76 (0·7–0·84)	104 681 (95 942–114 456)	0·76 (0·7–0·84)
	Hungary	0·28 (0·12–0·56)	21·7 (18·7–25·3)	47·0 (40·0–55·3)	82·4 (75·7–89·7)	86·4 (77·5–96·4)	39·9 (33·2–47·7)	7·2 (5·6–9·5)	0·22 (0·15–0·3)	0·0 (0·0–0·0)	1·4 (1·3–1·6)	0·35 (0·29–0·4)	0·67 (0·59–0·76)	86 143 (76 294–97 319)	0·69 (0·61–0·78)
	Macedonia	0·27 (0·12–0·54)	15·9 (13·7–18·5)	64·9 (58·5–71·7)	104·8 (99·2–110·7)	81·5 (75·4–87·9)	29·4 (25·1–34·1)	4·2 (3·1–5·6)	0·26 (0·17–0·38)	0·01 (0·0–0·01)	1·5 (1·4–1·6)	0·41 (0·37–0·44)	0·58 (0·54–0·62)	23 593 (22 076–25 167)	0·71 (0·67–0·76)
	Montenegro	0·12 (0·05–0·24)	11·3 (9·6–13·5)	63·3 (56·4–71·9)	112·5 (106·2–119·1)	95·1 (88·3–102·2)	42·9 (37·2–49·4)	8·8 (6·9–11·4)	0·45 (0·29–0·69)	0·01 (0·01–0·01)	1·7 (1·6–1·8)	0·37 (0·33–0·43)	0·74 (0·69–0·79)	7069 (6742–7432)	0·79 (0·76–0·84)
	Poland	0·05 (0·02–0·11)	12·7 (10·9–14·9)	49·8 (42·6–58·2)	89·9 (83·1–97·3)	73·5 (65·7–82·2)	29·7 (24·5–35·8)	5·7 (4·5–7·1)	0·23 (0·17–0·31)	0·0 (0·0–0·0)	1·3 (1·2–1·5)	0·31 (0·27–0·37)	0·55 (0·47–0·63)	355 970 (315 476–402 395)	0·63 (0·56–0·71)
	Romania	0·38 (0·17–0·79)	34·9 (30·5–40·6)	71·9 (61·9–83·5)	97·4 (90·0–105·5)	73·7 (65·7–82·8)	28·9 (23·6–35·3)	4·7 (3·6–6·1)	0·22 (0·16–0·29)	0·0 (0·0–0·0)	1·6 (1·4–1·7)	0·54 (0·48–0·6)	0·54 (0·47–0·62)	177 010 (158 216–198 220)	0·75 (0·67–0·84)
	Serbia	0·2 (0·09–0·4)	15·4 (13·3–18·0)	60·1 (51·0–70·7)	90·8 (83·4–99·0)	74·7 (66·3–84·2)	28·5 (23·4–34·6)	4·4 (3·4–5·7)	0·32 (0·22–0·45)	0·01 (0·01–0·01)	1·4 (1·2–1·6)	0·38 (0·32–0·44)	0·54 (0·47–0·62)	80 547 (71 021–91 372)	0·66 (0·58–0·75)
	Slovakia	0·13 (0·06–0·27)	23·1 (20·2–26·5)	54·5 (46·4–63·9)	87·0 (79·9–94·8)	76·7 (68·2–86·3)	31·8 (26·1–38·6)	5·3 (4·1–6·7)	0·21 (0·15–0·28)	0·0 (0·0–0·0)	1·4 (1·2–1·6)	0·39 (0·33–0·45)	0·57 (0·5–0·65)	52 596 (46 603–59 441)	0·67 (0·59–0·76)
	Slovenia	0·03 (0·01–0·07)	5·0 (4·1–6·0)	41·5 (34·9–49·4)	108·1 (100·3–117·2)	103·0 (93·7–114·0)	39·7 (33·0–48·5)	5·9 (4·3–8·0)	0·23 (0·14–0·36)	0·0 (0·0–0·0)	1·5 (1·4–1·7)	0·23 (0·2–0·28)	0·74 (0·66–0·84)	19 132 (17 463–21 101)	0·73 (0·67–0·8)
Eastern Europe		0·03 (0·01–0·07)	25·3 (21·8–29·4)	80·1 (68·1–94·0)	98·6 (90·2–107·8)	70·6 (61·7–80·6)	30·4 (24·2–37·8)	5·6 (4·3–7·3)	0·23 (0·17–0·3)	0·0 (0·0–0·0)	1·6 (1·4–1·8)	0·53 (0·45–0·62)	0·53 (0·45–0·63)	2 246 857 (1 958 844–2 577 202)	0·74 (0·64–0·85)
	Belarus	0·02 (0·01–0·03)	19·2 (16·5–22·2)	84·7 (74·5–96·3)	104·3 (95·9–113·6)	72·3 (64·1–81·4)	29·3 (24·3–35·3)	4·8 (3·8–6·1)	0·16 (0·11–0·23)	0·0 (0·0–0·0)	1·6 (1·4–1·8)	0·52 (0·46–0·59)	0·53 (0·46–0·62)	101 939 (90 523–114 916)	0·75 (0·67–0·85)
	Estonia	0·04 (0·02–0·07)	14·1 (11·8–16·9)	52·8 (44·6–62·5)	98·6 (91·1–106·9)	90·7 (81·6–100·8)	46·9 (38·9–56·2)	10·1 (7·5–13·5)	0·3 (0·18–0·46)	0·01 (0·01–0·01)	1·6 (1·4–1·8)	0·33 (0·28–0·4)	0·74 (0·64–0·85)	13 446 (11 863–15 268)	0·75 (0·66–0·86)
	Latvia	0·03 (0·01–0·06)	18·7 (15·9–22·0)	62·8 (53·0–74·2)	100·2 (92·2–109·0)	84·4 (74·8–95·1)	41·2 (33·8–50·0)	8·2 (6·4–10·3)	0·32 (0·22–0·43)	0·01 (0·01–0·01)	1·6 (1·4–1·8)	0·41 (0·34–0·48)	0·67 (0·59–0·77)	19 399 (17 182–21 920)	0·76 (0·67–0·86)
	Lithuania	0·03 (0·02–0·07)	15·8 (13·6–18·5)	61·1 (51·8–72·0)	114·5 (105·7–124·2)	89·8 (80·0–100·8)	36·2 (29·7–43·8)	6·3 (5·0–7·7)	0·24 (0·17–0·31)	0·0 (0·0–0·0)	1·6 (1·4–1·8)	0·39 (0·33–0·45)	0·66 (0·58–0·75)	29 108 (25 844–32 717)	0·78 (0·69–0·88)
	Moldova	0·05 (0·02–0·1)	23·5 (20·3–27·3)	75·7 (65·1–89·2)	82·2 (74·9–91·0)	53·7 (47·3–61·6)	21·6 (17·9–26·5)	4·1 (3·4–5·2)	0·13 (0·09–0·2)	0·0 (0·0–0·0)	1·3 (1·2–1·5)	0·5 (0·44–0·56)	0·4 (0·34–0·47)	35 612 (31 581–40 581)	0·62 (0·55–0·71)
	Russia	0·03 (0·01–0·07)	25·6 (22·0–29·9)	81·7 (68·9–96·4)	101·1 (92·5–110·7)	74·1 (64·7–84·8)	32·3 (25·6–40·5)	6·0 (4·4–7·9)	0·23 (0·15–0·33)	0·0 (0·0–0·0)	1·6 (1·4–1·9)	0·54 (0·46–0·63)	0·56 (0·48–0·67)	1 622 870 (1 410 393–1 868 353)	0·77 (0·66–0·88)
	Ukraine	0·03 (0·01–0·06)	26·8 (23·3–30·9)	77·7 (66·2–90·9)	89·4 (81·5–98·1)	57·8 (50·3–66·5)	23·7 (19·0–29·5)	4·6 (3·5–5·8)	0·24 (0·17–0·34)	0·0 (0·0–0·0)	1·4 (1·2–1·6)	0·52 (0·45–0·61)	0·43 (0·37–0·51)	424 480 (369 821–487 645)	0·66 (0·58–0·76)
**High income**		**0·36 (0·16–0·73)**	**16·2 (14·8–17·8)**	**53·5 (47·9–60·1)**	**91·0 (85·6–96·9)**	**104·4 (95·9–113·6)**	**56·1 (48·6–64·8)**	**11·6 (9·6–14·1)**	**0·65 (0·57–0·75)**	**0·01 (0·01–0·01)**	**1·7 (1·5–1·8)**	**0·35 (0·32–0·39)**	**0·86 (0·77–0·97)**	**11 470 352 (10 419 059–12 658 766)**	**0·81 (0·73–0·89)**
Australasia		0·22 (0·1–0·45)	14·5 (12·8–16·5)	51·9 (45·4–59·2)	101·1 (94·5–108·1)	125·9 (116·1–136·3)	69·8 (60·6–80·0)	14·4 (11·7–17·8)	0·81 (0·51–1·31)	0·02 (0·01–0·02)	1·9 (1·7–2·1)	0·33 (0·29–0·38)	1·1 (0·9–1·2)	373 680 (338 110–413 048)	0·91 (0·83–1·01)
	Australia	0·15 (0·06–0·3)	13·3 (11·3–15·6)	49·2 (41·8–57·8)	98·9 (91·5–107·1)	125·0 (113·8–137·1)	69·4 (58·9–81·4)	14·4 (11·1–18·4)	0·82 (0·51–1·33)	0·02 (0·02–0·02)	1·9 (1·6–2·1)	0·31 (0·27–0·37)	1·0 (0·9–1·2)	313 630 (278 661–353 002)	0·89 (0·79–1·01)
	New Zealand	0·59 (0·25–1·2)	20·1 (17·2–23·9)	66·4 (58·4–76·4)	114·1 (106·9–122·5)	131·7 (122·3–142·7)	72·0 (63·0–83·0)	14·9 (11·7–18·6)	0·74 (0·46–1·2)	0·01 (0·01–0·01)	2·1 (1·9–2·3)	0·44 (0·38–0·5)	1·1 (1·0–1·2)	60 050 (54 692–66 222)	1·01 (0·92–1·12)
High-income Asia Pacific		0·01 (0·01–0·03)	3·4 (2·9–4·0)	23·8 (19·5–29·1)	74·1 (67·8–80·8)	103·3 (93·5–114·1)	47·4 (39·7–56·5)	7·5 (5·8–9·8)	0·22 (0·15–0·31)	0·0 (0·0–0·0)	1·3 (1·2–1·5)	0·14 (0·11–0·17)	0·79 (0·69–0·9)	1 427 130 (1 260 959–1 623 740)	0·63 (0·56–0·71)
	Brunei	0·28 (0·12–0·57)	12·4 (10·3–14·9)	53·7 (43·8–65·4)	107·4 (97·8–117·5)	111·8 (98·6–125·8)	70·1 (56·8–87·0)	19·8 (14·6–26·3)	0·55 (0·34–0·86)	0·01 (0·01–0·01)	1·9 (1·7–2·0)	0·33 (0·27–0·4)	1·0 (0·9–1·1)	7093 (6568–7631)	0·89 (0·82–0·96)
	Japan	0·01 (0·0–0·01)	4·0 (3·3–4·9)	29·6 (23·1–37·6)	81·9 (72·7–92·2)	96·7 (82·6–112·5)	46·3 (35·2–59·9)	8·0 (5·6–11·1)	0·21 (0·13–0·31)	0·0 (0·0–0·0)	1·3 (1·1–1·6)	0·17 (0·13–0·21)	0·76 (0·62–0·92)	922 225 (767 131–1 106 046)	0·65 (0·54–0·77)
	Singapore	0·08 (0·03–0·16)	4·4 (3·6–5·3)	20·4 (15·8–26·0)	65·0 (57·4–73·7)	101·4 (86·9–117·8)	51·7 (39·5–66·5)	9·6 (6·8–13·3)	0·37 (0·23–0·6)	0·01 (0·01–0·01)	1·3 (1·1–1·5)	0·12 (0·1–0·16)	0·82 (0·67–0·99)	64 836 (54 182–77 378)	0·61 (0·51–0·73)
	South Korea	0·02 (0·01–0·04)	1·7 (1·4–2·1)	13·5 (11·7–15·5)	60·6 (57·3–64·1)	117·4 (111·6–123·3)	48·9 (44·2–53·8)	6·1 (4·9–7·4)	0·24 (0·15–0·39)	0·0 (0·0–0·0)	1·2 (1·2–1·3)	0·08 (0·07–0·09)	0·86 (0·81–0·92)	432 974 (412 109–453 553)	0·6 (0·57–0·63)
High-income North America		0·55 (0·24–1·11)	20·7 (18·8–22·7)	70·7 (64·2–77·9)	99·4 (94·4–104·8)	103·3 (96·4–110·8)	52·5 (46·5–59·2)	11·0 (9·3–13·1)	0·74 (0·55–0·99)	0·01 (0·01–0·01)	1·8 (1·7–1·9)	0·46 (0·42–0·51)	0·84 (0·76–0·92)	4 314 373 (3 982 175–4 683 089)	0·86 (0·8–0·94)
	Canada	0·15 (0·07–0·31)	12·8 (10·7–15·3)	46·8 (37·8–57·7)	99·2 (89·7–109·7)	111·9 (98·2–127·0)	51·8 (40·8–64·8)	9·5 (7·0–12·7)	0·42 (0·28–0·62)	0·01 (0·01–0·01)	1·7 (1·4–1·9)	0·3 (0·24–0·37)	0·87 (0·73–1·02)	390 262 (334 379–455 010)	0·8 (0·69–0·94)
	Greenland	0·62 (0·27–1·28)	42·5 (35·8–51·1)	104·5 (87·6–123·9)	119·1 (107·8–132·7)	87·0 (74·8–100·8)	42·9 (33·6–54·2)	6·5 (4·6–9·2)	0·05 (0·03–0·08)	0·0 (0·0–0·0)	2·0 (1·8–2·3)	0·74 (0·65–0·84)	0·68 (0·57–0·81)	817 (728–910)	0·94 (0·84–1·06)
	USA	0·58 (0·25–1·19)	21·4 (19·6–23·4)	73·1 (66·9–80·0)	99·4 (94·9–104·3)	102·3 (96·2–108·9)	52·5 (47·2–58·5)	11·2 (9·6–13·2)	0·78 (0·56–1·05)	0·01 (0·01–0·02)	1·8 (1·7–1·9)	0·48 (0·43–0·52)	0·83 (0·77–0·91)	3 923 218 (3 646 761–4 226 835)	0·87 (0·81–0·94)
Southern Latin America		1·5 (0·7–3·2)	53·7 (49·0–59·3)	92·0 (82·4–102·6)	96·7 (91·3–102·8)	90·6 (81·3–100·9)	61·8 (52·1–72·9)	15·6 (12·2–19·8)	0·99 (0·64–1·49)	0·02 (0·02–0·02)	2·1 (1·9–2·2)	0·74 (0·68–0·79)	0·84 (0·73–0·98)	1 041 669 (958 720–1 130 812)	1·0 (0·91–1·08)
	Argentina	1·7 (0·7–3·5)	58·2 (51·9–66·0)	100·2 (87·8–113·6)	101·3 (93·9–110·2)	92·0 (82·7–102·2)	63·8 (54·1–75·0)	15·8 (12·4–19·8)	1·0 (0·7–1·6)	0·02 (0·02–0·02)	2·2 (2·0–2·3)	0·8 (0·73–0·88)	0·86 (0·75–0·99)	747 539 (695 353–801 816)	1·04 (0·97–1·12)
	Chile	1·2 (0·5–2·4)	40·9 (35·9–46·6)	72·3 (62·3–83·9)	86·3 (79·5–93·9)	87·5 (78·2–97·8)	57·4 (48·0–68·3)	15·6 (12·1–19·9)	0·88 (0·55–1·36)	0·02 (0·02–0·02)	1·8 (1·6–2·1)	0·57 (0·5–0·66)	0·81 (0·69–0·94)	245 912 (215 928–279 946)	0·88 (0·77–1·0)
	Uruguay	1·2 (0·5–2·6)	53·9 (47·1–61·6)	86·9 (74·5–101·2)	93·6 (85·6–102·5)	87·8 (77·7–99·2)	56·1 (46·0–67·9)	14·1 (10·5–18·6)	1·0 (0·7–1·5)	0·02 (0·02–0·02)	2·0 (1·7–2·3)	0·71 (0·61–0·82)	0·8 (0·68–0·93)	48 170 (41 869–55 322)	0·95 (0·82–1·09)
Western Europe		0·05 (0·02–0·1)	8·7 (7·5–10·0)	40·6 (35·0–47·2)	87·8 (81·6–94·5)	106·6 (97·6–116·6)	61·2 (52·7–70·8)	13·3 (11·1–15·8)	0·73 (0·64–0·82)	0·01 (0·01–0·01)	1·6 (1·4–1·8)	0·25 (0·21–0·29)	0·91 (0·81–1·02)	4 313 498 (3 871 044–4 807 568)	0·77 (0·69–0·86)
	Andorra	0·22 (0·09–0·45)	4·9 (4·2–5·6)	26·7 (22·6–31·3)	57·2 (52·2–62·6)	85·1 (76·5–94·1)	52·1 (45·5–59·3)	12·6 (10·4–15·1)	0·96 (0·59–1·45)	0·02 (0·02–0·02)	1·2 (1·1–1·3)	0·16 (0·13–0·19)	0·75 (0·67–0·85)	642 (567–724)	0·58 (0·51–0·65)
	Austria	0·05 (0·02–0·11)	9·1 (7·7–10·6)	42·8 (37·4–49·0)	87·4 (81·8–93·4)	99·3 (91·8–107·5)	53·5 (46·7–61·2)	10·1 (8·2–12·4)	0·46 (0·32–0·63)	0·01 (0·01–0·01)	1·5 (1·4–1·7)	0·26 (0·23–0·3)	0·82 (0·75–0·9)	86 756 (79 382–94 860)	0·73 (0·67–0·8)
	Belgium	0·04 (0·02–0·07)	7·6 (6·4–9·1)	43·6 (36·9–51·5)	114·3 (106·5–122·7)	111·8 (101·9–122·7)	49·7 (41·7–59·0)	9·7 (7·8–12·0)	0·51 (0·36–0·71)	0·01 (0·01–0·01)	1·7 (1·5–1·9)	0·26 (0·22–0·3)	0·86 (0·77–0·96)	121 588 (109 546–134 907)	0·82 (0·74–0·91)
	Cyprus	0·03 (0·01–0·06)	4·0 (3·3–4·8)	24·5 (19·6–30·6)	58·8 (52·8–65·5)	68·3 (59·7–78·1)	35·9 (28·7–44·6)	9·3 (6·9–12·6)	1·1 (0·7–1·6)	0·02 (0·02–0·02)	1·0 (0·9–1·2)	0·14 (0·11–0·18)	0·57 (0·49–0·67)	10 788 (9310–12 496)	0·48 (0·42–0·56)
	Denmark	0·01 (0·01–0·03)	4·6 (3·9–5·6)	35·7 (29·6–43·0)	111·4 (103·0–120·6)	128·2 (116·8–140·5)	58·3 (49·4–68·6)	11·0 (8·8–13·6)	0·49 (0·33–0·7)	0·01 (0·01–0·01)	1·7 (1·6–1·9)	0·2 (0·17–0·24)	0·99 (0·89–1·1)	60 724 (54 681–67 563)	0·84 (0·76–0·94)
	Finland	0·02 (0·01–0·03)	7·0 (5·9–8·3)	45·0 (38·5–52·6)	95·5 (88·2–103·5)	109·9 (99·5–121·3)	57·1 (48·1–67·3)	12·8 (10·3–15·8)	0·69 (0·51–0·93)	0·01 (0·01–0·01)	1·6 (1·5–1·8)	0·26 (0·22–0·3)	0·9 (0·81–1·01)	55 235 (49 589–61 618)	0·8 (0·71–0·89)
	France	0·03 (0·01–0·06)	7·6 (6·6–8·8)	47·4 (41·0–54·8)	117·2 (109·7–125·4)	120·6 (110·6–131·4)	61·5 (52·6–71·7)	13·8 (11·1–17·1)	0·77 (0·55–1·03)	0·01 (0·01–0·02)	1·8 (1·7–2·0)	0·28 (0·24–0·32)	0·98 (0·88–1·1)	737 405 (664 102–819 651)	0·89 (0·81–0·99)
	Germany	0·04 (0·02–0·07)	8·0 (6·9–9·3)	34·3 (29·4–40·1)	75·2 (69·5–81·5)	97·3 (88·5–107·1)	53·6 (45·4–63·0)	9·9 (7·9–12·3)	0·4 (0·28–0·53)	0·01 (0·01–0·01)	1·4 (1·2–1·6)	0·21 (0·18–0·25)	0·81 (0·71–0·91)	710 634 (633 455–798 663)	0·67 (0·6–0·76)
	Greece	0·14 (0·06–0·29)	9·1 (7·9–10·6)	32·1 (27·0–38·2)	74·5 (68·4–81·1)	100·8 (91·0–111·5)	55·6 (46·7–65·8)	11·2 (8·6–14·6)	1·3 (0·9–1·9)	0·03 (0·02–0·03)	1·4 (1·3–1·6)	0·21 (0·18–0·24)	0·84 (0·75–0·95)	89 713 (79 740–100 854)	0·69 (0·61–0·77)
	Iceland	0·07 (0·03–0·14)	9·6 (8·1–11·3)	54·5 (46·5–63·9)	109·1 (100·9–118·1)	112·7 (102·0–124·6)	64·9 (55·2–75·5)	15·3 (12·2–19·2)	0·5 (0·31–0·81)	0·01 (0·01–0·01)	1·8 (1·7–2·0)	0·32 (0·28–0·37)	0·97 (0·88–1·05)	4250 (3897–4639)	0·89 (0·82–0·98)
	Ireland	0·05 (0·02–0·09)	10·6 (9·2–12·6)	40·5 (34·4–47·6)	77·1 (71·0–84·0)	123·4 (112·4–135·4)	93·4 (80·9–107·2)	22·0 (17·6–27·2)	1·2 (0·8–1·7)	0·02 (0·02–0·02)	1·8 (1·6–2·1)	0·26 (0·22–0·29)	1·2 (1·1–1·4)	64 902 (57 702–72 962)	0·89 (0·79–1·0)
	Israel	0·03 (0·01–0·06)	11·3 (9·6–13·5)	101·1 (88·1–115·8)	171·3 (161·6–181·8)	169·7 (157·9–182·1)	100·8 (88·7–114·0)	24·1 (19·8–29·1)	1·7 (1·2–2·3)	0·03 (0·03–0·03)	2·9 (2·6–3·2)	0·56 (0·49–0·65)	1·5 (1·3–1·6)	177 148 (161 025–194 812)	1·4 (1·27–1·54)
	Italy	0·02 (0·01–0·03)	5·6 (4·7–6·6)	28·4 (24·3–33·2)	66·2 (61·0–71·9)	91·6 (83·0–101·1)	58·5 (49·8–68·5)	14·9 (12·0–18·5)	0·92 (0·64–1·27)	0·02 (0·02–0·02)	1·3 (1·2–1·5)	0·17 (0·15–0·2)	0·83 (0·73–0·94)	464 442 (410 461–526 027)	0·64 (0·57–0·73)
	Luxembourg	0·08 (0·04–0·17)	6·2 (5·1–7·4)	34·4 (28·3–41·8)	75·7 (69·3–82·8)	103·8 (94·0–114·6)	62·2 (52·7–72·6)	13·4 (10·5–17·0)	0·61 (0·38–0·92)	0·01 (0·01–0·01)	1·5 (1·4–1·6)	0·2 (0·17–0·24)	0·9 (0·83–0·97)	6407 (5861–6965)	0·72 (0·65–0·78)
	Malta	0·19 (0·08–0·39)	12·6 (10·9–14·4)	38·1 (32·2–45·2)	89·2 (82·0–97·0)	101·3 (90·6–113·2)	47·0 (38·6–56·9)	9·0 (7·0–11·5)	0·44 (0·3–0·65)	0·01 (0·01–0·01)	1·5 (1·3–1·7)	0·25 (0·22–0·29)	0·79 (0·68–0·91)	4311 (3812–4880)	0·71 (0·63–0·81)
	Netherlands	0·02 (0·01–0·05)	4·1 (3·4–4·9)	30·0 (25·1–35·7)	98·2 (91·1–105·9)	129·2 (118·1–141·3)	60·5 (51·0–71·4)	9·7 (7·5–12·9)	0·43 (0·29–0·59)	0·01 (0·01–0·01)	1·7 (1·5–1·8)	0·17 (0·14–0·2)	1·0 (0·9–1·11)	172 472 (155 190–191 686)	0·8 (0·72–0·89)
	Norway	0·01 (0·01–0·03)	5·7 (4·7–6·9)	42·2 (36·3–49·0)	106·9 (100·2–114·1)	120·4 (111·6–129·9)	59·9 (51·9–68·9)	12·0 (9·2–15·4)	0·64 (0·4–0·98)	0·01 (0·01–0·01)	1·7 (1·6–1·9)	0·24 (0·21–0·28)	0·96 (0·88–1·05)	60 329 (55 230–66 015)	0·84 (0·77–0·92)
	Portugal	0·12 (0·05–0·25)	10·2 (8·7–12·0)	31·2 (26·0–37·3)	64·2 (58·8–70·2)	89·9 (80·9–99·9)	51·5 (43·2–61·2)	11·1 (8·8–14·0)	0·65 (0·45–0·88)	0·01 (0·01–0·01)	1·3 (1·1–1·5)	0·21 (0·17–0·25)	0·77 (0·67–0·88)	85 589 (74 860–97 921)	0·63 (0·55–0·72)
	Spain	0·08 (0·04–0·17)	8·3 (7·0–9·8)	26·0 (22·7–29·8)	57·8 (54·1–61·9)	95·1 (88·7–102·1)	66·5 (59·8–73·9)	15·7 (13·2–18·6)	0·87 (0·6–1·23)	0·02 (0·02–0·02)	1·4 (1·2–1·5)	0·17 (0·15–0·2)	0·89 (0·81–0·98)	407 088 (370 674–447 913)	0·65 (0·59–0·72)
	Sweden	0·02 (0·01–0·04)	5·2 (4·4–6·3)	43·6 (38·1–49·9)	108·8 (102·7–115·5)	125·7 (117·3–134·7)	68·7 (60·7–77·6)	14·4 (11·4–17·8)	0·73 (0·46–1·09)	0·01 (0·01–0·01)	1·8 (1·7–2·0)	0·24 (0·21–0·28)	1·0 (1·0–1·1)	118 087 (109 169–127 819)	0·88 (0·82–0·96)
	Switzerland	0·01 (0·01–0·03)	3·4 (2·8–4·1)	28·3 (23·3–34·3)	77·0 (70·4–84·3)	111·8 (101·6–123·0)	65·1 (55·4–76·2)	13·1 (10·6–16·1)	0·64 (0·45–0·85)	0·01 (0·01–0·01)	1·5 (1·3–1·7)	0·16 (0·13–0·19)	0·95 (0·85–1·06)	87 282 (78 003–97 459)	0·72 (0·64–0·81)
	UK	0·11 (0·05–0·21)	15·3 (13·5–17·4)	54·1 (46·8–62·4)	91·0 (84·7–97·9)	107·1 (97·8–117·3)	64·7 (55·9–74·7)	13·5 (11·0–16·5)	0·8 (0·58–1·07)	0·02 (0·01–0·02)	1·7 (1·6–1·9)	0·35 (0·3–0·4)	0·93 (0·83–1·05)	783 225 (703 221–873 164)	0·84 (0·75–0·93)
	England	0·1 (0·05–0·21)	14·9 (13·1–17·0)	54·7 (47·5–63·0)	92·4 (86·2–99·3)	108·5 (99·2–118·7)	66·1 (57·2–76·2)	14·0 (11·2–17·2)	0·82 (0·61–1·09)	0·02 (0·02–0·02)	1·8 (1·6–2·0)	0·35 (0·3–0·4)	0·95 (0·84–1·06)	672 857 (604 801–749 278)	0·85 (0·76–0·95)
	Northern Ireland	0·13 (0·06–0·27)	15·2 (13·1–17·6)	53·4 (44·9–63·4)	95·7 (87·9–104·3)	114·6 (103·4–126·9)	67·3 (56·7–79·4)	13·0 (9·7–16·9)	0·59 (0·31–1·04)	0·01 (0·01–0·01)	1·8 (1·6–2·0)	0·34 (0·29–0·41)	0·98 (0·85–1·12)	23 589 (20 766–26 808)	0·87 (0·76–0·99)
	Scotland	0·11 (0·05–0·22)	18·9 (16·9–21·2)	44·8 (38·8–51·8)	72·1 (66·9–77·8)	93·1 (84·6–102·5)	55·4 (47·8–64·0)	10·1 (8·1–12·4)	0·62 (0·31–1·16)	0·01 (0·01–0·01)	1·5 (1·3–1·6)	0·32 (0·28–0·37)	0·8 (0·72–0·88)	53 451 (48 281–59 123)	0·71 (0·64–0·79)
	Wales	0·11 (0·05–0·22)	16·1 (14·2–18·3)	59·8 (51·0–70·0)	96·1 (88·7–104·2)	100·2 (90·1–111·4)	52·4 (43·9–62·3)	10·0 (7·8–12·6)	0·76 (0·47–1·16)	0·01 (0·01–0·02)	1·7 (1·5–1·9)	0·38 (0·33–0·44)	0·82 (0·71–0·94)	33 470 (29 639–37 854)	0·81 (0·72–0·92)
**Latin America and Caribbean**		**2·0 (0·9–4·2)**	**63·5 (57·1–71·0)**	**112·9 (100·1–127·8)**	**104·2 (96·7–112·6)**	**85·7 (79·2–92·9)**	**51·5 (45·2–58·3)**	**15·5 (13·2–18·1)**	**1·3 (1·1–1·5)**	**0·02 (0·02–0·02)**	**2·2 (2·0–2·4)**	**0·89 (0·8–1·0)**	**0·77 (0·69–0·85)**	**10 393 604 (9 469 048–11 430 456)**	**1·04 (0·95–1·14)**
Andean Latin America		1·4 (0·6–2·9)	71·4 (63·9–79·5)	138·2 (121·6–157·2)	132·8 (123·2–143·6)	114·3 (102·9–127·4)	76·8 (65·8–88·4)	27·0 (22·1–32·5)	2·7 (2·1–3·4)	0·05 (0·05–0·05)	2·8 (2·6–3·1)	1·1 (0·9–1·2)	1·1 (1·0–1·2)	1 386 395 (1 260 285–1 526 533)	1·34 (1·22–1·47)
	Bolivia	2·2 (1·0–4·6)	71·9 (61·8–83·4)	156·2 (132·9–184·4)	154·6 (141·1–170·5)	136·2 (119·8–155·6)	92·4 (75·8–111·0)	30·9 (23·9–39·4)	4·3 (2·9–6·3)	0·08 (0·08–0·09)	3·2 (2·9–3·6)	1·2 (1·0–1·3)	1·3 (1·2–1·5)	301 119 (271 239–334 416)	1·52 (1·37–1·69)
	Ecuador	0·76 (0·33–1·58)	60·5 (51·5–70·9)	134·3 (112·3–159·1)	112·9 (101·6–125·3)	86·2 (73·8–100·2)	46·3 (36·0–58·7)	12·5 (9·3–16·6)	1·2 (0·8–1·8)	0·02 (0·02–0·02)	2·3 (1·9–2·7)	0·98 (0·82–1·15)	0·73 (0·6–0·89)	315 984 (268 796–370 024)	1·08 (0·92–1·26)
	Peru	1·5 (0·6–3·1)	78·0 (67·0–91·9)	133·5 (113·1–158·8)	135·0 (123·0–149·3)	120·9 (106·5–138·2)	87·0 (72·2–103·1)	32·9 (25·8–41·0)	3·0 (2·0–4·2)	0·06 (0·05–0·06)	3·0 (2·6–3·3)	1·1 (0·9–1·3)	1·2 (1·1–1·4)	769 292 (687 072–866 600)	1·42 (1·26–1·59)
Caribbean		1·2 (0·5–2·5)	58·1 (51·1–66·0)	119·3 (108·3–131·3)	112·8 (105·8–120·1)	86·0 (78·5–93·7)	52·7 (46·0–60·0)	15·3 (12·6–18·1)	1·9 (1·5–2·3)	0·03 (0·03–0·03)	2·2 (2·0–2·4)	0·89 (0·81–0·99)	0·78 (0·7–0·86)	815 882 (746 824–889 894)	1·04 (0·95–1·13)
	Antigua and Barbuda	2·5 (1·1–5·1)	50·1 (43·2–58·2)	81·5 (67·2–98·3)	75·7 (67·8–84·6)	57·8 (49·2–67·8)	27·5 (21·1–35·5)	6·9 (5·1–9·3)	0·08 (0·05–0·13)	0·0 (0·0–0·0)	1·5 (1·3–1·8)	0·67 (0·56–0·79)	0·46 (0·38–0·56)	1071 (905–1261)	0·73 (0·62–0·86)
	The Bahamas	0·76 (0·33–1·56)	33·5 (28·2–39·9)	73·4 (59·3–90·2)	82·4 (73·3–92·6)	65·8 (55·4–77·8)	41·5 (31·7–53·5)	10·8 (7·9–14·6)	0·37 (0·24–0·56)	0·01 (0·01–0·01)	1·5 (1·3–1·9)	0·54 (0·44–0·65)	0·59 (0·48–0·73)	4679 (3895–5611)	0·74 (0·62–0·88)
	Barbados	1·2 (0·5–2·4)	40·2 (34·3–47·1)	75·6 (61·9–91·8)	74·7 (66·6–83·7)	58·2 (49·2–68·7)	27·9 (21·2–36·2)	8·6 (6·3–11·6)	0·17 (0·11–0·25)	0·0 (0·0–0·0)	1·4 (1·2–1·7)	0·58 (0·48–0·7)	0·47 (0·38–0·58)	2850 (2393–3388)	0·68 (0·58–0·81)
	Belize	1·1 (0·5–2·3)	59·0 (51·1–68·2)	132·5 (115·2–151·8)	112·5 (102·9–123·1)	81·3 (72·5–91·2)	46·8 (39·2–55·6)	11·3 (9·4–13·5)	1·3 (1·1–1·5)	0·03 (0·02–0·03)	2·2 (2·0–2·5)	0·96 (0·83–1·1)	0·7 (0·61–0·81)	7843 (6904–8895)	1·06 (0·94–1·2)
	Bermuda	0·42 (0·18–0·86)	8·5 (7·3–9·8)	34·9 (30·0–40·6)	58·6 (53·5–64·4)	83·0 (74·8–92·2)	58·1 (49·8–67·6)	16·1 (13·3–19·7)	0·96 (0·61–1·47)	0·02 (0·02–0·02)	1·3 (1·2–1·5)	0·22 (0·19–0·25)	0·79 (0·71–0·89)	562 (502–631)	0·63 (0·57–0·71)
	Cuba	1·6 (0·7–3·3)	45·5 (41·1–51·0)	91·2 (82·5–100·4)	84·1 (78·8–90·4)	52·7 (47·5–58·4)	23·3 (19·3–28·0)	4·1 (3·1–5·5)	0·15 (0·1–0·24)	0·0 (0·0–0·0)	1·5 (1·4–1·6)	0·69 (0·64–0·75)	0·4 (0·35–0·46)	109 664 (103 731–116 193)	0·72 (0·68–0·77)
	Dominica	1·4 (0·6–3·0)	44·6 (38·0–53·2)	84·3 (68·6–104·7)	79·4 (70·7–90·2)	66·6 (56·4–79·5)	34·5 (27·7–43·3)	8·0 (6·0–10·6)	0·23 (0·15–0·36)	0·0 (0·0–0·0)	1·6 (1·3–1·9)	0·65 (0·54–0·8)	0·55 (0·45–0·67)	801 (677–960)	0·75 (0·63–0·89)
	Dominican Republic	0·91 (0·4–1·92)	90·9 (78·3–105·1)	153·6 (131·2–178·5)	123·0 (111·4–135·8)	69·6 (58·8–82·1)	29·5 (22·5–38·3)	6·2 (4·5–8·4)	0·65 (0·44–0·92)	0·01 (0·01–0·01)	2·4 (2·0–2·7)	1·2 (1·1–1·4)	0·53 (0·43–0·65)	216 514 (186 677–250 296)	1·12 (0·96–1·29)
	Grenada	0·92 (0·41–1·91)	48·2 (41·8–55·7)	88·0 (72·1–106·7)	84·3 (75·2–94·6)	85·3 (73·2–99·2)	53·3 (41·7–67·1)	16·1 (12·2–21·1)	0·69 (0·43–1·06)	0·01 (0·01–0·01)	1·9 (1·6–2·2)	0·69 (0·57–0·82)	0·78 (0·64–0·94)	1514 (1279–1786)	0·9 (0·76–1·06)
	Guyana	1·9 (0·8–3·9)	67·3 (58·2–77·8)	153·7 (131·8–178·1)	126·2 (114·6–138·9)	89·6 (77·8–102·8)	47·9 (38·4–59·1)	12·4 (9·6–15·9)	1·2 (0·8–1·7)	0·02 (0·02–0·02)	2·5 (2·2–2·9)	1·1 (1·0–1·3)	0·76 (0·63–0·9)	15 719 (13 597–18 087)	1·18 (1·02–1·35)
	Haiti	1·3 (0·6–2·6)	49·9 (42·6–58·3)	128·6 (108·7–153·2)	147·9 (135·1–163·1)	139·7 (123·9–158·4)	107·9 (90·8–128·2)	43·5 (34·9–53·0)	8·3 (6·3–10·7)	0·16 (0·15–0·17)	3·1 (2·8–3·5)	0·9 (0·79–1·02)	1·5 (1·3–1·7)	325 281 (290 528–365 513)	1·41 (1·25–1·59)
	Jamaica	1·1 (0·5–2·2)	41·0 (35·9–46·9)	81·8 (70·6–94·5)	75·7 (69·2–82·9)	62·7 (55·5–70·9)	39·2 (33·7–45·5)	12·8 (10·6–15·5)	0·93 (0·7–1·22)	0·02 (0·02–0·02)	1·6 (1·4–1·8)	0·62 (0·54–0·71)	0·58 (0·5–0·67)	38 063 (33 491–43 222)	0·76 (0·67–0·86)
	Puerto Rico	0·74 (0·32–1·51)	30·9 (26·9–35·5)	75·5 (66·6–86·5)	65·2 (59·9–71·2)	43·5 (38·1–49·6)	21·0 (16·8–26·2)	4·7 (3·4–6·4)	0·15 (0·1–0·21)	0·0 (0·0–0·0)	1·2 (1·1–1·3)	0·54 (0·49–0·59)	0·35 (0·29–0·41)	29 896 (27 172–32 946)	0·57 (0·52–0·63)
	Saint Lucia	0·94 (0·41–1·95)	44·5 (37·8–52·3)	84·2 (68·5–102·8)	71·9 (63·8–81·0)	60·4 (51·0–71·2)	34·6 (26·7–44·4)	10·3 (7·5–14·0)	0·52 (0·35–0·77)	0·01 (0·01–0·01)	1·5 (1·3–1·8)	0·65 (0·53–0·78)	0·53 (0·43–0·65)	2102 (1753–2513)	0·74 (0·62–0·88)
	Saint Vincent and the Grenadines	1·5 (0·6–3·1)	60·1 (51·4–70·3)	94·6 (77·7–114·3)	88·9 (79·4–99·5)	70·6 (59·8–83·1)	43·4 (33·3–55·8)	11·2 (8·4–14·7)	0·81 (0·5–1·3)	0·02 (0·01–0·02)	1·9 (1·6–2·2)	0·78 (0·65–0·93)	0·63 (0·51–0·77)	1551 (1302–1838)	0·88 (0·74–1·05)
	Suriname	3·4 (1·5–7·1)	55·6 (48·1–64·1)	112·7 (95·7–132·1)	115·0 (104·8–126·2)	91·2 (80·1–103·6)	49·7 (40·5–60·6)	12·3 (9·5–15·8)	0·84 (0·68–1·03)	0·02 (0·02–0·02)	2·2 (1·9–2·5)	0·86 (0·73–1·0)	0·77 (0·65–0·9)	9614 (8337–11 018)	1·04 (0·9–1·18)
	Trinidad and Tobago	0·74 (0·33–1·54)	39·4 (34·2–45·3)	96·7 (82·8–112·5)	92·3 (84·3–101·1)	68·8 (61·0–77·5)	34·6 (28·8–41·4)	7·9 (6·4–9·6)	0·49 (0·37–0·65)	0·01 (0·01–0·01)	1·7 (1·5–1·9)	0·68 (0·59–0·79)	0·56 (0·48–0·64)	17 521 (15 376–19 960)	0·81 (0·71–0·92)
	Virgin Islands	1·6 (0·7–3·4)	52·8 (44·9–62·0)	121·5 (100·8–145·1)	113·0 (101·8–125·3)	77·3 (66·0–90·1)	36·3 (27·9–46·7)	4·8 (3·6–6·3)	0·07 (0·04–0·1)	0·0 (0·0–0·0)	2·0 (1·7–2·4)	0·88 (0·73–1·04)	0·59 (0·49–0·71)	1287 (1097–1507)	0·98 (0·84–1·15)
Central Latin America		1·8 (0·8–3·7)	72·5 (65·4–80·4)	129·6 (115·3–145·5)	118·0 (110·1–126·5)	87·4 (78·9–97·1)	47·9 (40·7–56·2)	12·3 (10·0–15·1)	1·4 (1·0–1·7)	0·03 (0·02–0·03)	2·4 (2·1–2·6)	1·0 (0·9–1·1)	0·75 (0·65–0·85)	5 004 522 (4 502 598–5 583 565)	1·12 (1·01–1·25)
	Colombia	1·2 (0·5–2·5)	64·1 (55·2–74·4)	114·6 (97·0–134·7)	104·7 (94·9–115·7)	80·9 (70·3–92·9)	45·4 (36·7–55·9)	10·9 (8·4–14·0)	1·3 (0·9–1·8)	0·02 (0·02–0·03)	2·1 (1·8–2·5)	0·9 (0·77–1·05)	0·69 (0·58–0·82)	851 115 (733 293–985 161)	1·01 (0·87–1·17)
	Costa Rica	1·3 (0·6–2·6)	53·8 (48·4–60·5)	92·1 (82·7–103·5)	86·6 (80·7–93·7)	69·7 (63·0–77·8)	37·0 (31·1–43·9)	9·6 (7·2–12·6)	0·71 (0·51–0·99)	0·01 (0·01–0·01)	1·8 (1·6–1·9)	0·74 (0·66–0·83)	0·58 (0·53–0·64)	69 820 (64 085–76 591)	0·85 (0·78–0·93)
	El Salvador	1·1 (0·5–2·2)	63·6 (55·9–72·3)	105·5 (90·6–122·3)	93·7 (85·4–103·0)	72·4 (64·1–81·7)	40·1 (33·2–48·2)	11·7 (9·5–14·4)	1·0 (0·72–1·36)	0·02 (0·02–0·02)	1·9 (1·7–2·2)	0·85 (0·74–0·98)	0·63 (0·54–0·73)	107 660 (94 215–122 854)	0·93 (0·81–1·06)
	Guatemala	1·5 (0·7–3·2)	75·9 (65·7–87·5)	138·9 (118·3–161·9)	134·4 (122·4–147·5)	105·7 (92·1–120·9)	72·4 (58·5–88·4)	26·4 (20·7–33·3)	4·7 (3·3–6·6)	0·09 (0·09–0·09)	2·8 (2·4–3·2)	1·1 (0·9–1·3)	1·0 (0·9–1·2)	430 775 (372 362–496 213)	1·33 (1·15–1·53)
	Honduras	2·8 (1·2–6·0)	86·7 (75·5–100·7)	148·5 (127·8–173·7)	130·7 (119·4–144·2)	109·0 (95·7–125·1)	72·8 (59·4–89·5)	25·1 (19·6–32·3)	3·3 (2·3–4·7)	0·06 (0·06–0·07)	2·9 (2·5–3·4)	1·2 (1·0–1·4)	1·1 (0·9–1·3)	244 568 (212 956–283 074)	1·37 (1·19–1·59)
	Mexico	1·7 (0·8–3·6)	70·0 (59·9–82·8)	137·1 (115·2–164·1)	125·9 (113·7–140·5)	90·0 (77·1–105·9)	46·9 (36·5–60·7)	11·0 (7·9–15·4)	1·1 (0·8–1·5)	0·02 (0·02–0·02)	2·4 (2·1–2·9)	1·0 (0·9–1·2)	0·74 (0·61–0·91)	2 518 031 (2 153 263–2 972 894)	1·16 (0·99–1·37)
	Nicaragua	1·5 (0·6–3·0)	82·8 (71·5–95·6)	129·6 (108·9–153·1)	113·9 (102·7–126·3)	96·6 (83·1–111·8)	53·5 (41·8–67·5)	13·1 (9·7–17·5)	1·5 (1·0–2·2)	0·03 (0·03–0·03)	2·5 (2·1–2·9)	1·1 (0·9–1·2)	0·82 (0·68–1·0)	137 802 (117 837–160 442)	1·18 (1·01–1·37)
	Panama	2·4 (1·1–5·1)	77·1 (66·1–89·7)	126·4 (105·5–150·2)	113·5 (102·8–125·4)	85·3 (73·8–98·2)	44·7 (35·5–55·7)	11·3 (8·6–14·7)	0·78 (0·54–1·11)	0·01 (0·01–0·02)	2·3 (2·0–2·7)	1·0 (0·9–1·2)	0·71 (0·59–0·85)	69 684 (59 588–81 021)	1·1 (0·94–1·27)
	Venezuela	2·7 (1·2–5·8)	92·1 (83·1–102·1)	122·2 (107·4–138·7)	105·2 (97·4–113·7)	75·2 (66·8–84·5)	38·8 (31·9–47·1)	10·7 (8·2–13·9)	1·1 (0·8–1·7)	0·02 (0·02–0·02)	2·2 (2·0–2·5)	1·1 (1·0–1·2)	0·63 (0·54–0·74)	575 062 (511 153–645 003)	1·06 (0·95–1·19)
Tropical Latin America		2·8 (1·2–5·8)	50·4 (43·8–58·9)	82·8 (70·9–98·1)	78·0 (70·8–86·7)	76·7 (67·2–87·2)	48·9 (39·5–59·9)	16·2 (12·7–20·3)	0·73 (0·54–0·96)	0·01 (0·01–0·01)	1·8 (1·6–2·0)	0·68 (0·59–0·8)	0·71 (0·6–0·84)	3 186 804 (2 886 839–3 554 625)	0·85 (0·77–0·95)
	Brazil	2·9 (1·3–6·0)	49·9 (43·0–58·6)	81·6 (69·2–97·4)	76·4 (69·0–85·4)	75·7 (66·0–86·2)	48·3 (38·7–59·4)	16·1 (12·5–20·2)	0·72 (0·53–0·97)	0·01 (0·01–0·01)	1·8 (1·6–2·0)	0·67 (0·57–0·79)	0·7 (0·59–0·83)	3 040 969 (2 742 060–3 409 928)	0·84 (0·75–0·94)
	Paraguay	0·7 (0·31–1·45)	64·1 (54·8–74·8)	115·4 (96·9–136·6)	124·0 (112·3–137·0)	110·9 (96·6–126·8)	70·8 (57·1–86·8)	22·2 (16·9–28·8)	0·99 (0·65–1·47)	0·02 (0·02–0·02)	2·5 (2·2–3·0)	0·9 (0·76–1·06)	1·0 (0·9–1·2)	145 834 (125 161–169 315)	1·2 (1·03–1·4)
**North Africa and Middle East**		**0·28 (0·13–0·59)**	**47·3 (42·3–53·0)**	**131·6 (121·0–143·2)**	**138·9 (131·4–147·7)**	**117·7 (108·3–128·6)**	**72·7 (63·1–83·1)**	**28·2 (25·1–31·8)**	**5·1 (4·4–5·9)**	**0·09 (0·09–0·09)**	**2·7 (2·5–2·9)**	**0·9 (0·82–0·98)**	**1·1 (1·0–1·2)**	**13 008 474 (12 060 286–14 103 277)**	**1·26 (1·17–1·37)**
	Afghanistan	0·38 (0·16–0·8)	97·5 (84·5–113·6)	279·2 (255·7–302·0)	314·6 (304·2–324·5)	248·6 (234·8–261·8)	166·7 (150·2–182·4)	70·9 (61·0–80·8)	24·0 (20·1–27·9)	0·46 (0·44–0·48)	6·0 (5·7–6·3)	1·9 (1·7–2·0)	2·6 (2·4–2·7)	1 376 280 (1 303 953–1 448 446)	2·64 (2·52–2·76)
	Algeria	0·05 (0·02–0·09)	9·8 (8·2–11·6)	70·9 (59·1–84·3)	121·3 (109·5–133·9)	162·4 (145·4–179·9)	132·8 (115·8–149·9)	58·4 (49·7–67·4)	5·3 (4·3–6·5)	0·1 (0·1–0·11)	2·8 (2·5–3·1)	0·4 (0·34–0·48)	1·8 (1·6–2·0)	963 291 (855 732–1 073 343)	1·32 (1·17–1·47)
	Bahrain	0·2 (0·09–0·4)	15·1 (12·7–18·4)	95·6 (83·7–108·6)	127·7 (118·9–136·8)	87·0 (78·0–96·6)	59·9 (50·6–70·2)	20·6 (16·2–25·7)	3·2 (2·3–4·4)	0·06 (0·06–0·06)	2·0 (1·9–2·2)	0·55 (0·49–0·62)	0·85 (0·76–0·95)	19 881 (18 264–21 611)	0·99 (0·9–1·07)
	Egypt	0·3 (0·13–0·62)	61·8 (52·7–72·3)	171·9 (147·3–198·8)	136·6 (124·0–151·6)	102·0 (88·2–118·9)	46·7 (37·0–57·8)	11·0 (8·3–14·8)	2·0 (1·4–2·8)	0·04 (0·04–0·04)	2·7 (2·4–2·9)	1·2 (1·0–1·4)	0·81 (0·71–0·93)	2 127 960 (1 940 392–2 330 506)	1·26 (1·15–1·37)
	Iran	0·52 (0·22–1·06)	26·4 (22·1–32·0)	77·0 (62·4–93·6)	100·2 (89·8–111·3)	79·9 (68·1–92·8)	46·2 (36·0–58·1)	14·3 (10·7–18·7)	1·2 (0·8–1·7)	0·02 (0·02–0·02)	1·7 (1·5–2·0)	0·52 (0·44–0·61)	0·71 (0·58–0·86)	1 274 094 (1 085 203–1 494 227)	0·82 (0·7–0·96)
	Iraq	0·29 (0·13–0·61)	59·7 (50·8–71·0)	173·8 (149·3–203·1)	186·2 (172·4–202·2)	175·3 (159·5–193·5)	113·3 (97·7–129·5)	37·4 (30·6–44·9)	5·5 (4·0–7·2)	0·1 (0·1–0·11)	3·8 (3·4–4·1)	1·2 (1·0–1·4)	1·7 (1·5–1·8)	1 255 056 (1 135 149–1 393 958)	1·75 (1·59–1·93)
	Jordan	0·09 (0·04–0·18)	26·1 (21·9–31·2)	126·5 (106·5–151·4)	180·3 (165·9–197·0)	157·5 (140·7–174·8)	96·7 (81·0–113·4)	22·1 (16·7–28·6)	1·5 (1·0–2·1)	0·03 (0·03–0·03)	3·1 (2·8–3·4)	0·76 (0·66–0·89)	1·4 (1·2–1·6)	243 217 (223 920–266 880)	1·46 (1·34–1·6)
	Kuwait	0·03 (0·01–0·06)	8·3 (6·9–10·2)	62·3 (52·6–73·2)	78·4 (71·8–85·5)	68·9 (60·6–78·0)	44·8 (36·4–54·3)	18·8 (14·3–24·0)	2·8 (1·8–4·1)	0·05 (0·05–0·06)	1·4 (1·3–1·6)	0·35 (0·3–0·41)	0·68 (0·59–0·77)	60 885 (55 064–67 021)	0·68 (0·62–0·75)
	Lebanon	0·29 (0·13–0·6)	57·6 (48·9–68·8)	117·0 (96·9–142·3)	138·8 (125·9–154·1)	106·1 (91·9–123·3)	51·1 (40·5–64·9)	7·2 (5·6–9·2)	1·3 (0·9–1·7)	0·02 (0·02–0·03)	2·4 (2·1–2·8)	0·87 (0·73–1·06)	0·83 (0·7–0·98)	186 159 (160 797–217 399)	1·15 (0·99–1·34)
	Libya	0·13 (0·06–0·27)	13·3 (11·0–16·3)	50·8 (40·2–65·0)	114·5 (102·8–128·6)	122·3 (106·1–141·8)	77·7 (61·4–98·4)	36·8 (28·1–48·1)	8·0 (5·4–11·7)	0·15 (0·15–0·16)	2·1 (1·8–2·6)	0·32 (0·26–0·41)	1·2 (1·0–1·5)	122 256 (102 820–146 859)	0·99 (0·84–1·19)
	Morocco	0·2 (0·09–0·42)	20·0 (16·6–24·0)	73·3 (58·9–90·4)	97·3 (86·9–108·8)	106·8 (91·8–125·1)	80·8 (64·1–101·8)	43·3 (33·6–55·7)	6·2 (4·1–9·5)	0·12 (0·12–0·12)	2·1 (1·9–2·4)	0·47 (0·38–0·57)	1·2 (1·0–1·5)	601 214 (528 391–683 943)	1·01 (0·89–1·15)
	Oman	0·12 (0·05–0·25)	12·4 (10·7–14·3)	83·3 (69·6–98·7)	142·8 (131·2–154·8)	133·3 (118·8–148·4)	92·6 (78·5–107·8)	39·1 (31·7–47·2)	6·1 (4·8–7·5)	0·12 (0·11–0·12)	2·5 (2·3–2·8)	0·48 (0·41–0·56)	1·4 (1·2–1·5)	80 314 (72 628–88 378)	1·23 (1·11–1·35)
	Palestine	0·05 (0·02–0·11)	77·9 (67·5–91·0)	201·0 (176·8–226·0)	184·9 (171·1–199·0)	131·4 (116·6–146·9)	75·8 (63·4–89·4)	25·6 (21·2–30·6)	2·0 (1·5–2·5)	0·04 (0·04–0·04)	3·5 (3·2–3·9)	1·4 (1·3–1·5)	1·2 (1·0–1·3)	138 165 (125 084–152 033)	1·66 (1·51–1·83)
	Qatar	0·18 (0·08–0·37)	11·2 (9·5–13·1)	75·5 (64·1–88·1)	118·4 (109·1–128·1)	108·6 (97·6–120·1)	66·8 (57·6–76·8)	24·9 (20·3–30·0)	2·4 (1·8–3·2)	0·05 (0·04–0·05)	2·0 (1·9–2·2)	0·43 (0·38–0·5)	1·0 (0·9–1·1)	30 253 (27 787–32 803)	0·99 (0·9–1·07)
	Saudi Arabia	0·11 (0·05–0·23)	9·7 (8·1–11·4)	59·6 (48·8–72·1)	82·9 (75·5–90·8)	85·4 (74·4–97·2)	63·0 (50·5–77·0)	29·6 (23·3–36·9)	3·5 (2·4–4·9)	0·07 (0·06–0·07)	1·7 (1·5–1·9)	0·35 (0·29–0·42)	0·91 (0·78–1·05)	502 343 (444 354–565 465)	0·8 (0·7–0·9)
	Sudan	0·35 (0·15–0·74)	85·5 (74·2–99·7)	185·2 (162·3–212·3)	204·6 (190·7–220·6)	186·7 (170·1–205·5)	116·6 (99·2–134·6)	52·1 (42·9–62·0)	11·7 (8·6–15·4)	0·23 (0·22–0·23)	4·2 (3·9–4·6)	1·4 (1·2–1·6)	1·8 (1·7–2·0)	1 336 735 (1 216 996–1 472 638)	1·93 (1·78–2·1)
	Syria	0·22 (0·1–0·46)	35·2 (29·9–42·0)	98·7 (82·1–117·2)	113·6 (103·1–124·7)	103·5 (90·2–117·7)	60·3 (47·9–74·5)	19·1 (14·3–24·9)	3·8 (2·5–5·9)	0·07 (0·07–0·08)	2·2 (1·9–2·5)	0·67 (0·58–0·77)	0·93 (0·78–1·11)	276 298 (239 261–317 552)	0·99 (0·85–1·14)
	Tunisia	0·44 (0·19–0·91)	5·9 (4·9–7·3)	47·2 (37·8–59·8)	97·8 (87·8–109·8)	106·5 (93·5–122·3)	72·0 (59·7–87·5)	22·8 (18·2–28·7)	1·6 (1·2–2·3)	0·03 (0·03–0·03)	1·8 (1·5–2·1)	0·27 (0·22–0·34)	1·0 (0·9–1·2)	167 745 (144 002–197 624)	0·84 (0·72–0·99)
	Turkey	0·19 (0·08–0·38)	25·9 (21·9–30·6)	90·4 (76·2–108·5)	106·3 (96·4–118·2)	81·1 (71·3–93·1)	42·0 (35·1–49·7)	11·0 (8·9–13·5)	1·1 (0·8–1·4)	0·02 (0·02–0·02)	1·8 (1·6–2·0)	0·58 (0·51–0·67)	0·68 (0·61–0·75)	1 116 714 (1 004 994–1 245 930)	0·85 (0·77–0·95)
	United Arab Emirates	0·44 (0·19–0·89)	12·1 (10·2–14·7)	58·5 (46·9–73·9)	70·2 (62·1–80·2)	66·5 (56·6–77·3)	36·8 (29·6–45·1)	16·3 (12·9–20·3)	1·6 (1·1–2·1)	0·03 (0·03–0·03)	1·3 (1·2–1·5)	0·36 (0·29–0·45)	0·61 (0·52–0·7)	71 039 (62 614–80 613)	0·63 (0·56–0·71)
	Yemen	0·35 (0·15–0·73)	82·1 (71·0–96·0)	205·3 (180·0–234·7)	221·3 (206·8–237·8)	177·9 (160·4–197·8)	124·5 (105·8–143·5)	64·9 (54·4–75·7)	29·0 (24·1–33·7)	0·56 (0·54–0·58)	4·5 (4·2–5·0)	1·4 (1·3–1·7)	2·0 (1·8–2·2)	1 046 417 (953 260–1 151 663)	2·05 (1·89–2·23)
**South Asia**		**0·43 (0·19–0·88)**	**32·6 (28·4–37·8)**	**159·3 (137·3–185·9)**	**138·0 (126·3–151·8)**	**78·0 (69·5–87·4)**	**32·9 (27·6–39·4)**	**9·8 (7·4–12·8)**	**3·4 (2·5–4·6)**	**0·06 (0·06–0·07)**	**2·3 (2·0–2·5)**	**0·96 (0·83–1·12)**	**0·62 (0·54–0·72)**	**33 968 926 (30 525 169–38 074 532)**	**1·01 (0·92–1·13)**
	Bangladesh	0·97 (0·42–2·02)	70·9 (60·8–82·0)	133·1 (115·0–155·3)	99·5 (90·3–110·5)	56·2 (48·6–64·6)	28·2 (22·2–35·3)	6·9 (5·2–9·0)	3·4 (2·3–4·7)	0·06 (0·06–0·07)	2·0 (1·8–2·2)	1·0 (0·9–1·1)	0·47 (0·39–0·57)	2 858 475 (2 598 018–3 180 667)	0·93 (0·84–1·04)
	Bhutan	0·44 (0·2–0·92)	35·4 (29·6–42·9)	125·3 (103·6–152·4)	113·5 (101·8–127·6)	71·1 (59·9–83·5)	38·4 (29·1–49·6)	9·6 (6·8–13·1)	3·1 (2·0–4·6)	0·06 (0·06–0·06)	2·0 (1·8–2·3)	0·81 (0·67–0·98)	0·61 (0·49–0·75)	17 338 (15 394–19 757)	0·93 (0·82–1·06)
	India	0·37 (0·16–0·76)	25·4 (21·2–30·8)	162·3 (140·1–189·1)	133·7 (122·1–147·4)	70·0 (60·5–80·3)	26·3 (20·7–32·8)	8·0 (5·9–10·4)	2·6 (1·9–3·6)	0·05 (0·05–0·05)	2·1 (1·9–2·4)	0·94 (0·81–1·1)	0·53 (0·45–0·63)	24 568 864 (22 072 577–27 481 958)	0·96 (0·87–1·06)
	Nepal	0·58 (0·25–1·21)	59·0 (50·7–68·5)	156·9 (134·6–183·9)	114·0 (102·9–127·4)	67·7 (57·7–80·3)	30·8 (24·1–39·9)	9·9 (7·5–12·8)	3·3 (2·3–4·6)	0·06 (0·06–0·07)	2·2 (2·0–2·5)	1·1 (1·0–1·2)	0·56 (0·48–0·67)	632 646 (560 875–717 437)	1·03 (0·91–1·17)
	Pakistan	0·34 (0·15–0·71)	43·6 (36·8–52·4)	161·5 (137·5–190·5)	200·4 (185·8–217·2)	152·9 (135·6–173·1)	85·5 (69·1–105·8)	26·2 (19·7–33·8)	9·7 (6·7–14·0)	0·19 (0·18–0·19)	3·4 (3·0–3·9)	1·0 (0·9–1·2)	1·4 (1·2–1·6)	5 891 600 (5 173 076–6 733 145)	1·48 (1·3–1·69)
**Southeast Asia, east Asia, and Oceania**		**0·15 (0·07–0·31)**	**18·9 (17·3–20·8)**	**93·6 (86·4–101·6)**	**117·7 (113·6–121·9)**	**71·9 (68·4–75·9)**	**32·1 (29·5–35·3)**	**9·0 (8·1–10·1)**	**1·1 (0·9–1·3)**	**0·02 (0·02–0·02)**	**1·7 (1·6–1·8)**	**0·56 (0·52–0·61)**	**0·57 (0·54–0·61)**	**28 562 870 (27 037 176–30 297 827)**	**0·79 (0·75–0·84)**
East Asia		0·1 (0·04–0·21)	8·7 (8·0–9·4)	89·4 (82·8–96·2)	116·8 (112·3–121·4)	61·8 (57·9–65·7)	20·5 (18·4–22·7)	5·5 (4·8–6·2)	0·8 (0·61–1·07)	0·02 (0·01–0·02)	1·5 (1·4–1·6)	0·49 (0·45–0·53)	0·44 (0·41–0·48)	17 180 872 (16 167 753–18 210 317)	0·69 (0·65–0·73)
	China	0·1 (0·05–0·21)	8·9 (8·2–9·7)	91·5 (84·5–98·6)	117·6 (112·9–122·2)	61·4 (57·4–65·4)	20·2 (18·0–22·5)	5·5 (4·8–6·3)	0·82 (0·62–1·1)	0·02 (0·02–0·02)	1·5 (1·4–1·6)	0·5 (0·46–0·54)	0·44 (0·4–0·48)	16 469 641 (15 475 992–17 488 048)	0·69 (0·65–0·74)
	North Korea	0·05 (0·02–0·09)	1·8 (1·5–2·3)	49·2 (39·0–61·3)	112·8 (101·2–126·9)	71·8 (60·5–84·4)	23·3 (17·2–30·7)	5·4 (3·8–7·8)	0·43 (0·26–0·7)	0·01 (0·01–0·01)	1·3 (1·2–1·5)	0·26 (0·2–0·32)	0·5 (0·42–0·61)	258 789 (228 429–295 066)	0·62 (0·55–0·71)
	Taiwan (province of China)	0·08 (0·03–0·17)	4·0 (3·4–4·9)	23·5 (19·4–28·4)	63·5 (58·0–69·7)	78·8 (70·3–88·2)	34·1 (28·0–41·5)	4·4 (3·2–6·0)	0·03 (0·02–0·04)	0·0 (0·0–0·0)	1·0 (0·9–1·2)	0·14 (0·11–0·17)	0·59 (0·51–0·67)	175 666 (154 235–200 219)	0·5 (0·44–0·57)
Oceania		0·54 (0·24–1·13)	56·3 (48·0–65·8)	194·1 (170·4–219·3)	194·9 (182·0–208·4)	171·6 (155·8–189·1)	123·9 (106·5–143·3)	49·6 (40·7–60·2)	12·4 (9·1–16·7)	0·23 (0·22–0·24)	4·0 (3·7–4·4)	1·3 (1·1–1·4)	1·8 (1·6–2·0)	398 611 (364 718–433 764)	1·77 (1·62–1·93)
	American Samoa	0·72 (0·32–1·49)	35·3 (29·7–41·9)	142·8 (122·6–165·4)	171·4 (157·9–185·8)	136·3 (120·1–153·8)	80·4 (65·8–97·0)	16·1 (12·1–21·0)	0·91 (0·57–1·4)	0·02 (0·02–0·02)	2·9 (2·5–3·3)	0·89 (0·76–1·04)	1·2 (1·0–1·4)	1134 (987–1298)	1·37 (1·2–1·57)
	Federated States of Micronesia	1·8 (0·8–3·6)	36·0 (30·5–42·6)	127·9 (106·5–154·5)	146·1 (133·0–161·7)	127·2 (110·9–146·9)	82·6 (66·6–100·1)	18·0 (13·1–24·9)	4·3 (2·7–6·7)	0·08 (0·08–0·09)	2·7 (2·4–3·1)	0·83 (0·72–0·96)	1·2 (1·0–1·3)	2118 (1891–2392)	1·26 (1·12–1·43)
	Fiji	0·07 (0·03–0·14)	34·4 (29·2–40·4)	149·8 (129·9–172·0)	156·6 (144·6–169·6)	110·6 (98·2–124·4)	57·2 (47·9–67·9)	12·5 (9·8–15·9)	1·2 (0·9–1·6)	0·02 (0·02–0·02)	2·6 (2·3–3·0)	0·92 (0·8–1·06)	0·91 (0·78–1·05)	18 373 (16 204–20 801)	1·21 (1·07–1·37)
	Guam	0·65 (0·29–1·35)	44·8 (38·1–52·7)	146·7 (128·8–166·5)	165·2 (153·0–179·3)	141·2 (127·1–156·5)	74·3 (61·0–91·1)	15·5 (11·5–20·3)	0·56 (0·36–0·85)	0·01 (0·01–0·01)	2·9 (2·7–3·2)	0·96 (0·84–1·1)	1·2 (1·1–1·3)	3350 (3080–3638)	1·38 (1·28–1·5)
	Kiribati	0·33 (0·15–0·68)	38·8 (32·8–45·7)	188·0 (162·6–215·5)	184·8 (170·7–199·9)	175·3 (158·1–193·3)	117·1 (98·6–136·9)	29·9 (23·3–37·8)	7·6 (5·5–10·4)	0·15 (0·14–0·15)	3·7 (3·3–4·2)	1·1 (1·0–1·3)	1·7 (1·4–1·9)	3544 (3124–4001)	1·66 (1·46–1·86)
	Marshall Islands	0·82 (0·36–1·71)	66·1 (57·8–76·5)	177·1 (154·4–201·0)	148·8 (136·4–161·7)	109·1 (96·0–123·1)	55·0 (44·7–66·6)	14·9 (11·5–18·8)	1·1 (0·7–1·6)	0·02 (0·02–0·02)	2·9 (2·5–3·2)	1·2 (1·1–1·4)	0·9 (0·76–1·05)	1298 (1159–1448)	1·32 (1·17–1·47)
	Northern Mariana Islands	0·91 (0·4–1·89)	38·5 (33·6–44·0)	102·3 (85·3–121·1)	107·1 (96·4–118·4)	104·8 (91·1–119·6)	49·5 (40·3–60·0)	9·2 (6·7–12·3)	0·11 (0·07–0·17)	0·0 (0·0–0·0)	2·1 (1·8–2·3)	0·71 (0·61–0·81)	0·82 (0·72–0·92)	547 (483–612)	0·98 (0·86–1·09)
	Papua New Guinea	0·57 (0·25–1·2)	59·4 (50·4–70·0)	200·1 (173·1–229·1)	198·5 (183·9–213·9)	178·4 (160·3–199·0)	133·2 (113·1–156·0)	56·1 (45·0–69·5)	14·9 (10·6–20·5)	0·29 (0·28–0·3)	4·2 (3·8–4·6)	1·3 (1·1–1·5)	1·9 (1·6–2·2)	309 184 (282 124–336 719)	1·83 (1·67–2·0)
	Samoa	0·17 (0·07–0·35)	42·9 (36·5–51·1)	207·9 (182·2–237·7)	244·8 (230·5–260·6)	222·1 (205·2–240·2)	153·5 (134·7–173·9)	56·0 (46·0–68·0)	11·0 (8·1–14·4)	0·21 (0·2–0·22)	4·7 (4·2–5·2)	1·3 (1·1–1·4)	2·2 (2·0–2·5)	6070 (5464–6756)	2·18 (1·96–2·42)
	Solomon Islands	0·59 (0·26–1·22)	62·2 (52·9–72·9)	207·0 (180·4–237·8)	211·7 (196·9–228·6)	176·0 (158·0–196·6)	124·1 (104·4–146·7)	46·9 (37·0–57·7)	11·1 (7·7–15·4)	0·21 (0·21–0·22)	4·2 (3·8–4·6)	1·3 (1·2–1·5)	1·8 (1·6–2·0)	20 410 (18 459–22 554)	1·91 (1·72–2·12)
	Tonga	0·4 (0·17–0·8)	17·1 (14·3–20·5)	108·3 (89·8–129·6)	176·1 (162·2–191·1)	170·7 (153·8–188·6)	121·8 (103·5–141·3)	36·6 (28·7–45·9)	2·8 (2·0–3·8)	0·05 (0·05–0·05)	3·2 (2·8–3·6)	0·63 (0·52–0·75)	1·7 (1·4–1·9)	2184 (1908–2489)	1·49 (1·3–1·68)
	Vanuatu	0·53 (0·23–1·1)	51·2 (43·4–61·2)	190·1 (164·6–220·0)	187·2 (172·7–204·0)	158·8 (141·5–176·7)	109·5 (91·1–128·8)	41·3 (32·3–51·3)	8·0 (5·3–11·3)	0·15 (0·15–0·16)	3·7 (3·4–4·1)	1·2 (1·0–1·4)	1·6 (1·4–1·8)	8428 (7704–9314)	1·71 (1·56–1·88)
Southeast Asia		0·21 (0·09–0·43)	32·0 (28·5–36·3)	98·6 (83·7–117·5)	118·2 (111·0–127·0)	94·0 (86·3–103·2)	54·1 (47·9–61·5)	17·1 (14·8–19·9)	1·8 (1·4–2·2)	0·03 (0·03–0·03)	2·1 (1·9–2·3)	0·65 (0·56–0·77)	0·84 (0·75–0·93)	10 983 387 (9 949 746–12 233 978)	0·97 (0·88–1·08)
	Cambodia	0·34 (0·15–0·7)	43·6 (37·0–52·2)	133·8 (114·2–157·9)	150·7 (138·2–165·5)	118·5 (104·6–133·2)	75·4 (61·4–90·7)	20·4 (16·0–25·5)	4·1 (2·9–5·6)	0·08 (0·08–0·08)	2·7 (2·5–3·0)	0·89 (0·76–1·05)	1·1 (0·9–1·3)	377 406 (343 642–419 172)	1·28 (1·17–1·42)
	Indonesia	0·15 (0·06–0·3)	27·7 (23·5–32·6)	92·8 (76·9–113·1)	113·7 (103·0–126·6)	89·5 (77·7–104·1)	51·7 (41·7–64·8)	16·4 (12·7–21·5)	1·8 (1·2–2·5)	0·03 (0·03–0·04)	2·0 (1·7–2·3)	0·6 (0·52–0·7)	0·8 (0·67–0·96)	4 032 914 (3 491 800–4 705 431)	0·92 (0·8–1·08)
	Laos	0·41 (0·18–0·85)	62·9 (54·0–74·2)	164·6 (143·4–190·0)	147·0 (135·4–159·0)	109·1 (96·9–122·1)	65·9 (54·4–78·6)	23·1 (18·1–29·0)	7·7 (5·4–10·5)	0·15 (0·14–0·15)	2·9 (2·6–3·2)	1·1 (1·0–1·3)	1·0 (0·9–1·2)	176 836 (161 828–194 522)	1·32 (1·21–1·45)
	Malaysia	0·22 (0·09–0·44)	11·1 (9·4–13·1)	51·7 (44·2–60·2)	122·2 (113·9–130·8)	125·1 (114·1–136·6)	72·6 (61·9–84·2)	20·7 (16·4–25·6)	1·4 (0·9–2·2)	0·03 (0·03–0·03)	2·0 (1·8–2·3)	0·32 (0·28–0·36)	1·1 (1·0–1·2)	508 960 (457 674–566 097)	0·96 (0·86–1·07)
	Maldives	0·12 (0·05–0·24)	17·6 (14·7–21·0)	96·6 (83·8–110·2)	112·9 (104·0–121·9)	83·1 (73·3–93·7)	46·0 (36·9–58·1)	15·8 (11·7–21·1)	1·9 (1·2–2·9)	0·04 (0·04–0·04)	1·9 (1·7–2·0)	0·57 (0·5–0·64)	0·73 (0·66–0·82)	6844 (6312–7356)	0·9 (0·83–0·97)
	Mauritius	0·46 (0·2–0·93)	23·0 (19·6–26·9)	61·0 (52·3–70·6)	82·1 (75·2–90·5)	63·0 (55·5–72·2)	28·0 (22·4–34·8)	6·8 (4·9–9·4)	0·31 (0·19–0·49)	0·01 (0·01–0·01)	1·3 (1·2–1·4)	0·42 (0·36–0·49)	0·49 (0·43–0·55)	12 416 (11 454–13 507)	0·64 (0·59–0·69)
	Myanmar	0·25 (0·11–0·51)	24·8 (20·9–29·8)	84·7 (70·9–102·3)	105·4 (95·7–115·6)	94·6 (83·1–106·9)	65·3 (53·9–77·9)	25·5 (20·1–31·8)	3·0 (2·1–4·1)	0·06 (0·06–0·06)	2·0 (1·9–2·2)	0·55 (0·46–0·66)	0·94 (0·84–1·06)	876 249 (804 806–959 636)	0·92 (0·85–1·01)
	Philippines	0·22 (0·1–0·47)	54·2 (46·1–64·6)	137·3 (116·6–162·7)	162·4 (149·5–177·5)	141·5 (126·5–157·0)	92·2 (77·4–107·9)	32·2 (26·0–39·1)	3·5 (2·5–4·7)	0·07 (0·06–0·07)	3·1 (2·8–3·4)	0·96 (0·81–1·14)	1·3 (1·2–1·5)	2 526 359 (2 304 451–2 800 024)	1·44 (1·31–1·59)
	Sri Lanka	0·13 (0·06–0·27)	18·5 (15·4–22·2)	67·3 (54·0–83·3)	109·6 (98·6–121·7)	97·2 (84·2–111·8)	51·3 (40·9–63·7)	14·3 (10·9–18·6)	0·81 (0·56–1·17)	0·02 (0·02–0·02)	1·8 (1·5–2·1)	0·43 (0·35–0·53)	0·82 (0·68–0·98)	292 833 (248 351–344 723)	0·86 (0·73–1·01)
	Seychelles	1·1 (0·5–2·4)	58·0 (50·5–66·6)	114·4 (98·8–132·0)	111·8 (102·4–122·0)	81·8 (73·8–90·7)	48·8 (42·0–56·6)	13·6 (11·1–16·7)	0·5 (0·31–0·78)	0·01 (0·01–0·01)	2·1 (1·9–2·4)	0·87 (0·75–1·0)	0·72 (0·64–0·82)	1497 (1322–1693)	1·03 (0·91–1·16)
	Thailand	0·36 (0·16–0·73)	32·4 (27·1–39·3)	64·6 (52·1–81·1)	66·0 (58·6–74·0)	48·6 (41·2–57·0)	21·8 (16·8–27·7)	7·6 (5·8–9·9)	0·49 (0·34–0·69)	0·01 (0·01–0·01)	1·2 (1·1–1·4)	0·49 (0·4–0·6)	0·39 (0·32–0·48)	613 237 (539 713–701 506)	0·58 (0·51–0·66)
	Timor-Leste	0·4 (0·18–0·84)	61·1 (51·9–71·8)	176·0 (150·4–204·0)	209·8 (195·1–225·2)	194·0 (176·2–212·3)	123·2 (103·5–144·2)	52·5 (41·9–64·4)	10·7 (7·4–14·8)	0·21 (0·2–0·21)	4·1 (3·6–4·7)	1·2 (1·0–1·4)	1·9 (1·6–2·2)	38 826 (34 156–43 904)	1·92 (1·69–2·17)
	Vietnam	0·25 (0·11–0·51)	24·7 (20·9–29·6)	107·8 (91·8–127·8)	114·7 (105·5–124·5)	77·0 (67·9–86·8)	36·7 (30·4–43·9)	8·8 (7·0–10·8)	0·48 (0·34–0·65)	0·01 (0·01–0·01)	1·9 (1·7–2·0)	0·66 (0·56–0·79)	0·61 (0·53–0·71)	1 504 552 (1 372 351–1 660 292)	0·88 (0·8–0·97)
**Sub-Saharan Africa**		**2·1 (0·9–4·3)**	**93·7 (84·2–105·2)**	**199·0 (184·9–215·8)**	**206·3 (198·2–215·8)**	**190·1 (179·9–201·1)**	**137·5 (125·6–149·2)**	**71·2 (64·2–78·1)**	**23·3 (21·2–25·3)**	**0·44 (0·43–0·46)**	**4·6 (4·3–4·9)**	**1·5 (1·4–1·6)**	**2·1 (2·0–2·3)**	**36 181 702 (34 016 504–38 650 498)**	**2·02 (1·91–2·14)**
Central sub-Saharan Africa		1·5 (0·6–3·1)	94·1 (85·6–103·0)	195·3 (178·9–212·1)	209·5 (199·7–219·6)	214·4 (202·8–225·9)	159·2 (147·9–169·6)	81·9 (75·8–87·8)	19·0 (16·3–21·9)	0·37 (0·35–0·38)	4·9 (4·6–5·1)	1·5 (1·3–1·6)	2·4 (2·3–2·5)	4 318 103 (4 060 044–4 568 273)	2·14 (2·04–2·23)
	Angola	1·7 (0·7–3·5)	120·6 (105·7–137·3)	206·1 (179·5–234·4)	212·7 (198·1–228·0)	209·3 (192·1–226·9)	162·3 (143·3–182·6)	91·6 (81·2–102·5)	20·0 (16·0–24·9)	0·39 (0·37–0·4)	5·1 (4·7–5·5)	1·6 (1·4–1·9)	2·4 (2·2–2·6)	1 052 695 (962 954–1 146 611)	2·27 (2·09–2·46)
	Central African Republic	1·4 (0·6–3·0)	87·9 (75·6–101·8)	162·5 (137·3–192·9)	127·3 (114·8–142·3)	150·2 (132·6–170·9)	104·0 (85·0–126·8)	55·9 (45·0–67·4)	22·9 (17·9–28·1)	0·44 (0·42–0·46)	3·6 (3·2–4·0)	1·3 (1·1–1·4)	1·7 (1·5–1·9)	133 353 (119 271–149 763)	1·44 (1·31–1·58)
	Congo (Brazzaville)	1·2 (0·5–2·6)	69·8 (60·5–81·5)	130·4 (111·1–154·2)	137·4 (125·8–151·2)	157·4 (142·7–172·5)	111·7 (92·5–131·7)	41·4 (32·2–51·6)	11·0 (7·8–14·9)	0·21 (0·2–0·22)	3·3 (3·0–3·7)	1·0 (0·9–1·2)	1·6 (1·4–1·9)	131 030 (119 585–145 001)	1·48 (1·35–1·63)
	Democratic Republic of the Congo	1·4 (0·6–2·9)	87·4 (77·7–97·9)	200·0 (178·9–221·6)	221·3 (208·0–234·6)	227·4 (213·0–241·5)	168·1 (153·2–182·2)	85·1 (76·9–93·1)	19·5 (15·8–23·5)	0·38 (0·36–0·39)	5·1 (4·7–5·4)	1·4 (1·3–1·6)	2·5 (2·4–2·6)	2 920 848 (2 712 396–3 123 108)	2·21 (2·09–2·32)
	Equatorial Guinea	1·6 (0·7–3·3)	107·7 (93·4–123·7)	167·1 (141·7–195·1)	157·1 (143·3–172·0)	161·5 (143·6–180·4)	108·8 (89·5–129·8)	58·0 (46·8–70·2)	13·7 (9·7–19·0)	0·26 (0·25–0·27)	3·9 (3·4–4·4)	1·4 (1·2–1·6)	1·7 (1·5–2·0)	39 049 (33 922–44 596)	1·74 (1·51–1·98)
	Gabon	1·2 (0·5–2·5)	62·9 (53·4–75·0)	112·7 (92·5–138·4)	123·9 (111·6–138·7)	131·1 (114·4–151·2)	91·4 (73·7–110·5)	30·0 (22·6–38·6)	4·8 (3·2–7·0)	0·09 (0·09–0·1)	2·8 (2·5–3·2)	0·88 (0·73–1·07)	1·3 (1·1–1·5)	41 125 (36 192–47 105)	1·3 (1·15–1·48)
Eastern sub-Saharan Africa		1·9 (0·8–4·0)	92·8 (82·2–105·6)	209·7 (192·1–229·6)	203·1 (193·6–213·9)	186·7 (176·0–198·8)	138·2 (125·4–151·0)	73·1 (65·6–80·2)	23·9 (21·4–26·2)	0·46 (0·44–0·47)	4·6 (4·4–5·0)	1·5 (1·4–1·7)	2·1 (2·0–2·3)	13 995 648 (13 041 912–15 084 874)	2·07 (1·95–2·2)
	Burundi	1·4 (0·6–2·9)	52·6 (45·0–62·2)	206·7 (184·8–232·0)	238·1 (224·9–253·1)	243·2 (228·8–258·6)	198·9 (185·5–211·0)	96·7 (88·1–104·7)	23·0 (18·9–27·1)	0·44 (0·43–0·46)	5·3 (5·0–5·6)	1·3 (1·2–1·5)	2·8 (2·7–2·9)	406 276 (379 690–436 338)	2·32 (2·2–2·46)
	Comoros	1·3 (0·6–2·7)	43·7 (37·0–51·6)	123·3 (102·5–147·0)	144·1 (131·0–158·4)	169·2 (151·5–187·9)	120·1 (100·7–140·8)	49·5 (39·4–60·9)	25·5 (20·4–30·8)	0·49 (0·47–0·51)	3·4 (2·9–3·9)	0·84 (0·7–1·0)	1·8 (1·6–2·1)	18 191 (15 712–20 908)	1·54 (1·34–1·77)
	Djibouti	1·4 (0·6–2·8)	49·0 (41·3–58·0)	135·0 (112·3–160·8)	147·7 (134·3–162·3)	200·5 (182·8–218·7)	128·1 (108·0–149·3)	61·0 (49·6–73·4)	39·8 (36·6–42·5)	0·77 (0·74–0·79)	3·8 (3·3–4·3)	0·93 (0·77–1·1)	2·2 (1·9–2·4)	34 700 (30 383–39 346)	1·72 (1·5–1·95)
	Eritrea	1·3 (0·6–2·8)	48·2 (40·6–58·1)	146·7 (122·7–176·3)	128·6 (116·0–143·7)	185·8 (167·6–206·4)	167·4 (148·5–187·4)	82·5 (70·8–95·1)	43·8 (41·8–45·5)	0·84 (0·81–0·88)	4·0 (3·6–4·6)	0·98 (0·82–1·18)	2·4 (2·1–2·7)	177 412 (155 541–203 011)	1·82 (1·61–2·04)
	Ethiopia	1·9 (0·8–3·9)	89·0 (77·4–103·5)	202·7 (178·6–230·7)	207·2 (193·0–223·4)	200·2 (183·4–218·9)	151·3 (133·3–168·9)	75·3 (65·7–84·9)	29·4 (25·1–33·4)	0·57 (0·54–0·59)	4·8 (4·4–5·2)	1·5 (1·3–1·7)	2·3 (2·1–2·5)	3 714 299 (3 402 189–4 069 663)	2·15 (2·0–2·32)
	Kenya	1·5 (0·6–3·1)	70·9 (60·6–83·9)	184·0 (158·3–211·0)	153·7 (140·2–167·8)	139·0 (122·1–156·5)	85·0 (68·2–103·3)	35·2 (27·0–44·6)	6·4 (4·3–8·9)	0·12 (0·12–0·13)	3·4 (3·0–3·8)	1·3 (1·1–1·4)	1·3 (1·1–1·6)	1 365 160 (1 208 543–1 535 478)	1·54 (1·34–1·75)
	Madagascar	2·4 (1·0–5·0)	128·0 (113·1–144·4)	230·2 (204·3–257·3)	215·6 (201·2–230·7)	175·1 (157·7–193·5)	134·5 (115·5–154·3)	75·9 (65·5–86·6)	16·8 (13·2–21·0)	0·32 (0·31–0·34)	4·9 (4·4–5·5)	1·8 (1·6–2·0)	2·0 (1·8–2·3)	975 570 (871 322–1 083 828)	2·16 (1·93–2·4)
	Malawi	2·7 (1·2–5·6)	110·3 (98·7–124·4)	220·2 (199·4–244·0)	191·9 (179·5–206·2)	154·1 (140·3–168·2)	107·7 (93·9–122·2)	69·9 (60·9–78·9)	33·9 (30·3–37·2)	0·65 (0·63–0·68)	4·5 (4·2–4·8)	1·7 (1·5–1·9)	1·8 (1·6–2·0)	612 862 (571 079–660 504)	1·99 (1·86–2·12)
	Mozambique	2·0 (0·8–4·1)	98·2 (87·6–111·0)	187·4 (167·5–208·0)	160·7 (149·2–172·6)	157·7 (143·6–172·0)	114·7 (99·8–130·2)	74·4 (65·4–83·3)	36·5 (33·0–39·6)	0·7 (0·68–0·73)	4·2 (3·8–4·5)	1·4 (1·3–1·6)	1·9 (1·8–2·1)	988 056 (912 263–1 068 141)	1·78 (1·66–1·91)
	Rwanda	1·1 (0·5–2·2)	30·1 (25·5–36·2)	167·6 (143·9–196·1)	205·2 (190·6–222·0)	219·1 (202·2–237·6)	158·4 (139·9–178·5)	83·4 (72·8–93·5)	20·9 (16·1–26·7)	0·4 (0·39–0·42)	4·4 (4·0–4·9)	0·99 (0·85–1·17)	2·4 (2·2–2·6)	423 424 (381 994–470 006)	2·03 (1·85–2·24)
	Somalia	1·9 (0·8–4·1)	96·4 (83·1–112·9)	248·7 (221·2–279·3)	273·3 (259·9–288·1)	251·7 (236·9–267·4)	202·6 (188·7–214·9)	101·6 (92·0–110·1)	42·3 (39·8–44·3)	0·81 (0·78–0·85)	6·1 (5·7–6·5)	1·7 (1·5–2·0)	3·0 (2·8–3·1)	685 515 (638 214–737 921)	2·56 (2·44–2·69)
	South Sudan	2·3 (1·0–4·8)	129·6 (113·5–149·1)	271·7 (245·0–300·8)	262·4 (248·5–277·7)	251·7 (237·0–265·6)	163·5 (144·6–181·5)	78·4 (66·6–90·0)	25·7 (20·4–31·2)	0·49 (0·48–0·51)	5·9 (5·6–6·3)	2·0 (1·8–2·3)	2·6 (2·3–2·8)	413 783 (387 551–444 396)	2·5 (2·36–2·66)
	Tanzania	2·0 (0·9–4·1)	99·2 (87·4–113·8)	220·7 (197·7–247·1)	211·7 (198·2–227·1)	183·2 (167·3–201·4)	138·7 (122·5–155·0)	79·4 (71·0–87·8)	21·9 (17·9–26·0)	0·42 (0·41–0·44)	4·8 (4·4–5·2)	1·6 (1·4–1·8)	2·1 (2·0–2·3)	1 986 281 (1 828 505–2 163 767)	2·16 (2·02–2·32)
	Uganda	2·1 (0·9–4·4)	108·5 (97·4–121·9)	246·8 (226·3–269·9)	246·1 (234·2–257·9)	198·9 (185·1–212·7)	146·0 (131·7–160·0)	80·2 (73·0–87·4)	19·2 (15·7–23·0)	0·37 (0·36–0·38)	5·2 (5·0–5·5)	1·8 (1·6–2·0)	2·2 (2·1–2·4)	1 550 366 (1 471 798–1 638 382)	2·37 (2·26–2·48)
	Zambia	2·0 (0·9–4·3)	104·8 (91·1–121·6)	206·2 (180·0–236·7)	199·3 (184·8–216·1)	183·5 (165·8–203·6)	136·6 (116·9–158·8)	81·0 (69·8–91·8)	23·1 (18·0–28·3)	0·44 (0·43–0·46)	4·7 (4·2–5·2)	1·6 (1·4–1·8)	2·1 (1·9–2·3)	634 965 (568 251–710 865)	2·1 (1·9–2·31)
Southern sub-Saharan Africa		0·77 (0·34–1·61)	69·2 (59·8–81·1)	124·3 (109·2–143·1)	137·5 (127·6–149·0)	100·3 (88·0–113·7)	66·1 (54·1–79·6)	22·8 (18·7–27·5)	2·9 (2·1–3·8)	0·05 (0·05–0·06)	2·6 (2·4–2·9)	0·97 (0·85–1·12)	0·96 (0·82–1·12)	1 748 266 (1 595 640–1 938 810)	1·19 (1·09–1·32)
	Botswana	0·5 (0·22–1·03)	45·8 (39·3–54·0)	115·1 (102·5–130·6)	119·0 (111·2–127·2)	95·3 (87·3–103·8)	66·5 (58·7–74·8)	24·9 (21·5–28·7)	4·3 (3·3–5·5)	0·08 (0·08–0·09)	2·4 (2·2–2·5)	0·81 (0·71–0·92)	0·96 (0·87–1·04)	48 644 (45 386–52 258)	1·1 (1·03–1·19)
	Lesotho	1·1 (0·5–2·2)	70·2 (61·2–81·3)	150·9 (132·5–173·1)	123·4 (113·2–135·7)	108·8 (97·0–121·3)	73·9 (61·8–87·2)	38·4 (32·1–45·3)	6·5 (4·5–8·9)	0·12 (0·12–0·13)	2·9 (2·6–3·2)	1·1 (1·0–1·3)	1·1 (1·0–1·3)	48 751 (44 699–53 717)	1·23 (1·12–1·36)
	Namibia	3·1 (1·4–6·4)	54·5 (47·6–63·1)	129·4 (114·1–147·9)	146·9 (136·8–157·4)	128·9 (116·4–142·0)	95·5 (82·0–109·8)	37·3 (30·6–44·8)	7·0 (4·9–9·8)	0·14 (0·13–0·14)	3·0 (2·8–3·3)	0·93 (0·82–1·07)	1·3 (1·2–1·5)	59 520 (54 862–64 829)	1·4 (1·28–1·52)
	South Africa	0·59 (0·26–1·24)	59·3 (50·2–70·9)	102·9 (84·1–126·9)	129·6 (117·0–144·8)	89·0 (76·0–103·3)	56·7 (44·2–71·1)	18·1 (13·4–23·7)	1·9 (1·3–2·8)	0·04 (0·04–0·04)	2·3 (2·0–2·6)	0·81 (0·67–0·99)	0·83 (0·68–1·0)	1 091 574 (976 081–1 238 233)	1·05 (0·94–1·19)
	Swaziland (eSwatini)	1·2 (0·5–2·4)	73·3 (63·7–85·2)	153·0 (133·0–177·1)	129·3 (117·9–143·0)	125·5 (111·1–142·7)	83·8 (69·3–101·8)	35·8 (28·4–45·3)	6·5 (4·4–9·1)	0·12 (0·12–0·13)	3·0 (2·7–3·5)	1·1 (1·0–1·3)	1·3 (1·1–1·5)	30 680 (26 863–35 270)	1·36 (1·18–1·56)
	Zimbabwe	0·86 (0·38–1·82)	100·7 (89·6–114·2)	192·2 (176·1–208·8)	176·1 (166·7–185·8)	143·4 (132·6–154·6)	98·3 (87·1–109·9)	39·0 (32·7–45·8)	6·1 (4·8–7·8)	0·12 (0·11–0·12)	3·8 (3·5–4·0)	1·5 (1·4–1·6)	1·4 (1·3–1·6)	469 094 (438 268–501 128)	1·7 (1·6–1·81)
Western sub-Saharan Africa		2·5 (1·1–5·3)	98·0 (86·9–111·6)	203·0 (187·9–219·9)	222·8 (214·1–232·4)	207·2 (196·6–218·2)	147·2 (133·7–160·6)	78·0 (69·4–86·6)	29·1 (26·2–31·8)	0·56 (0·54–0·58)	4·9 (4·6–5·3)	1·5 (1·4–1·7)	2·3 (2·1–2·5)	16 119 684 (15 142 476–17 204 806)	2·12 (2·01–2·25)
	Benin	6·2 (2·7–12·9)	79·3 (69·3–91·6)	204·3 (182·9–229·0)	224·2 (210·5–239·8)	203·1 (186·6–221·6)	134·7 (116·5–153·0)	74·0 (63·7–84·3)	28·9 (23·9–33·7)	0·56 (0·54–0·58)	4·8 (4·4–5·2)	1·4 (1·3–1·6)	2·2 (2·0–2·4)	420 926 (387 743–457 500)	2·09 (1·96–2·24)
	Burkina Faso	2·4 (1·0–5·0)	98·0 (86·9–111·5)	235·6 (214·1–260·0)	247·3 (234·4–261·7)	217·2 (201·6–234·4)	164·4 (148·0–180·2)	85·9 (76·6–94·9)	29·0 (25·1–32·7)	0·56 (0·54–0·58)	5·4 (5·1–5·8)	1·7 (1·5–1·9)	2·5 (2·3–2·7)	850 128 (792 781–913 615)	2·31 (2·2–2·44)
	Cameroon	2·3 (1·0–4·9)	91·9 (80·0–106·7)	172·9 (150·4–199·7)	164·5 (151·4–179·8)	166·3 (149·8–185·4)	115·4 (97·5–136·4)	55·3 (45·8–65·5)	18·0 (14·4–21·9)	0·35 (0·33–0·36)	3·9 (3·5–4·4)	1·3 (1·2–1·5)	1·8 (1·6–2·0)	860 875 (767 156–970 019)	1·73 (1·54–1·94)
	Cape Verde	1·4 (0·6–2·9)	35·3 (29·6–42·1)	88·8 (71·9–108·7)	113·3 (101·8–126·1)	89·1 (75·8–104·2)	63·4 (49·3–80·1)	26·2 (19·5–34·6)	19·4 (14·4–25·0)	0·37 (0·36–0·39)	2·2 (1·8–2·6)	0·63 (0·51–0·76)	0·99 (0·8–1·22)	9895 (8296–11 738)	1·04 (0·86–1·24)
	Chad	3·2 (1·4–6·9)	172·7 (155·9–192·3)	294·7 (271·9–319·2)	306·6 (295·9–317·0)	270·3 (258·4–281·4)	188·0 (173·9–201·0)	84·4 (75·2–93·3)	24·2 (20·2–28·2)	0·47 (0·45–0·48)	6·7 (6·4–7·0)	2·4 (2·2–2·6)	2·8 (2·7–3·0)	716 150 (684 354–753 893)	2·81 (2·71–2·92)
	Côte d'Ivoire	3·1 (1·4–6·6)	99·1 (87·1–113·9)	187·7 (163·1–216·8)	184·2 (169·9–200·9)	183·6 (166·2–203·5)	136·2 (117·3–155·2)	77·9 (67·8–87·9)	28·1 (23·4–32·6)	0·54 (0·52–0·56)	4·5 (4·1–4·9)	1·4 (1·3–1·7)	2·1 (1·9–2·3)	863 669 (785 916–951 705)	1·97 (1·8–2·16)
	The Gambia	2·0 (0·9–4·3)	72·1 (61·5–85·5)	163·5 (138·3–193·9)	168·6 (154·4–185·4)	181·9 (163·8–202·5)	136·7 (116·6–159·4)	73·9 (62·4–85·5)	29·4 (24·3–34·3)	0·57 (0·54–0·59)	4·1 (3·7–4·7)	1·2 (1·0–1·4)	2·1 (1·9–2·3)	68 878 (60 884–78 170)	1·88 (1·68–2·09)
	Ghana	1·2 (0·5–2·4)	49·5 (41·8–58·4)	131·5 (109·5–156·4)	160·4 (146·5–175·3)	160·0 (142·4–178·9)	111·6 (92·4–132·5)	55·1 (44·2–67·1)	25·3 (20·2–31·0)	0·49 (0·47–0·51)	3·5 (3·0–4·0)	0·91 (0·76–1·08)	1·8 (1·5–2·0)	876 967 (760 322–1 005 644)	1·56 (1·36–1·79)
	Guinea	4·3 (1·9–9·0)	116·9 (105·2–131·1)	202·6 (182·7–223·0)	195·2 (182·1–210·2)	177·4 (162·0–193·0)	122·4 (106·1–138·9)	73·0 (64·2–81·8)	33·1 (29·6–36·3)	0·64 (0·61–0·66)	4·6 (4·4–4·9)	1·6 (1·5–1·7)	2·0 (1·9–2·2)	434 559 (409 818–461 664)	1·98 (1·88–2·08)
	Guinea-Bissau	2·3 (1·0–4·8)	90·5 (78·0–104·7)	189·0 (162·6–217·5)	185·6 (171·1–201·1)	191·3 (173·5–209·8)	141·5 (121·4–163·8)	83·7 (72·4–96·0)	40·8 (37·9–43·5)	0·79 (0·76–0·82)	4·6 (4·2–5·0)	1·4 (1·2–1·6)	2·3 (2·1–2·5)	68 623 (62 211–75 432)	2·01 (1·84–2·19)
	Liberia	1·3 (0·6–2·8)	99·9 (87·7–115·0)	183·5 (160·3–210·8)	165·9 (152·5–181·6)	168·1 (151·7–187·1)	125·4 (107·7–145·7)	76·0 (65·3–86·4)	28·5 (23·8–32·9)	0·55 (0·53–0·57)	4·2 (3·8–4·7)	1·4 (1·2–1·6)	2·0 (1·8–2·2)	154 182 (138 357–172 510)	1·85 (1·68–2·04)
	Mali	3·1 (1·3–6·6)	145·8 (132·4–161·6)	254·1 (232·4–278·4)	266·1 (253·6–279·9)	234·7 (220·5–248·4)	178·5 (164·1–192·1)	89·6 (81·0–97·9)	32·1 (28·2–35·6)	0·62 (0·59–0·64)	6·0 (5·7–6·4)	2·0 (1·8–2·2)	2·7 (2·5–2·9)	877 747 (829 520–932 043)	2·52 (2·41–2·63)
	Mauritania	2·0 (0·9–4·2)	69·7 (60·1–81·8)	155·0 (134·5–179·7)	164·2 (151·4–179·2)	192·3 (177·0–207·5)	146·2 (129·5–162·7)	68·9 (59·8–78·2)	32·1 (27·8–36·1)	0·62 (0·59–0·64)	4·2 (3·8–4·5)	1·1 (1·0–1·3)	2·2 (2·0–2·4)	118 860 (109 956–129 685)	1·91 (1·77–2·06)
	Niger	3·2 (1·4–6·9)	174·9 (158·1–194·4)	303·5 (282·6–326·0)	315·5 (305·2–326·6)	278·4 (267·0–290·3)	201·2 (188·6–212·6)	101·9 (92·9–110·0)	37·3 (33·7–40·5)	0·72 (0·69–0·75)	7·1 (6·8–7·4)	2·4 (2·2–2·6)	3·1 (3·0–3·2)	1 005 868 (952 540–1 063 380)	3·0 (2·9–3·1)
	Nigeria	2·3 (1·0–4·9)	91·5 (80·1–105·7)	202·4 (179·3–226·2)	239·9 (226·4–253·2)	219·2 (204·0–234·1)	152·4 (136·0–168·5)	82·5 (73·2–91·7)	30·4 (26·0–34·5)	0·58 (0·56–0·61)	5·1 (4·7–5·5)	1·5 (1·3–1·6)	2·4 (2·2–2·6)	7 798 484 (7 206 652–8 409 904)	2·17 (2·02–2·32)
	São Tomé and Príncipe	1·0 (0·4–2·1)	57·3 (49·8–66·0)	145·8 (126·3–167·4)	114·6 (103·7–126·5)	139·2 (123·5–156·2)	104·1 (88·0–121·7)	69·1 (58·1–80·6)	18·6 (14·8–22·8)	0·36 (0·34–0·37)	3·3 (2·8–3·7)	1·0 (0·9–1·2)	1·7 (1·4–1·9)	4948 (4317–5639)	1·52 (1·33–1·72)
	Senegal	2·1 (0·9–4·4)	74·9 (64·2–88·4)	182·9 (157·4–213·2)	198·4 (183·7–215·2)	201·3 (184·0–218·4)	151·3 (132·3–169·9)	80·1 (69·0–91·0)	23·3 (18·3–28·4)	0·45 (0·43–0·47)	4·6 (4·2–5·0)	1·3 (1·1–1·5)	2·3 (2·0–2·5)	496 713 (457 701–543 020)	2·1 (1·94–2·27)
	Sierra Leone	2·4 (1·0–5·0)	98·0 (85·6–113·3)	183·8 (159·9–208·7)	172·8 (159·1–186·9)	174·5 (157·6–191·7)	122·8 (104·8–141·3)	67·7 (57·4–78·2)	28·2 (23·7–32·5)	0·54 (0·52–0·56)	4·3 (3·8–4·7)	1·4 (1·3–1·6)	2·0 (1·7–2·2)	269 005 (243 337–296 085)	1·79 (1·64–1·95)
	Togo	1·7 (0·8–3·6)	51·8 (45·3–60·1)	149·0 (130·6–171·3)	151·0 (139·2–165·0)	177·5 (162·7–192·5)	131·0 (114·8–147·2)	63·1 (54·2–72·3)	37·3 (34·3–39·9)	0·72 (0·69–0·75)	3·8 (3·5–4·1)	1·0 (0·9–1·2)	2·0 (1·8–2·3)	223 039 (207 346–241 916)	1·69 (1·58–1·81)

95% uncertainty intervals are in parentheses. Data are presented to the number of decimal places as accuracy of these data allows. Super-regions, regions, and countries are listed alphabetically. Total fertility rate is the number of livebirths expected per woman in each age group if she were to survive through the reproductive years (10–54 years) under the age-specific fertility rates at that timepoint. Net reproductive rate is the number of female livebirths expected per woman, given the observed age-specific mortality and fertility rates. GBD=Global Burden of Diseases, Injuries, and Risk Factors Study. SDI=Socio-demographic Index.

**Figure 6:**
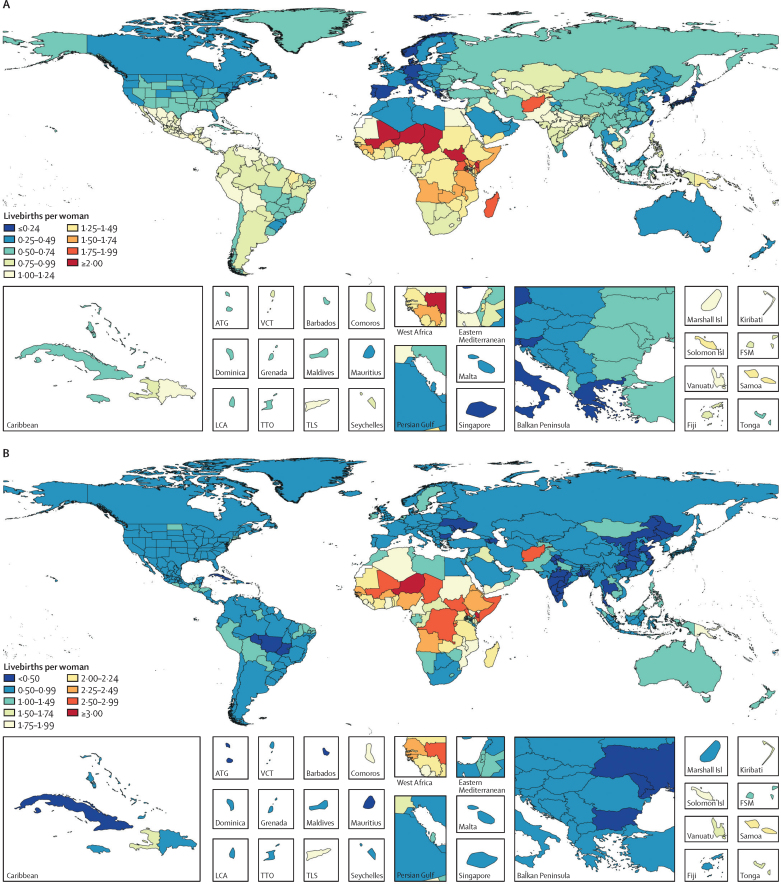
Total fertility rates under age 25 years (A) and total fertility rate over age 30 years (B), in 2017, by location Data are the number of livebirths expected for a hypothetical woman by age 25 years (A) or ageing from 30 to 54 years (B) who survived the age group and was exposed to current ASFRs. ATG=Antigua and Barbuda. FSM=Federated States of Micronesia. Isl=Islands. LCA=Saint Lucia. TLS=Timor-Leste. TTO=Trinidad and Tobago. VCT=Saint Vincent and the Grenadines.

In 2017, the TFU25 ranged from 0·08 livebirths (95% UI 0·07–0·09) in South Korea to 2·4 livebirths (2·2–2·6) in Niger (figure 6), which is 31 times higher. Countries and territories where the TFU25 was less than 0·25 livebirths included many in western Europe, Japan, South Korea, and Taiwan (province of China). TFU25 exceeded 1·5 livebirths in many parts of western, eastern, and central sub-Saharan Africa and in Afghanistan. Trends in TFO30 are more complex; decreases in fertility rate are observed at earlier stages of development, and there are sustained increases in fertility rate at higher levels of development due to women delaying childbearing. TFO30 ranged from a low of 0·3 livebirths (0·3–0·4) in Puerto Rico to a high of 3·1 livebirths (3·0–3·2) in Niger. In 2017, 145 countries showed higher fertility in women older than 30 years than in women younger than 25 years. The geographical pattern shows low fertility in women older than 30 years in disparate settings: central and eastern Europe, China, India, many parts of Latin America, and in some parts of the Middle East. North America, western Europe, central Europe, eastern Europe, Australasia, and high-income Asia Pacific had a higher TFO30 in 2017 than in 1975, with a mean of 60·2% higher TFO30 in these regions.

**Figure 7:**
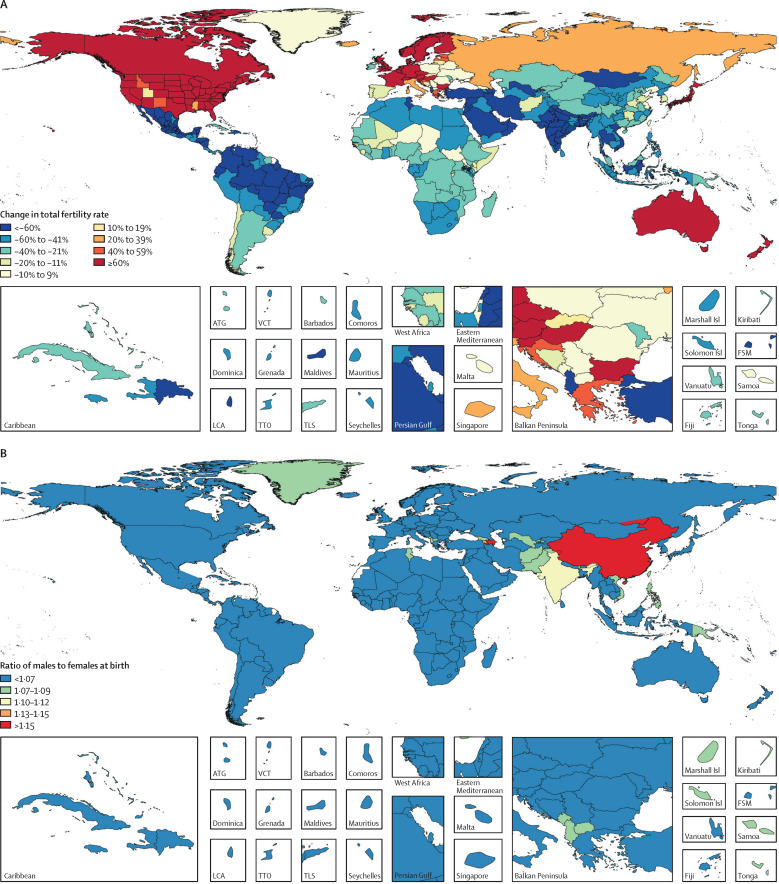
Percentage change in total fertility rates from 1975 to 2017 for women aged 30–54 years (A) and sex ratio at birth in 2017 (B), by location Data are the number of livebirths expected for a hypothetical woman ageing from 30 to 54 years who survived the age group and was exposed to current age-specific fertility rates (A) and the ratio of males to females at birth (B). ATG=Antigua and Barbuda. FSM=Federated States of Micronesia. Isl=Islands. LCA=Saint Lucia. TLS=Timor-Leste. TTO=Trinidad and Tobago. VCT=Saint Vincent and the Grenadines.

Figure 7 shows the areas where the TFO30 has been increasing since 1975; increases of more than 50% have been observed in most of western Europe, high-income North America, Australasia, and high-income Asia Pacific. The correlation of the ASFR over maternal age groups is shown in appendix 2. In 2017, 169 countries had a sex ratio of less than 1·07 males per female at birth. Countries with higher sex ratios at birth varied geographically (figure 7). For example, Greenland, Tunisia, and Afghanistan had sex ratios between 1·07 and 1·10 males per female at birth, and India had a sex ratio at birth of 1·10 males per female. Three countries had higher sex ratios at birth: Armenia (1·14 males per female), Azerbaijan (1·15 males per female), and China (1·17 males per female). High sex ratios at birth lower the effective net reproductive rate (the number of female livebirths expected per woman, given observed age-specific death and fertility rates) even more than the TFR. Estimates of the net reproductive rate are shown in table 1. Net reproductive rate in 2017 ranged from 0·48 female livebirths (0·42–0·56) expected per woman in Cyprus to 3·00 female livebirths (2·90–3·10) expected per woman in Niger. 95 countries had a net reproductive rate of less than 1 meaning that, without changes in fertility, death rates, or net immigration, populations in those countries will eventually decrease.

**Figure 8:**
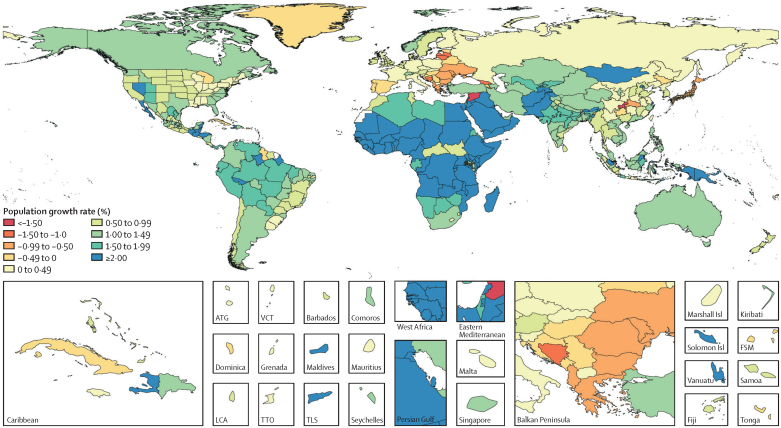
Population growth rate from 2010 to 2017, by location ATG=Antigua and Barbuda. FSM=Federated States of Micronesia. Isl=Islands. LCA=Saint Lucia. TLS=Timor-Leste. TTO=Trinidad and Tobago. VCT=Saint Vincent and the Grenadines.

The population growth rate from 2010 to 2017 is shown in figure 8. 33 countries had a negative population growth rate, most of which were located in central, eastern, and western Europe and the Caribbean. Outside Europe, negative growth rates were observed in 14 countries, and the largest negative growth rates were observed in Syria, the Northern Mariana Islands, Georgia, Puerto Rico, and the Virgin Islands. Cyprus (which has a growth rate of 1·7%), Israel (1·9%), and Luxembourg (2·3%) are notable in the GBD western Europe region because they are the only countries with a growth rate greater than 1·2%. Population growth rates in North America, Latin America, and the Caribbean ranged from −0·5% in Puerto Rico to 2·6% in Belize. Population growth rates of more than 2·0% were seen in 33 of 46 countries in sub-Saharan Africa. The Persian Gulf states, with the exception of the United Arab Emirates, all had growth rates of more than 2·2%, mostly due to the migration of workers, not fertility rates. Australia is of note among the GBD high-income super-region in the southern hemisphere, with a high population growth rate of 1·5%.

**Figure 9:**
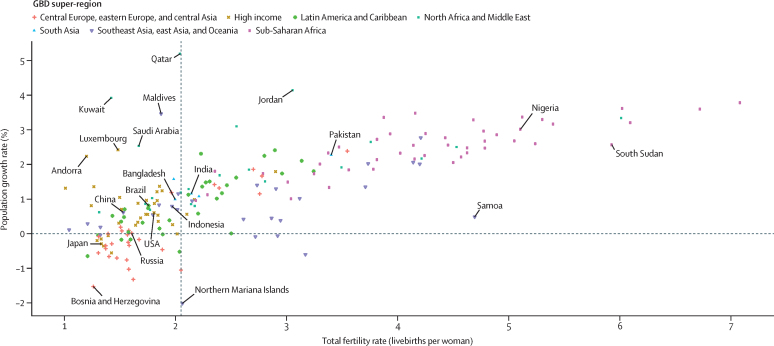
Relationship between total fertility rates and the population growth rate, 2017 Total fertility rate is the average number of children a woman would bear if she survived through the end of the reproductive age span (age 10–54 years) and experienced at each age a particular set of age-specific fertility rates observed in the year of interest. Each dot represents a single country or territory. A vertical line is shown at the total fertility rate of 2·05, representing the replacement value, and a horizontal line is shown at a population growth rate of zero.

Even when countries have a TFR of less than the replacement value (the TFR at which a population replaces itself from generation to generation, assuming no migration; generally estimated to be 2·05),^51^ populations can continue to grow because of population momentum: the phenomenon by which the past growth of birth cohorts leads to more women of childbearing age and increased births relative to deaths, even though the TFR for a time period is less than the replacement value.^52^ Populations can also grow due to immigration, as observed in many Persian Gulf nations. A comparison of the 2017 population growth rate versus the TFR is shown in figure 9, which highlights countries in which the TFR is less than the replacement value but where the population is still growing. The countries where the population is declining are also shown. Countries fall into four quadrants, defined as a TFR of more than or less than the replacement value and a population growth rate of more than or less than zero. Divergence between these two measures, as noted, is a function of lags between period TFR and growth rate (population momentum) or net migration.

Population estimates by country since 1950 are shown in table 2. Age-sex-specific detail for these same years is provided in appendix 2. Single-year, single-age population estimates for the entire period of 1950–2017 are available from the Global Health Data Exchange.

**Table 2:** The global population and the populations of SDI groups, GBD regions and super-regions, countries, and territories, 1950–2017

		**1950**	**1960**	**1970**	**1980**	**1990**	**2000**	**2010**	**2017**
**Global**		**2 571 129 (2 518 739–2 623 555)**	**3 097 198 (3 016 341–3 175 666)**	**3 775 519 (3 666 324–3 878 482)**	**4 546 838 (4 435 753–4 651 568)**	**5 394 707 (5 276 054–5 506 245)**	**6 189 102 (6 054 565–6 317 018)**	**7 032 925 (6 888 938–7 176 044)**	**7 640 466 (7 394 579–7 863 850)**
Low SDI		286 098 (274 890–297 338)	349 546 (336 103–363 341)	437 907 (422 312–454 314)	550 926 (532 322–569 634)	697 444 (674 783–720 903)	884 141 (854 560–913 871)	1 111 397 (1 073 601–1 150 583)	1 289 721 (1 232 696–1 350 886)
Low-middle SDI		428 432 (409 330–447 205)	525 533 (502 817–547 923)	664 708 (640 177–690 260)	842 355 (812 779–870 675)	1 044 178 (1 009 021–1 079 083)	1 267 751 (1 225 483–1 309 059)	1 512 969 (1 462 697–1 561 490)	1 704 731 (1 638 487–1 773 613)
Middle SDI		621 890 (600 571–645 428)	777 263 (742 412–812 838)	999 618 (947 545–1 048 416)	1 265 828 (1 216 449–1 313 285)	1 551 201 (1 501 707–1 605 269)	1 769 031 (1 711 246–1 829 574)	1 962 750 (1 906 995–2 020 809)	2 090 439 (1 993 635–2 188 823)
High-middle SDI		565 495 (547 267–585 066)	682 199 (652 794–713 287)	819 425 (777 362–859 588)	960 873 (923 189–999 256)	1 111 992 (1 075 191–1 151 041)	1 217 799 (1 176 410–1 260 107)	1 319 712 (1 279 281–1 364 508)	1 387 317 (1 310 630–1 462 683)
High SDI		660 034 (642 623–675 897)	749 699 (733 577–766 867)	836 408 (818 207–855 201)	905 978 (885 242–926 388)	965 963 (945 156–987 885)	1 024 486 (1 001 540–1 048 430)	1 098 420 (1 074 557–1 122 783)	1 139 825 (1 098 829–1 181 331)
**Central Europe, eastern Europe, and central Asia**		**279 682 (271 221–287 601)**	**321 818 (311 502–331 710)**	**360 299 (349 492–370 047)**	**392 771 (380 737–404 444)**	**420 814 (407 203–433 044)**	**416 949 (402 995–430 201)**	**411 243 (397 887–423 691)**	**415 928 (395 177–435 487)**
Central Asia		28 227 (27 431–29 009)	35 702 (34 598–36 847)	47 868 (46 467–49 262)	58 719 (56 970–60 513)	69 756 (67 616–71 961)	74 835 (71 158–78 628)	82 351 (76 500–88 059)	90 925 (83 164–99 015)
	Armenia	1453 (1352–1554)	1888 (1750–2024)	2571 (2408–2743)	3171 (2936–3426)	3419 (3161–3672)	3321 (3071–3555)	3105 (2872–3340)	3027 (2705–3349)
	Azerbaijan	3134 (2934–3343)	3946 (3651–4245)	5273 (4910–5604)	6292 (5851–6752)	7330 (6767–7855)	8245 (7597–8878)	9300 (8577–9979)	10 225 (8964–11 430)
	Georgia	3698 (3444–3957)	4225 (3904–4523)	4807 (4478–5152)	5171 (4766–5548)	5508 (5117–5908)	4691 (4326–5071)	3971 (3600–4344)	3691 (3373–4045)
	Kazakhstan	7859 (7340–8388)	9966 (9216–10 796)	13 419 (12 438–14 356)	15 318 (14 126–16 430)	16 843 (15 523–18 040)	15 357 (14 214–16 541)	16 204 (16 114–16 287)	17 904 (16 485–19 230)
	Kyrgyzstan	1765 (1641–1884)	2215 (2049–2378)	3029 (2811–3249)	3700 (3433–3979)	4462 (4138–4795)	5024 (4639–5413)	5639 (5251–6040)	6368 (5587–7101)
	Mongolia	809 (758–860)	967 (883–1050)	1276 (1182–1370)	1693 (1572–1814)	2152 (1999–2314)	2440 (2269–2607)	2826 (2638–3023)	3251 (2870–3619)
	Tajikistan	1667 (1558–1776)	2133 (1972–2288)	3015 (2809–3221)	4074 (3766–4359)	5376 (4988–5804)	6365 (5933–6844)	7818 (7339–8327)	9243 (8191–10 251)
	Turkmenistan	1252 (1171–1332)	1619 (1496–1746)	2228 (2080–2377)	2920 (2714–3137)	3701 (3426–3980)	4202 (3659–4764)	4559 (4096–5030)	4976 (4563–5397)
	Uzbekistan	6588 (6129–7015)	8738 (8063–9404)	12 248 (11 433–13 117)	16 375 (15 242–17 475)	20 961 (19 367–22 595)	25 186 (21 683–28 853)	28 925 (23 041–34 641)	32 236 (24 584–39 887)
Central Europe		88 946 (86 759–91 285)	101 568 (98 788–104 692)	110 731 (107 678–114 171)	120 005 (116 244–124 011)	124 127 (120 615–128 090)	121 176 (117 460–125 149)	117 167 (115 229–119 104)	114 803 (112 042–117 477)
	Albania	1268 (1186–1359)	1688 (1576–1807)	2196 (2035–2357)	2737 (2531–2941)	3307 (3048–3568)	3192 (2968–3432)	2889 (2674–3108)	2766 (2469–3068)
	Bosnia and Herzegovina	2831 (2636–3025)	3352 (3101–3613)	3819 (3536–4087)	4230 (3925–4531)	4509 (4160–4853)	4085 (3584–4617)	3768 (3427–4101)	3399 (3089–3720)
	Bulgaria	7348 (6835–7871)	8150 (7389–8939)	8741 (7893–9674)	9160 (8212–10 013)	8914 (8183–9640)	7965 (7422–8598)	7442 (7396–7486)	7052 (6530–7576)
	Croatia	3904 (3625–4192)	4227 (3905–4550)	4513 (4143–4853)	4856 (4497–5199)	4898 (4527–5281)	4560 (4235–4888)	4364 (4058–4676)	4275 (3838–4725)
	Czech Republic	8850 (8186–9456)	9495 (8814–10 191)	9802 (9168–10 485)	10 275 (9535–11 013)	10 279 (9458–11 050)	10 216 (10 145–10 288)	10 470 (10 397–10 548)	10 592 (10 516–10 668)
	Hungary	9325 (8708–9957)	10 021 (9354–10 715)	10 302 (9603–11 011)	10 638 (9973–11 385)	10 457 (9702–11 197)	10 195 (9432–10 949)	9930 (9176–10 656)	9727 (8739–10 785)
	Macedonia	1311 (1223–1406)	1434 (1324–1541)	1666 (1547–1788)	1943 (1793–2083)	2010 (1836–2200)	2021 (1863–2186)	2130 (1870–2379)	2174 (1825–2523)
	Montenegro	410 (380–438)	478 (445–510)	537 (497–575)	592 (549–633)	625 (582–673)	635 (578–693)	631 (583–678)	626 (558–693)
	Poland	25 291 (23 602–26 937)	30 308 (28 285–32 322)	33 452 (31 175–35 829)	36 651 (33 547–39 682)	39 059 (35 959–42 058)	38 898 (35 689–41 955)	38 439 (38 177–38 707)	38 393 (38 118–38 672)
	Romania	16 508 (15 328–17 597)	18 917 (17 237–20 689)	20 767 (18 850–22 784)	22 690 (20 777–24 679)	23 394 (21 570–25 252)	22 389 (20 571–24 271)	20 649 (19 122–22 276)	19 433 (17 350–21 520)
	Serbia	6946 (6491–7434)	7795 (7188–8364)	8627 (7948–9310)	9324 (8636–9966)	9400 (8633–10 120)	9642 (8860–10 444)	9010 (8348–9664)	8874 (7849–9837)
	Slovakia	3436 (3420–3451)	4073 (4055–4091)	4540 (4518–4561)	4980 (4956–5005)	5277 (5248–5303)	5385 (5352–5418)	5402 (5364–5442)	5419 (5006–5820)
	Slovenia	1513 (1406–1613)	1623 (1496–1747)	1764 (1639–1905)	1922 (1775–2083)	1991 (1776–2203)	1989 (1976–2003)	2036 (2020–2052)	2068 (2053–2085)
Eastern Europe		162 508 (154 850–170 367)	184 547 (175 249–194 678)	201 699 (191 753–211 238)	214 047 (203 174–225 685)	226 929 (214 331–239 070)	220 936 (208 467–233 519)	211 724 (200 353–222 893)	210 199 (192 574–228 244)
	Belarus	7418 (6920–7900)	8422 (7787–9053)	9277 (8680–9865)	9857 (9137–10 563)	10 455 (9656–11 248)	10 225 (9467–10 988)	9658 (8899–10 409)	9491 (8380–10 549)
	Estonia	1031 (1026–1035)	1204 (1198–1210)	1352 (1345–1359)	1472 (1465–1480)	1568 (1559–1576)	1393 (1385–1402)	1332 (1322–1341)	1314 (1304–1324)
	Latvia	1952 (1817–2084)	2178 (2014–2333)	2424 (2256–2581)	2582 (2393–2780)	2718 (2518–2922)	2431 (2250–2592)	2117 (2103–2131)	1945 (1931–1959)
	Lithuania	2473 (2299–2649)	2825 (2629–3034)	3207 (2994–3419)	3497 (3245–3756)	3752 (3473–4030)	3593 (3328–3853)	3136 (2882–3359)	2847 (2828–2870)
	Moldova	2520 (2346–2691)	3056 (2824–3277)	3684 (3451–3937)	4112 (3825–4411)	4463 (4140–4790)	4202 (3802–4597)	3870 (3450–4290)	3721 (3151–4276)
	Russia	108 890 (101 648–116 491)	123 122 (114 311–132 472)	133 296 (123 706–142 273)	141 389 (131 139–152 459)	151 280 (139 269–162 850)	149 335 (137 504–161 416)	145 342 (135 464–155 198)	146 189 (129 997–162 390)
	Ukraine	38 222 (35 486–40 820)	43 737 (40 535–46 886)	48 457 (45 206–51 901)	51 135 (47 072–54 769)	52 691 (48 740–56 440)	49 754 (46 128–53 518)	46 266 (40 680–51 959)	44 689 (37 188–51 843)
**High income**		**624 261 (607 829–640 001)**	**704 358 (687 585–721 417)**	**784 499 (765 553–803 595)**	**852 184 (830 617–872 817)**	**909 777 (888 581–930 669)**	**968 090 (945 346–991 026)**	**1 036 657 (1 012 835–1 060 283)**	**1 074 889 (1 033 559–1 116 701)**
Australasia		10 593 (9938–11 222)	12 947 (12 097–13 753)	15 656 (14 634–16 627)	17 897 (16 758–19 054)	20 271 (18 932–21 552)	22 664 (21 155–24 069)	25 864 (24 172–27 407)	28 391 (26 427–30 166)
	Australia	8636 (8016–9252)	10 511 (9697–11 300)	12 761 (11 805–13 698)	14 651 (13 589–15 804)	16 854 (15 599–18 087)	18 878 (17 440–20 289)	21 598 (20 005–23 097)	23 943 (22 091–25 629)
	New Zealand	1957 (1827–2087)	2435 (2254–2613)	2895 (2687–3099)	3245 (3009–3468)	3417 (3159–3674)	3785 (3504–4067)	4265 (3899–4656)	4448 (4042–4847)
High-income Asia Pacific		107 077 (100 965–112 694)	123 516 (116 754–130 199)	141 788 (133 926–149 440)	160 667 (151 955–169 179)	173 560 (164 314–182 570)	180 344 (170 747–189 636)	184 713 (174 519–194 370)	187 034 (175 679–198 805)
	Brunei	62 (58–66)	86 (80–92)	134 (124–144)	191 (177–205)	258 (239–277)	331 (305–356)	394 (363–424)	432 (388–477)
	Japan	85 811 (79 862–91 233)	95 915 (89 659–102 233)	106 925 (99 115–114 411)	119 572 (111 399–127 742)	125 857 (117 086–134 191)	129 002 (120 122–137 746)	129 954 (120 333–138 917)	128 363 (118 345–139 043)
	Singapore	1183 (1104–1264)	1666 (1517–1806)	2132 (1985–2270)	2474 (2319–2633)	3175 (2972–3390)	4167 (3873–4449)	5020 (4674–5367)	5568 (4906–6188)
	South Korea	20 019 (18 685–21 355)	25 848 (24 052–27 742)	32 595 (30 313–34 885)	38 429 (35 734–40 903)	44 268 (41 115–47 098)	46 842 (43 520–49 950)	49 343 (45 894–52 866)	52 670 (48 472–56 781)
High-income North America		167 071 (156 028–177 729)	200 987 (188 815–213 853)	230 418 (216 084–244 897)	253 712 (237 019–269 916)	280 718 (263 127–298 908)	310 870 (291 015–330 560)	342 507 (321 270–364 211)	360 884 (324 630–398 446)
	Canada	14 028 (13 129–14 957)	18 300 (16 943–19 638)	21 732 (20 163–23 312)	24 473 (22 762–26 217)	27 242 (25 184–29 399)	30 301 (28 135–32 397)	33 563 (30 968–35 916)	35 982 (33 302–38 581)
	Greenland	24 (22–25)	34 (31–36)	47 (44–50)	49 (49–50)	55 (55–55)	56 (55–56)	56 (56–56)	56 (55–56)
	USA	153 014 (141 989–163 572)	182 647 (170 786–195 050)	208 632 (194 348–222 510)	229 183 (212 518–245 136)	253 413 (236 114–271 078)	280 506 (260 887–299 946)	308 881 (287 626–330 134)	324 839 (288 772–362 239)
Southern Latin America		25 759 (24 521–27 013)	30 864 (29 278–32 252)	36 133 (34 286–37 829)	42 943 (40 694–45 029)	49 550 (46 839–52 051)	55 204 (52 208–58 135)	61 228 (58 049–64 578)	65 608 (60 307–70 557)
	Argentina	17 644 (16 522–18 909)	20 665 (19 199–21 994)	24 120 (22 465–25 726)	28 791 (26 865–30 710)	33 125 (30 785–35 434)	36 784 (34 178–39 630)	41 101 (38 531–43 887)	44 265 (39 144–49 229)
	Chile	5865 (5464–6251)	7614 (7135–8153)	9203 (8564–9843)	11 194 (10 266–12 121)	13 282 (12 242–14 355)	15 120 (13 857–16 323)	16 762 (14 885–18 566)	17 918 (16 679–19 069)
	Uruguay	2246 (2088–2398)	2582 (2353–2818)	2806 (2530–3094)	2955 (2663–3243)	3139 (2824–3483)	3297 (2988–3604)	3360 (3106–3600)	3421 (3059–3767)
Western Europe		313 759 (302 436–325 156)	336 042 (327 368–344 888)	360 501 (351 707–369 451)	376 964 (367 690–386 532)	385 678 (378 299–393 326)	399 006 (391 457–406 653)	422 344 (416 202–428 409)	432 969 (421 014–445 856)
	Andorra	5 (5–5)	9 (8–10)	18 (17–20)	34 (31–37)	54 (53–54)	65 (65–66)	84 (83–84)	79 (79–80)
	Austria	6922 (6482–7400)	7044 (6537–7546)	7431 (6891–7997)	7541 (7017–8108)	7765 (7224–8321)	8017 (7447–8594)	8368 (8301–8430)	8793 (8730–8855)
	Belgium	8663 (8082–9214)	9127 (8464–9784)	9649 (9004–10 304)	9832 (9080–10 615)	9977 (9169–10 739)	10 252 (9503–11 028)	10 861 (10 784–10 942)	11 319 (11 226–11 408)
	Cyprus	488 (453–521)	590 (550–633)	641 (557–724)	669 (612–726)	775 (716–836)	915 (848–980)	1120 (1033–1205)	1262 (1138–1391)
	Denmark	4270 (3976–4562)	4587 (4281–4909)	4934 (4585–5269)	5115 (5080–5148)	5139 (5101–5177)	5329 (5288–5372)	5529 (5483–5574)	5732 (5682–5779)
	Finland	4028 (3743–4316)	4433 (4132–4726)	4629 (4605–4656)	4796 (4764–4827)	5001 (4969–5034)	5182 (5146–5220)	5375 (5335–5416)	5517 (5474–5561)
	France	43 137 (40 160–46 060)	46 780 (43 056–50 537)	51 885 (47 890–56 018)	54 904 (50 517–59 115)	57 712 (53 593–61 378)	59 846 (55 427–64 284)	63 693 (59 476–67 922)	65 712 (59 712–71 552)
	Germany	71 934 (62 172–82 007)	75 192 (69 750–80 580)	79 263 (73 661–84 235)	80 311 (75 192–85 827)	80 041 (79 562–80 550)	82 317 (81 737–82 927)	81 692 (81 091–82 343)	83 294 (74 704–91 872)
	Greece	7766 (7251–8272)	8583 (7943–9231)	8930 (8259–9581)	9841 (9137–10 578)	10 418 (9642–11 205)	11 073 (10 256–11 901)	11 034 (10 265–11 774)	10 402 (9301–11 460)
	Iceland	141 (140–141)	173 (172–174)	203 (202–204)	227 (225–228)	253 (252–255)	279 (277–281)	318 (315–320)	337 (334–340)
	Ireland	3048 (2852–3245)	2900 (2684–3118)	3030 (2801–3274)	3487 (3215–3754)	3599 (3331–3858)	3862 (3555–4164)	4595 (4230–4972)	4860 (4519–5217)
	Israel	1556 (1451–1667)	2168 (1999–2324)	3037 (2802–3282)	3875 (3561–4213)	4963 (4474–5456)	6388 (5759–7071)	7841 (7191–8497)	8949 (7824–10 109)
	Italy	46 697 (43 475–49 705)	50 891 (46 782–54 804)	53 853 (49 819–57 792)	56 424 (52 179–60 406)	56 799 (52 808–60 687)	56 661 (52 418–60 671)	60 328 (59 854–60 768)	60 597 (60 155–61 024)
	Luxembourg	307 (286–327)	322 (300–343)	347 (324–370)	368 (339–396)	387 (357–414)	433 (401–466)	502 (498–506)	590 (585–595)
	Malta	333 (311–355)	328 (301–357)	321 (293–351)	339 (306–373)	369 (331–407)	400 (361–440)	422 (389–453)	434 (392–480)
	Netherlands	10 035 (9980–10 086)	11 414 (11 353–11 475)	12 972 (12 903–13 048)	14 083 (13 985–14 174)	14 914 (14 810–15 021)	15 875 (15 751–16 002)	16 585 (16 442–16 731)	17 029 (16 889–17 177)
	Norway	3277 (3060–3501)	3590 (3344–3820)	3885 (3621–4154)	4094 (3840–4381)	4233 (4205–4262)	4472 (4439–4507)	4858 (4821–4899)	5263 (5219–5310)
	Portugal	8749 (8131–9348)	9189 (8582–9837)	8894 (8270–9519)	10 007 (9248–10 726)	10 123 (9342–10 866)	10 518 (9764–11 278)	10 771 (10 010–11 517)	10 681 (9534–11 855)
	Spain	28 823 (26 809–30 811)	31 464 (29 402–33 634)	35 014 (32 739–37 502)	38 402 (35 587–41 263)	39 659 (37 010–42 676)	40 803 (40 523–41 063)	46 980 (46 656–47 300)	46 389 (42 868–49 868)
	Sweden	7038 (6547–7532)	7500 (7009–8008)	8046 (8000–8089)	8304 (8256–8355)	8575 (8521–8630)	8892 (8827–8957)	9404 (9331–9468)	10 044 (9340–10 726)
	Switzerland	4812 (4468–5149)	5536 (5148–5914)	6374 (5930–6794)	6494 (6069–6939)	6971 (6517–7430)	7401 (6916–7870)	7950 (7887–8009)	8593 (7909–9209)
	UK	51 455 (48 480–54 194)	53 936 (50 656–57 264)	56 820 (53 576–60 255)	57 464 (53 834–60 763)	57 567 (53 983–61 179)	59 617 (55 956–63 260)	63 595 (59 545–67 590)	66 635 (60 812–72 583)
	England	42 108 (39 171–44 851)	44 433 (41 093–47 763)	47 051 (43 853–50 449)	47 867 (44 155–51 133)	47 955 (44 409–51 589)	49 796 (46 122–53 444)	53 318 (49 243–57 349)	56 042 (50 152–61 990)
	Northern Ireland	1408 (1312–1500)	1461 (1350–1566)	1568 (1450–1692)	1526 (1420–1647)	1596 (1484–1715)	1705 (1575–1845)	1826 (1693–1962)	1914 (1711–2112)
	Scotland	5257 (4919–5591)	5313 (4922–5705)	5392 (4985–5808)	5186 (4812–5570)	5112 (4718–5475)	5159 (4778–5536)	5362 (4954–5775)	5501 (4880–6075)
	Wales	2681 (2493–2853)	2727 (2527–2939)	2807 (2591–3016)	2885 (2664–3103)	2902 (2681–3116)	2956 (2738–3188)	3087 (2855–3326)	3176 (2865–3512)
**Latin America and Caribbean**		**141 013 (136 721–145 145)**	**187 699 (181 895–193 122)**	**249 570 (242 059–256 807)**	**320 251 (310 191–329 706)**	**391 272 (378 561–403 097)**	**465 311 (451 038–478 794)**	**534 453 (517 913–550 186)**	**581 946 (553 278–607 679)**
Andean Latin America		13 876 (13 314–14 475)	18 187 (17 282–19 164)	23 910 (22 641–25 227)	30 722 (29 309–32 279)	38 359 (36 434–40 371)	46 462 (43 869–49 307)	53 990 (51 448–56 723)	61 448 (59 143–63 649)
	Bolivia	2850 (2648–3046)	3518 (3079–3973)	4329 (3849–4806)	5241 (4767–5738)	6455 (5906–6982)	8384 (7758–9008)	10 124 (9280–10 974)	11 542 (10 295–12 716)
	Ecuador	3301 (3059–3523)	4436 (4087–4795)	6012 (5453–6537)	7818 (7186–8436)	10 022 (9345–10 688)	12 377 (11 445–13 332)	14 906 (13 941–15 882)	16 686 (14 871–18 474)
	Peru	7725 (7207–8238)	10 232 (9441–10 956)	13 568 (12 427–14 616)	17 662 (16 407–18 988)	21 882 (19 972–23 707)	25 700 (23 134–28 464)	28 959 (26 635–31 236)	33 219 (33 065–33 364)
Caribbean		17 715 (17 167–18 255)	21 587 (20 614–22 550)	26 151 (25 327–26 952)	30 749 (29 698–31 808)	35 316 (33 544–37 048)	40 172 (38 761–41 590)	43 926 (42 256–45 624)	46 265 (43 663–48 895)
	Antigua and Barbuda	46 (43–49)	56 (52–60)	64 (60–69)	60 (52–68)	60 (55–64)	76 (70–82)	86 (79–92)	88 (79–98)
	The Bahamas	79 (74–85)	118 (108–129)	169 (158–180)	212 (197–228)	257 (239–275)	310 (290–332)	354 (330–380)	375 (331–415)
	Barbados	233 (216–248)	240 (225–256)	243 (226–259)	251 (234–268)	253 (236–271)	256 (240–273)	281 (262–299)	295 (264–330)
	Belize	69 (64–74)	94 (88–100)	124 (116–133)	150 (140–160)	188 (175–202)	239 (222–256)	329 (308–351)	394 (348–439)
	Bermuda	37 (34–40)	44 (41–47)	53 (50–57)	55 (51–59)	59 (54–63)	63 (59–67)	65 (60–69)	65 (58–73)
	Cuba	5704 (5330–6068)	6873 (6156–7637)	8630 (8064–9196)	9952 (9226–10 687)	10 836 (9518–12 097)	11 377 (10 476–12 256)	11 435 (10 572–12 351)	11 376 (10 251–12 434)
	Dominica	53 (49–56)	62 (57–66)	71 (66–75)	75 (69–80)	73 (68–79)	70 (65–75)	69 (64–75)	68 (61–76)
	Dominican Republic	2301 (2137–2457)	3201 (2984–3425)	4251 (3970–4548)	5730 (5328–6143)	7201 (6555–7836)	8659 (7953–9316)	9752 (9076–10 389)	10 451 (9310–11 553)
	Grenada	87 (81–93)	92 (86–97)	95 (89–102)	94 (87–101)	86 (80–93)	102 (94–110)	110 (102–118)	110 (98–122)
	Guyana	429 (400–457)	581 (542–620)	728 (678–777)	795 (741–848)	779 (721–831)	781 (721–844)	752 (695–812)	742 (670–823)
	Haiti	3282 (3053–3521)	3906 (3400–4444)	4455 (4116–4804)	5063 (4651–5478)	6376 (5598–7140)	8203 (7482–8886)	10 263 (9170–11 395)	11 824 (9880–13 736)
	Jamaica	1453 (1357–1550)	1668 (1549–1778)	1868 (1745–2001)	2216 (2036–2397)	2372 (2195–2552)	2641 (2457–2847)	2766 (2568–2977)	2779 (2466–3081)
	Puerto Rico	2209 (2058–2360)	2426 (2269–2585)	2792 (2599–2987)	3280 (3070–3491)	3612 (3356–3875)	3876 (3613–4125)	3799 (3540–4062)	3665 (3246–4091)
	Saint Lucia	77 (71–82)	90 (84–96)	102 (95–109)	118 (110–126)	136 (126–146)	155 (144–166)	169 (158–181)	176 (156–197)
	Saint Vincent and the Grenadines	75 (70–80)	83 (78–89)	89 (83–95)	101 (95–108)	110 (101–118)	110 (102–118)	112 (103–120)	114 (102–125)
	Suriname	193 (181–206)	287 (259–314)	390 (362–419)	367 (322–409)	388 (339–433)	449 (418–479)	537 (493–579)	572 (516–627)
	Trinidad and Tobago	671 (626–715)	860 (804–918)	969 (904–1030)	1087 (1012–1157)	1206 (1124–1287)	1296 (1208–1383)	1351 (1246–1453)	1391 (1241–1546)
	Virgin Islands	27 (25–29)	33 (31–35)	66 (62–71)	99 (92–106)	106 (99–112)	111 (104–118)	108 (101–115)	104 (93–117)
Central Latin America		53 305 (51 222–55 418)	72 777 (69 750–75 647)	100 359 (96 254–104 267)	132 448 (126 681–137 827)	164 144 (157 392–170 819)	199 489 (191 315–207 476)	232 490 (223 115–241 788)	255 488 (238 702–271 354)
	Colombia	11 518 (10 713–12 274)	16 035 (14 502–17 505)	22 096 (20 140–23 979)	26 989 (24 200–29 733)	32 643 (29 711–35 561)	39 822 (35 746–43 843)	46 396 (42 095–51 038)	50 606 (43 109–58 074)
	Costa Rica	845 (785–902)	1242 (1134–1347)	1789 (1639–1932)	2273 (2057–2487)	3041 (2732–3356)	3914 (3646–4170)	4398 (4093–4732)	4653 (4190–5146)
	El Salvador	1920 (1789–2050)	2581 (2399–2774)	3610 (3351–3881)	4622 (4048–5164)	5243 (4825–5659)	5793 (5178–6471)	5957 (5422–6523)	6086 (5315–6826)
	Guatemala	2963 (2763–3175)	4030 (3679–4398)	5115 (4688–5547)	6327 (5859–6800)	8007 (7215–8749)	10 939 (10 111–11 825)	14 427 (12 778–16 107)	16 924 (14 243–19 628)
	Honduras	1463 (1364–1561)	1910 (1772–2050)	2497 (2271–2746)	3413 (3049–3754)	4706 (4334–5095)	6191 (5739–6687)	7996 (7316–8682)	9498 (8567–10 397)
	Mexico	27 378 (25 506–29 357)	36 830 (34 236–39 260)	51 009 (47 429–54 370)	69 563 (64 691–73 998)	85 439 (79 728–91 534)	101 772 (94 994–108 972)	116 291 (108 390–123 903)	126 569 (112 520–141 480)
	Nicaragua	1120 (1047–1198)	1486 (1354–1617)	1953 (1810–2100)	2741 (2383–3078)	3893 (3496–4268)	4951 (4482–5429)	5781 (5208–6370)	6396 (5487–7334)
	Panama	815 (762–871)	1110 (1033–1189)	1483 (1374–1578)	1876 (1748–2000)	2387 (2209–2545)	2907 (2723–3115)	3491 (3256–3735)	3921 (3485–4377)
	Venezuela	5280 (4906–5656)	7550 (6970–8118)	10 803 (10 017–11 580)	14 640 (13 601–15 719)	18 781 (17 422–20 136)	23 197 (21 380–24 971)	27 749 (25 774–29 771)	30 831 (27 589–34 127)
Tropical Latin America		56 114 (52 441–59 899)	75 146 (70 400–80 193)	99 149 (92 501–105 855)	126 331 (117 933–134 844)	153 452 (142 917–164 089)	179 186 (167 179–191 447)	204 046 (190 090–218 128)	218 743 (195 334–242 050)
	Brazil	54 761 (51 039–58 521)	73 360 (68 585–78 366)	96 804 (90 169–103 453)	123 307 (114 851–131 752)	149 420 (138 774–159 951)	174 058 (161 715–186 328)	197 908 (183 737–211 808)	211 812 (187 982–234 855)
	Paraguay	1353 (1262–1446)	1786 (1640–1921)	2345 (2157–2533)	3024 (2790–3260)	4031 (3682–4369)	5128 (4711–5564)	6138 (5381–6897)	6931 (5885–8046)
**North Africa and Middle East**		**115 959 (112 279–119 565)**	**148 453 (143 729–153 233)**	**193 718 (187 700–199 829)**	**257 208 (249 717–264 577)**	**340 904 (330 888–350 735)**	**426 468 (412 356–440 350)**	**527 903 (512 116–544 418)**	**600 182 (579 215–621 820)**
	Afghanistan	7681 (5541–9575)	9465 (7772–11 203)	11 629 (10 087–13 133)	12 052 (11 180–12 917)	10 006 (8643–11 335)	17 928 (14 299–21 554)	26 294 (19 416–33 390)	32 854 (22 892–42 005)
	Algeria	8799 (8222–9375)	11 234 (10 036–12 402)	13 781 (12 541–15 031)	18 525 (16 936–20 235)	25 463 (23 280–27 514)	31 508 (29 092–33 981)	36 293 (33 467–39 148)	40 463 (35 851–45 748)
	Bahrain	116 (107–124)	155 (144–166)	216 (199–232)	345 (320–368)	507 (471–545)	651 (606–700)	1257 (1170–1344)	1470 (1305–1638)
	Egypt	20 786 (19 371–22 122)	27 091 (25 383–28 856)	34 251 (31 139–37 552)	43 063 (39 177–46 961)	54 991 (49 913–60 135)	66 897 (61 131–72 575)	83 106 (75 937–90 743)	96 484 (90 094–102 841)
	Iran	16 731 (15 621–17 904)	21 780 (19 732–23 814)	29 030 (26 396–31 568)	40 335 (36 967–44 296)	57 866 (52 672–62 812)	67 498 (61 587–73 597)	76 594 (71 133–82 082)	82 176 (75 839–88 022)
	Iraq	5377 (5048–5724)	7156 (6535–7761)	9710 (8716–10 707)	13 627 (12 253–14 787)	17 444 (15 844–19 013)	26 408 (22 685–30 551)	34 359 (26 137–41 960)	43 304 (31 839–54 011)
	Jordan	441 (335–550)	736 (602–871)	1300 (1133–1475)	2282 (2116–2453)	3739 (3401–4095)	4849 (4413–5301)	7534 (6787–8274)	10 648 (9754–11 559)
	Kuwait	94 (84–104)	283 (263–305)	772 (720–824)	1403 (1312–1495)	1773 (1591–1959)	1978 (1776–2176)	3010 (2780–3238)	4262 (3821–4708)
	Lebanon	1335 (1243–1421)	1750 (1538–1967)	2285 (2128–2449)	3202 (2787–3626)	4109 (3347–4867)	5270 (4041–6636)	6510 (4425–8615)	8511 (5685–11 791)
	Libya	1070 (994–1142)	1427 (1294–1568)	1915 (1742–2079)	3078 (2787–3357)	4184 (3769–4614)	5035 (4540–5535)	6188 (5601–6770)	6908 (5974–7823)
	Morocco	9176 (8574–9848)	11 890 (11 090–12 712)	15 497 (14 336–16 617)	20 157 (18 632–21 698)	25 207 (22 885–27 584)	29 532 (26 635–32 424)	33 167 (30 016–36 275)	35 488 (32 624–38 856)
	Oman	442 (290–590)	614 (451–776)	897 (705–1087)	1343 (1145–1550)	1917 (1747–2092)	2301 (2095–2500)	2850 (2664–3039)	4535 (4508–4563)
	Palestine	926 (777–1083)	973 (865–1083)	1102 (1005–1203)	1430 (1229–1635)	2037 (1810–2269)	3036 (2768–3312)	4175 (3822–4524)	4852 (4536–5156)
	Qatar	26 (18–33)	56 (43–69)	131 (109–152)	273 (243–301)	443 (401–483)	592 (538–643)	1741 (1622–1859)	2747 (2525–2976)
	Saudi Arabia	4329 (4036–4638)	4644 (4032–5254)	5956 (5386–6526)	9691 (8731–10 787)	16 386 (14 964–17 729)	21 143 (19 108–23 200)	28 053 (26 153–30 133)	34 444 (30 598–38 365)
	Sudan	6013 (5610–6390)	7146 (6463–7843)	10 351 (9412–11 273)	14 602 (13 374–15 958)	20 209 (18 414–21 941)	27 119 (24 040–30 238)	34 285 (31 632–37 135)	40 255 (34 770–45 494)
	Syria	3400 (3173–3633)	4708 (4377–5039)	6530 (6094–6946)	9087 (8429–9740)	12 687 (11 444–13 866)	16 588 (14 961–18 057)	22 738 (20 396–25 034)	18 131 (15 317–20 564)
	Tunisia	3691 (3431–3942)	4302 (3922–4704)	5117 (4656–5619)	6562 (5955–7194)	8412 (7628–9214)	9901 (8986–10 817)	10 810 (9827–11 809)	11 442 (10 350–12 472)
	Turkey	21 175 (19 749–22 566)	27 605 (25 702–29 512)	36 107 (33 578–38 511)	45 410 (42 172–48 627)	57 681 (53 805–61 370)	65 949 (58 509–73 185)	74 297 (73 904–74 694)	80 456 (80 023–80 937)
	United Arab Emirates	73 (59–86)	105 (93–117)	250 (229–270)	1075 (1004–1146)	1887 (1706–2073)	3251 (2922–3575)	8958 (8048–9894)	9734 (8433–11 170)
	Yemen	4254 (2729–5807)	5291 (3764–6756)	6804 (5262–8251)	9499 (8020–10 922)	13 726 (12 427–14 966)	18 706 (17 088–20 302)	25 182 (22 469–27 784)	30 449 (25 793–35 167)
**South Asia**		**457 107 (430 732–483 061)**	**552 631 (517 605–586 189)**	**698 004 (656 913–739 771)**	**891 598 (838 523–941 440)**	**1108 770 (1043 283–1175 270)**	**1346 782 (1265 595–1426 290)**	**1605 324 (1508 063–1700 357)**	**1782 677 (1638 317–1941 429)**
	Bangladesh	41 397 (38 577–44 053)	48 333 (44 678–51 917)	65 862 (59 840–71 907)	83 984 (77 577–90 506)	108 900 (101 213–116 979)	128 604 (119 080–137 940)	145 626 (134 711–156 550)	156 981 (140 228–173 145)
	Bhutan	181 (169–194)	221 (193–249)	293 (237–350)	404 (315–489)	562 (475–649)	603 (543–665)	789 (715–869)	957 (826–1094)
	India	372 174 (346 875–397 889)	454 421 (420 507–487 432)	561 030 (520 907–600 806)	708 230 (657 702–757 375)	871 428 (805 834–934 597)	1052 960 (971 762–1131 565)	1249 523 (1156 683–1341 804)	1380 560 (1236 095–1534 340)
	Nepal	8346 (7781–8884)	9837 (9139–10 572)	11 976 (11 092–12 844)	15 574 (14 479–16 722)	19 373 (17 882–20 852)	23 878 (22 183–25 498)	27 649 (25 630–29 701)	29 891 (26 626–32 797)
	Pakistan	35 007 (32 485–37 379)	39 815 (36 728–42 755)	58 840 (54 193–63 438)	83 404 (77 107–89 378)	108 505 (96 417–120 410)	140 735 (129 490–152 140)	181 734 (161 683–201 652)	214 287 (199 020–228 949)
**Southeast Asia, east Asia, and Oceania**		**774 843 (736 072–814 983)**	**957 155 (890 929–1021 806)**	**1201 660 (1101 819–1287 271)**	**1460 435 (1369 642–1543 503)**	**1731 863 (1642 563–1818 200)**	**1921 127 (1821 758–2016 695)**	**2068 109 (1975 307–2162 325)**	**2158 800 (1981 518–2320 037)**
East Asia		583 744 (547 376–625 484)	712 646 (650 162–777 420)	891 338 (796 510–980 025)	1073 817 (986 653–1157 549)	1258 648 (1176 009–1347 979)	1366 510 (1275 694–1459 970)	1439 061 (1351 366–1531 406)	1485 714 (1316 627–1646 304)
	China	557 744 (520 768–597 524)	678 243 (616 756–742 010)	846 255 (752 128–933 401)	1019 880 (933 340–1101 322)	1196 979 (1115 557–1286 245)	1298 681 (1208 608–1389 466)	1367 214 (1280 251–1457 810)	1412 480 (1245 008–1569 141)
	North Korea	10 681 (7186–14 481)	12 431 (9222–15 787)	15 201 (12 024–18 317)	17 633 (14 984–20 053)	20 296 (18 578–22 146)	23 188 (20 485–25 862)	25 160 (23 167–27 154)	25 716 (22 826–28 768)
	Taiwan (province of China)	7575 (7535–7617)	10 805 (10 751–10 858)	14 617 (14 553–14 681)	17 908 (17 828–17 986)	20 402 (20 294–20 517)	22 286 (22 152–22 417)	23 191 (23 025–23 360)	23 583 (23 397–23 769)
Oceania		2656 (2330–2976)	3236 (3024–3465)	4072 (3870–4281)	5115 (4879–5339)	6457 (5883–7021)	8325 (7924–8715)	10 685 (10 105–11 292)	12 602 (11 585–13 653)
	American Samoa	19 (18–20)	20 (19–22)	27 (25–29)	33 (30–35)	48 (45–51)	58 (54–62)	56 (52–60)	55 (49–61)
	Federated States of Micronesia	39 (36–41)	50 (44–57)	65 (52–78)	84 (72–98)	103 (94–114)	109 (102–116)	105 (98–112)	103 (93–115)
	Fiji	297 (276–317)	408 (372–446)	542 (489–594)	659 (599–722)	762 (693–833)	818 (741–895)	875 (798–949)	906 (846–970)
	Guam	61 (57–65)	69 (65–73)	87 (81–93)	108 (101–115)	136 (127–146)	159 (148–169)	163 (153–175)	167 (148–186)
	Kiribati	30 (27–32)	36 (33–40)	46 (42–50)	62 (57–67)	74 (69–79)	87 (81–94)	107 (100–114)	118 (108–128)
	Marshall Islands	11 (7–14)	16 (12–20)	23 (19–27)	34 (30–38)	45 (42–49)	52 (48–56)	54 (50–58)	56 (50–62)
	Northern Mariana Islands	4 (4–4)	6 (5–7)	9 (8–10)	16 (15–18)	45 (42–48)	72 (67–77)	54 (51–58)	44 (40–49)
	Papua New Guinea	1726 (1417–2022)	2031 (1833–2241)	2499 (2321–2693)	3156 (2956–3362)	4064 (3520–4594)	5525 (5151–5884)	7543 (7002–8110)	9227 (8264–10 220)
	Samoa	86 (80–92)	114 (106–122)	147 (136–157)	160 (148–173)	163 (151–175)	178 (164–190)	192 (178–206)	198 (183–212)
	Solomon Islands	105 (98–112)	135 (125–145)	170 (159–181)	233 (212–254)	337 (307–369)	444 (411–479)	552 (509–593)	637 (565–710)
	Tonga	49 (46–53)	66 (59–72)	85 (77–93)	95 (86–104)	96 (87–105)	100 (91–110)	106 (98–113)	102 (95–110)
	Vanuatu	49 (46–53)	64 (57–72)	88 (80–95)	118 (109–126)	150 (139–161)	192 (178–205)	247 (229–265)	287 (266–308)
Southeast Asia		188 442 (182 191–194 754)	241 272 (232 260–251 232)	306 249 (295 068–318 483)	381 501 (369 792–393 749)	466 758 (451 577–483 000)	546 290 (515 395–576 571)	618 362 (598 861–638 911)	660 484 (625 637–694 223)
	Cambodia	4438 (4137–4750)	5901 (5400–6403)	7554 (6695–8435)	7938 (6417–9346)	10 428 (9236–11 681)	12 634 (11 624–13 711)	14 560 (13 337–15 756)	16 122 (14 157–18 177)
	Indonesia	79 537 (74 213–84 967)	98 406 (91 399–105 742)	123 056 (113 430–132 056)	153 254 (143 916–162 920)	185 784 (173 237–198 423)	213 339 (184 326–242 359)	241 532 (225 765–257 592)	258 134 (228 486–286 754)
	Laos	1694 (1214–2166)	2107 (1651–2593)	2632 (2232–3062)	3302 (2966–3630)	4136 (3704–4539)	5330 (4800–5868)	6360 (5725–6943)	6970 (6442–7469)
	Malaysia	6249 (5441–7015)	8316 (7729–8849)	10 703 (9952–11 389)	13 557 (12 638–14 483)	17 639 (16 264–18 971)	23 837 (22 268–25 477)	28 119 (26 310–30 148)	30 639 (27 083–34 101)
	Maldives	77 (72–83)	92 (85–100)	120 (110–130)	162 (148–176)	219 (204–234)	278 (259–298)	352 (320–385)	458 (420–497)
	Mauritius	490 (460–524)	668 (614–723)	837 (770–902)	991 (902–1083)	1098 (1028–1173)	1213 (1128–1300)	1267 (1176–1365)	1272 (1147–1397)
	Myanmar	19 282 (17 833–20 583)	22 719 (19 724–25 734)	27 646 (25 258–30 089)	33 907 (31 033–36 686)	40 438 (36 067–44 754)	45 959 (38 921–53 049)	50 146 (45 580–55 132)	52 795 (48 406–57 281)
	Philippines	20 331 (18 972–21 688)	28 707 (26 687–30 602)	38 593 (36 063–41 123)	49 864 (46 687–52 939)	63 333 (59 158–67 655)	79 807 (74 205–85 456)	95 885 (89 486–102 745)	103 470 (94 554–111 888)
	Sri Lanka	7860 (7357–8423)	10 193 (9265–11 080)	12 930 (11 976–13 919)	15 187 (14 082–16 304)	17 179 (14 962–19 266)	18 798 (16 243–21 314)	20 524 (18 983–22 141)	21 596 (19 459–23 802)
	Seychelles	34 (32–37)	43 (40–46)	54 (50–58)	66 (60–72)	73 (66–79)	81 (74–88)	93 (87–99)	100 (90–112)
	Thailand	20 403 (18 913–21 794)	27 525 (25 618–29 354)	35 509 (33 009–37 896)	46 425 (43 256–49 679)	57 028 (53 286–60 983)	62 993 (58 922–67 354)	67 779 (63 187–72 386)	70 626 (62 645–78 551)
	Timor-Leste	413 (360–467)	543 (505–579)	560 (491–630)	580 (541–622)	781 (726–835)	912 (832–996)	1109 (1034–1180)	1287 (1188–1391)
	Vietnam	27 356 (25 495–29 238)	35 681 (31 167–40 285)	45 566 (39 978–51 388)	55 740 (51 473–59 718)	67 997 (62 530–73 389)	80 359 (74 668–86 543)	89 793 (83 334–96 170)	96 140 (84 738–108 043)
**Sub-Saharan Africa**		**178 260 (164 732–191 802)**	**225 081 (211 487–239 434)**	**287 767 (275 293–299 920)**	**372 388 (360 384–384 066)**	**491 304 (479 290–502 499)**	**644 373 (625 722–662 472)**	**849 233 (824 168–875 493)**	**1026 040 (988 588–1062 587)**
Central sub-Saharan Africa		19 588 (18 634–20 532)	25 453 (23 155–27 713)	32 835 (31 174–34 531)	41 915 (38 838–44 872)	55 023 (50 322–59 723)	73 396 (65 208–82 601)	99 517 (84 702–115 702)	121 670 (99 121–143 192)
	Angola	4393 (4097–4705)	5152 (4780–5526)	5934 (5534–6338)	7508 (6519–8450)	10 246 (8354–12 310)	14 687 (12 582–16 858)	21 784 (19 754–24 078)	28 202 (25 993–30 710)
	Central African Republic	1348 (1048–1648)	1630 (1378–1902)	2062 (1856–2266)	2294 (2078–2515)	2734 (2521–2971)	3612 (3317–3931)	4404 (3944–4879)	4622 (3945–5323)
	Congo (Brazzaville)	821 (644–1015)	1034 (878–1190)	1322 (1198–1449)	1768 (1598–1929)	2428 (2157–2683)	3173 (2811–3475)	4185 (3840–4520)	4913 (4244–5607)
	Democratic Republic of the Congo	12 459 (11 684–13 238)	16 949 (14 650–19 201)	22 683 (21 041–24 305)	29 288 (26 433–32 003)	38 211 (34 046–42 323)	50 035 (42 266–58 951)	66 608 (51 014–83 629)	80 884 (57 964–102 607)
	Equatorial Guinea	196 (183–210)	217 (189–243)	245 (210–281)	300 (274–328)	423 (378–470)	653 (543–757)	1034 (932–1138)	1345 (1236–1454)
	Gabon	369 (342–393)	470 (438–501)	587 (513–662)	754 (645–869)	980 (897–1076)	1233 (1092–1376)	1500 (1372–1624)	1702 (1546–1857)
Eastern sub-Saharan Africa		63 017 (57 728–68 369)	81 437 (76 298–87 231)	107 317 (102 585–112 025)	142 590 (138 124–147 178)	191 563 (185 668–197 939)	248 306 (240 183–257 027)	326 270 (315 878–336 860)	393 180 (375 866–410 737)
	Burundi	2391 (1831–3025)	3015 (2482–3612)	3632 (3181–4089)	4439 (4094–4783)	5500 (5136–5863)	6265 (5496–6987)	8976 (8277–9684)	10 905 (9535–12 329)
	Comoros	158 (132–184)	189 (169–209)	254 (231–277)	355 (330–379)	462 (429–495)	551 (503–599)	650 (575–731)	718 (608–828)
	Djibouti	62 (46–76)	97 (76–118)	156 (132–179)	299 (272–326)	498 (443–553)	648 (571–729)	902 (838–970)	1113 (984–1234)
	Eritrea	1114 (786–1436)	1467 (1142–1783)	1938 (1639–2239)	2568 (2326–2805)	2893 (2577–3200)	3499 (2958–4084)	5191 (3910–6431)	5859 (4233–7490)
	Ethiopia	17 731 (12 350–22 674)	22 150 (17 344–27 306)	27 867 (23 496–32 244)	34 702 (31 667–38 187)	51 404 (47 067–56 618)	68 429 (61 781–75 440)	86 259 (78 817–93 383)	102 883 (89 646–116 198)
	Kenya	5537 (5181–5896)	7901 (7298–8545)	11 965 (11 037–12 807)	16 750 (15 514–18 068)	23 198 (21 587–24 928)	30 893 (28 565–33 142)	40 694 (37 600–43 784)	48 326 (42 513–53 790)
	Madagascar	4302 (3997–4576)	5483 (4766–6173)	7099 (6407–7831)	9269 (8376–10 167)	11 955 (10 899–12 981)	15 858 (14 259–17 526)	21 285 (17 762–24 979)	26 108 (20 426–31 770)
	Malawi	2941 (2736–3146)	3705 (3315–4097)	4776 (4343–5210)	6416 (5840–6967)	9667 (8850–10 466)	11 168 (10 248–12 018)	14 338 (13 150–15 501)	17 191 (14 949–19 275)
	Mozambique	6069 (5643–6494)	7218 (6746–7709)	9096 (8487–9740)	12 285 (11 388–13 163)	14 401 (12 777–16 071)	17 315 (15 781–18 860)	23 491 (21 500–25 621)	30 035 (27 827–31 998)
	Rwanda	2515 (2350–2695)	3173 (2753–3574)	4067 (3618–4519)	5341 (4914–5777)	7266 (6758–7812)	8139 (7443–8811)	10 374 (9574–11 208)	12 554 (11 271–13 772)
	Somalia	2336 (2176–2488)	2906 (2519–3290)	3829 (3456–4206)	6424 (5728–7071)	7175 (6579–7781)	9738 (8336–11 203)	13 574 (10 638–16 499)	16 880 (12 489–21 415)
	South Sudan	2617 (2347–2884)	3169 (2887–3481)	3931 (3315–4535)	4861 (4437–5260)	5883 (5198–6573)	7288 (6440–8110)	9497 (8689–10 238)	9941 (8738–11 240)
	Tanzania	7566 (7030–8058)	10 278 (9380–11 168)	13 870 (12 628–15 021)	19 434 (17 797–21 093)	25 888 (23 767–27 993)	34 172 (31 362–36 995)	44 584 (41 315–47 884)	53 973 (48 580–59 610)
	Uganda	5291 (4920–5642)	7368 (6864–7877)	10 330 (9552–11 147)	13 374 (11 491–15 263)	17 349 (16 088–18 628)	24 305 (22 203–26 327)	32 574 (29 492–35 541)	39 078 (35 694–42 446)
	Zambia	2368 (2218–2525)	3285 (2919–3697)	4463 (4126–4793)	6010 (5628–6376)	7919 (7360–8483)	9881 (9175–10 573)	13 670 (12 838–14 542)	17 364 (15 312–19 457)
Southern sub-Saharan Africa		17 644 (16 546–18 863)	22 982 (21 717–24 414)	30 803 (29 257–32 561)	40 678 (36 735–44 712)	52 481 (48 570–56 500)	64 122 (60 418–67 632)	70 987 (67 220–74 904)	77 373 (71 350–83 396)
	Botswana	392 (366–419)	515 (467–562)	666 (595–739)	920 (850–991)	1310 (1211–1404)	1692 (1575–1815)	2008 (1859–2159)	2281 (2052–2527)
	Lesotho	576 (537–616)	762 (688–831)	1045 (943–1150)	1450 (1306–1586)	1806 (1644–1967)	1978 (1795–2177)	1919 (1745–2095)	1947 (1675–2215)
	Namibia	448 (418–477)	574 (536–612)	777 (720–833)	1049 (913–1188)	1415 (1310–1523)	1844 (1713–1972)	2118 (1964–2275)	2353 (2114–2595)
	South Africa	13 151 (12 211–14 119)	16 925 (15 767–18 024)	22 606 (21 186–24 070)	29 233 (25 330–33 144)	36 773 (32 941–40 675)	45 632 (42 015–49 033)	50 861 (47 239–54 509)	54 952 (49 033–60 617)
	Swaziland (eSwatini)	254 (236–271)	334 (299–369)	437 (398–481)	587 (533–639)	807 (732–885)	1011 (922–1102)	1068 (978–1165)	1124 (1047–1201)
	Zimbabwe	2821 (2014–3579)	3870 (3104–4685)	5269 (4550–5981)	7437 (6855–8030)	10 366 (9528–11 160)	11 961 (10 986–12 887)	13 011 (11 915–14 005)	14 713 (13 330–16 032)
Western sub-Saharan Africa		78 009 (65 663–90 262)	95 207 (82 616–108 235)	116 810 (105 261–127 661)	147 204 (137 869–157 367)	192 235 (184 599–199 575)	258 547 (245 286–271 665)	352 458 (336 581–367 819)	433 815 (413 644–453 718)
	Benin	2288 (1723–2882)	2413 (1926–2838)	2718 (2400–3048)	3459 (3209–3717)	4842 (4456–5242)	6698 (6148–7243)	9333 (8506–10 124)	11 585 (10 516–12 737)
	Burkina Faso	4325 (3282–5330)	4758 (3933–5616)	5482 (4907–6024)	7164 (6481–7830)	9562 (8610–10 525)	12 301 (11 148–13 532)	16 868 (15 293–18 437)	21 121 (18 146–24 118)
	Cameroon	4563 (3495–5578)	5571 (4638–6460)	6691 (5980–7402)	8017 (7253–8752)	10 355 (9454–11 280)	14 965 (13 507–16 519)	22 201 (19 903–24 366)	27 769 (23 792–31 860)
	Cape Verde	155 (145–164)	214 (199–228)	283 (264–303)	301 (281–323)	351 (326–376)	448 (418–479)	508 (472–542)	545 (484–606)
	Chad	2559 (1731–3399)	3093 (2239–3948)	3703 (2909–4507)	4621 (3926–5314)	6037 (5517–6569)	8267 (7338–9195)	11 803 (10 925–12 663)	15 222 (13 380–17 036)
	Côte d'Ivoire	3101 (2883–3309)	4147 (3600–4660)	5864 (5263–6443)	8286 (7495–9127)	12 251 (11 216–13 262)	17 112 (15 825–18 420)	21 621 (19 620–23 447)	24 965 (22 783–27 055)
	The Gambia	264 (247–280)	303 (275–327)	456 (418–495)	664 (606–720)	986 (903–1071)	1347 (1237–1455)	1765 (1607–1924)	2132 (1932–2334)
	Ghana	5099 (4782–5444)	6894 (6446–7342)	8985 (8378–9584)	11 690 (10 660–12 748)	14 936 (13 332–16 513)	19 143 (17 828–20 371)	25 227 (23 528–26 958)	30 205 (26 660–33 569)
	Guinea	2945 (2747–3149)	3369 (2978–3764)	3967 (3593–4331)	4655 (4258–5057)	6148 (5475–6817)	8121 (7407–8826)	9983 (9053–10 971)	11 819 (10 848–12 828)
	Guinea-Bissau	536 (498–571)	570 (531–611)	647 (571–729)	813 (751–869)	1009 (936–1083)	1248 (1085–1411)	1571 (1450–1685)	1855 (1636–2071)
	Liberia	909 (778–1043)	1079 (999–1166)	1412 (1283–1535)	1965 (1779–2138)	1985 (1776–2196)	2928 (2573–3288)	4051 (3722–4404)	4722 (4138–5272)
	Mali	3847 (3584–4118)	4708 (4136–5331)	5939 (5316–6580)	7233 (6536–7897)	8662 (7915–9459)	11 028 (10 142–11 941)	15 896 (14 642–17 132)	20 253 (17 822–22 672)
	Mauritania	659 (504–815)	874 (720–1019)	1150 (1019–1278)	1551 (1408–1684)	2071 (1903–2244)	2613 (2437–2792)	3336 (3058–3620)	3913 (3560–4285)
	Niger	2562 (1964–3134)	3359 (2785–3919)	4476 (3984–4964)	5955 (5447–6466)	8025 (7371–8642)	11 245 (10 391–12 091)	16 397 (15 090–17 678)	21 375 (19 349–23 648)
	Nigeria	38 269 (25 767–50 494)	46 573 (34 104–59 648)	55 844 (44 421–66 586)	69 128 (60 148–79 038)	89 790 (82 940–96 408)	121 832 (109 542–134 557)	166 431 (152 067–181 236)	206 087 (188 405–224 287)
	São Tomé and Principe	62 (58–66)	68 (63–74)	76 (71–81)	96 (88–103)	121 (112–130)	142 (132–153)	174 (160–188)	200 (180–219)
	Senegal	2529 (1953–3162)	3397 (2851–3959)	4523 (4020–5024)	5860 (5341–6380)	7624 (7011–8229)	9910 (9164–10 652)	12 556 (11 482–13 626)	14 688 (13 261–16 099)
	Sierra Leone	1923 (1640–2196)	2193 (2009–2385)	2621 (2378–2874)	3070 (2770–3384)	3781 (3413–4161)	4311 (3917–4728)	6348 (5717–6990)	7829 (7207–8482)
	Togo	1401 (1245–1567)	1609 (1477–1734)	1959 (1823–2091)	2662 (2483–2861)	3685 (3262–4111)	4874 (4289–5493)	6375 (5963–6813)	7516 (6726–8351)

Data are thousands of people (95% uncertainty intervals) for all ages and both sexes. Super-regions, regions, and countries are listed alphabetically. Estimates are de-facto population estimates. GBD=Global Burden of Diseases, Injuries, and Risk Factors Study. SDI=Socio-demographic Index.

**Figure 10:**
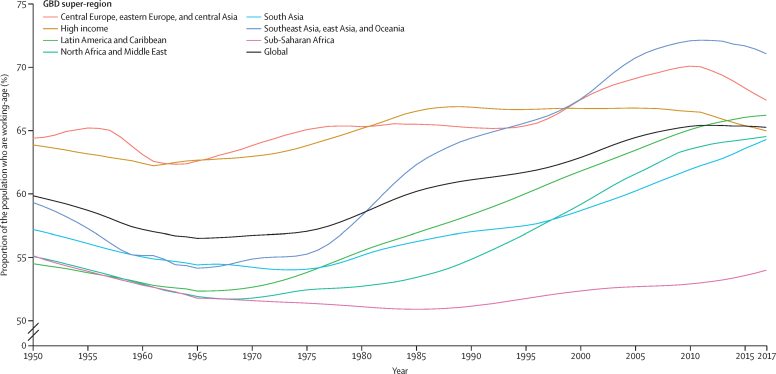
Proportion of the population that is of working age, globally and for GBD super-regions, 1950–2017 Working age is defined as 15–64 years. Data are for both sexes combined from 1950 to 2017. GBD= Global Burden of Diseases, Injuries, and Risk Factors Study.

The proportion of the population that was of working age from 1950 to 2017 by GBD super-region is shown in figure 10. Studies of economic growth have identified the potential for a demographic dividend when the proportion of the population that is of working age reaches more than 65%.^53^ In high-income countries, the proportion of the population that is of working age increased from the 1960s, crossed the 65% threshold in the late 1970s, and was relatively constant during the 1980s and 1990s. In 2005, this proportion began to decrease and was only just more than the 65% threshold in 2017. 12 of 34 high-income countries now have a proportion of the population of working age that is less than 65%, and Japan has a working-age proportion of less than 60%. Other than sub-Saharan Africa and high-income countries, the GBD super-regions have had a substantially increasing proportion of the population of working age from the mid-1960s to the present day; in 2017, Latin American and the Caribbean, north Africa and the Middle East, south Asia, and central Europe, eastern Europe, and central Asia all had proportions of the population that are of working age between 64% and 71%. The most pronounced increase in the working-age population occurred in southeast Asia, east Asia, and Oceania, which increased from 54·2% of the population in 1965 to 72·2% in 2011. Sub-Saharan Africa is the clear outlier among GBD super-regions; the proportion of the population of working age in this region has remained at or less than 55% during the entire time period, although this proportion has more recently increased. In sub-Saharan Africa, the proportion of the population that is of working age was less than 50% in Mali (49·7%), Chad (46·6%), and Niger (46·1%) in 2017.

## Discussion

### Main findings

To our knowledge, this study presents the first estimates of population by location from 1950 to 2017 that are based on transparent data and replicable analytical code. Annual population estimates are provided for single calendar years and single-year age groups compared with previous assessments that reported results for 5-year age groups.^4^ The global population increased nearly three-fold between 1950 and 2017, from 2·6 billion (2·5–2·6) people in 1950 to 7·6 billion (7·4–7·9) people in 2017. Although global population growth rates have declined from a peak of 2·0% in 1964 to 1·1% in 2017, the size of the global population has steadily been increasing by more than 80 million people annually since 1985. These global estimates mask huge country variation, with 35 countries showing decreasing populations in 2017 whereas 57 countries had population growth at a rate higher than 2·0%. Country variation in population growth rates is driven to a large extent by wide variations in fertility rates and to a lesser extent by migration rates.

Of the 59 countries with a TFR of more than three livebirths per woman in 2017 (figure 9), 41 are in sub-Saharan Africa. Of the remainder, six countries are in north Africa and the Middle East. These continuous high rates of total fertility are associated with high rates of population growth in sub-Saharan Africa and north Africa and the Middle East. The proportion of women whose contraceptive needs are being met through the provision of reproductive health services is 46·5% (95% UI 45·2–47·6) in sub-Saharan Africa and 69·0% (67·5–70·5) in north Africa and the Middle East.^54^ Given that the economic benefits of the demographic dividend are estimated to occur when the working-age population represents more than 65% of the population,^53^ government action to meet the need for family planning and to raise the educational attainment of women are two potential pathways towards faster economic growth. Notably, less than 55% of the population in sub-Saharan Africa, on average, are of working age, and this proportion is only slowly increasing. Fast economic growth in sub-Saharan Africa from 2002 to 2014 shows the potential for economic transition in the region; capitalising on the demographic dividend might add to this potential in the future. Policy options that focus on educating young girls, providing access to reproductive health services, and continued scale-up of effective interventions for child mortality are available to accelerate decreases in TFR and demographic change.

By contrast, 33 countries are in overall population decline since 2010, including Estonia, Ukraine, Belarus, Greece, Georgia, Bulgaria, Romania, and Spain. Many other countries are also likely to have decreasing populations as the size of their birth cohorts reduces. Population decline and the associated shift to an older population has profound cultural, economic, and social implications. One early measure of this trend is the percentage change in the number of livebirths over time; in 89 countries, the size of the birth cohort has decreased since 2000. The options in these countries to deal with the social and economic consequences of population decline include pro-natalist policies, liberal immigration policies, and increasing the retirement age. Pro-natalist policies have been pursued in more than a dozen countries but the effects on fertility rates have not been large.^55^, ^56^, ^57^, ^58^ Liberal immigration policies have been effective in sustaining population numbers in several countries, but such policies have been accompanied by social and political challenges in some. Dealing with population decline will be a central policy challenge for a substantial number of countries over the next few decades.

In high-income countries, the proportion of the population that is of working age has also decreased in the past 5 years, and this trend is likely to continue for the foreseeable future. This demographic shift toward an older population has a broad range of consequences, from reductions in economic growth, decreasing tax revenue, greater use of social security with fewer contributors, and increasing health-care and other demands prompted by an ageing population.^59^, ^60^, ^61^, ^62^, ^63^, ^64^, ^65^ This shift is advanced in several high-income countries, with one of the earliest examples being Japan.^66^ Our estimates show that more than 20% of the population is older than 65 years in eight countries, implying that the challenges of dealing effectively with ageing populations have already advanced in these settings. Similarly to overall population decline, several policy options have been debated and implemented, ranging from immigration, increasing retirement ages, pension reform, a focus on disease prevention, and investments in human capital, such as higher-level skill and knowledge building in a shrinking workforce.^63^, ^64^ In these same regions, the effects of decreases in the proportion of the population aged 15–64 years on economic productivity could be mitigated by individuals working far beyond age 65 years. This shift to later retirement is already occurring in many countries, including the USA, Australia, and Japan.^67^, ^68^, ^69^, ^70^, ^71^, ^72^

The fertility rates in children and adolescents aged 10–19 years is an SDG indicator for goal 3, target 3.7. To our knowledge, our analysis provides the first annual time series of fertility rates in these age groups. Fertility rates in ages 15–19 years typically decrease with a country's development but the trends in those aged 10–14 years are less clear. In addition to the global patterns in fertility rates in children and adolescents, there are marked variations across countries at similar levels of development. Within SDI bands, the ratio of highest to lowest adolescent fertility rates is often more than an order of magnitude, highlighting that many factors other than development status contribute to the fertility rate in children and adolescents. Some countries have been able to reduce adolescent fertility rates faster than expected. A detailed analysis of the determinants of the variation in fertility rate among children and adolescents across SDI bands, including policy factors, is beyond the scope of this study, but this finding suggests that such research is urgently needed.

The population decline that we found in Syria indicates the potentially important role of conflict on both fertility and migration rates. Conflict in some settings, such as in Kuwait during the first Persian Gulf War, can reduce fertility rates, but other examples have been found where conflict has led to younger marriage and increased fertility rates.^73^ We explored adding the death rate from conflict as a covariate to the fertility estimation model but we found that this variable, on average, did not predict changes in fertility; this finding is consistent with examples of increases and decreases in fertility in individual countries. Conflict is also associated with large migration flows; many of these are captured in the UNHCR migrant stock and derived flow data. Given the large-scale migration seen during the conflict in Syria, a deeper understanding of what determines the magnitude of migration before, during, and after conflict would be useful in planning public health, social, and policy interventions to ameliorate the effects of migration on individuals and families.

Sex ratios in most countries remain in the narrow band of 1·03–1·07 male livebirths for every female livebirth. We found in some countries, most notably India and China, that since the availability of ultrasonography in the early 1980s, the ratio of males to females has increased. In China, the sex ratios in 2017 were in excess of 1·16 males for every female. These ratios imply very substantial sex-selective abortion and even the possibility of female infanticide. The effect of such pronounced sex ratios on patterns of social interaction might be substantial in future generations. From the perspective of demographic growth, high sex ratios at birth reduce the net reproductive rate to below that predicted from the TFR alone. In China, low TFR and high sex ratios led to a net reproductive rate of 0·69 female livebirths expected per woman.

### Cross-cutting themes

An important debate in the medical literature about the decreases in fertility has been regarding the relative contribution of declines in the under-5 mortality rate, women's educational attainment, and the availability of reproductive health services, particularly modern contraception methods.^74^, ^75^, ^76^, ^77^, ^78^, ^79^ There is a strong correlation between estimated TFR and maternal education (*r*=–0·886), the met contraceptive need (*r*=–0·799), and the under-5 mortality rate (*r*=0·800), which are consistent over decades and across SDI quintiles. Nevertheless, use of time series of cross-sectional data to estimate causal relationships is particularly challenging given that all three of these measures are highly correlated. Understanding the magnitude of these different drivers and their complex interconnections is important to understand the future trajectory of ASFR. Fertility over the next few decades is hard to forecast in regions such as western sub-Saharan Africa, where fertility rates remain high, progress on educational attainment has been relatively modest, met need for contraception remains low (despite some recent improvements), and under-5 mortality has considerably decreased. Our more detailed time series of these drivers could provide opportunities for future studies to disentangle the contribution of these different factors.

Many factors other than maternal education, reproductive health services, and under-5 mortality rates influence annual fertility rates. The data compiled for our study show that there has been marked variation in fertility rates annually or over shorter durations in response to events with cultural significance or policy change. For example, the TFR in Singapore increased from 2·01 livebirths in 1999 to 2·39 livebirths in 2000, whereas in Japan in 1966—the year of the Fire Horse, during which giving birth to females was deemed unlucky^80^—the TFR decreased by 13% in a single year. Local legislation can also lead to an abrupt increase in the TFR: the introduction of a ban on abortion in Romania in 1966 increased TFR from 2·72 livebirths to 3·53 livebirths in the year following the ban. This ban on abortion also led to increases in the maternal mortality rate. The recent change from the one-child policy in China to a policy that allows second births was associated with an 11·7% increase in total livebirths from 2014 to 2017. These abrupt variations in fertility rates highlight the importance of understanding the magnitude of policy changes on fertility rates, especially in settings where fertility rates might have decreased far below the replacement value.

Over the past 25 years, annual livebirths globally have remained between 133·5 million and 141·7 million livebirths per year. This comparative stability has occurred even during marked changes in the population of women of reproductive age and highly heterogeneous trends in fertility rates. With each year, a larger proportion of the birth cohort is represented in regions with lower incomes and lower educational attainment because of different speeds of changing fertility in different locations, creating a phenomenon known as demographic headwinds.^61^, ^64^, ^65^ As more births occur in increasingly difficult circumstances, the challenge of meeting the ambitious SDG targets will become more difficult. We would expect the pace of reductions in the global under-5 mortality rate to slow due to the changes in the birth cohort, and similar global slowing might be expected for other indicators such as childhood vaccination. Other changes, such as the slower rates of decrease in neonatal mortality than in mortality in post-neonatal infants (age 28–365 days) and children aged 1–4 years, might slow the decrease in overall child mortality. Evaluating global progress will need to take into account these important compositional shifts in the global birth cohort in terms of income and educational attainment.

### Estimation challenges

The biggest challenge in creating population estimates that are consistent with observed population counts and with data on ASFR and age-specific mortality is the poor data available in many countries regarding net migration. We used the GBD Bayesian demographic balancing model to effectively infer net migration from the difference between the population expected from fertility and mortality rates and that observed in census or registry data. For some countries, the model has been informed with reported data on documented migration and UNHCR data on stocks and flows of refugees. Nevertheless, the only data that are increasingly available for many low-income and middle-income countries are stocks of migrants reported at the time of the census. Although these data are clearly useful, different assumptions about mortality rates and the timing of migration can lead to very different estimates of past migration flows, leading to the same observed stock of migrants in each country being estimated for. Even within these data, some temporary migrants who move for employment opportunities might not be recorded. More transparent estimates of population with standardised methods, such as the methods that we have presented, will hopefully drive a more extensive debate on data sources for assessing migration and how to improve them in the future.

We identified and extracted results from national PESs in only 165 censuses, although it is likely that many more have been done but their results have not been publicly released. PESs use direct or indirect methods: direct PESs match the records of individuals with actual census records to estimate census completeness, whereas indirect methods ask PES respondents if they participated in the census. Direct matching is more reliable but much harder to conduct. Censuses and PESs can miss certain populations such as homeless people in some countries or excluded minorities. The absence of PESs for most censuses in most countries means that the actual population count in many countries is uncertain. To avoid systematic bias, we estimated census completeness in all countries. The issue of census completeness remains a major challenge and one that cannot easily be addressed for past censuses. It is unlikely, for example, that we will empirically resolve debates on census completeness for many censuses in the 1950s–2000s. At best, we can adequately represent this uncertainty in our results. Moving forward, standardising the reporting of PES results so that some form of systematic analysis can be done will aid in future assessments.

Age misreporting, including age heaping, is a substantial challenge in use of data from many censuses, particularly in locations where numeracy of the respondents is relatively low.^35^, ^37^ In fact, some education research has used age heaping as a proxy measure of the quality of mathematics education in a country.^81^ We detected age misreporting in many earlier censuses in many countries, often manifested by implausible immigration rates required to match census counts in the oldest age groups. We mitigated the effect of age misreporting by excluding some data in the oldest age groups so that the estimates are driven by census data at younger age groups and mortality estimates, and by increasing the variance of population counts at older ages, but this approach does not remove all the effects of systematic age misreporting. For age heaping, we used the Feeney, Arriaga, and Arriaga strong corrections, dependent on the details of age group available and the degree of age heaping. These approaches have helped to mitigate age misreporting and age-heaping issues, but further work on how to analyse these complex error patterns in the data will be helpful to improve future estimates.

Demographers have long recognised that population estimates are necessary for planning, regardless of the availability and quality of the data. The challenge for demographers is to produce the most plausible estimates of population that can be used, rather than simply cataloguing all the limitations of the available data or the potential for error. This approach was part of the original inspiration for GBD. However, demographic estimation has also remained quite operator dependent: analytical choices by different demographers can lead to considerable differences in estimates for the same country. The differences between UNPOP estimates, US Census Bureau estimates, and national government estimates for many countries is one illustration of this analyst dependence. Demographic estimation has only recently started to examine statistical methods that generate uncertainty intervals,^47^, ^48^, ^49^, ^82^, ^83^, ^84^, ^85^ but these have not been widely used by UNPOP, the US Census Bureau, or by most national authorities for population estimation, and these methods remain primarily a research interest. To our knowledge, we have generated the first complete time series of the population size (with uncertainty intervals) for all countries by use of such methods; however, there are still many analytical choices that have been made that could arguably be changed in future efforts. These might include the choice of age-heaping smoother, the decision to exclude some census counts as outliers, or inclusion of documented migration estimates from various sources. We hope that this effort will stimulate vigorous debate on the analysis of population size for different countries.

### Limitations

This study has many limitations, some of which—including the paucity of direct measurement of net migration—have already been identified, whereas others need to be articulated. First, the GBD Bayesian demographic balancing model for population and migration estimation includes a number of hyperpriors. The results of the estimation are sensitive to the choice of these hyperpriors, such as the correlation of migration over time. We have largely used the same hyperpriors for all locations, but we have modified the hyperpriors in some locations to improve the fit of the model. Second, we sought to estimate de-facto population counts, but in some low-income and middle-income locations, only de-jure counts were available as inputs. De-jure counts could, in some countries, exclude temporary migrants; we identified and included migration data in locations where large labour migration is known to occur, but the use of de-jure counts in other settings could overestimate or underestimate de-facto counts. Third, we assume that the estimates of age-specific mortality from the GBD study and ASFR from this study are accurate. Any systematic errors in either would affect our estimates of migration and of population in years that are further from a census. Fourth, the estimation method requires a baseline estimate of the population in 1950 for detailed age groups, and any errors in this baseline based on a backwards cohort-component method of population projection will have a sustained effect on the population estimates from the baseline until at least the first census after 1950. Major errors in the baseline can also have an effect after the first census. Fifth, we were unable to obtain census counts by sex from ten known censuses and could not obtain age-specific population data in 62 censuses. Inclusion of this unpublished information could substantially change the results for those locations. Sixth, uncertainty in our current results is based on the uncertainty in population counts and the time since the last population count and, implicitly, errors in fertility and mortality estimation. We used an out-of-sample approach to estimate uncertainty in the population size in years without a census count, and we used uncertainty in the PES model prediction of completeness to estimate uncertainty in the years with and without a census count. The out-of-sample method provides a robust approach to estimating uncertainty but does not provide draws of migration, fertility, and mortality associated with each draw of population. We also assumed that years where registry counts are available only have uncertainty in the PES model prediction of completeness and zero uncertainty from the out-of-sample approach. This approach to estimating population uncertainty also does not incorporate any spatial correlation of uncertainty across countries and assumes complete correlation of uncertainty by age. Uncertainty at the country level could be exaggerated by this approach. Seventh, age-specific migration estimates can be affected by age-specific variation in census completeness. In our analysis, we have included the average age pattern of enumeration completeness, as detected in our analysis of PESs, but country-specific variation in the age pattern of enumeration is possible. Eighth, refugee flows might be misenumerated by UNHCR in some settings, leading to underestimates of migrants. Ninth, alternative hyperparameters could be selected and could change the results, although we believe that our selection of hyperparameters, which were based on several rounds of testing, provide sensible results. Tenth, we analysed each location independently, without imposing global constraints on global net migration. As a consequence, in some years, our estimates imply global net migration, which is not possible. For example, in 2015, our estimate of global net migration was 14 709 people. Finally, our model for fertility in girls aged 10–14 years is based on a simple linear regression of the ratio of fertility in those aged 10–14 years versus those aged 15–19 years, on the fertility rate in those aged 15–19 years and 50–54 years was estimated as a fixed fraction of the fertility rate in women aged 45–49 years because, even in the linear regression, the coefficient was not significant. This regression is based on locations with complete vital registration data, which tend to be high-SDI and middle-SDI countries. Other factors might drive fertility at these extreme ages that are not captured in our models or the available data.

### Future directions

There are many ways in which our estimation of population by age, sex, location, and year can be improved and made more useful for diverse applications. We currently use the GBD Bayesian demographic balancing model to estimate age-sex-year-specific migration, consistent with our estimated fertility and mortality rates and observed population numbers. In settings where direct measurement of migration is possible, it could be useful to use a version of the same model that allows the posterior values for fertility, mortality, and migration to change relative to the prior. This approach is conceptually appealing, allowing inconsistencies between fertility, mortality, and migration to be resolved through shifts in some or all of these inputs. However, our early testing of this approach showed considerable instability given that the same observed population count can be exactly explained by an infinite set of combinations of deaths and migration. This instability in the full Bayesian model led to estimates of implausible shifts in the age and time pattern of mortality. In some settings, it might be possible to provide more information on the credible age structure of death and migration to stabilise such a version of the model. A second improvement in the modelling approach would be to address how to ensure that the global net migration in any age-sex-year group is zero. Joint estimation of all locations simultaneously is unlikely to be computationally feasible given the complexity of the model for just one location at a time. Two-stage processes can be explored that might accommodate the logical requirement for global net migration to be zero. Another avenue that warrants investigation is the inclusion in the analysis of household age structure from household surveys; there is a very wide array of these surveys, and methods to use this information with appropriately wider data variance than a census could improve estimation in census-poor locations. We currently adjust data for age heaping with the three correction methods (Feeney, Arriaga, and Arriaga strong), but there could be other ways to incorporate age-heaping corrections directly into the GBD Bayesian demographic balancing model likelihood. In future analyses of fertility and population, the important role of urbanisation should be explored. Given the drive in many GBD-related analyses toward 5 × 5 km estimation,^86^, ^87^ the logical extension of our analysis will be to generate population estimates at a detailed local level. Such efforts will need to leverage similarly fine-grained assessments of fertility, mortality, and available population counts, supplemented with satellite imagery where feasible.

### Conclusion

Population size and age structure have substantial consequences on every aspect of social and economic life in every location. Over the past 70 years, there have been huge changes in ASFR, mortality, and migration that have reshaped population structures. Trends have not been homogeneous across and within countries and, although global population growth rates have decreased, the absolute increase in global population every year has remained notably constant for many decades. Linear growth in the global population is occurring despite population decreases in some parts of the world, particularly eastern Europe, and large population increases in sub-Saharan Africa. Demographic changes will continue to have substantial social and economic effects, highlighting the importance of close monitoring and analysis of fertility and population at the local level. The statistical methods for estimation that we present will hopefully facilitate this need, providing the essential demographic intelligence for countries to reliably inform their health and social development strategies.

Correspondence to: Prof Christopher J L Murray, Institute for Health Metrics and Evaluation, Seattle, WA 98121, USA cjlm@uw.edu

## Data sharing

To download the data used in these analyses, please visit the Global Health Data Exchange at http://ghdx.healthdata.org/gbd–2017.
