# Department of Error

**DOI:** 10.1016/S0140-6736(19)31045-1

**Published:** 2019-06-22

*GBD 2017 Population and Fertility Collaborators. Population and fertility by age and sex for 195 countries and territories, 1950–2017: a systematic analysis for the Global Burden of Disease Study 2017.* Lancet *2018; **392:** 1995–2051*—In this Global Health Metrics paper, Haley Comfort has been added to the collaborators list, Mohammad Reza Salahshoor's name has been corrected, affiliation details have been amended for Mohammad Reza Salahshoor and Yasin Jemal Yasin, and the declaration of interests statement has been amended for Boris Bikbov. These corrections have been made to the online version as of June 20, 2019.

