# Supplementary material for: Population and fertility by age and sex for 195 countries and territories, 1950–2017: a systematic analysis for the Global Burden of Disease Study 2017
Source: Lancet. 2018 Nov 10;392(10159):1995–2051. doi: 10.1016/S0140-6736(18)32278-5 (PMC6227915; doi:10.1016/S0140-6736(18)32278-5)
Supplement: Supplementary appendix 1 [file mmc1.pdf]

THE LANCET

Supplementary appendix 1

This appendix formed part of the original submission and has been peer reviewed. We post it as supplied by the authors.

Supplement to: GBD 2017 Population and Fertility Collaborators. Population and fertility by age and sex for 195 countries and territories, 1950–2017: a systematic analysis for the Global Burden of Disease Study 2017. *Lancet* 2018; **392:** 1995–2051.

Methods Appendix to Population and fertility by age and sex for 195 countries and territories 1950–2017: a systematic analysis for the Global Burden of Disease 2017

This appendix provides further methodical detail, supplemental figures, and more detailed results for Population and fertility by age and sex for 195 countries and territories 1950–2017: a systematic analysis for GBD 2017. This appendix is organised into sections that follow the structure of the main paper.

Supplementary results for Population and fertility by age and sex for 195 countries and territories 1950–2017: a systematic analysis for GBD 2017 are presented separately online.

Background

This appendix provides further methodological detail and more detailed results for Population and fertility by age and sex for 195 countries and territories 1950-2017: a systematic analysis for the GBD 2017. This study complies with the Guidelines for Accurate and Transparent Health Estimates Reporting (GATHER) recommendations. It includes detailed tables and information on data in an effort to maximize transparency in our estimation processes and provide a comprehensive description of analytical steps.

Table of Contents

Authors' contributions........ 5

Section 1. GBD Overview........ 14

Section 1.1. Geographic Units of the Analysis........ 14

Section 1.2. Time Periods of the Analysis........ 14

Section 1.3. Statement of GATHER Compliance........ 14

Section 1.4. List of Abbreviations........ 14

Section 1.5. GBD results overview........ 15

Section 1.6. Data input sources overview........ 16

Section 1.7. Funding Sources........ 16

Section 2. Population and Fertility Estimation Process........ 16

Section 2.1. Overview........ 16

Section 2.2. Fertility........ 17

Data Sources........ 17

Modelling Approach........ 19

Section 2.3 Population........ 26

Section 3. Socio-demographic Index (SDI) Analysis........ 37

Section 3.1. Overview........ 37

Section 3.2 Development of revised SDI indicator........ 37

Section 4. References........ 39

Section 5. Figures and Tables........ 41

Appendix Figure 1a. Analytical flowchart for the GBD 2017 population estimation process........ 42

Appendix Figure 1b. Analytical flowchart for the GBD 2017 fertility estimation process... 43

Appendix Figure 2. Census and registry availability by location and year........ 44

Appendix Table 1. GBD location hierarchy with levels........ 51

Appendix Table 2. GATHER checklist........ 65

Appendix Table 3. Number of sources used for the analysis of age-specific fertility (VR, CBH-survey, SBH-survey, SBH-census) for each location........ 68

Appendix Table 4. Number of sources used for the analysis of age-specific fertility by year (VR, CBH-survey, SBH-survey, SBH-census)........ 72

Appendix Table 5. List of all confirmed censuses by location and year........ 74

Appendix Table 6. World population standard........ 104

Appendix Table 7. SDI groupings by geography, based on 2017 values………………………… 105

Appendix Table 8. SDI values by location, 1950–1969…………………………………………………. 119

Appendix Table 9. SDI values by location, 1970–1989…………………………………………………. 129

Appendix Table 10. SDI values by location, 1990–2017……………………………………………….. 138

Appendix Table 11. Correlations of fertility by decade and by Socio-demographic Index (SDI) quintile for under-5 mortality, demand for contraception satisfied with modern methods, and female education……………………………………………………………………………………… 143

Authors' Contributions

Managing the estimation process

Ashkan Afshin, Elizabeth Cromwell, Lalit Dandona, Rakhi Dandona, Louisa Degenhardt, Samath Dharmaratne, Daniel Dicker, Charbel El Bcheraoui, Kara Estep, Valery Feigin, Nancy Fullman, Emmanuela Gakidou, Simon Hay, Spencer James, Nicholas Kassebaum, Ibrahim Khalil, Stephen Lim, Alan Lopez, Raphael Lozano, Felix Masiye, Awoke Misganaw, Ali Mokdad, Christopher Murray, Mohsen Naghavi, David Pigott, Robert Reiner, Joseph Salama, Katya Shackelford, Caitlyn Steiner, Stein Emil Vollset, Theo Vos, and Harvey Whiteford

Writing the first draft of the manuscript

Charlton Callender, Leslie Cornaby, Kara Estep, Thomas Hsiao, Xie Rachel Kulikoff, James Lee, Stephen Lim, Molly Miller-Petrie, Christopher Murray, Paulami Naik, Joseph Salama, Vinay Srinivasan, and Leo Zoeckler

Providing data or critical feedback on data sources

Kalkidan Hassen Abate, Aberash Abay, Solomon Mequanente Abay, Tarek Abd Elaziz, Alireza Abdi, Ibrahim Abdollahpour, Molla Abebe, Zegeye Abebe, Victor Aboyans, Aklilu Abrham Roba, Laith Abu-Raddad, Niveen Abu-Rmeileh, Manfred Accrombessi, Oladimeji Adebayo, Isaac Adedeji, Olatunji Adetokunboh, Tara Ballav Adhikari, Mina Adib, Mohsen Afarideh, Sargis Aghayan, Sutapa Agrawal, Mehdi Ahmadi, Muktar Ahmed, Ali Shafqat Akanda, Mohammadesmaeil Akbari, Tomi Akinyemiju, Nadia Akseer, Fares Alahdab, Khurshid Alam, Animut Alebel, Alicia Aleman, Kefyalew Addis Alene, Raghib Ali, Syed Mohamed Aljunid, François Alla, Peter Allebeck, Ali Almasi, Rajaa Al-Raddadi, Ubai Alsharif, Nelson Alvis-Guzman, Azmeraw T. Amare, Walid Ammar, Catalina Liliana Andrei, Hossein Ansari, Mustafa Geleto Ansha, Carl Abelardo Antonio, Olatunde Aremu, Al Artaman, Krishna K Aryal, Solomon Weldegebreal Asgedom, Reza Assadi, Suleman Atique, Marcel Ausloos, Ashish Awasthi, Beatriz Paulina Ayala Quintanilla, Rakesh Ayer, Peter Azzopardi, Arefeh Babazadeh, Hamid Badali, Alaa Badawi, Maciej Banach, Till Bärnighausen, Lope Barrero, Masoud Behzadifar, Bayu Begashaw Bekele, Saba Abraham Belay, Yihalem Abebe Belay, Aminu Bello, Derrick Bennett, Isabela Bensenor, Adugnaw Berhane, Adam Berman, Robert Bernstein, Tambe Betrand Ayuk, Mircea Beuran, Soumyadeep Bhaumik, Nigus Bililign, Sait Mentes Birlik, Charles Birungi, Nicola Luigi Bragazzi, Alexandra Brazinova, Nicholas Breitborde, Gabrielle Britton, Charlton Callender, Ismael Campos-Nonato, Mate Car, Juan Jesus Carrero, Carlos Castañeda-Orjuela, Franz Castro, Ferrán Catalá-López, Alanur Cavlin, Hsing-Yi Chang, Jung-Chen Chang, Peggy Pei-Chia Chiang, Abdulaal Chitheer, Rajiv Chowdhury, Flavia Cicuttini, Massimo Cirillo, Maria Magdalena Constantin, Cyrus Cooper, Paolo Angelo Cortesi, Alexandra Cucu, Petra Čukelj, Lalit Dandona, Rakhi Dandona, Paul I Dargan, Ahmad Daryani, Rajat Das Gupta, José Das Neves, Kairat Davletov, Gebre Demoz, Kebede Deribe, Nikolaos Dervenis, Don Des Jarlais, Mengistu Desalegn Tadese, Getenet Dessie, Samath Dharmaratne, Meghnath Dhimal, Eric L. Ding, Girmaye Dinsa, Klara Dokova, David Teye Doku, Kerrie Doyle, Manisha Dubey, Soheil Ebrahimpour, Eyasu Ejeta, Iqbal Elyazar, Aman Endries, Sergey Ermakov, Babak Eshrati, Sharareh Eskandarieh, Alireza Esteghamati, Sadaf Esteghamati, Hamed Fakhim, Mohammad Fareed, Carla Farinha, Andre Faro, Farshad Farzadfar, Mohammad Hosein Farzaei, Valery Feigin, Andrea B. Feigl, Fariba Feizy, Seyed-Mohammad Fereshtehnejad, Irina Filip, Nataliya Foigt, Takeshi Fukumoto, Adriana Galan, Gbetoho Gankpe, Gregory "Manny" M Garcia, M.A. Garcia-Gordillo, Tigist Gashaw, Teshome Gebre, Tsegaye Gebrehiwot, Amanuel Tesfay Gebremedhin, Tilayie Gelano, Johanna Geleijnse, Ayele Geleto, Ricard Genova-Maleras, Kebede Embaye Gezae, Keyghobad Ghadiri,

Hesam Ghiasvand, Mamata Ghimire, Srinivas Goli, Hector Gomez-Dantes, Sameer Gopalani, Bárbara Goulart, Ayman Grada, Giuseppe Grosso, Andre Guimaraes, Prakash Gupta, Rahul Gupta, Rajeev Gupta, Tanush Gupta, Juanita Haagsma, Nima Hafezi Nejad, Tekleberhan Beyene Hagos, Arya Haj-Mirzaian, Randah Hamadeh, Hilda Harb, Josep Maria Haro, Mehedi Hasan, Hadi Hassankhani, Hamid Y. Hassen, Rasmus Havmoeller, Akbar Hedayatizadeh-Omran, Mohamed Hegazy, Behzad Heibati, Behnam Heidari, Delia Hendrie, Claudiu Herteliu, Fatemeh Heydarpour, Long Hoang Nguyen, Michael Hole, Enayatollah Homaie Rad, Praveen Hoogar, H Dean Hosgood, Meimanat Hosseini-Chavoshi, Mehdi Hosseinzadeh, Mohamed Hsairi, Trang Huyen Nguyen, Kim Moesgaard Iburg, Ehimario Igumbor, Asnake Ararsa Irenso, Sheikh Mohammed Shariful Islam, Nader Jahanmehr, Mihajlo Jakovljevic, Moti Jalu , Simerjot Jassal, Mehdi Javanbakht, Achala Jayatilleke, Panniyammakal Jeemon, Vivekanand Jha, Jost B. Jonas, Jacek Jozwiak, Mikk Jürisson, Alemneh Kabeta, Zubair Kabir, Rajendra Kadel, Amaha Kahsay, Rizwan Kalani, Umesh Kapil, Manoochehr Karami, Corine Karema, Seyed M. Karimi, Amir Kasaeian, Getachew Mullu Kassa, Nicholas J Kassebaum, Anil Kaul, Norito Kawakami, Peter Keiyoro, Yousef Khader, Morteza Abdullatif Khafaie, Ibrahim Khalil, Young-Ho Khang, Alireza Khatony, Abdullah T. Khoja, Ardeshir Khosravi, Mohammad Hossein Khosravi, Daniel Kiirithio, Daniel Kim, Jun Kim, Young-Eun Kim, Adnan Kisa, Mika Kivimaki, Yoshihiro Kokubo, Soewarta Kosen, Parvaiz Koul, Ai Koyanagi, Kewal Krishan, Sanjay Krishnaswami, Kristopher Krohn, Barthelemy Kuate Defo, Burcu Kucuk Bicer, G Anil Kumar, Manasi Kumar, Pushpendra Kumar, Sudhir Kumar Jain, Michael Kutz, Sheetal Lad, Alessandra Lafranconi, Dharmesh Lal, Ratilal Lalloo, Hilton Lam, Faris Lami, Van Lansingh, Dennis Laryea, Misgan Legesse Liben, Cheru T Leshargie, Xiaohong Li, Yichong Li, Juan Liang, Xiaofeng Liang, Lee-Ling Lim, Shiwei Liu, Alan Lopez, Paulo Lotufo, Erlyn Rachelle Macarayan, Marek Majdan, Reza Majdzadeh, Azeem Majeed, Mohammad Ali Mansournia, Joemer Maravilla, Francisco Rogerlândio Martins-Melo, Winfried März, Melvin Marzan, Felix Masiye, Mohsen Mazidi, John Mcgrath, Varshil Mehta, Hagazi Gebre Meles, Kidanu Meles, Addisu Melese, Mulugeta Melku, Peter Memiah, Walter Mendoza, Melkamu Merid Mengesha, Getnet Mengistu, Zerihun Menlkalew Zenebe , Beyene Meressa, Atte Meretoja, Tuomo Meretoja, Tomislav Mestrovic, Haftay Berhane Mezgebe, Tomasz Miazgowski, Erkin Mirrakhimov, Babak Moazen, Karzan Mohammad, Moslem Mohammadi, Shafiu Mohammed, Ali H. Mokdad, Glen Mola, Lorenzo Monasta, Julio Montañez, Ghobad Moradi, Maziar Moradi-Lakeh, Mehdi Moradinazar, Joana Morgado-Da-Costa, Rintaro Mori, Shane Morrison, Marilita Moschos, Seyyed Meysam Mousavi, Achenef Muche, Kamarul Imran Musa, Ghulam Mustafa, Jean Nachega, Gabriele Nagel, Mohsen Naghavi, Seyed Sina Naghibi Irvani, Gurudatta Naik, Luigi Naldi, Bruno Nascimento, Haseeb Nawaz, Ionut Negoi, Ruxandra Irina Negoi, Charles Newton, Josephine Ngunjiri, Ana Maria Nogales Vasconcelos, Shuhei Nomura, Mehdi Noroozi, Jean Jacques Noubiap, Hamid Reza Nouri, Malihe Nourollahpour, Mohammad Reza Nowroozi, Okechukwu Ogah, Felix Ogbo, In-Hwan Oh, Anselm Okoro, Olanrewaju Oladimeji, Andrew T. Olagunju, Tinuke Olagunju, Bolajoko Olusanya, Jacob Olusanya, Sok King Ong, Stanislav S. Otstavnov, Mayowa Owolabi, Mahesh P A, Smita Pakhale, Adrian Pana, Basant Kumar Panda, Songhomitra Panda-Jonas, Eun-Kee Park, Shanti Patel, Snehal Patil, Ajay Patle, Deepak Paudel, Marcel Pedroso, David Pereira, William Petri, Max Petzold, Huyen Phuc Do, David Pigott, Julian Pillay, Meghdad Pirsaheb, Akram Pourshams, Hossein Poustchi, Swayam Prakash, Mostafa Qorbani, Amir Radfar, Anwar Rafay, Alireza Rafiei, Fakher Rahim, Afarin Rahimi-Movaghar, Vafa Rahimi-Movaghar, Mahfuzar Rahman, Mohammad Hifz Ur Rahman, Sajjad Rahman, Fatemeh Rajati, Sasa Rajsic, Chhani Lal Ranabhat, Prabhat Ranjan, Paturi Rao, David Rawaf, Salman Rawaf, Christian Razo, Andre Renzaho, Shahab Rezaeian, Seyed Mohammad Riahi, Maria Jesús Ríos Blancos, Leonardo Roever, Luca Ronfani, Gholamreza Roshandel, Ali Rostami, Enrico Rubagotti, Hosein Safari, Yahya Safari, Saeid Safiri, Mohammad Ali Sahraian,

Mohamadreza Salahshoor, Nasir Salam, Joseph Salama, Payman Salamati, Yahya Salimi, Hamideh Salimzadeh, Evanson Z Sambala, Abdallah M. Samy, Juan Sanabria, Milena Santric Mlicevic, Bruno Sao Jose, Muthupandian Saravanan, Mayank Sardana, Rodrigo Sarmiento-Suarez, Benn Sartorius, Shahabeddin Sarvi, Maheswar Satpathy, Arundhati Sawant, Monika Sawhney, Sonia Saxena, Elke Schaeffner, David C Schwebel, Falk Schwendicke, Mario Šekerija, Sadaf Sepanlou, Edson Serván-Mori, Amira Shaheen, Masood Ali Shaikh, Mehran Shams-Beyranvand, Morteza Shamsizadeh, Kiomars Sharafi, Mehdi Sharif, Jayendra Sharma, Jun She, Aziz Sheikh, Peilin Shi, Ivy Shiue, Farhad Shokraneh, Soraya Siabani, Abla Sibai, Diego Augusto Santos Silva, Dayane Silveira, Jasvinder Singh, Virendra Singh, Adauto Martins Soares Filho, Soheila Sobhani, Moslem Soofi, Luisa Sorio Flor, Chandrashekhar T Sreeramareddy, Mark Stokes, Muawiyyah Babale Sufiyan, Rizwan Suliankatchi Abdulkader, Bryan L. Sykes, Dillon Sylte, Cassandra Szoeke, Rafael Tabarés-Seisdedos, Karen Tabb, Segen Tassew, Nuno Taveira, Mohamad-Hani Temsah, Abdullah Terkawi, Belay Tessema, Mebrahtu Teweldemedhin, Js Thakur, Nu Thi Truong, Nihal Thomas, Binyam Tilahun, Marcello Tonelli, Miguel Tortajada-Girbés, Marcos Roberto Tovani-Palone, Hideaki Toyoshima, Bach Tran, Khanh Bao Tran, Thomas Truelsen, Nikolaos Tsilimparis, Kingsley N. Ukwaja, Irfan Ullah, Muhammad Shariq Usman, Olalekan Uthman, Muthiah Vaduganathan, Afsane Vaezi, Gaurang Vaidya, Pascual Valdez, Tommi Vasankari, Narayanaswamy Venketasubramanian, Sergey Vladimirov, Vasiliy Vlassov, Fasil Wagnew, Yasir Waheed, Walson Walson, Yanping Wang, Yuan-Pang Wang, Elisabete Weiderpass, Robert Weintraub, Inbal Weiss Salz, Andrea Werdecker, Ronny Westerman, Justyna Widecka, Katarzyna Widecka, Tissa Wijeratne, Charles Shey Wiysonge, Charles Wolfe, Shouling Wu, Grant Wyper, Gelin Xu, Tomohide Yamada, Yasin Jemal Yasin, Pengpeng Ye, Alex Yeshnaeh, Manaye Yihune, Ebrahim M. Yimer, Naohiro Yonemoto, Seok-Jun Yoon, Marcel Yotebieng, Mustafa Younis, Vesna Zadnik, Zoubida Zaidi, Sojib Bin Zaman, Mohammad Zamani, Zohreh Zare, Xueying Zhang, Jun Zhu, Stephanie Zimsen, and Sanjay Zodpey.

Developing methods or computational machinery

Tarek Abd Elaziz, Ibrahim Abdollahpour, Isaac Adedeji, Sutapa Agrawal, Mehdi Ahmadi, Mohammadesmaeil Akbari, Mehran Alijanzadeh, Suleman Atique, Habtamu Wondifraw Baynes, Sait Mentes Birlik, Roy Burstein, Charlton Callender, Pankaj Chaturvedi, Ahmad Daryani, Daniel Dicker, Hamed Fakhim, Mohammad Fareed, Farshad Farzadfar, Samuel Finegold, Kyle Foreman, Keyghobad Ghadiri, Akbar Hedayatizadeh-Omran, Meimanat Hosseini-Chavoshi, Mehdi Hosseinzadeh, Thomas Hsiao, Umesh Kapil, André Karch, Nicholas J Kassebaum, Ibrahim Khalil, Muhammad Shahzeb Khan, Adnan Kisa, Xie Rachel Kulikoff, Michael Kutz, Misgan Legesse Liben, Tefera Chane Mekonnen, Hagazi Gebre Meles, Kidanu Meles, Melkamu Merid Mengesha, Christopher J L Murray, Ayenew Negesse, Grant Nguyen, Bolajoko Olusanya, Jacob Olusanya, Aaron Osgood-Zimmerman, Mayowa Owolabi, David Pigott, Chhani Lal Ranabhat, Robert Reiner, Nicholas Roberts, Mohamadreza Salahshoor, Abdallah M. Samy, Shahabeddin Sarvi, Maheswar Satpathy, Kathryn Schelonka, David C Schwebel, Mehdi Sharif, Tariq Jamal Siddiqi, Naris Silpakit, Vinay Srinivasan, Leo Stewart, Ipsita Sutradhar, Bryan L. Sykes, Dillon Sylte, Segen Tassew, Irfan Ullah, Muhammad Shariq Usman, Tommi Vasankari, Ronny Westerman, Naohiro Yonemoto, and Leo Zoeckler.

Applying analytical methods to produce estimates

Tarek Abd Elaziz, Daisy Maria Xavier Abreu, Aklilu Abrham Roba, Sutapa Agrawal, Muktar Ahmed, Sayem Ahmed, Amani Nidhal Aichour, Ibtihel Aichour, Miloud Taki Eddine Aichour, Syed Mohamed Aljunid, Olatunde Aremu, Solomon Weldegebreal Asgedom, Arindam Basu, Masoud Behzadifar, Bayu Begashaw Bekele, Gregory Bertolacci, Sait Mentes Birlik, Alexandra Brazinova, Charlton Callender,

Devasahayam Jesudas Christopher, Matthew Cunningham, Ahmad Daryani, Don Des Jarlais, Daniel Dicker, Tim Driscoll, Manisha Dubey, Aman Endries, Sergey Ermakov, Alireza Esteghamati, Fariba Feizy, Samuel Finegold, Florian Fischer, Ayele Geleto, Keyghobad Ghadiri, Nima Hafezi Nejad, Dessalegn Haile, Hamid Y. Hassen, Akbar Hedayatizadeh-Omran, Nathaniel Henry, Long Hoang Nguyen, Meimanat Hosseini-Chavoshi, Thomas Hsiao, Trang Huyen Nguyen, Moti Jalu , Manoochehr Karami, Barthelemy Kuate Defo, Xie Rachel Kulikoff, Van Lansingh, James Lee, Misgan Legesse Liben, James Leigh, Samson Leta, Xiaofeng Liang, Reza Malekzadeh, Melvin Marzan, Mohsen Mazidi, Sanjay Mehendale, Tefera Chane Mekonnen, Hagazi Gebre Meles, Melkamu Merid Mengesha, Shafiu Mohammed, Haseeb Nawaz, Ayenew Negesse, Grant Nguyen, Jacob Olusanya, Huyen Phuc Do, David Pigott, Caroline Purcell, Alireza Rafiei, Fakher Rahim, Mahfuzar Rahman, Fatemeh Rajati, Chhani Lal Ranabhat, Seyed Mohammad Riahi, Kedir Teji Roba, Mohamadreza Salahshoor, Abdallah M. Samy, Shahabeddin Sarvi, Maheswar Satpathy, Arundhati Sawant, Masood Ali Shaikh, Mehdi Sharif, Jun She, Diego Augusto Santos Silva, Chandrashekhar T Sreeramareddy, Vinay Srinivasan, Dillon Sylte, Nu Thi Truong, Miguel Tortajada-Girbés, Bach Tran, Khanh Bao Tran, Kingsley N. Ukwaja, Ronny Westerman, Tissa Wijeratne, Mustafa Younis, Chuanhua Yu, Zoubida Zaidi, and Leo Zoeckler.

Providing critical feedback on methods or results

Degu Abate, Kalkidan Hassen Abate, Aberash Abay, Solomon Mequanente Abay, Nooshin Abbasi, Hedayat Abbastabar, Tarek Abd Elaziz, Jemal Abdela, Omar Abdel-Rahman, Alireza Abdi, Ibrahim Abdollahpour, Haftom Abebe, Molla Abebe, Zegeye Abebe, Teshome Abebo, Haftom Abraha, Daisy Maria Xavier Abreu, Aklilu Abrham Roba, Niveen Abu-Rmeileh, Pawan Acharya, Abdu Adamu, Oladimeji Adebayo, Isaac Adedeji, Victor Adekanmbi, Olatunji Adetokunboh, Tara Ballav Adhikari, Mina Adib, Kouablan Arsène Adou, José C. Adsuar, Mohsen Afarideh, Ashkan Afshin, Gina Agarwal, Sargis Aghayan, Sutapa Agrawal, Manzoor Ahmad, Alireza Ahmadi, Mehdi Ahmadi, Muktar Ahmed, Sayem Ahmed, Amani Nidhal Aichour, Ibtihel Aichour, Miloud Taki Eddine Aichour, Ali Shafqat Akanda, Mohammadesmaeil Akbari, Mohammed Akibu, Rufus Akinyemi, Tomi Akinyemiju, Nadia Akseer, Fares Alahdab, Ziyad Al-Aly, Khurshid Alam, Animut Alebel, Kefyalew Addis Alene, Ayman Al-Eyadhy, Mehran Alijanzadeh, Reza Alizadeh-Navaei, Syed Mohamed Aljunid, Ala'a Alkerwi, Peter Allebeck, Ali Almasi, Jordi Alonso, Rajaa Al-Raddadi, Ubai Alsharif, Khalid Altirkawi, Nelson Alvis-Guzman, Azmeraw T. Amare, Walid Ammar, Catalina Liliana Andrei, Sofia Androudi, Hossein Ansari, Carl Abelardo Antonio, Seth Christopher Yaw Appiah, Olatunde Aremu, Johan Ärnlöv, Al Artaman, Krishna K Aryal, Hamid Asayesh, Ephrem Tsegay Asfaw, Solomon Weldegebreal Asgedom, Reza Assadi, Tesfay Mehari Atey, Suleman Atique, Madhu S. Atteraya, Marcel Ausloos, Euripide Avokpaho, Ashish Awasthi, Beatriz Paulina Ayala Quintanilla, Wondimu Ayele, Rakesh Ayer, Peter Azzopardi, Tesleem Babalola, Arefeh Babazadeh, Alaa Badawi, Suzanne Barker-Collo, Till Bärnighausen, Lope Barrero, Huda Basaleem, Quique Bassat, Arindam Basu, Bernhard Baune, Habtamu Wondifraw Baynes, Ettore Beghi, Meysam Behzadifar, Abate Bekele, Bayu Begashaw Bekele, Ezra Belay, Saba Abraham Belay, Yihalem Abebe Belay, Michelle Bell, Aminu Bello, Derrick Bennett, Isabela Bensenor, Gilles Bergeron, Adugnaw Berhane, Adam Berman, Eduardo Bernabe, Robert Bernstein, Balem Demtsu Betsu, Mircea Beuran, Suraj Bhattarai, Soumyadeep Bhaumik, Belete Biadgo, Ali Bijani, Boris Bikbov, Nigus Bililign, Muhammad Shahdaat Bin Sayeed, Sait Mentes Birlik, Charles Birungi, Tuhin Biswas, Hailemichael Bizuneh, Archie Bleyer, Cristina Bosetti, Soufiane Boufous, Oliver Brady, Nicola Luigi Bragazzi, Michael Brainin, Nicholas Breitborde, Hermann Brenner, Gabrielle Britton, Roy Burstein, Reinhard Busse, Zahid Butt, Lucero Cahuana-Hurtado, Charlton Callender, Jorge Cano, Mate Car, Rosario Cárdenas, Juan Jesus Carrero, Felix Carvalho, Carlos Castañeda-Orjuela, Franz Castro, Ferrán Catalá-López, Ester Cerin, Jung-Chen Chang, Aparajita Chattopadhyay,

Pankaj Chaturvedi, Peggy Pei-Chia Chiang, Ken Chin, Vesper Chisumpa, Jee-Young Choi, Devasahayam Jesudas Christopher, Liliana G Ciobanu, Rafael Claro, Daniel Collado-Mateo, Maria Magdalena Constantin, Sara Conti, Cyrus Cooper, Paolo Angelo Cortesi, Monica Cortinovis, Megan Costa, Christopher Crowe, Alexandra Cucu, Berihun Dachew, Lalit Dandona, Rakhi Dandona, Paul I Dargan, Ahmad Daryani, Rajat Das Gupta, José Das Neves, Dragos Davitoiu, Diego De Leo, Jan-Walter De Neve, Megbaru Debalkie, Tizta Degfie, Gebre Demoz, Edgar Denova-Gutiérrez, Kebede Deribe, Nikolaos Dervenis, Mengistu Desalegn Tadese, Getenet Dessie, Samath Dharmaratne, Meghnath Dhimal, Daniel Dicker, Eric L. Ding, Girmaye Dinsa, Shirin Djalalinia, Klara Dokova, David Teye Doku, Kerrie Doyle, Tim Driscoll, Manisha Dubey, Eleonora Dubljanin, Andre Duraes, Soheil Ebrahimpour, David Edvardsson, Eyasu Ejeta, Ziad El-Khatib, Ahmadali Enayati, Aman Endries, Sergey Ermakov, Babak Eshrati, Sharareh Eskandarieh, Alireza Esteghamati, Sadaf Esteghamati, Hamed Fakhim, Tamer Farag, Mahbobeh Faramarzi, Carla Farinha, Andre Faro, Farshad Farzadfar, Mohammad Hosein Farzaei, Mir Sohail Fazeli, Valery Feigin, Andrea B. Feigl, Fariba Feizy, Ama Fenny, Netsanet Fentahun, Seyed-Mohammad Fereshtehnejad, Eduarda Fernandes, Garumma Tolu Feyissa, Irina Filip, Samuel Finegold, Florian Fischer, Nataliya Foigt, Kyle Foreman, Carla Fornari, Takeshi Fukumoto, Nancy Fullman, Adriana Galan, Silvano Gallus, Amiran Gamkrelidze, Morsaleh Ganji, Tigist Gashaw, Abadi Kahsu Gebre, Teshome Gebre, Gebremedhin Berhe Gebregergs, Tsegaye Gebrehiwot, Merhawi Gebremedhin, Afewerki Gebremeskel, Aregawi Gebreyesus Belay, Tilayie Gelano, Yalemzewod Gelaw, Johanna Geleijnse, Ayele Geleto, Ricard Genova-Maleras, Kebede Embaye Gezae, Reza Ghadimi, Keyghobad Ghadiri, Maryam Ghasemi-Kasman, Hesam Ghiasvand, Mamata Ghimire, Aloke Gopal Ghoshal, Kidu Gidey, Paramjit Gill, Tiffany Gill, Meaza Girma, Giorgia Giussani, Srinivas Goli, Philimon Gona, Amador Goodridge, Sameer Gopalani, Bárbara Goulart, Ayman Grada, Giuseppe Grosso, Harish Gugnani, Yuming Guo, Rahul Gupta, Rajeev Gupta, Tanush Gupta, Juanita Haagsma, Nima Hafezi Nejad, Tekleberhan Beyene Hagos, Dessalegn Haile, Gessessew Bugssa Hailu, Arvin Haj-Mirzaian, Arya Haj-Mirzaian, Randah Hamadeh, Samer Hamidi, Graeme Hankey, Yuantao Hao, Hilda Harb, Habtamu Hareri, Hamidreza Haririan, Josep Maria Haro, Mehedi Hasan, Hadi Hassankhani, Hamid Y. Hassen, Rasmus Havmoeller, Akbar Hedayatizadeh-Omran, Mohamed Hegazy, Behzad Heibati, Behnam Heidari, Delia Hendrie, Andualem Henok, Claudiu Herteliu, Fatemeh Heydarpour, Desalegn Hibstu, Long Hoang Nguyen, Michael Hole, Enayatollah Homaie Rad, Praveen Hoogar, H Dean Hosgood, Mostafa Hosseini, Mihaela Hostiuc, Sorin Hostiuc, Guoqing Hu, John Huang, Trang Huyen Nguyen, Kim Moesgaard Iburg, Ehimario Igumbor, Olayinka Ilesanmi, Usman Iqbal, Asnake Ararsa Irenso, Sheikh Mohammed Shariful Islam, Nader Jahanmehr, Mihajlo Jakovljevic, Moti Jalu , Spencer James, Mehdi Javanbakht, Panniyammakal Jeemon, Ravi Prakash Jha, Vivekanand Jha, Jost B. Jonas, Jacek Jozwiak, Suresh Jungari, Mikk Jürisson, Alemneh Kabeta, Zubair Kabir, Rajendra Kadel, Amaha Kahsay, Rizwan Kalani, Manoochehr Karami, André Karch, Corine Karema, Seyed M. Karimi, Amir Kasaeian, Getachew Mullu Kassa, Tesfaye Kassa, Nicholas J Kassebaum, Anshul Kastor, Vittal Katikireddi , Peter Keiyoro, Andre P Kengne, Andre Keren, Maia Kereselidze, Yousef Khader, Morteza Abdullatif Khafaie, Nauman Khalid, Ejaz Khan, Muhammad Shahzeb Khan, Young-Ho Khang, Tripti Khanna, Mona Khater, Zahra Khazaeipour, Habibolah Khazaie, Abdullah T. Khoja, Ardeshir Khosravi, Mohammad Hossein Khosravi, Getiye Kibret, Daniel Kiirithio, Paul Kilgore, Daniel Kim, Yun Jin Kim, Ruth Kimokoti, Yohannes Kinfu, Sanjay Kinra, Adnan Kisa, Mika Kivimaki, Sonali Kochhar, Tufa Kolola, Jacek Kopec, Margaret Kosek, Parvaiz Koul, Ai Koyanagi, Kewal Krishan, Kristopher Krohn, Barthelemy Kuate Defo, Xie Rachel Kulikoff, G Anil Kumar, Manasi Kumar, Pushpendra Kumar, Fekede Asefa Kumsa, Sheetal Lad, Alessandra Lafranconi, Dharmesh Lal, Ratilal Lalloo, Hilton Lam, Faris Lami, Dennis Laryea, Zohra Lassi, Avula Laxmaiah, Jeffrey Lazarus, Paul Lee, Misgan Legesse Liben, James Leigh, Cheru T Leshargie,

Samson Leta, Miriam Levi, Shanshan Li, Lee-Ling Lim, Miteku Limenih, Shai Linn, Alan Lopez, Stefan Lorkowski, Paulo Lotufo, Raimundas Lunevicius, Crispin Mabika Mabika, Erlyn Rachelle Macarayan, Mark Mackay, Fabiana Madotto, Marek Majdan, Reza Majdzadeh, Azeem Majeed, Reza Malekzadeh, Abdullah Mamun, Srikanth Mangalam, Mohammad Ali Mansournia, Lorenzo Mantovani, Chabila Mapoma, Dadi Marami, Joemer Maravilla, Francisco Rogerlândio Martins-Melo, Melvin Marzan, Tivani Mashamba-Thompson, Felix Masiye, Amanda J. Mason-Jones, Benjamin Massenburg, Manu Mathur, Pallab K Maulik, Mohsen Mazidi, John Mcgrath, Suresh Mehata, Sanjay Mehendale, Man Mohan Mehndiratta, Ravi Mehrotra, Saeed Mehrzadi, Kala M. Mehta, Varshil Mehta, Tefera Chane Mekonnen, Hagazi Gebre Meles, Kidanu Meles, Addisu Melese, Mulugeta Melku, Peter Memiah, Ziad Memish, Walter Mendoza, Melkamu Merid Mengesha, Getnet Mengistu, Zerihun Menlkalew Zenebe , George Mensah, Beyene Meressa, Seid Tiku Mereta, Atte Meretoja, Tuomo Meretoja, Tomislav Mestrovic, Haftay Berhane Mezgebe, Bartosz Miazgowski, Tomasz Miazgowski, Ted R Miller, Molly Miller-Petrie, Parvaneh Mirabi, Andreea Mirica, Erkin Mirrakhimov, Babak Moazen, Moslem Mohammadi, Mohammed Mohammed, Shafiu Mohammed, Ali H. Mokdad, Mulugeta Molla, Mariam Molokhia, Lorenzo Monasta, Ghobad Moradi, Mahmoudreza Moradi, Maziar Moradi-Lakeh, Mehdi Moradinazar, Paula Moraga, Joana Morgado-Da-Costa, Shane Morrison, Marilita Moschos, Achenef Muche, Kindie Fentahun Muchie, Ulrich Mueller, Tasha Murphy, Jonah Musa, Kamarul Imran Musa, Jean Nachega, Gabriele Nagel, Seyed Sina Naghibi Irvani, Aliya Naheed, Azin Nahvijou, Gurudatta Naik, Farid Najafi, Vinay Nangia, Jobert Richie Nansseu, Bruno Nascimento, Haseeb Nawaz, Busisiwe Ncama, Ayenew Negesse, Ionut Negoi, Ruxandra Irina Negoi, Subas Neupane, Charles Newton, Frida Ngalesoni, Josephine Ngunjiri, Grant Nguyen, Muhammad Imran Nisar, Ana Maria Nogales Vasconcelos, Mehdi Noroozi, Hamid Reza Nouri, Malihe Nourollahpour, Dina Nur Anggraini Ningrum, Alypio Nyandwi, Peter Nyasulu, Richard Ofori-Asenso, Okechukwu Ogah, Felix Ogbo, In-Hwan Oh, Anselm Okoro, Olanrewaju Oladimeji, Andrew T. Olagunju, Tinuke Olagunju, Pedro Olivares, Bolajoko Olusanya, Jacob Olusanya, Alberto Ortiz, Aaron Osgood-Zimmerman, Erika Ota, Brenda Otieno, Stanislav S. Otstavnov, Mayowa Owolabi, Abayomi Oyekale, Mahesh P A, Smita Pakhale, Adrian Pana, Basant Kumar Panda, Songhomitra Panda-Jonas, Eun-Kee Park, Hadi Parsian, Shanti Patel, Snehal Patil, George Patton, Deepak Paudel, David Pereira, Norberto Perico, Konrad Pesudovs, Huyen Phuc Do, Julian Pillay, Guilherme Polanczyk, Maarten Postma, Farshad Pourmalek, Akram Pourshams, Hossein Poustchi, Swayam Prakash, Narayan Prasad, Manorama Purwar, Mostafa Qorbani, Reginald Quansah, Amir Radfar, Anwar Rafay, Fakher Rahim, Afarin Rahimi-Movaghar, Vafa Rahimi-Movaghar, Mahfuzar Rahman, Md Shafiur Rahman, Muhammad Aziz Rahman, Sajjad Rahman, Rajesh Kumar Rai, Fatemeh Rajati, Usha Ram, Chhani Lal Ranabhat, Prabhat Ranjan, David Rawaf, Salman Rawaf, Sarah Ray, Christian Razo, Robert Reiner, Cesar Reis, Giuseppe Remuzzi, Andre Renzaho, Serge Resnikoff, Satar Rezaei, Shahab Rezaeian, Mohammad Sadegh Rezai, Seyed Mohammad Riahi, Maria Jesús Ríos Blancos, Kedir Teji Roba, Nicholas Roberts, Leonardo Roever, Luca Ronfani, Gholamreza Roshandel, Ali Rostami, George Ruhago, Yogesh Sabde, Perminder Sachdev, Basema Saddik, Hosein Safari, Yahya Safari, Roya Safari-Faramani, Mahdi Safdarian, Saeid Safiri, Rajesh Sagar, Amirhossein Sahebkar, Mohammad Ali Sahraian, Haniye Sadat Sajadi, Mohamadreza Salahshoor, Raphael Saldanha, Zikria Saleem, Yahya Salimi, Hamideh Salimzadeh, Joshua A Salomon, Sundeep Salvi, Evanson Z Sambala, Abdallah M. Samy, Juan Sanabria, Maria Dolores Sanchez-Niño, Ricardo Santiago, Itamar Santos, Milena Santric Mlicevic, Bruno Sao Jose, Muthupandian Saravanan, Mayank Sardana, Abdur Razzaque Sarker, Rodrigo Sarmiento-Suarez, Satish Saroshe, Benn Sartorius, Shahabeddin Sarvi, Brijesh Sathian, Thirunavukkarasu Sathish, Maheswar Satpathy, Arundhati Sawant, Monika Sawhney, Sonia Saxena, Elke Schaeffner, Ione Schneider, David C Schwebel, Falk Schwendicke, Soraya Seedat,

Sadaf Sepanlou, Edson Serván-Mori, Hosein Shabaninejad, Azadeh Shafieesabet, Amira Shaheen, Masood Ali Shaikh, Marina Shakhnazarova, Mehran Shams-Beyranvand, Mohammadbagher Shamsi, Heidar Sharafi, Kiomars Sharafi, Mehdi Sharif, Mahdi Sharif-Alhoseini, Rajesh Sharma, Aziz Sheikh, Kenji Shibuya, Mika Shigematsu, Rahman Shiri, Ivy Shiue, Sharvari Shukla, Si Si, Soraya Siabani, Tariq Jamal Siddiqi, Inga Dora Sigfusdottir, Rannveig Sigurvinsdottir, Diego Augusto Santos Silva, João Pedro Silva, Dayane Silveira, Narayana Sarma Singam, Jasvinder Singh, Narinder Pal Singh, Dhirendra Narain Sinha, Badr Sobaih, Soheila Sobhani, Moslem Soofi, Ireneous Soyiri, Chandrashekhar T Sreeramareddy, Vladimir Starodubov, Caitlyn Steiner, Mark Stokes, Mark Strong, Muawiyyah Babale Sufiyan, Rizwan Suliankatchi Abdulkader, Gerhard Sulo, Bruno Sunguya, Ipsita Sutradhar, Bryan L. Sykes, Pn Sylaja, Dillon Sylte, Cassandra Szoeke, Karen Tabb, Santosh Tadakamadla, Nikhil Tandon, Segen Tassew, Nuno Taveira, Arash Tehrani-Banihashemi, Zelalem Teklemariam, Mohamad-Hani Temsah, Tewodros Tesfa, Belay Tessema, Mebrahtu Teweldemedhin, Js Thakur, Kavumpurathu Thankappan, Nu Thi Truong, Nihal Thomas, Alan Thomson, Binyam Tilahun, Quyen To, Marcello Tonelli, Roman Topor-Madry, Miguel Tortajada-Girbés, Marcos Roberto Tovani-Palone, Bach Tran, Khanh Bao Tran, Srikanth Tripathy, Thomas Truelsen, Nikolaos Tsilimparis, Lorainne Tudor Car, Kingsley N. Ukwaja, Irfan Ullah, Muhammad Shariq Usman, Olalekan Uthman, Muthiah Vaduganathan, Afsane Vaezi, Pascual Valdez, Elena Varavikova, Santosh Varughese, Tommi Vasankari, Narayanaswamy Venketasubramanian, Santos Villafaina, Francesco S Violante, Vasiliy Vlassov, Stein Emil Vollset, Theo Vos, Kia Vosoughi, Isidora Vujcic, Fasil Wagnew, Yasir Waheed, Walson Walson, Yuan-Pang Wang, Elisabete Weiderpass, Robert Weintraub, Seifu Kebede Weldegiorgis, Andrea Werdecker, Ronny Westerman, Tissa Wijeratne, Andrea Sylvia Winkler, Charles Shey Wiysonge, Charles Wolfe, Grant Wyper, Gelin Xu, Tomohide Yamada, Yuichiro Yano, Mehdi Yaseri, Yasin Jemal Yasin, Pengpeng Ye, Alex Yeshnaeh, Manaye Yihune, Ebrahim M. Yimer, Nega Yimer, Engida Yisma, Miangotar Yode, Zemenu Yohannes, Naohiro Yonemoto, Seok-Jun Yoon, Marcel Yotebieng, Mustafa Younis, Mahmoud Yousefifard, Chuanhua Yu, Zoubida Zaidi, Sojib Bin Zaman, Mohammad Zamani, Zohreh Zare, Taddese Zerfu, and Xiu-Ju Zhao.

Drafting the work or revising is critically for important intellectual content

Tarek Abd Elaziz, Nasrin Abdoli, Isaac Adedeji, Olatunji Adetokunboh, Mohsen Afarideh, Sutapa Agrawal, Mohammadesmaeil Akbari, Tomi Akinyemiju, Fares Alahdab, Reza Alizadeh-Navaei, Nahla Anber, Reza Assadi, Ashish Awasthi, Peter Azzopardi, Hamid Badali, Suzanne Barker-Collo, Bernhard Baune, Masoud Behzadifar, Yihalem Abebe Belay, Adam Berman, Sait Mentes Birlik, Oliver Brady, Alessandra C Goulart, Charlton Callender, Julio Cesar Campuzano, Franz Castro, Devasahayam Jesudas Christopher, Flavia Cicuttini, Cyrus Cooper, Leslie Cornaby, Ahmad Daryani, Rajat Das Gupta, Nicole Davis Weaver, Dragos Davitoiu, Jan-Walter De Neve, Selina Deiparine, Getenet Dessie, Kate Dolan, Andre Duraes, David Edvardsson, Reza Esmaeili, Sadaf Esteghamati, Kara Estep, Mohammad Hosein Farzaei, Nataliya Foigt, Morsaleh Ganji, Aregawi Gebreyesus Belay, Ayele Geleto, Ricard Genova-Maleras, Keyghobad Ghadiri, Maryam Ghasemi-Kasman, Amador Goodridge, Mehedi Hasan, Hadi Hassankhani, Akbar Hedayatizadeh-Omran, Behnam Heidari, Long Hoang Nguyen, Enayatollah Homaie Rad, Thomas Hsiao, Asnake Ararsa Irenso, Sheikh Mohammed Shariful Islam, Ravi Prakash Jha, Manoochehr Karami, Getachew Mullu Kassa, Nicholas J Kassebaum, Grant Kemp, Morteza Abdullatif Khafaie, Alireza Khajavi, Mona Khater, Daniel Kim, Yun Jin Kim, Mika Kivimaki, Dharmesh Lal, Hilton Lam, James Lee, Misgan Legesse Liben, Alan Lopez, Crispin Mabika Mabika, Azeem Majeed, Reza Malekzadeh, Mohammad Ali Mansournia, Joemer Maravilla, Francisco Rogerlândio Martins-Melo, Benjamin Massenburg, Sanjay Mehendale, Varshil Mehta, Kidanu Meles, Melkamu Merid Mengesha, Tuomo Meretoja, Molly Miller-Petrie, Karzan Mohammad, Maryam Mohammadi-Khanaposhtani, Mehdi Moradinazar, Abbas

Mosapour, Christopher Murray, Kamarul Imran Musa, Ghulam Mustafa, Paulami Naik, Nahid Neamati, Molly Nixon, Andrew T. Olagunju, Tinuke Olagunju, Hadi Parsian, David Pereira, Konrad Pesudovs, Julian Pillay, Vafa Rahimi-Movaghar, Muhammad Aziz Rahman, Chhani Lal Ranabhat, Satar Rezaei, Seyed Mohammad Riahi, Hosein Safari, Sare Safi, Saeid Safiri, Mohamadreza Salahshoor, Juan Sanabria, Milena Santric Mlicevic, Bruno Sao Jose, Muthupandian Saravanan, Shahabeddin Sarvi, Maheswar Satpathy, Hosein Shabaninejad, Katya Shackelford, Mehdi Sharif, Mika Shigematsu, Inga Dora Sigfusdottir, Rannveig Sigurvinsdottir, Dhirendra Narain Sinha, Vinay Srinivasan, Vladimir Starodubov, Caitlyn Steiner, Leo Stewart, Michelle Subart, Ipsita Sutradhar, Segen Tassew, Nuno Taveira, Nu Thi Truong, Nihal Thomas, Marcos Roberto Tovani-Palone, Bach Tran, Olalekan Uthman, Elena Varavikova, Walson Walson, Yuan-Pang Wang, Robert Weintraub, Manaye Yihune, and Miangotar Yode.

Extracting, cleaning, or cataloging data; designing or coding figures and tables

Hedayat Abbastabar, Tarek Abd Elaziz, Ibrahim Abdollahpour, Daisy Maria Xavier Abreu, Kareha Agesa, Alireza Ahmadi, Muktar Ahmed, Syed Mohamed Aljunid, Mustafa Geleto Ansha, Seth Christopher Yaw Appiah, Nicholas Arian, Ashish Awasthi, Bayu Begashaw Bekele, Gregory Bertolacci, Muhammad Shahdaat Bin Sayeed, Sait Mentes Birlik, Paul Briant, Charlton Callender, Julio Cesar Campuzano, Alanur Cavlin, Haley Comfort, Leslie Cornaby, Matthew Cunningham, Ahmad Daryani, Nikolaos Dervenis, Mengistu Desalegn Tadese, Girmaye Dinsa, Manisha Dubey, Sarah Duncan, Iqbal Elyazar, Sergey Ermakov, Hamed Fakhim, Kairsetn Fay, Takeshi Fukumoto, John Fuller, Amiran Gamkrelidze, Gbetoho Gankpe, Gregory "Manny" M Garcia, Aregawi Gebreyesus Belay, Ayele Geleto, Keyghobad Ghadiri, Jingwen Guo, Yuming Guo, Mehedi Hasan, Yihua He, Akbar Hedayatizadeh-Omran, Behzad Heibati, Nathaniel Henry, Long Hoang Nguyen, Mehdi Hosseinzadeh, Thomas Hsiao, Trang Huyen Nguyen, Chad Ikeda, Panniyammakal Jeemon, Vivekanand Jha, Manoochehr Karami, André Karch, Tesfaye Kassa, Vittal Katikireddi , Norito Kawakami, Grant Kemp, Andre Keren, Maia Kereselidze, Nauman Khalid, Mohammad Hossein Khosravi, Jun Kim, Young-Eun Kim, Yun Jin Kim, Adnan Kisa, Barthelemy Kuate Defo, Xie Rachel Kulikoff, Michael Kutz, James Lee, Misgan Legesse Liben, Shanshan Li, Shiwei Liu, Reza Malekzadeh, Mohammad Ali Mansournia, Melvin Marzan, Benjamin Massenburg, Mohsen Mazidi, Sanjay Mehendale, Saeed Mehrzadi, Hagazi Gebre Meles, Melkamu Merid Mengesha, Zerihun Menlkalew Zenebe , Ted R Miller, Mulugeta Molla, Lorenzo Monasta, Maziar Moradi-Lakeh, Rintaro Mori, Abbas Mosapour, Ghulam Mustafa, Mohsen Naghavi, Paulami Naik, Mohammad Reza Nowroozi, In-Hwan Oh, Ajay Patle, David Pereira, William Petri, Max Petzold, Huyen Phuc Do, Maxwell Pierce, Hossein Poustchi, Narayan Prasad, Caroline Purcell, Anwar Rafay, Alireza Rafiei, Fatemeh Rajati, Seyed Mohammad Riahi, Nicholas Roberts, Luca Ronfani, Enrico Rubagotti, Hosein Safari, Saeid Safiri, Payman Salamati, Abdallah M. Samy, Muthupandian Saravanan, Shahabeddin Sarvi, Maheswar Satpathy, Monika Sawhney, Kathryn Schelonka, Sadaf Sepanlou, Marina Shakhnazarova, Mehdi Sharif, Peilin Shi, Farhad Shokraneh, Soraya Siabani, Dhirendra Narain Sinha, Luisa Sorio Flor, Vinay Srinivasan, Leo Stewart, Michelle Subart, Patrick Sur, Ipsita Sutradhar, Dillon Sylte, Cassandra Szoeke, Nu Thi Truong, Anna Torre, Miguel Tortajada-Girbés, Bach Tran, Kingsley N. Ukwaja, Irfan Ullah, Gaurang Vaidya, Tommi Vasankari, Inbal Weiss Salz, Mehdi Yaseri, Nega Yimer, Naohiro Yonemoto, Seok-Jun Yoon, Stephanie Zimsen, and Leo Zoeckler.

Managing the overall research enterprise

Ashkan Afshin, Peter Allebeck, Elizabeth Cromwell, Lalit Dandona, Rakhi Dandona, Louisa Degenhardt, Samath Dharmaratne, Daniel Dicker, Charbel EL Bcheraoui, Kara Estep, Tamer Farag, Valery Feigin, Kyle Foreman, Nancy Fullman, Emmanuela Gakidou, Tsegaye Gebrehiwot, Simon Hay, Spencer James, Nicholas Kassebaum, Ibrahim Khalil, Kris Krohn, Xiaofeng Liang, Stephen Lim, Alan Lopez, Rafael Lozano,

Felix Masiye, George Mensah, Molly Miller-Petrie, Awoke Misganaw, Ali Mokdad, Kate Muller, Christopher Murray, Mohsen Naghavi, Molly Nixon, David Pigott, Robert Reiner, Joseph Salama, Joshua A Salomon, Benn Sartorius, Katya Shackelford, Caitlyn Steiner, Roman Topor-Madry, Stein Emil Vollset, Theo Vos, Andrea Werdecker, and Harvey Whiteford.

Did not provide contribution information

Dash A P, Berrak Bora Basara, Jacqueline Castillo Rivas, Leslie Cooper, Tamirat Dasa, Maryam S. Farvid, Mohammad Rasoul Ghadami, Vladimir Hachinski, Alexis Handal, Oluwaseyi Isehunwa, John Ji, Behzad Karami Matin, Ali Kazemi Karyani, Arman Latifi, Yirga Legesse, Ana Laura Manda, Wagner Marcenes, Gk Mini, Noushin Mohammadifard, Satinath Mukhopadhyay, GVS Murthy, Abhijit Pakhare, Achyut Raj Pandey, Emmanuel Peprah, Sahar Saeedi Moghaddam, Nizal Sarrafzadegan, Araad Shakir, Reza Shirkoohi, Karen Sliwa, Joan B Soriano, Amanuel T. Tsegay, Selen Uzun, Yohanes Ayele Wondimkun, Gokalp Yentur, and Paul Yip.

Section 1. GBD Overview

Section 1.1. Geographic Units of the Analysis

The locations included in GBD 2017 have been arranged into a set of hierarchical categories composed of seven super-regions and a further nested set of 21 regions containing 195 countries and territories (Appendix Table 1). Subnational estimation in GBD 2017 includes Brazil, China, India, Indonesia, Japan, Kenya, Mexico, South Africa, Sweden, the United Kingdom, and the United States, and new subnational assessments at the administrative one level for Ethiopia, Iran, Norway, and Russia and by Maori ethnicity for New Zealand. Subnational 2017 GBD data will be released at a future time.

Section 1.2. Time Periods of the Analysis

A complete set of population and fertility numbers and rates were computed explicitly for the following years: 1950-2017.

Data and underlying code used for this analysis are available at http://ghdx.healthdata.org/gbd-2017. Results for all GBD metrics are available at http://www.healthdata.org/results/data-visualizations.

Section 1.3. Statement of GATHER Compliance

This study complies with the Guidelines for Accurate and Transparent Health Estimates Reporting (GATHER) recommendations. We have documented the steps involved in our analytical procedures and detailed the data sources. See Appendix Table 2 for GATHER checklist.

Section 1.4. List of Abbreviations

ASFR: age-specific fertility rate
CBH: complete birth history
CCMPP: cohort component method of population projection
CEB: children ever born
DALY: disability-adjusted life-year
DHS: Demographic and Health Surveys
DYB: Demographic Yearbook
EDU15+: mean education for those aged 15 and older
GATHER: Guidelines for Accurate and Transparent Health Estimates Reporting
GBD: Global Burden of Disease
GHDx: Global Health Data Exchange
GLMM: Gulf Labour Markets, Migration and Population
GPR: Gaussian process regression
HDI: Human Development Index
HFC: Human Fertility Collection

HMD: Human Mortality Database
IPUMS: Integrated Public Use Microdata Series
LDI: lag-distributed income per capita
LSMS: Living Standards and Measurement Surveys
MICS: Multiple Indicator Cluster Surveys
MPIDR: Max Planck Institute for Demographic Research
PES: post-enumeration survey
RHS: Reproductive Health Surveys
SBH: summary birth history
SDI: Socio-demographic Index
SRS: Sample Registration System
ST-GPR: Spatiotemporal Gaussian process regression
TFR: total fertility rate
TFO30: total fertility over age 30
TFU25: total fertility up to age 25
TMB: template model builder
UN: United Nations
UNHCR: United National High Commissioner for Refugees
UNPOP: United Nations Population Division
UNSTAT: United Nations Statistical Commission
VR: vital registration
WFS: World Fertility Surveys
WPP: World Population Prospects
YLD: years lived with disability
YLL: years of life lost

Section 1.5. GBD results overview

Results from the Global Burden of Disease Study (GBD) are now measured in terabytes. Results are available in an interactive data downloading tool on the Global Health Data exchange (GHDx). In the GBD 2017 version, the GHDx tool also contains measures such as prevalence and incidence as well as rate of change data. Data above a certain size cannot be viewed online but can be downloaded. Depending on the size of the download, users may need to enter an email address; a download location will be sent to them when the files are prepared.

The current version of the data download tool is available in the GHDx and contains core summary results for GBD 2017: http://ghdx.healthdata.org/gbd-results-tool. The core summary results include deaths, years of life lost (YLLs), years lived with disability (YLDs), and disability-adjusted life-years (DALYs). The GHDx includes data for causes, risks, cause-risk attribution, aetiologies, and impairments.

Section 1.6. Data input sources overview

GBD 2017 incorporated a large number and wide variety of input sources to estimate mortality, population, fertility, causes of death and illness, and risk factors for 195 countries and territories from 1990 to 2017. These input sources are accessible through an interactive citation tool available in the GHDx. Users can retrieve citations for a specific GBD component, cause or risk, and geography by choosing from the available selection boxes. They can then view and access GHDx records for input sources and export a CSV file that includes the GHDx metadata, citations, and information about where the data were used in GBD. Additional metadata for each input source are available through the citation tool, as required by the GATHER statement.

The citation tool is accessible through the GHDx at http://ghdx.healthdata.org/gbd-2017

Section 1.7. Funding Sources

Research reported in this publication was supported by the Bill & Melinda Gates Foundation, the National Institute on Aging of the National Institutes of Health (award P30AG047845), and the National Institute of Mental Health of the National Institutes of Health (award R01MH110163). The content is solely the responsibility of the authors and does not necessarily represent the official views of the Bill & Melinda Gates Foundation or the National Institutes of Health.

Section 2. Population and Fertility Estimation Process

Section 2.1. Overview

Previous GBD cycles have produced total fertility rate estimates accompanied by age-specific, single-year mortality estimates.[1,2] Previous GBD analyses incorporated population estimates produced by the United Nations Population Division[3] and the Human Mortality Database (HMD);[4] however, these population estimates were not consistent with GBD mortality estimates. This discrepancy was especially pronounced in older age groups and in countries without high-quality vital and civil registration systems. The goal of the GBD 2017 population and fertility estimation process is to produce internally consistent fertility, mortality, and population estimates.

If all countries had complete and accurate civil registration systems, estimating age-specific population and fertility would be a straightforward task. However, countries at lower sociodemographic levels often have incomplete information systems, and multiple sources must be used to create estimates. The analytical process to produce these estimates is outlined in Appendix Figure 1. Further details are provided below for each step in the population and fertility estimation process. Details on the mortality estimation process are provided in a separate manuscript.[5]

Section 2.2. Fertility

Overview

For iterations of GBD through 2015 we utilised UN WPP fertility estimates for all available national locations.[1] For GBD 2016, we estimated total fertility rate (TFR) based on a systematic synthesis of all available data for all GBD 2016 locations and used the age-specific fertility pattern from WPP.[2] For the GBD 2017 cycle, we expanded our previous work by estimating age-specific fertility rates for ages 10 to 54 years of age based on a systematic synthesis of all available data for each GBD location. TFR was computed as a function of the age-specific fertility rates.

Section 2.2.1 Data Sources

We used three primary source types for the fertility analysis: (1) the number of live births by age of mother reported through vital registration (VR) systems; (2) complete birth histories (CBH); and (3) summary birth histories (SBH). In total, we compiled 9548 unique country-source-years of data for women aged 10 to 54 for the 1950–2017 period. Including additional data generated from post-processing of CBH and SBH, this number grows to 15,309 unique country-years. Numbers of sources by location and year are provided in Appendix Table 3 and Appendix Table 4, respectively. Below we present a brief description of each of these source types and the results of data synthesis.

Fertility Data Source Types

Accurate and complete registration data of live births by age of the mother are typically regarded as the gold standard source of information on fertility; in theory, these regular (usually annual) reports should capture all births in a given country or subnational unit within a given year. High-income countries tended to have high-quality VR systems that contained the date and location of the birth, as well as detailed demographic characteristics of the mother and the date and location of birth. In lower-income countries, however, birth registration systems tended to suffer from interrupted and/or delayed reporting and incomplete coverage. Birth registries provided almost all of the fertility information pertaining to women aged 10-14 and 50-54, as the overwhelming majority of household surveys only collected birth histories from women aged 15-49 at the time of survey.

In cases where the completeness and quality of birth registration data were poor, we relied heavily on other types of data sources (namely household surveys and censuses) to triangulate the level and age-pattern of fertility. Fertility information in household surveys and censuses was predominantly in two forms – complete birth histories (CBH) and summary birth histories (SBH). CBHs, which grew to prominence with the World Fertility Surveys (WFS) administered in the 1970s and 1980s, collected information about a surveyed mother's date of birth, as well as the dates of birth and death of all children she can recall bearing in her lifetime. Since each birth could be linked to the time of birth and the mother's concurrent age, this permitted the calculation of period and age-specific fertility rates in the years prior to the survey under assumptions of no survivor, migrant, or recall bias. Many major survey programs contain CBH modules, including the Demographic and Health Surveys (DHS), Multiple Indicator Cluster Surveys (MICS), and the Reproductive Health Surveys (RHS).

SBHs, on the other hand, collected no information about the dates of birth of children, but instead only recorded the total number of children ever born (CEB) to a woman over her lifetime. Also collected is the mother's date of birth or age at the time of interview. These data provided valuable information about the overall level of fertility experienced by cohorts over time but could not be used by themselves to estimate period and age-specific fertility. Using assumptions similar to CBH (no survivor or migrant bias), cohort age patterns of fertility derived from other data sources could be used to split CEB information into period age-specific fertility rates. Availability of SBHs far surpassed that of CBHs, largely due to their relative simplicity but also their precedence. CEB questions have been featured in censuses since the turn of the 20th century and thus comprise a large share of information about fertility in low-income settings prior to the mid 1970s. In addition to censuses, a number of other survey families featured SBH, including a subset of MICS, the Living Standards and Measurement Surveys (LSMS), and a variety of country-specific surveys.

Fertility Data Identification and Synthesis

Registry data were identified through the UN Demographic Yearbook (DYB; UN Statistical Division [UNSTAT]),[6] the Human Fertility Collection (HFC); Max Planck Institute for Demographic Research (MPIDR),[7] the WHO mortality database, official publications, online data portals of national statistical offices, and international collaborators. The DYB and HFC compile registry-based fertility data as reported by national statistical offices and country research institutes. DYB reports of live births by age of mother were extracted for every year available from 1948, and the complete set of age-specific HFC data were downloaded in October 2017. Estimates provided to HFC by individual researchers were excluded from our analyses (ie, we incorporated only empirical data, per MPIDR's source categorisations); country-year-ages already covered by the DYB were also excluded. We also extracted data from sample registration systems (SRS) typical of South Asian countries, including India, Pakistan, and Bangladesh. In total, we compiled 7,817 unique country-source-years of VR data, with 2,421 of them coming prior to 1970 and 1,755 of them coming after 2000. We included 31 unique country-source-years of data from SRS, the majority of which cover India.

Fertility data from household surveys and censuses were initially identified using the Global Health Data Exchange (GHDx). Records classified as "survey" or "census" and that contained any of the keywords "complete birth history," "summary birth history," or "fertility" were then compiled and reviewed by research team members to verify that they contained sufficient detail for inclusion in GBD analysis. Additional seeking was conducted for identified gaps in data, primarily through country statistical office websites as well as major survey families such as DHS, MICS, WFS, and RHS. In cases where sufficiently detailed data were not publicly available, in-country collaborators assisted in its procurement. Fertility data from the 1950s and 1960s in low-income settings (particularly sub-Saharan Africa) were specifically sought in colonial censuses containing SBH information. Where sources provided microdata, we standardised and processed CBHs to compute period age-specific fertility rates (ASFR) every three years over a 15-year recall and collapsed SBHs to tabulations of average children ever born by mother's age to be later split by cohort age patterns from the first modelling stage (see Section 2.2.2 for further details). Where microdata were unavailable, we extracted period ASFRs or average CEB by mother's age as documented in reports or other publications. In total, we extracted and processed 429 CBHs and 977

SBHs, out of 4,259 identified surveys and censuses. Occasionally, the recall type of a survey for which tabulated period ASFRs were available was unable to be identified from the report or available documentation. These accounted for only 81 country-source-years. Detailed information about the nature and quantity of identified sources in this analysis can be found in Appendix Tables 3 and 4.

Section 2.2.2 Modelling strategy

Age-specific fertility rate estimation

Using all the data described above, we estimated age-specific fertility rates by five-year age groups from ages 10 to 54 years in two broad steps. First, we estimated age-specific fertility rates for 15 to 49 years of age using spatiotemporal Gaussian process regression (ST-GPR). Next, we estimated fertility rates for 10- to 14-year-olds as a function of estimated fertility in 15- to 19-year-olds and fertility rates for 50- to 54-year-olds as a function of estimated fertility in 45- to 49-year-olds. The sections below provide further estimation process details. Summary metrics of fertility, including TFR, total fertility up to age 25 (TFU25) and total fertility over age 30 (TFO30) were computed as a function of the relevant age-specific fertility rates.

Age-specific fertility rate estimation for 15 to 49 years

ASFR for age groups 15-19, 20-24, 25-29, 30-34, 35-39, 40-44, and 45-49 were estimated using ST-GPR, which has been covered in detail elsewhere.[1,2] The estimation of ASFR involved the following sequential steps: (1) Estimation of ASFR 20-24 using age-specific data from CBH and VR and using mean years of education in 20- to 24-year-olds as a predictor; (2) Estimation of ASFR for the remaining age groups using age-specific data from CBH and VR and using age-specific mean years of education and estimated ASFR 20-24; (3) Split SBH data by age and period using the estimated location, time and age-specific estimates of ASFR; (iv) Re-estimate ASFR 20-24 using CBH, VR, and the period-age-split SBH data; and (v) Re-estimate ASFR using CBH, VR, and the period-age-split SBH data.

The ST-GPR models for ASFR were implemented as follows. The first stage mixed effect regression was fit in bounded logit space:

$$Logit\left(\frac{ASFR\ data - lower\ bound_{age}}{upper\ bound_{age} - lower\ bound_{age}}\right)$$

The lower bound was the minimum fertility by age across time and geography, and the upper bound was the 99.3 percentile of fertility by age across time and geography, after dropping implausibly high ASFRs above 0.5. This upper bound on ASFR data produced an implied maximum TFR of 10.5.

The specifications of the mixed effects regression are below.

$$logit_{bound}(ASFR_{20-24}) = \beta_1 + \beta_2 * female\ education_{c.y} + \gamma_{locsource}$$

$$logit_{bound}(ASFR_{n-n+5}) = \beta_1 + \beta_2 * female\ education_{c,y} + spline(ASFR_{20-24,c,y}) + \gamma_{locsource}$$

Where n is between 25 and 45, β_1 is the intercept, β_2 is the coefficient on female education, female education and the ASFR 20-24 estimates are specific by country and year, and $\gamma_{locsource}$ is a location-source random intercept.

In the age groups other than 20-24, female education was not used as a covariate in high-income locations. Separate models were fit for high-income, sub-Saharan Africa, Central Europe, Eastern Europe, and Central Asia to account for the differences in the relationships between ASFR 20-24 and the other age groups. The knots in the linear spline (in logit space) chosen by age group and super-region are shown in Table A.

Table A: Knots on ASFR 20-24

Region	Age	Knot
Central Europe, Eastern Europe, and Central Asia	15	NA
Central Europe, Eastern Europe, and Central Asia	25	-1.5
Central Europe, Eastern Europe, and Central Asia	30	-2
Central Europe, Eastern Europe, and Central Asia	35	-1.75
Central Europe, Eastern Europe, and Central Asia	40	-1.75
Central Europe, Eastern Europe, and Central Asia	45	-2
High-income	15	NA
High-income	25	NA
High-income	30	-2.25
High-income	35	-2
High-income	40	-2.25
High-income	45	-2.25
Others	15	NA
Others	25	-1.5
Others	30	-1.3
Others	35	-1.3
Others	40	-2
Others	45	-2.5
Sub-Saharan Africa	15	NA
Sub-Saharan Africa	25	-1.75
Sub-Saharan Africa	30	-1.25
Sub-Saharan Africa	35	-1.3
Sub-Saharan Africa	40	-1.5
Sub-Saharan Africa	45	-1.75

We outliered the following categories of data: (1) implausibly high ASFR, defined as an ASFR over 0.5; (2) 0 values due to sampling error, especially in age group 45-49; (3) data with known undercounting of

births, when no other sources could be used to adjust data; and (4) an implausible level or trend as compared to more robust complete VR or CBH sources.

Data Source Adjustment

After the mixed-effects model was computed, the random intercept on the concatenation of location and source was used to adjust data to a reference or standard source. The adjustment factor was the sum of the difference between the reference source fixed and random effects, and the fixed and random effects on the data point for the specific source, as below, and was then added to the data to derive an adjusted value.

$$Adjustment\ Factor = (Location\ Source\ RE_{ref} - Location\ Source\ RE_{data\ point})$$

Where RE represents a random intercept of either a reference source or a data-point specific location-source. When more than one reference source was selected in a single location, the values of the location source random effects for each reference source were averaged to produce the first term of the equation.

Reference sources were initially chosen as: (1) complete VR for locations with complete VR, (2) an average of complete birth history sources for locations with one or more complete birth history, (3) and agnostically (as an average of all the sources for each location) for locations with neither complete VR nor complete birth histories. VR was designated as complete for a country if the median of child death registration completeness for the location over all available years was over 95%.[5] For some locations, reference sources were chosen based on expert judgement. For example, data from past censuses (1950s and 1960s) in sub-Saharan Africa were often chosen as reference sources to accurately capture the depressed fertility during that time period.

Hyper-parameter Selection

The residual smoothing and GPR stages of ST-GPR were implemented using the output of the mixed effects regression and data source adjustments. Hyper-parameters for the residual smoothing and GPR stages were chosen based on a location- and age-specific data density score. For locations with VR, the VR component of the score was calculated as the sum of the years for which VR data were available, and then down-weighted if the number of births in the age group was less than 100. Incomplete VR was down-weighted by 0.5. For non-VR sources, the number of sources was counted instead of the number of years. For example, one DHS survey would count as one source, even if it contributed more than one data point.

The data density score was calculated as follows:

$$\begin{aligned} DD\ Score_{loc,age} &= Complete\ VR\ years_{loc,age} + (2 * Number\ CBH\ Sources_{loc,age}) \\ &+ (0.25 * Number\ SBH\ sources_{loc,age}) + (0.5 * Incomplete\ VR\ years_{loc,age}) \\ &+ Number\ Other\ Sources_{loc,age} \end{aligned}$$

Where DD stands for data density, CBH is complete birth history, SBH is summary birth history, and all elements of the equation incorporate year and age.

ST-GPR hyper-parameters λ, ξ, and scale were designated by categories of data density, as shown in Table B below.

Table B: Hyper-parameter Values by Data Density

Data density	Lambda	Zeta	Scale
Over 50	0.2	0.99	5
Between 30 and 50	0.4	0.9	10
Between 20 and 30	0.6	0.8	15
Between 10 and 20	0.8	0.7	15
Under 10	1	0.6	15

For non-complete VR sources, data variance was calculated as the variance between the spatiotemporal prediction and the unadjusted data. For location-ages with fewer than five data points, the maximum data variance in the associated GBD region was used. For complete VR sources, we assumed that non-sampling variance was 0 and calculated sampling variance using the binomial equation shown below:

$$Sampling\ Variance = \frac{ASFR * (1 - ASFR)}{Births}$$

To calculate amplitude, we computed the mean of the location-specific standard deviation of the difference between the first-stage mixed-effect regression and the second-stage spatiotemporal smoothing, restricted to national locations with a data density score of over 50 in the years between 1990 to 2017. This amplitude was applied to all locations.

SBH Methods

SBHs that collected CEB data were more frequently available at early time points than CBHs. For example, questions about CEB were often included in early colonial censuses in Africa. Multiple techniques exist to compute period- and age-specific fertility from SBH information, the most widely used of which is the Brass Parity/Fertility ratio method. This method assumes, however, that age-specific fertility remains constant over time. To relax this assumption, we used the estimates of age-specific fertility based on all available CBH and registry data from the first full run-through of ST-GPR described above – which provided a dynamic measure of cohort age patterns over time – to split SBH into period – ASFR. From the SBH-naïve estimates, we first calculated implied annualised fertility for all five-year birth cohorts represented in a given SBH from age 10 up until either age 54 or the year of survey, whichever came sooner. Because ST-GPR only produces annual estimates for five-year age groups, in doing so we needed to account for fertility experienced in years where some proportion of them had graduated into the next five-year age group. To do this, we computed the weighted average of the estimated ASFRs in the lower and upper bounding age groups as the fertility experienced by that hypothetical cohort in that year, assuming a uniform age distribution within the group. For example, for a cohort of women aged 15 to 19

in 1971 with ASFR *F*, we compute the ASFR experienced by this cohort as women aged 16-20 in 1972 as $.8 *_5 F_{15}^{1971} + .2 *_5 F_{20}^{1976}$, since 20% of this cohort has now aged into the 20-24 group.

From our implied annualised cohort ASFR, we then calculated cumulative cohort fertility up to age of each cohort at the time of survey. On a cohort-by-cohort basis this measure of implied cumulative fertility was compared to the observed cumulative fertility (average CEB from SBH) to derive a scaling factor. We applied this scaling factor to the original implied cohort age pattern to distribute CEB back across time and age. Our method of utilising SBH only covered birth cohorts between 1940 (who began to experience AFSR 10-14 in 1950 at the beginning of our estimation period) and 2007 (who began to experience ASFR 10-14 in 2017 at the end of our estimation period).

Splitting of total birth and historic location aggregate data

Analogous to the cumulative cohort fertility provided by SBH, a large amount of data were only available as age aggregates (eg, total live births, rather than by mother's age) and/or location aggregates (eg, former USSR prior to its dissolution). In these situations, we split these data using the age and location proportions informed by the first ST-GPR run-through using only CBH and registry data. Once these data were split, we reran the entire estimation process described above, incorporating all CBH, registry data, period and age-split SBH data, and location and age-split miscellany. This approach provided additional information about aggregate levels of fertility over time and represented a large increase in the availability of past data.

Age-specific fertility rate estimation for 10- to 14-year-olds and 50- to 54-year-olds

ASFR for 10-14 and 50- to 54-year-olds was estimated separately, given the paucity of the data for those age groups in any locations without a vital registration system. In both models, we leverage the relationship between ASFR in one age group and the neighbouring age group. In age 10-14, we ran a mixed effects regression on the log of the ratio of ASFR 10-15 over ASFR 15-19, and used ASFR 15-19 as a predictor along with nested random intercepts by super-region, region, and location, as follows:

$$\log\left(\frac{ASFR\ 10-14}{ASFR\ 15-19}\right) = \beta_1 + \beta_2 \log(\mathrm{ASFR}\ 15-19) + \gamma_{\mathrm{sr}} + \gamma_r + \gamma_{\mathrm{loc}}$$

Where β_1 is the intercept and γ_{sr}, γ_r, and γ_{loc} are nested super-region, region, and location random intercepts.

For ASFR 50-54, we did not observe a clear relationship between the log ratio of ASFR 50-54 over ASFR 45-49 and ASFR 45-49. Instead, we estimated a regression on this ratio with a constant. We computed uncertainty by generating 1,000 draws from the variance-covariance matrix from the regression.

Fertility metrics

TFR was calculated as the time-weighted sum of the ASFRs; in our case, this was the sum of ASFR 10-14, 15-19, 20-24, 25-29, 30-34, 35-39, 40-44, 45-49, and 50-54 multiplied by the 5 years spent in each age

bin. TFU25 and TFO30 were calculated equivalently, with TFU25 being the sum of ASFR 10-14, 15-19, and 20-24 multiplied by the 5 years in each age bin, and TFO30 being the sum of ASFR 30-34, 35-39, 40-44, 45-49, and 50-54 multiplied by the 5 years in each age bin. Live births were calculated as the sum of ASFR multiplied by the age specific female population from the population model, described below in Section 2.3.

Estimation of education as a covariate

Estimates of average years of education were based on a compilation of 2,522 censuses and household surveys. These data and the methods hereafter build on an approach used to produce a previously published dataset of international educational attainment.[8] Each data source included information on the distribution of educational attainment by country, year, sex, and five- or ten-year age group. Where years of schooling data were available only for multi-year bins, eg, the fraction of the population with between six and nine years of completed education, we utilised a database of 1,792 sources reporting single years of completed schooling to split these binned data into single-year distributions from 0 to 18 years based on the average of the 12 closest distributions in terms of geographic proximity and year. From each of the subsequent data sources, we calculated the mean years of schooling by age and sex.

In the next step, age-cohort imputation was used to project observed cohorts through time, exploiting the relative constancy of education levels after age 25. For any data point representing a cohort aged 25 or older, we extrapolated the data forward and backward so that it was represented in all year-age combinations for that cohort. For example, a data point reflecting a cohort aged 35-39 in 2000 was projected forward for 40- to 44-year-olds in 2005, 45- to 49-year-olds in 2010, and so on. It was also projected backward for 30- to 34-year-olds in 1995 and 25- to 29-year-olds in 1990. Post-imputation, age-period models were fit on all original input data, as well as the imputed cohort data, in order to estimate a complete single-year series of educational attainment from 1950 through 2016 by age, sex, and location. Separately for each sex and GBD region, the mean level of educational attainment of the country-age-year-specific population, $Edu_{c,a,s,t}$, was estimated as:

$$logit\left(\frac{Edu_{c,a,s,t}}{Edu_{max_a}}\right) = \beta_{s,r}Year + \delta_{s,r}\,Age + I_{s,r} + \propto_{c,s},$$

where:

Edu_{max_a} is the maximum mean educational attainment for each age group, defined as 3 for ages 5-9, 8 for ages 10-14, 13 for ages 15-19, and 18 for all age groups 20-24 and up;

$\beta_{s,r}$is a sex- and region-specific intercept;

$\delta_{s,r}$ captures the linear secular trend for each sex and region;

$I_{s,r}$is a natural spline on age to capture the non-linear age pattern by sex and region, with knots at 15 and 25 years of age; and

$\propto_{c,s}$ is a country-sex-specific random intercept.

Finally, Gaussian process regression (GPR) was used to smooth the residuals from the age-period model, accounting for uncertainty in each data point. GPR also synthesises both data and model uncertainty to estimate uncertainty intervals.

Section 2.2.3 Sex Ratio at Birth

Overview

Another key component of population dynamics is the sex ratio at birth (SRB), as it has major implications for both future population structure and overall reproductive capacity. As such, SRB is a critical component for CCMPP and its variants. For the GBD analysis, we defined SRB as the ratio of total male to total female live births in a given calendar year. Some other publications such as the India SRS report the reciprocal.

The natural equilibrium for SRB generally hovers around 1.05 males per female, with some country-specific deviations.[9] Prior to the advent of ultrasound technologies and feasibility of sex-selective abortion, SRB remained stable over time. Since this period, however, remarkable shifts in SRB have manifested through systematic sex preferences for children, especially in the Caucasus, South Asia, and East Asia. To capture both historic equilibria and recent shifts, we developed a model to estimate SRB in all national GBD locations, Hong Kong, and Macau from 1950 to 2017.

Data Sources

As with estimation of fertility, registered live births by sex are the gold standard source for deriving SRB; however, their quality in some settings from may suffer from sex-differentials in registration or reporting. We extracted total live births by sex from all UN DYB reports back to 1950 and the most recent update to the WHO mortality database. Where VR data are poor or nonexistent, precedent exists to use other source types of SRB, including surveys, censuses, and population registries.[10] To supplement registered births, we extracted sex ratios from all complete birth histories identified for the fertility analysis using a 25-year recall and averaging over five-year windows. We also extracted under-1 counts by sex from censuses and population registries, and where the former were unavailable, under-5 census counts by sex from censuses and population registries. For India and China, respectively, where there have been documented periods of male preference but poor registry data, we utilised information available from the SRS and 1% Population Sample Survey. In total, we extracted 4690 unique location years of registered births by sex, 1457 of under-1 counts by sex and 299 of under-5 counts by sex from censuses and population registries, and 2490 unique location-years from 446 complete birth histories.

Modelling Approach

We estimated time trends in SRB using the GBD ST-GPR framework. Data variance for all source types was calculated using a binomial distributional assumption. We then specified a linear prior for the mean as follows:

$$logit(m_{ly}) = \alpha_r + \epsilon_{ly}$$

$$\alpha_r \sim N(\gamma_{sr}, \sigma_\alpha^2)$$

$$\gamma_{sr} \sim N(0, \sigma_\gamma^2)$$

Where m_{ly} is the proportion of live births that are male in location *l* and year *y* and γ_{sr} and α_r are nested random intercepts by GBD super-region and region. We chose to model the proportion of male live births in lieu of the mathematically equivalent SRB to take advantage of the stability of estimation in transformed logit space. Additionally, we used a flat prior, as we would not expect SRB to change over time except in clear cases of sex-preference. We refined this prior with spatiotemporal smoothing and subsequently passed it to the GPR to generate final estimates of mean SRB. Hyperparameters for spatiotemporal smoothing and GPR for a location *l* were chosen on the basis of a data density score derived as follows:

$$dd_l = .5 * sourceYears_{l,CBH} + sourceYears_{l,CENSUS} + sourceYears_{l,VR}$$

We down-weighted CBH relative to other source types due to the comparative magnitude of noise in the data apparent as a result of smaller sample sizes. Score thresholds for hyperparameter selection were chosen from their empirical distribution. Expert judgement was used to override automated hyperparameter selection, mostly in countries with small populations and medium data density. In these cases, we took a more conservative estimation approach since small denominators introduced additional volatility in the data.

Table C: Hyper-parameter Values by Data Density for Sex Ratio at Birth

Density Category (dd score range)	ζ	λ	GPR Scale
Low (0 -10)	.05	.04	20
Medium (10.5 – 30)	.025	.15	10
High (30+)	.01	.25	10

Due to the scope of additional data seeking and extraction, we did not extend this analysis to subnational units for GBD 2017; instead, we assume subnational SRB to equal that of the national. For future cycles, estimation will cover all GBD locations.

Section 2.3 Population

Overview

The demographic balancing equation defines how a population (N) changes from one time point to another.

$$N(T) = N(0) + B[0,T] - D[0,T] + I[0,T] - O[0,T]$$

People can only enter a population through birth (B) or immigration (I) and exit through death (D) or emigration (O). For this analysis we collapse immigration and emigration into net migration (G) where a positive value indicates net immigration and a negative value indicates net emigration.

$$N(T) = N(0) + B[0,T] - D[0,T] + G[0,T]$$

In order to estimate population size over a period of time we need to know the initial size of the population and how many people are entering and leaving the population through births, deaths, and migration. In short, the Bayesian population model used in this study reconciles GBD estimates of fertility and mortality with data on population size obtained through population censuses and registries. We will first describe each of the data sources used as inputs to the model and then describe the actual modelling process used to estimate migration and population. The calculated GBD world population standard is show in Appendix Table 6.

Section 2.3.1 Data sources and processing

Census and registry lists

Population censuses, conducted in most countries usually every ten years, are the primary source of data on population size by age and sex. In some countries continuous population registries are also maintained. We synthesised population census data by first compiling a list of censuses as documented by UNSTAT,[11] UNPOP,[3] UN DYB,[6] IPUMS,[12] and the Population Research Center at The University of Texas at Austin.[13] This compiled list is shown in appendix table 3; we also show all censuses and registries found for each country in Appendix Figure 2. A list of all confirmed censuses is included in Appendix Table 5.

Population data extraction

We extracted age-and-sex-specific population census and registry counts from the UN DYB,[6] IPUMS,[12] national statistic websites, and by searching the WorldCat catalog.[14] In several cases, multiple sources of population counts were available for a given census or registry-year. Preference was given to registry over census data, data for which population counts were reported de facto (people were counted based on place of enumeration) rather than de jure (people were counted based on place of usual residence), and data reporting population counts by more detailed age-and-sex groups. In some instances, detailed population count data by age and sex were only provided for a random sample of the total census population (ranging from 1% to 20% of the full census). In these cases, we assumed that the age and sex structure found in the sub-sample was the same as that for the total census, and the sub-sample counts were scaled up using the total census population counts.

In total, we extracted data for 1240 censuses out of 1249 censuses known to have occurred. The nine un-extracted censuses included those that were never released by the coordinating organization. Of the censuses extracted, 635 were de facto, 547 were de jure, and for 58 we were unable to determine whether the data were de facto or de jure. In total we extracted 756 location-years of registry data for 26 locations.

We also noted any published limitations of the census, including both limitations provided by the country's statistical division upon publication of the results and limitations identified by media and independent experts. In some excluded censuses, countries may have artificially inflated population counts or undercounted minorities for political reasons. Other censuses were excluded because they failed to enumerate the non-white population (ie, Rwanda 1953, Burundi 1952 and 1958). We excluded 75 censuses that were representative of only a population subset or utilised questionable methods; 20 censuses were excluded because they were inconsistent with adjacent data.

Population data processing

Population data, as initially extracted, could be inaccurate due to age misreporting, representativeness of the de facto population, and under/overenumeration. In identifying census data, we preferentially utilised raw census and registry population counts and applied a standardised set of methods to correct for these issues.

The first step was to distribute counts of individuals for whom age and/or sex were unknown. We used the age-sex structure of the remainder of the data from that particular census to distribute the counts of this group of individuals.

The second step was to adjust for age heaping that occurred when individuals reported their age as a round number (most often ending in 0 or 5) rather than their exact age. Population totals were first aggregated to the largest age interval length originally present in the data so that each population count was in standard-sized age groups and the 26 censuses that had unusually wide age groups were collapsed to total population by sex only. For each census we calculated the sex ratio score, age ratio score for males and females, and the joint score (JS); these measures are explained in detail in the US Census Bureau Population Analysis System documentation.[15]

Many age heaping corrections methods have been developed, and we use a combination of three for this analysis. The Feeney correction is applicable to census counts in single-year age groups and redistributes people reporting their age as a multiple of five to the eight adjacent single-year age groups so that the corrected counts in the adjacent age groups are proportional to the original counts and so that the corrected count and the eight adjacent age groups form a linear progression; the corrected counts are then reported in five-year age groups. The Arriaga correction takes as input census counts in five-year age groups and smooths by combining the three nearest ten-year age groups with a second-degree polynomial, the advantage of this technique over similar methods is that it also smooths the youngest and oldest age groups. The Arriaga strong method addresses more severe age misreporting by averaging three consecutive 10-year age groups. These three methods were used in countries outside of high-income and Central Europe, Eastern Europe, and Central Asia according to the following rules based on the age group length of the population counts and the JS.

Table D: Age heaping corrections

Age group length	Joint score (JS)	Age heaping correction
1	JS <= 20	None

1	JS > 20	Feeney
5	JS <= 20	None
5	20 < JS >= 40	Arriaga
5	JS > 40	Arriaga strong
10	JS <= 20	None
10	JS > 20	Arriaga strong
Total population	Not available	None

33 censuses were identified via visual inspection as being incorrectly scored or poor-quality and we did not correct the census data. This was predominantly because of sex ratio scores that were higher than normal but thought to be a real phenomenon, for example the Persian Gulf states. 21 censuses were also identified as exhibiting age heaping but were not being corrected due to being in the high-income or Central Europe, Eastern Europe, and Central Asia super-regions. After applying the age heaping corrections, we aggregated all single year age group population counts conducted outside of the high-income and Central Europe, Eastern Europe, and Central Asia super-regions into five-year age groups.

The third step facilitated utilisation of data that were not representative of the de facto population of interest, either in terms of the geographical area covered, the subpopulations covered, or a combination of both. A number of countries that exist in 2017 are amalgamations of two or more countries that existed in the past (eg, Germany), others have had semi-autonomous territories conduct separate censuses (eg, Transnistria), and other contemporary states are parts of historical aggregates (eg, states that were previously part of Yugoslavia). Oftentimes, censuses for fragmented or semi-autonomous areas occurred in different years and collected information about different age groups.

We estimated the population of the complete modern geographic unit for which we had a time series of separate censuses from all constituent historic or disputed territories. First, we collapsed all available censuses for the constituent parts to the most granular set of common age groups. After standardisation, we interpolated age-and-sex-specific populations using the age-and-sex-specific annual rate of change to generate annual time series spanning the available census data for each of the constituents. Finally, for all original census years corresponding to the largest constituent where there was overlap with the estimated populations from the smaller constituents, we summed the populations of all the constituents at the age-sex level to produce a census estimate representative of the entire modern geography. This method was used to combine censuses from East and West Germany, Moldova and Transnistria, Cyprus and the Turkish Republic of Northern Cyprus, Serbia and Kosovo, and Malaysia Peninsular, Sarawak, and Sabah. For modern countries that were formerly part of Yugoslavia, we were only able to extract historical census data that were reported according to ethnic (rather than geographic) affiliation. In this case, we applied the estimated age-sex proportions of the total historical aggregate population from the previous iteration of the Bayesian demographic balancing model to split the historic census data into modern geographies. These split data were used in Serbia; we obtained census data for the five other modern countries that were formerly part of Yugoslavia.

Singapore censuses only contained detailed age and sex population counts of residents, when non-residents accounted for a sizeable portion of the de facto population. Using available empirical death

counts in residents and non-residents, and assuming similar mortality between the two subpopulations, we utilised the relative age-pattern in the death counts to scale the resident population to total population.

A fourth step was used to correct for improper enumeration. Many censuses confront problems of under-enumeration, as in all but the smallest and most navigable countries reaching every single member of the population is logistically impossible. To address this issue, national statistical offices often conducted post-enumeration surveys (PES) soon after the completion of censuses to assess the degree to which individuals were either missed or double-counted by the census. The PES could thus quantify the overall bias of a given census, and those results could be used to adjust the population totals. We searched for PES based on the UNSTAT division's list of known or planned PES efforts in the 2000 and 2010 census rounds. We also looked for PES corresponding to any known census. Sources included country statistical websites, WorldCat catalog, data presented by countries during UN Stats symposia on the topic of PES, and academic publications. We identified a total of 165 PES that reported a net underenumeration percent, of which 63 reported underenumeration percent by age and/or sex. As most of our census data were unadjusted and for many we were unable to identify corresponding PES, we used a simple linear regression to predict percent adjustment for all ages and both sexes on the basis of the Socio-demographic Index (SDI) (Appendix Section 3).

$$totalPctAdjust = \beta_0 + \beta_1 SDI + \epsilon$$

The variance-covariance matrix from this model was used to simulate 1000 draws of the predicted total percent adjustment ($totalPctAdjust$) for each possible value of SDI. The variance of the 1000 draws simulated from the model was incorporated into the overall calculation of population uncertainty (See Appendix Section 2.3.2)

Given that under- and overenumeration varies significantly by age and sex, we used the 63 PES that reported underenumeration by age and/or sex to obtain a global age pattern of underenumeration. PES report inconsistent age groups; to address this we used DisMod-MR, an age-integrating Bayesian meta-regression tool that is widely used in the GBD.[16,17] We used SDI as a predictor in the DisMod-MR model. The age-sex pattern was shifted up or down to equal the predicted mean total percent adjustment and was applied to all census counts for which we had no documentation of prior adjustment.

As the final step of data processing, the Bayesian demographic balancing model and cohort component method of population projection (CCMPP) require a starting population as an input from which it can project populations forward in time. Where post-processed censuses were available for the year 1950 with single-year age groups up to the age group 95+, we used these directly. In all other cases we first split aggregate age groups in the oldest census available with person years lived (${}_nL_x$) in single-year age intervals using lifetables from the GBD 2017 mortality analyses[5]. This age pattern was then smoothed using a local first degree polynomial with bandwidth equal to 2 if the original age groups were in age interval lengths of 1 or 5 and bandwidth equal to 5 if in larger age.[18] If this oldest census was not in 1950 we projected the census backward using an inverse version of CCMPP with the assumption of zero migration. Normal CCMPP is described briefly in section 2.3.2; backward CCMPP simply solves for the population at the previous time point rather than the projecting to the next time point.

$$ {}_{1}N_{x-1}(t) = {}_{1}N_{x}\ (t+1) \cdot \frac{{}_{1}L_{x-1}(t)}{{}_{1}L_{x}\,(t)} $$

The equation for the open-ended age group is indeterminate and leaves what we call the upper missing triangle as the backward projection goes further back in time. These missing age groups are estimated using the 1950 ${}_{n}L_{x}$ age pattern from the GBD 2017 life tables. In some locations, as the census counts are projected backward, age misreporting in the oldest ages becomes extremely evident and leads to implausible age patterns; this is addressed by collapsing the original census to a lower open-ended age group so that the oldest age groups in the original census are ignored. In 33 countries we also used annualised rate of change between the oldest two censuses to back-project the total population and scaled the back projected age-specific population to the back-projected total population. Also, in 35 countries the assumption of zero migration used in backward CCMPP is not appropriate or the oldest census was too far from 1950 to back-project a reasonable age pattern; in these countries we have instead used GBD 2016 populations in 1950 as the baseline population. These 1950 populations were used as the prior for the Bayesian demographic balancing model but were input with considerable uncertainty to account for the fact that in most countries a census did not occur in 1950.

Migration

Population, births, deaths, and migration are the four main components of the demographic balancing equation. Of these four, migration was the least well measured component, especially outside of high-income countries. CCMPP can use as input either the net number of migrants by sex, age, and year or the net migration proportion by sex, age, and year. In locations where censuses had been frequently conducted we input a prior of zero net migration into the Bayesian population model described in more detail below. However, this zero prior fails in locations with large migration between censuses or in cases where there has been a large amount of migration after the most recent census. This section describes the identification and extraction of migration data to replace the zero prior in certain locations.

The United Nations High Commissioner for Refugees (UNHCR)[19] provided data on the number of refugees in each country at the end of every year (EOY) by country of origin. This refugee stock data was used to derive net flows of refugees into, and out of, countries. For example, the change in the number of refugees in Afghanistan during the year 1991 was calculated as the difference between the number of refugees in Afghanistan EOY in 1991 $D_{AFG}(EOY\ 1991)$ and EOY in 1990 $D_{AFG}(EOY\ 1990)$.

$$ \Delta D_{AFG}[1991] = D_{AFG}(EOY\ 1991) - D_{AFG}(EOY\ 1990) $$

Similarly, the change in the number of refugees originating from Afghanistan and moving to other countries during the year 1991 was calculated as the difference between the number of refugees originating from Afghanistan EOY in 1991 $O_{AFG}(EOY\ 1991)$ and EOY in 1990 $O_{AFG}(EOY\ 1990)$.

$$ \Delta O_{AFG}[1991] = O_{AFG}(EOY\ 1991) - O_{AFG}(EOY\ 1990) $$

The net refugee migration into or out of Afghanistan during the year 1991 was then calculated as

$$ \text{Net refugee migration}[1991] = (\Delta D_{AFG}[1991] - \Delta O_{AFG}[1991]) $$

This was then converted to migration totals between midyear points by averaging adjacent year net refugee migration totals. The UNHCR data included separate totals for internally displaced persons but we excluded these from the analysis. In cases where a refugee crisis was occurring, the large influx or outflux of people made up a large proportion of the total number of migrants in a given year, allowing the use of UNHCR data in place of a zero prior.

In some countries like Germany, the Czech Republic, Romania, the United Arab Emirates, and Bahrain, large non-refugee migrations have occurred recently that the Bayesian population model could not estimate because of a lack of recent census data. In these locations we sought out migration data from EUROSTAT,[20] the Gulf Labour Markets, Migration and Population (GLMM) programme,[21] and various national statistics websites.

Only EUROSTAT provided migration data by age and sex; all other sources of data were listed as total number of migrants over some period of time. In order to prep these totals to be age- and sex-specific migration inputs into CCMPP, we calculated an age-sex pattern of migration that was then scaled up or down to equal the input net migrant totals. In most locations we used the aggregate EUROSTAT age and sex pattern. Where most of the recent migration has been comprised of young adult male temporary workers (Saudi Arabia, Bahrain, United Arab Emirates, Oman, and Kuwait), we used the age and sex pattern from the Bayesian demographic balancing model fit in Qatar. The most recent census in Qatar was conducted in 2015, allowing us to capture the uptick in recent migration. In Rwanda we assumed that the migration proportion age pattern was uniform across age and sex because migration during the 1994 genocide likely did not follow the typical migrant age pattern that was present in the European Union or the migratory worker age pattern present in Qatar.

Mortality

The GBD mortality process produced yearly complete period life tables with single-year age groups up to 95+.[5] These life tables were used to calculate the survivorship ratio, which is a key input to CCMPP calculations and is shown briefly below in Appendix Section 2.3.2.

Fertility

The fertility model presented in this paper estimated ASFR in five-year age groups between the ages of 10-14 and 50-54. In order to project populations forward in time using CCMPP, we needed yearly single-year age group ASFR estimates. We assigned each five-year age group ASFR value to the middle of its age group (20-24 age group would be assigned the age 22.5) and used an interpolating spline to derive complete ASFR single-year age patterns for each location-year-draw combination. Each complete single-year age pattern was then raked to the input five-year age pattern for consistency.

Sex ratio at birth

The sex ratio at birth (srb) model presented in this paper produces yearly estimates of srb. These values are used in CCMPP to split live birth totals into sex-specific birth totals.

Section 2.3.2 Modelling Strategy

CCMPP

The demographic balancing equation defines how the total population changes over a time interval. CCMPP is commonly used for projecting populations forward in discrete time intervals at age- and sex-specific levels. It requires splitting the population into sex-specific cohorts and exposing these cohorts to fertility, mortality, and migration. This method is described in detail in Preston;[22] we will only be describing the fundamental equation here and one modification we made when incorporating migration.

$${}_1N_x\ (t+1) = \left[\left({}_1N_{x-1}(t) + \frac{{}_1G_{x-1}[t,t+1]}{2}\right) \cdot \frac{{}_1L_x(t)}{{}_1L_{x-1}(t)}\right] + \frac{{}_1G_{x-1}[t,t+1]}{2}$$

For any age group, except for the first and terminal age group, the population can be survived forward one-time interval with the survivorship ratio $\frac{{}_1L_x}{{}_1L_{x-1}}$ derived from the GBD period life tables. A key assumption used throughout this method was that half the migrants during a time interval migrate at the beginning of the time interval and half migrate at the end of the interval. Preston defined G as the net flow of migrants during the projection period in the age interval x - 1 to x, a period measure;[22] because we estimated migration with the GBD Bayesian Demographic Balancing Model (described below), we instead defined ${}_1G_{x-1}$ as the net flow of migrants during the projection period for the cohort initially between age x - 1 and x at the beginning of the projection period. This model estimated net migration proportions instead of counts, which we defined as the ratio of net migrants in a cohort to the population at the beginning of the projection period.

$${}_1g_x = \frac{{}_1G_x}{{}_1N_x}$$

This version of CCMPP was implemented in the template model builder (TMB)[23] code for the Bayesian Demographic Balancing Model and was implemented in R to calculate final posterior estimates of population.

Bayesian Demographic Balancing Model

Wheldon and colleagues have previously developed a Bayesian hierarchical model (popReconstruct) to reconstruct human populations back in time by age and sex.[24] The popReconstruct model simultaneously estimates baseline population counts, fertility, mortality, migration, and sex ratio at birth in order to make population projections from CCMPP consistent with later census data points. Wheldon used initial bias-reduced inputs from the UN for all the key components of population change, including net migration. GBD provided initial estimates of fertility and mortality derived using replicable methods but did not produce estimates of migration due to a lack of data on migration for most locations/years and the difficulty in modelling migration directly. Instead, we used a modified version of the popReconstruct model, the GBD Bayesian Demographic Balancing Model, to indirectly estimate net migration.

Model description

Both popReconstruct and the Demographic Balancing Model embedded CCMPP in a Bayesian hierarchical model. Consistent with the popReconstruct model, we used n, g, s, f, and srb to symbolise population counts, net migration proportion, survival ratio, age-specific fertility rate and sex-ratio at birth, respectively. These parameters were indexed by sex (l), single-year age groups (a) and single calendar years (t). We used an asterisk ($*$) to indicate initial values of parameters.

Level 1 modelled the percent difference between non-baseline sex-age-year-specific census counts ($n^*_{l,a,t}$) and the corresponding projected sex-age-year-specific populations counts ($n_{l,a,t}$) from CCMPP in Level 2. We modelled this as the percent difference, so that the variance ($\sigma^2_{n^*_{l,a,t}}$) associated with each census data point was on the same scale across locations and age groups of various population magnitudes.

$$Level\ 1\colon \frac{n^*_{l,a,t} - n_{l,a,t}}{n_{l,a,t}} \sim Normal(0, \sigma^2_{n^*_{l,a,t}})$$

Level 2 transformed the model inputs to projected sex-age-year-specific population counts ($n_{l,a,t}$) from input values.

$$Level\ 2\colon n_{l,a,t} = CCMPP(n_{l,a,t_0}, g_{l,a,t}, s^*_{l,a,t}, f^*_{l,a,t}, srb^*_t)$$

At Level 3 we modelled the initial estimates of migration ($g^*_{l,a,t}$) and the baseline 1950 population (n^*_{l,a,t_0}). Similar to level 1, we modelled the baseline population as the percent difference between the estimated baseline 1950 population and the input values. We chose to model the net migration proportion as an autoregressive process over both age and time in order to avoid extreme discontinuities in the age pattern and time series for migration. Unlike the popReconstruct model, we did not model ASFR ($f^*_{l,a,t}$), survival ratios ($s^*_{l,a,t}$) or sex ratio at birth (srb^*_t) and instead kept them fixed at their input values. With the zero prior that we often used for migration, the model could not distinguish between changing fertility, mortality, or migration to match the census counts.

$$Level\ 3\colon \frac{n_{l,a,t_0} - n^*_{l,a,t_0}}{n^*_{l,a,t_0}} \sim Normal\left(0, \sigma^2_{n^*_{l,a,t_0}}\right)$$

$$g_{l,a,t} - g^*_{l,a,t} \sim AR1\colon AR1(\sigma_g, \rho_{g_a}, \rho_{g_t})$$

Level 4 defined various hyper-priors. We specified the prior distribution on the correlation parameters for migration to have greater correlation over time than age.

$$Level\ 4\colon \log(\sigma_g) \sim Normal(-5, 9)$$

$$logit(\rho_{g_a}) \sim Normal(-3, 0.01\ )$$

$$logit(\rho_{g_t}) \sim Normal(3, 0.01)$$

Instead of estimating a single variance term for all census counts, we set the initial standard deviation associated with each sex-age-year-specific census data point ($\sigma_{n^*_{l,a,t}}$) to 0.01 if the location was in the high-income super-region, to 0.03 if the location was in Central Europe, Eastern Europe, or Central Asia, and to 0.05 for all other super-regions. This standard deviation could be multiplied by scalars to increase or decrease the weight of a data point, and we used this to address multiple issues: 1) Our method to estimate baseline populations produced rough estimates of population in 1950 that were input into the model, but the method could lead to extreme migration in the 1950s. Because of the considerable uncertainty in the baseline population, we multiplied the standard deviation of the baseline counts by two so that the estimated baseline population could change considerably. 2) We implemented the model such that census counts that were not in single-year age groups were compared to the projected counts aggregated to the same age groups. This led to fewer data points present in censuses with non-single-year age groups or terminal age groups below 95+; the model did not fit the census as well with less granular age groups. To address this, we divided each census data point's standard deviation by the width of the age interval, so that in total each census was given similar weight in the model. 3) To address age misreporting at older ages, we multiplied the standard deviation by a scalar that linearly increased from 1 at age 50 to 3 for the terminal age group 95+. 4) To address under enumeration in the under-5 age groups, we multiplied the standard deviation of the population counts by a scalar that linearly increased from 1 for age 5 to 3 for age 0. For locations outside of the high-income and Central Europe, Eastern Europe, and Central Asia super-regions, we did not input non-baseline under-5 population counts for all censuses. This made the under-5 populations dependent on fertility, under-5 mortality, and census counts at later age groups.

Older age misreporting was often evident, as the model predicted extreme immigration in older ages to match implausibly high counts of older individuals. The method described above gave less weight to the older census counts, and in addition we ran the model multiple times for different maximum ages between 55 and 95. For each model version we excluded from the model fitting process any population data points that included an age group that started above the maximum age. For each model version for a given location we then calculated 1) the mean average percent error (MAPE) between the input census data and the estimated population for each of the data points that were included in the model and 2) the weighted mean of the absolute value of the migration proportions for five-year age groups between 55 and 95 with a weight of 1 given to age group 55, 2 to age group 60 and so on, up to a weight of 9 for age group 95+. We then selected the highest maximum age group that was within 5% of the minimum MAPE value across the model versions and within 0.005 of the minimum weighted mean of the older age migration proportions. This method balanced lowering migration in the oldest age groups and maintaining a good fit to the census data included in the model.

The Bayesian Demographic Balancing model was fit separately by location for the years 1950–2017, with single-year age groups up to a terminal age group of 95+. Final population estimates were then derived using CCMPP with posterior estimates of the baseline population and net migration proportion, in addition to GBD estimates of fertility, mortality, and sex ratio at birth as inputs.

Population uncertainty

After estimating a complete time series of age- and sex-specific population using CCMPP, we estimated population uncertainty using a combination of out of sample predictive validity and uncertainty in the adjustment for census completeness as described in Appendix Section 2.3.1.

There was considerable uncertainty associated with the PES results and our model of PES adjustments. This uncertainty was incorporated into the uncertainty around population in census years and in years before, between, and after; censuses inform the size of the population in non-census years as well as census years. From the model of census completeness, we calculated the variance of the percent adjustment for completeness ($Var_{completeness}$) associated with each location-year based on the SDI value.

Additional uncertainty in population between censuses was a function of the number of years to the closest census and our estimates of migration, mortality, and fertility. To quantify this uncertainty, we held out census data from the GBD Bayesian Demographic Balancing Model in the 143 national locations where we had at least five censuses that had more than just total population by sex available after processing of the census data described in Appendix Section 2.3.1. In these locations we fit the model 20 times per location with a random subset of between one and five censuses used to fit the model and the rest held out. We calculated the percent error between the projected posterior estimates of population and the held-out census data and compiled these percent errors across locations by the number of years to the closest census. We then fit a linear regression on the root mean squared percent error ($RMSPE$) as a function of the number of years to the closest census ($YearsToCensus$).

$$RMSPE = \beta_0 + \beta_1 YearsToCensus + \varepsilon_i$$

For each location-year we then had the predicted variance of the percent adjustment for completeness ($Var_{completeness}$) based on the SDI in that year and the predicted out of sample percent error based on the distance from a census ($RMSPE$). We combined these sources of uncertainty in variance space for each year.

$$Var_{pop} = Var_{completeness} + RMSPE^2$$

This total variance for population was then used to simulate draws of population percent error ($PopPctError$) by location-year.

$$PopPctError \sim Normal(0, Var_{pop})$$

Finally, these percent errors were used to calculate draws of population that incorporated both the uncertainty in the PES adjustment and the out of sample variance associated with errors in migration, fertility, and mortality estimates and the distance from a census.

$$Pop_{draw} = Pop_{mean} + (Pop_{mean} * PopPctError_{draw})$$

GBD world population age standard

Age-standardized populations in the GBD were calculated using the GBD world population age standard. For GBD 2013, GBD 2015 and GBD 2016, the age-specific proportional distributions of all national locations from the UNPOP World Population Prospects 2012 revision for all years from 2010 to 2035 were used to generate a standard population age structure using the non-weighted mean across all the aforementioned country-years. For GBD 2017 we have used the non-weighted mean of 2017 age-specific proportional distributions from the estimates produced in this study for all national locations with a population greater than 5 million people in 2017 to generate an updated standard population age structure.

Section 3. Socio-demographic Index (SDI) Analysis

Section 3.1. Overview

The Socio-demographic Index (SDI) is a composite indicator of development status strongly correlated with health outcomes. In short, it is the geometric mean of 0 to 1 indices of TFU25, mean education for those aged 15 and older (EDU15+), and lag-distributed income (LDI) per capita.

Section 3.2 Development of revised SDI indicator

SDI was originally constructed for GBD 2015 using the Human Development Index (HDI) methodology, wherein a 0 to 1 index value was determined for each of the original three covariate inputs (TFR in ages 15 to 49, EDU15+, and LDI per capita) using the observed minima and maxima over the estimation period to set the scales.[1]

In response to feedback from collaborators and the evolution of the GBD, we have refined the indicator with each GBD cycle. For GBD 2017, in conjunction with our expanded estimation of age-specific fertility, we replaced TFR with TFU25 as one of the three component indices. The TFU25 provides a better measure of women's status in society, as it focuses on ages where childbearing disrupts the pursuit of education and entrance into the workforce. In addition, we observe that in highly developed countries the TFU25 has tended to decline consistently over time, even amid rebounds in TFR driven by increasing fertility in older ages. SDI calculated with TFU25 showed slightly higher correlation with U5MR (-.940; TFR: -.936) and life expectancy at birth (.904; TFR: .897). The concordance correlation coefficient between SDI using the GBD 2016 method and the updated method for GBD 2017 was 0.981.

During GBD 2016 we moved from using relative index scales to absolute scales to enhance the stability of SDI's interpretation over time, as we noticed that the measure was highly sensitive to the addition of subnational units that tended to stretch the empirical minima and maxima.[2] We selected the minima and maxima of the scales by examining the relationships each of the inputs had with life expectancy at birth and under-5 mortality and identifying points of limiting returns at both high and low values, if they occurred prior to theoretical limits (eg, a TFU25 of 0).

Thus, an index score of 0 represents the minimum level of each covariate input past which selected health outcomes can get no worse, while an index score of 1 represents the maximum level of each covariate input past which selected health outcomes cease to improve. As a composite, a location with an SDI of 0 would have a theoretical minimum level of development relevant to these health outcomes, while a location with an SDI of 1 would have a theoretical maximum level of development relevant to these health outcomes.

The final scales for GBD 2017 are summarized in table E below.

Table E. Final SDI scales

Input	Lower Bound	Upper Bound
TFU25	0	3
LDI per capita	250 USD (5.52 log USD)[a]	60,000 USD (11.00 log USD)
EDU15+	0 years	17 years

[a] The minimum for the LDI scale was originally set at the theoretical limit of 0 USD, as we did not observe an asymptotic relationship between log(LDI) and E_0 or 5q0 at lower values of log(LDI). Empirically, however, we also did not observe an LDI below 350 USD (5.86 log USD) for the estimation period 1970-2016. In log-space, this meant that approximately half of our scale was not being utilised, compressing the observed variation in LDI and diminishing its meaningful contribution to SDI. Accordingly, we set the lower limit on LDI to 250 USD (5.52 log USD) to ensure we were fully utilising the range of the scale to capture its variation across space and time, as is the case with the other two inputs.

Using scales described above, we computed the index scores underlying SDI as follows:

$$I_{Cly} = \frac{(C_{ly} - C_{low})}{(C_{high} - C_{low})}$$

Where I_{cly} – the index for covariate *C*, location *l*, and year *y* – is equal to the difference between the value of that covariate in that location-year and the lower bound of the covariate divided by the difference between the upper and lower bounds for that covariate. If the values of input covariates fell outside the upper or lower bounds (eg, LDI per capita greater than 60,000 USD), they were mapped to the respective upper or lower bounds. The index value for TFU25 was computed as $1 - I_{TFU25ly}$, as lower TFU25s correspond to higher levels of development, and thus higher index scores. For GBD 2017 we expanded the computation of SDI to 890 national and subnational locations spanning the time period 1950–2017.

The composite SDI was the geometric mean of these three indices for a given location-year. The cutoff values used to determine quintiles for analysis were then computed using country-level estimates of SDI for the year 2017, excluding countries with populations less than 1 million. SDI groupings by geography are provided in Appendix Table 7; SDI values by location are provided in Appendix Tables 8-10.

Example Calculation

Below we present the calculation of SDI for a hypothetical country in the year 2010

$$TFU25 = 1.09;\ Mean\ educ\ yrs\ pc = 8.23;\ lnLDI = 9.60$$

$$I_{TFU25} = 1 - \frac{1.09 - 0}{3 - 0} = .637$$

$$I_{Educ} = \frac{8.23 - 0}{17 - 0} = .484$$

$$I_{lnLDI} = \frac{9.60 - 5.52}{11.00 - 5.52} = .744$$

$$SDI = \sqrt[3]{I_{TFU25} * I_{Educ} * I_{lnLDI}} = \sqrt[3]{.637 * .484 * .744} = .611$$

Section 4. References

1 Wang H, Naghavi M, Allen C, *et al.* Global, regional, and national life expectancy, all-cause mortality, and cause-specific mortality for 249 causes of death, 1980–2015: a systematic analysis for the Global Burden of Disease Study 2015. *The Lancet* 2016; **388**: 1459–544.

2 Wang H, Abajobir AA, Abate KH, *et al.* Global, regional, and national under-5 mortality, adult mortality, age-specific mortality, and life expectancy, 1970–2016: a systematic analysis for the Global Burden of Disease Study 2016. *The Lancet* 2017; **390**: 1084–150.

3 United Nations Population Division | Department of Economic and Social Affairs. http://www.un.org/en/development/desa/population/

4 Human Mortality Database. http://www.mortality.org

5 Gakidou E. Global, regional, and national under-5 mortality, adult mortality, age-specific mortality, and life expectancy, 1950-2017: a systematic analysis for the Global Burden of Disease Study 2017. Under Review.

6 UNSD — Demographic and Social Statistics. https://unstats.un.org/unsd/demographic-social/products/dyb/

7 Max Planck Institute for Demographic Research (Germany) and Vienna Institute of Demography (Austria). Human Fertility Collection. www.fertilitydata.org

8 UNSD — Welcome to UNSD. https://unstats.un.org/home/

9 IPUMS USA. https://usa.ipums.org/usa/

10 UT College of Liberal Arts: https://liberalarts.utexas.edu/prc/

11 WorldCat.org: The World's Largest Library Catalog. https://www.worldcat.org/

12 Shryock HS, Siegel JS, Larmon EA. The Methods and Materials of Demography. U.S. Bureau of the Census, 1973.

13 Feeney G. A technique for correcting age distributions for heaping on multiples of five. *Asian Pac Census Forum* 1979; **5**: 12–4.

14 UNHCR - The UN Refugee Agency. http://www.unhcr.org/en-us/

15 Home - Eurostat. http://ec.europa.eu/eurostat (accessed April 8, 2018).

16 GLMM - Gulf Labour Markets and Migration. GLMM. http://gulfmigration.eu/

17 Preston SH. The Changing Relation between Mortality and Level of Economic Development. *Popul Stud* 1975; **29**: 231.

18 Wheldon MC, Raftery AE, Clark SJ, Gerland P. Reconstructing Past Populations With Uncertainty From Fragmentary Data. *J Am Stat Assoc* 2013; **108**: 96–110.

Section 5. Figures and Tables

Appendix Figure 1a. Analytical Flowchart for the GBD 2017 Population Estimation Process

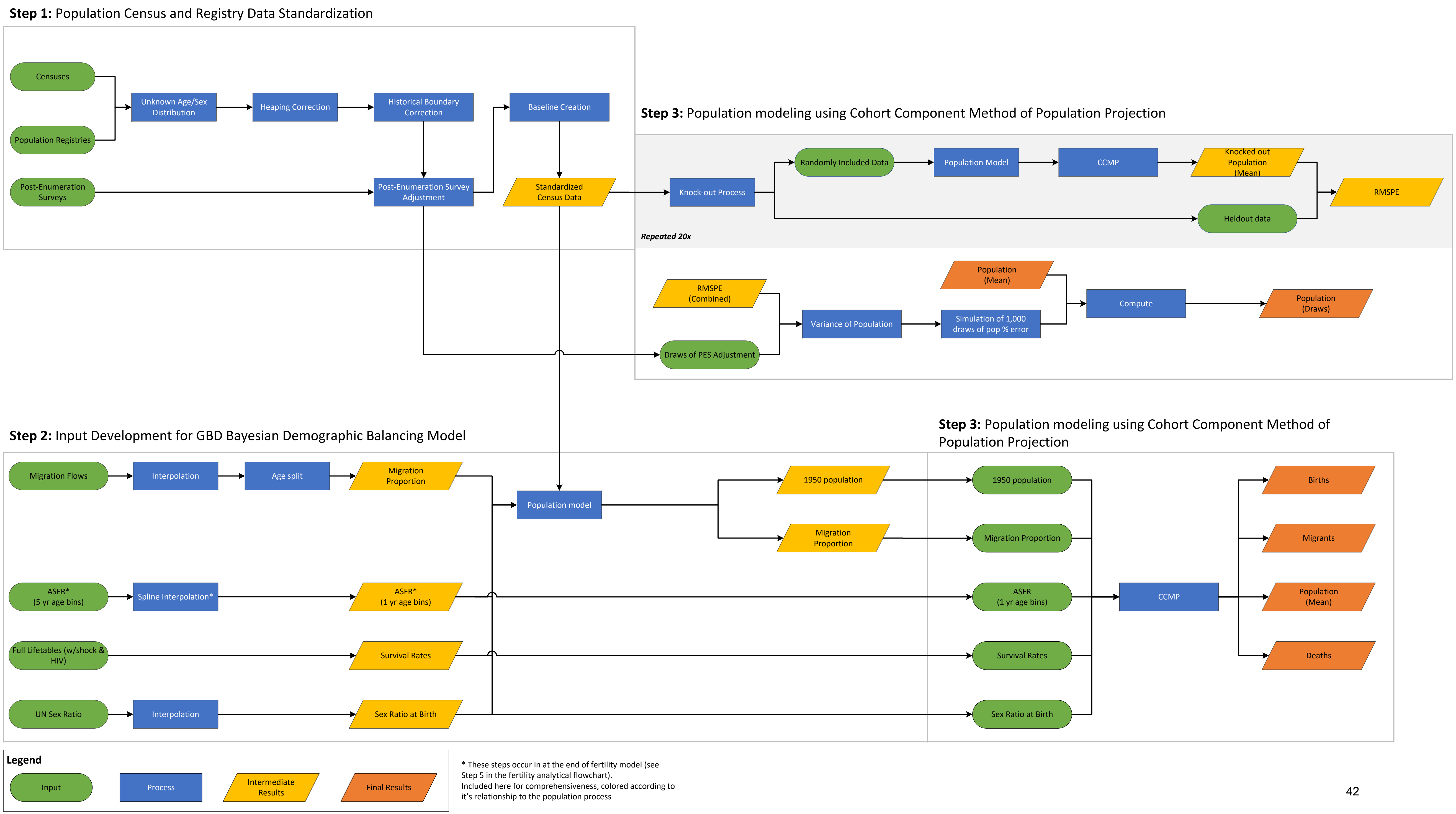

* These steps occur in at the end of fertility model (see Step 5 in the fertility analytical flowchart). Included here for comprehensiveness, colored according to it's relationship to the population process

Appendix Figure 1b. Analytical Flowchart for the GBD 2017 Fertility Estimation Process

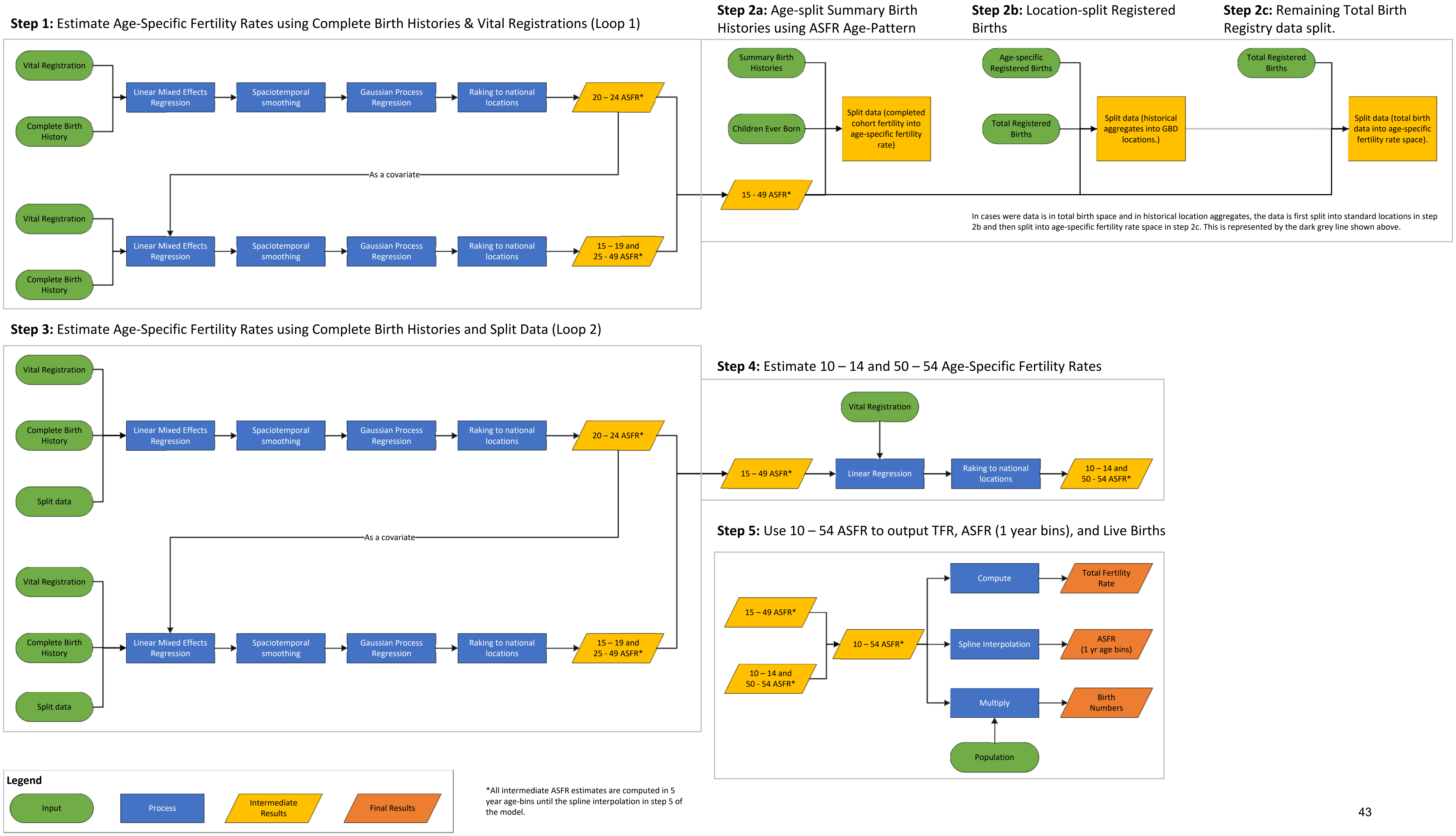

Appendix Figure 2. Census and registry availability by location and year
Central Europe, Eastern Europe, and Central Asia

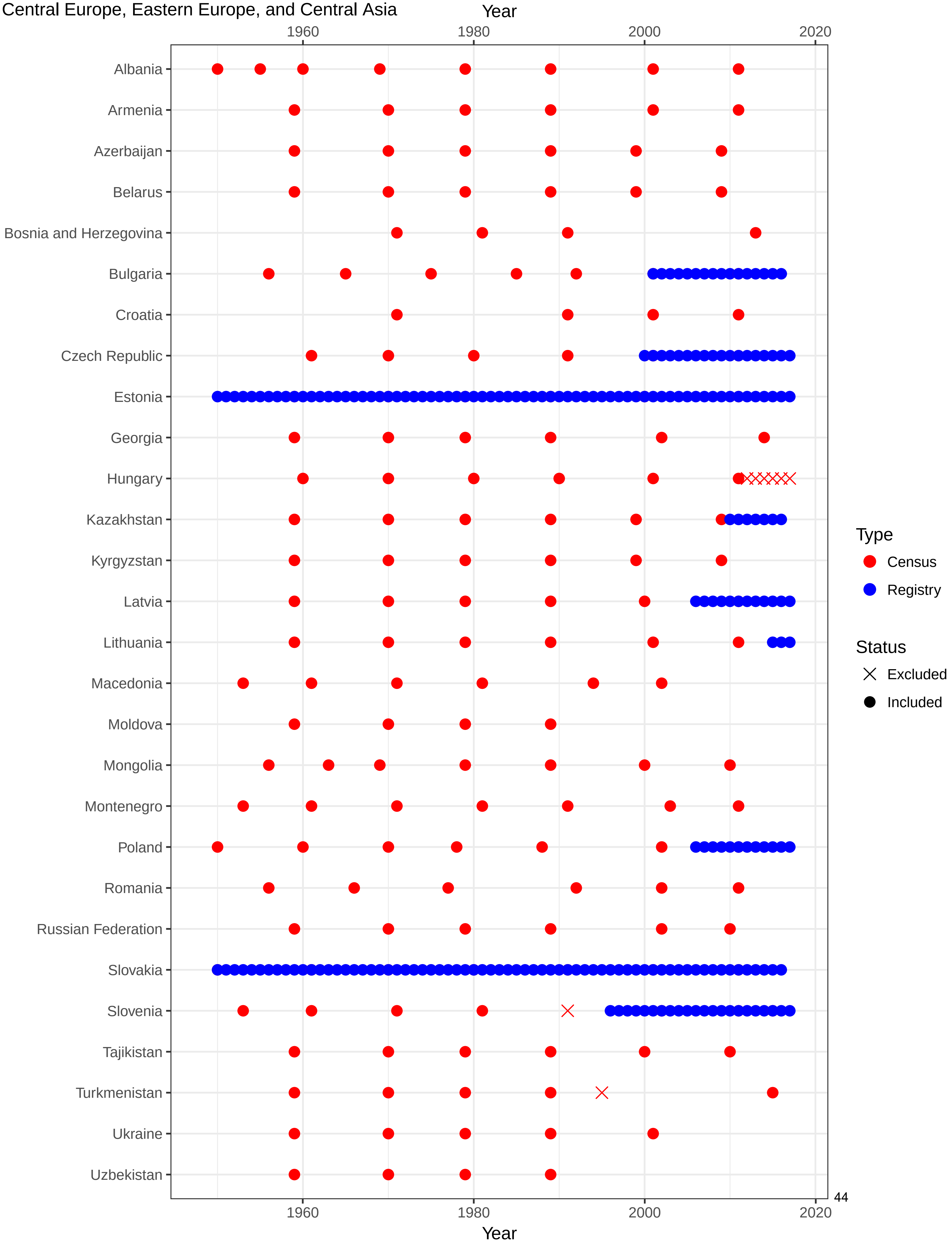

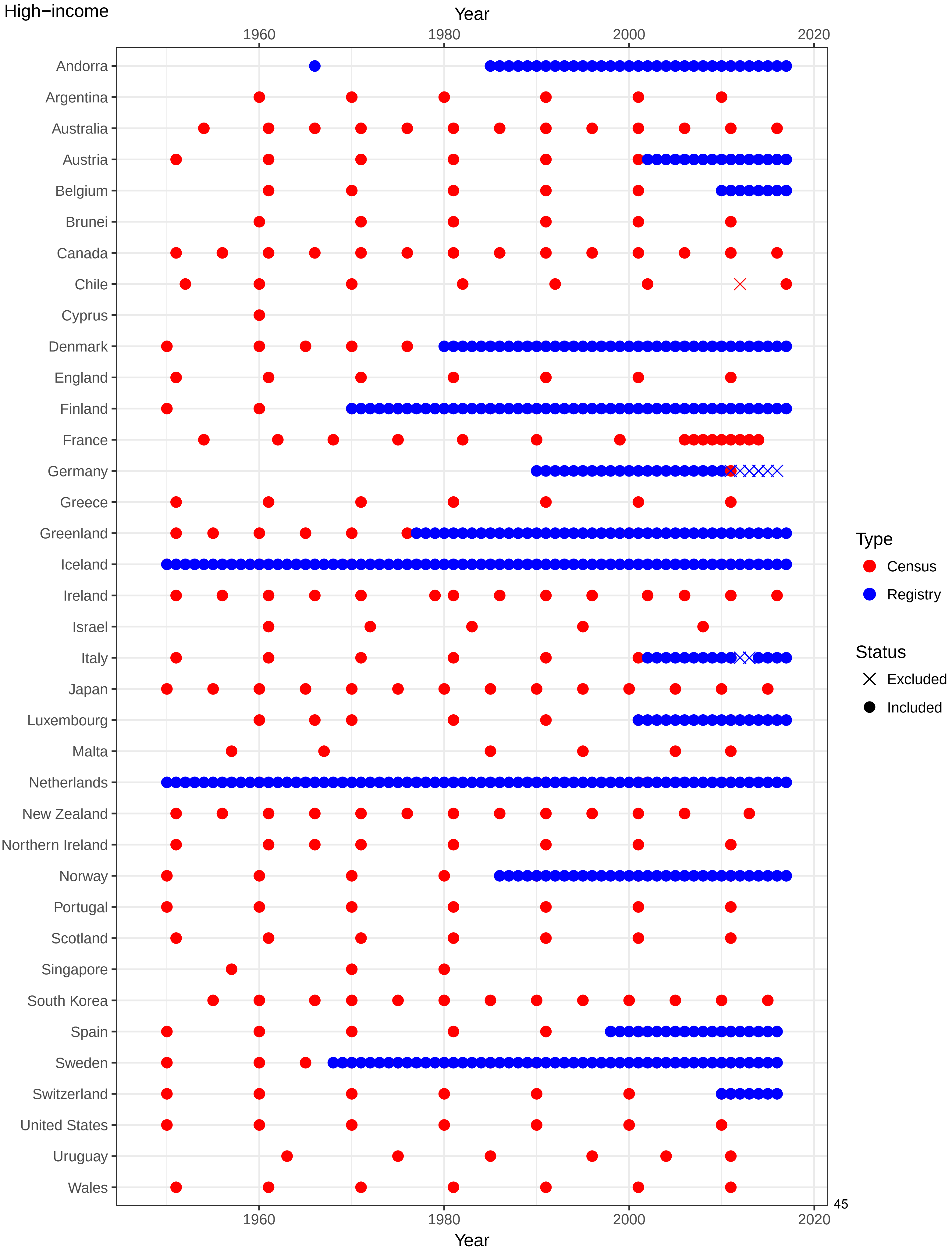

High−income
Year
1960
1980
2000
2020
Andorra
Argentina
Australia
Austria
Belgium
Brunei
Canada
Chile
Cyprus
Denmark
England
Finland
France
Germany
Greece
Greenland
Iceland
Ireland
Israel
Italy
Japan
Luxembourg
Malta
Netherlands
New Zealand
Northern Ireland
Norway
Portugal
Scotland
Singapore
South Korea
Spain
Sweden
Switzerland
United States
Uruguay
Wales
Type
Census
Registry
Status
Excluded
Included

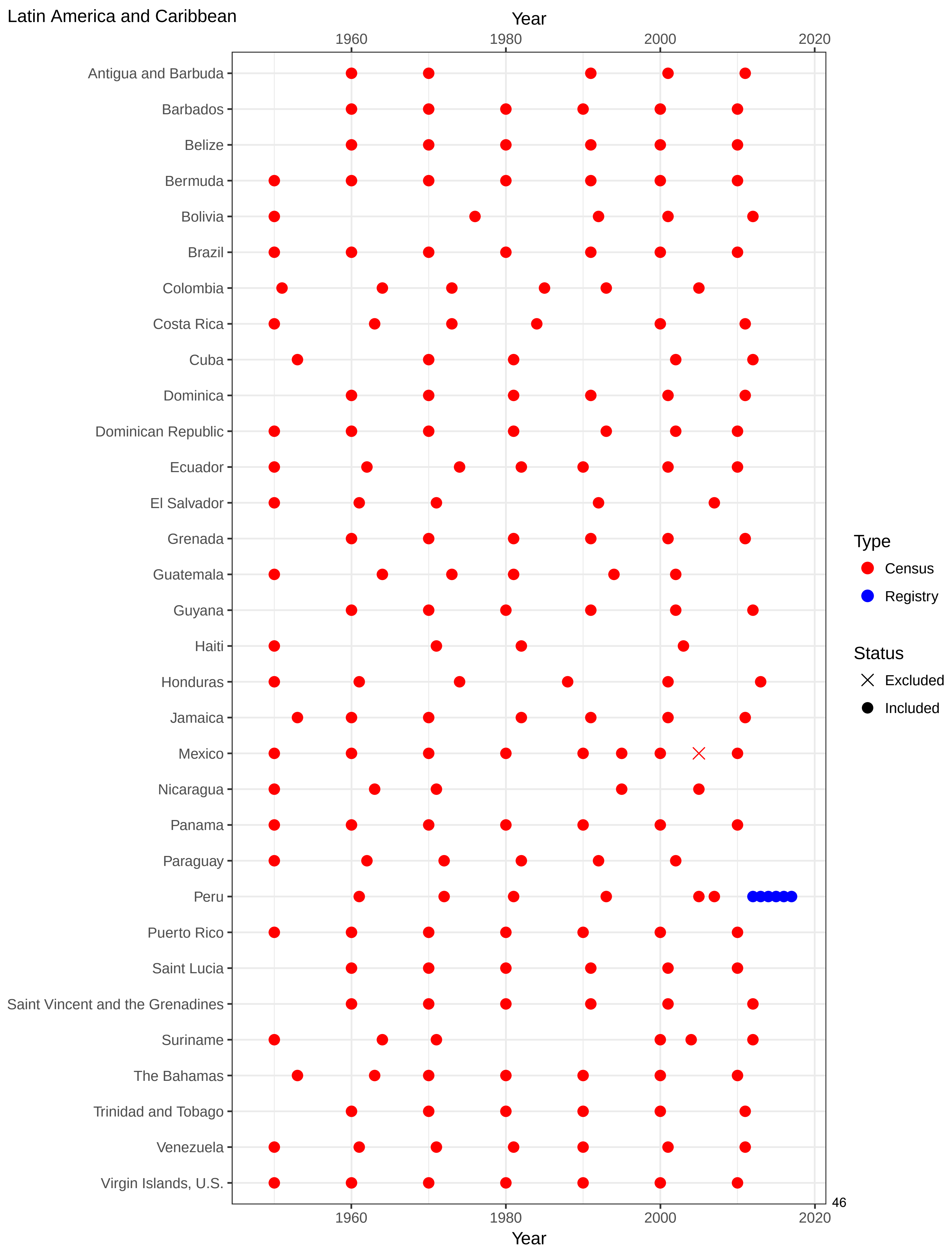

Latin America and Caribbean
Year
1960
1980
2000
2020
Antigua and Barbuda
Barbados
Belize
Bermuda
Bolivia
Brazil
Colombia
Costa Rica
Cuba
Dominica
Dominican Republic
Ecuador
El Salvador
Grenada
Guatemala
Guyana
Haiti
Honduras
Jamaica
Mexico
Nicaragua
Panama
Paraguay
Peru
Puerto Rico
Saint Lucia
Saint Vincent and the Grenadines
Suriname
The Bahamas
Trinidad and Tobago
Venezuela
Virgin Islands, U.S.
Type
Census
Registry
Status
Excluded
Included

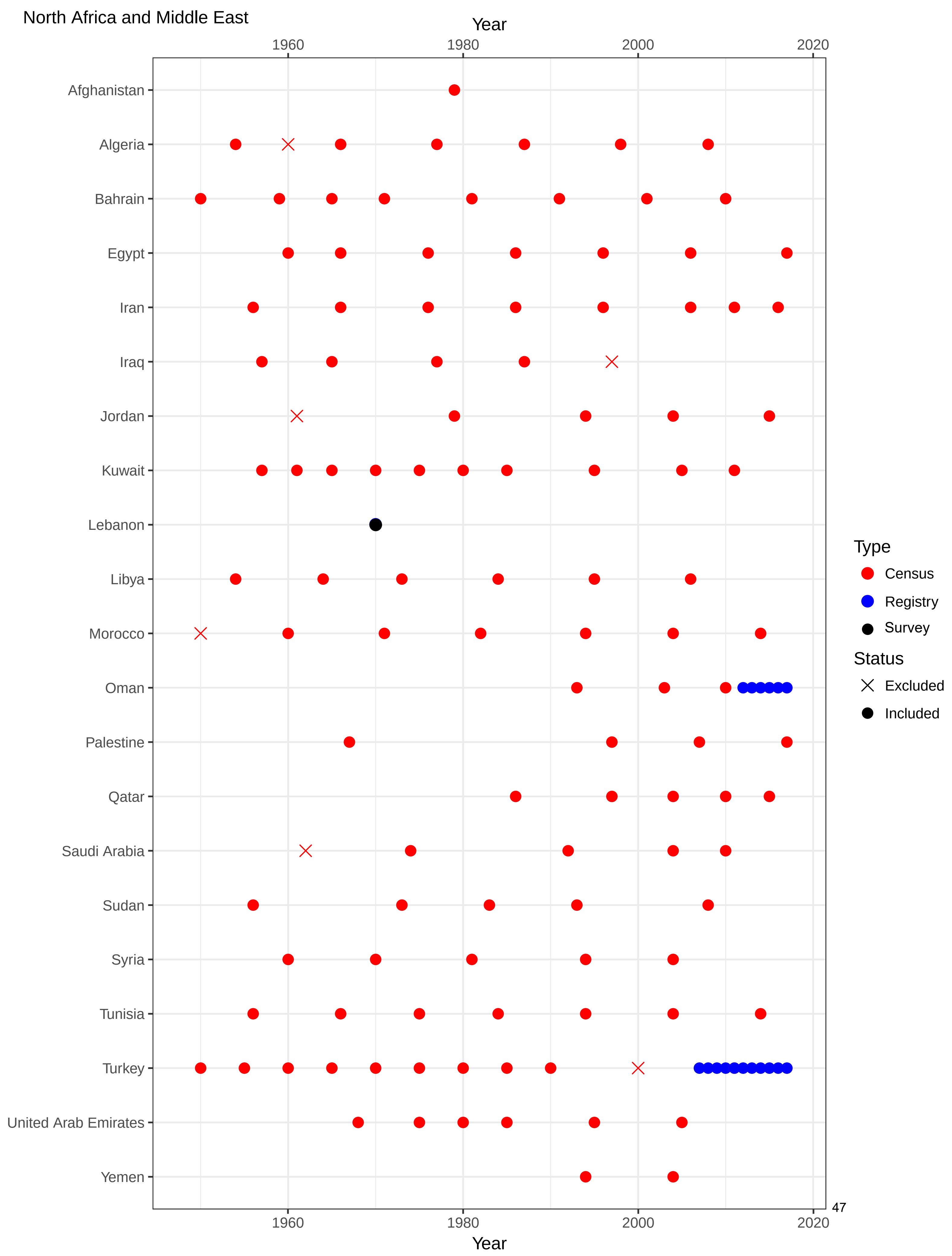
North Africa and Middle East
Year
1960
1980
2000
2020
Afghanistan
Algeria
Bahrain
Egypt
Iran
Iraq
Jordan
Kuwait
Lebanon
Libya
Morocco
Oman
Palestine
Qatar
Saudi Arabia
Sudan
Syria
Tunisia
Turkey
United Arab Emirates
Yemen
Type
Census
Registry
Survey
Status
Excluded
Included

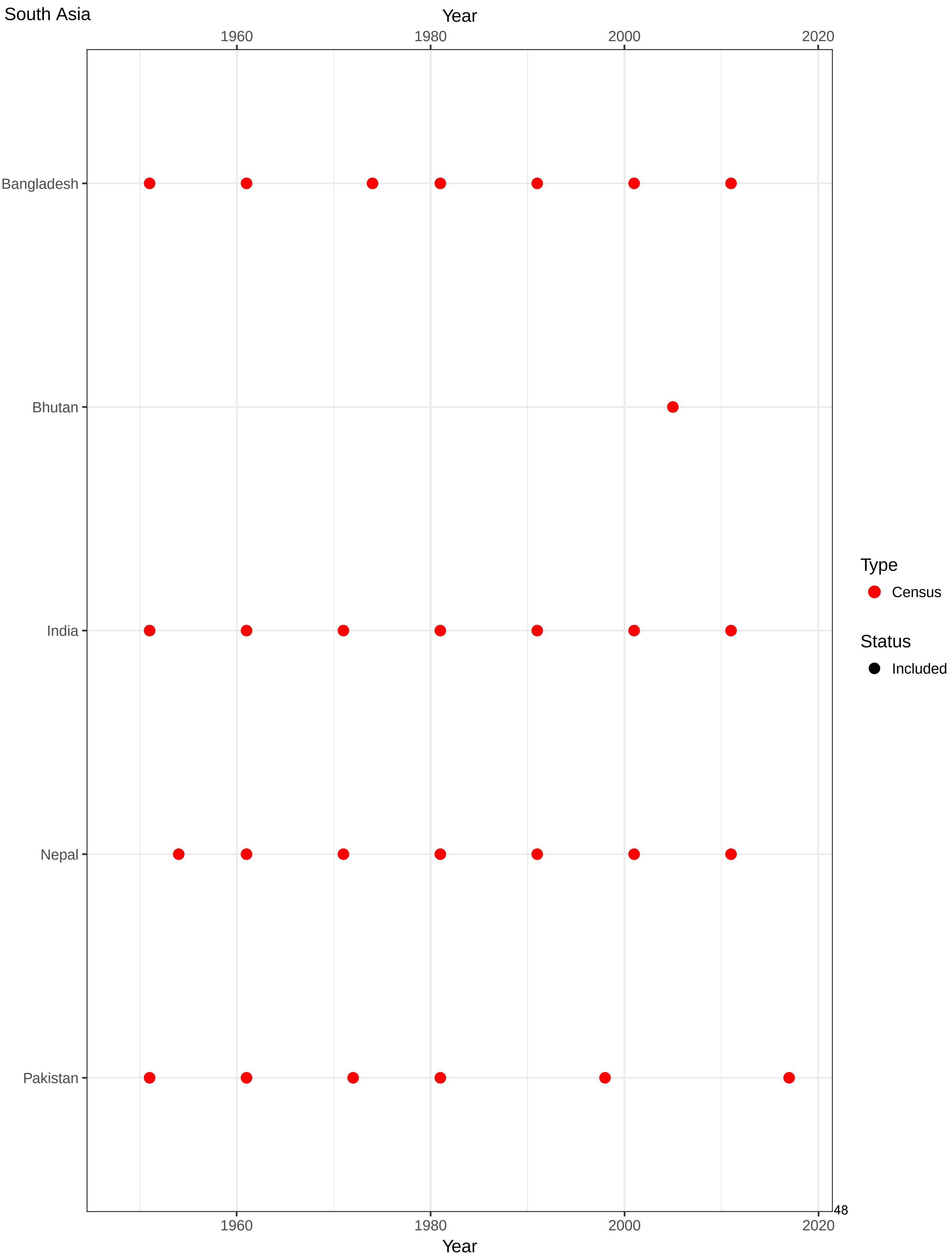
South Asia
Year
1960
1980
2000
2020
Bangladesh
Bhutan
India
Nepal
Pakistan
Type
Census
Status
Included

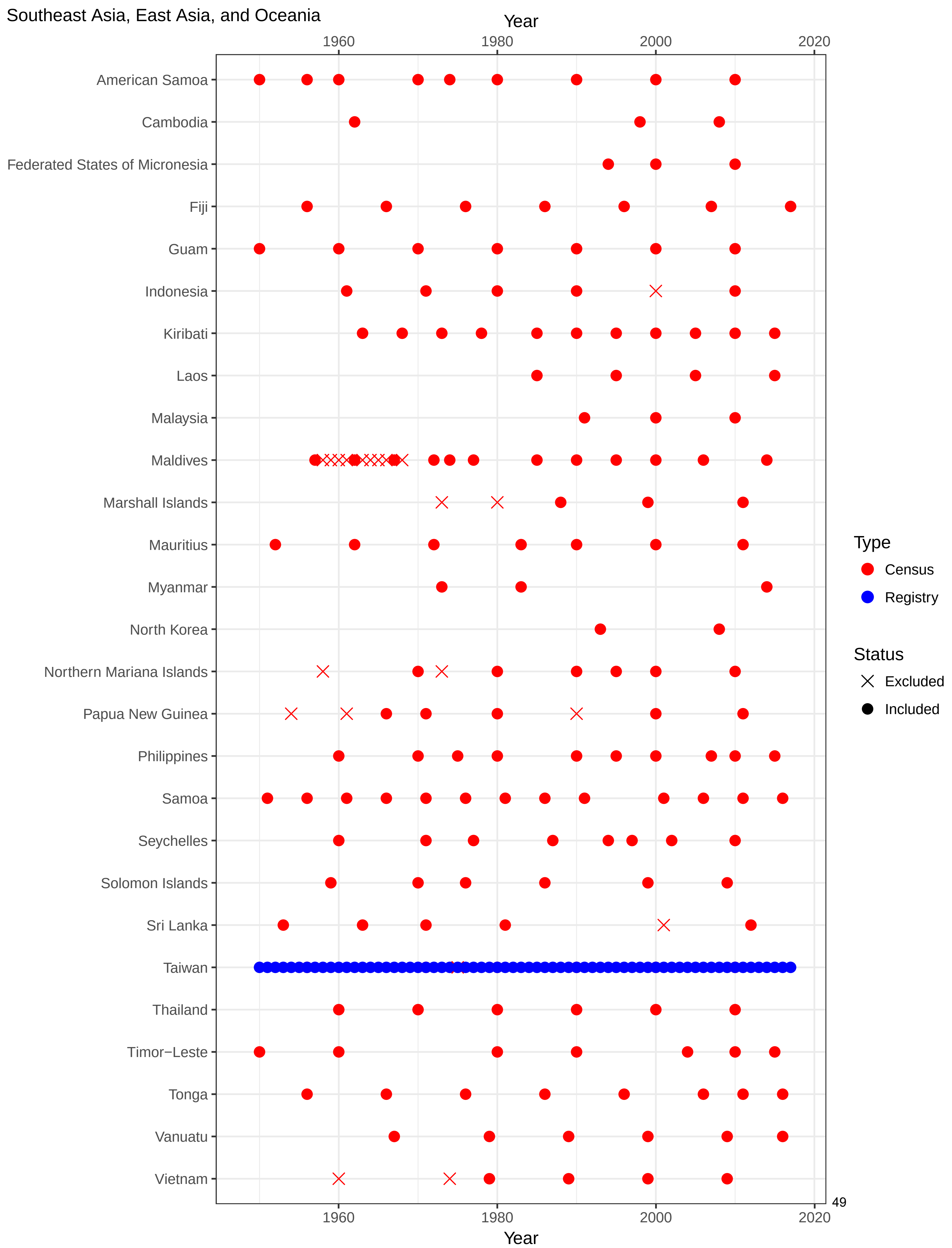

Southeast Asia, East Asia, and Oceania
Year
1960
1980
2000
2020
American Samoa
Cambodia
Federated States of Micronesia
Fiji
Guam
Indonesia
Kiribati
Laos
Malaysia
Maldives
Marshall Islands
Mauritius
Myanmar
North Korea
Northern Mariana Islands
Papua New Guinea
Philippines
Samoa
Seychelles
Solomon Islands
Sri Lanka
Taiwan
Thailand
Timor−Leste
Tonga
Vanuatu
Vietnam
Type
Census
Registry
Status
Excluded
Included

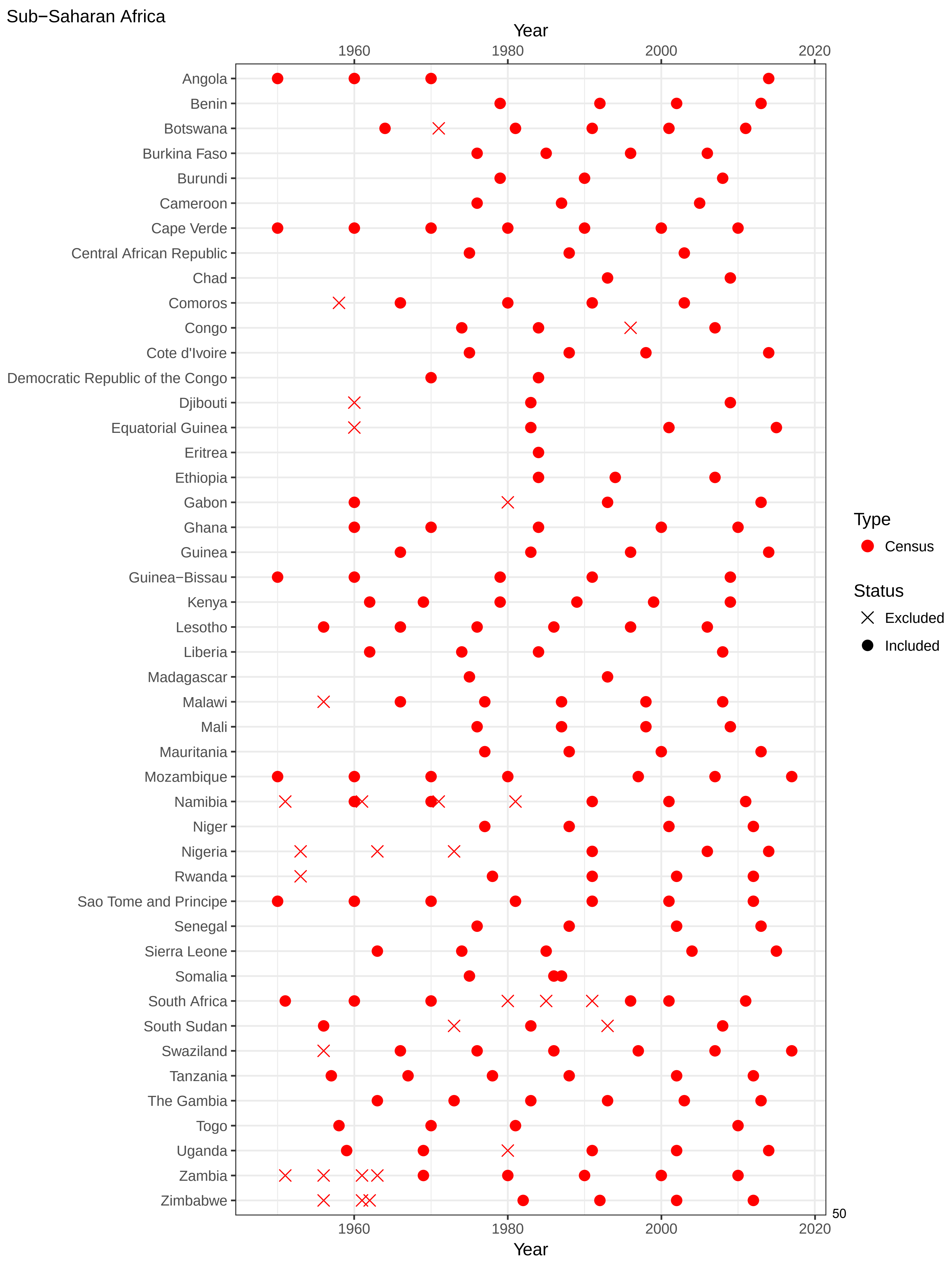

Sub−Saharan Africa
Year
1960
1980
2000
2020
Angola
Benin
Botswana
Burkina Faso
Burundi
Cameroon
Cape Verde
Central African Republic
Chad
Comoros
Congo
Cote d'Ivoire
Democratic Republic of the Congo
Djibouti
Equatorial Guinea
Eritrea
Ethiopia
Gabon
Ghana
Guinea
Guinea−Bissau
Kenya
Lesotho
Liberia
Madagascar
Malawi
Mali
Mauritania
Mozambique
Namibia
Niger
Nigeria
Rwanda
Sao Tome and Principe
Senegal
Sierra Leone
Somalia
South Africa
South Sudan
Swaziland
Tanzania
The Gambia
Togo
Uganda
Zambia
Zimbabwe
1960
1980
2000
2020
Year
Type
Census
Status
Excluded
Included

Appendix Table 1. GBD location hierarchy with levels

Geography	Level
Global	0
Low SDI	1
Low-middle SDI	1
Middle SDI	1
High-middle SDI	1
High SDI	1
Central Europe, Eastern Europe, and Central Asia	1
Central Asia	2
Armenia	3
Azerbaijan	3
Georgia	3
Kazakhstan	3
Kyrgyzstan	3
Mongolia	3
Tajikistan	3
Turkmenistan	3
Uzbekistan	3
Central Europe	2
Albania	3
Bosnia and Herzegovina	3
Bulgaria	3
Croatia	3
Czech Republic	3
Hungary	3
Macedonia	3
Montenegro	3
Poland	3
Romania	3
Serbia	3
Slovakia	3
Slovenia	3
Eastern Europe	2
Belarus	3
Estonia	3
Latvia	3
Lithuania	3
Moldova	3
Russian Federation	3
Ukraine	3
High-income	1
Australasia	2
Australia	3
New Zealand	3
High-income Asia Pacific	2

Appendix Table 1. GBD location hierarchy with levels	
**Geography**	**Level**
Brunei	3
Japan	3
Aichi	4
Akita	4
Aomori	4
Chiba	4
Ehime	4
Fukui	4
Fukuoka	4
Fukushima	4
Gifu	4
Gunma	4
Hiroshima	4
Hokkaidō	4
Hyōgo	4
Ibaraki	4
Ishikawa	4
Iwate	4
Kagawa	4
Kagoshima	4
Kanagawa	4
Kōchi	4
Kumamoto	4
Kyōto	4
Mie	4
Miyagi	4
Miyazaki	4
Nagano	4
Nagasaki	4
Nara	4
Niigata	4
Ōita	4
Okayama	4
Okinawa	4
Ōsaka	4
Saga	4
Saitama	4
Shiga	4
Shimane	4
Shizuoka	4
Tochigi	4
Tokushima	4
Tōkyō	4
Tottori	4
Toyama	4

Appendix Table 1. GBD location hierarchy with levels	
**Geography**	**Level**
Wakayama	4
Yamagata	4
Yamaguchi	4
Yamanashi	4
South Korea	3
Singapore	3
High-income North America	2
Canada	3
Greenland	3
United States	3
Alabama	4
Alaska	4
Arizona	4
Arkansas	4
California	4
Colorado	4
Connecticut	4
Delaware	4
District of Columbia	4
Florida	4
Georgia	4
Hawaii	4
Idaho	4
Illinois	4
Indiana	4
Iowa	4
Kansas	4
Kentucky	4
Louisiana	4
Maine	4
Maryland	4
Massachusetts	4
Michigan	4
Minnesota	4
Mississippi	4
Missouri	4
Montana	4
Nebraska	4
Nevada	4
New Hampshire	4
New Jersey	4
New Mexico	4
New York	4
North Carolina	4
North Dakota	4

Appendix Table 1. GBD location hierarchy with levels	
**Geography**	**Level**
Ohio	4
Oklahoma	4
Oregon	4
Pennsylvania	4
Rhode Island	4
South Carolina	4
South Dakota	4
Tennessee	4
Texas	4
Utah	4
Vermont	4
Virginia	4
Washington	4
West Virginia	4
Wisconsin	4
Wyoming	4
Southern Latin America	2
Argentina	3
Chile	3
Uruguay	3
Western Europe	2
Andorra	3
Austria	3
Belgium	3
Cyprus	3
Denmark	3
Finland	3
France	3
Germany	3
Greece	3
Iceland	3
Ireland	3
Israel	3
Italy	3
Luxembourg	3
Malta	3
Netherlands	3
Norway	3
Portugal	3
Spain	3
Sweden	3
Stockholm	4
Sweden except Stockholm	4
Switzerland	3
United Kingdom	3

Appendix Table 1. GBD location hierarchy with levels	
**Geography**	**Level**
England	4
East Midlands	5
Derby	6
Derbyshire	6
Leicester	6
Leicestershire	6
Lincolnshire	6
Northamptonshire	6
Nottingham	6
Nottinghamshire	6
Rutland	6
East of England	5
Bedford	6
Cambridgeshire	6
Central Bedfordshire	6
Essex	6
Hertfordshire	6
Luton	6
Norfolk	6
Peterborough	6
Southend-on-Sea	6
Suffolk	6
Thurrock	6
Greater London	5
Barking and Dagenham	6
Barnet	6
Bexley	6
Brent	6
Bromley	6
Camden	6
Croydon	6
Ealing	6
Enfield	6
Greenwich	6
Hackney	6
Hammersmith and Fulham	6
Haringey	6
Harrow	6
Havering	6
Hillingdon	6
Hounslow	6
Islington	6
Kensington and Chelsea	6
Kingston upon Thames	6
Lambeth	6

Appendix Table 1. GBD location hierarchy with levels	
**Geography**	**Level**
Lewisham	6
Merton	6
Newham	6
Redbridge	6
Richmond upon Thames	6
Southwark	6
Sutton	6
Tower Hamlets	6
Waltham Forest	6
Wandsworth	6
Westminster	6
North East England	5
County Durham	6
Darlington	6
Gateshead	6
Hartlepool	6
Middlesbrough	6
Newcastle upon Tyne	6
North Tyneside	6
Northumberland	6
Redcar and Cleveland	6
South Tyneside	6
Stockton-on-Tees	6
Sunderland	6
North West England	5
Blackburn with Darwen	6
Blackpool	6
Bolton	6
Bury	6
Cheshire East	6
Cheshire West and Chester	6
Cumbria	6
Halton	6
Knowsley	6
Lancashire	6
Liverpool	6
Manchester	6
Oldham	6
Rochdale	6
Salford	6
Sefton	6
St Helens	6
Stockport	6
Tameside	6
Trafford	6

Appendix Table 1. GBD location hierarchy with levels	
**Geography**	**Level**
Warrington	6
Wigan	6
Wirral	6
South East England	5
Bracknell Forest	6
Brighton and Hove	6
Buckinghamshire	6
East Sussex	6
Hampshire	6
Isle of Wight	6
Kent	6
Medway	6
Milton Keynes	6
Oxfordshire	6
Portsmouth	6
Reading	6
Slough	6
Southampton	6
Surrey	6
West Berkshire	6
West Sussex	6
Windsor and Maidenhead	6
Wokingham	6
South West England	5
Bath and North East Somerset	6
Bournemouth	6
Bristol, City of	6
Cornwall	6
Devon	6
Dorset	6
Gloucestershire	6
North Somerset	6
Plymouth	6
Poole	6
Somerset	6
South Gloucestershire	6
Swindon	6
Torbay	6
Wiltshire	6
West Midlands	5
Birmingham	6
Coventry	6
Dudley	6
Herefordshire, County of	6
Sandwell	6

Appendix Table 1. GBD location hierarchy with levels	
**Geography**	**Level**
Shropshire	6
Solihull	6
Staffordshire	6
Stoke-on-Trent	6
Telford and Wrekin	6
Walsall	6
Warwickshire	6
Wolverhampton	6
Worcestershire	6
Yorkshire and the Humber	5
Barnsley	6
Bradford	6
Calderdale	6
Doncaster	6
East Riding of Yorkshire	6
Kingston upon Hull, City of	6
Kirklees	6
Leeds	6
North East Lincolnshire	6
North Lincolnshire	6
North Yorkshire	6
Rotherham	6
Sheffield	6
Wakefield	6
York	6
Northern Ireland	4
Scotland	4
Wales	4
Latin America and Caribbean	1
Andean Latin America	2
Bolivia	3
Ecuador	3
Peru	3
Caribbean	2
Antigua and Barbuda	3
The Bahamas	3
Barbados	3
Belize	3
Bermuda	3
Cuba	3
Dominica	3
Dominican Republic	3
Grenada	3
Guyana	3
Haiti	3

Appendix Table 1. GBD location hierarchy with levels	
**Geography**	**Level**
Jamaica	3
Puerto Rico	3
Saint Lucia	3
Saint Vincent and the Grenadines	3
Suriname	3
Trinidad and Tobago	3
Virgin Islands, U.S.	3
Central Latin America	2
Colombia	3
Costa Rica	3
El Salvador	3
Guatemala	3
Honduras	3
Mexico	3
Aguascalientes	4
Baja California	4
Baja California Sur	4
Campeche	4
Chiapas	4
Chihuahua	4
Coahuila	4
Colima	4
Mexico City	4
Durango	4
Guanajuato	4
Guerrero	4
Hidalgo	4
Jalisco	4
México	4
Michoacán de Ocampo	4
Morelos	4
Nayarit	4
Nuevo León	4
Oaxaca	4
Puebla	4
Querétaro	4
Quintana Roo	4
San Luis Potosí	4
Sinaloa	4
Sonora	4
Tabasco	4
Tamaulipas	4
Tlaxcala	4
Veracruz de Ignacio de la Llave	4
Yucatán	4

Appendix Table 1. GBD location hierarchy with levels	
**Geography**	**Level**
Zacatecas	4
Nicaragua	3
Panama	3
Venezuela	3
Tropical Latin America	2
Brazil	3
Acre	4
Alagoas	4
Amapá	4
Amazonas	4
Bahia	4
Ceará	4
Distrito Federal	4
Espírito Santo	4
Goiás	4
Maranhão	4
Mato Grosso	4
Mato Grosso do Sul	4
Minas Gerais	4
Pará	4
Paraíba	4
Paraná	4
Pernambuco	4
Piaui	4
Rio de Janeiro	4
Rio Grande do Norte	4
Rio Grande do Sul	4
Rondônia	4
Roraima	4
Santa Catarina	4
São Paulo	4
Sergipe	4
Tocantins	4
Paraguay	3
North Africa and Middle East	1
North Africa and Middle East	2
Afghanistan	3
Algeria	3
Bahrain	3
Egypt	3
Iran	3
Iraq	3
Jordan	3
Kuwait	3
Lebanon	3

Appendix Table 1. GBD location hierarchy with levels

Geography	Level
Libya	3
Morocco	3
Palestine	3
Oman	3
Qatar	3
Saudi Arabia	3
Sudan	3
Syria	3
Tunisia	3
Turkey	3
United Arab Emirates	3
Yemen	3
South Asia	1
South Asia	2
Bangladesh	3
Bhutan	3
India	3
Andhra Pradesh	4
Arunachal Pradesh	4
Assam	4
Bihar	4
Chhattisgarh	4
Delhi	4
Goa	4
Gujarat	4
Haryana	4
Himachal Pradesh	4
Jammu and Kashmir	4
Jharkhand	4
Karnataka	4
Kerala	4
Madhya Pradesh	4
Maharashtra	4
Manipur	4
Meghalaya	4
Mizoram	4
Nagaland	4
Odisha	4
Punjab	4
Rajasthan	4
Sikkim	4
Tamil Nadu	4
Telangana	4
Tripura	4
Uttar Pradesh	4

Appendix Table 1. GBD location hierarchy with levels

Geography	Level
Uttarakhand	4
West Bengal	4
Union Territories other than Delhi	4
Nepal	3
Pakistan	3
Southeast Asia, East Asia, and Oceania	1
East Asia	2
China	3
North Korea	3
Taiwan	3
Oceania	2
American Samoa	3
Federated States of Micronesia	3
Fiji	3
Guam	3
Kiribati	3
Marshall Islands	3
Northern Mariana Islands	3
Papua New Guinea	3
Samoa	3
Solomon Islands	3
Tonga	3
Vanuatu	3
Southeast Asia	2
Cambodia	3
Indonesia	3
Laos	3
Malaysia	3
Maldives	3
Mauritius	3
Myanmar	3
Philippines	3
Sri Lanka	3
Seychelles	3
Thailand	3
Timor-Leste	3
Vietnam	3
Sub-Saharan Africa	1
Central Sub-Saharan Africa	2
Angola	3
Central African Republic	3
Congo	3
Democratic Republic of the Congo	3
Equatorial Guinea	3
Gabon	3

Appendix Table 1. GBD location hierarchy with levels	
**Geography**	**Level**
Eastern Sub-Saharan Africa	2
Burundi	3
Comoros	3
Djibouti	3
Eritrea	3
Ethiopia	3
Kenya	3
Baringo	4
Bomet	4
Bungoma	4
Busia	4
Elgeyo-Marakwet	4
Embu	4
Garissa	4
HomaBay	4
Isiolo	4
Kajiado	4
Kakamega	4
Kericho	4
Kiambu	4
Kilifi	4
Kirinyaga	4
Kisii	4
Kisumu	4
Kitui	4
Kwale	4
Laikipia	4
Lamu	4
Machakos	4
Makueni	4
Mandera	4
Marsabit	4
Meru	4
Migori	4
Mombasa	4
Murang’a	4
Nairobi	4
Nakuru	4
Nandi	4
Narok	4
Nyamira	4
Nyandarua	4
Nyeri	4
Samburu	4
Siaya	4

Appendix Table 1. GBD location hierarchy with levels	
**Geography**	**Level**
TaitaTaveta	4
TanaRiver	4
TharakaNithi	4
TransNzoia	4
Turkana	4
UasinGishu	4
Vihiga	4
Wajir	4
WestPokot	4
Madagascar	3
Malawi	3
Mozambique	3
Rwanda	3
Somalia	3
South Sudan	3
Tanzania	3
Uganda	3
Zambia	3
Southern Sub-Saharan Africa	2
Botswana	3
Lesotho	3
Namibia	3
South Africa	3
Swaziland	3
Zimbabwe	3
Western Sub-Saharan Africa	2
Benin	3
Burkina Faso	3
Cameroon	3
Cape Verde	3
Chad	3
Cote d'Ivoire	3
The Gambia	3
Ghana	3
Guinea	3
Guinea-Bissau	3
Liberia	3
Mali	3
Mauritania	3
Niger	3
Nigeria	3
Sao Tome and Principe	3
Senegal	3
Sierra Leone	3
Togo	3

Appendix Table 2. GATHER checklist of information that should be included in reports of global health estimates, with description of compliance and location of information for GBD 2017.

#	GATHER checklist item	Description of compliance	Reference
**Objectives and funding**			
1	Define the indicators, populations, and time periods for which estimates were made.	Narrative provided in paper and methods appendix describing indicators, definitions, and populations	Main text (Methods—Overview, Geographical units and time periods) and methods appendix
2	List the funding sources for the work.	Funding sources listed in paper	Main text (Summary)
**Data Inputs**			
For all data inputs from multiple sources that are synthesized as part of the study:			
3	Describe how the data were identified and how the data were accessed.	Narrative provided in paper and methods appendix describing data seeking methods	Main text (Methods) and methods appendix
4	Specify the inclusion and exclusion criteria. Identify all ad-hoc exclusions.	Narrative provided in paper and methods appendix describing inclusion and exclusion type	Main text (Methods) and methods appendix
5	Provide information on all included data sources and their main characteristics. For each data source used, report reference information or contact name/institution, population represented, data collection method, year(s) of data collection, sex and age range, diagnostic criteria or measurement method, and sample size, as relevant.	Metadata for data sources by component, geography, cause, risk, or impairment is available through an interactive, online data source tool	Online data citation tools, http://ghdx.healthdata.org/gbd-2017
6	Identify and describe any categories of input data that have potentially important biases (e.g., based on characteristics listed in item 5).	Summary of known biases included in methods appendix	Methods appendix
For data inputs that contribute to the analysis but were not synthesized as part of the study:			
7	Describe and give sources for any other data inputs.	Included in online data source tool	Online data citation tools, http://ghdx.healthdata.org/gbd-2017
For all data inputs:			
8	Provide all data inputs in a file format from which data can be efficiently extracted (e.g., a spreadsheet as opposed to a PDF), including all relevant meta-data listed in item 5. For any data inputs that cannot be shared due to ethical or legal reasons, such as third-party ownership, provide a contact	Downloads of input data available through online tools, including data visualization tools	Online data visualization tools, data query tools, and the Global Health Data Exchange,

	name or the name of the institution that retains the right to the data.	and data query tools; input data not available in tools will be made available upon request	http://ghdx.healthdata.org
**Data analysis**			
9	Provide a conceptual overview of the data analysis method. A diagram may be helpful.	Flow diagrams of the overall methodological processes, as well as cause-specific modelling processes, have been provided	Main text (Methods) and methods appendix
10	Provide a detailed description of all steps of the analysis, including mathematical formulae. This description should cover, as relevant, data cleaning, data pre-processing, data adjustments and weighting of data sources, and mathematical or statistical model(s).	Flow diagrams and corresponding methodological write-ups have been provided	Main text (Methods) and methods appendix
11	Describe how candidate models were evaluated and how the final model(s) were selected.	Details on evaluation of model performance have been provided	Methods appendix
12	Provide the results of an evaluation of model performance, if done, as well as the results of any relevant sensitivity analysis.	Details on evaluation of model performance have been provided	Methods appendix
13	Describe methods for calculating uncertainty of the estimates. State which sources of uncertainty were, and were not, accounted for in the uncertainty analysis.	Details on uncertainty calculations have been provide	Methods appendix
14	State how analytic or statistical source code used to generate estimates can be accessed.	Access statement provided	Code is provided in an online repository, http://ghdx.healthdata.org/gbd-2017
**Results and Discussion**			
15	Provide published estimates in a file format from which data can be efficiently extracted.	Results are available through online data visualization tools, the Global Health Data Exchange, and the online data query tool	Online data tools (data visualization tools, data query tools, and the Global Health Data Exchange, http://ghdx.healthdata.org/gbd-2017)
16	Report a quantitative measure of the uncertainty of the estimates (e.g. uncertainty intervals).	Uncertainty intervals are provided with all results	Main text, methods appendix, and online data tools (data visualization tools, data query tools, and the Global Health Data Exchange, http://ghdx.healthdata.org/gbd-2017)

17	Interpret results in light of existing evidence. If updating a previous set of estimates, describe the reasons for changes in estimates.	Discussion of methodological differences between GBD estimates and other available evidence provided in the paper and methods appendix	Main text (Methods and Discussion) and methods appendix
18	Discuss limitations of the estimates. Include a discussion of any modelling assumptions or data limitations that affect interpretation of the estimates.	Discussion of limitations provided was provided	Main text (Limitations) and methods appendix

**Appendix Table 3. Number of unique sources used for the analysis of age-specific fertility for each location**						
**Reference sources are bolded and italicized**						
**Location**	**Vital Registrations**	**Complete Birth Histories**	**Summary Birth Histories**	**Censues**	**Other**	**Sample Registrations**
Afghanistan	1	***2***	5	1	0	0
Albania	60	***2***	4	2	0	0
Algeria	***50***	***2***	3	0	0	0
American Samoa	***56***	0	0	***1***	0	0
Andorra	***43***	0	0	0	0	0
Angola	22	***3***	4	0	0	0
Antigua and Barbuda	***50***	0	0	0	0	0
Argentina	***59***	0	2	4	0	0
Armenia	***57***	***4***	2	2	0	0
Australia	***66***	0	0	2	0	0
Austria	***67***	0	0	2	0	0
Azerbaijan	***58***	***1***	4	2	0	0
Bahrain	***48***	0	2	2	1	0
Bangladesh	***35***	***10***	9	2	33	0
Barbados	***63***	0	1	1	0	0
Belarus	***67***	0	***3***	***1***	0	0
Belgium	***67***	0	0	1	0	0
Belize	***64***	0	***4***	***3***	0	0
Benin	2	***5***	***4***	1	0	0
Bermuda	***58***	0	0	***3***	0	0
Bhutan	***1***	0	***1***	***1***	0	0
Bolivia	48	***5***	7	4	1	0
Bosnia and Herzegovina	***28***	1	1	0	1	0
Botswana	***16***	***1***	***7***	***3***	0	0
Brazil	53	***4***	***15***	***3***	0	0
Brunei	***57***	0	0	1	0	0
Bulgaria	***67***	0	2	1	0	0
Burkina Faso	0	***5***	***7***	***4***	0	0
Burundi	5	***2***	8	***1***	0	0
Cambodia	1	***5***	5	2	0	0
Cameroon	0	***7***	***3***	2	0	0
Canada	***65***	0	0	2	0	0
Cape Verde	***42***	0	***2***	***1***	0	0
Central African Republic	0	***1***	4	***2***	0	0
Chad	0	***3***	***3***	0	0	0
Chile	***66***	0	1	4	0	0
China	***40***	9	2	***4***	***22***	0
Colombia	59	***8***	6	4	0	0
Comoros	9	***2***	2	1	0	0
Congo (Brazzaville)	0	***2***	***3***	0	0	0
Costa Rica	***62***	1	6	4	0	0
Cote d'Ivoire	1	***6***	5	***1***	0	1
Croatia	***36***	0	0	1	1	0
Cuba	***65***	0	3	1	0	0
Cyprus	***67***	0	1	2	39	0
Czech Republic	***67***	0	2	1	0	0
Denmark	***67***	0	0	0	16	0
Djibouti	28	***1***	3	0	0	0
Dominica	***50***	0	0	2	0	0
Dominican Republic	52	***10***	5	4	0	0
DR Congo	0	***2***	***7***	0	0	0
Ecuador	***58***	6	***10***	***5***	0	0
Egypt	***62***	9	8	2	0	0
El Salvador	***58***	***6***	4	3	0	0
Equatorial Guinea	17	***1***	1	0	0	0
Eritrea	0	***2***	2	0	0	0
Estonia	***67***	0	1	1	0	0
Ethiopia	0	***5***	4	3	0	0
Federated States of Micronesia	***8***	0	***1***	***3***	0	0
Fiji	***59***	***1***	3	4	0	0

Appendix Table 3. Number of unique sources used for the analysis of age-specific fertility for each location

Reference sources are bolded and italicized

Location	Vital Registrations	Complete Birth Histories	Summary Birth Histories	Censuses	Other	Sample Registrations
Finland	***67***	0	0	1	0	0
France	***67***	0	0	0	0	0
Gabon	0	***2***	1	***1***	0	0
Georgia	***51***	***1***	***3***	0	26	0
Germany	***67***	0	0	0	0	0
Ghana	28	***10***	13	2	0	0
Greece	***67***	0	1	1	4	0
Greenland	***67***	0	0	0	0	0
Grenada	***44***	0	0	1	0	0
Guam	***67***	0	0	1	0	0
Guatemala	***58***	6	5	1	0	0
Guinea	2	***3***	2	***2***	0	1
Guinea-Bissau	21	***1***	3	0	0	0
Guyana	34	***5***	7	1	0	0
Haiti	0	***5***	3	2	0	0
Honduras	36	***4***	7	2	0	0
Hong Kong Special Administrative Region of China	***67***	0	0	1	0	0
Hungary	***67***	0	1	4	13	0
Iceland	***67***	0	0	1	0	0
India	***65***	6	9	4	22	***24***
Indonesia	***15***	***8***	***10***	***6***	***5***	0
Iran	***55***	0	***5***	***4***	0	0
Iraq	39	***4***	6	1	0	0
Ireland	***67***	0	0	2	0	0
Israel	***67***	0	0	1	0	0
Italy	***67***	0	0	0	0	0
Jamaica	***56***	***1***	4	2	0	0
Japan	***67***	0	0	1	0	0
Jordan	48	***6***	6	1	9	0
Kazakhstan	***52***	***2***	4	2	0	0
Kenya	***49***	***8***	***11***	***5***	***3***	0
Kiribati	***18***	0	***1***	***3***	0	0
Kuwait	***57***	0	1	1	1	0
Kyrgyzstan	***53***	***3***	5	1	0	0
Laos	0	***2***	4	1	0	0
Latvia	***67***	0	1	1	0	0
Lebanon	27	***1***	4	0	0	0
Lesotho	2	***3***	6	1	1	0
Liberia	5	***4***	4	***4***	2	0
Libya	***46***	0	***2***	***1***	0	0
Lithuania	***67***	0	0	1	0	0
Luxembourg	***67***	0	0	2	0	0
Macao Special Administrative Region of China	***56***	0	1	1	0	0
Macedonia	***31***	0	2	1	1	0
Madagascar	24	***6***	4	0	0	0
Malawi	5	***7***	9	4	3	0
Malaysia	***58***	0	2	1	0	0
Maldives	***47***	***1***	***3***	3	0	0
Mali	3	***6***	3	3	0	0
Malta	***67***	0	0	0	0	0
Marshall Islands	***26***	0	***1***	***2***	0	0
Mauritania	1	***2***	5	1	0	0
Mauritius	***67***	0	0	1	0	0
Mexico	66	1	***11***	5	0	0
Moldova	***52***	***2***	4	0	0	0
Mongolia	***44***	***2***	9	0	0	0
Montenegro	***26***	0	1	0	1	0
Morocco	18	***5***	5	3	0	0
Mozambique	28	***4***	5	***3***	0	0

Appendix Table 3. Number of unique sources used for the analysis of age-specific fertility for each location

Reference sources are bolded and italicized

Location	Vital Registrations	Complete Birth Histories	Summary Birth Histories	Censuses	Other	Sample Registrations
Myanmar	6	***2***	3	2	0	0
Namibia	2	***4***	1	***3***	0	0
Nepal	0	***8***	***6***	2	0	0
Netherlands	***67***	0	0	1	0	0
New Zealand	***67***	0	0	2	0	0
Nicaragua	44	***5***	***6***	***3***	0	0
Niger	0	***4***	2	***1***	0	0
Nigeria	2	***8***	11	3	0	0
North Korea	***2***	0	0	0	0	0
Northern Mariana Islands	***8***	0	0	0	0	0
Norway	***67***	0	0	1	0	0
Oman	***11***	0	0	***1***	***8***	0
Pakistan	16	***6***	10	1	19	1
Palestine	20	***3***	6	0	0	0
Panama	***66***	1	5	5	0	0
Papua New Guinea	***4***	0	***2***	***3***	0	0
Paraguay	42	***6***	10	4	0	0
Peru	62	***14***	7	2	0	0
Philippines	66	***6***	4	3	***11***	0
Poland	***67***	0	0	1	6	0
Portugal	***67***	1	1	2	0	0
Puerto Rico	***58***	0	1	2	0	0
Qatar	***47***	0	0	***1***	***1***	0
Romania	***67***	0	3	3	0	0
Russian Federation	***66***	0	1	1	0	0
Rwanda	19	***8***	***8***	4	0	0
Saint Lucia	***47***	0	0	2	0	0
Saint Vincent and the Grenadines	***54***	0	0	2	0	0
Samoa	38	0	0	***1***	0	***3***
Sao Tome and Principe	***41***	***1***	5	***2***	0	0
Saudi Arabia	***8***	0	***2***	0	0	0
Senegal	3	***13***	4	***3***	0	0
Serbia	***67***	0	3	0	1	0
Seychelles	***67***	0	0	***3***	0	0
Sierra Leone	12	***3***	5	1	0	0
Singapore	***67***	0	0	2	0	0
Slovakia	***67***	0	1	1	0	0
Slovenia	***64***	0	0	1	1	0
Solomon Islands	1	***1***	0	3	0	0
Somalia	0	***1***	***3***	0	0	0
South Africa	27	***4***	5	1	0	0
South Korea	***43***	***1***	***2***	2	22	0
South Sudan	0	0	***2***	***1***	0	0
Spain	***67***	0	0	1	0	0
Sri Lanka	***66***	2	3	2	5	0
Sudan	4	***5***	***5***	3	0	0
Suriname	***51***	0	***2***	***1***	0	0
Swaziland	3	***3***	3	***3***	0	0
Sweden	***67***	0	1	0	0	0
Switzerland	***67***	0	0	2	0	0
Syria	***57***	2	6	2	0	0
Taiwan (Province of China)	***66***	0	0	0	0	0
Tajikistan	44	***2***	6	1	0	0
Tanzania	1	***7***	7	2	0	0
Thailand	***67***	1	8	3	6	0
The Bahamas	***65***	0	0	2	0	0
The Gambia	0	***1***	5	***2***	0	0
Timor-Leste	19	***5***	***12***	0	0	0
Togo	1	***4***	***3***	2	1	0
Tonga	***46***	0	***2***	***2***	0	0

**Appendix Table 3. Number of unique sources used for the analysis of age-specific fertility for each location**						
**Reference sources are bolded and italicized**						
**Location**	**Vital Registrations**	**Complete Birth Histories**	**Summary Birth Histories**	**Censuses**	**Other**	**Sample Registrations**
Trinidad and Tobago	***64***	***2***	***6***	2	0	0
Tunisia	***66***	***4***	7	1	0	0
Turkey	***25***	***4***	***9***	***2***	***4***	0
Turkmenistan	35	***1***	3	0	0	0
Uganda	2	***8***	10	2	0	1
Ukraine	***66***	***1***	3	1	0	0
United Arab Emirates	***32***	0	***1***	***1***	0	0
United Kingdom	***67***	0	0	0	0	0
Uruguay	***57***	0	3	5	0	0
USA	***67***	0	0	1	0	0
Uzbekistan	46	***2***	4	0	0	0
Vanuatu	0	0	***2***	***4***	0	0
Venezuela	***65***	1	3	3	0	0
Vietnam	5	***3***	***15***	3	4	0
Virgin Islands	***46***	0	0	1	0	0
Yemen	4	***3***	***7***	1	0	0
Zambia	3	***5***	0	0	0	0
Zimbabwe	1	***8***	2	2	0	0

Appendix Table 4. Number of unique sources used for the analysis of age-specific fertility by year						
Year	Vital Registrations	Complete Birth Histories	Summary Birth Histories	Censues	Other	Sample Registrations
1950	119	0	0	0	1	0
1951	119	1	0	0	1	0
1952	118	0	0	0	0	0
1953	119	0	0	0	0	0
1954	118	0	0	1	0	0
1955	122	0	0	0	1	0
1956	122	0	0	0	0	0
1957	122	0	0	0	0	0
1958	123	1	0	0	2	0
1959	124	0	0	0	0	0
1960	127	0	0	11	3	0
1961	125	0	0	2	3	0
1962	123	0	0	0	3	0
1963	126	0	0	0	3	0
1964	126	0	0	1	4	0
1965	122	0	0	0	4	0
1966	118	0	0	2	4	0
1967	115	0	0	1	4	0
1968	116	0	0	0	3	0
1969	117	0	0	1	2	0
1970	124	0	0	9	7	0
1971	121	0	3	10	5	1
1972	114	1	1	1	8	0
1973	111	3	1	7	2	0
1974	115	8	6	4	4	0
1975	114	3	5	4	5	0
1976	118	8	7	6	6	1
1977	113	4	6	4	3	0
1978	116	5	9	2	3	0
1979	124	0	5	3	3	0
1980	127	0	6	30	6	0
1981	125	0	8	18	5	1
1982	122	1	2	7	5	0
1983	121	1	3	4	4	0
1984	124	4	5	4	6	0
1985	127	8	5	3	5	0
1986	127	8	6	5	7	1
1987	127	7	11	4	7	0
1988	122	5	9	6	5	0
1989	123	4	9	4	5	0
1990	127	11	13	11	6	0
1991	124	11	20	16	6	1
1992	125	9	11	9	3	0
1993	119	9	15	4	6	0
1994	114	10	10	5	4	0
1995	109	10	12	3	5	0
1996	110	16	13	7	8	1
1997	96	9	13	3	8	0
1998	97	10	13	3	4	1
1999	100	15	20	8	7	1
2000	105	6	51	21	6	1
2001	98	14	21	21	6	1

Appendix Table 4. Number of unique sources used for the analysis of age-specific fertility by year

Year	Vital Registrations	Complete Birth Histories	Summary Birth Histories	Censues	Other	Sample Registrations
2002	109	12	14	10	6	1
2003	110	14	15	1	8	1
2004	107	20	14	2	5	1
2005	105	22	25	6	7	2
2006	108	15	36	3	7	1
2007	112	15	19	5	9	1
2008	111	13	22	8	5	1
2009	108	19	23	7	7	2
2010	104	22	30	10	5	1
2011	105	15	28	11	8	1
2012	137	21	35	4	7	1
2013	132	15	21	0	5	1
2014	126	17	26	1	4	2
2015	109	6	10	0	3	1
2016	73	1	6	1	0	4
2017	1	0	0	0	0	0

Appendix Table 5. List of all confirmed censuses by location and year

Location	Year	Type	Age detail	Enumeration method	Included or excluded from estimation
Afghanistan	1979	Census	5-year age groups	De facto	Included
Albania	1950	Census	5-year age groups	De facto	Included
Albania	1955	Census	5-year age groups	De facto	Included
Albania	1960	Census	5-year age groups	De facto	Included
Albania	1969	Census	all ages only	De facto	Included
Albania	1979	Census	all ages only	De jure	Included
Albania	1989	Census	all ages only	De jure	Included
Albania	2001	Census	5-year age groups	De facto	Included
Albania	2011	Census	5-year age groups	De jure	Included
Algeria	1954	Census	non-std age groups	Unknown	Included
Algeria	1960	Census	Not Available	Unknown	Excluded
Algeria	1966	Census	5-year age groups	De jure	Included
Algeria	1977	Census	5-year age groups	De jure	Included
Algeria	1987	Census	5-year age groups	De jure	Included
Algeria	1998	Census	5-year age groups	De jure	Included
Algeria	2008	Census	1-year age groups	De jure	Included
American Samoa	1950	Census	5-year age groups	De jure	Included
American Samoa	1956	Census	1-year age groups	De jure	Included
American Samoa	1960	Census	5-year age groups	De jure	Included
American Samoa	1970	Census	5-year age groups	De jure	Included
American Samoa	1974	Census	5-year age groups	De jure	Included
American Samoa	1980	Census	1-year age groups	De jure	Included
American Samoa	1990	Census	5-year age groups	De jure	Included
American Samoa	2000	Census	5-year age groups	De jure	Included
American Samoa	2010	Census	5-year age groups	De jure	Included
Andorra	1966	Registry	non-std age groups	NA	Included
Andorra	1985	Registry	1-year age groups	NA	Included
Andorra	1986	Registry	1-year age groups	NA	Included
Andorra	1987	Registry	1-year age groups	NA	Included
Andorra	1988	Registry	1-year age groups	NA	Included
Andorra	1989	Census	Not Available	Unknown	Excluded
Andorra	1989	Registry	1-year age groups	NA	Included
Andorra	1990	Registry	1-year age groups	NA	Included
Andorra	1991	Registry	1-year age groups	NA	Included
Andorra	1992	Registry	1-year age groups	NA	Included
Andorra	1993	Registry	1-year age groups	NA	Included
Andorra	1994	Registry	1-year age groups	NA	Included
Andorra	1995	Registry	1-year age groups	NA	Included
Andorra	1996	Registry	1-year age groups	NA	Included
Andorra	1997	Registry	1-year age groups	NA	Included
Andorra	1998	Registry	1-year age groups	NA	Included
Andorra	1999	Registry	1-year age groups	NA	Included
Andorra	2000	Registry	1-year age groups	NA	Included
Andorra	2001	Registry	1-year age groups	NA	Included
Andorra	2002	Registry	1-year age groups	NA	Included
Andorra	2003	Registry	1-year age groups	NA	Included
Andorra	2004	Registry	1-year age groups	NA	Included
Andorra	2005	Registry	1-year age groups	NA	Included
Andorra	2006	Registry	1-year age groups	NA	Included
Andorra	2007	Registry	1-year age groups	NA	Included
Andorra	2008	Registry	1-year age groups	NA	Included
Andorra	2009	Registry	1-year age groups	NA	Included
Andorra	2010	Registry	1-year age groups	NA	Included
Andorra	2011	Census	Not Available	Unknown	Included
Andorra	2011	Registry	1-year age groups	NA	Included
Andorra	2012	Registry	1-year age groups	NA	Included
Andorra	2013	Registry	1-year age groups	NA	Included
Andorra	2014	Registry	1-year age groups	NA	Included
Andorra	2015	Registry	1-year age groups	NA	Included
Andorra	2016	Registry	1-year age groups	NA	Included
Andorra	2017	Registry	1-year age groups	NA	Included
Angola	1950	Census	5-year age groups	De facto	Included
Angola	1960	Census	5-year age groups	De jure	Included
Angola	1970	Census	all ages only	Unknown	Included
Angola	2014	Census	1-year age groups	De facto	Included
Antigua and Barbuda	1960	Census	1-year age groups	De facto	Included
Antigua and Barbuda	1970	Census	5-year age groups	De jure	Included
Antigua and Barbuda	1991	Census	1-year age groups	De jure	Included
Antigua and Barbuda	2001	Census	1-year age groups	De facto	Included
Antigua and Barbuda	2011	Census	5-year age groups	De jure	Included
Argentina	1960	Census	1-year age groups	De facto	Included
Argentina	1970	Census	1-year age groups	De facto	Included
Argentina	1980	Census	1-year age groups	De facto	Included
Argentina	1991	Census	1-year age groups	De facto	Included
Argentina	2001	Census	1-year age groups	De facto	Included

Appendix Table 5. List of all confirmed censuses by location and year

Location	Year	Type	Age detail	Enumeration method	Included or excluded from estimation
Argentina	2010	Census	1-year age groups	De facto	Included
Armenia	1959	Census	non-std age groups	De facto	Included
Armenia	1970	Census	non-std age groups	De facto	Included
Armenia	1979	Census	5-year age groups	De facto	Included
Armenia	1989	Census	5-year age groups	De jure	Included
Armenia	2001	Census	1-year age groups	De jure	Included
Armenia	2011	Census	1-year age groups	De jure	Included
Australia	1954	Census	5-year age groups	De facto	Included
Australia	1961	Census	1-year age groups	De facto	Included
Australia	1966	Census	5-year age groups	De facto	Included
Australia	1971	Census	1-year age groups	De facto	Included
Australia	1976	Census	5-year age groups	De facto	Included
Australia	1981	Census	5-year age groups	De facto	Included
Australia	1986	Census	1-year age groups	De facto	Included
Australia	1991	Census	1-year age groups	De facto	Included
Australia	1996	Census	1-year age groups	De facto	Included
Australia	2001	Census	1-year age groups	De jure	Included
Australia	2006	Census	1-year age groups	De jure	Included
Australia	2011	Census	1-year age groups	De jure	Included
Australia	2016	Census	1-year age groups	De jure	Included
Austria	1951	Census	1-year age groups	De jure	Included
Austria	1961	Census	1-year age groups	De jure	Included
Austria	1971	Census	1-year age groups	De jure	Included
Austria	1981	Census	1-year age groups	De jure	Included
Austria	1991	Census	1-year age groups	De jure	Included
Austria	2001	Census	1-year age groups	De jure	Included
Austria	2002	Registry	5-year age groups	NA	Included
Austria	2003	Registry	5-year age groups	NA	Included
Austria	2004	Registry	5-year age groups	NA	Included
Austria	2005	Registry	5-year age groups	NA	Included
Austria	2006	Registry	5-year age groups	NA	Included
Austria	2007	Registry	5-year age groups	NA	Included
Austria	2008	Registry	5-year age groups	NA	Included
Austria	2009	Registry	5-year age groups	NA	Included
Austria	2010	Registry	5-year age groups	NA	Included
Austria	2011	Census	1-year age groups	De jure	Included
Austria	2011	Registry	5-year age groups	NA	Included
Austria	2012	Registry	5-year age groups	NA	Included
Austria	2013	Registry	5-year age groups	NA	Included
Austria	2014	Registry	5-year age groups	NA	Included
Austria	2015	Registry	5-year age groups	NA	Included
Austria	2016	Registry	5-year age groups	NA	Included
Austria	2017	Registry	5-year age groups	NA	Included
Azerbaijan	1959	Census	non-std age groups	De facto	Included
Azerbaijan	1970	Census	non-std age groups	De facto	Included
Azerbaijan	1979	Census	5-year age groups	De facto	Included
Azerbaijan	1989	Census	5-year age groups	De jure	Included
Azerbaijan	1999	Census	1-year age groups	De jure	Included
Azerbaijan	2009	Census	1-year age groups	De jure	Included
Bahrain	1950	Census	all ages only	De facto	Included
Bahrain	1959	Census	non-std age groups	De facto	Included
Bahrain	1965	Census	non-std age groups	De facto	Included
Bahrain	1971	Census	1-year age groups	De facto	Included
Bahrain	1981	Census	5-year age groups	De facto	Included
Bahrain	1991	Census	1-year age groups	De facto	Included
Bahrain	2001	Census	non-std age groups	De jure	Included
Bahrain	2010	Census	1-year age groups	De jure	Included
Bangladesh	1951	Census	non-std age groups	De jure	Included
Bangladesh	1961	Census	non-std age groups	De jure	Included
Bangladesh	1974	Census	5-year age groups	De facto	Included
Bangladesh	1981	Census	5-year age groups	De facto	Included
Bangladesh	1991	Census	non-std age groups	De facto	Included
Bangladesh	2001	Census	1-year age groups	De facto	Included
Bangladesh	2011	Census	5-year age groups	De facto	Included
Barbados	1960	Census	1-year age groups	De facto	Included
Barbados	1970	Census	1-year age groups	De facto	Included
Barbados	1980	Census	1-year age groups	De facto	Included
Barbados	1990	Census	5-year age groups	De jure	Included
Barbados	2000	Census	1-year age groups	De facto	Included
Barbados	2010	Census	5-year age groups	De jure	Included
Belarus	1959	Census	non-std age groups	De facto	Included
Belarus	1970	Census	non-std age groups	De facto	Included
Belarus	1979	Census	5-year age groups	De facto	Included
Belarus	1989	Census	1-year age groups	De jure	Included
Belarus	1999	Census	1-year age groups	De jure	Included

Appendix Table 5. List of all confirmed censuses by location and year					
**Location**	**Year**	**Type**	**Age detail**	**Enumeration method**	**Included or excluded from estimation**
Belarus	2009	Census	1-year age groups	De jure	Included
Belgium	1961	Census	1-year age groups	De jure	Included
Belgium	1970	Census	1-year age groups	De jure	Included
Belgium	1981	Census	1-year age groups	De jure	Included
Belgium	1991	Census	5-year age groups	De jure	Included
Belgium	2001	Census	1-year age groups	De jure	Included
Belgium	2010	Registry	1-year age groups	NA	Included
Belgium	2011	Census	1-year age groups	De jure	Included
Belgium	2011	Registry	1-year age groups	NA	Included
Belgium	2012	Registry	1-year age groups	NA	Included
Belgium	2013	Registry	1-year age groups	NA	Included
Belgium	2014	Registry	1-year age groups	NA	Included
Belgium	2015	Registry	1-year age groups	NA	Included
Belgium	2016	Registry	1-year age groups	NA	Included
Belgium	2017	Registry	1-year age groups	NA	Included
Belize	1960	Census	5-year age groups	De facto	Included
Belize	1970	Census	1-year age groups	De facto	Included
Belize	1980	Census	1-year age groups	De facto	Included
Belize	1991	Census	1-year age groups	De facto	Included
Belize	2000	Census	non-std age groups	De jure	Included
Belize	2010	Census	5-year age groups	De jure	Included
Benin	1951	Census	Not Available	Unknown	Excluded
Benin	1979	Census	5-year age groups	De facto	Included
Benin	1992	Census	1-year age groups	De facto	Included
Benin	2002	Census	1-year age groups	De jure	Included
Benin	2013	Census	5-year age groups	De jure	Included
Bermuda	1950	Census	1-year age groups	De jure	Included
Bermuda	1960	Census	1-year age groups	De jure	Included
Bermuda	1970	Census	1-year age groups	De jure	Included
Bermuda	1980	Census	1-year age groups	De jure	Included
Bermuda	1991	Census	1-year age groups	De jure	Included
Bermuda	2000	Census	1-year age groups	De jure	Included
Bermuda	2010	Census	1-year age groups	De jure	Included
Bhutan	1969	Census	Not Available	Unknown	Excluded
Bhutan	1980	Census	Not Available	Unknown	Excluded
Bhutan	2005	Census	1-year age groups	De facto	Included
Bhutan	2017	Census	Not Available	Unknown	Excluded
Bolivia	1950	Census	1-year age groups	De facto	Included
Bolivia	1976	Census	5-year age groups	De facto	Included
Bolivia	1992	Census	1-year age groups	De facto	Included
Bolivia	2001	Census	1-year age groups	De facto	Included
Bolivia	2012	Census	1-year age groups	De facto	Included
Bosnia and Herzegovina	1971	Census	1-year age groups	De facto	Included
Bosnia and Herzegovina	1981	Census	5-year age groups	De jure	Included
Bosnia and Herzegovina	1991	Census	1-year age groups	De jure	Included
Bosnia and Herzegovina	2013	Census	1-year age groups	De jure	Included
Botswana	1964	Census	1-year age groups	De facto	Included
Botswana	1971	Census	1-year age groups	De facto	Excluded
Botswana	1981	Census	1-year age groups	De facto	Included
Botswana	1991	Census	1-year age groups	De facto	Included
Botswana	2001	Census	5-year age groups	De facto	Included
Botswana	2011	Census	1-year age groups	De facto	Included
Brazil	1950	Census	1-year age groups	De facto	Included
Brazil	1960	Census	1-year age groups	De facto	Included
Brazil	1970	Census	1-year age groups	De facto	Included
Brazil	1980	Census	1-year age groups	De jure	Included
Brazil	1991	Census	1-year age groups	De jure	Included
Brazil	2000	Census	1-year age groups	De jure	Included
Brazil	2010	Census	1-year age groups	De jure	Included
Brunei	1960	Census	5-year age groups	De facto	Included
Brunei	1971	Census	1-year age groups	De facto	Included
Brunei	1981	Census	1-year age groups	De facto	Included
Brunei	1991	Census	1-year age groups	De facto	Included
Brunei	2001	Census	5-year age groups	De facto	Included
Brunei	2011	Census	1-year age groups	De jure	Included
Bulgaria	1956	Census	5-year age groups	De facto	Included
Bulgaria	1965	Census	5-year age groups	De facto	Included
Bulgaria	1975	Census	5-year age groups	De facto	Included
Bulgaria	1985	Census	5-year age groups	De jure	Included
Bulgaria	1992	Census	5-year age groups	De jure	Included
Bulgaria	2001	Census	5-year age groups	De jure	Included
Bulgaria	2001	Registry	5-year age groups	NA	Included
Bulgaria	2002	Registry	5-year age groups	NA	Included
Bulgaria	2003	Registry	5-year age groups	NA	Included
Bulgaria	2004	Registry	5-year age groups	NA	Included

Appendix Table 5. List of all confirmed censuses by location and year					
**Location**	**Year**	**Type**	**Age detail**	**Enumeration method**	**Included or excluded from estimation**
Bulgaria	2005	Registry	5-year age groups	NA	Included
Bulgaria	2006	Registry	5-year age groups	NA	Included
Bulgaria	2007	Registry	5-year age groups	NA	Included
Bulgaria	2008	Registry	5-year age groups	NA	Included
Bulgaria	2009	Registry	5-year age groups	NA	Included
Bulgaria	2010	Registry	5-year age groups	NA	Included
Bulgaria	2011	Census	1-year age groups	De jure	Included
Bulgaria	2011	Registry	5-year age groups	NA	Included
Bulgaria	2012	Registry	5-year age groups	NA	Included
Bulgaria	2013	Registry	5-year age groups	NA	Included
Bulgaria	2014	Registry	5-year age groups	NA	Included
Bulgaria	2015	Registry	5-year age groups	NA	Included
Bulgaria	2016	Registry	5-year age groups	NA	Included
Burkina Faso	1951	Census	Not Available	Unknown	Excluded
Burkina Faso	1976	Census	non-std age groups	Unknown	Included
Burkina Faso	1985	Census	1-year age groups	De jure	Included
Burkina Faso	1996	Census	1-year age groups	De jure	Included
Burkina Faso	2006	Census	1-year age groups	De jure	Included
Burkina Faso	2016	Census	Not Available	Unknown	Excluded
Burundi	1952	Census	Not Available	Unknown	Excluded
Burundi	1958	Census	Not Available	Unknown	Excluded
Burundi	1979	Census	5-year age groups	De jure	Included
Burundi	1990	Census	1-year age groups	De jure	Included
Burundi	2008	Census	1-year age groups	De jure	Included
Cambodia	1962	Census	1-year age groups	De facto	Included
Cambodia	1998	Census	1-year age groups	De facto	Included
Cambodia	2008	Census	1-year age groups	De facto	Included
Cameroon	1951	Census	Not Available	Unknown	Excluded
Cameroon	1957	Census	Not Available	Unknown	Excluded
Cameroon	1976	Census	5-year age groups	De jure	Included
Cameroon	1987	Census	1-year age groups	Unknown	Included
Cameroon	2005	Census	1-year age groups	De jure	Included
Canada	1951	Census	5-year age groups	De jure	Included
Canada	1956	Census	1-year age groups	De jure	Included
Canada	1961	Census	5-year age groups	De jure	Included
Canada	1966	Census	5-year age groups	De jure	Included
Canada	1971	Census	5-year age groups	De jure	Included
Canada	1976	Census	5-year age groups	De jure	Included
Canada	1981	Census	1-year age groups	De jure	Included
Canada	1986	Census	5-year age groups	De jure	Included
Canada	1991	Census	1-year age groups	De jure	Included
Canada	1996	Census	1-year age groups	De jure	Included
Canada	2001	Census	1-year age groups	De jure	Included
Canada	2006	Census	1-year age groups	De jure	Included
Canada	2011	Census	1-year age groups	De jure	Included
Canada	2016	Census	1-year age groups	De jure	Included
Cape Verde	1950	Census	non-std age groups	De facto	Included
Cape Verde	1960	Census	non-std age groups	De facto	Included
Cape Verde	1970	Census	5-year age groups	De facto	Included
Cape Verde	1980	Census	5-year age groups	De jure	Included
Cape Verde	1990	Census	1-year age groups	De facto	Included
Cape Verde	2000	Census	5-year age groups	De facto	Included
Cape Verde	2010	Census	1-year age groups	De jure	Included
Central African Republic	1951	Census	Not Available	Unknown	Excluded
Central African Republic	1956	Census	Not Available	Unknown	Excluded
Central African Republic	1975	Census	5-year age groups	De facto	Included
Central African Republic	1988	Census	1-year age groups	De facto	Included
Central African Republic	2003	Census	5-year age groups	De facto	Included
Chad	1951	Census	Not Available	Unknown	Excluded
Chad	1993	Census	5-year age groups	De jure	Included
Chad	2009	Census	5-year age groups	De jure	Included
Chile	1952	Census	5-year age groups	De facto	Included
Chile	1960	Census	5-year age groups	De facto	Included
Chile	1970	Census	1-year age groups	De facto	Included
Chile	1982	Census	1-year age groups	De facto	Included
Chile	1992	Census	1-year age groups	De facto	Included
Chile	2002	Census	5-year age groups	De facto	Included
Chile	2012	Census	1-year age groups	De jure	Excluded
Chile	2017	Census	5-year age groups	De facto	Included
China (without Hong Kong and Macao)	1953	Census	1-year age groups	De jure	Included
China (without Hong Kong and Macao)	1964	Census	1-year age groups	De jure	Included
China (without Hong Kong and Macao)	1982	Census	1-year age groups	De facto	Included
China (without Hong Kong and Macao)	1990	Census	1-year age groups	De jure	Included
China (without Hong Kong and Macao)	2000	Census	1-year age groups	De jure	Included
China (without Hong Kong and Macao)	2010	Census	1-year age groups	De jure	Included

Appendix Table 5. List of all confirmed censuses by location and year

Location	Year	Type	Age detail	Enumeration method	Included or excluded from estimation
Colombia	1951	Census	5-year age groups	De facto	Included
Colombia	1964	Census	1-year age groups	De facto	Included
Colombia	1973	Census	1-year age groups	De facto	Included
Colombia	1985	Census	1-year age groups	De facto	Included
Colombia	1993	Census	1-year age groups	De facto	Included
Colombia	2005	Census	1-year age groups	De facto	Included
Comoros	1958	Census	5-year age groups	De facto	Excluded
Comoros	1966	Census	non-std age groups	De jure	Included
Comoros	1980	Census	1-year age groups	De facto	Included
Comoros	1991	Census	5-year age groups	De jure	Included
Comoros	2003	Census	non-std age groups	De jure	Included
Comoros	2016	Census	Not Available	Unknown	Excluded
Congo	1951	Census	Not Available	Unknown	Excluded
Congo	1954	Census	Not Available	Unknown	Excluded
Congo	1956	Census	Not Available	Unknown	Excluded
Congo	1967	Census	Not Available	Unknown	Excluded
Congo	1974	Census	5-year age groups	De jure	Included
Congo	1984	Census	1-year age groups	De jure	Included
Congo	1996	Census	Not Available	Unknown	Excluded
Congo	2007	Census	1-year age groups	De facto	Included
Congo	2017	Census	Not Available	Unknown	Excluded
Costa Rica	1950	Census	1-year age groups	De jure	Included
Costa Rica	1963	Census	1-year age groups	De jure	Included
Costa Rica	1973	Census	1-year age groups	De jure	Included
Costa Rica	1984	Census	1-year age groups	De jure	Included
Costa Rica	2000	Census	5-year age groups	De jure	Included
Costa Rica	2011	Census	1-year age groups	De jure	Included
Cote d'Ivoire	1975	Census	5-year age groups	De facto	Included
Cote d'Ivoire	1988	Census	1-year age groups	De facto	Included
Cote d'Ivoire	1998	Census	5-year age groups	De jure	Included
Cote d'Ivoire	2014	Census	1-year age groups	De jure	Included
Croatia	1971	Census	1-year age groups	De facto	Included
Croatia	1991	Census	1-year age groups	De facto	Included
Croatia	2001	Census	1-year age groups	De jure	Included
Croatia	2011	Census	1-year age groups	De jure	Included
Cuba	1953	Census	5-year age groups	De jure	Included
Cuba	1970	Census	5-year age groups	De jure	Included
Cuba	1981	Census	1-year age groups	De jure	Included
Cuba	2002	Census	1-year age groups	De jure	Included
Cuba	2012	Census	1-year age groups	De jure	Included
Cyprus	1960	Census	non-std age groups	De jure	Included
Cyprus (Republic of)	1976	Census	5-year age groups	De jure	Included
Cyprus (Republic of)	1982	Census	5-year age groups	De jure	Included
Cyprus (Republic of)	1992	Census	1-year age groups	De jure	Included
Cyprus (Republic of)	2001	Census	1-year age groups	De jure	Included
Cyprus (Republic of)	2011	Census	1-year age groups	De jure	Included
Cyprus (Turkish Federated State of)	1978	Census	5-year age groups	De jure	Included
Cyprus (Turkish Federated State of)	1996	Census	5-year age groups	De jure	Included
Cyprus (Turkish Federated State of)	2006	Census	5-year age groups	De jure	Included
Cyprus (Turkish Federated State of)	2011	Census	5-year age groups	De jure	Included
Czech Republic	1961	Census	1-year age groups	De facto	Included
Czech Republic	1970	Census	1-year age groups	De jure	Included
Czech Republic	1980	Census	1-year age groups	De jure	Included
Czech Republic	1991	Census	1-year age groups	De jure	Included
Czech Republic	2000	Registry	1-year age groups	NA	Included
Czech Republic	2001	Census	1-year age groups	De jure	Included
Czech Republic	2001	Registry	1-year age groups	NA	Included
Czech Republic	2002	Registry	1-year age groups	NA	Included
Czech Republic	2003	Registry	1-year age groups	NA	Included
Czech Republic	2004	Registry	1-year age groups	NA	Included
Czech Republic	2005	Registry	1-year age groups	NA	Included
Czech Republic	2006	Registry	1-year age groups	NA	Included
Czech Republic	2007	Registry	1-year age groups	NA	Included
Czech Republic	2008	Registry	1-year age groups	NA	Included
Czech Republic	2009	Registry	1-year age groups	NA	Included
Czech Republic	2010	Registry	1-year age groups	NA	Included
Czech Republic	2011	Census	1-year age groups	De jure	Included
Czech Republic	2011	Registry	1-year age groups	NA	Included
Czech Republic	2012	Registry	1-year age groups	NA	Included
Czech Republic	2013	Registry	1-year age groups	NA	Included
Czech Republic	2014	Registry	1-year age groups	NA	Included
Czech Republic	2015	Registry	1-year age groups	NA	Included
Czech Republic	2016	Registry	1-year age groups	NA	Included
Czech Republic	2017	Registry	1-year age groups	NA	Included
Democratic Republic of the Congo	1952	Census	Not Available	Unknown	Excluded

Appendix Table 5. List of all confirmed censuses by location and year					
**Location**	**Year**	**Type**	**Age detail**	**Enumeration method**	**Included or excluded from estimation**
Democratic Republic of the Congo	1955	Census	Not Available	Unknown	Excluded
Democratic Republic of the Congo	1958	Census	Not Available	Unknown	Excluded
Democratic Republic of the Congo	1970	Census	all ages only	De jure	Included
Democratic Republic of the Congo	1984	Census	1-year age groups	Unknown	Included
Denmark	1950	Census	1-year age groups	De jure	Included
Denmark	1960	Census	1-year age groups	De jure	Included
Denmark	1965	Census	5-year age groups	De jure	Included
Denmark	1970	Census	1-year age groups	De jure	Included
Denmark	1976	Census	1-year age groups	De jure	Included
Denmark	1980	Registry	1-year age groups	NA	Included
Denmark	1981	Census	Not Available	Unknown	Excluded
Denmark	1981	Registry	1-year age groups	NA	Included
Denmark	1982	Registry	1-year age groups	NA	Included
Denmark	1983	Registry	1-year age groups	NA	Included
Denmark	1984	Registry	1-year age groups	NA	Included
Denmark	1985	Registry	1-year age groups	NA	Included
Denmark	1986	Registry	1-year age groups	NA	Included
Denmark	1987	Registry	1-year age groups	NA	Included
Denmark	1988	Registry	1-year age groups	NA	Included
Denmark	1989	Registry	1-year age groups	NA	Included
Denmark	1990	Registry	1-year age groups	NA	Included
Denmark	1991	Census	1-year age groups	De jure	Included
Denmark	1991	Registry	1-year age groups	NA	Included
Denmark	1992	Registry	1-year age groups	NA	Included
Denmark	1993	Registry	1-year age groups	NA	Included
Denmark	1994	Registry	1-year age groups	NA	Included
Denmark	1995	Registry	1-year age groups	NA	Included
Denmark	1996	Registry	1-year age groups	NA	Included
Denmark	1997	Registry	1-year age groups	NA	Included
Denmark	1998	Registry	1-year age groups	NA	Included
Denmark	1999	Registry	1-year age groups	NA	Included
Denmark	2000	Registry	1-year age groups	NA	Included
Denmark	2001	Census	Not Available	Unknown	Excluded
Denmark	2001	Registry	1-year age groups	NA	Included
Denmark	2002	Registry	1-year age groups	NA	Included
Denmark	2003	Registry	1-year age groups	NA	Included
Denmark	2004	Registry	1-year age groups	NA	Included
Denmark	2005	Registry	1-year age groups	NA	Included
Denmark	2006	Registry	1-year age groups	NA	Included
Denmark	2007	Registry	1-year age groups	NA	Included
Denmark	2008	Registry	1-year age groups	NA	Included
Denmark	2009	Registry	1-year age groups	NA	Included
Denmark	2010	Registry	1-year age groups	NA	Included
Denmark	2011	Census	Not Available	Unknown	Excluded
Denmark	2011	Registry	1-year age groups	NA	Included
Denmark	2012	Registry	1-year age groups	NA	Included
Denmark	2013	Registry	1-year age groups	NA	Included
Denmark	2014	Registry	1-year age groups	NA	Included
Denmark	2015	Registry	1-year age groups	NA	Included
Denmark	2016	Registry	1-year age groups	NA	Included
Denmark	2017	Registry	1-year age groups	NA	Included
Djibouti	1951	Census	Not Available	Unknown	Excluded
Djibouti	1956	Census	Not Available	Unknown	Excluded
Djibouti	1960	Census	Not Available	Unknown	Excluded
Djibouti	1983	Census	5-year age groups	De jure	Included
Djibouti	2009	Census	5-year age groups	De jure	Included
Dominica	1960	Census	5-year age groups	De facto	Included
Dominica	1970	Census	5-year age groups	De facto	Included
Dominica	1981	Census	1-year age groups	De jure	Included
Dominica	1991	Census	5-year age groups	De facto	Included
Dominica	2001	Census	5-year age groups	De facto	Included
Dominica	2011	Census	5-year age groups	De facto	Included
Dominican Republic	1950	Census	1-year age groups	De jure	Included
Dominican Republic	1960	Census	1-year age groups	De jure	Included
Dominican Republic	1970	Census	1-year age groups	De jure	Included
Dominican Republic	1981	Census	5-year age groups	De jure	Included
Dominican Republic	1993	Census	1-year age groups	De jure	Included
Dominican Republic	2002	Census	1-year age groups	De jure	Included
Dominican Republic	2010	Census	1-year age groups	De jure	Included
Ecuador	1950	Census	1-year age groups	De facto	Included
Ecuador	1962	Census	1-year age groups	De facto	Included
Ecuador	1974	Census	5-year age groups	De facto	Included
Ecuador	1982	Census	1-year age groups	De facto	Included
Ecuador	1990	Census	1-year age groups	De facto	Included
Ecuador	2001	Census	1-year age groups	De facto	Included

Appendix Table 5. List of all confirmed censuses by location and year					
**Location**	**Year**	**Type**	**Age detail**	**Enumeration method**	**Included or excluded from estimation**
Ecuador	2010	Census	1-year age groups	De facto	Included
Egypt	1960	Census	5-year age groups	De facto	Included
Egypt	1966	Census	all ages only	De facto	Included
Egypt	1976	Census	5-year age groups	De facto	Included
Egypt	1986	Census	5-year age groups	De facto	Included
Egypt	1996	Census	5-year age groups	De facto	Included
Egypt	2006	Census	1-year age groups	De facto	Included
Egypt	2017	Census	5-year age groups	De facto	Included
El Salvador	1950	Census	1-year age groups	De facto	Included
El Salvador	1961	Census	1-year age groups	De facto	Included
El Salvador	1971	Census	1-year age groups	De facto	Included
El Salvador	1992	Census	1-year age groups	De facto	Included
El Salvador	2007	Census	1-year age groups	De jure	Included
England	1951	Census	5-year age groups	De facto	Included
England	1961	Census	5-year age groups	Unknown	Included
England	1971	Census	5-year age groups	Unknown	Included
England	1981	Census	5-year age groups	Unknown	Included
England	1991	Census	non-std age groups	De jure	Included
England	2001	Census	5-year age groups	De jure	Included
England	2011	Census	1-year age groups	De jure	Included
Equatorial Guinea	1950	Census	Not Available	Unknown	Excluded
Equatorial Guinea	1960	Census	Not Available	Unknown	Excluded
Equatorial Guinea	1983	Census	5-year age groups	De facto	Included
Equatorial Guinea	1994	Census	Not Available	Unknown	Excluded
Equatorial Guinea	2001	Census	Not Available	Unknown	Included
Equatorial Guinea	2015	Census	all ages only	Unknown	Included
Eritrea	1984	Census	all ages only	De jure	Included
Estonia	1950	Registry	5-year age groups	NA	Included
Estonia	1951	Registry	5-year age groups	NA	Included
Estonia	1952	Registry	5-year age groups	NA	Included
Estonia	1953	Registry	5-year age groups	NA	Included
Estonia	1954	Registry	5-year age groups	NA	Included
Estonia	1955	Registry	5-year age groups	NA	Included
Estonia	1956	Registry	5-year age groups	NA	Included
Estonia	1957	Registry	5-year age groups	NA	Included
Estonia	1958	Registry	5-year age groups	NA	Included
Estonia	1959	Census	non-std age groups	De facto	Included
Estonia	1959	Registry	5-year age groups	NA	Included
Estonia	1960	Registry	5-year age groups	NA	Included
Estonia	1961	Registry	5-year age groups	NA	Included
Estonia	1962	Registry	5-year age groups	NA	Included
Estonia	1963	Registry	5-year age groups	NA	Included
Estonia	1964	Registry	5-year age groups	NA	Included
Estonia	1965	Registry	5-year age groups	NA	Included
Estonia	1966	Registry	5-year age groups	NA	Included
Estonia	1967	Registry	5-year age groups	NA	Included
Estonia	1968	Registry	5-year age groups	NA	Included
Estonia	1969	Registry	5-year age groups	NA	Included
Estonia	1970	Census	non-std age groups	De facto	Included
Estonia	1970	Registry	5-year age groups	NA	Included
Estonia	1971	Registry	5-year age groups	NA	Included
Estonia	1972	Registry	5-year age groups	NA	Included
Estonia	1973	Registry	5-year age groups	NA	Included
Estonia	1974	Registry	5-year age groups	NA	Included
Estonia	1975	Registry	5-year age groups	NA	Included
Estonia	1976	Registry	5-year age groups	NA	Included
Estonia	1977	Registry	5-year age groups	NA	Included
Estonia	1978	Registry	5-year age groups	NA	Included
Estonia	1979	Census	5-year age groups	De facto	Included
Estonia	1979	Registry	5-year age groups	NA	Included
Estonia	1980	Registry	5-year age groups	NA	Included
Estonia	1981	Registry	5-year age groups	NA	Included
Estonia	1982	Registry	5-year age groups	NA	Included
Estonia	1983	Registry	5-year age groups	NA	Included
Estonia	1984	Registry	5-year age groups	NA	Included
Estonia	1985	Registry	5-year age groups	NA	Included
Estonia	1986	Registry	5-year age groups	NA	Included
Estonia	1987	Registry	5-year age groups	NA	Included
Estonia	1988	Registry	5-year age groups	NA	Included
Estonia	1989	Census	5-year age groups	De facto	Included
Estonia	1989	Registry	5-year age groups	NA	Included
Estonia	1990	Registry	5-year age groups	NA	Included
Estonia	1991	Registry	5-year age groups	NA	Included
Estonia	1992	Registry	5-year age groups	NA	Included
Estonia	1993	Registry	5-year age groups	NA	Included

Appendix Table 5. List of all confirmed censuses by location and year					
**Location**	**Year**	**Type**	**Age detail**	**Enumeration method**	**Included or excluded from estimation**
Estonia	1994	Registry	5-year age groups	NA	Included
Estonia	1995	Registry	5-year age groups	NA	Included
Estonia	1996	Registry	5-year age groups	NA	Included
Estonia	1997	Registry	5-year age groups	NA	Included
Estonia	1998	Registry	5-year age groups	NA	Included
Estonia	1999	Registry	5-year age groups	NA	Included
Estonia	2000	Census	1-year age groups	De jure	Included
Estonia	2000	Registry	5-year age groups	NA	Included
Estonia	2001	Registry	5-year age groups	NA	Included
Estonia	2002	Registry	5-year age groups	NA	Included
Estonia	2003	Registry	5-year age groups	NA	Included
Estonia	2004	Registry	5-year age groups	NA	Included
Estonia	2005	Registry	5-year age groups	NA	Included
Estonia	2006	Registry	5-year age groups	NA	Included
Estonia	2007	Registry	5-year age groups	NA	Included
Estonia	2008	Registry	5-year age groups	NA	Included
Estonia	2009	Registry	5-year age groups	NA	Included
Estonia	2010	Registry	5-year age groups	NA	Included
Estonia	2011	Census	1-year age groups	De jure	Included
Estonia	2011	Registry	5-year age groups	NA	Included
Estonia	2012	Registry	5-year age groups	NA	Included
Estonia	2013	Registry	5-year age groups	NA	Included
Estonia	2014	Registry	5-year age groups	NA	Included
Estonia	2015	Registry	5-year age groups	NA	Included
Estonia	2016	Registry	5-year age groups	NA	Included
Estonia	2017	Registry	5-year age groups	NA	Included
Ethiopia	1984	Census	5-year age groups	De facto	Included
Ethiopia	1994	Census	1-year age groups	Unknown	Included
Ethiopia	2007	Census	1-year age groups	Unknown	Included
Federated States of Micronesia	1994	Census	5-year age groups	De jure	Included
Federated States of Micronesia	2000	Census	non-std age groups	De jure	Included
Federated States of Micronesia	2010	Census	5-year age groups	De facto	Included
Fiji	1956	Census	5-year age groups	De facto	Included
Fiji	1966	Census	5-year age groups	De facto	Included
Fiji	1976	Census	5-year age groups	De facto	Included
Fiji	1986	Census	1-year age groups	De facto	Included
Fiji	1996	Census	1-year age groups	De facto	Included
Fiji	2007	Census	5-year age groups	De facto	Included
Fiji	2017	Census	5-year age groups	De jure	Included
Finland	1950	Census	1-year age groups	De jure	Included
Finland	1960	Census	1-year age groups	De jure	Included
Finland	1970	Census	1-year age groups	De jure	Included
Finland	1970	Registry	1-year age groups	NA	Included
Finland	1971	Registry	1-year age groups	NA	Included
Finland	1972	Registry	1-year age groups	NA	Included
Finland	1973	Registry	1-year age groups	NA	Included
Finland	1974	Registry	1-year age groups	NA	Included
Finland	1975	Census	5-year age groups	De jure	Included
Finland	1975	Registry	1-year age groups	NA	Included
Finland	1976	Registry	1-year age groups	NA	Included
Finland	1977	Registry	1-year age groups	NA	Included
Finland	1978	Registry	1-year age groups	NA	Included
Finland	1979	Registry	1-year age groups	NA	Included
Finland	1980	Census	non-std age groups	De jure	Included
Finland	1980	Registry	1-year age groups	NA	Included
Finland	1981	Registry	1-year age groups	NA	Included
Finland	1982	Registry	1-year age groups	NA	Included
Finland	1983	Registry	1-year age groups	NA	Included
Finland	1984	Registry	1-year age groups	NA	Included
Finland	1985	Census	1-year age groups	De jure	Included
Finland	1985	Registry	1-year age groups	NA	Included
Finland	1986	Registry	1-year age groups	NA	Included
Finland	1987	Registry	1-year age groups	NA	Included
Finland	1988	Registry	1-year age groups	NA	Included
Finland	1989	Registry	1-year age groups	NA	Included
Finland	1990	Census	1-year age groups	De jure	Included
Finland	1990	Registry	1-year age groups	NA	Included
Finland	1991	Registry	1-year age groups	NA	Included
Finland	1992	Registry	1-year age groups	NA	Included
Finland	1993	Registry	1-year age groups	NA	Included
Finland	1994	Registry	1-year age groups	NA	Included
Finland	1995	Census	non-std age groups	De jure	Included
Finland	1995	Registry	1-year age groups	NA	Included
Finland	1996	Registry	1-year age groups	NA	Included
Finland	1997	Registry	1-year age groups	NA	Included

Appendix Table 5. List of all confirmed censuses by location and year

Location	Year	Type	Age detail	Enumeration method	Included or excluded from estimation
Finland	1998	Registry	1-year age groups	NA	Included
Finland	1999	Registry	1-year age groups	NA	Included
Finland	2000	Census	1-year age groups	De jure	Included
Finland	2000	Registry	1-year age groups	NA	Included
Finland	2001	Registry	1-year age groups	NA	Included
Finland	2002	Registry	1-year age groups	NA	Included
Finland	2003	Registry	1-year age groups	NA	Included
Finland	2004	Registry	1-year age groups	NA	Included
Finland	2005	Registry	1-year age groups	NA	Included
Finland	2006	Registry	1-year age groups	NA	Included
Finland	2007	Registry	1-year age groups	NA	Included
Finland	2008	Registry	1-year age groups	NA	Included
Finland	2009	Registry	1-year age groups	NA	Included
Finland	2010	Census	1-year age groups	De jure	Included
Finland	2010	Registry	1-year age groups	NA	Included
Finland	2011	Registry	1-year age groups	NA	Included
Finland	2012	Registry	1-year age groups	NA	Included
Finland	2013	Registry	1-year age groups	NA	Included
Finland	2014	Registry	1-year age groups	NA	Included
Finland	2015	Registry	1-year age groups	NA	Included
Finland	2016	Registry	1-year age groups	NA	Included
Finland	2017	Registry	1-year age groups	NA	Included
France	1954	Census	5-year age groups	De jure	Included
France	1962	Census	5-year age groups	De jure	Included
France	1968	Census	5-year age groups	De jure	Included
France	1975	Census	5-year age groups	De jure	Included
France	1982	Census	5-year age groups	De jure	Included
France	1990	Census	5-year age groups	De jure	Included
France	1999	Census	5-year age groups	De jure	Included
France	2006	Census	1-year age groups	De jure	Included
France	2007	Census	1-year age groups	De jure	Included
France	2008	Census	1-year age groups	De jure	Included
France	2009	Census	1-year age groups	De jure	Included
France	2010	Census	1-year age groups	De jure	Included
France	2011	Census	1-year age groups	De jure	Included
France	2012	Census	1-year age groups	De jure	Included
France	2013	Census	1-year age groups	De jure	Included
France	2014	Census	1-year age groups	De jure	Included
France	2015	Census	Not Available	Unknown	Excluded
Gabon	1951	Census	Not Available	Unknown	Excluded
Gabon	1956	Census	Not Available	Unknown	Excluded
Gabon	1960	Census	5-year age groups	De facto	Included
Gabon	1969	Census	Not Available	Unknown	Excluded
Gabon	1980	Census	5-year age groups	De jure	Excluded
Gabon	1993	Census	1-year age groups	De facto	Included
Gabon	2003	Census	Not Available	Unknown	Excluded
Gabon	2013	Census	non-std age groups	De jure	Included
Georgia	1959	Census	non-std age groups	De facto	Included
Georgia	1970	Census	non-std age groups	De facto	Included
Georgia	1979	Census	5-year age groups	De facto	Included
Georgia	1989	Census	5-year age groups	De jure	Included
Georgia	1989	Census	5-year age groups	De jure	Included
Georgia	2002	Census	1-year age groups	De jure	Included
Georgia	2014	Census	1-year age groups	De jure	Included
Germany	1990	Registry	1-year age groups	NA	Included
Germany	1991	Registry	1-year age groups	NA	Included
Germany	1992	Registry	1-year age groups	NA	Included
Germany	1993	Registry	1-year age groups	NA	Included
Germany	1994	Registry	1-year age groups	NA	Included
Germany	1995	Registry	1-year age groups	NA	Included
Germany	1996	Registry	1-year age groups	NA	Included
Germany	1997	Registry	1-year age groups	NA	Included
Germany	1998	Registry	1-year age groups	NA	Included
Germany	1999	Registry	1-year age groups	NA	Included
Germany	2000	Registry	1-year age groups	NA	Included
Germany	2001	Registry	1-year age groups	NA	Included
Germany	2002	Registry	1-year age groups	NA	Included
Germany	2003	Registry	1-year age groups	NA	Included
Germany	2004	Registry	1-year age groups	NA	Included
Germany	2005	Registry	1-year age groups	NA	Included
Germany	2006	Registry	1-year age groups	NA	Included
Germany	2007	Registry	1-year age groups	NA	Included
Germany	2008	Registry	1-year age groups	NA	Included
Germany	2009	Registry	1-year age groups	NA	Included
Germany	2010	Registry	1-year age groups	NA	Included

Appendix Table 5. List of all confirmed censuses by location and year					
Location	Year	Type	Age detail	Enumeration method	Included or excluded from estimation
Germany	2011	Census	1-year age groups	De jure	Included
Germany	2011	Registry	1-year age groups	NA	Excluded
Germany	2012	Registry	1-year age groups	NA	Excluded
Germany	2013	Registry	1-year age groups	NA	Excluded
Germany	2014	Registry	1-year age groups	NA	Excluded
Germany	2015	Registry	1-year age groups	NA	Excluded
Germany	2016	Registry	1-year age groups	NA	Excluded
Germany (Democratic Republic)	1950	Census	5-year age groups	De jure	Included
Germany (Democratic Republic)	1964	Census	5-year age groups	De jure	Included
Germany (Democratic Republic)	1971	Census	1-year age groups	De jure	Included
Germany (Democratic Republic)	1981	Census	1-year age groups	De jure	Included
Germany (Federal Republic of)	1950	Census	all ages only	De jure	Included
Germany (Federal Republic of)	1961	Census	5-year age groups	De jure	Included
Germany (Federal Republic of)	1970	Census	1-year age groups	De jure	Included
Germany (Federal Republic of)	1970	Registry	1-year age groups	NA	Included
Germany (Federal Republic of)	1971	Registry	1-year age groups	NA	Included
Germany (Federal Republic of)	1972	Registry	1-year age groups	NA	Included
Germany (Federal Republic of)	1973	Registry	1-year age groups	NA	Included
Germany (Federal Republic of)	1974	Registry	1-year age groups	NA	Included
Germany (Federal Republic of)	1975	Registry	1-year age groups	NA	Included
Germany (Federal Republic of)	1976	Registry	1-year age groups	NA	Included
Germany (Federal Republic of)	1977	Registry	1-year age groups	NA	Included
Germany (Federal Republic of)	1978	Registry	1-year age groups	NA	Included
Germany (Federal Republic of)	1979	Registry	1-year age groups	NA	Included
Germany (Federal Republic of)	1980	Registry	1-year age groups	NA	Included
Germany (Federal Republic of)	1981	Registry	1-year age groups	NA	Included
Germany (Federal Republic of)	1982	Registry	1-year age groups	NA	Included
Germany (Federal Republic of)	1983	Registry	1-year age groups	NA	Included
Germany (Federal Republic of)	1984	Registry	1-year age groups	NA	Included
Germany (Federal Republic of)	1985	Registry	1-year age groups	NA	Included
Germany (Federal Republic of)	1986	Registry	1-year age groups	NA	Included
Germany (Federal Republic of)	1987	Census	1-year age groups	De jure	Included
Germany (Federal Republic of)	1987	Registry	1-year age groups	NA	Included
Germany (Federal Republic of)	1988	Registry	1-year age groups	NA	Included
Germany (Federal Republic of)	1989	Registry	1-year age groups	NA	Included
Ghana	1960	Census	1-year age groups	De facto	Included
Ghana	1970	Census	1-year age groups	De facto	Included
Ghana	1984	Census	1-year age groups	De facto	Included
Ghana	2000	Census	5-year age groups	De facto	Included
Ghana	2010	Census	1-year age groups	De facto	Included
Greece	1951	Census	5-year age groups	De facto	Included
Greece	1961	Census	5-year age groups	De facto	Included
Greece	1971	Census	5-year age groups	De facto	Included
Greece	1981	Census	1-year age groups	De facto	Included
Greece	1991	Census	1-year age groups	De facto	Included
Greece	2001	Census	1-year age groups	De jure	Included
Greece	2011	Census	1-year age groups	De facto	Included
Greenland	1951	Census	5-year age groups	De jure	Included
Greenland	1955	Census	5-year age groups	De jure	Included
Greenland	1960	Census	non-std age groups	De jure	Included
Greenland	1965	Census	1-year age groups	De jure	Included
Greenland	1970	Census	1-year age groups	De jure	Included
Greenland	1976	Census	5-year age groups	De jure	Included
Greenland	1977	Registry	1-year age groups	NA	Included
Greenland	1978	Registry	1-year age groups	NA	Included
Greenland	1979	Registry	1-year age groups	NA	Included
Greenland	1980	Registry	1-year age groups	NA	Included
Greenland	1981	Registry	1-year age groups	NA	Included
Greenland	1982	Registry	1-year age groups	NA	Included
Greenland	1983	Registry	1-year age groups	NA	Included
Greenland	1984	Registry	1-year age groups	NA	Included
Greenland	1985	Registry	1-year age groups	NA	Included
Greenland	1986	Registry	1-year age groups	NA	Included
Greenland	1987	Registry	1-year age groups	NA	Included
Greenland	1988	Registry	1-year age groups	NA	Included
Greenland	1989	Registry	1-year age groups	NA	Included
Greenland	1990	Registry	1-year age groups	NA	Included
Greenland	1991	Registry	1-year age groups	NA	Included
Greenland	1992	Registry	1-year age groups	NA	Included
Greenland	1993	Registry	1-year age groups	NA	Included
Greenland	1994	Registry	1-year age groups	NA	Included
Greenland	1995	Registry	1-year age groups	NA	Included
Greenland	1996	Registry	1-year age groups	NA	Included
Greenland	1997	Registry	1-year age groups	NA	Included
Greenland	1998	Registry	1-year age groups	NA	Included

Appendix Table 5. List of all confirmed censuses by location and year

Location	Year	Type	Age detail	Enumeration method	Included or excluded from estimation
Greenland	1999	Registry	1-year age groups	NA	Included
Greenland	2000	Census	5-year age groups	De jure	Included
Greenland	2000	Registry	1-year age groups	NA	Included
Greenland	2001	Registry	1-year age groups	NA	Included
Greenland	2002	Registry	1-year age groups	NA	Included
Greenland	2003	Registry	1-year age groups	NA	Included
Greenland	2004	Registry	1-year age groups	NA	Included
Greenland	2005	Registry	1-year age groups	NA	Included
Greenland	2006	Registry	1-year age groups	NA	Included
Greenland	2007	Registry	1-year age groups	NA	Included
Greenland	2008	Registry	1-year age groups	NA	Included
Greenland	2009	Registry	1-year age groups	NA	Included
Greenland	2010	Census	Not Available	Unknown	Excluded
Greenland	2010	Registry	1-year age groups	NA	Included
Greenland	2011	Registry	1-year age groups	NA	Included
Greenland	2012	Registry	1-year age groups	NA	Included
Greenland	2013	Registry	1-year age groups	NA	Included
Greenland	2014	Registry	1-year age groups	NA	Included
Greenland	2015	Registry	1-year age groups	NA	Included
Greenland	2016	Registry	1-year age groups	NA	Included
Greenland	2017	Registry	1-year age groups	NA	Included
Grenada	1960	Census	5-year age groups	De facto	Included
Grenada	1970	Census	5-year age groups	De facto	Included
Grenada	1981	Census	1-year age groups	De facto	Included
Grenada	1991	Census	5-year age groups	De facto	Included
Grenada	2001	Census	5-year age groups	De facto	Included
Grenada	2011	Census	5-year age groups	De jure	Included
Guam	1950	Census	non-std age groups	De jure	Included
Guam	1960	Census	non-std age groups	De jure	Included
Guam	1970	Census	5-year age groups	De jure	Included
Guam	1980	Census	1-year age groups	De jure	Included
Guam	1990	Census	non-std age groups	De jure	Included
Guam	2000	Census	1-year age groups	De jure	Included
Guam	2010	Census	5-year age groups	De jure	Included
Guatemala	1950	Census	1-year age groups	De jure	Included
Guatemala	1964	Census	1-year age groups	De jure	Included
Guatemala	1973	Census	1-year age groups	De jure	Included
Guatemala	1981	Census	1-year age groups	De jure	Included
Guatemala	1994	Census	1-year age groups	De jure	Included
Guatemala	2002	Census	all ages only	De jure	Included
Guinea	1951	Census	Not Available	Unknown	Excluded
Guinea	1955	Census	Not Available	Unknown	Excluded
Guinea	1966	Census	all ages only	Unknown	Included
Guinea	1983	Census	1-year age groups	Unknown	Included
Guinea	1996	Census	5-year age groups	De facto	Included
Guinea	2014	Census	1-year age groups	De facto	Included
Guinea-Bissau	1950	Census	5-year age groups	De facto	Included
Guinea-Bissau	1960	Census	5-year age groups	Unknown	Included
Guinea-Bissau	1979	Census	5-year age groups	De jure	Included
Guinea-Bissau	1991	Census	non-std age groups	De facto	Included
Guinea-Bissau	2009	Census	1-year age groups	De jure	Included
Guyana	1960	Census	5-year age groups	De facto	Included
Guyana	1970	Census	1-year age groups	De facto	Included
Guyana	1980	Census	1-year age groups	De facto	Included
Guyana	1991	Census	5-year age groups	De facto	Included
Guyana	2002	Census	5-year age groups	De facto	Included
Guyana	2012	Census	5-year age groups	De jure	Included
Haiti	1950	Census	1-year age groups	De jure	Included
Haiti	1971	Census	1-year age groups	De jure	Included
Haiti	1982	Census	1-year age groups	De jure	Included
Haiti	2003	Census	1-year age groups	Unknown	Included
Honduras	1950	Census	5-year age groups	De facto	Included
Honduras	1961	Census	5-year age groups	De facto	Included
Honduras	1974	Census	5-year age groups	De facto	Included
Honduras	1988	Census	1-year age groups	De facto	Included
Honduras	2001	Census	1-year age groups	De jure	Included
Honduras	2013	Census	1-year age groups	De facto	Included
Hong Kong Special Administrative Region of China	1961	Census	5-year age groups	De facto	Included
Hong Kong Special Administrative Region of China	1971	Census	1-year age groups	De facto	Included
Hong Kong Special Administrative Region of China	1981	Census	1-year age groups	De facto	Included
Hong Kong Special Administrative Region of China	1986	Census	5-year age groups	De facto	Included
Hong Kong Special Administrative Region of China	1991	Census	5-year age groups	De facto	Included
Hong Kong Special Administrative Region of China	1996	Census	1-year age groups	De facto	Included
Hong Kong Special Administrative Region of China	2001	Census	1-year age groups	De jure	Included
Hong Kong Special Administrative Region of China	2006	Census	1-year age groups	De jure	Included

Appendix Table 5. List of all confirmed censuses by location and year					
**Location**	**Year**	**Type**	**Age detail**	**Enumeration method**	**Included or excluded from estimation**
Hong Kong Special Administrative Region of China	2011	Census	1-year age groups	De jure	Included
Hong Kong Special Administrative Region of China	2016	Census	1-year age groups	De jure	Included
Hungary	1960	Census	1-year age groups	De facto	Included
Hungary	1970	Census	1-year age groups	De facto	Included
Hungary	1980	Census	1-year age groups	Unknown	Included
Hungary	1990	Census	1-year age groups	Unknown	Included
Hungary	2001	Census	1-year age groups	Unknown	Included
Hungary	2011	Census	1-year age groups	De facto	Included
Hungary	2012	Census	1-year age groups	Unknown	Excluded
Hungary	2013	Census	1-year age groups	Unknown	Excluded
Hungary	2014	Census	1-year age groups	Unknown	Excluded
Hungary	2015	Census	1-year age groups	Unknown	Excluded
Hungary	2016	Census	1-year age groups	Unknown	Excluded
Hungary	2017	Census	1-year age groups	Unknown	Excluded
Iceland	1950	Census	5-year age groups	De jure	Included
Iceland	1950	Registry	1-year age groups	NA	Included
Iceland	1951	Registry	1-year age groups	NA	Included
Iceland	1952	Registry	1-year age groups	NA	Included
Iceland	1953	Registry	1-year age groups	NA	Included
Iceland	1954	Registry	1-year age groups	NA	Included
Iceland	1955	Registry	1-year age groups	NA	Included
Iceland	1956	Registry	1-year age groups	NA	Included
Iceland	1957	Registry	1-year age groups	NA	Included
Iceland	1958	Registry	1-year age groups	NA	Included
Iceland	1959	Registry	1-year age groups	NA	Included
Iceland	1960	Census	1-year age groups	De jure	Included
Iceland	1960	Registry	1-year age groups	NA	Included
Iceland	1961	Registry	1-year age groups	NA	Included
Iceland	1962	Registry	1-year age groups	NA	Included
Iceland	1963	Registry	1-year age groups	NA	Included
Iceland	1964	Registry	1-year age groups	NA	Included
Iceland	1965	Registry	1-year age groups	NA	Included
Iceland	1966	Registry	1-year age groups	NA	Included
Iceland	1967	Registry	1-year age groups	NA	Included
Iceland	1968	Registry	1-year age groups	NA	Included
Iceland	1969	Registry	1-year age groups	NA	Included
Iceland	1970	Census	5-year age groups	De jure	Included
Iceland	1970	Registry	1-year age groups	NA	Included
Iceland	1971	Registry	1-year age groups	NA	Included
Iceland	1972	Registry	1-year age groups	NA	Included
Iceland	1973	Registry	1-year age groups	NA	Included
Iceland	1974	Registry	1-year age groups	NA	Included
Iceland	1975	Registry	1-year age groups	NA	Included
Iceland	1976	Registry	1-year age groups	NA	Included
Iceland	1977	Registry	1-year age groups	NA	Included
Iceland	1978	Registry	1-year age groups	NA	Included
Iceland	1979	Registry	1-year age groups	NA	Included
Iceland	1980	Registry	1-year age groups	NA	Included
Iceland	1981	Census	Not Available	Unknown	Excluded
Iceland	1981	Registry	1-year age groups	NA	Included
Iceland	1982	Registry	1-year age groups	NA	Included
Iceland	1983	Registry	1-year age groups	NA	Included
Iceland	1984	Registry	1-year age groups	NA	Included
Iceland	1985	Registry	1-year age groups	NA	Included
Iceland	1986	Registry	1-year age groups	NA	Included
Iceland	1987	Registry	1-year age groups	NA	Included
Iceland	1988	Registry	1-year age groups	NA	Included
Iceland	1989	Registry	1-year age groups	NA	Included
Iceland	1990	Registry	1-year age groups	NA	Included
Iceland	1991	Registry	1-year age groups	NA	Included
Iceland	1992	Registry	1-year age groups	NA	Included
Iceland	1993	Registry	1-year age groups	NA	Included
Iceland	1994	Registry	1-year age groups	NA	Included
Iceland	1995	Registry	1-year age groups	NA	Included
Iceland	1996	Registry	1-year age groups	NA	Included
Iceland	1997	Registry	1-year age groups	NA	Included
Iceland	1998	Registry	1-year age groups	NA	Included
Iceland	1999	Registry	1-year age groups	NA	Included
Iceland	2000	Registry	1-year age groups	NA	Included
Iceland	2001	Registry	1-year age groups	NA	Included
Iceland	2002	Registry	1-year age groups	NA	Included
Iceland	2003	Registry	1-year age groups	NA	Included
Iceland	2004	Registry	1-year age groups	NA	Included
Iceland	2005	Registry	1-year age groups	NA	Included
Iceland	2006	Registry	1-year age groups	NA	Included

Appendix Table 5. List of all confirmed censuses by location and year					
Location	Year	Type	Age detail	Enumeration method	Included or excluded from estimation
Iceland	2007	Registry	1-year age groups	NA	Included
Iceland	2008	Registry	1-year age groups	NA	Included
Iceland	2009	Registry	1-year age groups	NA	Included
Iceland	2010	Registry	1-year age groups	NA	Included
Iceland	2011	Census	5-year age groups	De jure	Included
Iceland	2011	Registry	1-year age groups	NA	Included
Iceland	2012	Registry	1-year age groups	NA	Included
Iceland	2013	Registry	1-year age groups	NA	Included
Iceland	2014	Registry	1-year age groups	NA	Included
Iceland	2015	Registry	1-year age groups	NA	Included
Iceland	2016	Registry	1-year age groups	NA	Included
Iceland	2017	Registry	1-year age groups	NA	Included
India	1951	Census	1-year age groups	De facto	Included
India	1961	Census	1-year age groups	Unknown	Included
India	1971	Census	5-year age groups	Unknown	Included
India	1981	Census	1-year age groups	Unknown	Included
India	1991	Census	5-year age groups	De facto	Included
India	2001	Census	5-year age groups	De facto	Included
India	2011	Census	1-year age groups	De facto	Included
Indonesia	1961	Census	non-std age groups	De facto	Included
Indonesia	1971	Census	1-year age groups	De facto	Included
Indonesia	1980	Census	1-year age groups	Unknown	Included
Indonesia	1990	Census	1-year age groups	De jure	Included
Indonesia	2000	Census	1-year age groups	De jure	Excluded
Indonesia	2010	Census	1-year age groups	De jure	Included
Iran	1956	Census	5-year age groups	De facto	Included
Iran	1966	Census	1-year age groups	De facto	Included
Iran	1976	Census	5-year age groups	De facto	Included
Iran	1986	Census	5-year age groups	De facto	Included
Iran	1996	Census	1-year age groups	De jure	Included
Iran	2006	Census	1-year age groups	De jure	Included
Iran	2011	Census	1-year age groups	De jure	Included
Iran	2016	Census	1-year age groups	De jure	Included
Iraq	1957	Census	non-std age groups	De facto	Included
Iraq	1965	Census	1-year age groups	De facto	Included
Iraq	1977	Census	5-year age groups	De facto	Included
Iraq	1987	Census	5-year age groups	De facto	Included
Iraq	1997	Census	5-year age groups	De facto	Excluded
Ireland	1951	Census	1-year age groups	De facto	Included
Ireland	1956	Census	5-year age groups	De jure	Included
Ireland	1961	Census	5-year age groups	De facto	Included
Ireland	1966	Census	5-year age groups	De facto	Included
Ireland	1971	Census	1-year age groups	De facto	Included
Ireland	1979	Census	5-year age groups	De facto	Included
Ireland	1981	Census	5-year age groups	De facto	Included
Ireland	1986	Census	5-year age groups	De facto	Included
Ireland	1991	Census	1-year age groups	De facto	Included
Ireland	1996	Census	1-year age groups	De facto	Included
Ireland	2002	Census	1-year age groups	De facto	Included
Ireland	2006	Census	1-year age groups	De facto	Included
Ireland	2011	Census	1-year age groups	De facto	Included
Ireland	2016	Census	1-year age groups	De jure	Included
Israel	1961	Census	non-std age groups	De jure	Included
Israel	1972	Census	5-year age groups	De jure	Included
Israel	1983	Census	5-year age groups	De jure	Included
Israel	1995	Census	1-year age groups	De facto	Included
Israel	2008	Census	1-year age groups	De facto	Included
Italy	1951	Census	5-year age groups	De jure	Included
Italy	1961	Census	non-std age groups	De jure	Included
Italy	1971	Census	1-year age groups	De jure	Included
Italy	1981	Census	non-std age groups	De facto	Included
Italy	1991	Census	non-std age groups	De facto	Included
Italy	2001	Census	1-year age groups	De jure	Included
Italy	2002	Registry	1-year age groups	NA	Included
Italy	2003	Registry	1-year age groups	NA	Included
Italy	2004	Registry	1-year age groups	NA	Included
Italy	2005	Registry	1-year age groups	NA	Included
Italy	2006	Registry	1-year age groups	NA	Included
Italy	2007	Registry	1-year age groups	NA	Included
Italy	2008	Registry	1-year age groups	NA	Included
Italy	2009	Registry	1-year age groups	NA	Included
Italy	2010	Registry	1-year age groups	NA	Included
Italy	2011	Census	1-year age groups	De jure	Included
Italy	2011	Registry	1-year age groups	NA	Included
Italy	2012	Registry	1-year age groups	NA	Excluded

Appendix Table 5. List of all confirmed censuses by location and year					
**Location**	**Year**	**Type**	**Age detail**	**Enumeration method**	**Included or excluded from estimation**
Italy	2013	Registry	1-year age groups	NA	Excluded
Italy	2014	Registry	1-year age groups	NA	Included
Italy	2015	Registry	1-year age groups	NA	Included
Italy	2016	Registry	1-year age groups	NA	Included
Italy	2017	Registry	1-year age groups	NA	Included
Jamaica	1953	Census	5-year age groups	De jure	Included
Jamaica	1960	Census	5-year age groups	De jure	Included
Jamaica	1970	Census	5-year age groups	De jure	Included
Jamaica	1982	Census	1-year age groups	De jure	Included
Jamaica	1991	Census	1-year age groups	De jure	Included
Jamaica	2001	Census	1-year age groups	De jure	Included
Jamaica	2011	Census	1-year age groups	De jure	Included
Japan	1950	Census	1-year age groups	De facto	Included
Japan	1955	Census	5-year age groups	De facto	Included
Japan	1960	Census	5-year age groups	De facto	Included
Japan	1965	Census	1-year age groups	De facto	Included
Japan	1970	Census	1-year age groups	De facto	Included
Japan	1975	Census	5-year age groups	De facto	Included
Japan	1980	Census	1-year age groups	De facto	Included
Japan	1985	Census	5-year age groups	De facto	Included
Japan	1990	Census	1-year age groups	De facto	Included
Japan	1995	Census	1-year age groups	De facto	Included
Japan	2000	Census	1-year age groups	De jure	Included
Japan	2005	Census	1-year age groups	De jure	Included
Japan	2010	Census	1-year age groups	De jure	Included
Japan	2015	Census	1-year age groups	De jure	Included
Jordan	1961	Census	non-std age groups	De facto	Excluded
Jordan	1979	Census	5-year age groups	De facto	Included
Jordan	1994	Census	5-year age groups	De facto	Included
Jordan	2004	Census	5-year age groups	De facto	Included
Jordan	2015	Census	1-year age groups	De facto	Included
Kazakhstan	1959	Census	non-std age groups	De facto	Included
Kazakhstan	1970	Census	non-std age groups	De facto	Included
Kazakhstan	1979	Census	5-year age groups	De facto	Included
Kazakhstan	1989	Census	1-year age groups	De facto	Included
Kazakhstan	1999	Census	1-year age groups	De jure	Included
Kazakhstan	2009	Census	5-year age groups	De facto	Included
Kazakhstan	2010	Registry	all ages only	NA	Included
Kazakhstan	2011	Registry	all ages only	NA	Included
Kazakhstan	2012	Registry	all ages only	NA	Included
Kazakhstan	2013	Registry	all ages only	NA	Included
Kazakhstan	2014	Registry	all ages only	NA	Included
Kazakhstan	2015	Registry	all ages only	NA	Included
Kazakhstan	2016	Registry	all ages only	NA	Included
Kenya	1962	Census	1-year age groups	De facto	Included
Kenya	1969	Census	1-year age groups	De facto	Included
Kenya	1979	Census	5-year age groups	De facto	Included
Kenya	1989	Census	1-year age groups	De jure	Included
Kenya	1999	Census	5-year age groups	De facto	Included
Kenya	2009	Census	1-year age groups	De facto	Included
Kiribati	1963	Census	5-year age groups	De facto	Included
Kiribati	1968	Census	5-year age groups	De facto	Included
Kiribati	1973	Census	5-year age groups	De facto	Included
Kiribati	1978	Census	5-year age groups	De facto	Included
Kiribati	1985	Census	5-year age groups	De facto	Included
Kiribati	1990	Census	5-year age groups	De facto	Included
Kiribati	1995	Census	5-year age groups	De facto	Included
Kiribati	2000	Census	5-year age groups	De facto	Included
Kiribati	2005	Census	5-year age groups	De facto	Included
Kiribati	2010	Census	1-year age groups	De facto	Included
Kiribati	2015	Census	5-year age groups	De jure	Included
Kuwait	1957	Census	5-year age groups	De facto	Included
Kuwait	1961	Census	non-std age groups	De facto	Included
Kuwait	1965	Census	1-year age groups	De facto	Included
Kuwait	1970	Census	1-year age groups	De facto	Included
Kuwait	1975	Census	5-year age groups	De facto	Included
Kuwait	1980	Census	5-year age groups	De facto	Included
Kuwait	1985	Census	1-year age groups	De facto	Included
Kuwait	1995	Census	5-year age groups	De facto	Included
Kuwait	2005	Census	5-year age groups	De facto	Included
Kuwait	2011	Census	1-year age groups	De facto	Included
Kyrgyzstan	1959	Census	non-std age groups	De facto	Included
Kyrgyzstan	1970	Census	non-std age groups	De facto	Included
Kyrgyzstan	1979	Census	5-year age groups	De facto	Included
Kyrgyzstan	1989	Census	1-year age groups	De jure	Included

Appendix Table 5. List of all confirmed censuses by location and year					
**Location**	**Year**	**Type**	**Age detail**	**Enumeration method**	**Included or excluded from estimation**
Kyrgyzstan	1999	Census	1-year age groups	De jure	Included
Kyrgyzstan	2009	Census	1-year age groups	De jure	Included
Laos	1985	Census	5-year age groups	De jure	Included
Laos	1995	Census	5-year age groups	De facto	Included
Laos	2005	Census	1-year age groups	De jure	Included
Laos	2015	Census	1-year age groups	De jure	Included
Latvia	1959	Census	non-std age groups	De facto	Included
Latvia	1970	Census	non-std age groups	De facto	Included
Latvia	1979	Census	5-year age groups	De jure	Included
Latvia	1989	Census	5-year age groups	De jure	Included
Latvia	2000	Census	5-year age groups	De jure	Included
Latvia	2006	Registry	1-year age groups	NA	Included
Latvia	2007	Registry	1-year age groups	NA	Included
Latvia	2008	Registry	1-year age groups	NA	Included
Latvia	2009	Registry	1-year age groups	NA	Included
Latvia	2010	Registry	1-year age groups	NA	Included
Latvia	2011	Census	1-year age groups	De jure	Included
Latvia	2011	Registry	1-year age groups	NA	Included
Latvia	2012	Registry	1-year age groups	NA	Included
Latvia	2013	Registry	1-year age groups	NA	Included
Latvia	2014	Registry	1-year age groups	NA	Included
Latvia	2015	Registry	1-year age groups	NA	Included
Latvia	2016	Registry	1-year age groups	NA	Included
Latvia	2017	Registry	1-year age groups	NA	Included
Lebanon	1970	Survey	5-year age groups	NA	Included
Lesotho	1956	Census	non-std age groups	De facto	Included
Lesotho	1966	Census	1-year age groups	De facto	Included
Lesotho	1976	Census	5-year age groups	De jure	Included
Lesotho	1986	Census	5-year age groups	De jure	Included
Lesotho	1996	Census	5-year age groups	De jure	Included
Lesotho	2006	Census	1-year age groups	De jure	Included
Lesotho	2016	Census	Not Available	Unknown	Excluded
Liberia	1962	Census	5-year age groups	De facto	Included
Liberia	1974	Census	5-year age groups	De facto	Included
Liberia	1984	Census	1-year age groups	De facto	Included
Liberia	2008	Census	1-year age groups	De facto	Included
Libya	1954	Census	5-year age groups	De facto	Included
Libya	1964	Census	non-std age groups	De facto	Included
Libya	1973	Census	1-year age groups	De jure	Included
Libya	1984	Census	1-year age groups	De facto	Included
Libya	1995	Census	all ages only	Unknown	Included
Libya	2006	Census	5-year age groups	De facto	Included
Lithuania	1959	Census	non-std age groups	De facto	Included
Lithuania	1970	Census	non-std age groups	De facto	Included
Lithuania	1979	Census	5-year age groups	De facto	Included
Lithuania	1989	Census	1-year age groups	De jure	Included
Lithuania	2001	Census	5-year age groups	De jure	Included
Lithuania	2011	Census	1-year age groups	De jure	Included
Lithuania	2015	Registry	1-year age groups	NA	Included
Lithuania	2016	Registry	1-year age groups	NA	Included
Lithuania	2017	Registry	1-year age groups	NA	Included
Luxembourg	1960	Census	1-year age groups	De jure	Included
Luxembourg	1966	Census	5-year age groups	De jure	Included
Luxembourg	1970	Census	1-year age groups	De jure	Included
Luxembourg	1981	Census	1-year age groups	De jure	Included
Luxembourg	1991	Census	5-year age groups	De jure	Included
Luxembourg	2001	Census	1-year age groups	De jure	Included
Luxembourg	2001	Registry	5-year age groups	NA	Included
Luxembourg	2002	Registry	5-year age groups	NA	Included
Luxembourg	2003	Registry	5-year age groups	NA	Included
Luxembourg	2004	Registry	5-year age groups	NA	Included
Luxembourg	2005	Registry	5-year age groups	NA	Included
Luxembourg	2006	Registry	5-year age groups	NA	Included
Luxembourg	2007	Registry	5-year age groups	NA	Included
Luxembourg	2008	Registry	5-year age groups	NA	Included
Luxembourg	2009	Registry	5-year age groups	NA	Included
Luxembourg	2010	Registry	5-year age groups	NA	Included
Luxembourg	2011	Census	1-year age groups	De jure	Included
Luxembourg	2011	Registry	5-year age groups	NA	Included
Luxembourg	2012	Registry	5-year age groups	NA	Included
Luxembourg	2013	Registry	5-year age groups	NA	Included
Luxembourg	2014	Registry	5-year age groups	NA	Included
Luxembourg	2015	Registry	5-year age groups	NA	Included
Luxembourg	2016	Registry	5-year age groups	NA	Included
Luxembourg	2017	Registry	5-year age groups	NA	Included

Appendix Table 5. List of all confirmed censuses by location and year					
**Location**	**Year**	**Type**	**Age detail**	**Enumeration method**	**Included or excluded from estimation**
Macao Special Administrative Region of China	1950	Census	5-year age groups	De facto	Included
Macao Special Administrative Region of China	1960	Census	1-year age groups	De facto	Included
Macao Special Administrative Region of China	1970	Census	5-year age groups	De jure	Included
Macao Special Administrative Region of China	1981	Census	5-year age groups	De jure	Included
Macao Special Administrative Region of China	1991	Census	1-year age groups	De jure	Included
Macao Special Administrative Region of China	2001	Census	1-year age groups	De jure	Included
Macao Special Administrative Region of China	2006	Census	5-year age groups	De jure	Included
Macao Special Administrative Region of China	2011	Census	1-year age groups	De jure	Included
Macao Special Administrative Region of China	2016	Census	5-year age groups	De jure	Included
Macedonia	1953	Census	5-year age groups	De jure	Included
Macedonia	1961	Census	5-year age groups	De jure	Included
Macedonia	1971	Census	5-year age groups	De jure	Included
Macedonia	1981	Census	5-year age groups	De jure	Included
Macedonia	1994	Census	5-year age groups	De jure	Included
Macedonia	2002	Census	1-year age groups	De jure	Included
Madagascar	1951	Census	Not Available	Unknown	Excluded
Madagascar	1975	Census	5-year age groups	De facto	Included
Madagascar	1993	Census	5-year age groups	De facto	Included
Malawi	1956	Census	Not Available	Unknown	Excluded
Malawi	1961	Census	Not Available	Unknown	Excluded
Malawi	1966	Census	5-year age groups	De facto	Included
Malawi	1977	Census	5-year age groups	De facto	Included
Malawi	1987	Census	1-year age groups	De facto	Included
Malawi	1998	Census	1-year age groups	De facto	Included
Malawi	2008	Census	1-year age groups	De facto	Included
Malaysia	1991	Census	1-year age groups	De facto	Included
Malaysia	2000	Census	5-year age groups	De jure	Included
Malaysia	2010	Census	1-year age groups	De jure	Included
Malaysia (Penisular Malaya)	1957	Census	5-year age groups	De facto	Included
Malaysia (Penisular Malaya)	1970	Census	5-year age groups	De facto	Included
Malaysia (Penisular Malaya)	1980	Census	5-year age groups	De facto	Included
Malaysia (Sabah)	1960	Census	all ages only	De facto	Included
Malaysia (Sabah)	1970	Census	all ages only	De facto	Included
Malaysia (Sabah)	1980	Census	all ages only	De facto	Included
Malaysia (Sarawak)	1960	Census	all ages only	De facto	Included
Malaysia (Sarawak)	1970	Census	all ages only	De facto	Included
Malaysia (Sarawak)	1980	Census	all ages only	De facto	Included
Maldives	1953	Census	all ages only	De jure	Included
Maldives	1957	Census	all ages only	De jure	Included
Maldives	1962	Census	all ages only	Unknown	Included
Maldives	1965	Census	non-std age groups	De facto	Excluded
Maldives	1967	Census	1-year age groups	De facto	Included
Maldives	1972	Census	all ages only	De jure	Included
Maldives	1974	Census	5-year age groups	De facto	Included
Maldives	1977	Census	5-year age groups	De facto	Included
Maldives	1985	Census	1-year age groups	De facto	Included
Maldives	1990	Census	1-year age groups	De facto	Included
Maldives	1995	Census	1-year age groups	De facto	Included
Maldives	2000	Census	1-year age groups	De facto	Included
Maldives	2006	Census	1-year age groups	De facto	Included
Maldives	2014	Census	1-year age groups	De facto	Included
Mali	1951	Census	Not Available	Unknown	Excluded
Mali	1976	Census	5-year age groups	De facto	Included
Mali	1987	Census	1-year age groups	De jure	Included
Mali	1998	Census	5-year age groups	De jure	Included
Mali	2009	Census	1-year age groups	De facto	Included
Malta	1957	Census	5-year age groups	De facto	Included
Malta	1967	Census	5-year age groups	De facto	Included
Malta	1985	Census	5-year age groups	De facto	Included
Malta	1995	Census	1-year age groups	De jure	Included
Malta	2005	Census	1-year age groups	De jure	Included
Malta	2011	Census	1-year age groups	De facto	Included
Marshall Islands	1973	Census	5-year age groups	De facto	Excluded
Marshall Islands	1980	Census	non-std age groups	De jure	Excluded
Marshall Islands	1988	Census	1-year age groups	De facto	Included
Marshall Islands	1999	Census	1-year age groups	De facto	Included
Marshall Islands	2011	Census	all ages only	De jure	Included
Mauritania	1951	Census	Not Available	Unknown	Excluded
Mauritania	1977	Census	5-year age groups	De facto	Included
Mauritania	1988	Census	5-year age groups	De facto	Included
Mauritania	2000	Census	5-year age groups	De facto	Included
Mauritania	2013	Census	5-year age groups	De jure	Included
Mauritius	1952	Census	1-year age groups	De facto	Included
Mauritius	1962	Census	1-year age groups	De facto	Included
Mauritius	1972	Census	1-year age groups	De facto	Included

Appendix Table 5. List of all confirmed censuses by location and year					
Location	Year	Type	Age detail	Enumeration method	Included or excluded from estimation
Mauritius	1983	Census	all ages only	De facto	Included
Mauritius	1990	Census	1-year age groups	De facto	Included
Mauritius	2000	Census	1-year age groups	De jure	Included
Mauritius	2011	Census	1-year age groups	De jure	Included
Mexico	1950	Census	non-std age groups	De jure	Included
Mexico	1960	Census	5-year age groups	De jure	Included
Mexico	1970	Census	1-year age groups	De jure	Included
Mexico	1980	Census	1-year age groups	De jure	Included
Mexico	1990	Census	1-year age groups	De jure	Included
Mexico	1995	Census	5-year age groups	Unknown	Included
Mexico	2000	Census	1-year age groups	De jure	Included
Mexico	2005	Census	1-year age groups	De jure	Excluded
Mexico	2010	Census	1-year age groups	De facto	Included
Moldova	1959	Census	non-std age groups	De facto	Included
Moldova	1970	Census	non-std age groups	De facto	Included
Moldova	1979	Census	5-year age groups	De facto	Included
Moldova	1989	Census	1-year age groups	De jure	Included
Mongolia	1956	Census	non-std age groups	De facto	Included
Mongolia	1963	Census	5-year age groups	De jure	Included
Mongolia	1969	Census	5-year age groups	De facto	Included
Mongolia	1979	Census	5-year age groups	De facto	Included
Mongolia	1989	Census	5-year age groups	De facto	Included
Mongolia	2000	Census	1-year age groups	De facto	Included
Mongolia	2010	Census	5-year age groups	De facto	Included
Montenegro	1953	Census	5-year age groups	De jure	Included
Montenegro	1961	Census	5-year age groups	De jure	Included
Montenegro	1971	Census	5-year age groups	De jure	Included
Montenegro	1981	Census	5-year age groups	De jure	Included
Montenegro	1991	Census	5-year age groups	De facto	Included
Montenegro	2003	Census	1-year age groups	De jure	Included
Montenegro	2011	Census	1-year age groups	De jure	Included
Morocco	1950	Census	all ages only	De jure	Excluded
Morocco	1960	Census	5-year age groups	De jure	Included
Morocco	1971	Census	1-year age groups	De jure	Included
Morocco	1982	Census	5-year age groups	De facto	Included
Morocco	1994	Census	5-year age groups	De facto	Included
Morocco	2004	Census	1-year age groups	De facto	Included
Morocco	2014	Census	5-year age groups	De jure	Included
Mozambique	1950	Census	5-year age groups	De facto	Included
Mozambique	1960	Census	5-year age groups	De jure	Included
Mozambique	1970	Census	5-year age groups	De facto	Included
Mozambique	1980	Census	1-year age groups	De facto	Included
Mozambique	1997	Census	1-year age groups	De jure	Included
Mozambique	2007	Census	1-year age groups	De facto	Included
Mozambique	2017	Census	all ages only	Unknown	Included
Myanmar	1973	Census	5-year age groups	De jure	Included
Myanmar	1983	Census	1-year age groups	De facto	Included
Myanmar	2014	Census	1-year age groups	De facto	Included
Namibia	1951	Census	5-year age groups	De facto	Excluded
Namibia	1960	Census	5-year age groups	De facto	Included
Namibia	1961	Census	Not Available	Unknown	Excluded
Namibia	1970	Census	all ages only	De jure	Included
Namibia	1971	Census	Not Available	Unknown	Excluded
Namibia	1981	Census	Not Available	Unknown	Excluded
Namibia	1991	Census	1-year age groups	De facto	Included
Namibia	2001	Census	1-year age groups	De facto	Included
Namibia	2011	Census	1-year age groups	De facto	Included
Nepal	1954	Census	5-year age groups	De facto	Included
Nepal	1961	Census	5-year age groups	De facto	Included
Nepal	1971	Census	non-std age groups	De jure	Included
Nepal	1981	Census	1-year age groups	De jure	Included
Nepal	1991	Census	1-year age groups	De jure	Included
Nepal	2001	Census	1-year age groups	De jure	Included
Nepal	2011	Census	1-year age groups	De jure	Included
Netherlands	1950	Registry	1-year age groups	NA	Included
Netherlands	1951	Registry	1-year age groups	NA	Included
Netherlands	1952	Registry	1-year age groups	NA	Included
Netherlands	1953	Registry	1-year age groups	NA	Included
Netherlands	1954	Registry	1-year age groups	NA	Included
Netherlands	1955	Registry	1-year age groups	NA	Included
Netherlands	1956	Registry	1-year age groups	NA	Included
Netherlands	1957	Registry	1-year age groups	NA	Included
Netherlands	1958	Registry	1-year age groups	NA	Included
Netherlands	1959	Registry	1-year age groups	NA	Included
Netherlands	1960	Census	1-year age groups	De jure	Included

Appendix Table 5. List of all confirmed censuses by location and year

Location	Year	Type	Age detail	Enumeration method	Included or excluded from estimation
Netherlands	1960	Registry	1-year age groups	NA	Included
Netherlands	1961	Registry	1-year age groups	NA	Included
Netherlands	1962	Registry	1-year age groups	NA	Included
Netherlands	1963	Registry	1-year age groups	NA	Included
Netherlands	1964	Registry	1-year age groups	NA	Included
Netherlands	1965	Registry	1-year age groups	NA	Included
Netherlands	1966	Registry	1-year age groups	NA	Included
Netherlands	1967	Registry	1-year age groups	NA	Included
Netherlands	1968	Registry	1-year age groups	NA	Included
Netherlands	1969	Registry	1-year age groups	NA	Included
Netherlands	1970	Registry	1-year age groups	NA	Included
Netherlands	1971	Census	Not Available	Unknown	Excluded
Netherlands	1971	Registry	1-year age groups	NA	Included
Netherlands	1972	Registry	1-year age groups	NA	Included
Netherlands	1973	Registry	1-year age groups	NA	Included
Netherlands	1974	Registry	1-year age groups	NA	Included
Netherlands	1975	Registry	1-year age groups	NA	Included
Netherlands	1976	Registry	1-year age groups	NA	Included
Netherlands	1977	Registry	1-year age groups	NA	Included
Netherlands	1978	Registry	1-year age groups	NA	Included
Netherlands	1979	Registry	1-year age groups	NA	Included
Netherlands	1980	Census	Not Available	Unknown	Excluded
Netherlands	1980	Registry	1-year age groups	NA	Included
Netherlands	1981	Registry	1-year age groups	NA	Included
Netherlands	1982	Registry	1-year age groups	NA	Included
Netherlands	1983	Registry	1-year age groups	NA	Included
Netherlands	1984	Registry	1-year age groups	NA	Included
Netherlands	1985	Registry	1-year age groups	NA	Included
Netherlands	1986	Registry	1-year age groups	NA	Included
Netherlands	1987	Registry	1-year age groups	NA	Included
Netherlands	1988	Registry	1-year age groups	NA	Included
Netherlands	1989	Registry	1-year age groups	NA	Included
Netherlands	1990	Registry	1-year age groups	NA	Included
Netherlands	1991	Census	5-year age groups	De jure	Included
Netherlands	1991	Registry	1-year age groups	NA	Included
Netherlands	1992	Registry	1-year age groups	NA	Included
Netherlands	1993	Registry	1-year age groups	NA	Included
Netherlands	1994	Registry	1-year age groups	NA	Included
Netherlands	1995	Registry	1-year age groups	NA	Included
Netherlands	1996	Registry	1-year age groups	NA	Included
Netherlands	1997	Registry	1-year age groups	NA	Included
Netherlands	1998	Registry	1-year age groups	NA	Included
Netherlands	1999	Registry	1-year age groups	NA	Included
Netherlands	2000	Registry	1-year age groups	NA	Included
Netherlands	2001	Census	Not Available	Unknown	Included
Netherlands	2001	Registry	1-year age groups	NA	Included
Netherlands	2002	Census	1-year age groups	De jure	Included
Netherlands	2002	Registry	1-year age groups	NA	Included
Netherlands	2003	Registry	1-year age groups	NA	Included
Netherlands	2004	Registry	1-year age groups	NA	Included
Netherlands	2005	Registry	1-year age groups	NA	Included
Netherlands	2006	Registry	1-year age groups	NA	Included
Netherlands	2007	Registry	1-year age groups	NA	Included
Netherlands	2008	Registry	1-year age groups	NA	Included
Netherlands	2009	Registry	1-year age groups	NA	Included
Netherlands	2010	Registry	1-year age groups	NA	Included
Netherlands	2011	Census	1-year age groups	De jure	Included
Netherlands	2011	Registry	1-year age groups	NA	Included
Netherlands	2012	Registry	1-year age groups	NA	Included
Netherlands	2013	Registry	1-year age groups	NA	Included
Netherlands	2014	Registry	1-year age groups	NA	Included
Netherlands	2015	Registry	1-year age groups	NA	Included
Netherlands	2016	Registry	1-year age groups	NA	Included
Netherlands	2017	Registry	1-year age groups	NA	Included
New Zealand	1951	Census	1-year age groups	De facto	Included
New Zealand	1956	Census	5-year age groups	De jure	Included
New Zealand	1961	Census	1-year age groups	De jure	Included
New Zealand	1966	Census	5-year age groups	De facto	Included
New Zealand	1971	Census	5-year age groups	De facto	Included
New Zealand	1976	Census	5-year age groups	De facto	Included
New Zealand	1981	Census	1-year age groups	De jure	Included
New Zealand	1986	Census	1-year age groups	De jure	Included
New Zealand	1991	Census	1-year age groups	De facto	Included
New Zealand	1996	Census	1-year age groups	De jure	Included
New Zealand	2001	Census	5-year age groups	De jure	Included

Appendix Table 5. List of all confirmed censuses by location and year					
**Location**	**Year**	**Type**	**Age detail**	**Enumeration method**	**Included or excluded from estimation**
New Zealand	2006	Census	1-year age groups	De jure	Included
New Zealand	2013	Census	1-year age groups	De jure	Included
New Zealand	2018	Census	Not Available	Unknown	Excluded
Nicaragua	1950	Census	1-year age groups	De jure	Included
Nicaragua	1963	Census	1-year age groups	De jure	Included
Nicaragua	1971	Census	1-year age groups	De jure	Included
Nicaragua	1995	Census	1-year age groups	Unknown	Included
Nicaragua	2005	Census	1-year age groups	De jure	Included
Niger	1951	Census	Not Available	Unknown	Excluded
Niger	1977	Census	5-year age groups	De jure	Included
Niger	1988	Census	5-year age groups	De facto	Included
Niger	2001	Census	1-year age groups	De jure	Included
Niger	2012	Census	1-year age groups	De jure	Included
Nigeria	1953	Census	non-std age groups	De facto	Excluded
Nigeria	1963	Census	1-year age groups	De facto	Excluded
Nigeria	1973	Census	Not Available	Unknown	Excluded
Nigeria	1991	Census	1-year age groups	De facto	Included
Nigeria	2006	Census	5-year age groups	De facto	Included
Nigeria	2014	Census	5-year age groups	Micro-census	Included
North Korea	1965	Census	Not Available	Unknown	Excluded
North Korea	1967	Census	Not Available	Unknown	Excluded
North Korea	1968	Census	Not Available	Unknown	Excluded
North Korea	1993	Census	1-year age groups	De jure	Included
North Korea	2008	Census	1-year age groups	De jure	Included
Northern Ireland	1951	Census	1-year age groups	Unknown	Included
Northern Ireland	1961	Census	1-year age groups	Unknown	Included
Northern Ireland	1966	Census	1-year age groups	Unknown	Included
Northern Ireland	1971	Census	1-year age groups	Unknown	Included
Northern Ireland	1981	Census	1-year age groups	Unknown	Included
Northern Ireland	1991	Census	1-year age groups	De jure	Included
Northern Ireland	2001	Census	1-year age groups	De jure	Included
Northern Ireland	2011	Census	1-year age groups	De jure	Included
Northern Mariana Islands	1958	Census	5-year age groups	De jure	Excluded
Northern Mariana Islands	1970	Census	non-std age groups	De jure	Included
Northern Mariana Islands	1973	Census	1-year age groups	De facto	Excluded
Northern Mariana Islands	1980	Census	5-year age groups	De facto	Included
Northern Mariana Islands	1990	Census	non-std age groups	De facto	Included
Northern Mariana Islands	1995	Census	5-year age groups	De facto	Included
Northern Mariana Islands	2000	Census	non-std age groups	De jure	Included
Northern Mariana Islands	2010	Census	5-year age groups	De facto	Included
Norway	1950	Census	1-year age groups	De jure	Included
Norway	1960	Census	1-year age groups	De jure	Included
Norway	1970	Census	1-year age groups	De jure	Included
Norway	1980	Census	1-year age groups	De jure	Included
Norway	1986	Registry	1-year age groups	NA	Included
Norway	1987	Registry	1-year age groups	NA	Included
Norway	1988	Registry	1-year age groups	NA	Included
Norway	1989	Registry	1-year age groups	NA	Included
Norway	1990	Census	1-year age groups	De jure	Included
Norway	1990	Registry	1-year age groups	NA	Included
Norway	1991	Registry	1-year age groups	NA	Included
Norway	1992	Registry	1-year age groups	NA	Included
Norway	1993	Registry	1-year age groups	NA	Included
Norway	1994	Registry	1-year age groups	NA	Included
Norway	1995	Registry	1-year age groups	NA	Included
Norway	1996	Registry	1-year age groups	NA	Included
Norway	1997	Registry	1-year age groups	NA	Included
Norway	1998	Registry	1-year age groups	NA	Included
Norway	1999	Registry	1-year age groups	NA	Included
Norway	2000	Registry	1-year age groups	NA	Included
Norway	2001	Census	1-year age groups	De jure	Included
Norway	2001	Registry	1-year age groups	NA	Included
Norway	2002	Registry	1-year age groups	NA	Included
Norway	2003	Registry	1-year age groups	NA	Included
Norway	2004	Registry	1-year age groups	NA	Included
Norway	2005	Registry	1-year age groups	NA	Included
Norway	2006	Registry	1-year age groups	NA	Included
Norway	2007	Registry	1-year age groups	NA	Included
Norway	2008	Registry	1-year age groups	NA	Included
Norway	2009	Registry	1-year age groups	NA	Included
Norway	2010	Registry	1-year age groups	NA	Included
Norway	2011	Census	1-year age groups	De jure	Included
Norway	2011	Registry	1-year age groups	NA	Included
Norway	2012	Registry	1-year age groups	NA	Included
Norway	2013	Registry	1-year age groups	NA	Included

Appendix Table 5. List of all confirmed censuses by location and year					
**Location**	**Year**	**Type**	**Age detail**	**Enumeration method**	**Included or excluded from estimation**
Norway	2014	Registry	1-year age groups	NA	Included
Norway	2015	Registry	1-year age groups	NA	Included
Norway	2016	Registry	1-year age groups	NA	Included
Norway	2017	Registry	1-year age groups	NA	Included
Oman	1993	Census	5-year age groups	De facto	Included
Oman	2003	Census	1-year age groups	De facto	Included
Oman	2010	Census	5-year age groups	De facto	Included
Oman	2012	Registry	5-year age groups	NA	Included
Oman	2013	Registry	5-year age groups	NA	Included
Oman	2014	Registry	5-year age groups	NA	Included
Oman	2015	Registry	5-year age groups	NA	Included
Oman	2016	Registry	5-year age groups	NA	Included
Oman	2017	Registry	5-year age groups	NA	Included
Pakistan	1951	Census	non-std age groups	De jure	Included
Pakistan	1961	Census	non-std age groups	De jure	Included
Pakistan	1972	Census	1-year age groups	De jure	Included
Pakistan	1981	Census	1-year age groups	De facto	Included
Pakistan	1998	Census	5-year age groups	De facto	Included
Pakistan	2017	Census	all ages only	Unknown	Included
Palestine	1967	Census	5-year age groups	De jure	Included
Palestine	1997	Census	1-year age groups	De facto	Included
Palestine	2007	Census	1-year age groups	De facto	Included
Palestine	2017	Census	all ages only	Unknown	Included
Panama	1950	Census	1-year age groups	De facto	Included
Panama	1960	Census	1-year age groups	De facto	Included
Panama	1970	Census	5-year age groups	De facto	Included
Panama	1980	Census	1-year age groups	De facto	Included
Panama	1990	Census	1-year age groups	De facto	Included
Panama	2000	Census	1-year age groups	De facto	Included
Panama	2010	Census	5-year age groups	De facto	Included
Papua New Guinea	1954	Census	Not Available	Unknown	Excluded
Papua New Guinea	1961	Census	Not Available	Unknown	Excluded
Papua New Guinea	1966	Census	5-year age groups	De facto	Included
Papua New Guinea	1971	Census	1-year age groups	De facto	Included
Papua New Guinea	1980	Census	1-year age groups	De facto	Included
Papua New Guinea	1990	Census	5-year age groups	De facto	Excluded
Papua New Guinea	2000	Census	1-year age groups	De facto	Included
Papua New Guinea	2011	Census	5-year age groups	De facto	Included
Paraguay	1950	Census	non-std age groups	De facto	Included
Paraguay	1962	Census	1-year age groups	De facto	Included
Paraguay	1972	Census	1-year age groups	De facto	Included
Paraguay	1982	Census	1-year age groups	De facto	Included
Paraguay	1992	Census	1-year age groups	De facto	Included
Paraguay	2002	Census	1-year age groups	De facto	Included
Peru	1961	Census	1-year age groups	De facto	Included
Peru	1972	Census	1-year age groups	De facto	Included
Peru	1981	Census	1-year age groups	De facto	Included
Peru	1993	Census	1-year age groups	Unknown	Included
Peru	2005	Census	1-year age groups	De facto	Included
Peru	2007	Census	1-year age groups	De facto	Included
Peru	2012	Registry	all ages only	NA	Included
Peru	2013	Registry	all ages only	NA	Included
Peru	2014	Registry	all ages only	NA	Included
Peru	2015	Registry	all ages only	NA	Included
Peru	2016	Registry	all ages only	NA	Included
Peru	2017	Census	Not Available	Unknown	Excluded
Peru	2017	Registry	all ages only	NA	Included
Philippines	1960	Census	1-year age groups	De jure	Included
Philippines	1970	Census	1-year age groups	De jure	Included
Philippines	1975	Census	5-year age groups	De jure	Included
Philippines	1980	Census	5-year age groups	De jure	Included
Philippines	1990	Census	1-year age groups	De jure	Included
Philippines	1995	Census	1-year age groups	De jure	Included
Philippines	2000	Census	1-year age groups	De jure	Included
Philippines	2007	Census	1-year age groups	De jure	Included
Philippines	2010	Census	5-year age groups	De jure	Included
Philippines	2015	Census	1-year age groups	De jure	Included
Poland	1950	Census	5-year age groups	De facto	Included
Poland	1960	Census	5-year age groups	De facto	Included
Poland	1970	Census	1-year age groups	De facto	Included
Poland	1978	Census	5-year age groups	De facto	Included
Poland	1988	Census	1-year age groups	De facto	Included
Poland	2002	Census	1-year age groups	De jure	Included
Poland	2006	Registry	1-year age groups	NA	Included
Poland	2007	Registry	1-year age groups	NA	Included

Appendix Table 5. List of all confirmed censuses by location and year					
**Location**	**Year**	**Type**	**Age detail**	**Enumeration method**	**Included or excluded from estimation**
Poland	2008	Registry	1-year age groups	NA	Included
Poland	2009	Registry	1-year age groups	NA	Included
Poland	2010	Registry	1-year age groups	NA	Included
Poland	2011	Census	1-year age groups	De jure	Included
Poland	2011	Registry	1-year age groups	NA	Included
Poland	2012	Registry	1-year age groups	NA	Included
Poland	2013	Registry	1-year age groups	NA	Included
Poland	2014	Registry	1-year age groups	NA	Included
Poland	2015	Registry	1-year age groups	NA	Included
Poland	2016	Registry	1-year age groups	NA	Included
Poland	2017	Registry	1-year age groups	NA	Included
Portugal	1950	Census	1-year age groups	De facto	Included
Portugal	1960	Census	5-year age groups	De facto	Included
Portugal	1970	Census	1-year age groups	De facto	Included
Portugal	1981	Census	1-year age groups	De facto	Included
Portugal	1991	Census	1-year age groups	De facto	Included
Portugal	2001	Census	5-year age groups	De facto	Included
Portugal	2011	Census	1-year age groups	De jure	Included
Pridnestrovian Moldavian Republic	2004	Census	all ages only	De jure	Included
Pridnestrovian Moldavian Republic	2015	Census	all ages only	De jure	Included
Puerto Rico	1950	Census	non-std age groups	De jure	Included
Puerto Rico	1960	Census	non-std age groups	De jure	Included
Puerto Rico	1970	Census	1-year age groups	De jure	Included
Puerto Rico	1980	Census	1-year age groups	De jure	Included
Puerto Rico	1990	Census	1-year age groups	De jure	Included
Puerto Rico	2000	Census	1-year age groups	De jure	Included
Puerto Rico	2010	Census	1-year age groups	De jure	Included
Qatar	1986	Census	1-year age groups	De facto	Included
Qatar	1997	Census	5-year age groups	De facto	Included
Qatar	2004	Census	5-year age groups	De facto	Included
Qatar	2010	Census	5-year age groups	De facto	Included
Qatar	2015	Census	5-year age groups	De facto	Included
Republic of Kosovo	1953	Census	5-year age groups	Unknown	Included
Republic of Kosovo	1961	Census	5-year age groups	Unknown	Included
Republic of Kosovo	1971	Census	5-year age groups	Unknown	Included
Republic of Kosovo	1981	Census	5-year age groups	Unknown	Included
Republic of Kosovo	1991	Census	5-year age groups	Unknown	Included
Republic of Kosovo	2011	Census	5-year age groups	Unknown	Included
Republic of Moldova	2004	Census	1-year age groups	De facto	Included
Republic of Moldova	2014	Census	1-year age groups	De jure	Included
Republic of Serbia	1971	Census	1-year age groups	De facto	Included
Republic of Serbia	1991	Census	5-year age groups	De jure	Included
Republic of Serbia	2002	Census	1-year age groups	De jure	Included
Republic of Serbia	2011	Census	1-year age groups	De jure	Included
Romania	1956	Census	5-year age groups	De jure	Included
Romania	1966	Census	1-year age groups	De jure	Included
Romania	1977	Census	5-year age groups	De facto	Included
Romania	1992	Census	1-year age groups	De jure	Included
Romania	2002	Census	1-year age groups	De jure	Included
Romania	2011	Census	1-year age groups	De jure	Included
Russian Federation	1959	Census	1-year age groups	Unknown	Included
Russian Federation	1970	Census	5-year age groups	Unknown	Included
Russian Federation	1979	Census	1-year age groups	Unknown	Included
Russian Federation	1989	Census	1-year age groups	Unknown	Included
Russian Federation	2002	Census	5-year age groups	De jure	Included
Russian Federation	2010	Census	1-year age groups	De jure	Included
Rwanda	1953	Census	Not Available	Unknown	Excluded
Rwanda	1978	Census	5-year age groups	De facto	Included
Rwanda	1991	Census	5-year age groups	De facto	Included
Rwanda	2002	Census	1-year age groups	De jure	Included
Rwanda	2012	Census	1-year age groups	De jure	Included
Saint Lucia	1960	Census	5-year age groups	De facto	Included
Saint Lucia	1970	Census	5-year age groups	De facto	Included
Saint Lucia	1980	Census	1-year age groups	De facto	Included
Saint Lucia	1991	Census	1-year age groups	De facto	Included
Saint Lucia	2001	Census	1-year age groups	De facto	Included
Saint Lucia	2010	Census	5-year age groups	De jure	Included
Saint Vincent and the Grenadines	1960	Census	5-year age groups	De facto	Included
Saint Vincent and the Grenadines	1970	Census	5-year age groups	De facto	Included
Saint Vincent and the Grenadines	1980	Census	1-year age groups	De facto	Included
Saint Vincent and the Grenadines	1991	Census	1-year age groups	De facto	Included
Saint Vincent and the Grenadines	2001	Census	1-year age groups	De facto	Included
Saint Vincent and the Grenadines	2012	Census	5-year age groups	De facto	Included
Samoa	1951	Census	5-year age groups	De facto	Included
Samoa	1956	Census	1-year age groups	De facto	Included

Appendix Table 5. List of all confirmed censuses by location and year					
Location	Year	Type	Age detail	Enumeration method	Included or excluded from estimation
Samoa	1961	Census	5-year age groups	De facto	Included
Samoa	1966	Census	5-year age groups	De facto	Included
Samoa	1971	Census	1-year age groups	De facto	Included
Samoa	1976	Census	5-year age groups	De facto	Included
Samoa	1981	Census	5-year age groups	De facto	Included
Samoa	1986	Census	5-year age groups	De facto	Included
Samoa	1991	Census	5-year age groups	De facto	Included
Samoa	2001	Census	1-year age groups	De facto	Included
Samoa	2006	Census	5-year age groups	De facto	Included
Samoa	2011	Census	5-year age groups	De facto	Included
Samoa	2016	Census	all ages only	Unknown	Included
Sao Tome and Principe	1950	Census	all ages only	Unknown	Included
Sao Tome and Principe	1960	Census	5-year age groups	De jure	Included
Sao Tome and Principe	1970	Census	5-year age groups	De facto	Included
Sao Tome and Principe	1981	Census	1-year age groups	De facto	Included
Sao Tome and Principe	1991	Census	1-year age groups	De facto	Included
Sao Tome and Principe	2001	Census	5-year age groups	De facto	Included
Sao Tome and Principe	2012	Census	1-year age groups	De jure	Included
Saudi Arabia	1962	Census	Not Available	Unknown	Excluded
Saudi Arabia	1974	Census	all ages only	De facto	Included
Saudi Arabia	1992	Census	5-year age groups	De facto	Included
Saudi Arabia	2004	Census	5-year age groups	De facto	Included
Saudi Arabia	2010	Census	5-year age groups	De facto	Included
Scotland	1951	Census	all ages only	Unknown	Included
Scotland	1961	Census	5-year age groups	Unknown	Included
Scotland	1971	Census	5-year age groups	Unknown	Included
Scotland	1981	Census	5-year age groups	De jure	Included
Scotland	1991	Census	non-std age groups	De jure	Included
Scotland	2001	Census	5-year age groups	De jure	Included
Scotland	2011	Census	1-year age groups	De jure	Included
Senegal	1951	Census	Not Available	Unknown	Excluded
Senegal	1976	Census	5-year age groups	De jure	Included
Senegal	1988	Census	5-year age groups	De jure	Included
Senegal	2002	Census	5-year age groups	De jure	Included
Senegal	2013	Census	5-year age groups	De facto	Included
Seychelles	1960	Census	5-year age groups	De facto	Included
Seychelles	1971	Census	1-year age groups	De jure	Included
Seychelles	1977	Census	5-year age groups	De facto	Included
Seychelles	1987	Census	1-year age groups	De facto	Included
Seychelles	1994	Census	non-std age groups	De facto	Included
Seychelles	1997	Census	1-year age groups	De facto	Included
Seychelles	2002	Census	1-year age groups	De jure	Included
Seychelles	2010	Census	1-year age groups	De facto	Included
Sierra Leone	1963	Census	5-year age groups	De facto	Included
Sierra Leone	1974	Census	5-year age groups	De facto	Included
Sierra Leone	1985	Census	5-year age groups	De facto	Included
Sierra Leone	2004	Census	1-year age groups	Unknown	Included
Sierra Leone	2015	Census	5-year age groups	De facto	Included
Singapore	1957	Census	5-year age groups	De facto	Included
Singapore	1970	Census	1-year age groups	De facto	Included
Singapore	1980	Census	1-year age groups	De facto	Included
Singapore (Residents Only)	1990	Census	5-year age groups	De facto	Included
Singapore (Residents Only)	2000	Census	1-year age groups	De jure	Included
Singapore (Residents Only)	2000	Registry	5-year age groups	NA	Excluded
Singapore (Residents Only)	2001	Registry	5-year age groups	NA	Excluded
Singapore (Residents Only)	2002	Registry	5-year age groups	NA	Excluded
Singapore (Residents Only)	2003	Registry	5-year age groups	NA	Excluded
Singapore (Residents Only)	2004	Registry	5-year age groups	NA	Excluded
Singapore (Residents Only)	2005	Registry	5-year age groups	NA	Excluded
Singapore (Residents Only)	2006	Registry	5-year age groups	NA	Excluded
Singapore (Residents Only)	2007	Registry	5-year age groups	NA	Excluded
Singapore (Residents Only)	2008	Registry	5-year age groups	NA	Excluded
Singapore (Residents Only)	2009	Registry	5-year age groups	NA	Excluded
Singapore (Residents Only)	2010	Census	1-year age groups	De jure	Included
Singapore (Residents Only)	2010	Registry	5-year age groups	NA	Excluded
Singapore (Residents Only)	2011	Registry	5-year age groups	NA	Excluded
Singapore (Residents Only)	2012	Registry	5-year age groups	NA	Excluded
Singapore (Residents Only)	2013	Registry	5-year age groups	NA	Excluded
Singapore (Residents Only)	2014	Registry	5-year age groups	NA	Excluded
Singapore (Residents Only)	2015	Registry	5-year age groups	NA	Excluded
Singapore (Residents Only)	2016	Registry	5-year age groups	NA	Excluded
Singapore (Residents Only)	2017	Registry	5-year age groups	NA	Excluded
Slovakia	1950	Census	5-year age groups	De jure	Included
Slovakia	1950	Registry	1-year age groups	NA	Included
Slovakia	1951	Registry	1-year age groups	NA	Included

Appendix Table 5. List of all confirmed censuses by location and year

Location	Year	Type	Age detail	Enumeration method	Included or excluded from estimation
Slovakia	1952	Registry	1-year age groups	NA	Included
Slovakia	1953	Registry	1-year age groups	NA	Included
Slovakia	1954	Registry	1-year age groups	NA	Included
Slovakia	1955	Registry	1-year age groups	NA	Included
Slovakia	1956	Registry	1-year age groups	NA	Included
Slovakia	1957	Registry	1-year age groups	NA	Included
Slovakia	1958	Registry	1-year age groups	NA	Included
Slovakia	1959	Registry	1-year age groups	NA	Included
Slovakia	1960	Registry	1-year age groups	NA	Included
Slovakia	1961	Census	1-year age groups	De jure	Included
Slovakia	1961	Registry	1-year age groups	NA	Included
Slovakia	1962	Registry	1-year age groups	NA	Included
Slovakia	1963	Registry	1-year age groups	NA	Included
Slovakia	1964	Registry	1-year age groups	NA	Included
Slovakia	1965	Registry	1-year age groups	NA	Included
Slovakia	1966	Registry	1-year age groups	NA	Included
Slovakia	1967	Registry	1-year age groups	NA	Included
Slovakia	1968	Registry	1-year age groups	NA	Included
Slovakia	1969	Registry	1-year age groups	NA	Included
Slovakia	1970	Census	all ages only	De jure	Included
Slovakia	1970	Registry	1-year age groups	NA	Included
Slovakia	1971	Registry	1-year age groups	NA	Included
Slovakia	1972	Registry	1-year age groups	NA	Included
Slovakia	1973	Registry	1-year age groups	NA	Included
Slovakia	1974	Registry	1-year age groups	NA	Included
Slovakia	1975	Registry	1-year age groups	NA	Included
Slovakia	1976	Registry	1-year age groups	NA	Included
Slovakia	1977	Registry	1-year age groups	NA	Included
Slovakia	1978	Registry	1-year age groups	NA	Included
Slovakia	1979	Registry	1-year age groups	NA	Included
Slovakia	1980	Census	5-year age groups	De jure	Included
Slovakia	1980	Registry	1-year age groups	NA	Included
Slovakia	1981	Registry	1-year age groups	NA	Included
Slovakia	1982	Registry	1-year age groups	NA	Included
Slovakia	1983	Registry	1-year age groups	NA	Included
Slovakia	1984	Registry	1-year age groups	NA	Included
Slovakia	1985	Registry	1-year age groups	NA	Included
Slovakia	1986	Registry	1-year age groups	NA	Included
Slovakia	1987	Registry	1-year age groups	NA	Included
Slovakia	1988	Registry	1-year age groups	NA	Included
Slovakia	1989	Registry	1-year age groups	NA	Included
Slovakia	1990	Registry	1-year age groups	NA	Included
Slovakia	1991	Census	1-year age groups	De jure	Included
Slovakia	1991	Registry	1-year age groups	NA	Included
Slovakia	1992	Registry	1-year age groups	NA	Included
Slovakia	1993	Registry	1-year age groups	NA	Included
Slovakia	1994	Registry	1-year age groups	NA	Included
Slovakia	1995	Registry	1-year age groups	NA	Included
Slovakia	1996	Registry	1-year age groups	NA	Included
Slovakia	1997	Registry	1-year age groups	NA	Included
Slovakia	1998	Registry	1-year age groups	NA	Included
Slovakia	1999	Registry	1-year age groups	NA	Included
Slovakia	2000	Registry	1-year age groups	NA	Included
Slovakia	2001	Census	1-year age groups	De jure	Included
Slovakia	2001	Registry	1-year age groups	NA	Included
Slovakia	2002	Registry	1-year age groups	NA	Included
Slovakia	2003	Registry	1-year age groups	NA	Included
Slovakia	2004	Registry	1-year age groups	NA	Included
Slovakia	2005	Registry	1-year age groups	NA	Included
Slovakia	2006	Registry	1-year age groups	NA	Included
Slovakia	2007	Registry	1-year age groups	NA	Included
Slovakia	2008	Registry	1-year age groups	NA	Included
Slovakia	2009	Registry	1-year age groups	NA	Included
Slovakia	2010	Registry	1-year age groups	NA	Included
Slovakia	2011	Census	1-year age groups	De jure	Included
Slovakia	2011	Registry	1-year age groups	NA	Included
Slovakia	2012	Registry	1-year age groups	NA	Included
Slovakia	2013	Registry	1-year age groups	NA	Included
Slovakia	2014	Registry	1-year age groups	NA	Included
Slovakia	2015	Registry	1-year age groups	NA	Included
Slovakia	2016	Registry	1-year age groups	NA	Included
Slovenia	1953	Census	all ages only	Unknown	Included
Slovenia	1961	Census	all ages only	Unknown	Included
Slovenia	1971	Census	1-year age groups	De facto	Included
Slovenia	1981	Census	all ages only	Unknown	Included

Appendix Table 5. List of all confirmed censuses by location and year					
**Location**	**Year**	**Type**	**Age detail**	**Enumeration method**	**Included or excluded from estimation**
Slovenia	1991	Census	1-year age groups	De jure	Excluded
Slovenia	1996	Registry	1-year age groups	NA	Included
Slovenia	1997	Registry	1-year age groups	NA	Included
Slovenia	1998	Registry	1-year age groups	NA	Included
Slovenia	1999	Registry	1-year age groups	NA	Included
Slovenia	2000	Registry	1-year age groups	NA	Included
Slovenia	2001	Registry	1-year age groups	NA	Included
Slovenia	2002	Census	1-year age groups	De jure	Included
Slovenia	2002	Registry	1-year age groups	NA	Included
Slovenia	2003	Registry	1-year age groups	NA	Included
Slovenia	2004	Registry	1-year age groups	NA	Included
Slovenia	2005	Registry	1-year age groups	NA	Included
Slovenia	2006	Registry	1-year age groups	NA	Included
Slovenia	2007	Registry	1-year age groups	NA	Included
Slovenia	2008	Registry	1-year age groups	NA	Included
Slovenia	2009	Registry	1-year age groups	NA	Included
Slovenia	2010	Registry	1-year age groups	NA	Included
Slovenia	2011	Census	1-year age groups	De jure	Included
Slovenia	2011	Registry	1-year age groups	NA	Included
Slovenia	2012	Registry	1-year age groups	NA	Included
Slovenia	2013	Registry	1-year age groups	NA	Included
Slovenia	2014	Registry	1-year age groups	NA	Included
Slovenia	2015	Census	1-year age groups	De jure	Included
Slovenia	2015	Registry	1-year age groups	NA	Included
Slovenia	2016	Registry	1-year age groups	NA	Included
Slovenia	2017	Registry	1-year age groups	NA	Included
Solomon Islands	1959	Census	5-year age groups	De facto	Included
Solomon Islands	1970	Census	5-year age groups	De facto	Included
Solomon Islands	1976	Census	5-year age groups	De facto	Included
Solomon Islands	1986	Census	5-year age groups	De facto	Included
Solomon Islands	1999	Census	5-year age groups	De facto	Included
Solomon Islands	2009	Census	5-year age groups	De facto	Included
Somalia	1953	Census	Not Available	Unknown	Excluded
Somalia	1975	Census	5-year age groups	De facto	Included
Somalia	1986	Census	Not Available	Unknown	Included
Somalia	1987	Census	all ages only	Unknown	Included
South Africa	1951	Census	5-year age groups	De facto	Included
South Africa	1960	Census	1-year age groups	De facto	Included
South Africa	1970	Census	1-year age groups	De facto	Included
South Africa	1980	Census	5-year age groups	De facto	Excluded
South Africa	1985	Census	1-year age groups	De facto	Excluded
South Africa	1991	Census	1-year age groups	De facto	Excluded
South Africa	1996	Census	1-year age groups	De facto	Included
South Africa	2001	Census	5-year age groups	De facto	Included
South Africa	2011	Census	5-year age groups	De facto	Included
South Korea	1955	Census	5-year age groups	De facto	Included
South Korea	1960	Census	5-year age groups	De facto	Included
South Korea	1966	Census	1-year age groups	De facto	Included
South Korea	1970	Census	1-year age groups	De facto	Included
South Korea	1975	Census	5-year age groups	De facto	Included
South Korea	1980	Census	1-year age groups	De facto	Included
South Korea	1985	Census	1-year age groups	De facto	Included
South Korea	1990	Census	1-year age groups	De facto	Included
South Korea	1995	Census	1-year age groups	De jure	Included
South Korea	2000	Census	1-year age groups	De jure	Included
South Korea	2005	Census	1-year age groups	De jure	Included
South Korea	2010	Census	5-year age groups	De jure	Included
South Korea	2015	Census	1-year age groups	De jure	Included
South Sudan	1956	Census	5-year age groups	De jure	Included
South Sudan	1973	Census	5-year age groups	De facto	Excluded
South Sudan	1983	Census	all ages only	De jure	Included
South Sudan	1993	Census	Not Available	Unknown	Excluded
South Sudan	2008	Census	1-year age groups	De facto	Included
Spain	1950	Census	1-year age groups	De facto	Included
Spain	1960	Census	1-year age groups	De jure	Included
Spain	1970	Census	5-year age groups	Unknown	Included
Spain	1981	Census	5-year age groups	De jure	Included
Spain	1991	Census	1-year age groups	De jure	Included
Spain	1998	Registry	1-year age groups	NA	Included
Spain	1999	Registry	1-year age groups	NA	Included
Spain	2000	Registry	1-year age groups	NA	Included
Spain	2001	Census	1-year age groups	De facto	Included
Spain	2001	Registry	1-year age groups	NA	Included
Spain	2002	Registry	1-year age groups	NA	Included
Spain	2003	Registry	1-year age groups	NA	Included

Appendix Table 5. List of all confirmed censuses by location and year					
**Location**	**Year**	**Type**	**Age detail**	**Enumeration method**	**Included or excluded from estimation**
Spain	2004	Registry	1-year age groups	NA	Included
Spain	2005	Registry	1-year age groups	NA	Included
Spain	2006	Registry	1-year age groups	NA	Included
Spain	2007	Registry	1-year age groups	NA	Included
Spain	2008	Registry	1-year age groups	NA	Included
Spain	2009	Registry	1-year age groups	NA	Included
Spain	2010	Registry	1-year age groups	NA	Included
Spain	2011	Census	1-year age groups	De jure	Included
Spain	2011	Registry	1-year age groups	NA	Included
Spain	2012	Registry	1-year age groups	NA	Included
Spain	2013	Registry	1-year age groups	NA	Included
Spain	2014	Registry	1-year age groups	NA	Included
Spain	2015	Registry	1-year age groups	NA	Included
Spain	2016	Registry	1-year age groups	NA	Included
Sri Lanka	1953	Census	5-year age groups	De facto	Included
Sri Lanka	1963	Census	1-year age groups	De facto	Included
Sri Lanka	1971	Census	1-year age groups	De facto	Included
Sri Lanka	1981	Census	1-year age groups	De facto	Included
Sri Lanka	2001	Census	1-year age groups	De facto	Excluded
Sri Lanka	2012	Census	1-year age groups	De jure	Included
Sudan	1956	Census	5-year age groups	De jure	Included
Sudan	1973	Census	5-year age groups	De facto	Included
Sudan	1983	Census	all ages only	De jure	Included
Sudan	1993	Census	5-year age groups	De jure	Included
Sudan	2008	Census	1-year age groups	Unknown	Included
Suriname	1950	Census	1-year age groups	De facto	Included
Suriname	1964	Census	5-year age groups	De facto	Included
Suriname	1971	Census	all ages only	De jure	Included
Suriname	1980	Census	Not Available	Unknown	Excluded
Suriname	2000	Census	all ages only	Unknown	Included
Suriname	2004	Census	1-year age groups	De jure	Included
Suriname	2012	Census	1-year age groups	De jure	Included
Swaziland	1956	Census	non-std age groups	De facto	Excluded
Swaziland	1966	Census	1-year age groups	De facto	Included
Swaziland	1976	Census	5-year age groups	De facto	Included
Swaziland	1986	Census	1-year age groups	De facto	Included
Swaziland	1997	Census	1-year age groups	De facto	Included
Swaziland	2007	Census	1-year age groups	De jure	Included
Swaziland	2017	Census	all ages only	Unknown	Included
Sweden	1950	Census	1-year age groups	De jure	Included
Sweden	1960	Census	5-year age groups	De jure	Included
Sweden	1965	Census	5-year age groups	De jure	Included
Sweden	1968	Registry	1-year age groups	NA	Included
Sweden	1969	Registry	1-year age groups	NA	Included
Sweden	1970	Census	5-year age groups	De jure	Included
Sweden	1970	Registry	1-year age groups	NA	Included
Sweden	1971	Registry	1-year age groups	NA	Included
Sweden	1972	Registry	1-year age groups	NA	Included
Sweden	1973	Registry	1-year age groups	NA	Included
Sweden	1974	Registry	1-year age groups	NA	Included
Sweden	1975	Census	5-year age groups	De jure	Included
Sweden	1975	Registry	1-year age groups	NA	Included
Sweden	1976	Registry	1-year age groups	NA	Included
Sweden	1977	Registry	1-year age groups	NA	Included
Sweden	1978	Registry	1-year age groups	NA	Included
Sweden	1979	Registry	1-year age groups	NA	Included
Sweden	1980	Census	1-year age groups	De jure	Included
Sweden	1980	Registry	1-year age groups	NA	Included
Sweden	1981	Registry	1-year age groups	NA	Included
Sweden	1982	Registry	1-year age groups	NA	Included
Sweden	1983	Registry	1-year age groups	NA	Included
Sweden	1984	Registry	1-year age groups	NA	Included
Sweden	1985	Census	5-year age groups	De jure	Included
Sweden	1985	Registry	1-year age groups	NA	Included
Sweden	1986	Registry	1-year age groups	NA	Included
Sweden	1987	Registry	1-year age groups	NA	Included
Sweden	1988	Registry	1-year age groups	NA	Included
Sweden	1989	Registry	1-year age groups	NA	Included
Sweden	1990	Census	1-year age groups	De jure	Included
Sweden	1990	Registry	1-year age groups	NA	Included
Sweden	1991	Registry	1-year age groups	NA	Included
Sweden	1992	Registry	1-year age groups	NA	Included
Sweden	1993	Registry	1-year age groups	NA	Included
Sweden	1994	Registry	1-year age groups	NA	Included
Sweden	1995	Registry	1-year age groups	NA	Included

Appendix Table 5. List of all confirmed censuses by location and year					
**Location**	**Year**	**Type**	**Age detail**	**Enumeration method**	**Included or excluded from estimation**
Sweden	1996	Registry	1-year age groups	NA	Included
Sweden	1997	Registry	1-year age groups	NA	Included
Sweden	1998	Registry	1-year age groups	NA	Included
Sweden	1999	Registry	1-year age groups	NA	Included
Sweden	2000	Registry	1-year age groups	NA	Included
Sweden	2001	Registry	1-year age groups	NA	Included
Sweden	2002	Registry	1-year age groups	NA	Included
Sweden	2003	Census	1-year age groups	De jure	Included
Sweden	2003	Registry	1-year age groups	NA	Included
Sweden	2004	Registry	1-year age groups	NA	Included
Sweden	2005	Registry	1-year age groups	NA	Included
Sweden	2006	Registry	1-year age groups	NA	Included
Sweden	2007	Registry	1-year age groups	NA	Included
Sweden	2008	Registry	1-year age groups	NA	Included
Sweden	2009	Registry	1-year age groups	NA	Included
Sweden	2010	Registry	1-year age groups	NA	Included
Sweden	2011	Census	1-year age groups	De jure	Included
Sweden	2011	Registry	1-year age groups	NA	Included
Sweden	2012	Registry	1-year age groups	NA	Included
Sweden	2013	Registry	1-year age groups	NA	Included
Sweden	2014	Registry	1-year age groups	NA	Included
Sweden	2015	Registry	1-year age groups	NA	Included
Sweden	2016	Registry	1-year age groups	NA	Included
Switzerland	1950	Census	1-year age groups	De jure	Included
Switzerland	1960	Census	5-year age groups	De jure	Included
Switzerland	1970	Census	1-year age groups	De jure	Included
Switzerland	1980	Census	1-year age groups	De jure	Included
Switzerland	1990	Census	1-year age groups	De jure	Included
Switzerland	2000	Census	5-year age groups	De facto	Included
Switzerland	2010	Census	Not Available	Unknown	Included
Switzerland	2010	Registry	1-year age groups	NA	Included
Switzerland	2011	Census	1-year age groups	De jure	Included
Switzerland	2011	Registry	1-year age groups	NA	Included
Switzerland	2012	Registry	1-year age groups	NA	Included
Switzerland	2013	Registry	1-year age groups	NA	Included
Switzerland	2014	Registry	1-year age groups	NA	Included
Switzerland	2015	Registry	1-year age groups	NA	Included
Switzerland	2016	Registry	1-year age groups	NA	Included
Syria	1960	Census	5-year age groups	De facto	Included
Syria	1970	Census	1-year age groups	De facto	Included
Syria	1981	Census	1-year age groups	De facto	Included
Syria	1994	Census	1-year age groups	De facto	Included
Syria	2004	Census	5-year age groups	De facto	Included
Taiwan	1950	Registry	1-year age groups	NA	Included
Taiwan	1951	Registry	1-year age groups	NA	Included
Taiwan	1952	Registry	1-year age groups	NA	Included
Taiwan	1953	Registry	1-year age groups	NA	Included
Taiwan	1954	Registry	1-year age groups	NA	Included
Taiwan	1955	Registry	1-year age groups	NA	Included
Taiwan	1956	Census	5-year age groups	De jure	Included
Taiwan	1956	Registry	1-year age groups	NA	Included
Taiwan	1957	Registry	1-year age groups	NA	Included
Taiwan	1958	Registry	1-year age groups	NA	Included
Taiwan	1959	Registry	1-year age groups	NA	Included
Taiwan	1960	Registry	1-year age groups	NA	Included
Taiwan	1961	Registry	1-year age groups	NA	Included
Taiwan	1962	Registry	1-year age groups	NA	Included
Taiwan	1963	Registry	1-year age groups	NA	Included
Taiwan	1964	Registry	1-year age groups	NA	Included
Taiwan	1965	Registry	5-year age groups	NA	Included
Taiwan	1966	Census	Not Available	Unknown	Excluded
Taiwan	1966	Registry	5-year age groups	NA	Included
Taiwan	1967	Registry	5-year age groups	NA	Included
Taiwan	1968	Registry	5-year age groups	NA	Included
Taiwan	1969	Registry	non-std age groups	NA	Included
Taiwan	1970	Registry	1-year age groups	NA	Included
Taiwan	1971	Census	5-year age groups	De jure	Included
Taiwan	1971	Registry	1-year age groups	NA	Included
Taiwan	1972	Registry	1-year age groups	NA	Included
Taiwan	1973	Registry	1-year age groups	NA	Included
Taiwan	1974	Registry	1-year age groups	NA	Included
Taiwan	1975	Census	Not Available	Unknown	Excluded
Taiwan	1975	Registry	1-year age groups	NA	Included
Taiwan	1976	Registry	1-year age groups	NA	Included
Taiwan	1977	Registry	1-year age groups	NA	Included

Appendix Table 5. List of all confirmed censuses by location and year					
Location	Year	Type	Age detail	Enumeration method	Included or excluded from estimation
Taiwan	1978	Registry	1-year age groups	NA	Included
Taiwan	1979	Registry	1-year age groups	NA	Included
Taiwan	1980	Census	5-year age groups	Unknown	Included
Taiwan	1980	Registry	1-year age groups	NA	Included
Taiwan	1981	Registry	1-year age groups	NA	Included
Taiwan	1982	Registry	1-year age groups	NA	Included
Taiwan	1983	Registry	1-year age groups	NA	Included
Taiwan	1984	Registry	1-year age groups	NA	Included
Taiwan	1985	Registry	1-year age groups	NA	Included
Taiwan	1986	Registry	1-year age groups	NA	Included
Taiwan	1987	Registry	1-year age groups	NA	Included
Taiwan	1988	Registry	1-year age groups	NA	Included
Taiwan	1989	Registry	1-year age groups	NA	Included
Taiwan	1990	Census	1-year age groups	De facto	Included
Taiwan	1990	Registry	1-year age groups	NA	Included
Taiwan	1991	Registry	1-year age groups	NA	Included
Taiwan	1992	Registry	1-year age groups	NA	Included
Taiwan	1993	Registry	1-year age groups	NA	Included
Taiwan	1994	Registry	1-year age groups	NA	Included
Taiwan	1995	Registry	1-year age groups	NA	Included
Taiwan	1996	Registry	1-year age groups	NA	Included
Taiwan	1997	Registry	1-year age groups	NA	Included
Taiwan	1998	Registry	1-year age groups	NA	Included
Taiwan	1999	Registry	1-year age groups	NA	Included
Taiwan	2000	Census	1-year age groups	De facto	Included
Taiwan	2000	Registry	1-year age groups	NA	Included
Taiwan	2001	Registry	1-year age groups	NA	Included
Taiwan	2002	Registry	1-year age groups	NA	Included
Taiwan	2003	Registry	1-year age groups	NA	Included
Taiwan	2004	Registry	1-year age groups	NA	Included
Taiwan	2005	Registry	1-year age groups	NA	Included
Taiwan	2006	Registry	1-year age groups	NA	Included
Taiwan	2007	Registry	1-year age groups	NA	Included
Taiwan	2008	Registry	1-year age groups	NA	Included
Taiwan	2009	Registry	1-year age groups	NA	Included
Taiwan	2010	Census	5-year age groups	De facto	Included
Taiwan	2010	Registry	1-year age groups	NA	Included
Taiwan	2011	Registry	1-year age groups	NA	Included
Taiwan	2012	Registry	1-year age groups	NA	Included
Taiwan	2013	Registry	1-year age groups	NA	Included
Taiwan	2014	Registry	1-year age groups	NA	Included
Taiwan	2015	Registry	1-year age groups	NA	Included
Taiwan	2016	Registry	1-year age groups	NA	Included
Taiwan	2017	Registry	1-year age groups	NA	Included
Tajikistan	1959	Census	non-std age groups	De facto	Included
Tajikistan	1970	Census	non-std age groups	De facto	Included
Tajikistan	1979	Census	5-year age groups	De facto	Included
Tajikistan	1989	Census	1-year age groups	De jure	Included
Tajikistan	2000	Census	1-year age groups	De facto	Included
Tajikistan	2010	Census	1-year age groups	De facto	Included
Tanzania	1957	Census	5-year age groups	De jure	Included
Tanzania	1967	Census	1-year age groups	De facto	Included
Tanzania	1978	Census	5-year age groups	De facto	Included
Tanzania	1988	Census	1-year age groups	De facto	Included
Tanzania	2002	Census	5-year age groups	De facto	Included
Tanzania	2012	Census	1-year age groups	De facto	Included
Thailand	1960	Census	5-year age groups	De jure	Included
Thailand	1970	Census	5-year age groups	De jure	Included
Thailand	1980	Census	1-year age groups	De jure	Included
Thailand	1990	Census	5-year age groups	De jure	Included
Thailand	2000	Census	1-year age groups	De jure	Included
Thailand	2010	Census	1-year age groups	De jure	Included
The Bahamas	1953	Census	5-year age groups	De facto	Included
The Bahamas	1963	Census	non-std age groups	Unknown	Included
The Bahamas	1970	Census	1-year age groups	De facto	Included
The Bahamas	1980	Census	1-year age groups	De facto	Included
The Bahamas	1990	Census	1-year age groups	De facto	Included
The Bahamas	2000	Census	1-year age groups	De facto	Included
The Bahamas	2010	Census	1-year age groups	De jure	Included
The Gambia	1951	Census	Not Available	Unknown	Excluded
The Gambia	1963	Census	non-std age groups	De facto	Included
The Gambia	1973	Census	1-year age groups	De facto	Included
The Gambia	1983	Census	1-year age groups	De facto	Included
The Gambia	1993	Census	1-year age groups	De facto	Included
The Gambia	2003	Census	5-year age groups	De facto	Included

Appendix Table 5. List of all confirmed censuses by location and year					
**Location**	**Year**	**Type**	**Age detail**	**Enumeration method**	**Included or excluded from estimation**
The Gambia	2013	Census	5-year age groups	De facto	Included
Timor-Leste	1950	Census	all ages only	Unknown	Included
Timor-Leste	1960	Census	all ages only	Unknown	Included
Timor-Leste	1970	Census	Not Available	Unknown	Excluded
Timor-Leste	1980	Census	all ages only	Unknown	Included
Timor-Leste	1990	Census	1-year age groups	De jure	Included
Timor-Leste	2004	Census	5-year age groups	De facto	Included
Timor-Leste	2010	Census	1-year age groups	De facto	Included
Timor-Leste	2015	Census	5-year age groups	De facto	Included
Togo	1951	Census	Not Available	Unknown	Excluded
Togo	1958	Census	1-year age groups	De facto	Included
Togo	1970	Census	5-year age groups	De jure	Included
Togo	1981	Census	5-year age groups	De facto	Included
Togo	2010	Census	5-year age groups	De jure	Included
Tonga	1956	Census	5-year age groups	De facto	Included
Tonga	1966	Census	5-year age groups	De facto	Included
Tonga	1976	Census	5-year age groups	De jure	Included
Tonga	1986	Census	5-year age groups	De facto	Included
Tonga	1996	Census	5-year age groups	De facto	Included
Tonga	2006	Census	1-year age groups	De jure	Included
Tonga	2011	Census	non-std age groups	De jure	Included
Tonga	2016	Census	all ages only	De jure	Included
Trinidad and Tobago	1960	Census	1-year age groups	De jure	Included
Trinidad and Tobago	1970	Census	1-year age groups	De facto	Included
Trinidad and Tobago	1980	Census	1-year age groups	De facto	Included
Trinidad and Tobago	1990	Census	5-year age groups	De facto	Included
Trinidad and Tobago	2000	Census	5-year age groups	De facto	Included
Trinidad and Tobago	2011	Census	1-year age groups	De jure	Included
Tunisia	1956	Census	non-std age groups	De facto	Included
Tunisia	1966	Census	1-year age groups	De facto	Included
Tunisia	1975	Census	5-year age groups	De facto	Included
Tunisia	1984	Census	1-year age groups	De facto	Included
Tunisia	1994	Census	1-year age groups	De facto	Included
Tunisia	2004	Census	5-year age groups	De facto	Included
Tunisia	2014	Census	5-year age groups	De facto	Included
Turkey	1950	Census	5-year age groups	De facto	Included
Turkey	1955	Census	5-year age groups	De facto	Included
Turkey	1960	Census	5-year age groups	De facto	Included
Turkey	1965	Census	5-year age groups	De facto	Included
Turkey	1970	Census	1-year age groups	De facto	Included
Turkey	1975	Census	5-year age groups	De facto	Included
Turkey	1980	Census	5-year age groups	De facto	Included
Turkey	1985	Census	1-year age groups	De facto	Included
Turkey	1990	Census	1-year age groups	De facto	Included
Turkey	2000	Census	5-year age groups	De facto	Excluded
Turkey	2007	Registry	5-year age groups	NA	Included
Turkey	2008	Registry	5-year age groups	NA	Included
Turkey	2009	Registry	5-year age groups	NA	Included
Turkey	2010	Registry	5-year age groups	NA	Included
Turkey	2011	Census	1-year age groups	De jure	Included
Turkey	2011	Registry	5-year age groups	NA	Included
Turkey	2012	Registry	5-year age groups	NA	Included
Turkey	2013	Registry	5-year age groups	NA	Included
Turkey	2014	Registry	5-year age groups	NA	Included
Turkey	2015	Registry	5-year age groups	NA	Included
Turkey	2016	Registry	5-year age groups	NA	Included
Turkey	2017	Registry	5-year age groups	NA	Included
Turkmenistan	1959	Census	non-std age groups	De facto	Included
Turkmenistan	1970	Census	non-std age groups	De facto	Included
Turkmenistan	1979	Census	5-year age groups	De facto	Included
Turkmenistan	1989	Census	5-year age groups	De facto	Included
Turkmenistan	1995	Census	5-year age groups	De facto	Excluded
Turkmenistan	2012	Census	Not Available	Unknown	Excluded
Turkmenistan	2015	Census	all ages only	Unknown	Included
Uganda	1959	Census	5-year age groups	Unknown	Included
Uganda	1969	Census	1-year age groups	De facto	Included
Uganda	1980	Census	all ages only	De jure	Excluded
Uganda	1991	Census	1-year age groups	De facto	Included
Uganda	2002	Census	1-year age groups	De facto	Included
Uganda	2014	Census	1-year age groups	De facto	Included
Ukraine	1959	Census	non-std age groups	De facto	Included
Ukraine	1970	Census	non-std age groups	De facto	Included
Ukraine	1979	Census	5-year age groups	De facto	Included
Ukraine	1989	Census	5-year age groups	De jure	Included
Ukraine	2001	Census	1-year age groups	De facto	Included

Appendix Table 5. List of all confirmed censuses by location and year					
**Location**	**Year**	**Type**	**Age detail**	**Enumeration method**	**Included or excluded from estimation**
United Arab Emirates	1968	Census	non-std age groups	De facto	Included
United Arab Emirates	1975	Census	5-year age groups	De facto	Included
United Arab Emirates	1980	Census	5-year age groups	De jure	Included
United Arab Emirates	1985	Census	5-year age groups	De facto	Included
United Arab Emirates	1995	Census	5-year age groups	De facto	Included
United Arab Emirates	2005	Census	5-year age groups	De facto	Included
United States	1950	Census	1-year age groups	De jure	Included
United States	1960	Census	5-year age groups	De jure	Included
United States	1970	Census	1-year age groups	De jure	Included
United States	1980	Census	1-year age groups	De jure	Included
United States	1990	Census	1-year age groups	De jure	Included
United States	2000	Census	1-year age groups	De jure	Included
United States	2010	Census	1-year age groups	De jure	Included
Uruguay	1963	Census	1-year age groups	De facto	Included
Uruguay	1975	Census	5-year age groups	De facto	Included
Uruguay	1985	Census	1-year age groups	De facto	Included
Uruguay	1996	Census	1-year age groups	De facto	Included
Uruguay	2004	Census	1-year age groups	De facto	Included
Uruguay	2011	Census	1-year age groups	De jure	Included
Uzbekistan	1959	Census	non-std age groups	De facto	Included
Uzbekistan	1970	Census	non-std age groups	De facto	Included
Uzbekistan	1979	Census	5-year age groups	De facto	Included
Uzbekistan	1989	Census	5-year age groups	De jure	Included
Vanuatu	1967	Census	5-year age groups	De facto	Included
Vanuatu	1979	Census	5-year age groups	De facto	Included
Vanuatu	1989	Census	1-year age groups	De jure	Included
Vanuatu	1999	Census	5-year age groups	De facto	Included
Vanuatu	2009	Census	1-year age groups	De jure	Included
Vanuatu	2016	Census	1-year age groups	De jure	Included
Venezuela	1950	Census	1-year age groups	De facto	Included
Venezuela	1961	Census	1-year age groups	De facto	Included
Venezuela	1971	Census	5-year age groups	De facto	Included
Venezuela	1981	Census	1-year age groups	De facto	Included
Venezuela	1990	Census	1-year age groups	De facto	Included
Venezuela	2001	Census	non-std age groups	De facto	Included
Venezuela	2011	Census	1-year age groups	De jure	Included
Vietnam	1960	Census	Not Available	Unknown	Excluded
Vietnam	1974	Census	Not Available	Unknown	Excluded
Vietnam	1979	Census	5-year age groups	De facto	Included
Vietnam	1989	Census	1-year age groups	De jure	Included
Vietnam	1999	Census	1-year age groups	De jure	Included
Vietnam	2009	Census	1-year age groups	De jure	Included
Virgin Islands, U.S.	1950	Census	5-year age groups	De jure	Included
Virgin Islands, U.S.	1960	Census	5-year age groups	De jure	Included
Virgin Islands, U.S.	1970	Census	5-year age groups	De jure	Included
Virgin Islands, U.S.	1980	Census	1-year age groups	De jure	Included
Virgin Islands, U.S.	1990	Census	5-year age groups	De jure	Included
Virgin Islands, U.S.	2000	Census	non-std age groups	De jure	Included
Virgin Islands, U.S.	2010	Census	5-year age groups	De jure	Included
Wales	1951	Census	5-year age groups	De jure	Included
Wales	1961	Census	5-year age groups	De jure	Included
Wales	1971	Census	5-year age groups	De jure	Included
Wales	1981	Census	5-year age groups	De jure	Included
Wales	1991	Census	non-std age groups	De jure	Included
Wales	2001	Census	5-year age groups	De jure	Included
Wales	2011	Census	1-year age groups	De jure	Included
Yemen	1994	Census	1-year age groups	De facto	Included
Yemen	2004	Census	5-year age groups	De facto	Included
Yemen (Arab Republic)	1975	Census	5-year age groups	Unknown	Included
Yemen (Arab Republic)	1986	Census	all ages only	Unknown	Included
Yemen (People's Democratic Republic of)	1973	Census	all ages only	De facto	Included
Yugoslavia	1953	Census	5-year age groups	De jure	Included
Yugoslavia	1961	Census	5-year age groups	De jure	Included
Yugoslavia	1971	Census	5-year age groups	De jure	Included
Yugoslavia	1981	Census	5-year age groups	De jure	Included
Zambia	1951	Census	Not Available	Unknown	Excluded
Zambia	1956	Census	Not Available	Unknown	Excluded
Zambia	1961	Census	Not Available	Unknown	Excluded
Zambia	1963	Census	Not Available	Unknown	Excluded
Zambia	1969	Census	1-year age groups	De facto	Included
Zambia	1980	Census	1-year age groups	De facto	Included
Zambia	1990	Census	1-year age groups	De facto	Included
Zambia	2000	Census	1-year age groups	De facto	Included
Zambia	2010	Census	1-year age groups	De jure	Included
Zimbabwe	1956	Census	Not Available	Unknown	Excluded

Appendix Table 5. List of all confirmed censuses by location and year					
**Location**	**Year**	**Type**	**Age detail**	**Enumeration method**	**Included or excluded from estimation**
Zimbabwe	1961	Census	Not Available	Unknown	Excluded
Zimbabwe	1962	Census	non-std age groups	De jure	Excluded
Zimbabwe	1969	Census	Not Available	Unknown	Excluded
Zimbabwe	1982	Census	1-year age groups	De facto	Included
Zimbabwe	1992	Census	1-year age groups	De facto	Included
Zimbabwe	2002	Census	1-year age groups	De facto	Included
Zimbabwe	2012	Census	1-year age groups	De facto	Included

Appendix Table 6. GBD world population age standard		
**Age group**	**Percent of Population**	**Rounded**
ENN	0.040802107	0.04
LNN	0.121276112	0.12
PNN	1.916163425	1.92
0-1	2.078241644	2.08
1-4	8.102445249	8.1
5-9	9.677325318	9.68
10-14	8.952609678	8.95
15-19	8.382858071	8.38
20-24	8.01707612	8.02
25-29	7.778732811	7.78
30-34	7.331586983	7.33
35-39	6.775951151	6.78
40-44	6.089034035	6.09
45-49	5.465811837	5.47
50-54	4.873621621	4.87
55-59	4.251640217	4.25
60-64	3.5961672	3.6
65-69	2.914902912	2.91
70-74	2.130279933	2.13
75-79	1.608333391	1.61
80-84	1.078423497	1.08
85-89	0.603162091	0.6
90-94	0.234261019	0.23
95+	0.057535221	0.06

Appendix Table 7. Socio-demographic Index groupings by geography, based on 2017 values		
**Geography**	**2017 SDI**	**SDI Quintile**
Global	0.652	
Central Europe, Eastern Europe, and Central Asia	0.766	
Central Asia	0.673	
Armenia	0.702	High-middle SDI
Azerbaijan	0.701	High-middle SDI
Georgia	0.7	High-middle SDI
Kazakhstan	0.735	High-middle SDI
Kyrgyzstan	0.607	Low-middle SDI
Mongolia	0.662	Middle SDI
Tajikistan	0.523	Low-middle SDI
Turkmenistan	0.696	Middle SDI
Uzbekistan	0.63	Middle SDI
Central Europe	0.814	
Albania	0.685	Middle SDI
Bosnia and Herzegovina	0.713	High-middle SDI
Bulgaria	0.792	High-middle SDI
Croatia	0.825	High SDI
Czech Republic	0.851	High SDI
Hungary	0.817	High-middle SDI
Macedonia	0.754	High-middle SDI
Montenegro	0.788	High-middle SDI
Poland	0.844	High SDI
Romania	0.784	High-middle SDI
Serbia	0.752	High-middle SDI
Slovakia	0.842	High SDI
Slovenia	0.86	High SDI
Eastern Europe	0.785	
Belarus	0.773	High-middle SDI
Estonia	0.858	High SDI
Latvia	0.825	High SDI
Lithuania	0.841	High SDI
Moldova	0.676	Middle SDI
Russian Federation	0.792	High-middle SDI
Ukraine	0.74	High-middle SDI
High-income	0.854	
Australasia	0.869	
Australia	0.873	High SDI
New Zealand	0.842	High SDI
High-income Asia-Pacific	0.869	
Brunei	0.856	High SDI
Japan	0.865	High SDI
Aichi	0.875	High SDI
Akita	0.829	High SDI
Aomori	0.825	High SDI

Appendix Table 7. Socio-demographic Index groupings by geography, based on 2017 values		
**Geography**	**2017 SDI**	**SDI Quintile**
Chiba	0.859	High SDI
Ehime	0.838	High SDI
Fukui	0.852	High SDI
Fukuoka	0.855	High SDI
Fukushima	0.831	High SDI
Gifu	0.849	High SDI
Gunma	0.851	High SDI
Hiroshima	0.863	High SDI
Hokkaidō	0.842	High SDI
Hyōgo	0.86	High SDI
Ibaraki	0.851	High SDI
Ishikawa	0.856	High SDI
Iwate	0.825	High SDI
Kagawa	0.85	High SDI
Kagoshima	0.83	High SDI
Kanagawa	0.875	High SDI
Kōchi	0.825	High SDI
Kumamoto	0.832	High SDI
Kyōto	0.873	High SDI
Mie	0.854	High SDI
Miyagi	0.85	High SDI
Miyazaki	0.823	High SDI
Nagano	0.851	High SDI
Nagasaki	0.826	High SDI
Nara	0.848	High SDI
Niigata	0.843	High SDI
Ōita	0.846	High SDI
Okayama	0.856	High SDI
Okinawa	0.818	High SDI
Ōsaka	0.872	High SDI
Saga	0.834	High SDI
Saitama	0.852	High SDI
Shiga	0.871	High SDI
Shimane	0.831	High SDI
Shizuoka	0.859	High SDI
Tochigi	0.853	High SDI
Tokushima	0.845	High SDI
Tōkyō	0.924	High SDI
Tottori	0.834	High SDI
Toyama	0.86	High SDI
Wakayama	0.84	High SDI
Yamagata	0.832	High SDI
Yamaguchi	0.849	High SDI
Yamanashi	0.854	High SDI
South Korea	0.872	High SDI

Appendix Table 7. Socio-demographic Index groupings by geography, based on 2017 values		
**Geography**	**2017 SDI**	**SDI Quintile**
Singapore	0.872	High SDI
High-income North America	0.868	
Canada	0.882	High SDI
Greenland	0.76	High-middle SDI
USA	0.867	High SDI
Alabama	0.837	High SDI
Alaska	0.861	High SDI
Arizona	0.845	High SDI
Arkansas	0.826	High SDI
California	0.872	High SDI
Colorado	0.882	High SDI
Connecticut	0.906	High SDI
Delaware	0.874	High SDI
Washington, DC	0.89	High SDI
Florida	0.864	High SDI
Georgia	0.848	High SDI
Hawaii	0.872	High SDI
Idaho	0.841	High SDI
Illinois	0.879	High SDI
Indiana	0.848	High SDI
Iowa	0.87	High SDI
Kansas	0.864	High SDI
Kentucky	0.831	High SDI
Louisiana	0.835	High SDI
Maine	0.872	High SDI
Maryland	0.896	High SDI
Massachusetts	0.913	High SDI
Michigan	0.868	High SDI
Minnesota	0.893	High SDI
Mississippi	0.819	High SDI
Missouri	0.853	High SDI
Montana	0.863	High SDI
Nebraska	0.873	High SDI
Nevada	0.847	High SDI
New Hampshire	0.904	High SDI
New Jersey	0.899	High SDI
New Mexico	0.835	High SDI
New York	0.893	High SDI
North Carolina	0.85	High SDI
North Dakota	0.88	High SDI
Ohio	0.858	High SDI
Oklahoma	0.838	High SDI
Oregon	0.871	High SDI
Pennsylvania	0.879	High SDI
Rhode Island	0.89	High SDI

Appendix Table 7. Socio-demographic Index groupings by geography, based on 2017 values		
**Geography**	**2017 SDI**	**SDI Quintile**
South Carolina	0.846	High SDI
South Dakota	0.86	High SDI
Tennessee	0.837	High SDI
Texas	0.838	High SDI
Utah	0.856	High SDI
Vermont	0.896	High SDI
Virginia	0.885	High SDI
Washington	0.884	High SDI
West Virginia	0.825	High SDI
Wisconsin	0.878	High SDI
Wyoming	0.869	High SDI
Southern Latin America	0.72	
Argentina	0.71	High-middle SDI
Chile	0.748	High-middle SDI
Uruguay	0.707	High-middle SDI
Western Europe	0.857	
Andorra	0.902	High SDI
Austria	0.866	High SDI
Belgium	0.886	High SDI
Cyprus	0.865	High SDI
Denmark	0.918	High SDI
Finland	0.893	High SDI
France	0.865	High SDI
Germany	0.87	High SDI
Greece	0.817	High SDI
Iceland	0.907	High SDI
Ireland	0.882	High SDI
Israel	0.816	High-middle SDI
Italy	0.843	High SDI
Luxembourg	0.916	High SDI
Malta	0.836	High SDI
Netherlands	0.912	High SDI
Norway	0.911	High SDI
Portugal	0.778	High-middle SDI
Spain	0.825	High SDI
Sweden	0.883	High SDI
Stockholm	0.914	High SDI
Sweden except Stockholm	0.873	High SDI
Switzerland	0.889	High SDI
United Kingdom	0.843	High SDI
England	0.849	High SDI
East Midlands	0.83	High SDI
Derby	0.846	High SDI
Derbyshire	0.817	High SDI
Leicester	0.839	High SDI

Appendix Table 7. Socio-demographic Index groupings by geography, based on 2017 values		
**Geography**	**2017 SDI**	**SDI Quintile**
Leicestershire	0.846	High SDI
Lincolnshire	0.812	High SDI
Northamptonshire	0.829	High SDI
Nottingham	0.863	High SDI
Nottinghamshire	0.814	High SDI
Rutland	0.833	High SDI
East of England	0.84	High SDI
Bedford	0.838	High SDI
Cambridgeshire	0.871	High SDI
Central Bedfordshire	0.834	High SDI
Essex	0.832	High SDI
Hertfordshire	0.87	High SDI
Luton	0.833	High SDI
Norfolk	0.826	High SDI
Peterborough	0.818	High SDI
Southend-on-Sea	0.811	High SDI
Suffolk	0.821	High SDI
Thurrock	0.807	High SDI
Greater London	0.894	High SDI
Barking and Dagenham	0.802	High SDI
Barnet	0.865	High SDI
Bexley	0.826	High SDI
Brent	0.849	High SDI
Bromley	0.848	High SDI
Camden	0.93	High SDI
Croydon	0.833	High SDI
Ealing	0.865	High SDI
Enfield	0.839	High SDI
Greenwich	0.833	High SDI
Hackney	0.887	High SDI
Hammersmith and Fulham	0.927	High SDI
Haringey	0.854	High SDI
Harrow	0.848	High SDI
Havering	0.824	High SDI
Hillingdon	0.882	High SDI
Hounslow	0.879	High SDI
Islington	0.922	High SDI
Kensington and Chelsea	0.932	High SDI
Kingston upon Thames	0.89	High SDI
Lambeth	0.9	High SDI
Lewisham	0.843	High SDI
Merton	0.873	High SDI
Newham	0.838	High SDI
Redbridge	0.831	High SDI
Richmond upon Thames	0.902	High SDI

Appendix Table 7. Socio-demographic Index groupings by geography, based on 2017 values		
**Geography**	**2017 SDI**	**SDI Quintile**
Southwark	0.912	High SDI
Sutton	0.843	High SDI
Tower Hamlets	0.905	High SDI
Waltham Forest	0.819	High SDI
Wandsworth	0.911	High SDI
Westminster	0.927	High SDI
North East England	0.821	High SDI
County Durham	0.81	High SDI
Darlington	0.825	High SDI
Gateshead	0.826	High SDI
Hartlepool	0.793	High SDI
Middlesbrough	0.808	High SDI
Newcastle upon Tyne	0.872	High SDI
North Tyneside	0.825	High SDI
Northumberland	0.808	High SDI
Redcar and Cleveland	0.79	High SDI
South Tyneside	0.794	High SDI
Stockton-on-Tees	0.823	High SDI
Sunderland	0.815	High SDI
North West England	0.834	High SDI
Blackburn with Darwen	0.802	High SDI
Blackpool	0.781	High SDI
Bolton	0.805	High SDI
Bury	0.815	High SDI
Cheshire East	0.864	High SDI
Cheshire West and Chester	0.855	High SDI
Cumbria	0.828	High SDI
Halton	0.824	High SDI
Knowsley	0.816	High SDI
Lancashire	0.831	High SDI
Liverpool	0.852	High SDI
Manchester	0.885	High SDI
Oldham	0.79	High SDI
Rochdale	0.795	High SDI
Salford	0.838	High SDI
Sefton	0.812	High SDI
St Helens	0.803	High SDI
Stockport	0.843	High SDI
Tameside	0.797	High SDI
Trafford	0.873	High SDI
Warrington	0.86	High SDI
Wigan	0.798	High SDI
Wirral	0.803	High SDI
South East England	0.856	High SDI
Bracknell Forest	0.869	High SDI

Appendix Table 7. Socio-demographic Index groupings by geography, based on 2017 values		
**Geography**	**2017 SDI**	**SDI Quintile**
Brighton and Hove	0.885	High SDI
Buckinghamshire	0.865	High SDI
East Sussex	0.814	High SDI
Hampshire	0.85	High SDI
Isle of Wight	0.814	High SDI
Kent	0.828	High SDI
Medway	0.809	High SDI
Milton Keynes	0.86	High SDI
Oxfordshire	0.879	High SDI
Portsmouth	0.86	High SDI
Reading	0.895	High SDI
Slough	0.859	High SDI
Southampton	0.858	High SDI
Surrey	0.883	High SDI
West Berkshire	0.872	High SDI
West Sussex	0.843	High SDI
Windsor and Maidenhead	0.889	High SDI
Wokingham	0.885	High SDI
South West England	0.841	High SDI
Bath and North East Somerset	0.875	High SDI
Bournemouth	0.858	High SDI
Bristol, City of	0.884	High SDI
Cornwall	0.817	High SDI
Devon	0.837	High SDI
Dorset	0.825	High SDI
Gloucestershire	0.85	High SDI
North Somerset	0.832	High SDI
Plymouth	0.836	High SDI
Poole	0.842	High SDI
Somerset	0.816	High SDI
South Gloucestershire	0.867	High SDI
Swindon	0.847	High SDI
Torbay	0.79	High SDI
Wiltshire	0.829	High SDI
West Midlands	0.829	High SDI
Birmingham	0.84	High SDI
Coventry	0.848	High SDI
Dudley	0.799	High SDI
Herefordshire, County of	0.828	High SDI
Sandwell	0.797	High SDI
Shropshire	0.832	High SDI
Solihull	0.855	High SDI
Staffordshire	0.826	High SDI
Stoke-on-Trent	0.804	High SDI
Telford and Wrekin	0.822	High SDI

Appendix Table 7. Socio-demographic Index groupings by geography, based on 2017 values		
**Geography**	**2017 SDI**	**SDI Quintile**
Walsall	0.791	High SDI
Warwickshire	0.857	High SDI
Wolverhampton	0.811	High SDI
Worcestershire	0.833	High SDI
Yorkshire and the Humber	0.83	High SDI
Barnsley	0.787	High SDI
Bradford	0.807	High SDI
Calderdale	0.827	High SDI
Doncaster	0.791	High SDI
East Riding of Yorkshire	0.822	High SDI
Kingston upon Hull, City of	0.813	High SDI
Kirklees	0.816	High SDI
Leeds	0.868	High SDI
North East Lincolnshire	0.804	High SDI
North Lincolnshire	0.811	High SDI
North Yorkshire	0.839	High SDI
Rotherham	0.796	High SDI
Sheffield	0.853	High SDI
Wakefield	0.806	High SDI
York	0.879	High SDI
Northern Ireland	0.835	High SDI
Scotland	0.805	High SDI
Wales	0.806	High SDI
Latin America and Caribbean	0.64	
Andean Latin America	0.628	
Bolivia	0.587	Low-middle SDI
Ecuador	0.636	Middle SDI
Peru	0.636	Middle SDI
Caribbean	0.638	
Antigua and Barbuda	0.715	High-middle SDI
The Bahamas	0.756	High-middle SDI
Barbados	0.739	High-middle SDI
Belize	0.602	Low-middle SDI
Bermuda	0.805	High-middle SDI
Cuba	0.688	Middle SDI
Dominica	0.687	Middle SDI
Dominican Republic	0.593	Low-middle SDI
Grenada	0.64	Middle SDI
Guyana	0.584	Low-middle SDI
Haiti	0.442	Low SDI
Jamaica	0.679	Middle SDI
Puerto Rico	0.813	High-middle SDI
Saint Lucia	0.653	Middle SDI
Saint Vincent and the Grenadines	0.608	Middle SDI
Suriname	0.641	Middle SDI

Appendix Table 7. Socio-demographic Index groupings by geography, based on 2017 values		
**Geography**	**2017 SDI**	**SDI Quintile**
Trinidad and Tobago	0.698	Middle SDI
Virgin Islands	0.807	High-middle SDI
Central Latin America	0.623	
Colombia	0.634	Middle SDI
Costa Rica	0.662	Middle SDI
El Salvador	0.593	Low-middle SDI
Guatemala	0.524	Low-middle SDI
Honduras	0.512	Low-middle SDI
Mexico	0.628	Middle SDI
Aguascalientes	0.659	Middle SDI
Baja California	0.657	Middle SDI
Baja California Sur	0.659	Middle SDI
Campeche	0.616	Middle SDI
Chiapas	0.533	Middle SDI
Chihuahua	0.639	Middle SDI
Coahuila	0.645	Middle SDI
Colima	0.654	Middle SDI
Mexico City	0.716	Middle SDI
Durango	0.624	Middle SDI
Guanajuato	0.621	Middle SDI
Guerrero	0.562	Middle SDI
Hidalgo	0.587	Middle SDI
Jalisco	0.649	Middle SDI
México	0.635	Middle SDI
Michoacán de Ocampo	0.586	Middle SDI
Morelos	0.635	Middle SDI
Nayarit	0.62	Middle SDI
Nuevo León	0.677	Middle SDI
Oaxaca	0.561	Middle SDI
Puebla	0.584	Middle SDI
Querétaro	0.639	Middle SDI
Quintana Roo	0.626	Middle SDI
San Luis Potosí	0.621	Middle SDI
Sinaloa	0.649	Middle SDI
Sonora	0.65	Middle SDI
Tabasco	0.611	Middle SDI
Tamaulipas	0.647	Middle SDI
Tlaxcala	0.604	Middle SDI
Veracruz de Ignacio de la Llave	0.592	Middle SDI
Yucatán	0.63	Middle SDI
Zacatecas	0.608	Middle SDI
Nicaragua	0.53	Low-middle SDI
Panama	0.677	Middle SDI
Venezuela	0.655	Middle SDI
Tropical Latin America	0.662	

Appendix Table 7. Socio-demographic Index groupings by geography, based on 2017 values		
**Geography**	**2017 SDI**	**SDI Quintile**
Brazil	0.663	Middle SDI
Acre	0.602	Low-middle SDI
Alagoas	0.556	Low-middle SDI
Amapá	0.659	Middle SDI
Amazonas	0.629	Middle SDI
Bahia	0.591	Low-middle SDI
Ceará	0.6	Low-middle SDI
Distrito Federal	0.792	High-middle SDI
Espírito Santo	0.677	Middle SDI
Goiás	0.65	Middle SDI
Maranhão	0.507	Low-middle SDI
Mato Grosso	0.662	Middle SDI
Mato Grosso do Sul	0.65	Middle SDI
Minas Gerais	0.661	Middle SDI
Pará	0.579	Low-middle SDI
Paraíba	0.574	Low-middle SDI
Paraná	0.682	Middle SDI
Pernambuco	0.594	Low-middle SDI
Piauí	0.552	Low-middle SDI
Rio de Janeiro	0.709	High-middle SDI
Rio Grande do Norte	0.605	Low-middle SDI
Rio Grande do Sul	0.693	Middle SDI
Rondônia	0.622	Middle SDI
Roraima	0.646	Middle SDI
Santa Catarina	0.702	High-middle SDI
São Paulo	0.72	High-middle SDI
Sergipe	0.616	Middle SDI
Tocantins	0.611	Middle SDI
Paraguay	0.619	Middle SDI
North Africa and Middle East	0.639	
North Africa and Middle East	0.639	
Afghanistan	0.29	Low SDI
Algeria	0.696	Middle SDI
Bahrain	0.712	High-middle SDI
Egypt	0.604	Low-middle SDI
Iran	0.7	High-middle SDI
Iraq	0.585	Low-middle SDI
Jordan	0.697	Middle SDI
Kuwait	0.786	High-middle SDI
Lebanon	0.73	High-middle SDI
Libya	0.761	High-middle SDI
Morocco	0.579	Low-middle SDI
Palestine	0.541	Low-middle SDI
Oman	0.744	High-middle SDI
Qatar	0.766	High-middle SDI

Appendix Table 7. Socio-demographic Index groupings by geography, based on 2017 values		
**Geography**	**2017 SDI**	**SDI Quintile**
Saudi Arabia	0.779	High-middle SDI
Sudan	0.478	Low-middle SDI
Syria	0.611	Middle SDI
Tunisia	0.675	Middle SDI
Turkey	0.729	High-middle SDI
United Arab Emirates	0.795	High-middle SDI
Yemen	0.43	Low SDI
South Asia	0.534	
South Asia	0.534	
Bangladesh	0.458	Low SDI
Bhutan	0.57	Low-middle SDI
India	0.55	Low-middle SDI
Andhra Pradesh	0.536	Low-middle SDI
Arunachal Pradesh	0.556	Low-middle SDI
Assam	0.53	Low-middle SDI
Bihar	0.433	Low SDI
Chhattisgarh	0.512	Low-middle SDI
Delhi	0.715	High-middle SDI
Goa	0.74	High-middle SDI
Gujarat	0.584	Low-middle SDI
Haryana	0.6	Low-middle SDI
Himachal Pradesh	0.633	Middle SDI
Jammu and Kashmir	0.59	Low-middle SDI
Jharkhand	0.487	Low-middle SDI
Karnataka	0.574	Low-middle SDI
Kerala	0.659	Middle SDI
Madhya Pradesh	0.487	Low-middle SDI
Maharashtra	0.618	Middle SDI
Manipur	0.59	Low-middle SDI
Meghalaya	0.565	Low-middle SDI
Mizoram	0.616	Middle SDI
Nagaland	0.633	Middle SDI
Odisha	0.524	Low-middle SDI
Punjab	0.622	Middle SDI
Rajasthan	0.492	Low-middle SDI
Sikkim	0.628	Middle SDI
Tamil Nadu	0.615	Middle SDI
Telangana	0.575	Low-middle SDI
Tripura	0.543	Low-middle SDI
Uttar Pradesh	0.488	Low-middle SDI
Uttarakhand	0.607	Middle SDI
West Bengal	0.538	Low-middle SDI
Union Territories other than Delhi	0.653	Middle SDI
Nepal	0.429	Low SDI
Pakistan	0.492	Low-middle SDI

Appendix Table 7. Socio-demographic Index groupings by geography, based on 2017 values		
**Geography**	**2017 SDI**	**SDI Quintile**
Southeast Asia, East Asia, and Oceania	0.685	
East Asia	0.709	
China	0.707	High-middle SDI
North Korea	0.538	Low-middle SDI
Taiwan (Province of China)	0.864	High SDI
Oceania	0.471	
American Samoa	0.702	High-middle SDI
Federated States of Micronesia	0.575	Low-middle SDI
Fiji	0.641	Middle SDI
Guam	0.794	High-middle SDI
Kiribati	0.427	Low SDI
Marshall Islands	0.55	Low-middle SDI
Northern Mariana Islands	0.758	High-middle SDI
Papua New Guinea	0.419	Low SDI
Samoa	0.576	Low-middle SDI
Solomon Islands	0.425	Low SDI
Tonga	0.625	Middle SDI
Vanuatu	0.475	Low-middle SDI
Southeast Asia	0.641	
Cambodia	0.482	Low-middle SDI
Indonesia	0.648	Middle SDI
Laos	0.519	Low-middle SDI
Malaysia	0.759	High-middle SDI
Maldives	0.655	Middle SDI
Mauritius	0.72	High-middle SDI
Myanmar	0.556	Low-middle SDI
Philippines	0.617	Middle SDI
Sri Lanka	0.68	Middle SDI
Seychelles	0.692	Middle SDI
Thailand	0.684	Middle SDI
Timor-Leste	0.505	Low-middle SDI
Vietnam	0.607	Middle SDI
Sub-Saharan Africa	0.446	
Central sub-Saharan Africa	0.457	
Angola	0.461	Low-middle SDI
Central African Republic	0.334	Low SDI
Congo (Brazzaville)	0.574	Low-middle SDI
DR Congo	0.364	Low SDI
Equatorial Guinea	0.625	Middle SDI
Gabon	0.651	Middle SDI
Eastern sub-Saharan Africa	0.387	
Burundi	0.31	Low SDI
Comoros	0.434	Low SDI
Djibouti	0.485	Low-middle SDI
Eritrea	0.409	Low SDI

Appendix Table 7. Socio-demographic Index groupings by geography, based on 2017 values		
**Geography**	**2017 SDI**	**SDI Quintile**
Ethiopia	0.334	Low SDI
Kenya	0.499	Low-middle SDI
Baringo	0.444	Low-middle SDI
Bomet	0.496	Low-middle SDI
Bungoma	0.463	Low-middle SDI
Busia	0.438	Low-middle SDI
Elgeyo Marakwet	0.496	Low-middle SDI
Embu	0.533	Low-middle SDI
Garissa	0.334	Low-middle SDI
Homa Bay	0.425	Low-middle SDI
Isiolo	0.385	Low-middle SDI
Kajiado	0.534	Low-middle SDI
Kakamega	0.45	Low-middle SDI
Kericho	0.5	Low-middle SDI
Kiambu	0.58	Low-middle SDI
Kilifi	0.456	Low-middle SDI
Kirinyaga	0.533	Low-middle SDI
Kisii	0.522	Low-middle SDI
Kisumu	0.503	Low-middle SDI
Kitui	0.461	Low-middle SDI
Kwale	0.457	Low-middle SDI
Laikipia	0.556	Low-middle SDI
Lamu	0.453	Low-middle SDI
Machakos	0.518	Low-middle SDI
Makueni	0.469	Low-middle SDI
Mandera	0.295	Low-middle SDI
Marsabit	0.34	Low-middle SDI
Meru	0.508	Low-middle SDI
Migori	0.419	Low-middle SDI
Mombasa	0.568	Low-middle SDI
Murang’a	0.528	Low-middle SDI
Nairobi	0.674	Low-middle SDI
Nakuru	0.545	Low-middle SDI
Nandi	0.501	Low-middle SDI
Narok	0.402	Low-middle SDI
Nyamira	0.544	Low-middle SDI
Nyandarua	0.534	Low-middle SDI
Nyeri	0.554	Low-middle SDI
Samburu	0.308	Low-middle SDI
Siaya	0.46	Low-middle SDI
Taita Taveta	0.529	Low-middle SDI
Tana River	0.379	Low-middle SDI
Tharaka Nithi	0.528	Low-middle SDI
Trans Nzoia	0.496	Low-middle SDI
Turkana	0.295	Low-middle SDI

Appendix Table 7. Socio-demographic Index groupings by geography, based on 2017 values		
**Geography**	**2017 SDI**	**SDI Quintile**
Uasin Gishu	0.545	Low-middle SDI
Vihiga	0.477	Low-middle SDI
Wajir	0.243	Low-middle SDI
West Pokot	0.382	Low-middle SDI
Madagascar	0.331	Low SDI
Malawi	0.349	Low SDI
Mozambique	0.34	Low SDI
Rwanda	0.407	Low SDI
Somalia	0.235	Low SDI
South Sudan	0.275	Low SDI
Tanzania	0.412	Low SDI
Uganda	0.388	Low SDI
Zambia	0.472	Low-middle SDI
Southern sub-Saharan Africa	0.64	
Botswana	0.663	Middle SDI
Lesotho	0.493	Low-middle SDI
Namibia	0.616	Middle SDI
South Africa	0.677	Middle SDI
Swaziland	0.578	Low-middle SDI
Zimbabwe	0.463	Low-middle SDI
Western sub-Saharan Africa	0.441	
Benin	0.373	Low SDI
Burkina Faso	0.284	Low SDI
Cameroon	0.482	Low-middle SDI
Cape Verde	0.549	Low-middle SDI
Chad	0.253	Low SDI
Cote d'Ivoire	0.412	Low SDI
The Gambia	0.405	Low SDI
Ghana	0.537	Low-middle SDI
Guinea	0.325	Low SDI
Guinea-Bissau	0.349	Low SDI
Liberia	0.328	Low SDI
Mali	0.267	Low SDI
Mauritania	0.471	Low-middle SDI
Niger	0.191	Low SDI
Nigeria	0.493	Low-middle SDI
Sao Tome and Principe	0.488	Low-middle SDI
Senegal	0.373	Low SDI
Sierra Leone	0.357	Low SDI
Togo	0.413	Low SDI

Appendix Table 8. Socio-Demographic Index values for all estimated GBD 2017 locations, 1950-1969																				
**Location**	**1950**	**1951**	**1952**	**1953**	**1954**	**1955**	**1956**	**1957**	**1958**	**1959**	**1960**	**1961**	**1962**	**1963**	**1964**	**1965**	**1966**	**1967**	**1968**	**1969**
Global	0.359	0.361	0.364	0.366	0.368	0.371	0.372	0.373	0.374	0.376	0.378	0.381	0.385	0.389	0.393	0.398	0.402	0.406	0.411	0.417
Central Europe, Eastern Europe, and Central Asia	0.53	0.533	0.539	0.543	0.545	0.549	0.551	0.552	0.553	0.554	0.557	0.563	0.57	0.577	0.584	0.59	0.591	0.591	0.593	0.594
Central Asia	0.404	0.408	0.412	0.415	0.418	0.421	0.424	0.427	0.429	0.431	0.435	0.44	0.446	0.45	0.455	0.46	0.464	0.469	0.474	0.48
Armenia	0.378	0.381	0.386	0.391	0.396	0.402	0.409	0.416	0.422	0.428	0.436	0.445	0.452	0.457	0.464	0.471	0.477	0.484	0.489	0.495
Azerbaijan	0.381	0.384	0.39	0.395	0.4	0.406	0.413	0.419	0.425	0.43	0.436	0.444	0.451	0.456	0.465	0.473	0.481	0.488	0.496	0.503
Georgia	0.477	0.481	0.487	0.493	0.499	0.506	0.513	0.519	0.524	0.528	0.534	0.539	0.545	0.548	0.553	0.558	0.562	0.566	0.569	0.573
Kazakhstan	0.437	0.442	0.447	0.45	0.451	0.453	0.454	0.455	0.456	0.457	0.46	0.465	0.473	0.481	0.49	0.497	0.504	0.511	0.517	0.522
Kyrgyzstan	0.43	0.433	0.437	0.44	0.443	0.446	0.45	0.452	0.455	0.456	0.46	0.464	0.469	0.471	0.474	0.477	0.479	0.481	0.483	0.484
Mongolia	0.323	0.33	0.336	0.343	0.349	0.354	0.356	0.358	0.359	0.361	0.364	0.367	0.37	0.373	0.378	0.384	0.39	0.397	0.403	0.41
Tajikistan	0.271	0.273	0.276	0.278	0.281	0.284	0.288	0.292	0.295	0.297	0.301	0.306	0.309	0.309	0.312	0.316	0.32	0.322	0.324	0.326
Turkmenistan	0.421	0.424	0.428	0.431	0.434	0.437	0.44	0.442	0.444	0.445	0.447	0.45	0.453	0.457	0.463	0.469	0.475	0.481	0.486	0.491
Uzbekistan	0.331	0.332	0.334	0.336	0.337	0.338	0.34	0.341	0.343	0.344	0.347	0.352	0.355	0.358	0.362	0.367	0.371	0.376	0.38	0.384
Central Europe	0.519	0.523	0.526	0.527	0.528	0.531	0.534	0.536	0.54	0.544	0.549	0.555	0.56	0.564	0.57	0.576	0.575	0.573	0.579	0.585
Albania	0.369	0.372	0.371	0.371	0.371	0.373	0.376	0.378	0.38	0.382	0.385	0.389	0.394	0.4	0.407	0.413	0.417	0.42	0.423	0.427
Bosnia and Herzegovina	0.259	0.264	0.27	0.274	0.276	0.284	0.287	0.292	0.297	0.306	0.315	0.323	0.329	0.337	0.345	0.353	0.36	0.367	0.373	0.38
Bulgaria	0.48	0.496	0.498	0.503	0.504	0.504	0.505	0.509	0.511	0.512	0.516	0.523	0.53	0.534	0.542	0.551	0.556	0.553	0.552	0.558
Croatia	0.505	0.509	0.513	0.518	0.523	0.53	0.535	0.542	0.549	0.555	0.563	0.57	0.576	0.583	0.591	0.597	0.604	0.61	0.616	0.622
Czech Republic	0.598	0.6	0.605	0.609	0.611	0.612	0.613	0.618	0.625	0.629	0.63	0.63	0.627	0.626	0.633	0.644	0.652	0.658	0.66	0.662
Hungary	0.516	0.521	0.521	0.515	0.513	0.518	0.525	0.532	0.538	0.544	0.55	0.558	0.566	0.57	0.574	0.575	0.573	0.572	0.576	0.582
Macedonia	0.444	0.447	0.451	0.455	0.458	0.464	0.469	0.475	0.481	0.486	0.492	0.498	0.502	0.508	0.517	0.523	0.53	0.535	0.541	0.546
Montenegro	0.56	0.564	0.569	0.574	0.578	0.583	0.588	0.592	0.596	0.6	0.604	0.608	0.612	0.616	0.621	0.625	0.629	0.634	0.638	0.643
Poland	0.537	0.538	0.538	0.538	0.536	0.536	0.535	0.535	0.539	0.546	0.554	0.561	0.567	0.574	0.581	0.588	0.597	0.603	0.607	0.61
Romania	0.462	0.468	0.473	0.478	0.483	0.488	0.492	0.496	0.5	0.505	0.511	0.518	0.524	0.528	0.537	0.542	0.513	0.482	0.499	0.519
Serbia	0.52	0.524	0.527	0.527	0.53	0.538	0.545	0.547	0.545	0.541	0.539	0.542	0.545	0.547	0.547	0.549	0.553	0.556	0.56	0.566
Slovakia	0.544	0.546	0.55	0.554	0.555	0.555	0.556	0.561	0.565	0.567	0.571	0.576	0.581	0.585	0.592	0.6	0.609	0.617	0.621	0.623
Slovenia	0.571	0.578	0.584	0.589	0.594	0.597	0.601	0.606	0.611	0.615	0.619	0.621	0.622	0.623	0.623	0.625	0.631	0.638	0.645	0.648
Eastern Europe	0.545	0.549	0.558	0.563	0.566	0.572	0.574	0.573	0.573	0.575	0.576	0.583	0.593	0.601	0.611	0.617	0.62	0.622	0.621	0.618
Belarus	0.487	0.49	0.495	0.499	0.502	0.506	0.51	0.514	0.518	0.522	0.527	0.532	0.537	0.54	0.544	0.548	0.55	0.552	0.554	0.556
Estonia	0.606	0.61	0.615	0.621	0.626	0.631	0.637	0.64	0.644	0.646	0.649	0.654	0.66	0.665	0.669	0.671	0.672	0.672	0.67	0.669
Latvia	0.578	0.584	0.59	0.595	0.598	0.601	0.605	0.609	0.613	0.616	0.62	0.624	0.631	0.637	0.643	0.647	0.649	0.65	0.651	0.651
Lithuania	0.565	0.572	0.581	0.589	0.597	0.606	0.614	0.619	0.624	0.627	0.631	0.636	0.643	0.649	0.653	0.656	0.658	0.659	0.659	0.658
Moldova	0.419	0.424	0.43	0.435	0.441	0.446	0.451	0.457	0.463	0.469	0.476	0.483	0.49	0.495	0.499	0.504	0.508	0.513	0.517	0.521
Russian Federation	0.555	0.559	0.57	0.575	0.578	0.585	0.586	0.584	0.582	0.582	0.582	0.589	0.602	0.612	0.624	0.632	0.635	0.635	0.632	0.628
Ukraine	0.515	0.52	0.525	0.529	0.533	0.537	0.54	0.542	0.545	0.548	0.552	0.558	0.564	0.569	0.575	0.58	0.584	0.586	0.589	0.59
High-income	0.598	0.601	0.603	0.606	0.607	0.609	0.61	0.611	0.612	0.614	0.616	0.619	0.623	0.627	0.633	0.639	0.645	0.651	0.657	0.661
Australasia	0.621	0.616	0.612	0.612	0.611	0.609	0.607	0.606	0.606	0.607	0.608	0.612	0.621	0.633	0.642	0.649	0.654	0.656	0.658	0.661
Australia	0.628	0.622	0.618	0.617	0.616	0.614	0.612	0.611	0.611	0.613	0.614	0.618	0.626	0.636	0.647	0.654	0.659	0.66	0.662	0.665
New Zealand	0.589	0.588	0.587	0.586	0.585	0.585	0.585	0.584	0.583	0.581	0.579	0.583	0.599	0.616	0.622	0.623	0.628	0.634	0.639	0.643
High-income Asia-Pacific	0.532	0.543	0.551	0.558	0.563	0.569	0.575	0.58	0.582	0.587	0.594	0.602	0.608	0.615	0.619	0.628	0.635	0.639	0.65	0.658
Brunei	0.436	0.436	0.434	0.428	0.421	0.417	0.416	0.415	0.417	0.426	0.444	0.466	0.486	0.5	0.512	0.523	0.532	0.541	0.551	0.561
Japan	0.555	0.568	0.577	0.585	0.591	0.597	0.605	0.609	0.613	0.618	0.627	0.635	0.642	0.649	0.652	0.662	0.67	0.674	0.685	0.693
Aichi	0.563	0.576	0.586	0.593	0.599	0.605	0.613	0.617	0.62	0.625	0.633	0.641	0.648	0.654	0.656	0.667	0.674	0.676	0.689	0.696
Akita	0.527	0.539	0.548	0.555	0.56	0.565	0.573	0.577	0.579	0.584	0.592	0.599	0.606	0.613	0.615	0.626	0.633	0.636	0.648	0.656
Aomori	0.517	0.53	0.54	0.547	0.553	0.558	0.566	0.57	0.572	0.577	0.585	0.593	0.6	0.606	0.608	0.619	0.626	0.629	0.641	0.648
Chiba	0.55	0.562	0.572	0.579	0.585	0.592	0.599	0.604	0.607	0.613	0.621	0.63	0.637	0.644	0.647	0.657	0.665	0.668	0.68	0.688
Ehime	0.528	0.541	0.55	0.558	0.564	0.569	0.577	0.581	0.584	0.59	0.598	0.606	0.613	0.62	0.622	0.633	0.641	0.644	0.657	0.664
Fukui	0.532	0.545	0.555	0.562	0.568	0.574	0.581	0.586	0.588	0.593	0.601	0.609	0.616	0.622	0.624	0.635	0.643	0.645	0.658	0.666
Fukuoka	0.55	0.562	0.572	0.579	0.585	0.591	0.599	0.603	0.607	0.612	0.62	0.629	0.636	0.643	0.647	0.657	0.665	0.669	0.681	0.688
Fukushima	0.527	0.54	0.549	0.557	0.562	0.568	0.575	0.58	0.582	0.587	0.595	0.602	0.61	0.616	0.619	0.63	0.637	0.64	0.653	0.66
Gifu	0.535	0.547	0.556	0.564	0.569	0.575	0.583	0.587	0.59	0.596	0.604	0.612	0.619	0.625	0.628	0.639	0.646	0.649	0.661	0.669
Gunma	0.541	0.554	0.563	0.571	0.576	0.582	0.59	0.595	0.598	0.603	0.612	0.62	0.627	0.634	0.637	0.648	0.656	0.66	0.672	0.679
Hiroshima	0.55	0.563	0.573	0.581	0.587	0.593	0.6	0.605	0.607	0.613	0.621	0.629	0.636	0.642	0.644	0.655	0.662	0.665	0.678	0.685
Hokkaidō	0.541	0.553	0.562	0.569	0.575	0.581	0.588	0.593	0.596	0.601	0.609	0.617	0.624	0.63	0.633	0.643	0.651	0.654	0.666	0.673
Hyōgo	0.55	0.562	0.571	0.579	0.585	0.591	0.598	0.603	0.606	0.612	0.62	0.628	0.635	0.642	0.645	0.655	0.663	0.667	0.678	0.686
Ibaraki	0.538	0.551	0.56	0.568	0.574	0.58	0.588	0.593	0.595	0.601	0.609	0.617	0.624	0.631	0.634	0.645	0.652	0.656	0.668	0.676
Ishikawa	0.533	0.546	0.556	0.564	0.57	0.576	0.584	0.588	0.59	0.595	0.603	0.611	0.618	0.625	0.626	0.637	0.645	0.647	0.66	0.667
Iwate	0.517	0.53	0.539	0.546	0.552	0.558	0.565	0.569	0.571	0.577	0.585	0.592	0.599	0.606	0.608	0.619	0.626	0.629	0.641	0.648
Kagawa	0.535	0.548	0.558	0.566	0.572	0.578	0.586	0.59	0.593	0.598	0.607	0.615	0.622	0.628	0.631	0.642	0.649	0.652	0.665	0.673
Kagoshima	0.522	0.534	0.542	0.549	0.555	0.56	0.567	0.572	0.575	0.58	0.588	0.596	0.603	0.611	0.614	0.625	0.633	0.636	0.648	0.656
Kanagawa	0.572	0.584	0.593	0.601	0.607	0.613	0.62	0.625	0.628	0.634	0.642	0.65	0.657	0.664	0.668	0.677	0.685	0.689	0.7	0.708
Kōchi	0.505	0.519	0.528	0.536	0.542	0.548	0.556	0.561	0.563	0.569	0.577	0.585	0.593	0.6	0.603	0.614	0.622	0.625	0.638	0.645
Kumamoto	0.522	0.534	0.543	0.55	0.556	0.562	0.569	0.574	0.577	0.582	0.59	0.598	0.606	0.613	0.616	0.627	0.634	0.638	0.65	0.658
Kyōto	0.568	0.58	0.588	0.596	0.602	0.608	0.615	0.62	0.624	0.629	0.638	0.646	0.653	0.66	0.665	0.674	0.682	0.687	0.698	0.706
Mie	0.535	0.548	0.557	0.565	0.571	0.577	0.585	0.589	0.592	0.597	0.605	0.613	0.62	0.627	0.629	0.64	0.648	0.65	0.663	0.67
Miyagi	0.541	0.553	0.562	0.57	0.575	0.581	0.589	0.593	0.596	0.601	0.609	0.617	0.625	0.632	0.635	0.645	0.653	0.657	0.669	0.677

Appendix Table 8. Socio-Demographic Index values for all estimated GBD 2017 locations, 1950-1969

Location	1950	1951	1952	1953	1954	1955	1956	1957	1958	1959	1960	1961	1962	1963	1964	1965	1966	1967	1968	1969
Miyazaki	0.515	0.528	0.538	0.545	0.551	0.556	0.564	0.568	0.571	0.576	0.584	0.592	0.599	0.606	0.608	0.619	0.627	0.63	0.642	0.65
Nagano	0.55	0.56	0.569	0.576	0.581	0.587	0.594	0.599	0.603	0.609	0.617	0.625	0.632	0.64	0.644	0.654	0.662	0.667	0.678	0.686
Nagasaki	0.525	0.537	0.546	0.553	0.558	0.564	0.571	0.575	0.578	0.584	0.592	0.599	0.607	0.614	0.617	0.627	0.635	0.639	0.651	0.658
Nara	0.535	0.546	0.555	0.563	0.569	0.575	0.582	0.587	0.591	0.597	0.606	0.614	0.622	0.629	0.634	0.644	0.652	0.656	0.668	0.676
Niigata	0.534	0.546	0.555	0.562	0.568	0.573	0.581	0.585	0.588	0.593	0.601	0.608	0.616	0.622	0.625	0.636	0.643	0.646	0.658	0.666
Ōita	0.541	0.553	0.562	0.569	0.575	0.581	0.588	0.593	0.596	0.602	0.61	0.618	0.625	0.632	0.635	0.645	0.653	0.657	0.669	0.676
Okayama	0.537	0.551	0.561	0.568	0.574	0.58	0.588	0.593	0.595	0.601	0.609	0.617	0.624	0.631	0.633	0.644	0.651	0.654	0.667	0.674
Okinawa	0.505	0.519	0.53	0.538	0.543	0.549	0.557	0.562	0.564	0.569	0.577	0.584	0.592	0.598	0.6	0.612	0.619	0.621	0.634	0.641
Ōsaka	0.572	0.585	0.594	0.602	0.608	0.614	0.621	0.626	0.629	0.634	0.642	0.65	0.657	0.664	0.667	0.677	0.684	0.687	0.699	0.706
Saga	0.527	0.539	0.548	0.555	0.561	0.566	0.574	0.579	0.582	0.587	0.595	0.603	0.611	0.618	0.621	0.632	0.64	0.644	0.656	0.664
Saitama	0.543	0.555	0.564	0.572	0.578	0.584	0.591	0.596	0.6	0.605	0.614	0.622	0.629	0.636	0.639	0.649	0.657	0.66	0.672	0.68
Shiga	0.552	0.565	0.574	0.582	0.588	0.594	0.602	0.606	0.609	0.615	0.623	0.631	0.638	0.645	0.648	0.659	0.667	0.67	0.682	0.69
Shimane	0.518	0.53	0.54	0.547	0.553	0.558	0.566	0.57	0.573	0.579	0.587	0.594	0.602	0.609	0.612	0.623	0.63	0.634	0.646	0.654
Shizuoka	0.553	0.565	0.575	0.582	0.588	0.593	0.601	0.605	0.608	0.613	0.621	0.629	0.636	0.642	0.644	0.655	0.662	0.665	0.677	0.684
Tochigi	0.543	0.556	0.565	0.573	0.579	0.584	0.592	0.596	0.599	0.604	0.612	0.62	0.627	0.634	0.637	0.648	0.655	0.659	0.671	0.678
Tokushima	0.529	0.542	0.551	0.559	0.564	0.57	0.578	0.582	0.585	0.59	0.598	0.606	0.613	0.62	0.623	0.634	0.641	0.645	0.657	0.665
Tōkyō	0.642	0.652	0.661	0.667	0.673	0.678	0.685	0.69	0.694	0.699	0.707	0.714	0.721	0.728	0.732	0.74	0.747	0.752	0.762	0.77
Tottori	0.521	0.534	0.543	0.551	0.557	0.563	0.57	0.575	0.578	0.584	0.592	0.6	0.607	0.614	0.617	0.628	0.636	0.64	0.652	0.66
Toyama	0.541	0.554	0.563	0.571	0.576	0.582	0.59	0.594	0.596	0.601	0.609	0.617	0.624	0.63	0.632	0.643	0.65	0.652	0.665	0.672
Wakayama	0.524	0.538	0.547	0.555	0.561	0.567	0.575	0.58	0.582	0.588	0.596	0.604	0.611	0.618	0.62	0.631	0.639	0.642	0.655	0.662
Yamagata	0.524	0.536	0.545	0.552	0.557	0.563	0.57	0.575	0.577	0.582	0.59	0.598	0.605	0.612	0.615	0.626	0.634	0.637	0.649	0.657
Yamaguchi	0.545	0.558	0.567	0.575	0.581	0.586	0.594	0.599	0.601	0.607	0.615	0.623	0.63	0.637	0.639	0.65	0.658	0.661	0.673	0.681
Yamanashi	0.55	0.56	0.569	0.576	0.581	0.587	0.594	0.599	0.603	0.609	0.617	0.625	0.632	0.64	0.644	0.654	0.662	0.667	0.678	0.686
South Korea	0.39	0.389	0.392	0.401	0.408	0.414	0.418	0.423	0.427	0.431	0.435	0.438	0.442	0.445	0.448	0.451	0.457	0.464	0.472	0.482
Singapore	0.419	0.414	0.406	0.403	0.407	0.412	0.416	0.422	0.428	0.443	0.458	0.467	0.476	0.486	0.499	0.509	0.518	0.533	0.548	0.559
High-income North America	0.619	0.614	0.612	0.607	0.603	0.598	0.59	0.59	0.592	0.593	0.596	0.603	0.617	0.63	0.647	0.662	0.673	0.685	0.692	0.695
Canada	0.632	0.627	0.622	0.617	0.615	0.614	0.611	0.608	0.608	0.608	0.612	0.617	0.624	0.633	0.647	0.663	0.673	0.681	0.689	0.694
Greenland	0.48	0.487	0.49	0.488	0.483	0.479	0.476	0.473	0.469	0.465	0.461	0.459	0.457	0.451	0.444	0.445	0.458	0.481	0.512	0.543
USA	0.617	0.612	0.611	0.606	0.601	0.596	0.587	0.588	0.59	0.591	0.593	0.601	0.616	0.629	0.646	0.662	0.673	0.685	0.692	0.695
Alabama	0.543	0.537	0.536	0.529	0.523	0.517	0.506	0.507	0.511	0.513	0.517	0.528	0.547	0.563	0.584	0.603	0.615	0.629	0.637	0.638
Alaska	0.609	0.601	0.599	0.588	0.578	0.565	0.544	0.54	0.539	0.535	0.535	0.545	0.568	0.587	0.614	0.637	0.652	0.669	0.679	0.68
Arizona	0.592	0.586	0.585	0.577	0.57	0.562	0.55	0.55	0.552	0.553	0.557	0.568	0.588	0.604	0.626	0.645	0.657	0.67	0.677	0.677
Arkansas	0.529	0.522	0.52	0.511	0.504	0.497	0.484	0.484	0.487	0.489	0.493	0.505	0.526	0.544	0.567	0.587	0.601	0.617	0.626	0.627
California	0.649	0.644	0.644	0.639	0.635	0.63	0.621	0.621	0.623	0.623	0.625	0.632	0.645	0.657	0.674	0.689	0.699	0.711	0.719	0.723
Colorado	0.64	0.636	0.636	0.63	0.625	0.62	0.611	0.611	0.612	0.612	0.614	0.621	0.635	0.647	0.664	0.679	0.69	0.702	0.71	0.713
Connecticut	0.672	0.669	0.669	0.666	0.663	0.66	0.654	0.653	0.653	0.653	0.654	0.658	0.668	0.677	0.691	0.704	0.714	0.726	0.734	0.74
Delaware	0.647	0.64	0.639	0.632	0.627	0.622	0.613	0.612	0.613	0.612	0.613	0.619	0.633	0.645	0.662	0.678	0.688	0.701	0.71	0.713
Washington, DC	0.663	0.657	0.654	0.647	0.641	0.633	0.621	0.62	0.621	0.62	0.62	0.627	0.643	0.657	0.674	0.69	0.702	0.716	0.727	0.73
Florida	0.604	0.596	0.596	0.59	0.585	0.58	0.57	0.572	0.575	0.578	0.581	0.591	0.607	0.621	0.639	0.654	0.664	0.675	0.681	0.68
Georgia	0.553	0.547	0.546	0.539	0.534	0.528	0.517	0.518	0.522	0.524	0.528	0.539	0.558	0.574	0.594	0.612	0.624	0.638	0.645	0.645
Hawaii	0.621	0.616	0.616	0.61	0.605	0.599	0.589	0.588	0.589	0.59	0.594	0.603	0.619	0.634	0.654	0.672	0.684	0.699	0.708	0.711
Idaho	0.588	0.579	0.577	0.567	0.558	0.547	0.532	0.53	0.53	0.528	0.53	0.54	0.56	0.578	0.602	0.624	0.638	0.654	0.663	0.665
Illinois	0.629	0.624	0.623	0.618	0.615	0.61	0.602	0.603	0.605	0.607	0.61	0.618	0.633	0.645	0.662	0.677	0.687	0.698	0.704	0.704
Indiana	0.608	0.603	0.602	0.596	0.591	0.585	0.575	0.575	0.577	0.577	0.58	0.588	0.604	0.617	0.636	0.652	0.663	0.674	0.681	0.681
Iowa	0.633	0.627	0.626	0.619	0.614	0.607	0.596	0.595	0.595	0.593	0.594	0.599	0.612	0.623	0.641	0.657	0.669	0.682	0.691	0.696
Kansas	0.625	0.619	0.619	0.613	0.608	0.601	0.59	0.588	0.589	0.588	0.589	0.596	0.61	0.623	0.641	0.658	0.67	0.684	0.693	0.698
Kentucky	0.551	0.546	0.546	0.539	0.534	0.528	0.518	0.519	0.522	0.524	0.527	0.537	0.555	0.571	0.59	0.608	0.62	0.633	0.641	0.642
Louisiana	0.545	0.537	0.535	0.527	0.519	0.51	0.496	0.495	0.496	0.495	0.497	0.505	0.524	0.541	0.564	0.584	0.599	0.616	0.628	0.633
Maine	0.6	0.593	0.592	0.585	0.579	0.573	0.562	0.561	0.562	0.561	0.563	0.571	0.587	0.601	0.62	0.638	0.65	0.663	0.671	0.673
Maryland	0.641	0.636	0.636	0.632	0.627	0.622	0.614	0.614	0.615	0.615	0.617	0.623	0.636	0.647	0.663	0.678	0.689	0.702	0.711	0.717
Massachusetts	0.676	0.673	0.673	0.67	0.668	0.665	0.66	0.66	0.661	0.662	0.664	0.67	0.679	0.688	0.701	0.713	0.721	0.731	0.738	0.742
Michigan	0.627	0.622	0.621	0.616	0.612	0.607	0.597	0.597	0.597	0.597	0.598	0.604	0.617	0.629	0.646	0.662	0.672	0.684	0.691	0.693
Minnesota	0.645	0.641	0.64	0.635	0.632	0.627	0.62	0.619	0.619	0.619	0.62	0.626	0.637	0.648	0.663	0.678	0.689	0.702	0.711	0.717
Mississippi	0.51	0.5	0.498	0.488	0.48	0.471	0.456	0.455	0.457	0.458	0.462	0.474	0.497	0.516	0.541	0.562	0.576	0.592	0.602	0.602
Missouri	0.601	0.595	0.594	0.588	0.584	0.578	0.569	0.569	0.571	0.572	0.574	0.582	0.598	0.612	0.63	0.647	0.658	0.671	0.68	0.683
Montana	0.621	0.614	0.611	0.604	0.597	0.59	0.578	0.577	0.578	0.577	0.579	0.587	0.603	0.618	0.637	0.655	0.667	0.68	0.687	0.687
Nebraska	0.634	0.626	0.625	0.618	0.613	0.607	0.596	0.596	0.597	0.596	0.598	0.605	0.619	0.632	0.649	0.666	0.677	0.69	0.698	0.701
Nevada	0.627	0.619	0.618	0.611	0.604	0.597	0.583	0.581	0.582	0.58	0.58	0.588	0.606	0.622	0.643	0.662	0.674	0.688	0.697	0.7
New Hampshire	0.633	0.628	0.627	0.622	0.618	0.613	0.604	0.604	0.604	0.604	0.605	0.612	0.625	0.636	0.653	0.669	0.679	0.692	0.7	0.704
New Jersey	0.655	0.651	0.651	0.648	0.645	0.641	0.635	0.635	0.637	0.637	0.64	0.645	0.657	0.667	0.682	0.695	0.704	0.714	0.721	0.724
New Mexico	0.574	0.565	0.563	0.554	0.546	0.537	0.522	0.521	0.523	0.523	0.525	0.536	0.556	0.573	0.597	0.618	0.631	0.647	0.656	0.657
New York	0.654	0.65	0.649	0.646	0.643	0.64	0.634	0.636	0.638	0.639	0.643	0.65	0.662	0.672	0.687	0.7	0.708	0.718	0.724	0.726
North Carolina	0.57	0.564	0.563	0.556	0.552	0.546	0.537	0.538	0.541	0.542	0.546	0.556	0.573	0.587	0.606	0.622	0.634	0.647	0.655	0.656
North Dakota	0.613	0.605	0.6	0.593	0.586	0.58	0.57	0.569	0.569	0.568	0.571	0.577	0.593	0.607	0.625	0.643	0.656	0.669	0.677	0.681
Ohio	0.624	0.62	0.62	0.616	0.612	0.608	0.6	0.601	0.603	0.605	0.608	0.616	0.629	0.641	0.657	0.671	0.68	0.69	0.695	0.695

Appendix Table 8. Socio-Demographic Index values for all estimated GBD 2017 locations, 1950-1969

Location	1950	1951	1952	1953	1954	1955	1956	1957	1958	1959	1960	1961	1962	1963	1964	1965	1966	1967	1968	1969
Oklahoma	0.575	0.569	0.568	0.561	0.556	0.549	0.538	0.538	0.541	0.542	0.545	0.556	0.574	0.59	0.611	0.629	0.641	0.655	0.664	0.666
Oregon	0.645	0.639	0.638	0.632	0.627	0.621	0.611	0.609	0.609	0.608	0.609	0.615	0.628	0.639	0.657	0.673	0.684	0.698	0.707	0.712
Pennsylvania	0.636	0.632	0.632	0.629	0.626	0.622	0.617	0.618	0.621	0.622	0.626	0.633	0.645	0.656	0.67	0.683	0.691	0.701	0.706	0.706
Rhode Island	0.642	0.638	0.637	0.633	0.629	0.625	0.617	0.617	0.617	0.617	0.618	0.624	0.635	0.646	0.661	0.675	0.686	0.698	0.706	0.71
South Carolina	0.545	0.54	0.54	0.533	0.526	0.518	0.506	0.505	0.508	0.509	0.512	0.523	0.542	0.558	0.58	0.599	0.613	0.628	0.637	0.639
South Dakota	0.601	0.596	0.592	0.584	0.579	0.57	0.559	0.559	0.56	0.559	0.562	0.571	0.588	0.603	0.622	0.641	0.653	0.667	0.676	0.678
Tennessee	0.563	0.557	0.556	0.55	0.545	0.54	0.53	0.531	0.534	0.536	0.539	0.549	0.566	0.58	0.599	0.616	0.628	0.642	0.651	0.653
Texas	0.572	0.565	0.564	0.555	0.549	0.541	0.528	0.528	0.53	0.53	0.532	0.542	0.56	0.576	0.597	0.616	0.629	0.643	0.652	0.655
Utah	0.608	0.6	0.598	0.589	0.581	0.573	0.56	0.559	0.56	0.559	0.563	0.574	0.593	0.61	0.634	0.654	0.667	0.682	0.69	0.689
Vermont	0.627	0.623	0.622	0.617	0.612	0.607	0.598	0.598	0.598	0.598	0.6	0.607	0.62	0.631	0.648	0.664	0.675	0.688	0.697	0.7
Virginia	0.607	0.603	0.603	0.597	0.593	0.588	0.579	0.58	0.582	0.583	0.586	0.594	0.609	0.622	0.64	0.656	0.667	0.68	0.689	0.692
Washington	0.655	0.648	0.647	0.641	0.636	0.629	0.618	0.616	0.615	0.613	0.612	0.617	0.629	0.64	0.657	0.673	0.684	0.699	0.71	0.718
West Virginia	0.569	0.563	0.562	0.555	0.55	0.544	0.534	0.535	0.538	0.54	0.544	0.553	0.569	0.584	0.603	0.62	0.631	0.644	0.651	0.65
Wisconsin	0.638	0.634	0.634	0.629	0.624	0.619	0.612	0.611	0.612	0.611	0.613	0.619	0.63	0.64	0.656	0.671	0.681	0.693	0.701	0.705
Wyoming	0.609	0.6	0.597	0.587	0.578	0.569	0.554	0.552	0.552	0.55	0.551	0.56	0.579	0.596	0.619	0.639	0.651	0.666	0.676	0.678
Southern Latin America	0.464	0.468	0.47	0.472	0.474	0.475	0.477	0.481	0.485	0.486	0.489	0.492	0.494	0.496	0.501	0.507	0.513	0.517	0.516	0.517
Argentina	0.472	0.475	0.477	0.479	0.482	0.485	0.488	0.492	0.497	0.501	0.504	0.506	0.508	0.51	0.515	0.521	0.527	0.53	0.525	0.524
Chile	0.436	0.441	0.443	0.445	0.446	0.445	0.443	0.446	0.449	0.446	0.448	0.455	0.456	0.458	0.464	0.471	0.477	0.485	0.494	0.498
Uruguay	0.479	0.484	0.484	0.484	0.484	0.483	0.484	0.487	0.489	0.49	0.49	0.491	0.495	0.5	0.504	0.507	0.511	0.514	0.514	0.515
Western Europe	0.597	0.6	0.602	0.605	0.607	0.609	0.61	0.612	0.613	0.614	0.615	0.616	0.617	0.619	0.622	0.624	0.629	0.634	0.637	0.643
Andorra	0.721	0.724	0.728	0.733	0.737	0.741	0.745	0.75	0.754	0.758	0.762	0.767	0.771	0.776	0.781	0.786	0.79	0.794	0.798	0.801
Austria	0.651	0.654	0.654	0.656	0.656	0.655	0.654	0.653	0.652	0.647	0.645	0.649	0.652	0.655	0.658	0.659	0.658	0.657	0.659	0.665
Belgium	0.657	0.659	0.66	0.66	0.66	0.66	0.66	0.66	0.66	0.66	0.66	0.66	0.66	0.661	0.665	0.67	0.676	0.682	0.686	0.689
Cyprus	0.544	0.55	0.556	0.561	0.563	0.562	0.561	0.561	0.562	0.564	0.567	0.571	0.575	0.579	0.581	0.584	0.587	0.591	0.595	0.6
Denmark	0.693	0.692	0.689	0.688	0.688	0.688	0.689	0.691	0.694	0.697	0.7	0.701	0.702	0.704	0.705	0.706	0.714	0.73	0.744	0.753
Finland	0.633	0.637	0.637	0.637	0.638	0.64	0.643	0.648	0.652	0.653	0.656	0.658	0.659	0.663	0.671	0.678	0.682	0.688	0.697	0.707
France	0.561	0.564	0.567	0.569	0.571	0.574	0.577	0.58	0.58	0.581	0.582	0.584	0.583	0.582	0.585	0.589	0.595	0.603	0.61	0.615
Germany	0.615	0.62	0.623	0.631	0.638	0.642	0.644	0.647	0.648	0.649	0.65	0.651	0.652	0.655	0.659	0.662	0.667	0.674	0.672	0.68
Greece	0.55	0.553	0.554	0.556	0.559	0.563	0.568	0.571	0.572	0.574	0.578	0.581	0.582	0.583	0.583	0.582	0.579	0.576	0.58	0.588
Iceland	0.629	0.627	0.623	0.62	0.618	0.617	0.617	0.62	0.627	0.635	0.636	0.632	0.629	0.628	0.631	0.639	0.651	0.664	0.674	0.68
Ireland	0.609	0.611	0.615	0.62	0.623	0.626	0.628	0.629	0.629	0.629	0.629	0.628	0.628	0.629	0.631	0.634	0.637	0.639	0.641	0.641
Israel	0.525	0.526	0.527	0.531	0.533	0.536	0.542	0.551	0.561	0.566	0.571	0.578	0.583	0.587	0.592	0.6	0.61	0.619	0.623	0.624
Italy	0.578	0.583	0.587	0.588	0.59	0.592	0.595	0.598	0.601	0.604	0.607	0.609	0.611	0.617	0.619	0.617	0.621	0.625	0.629	0.633
Luxembourg	0.706	0.71	0.712	0.711	0.71	0.709	0.71	0.71	0.711	0.712	0.713	0.714	0.715	0.715	0.717	0.719	0.721	0.725	0.729	0.735
Malta	0.494	0.501	0.508	0.515	0.522	0.526	0.53	0.532	0.534	0.536	0.542	0.55	0.56	0.571	0.583	0.594	0.602	0.608	0.613	0.618
Netherlands	0.683	0.683	0.685	0.688	0.689	0.691	0.692	0.692	0.691	0.691	0.692	0.692	0.692	0.692	0.694	0.696	0.698	0.702	0.705	0.71
Norway	0.684	0.684	0.68	0.677	0.674	0.672	0.67	0.67	0.67	0.67	0.668	0.667	0.668	0.669	0.67	0.673	0.675	0.677	0.68	0.684
Portugal	0.454	0.456	0.458	0.461	0.462	0.463	0.464	0.466	0.467	0.468	0.469	0.47	0.474	0.478	0.481	0.485	0.488	0.493	0.5	0.507
Spain	0.533	0.536	0.537	0.537	0.537	0.537	0.537	0.538	0.54	0.543	0.547	0.55	0.553	0.555	0.558	0.563	0.568	0.57	0.572	0.577
Sweden	0.645	0.647	0.647	0.648	0.649	0.65	0.652	0.656	0.66	0.664	0.668	0.669	0.67	0.669	0.67	0.673	0.678	0.687	0.697	0.702
Stockholm	0.697	0.699	0.699	0.701	0.702	0.703	0.706	0.71	0.714	0.719	0.722	0.724	0.726	0.726	0.728	0.73	0.734	0.741	0.748	0.75
Sweden except Stockholm	0.634	0.636	0.636	0.637	0.638	0.638	0.64	0.644	0.648	0.652	0.655	0.657	0.657	0.656	0.656	0.659	0.664	0.674	0.684	0.69
Switzerland	0.761	0.765	0.766	0.767	0.768	0.768	0.768	0.768	0.768	0.769	0.768	0.766	0.763	0.761	0.763	0.767	0.771	0.774	0.778	0.781
United Kingdom	0.572	0.574	0.575	0.576	0.576	0.576	0.575	0.574	0.573	0.571	0.57	0.569	0.568	0.569	0.572	0.576	0.581	0.587	0.593	0.599
England	0.587	0.589	0.59	0.591	0.592	0.591	0.59	0.588	0.587	0.585	0.584	0.582	0.582	0.582	0.585	0.589	0.594	0.6	0.606	0.611
East Midlands	0.552	0.555	0.556	0.557	0.558	0.558	0.557	0.556	0.555	0.554	0.553	0.552	0.551	0.553	0.555	0.56	0.565	0.571	0.577	0.583
Derby	0.559	0.561	0.562	0.563	0.564	0.563	0.562	0.561	0.559	0.557	0.556	0.554	0.553	0.554	0.557	0.561	0.566	0.572	0.578	0.584
Derbyshire	0.542	0.545	0.546	0.547	0.548	0.548	0.547	0.546	0.545	0.544	0.543	0.542	0.542	0.543	0.546	0.55	0.556	0.562	0.568	0.574
Leicester	0.54	0.542	0.543	0.545	0.545	0.545	0.544	0.543	0.542	0.541	0.539	0.538	0.538	0.539	0.542	0.546	0.552	0.558	0.564	0.57
Leicestershire	0.57	0.572	0.573	0.574	0.575	0.575	0.575	0.574	0.573	0.572	0.571	0.571	0.571	0.572	0.575	0.579	0.585	0.591	0.597	0.603
Lincolnshire	0.546	0.548	0.549	0.55	0.551	0.551	0.55	0.549	0.548	0.547	0.546	0.545	0.545	0.546	0.549	0.553	0.558	0.564	0.571	0.577
Northamptonshire	0.555	0.557	0.558	0.559	0.56	0.56	0.559	0.558	0.557	0.556	0.554	0.553	0.553	0.554	0.557	0.561	0.567	0.573	0.579	0.585
Nottingham	0.568	0.57	0.571	0.572	0.573	0.573	0.572	0.572	0.571	0.57	0.569	0.568	0.568	0.569	0.572	0.577	0.582	0.588	0.594	0.6
Nottinghamshire	0.541	0.543	0.544	0.546	0.546	0.546	0.546	0.545	0.544	0.543	0.541	0.541	0.541	0.542	0.545	0.549	0.554	0.56	0.566	0.572
Rutland	0.57	0.573	0.574	0.575	0.576	0.576	0.575	0.574	0.573	0.572	0.571	0.57	0.57	0.571	0.574	0.579	0.584	0.59	0.596	0.602
East of England	0.567	0.57	0.571	0.572	0.573	0.573	0.572	0.572	0.571	0.57	0.568	0.568	0.568	0.569	0.572	0.576	0.581	0.587	0.594	0.6
Bedford	0.57	0.573	0.574	0.575	0.576	0.576	0.575	0.574	0.573	0.572	0.571	0.57	0.569	0.571	0.573	0.578	0.583	0.589	0.595	0.601
Cambridgeshire	0.589	0.592	0.593	0.594	0.595	0.595	0.595	0.594	0.594	0.593	0.593	0.592	0.593	0.594	0.597	0.601	0.607	0.613	0.619	0.625
Central Bedfordshire	0.563	0.565	0.567	0.568	0.569	0.569	0.568	0.567	0.566	0.565	0.564	0.563	0.562	0.563	0.566	0.571	0.576	0.582	0.588	0.595
Essex	0.557	0.559	0.561	0.562	0.563	0.563	0.562	0.562	0.561	0.56	0.559	0.558	0.558	0.56	0.563	0.567	0.572	0.578	0.584	0.591
Hertfordshire	0.592	0.594	0.595	0.597	0.598	0.598	0.597	0.596	0.595	0.594	0.593	0.593	0.592	0.594	0.597	0.601	0.606	0.612	0.618	0.624
Luton	0.551	0.553	0.554	0.556	0.556	0.556	0.555	0.554	0.552	0.55	0.549	0.547	0.546	0.547	0.55	0.554	0.56	0.566	0.573	0.579
Norfolk	0.555	0.558	0.559	0.56	0.561	0.561	0.561	0.56	0.559	0.558	0.557	0.557	0.557	0.558	0.561	0.565	0.571	0.577	0.583	0.589
Peterborough	0.557	0.559	0.56	0.562	0.562	0.562	0.561	0.559	0.558	0.556	0.554	0.553	0.552	0.552	0.555	0.56	0.565	0.572	0.578	0.584
Southend-on-Sea	0.534	0.536	0.538	0.539	0.54	0.54	0.539	0.539	0.538	0.537	0.536	0.535	0.535	0.537	0.54	0.544	0.55	0.556	0.562	0.568

Appendix Table 8. Socio-Demographic Index values for all estimated GBD 2017 locations, 1950-1969																				
**Location**	**1950**	**1951**	**1952**	**1953**	**1954**	**1955**	**1956**	**1957**	**1958**	**1959**	**1960**	**1961**	**1962**	**1963**	**1964**	**1965**	**1966**	**1967**	**1968**	**1969**
Suffolk	0.556	0.559	0.56	0.561	0.562	0.562	0.561	0.56	0.559	0.557	0.556	0.555	0.554	0.555	0.558	0.562	0.568	0.574	0.58	0.586
Thurrock	0.56	0.562	0.563	0.564	0.564	0.564	0.563	0.562	0.561	0.559	0.558	0.557	0.556	0.557	0.56	0.564	0.57	0.576	0.582	0.588
Greater London	0.635	0.638	0.639	0.639	0.64	0.64	0.638	0.637	0.636	0.634	0.633	0.631	0.631	0.632	0.634	0.638	0.643	0.648	0.653	0.659
Barking and Dagenham	0.564	0.566	0.567	0.567	0.568	0.567	0.566	0.565	0.563	0.561	0.559	0.558	0.557	0.557	0.56	0.564	0.568	0.574	0.58	0.586
Barnet	0.609	0.611	0.612	0.613	0.614	0.614	0.613	0.613	0.612	0.611	0.61	0.609	0.609	0.61	0.612	0.616	0.621	0.626	0.632	0.637
Bexley	0.574	0.577	0.578	0.579	0.58	0.58	0.579	0.578	0.577	0.576	0.575	0.575	0.574	0.575	0.578	0.582	0.587	0.593	0.598	0.604
Brent	0.597	0.599	0.6	0.601	0.602	0.602	0.601	0.6	0.599	0.597	0.596	0.595	0.594	0.595	0.598	0.602	0.607	0.613	0.618	0.624
Bromley	0.606	0.608	0.609	0.611	0.611	0.611	0.611	0.61	0.609	0.608	0.607	0.606	0.606	0.607	0.609	0.613	0.618	0.624	0.629	0.635
Camden	0.709	0.712	0.713	0.714	0.715	0.715	0.715	0.715	0.714	0.714	0.714	0.713	0.714	0.715	0.718	0.722	0.726	0.731	0.735	0.739
Croydon	0.591	0.593	0.594	0.596	0.596	0.596	0.595	0.594	0.592	0.591	0.589	0.588	0.588	0.588	0.591	0.595	0.6	0.606	0.612	0.618
Ealing	0.609	0.611	0.612	0.613	0.614	0.614	0.613	0.611	0.61	0.609	0.607	0.606	0.605	0.606	0.608	0.612	0.617	0.623	0.628	0.634
Enfield	0.585	0.588	0.589	0.59	0.591	0.591	0.59	0.589	0.588	0.587	0.585	0.584	0.584	0.585	0.587	0.591	0.596	0.602	0.608	0.614
Greenwich	0.577	0.579	0.58	0.581	0.581	0.581	0.58	0.579	0.577	0.576	0.574	0.573	0.572	0.573	0.575	0.579	0.584	0.59	0.595	0.601
Hackney	0.65	0.652	0.652	0.652	0.651	0.649	0.645	0.642	0.638	0.634	0.629	0.625	0.622	0.62	0.621	0.624	0.629	0.635	0.641	0.647
Hammersmith and Fulham	0.665	0.668	0.669	0.67	0.671	0.671	0.671	0.67	0.669	0.669	0.668	0.668	0.668	0.67	0.672	0.676	0.68	0.686	0.691	0.696
Haringey	0.597	0.599	0.6	0.602	0.603	0.602	0.602	0.601	0.6	0.598	0.597	0.596	0.596	0.597	0.599	0.603	0.608	0.614	0.62	0.626
Harrow	0.597	0.599	0.6	0.602	0.602	0.602	0.602	0.601	0.6	0.598	0.597	0.596	0.596	0.597	0.599	0.603	0.608	0.614	0.62	0.625
Havering	0.579	0.581	0.582	0.584	0.584	0.584	0.583	0.582	0.582	0.58	0.579	0.579	0.578	0.579	0.582	0.586	0.591	0.596	0.602	0.607
Hillingdon	0.631	0.634	0.635	0.636	0.637	0.636	0.635	0.634	0.633	0.632	0.63	0.629	0.629	0.63	0.632	0.636	0.641	0.647	0.653	0.658
Hounslow	0.615	0.617	0.619	0.62	0.62	0.62	0.619	0.618	0.616	0.615	0.613	0.612	0.612	0.612	0.615	0.619	0.624	0.63	0.636	0.641
Islington	0.669	0.672	0.673	0.674	0.674	0.674	0.673	0.671	0.67	0.669	0.668	0.667	0.666	0.667	0.67	0.673	0.678	0.684	0.69	0.695
Kensington and Chelsea	0.688	0.69	0.691	0.693	0.694	0.694	0.695	0.695	0.695	0.695	0.695	0.695	0.696	0.698	0.7	0.704	0.708	0.713	0.719	0.724
Kingston upon Thames	0.637	0.639	0.641	0.642	0.643	0.643	0.643	0.643	0.642	0.642	0.642	0.641	0.642	0.643	0.646	0.649	0.654	0.659	0.664	0.669
Lambeth	0.616	0.618	0.619	0.62	0.621	0.62	0.619	0.617	0.616	0.614	0.613	0.611	0.611	0.612	0.614	0.618	0.623	0.628	0.634	0.64
Lewisham	0.579	0.581	0.582	0.583	0.584	0.584	0.583	0.582	0.581	0.579	0.578	0.577	0.577	0.578	0.58	0.584	0.589	0.594	0.6	0.605
Merton	0.607	0.609	0.61	0.611	0.612	0.612	0.61	0.609	0.608	0.607	0.605	0.604	0.604	0.605	0.607	0.611	0.616	0.621	0.627	0.633
Newham	0.564	0.566	0.567	0.567	0.568	0.567	0.565	0.564	0.562	0.56	0.557	0.555	0.554	0.555	0.557	0.561	0.567	0.573	0.579	0.585
Redbridge	0.581	0.583	0.584	0.585	0.586	0.586	0.585	0.584	0.583	0.582	0.581	0.58	0.58	0.581	0.583	0.587	0.592	0.597	0.603	0.609
Richmond upon Thames	0.648	0.65	0.651	0.653	0.653	0.654	0.653	0.653	0.652	0.651	0.651	0.651	0.651	0.652	0.654	0.658	0.662	0.667	0.673	0.678
Southwark	0.637	0.639	0.64	0.64	0.641	0.64	0.638	0.637	0.635	0.633	0.631	0.63	0.629	0.629	0.631	0.635	0.64	0.646	0.652	0.657
Sutton	0.584	0.587	0.588	0.589	0.59	0.59	0.589	0.589	0.588	0.587	0.585	0.585	0.584	0.586	0.588	0.592	0.597	0.603	0.609	0.615
Tower Hamlets	0.636	0.638	0.639	0.639	0.638	0.637	0.634	0.631	0.628	0.625	0.621	0.618	0.615	0.614	0.615	0.618	0.622	0.628	0.633	0.639
Waltham Forest	0.558	0.56	0.561	0.563	0.563	0.563	0.562	0.561	0.56	0.559	0.557	0.556	0.555	0.556	0.559	0.562	0.568	0.573	0.579	0.585
Wandsworth	0.634	0.636	0.637	0.639	0.639	0.639	0.638	0.638	0.637	0.636	0.635	0.634	0.634	0.635	0.637	0.641	0.645	0.651	0.656	0.661
Westminster	0.72	0.722	0.723	0.723	0.723	0.722	0.721	0.72	0.718	0.717	0.715	0.714	0.713	0.714	0.715	0.718	0.721	0.725	0.73	0.734
North East England	0.536	0.538	0.539	0.541	0.542	0.542	0.541	0.539	0.538	0.537	0.535	0.534	0.534	0.535	0.538	0.542	0.548	0.554	0.56	0.566
County Durham	0.529	0.531	0.533	0.534	0.535	0.535	0.534	0.533	0.532	0.531	0.53	0.529	0.529	0.53	0.533	0.537	0.543	0.549	0.555	0.561
Darlington	0.547	0.55	0.55	0.552	0.552	0.552	0.551	0.55	0.548	0.546	0.545	0.543	0.542	0.543	0.546	0.55	0.556	0.563	0.569	0.576
Gateshead	0.542	0.544	0.545	0.546	0.547	0.547	0.546	0.545	0.543	0.541	0.54	0.539	0.538	0.539	0.542	0.546	0.551	0.558	0.564	0.57
Hartlepool	0.522	0.524	0.525	0.527	0.527	0.527	0.525	0.524	0.522	0.52	0.518	0.516	0.515	0.516	0.519	0.523	0.529	0.535	0.542	0.548
Middlesbrough	0.526	0.528	0.529	0.53	0.531	0.531	0.529	0.528	0.526	0.524	0.522	0.521	0.52	0.521	0.523	0.528	0.534	0.54	0.547	0.553
Newcastle upon Tyne	0.566	0.569	0.57	0.571	0.572	0.573	0.572	0.572	0.571	0.571	0.57	0.57	0.57	0.572	0.575	0.579	0.585	0.591	0.597	0.603
North Tyneside	0.539	0.541	0.542	0.544	0.545	0.544	0.543	0.542	0.541	0.54	0.538	0.537	0.537	0.538	0.541	0.545	0.55	0.557	0.563	0.569
Northumberland	0.535	0.538	0.539	0.54	0.541	0.541	0.541	0.539	0.538	0.537	0.536	0.534	0.534	0.535	0.538	0.542	0.548	0.554	0.561	0.567
Redcar and Cleveland	0.513	0.516	0.517	0.518	0.519	0.518	0.517	0.516	0.514	0.513	0.511	0.51	0.509	0.509	0.512	0.517	0.522	0.529	0.535	0.541
South Tyneside	0.507	0.509	0.51	0.512	0.513	0.512	0.511	0.51	0.509	0.508	0.506	0.505	0.505	0.506	0.509	0.513	0.519	0.525	0.531	0.538
Stockton-on-Tees	0.546	0.549	0.55	0.551	0.551	0.551	0.55	0.548	0.547	0.545	0.543	0.541	0.54	0.541	0.544	0.548	0.554	0.56	0.567	0.573
Sunderland	0.528	0.53	0.531	0.532	0.533	0.533	0.532	0.53	0.529	0.527	0.525	0.524	0.523	0.523	0.526	0.531	0.536	0.543	0.549	0.556
North West England	0.567	0.57	0.571	0.572	0.572	0.572	0.571	0.57	0.568	0.567	0.565	0.564	0.563	0.564	0.567	0.571	0.576	0.582	0.588	0.594
Blackburn with Darwen	0.539	0.541	0.542	0.544	0.544	0.543	0.542	0.54	0.538	0.536	0.533	0.531	0.53	0.53	0.533	0.537	0.542	0.549	0.555	0.562
Blackpool	0.53	0.532	0.534	0.535	0.536	0.536	0.535	0.534	0.533	0.532	0.531	0.53	0.53	0.531	0.534	0.538	0.544	0.55	0.556	0.562
Bolton	0.551	0.553	0.554	0.555	0.556	0.556	0.554	0.553	0.551	0.549	0.548	0.546	0.545	0.546	0.548	0.552	0.558	0.564	0.57	0.576
Bury	0.555	0.558	0.559	0.56	0.561	0.56	0.559	0.558	0.557	0.555	0.554	0.552	0.552	0.553	0.555	0.559	0.565	0.571	0.577	0.583
Cheshire East	0.602	0.604	0.605	0.606	0.607	0.607	0.606	0.604	0.603	0.602	0.6	0.599	0.598	0.599	0.602	0.606	0.611	0.617	0.623	0.628
Cheshire West and Chester	0.594	0.596	0.597	0.598	0.599	0.598	0.597	0.596	0.594	0.593	0.591	0.59	0.589	0.59	0.592	0.596	0.602	0.607	0.613	0.619
Cumbria	0.572	0.575	0.576	0.577	0.578	0.577	0.576	0.575	0.574	0.572	0.571	0.569	0.569	0.57	0.573	0.577	0.582	0.588	0.594	0.6
Halton	0.557	0.559	0.56	0.561	0.562	0.561	0.559	0.557	0.556	0.553	0.551	0.549	0.548	0.549	0.551	0.555	0.561	0.567	0.573	0.579
Knowsley	0.547	0.549	0.55	0.551	0.551	0.55	0.549	0.547	0.545	0.543	0.541	0.54	0.539	0.539	0.542	0.546	0.551	0.557	0.562	0.568
Lancashire	0.569	0.571	0.572	0.573	0.574	0.574	0.573	0.572	0.57	0.568	0.567	0.566	0.565	0.566	0.569	0.573	0.578	0.584	0.59	0.596
Liverpool	0.567	0.569	0.57	0.571	0.572	0.572	0.571	0.57	0.569	0.568	0.567	0.566	0.566	0.567	0.57	0.574	0.579	0.585	0.591	0.596
Manchester	0.59	0.592	0.593	0.594	0.595	0.594	0.593	0.592	0.591	0.589	0.588	0.587	0.586	0.587	0.59	0.594	0.599	0.605	0.611	0.617
Oldham	0.538	0.541	0.542	0.543	0.543	0.543	0.541	0.54	0.538	0.536	0.534	0.532	0.531	0.531	0.534	0.538	0.543	0.549	0.556	0.562
Rochdale	0.54	0.542	0.543	0.544	0.545	0.544	0.543	0.541	0.539	0.538	0.536	0.534	0.533	0.533	0.536	0.54	0.545	0.552	0.558	0.564
Salford	0.562	0.564	0.565	0.566	0.566	0.565	0.564	0.562	0.561	0.559	0.557	0.555	0.554	0.555	0.557	0.561	0.567	0.573	0.579	0.585
Sefton	0.559	0.561	0.562	0.563	0.564	0.564	0.563	0.562	0.561	0.559	0.558	0.557	0.557	0.558	0.561	0.565	0.57	0.575	0.581	0.587

Appendix Table 8. Socio-Demographic Index values for all estimated GBD 2017 locations, 1950-1969																				
**Location**	**1950**	**1951**	**1952**	**1953**	**1954**	**1955**	**1956**	**1957**	**1958**	**1959**	**1960**	**1961**	**1962**	**1963**	**1964**	**1965**	**1966**	**1967**	**1968**	**1969**
St Helens	0.539	0.541	0.542	0.543	0.544	0.543	0.542	0.541	0.54	0.538	0.537	0.535	0.535	0.535	0.538	0.542	0.547	0.553	0.559	0.565
Stockport	0.581	0.583	0.584	0.585	0.586	0.585	0.584	0.583	0.581	0.58	0.578	0.577	0.576	0.577	0.58	0.584	0.589	0.594	0.6	0.606
Tameside	0.544	0.547	0.547	0.549	0.549	0.549	0.548	0.546	0.545	0.543	0.542	0.54	0.539	0.54	0.543	0.547	0.552	0.558	0.564	0.57
Trafford	0.606	0.608	0.609	0.61	0.61	0.61	0.608	0.607	0.605	0.603	0.601	0.6	0.599	0.6	0.602	0.606	0.611	0.617	0.623	0.628
Warrington	0.593	0.596	0.596	0.597	0.598	0.597	0.596	0.594	0.592	0.59	0.588	0.587	0.586	0.586	0.589	0.593	0.598	0.604	0.61	0.616
Wigan	0.543	0.545	0.546	0.547	0.548	0.547	0.546	0.545	0.543	0.542	0.54	0.539	0.538	0.539	0.541	0.546	0.551	0.557	0.563	0.569
Wirral	0.549	0.551	0.552	0.553	0.554	0.553	0.552	0.551	0.549	0.547	0.546	0.544	0.544	0.544	0.547	0.551	0.556	0.562	0.568	0.574
South East England	0.608	0.611	0.611	0.612	0.613	0.613	0.611	0.61	0.609	0.607	0.605	0.604	0.603	0.604	0.607	0.611	0.616	0.621	0.627	0.633
Bracknell Forest	0.62	0.622	0.622	0.623	0.623	0.623	0.621	0.619	0.617	0.615	0.613	0.612	0.611	0.611	0.613	0.617	0.622	0.628	0.633	0.639
Brighton and Hove	0.616	0.618	0.619	0.62	0.621	0.622	0.622	0.621	0.621	0.621	0.62	0.62	0.621	0.622	0.625	0.629	0.633	0.638	0.643	0.648
Buckinghamshire	0.628	0.63	0.631	0.631	0.632	0.631	0.63	0.628	0.626	0.625	0.623	0.621	0.62	0.621	0.623	0.627	0.632	0.637	0.643	0.649
East Sussex	0.568	0.571	0.572	0.573	0.574	0.574	0.573	0.572	0.571	0.569	0.568	0.566	0.566	0.567	0.57	0.574	0.579	0.585	0.591	0.597
Hampshire	0.605	0.607	0.608	0.609	0.609	0.609	0.607	0.606	0.604	0.602	0.6	0.599	0.598	0.598	0.601	0.605	0.61	0.616	0.621	0.627
Isle of Wight	0.565	0.568	0.569	0.57	0.571	0.57	0.569	0.567	0.566	0.564	0.562	0.56	0.559	0.56	0.562	0.566	0.572	0.578	0.584	0.59
Kent	0.582	0.585	0.586	0.587	0.587	0.587	0.585	0.584	0.582	0.581	0.579	0.577	0.577	0.577	0.58	0.584	0.589	0.595	0.601	0.607
Medway	0.56	0.563	0.564	0.565	0.565	0.564	0.563	0.561	0.559	0.557	0.555	0.554	0.553	0.553	0.556	0.56	0.565	0.571	0.577	0.583
Milton Keynes	0.602	0.605	0.606	0.607	0.608	0.607	0.605	0.603	0.601	0.599	0.596	0.594	0.592	0.593	0.595	0.599	0.605	0.611	0.618	0.625
Oxfordshire	0.63	0.632	0.633	0.634	0.634	0.634	0.633	0.631	0.63	0.629	0.627	0.626	0.626	0.627	0.629	0.633	0.638	0.643	0.649	0.654
Portsmouth	0.593	0.596	0.597	0.598	0.599	0.599	0.598	0.597	0.596	0.595	0.594	0.593	0.592	0.593	0.596	0.601	0.606	0.612	0.619	0.625
Reading	0.635	0.637	0.638	0.639	0.639	0.639	0.638	0.636	0.635	0.633	0.632	0.631	0.63	0.631	0.634	0.637	0.643	0.648	0.654	0.66
Slough	0.625	0.627	0.628	0.628	0.628	0.627	0.624	0.621	0.619	0.616	0.612	0.609	0.607	0.607	0.609	0.613	0.618	0.624	0.631	0.637
Southampton	0.609	0.611	0.612	0.613	0.613	0.613	0.612	0.61	0.609	0.608	0.606	0.605	0.605	0.605	0.608	0.612	0.617	0.623	0.628	0.634
Surrey	0.636	0.638	0.639	0.64	0.641	0.64	0.639	0.638	0.637	0.635	0.634	0.633	0.633	0.634	0.636	0.64	0.645	0.65	0.655	0.661
West Berkshire	0.631	0.633	0.634	0.635	0.635	0.634	0.633	0.631	0.629	0.627	0.624	0.623	0.621	0.622	0.624	0.628	0.634	0.64	0.646	0.652
West Sussex	0.596	0.598	0.599	0.601	0.601	0.601	0.6	0.599	0.597	0.596	0.594	0.593	0.593	0.593	0.596	0.6	0.605	0.611	0.617	0.623
Windsor and Maidenhead	0.642	0.644	0.645	0.646	0.646	0.646	0.644	0.643	0.641	0.639	0.637	0.636	0.635	0.636	0.638	0.642	0.647	0.652	0.658	0.664
Wokingham	0.642	0.643	0.644	0.645	0.645	0.644	0.643	0.642	0.64	0.639	0.637	0.636	0.635	0.636	0.638	0.642	0.647	0.652	0.658	0.664
South West England	0.577	0.58	0.581	0.582	0.583	0.583	0.582	0.581	0.58	0.579	0.578	0.577	0.577	0.578	0.58	0.585	0.59	0.596	0.602	0.608
Bath and North East Somerset	0.597	0.599	0.601	0.602	0.603	0.604	0.604	0.604	0.604	0.603	0.604	0.604	0.605	0.606	0.609	0.613	0.618	0.624	0.629	0.634
Bournemouth	0.573	0.576	0.577	0.579	0.58	0.581	0.581	0.581	0.581	0.581	0.581	0.582	0.583	0.585	0.588	0.592	0.597	0.603	0.608	0.614
Bristol, City of	0.607	0.609	0.61	0.611	0.612	0.612	0.611	0.61	0.61	0.609	0.608	0.607	0.607	0.608	0.611	0.615	0.62	0.626	0.632	0.637
Cornwall	0.548	0.551	0.552	0.553	0.554	0.554	0.553	0.552	0.551	0.549	0.548	0.547	0.546	0.547	0.55	0.554	0.56	0.566	0.572	0.578
Devon	0.566	0.568	0.57	0.571	0.572	0.572	0.572	0.571	0.57	0.57	0.569	0.568	0.568	0.57	0.573	0.577	0.582	0.588	0.594	0.6
Dorset	0.568	0.57	0.571	0.573	0.573	0.573	0.573	0.572	0.571	0.569	0.568	0.567	0.567	0.568	0.571	0.575	0.58	0.586	0.593	0.599
Gloucestershire	0.584	0.587	0.588	0.589	0.59	0.59	0.589	0.587	0.586	0.585	0.584	0.583	0.582	0.583	0.586	0.59	0.595	0.601	0.607	0.613
North Somerset	0.561	0.563	0.564	0.566	0.566	0.566	0.566	0.565	0.564	0.562	0.561	0.56	0.56	0.561	0.564	0.568	0.573	0.579	0.585	0.591
Plymouth	0.56	0.563	0.564	0.566	0.567	0.567	0.566	0.566	0.565	0.564	0.563	0.562	0.562	0.563	0.566	0.571	0.576	0.582	0.588	0.594
Poole	0.575	0.578	0.579	0.58	0.581	0.581	0.58	0.579	0.578	0.576	0.575	0.574	0.574	0.575	0.578	0.582	0.587	0.593	0.6	0.606
Somerset	0.564	0.567	0.568	0.569	0.57	0.57	0.569	0.568	0.566	0.565	0.563	0.562	0.562	0.563	0.565	0.57	0.575	0.581	0.587	0.593
South Gloucestershire	0.599	0.601	0.602	0.603	0.604	0.604	0.603	0.601	0.6	0.599	0.597	0.596	0.596	0.597	0.6	0.604	0.609	0.615	0.621	0.627
Swindon	0.598	0.6	0.601	0.602	0.603	0.602	0.601	0.599	0.597	0.595	0.593	0.591	0.59	0.591	0.593	0.597	0.602	0.609	0.615	0.621
Torbay	0.542	0.545	0.546	0.548	0.549	0.549	0.549	0.548	0.548	0.547	0.546	0.545	0.545	0.546	0.549	0.554	0.559	0.565	0.571	0.578
Wiltshire	0.58	0.582	0.584	0.585	0.585	0.585	0.583	0.582	0.58	0.579	0.577	0.575	0.574	0.575	0.578	0.582	0.587	0.593	0.599	0.605
West Midlands	0.555	0.557	0.558	0.559	0.56	0.559	0.558	0.557	0.555	0.554	0.552	0.551	0.55	0.551	0.553	0.557	0.563	0.569	0.575	0.581
Birmingham	0.552	0.554	0.555	0.555	0.556	0.555	0.554	0.552	0.551	0.549	0.547	0.545	0.544	0.545	0.547	0.551	0.557	0.563	0.569	0.575
Coventry	0.57	0.572	0.572	0.573	0.574	0.573	0.572	0.57	0.569	0.567	0.565	0.564	0.563	0.564	0.567	0.571	0.576	0.582	0.588	0.594
Dudley	0.543	0.545	0.546	0.547	0.548	0.547	0.546	0.545	0.543	0.542	0.54	0.539	0.538	0.539	0.541	0.546	0.551	0.557	0.563	0.569
Herefordshire, County of	0.557	0.559	0.56	0.561	0.562	0.562	0.56	0.559	0.558	0.556	0.555	0.553	0.553	0.554	0.556	0.56	0.566	0.572	0.578	0.584
Sandwell	0.535	0.537	0.538	0.539	0.539	0.539	0.537	0.535	0.534	0.531	0.529	0.527	0.526	0.527	0.529	0.533	0.538	0.544	0.55	0.556
Shropshire	0.558	0.56	0.561	0.562	0.563	0.563	0.562	0.561	0.56	0.558	0.557	0.556	0.555	0.556	0.559	0.563	0.569	0.575	0.581	0.587
Solihull	0.59	0.592	0.593	0.594	0.595	0.594	0.593	0.591	0.59	0.588	0.586	0.585	0.584	0.585	0.587	0.591	0.596	0.602	0.608	0.614
Staffordshire	0.557	0.559	0.56	0.561	0.562	0.562	0.561	0.559	0.558	0.557	0.555	0.554	0.554	0.555	0.557	0.562	0.567	0.573	0.579	0.585
Stoke-on-Trent	0.538	0.54	0.54	0.541	0.542	0.541	0.54	0.539	0.537	0.536	0.534	0.532	0.532	0.532	0.535	0.539	0.544	0.55	0.556	0.562
Telford and Wrekin	0.561	0.564	0.564	0.565	0.566	0.565	0.564	0.562	0.56	0.558	0.556	0.554	0.553	0.554	0.556	0.56	0.566	0.572	0.579	0.585
Walsall	0.534	0.536	0.537	0.538	0.539	0.538	0.537	0.535	0.533	0.532	0.53	0.528	0.527	0.528	0.53	0.534	0.54	0.546	0.552	0.558
Warwickshire	0.582	0.584	0.585	0.586	0.586	0.586	0.585	0.584	0.582	0.581	0.579	0.578	0.577	0.578	0.581	0.585	0.59	0.596	0.602	0.608
Wolverhampton	0.544	0.546	0.547	0.547	0.548	0.547	0.546	0.544	0.542	0.541	0.539	0.537	0.536	0.536	0.539	0.543	0.548	0.554	0.56	0.566
Worcestershire	0.556	0.558	0.559	0.56	0.561	0.561	0.56	0.559	0.557	0.556	0.555	0.554	0.553	0.554	0.557	0.561	0.567	0.573	0.579	0.585
Yorkshire and the Humber	0.554	0.556	0.557	0.559	0.559	0.559	0.558	0.557	0.556	0.555	0.553	0.552	0.552	0.553	0.556	0.56	0.565	0.571	0.577	0.584
Barnsley	0.524	0.526	0.527	0.528	0.529	0.528	0.527	0.526	0.525	0.523	0.522	0.52	0.52	0.521	0.524	0.528	0.533	0.539	0.546	0.552
Bradford	0.534	0.537	0.538	0.539	0.539	0.539	0.537	0.536	0.534	0.532	0.53	0.528	0.526	0.527	0.53	0.534	0.54	0.546	0.553	0.559
Calderdale	0.55	0.552	0.553	0.555	0.555	0.555	0.554	0.552	0.551	0.549	0.547	0.546	0.545	0.545	0.548	0.553	0.558	0.565	0.571	0.578
Doncaster	0.524	0.526	0.527	0.528	0.529	0.529	0.527	0.526	0.524	0.522	0.521	0.519	0.518	0.519	0.522	0.526	0.531	0.538	0.544	0.55
East Riding of Yorkshire	0.559	0.562	0.563	0.564	0.565	0.565	0.564	0.563	0.562	0.561	0.56	0.559	0.559	0.56	0.563	0.567	0.572	0.579	0.585	0.591
Kingston upon Hull, City of	0.532	0.534	0.535	0.536	0.537	0.537	0.535	0.534	0.532	0.531	0.529	0.528	0.527	0.528	0.531	0.535	0.541	0.547	0.554	0.56

Appendix Table 8. Socio-Demographic Index values for all estimated GBD 2017 locations, 1950-1969

Location	1950	1951	1952	1953	1954	1955	1956	1957	1958	1959	1960	1961	1962	1963	1964	1965	1966	1967	1968	1969
Kirklees	0.543	0.545	0.546	0.548	0.548	0.548	0.547	0.546	0.545	0.543	0.542	0.54	0.54	0.541	0.544	0.548	0.553	0.56	0.566	0.572
Leeds	0.579	0.581	0.582	0.583	0.584	0.584	0.583	0.583	0.582	0.581	0.58	0.579	0.579	0.58	0.583	0.587	0.592	0.598	0.604	0.61
North East Lincolnshire	0.547	0.549	0.55	0.551	0.552	0.551	0.549	0.547	0.545	0.543	0.541	0.538	0.537	0.538	0.54	0.545	0.55	0.557	0.564	0.57
North Lincolnshire	0.554	0.556	0.557	0.558	0.558	0.558	0.556	0.555	0.553	0.551	0.549	0.547	0.546	0.547	0.549	0.553	0.559	0.565	0.572	0.578
North Yorkshire	0.569	0.571	0.573	0.574	0.575	0.575	0.574	0.573	0.572	0.571	0.569	0.568	0.568	0.569	0.572	0.576	0.582	0.588	0.594	0.6
Rotherham	0.531	0.533	0.534	0.535	0.536	0.536	0.534	0.533	0.532	0.53	0.528	0.527	0.526	0.527	0.53	0.534	0.539	0.546	0.552	0.558
Sheffield	0.569	0.571	0.572	0.573	0.574	0.574	0.574	0.573	0.573	0.572	0.571	0.571	0.571	0.572	0.575	0.58	0.585	0.591	0.596	0.602
Wakefield	0.539	0.542	0.543	0.544	0.545	0.544	0.543	0.542	0.54	0.539	0.537	0.536	0.535	0.536	0.539	0.543	0.548	0.555	0.561	0.567
York	0.601	0.603	0.605	0.606	0.607	0.607	0.606	0.606	0.605	0.605	0.604	0.604	0.604	0.606	0.609	0.613	0.618	0.623	0.629	0.635
Northern Ireland	0.502	0.505	0.506	0.508	0.508	0.509	0.509	0.511	0.512	0.513	0.514	0.515	0.517	0.52	0.525	0.533	0.541	0.549	0.557	0.565
Scotland	0.508	0.51	0.51	0.51	0.511	0.511	0.509	0.508	0.508	0.509	0.509	0.509	0.509	0.511	0.514	0.519	0.523	0.527	0.533	0.538
Wales	0.465	0.467	0.468	0.469	0.47	0.47	0.469	0.468	0.467	0.466	0.464	0.463	0.463	0.464	0.467	0.471	0.477	0.483	0.489	0.496
Latin America and Caribbean	0.28	0.284	0.287	0.29	0.293	0.296	0.3	0.303	0.306	0.308	0.312	0.316	0.321	0.326	0.331	0.338	0.345	0.352	0.359	0.366
Andean Latin America	0.256	0.264	0.272	0.278	0.28	0.282	0.285	0.288	0.289	0.291	0.293	0.296	0.301	0.306	0.312	0.32	0.329	0.337	0.344	0.349
Bolivia	0.235	0.245	0.251	0.255	0.258	0.264	0.268	0.265	0.258	0.253	0.252	0.253	0.256	0.26	0.265	0.272	0.279	0.285	0.29	0.295
Ecuador	0.288	0.3	0.313	0.323	0.325	0.321	0.319	0.322	0.326	0.329	0.327	0.325	0.328	0.333	0.339	0.35	0.362	0.37	0.38	0.385
Peru	0.244	0.248	0.252	0.255	0.259	0.263	0.268	0.272	0.277	0.281	0.286	0.293	0.299	0.305	0.31	0.316	0.324	0.332	0.338	0.341
Caribbean	0.344	0.348	0.35	0.35	0.35	0.351	0.353	0.355	0.355	0.354	0.354	0.355	0.357	0.359	0.364	0.374	0.387	0.398	0.403	0.407
Antigua and Barbuda	0.331	0.334	0.335	0.336	0.336	0.337	0.341	0.346	0.352	0.359	0.367	0.375	0.384	0.392	0.402	0.411	0.421	0.433	0.444	0.456
The Bahamas	0.446	0.45	0.454	0.46	0.468	0.475	0.481	0.489	0.498	0.504	0.504	0.502	0.501	0.505	0.512	0.519	0.526	0.534	0.543	0.553
Barbados	0.433	0.428	0.422	0.416	0.415	0.42	0.427	0.431	0.432	0.434	0.442	0.455	0.464	0.47	0.473	0.479	0.489	0.499	0.507	0.515
Belize	0.274	0.276	0.276	0.275	0.274	0.272	0.27	0.267	0.264	0.261	0.258	0.255	0.253	0.252	0.252	0.254	0.259	0.264	0.269	0.273
Bermuda	0.451	0.458	0.463	0.468	0.473	0.478	0.484	0.489	0.496	0.502	0.508	0.514	0.522	0.533	0.545	0.556	0.568	0.58	0.591	0.6
Cuba	0.412	0.417	0.418	0.415	0.413	0.414	0.414	0.414	0.41	0.402	0.392	0.382	0.376	0.375	0.38	0.393	0.412	0.428	0.429	0.428
Dominica	0.322	0.322	0.319	0.314	0.307	0.303	0.299	0.295	0.294	0.294	0.298	0.304	0.31	0.312	0.312	0.314	0.318	0.322	0.329	0.339
Dominican Republic	0.208	0.213	0.215	0.215	0.214	0.217	0.221	0.225	0.227	0.228	0.234	0.244	0.25	0.251	0.253	0.258	0.267	0.275	0.281	0.285
Grenada	0.227	0.225	0.222	0.217	0.211	0.207	0.206	0.21	0.22	0.232	0.246	0.26	0.273	0.284	0.295	0.303	0.311	0.317	0.323	0.329
Guyana	0.28	0.282	0.284	0.285	0.285	0.285	0.285	0.285	0.286	0.287	0.291	0.297	0.305	0.313	0.323	0.335	0.348	0.362	0.374	0.384
Haiti	0.206	0.208	0.211	0.214	0.217	0.22	0.223	0.226	0.23	0.233	0.237	0.24	0.244	0.248	0.251	0.254	0.258	0.261	0.264	0.267
Jamaica	0.402	0.404	0.404	0.404	0.402	0.399	0.396	0.394	0.39	0.386	0.382	0.377	0.374	0.372	0.37	0.372	0.377	0.385	0.393	0.399
Puerto Rico	0.401	0.409	0.419	0.425	0.423	0.421	0.426	0.433	0.44	0.448	0.457	0.465	0.471	0.476	0.485	0.501	0.522	0.54	0.552	0.559
Saint Lucia	0.317	0.321	0.322	0.32	0.316	0.312	0.307	0.303	0.3	0.3	0.302	0.304	0.306	0.308	0.309	0.311	0.314	0.316	0.319	0.324
Saint Vincent and the Grenadines	0.26	0.258	0.252	0.238	0.22	0.199	0.18	0.171	0.169	0.173	0.185	0.202	0.213	0.216	0.218	0.226	0.24	0.255	0.266	0.271
Suriname	0.314	0.311	0.309	0.308	0.308	0.309	0.309	0.307	0.306	0.306	0.309	0.318	0.335	0.349	0.356	0.361	0.366	0.371	0.377	0.385
Trinidad and Tobago	0.363	0.366	0.366	0.365	0.365	0.366	0.367	0.368	0.371	0.374	0.38	0.388	0.397	0.409	0.423	0.436	0.45	0.463	0.476	0.487
Virgin Islands	0.453	0.456	0.457	0.453	0.446	0.439	0.431	0.424	0.419	0.419	0.424	0.429	0.433	0.437	0.442	0.45	0.46	0.471	0.485	0.499
Central Latin America	0.275	0.277	0.28	0.282	0.285	0.288	0.292	0.294	0.296	0.298	0.301	0.306	0.311	0.316	0.322	0.329	0.336	0.344	0.353	0.36
Colombia	0.28	0.281	0.283	0.285	0.287	0.287	0.287	0.287	0.287	0.287	0.288	0.291	0.296	0.301	0.308	0.315	0.324	0.333	0.342	0.351
Costa Rica	0.308	0.313	0.317	0.316	0.312	0.313	0.315	0.314	0.307	0.303	0.306	0.314	0.325	0.339	0.352	0.364	0.376	0.39	0.405	0.417
El Salvador	0.22	0.222	0.224	0.226	0.228	0.23	0.233	0.236	0.239	0.242	0.245	0.249	0.253	0.257	0.262	0.267	0.272	0.278	0.284	0.29
Guatemala	0.203	0.208	0.211	0.213	0.219	0.226	0.228	0.229	0.23	0.229	0.23	0.235	0.239	0.238	0.244	0.253	0.259	0.264	0.269	0.273
Honduras	0.17	0.173	0.176	0.18	0.182	0.185	0.189	0.193	0.198	0.202	0.204	0.206	0.207	0.208	0.211	0.215	0.219	0.224	0.23	0.236
Mexico	0.271	0.274	0.278	0.281	0.284	0.289	0.293	0.297	0.301	0.306	0.31	0.315	0.32	0.325	0.331	0.337	0.344	0.351	0.358	0.365
Aguascalientes	0.29	0.294	0.297	0.3	0.303	0.308	0.312	0.317	0.321	0.326	0.332	0.339	0.345	0.351	0.356	0.363	0.371	0.379	0.387	0.394
Baja California	0.297	0.302	0.306	0.309	0.313	0.318	0.322	0.327	0.331	0.336	0.341	0.347	0.352	0.359	0.366	0.373	0.381	0.39	0.399	0.408
Baja California Sur	0.303	0.307	0.31	0.313	0.317	0.321	0.325	0.329	0.332	0.336	0.34	0.345	0.35	0.356	0.361	0.368	0.375	0.383	0.392	0.4
Campeche	0.267	0.271	0.275	0.278	0.282	0.286	0.29	0.294	0.297	0.301	0.304	0.308	0.311	0.315	0.32	0.324	0.33	0.335	0.341	0.346
Chiapas	0.22	0.223	0.226	0.229	0.232	0.236	0.239	0.243	0.246	0.25	0.253	0.257	0.261	0.265	0.27	0.275	0.28	0.286	0.291	0.297
Chihuahua	0.271	0.275	0.279	0.282	0.286	0.29	0.294	0.299	0.303	0.307	0.312	0.318	0.323	0.329	0.335	0.342	0.349	0.357	0.365	0.373
Coahuila	0.295	0.299	0.303	0.307	0.311	0.315	0.319	0.324	0.328	0.333	0.338	0.343	0.348	0.354	0.36	0.367	0.373	0.38	0.387	0.394
Colima	0.272	0.275	0.279	0.282	0.286	0.291	0.295	0.299	0.304	0.308	0.314	0.319	0.325	0.33	0.337	0.344	0.351	0.358	0.366	0.373
Mexico City	0.313	0.318	0.322	0.325	0.33	0.335	0.34	0.345	0.351	0.357	0.362	0.368	0.374	0.381	0.389	0.397	0.404	0.415	0.426	0.436
Durango	0.253	0.256	0.259	0.262	0.265	0.269	0.273	0.277	0.281	0.285	0.29	0.296	0.301	0.306	0.312	0.319	0.326	0.333	0.339	0.346
Guanajuato	0.244	0.247	0.25	0.252	0.256	0.26	0.264	0.268	0.272	0.276	0.281	0.286	0.291	0.297	0.302	0.308	0.315	0.321	0.328	0.334
Guerrero	0.203	0.207	0.21	0.213	0.216	0.22	0.224	0.228	0.232	0.236	0.24	0.244	0.249	0.253	0.259	0.264	0.27	0.276	0.282	0.287
Hidalgo	0.192	0.195	0.198	0.2	0.204	0.207	0.21	0.214	0.218	0.223	0.227	0.23	0.235	0.24	0.246	0.252	0.257	0.263	0.269	0.275
Jalisco	0.278	0.282	0.286	0.289	0.293	0.298	0.303	0.307	0.312	0.317	0.323	0.329	0.335	0.341	0.347	0.354	0.361	0.368	0.375	0.382
México	0.272	0.276	0.279	0.282	0.286	0.291	0.295	0.3	0.305	0.31	0.315	0.321	0.326	0.332	0.339	0.345	0.35	0.356	0.363	0.369
Michoacán de Ocampo	0.226	0.229	0.232	0.235	0.239	0.243	0.247	0.251	0.255	0.26	0.265	0.27	0.275	0.281	0.287	0.293	0.3	0.307	0.313	0.319
Morelos	0.279	0.283	0.287	0.291	0.295	0.3	0.304	0.309	0.313	0.317	0.322	0.327	0.331	0.336	0.341	0.347	0.353	0.359	0.366	0.372
Nayarit	0.236	0.239	0.242	0.244	0.247	0.251	0.255	0.259	0.263	0.267	0.272	0.278	0.283	0.288	0.294	0.301	0.308	0.315	0.322	0.329
Nuevo León	0.317	0.321	0.325	0.328	0.332	0.337	0.341	0.345	0.349	0.354	0.359	0.364	0.37	0.376	0.383	0.39	0.398	0.407	0.416	0.425
Oaxaca	0.206	0.21	0.213	0.216	0.219	0.223	0.227	0.231	0.235	0.239	0.243	0.247	0.251	0.255	0.26	0.265	0.271	0.276	0.282	0.287
Puebla	0.217	0.22	0.223	0.226	0.23	0.234	0.237	0.242	0.246	0.251	0.255	0.259	0.264	0.27	0.276	0.282	0.287	0.293	0.3	0.307
Querétaro	0.253	0.256	0.259	0.262	0.266	0.27	0.274	0.279	0.283	0.288	0.293	0.298	0.304	0.31	0.316	0.323	0.33	0.337	0.345	0.353

Appendix Table 8. Socio-Demographic Index values for all estimated GBD 2017 locations, 1950-1969

Location	1950	1951	1952	1953	1954	1955	1956	1957	1958	1959	1960	1961	1962	1963	1964	1965	1966	1967	1968	1969
Quintana Roo	0.301	0.305	0.309	0.313	0.318	0.322	0.326	0.33	0.334	0.339	0.344	0.349	0.354	0.359	0.365	0.371	0.378	0.385	0.391	0.395
San Luis Potosí	0.235	0.239	0.242	0.245	0.249	0.253	0.257	0.261	0.266	0.27	0.275	0.28	0.285	0.29	0.296	0.302	0.309	0.316	0.323	0.329
Sinaloa	0.265	0.268	0.271	0.274	0.277	0.281	0.285	0.288	0.292	0.296	0.301	0.306	0.311	0.316	0.322	0.328	0.335	0.342	0.348	0.355
Sonora	0.279	0.283	0.286	0.289	0.293	0.298	0.302	0.306	0.31	0.315	0.32	0.326	0.331	0.338	0.345	0.352	0.36	0.368	0.377	0.386
Tabasco	0.234	0.237	0.241	0.244	0.247	0.251	0.255	0.259	0.262	0.266	0.27	0.274	0.278	0.282	0.287	0.292	0.299	0.305	0.311	0.316
Tamaulipas	0.286	0.29	0.293	0.297	0.301	0.306	0.31	0.315	0.319	0.324	0.329	0.335	0.34	0.346	0.353	0.36	0.368	0.376	0.384	0.393
Tlaxcala	0.225	0.228	0.232	0.235	0.239	0.243	0.247	0.252	0.257	0.262	0.267	0.272	0.278	0.283	0.289	0.295	0.302	0.308	0.315	0.321
Veracruz de Ignacio de la Llave	0.236	0.24	0.244	0.247	0.25	0.255	0.258	0.263	0.267	0.271	0.275	0.279	0.283	0.288	0.294	0.299	0.305	0.311	0.317	0.323
Yucatán	0.274	0.278	0.281	0.284	0.288	0.293	0.297	0.301	0.306	0.311	0.315	0.318	0.322	0.327	0.332	0.337	0.341	0.346	0.352	0.357
Zacatecas	0.221	0.225	0.227	0.23	0.233	0.237	0.24	0.244	0.248	0.252	0.258	0.263	0.269	0.274	0.28	0.287	0.294	0.301	0.307	0.313
Nicaragua	0.217	0.219	0.221	0.221	0.22	0.219	0.218	0.216	0.215	0.214	0.215	0.217	0.221	0.224	0.227	0.232	0.237	0.243	0.249	0.257
Panama	0.323	0.322	0.322	0.324	0.327	0.33	0.332	0.337	0.342	0.346	0.347	0.348	0.349	0.351	0.354	0.36	0.367	0.374	0.382	0.391
Venezuela	0.342	0.34	0.337	0.333	0.336	0.344	0.351	0.353	0.345	0.334	0.334	0.345	0.356	0.362	0.37	0.379	0.384	0.396	0.412	0.422
Tropical Latin America	0.261	0.265	0.269	0.273	0.277	0.281	0.285	0.289	0.294	0.298	0.303	0.308	0.314	0.32	0.325	0.331	0.336	0.342	0.348	0.355
Brazil	0.26	0.264	0.268	0.272	0.276	0.28	0.284	0.289	0.293	0.297	0.302	0.308	0.314	0.319	0.325	0.33	0.336	0.342	0.348	0.354
Acre	0.172	0.175	0.177	0.18	0.182	0.185	0.187	0.189	0.192	0.195	0.199	0.203	0.208	0.213	0.218	0.223	0.228	0.232	0.237	0.242
Alagoas	0.149	0.151	0.154	0.156	0.159	0.161	0.163	0.166	0.168	0.171	0.174	0.177	0.181	0.184	0.188	0.191	0.195	0.2	0.205	0.209
Amapá	0.234	0.238	0.242	0.245	0.249	0.253	0.257	0.26	0.264	0.269	0.274	0.279	0.285	0.29	0.295	0.3	0.305	0.309	0.313	0.318
Amazonas	0.189	0.192	0.195	0.198	0.201	0.203	0.206	0.208	0.211	0.214	0.218	0.223	0.229	0.234	0.24	0.245	0.251	0.256	0.262	0.268
Bahia	0.184	0.187	0.19	0.193	0.196	0.2	0.203	0.206	0.209	0.213	0.217	0.222	0.226	0.231	0.235	0.24	0.244	0.248	0.252	0.256
Ceará	0.194	0.197	0.2	0.202	0.206	0.209	0.212	0.216	0.219	0.223	0.228	0.233	0.238	0.243	0.248	0.253	0.258	0.263	0.268	0.273
Distrito Federal	0.372	0.376	0.382	0.387	0.392	0.397	0.403	0.408	0.413	0.419	0.426	0.432	0.439	0.446	0.454	0.461	0.468	0.475	0.483	0.491
Espírito Santo	0.257	0.26	0.263	0.267	0.27	0.274	0.278	0.281	0.286	0.29	0.296	0.303	0.31	0.316	0.323	0.33	0.337	0.344	0.351	0.358
Goiás	0.215	0.218	0.222	0.225	0.228	0.232	0.235	0.239	0.243	0.246	0.251	0.256	0.261	0.266	0.271	0.276	0.282	0.288	0.294	0.3
Maranhão	0.127	0.129	0.132	0.134	0.136	0.138	0.14	0.142	0.144	0.147	0.15	0.154	0.158	0.162	0.167	0.17	0.173	0.176	0.179	0.183
Mato Grosso	0.232	0.235	0.238	0.242	0.245	0.248	0.252	0.255	0.258	0.262	0.266	0.27	0.275	0.28	0.285	0.29	0.296	0.302	0.31	0.318
Mato Grosso do Sul	0.229	0.232	0.235	0.237	0.24	0.243	0.246	0.249	0.253	0.256	0.262	0.268	0.274	0.28	0.286	0.292	0.299	0.306	0.314	0.321
Minas Gerais	0.26	0.264	0.268	0.272	0.276	0.28	0.284	0.288	0.293	0.297	0.302	0.307	0.313	0.318	0.323	0.329	0.334	0.339	0.345	0.351
Pará	0.204	0.207	0.21	0.214	0.217	0.22	0.224	0.227	0.23	0.234	0.239	0.244	0.249	0.254	0.259	0.263	0.268	0.273	0.277	0.282
Paraíba	0.172	0.175	0.178	0.181	0.184	0.187	0.19	0.192	0.195	0.198	0.202	0.206	0.21	0.214	0.218	0.221	0.225	0.229	0.234	0.238
Paraná	0.267	0.27	0.274	0.278	0.282	0.286	0.29	0.294	0.298	0.302	0.306	0.311	0.315	0.32	0.324	0.328	0.333	0.339	0.345	0.351
Pernambuco	0.19	0.193	0.196	0.199	0.202	0.205	0.209	0.212	0.215	0.219	0.223	0.228	0.232	0.237	0.242	0.246	0.251	0.256	0.262	0.267
Piauí	0.162	0.164	0.167	0.169	0.171	0.174	0.176	0.179	0.182	0.185	0.189	0.193	0.198	0.202	0.206	0.21	0.214	0.218	0.222	0.227
Rio de Janeiro	0.342	0.347	0.352	0.357	0.362	0.367	0.373	0.378	0.383	0.389	0.395	0.402	0.409	0.416	0.422	0.429	0.436	0.442	0.449	0.456
Rio Grande do Norte	0.184	0.187	0.19	0.193	0.196	0.199	0.202	0.205	0.208	0.211	0.215	0.219	0.223	0.227	0.231	0.235	0.24	0.244	0.25	0.254
Rio Grande do Sul	0.314	0.318	0.323	0.327	0.331	0.336	0.34	0.345	0.349	0.354	0.36	0.366	0.372	0.378	0.384	0.391	0.398	0.405	0.412	0.419
Rondônia	0.199	0.202	0.205	0.207	0.21	0.214	0.216	0.219	0.222	0.226	0.23	0.235	0.241	0.246	0.251	0.256	0.262	0.267	0.272	0.278
Roraima	0.205	0.208	0.212	0.215	0.219	0.222	0.225	0.228	0.232	0.236	0.241	0.246	0.252	0.258	0.263	0.269	0.275	0.28	0.285	0.29
Santa Catarina	0.296	0.3	0.304	0.308	0.312	0.316	0.32	0.325	0.329	0.334	0.34	0.347	0.353	0.359	0.365	0.371	0.378	0.384	0.39	0.397
São Paulo	0.323	0.327	0.332	0.337	0.341	0.346	0.351	0.356	0.361	0.366	0.372	0.378	0.384	0.39	0.397	0.403	0.409	0.416	0.423	0.429
Sergipe	0.196	0.199	0.202	0.205	0.208	0.212	0.215	0.218	0.221	0.225	0.229	0.233	0.238	0.242	0.246	0.251	0.255	0.259	0.264	0.269
Tocantins	0.175	0.178	0.181	0.185	0.188	0.191	0.193	0.196	0.199	0.202	0.205	0.209	0.214	0.218	0.222	0.226	0.229	0.232	0.236	0.239
Paraguay	0.285	0.293	0.3	0.307	0.312	0.317	0.319	0.32	0.32	0.318	0.318	0.319	0.321	0.323	0.326	0.33	0.334	0.338	0.341	0.346
North Africa and Middle East	0.182	0.186	0.19	0.195	0.199	0.204	0.209	0.214	0.219	0.224	0.23	0.236	0.242	0.248	0.255	0.261	0.269	0.276	0.283	0.29
North Africa and Middle East	0.182	0.186	0.19	0.195	0.199	0.204	0.209	0.214	0.219	0.224	0.23	0.236	0.242	0.248	0.255	0.261	0.269	0.276	0.283	0.29
Afghanistan	0.078	0.08	0.081	0.083	0.085	0.087	0.089	0.09	0.092	0.094	0.096	0.098	0.1	0.101	0.103	0.105	0.106	0.108	0.11	0.111
Algeria	0.194	0.198	0.202	0.207	0.212	0.217	0.222	0.228	0.234	0.241	0.249	0.255	0.26	0.265	0.271	0.278	0.283	0.288	0.293	0.298
Bahrain	0.226	0.232	0.238	0.244	0.25	0.257	0.264	0.272	0.28	0.289	0.298	0.309	0.321	0.334	0.347	0.36	0.372	0.384	0.393	0.404
Egypt	0.178	0.182	0.186	0.189	0.191	0.193	0.196	0.198	0.201	0.204	0.209	0.215	0.221	0.227	0.234	0.242	0.25	0.259	0.267	0.275
Iran	0.166	0.17	0.174	0.178	0.182	0.186	0.192	0.199	0.206	0.213	0.222	0.235	0.246	0.254	0.261	0.27	0.281	0.293	0.302	0.311
Iraq	0.151	0.155	0.159	0.165	0.172	0.177	0.183	0.188	0.194	0.2	0.207	0.214	0.221	0.227	0.234	0.241	0.247	0.254	0.26	0.267
Jordan	0.054	0.056	0.057	0.059	0.06	0.077	0.106	0.129	0.148	0.16	0.167	0.177	0.195	0.214	0.228	0.24	0.252	0.263	0.276	0.289
Kuwait	0.278	0.286	0.294	0.303	0.311	0.319	0.328	0.334	0.337	0.336	0.336	0.332	0.324	0.321	0.327	0.331	0.33	0.328	0.345	0.38
Lebanon	0.154	0.157	0.161	0.166	0.172	0.177	0.183	0.188	0.194	0.199	0.204	0.21	0.217	0.224	0.232	0.242	0.251	0.261	0.272	0.283
Libya	0.183	0.19	0.198	0.204	0.211	0.219	0.227	0.236	0.243	0.25	0.259	0.267	0.275	0.281	0.288	0.296	0.304	0.312	0.32	0.323
Morocco	0.136	0.138	0.141	0.144	0.146	0.149	0.152	0.154	0.157	0.16	0.163	0.167	0.172	0.177	0.183	0.189	0.195	0.202	0.209	0.216
Palestine	0.097	0.101	0.105	0.109	0.114	0.118	0.123	0.129	0.134	0.14	0.147	0.153	0.16	0.167	0.175	0.183	0.191	0.198	0.206	0.215
Oman	0.141	0.144	0.148	0.152	0.157	0.162	0.166	0.171	0.176	0.182	0.187	0.193	0.2	0.206	0.213	0.219	0.225	0.234	0.247	0.259
Qatar	0.231	0.238	0.246	0.254	0.262	0.271	0.28	0.29	0.3	0.311	0.321	0.331	0.342	0.352	0.363	0.374	0.385	0.396	0.407	0.418
Saudi Arabia	0.14	0.143	0.147	0.15	0.154	0.157	0.161	0.165	0.168	0.172	0.177	0.181	0.186	0.19	0.194	0.2	0.205	0.21	0.215	0.22
Sudan	0.072	0.074	0.077	0.079	0.082	0.085	0.09	0.096	0.101	0.105	0.109	0.113	0.116	0.119	0.121	0.124	0.125	0.127	0.129	0.131
Syria	0.154	0.157	0.163	0.168	0.174	0.179	0.185	0.191	0.196	0.201	0.206	0.211	0.217	0.222	0.227	0.233	0.237	0.241	0.243	0.245
Tunisia	0.154	0.158	0.162	0.166	0.171	0.174	0.179	0.183	0.187	0.191	0.196	0.201	0.207	0.212	0.219	0.225	0.232	0.239	0.246	0.253
Turkey	0.237	0.244	0.25	0.257	0.262	0.268	0.274	0.281	0.287	0.293	0.298	0.303	0.308	0.313	0.319	0.324	0.329	0.334	0.339	0.343
United Arab Emirates	0.205	0.212	0.219	0.228	0.237	0.244	0.251	0.26	0.273	0.282	0.284	0.283	0.287	0.298	0.307	0.306	0.301	0.302	0.316	0.33

Appendix Table 8. Socio-Demographic Index values for all estimated GBD 2017 locations, 1950-1969

Location	1950	1951	1952	1953	1954	1955	1956	1957	1958	1959	1960	1961	1962	1963	1964	1965	1966	1967	1968	1969
Yemen	0.075	0.077	0.079	0.081	0.083	0.085	0.088	0.09	0.093	0.095	0.097	0.099	0.102	0.104	0.106	0.108	0.111	0.114	0.116	0.118
South Asia	0.171	0.173	0.175	0.177	0.18	0.182	0.183	0.184	0.185	0.186	0.188	0.19	0.192	0.195	0.197	0.199	0.202	0.204	0.208	0.214
South Asia	0.171	0.173	0.175	0.177	0.18	0.182	0.183	0.184	0.185	0.186	0.188	0.19	0.192	0.195	0.197	0.199	0.202	0.204	0.208	0.214
Bangladesh	0.045	0.046	0.046	0.047	0.047	0.047	0.048	0.048	0.048	0.049	0.049	0.05	0.05	0.051	0.051	0.052	0.053	0.053	0.053	0.054
Bhutan	0.112	0.115	0.118	0.122	0.126	0.13	0.134	0.138	0.142	0.146	0.151	0.156	0.161	0.165	0.17	0.175	0.181	0.186	0.191	0.197
India	0.175	0.177	0.18	0.182	0.185	0.187	0.189	0.189	0.19	0.191	0.194	0.196	0.199	0.201	0.204	0.206	0.209	0.212	0.215	0.22
Andhra Pradesh	0.14	0.142	0.143	0.145	0.147	0.148	0.148	0.146	0.144	0.143	0.143	0.145	0.146	0.146	0.146	0.146	0.148	0.149	0.152	0.158
Arunachal Pradesh	0.145	0.147	0.149	0.151	0.153	0.155	0.155	0.153	0.152	0.15	0.151	0.153	0.155	0.157	0.159	0.16	0.163	0.167	0.173	0.179
Assam	0.17	0.172	0.175	0.178	0.181	0.183	0.185	0.186	0.186	0.188	0.191	0.194	0.197	0.201	0.205	0.209	0.212	0.213	0.215	0.218
Bihar	0.153	0.155	0.157	0.159	0.162	0.164	0.166	0.167	0.168	0.17	0.172	0.174	0.177	0.179	0.181	0.183	0.185	0.187	0.189	0.193
Chhattisgarh	0.168	0.17	0.172	0.173	0.175	0.177	0.178	0.178	0.177	0.177	0.178	0.18	0.181	0.183	0.183	0.184	0.186	0.188	0.191	0.197
Delhi	0.295	0.3	0.304	0.31	0.315	0.319	0.323	0.324	0.326	0.329	0.334	0.34	0.346	0.351	0.356	0.361	0.367	0.374	0.381	0.387
Goa	0.278	0.282	0.285	0.291	0.295	0.299	0.302	0.305	0.308	0.311	0.316	0.32	0.324	0.328	0.332	0.336	0.34	0.346	0.352	0.359
Gujarat	0.189	0.192	0.194	0.197	0.2	0.202	0.204	0.206	0.207	0.208	0.211	0.214	0.217	0.22	0.223	0.226	0.229	0.231	0.235	0.239
Haryana	0.192	0.194	0.197	0.199	0.202	0.204	0.206	0.207	0.208	0.209	0.212	0.214	0.216	0.217	0.217	0.219	0.222	0.226	0.231	0.237
Himachal Pradesh	0.168	0.17	0.172	0.175	0.177	0.179	0.181	0.182	0.183	0.184	0.186	0.188	0.189	0.191	0.192	0.193	0.195	0.198	0.202	0.207
Jammu and Kashmir	0.176	0.178	0.181	0.183	0.186	0.189	0.191	0.193	0.195	0.197	0.2	0.203	0.205	0.208	0.21	0.212	0.215	0.218	0.222	0.228
Jharkhand	0.171	0.173	0.175	0.177	0.179	0.181	0.181	0.181	0.181	0.181	0.182	0.184	0.185	0.187	0.188	0.188	0.19	0.192	0.195	0.2
Karnataka	0.167	0.169	0.171	0.174	0.176	0.178	0.18	0.18	0.18	0.18	0.183	0.185	0.187	0.19	0.192	0.193	0.196	0.201	0.207	0.214
Kerala	0.231	0.235	0.238	0.244	0.248	0.252	0.256	0.259	0.263	0.267	0.272	0.277	0.281	0.286	0.291	0.296	0.3	0.305	0.308	0.312
Madhya Pradesh	0.148	0.15	0.152	0.154	0.156	0.158	0.159	0.158	0.158	0.157	0.159	0.161	0.162	0.163	0.163	0.165	0.166	0.167	0.168	0.172
Maharashtra	0.198	0.2	0.203	0.206	0.209	0.211	0.213	0.213	0.214	0.214	0.217	0.219	0.222	0.225	0.227	0.229	0.233	0.238	0.244	0.252
Manipur	0.184	0.187	0.19	0.193	0.196	0.199	0.201	0.203	0.205	0.207	0.21	0.214	0.218	0.222	0.225	0.229	0.232	0.236	0.24	0.246
Meghalaya	0.168	0.171	0.174	0.177	0.18	0.183	0.185	0.186	0.187	0.188	0.191	0.195	0.198	0.201	0.204	0.207	0.21	0.214	0.218	0.224
Mizoram	0.194	0.197	0.201	0.205	0.209	0.212	0.215	0.217	0.219	0.222	0.226	0.231	0.235	0.239	0.244	0.249	0.252	0.255	0.258	0.264
Nagaland	0.203	0.206	0.21	0.213	0.217	0.221	0.225	0.227	0.23	0.233	0.238	0.243	0.248	0.253	0.258	0.263	0.267	0.27	0.273	0.278
Odisha	0.147	0.149	0.151	0.153	0.155	0.157	0.158	0.159	0.16	0.16	0.162	0.165	0.167	0.169	0.171	0.173	0.175	0.178	0.181	0.185
Punjab	0.218	0.221	0.224	0.228	0.232	0.235	0.238	0.241	0.244	0.247	0.251	0.255	0.258	0.261	0.265	0.269	0.273	0.278	0.283	0.289
Rajasthan	0.131	0.132	0.134	0.136	0.138	0.14	0.14	0.14	0.14	0.139	0.141	0.143	0.144	0.146	0.147	0.147	0.149	0.152	0.156	0.161
Sikkim	0.16	0.163	0.165	0.167	0.17	0.171	0.173	0.173	0.173	0.173	0.176	0.178	0.181	0.183	0.186	0.188	0.191	0.194	0.198	0.204
Tamil Nadu	0.189	0.192	0.194	0.198	0.201	0.203	0.205	0.207	0.208	0.21	0.213	0.216	0.219	0.222	0.225	0.228	0.23	0.233	0.238	0.244
Telangana	0.151	0.153	0.155	0.157	0.16	0.161	0.162	0.163	0.163	0.163	0.165	0.167	0.168	0.17	0.171	0.172	0.174	0.176	0.179	0.184
Tripura	0.164	0.167	0.17	0.173	0.176	0.178	0.179	0.178	0.177	0.176	0.179	0.182	0.185	0.189	0.192	0.194	0.198	0.201	0.206	0.212
Uttar Pradesh	0.152	0.154	0.156	0.158	0.161	0.163	0.164	0.165	0.166	0.168	0.17	0.172	0.175	0.177	0.179	0.181	0.183	0.185	0.188	0.192
Uttarakhand	0.183	0.185	0.187	0.189	0.191	0.193	0.194	0.194	0.194	0.195	0.196	0.198	0.2	0.201	0.202	0.203	0.204	0.206	0.21	0.215
West Bengal	0.19	0.193	0.196	0.199	0.202	0.204	0.205	0.205	0.204	0.204	0.207	0.21	0.213	0.217	0.22	0.223	0.226	0.226	0.227	0.231
Union Territories other than Delhi	0.236	0.24	0.243	0.245	0.249	0.252	0.253	0.254	0.253	0.253	0.256	0.259	0.262	0.265	0.267	0.269	0.273	0.277	0.283	0.291
Nepal	0.118	0.12	0.122	0.124	0.126	0.127	0.129	0.131	0.133	0.135	0.137	0.139	0.141	0.142	0.145	0.147	0.149	0.151	0.153	0.154
Pakistan	0.185	0.186	0.188	0.19	0.192	0.194	0.196	0.198	0.2	0.202	0.205	0.207	0.21	0.213	0.217	0.221	0.225	0.229	0.233	0.237
Southeast Asia, East Asia, and Oceania	0.19	0.194	0.199	0.204	0.21	0.215	0.219	0.223	0.227	0.232	0.236	0.239	0.242	0.247	0.253	0.259	0.264	0.269	0.275	0.284
East Asia	0.17	0.175	0.182	0.188	0.194	0.199	0.203	0.208	0.212	0.217	0.222	0.222	0.224	0.227	0.233	0.239	0.245	0.249	0.255	0.265
China	0.165	0.171	0.177	0.183	0.189	0.194	0.198	0.202	0.207	0.212	0.216	0.216	0.218	0.221	0.226	0.233	0.238	0.242	0.248	0.258
North Korea	0.259	0.257	0.26	0.268	0.276	0.283	0.29	0.297	0.304	0.311	0.318	0.325	0.331	0.339	0.345	0.352	0.361	0.37	0.379	0.389
Taiwan (Province of China)	0.345	0.345	0.357	0.366	0.368	0.371	0.381	0.39	0.391	0.392	0.397	0.4	0.406	0.418	0.43	0.443	0.456	0.467	0.474	0.479
Oceania	0.253	0.257	0.26	0.263	0.266	0.27	0.273	0.276	0.279	0.283	0.286	0.29	0.294	0.298	0.303	0.307	0.312	0.316	0.32	0.325
American Samoa	0.452	0.458	0.465	0.471	0.477	0.483	0.488	0.491	0.493	0.495	0.497	0.497	0.497	0.497	0.498	0.501	0.505	0.512	0.52	0.529
Federated States of Micronesia	0.249	0.251	0.254	0.257	0.26	0.262	0.265	0.269	0.272	0.275	0.278	0.282	0.285	0.289	0.294	0.298	0.303	0.308	0.313	0.319
Fiji	0.313	0.315	0.317	0.32	0.321	0.323	0.324	0.326	0.328	0.331	0.335	0.341	0.348	0.358	0.369	0.377	0.386	0.394	0.402	0.409
Guam	0.551	0.549	0.542	0.531	0.517	0.501	0.487	0.478	0.467	0.458	0.463	0.482	0.505	0.526	0.544	0.559	0.573	0.583	0.59	0.592
Kiribati	0.249	0.251	0.253	0.255	0.257	0.259	0.261	0.264	0.266	0.268	0.27	0.274	0.277	0.281	0.285	0.289	0.293	0.297	0.301	0.306
Marshall Islands	0.221	0.224	0.227	0.229	0.232	0.235	0.237	0.24	0.242	0.244	0.247	0.249	0.252	0.254	0.256	0.258	0.26	0.262	0.264	0.267
Northern Mariana Islands	0.457	0.463	0.47	0.476	0.483	0.489	0.496	0.503	0.51	0.517	0.524	0.531	0.538	0.544	0.549	0.554	0.56	0.566	0.572	0.578
Papua New Guinea	0.186	0.189	0.192	0.195	0.199	0.202	0.205	0.209	0.212	0.215	0.218	0.221	0.224	0.227	0.231	0.234	0.238	0.242	0.246	0.25
Samoa	0.311	0.315	0.318	0.32	0.322	0.324	0.327	0.331	0.335	0.34	0.346	0.353	0.361	0.368	0.374	0.38	0.386	0.393	0.398	0.403
Solomon Islands	0.181	0.183	0.185	0.187	0.19	0.192	0.195	0.197	0.2	0.203	0.206	0.209	0.212	0.216	0.22	0.223	0.227	0.231	0.235	0.238
Tonga	0.278	0.282	0.287	0.292	0.297	0.302	0.307	0.313	0.318	0.322	0.328	0.333	0.339	0.344	0.349	0.354	0.359	0.363	0.368	0.372
Vanuatu	0.216	0.218	0.221	0.224	0.227	0.23	0.234	0.237	0.241	0.244	0.248	0.251	0.256	0.26	0.265	0.269	0.274	0.278	0.283	0.287
Southeast Asia	0.216	0.22	0.224	0.229	0.233	0.237	0.241	0.245	0.25	0.255	0.26	0.265	0.27	0.276	0.282	0.287	0.293	0.298	0.304	0.31
Cambodia	0.15	0.152	0.154	0.157	0.16	0.162	0.165	0.169	0.172	0.176	0.18	0.183	0.187	0.191	0.194	0.197	0.2	0.203	0.206	0.209
Indonesia	0.194	0.198	0.202	0.205	0.209	0.213	0.217	0.221	0.225	0.229	0.233	0.237	0.241	0.245	0.25	0.253	0.257	0.262	0.267	0.273
Laos	0.146	0.148	0.151	0.153	0.156	0.158	0.16	0.163	0.166	0.168	0.171	0.174	0.177	0.18	0.183	0.186	0.189	0.192	0.195	0.198
Malaysia	0.275	0.278	0.281	0.284	0.287	0.288	0.289	0.292	0.299	0.309	0.31	0.306	0.318	0.332	0.345	0.355	0.368	0.386	0.397	0.405
Maldives	0.184	0.186	0.188	0.191	0.193	0.197	0.201	0.206	0.211	0.216	0.221	0.228	0.234	0.238	0.237	0.235	0.234	0.234	0.233	0.233
Mauritius	0.247	0.252	0.259	0.265	0.269	0.272	0.275	0.28	0.284	0.288	0.29	0.294	0.298	0.306	0.316	0.329	0.343	0.357	0.369	0.38
Myanmar	0.093	0.104	0.108	0.112	0.113	0.117	0.121	0.126	0.13	0.136	0.142	0.147	0.153	0.159	0.164	0.169	0.173	0.175	0.179	0.182

Appendix Table 8. Socio-Demographic Index values for all estimated GBD 2017 locations, 1950-1969																				
**Location**	**1950**	**1951**	**1952**	**1953**	**1954**	**1955**	**1956**	**1957**	**1958**	**1959**	**1960**	**1961**	**1962**	**1963**	**1964**	**1965**	**1966**	**1967**	**1968**	**1969**
Philippines	0.298	0.304	0.31	0.317	0.324	0.33	0.336	0.343	0.352	0.36	0.367	0.374	0.382	0.388	0.393	0.398	0.402	0.406	0.41	0.414
Sri Lanka	0.28	0.286	0.29	0.295	0.3	0.305	0.309	0.313	0.316	0.32	0.324	0.328	0.333	0.339	0.345	0.352	0.36	0.368	0.375	0.38
Seychelles	0.297	0.302	0.305	0.307	0.309	0.31	0.311	0.313	0.314	0.315	0.316	0.316	0.318	0.321	0.325	0.329	0.333	0.339	0.345	0.351
Thailand	0.245	0.249	0.252	0.256	0.259	0.263	0.267	0.271	0.276	0.281	0.286	0.291	0.296	0.302	0.309	0.315	0.322	0.328	0.333	0.338
Timor-Leste	0.082	0.085	0.089	0.092	0.095	0.098	0.102	0.105	0.108	0.113	0.117	0.12	0.125	0.129	0.134	0.139	0.144	0.149	0.155	0.161
Vietnam	0.194	0.197	0.201	0.205	0.209	0.213	0.217	0.221	0.226	0.23	0.235	0.24	0.246	0.252	0.258	0.264	0.269	0.273	0.276	0.28
Sub-Saharan Africa	0.14	0.143	0.146	0.149	0.152	0.155	0.158	0.161	0.165	0.168	0.172	0.176	0.18	0.184	0.188	0.191	0.195	0.199	0.203	0.207
Central sub-Saharan Africa	0.118	0.121	0.123	0.126	0.128	0.13	0.132	0.135	0.137	0.139	0.142	0.144	0.148	0.151	0.154	0.158	0.163	0.167	0.172	0.176
Angola	0.11	0.111	0.112	0.113	0.112	0.112	0.111	0.11	0.11	0.11	0.11	0.11	0.111	0.111	0.111	0.111	0.113	0.115	0.118	0.122
Central African Republic	0.107	0.11	0.112	0.114	0.116	0.118	0.121	0.122	0.124	0.126	0.128	0.131	0.134	0.135	0.137	0.139	0.143	0.145	0.147	0.149
Congo (Brazzaville)	0.137	0.14	0.144	0.147	0.151	0.155	0.158	0.162	0.166	0.17	0.174	0.179	0.183	0.188	0.193	0.197	0.202	0.207	0.211	0.216
DR Congo	0.111	0.114	0.117	0.12	0.123	0.126	0.129	0.132	0.135	0.138	0.141	0.143	0.147	0.151	0.154	0.158	0.163	0.167	0.172	0.177
Equatorial Guinea	0.055	0.057	0.057	0.057	0.056	0.053	0.049	0.04	0.032	0.033	0.034	0.036	0.037	0.039	0.04	0.042	0.043	0.045	0.046	0.048
Gabon	0.146	0.149	0.153	0.156	0.159	0.162	0.165	0.169	0.173	0.177	0.182	0.188	0.194	0.201	0.208	0.216	0.226	0.234	0.24	0.245
Eastern sub-Saharan Africa	0.099	0.102	0.103	0.105	0.107	0.11	0.112	0.115	0.118	0.12	0.123	0.126	0.129	0.133	0.135	0.138	0.141	0.144	0.148	0.152
Burundi	0.081	0.084	0.086	0.088	0.09	0.093	0.095	0.098	0.101	0.104	0.107	0.11	0.113	0.116	0.119	0.122	0.125	0.129	0.133	0.136
Comoros	0.131	0.133	0.134	0.135	0.136	0.137	0.137	0.137	0.138	0.139	0.14	0.141	0.143	0.145	0.147	0.148	0.149	0.15	0.151	0.153
Djibouti	0.14	0.142	0.145	0.147	0.15	0.154	0.158	0.163	0.167	0.17	0.172	0.174	0.176	0.178	0.18	0.181	0.184	0.187	0.19	0.194
Eritrea	0.053	0.056	0.058	0.06	0.063	0.066	0.069	0.071	0.074	0.077	0.08	0.084	0.087	0.09	0.094	0.097	0.101	0.104	0.108	0.112
Ethiopia	0.053	0.055	0.057	0.059	0.06	0.062	0.064	0.066	0.067	0.069	0.071	0.073	0.076	0.078	0.079	0.08	0.082	0.084	0.086	0.087
Kenya	0.08	0.085	0.089	0.094	0.099	0.104	0.11	0.116	0.122	0.126	0.13	0.134	0.14	0.146	0.151	0.155	0.161	0.169	0.179	0.19
Baringo	0.052	0.058	0.063	0.069	0.075	0.081	0.088	0.094	0.099	0.102	0.104	0.107	0.111	0.116	0.117	0.116	0.118	0.125	0.135	0.145
Bomet	0.047	0.048	0.049	0.05	0.051	0.052	0.053	0.054	0.055	0.056	0.057	0.058	0.059	0.06	0.061	0.062	0.063	0.087	0.112	0.133
Bungoma	0.047	0.049	0.05	0.05	0.052	0.053	0.055	0.071	0.082	0.088	0.094	0.101	0.109	0.117	0.123	0.127	0.133	0.143	0.156	0.169
Busia	0.049	0.05	0.051	0.052	0.053	0.054	0.055	0.056	0.066	0.071	0.074	0.08	0.088	0.101	0.111	0.12	0.129	0.143	0.159	0.175
Elgeyo Marakwet	0.057	0.061	0.065	0.069	0.074	0.078	0.084	0.089	0.094	0.097	0.1	0.103	0.108	0.112	0.116	0.118	0.123	0.131	0.141	0.152
Embu	0.095	0.1	0.103	0.107	0.111	0.116	0.12	0.126	0.131	0.136	0.141	0.146	0.151	0.157	0.163	0.168	0.174	0.182	0.191	0.2
Garissa	0.042	0.044	0.045	0.046	0.048	0.05	0.052	0.054	0.055	0.057	0.058	0.06	0.062	0.064	0.066	0.068	0.07	0.075	0.079	0.085
Homa Bay	0.041	0.042	0.043	0.044	0.045	0.046	0.047	0.048	0.049	0.049	0.05	0.051	0.052	0.053	0.054	0.055	0.061	0.09	0.113	0.132
Isiolo	0.101	0.104	0.106	0.109	0.112	0.115	0.118	0.122	0.126	0.129	0.132	0.135	0.139	0.143	0.148	0.152	0.157	0.164	0.172	0.18
Kajiado	0.149	0.155	0.159	0.163	0.168	0.173	0.179	0.184	0.19	0.194	0.198	0.202	0.207	0.212	0.215	0.218	0.222	0.228	0.235	0.243
Kakamega	0.047	0.049	0.049	0.05	0.051	0.052	0.066	0.077	0.086	0.092	0.097	0.104	0.111	0.119	0.126	0.131	0.138	0.149	0.162	0.175
Kericho	0.041	0.043	0.043	0.044	0.045	0.046	0.047	0.052	0.062	0.066	0.069	0.074	0.081	0.088	0.092	0.095	0.1	0.112	0.125	0.137
Kiambu	0.099	0.106	0.112	0.118	0.125	0.133	0.141	0.149	0.156	0.163	0.169	0.176	0.183	0.191	0.197	0.203	0.21	0.22	0.231	0.244
Kilifi	0.098	0.102	0.104	0.107	0.11	0.113	0.117	0.121	0.124	0.126	0.129	0.132	0.135	0.139	0.141	0.143	0.146	0.151	0.158	0.165
Kirinyaga	0.072	0.077	0.081	0.084	0.089	0.094	0.1	0.106	0.111	0.115	0.12	0.125	0.13	0.137	0.144	0.151	0.159	0.17	0.183	0.196
Kisii	0.058	0.062	0.066	0.07	0.075	0.08	0.086	0.092	0.098	0.102	0.107	0.112	0.118	0.125	0.131	0.135	0.142	0.152	0.163	0.175
Kisumu	0.043	0.045	0.046	0.046	0.047	0.048	0.049	0.058	0.066	0.067	0.069	0.073	0.08	0.088	0.094	0.097	0.105	0.119	0.135	0.15
Kitui	0.063	0.067	0.069	0.072	0.075	0.079	0.083	0.087	0.091	0.094	0.098	0.102	0.107	0.113	0.119	0.124	0.13	0.139	0.149	0.158
Kwale	0.08	0.083	0.085	0.087	0.089	0.092	0.095	0.098	0.101	0.104	0.106	0.109	0.112	0.116	0.12	0.124	0.129	0.136	0.144	0.154
Laikipia	0.038	0.04	0.041	0.045	0.053	0.06	0.067	0.074	0.08	0.083	0.087	0.091	0.096	0.103	0.108	0.111	0.118	0.129	0.142	0.155
Lamu	0.04	0.041	0.044	0.049	0.054	0.059	0.064	0.07	0.075	0.076	0.077	0.079	0.083	0.088	0.092	0.092	0.097	0.108	0.121	0.134
Machakos	0.085	0.09	0.093	0.097	0.101	0.106	0.111	0.117	0.122	0.126	0.13	0.135	0.141	0.147	0.152	0.157	0.163	0.172	0.184	0.196
Makueni	0.075	0.078	0.081	0.085	0.089	0.094	0.099	0.104	0.109	0.113	0.117	0.122	0.128	0.135	0.142	0.148	0.155	0.166	0.178	0.19
Mandera	0.03	0.031	0.032	0.033	0.034	0.036	0.037	0.039	0.04	0.04	0.041	0.041	0.042	0.044	0.045	0.046	0.047	0.052	0.057	0.063
Marsabit	0.085	0.089	0.09	0.092	0.094	0.097	0.1	0.103	0.105	0.108	0.11	0.112	0.115	0.118	0.121	0.124	0.128	0.133	0.139	0.145
Meru	0.088	0.093	0.096	0.099	0.104	0.108	0.113	0.118	0.123	0.127	0.131	0.135	0.14	0.145	0.15	0.154	0.16	0.168	0.177	0.187
Migori	0.041	0.042	0.043	0.044	0.045	0.046	0.047	0.048	0.049	0.05	0.051	0.052	0.053	0.062	0.072	0.075	0.086	0.103	0.12	0.136
Mombasa	0.103	0.111	0.113	0.116	0.12	0.125	0.13	0.136	0.141	0.146	0.151	0.156	0.162	0.168	0.174	0.18	0.187	0.195	0.204	0.214
Murang’a	0.098	0.104	0.109	0.115	0.121	0.128	0.135	0.142	0.149	0.154	0.159	0.165	0.171	0.178	0.183	0.188	0.193	0.202	0.211	0.221
Nairobi	0.13	0.14	0.146	0.151	0.159	0.167	0.176	0.185	0.194	0.202	0.21	0.219	0.228	0.237	0.246	0.254	0.265	0.277	0.289	0.302
Nakuru	0.076	0.083	0.085	0.088	0.092	0.097	0.102	0.108	0.113	0.117	0.122	0.126	0.131	0.137	0.142	0.146	0.151	0.159	0.167	0.175
Nandi	0.066	0.072	0.077	0.083	0.089	0.095	0.102	0.109	0.115	0.118	0.122	0.126	0.131	0.136	0.139	0.14	0.143	0.151	0.16	0.17
Narok	0.041	0.042	0.043	0.044	0.045	0.046	0.047	0.047	0.048	0.049	0.05	0.051	0.052	0.053	0.053	0.054	0.055	0.056	0.057	0.076
Nyamira	0.042	0.043	0.044	0.05	0.058	0.065	0.073	0.081	0.087	0.091	0.096	0.102	0.11	0.118	0.125	0.131	0.141	0.153	0.167	0.181
Nyandarua	0.045	0.047	0.048	0.048	0.05	0.051	0.052	0.053	0.054	0.055	0.056	0.057	0.063	0.079	0.091	0.101	0.113	0.131	0.149	0.167
Nyeri	0.077	0.084	0.088	0.094	0.1	0.106	0.113	0.12	0.126	0.131	0.136	0.142	0.148	0.156	0.163	0.168	0.176	0.188	0.201	0.215
Samburu	0.043	0.045	0.047	0.05	0.053	0.057	0.061	0.066	0.07	0.071	0.072	0.074	0.078	0.082	0.085	0.086	0.09	0.098	0.108	0.117
Siaya	0.01	0.01	0.01	0.011	0.011	0.012	0.014	0.021	0.027	0.031	0.036	0.041	0.046	0.053	0.059	0.064	0.072	0.082	0.091	0.102
Taita Taveta	0.105	0.111	0.114	0.116	0.121	0.125	0.129	0.134	0.139	0.142	0.146	0.15	0.154	0.159	0.162	0.165	0.17	0.175	0.182	0.19
Tana River	0.041	0.045	0.046	0.047	0.048	0.051	0.053	0.056	0.058	0.06	0.062	0.065	0.068	0.072	0.076	0.079	0.084	0.091	0.099	0.107
Tharaka Nithi	0.078	0.084	0.086	0.088	0.092	0.096	0.1	0.105	0.109	0.113	0.118	0.123	0.128	0.134	0.14	0.146	0.153	0.162	0.172	0.183
Trans Nzoia	0.089	0.094	0.098	0.102	0.107	0.112	0.118	0.124	0.129	0.132	0.135	0.138	0.142	0.147	0.15	0.152	0.156	0.164	0.174	0.185
Turkana	0.101	0.104	0.106	0.108	0.11	0.113	0.115	0.118	0.121	0.123	0.125	0.128	0.13	0.133	0.136	0.138	0.14	0.145	0.15	0.155
Uasin Gishu	0.085	0.089	0.093	0.098	0.103	0.109	0.114	0.121	0.126	0.13	0.134	0.139	0.144	0.149	0.154	0.157	0.162	0.171	0.181	0.193

Appendix Table 8. Socio-Demographic Index values for all estimated GBD 2017 locations, 1950-1969																				
**Location**	**1950**	**1951**	**1952**	**1953**	**1954**	**1955**	**1956**	**1957**	**1958**	**1959**	**1960**	**1961**	**1962**	**1963**	**1964**	**1965**	**1966**	**1967**	**1968**	**1969**
Vihiga	0.064	0.071	0.078	0.085	0.092	0.099	0.107	0.114	0.121	0.126	0.13	0.136	0.142	0.148	0.154	0.158	0.164	0.173	0.184	0.195
Wajir	0.041	0.042	0.043	0.044	0.046	0.048	0.049	0.051	0.053	0.053	0.054	0.055	0.057	0.059	0.06	0.06	0.061	0.065	0.07	0.075
West Pokot	0.064	0.067	0.069	0.072	0.075	0.078	0.082	0.086	0.089	0.092	0.094	0.097	0.1	0.104	0.107	0.109	0.113	0.119	0.126	0.133
Madagascar	0.125	0.127	0.128	0.129	0.13	0.132	0.134	0.136	0.138	0.141	0.142	0.144	0.146	0.147	0.147	0.147	0.147	0.148	0.15	0.153
Malawi	0.067	0.068	0.069	0.071	0.072	0.074	0.076	0.078	0.081	0.083	0.085	0.088	0.091	0.093	0.096	0.099	0.103	0.107	0.111	0.115
Mozambique	0.054	0.056	0.057	0.059	0.061	0.063	0.064	0.066	0.069	0.071	0.074	0.076	0.079	0.082	0.085	0.088	0.09	0.093	0.096	0.1
Rwanda	0.113	0.115	0.118	0.12	0.123	0.126	0.128	0.131	0.134	0.138	0.142	0.146	0.152	0.157	0.159	0.16	0.16	0.162	0.165	0.17
Somalia	0.079	0.08	0.082	0.084	0.085	0.087	0.089	0.091	0.093	0.095	0.096	0.099	0.101	0.103	0.104	0.105	0.107	0.109	0.111	0.112
South Sudan	0.104	0.106	0.107	0.109	0.11	0.112	0.113	0.115	0.116	0.118	0.12	0.121	0.123	0.125	0.127	0.129	0.13	0.132	0.134	0.136
Tanzania	0.101	0.105	0.107	0.11	0.113	0.116	0.119	0.122	0.125	0.128	0.132	0.135	0.138	0.141	0.144	0.147	0.15	0.153	0.155	0.158
Uganda	0.085	0.085	0.084	0.083	0.083	0.082	0.082	0.082	0.083	0.084	0.084	0.085	0.087	0.089	0.091	0.093	0.094	0.097	0.099	0.101
Zambia	0.135	0.138	0.142	0.145	0.149	0.153	0.157	0.161	0.164	0.169	0.174	0.179	0.184	0.189	0.195	0.201	0.208	0.215	0.222	0.23
Southern sub-Saharan Africa	0.277	0.282	0.287	0.293	0.298	0.304	0.31	0.316	0.321	0.327	0.332	0.338	0.344	0.349	0.355	0.36	0.365	0.372	0.378	0.384
Botswana	0.166	0.17	0.174	0.178	0.182	0.187	0.191	0.196	0.2	0.205	0.209	0.213	0.217	0.221	0.225	0.228	0.232	0.237	0.242	0.247
Lesotho	0.126	0.131	0.135	0.139	0.143	0.147	0.152	0.156	0.16	0.165	0.17	0.174	0.179	0.185	0.192	0.197	0.202	0.208	0.212	0.217
Namibia	0.215	0.219	0.224	0.228	0.233	0.238	0.244	0.249	0.254	0.26	0.266	0.271	0.277	0.283	0.29	0.297	0.303	0.308	0.313	0.318
South Africa	0.31	0.316	0.321	0.326	0.332	0.339	0.345	0.351	0.356	0.362	0.368	0.374	0.38	0.385	0.391	0.396	0.402	0.409	0.416	0.422
Swaziland	0.166	0.171	0.175	0.179	0.184	0.188	0.193	0.198	0.203	0.208	0.215	0.222	0.231	0.239	0.248	0.257	0.264	0.269	0.274	0.278
Zimbabwe	0.152	0.157	0.161	0.166	0.17	0.175	0.18	0.185	0.19	0.195	0.201	0.206	0.211	0.216	0.22	0.225	0.229	0.233	0.237	0.241
Western sub-Saharan Africa	0.131	0.134	0.137	0.14	0.144	0.147	0.15	0.153	0.157	0.161	0.165	0.169	0.173	0.177	0.182	0.186	0.189	0.193	0.196	0.201
Benin	0.098	0.1	0.102	0.104	0.106	0.109	0.111	0.113	0.116	0.119	0.122	0.124	0.127	0.129	0.131	0.134	0.137	0.14	0.142	0.145
Burkina Faso	0.056	0.058	0.06	0.062	0.064	0.066	0.068	0.071	0.073	0.076	0.078	0.08	0.083	0.085	0.087	0.088	0.089	0.09	0.092	0.094
Cameroon	0.128	0.132	0.136	0.14	0.145	0.149	0.154	0.159	0.164	0.17	0.175	0.18	0.185	0.19	0.194	0.199	0.203	0.206	0.209	0.212
Cape Verde	0.123	0.124	0.125	0.125	0.126	0.127	0.128	0.128	0.13	0.133	0.136	0.14	0.144	0.147	0.152	0.157	0.163	0.17	0.177	0.185
Chad	0.064	0.065	0.067	0.068	0.07	0.071	0.073	0.075	0.076	0.078	0.08	0.081	0.083	0.085	0.086	0.088	0.089	0.091	0.092	0.093
Cote d'Ivoire	0.113	0.116	0.118	0.121	0.124	0.127	0.13	0.134	0.137	0.141	0.145	0.149	0.153	0.158	0.162	0.166	0.171	0.175	0.18	0.184
The Gambia	0.099	0.101	0.104	0.106	0.109	0.111	0.114	0.117	0.119	0.123	0.126	0.129	0.132	0.134	0.137	0.139	0.142	0.145	0.148	0.151
Ghana	0.225	0.229	0.231	0.234	0.237	0.239	0.239	0.241	0.243	0.246	0.249	0.252	0.255	0.26	0.264	0.268	0.271	0.272	0.274	0.277
Guinea	0.066	0.068	0.069	0.07	0.073	0.075	0.078	0.081	0.084	0.087	0.091	0.094	0.098	0.101	0.104	0.107	0.11	0.113	0.116	0.118
Guinea-Bissau	0.059	0.061	0.062	0.064	0.067	0.071	0.074	0.079	0.083	0.087	0.092	0.098	0.104	0.109	0.115	0.12	0.124	0.127	0.131	0.134
Liberia	0.103	0.106	0.108	0.111	0.114	0.116	0.119	0.122	0.126	0.129	0.132	0.135	0.139	0.141	0.144	0.145	0.148	0.15	0.152	0.155
Mali	0.059	0.06	0.061	0.063	0.064	0.065	0.067	0.068	0.07	0.072	0.073	0.075	0.077	0.078	0.08	0.081	0.082	0.084	0.085	0.087
Mauritania	0.124	0.128	0.131	0.135	0.138	0.142	0.146	0.15	0.154	0.159	0.164	0.168	0.172	0.175	0.18	0.185	0.189	0.193	0.196	0.2
Niger	0.056	0.057	0.058	0.059	0.06	0.061	0.062	0.063	0.065	0.066	0.067	0.068	0.07	0.071	0.071	0.072	0.073	0.074	0.075	0.076
Nigeria	0.144	0.147	0.151	0.154	0.158	0.162	0.166	0.17	0.174	0.179	0.184	0.189	0.194	0.199	0.205	0.21	0.215	0.22	0.225	0.231
Sao Tome and Principe	0.146	0.15	0.152	0.153	0.153	0.154	0.154	0.154	0.155	0.157	0.158	0.16	0.162	0.164	0.165	0.167	0.169	0.171	0.174	0.179
Senegal	0.108	0.111	0.114	0.117	0.12	0.124	0.128	0.132	0.135	0.137	0.14	0.144	0.146	0.146	0.146	0.147	0.149	0.152	0.154	0.155
Sierra Leone	0.093	0.096	0.098	0.1	0.102	0.105	0.107	0.11	0.112	0.115	0.118	0.121	0.124	0.127	0.13	0.133	0.136	0.139	0.142	0.145
Togo	0.103	0.105	0.108	0.11	0.112	0.115	0.117	0.12	0.122	0.125	0.128	0.131	0.134	0.137	0.14	0.143	0.147	0.151	0.155	0.159

Appendix Table 9. Socio-Demographic Index values for all estimated GBD 2017 locations, 1970–1989																				
**Location**	**1970**	**1971**	**1972**	**1973**	**1974**	**1975**	**1976**	**1977**	**1978**	**1979**	**1980**	**1981**	**1982**	**1983**	**1984**	**1985**	**1986**	**1987**	**1988**	**1989**
Global	0.423	0.43	0.436	0.443	0.45	0.457	0.462	0.467	0.472	0.477	0.482	0.486	0.488	0.491	0.494	0.498	0.501	0.507	0.513	0.518
Central Europe, Eastern Europe, and Central Asia	0.597	0.601	0.605	0.608	0.61	0.613	0.617	0.621	0.623	0.626	0.629	0.632	0.632	0.633	0.635	0.637	0.64	0.643	0.647	0.651
Central Asia	0.486	0.49	0.494	0.499	0.503	0.508	0.514	0.519	0.525	0.529	0.532	0.536	0.538	0.541	0.543	0.545	0.548	0.552	0.556	0.56
Armenia	0.501	0.507	0.512	0.518	0.524	0.529	0.534	0.538	0.541	0.544	0.546	0.546	0.546	0.546	0.546	0.545	0.546	0.547	0.549	0.552
Azerbaijan	0.511	0.519	0.526	0.534	0.542	0.55	0.559	0.567	0.574	0.579	0.584	0.589	0.593	0.596	0.599	0.601	0.604	0.606	0.608	0.61
Georgia	0.578	0.584	0.589	0.595	0.6	0.603	0.607	0.612	0.616	0.617	0.618	0.62	0.623	0.627	0.629	0.63	0.633	0.639	0.645	0.65
Kazakhstan	0.527	0.533	0.539	0.546	0.553	0.559	0.565	0.57	0.576	0.58	0.584	0.588	0.591	0.593	0.594	0.595	0.598	0.602	0.606	0.61
Kyrgyzstan	0.485	0.487	0.492	0.499	0.504	0.509	0.515	0.52	0.525	0.529	0.531	0.532	0.533	0.536	0.54	0.543	0.547	0.552	0.557	0.56
Mongolia	0.417	0.423	0.429	0.435	0.44	0.446	0.451	0.456	0.461	0.466	0.472	0.479	0.486	0.492	0.498	0.503	0.509	0.516	0.523	0.529
Tajikistan	0.327	0.328	0.326	0.322	0.324	0.34	0.36	0.375	0.384	0.393	0.403	0.412	0.419	0.425	0.43	0.436	0.444	0.455	0.464	0.469
Turkmenistan	0.497	0.502	0.507	0.513	0.518	0.523	0.528	0.533	0.537	0.542	0.546	0.55	0.554	0.558	0.562	0.565	0.569	0.574	0.579	0.584
Uzbekistan	0.388	0.393	0.396	0.401	0.405	0.41	0.416	0.422	0.428	0.433	0.437	0.442	0.446	0.45	0.454	0.458	0.463	0.467	0.472	0.477
Central Europe	0.59	0.595	0.598	0.598	0.598	0.601	0.605	0.609	0.613	0.617	0.622	0.626	0.629	0.631	0.633	0.637	0.641	0.646	0.651	0.658
Albania	0.433	0.441	0.451	0.46	0.469	0.478	0.486	0.495	0.503	0.511	0.519	0.526	0.532	0.535	0.537	0.541	0.546	0.549	0.549	0.549
Bosnia and Herzegovina	0.386	0.393	0.4	0.406	0.413	0.419	0.425	0.432	0.438	0.445	0.452	0.458	0.465	0.47	0.475	0.48	0.484	0.489	0.492	0.495
Bulgaria	0.565	0.571	0.572	0.567	0.568	0.572	0.575	0.579	0.583	0.589	0.596	0.601	0.605	0.61	0.617	0.621	0.624	0.628	0.635	0.646
Croatia	0.628	0.633	0.637	0.64	0.644	0.647	0.651	0.656	0.661	0.667	0.672	0.678	0.683	0.688	0.692	0.697	0.702	0.707	0.713	0.719
Czech Republic	0.664	0.664	0.657	0.648	0.644	0.646	0.65	0.652	0.656	0.666	0.677	0.681	0.683	0.686	0.688	0.692	0.697	0.701	0.704	0.708
Hungary	0.586	0.591	0.593	0.587	0.58	0.583	0.59	0.598	0.605	0.615	0.624	0.633	0.642	0.648	0.65	0.653	0.658	0.664	0.67	0.675
Macedonia	0.552	0.557	0.561	0.564	0.567	0.57	0.574	0.576	0.579	0.583	0.588	0.594	0.598	0.601	0.605	0.61	0.614	0.617	0.62	0.623
Montenegro	0.647	0.652	0.656	0.661	0.665	0.669	0.674	0.677	0.681	0.685	0.687	0.689	0.691	0.692	0.694	0.696	0.698	0.699	0.701	0.704
Poland	0.612	0.615	0.619	0.621	0.623	0.623	0.626	0.631	0.633	0.633	0.636	0.635	0.63	0.63	0.633	0.639	0.645	0.649	0.654	0.659
Romania	0.532	0.542	0.55	0.549	0.548	0.555	0.559	0.564	0.569	0.573	0.58	0.589	0.601	0.604	0.604	0.604	0.606	0.611	0.62	0.636
Serbia	0.571	0.574	0.577	0.58	0.582	0.584	0.587	0.591	0.593	0.595	0.601	0.604	0.604	0.605	0.608	0.612	0.615	0.617	0.622	0.627
Slovakia	0.628	0.631	0.63	0.628	0.63	0.633	0.636	0.638	0.64	0.645	0.652	0.655	0.657	0.658	0.659	0.663	0.669	0.674	0.679	0.681
Slovenia	0.651	0.654	0.657	0.661	0.664	0.665	0.668	0.671	0.675	0.68	0.685	0.691	0.697	0.704	0.71	0.714	0.718	0.724	0.73	0.736
Eastern Europe	0.619	0.622	0.628	0.633	0.637	0.641	0.644	0.648	0.65	0.653	0.655	0.658	0.657	0.658	0.66	0.662	0.664	0.666	0.671	0.674
Belarus	0.559	0.561	0.565	0.569	0.573	0.576	0.58	0.583	0.585	0.588	0.59	0.592	0.593	0.595	0.597	0.601	0.605	0.609	0.615	0.62
Estonia	0.668	0.671	0.676	0.682	0.684	0.684	0.684	0.686	0.69	0.692	0.69	0.689	0.69	0.694	0.696	0.695	0.692	0.692	0.699	0.705
Latvia	0.651	0.655	0.659	0.664	0.668	0.669	0.672	0.675	0.678	0.68	0.681	0.681	0.68	0.681	0.683	0.683	0.683	0.685	0.689	0.693
Lithuania	0.66	0.664	0.67	0.677	0.681	0.684	0.686	0.689	0.692	0.695	0.701	0.705	0.707	0.708	0.709	0.711	0.712	0.712	0.71	0.709
Moldova	0.524	0.528	0.531	0.536	0.54	0.545	0.549	0.552	0.554	0.556	0.558	0.558	0.558	0.559	0.56	0.562	0.564	0.566	0.569	0.571
Russian Federation	0.628	0.632	0.639	0.644	0.648	0.651	0.655	0.658	0.66	0.663	0.666	0.668	0.666	0.666	0.668	0.669	0.671	0.673	0.677	0.679
Ukraine	0.593	0.596	0.599	0.604	0.609	0.613	0.617	0.62	0.624	0.627	0.63	0.632	0.633	0.636	0.639	0.643	0.646	0.65	0.655	0.659
High-income	0.666	0.673	0.681	0.688	0.694	0.7	0.705	0.71	0.715	0.718	0.723	0.728	0.734	0.74	0.744	0.749	0.754	0.758	0.762	0.765
Australasia	0.66	0.664	0.677	0.687	0.697	0.707	0.716	0.724	0.731	0.736	0.739	0.743	0.747	0.752	0.758	0.762	0.767	0.772	0.776	0.779
Australia	0.664	0.667	0.68	0.69	0.699	0.709	0.718	0.725	0.732	0.737	0.741	0.744	0.748	0.754	0.759	0.764	0.77	0.775	0.779	0.783
New Zealand	0.645	0.651	0.662	0.675	0.687	0.697	0.706	0.716	0.723	0.728	0.733	0.738	0.743	0.747	0.751	0.754	0.756	0.757	0.759	0.762
High-income Asia-Pacific	0.663	0.667	0.672	0.68	0.688	0.695	0.702	0.708	0.713	0.718	0.722	0.726	0.732	0.738	0.745	0.752	0.758	0.764	0.771	0.777
Brunei	0.57	0.584	0.601	0.619	0.639	0.654	0.657	0.654	0.653	0.656	0.66	0.668	0.677	0.685	0.691	0.697	0.704	0.712	0.718	0.724
Japan	0.696	0.697	0.701	0.707	0.714	0.722	0.73	0.737	0.743	0.749	0.754	0.759	0.762	0.766	0.771	0.777	0.781	0.787	0.792	0.798
Aichi	0.698	0.698	0.701	0.706	0.715	0.724	0.733	0.74	0.747	0.754	0.76	0.764	0.768	0.771	0.777	0.782	0.787	0.793	0.8	0.806
Akita	0.658	0.658	0.66	0.665	0.671	0.679	0.686	0.693	0.7	0.707	0.714	0.719	0.723	0.728	0.734	0.74	0.745	0.75	0.756	0.762
Aomori	0.65	0.65	0.652	0.657	0.664	0.673	0.68	0.688	0.695	0.702	0.708	0.713	0.716	0.721	0.727	0.733	0.738	0.744	0.75	0.756
Chiba	0.691	0.692	0.696	0.701	0.709	0.718	0.726	0.734	0.741	0.747	0.753	0.757	0.761	0.765	0.77	0.775	0.78	0.786	0.792	0.798
Ehime	0.667	0.668	0.671	0.677	0.685	0.693	0.701	0.708	0.714	0.721	0.727	0.731	0.735	0.739	0.744	0.749	0.754	0.759	0.765	0.771
Fukui	0.667	0.667	0.669	0.674	0.682	0.691	0.698	0.706	0.713	0.72	0.727	0.732	0.737	0.741	0.748	0.754	0.759	0.765	0.772	0.779
Fukuoka	0.692	0.695	0.699	0.705	0.713	0.72	0.727	0.734	0.739	0.745	0.75	0.754	0.757	0.761	0.766	0.771	0.775	0.781	0.787	0.792
Fukushima	0.663	0.663	0.665	0.671	0.678	0.686	0.694	0.701	0.707	0.713	0.719	0.723	0.727	0.73	0.736	0.741	0.746	0.752	0.759	0.765
Gifu	0.672	0.673	0.676	0.681	0.689	0.697	0.705	0.712	0.719	0.725	0.732	0.737	0.741	0.745	0.751	0.757	0.762	0.768	0.774	0.78
Gunma	0.683	0.685	0.689	0.695	0.702	0.71	0.717	0.724	0.729	0.735	0.74	0.745	0.748	0.751	0.756	0.761	0.766	0.771	0.776	0.782
Hiroshima	0.688	0.688	0.691	0.697	0.705	0.715	0.723	0.731	0.738	0.744	0.75	0.755	0.758	0.762	0.768	0.773	0.778	0.783	0.789	0.795
Hokkaidō	0.676	0.677	0.68	0.686	0.693	0.702	0.71	0.718	0.725	0.731	0.737	0.742	0.745	0.749	0.754	0.758	0.763	0.768	0.773	0.779
Hyōgo	0.689	0.691	0.694	0.7	0.708	0.717	0.725	0.732	0.739	0.745	0.75	0.755	0.758	0.762	0.767	0.772	0.776	0.782	0.787	0.793
Ibaraki	0.679	0.68	0.683	0.689	0.697	0.706	0.713	0.72	0.726	0.732	0.738	0.742	0.745	0.749	0.755	0.76	0.765	0.771	0.777	0.783
Ishikawa	0.669	0.668	0.67	0.675	0.682	0.691	0.7	0.707	0.715	0.722	0.729	0.734	0.739	0.744	0.751	0.757	0.763	0.77	0.777	0.783
Iwate	0.65	0.65	0.652	0.657	0.665	0.674	0.681	0.688	0.695	0.701	0.707	0.712	0.716	0.721	0.727	0.733	0.738	0.744	0.749	0.755
Kagawa	0.675	0.676	0.678	0.684	0.692	0.701	0.709	0.716	0.722	0.729	0.735	0.74	0.743	0.748	0.754	0.759	0.764	0.769	0.775	0.782
Kagoshima	0.66	0.662	0.665	0.671	0.679	0.686	0.693	0.699	0.705	0.711	0.717	0.721	0.725	0.73	0.735	0.741	0.746	0.751	0.757	0.763
Kanagawa	0.711	0.713	0.717	0.723	0.731	0.739	0.747	0.754	0.761	0.767	0.772	0.777	0.78	0.784	0.789	0.793	0.797	0.803	0.808	0.814
Kōchi	0.648	0.649	0.652	0.658	0.665	0.674	0.681	0.688	0.695	0.701	0.708	0.712	0.716	0.72	0.726	0.732	0.737	0.742	0.748	0.754
Kumamoto	0.662	0.663	0.667	0.673	0.68	0.688	0.695	0.702	0.708	0.714	0.719	0.724	0.727	0.731	0.737	0.742	0.747	0.753	0.759	0.765
Kyōto	0.71	0.714	0.718	0.724	0.731	0.738	0.745	0.751	0.757	0.763	0.768	0.772	0.775	0.779	0.783	0.788	0.792	0.797	0.802	0.808
Mie	0.673	0.673	0.676	0.681	0.689	0.698	0.706	0.713	0.72	0.726	0.732	0.737	0.741	0.744	0.75	0.756	0.761	0.767	0.774	0.781
Miyagi	0.68	0.681	0.684	0.69	0.697	0.704	0.711	0.718	0.724	0.73	0.736	0.741	0.744	0.749	0.754	0.76	0.765	0.771	0.777	0.783
Miyazaki	0.652	0.653	0.655	0.661	0.668	0.677	0.684	0.691	0.698	0.704	0.71	0.715	0.719	0.724	0.73	0.736	0.741	0.747	0.753	0.759
Nagano	0.69	0.693	0.698	0.704	0.711	0.718	0.724	0.731	0.736	0.742	0.748	0.752	0.756	0.76	0.764	0.768	0.772	0.777	0.782	0.788
Nagasaki	0.662	0.663	0.667	0.673	0.68	0.688	0.695	0.701	0.706	0.712	0.718	0.722	0.726	0.73	0.736	0.741	0.745	0.75	0.756	0.761
Nara	0.681	0.684	0.688	0.694	0.701	0.709	0.716	0.723	0.729	0.735	0.741	0.745	0.749	0.753	0.758	0.763	0.767	0.773	0.778	0.784
Niigata	0.669	0.67	0.672	0.678	0.685	0.693	0.7	0.707	0.714	0.72	0.726	0.731	0.735	0.739	0.744	0.75	0.754	0.76	0.765	0.771

Appendix Table 9. Socio-Demographic Index values for all estimated GBD 2017 locations, 1970–1989

Location	1970	1971	1972	1973	1974	1975	1976	1977	1978	1979	1980	1981	1982	1983	1984	1985	1986	1987	1988	1989
Ōita	0.679	0.681	0.684	0.689	0.696	0.704	0.711	0.717	0.723	0.729	0.735	0.74	0.744	0.748	0.754	0.759	0.764	0.769	0.774	0.78
Okayama	0.677	0.677	0.68	0.685	0.694	0.703	0.711	0.719	0.726	0.732	0.737	0.742	0.745	0.749	0.755	0.761	0.766	0.772	0.778	0.784
Okinawa	0.643	0.643	0.644	0.649	0.657	0.665	0.673	0.68	0.686	0.692	0.698	0.703	0.707	0.711	0.717	0.723	0.729	0.735	0.742	0.748
Ōsaka	0.709	0.711	0.714	0.72	0.728	0.737	0.745	0.752	0.759	0.765	0.77	0.774	0.777	0.781	0.785	0.789	0.794	0.799	0.804	0.81
Saga	0.668	0.67	0.674	0.68	0.687	0.695	0.701	0.707	0.713	0.719	0.724	0.729	0.732	0.736	0.742	0.747	0.751	0.757	0.762	0.768
Saitama	0.683	0.685	0.688	0.694	0.702	0.711	0.719	0.726	0.733	0.74	0.746	0.75	0.754	0.757	0.763	0.767	0.772	0.777	0.783	0.788
Shiga	0.694	0.695	0.698	0.704	0.712	0.72	0.727	0.734	0.74	0.747	0.753	0.758	0.761	0.765	0.77	0.775	0.78	0.786	0.792	0.798
Shimane	0.658	0.659	0.662	0.668	0.676	0.684	0.69	0.697	0.702	0.708	0.713	0.718	0.722	0.725	0.731	0.736	0.74	0.746	0.751	0.757
Shizuoka	0.687	0.687	0.69	0.696	0.704	0.713	0.721	0.729	0.735	0.742	0.747	0.752	0.755	0.759	0.764	0.77	0.774	0.78	0.786	0.792
Tochigi	0.682	0.683	0.686	0.692	0.699	0.707	0.714	0.721	0.727	0.733	0.738	0.743	0.746	0.749	0.754	0.759	0.764	0.77	0.776	0.782
Tokushima	0.668	0.669	0.672	0.678	0.685	0.694	0.701	0.707	0.713	0.719	0.725	0.729	0.732	0.736	0.742	0.748	0.753	0.758	0.765	0.771
Tōkyō	0.774	0.777	0.782	0.788	0.795	0.803	0.809	0.815	0.821	0.826	0.83	0.834	0.837	0.84	0.844	0.848	0.852	0.856	0.861	0.866
Tottori	0.664	0.665	0.668	0.674	0.682	0.69	0.697	0.704	0.71	0.715	0.721	0.726	0.729	0.733	0.739	0.744	0.749	0.754	0.76	0.766
Toyama	0.674	0.673	0.675	0.68	0.687	0.696	0.704	0.712	0.719	0.726	0.733	0.738	0.743	0.747	0.753	0.759	0.765	0.771	0.778	0.785
Wakayama	0.665	0.667	0.67	0.676	0.685	0.694	0.702	0.709	0.714	0.718	0.721	0.723	0.726	0.73	0.738	0.744	0.75	0.757	0.763	0.77
Yamagata	0.66	0.661	0.663	0.669	0.675	0.683	0.689	0.696	0.702	0.709	0.715	0.72	0.725	0.729	0.734	0.74	0.744	0.75	0.756	0.761
Yamaguchi	0.684	0.685	0.688	0.694	0.702	0.71	0.717	0.724	0.73	0.736	0.742	0.746	0.75	0.753	0.759	0.764	0.768	0.774	0.78	0.786
Yamanashi	0.691	0.694	0.699	0.705	0.712	0.72	0.726	0.732	0.737	0.743	0.748	0.753	0.756	0.76	0.764	0.769	0.772	0.777	0.782	0.787
South Korea	0.494	0.508	0.523	0.542	0.557	0.568	0.578	0.587	0.595	0.601	0.605	0.611	0.623	0.636	0.647	0.659	0.671	0.682	0.692	0.702
Singapore	0.568	0.576	0.586	0.598	0.612	0.621	0.63	0.641	0.647	0.654	0.662	0.669	0.678	0.687	0.694	0.702	0.71	0.715	0.722	0.729
High-income North America	0.702	0.719	0.733	0.74	0.745	0.751	0.755	0.758	0.762	0.762	0.765	0.769	0.773	0.777	0.779	0.782	0.786	0.787	0.786	0.784
Canada	0.702	0.712	0.721	0.728	0.732	0.736	0.742	0.749	0.755	0.759	0.764	0.769	0.772	0.777	0.782	0.787	0.792	0.796	0.798	0.8
Greenland	0.57	0.592	0.609	0.622	0.631	0.636	0.642	0.648	0.653	0.657	0.662	0.666	0.671	0.674	0.675	0.676	0.676	0.675	0.673	0.673
USA	0.702	0.719	0.734	0.741	0.746	0.752	0.756	0.759	0.762	0.762	0.765	0.769	0.772	0.777	0.779	0.781	0.785	0.786	0.784	0.782
Alabama	0.643	0.659	0.675	0.684	0.69	0.699	0.705	0.71	0.716	0.718	0.723	0.729	0.733	0.739	0.742	0.745	0.749	0.75	0.748	0.745
Alaska	0.687	0.706	0.722	0.728	0.734	0.745	0.752	0.757	0.761	0.758	0.76	0.762	0.764	0.768	0.767	0.768	0.772	0.771	0.767	0.759
Arizona	0.679	0.694	0.708	0.715	0.723	0.734	0.741	0.746	0.75	0.748	0.75	0.751	0.752	0.755	0.754	0.755	0.759	0.759	0.757	0.753
Arkansas	0.631	0.646	0.659	0.667	0.673	0.683	0.689	0.695	0.701	0.702	0.706	0.711	0.716	0.721	0.723	0.726	0.729	0.729	0.727	0.723
California	0.733	0.752	0.765	0.769	0.77	0.773	0.775	0.777	0.779	0.777	0.779	0.781	0.782	0.786	0.786	0.786	0.788	0.786	0.782	0.775
Colorado	0.721	0.738	0.752	0.761	0.768	0.775	0.78	0.783	0.785	0.784	0.786	0.789	0.791	0.794	0.795	0.797	0.8	0.801	0.801	0.799
Connecticut	0.751	0.768	0.782	0.789	0.794	0.8	0.803	0.805	0.808	0.809	0.812	0.816	0.819	0.824	0.827	0.83	0.835	0.837	0.839	0.84
Delaware	0.722	0.738	0.752	0.76	0.765	0.772	0.775	0.778	0.78	0.779	0.782	0.785	0.787	0.791	0.793	0.795	0.799	0.8	0.8	0.8
Washington, DC	0.736	0.749	0.761	0.768	0.774	0.783	0.788	0.792	0.796	0.795	0.797	0.799	0.8	0.802	0.801	0.802	0.804	0.803	0.801	0.797
Florida	0.684	0.701	0.717	0.727	0.736	0.748	0.755	0.76	0.764	0.763	0.765	0.768	0.77	0.773	0.773	0.775	0.778	0.778	0.777	0.774
Georgia	0.651	0.668	0.685	0.696	0.704	0.714	0.72	0.726	0.73	0.731	0.734	0.738	0.742	0.747	0.75	0.753	0.757	0.758	0.757	0.755
Hawaii	0.717	0.731	0.744	0.75	0.756	0.764	0.768	0.772	0.775	0.774	0.776	0.778	0.779	0.782	0.782	0.783	0.787	0.787	0.786	0.782
Idaho	0.671	0.687	0.7	0.705	0.709	0.715	0.717	0.719	0.724	0.725	0.731	0.738	0.743	0.751	0.753	0.756	0.76	0.761	0.76	0.757
Illinois	0.711	0.728	0.742	0.75	0.755	0.761	0.764	0.767	0.77	0.769	0.772	0.776	0.779	0.784	0.787	0.789	0.793	0.793	0.791	0.788
Indiana	0.687	0.704	0.719	0.726	0.731	0.738	0.742	0.745	0.749	0.749	0.753	0.758	0.762	0.767	0.769	0.771	0.775	0.775	0.774	0.772
Iowa	0.706	0.724	0.738	0.746	0.75	0.755	0.756	0.758	0.762	0.763	0.767	0.772	0.777	0.783	0.785	0.789	0.793	0.794	0.794	0.793
Kansas	0.707	0.724	0.738	0.744	0.748	0.753	0.754	0.755	0.757	0.756	0.759	0.763	0.767	0.772	0.774	0.777	0.782	0.784	0.784	0.782
Kentucky	0.647	0.664	0.679	0.687	0.692	0.699	0.703	0.707	0.712	0.714	0.72	0.726	0.731	0.737	0.74	0.744	0.747	0.748	0.747	0.744
Louisiana	0.642	0.662	0.678	0.687	0.692	0.698	0.702	0.705	0.708	0.708	0.713	0.719	0.724	0.73	0.733	0.736	0.741	0.741	0.74	0.736
Maine	0.68	0.697	0.712	0.72	0.726	0.734	0.739	0.743	0.748	0.749	0.754	0.758	0.763	0.768	0.77	0.774	0.779	0.782	0.784	0.786
Maryland	0.727	0.746	0.762	0.771	0.778	0.785	0.788	0.791	0.794	0.794	0.796	0.798	0.8	0.804	0.806	0.808	0.812	0.813	0.813	0.812
Massachusetts	0.75	0.766	0.779	0.787	0.792	0.798	0.801	0.804	0.808	0.809	0.812	0.815	0.818	0.823	0.826	0.83	0.834	0.838	0.84	0.841
Michigan	0.701	0.719	0.735	0.743	0.749	0.756	0.76	0.763	0.766	0.766	0.77	0.773	0.777	0.782	0.784	0.787	0.791	0.792	0.79	0.788
Minnesota	0.728	0.744	0.756	0.761	0.765	0.769	0.77	0.772	0.776	0.777	0.781	0.786	0.791	0.797	0.801	0.806	0.811	0.814	0.816	0.816
Mississippi	0.607	0.625	0.642	0.653	0.661	0.672	0.679	0.685	0.691	0.692	0.698	0.704	0.709	0.716	0.718	0.721	0.725	0.725	0.723	0.719
Missouri	0.69	0.706	0.72	0.727	0.732	0.739	0.742	0.744	0.747	0.746	0.749	0.753	0.757	0.763	0.765	0.768	0.773	0.774	0.773	0.77
Montana	0.693	0.708	0.721	0.728	0.733	0.742	0.745	0.749	0.754	0.755	0.759	0.763	0.767	0.772	0.773	0.775	0.778	0.779	0.778	0.775
Nebraska	0.708	0.724	0.737	0.744	0.749	0.756	0.759	0.762	0.766	0.766	0.769	0.774	0.778	0.783	0.785	0.788	0.793	0.794	0.795	0.794
Nevada	0.708	0.726	0.742	0.75	0.756	0.765	0.769	0.772	0.774	0.772	0.773	0.774	0.775	0.778	0.777	0.776	0.778	0.775	0.771	0.764
New Hampshire	0.711	0.728	0.743	0.751	0.759	0.767	0.772	0.777	0.782	0.784	0.788	0.792	0.796	0.801	0.804	0.808	0.814	0.818	0.822	0.824
New Jersey	0.732	0.749	0.763	0.772	0.778	0.784	0.788	0.791	0.795	0.796	0.799	0.803	0.807	0.812	0.816	0.819	0.823	0.825	0.826	0.827
New Mexico	0.663	0.68	0.694	0.7	0.706	0.715	0.719	0.722	0.725	0.723	0.725	0.729	0.732	0.737	0.738	0.74	0.744	0.744	0.741	0.735
New York	0.735	0.753	0.767	0.773	0.776	0.781	0.784	0.787	0.792	0.793	0.796	0.8	0.802	0.806	0.808	0.811	0.814	0.815	0.816	0.815
North Carolina	0.662	0.678	0.693	0.703	0.711	0.72	0.727	0.733	0.739	0.741	0.745	0.749	0.752	0.757	0.759	0.761	0.764	0.765	0.764	0.762
North Dakota	0.687	0.703	0.717	0.728	0.735	0.743	0.745	0.748	0.753	0.753	0.756	0.761	0.765	0.771	0.774	0.778	0.783	0.787	0.788	0.788
Ohio	0.7	0.719	0.734	0.742	0.746	0.752	0.755	0.758	0.761	0.76	0.763	0.767	0.77	0.775	0.777	0.779	0.783	0.784	0.783	0.781
Oklahoma	0.672	0.688	0.703	0.71	0.716	0.723	0.726	0.729	0.732	0.73	0.734	0.738	0.742	0.748	0.75	0.752	0.757	0.758	0.755	0.751
Oregon	0.722	0.739	0.752	0.759	0.763	0.767	0.769	0.77	0.773	0.772	0.774	0.777	0.78	0.784	0.785	0.786	0.789	0.789	0.788	0.786
Pennsylvania	0.712	0.728	0.743	0.751	0.756	0.763	0.766	0.77	0.773	0.774	0.777	0.781	0.784	0.789	0.791	0.793	0.797	0.799	0.799	0.799
Rhode Island	0.719	0.735	0.749	0.758	0.764	0.771	0.775	0.779	0.782	0.783	0.786	0.79	0.793	0.797	0.799	0.802	0.807	0.809	0.811	0.812
South Carolina	0.644	0.661	0.676	0.686	0.695	0.706	0.714	0.72	0.726	0.728	0.732	0.737	0.741	0.746	0.748	0.75	0.753	0.754	0.753	0.751
South Dakota	0.684	0.698	0.711	0.719	0.724	0.731	0.733	0.735	0.739	0.74	0.743	0.748	0.752	0.757	0.76	0.763	0.767	0.769	0.769	0.768
Tennessee	0.659	0.676	0.69	0.699	0.706	0.714	0.719	0.723	0.728	0.73	0.734	0.738	0.742	0.747	0.749	0.751	0.754	0.755	0.753	0.75
Texas	0.663	0.683	0.7	0.709	0.716	0.724	0.729	0.731	0.733	0.73	0.732	0.735	0.738	0.743	0.744	0.747	0.751	0.752	0.75	0.745
Utah	0.693	0.707	0.717	0.719	0.717	0.719	0.716	0.714	0.716	0.714	0.72	0.727	0.734	0.743	0.746	0.751	0.758	0.761	0.761	0.76
Vermont	0.709	0.725	0.74	0.748	0.755	0.763	0.768	0.772	0.777	0.778	0.781	0.785	0.788	0.793	0.795	0.798	0.804	0.807	0.81	0.812
Virginia	0.7	0.717	0.731	0.739	0.745	0.754	0.759	0.765	0.77	0.772	0.777	0.781	0.785	0.789	0.791	0.794	0.798	0.8	0.8	0.799

Appendix Table 9. Socio-Demographic Index values for all estimated GBD 2017 locations, 1970–1989

Location	1970	1971	1972	1973	1974	1975	1976	1977	1978	1979	1980	1981	1982	1983	1984	1985	1986	1987	1988	1989
Washington	0.732	0.752	0.767	0.773	0.776	0.78	0.781	0.782	0.784	0.782	0.785	0.788	0.79	0.794	0.795	0.797	0.8	0.801	0.8	0.797
West Virginia	0.654	0.669	0.684	0.692	0.697	0.704	0.708	0.712	0.717	0.719	0.724	0.73	0.736	0.742	0.744	0.747	0.751	0.752	0.751	0.748
Wisconsin	0.715	0.732	0.745	0.752	0.755	0.761	0.764	0.767	0.77	0.77	0.773	0.777	0.781	0.785	0.788	0.791	0.796	0.799	0.8	0.8
Wyoming	0.685	0.703	0.717	0.723	0.728	0.736	0.738	0.739	0.741	0.739	0.742	0.746	0.75	0.755	0.755	0.758	0.765	0.767	0.767	0.765
Southern Latin America	0.522	0.527	0.529	0.531	0.534	0.537	0.54	0.542	0.545	0.549	0.554	0.56	0.566	0.572	0.576	0.577	0.579	0.583	0.586	0.591
Argentina	0.531	0.536	0.538	0.539	0.538	0.537	0.537	0.537	0.538	0.543	0.55	0.557	0.563	0.569	0.572	0.572	0.575	0.579	0.584	0.589
Chile	0.501	0.506	0.509	0.517	0.527	0.536	0.544	0.55	0.554	0.557	0.558	0.562	0.569	0.575	0.579	0.583	0.586	0.587	0.589	0.593
Uruguay	0.516	0.517	0.519	0.522	0.526	0.53	0.533	0.537	0.543	0.55	0.557	0.562	0.567	0.572	0.577	0.579	0.581	0.582	0.583	0.587
Western Europe	0.649	0.653	0.66	0.667	0.674	0.68	0.685	0.691	0.697	0.701	0.707	0.714	0.72	0.727	0.732	0.737	0.743	0.748	0.753	0.758
Andorra	0.803	0.805	0.807	0.81	0.812	0.815	0.818	0.82	0.822	0.824	0.826	0.829	0.832	0.835	0.838	0.841	0.843	0.844	0.846	0.847
Austria	0.674	0.68	0.688	0.697	0.704	0.712	0.72	0.726	0.73	0.732	0.733	0.736	0.741	0.748	0.754	0.759	0.763	0.767	0.771	0.774
Belgium	0.693	0.7	0.708	0.717	0.725	0.731	0.737	0.741	0.746	0.75	0.755	0.761	0.767	0.773	0.779	0.784	0.789	0.792	0.796	0.8
Cyprus	0.604	0.609	0.619	0.632	0.64	0.643	0.645	0.646	0.646	0.648	0.652	0.658	0.664	0.67	0.677	0.685	0.694	0.702	0.71	0.718
Denmark	0.756	0.758	0.764	0.769	0.771	0.777	0.785	0.791	0.796	0.802	0.809	0.815	0.821	0.825	0.828	0.832	0.836	0.839	0.841	0.843
Finland	0.715	0.723	0.731	0.736	0.739	0.743	0.748	0.754	0.759	0.765	0.769	0.772	0.776	0.782	0.789	0.795	0.799	0.803	0.807	0.811
France	0.62	0.626	0.63	0.64	0.657	0.667	0.673	0.679	0.686	0.692	0.698	0.705	0.713	0.721	0.728	0.735	0.742	0.75	0.756	0.763
Germany	0.693	0.699	0.71	0.717	0.722	0.727	0.73	0.735	0.741	0.744	0.747	0.752	0.757	0.763	0.768	0.768	0.771	0.777	0.78	0.781
Greece	0.596	0.602	0.607	0.61	0.611	0.614	0.617	0.62	0.623	0.627	0.634	0.643	0.651	0.66	0.67	0.68	0.689	0.696	0.703	0.711
Iceland	0.682	0.684	0.69	0.7	0.711	0.72	0.729	0.736	0.741	0.747	0.754	0.762	0.771	0.78	0.788	0.794	0.798	0.802	0.805	0.809
Ireland	0.641	0.642	0.645	0.65	0.656	0.663	0.669	0.675	0.68	0.684	0.69	0.698	0.706	0.713	0.721	0.727	0.733	0.74	0.746	0.751
Israel	0.626	0.631	0.637	0.64	0.641	0.642	0.648	0.657	0.664	0.67	0.676	0.679	0.682	0.689	0.696	0.705	0.713	0.719	0.724	0.729
Italy	0.636	0.639	0.643	0.647	0.653	0.662	0.666	0.675	0.688	0.694	0.701	0.71	0.717	0.724	0.731	0.738	0.744	0.75	0.756	0.762
Luxembourg	0.744	0.753	0.76	0.768	0.774	0.779	0.783	0.787	0.791	0.794	0.798	0.802	0.808	0.813	0.818	0.823	0.828	0.831	0.835	0.84
Malta	0.623	0.627	0.632	0.635	0.639	0.646	0.656	0.665	0.673	0.68	0.687	0.694	0.7	0.706	0.71	0.714	0.717	0.72	0.723	0.726
Netherlands	0.718	0.727	0.736	0.746	0.754	0.761	0.767	0.773	0.777	0.781	0.786	0.791	0.796	0.799	0.803	0.807	0.811	0.815	0.819	0.823
Norway	0.689	0.694	0.703	0.712	0.722	0.732	0.743	0.751	0.757	0.762	0.768	0.774	0.781	0.786	0.791	0.796	0.799	0.802	0.804	0.807
Portugal	0.512	0.517	0.526	0.535	0.535	0.527	0.526	0.536	0.547	0.556	0.564	0.571	0.577	0.584	0.592	0.601	0.609	0.616	0.624	0.633
Spain	0.581	0.582	0.586	0.591	0.596	0.599	0.601	0.608	0.617	0.625	0.634	0.644	0.653	0.661	0.668	0.676	0.683	0.691	0.698	0.707
Sweden	0.703	0.705	0.71	0.715	0.721	0.73	0.738	0.744	0.749	0.753	0.757	0.763	0.768	0.773	0.776	0.779	0.78	0.781	0.781	0.781
Stockholm	0.75	0.752	0.757	0.763	0.769	0.777	0.784	0.789	0.794	0.797	0.802	0.807	0.811	0.815	0.817	0.819	0.82	0.821	0.822	0.823
Sweden except Stockholm	0.691	0.694	0.698	0.703	0.709	0.718	0.726	0.733	0.738	0.742	0.746	0.751	0.757	0.762	0.765	0.768	0.77	0.771	0.77	0.771
Switzerland	0.785	0.79	0.796	0.801	0.806	0.812	0.817	0.821	0.821	0.821	0.822	0.825	0.827	0.829	0.832	0.835	0.837	0.839	0.84	0.841
United Kingdom	0.605	0.612	0.62	0.629	0.639	0.647	0.654	0.659	0.661	0.664	0.669	0.678	0.684	0.689	0.693	0.698	0.702	0.706	0.711	0.717
England	0.618	0.625	0.633	0.642	0.652	0.66	0.667	0.672	0.673	0.676	0.682	0.689	0.696	0.701	0.705	0.709	0.713	0.716	0.722	0.727
East Midlands	0.59	0.598	0.606	0.615	0.625	0.634	0.641	0.646	0.648	0.651	0.657	0.665	0.672	0.677	0.681	0.685	0.689	0.693	0.699	0.705
Derby	0.591	0.599	0.608	0.617	0.628	0.636	0.644	0.649	0.65	0.652	0.658	0.667	0.674	0.68	0.684	0.689	0.694	0.698	0.704	0.71
Derbyshire	0.581	0.588	0.597	0.606	0.616	0.625	0.632	0.637	0.639	0.642	0.648	0.656	0.663	0.668	0.672	0.676	0.679	0.683	0.688	0.693
Leicester	0.577	0.585	0.593	0.603	0.613	0.621	0.629	0.634	0.635	0.638	0.643	0.652	0.659	0.665	0.67	0.676	0.682	0.687	0.694	0.702
Leicestershire	0.609	0.616	0.624	0.634	0.643	0.651	0.658	0.663	0.666	0.669	0.675	0.683	0.689	0.694	0.698	0.702	0.706	0.709	0.715	0.721
Lincolnshire	0.584	0.591	0.6	0.609	0.619	0.628	0.635	0.64	0.643	0.646	0.652	0.66	0.666	0.67	0.674	0.678	0.681	0.684	0.689	0.695
Northamptonshire	0.593	0.6	0.609	0.619	0.629	0.638	0.646	0.651	0.653	0.656	0.662	0.671	0.677	0.682	0.686	0.69	0.693	0.697	0.702	0.708
Nottingham	0.607	0.614	0.622	0.632	0.641	0.649	0.657	0.662	0.664	0.667	0.673	0.681	0.688	0.694	0.7	0.705	0.71	0.715	0.721	0.728
Nottinghamshire	0.579	0.587	0.595	0.604	0.614	0.622	0.629	0.634	0.637	0.64	0.645	0.653	0.66	0.664	0.668	0.672	0.676	0.68	0.685	0.691
Rutland	0.609	0.617	0.625	0.634	0.644	0.652	0.659	0.663	0.665	0.667	0.673	0.681	0.687	0.692	0.697	0.702	0.708	0.712	0.719	0.725
East of England	0.606	0.614	0.622	0.632	0.642	0.65	0.658	0.663	0.665	0.667	0.673	0.681	0.688	0.693	0.697	0.701	0.705	0.708	0.714	0.719
Bedford	0.608	0.615	0.624	0.633	0.643	0.652	0.659	0.664	0.666	0.668	0.674	0.682	0.689	0.695	0.699	0.704	0.708	0.712	0.718	0.724
Cambridgeshire	0.631	0.638	0.646	0.655	0.665	0.673	0.68	0.685	0.688	0.691	0.697	0.704	0.711	0.715	0.72	0.724	0.728	0.732	0.738	0.744
Central Bedfordshire	0.602	0.609	0.618	0.628	0.638	0.647	0.655	0.66	0.661	0.663	0.669	0.678	0.684	0.689	0.694	0.698	0.702	0.707	0.712	0.718
Essex	0.597	0.605	0.613	0.623	0.632	0.641	0.648	0.653	0.656	0.658	0.664	0.672	0.678	0.683	0.687	0.691	0.694	0.698	0.703	0.708
Hertfordshire	0.631	0.638	0.646	0.655	0.665	0.673	0.681	0.686	0.688	0.691	0.697	0.705	0.711	0.716	0.72	0.725	0.729	0.732	0.738	0.743
Luton	0.586	0.594	0.603	0.613	0.624	0.633	0.641	0.646	0.648	0.65	0.655	0.664	0.671	0.677	0.681	0.685	0.689	0.693	0.699	0.705
Norfolk	0.596	0.603	0.612	0.621	0.631	0.64	0.647	0.652	0.654	0.657	0.663	0.671	0.677	0.682	0.686	0.69	0.694	0.697	0.702	0.707
Peterborough	0.592	0.6	0.609	0.619	0.63	0.64	0.648	0.653	0.654	0.656	0.662	0.671	0.678	0.684	0.688	0.693	0.697	0.7	0.705	0.71
Southend-on-Sea	0.575	0.583	0.591	0.601	0.611	0.619	0.627	0.632	0.634	0.637	0.643	0.652	0.658	0.663	0.667	0.671	0.676	0.68	0.686	0.691
Suffolk	0.593	0.601	0.61	0.62	0.63	0.639	0.647	0.651	0.653	0.655	0.661	0.669	0.676	0.681	0.685	0.69	0.694	0.697	0.703	0.708
Thurrock	0.595	0.603	0.611	0.621	0.631	0.64	0.647	0.652	0.654	0.656	0.662	0.67	0.676	0.68	0.684	0.688	0.691	0.695	0.7	0.705
Greater London	0.665	0.672	0.68	0.688	0.697	0.705	0.712	0.717	0.719	0.721	0.727	0.735	0.741	0.746	0.75	0.755	0.759	0.763	0.768	0.774
Barking and Dagenham	0.592	0.6	0.609	0.618	0.628	0.637	0.644	0.649	0.651	0.654	0.66	0.668	0.675	0.679	0.683	0.686	0.689	0.692	0.696	0.7
Barnet	0.643	0.651	0.658	0.667	0.676	0.684	0.691	0.696	0.698	0.701	0.707	0.714	0.721	0.726	0.73	0.735	0.739	0.743	0.748	0.754
Bexley	0.611	0.618	0.626	0.635	0.645	0.653	0.66	0.665	0.667	0.67	0.676	0.683	0.689	0.694	0.697	0.7	0.704	0.706	0.711	0.716
Brent	0.631	0.639	0.647	0.656	0.666	0.675	0.682	0.687	0.689	0.691	0.697	0.705	0.712	0.717	0.722	0.727	0.732	0.736	0.742	0.748
Bromley	0.641	0.648	0.656	0.665	0.674	0.682	0.688	0.693	0.696	0.699	0.705	0.712	0.718	0.723	0.726	0.73	0.733	0.736	0.741	0.747
Camden	0.744	0.75	0.755	0.762	0.769	0.776	0.781	0.785	0.787	0.79	0.794	0.8	0.806	0.81	0.814	0.818	0.822	0.825	0.829	0.834
Croydon	0.625	0.632	0.641	0.65	0.661	0.669	0.677	0.682	0.684	0.686	0.692	0.701	0.707	0.712	0.716	0.72	0.724	0.727	0.731	0.736
Ealing	0.641	0.648	0.657	0.666	0.676	0.684	0.691	0.696	0.698	0.7	0.706	0.714	0.721	0.727	0.732	0.738	0.743	0.748	0.754	0.759
Enfield	0.621	0.629	0.637	0.647	0.657	0.665	0.672	0.677	0.68	0.683	0.689	0.697	0.703	0.707	0.711	0.715	0.719	0.722	0.727	0.732
Greenwich	0.608	0.616	0.624	0.634	0.644	0.653	0.66	0.665	0.667	0.669	0.675	0.684	0.69	0.695	0.699	0.702	0.706	0.709	0.714	0.72
Hackney	0.654	0.662	0.671	0.682	0.693	0.703	0.711	0.716	0.716	0.716	0.722	0.731	0.737	0.741	0.745	0.747	0.75	0.752	0.756	0.762
Hammersmith and Fulham	0.702	0.709	0.716	0.724	0.732	0.739	0.745	0.75	0.753	0.756	0.762	0.769	0.776	0.781	0.787	0.793	0.799	0.805	0.811	0.818
Haringey	0.633	0.641	0.649	0.659	0.669	0.677	0.684	0.689	0.692	0.695	0.701	0.709	0.716	0.721	0.725	0.73	0.734	0.738	0.744	0.749
Harrow	0.632	0.639	0.647	0.656	0.666	0.674	0.681	0.686	0.688	0.691	0.697	0.704	0.71	0.715	0.719	0.723	0.727	0.731	0.736	0.742

Appendix Table 9. Socio-Demographic Index values for all estimated GBD 2017 locations, 1970–1989

Location	1970	1971	1972	1973	1974	1975	1976	1977	1978	1979	1980	1981	1982	1983	1984	1985	1986	1987	1988	1989
Havering	0.613	0.621	0.628	0.637	0.646	0.654	0.661	0.666	0.668	0.671	0.677	0.684	0.69	0.694	0.697	0.7	0.703	0.705	0.71	0.715
Hillingdon	0.665	0.672	0.681	0.69	0.699	0.708	0.715	0.72	0.722	0.725	0.731	0.739	0.746	0.751	0.755	0.759	0.763	0.766	0.771	0.776
Hounslow	0.648	0.656	0.664	0.674	0.684	0.693	0.7	0.705	0.707	0.71	0.716	0.724	0.731	0.736	0.74	0.744	0.748	0.752	0.757	0.764
Islington	0.702	0.709	0.717	0.726	0.736	0.744	0.751	0.755	0.756	0.757	0.762	0.769	0.775	0.779	0.784	0.789	0.793	0.796	0.801	0.806
Kensington and Chelsea	0.73	0.736	0.742	0.75	0.757	0.764	0.77	0.775	0.778	0.782	0.787	0.793	0.799	0.804	0.808	0.813	0.817	0.821	0.826	0.833
Kingston upon Thames	0.674	0.681	0.688	0.695	0.703	0.711	0.717	0.721	0.724	0.727	0.733	0.739	0.745	0.75	0.754	0.759	0.764	0.768	0.775	0.781
Lambeth	0.647	0.655	0.663	0.672	0.682	0.69	0.698	0.702	0.705	0.707	0.713	0.721	0.728	0.734	0.739	0.745	0.751	0.756	0.762	0.769
Lewisham	0.612	0.619	0.627	0.636	0.645	0.653	0.66	0.665	0.667	0.67	0.675	0.683	0.69	0.696	0.701	0.707	0.712	0.716	0.722	0.728
Merton	0.639	0.646	0.654	0.663	0.673	0.681	0.688	0.693	0.695	0.698	0.704	0.711	0.718	0.722	0.726	0.731	0.736	0.74	0.746	0.752
Newham	0.592	0.601	0.61	0.62	0.631	0.64	0.648	0.653	0.655	0.657	0.663	0.673	0.68	0.685	0.69	0.694	0.698	0.702	0.707	0.712
Redbridge	0.615	0.623	0.63	0.639	0.649	0.657	0.664	0.669	0.671	0.674	0.68	0.688	0.694	0.698	0.702	0.706	0.71	0.713	0.718	0.723
Richmond upon Thames	0.683	0.69	0.697	0.705	0.713	0.72	0.727	0.731	0.734	0.737	0.743	0.749	0.755	0.76	0.764	0.768	0.773	0.777	0.782	0.789
Southwark	0.664	0.672	0.68	0.69	0.7	0.708	0.715	0.72	0.722	0.725	0.731	0.74	0.746	0.753	0.758	0.763	0.769	0.774	0.78	0.787
Sutton	0.621	0.629	0.637	0.646	0.656	0.664	0.671	0.676	0.679	0.682	0.688	0.695	0.702	0.706	0.71	0.713	0.716	0.72	0.725	0.73
Tower Hamlets	0.645	0.653	0.662	0.672	0.683	0.692	0.7	0.704	0.703	0.703	0.708	0.717	0.724	0.729	0.734	0.739	0.745	0.749	0.755	0.761
Waltham Forest	0.592	0.6	0.608	0.618	0.628	0.636	0.643	0.648	0.65	0.653	0.659	0.667	0.673	0.679	0.683	0.688	0.692	0.696	0.702	0.707
Wandsworth	0.667	0.674	0.681	0.689	0.698	0.705	0.712	0.716	0.718	0.721	0.727	0.734	0.741	0.747	0.753	0.76	0.766	0.772	0.78	0.787
Westminster	0.739	0.745	0.751	0.758	0.766	0.772	0.778	0.782	0.784	0.787	0.791	0.797	0.802	0.806	0.808	0.811	0.814	0.817	0.821	0.826
North East England	0.573	0.581	0.59	0.6	0.61	0.619	0.627	0.632	0.634	0.637	0.643	0.651	0.658	0.663	0.667	0.671	0.676	0.679	0.685	0.691
County Durham	0.568	0.576	0.585	0.594	0.604	0.613	0.62	0.626	0.628	0.631	0.637	0.645	0.652	0.658	0.662	0.667	0.671	0.674	0.679	0.685
Darlington	0.583	0.591	0.6	0.611	0.622	0.631	0.639	0.644	0.646	0.649	0.655	0.664	0.671	0.676	0.68	0.684	0.687	0.689	0.693	0.698
Gateshead	0.577	0.585	0.594	0.604	0.614	0.623	0.631	0.636	0.638	0.64	0.646	0.655	0.661	0.666	0.67	0.674	0.679	0.682	0.688	0.694
Hartlepool	0.556	0.564	0.573	0.584	0.595	0.604	0.612	0.617	0.619	0.621	0.627	0.636	0.643	0.648	0.651	0.655	0.658	0.661	0.666	0.672
Middlesbrough	0.56	0.569	0.578	0.588	0.599	0.609	0.617	0.622	0.623	0.625	0.631	0.64	0.647	0.652	0.656	0.66	0.665	0.669	0.675	0.682
Newcastle upon Tyne	0.609	0.617	0.625	0.634	0.643	0.651	0.658	0.663	0.666	0.669	0.675	0.683	0.689	0.695	0.699	0.704	0.709	0.715	0.721	0.729
North Tyneside	0.576	0.584	0.592	0.602	0.612	0.621	0.629	0.634	0.636	0.639	0.645	0.653	0.659	0.664	0.668	0.671	0.675	0.679	0.685	0.691
Northumberland	0.574	0.582	0.591	0.601	0.611	0.62	0.628	0.633	0.635	0.638	0.644	0.653	0.66	0.665	0.67	0.674	0.678	0.681	0.687	0.692
Redcar and Cleveland	0.549	0.557	0.566	0.576	0.587	0.596	0.604	0.608	0.61	0.612	0.618	0.627	0.634	0.639	0.643	0.646	0.65	0.653	0.659	0.665
South Tyneside	0.545	0.553	0.562	0.572	0.582	0.591	0.599	0.604	0.606	0.609	0.615	0.623	0.629	0.633	0.636	0.639	0.643	0.646	0.652	0.659
Stockton-on-Tees	0.581	0.589	0.598	0.609	0.62	0.629	0.637	0.642	0.644	0.646	0.652	0.661	0.667	0.672	0.676	0.68	0.684	0.687	0.693	0.699
Sunderland	0.563	0.571	0.58	0.59	0.601	0.61	0.618	0.623	0.624	0.626	0.632	0.641	0.648	0.654	0.658	0.663	0.667	0.671	0.677	0.684
North West England	0.6	0.608	0.616	0.625	0.635	0.643	0.65	0.655	0.657	0.659	0.665	0.673	0.679	0.684	0.688	0.692	0.696	0.7	0.705	0.711
Blackburn with Darwen	0.569	0.577	0.586	0.597	0.608	0.617	0.625	0.63	0.631	0.633	0.639	0.648	0.655	0.661	0.665	0.669	0.673	0.676	0.681	0.687
Blackpool	0.569	0.576	0.585	0.594	0.604	0.612	0.62	0.625	0.628	0.631	0.637	0.645	0.651	0.655	0.658	0.661	0.663	0.666	0.671	0.676
Bolton	0.583	0.591	0.599	0.609	0.619	0.628	0.635	0.64	0.641	0.644	0.649	0.658	0.664	0.669	0.673	0.677	0.681	0.685	0.69	0.696
Bury	0.589	0.597	0.605	0.615	0.624	0.632	0.64	0.644	0.646	0.649	0.655	0.663	0.669	0.673	0.677	0.68	0.684	0.687	0.692	0.698
Cheshire East	0.635	0.642	0.65	0.659	0.669	0.677	0.684	0.688	0.691	0.693	0.699	0.707	0.712	0.717	0.721	0.725	0.728	0.732	0.737	0.743
Cheshire West and Chester	0.626	0.633	0.641	0.65	0.66	0.668	0.675	0.679	0.681	0.684	0.69	0.697	0.703	0.708	0.712	0.716	0.72	0.723	0.729	0.735
Cumbria	0.606	0.614	0.622	0.631	0.641	0.649	0.656	0.661	0.663	0.665	0.671	0.679	0.685	0.69	0.694	0.698	0.702	0.705	0.71	0.715
Halton	0.586	0.594	0.603	0.613	0.624	0.632	0.64	0.645	0.647	0.649	0.655	0.663	0.669	0.674	0.677	0.68	0.683	0.686	0.692	0.698
Knowsley	0.575	0.582	0.591	0.6	0.61	0.618	0.626	0.63	0.632	0.634	0.639	0.648	0.654	0.659	0.664	0.668	0.672	0.675	0.68	0.686
Lancashire	0.603	0.611	0.619	0.629	0.638	0.647	0.654	0.659	0.661	0.663	0.669	0.677	0.683	0.688	0.692	0.696	0.7	0.703	0.708	0.715
Liverpool	0.602	0.609	0.617	0.626	0.635	0.642	0.649	0.654	0.656	0.659	0.664	0.672	0.678	0.683	0.687	0.692	0.697	0.702	0.708	0.715
Manchester	0.623	0.63	0.638	0.647	0.657	0.665	0.672	0.677	0.679	0.682	0.688	0.696	0.703	0.709	0.713	0.718	0.723	0.728	0.734	0.74
Oldham	0.569	0.577	0.586	0.596	0.606	0.615	0.622	0.627	0.628	0.631	0.636	0.645	0.652	0.657	0.66	0.664	0.668	0.671	0.676	0.681
Rochdale	0.571	0.579	0.588	0.598	0.608	0.617	0.624	0.629	0.631	0.633	0.639	0.648	0.654	0.659	0.663	0.666	0.67	0.672	0.677	0.682
Salford	0.592	0.599	0.608	0.617	0.627	0.636	0.643	0.648	0.65	0.652	0.658	0.666	0.672	0.677	0.681	0.685	0.689	0.692	0.697	0.703
Sefton	0.593	0.601	0.608	0.617	0.627	0.634	0.641	0.646	0.648	0.651	0.656	0.664	0.67	0.674	0.678	0.682	0.686	0.689	0.694	0.701
St Helens	0.571	0.579	0.587	0.596	0.605	0.613	0.62	0.625	0.627	0.629	0.635	0.643	0.65	0.655	0.659	0.663	0.667	0.67	0.675	0.68
Stockport	0.613	0.62	0.628	0.637	0.646	0.654	0.661	0.666	0.668	0.67	0.676	0.683	0.69	0.694	0.698	0.702	0.706	0.71	0.715	0.722
Tameside	0.577	0.584	0.593	0.602	0.612	0.62	0.628	0.633	0.634	0.637	0.643	0.651	0.657	0.662	0.666	0.669	0.673	0.676	0.681	0.687
Trafford	0.635	0.642	0.65	0.659	0.669	0.677	0.684	0.689	0.691	0.693	0.699	0.706	0.712	0.717	0.721	0.725	0.73	0.734	0.739	0.745
Warrington	0.623	0.63	0.638	0.648	0.658	0.666	0.673	0.678	0.68	0.682	0.688	0.696	0.702	0.707	0.711	0.715	0.719	0.722	0.728	0.734
Wigan	0.575	0.583	0.591	0.601	0.61	0.619	0.626	0.631	0.632	0.635	0.64	0.648	0.655	0.66	0.664	0.668	0.672	0.676	0.681	0.687
Wirral	0.581	0.589	0.597	0.607	0.616	0.625	0.632	0.637	0.638	0.641	0.647	0.655	0.661	0.666	0.669	0.673	0.676	0.679	0.685	0.69
South East England	0.639	0.646	0.654	0.663	0.672	0.68	0.687	0.692	0.694	0.696	0.701	0.709	0.715	0.72	0.724	0.727	0.731	0.735	0.739	0.745
Bracknell Forest	0.645	0.653	0.66	0.669	0.679	0.687	0.694	0.698	0.699	0.701	0.707	0.715	0.721	0.727	0.732	0.736	0.74	0.744	0.749	0.755
Brighton and Hove	0.654	0.66	0.667	0.674	0.682	0.689	0.695	0.699	0.702	0.705	0.71	0.716	0.722	0.727	0.732	0.736	0.742	0.747	0.754	0.76
Buckinghamshire	0.655	0.662	0.67	0.679	0.688	0.696	0.702	0.707	0.708	0.711	0.716	0.724	0.73	0.735	0.739	0.743	0.747	0.75	0.754	0.759
East Sussex	0.604	0.611	0.62	0.629	0.639	0.647	0.654	0.659	0.661	0.663	0.669	0.676	0.682	0.686	0.69	0.693	0.696	0.699	0.704	0.708
Hampshire	0.634	0.641	0.649	0.659	0.668	0.676	0.683	0.688	0.689	0.691	0.697	0.704	0.71	0.715	0.719	0.723	0.726	0.73	0.735	0.74
Isle of Wight	0.596	0.604	0.612	0.622	0.632	0.64	0.647	0.652	0.653	0.655	0.66	0.668	0.674	0.678	0.681	0.684	0.688	0.691	0.695	0.7
Kent	0.614	0.622	0.63	0.639	0.649	0.658	0.665	0.67	0.671	0.674	0.68	0.687	0.693	0.698	0.701	0.704	0.707	0.71	0.714	0.719
Medway	0.59	0.598	0.606	0.616	0.626	0.635	0.642	0.646	0.648	0.65	0.655	0.663	0.669	0.673	0.677	0.68	0.684	0.687	0.692	0.698
Milton Keynes	0.632	0.64	0.65	0.66	0.672	0.681	0.689	0.694	0.695	0.697	0.702	0.711	0.717	0.722	0.726	0.73	0.734	0.738	0.744	0.749
Oxfordshire	0.66	0.667	0.674	0.683	0.691	0.699	0.705	0.71	0.712	0.714	0.72	0.727	0.733	0.739	0.743	0.747	0.751	0.755	0.76	0.765
Portsmouth	0.632	0.639	0.648	0.657	0.667	0.675	0.682	0.687	0.69	0.692	0.698	0.706	0.711	0.716	0.719	0.723	0.728	0.732	0.738	0.744
Reading	0.666	0.674	0.681	0.69	0.699	0.707	0.714	0.719	0.721	0.723	0.729	0.736	0.742	0.747	0.751	0.756	0.761	0.766	0.773	0.779
Slough	0.644	0.652	0.66	0.67	0.681	0.69	0.698	0.702	0.703	0.704	0.71	0.719	0.726	0.731	0.735	0.739	0.744	0.748	0.754	0.76
Southampton	0.64	0.647	0.655	0.664	0.673	0.68	0.687	0.692	0.694	0.696	0.702	0.709	0.715	0.72	0.724	0.728	0.732	0.736	0.741	0.747
Surrey	0.667	0.673	0.68	0.689	0.697	0.705	0.711	0.716	0.718	0.721	0.726	0.733	0.739	0.743	0.747	0.751	0.755	0.758	0.763	0.769

Appendix Table 9. Socio-Demographic Index values for all estimated GBD 2017 locations, 1970–1989

Location	1970	1971	1972	1973	1974	1975	1976	1977	1978	1979	1980	1981	1982	1983	1984	1985	1986	1987	1988	1989
West Berkshire	0.659	0.667	0.675	0.685	0.695	0.703	0.711	0.715	0.717	0.719	0.724	0.733	0.739	0.743	0.747	0.751	0.755	0.758	0.764	0.769
West Sussex	0.629	0.636	0.644	0.654	0.663	0.671	0.678	0.683	0.684	0.687	0.692	0.7	0.706	0.711	0.715	0.719	0.722	0.725	0.73	0.735
Windsor and Maidenhead	0.67	0.677	0.685	0.693	0.702	0.71	0.717	0.721	0.723	0.725	0.731	0.738	0.744	0.748	0.752	0.756	0.761	0.764	0.769	0.774
Wokingham	0.67	0.677	0.684	0.693	0.701	0.709	0.715	0.72	0.722	0.724	0.729	0.736	0.741	0.745	0.749	0.753	0.757	0.761	0.767	0.773
South West England	0.614	0.622	0.63	0.639	0.648	0.656	0.664	0.668	0.67	0.673	0.679	0.687	0.693	0.698	0.702	0.705	0.709	0.713	0.718	0.724
Bath and North East Somerset	0.64	0.647	0.654	0.662	0.67	0.676	0.683	0.688	0.691	0.694	0.699	0.706	0.712	0.717	0.721	0.725	0.73	0.734	0.74	0.746
Bournemouth	0.62	0.627	0.634	0.642	0.65	0.657	0.664	0.669	0.672	0.675	0.681	0.688	0.694	0.698	0.703	0.708	0.713	0.718	0.724	0.73
Bristol, City of	0.644	0.651	0.659	0.667	0.676	0.684	0.691	0.696	0.698	0.701	0.706	0.714	0.72	0.726	0.73	0.735	0.74	0.744	0.751	0.757
Cornwall	0.585	0.593	0.602	0.611	0.621	0.63	0.637	0.642	0.644	0.646	0.652	0.66	0.666	0.67	0.674	0.677	0.681	0.684	0.689	0.695
Devon	0.606	0.614	0.622	0.631	0.64	0.648	0.655	0.66	0.662	0.665	0.671	0.679	0.685	0.69	0.693	0.697	0.701	0.704	0.709	0.715
Dorset	0.605	0.613	0.621	0.63	0.64	0.648	0.655	0.66	0.662	0.665	0.671	0.679	0.685	0.689	0.692	0.696	0.699	0.702	0.707	0.712
Gloucestershire	0.62	0.627	0.635	0.644	0.654	0.662	0.669	0.674	0.676	0.679	0.684	0.692	0.699	0.704	0.708	0.712	0.716	0.719	0.724	0.73
North Somerset	0.598	0.605	0.614	0.623	0.633	0.641	0.648	0.653	0.655	0.657	0.663	0.671	0.677	0.682	0.687	0.691	0.694	0.698	0.704	0.71
Plymouth	0.601	0.608	0.616	0.626	0.636	0.644	0.651	0.656	0.658	0.66	0.666	0.674	0.681	0.687	0.692	0.697	0.702	0.706	0.713	0.719
Poole	0.613	0.62	0.629	0.639	0.648	0.657	0.664	0.669	0.671	0.674	0.68	0.687	0.693	0.697	0.7	0.704	0.708	0.712	0.717	0.723
Somerset	0.6	0.608	0.616	0.626	0.636	0.645	0.652	0.657	0.659	0.661	0.667	0.675	0.682	0.687	0.69	0.694	0.697	0.699	0.704	0.708
South Gloucestershire	0.633	0.641	0.649	0.658	0.667	0.676	0.683	0.688	0.69	0.693	0.698	0.706	0.712	0.717	0.721	0.725	0.729	0.732	0.738	0.743
Swindon	0.628	0.636	0.644	0.654	0.664	0.673	0.681	0.686	0.687	0.689	0.695	0.703	0.71	0.715	0.719	0.723	0.727	0.73	0.736	0.742
Torbay	0.584	0.592	0.601	0.61	0.62	0.628	0.636	0.641	0.643	0.646	0.652	0.66	0.665	0.669	0.672	0.676	0.68	0.683	0.689	0.694
Wiltshire	0.612	0.62	0.628	0.638	0.648	0.656	0.664	0.668	0.67	0.672	0.677	0.686	0.693	0.698	0.702	0.706	0.71	0.713	0.718	0.722
West Midlands	0.588	0.595	0.604	0.613	0.623	0.632	0.639	0.644	0.646	0.649	0.655	0.663	0.669	0.674	0.679	0.683	0.687	0.69	0.696	0.702
Birmingham	0.582	0.59	0.598	0.608	0.618	0.627	0.635	0.639	0.641	0.643	0.649	0.658	0.665	0.67	0.675	0.68	0.685	0.689	0.695	0.702
Coventry	0.601	0.608	0.617	0.626	0.636	0.644	0.652	0.656	0.658	0.661	0.666	0.674	0.681	0.686	0.69	0.694	0.698	0.702	0.709	0.716
Dudley	0.576	0.583	0.592	0.601	0.611	0.619	0.627	0.632	0.633	0.636	0.642	0.65	0.656	0.661	0.665	0.669	0.673	0.677	0.682	0.688
Herefordshire, County of	0.591	0.599	0.607	0.617	0.627	0.635	0.643	0.648	0.65	0.653	0.659	0.667	0.674	0.678	0.682	0.685	0.688	0.691	0.695	0.701
Sandwell	0.563	0.571	0.579	0.589	0.599	0.608	0.615	0.62	0.621	0.623	0.629	0.638	0.645	0.65	0.654	0.658	0.663	0.666	0.672	0.678
Shropshire	0.594	0.601	0.61	0.619	0.629	0.637	0.645	0.65	0.652	0.655	0.661	0.669	0.675	0.679	0.683	0.687	0.69	0.693	0.698	0.704
Solihull	0.621	0.629	0.637	0.647	0.656	0.665	0.672	0.677	0.679	0.682	0.688	0.695	0.701	0.706	0.709	0.712	0.715	0.718	0.723	0.728
Staffordshire	0.592	0.599	0.607	0.617	0.627	0.635	0.642	0.647	0.649	0.652	0.658	0.666	0.672	0.677	0.68	0.684	0.688	0.691	0.696	0.702
Stoke-on-Trent	0.569	0.576	0.584	0.594	0.604	0.612	0.62	0.624	0.626	0.628	0.634	0.643	0.649	0.655	0.659	0.664	0.668	0.672	0.677	0.684
Telford and Wrekin	0.592	0.6	0.609	0.619	0.63	0.639	0.647	0.652	0.653	0.655	0.661	0.67	0.677	0.683	0.687	0.691	0.695	0.698	0.703	0.709
Walsall	0.565	0.572	0.581	0.59	0.6	0.609	0.616	0.621	0.623	0.625	0.631	0.639	0.646	0.651	0.655	0.659	0.663	0.666	0.671	0.676
Warwickshire	0.615	0.622	0.63	0.64	0.65	0.658	0.665	0.67	0.672	0.675	0.681	0.689	0.695	0.7	0.704	0.707	0.711	0.715	0.72	0.726
Wolverhampton	0.573	0.581	0.589	0.599	0.609	0.618	0.625	0.63	0.631	0.634	0.64	0.648	0.655	0.66	0.664	0.668	0.672	0.675	0.68	0.686
Worcestershire	0.591	0.599	0.607	0.617	0.626	0.635	0.642	0.647	0.65	0.653	0.659	0.666	0.673	0.678	0.681	0.685	0.688	0.692	0.697	0.702
Yorkshire and the Humber	0.59	0.598	0.606	0.616	0.626	0.634	0.642	0.647	0.649	0.652	0.658	0.666	0.672	0.677	0.681	0.686	0.69	0.694	0.699	0.705
Barnsley	0.558	0.566	0.575	0.584	0.594	0.603	0.61	0.615	0.617	0.619	0.625	0.634	0.64	0.645	0.649	0.653	0.657	0.661	0.665	0.671
Bradford	0.567	0.575	0.584	0.595	0.605	0.615	0.623	0.627	0.629	0.631	0.637	0.646	0.653	0.658	0.663	0.668	0.672	0.676	0.682	0.688
Calderdale	0.585	0.593	0.602	0.612	0.623	0.632	0.64	0.645	0.647	0.649	0.655	0.664	0.67	0.675	0.679	0.683	0.687	0.691	0.697	0.703
Doncaster	0.557	0.566	0.574	0.584	0.595	0.603	0.611	0.616	0.618	0.62	0.626	0.635	0.641	0.646	0.649	0.653	0.657	0.66	0.665	0.67
East Riding of Yorkshire	0.598	0.605	0.614	0.623	0.633	0.641	0.648	0.653	0.656	0.659	0.665	0.673	0.679	0.684	0.687	0.691	0.694	0.698	0.703	0.709
Kingston upon Hull, City of	0.567	0.576	0.584	0.594	0.605	0.613	0.621	0.626	0.628	0.631	0.637	0.645	0.652	0.657	0.66	0.664	0.668	0.672	0.677	0.683
Kirklees	0.579	0.587	0.596	0.606	0.616	0.624	0.632	0.637	0.639	0.641	0.647	0.656	0.662	0.668	0.672	0.677	0.681	0.685	0.691	0.697
Leeds	0.617	0.624	0.632	0.641	0.65	0.657	0.664	0.669	0.672	0.675	0.681	0.688	0.695	0.7	0.705	0.709	0.714	0.719	0.725	0.732
North East Lincolnshire	0.578	0.586	0.595	0.606	0.617	0.626	0.634	0.639	0.641	0.643	0.649	0.658	0.664	0.669	0.672	0.675	0.679	0.682	0.687	0.693
North Lincolnshire	0.585	0.593	0.602	0.612	0.622	0.631	0.639	0.644	0.645	0.648	0.654	0.662	0.669	0.674	0.677	0.681	0.685	0.687	0.692	0.698
North Yorkshire	0.607	0.615	0.623	0.633	0.643	0.651	0.658	0.663	0.666	0.669	0.675	0.683	0.689	0.694	0.698	0.702	0.705	0.709	0.714	0.719
Rotherham	0.565	0.573	0.582	0.592	0.602	0.61	0.618	0.623	0.624	0.627	0.633	0.641	0.648	0.653	0.656	0.66	0.664	0.667	0.672	0.677
Sheffield	0.609	0.616	0.624	0.633	0.641	0.649	0.656	0.661	0.664	0.667	0.673	0.68	0.687	0.692	0.696	0.7	0.705	0.709	0.715	0.721
Wakefield	0.574	0.582	0.591	0.601	0.611	0.62	0.627	0.632	0.634	0.637	0.643	0.651	0.658	0.662	0.666	0.67	0.674	0.677	0.682	0.688
York	0.641	0.648	0.656	0.664	0.673	0.68	0.687	0.692	0.695	0.698	0.704	0.711	0.717	0.723	0.727	0.732	0.737	0.742	0.748	0.756
Northern Ireland	0.574	0.582	0.59	0.598	0.607	0.614	0.622	0.627	0.628	0.63	0.638	0.649	0.657	0.663	0.668	0.673	0.679	0.685	0.695	0.704
Scotland	0.541	0.547	0.556	0.563	0.571	0.58	0.59	0.596	0.598	0.601	0.607	0.616	0.624	0.631	0.636	0.642	0.648	0.653	0.66	0.667
Wales	0.503	0.51	0.519	0.529	0.54	0.549	0.557	0.563	0.566	0.569	0.576	0.586	0.593	0.6	0.605	0.61	0.615	0.62	0.627	0.635
Latin America and Caribbean	0.372	0.378	0.385	0.392	0.399	0.406	0.413	0.421	0.428	0.434	0.441	0.447	0.453	0.46	0.466	0.472	0.477	0.483	0.488	0.493
Andean Latin America	0.352	0.358	0.364	0.371	0.377	0.385	0.395	0.404	0.412	0.419	0.425	0.432	0.44	0.448	0.455	0.461	0.467	0.472	0.477	0.48
Bolivia	0.302	0.31	0.318	0.324	0.329	0.332	0.332	0.332	0.335	0.34	0.346	0.352	0.36	0.366	0.371	0.375	0.38	0.388	0.396	0.4
Ecuador	0.384	0.387	0.395	0.404	0.417	0.434	0.449	0.457	0.46	0.459	0.455	0.454	0.459	0.467	0.475	0.484	0.491	0.496	0.499	0.502
Peru	0.345	0.352	0.357	0.362	0.365	0.369	0.379	0.393	0.407	0.418	0.431	0.443	0.453	0.461	0.468	0.473	0.478	0.482	0.487	0.491
Caribbean	0.408	0.41	0.417	0.427	0.437	0.445	0.454	0.464	0.472	0.479	0.484	0.487	0.491	0.497	0.503	0.51	0.516	0.518	0.521	0.525
Antigua and Barbuda	0.467	0.479	0.489	0.497	0.502	0.505	0.508	0.512	0.516	0.524	0.531	0.539	0.548	0.556	0.564	0.572	0.58	0.588	0.595	0.603
The Bahamas	0.561	0.569	0.578	0.586	0.589	0.588	0.589	0.593	0.597	0.602	0.606	0.61	0.616	0.626	0.639	0.652	0.663	0.67	0.674	0.675
Barbados	0.522	0.53	0.539	0.55	0.562	0.573	0.582	0.588	0.592	0.596	0.599	0.603	0.607	0.613	0.621	0.629	0.637	0.643	0.646	0.648
Belize	0.277	0.281	0.286	0.29	0.294	0.298	0.302	0.308	0.313	0.319	0.326	0.333	0.342	0.349	0.356	0.363	0.371	0.378	0.385	0.392
Bermuda	0.609	0.619	0.629	0.639	0.648	0.657	0.665	0.673	0.68	0.686	0.691	0.695	0.699	0.703	0.708	0.712	0.716	0.719	0.723	0.727
Cuba	0.421	0.412	0.418	0.432	0.448	0.462	0.481	0.5	0.515	0.528	0.536	0.539	0.541	0.547	0.553	0.563	0.572	0.574	0.576	0.582
Dominica	0.349	0.361	0.379	0.404	0.428	0.446	0.462	0.474	0.483	0.487	0.489	0.493	0.499	0.505	0.512	0.517	0.522	0.525	0.529	0.532
Dominican Republic	0.288	0.295	0.307	0.319	0.33	0.339	0.349	0.359	0.366	0.368	0.368	0.37	0.38	0.395	0.408	0.417	0.421	0.424	0.43	0.437
Grenada	0.335	0.342	0.349	0.357	0.366	0.375	0.384	0.391	0.398	0.405	0.409	0.412	0.413	0.415	0.418	0.422	0.427	0.433	0.44	0.449
Guyana	0.393	0.401	0.409	0.416	0.422	0.429	0.435	0.438	0.44	0.441	0.441	0.443	0.444	0.443	0.441	0.439	0.439	0.44	0.44	0.44
Haiti	0.27	0.274	0.277	0.28	0.284	0.287	0.291	0.294	0.297	0.3	0.304	0.306	0.308	0.31	0.312	0.314	0.316	0.319	0.322	0.325

Appendix Table 9. Socio-Demographic Index values for all estimated GBD 2017 locations, 1970–1989																				
Location	1970	1971	1972	1973	1974	1975	1976	1977	1978	1979	1980	1981	1982	1983	1984	1985	1986	1987	1988	1989
Jamaica	0.406	0.416	0.429	0.442	0.453	0.465	0.476	0.486	0.493	0.498	0.502	0.508	0.513	0.519	0.524	0.531	0.537	0.54	0.542	0.543
Puerto Rico	0.564	0.572	0.579	0.586	0.593	0.596	0.597	0.6	0.61	0.62	0.628	0.637	0.648	0.657	0.664	0.668	0.672	0.675	0.677	0.68
Saint Lucia	0.333	0.342	0.352	0.362	0.373	0.383	0.395	0.406	0.417	0.426	0.432	0.436	0.44	0.445	0.451	0.461	0.471	0.482	0.491	0.5
Saint Vincent and the Grenadines	0.274	0.283	0.298	0.314	0.329	0.342	0.354	0.361	0.367	0.373	0.379	0.385	0.395	0.407	0.419	0.43	0.44	0.448	0.455	0.46
Suriname	0.395	0.406	0.415	0.422	0.43	0.436	0.438	0.443	0.45	0.455	0.457	0.458	0.461	0.468	0.478	0.491	0.504	0.512	0.517	0.523
Trinidad and Tobago	0.497	0.505	0.513	0.52	0.528	0.534	0.54	0.545	0.548	0.552	0.556	0.559	0.563	0.568	0.573	0.579	0.585	0.59	0.594	0.597
Virgin Islands	0.514	0.531	0.551	0.57	0.586	0.6	0.611	0.619	0.626	0.631	0.635	0.638	0.64	0.643	0.647	0.65	0.652	0.655	0.657	0.659
Central Latin America	0.368	0.376	0.384	0.391	0.398	0.405	0.413	0.422	0.429	0.436	0.442	0.45	0.457	0.463	0.47	0.475	0.479	0.483	0.487	0.49
Colombia	0.36	0.37	0.379	0.387	0.395	0.402	0.409	0.416	0.423	0.43	0.438	0.445	0.451	0.457	0.463	0.468	0.472	0.475	0.478	0.481
Costa Rica	0.427	0.436	0.445	0.451	0.454	0.455	0.457	0.459	0.464	0.471	0.477	0.483	0.489	0.494	0.497	0.5	0.503	0.506	0.51	0.516
El Salvador	0.296	0.3	0.304	0.309	0.315	0.322	0.329	0.339	0.349	0.357	0.363	0.37	0.377	0.383	0.388	0.392	0.395	0.397	0.4	0.402
Guatemala	0.273	0.273	0.277	0.284	0.289	0.284	0.271	0.265	0.265	0.264	0.263	0.266	0.274	0.282	0.287	0.293	0.303	0.303	0.302	0.308
Honduras	0.24	0.244	0.248	0.252	0.255	0.258	0.262	0.266	0.272	0.278	0.286	0.296	0.305	0.311	0.315	0.319	0.322	0.326	0.33	0.336
Mexico	0.372	0.379	0.387	0.396	0.404	0.413	0.424	0.435	0.443	0.449	0.456	0.463	0.47	0.477	0.483	0.489	0.494	0.499	0.504	0.509
Aguascalientes	0.402	0.412	0.422	0.432	0.442	0.453	0.464	0.475	0.484	0.491	0.498	0.506	0.513	0.519	0.526	0.532	0.538	0.543	0.549	0.554
Baja California	0.417	0.427	0.436	0.447	0.457	0.467	0.48	0.492	0.5	0.507	0.514	0.523	0.531	0.538	0.545	0.551	0.557	0.561	0.564	0.567
Baja California Sur	0.409	0.417	0.427	0.437	0.446	0.457	0.469	0.482	0.49	0.497	0.504	0.512	0.521	0.528	0.535	0.542	0.549	0.555	0.56	0.565
Campeche	0.352	0.358	0.365	0.372	0.379	0.387	0.398	0.408	0.415	0.42	0.426	0.434	0.441	0.446	0.451	0.458	0.464	0.47	0.475	0.48
Chiapas	0.302	0.309	0.315	0.321	0.327	0.334	0.344	0.352	0.358	0.362	0.367	0.372	0.377	0.38	0.384	0.388	0.391	0.394	0.397	0.4
Chihuahua	0.381	0.389	0.399	0.408	0.418	0.428	0.44	0.453	0.462	0.469	0.477	0.487	0.497	0.505	0.513	0.52	0.528	0.534	0.538	0.543
Coahuila	0.401	0.409	0.417	0.425	0.432	0.44	0.451	0.462	0.468	0.473	0.479	0.487	0.494	0.501	0.509	0.517	0.524	0.531	0.538	0.544
Colima	0.381	0.389	0.398	0.406	0.414	0.423	0.434	0.445	0.452	0.457	0.464	0.471	0.478	0.485	0.492	0.5	0.508	0.516	0.524	0.531
Mexico City	0.446	0.456	0.468	0.48	0.492	0.504	0.518	0.532	0.542	0.551	0.56	0.569	0.576	0.579	0.581	0.581	0.58	0.581	0.585	0.593
Durango	0.354	0.362	0.371	0.38	0.388	0.398	0.411	0.423	0.432	0.439	0.447	0.457	0.465	0.472	0.478	0.485	0.491	0.496	0.499	0.503
Guanajuato	0.34	0.348	0.356	0.364	0.372	0.381	0.391	0.401	0.408	0.413	0.419	0.424	0.43	0.436	0.442	0.451	0.459	0.468	0.477	0.486
Guerrero	0.294	0.3	0.308	0.315	0.322	0.33	0.34	0.351	0.358	0.364	0.371	0.379	0.386	0.392	0.398	0.404	0.409	0.414	0.418	0.423
Hidalgo	0.282	0.289	0.296	0.304	0.313	0.321	0.333	0.344	0.35	0.355	0.36	0.364	0.368	0.371	0.374	0.38	0.386	0.394	0.402	0.41
Jalisco	0.389	0.396	0.404	0.412	0.419	0.427	0.437	0.447	0.454	0.461	0.468	0.476	0.483	0.49	0.496	0.503	0.508	0.514	0.52	0.526
México	0.375	0.381	0.389	0.396	0.404	0.412	0.424	0.436	0.445	0.453	0.461	0.471	0.482	0.493	0.505	0.516	0.524	0.53	0.533	0.532
Michoacán de Ocampo	0.326	0.333	0.34	0.347	0.354	0.362	0.371	0.38	0.386	0.391	0.397	0.403	0.41	0.417	0.424	0.432	0.439	0.446	0.452	0.459
Morelos	0.378	0.385	0.393	0.4	0.408	0.415	0.425	0.435	0.441	0.446	0.451	0.458	0.465	0.472	0.479	0.486	0.495	0.503	0.51	0.518
Nayarit	0.337	0.346	0.354	0.363	0.372	0.382	0.394	0.406	0.414	0.421	0.429	0.438	0.446	0.453	0.459	0.466	0.472	0.478	0.484	0.49
Nuevo León	0.433	0.442	0.452	0.463	0.472	0.482	0.494	0.506	0.514	0.521	0.528	0.536	0.545	0.554	0.564	0.572	0.579	0.585	0.59	0.594
Oaxaca	0.292	0.299	0.305	0.312	0.318	0.326	0.335	0.345	0.352	0.357	0.364	0.372	0.38	0.387	0.393	0.4	0.407	0.413	0.419	0.425
Puebla	0.314	0.322	0.33	0.338	0.346	0.355	0.368	0.38	0.387	0.392	0.398	0.403	0.408	0.416	0.422	0.425	0.426	0.432	0.441	0.449
Querétaro	0.361	0.369	0.377	0.386	0.395	0.405	0.417	0.428	0.436	0.443	0.449	0.455	0.458	0.46	0.462	0.462	0.464	0.47	0.478	0.489
Quintana Roo	0.4	0.405	0.411	0.417	0.422	0.428	0.439	0.449	0.454	0.458	0.463	0.47	0.477	0.483	0.488	0.495	0.501	0.506	0.51	0.515
San Luis Potosí	0.336	0.344	0.351	0.359	0.366	0.375	0.386	0.396	0.404	0.41	0.418	0.426	0.434	0.44	0.447	0.454	0.46	0.465	0.471	0.477
Sinaloa	0.362	0.371	0.379	0.388	0.397	0.407	0.42	0.432	0.44	0.448	0.456	0.465	0.474	0.481	0.488	0.495	0.501	0.507	0.512	0.518
Sonora	0.395	0.403	0.413	0.423	0.432	0.442	0.454	0.465	0.473	0.48	0.488	0.495	0.503	0.512	0.52	0.527	0.533	0.538	0.543	0.549
Tabasco	0.323	0.33	0.338	0.345	0.352	0.361	0.373	0.384	0.392	0.398	0.405	0.413	0.422	0.428	0.435	0.442	0.449	0.456	0.462	0.468
Tamaulipas	0.401	0.41	0.419	0.428	0.436	0.445	0.456	0.467	0.473	0.478	0.483	0.49	0.497	0.504	0.511	0.519	0.525	0.532	0.538	0.544
Tlaxcala	0.328	0.335	0.343	0.351	0.358	0.367	0.378	0.389	0.395	0.399	0.403	0.407	0.412	0.415	0.419	0.427	0.437	0.449	0.46	0.47
Veracruz de Ignacio de la Llave	0.329	0.336	0.343	0.35	0.357	0.365	0.375	0.385	0.393	0.399	0.406	0.414	0.422	0.429	0.435	0.441	0.447	0.451	0.455	0.459
Yucatán	0.363	0.368	0.375	0.383	0.39	0.398	0.408	0.417	0.425	0.431	0.438	0.445	0.452	0.458	0.463	0.469	0.474	0.48	0.485	0.491
Zacatecas	0.32	0.328	0.337	0.344	0.352	0.362	0.373	0.384	0.392	0.399	0.407	0.416	0.425	0.432	0.439	0.447	0.455	0.462	0.469	0.476
Nicaragua	0.264	0.27	0.274	0.277	0.279	0.282	0.285	0.287	0.29	0.292	0.297	0.303	0.309	0.316	0.323	0.33	0.337	0.343	0.348	0.352
Panama	0.401	0.411	0.421	0.431	0.442	0.452	0.459	0.465	0.472	0.479	0.486	0.493	0.499	0.506	0.513	0.519	0.524	0.529	0.533	0.537
Venezuela	0.432	0.441	0.45	0.453	0.456	0.461	0.468	0.477	0.483	0.489	0.496	0.503	0.51	0.519	0.528	0.529	0.528	0.53	0.535	0.533
Tropical Latin America	0.361	0.367	0.373	0.38	0.387	0.393	0.4	0.407	0.413	0.42	0.426	0.432	0.438	0.444	0.45	0.457	0.465	0.472	0.48	0.487
Brazil	0.361	0.367	0.373	0.38	0.387	0.393	0.4	0.407	0.413	0.42	0.427	0.433	0.439	0.444	0.451	0.457	0.465	0.472	0.48	0.487
Acre	0.247	0.252	0.256	0.261	0.265	0.27	0.274	0.279	0.283	0.289	0.294	0.3	0.305	0.311	0.319	0.327	0.336	0.347	0.357	0.367
Alagoas	0.213	0.216	0.221	0.227	0.232	0.238	0.243	0.249	0.255	0.262	0.269	0.276	0.283	0.291	0.3	0.309	0.319	0.329	0.338	0.347
Amapá	0.322	0.327	0.332	0.338	0.343	0.349	0.356	0.362	0.369	0.377	0.384	0.392	0.399	0.406	0.414	0.422	0.431	0.44	0.45	0.459
Amazonas	0.272	0.277	0.282	0.288	0.293	0.298	0.303	0.308	0.315	0.323	0.33	0.339	0.348	0.358	0.369	0.381	0.393	0.405	0.416	0.427
Bahia	0.26	0.265	0.269	0.274	0.279	0.283	0.287	0.292	0.297	0.303	0.31	0.317	0.325	0.333	0.342	0.352	0.362	0.373	0.383	0.393
Ceará	0.278	0.284	0.289	0.294	0.299	0.304	0.308	0.313	0.319	0.325	0.332	0.339	0.347	0.354	0.362	0.37	0.379	0.388	0.396	0.404
Distrito Federal	0.498	0.506	0.513	0.52	0.526	0.533	0.54	0.547	0.554	0.561	0.568	0.574	0.58	0.585	0.591	0.596	0.603	0.61	0.617	0.623
Espírito Santo	0.364	0.371	0.379	0.387	0.394	0.401	0.408	0.414	0.42	0.426	0.431	0.436	0.442	0.447	0.454	0.46	0.468	0.475	0.483	0.491
Goiás	0.306	0.312	0.319	0.326	0.333	0.341	0.349	0.357	0.365	0.374	0.382	0.39	0.397	0.403	0.41	0.417	0.426	0.434	0.443	0.452
Maranhão	0.185	0.188	0.191	0.193	0.196	0.198	0.201	0.204	0.208	0.213	0.219	0.225	0.232	0.24	0.249	0.259	0.27	0.282	0.293	0.303
Mato Grosso	0.326	0.334	0.342	0.351	0.359	0.366	0.374	0.38	0.386	0.391	0.397	0.402	0.408	0.414	0.421	0.429	0.438	0.447	0.456	0.466
Mato Grosso do Sul	0.329	0.336	0.344	0.351	0.358	0.364	0.37	0.375	0.38	0.385	0.39	0.396	0.402	0.408	0.415	0.423	0.431	0.44	0.448	0.457
Minas Gerais	0.357	0.364	0.371	0.378	0.386	0.393	0.4	0.407	0.414	0.421	0.428	0.434	0.44	0.446	0.451	0.457	0.464	0.471	0.477	0.484
Pará	0.286	0.29	0.295	0.3	0.304	0.309	0.314	0.319	0.324	0.329	0.335	0.34	0.346	0.352	0.358	0.366	0.375	0.384	0.393	0.402
Paraíba	0.243	0.248	0.254	0.261	0.268	0.276	0.283	0.292	0.3	0.308	0.316	0.324	0.332	0.34	0.348	0.356	0.365	0.375	0.383	0.391
Paraná	0.357	0.364	0.372	0.38	0.389	0.399	0.408	0.418	0.428	0.438	0.447	0.455	0.463	0.47	0.476	0.482	0.489	0.495	0.501	0.507
Pernambuco	0.272	0.278	0.284	0.29	0.297	0.303	0.309	0.315	0.322	0.329	0.336	0.343	0.351	0.358	0.366	0.375	0.384	0.393	0.401	0.409
Piauí	0.231	0.236	0.241	0.247	0.252	0.258	0.264	0.271	0.277	0.284	0.29	0.297	0.304	0.311	0.318	0.326	0.334	0.342	0.35	0.358
Rio de Janeiro	0.462	0.469	0.476	0.484	0.491	0.497	0.505	0.511	0.518	0.524	0.529	0.534	0.539	0.543	0.547	0.551	0.556	0.561	0.567	0.572
Rio Grande do Norte	0.259	0.265	0.271	0.279	0.286	0.294	0.302	0.31	0.318	0.326	0.335	0.343	0.351	0.359	0.367	0.375	0.383	0.392	0.4	0.408

Appendix Table 9. Socio-Demographic Index values for all estimated GBD 2017 locations, 1970–1989																				
Location	1970	1971	1972	1973	1974	1975	1976	1977	1978	1979	1980	1981	1982	1983	1984	1985	1986	1987	1988	1989
Rio Grande do Sul	0.426	0.433	0.44	0.447	0.454	0.46	0.466	0.472	0.477	0.483	0.488	0.493	0.498	0.502	0.507	0.513	0.519	0.526	0.532	0.538
Rondônia	0.283	0.288	0.294	0.3	0.305	0.31	0.315	0.32	0.326	0.331	0.337	0.343	0.35	0.357	0.365	0.374	0.384	0.394	0.404	0.414
Roraima	0.294	0.299	0.303	0.307	0.312	0.316	0.32	0.324	0.328	0.333	0.338	0.343	0.349	0.356	0.364	0.374	0.384	0.395	0.407	0.417
Santa Catarina	0.403	0.409	0.415	0.422	0.429	0.435	0.443	0.45	0.458	0.466	0.473	0.481	0.488	0.494	0.5	0.507	0.514	0.521	0.528	0.535
São Paulo	0.435	0.441	0.447	0.453	0.459	0.465	0.471	0.477	0.482	0.488	0.494	0.5	0.505	0.51	0.516	0.522	0.529	0.537	0.544	0.551
Sergipe	0.274	0.28	0.286	0.293	0.3	0.307	0.314	0.321	0.328	0.336	0.343	0.351	0.358	0.365	0.373	0.381	0.39	0.399	0.408	0.417
Tocantins	0.242	0.246	0.25	0.254	0.259	0.265	0.272	0.279	0.287	0.295	0.304	0.313	0.322	0.33	0.34	0.349	0.36	0.37	0.379	0.388
Paraguay	0.352	0.357	0.362	0.367	0.373	0.378	0.383	0.388	0.393	0.398	0.404	0.41	0.416	0.421	0.427	0.433	0.44	0.449	0.457	0.463
North Africa and Middle East	0.297	0.304	0.312	0.32	0.328	0.335	0.342	0.35	0.357	0.364	0.373	0.382	0.39	0.399	0.408	0.415	0.422	0.431	0.439	0.448
North Africa and Middle East	0.297	0.304	0.312	0.32	0.328	0.335	0.342	0.35	0.357	0.364	0.373	0.382	0.39	0.399	0.408	0.415	0.422	0.431	0.439	0.448
Afghanistan	0.113	0.115	0.116	0.118	0.119	0.121	0.123	0.125	0.127	0.129	0.13	0.13	0.132	0.135	0.138	0.14	0.143	0.145	0.147	0.148
Algeria	0.304	0.31	0.317	0.324	0.332	0.34	0.348	0.356	0.365	0.375	0.387	0.399	0.41	0.422	0.435	0.448	0.459	0.468	0.477	0.487
Bahrain	0.414	0.427	0.44	0.454	0.467	0.477	0.489	0.501	0.511	0.521	0.531	0.541	0.552	0.564	0.575	0.583	0.591	0.596	0.602	0.607
Egypt	0.282	0.29	0.297	0.303	0.308	0.313	0.32	0.327	0.334	0.342	0.351	0.359	0.367	0.374	0.381	0.389	0.399	0.41	0.422	0.432
Iran	0.319	0.329	0.339	0.348	0.357	0.363	0.37	0.376	0.379	0.382	0.384	0.388	0.401	0.423	0.443	0.453	0.459	0.468	0.48	0.493
Iraq	0.274	0.281	0.287	0.294	0.302	0.311	0.32	0.33	0.34	0.35	0.361	0.37	0.379	0.386	0.393	0.399	0.406	0.413	0.42	0.427
Jordan	0.302	0.316	0.329	0.343	0.357	0.371	0.385	0.399	0.414	0.429	0.446	0.463	0.477	0.49	0.503	0.515	0.526	0.535	0.542	0.547
Kuwait	0.41	0.423	0.424	0.423	0.427	0.436	0.452	0.472	0.49	0.504	0.517	0.531	0.546	0.562	0.579	0.593	0.603	0.618	0.634	0.649
Lebanon	0.295	0.307	0.32	0.332	0.351	0.363	0.372	0.383	0.383	0.395	0.407	0.419	0.429	0.439	0.452	0.466	0.48	0.494	0.505	0.512
Libya	0.318	0.31	0.308	0.326	0.357	0.39	0.423	0.456	0.489	0.519	0.545	0.563	0.58	0.594	0.604	0.611	0.616	0.622	0.628	0.636
Morocco	0.223	0.231	0.238	0.246	0.254	0.262	0.27	0.279	0.287	0.296	0.304	0.312	0.321	0.329	0.338	0.346	0.355	0.364	0.373	0.381
Palestine	0.223	0.231	0.24	0.248	0.257	0.266	0.276	0.285	0.295	0.304	0.312	0.32	0.326	0.332	0.338	0.343	0.348	0.352	0.355	0.358
Oman	0.27	0.28	0.289	0.297	0.305	0.314	0.323	0.332	0.34	0.349	0.358	0.367	0.378	0.39	0.403	0.416	0.428	0.439	0.45	0.462
Qatar	0.43	0.441	0.451	0.462	0.473	0.484	0.495	0.506	0.517	0.527	0.534	0.54	0.545	0.548	0.551	0.556	0.563	0.571	0.58	0.591
Saudi Arabia	0.226	0.232	0.238	0.245	0.251	0.257	0.261	0.266	0.271	0.276	0.28	0.285	0.292	0.303	0.318	0.334	0.352	0.371	0.393	0.415
Sudan	0.133	0.135	0.138	0.141	0.145	0.149	0.154	0.16	0.165	0.17	0.174	0.179	0.184	0.188	0.192	0.197	0.201	0.205	0.21	0.215
Syria	0.247	0.248	0.251	0.254	0.257	0.262	0.269	0.278	0.288	0.298	0.307	0.317	0.327	0.337	0.344	0.352	0.359	0.368	0.376	0.383
Tunisia	0.26	0.268	0.277	0.285	0.293	0.302	0.311	0.321	0.332	0.342	0.352	0.361	0.369	0.379	0.391	0.403	0.413	0.423	0.433	0.443
Turkey	0.349	0.355	0.362	0.369	0.377	0.385	0.393	0.402	0.411	0.419	0.426	0.434	0.441	0.448	0.455	0.462	0.47	0.479	0.489	0.498
United Arab Emirates	0.333	0.332	0.338	0.354	0.375	0.396	0.412	0.427	0.445	0.462	0.475	0.484	0.497	0.516	0.535	0.549	0.561	0.574	0.589	0.605
Yemen	0.12	0.124	0.128	0.132	0.136	0.139	0.142	0.146	0.149	0.154	0.158	0.162	0.167	0.174	0.181	0.187	0.191	0.194	0.197	0.199
South Asia	0.219	0.223	0.227	0.232	0.238	0.244	0.249	0.254	0.259	0.264	0.267	0.269	0.272	0.277	0.281	0.286	0.292	0.298	0.303	0.308
South Asia	0.219	0.223	0.227	0.232	0.238	0.244	0.249	0.254	0.259	0.264	0.267	0.269	0.272	0.277	0.281	0.286	0.292	0.298	0.303	0.308
Bangladesh	0.084	0.116	0.138	0.153	0.166	0.177	0.187	0.195	0.202	0.207	0.211	0.215	0.219	0.222	0.225	0.228	0.231	0.236	0.241	0.248
Bhutan	0.202	0.208	0.212	0.217	0.221	0.225	0.229	0.234	0.238	0.243	0.248	0.254	0.26	0.267	0.274	0.281	0.289	0.297	0.306	0.315
India	0.225	0.23	0.233	0.238	0.245	0.251	0.255	0.26	0.265	0.27	0.273	0.275	0.278	0.283	0.287	0.293	0.299	0.306	0.311	0.316
Andhra Pradesh	0.162	0.166	0.169	0.174	0.183	0.191	0.195	0.198	0.203	0.208	0.213	0.219	0.225	0.233	0.24	0.248	0.255	0.262	0.268	0.274
Arunachal Pradesh	0.185	0.19	0.196	0.202	0.209	0.214	0.216	0.221	0.228	0.234	0.236	0.24	0.247	0.254	0.261	0.269	0.279	0.288	0.298	0.305
Assam	0.223	0.228	0.231	0.235	0.241	0.245	0.248	0.25	0.255	0.259	0.261	0.264	0.269	0.276	0.283	0.289	0.296	0.306	0.314	0.321
Bihar	0.197	0.201	0.205	0.21	0.215	0.221	0.226	0.231	0.237	0.242	0.244	0.245	0.248	0.252	0.258	0.264	0.271	0.277	0.282	0.287
Chhattisgarh	0.202	0.207	0.211	0.216	0.224	0.232	0.238	0.243	0.249	0.254	0.258	0.261	0.264	0.268	0.272	0.276	0.282	0.286	0.288	0.29
Delhi	0.391	0.397	0.407	0.416	0.424	0.431	0.437	0.442	0.447	0.45	0.45	0.449	0.453	0.456	0.459	0.462	0.466	0.472	0.478	0.483
Goa	0.366	0.373	0.38	0.387	0.394	0.402	0.41	0.417	0.425	0.431	0.436	0.44	0.447	0.452	0.459	0.464	0.471	0.478	0.486	0.493
Gujarat	0.244	0.249	0.255	0.262	0.268	0.272	0.276	0.283	0.29	0.296	0.297	0.297	0.3	0.304	0.305	0.307	0.315	0.326	0.338	0.345
Haryana	0.243	0.248	0.253	0.26	0.269	0.277	0.283	0.288	0.293	0.297	0.299	0.299	0.302	0.305	0.307	0.311	0.316	0.323	0.332	0.337
Himachal Pradesh	0.213	0.218	0.224	0.23	0.238	0.245	0.251	0.259	0.267	0.275	0.279	0.283	0.287	0.291	0.296	0.302	0.31	0.317	0.326	0.333
Jammu and Kashmir	0.234	0.239	0.245	0.251	0.258	0.265	0.272	0.279	0.284	0.289	0.294	0.297	0.301	0.304	0.307	0.313	0.319	0.324	0.329	0.332
Jharkhand	0.205	0.21	0.213	0.219	0.226	0.233	0.238	0.243	0.249	0.253	0.256	0.258	0.26	0.262	0.265	0.269	0.272	0.275	0.278	0.279
Karnataka	0.22	0.223	0.226	0.231	0.237	0.242	0.245	0.249	0.253	0.257	0.259	0.261	0.266	0.271	0.277	0.283	0.291	0.3	0.309	0.316
Kerala	0.318	0.323	0.328	0.332	0.337	0.344	0.349	0.354	0.357	0.36	0.363	0.364	0.366	0.369	0.373	0.378	0.385	0.391	0.397	0.402
Madhya Pradesh	0.175	0.178	0.18	0.185	0.192	0.198	0.202	0.207	0.214	0.22	0.224	0.226	0.232	0.239	0.246	0.254	0.261	0.269	0.275	0.279
Maharashtra	0.258	0.262	0.266	0.272	0.279	0.284	0.288	0.292	0.298	0.303	0.305	0.306	0.309	0.314	0.318	0.323	0.33	0.34	0.349	0.357
Manipur	0.251	0.257	0.261	0.267	0.274	0.282	0.288	0.294	0.3	0.306	0.311	0.316	0.321	0.328	0.334	0.341	0.348	0.358	0.367	0.374
Meghalaya	0.229	0.234	0.239	0.244	0.25	0.256	0.259	0.263	0.268	0.272	0.276	0.279	0.283	0.288	0.293	0.299	0.306	0.314	0.32	0.327
Mizoram	0.27	0.275	0.28	0.284	0.29	0.297	0.303	0.307	0.313	0.318	0.322	0.325	0.329	0.333	0.34	0.351	0.363	0.376	0.384	0.389
Nagaland	0.283	0.287	0.291	0.295	0.301	0.307	0.312	0.316	0.321	0.325	0.329	0.333	0.339	0.345	0.351	0.358	0.366	0.375	0.383	0.39
Odisha	0.19	0.194	0.197	0.201	0.207	0.214	0.218	0.223	0.227	0.232	0.236	0.239	0.242	0.247	0.251	0.258	0.266	0.27	0.276	0.28
Punjab	0.294	0.299	0.303	0.308	0.313	0.318	0.324	0.33	0.334	0.339	0.341	0.342	0.345	0.348	0.351	0.355	0.359	0.367	0.374	0.38
Rajasthan	0.165	0.17	0.174	0.18	0.187	0.195	0.199	0.202	0.207	0.211	0.215	0.217	0.221	0.225	0.23	0.237	0.244	0.249	0.254	0.258
Sikkim	0.209	0.214	0.218	0.225	0.233	0.24	0.246	0.251	0.258	0.263	0.267	0.271	0.276	0.281	0.288	0.298	0.308	0.319	0.328	0.336
Tamil Nadu	0.249	0.253	0.257	0.263	0.268	0.274	0.279	0.283	0.288	0.293	0.297	0.3	0.304	0.308	0.315	0.321	0.328	0.335	0.341	0.347
Telangana	0.188	0.192	0.196	0.201	0.207	0.214	0.219	0.224	0.229	0.233	0.236	0.239	0.243	0.247	0.252	0.258	0.263	0.269	0.275	0.281
Tripura	0.217	0.223	0.228	0.236	0.245	0.253	0.257	0.26	0.264	0.269	0.274	0.28	0.286	0.292	0.297	0.303	0.31	0.316	0.323	0.329
Uttar Pradesh	0.196	0.2	0.204	0.208	0.213	0.218	0.221	0.226	0.231	0.235	0.238	0.238	0.241	0.244	0.247	0.251	0.256	0.261	0.265	0.268
Uttarakhand	0.22	0.226	0.23	0.236	0.243	0.251	0.257	0.263	0.269	0.275	0.279	0.28	0.282	0.285	0.288	0.291	0.295	0.298	0.3	0.302
West Bengal	0.235	0.238	0.24	0.244	0.252	0.259	0.263	0.266	0.272	0.278	0.283	0.287	0.292	0.296	0.301	0.307	0.314	0.319	0.321	0.322
Union Territories other than Delhi	0.299	0.306	0.313	0.322	0.332	0.343	0.35	0.357	0.365	0.371	0.376	0.378	0.381	0.385	0.389	0.392	0.396	0.399	0.402	0.404
Nepal	0.156	0.158	0.16	0.162	0.164	0.166	0.169	0.171	0.174	0.177	0.179	0.181	0.184	0.187	0.189	0.192	0.196	0.201	0.206	0.211
Pakistan	0.241	0.245	0.249	0.252	0.255	0.26	0.264	0.268	0.271	0.273	0.275	0.276	0.279	0.282	0.285	0.289	0.292	0.295	0.299	0.302
Southeast Asia, East Asia, and Oceania	0.294	0.304	0.315	0.326	0.338	0.349	0.358	0.366	0.374	0.382	0.39	0.397	0.401	0.405	0.411	0.417	0.424	0.434	0.446	0.456
East Asia	0.277	0.288	0.3	0.314	0.327	0.339	0.348	0.356	0.364	0.372	0.379	0.386	0.389	0.392	0.397	0.404	0.412	0.424	0.438	0.45

Appendix Table 9. Socio-Demographic Index values for all estimated GBD 2017 locations, 1970–1989																				
**Location**	**1970**	**1971**	**1972**	**1973**	**1974**	**1975**	**1976**	**1977**	**1978**	**1979**	**1980**	**1981**	**1982**	**1983**	**1984**	**1985**	**1986**	**1987**	**1988**	**1989**
China	0.269	0.28	0.292	0.305	0.318	0.33	0.339	0.347	0.355	0.362	0.37	0.377	0.38	0.383	0.389	0.396	0.404	0.417	0.431	0.443
North Korea	0.398	0.411	0.421	0.431	0.44	0.448	0.453	0.458	0.462	0.466	0.47	0.474	0.478	0.482	0.486	0.49	0.494	0.498	0.503	0.507
Taiwan (Province of China)	0.485	0.497	0.51	0.52	0.53	0.53	0.535	0.548	0.555	0.566	0.577	0.588	0.601	0.613	0.627	0.643	0.655	0.663	0.672	0.682
Oceania	0.329	0.333	0.338	0.343	0.349	0.353	0.358	0.362	0.366	0.37	0.374	0.377	0.38	0.384	0.387	0.39	0.393	0.397	0.4	0.403
American Samoa	0.537	0.546	0.557	0.567	0.575	0.58	0.586	0.59	0.595	0.599	0.6	0.6	0.599	0.597	0.597	0.597	0.599	0.601	0.604	0.607
Federated States of Micronesia	0.325	0.331	0.337	0.344	0.351	0.358	0.365	0.373	0.38	0.387	0.395	0.402	0.408	0.415	0.422	0.429	0.436	0.443	0.449	0.456
Fiji	0.416	0.424	0.433	0.444	0.454	0.463	0.471	0.477	0.483	0.488	0.491	0.495	0.499	0.503	0.507	0.511	0.516	0.52	0.525	0.529
Guam	0.589	0.586	0.592	0.609	0.631	0.651	0.669	0.684	0.695	0.703	0.707	0.708	0.709	0.71	0.71	0.71	0.71	0.708	0.706	0.702
Kiribati	0.31	0.314	0.318	0.323	0.332	0.34	0.345	0.348	0.352	0.354	0.354	0.354	0.353	0.352	0.352	0.351	0.351	0.351	0.352	0.354
Marshall Islands	0.269	0.271	0.273	0.279	0.287	0.296	0.303	0.31	0.318	0.328	0.337	0.345	0.35	0.355	0.361	0.366	0.375	0.384	0.394	0.403
Northern Mariana Islands	0.585	0.591	0.598	0.605	0.613	0.622	0.63	0.639	0.648	0.656	0.664	0.671	0.678	0.686	0.695	0.704	0.713	0.721	0.728	0.734
Papua New Guinea	0.255	0.259	0.264	0.268	0.272	0.276	0.279	0.282	0.285	0.288	0.29	0.293	0.295	0.298	0.3	0.303	0.306	0.309	0.312	0.315
Samoa	0.411	0.422	0.431	0.442	0.451	0.458	0.467	0.475	0.483	0.492	0.499	0.503	0.507	0.51	0.514	0.518	0.522	0.527	0.531	0.535
Solomon Islands	0.241	0.244	0.247	0.25	0.254	0.258	0.262	0.266	0.271	0.277	0.282	0.286	0.29	0.294	0.297	0.3	0.303	0.306	0.309	0.312
Tonga	0.376	0.38	0.384	0.388	0.392	0.397	0.402	0.408	0.414	0.42	0.428	0.436	0.446	0.455	0.469	0.481	0.492	0.502	0.51	0.517
Vanuatu	0.292	0.296	0.3	0.305	0.313	0.32	0.326	0.331	0.337	0.342	0.346	0.35	0.353	0.357	0.361	0.365	0.369	0.372	0.374	0.377
Southeast Asia	0.316	0.323	0.329	0.336	0.343	0.351	0.359	0.367	0.374	0.382	0.39	0.398	0.406	0.414	0.422	0.43	0.437	0.444	0.451	0.459
Cambodia	0.211	0.213	0.214	0.214	0.214	0.214	0.214	0.213	0.212	0.212	0.213	0.216	0.218	0.222	0.226	0.23	0.235	0.24	0.246	0.253
Indonesia	0.28	0.287	0.294	0.303	0.312	0.321	0.33	0.339	0.347	0.357	0.366	0.376	0.385	0.395	0.405	0.414	0.424	0.433	0.443	0.454
Laos	0.201	0.204	0.207	0.211	0.215	0.219	0.223	0.227	0.231	0.235	0.24	0.246	0.253	0.259	0.266	0.273	0.28	0.287	0.293	0.3
Malaysia	0.412	0.421	0.427	0.43	0.439	0.453	0.468	0.48	0.489	0.496	0.503	0.51	0.52	0.527	0.535	0.543	0.548	0.551	0.559	0.565
Maldives	0.237	0.241	0.245	0.249	0.254	0.259	0.264	0.269	0.273	0.278	0.285	0.292	0.3	0.306	0.314	0.325	0.337	0.349	0.361	0.373
Mauritius	0.391	0.401	0.41	0.416	0.42	0.426	0.436	0.446	0.456	0.467	0.481	0.495	0.507	0.517	0.525	0.532	0.538	0.542	0.544	0.547
Myanmar	0.195	0.206	0.215	0.223	0.23	0.238	0.245	0.251	0.258	0.264	0.271	0.277	0.284	0.292	0.3	0.307	0.314	0.319	0.323	0.326
Philippines	0.417	0.421	0.425	0.43	0.433	0.437	0.442	0.449	0.455	0.463	0.47	0.477	0.484	0.49	0.494	0.497	0.498	0.5	0.502	0.505
Sri Lanka	0.385	0.393	0.399	0.401	0.405	0.41	0.415	0.418	0.422	0.425	0.429	0.433	0.439	0.444	0.45	0.456	0.462	0.469	0.477	0.484
Seychelles	0.358	0.365	0.372	0.38	0.389	0.399	0.409	0.42	0.432	0.445	0.457	0.469	0.479	0.488	0.497	0.507	0.516	0.524	0.533	0.541
Thailand	0.346	0.355	0.363	0.371	0.379	0.387	0.395	0.403	0.409	0.413	0.42	0.429	0.437	0.444	0.454	0.464	0.473	0.48	0.485	0.492
Timor-Leste	0.167	0.174	0.18	0.186	0.193	0.199	0.206	0.212	0.218	0.225	0.23	0.234	0.239	0.243	0.247	0.252	0.258	0.263	0.267	0.272
Vietnam	0.284	0.288	0.292	0.297	0.301	0.306	0.312	0.319	0.326	0.333	0.339	0.347	0.354	0.361	0.369	0.375	0.382	0.388	0.394	0.4
Sub-Saharan Africa	0.212	0.216	0.22	0.225	0.23	0.235	0.24	0.245	0.25	0.255	0.26	0.265	0.27	0.275	0.28	0.284	0.288	0.292	0.296	0.3
Central sub-Saharan Africa	0.182	0.187	0.192	0.198	0.204	0.209	0.215	0.221	0.226	0.232	0.238	0.244	0.25	0.256	0.262	0.269	0.275	0.281	0.287	0.293
Angola	0.127	0.132	0.137	0.142	0.148	0.154	0.16	0.166	0.172	0.178	0.184	0.19	0.196	0.2	0.205	0.21	0.214	0.22	0.225	0.23
Central African Republic	0.153	0.156	0.159	0.162	0.163	0.167	0.171	0.175	0.178	0.18	0.183	0.187	0.189	0.192	0.194	0.198	0.201	0.205	0.21	0.215
Congo (Brazzaville)	0.221	0.227	0.232	0.239	0.245	0.251	0.258	0.264	0.271	0.278	0.286	0.296	0.306	0.316	0.326	0.336	0.346	0.354	0.364	0.373
DR Congo	0.182	0.187	0.192	0.198	0.204	0.209	0.213	0.218	0.223	0.229	0.234	0.24	0.246	0.251	0.258	0.265	0.271	0.277	0.283	0.289
Equatorial Guinea	0.049	0.05	0.05	0.054	0.071	0.083	0.094	0.106	0.117	0.128	0.137	0.146	0.154	0.162	0.168	0.174	0.179	0.184	0.189	0.195
Gabon	0.252	0.26	0.269	0.275	0.283	0.292	0.304	0.314	0.322	0.329	0.338	0.348	0.357	0.365	0.374	0.383	0.392	0.402	0.412	0.422
Eastern sub-Saharan Africa	0.155	0.158	0.161	0.164	0.166	0.169	0.173	0.176	0.18	0.183	0.187	0.192	0.196	0.2	0.205	0.209	0.213	0.217	0.222	0.226
Burundi	0.141	0.146	0.149	0.153	0.157	0.161	0.165	0.171	0.176	0.182	0.188	0.194	0.2	0.206	0.212	0.218	0.225	0.231	0.236	0.242
Comoros	0.157	0.161	0.165	0.171	0.176	0.18	0.184	0.187	0.191	0.196	0.201	0.207	0.212	0.217	0.221	0.227	0.237	0.247	0.256	0.265
Djibouti	0.198	0.202	0.206	0.208	0.21	0.215	0.222	0.229	0.238	0.245	0.25	0.256	0.262	0.267	0.272	0.28	0.288	0.296	0.303	0.308
Eritrea	0.116	0.12	0.124	0.128	0.133	0.136	0.14	0.145	0.149	0.153	0.158	0.162	0.166	0.17	0.174	0.178	0.183	0.187	0.192	0.197
Ethiopia	0.088	0.089	0.091	0.092	0.092	0.094	0.096	0.097	0.098	0.1	0.103	0.106	0.11	0.113	0.116	0.119	0.123	0.127	0.131	0.134
Kenya	0.201	0.206	0.205	0.207	0.213	0.221	0.228	0.231	0.235	0.239	0.246	0.255	0.264	0.272	0.279	0.287	0.297	0.309	0.321	0.331
Baringo	0.155	0.154	0.14	0.124	0.12	0.131	0.135	0.127	0.111	0.093	0.09	0.109	0.125	0.139	0.151	0.164	0.183	0.206	0.227	0.242
Bomet	0.15	0.154	0.144	0.138	0.145	0.16	0.167	0.168	0.167	0.168	0.174	0.189	0.204	0.216	0.226	0.235	0.248	0.265	0.281	0.295
Bungoma	0.181	0.184	0.179	0.176	0.181	0.192	0.2	0.204	0.207	0.213	0.22	0.23	0.239	0.247	0.253	0.259	0.268	0.281	0.294	0.306
Busia	0.19	0.197	0.195	0.195	0.201	0.21	0.216	0.218	0.22	0.222	0.227	0.234	0.239	0.244	0.247	0.251	0.258	0.269	0.28	0.289
Elgeyo Marakwet	0.162	0.166	0.164	0.164	0.17	0.179	0.185	0.187	0.187	0.189	0.193	0.201	0.21	0.218	0.226	0.233	0.244	0.257	0.27	0.282
Embu	0.209	0.214	0.215	0.219	0.227	0.236	0.243	0.248	0.255	0.263	0.272	0.282	0.293	0.303	0.312	0.321	0.332	0.344	0.355	0.366
Garissa	0.09	0.093	0.091	0.091	0.093	0.096	0.098	0.098	0.097	0.098	0.1	0.103	0.106	0.109	0.111	0.114	0.119	0.127	0.137	0.145
Homa Bay	0.148	0.153	0.148	0.145	0.151	0.162	0.169	0.168	0.164	0.161	0.16	0.163	0.165	0.164	0.162	0.16	0.165	0.178	0.193	0.205
Isiolo	0.188	0.192	0.192	0.193	0.197	0.203	0.207	0.208	0.21	0.213	0.216	0.22	0.224	0.227	0.229	0.232	0.237	0.244	0.251	0.258
Kajiado	0.251	0.256	0.258	0.262	0.269	0.278	0.285	0.29	0.295	0.3	0.307	0.315	0.322	0.329	0.335	0.341	0.35	0.359	0.368	0.377
Kakamega	0.187	0.192	0.188	0.187	0.192	0.2	0.206	0.206	0.206	0.208	0.213	0.221	0.228	0.233	0.237	0.242	0.25	0.262	0.275	0.286
Kericho	0.148	0.149	0.137	0.128	0.129	0.136	0.136	0.125	0.108	0.094	0.096	0.119	0.138	0.152	0.165	0.178	0.197	0.218	0.238	0.254
Kiambu	0.256	0.263	0.264	0.268	0.276	0.286	0.294	0.3	0.306	0.313	0.323	0.335	0.347	0.358	0.369	0.379	0.39	0.402	0.415	0.425
Kilifi	0.174	0.178	0.177	0.178	0.182	0.19	0.195	0.197	0.2	0.204	0.21	0.217	0.225	0.231	0.238	0.245	0.254	0.264	0.275	0.284
Kirinyaga	0.209	0.218	0.222	0.228	0.237	0.248	0.257	0.264	0.271	0.279	0.288	0.299	0.311	0.321	0.331	0.341	0.351	0.362	0.372	0.381
Kisii	0.186	0.191	0.189	0.19	0.196	0.205	0.211	0.215	0.218	0.224	0.231	0.24	0.25	0.26	0.269	0.278	0.29	0.303	0.317	0.329
Kisumu	0.165	0.17	0.166	0.165	0.173	0.184	0.193	0.196	0.198	0.204	0.212	0.222	0.231	0.239	0.245	0.252	0.263	0.277	0.292	0.305
Kitui	0.167	0.172	0.173	0.176	0.182	0.189	0.195	0.198	0.201	0.205	0.21	0.217	0.222	0.226	0.23	0.234	0.241	0.251	0.262	0.272
Kwale	0.164	0.169	0.171	0.173	0.179	0.188	0.194	0.198	0.202	0.208	0.214	0.223	0.23	0.237	0.244	0.25	0.258	0.268	0.278	0.286
Laikipia	0.168	0.175	0.173	0.173	0.18	0.193	0.203	0.209	0.214	0.22	0.229	0.242	0.255	0.267	0.278	0.288	0.301	0.314	0.326	0.337
Lamu	0.148	0.154	0.152	0.152	0.159	0.168	0.174	0.176	0.18	0.187	0.196	0.209	0.22	0.23	0.238	0.246	0.255	0.267	0.278	0.287
Machakos	0.207	0.214	0.216	0.219	0.226	0.235	0.242	0.246	0.251	0.257	0.264	0.272	0.28	0.286	0.292	0.299	0.308	0.319	0.331	0.342
Makueni	0.201	0.207	0.207	0.209	0.215	0.223	0.229	0.232	0.234	0.237	0.241	0.247	0.252	0.255	0.259	0.262	0.268	0.276	0.285	0.293
Mandera	0.068	0.069	0.066	0.063	0.064	0.066	0.066	0.063	0.057	0.053	0.052	0.055	0.057	0.058	0.057	0.055	0.061	0.073	0.086	0.096
Marsabit	0.151	0.155	0.156	0.157	0.161	0.166	0.17	0.173	0.175	0.178	0.182	0.186	0.19	0.193	0.196	0.198	0.202	0.206	0.211	0.216
Meru	0.197	0.204	0.208	0.212	0.219	0.228	0.235	0.24	0.246	0.252	0.258	0.265	0.272	0.279	0.285	0.292	0.301	0.31	0.32	0.329

Appendix Table 9. Socio-Demographic Index values for all estimated GBD 2017 locations, 1970–1989

Location	1970	1971	1972	1973	1974	1975	1976	1977	1978	1979	1980	1981	1982	1983	1984	1985	1986	1987	1988	1989
Migori	0.151	0.154	0.148	0.145	0.151	0.161	0.167	0.166	0.163	0.161	0.162	0.168	0.173	0.175	0.175	0.177	0.184	0.197	0.21	0.221
Mombasa	0.225	0.233	0.238	0.245	0.254	0.264	0.272	0.28	0.288	0.297	0.306	0.316	0.324	0.332	0.34	0.348	0.357	0.367	0.377	0.387
Murang’a	0.231	0.236	0.235	0.237	0.243	0.252	0.258	0.262	0.267	0.273	0.282	0.294	0.304	0.313	0.321	0.33	0.34	0.351	0.363	0.373
Nairobi	0.315	0.325	0.332	0.34	0.351	0.363	0.374	0.384	0.393	0.403	0.413	0.423	0.432	0.44	0.448	0.455	0.463	0.473	0.482	0.491
Nakuru	0.184	0.189	0.191	0.194	0.2	0.209	0.215	0.219	0.223	0.227	0.234	0.242	0.251	0.259	0.267	0.276	0.286	0.3	0.313	0.326
Nandi	0.179	0.181	0.172	0.166	0.168	0.177	0.182	0.181	0.179	0.178	0.184	0.196	0.21	0.224	0.236	0.249	0.264	0.281	0.297	0.311
Narok	0.093	0.086	0.061	0.062	0.063	0.064	0.065	0.066	0.067	0.068	0.069	0.07	0.071	0.072	0.101	0.121	0.144	0.169	0.191	0.206
Nyamira	0.195	0.202	0.204	0.207	0.216	0.228	0.238	0.245	0.251	0.257	0.266	0.275	0.285	0.293	0.301	0.308	0.318	0.329	0.341	0.351
Nyandarua	0.184	0.193	0.193	0.195	0.201	0.211	0.218	0.22	0.222	0.227	0.235	0.247	0.26	0.272	0.283	0.293	0.305	0.319	0.332	0.344
Nyeri	0.229	0.238	0.242	0.247	0.256	0.267	0.275	0.282	0.288	0.295	0.304	0.315	0.326	0.337	0.346	0.355	0.366	0.377	0.388	0.398
Samburu	0.126	0.128	0.122	0.117	0.12	0.127	0.13	0.128	0.124	0.12	0.122	0.13	0.139	0.146	0.152	0.158	0.167	0.179	0.19	0.199
Siaya	0.112	0.117	0.117	0.118	0.123	0.131	0.137	0.14	0.142	0.145	0.15	0.156	0.162	0.167	0.172	0.177	0.185	0.196	0.208	0.218
Taita Taveta	0.199	0.205	0.207	0.211	0.218	0.226	0.233	0.239	0.245	0.251	0.259	0.268	0.276	0.284	0.292	0.3	0.309	0.32	0.331	0.342
Tana River	0.116	0.122	0.124	0.128	0.134	0.143	0.149	0.154	0.158	0.163	0.169	0.177	0.183	0.188	0.192	0.196	0.202	0.209	0.217	0.224
Tharaka Nithi	0.193	0.201	0.207	0.213	0.221	0.23	0.238	0.244	0.251	0.259	0.266	0.274	0.281	0.288	0.294	0.301	0.309	0.317	0.326	0.334
Trans Nzoia	0.195	0.198	0.194	0.192	0.196	0.204	0.208	0.207	0.204	0.203	0.206	0.215	0.224	0.233	0.242	0.252	0.265	0.28	0.295	0.308
Turkana	0.16	0.162	0.161	0.161	0.164	0.168	0.17	0.171	0.17	0.171	0.172	0.176	0.179	0.182	0.185	0.187	0.191	0.196	0.202	0.206
Uasin Gishu	0.203	0.207	0.204	0.203	0.209	0.219	0.226	0.228	0.23	0.232	0.239	0.25	0.263	0.275	0.286	0.296	0.309	0.324	0.339	0.352
Vihiga	0.206	0.211	0.21	0.212	0.218	0.226	0.232	0.235	0.238	0.242	0.247	0.254	0.261	0.267	0.272	0.278	0.286	0.297	0.308	0.319
Wajir	0.08	0.08	0.074	0.069	0.068	0.069	0.067	0.059	0.049	0.037	0.038	0.038	0.041	0.044	0.043	0.042	0.053	0.071	0.086	0.097
West Pokot	0.14	0.142	0.138	0.134	0.137	0.143	0.147	0.145	0.141	0.137	0.136	0.14	0.146	0.152	0.156	0.161	0.17	0.183	0.195	0.205
Madagascar	0.158	0.163	0.169	0.175	0.18	0.185	0.19	0.196	0.202	0.207	0.212	0.219	0.226	0.233	0.238	0.244	0.249	0.253	0.257	0.26
Malawi	0.119	0.123	0.127	0.131	0.134	0.137	0.14	0.144	0.147	0.151	0.156	0.16	0.164	0.169	0.174	0.18	0.184	0.189	0.192	0.196
Mozambique	0.103	0.107	0.109	0.112	0.114	0.116	0.118	0.119	0.121	0.123	0.125	0.127	0.128	0.128	0.129	0.129	0.129	0.129	0.129	0.131
Rwanda	0.176	0.181	0.186	0.19	0.194	0.197	0.2	0.203	0.206	0.211	0.216	0.222	0.228	0.235	0.24	0.246	0.251	0.256	0.26	0.264
Somalia	0.113	0.115	0.117	0.119	0.12	0.123	0.125	0.128	0.13	0.132	0.134	0.134	0.136	0.137	0.138	0.14	0.141	0.144	0.147	0.15
South Sudan	0.139	0.141	0.143	0.145	0.148	0.15	0.153	0.155	0.158	0.16	0.162	0.165	0.167	0.169	0.17	0.172	0.173	0.175	0.176	0.178
Tanzania	0.16	0.163	0.167	0.171	0.176	0.18	0.186	0.193	0.2	0.208	0.216	0.222	0.229	0.235	0.241	0.248	0.254	0.259	0.265	0.27
Uganda	0.104	0.106	0.108	0.112	0.116	0.12	0.124	0.129	0.133	0.136	0.138	0.141	0.145	0.148	0.151	0.154	0.156	0.158	0.16	0.161
Zambia	0.236	0.238	0.24	0.242	0.244	0.246	0.249	0.252	0.257	0.263	0.268	0.274	0.28	0.285	0.29	0.294	0.299	0.303	0.306	0.309
Southern sub-Saharan Africa	0.388	0.393	0.4	0.409	0.417	0.422	0.428	0.436	0.445	0.452	0.457	0.463	0.471	0.48	0.487	0.492	0.496	0.503	0.511	0.517
Botswana	0.253	0.26	0.269	0.279	0.289	0.299	0.309	0.319	0.329	0.339	0.349	0.36	0.37	0.381	0.393	0.404	0.417	0.429	0.44	0.452
Lesotho	0.221	0.223	0.227	0.232	0.238	0.242	0.247	0.254	0.263	0.269	0.276	0.282	0.288	0.293	0.299	0.304	0.31	0.315	0.32	0.327
Namibia	0.324	0.329	0.335	0.342	0.349	0.356	0.364	0.371	0.379	0.386	0.393	0.401	0.408	0.414	0.421	0.427	0.432	0.438	0.444	0.449
South Africa	0.426	0.431	0.439	0.45	0.458	0.463	0.469	0.477	0.487	0.494	0.499	0.504	0.512	0.521	0.529	0.532	0.535	0.542	0.549	0.554
Swaziland	0.283	0.289	0.294	0.299	0.305	0.311	0.317	0.323	0.329	0.334	0.34	0.348	0.356	0.363	0.371	0.379	0.388	0.398	0.408	0.417
Zimbabwe	0.246	0.252	0.258	0.264	0.271	0.277	0.283	0.288	0.294	0.3	0.308	0.316	0.326	0.336	0.346	0.358	0.369	0.38	0.39	0.4
Western sub-Saharan Africa	0.206	0.211	0.216	0.221	0.227	0.233	0.239	0.244	0.25	0.255	0.259	0.264	0.268	0.271	0.275	0.279	0.282	0.284	0.287	0.29
Benin	0.147	0.15	0.153	0.156	0.159	0.162	0.165	0.168	0.171	0.174	0.178	0.182	0.185	0.189	0.193	0.197	0.201	0.206	0.21	0.214
Burkina Faso	0.095	0.095	0.096	0.098	0.1	0.101	0.102	0.103	0.105	0.108	0.111	0.113	0.115	0.117	0.119	0.123	0.126	0.129	0.132	0.136
Cameroon	0.215	0.218	0.222	0.225	0.229	0.234	0.238	0.244	0.25	0.256	0.261	0.267	0.273	0.28	0.288	0.295	0.303	0.311	0.318	0.326
Cape Verde	0.193	0.2	0.207	0.213	0.218	0.223	0.227	0.231	0.235	0.239	0.244	0.247	0.25	0.254	0.259	0.265	0.272	0.281	0.29	0.299
Chad	0.095	0.096	0.097	0.097	0.098	0.099	0.1	0.101	0.101	0.101	0.1	0.1	0.099	0.1	0.101	0.103	0.105	0.108	0.111	0.115
Cote d'Ivoire	0.19	0.195	0.199	0.204	0.208	0.213	0.217	0.219	0.222	0.224	0.226	0.228	0.23	0.233	0.236	0.24	0.245	0.252	0.259	0.266
The Gambia	0.153	0.155	0.158	0.161	0.165	0.169	0.173	0.177	0.181	0.185	0.189	0.192	0.196	0.202	0.208	0.214	0.221	0.227	0.233	0.238
Ghana	0.285	0.292	0.296	0.298	0.301	0.306	0.315	0.323	0.329	0.332	0.337	0.343	0.346	0.349	0.352	0.356	0.361	0.365	0.37	0.374
Guinea	0.12	0.122	0.123	0.125	0.128	0.131	0.133	0.136	0.138	0.14	0.143	0.145	0.147	0.148	0.15	0.152	0.155	0.157	0.159	0.161
Guinea-Bissau	0.137	0.138	0.14	0.142	0.143	0.145	0.148	0.149	0.15	0.152	0.153	0.155	0.156	0.158	0.16	0.163	0.167	0.171	0.174	0.178
Liberia	0.16	0.165	0.169	0.173	0.177	0.18	0.182	0.184	0.186	0.189	0.192	0.194	0.196	0.198	0.2	0.203	0.205	0.208	0.212	0.216
Mali	0.088	0.09	0.092	0.094	0.095	0.097	0.099	0.102	0.104	0.107	0.109	0.112	0.114	0.116	0.117	0.119	0.121	0.123	0.125	0.128
Mauritania	0.203	0.207	0.21	0.212	0.214	0.217	0.22	0.223	0.226	0.229	0.232	0.238	0.244	0.251	0.257	0.264	0.271	0.278	0.285	0.292
Niger	0.077	0.077	0.077	0.075	0.074	0.073	0.072	0.07	0.067	0.064	0.062	0.059	0.056	0.056	0.059	0.064	0.07	0.076	0.082	0.088
Nigeria	0.237	0.245	0.252	0.26	0.268	0.276	0.284	0.292	0.3	0.307	0.313	0.319	0.324	0.329	0.333	0.337	0.339	0.339	0.341	0.342
Sao Tome and Principe	0.184	0.19	0.197	0.204	0.209	0.215	0.221	0.228	0.234	0.24	0.244	0.249	0.253	0.257	0.26	0.264	0.268	0.273	0.278	0.283
Senegal	0.157	0.159	0.161	0.163	0.165	0.168	0.171	0.174	0.176	0.18	0.184	0.188	0.192	0.198	0.204	0.21	0.216	0.222	0.23	0.237
Sierra Leone	0.149	0.153	0.156	0.16	0.163	0.167	0.17	0.173	0.175	0.176	0.178	0.18	0.182	0.183	0.185	0.187	0.189	0.191	0.194	0.198
Togo	0.163	0.167	0.171	0.174	0.179	0.183	0.187	0.192	0.197	0.203	0.209	0.214	0.219	0.224	0.229	0.234	0.239	0.244	0.25	0.256

Appendix Table 10. Socio-Demographic Index values for all estimated GBD 2017 locations, 1990-2017

Location	1990	1991	1992	1993	1994	1995	1996	1997	1998	1999	2000	2001	2002	2003	2004	2005	2006	2007	2008	2009	2010	2011	2012	2013	2014	2015	2016	2017
Global	0.523	0.529	0.534	0.539	0.543	0.548	0.553	0.557	0.561	0.566	0.571	0.576	0.581	0.585	0.59	0.595	0.601	0.606	0.611	0.616	0.62	0.624	0.628	0.633	0.639	0.644	0.647	0.652
Central Europe, Eastern Europe, and Central Asia	0.656	0.662	0.67	0.674	0.677	0.682	0.686	0.689	0.691	0.694	0.698	0.701	0.705	0.709	0.715	0.72	0.725	0.73	0.735	0.739	0.743	0.747	0.75	0.753	0.757	0.76	0.763	0.766
Central Asia	0.563	0.567	0.57	0.573	0.575	0.577	0.578	0.579	0.58	0.582	0.585	0.588	0.593	0.598	0.603	0.609	0.615	0.621	0.627	0.633	0.639	0.644	0.649	0.654	0.659	0.664	0.669	0.673
Armenia	0.555	0.559	0.56	0.562	0.565	0.567	0.57	0.573	0.577	0.581	0.586	0.592	0.6	0.61	0.619	0.629	0.639	0.65	0.66	0.667	0.673	0.678	0.683	0.687	0.691	0.695	0.699	0.702
Azerbaijan	0.611	0.614	0.616	0.617	0.616	0.613	0.61	0.607	0.604	0.601	0.6	0.6	0.602	0.605	0.608	0.615	0.625	0.635	0.645	0.654	0.664	0.672	0.678	0.684	0.689	0.694	0.698	0.701
Georgia	0.654	0.66	0.661	0.658	0.653	0.645	0.639	0.634	0.63	0.625	0.621	0.62	0.622	0.624	0.628	0.633	0.638	0.644	0.649	0.654	0.659	0.665	0.671	0.676	0.682	0.688	0.695	0.7
Kazakhstan	0.613	0.615	0.619	0.625	0.632	0.638	0.643	0.645	0.646	0.647	0.651	0.656	0.661	0.666	0.671	0.677	0.683	0.689	0.696	0.702	0.705	0.707	0.708	0.711	0.716	0.723	0.73	0.735
Kyrgyzstan	0.565	0.571	0.576	0.578	0.577	0.572	0.569	0.567	0.564	0.562	0.56	0.559	0.56	0.562	0.565	0.566	0.567	0.569	0.572	0.575	0.576	0.581	0.584	0.589	0.594	0.598	0.603	0.607
Mongolia	0.537	0.545	0.55	0.555	0.559	0.564	0.569	0.573	0.577	0.581	0.585	0.589	0.594	0.598	0.603	0.608	0.614	0.619	0.624	0.628	0.632	0.636	0.641	0.646	0.65	0.654	0.658	0.662
Tajikistan	0.474	0.481	0.485	0.487	0.486	0.481	0.474	0.468	0.463	0.459	0.455	0.454	0.456	0.462	0.465	0.466	0.472	0.479	0.483	0.488	0.494	0.501	0.506	0.51	0.514	0.517	0.52	0.523
Turkmenistan	0.588	0.592	0.594	0.599	0.602	0.604	0.606	0.606	0.606	0.607	0.61	0.613	0.617	0.622	0.628	0.635	0.638	0.641	0.644	0.647	0.651	0.657	0.663	0.669	0.678	0.685	0.691	0.696
Uzbekistan	0.481	0.484	0.487	0.493	0.497	0.502	0.508	0.513	0.52	0.526	0.532	0.537	0.543	0.549	0.555	0.56	0.565	0.57	0.575	0.581	0.587	0.592	0.598	0.604	0.611	0.618	0.624	0.63
Central Europe	0.665	0.671	0.677	0.683	0.69	0.698	0.705	0.711	0.717	0.723	0.731	0.738	0.745	0.751	0.757	0.762	0.767	0.772	0.776	0.782	0.788	0.793	0.797	0.802	0.805	0.808	0.811	0.814
Albania	0.548	0.545	0.542	0.541	0.542	0.546	0.552	0.558	0.566	0.577	0.584	0.593	0.602	0.611	0.619	0.627	0.635	0.642	0.648	0.653	0.658	0.661	0.665	0.668	0.672	0.676	0.681	0.685
Bosnia and Herzegovina	0.497	0.499	0.5	0.5	0.501	0.507	0.525	0.549	0.571	0.592	0.607	0.619	0.63	0.639	0.647	0.654	0.66	0.667	0.673	0.679	0.685	0.69	0.694	0.699	0.703	0.706	0.71	0.713
Bulgaria	0.658	0.668	0.676	0.684	0.693	0.699	0.705	0.706	0.704	0.703	0.708	0.715	0.721	0.726	0.731	0.736	0.741	0.746	0.751	0.757	0.765	0.771	0.775	0.778	0.781	0.784	0.788	0.792
Croatia	0.725	0.73	0.732	0.732	0.731	0.731	0.732	0.737	0.743	0.749	0.755	0.762	0.768	0.773	0.778	0.782	0.787	0.792	0.797	0.801	0.805	0.809	0.813	0.816	0.818	0.821	0.823	0.825
Czech Republic	0.711	0.717	0.726	0.74	0.757	0.769	0.777	0.783	0.788	0.794	0.799	0.804	0.809	0.814	0.819	0.823	0.827	0.83	0.833	0.836	0.84	0.843	0.846	0.847	0.848	0.848	0.849	0.851
Hungary	0.678	0.683	0.691	0.699	0.707	0.716	0.724	0.732	0.739	0.745	0.751	0.758	0.764	0.77	0.776	0.781	0.786	0.791	0.795	0.799	0.803	0.806	0.807	0.808	0.809	0.811	0.814	0.817
Macedonia	0.626	0.629	0.63	0.631	0.632	0.635	0.64	0.647	0.654	0.661	0.665	0.67	0.677	0.685	0.693	0.699	0.704	0.709	0.715	0.719	0.724	0.729	0.734	0.739	0.744	0.748	0.751	0.754
Montenegro	0.705	0.706	0.705	0.701	0.698	0.696	0.696	0.698	0.7	0.703	0.706	0.711	0.716	0.721	0.726	0.731	0.737	0.743	0.75	0.756	0.761	0.767	0.771	0.775	0.779	0.782	0.785	0.788
Poland	0.662	0.668	0.678	0.686	0.697	0.707	0.714	0.724	0.733	0.741	0.75	0.759	0.767	0.773	0.779	0.784	0.789	0.792	0.797	0.804	0.811	0.818	0.823	0.829	0.833	0.837	0.841	0.844
Romania	0.652	0.66	0.663	0.666	0.671	0.678	0.682	0.685	0.689	0.694	0.7	0.707	0.713	0.718	0.724	0.73	0.734	0.739	0.745	0.751	0.758	0.763	0.768	0.772	0.774	0.777	0.78	0.784
Serbia	0.632	0.638	0.643	0.642	0.641	0.641	0.643	0.648	0.653	0.655	0.661	0.665	0.669	0.675	0.684	0.692	0.699	0.705	0.709	0.713	0.718	0.723	0.729	0.736	0.742	0.747	0.75	0.752
Slovakia	0.684	0.69	0.699	0.71	0.722	0.732	0.74	0.748	0.756	0.764	0.772	0.779	0.784	0.788	0.793	0.798	0.804	0.809	0.814	0.818	0.823	0.828	0.832	0.834	0.836	0.838	0.839	0.842
Slovenia	0.741	0.747	0.753	0.759	0.764	0.769	0.775	0.781	0.788	0.794	0.801	0.808	0.814	0.819	0.824	0.828	0.833	0.837	0.841	0.843	0.846	0.848	0.85	0.852	0.854	0.856	0.858	0.86
Eastern Europe	0.678	0.685	0.694	0.698	0.7	0.704	0.708	0.708	0.71	0.711	0.712	0.713	0.715	0.72	0.727	0.734	0.739	0.745	0.751	0.756	0.761	0.764	0.767	0.772	0.776	0.779	0.783	0.785
Belarus	0.625	0.631	0.636	0.641	0.645	0.647	0.65	0.654	0.657	0.661	0.665	0.67	0.676	0.682	0.689	0.696	0.704	0.712	0.72	0.727	0.733	0.74	0.747	0.753	0.759	0.764	0.769	0.773
Estonia	0.711	0.719	0.728	0.736	0.742	0.746	0.75	0.755	0.761	0.766	0.772	0.778	0.783	0.788	0.794	0.799	0.806	0.813	0.82	0.826	0.832	0.838	0.843	0.847	0.851	0.854	0.856	0.858
Latvia	0.696	0.703	0.712	0.721	0.727	0.731	0.733	0.734	0.735	0.738	0.741	0.745	0.75	0.757	0.763	0.769	0.776	0.783	0.792	0.8	0.806	0.81	0.814	0.816	0.817	0.819	0.822	0.825
Lithuania	0.707	0.71	0.717	0.725	0.728	0.731	0.733	0.736	0.74	0.746	0.753	0.76	0.765	0.772	0.779	0.785	0.79	0.796	0.802	0.808	0.815	0.822	0.828	0.833	0.836	0.838	0.839	0.841
Moldova	0.575	0.578	0.58	0.582	0.583	0.584	0.584	0.582	0.58	0.577	0.574	0.574	0.577	0.582	0.588	0.595	0.602	0.61	0.618	0.624	0.632	0.64	0.647	0.654	0.66	0.666	0.671	0.676
Russian Federation	0.683	0.692	0.704	0.708	0.708	0.714	0.718	0.719	0.72	0.722	0.722	0.722	0.724	0.728	0.734	0.742	0.747	0.752	0.757	0.763	0.768	0.77	0.772	0.777	0.781	0.785	0.789	0.792
Ukraine	0.664	0.667	0.67	0.673	0.675	0.676	0.676	0.675	0.673	0.672	0.672	0.673	0.675	0.68	0.687	0.694	0.7	0.707	0.714	0.717	0.721	0.725	0.729	0.732	0.735	0.736	0.738	0.74
High-income	0.769	0.774	0.779	0.783	0.787	0.792	0.796	0.798	0.801	0.804	0.807	0.811	0.814	0.817	0.82	0.822	0.823	0.826	0.829	0.832	0.836	0.839	0.842	0.845	0.848	0.851	0.853	0.854
Australasia	0.783	0.786	0.79	0.794	0.797	0.801	0.805	0.809	0.813	0.817	0.821	0.825	0.828	0.832	0.835	0.837	0.838	0.84	0.842	0.845	0.848	0.851	0.855	0.859	0.862	0.864	0.867	0.869
Australia	0.786	0.79	0.793	0.797	0.801	0.805	0.81	0.814	0.818	0.822	0.825	0.829	0.833	0.837	0.84	0.843	0.844	0.845	0.848	0.851	0.854	0.856	0.86	0.864	0.867	0.869	0.871	0.873
New Zealand	0.765	0.768	0.771	0.774	0.777	0.78	0.783	0.786	0.79	0.794	0.798	0.802	0.805	0.807	0.809	0.811	0.811	0.811	0.813	0.816	0.819	0.823	0.828	0.832	0.835	0.838	0.84	0.842
High-income Asia-Pacific	0.783	0.789	0.794	0.799	0.804	0.809	0.813	0.817	0.82	0.823	0.826	0.83	0.833	0.836	0.839	0.842	0.844	0.846	0.849	0.851	0.853	0.856	0.858	0.861	0.863	0.865	0.867	0.869
Brunei	0.728	0.733	0.739	0.745	0.751	0.757	0.763	0.769	0.774	0.779	0.784	0.789	0.795	0.802	0.808	0.814	0.819	0.824	0.828	0.831	0.835	0.838	0.842	0.845	0.848	0.851	0.854	0.856
Japan	0.803	0.807	0.812	0.816	0.82	0.823	0.826	0.829	0.831	0.833	0.834	0.836	0.838	0.84	0.842	0.844	0.846	0.847	0.849	0.851	0.853	0.855	0.857	0.859	0.861	0.862	0.863	0.865
Aichi	0.812	0.816	0.821	0.825	0.829	0.833	0.836	0.839	0.841	0.843	0.844	0.846	0.847	0.85	0.852	0.854	0.855	0.856	0.858	0.86	0.862	0.864	0.866	0.869	0.871	0.872	0.873	0.875
Akita	0.766	0.77	0.775	0.778	0.781	0.785	0.788	0.791	0.793	0.795	0.796	0.798	0.8	0.802	0.805	0.806	0.808	0.81	0.812	0.814	0.816	0.818	0.821	0.823	0.825	0.826	0.827	0.829
Aomori	0.761	0.765	0.77	0.773	0.777	0.78	0.783	0.786	0.788	0.79	0.791	0.793	0.795	0.798	0.801	0.803	0.805	0.807	0.809	0.81	0.812	0.815	0.817	0.819	0.821	0.822	0.823	0.825
Chiba	0.803	0.807	0.812	0.816	0.82	0.823	0.827	0.829	0.831	0.832	0.833	0.834	0.836	0.837	0.839	0.841	0.842	0.843	0.845	0.846	0.848	0.85	0.852	0.854	0.856	0.857	0.858	0.859
Ehime	0.776	0.78	0.785	0.788	0.792	0.796	0.799	0.802	0.804	0.806	0.808	0.809	0.811	0.814	0.816	0.818	0.819	0.82	0.822	0.824	0.825	0.828	0.83	0.832	0.834	0.835	0.836	0.838
Fukui	0.784	0.789	0.794	0.798	0.802	0.806	0.81	0.813	0.815	0.818	0.819	0.821	0.823	0.826	0.828	0.83	0.832	0.834	0.836	0.837	0.839	0.842	0.844	0.846	0.848	0.849	0.85	0.852
Fukuoka	0.797	0.801	0.806	0.809	0.813	0.816	0.819	0.822	0.824	0.826	0.828	0.83	0.832	0.834	0.836	0.837	0.838	0.839	0.841	0.842	0.843	0.845	0.847	0.849	0.851	0.853	0.854	0.855
Fukushima	0.769	0.773	0.778	0.781	0.784	0.788	0.791	0.793	0.794	0.795	0.797	0.798	0.8	0.803	0.807	0.809	0.811	0.813	0.814	0.816	0.819	0.821	0.823	0.825	0.827	0.828	0.829	0.831
Gifu	0.786	0.79	0.795	0.799	0.803	0.807	0.81	0.813	0.815	0.817	0.818	0.82	0.821	0.824	0.826	0.828	0.829	0.831	0.833	0.835	0.837	0.839	0.841	0.843	0.845	0.846	0.847	0.849
Gunma	0.787	0.791	0.796	0.8	0.803	0.807	0.81	0.813	0.815	0.816	0.818	0.819	0.821	0.824	0.827	0.829	0.831	0.833	0.835	0.837	0.839	0.841	0.843	0.845	0.847	0.848	0.849	0.851
Hiroshima	0.8	0.805	0.809	0.813	0.817	0.821	0.825	0.828	0.83	0.832	0.833	0.835	0.836	0.839	0.841	0.842	0.843	0.844	0.846	0.847	0.849	0.852	0.854	0.856	0.858	0.86	0.861	0.863
Hokkaidō	0.783	0.788	0.792	0.796	0.799	0.803	0.806	0.808	0.809	0.811	0.812	0.813	0.815	0.818	0.82	0.822	0.824	0.825	0.827	0.828	0.83	0.832	0.834	0.836	0.838	0.839	0.84	0.842
Hyōgo	0.798	0.803	0.807	0.811	0.815	0.819	0.822	0.825	0.827	0.829	0.831	0.833	0.835	0.837	0.839	0.841	0.842	0.843	0.845	0.846	0.848	0.85	0.852	0.854	0.856	0.857	0.858	0.86
Ibaraki	0.789	0.793	0.798	0.802	0.805	0.809	0.813	0.815	0.817	0.819	0.82	0.821	0.823	0.826	0.828	0.83	0.831	0.833	0.835	0.837	0.839	0.841	0.843	0.845	0.847	0.848	0.849	0.851
Ishikawa	0.789	0.795	0.8	0.804	0.808	0.812	0.815	0.818	0.82	0.822	0.824	0.826	0.828	0.831	0.833	0.835	0.837	0.839	0.84	0.842	0.844	0.846	0.848	0.85	0.852	0.853	0.854	0.856
Iwate	0.76	0.764	0.768	0.772	0.775	0.779	0.782	0.785	0.787	0.788	0.79	0.791	0.793	0.796	0.799	0.801	0.803	0.806	0.808	0.81	0.812	0.815	0.817	0.819	0.821	0.823	0.823	0.825
Kagawa	0.787	0.792	0.797	0.801	0.804	0.808	0.811	0.814	0.816	0.817	0.819	0.82	0.822	0.825	0.827	0.829	0.831	0.832	0.834	0.835	0.837	0.839	0.842	0.844	0.846	0.847	0.848	0.85
Kagoshima	0.768	0.772	0.777	0.781	0.784	0.788	0.791	0.793	0.795	0.797	0.798	0.799	0.801	0.804	0.806	0.808	0.809	0.81	0.812	0.814	0.816	0.818	0.82	0.823	0.825	0.827	0.828	0.83
Kanagawa	0.818	0.823	0.827	0.831	0.835	0.838	0.842	0.844	0.846	0.848	0.849	0.85	0.852	0.854	0.856	0.857	0.859	0.86	0.862	0.863	0.865	0.866	0.868	0.87	0.871	0.873	0.873	0.875
Kōchi	0.759	0.763	0.767	0.771	0.775	0.779	0.783	0.786	0.788	0.791	0.793	0.795	0.797	0.8	0.803	0.805	0.806	0.808	0.81	0.812	0.813	0.816	0.818	0.82	0.822	0.823	0.824	0.825
Kumamoto	0.77	0.774	0.778	0.782	0.785	0.789	0.792	0.794	0.796	0.798	0.799	0.801	0.803	0.805	0.808	0.81	0.811	0.812	0.814	0.815	0.817	0.82	0.822	0.825	0.827	0.829	0.83	0.832
Kyōto	0.813	0.817	0.822	0.826	0.83	0.833	0.837	0.839	0.841	0.843	0.845	0.847	0.849	0.851	0.853	0.854	0.856	0.857	0.859	0.86	0.862	0.863	0.865	0.867	0.869	0.87	0.871	0.873
Mie	0.787	0.792	0.797	0.801	0.805	0.81	0.813	0.815	0.818	0.819	0.821	0.823	0.825	0.827	0.83	0.832	0.833	0.835	0.836	0.838	0.84	0.842	0.845	0.847	0.849	0.851	0.852	0.854
Miyagi	0.788	0.793	0.798	0.801	0.805	0.809	0.812	0.814	0.816	0.817	0.818	0.819	0.821	0.824	0.827	0.829	0.831	0.833	0.835	0.836	0.838	0.841	0.843	0.845	0.847	0.848	0.849	0.85
Miyazaki	0.764	0.768	0.772	0.775	0.778	0.781	0.784	0.786	0.788	0.79	0.791	0.793	0.795	0.797	0.8	0.801	0.802	0.804	0.805	0.807	0.809	0.812	0.814	0.816	0.819	0.82	0.821	0.823
Nagano	0.792	0.797	0.801	0.805	0.808	0.811	0.814	0.817	0.818	0.82	0.821	0.823	0.824	0.827	0.829	0.831	0.832	0.834	0.836	0.838	0.839	0.842	0.844	0.845	0.847	0.849	0.849	0.851
Nagasaki	0.766	0.77	0.774	0.777	0.781	0.784	0.787	0.79	0.792	0.794	0.795	0.797	0.799	0.801	0.803	0.805	0.806	0.808	0.809	0.811	0.813	0.815	0.817	0.82	0.822	0.823	0.824	0.826
Nara	0.789	0.794	0.799	0.803	0.807	0.811	0.814	0.817	0.819	0.821	0.822	0.824	0.825	0.827	0.829	0.831	0.832	0.834	0.835	0.836	0.838	0.839	0.841	0.843	0.844	0.845	0.846	0.848
Niigata	0.776	0.78	0.784	0.788	0.792	0.795	0.799	0.801	0.804	0.806	0.808	0.81	0.813	0.815	0.818	0.82	0.822	0.824	0.826	0.828	0.83	0.832	0.835	0.837	0.839	0.84	0.842	0.843
Ōita	0.785	0.789	0.795	0.802	0.808	0.809	0.809	0.81	0.812	0.813	0.815	0.816	0.819	0.821	0.824	0.825	0.827	0.828	0.83	0.831	0.833	0.835	0.838	0.84	0.842	0.843	0.844	0.846
Okayama	0.79	0.794	0.799	0.803	0.807	0.811	0.814	0.817	0.819	0.821	0.823	0.824	0.827	0.829	0.832	0.834	0.836	0.837	0.839	0.841	0.843	0.846	0.848	0.85	0.852	0.853	0.854	0.856
Okinawa	0.753	0.757	0.764	0.772	0.778	0.779	0.777	0.777	0.778	0.78	0.781	0.783	0.786	0.789	0.793	0.795	0.797	0.799	0.8	0.802	0.804	0.807	0.809	0.812	0.814	0.815	0.816	0.818
Ōsaka	0.815	0.819	0.823	0.827	0.831	0.834	0.837	0.84	0.842	0.844	0.845	0.847	0.849	0.851	0.853	0.854	0.855	0.856	0.858	0.859	0.861	0.863	0.865	0.867	0.868	0.87	0.871	0.872
Saga	0.773	0.777	0.782	0.785	0.788	0.792	0.795	0.797	0.799	0.8	0.801	0.803	0.805	0.808	0.81	0.812	0.814	0.815	0.817	0.819	0.821	0.823	0.825	0.827	0.83	0.831	0.832	0.834
Saitama	0.793	0.798	0.802	0.806	0.81	0.814	0.818	0.82	0.822	0.824	0.825	0.826	0.827	0.829	0.831	0.833	0.835	0.836	0.838	0.839	0.841	0.843	0.845	0.847	0.848	0.849	0.85	0.852
Shiga	0.804	0.809	0.814	0.819	0.823	0.827	0.83	0.833	0.836	0.838	0.84	0.841	0.843	0.846	0.848	0.85	0.852	0.853	0.855	0.857	0.858	0.861	0.863	0.865	0.867	0.868	0.869	0.871
Shimane	0.762	0.766	0.771	0.775	0.779	0.783	0.787	0.79	0.793	0.795	0.797	0.798	0.801	0.803	0.806	0.808	0.81	0.812	0.814	0.816	0.818	0.82	0.823	0.825	0.827	0.828	0.829	0.831
Shizuoka	0.798	0.802	0.807	0.81	0.814	0.818	0.821	0.823	0.825	0.826	0.827	0.828	0.83	0.833	0.835	0.837	0.838	0.84	0.842	0.843	0.846	0.848	0.85	0.853	0.855	0.856	0.857	0.859
Tochigi	0.787	0.792	0.797	0.801	0.805	0.809	0.813	0.815	0.817	0.819	0.82	0.821	0.823	0.826	0.829	0.831	0.832	0.834	0.836	0.838	0.84	0.842	0.845	0.847	0.849	0.85	0.851	0.853
Tokushima	0.776	0.781	0.786	0.79	0.794	0.798	0.802	0.805	0.807	0.809	0.812	0.814	0.816	0.819	0.822	0.824	0.826	0.828	0.83	0.831	0.833	0.835	0.837	0.839	0.841	0.842	0.843	0.845
Tōkyō	0.87	0.875	0.879	0.883	0.887	0.892	0.896	0.899	0.901	0.903	0.905	0.906	0.908	0.91	0.911	0.913	0.914	0.915	0.916	0.917	0.918	0.919	0.92	0.921	0.922	0.923	0.923	0.924
Tottori	0.77	0.775	0.78	0.783	0.787	0.791	0.794	0.797	0.799	0.801	0.802	0.804	0.806	0.808	0.811	0.813	0.814	0.816	0.818	0.819	0.821	0.824	0.826	0.828	0.831	0.832	0.833	0.834
Toyama	0.79	0.795	0.8	0.805	0.808	0.812	0.816	0.818	0.821	0.823	0.825	0.827	0.829	0.832	0.835	0.837	0.839	0.841	0.843	0.845	0.847	0.849	0.852	0.854	0.856	0.857	0.858	0.86
Wakayama	0.775	0.78	0.785	0.789	0.792	0.796	0.8	0.802	0.805	0.806	0.808	0.81	0.812	0.815	0.817	0.819	0.821	0.822	0.824	0.825	0.827	0.829	0.831	0.833	0.836	0.837	0.838	0.84
Yamagata	0.766	0.77	0.775	0.778	0.782	0.785	0.788	0.791	0.792	0.794	0.795	0.796	0.799	0.802	0.805	0.807	0.809	0.812	0.814	0.816	0.819	0.821	0.824	0.826	0.828	0.829	0.83	0.832
Yamaguchi	0.79	0.794	0.799	0.802	0.806	0.809	0.812	0.815	0.817	0.819	0.82	0.822	0.824	0.826	0.828	0.83	0.831	0.832	0.834	0.835	0.837	0.839	0.841	0.843	0.846	0.847	0.848	0.849
Yamanashi	0.791	0.796	0.8	0.804	0.808	0.812	0.815	0.818	0.82	0.822	0.823	0.825	0.827	0.829	0.832	0.834	0.836	0.837	0.839	0.841	0.842	0.844	0.846	0.848	0.85	0.851	0.853	0.854
South Korea	0.713	0.724	0.733	0.742	0.751	0.76	0.768	0.777	0.783	0.79	0.799	0.806	0.814	0.82	0.825	0.83	0.834	0.839	0.843	0.846	0.85	0.853	0.857	0.86	0.864	0.867	0.869	0.872
Singapore	0.736	0.744	0.75	0.758	0.765	0.772	0.78	0.786	0.79	0.79	0.797	0.808	0.815	0.82	0.824	0.827	0.832	0.838	0.844	0.849	0.854	0.857	0.86	0.863	0.865	0.868	0.87	0.872
High-income North America	0.784	0.786	0.789	0.793	0.796	0.8	0.805	0.807	0.809	0.812	0.815	0.82	0.824	0.827	0.829	0.83	0.829	0.832	0.837	0.843	0.848	0.852	0.856	0.859	0.861	0.865	0.867	0.868
Canada	0.802	0.805	0.808	0.811	0.814	0.818	0.823	0.828	0.832	0.836	0.841	0.846	0.85	0.853	0.857	0.859	0.861	0.862	0.864	0.867	0.871	0.874	0.877	0.878	0.879	0.88	0.881	0.882
Greenland	0.671	0.67	0.67	0.671	0.672	0.675	0.679	0.683	0.687	0.691	0.695	0.699	0.703	0.708	0.713	0.717	0.722	0.726	0.731	0.737	0.743	0.747	0.751	0.754	0.756	0.757	0.759	0.76
USA	0.781	0.784	0.787	0.79	0.793	0.798	0.803	0.804	0.806	0.809	0.812	0.817	0.821	0.823	0.826	0.826	0.826	0.829	0.834	0.84	0.846	0.85	0.853	0.857	0.859	0.863	0.866	0.867
Alabama	0.745	0.749	0.753	0.757	0.76	0.765	0.769	0.769	0.771	0.773	0.777	0.783	0.787	0.791	0.794	0.794	0.793	0.796	0.801	0.809	0.816	0.82	0.824	0.827	0.83	0.834	0.837	0.837
Alaska	0.755	0.757	0.761	0.767	0.772	0.781	0.788	0.79	0.793	0.795	0.799	0.804	0.808	0.811	0.814	0.813	0.811	0.813	0.818	0.826	0.833	0.838	0.843	0.847	0.851	0.857	0.86	0.861
Arizona	0.751	0.753	0.755	0.758	0.76	0.764	0.769	0.77	0.771	0.772	0.775	0.781	0.784	0.787	0.79	0.791	0.791	0.797	0.806	0.815	0.823	0.828	0.832	0.836	0.839	0.842	0.845	0.845
Arkansas	0.723	0.727	0.733	0.738	0.741	0.746	0.751	0.751	0.753	0.754	0.757	0.763	0.767	0.77	0.773	0.772	0.77	0.774	0.78	0.789	0.797	0.802	0.807	0.811	0.816	0.821	0.825	0.826
California	0.771	0.771	0.773	0.776	0.78	0.787	0.794	0.798	0.803	0.807	0.812	0.819	0.823	0.826	0.829	0.83	0.831	0.835	0.84	0.846	0.852	0.856	0.859	0.863	0.865	0.869	0.871	0.872
Colorado	0.798	0.801	0.804	0.807	0.81	0.815	0.818	0.819	0.82	0.821	0.824	0.83	0.833	0.836	0.839	0.84	0.84	0.844	0.849	0.855	0.861	0.865	0.869	0.872	0.875	0.879	0.881	0.882
Connecticut	0.841	0.844	0.847	0.85	0.853	0.856	0.859	0.861	0.863	0.866	0.869	0.874	0.877	0.879	0.881	0.881	0.881	0.883	0.886	0.89	0.894	0.896	0.899	0.901	0.903	0.905	0.906	0.906
Delaware	0.801	0.804	0.808	0.811	0.813	0.816	0.819	0.819	0.82	0.822	0.825	0.83	0.833	0.836	0.838	0.839	0.838	0.841	0.846	0.852	0.858	0.861	0.864	0.866	0.868	0.871	0.873	0.874
Washington, DC	0.797	0.801	0.806	0.812	0.818	0.826	0.834	0.839	0.845	0.85	0.855	0.861	0.865	0.868	0.869	0.868	0.866	0.866	0.868	0.871	0.874	0.876	0.877	0.88	0.883	0.887	0.89	0.89
Florida	0.774	0.778	0.782	0.786	0.789	0.794	0.799	0.8	0.802	0.804	0.808	0.813	0.817	0.82	0.823	0.824	0.825	0.828	0.834	0.84	0.846	0.85	0.853	0.855	0.858	0.861	0.863	0.864
Georgia	0.755	0.759	0.764	0.769	0.772	0.778	0.782	0.783	0.784	0.785	0.788	0.793	0.796	0.798	0.801	0.801	0.8	0.804	0.81	0.818	0.825	0.83	0.834	0.837	0.841	0.845	0.848	0.848
Hawaii	0.781	0.784	0.788	0.792	0.797	0.805	0.811	0.813	0.815	0.817	0.82	0.824	0.828	0.83	0.833	0.833	0.832	0.834	0.839	0.845	0.851	0.854	0.858	0.861	0.864	0.868	0.871	0.872
Idaho	0.756	0.76	0.764	0.769	0.773	0.778	0.783	0.783	0.784	0.785	0.787	0.791	0.794	0.796	0.799	0.798	0.797	0.801	0.807	0.814	0.821	0.824	0.827	0.83	0.832	0.836	0.84	0.841
Illinois	0.787	0.789	0.792	0.796	0.799	0.805	0.81	0.812	0.816	0.818	0.823	0.828	0.833	0.837	0.84	0.841	0.841	0.845	0.85	0.855	0.861	0.864	0.867	0.87	0.873	0.876	0.879	0.879
Indiana	0.772	0.774	0.778	0.781	0.783	0.787	0.791	0.791	0.793	0.794	0.797	0.802	0.806	0.808	0.81	0.809	0.807	0.809	0.813	0.819	0.825	0.829	0.832	0.836	0.839	0.844	0.847	0.848
Iowa	0.794	0.797	0.8	0.803	0.806	0.809	0.813	0.814	0.816	0.818	0.821	0.825	0.828	0.829	0.832	0.831	0.83	0.832	0.837	0.843	0.848	0.852	0.856	0.86	0.863	0.867	0.87	0.87
Kansas	0.782	0.785	0.788	0.791	0.794	0.798	0.802	0.803	0.804	0.806	0.809	0.814	0.817	0.819	0.821	0.819	0.817	0.819	0.823	0.83	0.837	0.842	0.847	0.852	0.856	0.861	0.864	0.864
Kentucky	0.743	0.747	0.751	0.755	0.759	0.763	0.768	0.769	0.77	0.772	0.776	0.782	0.786	0.789	0.791	0.79	0.787	0.789	0.793	0.799	0.806	0.81	0.814	0.817	0.821	0.827	0.83	0.831
Louisiana	0.735	0.739	0.743	0.748	0.752	0.758	0.762	0.764	0.765	0.767	0.771	0.777	0.782	0.786	0.789	0.788	0.787	0.79	0.796	0.803	0.811	0.815	0.819	0.823	0.826	0.831	0.835	0.835
Maine	0.789	0.795	0.8	0.805	0.809	0.814	0.817	0.819	0.821	0.823	0.826	0.83	0.834	0.837	0.84	0.84	0.84	0.842	0.846	0.851	0.856	0.859	0.861	0.864	0.866	0.869	0.871	0.872
Maryland	0.813	0.817	0.821	0.825	0.828	0.833	0.836	0.838	0.839	0.841	0.845	0.85	0.854	0.857	0.86	0.861	0.861	0.864	0.868	0.874	0.879	0.883	0.886	0.889	0.891	0.894	0.896	0.896
Massachusetts	0.843	0.847	0.85	0.853	0.856	0.86	0.864	0.866	0.869	0.871	0.875	0.88	0.883	0.886	0.888	0.889	0.89	0.893	0.896	0.899	0.902	0.904	0.907	0.909	0.91	0.912	0.913	0.913
Michigan	0.788	0.791	0.795	0.799	0.803	0.808	0.813	0.815	0.817	0.82	0.823	0.829	0.832	0.835	0.838	0.838	0.837	0.839	0.841	0.845	0.849	0.852	0.854	0.857	0.86	0.864	0.867	0.868
Minnesota	0.818	0.821	0.825	0.828	0.83	0.834	0.837	0.839	0.841	0.843	0.845	0.849	0.852	0.855	0.857	0.858	0.858	0.86	0.865	0.87	0.874	0.877	0.881	0.884	0.886	0.89	0.892	0.893
Mississippi	0.719	0.722	0.727	0.732	0.736	0.741	0.746	0.747	0.748	0.75	0.753	0.758	0.762	0.764	0.765	0.764	0.76	0.763	0.77	0.779	0.789	0.794	0.8	0.804	0.809	0.814	0.818	0.819
Missouri	0.77	0.773	0.777	0.781	0.784	0.789	0.793	0.795	0.797	0.799	0.802	0.806	0.81	0.812	0.813	0.812	0.811	0.813	0.818	0.825	0.831	0.835	0.839	0.842	0.846	0.85	0.853	0.853
Montana	0.775	0.779	0.783	0.789	0.793	0.799	0.804	0.805	0.807	0.808	0.81	0.814	0.816	0.819	0.822	0.822	0.821	0.823	0.827	0.833	0.839	0.843	0.847	0.851	0.855	0.859	0.862	0.863
Nebraska	0.795	0.798	0.802	0.805	0.808	0.813	0.817	0.817	0.819	0.819	0.821	0.824	0.827	0.829	0.831	0.831	0.83	0.832	0.836	0.842	0.848	0.853	0.857	0.861	0.865	0.869	0.872	0.873
Nevada	0.76	0.761	0.762	0.765	0.767	0.773	0.779	0.781	0.784	0.787	0.791	0.798	0.802	0.805	0.808	0.809	0.81	0.814	0.82	0.827	0.833	0.836	0.838	0.84	0.842	0.845	0.847	0.847
New Hampshire	0.827	0.832	0.835	0.838	0.84	0.844	0.847	0.849	0.852	0.856	0.86	0.865	0.869	0.872	0.874	0.875	0.876	0.878	0.881	0.885	0.889	0.891	0.894	0.897	0.899	0.902	0.904	0.904
New Jersey	0.828	0.831	0.834	0.838	0.841	0.845	0.849	0.85	0.852	0.854	0.857	0.861	0.864	0.867	0.87	0.87	0.871	0.873	0.876	0.881	0.885	0.888	0.89	0.893	0.895	0.897	0.899	0.899
New Mexico	0.733	0.735	0.739	0.743	0.748	0.755	0.76	0.762	0.764	0.765	0.767	0.773	0.777	0.779	0.782	0.781	0.779	0.783	0.79	0.799	0.808	0.814	0.819	0.823	0.828	0.832	0.835	0.835
New York	0.816	0.819	0.822	0.825	0.828	0.832	0.836	0.838	0.841	0.843	0.847	0.852	0.855	0.858	0.86	0.861	0.861	0.864	0.868	0.872	0.877	0.88	0.883	0.886	0.889	0.892	0.893	0.893
North Carolina	0.763	0.766	0.77	0.774	0.777	0.781	0.785	0.785	0.786	0.788	0.791	0.796	0.8	0.802	0.805	0.805	0.805	0.809	0.815	0.822	0.829	0.833	0.836	0.84	0.843	0.846	0.849	0.85
North Dakota	0.79	0.792	0.796	0.799	0.802	0.807	0.813	0.816	0.82	0.823	0.827	0.83	0.833	0.834	0.835	0.835	0.833	0.835	0.839	0.844	0.85	0.854	0.859	0.864	0.87	0.875	0.879	0.88
Ohio	0.78	0.783	0.787	0.79	0.793	0.797	0.801	0.802	0.804	0.806	0.809	0.814	0.818	0.82	0.822	0.822	0.821	0.822	0.826	0.832	0.837	0.841	0.844	0.847	0.85	0.854	0.857	0.858

Appendix Table 10. Socio-Demographic Index values for all estimated GBD 2017 locations, 1990-2017

Location	1990	1991	1992	1993	1994	1995	1996	1997	1998	1999	2000	2001	2002	2003	2004	2005	2006	2007	2008	2009	2010	2011	2012	2013	2014	2015	2016	2017
Oklahoma	0.749	0.751	0.755	0.758	0.76	0.764	0.768	0.768	0.768	0.769	0.772	0.777	0.781	0.784	0.786	0.785	0.784	0.786	0.792	0.8	0.808	0.813	0.818	0.824	0.829	0.835	0.838	0.838
Oregon	0.785	0.788	0.791	0.794	0.797	0.802	0.806	0.808	0.811	0.814	0.818	0.824	0.827	0.83	0.833	0.833	0.833	0.836	0.841	0.847	0.852	0.855	0.858	0.861	0.864	0.867	0.87	0.871
Pennsylvania	0.8	0.804	0.808	0.812	0.816	0.821	0.824	0.826	0.828	0.83	0.833	0.837	0.84	0.842	0.845	0.845	0.845	0.847	0.851	0.856	0.86	0.863	0.866	0.869	0.871	0.875	0.878	0.879
Rhode Island	0.815	0.818	0.822	0.825	0.827	0.831	0.834	0.835	0.838	0.84	0.843	0.848	0.851	0.854	0.857	0.858	0.859	0.862	0.866	0.87	0.875	0.877	0.88	0.882	0.885	0.887	0.889	0.89
South Carolina	0.752	0.757	0.762	0.768	0.772	0.777	0.781	0.782	0.783	0.785	0.787	0.793	0.796	0.799	0.801	0.8	0.799	0.802	0.808	0.815	0.822	0.826	0.83	0.834	0.838	0.842	0.845	0.846
South Dakota	0.769	0.772	0.777	0.783	0.788	0.794	0.799	0.801	0.804	0.805	0.808	0.811	0.813	0.814	0.816	0.814	0.812	0.814	0.819	0.826	0.833	0.838	0.842	0.847	0.851	0.856	0.859	0.86
Tennessee	0.749	0.752	0.757	0.761	0.765	0.77	0.774	0.775	0.777	0.779	0.781	0.786	0.788	0.789	0.79	0.789	0.786	0.789	0.795	0.803	0.81	0.815	0.819	0.823	0.827	0.832	0.836	0.837
Texas	0.743	0.745	0.747	0.75	0.752	0.757	0.761	0.763	0.764	0.766	0.769	0.775	0.779	0.782	0.784	0.783	0.782	0.785	0.792	0.801	0.809	0.815	0.82	0.824	0.829	0.834	0.837	0.838
Utah	0.761	0.765	0.769	0.773	0.776	0.781	0.786	0.787	0.79	0.792	0.795	0.8	0.803	0.806	0.808	0.808	0.807	0.811	0.817	0.825	0.832	0.837	0.841	0.845	0.848	0.852	0.855	0.856
Vermont	0.815	0.819	0.823	0.827	0.83	0.833	0.837	0.839	0.841	0.844	0.848	0.853	0.857	0.86	0.864	0.866	0.866	0.869	0.872	0.876	0.88	0.882	0.885	0.887	0.89	0.893	0.895	0.896
Virginia	0.8	0.803	0.807	0.81	0.814	0.818	0.822	0.823	0.824	0.826	0.829	0.834	0.838	0.841	0.845	0.846	0.847	0.851	0.856	0.862	0.867	0.871	0.874	0.877	0.88	0.883	0.885	0.885
Washington	0.797	0.8	0.804	0.807	0.811	0.816	0.821	0.823	0.827	0.83	0.834	0.84	0.844	0.846	0.849	0.848	0.848	0.85	0.855	0.86	0.865	0.868	0.871	0.874	0.877	0.881	0.883	0.884
West Virginia	0.749	0.752	0.756	0.761	0.764	0.769	0.773	0.774	0.775	0.776	0.778	0.783	0.786	0.787	0.789	0.787	0.784	0.784	0.787	0.793	0.799	0.802	0.806	0.81	0.814	0.82	0.824	0.825
Wisconsin	0.801	0.804	0.808	0.812	0.815	0.819	0.823	0.825	0.826	0.828	0.831	0.835	0.839	0.841	0.843	0.843	0.843	0.845	0.849	0.853	0.858	0.862	0.865	0.868	0.871	0.874	0.877	0.878
Wyoming	0.766	0.771	0.777	0.782	0.786	0.792	0.796	0.797	0.799	0.801	0.804	0.809	0.813	0.816	0.818	0.819	0.819	0.823	0.831	0.838	0.846	0.851	0.855	0.858	0.862	0.866	0.869	0.869
Southern Latin America	0.594	0.6	0.607	0.613	0.619	0.626	0.632	0.638	0.643	0.648	0.652	0.655	0.658	0.662	0.667	0.673	0.677	0.679	0.682	0.685	0.69	0.695	0.7	0.704	0.707	0.713	0.717	0.72
Argentina	0.59	0.595	0.604	0.61	0.617	0.624	0.63	0.635	0.64	0.644	0.647	0.649	0.65	0.653	0.658	0.665	0.669	0.672	0.675	0.677	0.681	0.686	0.691	0.693	0.696	0.702	0.707	0.71
Chile	0.6	0.608	0.615	0.62	0.626	0.633	0.64	0.647	0.654	0.661	0.667	0.674	0.681	0.687	0.692	0.696	0.698	0.701	0.704	0.708	0.714	0.721	0.727	0.732	0.738	0.742	0.746	0.748
Uruguay	0.592	0.597	0.6	0.602	0.606	0.609	0.613	0.618	0.625	0.632	0.637	0.64	0.643	0.647	0.652	0.656	0.659	0.661	0.663	0.666	0.671	0.675	0.68	0.685	0.691	0.697	0.702	0.707
Western Europe	0.764	0.77	0.776	0.782	0.787	0.791	0.795	0.798	0.801	0.805	0.809	0.813	0.817	0.82	0.822	0.825	0.828	0.83	0.833	0.836	0.838	0.842	0.845	0.848	0.851	0.853	0.855	0.857
Andorra	0.85	0.854	0.856	0.857	0.858	0.859	0.86	0.863	0.866	0.868	0.871	0.873	0.875	0.878	0.881	0.883	0.885	0.886	0.888	0.89	0.891	0.894	0.896	0.897	0.899	0.9	0.901	0.902
Austria	0.776	0.778	0.78	0.785	0.79	0.795	0.8	0.805	0.809	0.813	0.818	0.822	0.825	0.828	0.831	0.834	0.838	0.841	0.845	0.847	0.85	0.854	0.857	0.859	0.862	0.863	0.865	0.866
Belgium	0.803	0.808	0.813	0.818	0.822	0.826	0.829	0.832	0.835	0.837	0.84	0.843	0.847	0.849	0.852	0.854	0.856	0.858	0.861	0.863	0.867	0.871	0.875	0.879	0.882	0.884	0.885	0.886
Cyprus	0.724	0.73	0.74	0.75	0.758	0.765	0.771	0.778	0.784	0.789	0.795	0.803	0.81	0.817	0.824	0.83	0.837	0.842	0.847	0.851	0.854	0.857	0.859	0.861	0.862	0.863	0.864	0.865
Denmark	0.846	0.849	0.852	0.855	0.858	0.862	0.866	0.87	0.874	0.877	0.881	0.884	0.888	0.891	0.893	0.895	0.897	0.898	0.9	0.902	0.904	0.907	0.91	0.912	0.914	0.915	0.916	0.918
Finland	0.813	0.813	0.814	0.817	0.821	0.825	0.828	0.831	0.835	0.84	0.844	0.847	0.851	0.854	0.857	0.859	0.862	0.865	0.869	0.871	0.875	0.878	0.881	0.884	0.887	0.889	0.891	0.893
France	0.769	0.776	0.783	0.79	0.793	0.795	0.802	0.806	0.808	0.813	0.816	0.819	0.824	0.827	0.83	0.833	0.836	0.838	0.84	0.842	0.845	0.848	0.851	0.854	0.857	0.86	0.863	0.865
Germany	0.787	0.796	0.801	0.805	0.809	0.811	0.812	0.813	0.813	0.814	0.818	0.823	0.827	0.829	0.832	0.835	0.838	0.842	0.846	0.848	0.851	0.855	0.858	0.861	0.864	0.866	0.868	0.87
Greece	0.717	0.723	0.731	0.738	0.744	0.75	0.755	0.761	0.767	0.773	0.778	0.782	0.787	0.792	0.796	0.8	0.803	0.806	0.809	0.812	0.815	0.818	0.819	0.82	0.819	0.818	0.817	0.817
Iceland	0.814	0.818	0.821	0.825	0.828	0.83	0.833	0.835	0.839	0.843	0.848	0.854	0.859	0.862	0.865	0.869	0.872	0.876	0.88	0.883	0.886	0.889	0.892	0.895	0.899	0.902	0.905	0.907
Ireland	0.756	0.762	0.768	0.774	0.779	0.785	0.79	0.795	0.802	0.808	0.814	0.821	0.827	0.834	0.84	0.844	0.846	0.849	0.851	0.855	0.858	0.862	0.865	0.867	0.87	0.874	0.878	0.882
Israel	0.734	0.738	0.743	0.748	0.752	0.757	0.76	0.764	0.768	0.772	0.776	0.78	0.783	0.786	0.789	0.793	0.796	0.798	0.798	0.799	0.801	0.803	0.805	0.808	0.81	0.812	0.814	0.816
Italy	0.767	0.772	0.778	0.783	0.788	0.793	0.797	0.8	0.804	0.807	0.81	0.814	0.817	0.819	0.821	0.823	0.825	0.827	0.829	0.83	0.832	0.834	0.836	0.838	0.839	0.841	0.842	0.843
Luxembourg	0.845	0.849	0.851	0.854	0.858	0.862	0.866	0.869	0.873	0.876	0.878	0.88	0.881	0.883	0.885	0.888	0.891	0.894	0.896	0.899	0.901	0.904	0.906	0.909	0.912	0.913	0.915	0.916
Malta	0.729	0.733	0.737	0.743	0.748	0.752	0.756	0.761	0.766	0.773	0.779	0.784	0.788	0.792	0.796	0.799	0.802	0.805	0.808	0.811	0.814	0.817	0.82	0.823	0.826	0.829	0.833	0.836
Netherlands	0.827	0.832	0.837	0.841	0.845	0.849	0.852	0.855	0.858	0.862	0.866	0.87	0.873	0.876	0.879	0.882	0.885	0.887	0.89	0.892	0.895	0.898	0.901	0.904	0.906	0.908	0.91	0.912
Norway	0.811	0.816	0.821	0.827	0.831	0.835	0.84	0.846	0.85	0.855	0.86	0.866	0.87	0.873	0.876	0.878	0.88	0.882	0.885	0.888	0.892	0.896	0.9	0.903	0.906	0.909	0.91	0.911
Portugal	0.642	0.65	0.659	0.667	0.675	0.682	0.688	0.694	0.699	0.705	0.71	0.716	0.722	0.727	0.732	0.736	0.741	0.744	0.748	0.751	0.755	0.76	0.764	0.768	0.771	0.773	0.775	0.778
Spain	0.715	0.723	0.731	0.738	0.745	0.752	0.758	0.763	0.768	0.773	0.778	0.782	0.786	0.79	0.794	0.797	0.799	0.802	0.805	0.809	0.812	0.815	0.818	0.819	0.82	0.822	0.823	0.825
Sweden	0.784	0.789	0.795	0.802	0.808	0.815	0.82	0.825	0.831	0.835	0.838	0.841	0.844	0.847	0.85	0.853	0.855	0.857	0.86	0.862	0.865	0.868	0.871	0.874	0.876	0.879	0.881	0.883
Stockholm	0.825	0.83	0.835	0.84	0.845	0.85	0.854	0.859	0.864	0.867	0.871	0.873	0.876	0.879	0.882	0.885	0.888	0.891	0.893	0.896	0.899	0.902	0.904	0.907	0.909	0.911	0.913	0.914
Sweden except Stockholm	0.773	0.778	0.785	0.792	0.798	0.805	0.811	0.816	0.821	0.825	0.829	0.832	0.834	0.838	0.841	0.843	0.845	0.848	0.85	0.852	0.854	0.858	0.86	0.863	0.866	0.868	0.87	0.873
Switzerland	0.841	0.842	0.844	0.846	0.847	0.848	0.849	0.85	0.851	0.852	0.854	0.857	0.859	0.86	0.862	0.863	0.865	0.868	0.871	0.873	0.875	0.877	0.88	0.882	0.884	0.886	0.887	0.889
United Kingdom	0.723	0.729	0.736	0.743	0.749	0.754	0.758	0.762	0.767	0.774	0.78	0.786	0.791	0.794	0.797	0.801	0.804	0.807	0.81	0.813	0.817	0.821	0.826	0.832	0.836	0.839	0.841	0.843
England	0.732	0.738	0.745	0.752	0.758	0.763	0.766	0.77	0.776	0.782	0.788	0.794	0.798	0.801	0.804	0.807	0.81	0.813	0.816	0.819	0.823	0.827	0.832	0.838	0.842	0.845	0.847	0.849
East Midlands	0.71	0.716	0.723	0.73	0.737	0.741	0.745	0.75	0.755	0.762	0.768	0.773	0.778	0.781	0.785	0.788	0.791	0.794	0.797	0.8	0.804	0.808	0.813	0.819	0.823	0.826	0.828	0.83
Derby	0.715	0.722	0.73	0.738	0.745	0.75	0.754	0.758	0.764	0.771	0.778	0.785	0.79	0.793	0.797	0.801	0.803	0.806	0.809	0.811	0.815	0.82	0.826	0.832	0.837	0.841	0.844	0.846
Derbyshire	0.698	0.703	0.71	0.716	0.723	0.727	0.731	0.735	0.74	0.747	0.753	0.758	0.762	0.765	0.768	0.771	0.774	0.777	0.779	0.782	0.785	0.79	0.796	0.803	0.808	0.812	0.815	0.817
Leicester	0.708	0.715	0.723	0.73	0.737	0.743	0.747	0.751	0.756	0.763	0.769	0.775	0.78	0.785	0.79	0.794	0.798	0.802	0.805	0.809	0.813	0.817	0.822	0.828	0.832	0.835	0.837	0.839
Leicestershire	0.727	0.733	0.74	0.748	0.754	0.759	0.763	0.767	0.773	0.778	0.784	0.789	0.794	0.798	0.802	0.806	0.81	0.814	0.817	0.82	0.823	0.827	0.831	0.836	0.839	0.842	0.844	0.846
Lincolnshire	0.699	0.705	0.711	0.718	0.724	0.729	0.732	0.737	0.742	0.748	0.754	0.76	0.764	0.768	0.77	0.772	0.774	0.776	0.779	0.781	0.784	0.788	0.793	0.8	0.804	0.808	0.81	0.812
Northamptonshire	0.714	0.72	0.728	0.735	0.741	0.746	0.749	0.753	0.757	0.764	0.77	0.775	0.779	0.781	0.784	0.787	0.79	0.792	0.796	0.798	0.802	0.806	0.812	0.818	0.822	0.825	0.828	0.829
Nottingham	0.735	0.742	0.749	0.757	0.763	0.769	0.774	0.78	0.786	0.793	0.8	0.806	0.812	0.817	0.822	0.826	0.829	0.832	0.835	0.839	0.842	0.847	0.852	0.856	0.859	0.861	0.862	0.863
Nottinghamshire	0.696	0.702	0.708	0.715	0.722	0.726	0.73	0.734	0.74	0.747	0.754	0.76	0.765	0.768	0.771	0.775	0.778	0.781	0.783	0.785	0.788	0.792	0.797	0.803	0.807	0.81	0.812	0.814
Rutland	0.73	0.735	0.741	0.746	0.752	0.756	0.759	0.762	0.767	0.772	0.777	0.781	0.784	0.787	0.79	0.793	0.797	0.8	0.804	0.806	0.81	0.814	0.818	0.823	0.826	0.829	0.831	0.833
East of England	0.724	0.73	0.737	0.744	0.75	0.755	0.759	0.763	0.768	0.774	0.78	0.786	0.79	0.793	0.796	0.799	0.802	0.805	0.808	0.811	0.814	0.818	0.824	0.83	0.834	0.837	0.839	0.84
Bedford	0.73	0.736	0.743	0.749	0.756	0.76	0.764	0.768	0.774	0.78	0.785	0.79	0.794	0.797	0.8	0.803	0.805	0.807	0.81	0.812	0.816	0.82	0.825	0.83	0.833	0.836	0.837	0.838
Cambridgeshire	0.75	0.757	0.764	0.771	0.777	0.782	0.786	0.791	0.797	0.803	0.809	0.815	0.82	0.823	0.827	0.83	0.833	0.836	0.84	0.842	0.846	0.85	0.855	0.86	0.864	0.867	0.869	0.871
Central Bedfordshire	0.723	0.729	0.736	0.743	0.749	0.753	0.757	0.76	0.765	0.771	0.776	0.781	0.785	0.787	0.79	0.793	0.795	0.797	0.8	0.803	0.806	0.81	0.816	0.822	0.827	0.83	0.833	0.834
Essex	0.713	0.719	0.726	0.734	0.74	0.745	0.748	0.753	0.758	0.764	0.77	0.775	0.78	0.783	0.787	0.79	0.793	0.796	0.8	0.802	0.806	0.81	0.816	0.821	0.825	0.828	0.83	0.832
Hertfordshire	0.749	0.755	0.762	0.769	0.776	0.781	0.784	0.789	0.795	0.802	0.81	0.816	0.821	0.825	0.829	0.832	0.835	0.837	0.84	0.843	0.846	0.85	0.855	0.86	0.863	0.866	0.868	0.87
Luton	0.71	0.716	0.723	0.731	0.737	0.742	0.745	0.749	0.754	0.76	0.766	0.771	0.775	0.778	0.781	0.785	0.79	0.795	0.8	0.805	0.809	0.814	0.819	0.825	0.828	0.83	0.832	0.833
Norfolk	0.712	0.717	0.724	0.731	0.737	0.741	0.745	0.749	0.754	0.761	0.767	0.772	0.776	0.779	0.781	0.784	0.787	0.79	0.792	0.795	0.798	0.803	0.808	0.814	0.819	0.822	0.824	0.826
Peterborough	0.713	0.718	0.724	0.731	0.737	0.741	0.743	0.746	0.751	0.757	0.761	0.765	0.768	0.77	0.772	0.774	0.776	0.778	0.779	0.781	0.784	0.788	0.795	0.803	0.808	0.813	0.816	0.818
Southend-on-Sea	0.696	0.701	0.707	0.714	0.72	0.724	0.727	0.731	0.736	0.743	0.75	0.755	0.761	0.765	0.768	0.77	0.773	0.775	0.778	0.781	0.785	0.79	0.795	0.801	0.805	0.808	0.81	0.811
Suffolk	0.713	0.719	0.726	0.732	0.738	0.742	0.745	0.748	0.753	0.758	0.763	0.767	0.771	0.773	0.775	0.777	0.78	0.782	0.785	0.788	0.792	0.797	0.803	0.81	0.814	0.817	0.819	0.821
Thurrock	0.71	0.716	0.723	0.73	0.737	0.741	0.745	0.749	0.754	0.759	0.763	0.767	0.769	0.77	0.772	0.774	0.775	0.776	0.778	0.781	0.784	0.789	0.793	0.799	0.802	0.804	0.806	0.807
Greater London	0.78	0.786	0.793	0.8	0.806	0.81	0.814	0.818	0.823	0.83	0.836	0.842	0.846	0.849	0.852	0.855	0.858	0.861	0.863	0.866	0.87	0.875	0.88	0.885	0.889	0.891	0.893	0.894
Barking and Dagenham	0.704	0.709	0.716	0.723	0.73	0.734	0.737	0.741	0.746	0.752	0.756	0.76	0.762	0.762	0.763	0.763	0.763	0.763	0.764	0.765	0.768	0.773	0.78	0.787	0.793	0.797	0.8	0.802
Barnet	0.759	0.765	0.771	0.777	0.783	0.788	0.792	0.796	0.802	0.809	0.814	0.82	0.824	0.827	0.829	0.831	0.834	0.835	0.838	0.84	0.843	0.847	0.852	0.856	0.859	0.862	0.864	0.865
Bexley	0.721	0.727	0.734	0.74	0.746	0.75	0.753	0.757	0.761	0.767	0.772	0.777	0.781	0.784	0.787	0.789	0.791	0.793	0.796	0.798	0.801	0.804	0.809	0.815	0.819	0.822	0.824	0.826
Brent	0.753	0.758	0.764	0.771	0.777	0.781	0.785	0.789	0.795	0.801	0.806	0.811	0.814	0.816	0.818	0.819	0.821	0.821	0.822	0.823	0.825	0.828	0.833	0.839	0.842	0.845	0.847	0.849
Bromley	0.751	0.756	0.762	0.768	0.774	0.778	0.782	0.786	0.791	0.798	0.804	0.809	0.814	0.817	0.82	0.822	0.825	0.827	0.829	0.83	0.831	0.833	0.837	0.84	0.843	0.845	0.847	0.848
Camden	0.838	0.843	0.848	0.854	0.858	0.862	0.866	0.869	0.873	0.88	0.885	0.889	0.892	0.895	0.898	0.901	0.904	0.906	0.908	0.911	0.913	0.917	0.92	0.924	0.926	0.927	0.929	0.93
Croydon	0.741	0.747	0.754	0.761	0.767	0.771	0.774	0.777	0.782	0.788	0.794	0.799	0.803	0.805	0.808	0.81	0.812	0.813	0.815	0.816	0.818	0.82	0.824	0.827	0.83	0.831	0.832	0.833
Ealing	0.764	0.77	0.776	0.783	0.789	0.794	0.797	0.801	0.807	0.814	0.82	0.825	0.828	0.831	0.833	0.834	0.835	0.836	0.837	0.837	0.839	0.843	0.848	0.853	0.857	0.86	0.863	0.865
Enfield	0.737	0.742	0.749	0.755	0.761	0.765	0.768	0.772	0.777	0.783	0.788	0.793	0.797	0.799	0.801	0.802	0.804	0.806	0.809	0.811	0.814	0.818	0.824	0.829	0.833	0.836	0.838	0.839
Greenwich	0.725	0.732	0.74	0.747	0.754	0.758	0.762	0.766	0.771	0.777	0.783	0.788	0.791	0.793	0.795	0.797	0.799	0.801	0.803	0.805	0.809	0.813	0.819	0.824	0.828	0.83	0.832	0.833
Hackney	0.767	0.774	0.781	0.789	0.795	0.798	0.8	0.801	0.805	0.811	0.817	0.822	0.825	0.828	0.831	0.834	0.838	0.843	0.848	0.853	0.858	0.864	0.871	0.877	0.882	0.884	0.886	0.887
Hammersmith and Fulham	0.825	0.833	0.839	0.845	0.851	0.856	0.86	0.864	0.869	0.875	0.879	0.883	0.887	0.889	0.892	0.895	0.898	0.901	0.904	0.907	0.91	0.914	0.917	0.921	0.923	0.924	0.925	0.927
Haringey	0.755	0.761	0.766	0.773	0.778	0.782	0.785	0.788	0.792	0.798	0.804	0.809	0.813	0.815	0.818	0.821	0.823	0.825	0.828	0.829	0.832	0.835	0.84	0.845	0.848	0.851	0.852	0.854
Harrow	0.747	0.754	0.76	0.767	0.773	0.777	0.78	0.784	0.79	0.796	0.802	0.808	0.812	0.815	0.819	0.821	0.824	0.825	0.827	0.829	0.83	0.832	0.835	0.839	0.842	0.844	0.846	0.848
Havering	0.719	0.725	0.731	0.737	0.743	0.747	0.75	0.754	0.759	0.765	0.77	0.775	0.78	0.783	0.786	0.789	0.792	0.794	0.797	0.8	0.802	0.805	0.809	0.814	0.817	0.82	0.823	0.824
Hillingdon	0.781	0.787	0.794	0.801	0.807	0.811	0.815	0.818	0.822	0.828	0.832	0.836	0.84	0.842	0.845	0.846	0.848	0.85	0.852	0.854	0.857	0.862	0.867	0.872	0.876	0.879	0.881	0.882
Hounslow	0.769	0.775	0.781	0.788	0.793	0.797	0.801	0.805	0.811	0.818	0.824	0.83	0.834	0.837	0.84	0.842	0.844	0.845	0.848	0.849	0.852	0.856	0.862	0.868	0.872	0.875	0.877	0.879
Islington	0.812	0.818	0.825	0.832	0.838	0.843	0.847	0.851	0.856	0.862	0.867	0.872	0.876	0.879	0.882	0.885	0.888	0.891	0.895	0.898	0.902	0.906	0.91	0.914	0.917	0.919	0.921	0.922
Kensington and Chelsea	0.839	0.845	0.851	0.857	0.861	0.865	0.869	0.873	0.877	0.884	0.889	0.894	0.897	0.899	0.902	0.905	0.907	0.909	0.912	0.915	0.918	0.921	0.924	0.927	0.929	0.93	0.931	0.932
Kingston upon Thames	0.787	0.794	0.8	0.807	0.812	0.817	0.821	0.826	0.831	0.838	0.844	0.849	0.854	0.858	0.861	0.864	0.867	0.869	0.872	0.874	0.876	0.879	0.882	0.885	0.887	0.888	0.889	0.89
Lambeth	0.775	0.783	0.791	0.797	0.804	0.808	0.812	0.816	0.821	0.828	0.835	0.84	0.845	0.848	0.852	0.856	0.86	0.864	0.869	0.873	0.877	0.882	0.887	0.892	0.895	0.897	0.899	0.9
Lewisham	0.733	0.74	0.746	0.753	0.759	0.763	0.766	0.77	0.775	0.781	0.787	0.793	0.797	0.799	0.802	0.804	0.806	0.808	0.811	0.813	0.816	0.821	0.826	0.832	0.836	0.839	0.841	0.843
Merton	0.758	0.765	0.771	0.779	0.785	0.79	0.795	0.799	0.805	0.812	0.819	0.824	0.828	0.83	0.833	0.835	0.837	0.839	0.841	0.843	0.845	0.849	0.855	0.86	0.865	0.868	0.871	0.873
Newham	0.716	0.722	0.728	0.734	0.74	0.743	0.746	0.748	0.753	0.76	0.765	0.771	0.775	0.777	0.78	0.781	0.783	0.785	0.788	0.792	0.799	0.806	0.815	0.823	0.829	0.833	0.836	0.838
Redbridge	0.728	0.733	0.739	0.746	0.751	0.756	0.759	0.763	0.768	0.774	0.78	0.785	0.789	0.791	0.794	0.796	0.798	0.801	0.803	0.805	0.807	0.811	0.816	0.821	0.825	0.827	0.83	0.831
Richmond upon Thames	0.794	0.801	0.807	0.813	0.819	0.824	0.827	0.832	0.837	0.844	0.85	0.856	0.86	0.864	0.867	0.87	0.872	0.874	0.876	0.878	0.88	0.883	0.887	0.891	0.895	0.897	0.9	0.902
Southwark	0.793	0.8	0.807	0.815	0.821	0.827	0.83	0.835	0.84	0.847	0.852	0.857	0.86	0.863	0.866	0.869	0.872	0.875	0.879	0.883	0.887	0.893	0.898	0.904	0.907	0.909	0.911	0.912
Sutton	0.735	0.74	0.747	0.754	0.761	0.765	0.769	0.774	0.78	0.787	0.793	0.798	0.802	0.805	0.808	0.809	0.811	0.813	0.815	0.817	0.819	0.823	0.828	0.833	0.836	0.839	0.841	0.843
Tower Hamlets	0.766	0.773	0.78	0.788	0.795	0.801	0.805	0.81	0.817	0.825	0.832	0.839	0.844	0.848	0.852	0.857	0.861	0.866	0.871	0.876	0.881	0.886	0.891	0.896	0.9	0.902	0.904	0.905
Waltham Forest	0.712	0.718	0.724	0.731	0.738	0.742	0.745	0.749	0.754	0.76	0.765	0.77	0.773	0.775	0.777	0.778	0.78	0.781	0.783	0.784	0.787	0.792	0.798	0.805	0.81	0.814	0.817	0.819
Wandsworth	0.793	0.8	0.807	0.814	0.82	0.825	0.83	0.835	0.84	0.848	0.855	0.862	0.867	0.87	0.873	0.876	0.879	0.881	0.885	0.887	0.889	0.893	0.897	0.901	0.905	0.907	0.909	0.911
Westminster	0.831	0.837	0.843	0.849	0.854	0.858	0.862	0.865	0.87	0.875	0.881	0.885	0.889	0.891	0.895	0.898	0.901	0.903	0.905	0.907	0.91	0.913	0.916	0.92	0.922	0.924	0.925	0.927
North East England	0.697	0.703	0.711	0.718	0.725	0.73	0.733	0.738	0.744	0.75	0.757	0.763	0.767	0.771	0.774	0.778	0.781	0.784	0.787	0.79	0.794	0.798	0.804	0.81	0.814	0.817	0.819	0.821
County Durham	0.69	0.696	0.703	0.71	0.717	0.721	0.725	0.73	0.735	0.742	0.748	0.754	0.758	0.762	0.765	0.767	0.77	0.772	0.776	0.778	0.782	0.786	0.792	0.798	0.802	0.806	0.808	0.81
Darlington	0.703	0.709	0.716	0.723	0.73	0.734	0.737	0.74	0.745	0.752	0.758	0.764	0.769	0.773	0.777	0.78	0.783	0.785	0.788	0.791	0.795	0.8	0.806	0.812	0.817	0.821	0.823	0.825
Gateshead	0.7	0.706	0.713	0.72	0.727	0.732	0.735	0.739	0.745	0.751	0.758	0.764	0.769	0.773	0.777	0.781	0.784	0.787	0.791	0.794	0.798	0.803	0.808	0.814	0.818	0.821	0.824	0.826
Hartlepool	0.677	0.683	0.69	0.697	0.703	0.707	0.71	0.714	0.719	0.726	0.732	0.737	0.74	0.742	0.744	0.747	0.749	0.752	0.755	0.758	0.762	0.767	0.773	0.78	0.785	0.788	0.791	0.793
Middlesbrough	0.688	0.695	0.702	0.709	0.716	0.72	0.723	0.727	0.732	0.74	0.746	0.751	0.755	0.758	0.762	0.766	0.769	0.772	0.775	0.778	0.781	0.786	0.792	0.797	0.801	0.804	0.806	0.808
Newcastle upon Tyne	0.736	0.743	0.751	0.759	0.765	0.771	0.776	0.781	0.787	0.794	0.801	0.808	0.814	0.82	0.825	0.831	0.835	0.84	0.843	0.846	0.85	0.854	0.858	0.863	0.866	0.869	0.87	0.872
North Tyneside	0.696	0.703	0.71	0.718	0.724	0.729	0.733	0.737	0.743	0.749	0.756	0.762	0.768	0.772	0.776	0.78	0.784	0.787	0.79	0.793	0.797	0.802	0.807	0.813	0.817	0.821	0.823	0.825
Northumberland	0.697	0.703	0.709	0.716	0.722	0.727	0.73	0.735	0.74	0.746	0.752	0.757	0.761	0.764	0.767	0.77	0.773	0.776	0.779	0.781	0.784	0.787	0.793	0.798	0.802	0.805	0.807	0.808
Redcar and Cleveland	0.67	0.675	0.682	0.689	0.696	0.701	0.704	0.708	0.714	0.721	0.727	0.732	0.736	0.738	0.741	0.743	0.745	0.747	0.75	0.752	0.756	0.76	0.767	0.775	0.78	0.784	0.787	0.79
South Tyneside	0.664	0.671	0.678	0.685	0.692	0.697	0.7	0.705	0.711	0.718	0.725	0.731	0.737	0.74	0.744	0.748	0.751	0.754	0.757	0.759	0.762	0.767	0.773	0.78	0.785	0.789	0.792	0.794
Stockton-on-Tees	0.705	0.712	0.72	0.728	0.735	0.74	0.744	0.748	0.754	0.761	0.767	0.772	0.775	0.777	0.779	0.781	0.784	0.785	0.788	0.791	0.794	0.798	0.804	0.811	0.815	0.818	0.821	0.823
Sunderland	0.689	0.696	0.704	0.713	0.72	0.725	0.729	0.733	0.739	0.746	0.752	0.758	0.763	0.766	0.77	0.773	0.776	0.779	0.782	0.784	0.787	0.791	0.797	0.803	0.808	0.811	0.813	0.815
North West England	0.716	0.723	0.729	0.736	0.742	0.747	0.751	0.755	0.76	0.766	0.772	0.778	0.782	0.786	0.789	0.792	0.795	0.798	0.801	0.804	0.807	0.812	0.817	0.823	0.827	0.83	0.832	0.834
Blackburn with Darwen	0.691	0.695	0.701	0.707	0.713	0.717	0.719	0.723	0.728	0.734	0.74	0.745	0.748	0.75	0.753	0.756	0.758	0.761	0.764	0.766	0.769	0.774	0.78	0.788	0.793	0.797	0.8	0.802
Blackpool	0.68	0.685	0.691	0.698	0.703	0.707	0.71	0.713	0.718	0.724	0.729	0.733	0.737	0.738	0.741	0.743	0.745	0.747	0.748	0.75	0.752	0.756	0.762	0.768	0.773	0.776	0.779	0.781
Bolton	0.701	0.707	0.714	0.72	0.726	0.73	0.733	0.736	0.741	0.746	0.751	0.756	0.759	0.761	0.763	0.765	0.768	0.769	0.772	0.774	0.777	0.781	0.787	0.794	0.798	0.801	0.803	0.805
Bury	0.703	0.709	0.716	0.723	0.73	0.734	0.738	0.742	0.747	0.752	0.758	0.762	0.766	0.768	0.771	0.774	0.777	0.779	0.781	0.783	0.786	0.791	0.797	0.803	0.807	0.81	0.813	0.815
Cheshire East	0.748	0.754	0.76	0.767	0.773	0.778	0.782	0.787	0.792	0.798	0.803	0.809	0.813	0.817	0.82	0.823	0.826	0.829	0.832	0.834	0.836	0.84	0.845	0.851	0.856	0.859	0.861	0.864
Cheshire West and Chester	0.74	0.747	0.754	0.761	0.768	0.772	0.776	0.78	0.785	0.792	0.797	0.803	0.807	0.811	0.815	0.818	0.822	0.825	0.828	0.831	0.834	0.838	0.842	0.847	0.85	0.852	0.854	0.855
Cumbria	0.719	0.724	0.731	0.737	0.743	0.748	0.751	0.755	0.76	0.765	0.77	0.774	0.777	0.779	0.782	0.784	0.787	0.789	0.792	0.794	0.798	0.802	0.808	0.815	0.819	0.823	0.826	0.828
Halton	0.702	0.708	0.715	0.723	0.729	0.734	0.737	0.74	0.745	0.75	0.756	0.762	0.766	0.769	0.772	0.775	0.778	0.78	0.784	0.787	0.791	0.797	0.803	0.81	0.815	0.819	0.822	0.824
Knowsley	0.691	0.696	0.702	0.708	0.714	0.718	0.722	0.726	0.731	0.737	0.743	0.749	0.754	0.757	0.76	0.764	0.767	0.77	0.774	0.777	0.782	0.787	0.793	0.8	0.806	0.81	0.813	0.816
Lancashire	0.72	0.726	0.734	0.741	0.747	0.752	0.756	0.76	0.765	0.77	0.775	0.78	0.784	0.786	0.789	0.792	0.794	0.797	0.799	0.802	0.805	0.809	0.815	0.82	0.824	0.827	0.829	0.831
Liverpool	0.721	0.728	0.735	0.742	0.748	0.753	0.757	0.762	0.768	0.775	0.783	0.789	0.795	0.801	0.806	0.811	0.815	0.819	0.823	0.828	0.831	0.835	0.84	0.844	0.847	0.849	0.851	0.852
Manchester	0.746	0.753	0.76	0.768	0.775	0.78	0.785	0.79	0.796	0.804	0.812	0.819	0.825	0.83	0.835	0.84	0.844	0.849	0.853	0.856	0.86	0.864	0.869	0.874	0.878	0.88	0.883	0.885
Oldham	0.685	0.69	0.697	0.703	0.708	0.711	0.713	0.715	0.719	0.724	0.729	0.733	0.737	0.739	0.741	0.744	0.746	0.749	0.752	0.754	0.758	0.763	0.77	0.778	0.782	0.786	0.788	0.79
Rochdale	0.686	0.692	0.698	0.705	0.711	0.715	0.717	0.721	0.726	0.732	0.738	0.742	0.746	0.748	0.75	0.753	0.755	0.757	0.76	0.762	0.765	0.77	0.777	0.783	0.787	0.791	0.793	0.795
Salford	0.708	0.714	0.721	0.728	0.735	0.74	0.743	0.747	0.753	0.761	0.768	0.775	0.781	0.785	0.79	0.794	0.797	0.8	0.803	0.806	0.809	0.814	0.819	0.826	0.83	0.834	0.836	0.838
Sefton	0.706	0.713	0.72	0.727	0.733	0.738	0.741	0.746	0.751	0.757	0.763	0.768	0.773	0.776	0.78	0.783	0.786	0.788	0.79	0.792	0.794	0.796	0.8	0.803	0.806	0.809	0.81	0.812

Appendix Table 10. Socio-Demographic Index values for all estimated GBD 2017 locations, 1990-2017

Location	1990	1991	1992	1993	1994	1995	1996	1997	1998	1999	2000	2001	2002	2003	2004	2005	2006	2007	2008	2009	2010	2011	2012	2013	2014	2015	2016	2017
St Helens	0.684	0.69	0.696	0.703	0.709	0.713	0.716	0.72	0.725	0.731	0.738	0.744	0.749	0.753	0.756	0.759	0.762	0.765	0.768	0.771	0.774	0.779	0.785	0.791	0.795	0.798	0.801	0.803
Stockport	0.727	0.734	0.741	0.748	0.755	0.759	0.763	0.767	0.772	0.779	0.785	0.791	0.796	0.799	0.803	0.806	0.809	0.812	0.814	0.817	0.82	0.823	0.828	0.833	0.837	0.839	0.841	0.843
Tameside	0.691	0.697	0.703	0.71	0.716	0.721	0.724	0.728	0.734	0.74	0.746	0.751	0.755	0.758	0.76	0.762	0.763	0.764	0.766	0.768	0.77	0.774	0.779	0.785	0.79	0.793	0.795	0.797
Trafford	0.751	0.757	0.764	0.771	0.778	0.782	0.786	0.79	0.795	0.802	0.809	0.815	0.82	0.824	0.829	0.832	0.836	0.839	0.842	0.844	0.848	0.852	0.856	0.862	0.865	0.868	0.871	0.873
Warrington	0.739	0.745	0.752	0.759	0.765	0.769	0.773	0.776	0.782	0.788	0.795	0.801	0.807	0.811	0.814	0.818	0.822	0.825	0.827	0.83	0.833	0.838	0.843	0.849	0.853	0.856	0.858	0.86
Wigan	0.691	0.697	0.703	0.71	0.716	0.72	0.723	0.727	0.731	0.737	0.742	0.747	0.75	0.753	0.755	0.757	0.76	0.762	0.764	0.766	0.769	0.774	0.78	0.786	0.79	0.793	0.796	0.798
Wirral	0.695	0.701	0.708	0.714	0.721	0.725	0.728	0.732	0.737	0.743	0.749	0.753	0.757	0.761	0.763	0.766	0.768	0.77	0.773	0.775	0.777	0.781	0.786	0.792	0.796	0.799	0.801	0.803
South East England	0.749	0.755	0.761	0.767	0.773	0.777	0.781	0.784	0.789	0.795	0.801	0.806	0.809	0.812	0.815	0.818	0.821	0.823	0.826	0.828	0.831	0.836	0.841	0.846	0.85	0.852	0.855	0.856
Bracknell Forest	0.759	0.764	0.77	0.776	0.781	0.785	0.788	0.791	0.797	0.804	0.811	0.817	0.822	0.825	0.829	0.832	0.835	0.838	0.841	0.843	0.845	0.849	0.854	0.859	0.862	0.865	0.867	0.869
Brighton and Hove	0.766	0.772	0.779	0.785	0.791	0.796	0.801	0.806	0.811	0.816	0.822	0.827	0.833	0.838	0.843	0.847	0.851	0.855	0.859	0.863	0.865	0.868	0.872	0.876	0.879	0.881	0.883	0.885
Buckinghamshire	0.764	0.769	0.775	0.782	0.788	0.792	0.795	0.799	0.804	0.81	0.815	0.82	0.824	0.826	0.829	0.832	0.834	0.836	0.838	0.84	0.842	0.846	0.851	0.855	0.859	0.861	0.863	0.865
East Sussex	0.712	0.718	0.724	0.73	0.736	0.74	0.742	0.745	0.749	0.754	0.759	0.763	0.766	0.769	0.771	0.773	0.776	0.778	0.781	0.783	0.787	0.791	0.797	0.803	0.807	0.81	0.812	0.814
Hampshire	0.744	0.75	0.756	0.762	0.768	0.772	0.775	0.778	0.782	0.788	0.793	0.798	0.802	0.804	0.807	0.81	0.812	0.815	0.818	0.82	0.824	0.828	0.834	0.839	0.843	0.846	0.848	0.85
Isle of Wight	0.704	0.709	0.715	0.722	0.727	0.732	0.735	0.739	0.744	0.749	0.756	0.761	0.765	0.767	0.768	0.771	0.773	0.776	0.779	0.781	0.784	0.788	0.794	0.801	0.806	0.809	0.812	0.814
Kent	0.723	0.728	0.734	0.74	0.746	0.75	0.752	0.756	0.76	0.765	0.77	0.774	0.777	0.779	0.782	0.785	0.787	0.79	0.793	0.796	0.8	0.805	0.811	0.817	0.822	0.824	0.826	0.828
Medway	0.703	0.709	0.715	0.722	0.728	0.731	0.734	0.737	0.742	0.747	0.752	0.756	0.76	0.762	0.765	0.768	0.771	0.773	0.776	0.778	0.781	0.785	0.791	0.797	0.802	0.805	0.807	0.809
Milton Keynes	0.754	0.76	0.767	0.774	0.78	0.784	0.786	0.789	0.793	0.798	0.802	0.806	0.81	0.812	0.815	0.817	0.819	0.821	0.823	0.825	0.829	0.834	0.84	0.847	0.852	0.856	0.859	0.86
Oxfordshire	0.769	0.775	0.781	0.788	0.794	0.798	0.801	0.805	0.81	0.816	0.822	0.827	0.831	0.835	0.838	0.841	0.844	0.847	0.849	0.852	0.855	0.859	0.864	0.869	0.872	0.875	0.878	0.879
Portsmouth	0.75	0.756	0.763	0.77	0.776	0.781	0.785	0.79	0.795	0.8	0.805	0.81	0.815	0.818	0.822	0.824	0.827	0.829	0.832	0.835	0.838	0.842	0.846	0.851	0.854	0.857	0.858	0.86
Reading	0.785	0.791	0.797	0.803	0.809	0.813	0.817	0.821	0.827	0.834	0.84	0.847	0.852	0.856	0.86	0.864	0.866	0.868	0.87	0.872	0.874	0.877	0.882	0.887	0.89	0.892	0.894	0.895
Slough	0.764	0.77	0.777	0.784	0.79	0.793	0.796	0.799	0.802	0.806	0.81	0.813	0.815	0.816	0.817	0.818	0.819	0.82	0.822	0.825	0.829	0.834	0.841	0.848	0.852	0.855	0.858	0.859
Southampton	0.752	0.758	0.765	0.772	0.779	0.784	0.789	0.794	0.8	0.805	0.81	0.815	0.819	0.823	0.826	0.829	0.831	0.834	0.836	0.837	0.839	0.842	0.845	0.849	0.852	0.855	0.856	0.858
Surrey	0.773	0.779	0.785	0.791	0.797	0.801	0.805	0.809	0.814	0.821	0.827	0.833	0.838	0.841	0.845	0.847	0.85	0.853	0.856	0.858	0.861	0.864	0.868	0.873	0.876	0.879	0.881	0.883
West Berkshire	0.774	0.78	0.786	0.793	0.799	0.803	0.805	0.808	0.813	0.819	0.824	0.829	0.832	0.835	0.836	0.836	0.837	0.838	0.84	0.842	0.846	0.851	0.857	0.863	0.867	0.869	0.871	0.872
West Sussex	0.74	0.745	0.751	0.757	0.763	0.767	0.77	0.773	0.777	0.783	0.788	0.793	0.796	0.799	0.802	0.804	0.807	0.809	0.812	0.814	0.818	0.822	0.827	0.833	0.837	0.84	0.842	0.843
Windsor and Maidenhead	0.778	0.783	0.789	0.795	0.8	0.805	0.808	0.811	0.816	0.823	0.829	0.835	0.839	0.843	0.847	0.851	0.854	0.857	0.86	0.863	0.866	0.87	0.874	0.88	0.883	0.885	0.887	0.889
Wokingham	0.778	0.784	0.79	0.797	0.802	0.806	0.81	0.814	0.82	0.826	0.832	0.837	0.842	0.845	0.849	0.853	0.856	0.858	0.861	0.863	0.865	0.868	0.871	0.876	0.879	0.882	0.883	0.885
South West England	0.729	0.735	0.741	0.748	0.754	0.758	0.762	0.766	0.771	0.777	0.783	0.788	0.792	0.796	0.799	0.802	0.805	0.807	0.81	0.813	0.816	0.82	0.825	0.831	0.835	0.838	0.84	0.841
Bath and North East Somerset	0.752	0.758	0.764	0.77	0.777	0.782	0.786	0.79	0.796	0.803	0.809	0.816	0.822	0.828	0.833	0.838	0.842	0.846	0.85	0.853	0.856	0.859	0.863	0.867	0.869	0.872	0.874	0.875
Bournemouth	0.736	0.743	0.75	0.757	0.763	0.768	0.773	0.778	0.784	0.79	0.797	0.803	0.808	0.812	0.816	0.821	0.824	0.828	0.831	0.834	0.836	0.839	0.843	0.848	0.851	0.853	0.856	0.858
Bristol, City of	0.763	0.77	0.777	0.784	0.791	0.796	0.8	0.805	0.81	0.817	0.823	0.828	0.833	0.836	0.839	0.843	0.846	0.849	0.853	0.856	0.859	0.863	0.868	0.873	0.877	0.88	0.882	0.884
Cornwall	0.7	0.706	0.713	0.721	0.727	0.731	0.734	0.738	0.743	0.749	0.755	0.76	0.764	0.768	0.771	0.774	0.777	0.78	0.783	0.786	0.789	0.793	0.799	0.806	0.81	0.813	0.815	0.817
Devon	0.72	0.726	0.733	0.74	0.746	0.75	0.753	0.757	0.762	0.769	0.775	0.78	0.785	0.789	0.793	0.796	0.8	0.803	0.806	0.808	0.811	0.816	0.821	0.826	0.83	0.833	0.835	0.837
Dorset	0.716	0.721	0.727	0.734	0.74	0.744	0.747	0.751	0.756	0.762	0.769	0.773	0.777	0.779	0.781	0.783	0.786	0.788	0.791	0.793	0.797	0.802	0.808	0.814	0.818	0.821	0.823	0.825
Gloucestershire	0.735	0.741	0.747	0.754	0.76	0.765	0.768	0.772	0.777	0.783	0.79	0.795	0.8	0.804	0.808	0.811	0.813	0.816	0.818	0.82	0.824	0.828	0.833	0.839	0.843	0.846	0.848	0.85
North Somerset	0.714	0.72	0.727	0.733	0.739	0.743	0.746	0.75	0.755	0.76	0.766	0.771	0.776	0.78	0.783	0.786	0.789	0.792	0.795	0.798	0.801	0.806	0.813	0.819	0.824	0.827	0.83	0.832
Plymouth	0.724	0.73	0.737	0.744	0.75	0.754	0.758	0.762	0.767	0.772	0.778	0.783	0.787	0.79	0.793	0.796	0.799	0.802	0.805	0.807	0.81	0.814	0.819	0.825	0.829	0.832	0.834	0.836
Poole	0.727	0.733	0.74	0.746	0.753	0.758	0.761	0.765	0.771	0.777	0.783	0.789	0.793	0.796	0.798	0.801	0.804	0.806	0.809	0.811	0.814	0.818	0.824	0.83	0.835	0.838	0.84	0.842
Somerset	0.713	0.718	0.724	0.731	0.737	0.741	0.744	0.748	0.752	0.757	0.763	0.767	0.77	0.772	0.775	0.777	0.78	0.782	0.785	0.787	0.789	0.794	0.799	0.805	0.809	0.812	0.814	0.816
South Gloucestershire	0.747	0.752	0.758	0.765	0.771	0.775	0.779	0.783	0.789	0.796	0.802	0.808	0.813	0.817	0.821	0.824	0.827	0.831	0.834	0.837	0.84	0.844	0.849	0.855	0.859	0.862	0.865	0.867
Swindon	0.747	0.753	0.76	0.767	0.773	0.776	0.778	0.781	0.786	0.792	0.797	0.801	0.805	0.806	0.807	0.809	0.811	0.813	0.815	0.818	0.82	0.825	0.831	0.837	0.841	0.844	0.846	0.847
Torbay	0.699	0.705	0.711	0.717	0.723	0.727	0.73	0.733	0.736	0.741	0.745	0.749	0.75	0.751	0.753	0.755	0.757	0.759	0.761	0.762	0.764	0.767	0.773	0.779	0.783	0.786	0.789	0.79
Wiltshire	0.726	0.731	0.737	0.743	0.749	0.753	0.756	0.759	0.763	0.769	0.775	0.78	0.784	0.786	0.789	0.791	0.793	0.795	0.797	0.799	0.803	0.808	0.813	0.819	0.823	0.826	0.828	0.829
West Midlands	0.707	0.713	0.72	0.727	0.734	0.739	0.743	0.747	0.752	0.759	0.765	0.771	0.776	0.779	0.783	0.786	0.789	0.792	0.795	0.798	0.802	0.806	0.812	0.818	0.822	0.825	0.828	0.829
Birmingham	0.707	0.714	0.721	0.728	0.735	0.739	0.743	0.747	0.753	0.76	0.766	0.773	0.778	0.782	0.787	0.791	0.794	0.798	0.803	0.807	0.811	0.817	0.823	0.829	0.834	0.837	0.839	0.84
Coventry	0.722	0.729	0.737	0.745	0.751	0.757	0.762	0.766	0.772	0.779	0.785	0.791	0.796	0.8	0.803	0.807	0.81	0.812	0.815	0.818	0.821	0.825	0.831	0.837	0.841	0.844	0.847	0.848
Dudley	0.692	0.698	0.704	0.71	0.716	0.721	0.724	0.728	0.733	0.739	0.746	0.751	0.756	0.759	0.762	0.765	0.767	0.77	0.772	0.774	0.776	0.779	0.784	0.789	0.793	0.795	0.797	0.799
Herefordshire, County of	0.706	0.712	0.719	0.726	0.733	0.738	0.742	0.747	0.752	0.759	0.766	0.771	0.776	0.779	0.783	0.786	0.789	0.792	0.795	0.798	0.801	0.806	0.812	0.818	0.822	0.825	0.827	0.828
Sandwell	0.682	0.688	0.695	0.702	0.708	0.712	0.715	0.719	0.724	0.729	0.735	0.74	0.744	0.747	0.75	0.752	0.754	0.756	0.759	0.761	0.765	0.769	0.776	0.783	0.788	0.792	0.795	0.797
Shropshire	0.708	0.714	0.721	0.728	0.734	0.739	0.743	0.747	0.752	0.759	0.766	0.771	0.776	0.781	0.785	0.789	0.793	0.795	0.799	0.802	0.806	0.811	0.816	0.821	0.825	0.828	0.83	0.832
Solihull	0.733	0.74	0.747	0.755	0.762	0.767	0.771	0.776	0.783	0.789	0.795	0.801	0.807	0.811	0.815	0.818	0.822	0.825	0.828	0.83	0.832	0.835	0.84	0.845	0.848	0.851	0.853	0.855
Staffordshire	0.708	0.714	0.722	0.729	0.736	0.74	0.744	0.749	0.755	0.761	0.767	0.772	0.777	0.78	0.783	0.787	0.79	0.793	0.796	0.799	0.802	0.806	0.811	0.816	0.819	0.822	0.824	0.826
Stoke-on-Trent	0.689	0.696	0.703	0.71	0.716	0.721	0.724	0.728	0.733	0.738	0.743	0.747	0.75	0.751	0.753	0.754	0.756	0.757	0.76	0.763	0.767	0.772	0.78	0.788	0.794	0.798	0.801	0.804
Telford and Wrekin	0.713	0.719	0.726	0.733	0.74	0.745	0.749	0.754	0.759	0.764	0.769	0.773	0.776	0.777	0.779	0.781	0.783	0.784	0.787	0.789	0.791	0.796	0.802	0.809	0.814	0.817	0.82	0.822
Walsall	0.681	0.686	0.692	0.698	0.704	0.707	0.709	0.712	0.717	0.723	0.729	0.734	0.738	0.74	0.743	0.746	0.749	0.752	0.755	0.757	0.761	0.766	0.772	0.779	0.783	0.787	0.789	0.791
Warwickshire	0.732	0.738	0.746	0.753	0.76	0.765	0.769	0.774	0.779	0.786	0.792	0.798	0.802	0.806	0.809	0.812	0.816	0.819	0.823	0.826	0.83	0.834	0.84	0.845	0.85	0.853	0.855	0.857
Wolverhampton	0.691	0.697	0.704	0.712	0.719	0.723	0.727	0.731	0.737	0.744	0.751	0.757	0.761	0.764	0.768	0.771	0.774	0.776	0.779	0.781	0.784	0.788	0.794	0.8	0.804	0.807	0.809	0.811
Worcestershire	0.707	0.713	0.72	0.727	0.733	0.738	0.742	0.747	0.754	0.762	0.771	0.778	0.782	0.786	0.791	0.794	0.798	0.8	0.803	0.805	0.808	0.811	0.816	0.822	0.826	0.829	0.831	0.833
Yorkshire and the Humber	0.711	0.717	0.724	0.731	0.737	0.742	0.745	0.75	0.755	0.762	0.768	0.773	0.778	0.781	0.785	0.788	0.791	0.794	0.797	0.8	0.803	0.808	0.813	0.819	0.823	0.826	0.828	0.83
Barnsley	0.675	0.68	0.686	0.692	0.698	0.702	0.705	0.709	0.715	0.721	0.726	0.731	0.735	0.738	0.741	0.743	0.746	0.748	0.75	0.752	0.755	0.759	0.765	0.772	0.778	0.781	0.785	0.787
Bradford	0.693	0.699	0.705	0.712	0.718	0.721	0.723	0.725	0.729	0.736	0.741	0.747	0.751	0.753	0.756	0.759	0.762	0.765	0.768	0.771	0.775	0.78	0.787	0.794	0.799	0.803	0.805	0.807
Calderdale	0.708	0.714	0.721	0.729	0.735	0.739	0.743	0.746	0.751	0.758	0.763	0.768	0.771	0.774	0.777	0.78	0.783	0.785	0.788	0.791	0.795	0.8	0.807	0.814	0.819	0.823	0.825	0.827
Doncaster	0.674	0.679	0.685	0.692	0.697	0.701	0.704	0.707	0.712	0.718	0.724	0.729	0.733	0.736	0.74	0.743	0.746	0.749	0.752	0.755	0.759	0.764	0.771	0.778	0.783	0.786	0.789	0.791
East Riding of Yorkshire	0.714	0.72	0.727	0.734	0.741	0.745	0.749	0.753	0.758	0.764	0.769	0.774	0.778	0.781	0.784	0.787	0.79	0.792	0.794	0.797	0.799	0.802	0.807	0.812	0.815	0.818	0.82	0.822
Kingston upon Hull, City of	0.689	0.695	0.702	0.71	0.717	0.722	0.726	0.73	0.736	0.742	0.749	0.755	0.76	0.763	0.767	0.77	0.773	0.776	0.779	0.782	0.785	0.789	0.795	0.801	0.806	0.809	0.811	0.813
Kirklees	0.703	0.709	0.716	0.723	0.73	0.734	0.737	0.741	0.746	0.752	0.758	0.763	0.767	0.77	0.773	0.776	0.779	0.781	0.783	0.785	0.788	0.793	0.798	0.805	0.809	0.812	0.814	0.816
Leeds	0.739	0.746	0.753	0.761	0.768	0.773	0.778	0.783	0.789	0.797	0.803	0.81	0.816	0.82	0.825	0.83	0.834	0.837	0.841	0.843	0.846	0.849	0.854	0.858	0.862	0.864	0.867	0.868
North East Lincolnshire	0.698	0.705	0.712	0.72	0.727	0.731	0.733	0.737	0.741	0.745	0.749	0.753	0.755	0.757	0.758	0.761	0.763	0.767	0.77	0.775	0.779	0.784	0.79	0.796	0.799	0.802	0.803	0.804
North Lincolnshire	0.702	0.707	0.714	0.72	0.727	0.731	0.734	0.737	0.742	0.748	0.752	0.757	0.76	0.762	0.764	0.766	0.768	0.77	0.773	0.776	0.78	0.785	0.792	0.799	0.803	0.807	0.809	0.811
North Yorkshire	0.724	0.73	0.737	0.743	0.75	0.754	0.758	0.762	0.767	0.774	0.78	0.785	0.79	0.794	0.798	0.801	0.803	0.806	0.808	0.81	0.813	0.817	0.822	0.827	0.831	0.834	0.836	0.839
Rotherham	0.681	0.687	0.693	0.7	0.707	0.711	0.714	0.717	0.722	0.728	0.734	0.739	0.744	0.747	0.75	0.753	0.756	0.759	0.761	0.763	0.766	0.77	0.776	0.782	0.787	0.791	0.794	0.796
Sheffield	0.727	0.733	0.74	0.747	0.753	0.758	0.762	0.767	0.773	0.78	0.786	0.792	0.798	0.803	0.807	0.811	0.815	0.819	0.823	0.826	0.83	0.834	0.839	0.843	0.847	0.849	0.852	0.853
Wakefield	0.692	0.698	0.705	0.712	0.718	0.722	0.726	0.729	0.734	0.741	0.747	0.752	0.755	0.758	0.76	0.763	0.765	0.766	0.768	0.769	0.772	0.777	0.784	0.791	0.797	0.801	0.804	0.806
York	0.762	0.769	0.776	0.783	0.789	0.795	0.799	0.804	0.81	0.816	0.822	0.829	0.834	0.839	0.844	0.848	0.851	0.854	0.857	0.859	0.862	0.865	0.868	0.872	0.874	0.876	0.878	0.879
Northern Ireland	0.71	0.718	0.726	0.735	0.742	0.747	0.751	0.755	0.761	0.767	0.774	0.78	0.786	0.79	0.795	0.798	0.801	0.805	0.808	0.812	0.815	0.818	0.822	0.825	0.828	0.831	0.833	0.835
Scotland	0.672	0.678	0.686	0.693	0.701	0.706	0.711	0.716	0.722	0.73	0.737	0.743	0.748	0.752	0.757	0.761	0.764	0.768	0.772	0.777	0.781	0.785	0.789	0.793	0.797	0.8	0.803	0.805
Wales	0.642	0.65	0.659	0.668	0.676	0.683	0.688	0.694	0.702	0.711	0.719	0.727	0.734	0.739	0.745	0.75	0.755	0.76	0.765	0.77	0.774	0.78	0.786	0.792	0.797	0.8	0.803	0.806
Latin America and Caribbean	0.497	0.502	0.508	0.514	0.521	0.527	0.534	0.54	0.546	0.552	0.557	0.563	0.567	0.571	0.576	0.581	0.587	0.592	0.598	0.603	0.608	0.613	0.618	0.624	0.628	0.633	0.637	0.64
Andean Latin America	0.483	0.486	0.49	0.495	0.501	0.507	0.512	0.518	0.526	0.533	0.539	0.546	0.552	0.558	0.563	0.567	0.572	0.577	0.581	0.585	0.59	0.595	0.601	0.606	0.612	0.618	0.624	0.628
Bolivia	0.403	0.408	0.416	0.424	0.431	0.438	0.447	0.456	0.466	0.475	0.485	0.494	0.504	0.513	0.52	0.526	0.532	0.536	0.539	0.543	0.547	0.551	0.555	0.559	0.565	0.573	0.581	0.587
Ecuador	0.506	0.51	0.515	0.52	0.524	0.524	0.522	0.521	0.526	0.532	0.539	0.548	0.557	0.564	0.57	0.575	0.581	0.585	0.588	0.59	0.594	0.603	0.611	0.618	0.623	0.628	0.632	0.636
Peru	0.493	0.495	0.497	0.501	0.509	0.517	0.526	0.534	0.542	0.549	0.555	0.56	0.564	0.568	0.571	0.575	0.578	0.584	0.59	0.594	0.6	0.604	0.608	0.614	0.62	0.626	0.632	0.636
Caribbean	0.531	0.536	0.539	0.543	0.546	0.549	0.552	0.555	0.559	0.563	0.568	0.573	0.578	0.583	0.589	0.596	0.602	0.607	0.61	0.613	0.617	0.621	0.624	0.626	0.628	0.631	0.635	0.638
Antigua and Barbuda	0.61	0.616	0.622	0.627	0.631	0.633	0.636	0.64	0.644	0.649	0.653	0.657	0.661	0.666	0.67	0.675	0.68	0.685	0.69	0.694	0.696	0.698	0.7	0.703	0.706	0.709	0.712	0.715
The Bahamas	0.674	0.669	0.665	0.667	0.671	0.676	0.681	0.687	0.695	0.703	0.71	0.715	0.718	0.721	0.723	0.727	0.732	0.737	0.742	0.746	0.751	0.753	0.755	0.755	0.755	0.755	0.755	0.756
Barbados	0.65	0.655	0.661	0.667	0.672	0.675	0.678	0.68	0.682	0.683	0.686	0.689	0.693	0.698	0.703	0.707	0.71	0.713	0.717	0.72	0.723	0.726	0.729	0.732	0.734	0.736	0.738	0.739
Belize	0.402	0.414	0.429	0.445	0.458	0.471	0.482	0.49	0.496	0.501	0.506	0.511	0.518	0.525	0.533	0.541	0.549	0.557	0.563	0.569	0.576	0.581	0.586	0.59	0.594	0.598	0.6	0.602
Bermuda	0.73	0.732	0.734	0.737	0.738	0.74	0.741	0.743	0.746	0.749	0.752	0.754	0.757	0.761	0.765	0.769	0.773	0.777	0.782	0.786	0.79	0.794	0.797	0.8	0.803	0.804	0.805	0.805
Cuba	0.592	0.6	0.604	0.605	0.604	0.603	0.601	0.6	0.601	0.604	0.607	0.611	0.617	0.623	0.629	0.636	0.644	0.646	0.647	0.649	0.652	0.656	0.663	0.668	0.671	0.676	0.683	0.688
Dominica	0.534	0.535	0.539	0.546	0.555	0.563	0.57	0.577	0.584	0.592	0.599	0.606	0.614	0.621	0.63	0.636	0.641	0.645	0.65	0.656	0.662	0.668	0.673	0.677	0.68	0.682	0.684	0.687
Dominican Republic	0.442	0.443	0.445	0.449	0.454	0.459	0.464	0.469	0.475	0.48	0.484	0.49	0.495	0.499	0.511	0.525	0.536	0.547	0.555	0.562	0.568	0.572	0.575	0.576	0.578	0.583	0.589	0.593
Grenada	0.46	0.472	0.485	0.497	0.508	0.519	0.529	0.54	0.55	0.558	0.566	0.573	0.578	0.584	0.589	0.594	0.599	0.604	0.608	0.612	0.615	0.618	0.62	0.624	0.628	0.632	0.637	0.64
Guyana	0.442	0.446	0.45	0.454	0.458	0.465	0.472	0.481	0.489	0.497	0.503	0.51	0.516	0.521	0.526	0.53	0.533	0.537	0.542	0.547	0.552	0.557	0.563	0.568	0.573	0.576	0.58	0.584
Haiti	0.328	0.332	0.336	0.339	0.342	0.345	0.35	0.355	0.361	0.367	0.373	0.379	0.384	0.389	0.393	0.397	0.401	0.405	0.409	0.413	0.416	0.42	0.423	0.427	0.431	0.435	0.439	0.442
Jamaica	0.547	0.553	0.56	0.565	0.571	0.578	0.585	0.591	0.596	0.6	0.607	0.615	0.621	0.626	0.63	0.635	0.639	0.644	0.649	0.653	0.658	0.663	0.667	0.671	0.673	0.676	0.677	0.679
Puerto Rico	0.684	0.689	0.693	0.698	0.703	0.708	0.714	0.719	0.724	0.729	0.736	0.745	0.752	0.756	0.759	0.762	0.766	0.77	0.774	0.778	0.782	0.786	0.791	0.797	0.805	0.811	0.813	0.813
Saint Lucia	0.509	0.516	0.524	0.532	0.54	0.549	0.558	0.566	0.573	0.58	0.585	0.589	0.593	0.598	0.603	0.606	0.609	0.613	0.618	0.623	0.628	0.633	0.636	0.64	0.643	0.646	0.65	0.653
Saint Vincent and the Grenadines	0.465	0.472	0.479	0.486	0.492	0.499	0.506	0.512	0.518	0.524	0.53	0.537	0.543	0.549	0.553	0.557	0.561	0.566	0.571	0.575	0.579	0.584	0.588	0.593	0.597	0.601	0.605	0.608
Suriname	0.529	0.534	0.538	0.541	0.543	0.543	0.544	0.547	0.551	0.555	0.56	0.568	0.576	0.585	0.592	0.597	0.601	0.604	0.608	0.612	0.616	0.62	0.625	0.628	0.632	0.636	0.639	0.641
Trinidad and Tobago	0.601	0.604	0.608	0.611	0.615	0.619	0.624	0.629	0.634	0.64	0.646	0.652	0.657	0.663	0.669	0.675	0.68	0.685	0.689	0.691	0.693	0.694	0.695	0.696	0.697	0.698	0.698	0.698
Virgin Islands	0.673	0.683	0.691	0.699	0.706	0.713	0.719	0.725	0.73	0.734	0.737	0.74	0.748	0.755	0.762	0.768	0.774	0.78	0.786	0.79	0.795	0.799	0.802	0.804	0.805	0.806	0.806	0.807
Central Latin America	0.492	0.496	0.501	0.507	0.515	0.521	0.528	0.534	0.54	0.545	0.551	0.557	0.561	0.564	0.567	0.571	0.577	0.583	0.588	0.593	0.597	0.601	0.606	0.61	0.614	0.617	0.621	0.623
Colombia	0.482	0.483	0.485	0.49	0.496	0.503	0.511	0.518	0.524	0.528	0.533	0.537	0.541	0.544	0.548	0.553	0.558	0.565	0.573	0.581	0.588	0.596	0.604	0.611	0.617	0.624	0.629	0.634
Costa Rica	0.524	0.53	0.536	0.54	0.545	0.55	0.555	0.56	0.566	0.573	0.581	0.589	0.594	0.598	0.603	0.607	0.609	0.612	0.616	0.623	0.629	0.634	0.64	0.645	0.649	0.654	0.658	0.662
El Salvador	0.406	0.409	0.413	0.418	0.424	0.431	0.44	0.449	0.459	0.47	0.481	0.491	0.5	0.508	0.515	0.523	0.531	0.539	0.545	0.55	0.556	0.561	0.567	0.573	0.578	0.584	0.589	0.593
Guatemala	0.313	0.318	0.326	0.338	0.353	0.363	0.371	0.381	0.397	0.411	0.42	0.427	0.433	0.44	0.449	0.458	0.465	0.47	0.476	0.482	0.489	0.496	0.501	0.507	0.513	0.517	0.521	0.524
Honduras	0.34	0.344	0.347	0.352	0.358	0.365	0.372	0.381	0.39	0.4	0.41	0.419	0.428	0.437	0.445	0.452	0.459	0.465	0.47	0.475	0.481	0.486	0.49	0.495	0.499	0.504	0.508	0.512
Mexico	0.513	0.517	0.521	0.529	0.537	0.543	0.549	0.555	0.561	0.566	0.572	0.577	0.581	0.586	0.59	0.595	0.598	0.602	0.606	0.608	0.611	0.613	0.616	0.619	0.622	0.624	0.626	0.628
Aguascalientes	0.558	0.561	0.565	0.571	0.577	0.583	0.591	0.597	0.603	0.606	0.609	0.612	0.615	0.621	0.626	0.632	0.636	0.64	0.643	0.644	0.645	0.647	0.649	0.651	0.653	0.655	0.657	0.659
Baja California	0.57	0.572	0.575	0.579	0.582	0.585	0.589	0.594	0.599	0.604	0.608	0.611	0.615	0.618	0.622	0.626	0.63	0.634	0.639	0.642	0.644	0.646	0.649	0.651	0.653	0.654	0.656	0.657
Baja California Sur	0.569	0.573	0.577	0.582	0.586	0.589	0.593	0.598	0.602	0.605	0.609	0.612	0.614	0.617	0.621	0.624	0.628	0.633	0.637	0.641	0.644	0.647	0.649	0.652	0.654	0.656	0.657	0.659
Campeche	0.485	0.49	0.496	0.504	0.511	0.517	0.525	0.534	0.543	0.551	0.558	0.564	0.569	0.573	0.577	0.581	0.585	0.589	0.593	0.595	0.597	0.599	0.602	0.605	0.608	0.611	0.614	0.616
Chiapas	0.403	0.407	0.413	0.422	0.43	0.436	0.444	0.452	0.459	0.465	0.47	0.475	0.48	0.484	0.489	0.494	0.498	0.503	0.508	0.512	0.514	0.517	0.52	0.522	0.525	0.528	0.531	0.533
Chihuahua	0.546	0.549	0.552	0.555	0.558	0.559	0.562	0.566	0.57	0.574	0.577	0.581	0.585	0.589	0.594	0.599	0.604	0.61	0.615	0.619	0.622	0.625	0.628	0.63	0.633	0.635	0.637	0.639
Coahuila	0.549	0.555	0.561	0.567	0.573	0.576	0.581	0.587	0.592	0.597	0.601	0.604	0.607	0.611	0.616	0.62	0.624	0.628	0.631	0.633	0.634	0.635	0.637	0.638	0.64	0.642	0.644	0.645
Colima	0.538	0.543	0.549	0.556	0.561	0.565	0.571	0.577	0.583	0.588	0.594	0.598	0.603	0.607	0.611	0.616	0.621	0.626	0.631	0.634	0.637	0.64	0.643	0.645	0.648	0.65	0.652	0.654
Mexico City	0.602	0.606	0.61	0.617	0.629	0.64	0.65	0.656	0.661	0.666	0.671	0.676	0.681	0.685	0.689	0.693	0.696	0.698	0.7	0.701	0.702	0.704	0.706	0.708	0.71	0.712	0.714	0.716
Durango	0.506	0.508	0.511	0.516	0.52	0.523	0.528	0.535	0.542	0.55	0.556	0.562	0.568	0.574	0.581	0.586	0.592	0.597	0.602	0.605	0.607	0.609	0.612	0.614	0.617	0.619	0.622	0.624
Guanajuato	0.494	0.499	0.504	0.511	0.519	0.527	0.534	0.541	0.546	0.551	0.558	0.565	0.571	0.575	0.579	0.583	0.587	0.591	0.595	0.598	0.601	0.604	0.607	0.611	0.614	0.616	0.619	0.621
Guerrero	0.426	0.429	0.433	0.44	0.445	0.45	0.456	0.465	0.473	0.48	0.487	0.492	0.498	0.505	0.511	0.517	0.523	0.529	0.535	0.539	0.542	0.545	0.548	0.552	0.555	0.558	0.56	0.562
Hidalgo	0.417	0.424	0.433	0.444	0.454	0.462	0.471	0.481	0.491	0.501	0.511	0.52	0.527	0.531	0.535	0.538	0.543	0.548	0.554	0.558	0.562	0.567	0.572	0.576	0.58	0.583	0.585	0.587
Jalisco	0.532	0.538	0.545	0.552	0.558	0.563	0.569	0.576	0.582	0.588	0.593	0.598	0.602	0.606	0.611	0.615	0.619	0.624	0.628	0.63	0.632	0.635	0.637	0.64	0.642	0.645	0.647	0.649
México	0.529	0.524	0.525	0.539	0.558	0.568	0.574	0.577	0.58	0.585	0.59	0.596	0.6	0.603	0.607	0.611	0.613	0.614	0.614	0.614	0.615	0.617	0.621	0.625	0.628	0.631	0.633	0.635
Michoacán de Ocampo	0.464	0.47	0.476	0.483	0.489	0.494	0.501	0.508	0.515	0.522	0.527	0.532	0.537	0.542	0.546	0.551	0.555	0.559	0.563	0.566	0.568	0.571	0.573	0.576	0.579	0.581	0.584	0.586
Morelos	0.525	0.532	0.539	0.547	0.553	0.558	0.564	0.571	0.577	0.582	0.587	0.591	0.595	0.598	0.601	0.605	0.608	0.612	0.615	0.617	0.619	0.622	0.624	0.627	0.629	0.631	0.634	0.635
Nayarit	0.495	0.5	0.506	0.512	0.518	0.523	0.529	0.537	0.545	0.553	0.56	0.566	0.571	0.576	0.581	0.585	0.59	0.594	0.598	0.6	0.602	0.604	0.607	0.61	0.613	0.615	0.618	0.62
Nuevo León	0.598	0.602	0.606	0.611	0.615	0.617	0.62	0.625	0.63	0.635	0.64	0.644	0.647	0.65	0.653	0.656	0.659	0.662	0.665	0.666	0.667	0.669	0.67	0.672	0.673	0.675	0.676	0.677
Oaxaca	0.431	0.435	0.441	0.447	0.452	0.455	0.459	0.466	0.473	0.479	0.485	0.49	0.496	0.501	0.507	0.512	0.517	0.523	0.529	0.533	0.537	0.541	0.545	0.549	0.552	0.555	0.558	0.561
Puebla	0.456	0.463	0.47	0.478	0.487	0.493	0.496	0.497	0.498	0.499	0.504	0.509	0.516	0.524	0.532	0.538	0.544	0.549	0.554	0.558	0.563	0.567	0.571	0.575	0.577	0.58	0.582	0.584
Querétaro	0.497	0.502	0.508	0.518	0.53	0.543	0.556	0.566	0.575	0.583	0.591	0.597	0.602	0.606	0.609	0.612	0.616	0.619	0.623	0.624	0.626	0.629	0.631	0.633	0.635	0.636	0.638	0.639

Appendix Table 10. Socio-Demographic Index values for all estimated GBD 2017 locations, 1990-2017

Location	1990	1991	1992	1993	1994	1995	1996	1997	1998	1999	2000	2001	2002	2003	2004	2005	2006	2007	2008	2009	2010	2011	2012	2013	2014	2015	2016	2017
Quintana Roo	0.52	0.526	0.533	0.541	0.548	0.555	0.562	0.57	0.577	0.582	0.587	0.591	0.594	0.596	0.599	0.601	0.605	0.608	0.612	0.614	0.616	0.618	0.62	0.621	0.623	0.624	0.625	0.626
San Luis Potosí	0.482	0.486	0.492	0.5	0.508	0.514	0.522	0.53	0.538	0.545	0.551	0.556	0.561	0.566	0.571	0.575	0.58	0.586	0.591	0.595	0.599	0.602	0.606	0.61	0.613	0.616	0.619	0.621
Sinaloa	0.523	0.528	0.533	0.539	0.544	0.549	0.555	0.562	0.57	0.577	0.583	0.589	0.594	0.599	0.604	0.609	0.614	0.619	0.623	0.627	0.63	0.633	0.636	0.639	0.642	0.644	0.646	0.649
Sonora	0.553	0.557	0.562	0.566	0.57	0.573	0.578	0.583	0.588	0.593	0.597	0.601	0.605	0.608	0.612	0.616	0.621	0.625	0.629	0.632	0.635	0.637	0.64	0.643	0.645	0.647	0.649	0.65
Tabasco	0.474	0.479	0.486	0.493	0.5	0.507	0.515	0.524	0.533	0.541	0.548	0.553	0.558	0.563	0.568	0.573	0.578	0.583	0.588	0.591	0.594	0.596	0.599	0.602	0.604	0.607	0.609	0.611
Tamaulipas	0.548	0.553	0.558	0.564	0.568	0.571	0.574	0.579	0.586	0.592	0.598	0.602	0.606	0.609	0.613	0.616	0.62	0.624	0.628	0.63	0.633	0.635	0.637	0.64	0.642	0.643	0.645	0.647
Tlaxcala	0.478	0.482	0.487	0.495	0.506	0.515	0.524	0.531	0.536	0.541	0.545	0.55	0.556	0.561	0.567	0.573	0.578	0.583	0.587	0.59	0.591	0.594	0.596	0.598	0.6	0.601	0.603	0.604
Veracruz de Ignacio de la Llave	0.461	0.463	0.467	0.472	0.477	0.48	0.485	0.492	0.5	0.509	0.517	0.523	0.529	0.534	0.54	0.546	0.551	0.557	0.563	0.567	0.571	0.574	0.578	0.581	0.584	0.587	0.59	0.592
Yucatán	0.497	0.502	0.508	0.516	0.524	0.531	0.54	0.548	0.556	0.562	0.569	0.575	0.581	0.587	0.592	0.596	0.599	0.602	0.605	0.608	0.61	0.613	0.617	0.62	0.623	0.626	0.628	0.63
Zacatecas	0.483	0.489	0.495	0.502	0.509	0.514	0.52	0.527	0.534	0.54	0.546	0.551	0.555	0.56	0.564	0.568	0.572	0.577	0.581	0.584	0.586	0.589	0.592	0.595	0.598	0.602	0.605	0.608
Nicaragua	0.357	0.363	0.368	0.374	0.381	0.389	0.397	0.406	0.415	0.424	0.432	0.439	0.446	0.453	0.46	0.466	0.47	0.475	0.481	0.486	0.492	0.499	0.504	0.509	0.514	0.52	0.525	0.53
Panama	0.542	0.546	0.55	0.555	0.56	0.565	0.569	0.573	0.578	0.583	0.589	0.595	0.6	0.604	0.608	0.611	0.614	0.618	0.622	0.626	0.63	0.635	0.641	0.648	0.656	0.664	0.671	0.677
Venezuela	0.528	0.536	0.553	0.559	0.566	0.578	0.587	0.592	0.591	0.587	0.591	0.602	0.602	0.588	0.576	0.579	0.594	0.61	0.621	0.625	0.629	0.633	0.64	0.646	0.648	0.651	0.654	0.655
Tropical Latin America	0.494	0.5	0.507	0.514	0.521	0.529	0.537	0.544	0.55	0.556	0.561	0.566	0.571	0.577	0.582	0.588	0.594	0.601	0.608	0.614	0.621	0.628	0.635	0.642	0.648	0.654	0.659	0.662
Brazil	0.494	0.501	0.508	0.515	0.522	0.53	0.537	0.545	0.551	0.556	0.562	0.567	0.572	0.577	0.583	0.589	0.595	0.602	0.608	0.615	0.622	0.629	0.636	0.643	0.649	0.655	0.66	0.663
Acre	0.376	0.386	0.395	0.405	0.415	0.424	0.435	0.445	0.453	0.46	0.466	0.472	0.479	0.485	0.492	0.5	0.508	0.517	0.527	0.536	0.546	0.556	0.565	0.575	0.583	0.591	0.597	0.602
Alagoas	0.355	0.363	0.371	0.379	0.387	0.395	0.404	0.412	0.419	0.425	0.431	0.436	0.442	0.448	0.455	0.462	0.47	0.478	0.487	0.496	0.505	0.514	0.523	0.531	0.539	0.546	0.552	0.556
Amapá	0.467	0.475	0.483	0.491	0.5	0.508	0.517	0.526	0.534	0.54	0.546	0.552	0.558	0.564	0.57	0.576	0.583	0.591	0.598	0.605	0.613	0.621	0.629	0.636	0.643	0.65	0.655	0.659
Amazonas	0.438	0.447	0.457	0.466	0.475	0.483	0.492	0.499	0.505	0.51	0.514	0.519	0.523	0.528	0.533	0.539	0.546	0.553	0.561	0.568	0.577	0.585	0.594	0.602	0.611	0.618	0.625	0.629
Bahia	0.402	0.41	0.419	0.427	0.435	0.443	0.451	0.459	0.465	0.47	0.475	0.48	0.485	0.491	0.496	0.503	0.51	0.518	0.526	0.534	0.542	0.551	0.559	0.567	0.575	0.582	0.587	0.591
Ceará	0.411	0.419	0.426	0.433	0.44	0.448	0.455	0.463	0.469	0.475	0.48	0.486	0.492	0.498	0.505	0.512	0.52	0.528	0.536	0.544	0.553	0.561	0.569	0.577	0.584	0.591	0.596	0.6
Distrito Federal	0.63	0.636	0.642	0.649	0.656	0.663	0.671	0.679	0.685	0.691	0.696	0.702	0.707	0.713	0.719	0.725	0.731	0.738	0.744	0.75	0.756	0.763	0.769	0.775	0.78	0.785	0.789	0.792
Espírito Santo	0.499	0.507	0.515	0.524	0.532	0.54	0.549	0.557	0.564	0.57	0.576	0.582	0.588	0.593	0.599	0.606	0.612	0.618	0.625	0.631	0.638	0.644	0.651	0.657	0.663	0.669	0.673	0.677
Goiás	0.46	0.468	0.476	0.484	0.493	0.501	0.51	0.518	0.526	0.532	0.538	0.545	0.551	0.558	0.564	0.571	0.579	0.586	0.594	0.601	0.608	0.616	0.623	0.63	0.636	0.642	0.647	0.65
Maranhão	0.313	0.322	0.33	0.339	0.347	0.355	0.364	0.371	0.377	0.38	0.383	0.386	0.389	0.392	0.396	0.402	0.409	0.418	0.427	0.436	0.446	0.456	0.467	0.477	0.486	0.495	0.502	0.507
Mato Grosso	0.475	0.484	0.492	0.501	0.509	0.518	0.527	0.535	0.543	0.548	0.554	0.559	0.564	0.57	0.576	0.582	0.589	0.596	0.604	0.611	0.618	0.626	0.633	0.641	0.648	0.654	0.659	0.662
Mato Grosso do Sul	0.465	0.473	0.481	0.489	0.497	0.506	0.515	0.523	0.531	0.537	0.543	0.549	0.555	0.56	0.566	0.573	0.58	0.587	0.594	0.6	0.607	0.614	0.622	0.629	0.636	0.642	0.647	0.65
Minas Gerais	0.491	0.498	0.506	0.513	0.521	0.53	0.538	0.545	0.551	0.557	0.562	0.567	0.573	0.579	0.585	0.591	0.598	0.604	0.611	0.618	0.624	0.631	0.637	0.643	0.649	0.654	0.658	0.661
Pará	0.41	0.418	0.425	0.432	0.44	0.447	0.454	0.461	0.466	0.47	0.473	0.476	0.48	0.483	0.488	0.493	0.499	0.506	0.514	0.521	0.529	0.538	0.546	0.554	0.562	0.569	0.575	0.579
Paraíba	0.399	0.406	0.413	0.42	0.427	0.434	0.441	0.447	0.453	0.457	0.461	0.465	0.469	0.474	0.48	0.486	0.493	0.501	0.509	0.517	0.526	0.535	0.543	0.551	0.559	0.565	0.571	0.574
Paraná	0.513	0.519	0.525	0.532	0.539	0.548	0.556	0.564	0.572	0.578	0.585	0.591	0.597	0.603	0.609	0.615	0.622	0.628	0.634	0.64	0.646	0.652	0.658	0.664	0.67	0.675	0.679	0.682
Pernambuco	0.416	0.423	0.43	0.437	0.444	0.451	0.458	0.466	0.472	0.477	0.481	0.486	0.492	0.497	0.503	0.51	0.517	0.525	0.533	0.54	0.548	0.556	0.564	0.572	0.579	0.585	0.59	0.594
Piauí	0.365	0.372	0.379	0.386	0.393	0.4	0.408	0.415	0.42	0.425	0.429	0.434	0.439	0.444	0.45	0.457	0.465	0.473	0.482	0.491	0.5	0.51	0.518	0.527	0.535	0.542	0.548	0.552
Rio de Janeiro	0.576	0.581	0.585	0.59	0.595	0.601	0.608	0.614	0.619	0.624	0.628	0.632	0.637	0.641	0.645	0.65	0.655	0.66	0.665	0.67	0.675	0.681	0.686	0.692	0.697	0.702	0.706	0.709
Rio Grande do Norte	0.415	0.422	0.429	0.436	0.444	0.451	0.46	0.467	0.474	0.48	0.485	0.491	0.497	0.503	0.509	0.516	0.524	0.532	0.541	0.549	0.558	0.567	0.575	0.583	0.59	0.597	0.602	0.605
Rio Grande do Sul	0.543	0.549	0.555	0.561	0.567	0.574	0.581	0.587	0.593	0.598	0.603	0.608	0.614	0.619	0.624	0.63	0.635	0.641	0.647	0.653	0.659	0.665	0.67	0.676	0.681	0.686	0.69	0.693
Rondônia	0.423	0.433	0.441	0.45	0.458	0.467	0.475	0.484	0.491	0.497	0.502	0.508	0.515	0.521	0.528	0.535	0.543	0.551	0.559	0.567	0.575	0.584	0.592	0.599	0.606	0.613	0.618	0.622
Roraima	0.428	0.438	0.447	0.456	0.465	0.474	0.483	0.492	0.499	0.504	0.509	0.514	0.521	0.527	0.534	0.543	0.552	0.562	0.572	0.581	0.591	0.601	0.611	0.62	0.628	0.636	0.642	0.646
Santa Catarina	0.541	0.548	0.554	0.56	0.567	0.574	0.582	0.589	0.595	0.601	0.606	0.612	0.618	0.623	0.629	0.635	0.641	0.647	0.653	0.659	0.665	0.672	0.678	0.684	0.69	0.695	0.699	0.702
São Paulo	0.558	0.565	0.572	0.579	0.587	0.595	0.603	0.611	0.618	0.624	0.63	0.636	0.641	0.646	0.652	0.657	0.663	0.669	0.674	0.68	0.685	0.691	0.697	0.703	0.708	0.713	0.717	0.72
Sergipe	0.425	0.433	0.441	0.448	0.456	0.464	0.473	0.481	0.488	0.494	0.5	0.506	0.512	0.518	0.524	0.531	0.538	0.546	0.554	0.562	0.57	0.578	0.586	0.594	0.601	0.607	0.612	0.616
Tocantins	0.396	0.404	0.412	0.42	0.428	0.436	0.445	0.453	0.46	0.466	0.471	0.477	0.484	0.491	0.498	0.507	0.517	0.527	0.537	0.547	0.558	0.568	0.577	0.586	0.594	0.601	0.607	0.611
Paraguay	0.467	0.471	0.475	0.48	0.485	0.491	0.497	0.504	0.512	0.519	0.525	0.532	0.538	0.544	0.548	0.553	0.558	0.562	0.566	0.569	0.573	0.577	0.582	0.589	0.598	0.606	0.613	0.619
North Africa and Middle East	0.456	0.464	0.472	0.478	0.485	0.492	0.499	0.506	0.513	0.521	0.531	0.539	0.547	0.553	0.561	0.568	0.574	0.582	0.589	0.596	0.602	0.607	0.612	0.617	0.622	0.628	0.635	0.639
North Africa and Middle East	0.456	0.464	0.472	0.478	0.485	0.492	0.499	0.506	0.513	0.521	0.531	0.539	0.547	0.553	0.561	0.568	0.574	0.582	0.589	0.596	0.602	0.607	0.612	0.617	0.622	0.628	0.635	0.639
Afghanistan	0.149	0.15	0.151	0.152	0.151	0.151	0.152	0.152	0.154	0.155	0.157	0.159	0.164	0.171	0.177	0.184	0.191	0.199	0.208	0.218	0.228	0.238	0.248	0.258	0.267	0.276	0.284	0.29
Algeria	0.495	0.504	0.513	0.522	0.531	0.54	0.549	0.558	0.567	0.574	0.583	0.591	0.599	0.608	0.617	0.625	0.633	0.641	0.648	0.653	0.659	0.665	0.67	0.675	0.68	0.685	0.691	0.696
Bahrain	0.612	0.618	0.624	0.63	0.635	0.639	0.644	0.648	0.653	0.657	0.662	0.668	0.673	0.677	0.68	0.684	0.687	0.69	0.693	0.695	0.696	0.697	0.698	0.7	0.702	0.706	0.709	0.712
Egypt	0.441	0.451	0.461	0.47	0.477	0.485	0.493	0.5	0.507	0.514	0.52	0.526	0.532	0.537	0.542	0.547	0.55	0.553	0.556	0.559	0.563	0.565	0.564	0.566	0.572	0.584	0.596	0.604
Iran	0.503	0.513	0.522	0.532	0.541	0.548	0.553	0.556	0.56	0.572	0.589	0.604	0.616	0.626	0.634	0.641	0.649	0.656	0.662	0.667	0.672	0.677	0.68	0.683	0.687	0.691	0.696	0.7
Iraq	0.433	0.433	0.437	0.441	0.444	0.446	0.45	0.455	0.463	0.473	0.481	0.488	0.494	0.496	0.502	0.507	0.512	0.517	0.522	0.528	0.535	0.542	0.551	0.559	0.566	0.572	0.58	0.585
Jordan	0.552	0.556	0.561	0.566	0.57	0.574	0.578	0.584	0.589	0.594	0.597	0.6	0.603	0.607	0.612	0.616	0.621	0.626	0.633	0.643	0.653	0.662	0.671	0.679	0.685	0.69	0.694	0.697
Kuwait	0.656	0.651	0.642	0.637	0.633	0.632	0.638	0.651	0.665	0.674	0.681	0.683	0.685	0.687	0.693	0.701	0.711	0.721	0.73	0.737	0.743	0.748	0.754	0.762	0.769	0.776	0.781	0.786
Lebanon	0.519	0.528	0.536	0.544	0.552	0.56	0.569	0.578	0.588	0.597	0.604	0.611	0.618	0.626	0.635	0.643	0.65	0.658	0.667	0.676	0.686	0.694	0.702	0.711	0.717	0.722	0.726	0.73
Libya	0.645	0.657	0.669	0.679	0.688	0.696	0.705	0.713	0.72	0.725	0.729	0.732	0.736	0.742	0.747	0.753	0.759	0.765	0.771	0.777	0.783	0.781	0.784	0.783	0.779	0.773	0.766	0.761
Morocco	0.389	0.397	0.404	0.411	0.419	0.426	0.434	0.442	0.449	0.456	0.463	0.47	0.477	0.484	0.49	0.496	0.503	0.51	0.517	0.525	0.532	0.54	0.547	0.554	0.561	0.567	0.574	0.579
Palestine	0.362	0.366	0.371	0.377	0.384	0.39	0.397	0.405	0.413	0.421	0.427	0.431	0.433	0.435	0.439	0.444	0.448	0.453	0.46	0.468	0.479	0.491	0.503	0.513	0.521	0.528	0.536	0.541
Oman	0.473	0.485	0.497	0.509	0.521	0.533	0.546	0.558	0.57	0.582	0.594	0.606	0.618	0.629	0.639	0.649	0.659	0.668	0.678	0.686	0.695	0.703	0.711	0.72	0.727	0.734	0.739	0.744
Qatar	0.603	0.615	0.626	0.637	0.647	0.656	0.665	0.674	0.681	0.688	0.695	0.701	0.706	0.712	0.717	0.721	0.725	0.728	0.731	0.735	0.738	0.743	0.747	0.751	0.755	0.759	0.762	0.766
Saudi Arabia	0.436	0.457	0.477	0.496	0.514	0.53	0.545	0.56	0.573	0.586	0.598	0.61	0.621	0.633	0.645	0.658	0.67	0.682	0.695	0.706	0.716	0.728	0.738	0.748	0.757	0.765	0.773	0.779
Sudan	0.221	0.228	0.236	0.244	0.252	0.26	0.267	0.275	0.283	0.291	0.3	0.308	0.317	0.326	0.336	0.346	0.357	0.367	0.378	0.389	0.401	0.414	0.425	0.437	0.448	0.46	0.47	0.478
Syria	0.389	0.394	0.401	0.409	0.419	0.429	0.439	0.448	0.458	0.466	0.474	0.481	0.489	0.496	0.506	0.518	0.527	0.536	0.545	0.556	0.566	0.576	0.584	0.589	0.595	0.601	0.606	0.611
Tunisia	0.453	0.463	0.473	0.482	0.491	0.501	0.511	0.521	0.532	0.543	0.553	0.563	0.572	0.582	0.592	0.6	0.609	0.617	0.625	0.632	0.639	0.645	0.65	0.656	0.661	0.666	0.671	0.675
Turkey	0.507	0.516	0.524	0.533	0.54	0.548	0.556	0.564	0.572	0.579	0.587	0.594	0.601	0.608	0.616	0.625	0.633	0.643	0.652	0.66	0.669	0.678	0.688	0.697	0.706	0.715	0.723	0.729
United Arab Emirates	0.621	0.637	0.651	0.663	0.675	0.686	0.696	0.707	0.716	0.726	0.734	0.742	0.749	0.755	0.76	0.765	0.77	0.776	0.781	0.784	0.788	0.79	0.791	0.793	0.791	0.794	0.794	0.795
Yemen	0.203	0.208	0.214	0.22	0.228	0.238	0.247	0.256	0.266	0.275	0.284	0.294	0.304	0.313	0.324	0.336	0.349	0.362	0.373	0.382	0.393	0.401	0.408	0.415	0.422	0.426	0.429	0.43
South Asia	0.312	0.317	0.324	0.331	0.338	0.345	0.352	0.358	0.363	0.371	0.38	0.388	0.395	0.403	0.412	0.421	0.43	0.438	0.446	0.455	0.462	0.468	0.476	0.489	0.504	0.517	0.526	0.534
South Asia	0.312	0.317	0.324	0.331	0.338	0.345	0.352	0.358	0.363	0.371	0.38	0.388	0.395	0.403	0.412	0.421	0.43	0.438	0.446	0.455	0.462	0.468	0.476	0.489	0.504	0.517	0.526	0.534
Bangladesh	0.256	0.264	0.272	0.281	0.289	0.298	0.306	0.314	0.322	0.329	0.335	0.34	0.345	0.35	0.356	0.362	0.369	0.377	0.385	0.393	0.401	0.41	0.418	0.427	0.435	0.443	0.451	0.458
Bhutan	0.324	0.333	0.343	0.352	0.362	0.372	0.382	0.392	0.402	0.412	0.422	0.432	0.442	0.452	0.461	0.47	0.479	0.489	0.498	0.507	0.516	0.525	0.534	0.542	0.549	0.557	0.564	0.57
India	0.32	0.324	0.331	0.339	0.346	0.353	0.36	0.365	0.37	0.378	0.387	0.395	0.403	0.412	0.422	0.431	0.44	0.448	0.457	0.465	0.472	0.477	0.485	0.5	0.517	0.532	0.542	0.55
Andhra Pradesh	0.28	0.286	0.294	0.303	0.312	0.322	0.331	0.337	0.344	0.352	0.363	0.374	0.384	0.395	0.406	0.415	0.424	0.434	0.442	0.451	0.459	0.466	0.473	0.487	0.503	0.517	0.528	0.536
Arunachal Pradesh	0.311	0.317	0.323	0.33	0.336	0.345	0.354	0.36	0.366	0.374	0.381	0.388	0.395	0.405	0.416	0.424	0.432	0.44	0.447	0.458	0.467	0.473	0.479	0.498	0.52	0.536	0.548	0.556
Assam	0.327	0.332	0.339	0.345	0.351	0.358	0.363	0.367	0.371	0.379	0.389	0.397	0.406	0.415	0.424	0.431	0.439	0.446	0.453	0.459	0.464	0.467	0.472	0.485	0.5	0.513	0.523	0.53
Bihar	0.29	0.293	0.297	0.298	0.298	0.298	0.298	0.297	0.292	0.291	0.292	0.294	0.296	0.299	0.303	0.308	0.316	0.324	0.334	0.342	0.349	0.355	0.365	0.382	0.4	0.414	0.425	0.433
Chhattisgarh	0.292	0.294	0.298	0.303	0.306	0.311	0.316	0.319	0.322	0.328	0.336	0.344	0.352	0.362	0.372	0.382	0.392	0.403	0.413	0.421	0.427	0.431	0.438	0.455	0.474	0.49	0.503	0.512
Delhi	0.486	0.493	0.504	0.516	0.522	0.526	0.531	0.535	0.537	0.545	0.555	0.565	0.575	0.584	0.593	0.602	0.612	0.62	0.628	0.637	0.644	0.649	0.656	0.67	0.686	0.7	0.709	0.715
Goa	0.499	0.505	0.512	0.519	0.526	0.534	0.542	0.55	0.56	0.571	0.58	0.588	0.597	0.606	0.616	0.626	0.634	0.643	0.655	0.665	0.674	0.683	0.691	0.702	0.714	0.724	0.733	0.74
Gujarat	0.35	0.353	0.361	0.37	0.379	0.388	0.396	0.402	0.409	0.418	0.426	0.433	0.438	0.447	0.457	0.469	0.478	0.487	0.496	0.506	0.515	0.521	0.528	0.541	0.556	0.569	0.577	0.584
Haryana	0.342	0.348	0.356	0.364	0.372	0.38	0.388	0.394	0.398	0.405	0.414	0.423	0.431	0.44	0.451	0.462	0.472	0.481	0.491	0.5	0.508	0.512	0.521	0.54	0.564	0.582	0.593	0.6
Himachal Pradesh	0.34	0.347	0.356	0.366	0.376	0.386	0.397	0.408	0.418	0.431	0.445	0.457	0.468	0.479	0.491	0.502	0.513	0.524	0.534	0.544	0.553	0.562	0.571	0.585	0.601	0.614	0.625	0.633
Jammu and Kashmir	0.334	0.337	0.341	0.349	0.356	0.363	0.37	0.38	0.389	0.402	0.417	0.43	0.442	0.454	0.466	0.476	0.485	0.493	0.503	0.511	0.519	0.527	0.535	0.547	0.562	0.574	0.582	0.59
Jharkhand	0.281	0.283	0.286	0.29	0.293	0.297	0.3	0.304	0.307	0.312	0.319	0.325	0.333	0.342	0.353	0.362	0.371	0.38	0.388	0.397	0.405	0.411	0.419	0.434	0.451	0.466	0.478	0.487
Karnataka	0.323	0.331	0.339	0.349	0.357	0.366	0.375	0.38	0.386	0.396	0.406	0.415	0.424	0.434	0.444	0.454	0.463	0.472	0.481	0.49	0.498	0.504	0.512	0.526	0.542	0.556	0.566	0.574
Kerala	0.404	0.408	0.418	0.429	0.438	0.447	0.456	0.463	0.472	0.483	0.496	0.506	0.515	0.525	0.536	0.548	0.557	0.565	0.574	0.581	0.587	0.592	0.599	0.612	0.629	0.642	0.652	0.659
Madhya Pradesh	0.284	0.288	0.293	0.299	0.303	0.309	0.315	0.317	0.319	0.326	0.334	0.342	0.348	0.355	0.363	0.369	0.373	0.378	0.384	0.39	0.394	0.398	0.406	0.425	0.447	0.465	0.477	0.487
Maharashtra	0.363	0.369	0.379	0.39	0.399	0.41	0.419	0.426	0.431	0.44	0.451	0.459	0.468	0.478	0.489	0.5	0.51	0.52	0.529	0.538	0.546	0.552	0.559	0.572	0.588	0.602	0.611	0.618
Manipur	0.381	0.387	0.393	0.4	0.405	0.411	0.417	0.421	0.427	0.436	0.445	0.454	0.461	0.469	0.479	0.49	0.498	0.505	0.512	0.52	0.526	0.532	0.539	0.549	0.561	0.572	0.582	0.59
Meghalaya	0.334	0.339	0.345	0.352	0.357	0.364	0.37	0.376	0.382	0.39	0.401	0.411	0.42	0.43	0.441	0.45	0.46	0.469	0.478	0.487	0.495	0.501	0.507	0.52	0.534	0.547	0.557	0.565
Mizoram	0.392	0.399	0.408	0.417	0.424	0.433	0.443	0.449	0.456	0.465	0.475	0.483	0.492	0.502	0.511	0.52	0.526	0.531	0.537	0.545	0.55	0.552	0.557	0.57	0.587	0.6	0.609	0.616
Nagaland	0.395	0.399	0.406	0.414	0.421	0.429	0.435	0.441	0.445	0.452	0.462	0.472	0.482	0.492	0.503	0.513	0.522	0.532	0.543	0.552	0.56	0.567	0.574	0.587	0.602	0.614	0.625	0.633
Odisha	0.283	0.287	0.292	0.299	0.305	0.314	0.32	0.325	0.331	0.34	0.349	0.357	0.364	0.373	0.384	0.393	0.404	0.415	0.425	0.434	0.441	0.447	0.456	0.472	0.49	0.504	0.515	0.524
Punjab	0.383	0.388	0.396	0.404	0.413	0.422	0.429	0.435	0.441	0.449	0.459	0.468	0.475	0.483	0.492	0.501	0.511	0.52	0.53	0.539	0.547	0.554	0.563	0.576	0.592	0.605	0.614	0.622
Rajasthan	0.263	0.268	0.274	0.28	0.287	0.295	0.303	0.309	0.314	0.322	0.332	0.342	0.349	0.359	0.369	0.377	0.385	0.391	0.398	0.404	0.41	0.415	0.421	0.438	0.458	0.473	0.484	0.492
Sikkim	0.343	0.349	0.355	0.362	0.368	0.375	0.382	0.388	0.395	0.403	0.413	0.421	0.431	0.442	0.453	0.463	0.473	0.482	0.492	0.512	0.528	0.541	0.555	0.574	0.593	0.608	0.619	0.628
Tamil Nadu	0.35	0.354	0.363	0.373	0.383	0.391	0.399	0.406	0.414	0.424	0.436	0.446	0.455	0.465	0.475	0.486	0.497	0.506	0.514	0.522	0.529	0.535	0.543	0.559	0.579	0.595	0.607	0.615
Telangana	0.288	0.294	0.3	0.307	0.315	0.323	0.331	0.337	0.344	0.352	0.363	0.373	0.383	0.395	0.407	0.418	0.429	0.441	0.454	0.465	0.478	0.488	0.5	0.517	0.536	0.552	0.565	0.575
Tripura	0.333	0.336	0.34	0.346	0.35	0.356	0.363	0.368	0.373	0.385	0.398	0.409	0.419	0.43	0.44	0.449	0.457	0.462	0.469	0.476	0.48	0.483	0.488	0.5	0.515	0.527	0.536	0.543
Uttar Pradesh	0.272	0.276	0.281	0.286	0.291	0.297	0.303	0.307	0.31	0.317	0.325	0.333	0.34	0.348	0.358	0.366	0.374	0.382	0.39	0.398	0.404	0.41	0.419	0.436	0.454	0.469	0.48	0.488
Uttarakhand	0.304	0.306	0.31	0.315	0.321	0.329	0.336	0.341	0.347	0.355	0.367	0.378	0.391	0.405	0.42	0.434	0.449	0.463	0.478	0.494	0.509	0.522	0.535	0.552	0.57	0.586	0.598	0.607
West Bengal	0.321	0.322	0.329	0.338	0.345	0.353	0.36	0.366	0.371	0.38	0.389	0.398	0.406	0.416	0.425	0.434	0.441	0.448	0.455	0.462	0.467	0.471	0.476	0.49	0.506	0.519	0.53	0.538
Union Territories other than Delhi	0.406	0.408	0.412	0.417	0.423	0.43	0.439	0.446	0.453	0.463	0.474	0.486	0.498	0.511	0.524	0.537	0.548	0.559	0.569	0.579	0.586	0.591	0.597	0.61	0.624	0.636	0.646	0.653
Nepal	0.216	0.222	0.228	0.235	0.242	0.249	0.257	0.265	0.273	0.281	0.29	0.298	0.307	0.315	0.323	0.331	0.339	0.347	0.355	0.364	0.373	0.382	0.39	0.399	0.407	0.415	0.422	0.429
Pakistan	0.305	0.31	0.314	0.319	0.324	0.33	0.337	0.343	0.35	0.359	0.367	0.375	0.382	0.389	0.396	0.404	0.412	0.42	0.428	0.436	0.443	0.449	0.458	0.466	0.474	0.48	0.486	0.492
Southeast Asia, East Asia, and Oceania	0.468	0.479	0.489	0.498	0.506	0.517	0.528	0.538	0.546	0.553	0.561	0.568	0.574	0.58	0.588	0.596	0.606	0.615	0.624	0.632	0.641	0.649	0.656	0.662	0.67	0.673	0.675	0.685
East Asia	0.463	0.476	0.486	0.495	0.504	0.516	0.528	0.54	0.55	0.559	0.568	0.577	0.584	0.591	0.599	0.61	0.621	0.632	0.642	0.652	0.663	0.672	0.679	0.685	0.694	0.696	0.696	0.709
China	0.456	0.469	0.479	0.489	0.498	0.51	0.523	0.535	0.545	0.554	0.563	0.573	0.58	0.587	0.595	0.606	0.618	0.63	0.64	0.65	0.661	0.671	0.678	0.683	0.693	0.695	0.694	0.707
North Korea	0.512	0.517	0.52	0.524	0.524	0.522	0.519	0.515	0.51	0.506	0.503	0.5	0.498	0.498	0.499	0.501	0.503	0.506	0.508	0.511	0.514	0.517	0.52	0.524	0.527	0.531	0.535	0.538
Taiwan (Province of China)	0.691	0.7	0.708	0.715	0.723	0.731	0.739	0.75	0.759	0.764	0.77	0.778	0.786	0.793	0.8	0.807	0.813	0.821	0.827	0.833	0.838	0.842	0.846	0.85	0.854	0.858	0.861	0.864
Oceania	0.406	0.408	0.41	0.412	0.415	0.418	0.421	0.423	0.426	0.428	0.43	0.431	0.432	0.433	0.435	0.436	0.438	0.44	0.442	0.444	0.446	0.449	0.452	0.455	0.459	0.463	0.467	0.471
American Samoa	0.609	0.612	0.615	0.62	0.624	0.629	0.633	0.636	0.639	0.643	0.647	0.651	0.655	0.659	0.662	0.666	0.668	0.671	0.673	0.675	0.678	0.682	0.687	0.691	0.694	0.697	0.7	0.702
Federated States of Micronesia	0.462	0.469	0.476	0.483	0.489	0.496	0.501	0.505	0.509	0.514	0.518	0.523	0.527	0.532	0.536	0.54	0.543	0.547	0.549	0.552	0.555	0.559	0.562	0.565	0.567	0.57	0.573	0.575
Fiji	0.533	0.537	0.542	0.547	0.552	0.558	0.563	0.568	0.574	0.58	0.585	0.589	0.594	0.598	0.602	0.604	0.607	0.61	0.611	0.613	0.615	0.617	0.619	0.623	0.627	0.632	0.637	0.641
Guam	0.698	0.695	0.693	0.694	0.698	0.703	0.709	0.717	0.727	0.739	0.75	0.759	0.765	0.768	0.77	0.77	0.769	0.77	0.771	0.774	0.776	0.78	0.784	0.788	0.792	0.794	0.794	0.794
Kiribati	0.355	0.357	0.359	0.361	0.363	0.365	0.368	0.37	0.373	0.376	0.379	0.382	0.385	0.388	0.39	0.393	0.395	0.397	0.398	0.399	0.401	0.403	0.406	0.41	0.414	0.418	0.423	0.427
Marshall Islands	0.413	0.421	0.429	0.436	0.444	0.451	0.457	0.462	0.465	0.469	0.473	0.477	0.482	0.485	0.489	0.493	0.498	0.503	0.507	0.512	0.518	0.523	0.528	0.533	0.538	0.542	0.547	0.55
Northern Mariana Islands	0.738	0.744	0.748	0.75	0.752	0.754	0.756	0.757	0.758	0.759	0.763	0.766	0.767	0.767	0.767	0.766	0.765	0.764	0.763	0.761	0.759	0.758	0.757	0.756	0.756	0.756	0.757	0.758
Papua New Guinea	0.318	0.321	0.324	0.33	0.335	0.34	0.344	0.348	0.351	0.354	0.356	0.358	0.36	0.361	0.363	0.365	0.368	0.371	0.375	0.379	0.383	0.388	0.393	0.398	0.404	0.409	0.415	0.419
Samoa	0.538	0.539	0.538	0.536	0.535	0.536	0.536	0.537	0.538	0.54	0.542	0.544	0.546	0.548	0.55	0.552	0.554	0.556	0.558	0.559	0.561	0.563	0.56	0.558	0.562	0.567	0.572	0.576
Solomon Islands	0.316	0.32	0.326	0.331	0.337	0.344	0.35	0.355	0.36	0.364	0.365	0.366	0.366	0.366	0.367	0.369	0.371	0.375	0.38	0.384	0.388	0.394	0.4	0.406	0.411	0.416	0.421	0.425
Tonga	0.522	0.528	0.533	0.538	0.544	0.549	0.553	0.557	0.56	0.564	0.566	0.569	0.573	0.577	0.581	0.584	0.587	0.589	0.592	0.595	0.599	0.603	0.607	0.61	0.614	0.617	0.621	0.625
Vanuatu	0.38	0.384	0.388	0.391	0.395	0.398	0.402	0.406	0.409	0.413	0.416	0.419	0.421	0.423	0.425	0.428	0.431	0.435	0.44	0.445	0.449	0.453	0.458	0.462	0.465	0.469	0.472	0.475
Southeast Asia	0.467	0.477	0.486	0.495	0.504	0.513	0.521	0.529	0.534	0.538	0.543	0.548	0.552	0.557	0.562	0.567	0.572	0.578	0.584	0.589	0.595	0.602	0.609	0.616	0.622	0.629	0.635	0.641
Cambodia	0.259	0.266	0.273	0.279	0.286	0.294	0.302	0.31	0.317	0.326	0.335	0.344	0.353	0.362	0.372	0.382	0.392	0.401	0.411	0.42	0.427	0.435	0.443	0.451	0.459	0.467	0.475	0.482
Indonesia	0.465	0.476	0.487	0.499	0.51	0.52	0.53	0.537	0.542	0.546	0.55	0.554	0.558	0.562	0.566	0.571	0.576	0.581	0.587	0.594	0.6	0.608	0.615	0.622	0.629	0.636	0.642	0.648
Laos	0.307	0.313	0.32	0.326	0.332	0.339	0.345	0.352	0.358	0.364	0.371	0.378	0.385	0.392	0.4	0.407	0.416	0.425	0.434	0.444	0.453	0.463	0.473	0.483	0.493	0.502	0.511	0.519
Malaysia	0.57	0.575	0.581	0.589	0.598	0.606	0.616	0.626	0.638	0.654	0.661	0.664	0.671	0.679	0.684	0.689	0.697	0.704	0.709	0.714	0.721	0.727	0.733	0.739	0.744	0.749	0.754	0.759
Maldives	0.386	0.399	0.412	0.424	0.435	0.446	0.46	0.477	0.493	0.507	0.518	0.528	0.537	0.545	0.554	0.56	0.567	0.575	0.584	0.593	0.602	0.611	0.62	0.629	0.636	0.643	0.65	0.655
Mauritius	0.554	0.561	0.568	0.576	0.585	0.595	0.603	0.609	0.613	0.616	0.621	0.627	0.633	0.639	0.646	0.651	0.657	0.663	0.669	0.675	0.68	0.687	0.694	0.701	0.708	0.713	0.717	0.72
Myanmar	0.33	0.333	0.337	0.341	0.347	0.353	0.36	0.367	0.375	0.383	0.392	0.402	0.412	0.423	0.434	0.446	0.458	0.47	0.481	0.492	0.501	0.51	0.518	0.527	0.535	0.542	0.549	0.556

Appendix Table 10. Socio-Demographic Index values for all estimated GBD 2017 locations, 1990-2017

Location	1990	1991	1992	1993	1994	1995	1996	1997	1998	1999	2000	2001	2002	2003	2004	2005	2006	2007	2008	2009	2010	2011	2012	2013	2014	2015	2016	2017
Philippines	0.511	0.516	0.521	0.525	0.53	0.534	0.539	0.542	0.545	0.547	0.55	0.553	0.555	0.557	0.559	0.561	0.563	0.566	0.568	0.569	0.572	0.579	0.586	0.593	0.599	0.605	0.612	0.617
Sri Lanka	0.49	0.495	0.501	0.508	0.516	0.524	0.532	0.54	0.547	0.553	0.559	0.565	0.571	0.578	0.584	0.59	0.597	0.604	0.611	0.618	0.626	0.634	0.642	0.65	0.658	0.666	0.673	0.68
Seychelles	0.549	0.557	0.565	0.573	0.582	0.589	0.597	0.605	0.613	0.62	0.626	0.631	0.636	0.64	0.643	0.646	0.65	0.653	0.656	0.658	0.66	0.663	0.667	0.671	0.675	0.68	0.686	0.692
Thailand	0.502	0.514	0.525	0.534	0.542	0.552	0.561	0.567	0.569	0.572	0.579	0.587	0.594	0.6	0.605	0.61	0.616	0.623	0.629	0.635	0.641	0.647	0.654	0.66	0.667	0.673	0.679	0.684
Timor-Leste	0.276	0.283	0.29	0.296	0.302	0.307	0.314	0.321	0.325	0.321	0.32	0.325	0.332	0.345	0.362	0.379	0.4	0.419	0.437	0.449	0.46	0.471	0.481	0.49	0.495	0.5	0.504	0.505
Vietnam	0.406	0.413	0.42	0.427	0.435	0.444	0.452	0.461	0.469	0.477	0.483	0.49	0.497	0.504	0.511	0.518	0.525	0.532	0.54	0.547	0.554	0.562	0.57	0.578	0.585	0.593	0.6	0.607
Sub-Saharan Africa	0.304	0.307	0.311	0.314	0.317	0.32	0.324	0.328	0.332	0.335	0.339	0.343	0.348	0.353	0.359	0.365	0.371	0.379	0.386	0.393	0.4	0.407	0.414	0.421	0.428	0.435	0.441	0.446
Central sub-Saharan Africa	0.298	0.303	0.307	0.309	0.311	0.313	0.316	0.318	0.32	0.323	0.325	0.328	0.332	0.336	0.341	0.348	0.355	0.364	0.373	0.382	0.391	0.402	0.413	0.423	0.433	0.443	0.452	0.457
Angola	0.235	0.24	0.245	0.249	0.253	0.258	0.263	0.269	0.276	0.282	0.288	0.293	0.299	0.305	0.312	0.32	0.329	0.34	0.351	0.363	0.375	0.389	0.401	0.414	0.428	0.441	0.453	0.461
Central African Republic	0.22	0.225	0.228	0.232	0.236	0.24	0.242	0.245	0.249	0.254	0.257	0.261	0.265	0.268	0.271	0.275	0.28	0.285	0.29	0.296	0.304	0.313	0.323	0.325	0.328	0.33	0.333	0.334
Congo (Brazzaville)	0.382	0.39	0.398	0.405	0.41	0.416	0.421	0.426	0.43	0.434	0.439	0.444	0.449	0.455	0.46	0.467	0.475	0.482	0.49	0.499	0.509	0.52	0.531	0.542	0.552	0.561	0.569	0.574
DR Congo	0.293	0.296	0.298	0.298	0.296	0.294	0.291	0.288	0.283	0.279	0.274	0.269	0.265	0.263	0.262	0.264	0.264	0.265	0.267	0.27	0.278	0.288	0.3	0.315	0.33	0.344	0.356	0.364
Equatorial Guinea	0.2	0.204	0.212	0.22	0.229	0.241	0.26	0.292	0.316	0.339	0.363	0.388	0.41	0.429	0.449	0.467	0.483	0.499	0.516	0.53	0.544	0.559	0.573	0.587	0.599	0.61	0.62	0.625
Gabon	0.433	0.443	0.453	0.462	0.472	0.481	0.49	0.498	0.506	0.514	0.522	0.529	0.535	0.542	0.549	0.556	0.562	0.569	0.576	0.582	0.589	0.598	0.607	0.616	0.625	0.634	0.644	0.651
Eastern sub-Saharan Africa	0.23	0.233	0.236	0.239	0.241	0.245	0.249	0.254	0.259	0.262	0.266	0.271	0.276	0.282	0.288	0.294	0.301	0.308	0.316	0.324	0.332	0.34	0.348	0.356	0.365	0.373	0.381	0.387
Burundi	0.247	0.252	0.257	0.258	0.263	0.265	0.265	0.266	0.268	0.268	0.268	0.267	0.268	0.269	0.271	0.272	0.274	0.276	0.278	0.282	0.286	0.29	0.295	0.299	0.303	0.306	0.308	0.31
Comoros	0.272	0.279	0.286	0.293	0.298	0.303	0.306	0.31	0.314	0.319	0.325	0.331	0.338	0.344	0.351	0.358	0.365	0.372	0.378	0.384	0.39	0.396	0.403	0.41	0.417	0.423	0.429	0.434
Djibouti	0.313	0.317	0.32	0.322	0.325	0.329	0.333	0.337	0.339	0.341	0.342	0.347	0.359	0.374	0.388	0.4	0.407	0.412	0.419	0.425	0.432	0.439	0.446	0.454	0.462	0.47	0.478	0.485
Eritrea	0.202	0.214	0.223	0.234	0.247	0.26	0.272	0.285	0.296	0.306	0.315	0.323	0.331	0.337	0.343	0.348	0.353	0.357	0.36	0.364	0.368	0.372	0.378	0.383	0.39	0.396	0.403	0.409
Ethiopia	0.138	0.141	0.143	0.146	0.148	0.15	0.155	0.161	0.166	0.169	0.172	0.177	0.183	0.189	0.195	0.202	0.21	0.221	0.233	0.245	0.257	0.268	0.28	0.292	0.303	0.314	0.325	0.334
Kenya	0.341	0.349	0.357	0.364	0.372	0.377	0.382	0.387	0.392	0.398	0.401	0.403	0.406	0.411	0.416	0.42	0.425	0.432	0.438	0.445	0.452	0.459	0.465	0.473	0.481	0.488	0.494	0.499
Baringo	0.254	0.266	0.278	0.289	0.299	0.307	0.313	0.319	0.326	0.333	0.338	0.341	0.345	0.352	0.358	0.362	0.368	0.376	0.384	0.393	0.401	0.408	0.414	0.421	0.428	0.434	0.439	0.444
Bomet	0.306	0.315	0.325	0.333	0.341	0.347	0.351	0.355	0.361	0.367	0.371	0.373	0.378	0.385	0.392	0.398	0.406	0.414	0.423	0.433	0.442	0.449	0.456	0.465	0.475	0.483	0.49	0.496
Bungoma	0.316	0.325	0.333	0.341	0.348	0.353	0.357	0.36	0.365	0.37	0.373	0.373	0.376	0.38	0.384	0.387	0.391	0.397	0.403	0.41	0.417	0.423	0.429	0.436	0.445	0.451	0.458	0.463
Busia	0.297	0.304	0.312	0.32	0.327	0.332	0.336	0.339	0.344	0.349	0.352	0.353	0.356	0.361	0.367	0.37	0.375	0.381	0.386	0.393	0.4	0.404	0.409	0.415	0.423	0.428	0.434	0.438
Elgeyo Marakwet	0.292	0.302	0.312	0.321	0.329	0.336	0.342	0.348	0.355	0.362	0.368	0.372	0.378	0.386	0.394	0.4	0.408	0.417	0.425	0.435	0.443	0.451	0.458	0.467	0.475	0.483	0.49	0.496
Embu	0.375	0.384	0.393	0.4	0.407	0.413	0.417	0.422	0.427	0.431	0.434	0.437	0.44	0.444	0.449	0.452	0.458	0.464	0.47	0.478	0.486	0.493	0.499	0.507	0.514	0.521	0.527	0.533
Garissa	0.153	0.16	0.168	0.177	0.184	0.19	0.195	0.201	0.207	0.213	0.217	0.219	0.223	0.228	0.233	0.237	0.242	0.249	0.255	0.263	0.272	0.28	0.288	0.298	0.309	0.318	0.326	0.334
Homa Bay	0.214	0.222	0.232	0.243	0.253	0.26	0.265	0.271	0.279	0.288	0.292	0.293	0.297	0.305	0.313	0.319	0.328	0.338	0.346	0.356	0.366	0.374	0.382	0.392	0.403	0.411	0.419	0.425
Isiolo	0.264	0.27	0.276	0.282	0.288	0.292	0.295	0.298	0.301	0.305	0.307	0.308	0.31	0.314	0.318	0.321	0.326	0.331	0.337	0.343	0.35	0.355	0.36	0.365	0.372	0.377	0.381	0.385
Kajiado	0.384	0.392	0.4	0.407	0.414	0.42	0.425	0.429	0.434	0.438	0.442	0.445	0.448	0.452	0.456	0.46	0.464	0.47	0.475	0.481	0.486	0.492	0.498	0.506	0.514	0.521	0.528	0.534
Kakamega	0.295	0.303	0.311	0.319	0.326	0.332	0.337	0.342	0.348	0.356	0.36	0.361	0.365	0.37	0.375	0.378	0.383	0.389	0.394	0.4	0.407	0.412	0.417	0.425	0.433	0.439	0.445	0.45
Kericho	0.266	0.277	0.288	0.299	0.309	0.317	0.324	0.331	0.339	0.348	0.353	0.356	0.362	0.37	0.378	0.385	0.394	0.404	0.414	0.425	0.436	0.445	0.454	0.464	0.475	0.485	0.493	0.5
Kiambu	0.435	0.443	0.45	0.457	0.464	0.469	0.473	0.476	0.48	0.484	0.487	0.489	0.492	0.496	0.5	0.504	0.509	0.516	0.521	0.528	0.535	0.541	0.548	0.555	0.562	0.569	0.575	0.58
Kilifi	0.292	0.3	0.307	0.314	0.321	0.327	0.331	0.336	0.34	0.346	0.348	0.349	0.352	0.357	0.361	0.365	0.371	0.378	0.385	0.392	0.4	0.408	0.415	0.424	0.434	0.442	0.45	0.456
Kirinyaga	0.389	0.396	0.402	0.407	0.411	0.415	0.418	0.422	0.425	0.429	0.432	0.434	0.437	0.442	0.447	0.451	0.457	0.464	0.471	0.479	0.486	0.493	0.5	0.507	0.514	0.521	0.527	0.533
Kisii	0.34	0.35	0.36	0.368	0.377	0.383	0.389	0.395	0.401	0.407	0.411	0.414	0.418	0.424	0.429	0.434	0.44	0.448	0.454	0.463	0.471	0.479	0.486	0.495	0.503	0.51	0.517	0.522
Kisumu	0.315	0.325	0.334	0.342	0.349	0.355	0.36	0.364	0.37	0.376	0.381	0.384	0.388	0.395	0.402	0.407	0.415	0.424	0.432	0.442	0.451	0.459	0.466	0.475	0.484	0.491	0.497	0.503
Kitui	0.28	0.288	0.297	0.304	0.311	0.317	0.322	0.327	0.332	0.338	0.343	0.346	0.351	0.357	0.363	0.369	0.376	0.383	0.391	0.399	0.408	0.416	0.423	0.432	0.44	0.448	0.455	0.461
Kwale	0.294	0.301	0.308	0.314	0.321	0.326	0.33	0.334	0.338	0.342	0.344	0.346	0.348	0.352	0.357	0.36	0.366	0.374	0.381	0.39	0.399	0.407	0.414	0.424	0.433	0.442	0.45	0.457
Laikipia	0.346	0.354	0.361	0.368	0.375	0.38	0.385	0.389	0.395	0.401	0.405	0.409	0.415	0.423	0.433	0.442	0.451	0.462	0.472	0.483	0.494	0.502	0.511	0.521	0.531	0.54	0.549	0.556
Lamu	0.295	0.303	0.312	0.321	0.328	0.334	0.338	0.343	0.347	0.352	0.355	0.356	0.359	0.364	0.369	0.373	0.378	0.384	0.39	0.397	0.405	0.411	0.417	0.425	0.434	0.441	0.448	0.453
Machakos	0.352	0.362	0.372	0.38	0.389	0.396	0.403	0.411	0.418	0.426	0.431	0.435	0.44	0.444	0.449	0.452	0.456	0.461	0.465	0.471	0.476	0.482	0.487	0.493	0.5	0.507	0.512	0.518
Makueni	0.3	0.307	0.314	0.322	0.328	0.333	0.336	0.339	0.343	0.348	0.35	0.351	0.354	0.36	0.367	0.373	0.381	0.391	0.401	0.413	0.423	0.432	0.439	0.447	0.454	0.46	0.465	0.469
Mandera	0.102	0.109	0.118	0.126	0.135	0.142	0.149	0.156	0.164	0.172	0.178	0.181	0.187	0.194	0.201	0.206	0.212	0.218	0.224	0.231	0.238	0.244	0.251	0.261	0.271	0.28	0.288	0.295
Marsabit	0.22	0.223	0.227	0.231	0.236	0.239	0.241	0.244	0.247	0.252	0.255	0.256	0.259	0.264	0.268	0.271	0.276	0.281	0.286	0.293	0.299	0.304	0.309	0.315	0.323	0.329	0.335	0.34
Meru	0.338	0.346	0.354	0.362	0.369	0.376	0.381	0.387	0.393	0.399	0.403	0.407	0.411	0.417	0.423	0.428	0.434	0.441	0.447	0.454	0.462	0.469	0.475	0.482	0.49	0.496	0.503	0.508
Migori	0.229	0.237	0.246	0.255	0.262	0.267	0.27	0.274	0.279	0.285	0.287	0.286	0.289	0.296	0.303	0.307	0.315	0.325	0.333	0.344	0.355	0.364	0.372	0.383	0.395	0.404	0.412	0.419
Mombasa	0.396	0.405	0.414	0.422	0.43	0.437	0.443	0.449	0.455	0.461	0.465	0.468	0.471	0.475	0.478	0.481	0.486	0.491	0.496	0.502	0.51	0.517	0.525	0.534	0.543	0.552	0.561	0.568
Murang'a	0.381	0.389	0.396	0.402	0.408	0.413	0.418	0.424	0.429	0.436	0.441	0.444	0.449	0.454	0.46	0.465	0.47	0.476	0.482	0.488	0.494	0.499	0.504	0.51	0.515	0.52	0.524	0.528
Nairobi	0.499	0.506	0.513	0.519	0.525	0.53	0.534	0.539	0.544	0.549	0.553	0.556	0.56	0.566	0.572	0.577	0.585	0.593	0.602	0.611	0.619	0.627	0.634	0.643	0.652	0.66	0.667	0.674
Nakuru	0.337	0.347	0.357	0.367	0.375	0.383	0.389	0.394	0.399	0.405	0.409	0.413	0.417	0.423	0.43	0.436	0.445	0.454	0.464	0.474	0.484	0.493	0.502	0.511	0.521	0.53	0.538	0.545
Nandi	0.323	0.333	0.344	0.354	0.362	0.369	0.375	0.381	0.388	0.394	0.399	0.402	0.406	0.412	0.418	0.422	0.428	0.435	0.441	0.449	0.456	0.462	0.468	0.475	0.483	0.49	0.496	0.501
Narok	0.217	0.225	0.234	0.242	0.25	0.255	0.259	0.264	0.272	0.28	0.284	0.285	0.289	0.297	0.305	0.31	0.318	0.326	0.333	0.342	0.35	0.356	0.362	0.371	0.381	0.389	0.396	0.402
Nyamira	0.361	0.37	0.379	0.387	0.395	0.401	0.406	0.41	0.414	0.419	0.421	0.423	0.426	0.431	0.437	0.443	0.451	0.46	0.468	0.478	0.488	0.496	0.504	0.513	0.523	0.531	0.538	0.544
Nyandarua	0.353	0.361	0.369	0.376	0.382	0.388	0.392	0.397	0.402	0.409	0.413	0.416	0.421	0.428	0.436	0.442	0.449	0.458	0.466	0.475	0.484	0.491	0.498	0.506	0.514	0.521	0.528	0.534
Nyeri	0.407	0.414	0.421	0.427	0.434	0.439	0.444	0.449	0.454	0.459	0.463	0.466	0.47	0.475	0.479	0.484	0.489	0.496	0.502	0.509	0.516	0.522	0.527	0.533	0.539	0.544	0.549	0.554
Samburu	0.205	0.21	0.215	0.221	0.225	0.228	0.23	0.232	0.235	0.239	0.24	0.239	0.24	0.243	0.247	0.249	0.253	0.258	0.262	0.267	0.273	0.277	0.28	0.287	0.294	0.299	0.304	0.308
Siaya	0.227	0.236	0.246	0.255	0.265	0.272	0.279	0.287	0.294	0.303	0.309	0.313	0.318	0.326	0.334	0.34	0.349	0.359	0.368	0.379	0.39	0.4	0.409	0.421	0.432	0.443	0.452	0.46
Taita Taveta	0.352	0.362	0.371	0.38	0.389	0.396	0.403	0.409	0.416	0.422	0.427	0.43	0.434	0.438	0.443	0.447	0.452	0.458	0.464	0.472	0.48	0.487	0.494	0.502	0.509	0.516	0.523	0.529
Tana River	0.231	0.237	0.244	0.25	0.255	0.26	0.263	0.267	0.27	0.274	0.276	0.276	0.278	0.281	0.285	0.288	0.292	0.298	0.304	0.311	0.319	0.326	0.333	0.342	0.353	0.362	0.371	0.379
Tharaka Nithi	0.342	0.35	0.357	0.364	0.371	0.377	0.382	0.387	0.392	0.397	0.402	0.405	0.41	0.417	0.423	0.429	0.437	0.445	0.453	0.463	0.472	0.48	0.488	0.497	0.506	0.514	0.522	0.528
Trans Nzoia	0.318	0.328	0.337	0.346	0.353	0.359	0.363	0.368	0.373	0.38	0.384	0.388	0.392	0.399	0.406	0.411	0.418	0.426	0.433	0.441	0.448	0.454	0.46	0.468	0.477	0.484	0.491	0.496
Turkana	0.211	0.216	0.221	0.226	0.231	0.234	0.237	0.239	0.242	0.245	0.247	0.247	0.248	0.25	0.252	0.252	0.253	0.255	0.257	0.26	0.264	0.266	0.269	0.274	0.281	0.286	0.291	0.295
Uasin Gishu	0.363	0.373	0.382	0.391	0.399	0.405	0.41	0.416	0.421	0.428	0.433	0.436	0.441	0.447	0.453	0.458	0.465	0.472	0.48	0.488	0.496	0.503	0.51	0.517	0.526	0.533	0.539	0.545
Vihiga	0.328	0.337	0.346	0.355	0.362	0.367	0.371	0.374	0.376	0.378	0.378	0.378	0.379	0.382	0.387	0.392	0.399	0.408	0.417	0.427	0.437	0.444	0.45	0.457	0.464	0.469	0.474	0.477
Wajir	0.104	0.111	0.12	0.128	0.136	0.142	0.146	0.151	0.156	0.162	0.165	0.166	0.168	0.172	0.176	0.178	0.181	0.185	0.189	0.195	0.201	0.206	0.21	0.218	0.226	0.233	0.238	0.243
West Pokot	0.213	0.219	0.226	0.233	0.24	0.245	0.249	0.253	0.258	0.265	0.268	0.27	0.274	0.28	0.286	0.291	0.298	0.305	0.312	0.319	0.327	0.333	0.34	0.349	0.359	0.368	0.376	0.382
Madagascar	0.262	0.264	0.265	0.265	0.265	0.265	0.266	0.268	0.27	0.272	0.274	0.278	0.282	0.286	0.291	0.294	0.295	0.295	0.298	0.3	0.302	0.303	0.306	0.31	0.315	0.321	0.326	0.331
Malawi	0.199	0.202	0.204	0.206	0.208	0.212	0.216	0.221	0.225	0.23	0.234	0.238	0.244	0.249	0.256	0.262	0.27	0.279	0.288	0.296	0.305	0.313	0.32	0.327	0.333	0.339	0.345	0.349
Mozambique	0.133	0.135	0.136	0.138	0.14	0.142	0.147	0.154	0.162	0.169	0.175	0.182	0.189	0.197	0.206	0.215	0.224	0.235	0.246	0.257	0.268	0.279	0.29	0.301	0.311	0.322	0.332	0.34
Rwanda	0.267	0.269	0.272	0.274	0.268	0.267	0.269	0.272	0.276	0.28	0.284	0.29	0.297	0.304	0.311	0.319	0.327	0.334	0.342	0.349	0.356	0.363	0.371	0.378	0.386	0.393	0.401	0.407
Somalia	0.153	0.156	0.157	0.159	0.16	0.161	0.162	0.163	0.163	0.164	0.165	0.167	0.169	0.172	0.176	0.179	0.183	0.188	0.193	0.198	0.203	0.208	0.212	0.217	0.222	0.227	0.231	0.235
South Sudan	0.179	0.18	0.182	0.183	0.184	0.186	0.187	0.189	0.191	0.194	0.197	0.2	0.204	0.207	0.211	0.215	0.22	0.225	0.229	0.234	0.239	0.244	0.249	0.254	0.259	0.265	0.27	0.275
Tanzania	0.275	0.28	0.285	0.289	0.291	0.294	0.297	0.3	0.304	0.307	0.311	0.315	0.32	0.325	0.33	0.334	0.339	0.344	0.35	0.355	0.36	0.366	0.373	0.381	0.388	0.397	0.405	0.412
Uganda	0.163	0.164	0.166	0.169	0.173	0.178	0.185	0.193	0.202	0.212	0.222	0.232	0.243	0.254	0.265	0.276	0.287	0.298	0.31	0.32	0.33	0.339	0.348	0.357	0.366	0.374	0.382	0.388
Zambia	0.312	0.314	0.316	0.319	0.321	0.324	0.326	0.329	0.332	0.335	0.338	0.343	0.348	0.353	0.358	0.365	0.373	0.382	0.392	0.402	0.412	0.421	0.431	0.44	0.449	0.458	0.466	0.472
Southern sub-Saharan Africa	0.521	0.525	0.53	0.535	0.539	0.543	0.546	0.55	0.555	0.56	0.565	0.569	0.574	0.578	0.582	0.585	0.59	0.596	0.603	0.607	0.612	0.616	0.619	0.622	0.627	0.633	0.637	0.64
Botswana	0.463	0.473	0.483	0.492	0.501	0.509	0.517	0.524	0.532	0.539	0.546	0.553	0.56	0.567	0.574	0.581	0.588	0.597	0.605	0.612	0.619	0.626	0.633	0.639	0.646	0.652	0.658	0.663
Lesotho	0.333	0.34	0.347	0.354	0.362	0.369	0.376	0.383	0.389	0.396	0.402	0.407	0.413	0.417	0.422	0.426	0.431	0.436	0.441	0.446	0.452	0.458	0.464	0.47	0.476	0.482	0.488	0.493
Namibia	0.454	0.459	0.465	0.471	0.477	0.483	0.489	0.495	0.501	0.507	0.512	0.516	0.521	0.525	0.53	0.534	0.54	0.546	0.552	0.558	0.565	0.572	0.579	0.587	0.595	0.603	0.61	0.616
South Africa	0.557	0.56	0.565	0.57	0.574	0.576	0.579	0.583	0.587	0.593	0.598	0.602	0.607	0.611	0.615	0.619	0.624	0.631	0.639	0.644	0.649	0.653	0.655	0.658	0.664	0.669	0.674	0.677
Swaziland	0.426	0.435	0.444	0.452	0.459	0.466	0.473	0.478	0.483	0.489	0.494	0.499	0.503	0.508	0.513	0.518	0.523	0.528	0.533	0.538	0.543	0.547	0.552	0.557	0.563	0.569	0.574	0.578
Zimbabwe	0.408	0.417	0.424	0.43	0.436	0.44	0.446	0.45	0.453	0.456	0.457	0.459	0.458	0.456	0.453	0.449	0.443	0.438	0.43	0.426	0.424	0.425	0.43	0.436	0.444	0.451	0.458	0.463
Western sub-Saharan Africa	0.293	0.296	0.299	0.303	0.306	0.309	0.313	0.316	0.32	0.324	0.328	0.333	0.337	0.343	0.35	0.357	0.365	0.373	0.381	0.389	0.396	0.403	0.411	0.417	0.424	0.43	0.437	0.441
Benin	0.218	0.223	0.228	0.233	0.238	0.243	0.248	0.253	0.258	0.263	0.268	0.273	0.278	0.284	0.29	0.296	0.301	0.307	0.313	0.32	0.326	0.332	0.338	0.345	0.353	0.36	0.367	0.373
Burkina Faso	0.139	0.143	0.147	0.151	0.154	0.158	0.163	0.168	0.173	0.179	0.184	0.19	0.195	0.201	0.208	0.214	0.22	0.226	0.232	0.237	0.242	0.248	0.253	0.259	0.266	0.272	0.279	0.284
Cameroon	0.332	0.338	0.343	0.347	0.35	0.352	0.355	0.357	0.359	0.36	0.361	0.361	0.361	0.362	0.366	0.373	0.38	0.388	0.396	0.405	0.414	0.423	0.433	0.443	0.453	0.464	0.474	0.482
Cape Verde	0.307	0.315	0.322	0.329	0.337	0.347	0.357	0.367	0.377	0.387	0.399	0.41	0.42	0.431	0.441	0.451	0.461	0.472	0.482	0.491	0.5	0.509	0.517	0.524	0.531	0.538	0.544	0.549
Chad	0.119	0.123	0.128	0.131	0.135	0.138	0.141	0.144	0.146	0.148	0.15	0.152	0.154	0.156	0.161	0.166	0.171	0.178	0.185	0.192	0.2	0.207	0.216	0.224	0.232	0.24	0.248	0.253
Cote d'Ivoire	0.273	0.28	0.287	0.292	0.297	0.301	0.306	0.312	0.317	0.322	0.326	0.33	0.334	0.338	0.342	0.346	0.349	0.353	0.357	0.361	0.367	0.371	0.377	0.384	0.391	0.398	0.406	0.412
The Gambia	0.245	0.251	0.257	0.263	0.269	0.275	0.282	0.288	0.294	0.3	0.307	0.314	0.319	0.325	0.332	0.338	0.343	0.349	0.355	0.361	0.367	0.372	0.378	0.384	0.389	0.395	0.401	0.405
Ghana	0.38	0.386	0.392	0.396	0.4	0.403	0.408	0.413	0.416	0.418	0.422	0.425	0.43	0.435	0.439	0.443	0.45	0.459	0.467	0.474	0.482	0.49	0.498	0.505	0.513	0.521	0.53	0.537
Guinea	0.164	0.168	0.171	0.175	0.178	0.183	0.188	0.194	0.2	0.206	0.211	0.217	0.222	0.228	0.234	0.24	0.246	0.252	0.26	0.266	0.273	0.281	0.288	0.295	0.303	0.31	0.318	0.325
Guinea-Bissau	0.183	0.189	0.194	0.199	0.205	0.211	0.217	0.224	0.229	0.235	0.241	0.246	0.252	0.257	0.263	0.268	0.274	0.28	0.286	0.293	0.3	0.308	0.315	0.322	0.329	0.336	0.343	0.349
Liberia	0.207	0.209	0.21	0.206	0.199	0.19	0.178	0.174	0.175	0.183	0.198	0.214	0.228	0.234	0.239	0.245	0.251	0.257	0.264	0.27	0.276	0.284	0.292	0.301	0.31	0.318	0.324	0.328
Mali	0.13	0.134	0.137	0.141	0.145	0.148	0.152	0.157	0.161	0.166	0.171	0.176	0.181	0.187	0.192	0.198	0.203	0.209	0.215	0.22	0.226	0.232	0.238	0.243	0.249	0.255	0.262	0.267
Mauritania	0.299	0.307	0.314	0.321	0.328	0.335	0.341	0.346	0.351	0.356	0.359	0.363	0.367	0.371	0.377	0.382	0.39	0.398	0.406	0.412	0.42	0.427	0.435	0.442	0.45	0.458	0.465	0.471
Niger	0.092	0.097	0.101	0.105	0.108	0.111	0.113	0.115	0.118	0.121	0.123	0.125	0.127	0.13	0.134	0.137	0.14	0.144	0.147	0.151	0.156	0.16	0.165	0.17	0.176	0.181	0.186	0.191
Nigeria	0.344	0.346	0.347	0.349	0.352	0.355	0.358	0.362	0.365	0.369	0.374	0.379	0.385	0.391	0.4	0.41	0.419	0.429	0.437	0.445	0.452	0.459	0.466	0.473	0.478	0.484	0.49	0.493
Sao Tome and Principe	0.287	0.292	0.296	0.3	0.305	0.309	0.314	0.32	0.326	0.332	0.338	0.345	0.353	0.362	0.371	0.381	0.39	0.399	0.408	0.416	0.426	0.435	0.444	0.453	0.463	0.472	0.481	0.488
Senegal	0.244	0.25	0.257	0.262	0.267	0.272	0.277	0.281	0.286	0.291	0.295	0.299	0.303	0.307	0.311	0.316	0.319	0.324	0.328	0.333	0.338	0.342	0.347	0.352	0.357	0.362	0.368	0.373
Sierra Leone	0.202	0.205	0.207	0.211	0.216	0.219	0.222	0.225	0.226	0.228	0.23	0.233	0.238	0.244	0.249	0.256	0.262	0.27	0.278	0.286	0.294	0.302	0.312	0.325	0.336	0.344	0.351	0.357
Togo	0.263	0.27	0.276	0.281	0.286	0.292	0.297	0.303	0.307	0.311	0.315	0.318	0.321	0.325	0.329	0.333	0.337	0.343	0.348	0.353	0.36	0.366	0.373	0.381	0.39	0.398	0.406	0.413

Appendix Table 11. Correlations of cumulative total fertility up to maternal age 25 (TFU25), cumulative total fertility from maternal age 30 to age 55 (TFO30), and age-specific fertility, for each Socio-demographic Index (SDI) quintile for under 5 mortality, demand for contraception satisfied with modern methods, and female education for 2017.

	Low SDI			Low-middle SDI			Middle SDI			High-middle SDI			High SDI		
	Under 5 mortality	Contraception	Education	Under 5 mortality	Contraception	Education	Under 5 mortality	Contraception	Education	Under 5 mortality	Contraception	Education	Under 5 mortality	Contraception	Education
TFR	0.66	-0.76	-0.72	0.68	-0.80	-0.73	0.77	-0.71	-0.83	0.64	-0.27	-0.79	0.72	0.05	-0.70
TFU25	0.68	-0.61	-0.76	0.65	-0.62	-0.65	0.73	-0.59	-0.77	0.61	-0.41	-0.76	0.65	-0.45	-0.65
TFO30	0.53	-0.76	-0.53	0.64	-0.84	-0.72	0.73	-0.62	-0.78	0.60	-0.13	-0.73	0.55	0.43	-0.49
ASFR 10-14	0.35	NA	-0.32	0.18	NA	-0.25	0.10	NA	-0.18	-0.02	NA	-0.07	0.13	NA	-0.17
ASFR 15-19	0.64	-0.52	-0.67	0.57	-0.46	-0.54	0.55	-0.26	-0.56	0.45	-0.19	-0.54	0.43	-0.36	-0.25
ASFR 20-24	0.66	-0.63	-0.78	0.64	-0.64	-0.65	0.75	-0.66	-0.79	0.65	-0.46	-0.82	0.69	-0.44	-0.76
ASFR 25-29	0.60	-0.70	-0.67	0.66	-0.74	-0.66	0.74	-0.71	-0.76	0.62	-0.30	-0.76	0.65	0.02	-0.72
ASFR 30-34	0.57	-0.77	-0.60	0.64	-0.82	-0.69	0.73	-0.65	-0.78	0.58	-0.14	-0.72	0.48	0.42	-0.47
ASFR 35-39	0.52	-0.75	-0.52	0.63	-0.83	-0.70	0.72	-0.59	-0.78	0.58	-0.12	-0.73	0.56	0.35	-0.50
ASFR 40-44	0.45	-0.72	-0.38	0.59	-0.83	-0.69	0.70	-0.60	-0.73	0.64	-0.19	-0.76	0.66	0.20	-0.59
ASFR 45-49	0.20	-0.51	-0.28	0.56	-0.71	-0.70	0.64	-0.50	-0.70	0.60	-0.26	-0.71	0.63	0.18	-0.53
ASFR 50-54	0.20	NA	-0.30	0.56	NA	-0.70	0.64	NA	-0.69	0.60	NA	-0.68	0.63	NA	-0.51
