# Supplementary material for: Population and fertility by age and sex for 195 countries and territories, 1950–2017: a systematic analysis for the Global Burden of Disease Study 2017
Source: Lancet. 2018 Nov 10;392(10159):1995–2051. doi: 10.1016/S0140-6736(18)32278-5 (PMC6227915; doi:10.1016/S0140-6736(18)32278-5)
Supplement: Supplementary appendix 2 [file mmc2.pdf]

THE LANCET

Supplementary appendix 2

This appendix formed part of the original submission and has been peer reviewed. We post it as supplied by the authors.

Supplement to: GBD 2017 Population and Fertility Collaborators. Population and fertility by age and sex for 195 countries and territories, 1950–2017: a systematic analysis for the Global Burden of Disease Study 2017. *Lancet* 2018; **392:** 1995–2051.

Supplementary results appendix for Population and fertility by age and sex for 195 countries and territories 1950-2017: a systematic analysis for the Global Burden of Disease 2017

This supplement provides additional figures and more detailed results for population and fertility.

Complete results from the Global Burden of Disease Study (GBD 2017) are now measured in terabytes. These results are available in an interactive data downloading tool in the Global Health Data Exchange (GHDx). The tool contains the complete set of results from all GBD 2017 papers; however, specialised tables from the papers will be available as separate entries in the GHDx. Access to the results and tables through the GHDx are available at http://ghdx.healthdata.org/gbd−2017.

Table of Contents

Supplemental Results Figure 1: Percent difference between GBD 2017 population estimates and UN WPP all-age population estimates, by location, by sex, 1950, 1975, 2000, 2015............. 3

Supplemental Results Figure 2: Percent difference between GBD 2017 population estimates and UN WPP estimates, by location, ages 5-9, 45-49, 70-74, by sex, 2017.................................... 4

Supplemental Results Figure 3: Age-specific fertility fitted trend lines by location, 1950-2017 5

Supplemental Results Figure 4: GBD Bayesian Demographic Balancing Model fit by location for (a) total population of males, and (b) total population of females, 1950−2017...................... 1016

Supplemental Results Figure 5: Population pyramids for females and males, by age and by location, for 1950, 1975, 2000, and 2017.. 1216

Supplemental Results Figure 6: Global net migrants.. 1412

Supplemental Results Table 1: Correlation of age-specific fertility rates across 195 countries or territories by age of mother, 2017.. 1413

Supplemental Results Table 2: Live births (in thousands) by location, both sexes combined, 1950, 1960, 1970, 1980, 1990, 2000, 2010, 2017.. 1414

Supplemental Results Table 3: Live births (in thousands) by location, males, 1950, 1960, 1970, 1980, 1990, 2000, 2010, 2017.. 1419

Supplemental Results Table 4: Live births (in thousands) by location, females, 1950, 1960, 1970, 1980, 1990, 2000, 2010, 2017... 1425

Supplemental Results Table 5: Age-specific fertility rates per 1000, cumulative total fertility up to maternal age 25, cumulative total fertility from maternal age 30 to age 55, and cumulative total fertility, by location, 1950-2017... 1413

Supplemental Results Table 6: Population (in thousands) by location, both sexes combined, 1950, 1960, 1970, 1980, 1990, 2000, 2010, 2017.. 2060

Supplemental Results Table 7: Population (in thousands) by location, males, 1950, 1960, 1970, 1980, 1990, 2000, 2010, 2017.. 2069

Supplemental Results Table 8: Population (in thousands) by location, females, 1950, 1960, 1970, 1980, 1990, 2000, 2010, 2017... 2074

Supplemental Results Table 9: Under-5 populations (in thousands) by location, both sexes combined, 1950, 1960, 1970, 1980, 1990, 2000, 2010, 2017.. 2083

Supplemental Results Table 10: Under-5 populations (in thousands) by location, males, 1950, 1960, 1970, 1980, 1990, 2000, 2010, 2017... 2088

Supplemental Results Table 11: Under-5 populations (in thousands) by location, females, 1950, 1960, 1970, 1980, 1990, 2000, 2010, 2017.. 2098

Supplemental Results Figure 1: Percent difference between GBD 2017 population estimates and UN WPP all−age population estimates, by location, by sex, 1950, 1975, 2000, 2015. Kuwait is not included in figure.

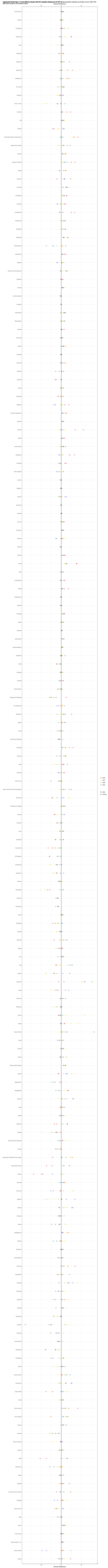

Supplemental Results Figure 2: Percent difference between GBD 2017 population estimates and UN WPP estimates, by location, ages 5−9, 45−49, 70−74, by sex, 2017.

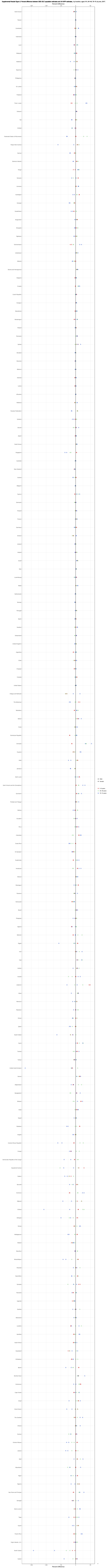

Supplemental Results Figure 3: Age-specific fertility and total fertility rate fitted trend lines by location, 1950-2017

Shaded areas are uncertainty intervals. Symbols with faded colors are unadjusted data while those which are not faded are adjusted.

CEB=Children ever born. CBH=Complete birth history. DHS=Demographic and Health Survey. GBD=Global Burden of Disease. MICS=Multiple Indicator Cluster Survey. SBH=Sibling Birth History. UN WPP=United Nations World Population Prospects. VR=Vital registration

Age−specific fertility fitted trend lines, 1950−2017: Afghanistan, TFR
Data points enclosed with a diamond are reference sources

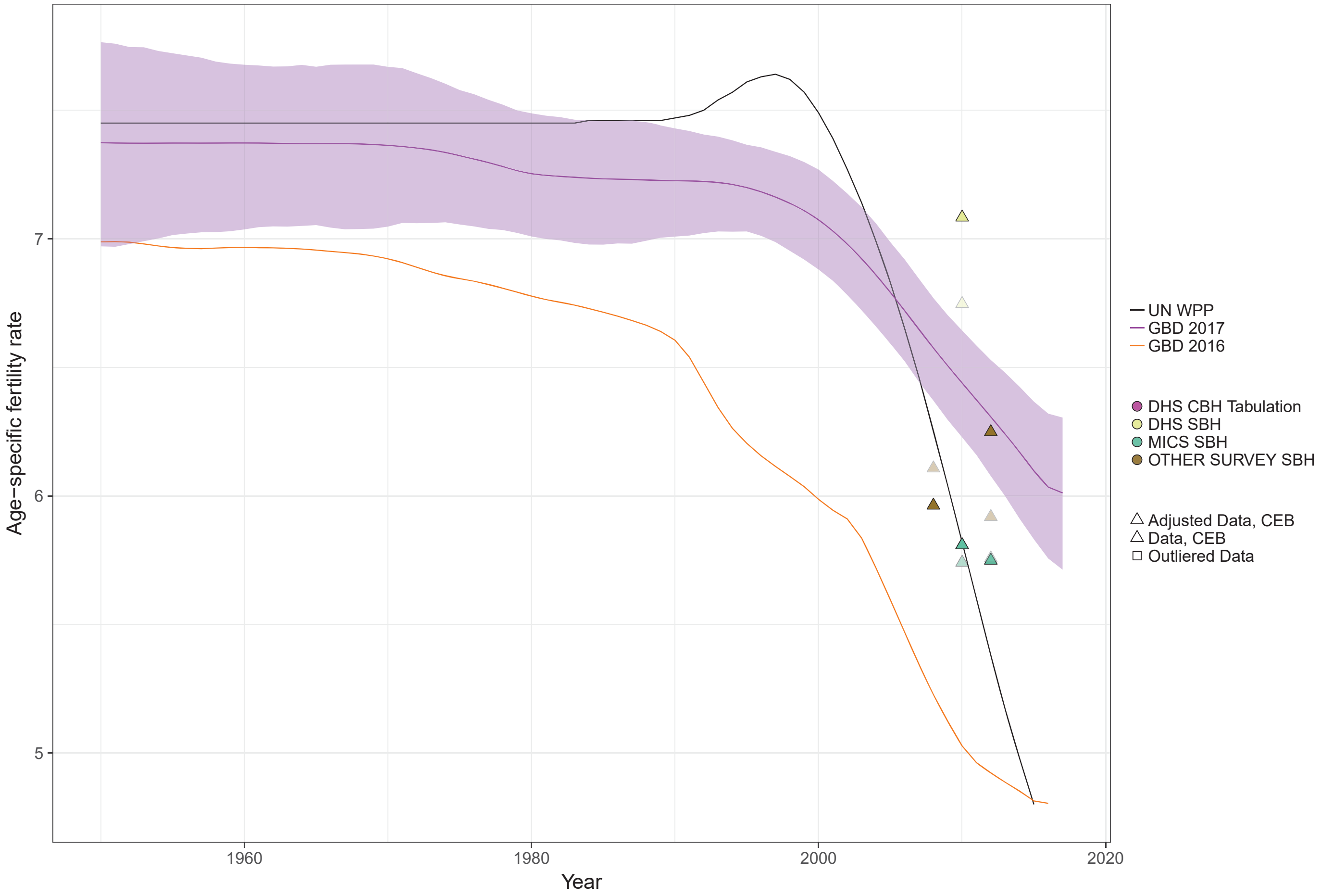

Age−specific fertility fitted trend lines, 1950−2017: Afghanistan, Ages: 10−14
Data points enclosed with a diamond are reference sources

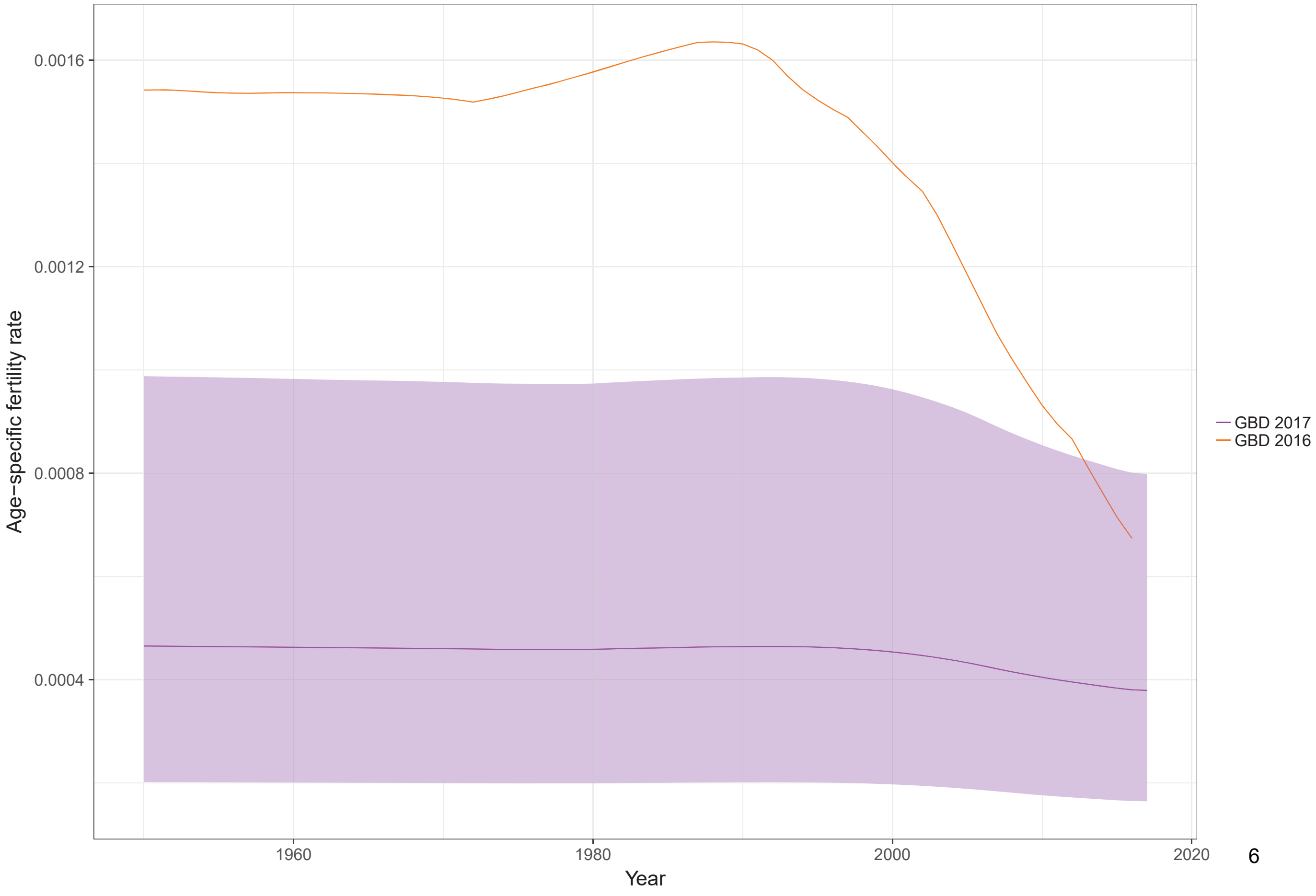

Age−specific fertility fitted trend lines, 1950−2017: Afghanistan, Ages: 15−19
Data points enclosed with a diamond are reference sources

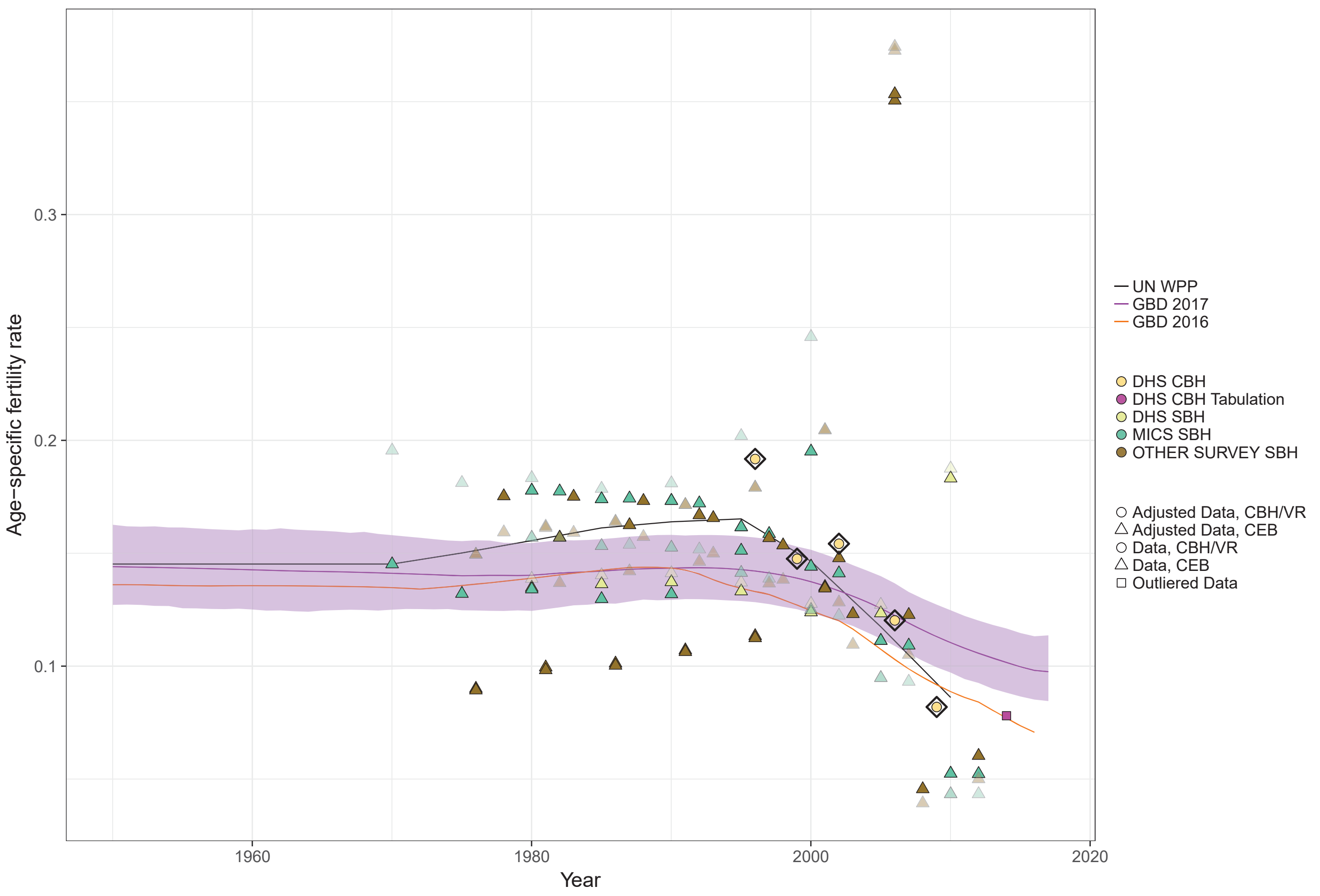

Age−specific fertility fitted trend lines, 1950−2017: Afghanistan, Ages: 20−24
Data points enclosed with a diamond are reference sources

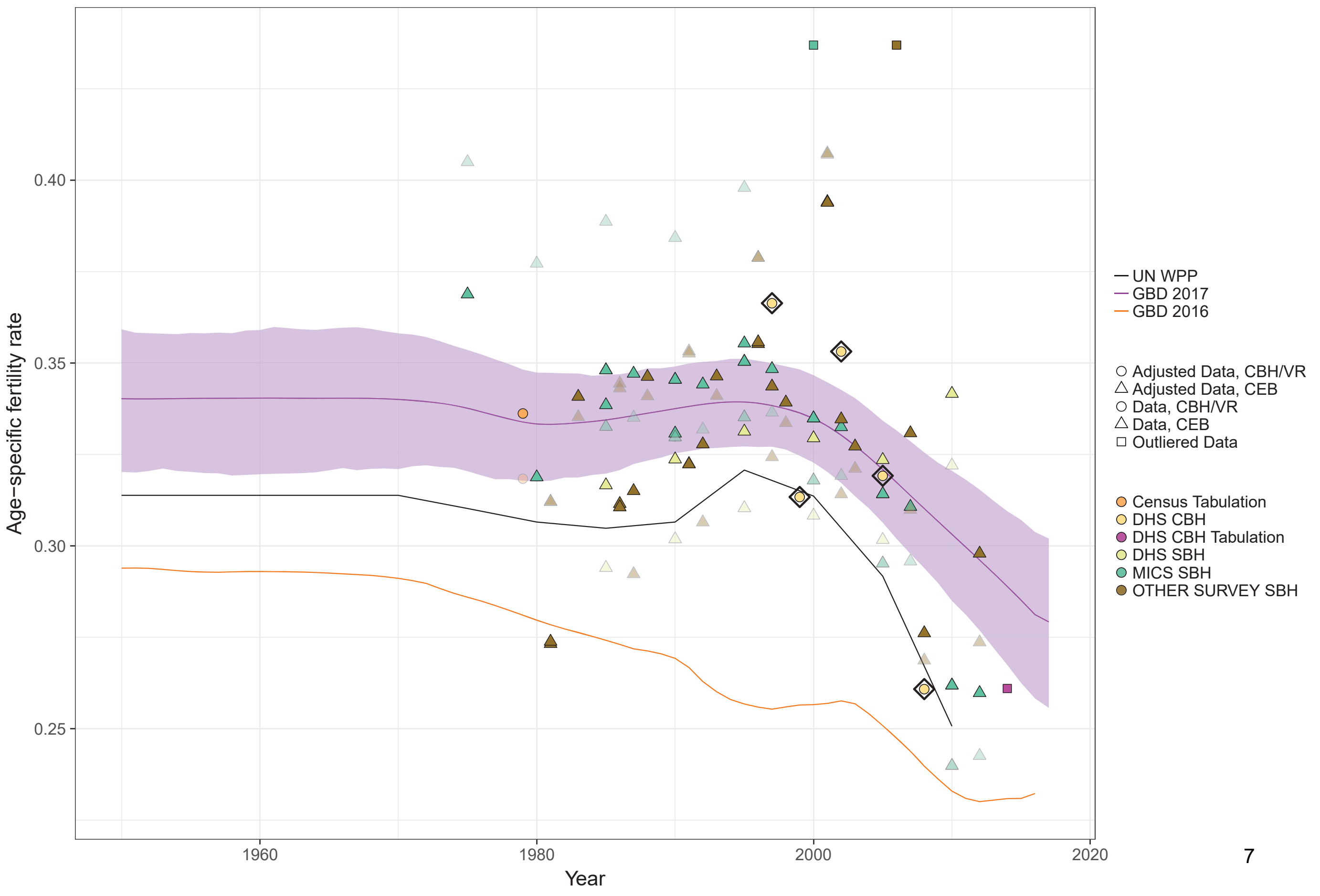

Age−specific fertility fitted trend lines, 1950−2017: Afghanistan, Ages: 25−29
Data points enclosed with a diamond are reference sources

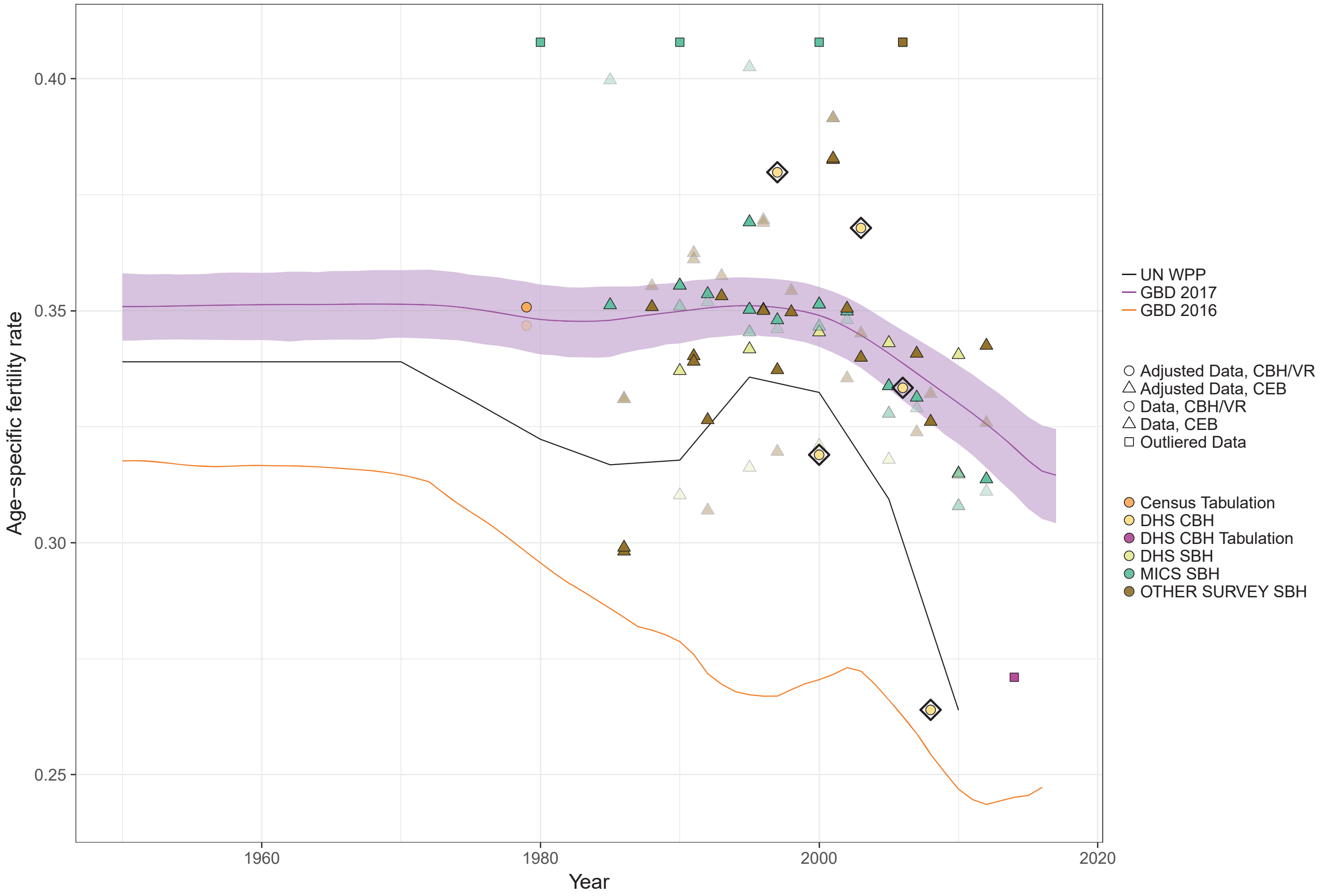

Age−specific fertility fitted trend lines, 1950−2017: Afghanistan, Ages: 30−34
Data points enclosed with a diamond are reference sources

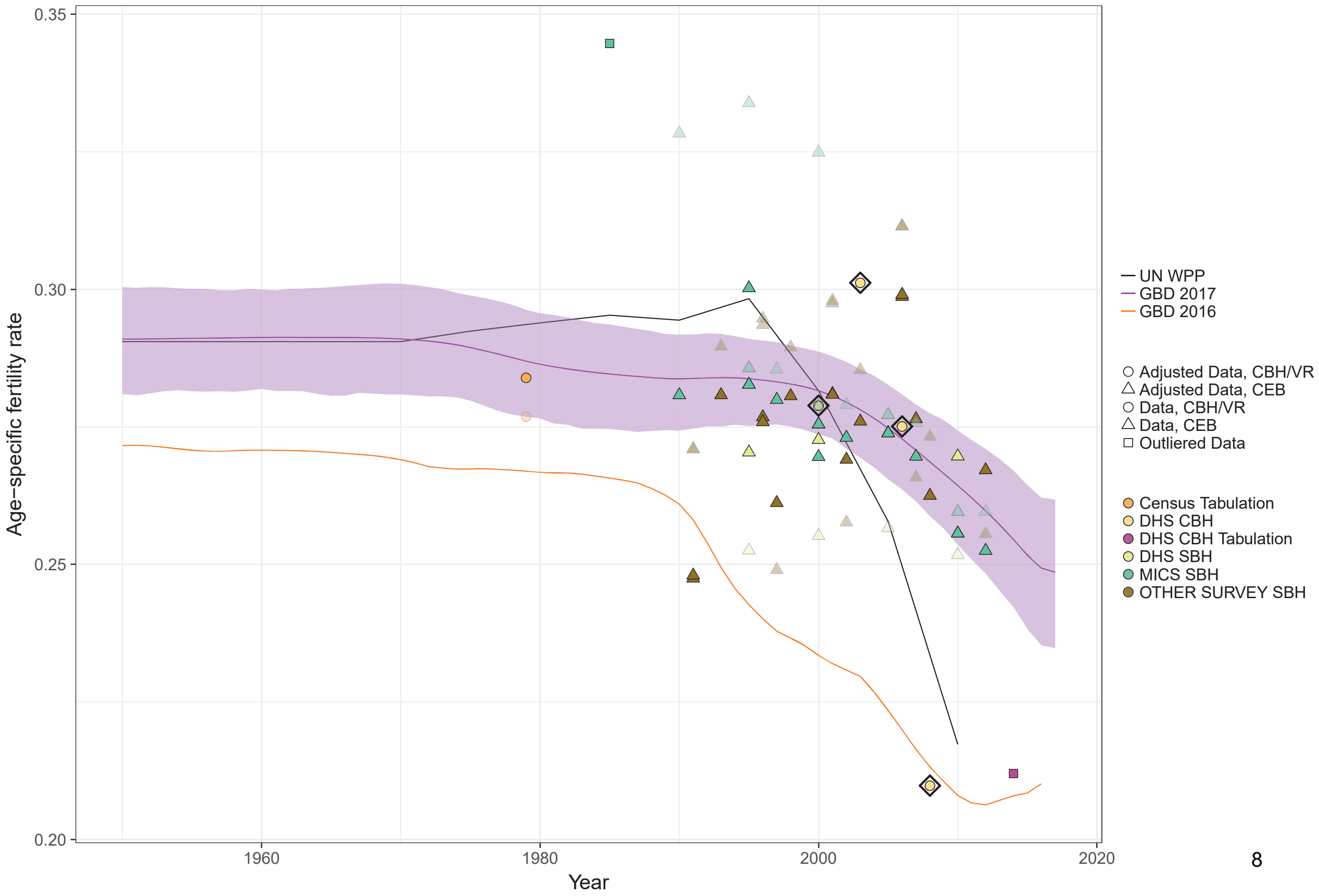

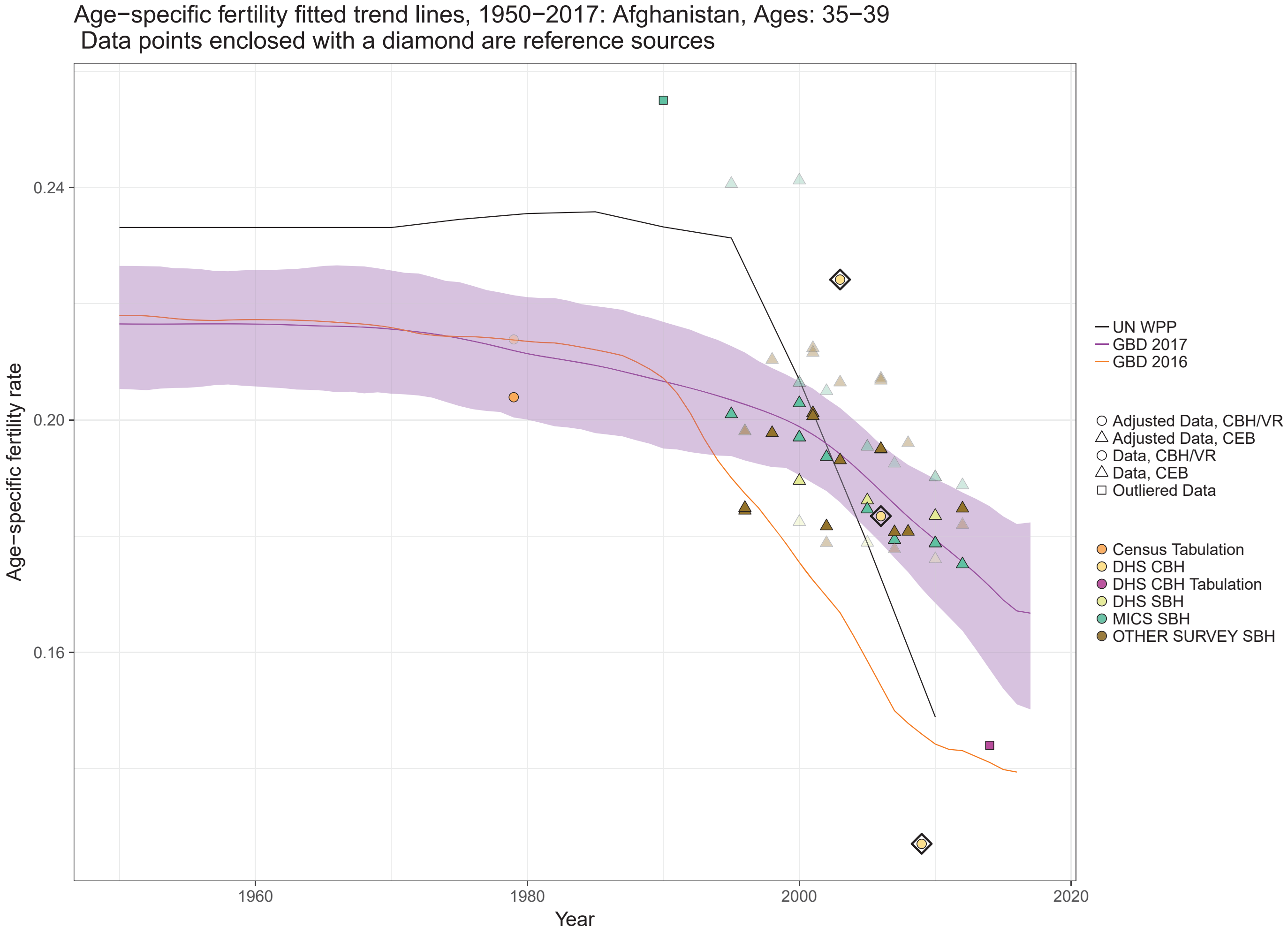
Age−specific fertility fitted trend lines, 1950−2017: Afghanistan, Ages: 35−39
Data points enclosed with a diamond are reference sources
Age−specific fertility rate
0.24
0.20
0.16
1960
1980
2000
2020
Year
UN WPP
GBD 2017
GBD 2016
Adjusted Data, CBH/VR
Adjusted Data, CEB
Data, CBH/VR
Data, CEB
Outliered Data
Census Tabulation
DHS CBH
DHS CBH Tabulation
DHS SBH
MICS SBH
OTHER SURVEY SBH

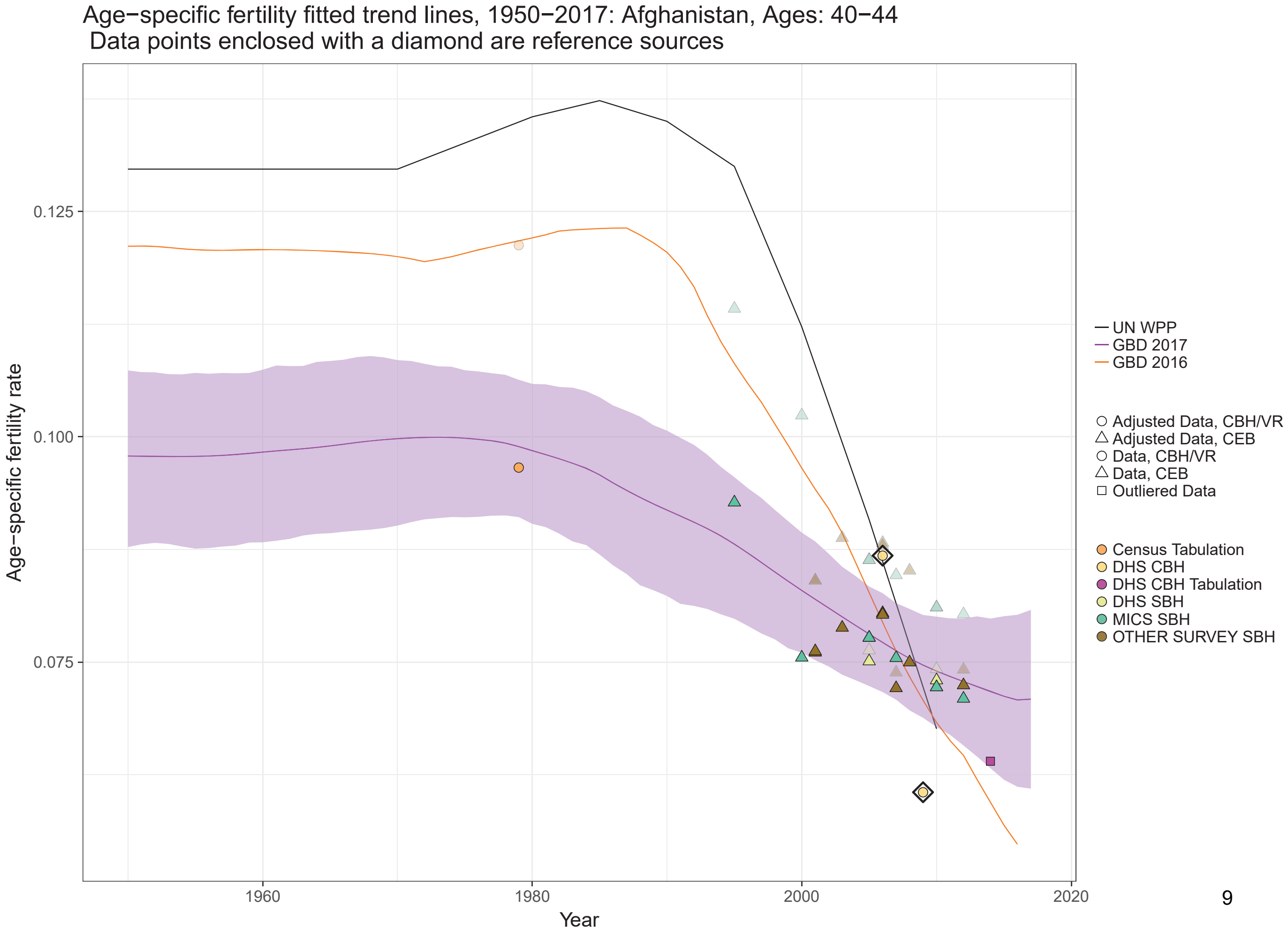
Age−specific fertility fitted trend lines, 1950−2017: Afghanistan, Ages: 40−44
Data points enclosed with a diamond are reference sources
Age−specific fertility rate
0.125
0.100
0.075
1960
1980
2000
2020
Year
UN WPP
GBD 2017
GBD 2016
Adjusted Data, CBH/VR
Adjusted Data, CEB
Data, CBH/VR
Data, CEB
Outliered Data
Census Tabulation
DHS CBH
DHS CBH Tabulation
DHS SBH
MICS SBH
OTHER SURVEY SBH

Age−specific fertility fitted trend lines, 1950−2017: Afghanistan, Ages: 45−49
Data points enclosed with a diamond are reference sources

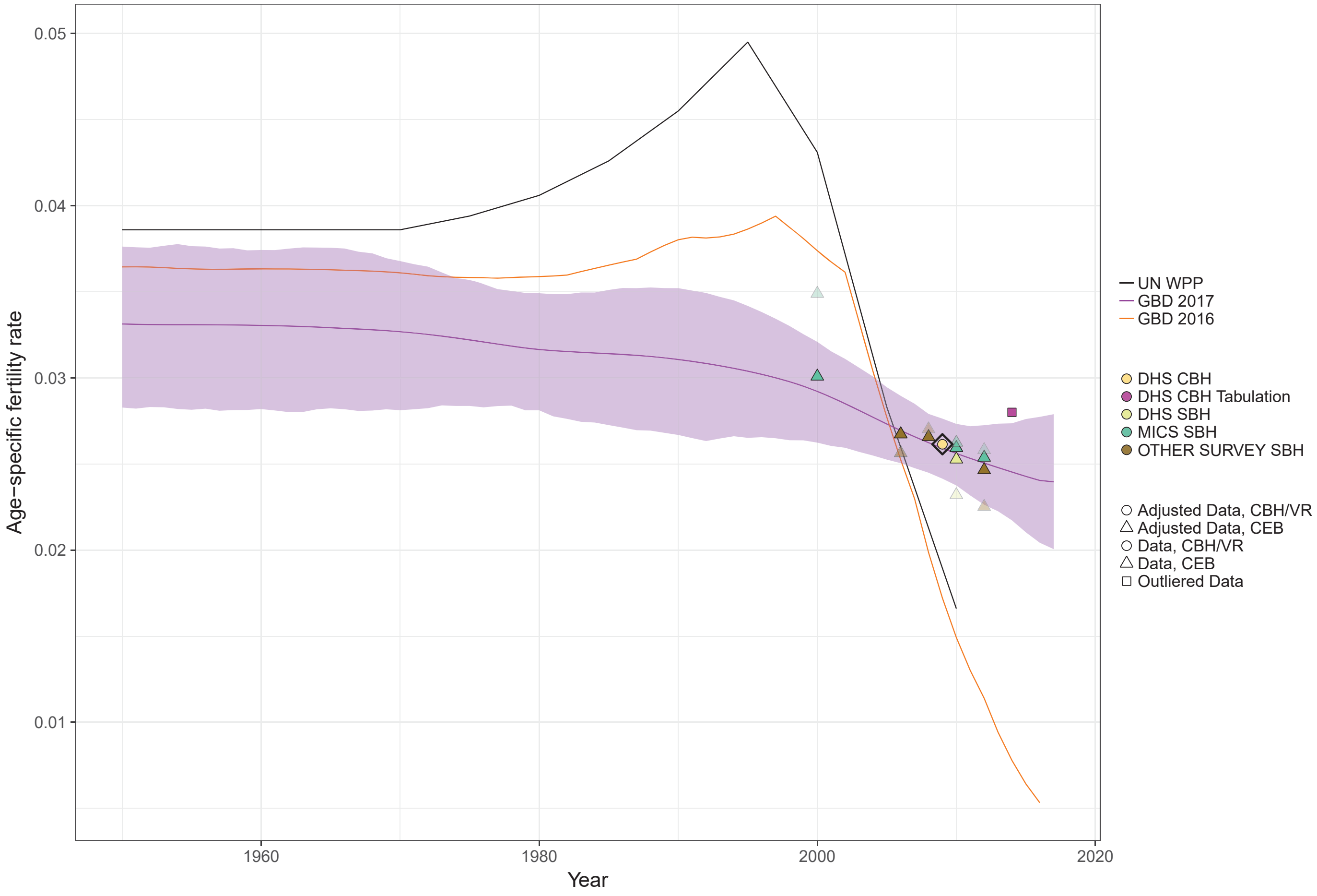

Age−specific fertility fitted trend lines, 1950−2017: Afghanistan, Ages: 50−54
Data points enclosed with a diamond are reference sources

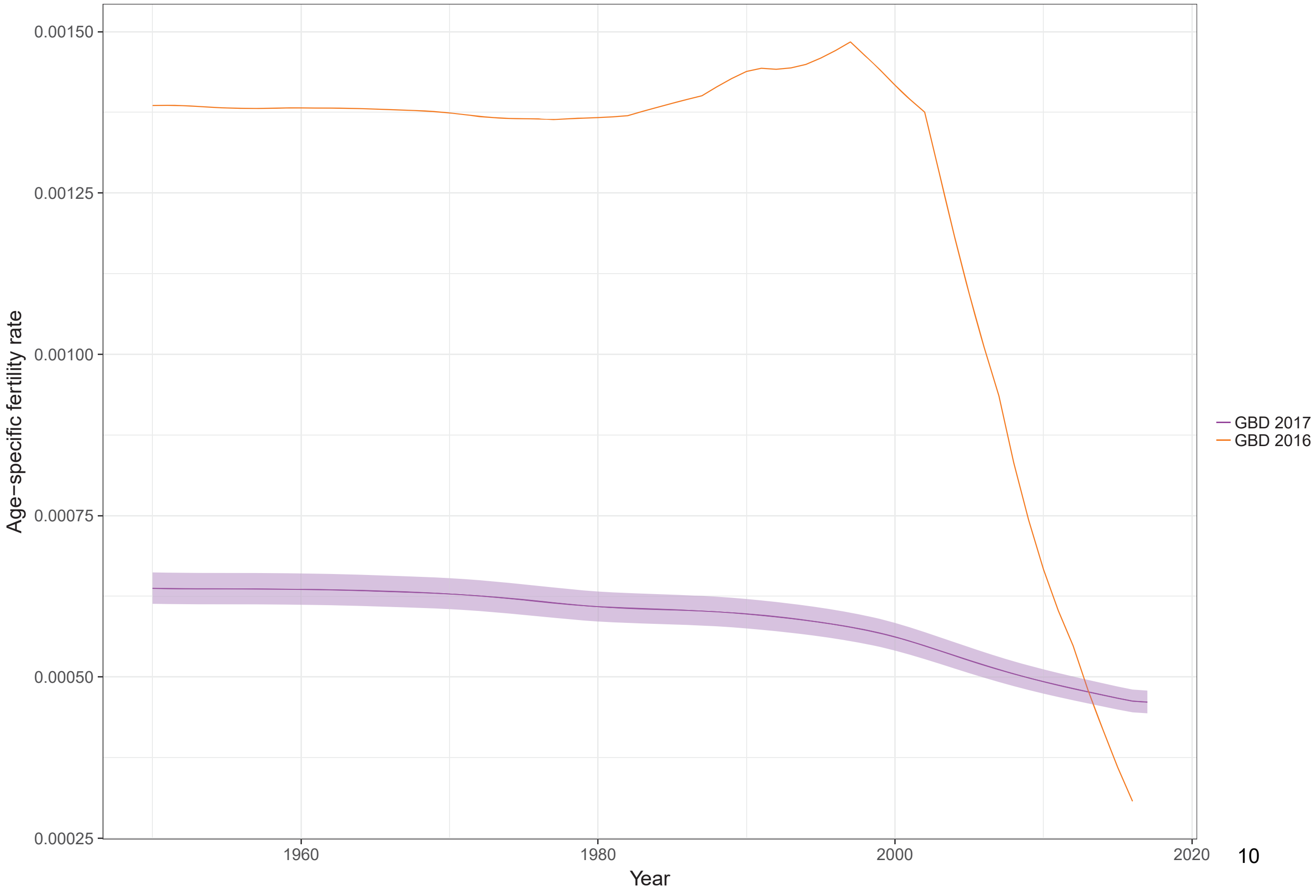

Age−specific fertility fitted trend lines, 1950−2017: Albania, TFR
Data points enclosed with a diamond are reference sources

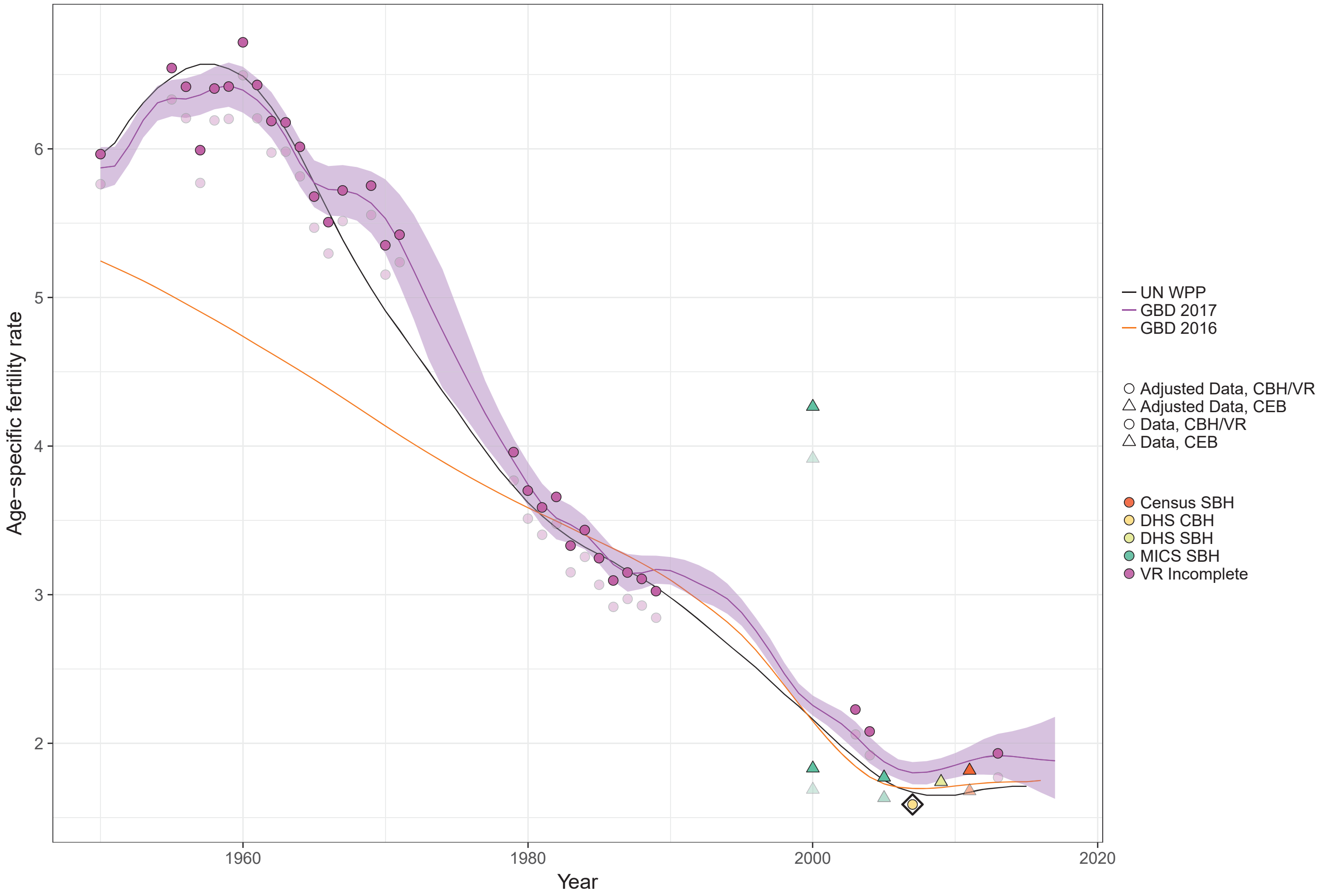

Age−specific fertility fitted trend lines, 1950−2017: Albania, Ages: 10−14
Data points enclosed with a diamond are reference sources

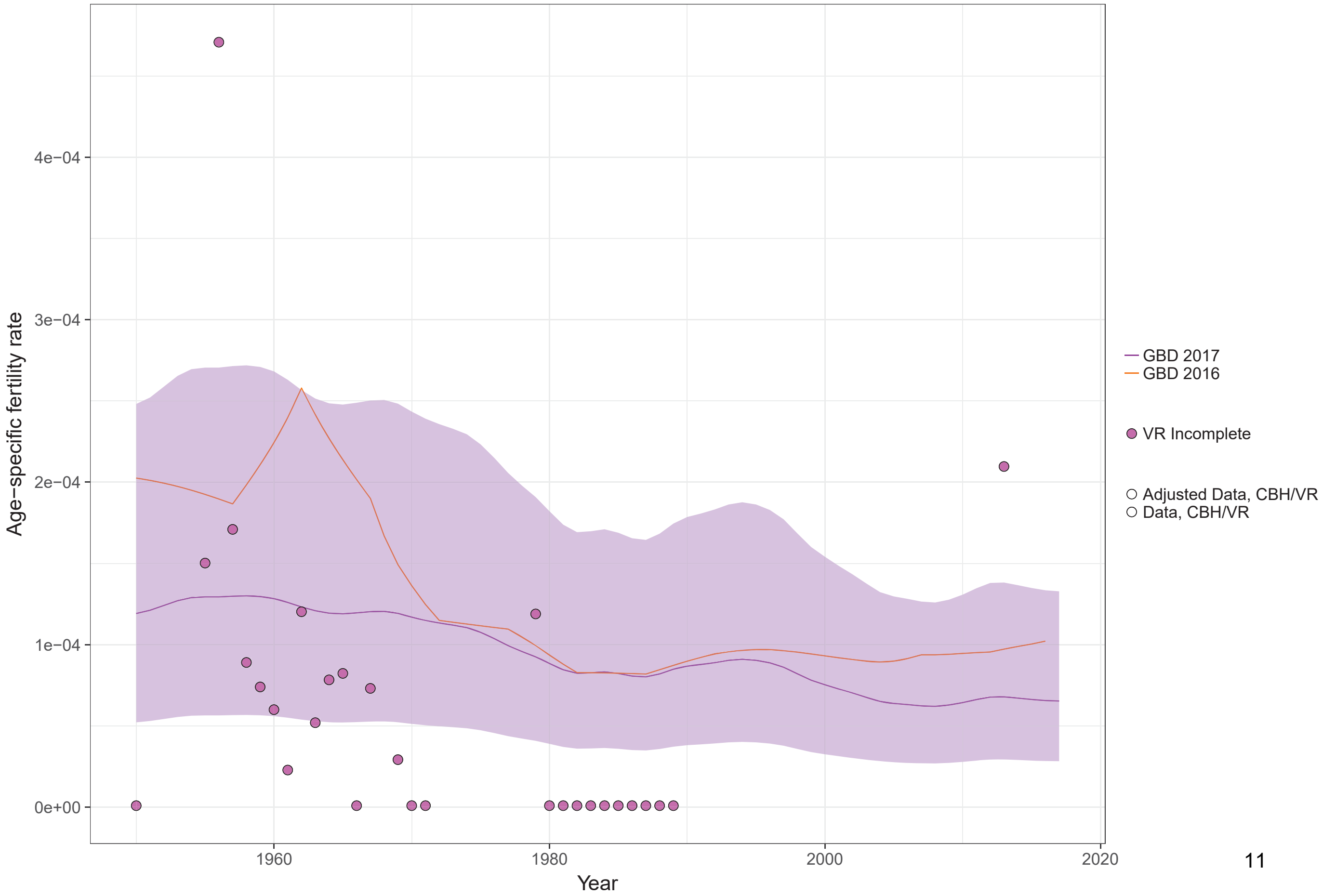

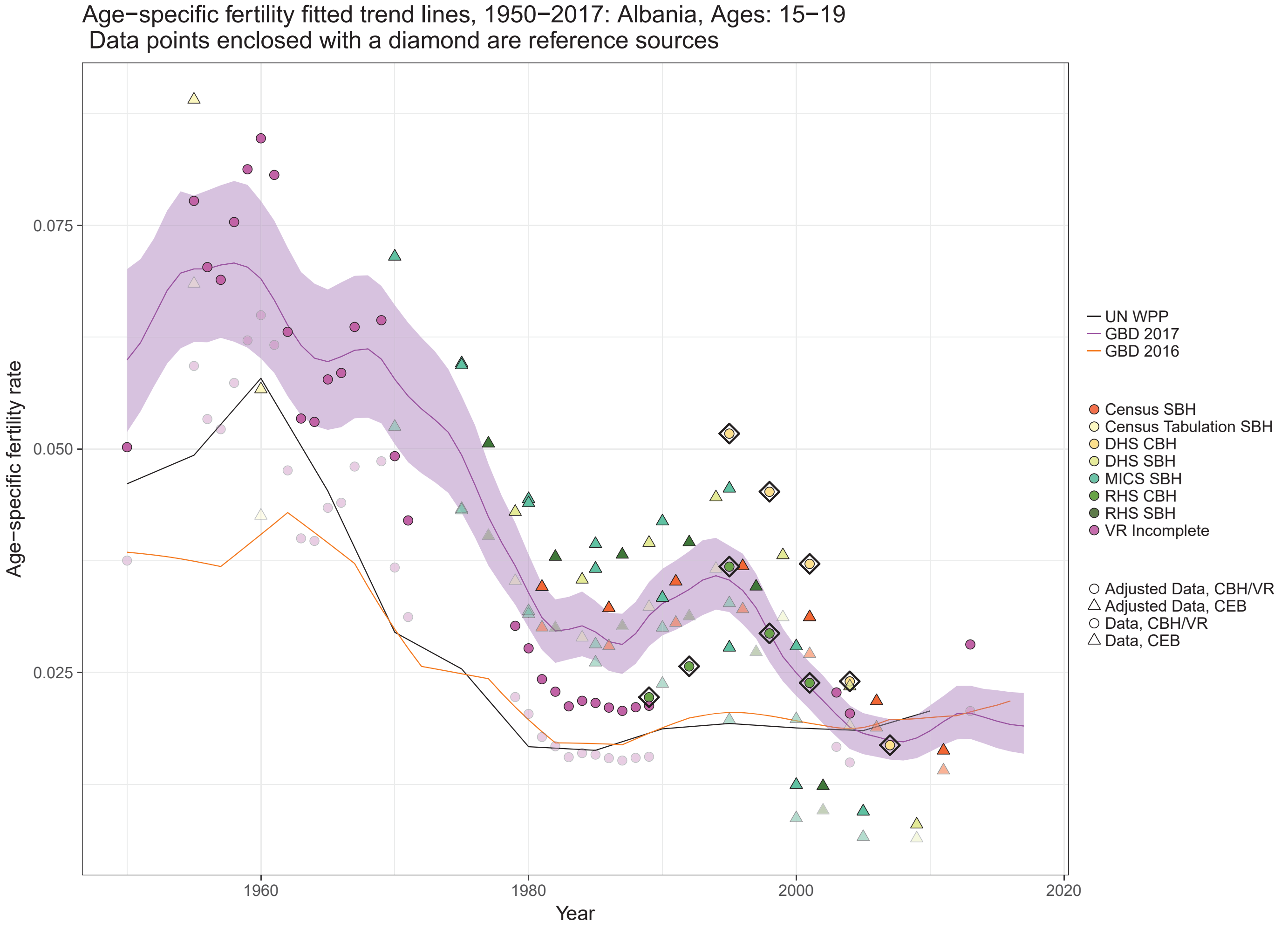

Age−specific fertility fitted trend lines, 1950−2017: Albania, Ages: 15−19
Data points enclosed with a diamond are reference sources
Age−specific fertility rate
0.025
0.050
0.075
1960
1980
2000
2020
Year
UN WPP
GBD 2017
GBD 2016
Census SBH
Census Tabulation SBH
DHS CBH
DHS SBH
MICS SBH
RHS CBH
RHS SBH
VR Incomplete
Adjusted Data, CBH/VR
Adjusted Data, CEB
Data, CBH/VR
Data, CEB

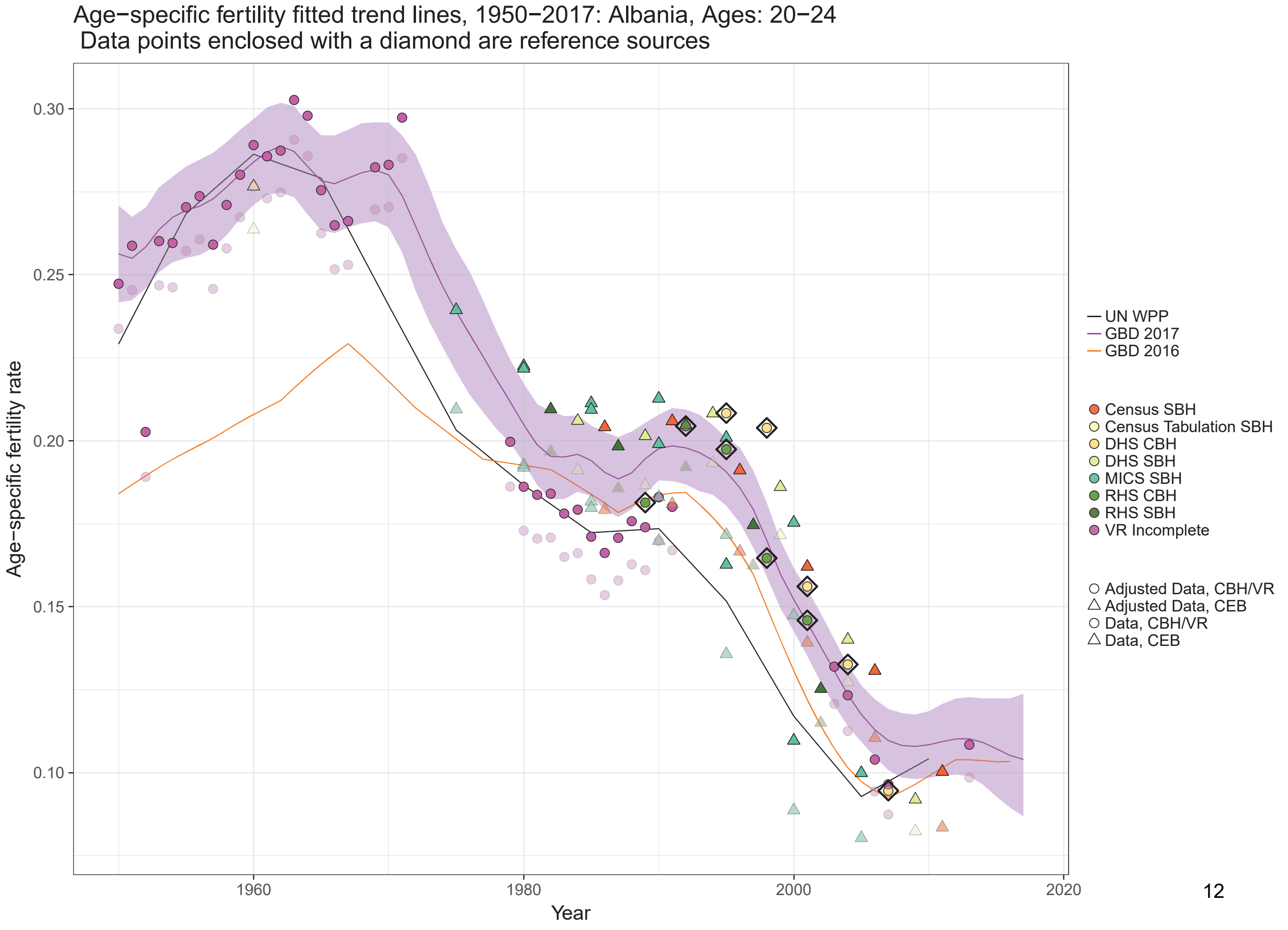

Age−specific fertility fitted trend lines, 1950−2017: Albania, Ages: 20−24
Data points enclosed with a diamond are reference sources
Age−specific fertility rate
0.10
0.15
0.20
0.25
0.30
1960
1980
2000
2020
Year
UN WPP
GBD 2017
GBD 2016
Census SBH
Census Tabulation SBH
DHS CBH
DHS SBH
MICS SBH
RHS CBH
RHS SBH
VR Incomplete
Adjusted Data, CBH/VR
Adjusted Data, CEB
Data, CBH/VR
Data, CEB

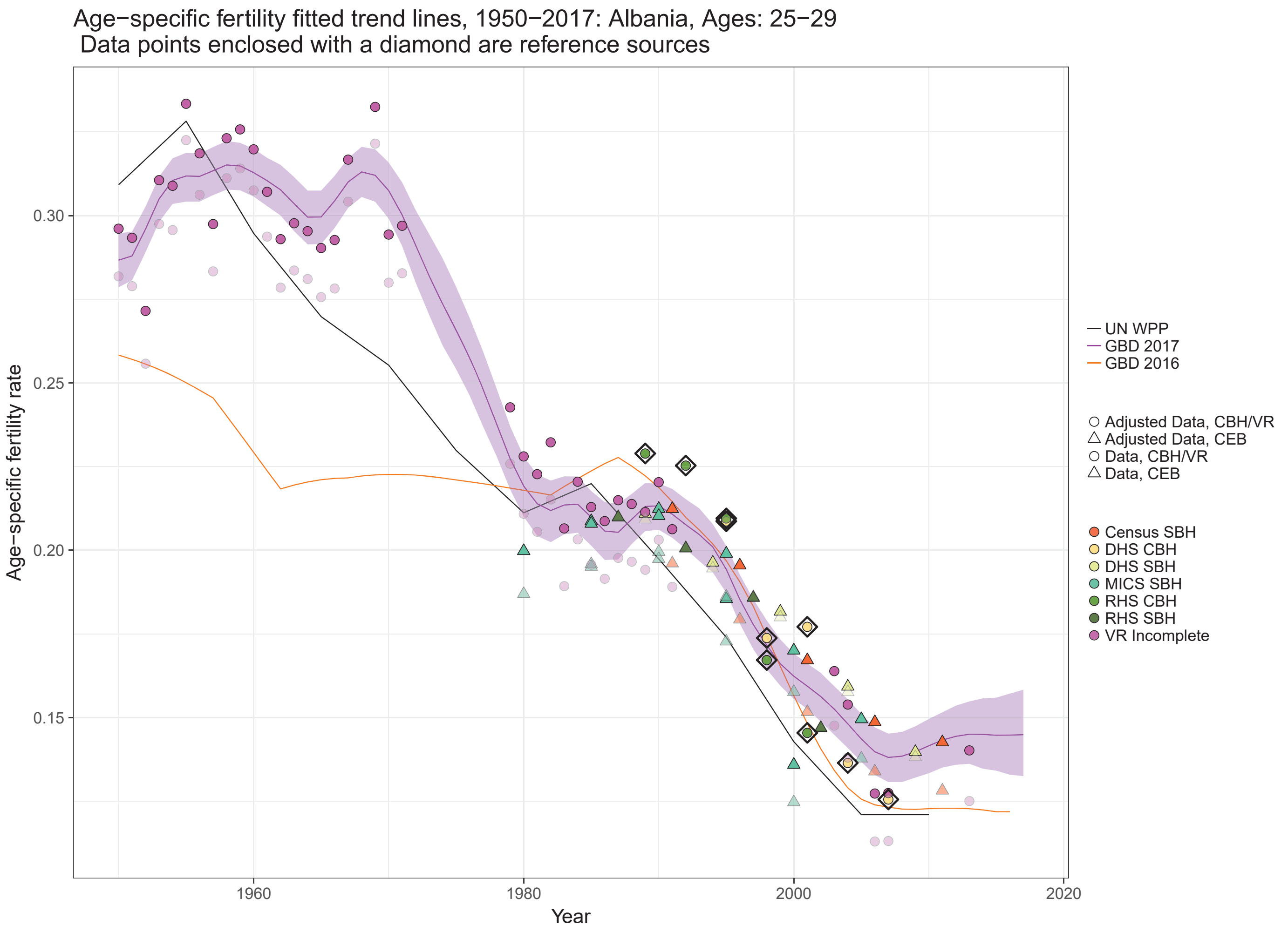

Age−specific fertility fitted trend lines, 1950−2017: Albania, Ages: 25−29
Data points enclosed with a diamond are reference sources
Age−specific fertility rate
0.30
0.25
0.20
0.15
1960
1980
2000
2020
Year
UN WPP
GBD 2017
GBD 2016
Adjusted Data, CBH/VR
Adjusted Data, CEB
Data, CBH/VR
Data, CEB
Census SBH
DHS CBH
DHS SBH
MICS SBH
RHS CBH
RHS SBH
VR Incomplete

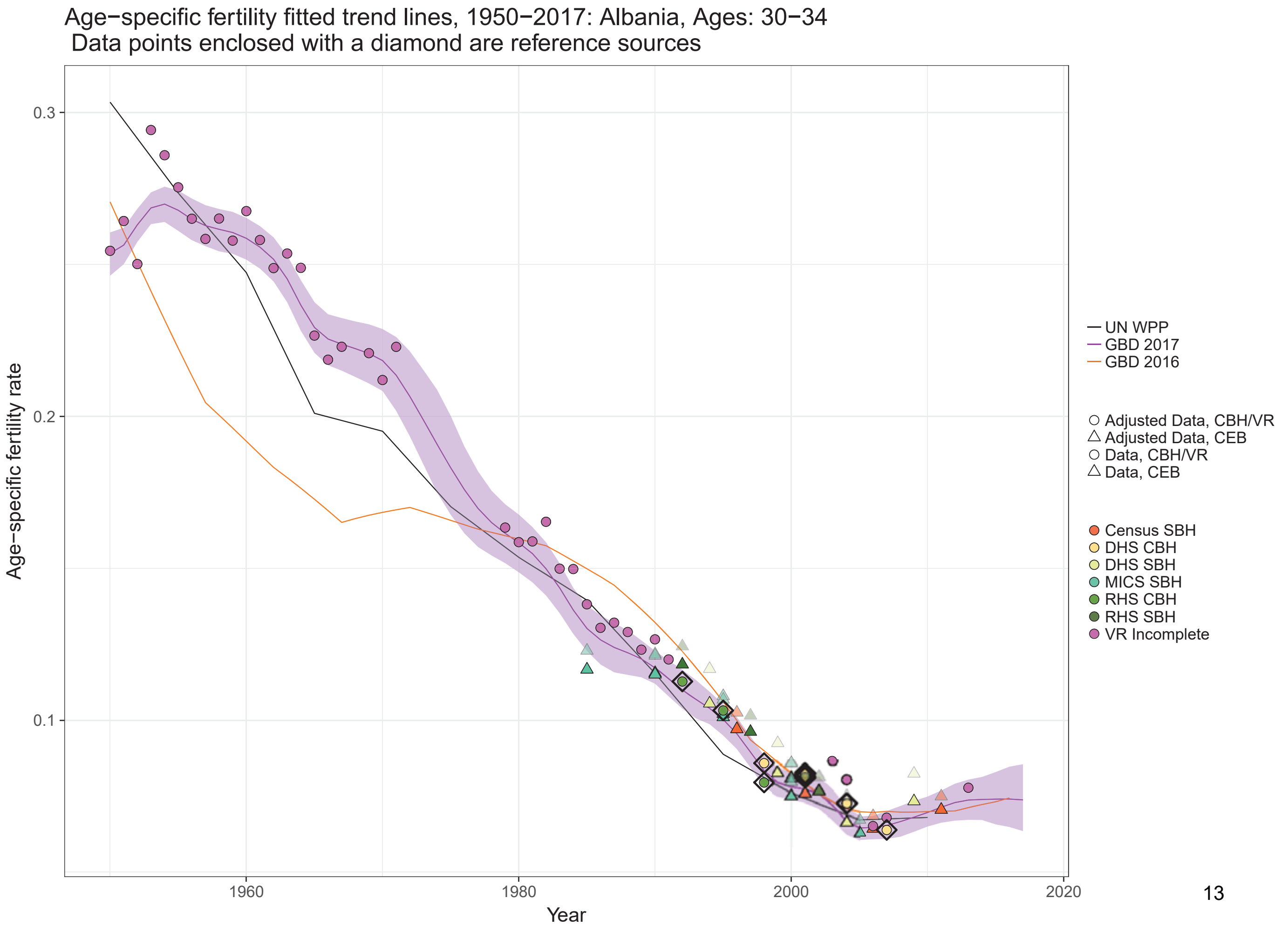

Age−specific fertility fitted trend lines, 1950−2017: Albania, Ages: 30−34
Data points enclosed with a diamond are reference sources
Age−specific fertility rate
0.3
0.2
0.1
1960
1980
2000
2020
Year
UN WPP
GBD 2017
GBD 2016
Adjusted Data, CBH/VR
Adjusted Data, CEB
Data, CBH/VR
Data, CEB
Census SBH
DHS CBH
DHS SBH
MICS SBH
RHS CBH
RHS SBH
VR Incomplete

Age−specific fertility fitted trend lines, 1950−2017: Albania, Ages: 35−39
Data points enclosed with a diamond are reference sources

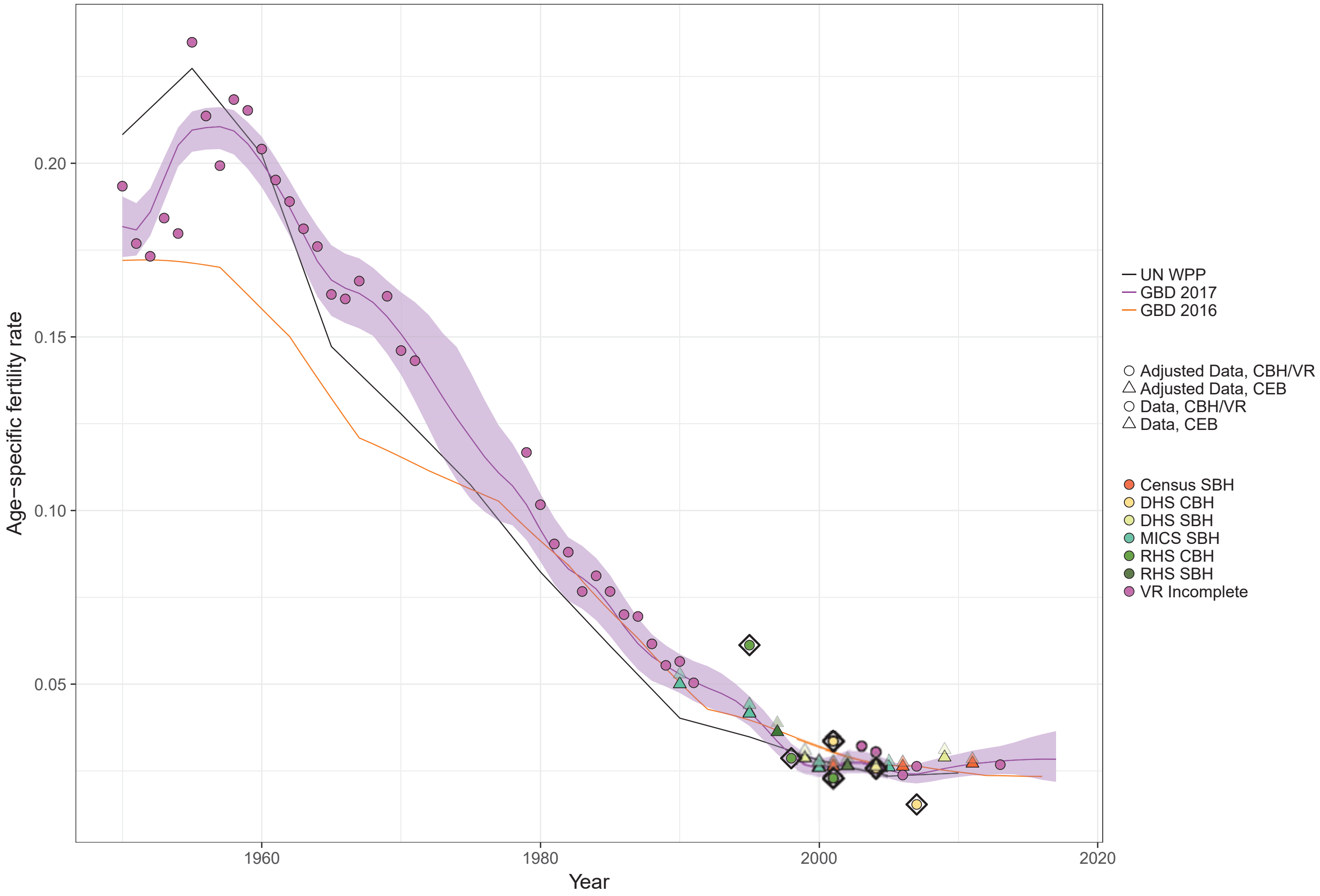

Age−specific fertility fitted trend lines, 1950−2017: Albania, Ages: 40−44
Data points enclosed with a diamond are reference sources

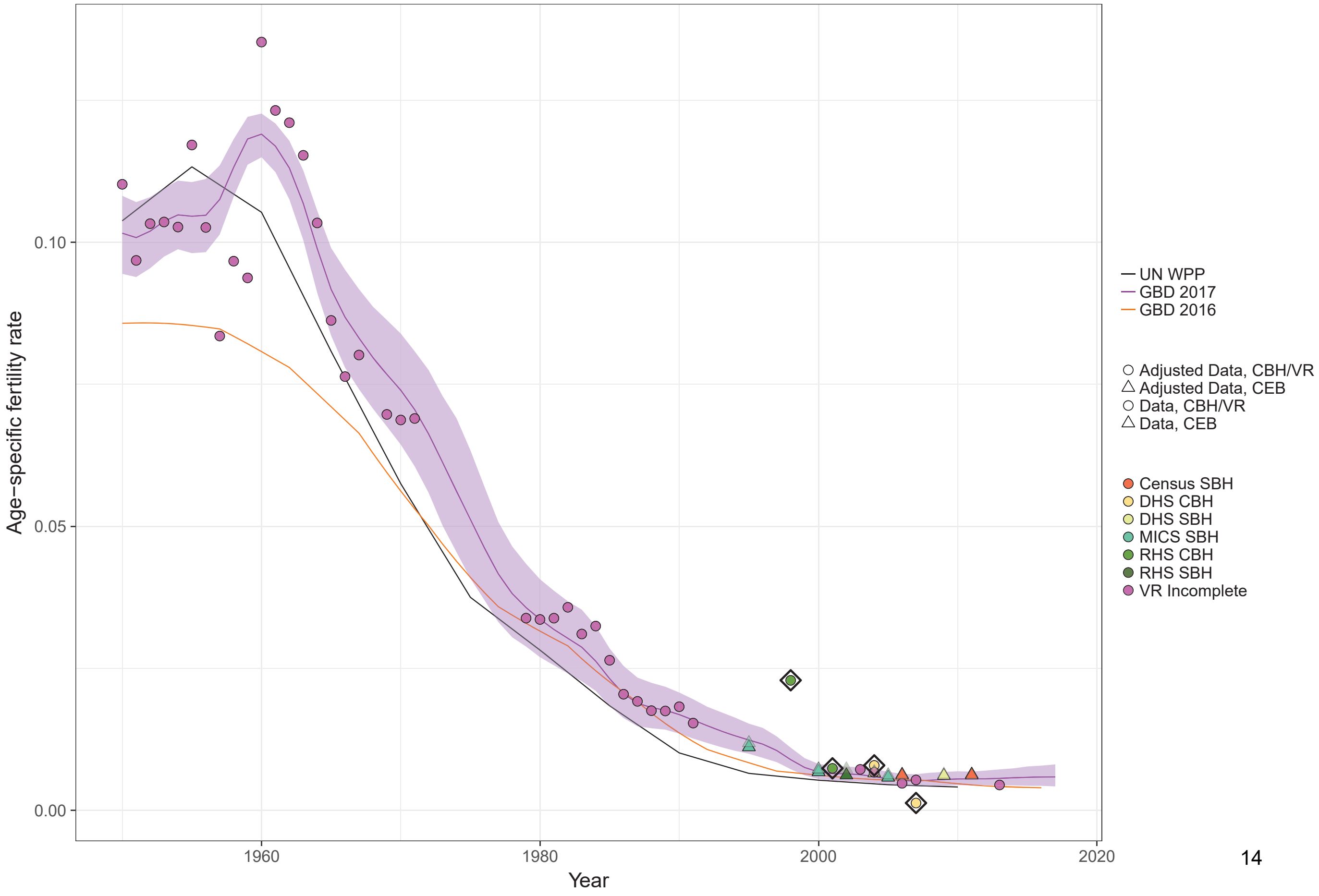

Age−specific fertility fitted trend lines, 1950−2017: Albania, Ages: 45−49
Data points enclosed with a diamond are reference sources

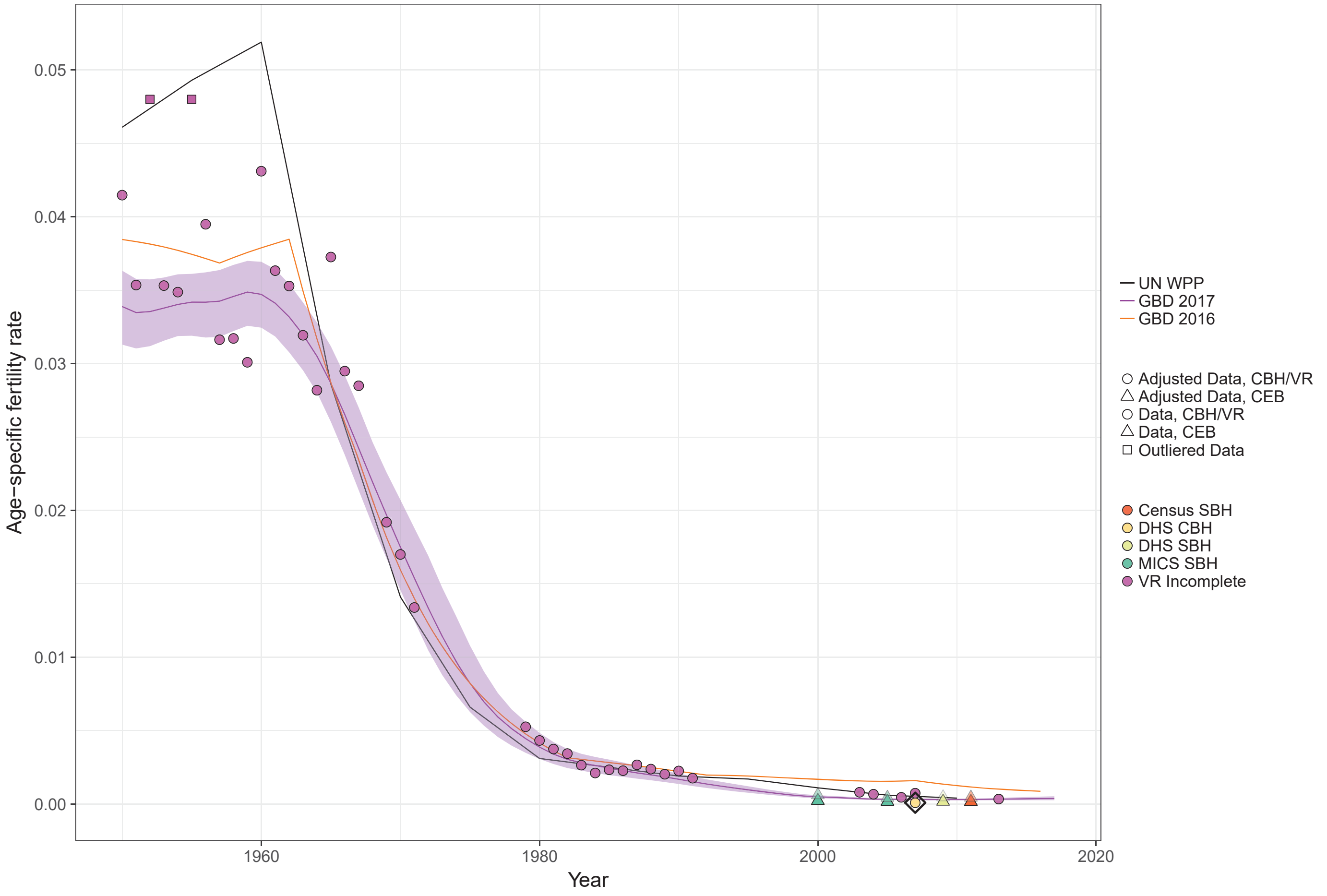

Age−specific fertility fitted trend lines, 1950−2017: Albania, Ages: 50−54
Data points enclosed with a diamond are reference sources

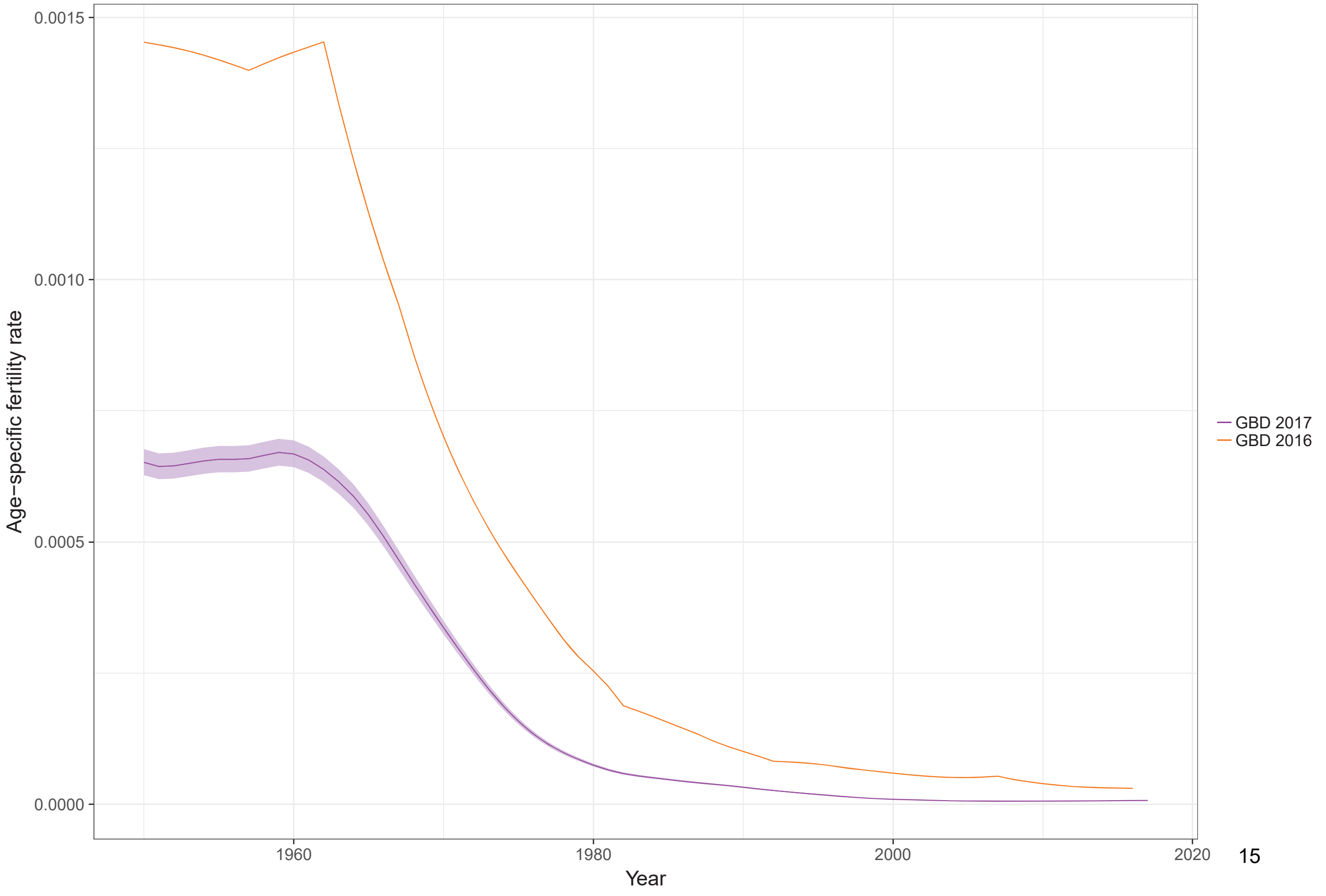

Age−specific fertility fitted trend lines, 1950−2017: Algeria, TFR
Data points enclosed with a diamond are reference sources

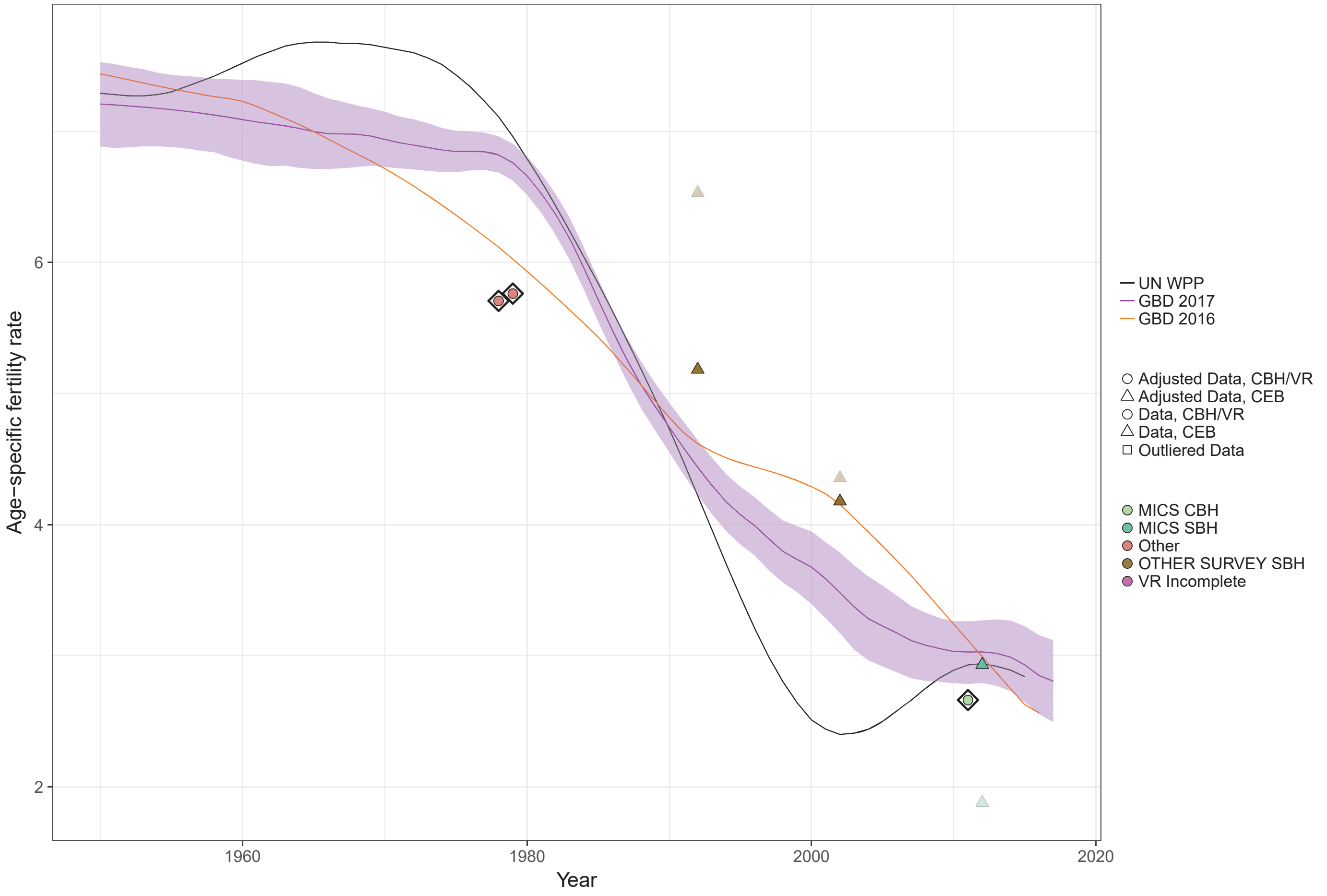

Age−specific fertility fitted trend lines, 1950−2017: Algeria, Ages: 10−14
Data points enclosed with a diamond are reference sources

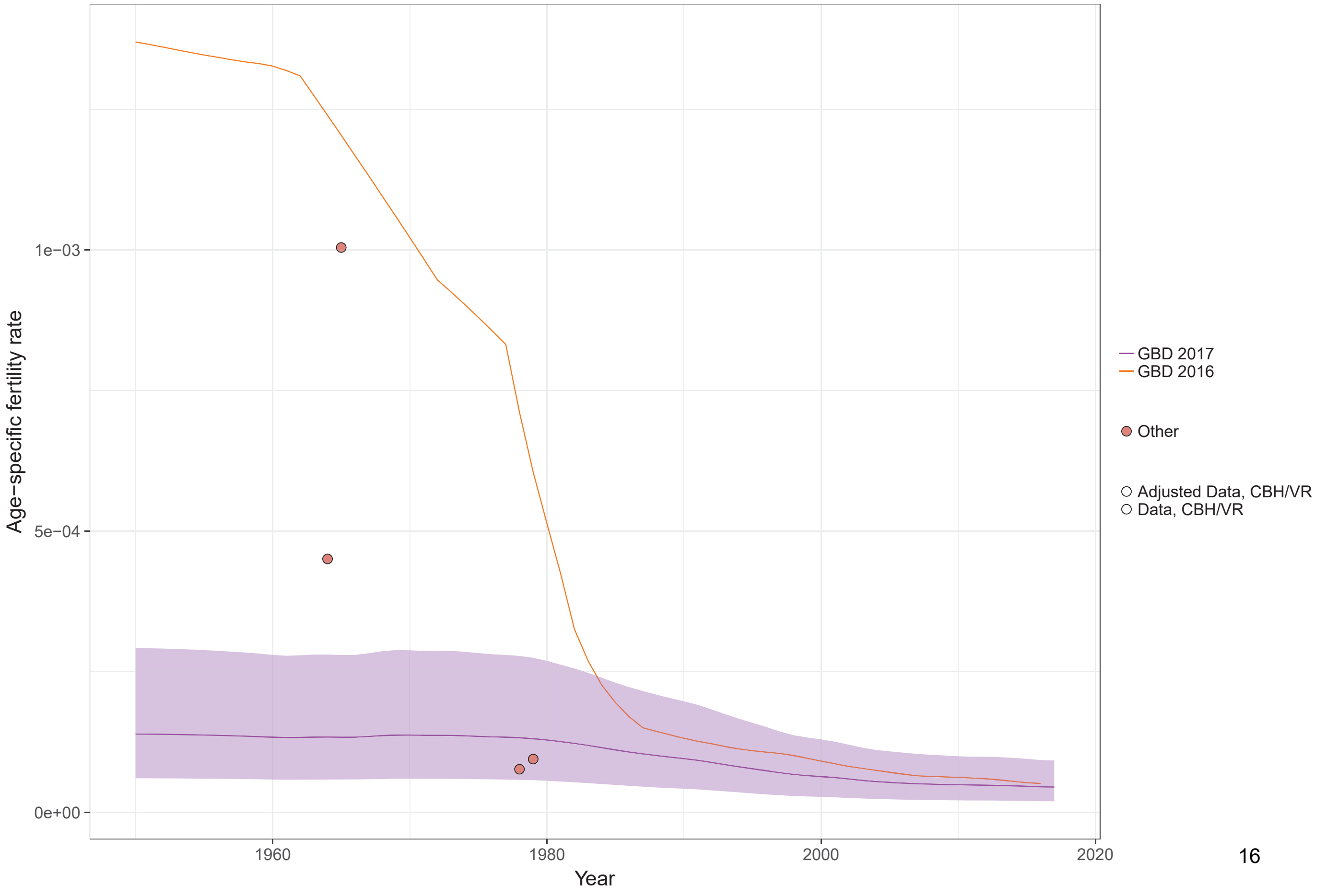

Age−specific fertility fitted trend lines, 1950−2017: Algeria, Ages: 15−19
Data points enclosed with a diamond are reference sources

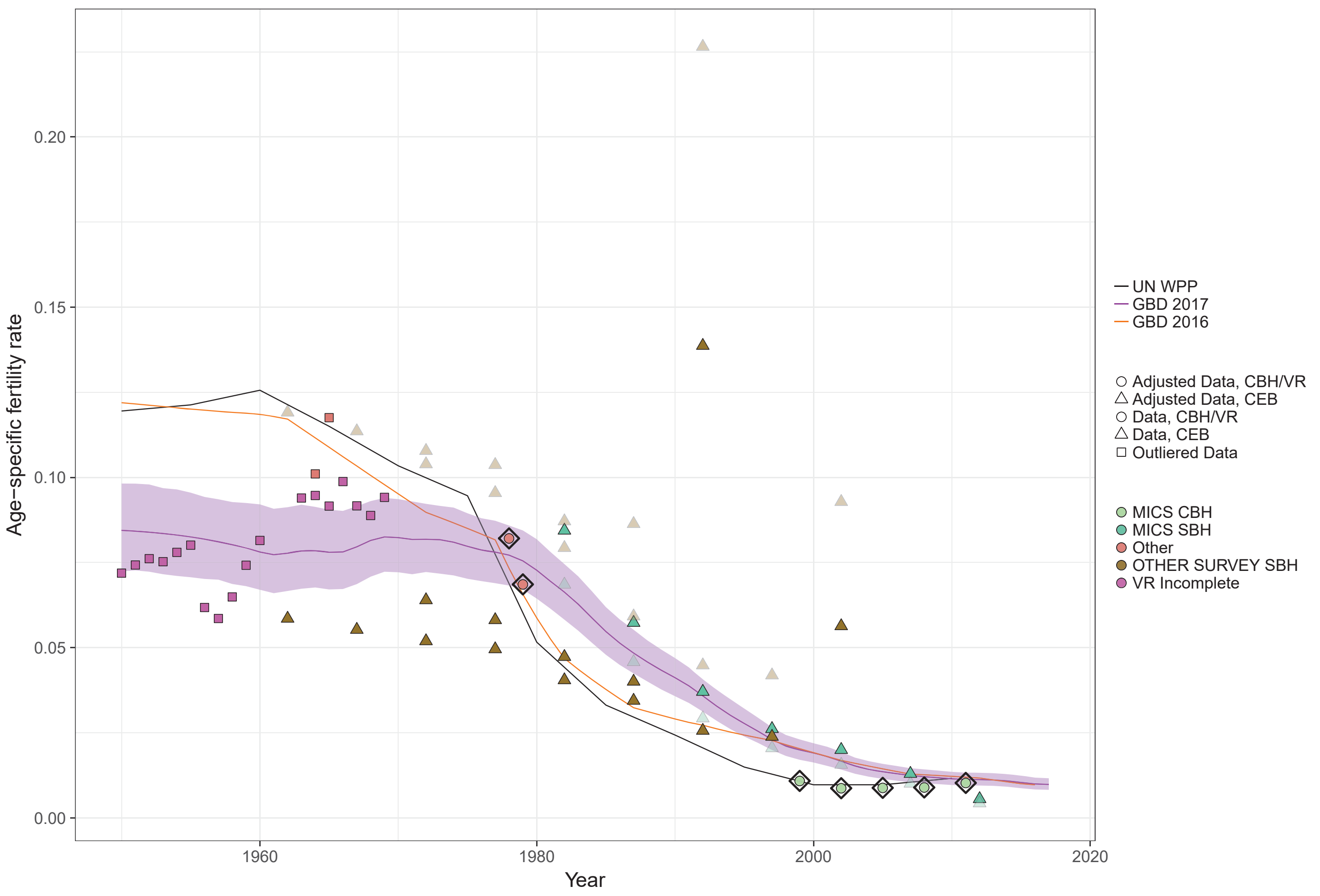

Age−specific fertility fitted trend lines, 1950−2017: Algeria, Ages: 20−24
Data points enclosed with a diamond are reference sources

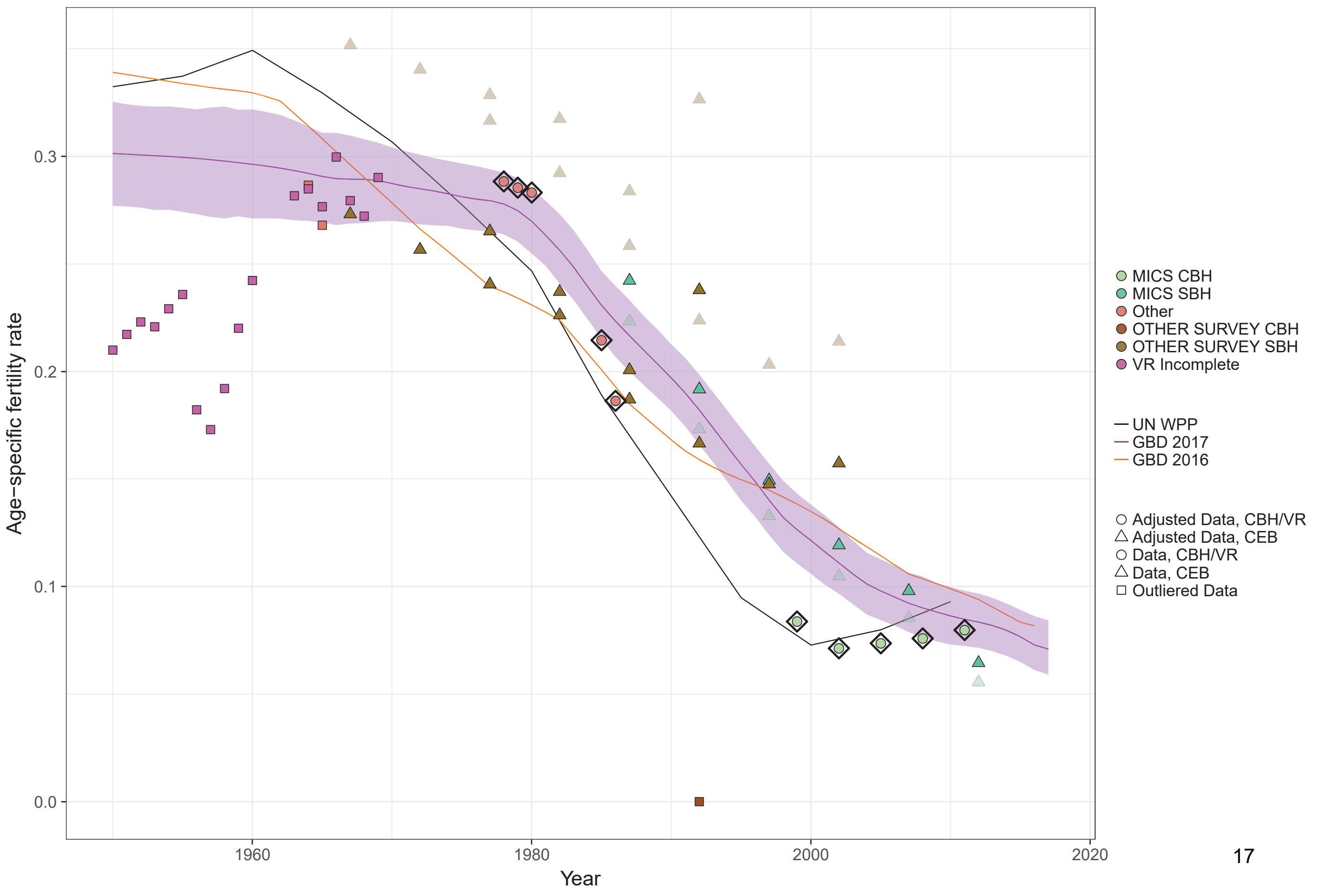

Age−specific fertility fitted trend lines, 1950−2017: Algeria, Ages: 25−29
Data points enclosed with a diamond are reference sources

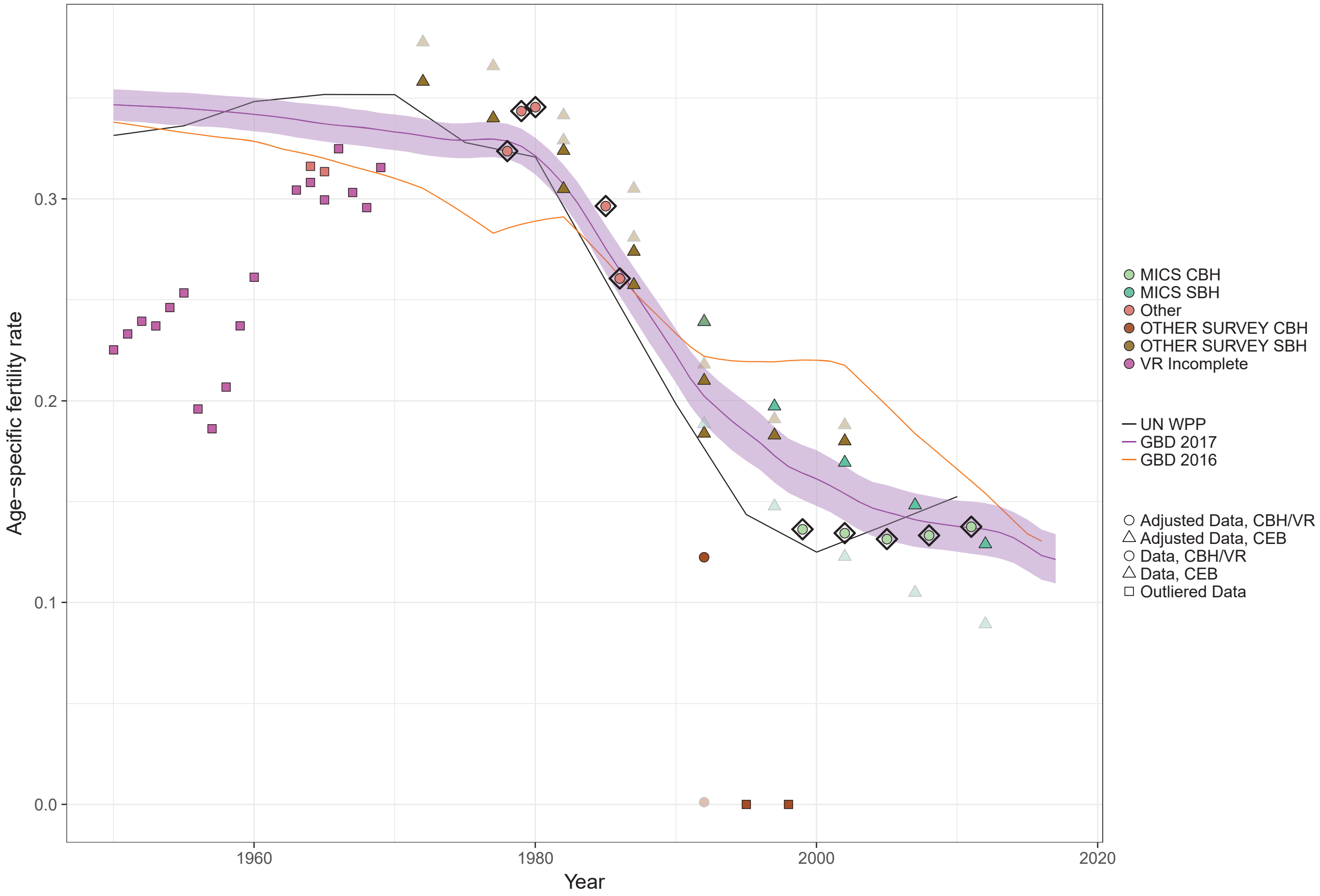

Age−specific fertility fitted trend lines, 1950−2017: Algeria, Ages: 30−34
Data points enclosed with a diamond are reference sources

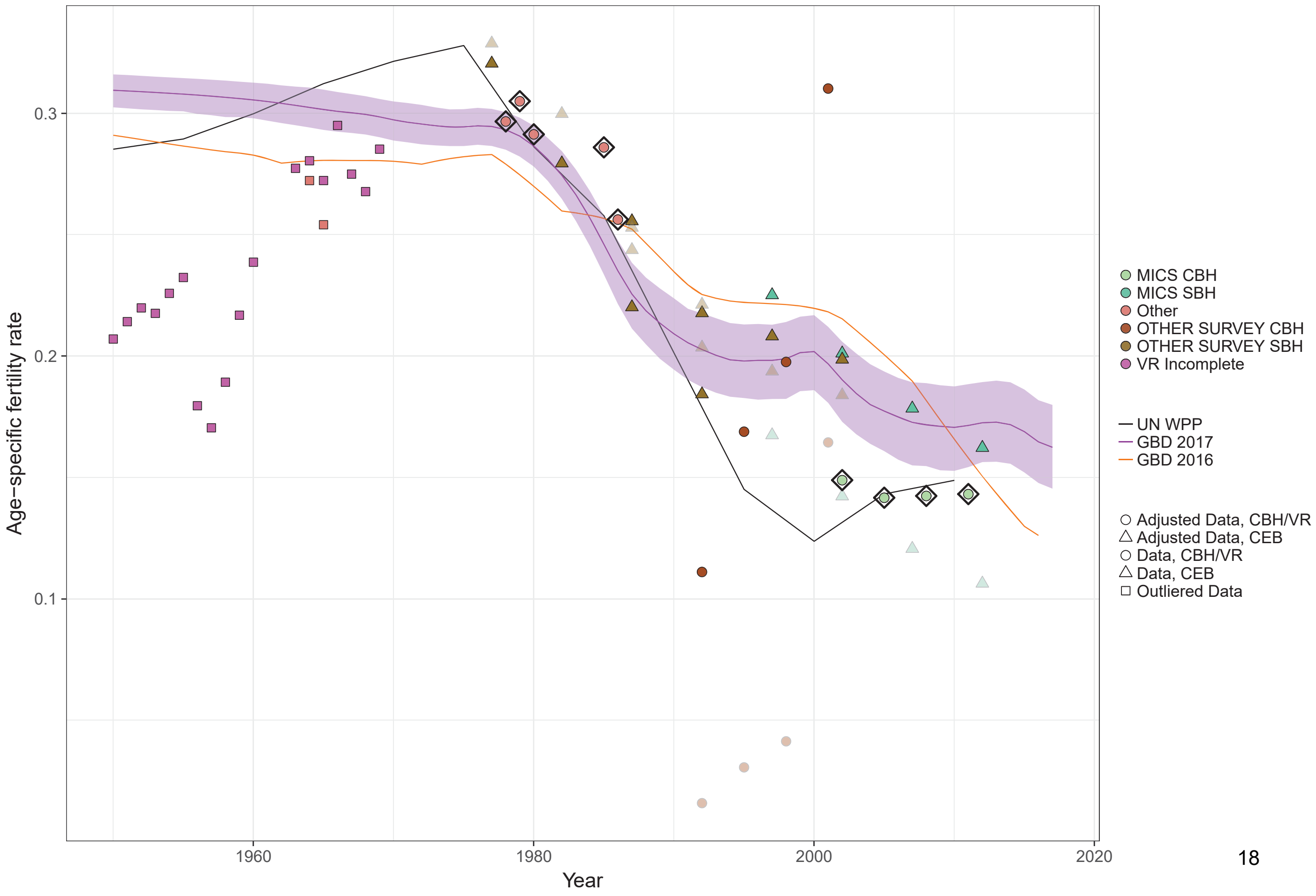

Age−specific fertility fitted trend lines, 1950−2017: Algeria, Ages: 35−39
Data points enclosed with a diamond are reference sources

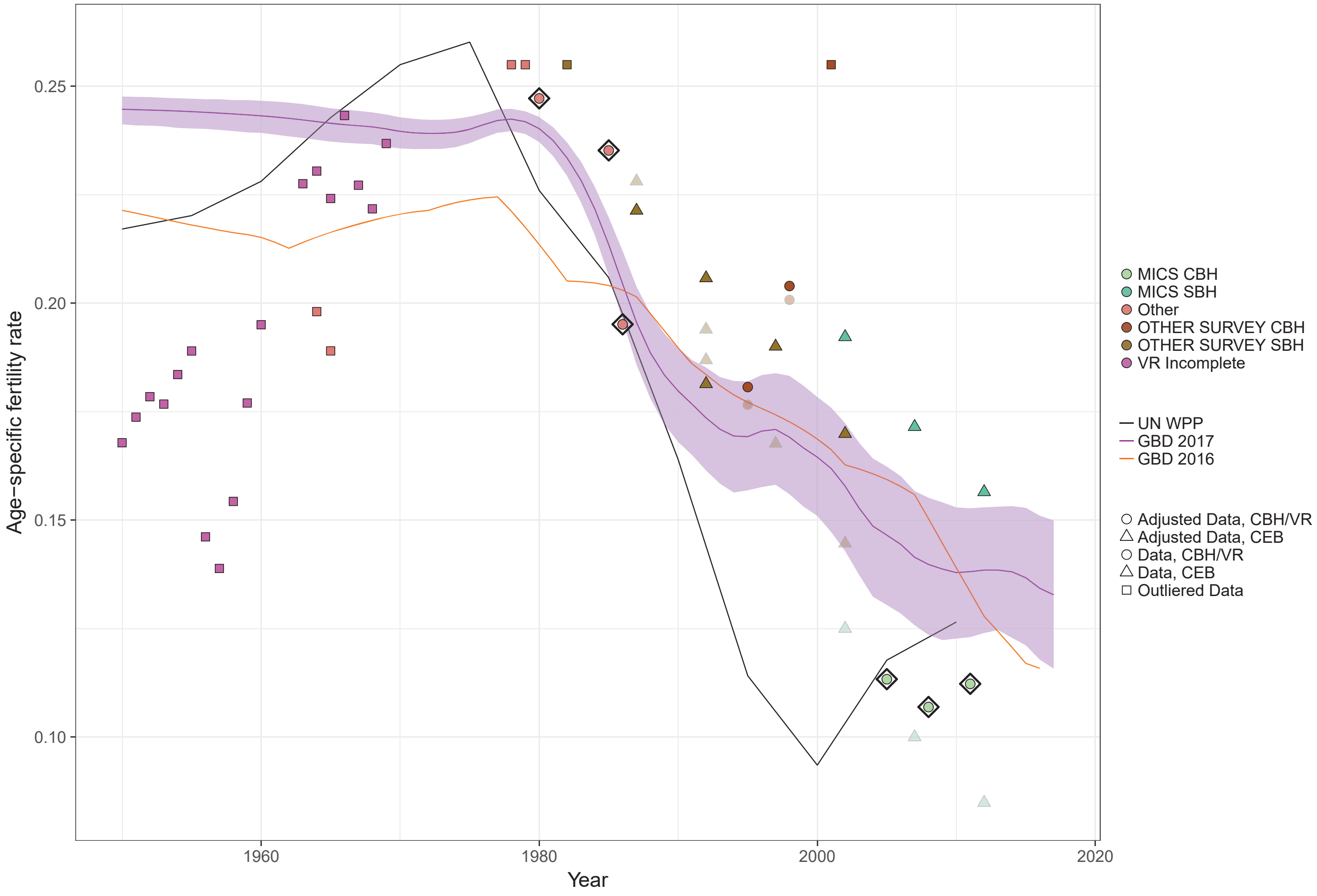

Age−specific fertility fitted trend lines, 1950−2017: Algeria, Ages: 40−44
Data points enclosed with a diamond are reference sources

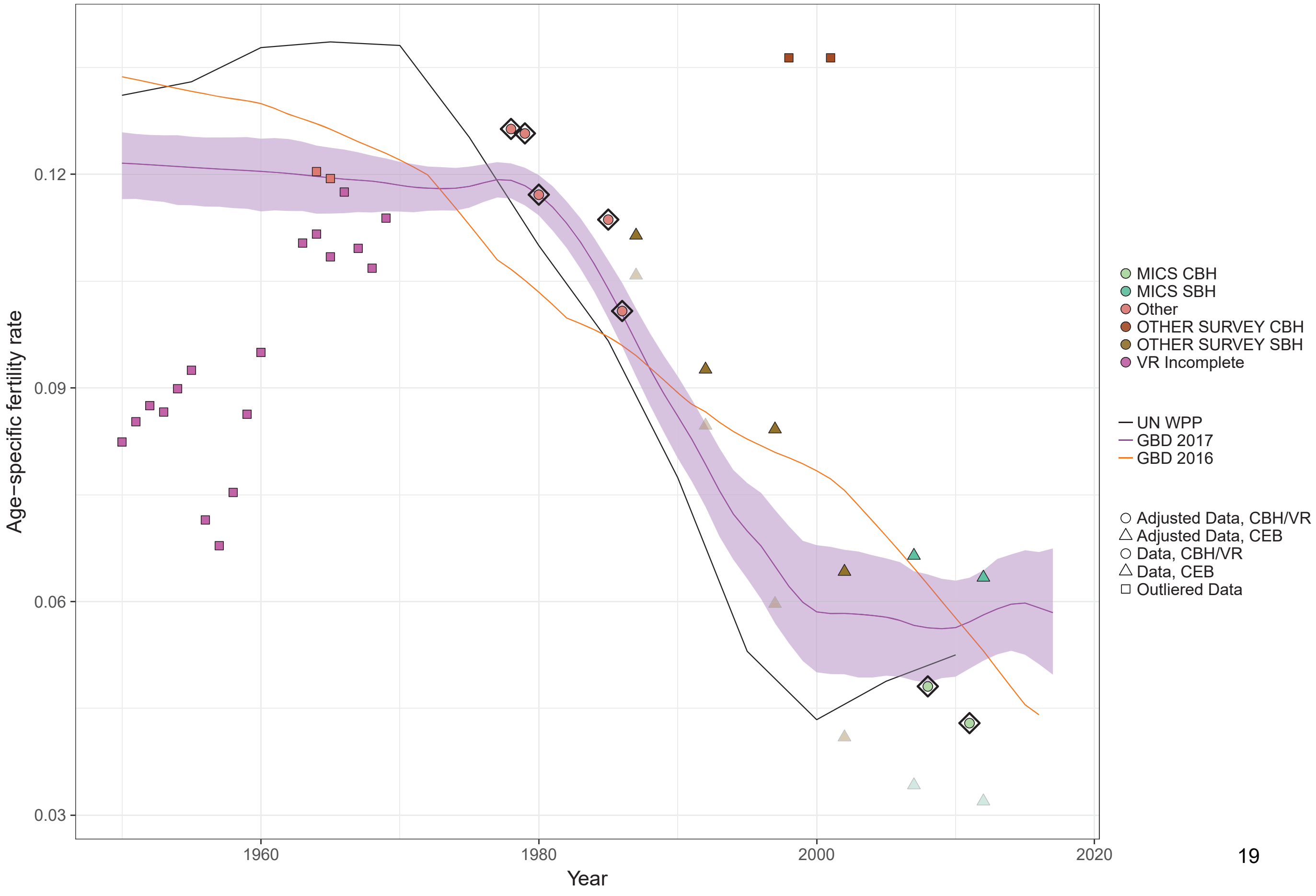

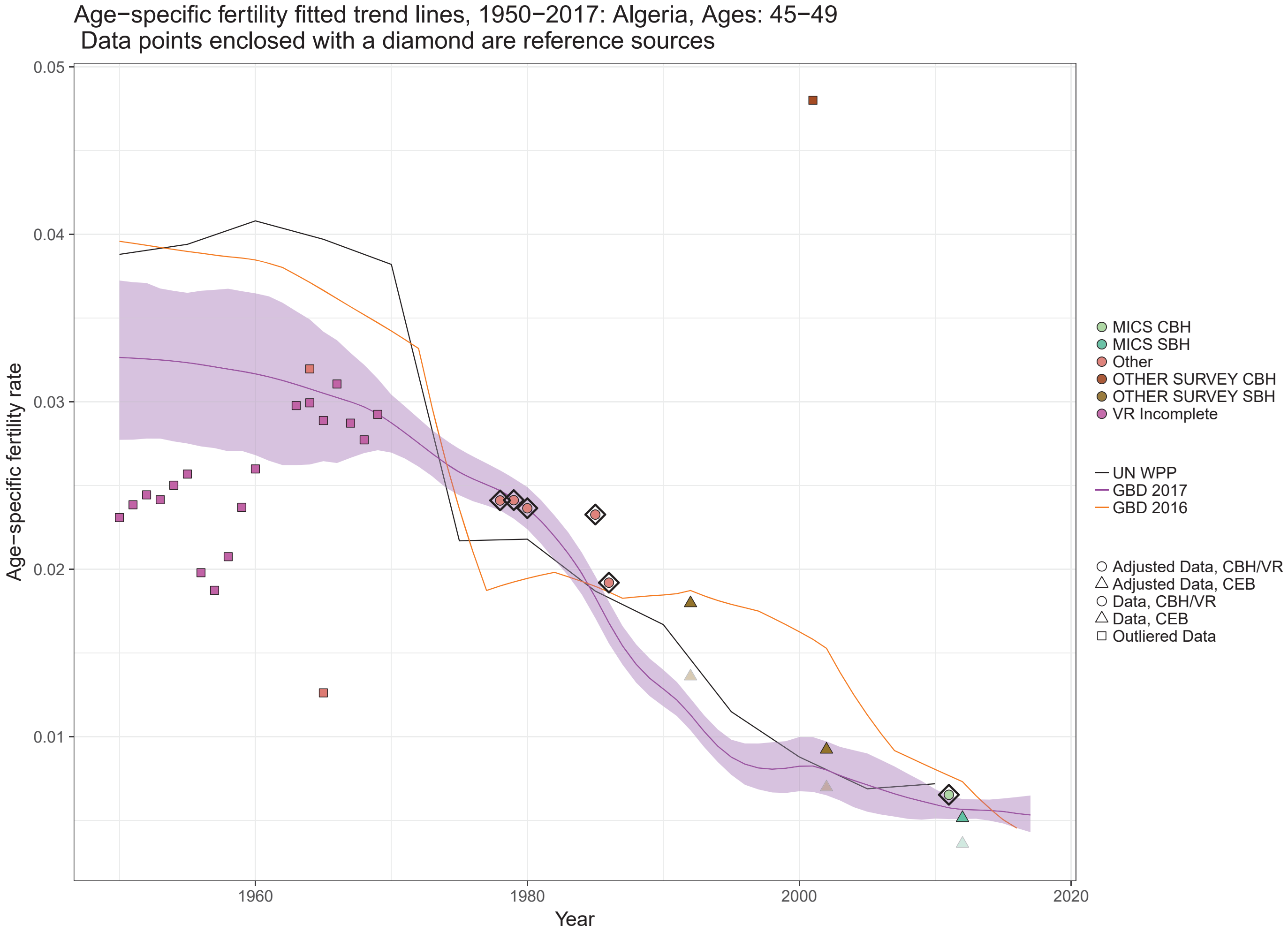

Age−specific fertility fitted trend lines, 1950−2017: Algeria, Ages: 45−49
Data points enclosed with a diamond are reference sources
Age−specific fertility rate
0.05
0.04
0.03
0.02
0.01
1960
1980
2000
2020
Year
MICS CBH
MICS SBH
Other
OTHER SURVEY CBH
OTHER SURVEY SBH
VR Incomplete
UN WPP
GBD 2017
GBD 2016
Adjusted Data, CBH/VR
Adjusted Data, CEB
Data, CBH/VR
Data, CEB
Outliered Data

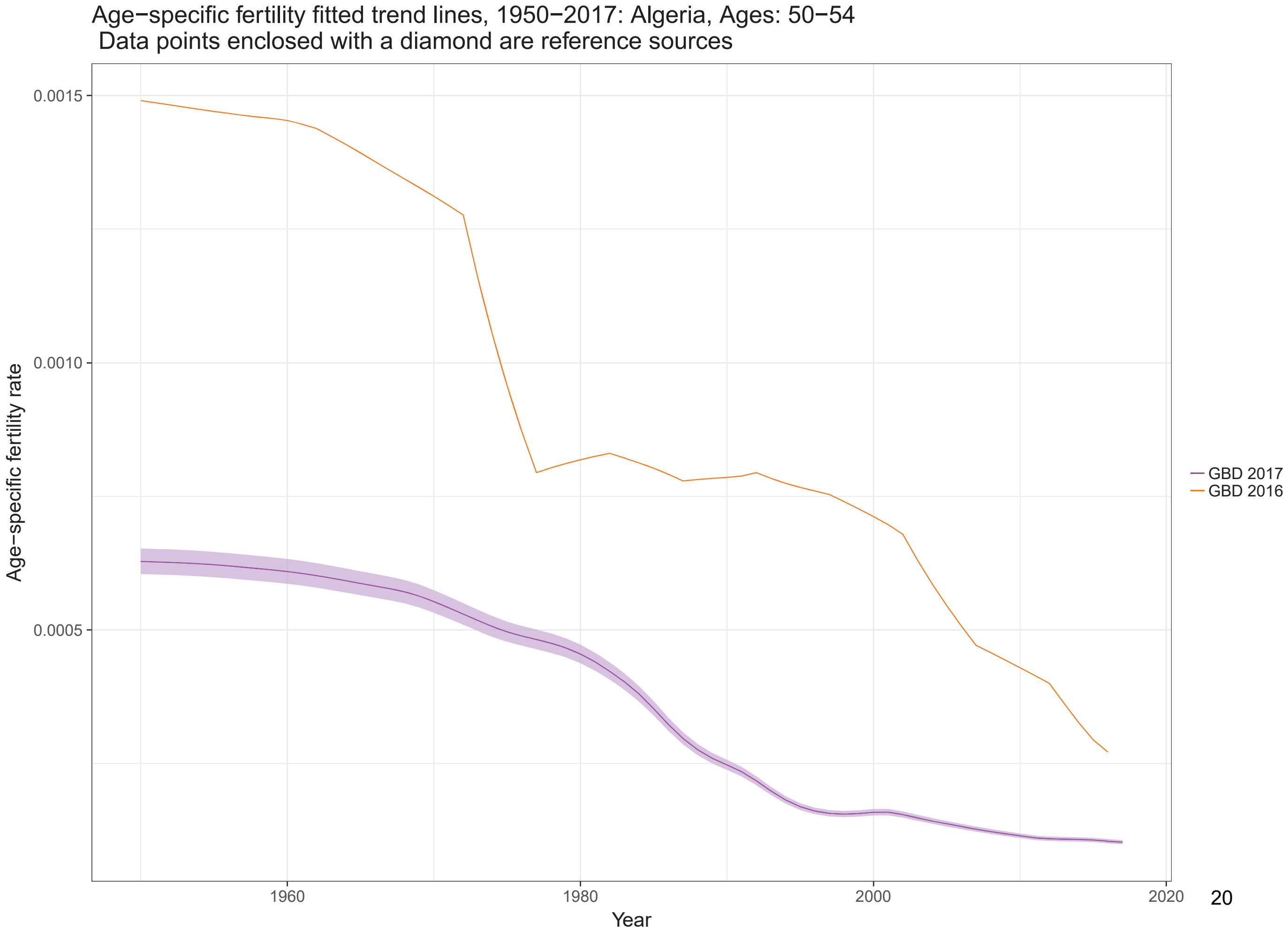

Age−specific fertility fitted trend lines, 1950−2017: Algeria, Ages: 50−54
Data points enclosed with a diamond are reference sources
Age−specific fertility rate
0.0015
0.0010
0.0005
1960
1980
2000
2020
Year
GBD 2017
GBD 2016

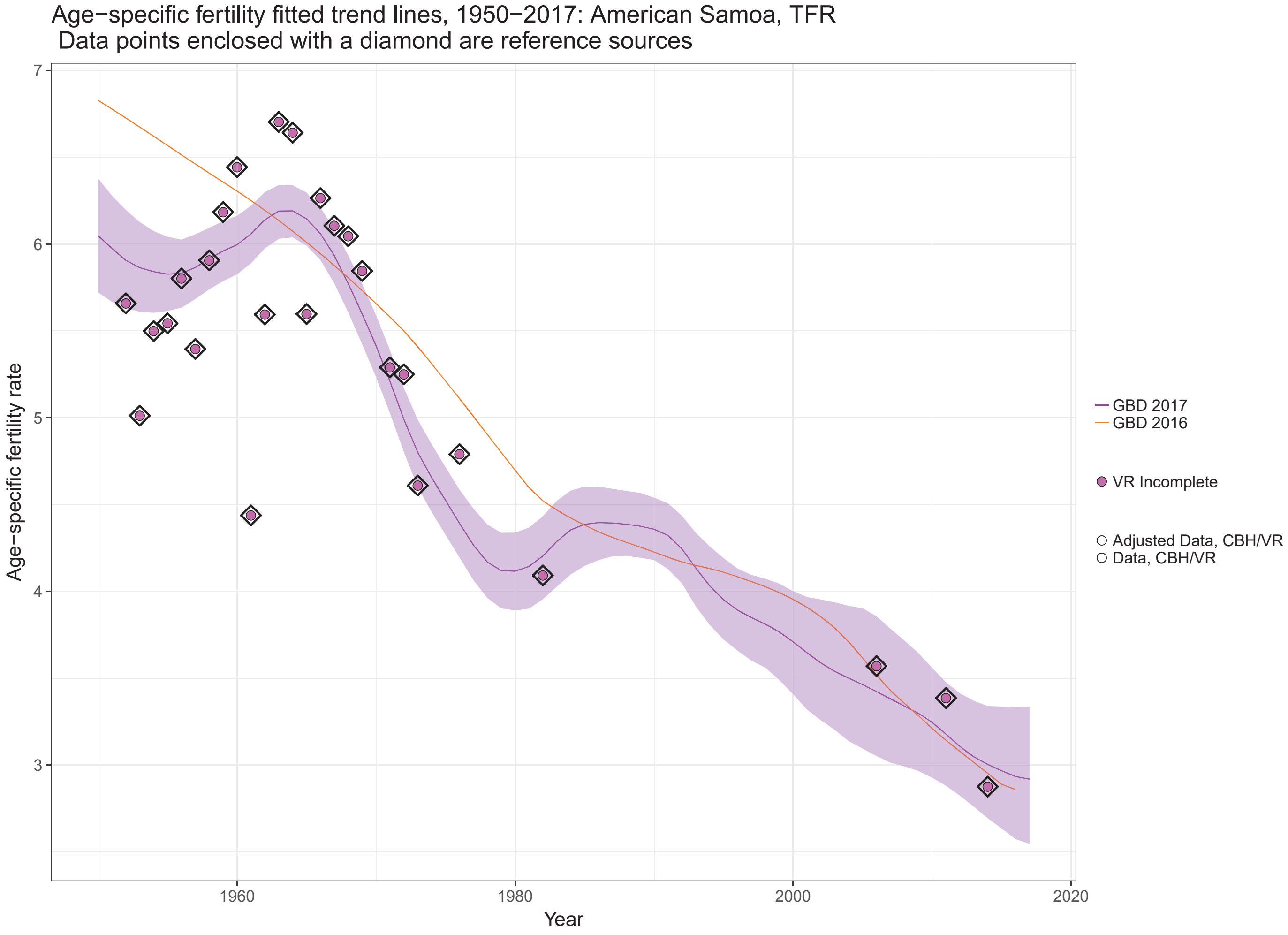
Age−specific fertility fitted trend lines, 1950−2017: American Samoa, TFR
Data points enclosed with a diamond are reference sources
Age−specific fertility rate
Year
GBD 2017
GBD 2016
VR Incomplete
Adjusted Data, CBH/VR
Data, CBH/VR

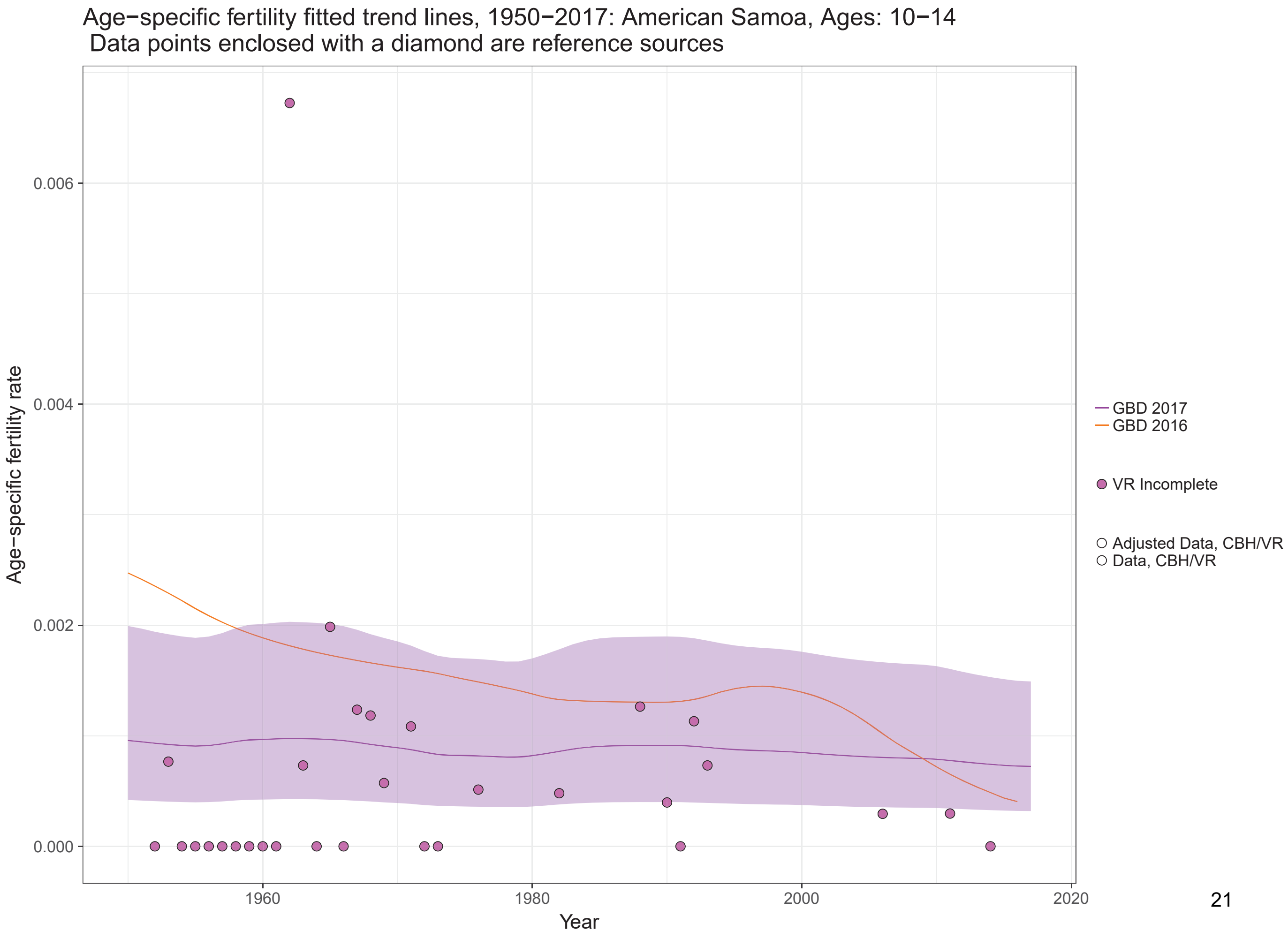
Age−specific fertility fitted trend lines, 1950−2017: American Samoa, Ages: 10−14
Data points enclosed with a diamond are reference sources
Age−specific fertility rate
Year
GBD 2017
GBD 2016
VR Incomplete
Adjusted Data, CBH/VR
Data, CBH/VR

Age−specific fertility fitted trend lines, 1950−2017: American Samoa, Ages: 15−19
Data points enclosed with a diamond are reference sources

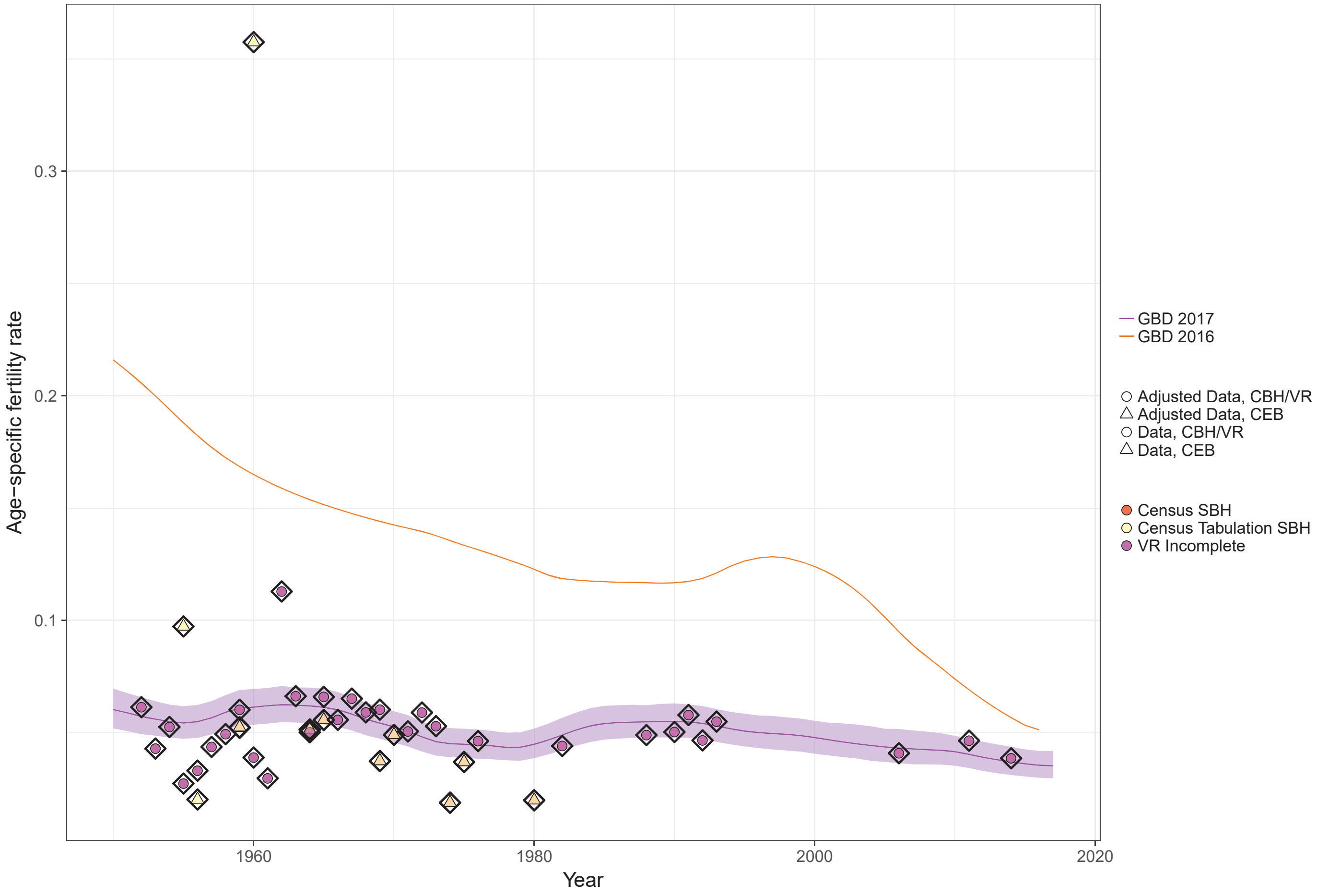

Age−specific fertility fitted trend lines, 1950−2017: American Samoa, Ages: 20−24
Data points enclosed with a diamond are reference sources

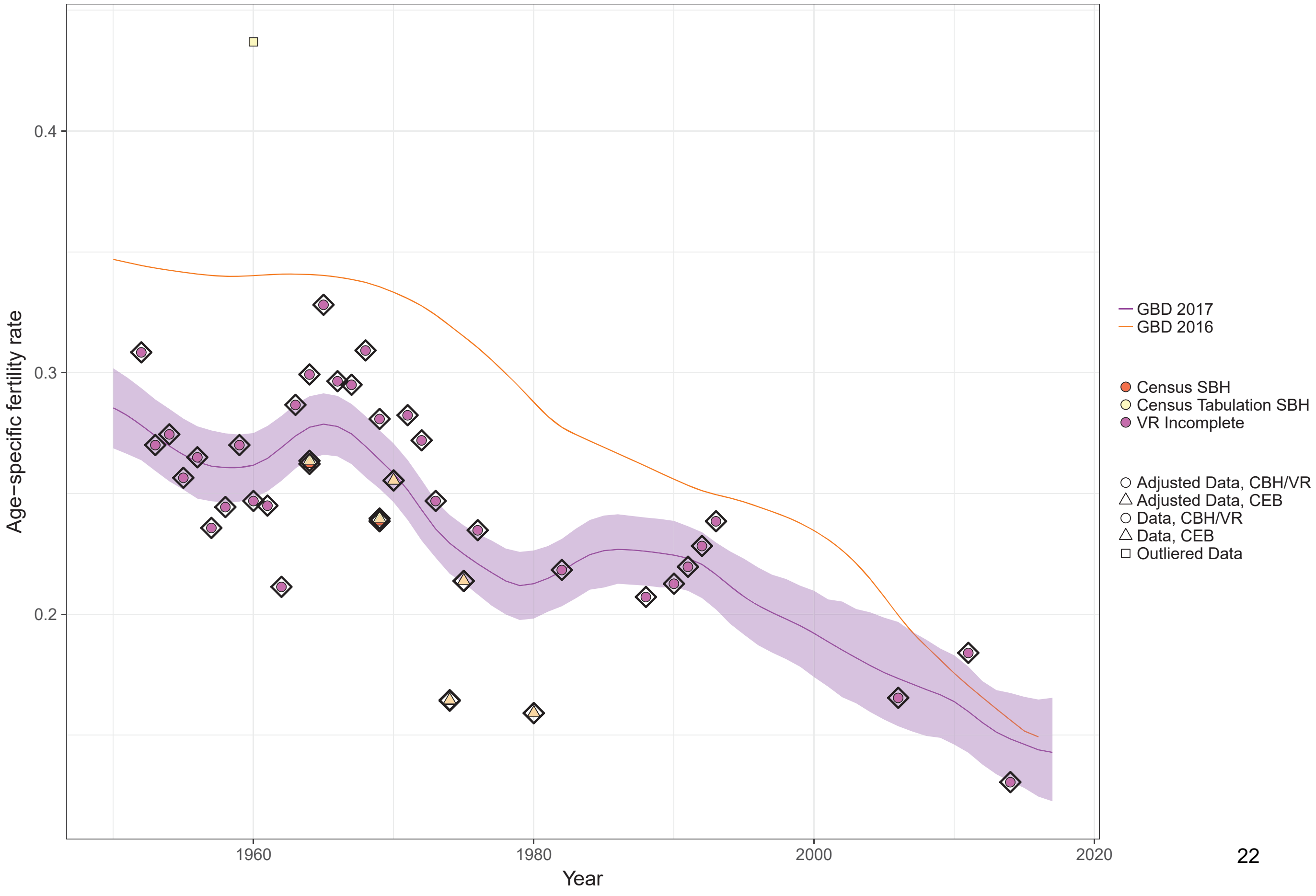

Age−specific fertility fitted trend lines, 1950−2017: American Samoa, Ages: 25−29
Data points enclosed with a diamond are reference sources

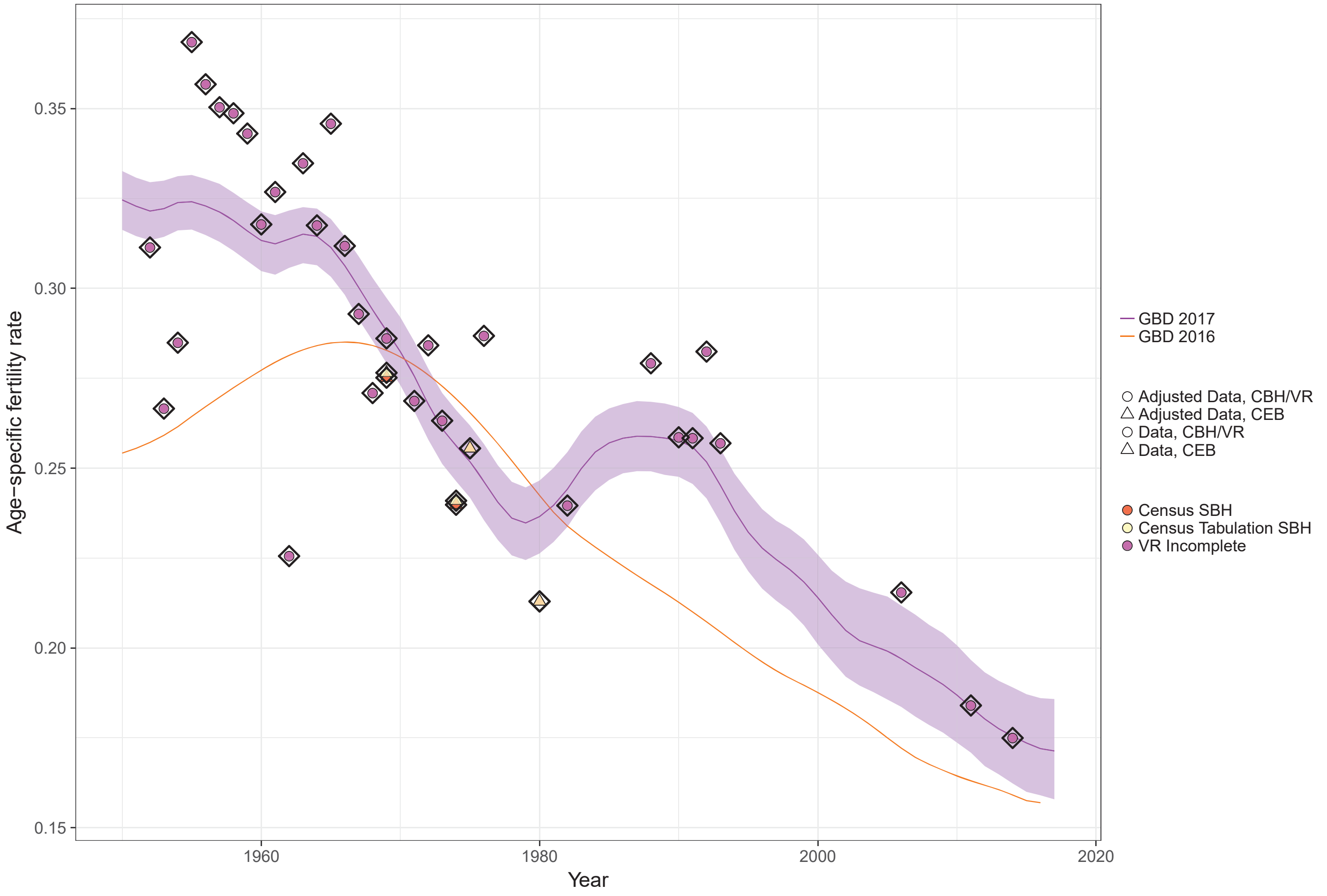

Age−specific fertility fitted trend lines, 1950−2017: American Samoa, Ages: 30−34
Data points enclosed with a diamond are reference sources

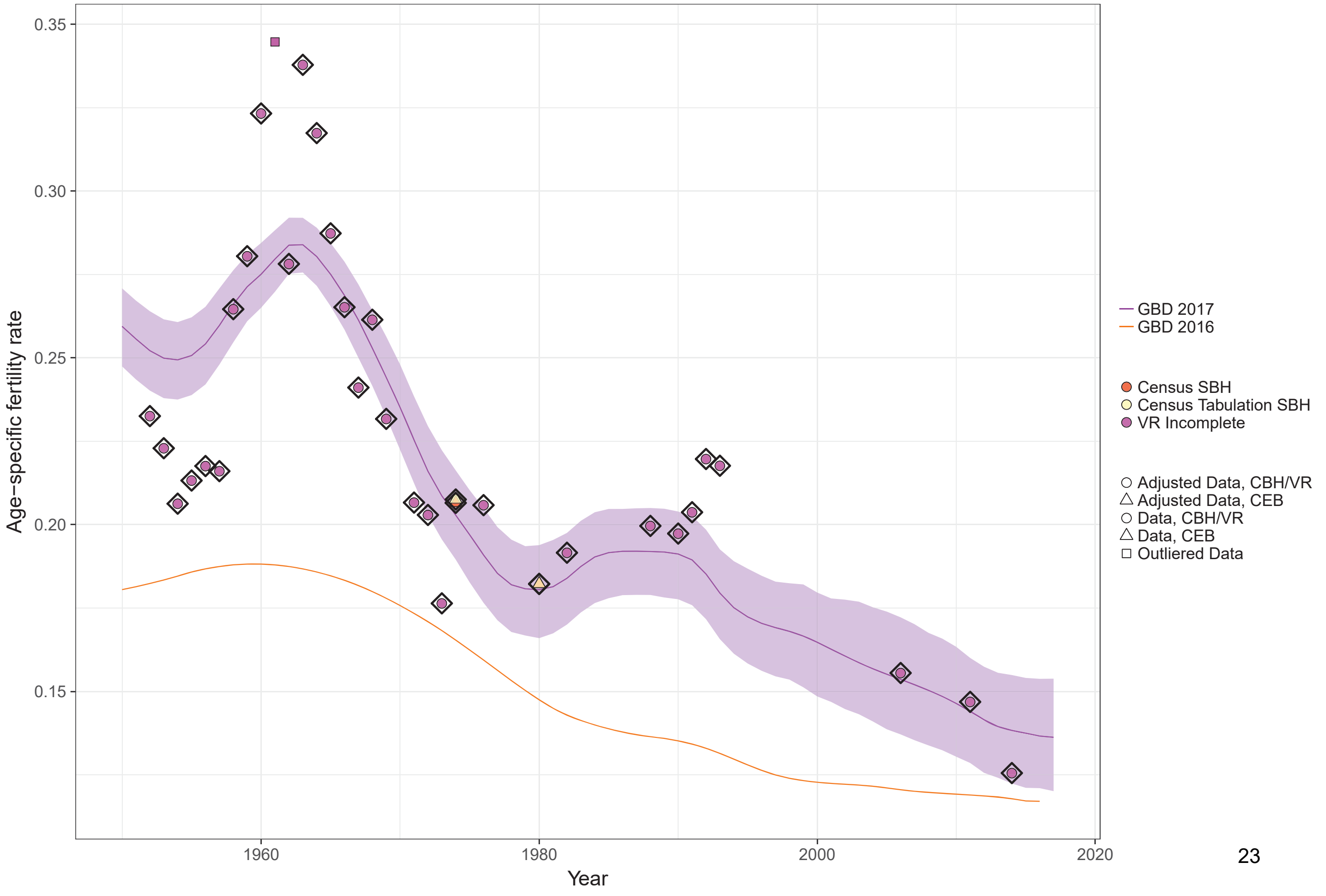

Age−specific fertility fitted trend lines, 1950−2017: American Samoa, Ages: 35−39
Data points enclosed with a diamond are reference sources

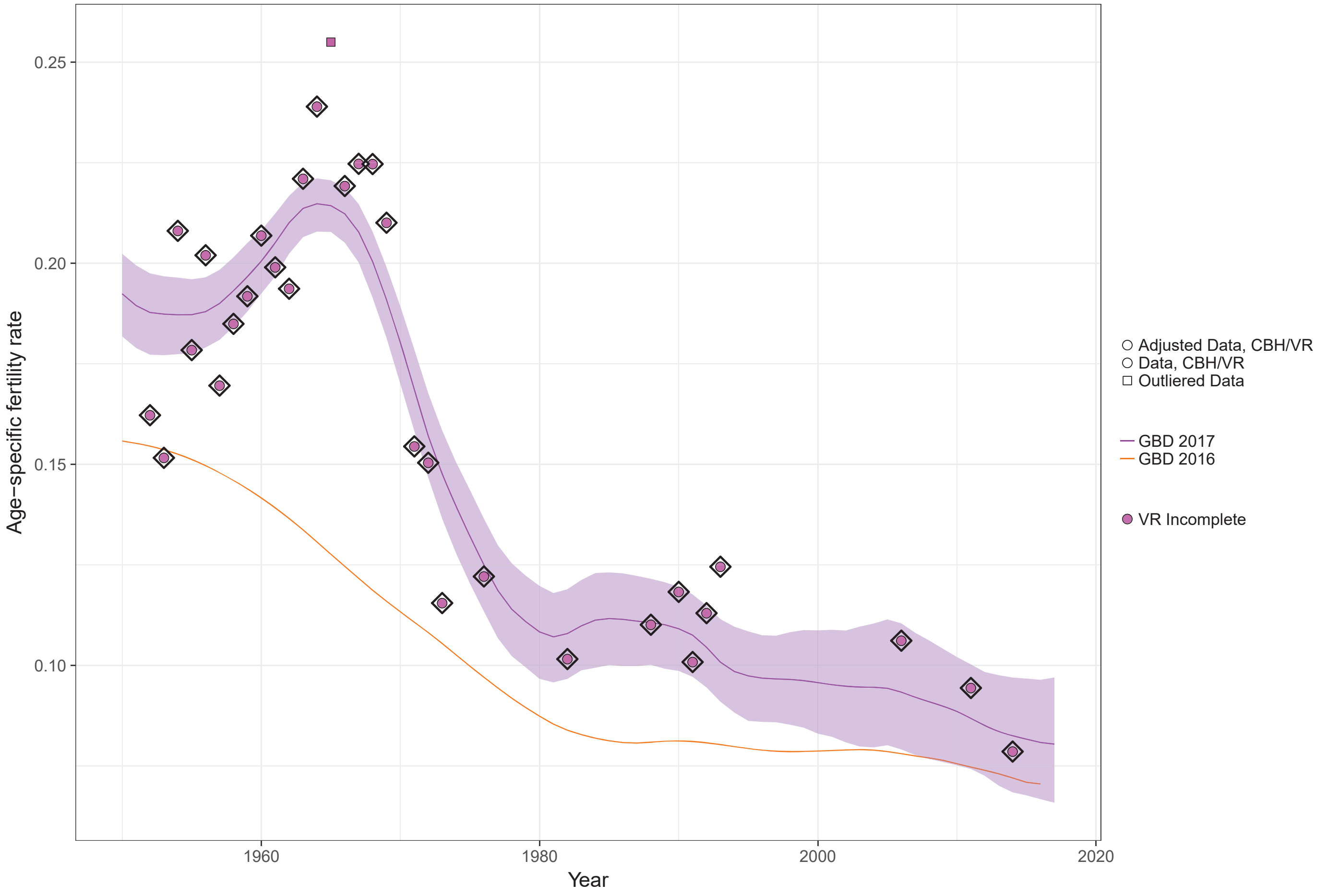

Age−specific fertility fitted trend lines, 1950−2017: American Samoa, Ages: 40−44
Data points enclosed with a diamond are reference sources

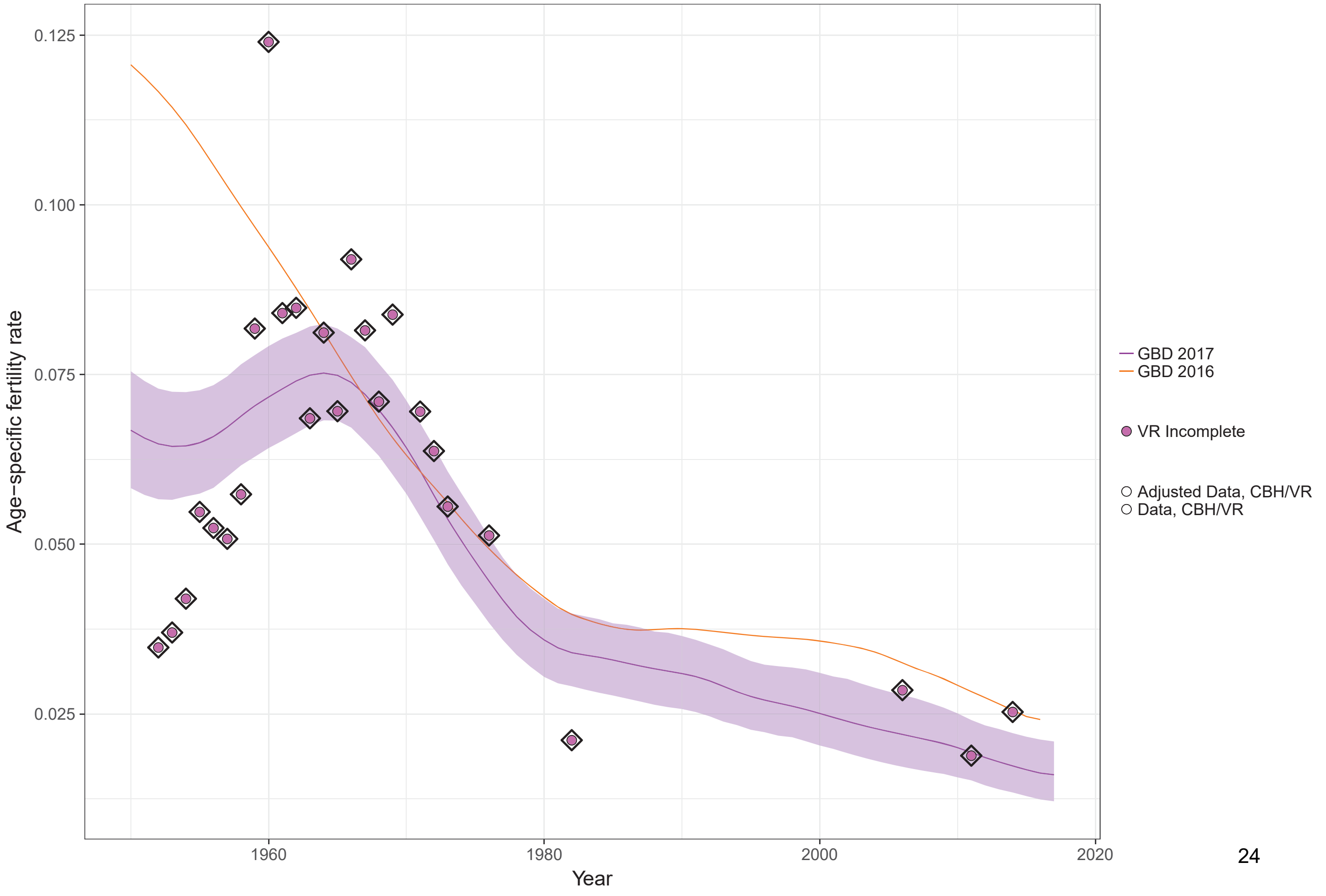

Age−specific fertility fitted trend lines, 1950−2017: American Samoa, Ages: 45−49
Data points enclosed with a diamond are reference sources

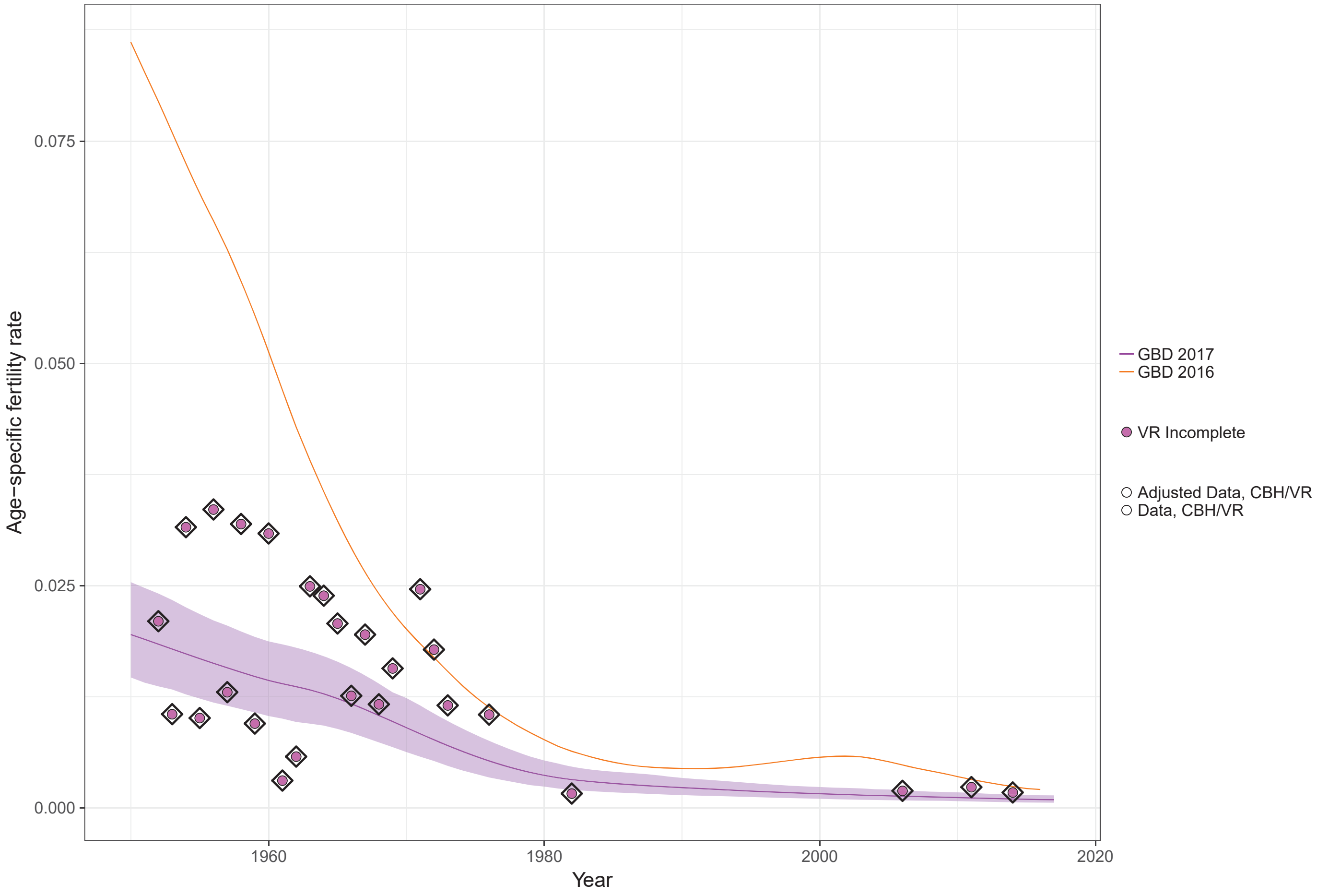

Age−specific fertility fitted trend lines, 1950−2017: American Samoa, Ages: 50−54
Data points enclosed with a diamond are reference sources

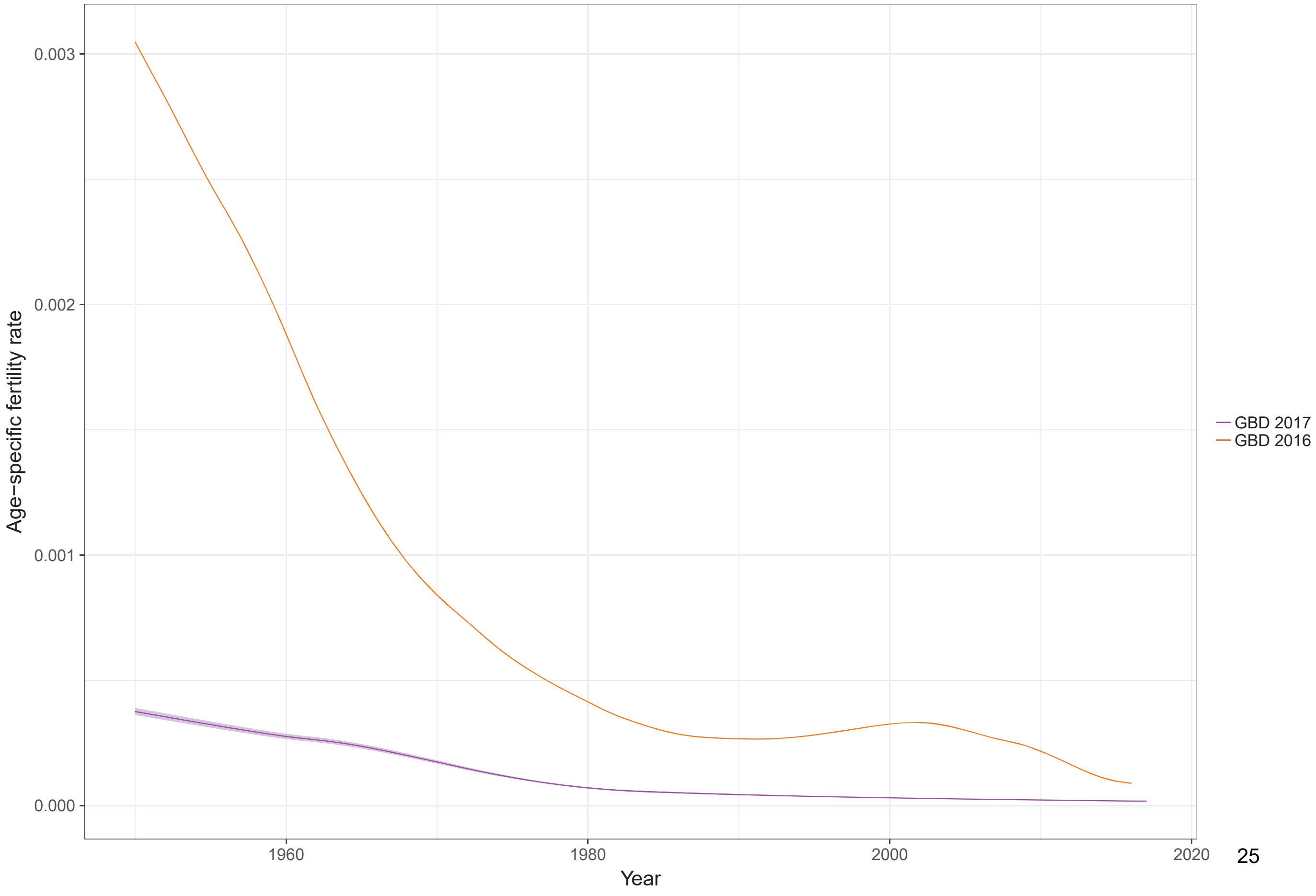

Age−specific fertility fitted trend lines, 1950−2017: Andorra, TFR
Data points enclosed with a diamond are reference sources

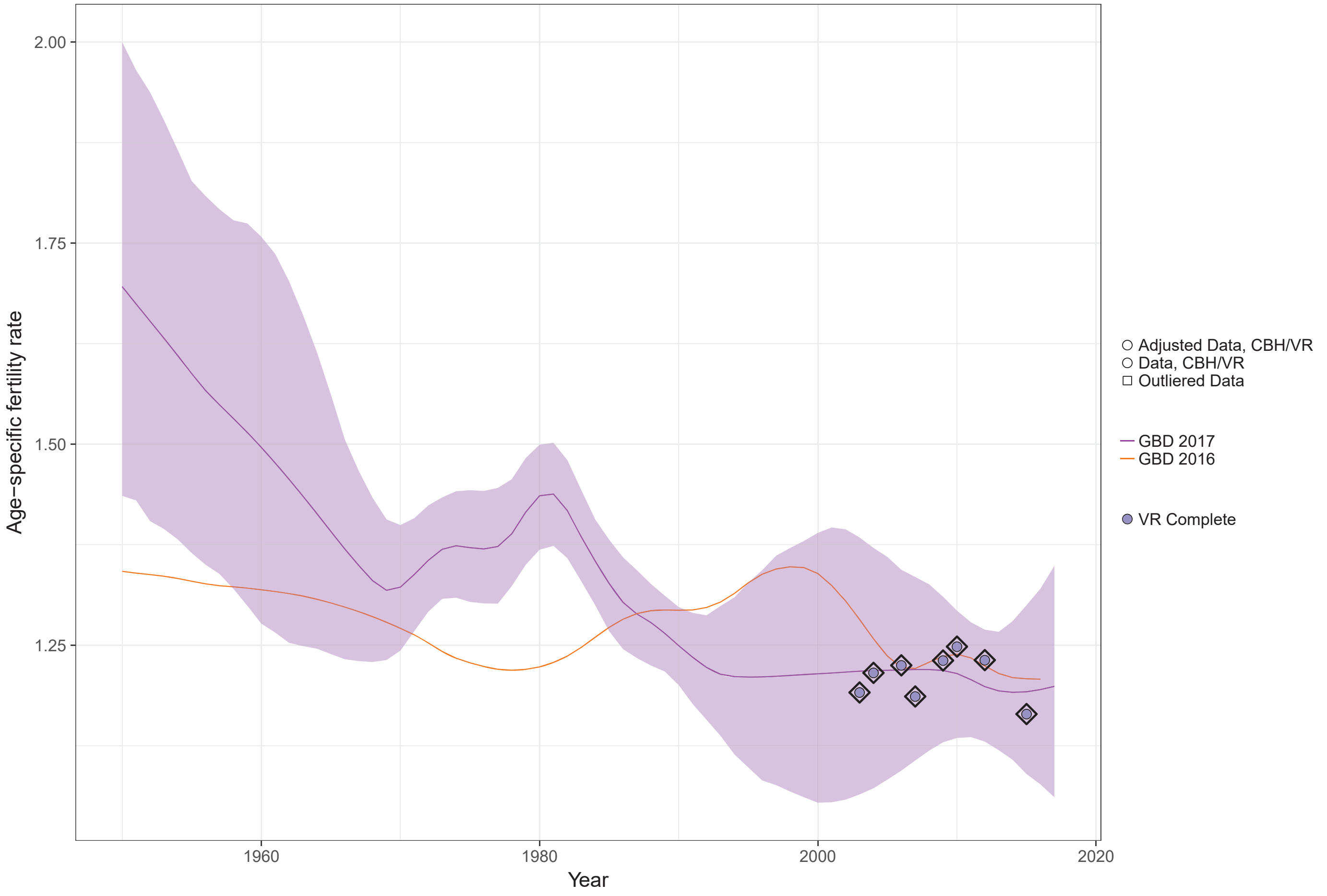

Age−specific fertility fitted trend lines, 1950−2017: Andorra, Ages: 10−14
Data points enclosed with a diamond are reference sources

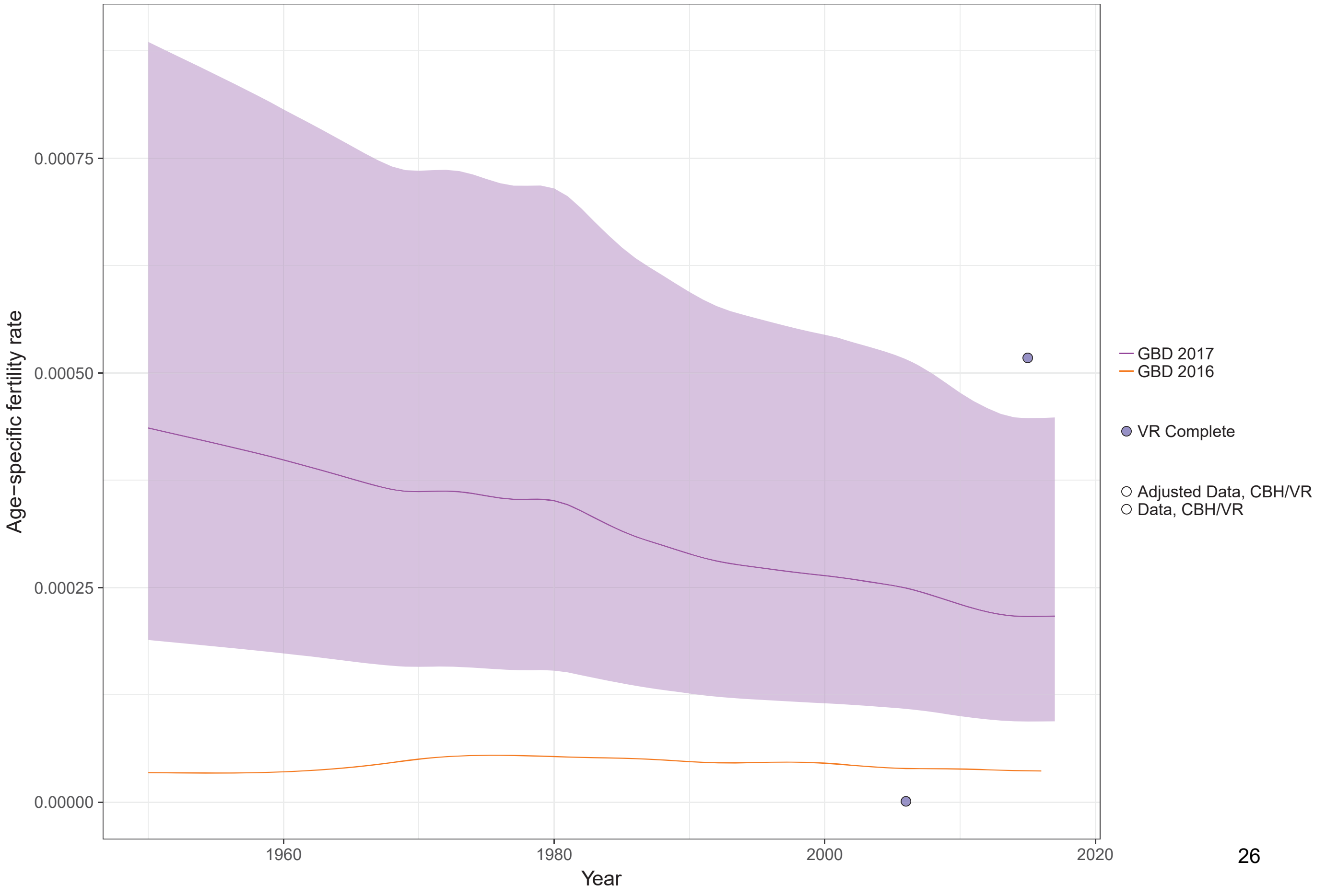

Age−specific fertility fitted trend lines, 1950−2017: Andorra, Ages: 15−19
Data points enclosed with a diamond are reference sources

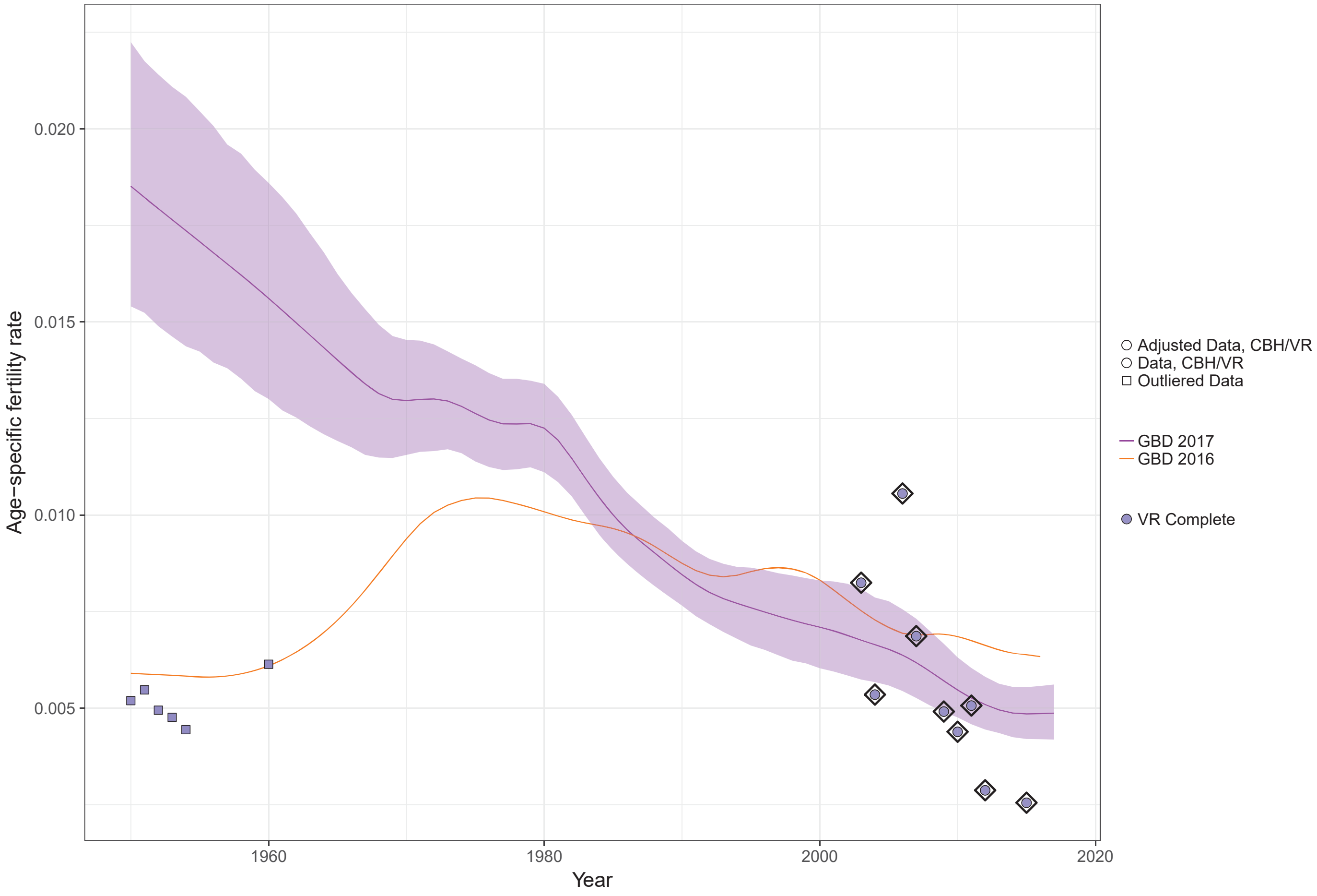

Age−specific fertility fitted trend lines, 1950−2017: Andorra, Ages: 20−24
Data points enclosed with a diamond are reference sources

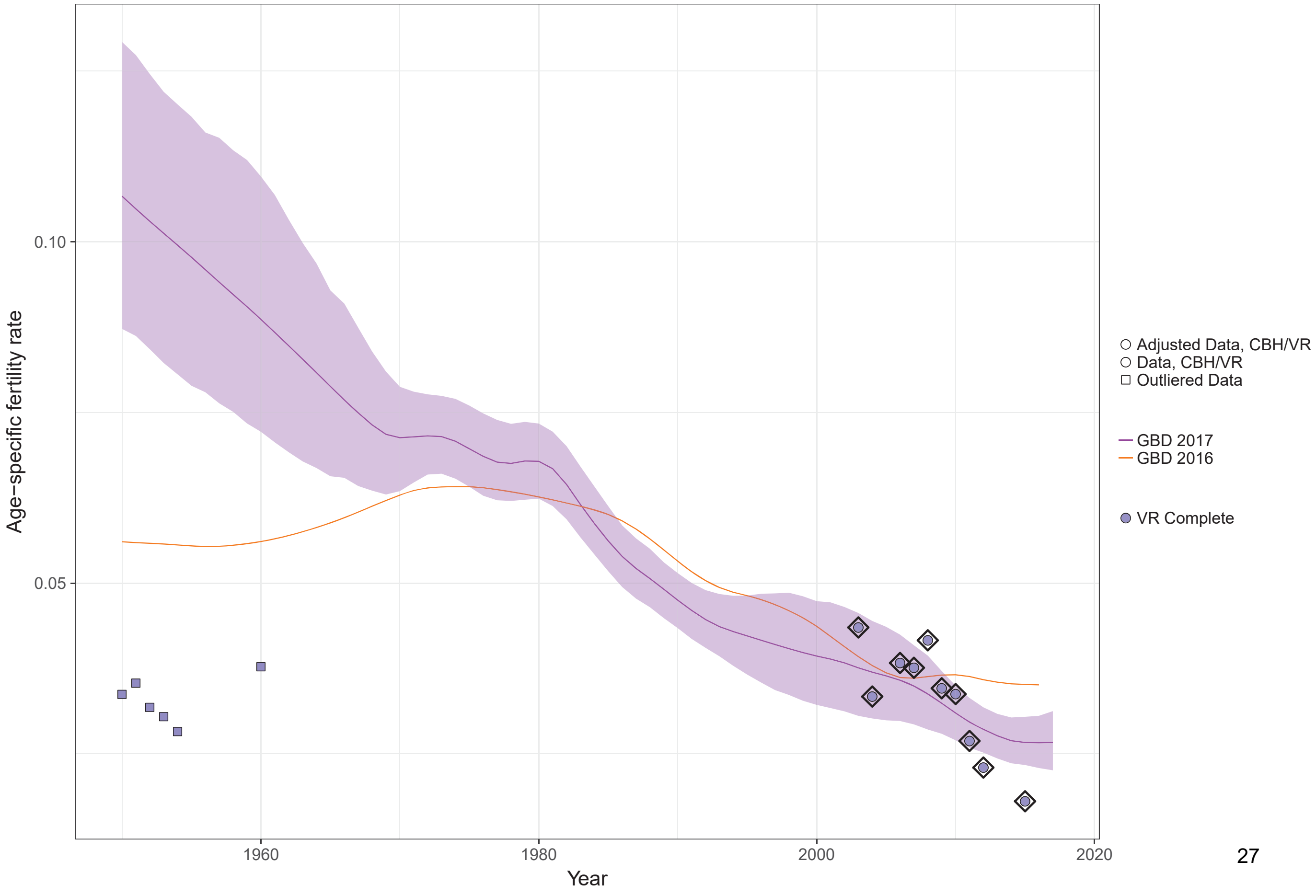

Age−specific fertility fitted trend lines, 1950−2017: Andorra, Ages: 25−29
Data points enclosed with a diamond are reference sources

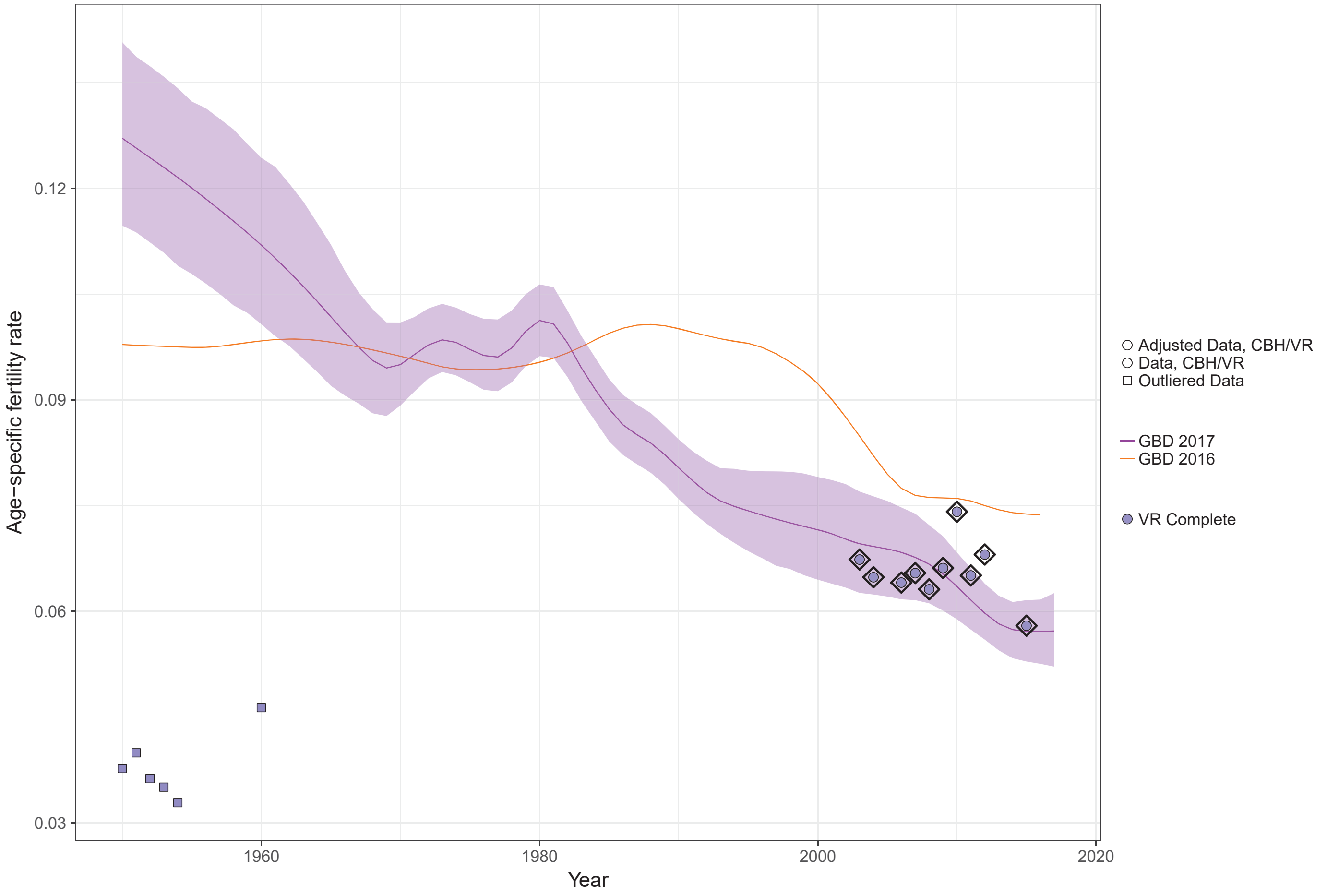

Age−specific fertility fitted trend lines, 1950−2017: Andorra, Ages: 30−34
Data points enclosed with a diamond are reference sources

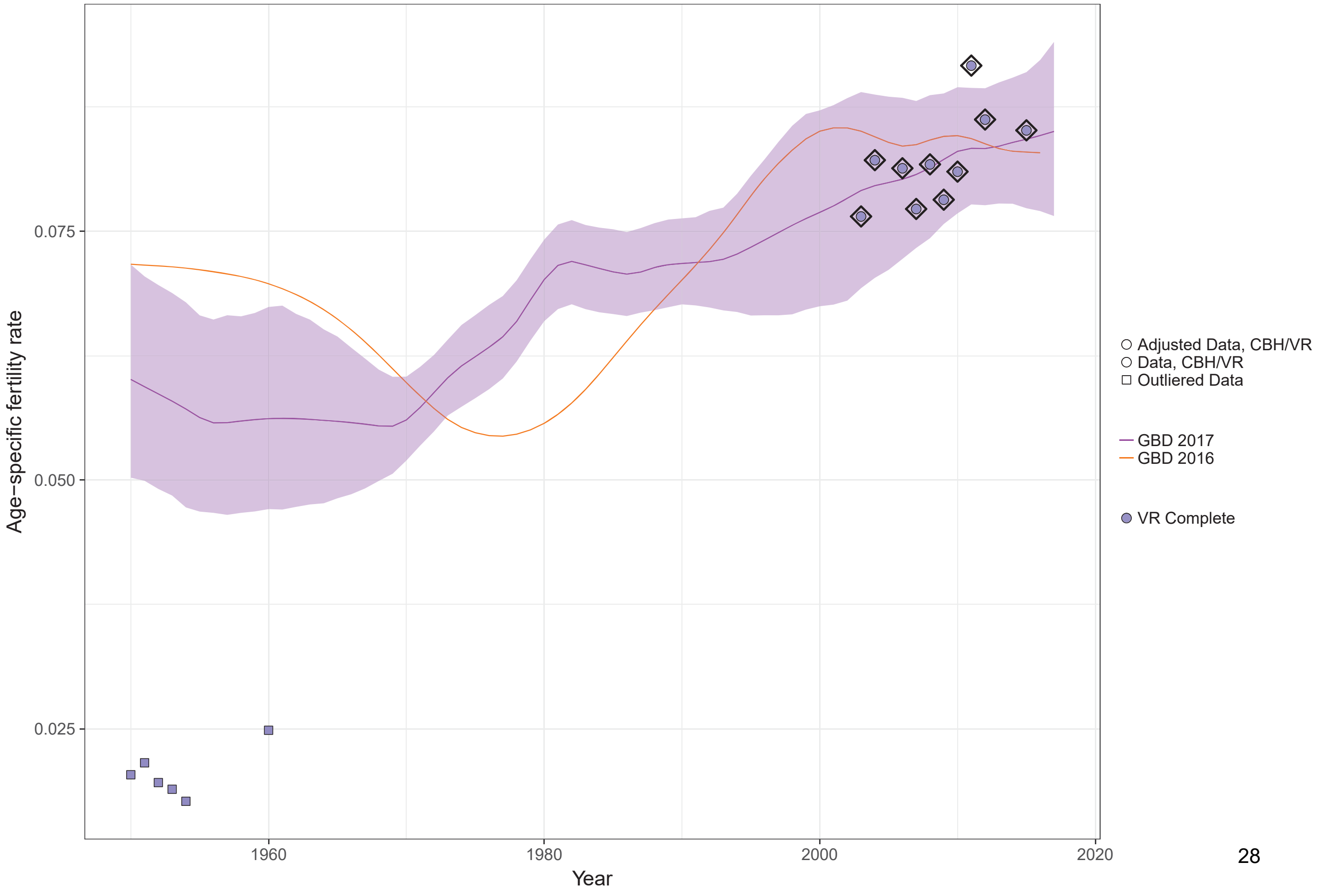

Age−specific fertility fitted trend lines, 1950−2017: Andorra, Ages: 35−39
Data points enclosed with a diamond are reference sources

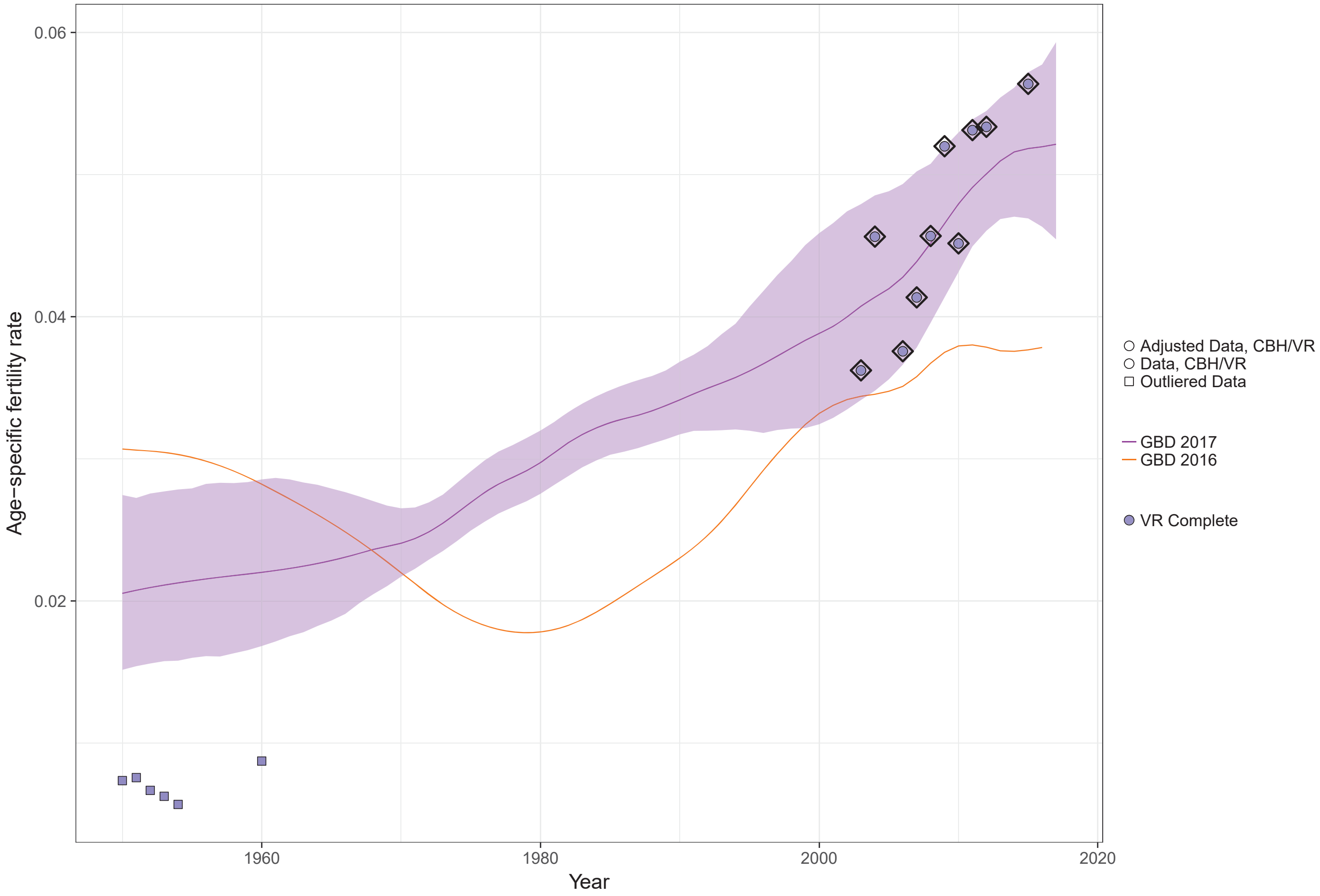

Age−specific fertility fitted trend lines, 1950−2017: Andorra, Ages: 40−44
Data points enclosed with a diamond are reference sources

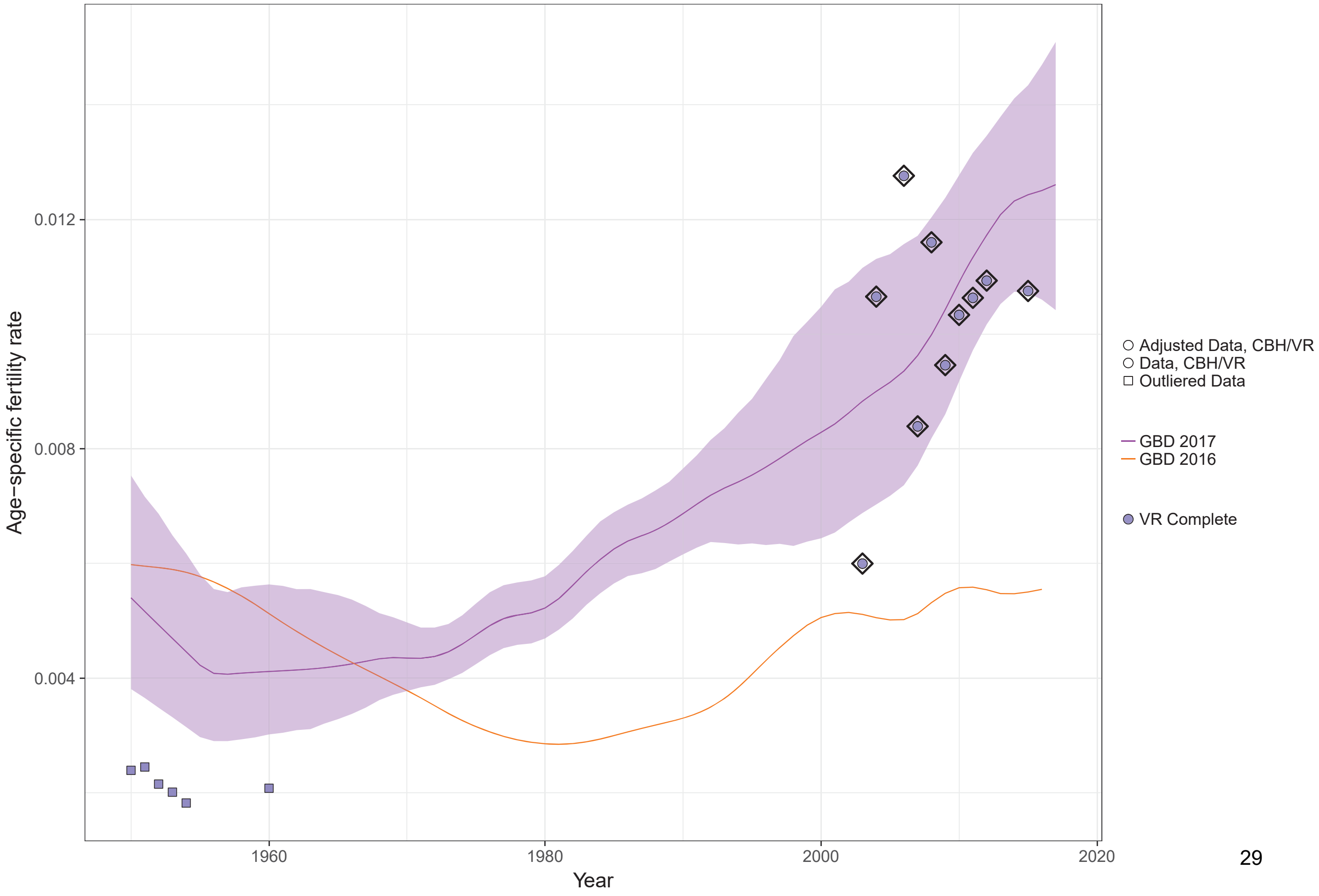

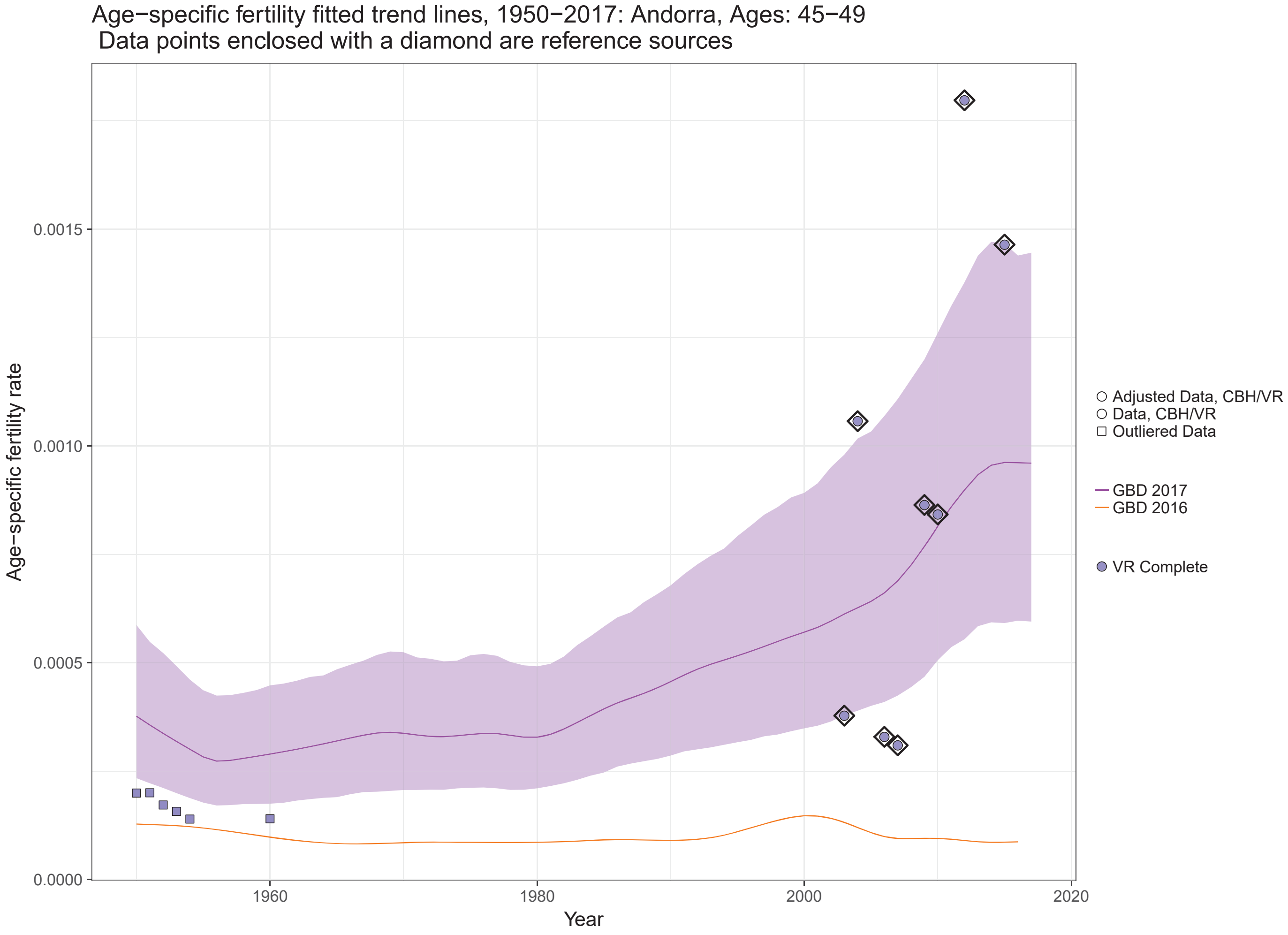
Age−specific fertility fitted trend lines, 1950−2017: Andorra, Ages: 45−49
Data points enclosed with a diamond are reference sources
0.0015
0.0010
0.0005
0.0000
Age−specific fertility rate
1960
1980
2000
2020
Year
Adjusted Data, CBH/VR
Data, CBH/VR
Outliered Data
GBD 2017
GBD 2016
VR Complete

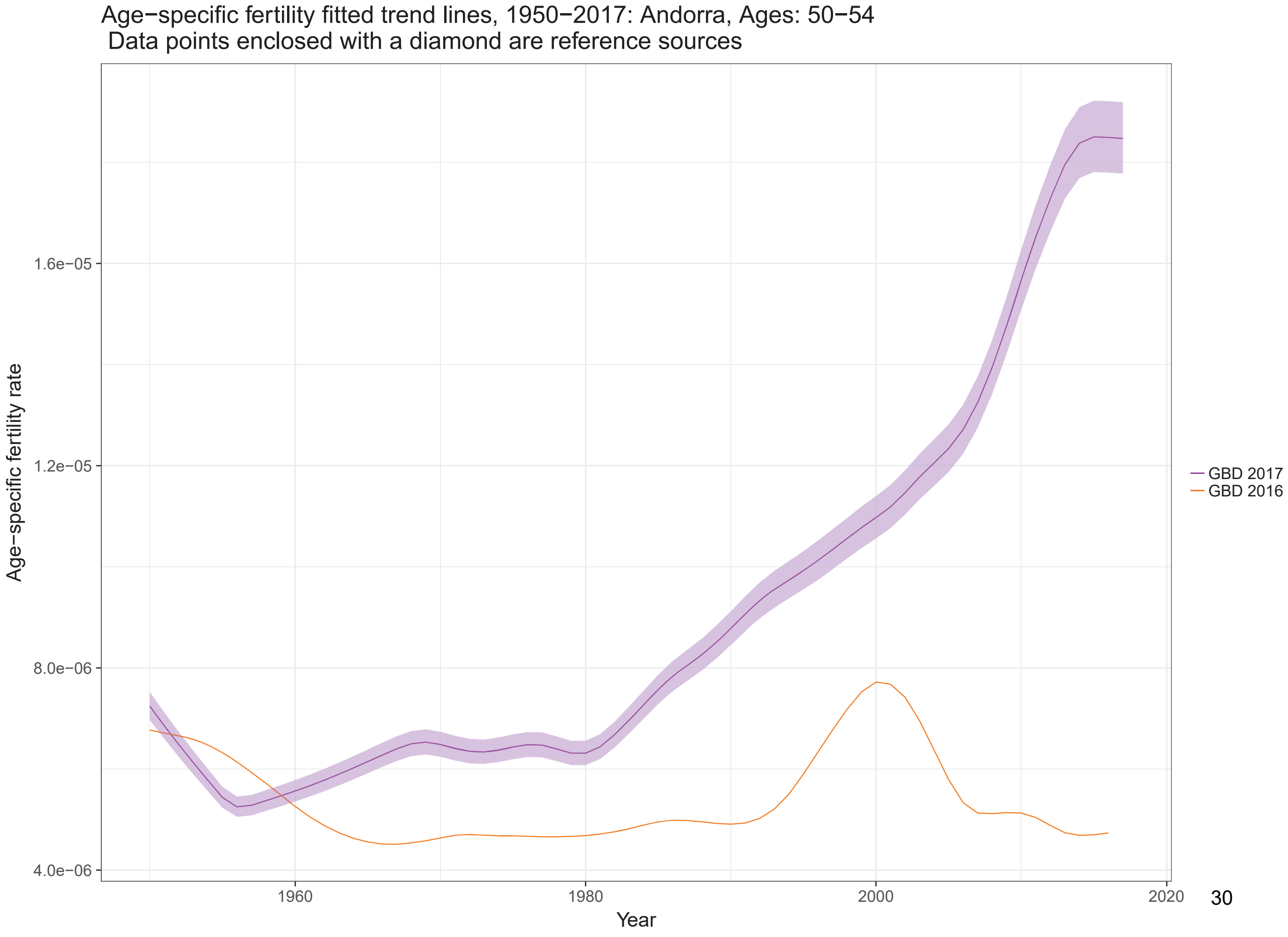
Age−specific fertility fitted trend lines, 1950−2017: Andorra, Ages: 50−54
Data points enclosed with a diamond are reference sources
1.6e−05
1.2e−05
8.0e−06
4.0e−06
Age−specific fertility rate
1960
1980
2000
2020
Year
GBD 2017
GBD 2016

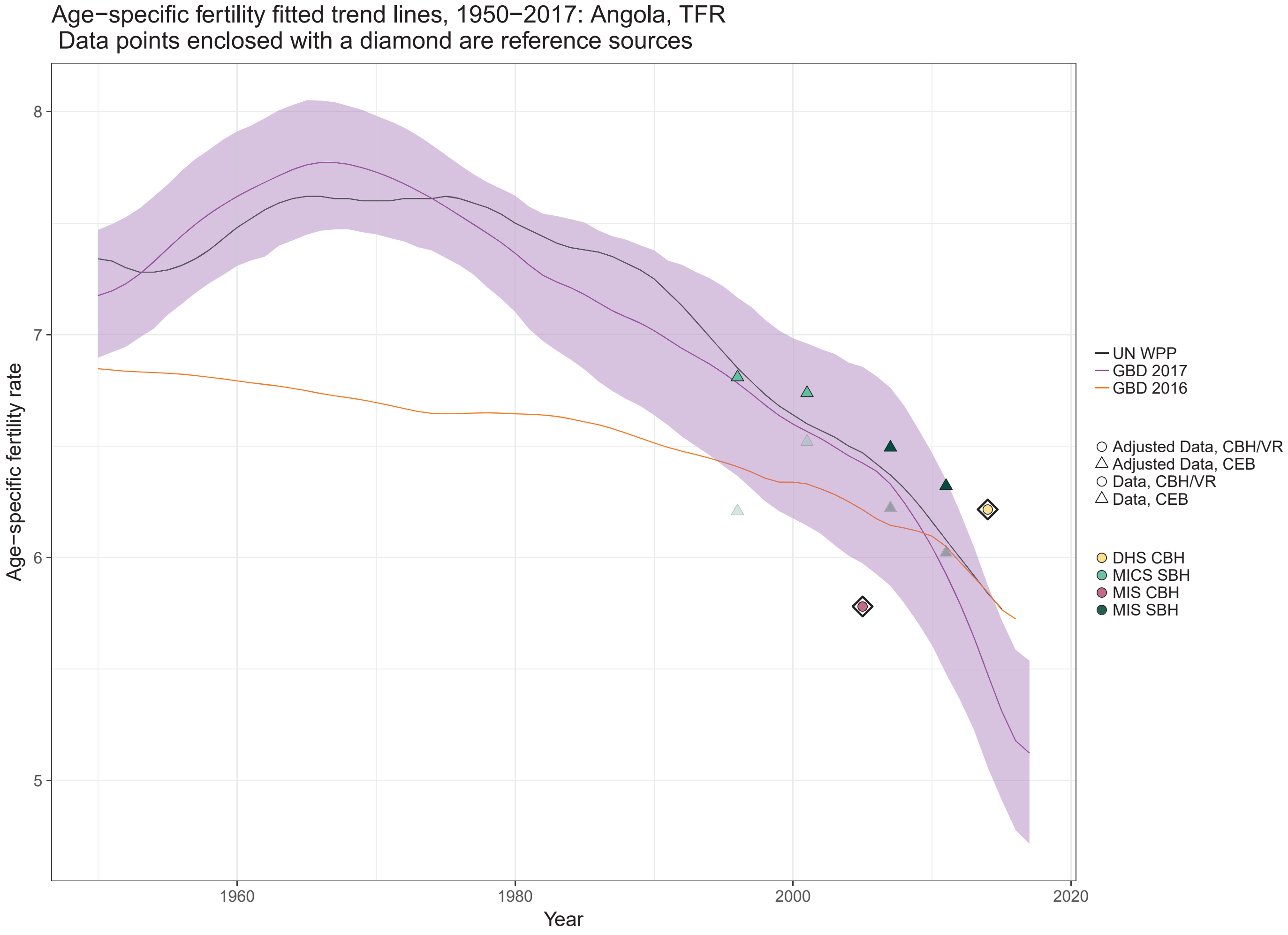
Age−specific fertility fitted trend lines, 1950−2017: Angola, TFR
Data points enclosed with a diamond are reference sources
Age−specific fertility rate
Year
8
7
6
5
1960
1980
2000
2020
UN WPP
GBD 2017
GBD 2016
Adjusted Data, CBH/VR
Adjusted Data, CEB
Data, CBH/VR
Data, CEB
DHS CBH
MICS SBH
MIS CBH
MIS SBH

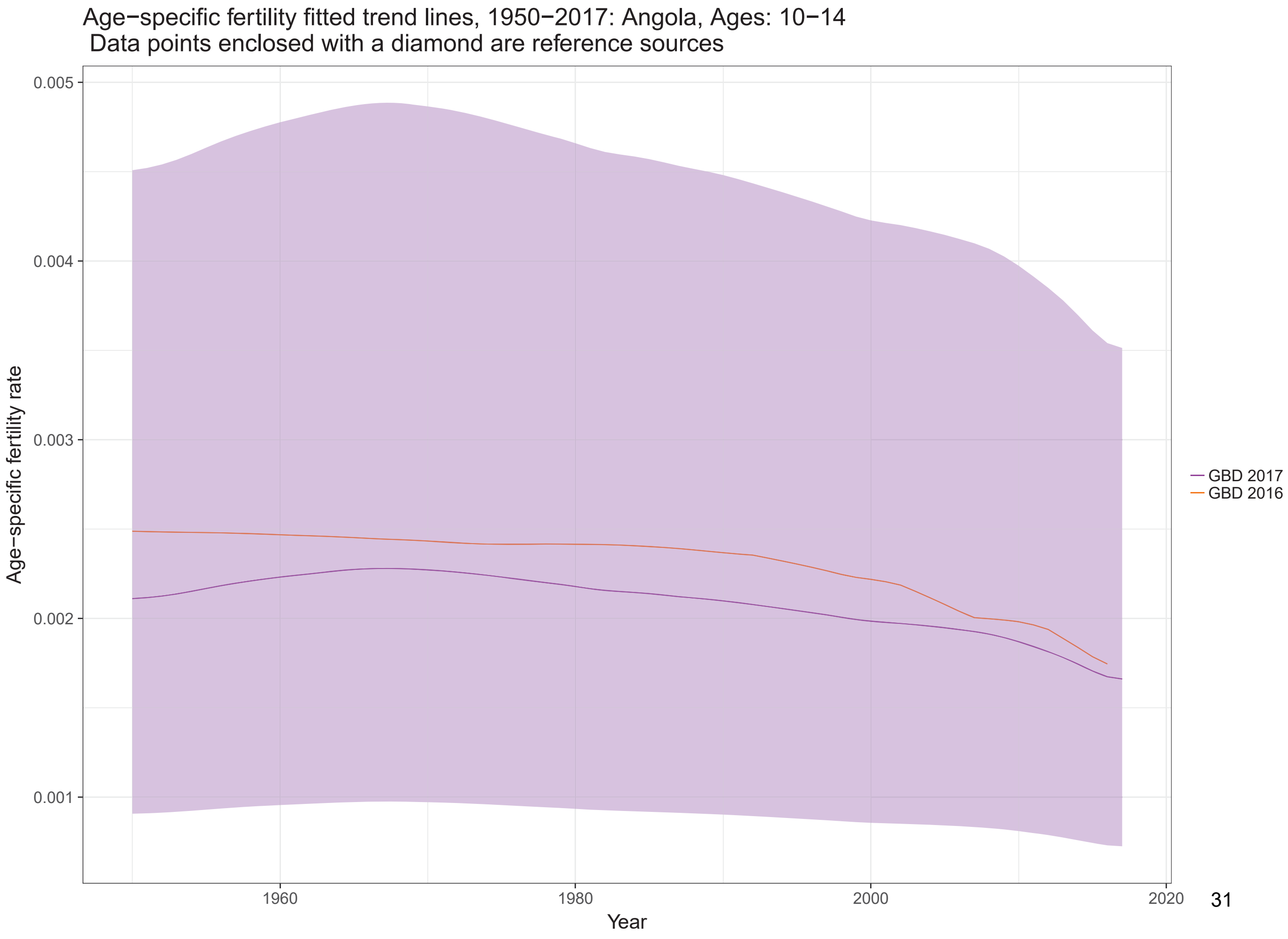
Age−specific fertility fitted trend lines, 1950−2017: Angola, Ages: 10−14
Data points enclosed with a diamond are reference sources
Age−specific fertility rate
Year
0.005
0.004
0.003
0.002
0.001
1960
1980
2000
2020
GBD 2017
GBD 2016

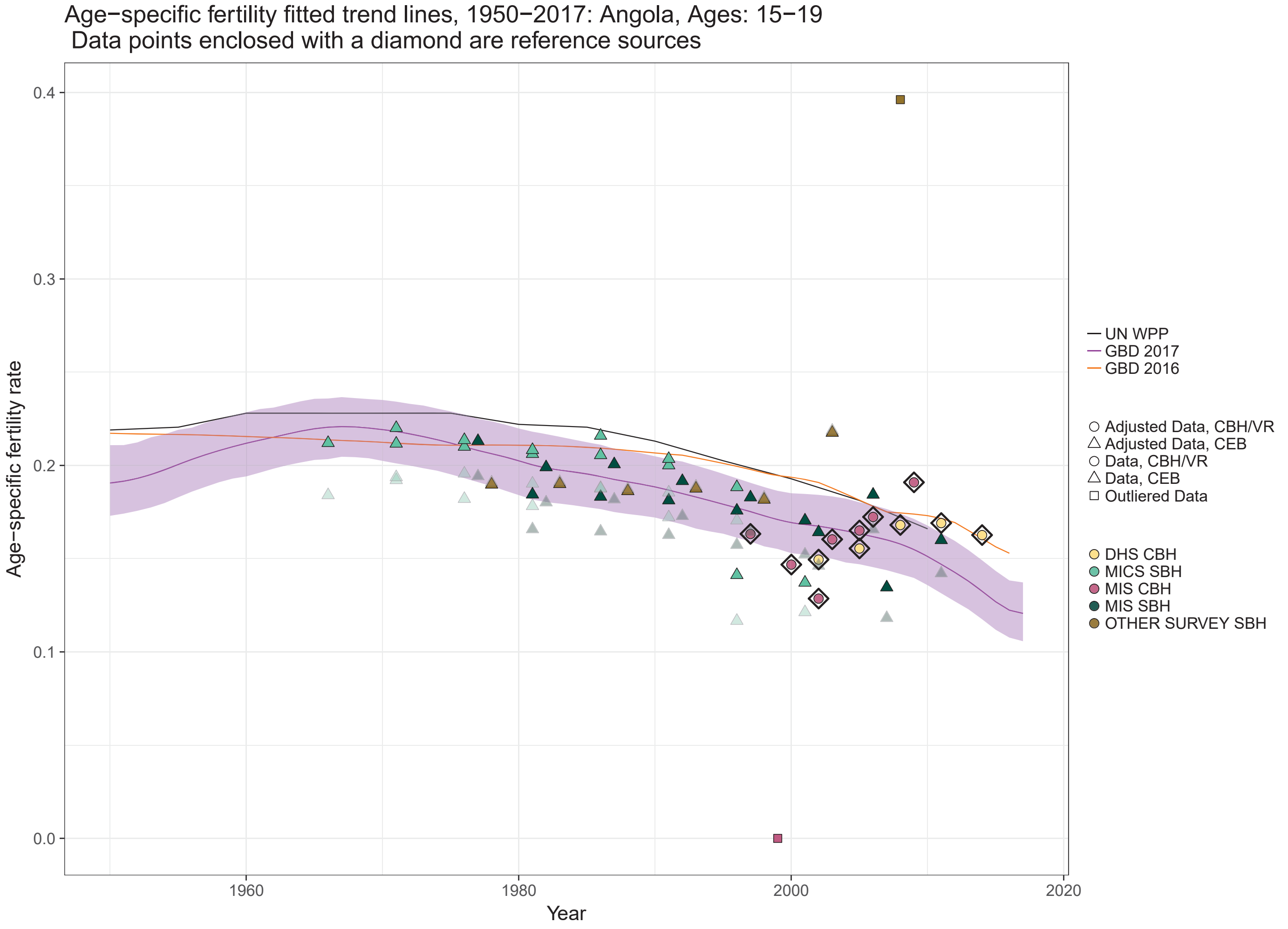

Age−specific fertility fitted trend lines, 1950−2017: Angola, Ages: 15−19
Data points enclosed with a diamond are reference sources
Age−specific fertility rate
Year
0.0
0.1
0.2
0.3
0.4
1960
1980
2000
2020
UN WPP
GBD 2017
GBD 2016
Adjusted Data, CBH/VR
Adjusted Data, CEB
Data, CBH/VR
Data, CEB
Outliered Data
DHS CBH
MICS SBH
MIS CBH
MIS SBH
OTHER SURVEY SBH

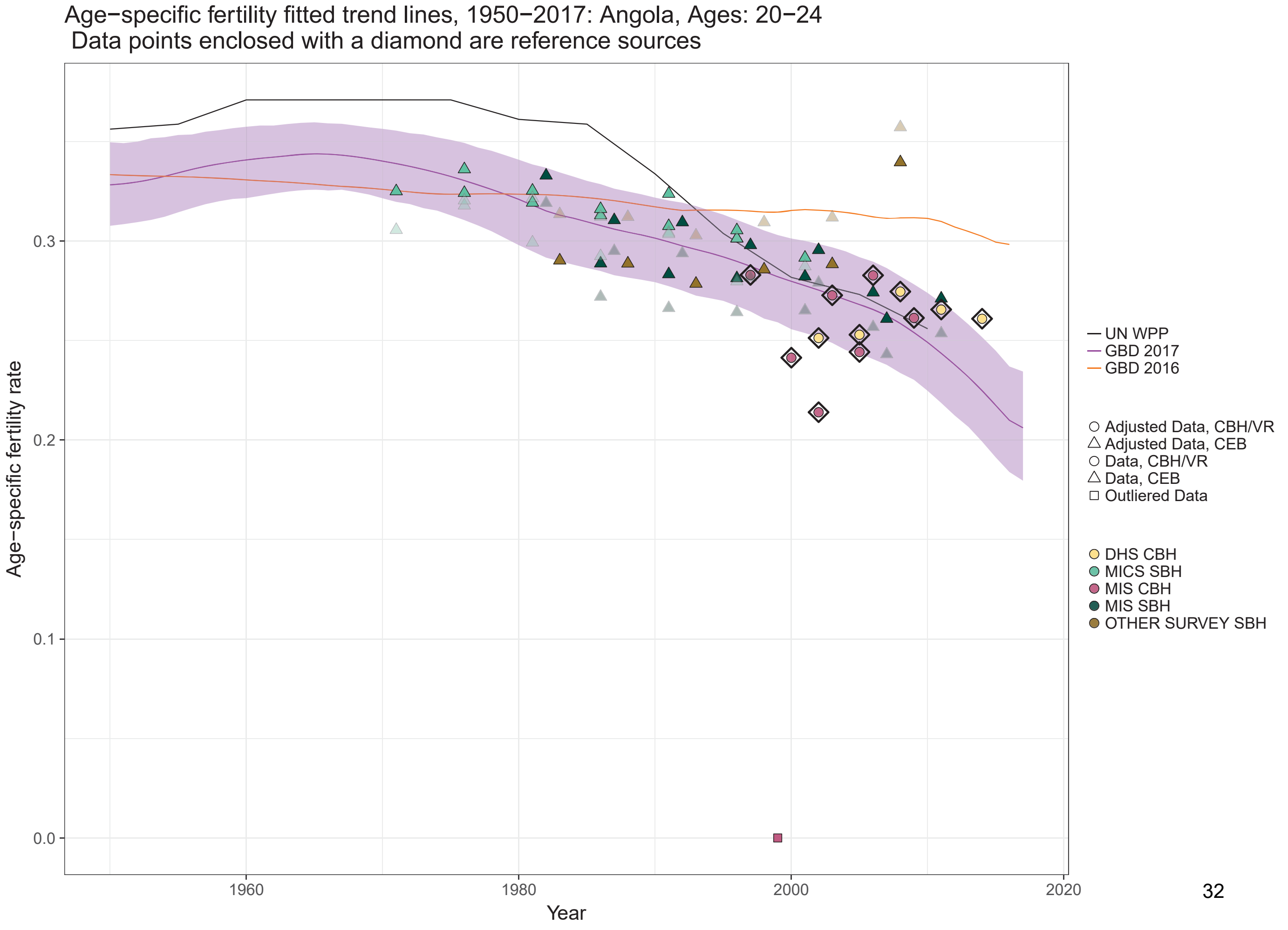

Age−specific fertility fitted trend lines, 1950−2017: Angola, Ages: 20−24
Data points enclosed with a diamond are reference sources
Age−specific fertility rate
Year
0.0
0.1
0.2
0.3
1960
1980
2000
2020
UN WPP
GBD 2017
GBD 2016
Adjusted Data, CBH/VR
Adjusted Data, CEB
Data, CBH/VR
Data, CEB
Outliered Data
DHS CBH
MICS SBH
MIS CBH
MIS SBH
OTHER SURVEY SBH

Age−specific fertility fitted trend lines, 1950−2017: Angola, Ages: 25−29
Data points enclosed with a diamond are reference sources

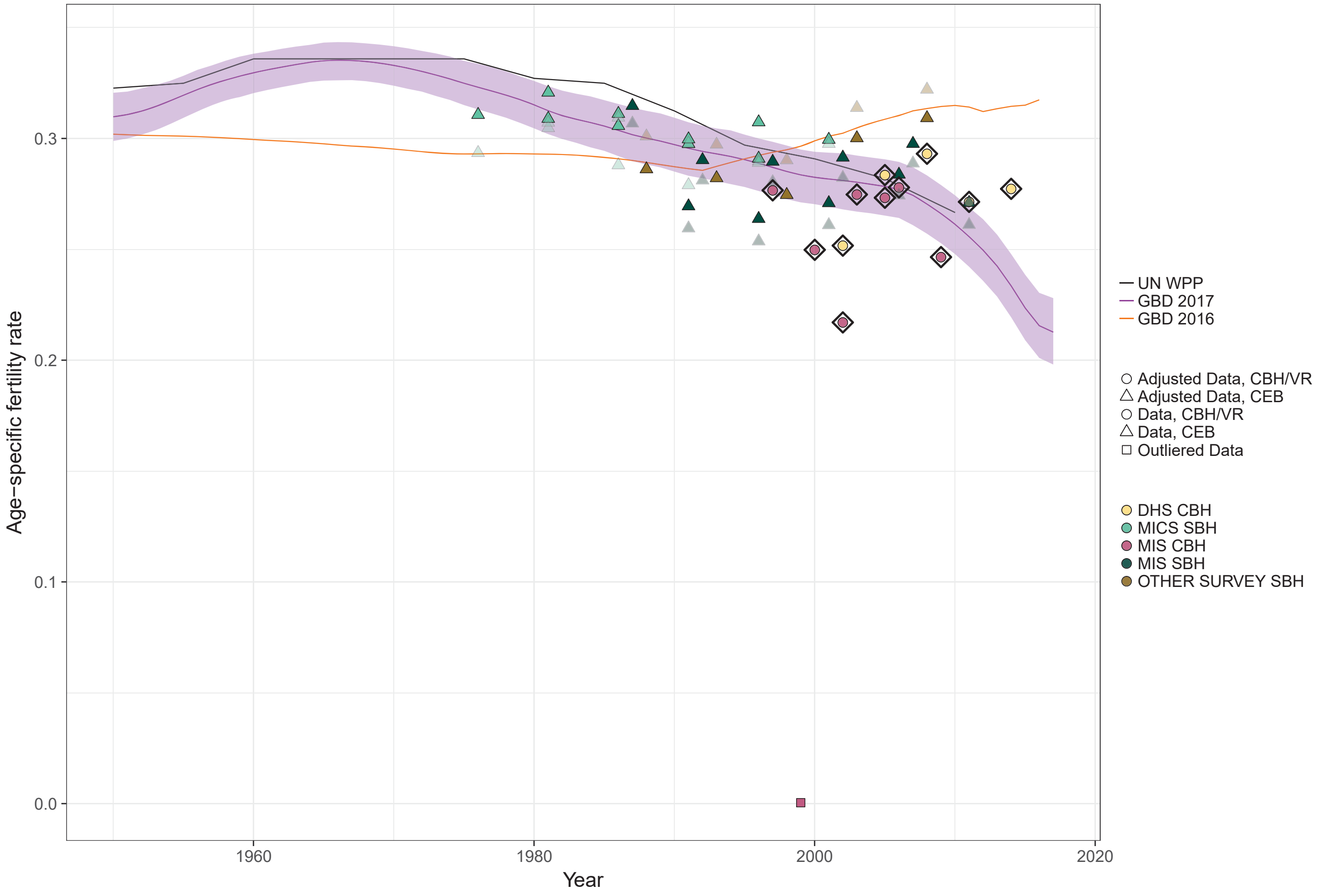

Age−specific fertility fitted trend lines, 1950−2017: Angola, Ages: 30−34
Data points enclosed with a diamond are reference sources

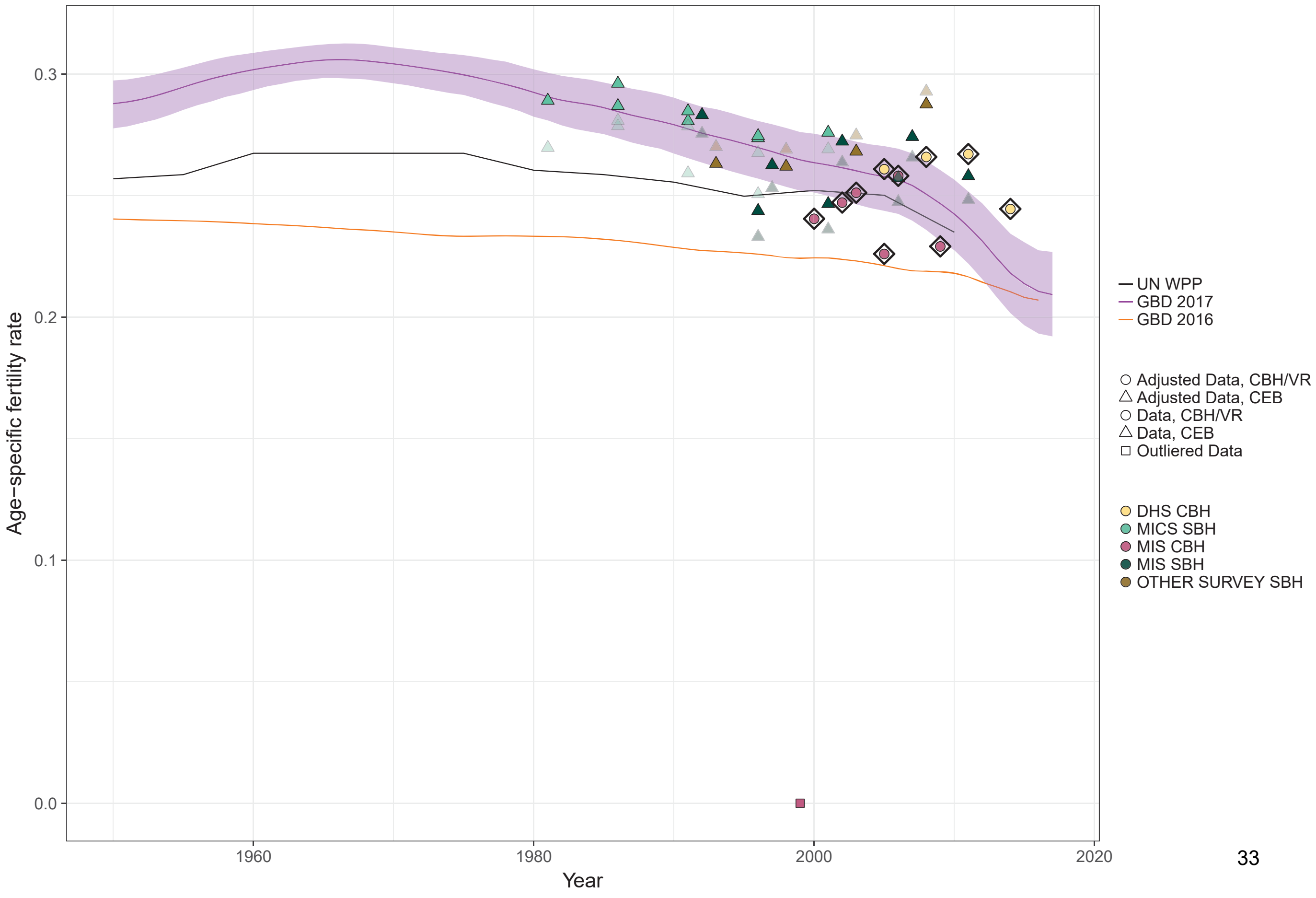

Age−specific fertility fitted trend lines, 1950−2017: Angola, Ages: 35−39
Data points enclosed with a diamond are reference sources

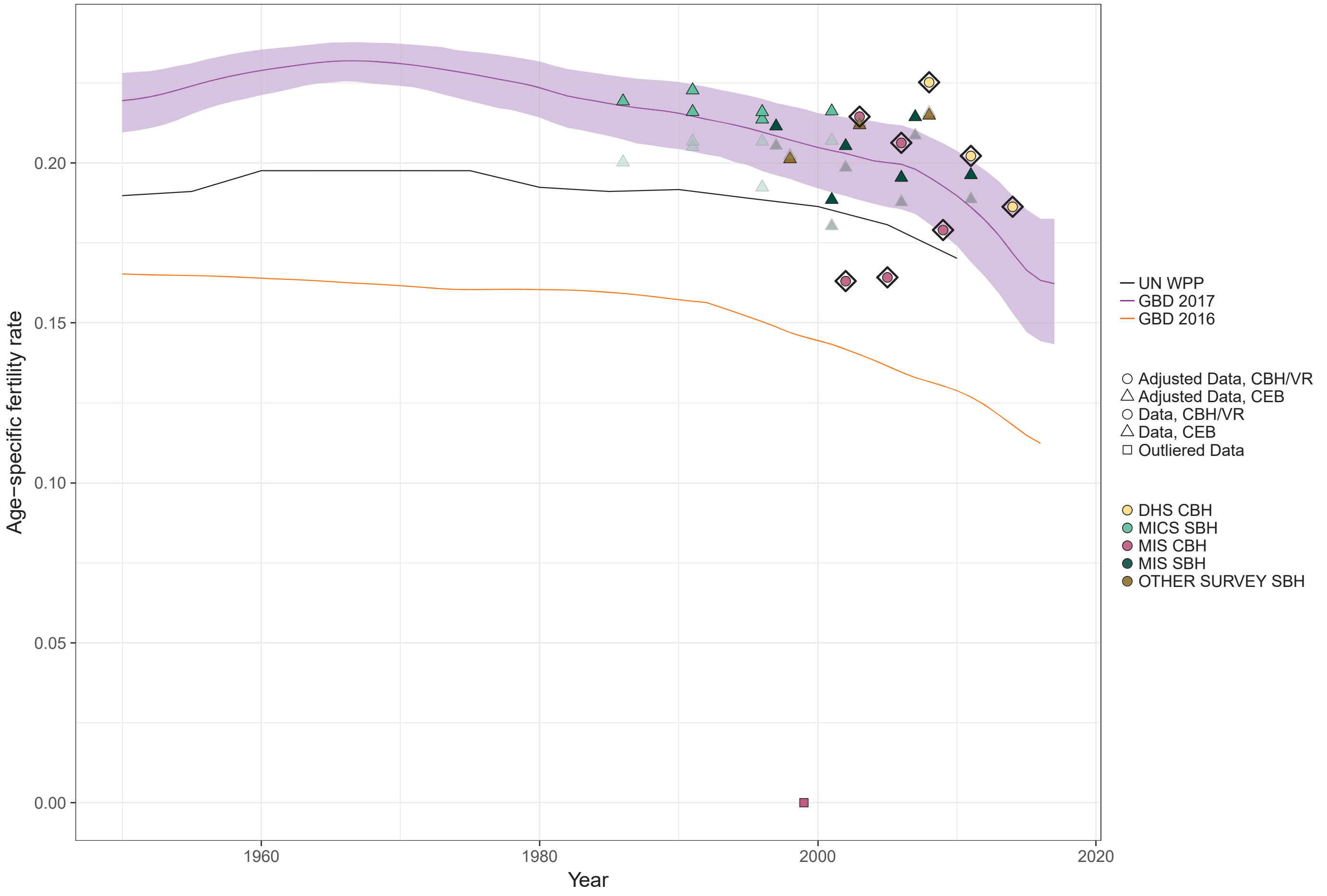

Age−specific fertility fitted trend lines, 1950−2017: Angola, Ages: 40−44
Data points enclosed with a diamond are reference sources

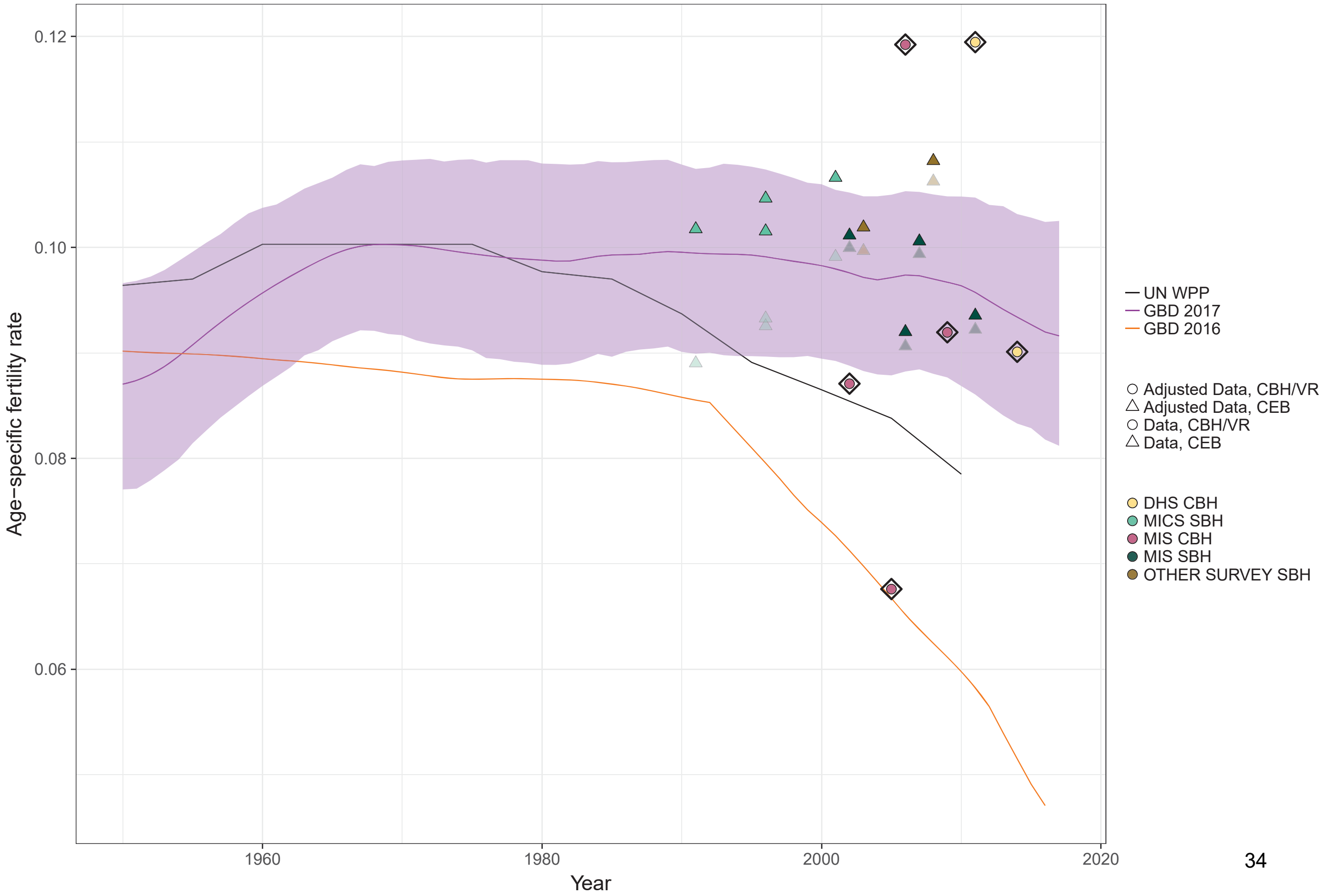

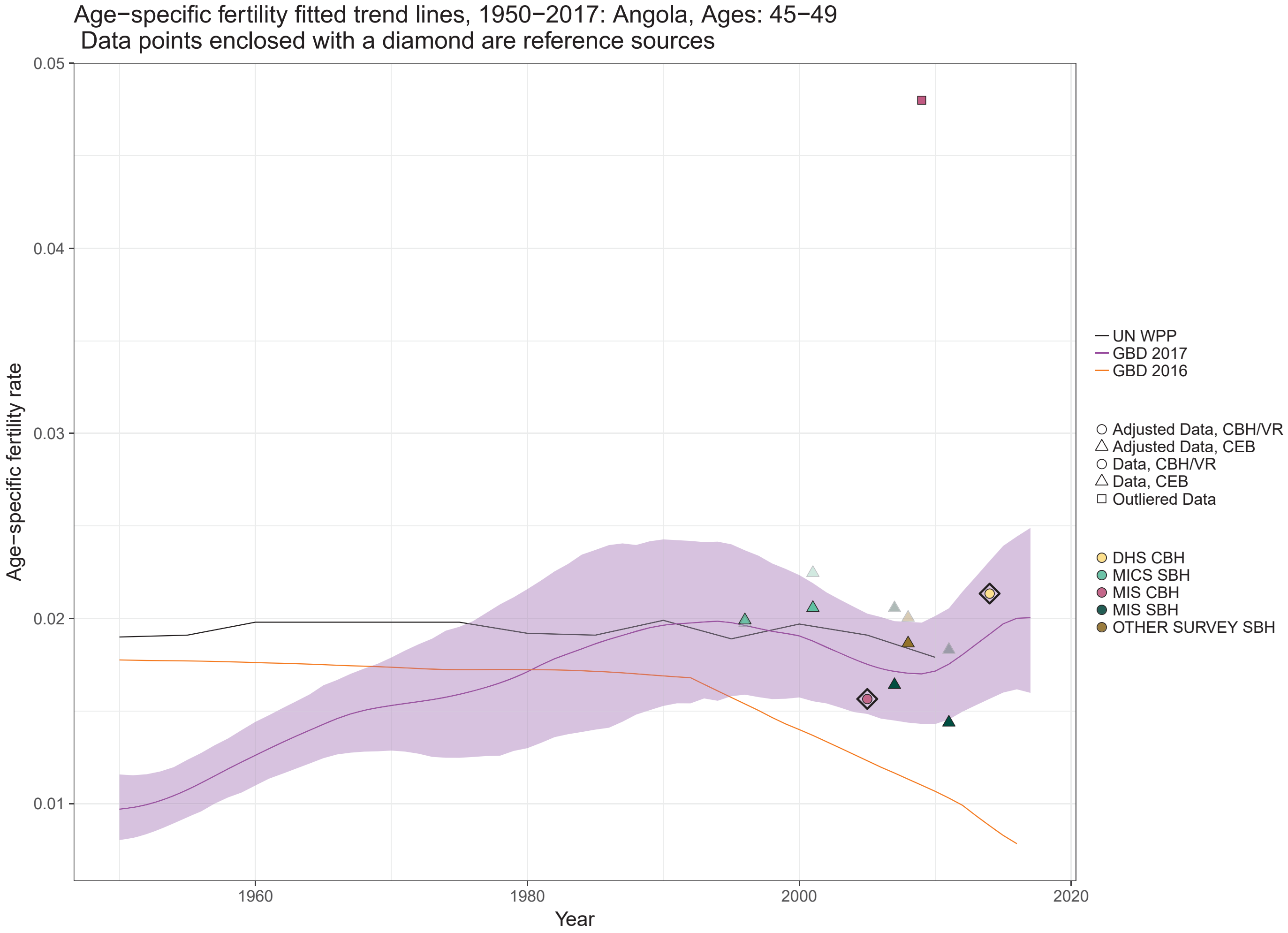

Age−specific fertility fitted trend lines, 1950−2017: Angola, Ages: 45−49
Data points enclosed with a diamond are reference sources
Age−specific fertility rate
0.05
0.04
0.03
0.02
0.01
1960
1980
2000
2020
Year
UN WPP
GBD 2017
GBD 2016
Adjusted Data, CBH/VR
Adjusted Data, CEB
Data, CBH/VR
Data, CEB
Outliered Data
DHS CBH
MICS SBH
MIS CBH
MIS SBH
OTHER SURVEY SBH

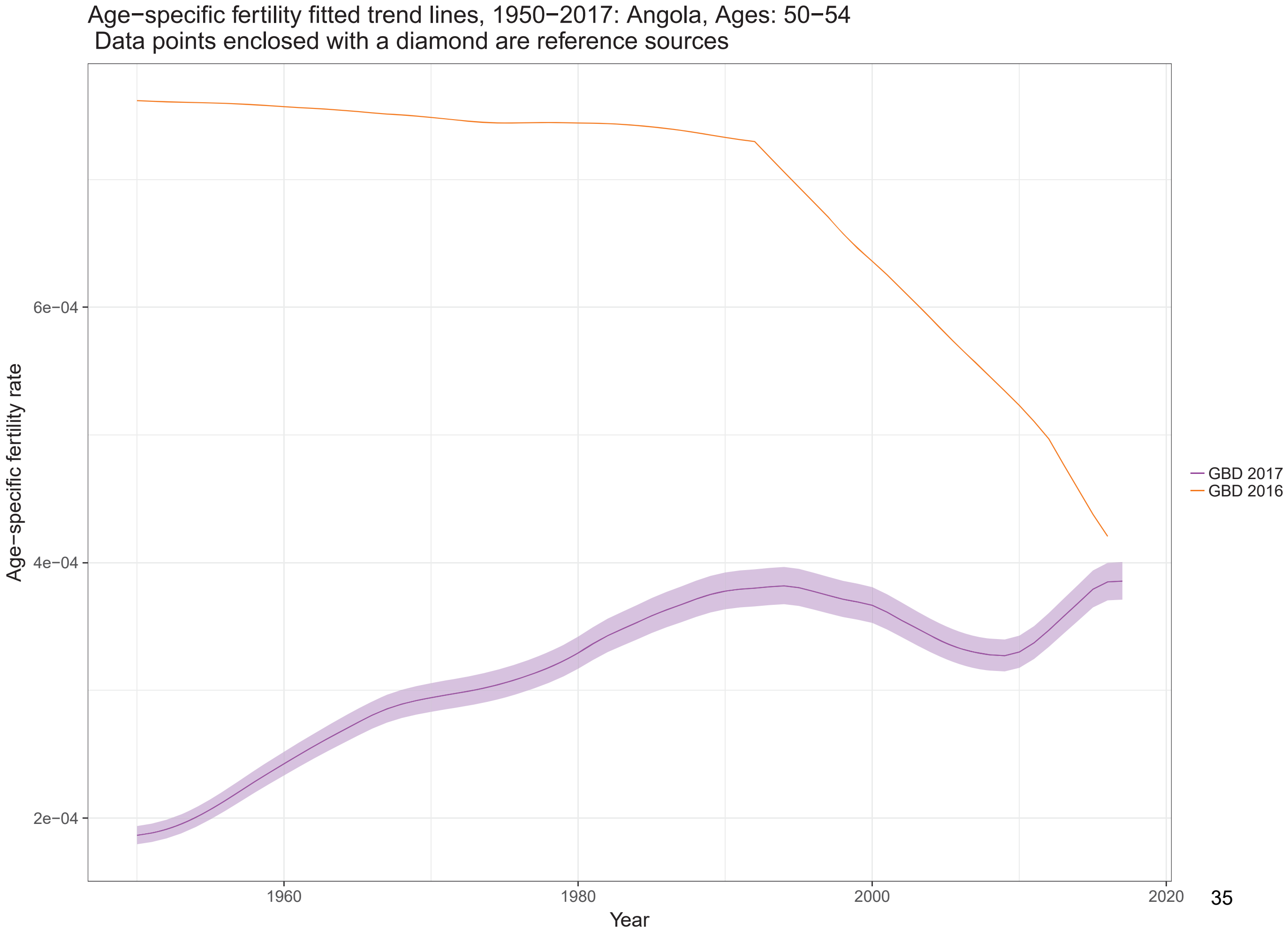

Age−specific fertility fitted trend lines, 1950−2017: Angola, Ages: 50−54
Data points enclosed with a diamond are reference sources
Age−specific fertility rate
6e−04
4e−04
2e−04
1960
1980
2000
2020
Year
GBD 2017
GBD 2016

Age−specific fertility fitted trend lines, 1950−2017: Antigua and Barbuda, TFR
Data points enclosed with a diamond are reference sources

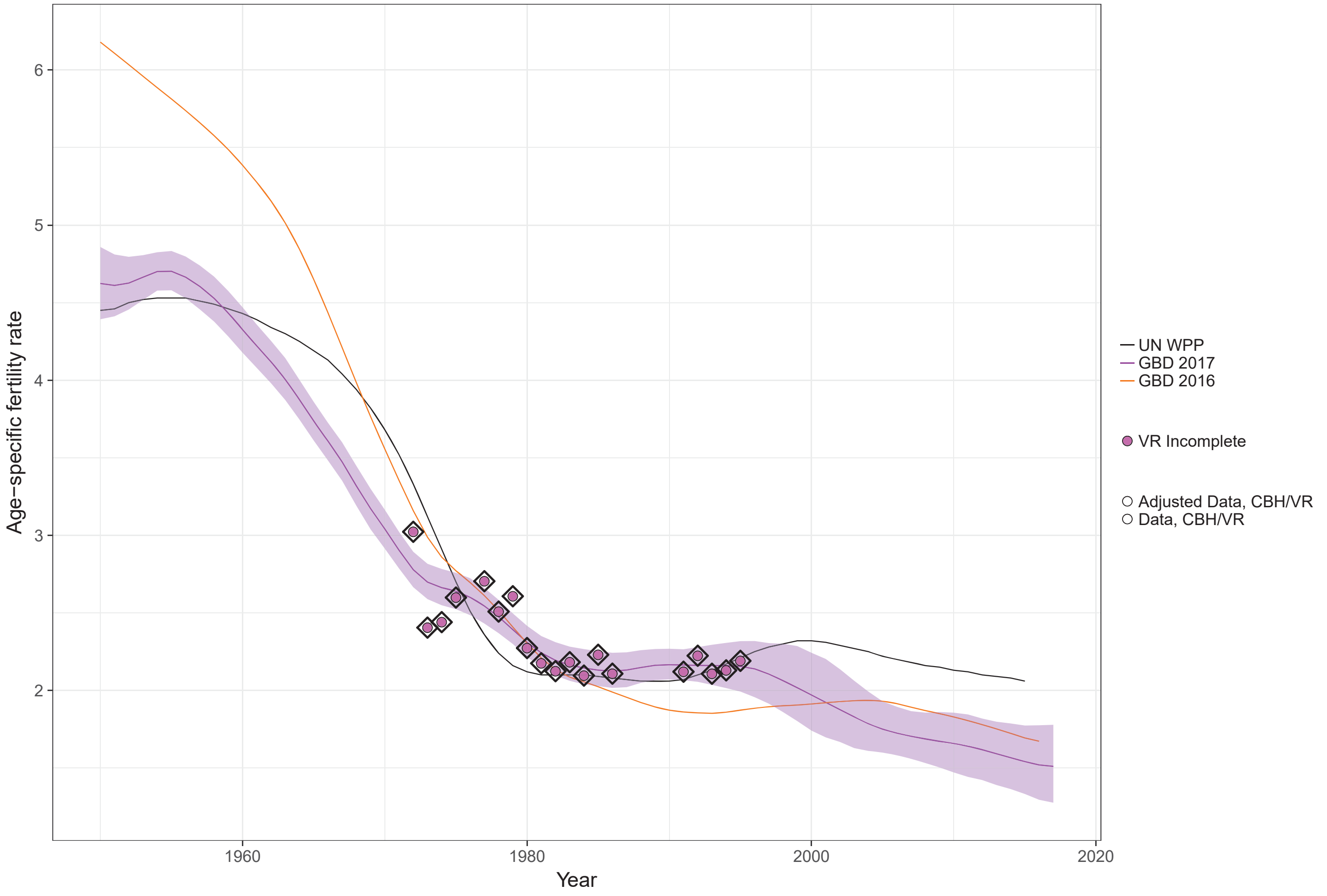

Age−specific fertility fitted trend lines, 1950−2017: Antigua and Barbuda, Ages: 10−14
Data points enclosed with a diamond are reference sources

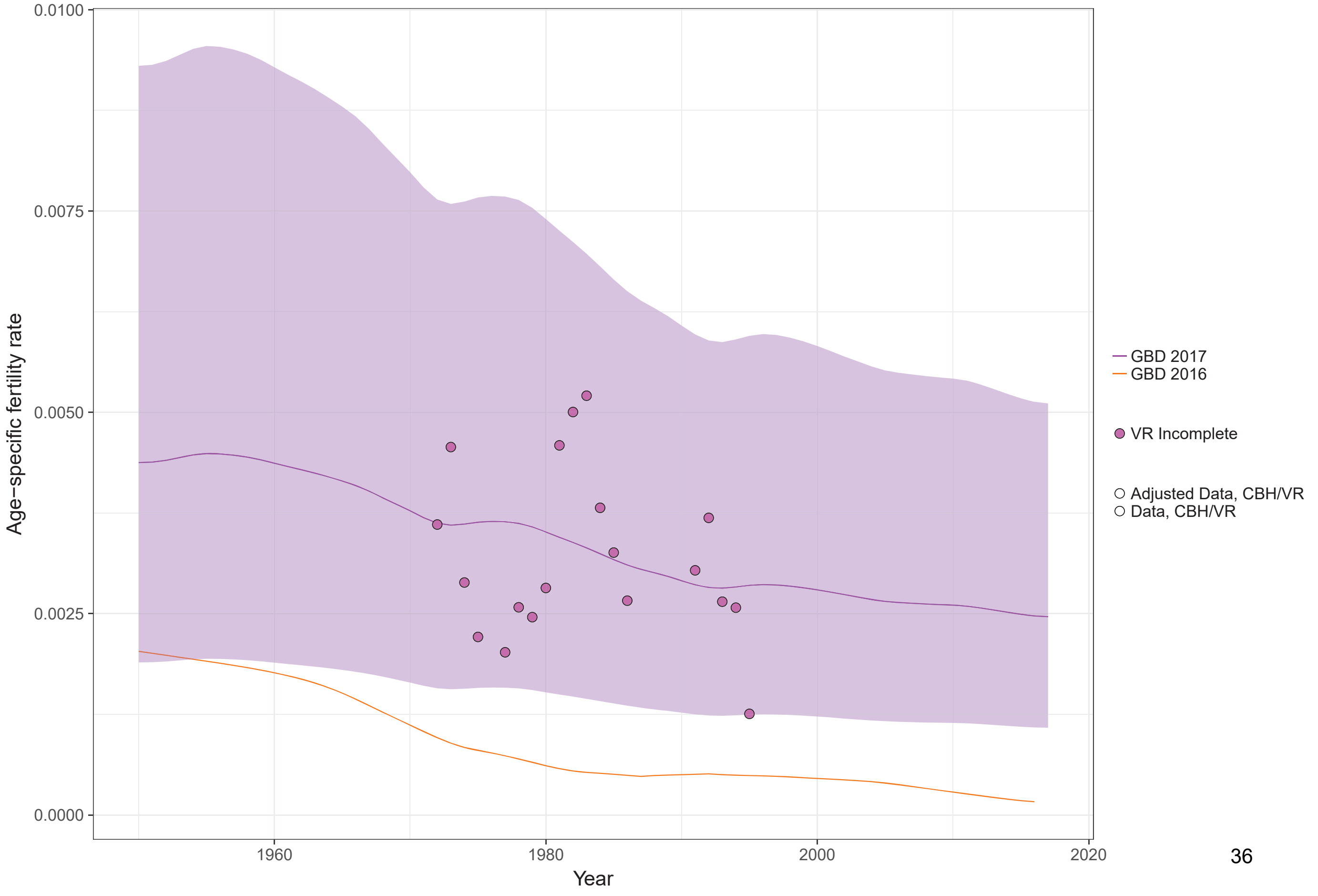

Age−specific fertility fitted trend lines, 1950−2017: Antigua and Barbuda, Ages: 15−19
Data points enclosed with a diamond are reference sources

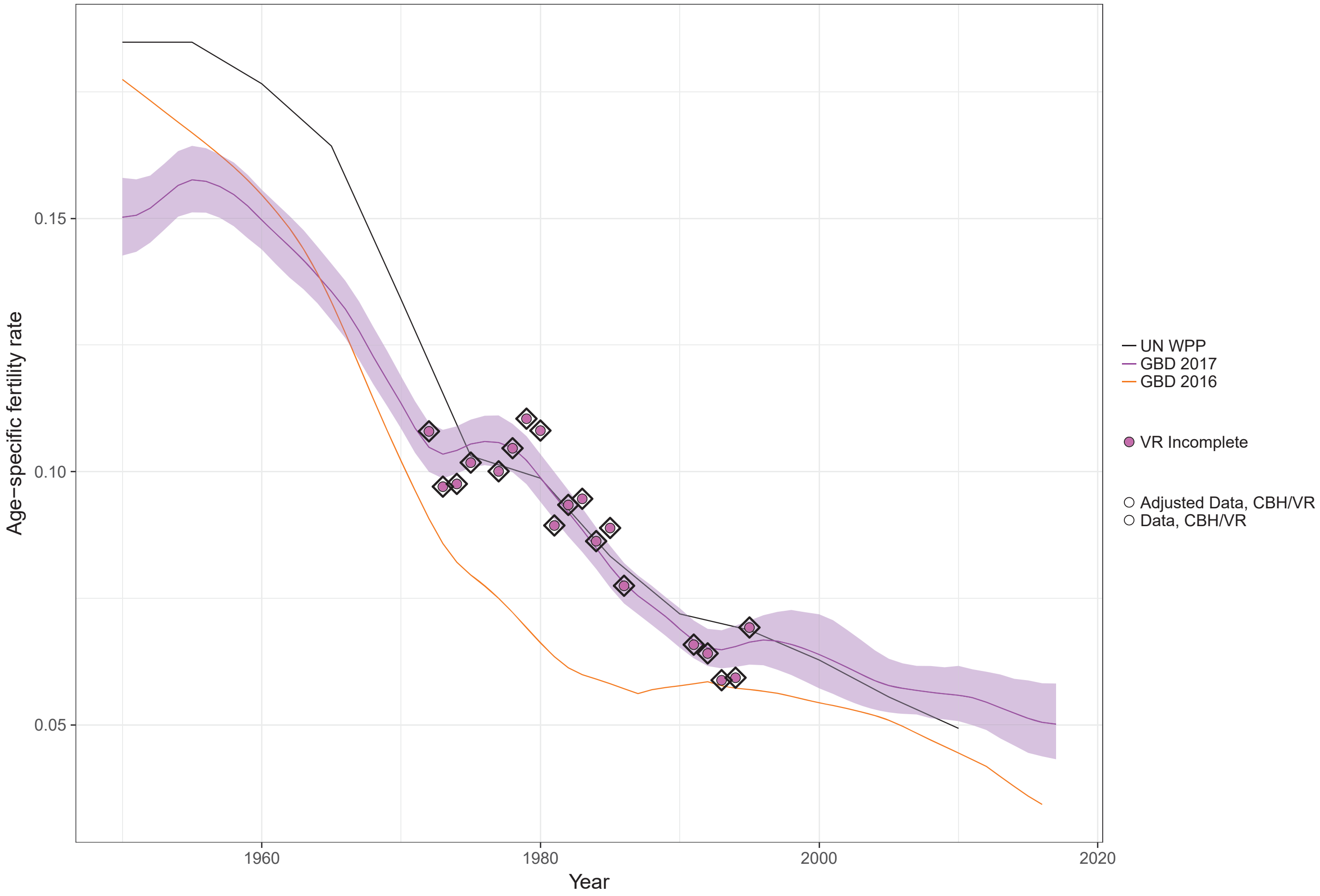

Age−specific fertility fitted trend lines, 1950−2017: Antigua and Barbuda, Ages: 20−24
Data points enclosed with a diamond are reference sources

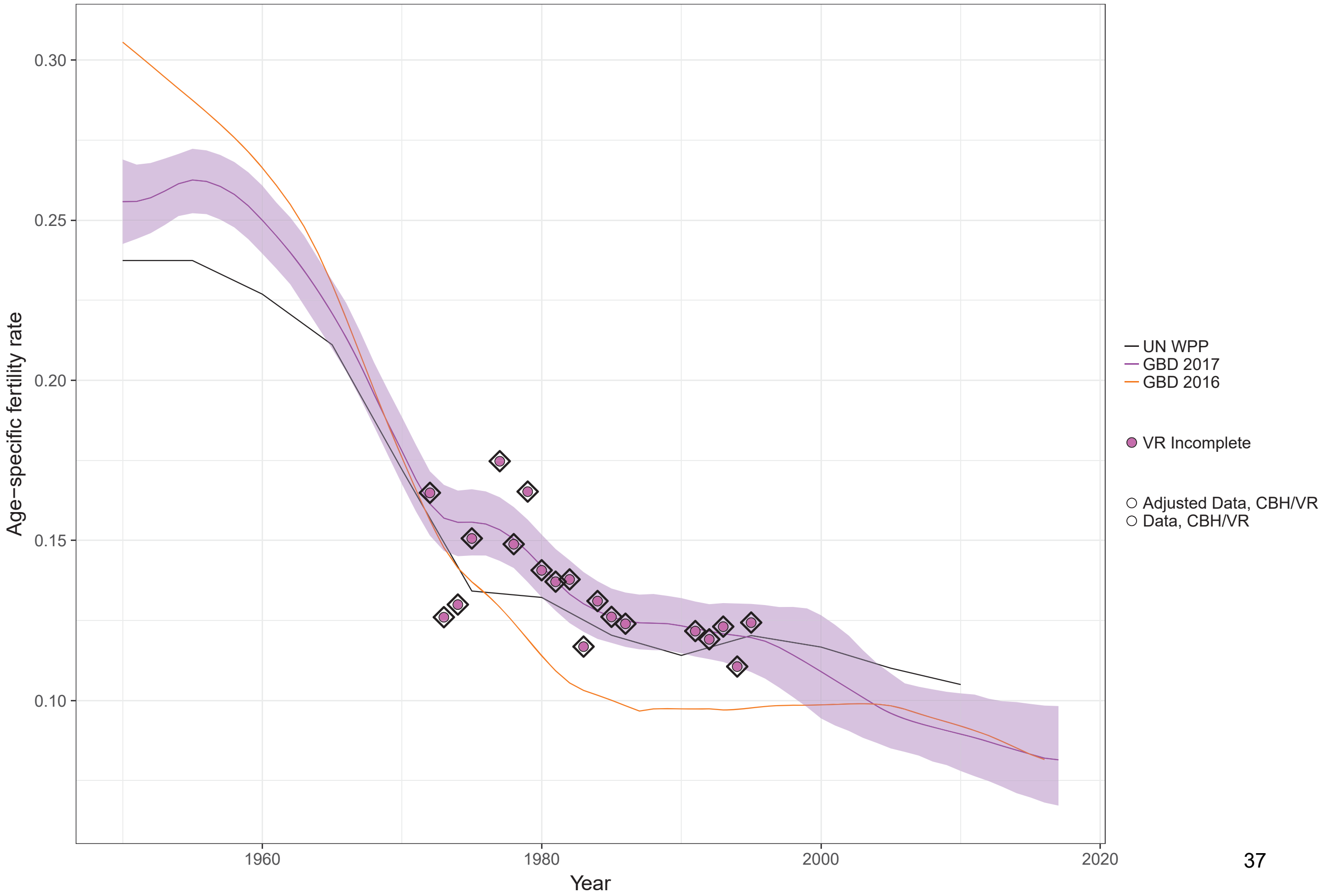

Age−specific fertility fitted trend lines, 1950−2017: Antigua and Barbuda, Ages: 25−29
Data points enclosed with a diamond are reference sources

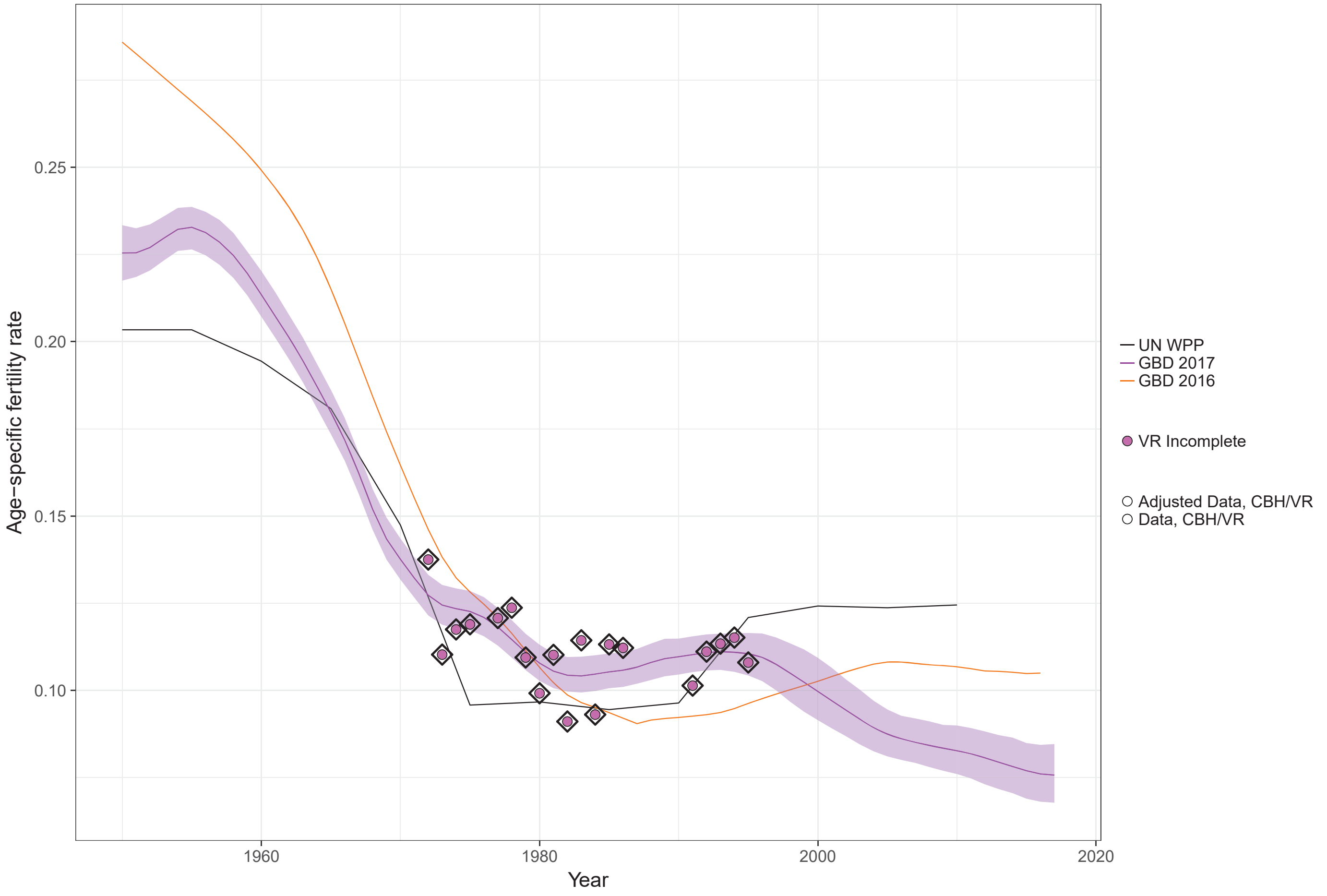

Age−specific fertility fitted trend lines, 1950−2017: Antigua and Barbuda, Ages: 30−34
Data points enclosed with a diamond are reference sources

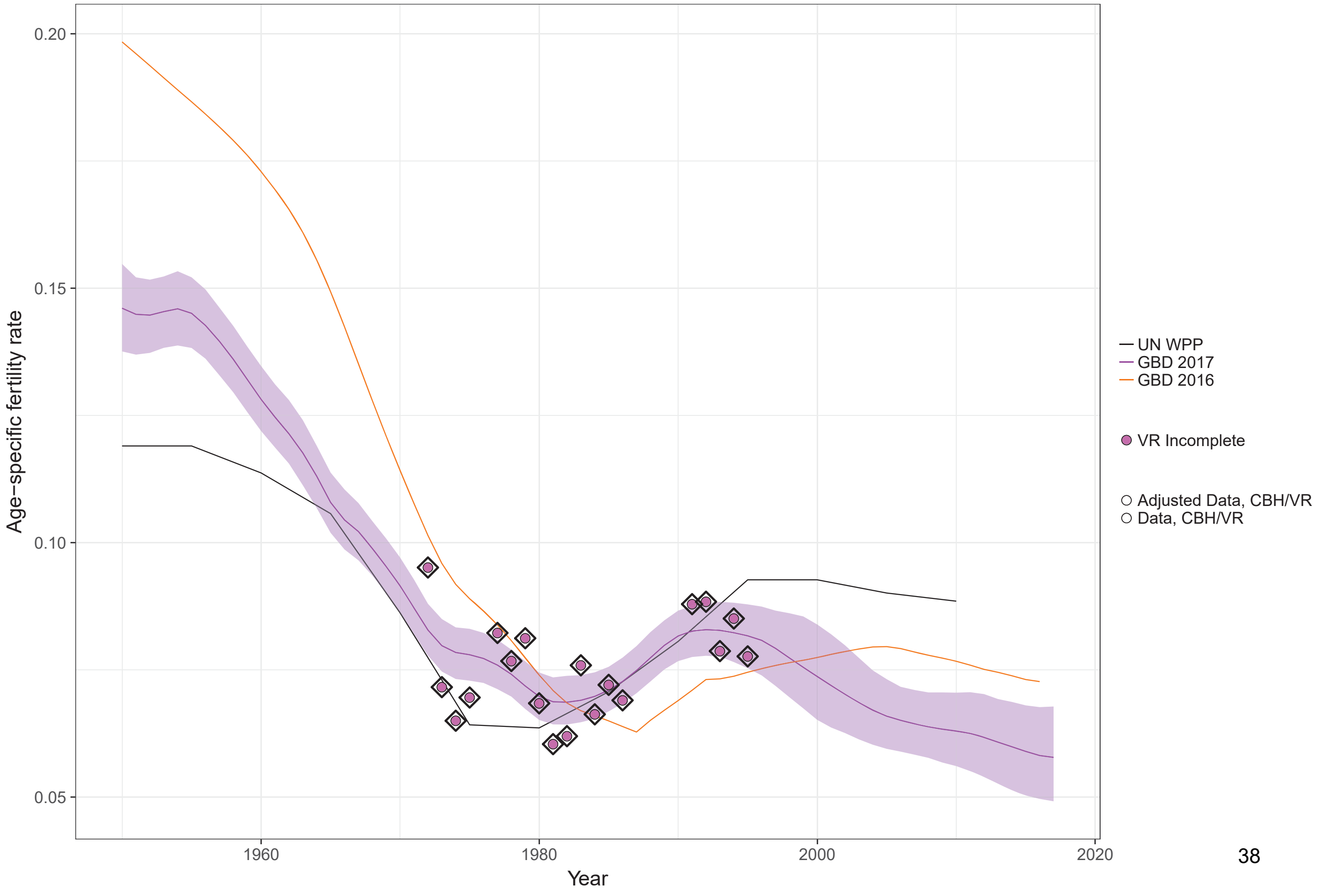

Age−specific fertility fitted trend lines, 1950−2017: Antigua and Barbuda, Ages: 35−39
Data points enclosed with a diamond are reference sources

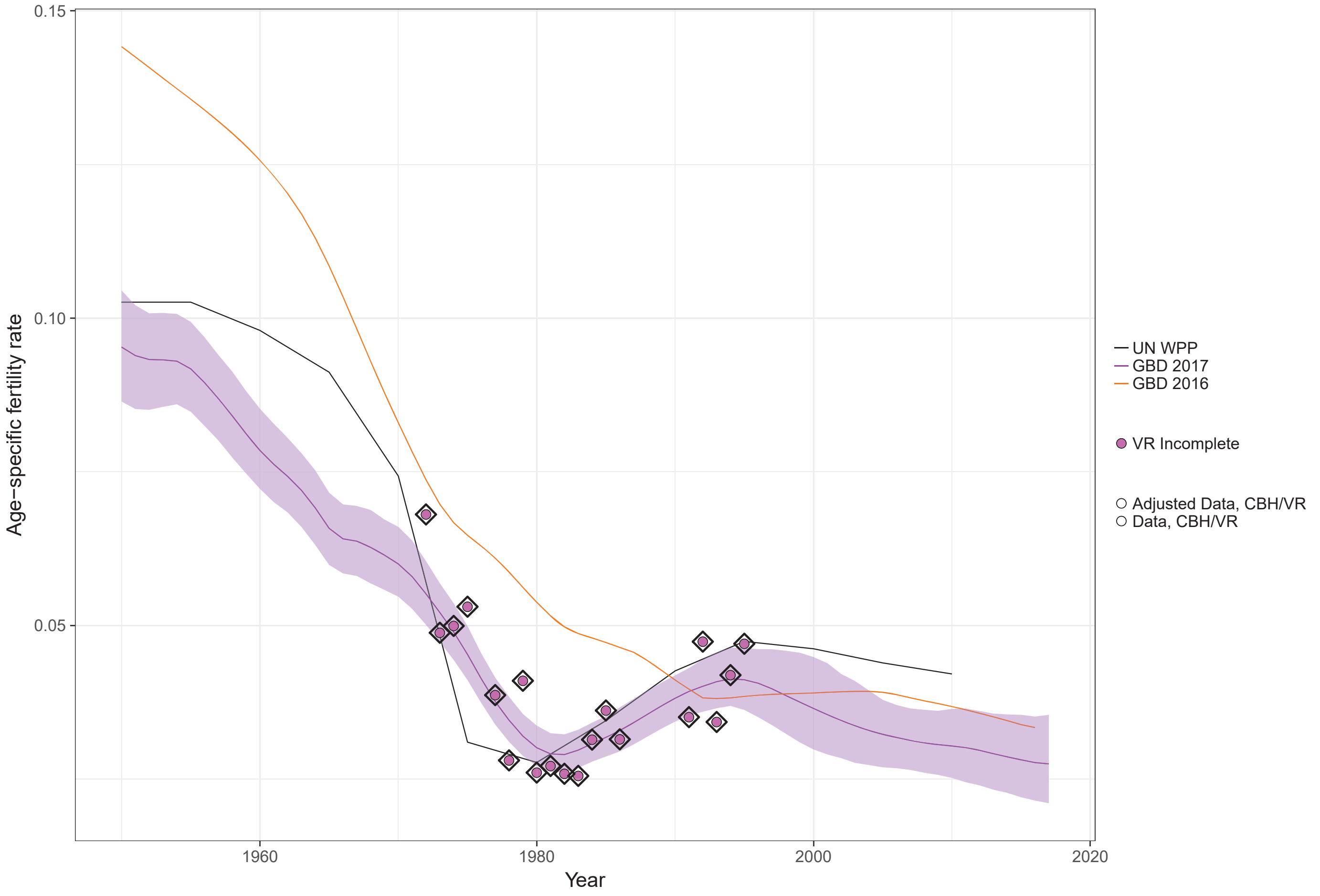

Age−specific fertility fitted trend lines, 1950−2017: Antigua and Barbuda, Ages: 40−44
Data points enclosed with a diamond are reference sources

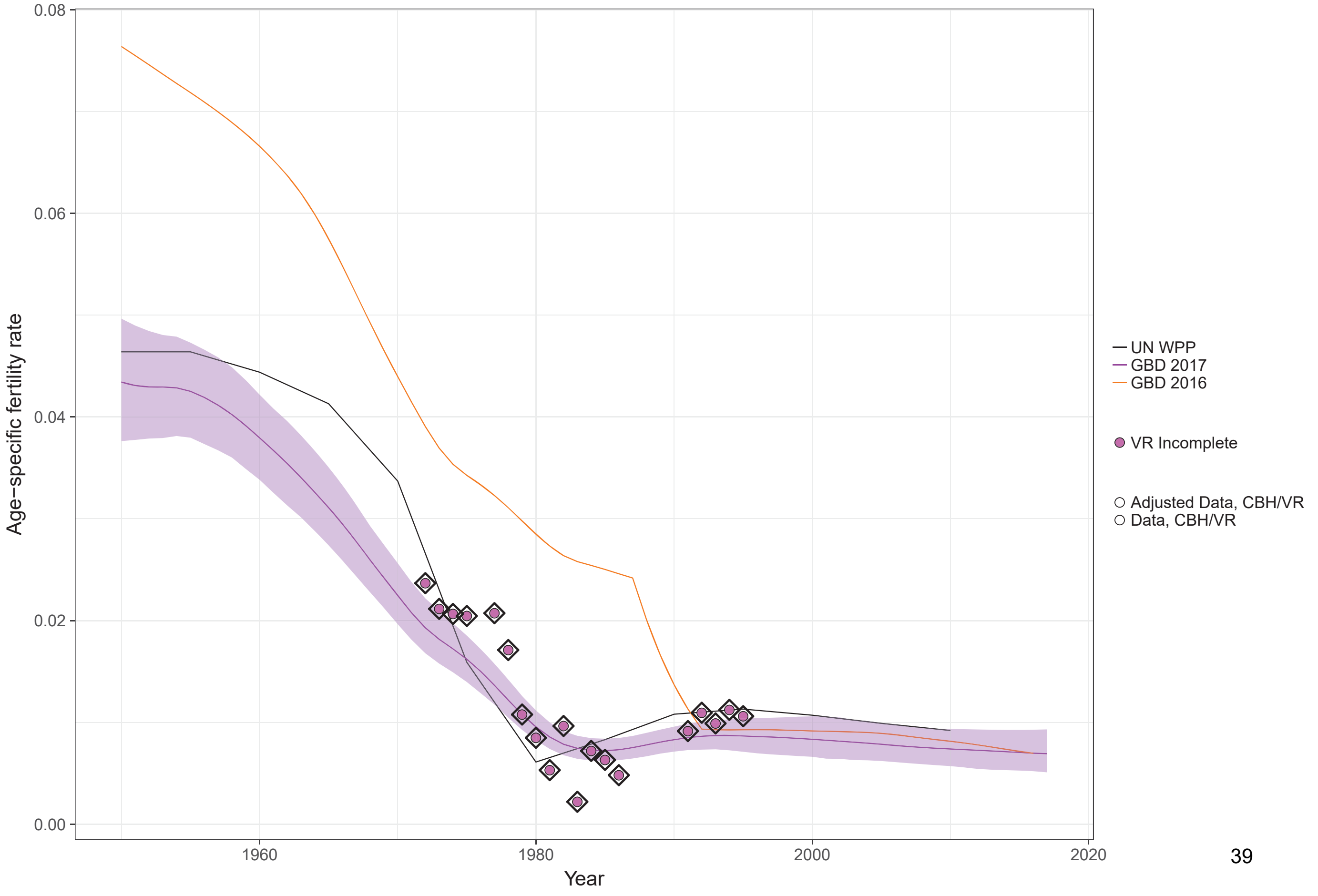

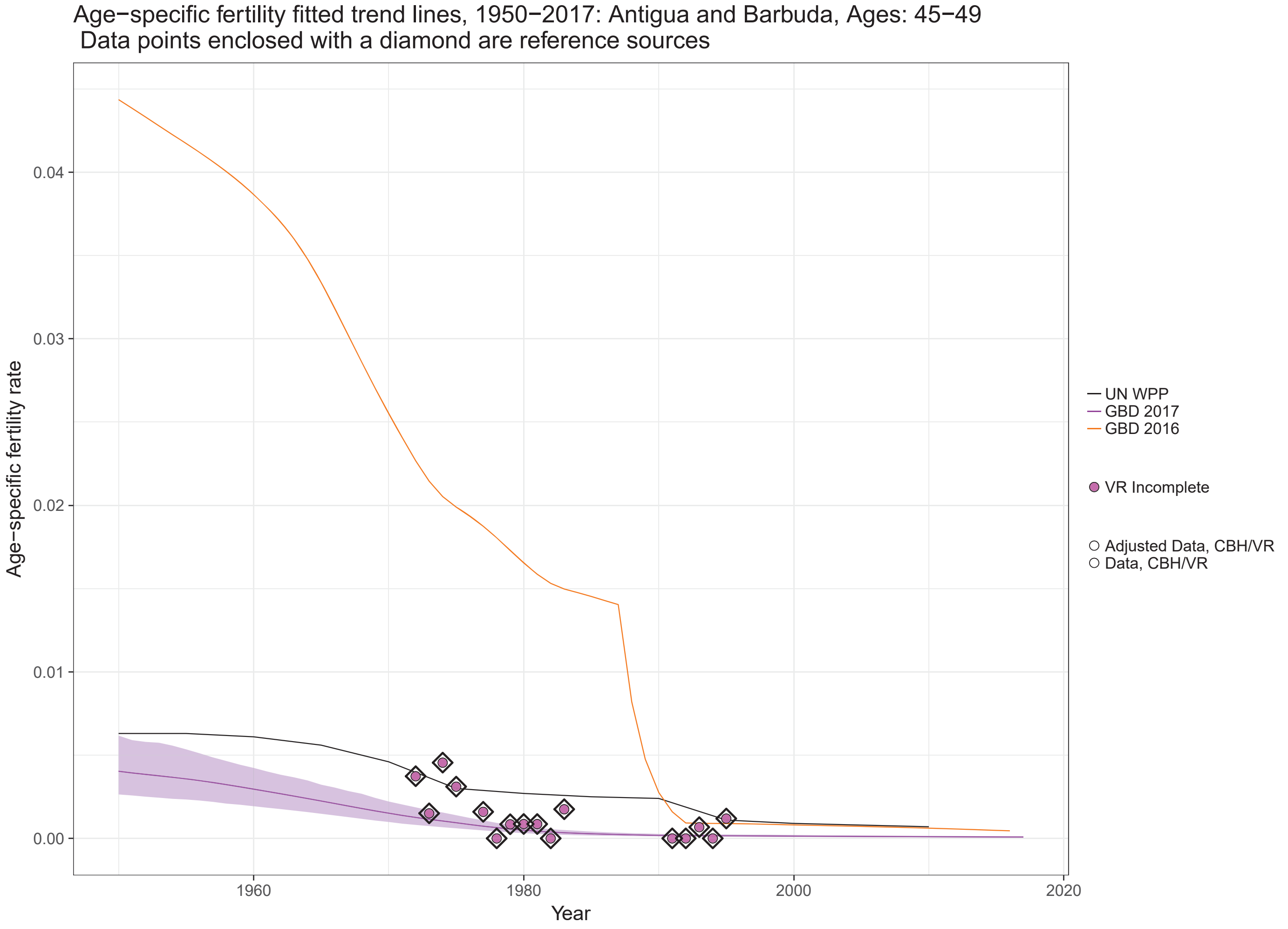
Age−specific fertility fitted trend lines, 1950−2017: Antigua and Barbuda, Ages: 45−49
Data points enclosed with a diamond are reference sources
Age−specific fertility rate
Year
UN WPP
GBD 2017
GBD 2016
VR Incomplete
Adjusted Data, CBH/VR
Data, CBH/VR

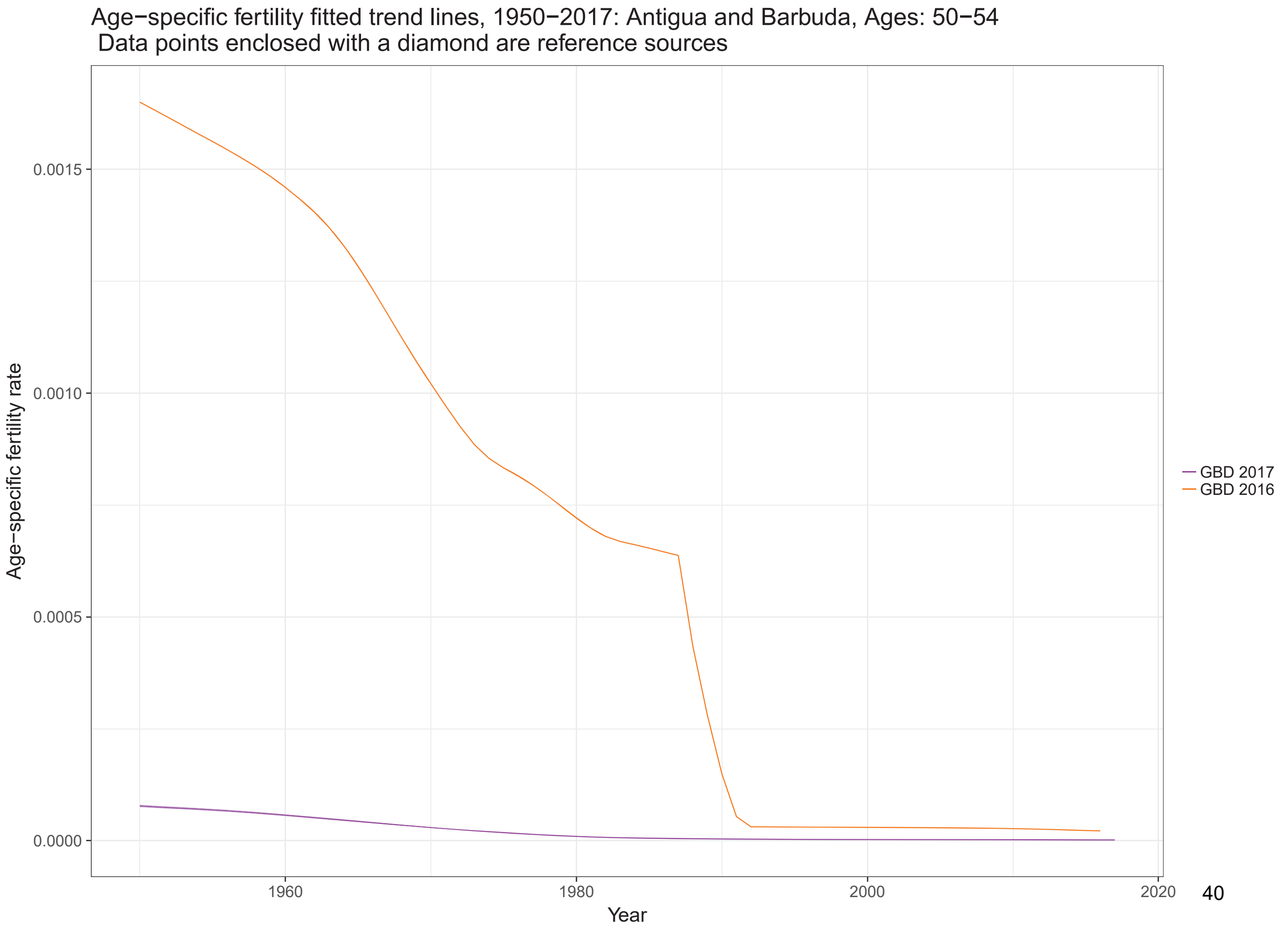
Age−specific fertility fitted trend lines, 1950−2017: Antigua and Barbuda, Ages: 50−54
Data points enclosed with a diamond are reference sources
Age−specific fertility rate
Year
GBD 2017
GBD 2016

Age−specific fertility fitted trend lines, 1950−2017: Argentina, TFR
Data points enclosed with a diamond are reference sources

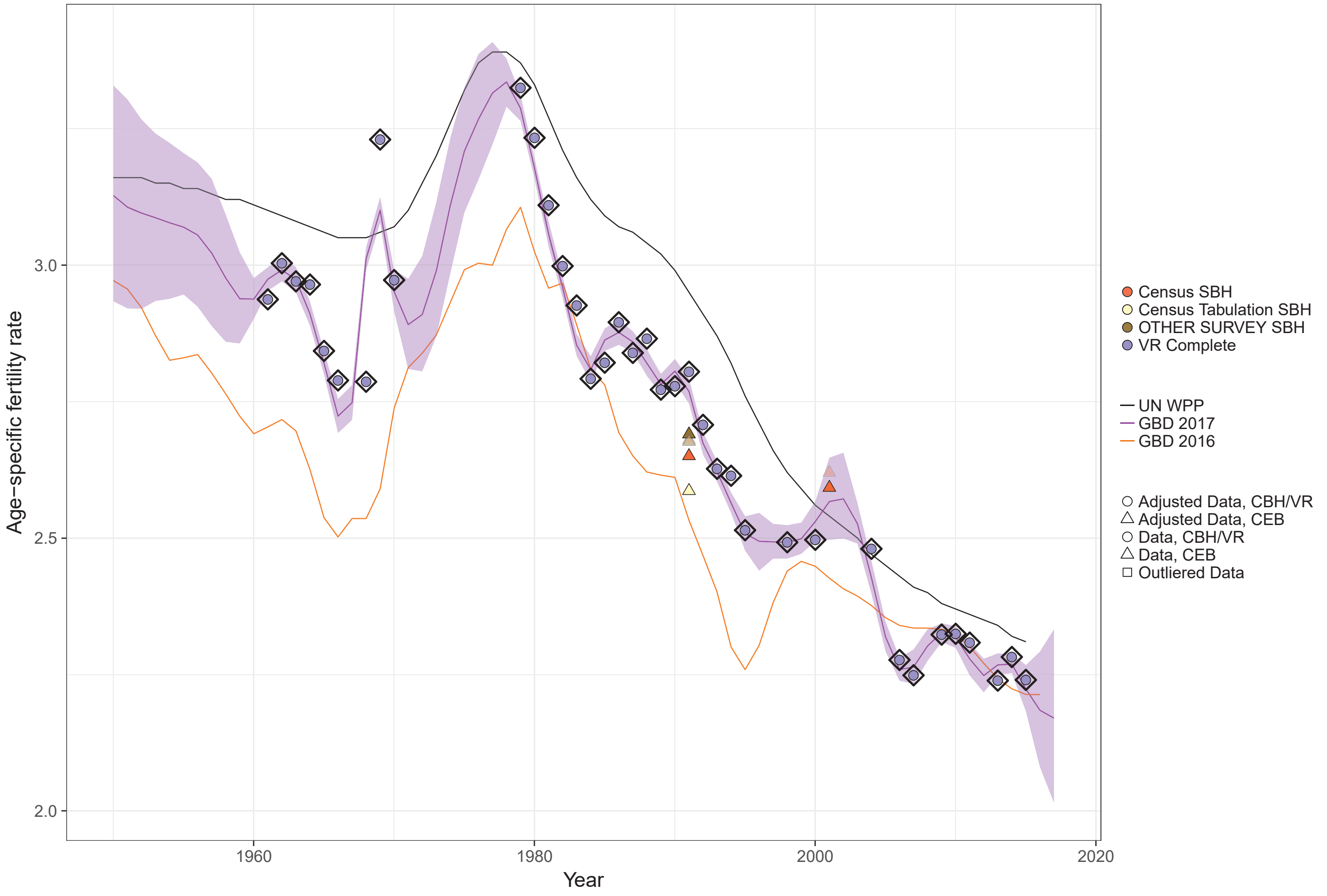

Age−specific fertility fitted trend lines, 1950−2017: Argentina, Ages: 10−14
Data points enclosed with a diamond are reference sources

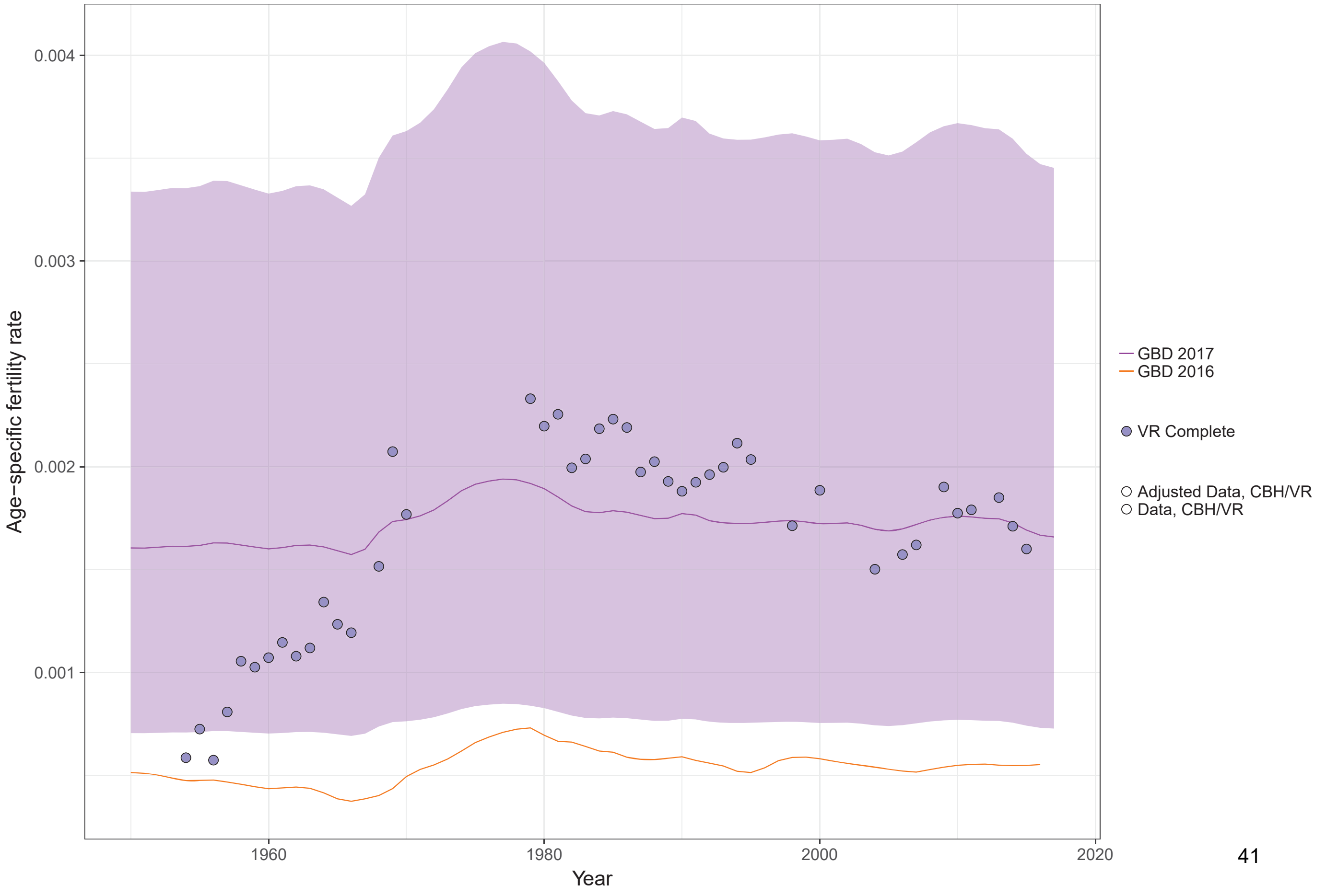

Age−specific fertility fitted trend lines, 1950−2017: Argentina, Ages: 15−19
Data points enclosed with a diamond are reference sources

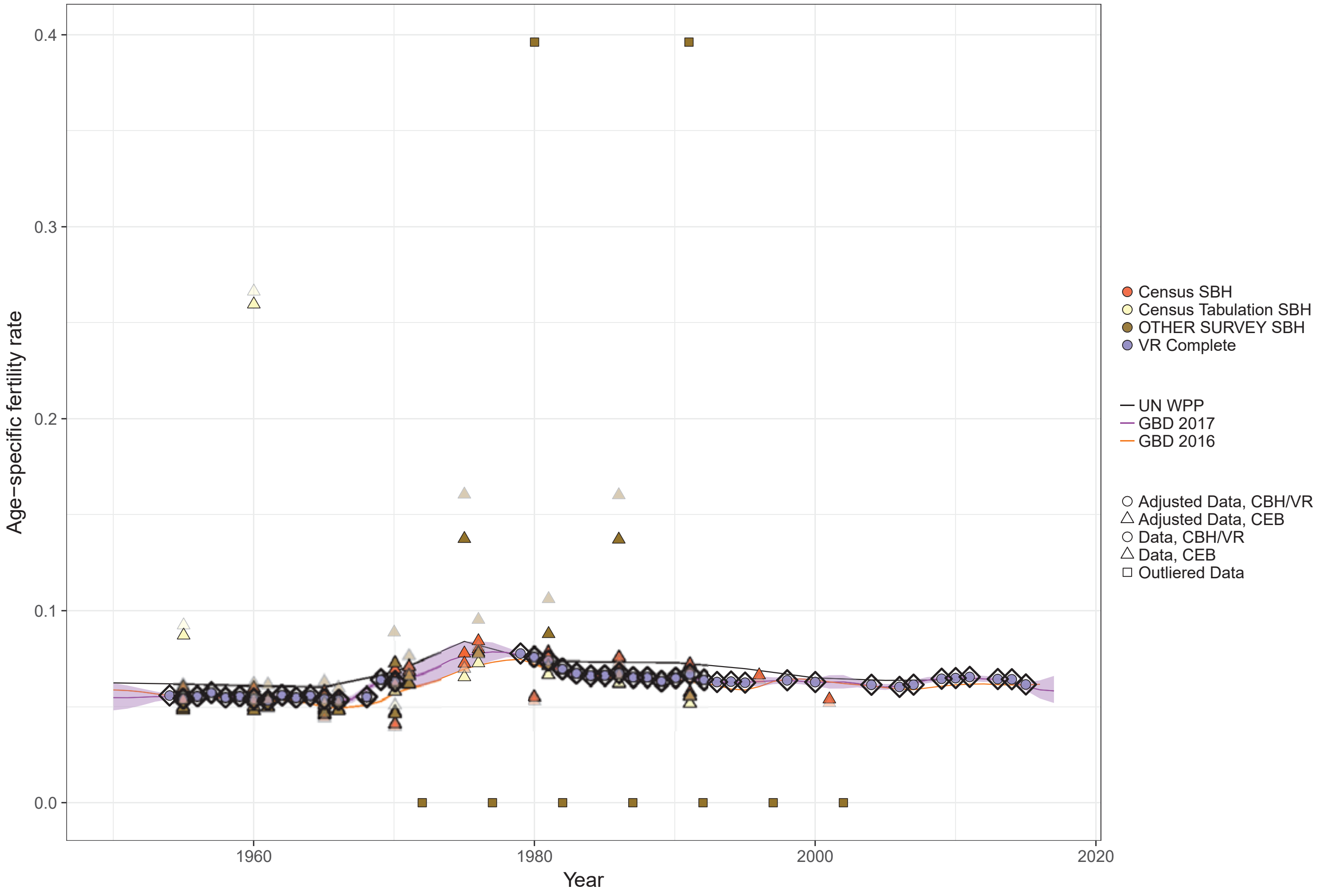

Age−specific fertility fitted trend lines, 1950−2017: Argentina, Ages: 20−24
Data points enclosed with a diamond are reference sources

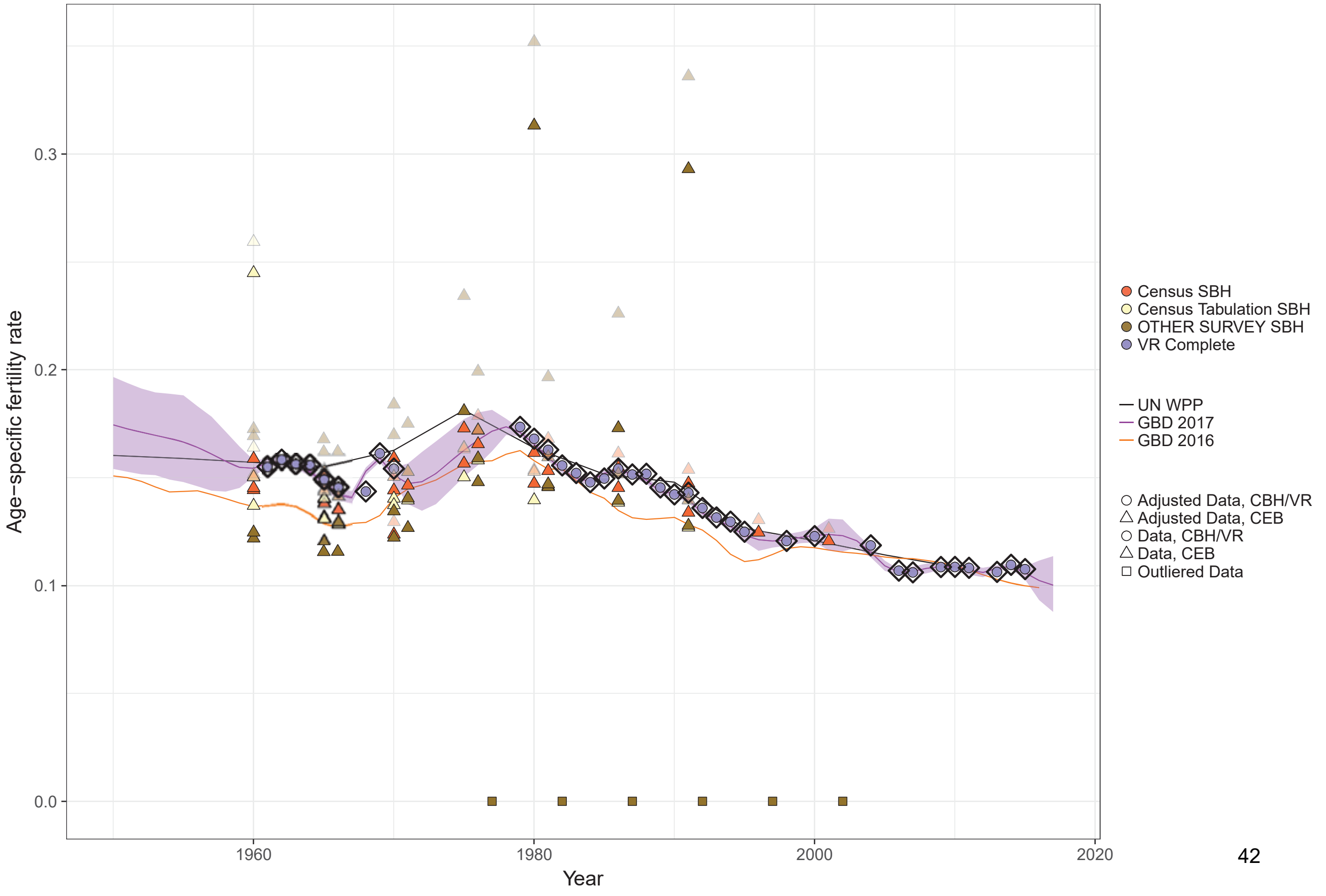

Age−specific fertility fitted trend lines, 1950−2017: Argentina, Ages: 25−29
Data points enclosed with a diamond are reference sources

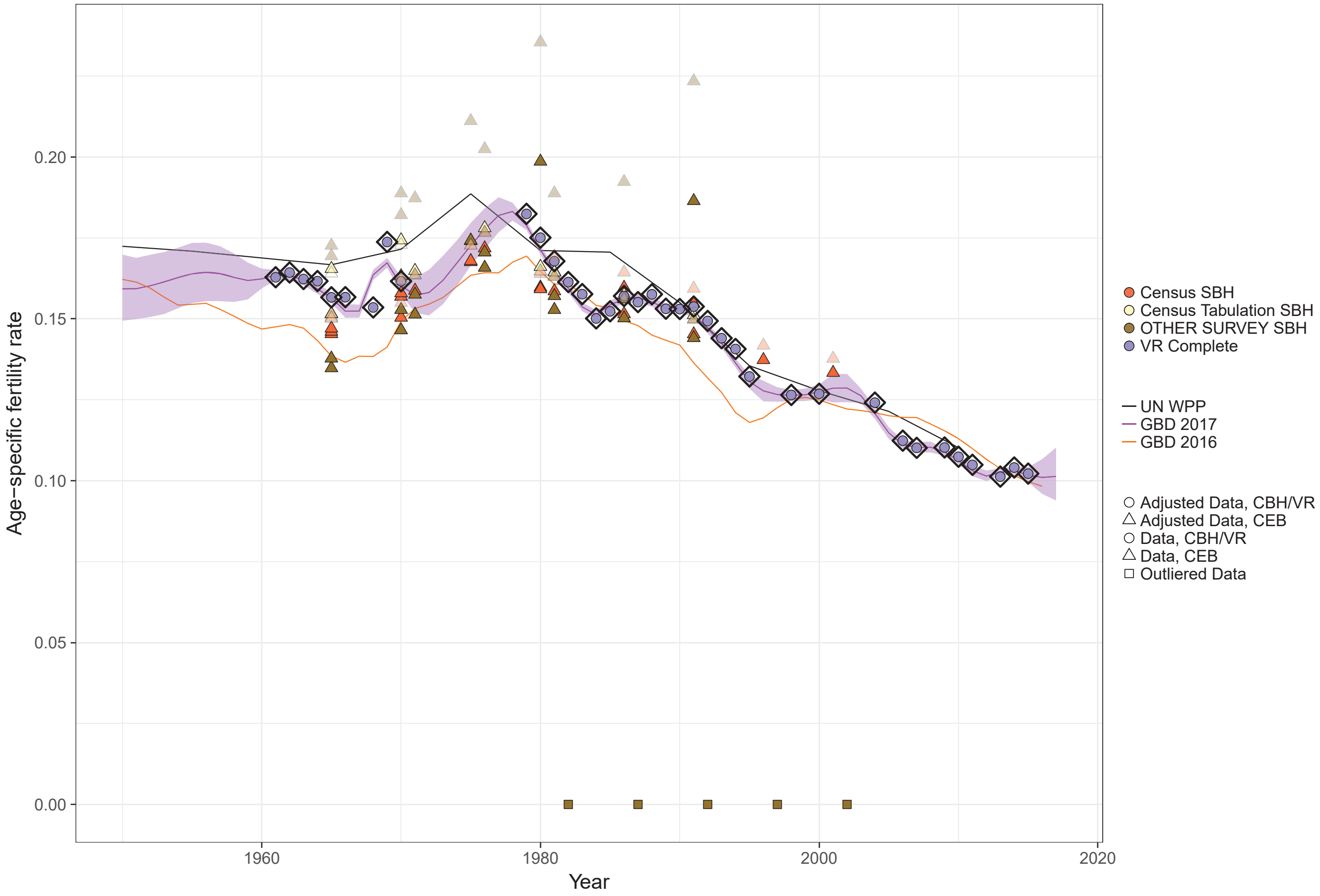

Age−specific fertility fitted trend lines, 1950−2017: Argentina, Ages: 30−34
Data points enclosed with a diamond are reference sources

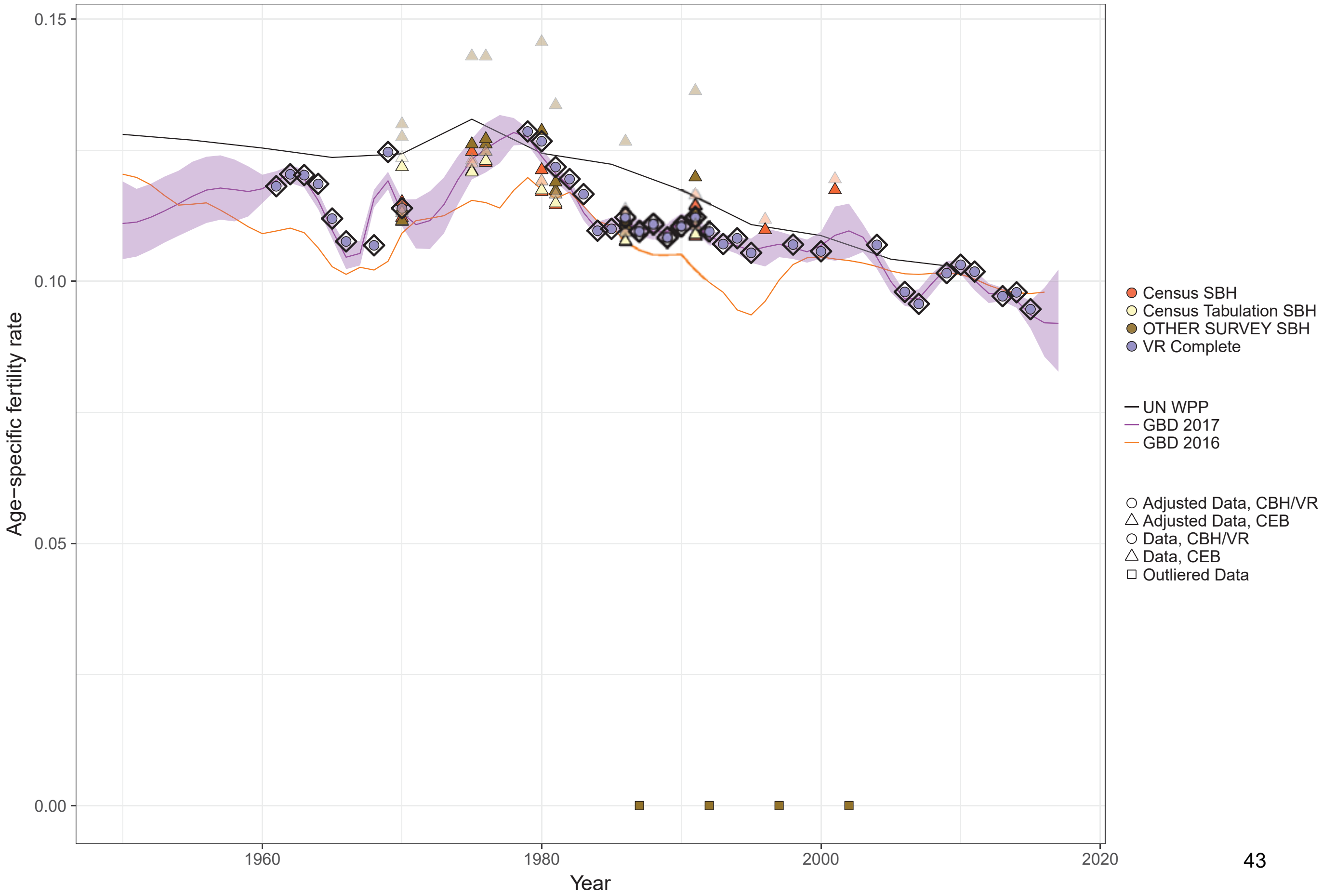

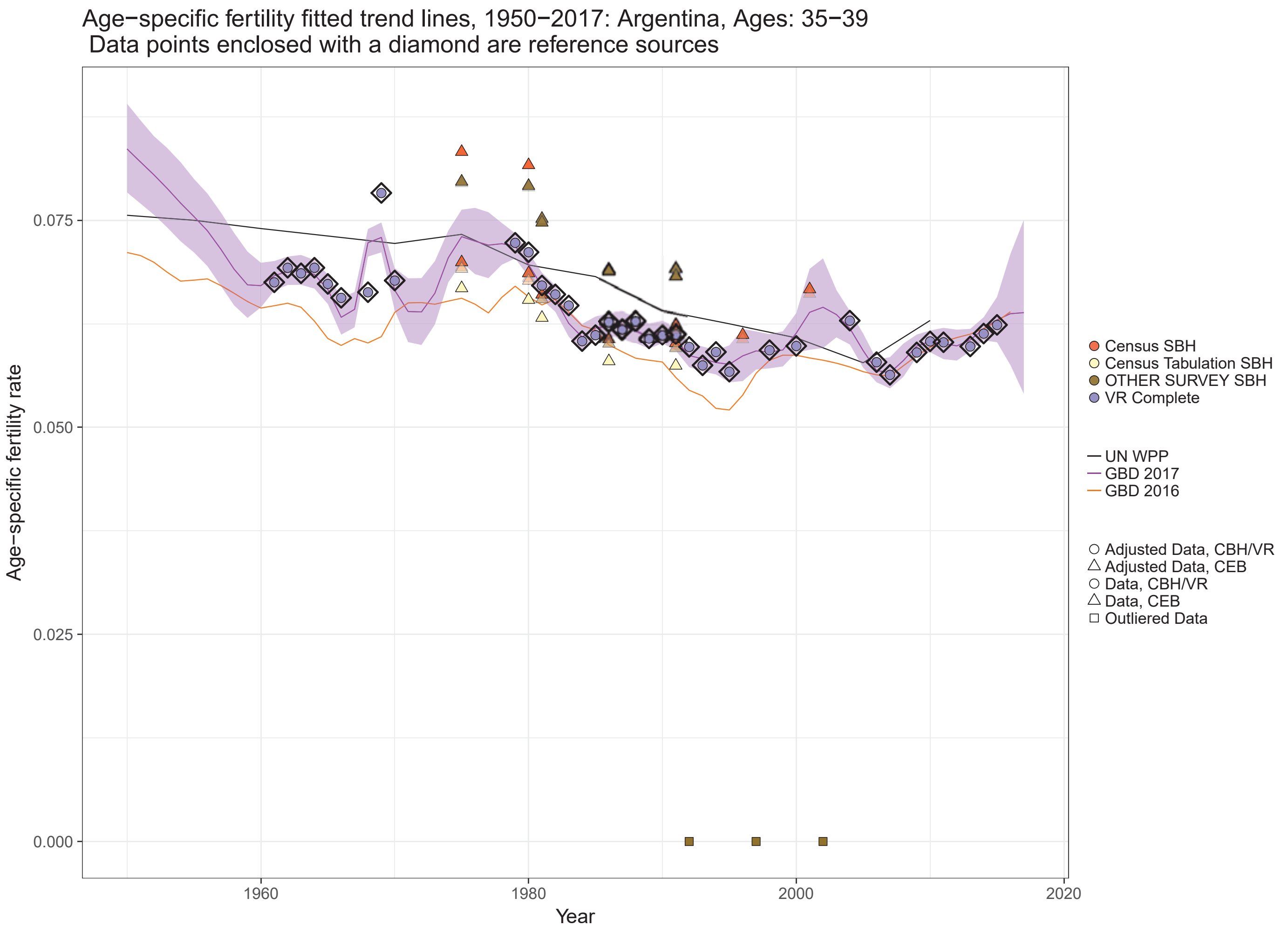

Age−specific fertility fitted trend lines, 1950−2017: Argentina, Ages: 35−39
Data points enclosed with a diamond are reference sources
Age−specific fertility rate
Year
0.000
0.025
0.050
0.075
1960
1980
2000
2020
Census SBH
Census Tabulation SBH
OTHER SURVEY SBH
VR Complete
UN WPP
GBD 2017
GBD 2016
Adjusted Data, CBH/VR
Adjusted Data, CEB
Data, CBH/VR
Data, CEB
Outliered Data

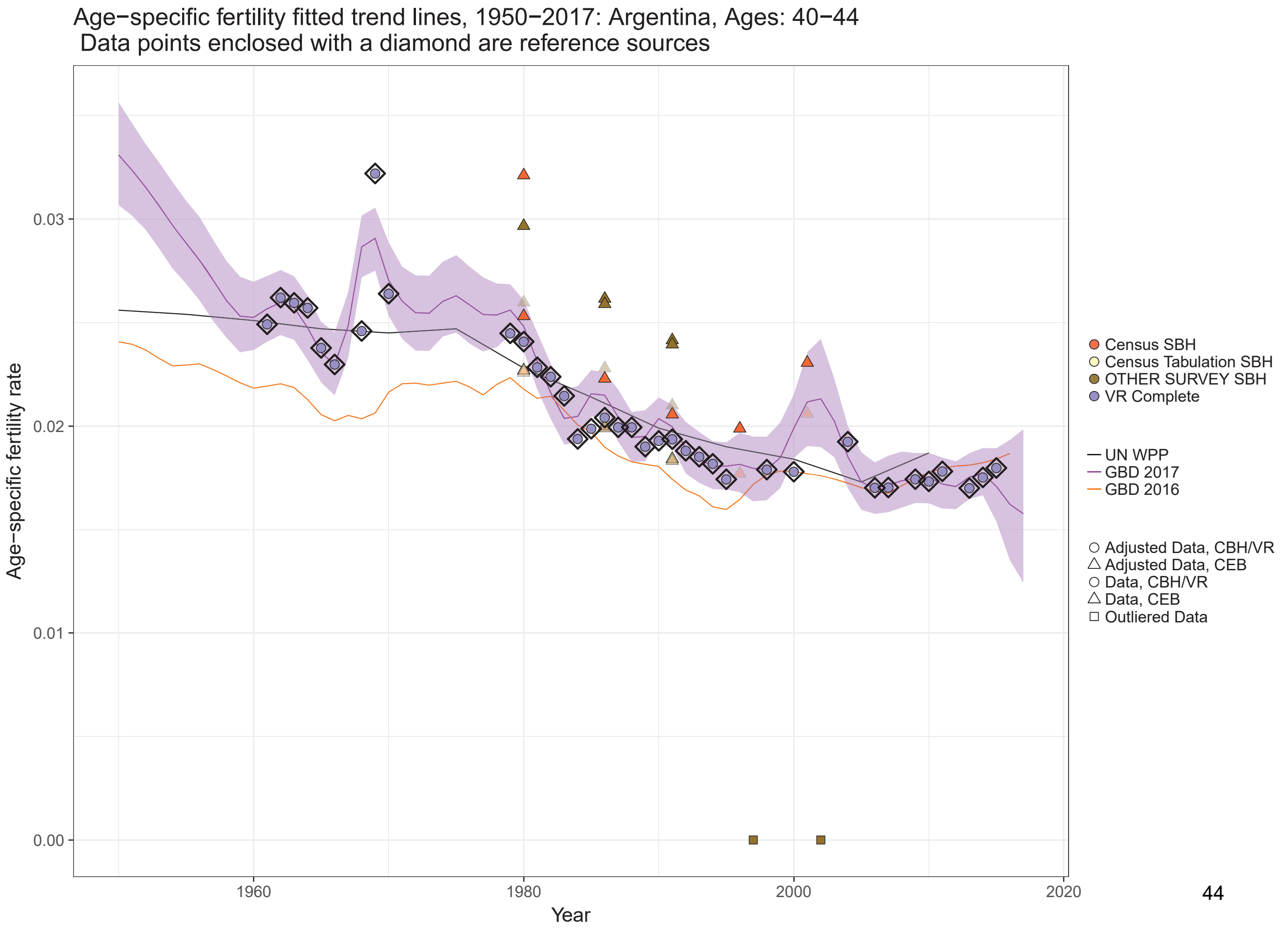

Age−specific fertility fitted trend lines, 1950−2017: Argentina, Ages: 40−44
Data points enclosed with a diamond are reference sources
Age−specific fertility rate
Year
0.00
0.01
0.02
0.03
1960
1980
2000
2020
Census SBH
Census Tabulation SBH
OTHER SURVEY SBH
VR Complete
UN WPP
GBD 2017
GBD 2016
Adjusted Data, CBH/VR
Adjusted Data, CEB
Data, CBH/VR
Data, CEB
Outliered Data

Age−specific fertility fitted trend lines, 1950−2017: Argentina, Ages: 45−49
Data points enclosed with a diamond are reference sources

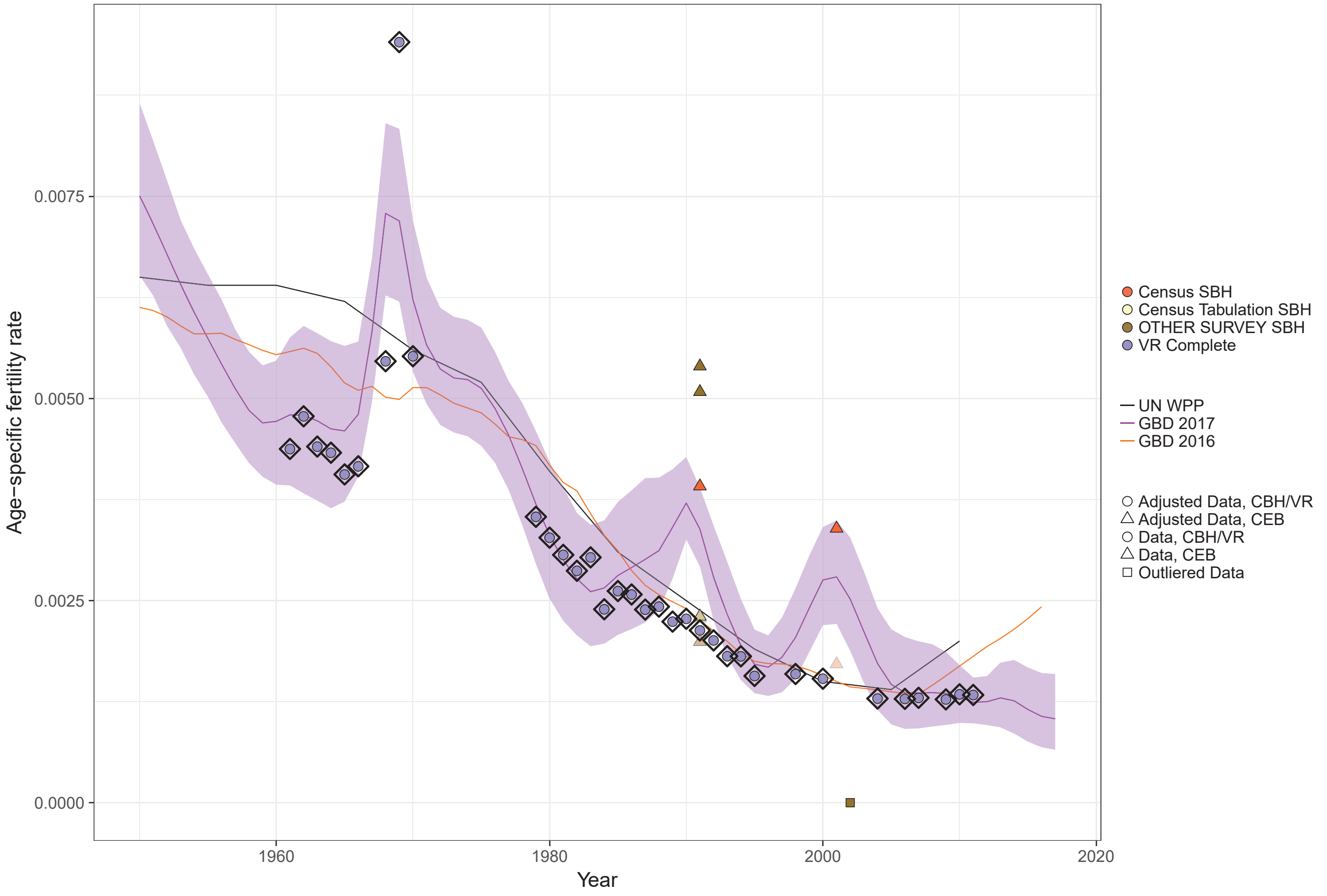

Age−specific fertility fitted trend lines, 1950−2017: Argentina, Ages: 50−54
Data points enclosed with a diamond are reference sources

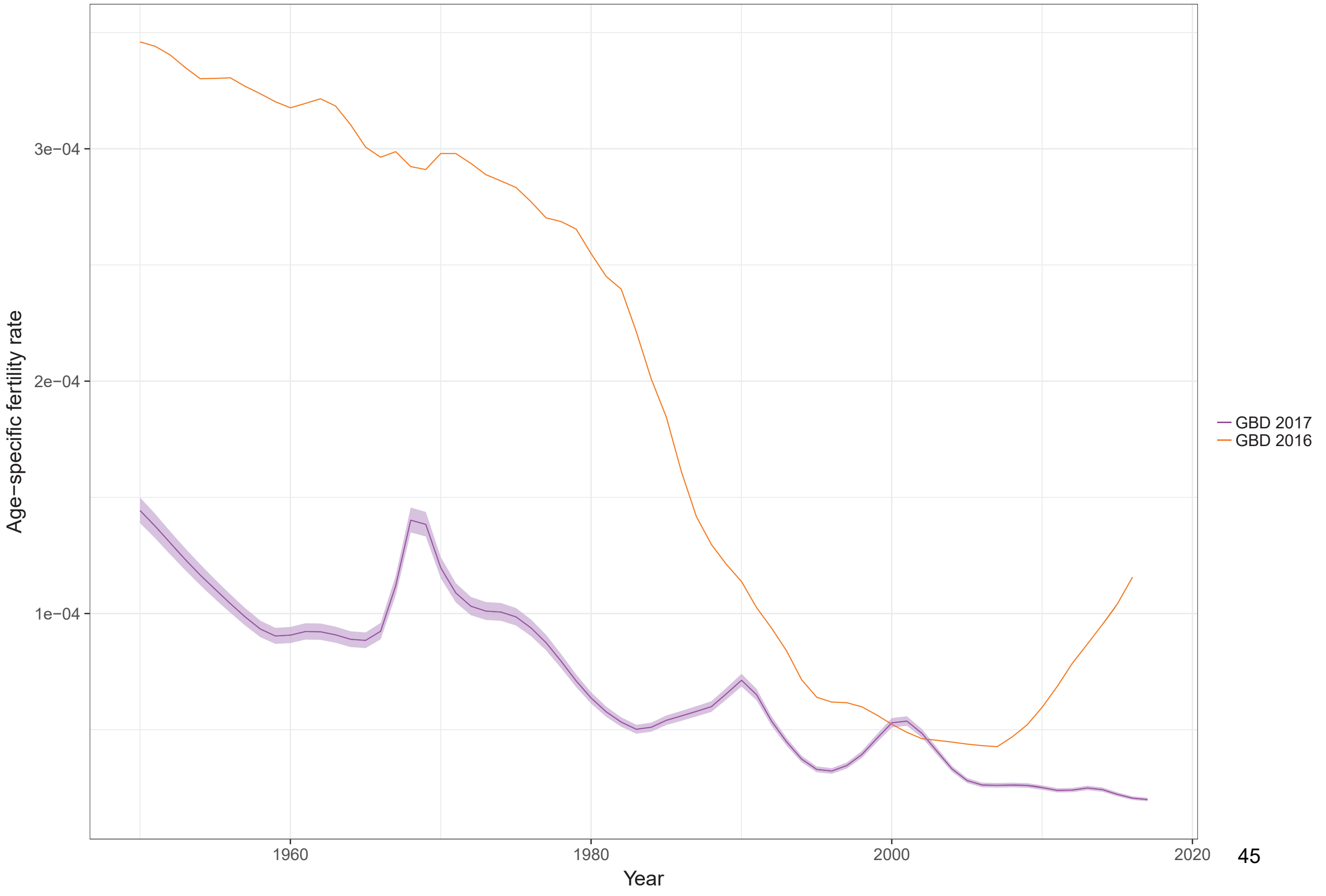

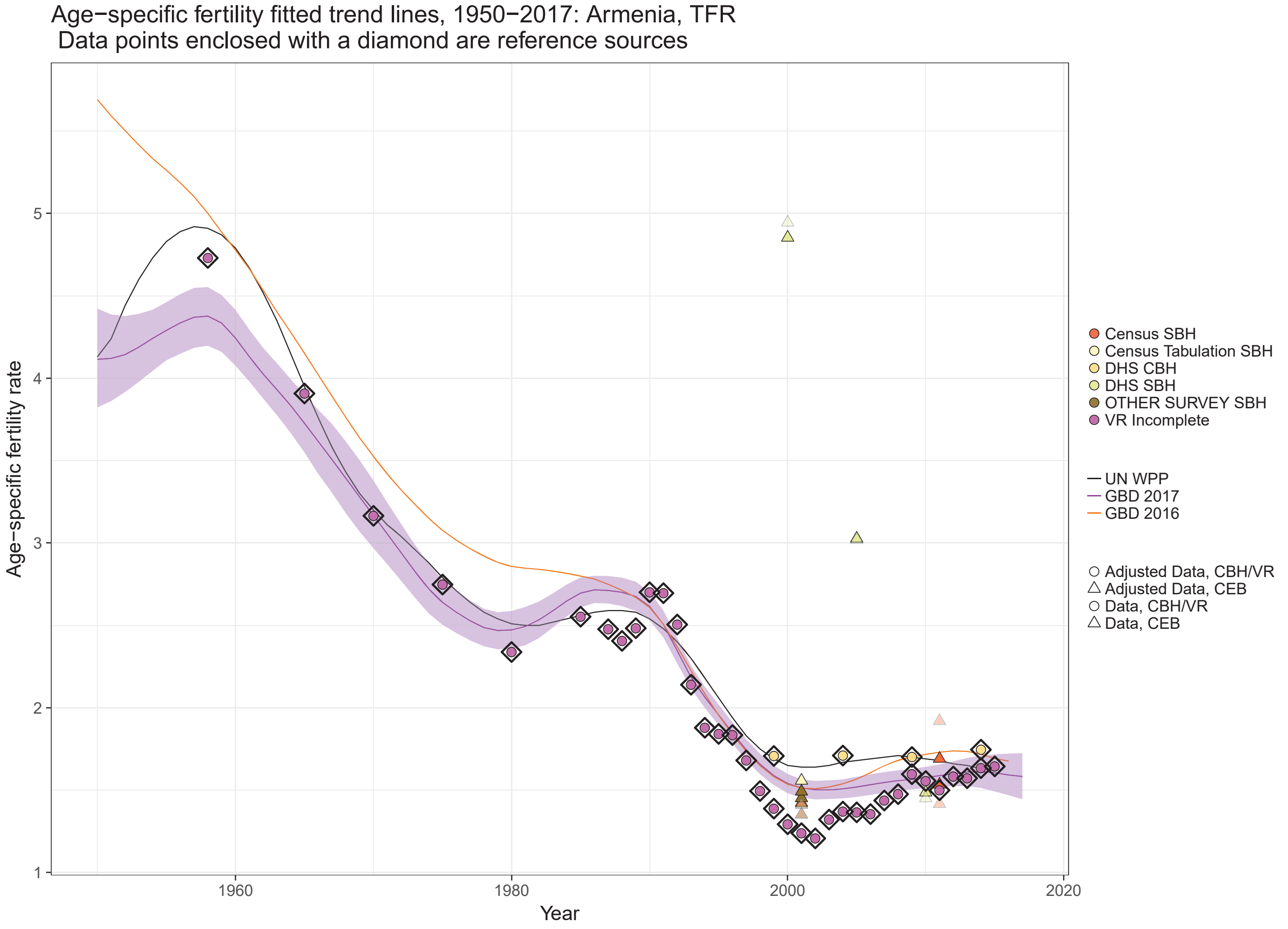
Age−specific fertility fitted trend lines, 1950−2017: Armenia, TFR
Data points enclosed with a diamond are reference sources
Age−specific fertility rate
Year
1960
1980
2000
2020
1
2
3
4
5
Census SBH
Census Tabulation SBH
DHS CBH
DHS SBH
OTHER SURVEY SBH
VR Incomplete
UN WPP
GBD 2017
GBD 2016
Adjusted Data, CBH/VR
Adjusted Data, CEB
Data, CBH/VR
Data, CEB

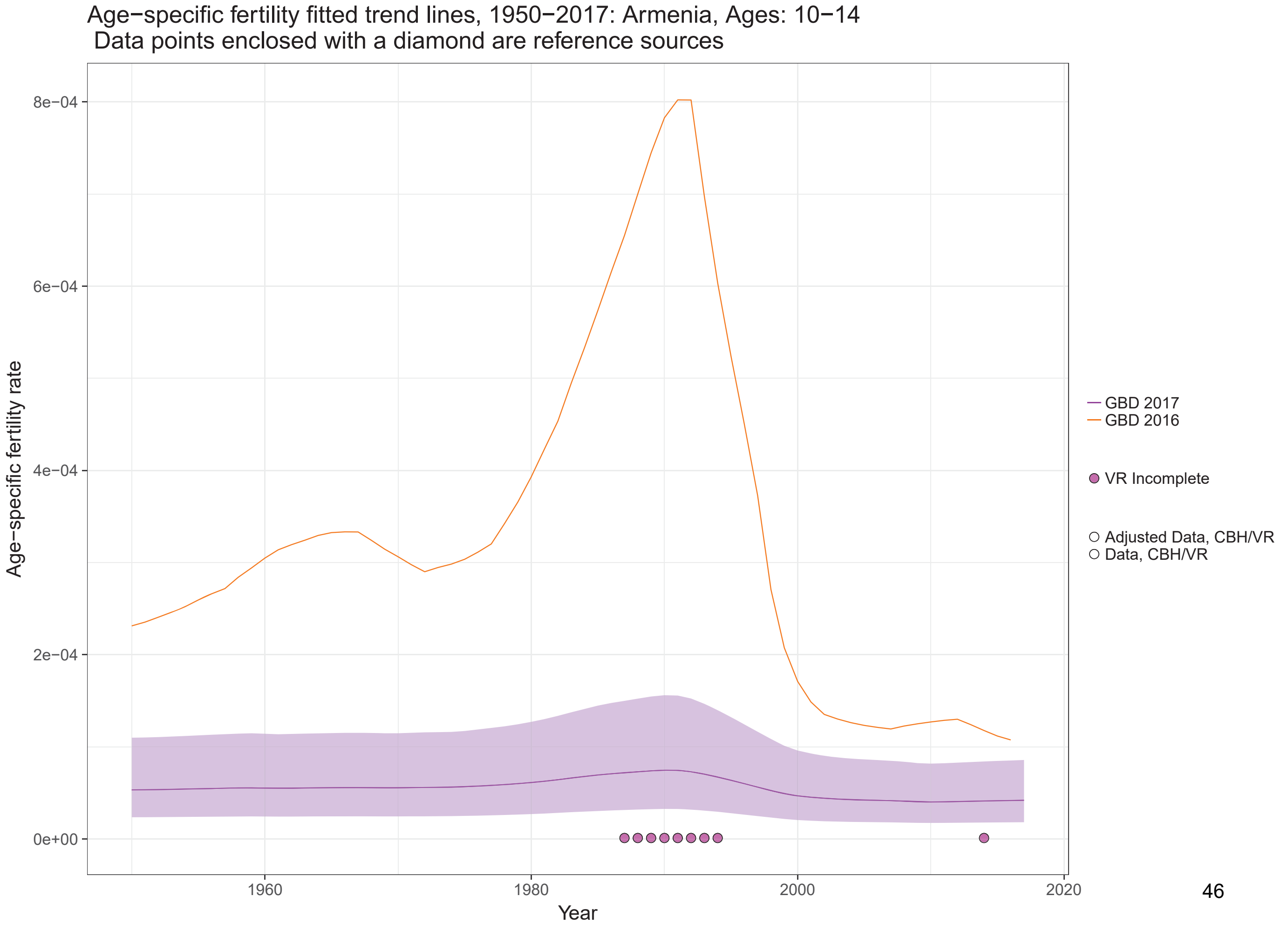
Age−specific fertility fitted trend lines, 1950−2017: Armenia, Ages: 10−14
Data points enclosed with a diamond are reference sources
Age−specific fertility rate
Year
0e+00
2e−04
4e−04
6e−04
8e−04
1960
1980
2000
2020
GBD 2017
GBD 2016
VR Incomplete
Adjusted Data, CBH/VR
Data, CBH/VR

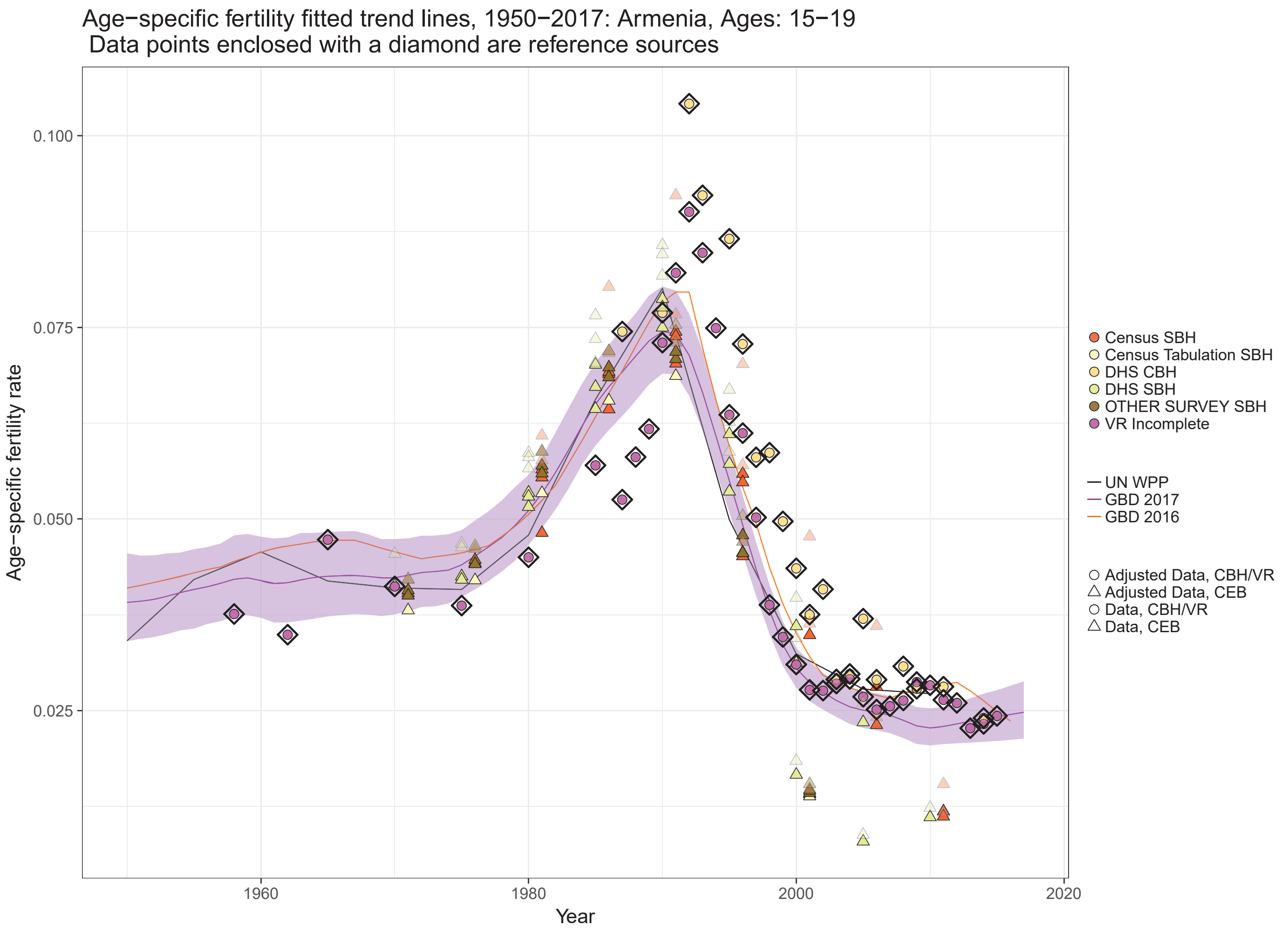
Age−specific fertility fitted trend lines, 1950−2017: Armenia, Ages: 15−19
Data points enclosed with a diamond are reference sources
Age−specific fertility rate
Year
0.100
0.075
0.050
0.025
1960
1980
2000
2020
Census SBH
Census Tabulation SBH
DHS CBH
DHS SBH
OTHER SURVEY SBH
VR Incomplete
UN WPP
GBD 2017
GBD 2016
Adjusted Data, CBH/VR
Adjusted Data, CEB
Data, CBH/VR
Data, CEB

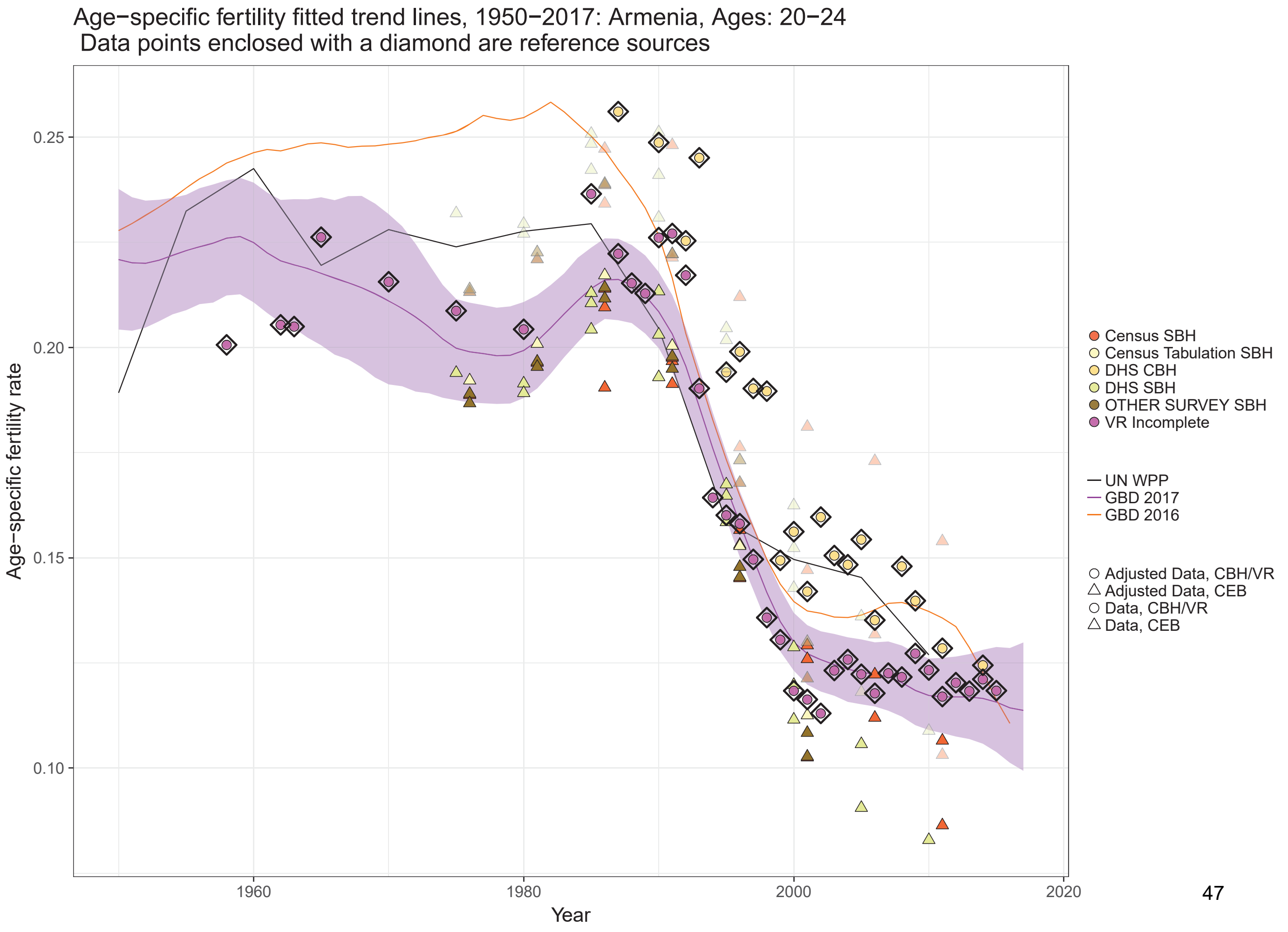
Age−specific fertility fitted trend lines, 1950−2017: Armenia, Ages: 20−24
Data points enclosed with a diamond are reference sources
Age−specific fertility rate
Year
0.25
0.20
0.15
0.10
1960
1980
2000
2020
Census SBH
Census Tabulation SBH
DHS CBH
DHS SBH
OTHER SURVEY SBH
VR Incomplete
UN WPP
GBD 2017
GBD 2016
Adjusted Data, CBH/VR
Adjusted Data, CEB
Data, CBH/VR
Data, CEB

Age−specific fertility fitted trend lines, 1950−2017: Armenia, Ages: 25−29
Data points enclosed with a diamond are reference sources

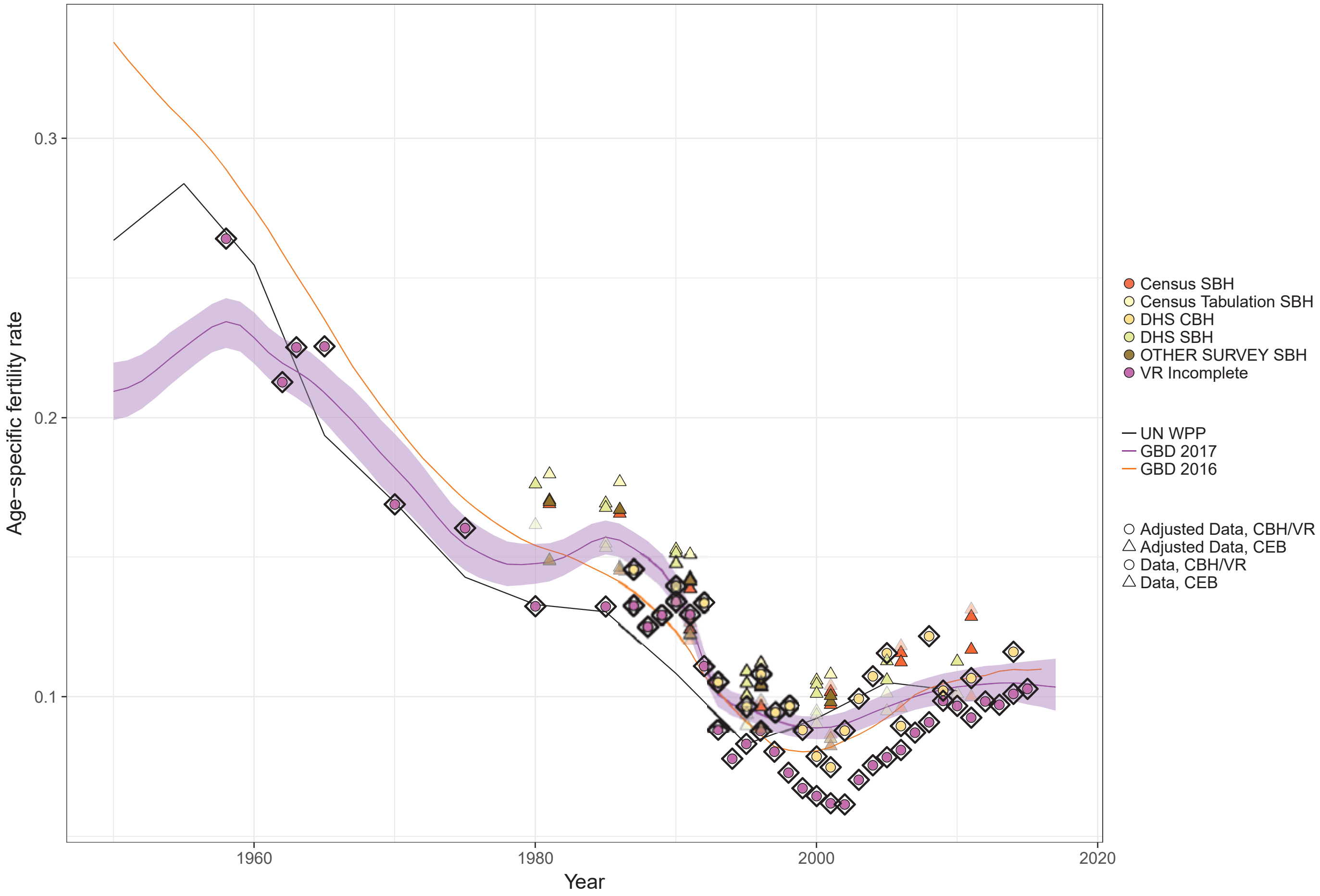

Age−specific fertility fitted trend lines, 1950−2017: Armenia, Ages: 30−34
Data points enclosed with a diamond are reference sources

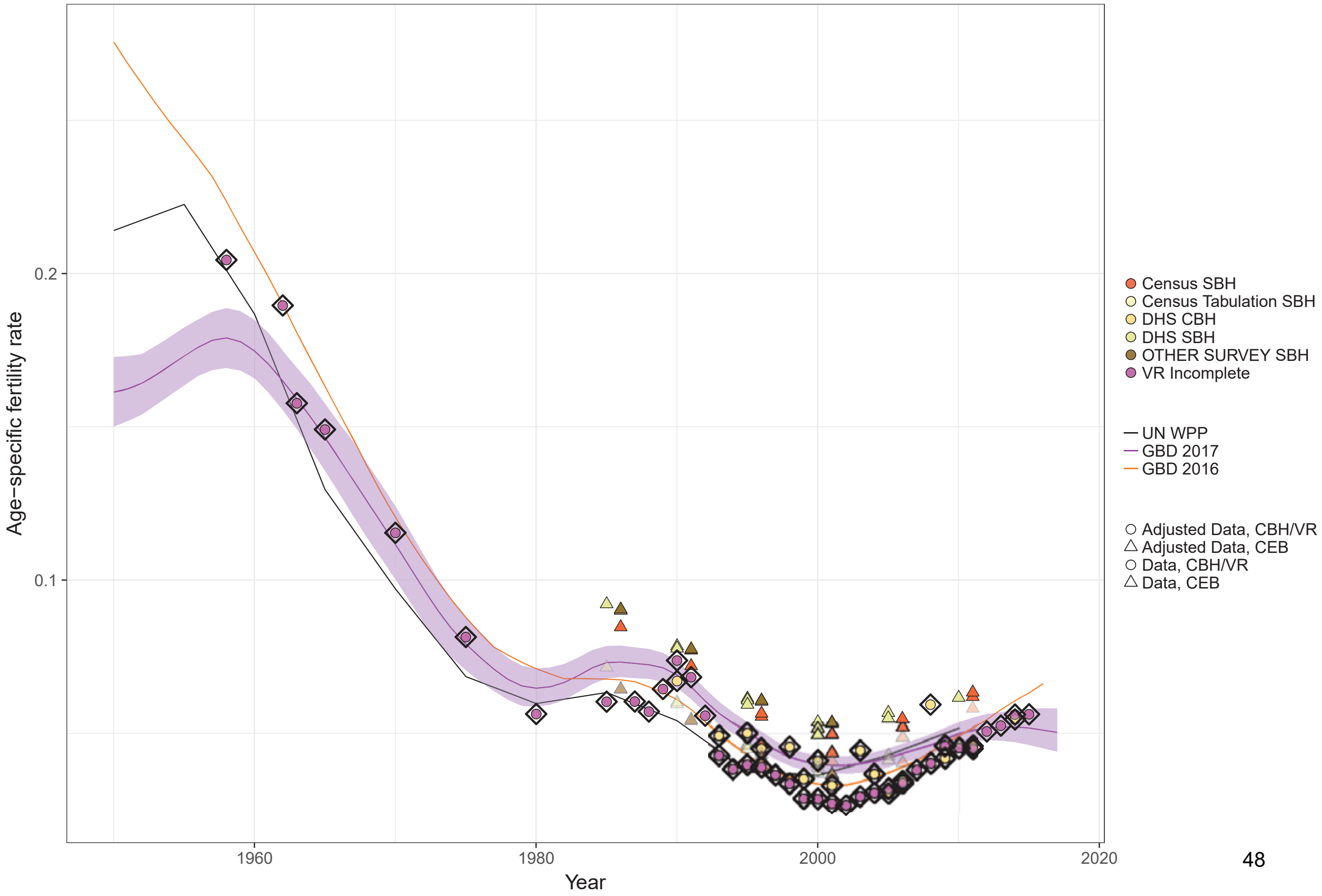

Age−specific fertility fitted trend lines, 1950−2017: Armenia, Ages: 35−39
Data points enclosed with a diamond are reference sources

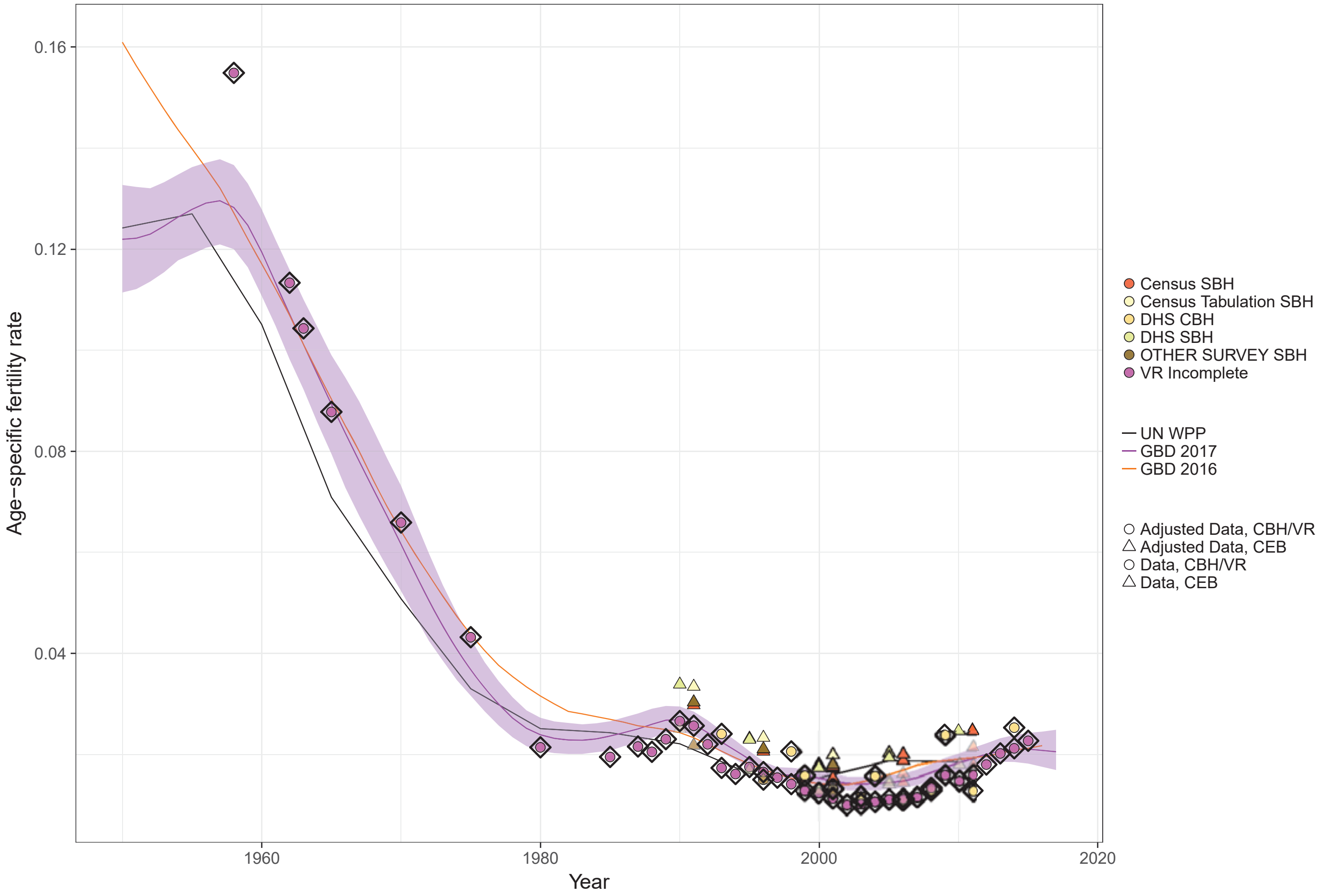

Age−specific fertility fitted trend lines, 1950−2017: Armenia, Ages: 40−44
Data points enclosed with a diamond are reference sources

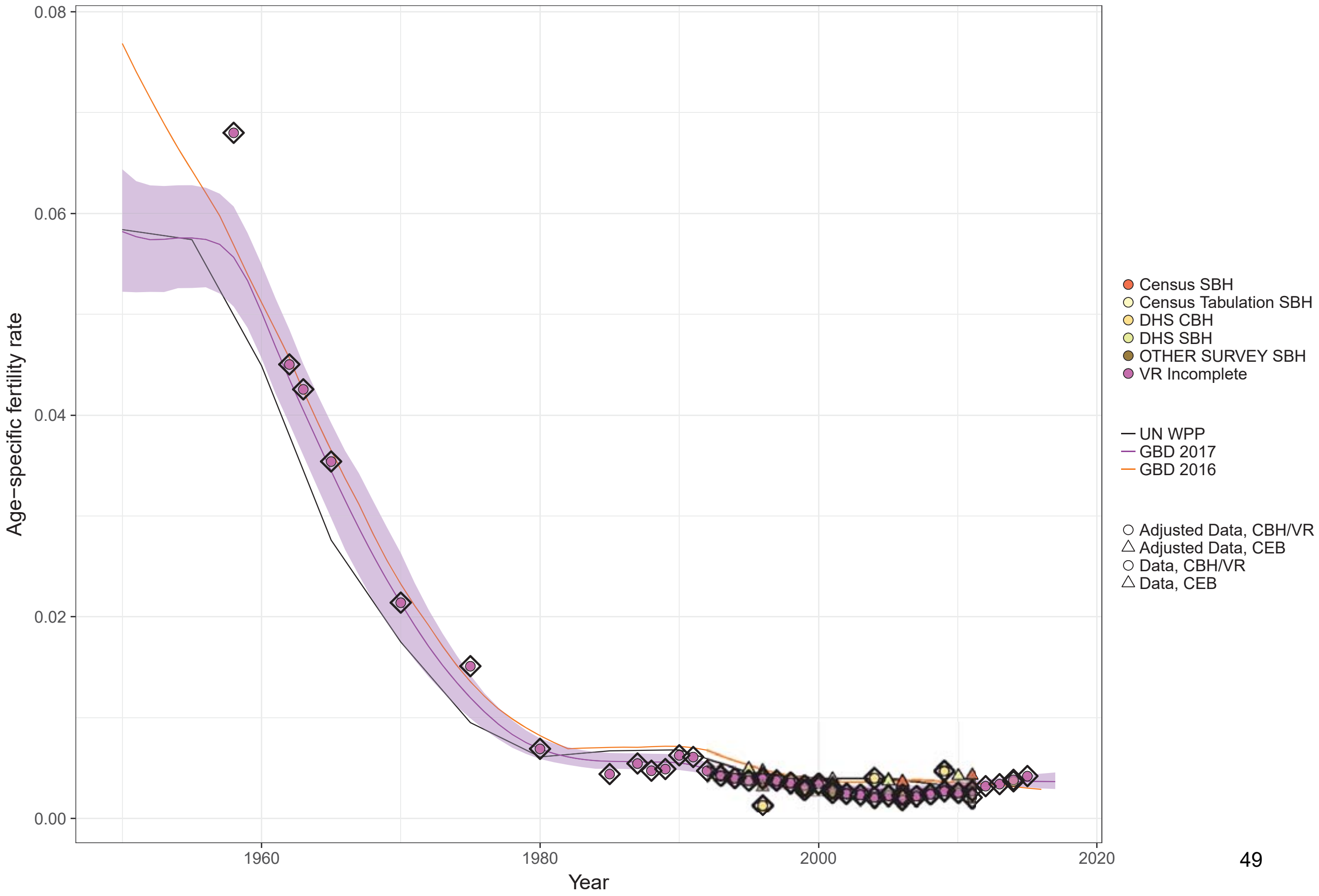

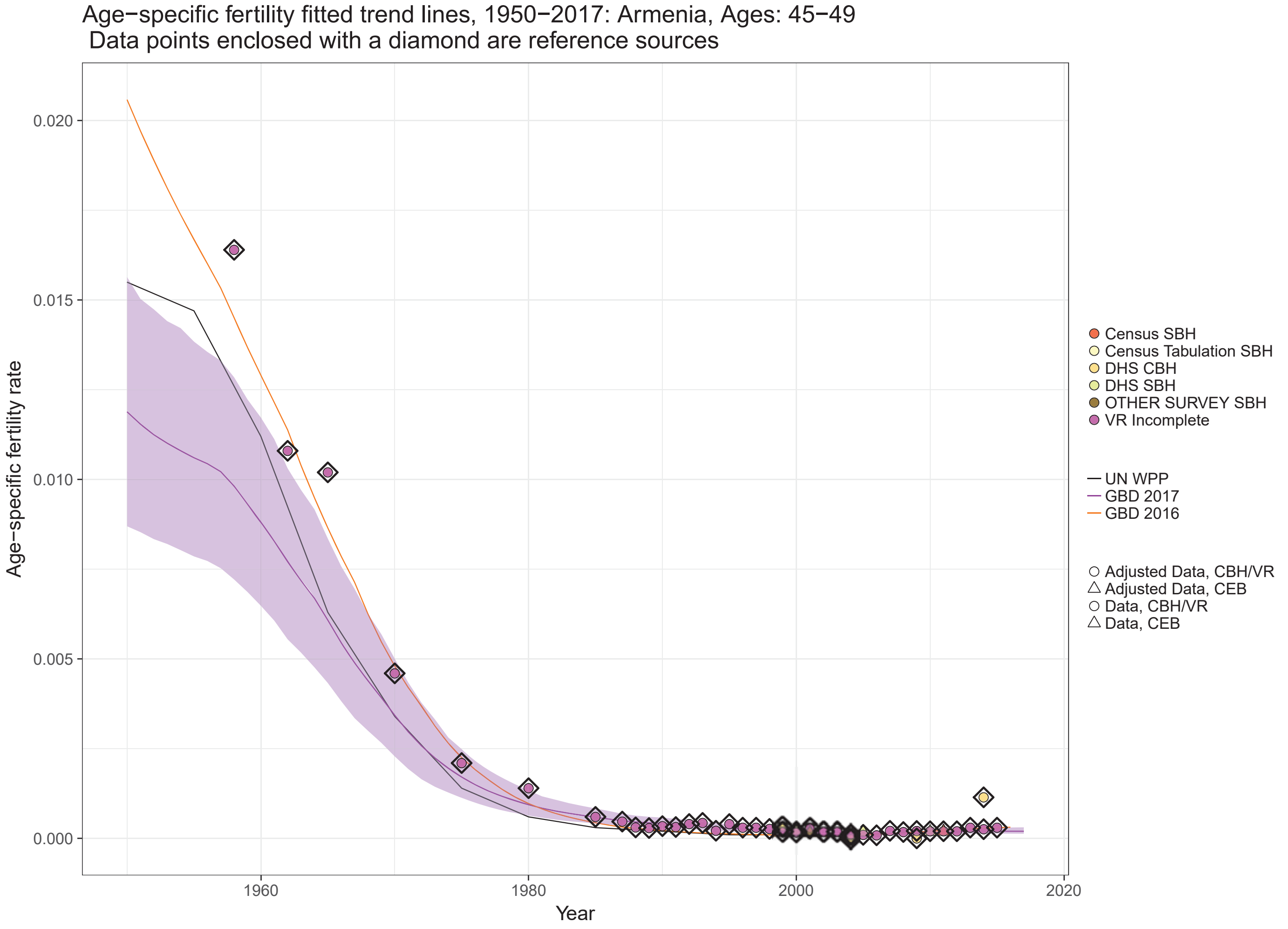
Age−specific fertility fitted trend lines, 1950−2017: Armenia, Ages: 45−49
Data points enclosed with a diamond are reference sources
Age−specific fertility rate
0.020
0.015
0.010
0.005
0.000
1960
1980
2000
2020
Year
Census SBH
Census Tabulation SBH
DHS CBH
DHS SBH
OTHER SURVEY SBH
VR Incomplete
UN WPP
GBD 2017
GBD 2016
Adjusted Data, CBH/VR
Adjusted Data, CEB
Data, CBH/VR
Data, CEB

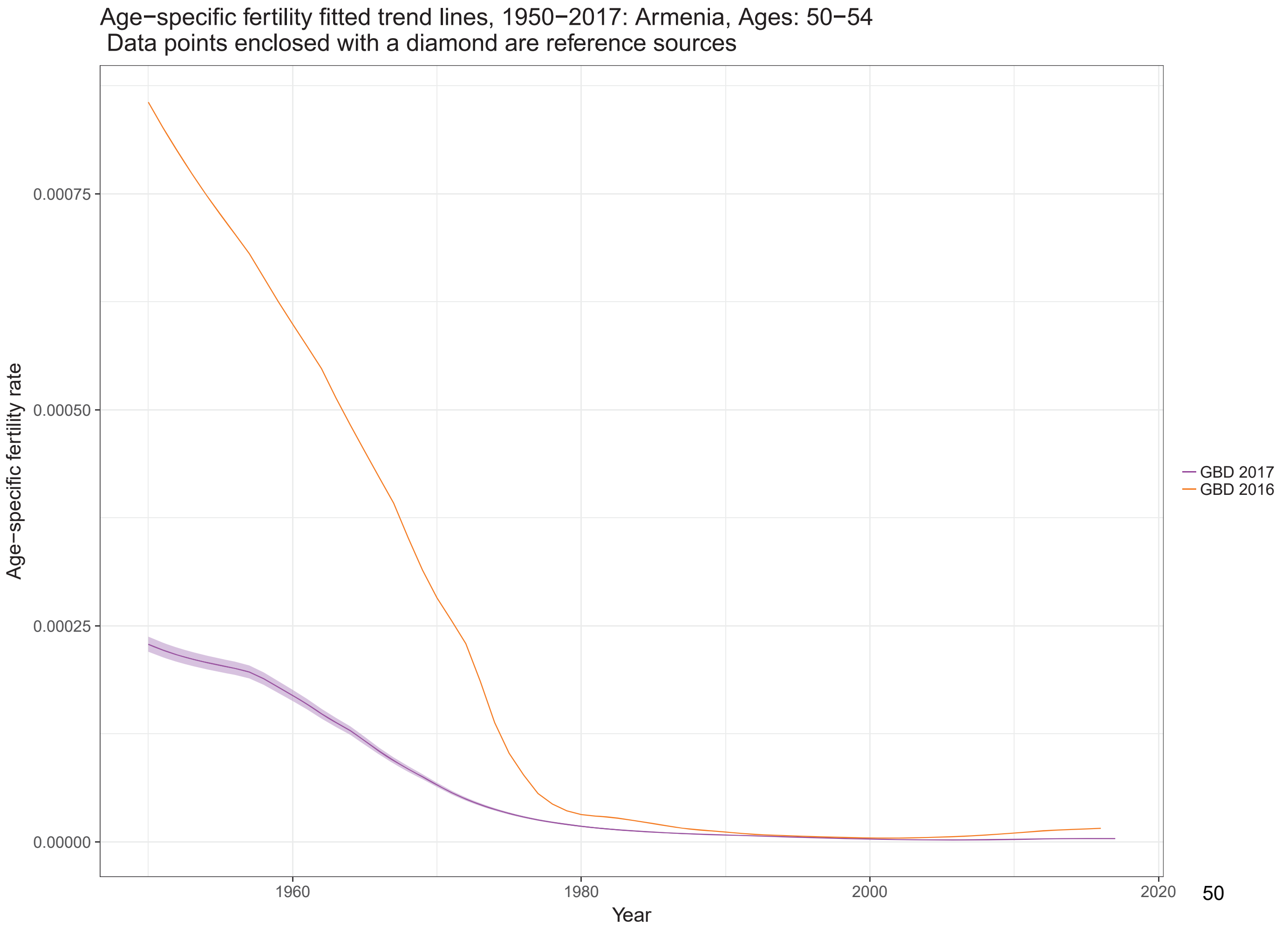
Age−specific fertility fitted trend lines, 1950−2017: Armenia, Ages: 50−54
Data points enclosed with a diamond are reference sources
Age−specific fertility rate
0.00075
0.00050
0.00025
0.00000
1960
1980
2000
2020
Year
GBD 2017
GBD 2016

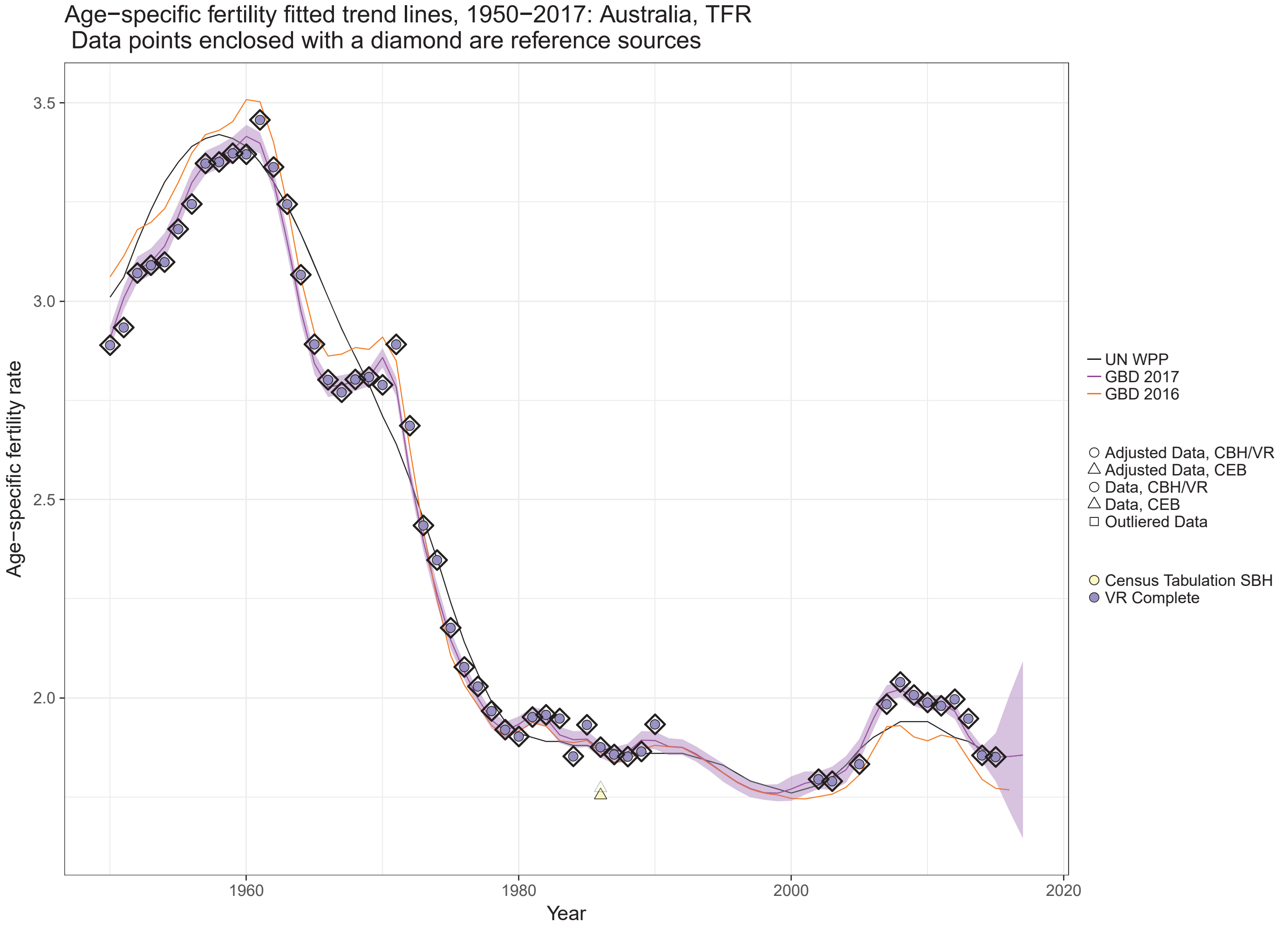

Age−specific fertility fitted trend lines, 1950−2017: Australia, TFR
Data points enclosed with a diamond are reference sources
Age−specific fertility rate
Year
3.5
3.0
2.5
2.0
1960
1980
2000
2020
UN WPP
GBD 2017
GBD 2016
Adjusted Data, CBH/VR
Adjusted Data, CEB
Data, CBH/VR
Data, CEB
Outliered Data
Census Tabulation SBH
VR Complete

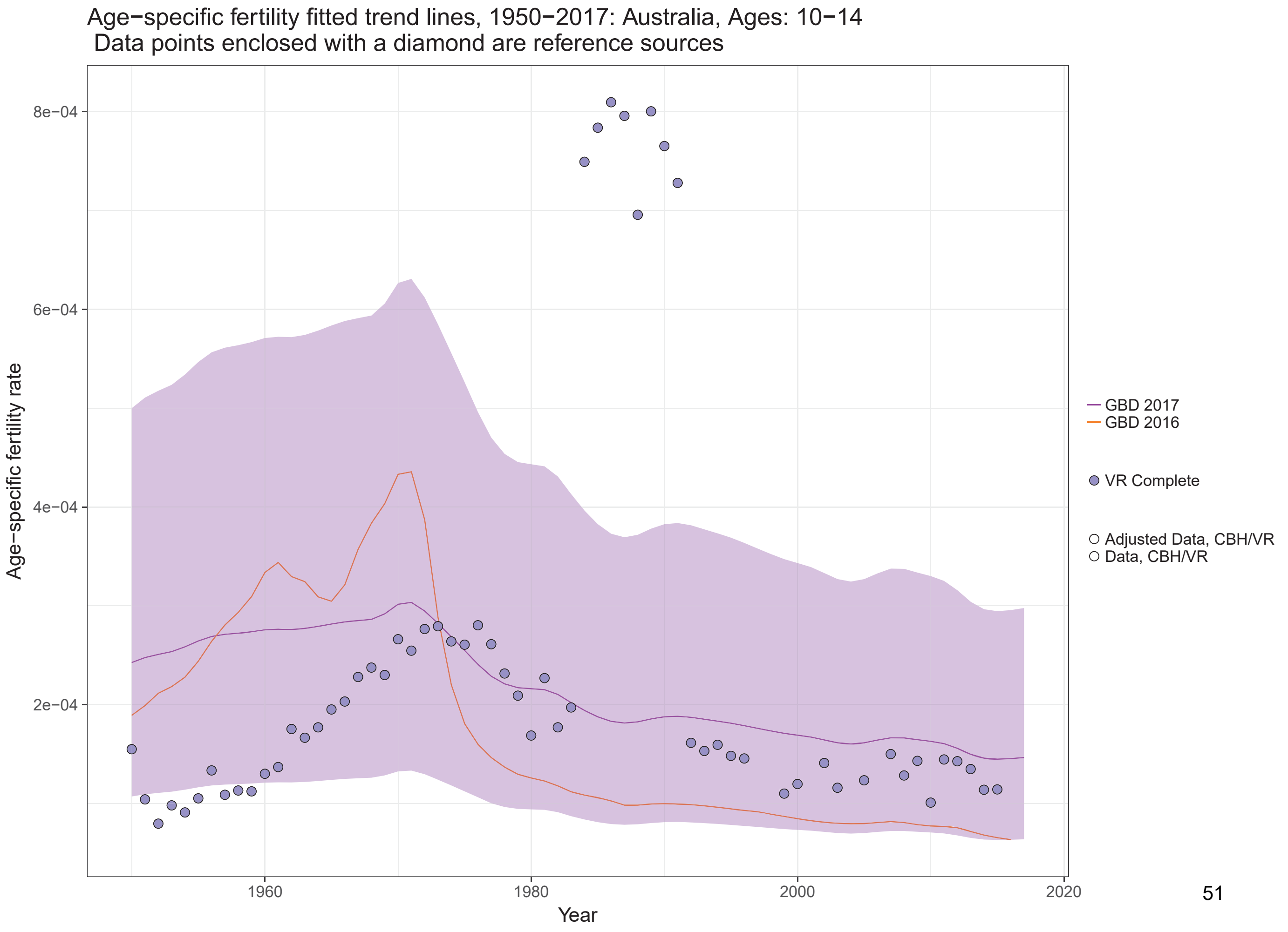

Age−specific fertility fitted trend lines, 1950−2017: Australia, Ages: 10−14
Data points enclosed with a diamond are reference sources
Age−specific fertility rate
Year
8e−04
6e−04
4e−04
2e−04
1960
1980
2000
2020
GBD 2017
GBD 2016
VR Complete
Adjusted Data, CBH/VR
Data, CBH/VR

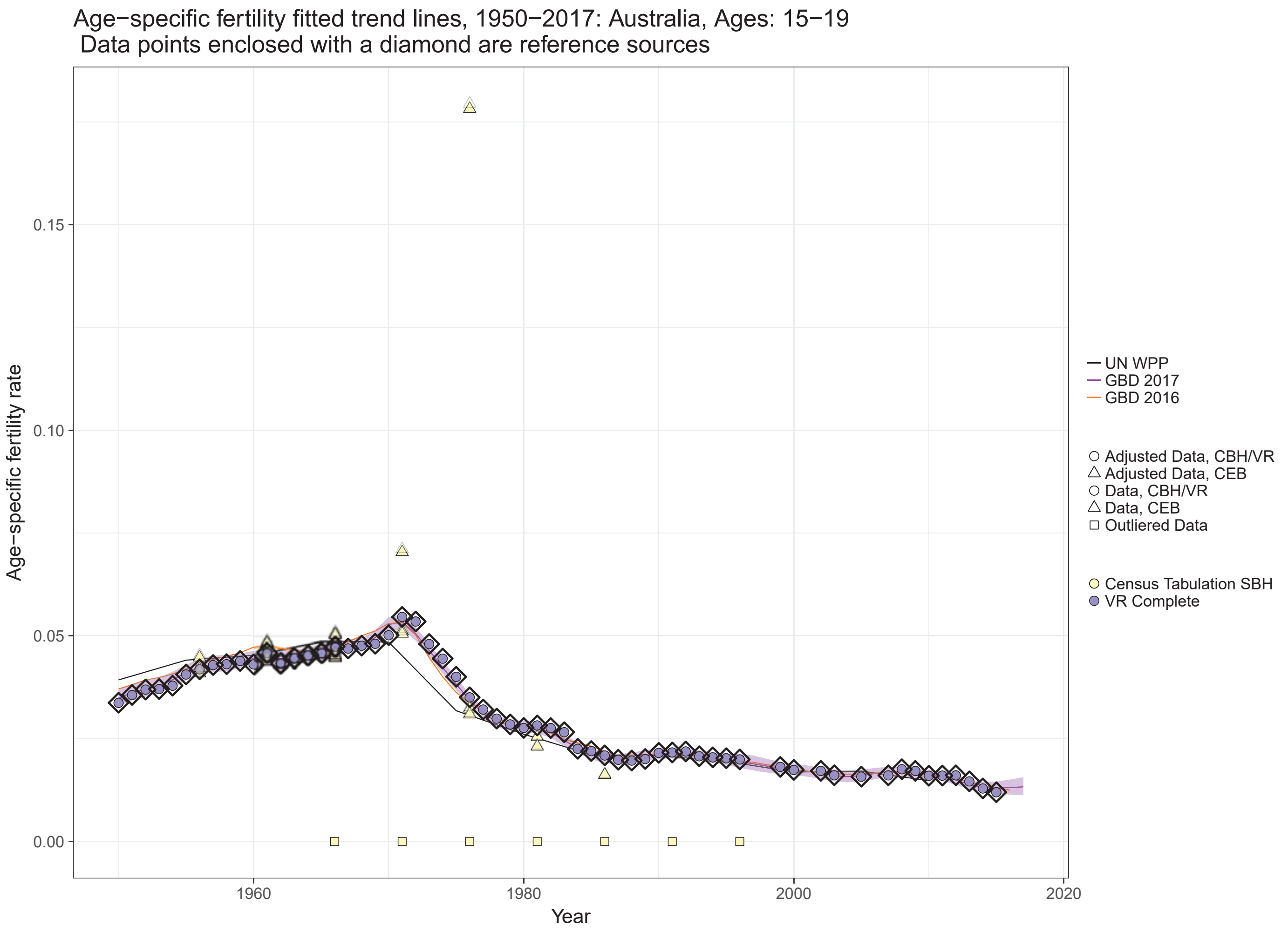
Age−specific fertility fitted trend lines, 1950−2017: Australia, Ages: 15−19
Data points enclosed with a diamond are reference sources
Age−specific fertility rate
Year
0.00
0.05
0.10
0.15
1960
1980
2000
2020
UN WPP
GBD 2017
GBD 2016
Adjusted Data, CBH/VR
Adjusted Data, CEB
Data, CBH/VR
Data, CEB
Outliered Data
Census Tabulation SBH
VR Complete

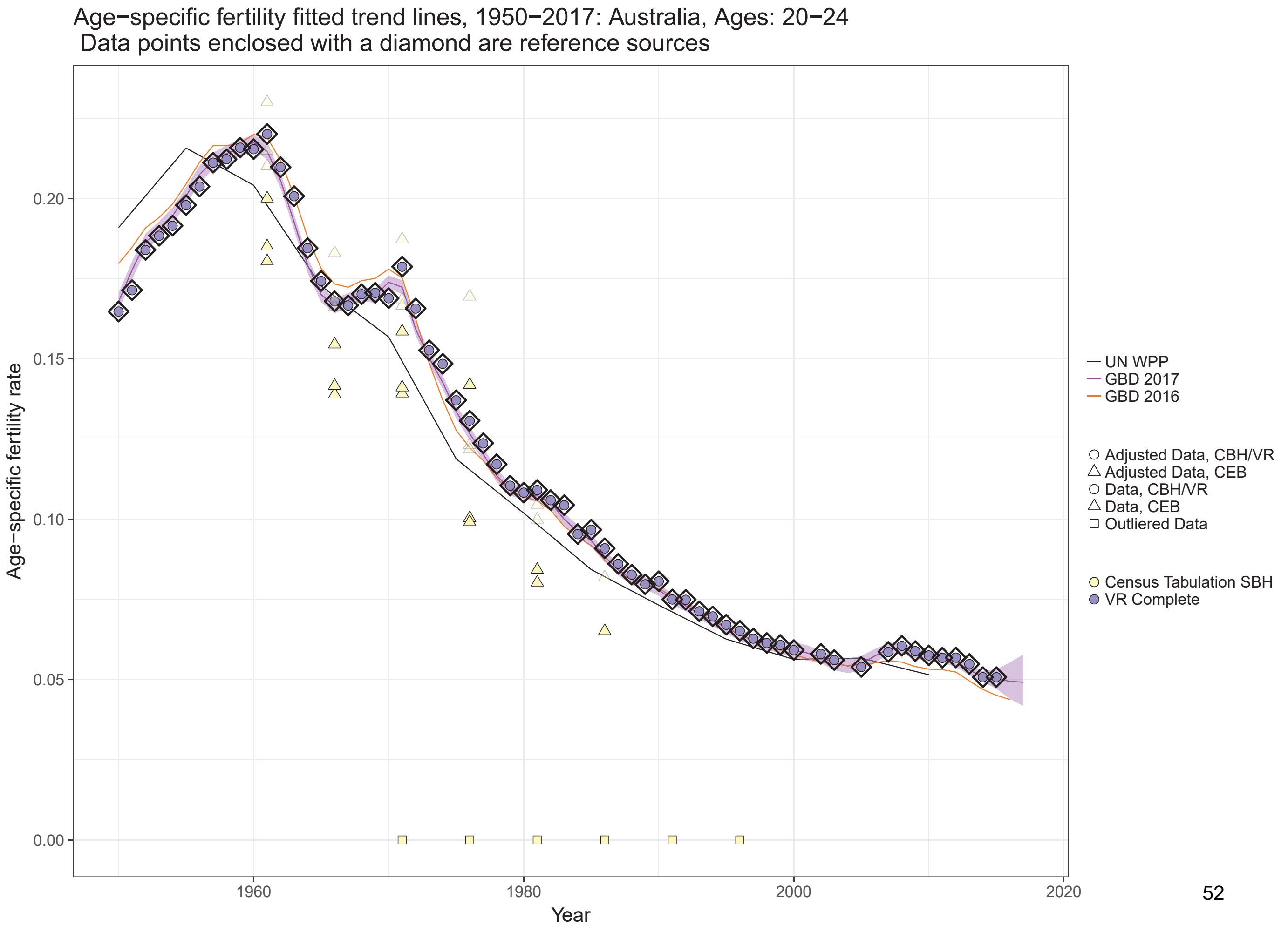
Age−specific fertility fitted trend lines, 1950−2017: Australia, Ages: 20−24
Data points enclosed with a diamond are reference sources
Age−specific fertility rate
Year
0.00
0.05
0.10
0.15
0.20
1960
1980
2000
2020
UN WPP
GBD 2017
GBD 2016
Adjusted Data, CBH/VR
Adjusted Data, CEB
Data, CBH/VR
Data, CEB
Outliered Data
Census Tabulation SBH
VR Complete

Age−specific fertility fitted trend lines, 1950−2017: Australia, Ages: 25−29
Data points enclosed with a diamond are reference sources

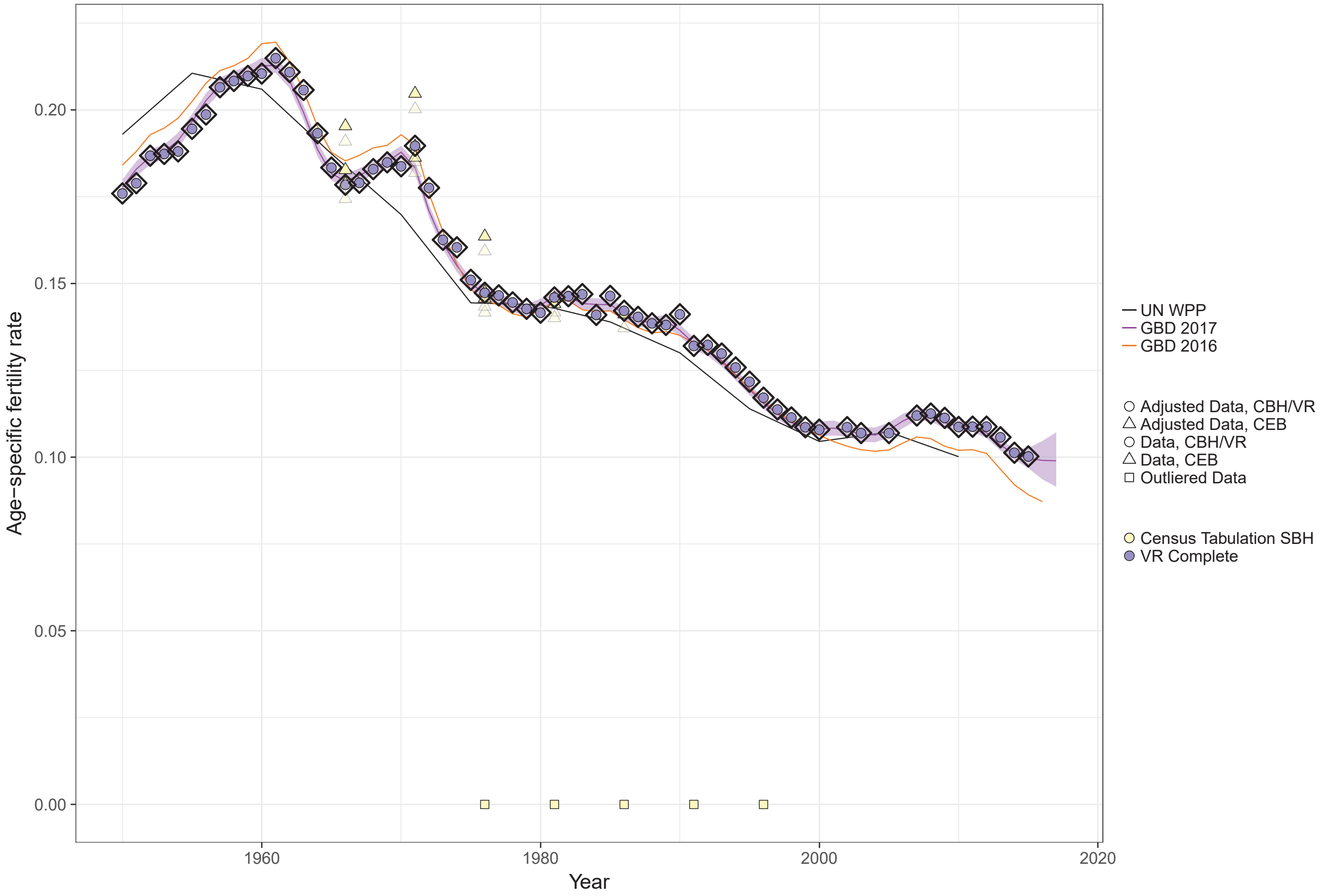

Age−specific fertility fitted trend lines, 1950−2017: Australia, Ages: 30−34
Data points enclosed with a diamond are reference sources

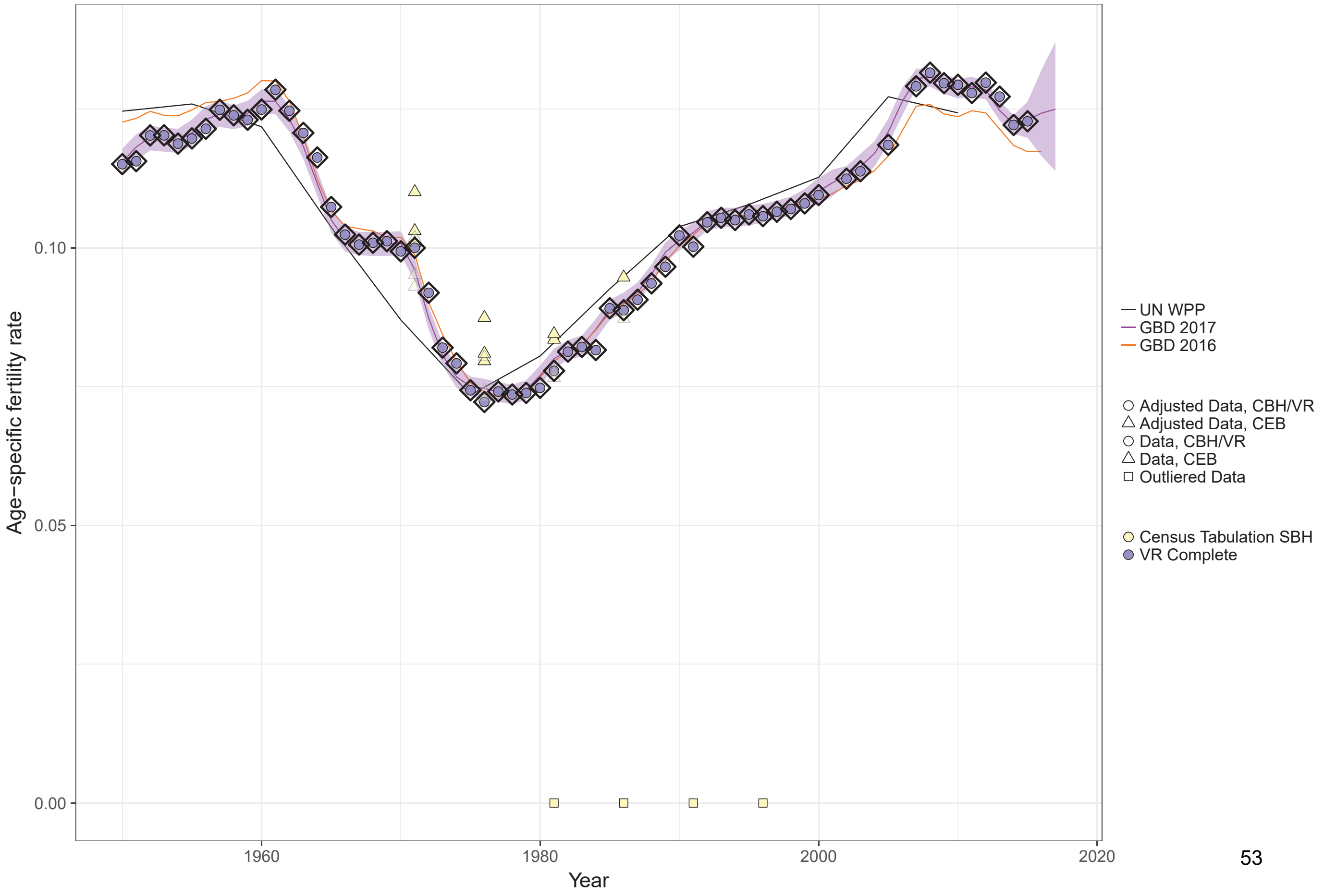

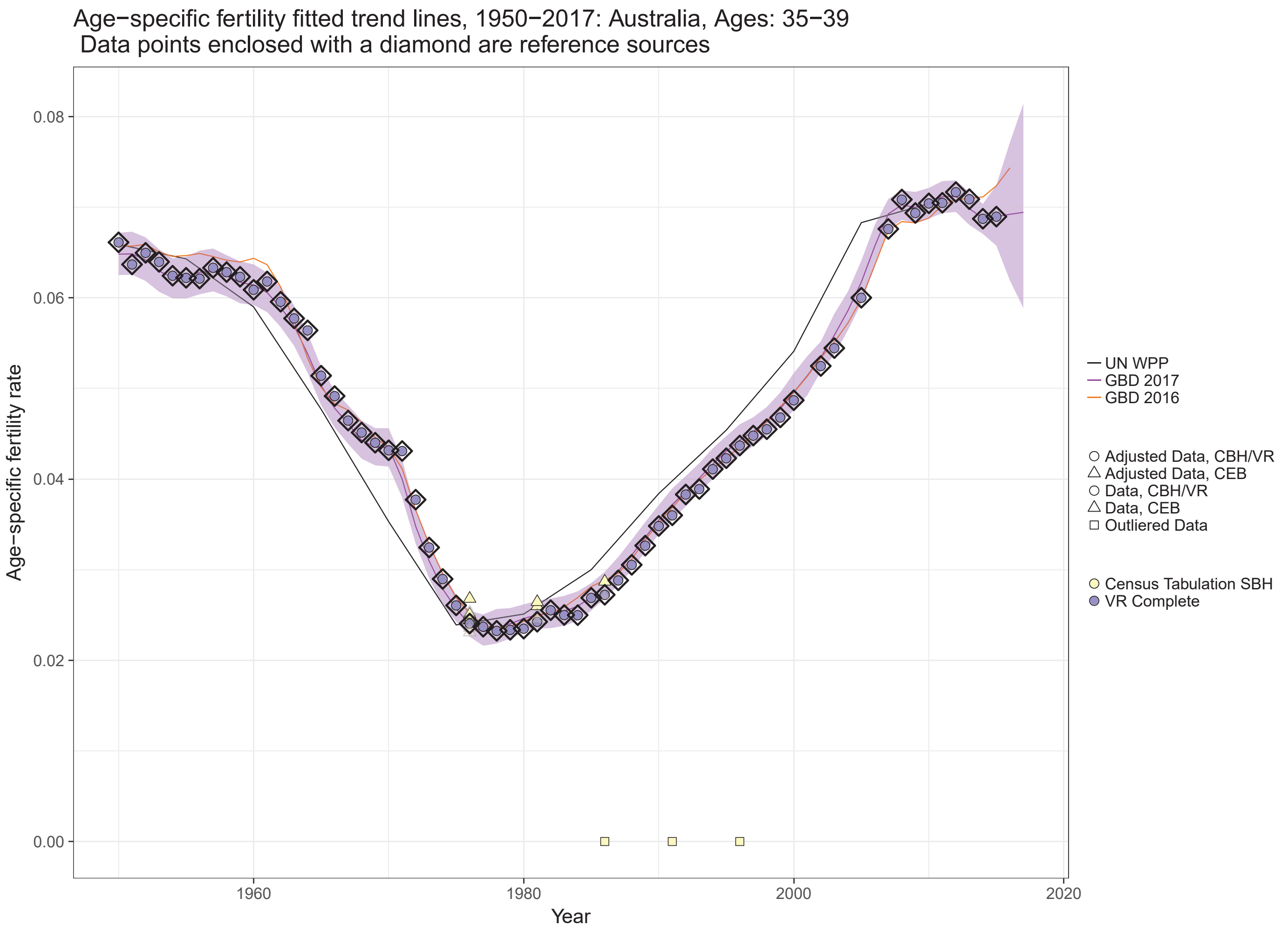
Age−specific fertility fitted trend lines, 1950−2017: Australia, Ages: 35−39
Data points enclosed with a diamond are reference sources
Age−specific fertility rate
Year
0.00
0.02
0.04
0.06
0.08
1960
1980
2000
2020
UN WPP
GBD 2017
GBD 2016
Adjusted Data, CBH/VR
Adjusted Data, CEB
Data, CBH/VR
Data, CEB
Outliered Data
Census Tabulation SBH
VR Complete

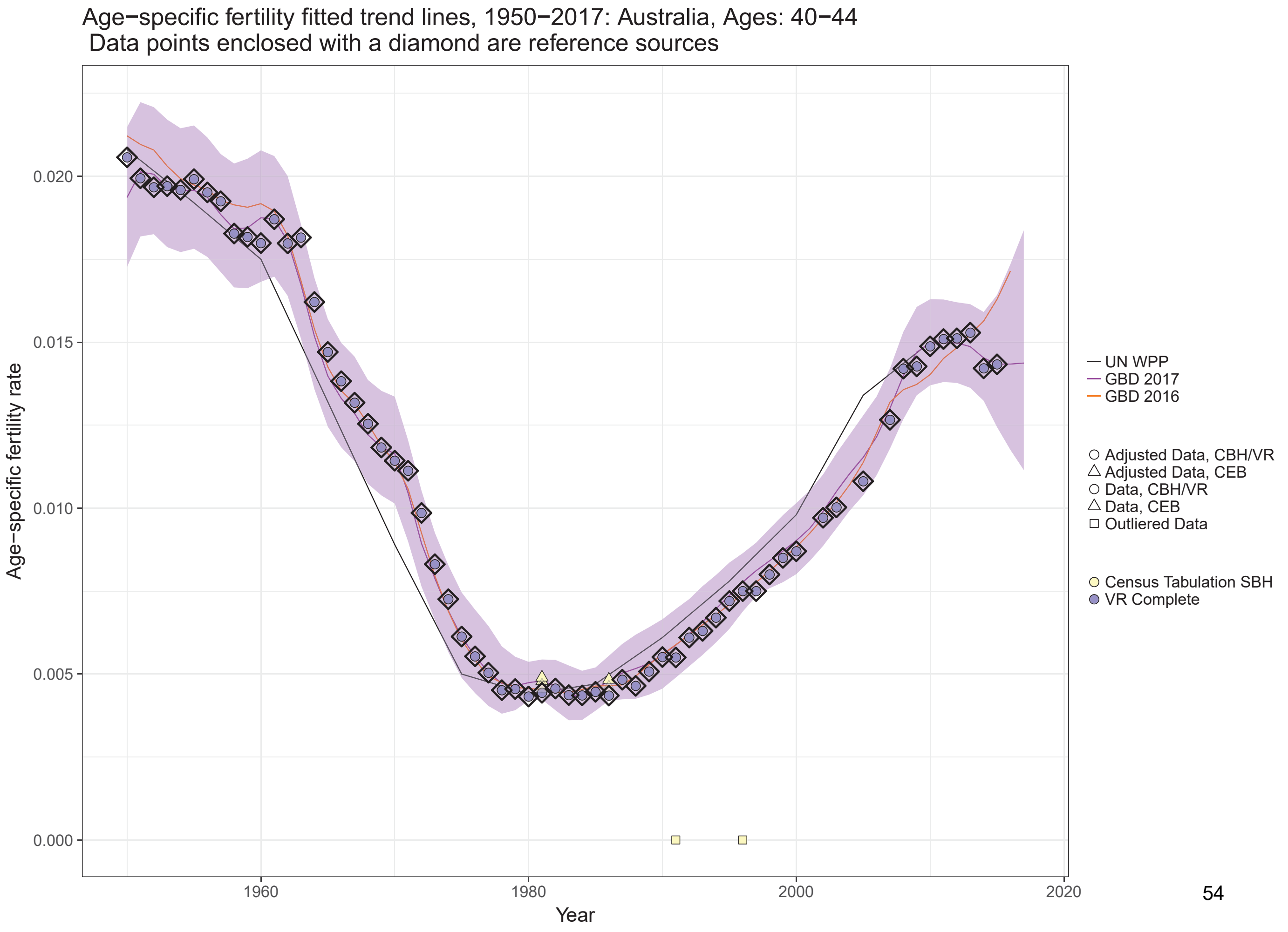
Age−specific fertility fitted trend lines, 1950−2017: Australia, Ages: 40−44
Data points enclosed with a diamond are reference sources
Age−specific fertility rate
Year
0.000
0.005
0.010
0.015
0.020
1960
1980
2000
2020
UN WPP
GBD 2017
GBD 2016
Adjusted Data, CBH/VR
Adjusted Data, CEB
Data, CBH/VR
Data, CEB
Outliered Data
Census Tabulation SBH
VR Complete

Age−specific fertility fitted trend lines, 1950−2017: Australia, Ages: 45−49
Data points enclosed with a diamond are reference sources

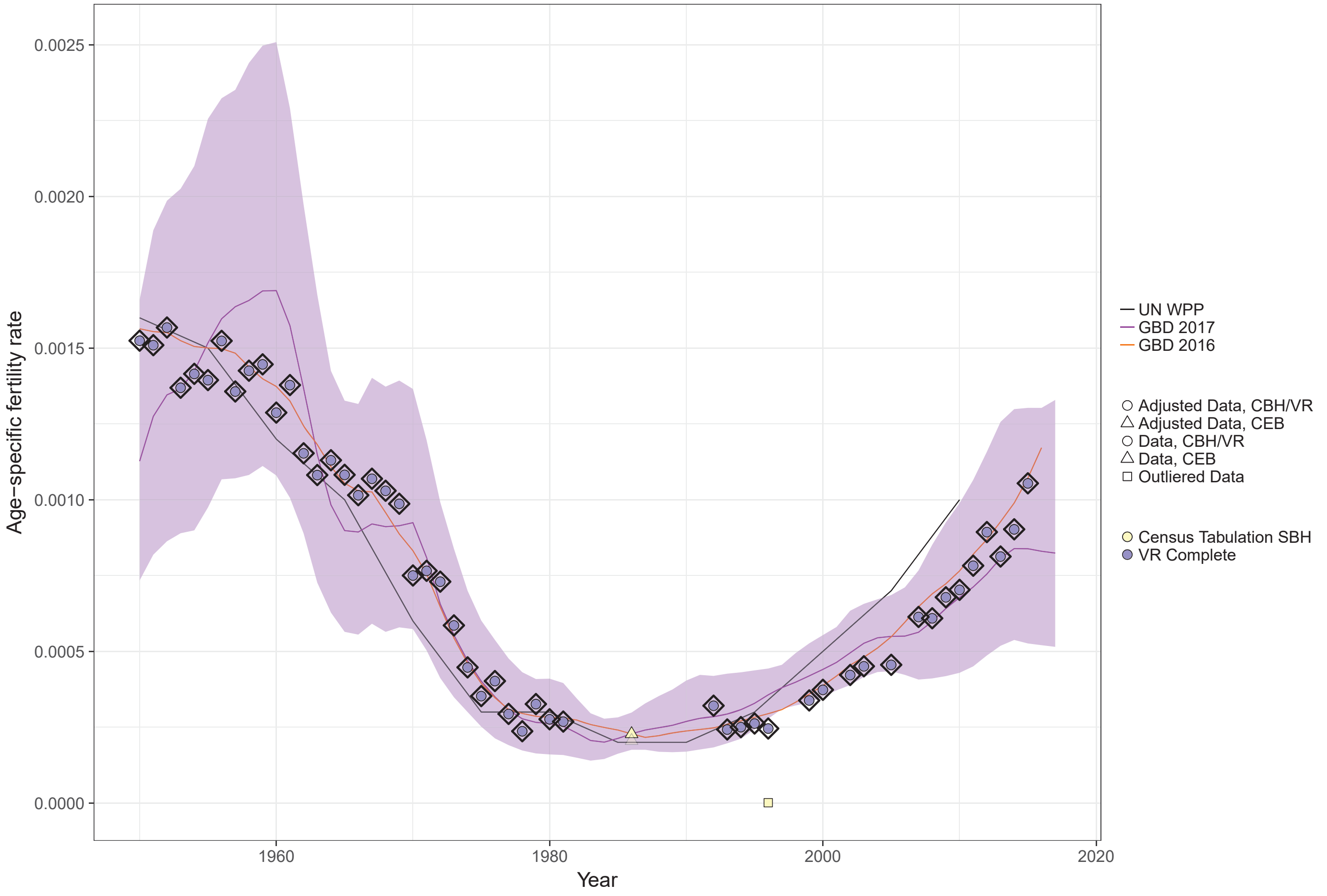

Age−specific fertility fitted trend lines, 1950−2017: Australia, Ages: 50−54
Data points enclosed with a diamond are reference sources

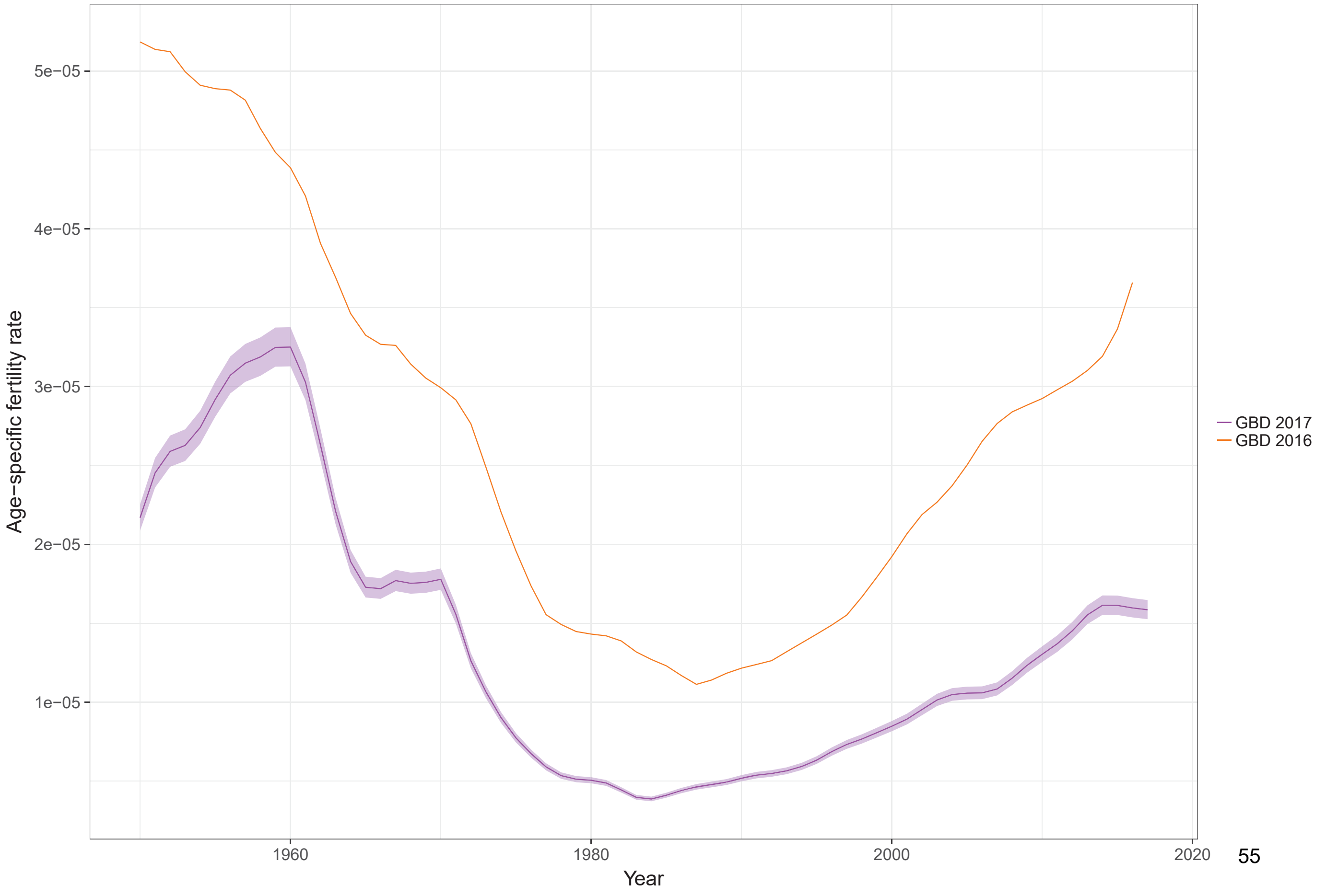

Age−specific fertility fitted trend lines, 1950−2017: Austria, TFR
Data points enclosed with a diamond are reference sources

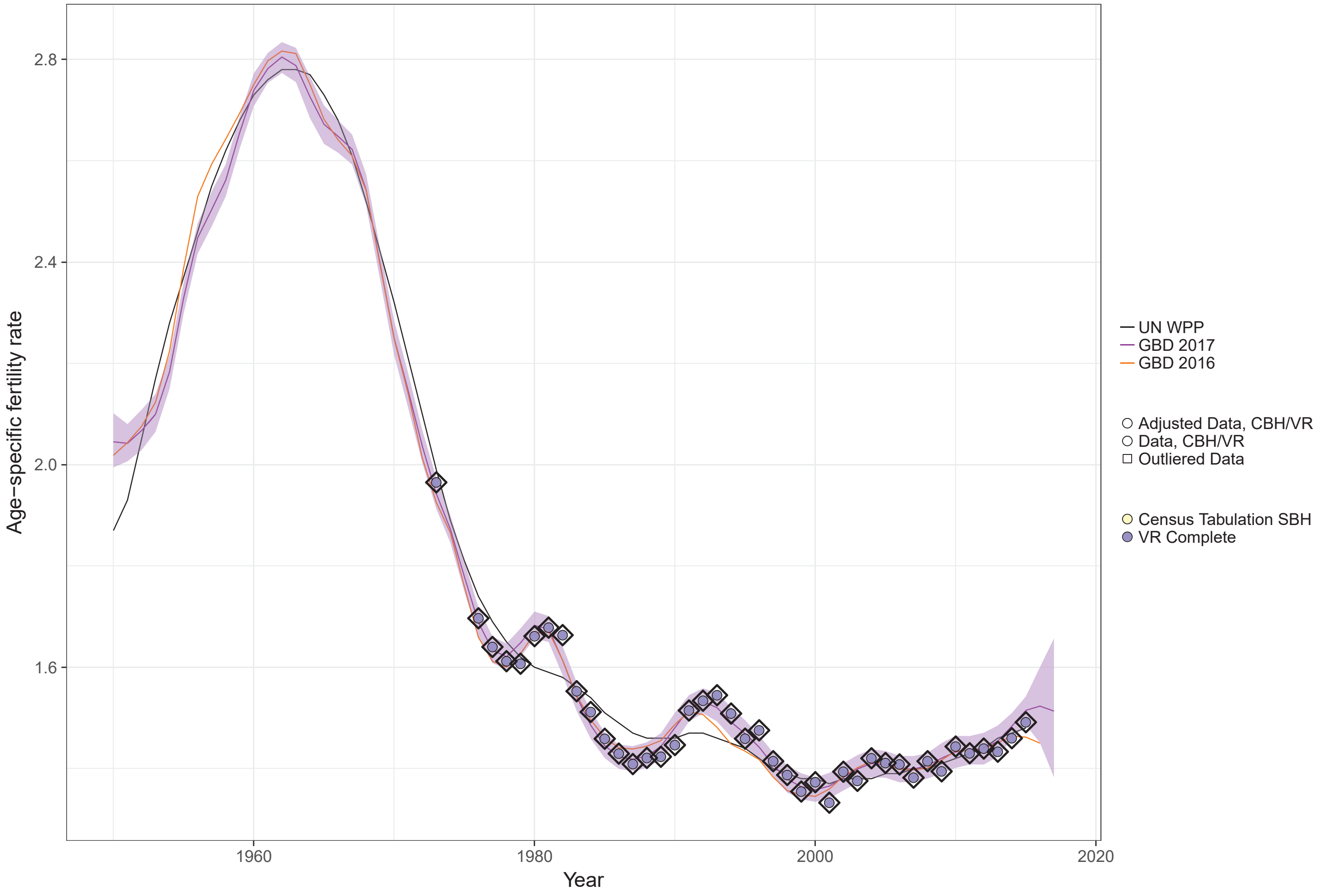

Age−specific fertility fitted trend lines, 1950−2017: Austria, Ages: 10−14
Data points enclosed with a diamond are reference sources

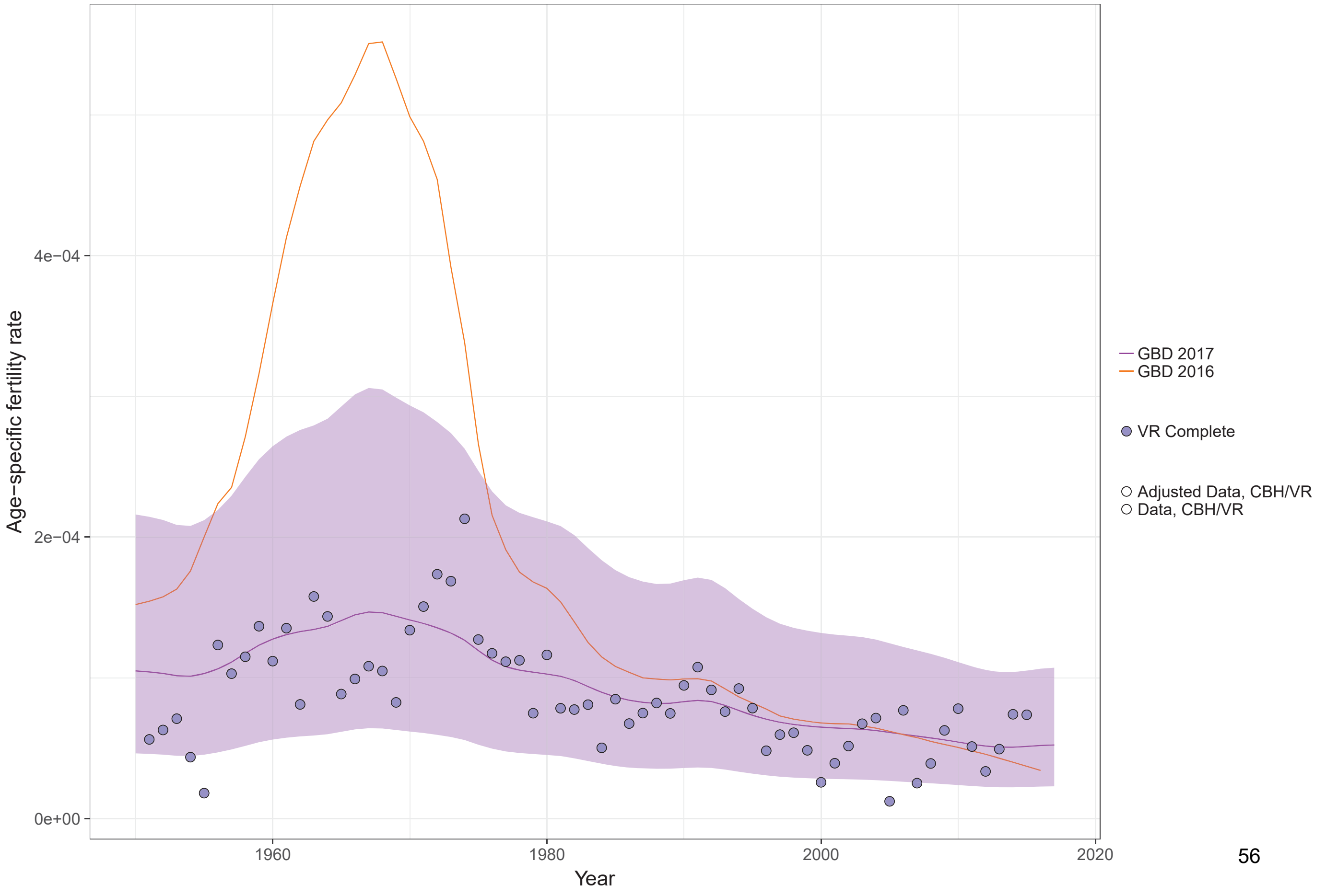

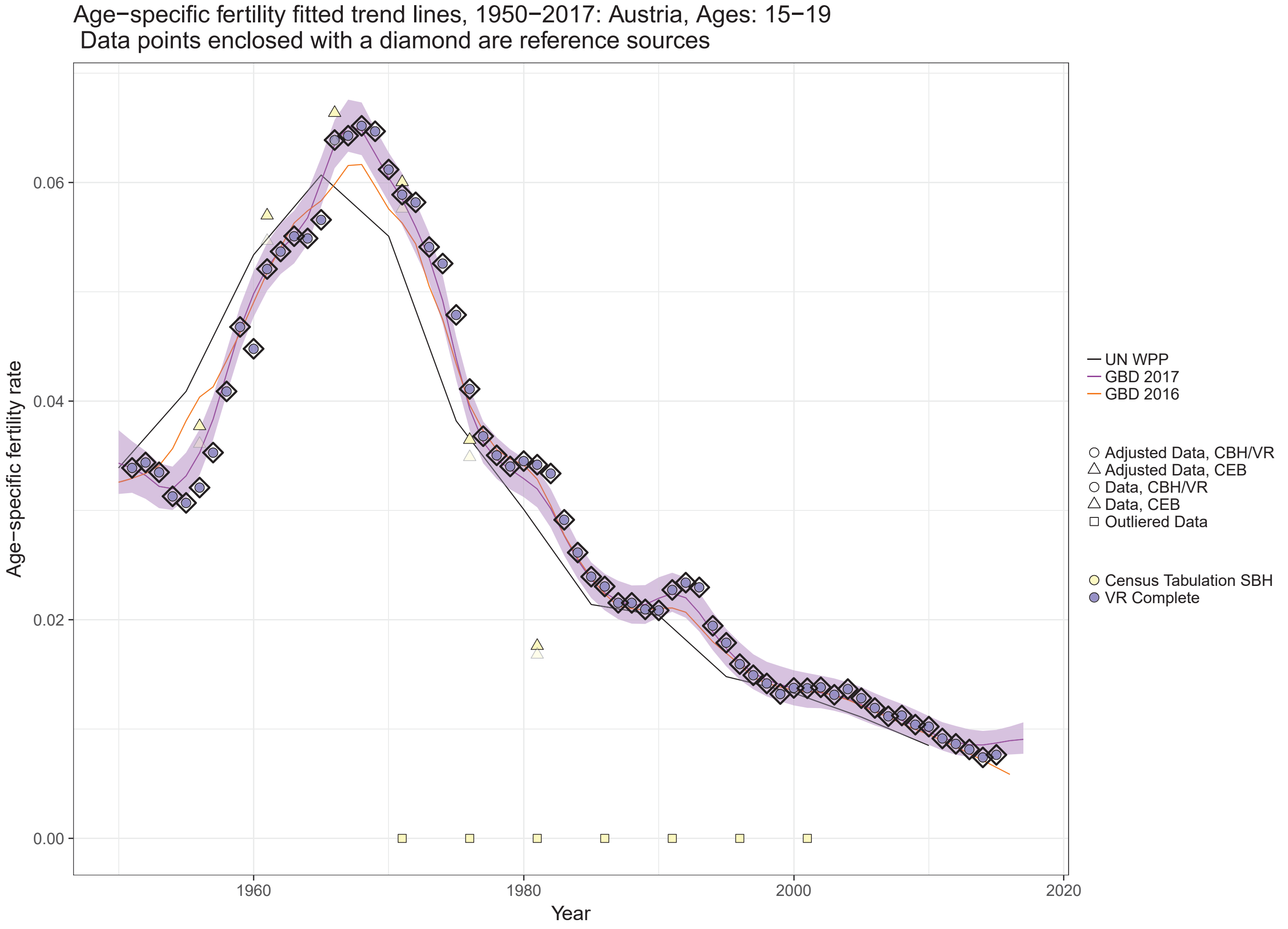
Age−specific fertility fitted trend lines, 1950−2017: Austria, Ages: 15−19
Data points enclosed with a diamond are reference sources
Age−specific fertility rate
0.00
0.02
0.04
0.06
1960
1980
2000
2020
Year
UN WPP
GBD 2017
GBD 2016
Adjusted Data, CBH/VR
Adjusted Data, CEB
Data, CBH/VR
Data, CEB
Outliered Data
Census Tabulation SBH
VR Complete

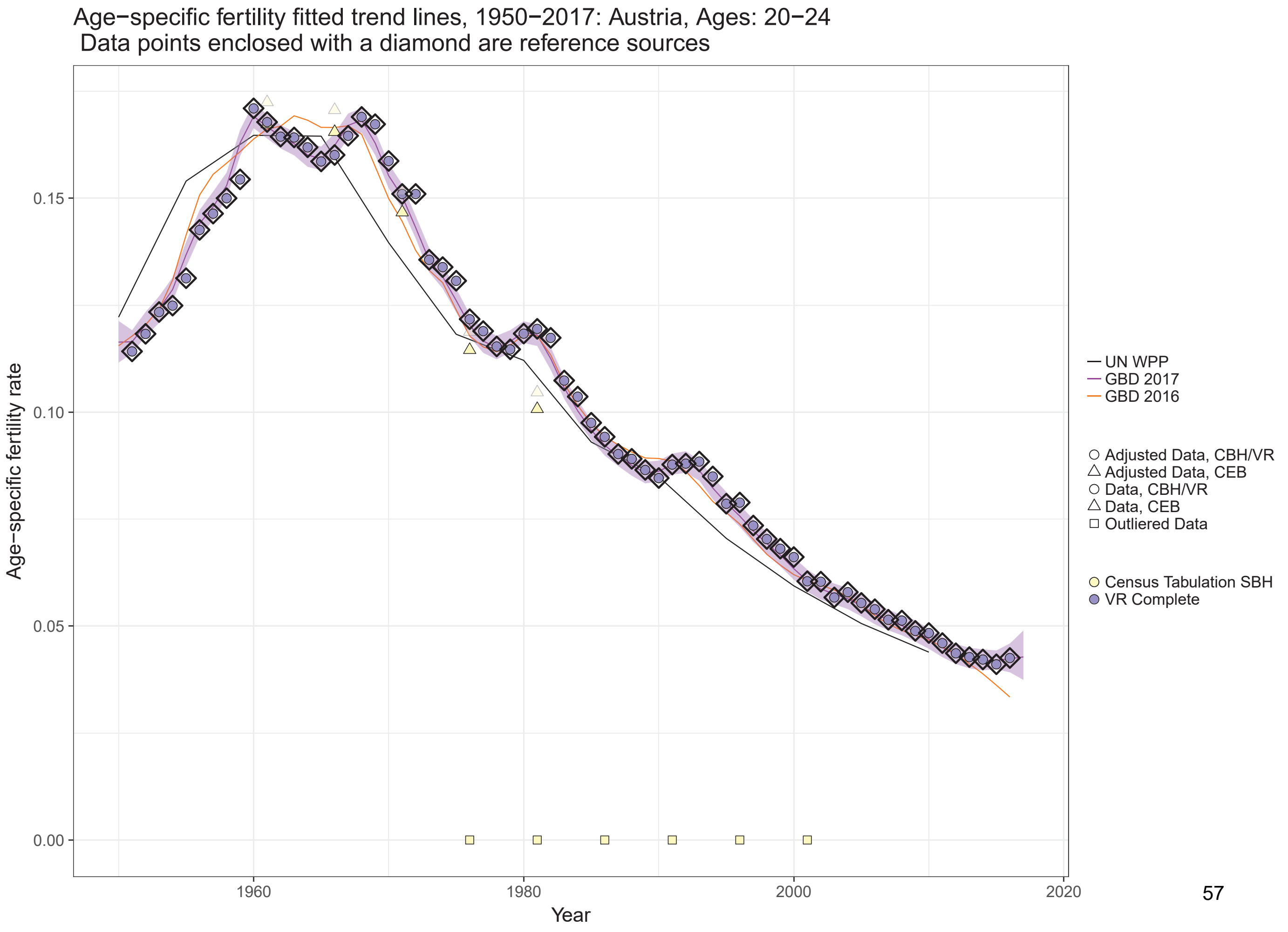
Age−specific fertility fitted trend lines, 1950−2017: Austria, Ages: 20−24
Data points enclosed with a diamond are reference sources
Age−specific fertility rate
0.00
0.05
0.10
0.15
1960
1980
2000
2020
Year
UN WPP
GBD 2017
GBD 2016
Adjusted Data, CBH/VR
Adjusted Data, CEB
Data, CBH/VR
Data, CEB
Outliered Data
Census Tabulation SBH
VR Complete

Age−specific fertility fitted trend lines, 1950−2017: Austria, Ages: 25−29
Data points enclosed with a diamond are reference sources

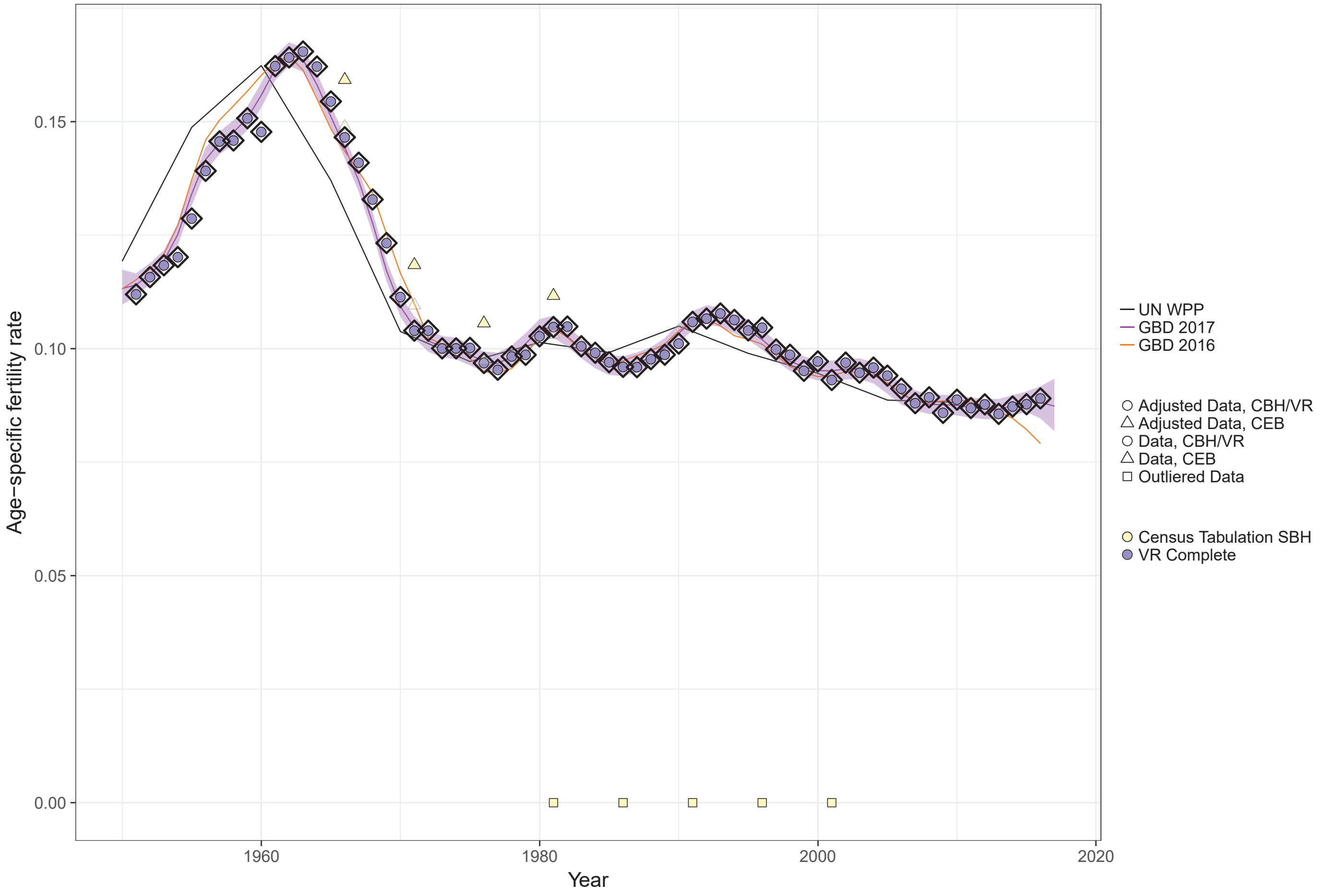

Age−specific fertility fitted trend lines, 1950−2017: Austria, Ages: 30−34
Data points enclosed with a diamond are reference sources

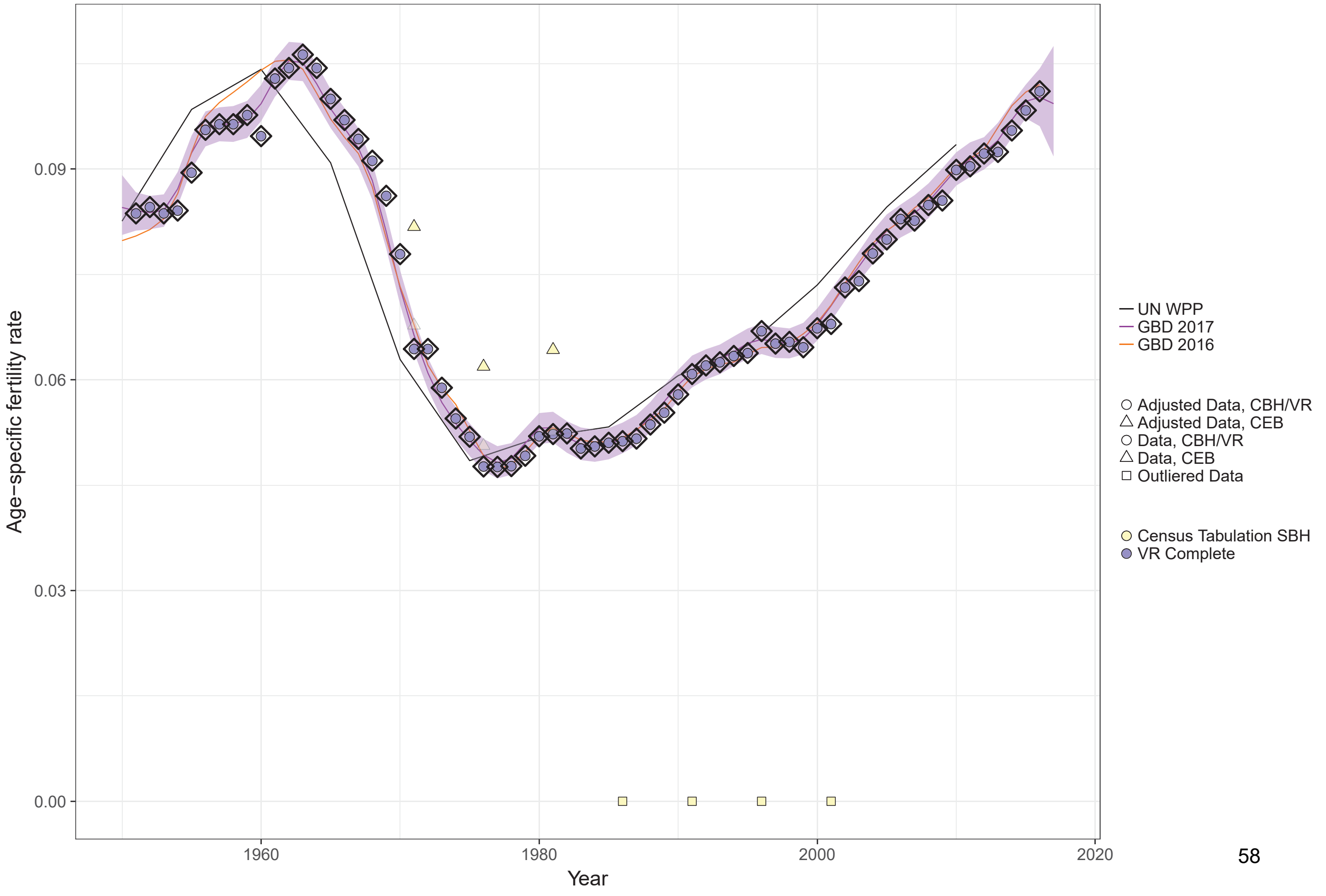

Age−specific fertility fitted trend lines, 1950−2017: Austria, Ages: 35−39
Data points enclosed with a diamond are reference sources

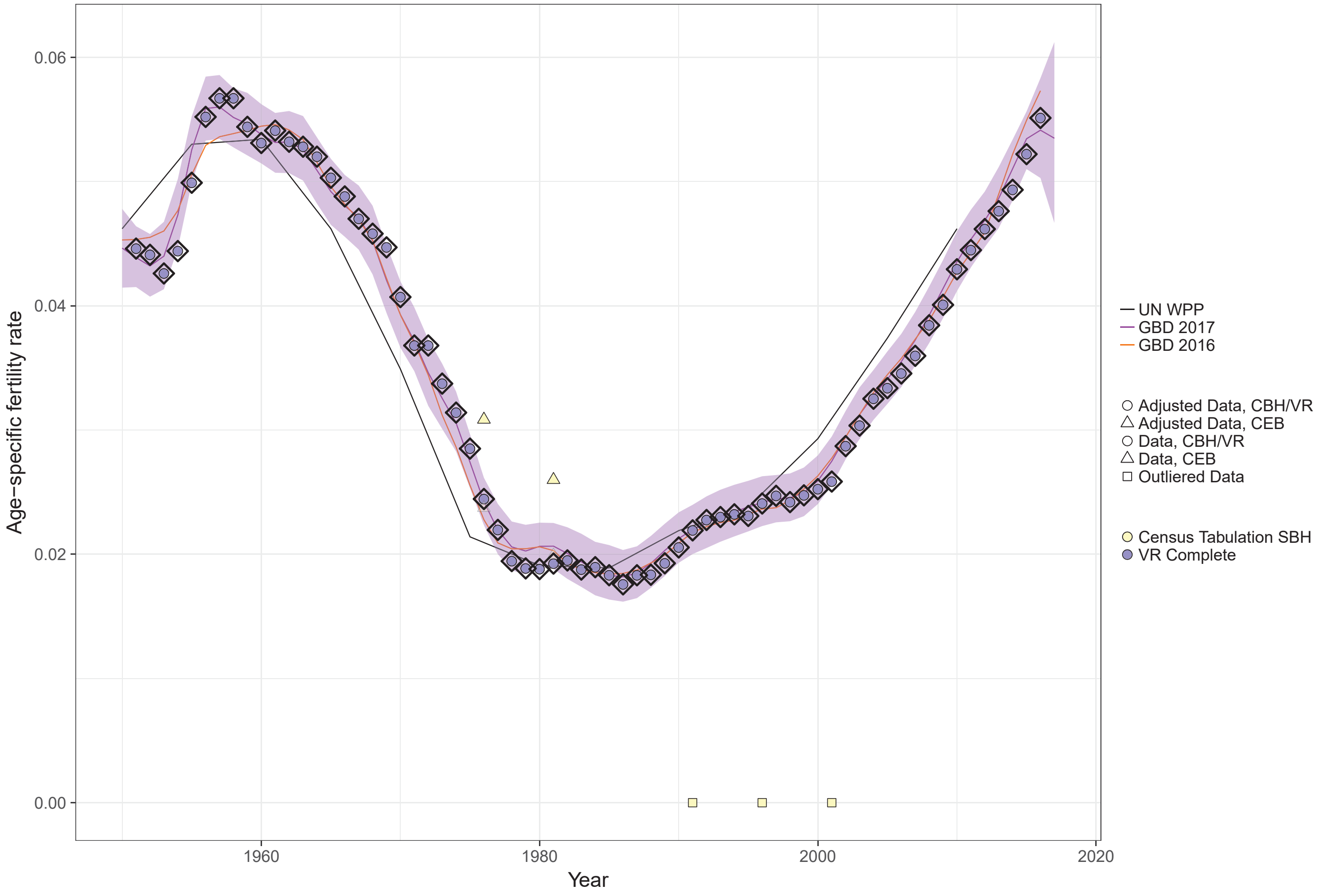

Age−specific fertility fitted trend lines, 1950−2017: Austria, Ages: 40−44
Data points enclosed with a diamond are reference sources

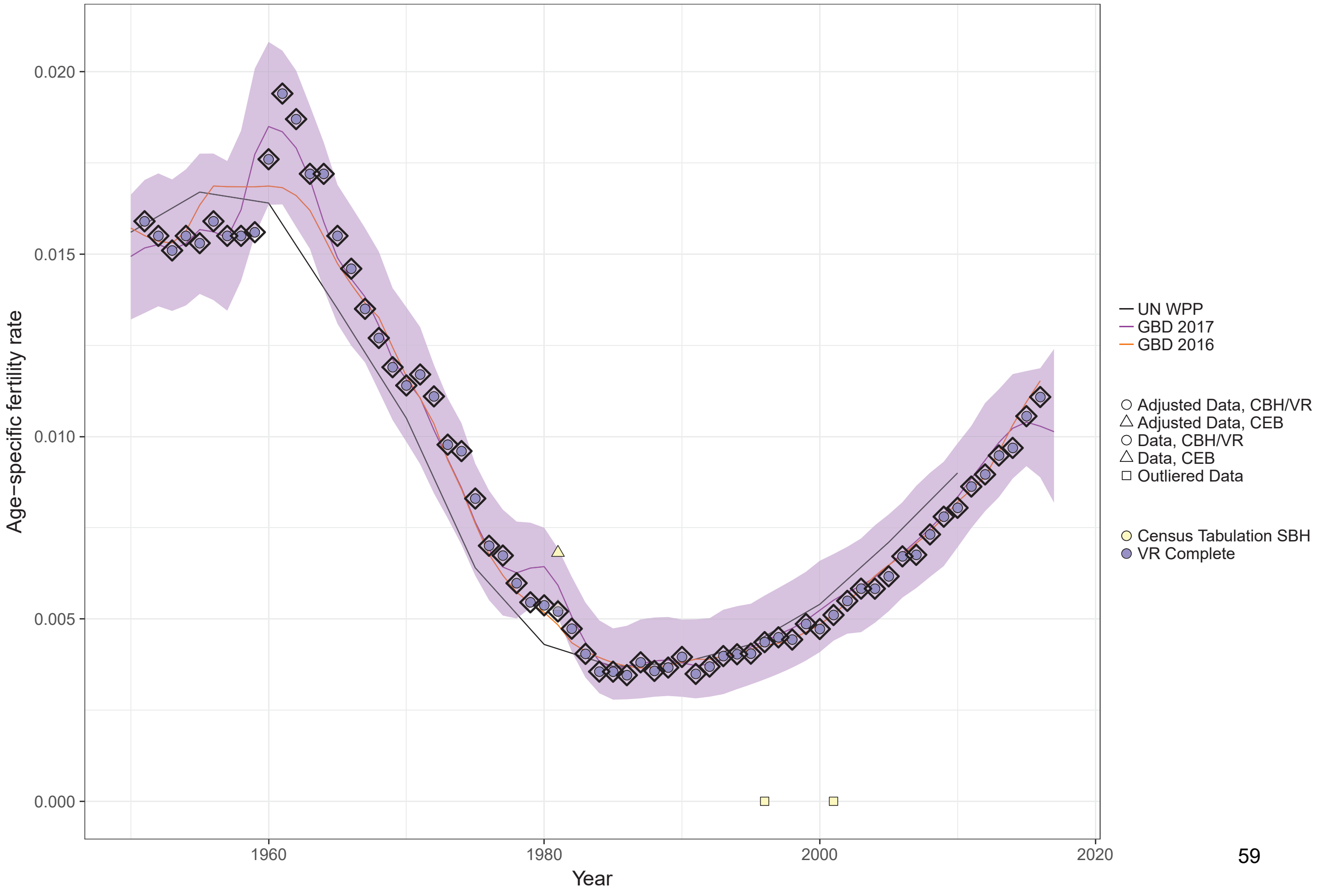

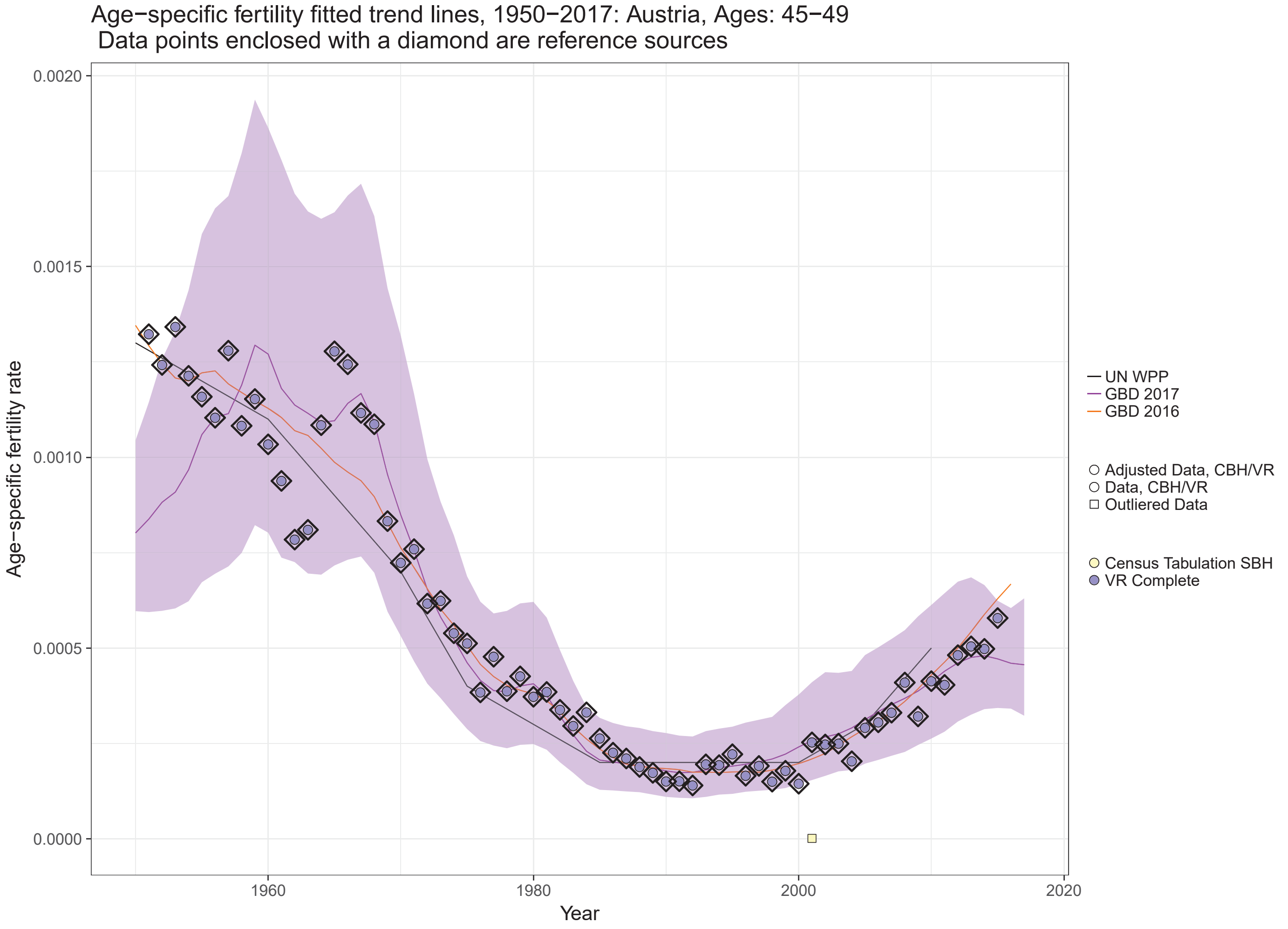

Age−specific fertility fitted trend lines, 1950−2017: Austria, Ages: 45−49
Data points enclosed with a diamond are reference sources
Age−specific fertility rate
0.0020
0.0015
0.0010
0.0005
0.0000
1960
1980
2000
2020
Year
UN WPP
GBD 2017
GBD 2016
Adjusted Data, CBH/VR
Data, CBH/VR
Outliered Data
Census Tabulation SBH
VR Complete

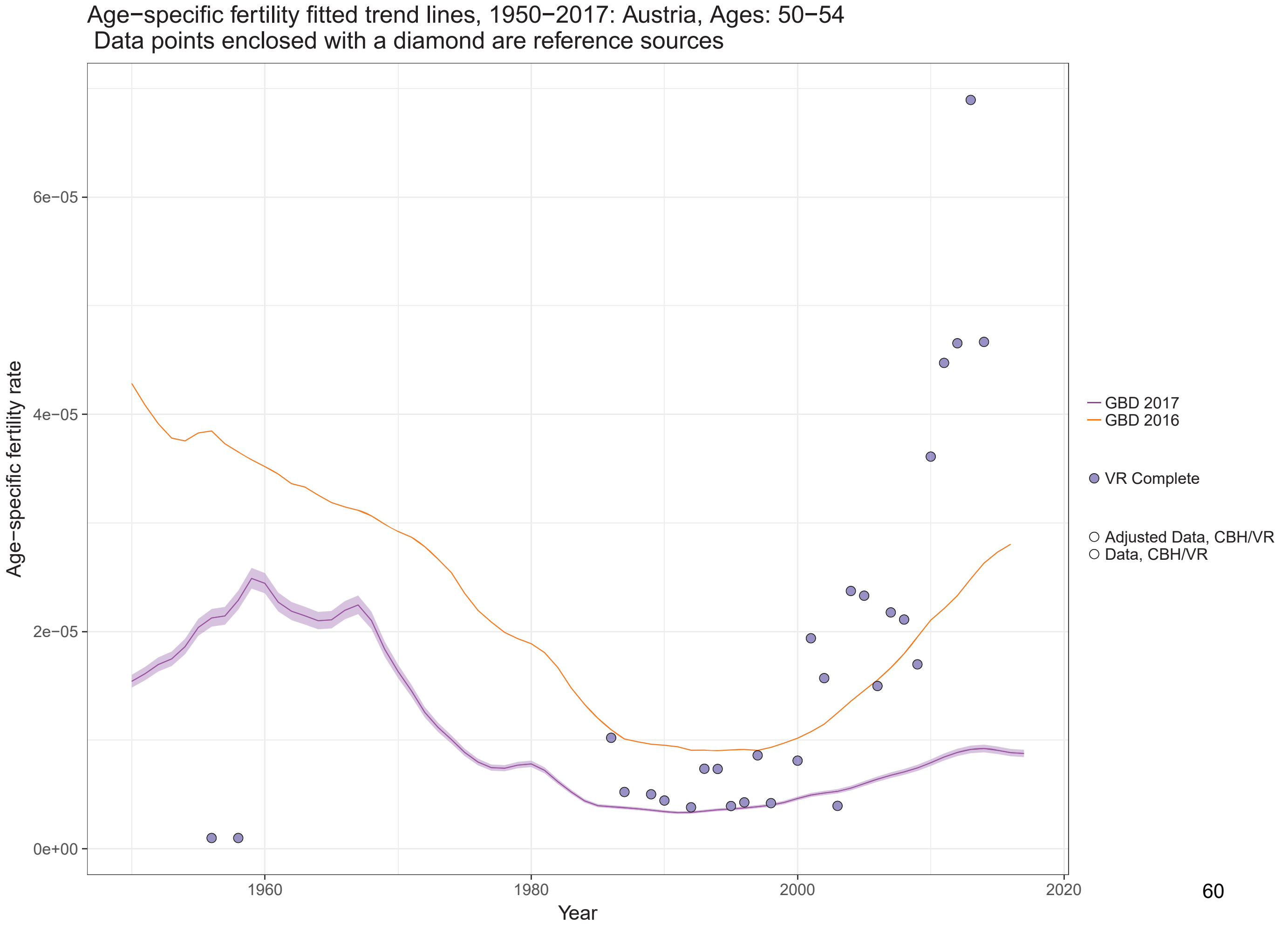

Age−specific fertility fitted trend lines, 1950−2017: Austria, Ages: 50−54
Data points enclosed with a diamond are reference sources
Age−specific fertility rate
6e−05
4e−05
2e−05
0e+00
1960
1980
2000
2020
Year
GBD 2017
GBD 2016
VR Complete
Adjusted Data, CBH/VR
Data, CBH/VR

Age−specific fertility fitted trend lines, 1950−2017: Azerbaijan, TFR
Data points enclosed with a diamond are reference sources

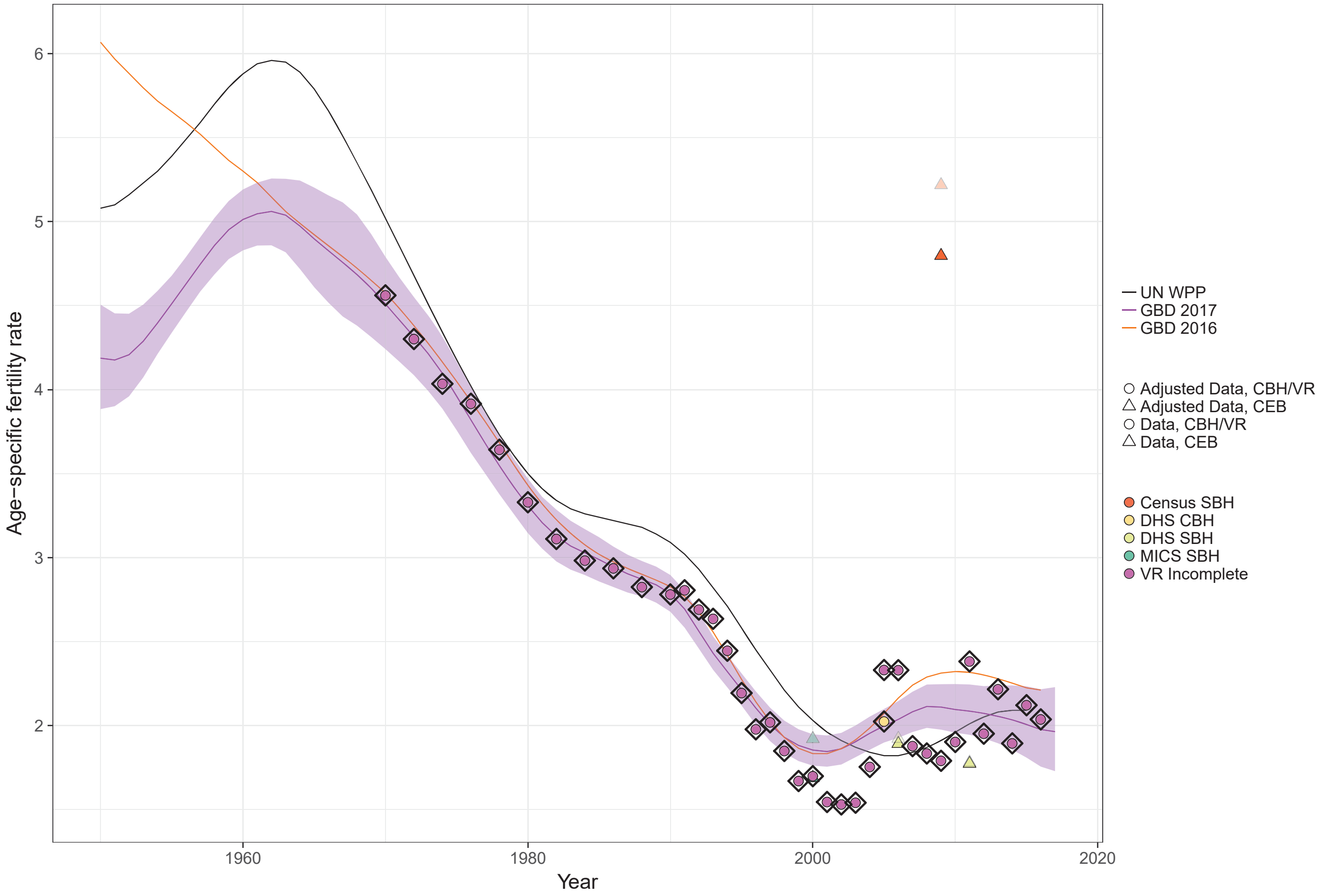

Age−specific fertility fitted trend lines, 1950−2017: Azerbaijan, Ages: 10−14
Data points enclosed with a diamond are reference sources

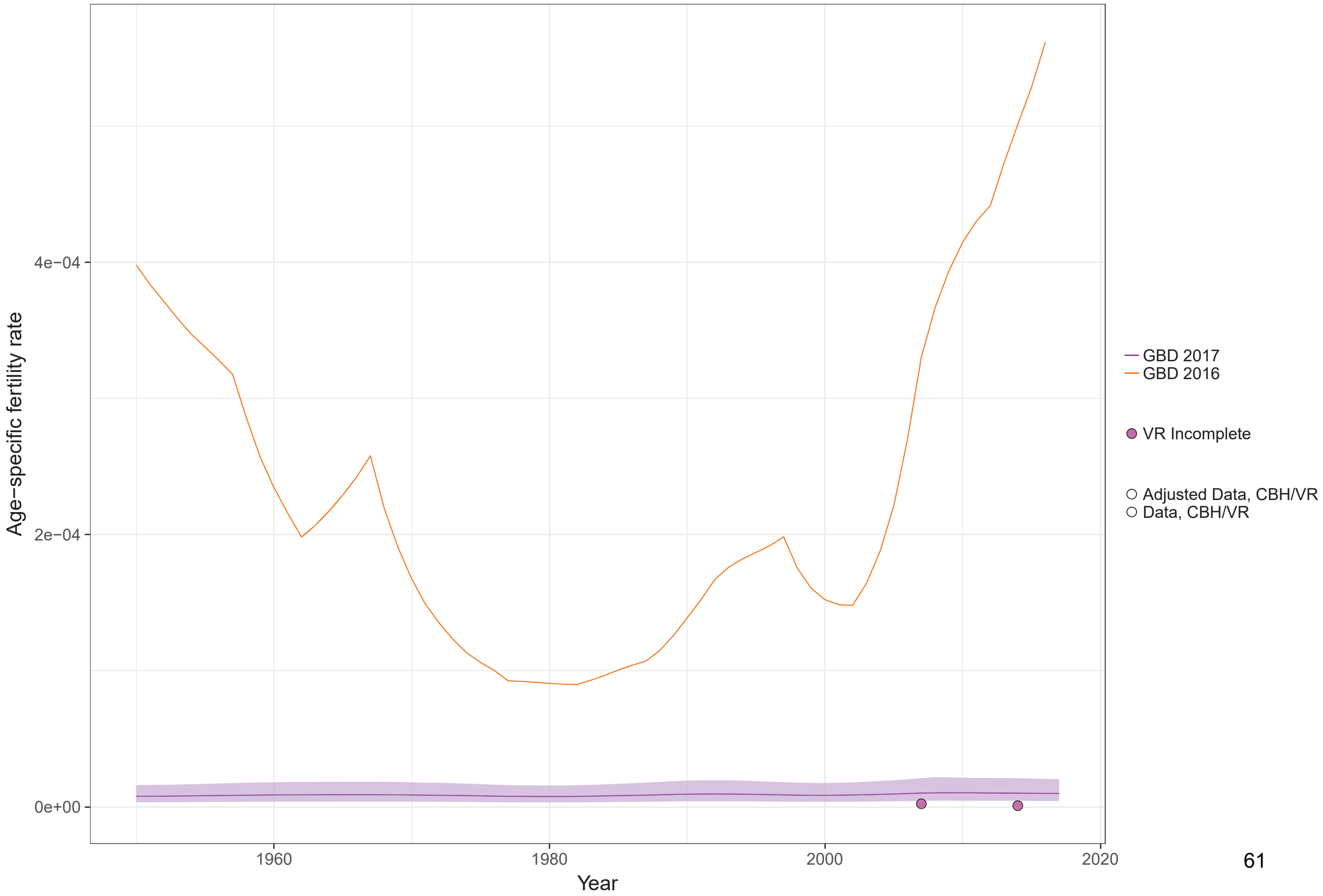

Age−specific fertility fitted trend lines, 1950−2017: Azerbaijan, Ages: 15−19
Data points enclosed with a diamond are reference sources

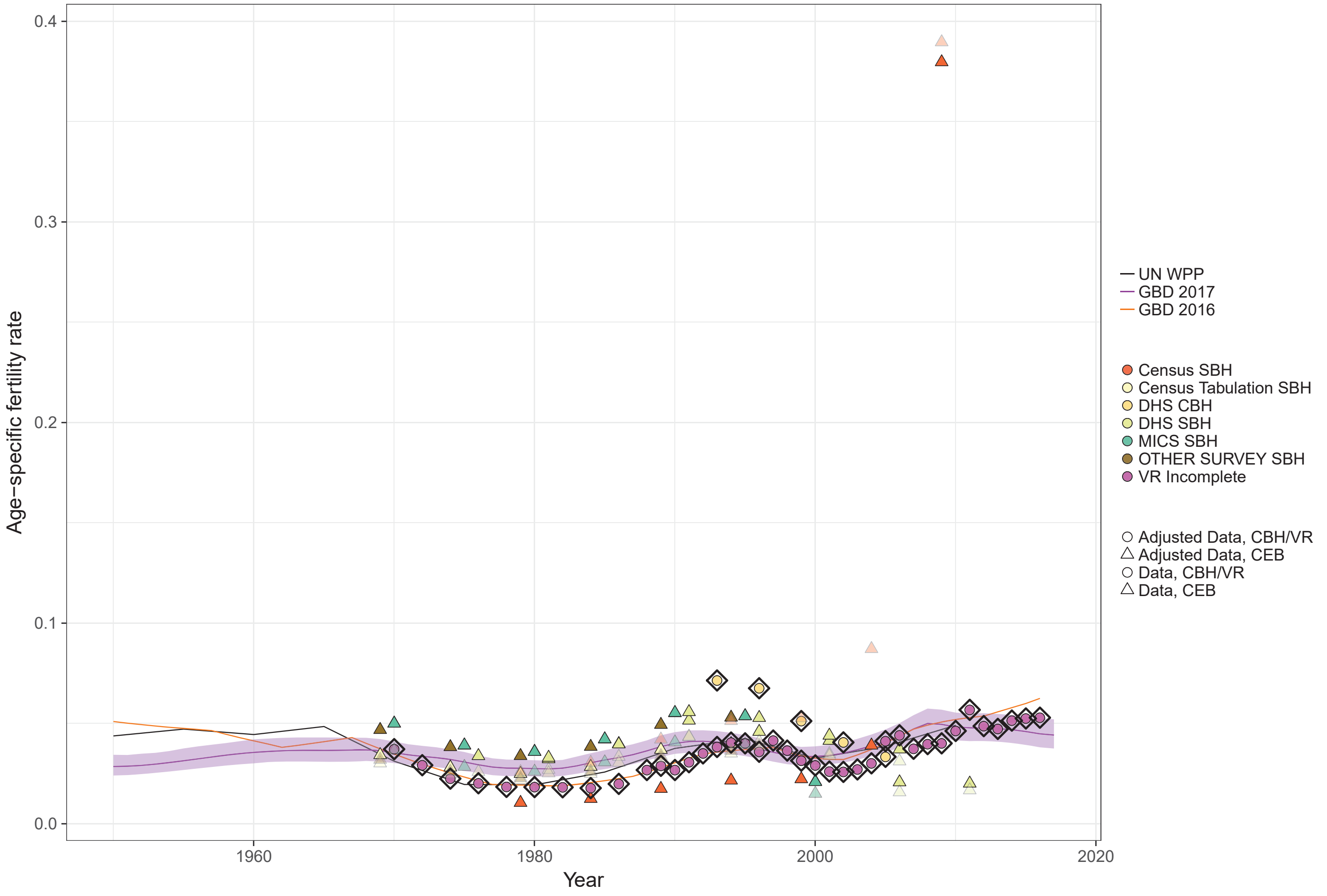

Age−specific fertility fitted trend lines, 1950−2017: Azerbaijan, Ages: 20−24
Data points enclosed with a diamond are reference sources

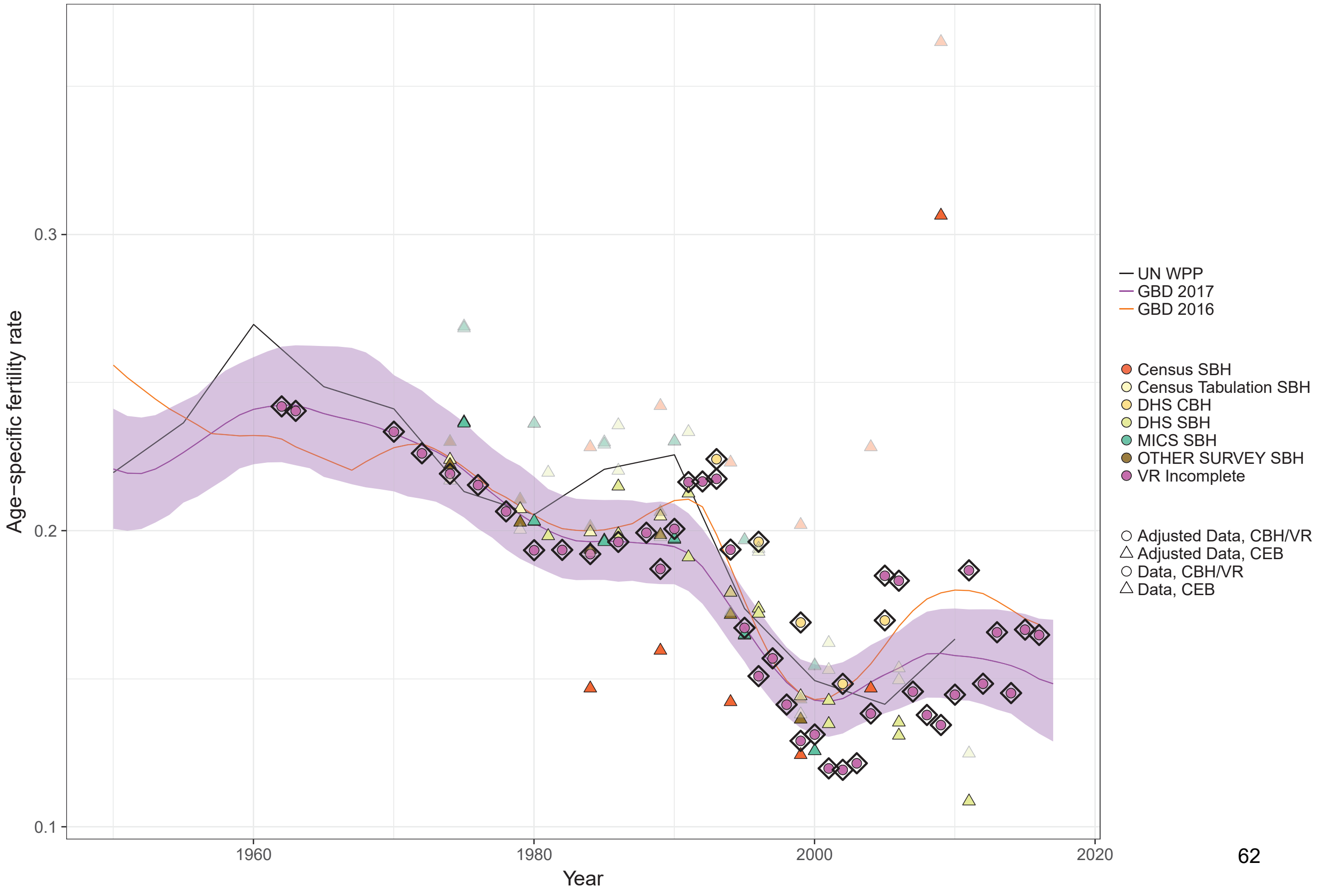

Age−specific fertility fitted trend lines, 1950−2017: Azerbaijan, Ages: 25−29
Data points enclosed with a diamond are reference sources

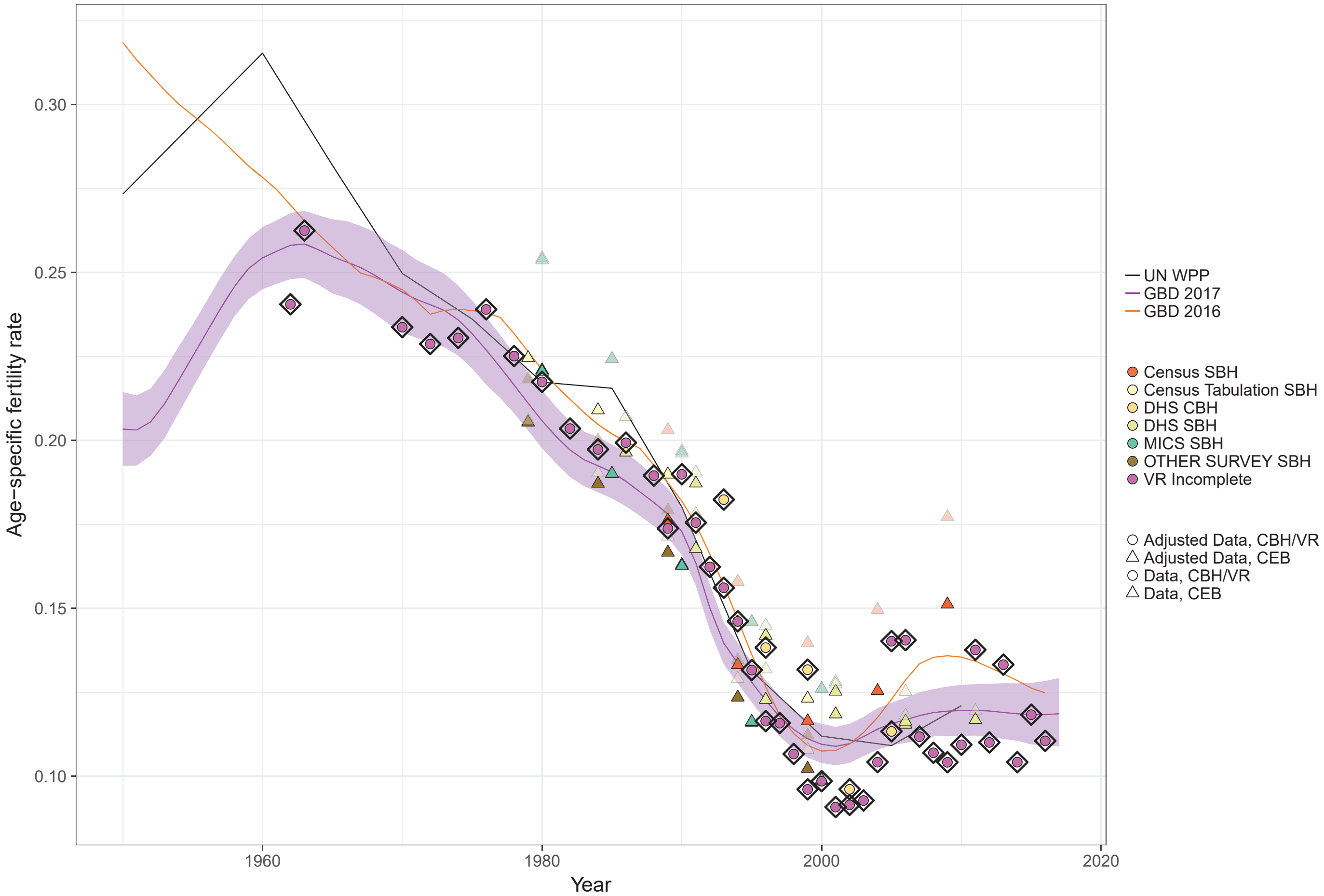

Age−specific fertility fitted trend lines, 1950−2017: Azerbaijan, Ages: 30−34
Data points enclosed with a diamond are reference sources

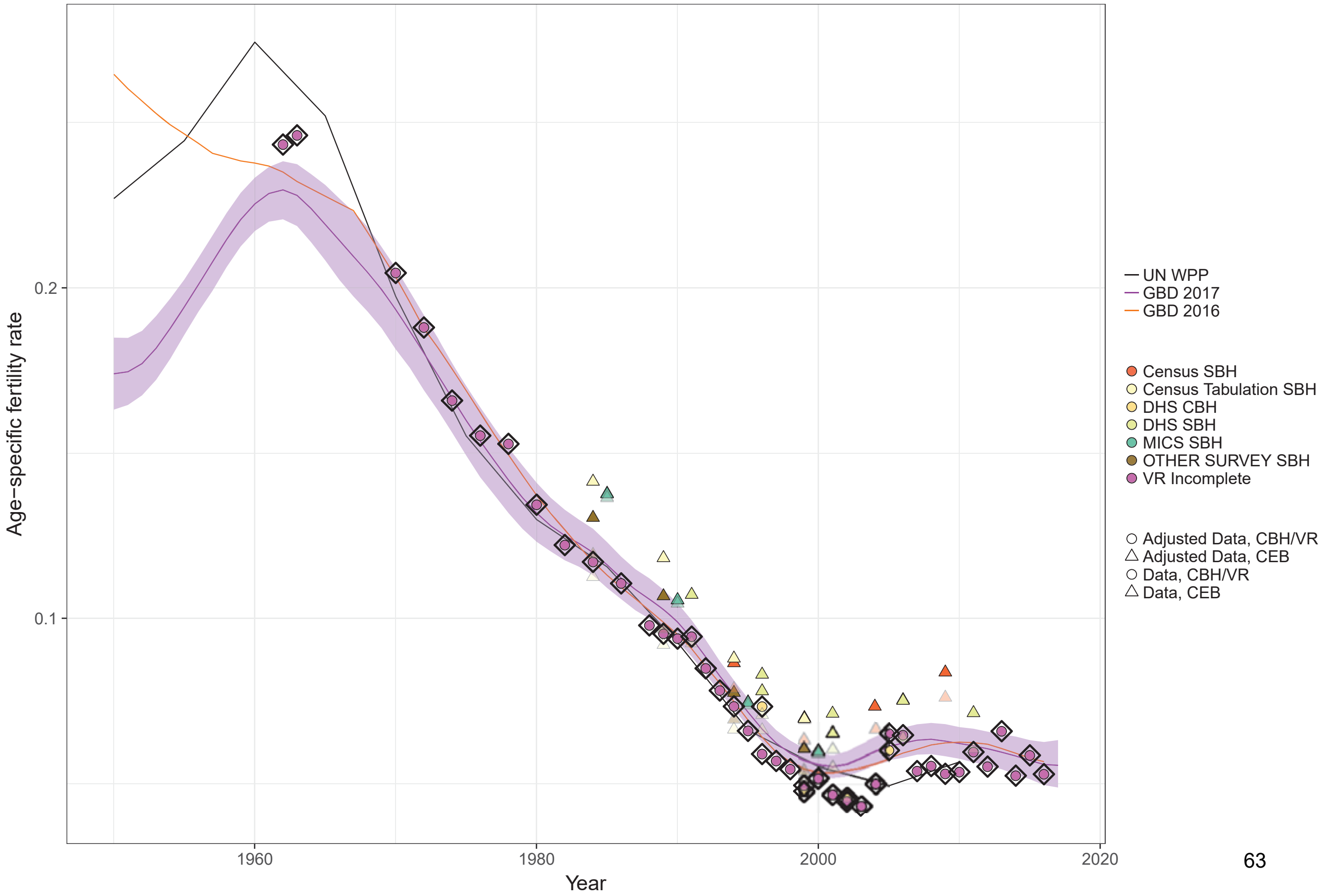

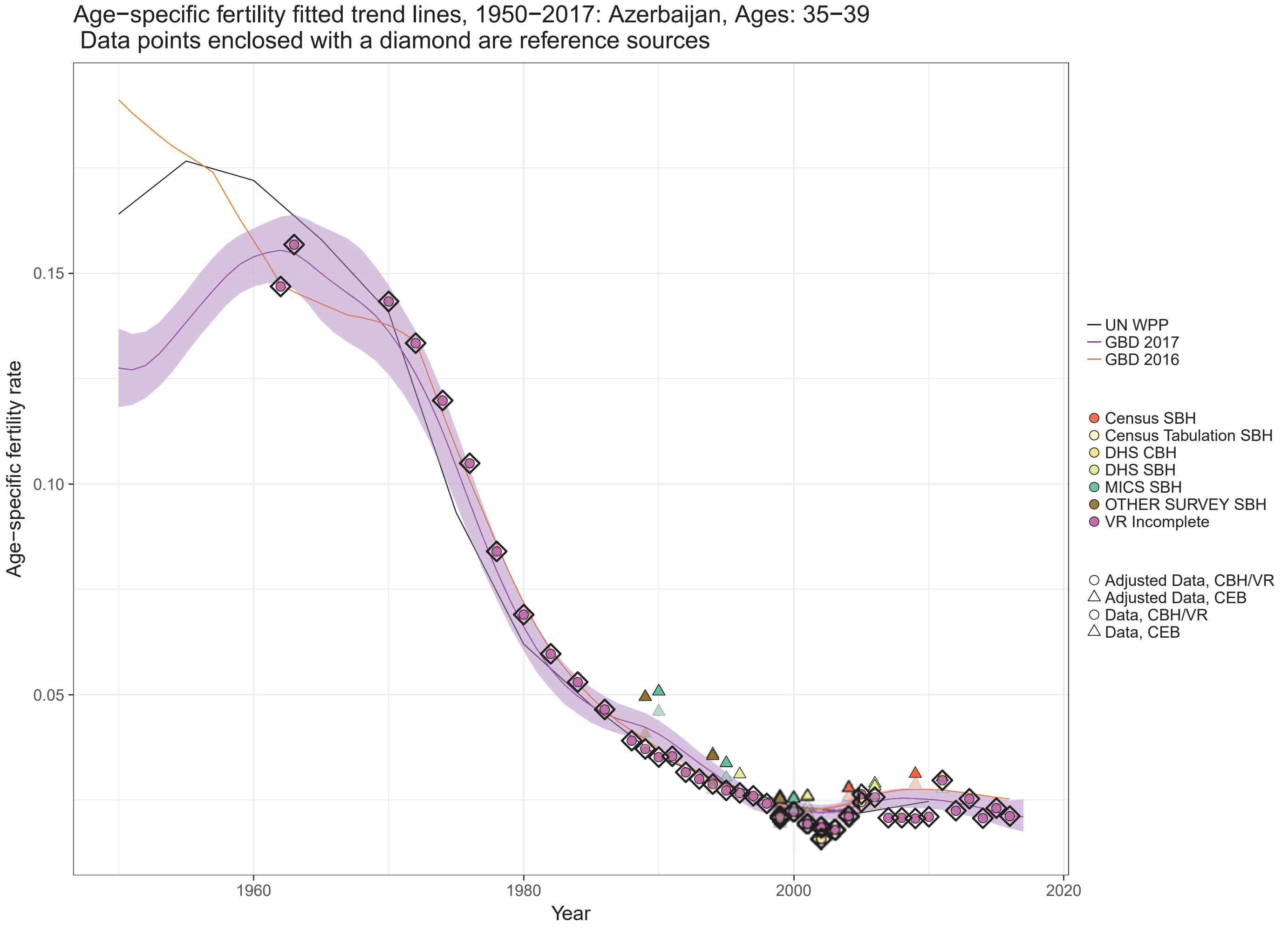

Age−specific fertility fitted trend lines, 1950−2017: Azerbaijan, Ages: 35−39
Data points enclosed with a diamond are reference sources
Age−specific fertility rate
0.15
0.10
0.05
1960
1980
2000
2020
Year
UN WPP
GBD 2017
GBD 2016
Census SBH
Census Tabulation SBH
DHS CBH
DHS SBH
MICS SBH
OTHER SURVEY SBH
VR Incomplete
Adjusted Data, CBH/VR
Adjusted Data, CEB
Data, CBH/VR
Data, CEB

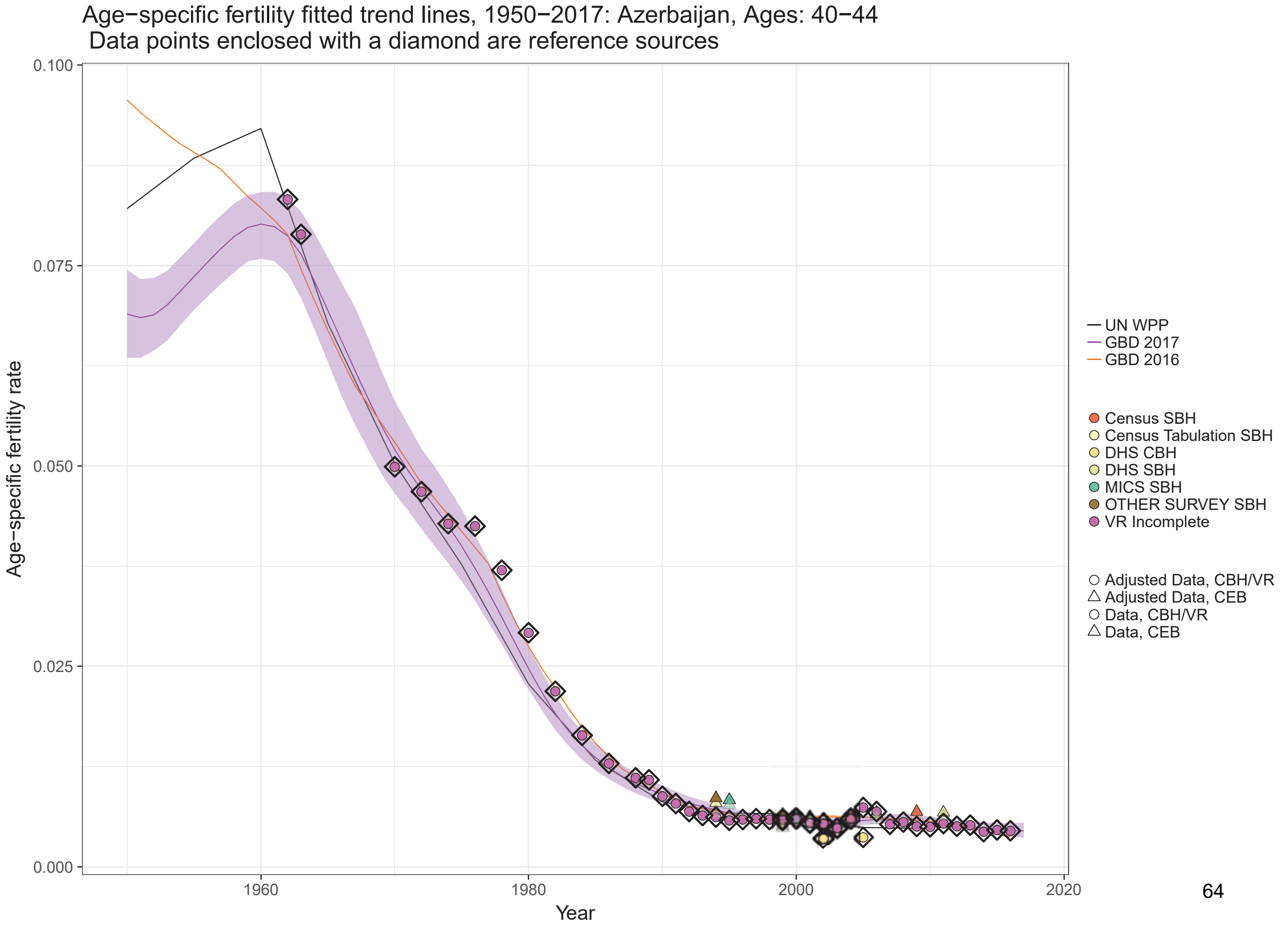

Age−specific fertility fitted trend lines, 1950−2017: Azerbaijan, Ages: 40−44
Data points enclosed with a diamond are reference sources
Age−specific fertility rate
0.100
0.075
0.050
0.025
0.000
1960
1980
2000
2020
Year
UN WPP
GBD 2017
GBD 2016
Census SBH
Census Tabulation SBH
DHS CBH
DHS SBH
MICS SBH
OTHER SURVEY SBH
VR Incomplete
Adjusted Data, CBH/VR
Adjusted Data, CEB
Data, CBH/VR
Data, CEB

Age−specific fertility fitted trend lines, 1950−2017: Azerbaijan, Ages: 45−49
Data points enclosed with a diamond are reference sources

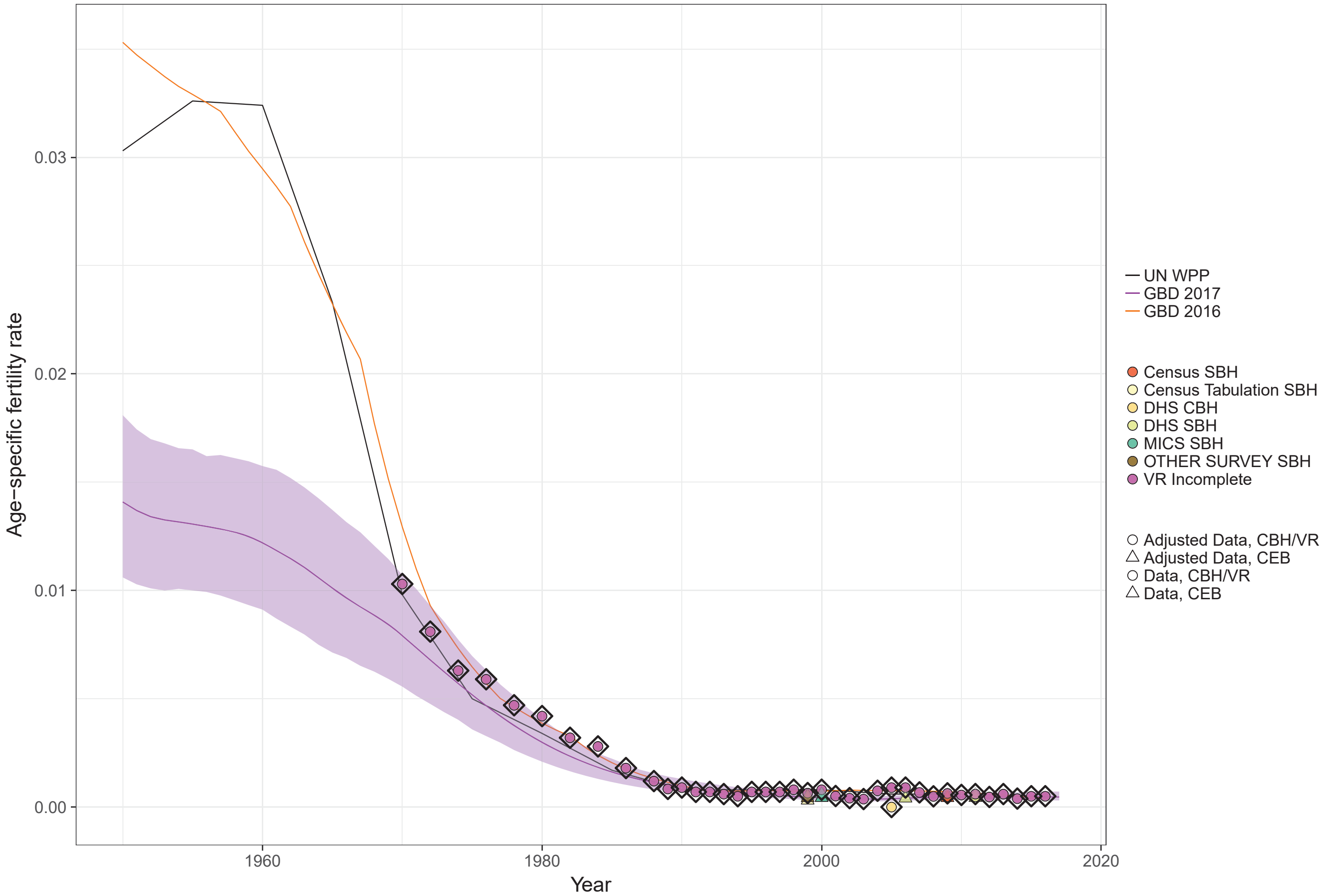

Age−specific fertility fitted trend lines, 1950−2017: Azerbaijan, Ages: 50−54
Data points enclosed with a diamond are reference sources

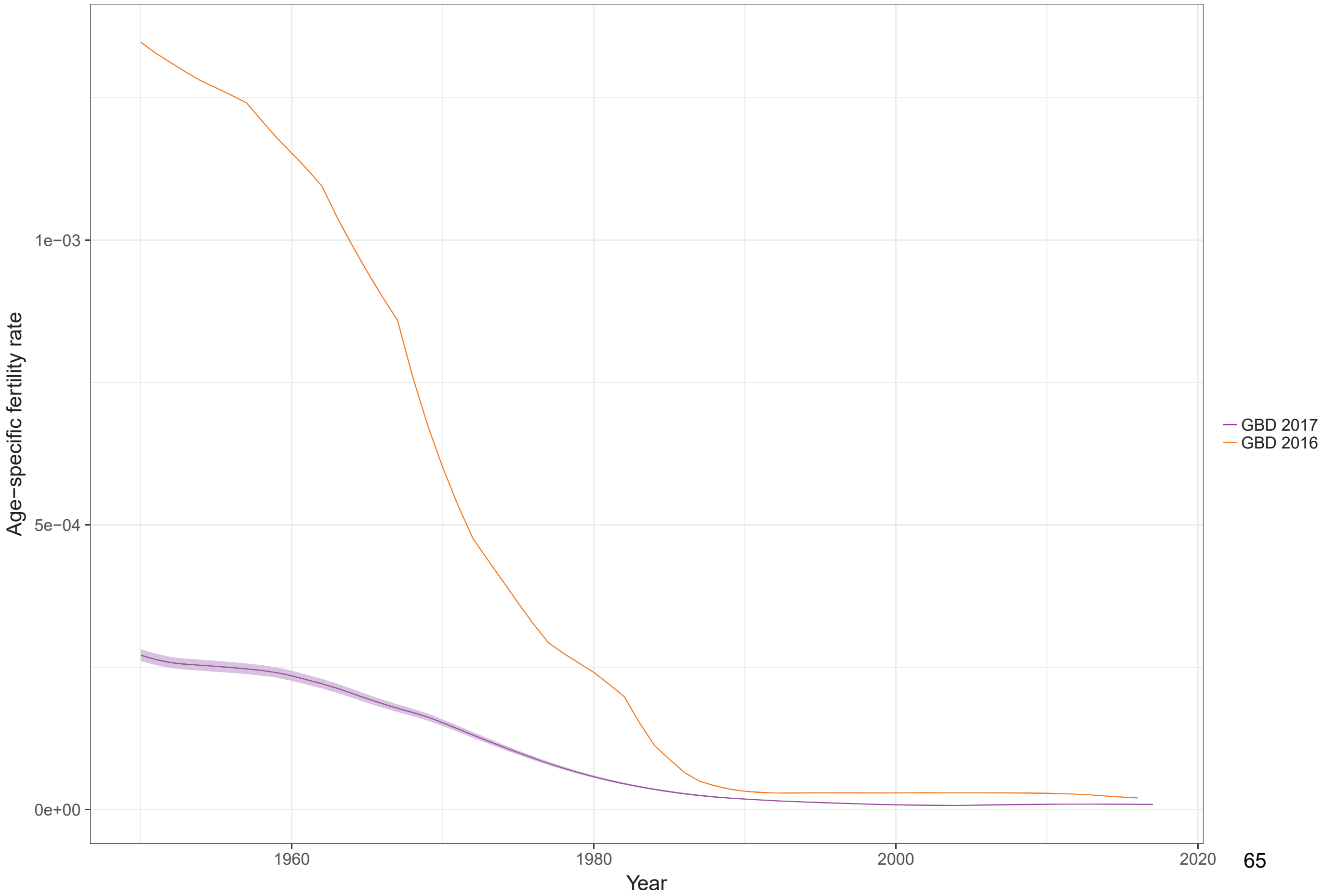

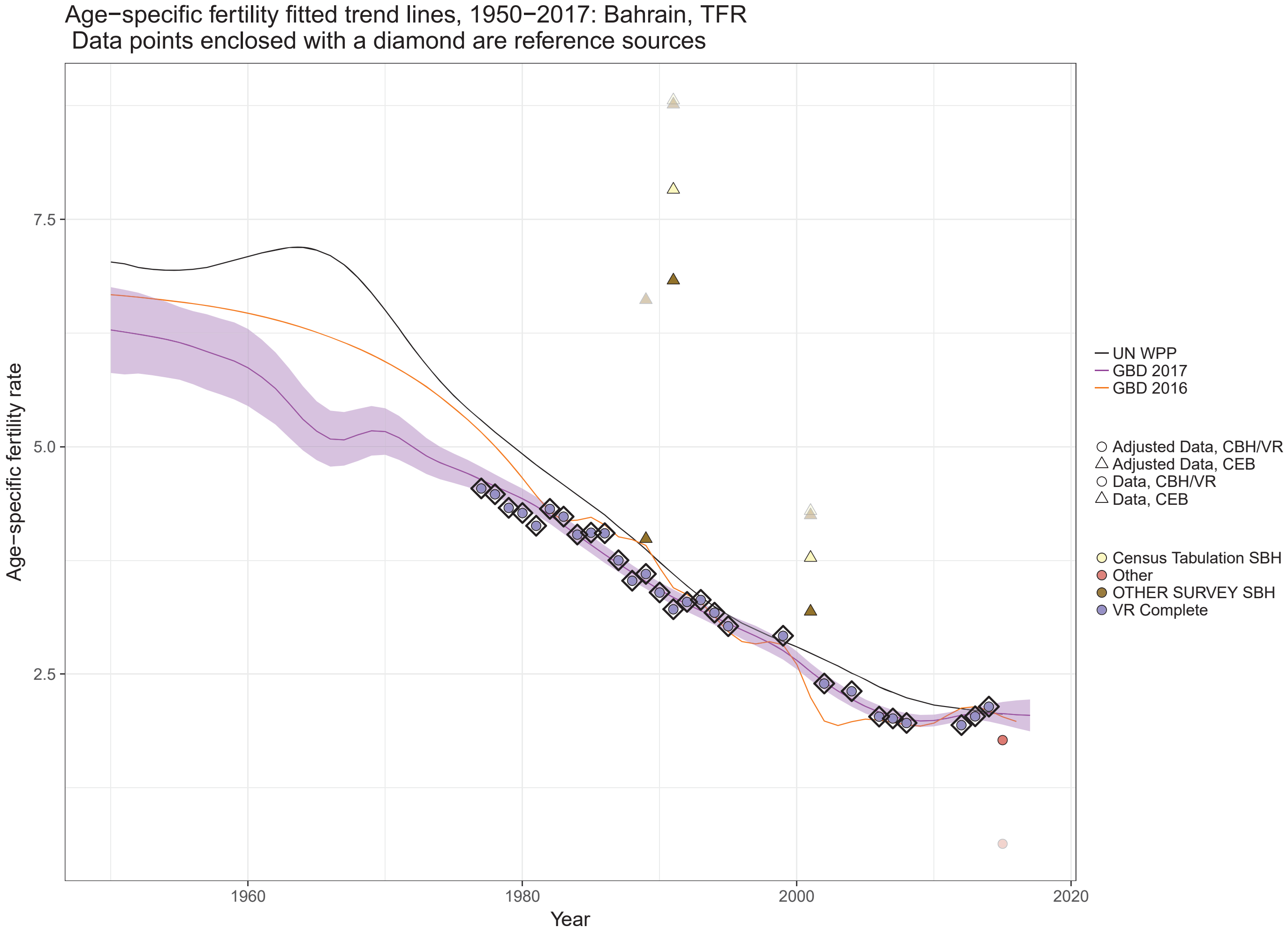
Age−specific fertility fitted trend lines, 1950−2017: Bahrain, TFR
Data points enclosed with a diamond are reference sources
Age−specific fertility rate
7.5
5.0
2.5
1960
1980
2000
2020
Year
UN WPP
GBD 2017
GBD 2016
Adjusted Data, CBH/VR
Adjusted Data, CEB
Data, CBH/VR
Data, CEB
Census Tabulation SBH
Other
OTHER SURVEY SBH
VR Complete

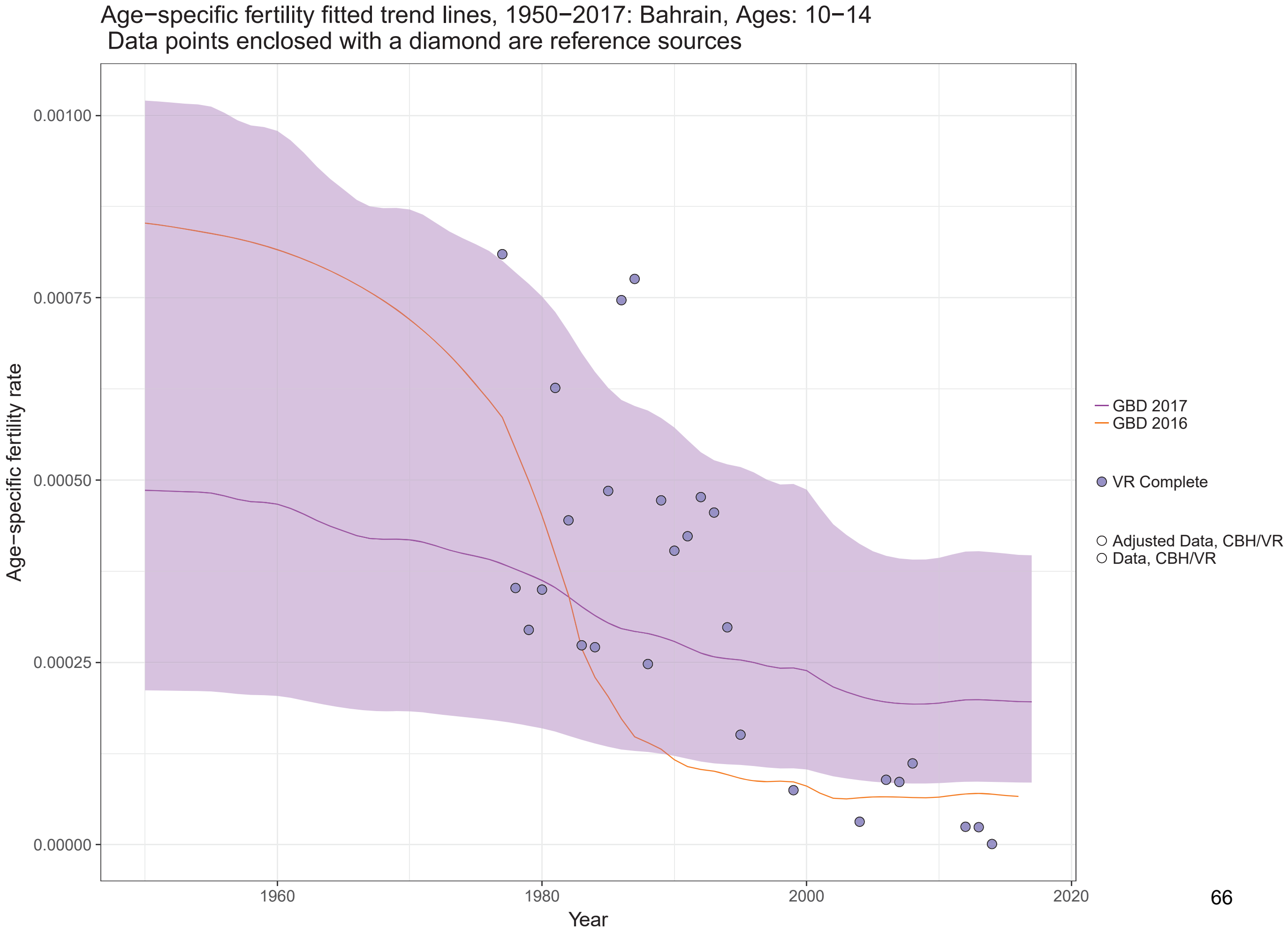
Age−specific fertility fitted trend lines, 1950−2017: Bahrain, Ages: 10−14
Data points enclosed with a diamond are reference sources
Age−specific fertility rate
0.00100
0.00075
0.00050
0.00025
0.00000
1960
1980
2000
2020
Year
GBD 2017
GBD 2016
VR Complete
Adjusted Data, CBH/VR
Data, CBH/VR

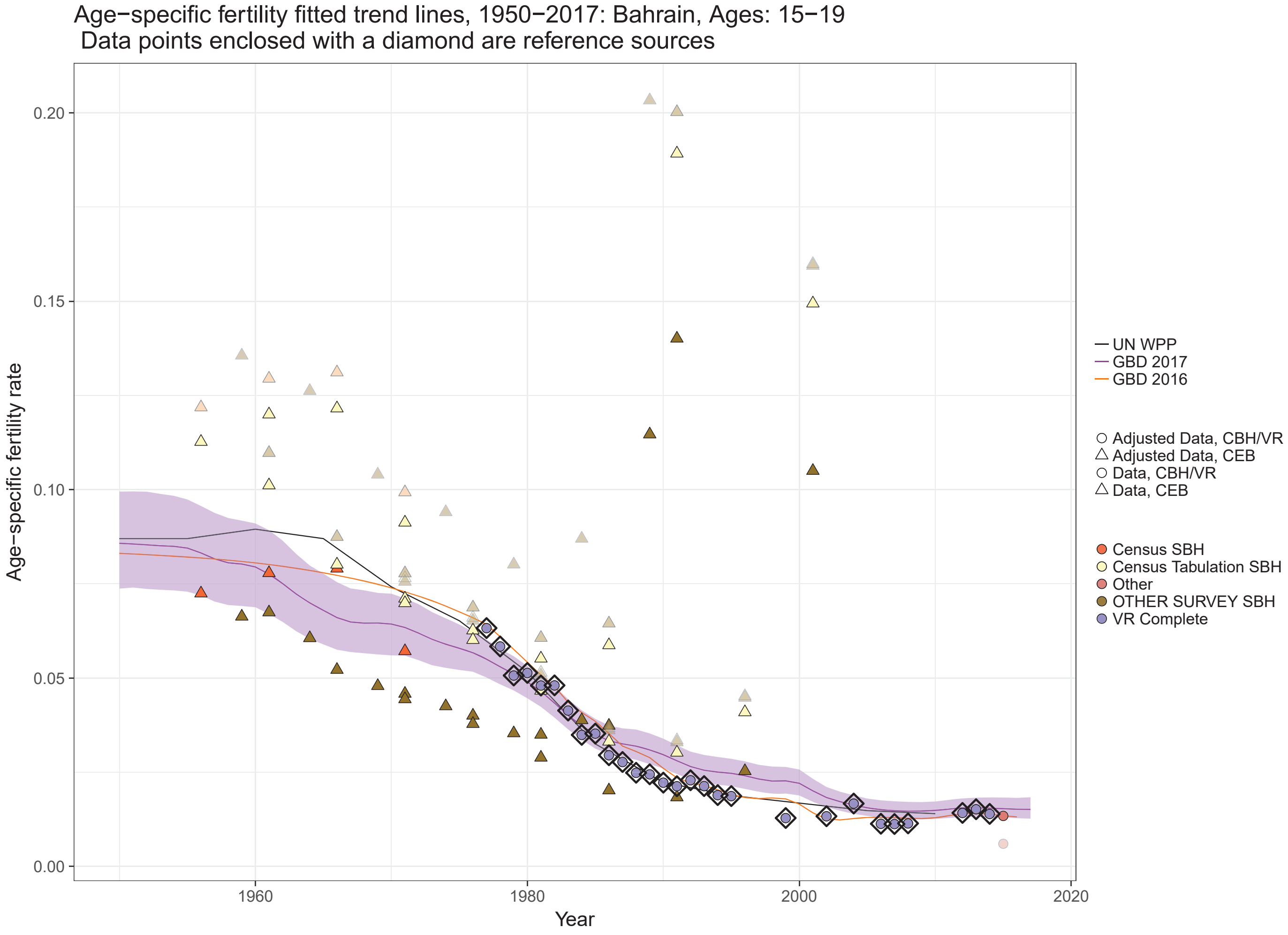
Age−specific fertility fitted trend lines, 1950−2017: Bahrain, Ages: 15−19
Data points enclosed with a diamond are reference sources
Age−specific fertility rate
Year
UN WPP
GBD 2017
GBD 2016
Adjusted Data, CBH/VR
Adjusted Data, CEB
Data, CBH/VR
Data, CEB
Census SBH
Census Tabulation SBH
Other
OTHER SURVEY SBH
VR Complete

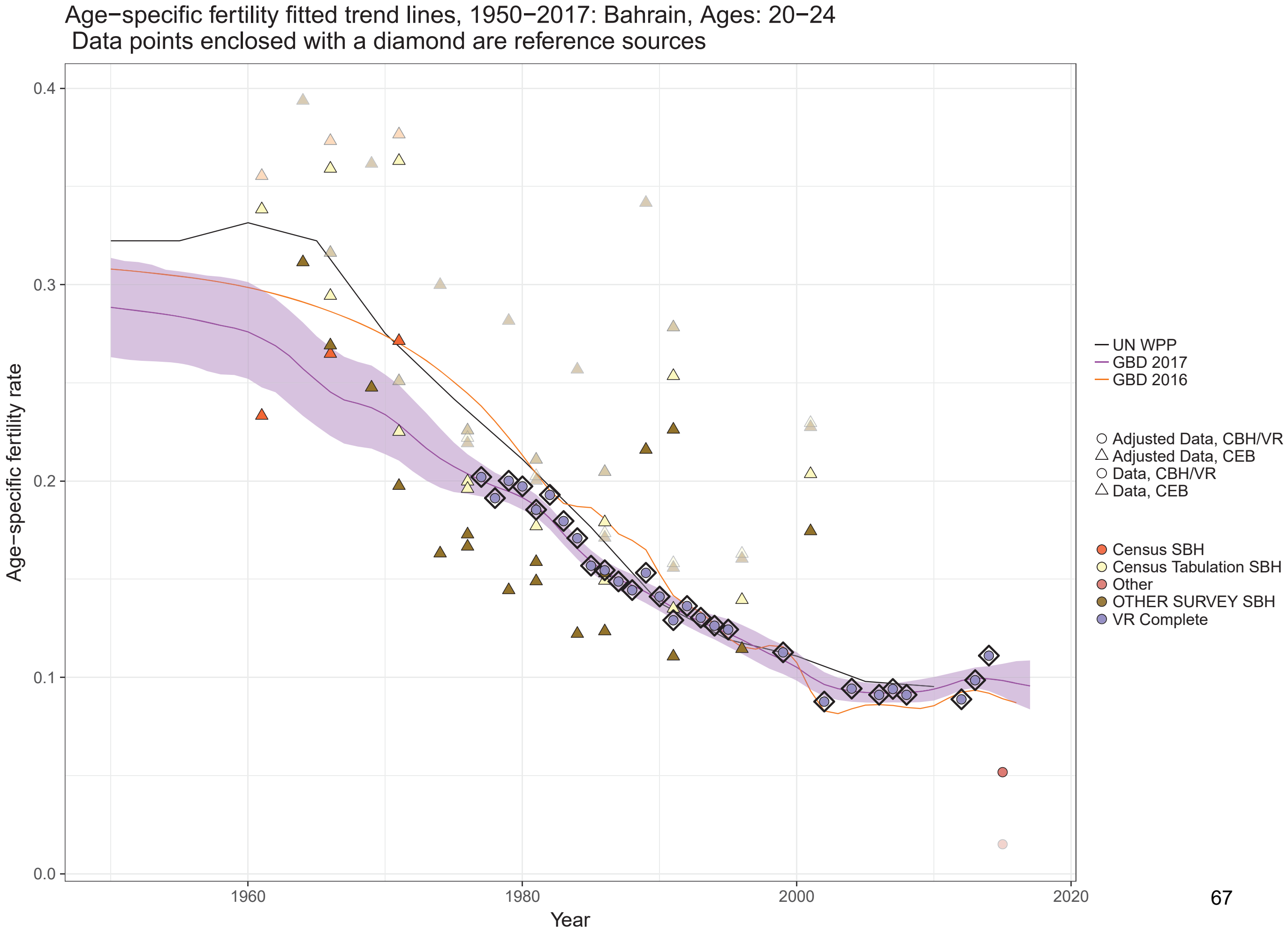
Age−specific fertility fitted trend lines, 1950−2017: Bahrain, Ages: 20−24
Data points enclosed with a diamond are reference sources
Age−specific fertility rate
Year
UN WPP
GBD 2017
GBD 2016
Adjusted Data, CBH/VR
Adjusted Data, CEB
Data, CBH/VR
Data, CEB
Census SBH
Census Tabulation SBH
Other
OTHER SURVEY SBH
VR Complete

Age−specific fertility fitted trend lines, 1950−2017: Bahrain, Ages: 25−29
Data points enclosed with a diamond are reference sources

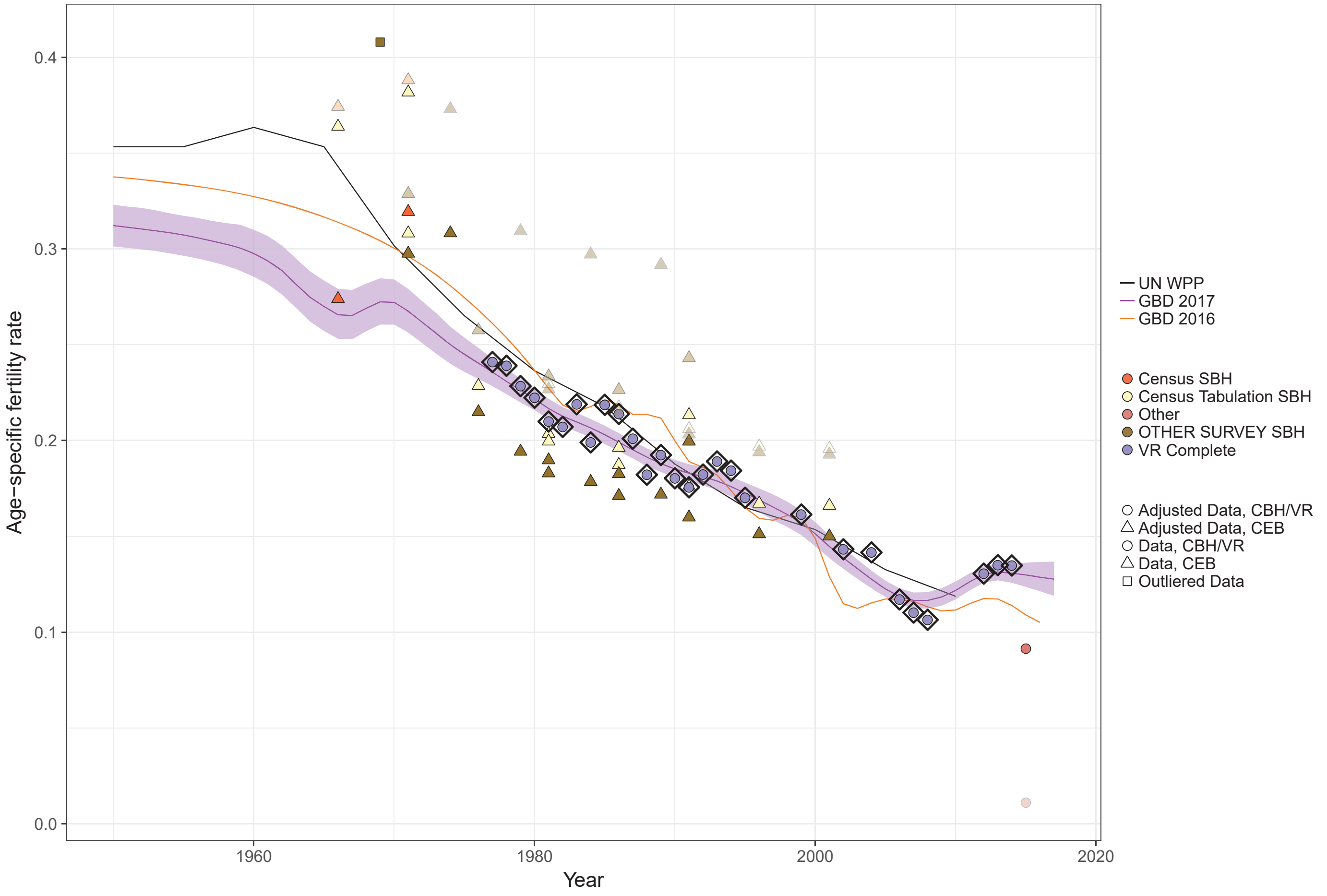

Age−specific fertility fitted trend lines, 1950−2017: Bahrain, Ages: 30−34
Data points enclosed with a diamond are reference sources

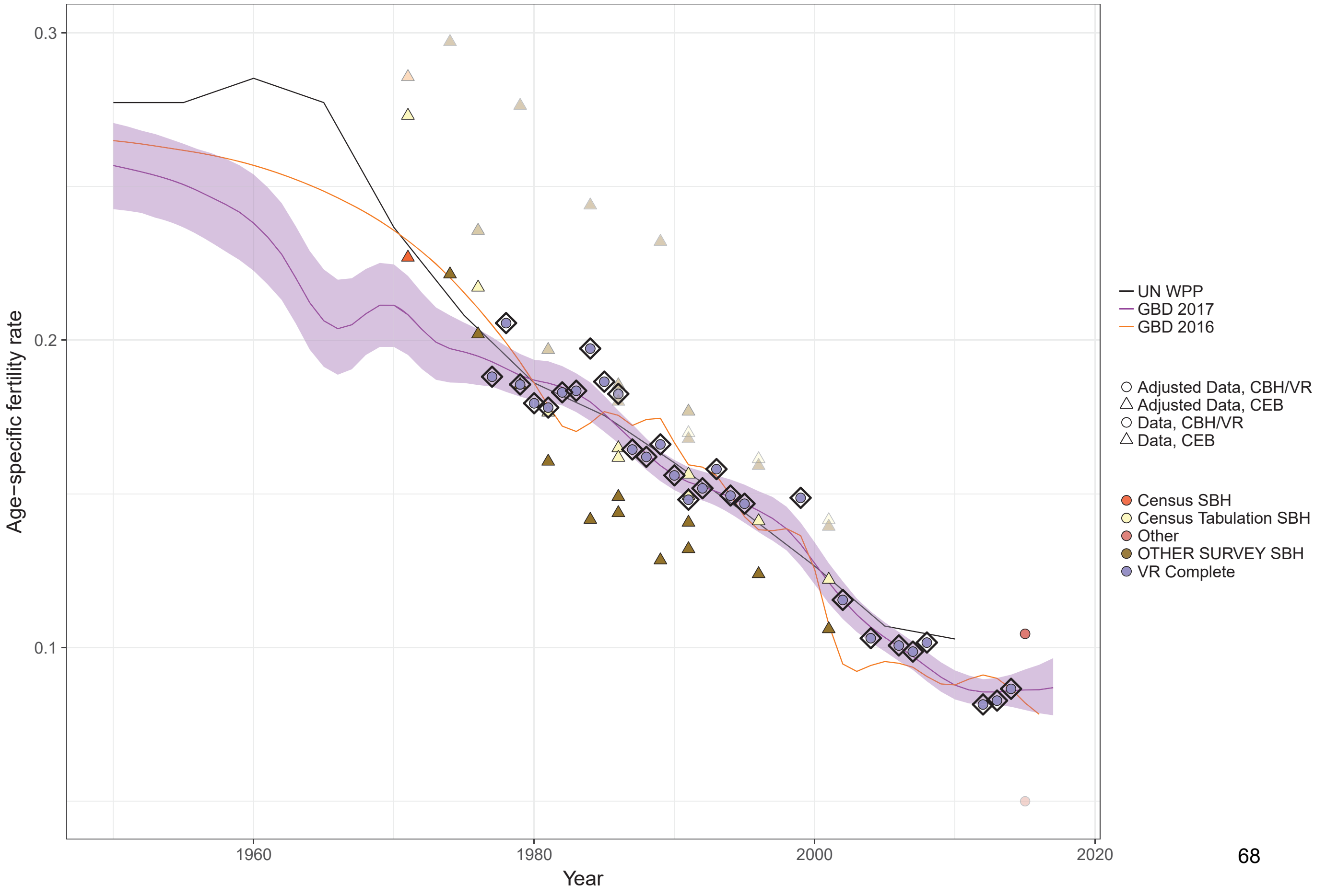

Age−specific fertility fitted trend lines, 1950−2017: Bahrain, Ages: 35−39
Data points enclosed with a diamond are reference sources

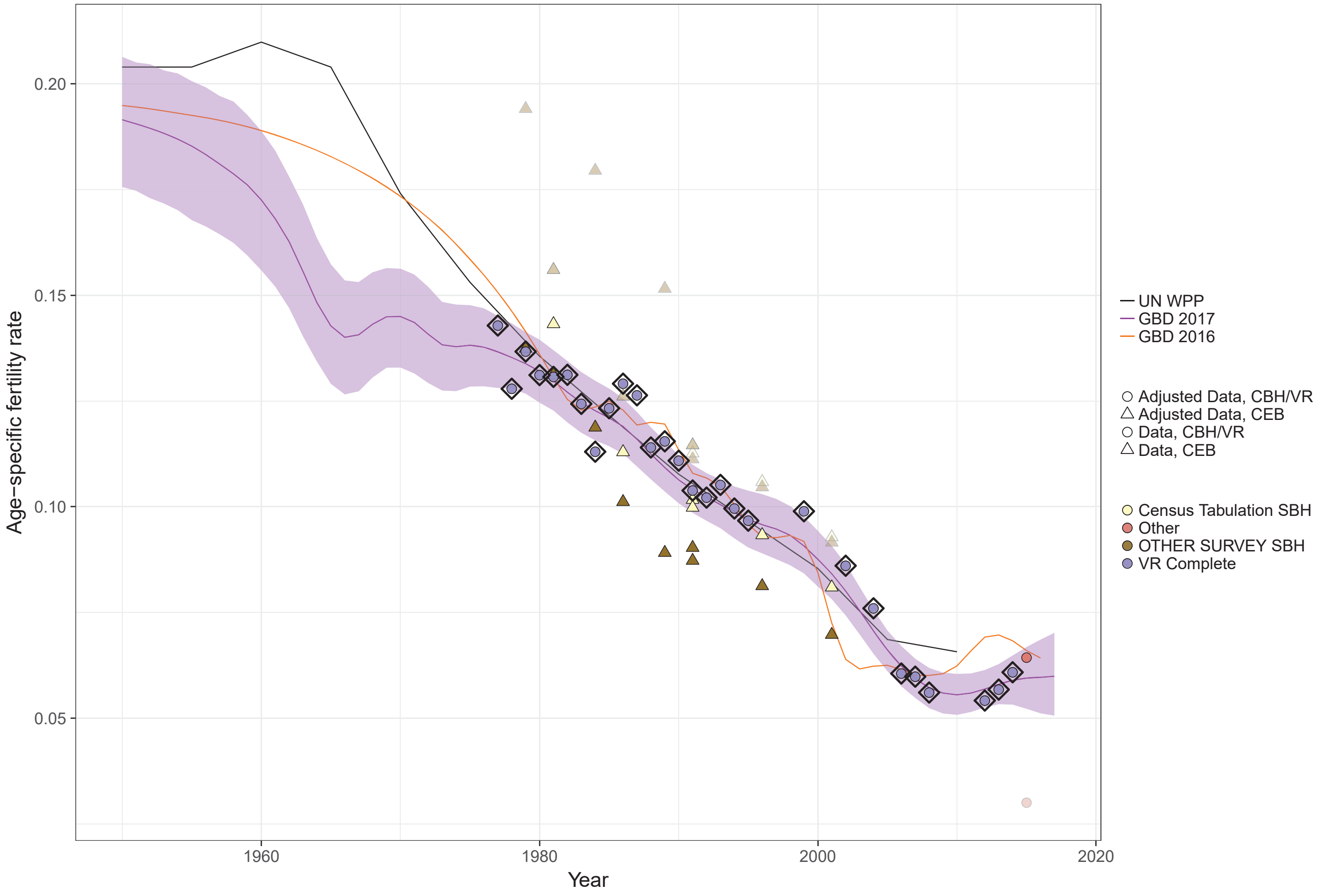

Age−specific fertility fitted trend lines, 1950−2017: Bahrain, Ages: 40−44
Data points enclosed with a diamond are reference sources

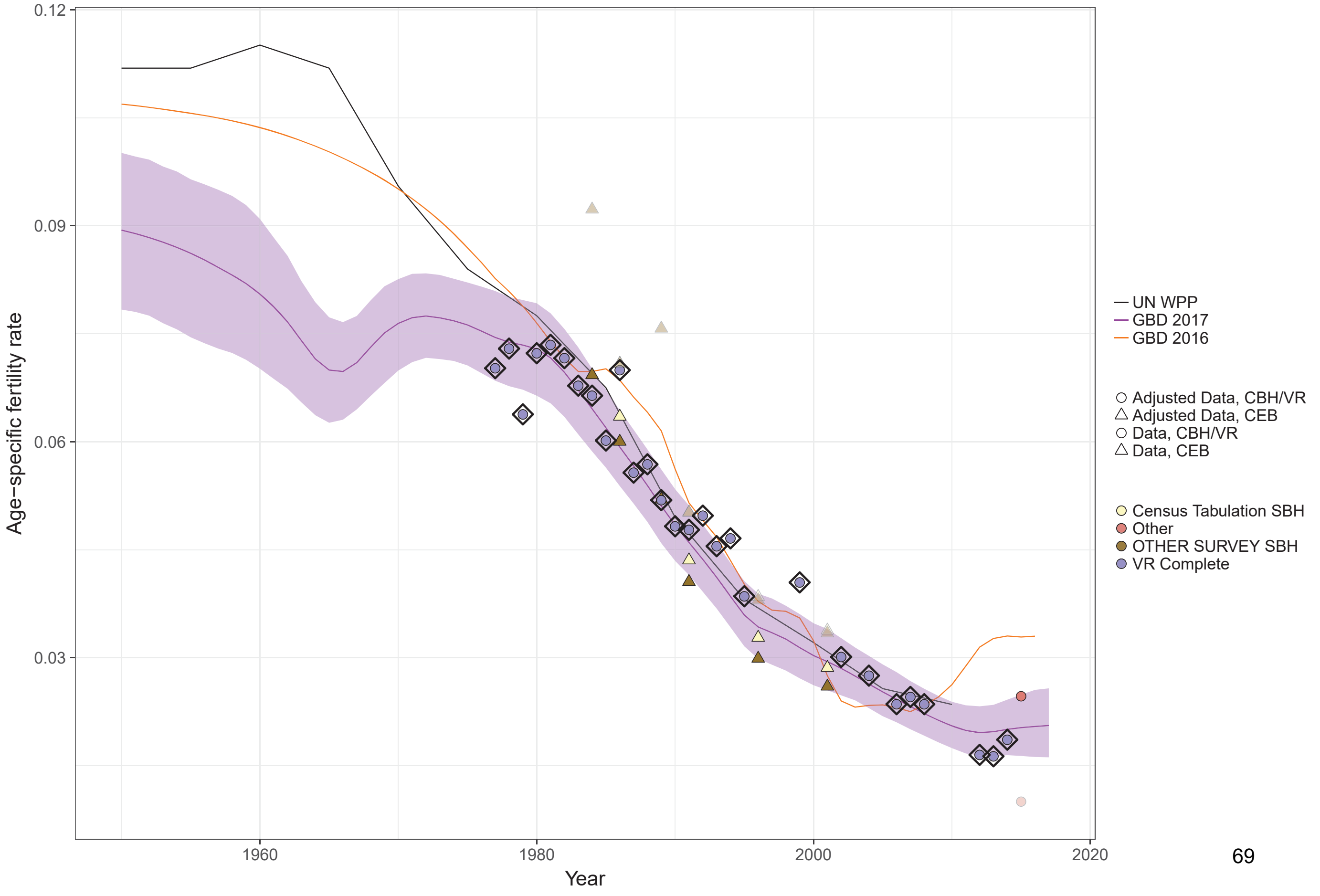

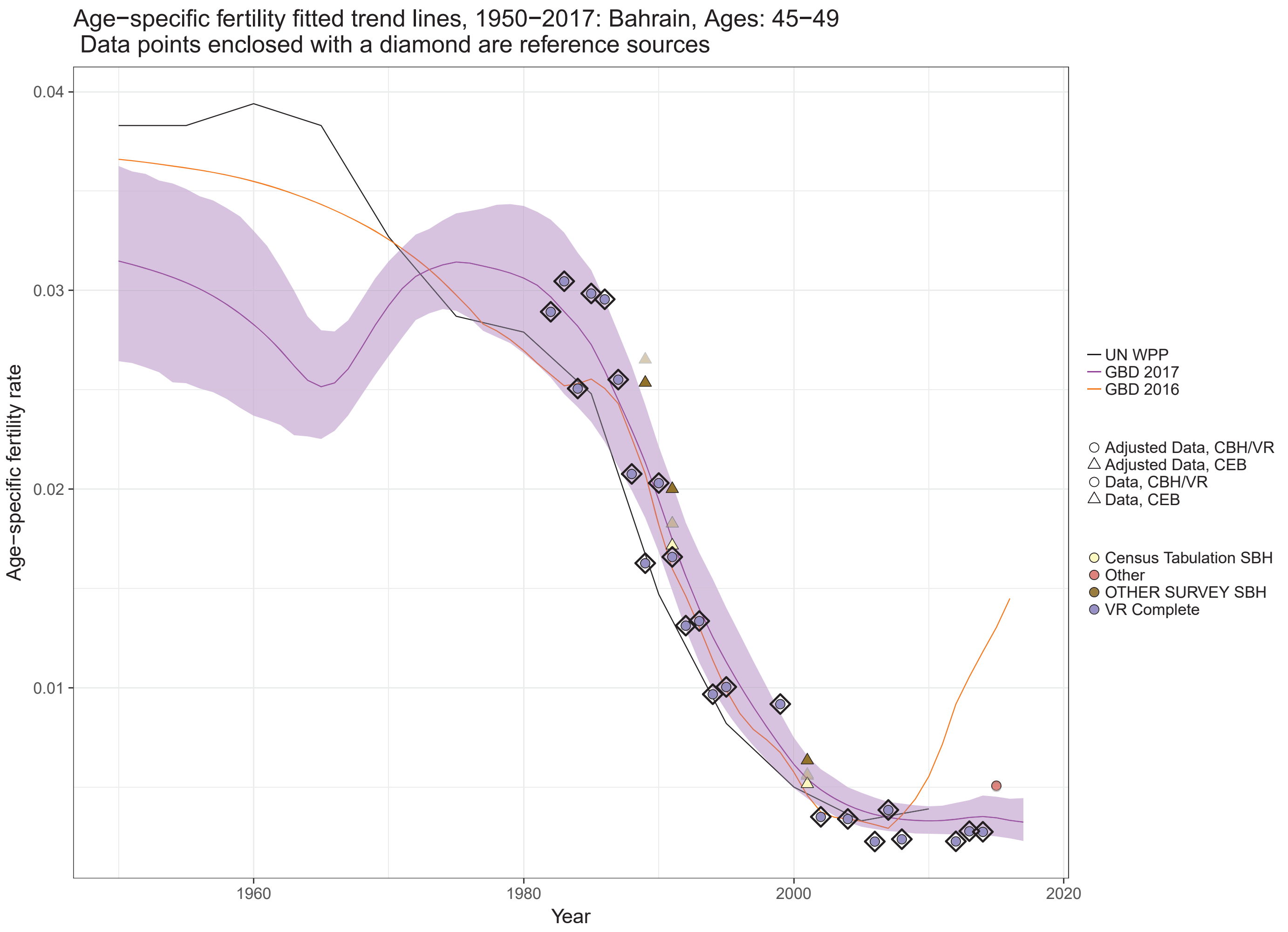

Age−specific fertility fitted trend lines, 1950−2017: Bahrain, Ages: 45−49
Data points enclosed with a diamond are reference sources
Age−specific fertility rate
0.04
0.03
0.02
0.01
1960
1980
2000
2020
Year
UN WPP
GBD 2017
GBD 2016
Adjusted Data, CBH/VR
Adjusted Data, CEB
Data, CBH/VR
Data, CEB
Census Tabulation SBH
Other
OTHER SURVEY SBH
VR Complete

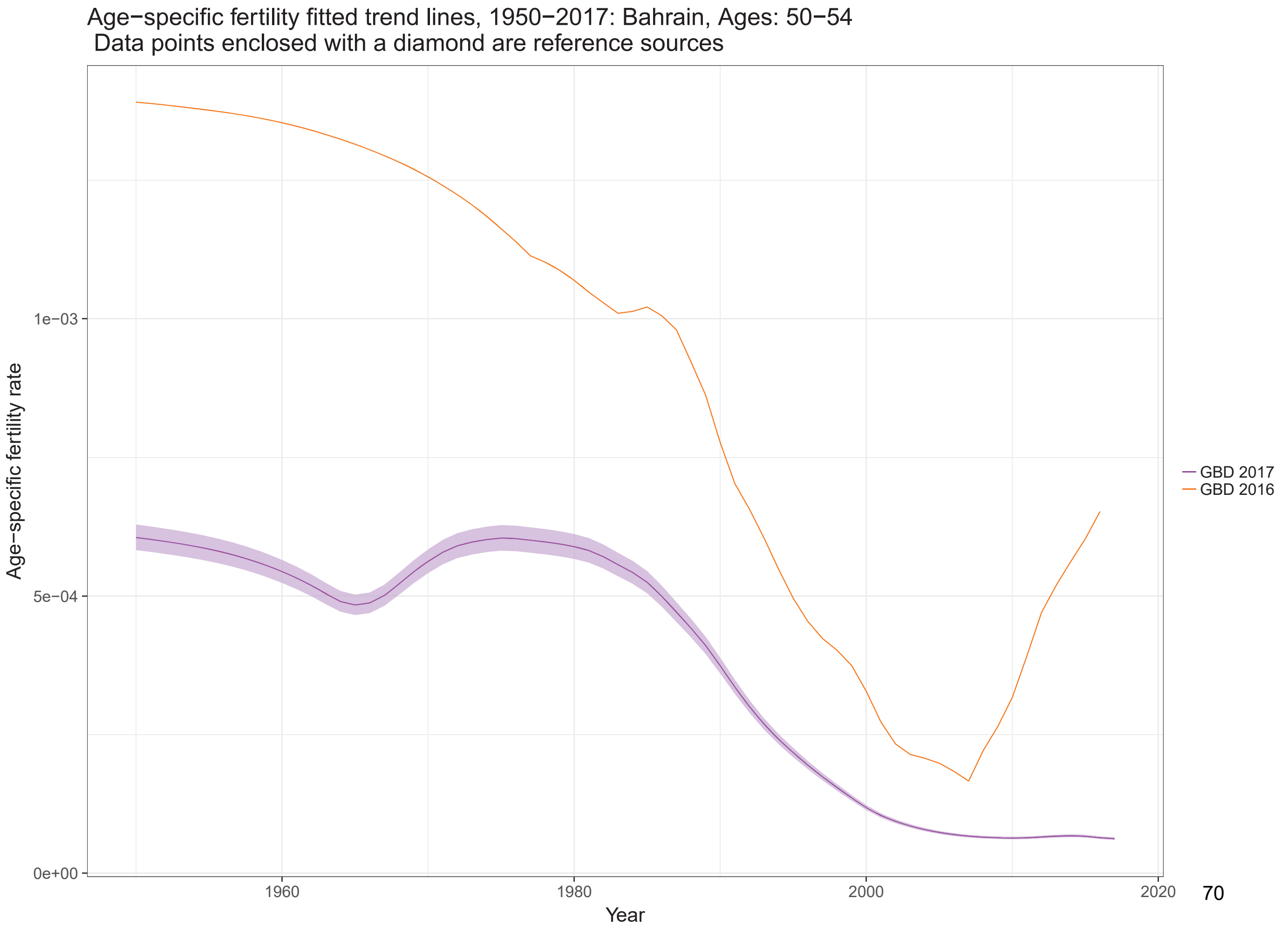

Age−specific fertility fitted trend lines, 1950−2017: Bahrain, Ages: 50−54
Data points enclosed with a diamond are reference sources
Age−specific fertility rate
1e−03
5e−04
0e+00
1960
1980
2000
2020
Year
GBD 2017
GBD 2016

Age−specific fertility fitted trend lines, 1950−2017: Bangladesh, TFR
Data points enclosed with a diamond are reference sources

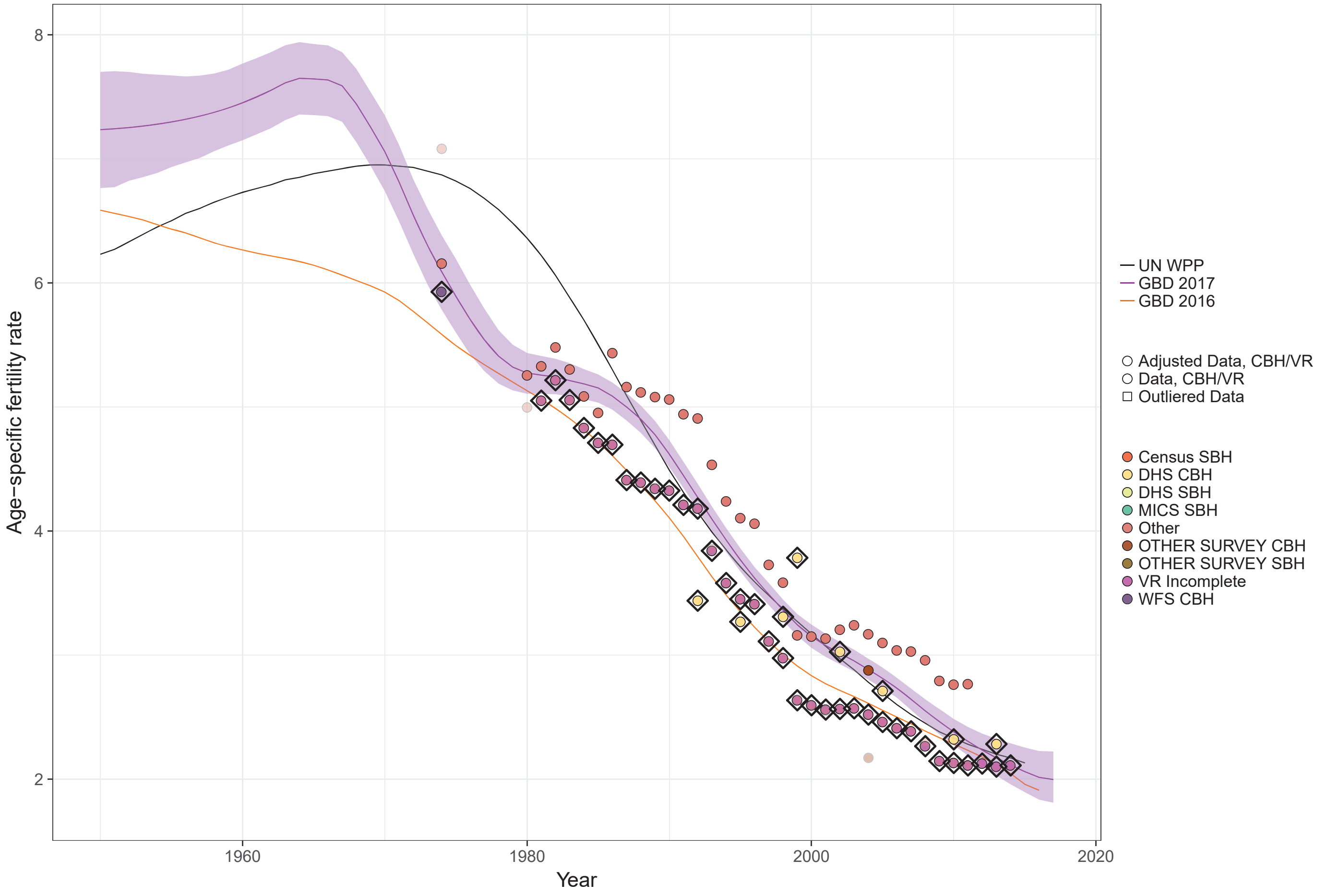

Age−specific fertility fitted trend lines, 1950−2017: Bangladesh, Ages: 10−14
Data points enclosed with a diamond are reference sources

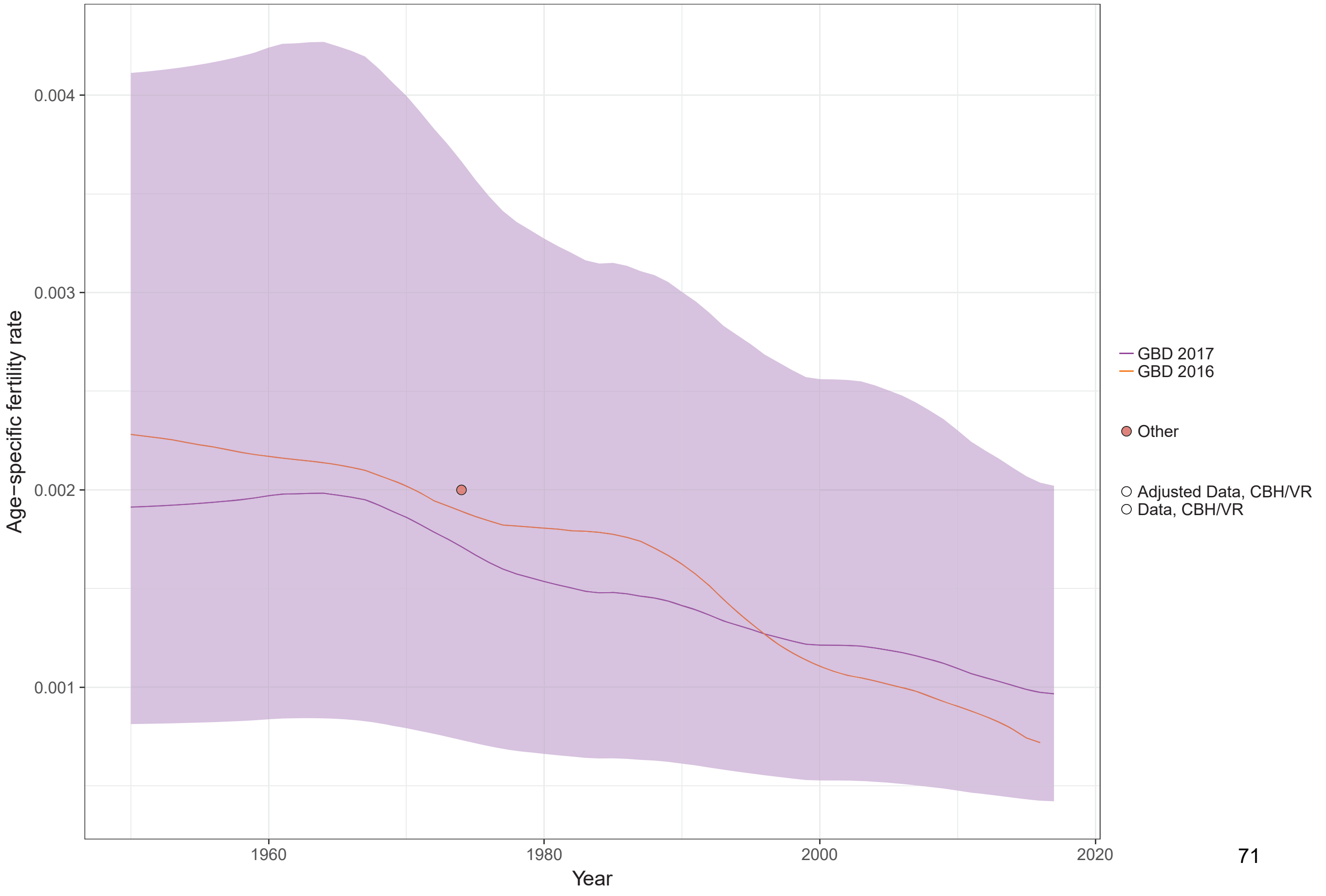

Age−specific fertility fitted trend lines, 1950−2017: Bangladesh, Ages: 15−19
Data points enclosed with a diamond are reference sources

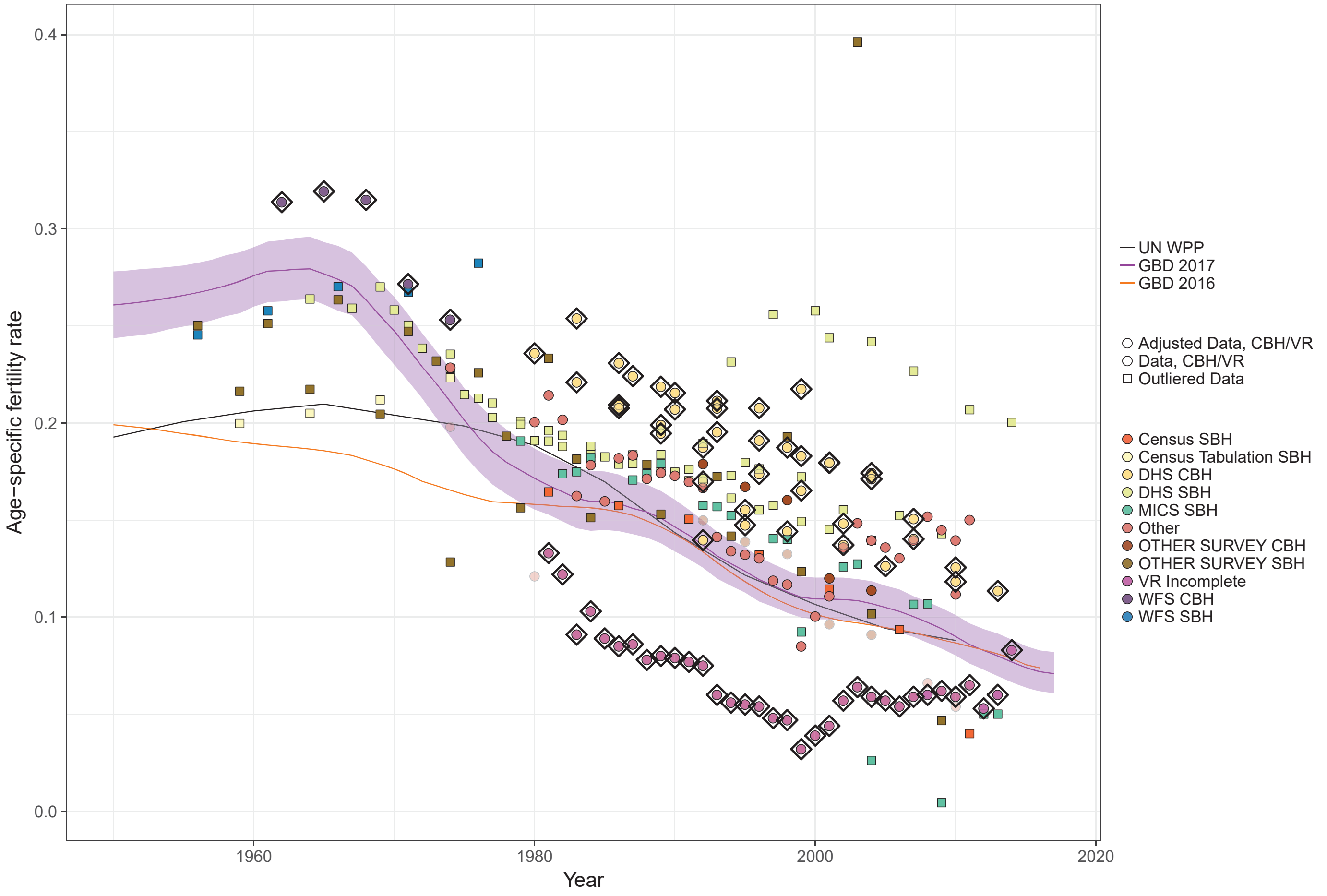

Age−specific fertility fitted trend lines, 1950−2017: Bangladesh, Ages: 20−24
Data points enclosed with a diamond are reference sources

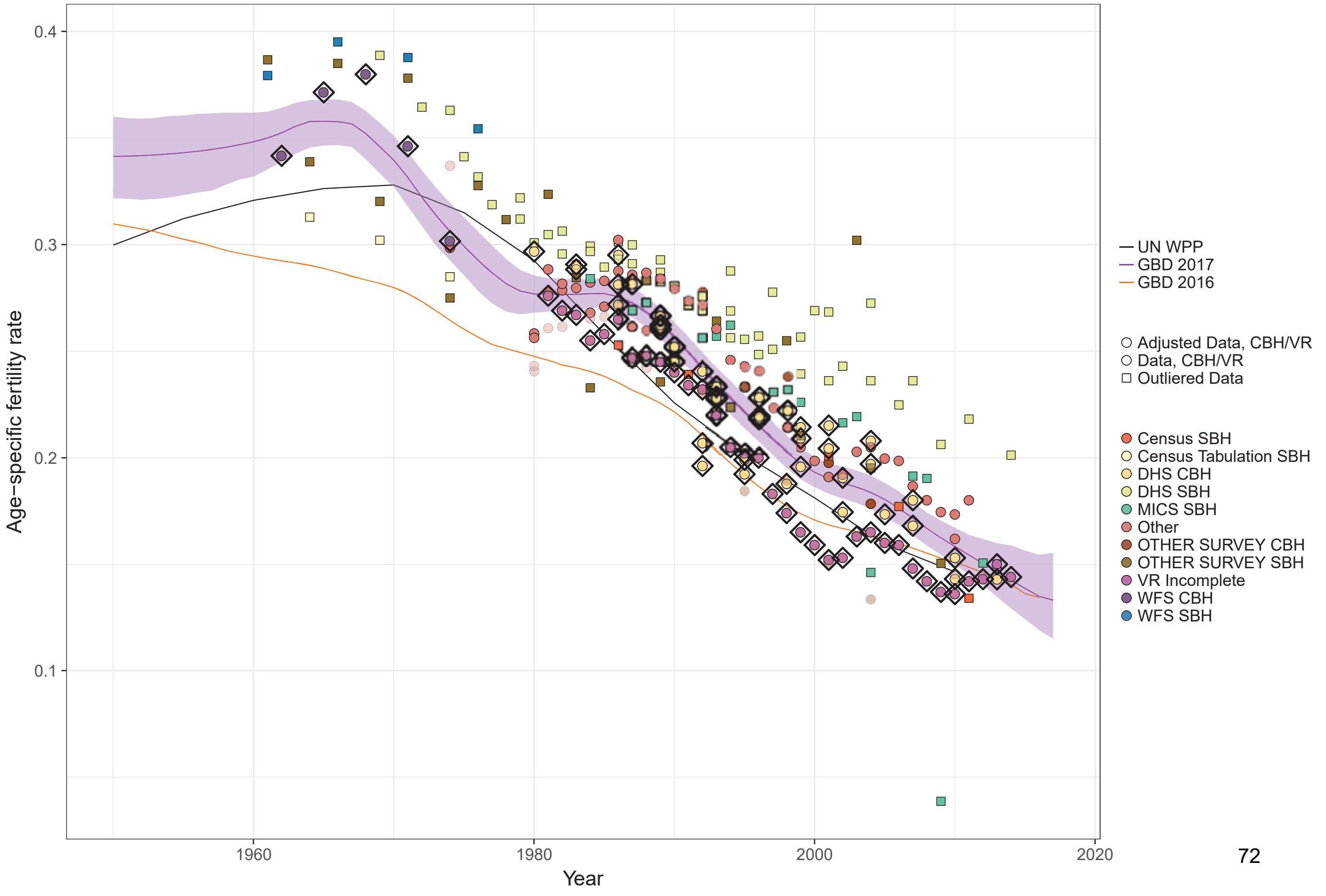

Age−specific fertility fitted trend lines, 1950−2017: Bangladesh, Ages: 25−29
Data points enclosed with a diamond are reference sources

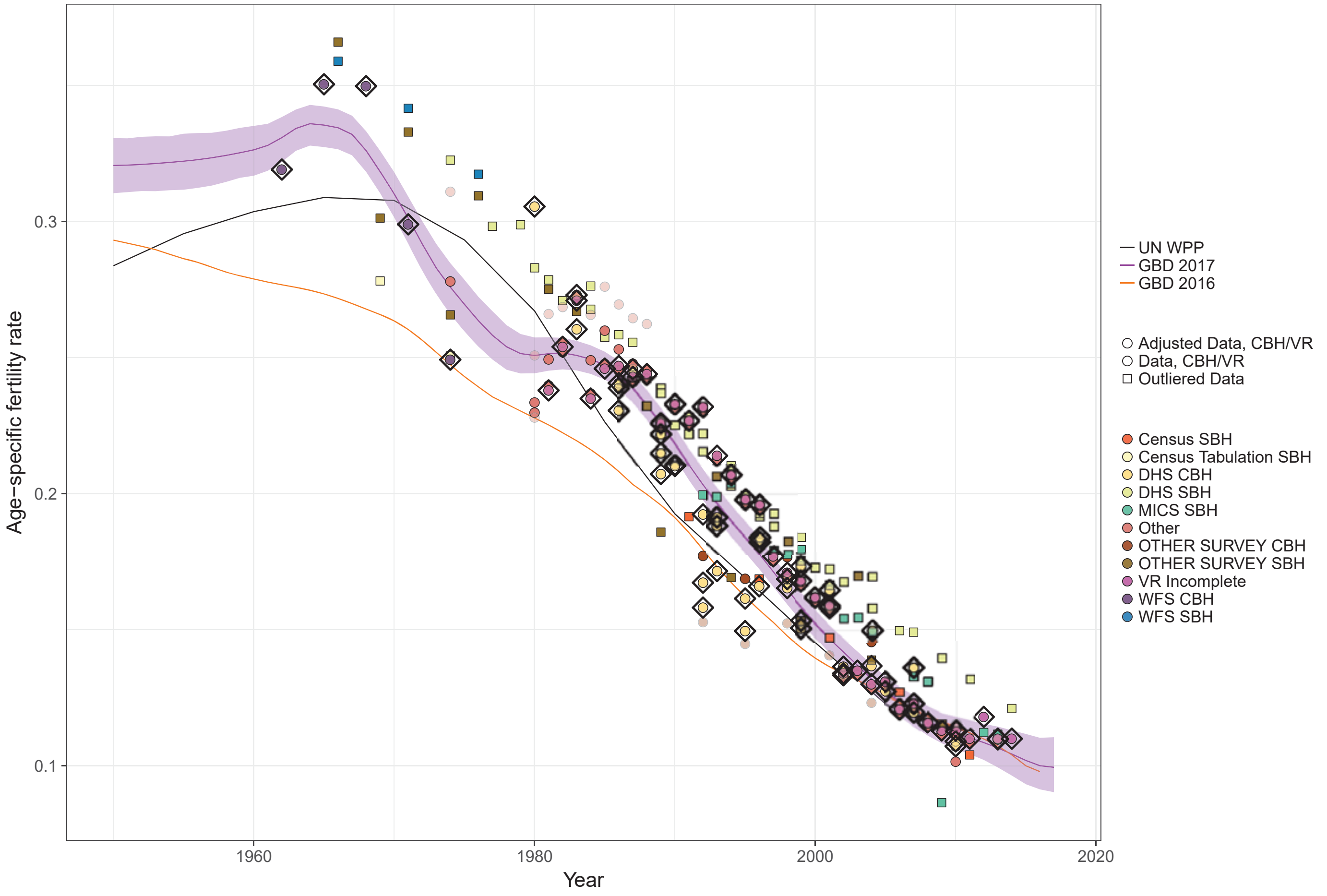

Age−specific fertility fitted trend lines, 1950−2017: Bangladesh, Ages: 30−34
Data points enclosed with a diamond are reference sources

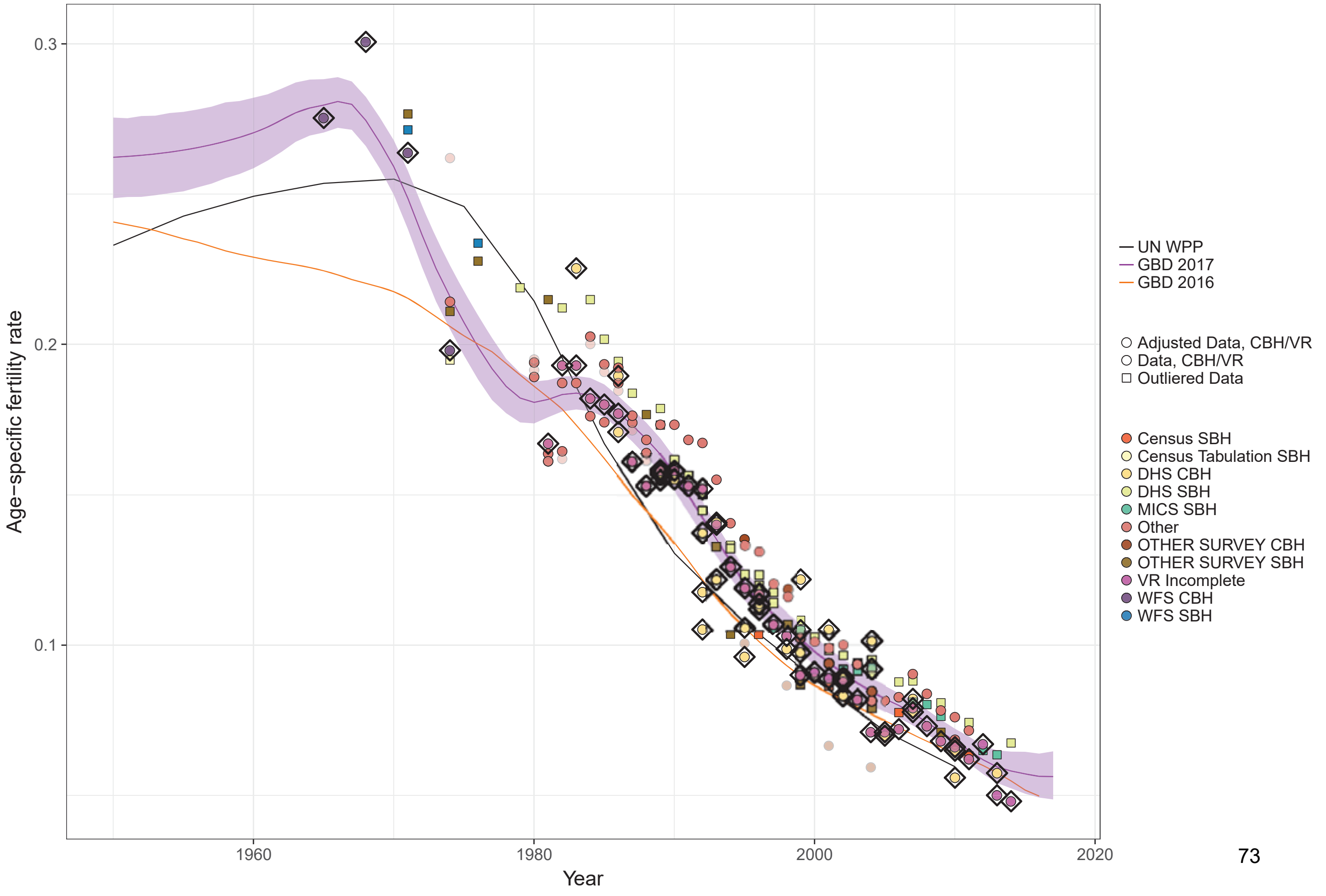

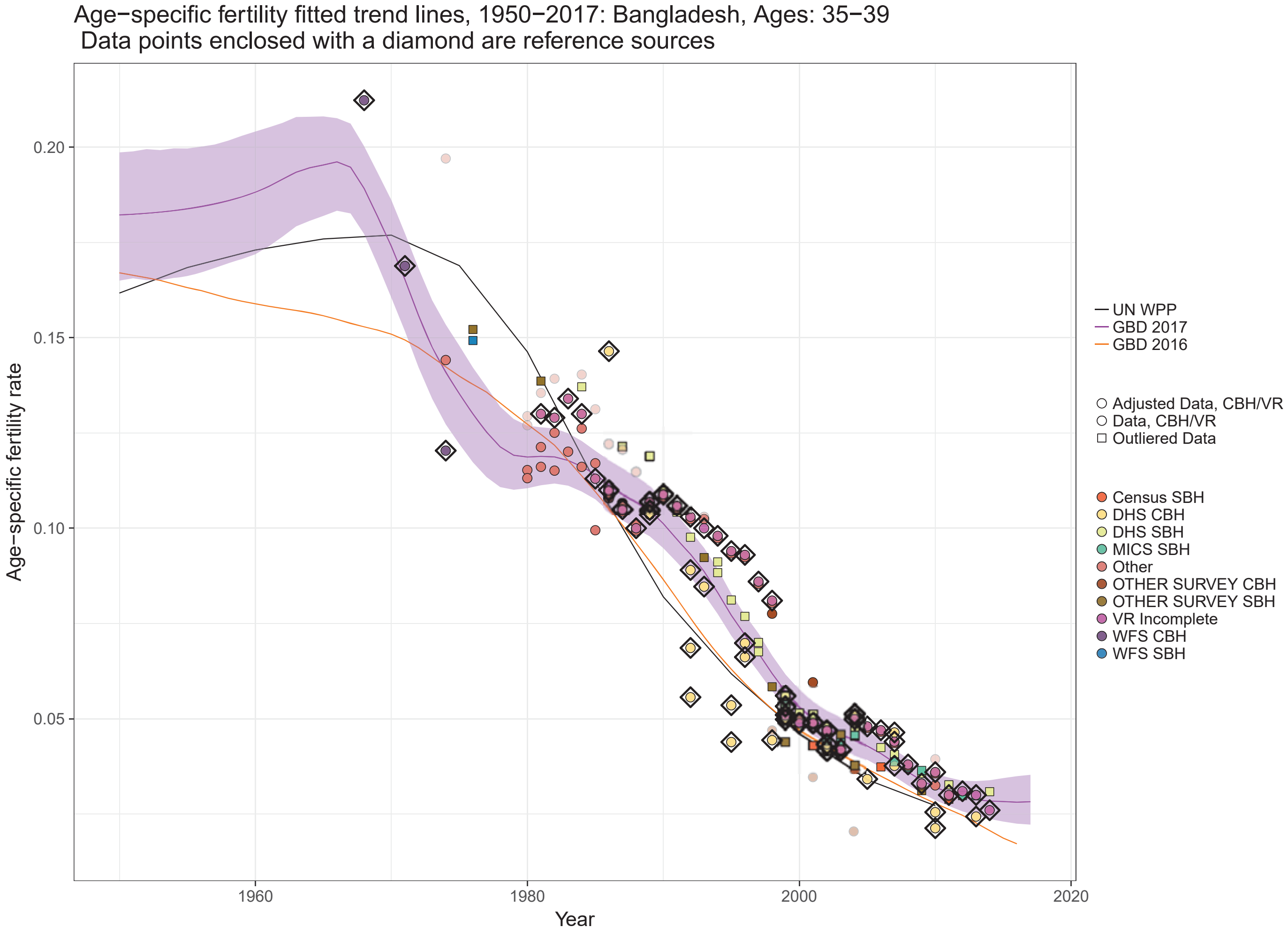

Age−specific fertility fitted trend lines, 1950−2017: Bangladesh, Ages: 35−39
Data points enclosed with a diamond are reference sources
Age−specific fertility rate
0.20
0.15
0.10
0.05
1960
1980
2000
2020
Year
UN WPP
GBD 2017
GBD 2016
Adjusted Data, CBH/VR
Data, CBH/VR
Outliered Data
Census SBH
DHS CBH
DHS SBH
MICS SBH
Other
OTHER SURVEY CBH
OTHER SURVEY SBH
VR Incomplete
WFS CBH
WFS SBH

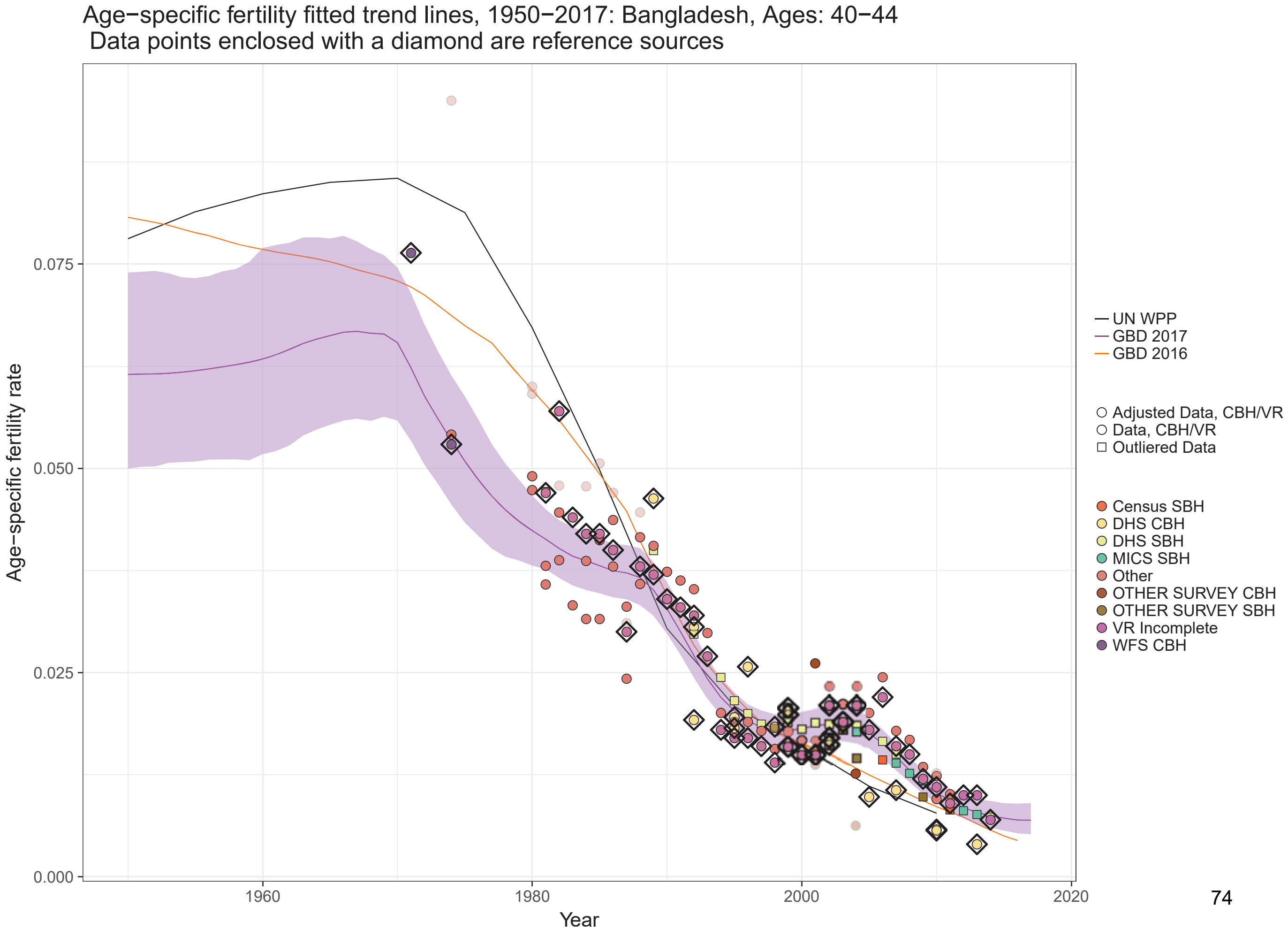

Age−specific fertility fitted trend lines, 1950−2017: Bangladesh, Ages: 40−44
Data points enclosed with a diamond are reference sources
Age−specific fertility rate
0.075
0.050
0.025
0.000
1960
1980
2000
2020
Year
UN WPP
GBD 2017
GBD 2016
Adjusted Data, CBH/VR
Data, CBH/VR
Outliered Data
Census SBH
DHS CBH
DHS SBH
MICS SBH
Other
OTHER SURVEY CBH
OTHER SURVEY SBH
VR Incomplete
WFS CBH

Age−specific fertility fitted trend lines, 1950−2017: Bangladesh, Ages: 45−49
Data points enclosed with a diamond are reference sources

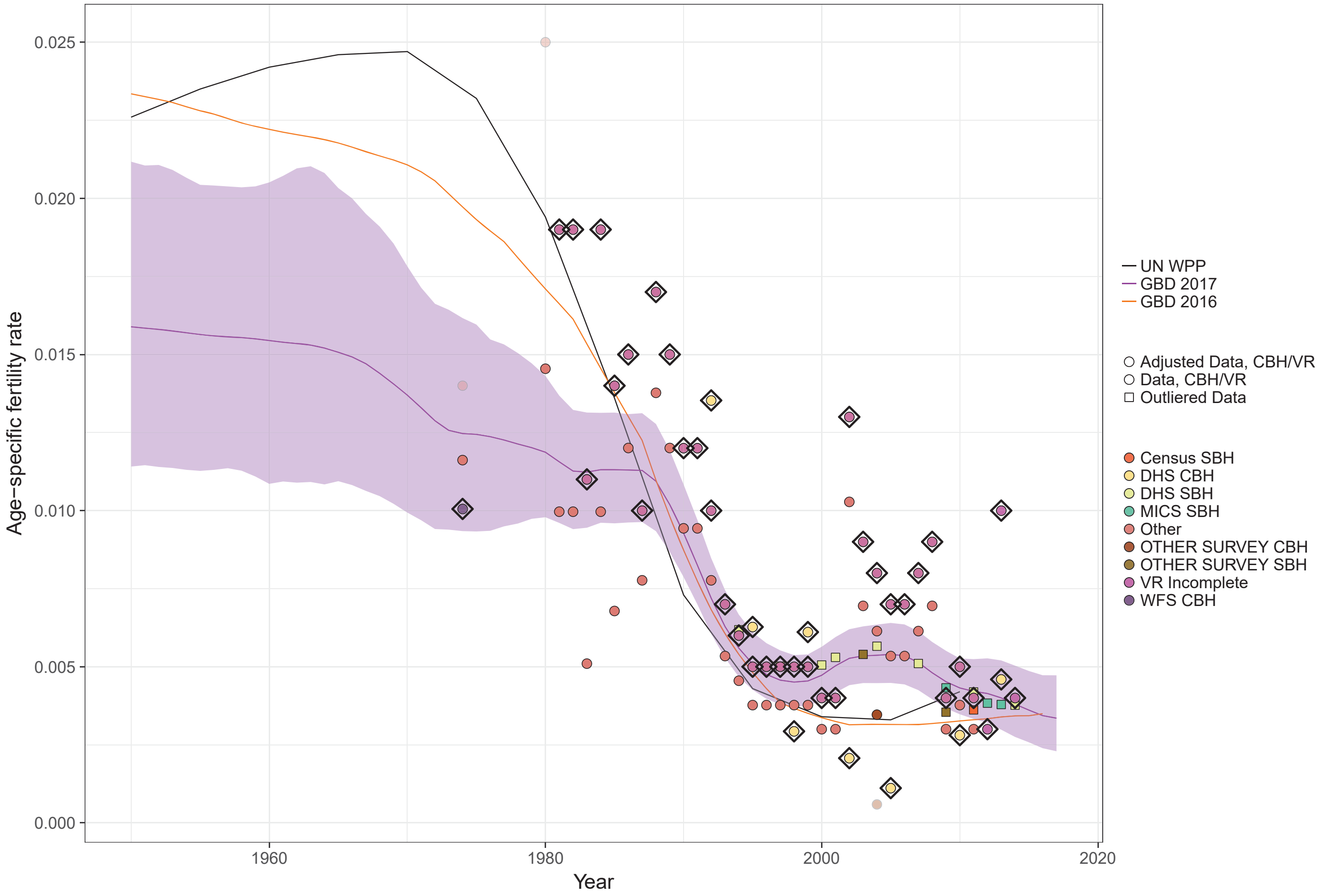

Age−specific fertility fitted trend lines, 1950−2017: Bangladesh, Ages: 50−54
Data points enclosed with a diamond are reference sources

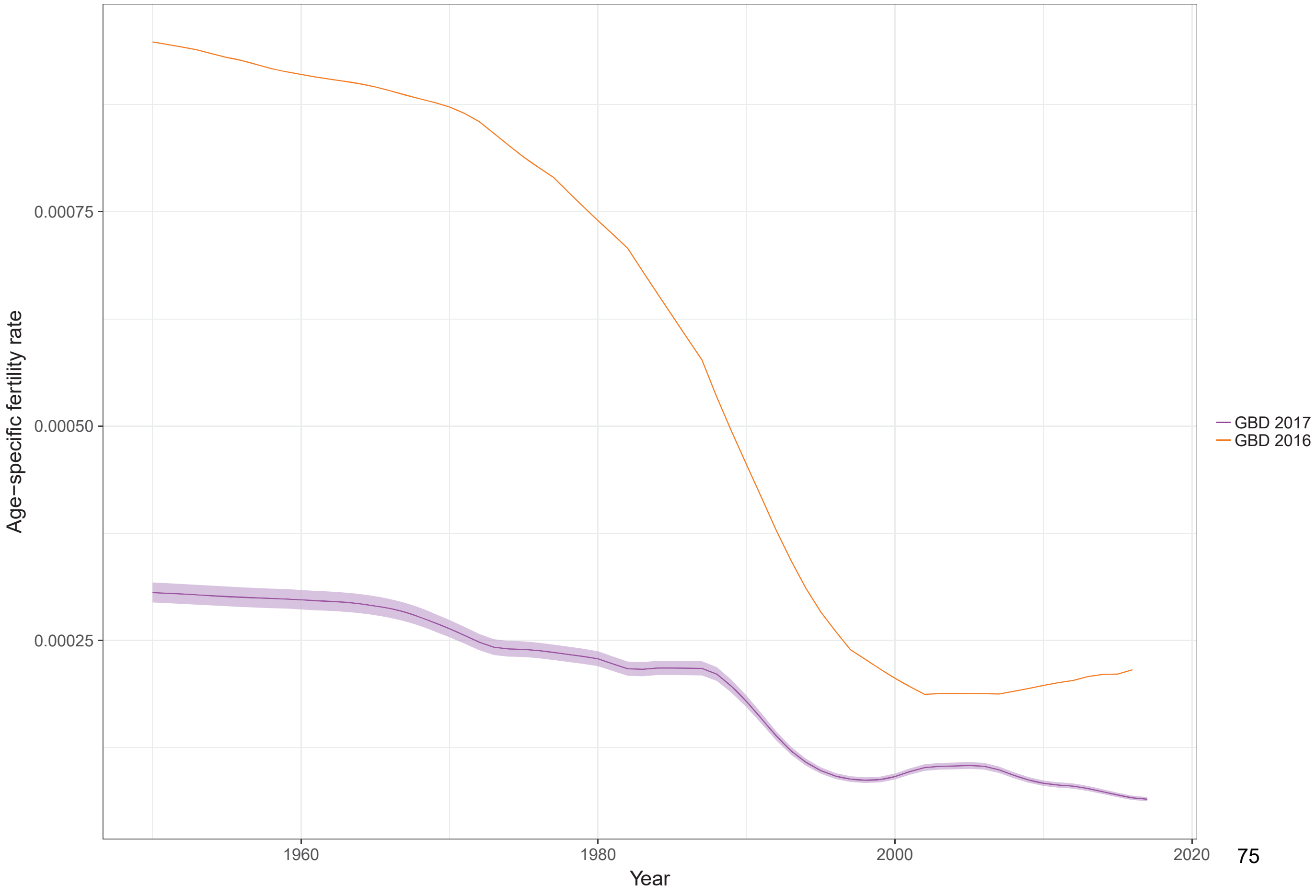

Age−specific fertility fitted trend lines, 1950−2017: Barbados, TFR
Data points enclosed with a diamond are reference sources

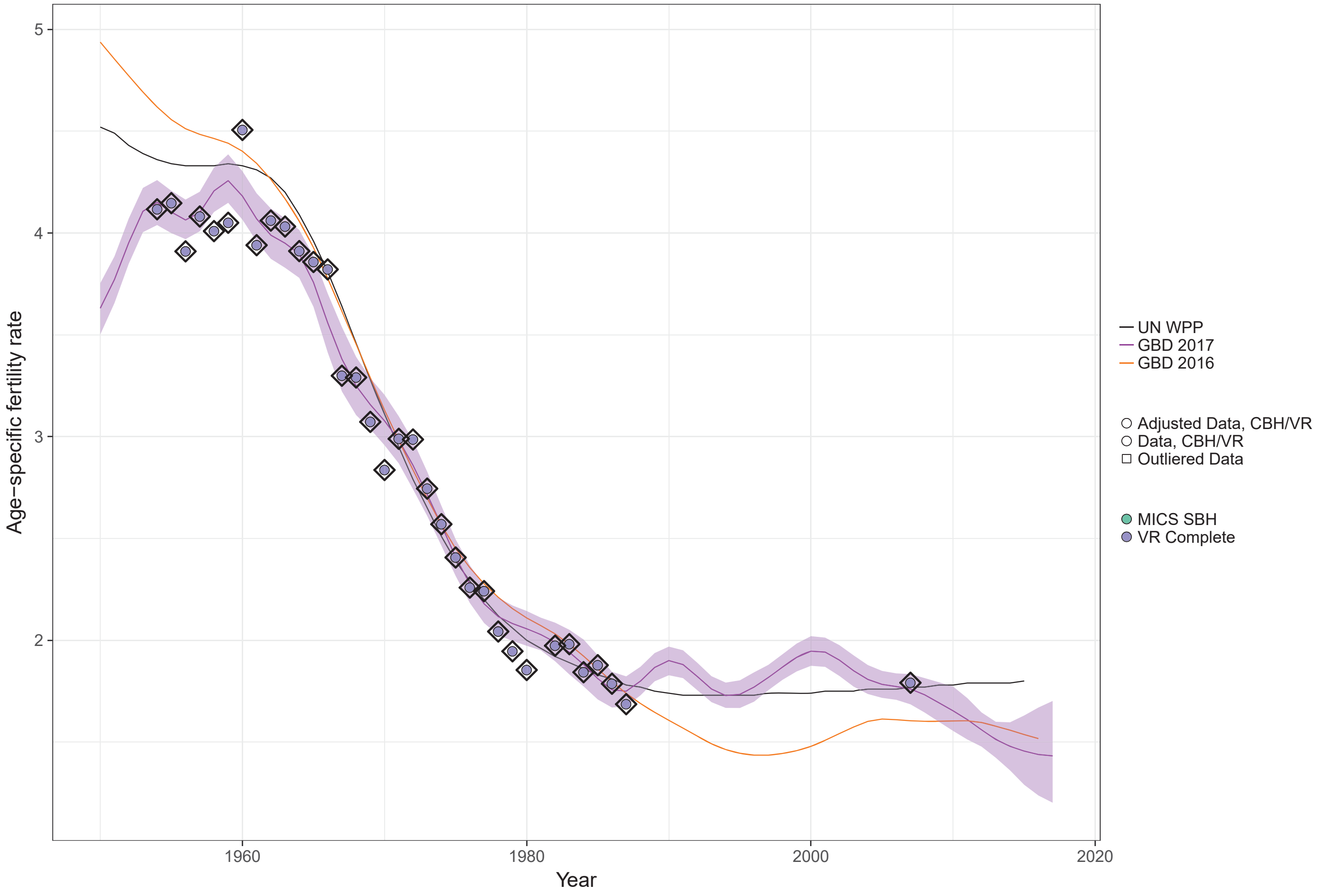

Age−specific fertility fitted trend lines, 1950−2017: Barbados, Ages: 10−14
Data points enclosed with a diamond are reference sources

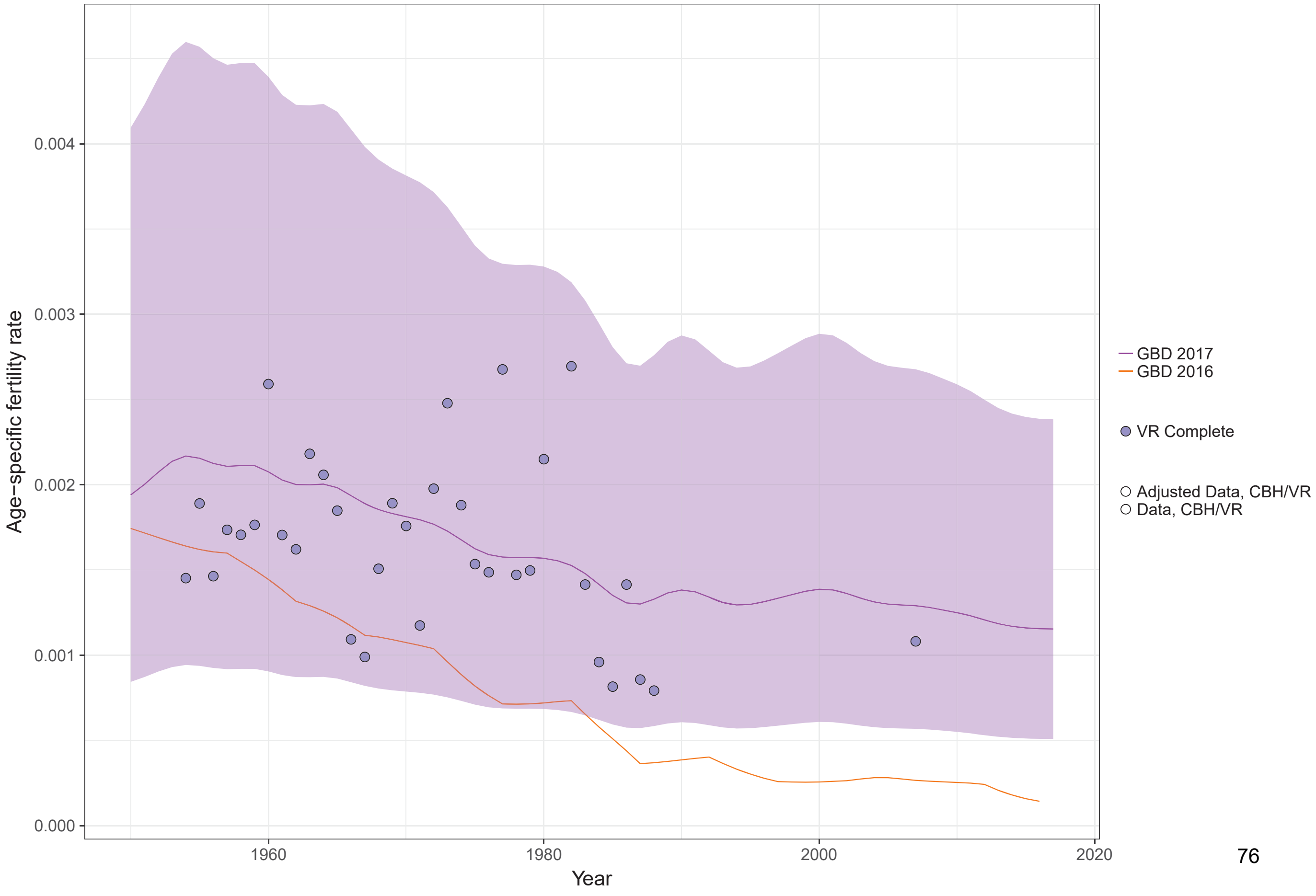

Age−specific fertility fitted trend lines, 1950−2017: Barbados, Ages: 15−19
Data points enclosed with a diamond are reference sources

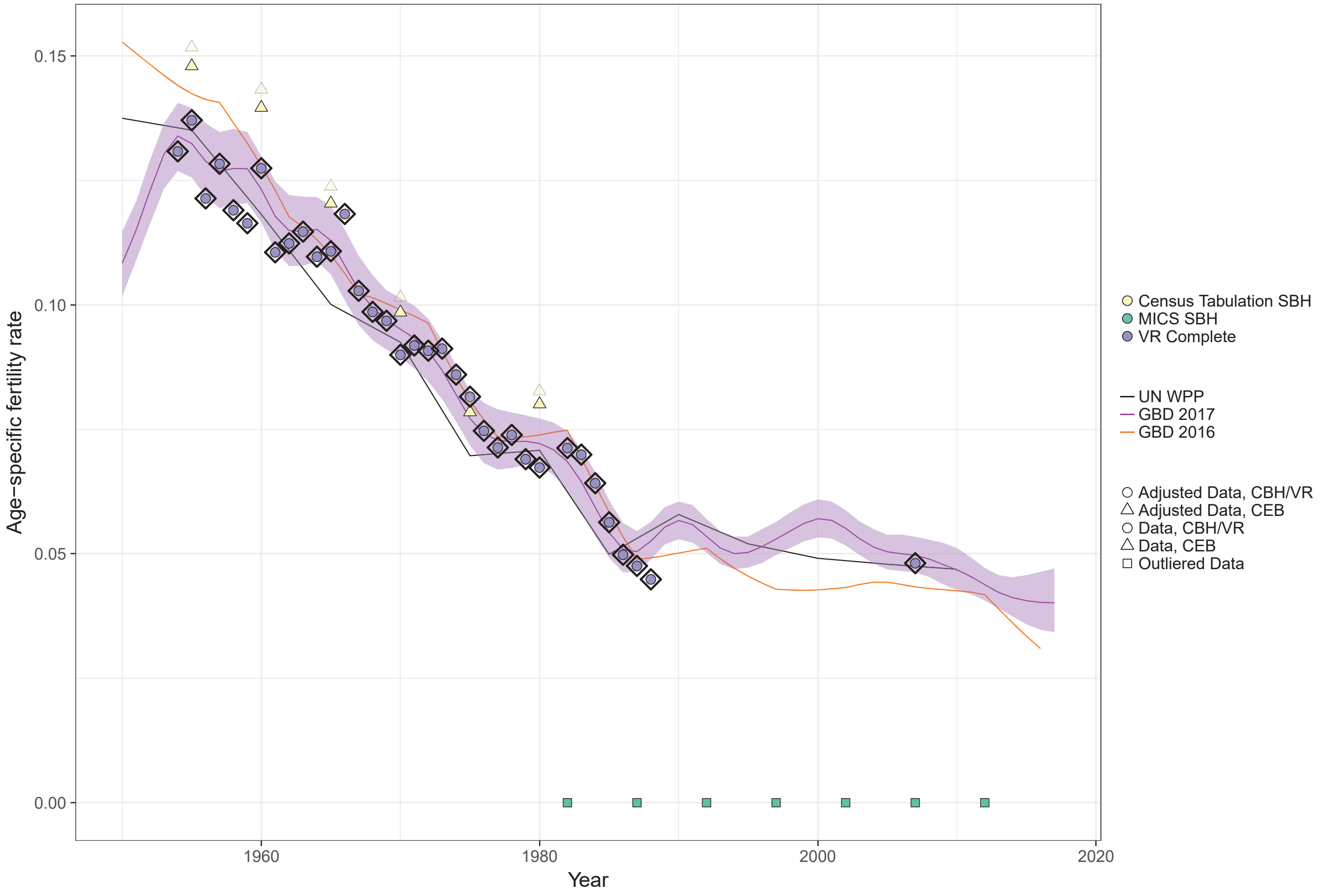

Age−specific fertility fitted trend lines, 1950−2017: Barbados, Ages: 20−24
Data points enclosed with a diamond are reference sources

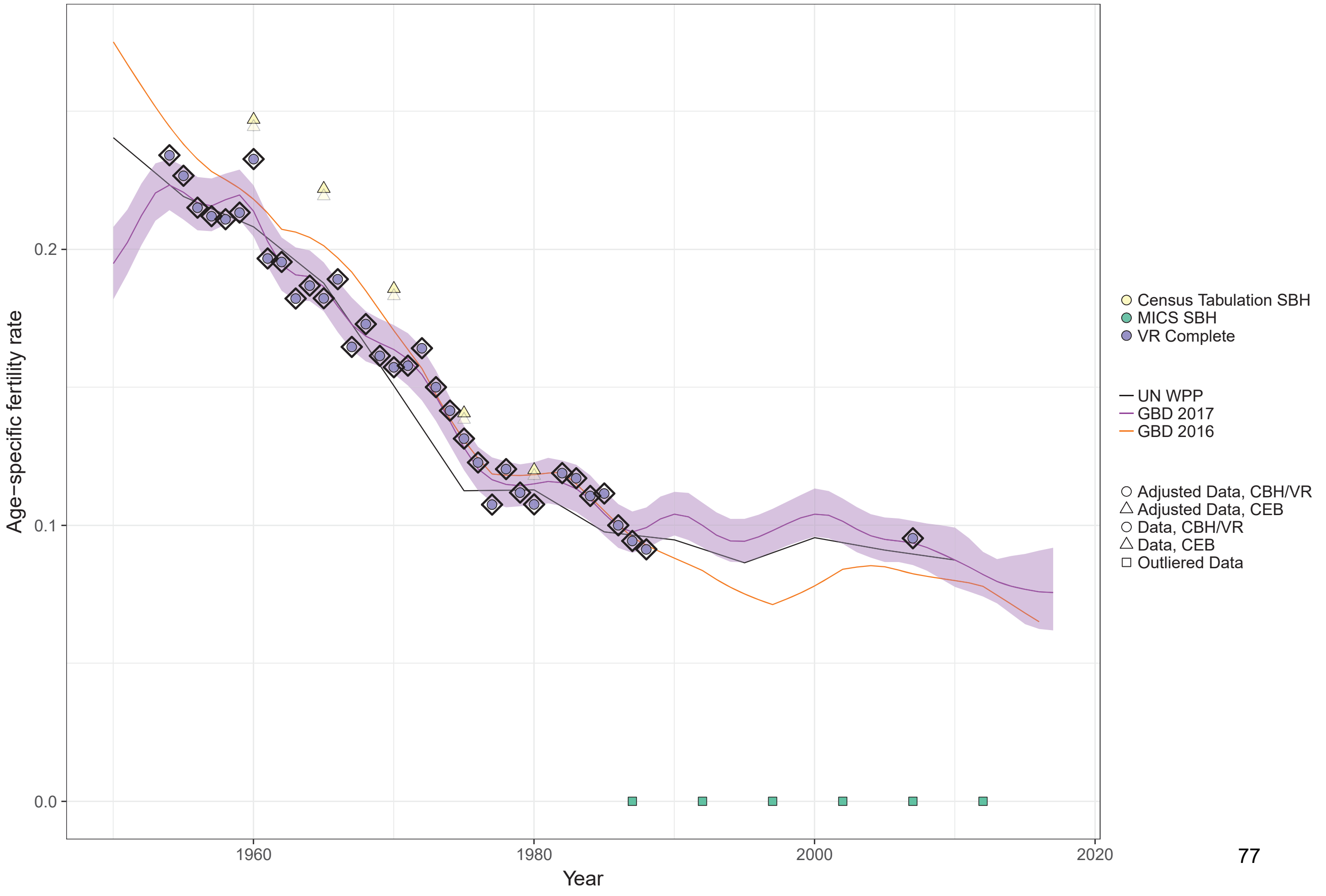

Age−specific fertility fitted trend lines, 1950−2017: Barbados, Ages: 25−29
Data points enclosed with a diamond are reference sources

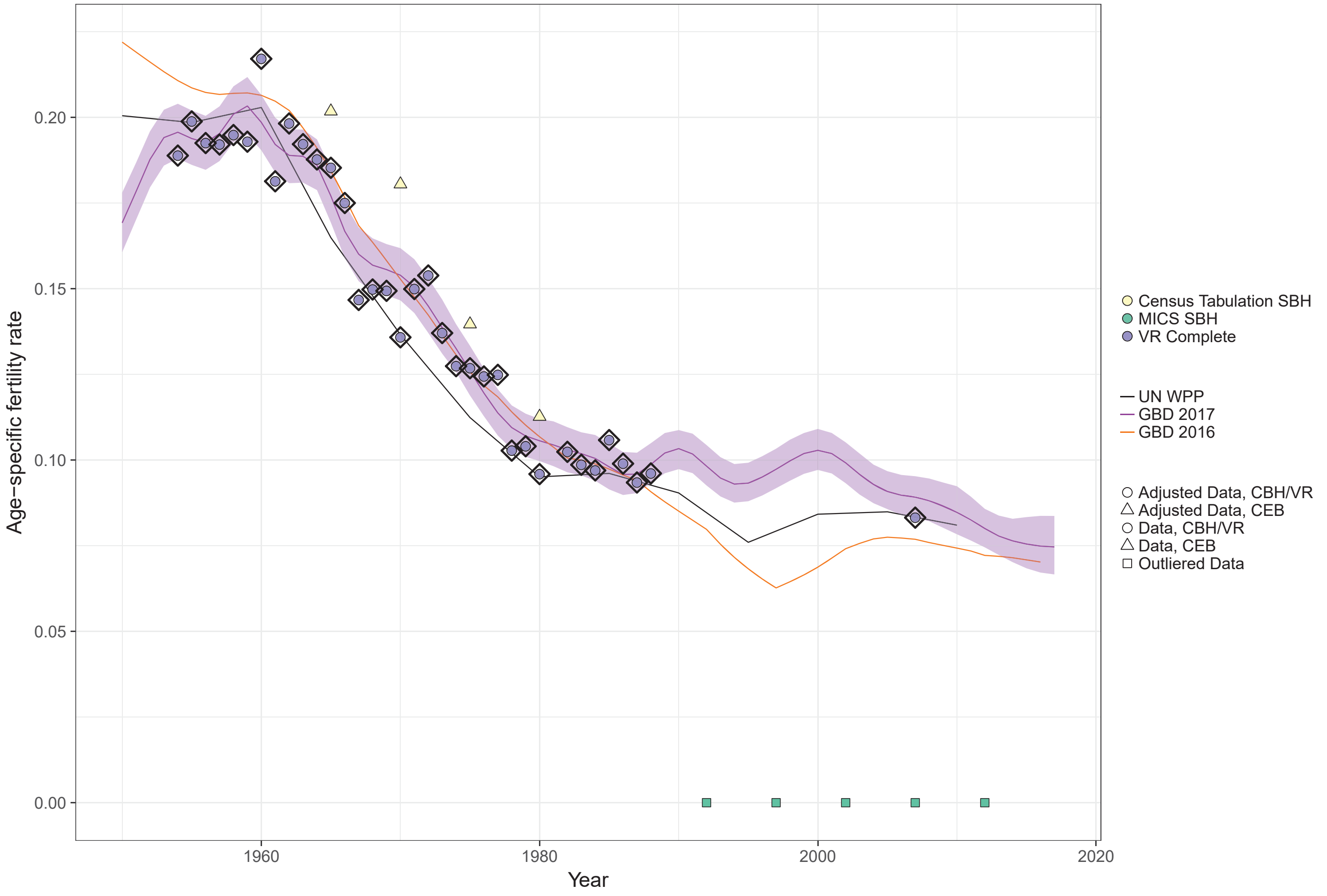

Age−specific fertility fitted trend lines, 1950−2017: Barbados, Ages: 30−34
Data points enclosed with a diamond are reference sources

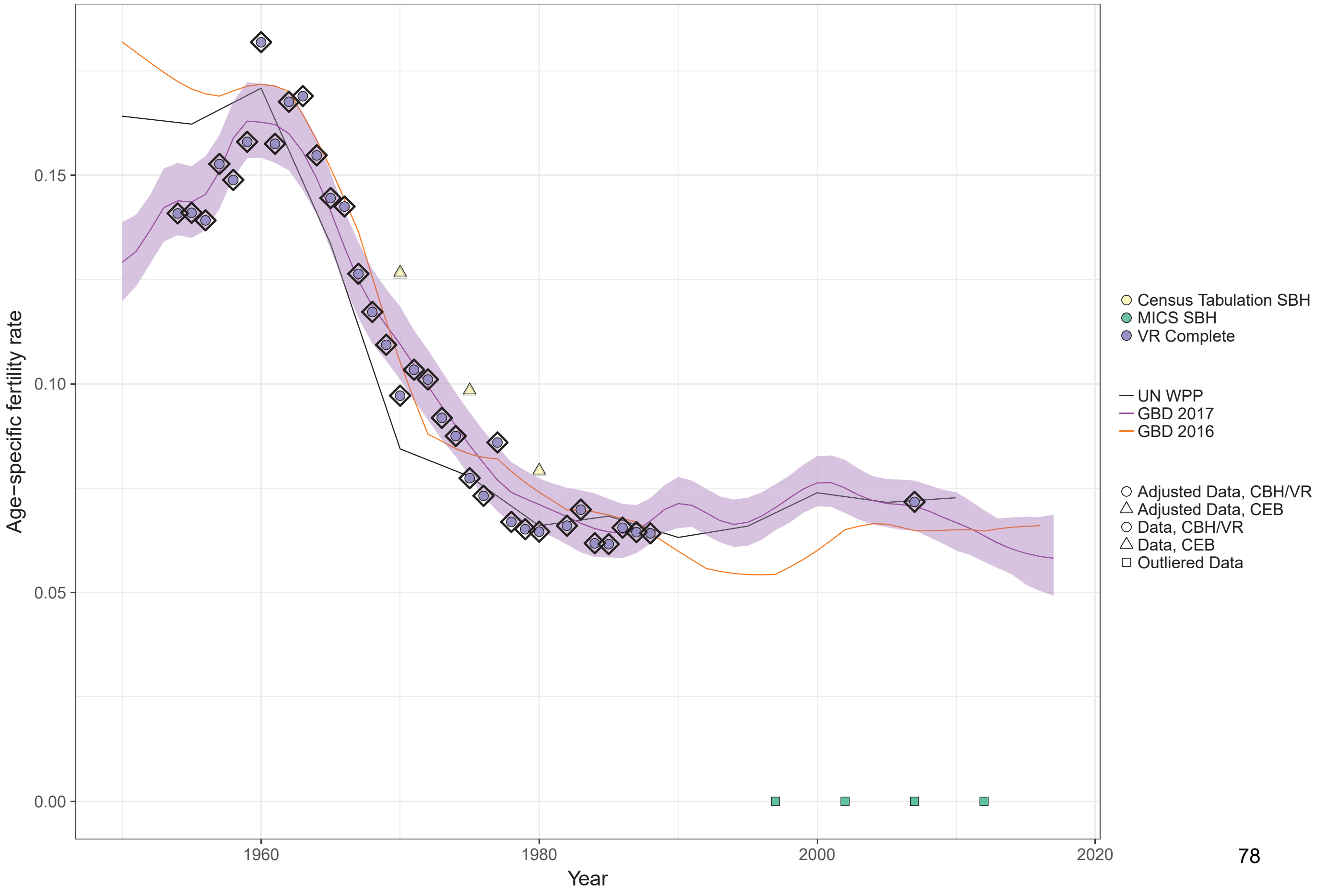

Age−specific fertility fitted trend lines, 1950−2017: Barbados, Ages: 35−39
Data points enclosed with a diamond are reference sources

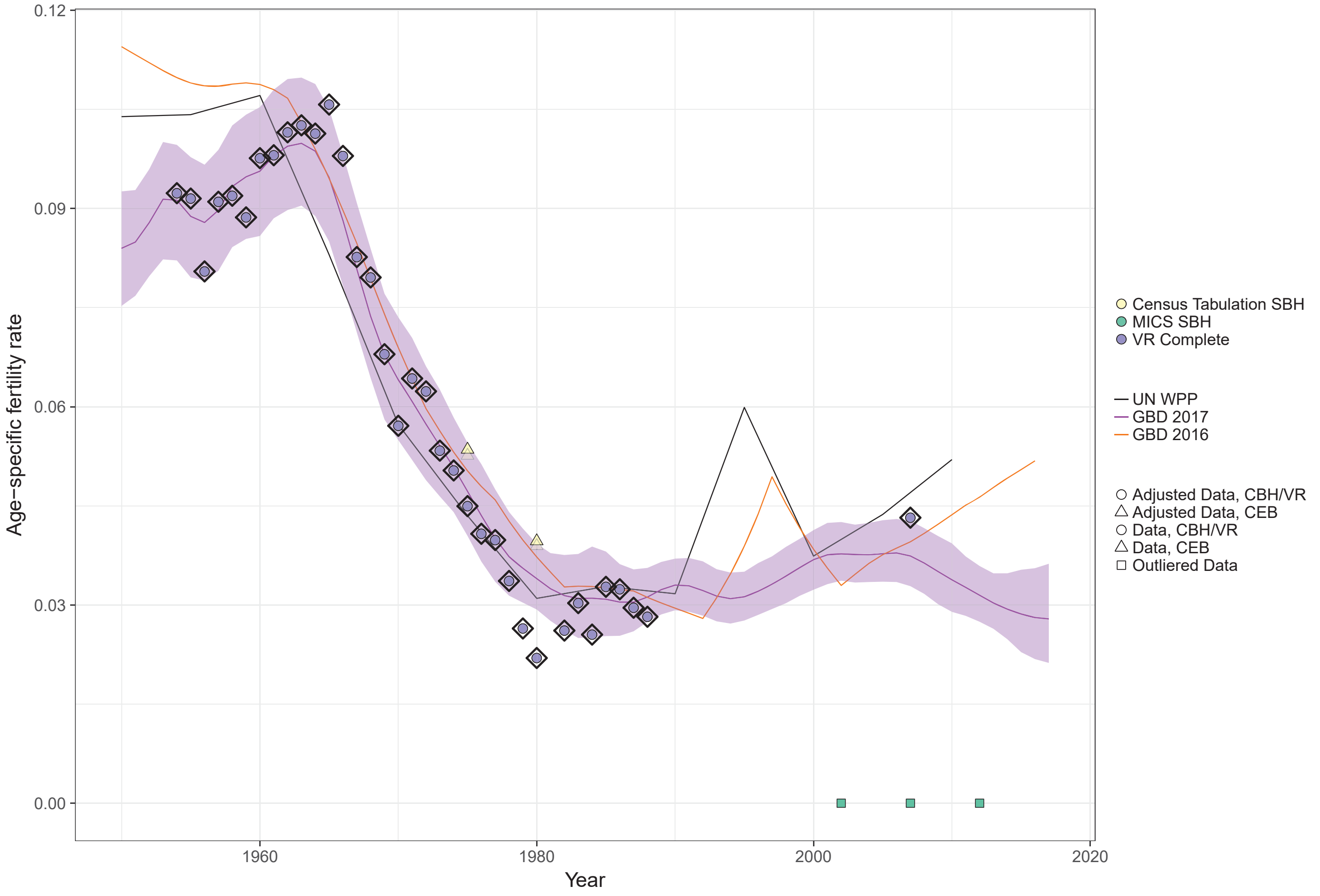

Age−specific fertility fitted trend lines, 1950−2017: Barbados, Ages: 40−44
Data points enclosed with a diamond are reference sources

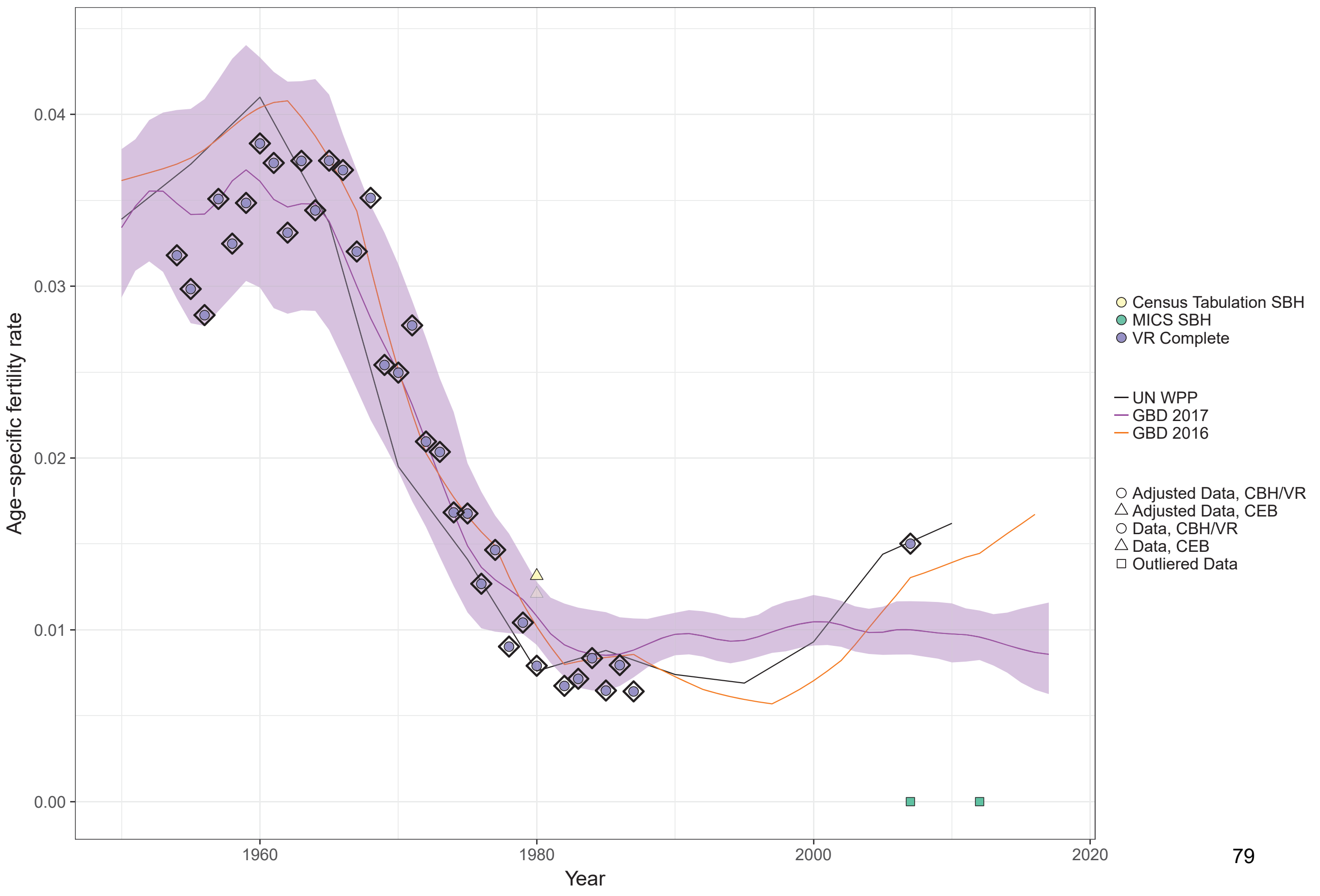

Age−specific fertility fitted trend lines, 1950−2017: Barbados, Ages: 45−49
Data points enclosed with a diamond are reference sources

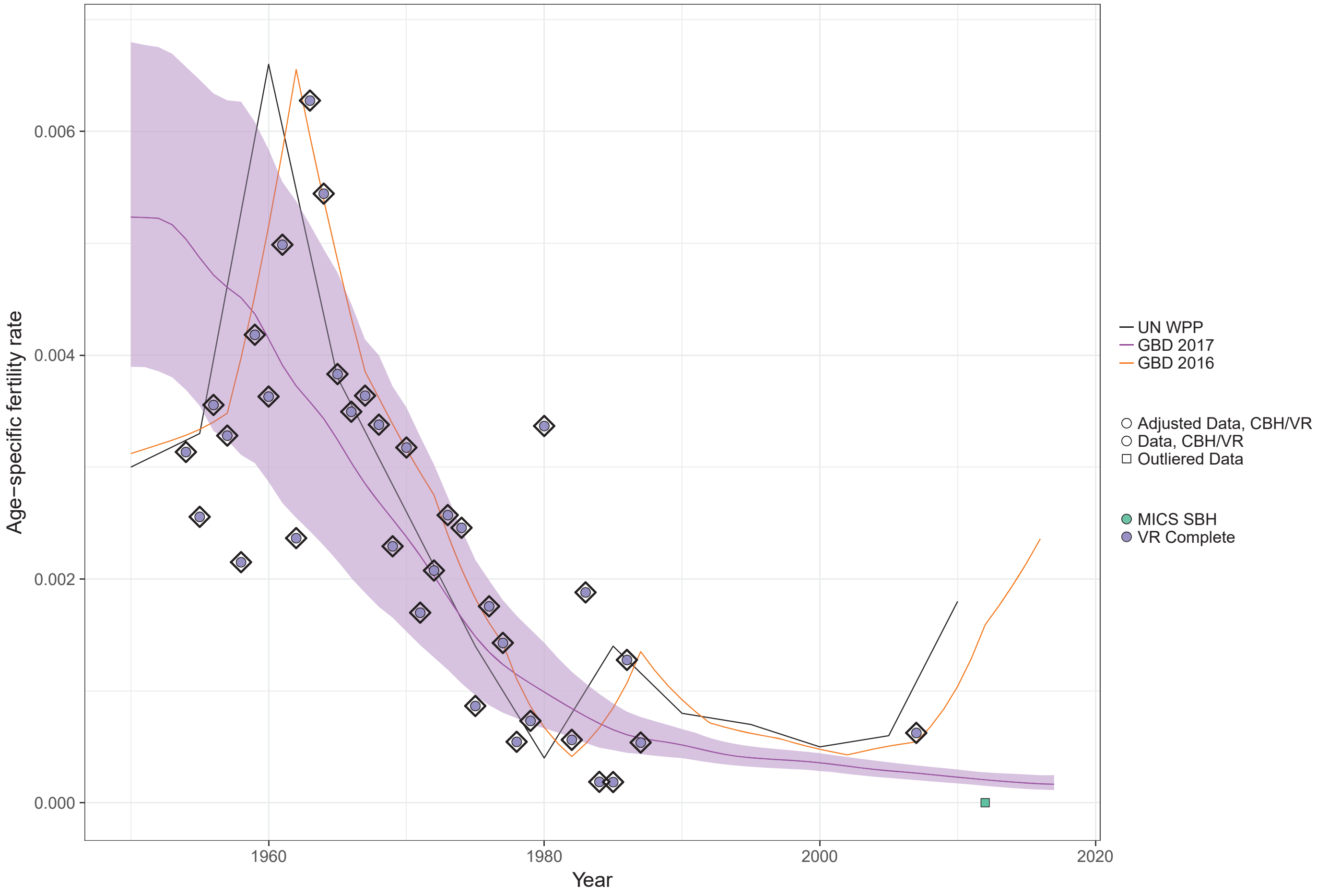

Age−specific fertility fitted trend lines, 1950−2017: Barbados, Ages: 50−54
Data points enclosed with a diamond are reference sources

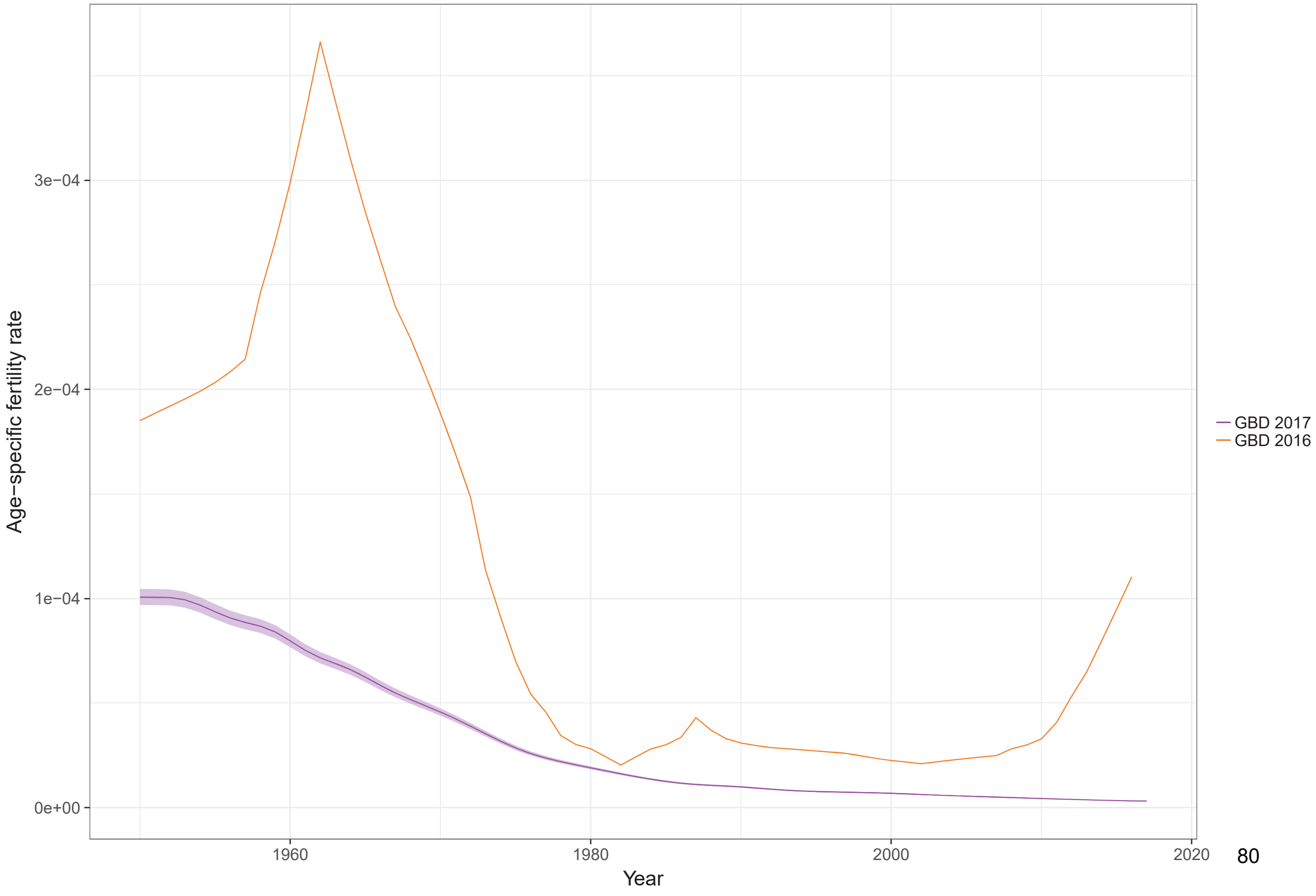

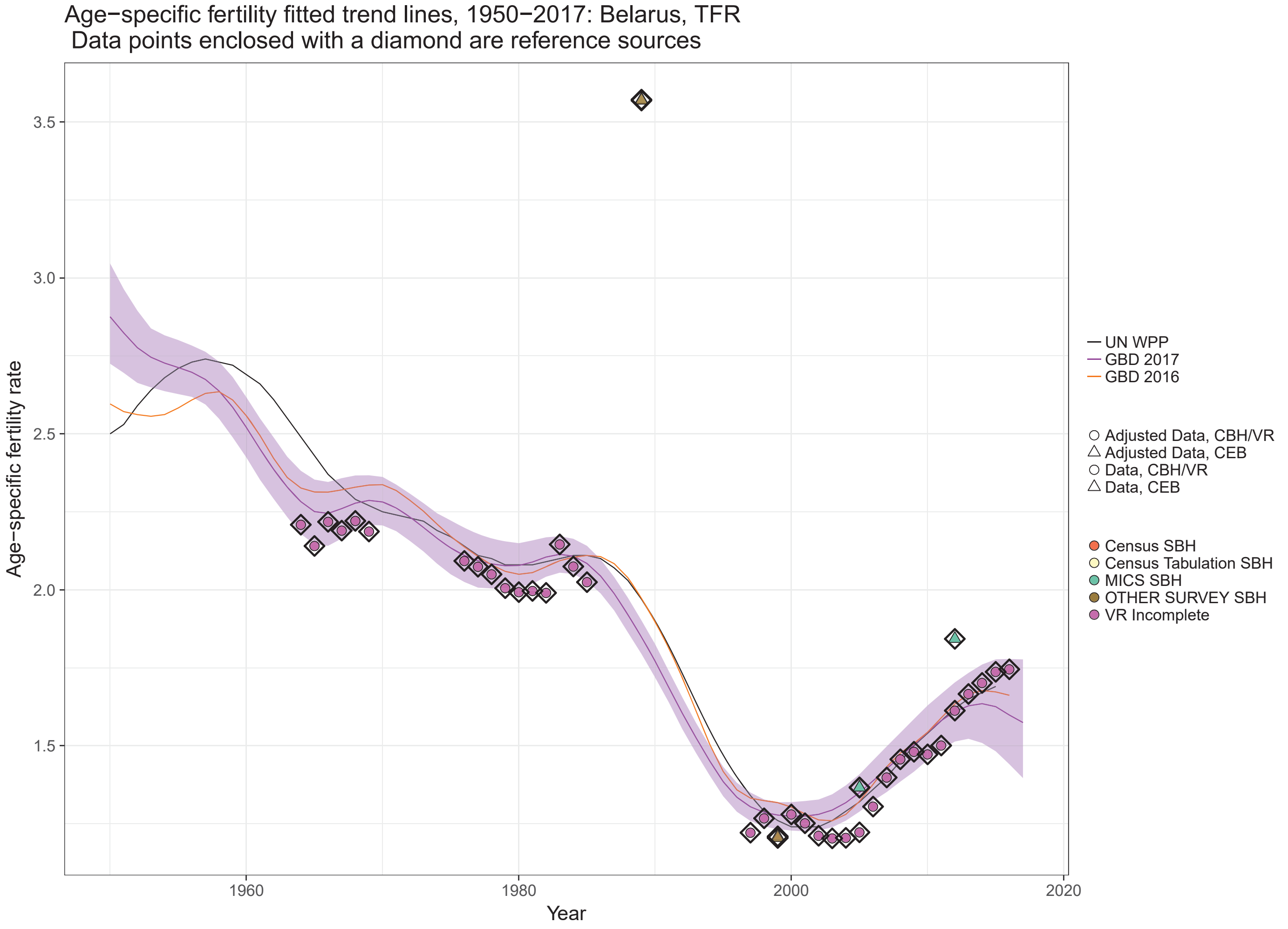
Age−specific fertility fitted trend lines, 1950−2017: Belarus, TFR
Data points enclosed with a diamond are reference sources
Age−specific fertility rate
Year
UN WPP
GBD 2017
GBD 2016
Adjusted Data, CBH/VR
Adjusted Data, CEB
Data, CBH/VR
Data, CEB
Census SBH
Census Tabulation SBH
MICS SBH
OTHER SURVEY SBH
VR Incomplete

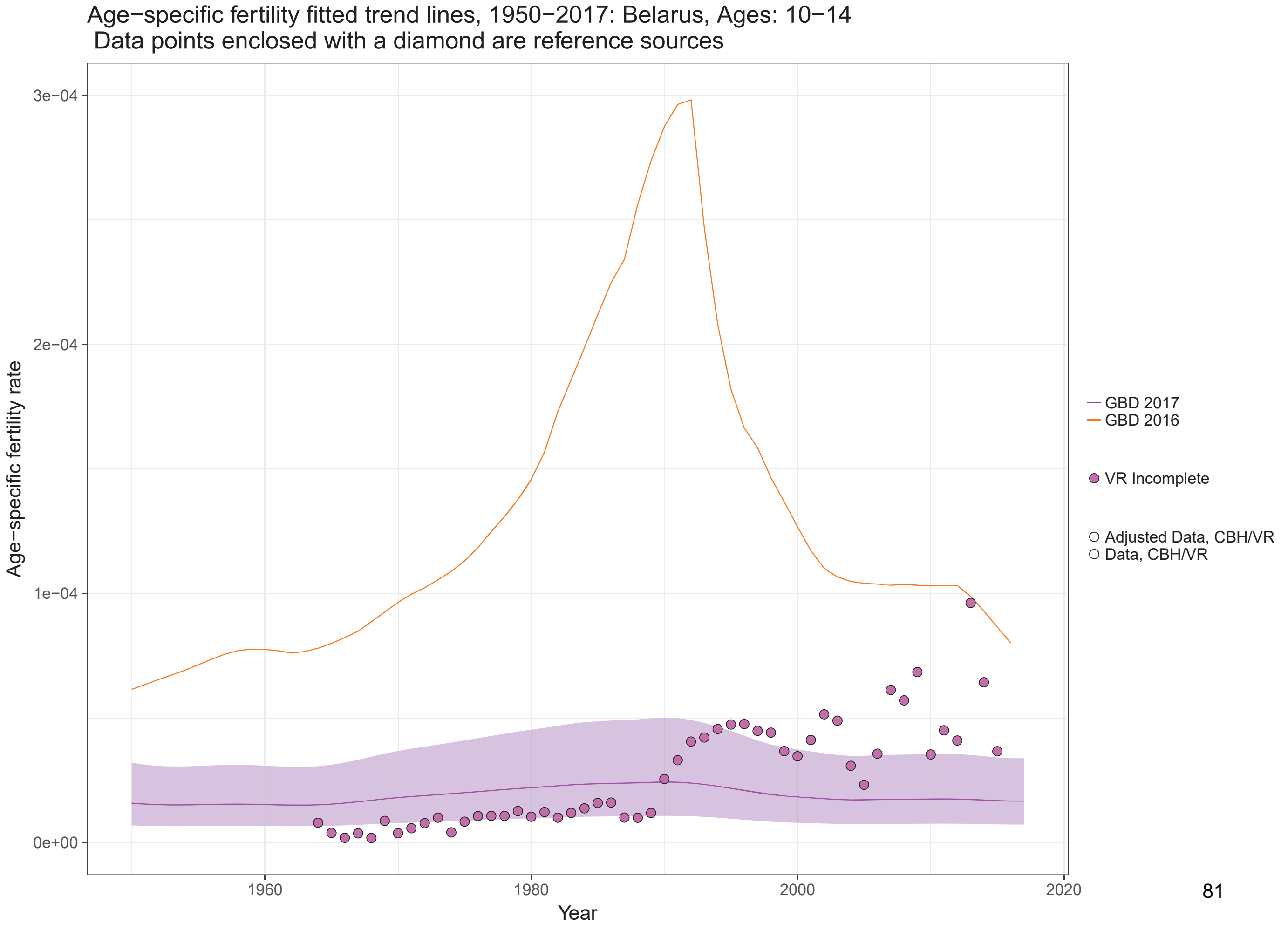
Age−specific fertility fitted trend lines, 1950−2017: Belarus, Ages: 10−14
Data points enclosed with a diamond are reference sources
Age−specific fertility rate
Year
GBD 2017
GBD 2016
VR Incomplete
Adjusted Data, CBH/VR
Data, CBH/VR

Age−specific fertility fitted trend lines, 1950−2017: Belarus, Ages: 15−19
Data points enclosed with a diamond are reference sources

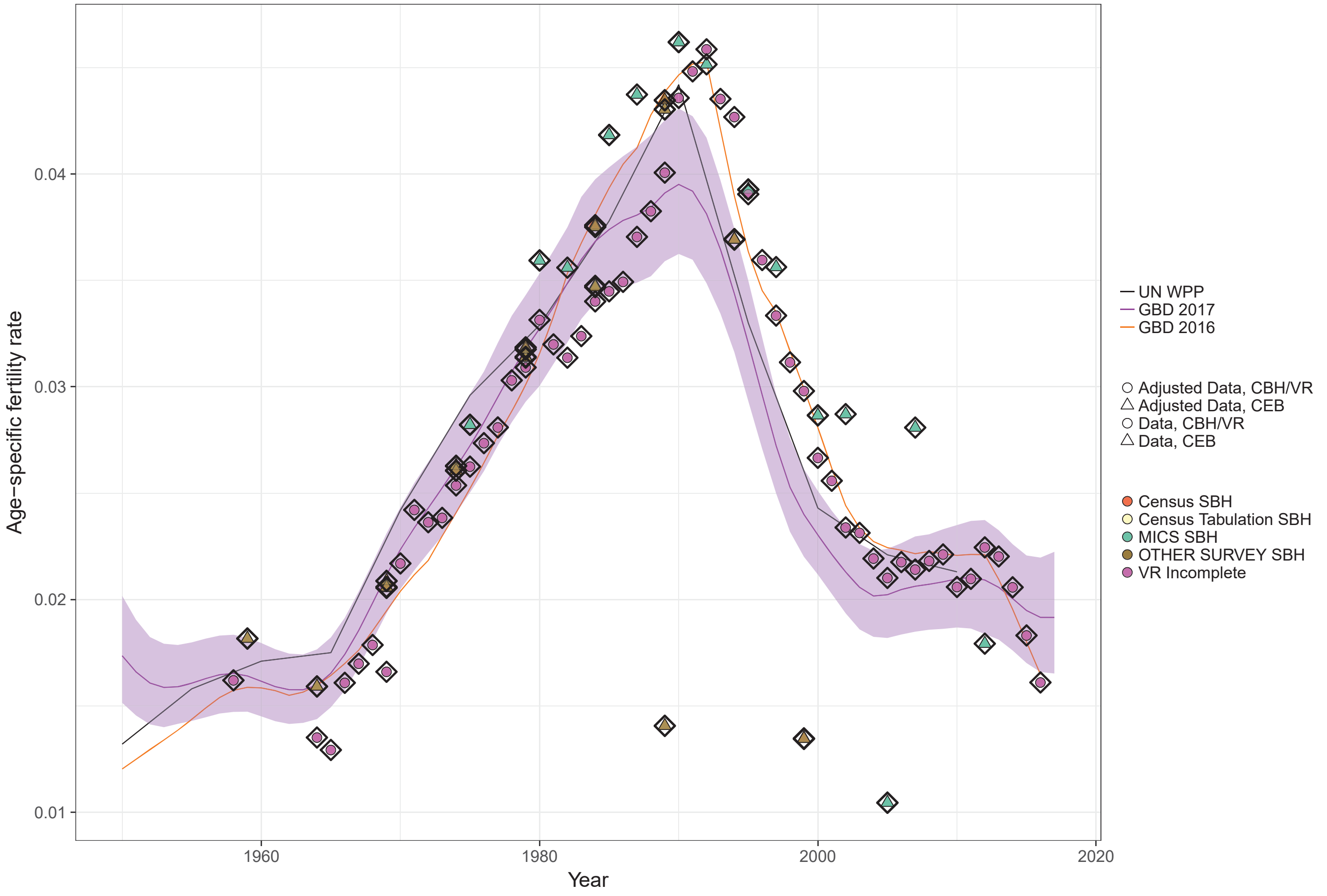

Age−specific fertility fitted trend lines, 1950−2017: Belarus, Ages: 20−24
Data points enclosed with a diamond are reference sources

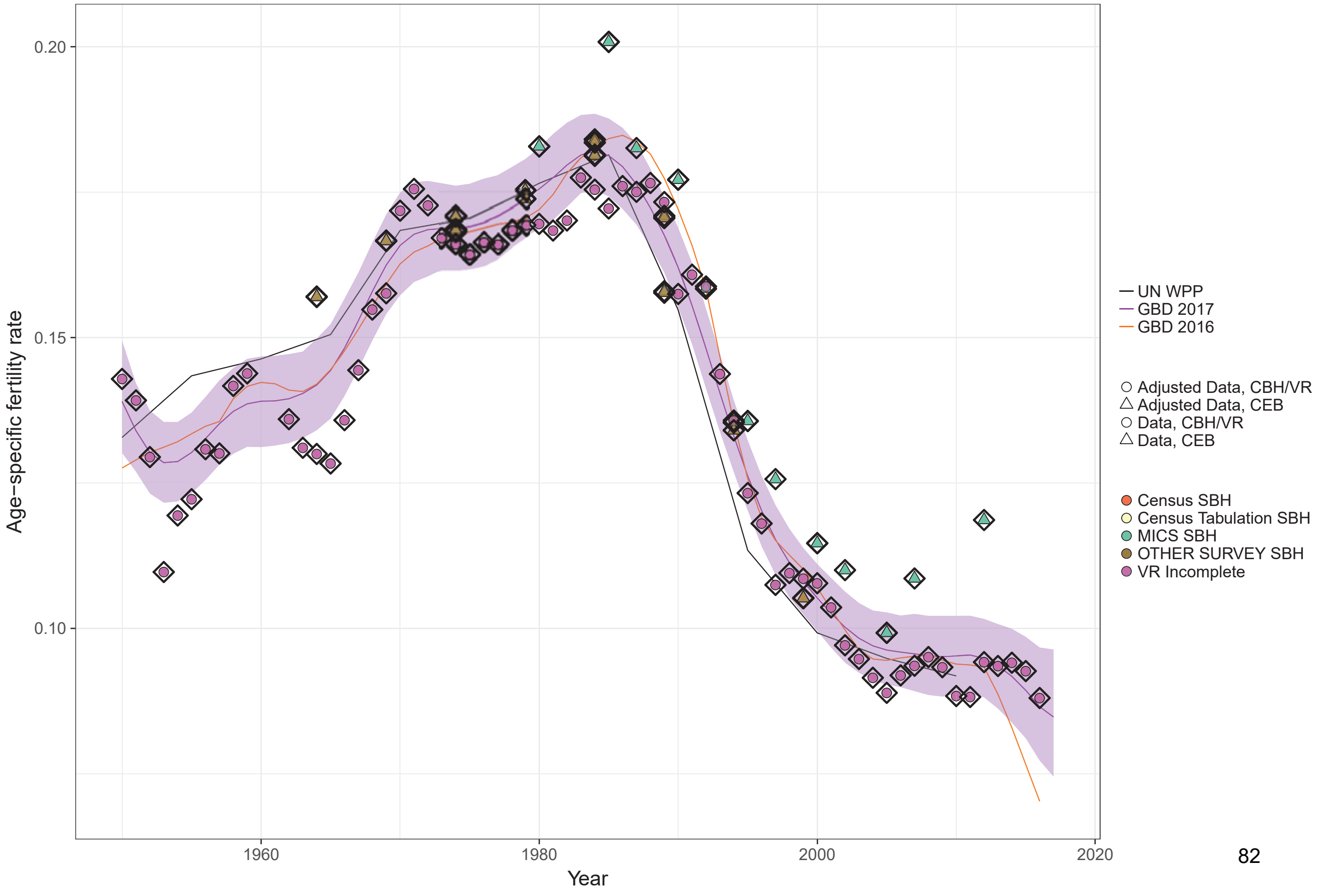

Age−specific fertility fitted trend lines, 1950−2017: Belarus, Ages: 25−29
Data points enclosed with a diamond are reference sources

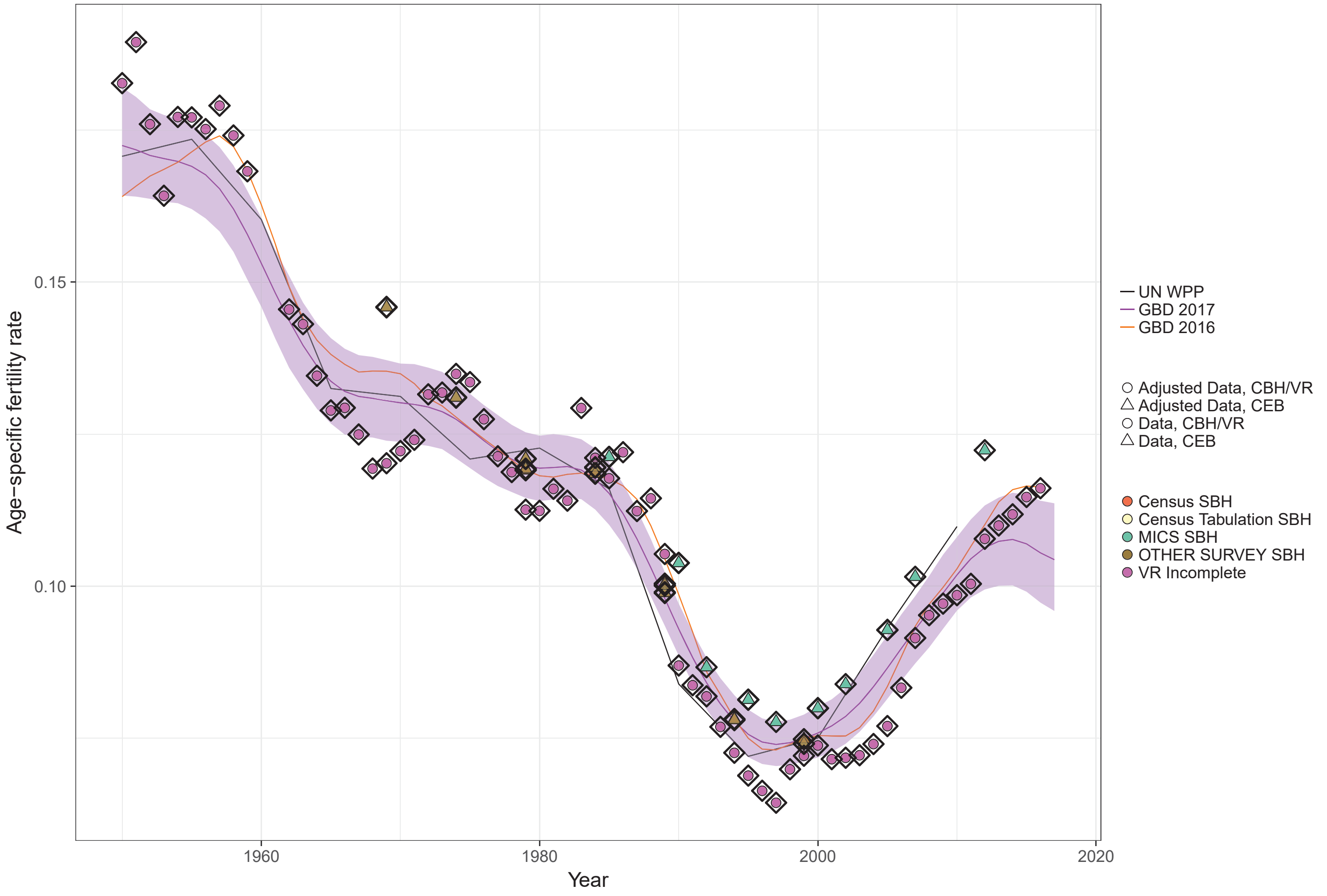

Age−specific fertility fitted trend lines, 1950−2017: Belarus, Ages: 30−34
Data points enclosed with a diamond are reference sources

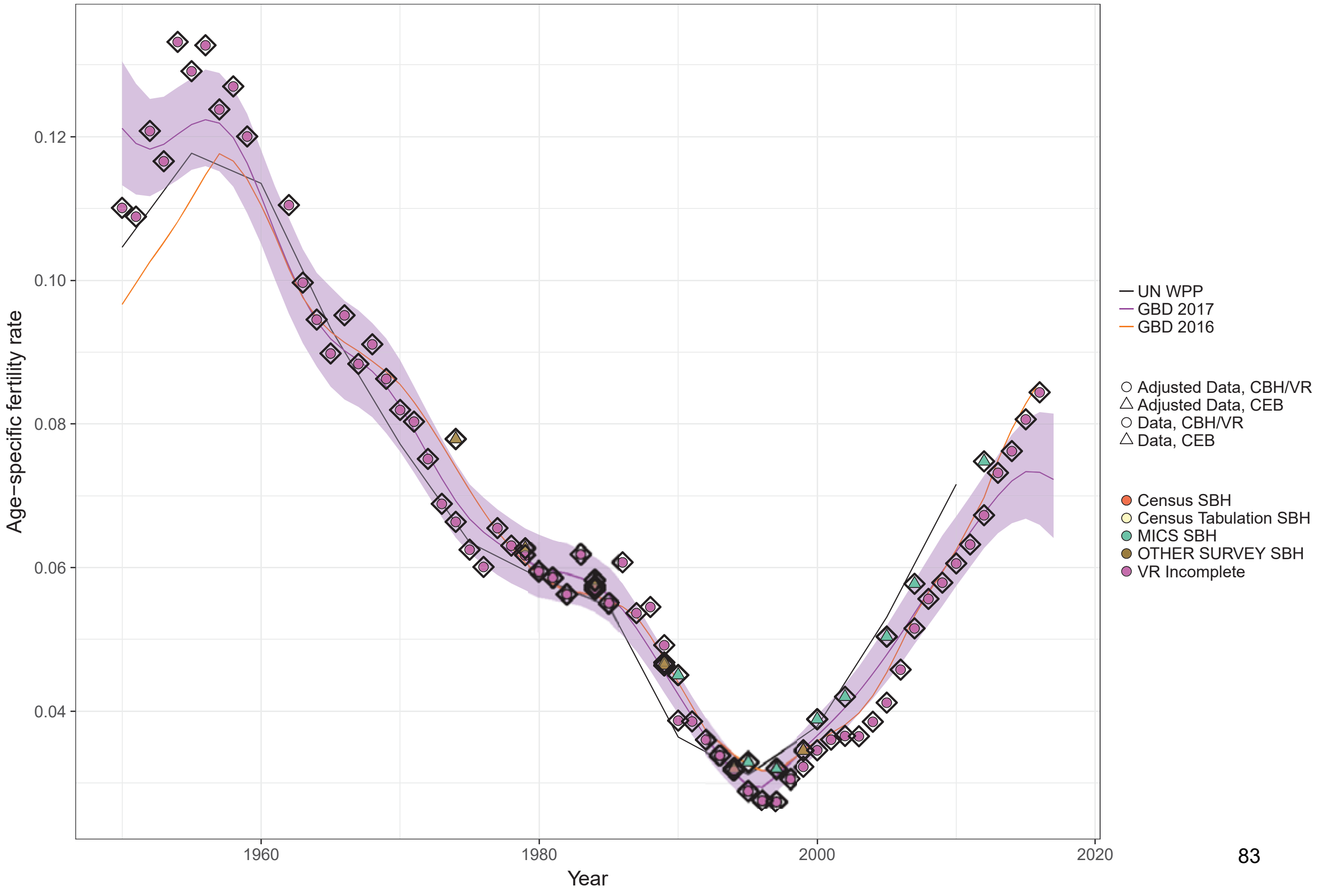

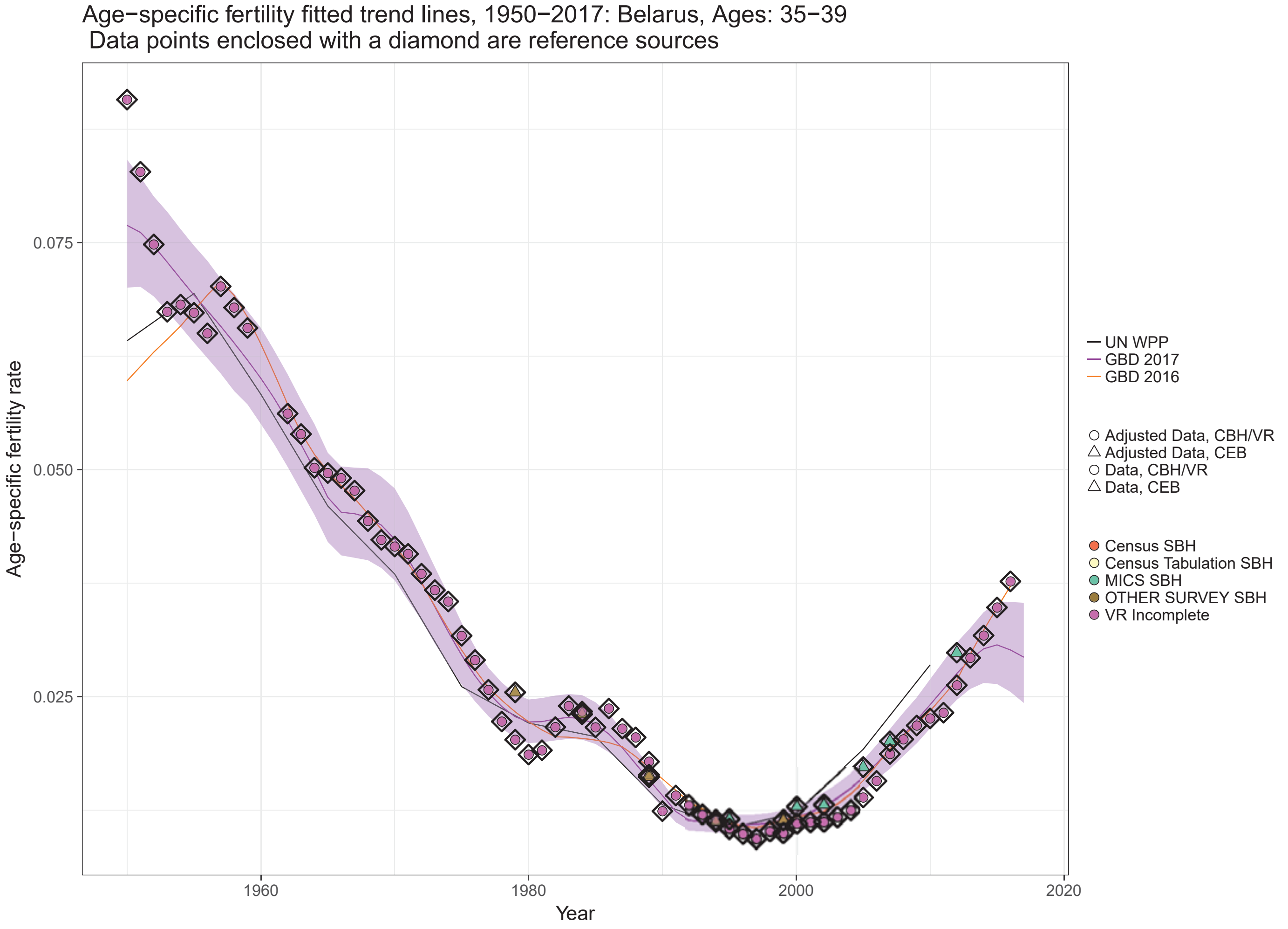

Age−specific fertility fitted trend lines, 1950−2017: Belarus, Ages: 35−39
Data points enclosed with a diamond are reference sources
Age−specific fertility rate
0.075
0.050
0.025
1960
1980
2000
2020
Year
UN WPP
GBD 2017
GBD 2016
Adjusted Data, CBH/VR
Adjusted Data, CEB
Data, CBH/VR
Data, CEB
Census SBH
Census Tabulation SBH
MICS SBH
OTHER SURVEY SBH
VR Incomplete

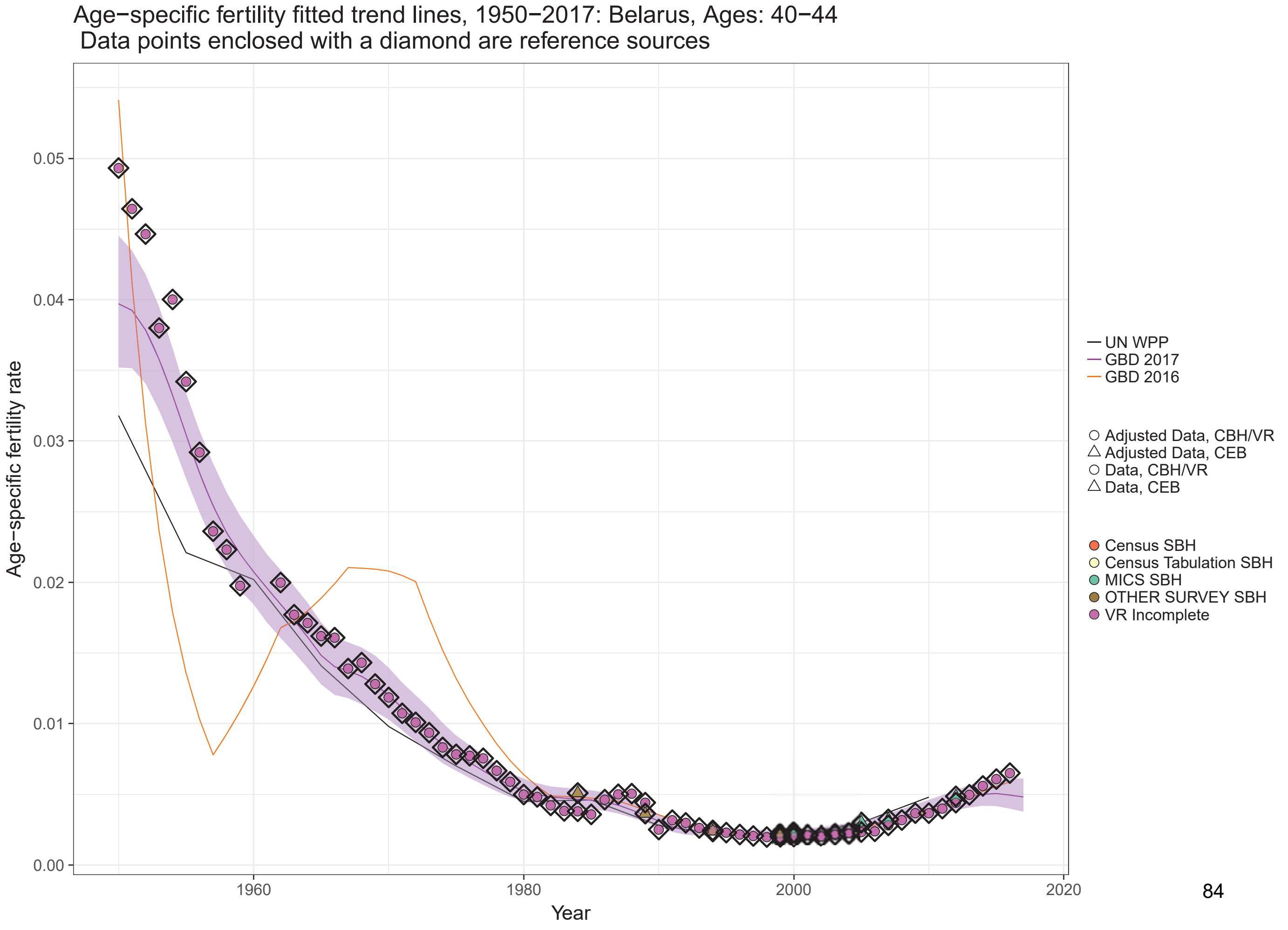

Age−specific fertility fitted trend lines, 1950−2017: Belarus, Ages: 40−44
Data points enclosed with a diamond are reference sources
Age−specific fertility rate
0.05
0.04
0.03
0.02
0.01
0.00
1960
1980
2000
2020
Year
UN WPP
GBD 2017
GBD 2016
Adjusted Data, CBH/VR
Adjusted Data, CEB
Data, CBH/VR
Data, CEB
Census SBH
Census Tabulation SBH
MICS SBH
OTHER SURVEY SBH
VR Incomplete

Age−specific fertility fitted trend lines, 1950−2017: Belarus, Ages: 45−49
Data points enclosed with a diamond are reference sources

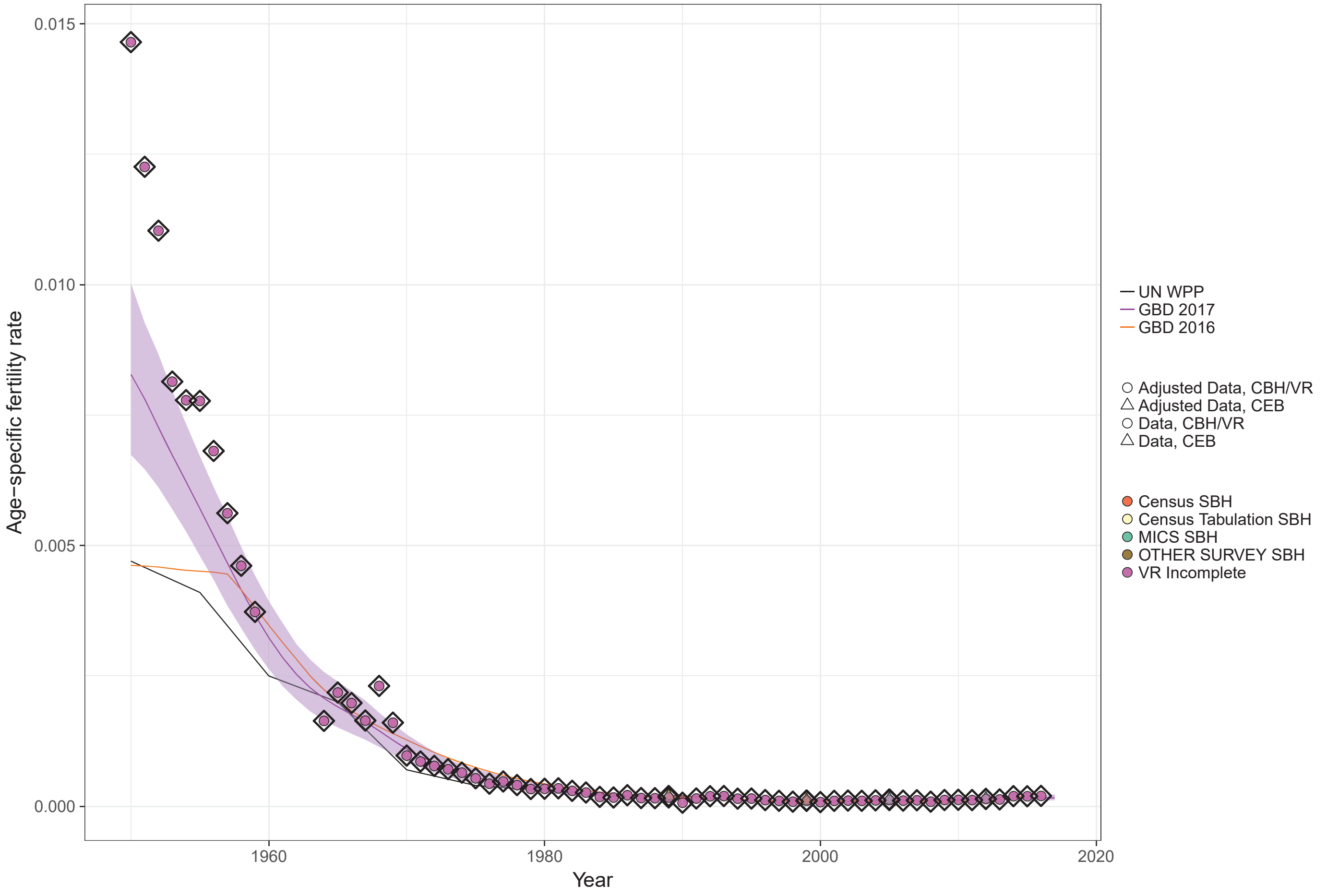

Age−specific fertility fitted trend lines, 1950−2017: Belarus, Ages: 50−54
Data points enclosed with a diamond are reference sources

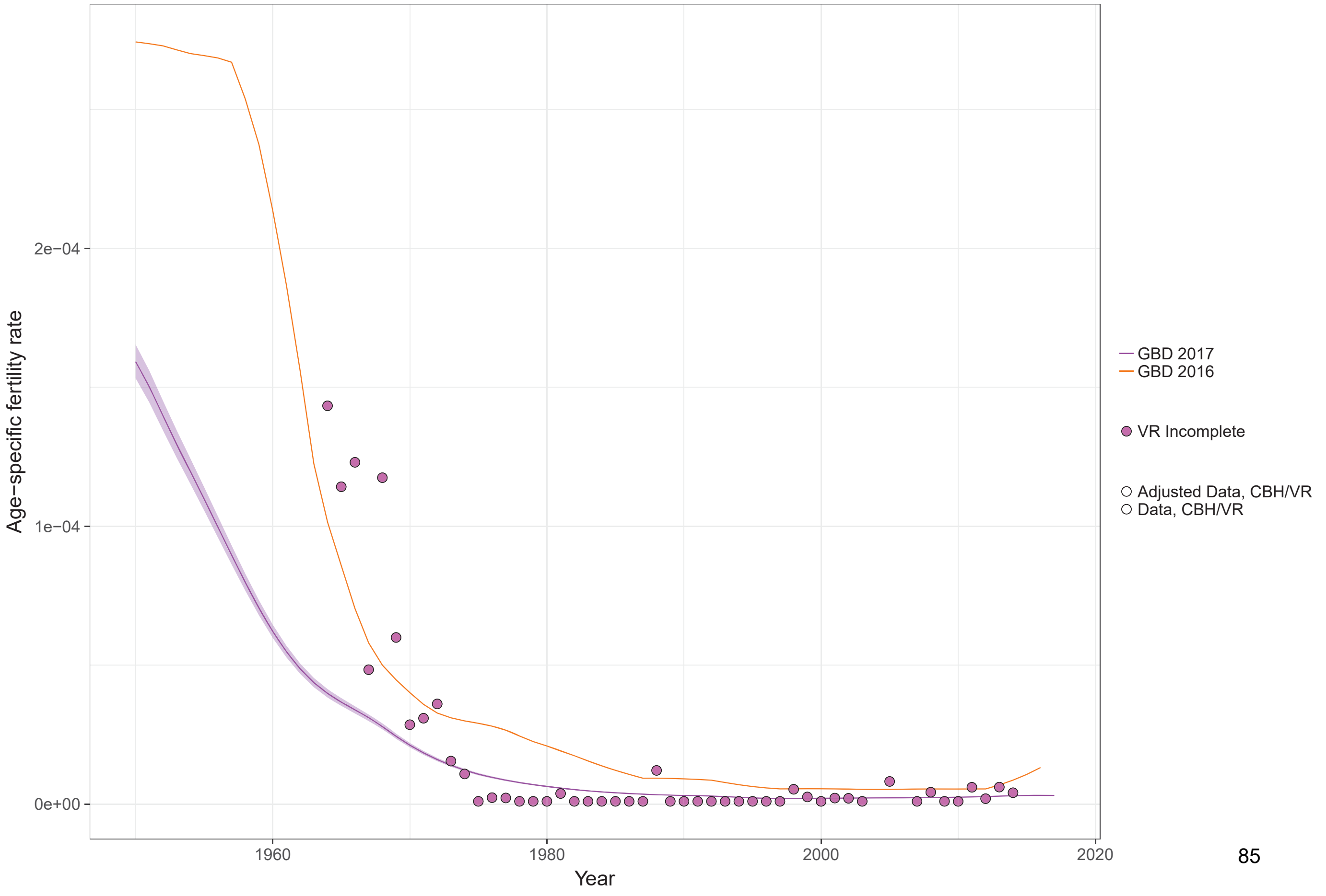

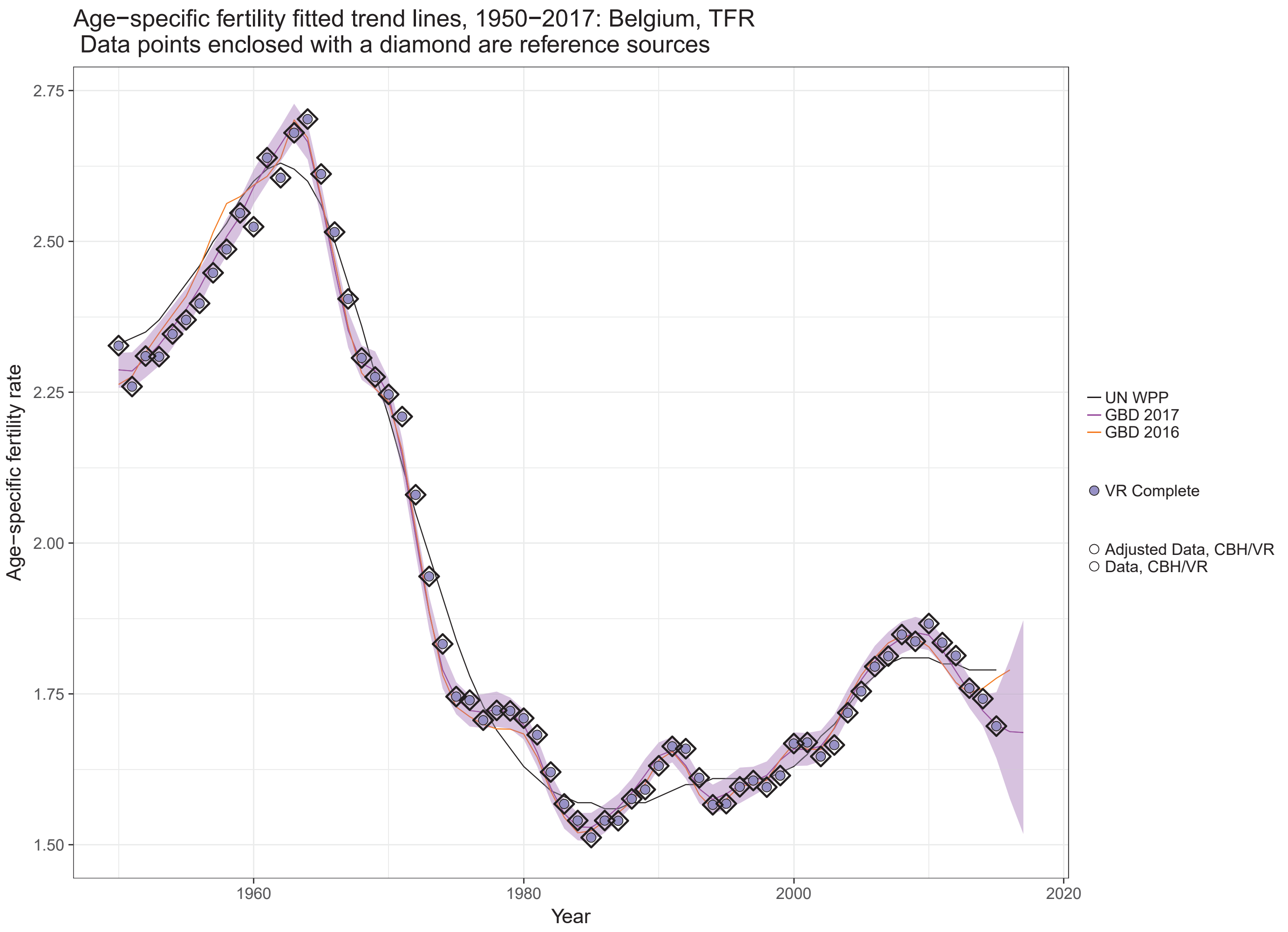
Age−specific fertility fitted trend lines, 1950−2017: Belgium, TFR
Data points enclosed with a diamond are reference sources
Age−specific fertility rate
Year
UN WPP
GBD 2017
GBD 2016
VR Complete
Adjusted Data, CBH/VR
Data, CBH/VR

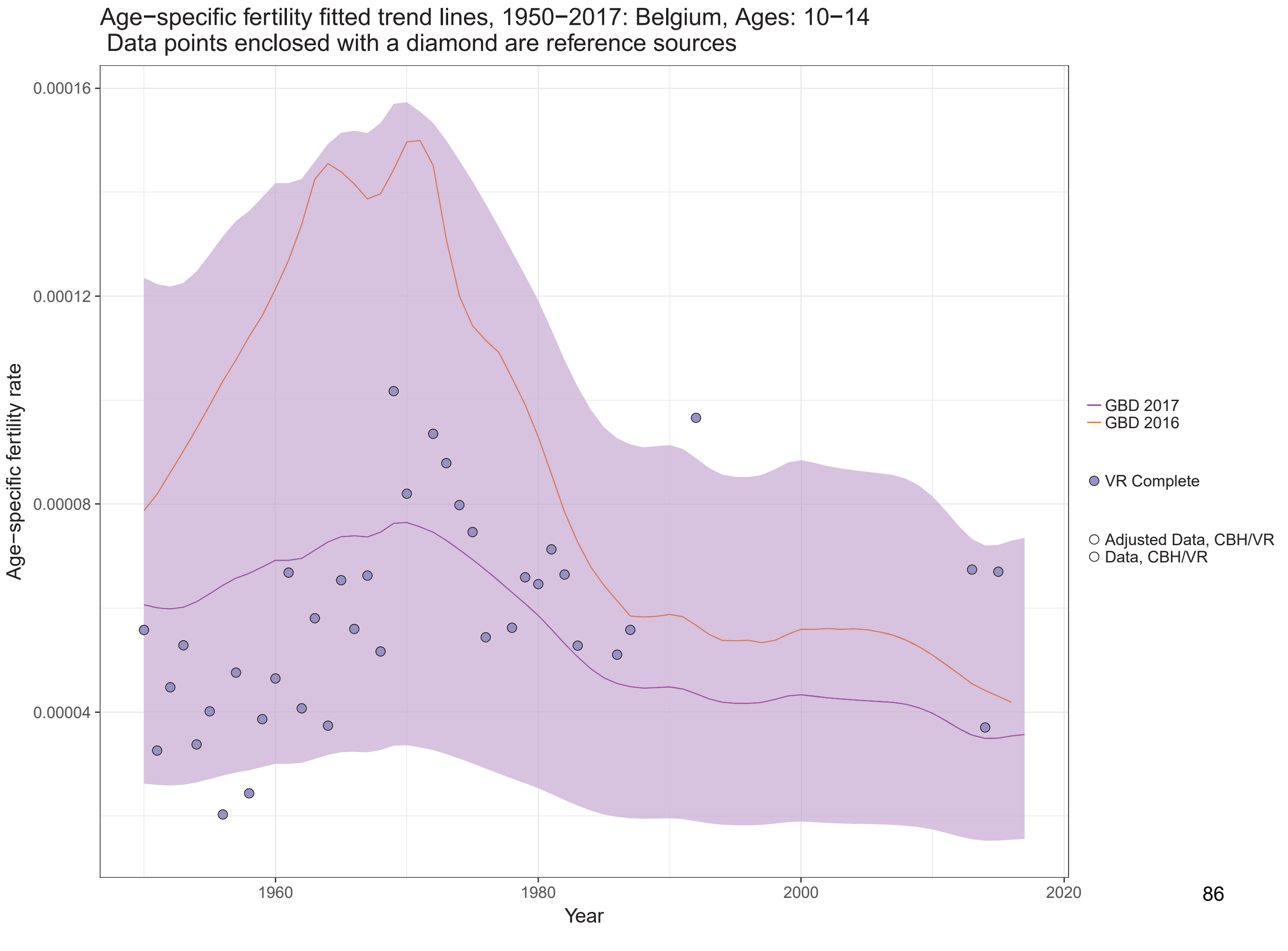
Age−specific fertility fitted trend lines, 1950−2017: Belgium, Ages: 10−14
Data points enclosed with a diamond are reference sources
Age−specific fertility rate
Year
GBD 2017
GBD 2016
VR Complete
Adjusted Data, CBH/VR
Data, CBH/VR

Age−specific fertility fitted trend lines, 1950−2017: Belgium, Ages: 15−19
Data points enclosed with a diamond are reference sources

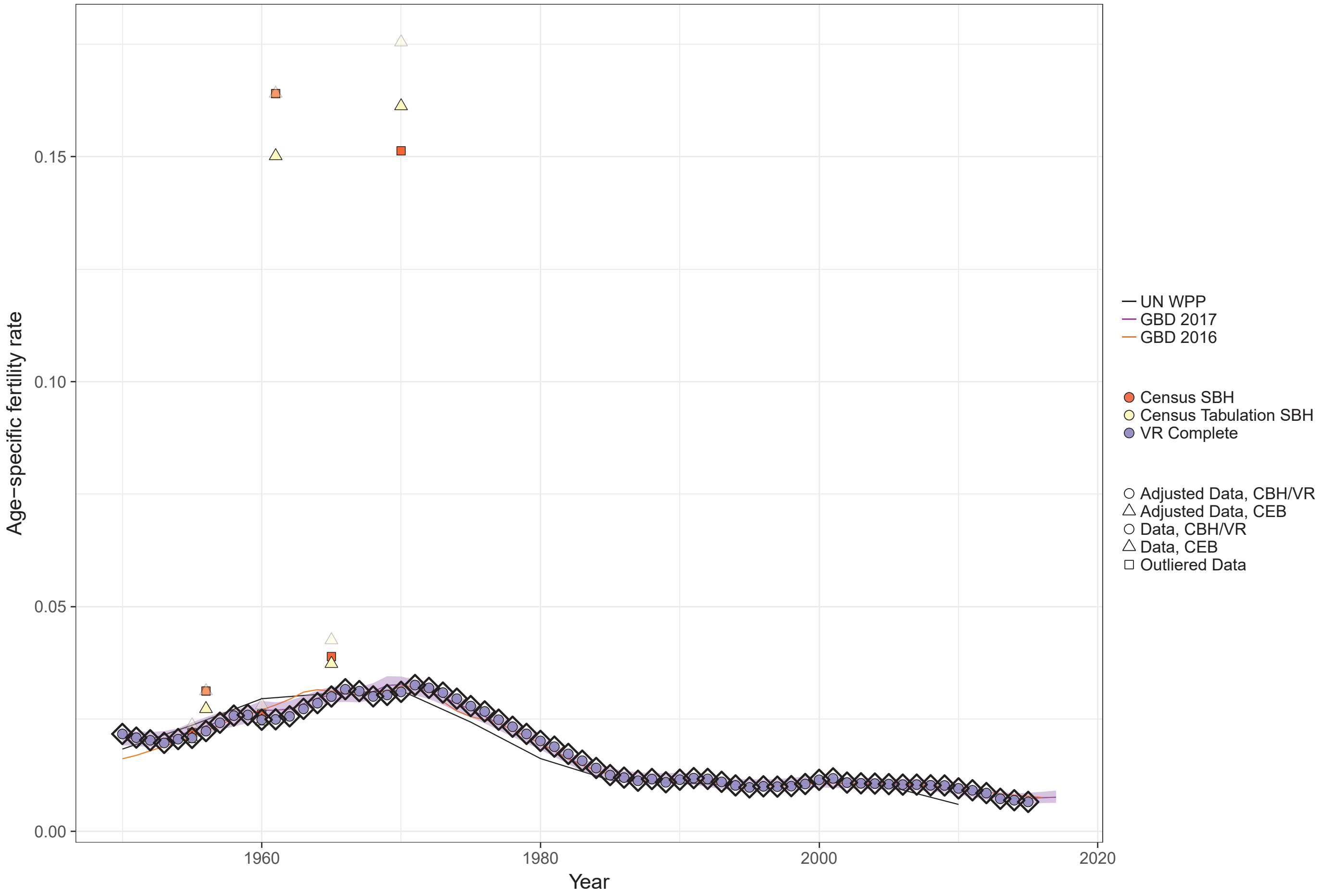

Age−specific fertility fitted trend lines, 1950−2017: Belgium, Ages: 20−24
Data points enclosed with a diamond are reference sources

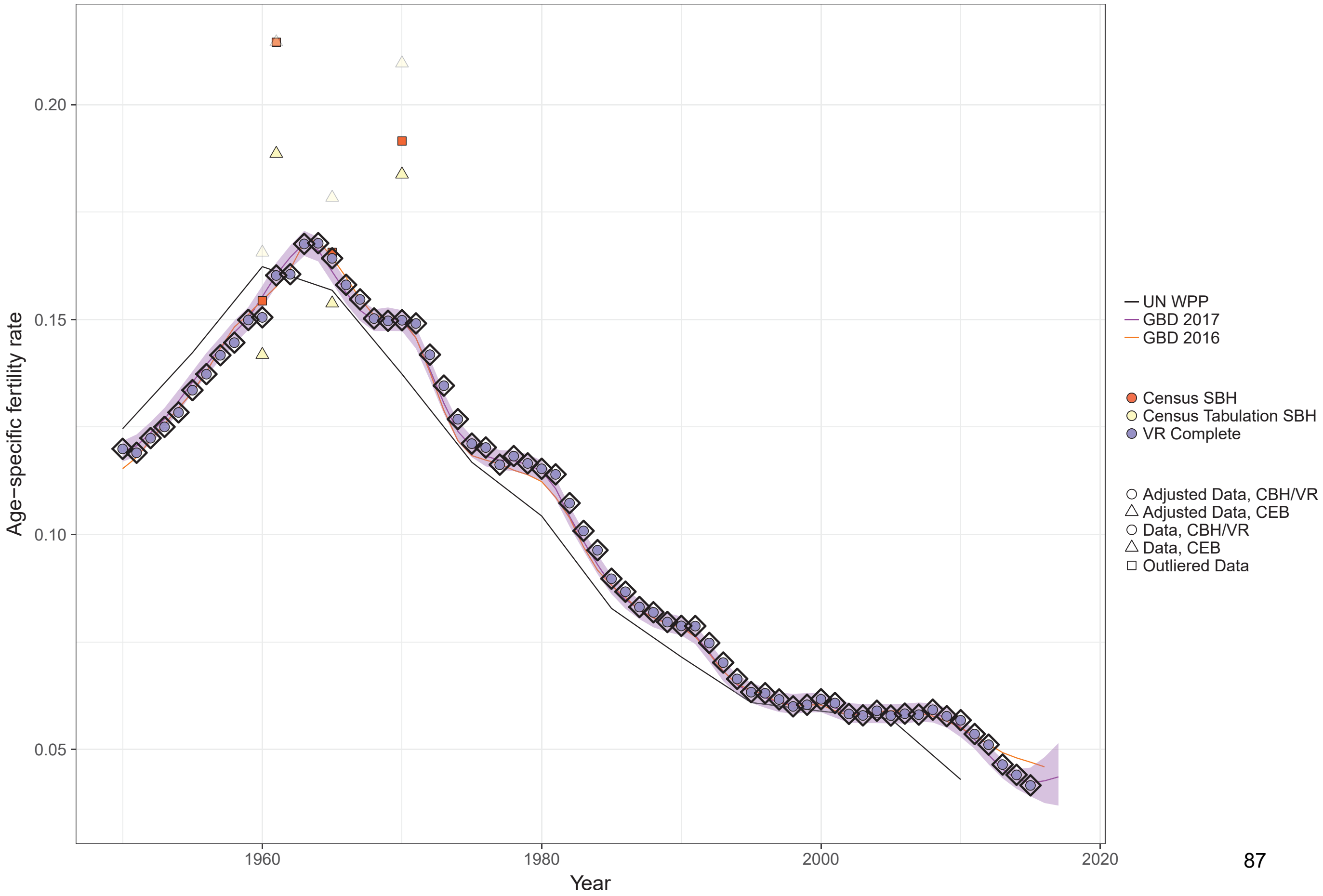

Age−specific fertility fitted trend lines, 1950−2017: Belgium, Ages: 25−29
Data points enclosed with a diamond are reference sources

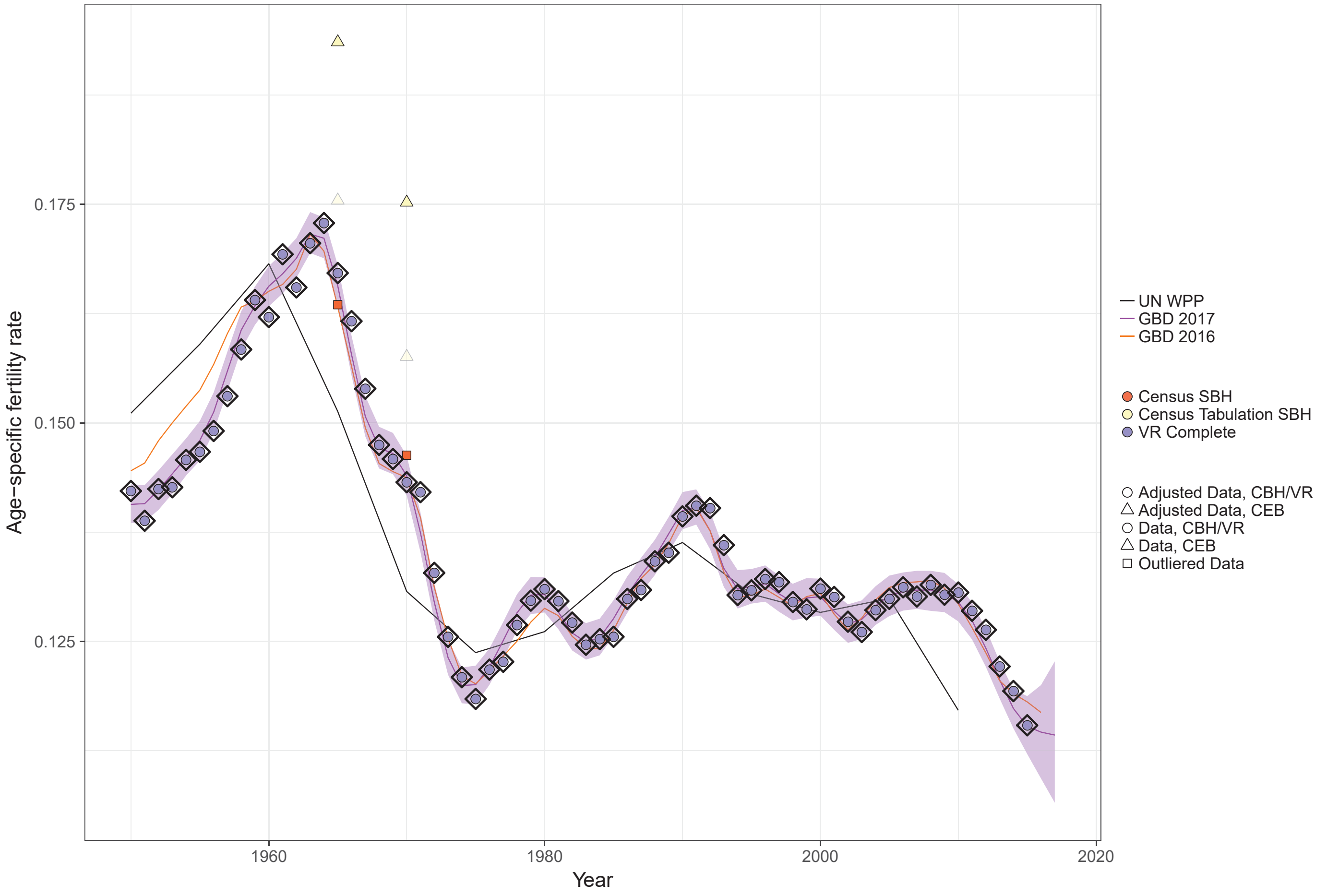

Age−specific fertility fitted trend lines, 1950−2017: Belgium, Ages: 30−34
Data points enclosed with a diamond are reference sources

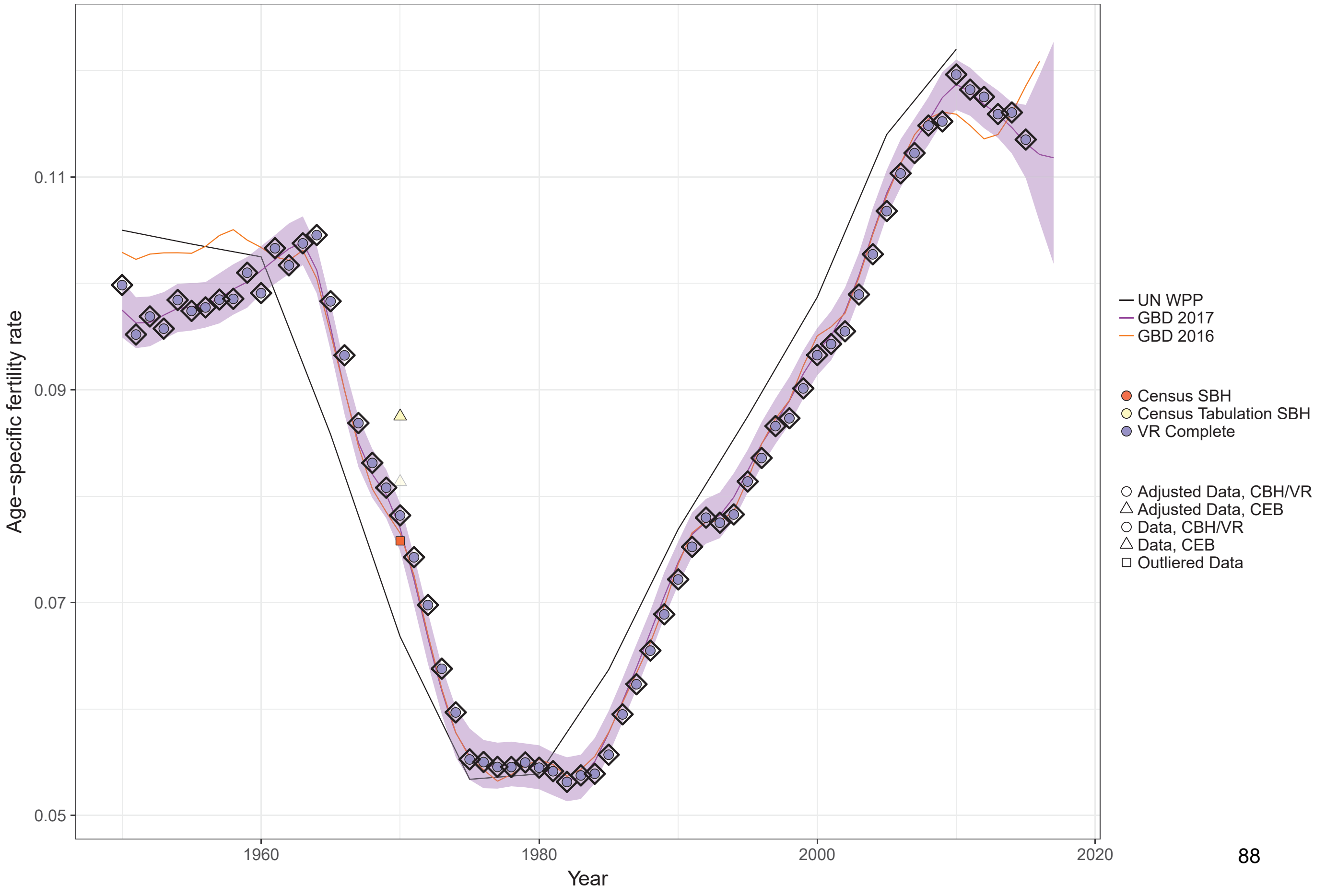

Age−specific fertility fitted trend lines, 1950−2017: Belgium, Ages: 35−39
Data points enclosed with a diamond are reference sources

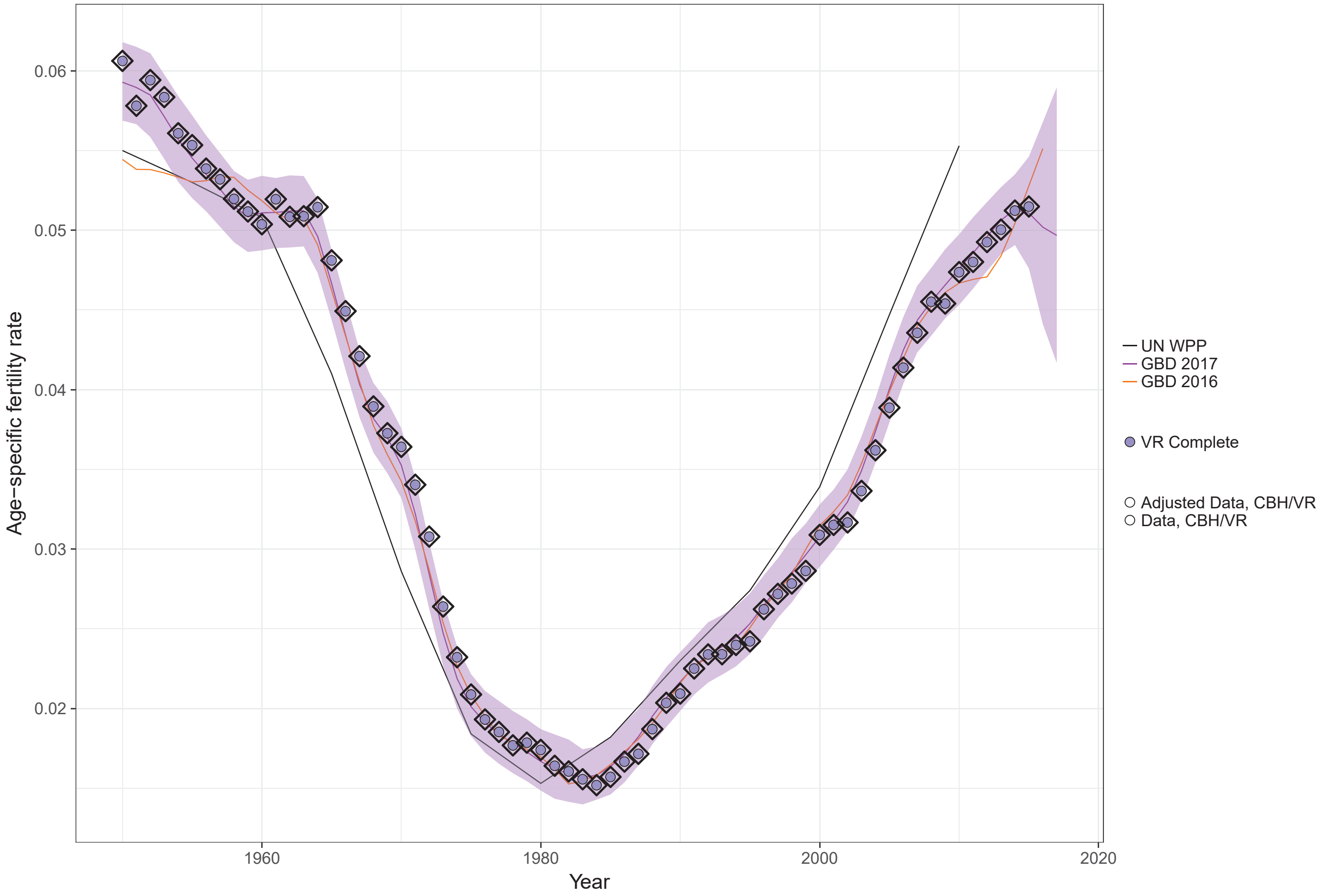

Age−specific fertility fitted trend lines, 1950−2017: Belgium, Ages: 40−44
Data points enclosed with a diamond are reference sources

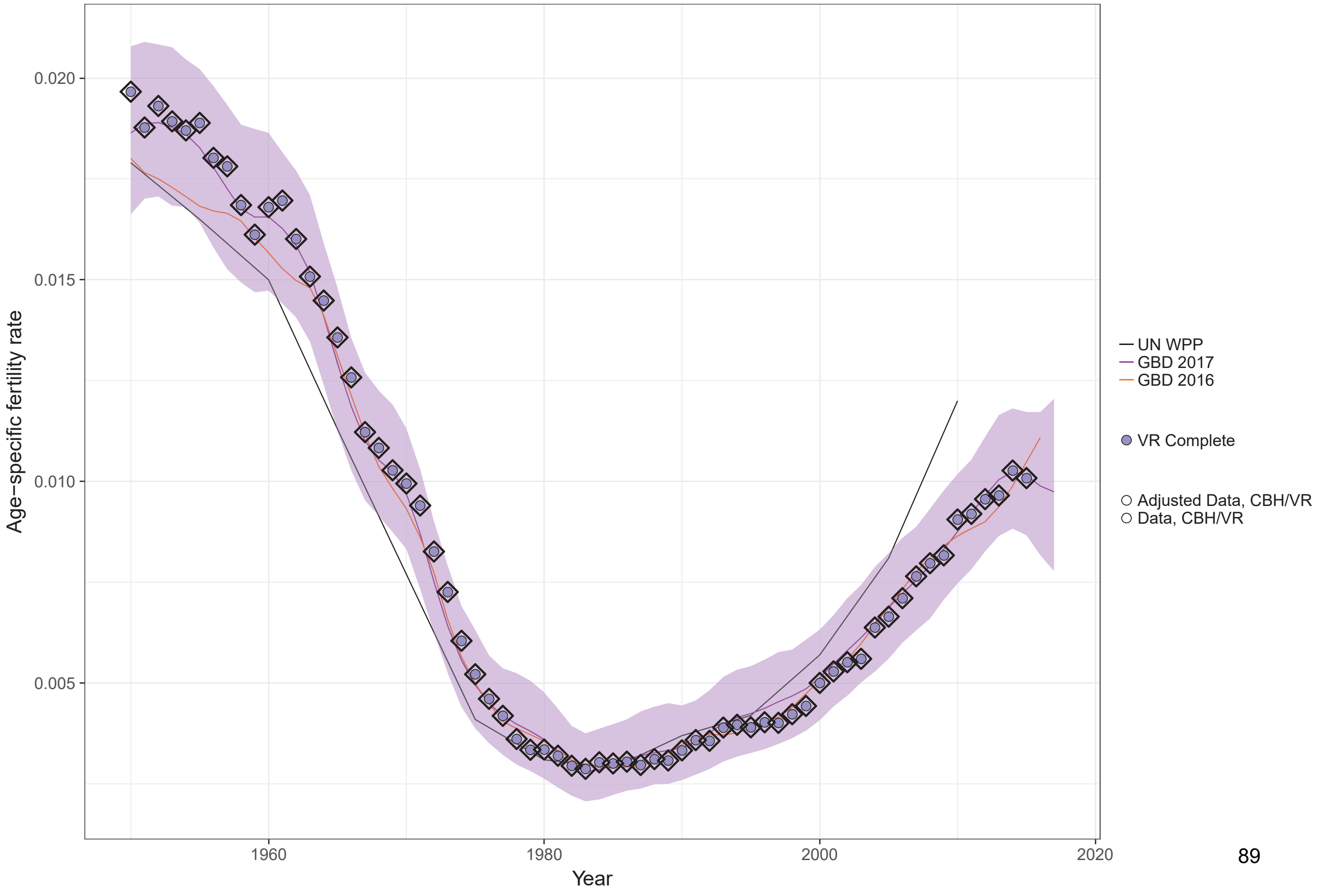

Age−specific fertility fitted trend lines, 1950−2017: Belgium, Ages: 45−49
Data points enclosed with a diamond are reference sources

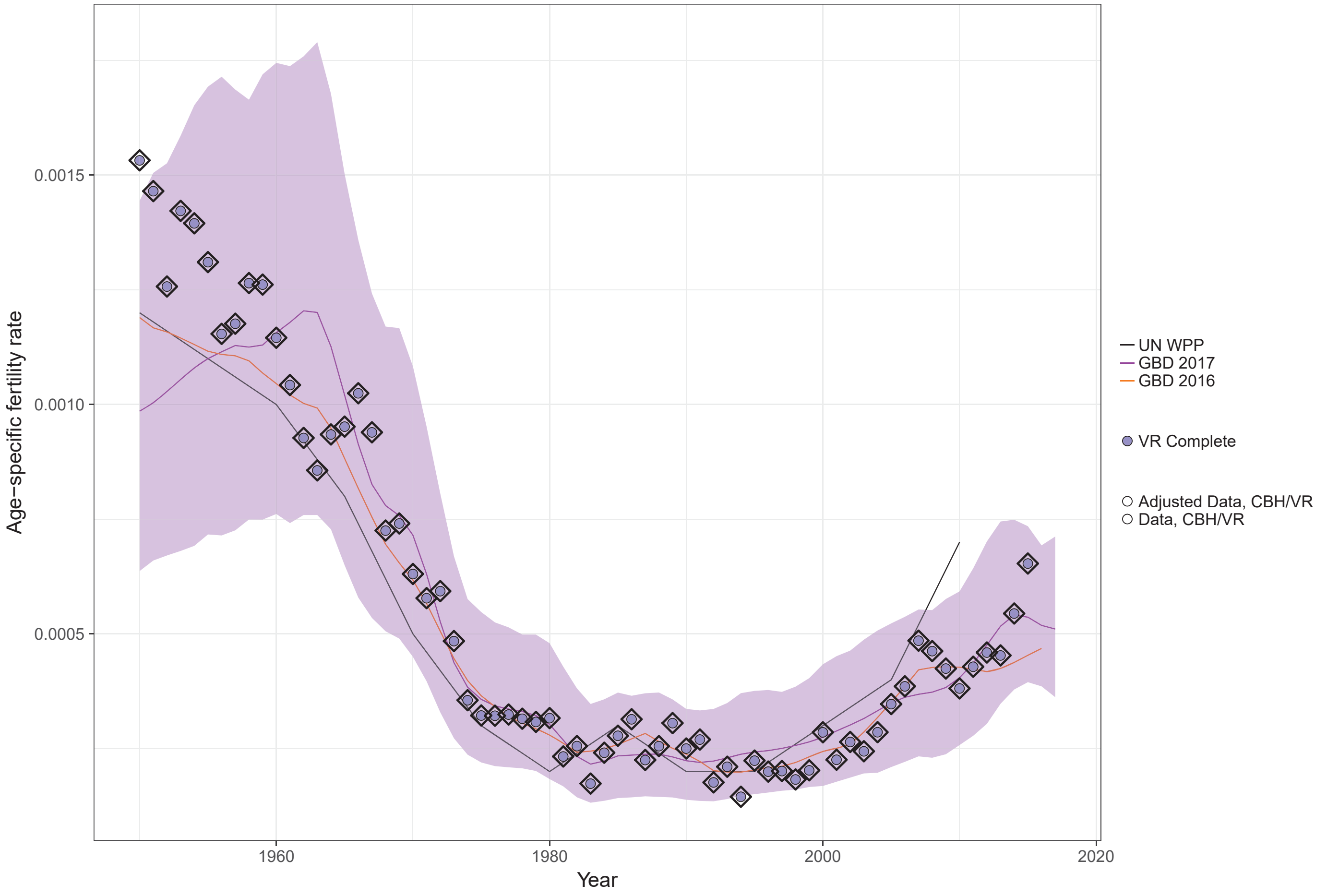

Age−specific fertility fitted trend lines, 1950−2017: Belgium, Ages: 50−54
Data points enclosed with a diamond are reference sources

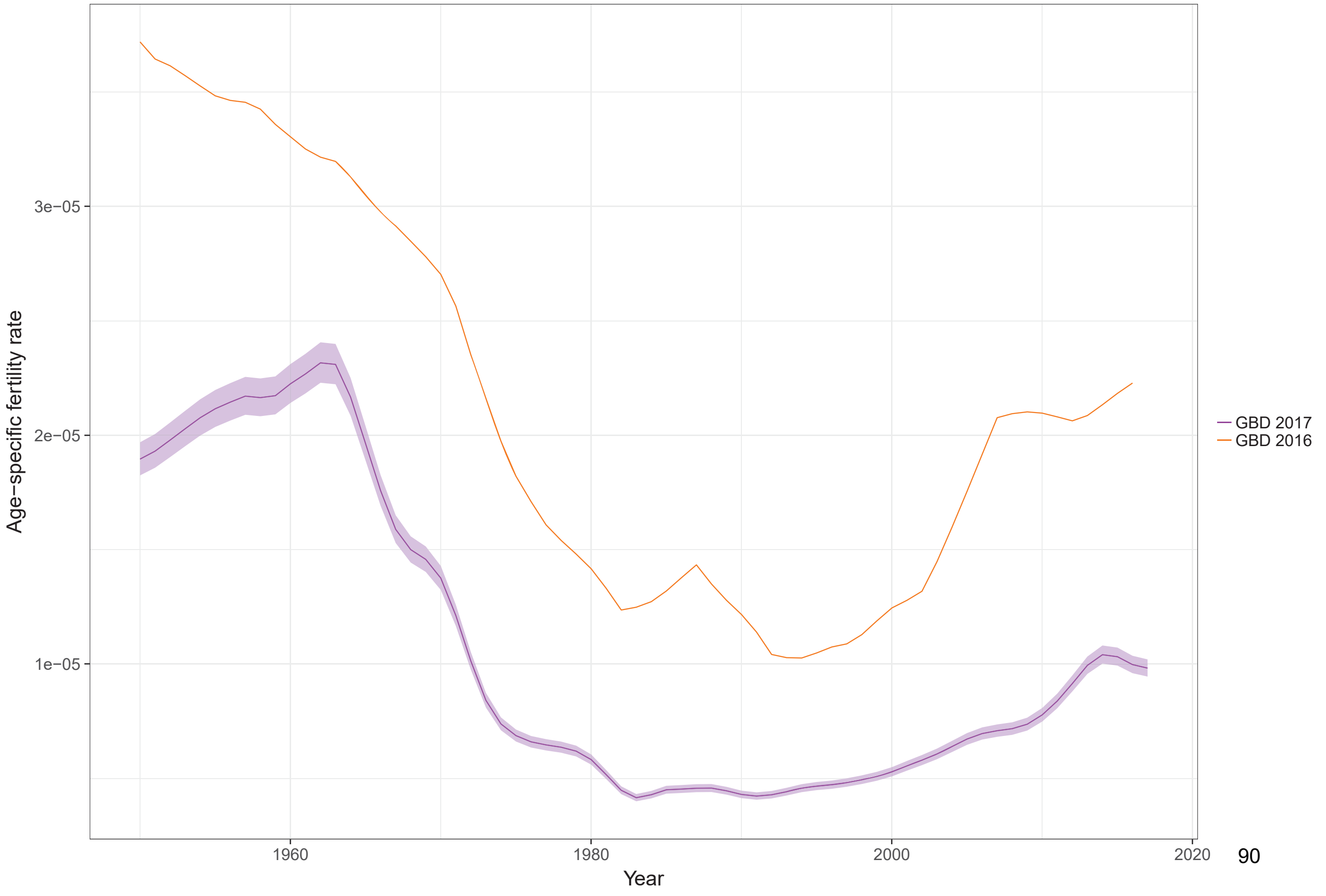

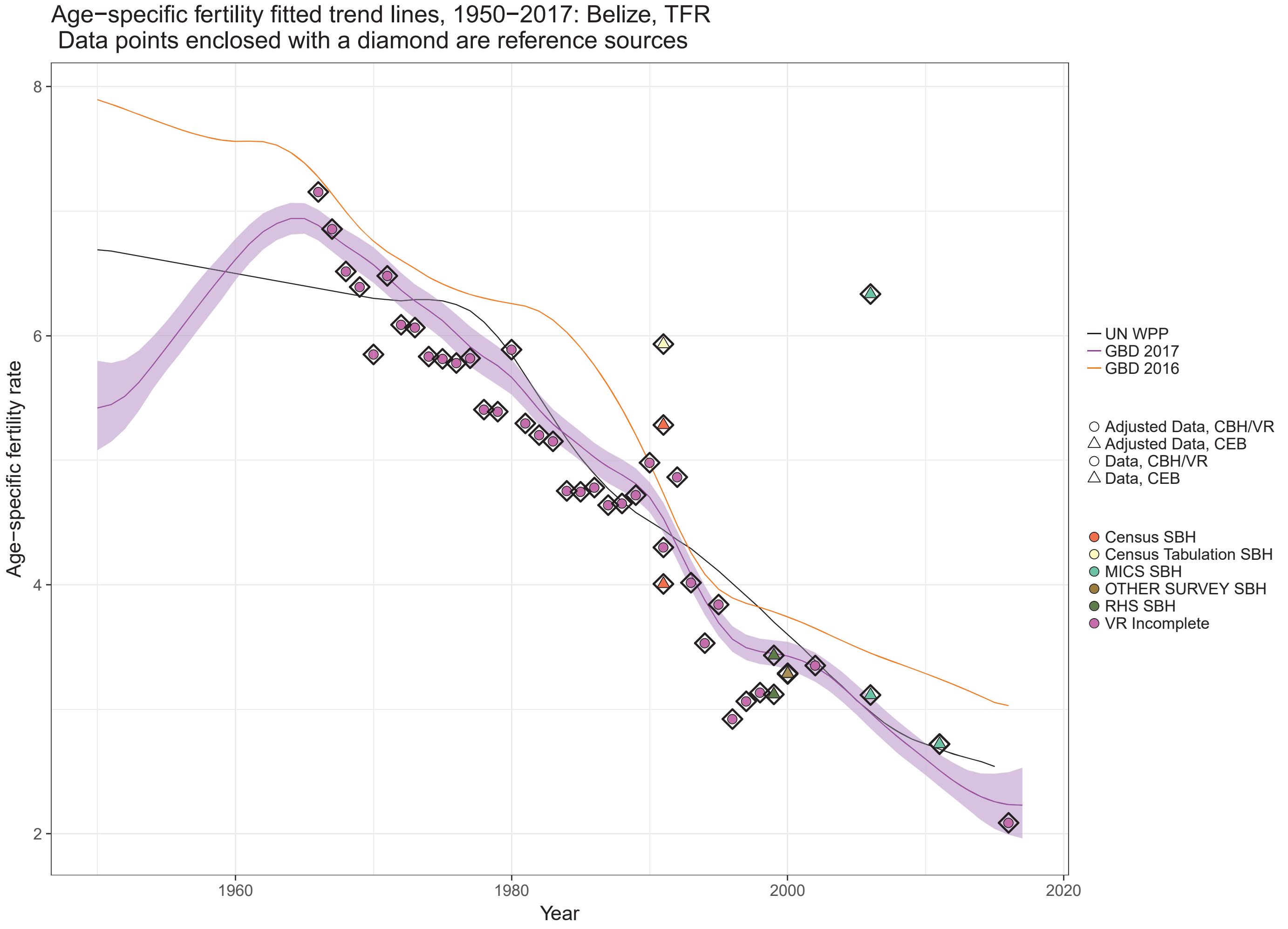
Age−specific fertility fitted trend lines, 1950−2017: Belize, TFR
Data points enclosed with a diamond are reference sources
Age−specific fertility rate
Year
UN WPP
GBD 2017
GBD 2016
Adjusted Data, CBH/VR
Adjusted Data, CEB
Data, CBH/VR
Data, CEB
Census SBH
Census Tabulation SBH
MICS SBH
OTHER SURVEY SBH
RHS SBH
VR Incomplete

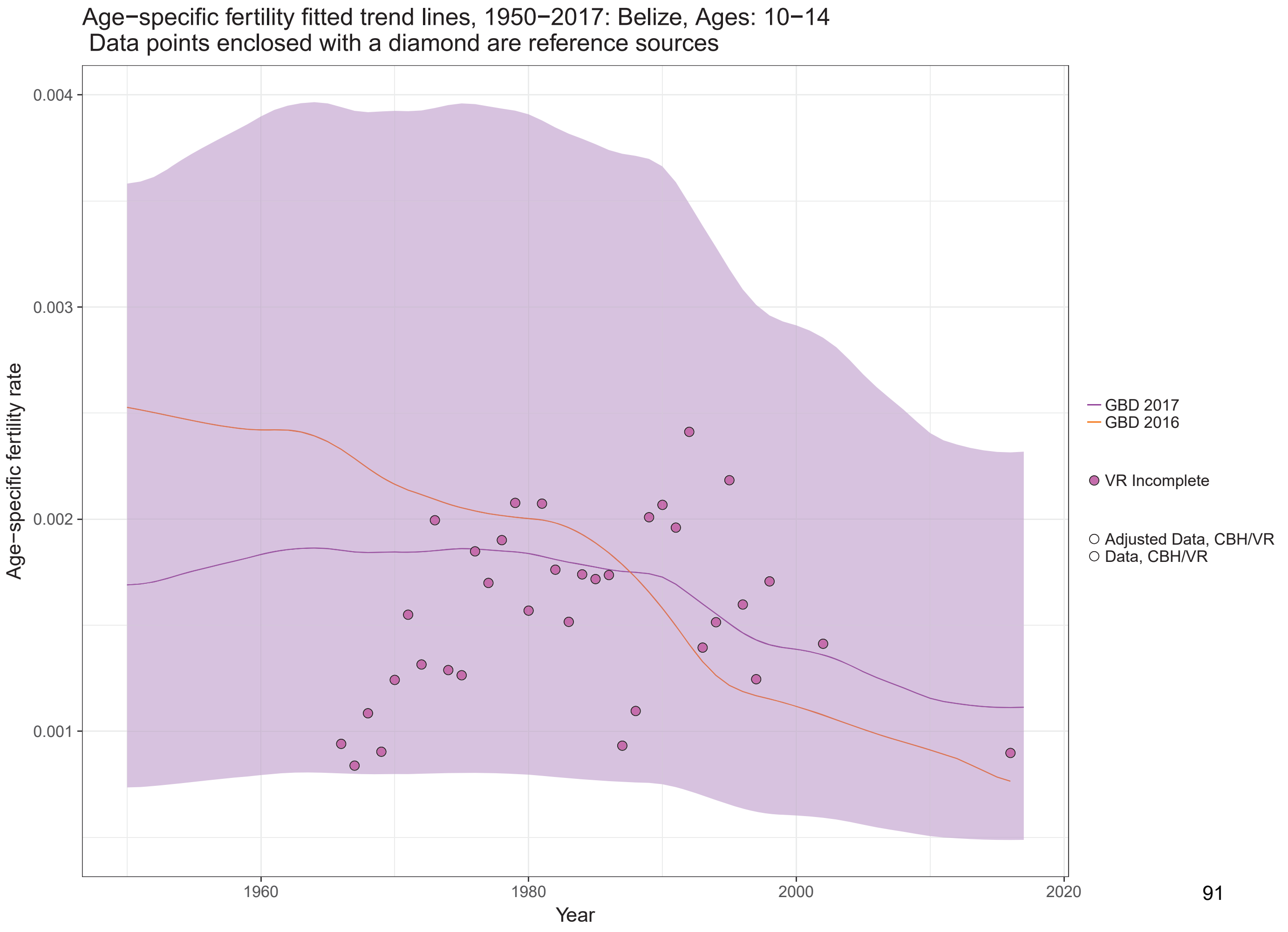
Age−specific fertility fitted trend lines, 1950−2017: Belize, Ages: 10−14
Data points enclosed with a diamond are reference sources
Age−specific fertility rate
Year
GBD 2017
GBD 2016
VR Incomplete
Adjusted Data, CBH/VR
Data, CBH/VR

Age−specific fertility fitted trend lines, 1950−2017: Belize, Ages: 15−19
Data points enclosed with a diamond are reference sources

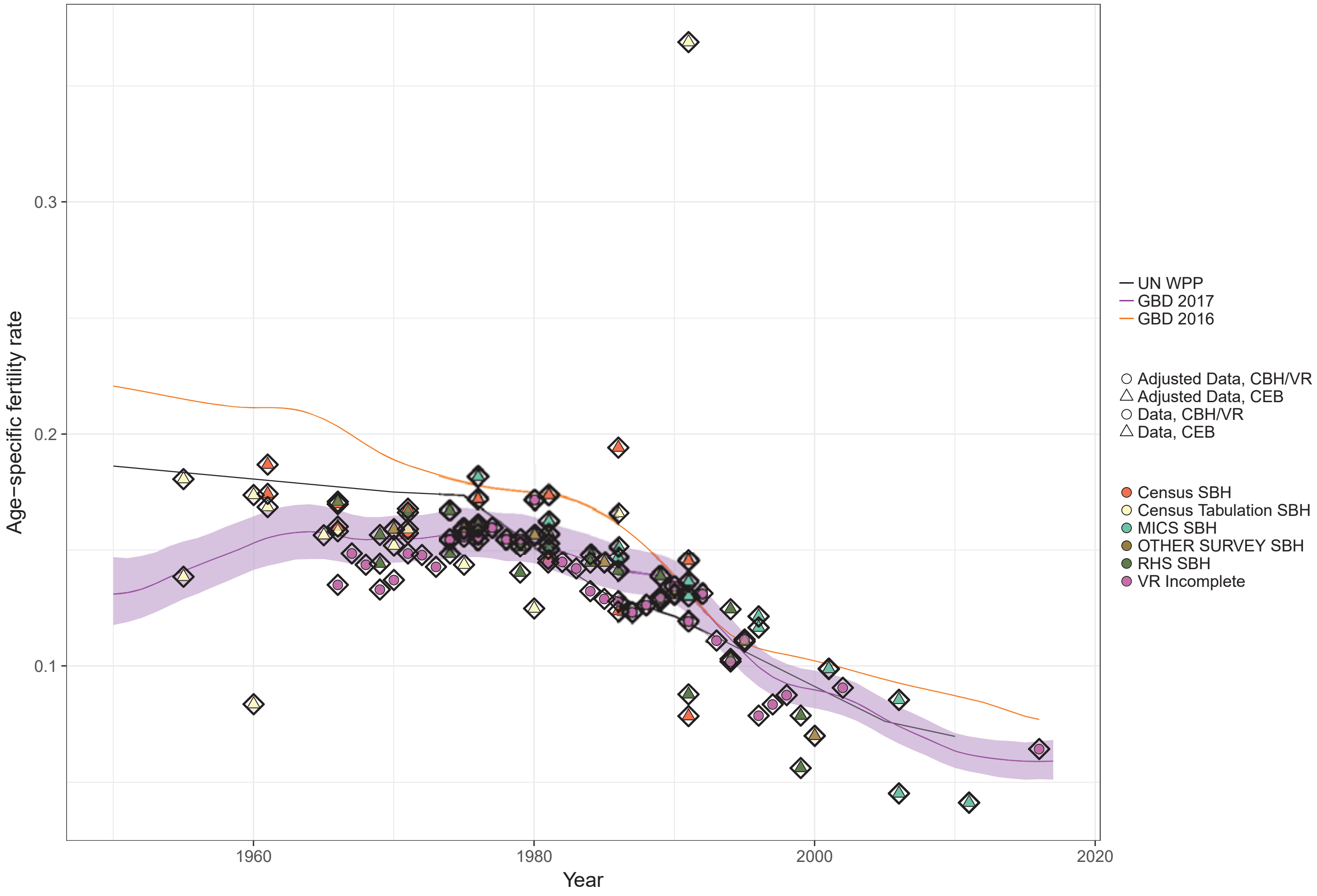

Age−specific fertility fitted trend lines, 1950−2017: Belize, Ages: 20−24
Data points enclosed with a diamond are reference sources

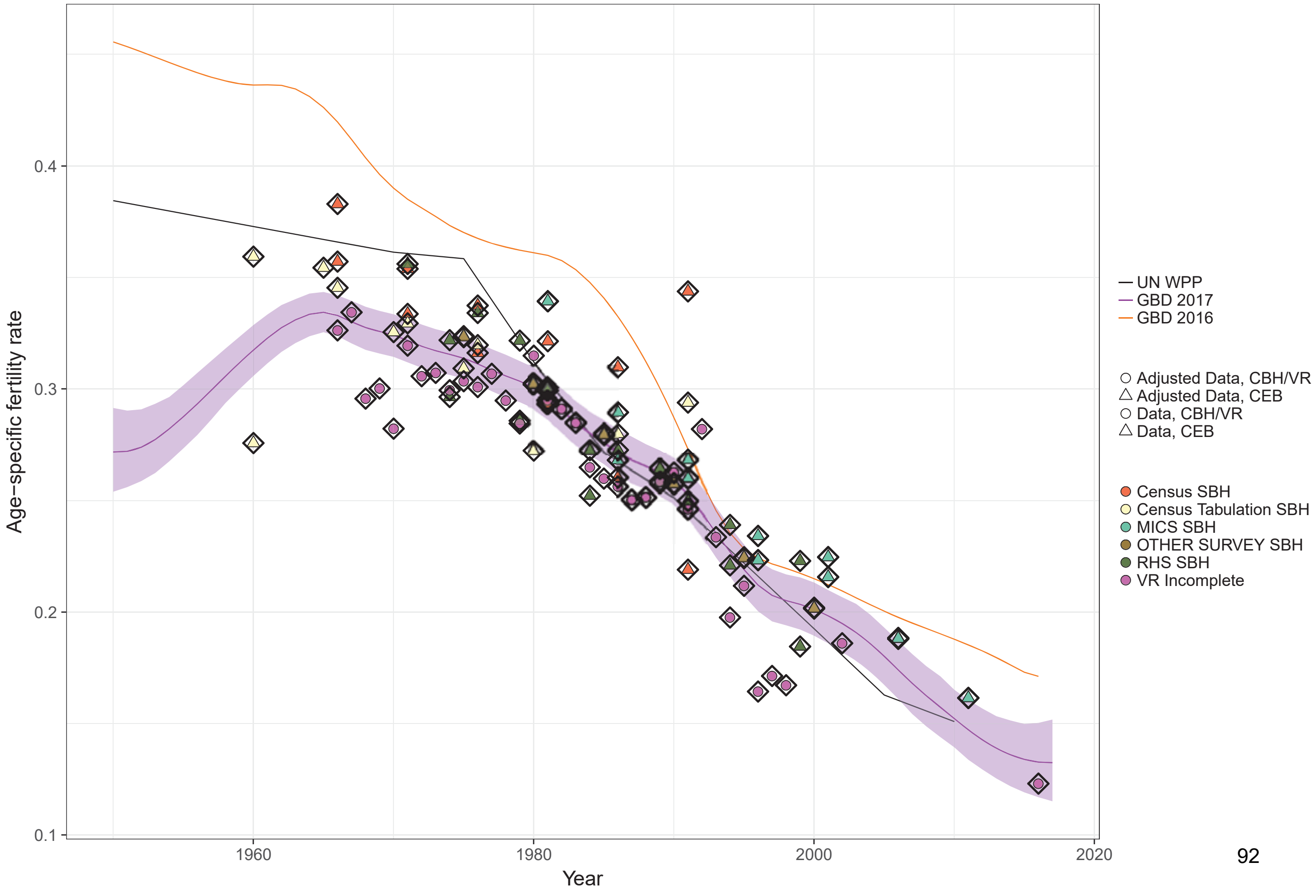

Age−specific fertility fitted trend lines, 1950−2017: Belize, Ages: 25−29
Data points enclosed with a diamond are reference sources

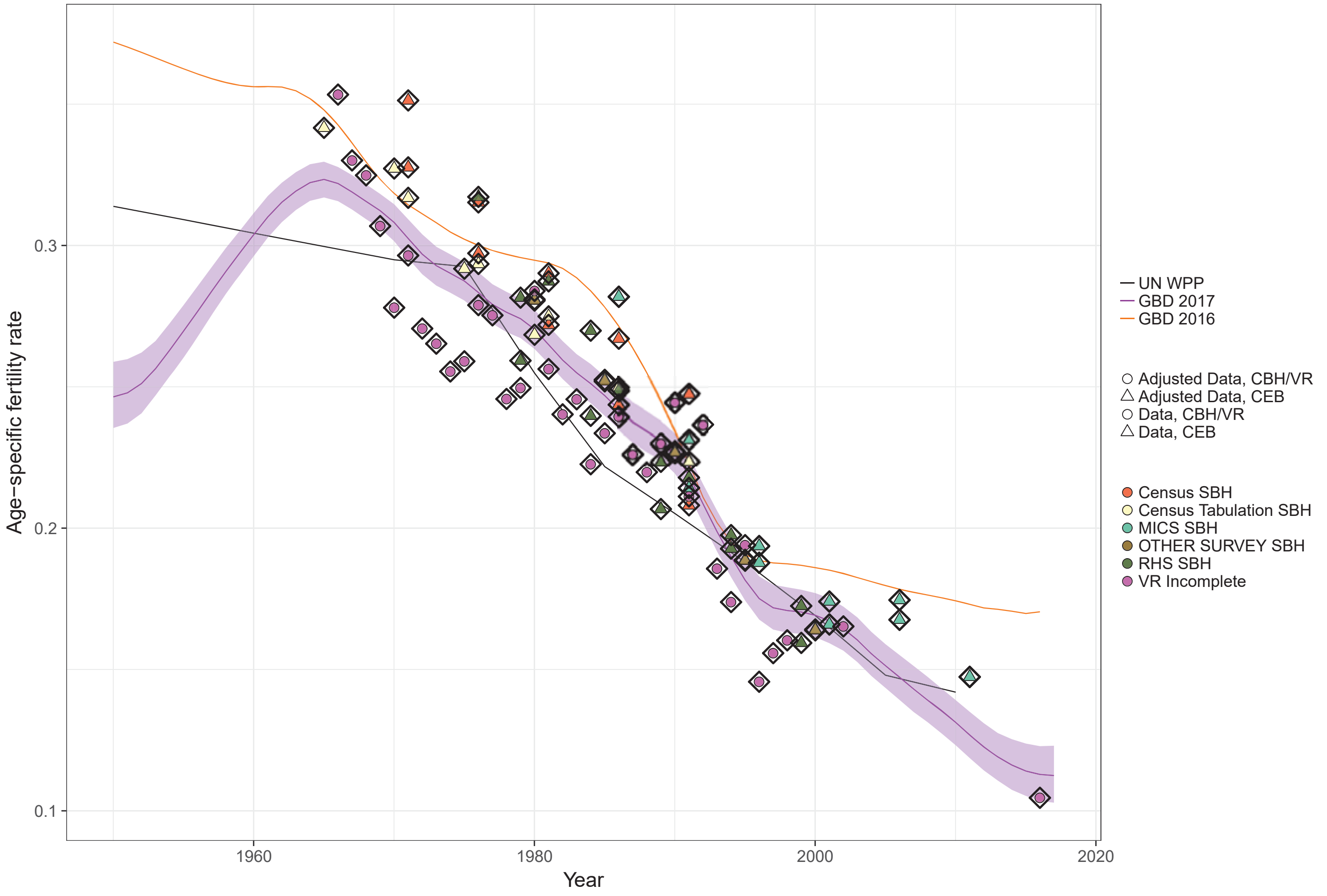

Age−specific fertility fitted trend lines, 1950−2017: Belize, Ages: 30−34
Data points enclosed with a diamond are reference sources

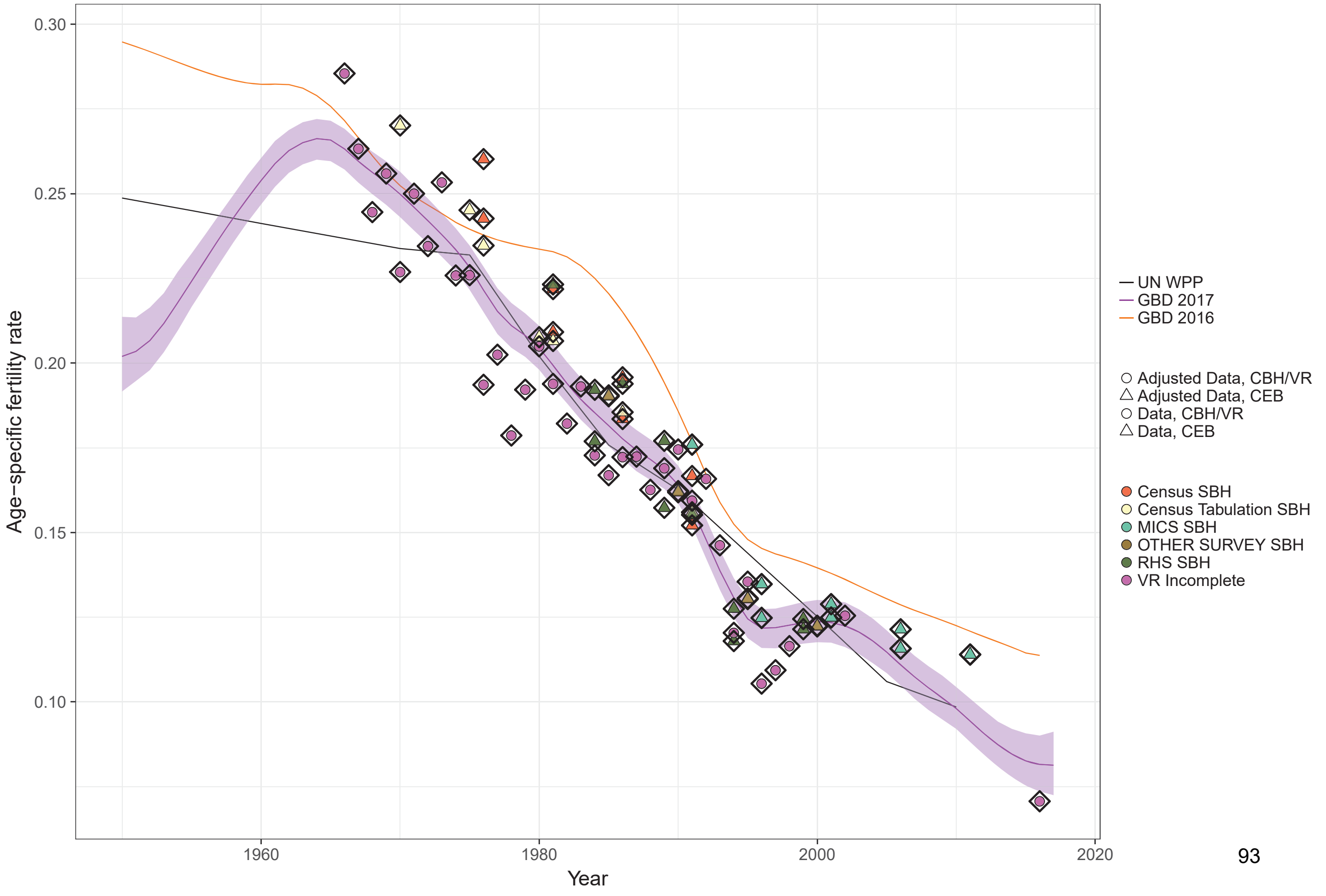

Age−specific fertility fitted trend lines, 1950−2017: Belize, Ages: 35−39
Data points enclosed with a diamond are reference sources

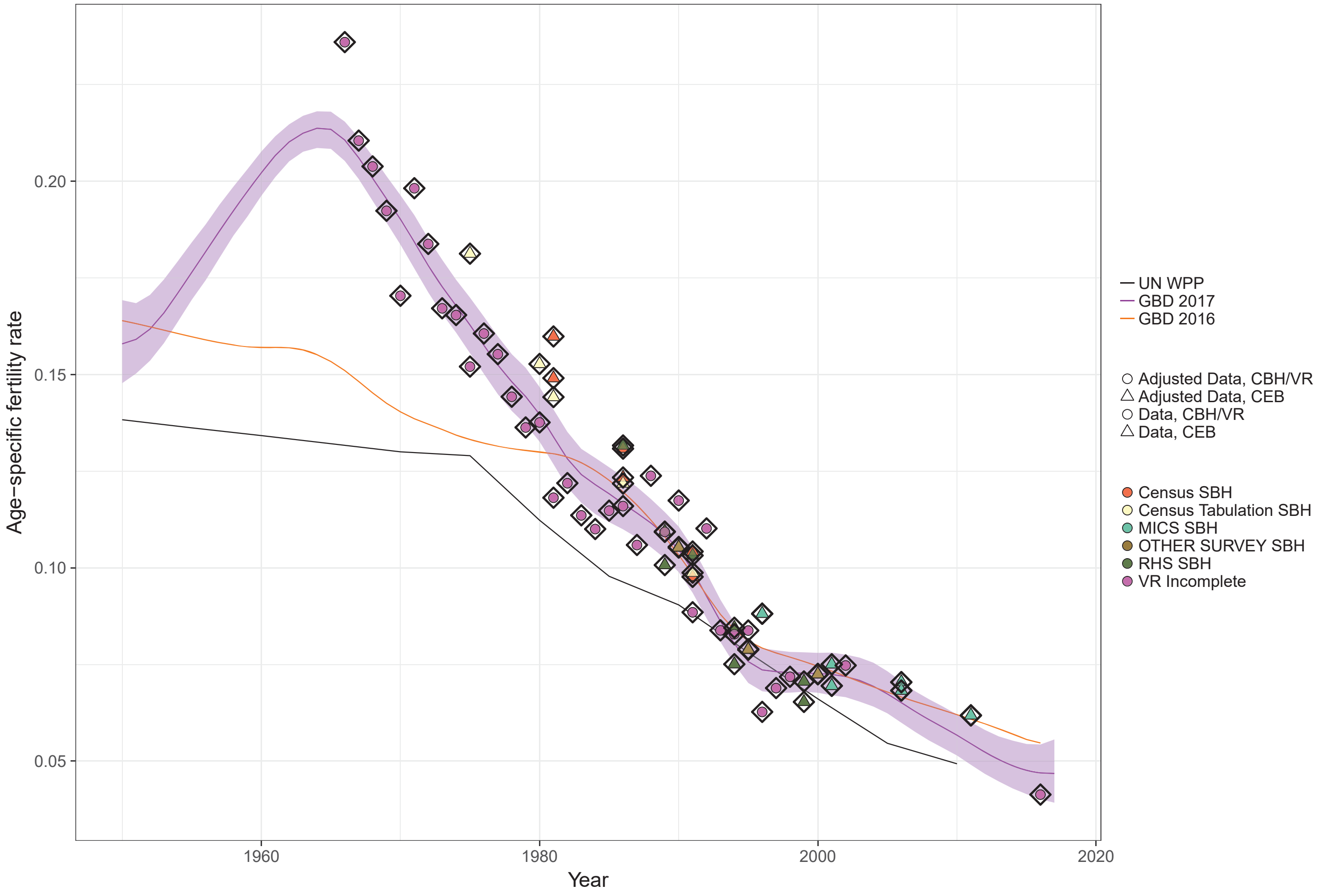

Age−specific fertility fitted trend lines, 1950−2017: Belize, Ages: 40−44
Data points enclosed with a diamond are reference sources

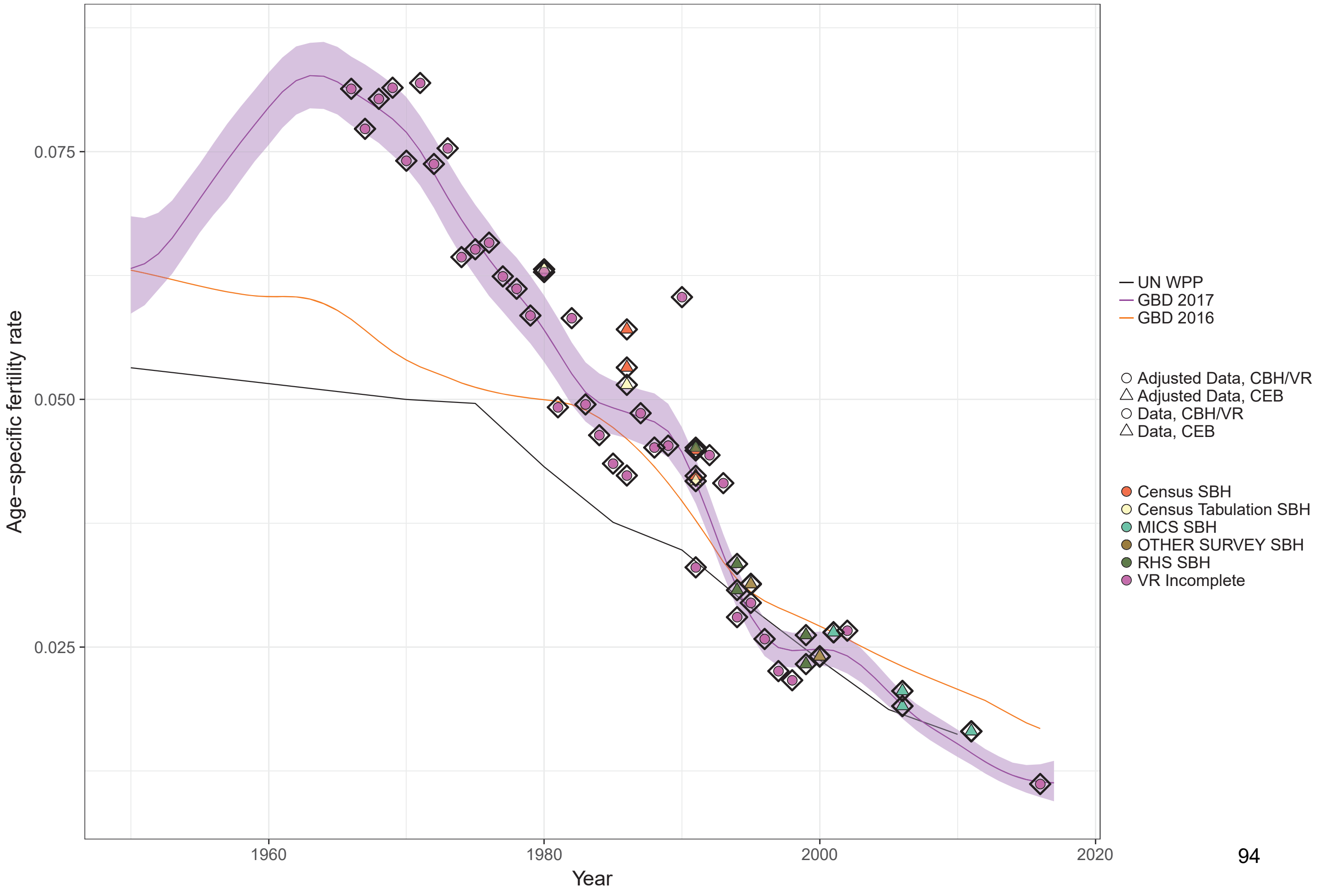

Age−specific fertility fitted trend lines, 1950−2017: Belize, Ages: 45−49
Data points enclosed with a diamond are reference sources

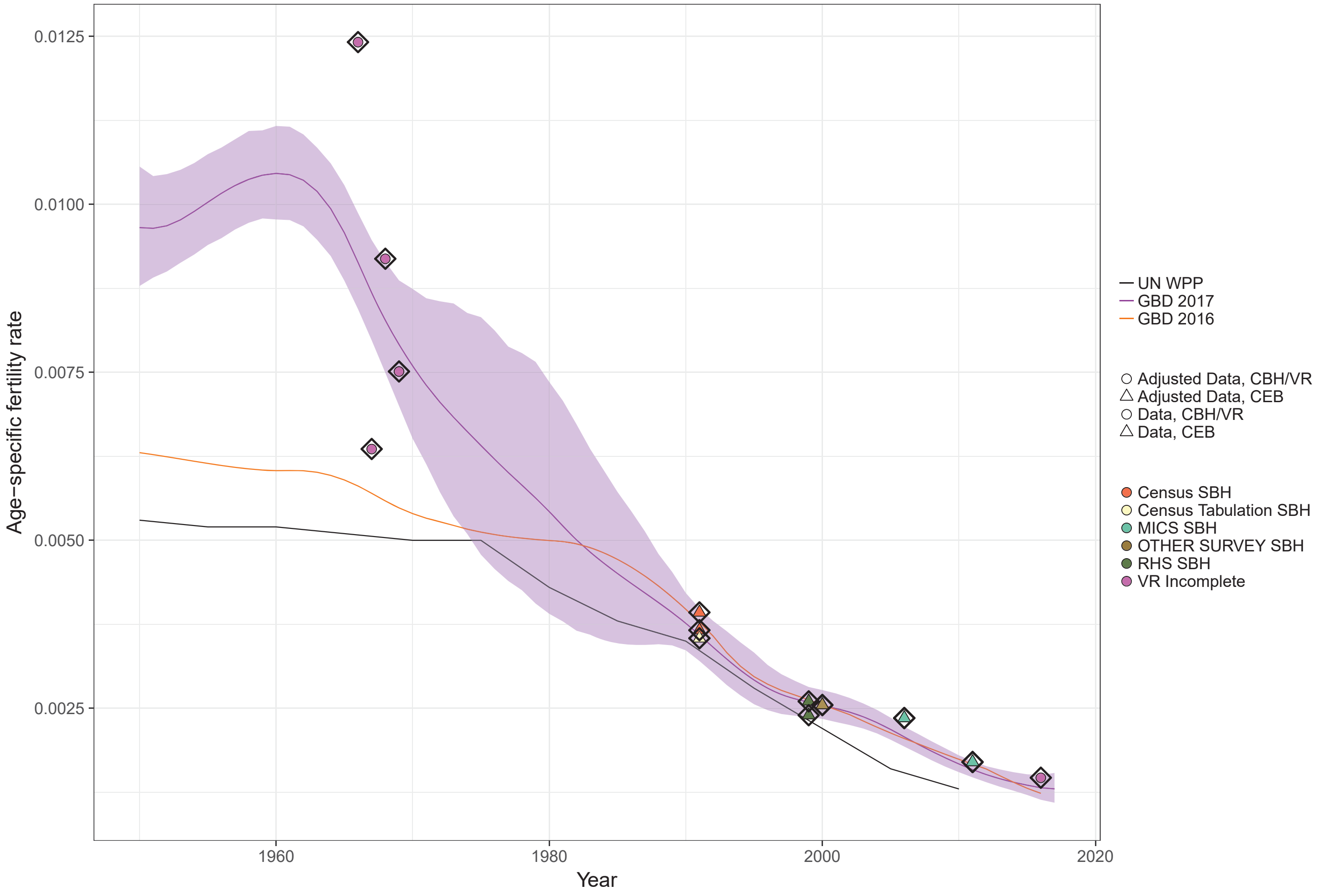

Age−specific fertility fitted trend lines, 1950−2017: Belize, Ages: 50−54
Data points enclosed with a diamond are reference sources

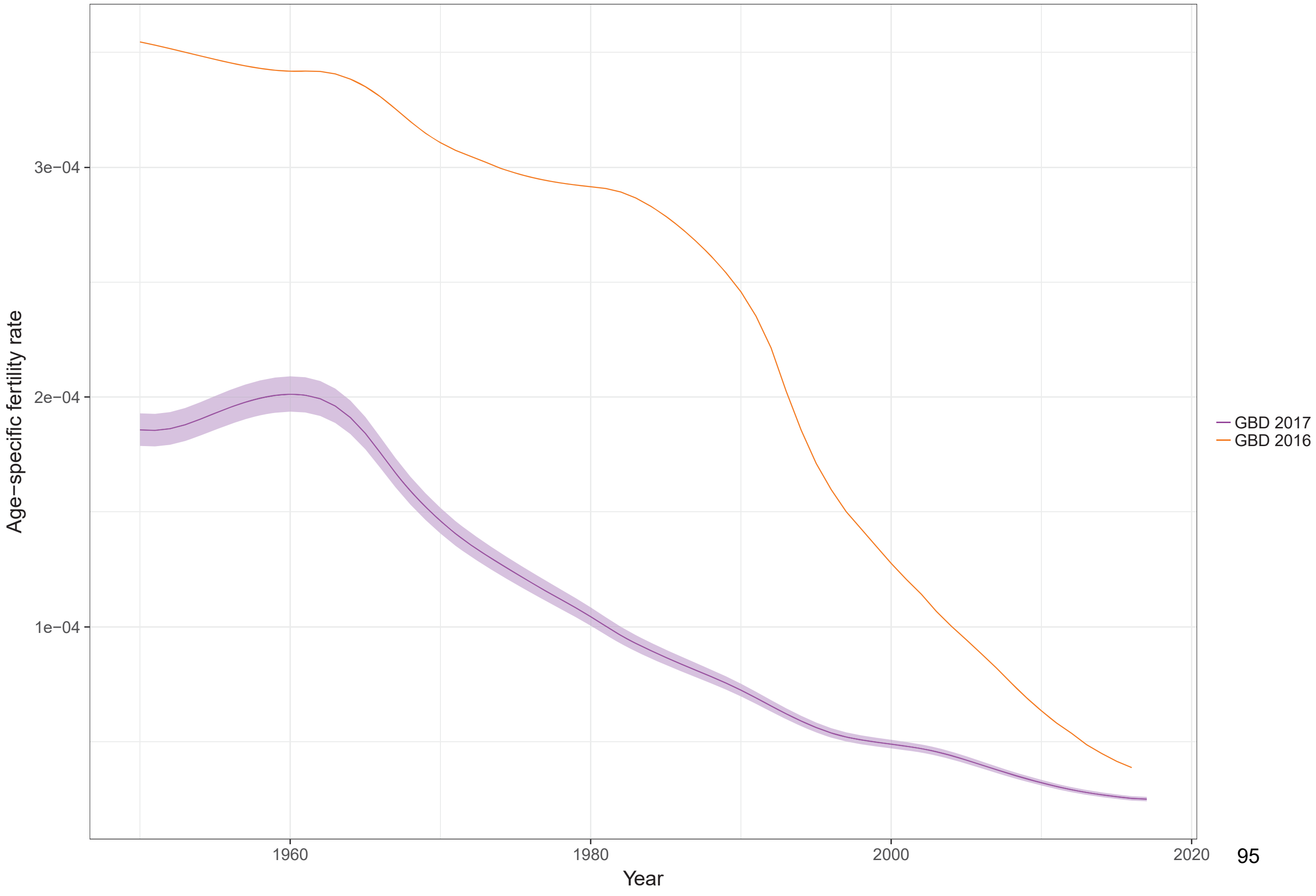

Age−specific fertility fitted trend lines, 1950−2017: Benin, TFR
Data points enclosed with a diamond are reference sources

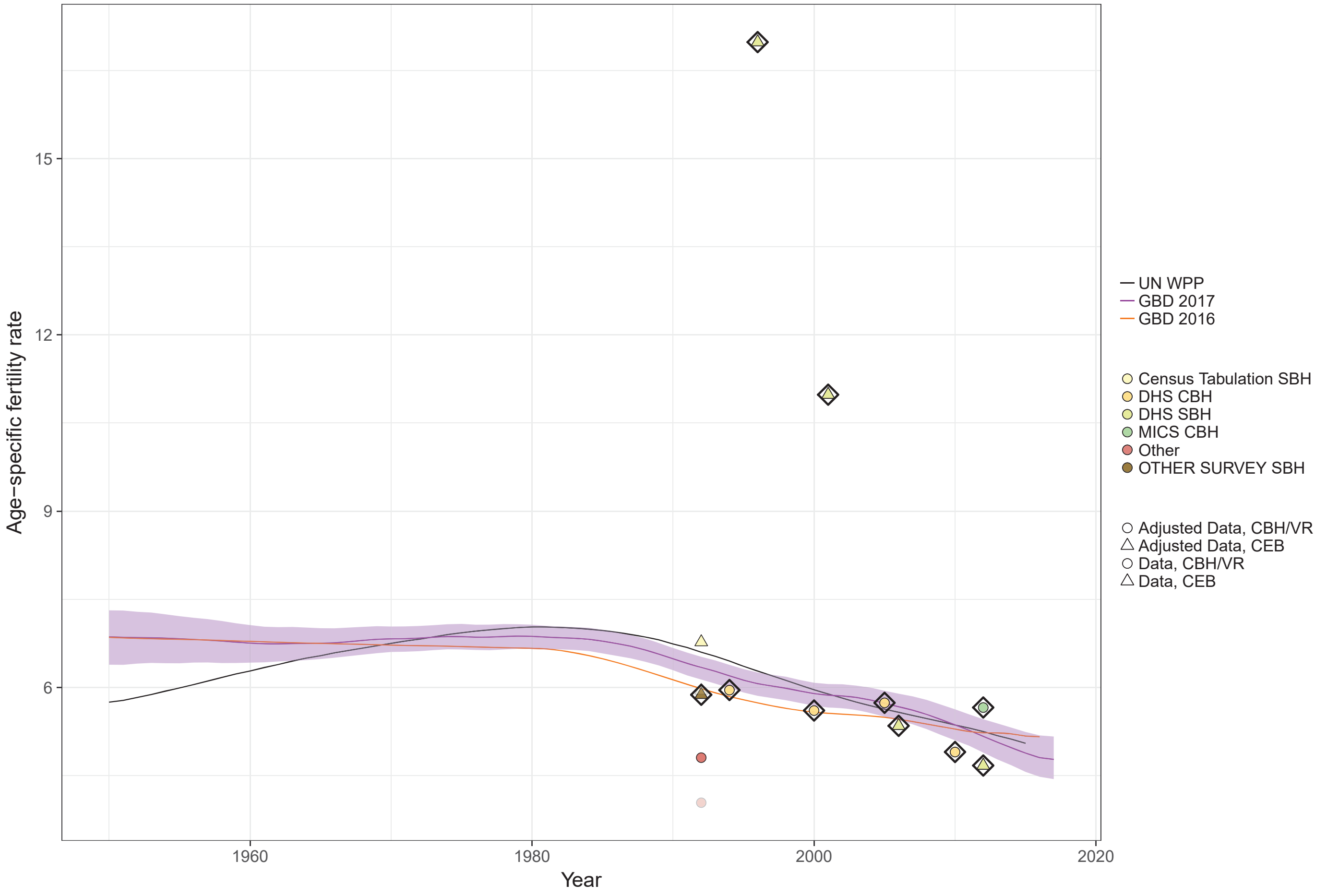

Age−specific fertility fitted trend lines, 1950−2017: Benin, Ages: 10−14
Data points enclosed with a diamond are reference sources

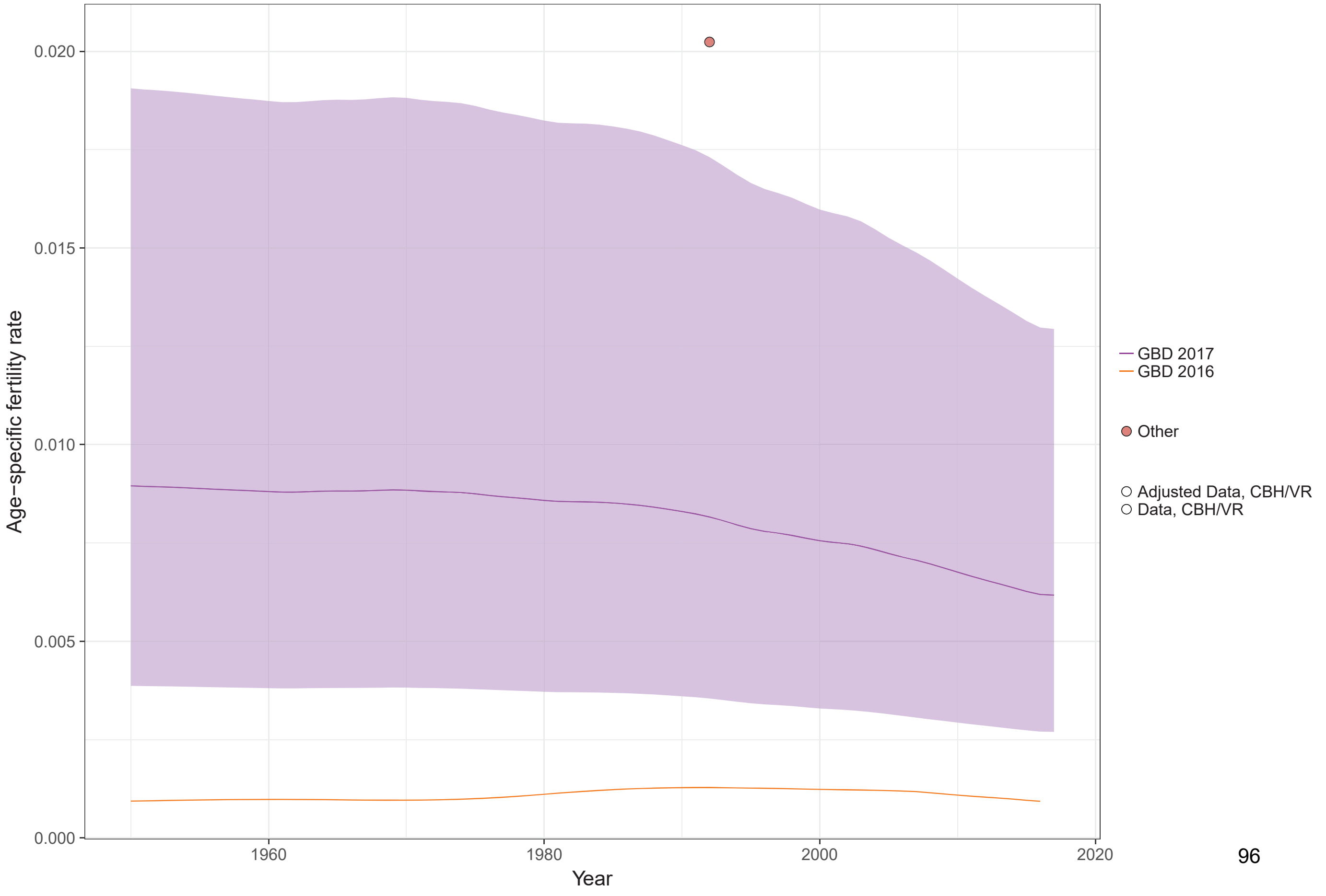

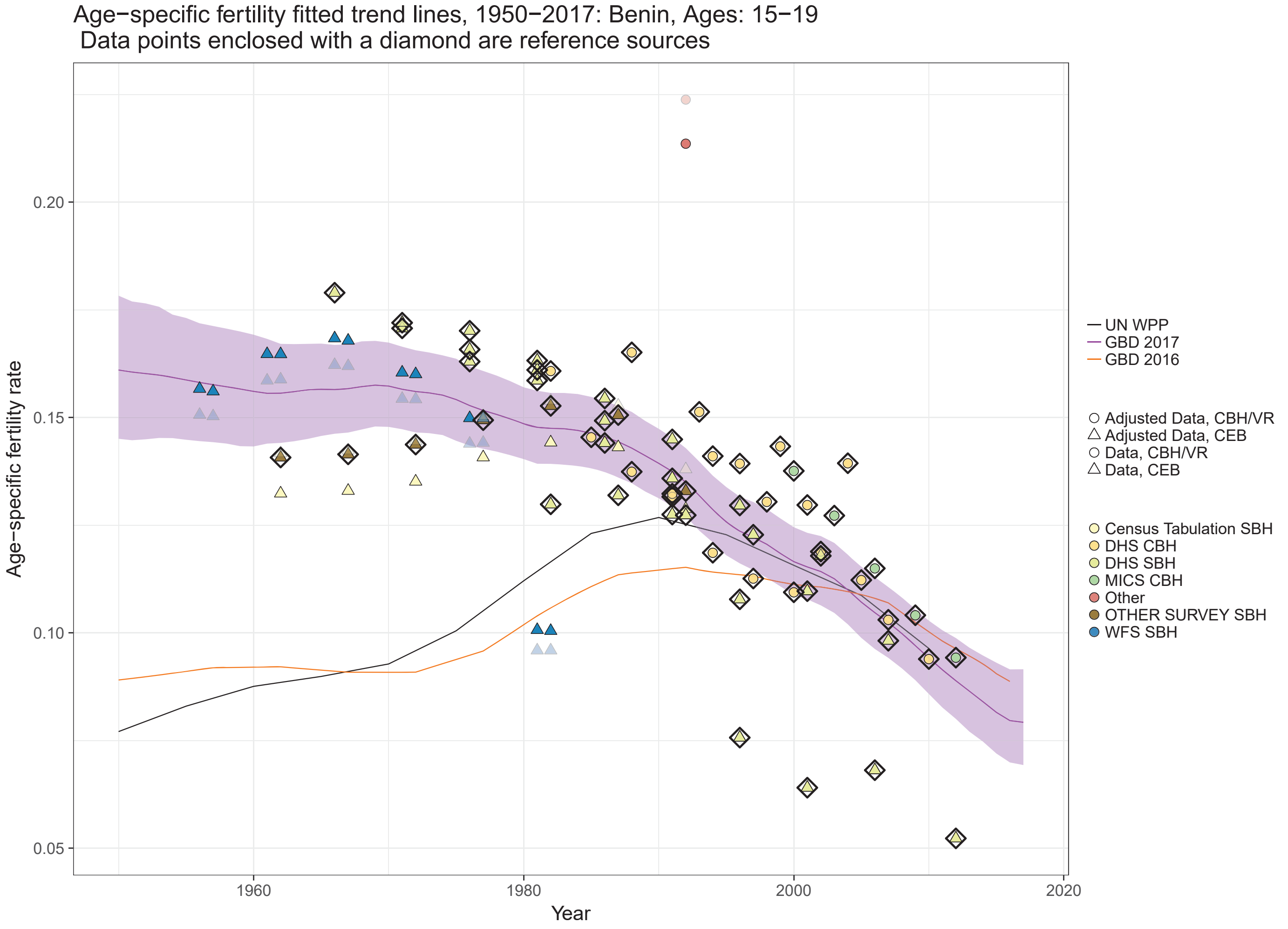
Age−specific fertility fitted trend lines, 1950−2017: Benin, Ages: 15−19
Data points enclosed with a diamond are reference sources
Age-specific fertility rate
Year
UN WPP
GBD 2017
GBD 2016
Adjusted Data, CBH/VR
Adjusted Data, CEB
Data, CBH/VR
Data, CEB
Census Tabulation SBH
DHS CBH
DHS SBH
MICS CBH
Other
OTHER SURVEY SBH
WFS SBH

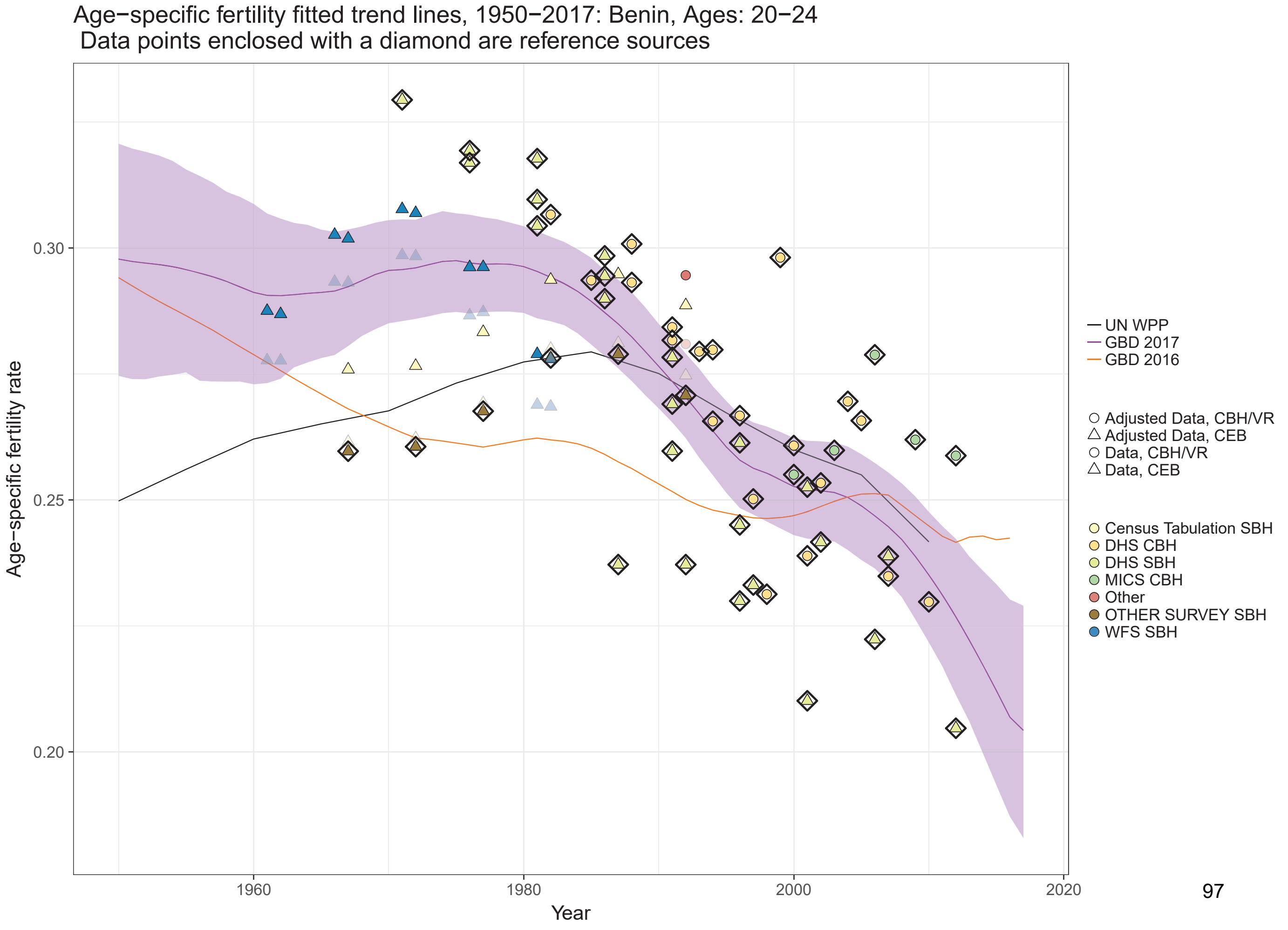
Age−specific fertility fitted trend lines, 1950−2017: Benin, Ages: 20−24
Data points enclosed with a diamond are reference sources
Age-specific fertility rate
Year
UN WPP
GBD 2017
GBD 2016
Adjusted Data, CBH/VR
Adjusted Data, CEB
Data, CBH/VR
Data, CEB
Census Tabulation SBH
DHS CBH
DHS SBH
MICS CBH
Other
OTHER SURVEY SBH
WFS SBH

Age−specific fertility fitted trend lines, 1950−2017: Benin, Ages: 25−29
Data points enclosed with a diamond are reference sources

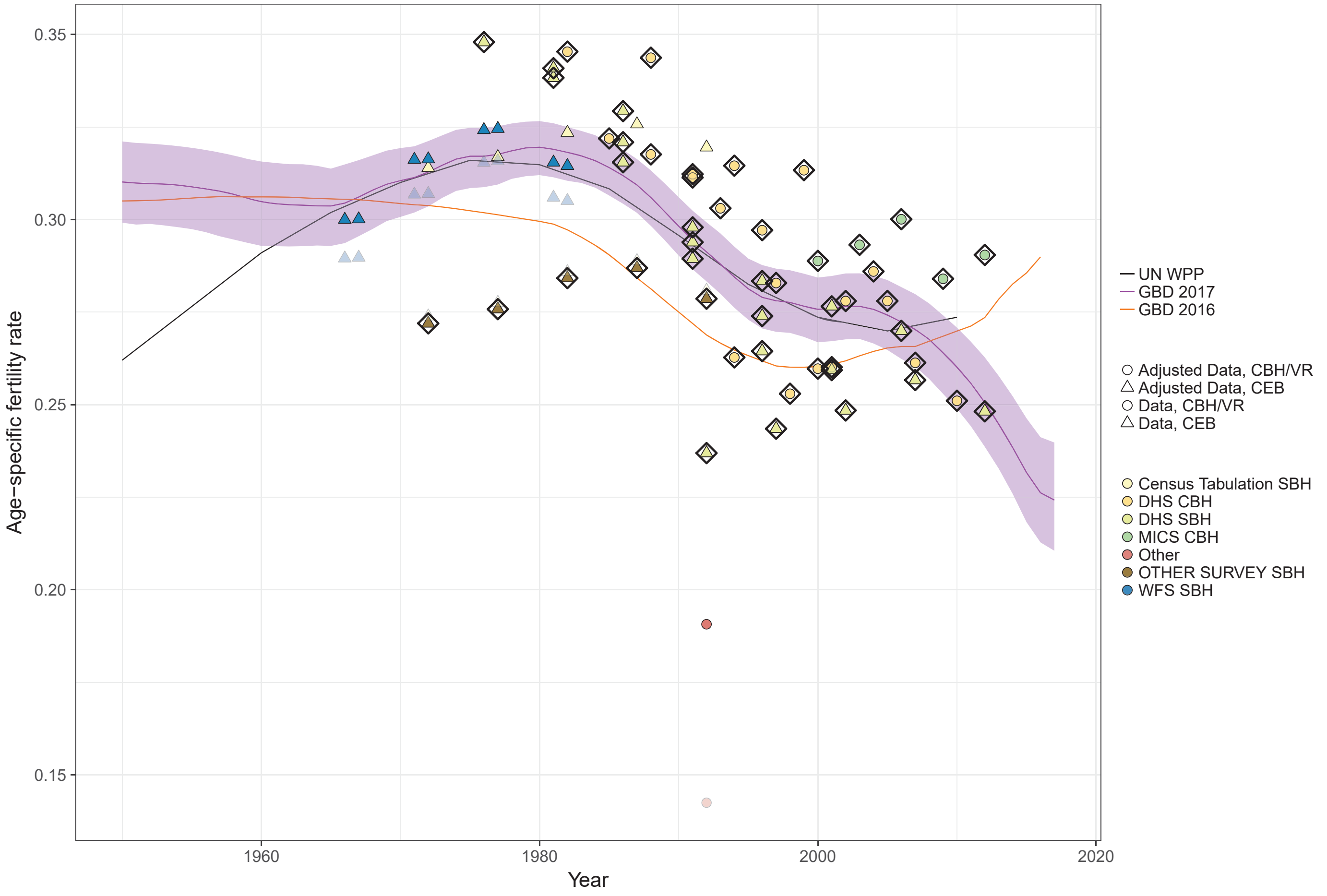

Age−specific fertility fitted trend lines, 1950−2017: Benin, Ages: 30−34
Data points enclosed with a diamond are reference sources

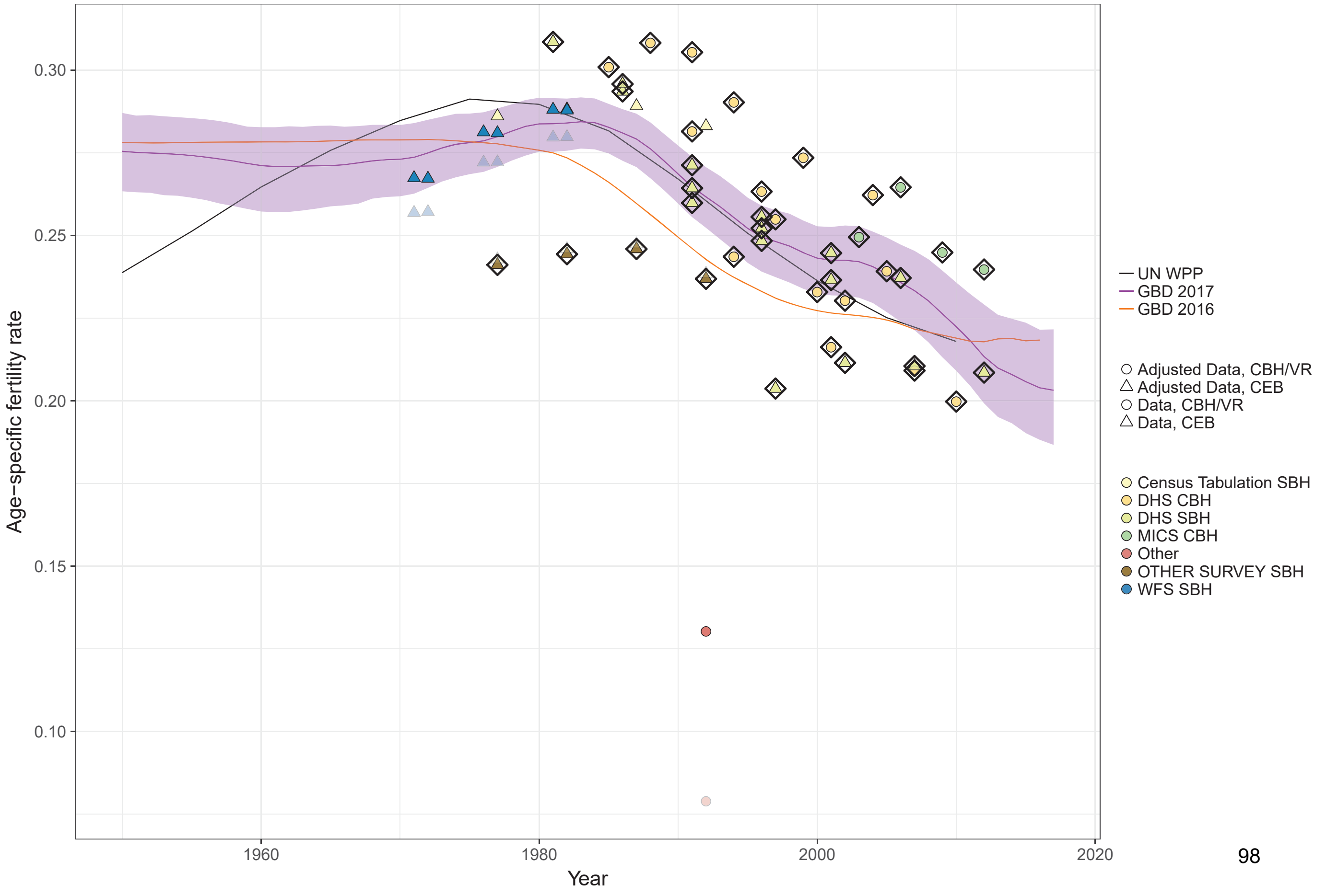

Age−specific fertility fitted trend lines, 1950−2017: Benin, Ages: 35−39
Data points enclosed with a diamond are reference sources

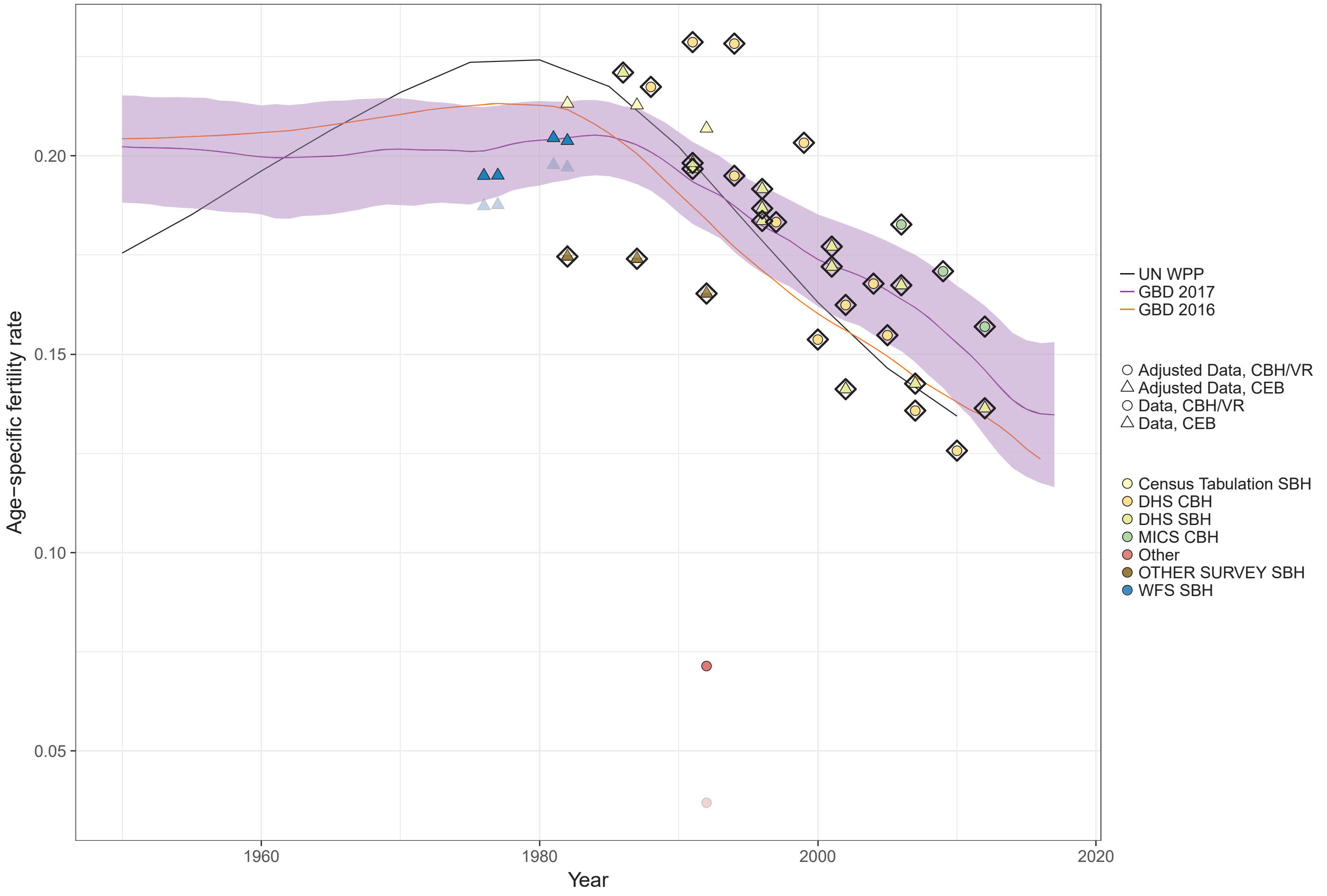

Age−specific fertility fitted trend lines, 1950−2017: Benin, Ages: 40−44
Data points enclosed with a diamond are reference sources

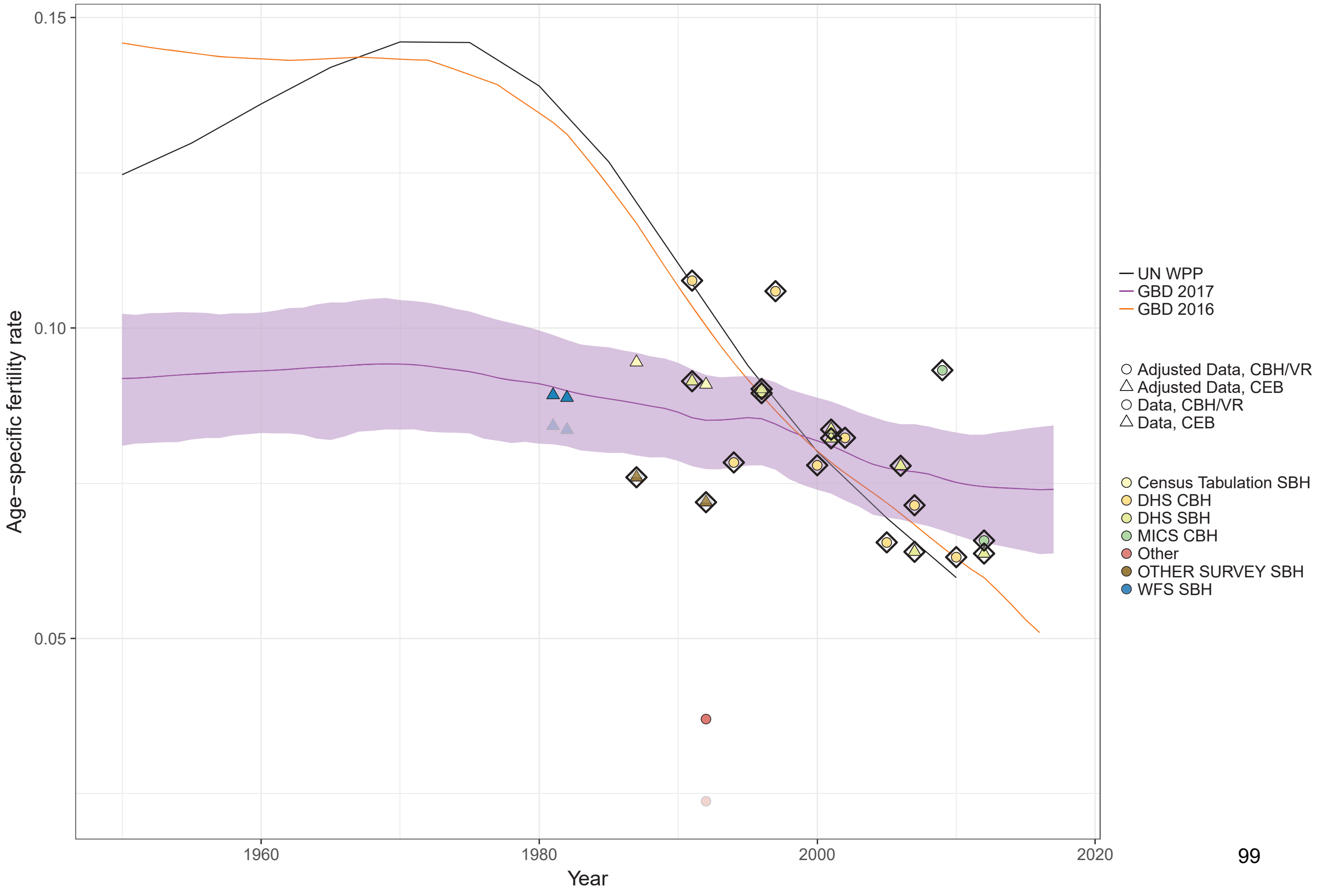

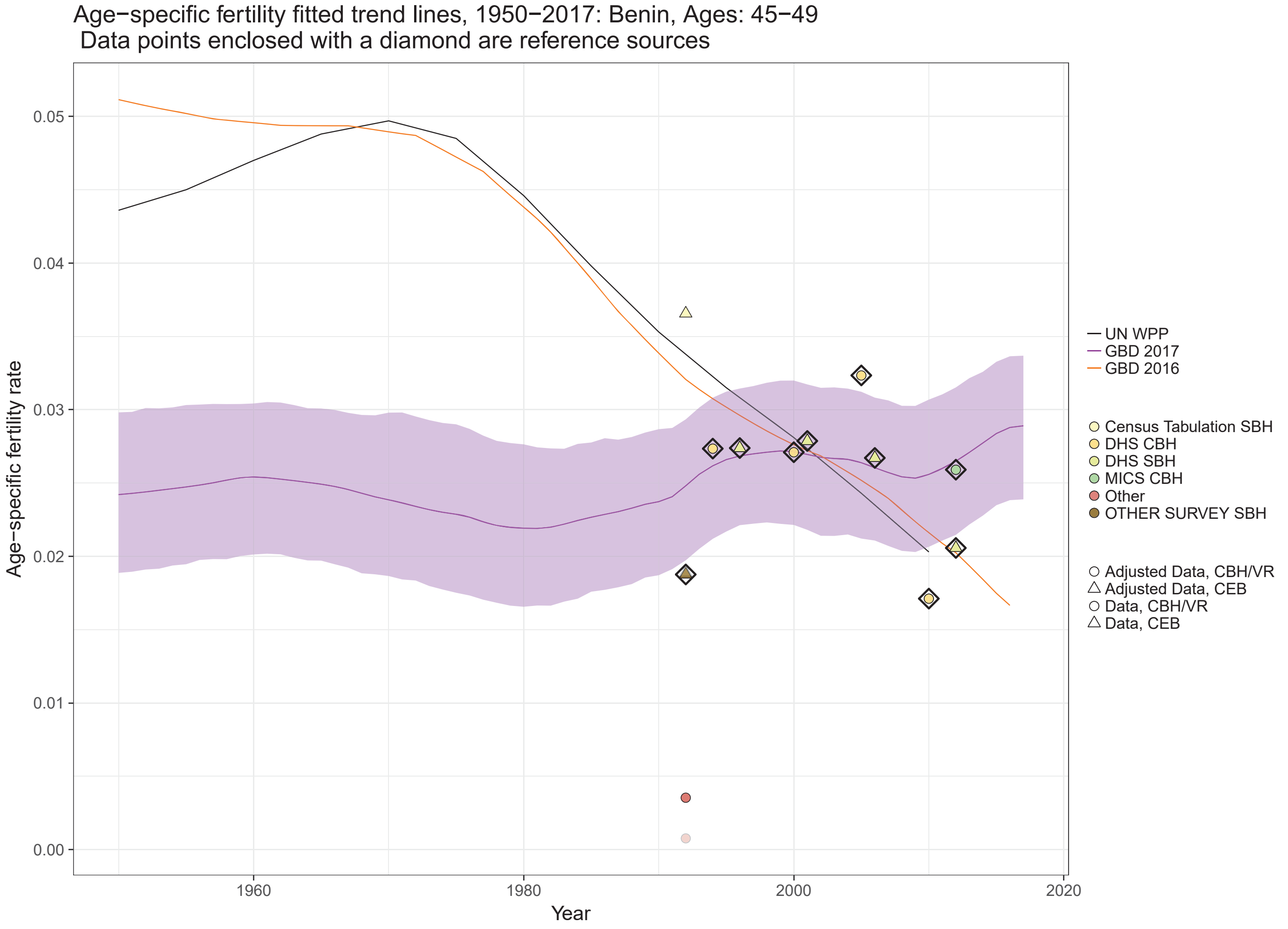

Age−specific fertility fitted trend lines, 1950−2017: Benin, Ages: 45−49
Data points enclosed with a diamond are reference sources
Age−specific fertility rate
0.00
0.01
0.02
0.03
0.04
0.05
1960
1980
2000
2020
Year
UN WPP
GBD 2017
GBD 2016
Census Tabulation SBH
DHS CBH
DHS SBH
MICS CBH
Other
OTHER SURVEY SBH
Adjusted Data, CBH/VR
Adjusted Data, CEB
Data, CBH/VR
Data, CEB

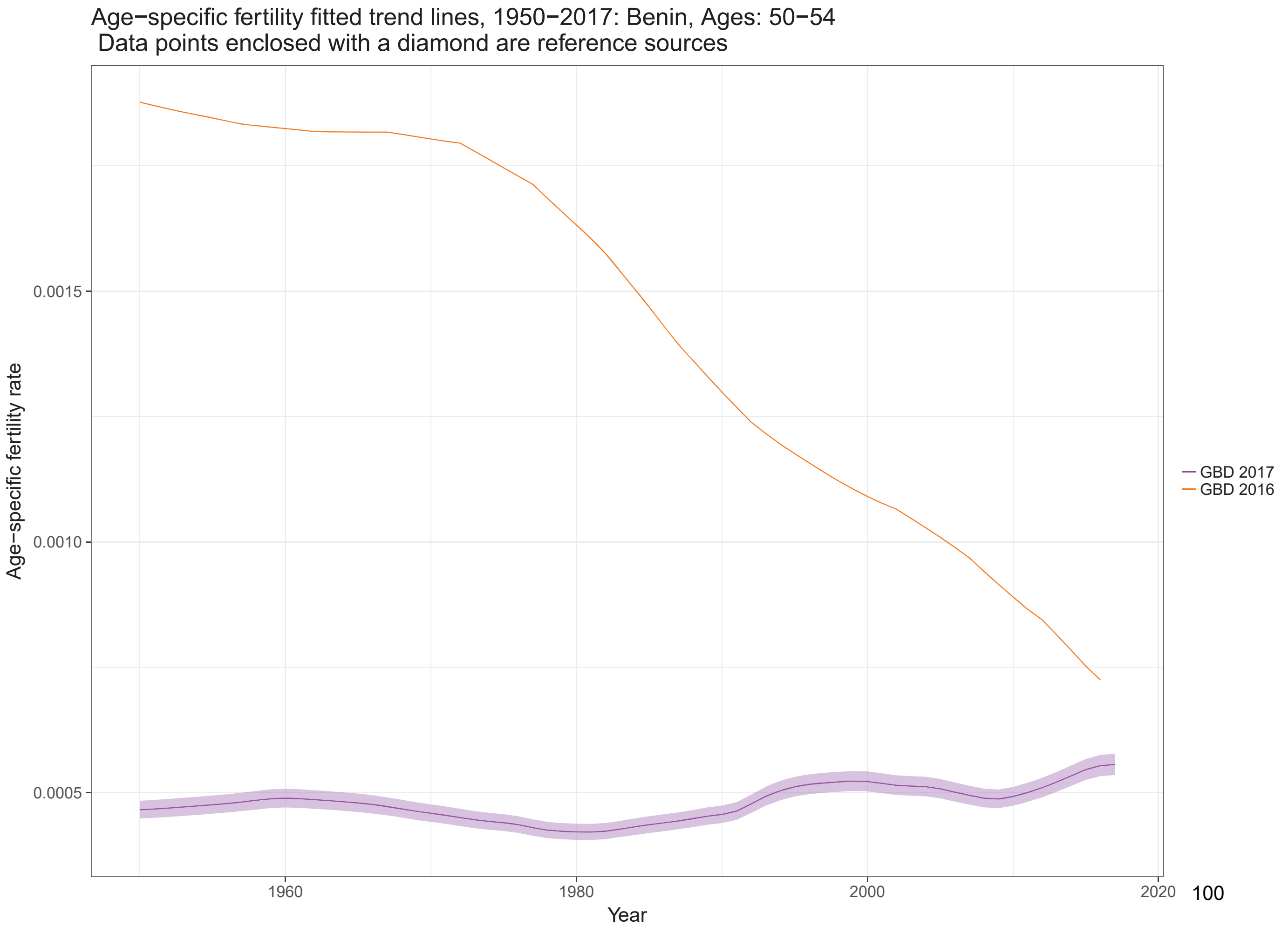

Age−specific fertility fitted trend lines, 1950−2017: Benin, Ages: 50−54
Data points enclosed with a diamond are reference sources
Age−specific fertility rate
0.0005
0.0010
0.0015
1960
1980
2000
2020
Year
GBD 2017
GBD 2016

Age−specific fertility fitted trend lines, 1950−2017: Bermuda, TFR
Data points enclosed with a diamond are reference sources

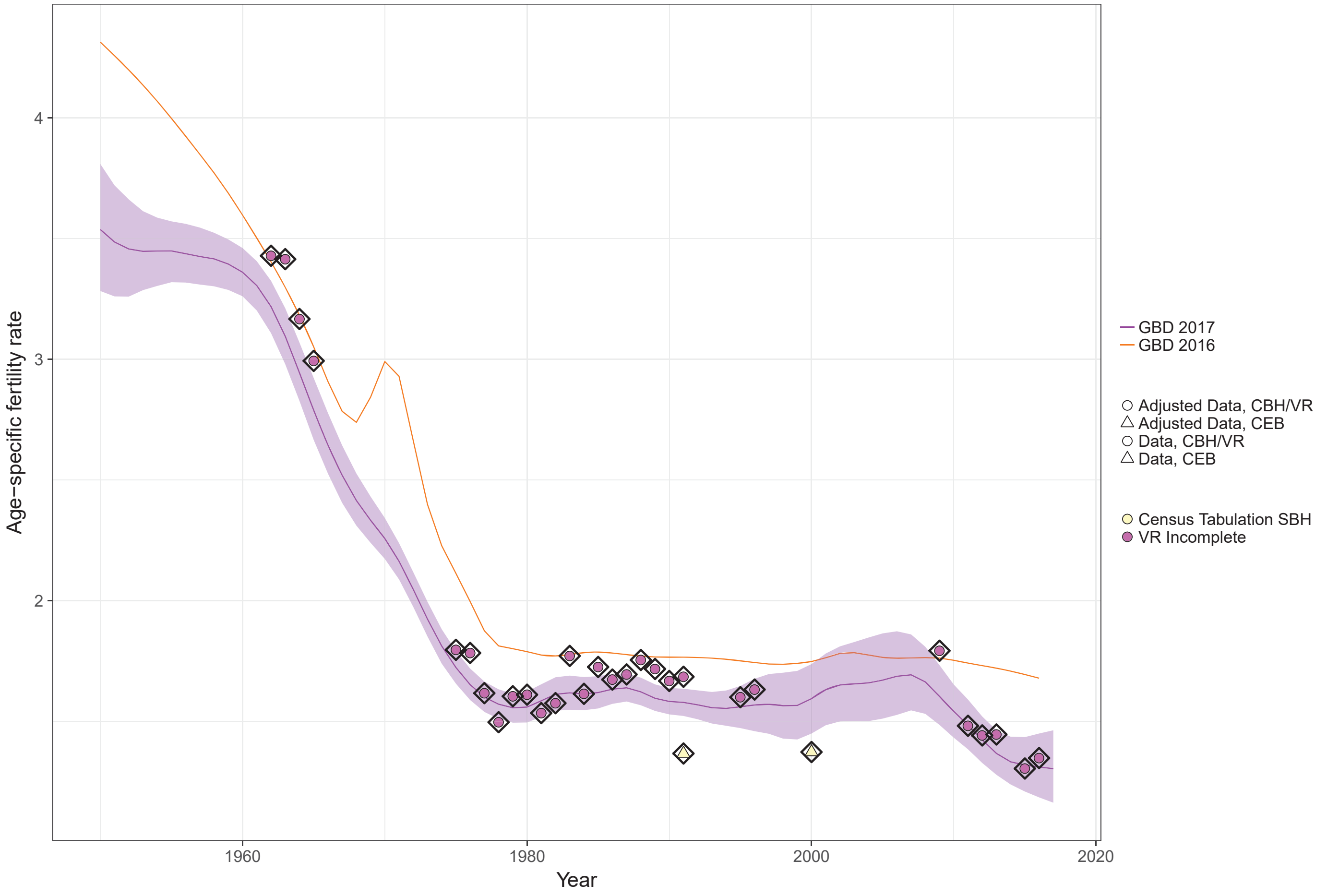

Age−specific fertility fitted trend lines, 1950−2017: Bermuda, Ages: 10−14
Data points enclosed with a diamond are reference sources

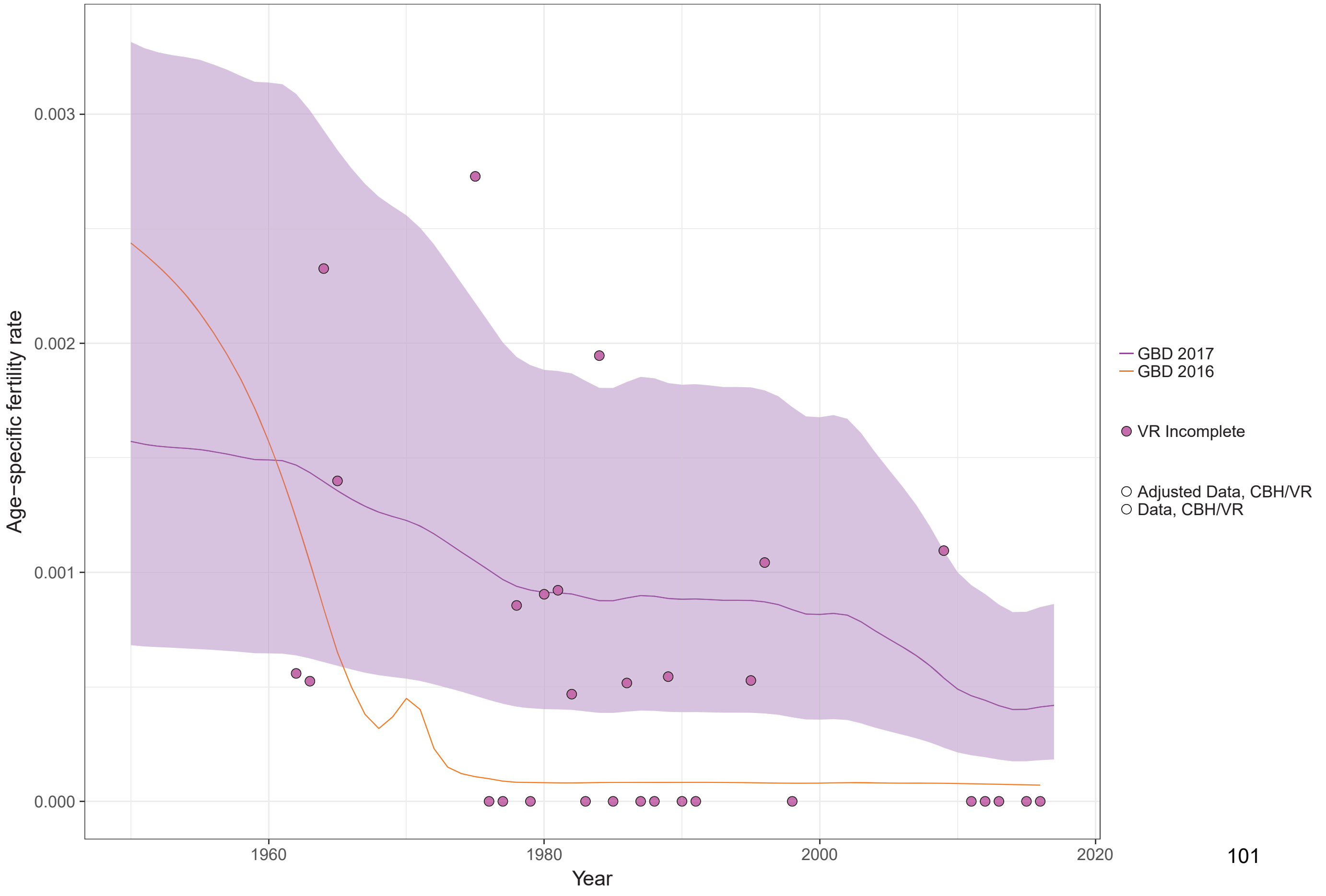

Age−specific fertility fitted trend lines, 1950−2017: Bermuda, Ages: 15−19
Data points enclosed with a diamond are reference sources

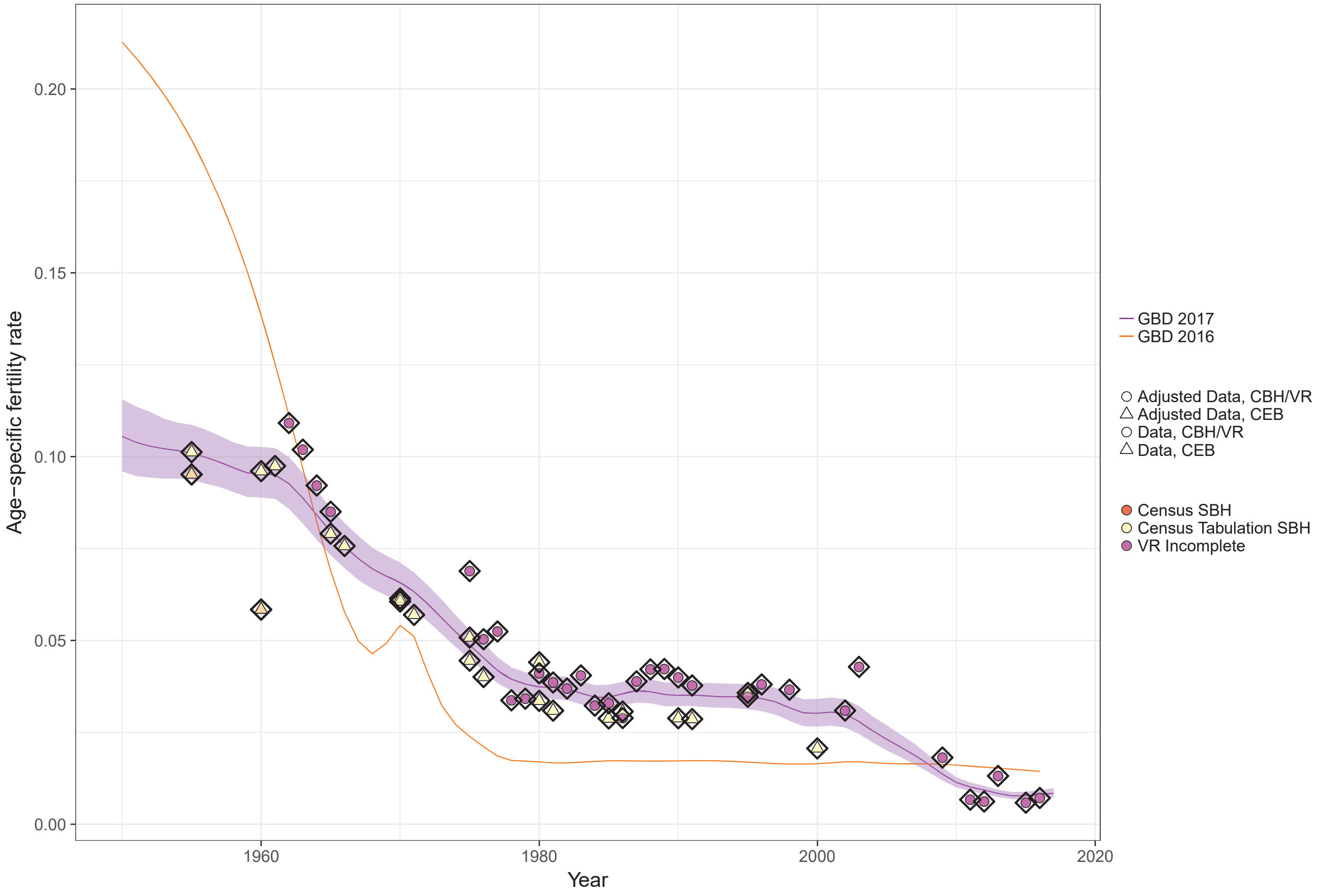

Age−specific fertility fitted trend lines, 1950−2017: Bermuda, Ages: 20−24
Data points enclosed with a diamond are reference sources

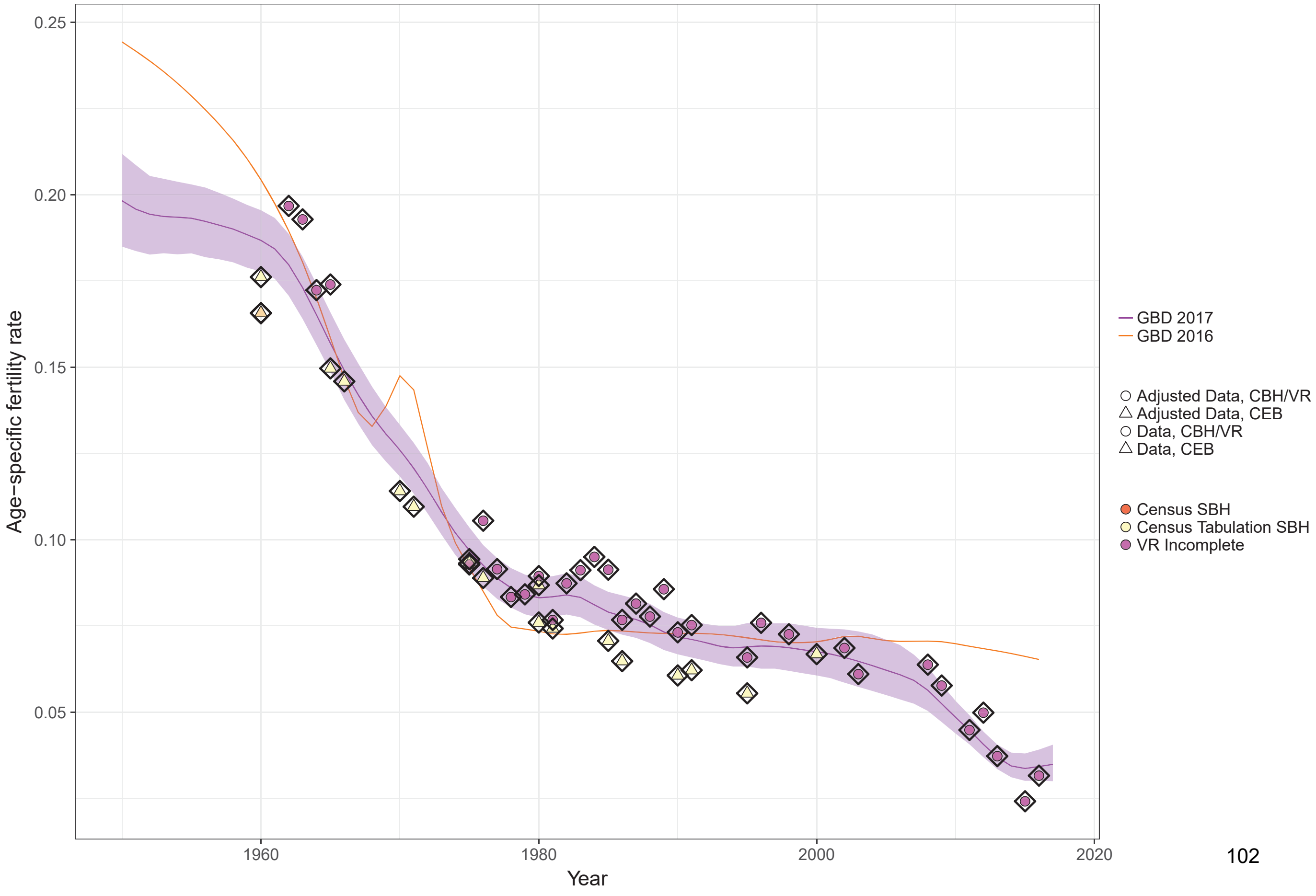

Age−specific fertility fitted trend lines, 1950−2017: Bermuda, Ages: 25−29
Data points enclosed with a diamond are reference sources

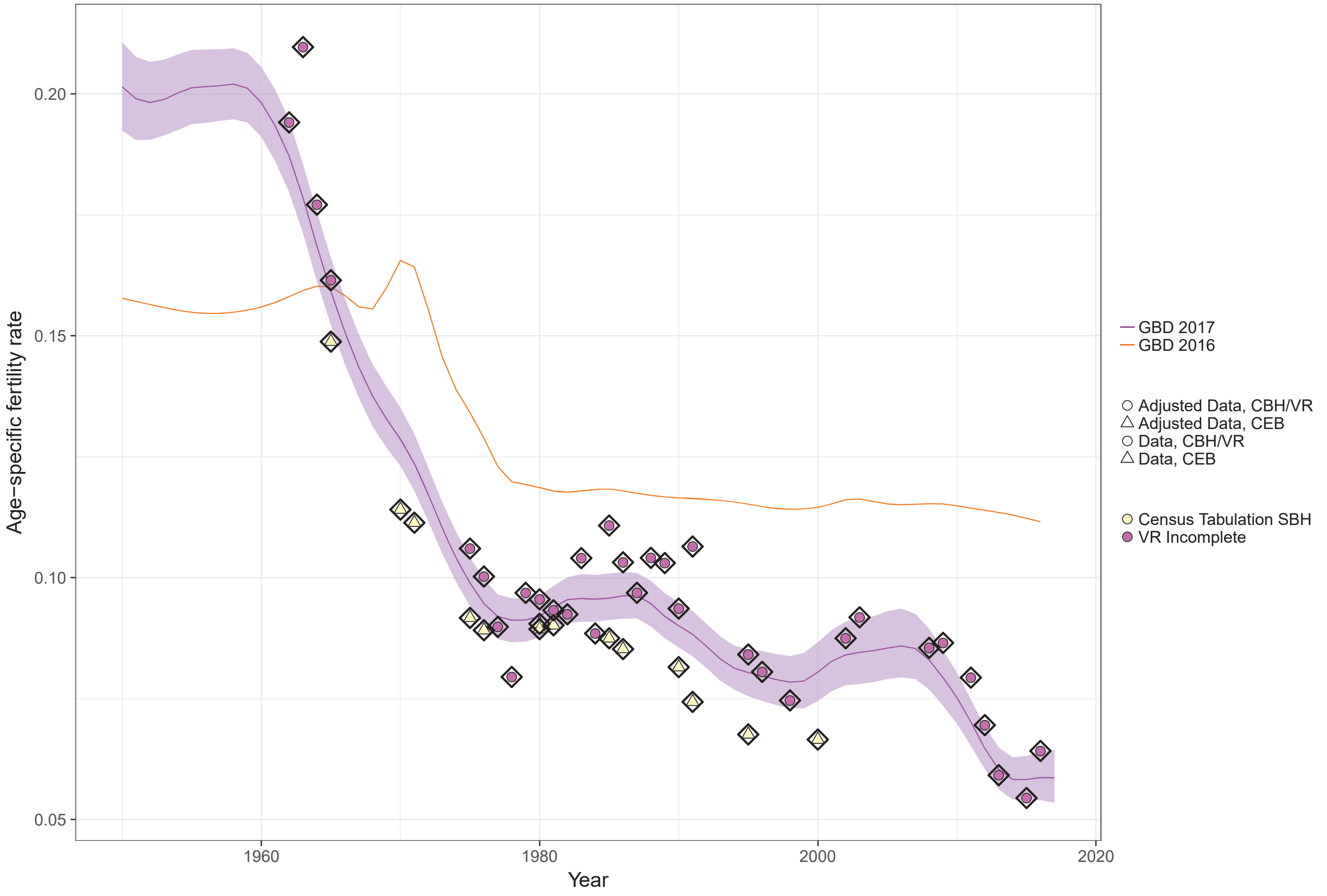

Age−specific fertility fitted trend lines, 1950−2017: Bermuda, Ages: 30−34
Data points enclosed with a diamond are reference sources

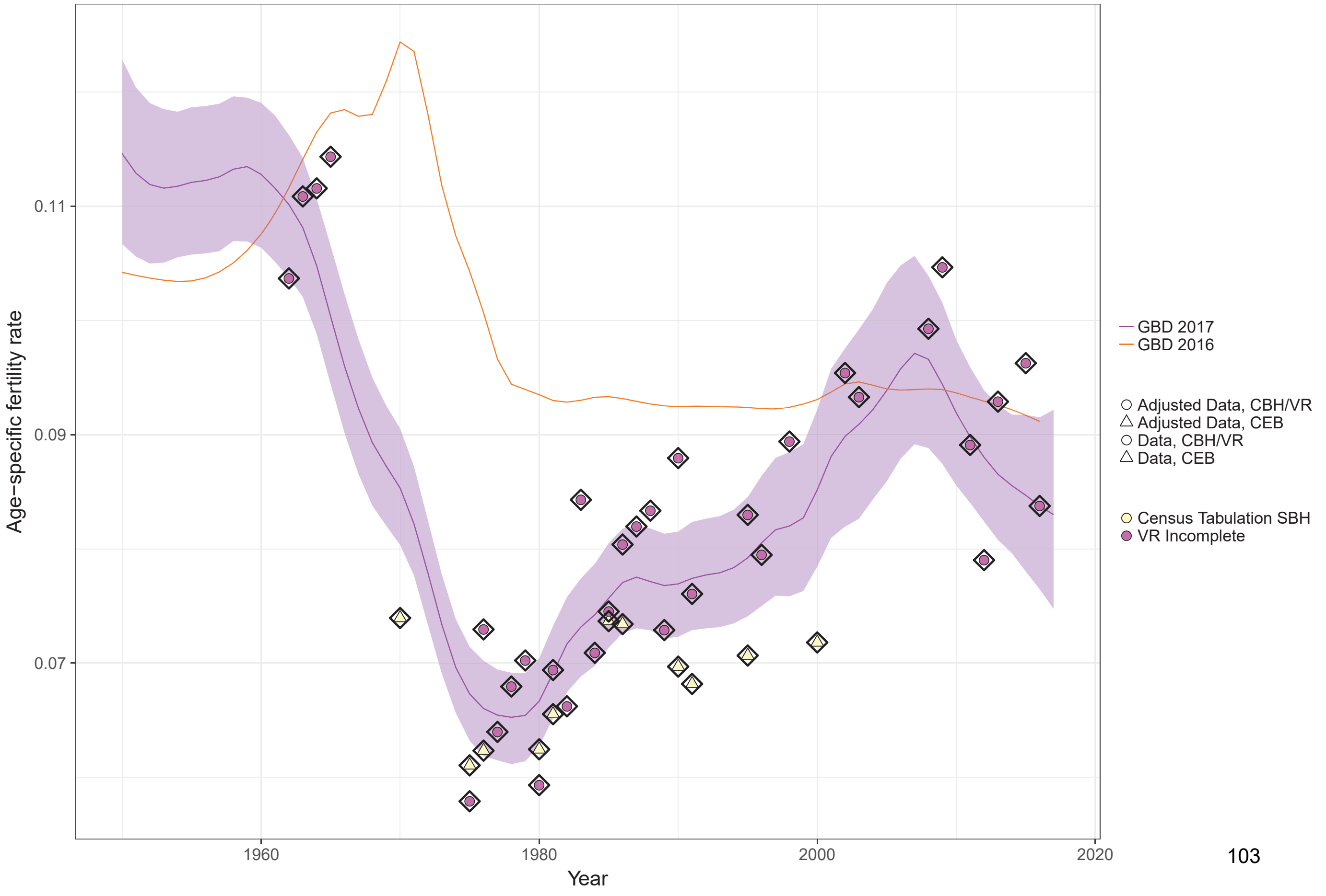

Age−specific fertility fitted trend lines, 1950−2017: Bermuda, Ages: 35−39
Data points enclosed with a diamond are reference sources

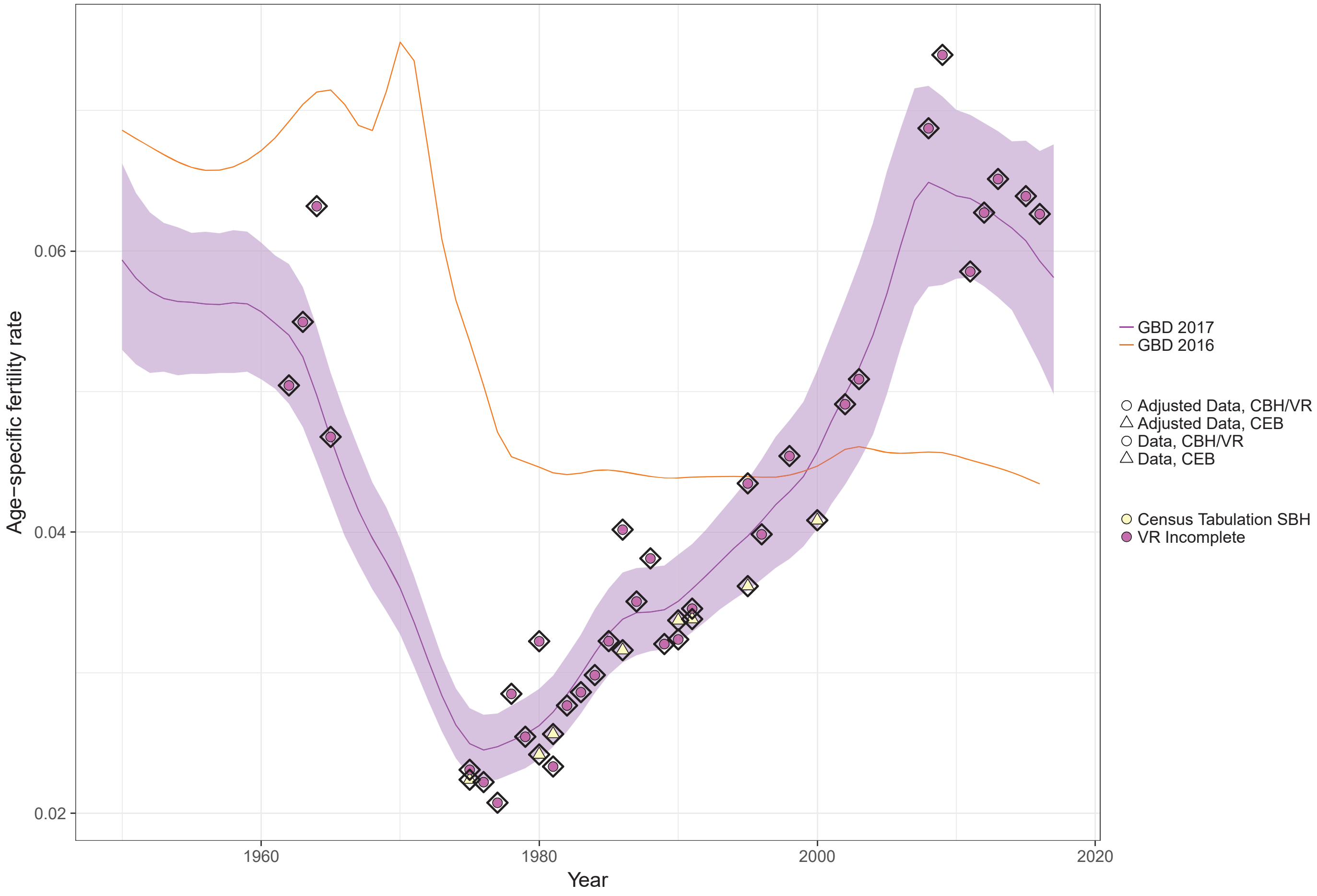

Age−specific fertility fitted trend lines, 1950−2017: Bermuda, Ages: 40−44
Data points enclosed with a diamond are reference sources

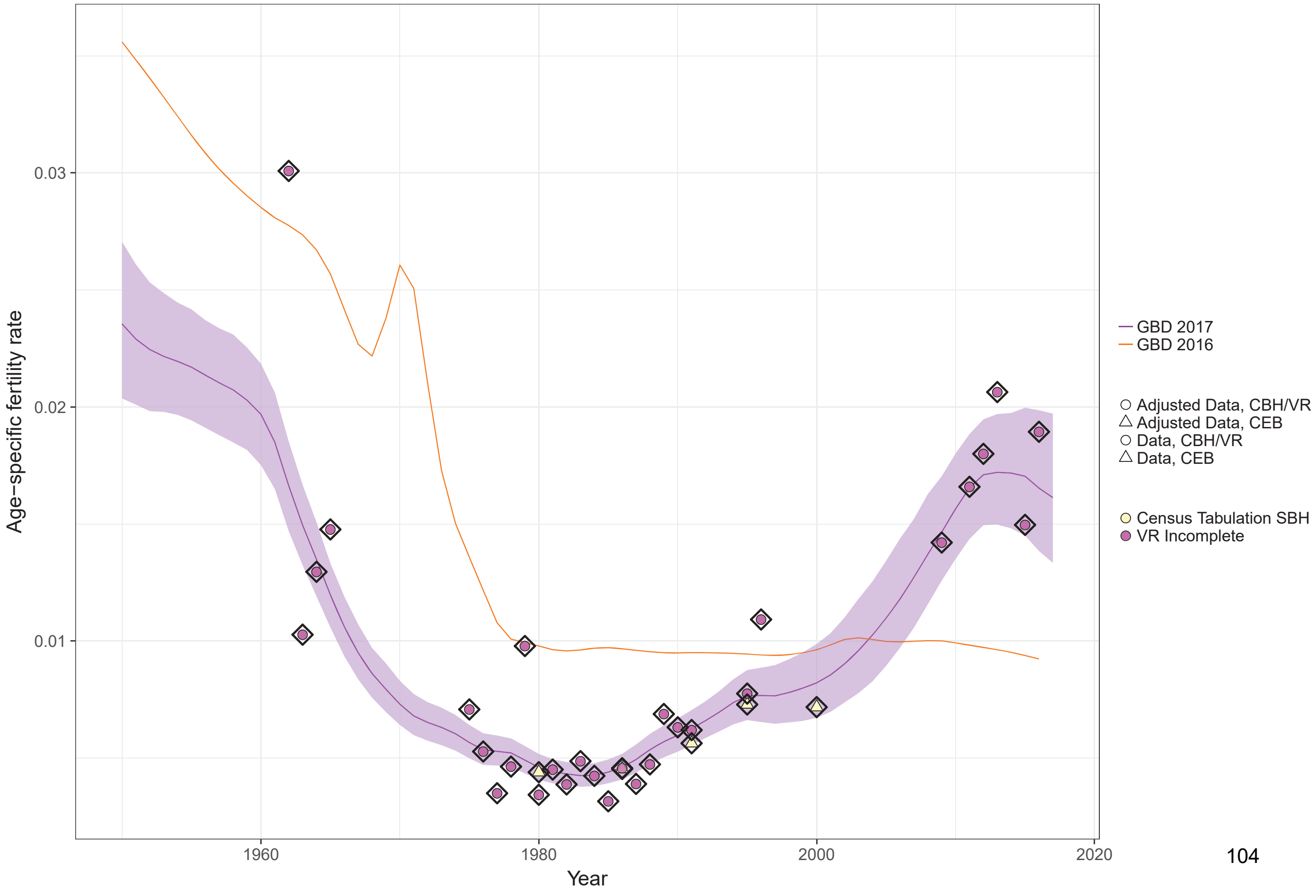

Age−specific fertility fitted trend lines, 1950−2017: Bermuda, Ages: 45−49
Data points enclosed with a diamond are reference sources

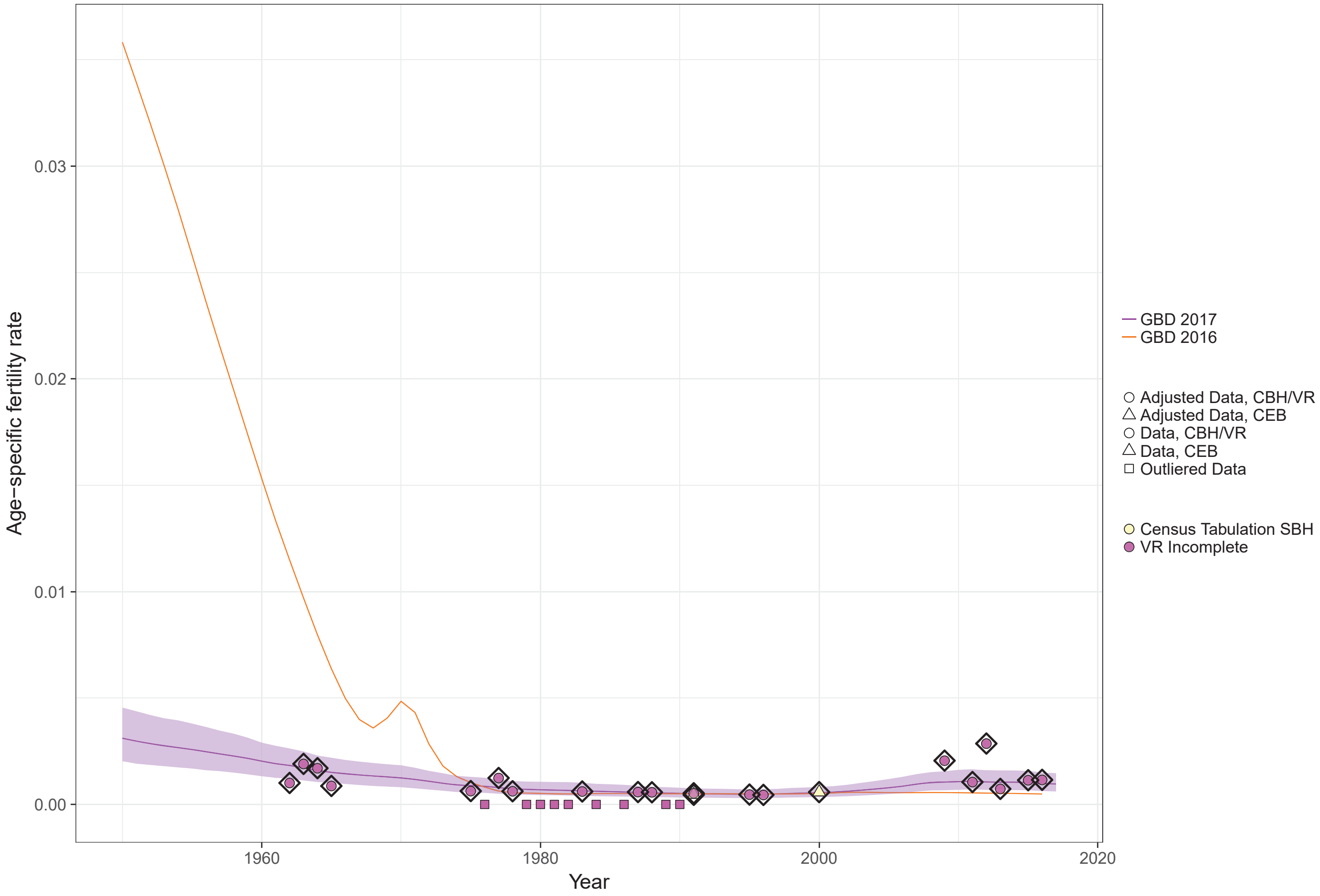

Age−specific fertility fitted trend lines, 1950−2017: Bermuda, Ages: 50−54
Data points enclosed with a diamond are reference sources

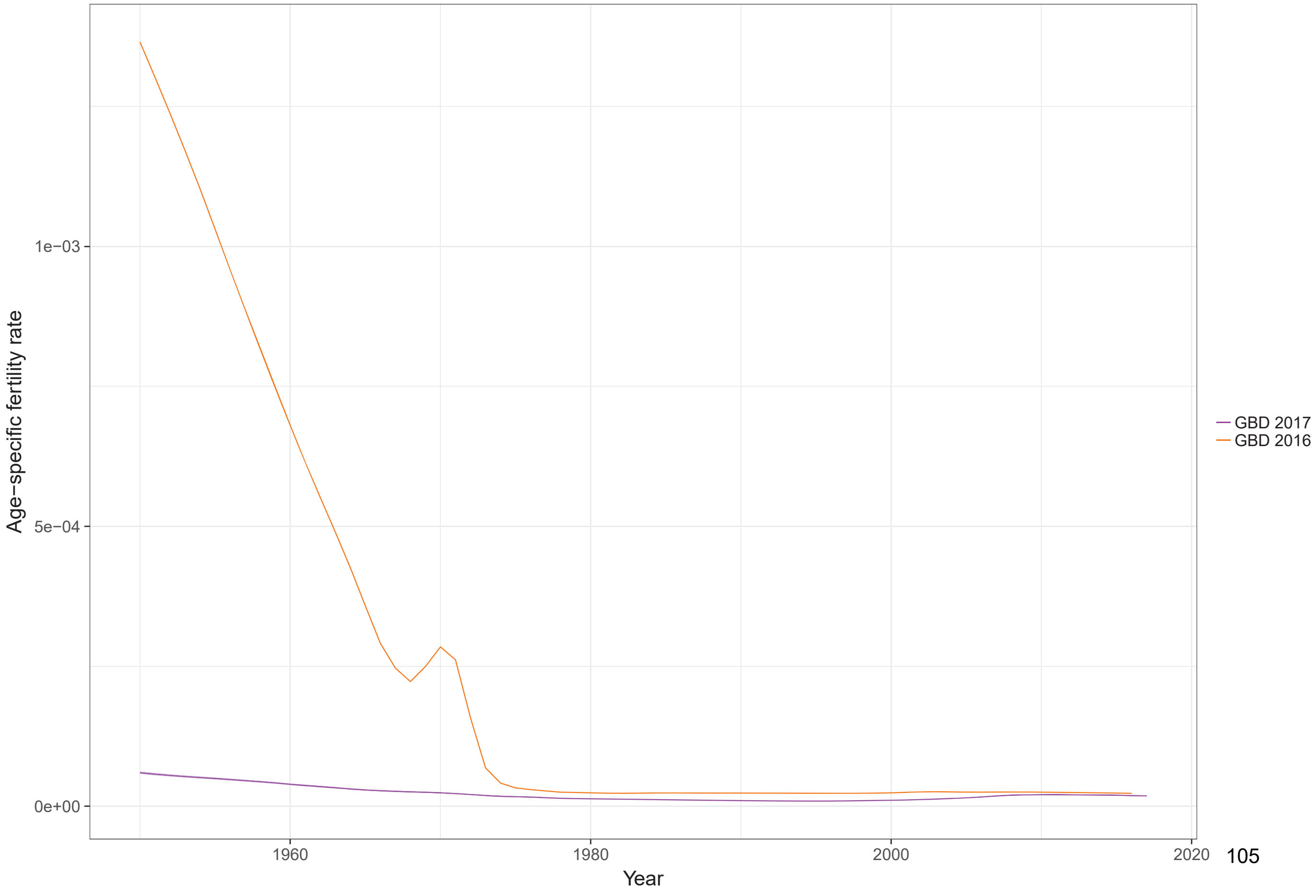

Age−specific fertility fitted trend lines, 1950−2017: Bhutan, TFR
Data points enclosed with a diamond are reference sources

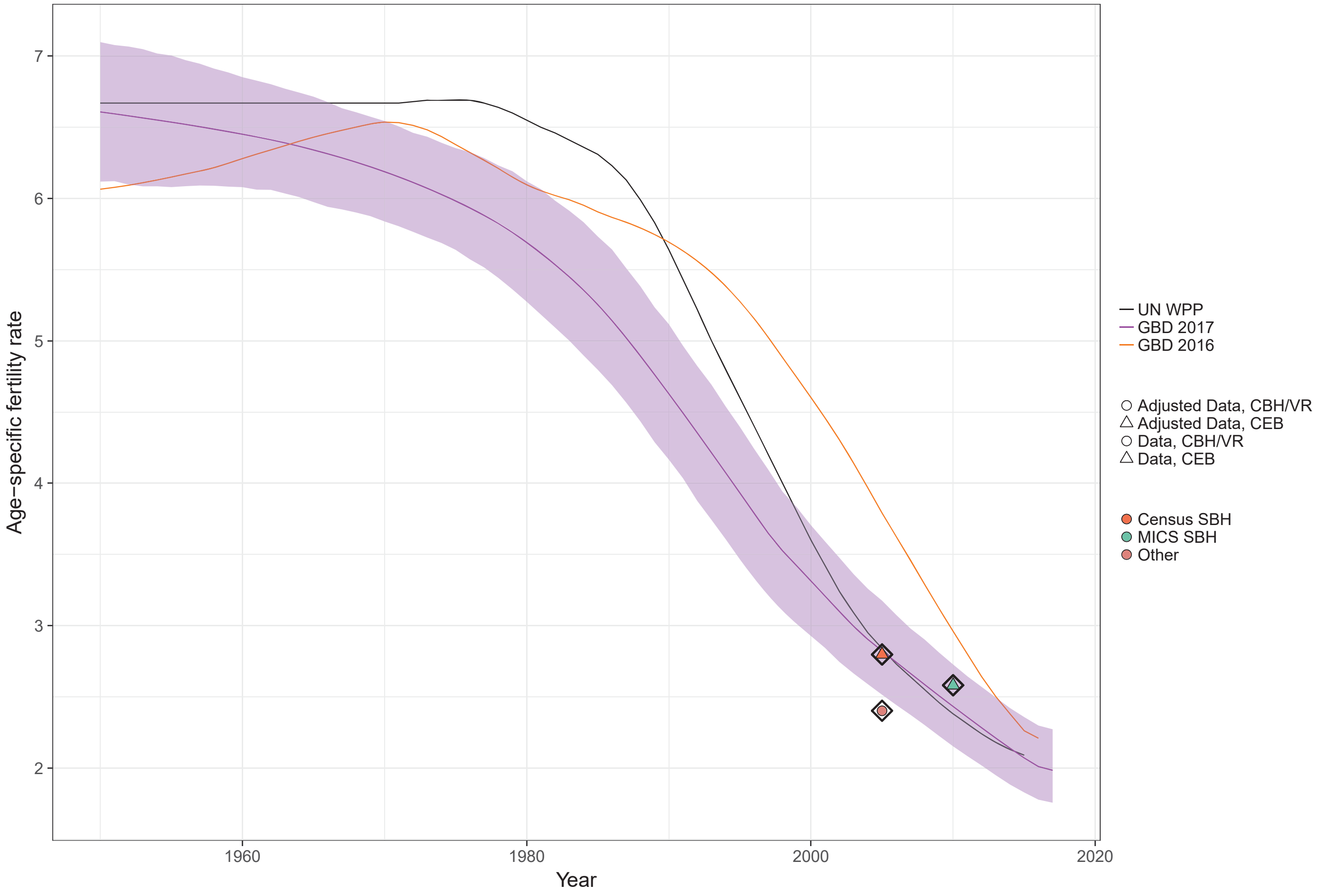

Age−specific fertility fitted trend lines, 1950−2017: Bhutan, Ages: 10−14
Data points enclosed with a diamond are reference sources

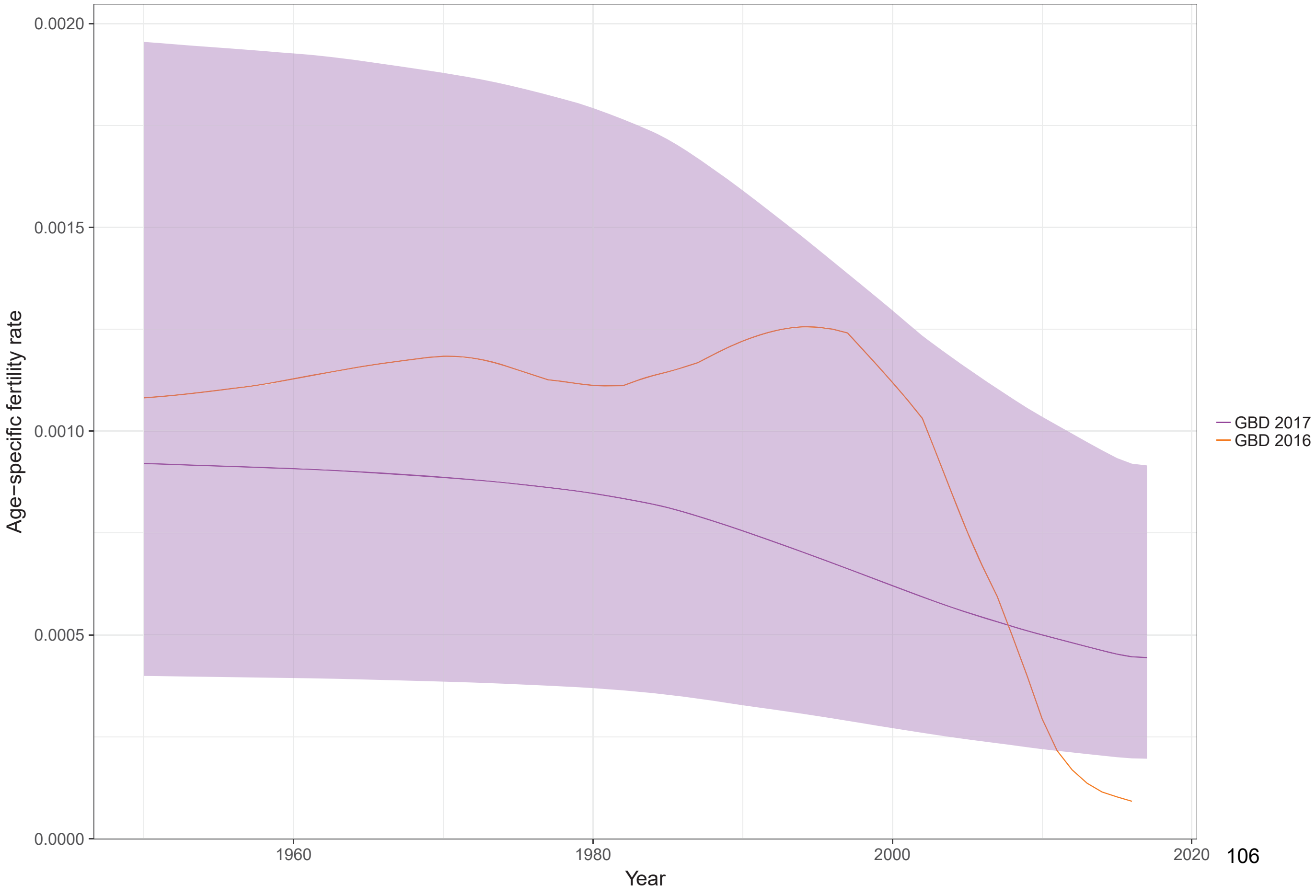

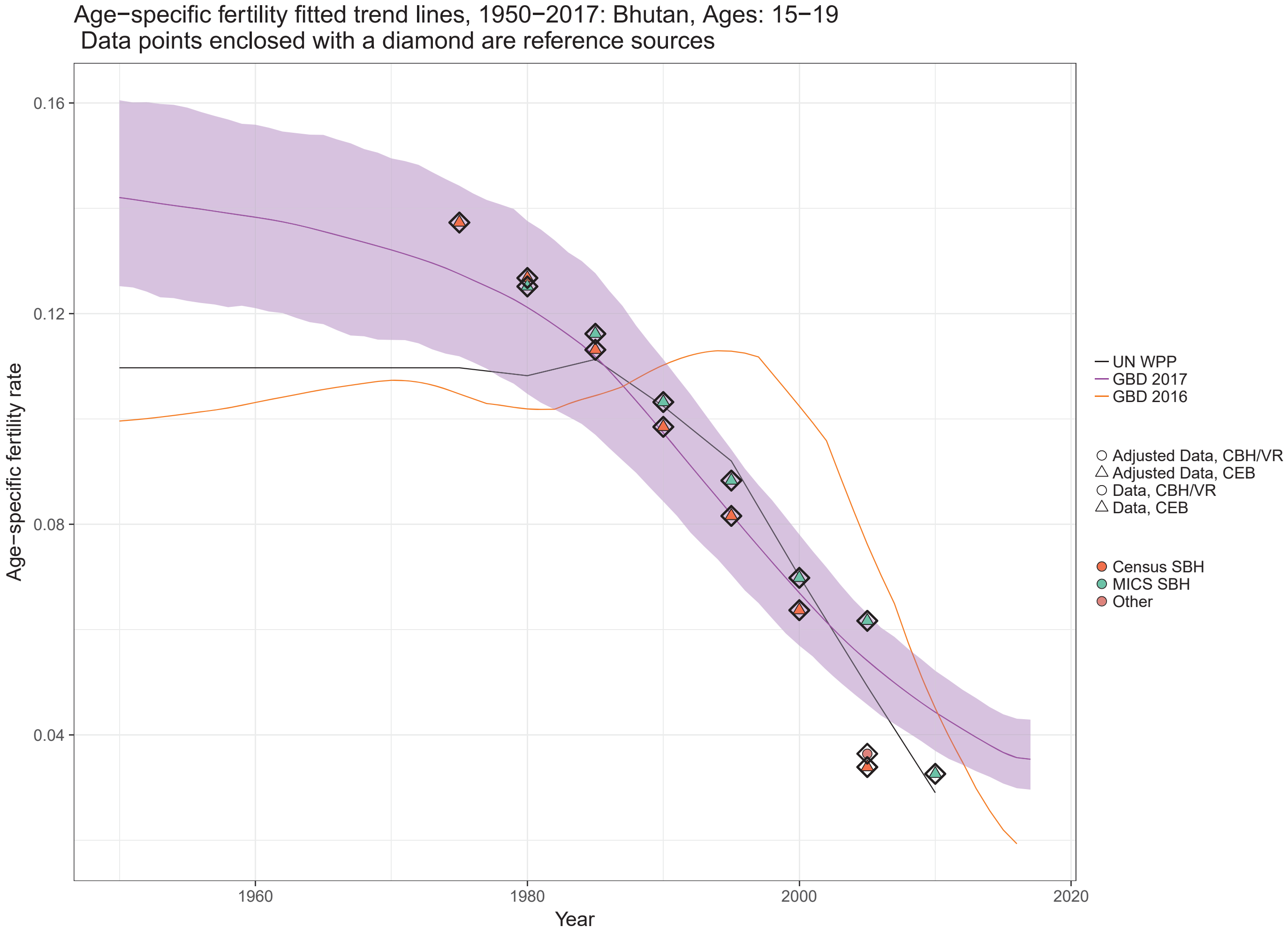

Age−specific fertility fitted trend lines, 1950−2017: Bhutan, Ages: 15−19
Data points enclosed with a diamond are reference sources
Age−specific fertility rate
0.16
0.12
0.08
0.04
1960
1980
2000
2020
Year
UN WPP
GBD 2017
GBD 2016
Adjusted Data, CBH/VR
Adjusted Data, CEB
Data, CBH/VR
Data, CEB
Census SBH
MICS SBH
Other

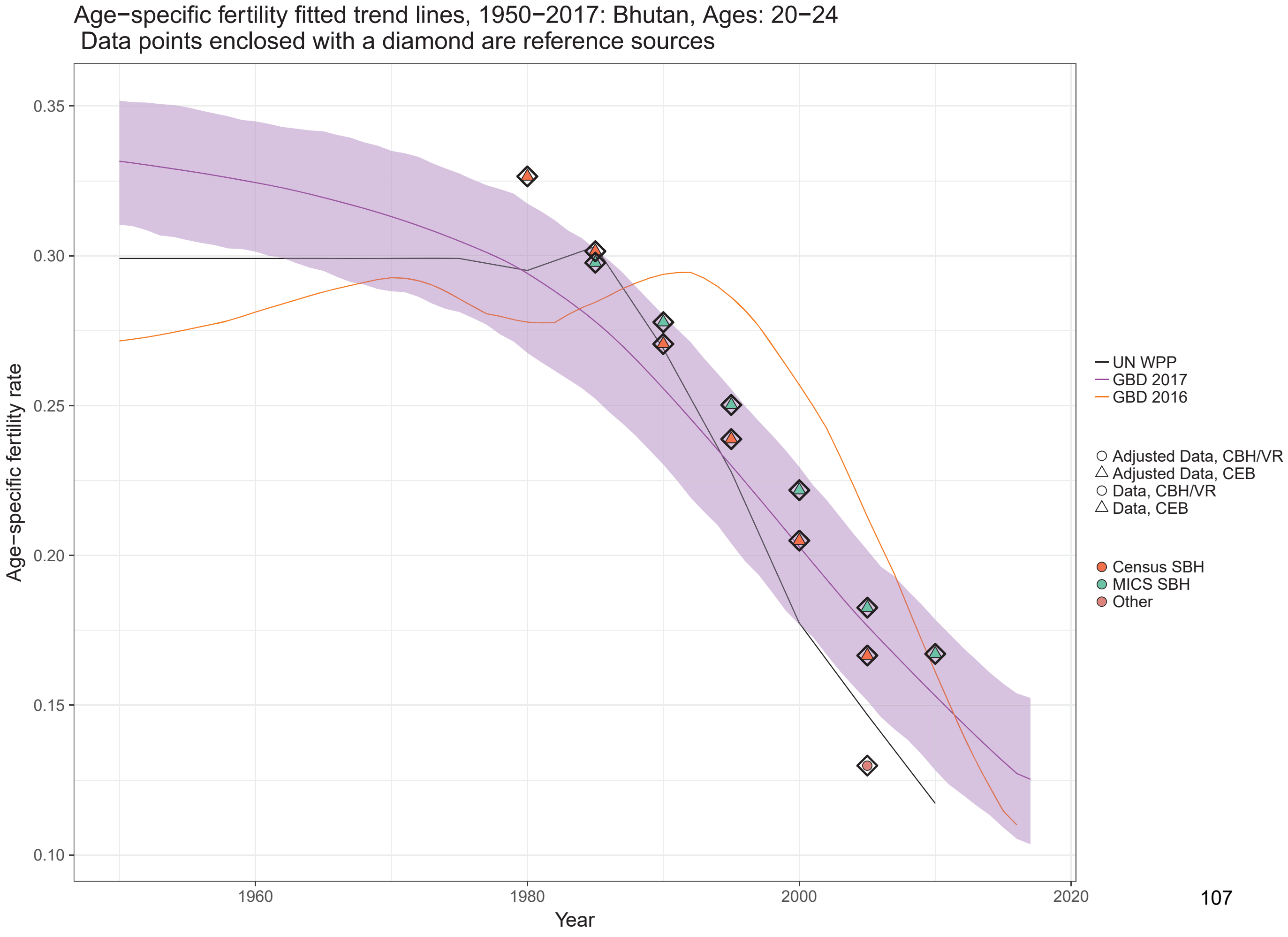

Age−specific fertility fitted trend lines, 1950−2017: Bhutan, Ages: 20−24
Data points enclosed with a diamond are reference sources
Age−specific fertility rate
0.35
0.30
0.25
0.20
0.15
0.10
1960
1980
2000
2020
Year
UN WPP
GBD 2017
GBD 2016
Adjusted Data, CBH/VR
Adjusted Data, CEB
Data, CBH/VR
Data, CEB
Census SBH
MICS SBH
Other

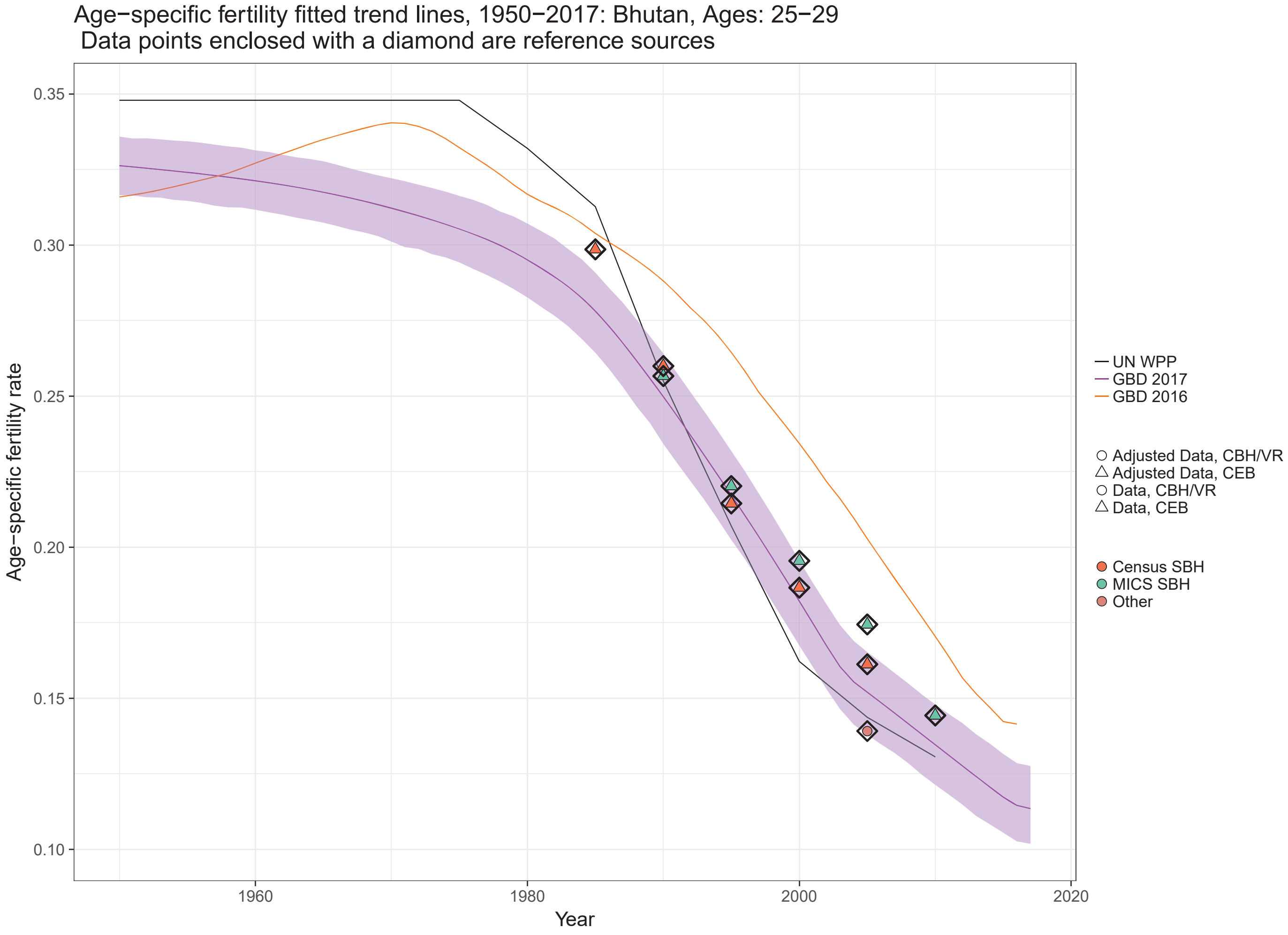
Age−specific fertility fitted trend lines, 1950−2017: Bhutan, Ages: 25−29
Data points enclosed with a diamond are reference sources
Age−specific fertility rate
Year
UN WPP
GBD 2017
GBD 2016
Adjusted Data, CBH/VR
Adjusted Data, CEB
Data, CBH/VR
Data, CEB
Census SBH
MICS SBH
Other

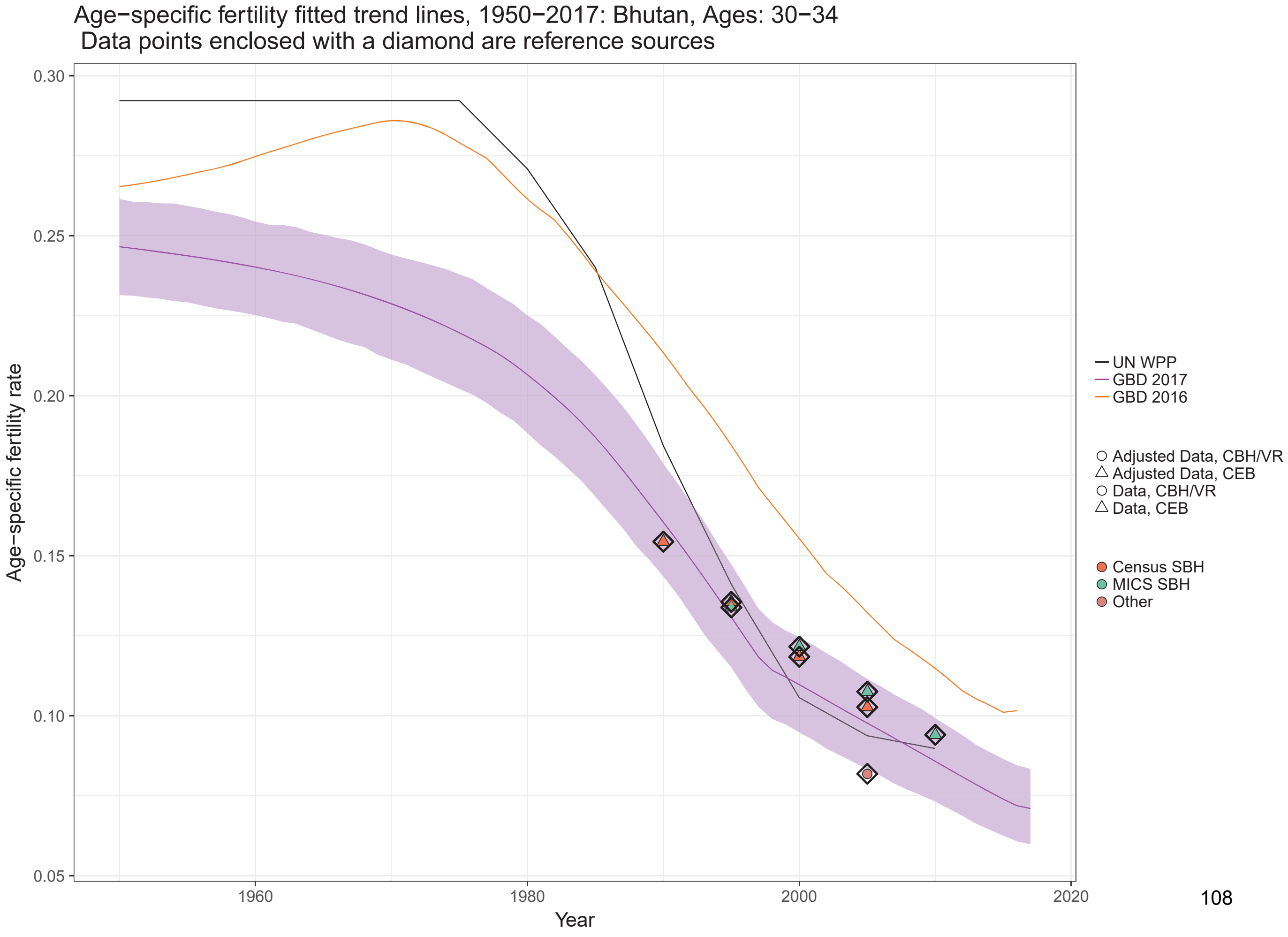
Age−specific fertility fitted trend lines, 1950−2017: Bhutan, Ages: 30−34
Data points enclosed with a diamond are reference sources
Age−specific fertility rate
Year
UN WPP
GBD 2017
GBD 2016
Adjusted Data, CBH/VR
Adjusted Data, CEB
Data, CBH/VR
Data, CEB
Census SBH
MICS SBH
Other

Age−specific fertility fitted trend lines, 1950−2017: Bhutan, Ages: 35−39
Data points enclosed with a diamond are reference sources

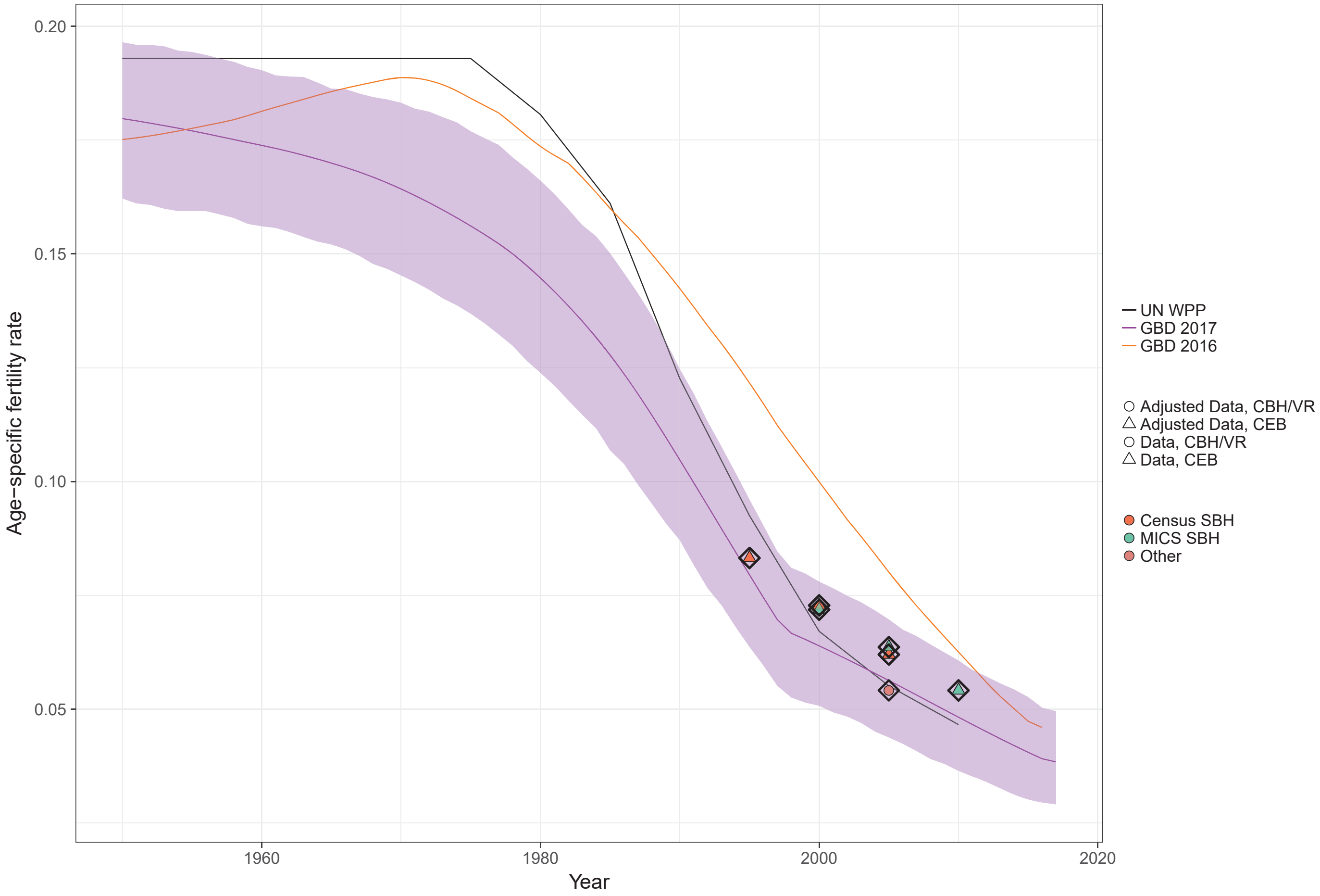

Age−specific fertility fitted trend lines, 1950−2017: Bhutan, Ages: 40−44
Data points enclosed with a diamond are reference sources

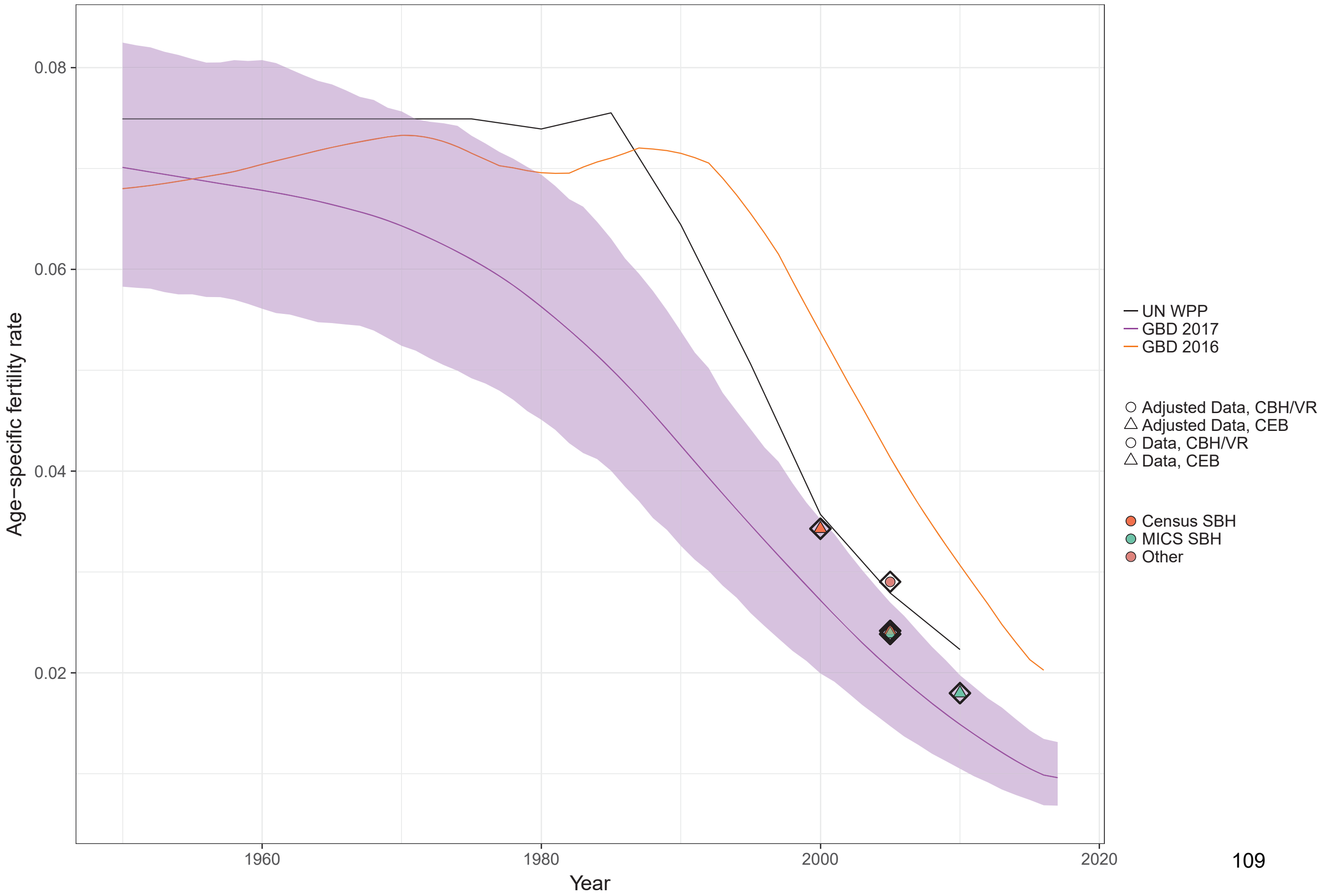

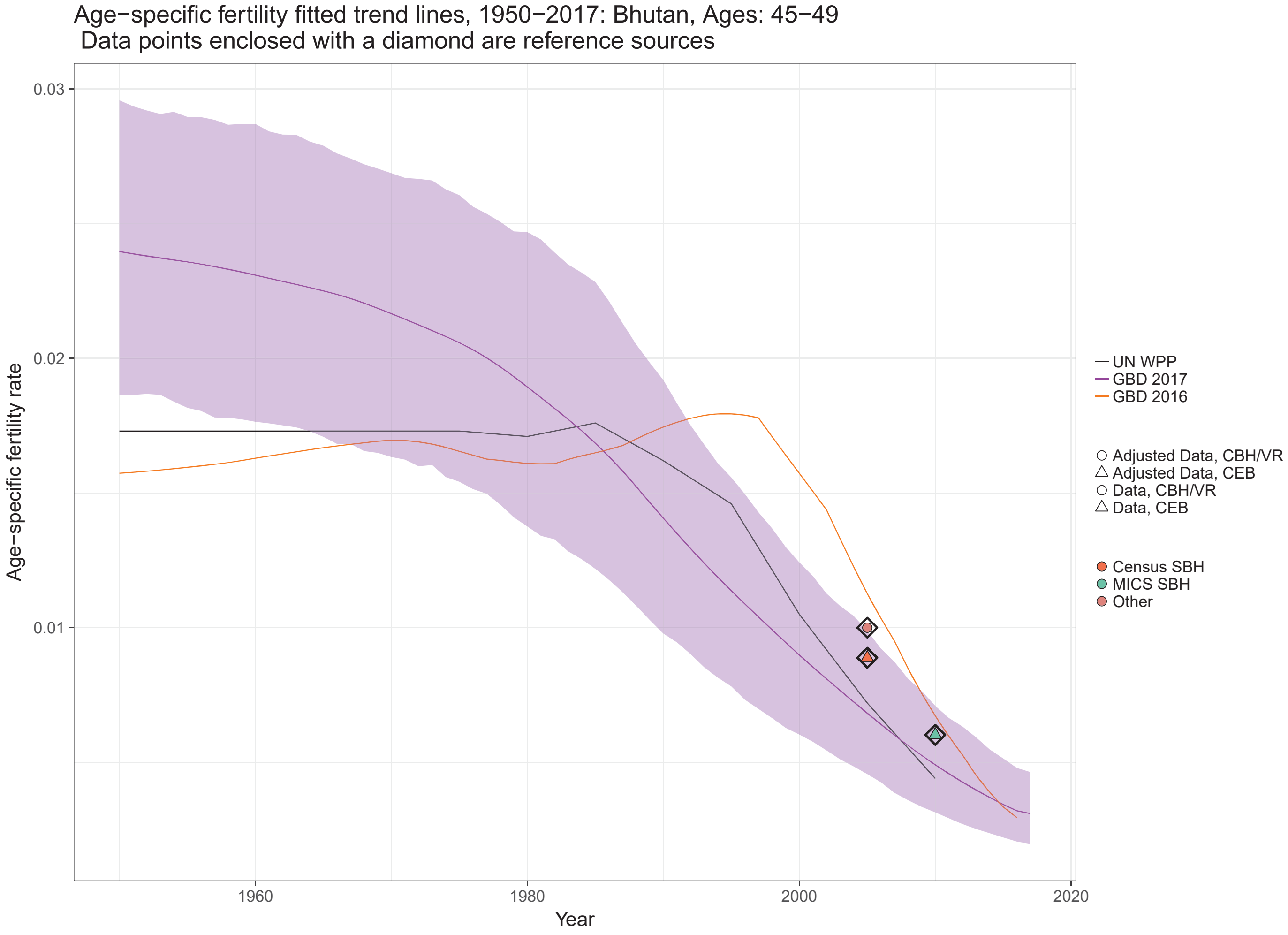

Age−specific fertility fitted trend lines, 1950−2017: Bhutan, Ages: 45−49
Data points enclosed with a diamond are reference sources
Age−specific fertility rate
0.03
0.02
0.01
1960
1980
2000
2020
Year
UN WPP
GBD 2017
GBD 2016
Adjusted Data, CBH/VR
Adjusted Data, CEB
Data, CBH/VR
Data, CEB
Census SBH
MICS SBH
Other

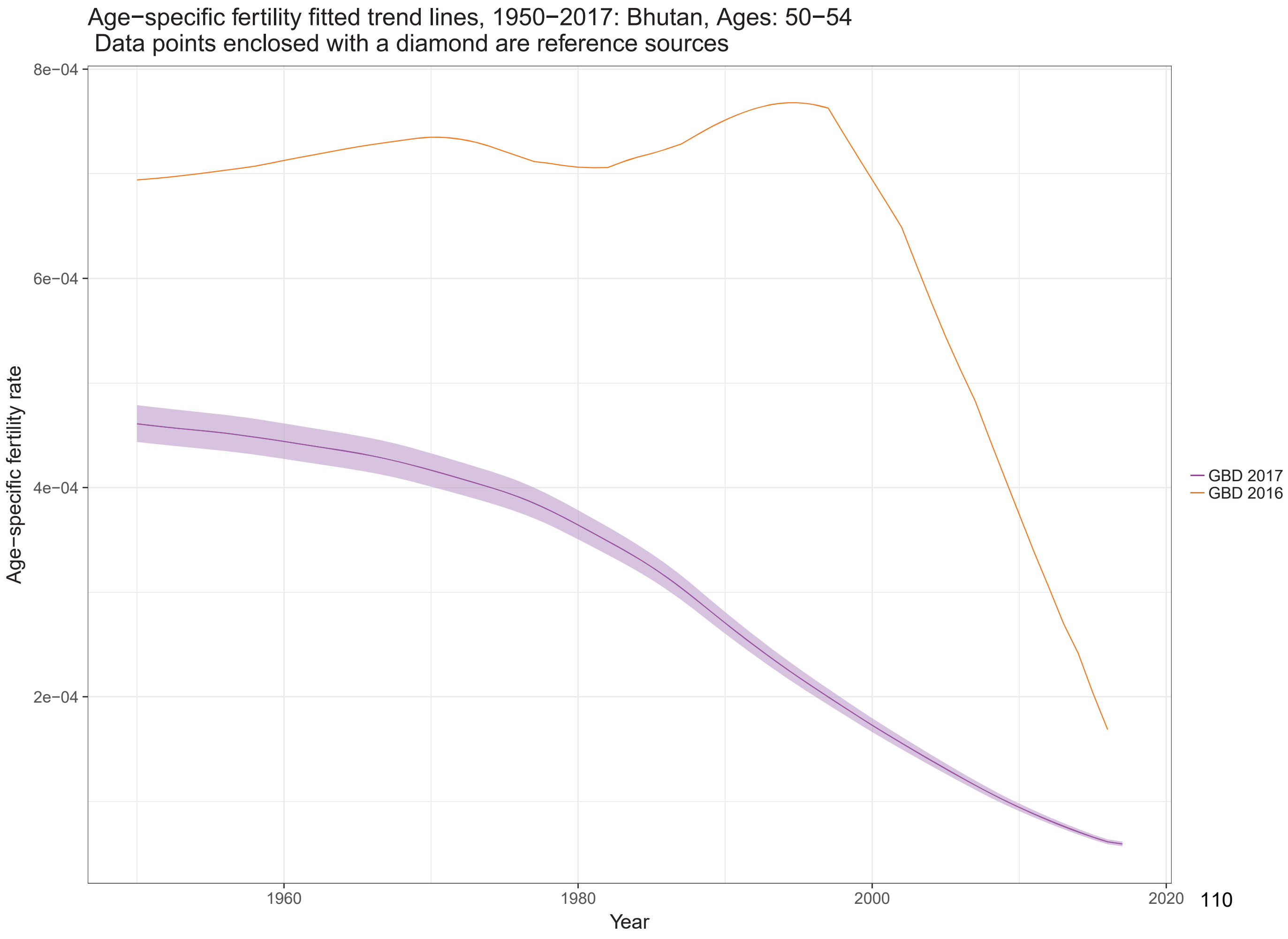

Age−specific fertility fitted trend lines, 1950−2017: Bhutan, Ages: 50−54
Data points enclosed with a diamond are reference sources
Age−specific fertility rate
8e−04
6e−04
4e−04
2e−04
1960
1980
2000
2020
Year
GBD 2017
GBD 2016

Age−specific fertility fitted trend lines, 1950−2017: Bolivia, TFR
Data points enclosed with a diamond are reference sources

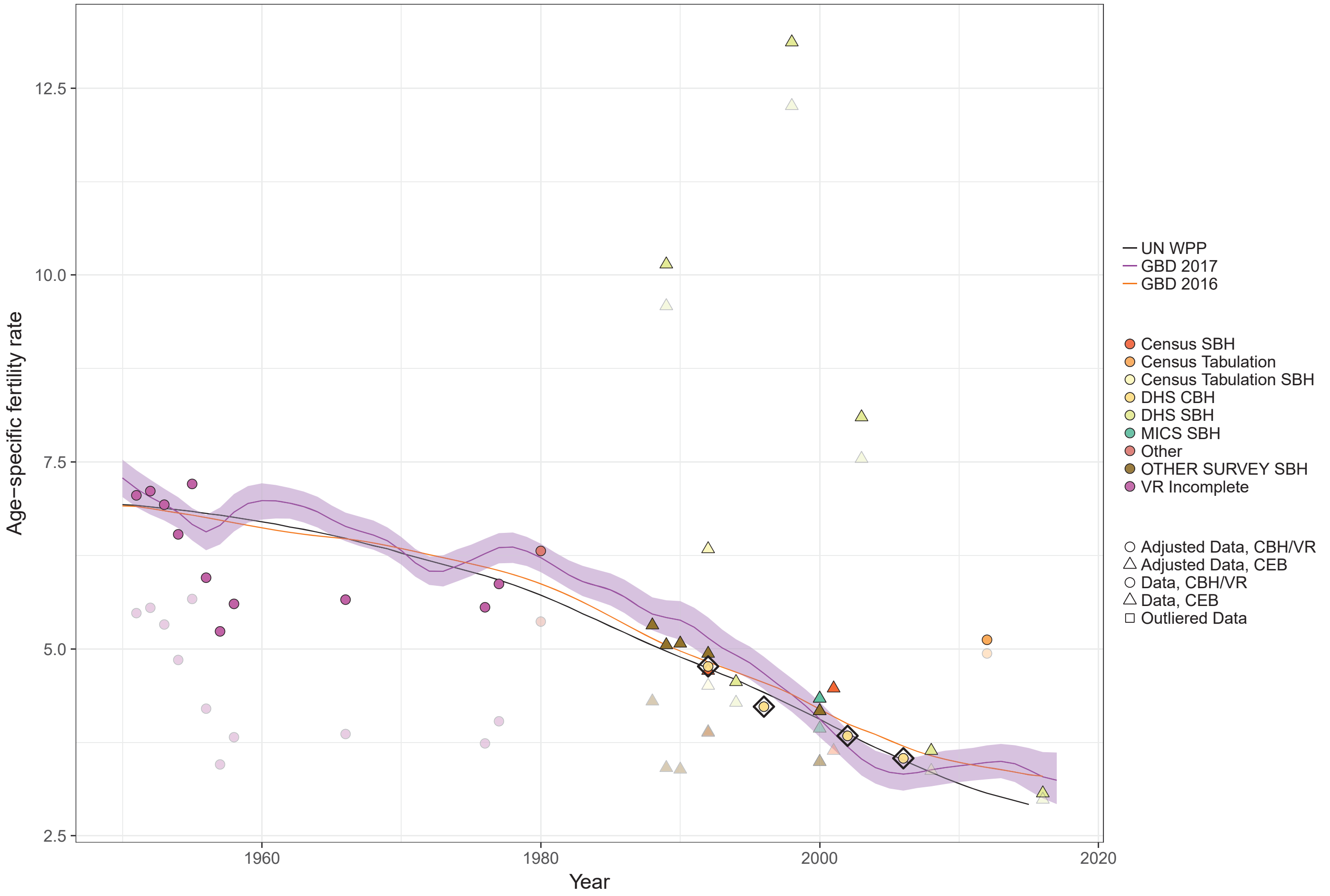

Age−specific fertility fitted trend lines, 1950−2017: Bolivia, Ages: 10−14
Data points enclosed with a diamond are reference sources

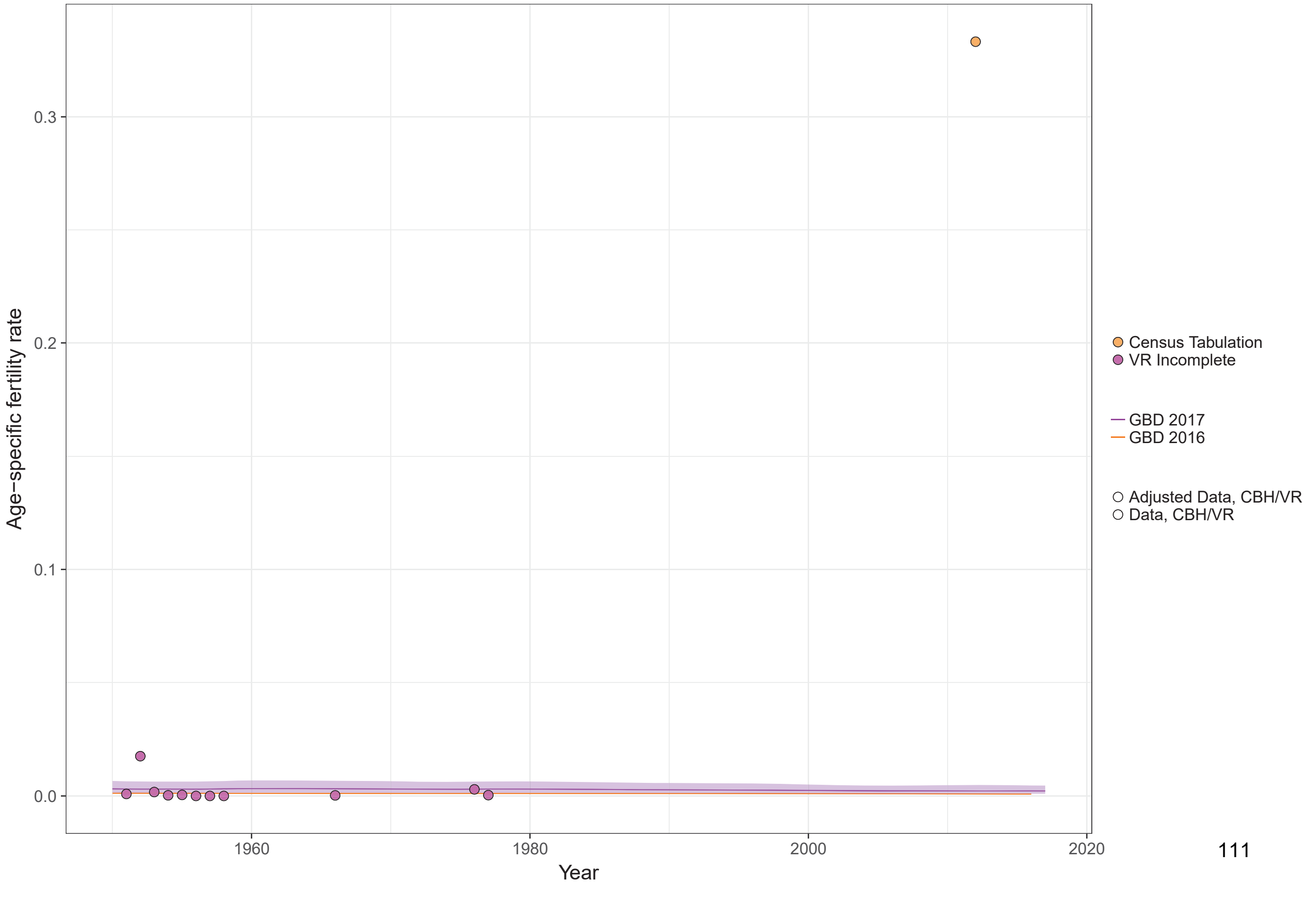

Age−specific fertility fitted trend lines, 1950−2017: Bolivia, Ages: 15−19
Data points enclosed with a diamond are reference sources

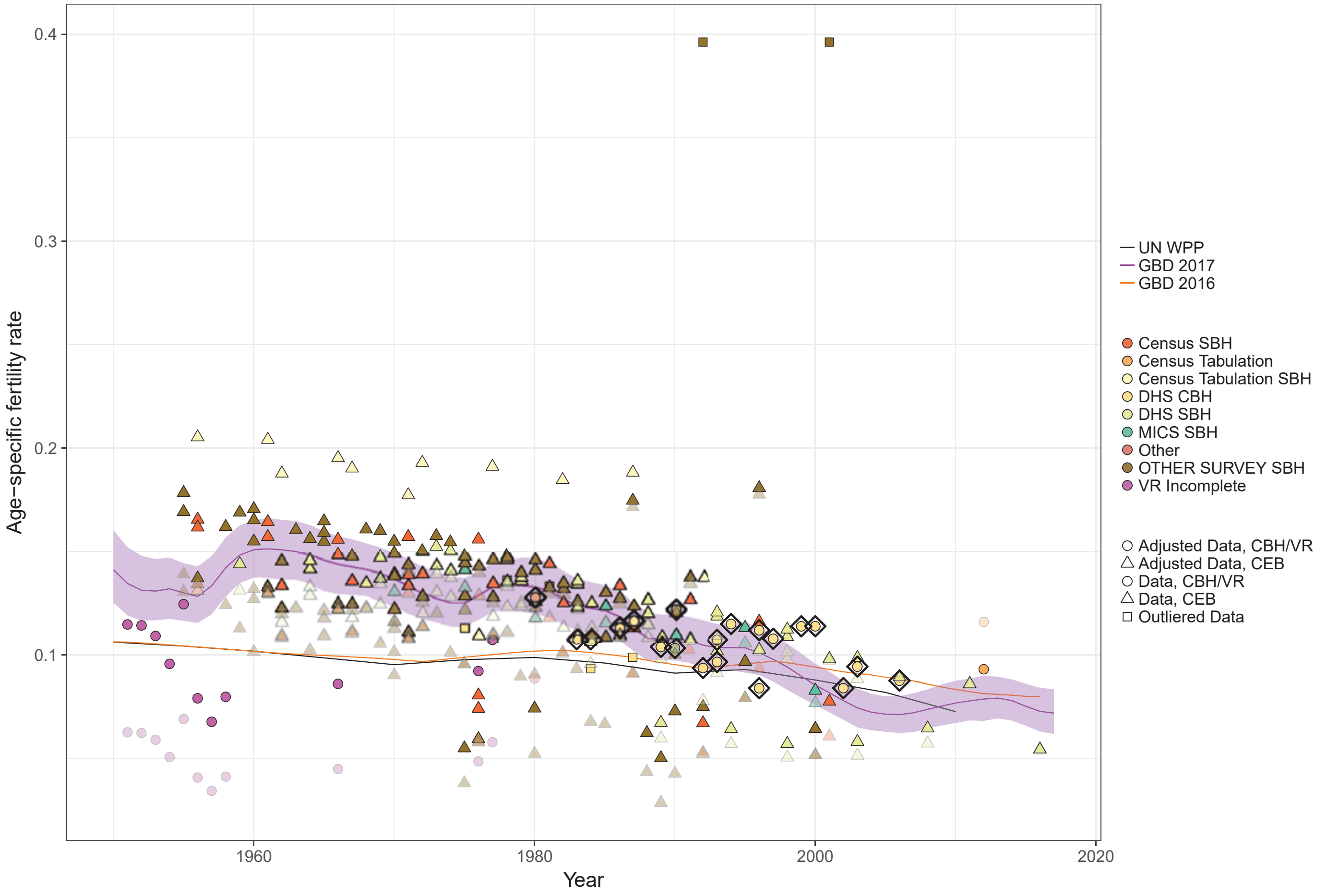

Age−specific fertility fitted trend lines, 1950−2017: Bolivia, Ages: 20−24
Data points enclosed with a diamond are reference sources

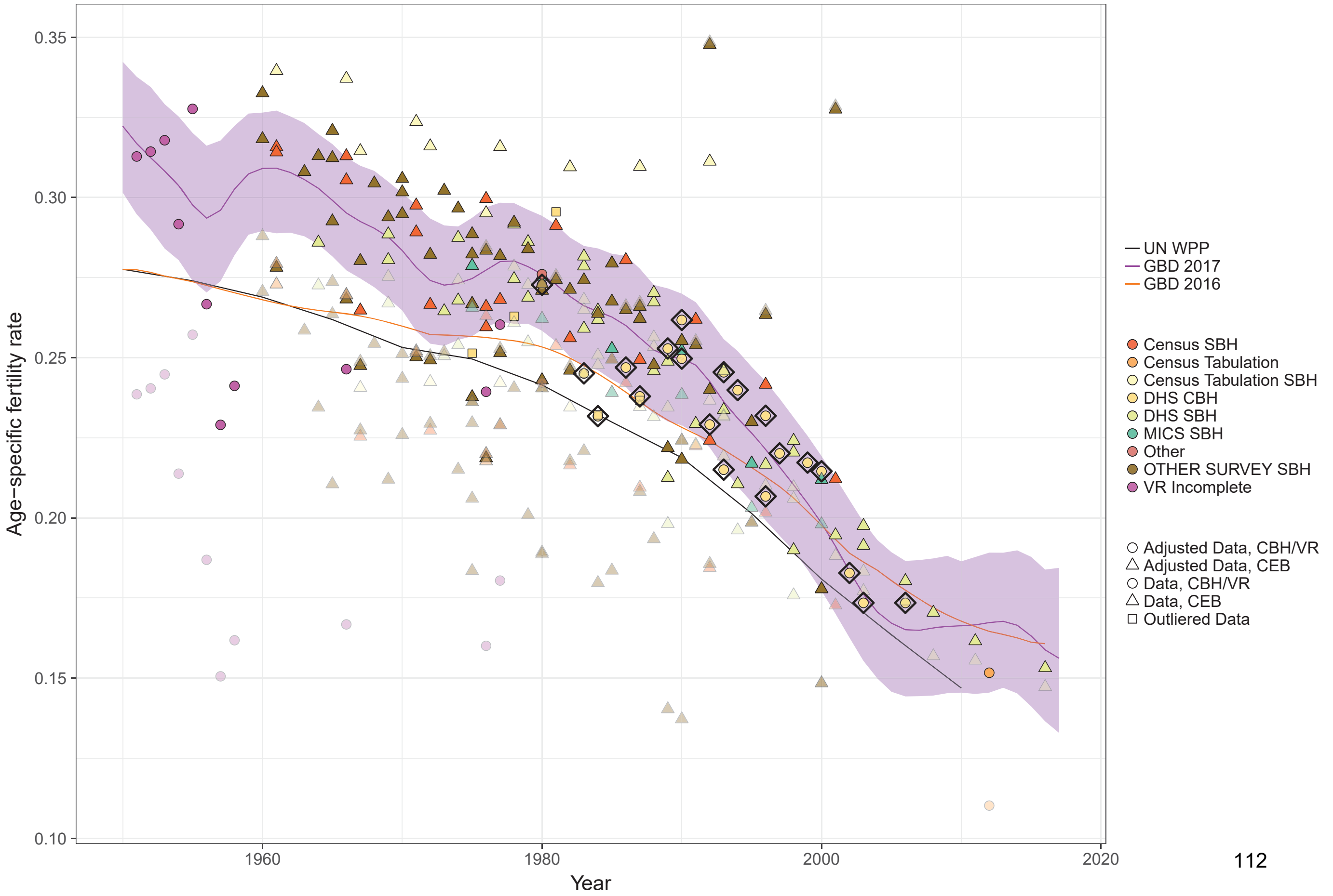

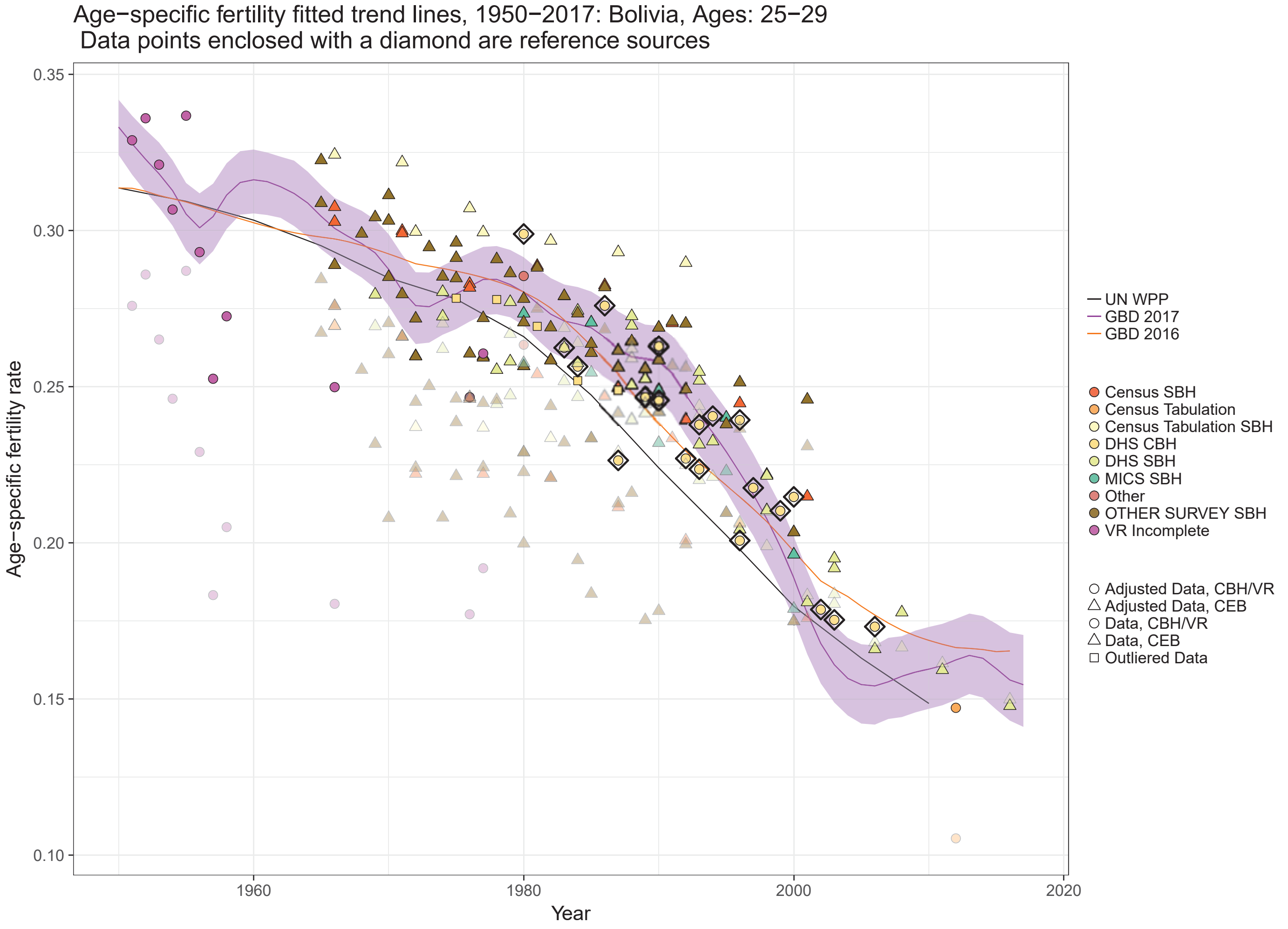

Age−specific fertility fitted trend lines, 1950−2017: Bolivia, Ages: 25−29
Data points enclosed with a diamond are reference sources
Age−specific fertility rate
0.35
0.30
0.25
0.20
0.15
0.10
1960
1980
2000
2020
Year
UN WPP
GBD 2017
GBD 2016
Census SBH
Census Tabulation
Census Tabulation SBH
DHS CBH
DHS SBH
MICS SBH
Other
OTHER SURVEY SBH
VR Incomplete
Adjusted Data, CBH/VR
Adjusted Data, CEB
Data, CBH/VR
Data, CEB
Outliered Data

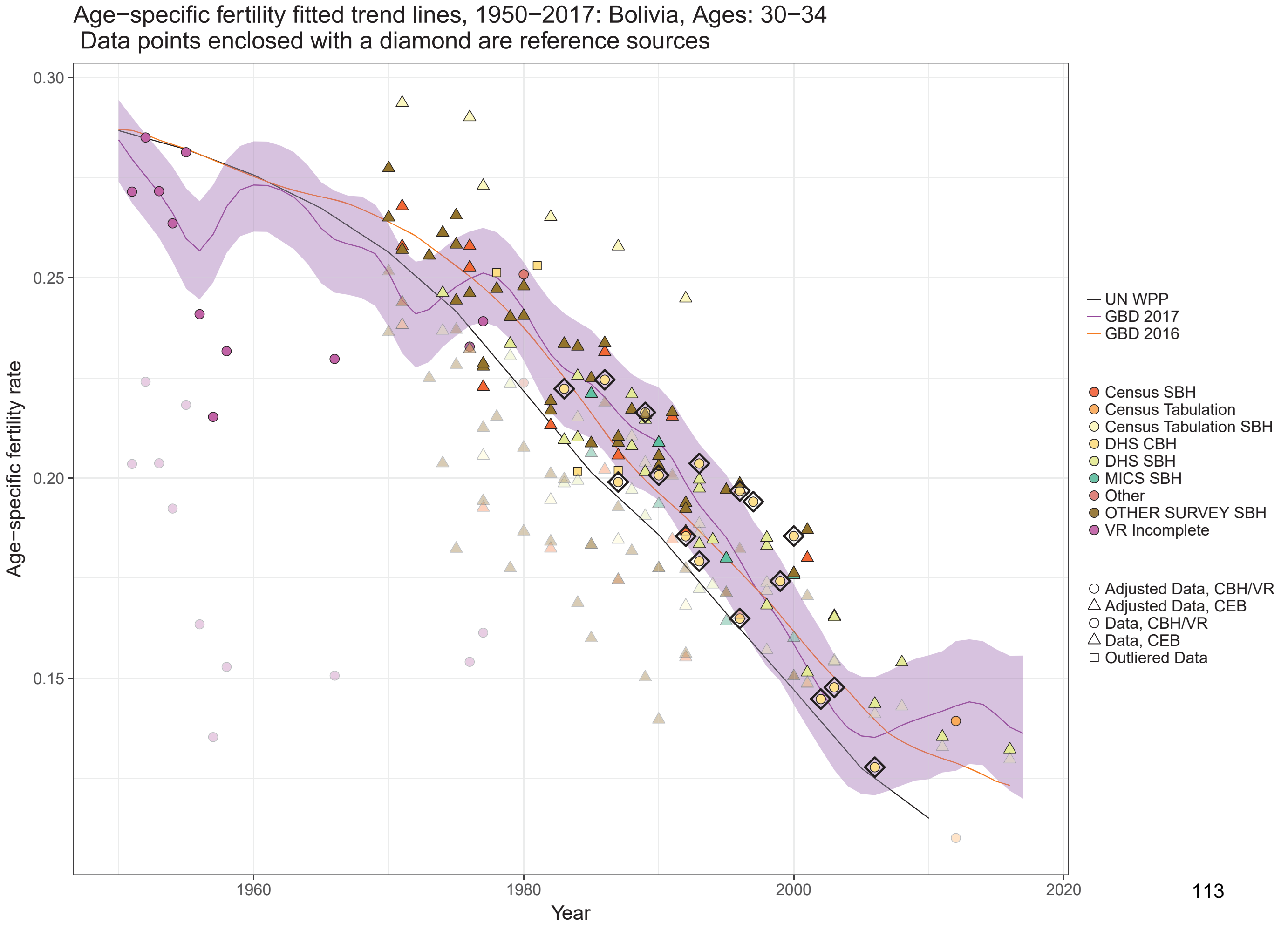

Age−specific fertility fitted trend lines, 1950−2017: Bolivia, Ages: 30−34
Data points enclosed with a diamond are reference sources
Age−specific fertility rate
0.30
0.25
0.20
0.15
1960
1980
2000
2020
Year
UN WPP
GBD 2017
GBD 2016
Census SBH
Census Tabulation
Census Tabulation SBH
DHS CBH
DHS SBH
MICS SBH
Other
OTHER SURVEY SBH
VR Incomplete
Adjusted Data, CBH/VR
Adjusted Data, CEB
Data, CBH/VR
Data, CEB
Outliered Data

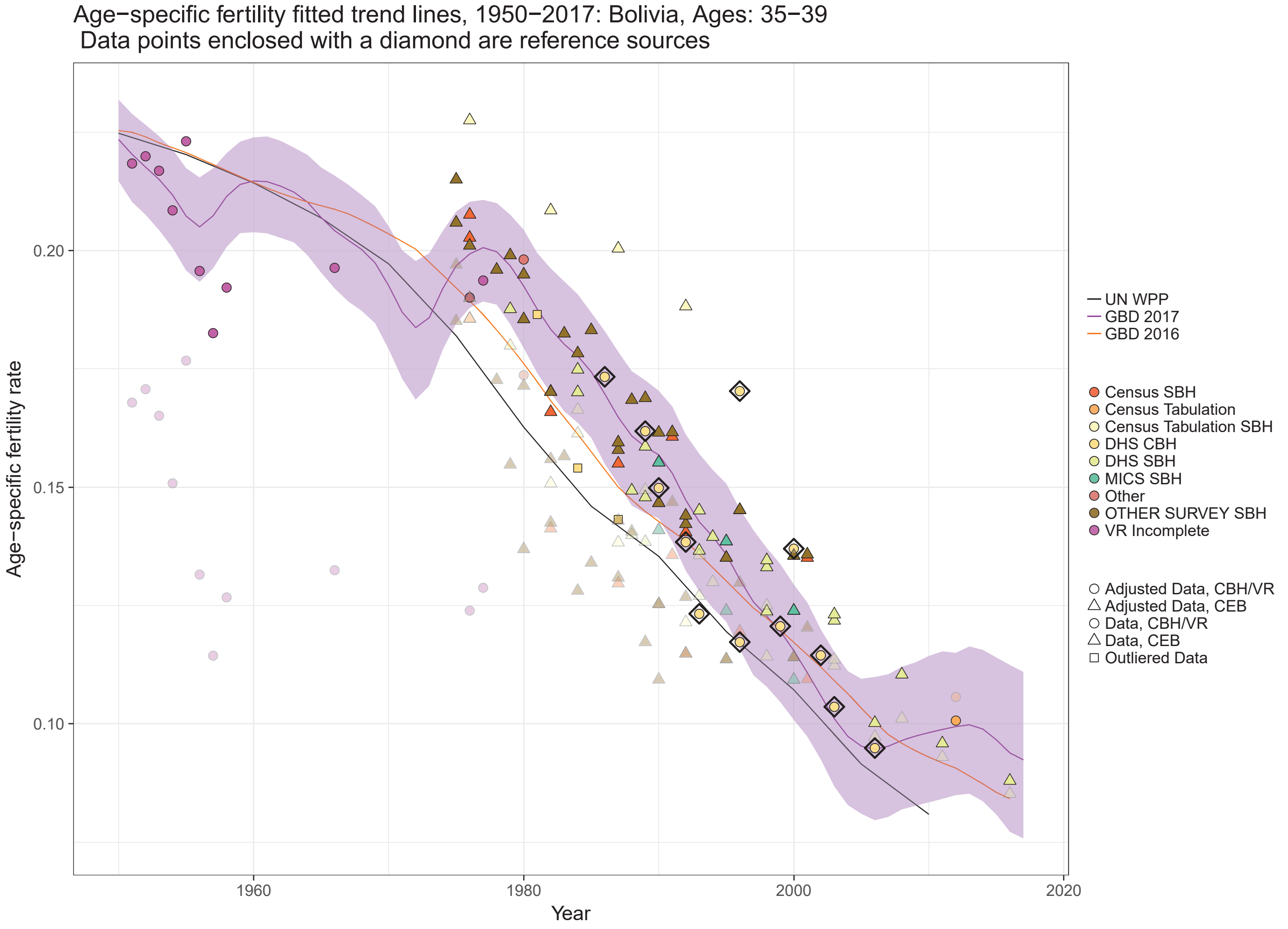
Age−specific fertility fitted trend lines, 1950−2017: Bolivia, Ages: 35−39
Data points enclosed with a diamond are reference sources
Age-specific fertility rate
Year
UN WPP
GBD 2017
GBD 2016
Census SBH
Census Tabulation
Census Tabulation SBH
DHS CBH
DHS SBH
MICS SBH
Other
OTHER SURVEY SBH
VR Incomplete
Adjusted Data, CBH/VR
Adjusted Data, CEB
Data, CBH/VR
Data, CEB
Outliered Data

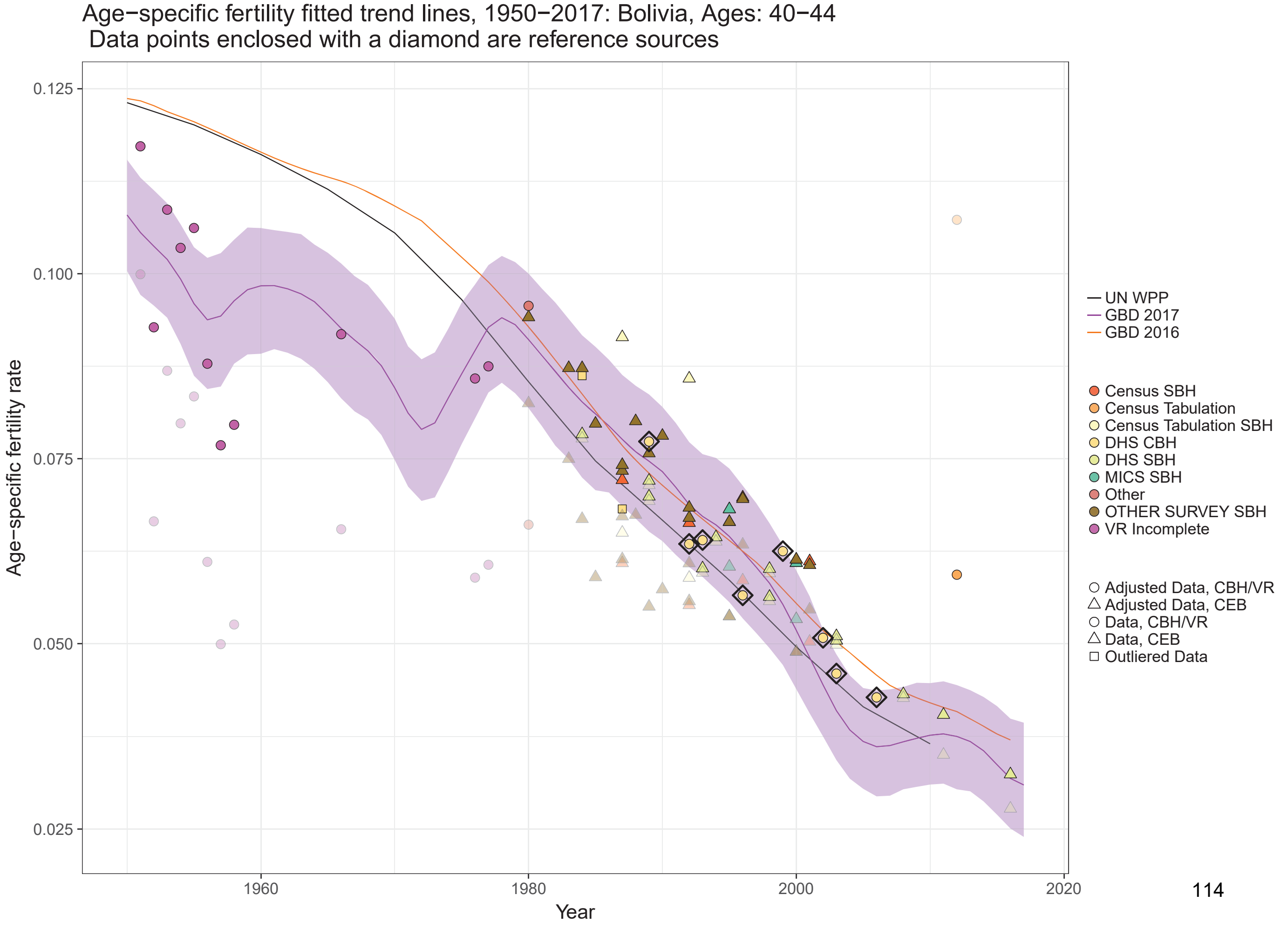
Age−specific fertility fitted trend lines, 1950−2017: Bolivia, Ages: 40−44
Data points enclosed with a diamond are reference sources
Age-specific fertility rate
Year
UN WPP
GBD 2017
GBD 2016
Census SBH
Census Tabulation
Census Tabulation SBH
DHS CBH
DHS SBH
MICS SBH
Other
OTHER SURVEY SBH
VR Incomplete
Adjusted Data, CBH/VR
Adjusted Data, CEB
Data, CBH/VR
Data, CEB
Outliered Data

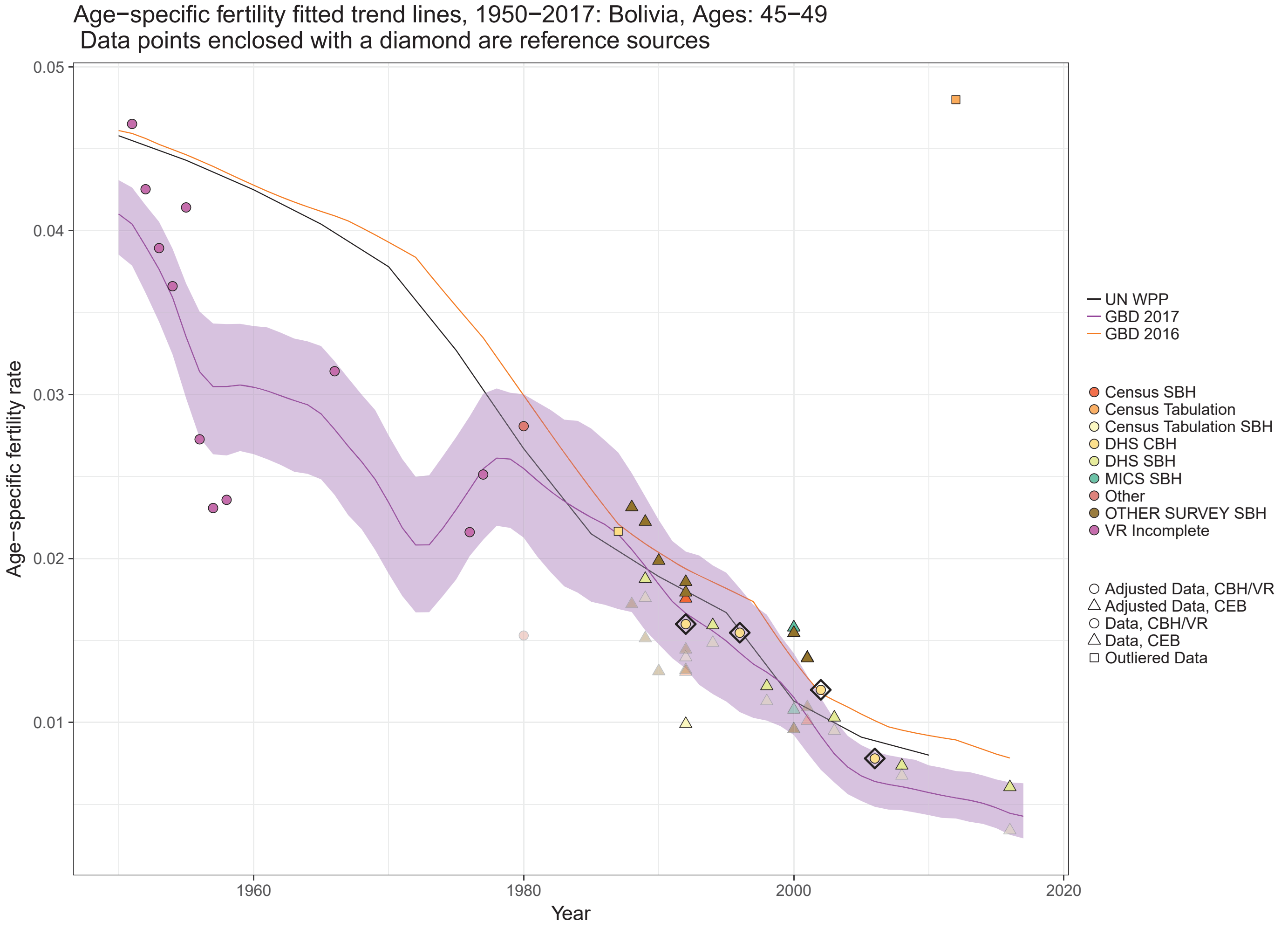
Age−specific fertility fitted trend lines, 1950−2017: Bolivia, Ages: 45−49
Data points enclosed with a diamond are reference sources
Age−specific fertility rate
Year
UN WPP
GBD 2017
GBD 2016
Census SBH
Census Tabulation
Census Tabulation SBH
DHS CBH
DHS SBH
MICS SBH
Other
OTHER SURVEY SBH
VR Incomplete
Adjusted Data, CBH/VR
Adjusted Data, CEB
Data, CBH/VR
Data, CEB
Outliered Data

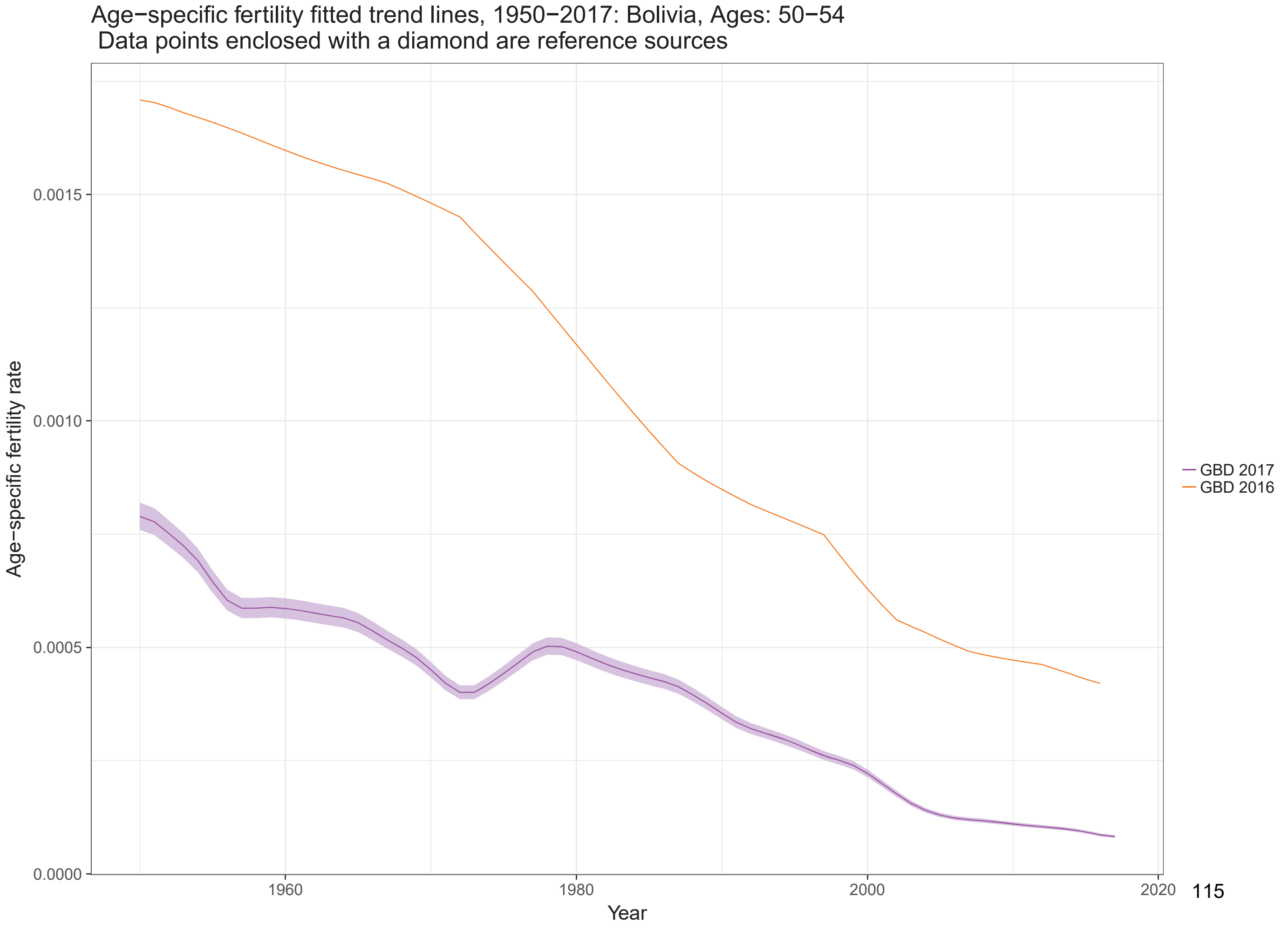
Age−specific fertility fitted trend lines, 1950−2017: Bolivia, Ages: 50−54
Data points enclosed with a diamond are reference sources
Age−specific fertility rate
Year
GBD 2017
GBD 2016

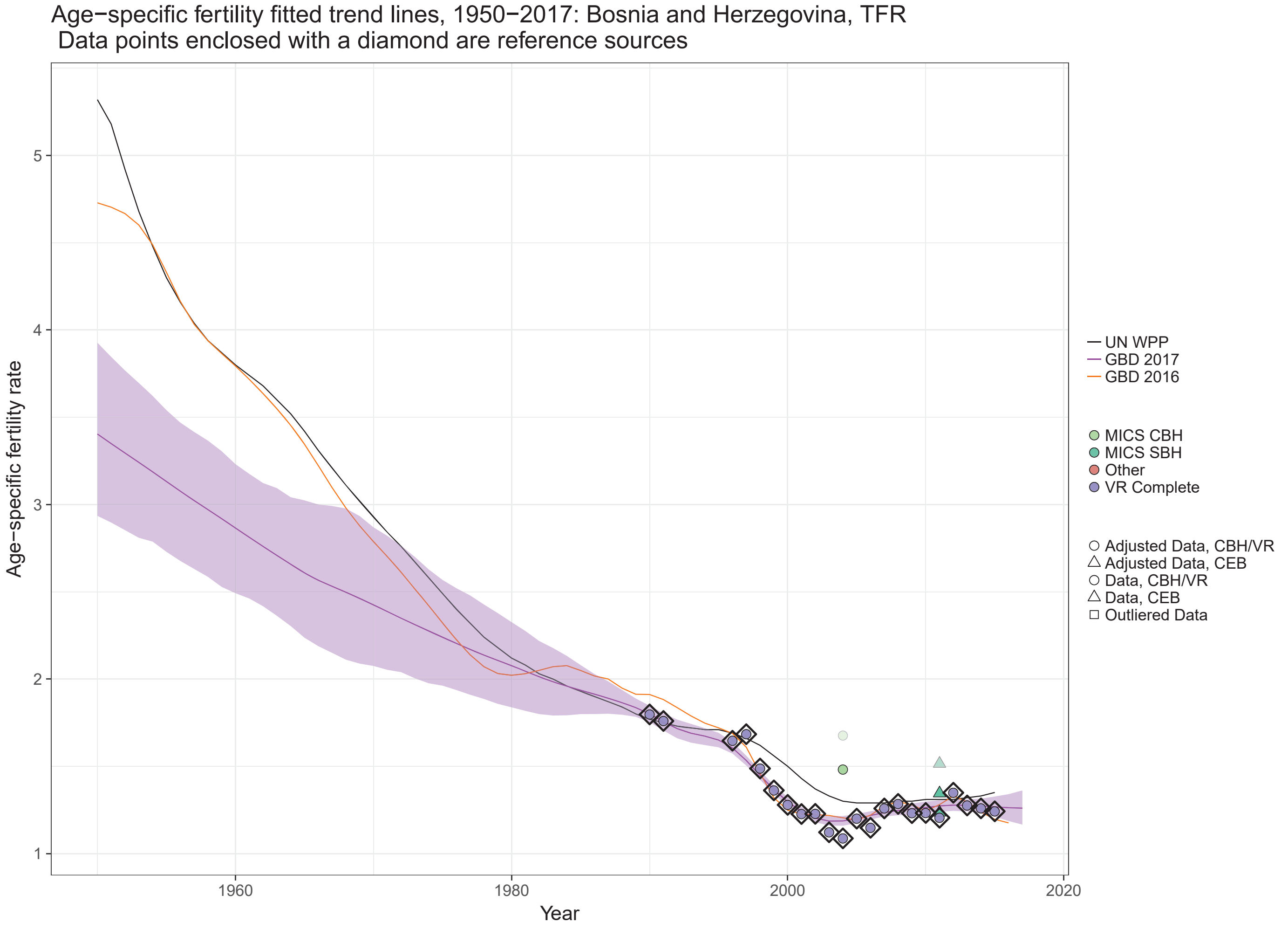

Age−specific fertility fitted trend lines, 1950−2017: Bosnia and Herzegovina, TFR
Data points enclosed with a diamond are reference sources
Age−specific fertility rate
Year
UN WPP
GBD 2017
GBD 2016
MICS CBH
MICS SBH
Other
VR Complete
Adjusted Data, CBH/VR
Adjusted Data, CEB
Data, CBH/VR
Data, CEB
Outliered Data

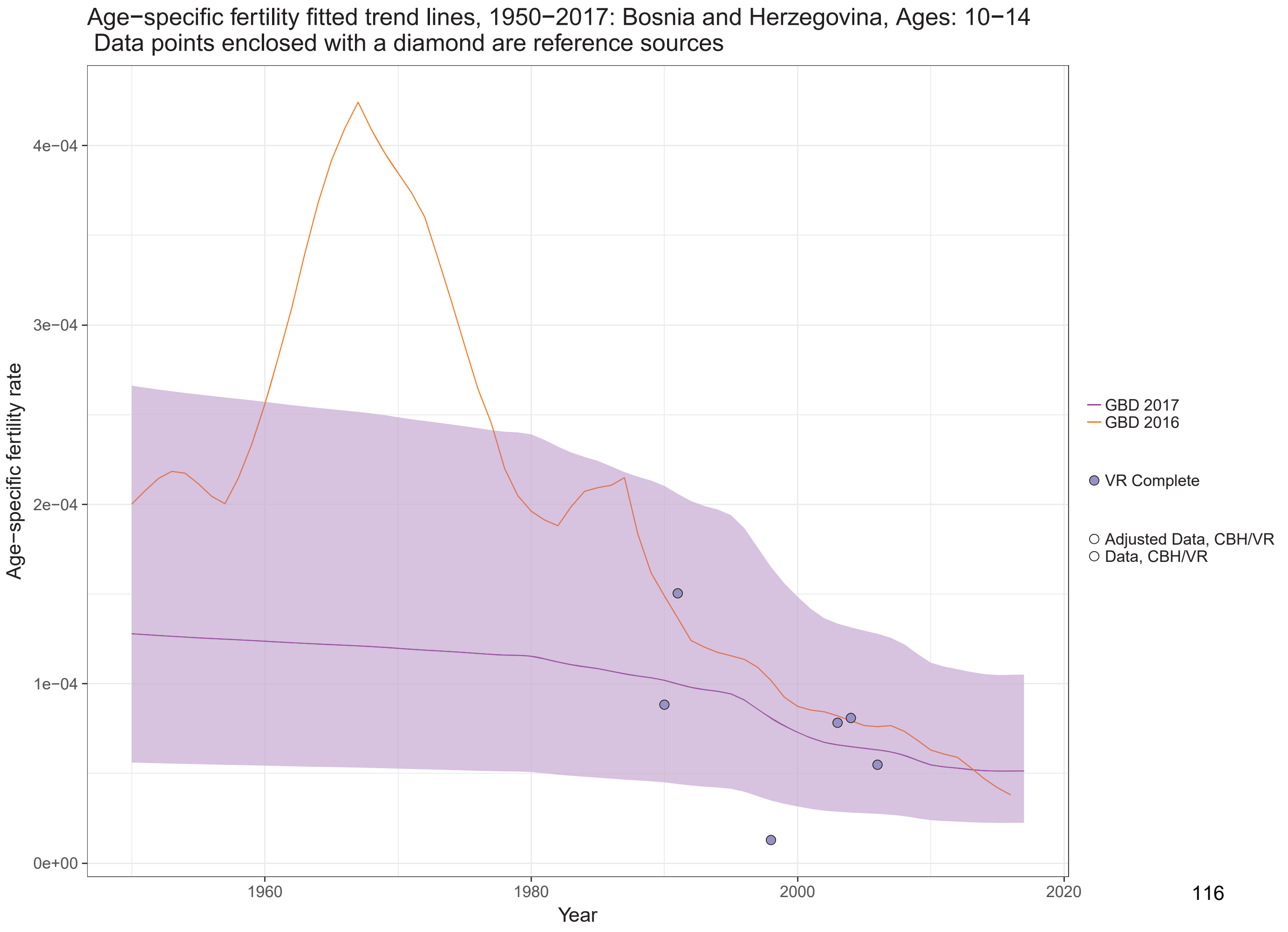

Age−specific fertility fitted trend lines, 1950−2017: Bosnia and Herzegovina, Ages: 10−14
Data points enclosed with a diamond are reference sources
Age−specific fertility rate
Year
GBD 2017
GBD 2016
VR Complete
Adjusted Data, CBH/VR
Data, CBH/VR

Age−specific fertility fitted trend lines, 1950−2017: Bosnia and Herzegovina, Ages: 15−19
Data points enclosed with a diamond are reference sources

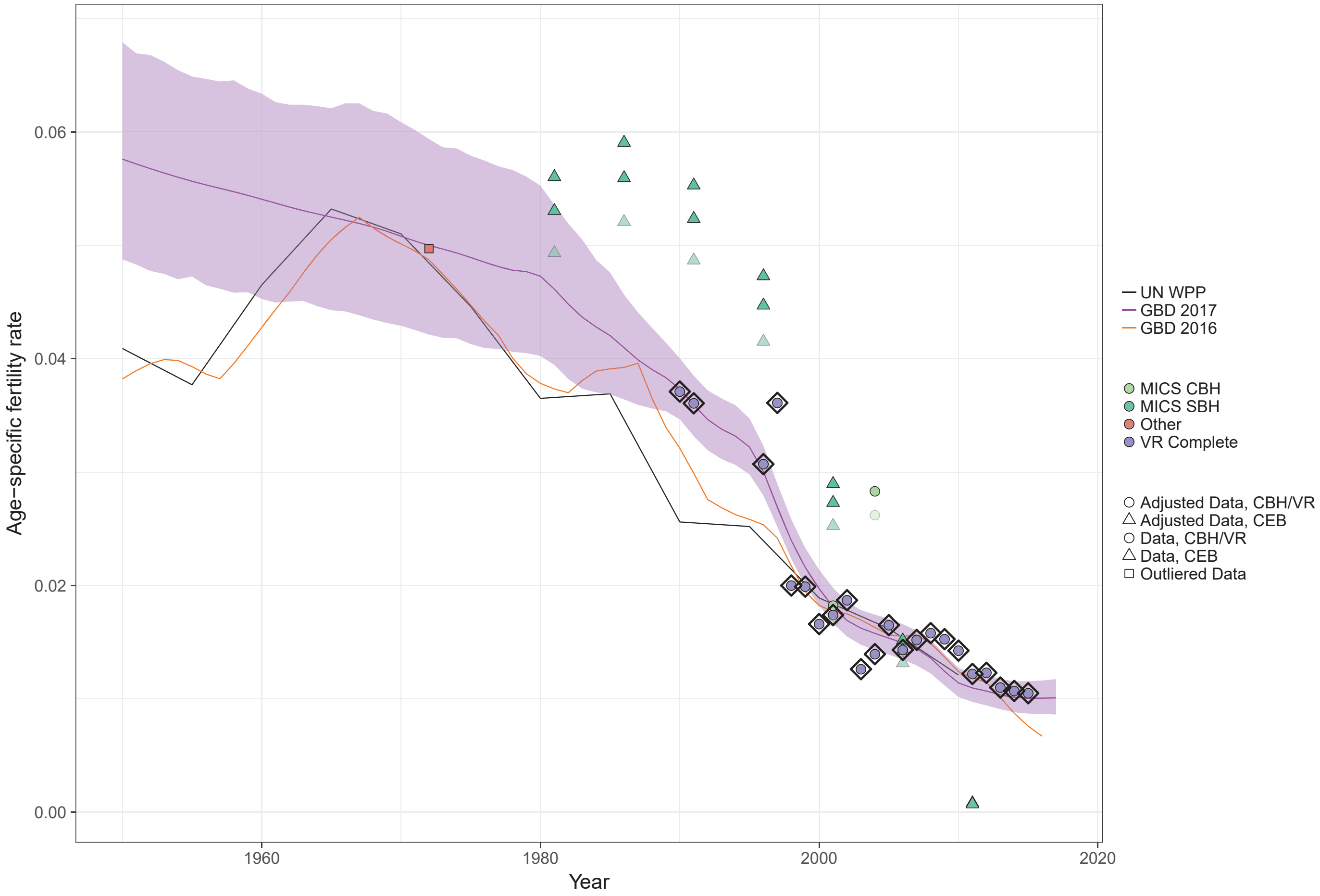

Age−specific fertility fitted trend lines, 1950−2017: Bosnia and Herzegovina, Ages: 20−24
Data points enclosed with a diamond are reference sources

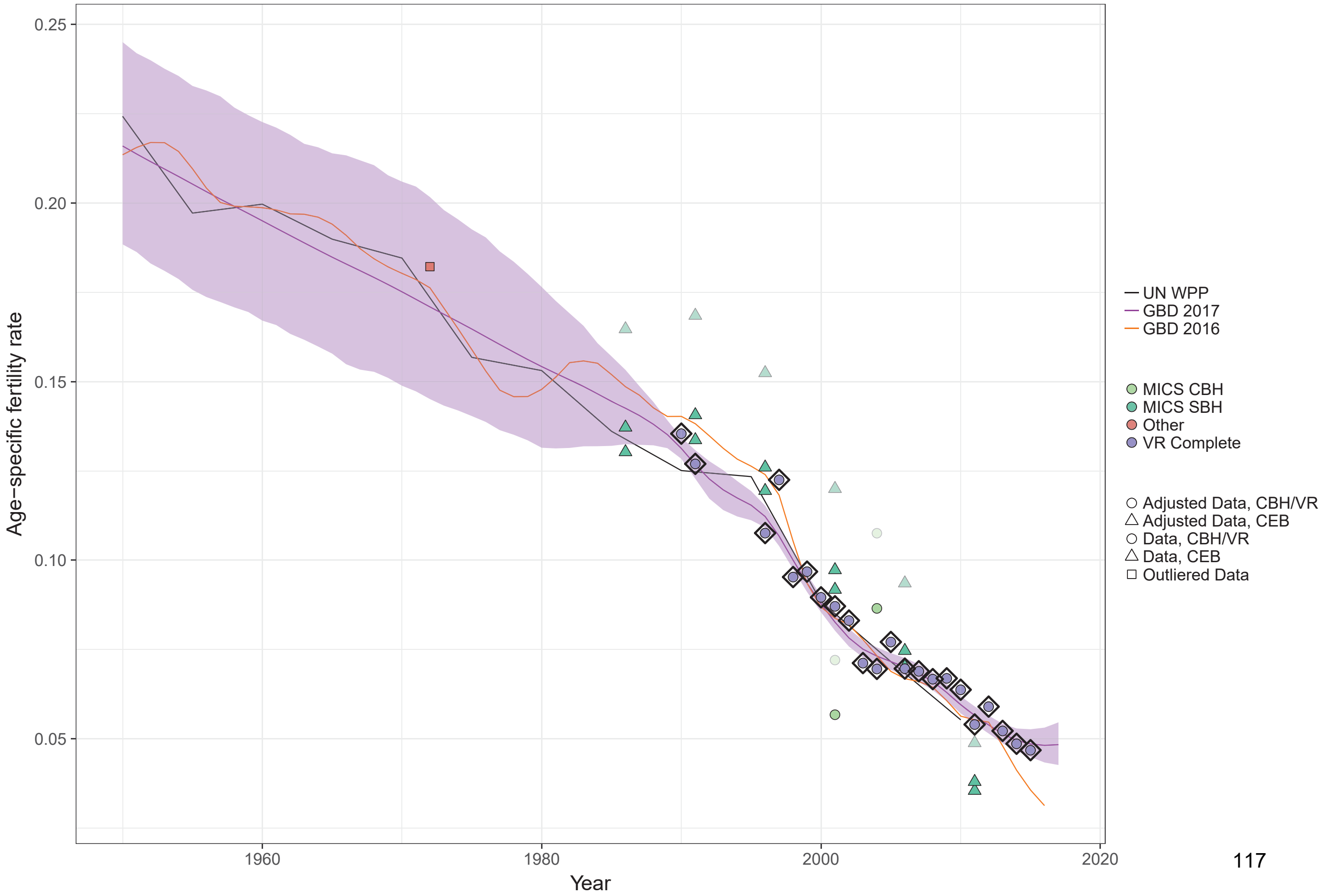

Age−specific fertility fitted trend lines, 1950−2017: Bosnia and Herzegovina, Ages: 25−29
Data points enclosed with a diamond are reference sources

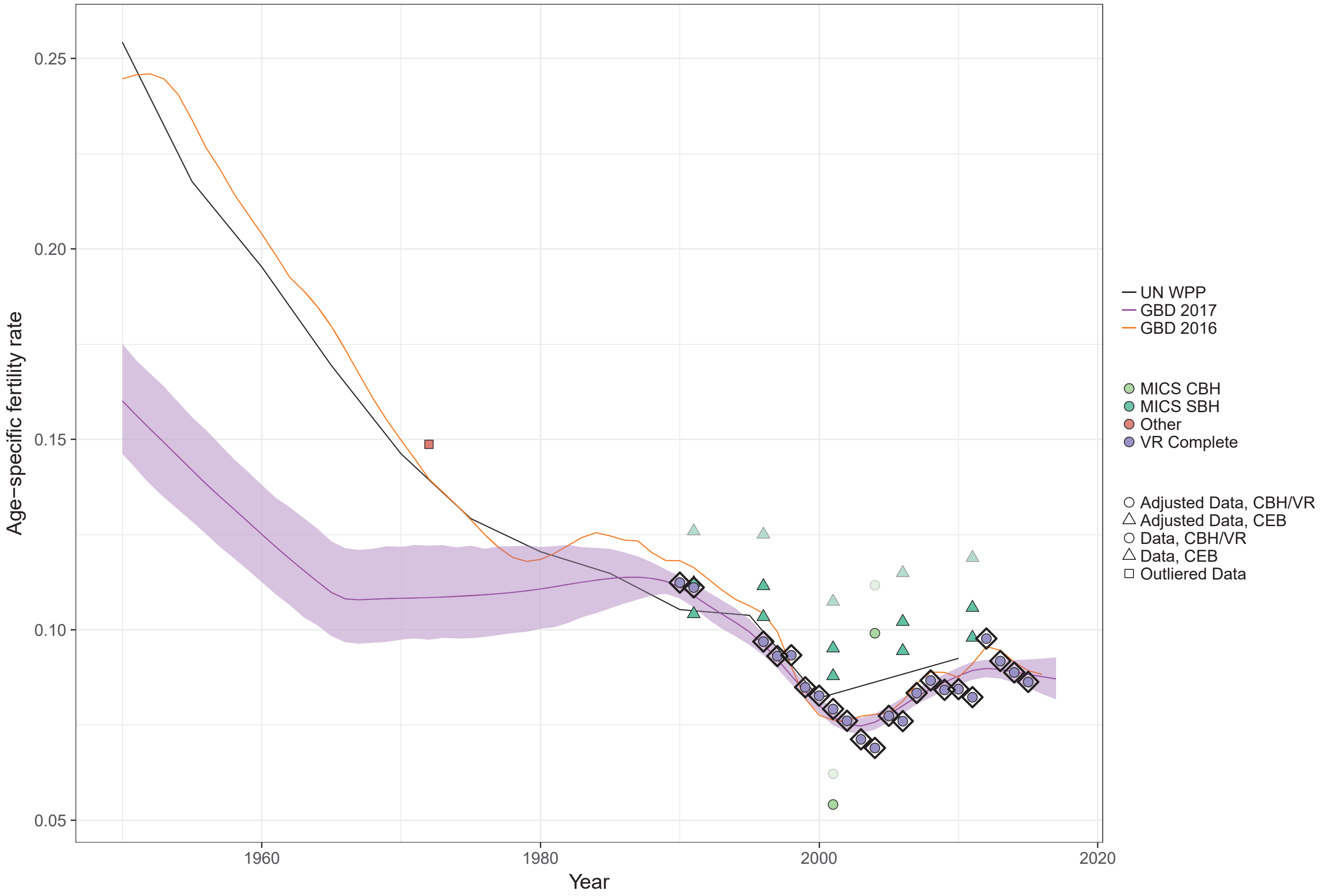

Age−specific fertility fitted trend lines, 1950−2017: Bosnia and Herzegovina, Ages: 30−34
Data points enclosed with a diamond are reference sources

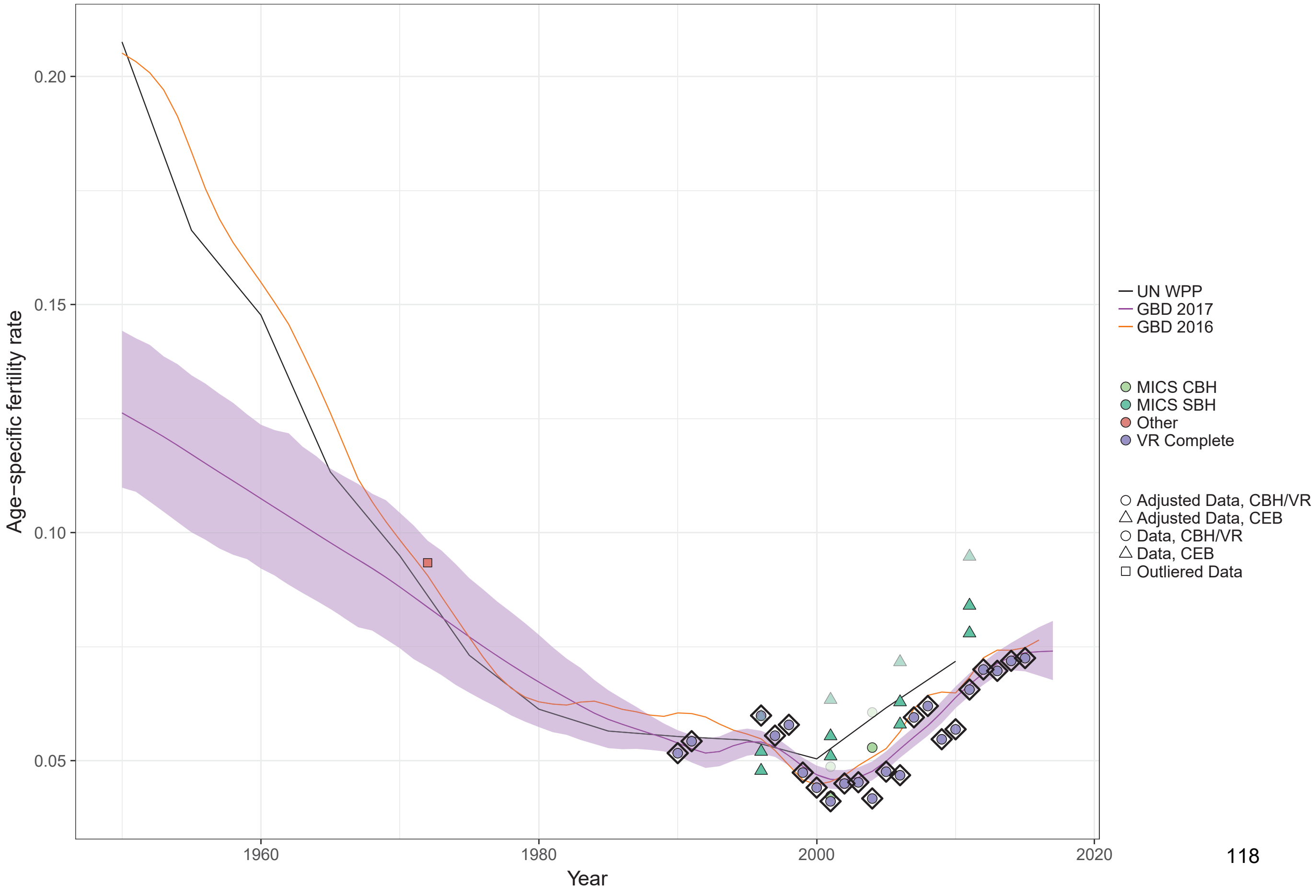

Age−specific fertility fitted trend lines, 1950−2017: Bosnia and Herzegovina, Ages: 35−39
Data points enclosed with a diamond are reference sources

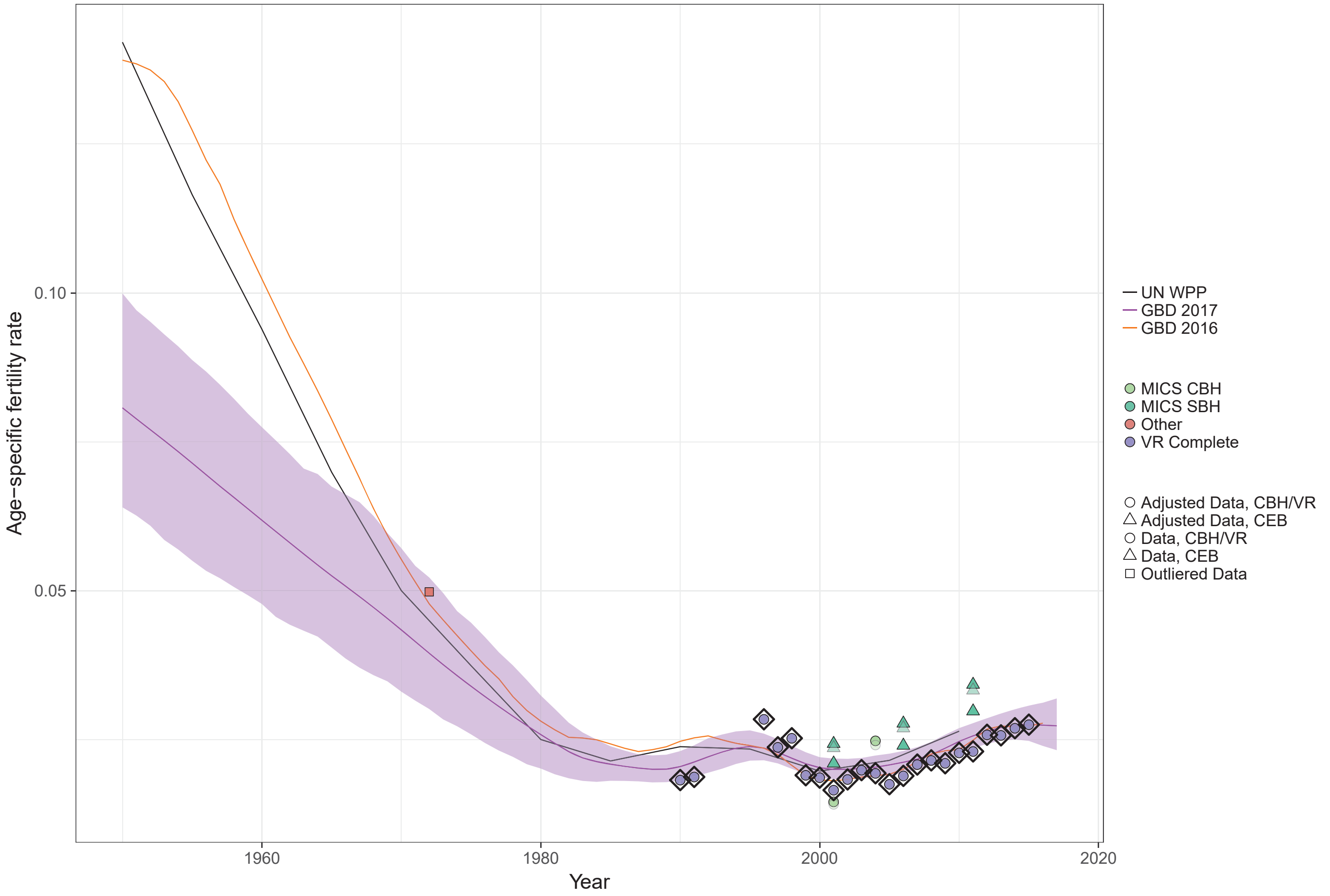

Age−specific fertility fitted trend lines, 1950−2017: Bosnia and Herzegovina, Ages: 40−44
Data points enclosed with a diamond are reference sources

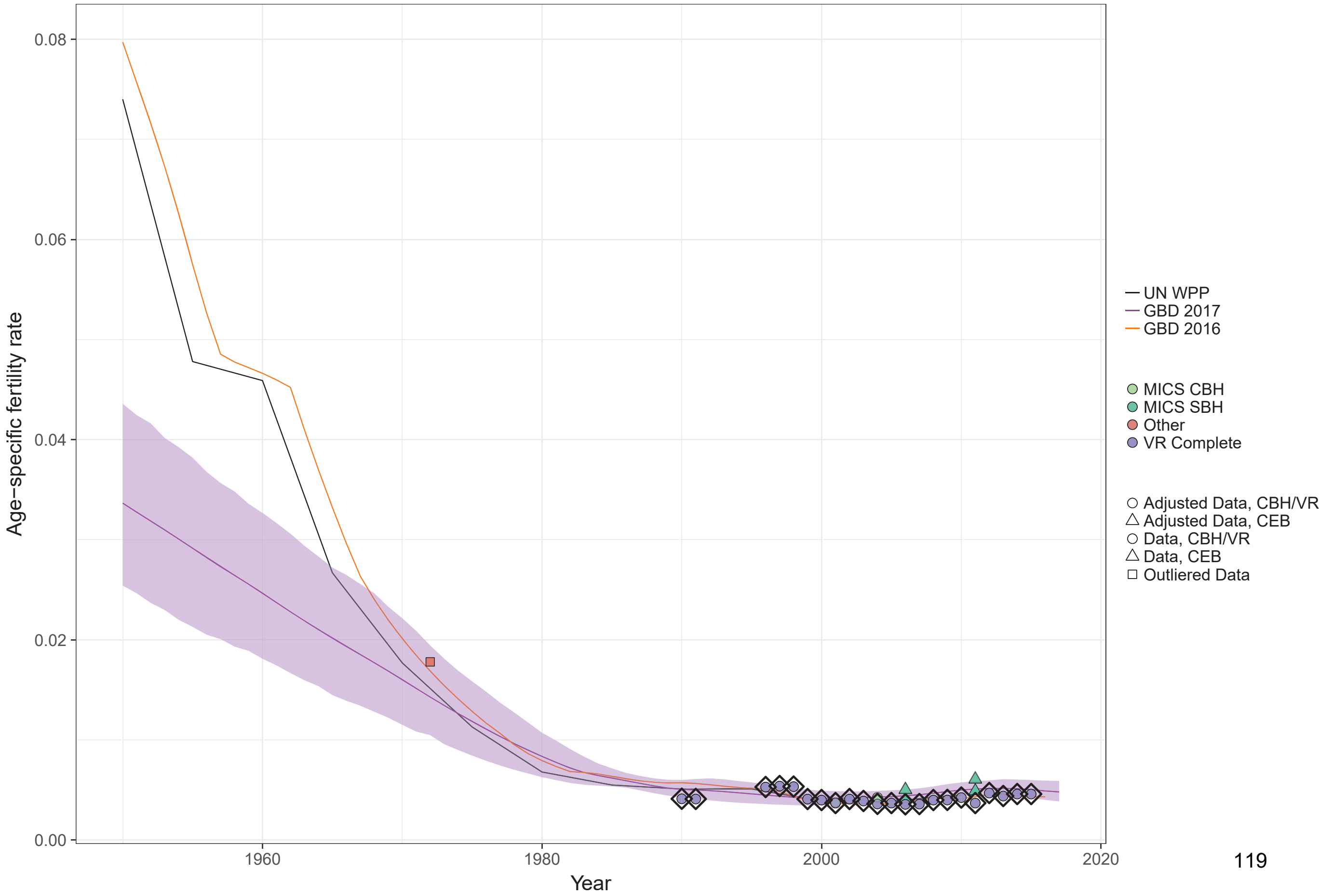

Age−specific fertility fitted trend lines, 1950−2017: Bosnia and Herzegovina, Ages: 45−49
Data points enclosed with a diamond are reference sources

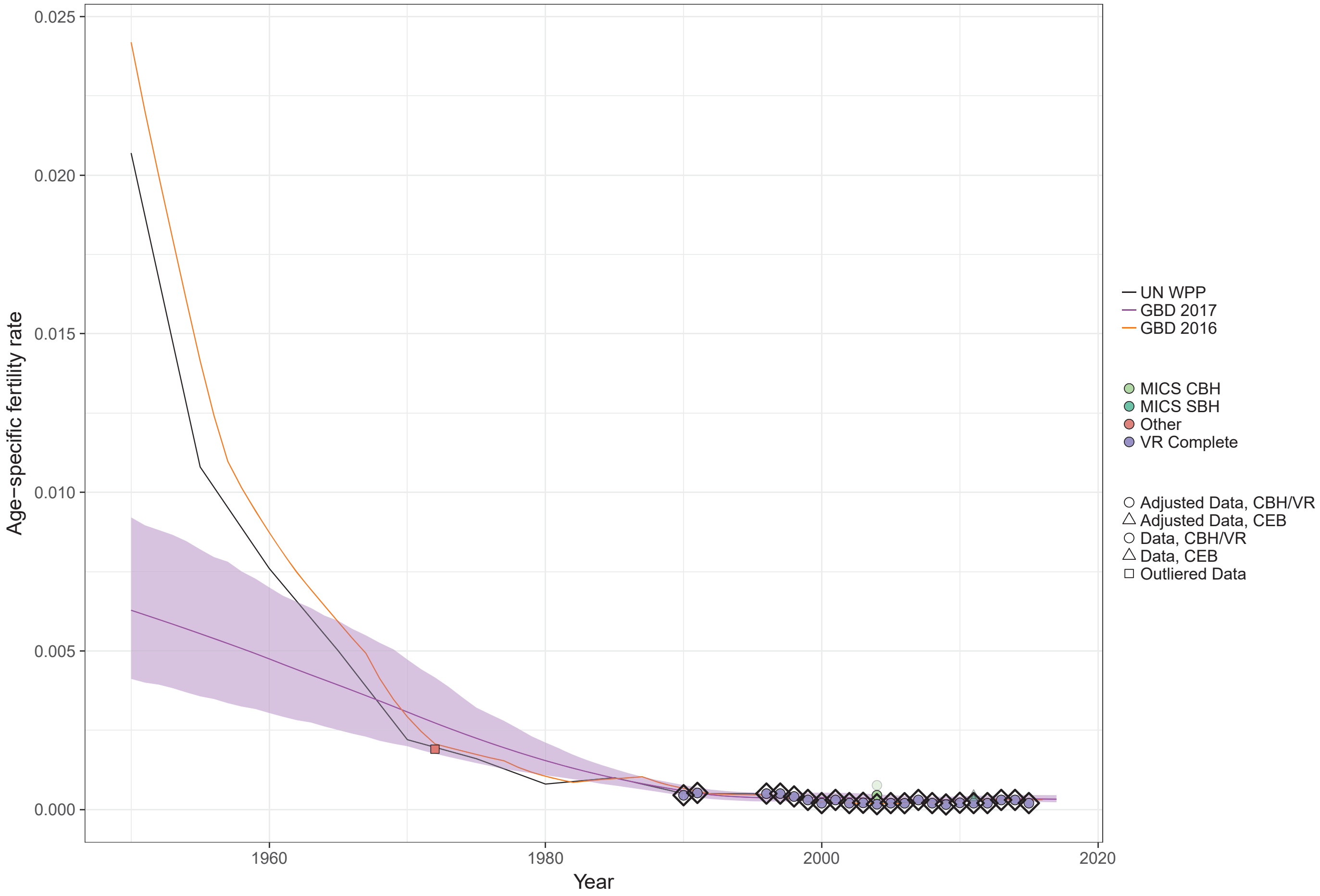

Age−specific fertility fitted trend lines, 1950−2017: Bosnia and Herzegovina, Ages: 50−54
Data points enclosed with a diamond are reference sources

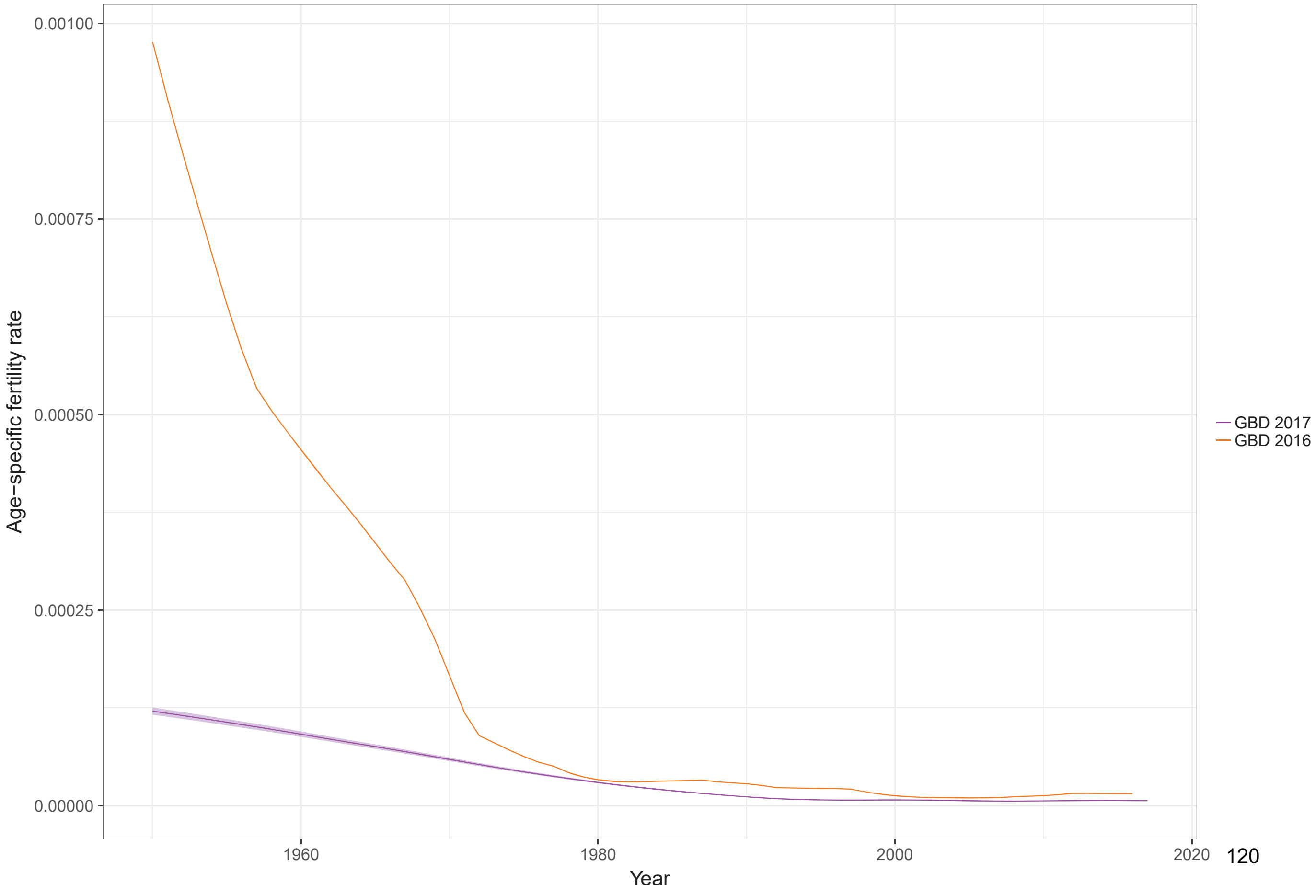

Age−specific fertility fitted trend lines, 1950−2017: Botswana, TFR
Data points enclosed with a diamond are reference sources

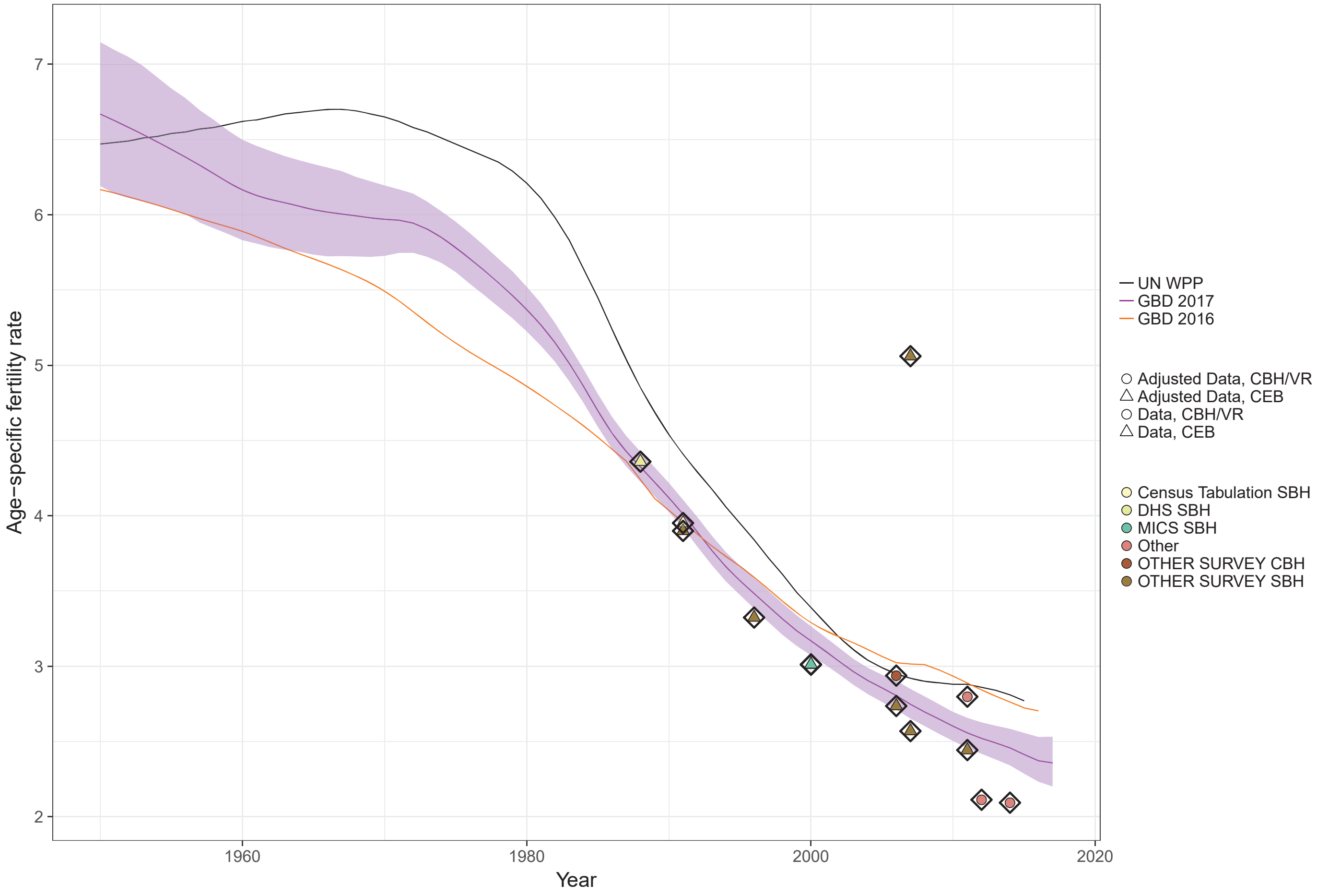

Age−specific fertility fitted trend lines, 1950−2017: Botswana, Ages: 10−14
Data points enclosed with a diamond are reference sources

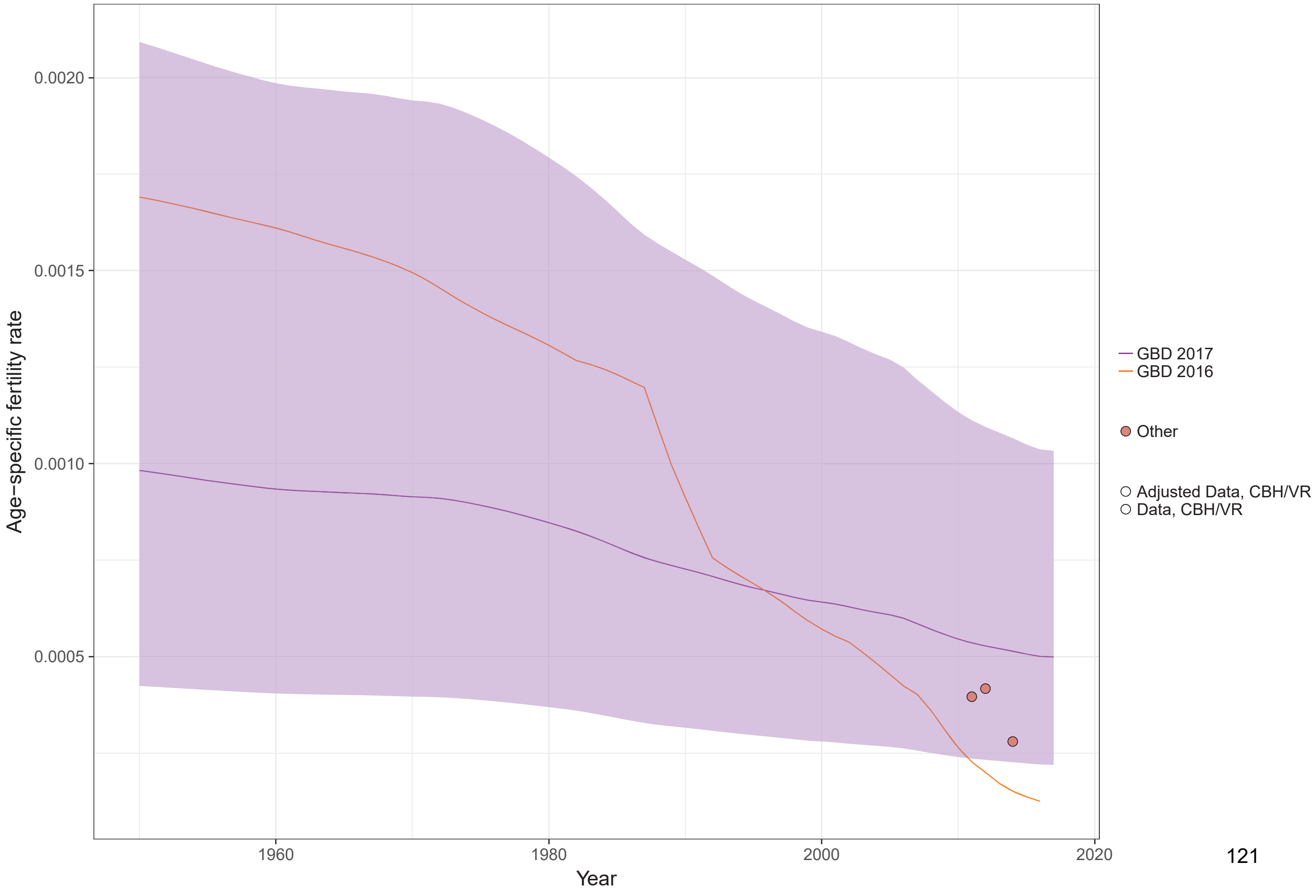

Age−specific fertility fitted trend lines, 1950−2017: Botswana, Ages: 15−19
Data points enclosed with a diamond are reference sources

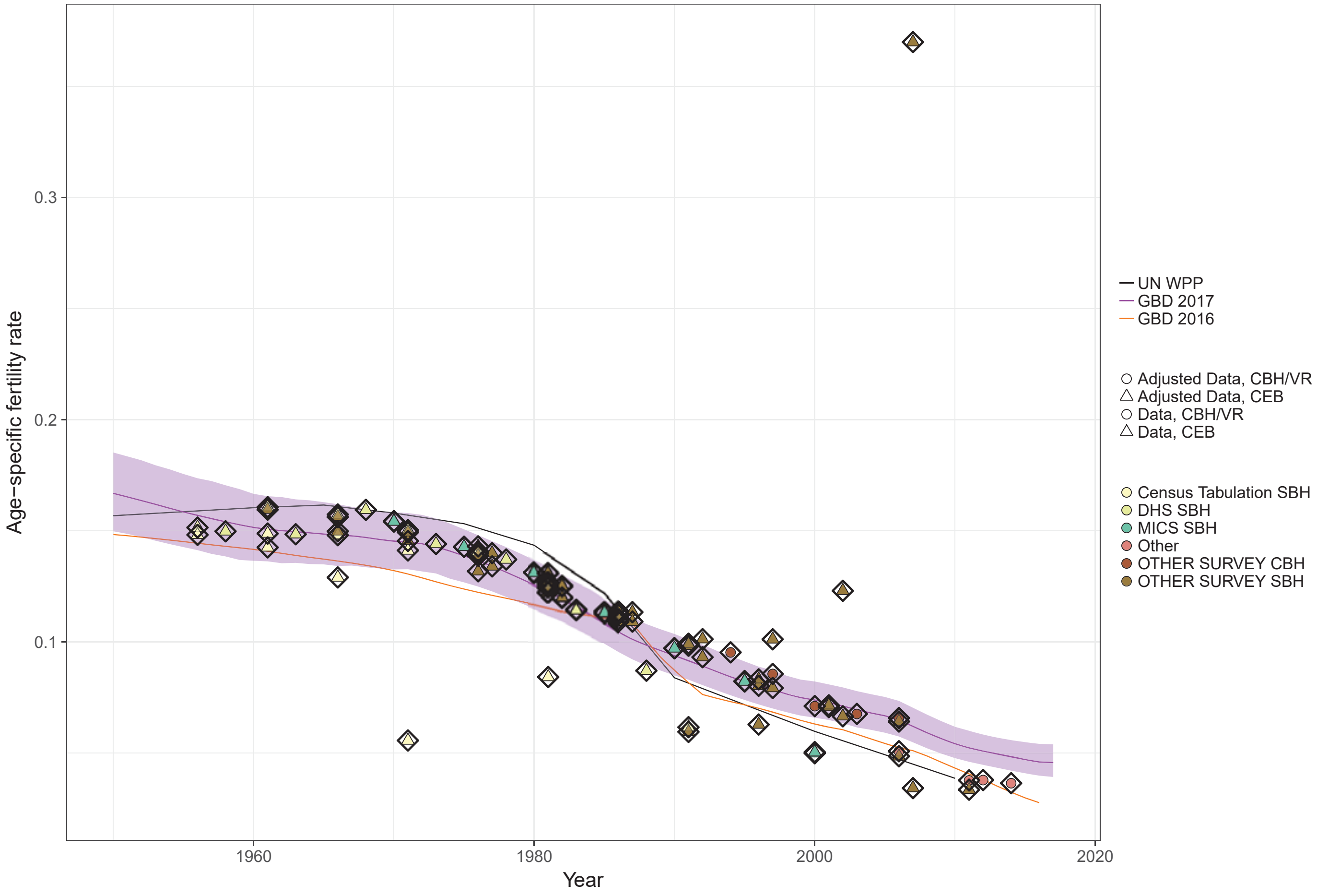

Age−specific fertility fitted trend lines, 1950−2017: Botswana, Ages: 20−24
Data points enclosed with a diamond are reference sources

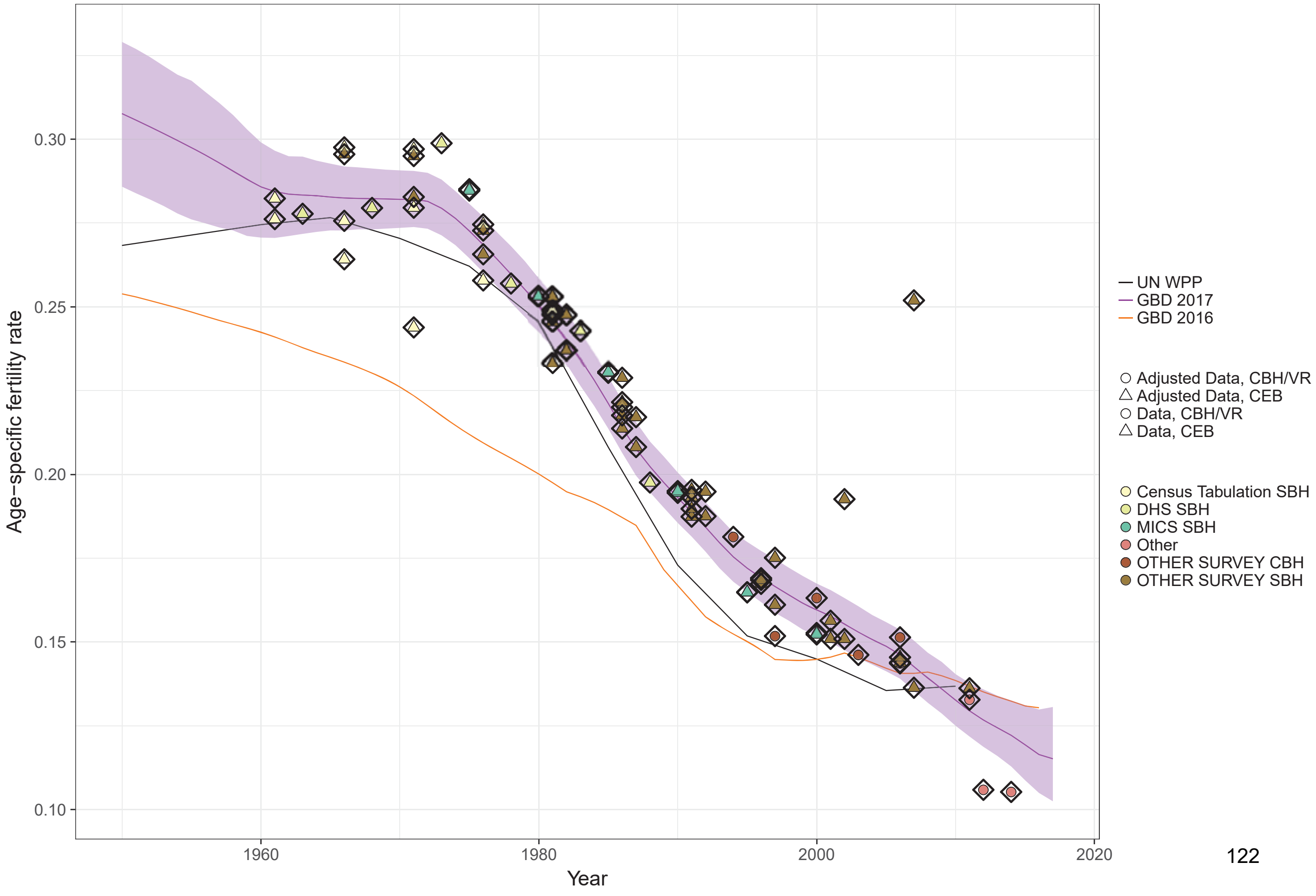

Age−specific fertility fitted trend lines, 1950−2017: Botswana, Ages: 25−29
Data points enclosed with a diamond are reference sources

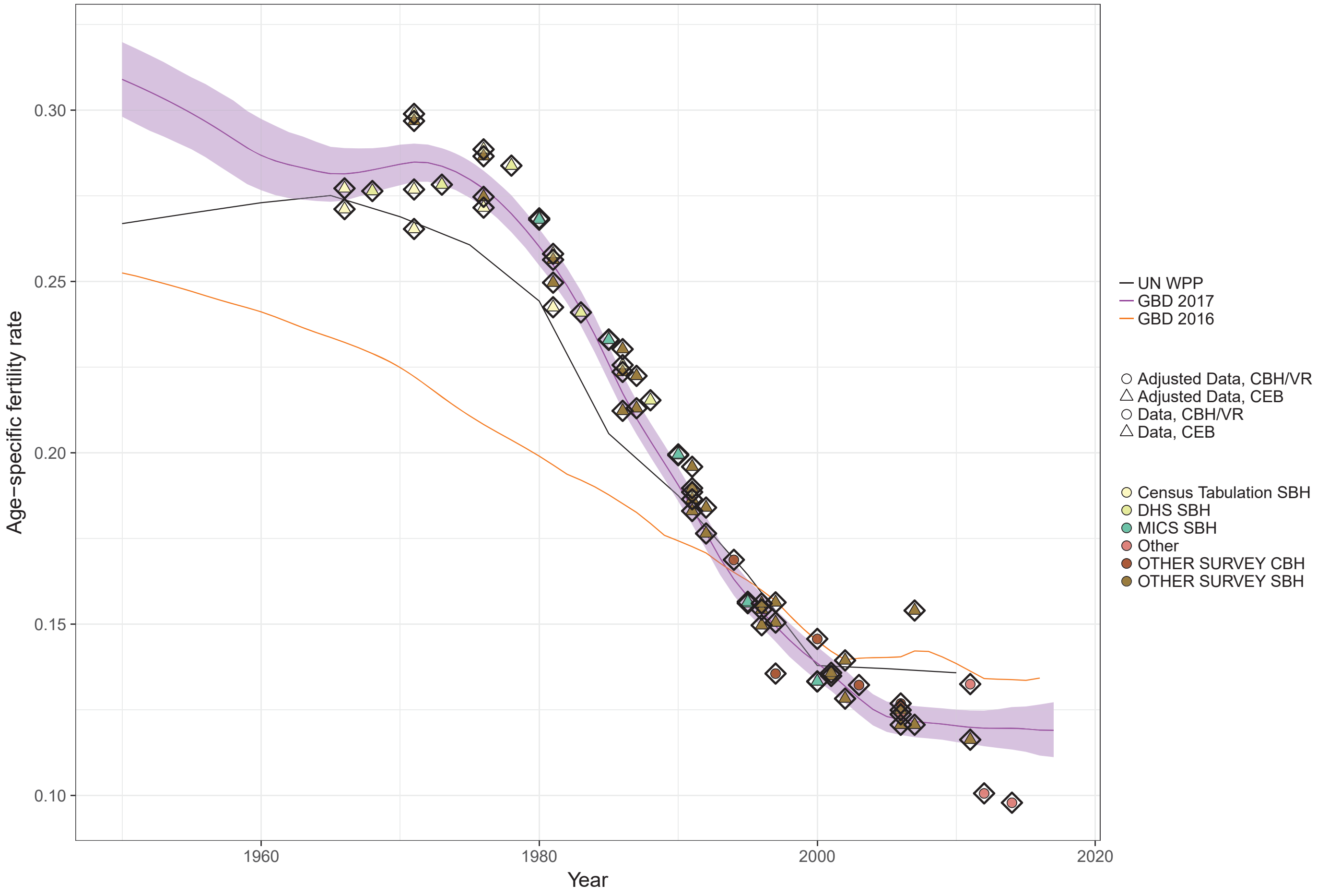

Age−specific fertility fitted trend lines, 1950−2017: Botswana, Ages: 30−34
Data points enclosed with a diamond are reference sources

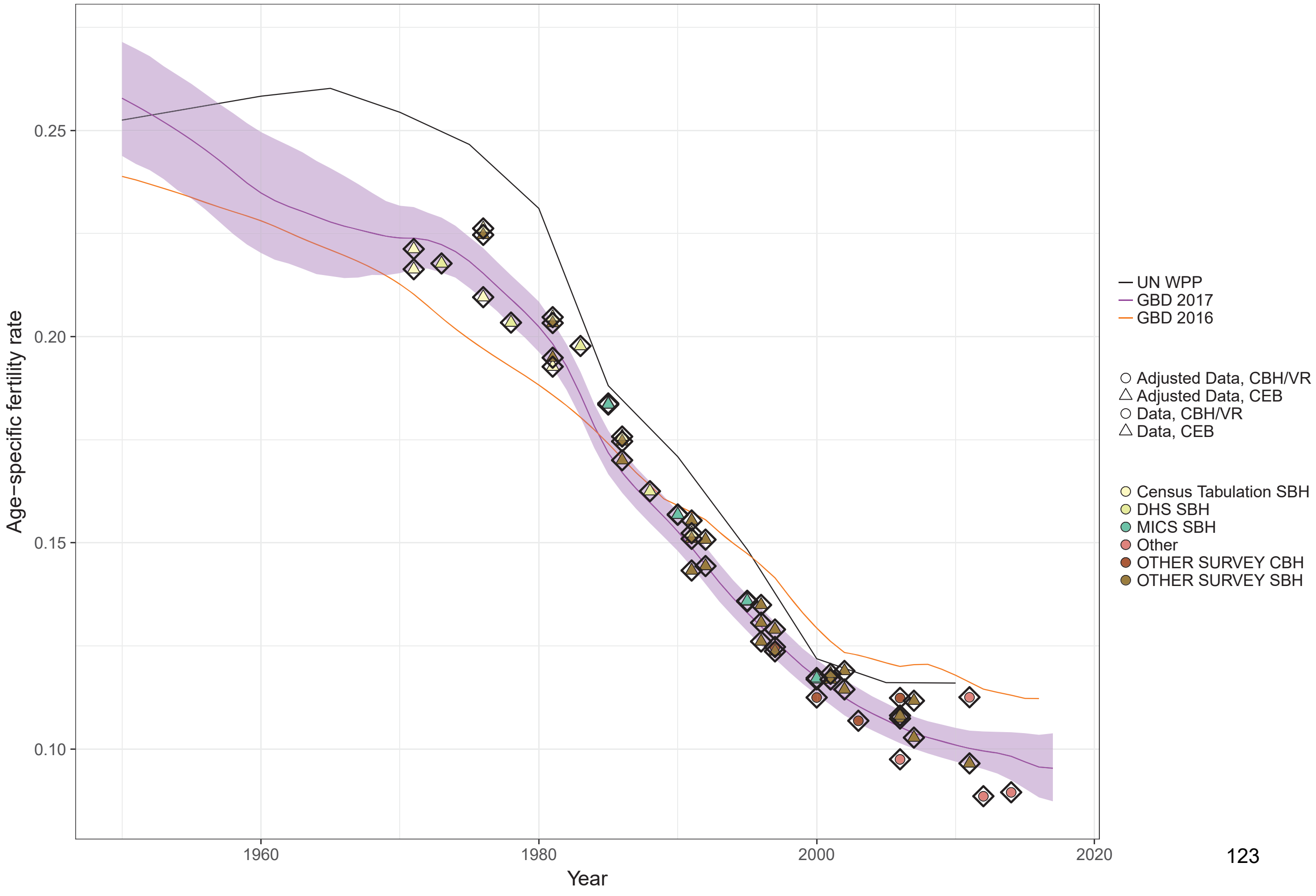

Age−specific fertility fitted trend lines, 1950−2017: Botswana, Ages: 35−39
Data points enclosed with a diamond are reference sources

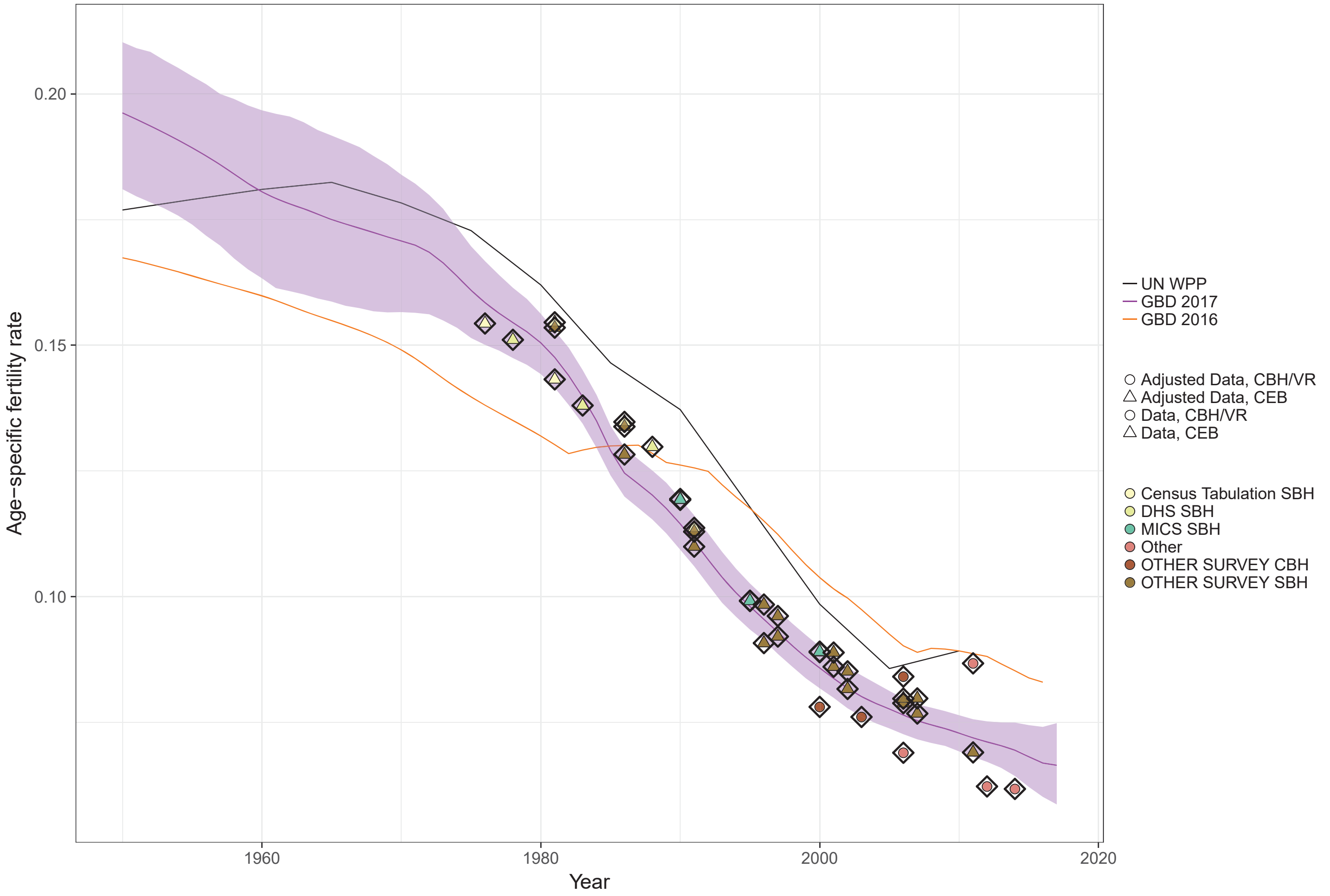

Age−specific fertility fitted trend lines, 1950−2017: Botswana, Ages: 40−44
Data points enclosed with a diamond are reference sources

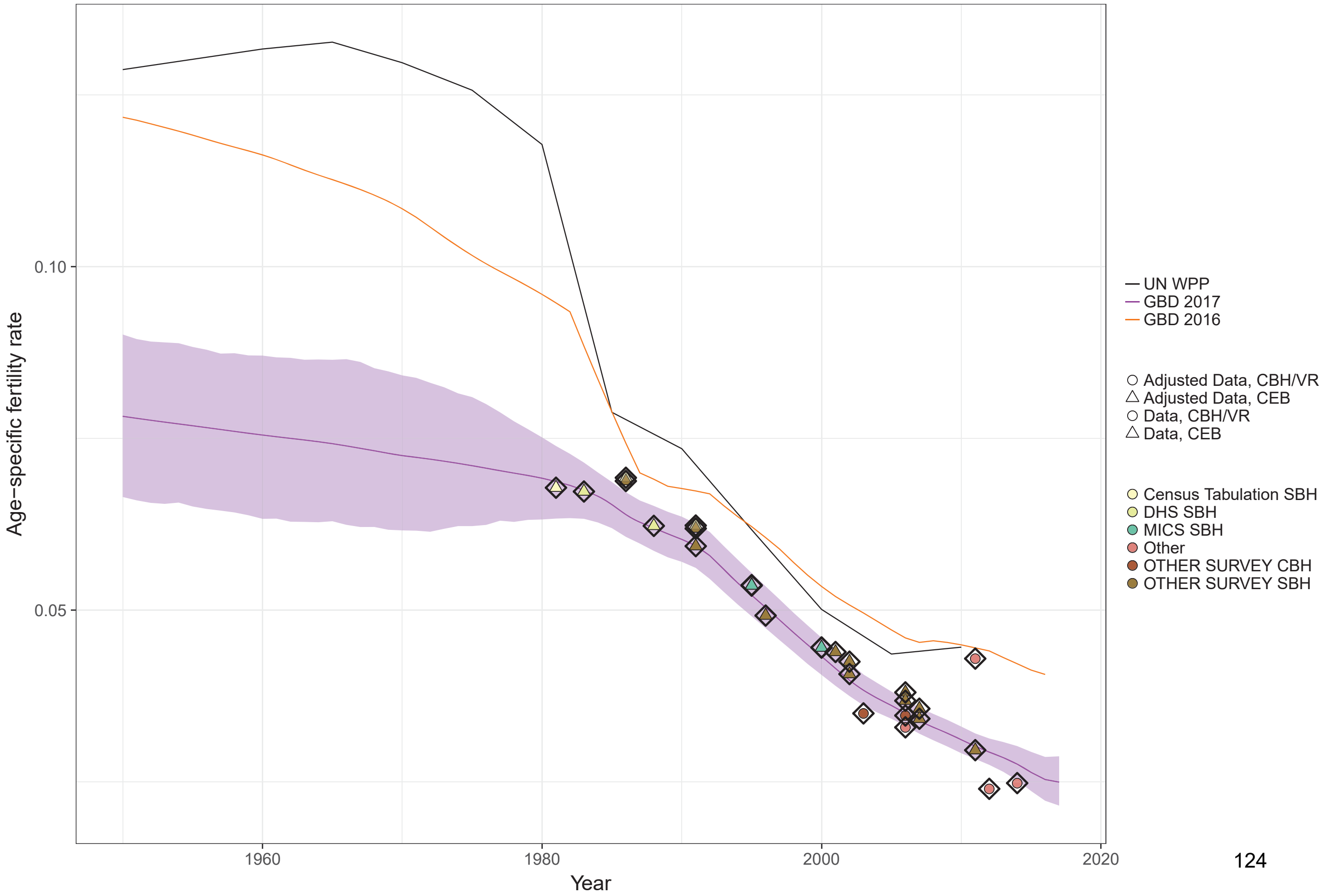

Age−specific fertility fitted trend lines, 1950−2017: Botswana, Ages: 45−49
Data points enclosed with a diamond are reference sources

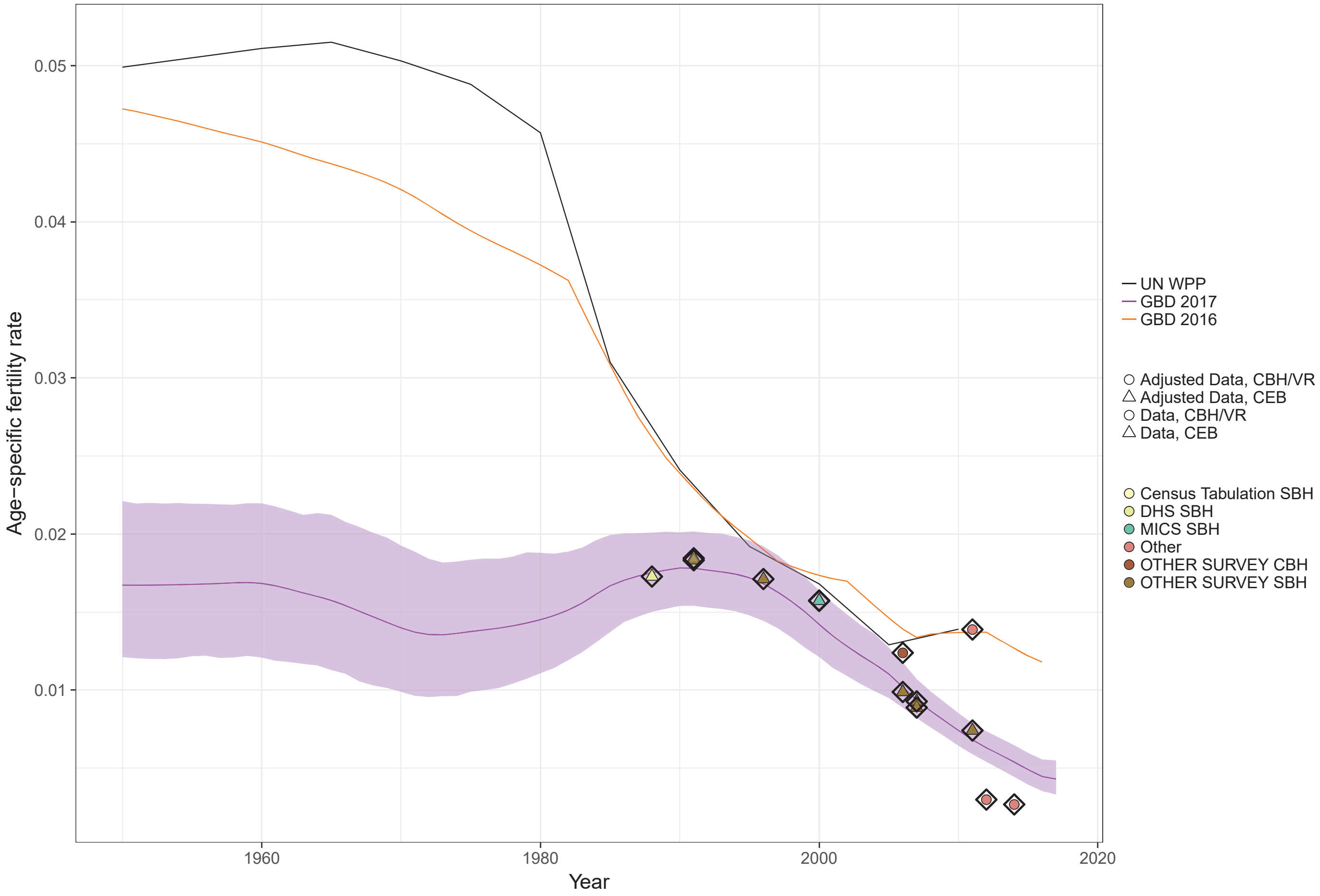

Age−specific fertility fitted trend lines, 1950−2017: Botswana, Ages: 50−54
Data points enclosed with a diamond are reference sources

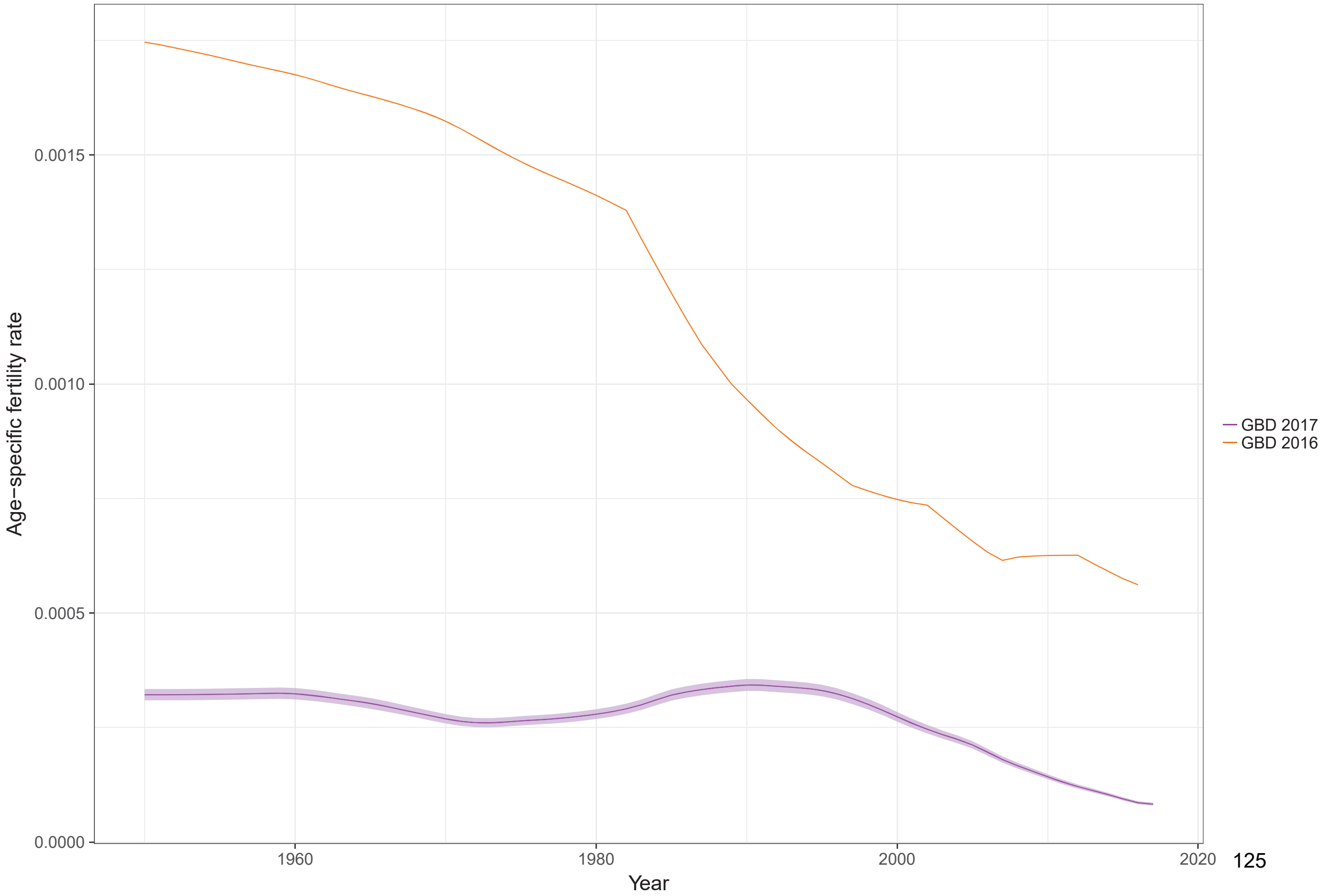

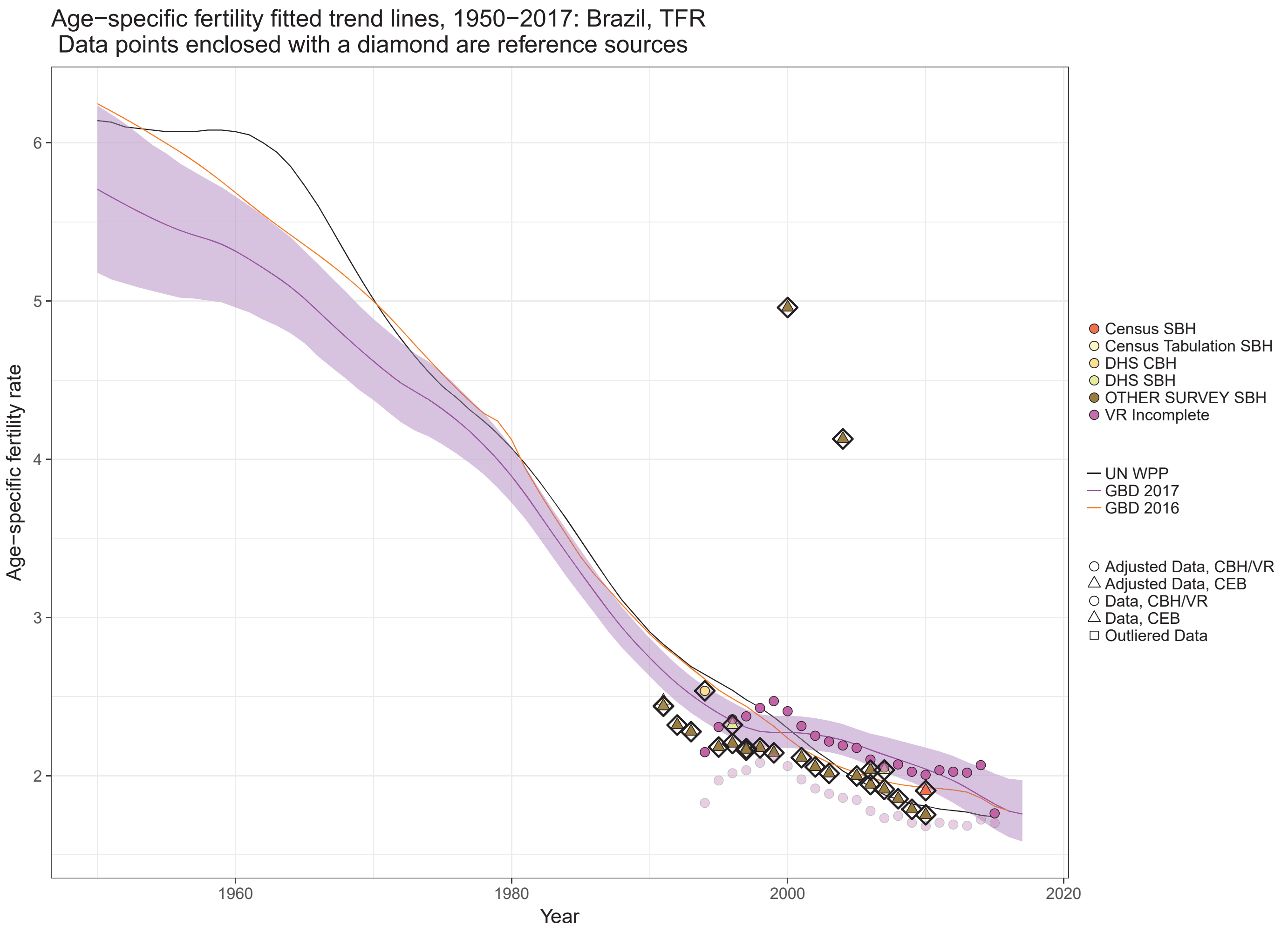

Age−specific fertility fitted trend lines, 1950−2017: Brazil, TFR
Data points enclosed with a diamond are reference sources
Age−specific fertility rate
Year
6
5
4
3
2
1960
1980
2000
2020
Census SBH
Census Tabulation SBH
DHS CBH
DHS SBH
OTHER SURVEY SBH
VR Incomplete
UN WPP
GBD 2017
GBD 2016
Adjusted Data, CBH/VR
Adjusted Data, CEB
Data, CBH/VR
Data, CEB
Outliered Data

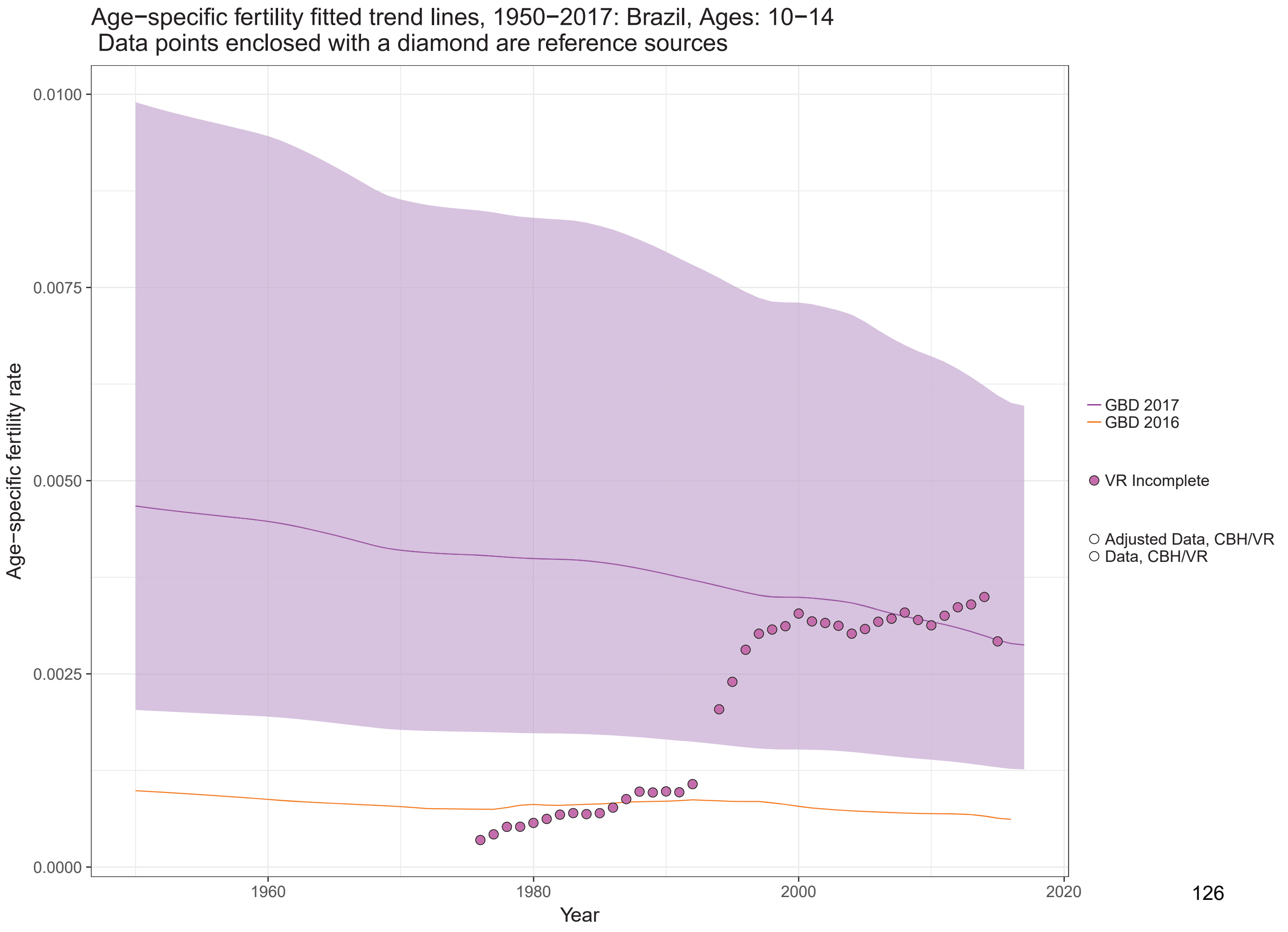

Age−specific fertility fitted trend lines, 1950−2017: Brazil, Ages: 10−14
Data points enclosed with a diamond are reference sources
Age−specific fertility rate
Year
0.0100
0.0075
0.0050
0.0025
0.0000
1960
1980
2000
2020
GBD 2017
GBD 2016
VR Incomplete
Adjusted Data, CBH/VR
Data, CBH/VR

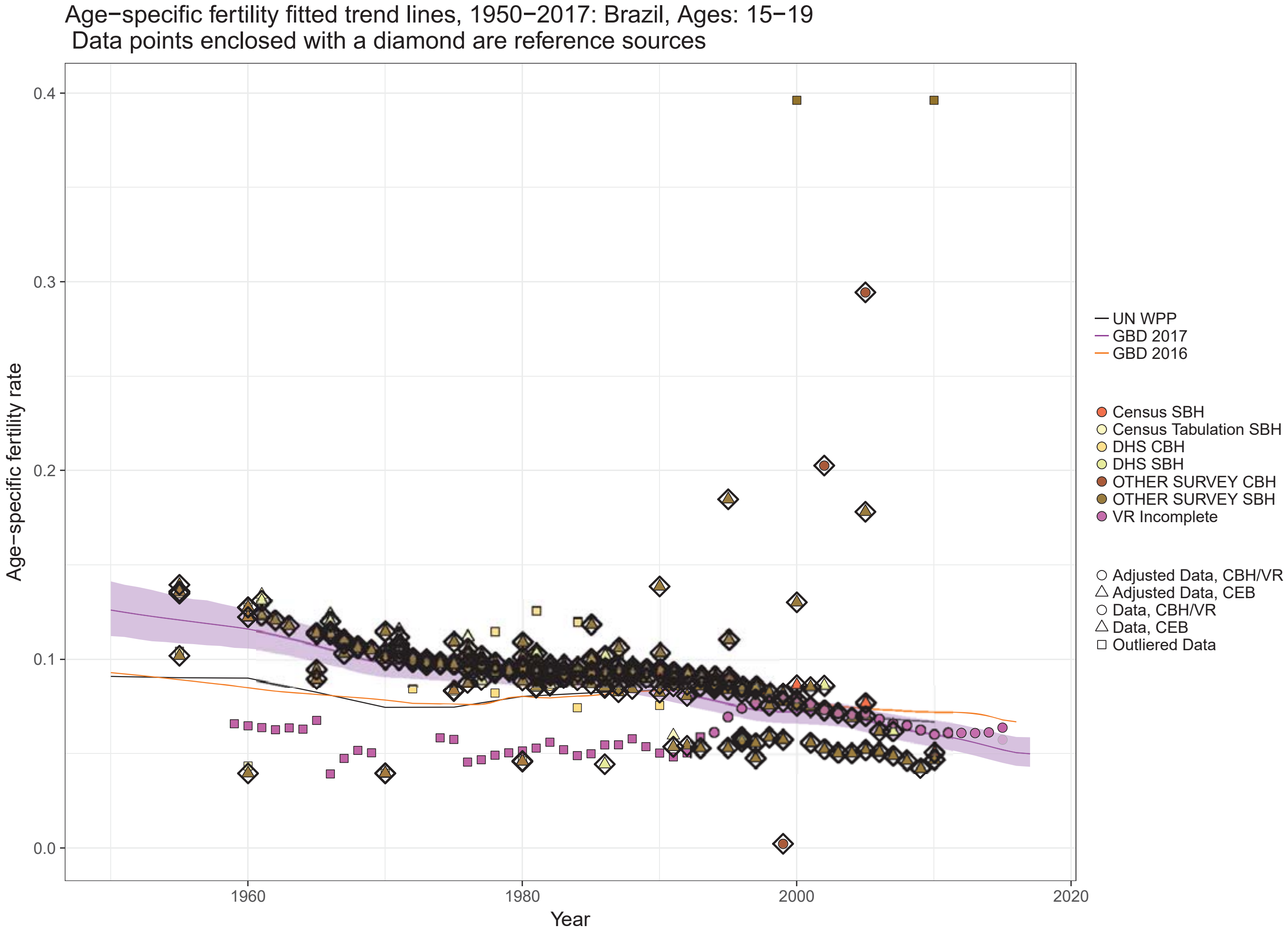
Age−specific fertility fitted trend lines, 1950−2017: Brazil, Ages: 15−19
Data points enclosed with a diamond are reference sources
Age−specific fertility rate
Year
0.0
0.1
0.2
0.3
0.4
1960
1980
2000
2020
UN WPP
GBD 2017
GBD 2016
Census SBH
Census Tabulation SBH
DHS CBH
DHS SBH
OTHER SURVEY CBH
OTHER SURVEY SBH
VR Incomplete
Adjusted Data, CBH/VR
Adjusted Data, CEB
Data, CBH/VR
Data, CEB
Outliered Data

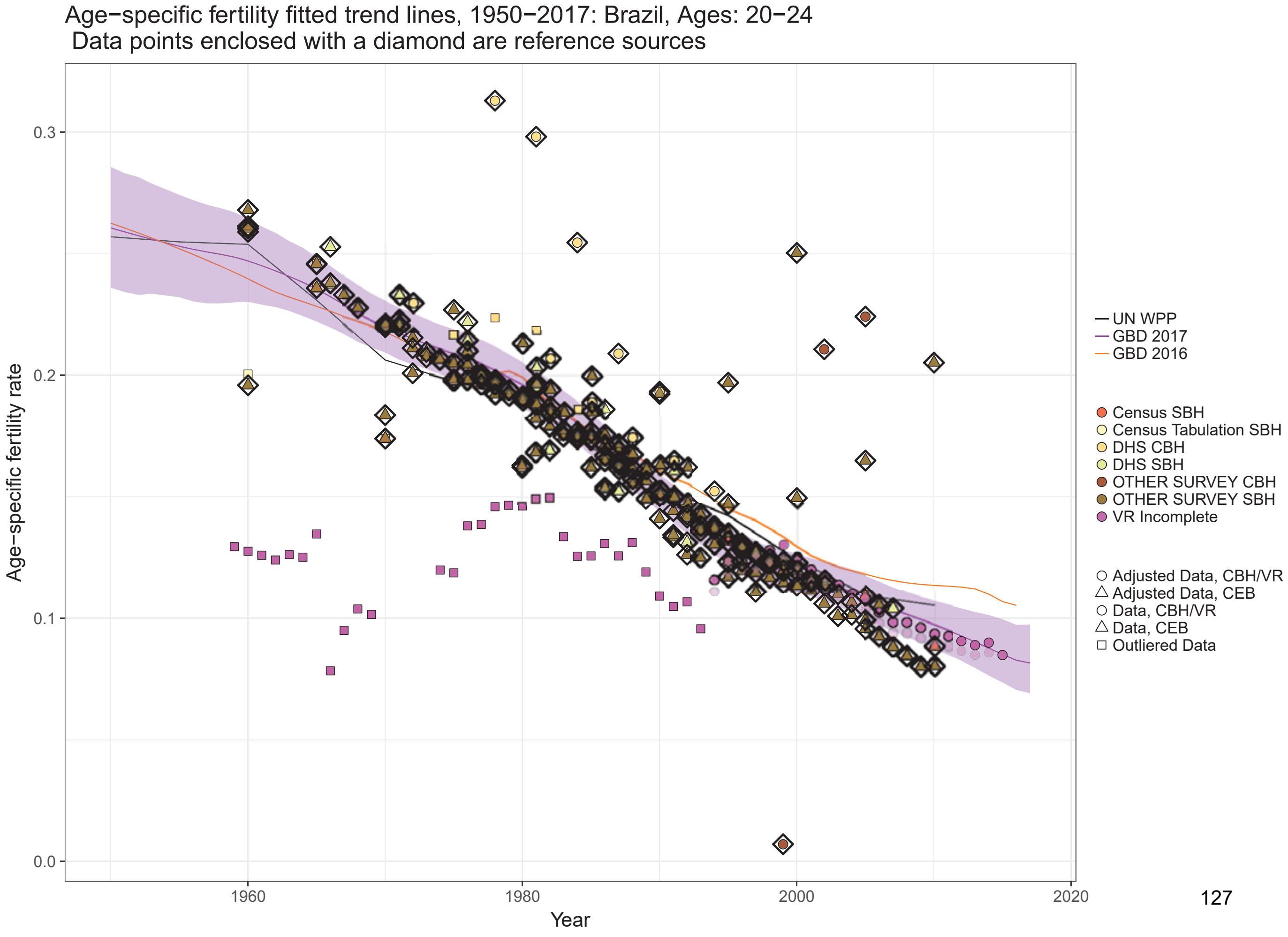
Age−specific fertility fitted trend lines, 1950−2017: Brazil, Ages: 20−24
Data points enclosed with a diamond are reference sources
Age−specific fertility rate
Year
0.0
0.1
0.2
0.3
1960
1980
2000
2020
UN WPP
GBD 2017
GBD 2016
Census SBH
Census Tabulation SBH
DHS CBH
DHS SBH
OTHER SURVEY CBH
OTHER SURVEY SBH
VR Incomplete
Adjusted Data, CBH/VR
Adjusted Data, CEB
Data, CBH/VR
Data, CEB
Outliered Data

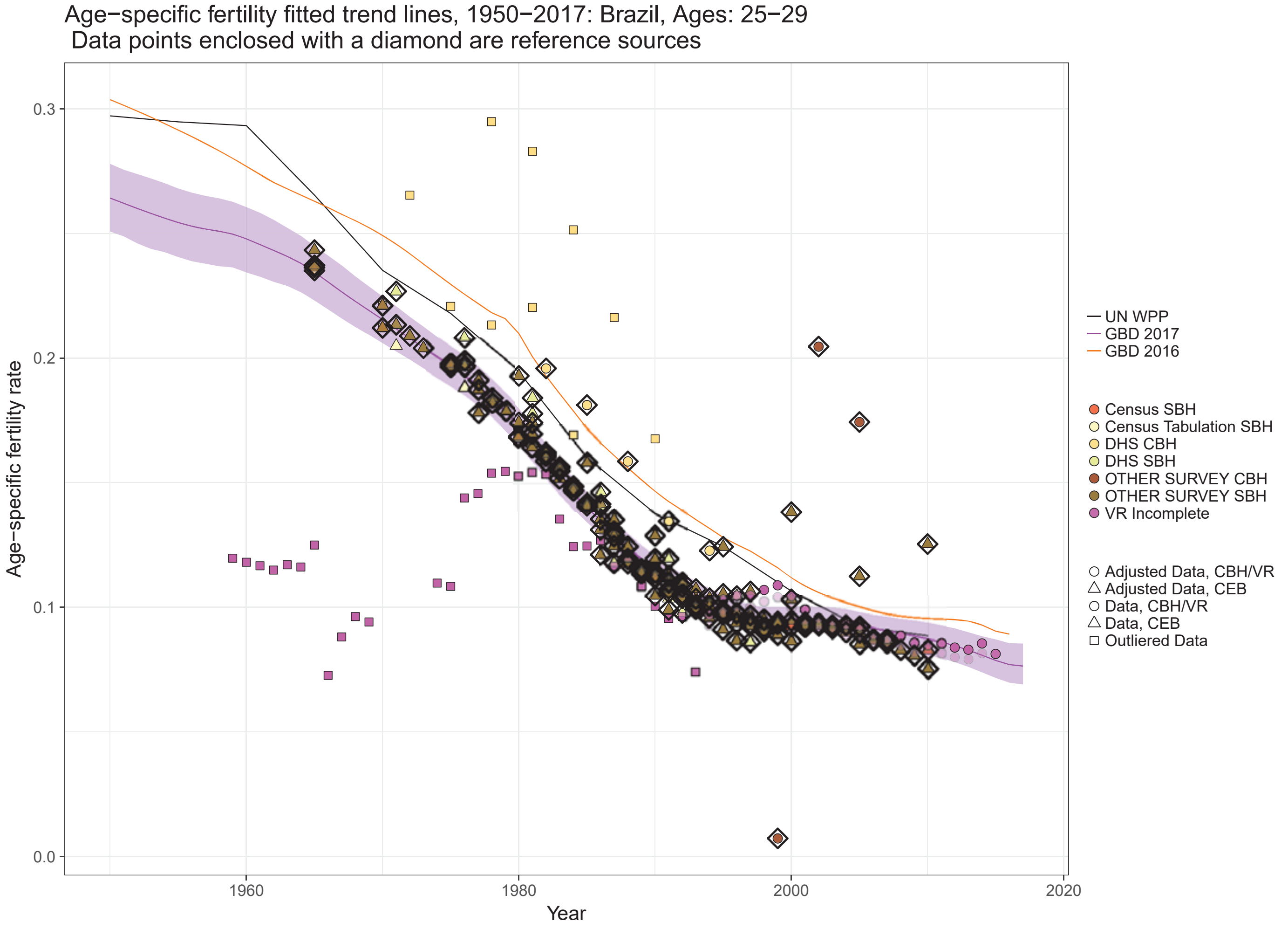

Age−specific fertility fitted trend lines, 1950−2017: Brazil, Ages: 25−29
Data points enclosed with a diamond are reference sources
Age-specific fertility rate
0.0
0.1
0.2
0.3
1960
1980
2000
2020
Year
UN WPP
GBD 2017
GBD 2016
Census SBH
Census Tabulation SBH
DHS CBH
DHS SBH
OTHER SURVEY CBH
OTHER SURVEY SBH
VR Incomplete
Adjusted Data, CBH/VR
Adjusted Data, CEB
Data, CBH/VR
Data, CEB
Outliered Data

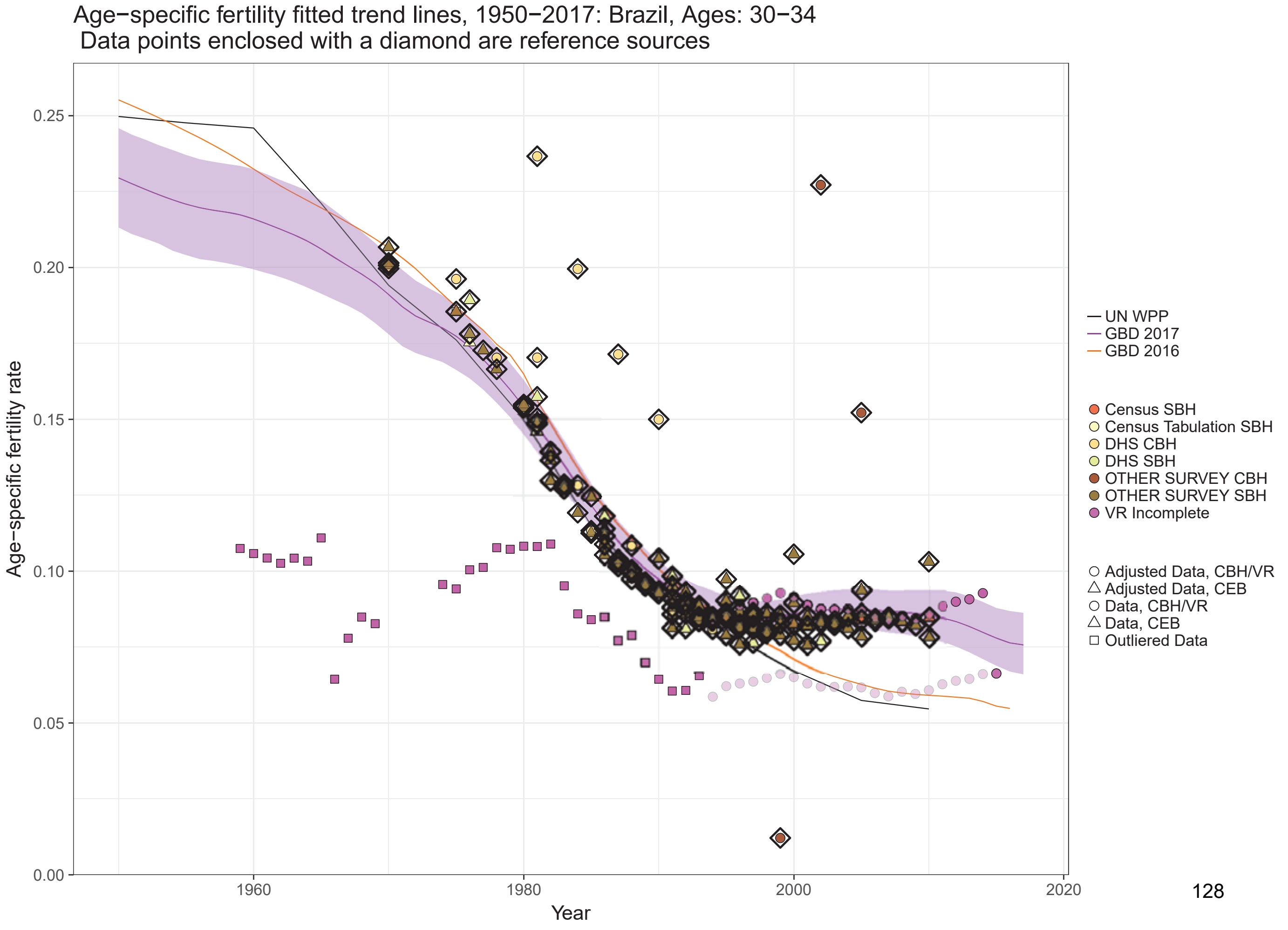

Age−specific fertility fitted trend lines, 1950−2017: Brazil, Ages: 30−34
Data points enclosed with a diamond are reference sources
Age-specific fertility rate
0.00
0.05
0.10
0.15
0.20
0.25
1960
1980
2000
2020
Year
UN WPP
GBD 2017
GBD 2016
Census SBH
Census Tabulation SBH
DHS CBH
DHS SBH
OTHER SURVEY CBH
OTHER SURVEY SBH
VR Incomplete
Adjusted Data, CBH/VR
Adjusted Data, CEB
Data, CBH/VR
Data, CEB
Outliered Data

Age−specific fertility fitted trend lines, 1950−2017: Brazil, Ages: 35−39
Data points enclosed with a diamond are reference sources

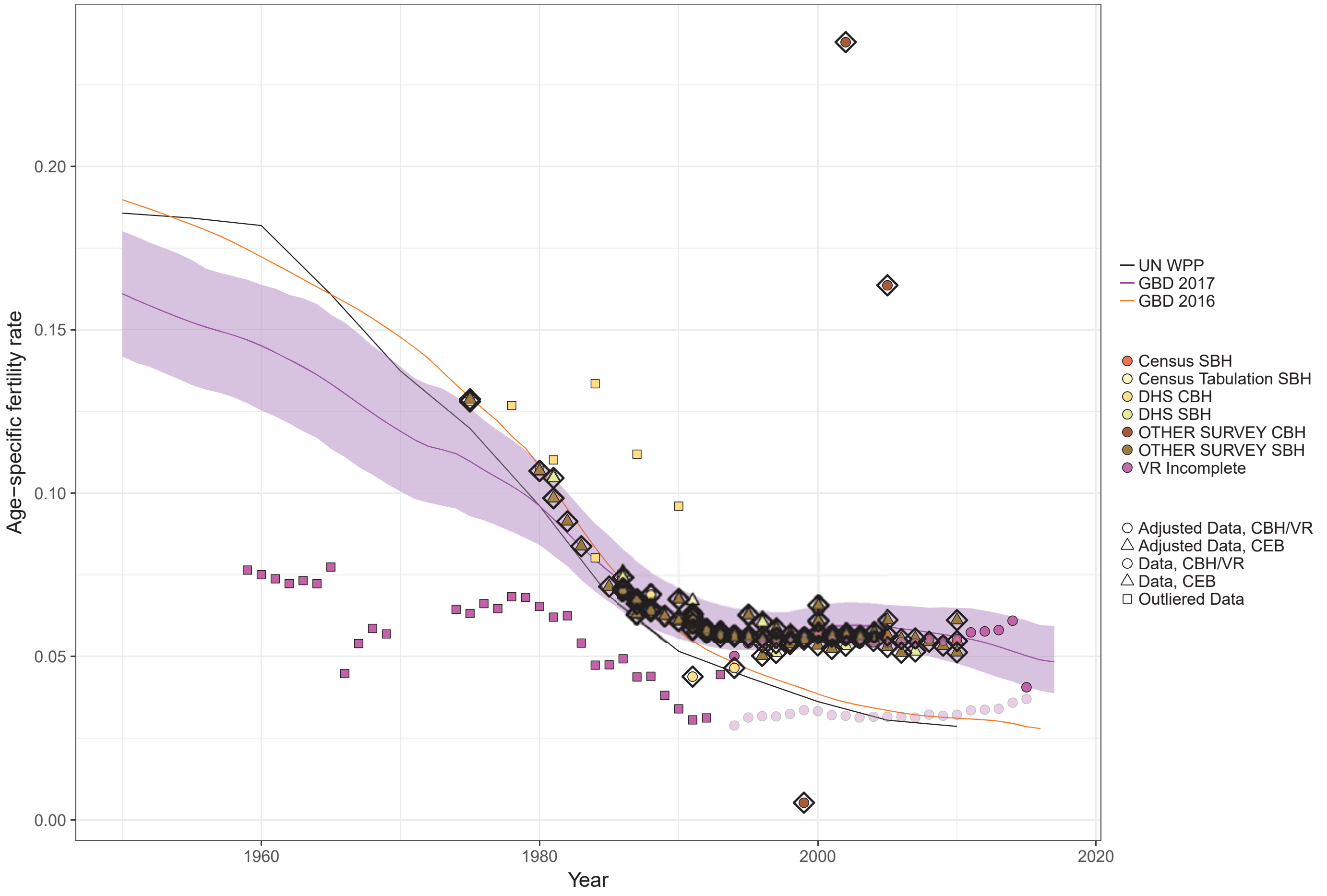

Age−specific fertility fitted trend lines, 1950−2017: Brazil, Ages: 40−44
Data points enclosed with a diamond are reference sources

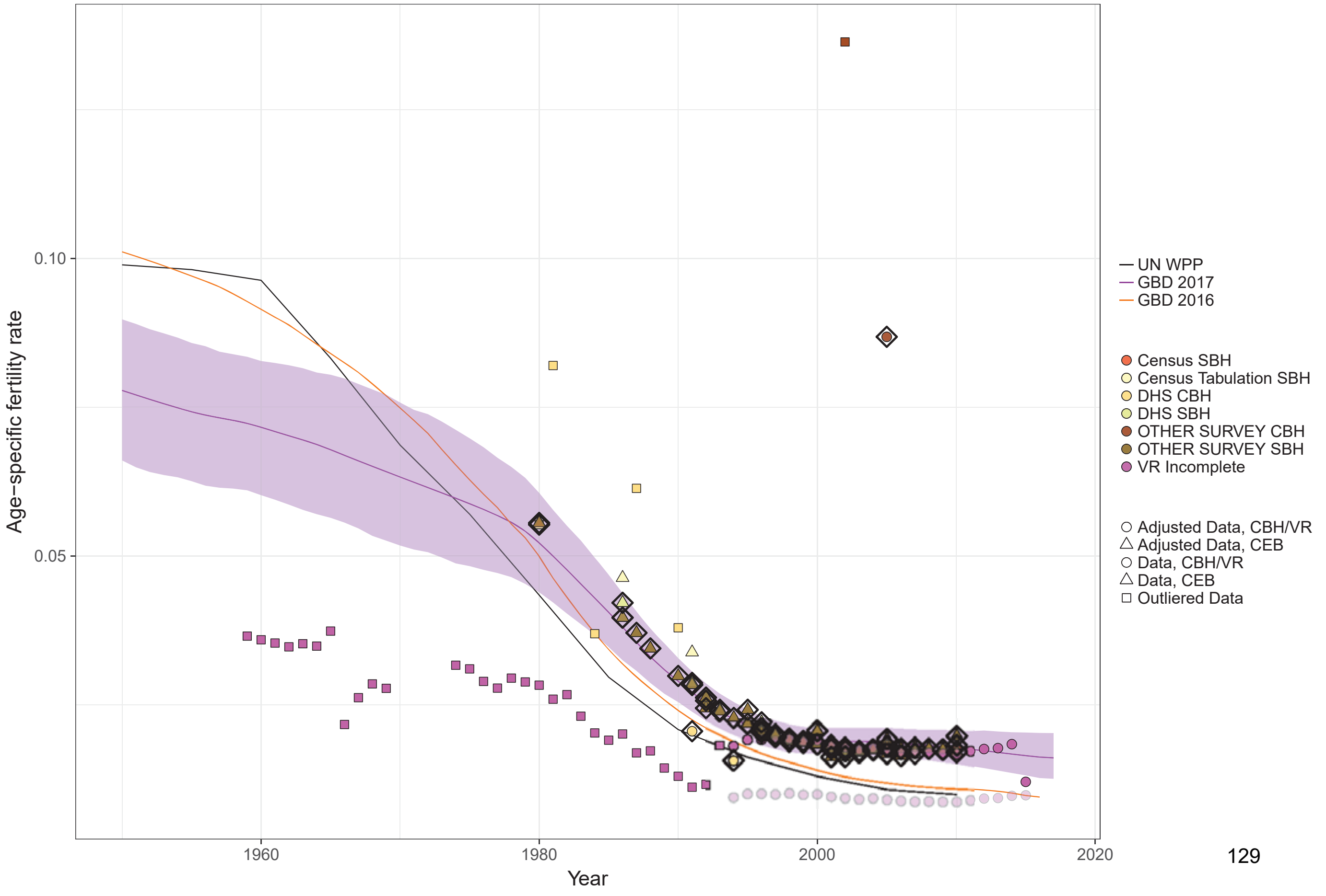

Age−specific fertility fitted trend lines, 1950−2017: Brazil, Ages: 45−49
Data points enclosed with a diamond are reference sources

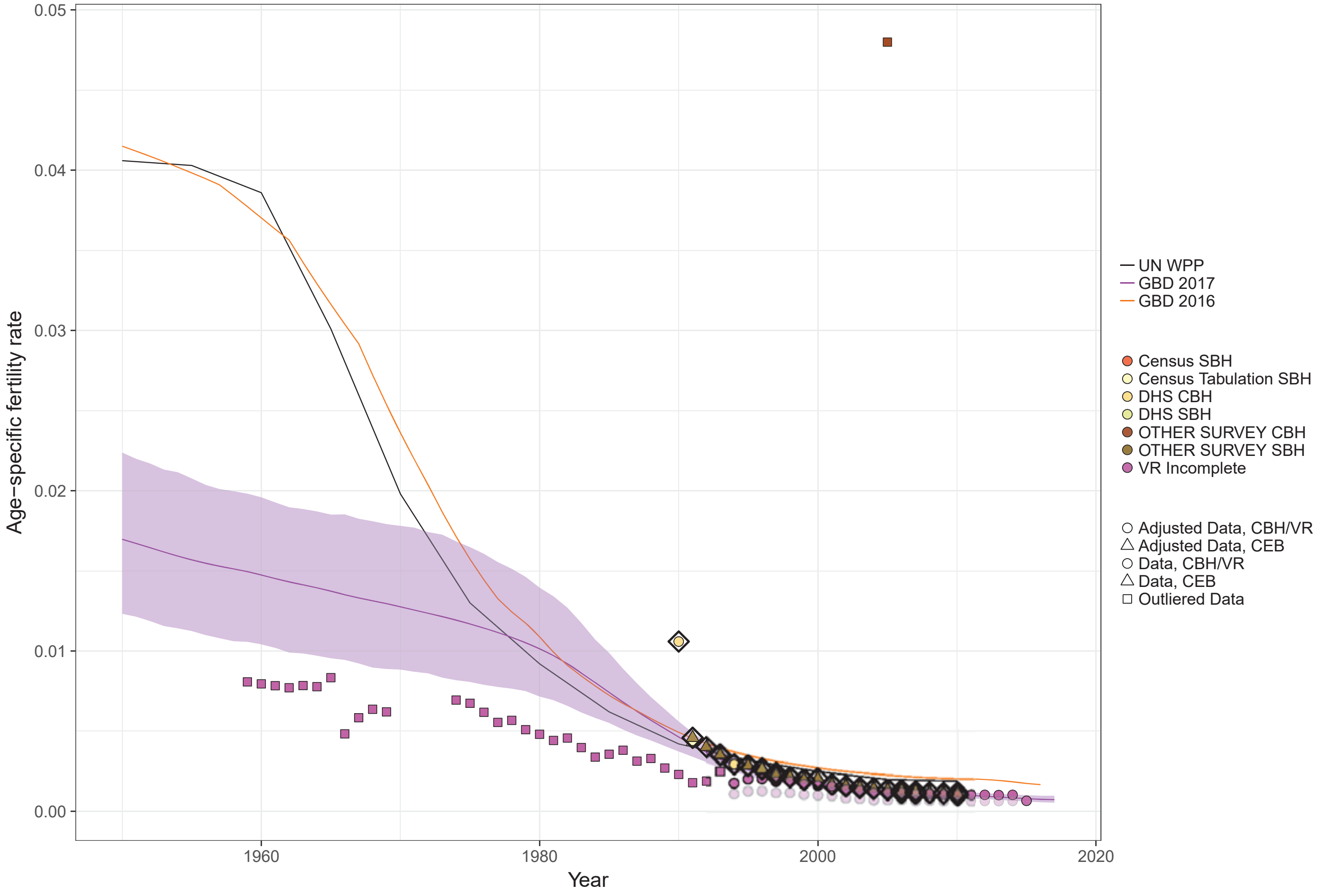

Age−specific fertility fitted trend lines, 1950−2017: Brazil, Ages: 50−54
Data points enclosed with a diamond are reference sources

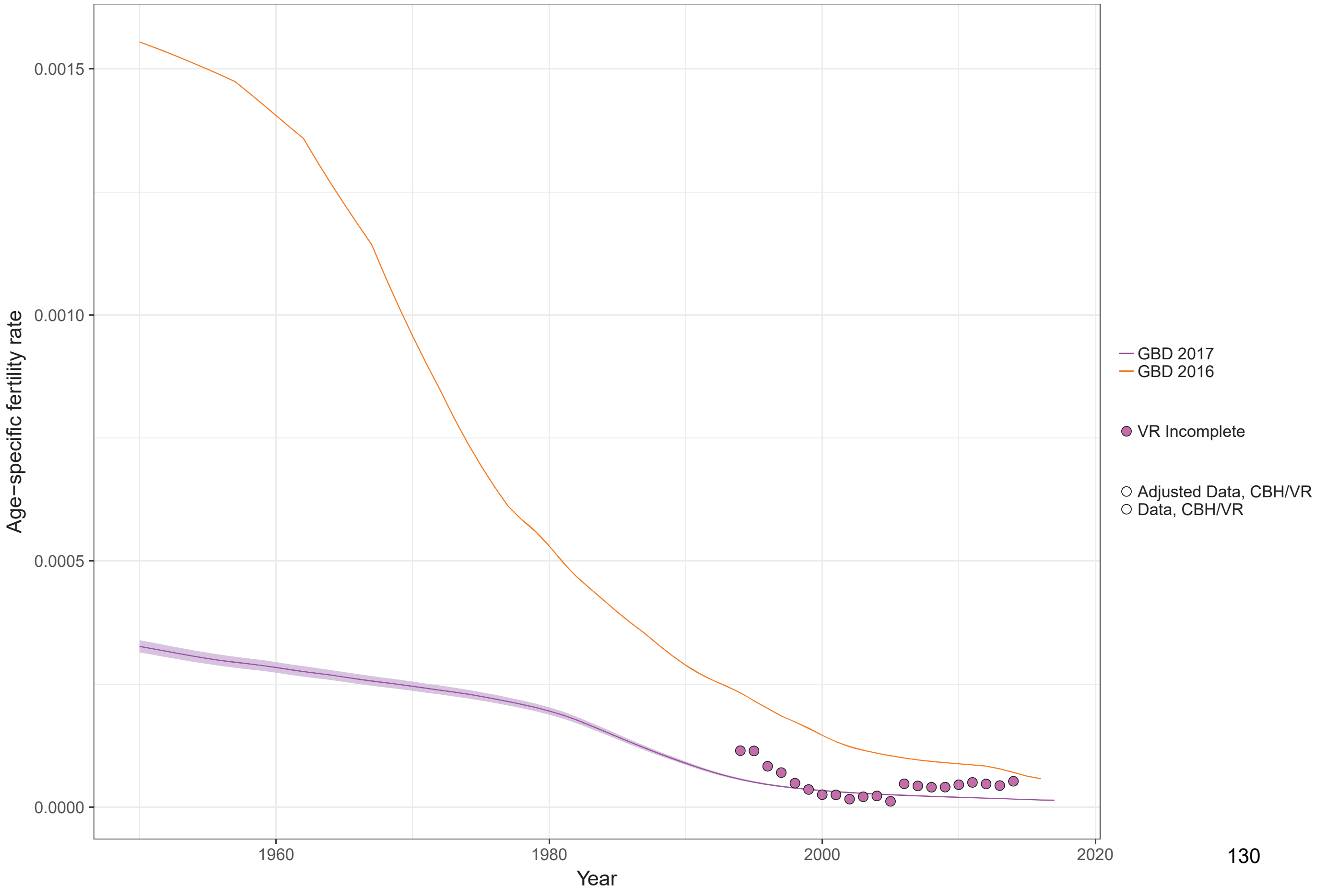

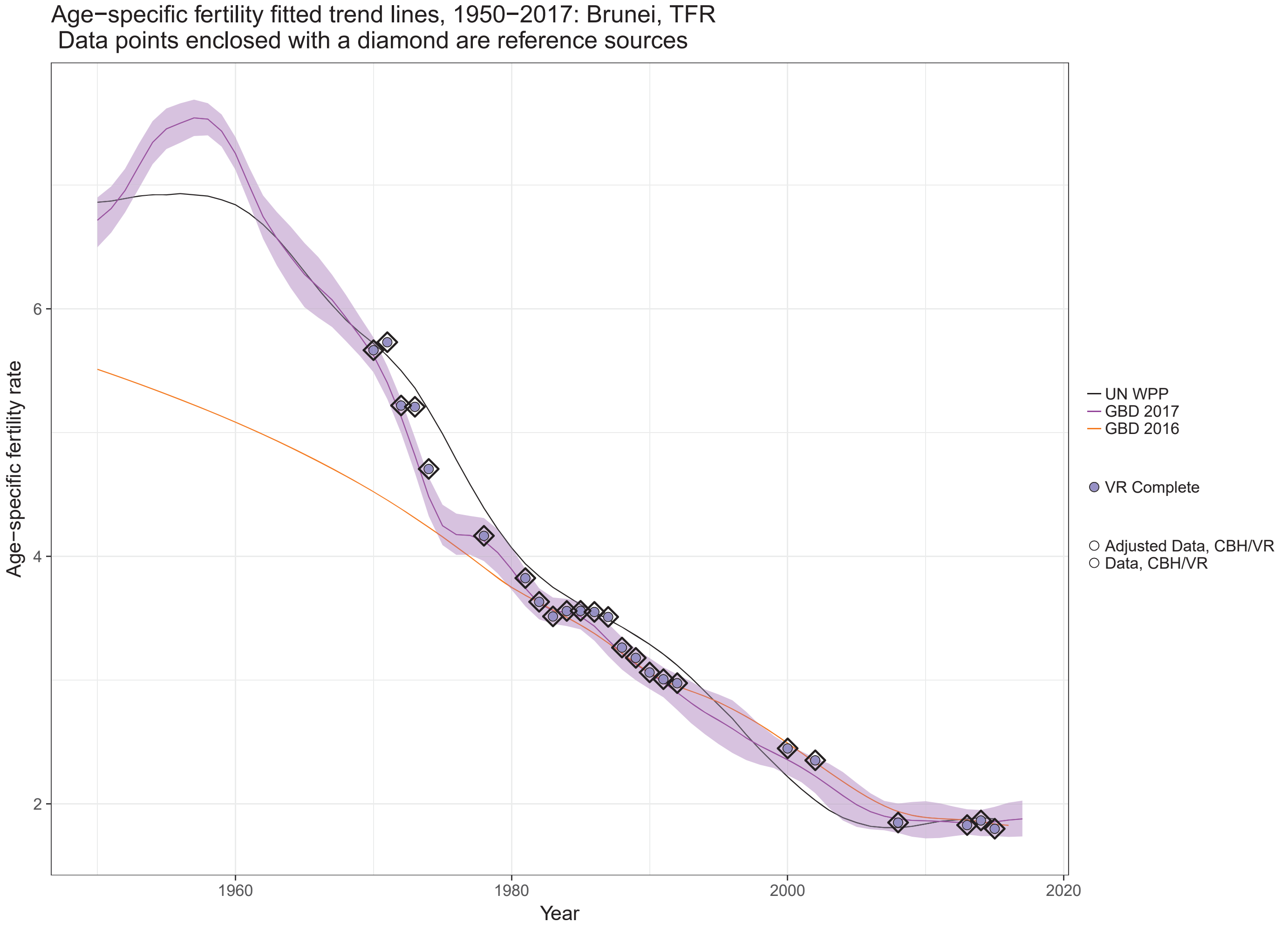

Age−specific fertility fitted trend lines, 1950−2017: Brunei, TFR
Data points enclosed with a diamond are reference sources
Age−specific fertility rate
Year
6
4
2
1960
1980
2000
2020
UN WPP
GBD 2017
GBD 2016
VR Complete
Adjusted Data, CBH/VR
Data, CBH/VR

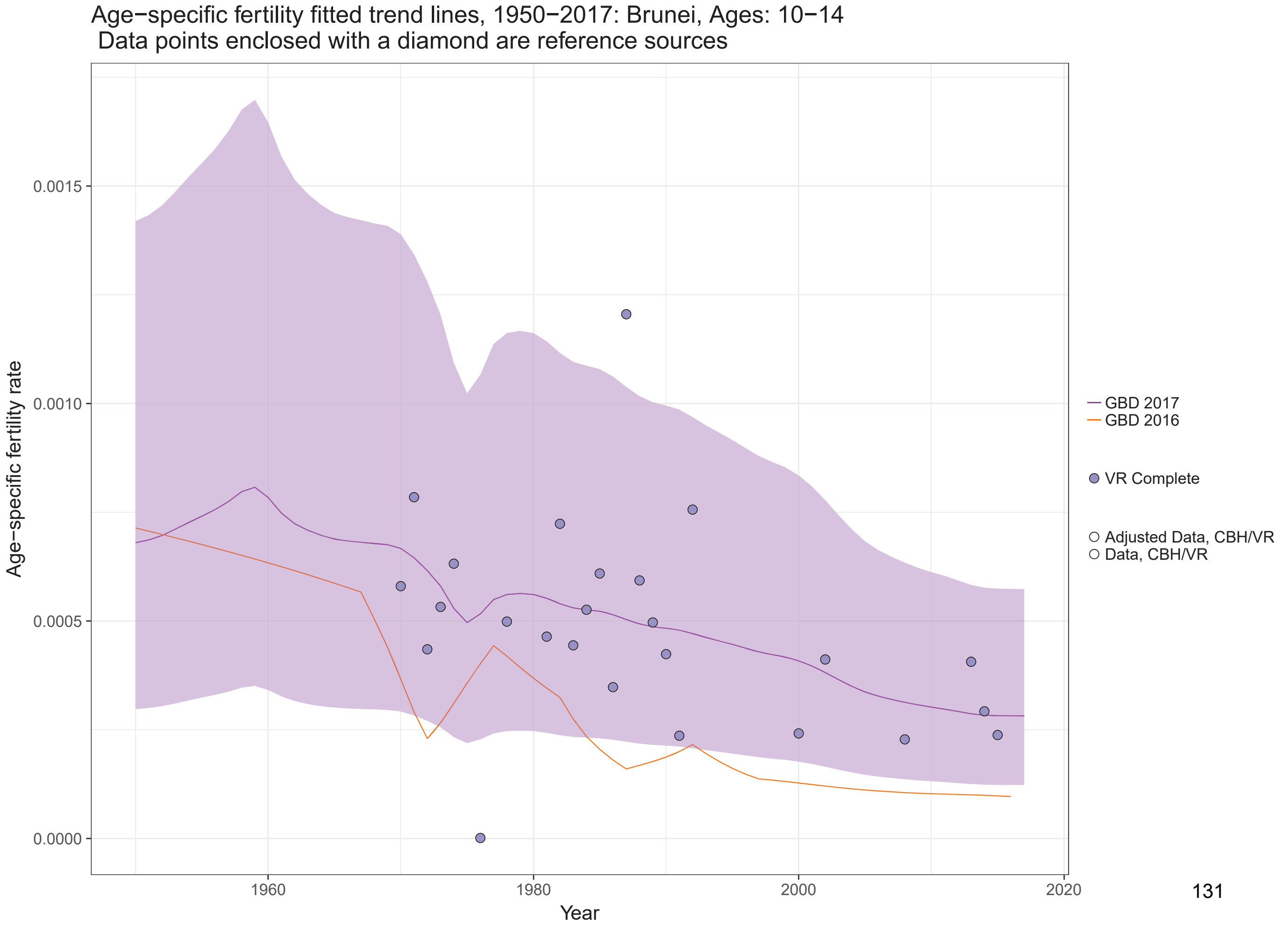

Age−specific fertility fitted trend lines, 1950−2017: Brunei, Ages: 10−14
Data points enclosed with a diamond are reference sources
Age−specific fertility rate
Year
0.0015
0.0010
0.0005
0.0000
1960
1980
2000
2020
GBD 2017
GBD 2016
VR Complete
Adjusted Data, CBH/VR
Data, CBH/VR

Age−specific fertility fitted trend lines, 1950−2017: Brunei, Ages: 15−19
Data points enclosed with a diamond are reference sources

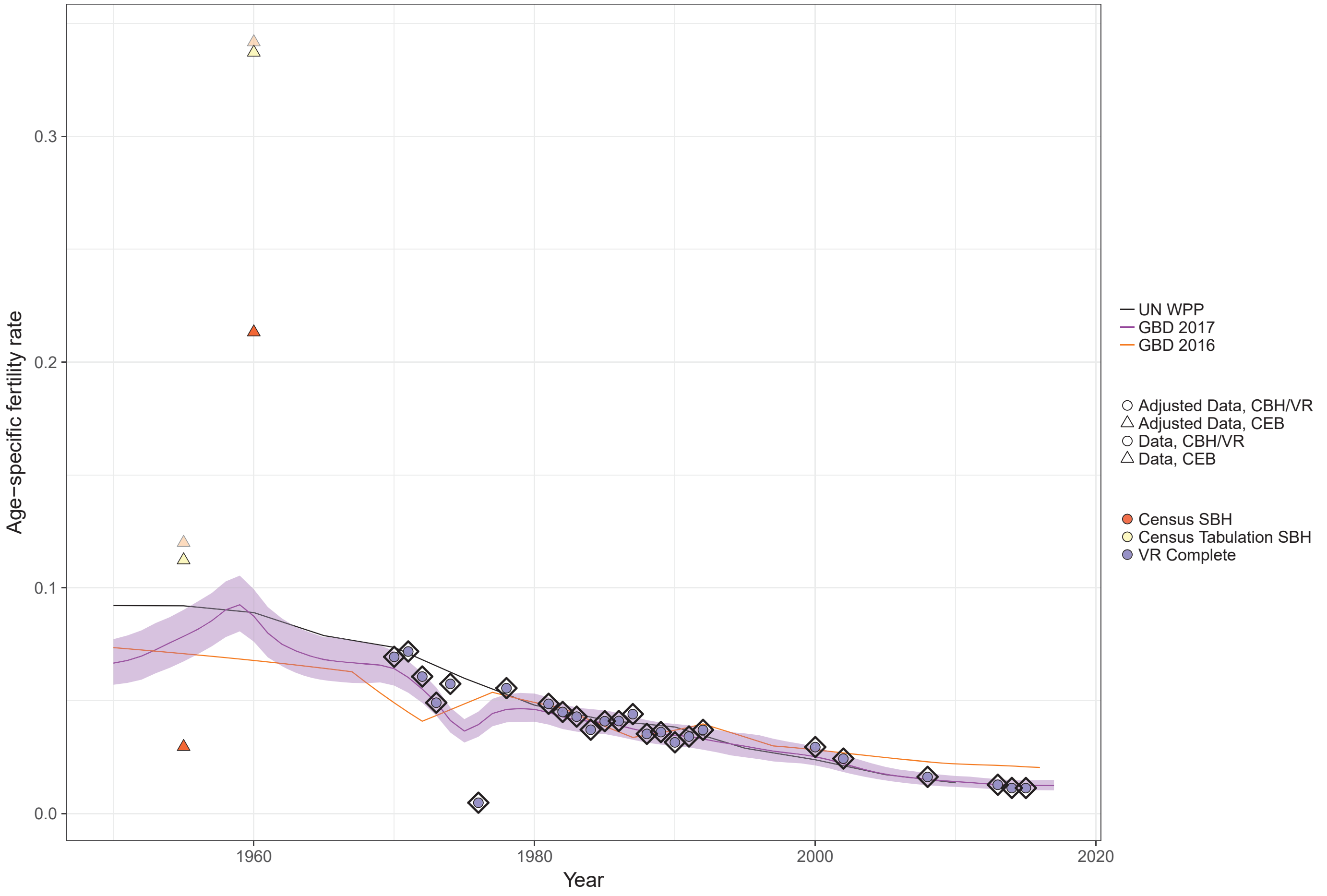

Age−specific fertility fitted trend lines, 1950−2017: Brunei, Ages: 20−24
Data points enclosed with a diamond are reference sources

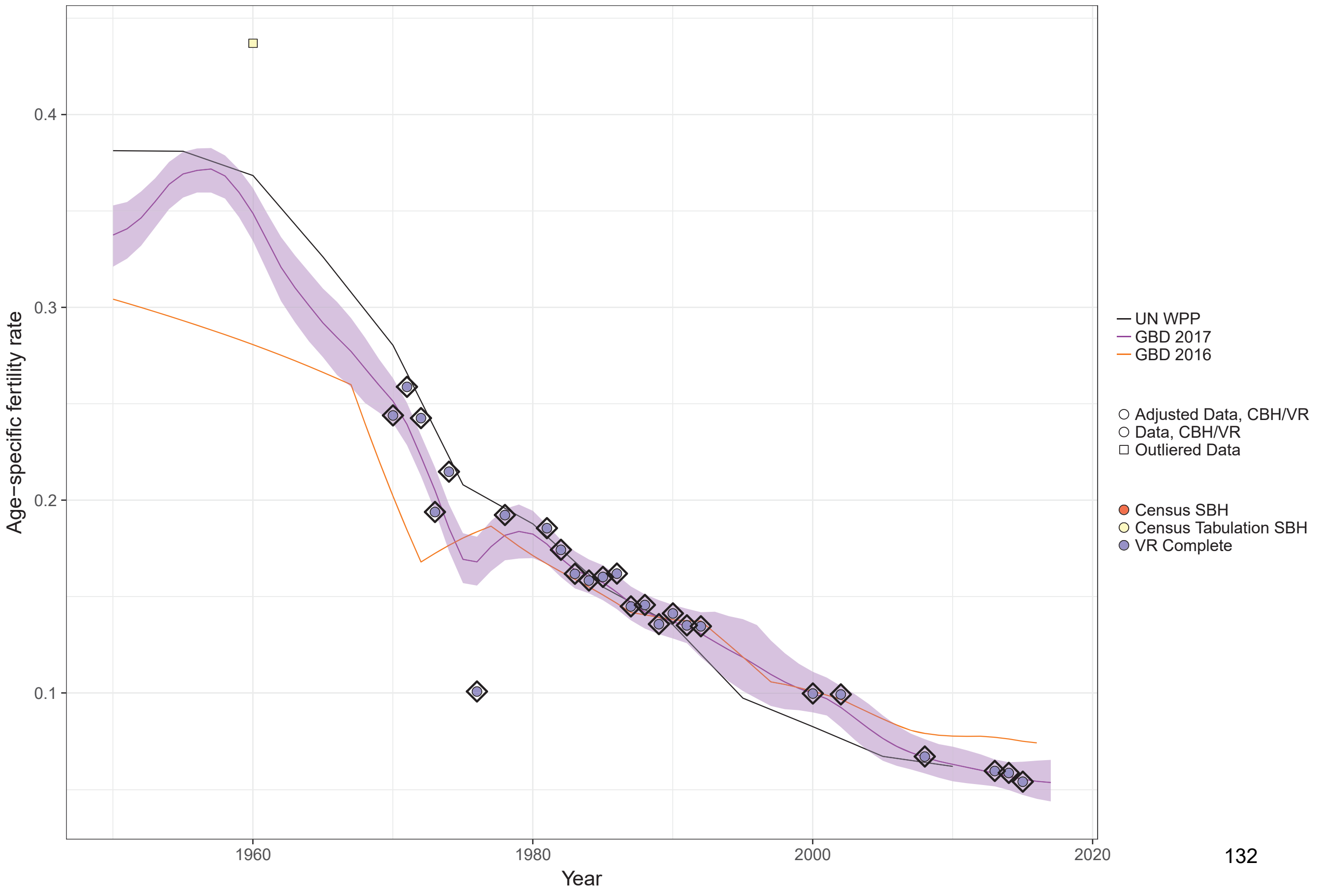

Age−specific fertility fitted trend lines, 1950−2017: Brunei, Ages: 25−29
Data points enclosed with a diamond are reference sources

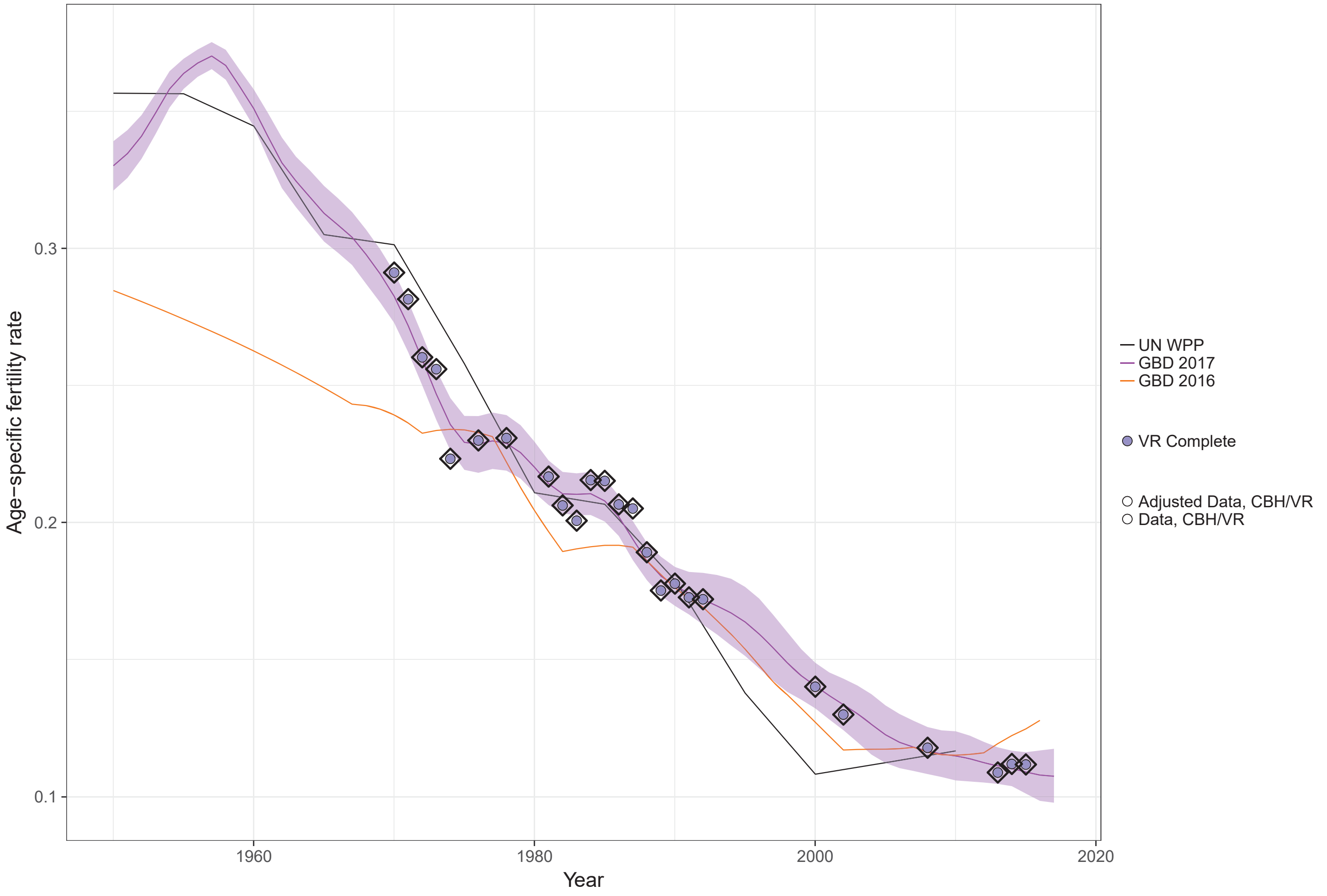

Age−specific fertility fitted trend lines, 1950−2017: Brunei, Ages: 30−34
Data points enclosed with a diamond are reference sources

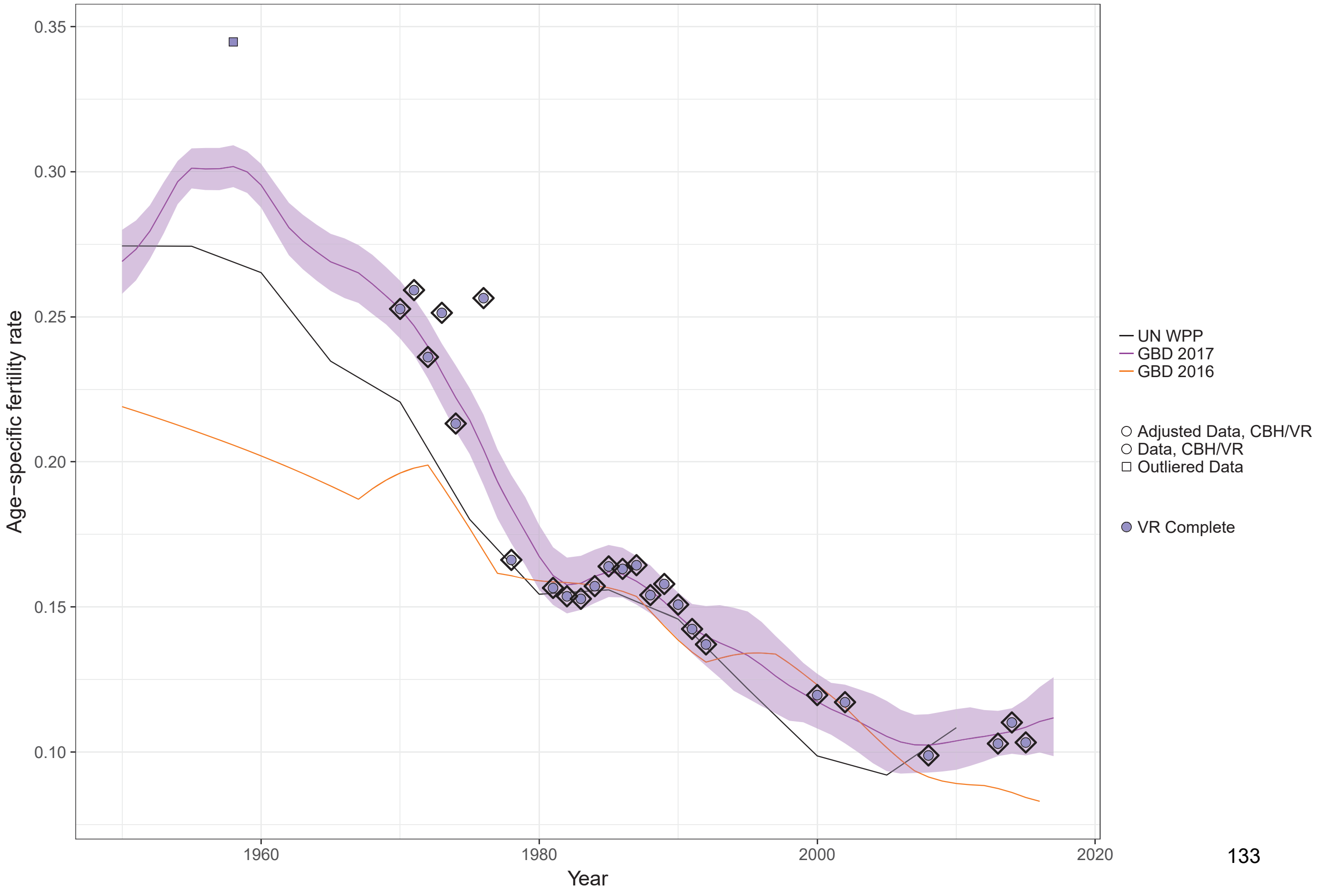

Age−specific fertility fitted trend lines, 1950−2017: Brunei, Ages: 35−39
Data points enclosed with a diamond are reference sources

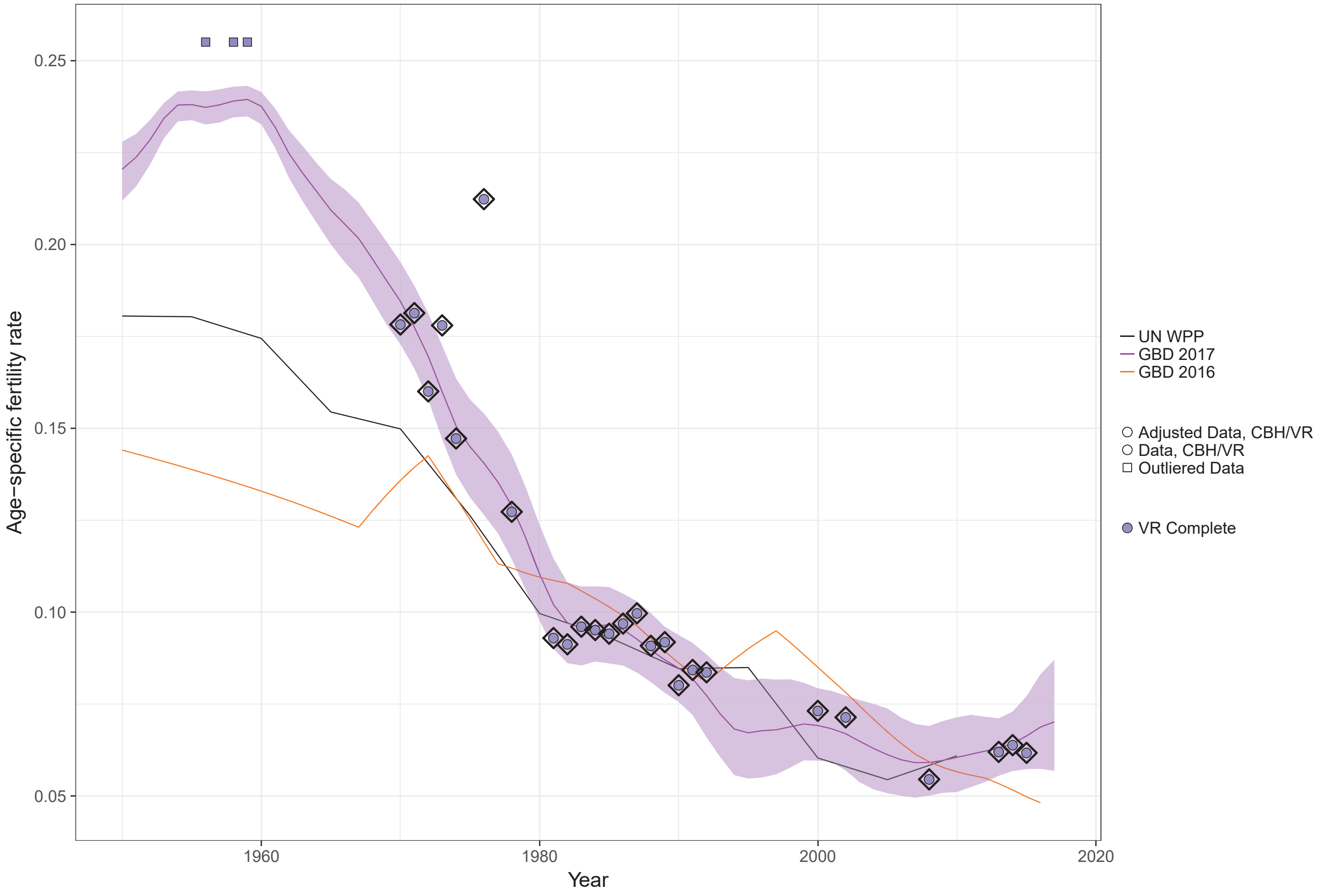

Age−specific fertility fitted trend lines, 1950−2017: Brunei, Ages: 40−44
Data points enclosed with a diamond are reference sources

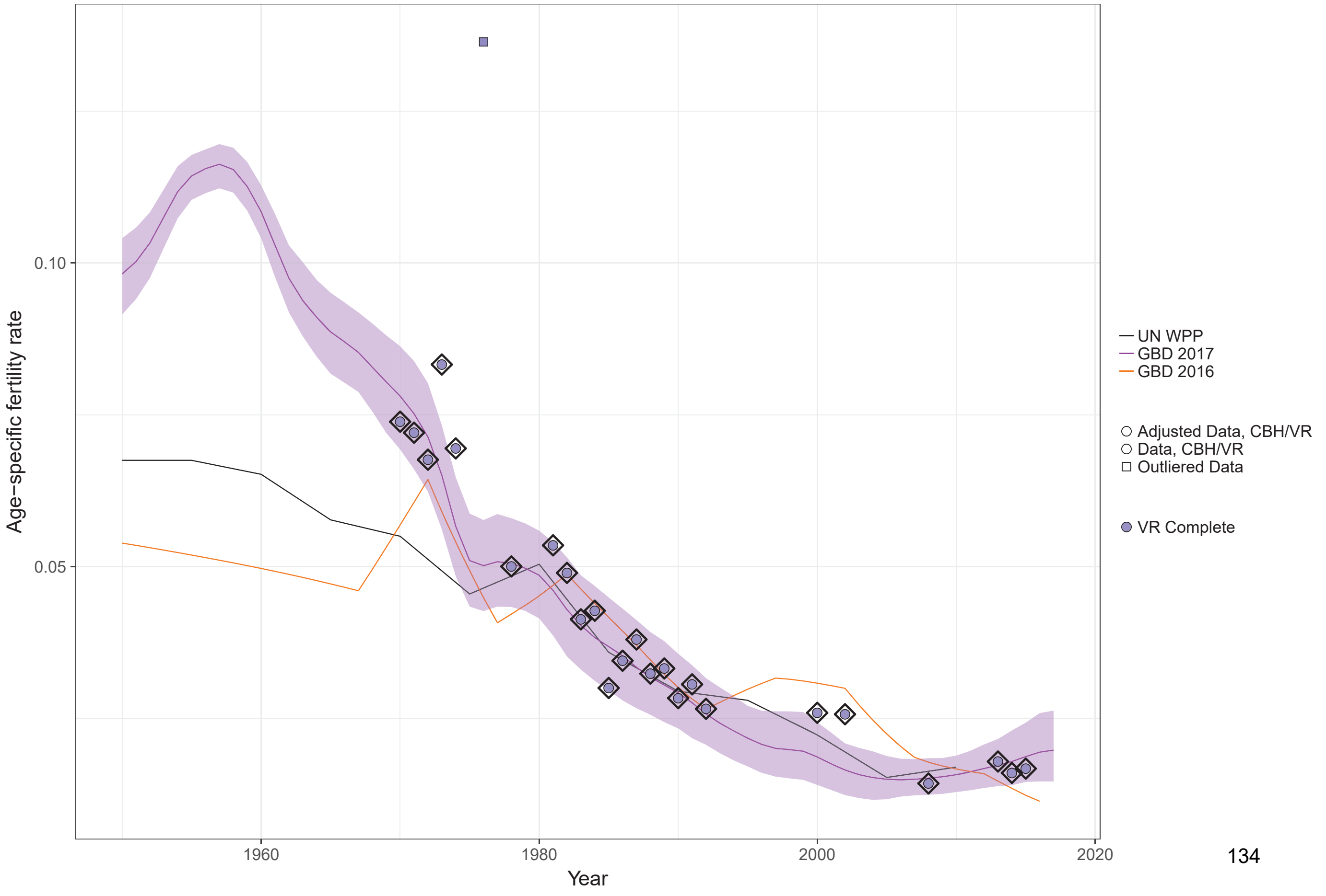

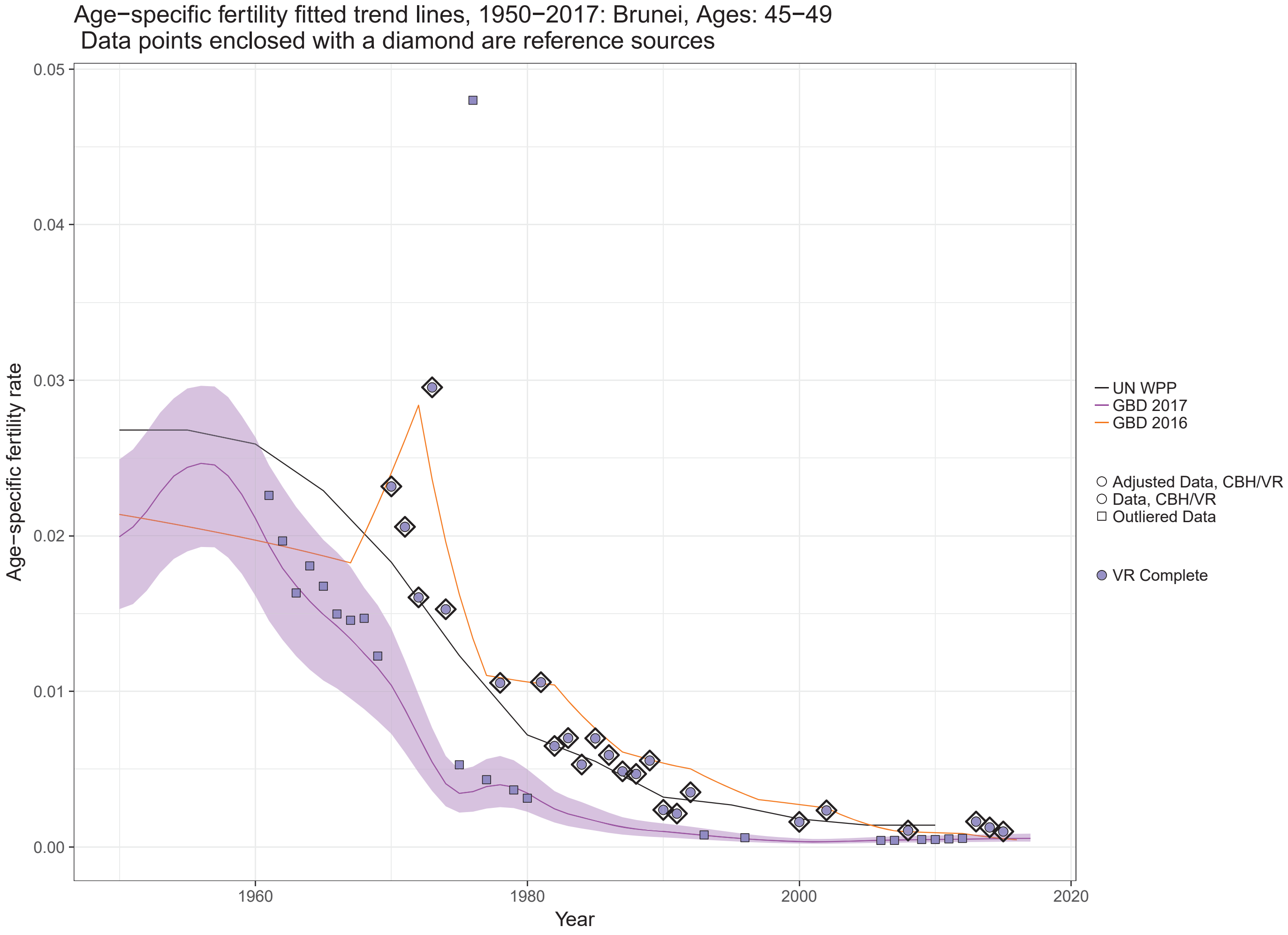
Age−specific fertility fitted trend lines, 1950−2017: Brunei, Ages: 45−49
Data points enclosed with a diamond are reference sources
Age−specific fertility rate
0.05
0.04
0.03
0.02
0.01
0.00
1960
1980
2000
2020
Year
UN WPP
GBD 2017
GBD 2016
Adjusted Data, CBH/VR
Data, CBH/VR
Outliered Data
VR Complete

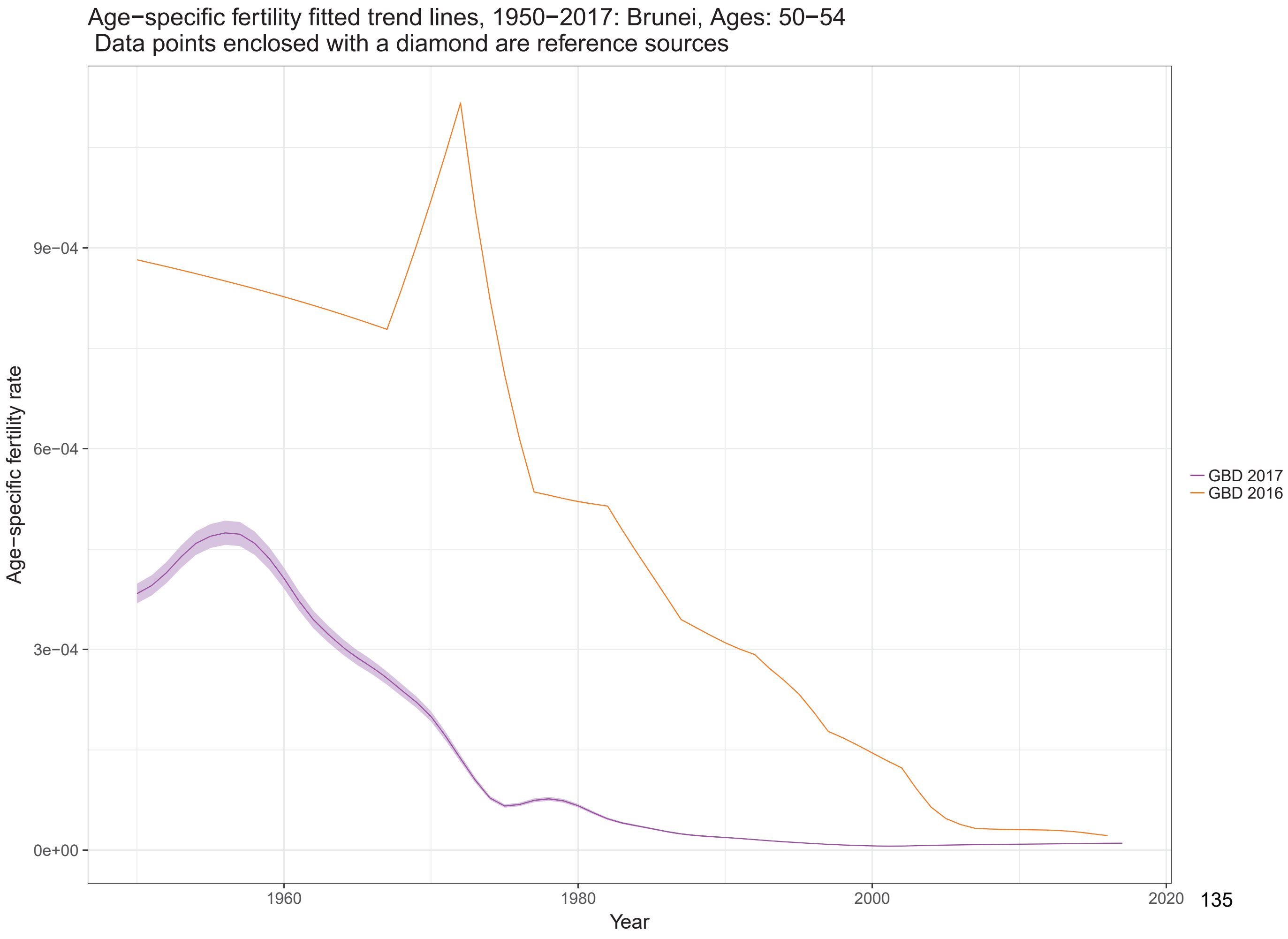
Age−specific fertility fitted trend lines, 1950−2017: Brunei, Ages: 50−54
Data points enclosed with a diamond are reference sources
Age−specific fertility rate
9e−04
6e−04
3e−04
0e+00
1960
1980
2000
2020
Year
GBD 2017
GBD 2016

Age−specific fertility fitted trend lines, 1950−2017: Bulgaria, TFR
Data points enclosed with a diamond are reference sources

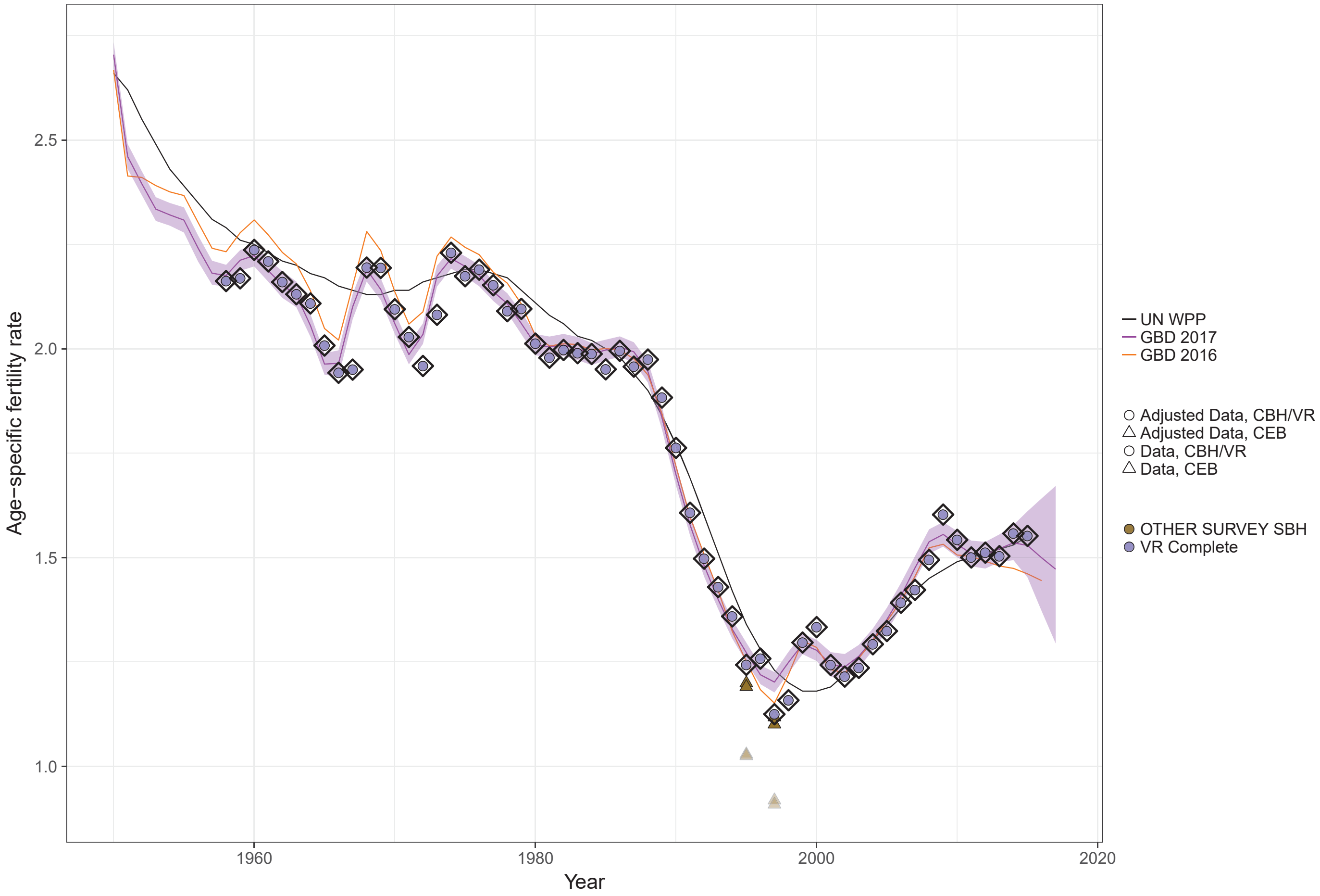

Age−specific fertility fitted trend lines, 1950−2017: Bulgaria, Ages: 10−14
Data points enclosed with a diamond are reference sources

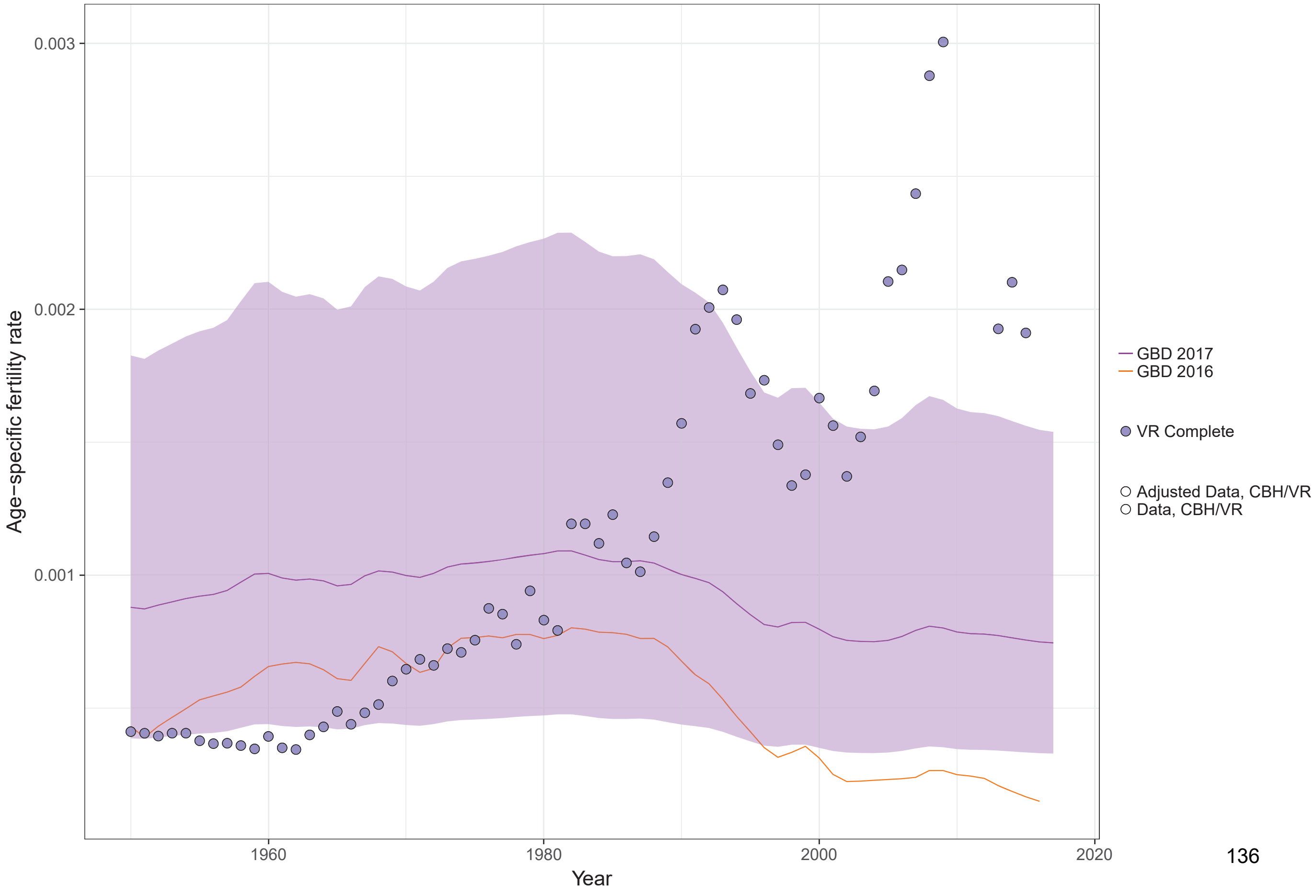

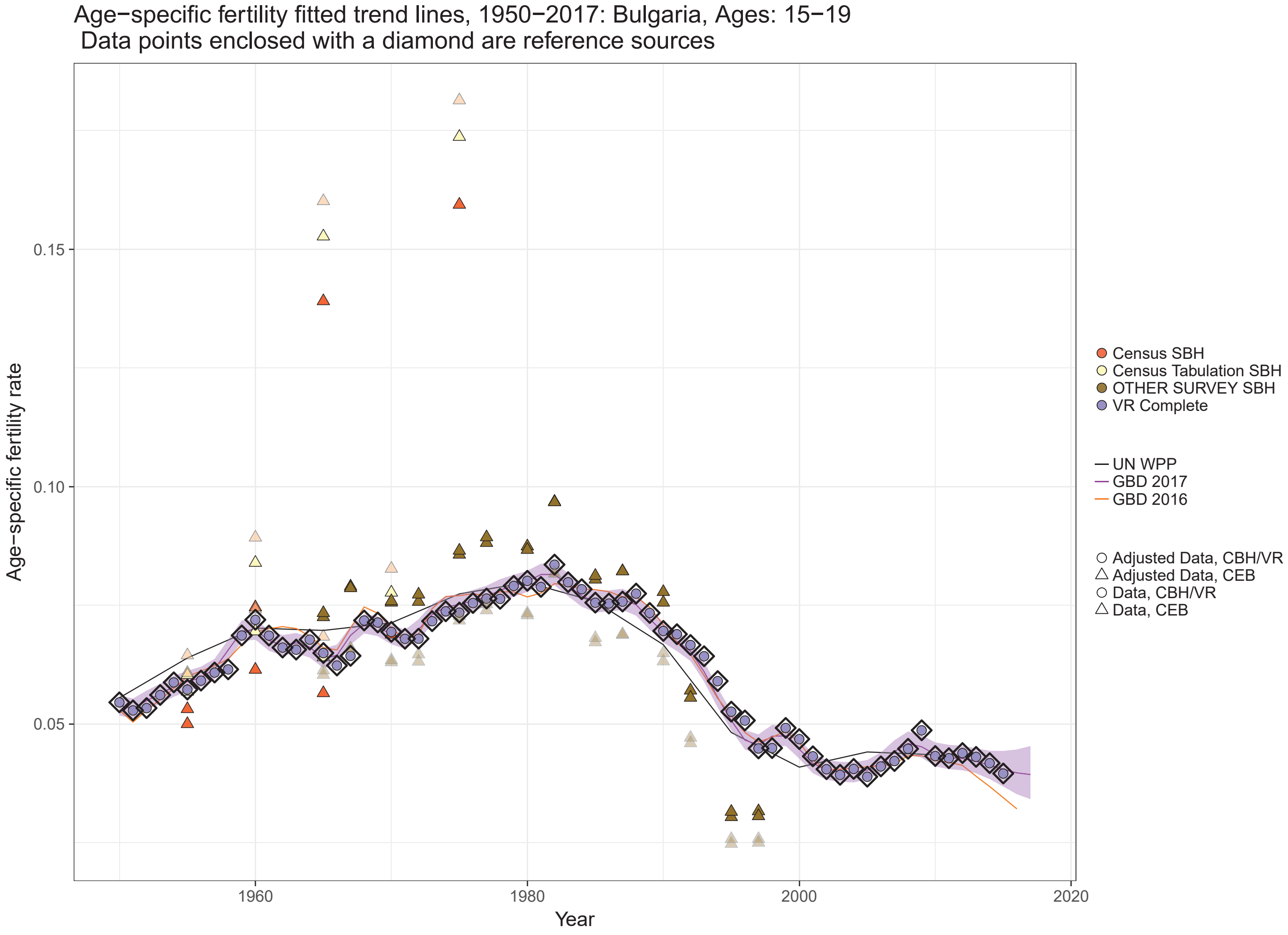

Age−specific fertility fitted trend lines, 1950−2017: Bulgaria, Ages: 15−19
Data points enclosed with a diamond are reference sources
Age−specific fertility rate
0.05
0.10
0.15
Year
1960
1980
2000
2020
Census SBH
Census Tabulation SBH
OTHER SURVEY SBH
VR Complete
UN WPP
GBD 2017
GBD 2016
Adjusted Data, CBH/VR
Adjusted Data, CEB
Data, CBH/VR
Data, CEB

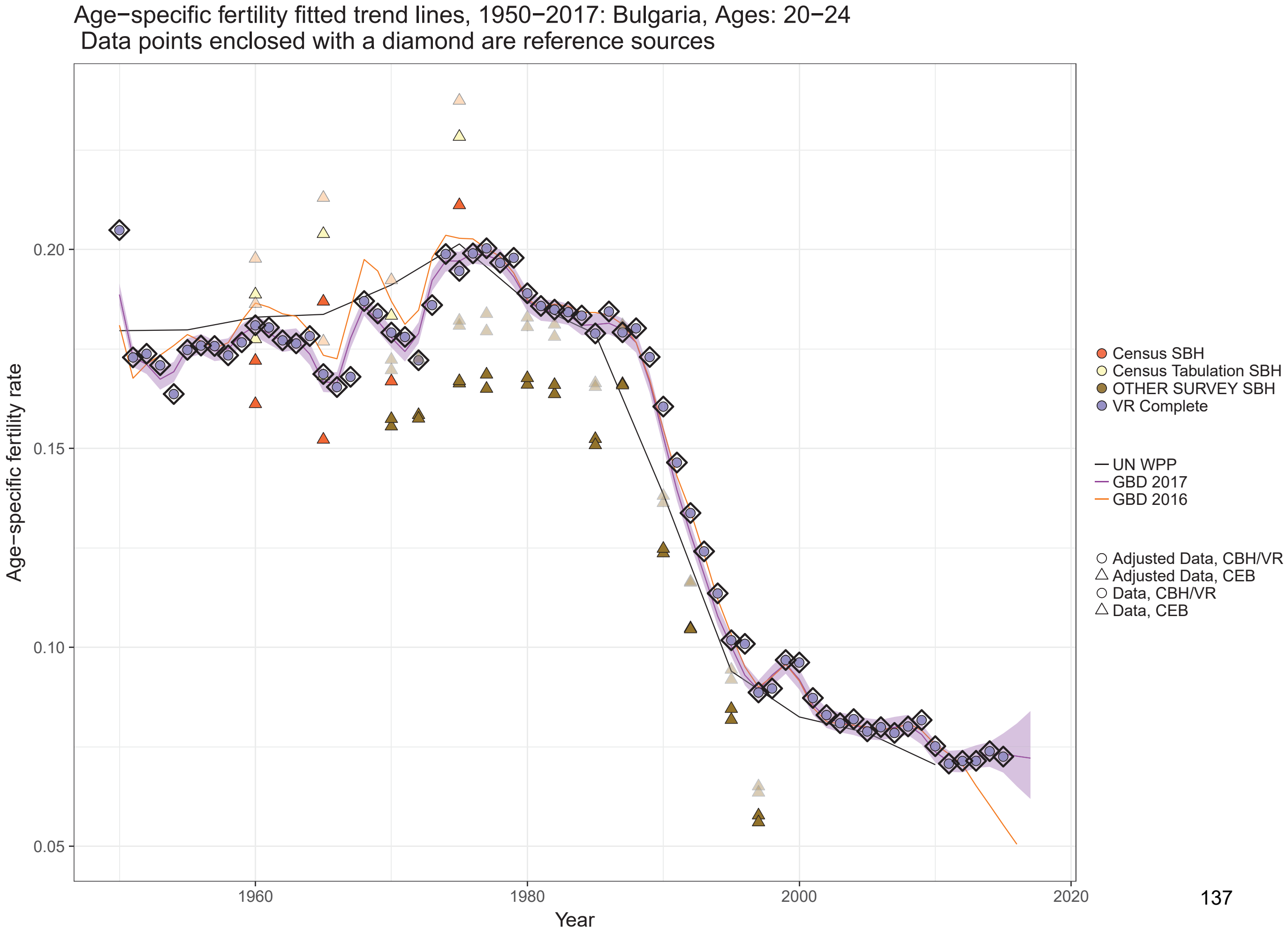

Age−specific fertility fitted trend lines, 1950−2017: Bulgaria, Ages: 20−24
Data points enclosed with a diamond are reference sources
Age−specific fertility rate
0.05
0.10
0.15
0.20
Year
1960
1980
2000
2020
Census SBH
Census Tabulation SBH
OTHER SURVEY SBH
VR Complete
UN WPP
GBD 2017
GBD 2016
Adjusted Data, CBH/VR
Adjusted Data, CEB
Data, CBH/VR
Data, CEB

Age−specific fertility fitted trend lines, 1950−2017: Bulgaria, Ages: 25−29
Data points enclosed with a diamond are reference sources

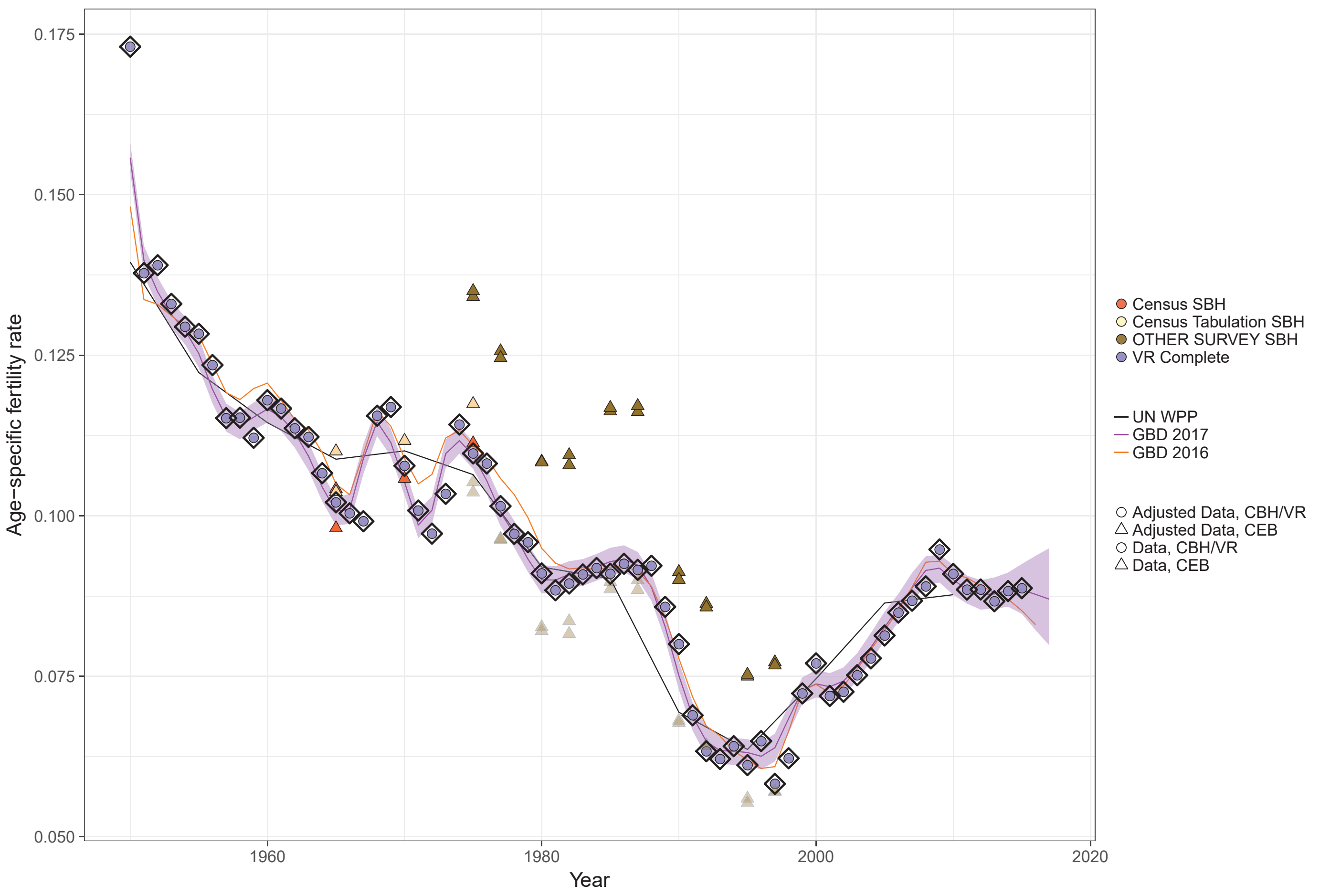

Age−specific fertility fitted trend lines, 1950−2017: Bulgaria, Ages: 30−34
Data points enclosed with a diamond are reference sources

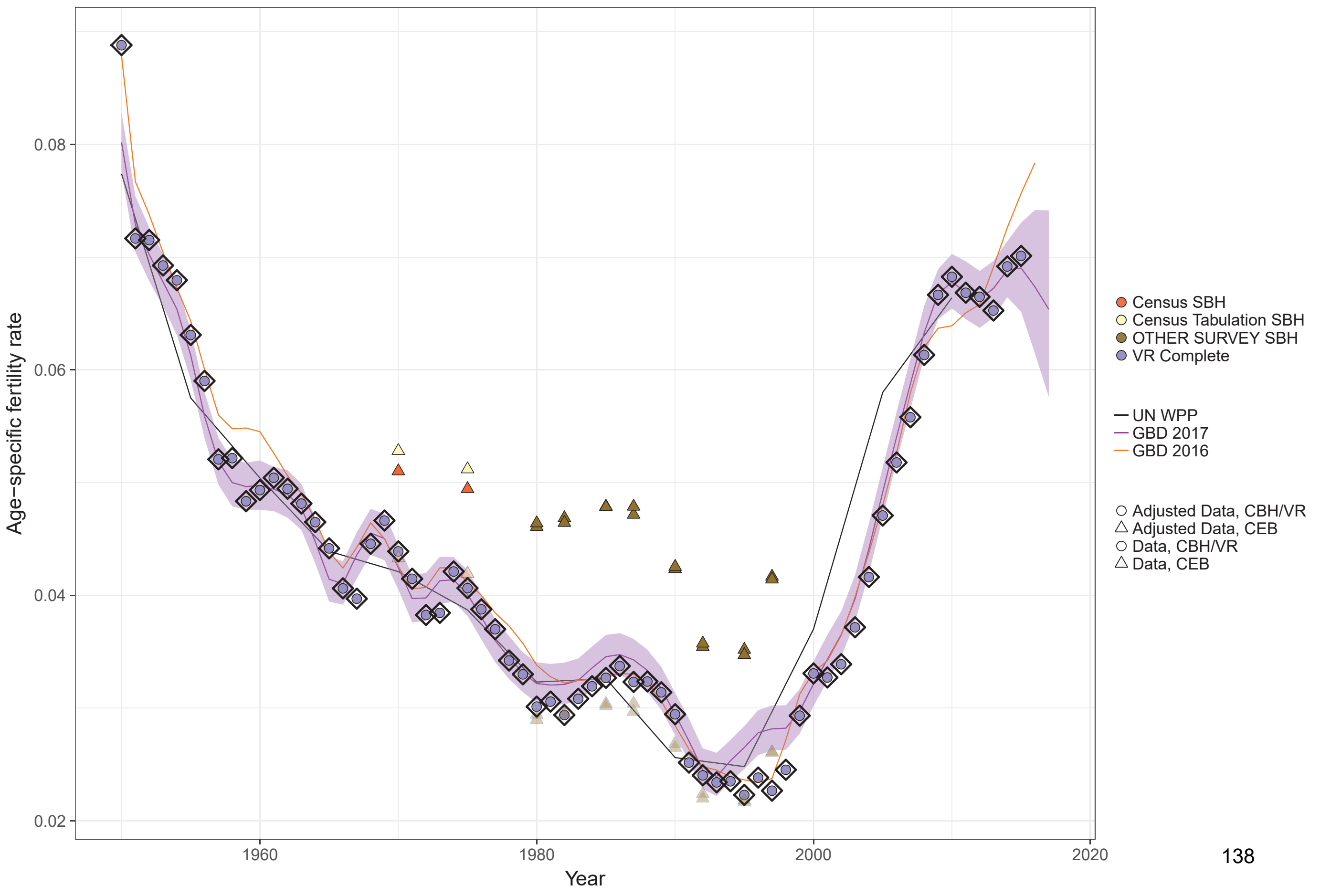

Age−specific fertility fitted trend lines, 1950−2017: Bulgaria, Ages: 35−39
Data points enclosed with a diamond are reference sources

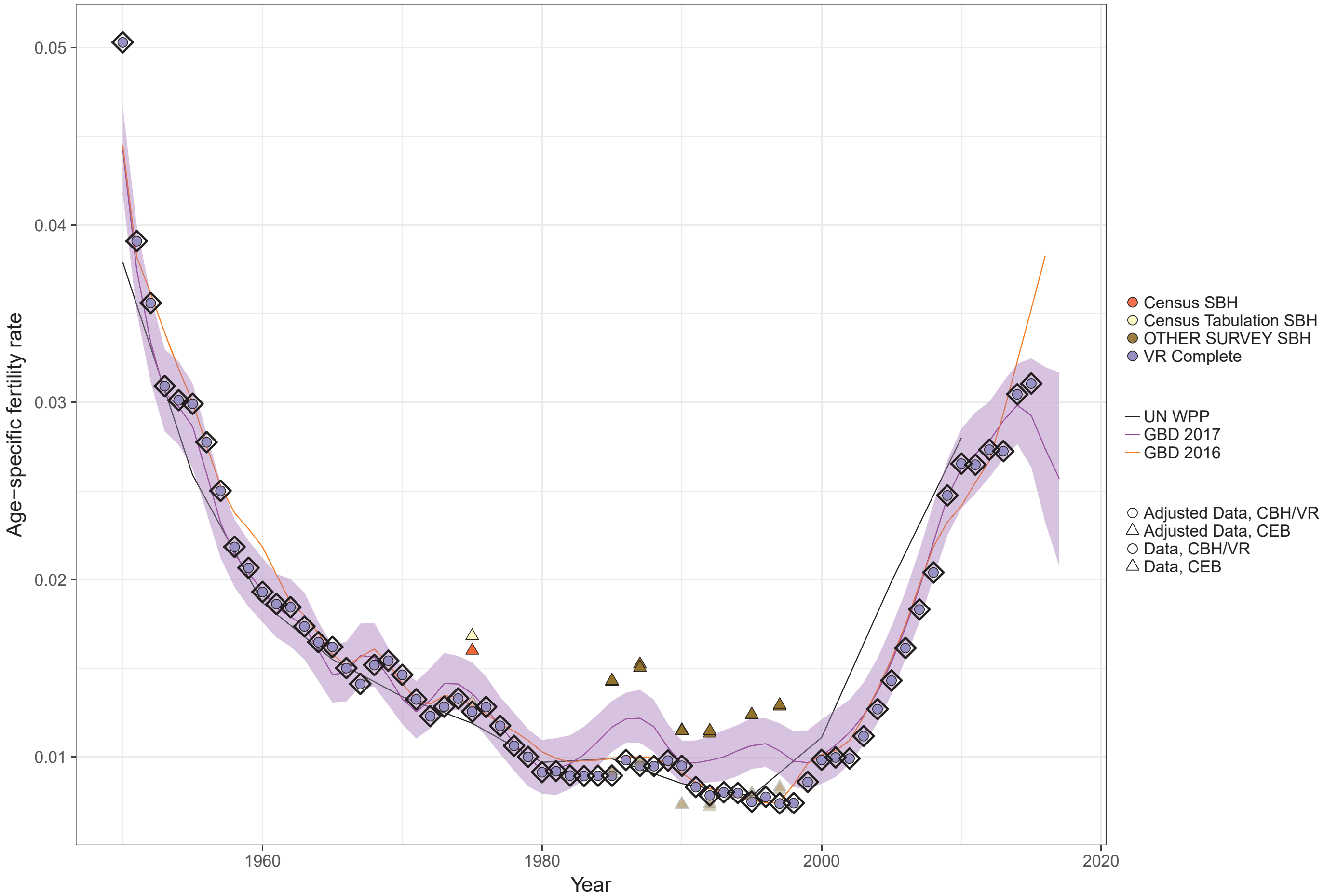

Age−specific fertility fitted trend lines, 1950−2017: Bulgaria, Ages: 40−44
Data points enclosed with a diamond are reference sources

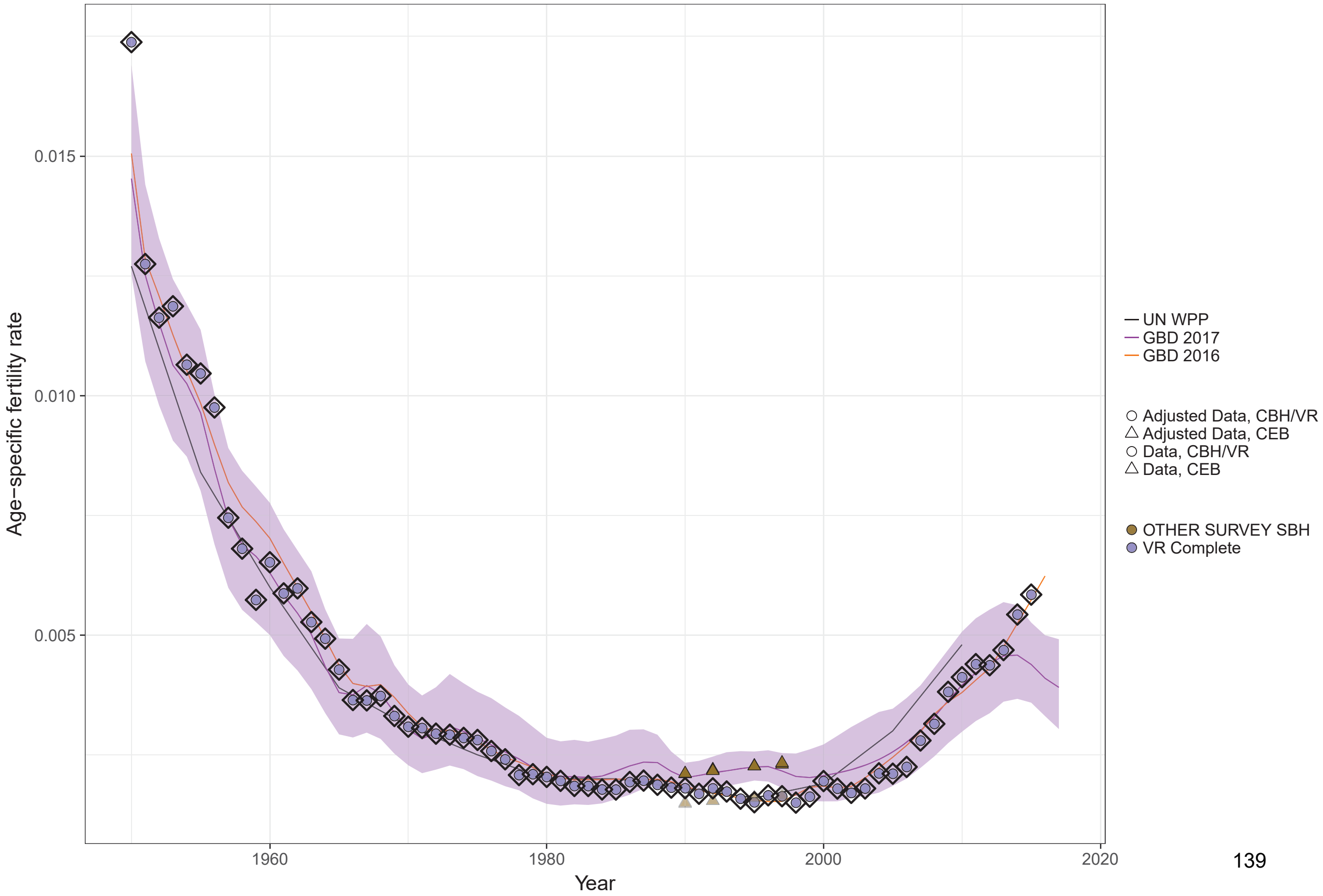

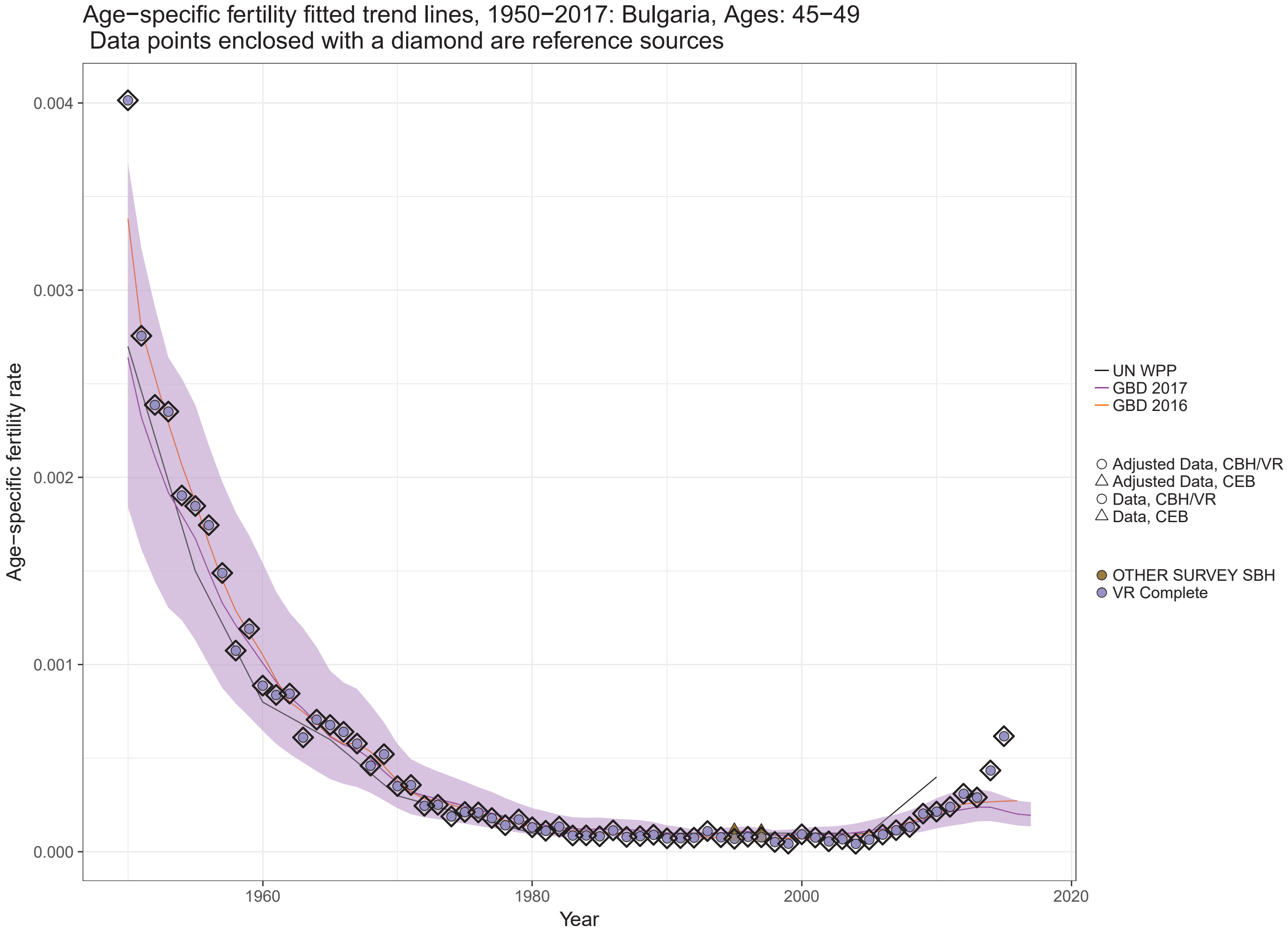
Age−specific fertility fitted trend lines, 1950−2017: Bulgaria, Ages: 45−49
Data points enclosed with a diamond are reference sources
Age−specific fertility rate
Year
UN WPP
GBD 2017
GBD 2016
Adjusted Data, CBH/VR
Adjusted Data, CEB
Data, CBH/VR
Data, CEB
OTHER SURVEY SBH
VR Complete

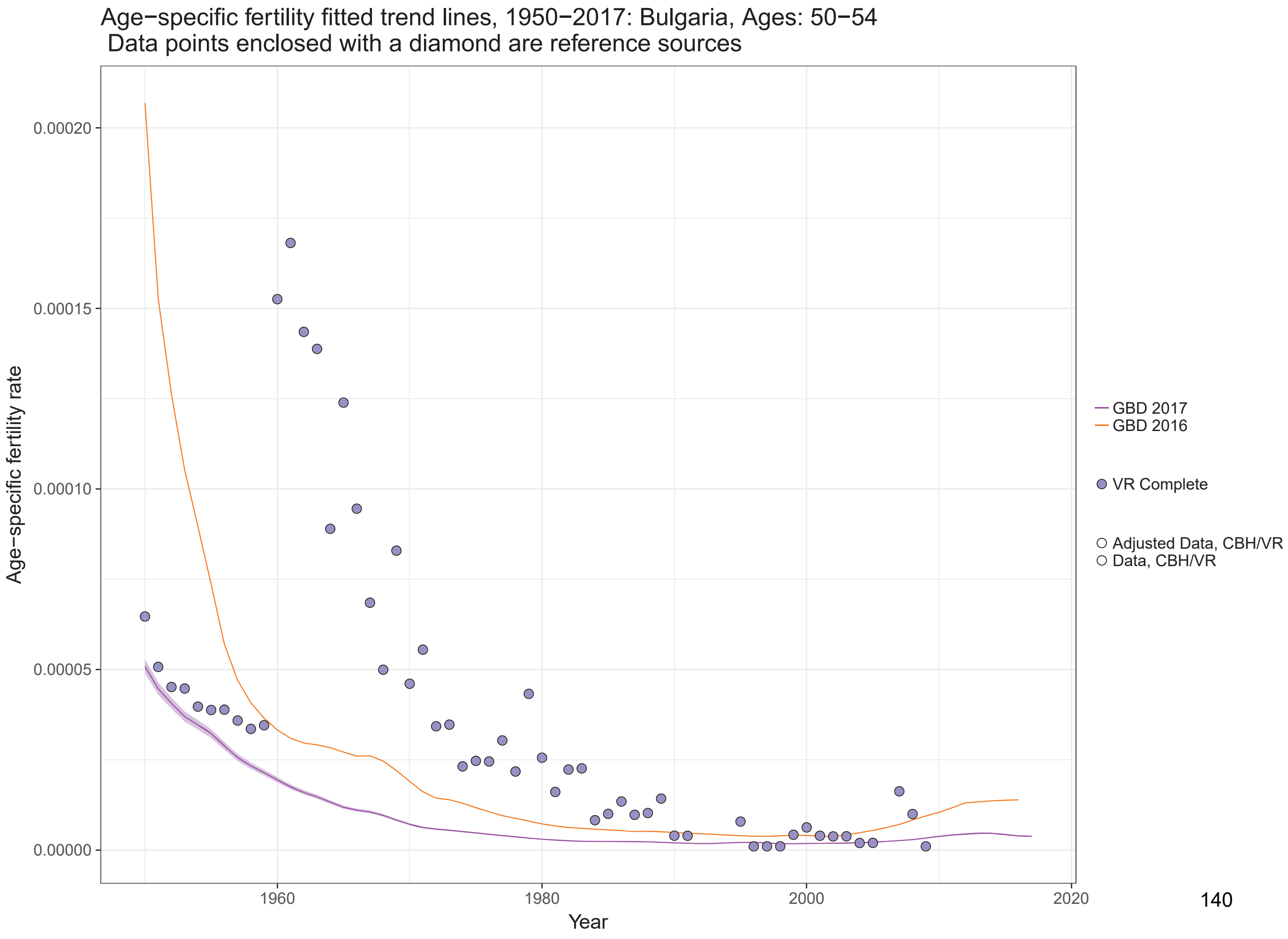
Age−specific fertility fitted trend lines, 1950−2017: Bulgaria, Ages: 50−54
Data points enclosed with a diamond are reference sources
Age−specific fertility rate
Year
GBD 2017
GBD 2016
VR Complete
Adjusted Data, CBH/VR
Data, CBH/VR

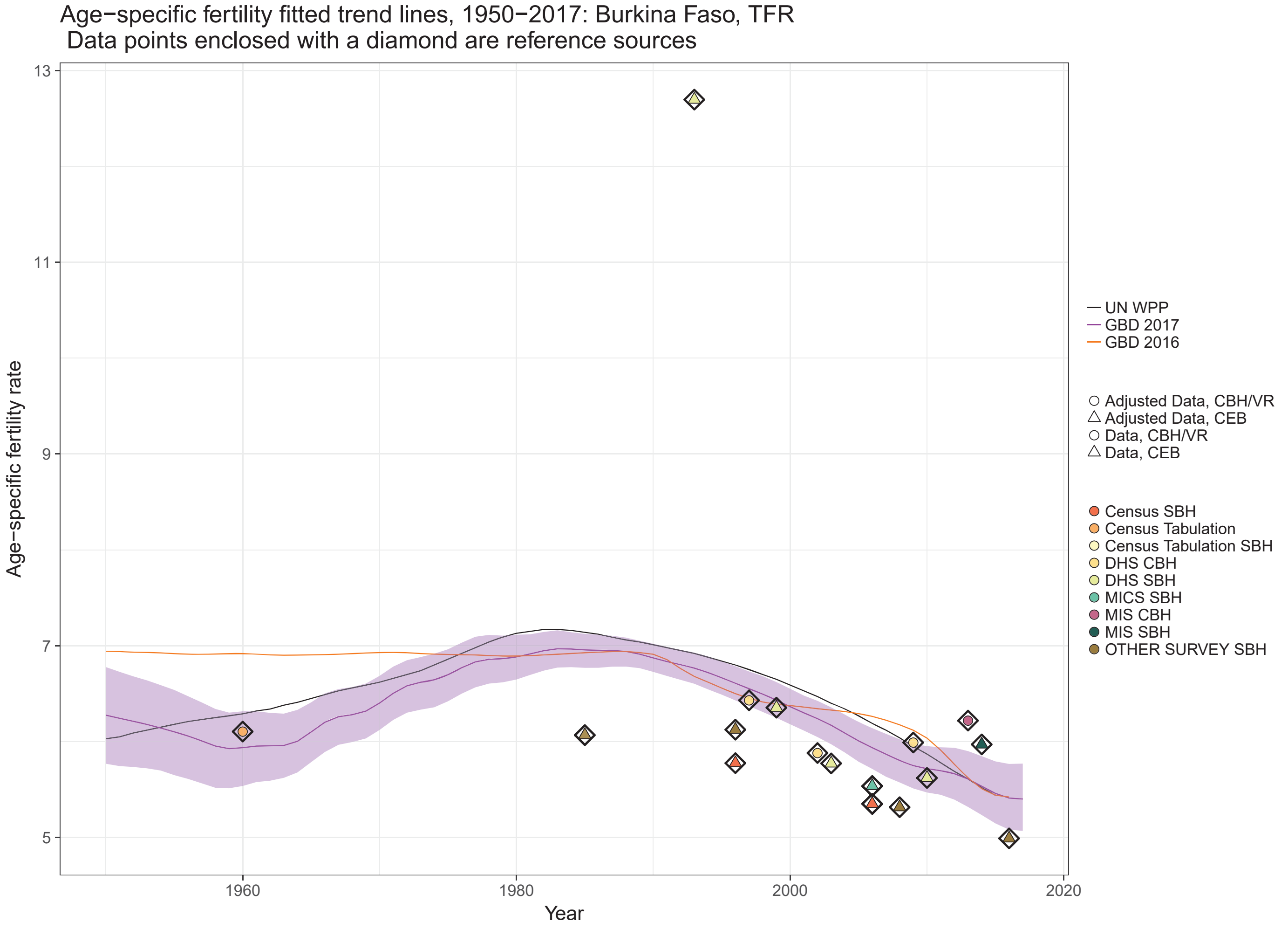
Age−specific fertility fitted trend lines, 1950−2017: Burkina Faso, TFR
Data points enclosed with a diamond are reference sources
Age−specific fertility rate
Year
13
11
9
7
5
1960
1980
2000
2020
UN WPP
GBD 2017
GBD 2016
Adjusted Data, CBH/VR
Adjusted Data, CEB
Data, CBH/VR
Data, CEB
Census SBH
Census Tabulation
Census Tabulation SBH
DHS CBH
DHS SBH
MICS SBH
MIS CBH
MIS SBH
OTHER SURVEY SBH

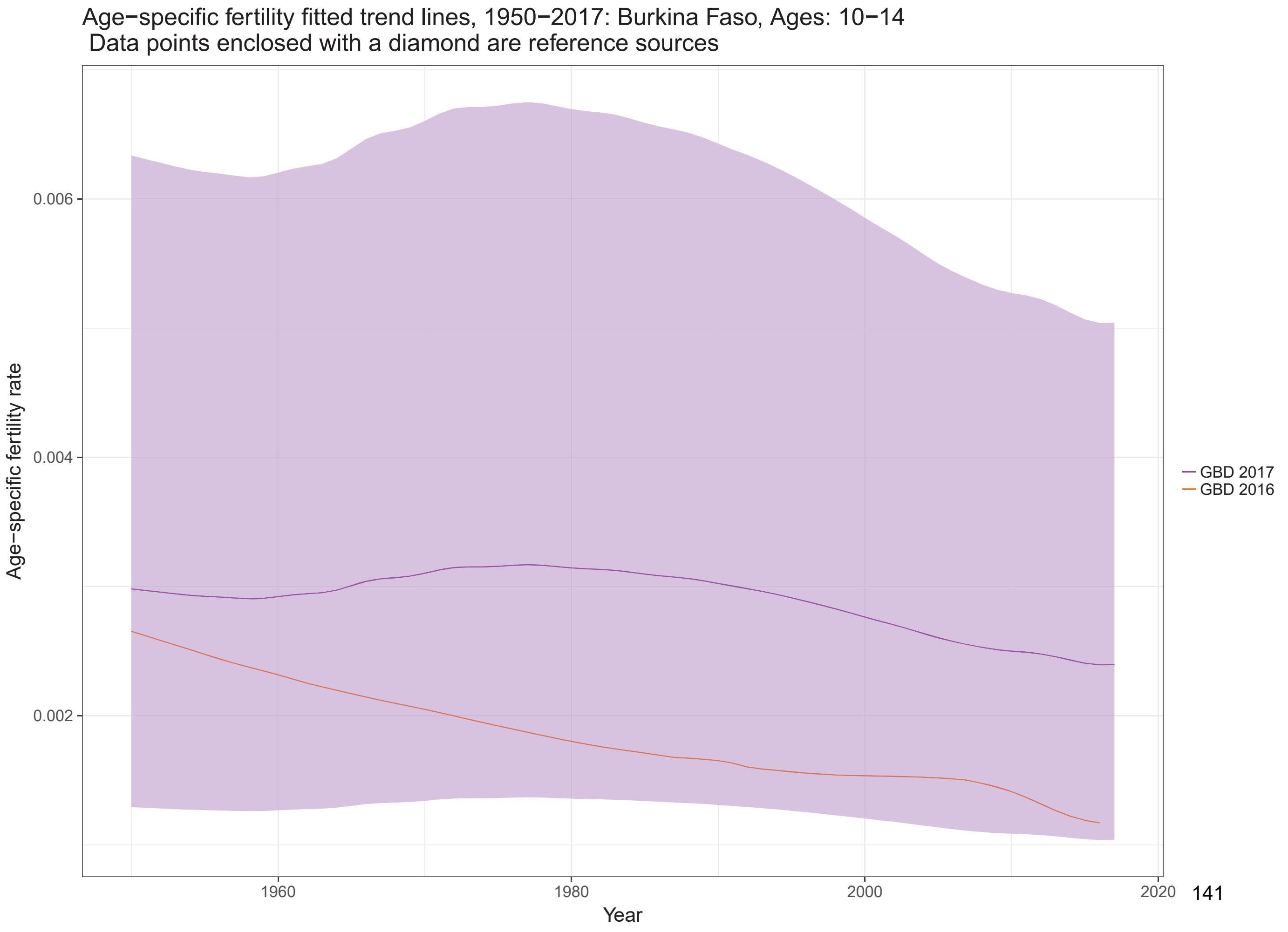
Age−specific fertility fitted trend lines, 1950−2017: Burkina Faso, Ages: 10−14
Data points enclosed with a diamond are reference sources
Age−specific fertility rate
Year
0.006
0.004
0.002
1960
1980
2000
2020
GBD 2017
GBD 2016

Age−specific fertility fitted trend lines, 1950−2017: Burkina Faso, Ages: 15−19
Data points enclosed with a diamond are reference sources

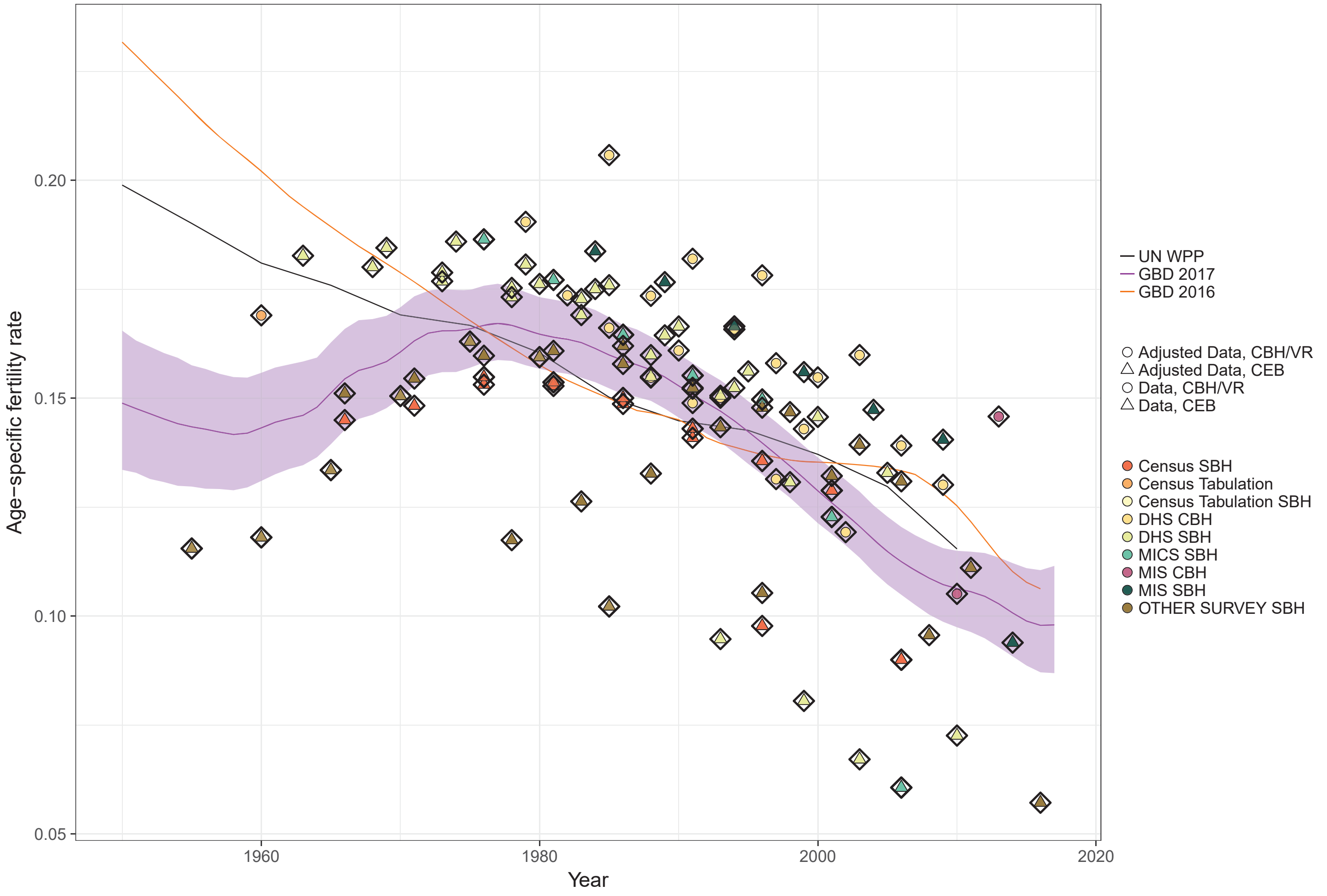

Age−specific fertility fitted trend lines, 1950−2017: Burkina Faso, Ages: 20−24
Data points enclosed with a diamond are reference sources

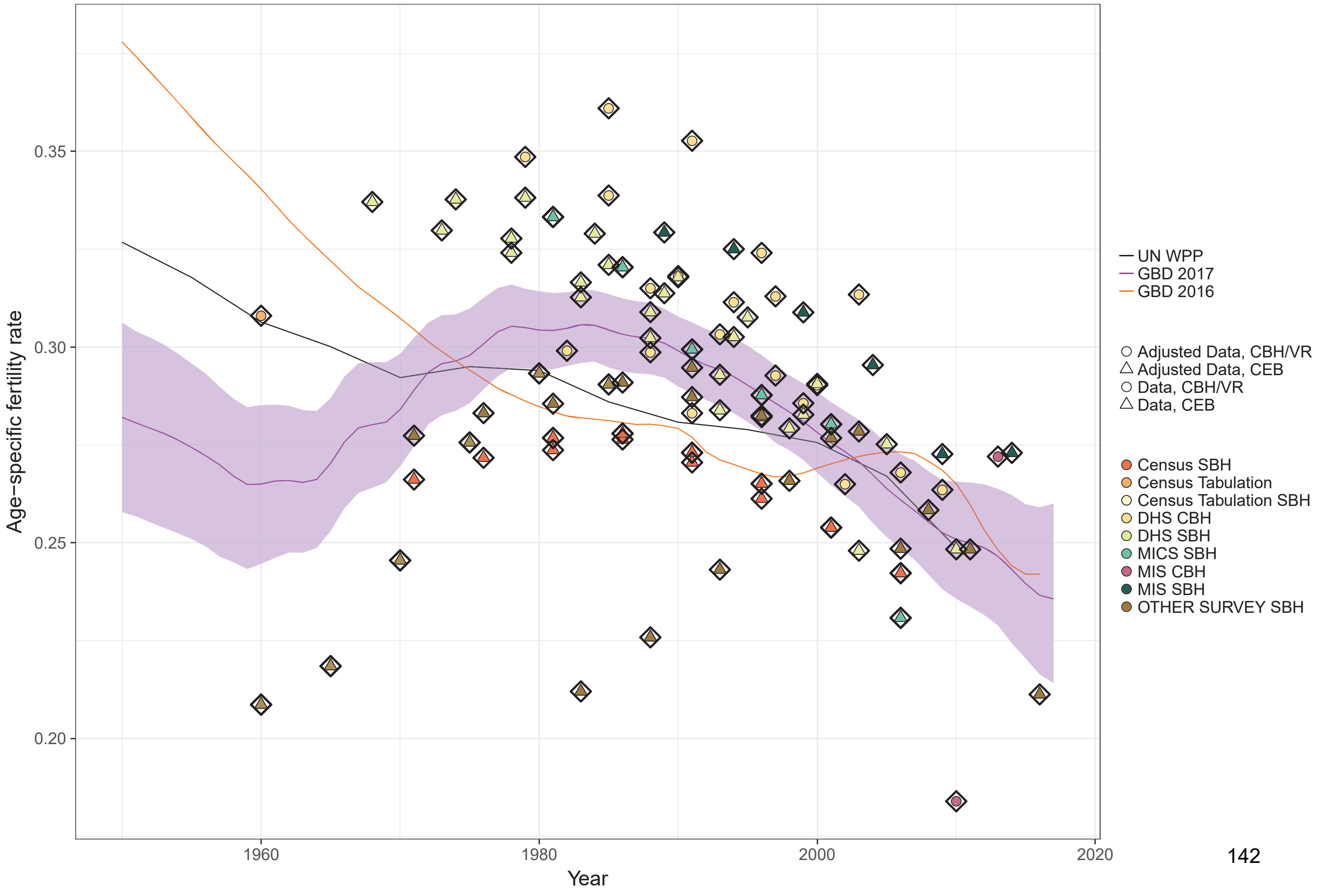

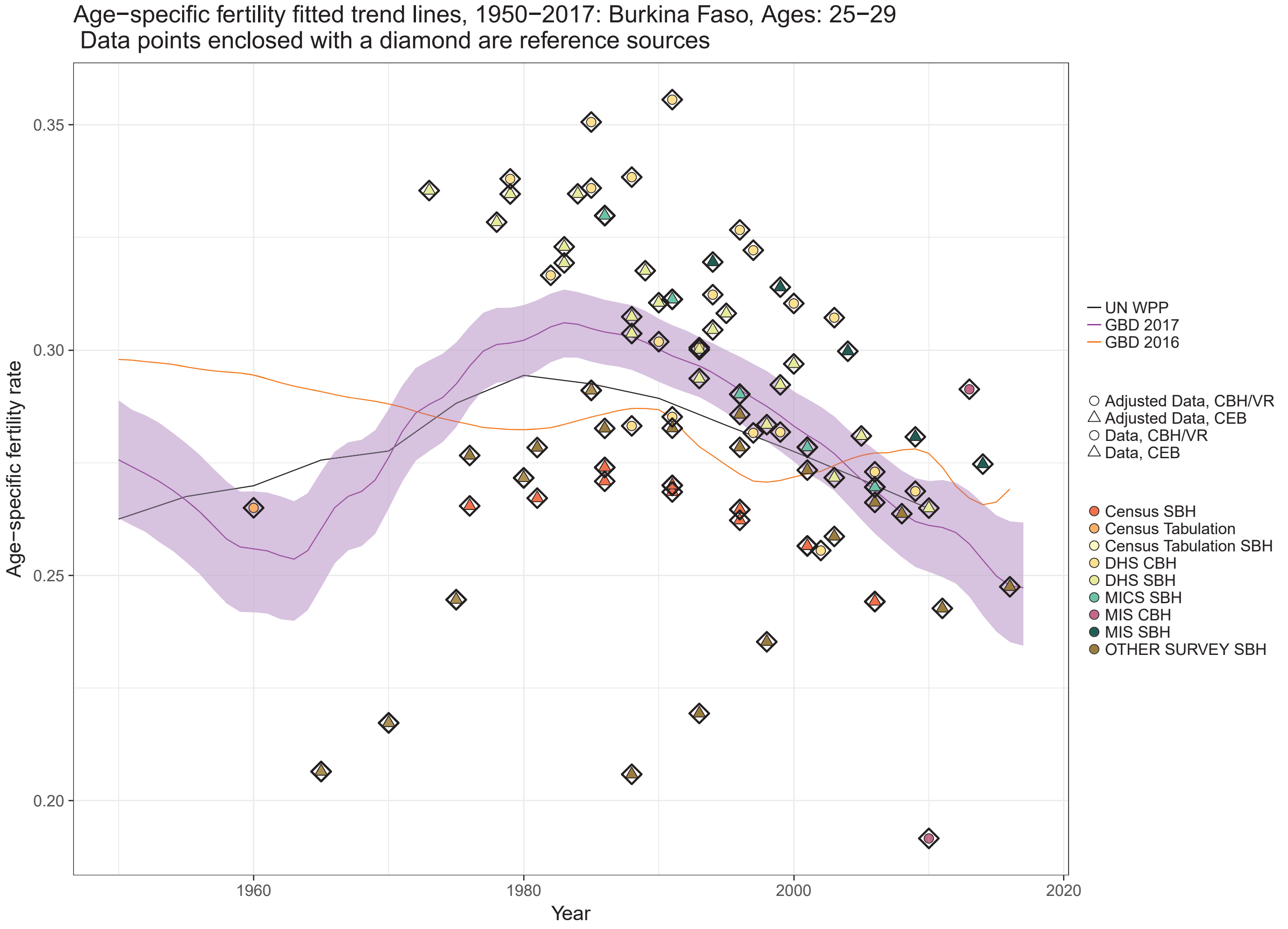
Age−specific fertility fitted trend lines, 1950−2017: Burkina Faso, Ages: 25−29
Data points enclosed with a diamond are reference sources
Age−specific fertility rate
Year
UN WPP
GBD 2017
GBD 2016
Adjusted Data, CBH/VR
Adjusted Data, CEB
Data, CBH/VR
Data, CEB
Census SBH
Census Tabulation
Census Tabulation SBH
DHS CBH
DHS SBH
MICS SBH
MIS CBH
MIS SBH
OTHER SURVEY SBH

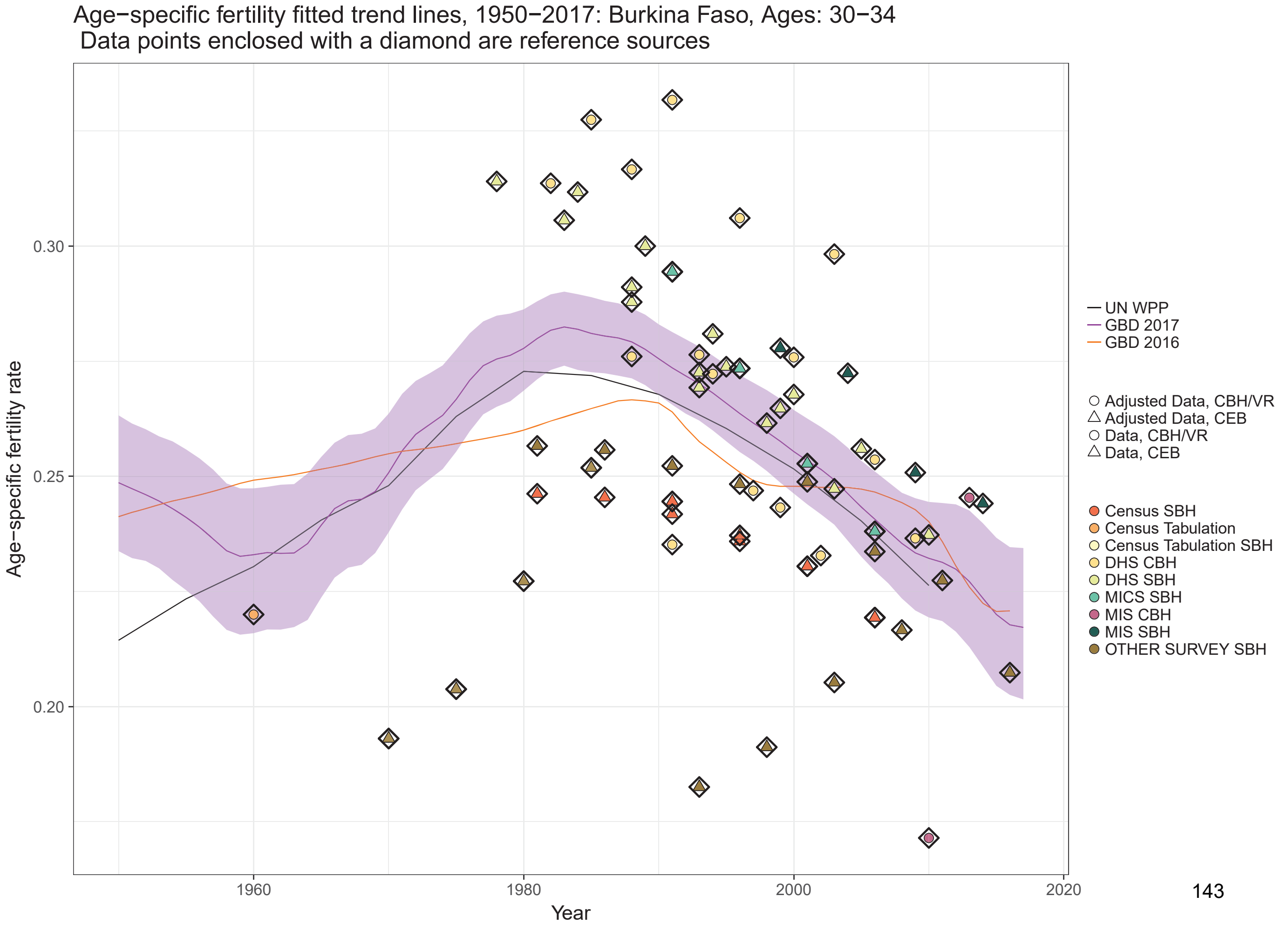
Age−specific fertility fitted trend lines, 1950−2017: Burkina Faso, Ages: 30−34
Data points enclosed with a diamond are reference sources
Age−specific fertility rate
Year
UN WPP
GBD 2017
GBD 2016
Adjusted Data, CBH/VR
Adjusted Data, CEB
Data, CBH/VR
Data, CEB
Census SBH
Census Tabulation
Census Tabulation SBH
DHS CBH
DHS SBH
MICS SBH
MIS CBH
MIS SBH
OTHER SURVEY SBH

Age−specific fertility fitted trend lines, 1950−2017: Burkina Faso, Ages: 35−39
Data points enclosed with a diamond are reference sources

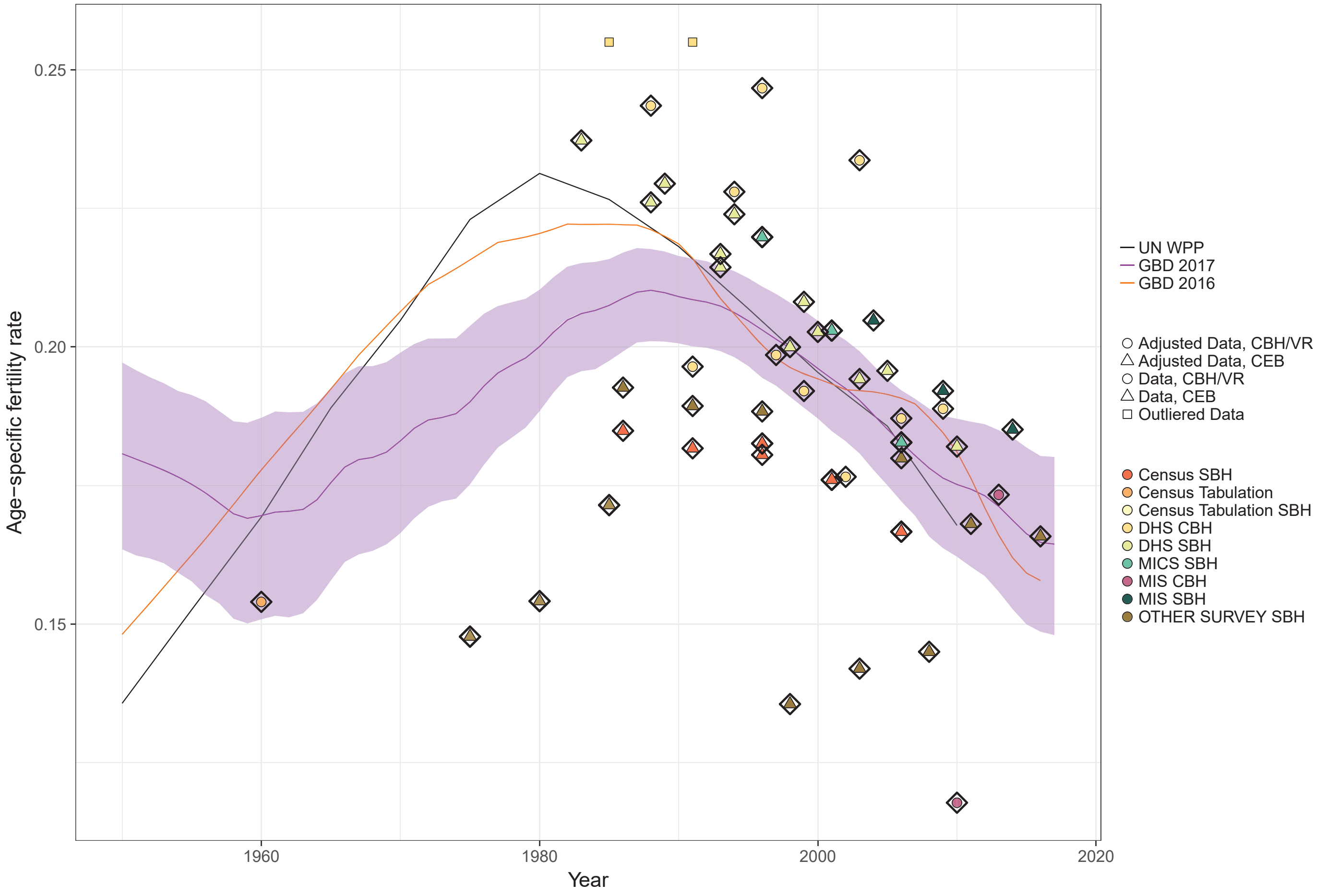

Age−specific fertility fitted trend lines, 1950−2017: Burkina Faso, Ages: 40−44
Data points enclosed with a diamond are reference sources

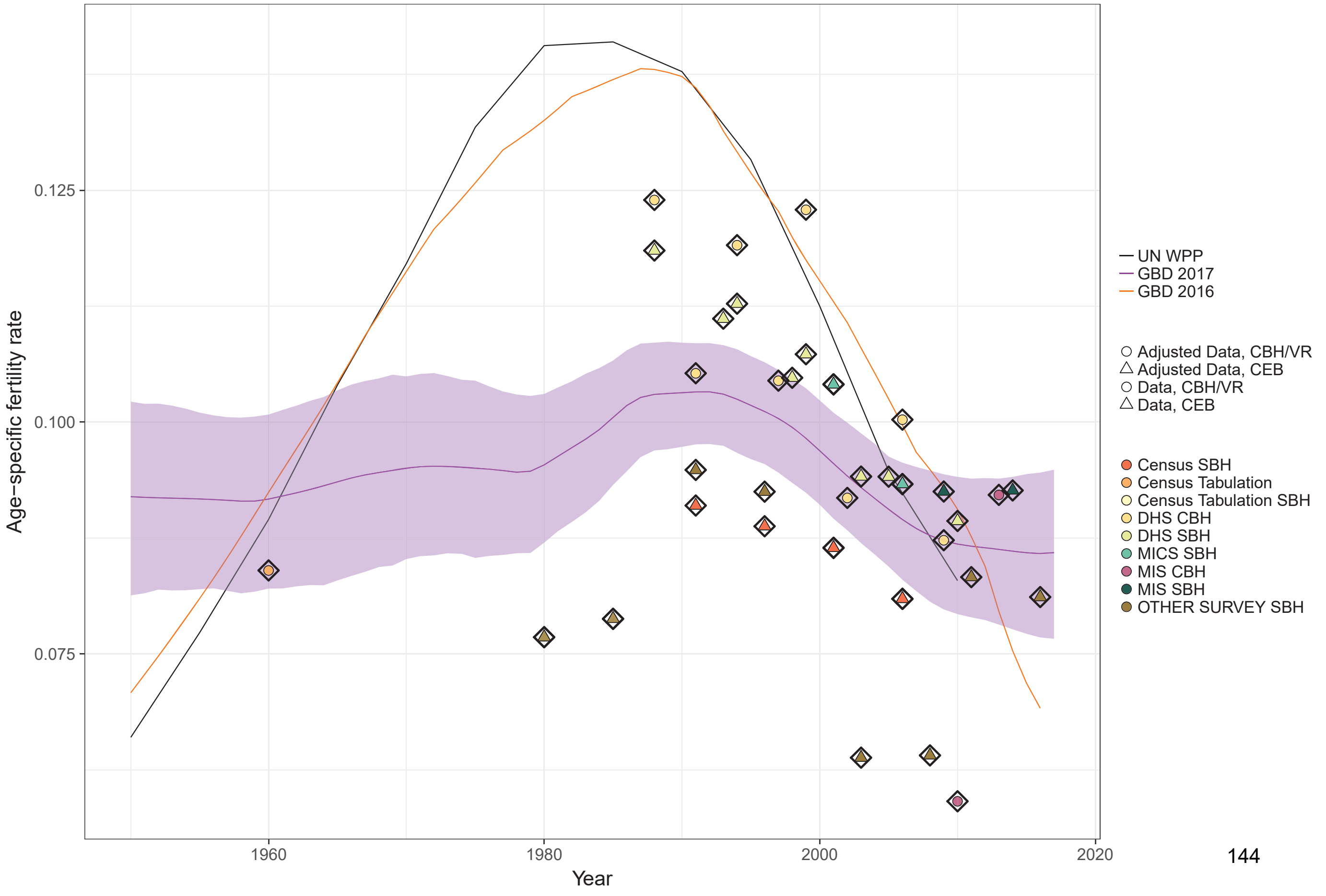

Age−specific fertility fitted trend lines, 1950−2017: Burkina Faso, Ages: 45−49
Data points enclosed with a diamond are reference sources

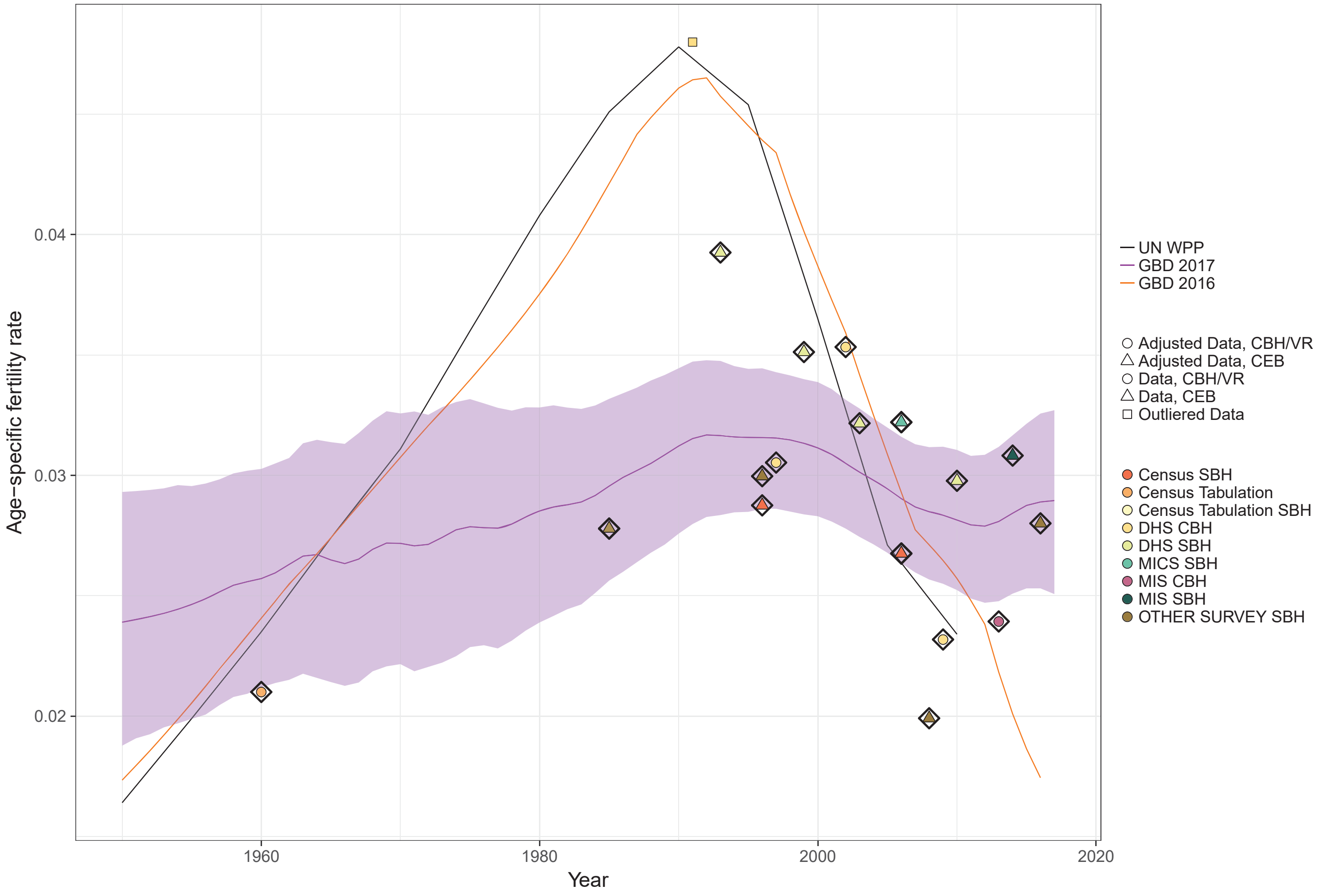

Age−specific fertility fitted trend lines, 1950−2017: Burkina Faso, Ages: 50−54
Data points enclosed with a diamond are reference sources

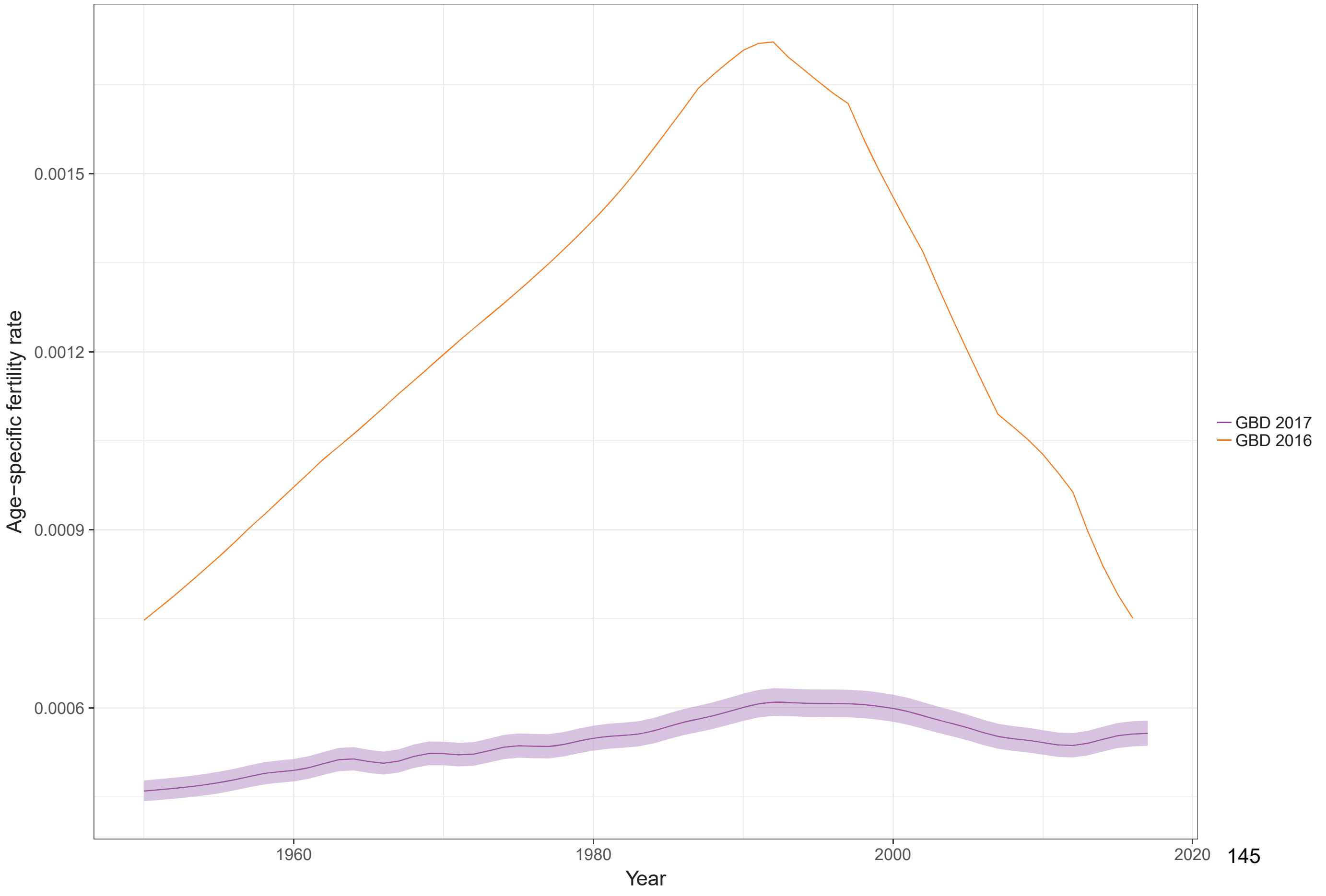

Age−specific fertility fitted trend lines, 1950−2017: Burundi, TFR
Data points enclosed with a diamond are reference sources

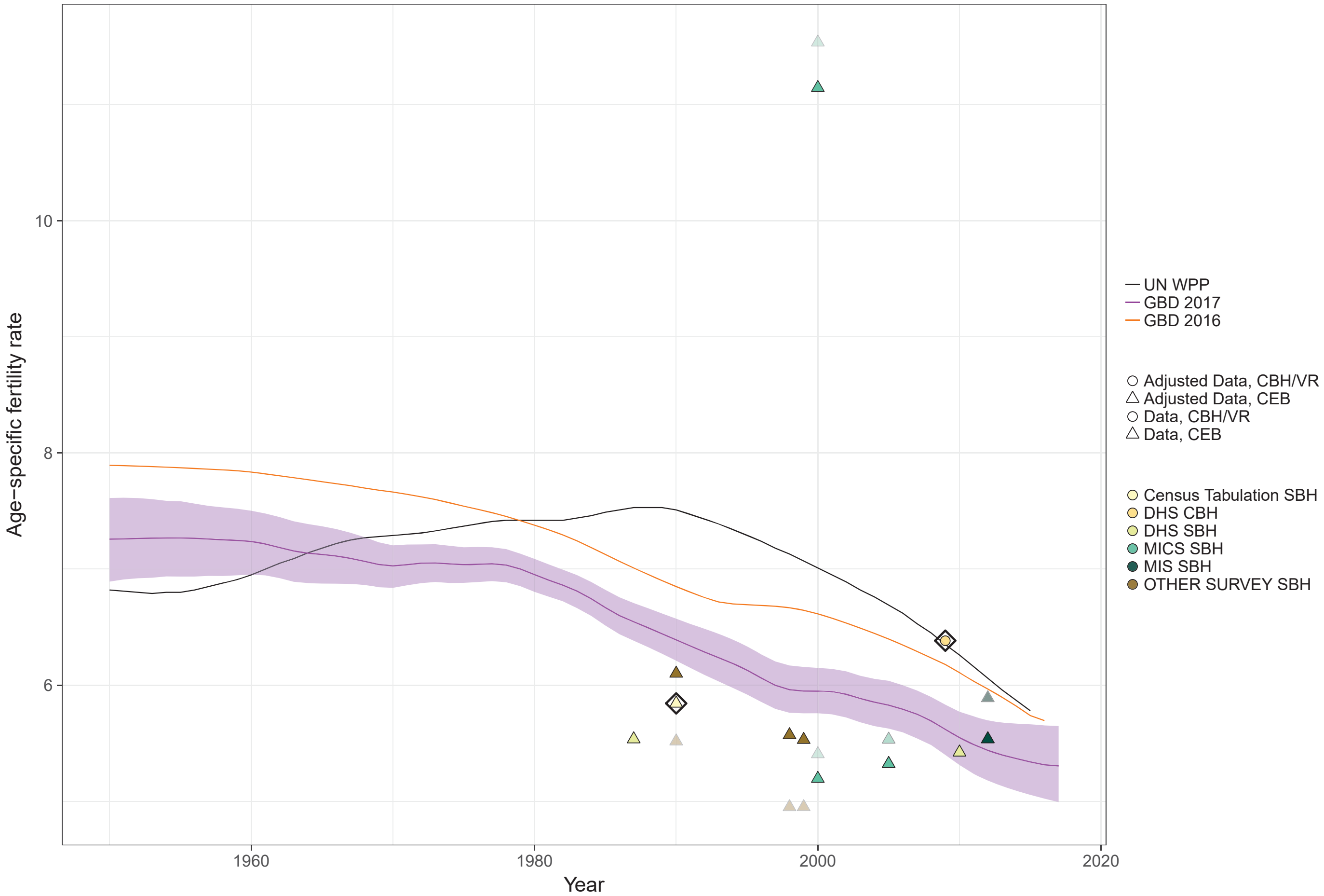

Age−specific fertility fitted trend lines, 1950−2017: Burundi, Ages: 10−14
Data points enclosed with a diamond are reference sources

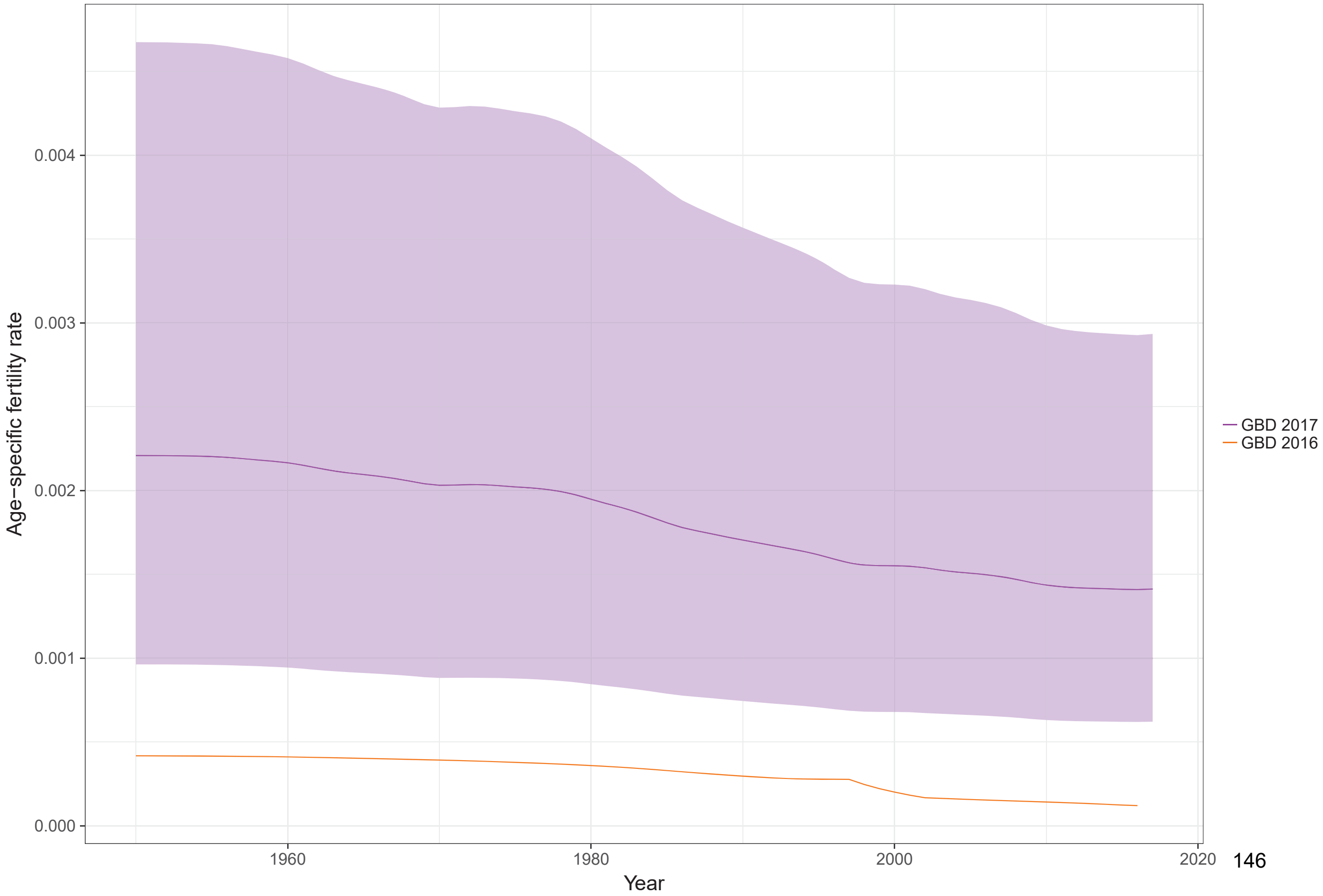

Age−specific fertility fitted trend lines, 1950−2017: Burundi, Ages: 15−19
Data points enclosed with a diamond are reference sources

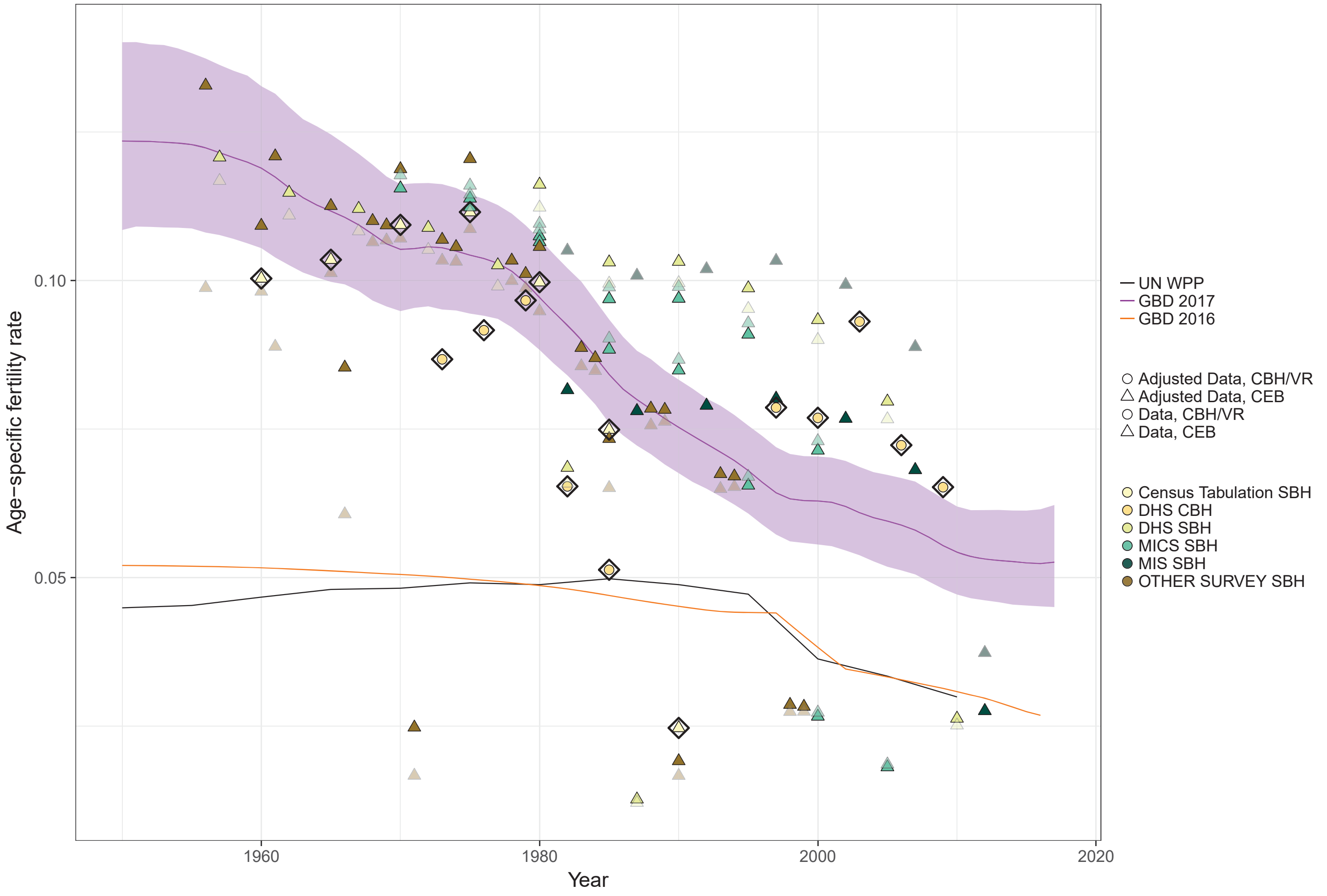

Age−specific fertility fitted trend lines, 1950−2017: Burundi, Ages: 20−24
Data points enclosed with a diamond are reference sources

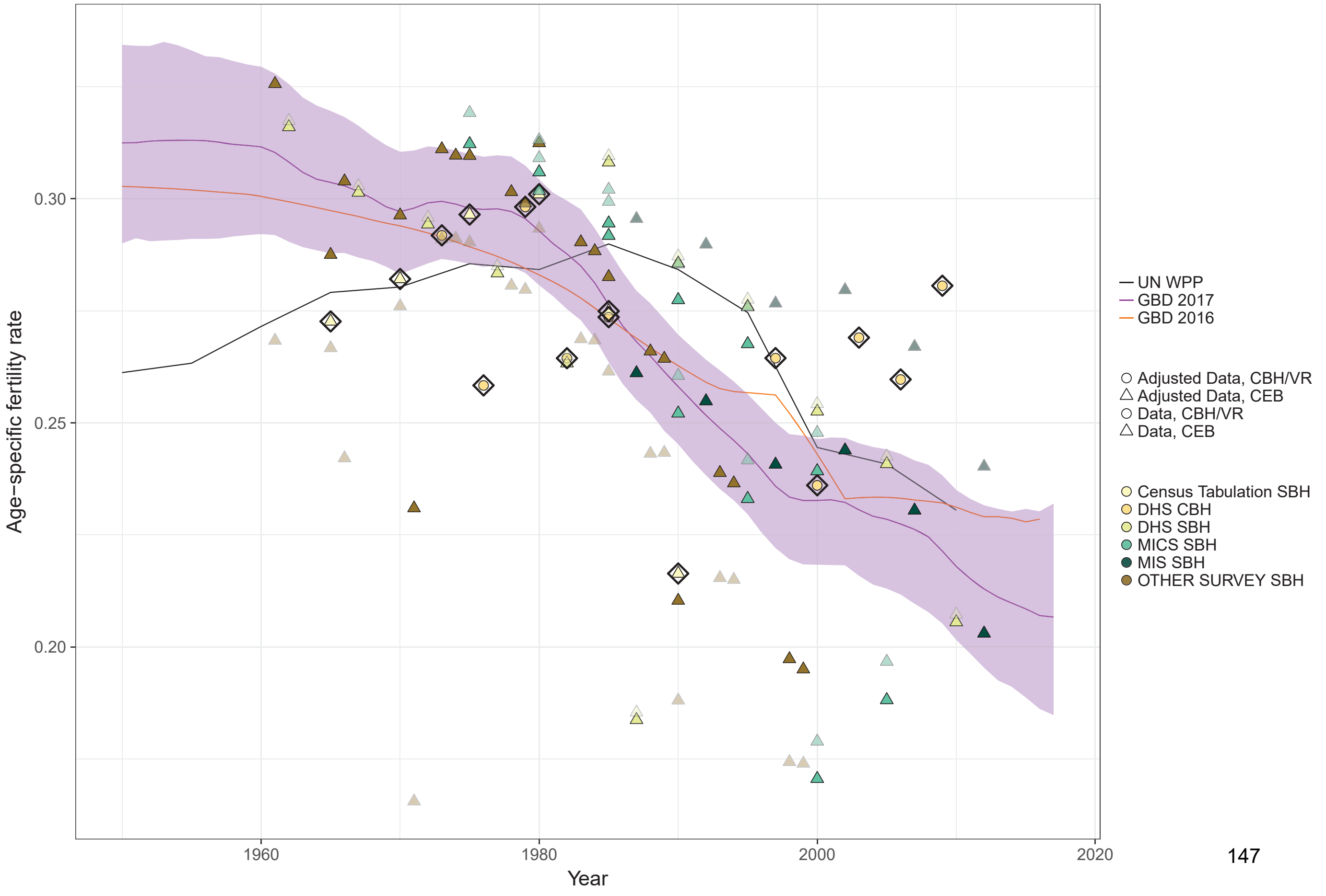

Age−specific fertility fitted trend lines, 1950−2017: Burundi, Ages: 25−29
Data points enclosed with a diamond are reference sources

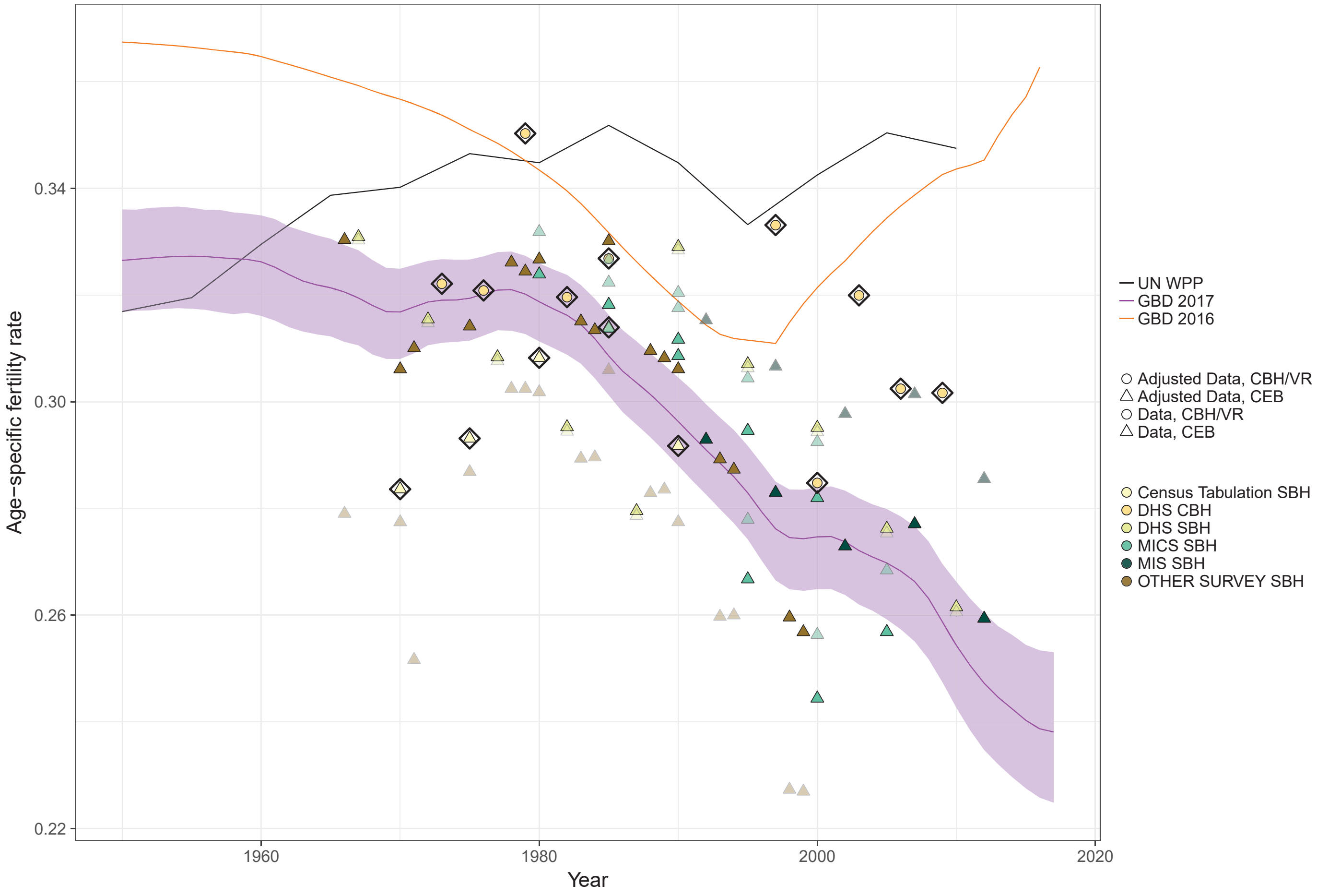

Age−specific fertility fitted trend lines, 1950−2017: Burundi, Ages: 30−34
Data points enclosed with a diamond are reference sources

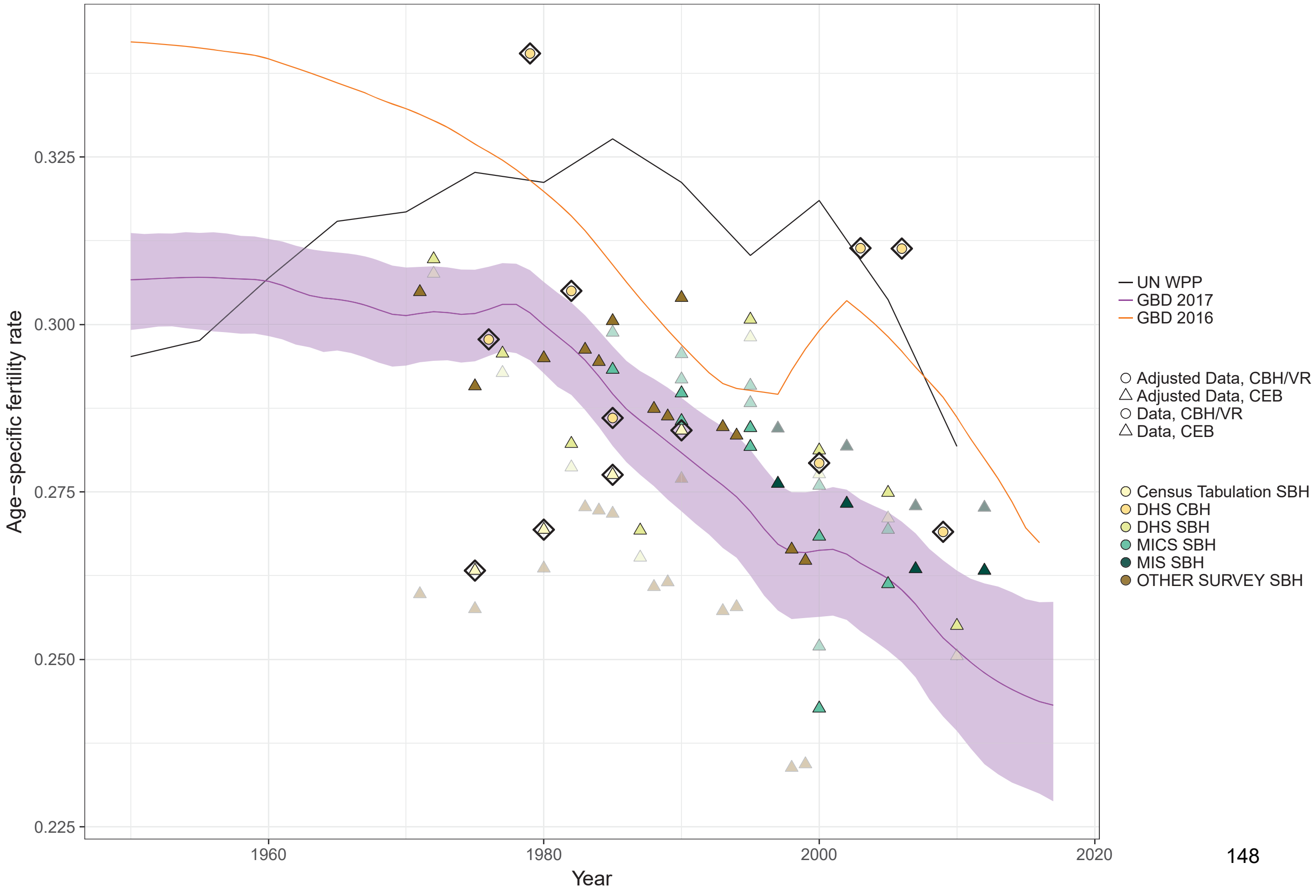

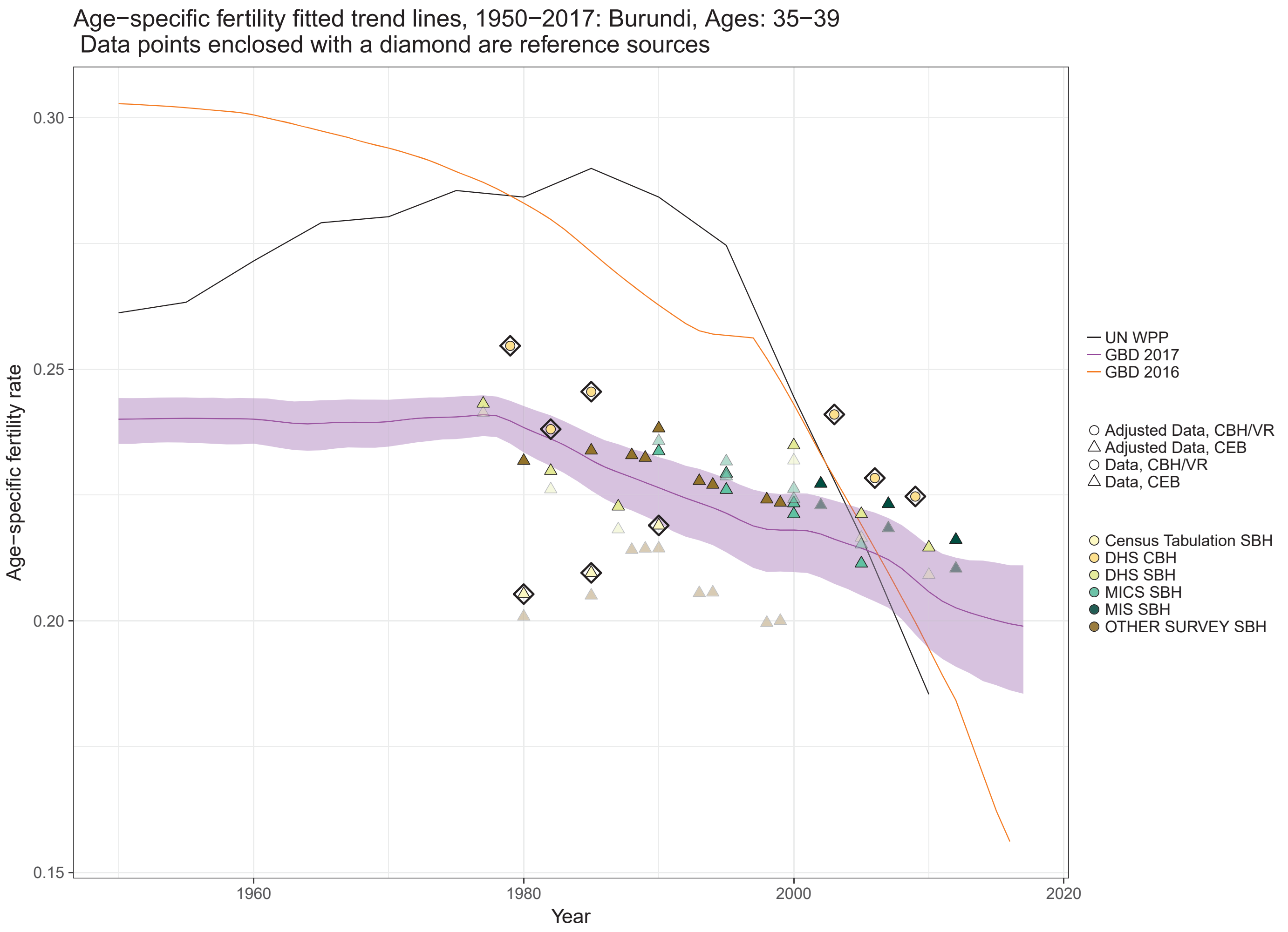

Age−specific fertility fitted trend lines, 1950−2017: Burundi, Ages: 35−39
Data points enclosed with a diamond are reference sources
Age−specific fertility rate
0.30
0.25
0.20
0.15
1960
1980
2000
2020
Year
UN WPP
GBD 2017
GBD 2016
Adjusted Data, CBH/VR
Adjusted Data, CEB
Data, CBH/VR
Data, CEB
Census Tabulation SBH
DHS CBH
DHS SBH
MICS SBH
MIS SBH
OTHER SURVEY SBH

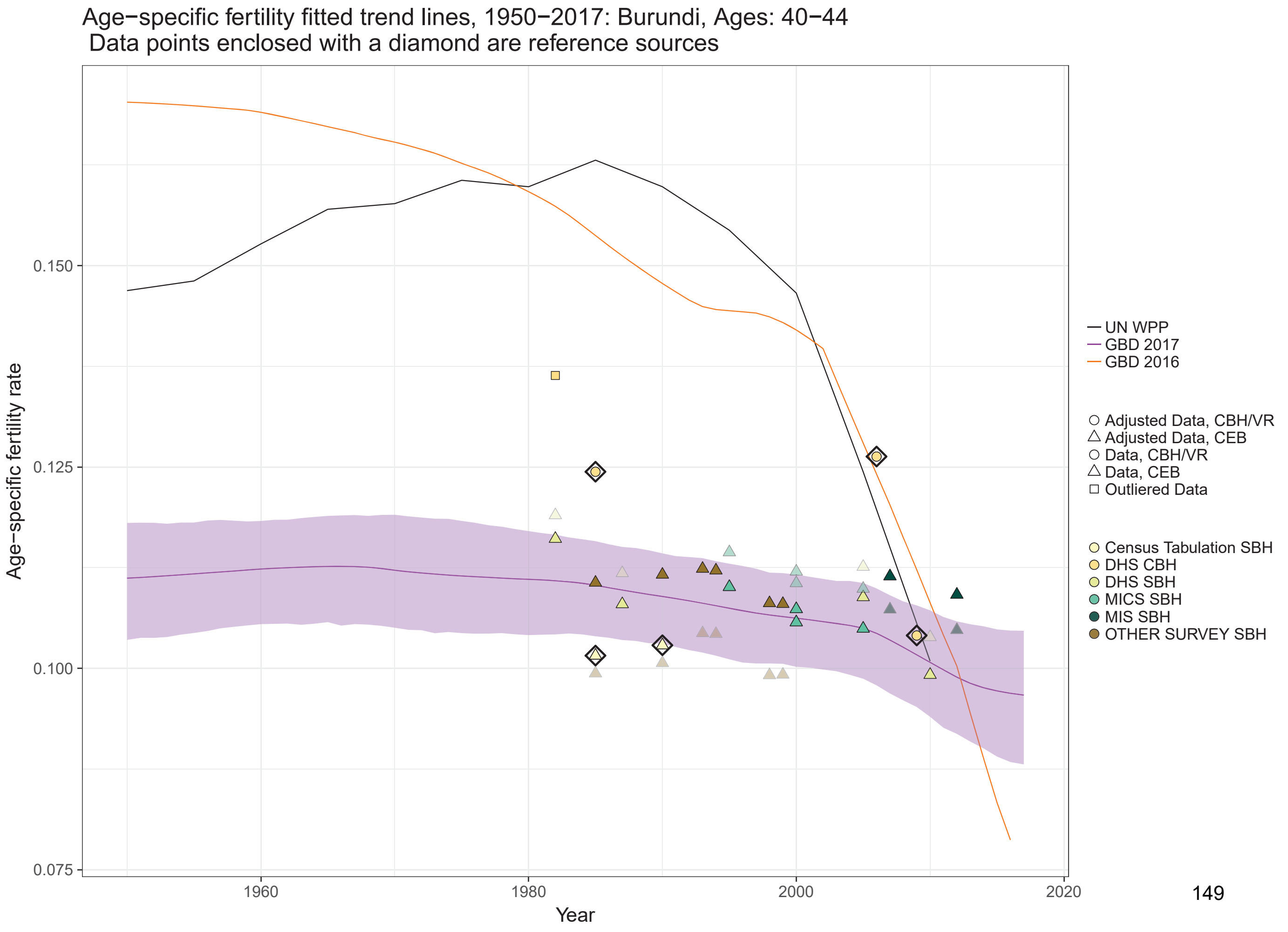

Age−specific fertility fitted trend lines, 1950−2017: Burundi, Ages: 40−44
Data points enclosed with a diamond are reference sources
Age−specific fertility rate
0.150
0.125
0.100
0.075
1960
1980
2000
2020
Year
UN WPP
GBD 2017
GBD 2016
Adjusted Data, CBH/VR
Adjusted Data, CEB
Data, CBH/VR
Data, CEB
Outliered Data
Census Tabulation SBH
DHS CBH
DHS SBH
MICS SBH
MIS SBH
OTHER SURVEY SBH

Age−specific fertility fitted trend lines, 1950−2017: Burundi, Ages: 45−49
Data points enclosed with a diamond are reference sources

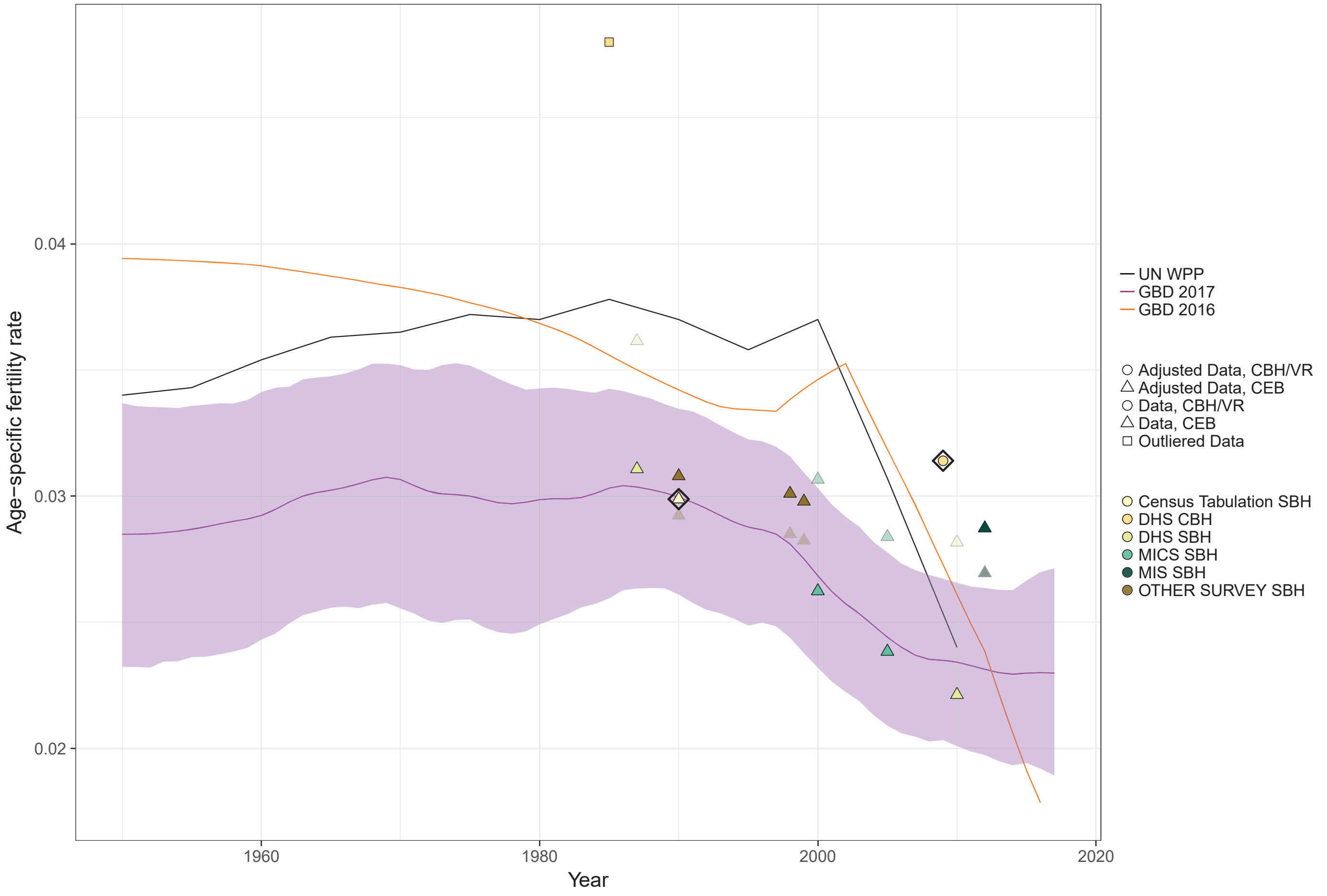

Age−specific fertility fitted trend lines, 1950−2017: Burundi, Ages: 50−54
Data points enclosed with a diamond are reference sources

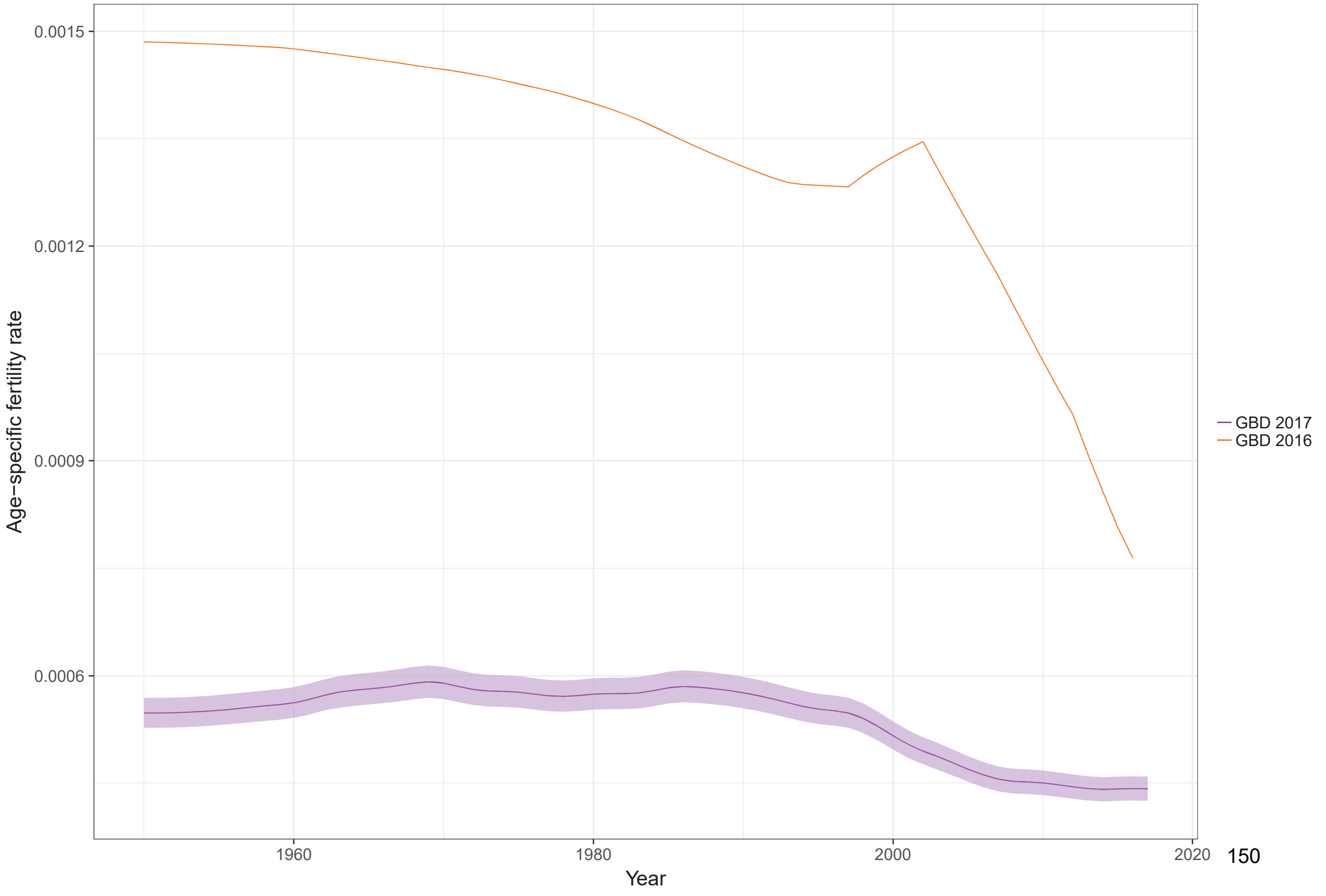

Age−specific fertility fitted trend lines, 1950−2017: Cambodia, TFR
Data points enclosed with a diamond are reference sources

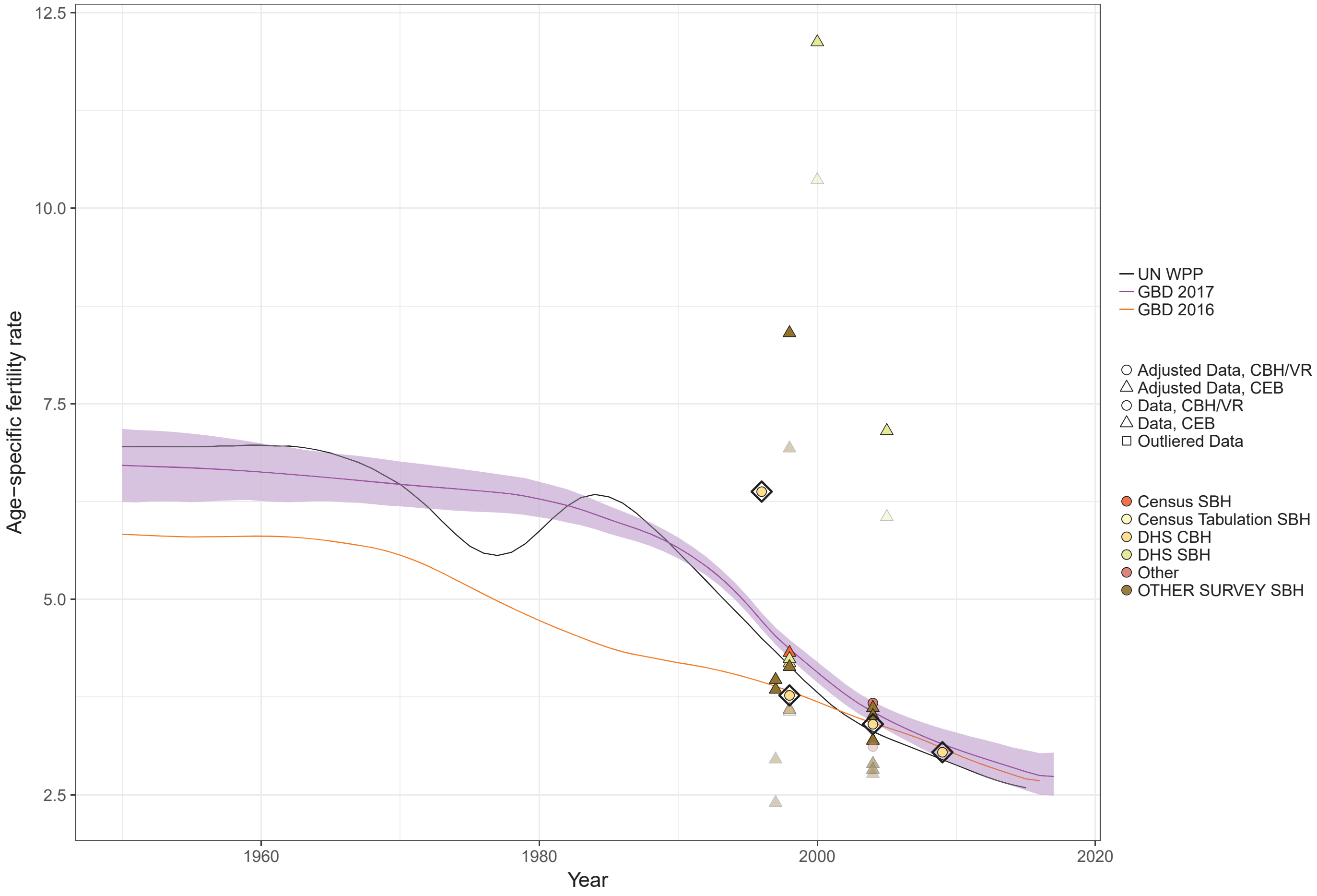

Age−specific fertility fitted trend lines, 1950−2017: Cambodia, Ages: 10−14
Data points enclosed with a diamond are reference sources

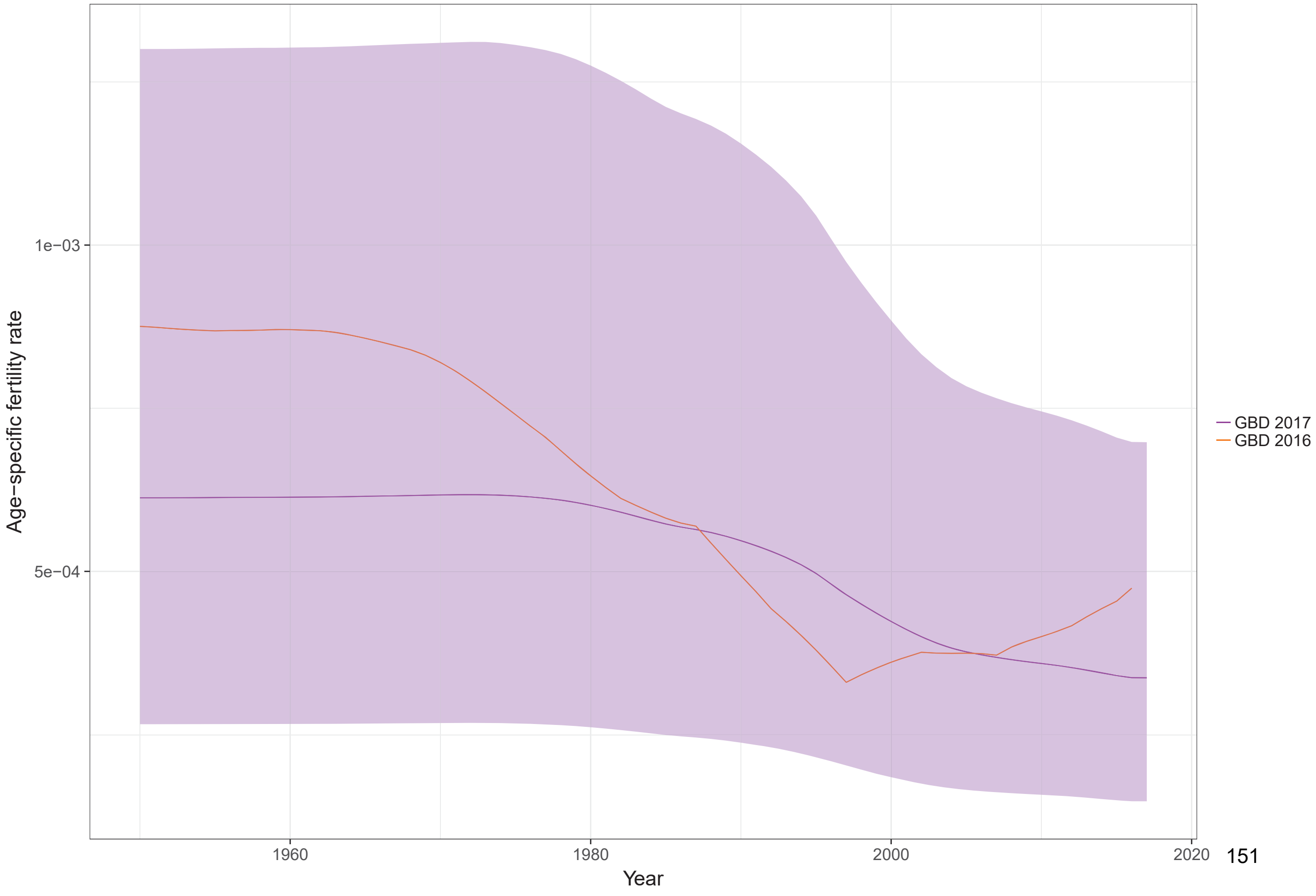

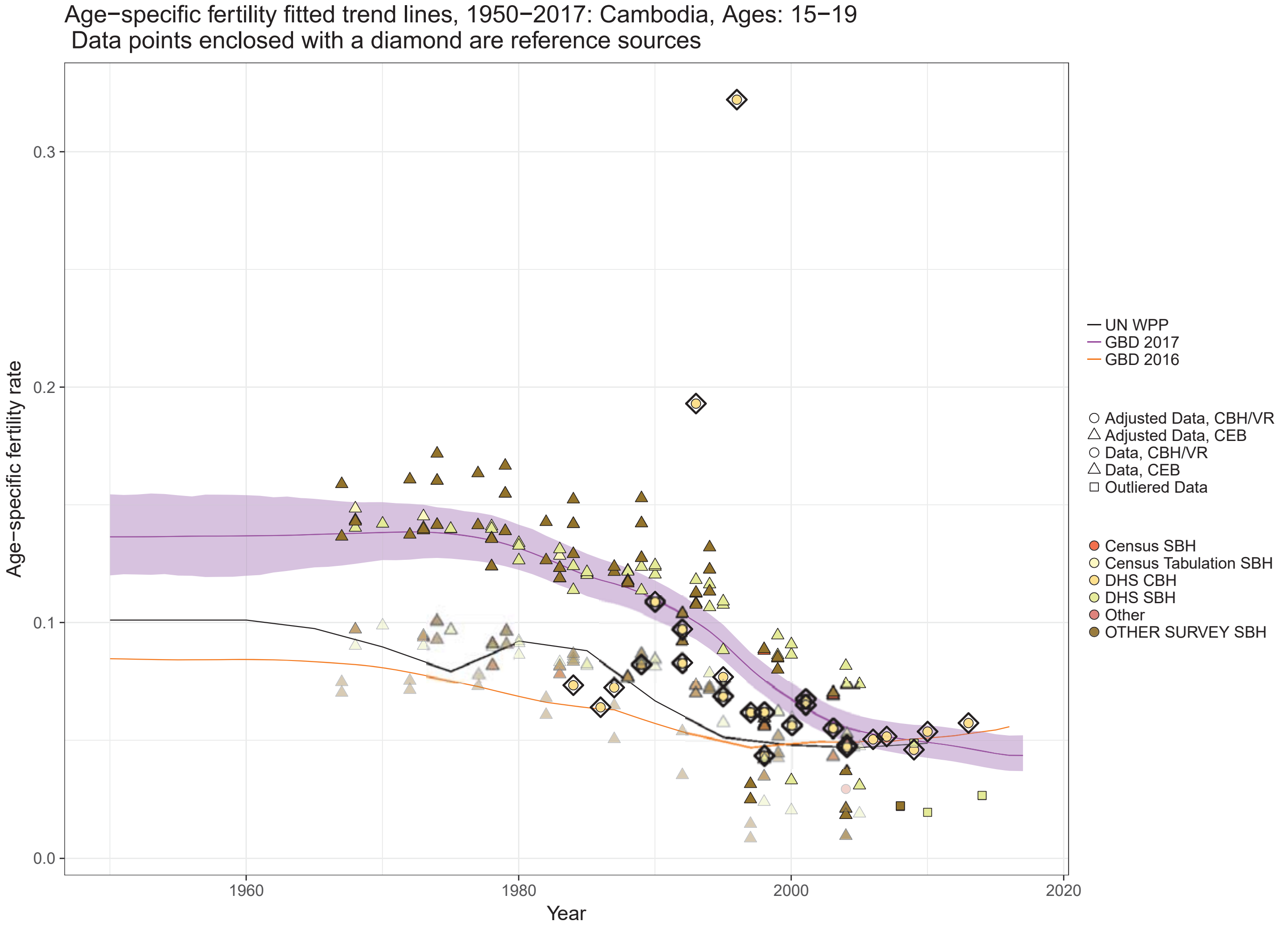
Age−specific fertility fitted trend lines, 1950−2017: Cambodia, Ages: 15−19
Data points enclosed with a diamond are reference sources
Age-specific fertility rate
0.0
0.1
0.2
0.3
1960
1980
2000
2020
Year
UN WPP
GBD 2017
GBD 2016
Adjusted Data, CBH/VR
Adjusted Data, CEB
Data, CBH/VR
Data, CEB
Outliered Data
Census SBH
Census Tabulation SBH
DHS CBH
DHS SBH
Other
OTHER SURVEY SBH

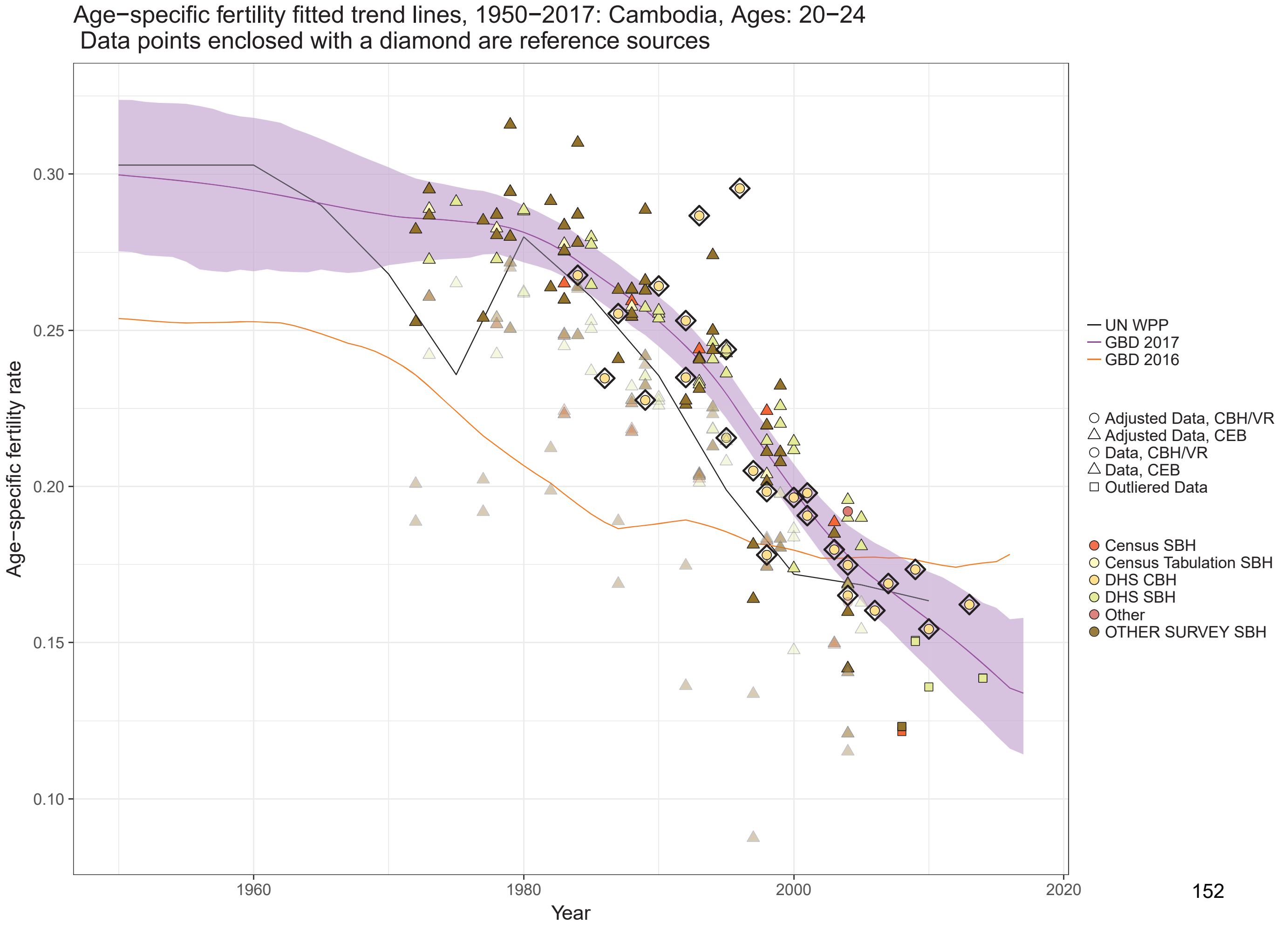
Age−specific fertility fitted trend lines, 1950−2017: Cambodia, Ages: 20−24
Data points enclosed with a diamond are reference sources
Age-specific fertility rate
0.10
0.15
0.20
0.25
0.30
1960
1980
2000
2020
Year
UN WPP
GBD 2017
GBD 2016
Adjusted Data, CBH/VR
Adjusted Data, CEB
Data, CBH/VR
Data, CEB
Outliered Data
Census SBH
Census Tabulation SBH
DHS CBH
DHS SBH
Other
OTHER SURVEY SBH

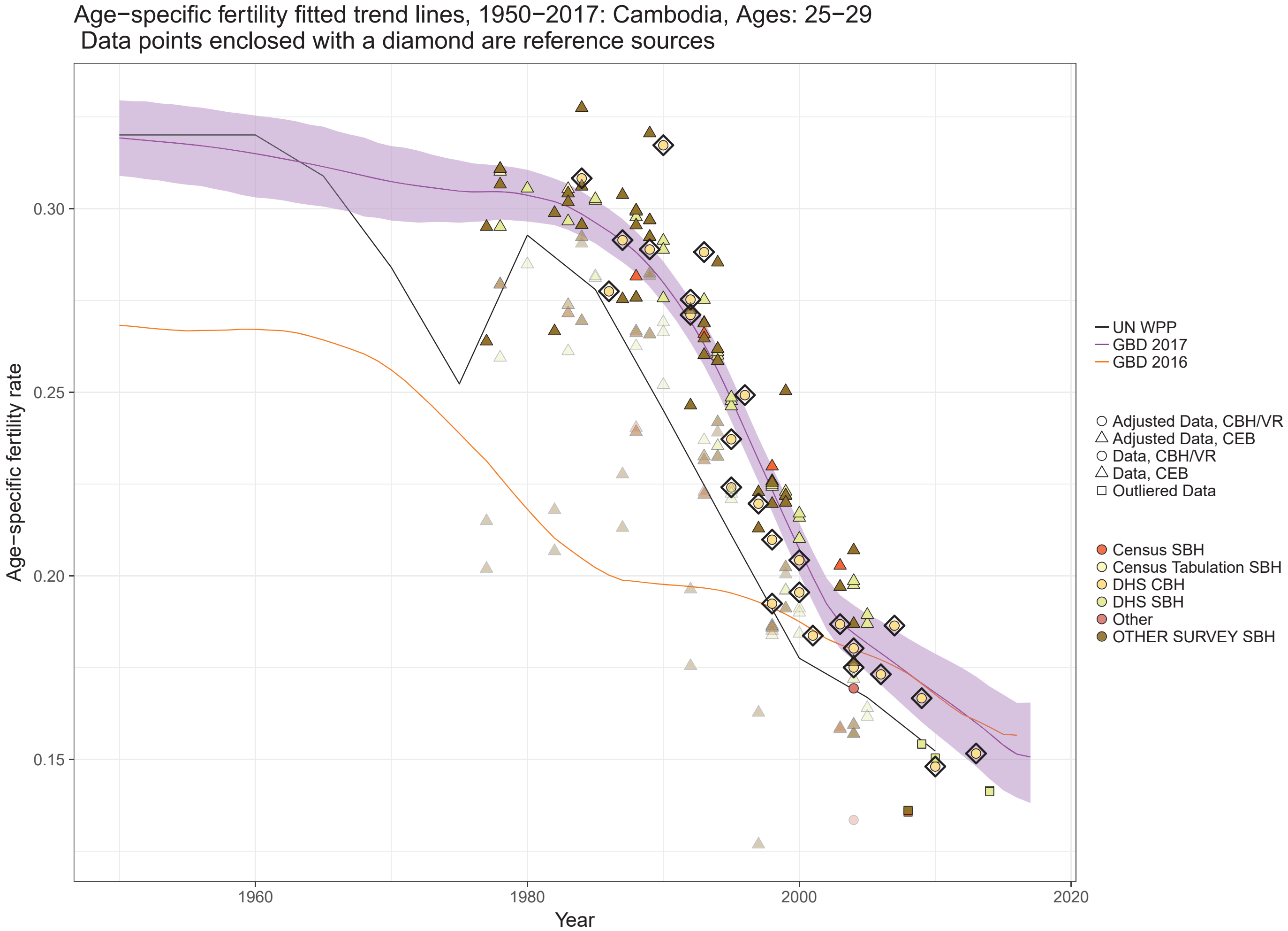
Age−specific fertility fitted trend lines, 1950−2017: Cambodia, Ages: 25−29
Data points enclosed with a diamond are reference sources
Age−specific fertility rate
Year
0.30
0.25
0.20
0.15
1960
1980
2000
2020
UN WPP
GBD 2017
GBD 2016
Adjusted Data, CBH/VR
Adjusted Data, CEB
Data, CBH/VR
Data, CEB
Outliered Data
Census SBH
Census Tabulation SBH
DHS CBH
DHS SBH
Other
OTHER SURVEY SBH

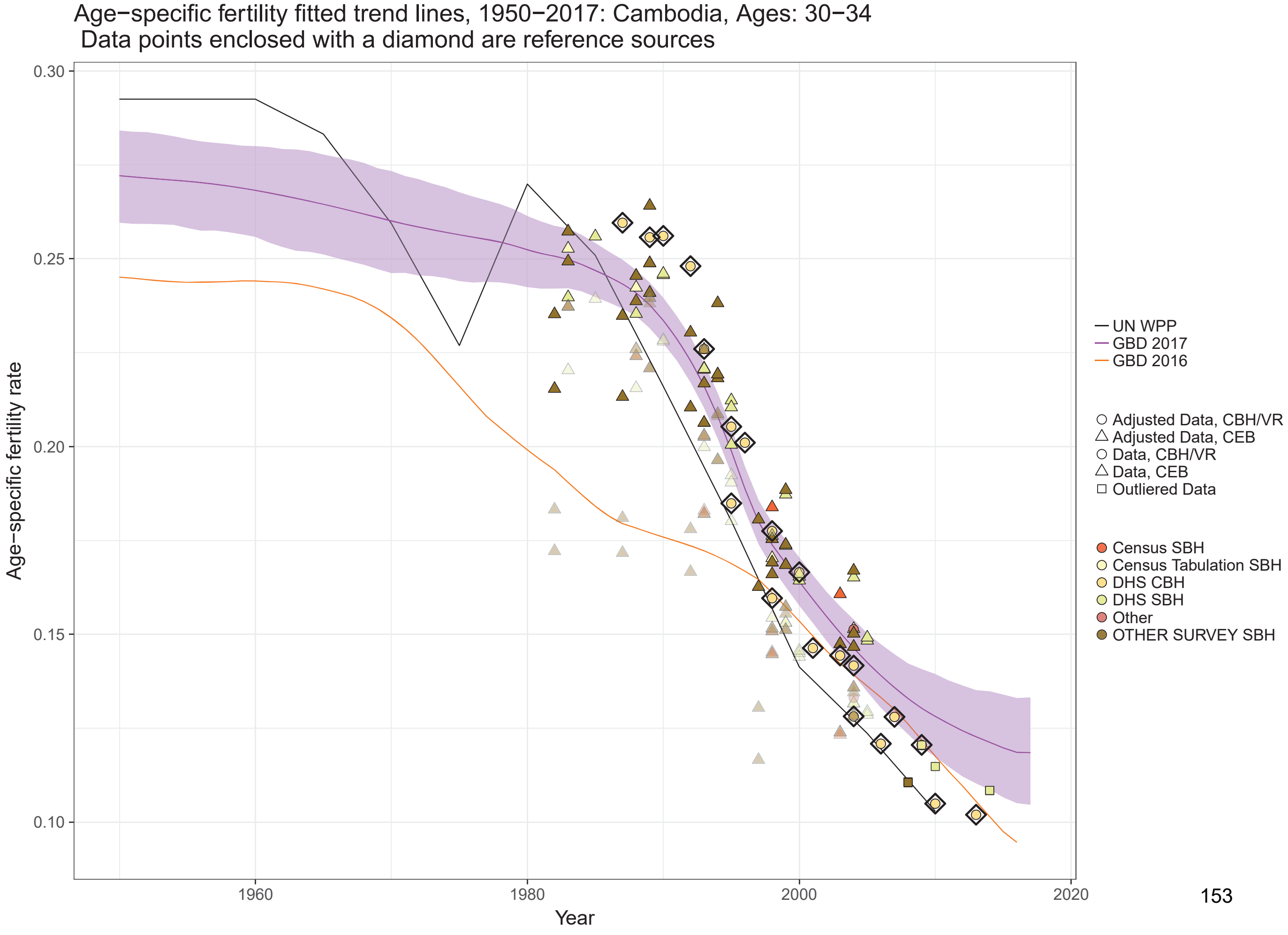
Age−specific fertility fitted trend lines, 1950−2017: Cambodia, Ages: 30−34
Data points enclosed with a diamond are reference sources
Age−specific fertility rate
Year
0.30
0.25
0.20
0.15
0.10
1960
1980
2000
2020
UN WPP
GBD 2017
GBD 2016
Adjusted Data, CBH/VR
Adjusted Data, CEB
Data, CBH/VR
Data, CEB
Outliered Data
Census SBH
Census Tabulation SBH
DHS CBH
DHS SBH
Other
OTHER SURVEY SBH

Age−specific fertility fitted trend lines, 1950−2017: Cambodia, Ages: 35−39
Data points enclosed with a diamond are reference sources

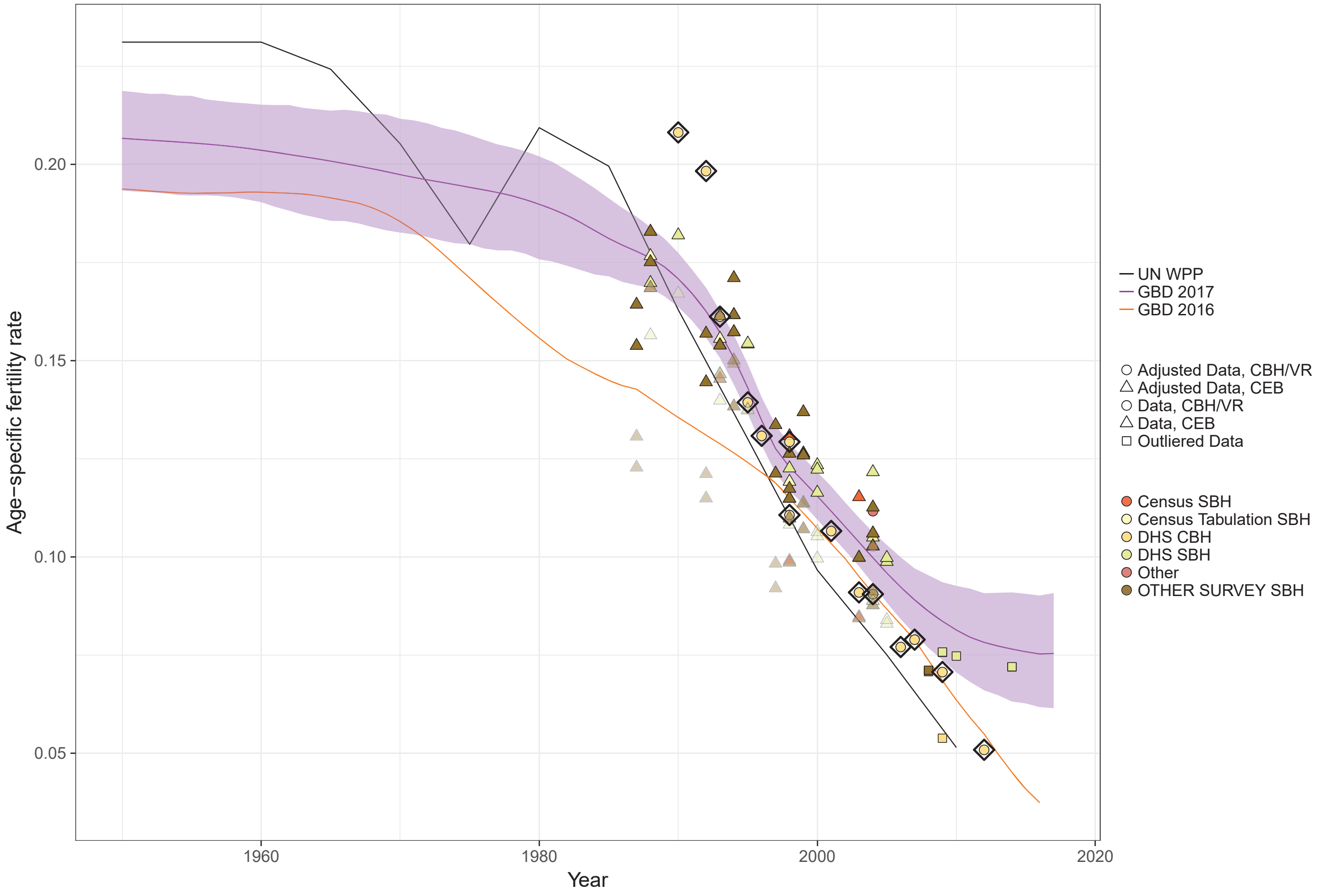

Age−specific fertility fitted trend lines, 1950−2017: Cambodia, Ages: 40−44
Data points enclosed with a diamond are reference sources

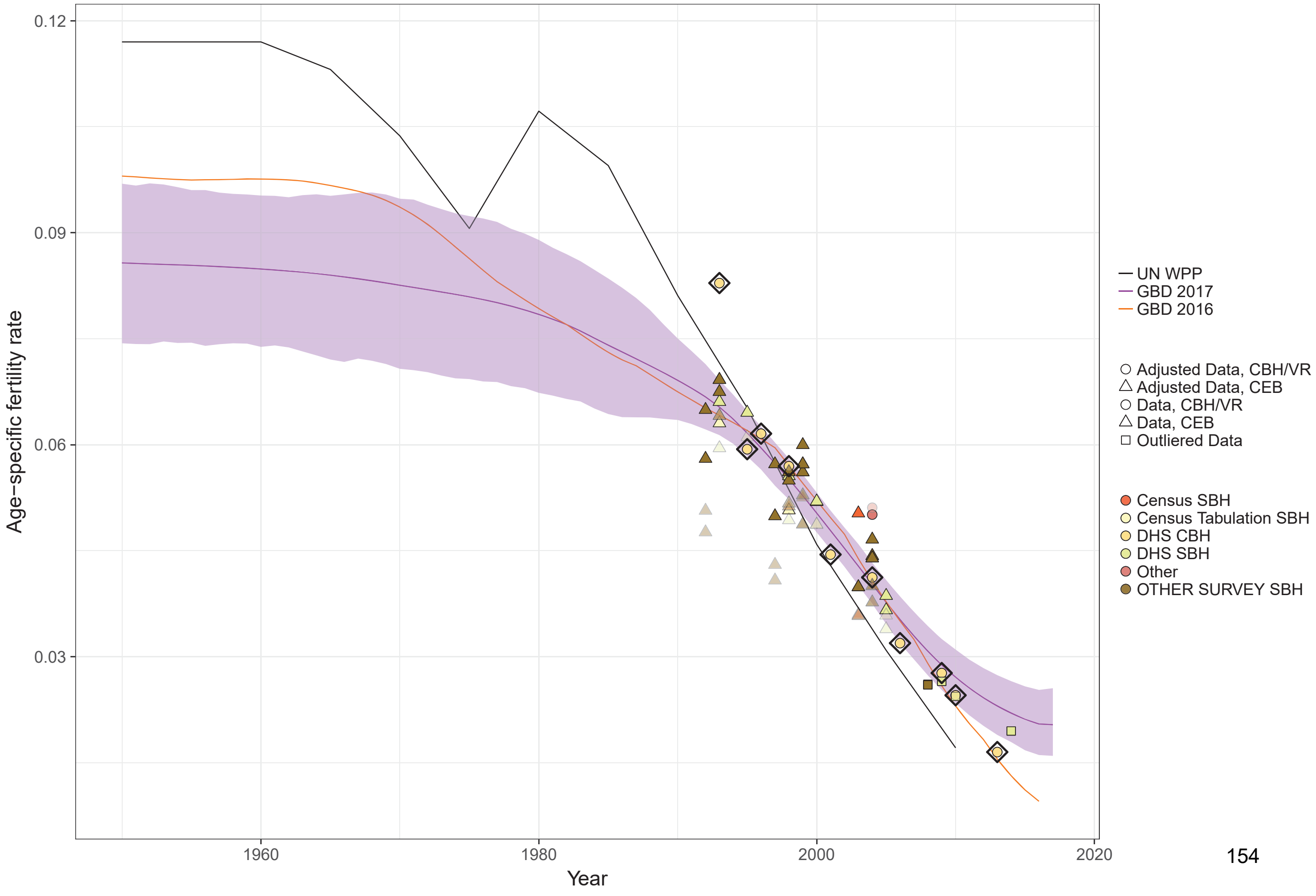

Age−specific fertility fitted trend lines, 1950−2017: Cambodia, Ages: 45−49
Data points enclosed with a diamond are reference sources

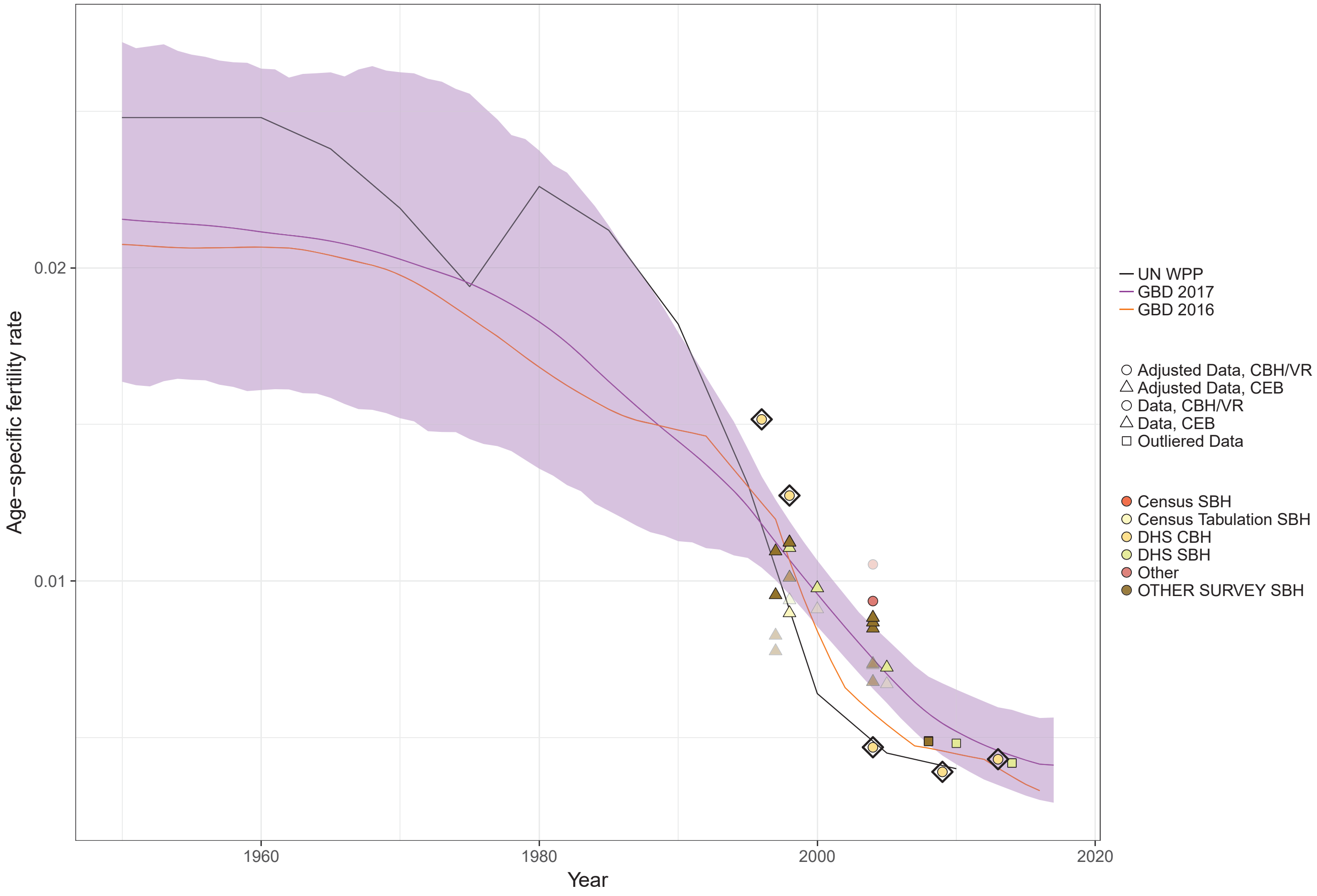

Age−specific fertility fitted trend lines, 1950−2017: Cambodia, Ages: 50−54
Data points enclosed with a diamond are reference sources

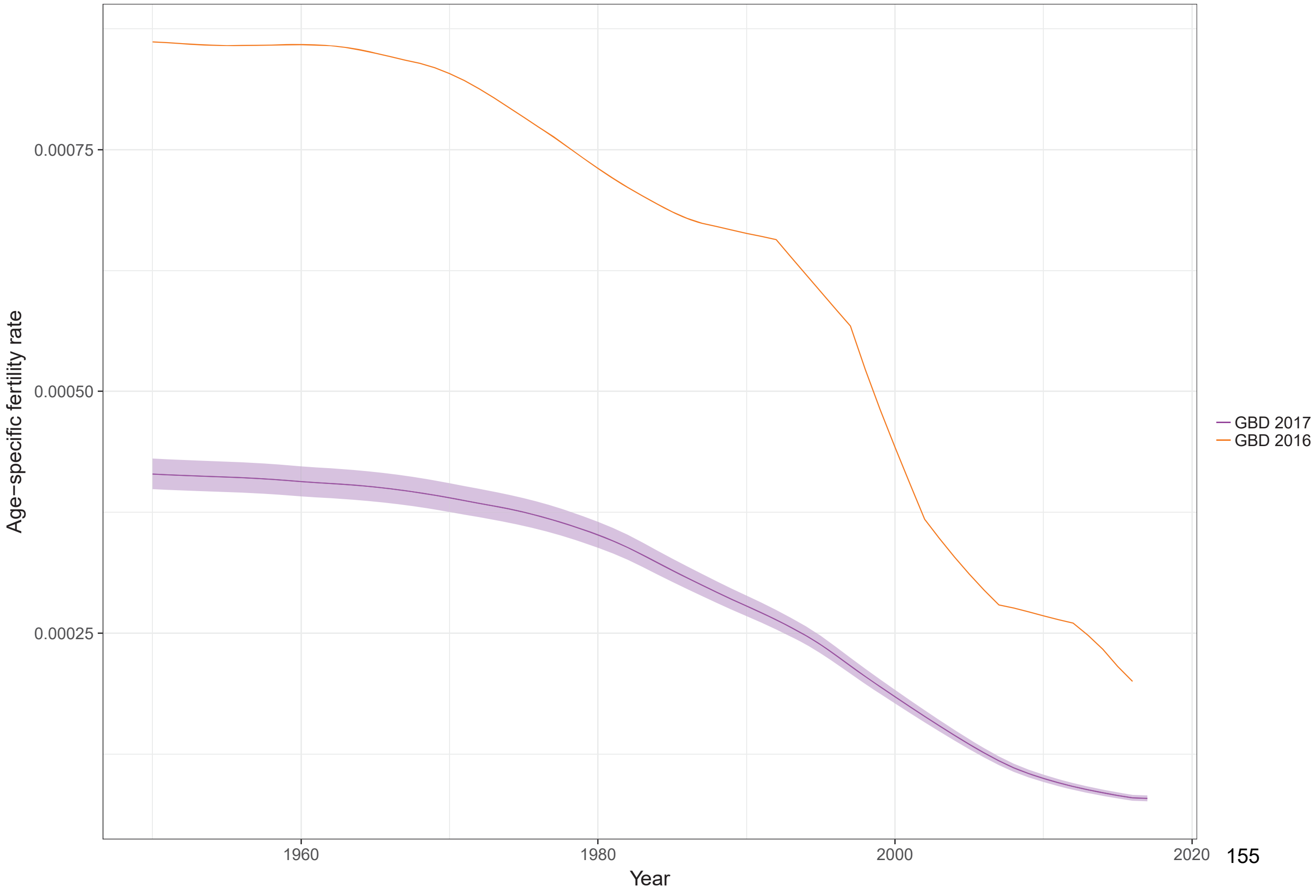

Age−specific fertility fitted trend lines, 1950−2017: Cameroon, TFR
Data points enclosed with a diamond are reference sources

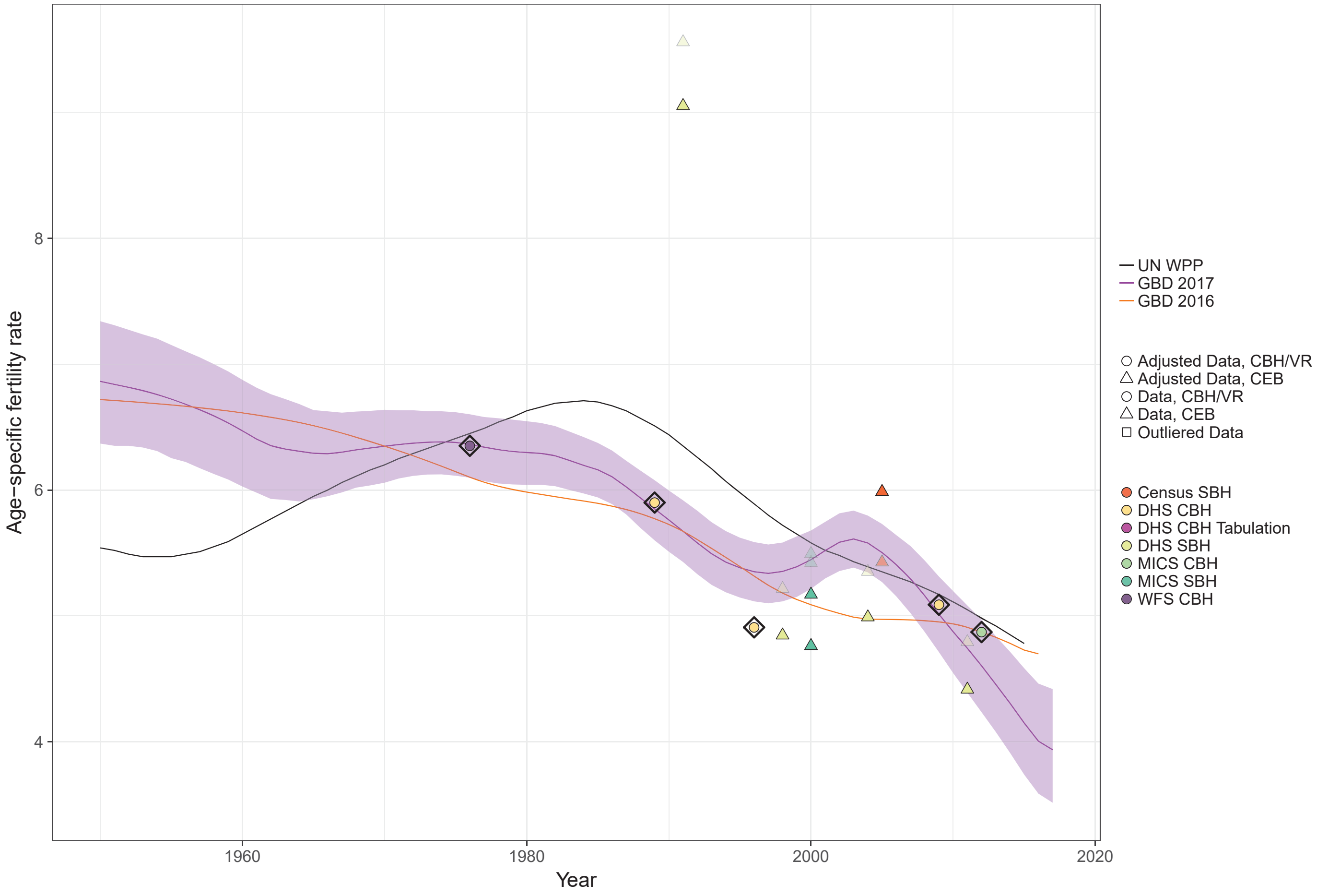

Age−specific fertility fitted trend lines, 1950−2017: Cameroon, Ages: 10−14
Data points enclosed with a diamond are reference sources

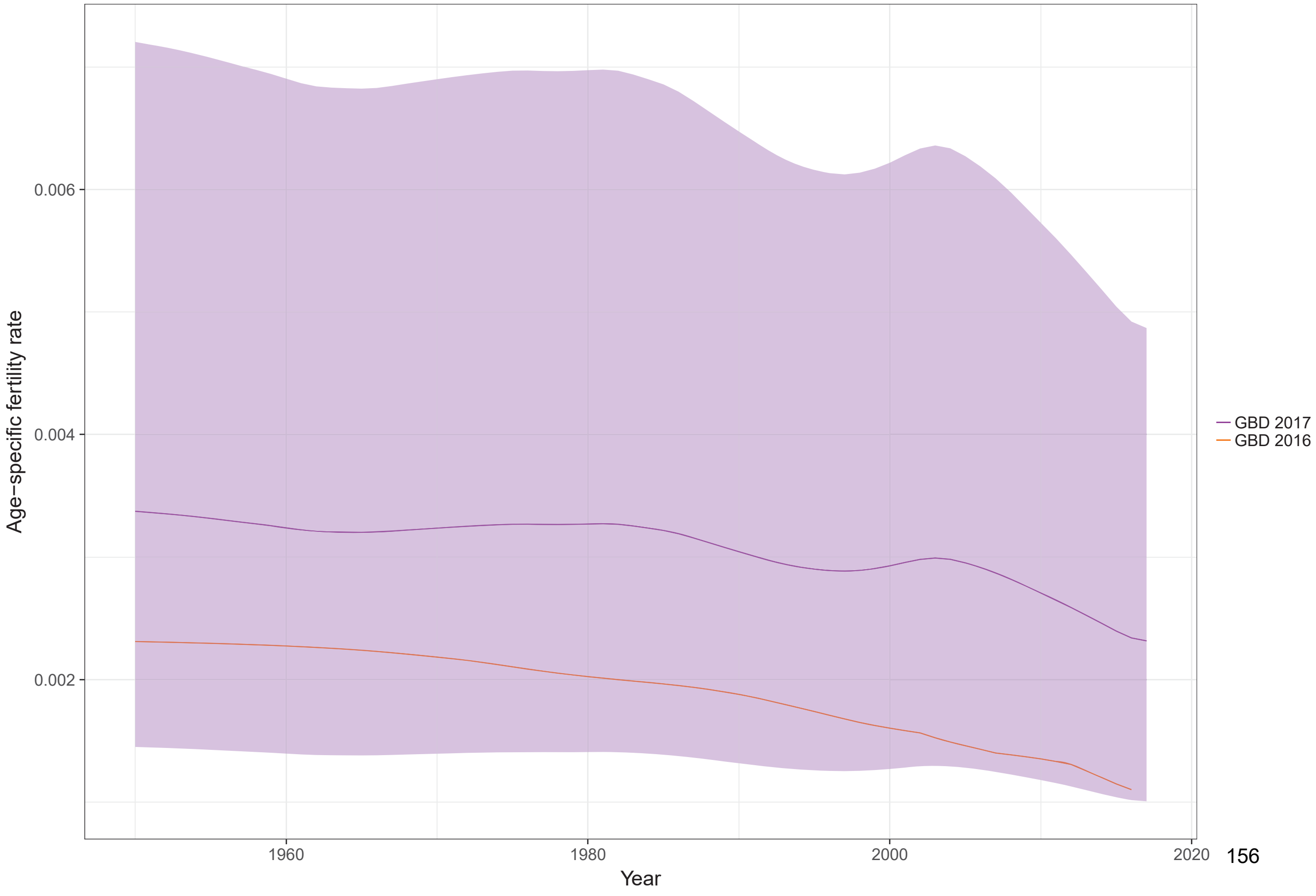

Age−specific fertility fitted trend lines, 1950−2017: Cameroon, Ages: 15−19
Data points enclosed with a diamond are reference sources

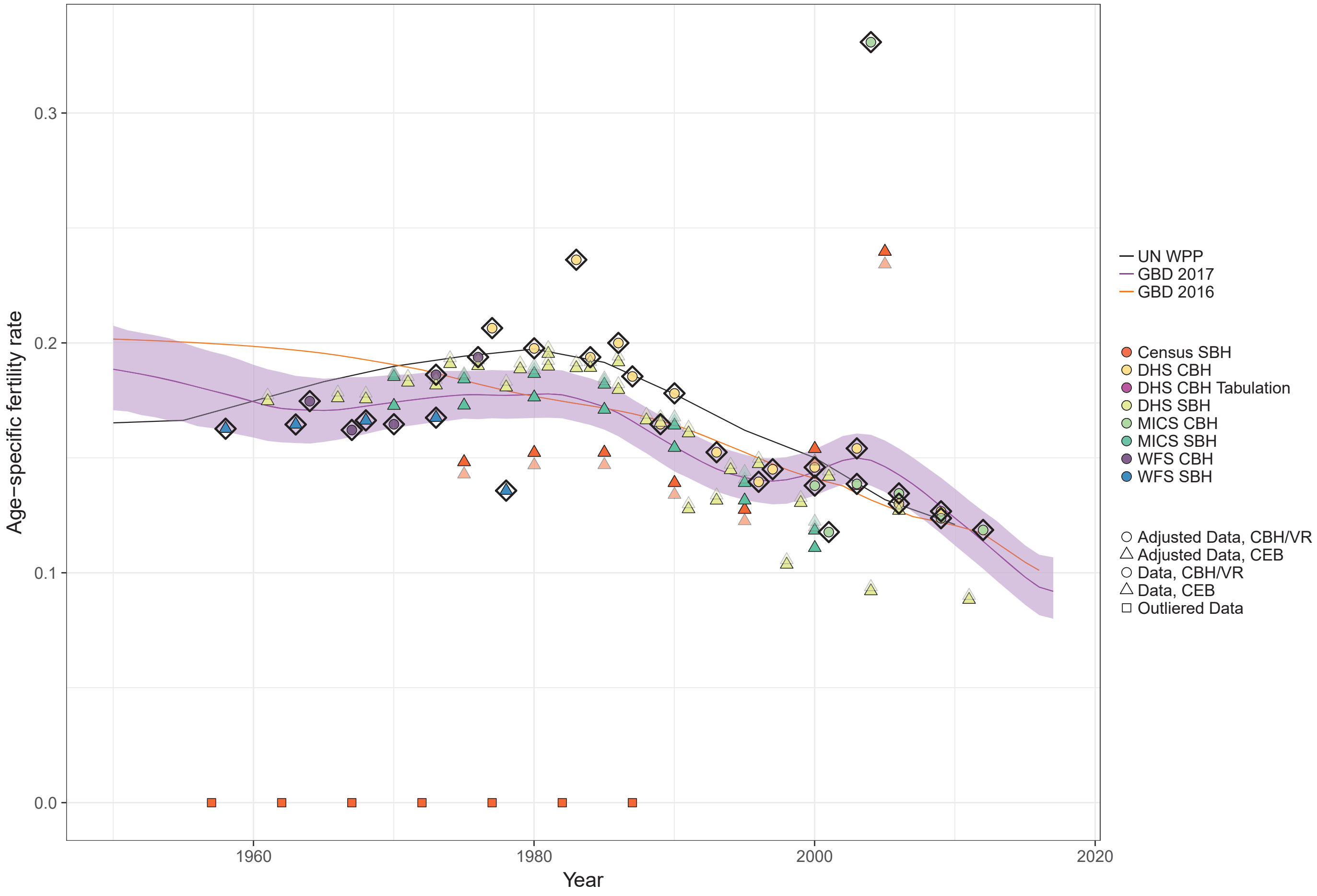

Age−specific fertility fitted trend lines, 1950−2017: Cameroon, Ages: 20−24
Data points enclosed with a diamond are reference sources

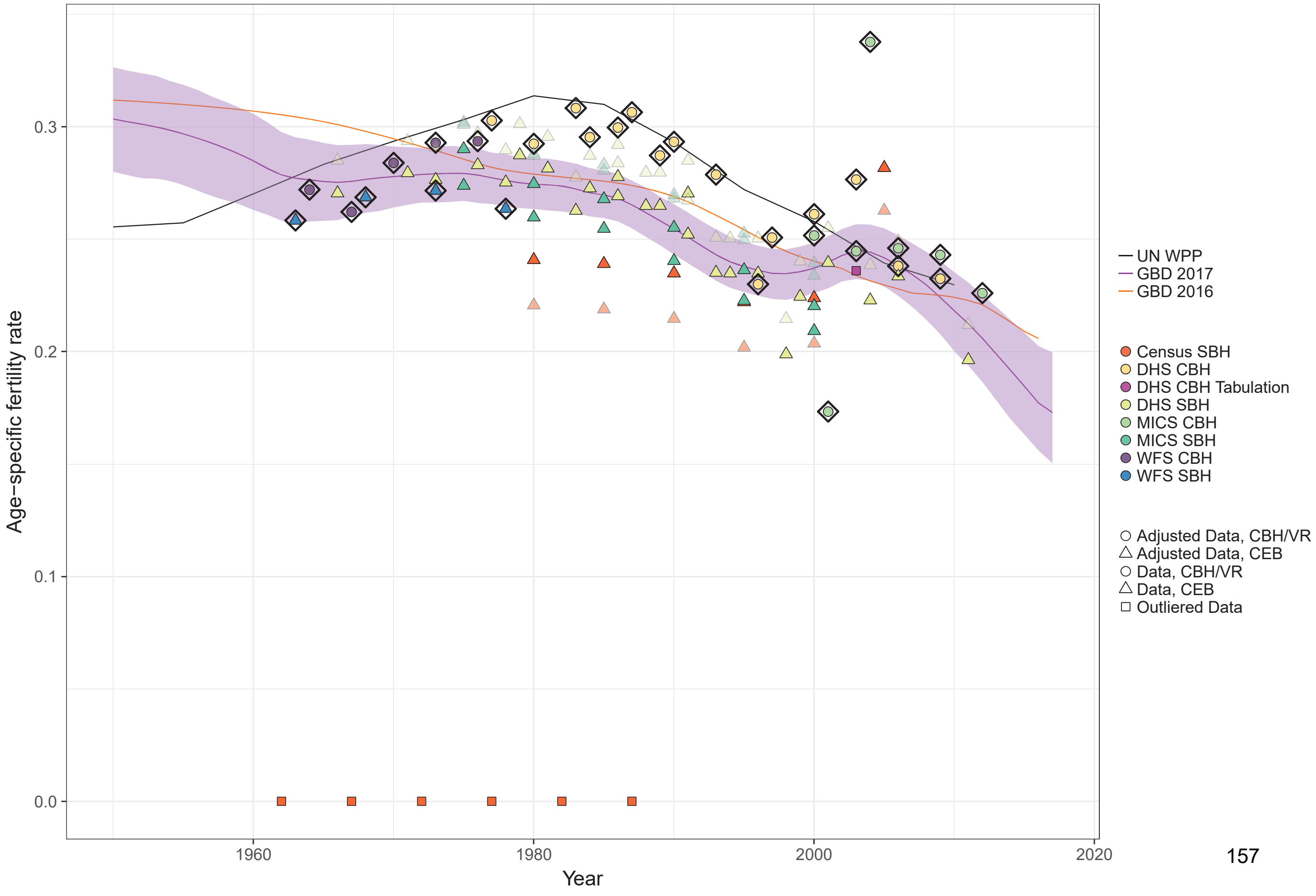

Age−specific fertility fitted trend lines, 1950−2017: Cameroon, Ages: 25−29
Data points enclosed with a diamond are reference sources

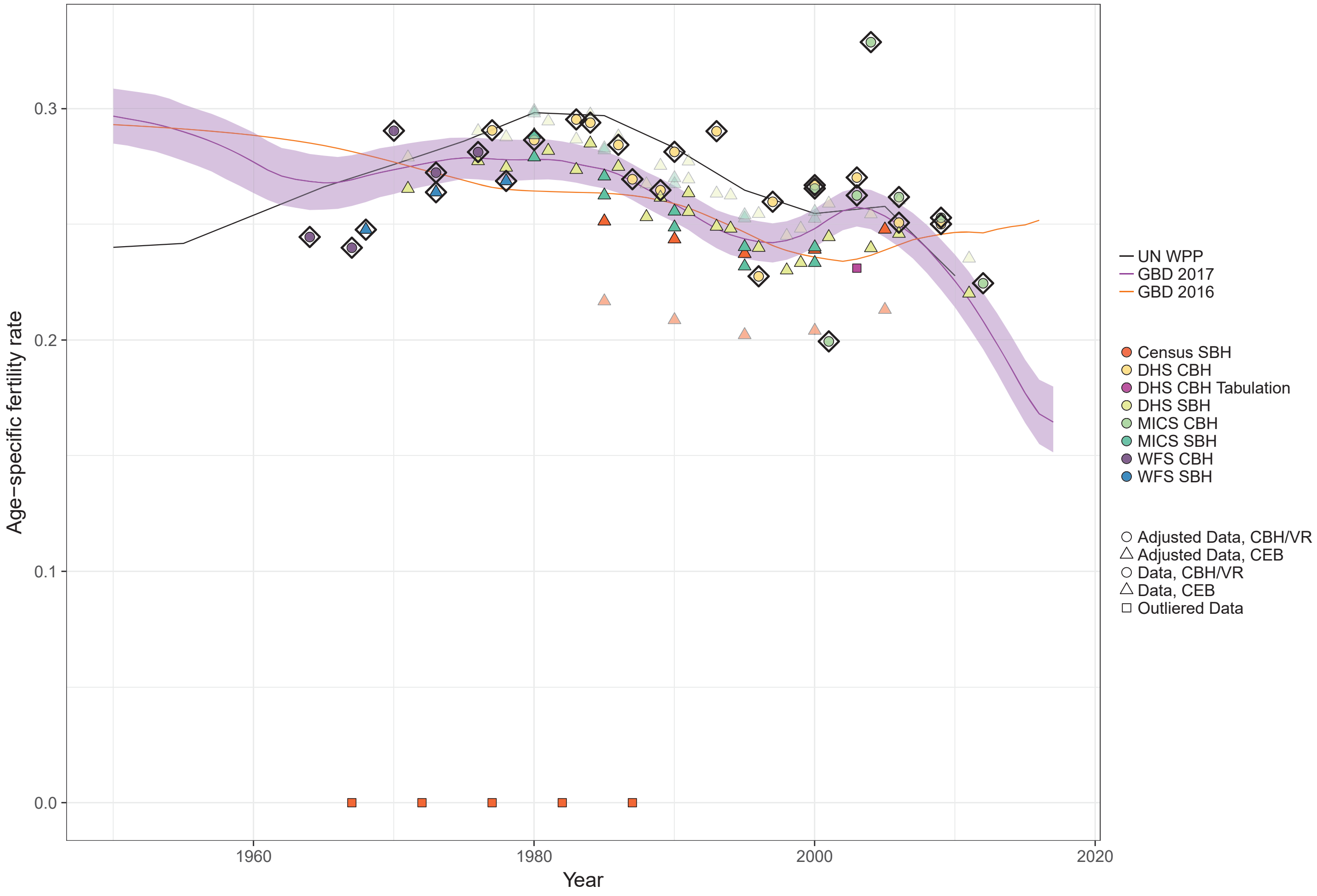

Age−specific fertility fitted trend lines, 1950−2017: Cameroon, Ages: 30−34
Data points enclosed with a diamond are reference sources

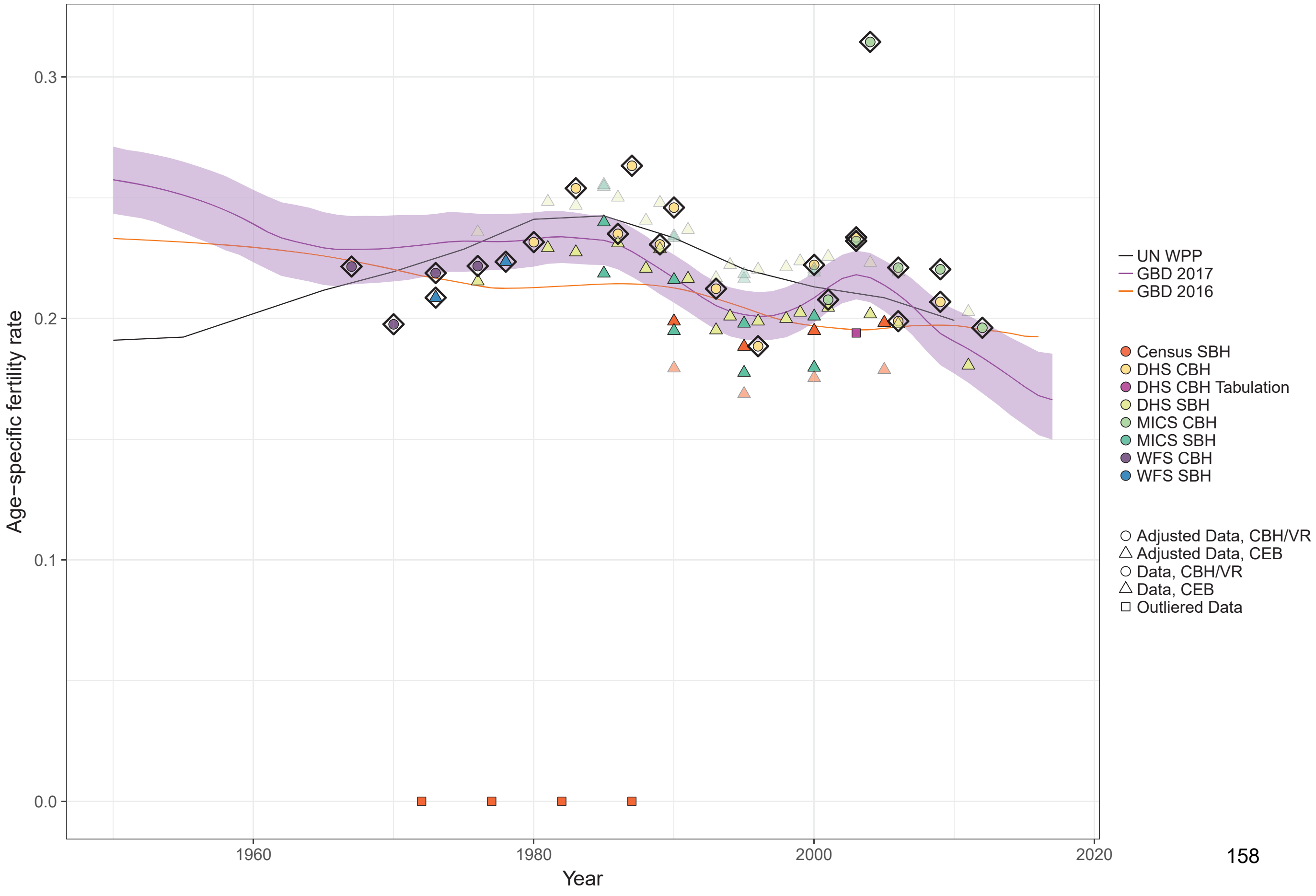

Age−specific fertility fitted trend lines, 1950−2017: Cameroon, Ages: 35−39
Data points enclosed with a diamond are reference sources

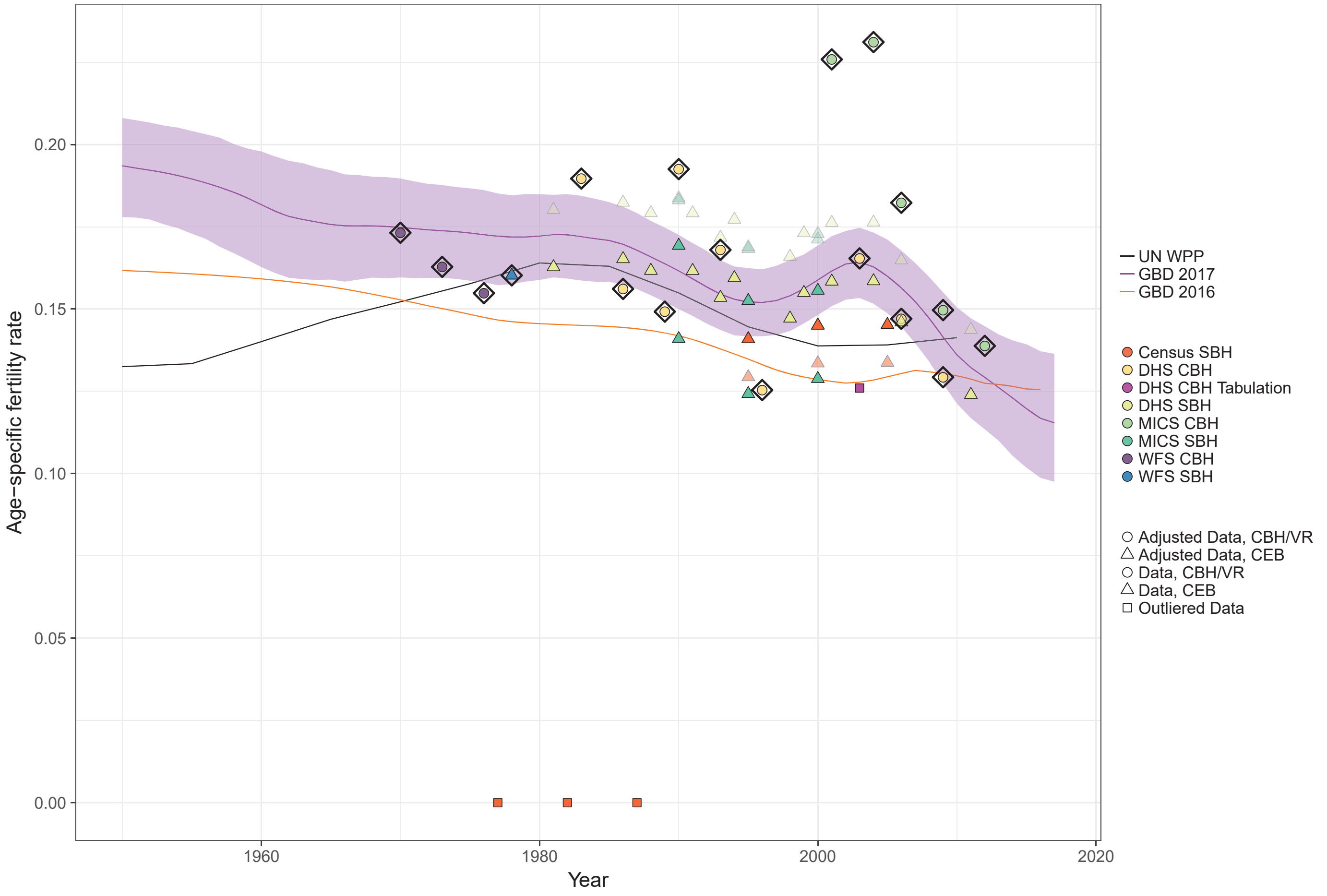

Age−specific fertility fitted trend lines, 1950−2017: Cameroon, Ages: 40−44
Data points enclosed with a diamond are reference sources

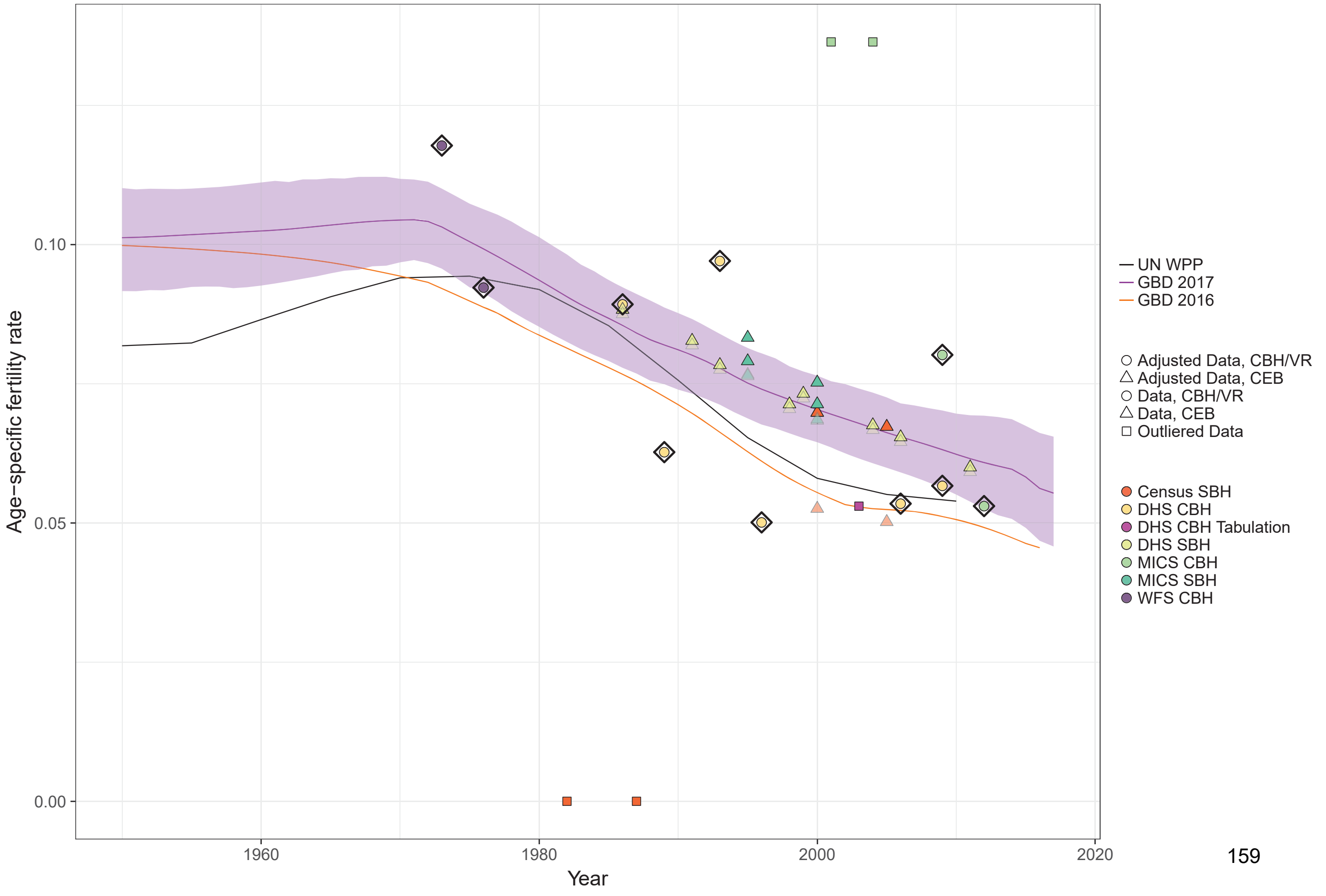

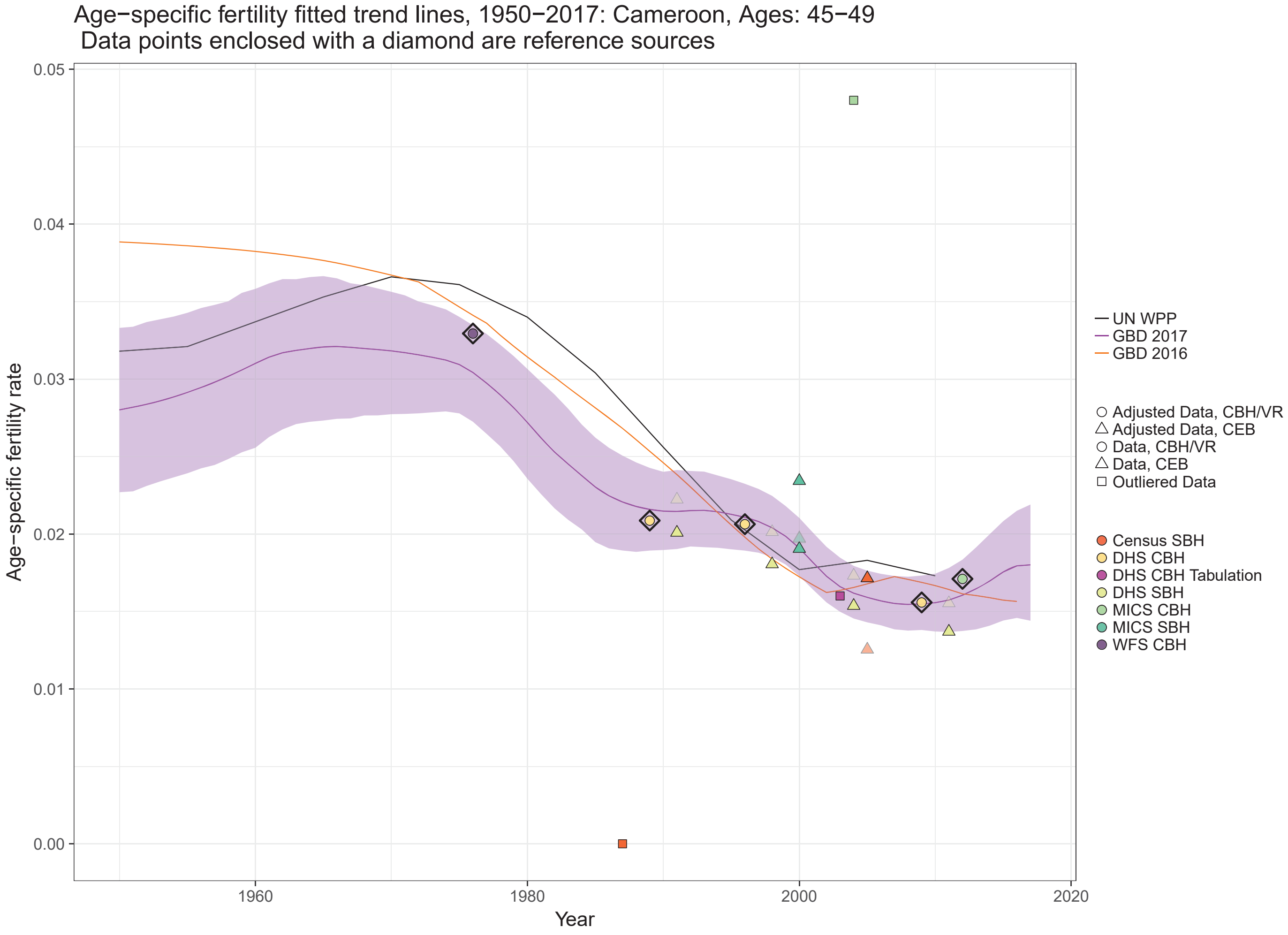
Age−specific fertility fitted trend lines, 1950−2017: Cameroon, Ages: 45−49
Data points enclosed with a diamond are reference sources
Age−specific fertility rate
Year
0.00
0.01
0.02
0.03
0.04
0.05
1960
1980
2000
2020
UN WPP
GBD 2017
GBD 2016
Adjusted Data, CBH/VR
Adjusted Data, CEB
Data, CBH/VR
Data, CEB
Outliered Data
Census SBH
DHS CBH
DHS CBH Tabulation
DHS SBH
MICS CBH
MICS SBH
WFS CBH

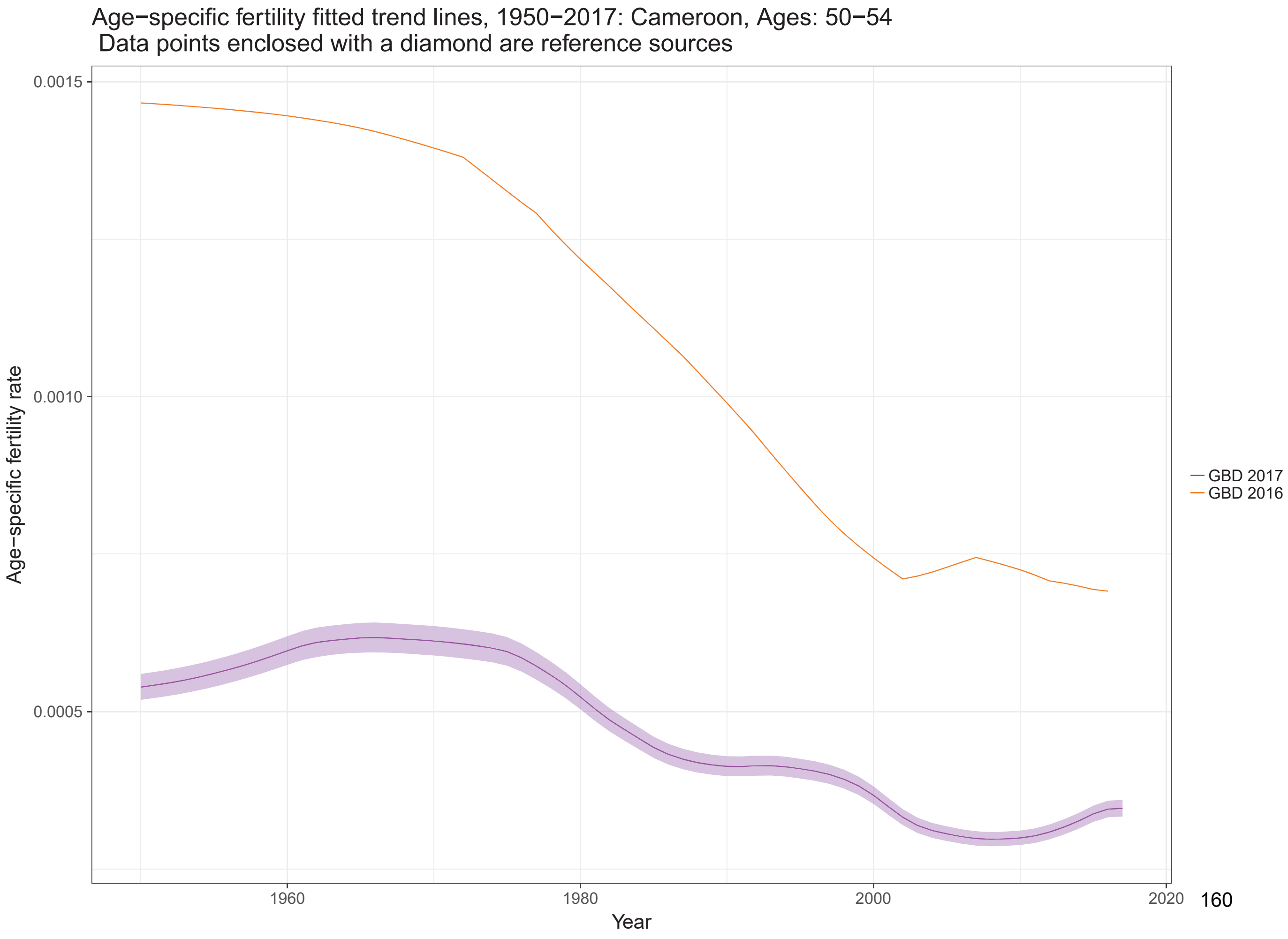
Age−specific fertility fitted trend lines, 1950−2017: Cameroon, Ages: 50−54
Data points enclosed with a diamond are reference sources
Age−specific fertility rate
Year
0.0005
0.0010
0.0015
1960
1980
2000
2020
GBD 2017
GBD 2016

Age−specific fertility fitted trend lines, 1950−2017: Canada, TFR
Data points enclosed with a diamond are reference sources

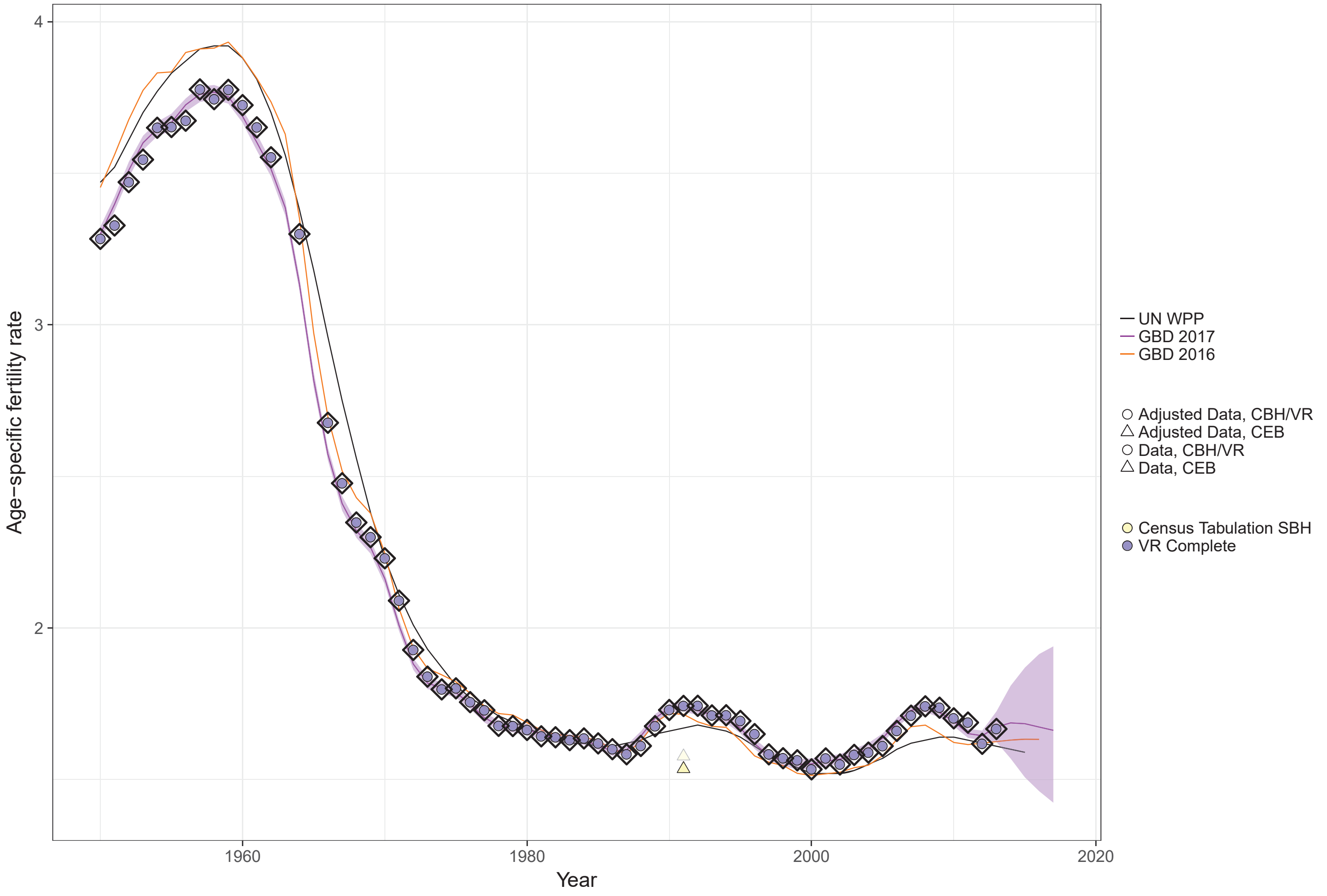

Age−specific fertility fitted trend lines, 1950−2017: Canada, Ages: 10−14
Data points enclosed with a diamond are reference sources

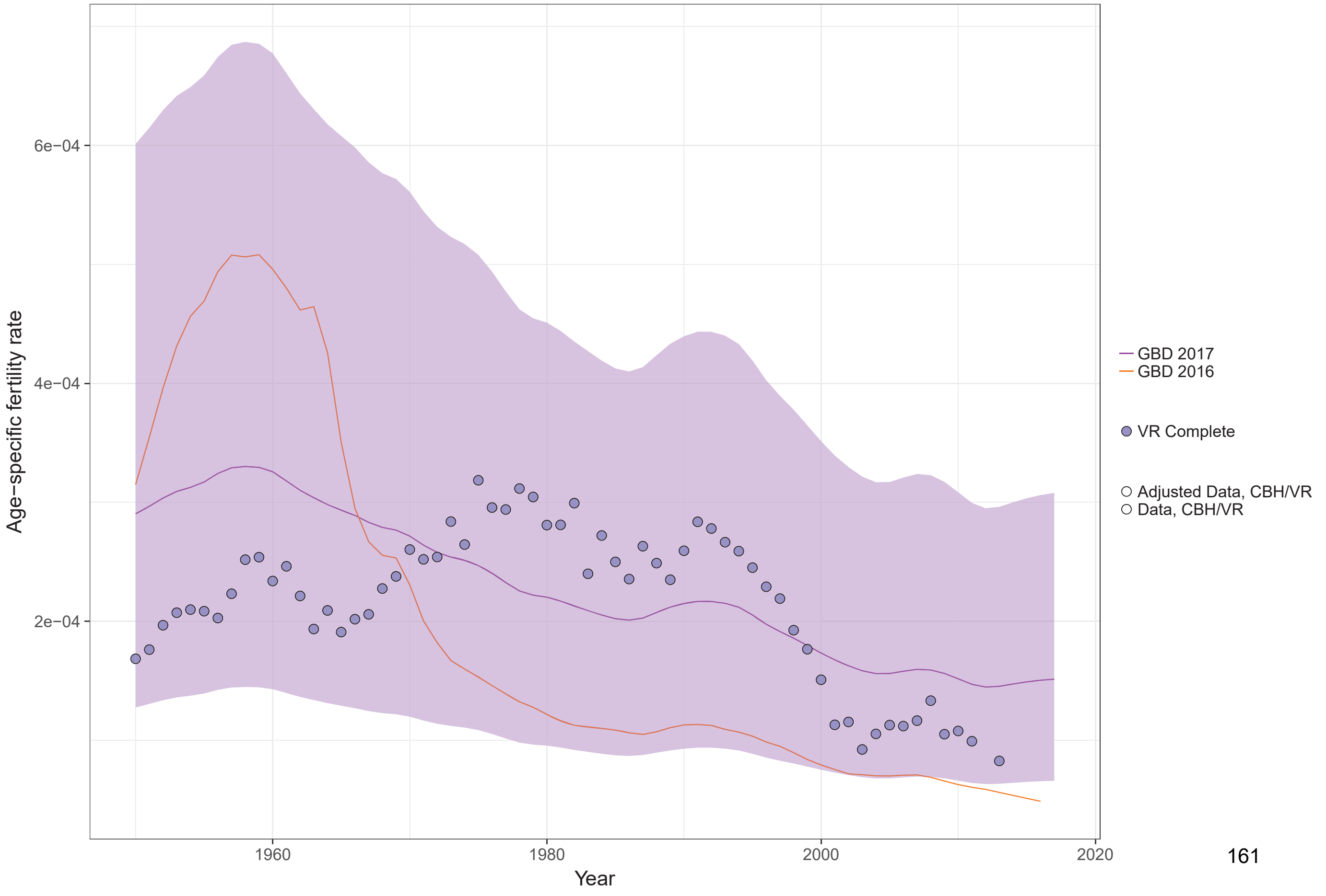

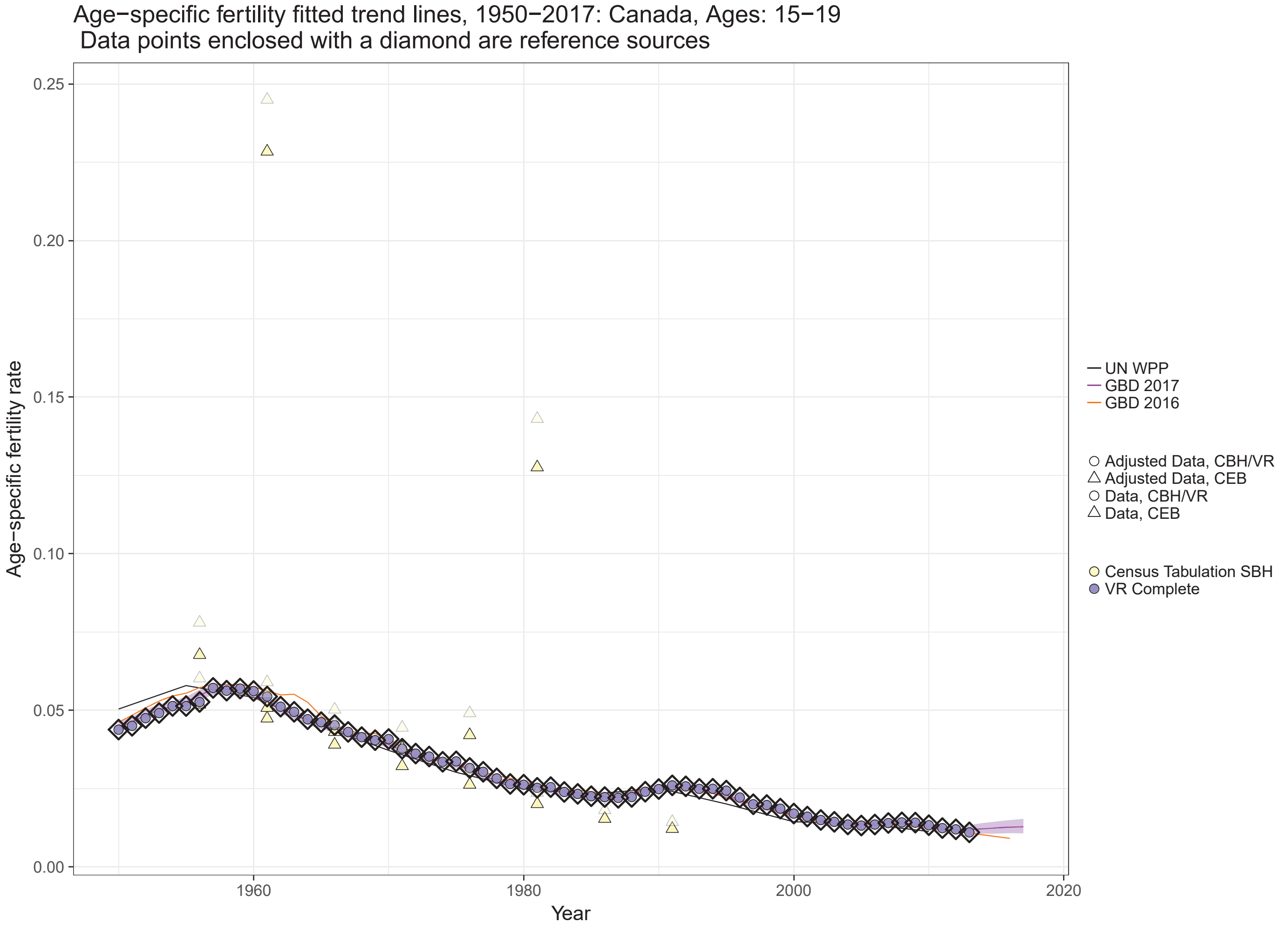
Age−specific fertility fitted trend lines, 1950−2017: Canada, Ages: 15−19
Data points enclosed with a diamond are reference sources
Age−specific fertility rate
0.25
0.20
0.15
0.10
0.05
0.00
1960
1980
2000
2020
Year
UN WPP
GBD 2017
GBD 2016
Adjusted Data, CBH/VR
Adjusted Data, CEB
Data, CBH/VR
Data, CEB
Census Tabulation SBH
VR Complete

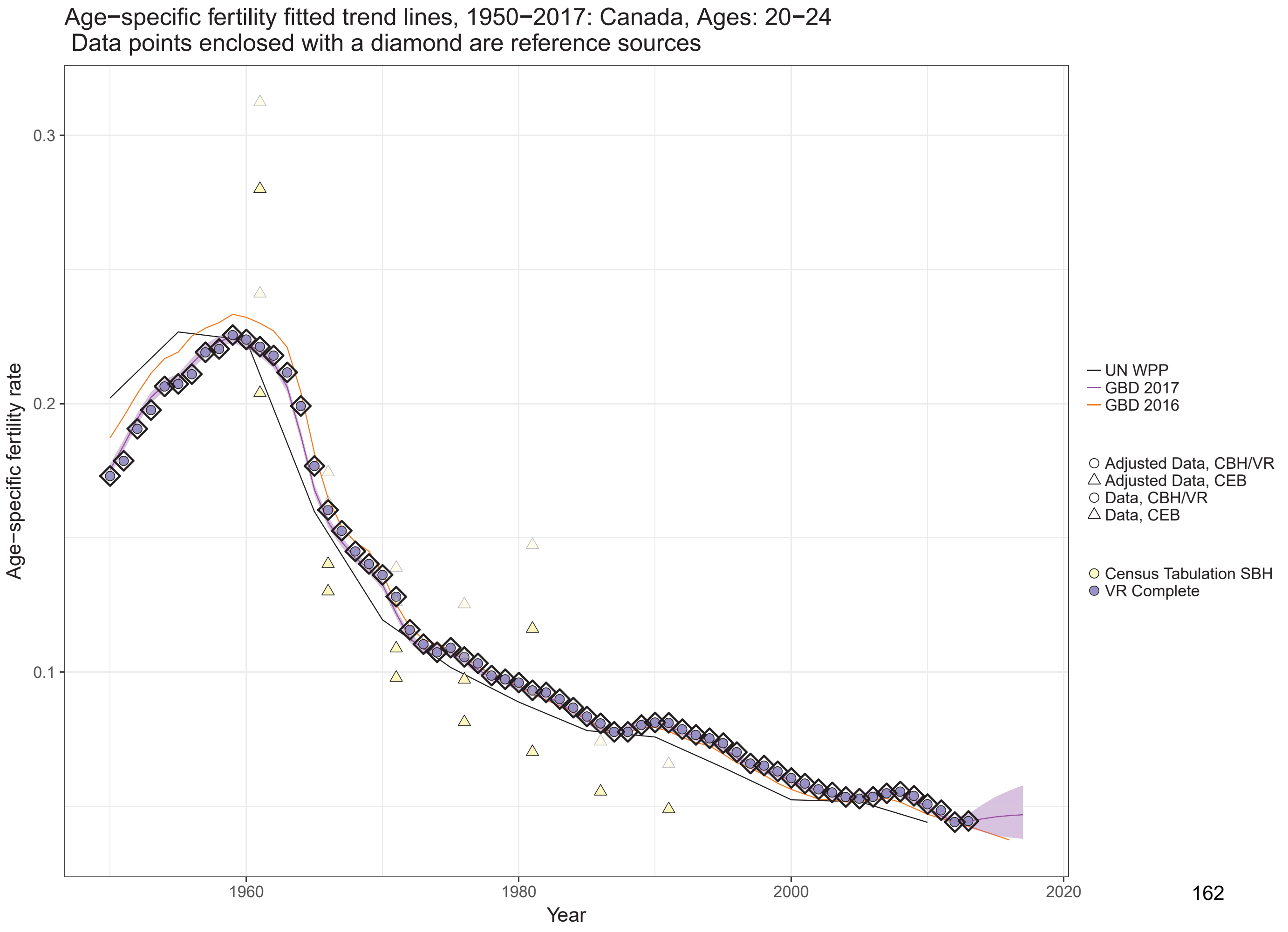
Age−specific fertility fitted trend lines, 1950−2017: Canada, Ages: 20−24
Data points enclosed with a diamond are reference sources
Age−specific fertility rate
0.3
0.2
0.1
1960
1980
2000
2020
Year
UN WPP
GBD 2017
GBD 2016
Adjusted Data, CBH/VR
Adjusted Data, CEB
Data, CBH/VR
Data, CEB
Census Tabulation SBH
VR Complete

Age−specific fertility fitted trend lines, 1950−2017: Canada, Ages: 25−29
Data points enclosed with a diamond are reference sources

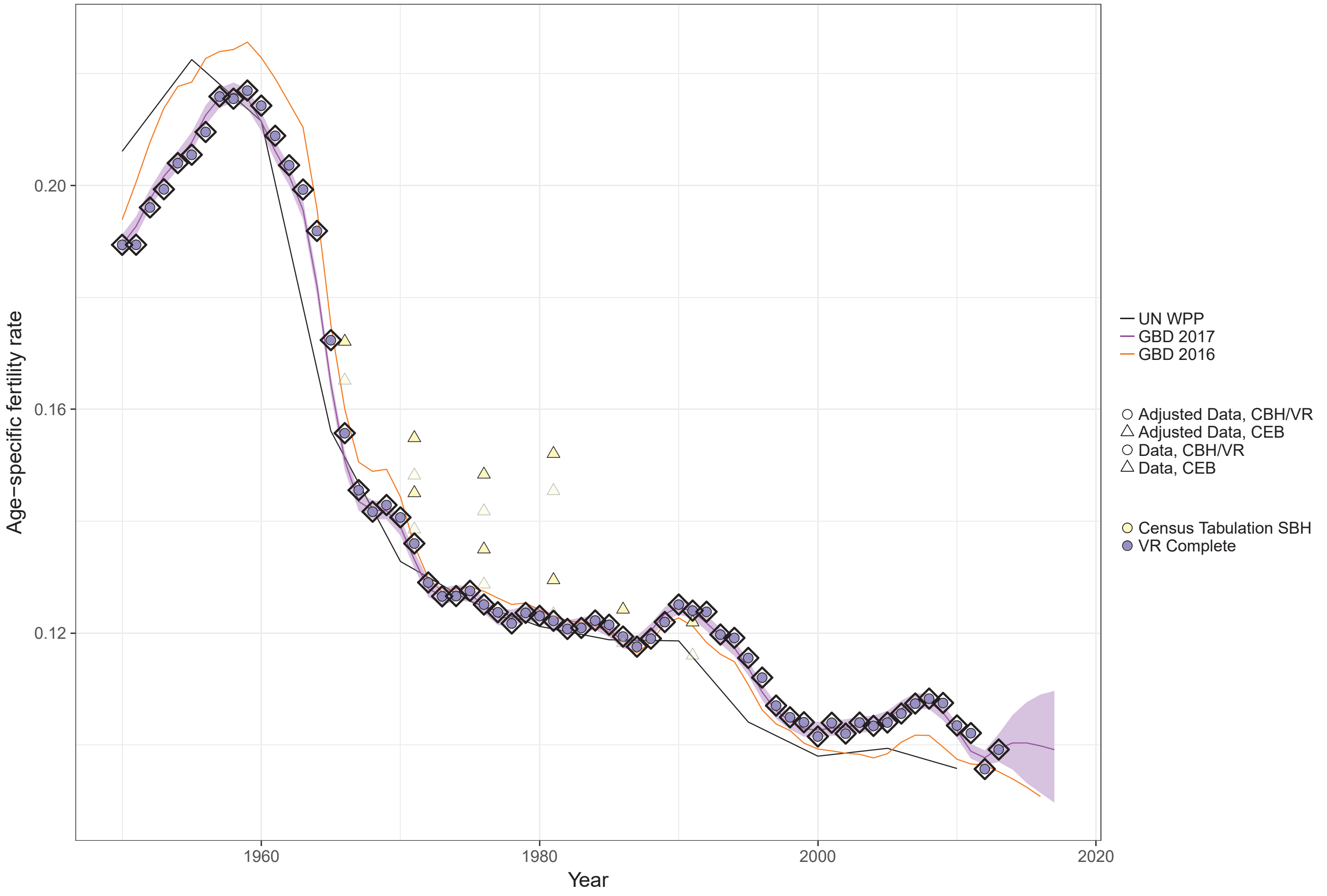

Age−specific fertility fitted trend lines, 1950−2017: Canada, Ages: 30−34
Data points enclosed with a diamond are reference sources

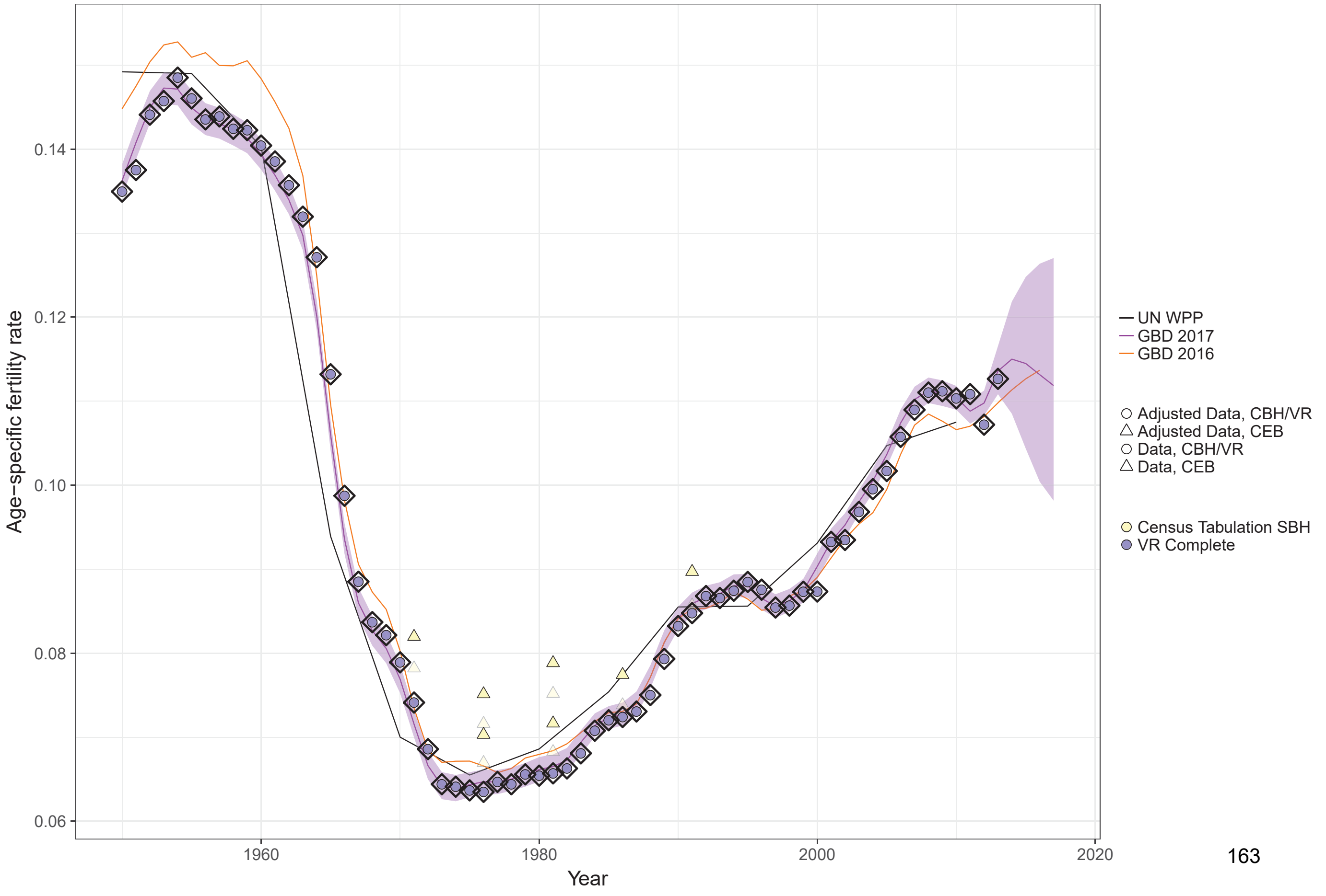

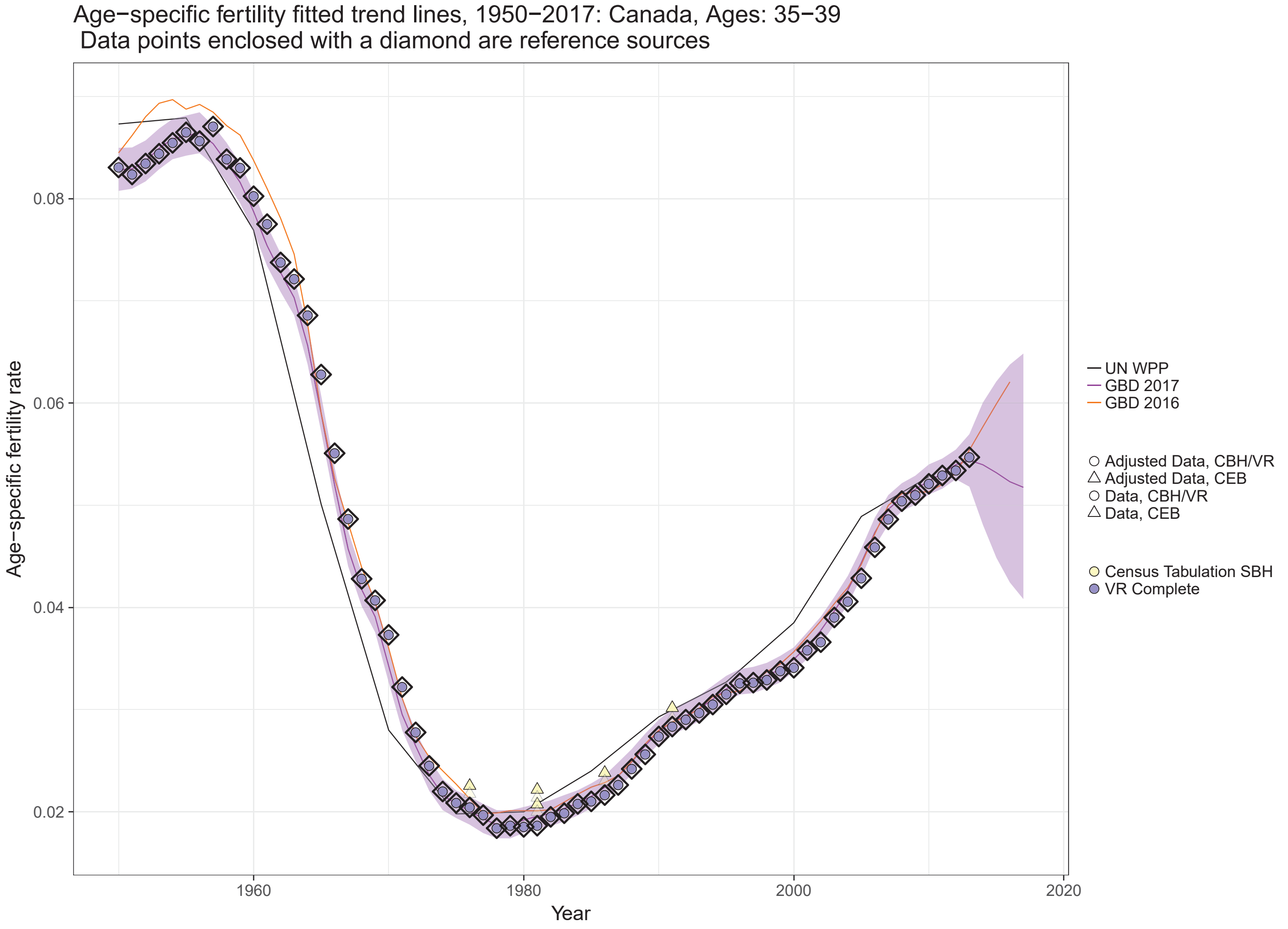

Age−specific fertility fitted trend lines, 1950−2017: Canada, Ages: 35−39
Data points enclosed with a diamond are reference sources
Age−specific fertility rate
0.02
0.04
0.06
0.08
1960
1980
2000
2020
Year
UN WPP
GBD 2017
GBD 2016
Adjusted Data, CBH/VR
Adjusted Data, CEB
Data, CBH/VR
Data, CEB
Census Tabulation SBH
VR Complete

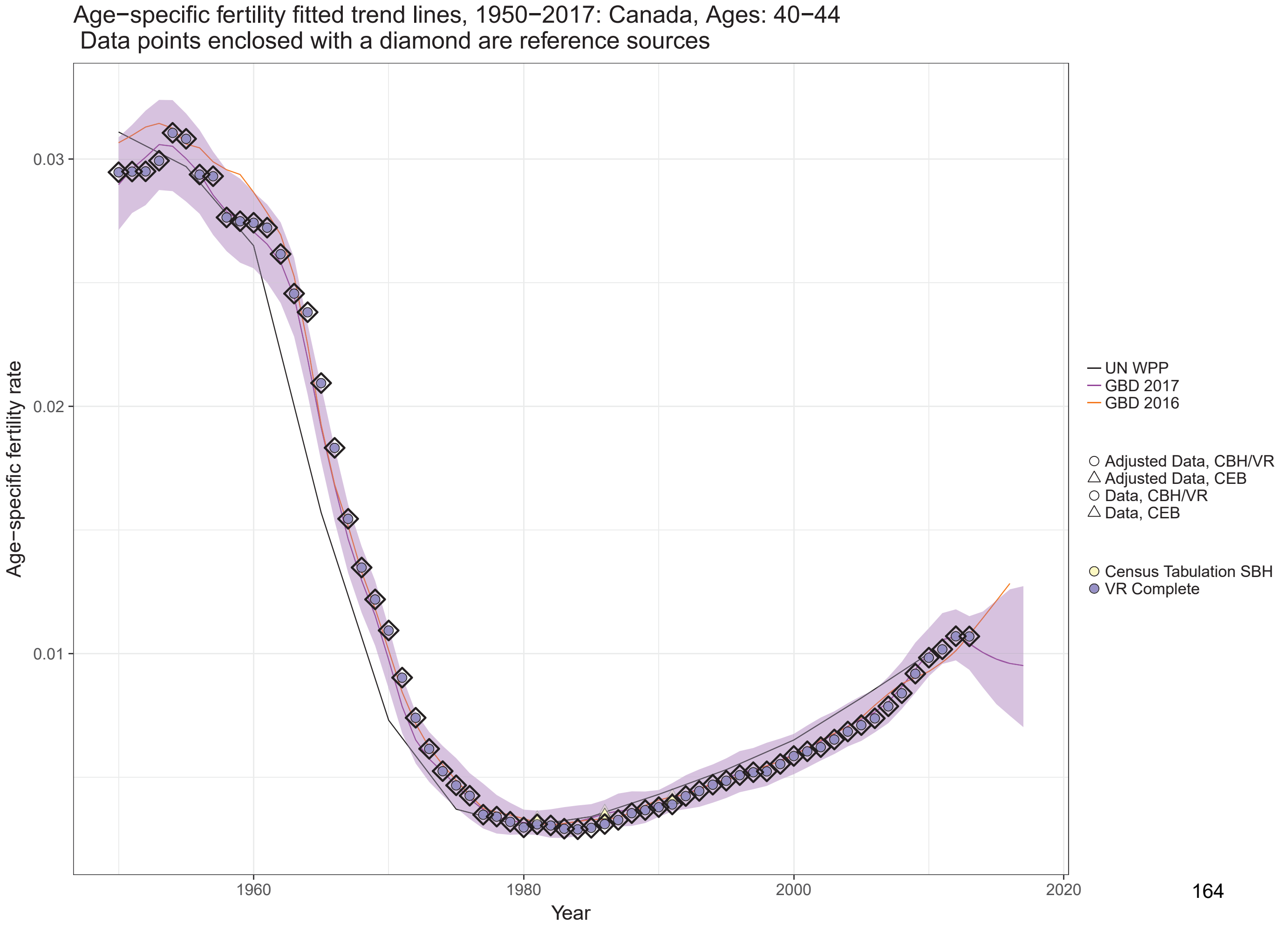

Age−specific fertility fitted trend lines, 1950−2017: Canada, Ages: 40−44
Data points enclosed with a diamond are reference sources
Age−specific fertility rate
0.01
0.02
0.03
1960
1980
2000
2020
Year
UN WPP
GBD 2017
GBD 2016
Adjusted Data, CBH/VR
Adjusted Data, CEB
Data, CBH/VR
Data, CEB
Census Tabulation SBH
VR Complete

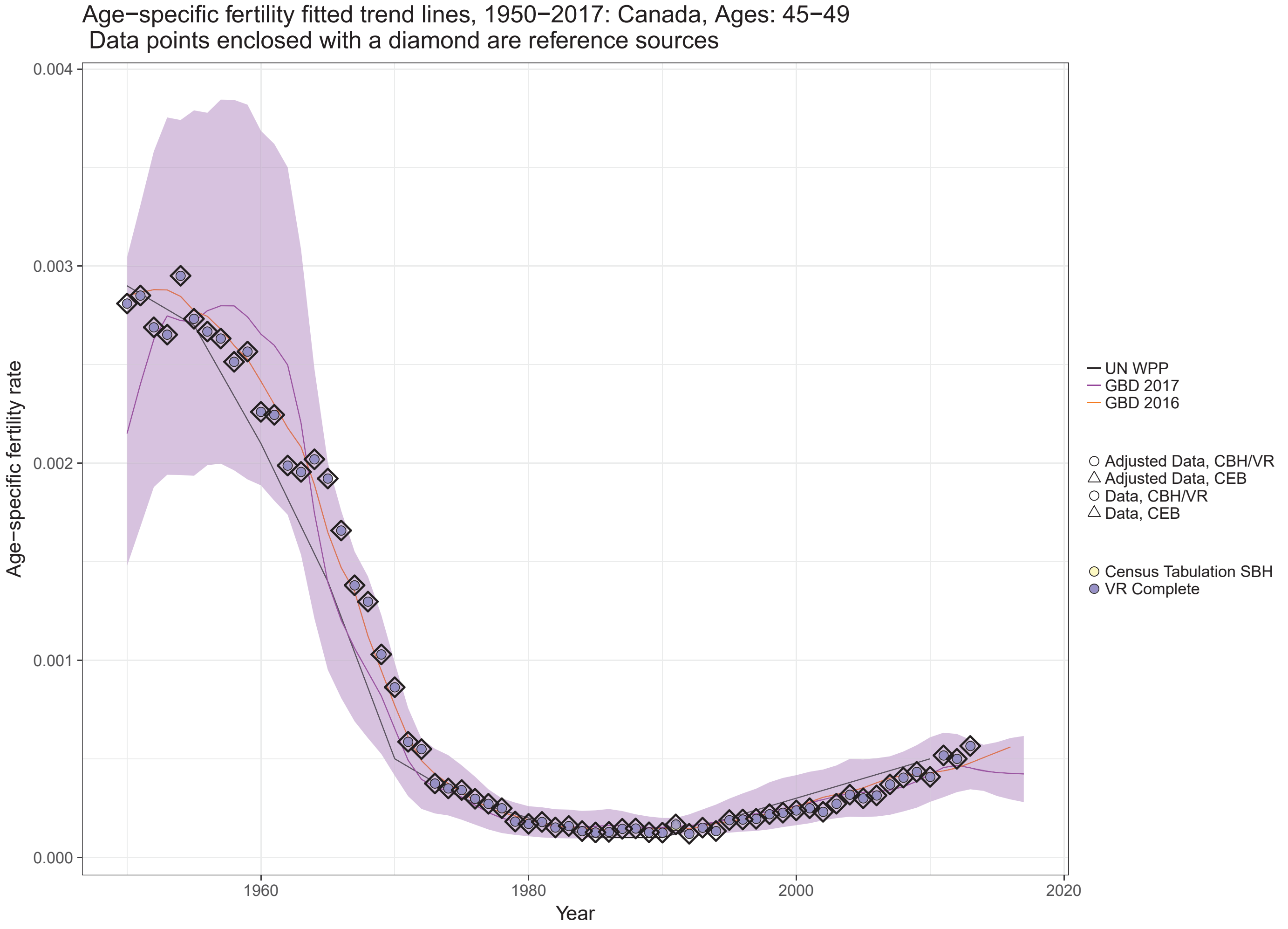
Age−specific fertility fitted trend lines, 1950−2017: Canada, Ages: 45−49
Data points enclosed with a diamond are reference sources
Age−specific fertility rate
Year
0.000
0.001
0.002
0.003
0.004
1960
1980
2000
2020
UN WPP
GBD 2017
GBD 2016
Adjusted Data, CBH/VR
Adjusted Data, CEB
Data, CBH/VR
Data, CEB
Census Tabulation SBH
VR Complete

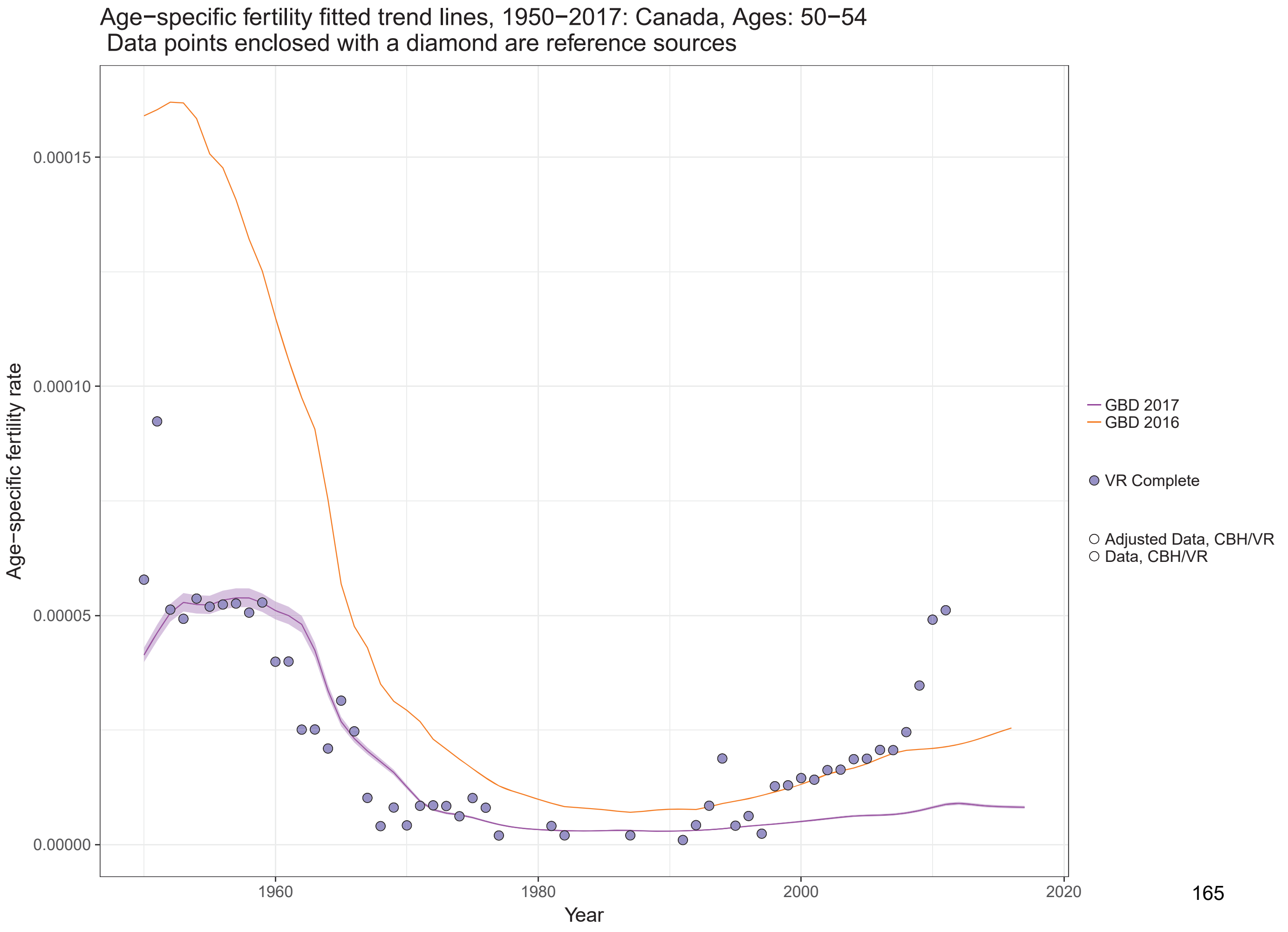
Age−specific fertility fitted trend lines, 1950−2017: Canada, Ages: 50−54
Data points enclosed with a diamond are reference sources
Age−specific fertility rate
Year
0.00000
0.00005
0.00010
0.00015
1960
1980
2000
2020
GBD 2017
GBD 2016
VR Complete
Adjusted Data, CBH/VR
Data, CBH/VR

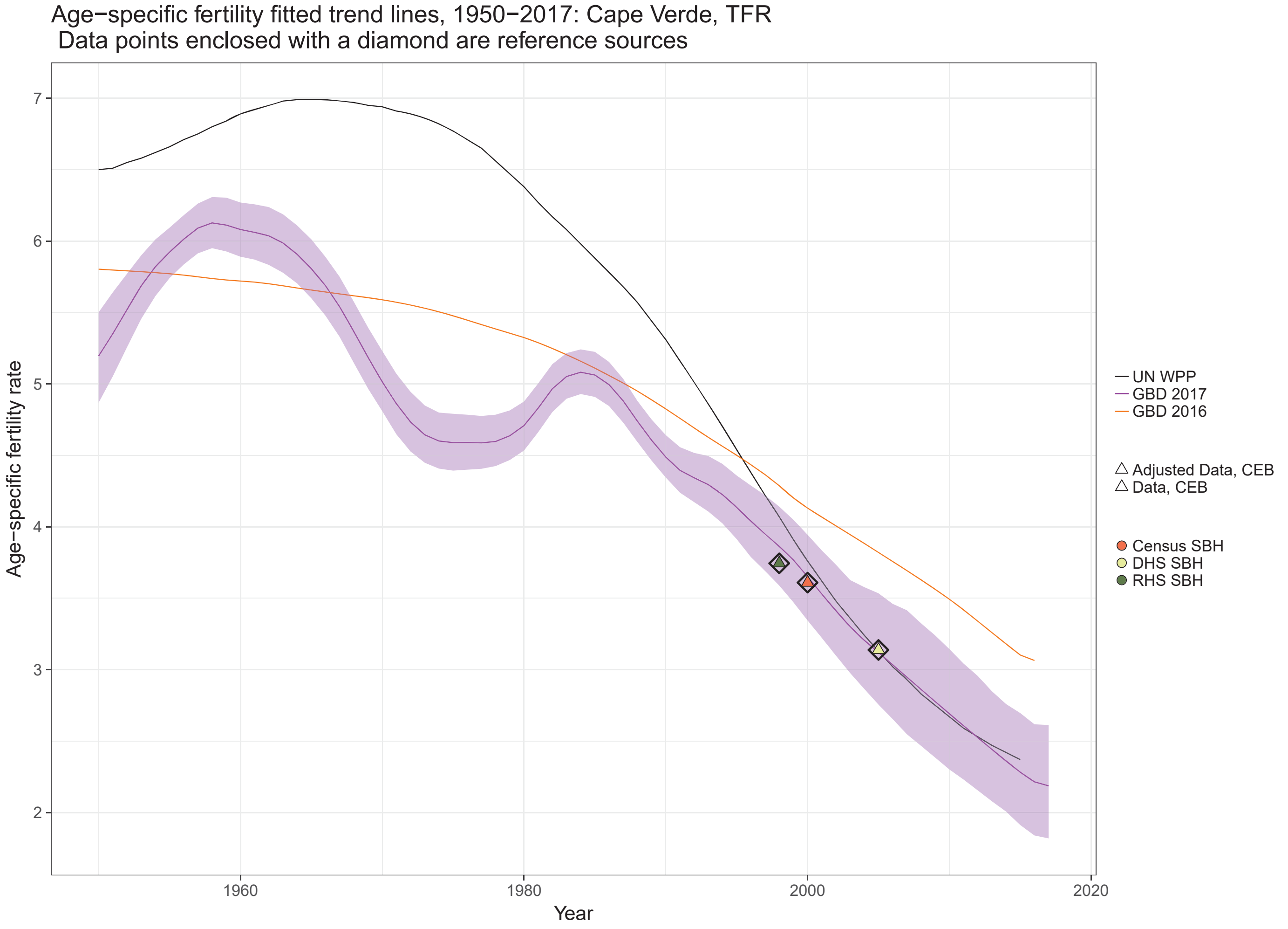
Age−specific fertility fitted trend lines, 1950−2017: Cape Verde, TFR
Data points enclosed with a diamond are reference sources
Age−specific fertility rate
Year
7
6
5
4
3
2
1960
1980
2000
2020
UN WPP
GBD 2017
GBD 2016
Adjusted Data, CEB
Data, CEB
Census SBH
DHS SBH
RHS SBH

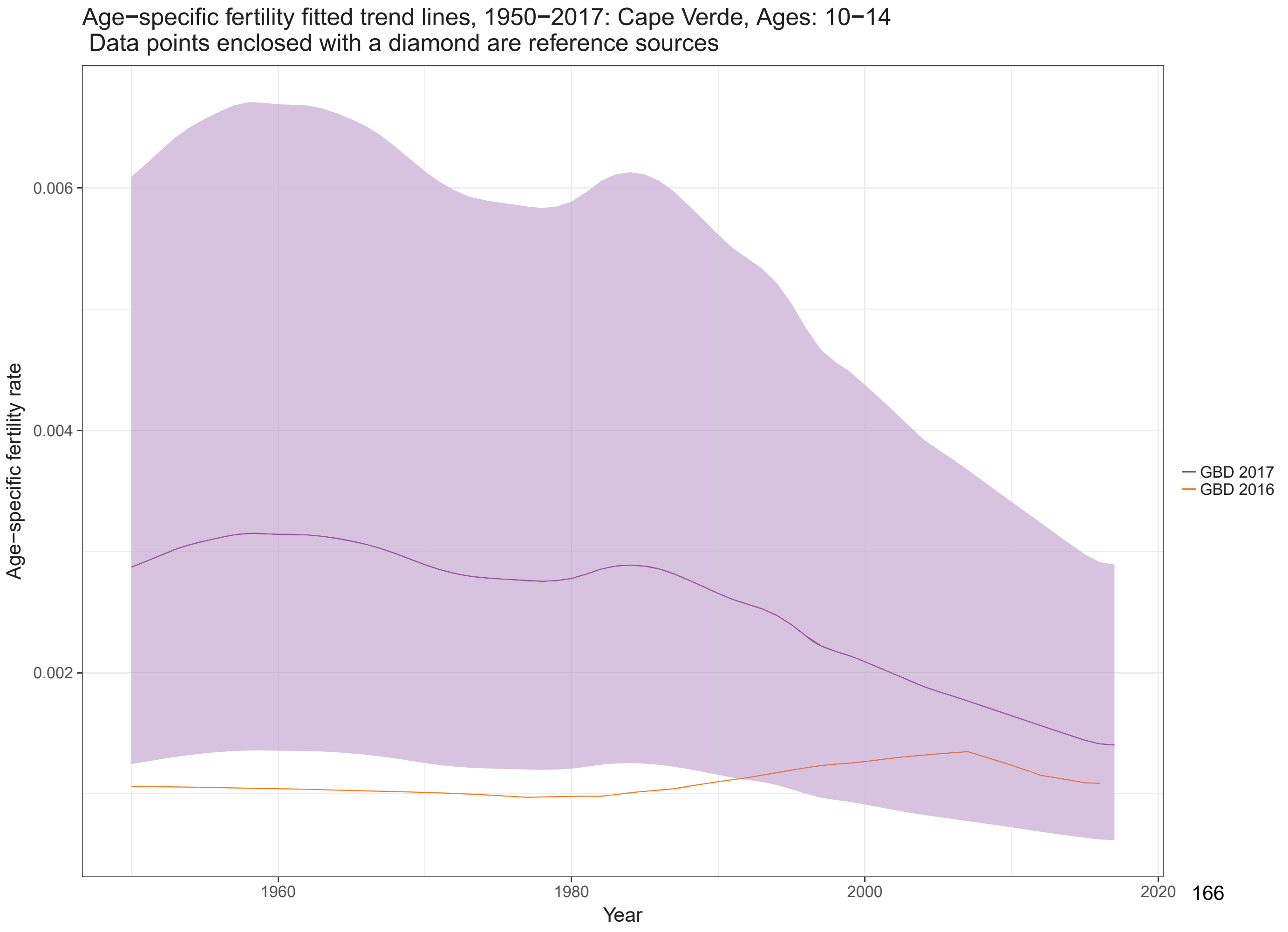
Age−specific fertility fitted trend lines, 1950−2017: Cape Verde, Ages: 10−14
Data points enclosed with a diamond are reference sources
Age−specific fertility rate
Year
0.006
0.004
0.002
1960
1980
2000
2020
GBD 2017
GBD 2016

Age−specific fertility fitted trend lines, 1950−2017: Cape Verde, Ages: 15−19
Data points enclosed with a diamond are reference sources

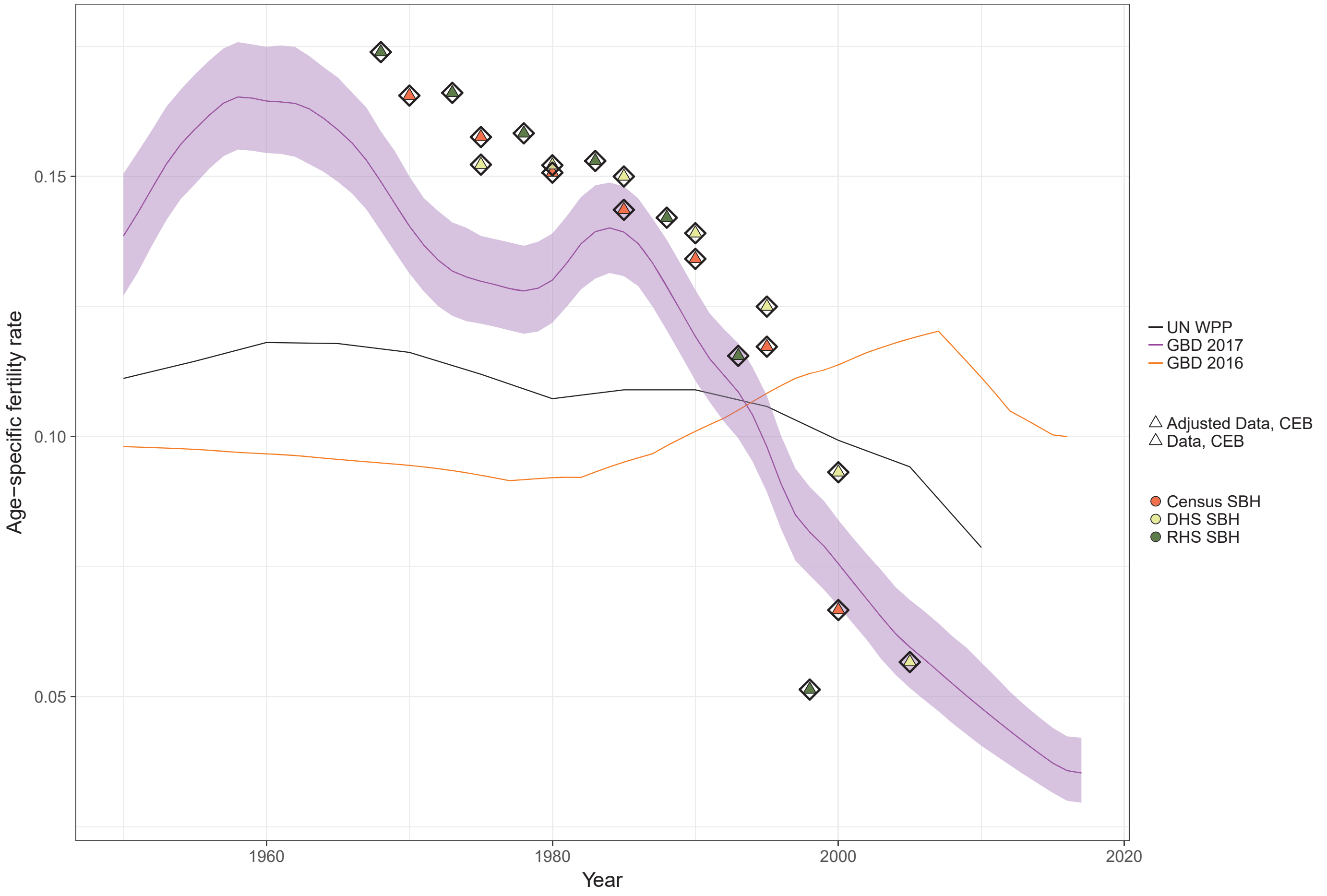

Age−specific fertility fitted trend lines, 1950−2017: Cape Verde, Ages: 20−24
Data points enclosed with a diamond are reference sources

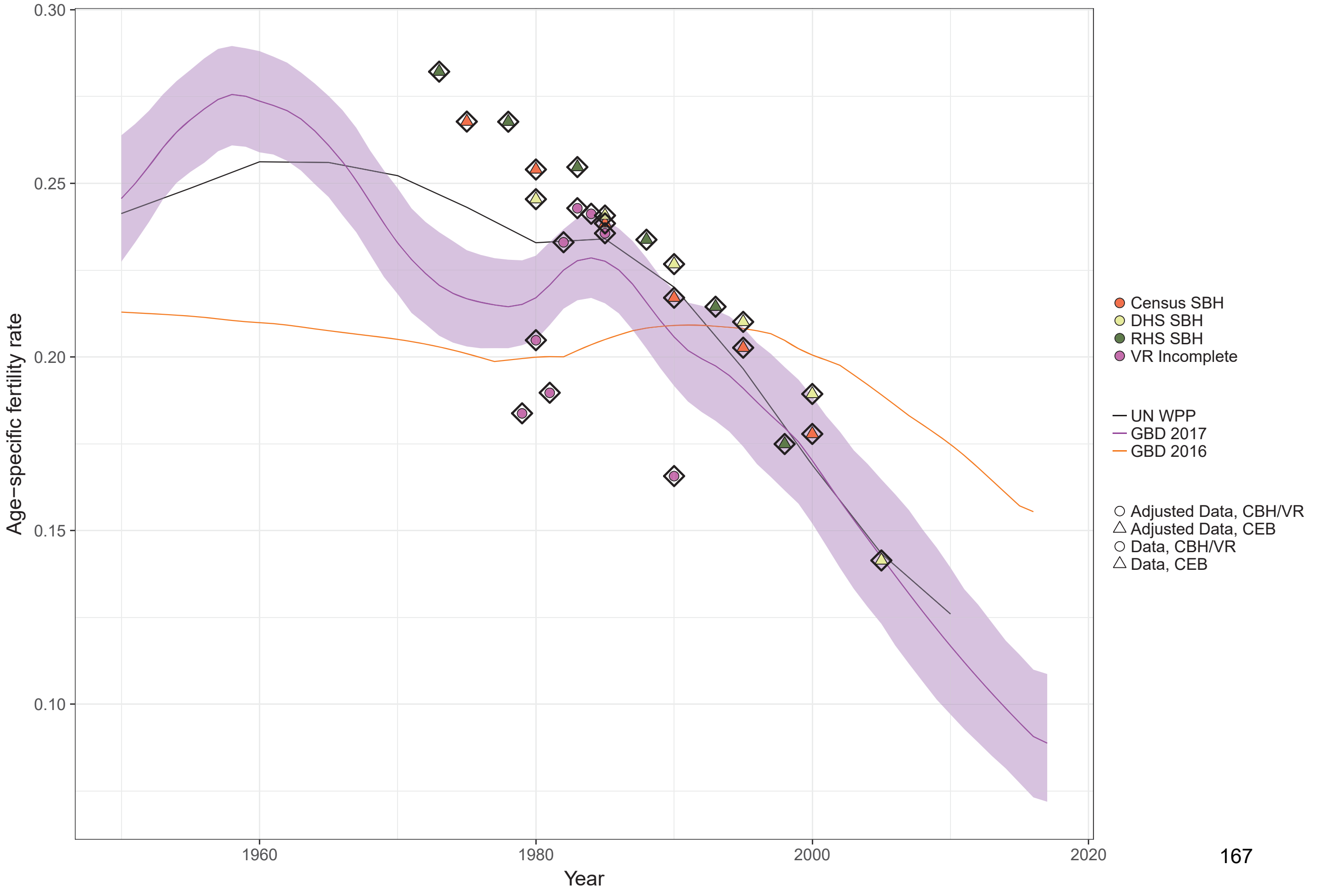

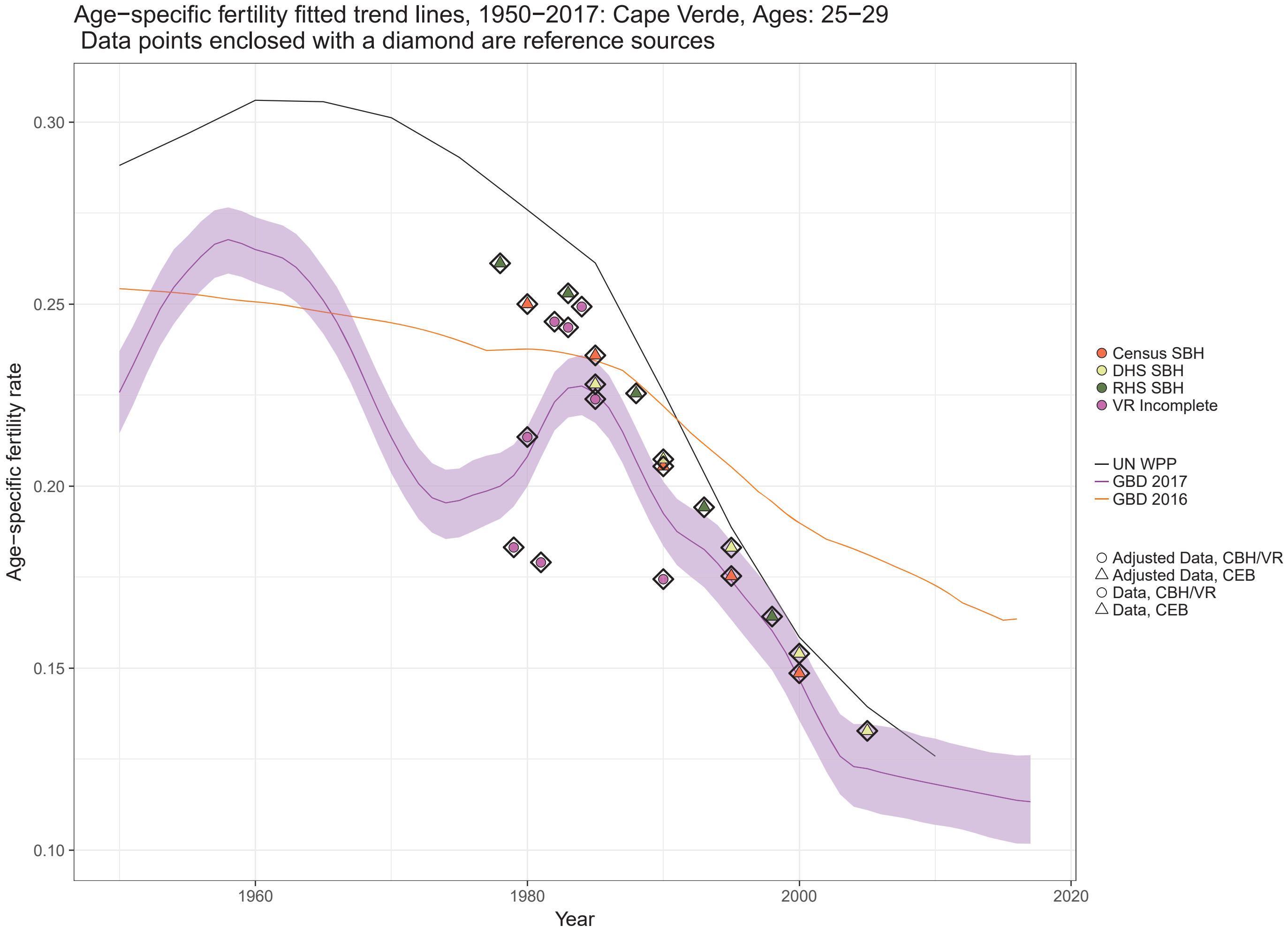
Age−specific fertility fitted trend lines, 1950−2017: Cape Verde, Ages: 25−29
Data points enclosed with a diamond are reference sources
Age−specific fertility rate
0.30
0.25
0.20
0.15
0.10
1960
1980
2000
2020
Year
Census SBH
DHS SBH
RHS SBH
VR Incomplete
UN WPP
GBD 2017
GBD 2016
Adjusted Data, CBH/VR
Adjusted Data, CEB
Data, CBH/VR
Data, CEB

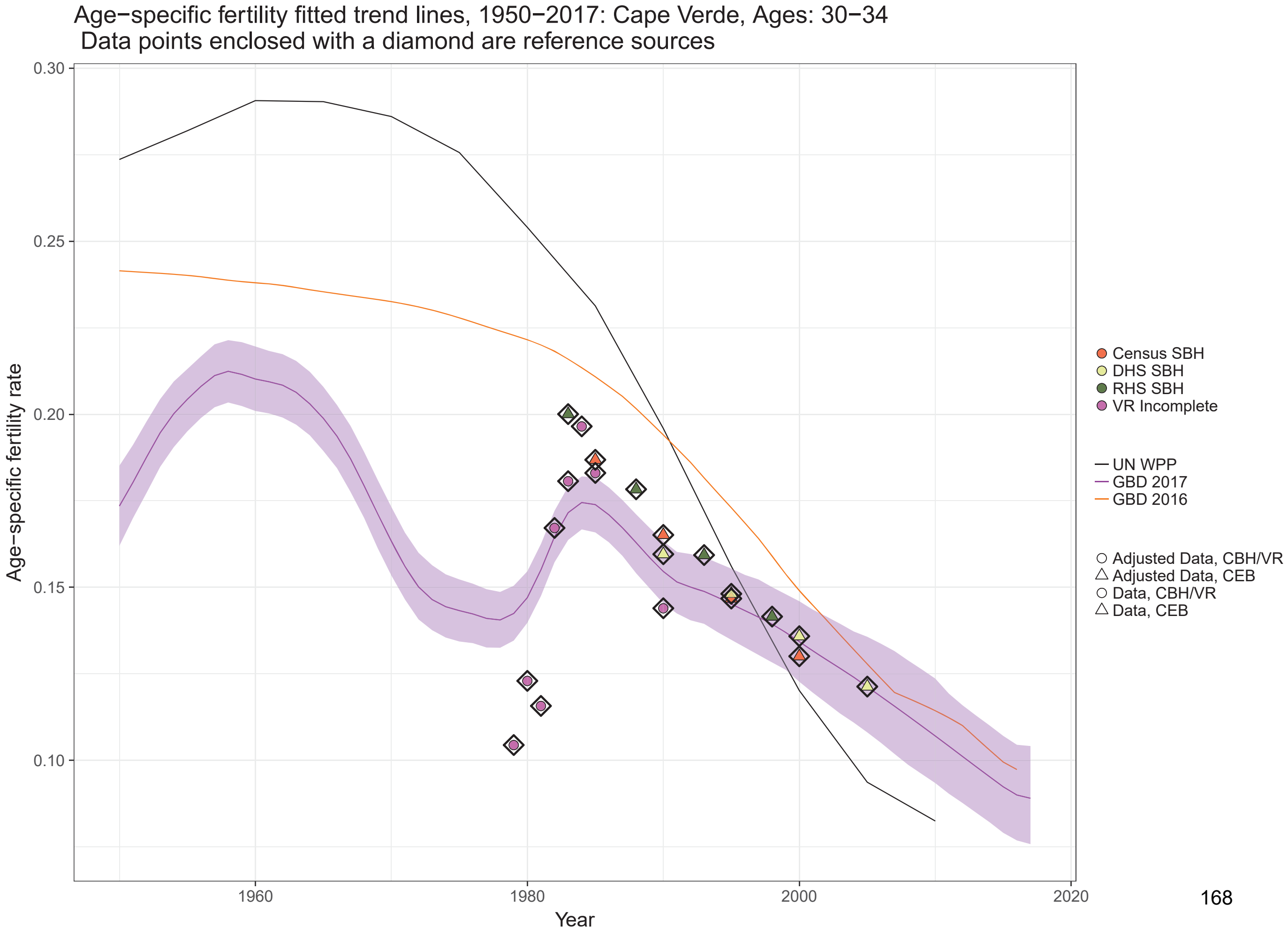
Age−specific fertility fitted trend lines, 1950−2017: Cape Verde, Ages: 30−34
Data points enclosed with a diamond are reference sources
Age−specific fertility rate
0.30
0.25
0.20
0.15
0.10
1960
1980
2000
2020
Year
Census SBH
DHS SBH
RHS SBH
VR Incomplete
UN WPP
GBD 2017
GBD 2016
Adjusted Data, CBH/VR
Adjusted Data, CEB
Data, CBH/VR
Data, CEB

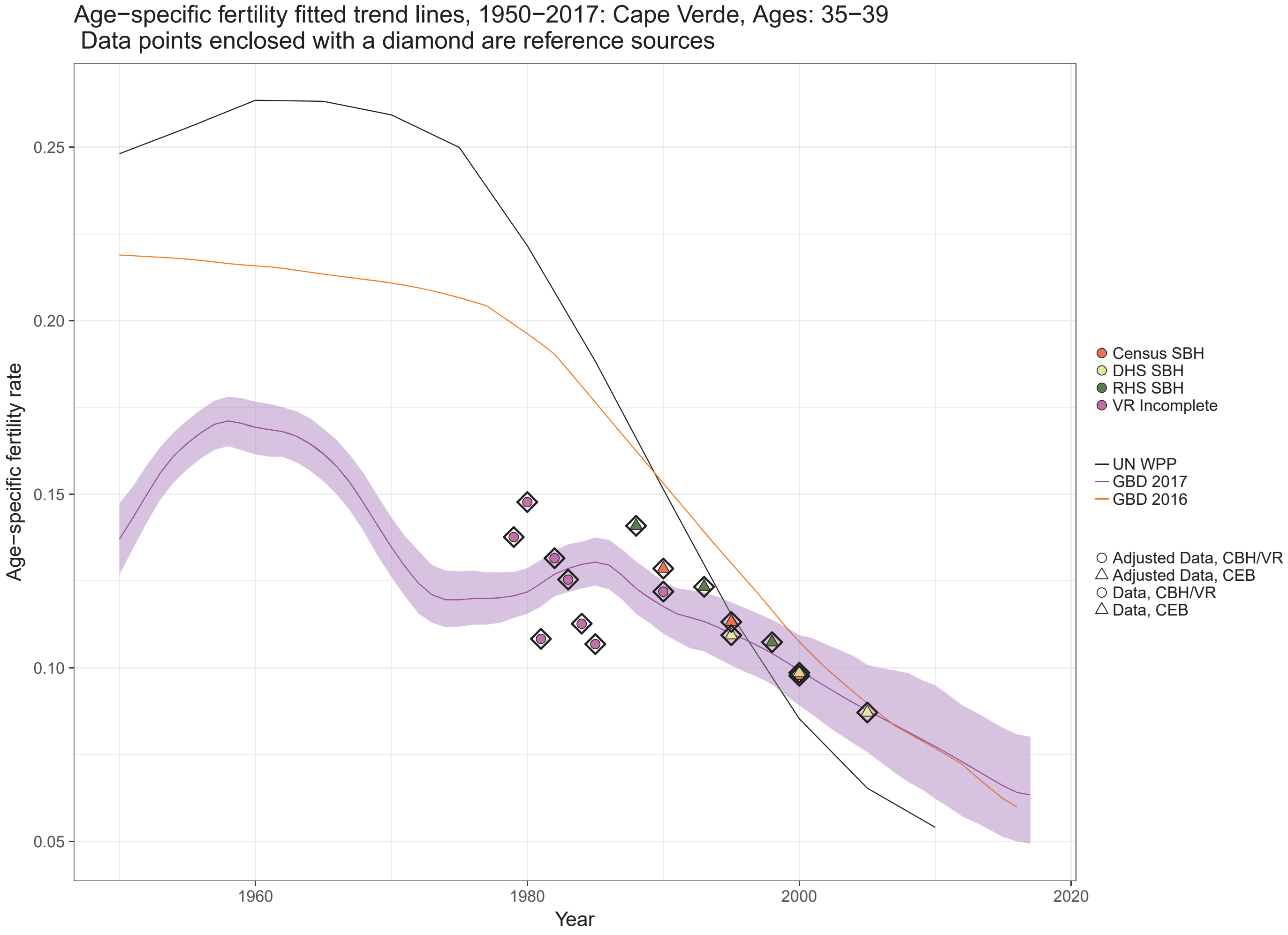
Age−specific fertility fitted trend lines, 1950−2017: Cape Verde, Ages: 35−39
Data points enclosed with a diamond are reference sources
Age−specific fertility rate
0.25
0.20
0.15
0.10
0.05
1960
1980
2000
2020
Year
Census SBH
DHS SBH
RHS SBH
VR Incomplete
UN WPP
GBD 2017
GBD 2016
Adjusted Data, CBH/VR
Adjusted Data, CEB
Data, CBH/VR
Data, CEB

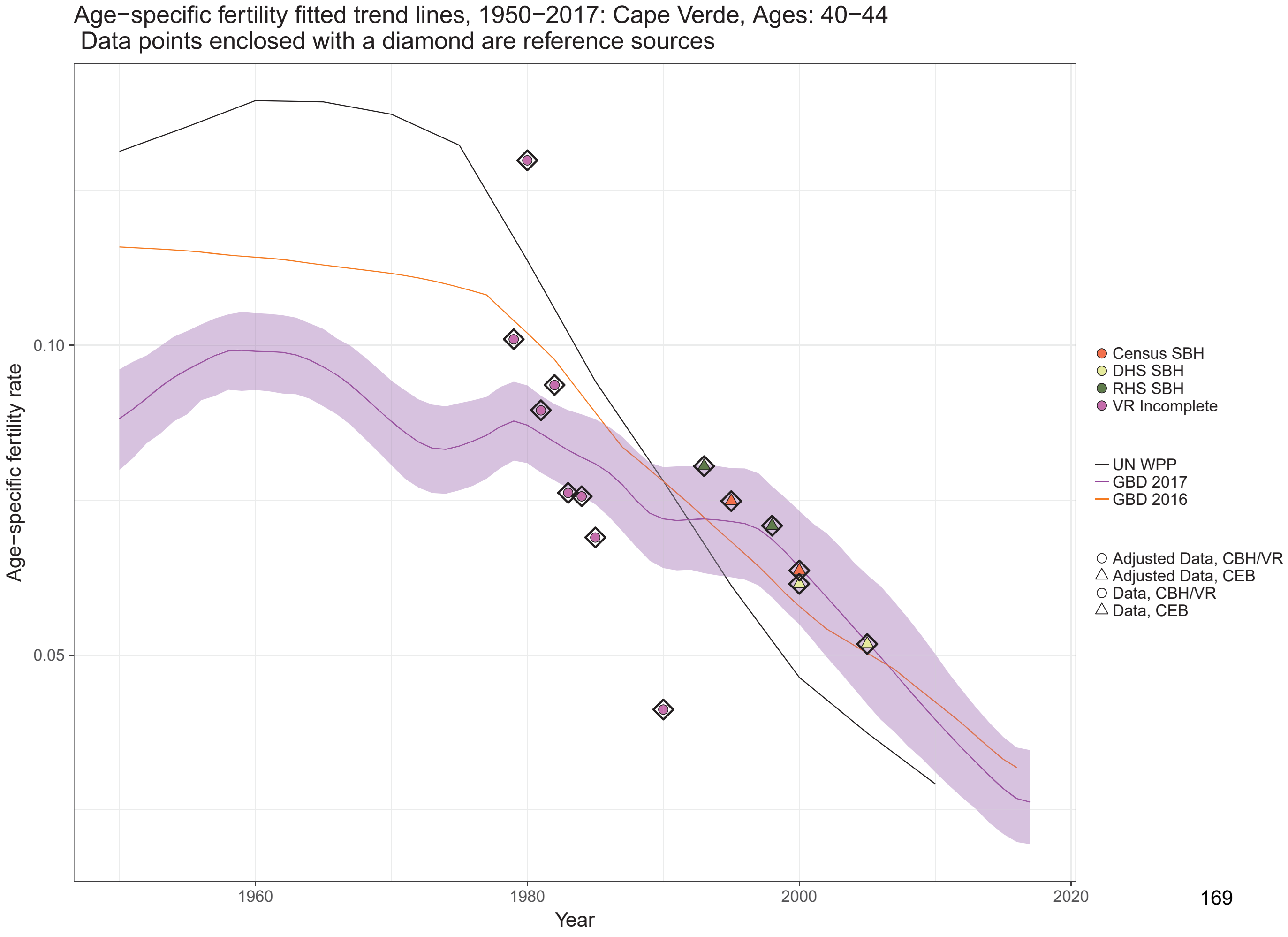
Age−specific fertility fitted trend lines, 1950−2017: Cape Verde, Ages: 40−44
Data points enclosed with a diamond are reference sources
Age−specific fertility rate
0.10
0.05
1960
1980
2000
2020
Year
Census SBH
DHS SBH
RHS SBH
VR Incomplete
UN WPP
GBD 2017
GBD 2016
Adjusted Data, CBH/VR
Adjusted Data, CEB
Data, CBH/VR
Data, CEB

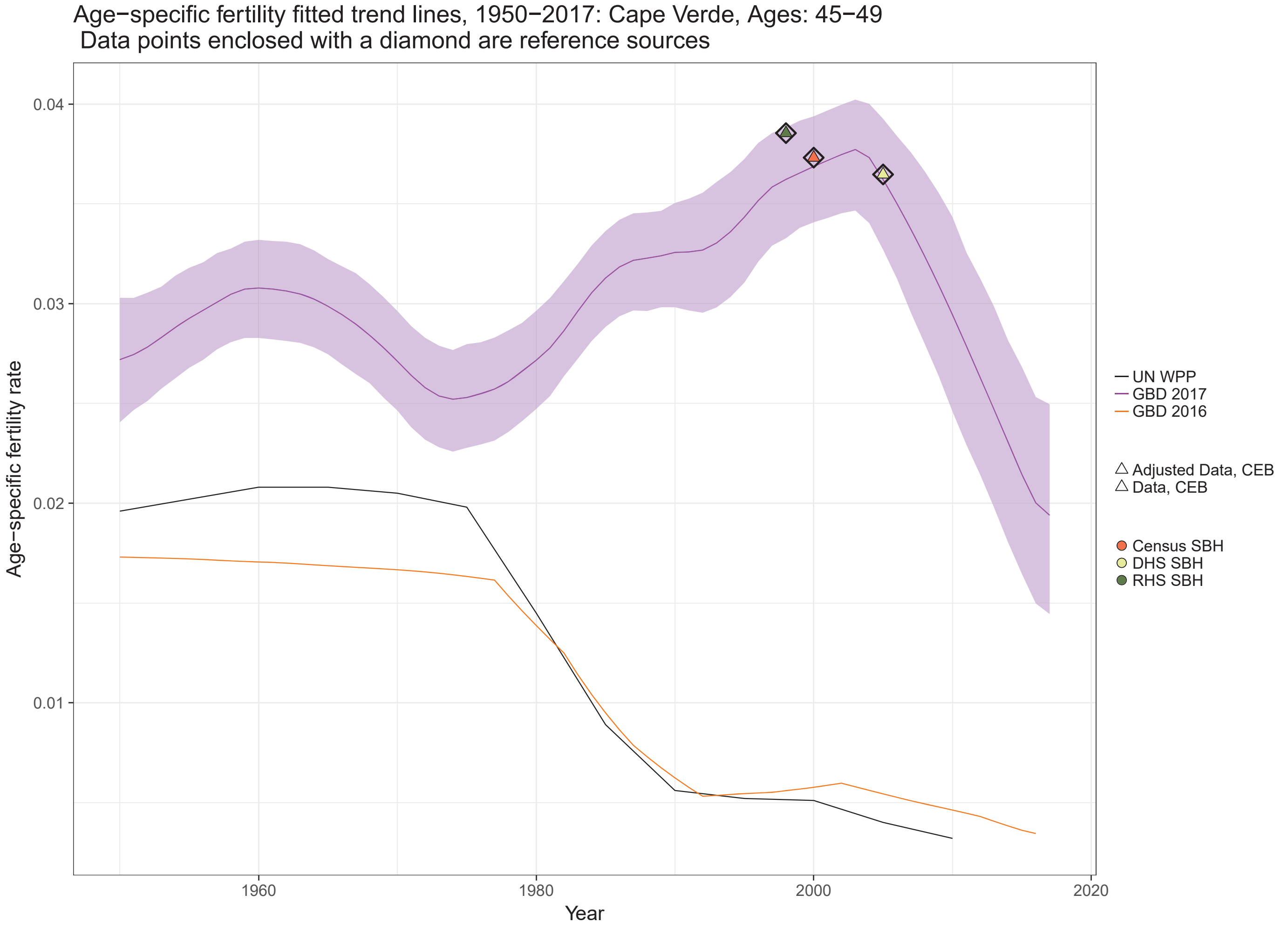
Age−specific fertility fitted trend lines, 1950−2017: Cape Verde, Ages: 45−49
Data points enclosed with a diamond are reference sources
Age−specific fertility rate
0.04
0.03
0.02
0.01
1960
1980
2000
2020
Year
UN WPP
GBD 2017
GBD 2016
Adjusted Data, CEB
Data, CEB
Census SBH
DHS SBH
RHS SBH

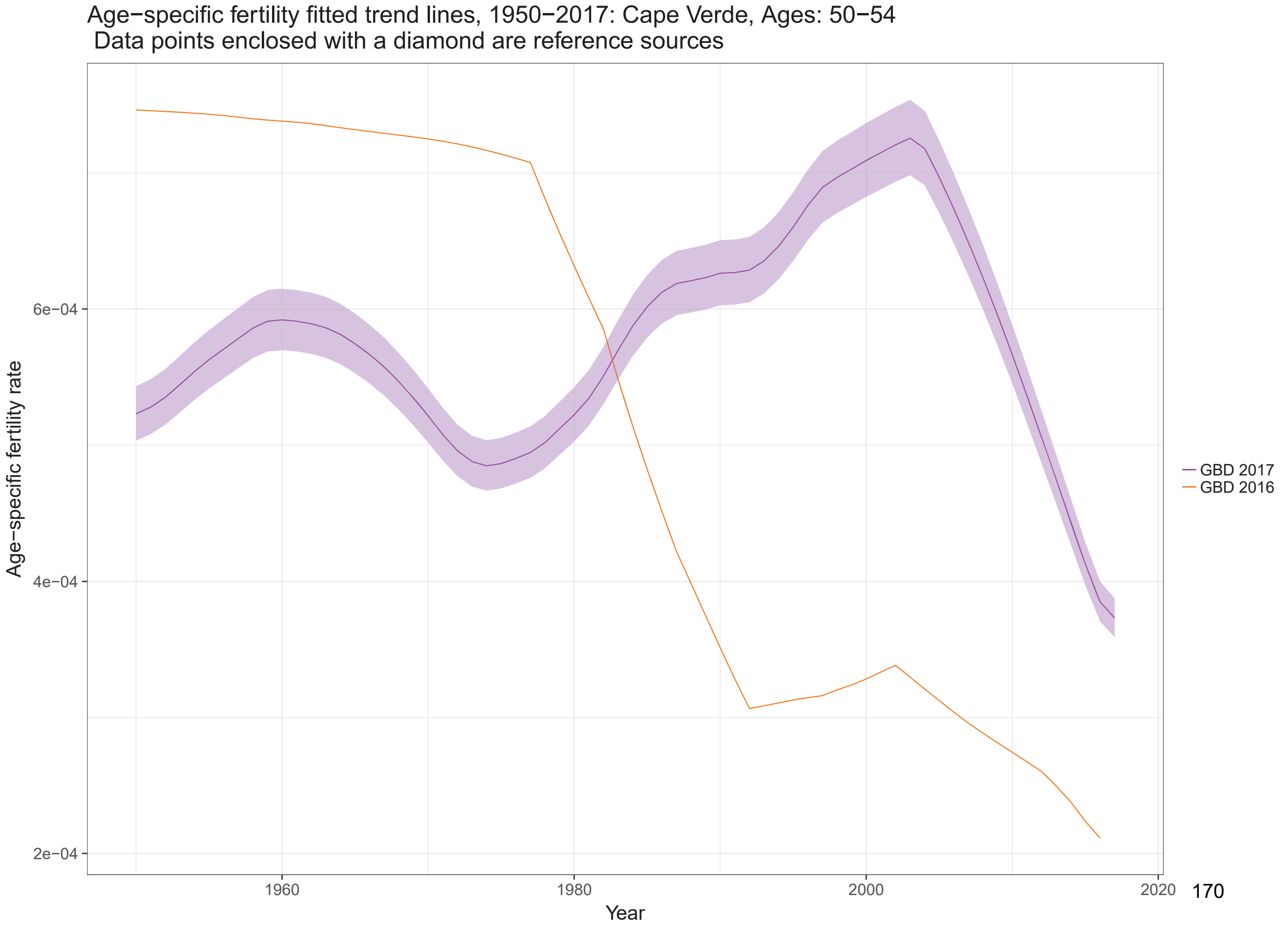
Age−specific fertility fitted trend lines, 1950−2017: Cape Verde, Ages: 50−54
Data points enclosed with a diamond are reference sources
Age−specific fertility rate
6e−04
4e−04
2e−04
1960
1980
2000
2020
Year
GBD 2017
GBD 2016

Age−specific fertility fitted trend lines, 1950−2017: Central African Republic, TFR
Data points enclosed with a diamond are reference sources

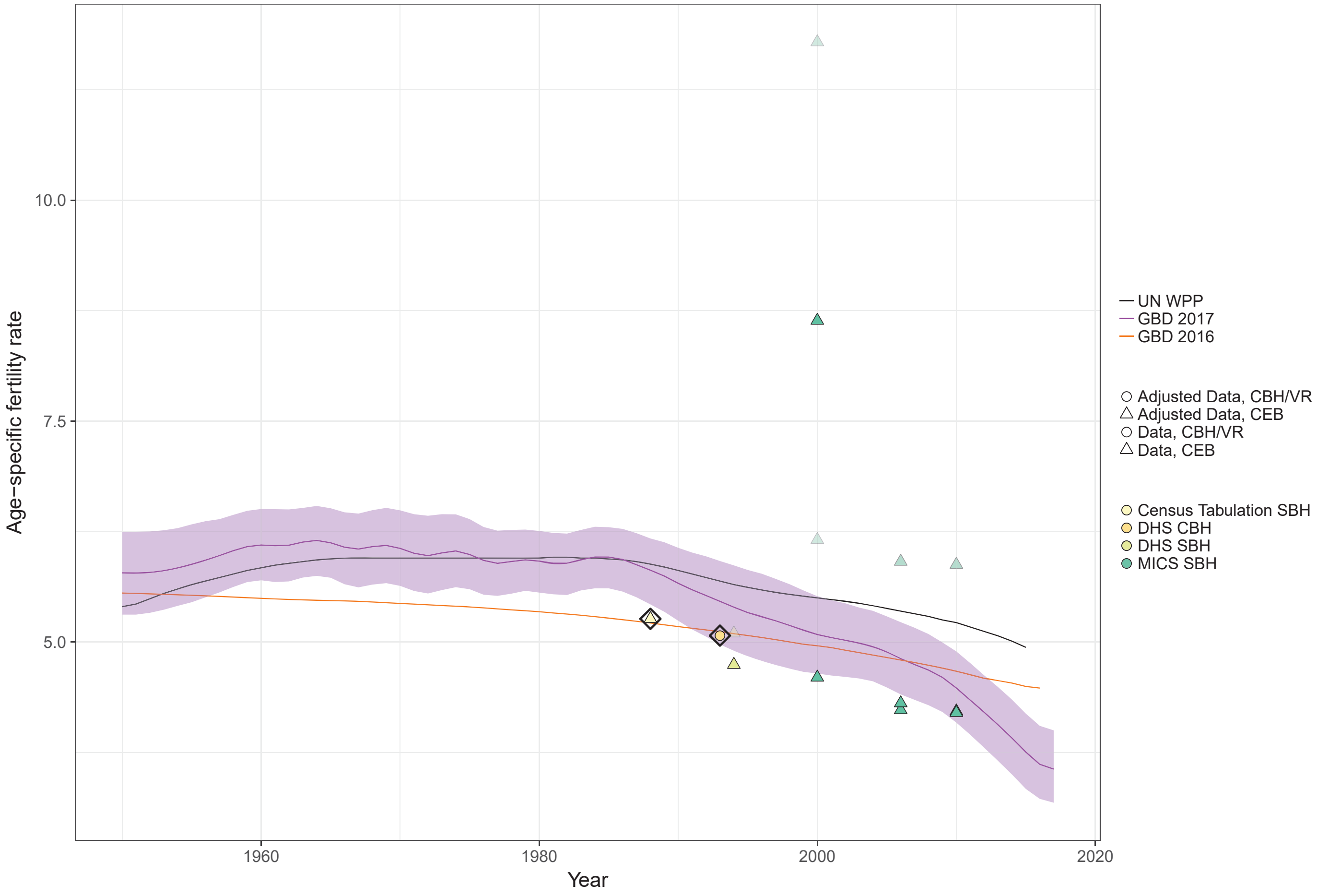

Age−specific fertility fitted trend lines, 1950−2017: Central African Republic, Ages: 10−14
Data points enclosed with a diamond are reference sources

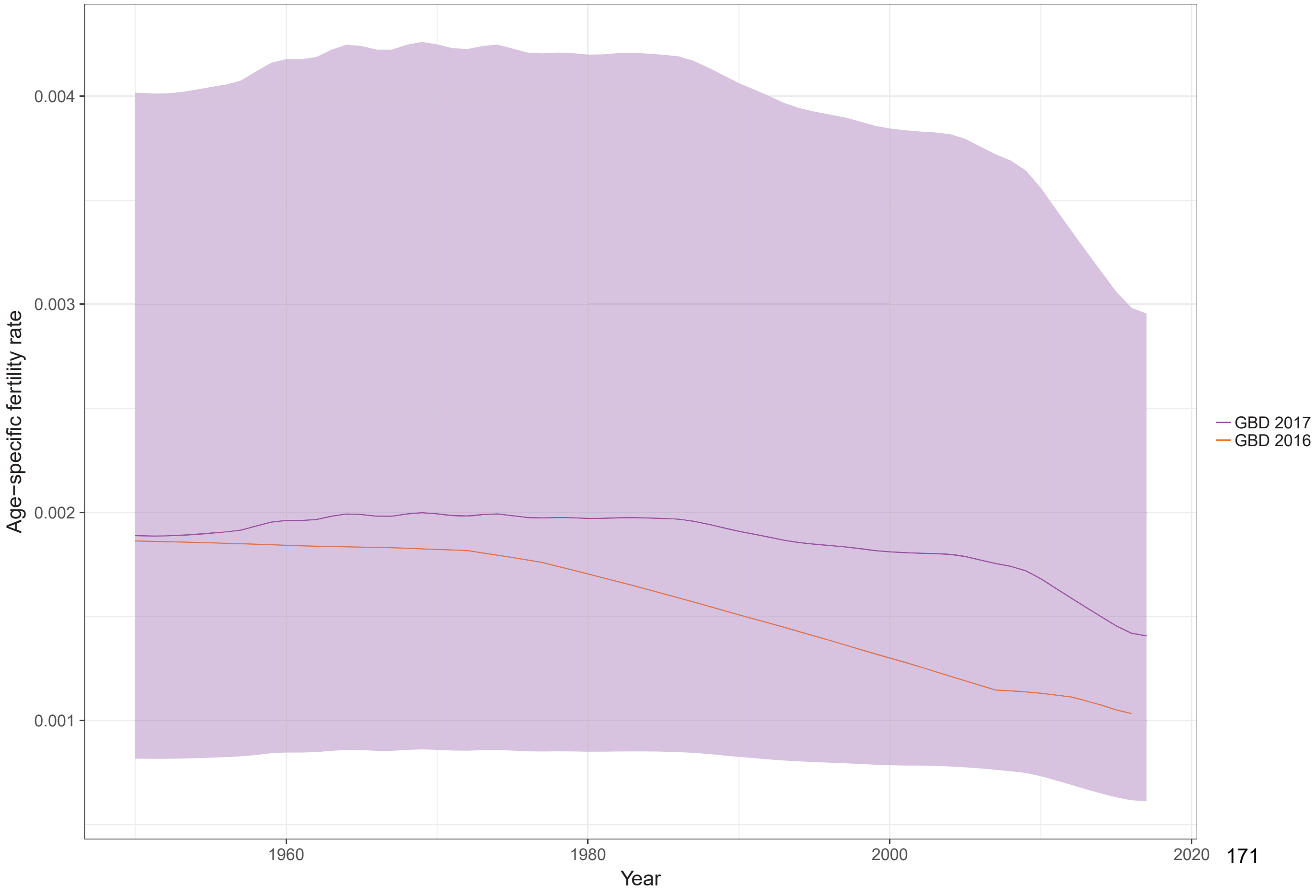

Age−specific fertility fitted trend lines, 1950−2017: Central African Republic, Ages: 15−19
Data points enclosed with a diamond are reference sources

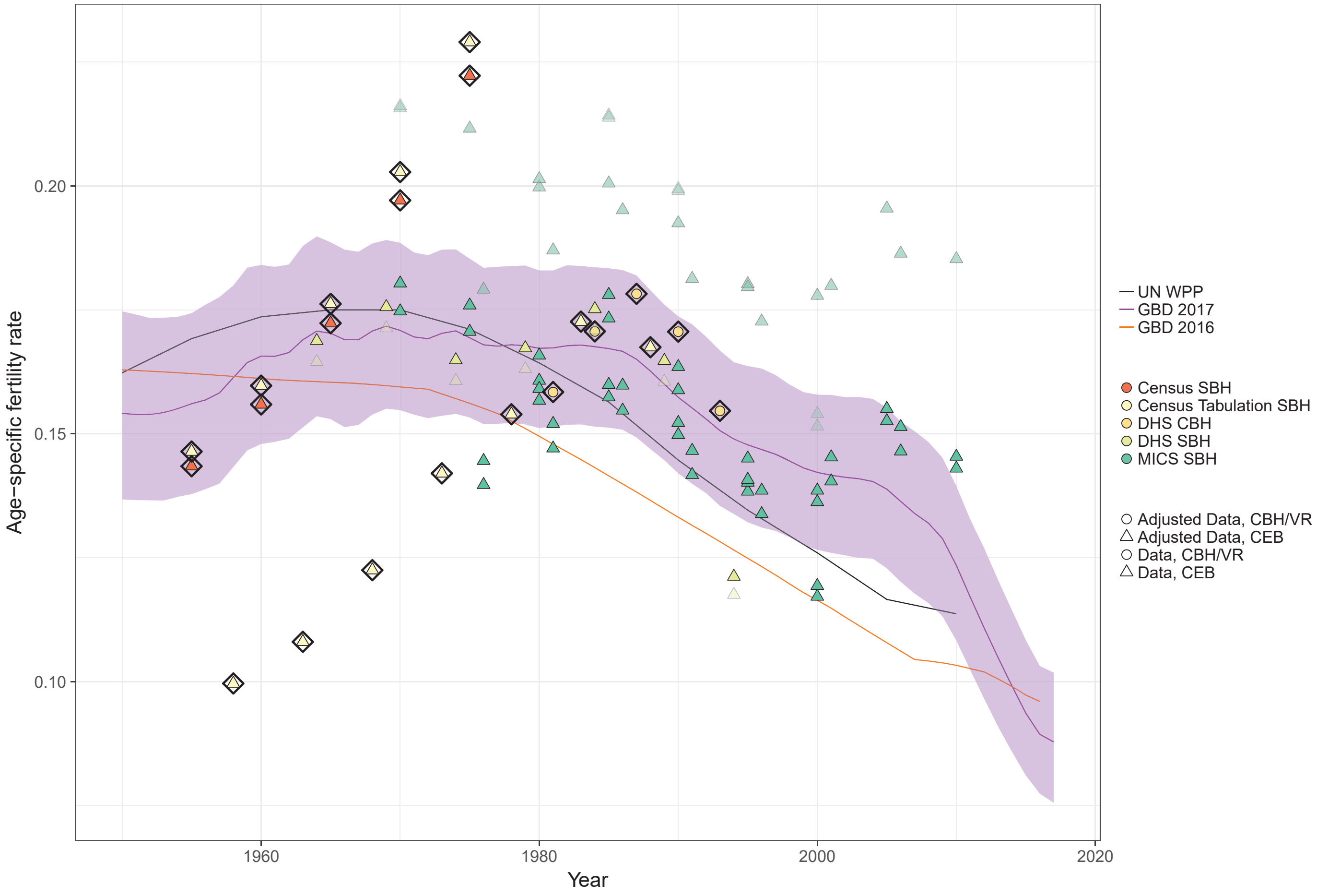

Age−specific fertility fitted trend lines, 1950−2017: Central African Republic, Ages: 20−24
Data points enclosed with a diamond are reference sources

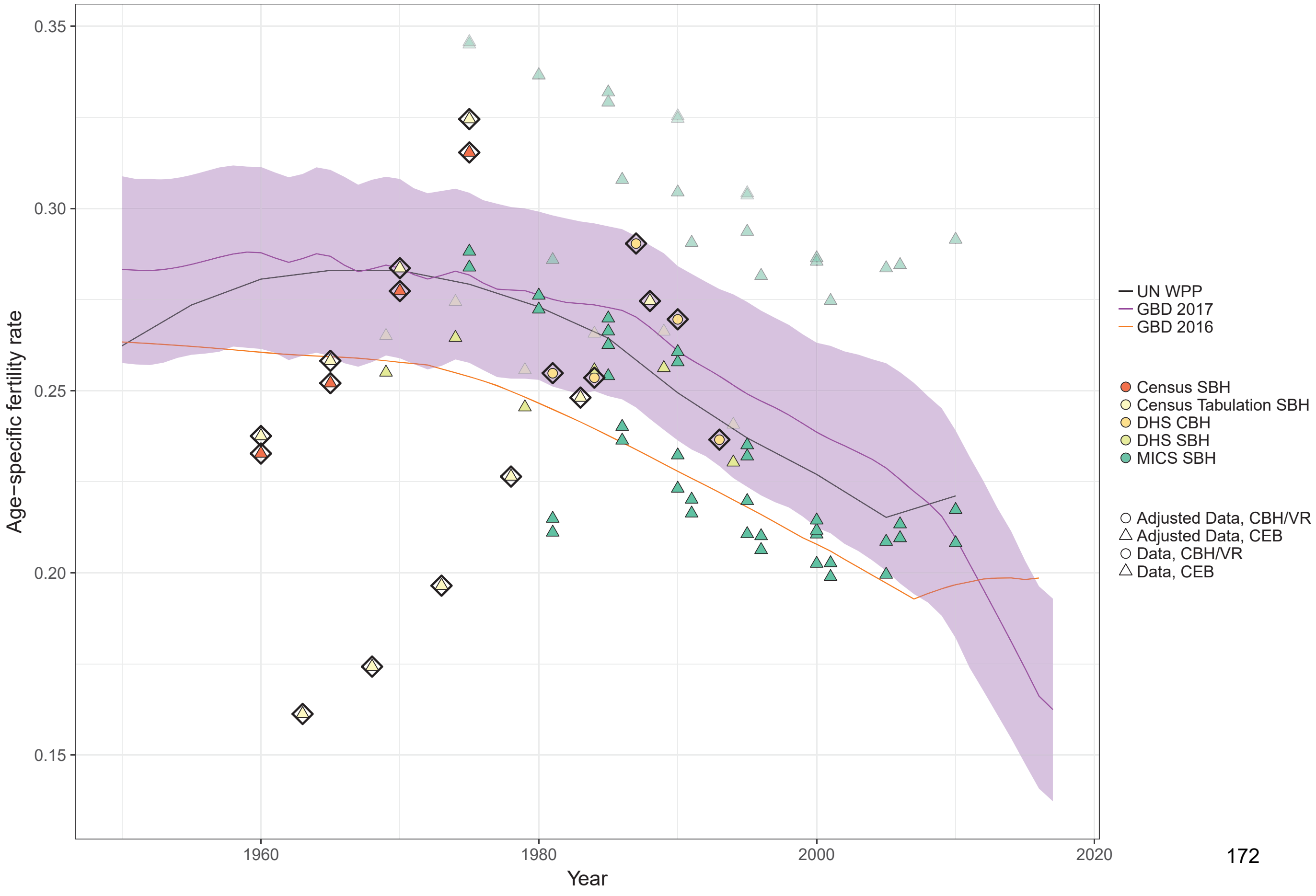

Age−specific fertility fitted trend lines, 1950−2017: Central African Republic, Ages: 25−29
Data points enclosed with a diamond are reference sources

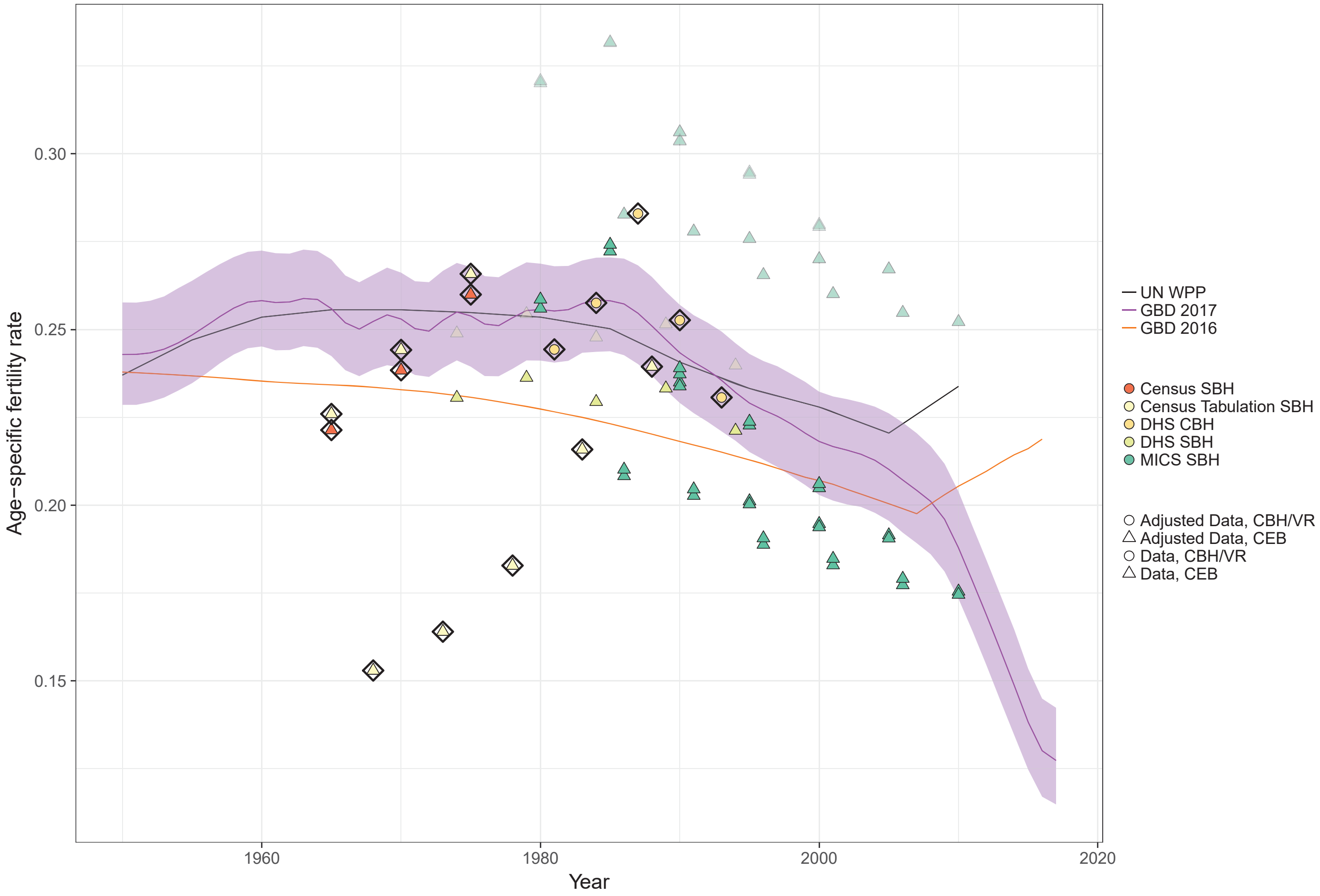

Age−specific fertility fitted trend lines, 1950−2017: Central African Republic, Ages: 30−34
Data points enclosed with a diamond are reference sources

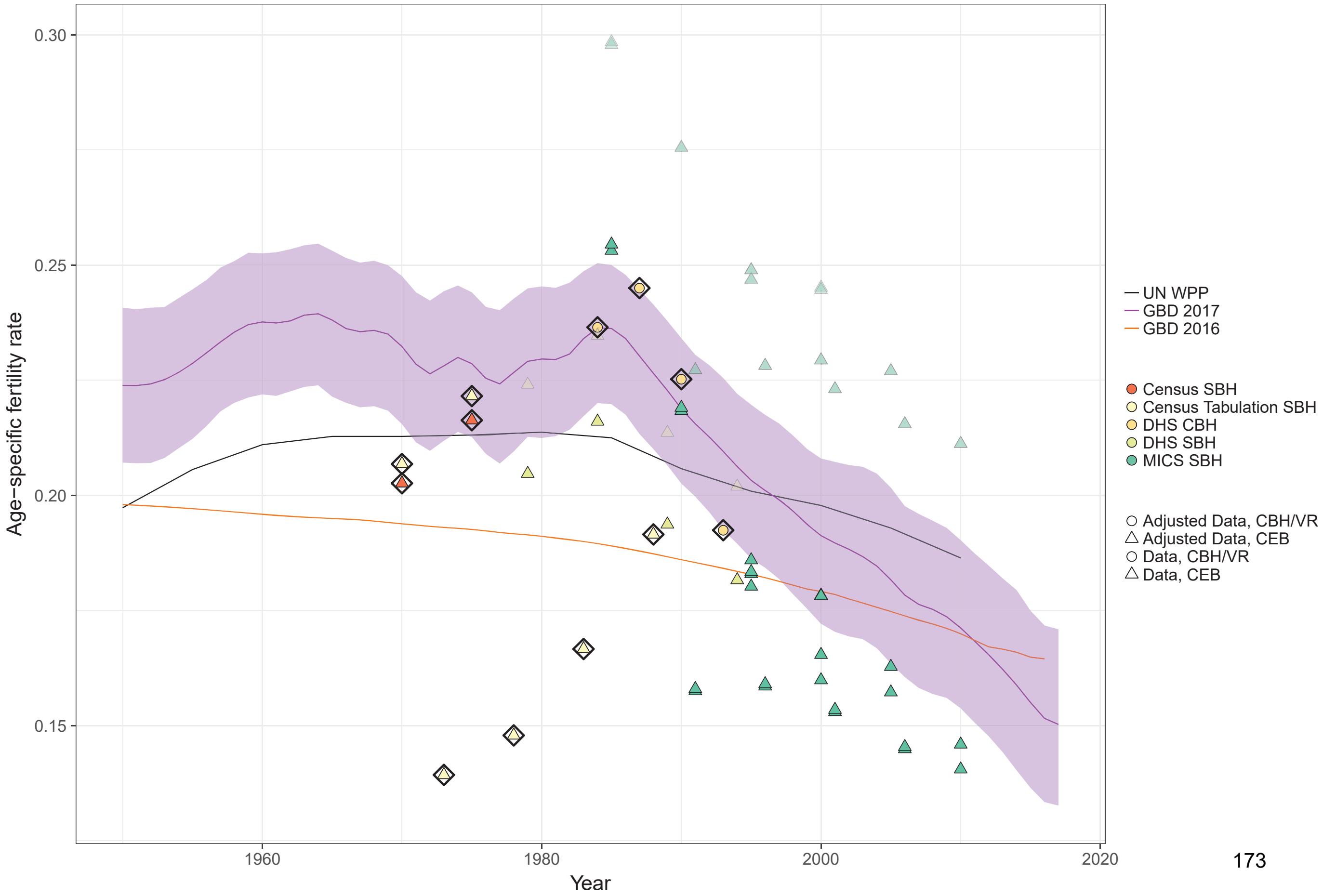

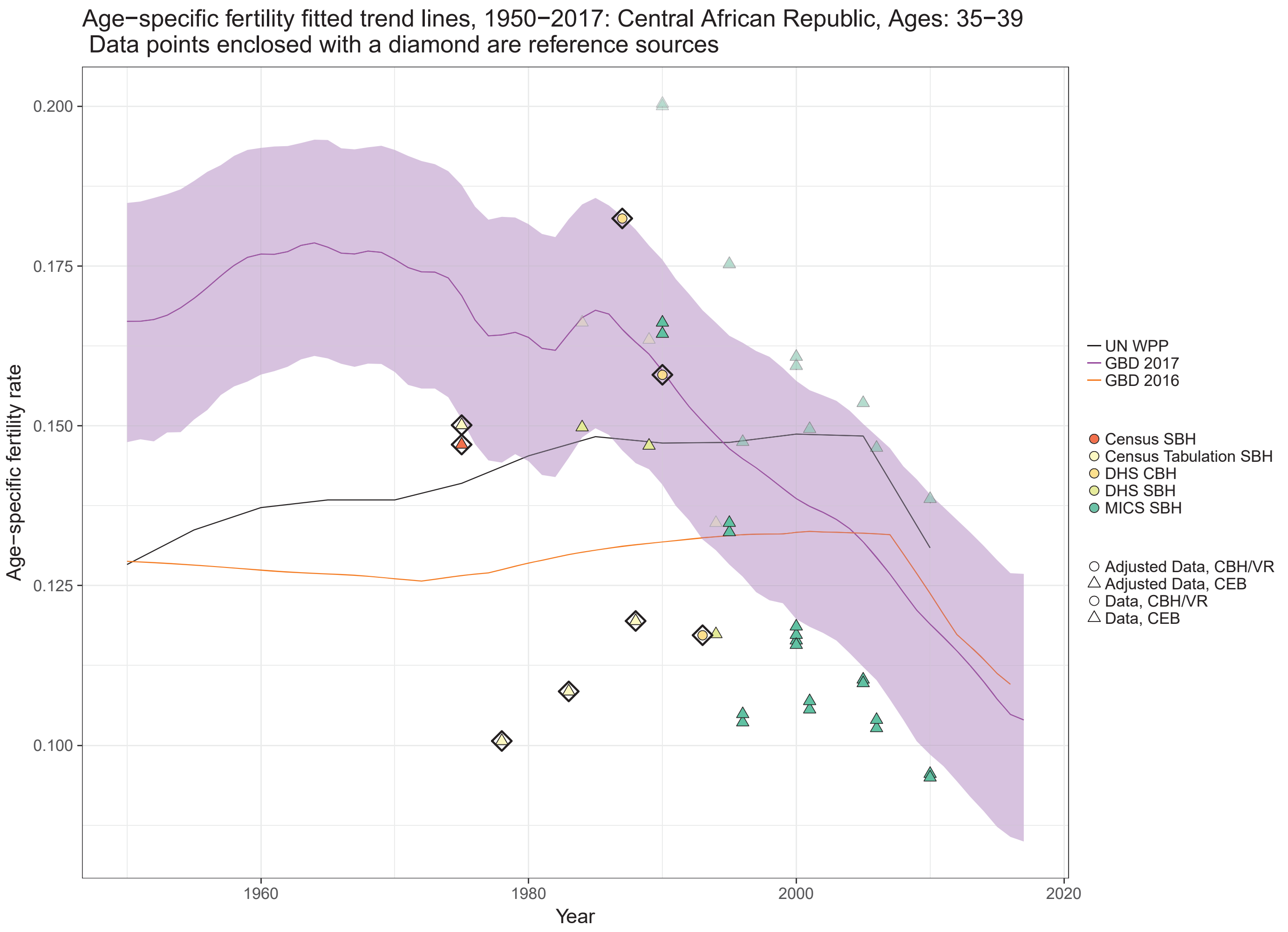

Age−specific fertility fitted trend lines, 1950−2017: Central African Republic, Ages: 35−39
Data points enclosed with a diamond are reference sources
Age−specific fertility rate
0.200
0.175
0.150
0.125
0.100
1960
1980
2000
2020
Year
UN WPP
GBD 2017
GBD 2016
Census SBH
Census Tabulation SBH
DHS CBH
DHS SBH
MICS SBH
Adjusted Data, CBH/VR
Adjusted Data, CEB
Data, CBH/VR
Data, CEB

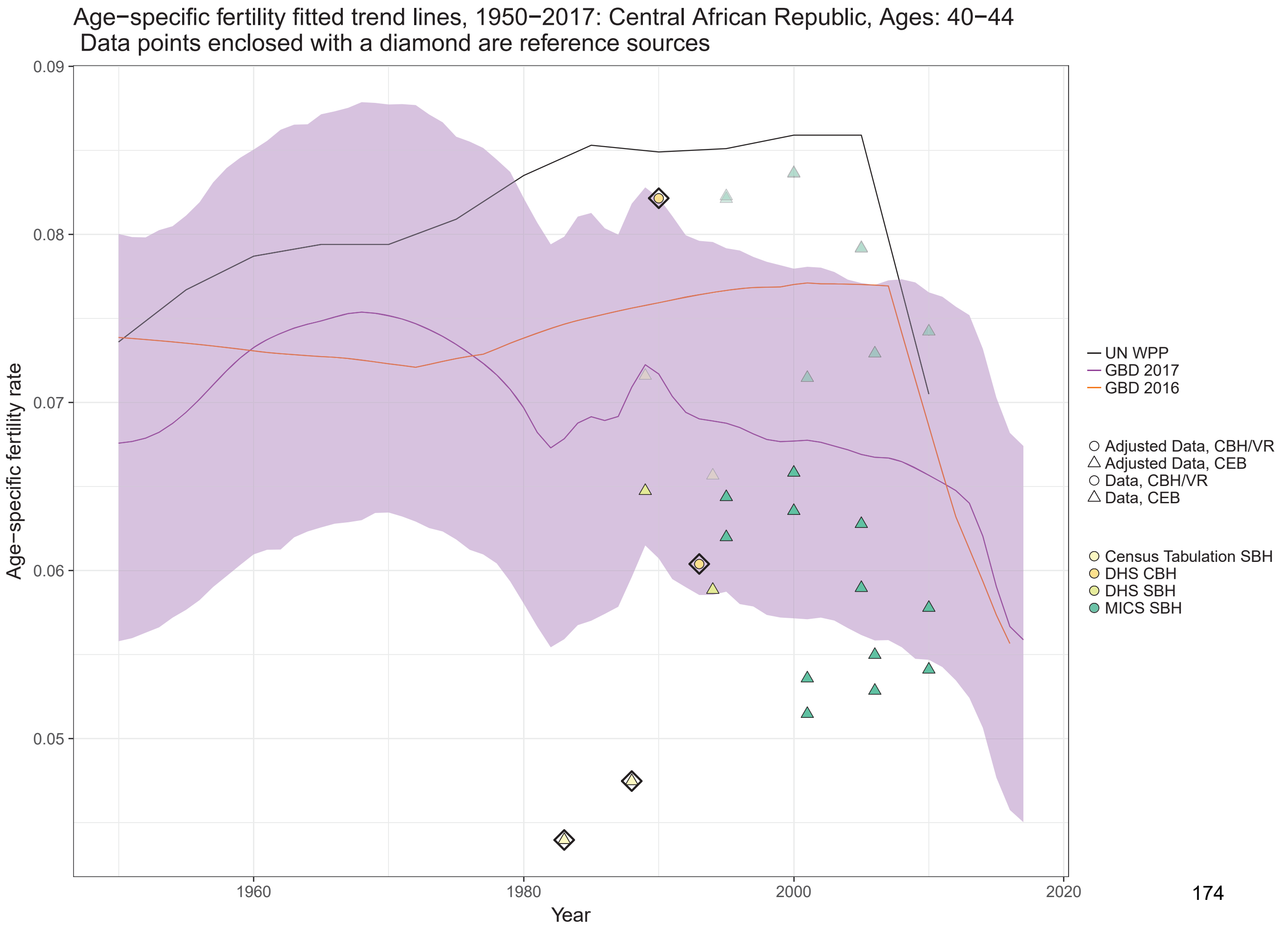

Age−specific fertility fitted trend lines, 1950−2017: Central African Republic, Ages: 40−44
Data points enclosed with a diamond are reference sources
Age−specific fertility rate
0.09
0.08
0.07
0.06
0.05
1960
1980
2000
2020
Year
UN WPP
GBD 2017
GBD 2016
Adjusted Data, CBH/VR
Adjusted Data, CEB
Data, CBH/VR
Data, CEB
Census Tabulation SBH
DHS CBH
DHS SBH
MICS SBH

Age−specific fertility fitted trend lines, 1950−2017: Central African Republic, Ages: 45−49
Data points enclosed with a diamond are reference sources

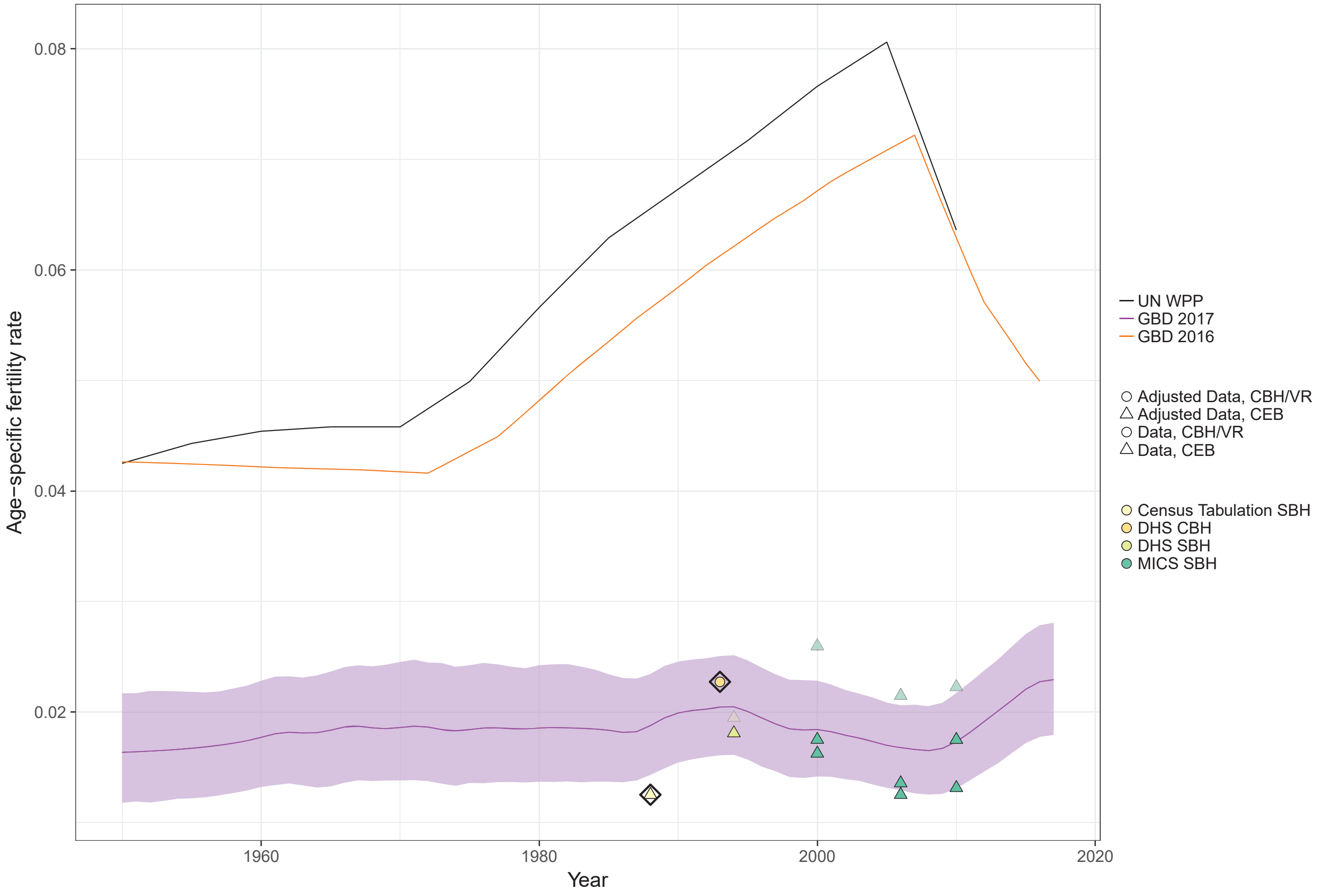

Age−specific fertility fitted trend lines, 1950−2017: Central African Republic, Ages: 50−54
Data points enclosed with a diamond are reference sources

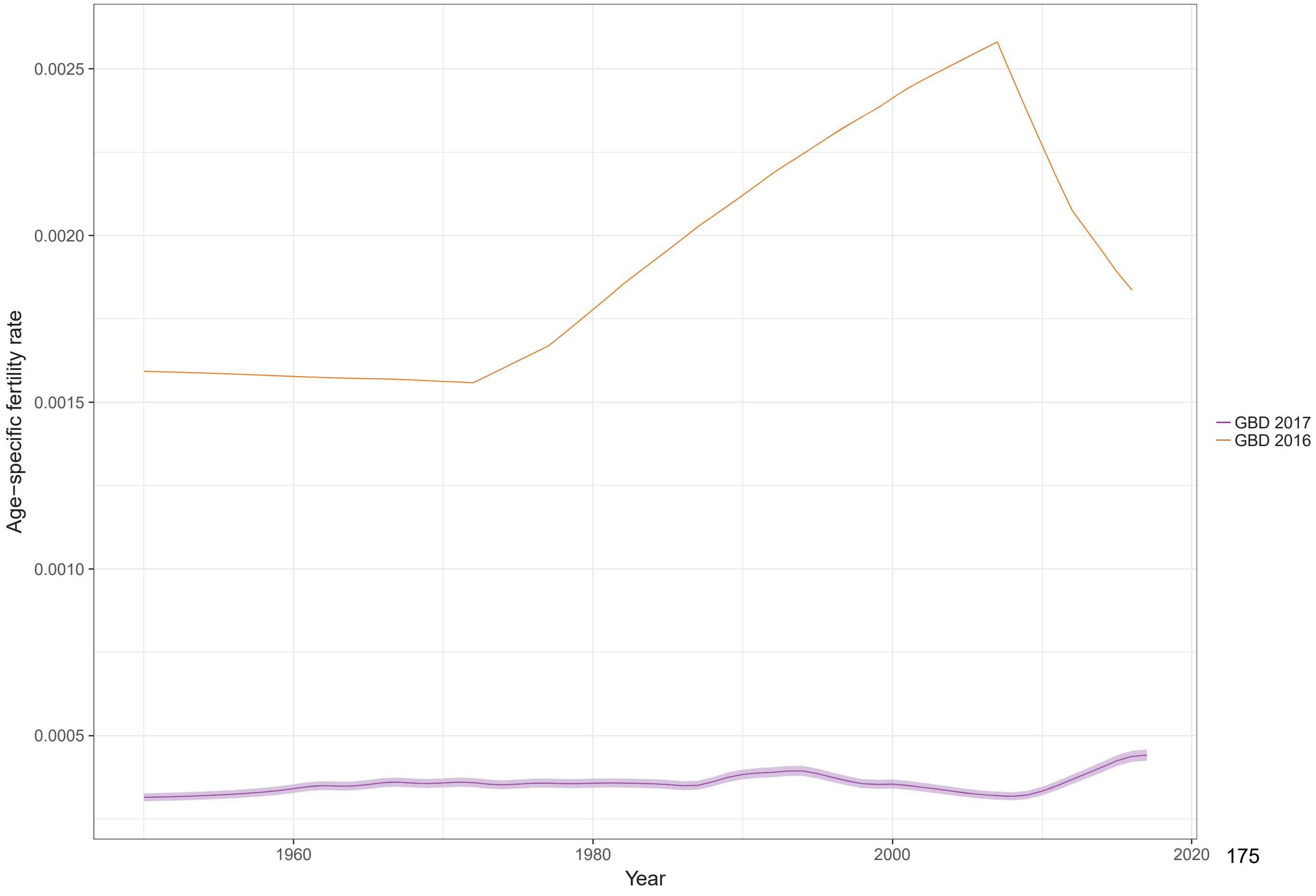

Age−specific fertility fitted trend lines, 1950−2017: Chad, TFR
Data points enclosed with a diamond are reference sources

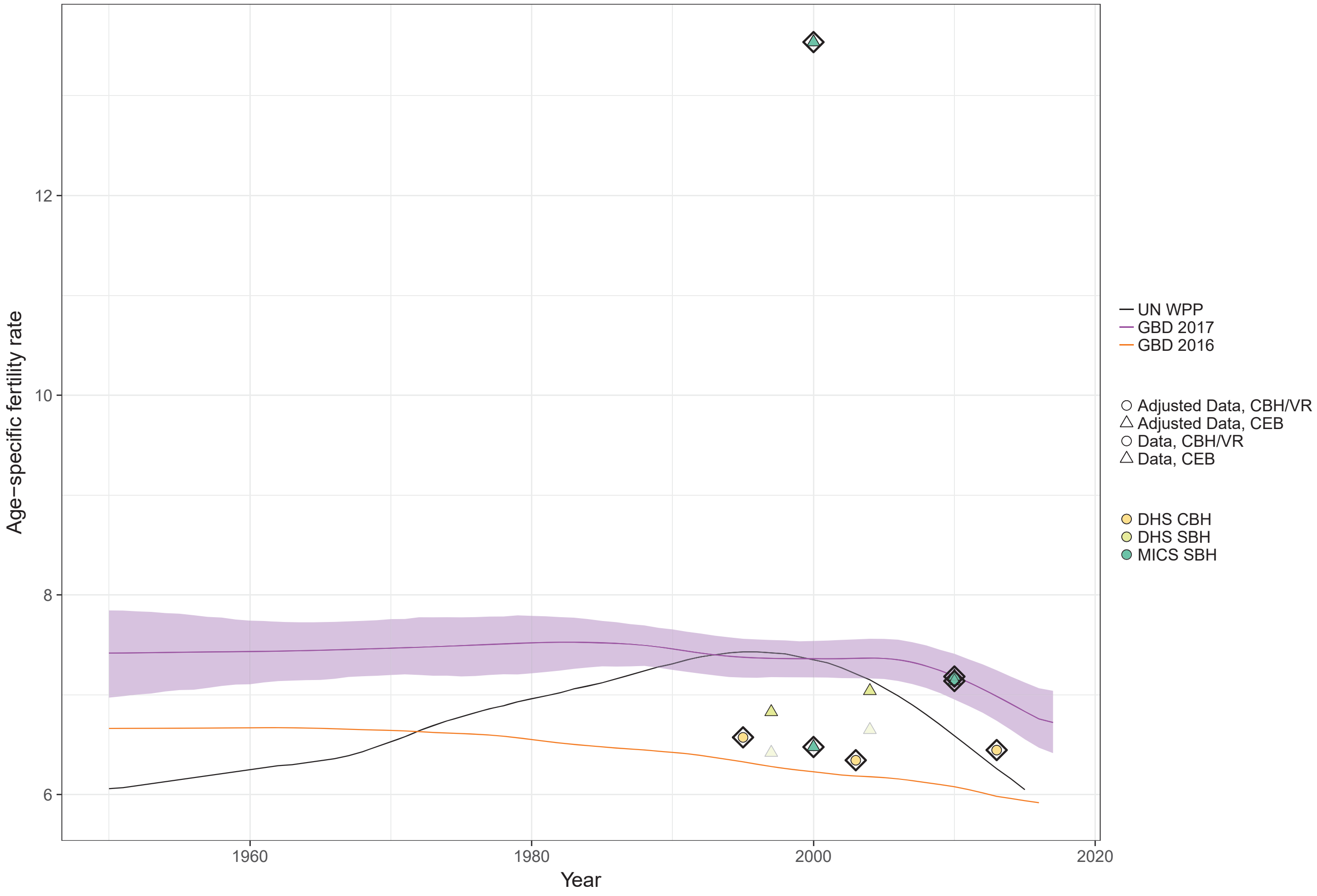

Age−specific fertility fitted trend lines, 1950−2017: Chad, Ages: 10−14
Data points enclosed with a diamond are reference sources

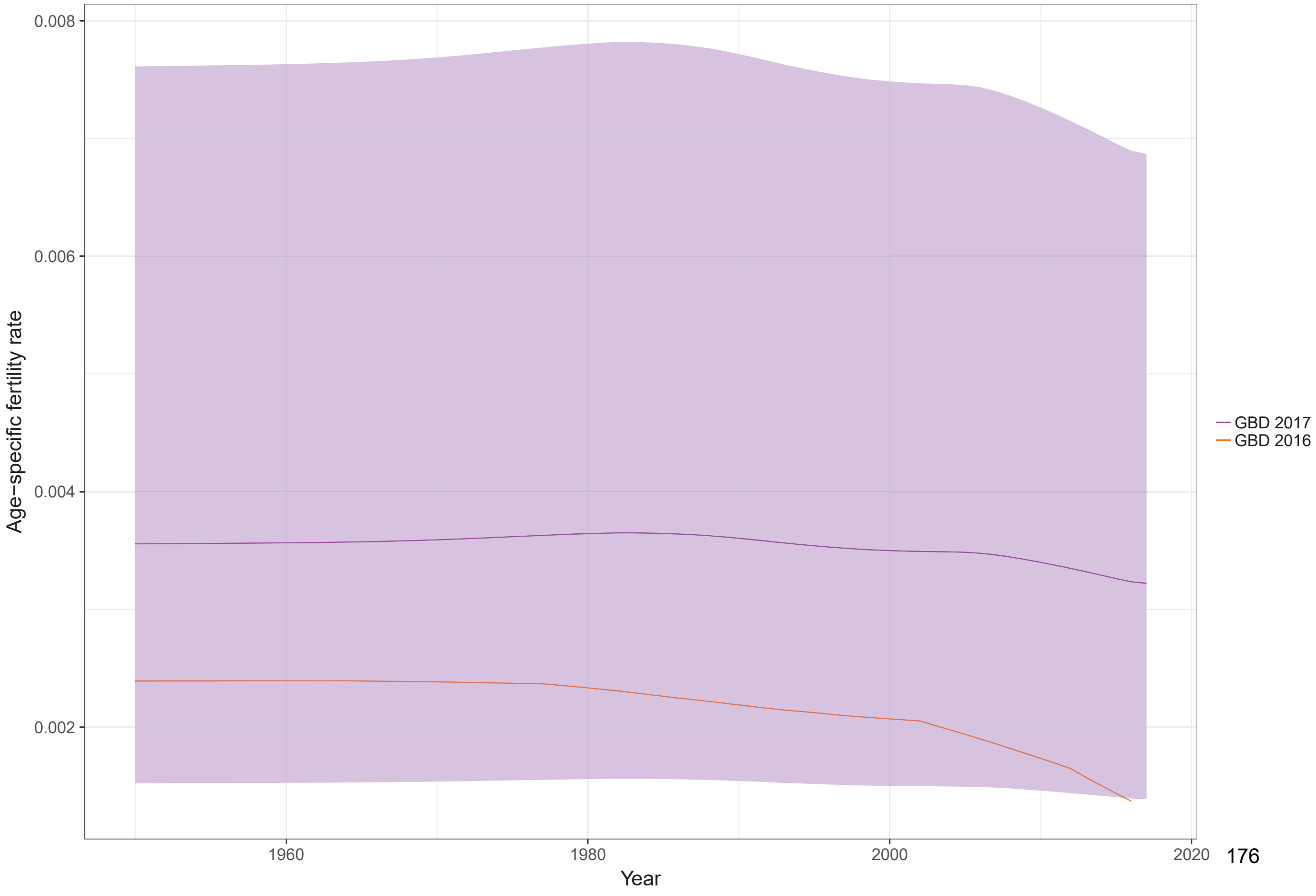

Age−specific fertility fitted trend lines, 1950−2017: Chad, Ages: 15−19
Data points enclosed with a diamond are reference sources

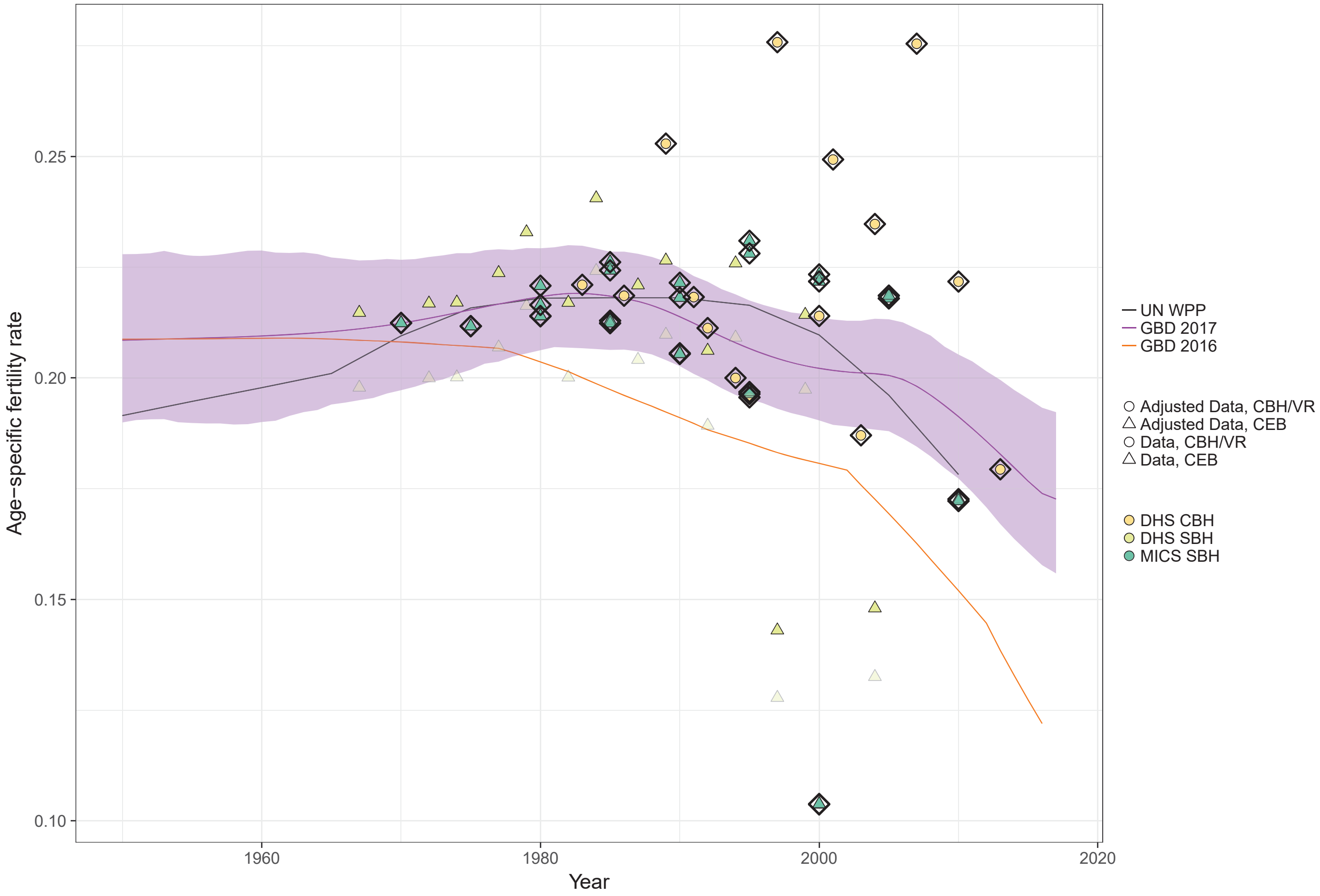

Age−specific fertility fitted trend lines, 1950−2017: Chad, Ages: 20−24
Data points enclosed with a diamond are reference sources

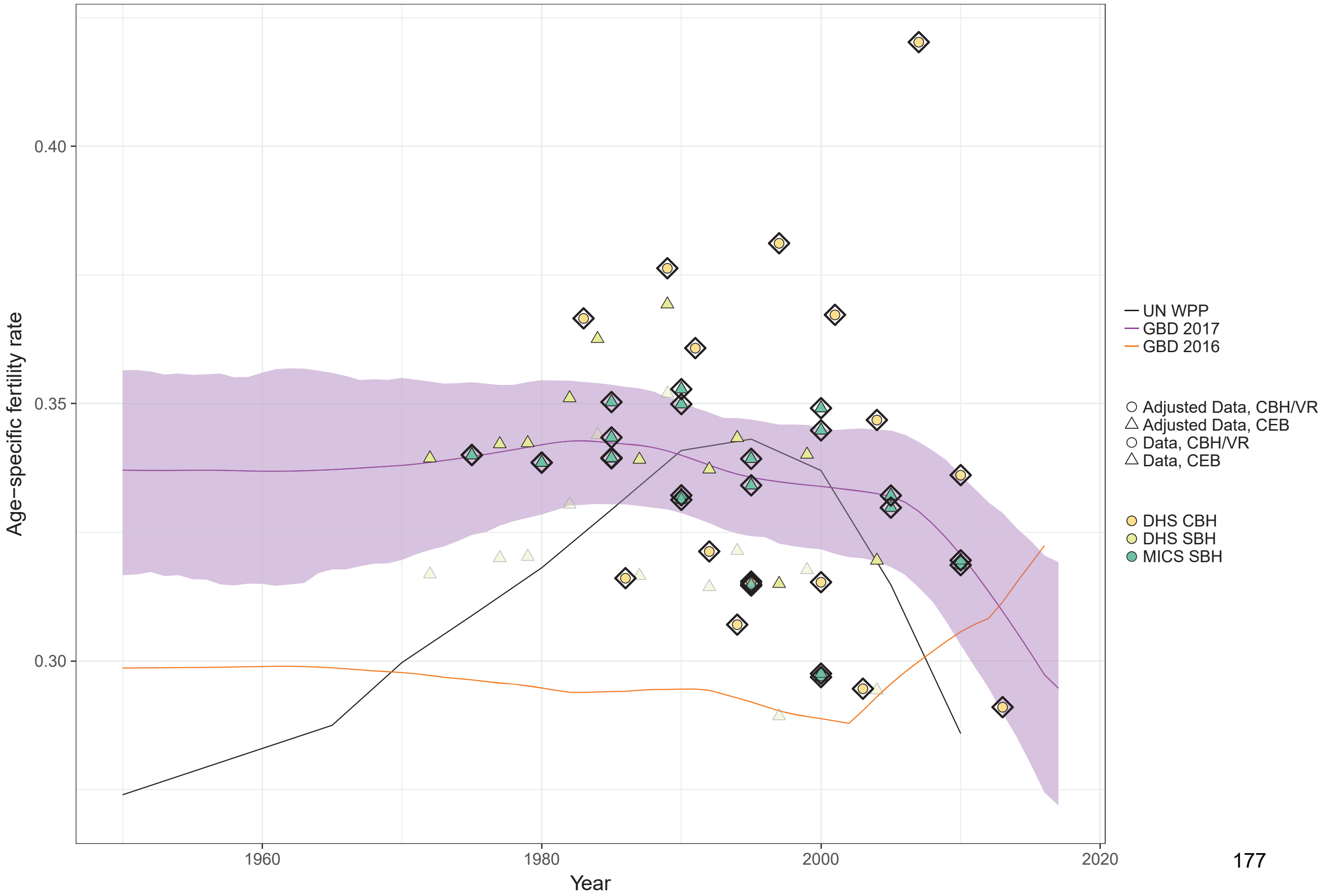

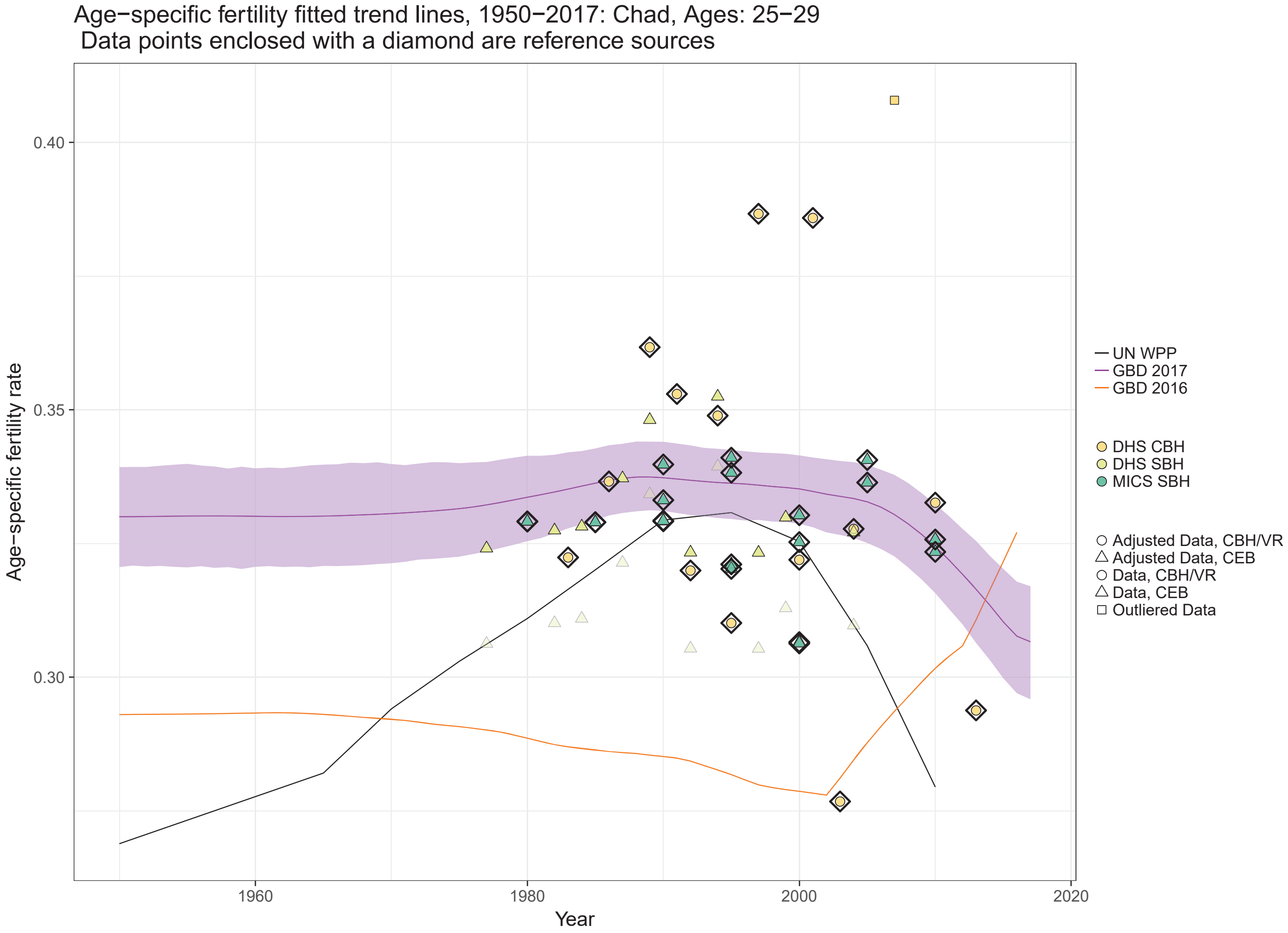

Age−specific fertility fitted trend lines, 1950−2017: Chad, Ages: 25−29
Data points enclosed with a diamond are reference sources
Age-specific fertility rate
0.30
0.35
0.40
1960
1980
2000
2020
Year
UN WPP
GBD 2017
GBD 2016
DHS CBH
DHS SBH
MICS SBH
Adjusted Data, CBH/VR
Adjusted Data, CEB
Data, CBH/VR
Data, CEB
Outliered Data

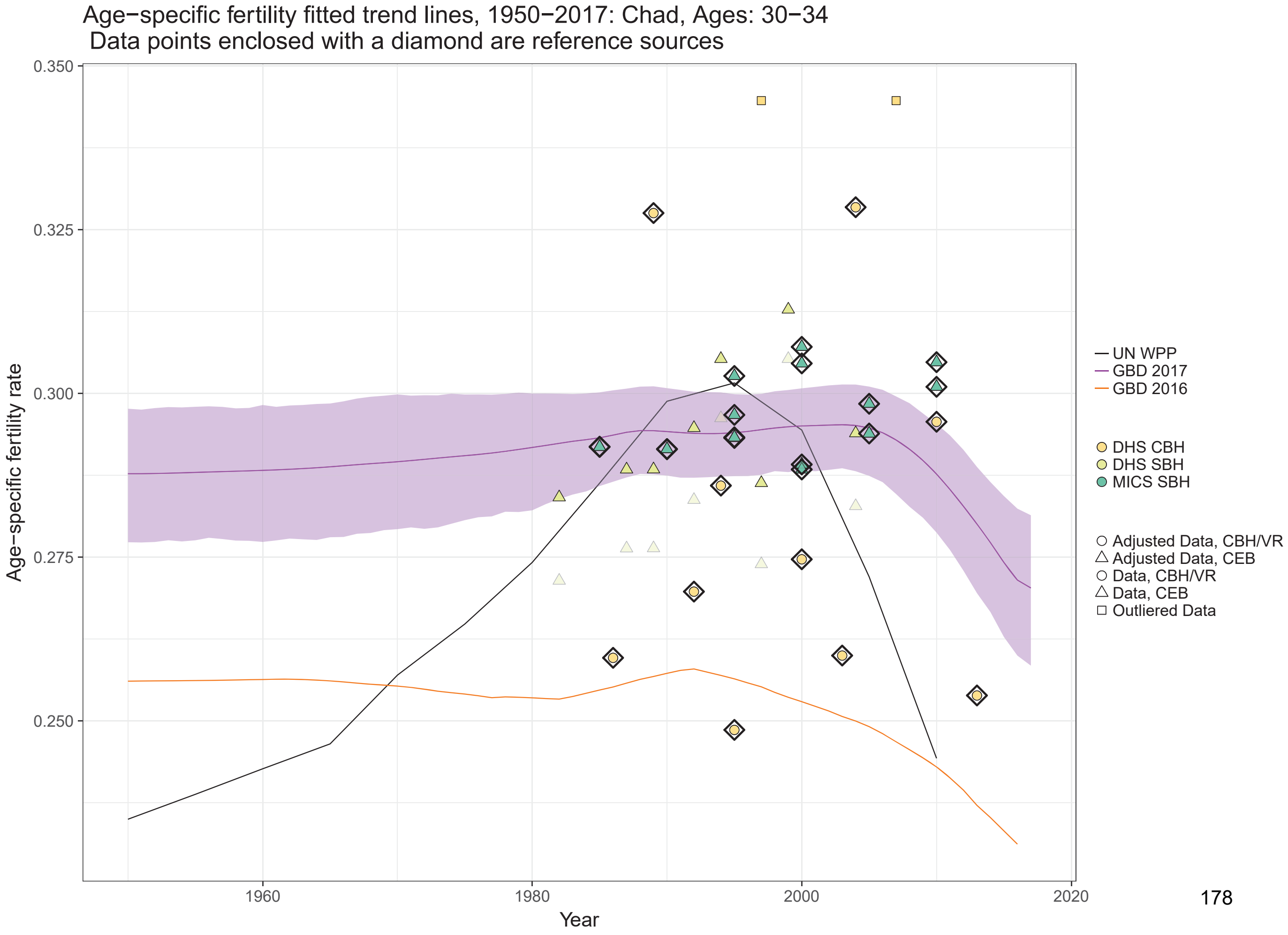

Age−specific fertility fitted trend lines, 1950−2017: Chad, Ages: 30−34
Data points enclosed with a diamond are reference sources
Age-specific fertility rate
0.250
0.275
0.300
0.325
0.350
1960
1980
2000
2020
Year
UN WPP
GBD 2017
GBD 2016
DHS CBH
DHS SBH
MICS SBH
Adjusted Data, CBH/VR
Adjusted Data, CEB
Data, CBH/VR
Data, CEB
Outliered Data

Age−specific fertility fitted trend lines, 1950−2017: Chad, Ages: 35−39
Data points enclosed with a diamond are reference sources

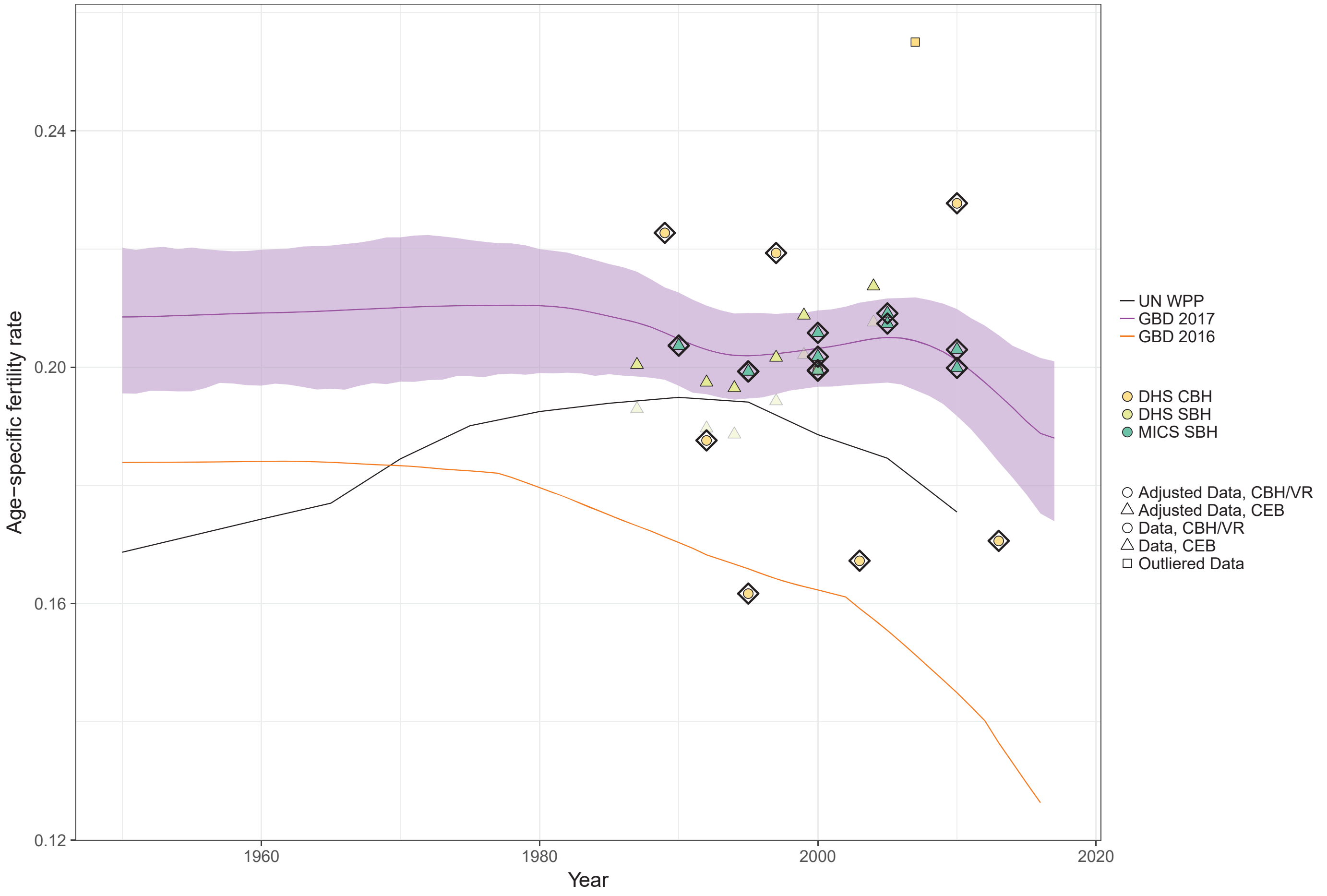

Age−specific fertility fitted trend lines, 1950−2017: Chad, Ages: 40−44
Data points enclosed with a diamond are reference sources

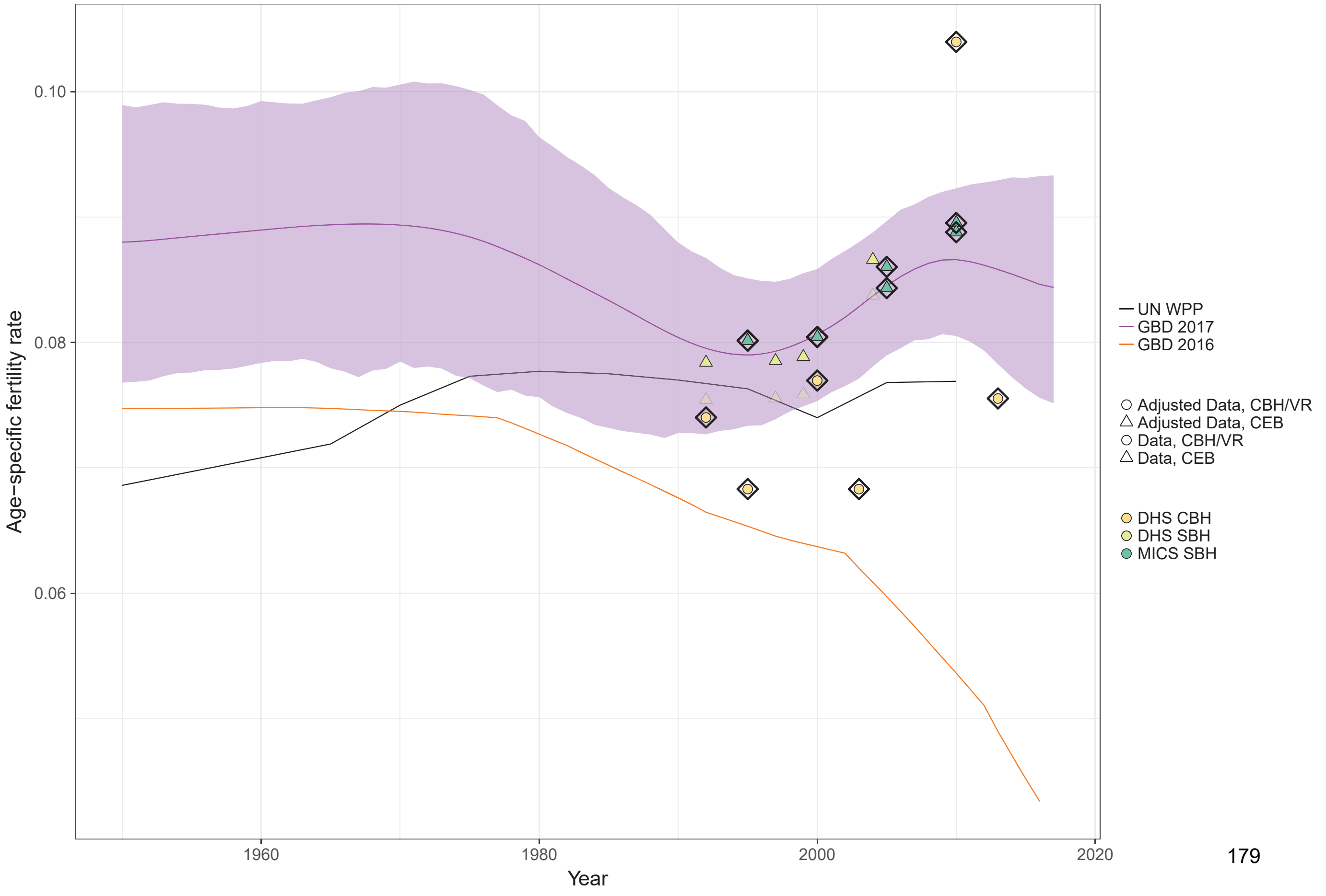

Age−specific fertility fitted trend lines, 1950−2017: Chad, Ages: 45−49
Data points enclosed with a diamond are reference sources

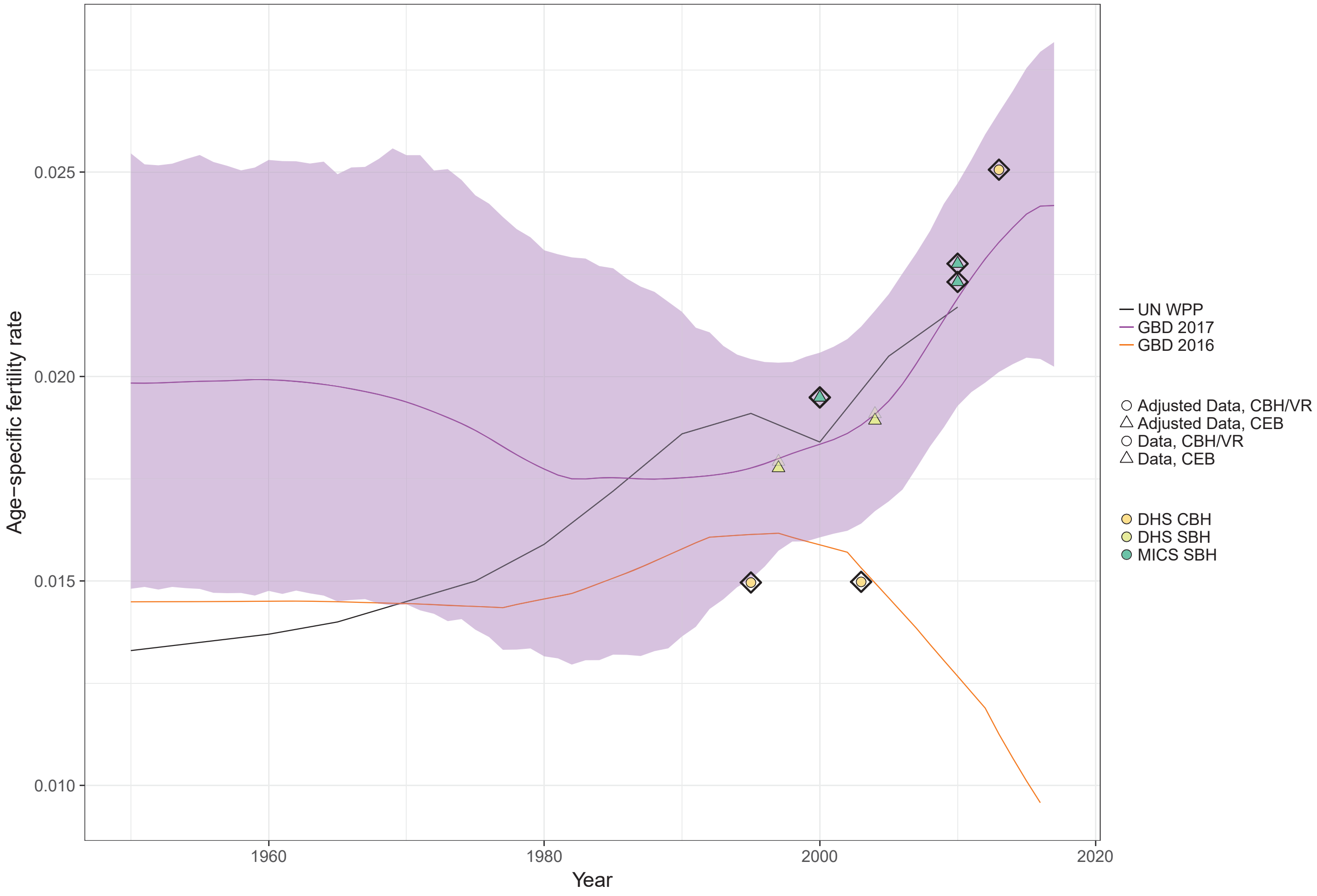

Age−specific fertility fitted trend lines, 1950−2017: Chad, Ages: 50−54
Data points enclosed with a diamond are reference sources

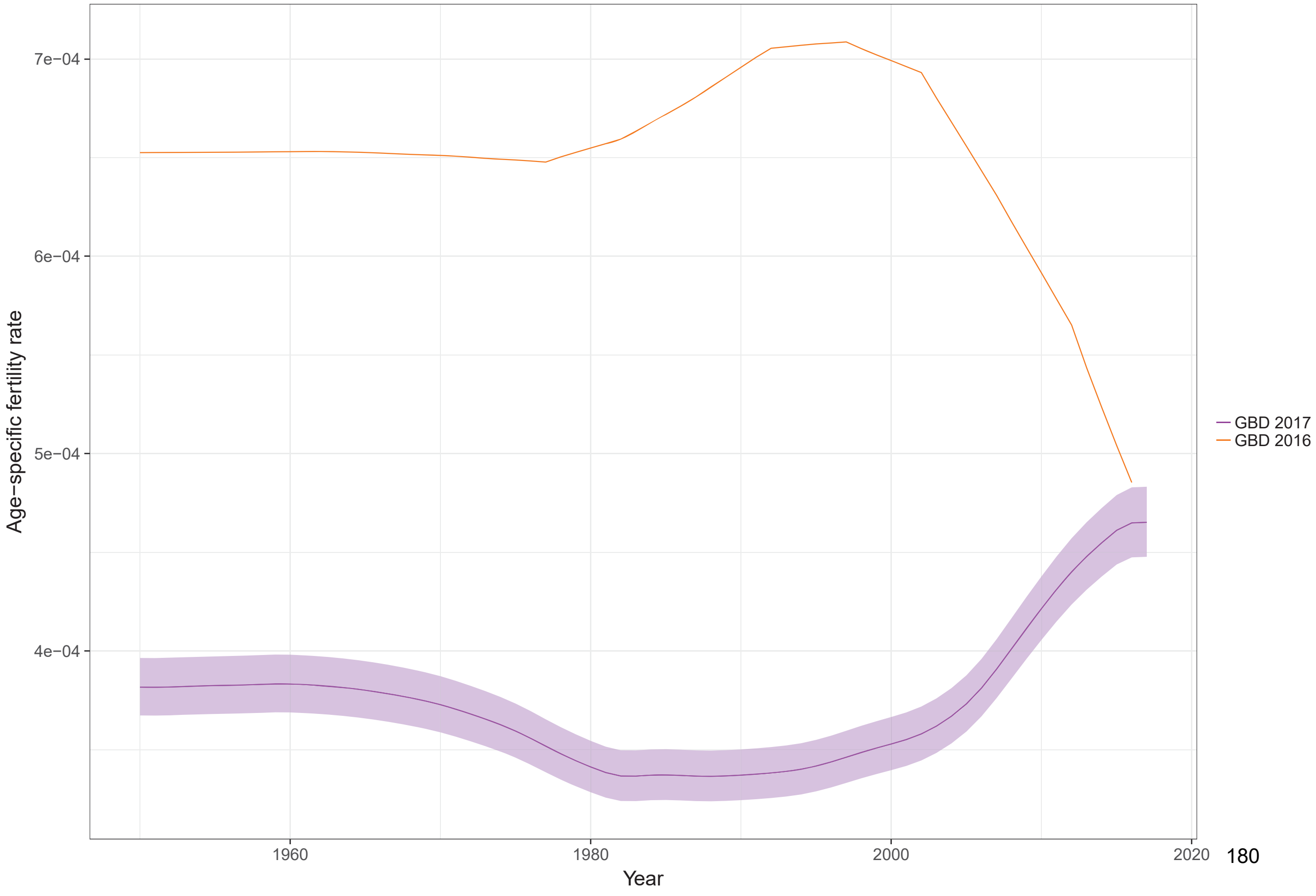

Age−specific fertility fitted trend lines, 1950−2017: Chile, TFR
Data points enclosed with a diamond are reference sources

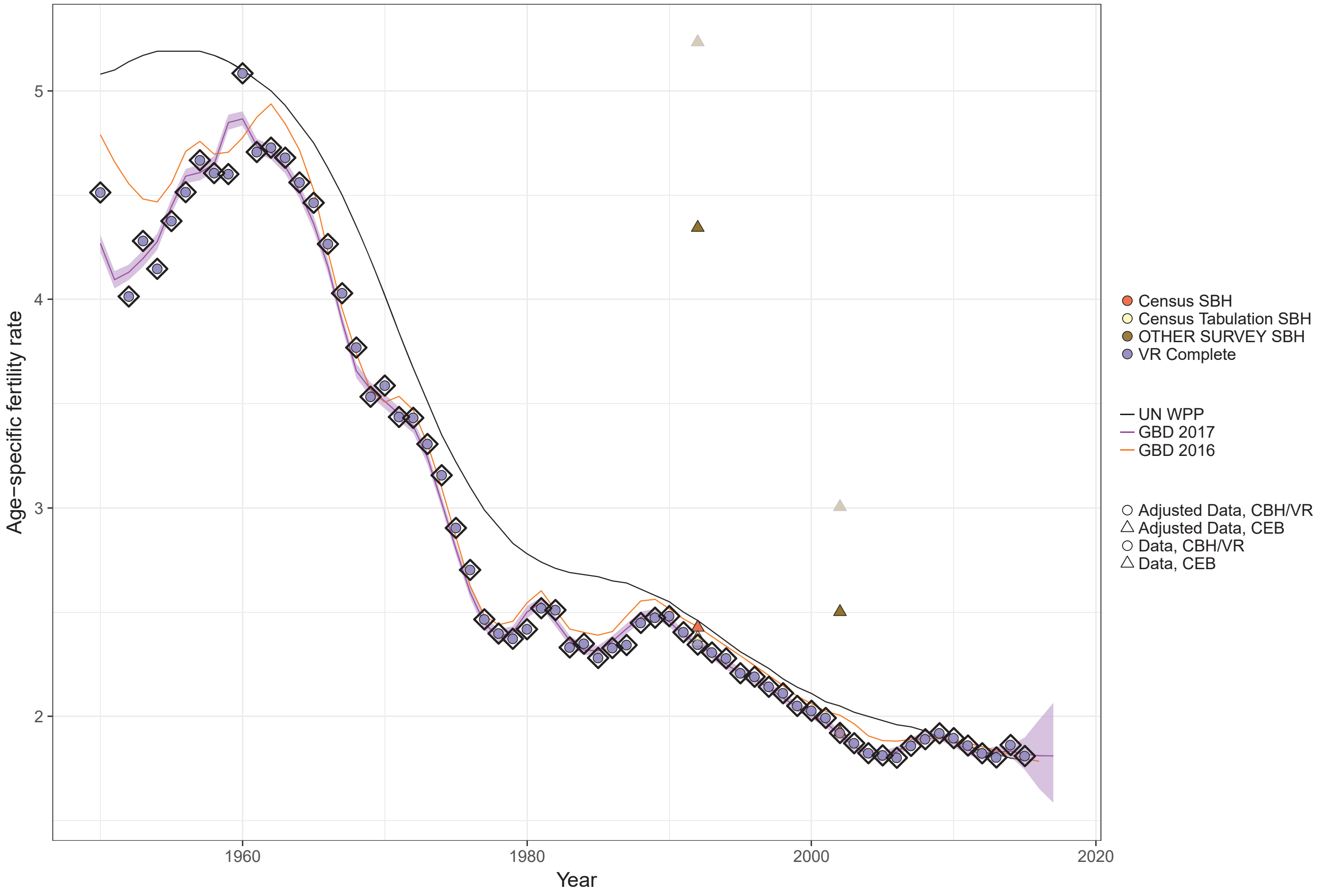

Age−specific fertility fitted trend lines, 1950−2017: Chile, Ages: 10−14
Data points enclosed with a diamond are reference sources

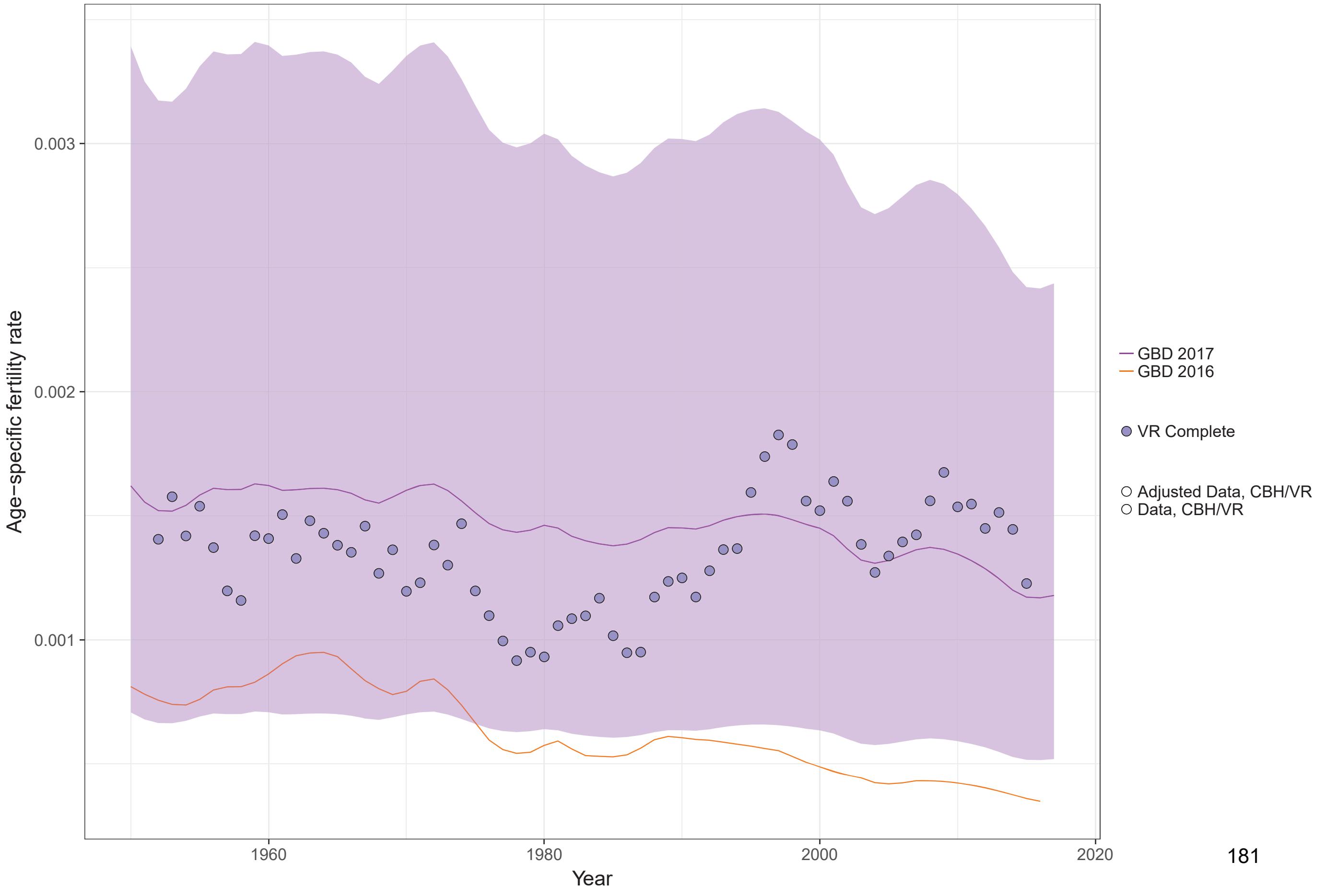

Age−specific fertility fitted trend lines, 1950−2017: Chile, Ages: 15−19
Data points enclosed with a diamond are reference sources

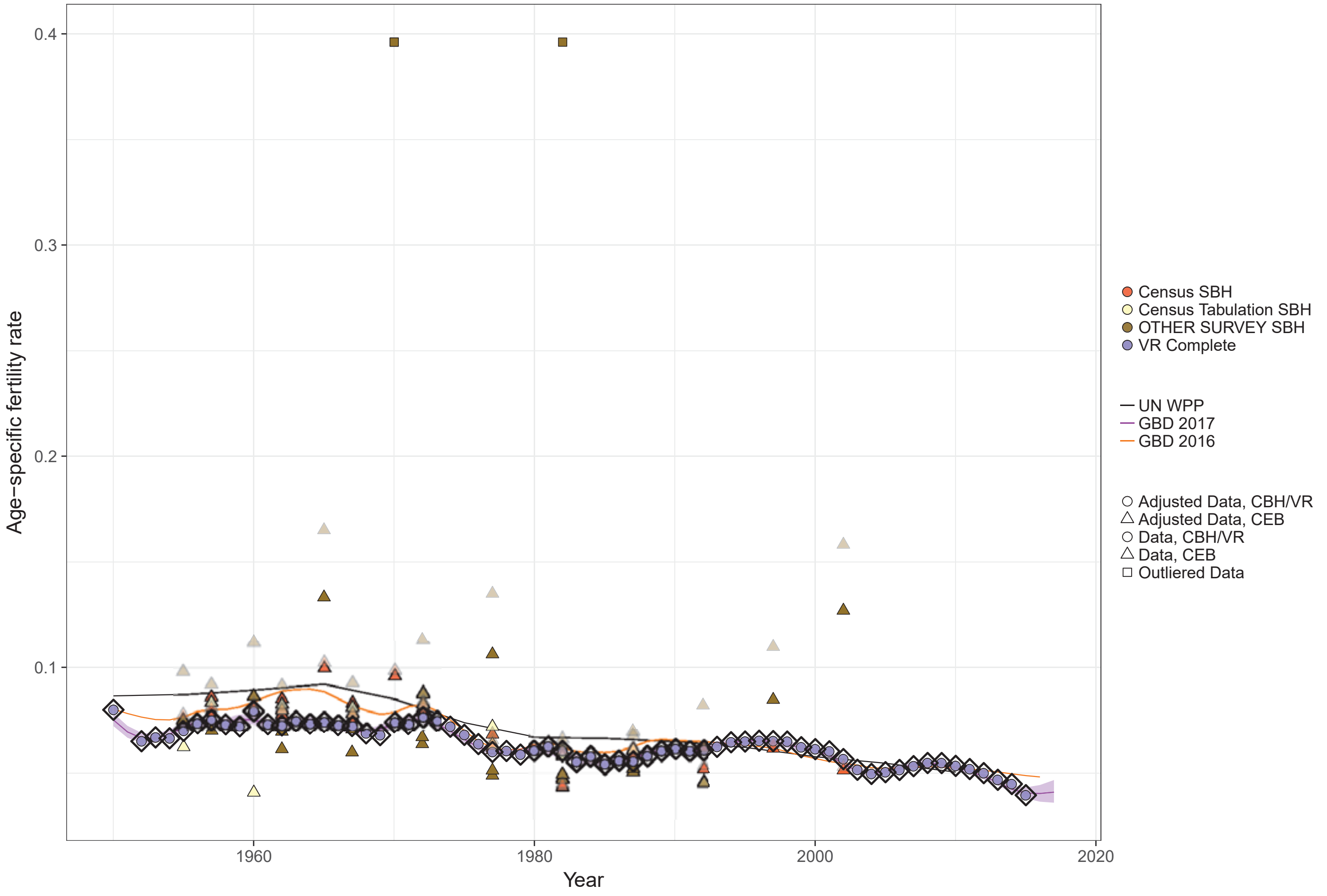

Age−specific fertility fitted trend lines, 1950−2017: Chile, Ages: 20−24
Data points enclosed with a diamond are reference sources

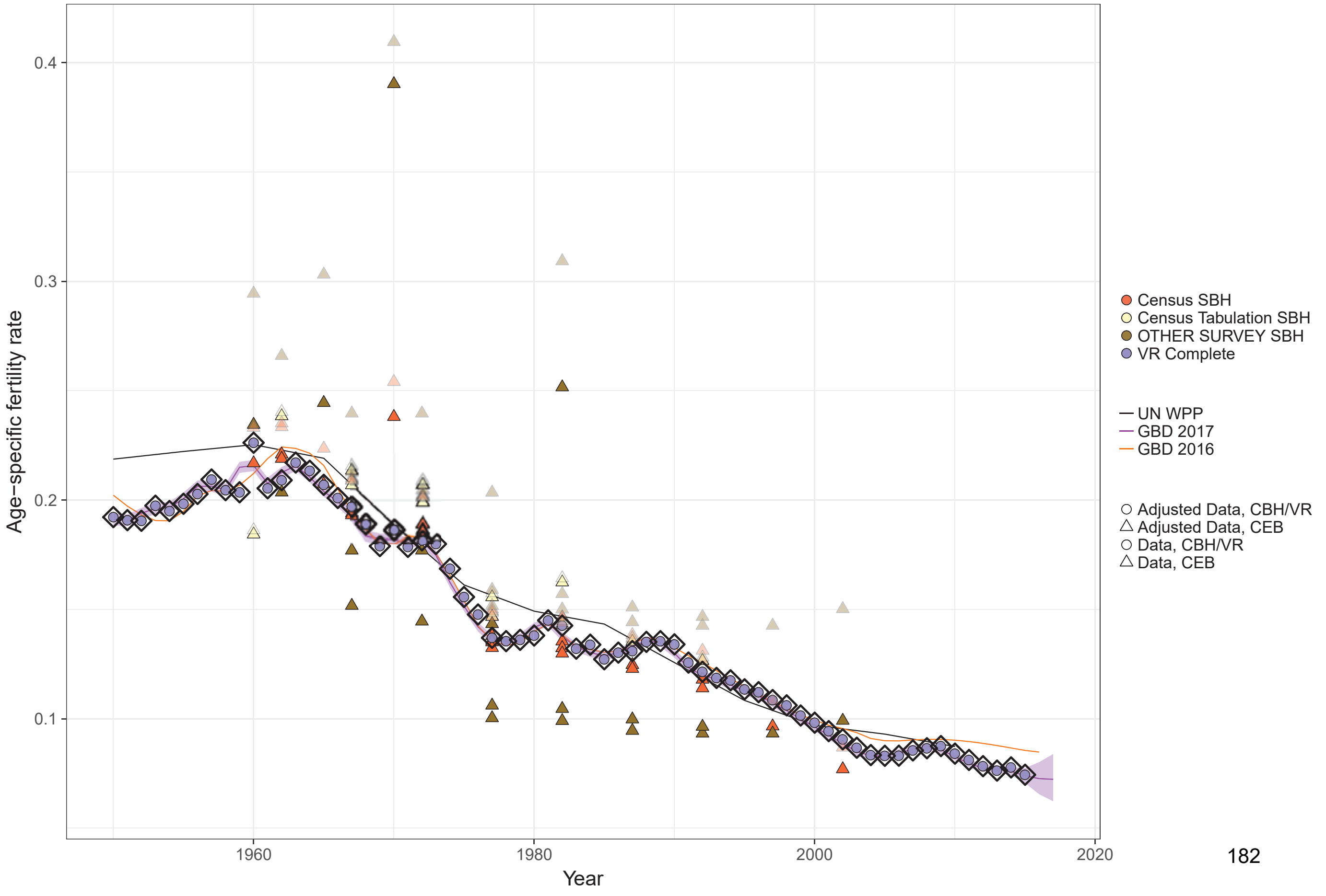

Age−specific fertility fitted trend lines, 1950−2017: Chile, Ages: 25−29
Data points enclosed with a diamond are reference sources

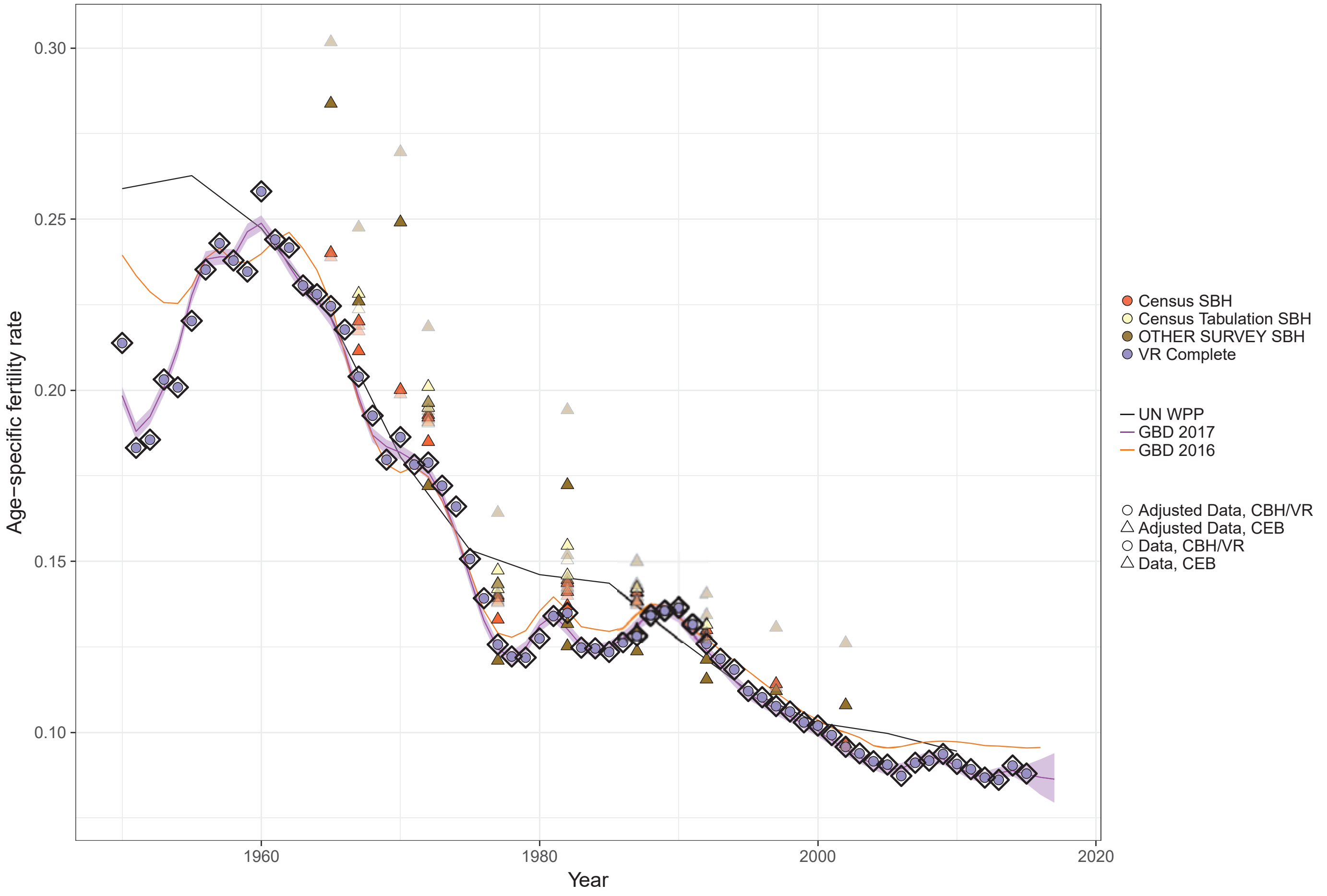

Age−specific fertility fitted trend lines, 1950−2017: Chile, Ages: 30−34
Data points enclosed with a diamond are reference sources

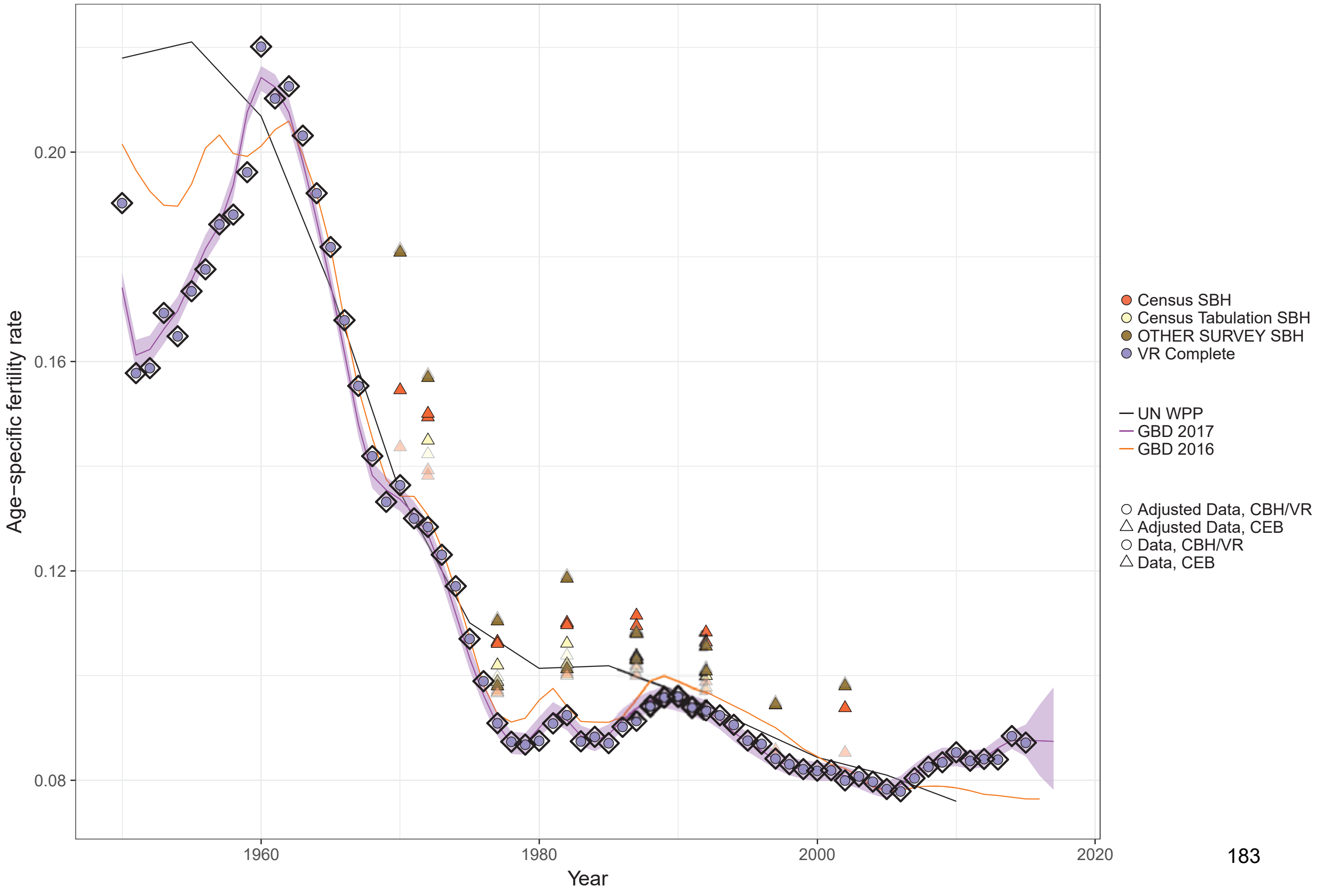

Age−specific fertility fitted trend lines, 1950−2017: Chile, Ages: 35−39
Data points enclosed with a diamond are reference sources

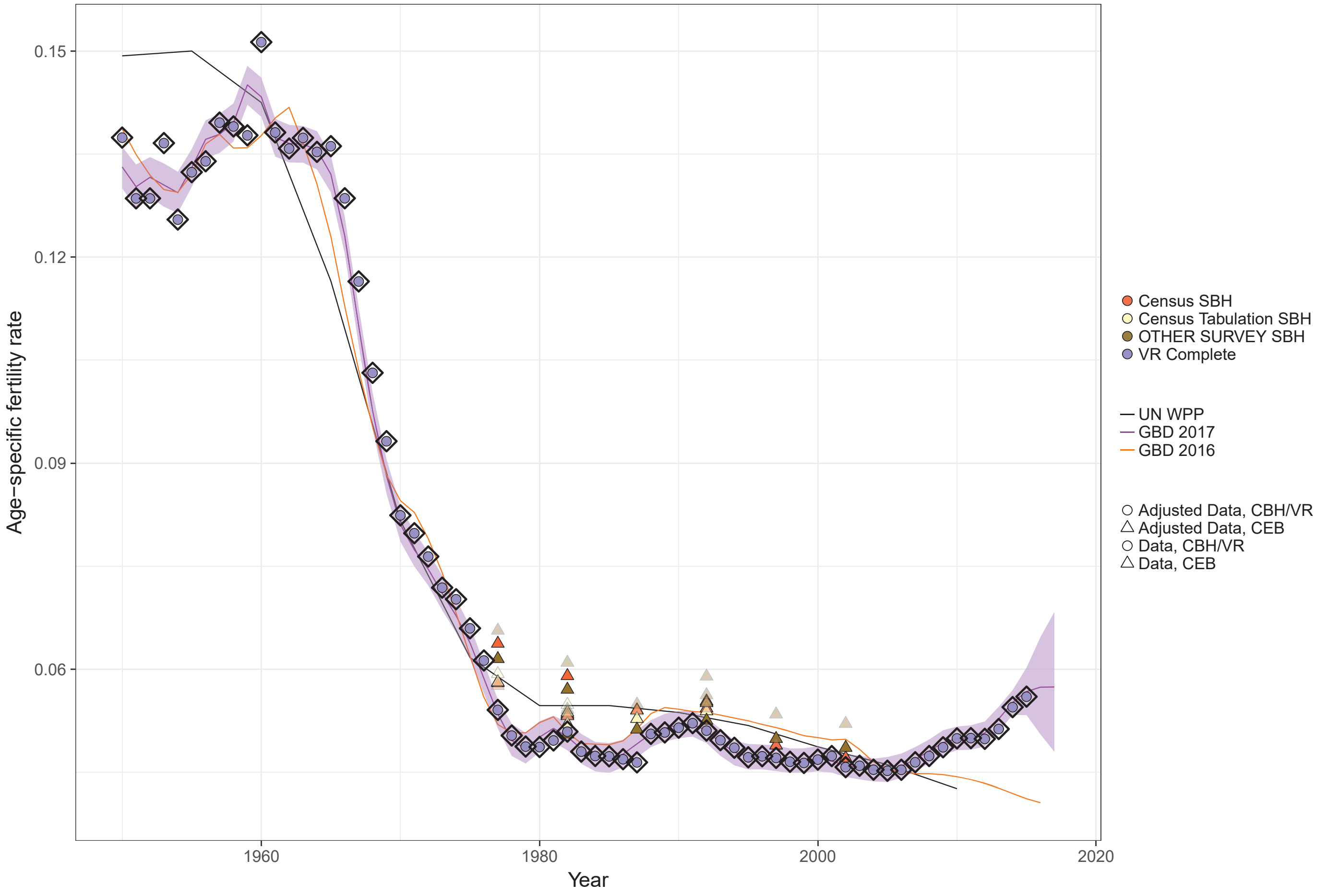

Age−specific fertility fitted trend lines, 1950−2017: Chile, Ages: 40−44
Data points enclosed with a diamond are reference sources

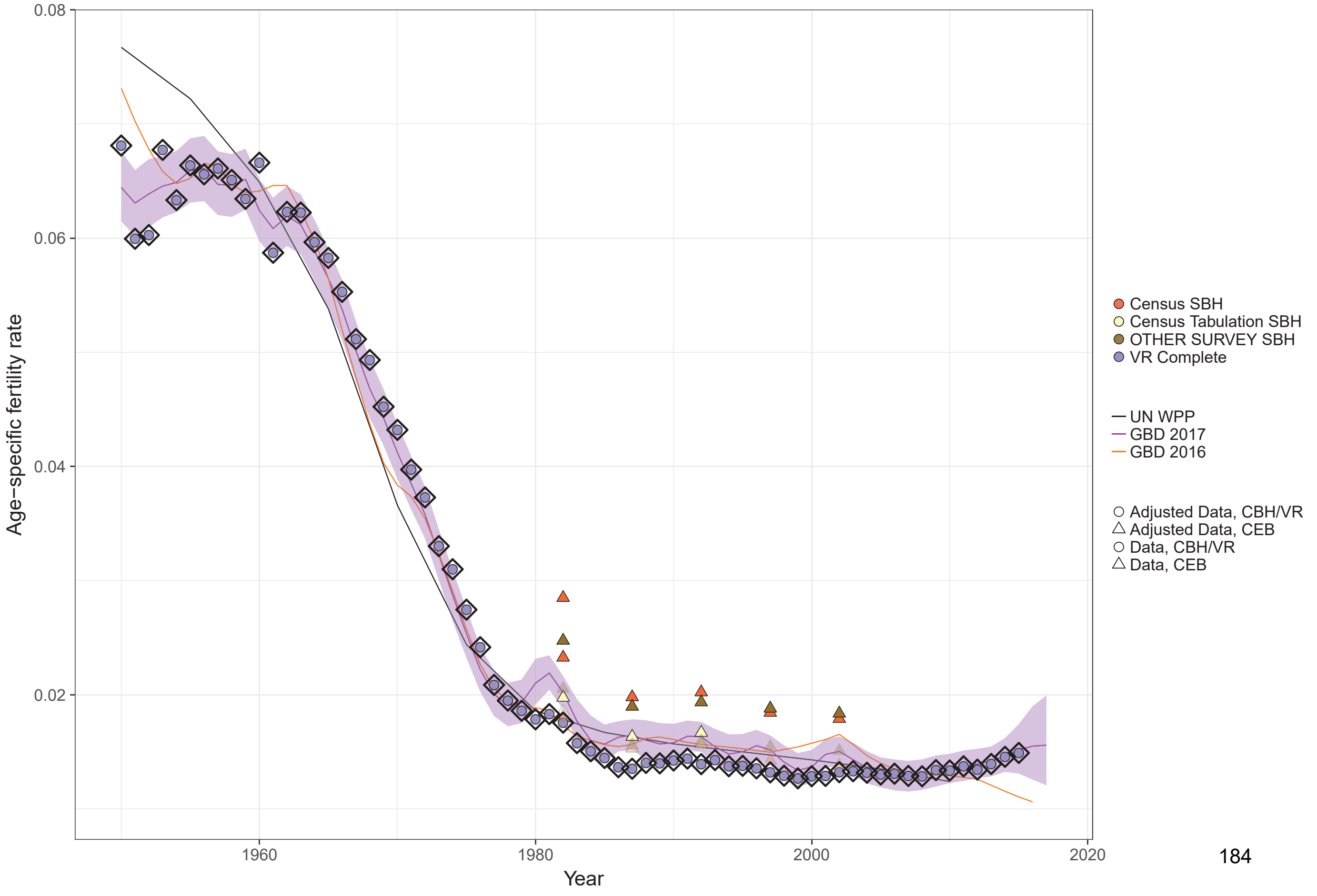

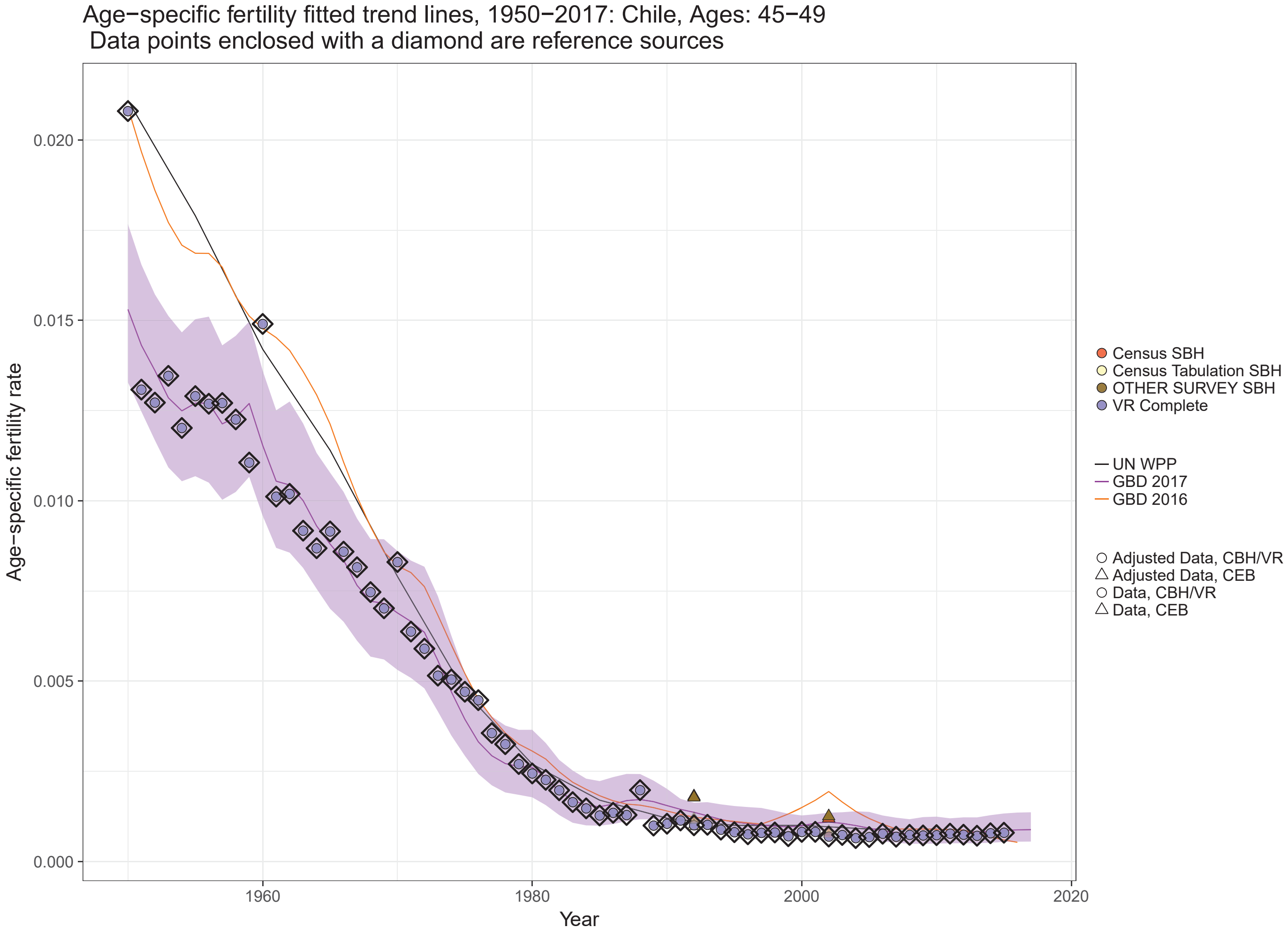

Age−specific fertility fitted trend lines, 1950−2017: Chile, Ages: 45−49
Data points enclosed with a diamond are reference sources
Age−specific fertility rate
0.000
0.005
0.010
0.015
0.020
1960
1980
2000
2020
Year
Census SBH
Census Tabulation SBH
OTHER SURVEY SBH
VR Complete
UN WPP
GBD 2017
GBD 2016
Adjusted Data, CBH/VR
Adjusted Data, CEB
Data, CBH/VR
Data, CEB

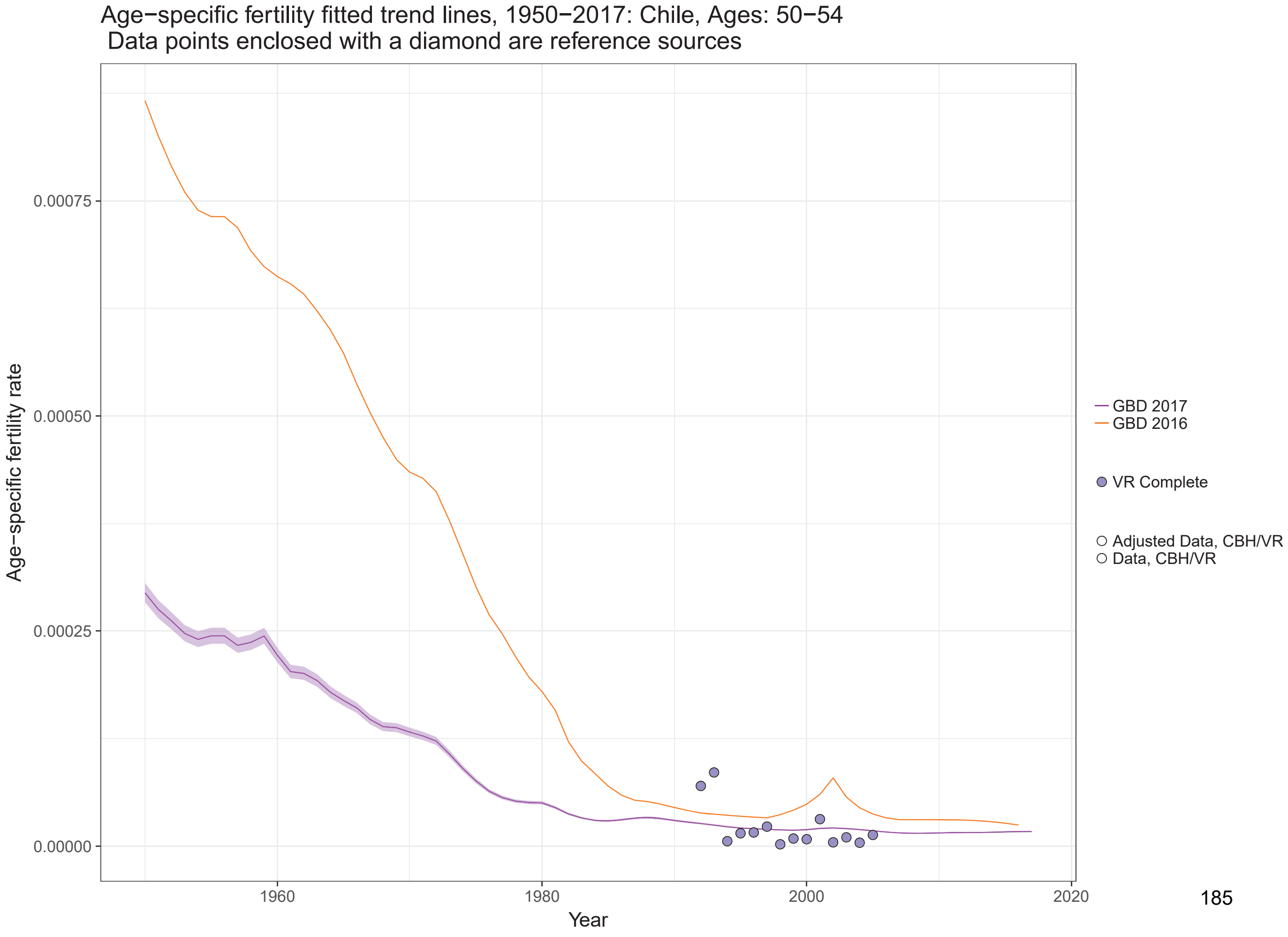

Age−specific fertility fitted trend lines, 1950−2017: Chile, Ages: 50−54
Data points enclosed with a diamond are reference sources
Age−specific fertility rate
0.00000
0.00025
0.00050
0.00075
1960
1980
2000
2020
Year
GBD 2017
GBD 2016
VR Complete
Adjusted Data, CBH/VR
Data, CBH/VR

Age−specific fertility fitted trend lines, 1950−2017: China, TFR
Data points enclosed with a diamond are reference sources

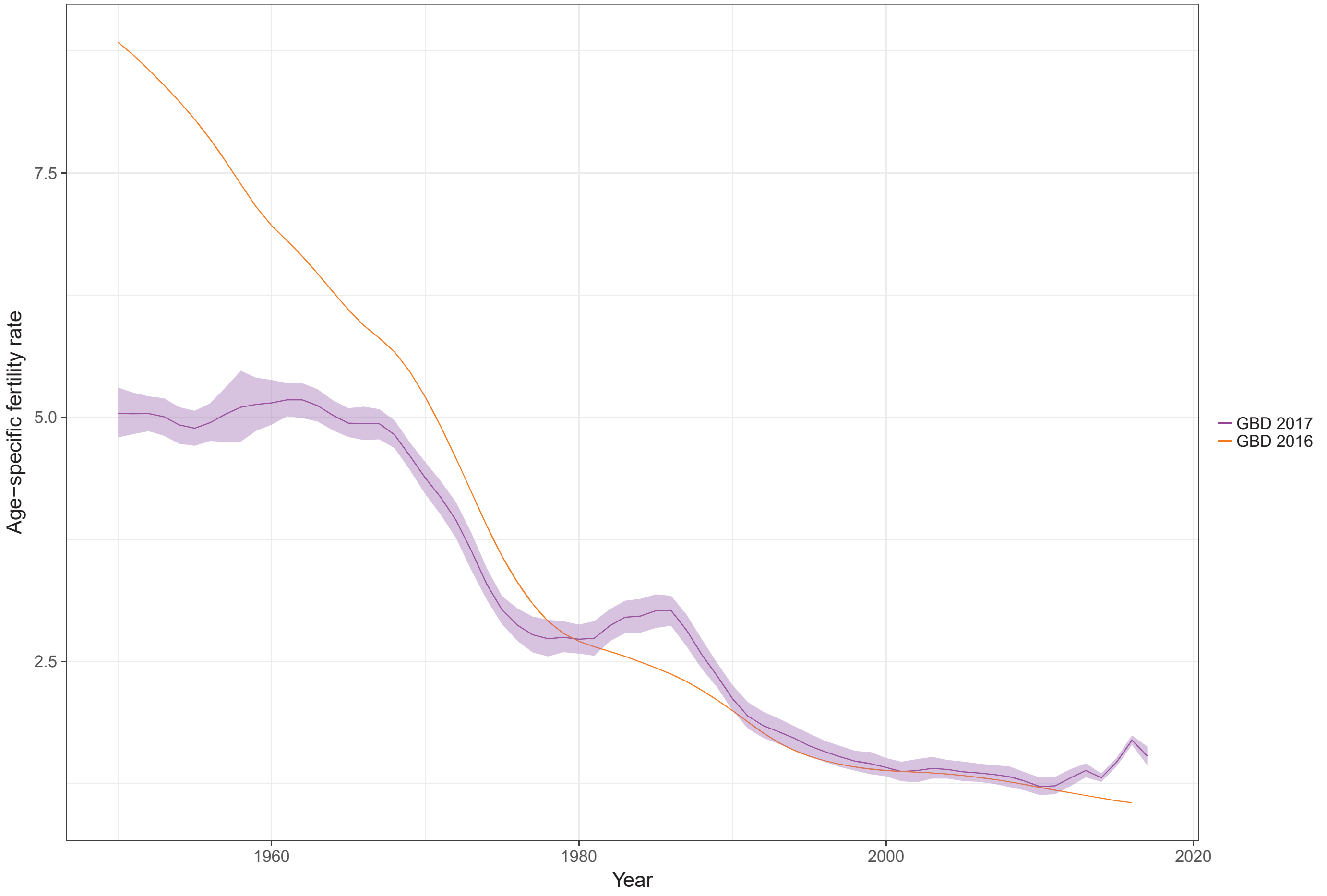

Age−specific fertility fitted trend lines, 1950−2017: China, Ages: 10−14
Data points enclosed with a diamond are reference sources

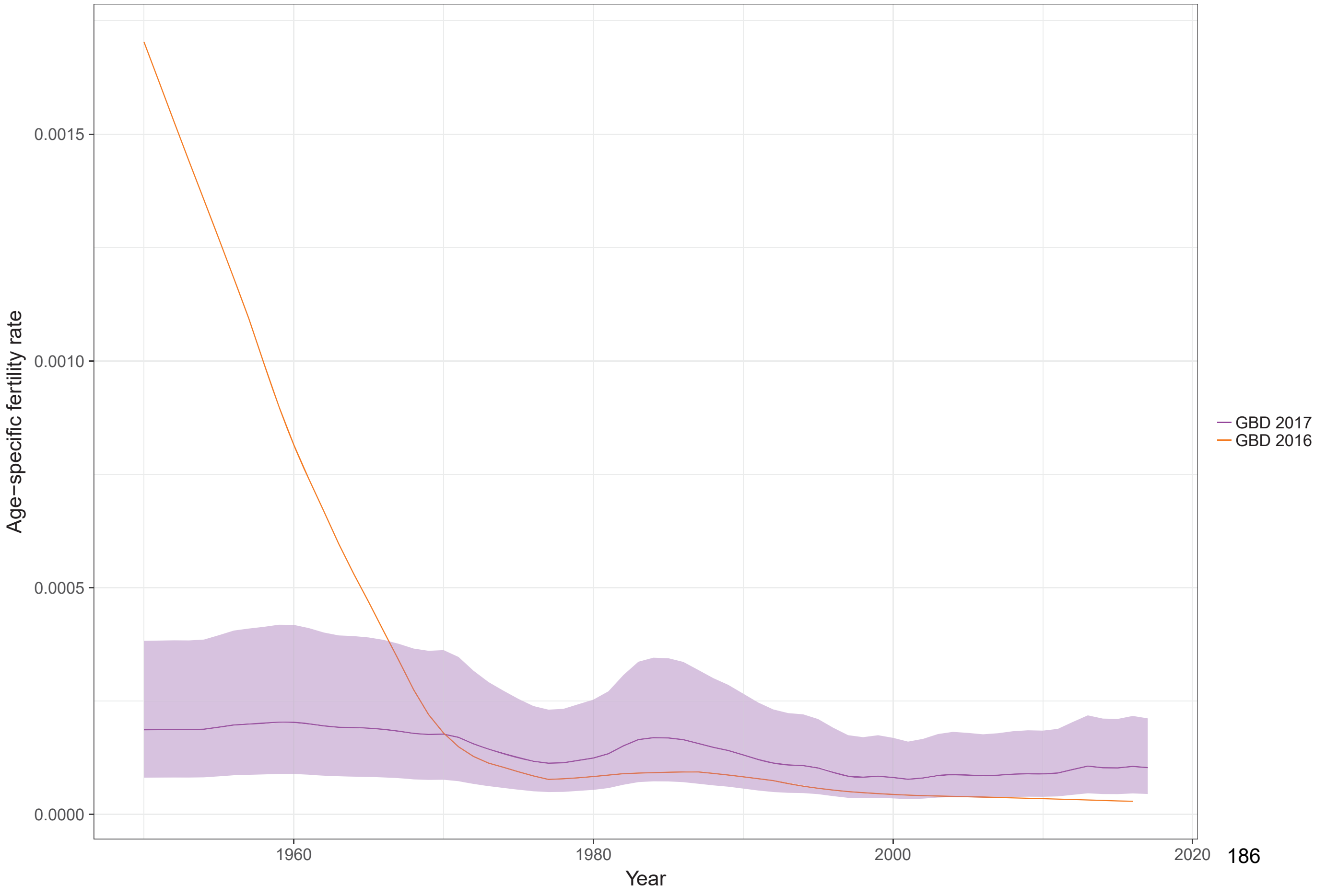

Age−specific fertility fitted trend lines, 1950−2017: China, Ages: 15−19
Data points enclosed with a diamond are reference sources

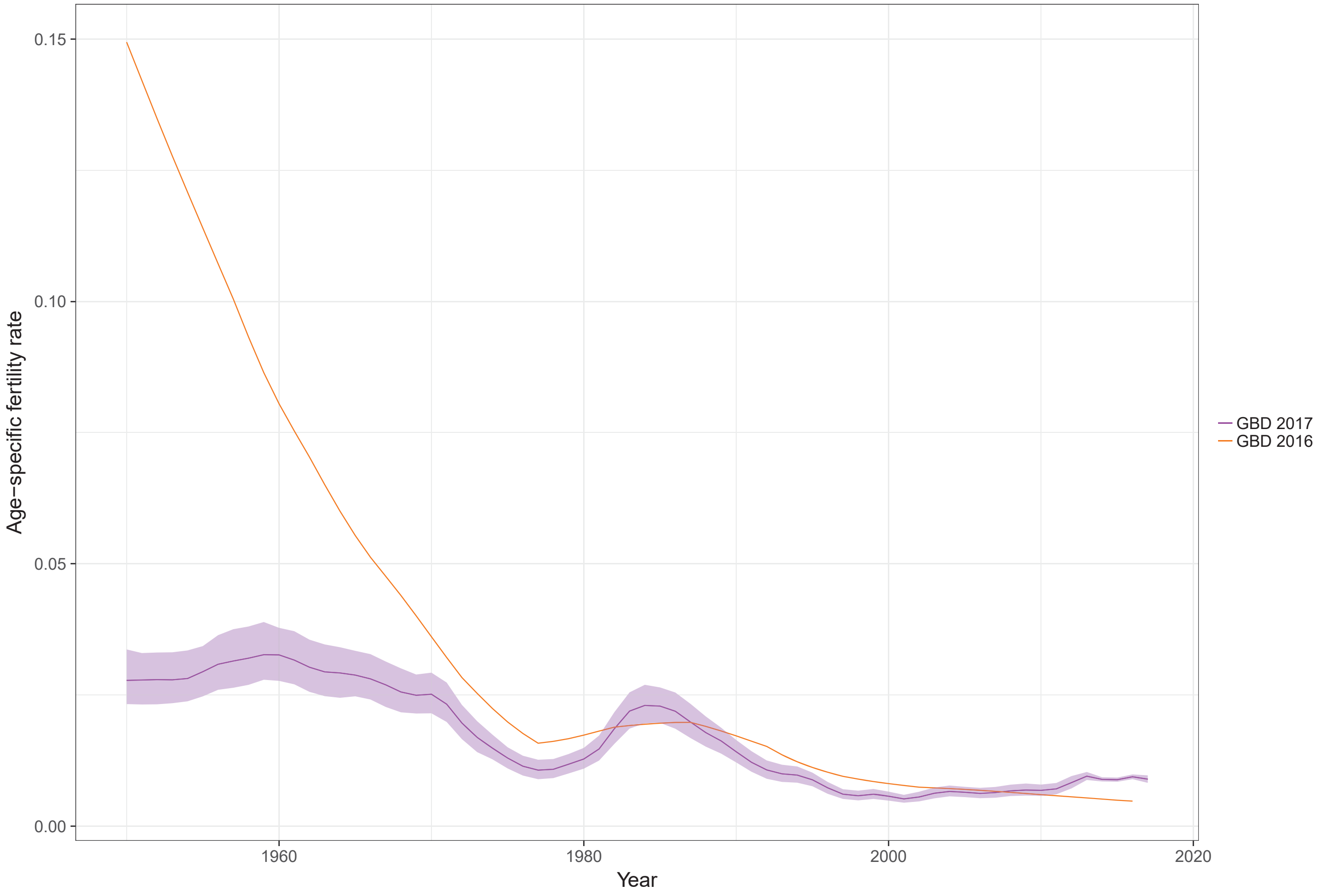

Age−specific fertility fitted trend lines, 1950−2017: China, Ages: 20−24
Data points enclosed with a diamond are reference sources

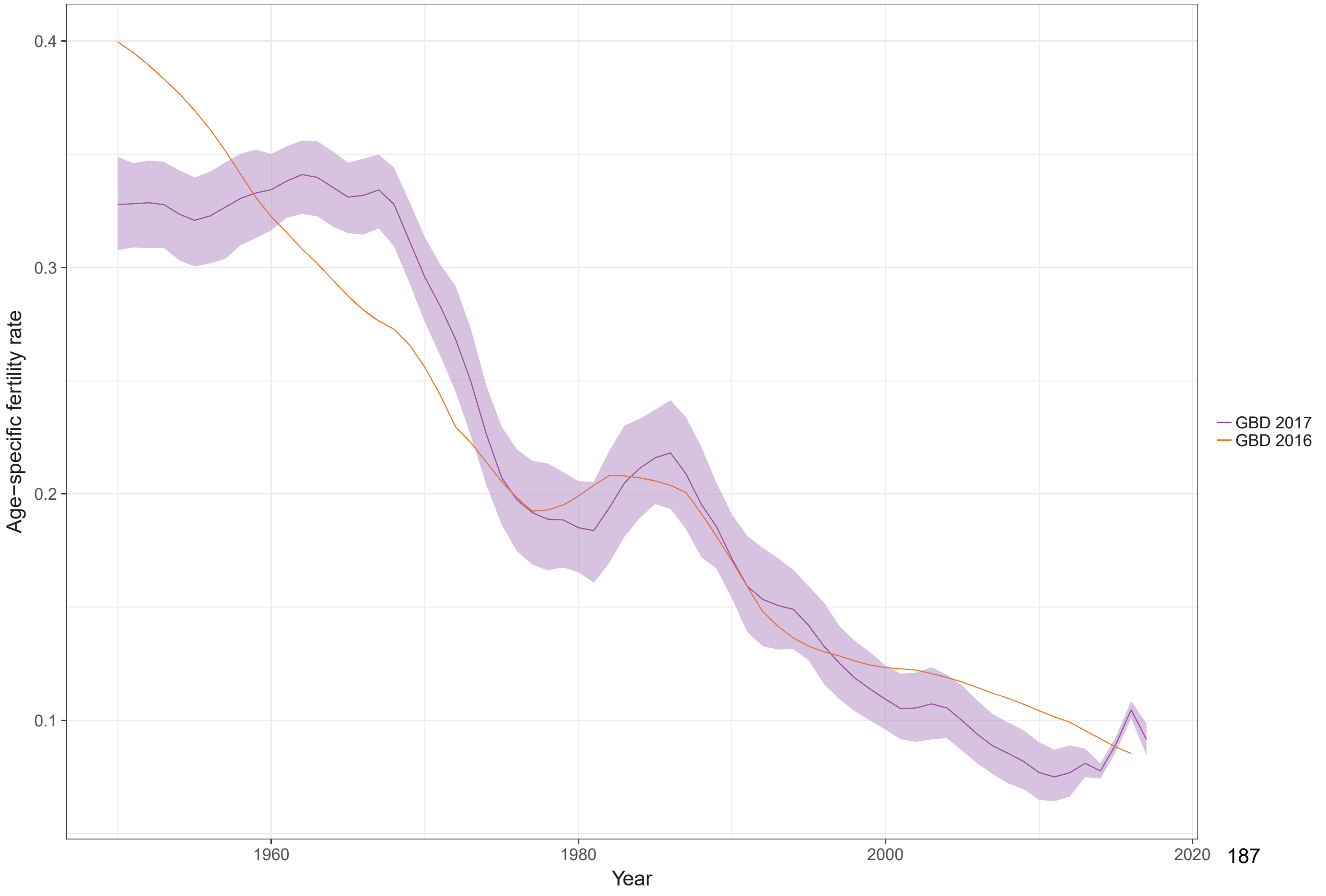

Age−specific fertility fitted trend lines, 1950−2017: China, Ages: 25−29
Data points enclosed with a diamond are reference sources

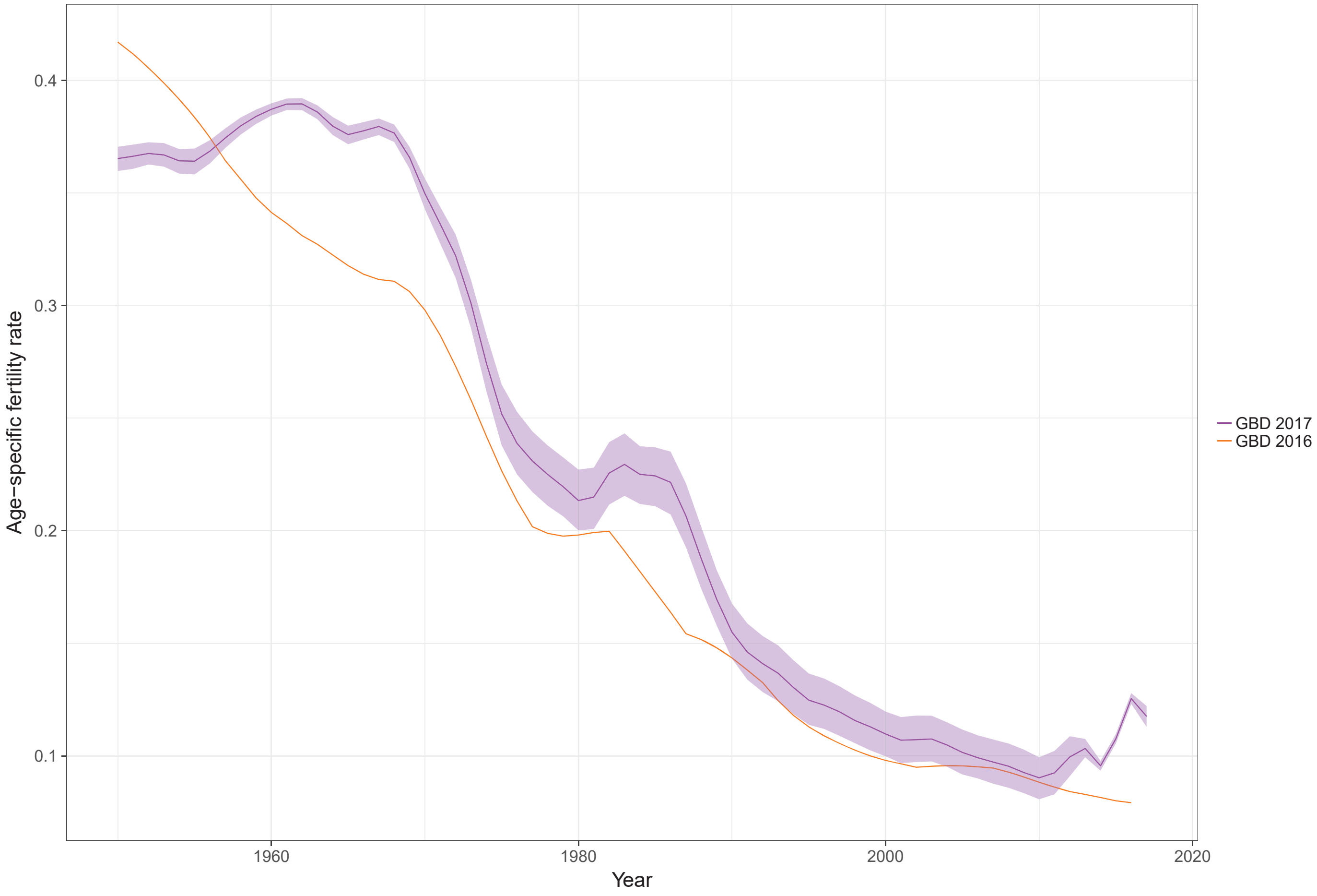

Age−specific fertility fitted trend lines, 1950−2017: China, Ages: 30−34
Data points enclosed with a diamond are reference sources

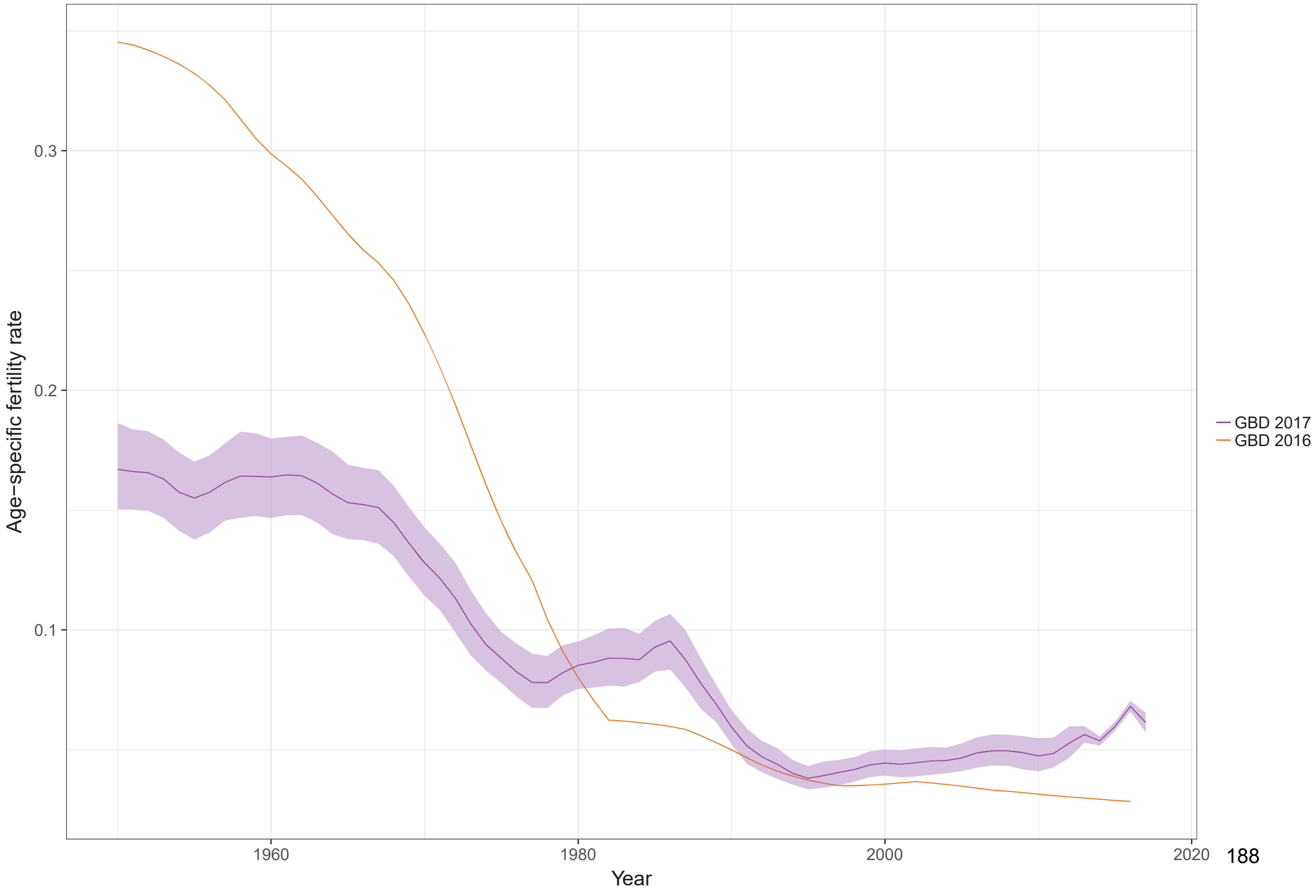

Age−specific fertility fitted trend lines, 1950−2017: China, Ages: 35−39
Data points enclosed with a diamond are reference sources

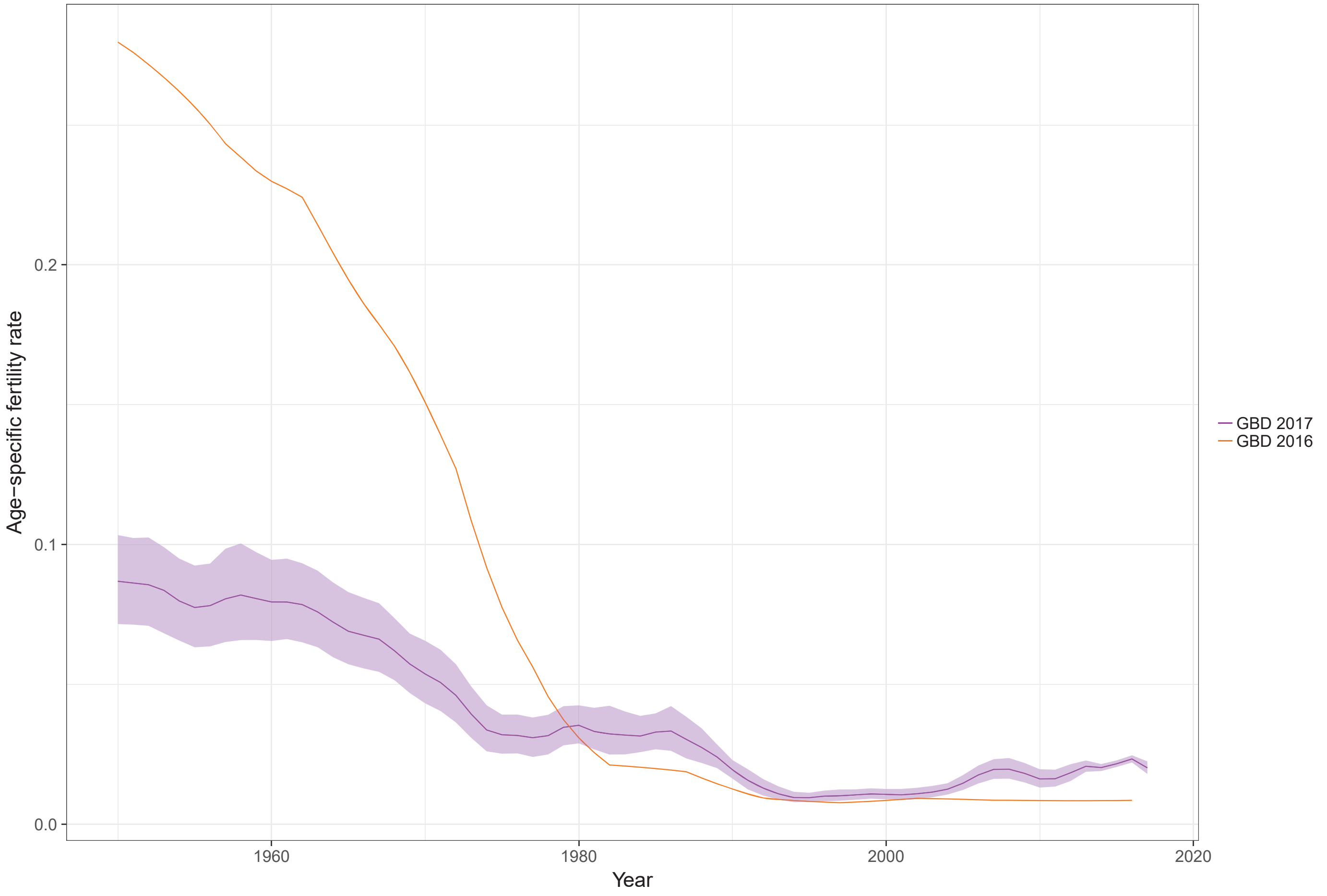

Age−specific fertility fitted trend lines, 1950−2017: China, Ages: 40−44
Data points enclosed with a diamond are reference sources

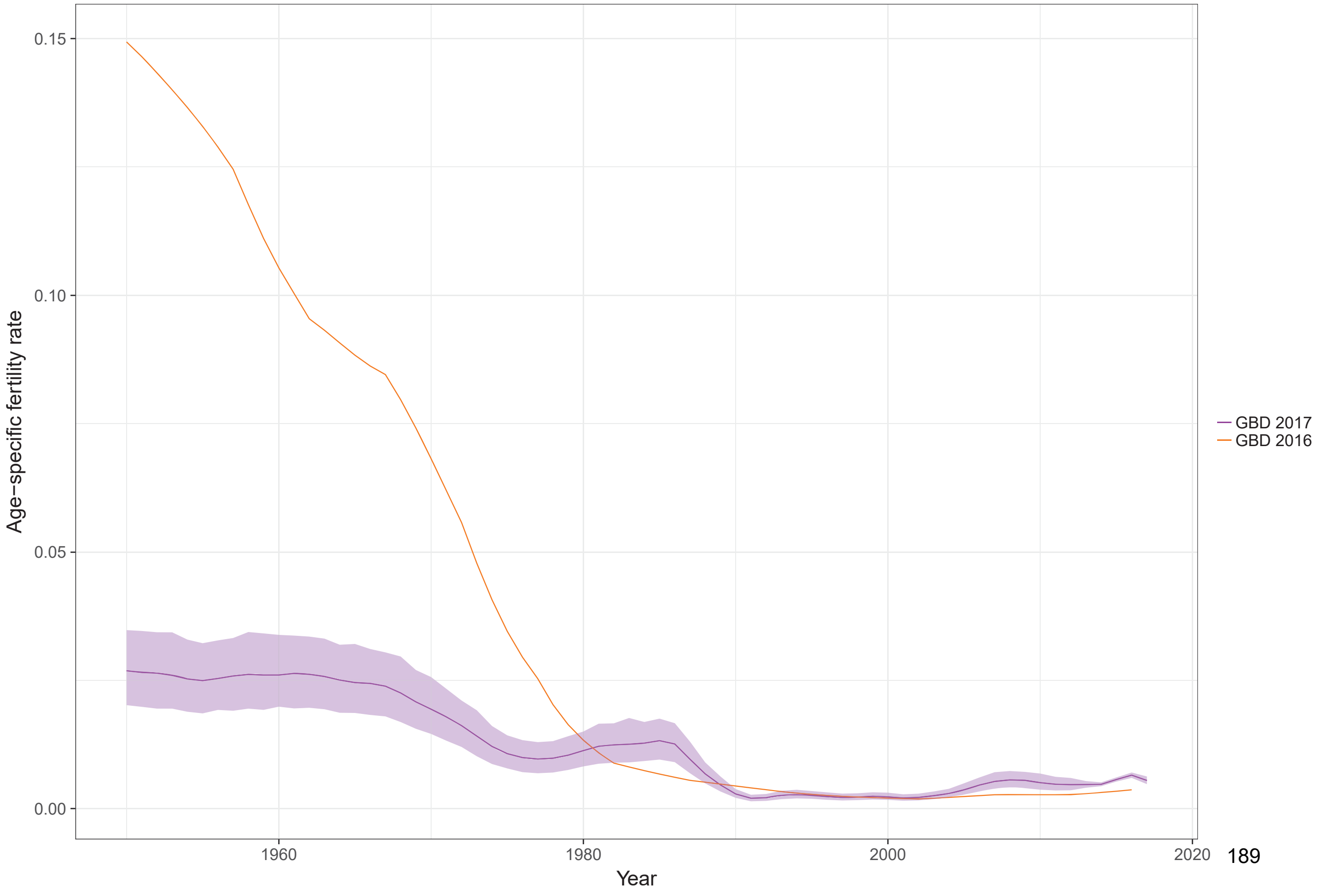

Age−specific fertility fitted trend lines, 1950−2017: China, Ages: 45−49
Data points enclosed with a diamond are reference sources

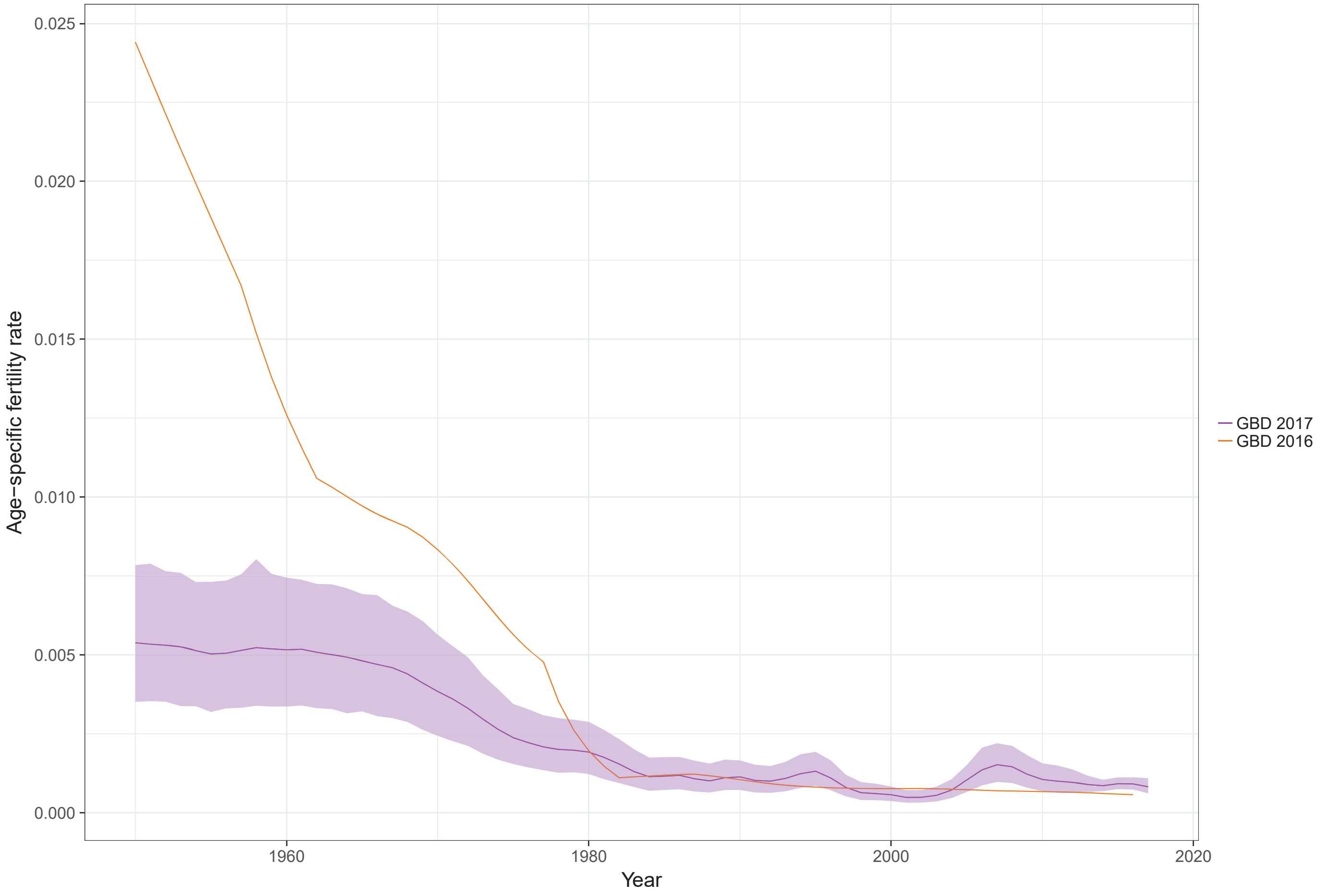

Age−specific fertility fitted trend lines, 1950−2017: China, Ages: 50−54
Data points enclosed with a diamond are reference sources

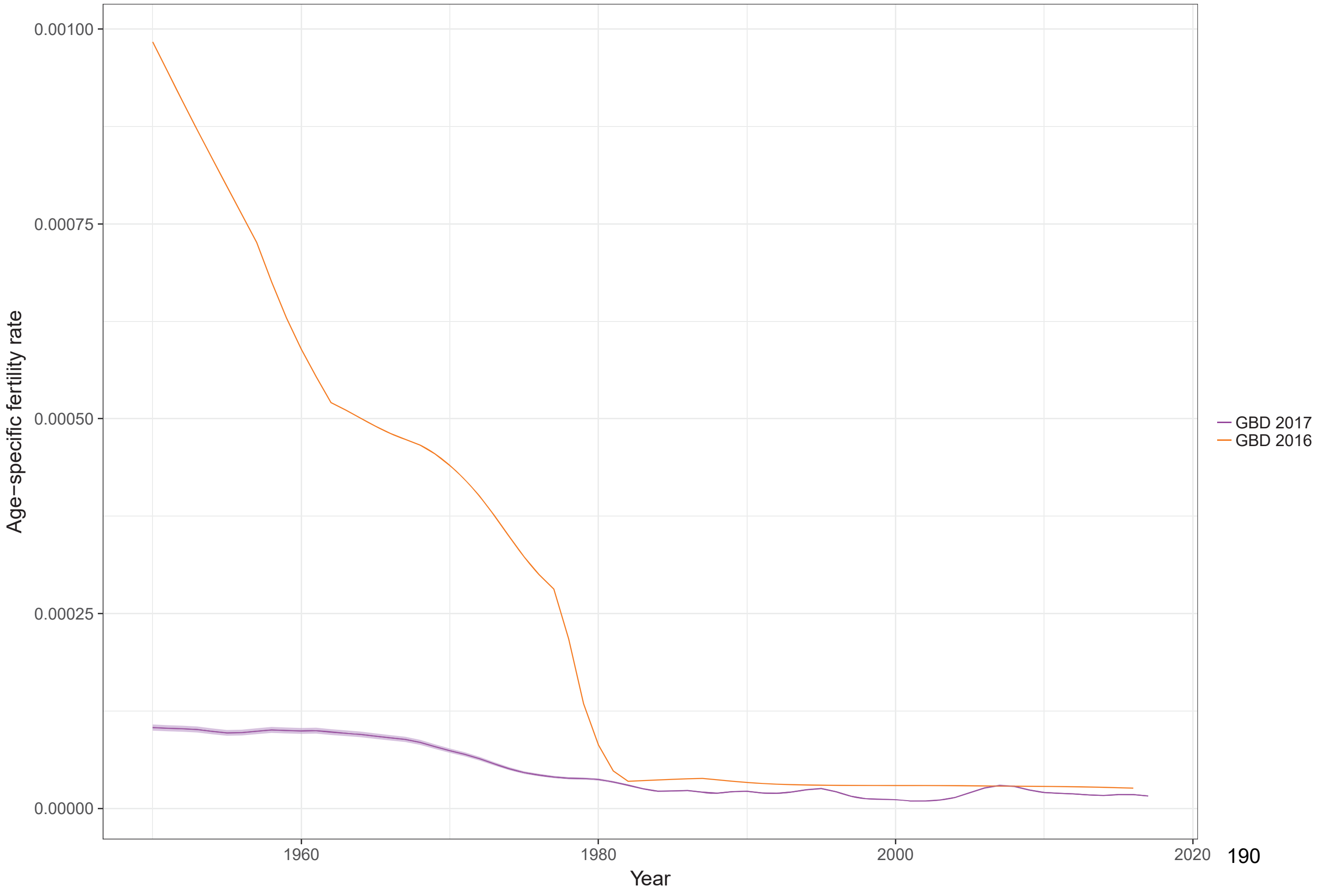

Age−specific fertility fitted trend lines, 1950−2017: China (without Hong Kong and Macao), TFR
Data points enclosed with a diamond are reference sources

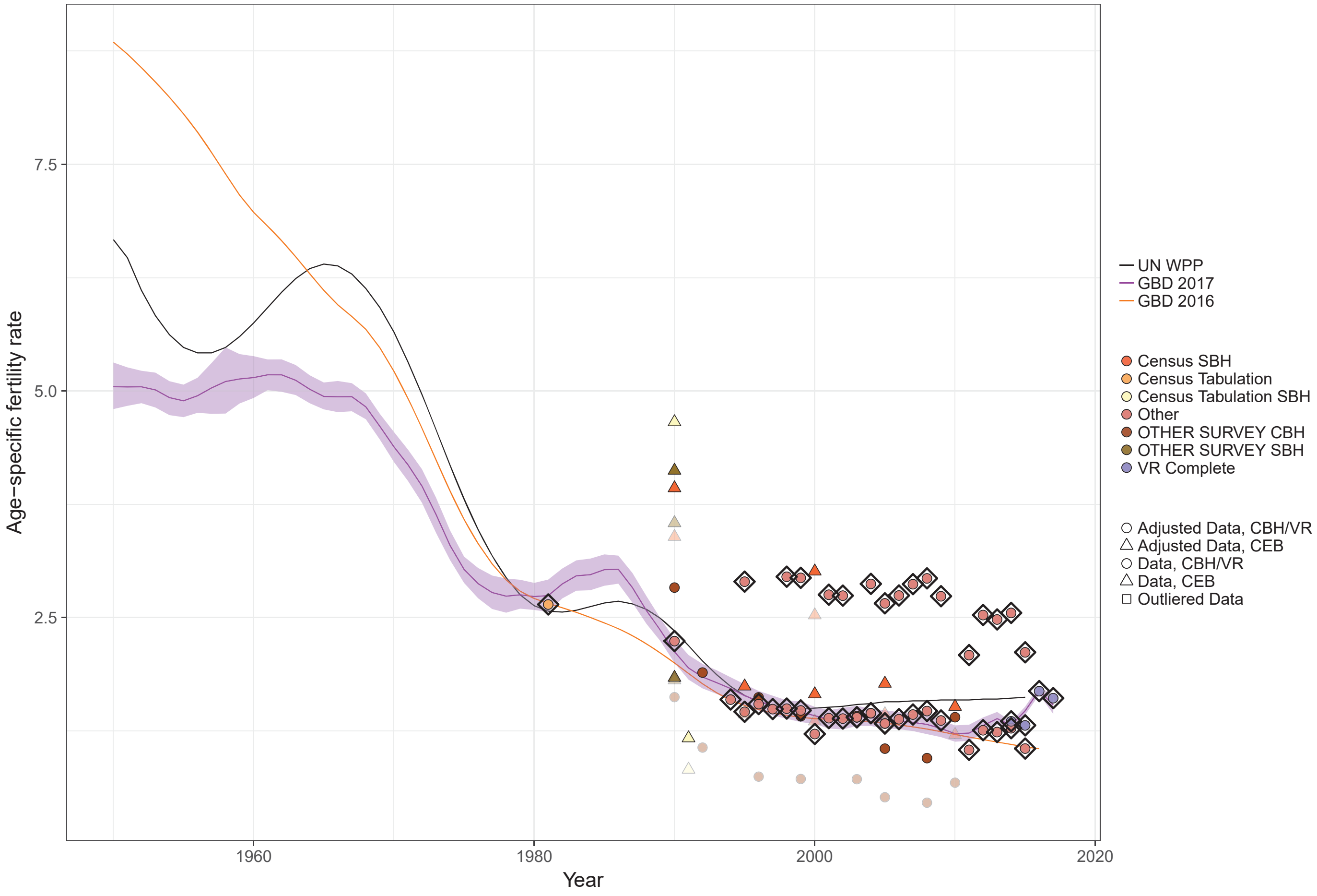

Age−specific fertility fitted trend lines, 1950−2017: China (without Hong Kong and Macao), Ages: 10−14
Data points enclosed with a diamond are reference sources

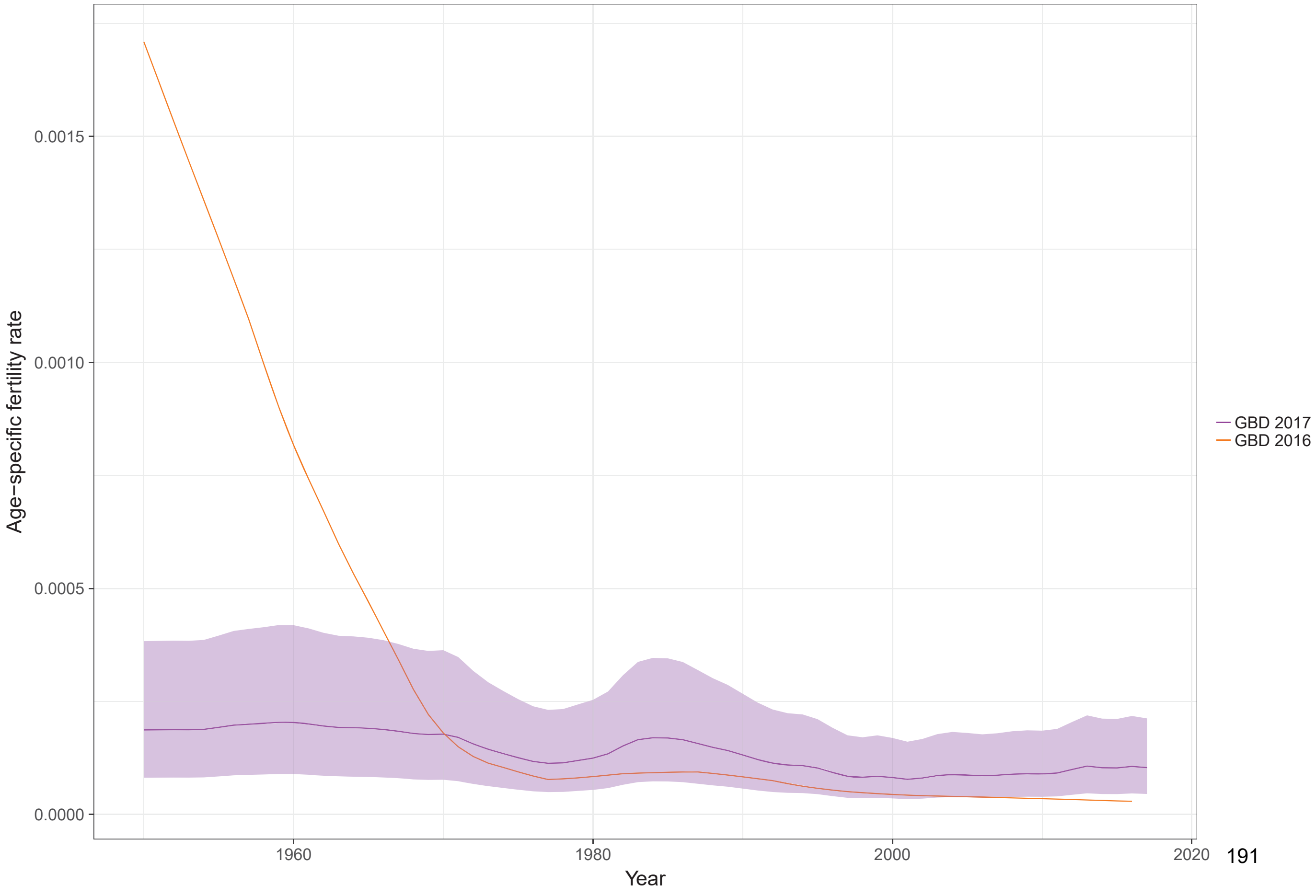

Age−specific fertility fitted trend lines, 1950−2017: China (without Hong Kong and Macao), Ages: 15−19
Data points enclosed with a diamond are reference sources

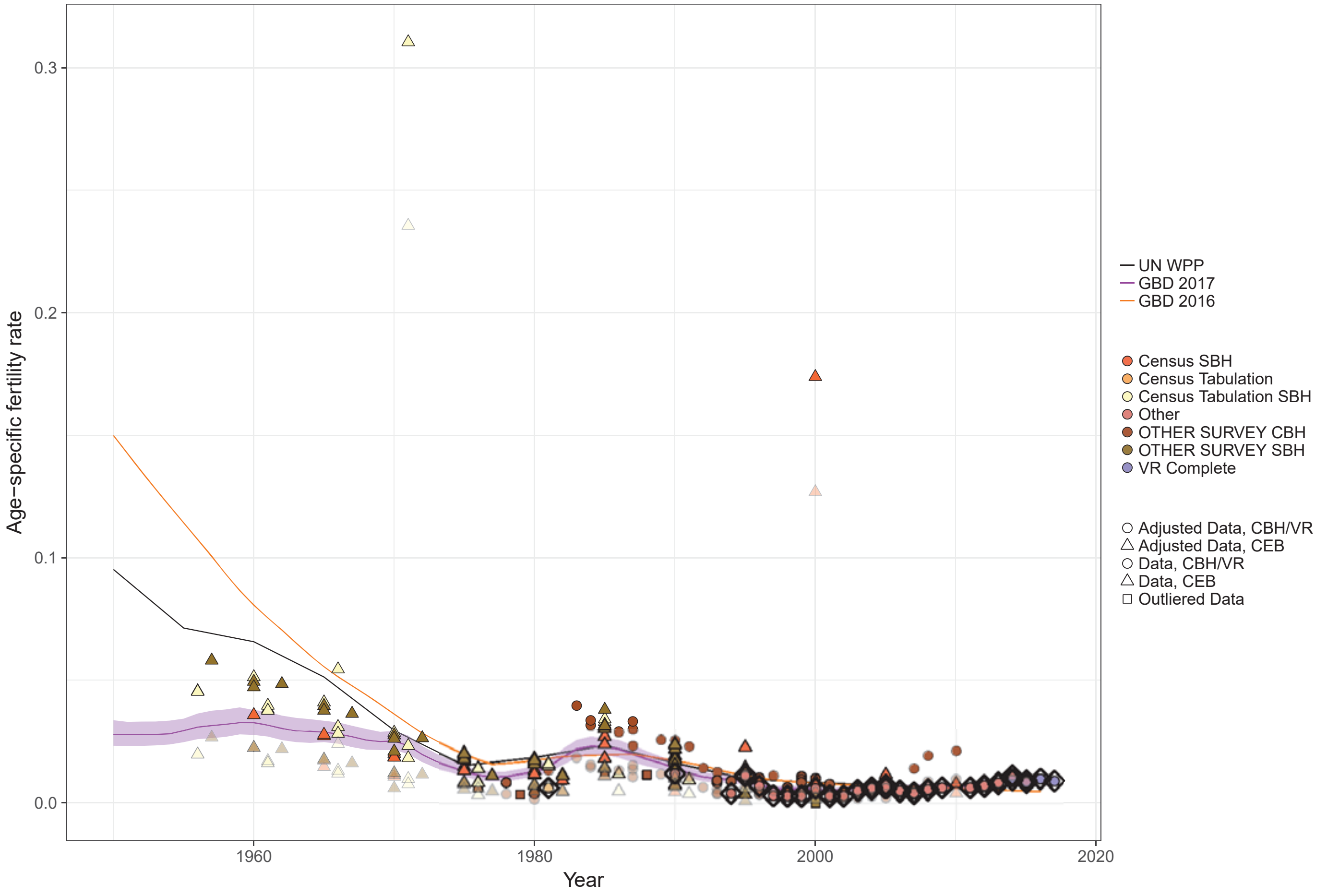

Age−specific fertility fitted trend lines, 1950−2017: China (without Hong Kong and Macao), Ages: 20−24
Data points enclosed with a diamond are reference sources

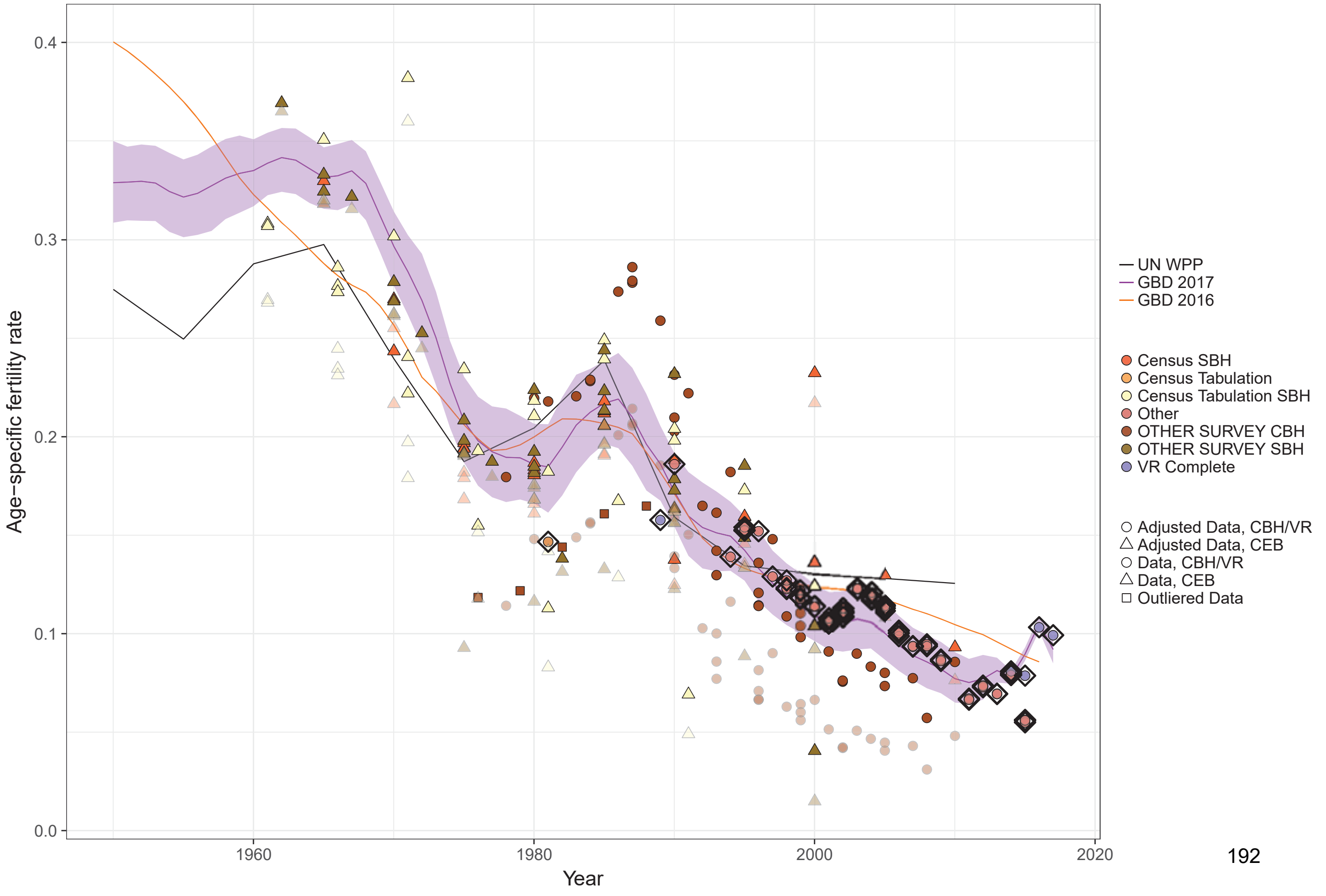

Age−specific fertility fitted trend lines, 1950−2017: China (without Hong Kong and Macao), Ages: 25−29
Data points enclosed with a diamond are reference sources

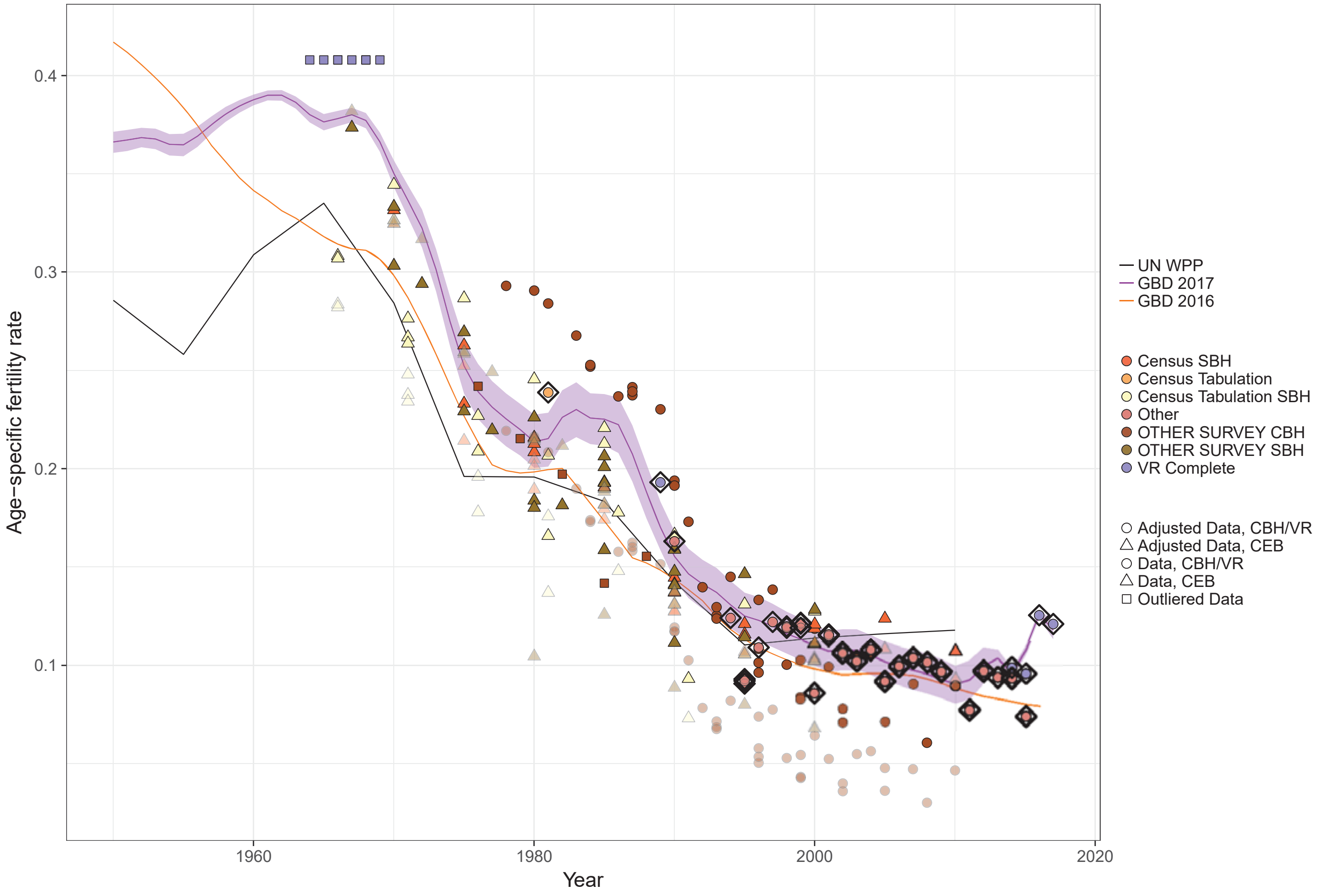

Age−specific fertility fitted trend lines, 1950−2017: China (without Hong Kong and Macao), Ages: 30−34
Data points enclosed with a diamond are reference sources

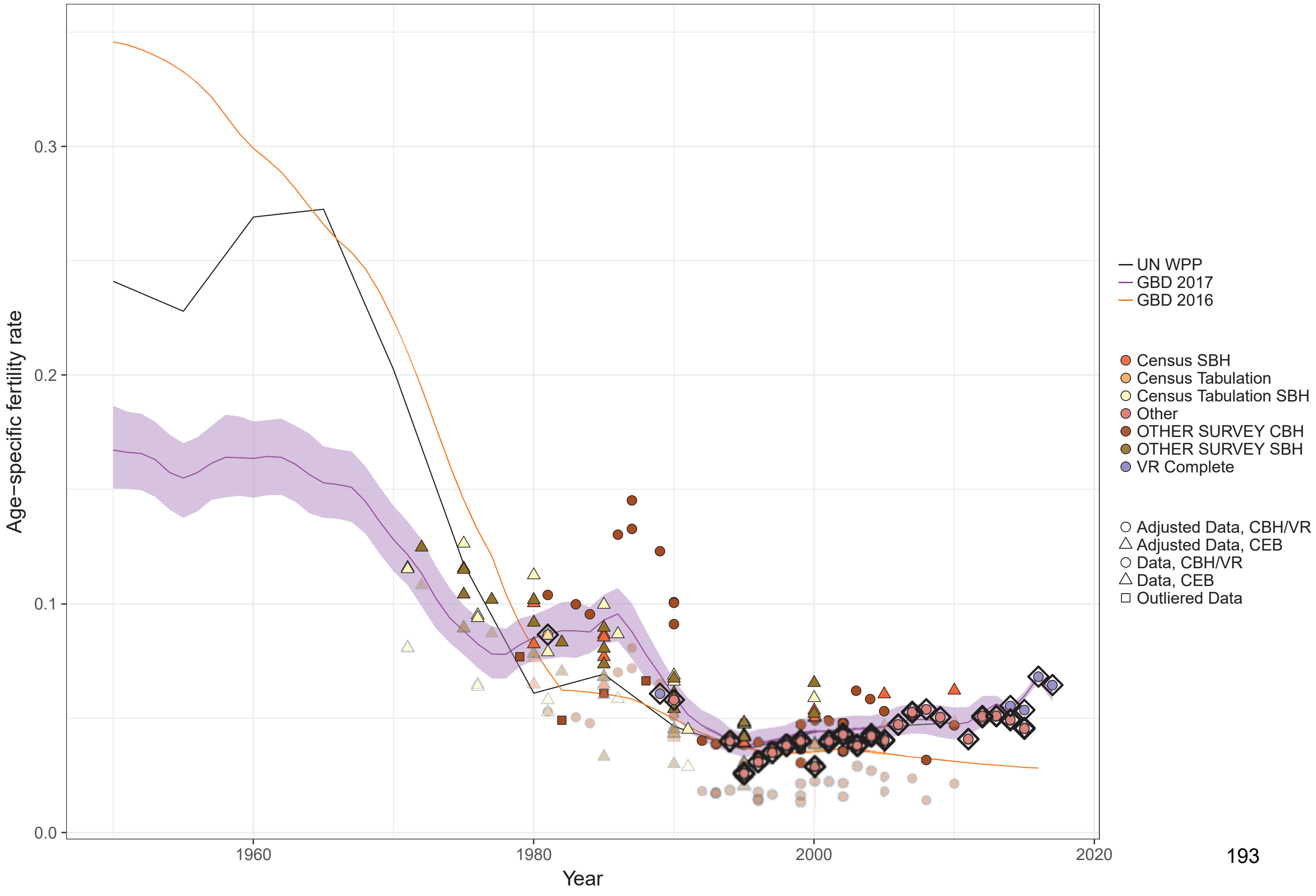

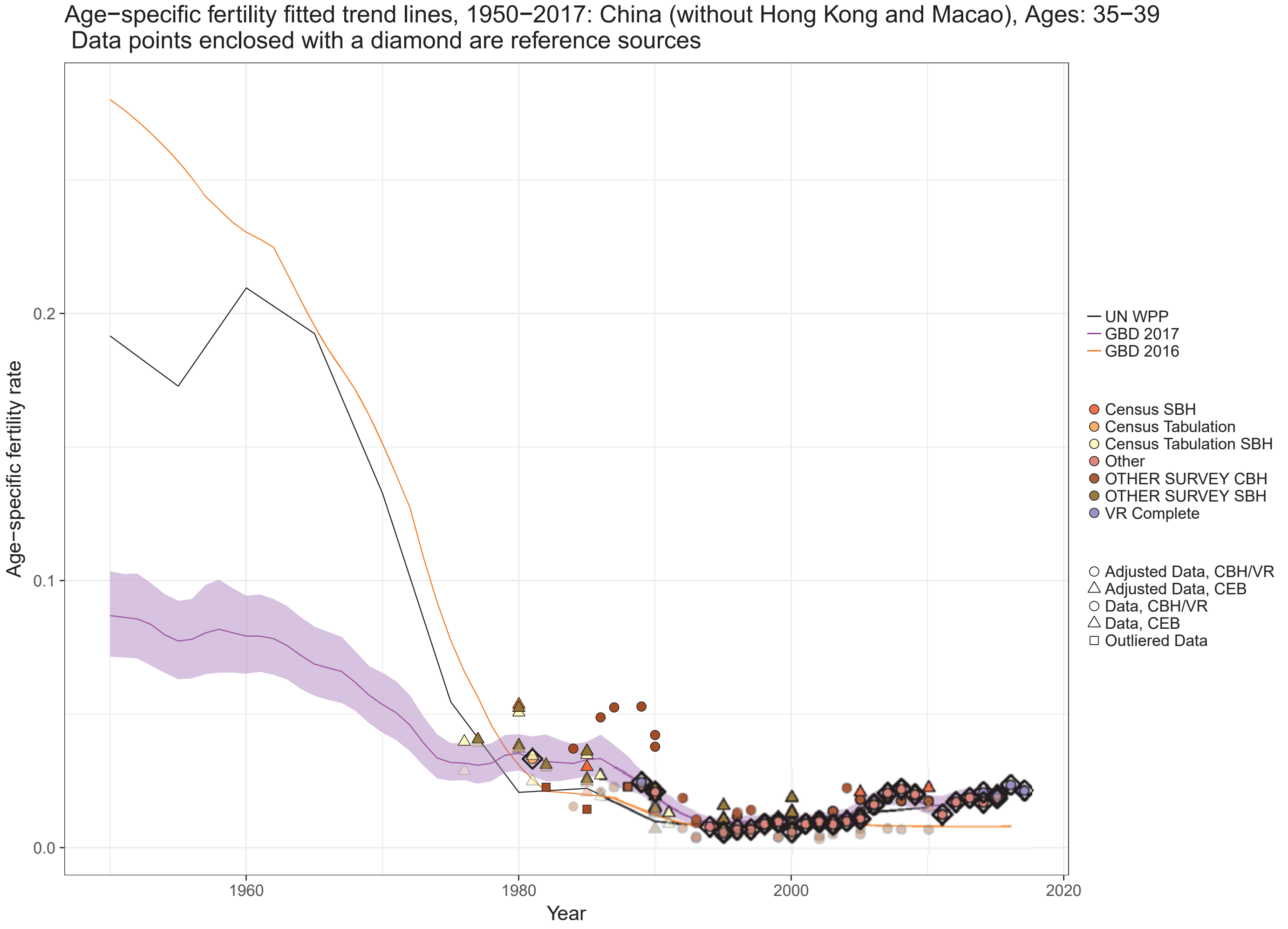
Age−specific fertility fitted trend lines, 1950−2017: China (without Hong Kong and Macao), Ages: 35−39
Data points enclosed with a diamond are reference sources
Age−specific fertility rate
0.0
0.1
0.2
1960
1980
2000
2020
Year
UN WPP
GBD 2017
GBD 2016
Census SBH
Census Tabulation
Census Tabulation SBH
Other
OTHER SURVEY CBH
OTHER SURVEY SBH
VR Complete
Adjusted Data, CBH/VR
Adjusted Data, CEB
Data, CBH/VR
Data, CEB
Outliered Data

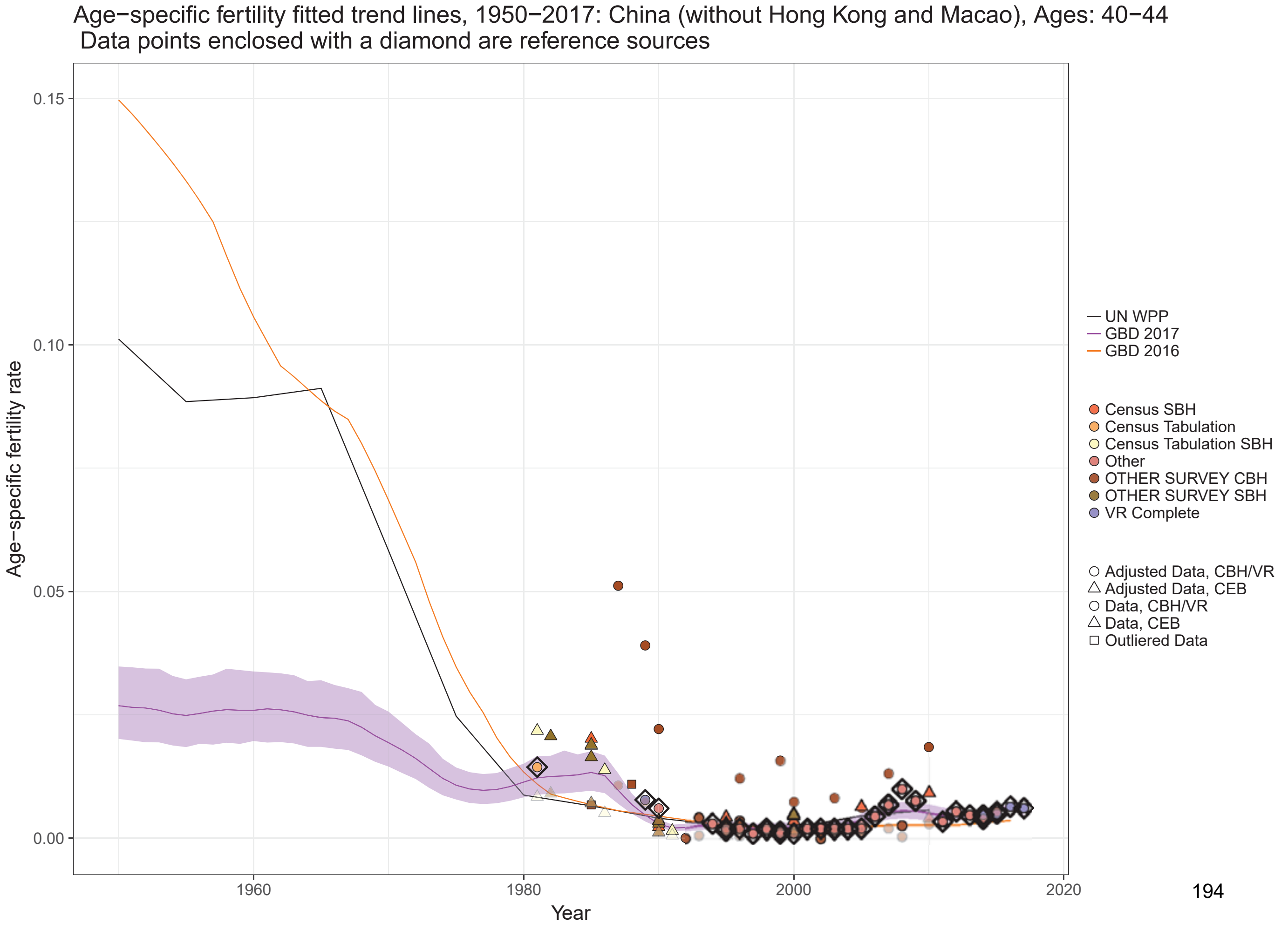
Age−specific fertility fitted trend lines, 1950−2017: China (without Hong Kong and Macao), Ages: 40−44
Data points enclosed with a diamond are reference sources
Age−specific fertility rate
0.00
0.05
0.10
0.15
1960
1980
2000
2020
Year
UN WPP
GBD 2017
GBD 2016
Census SBH
Census Tabulation
Census Tabulation SBH
Other
OTHER SURVEY CBH
OTHER SURVEY SBH
VR Complete
Adjusted Data, CBH/VR
Adjusted Data, CEB
Data, CBH/VR
Data, CEB
Outliered Data

Age−specific fertility fitted trend lines, 1950−2017: China (without Hong Kong and Macao), Ages: 45−49
Data points enclosed with a diamond are reference sources

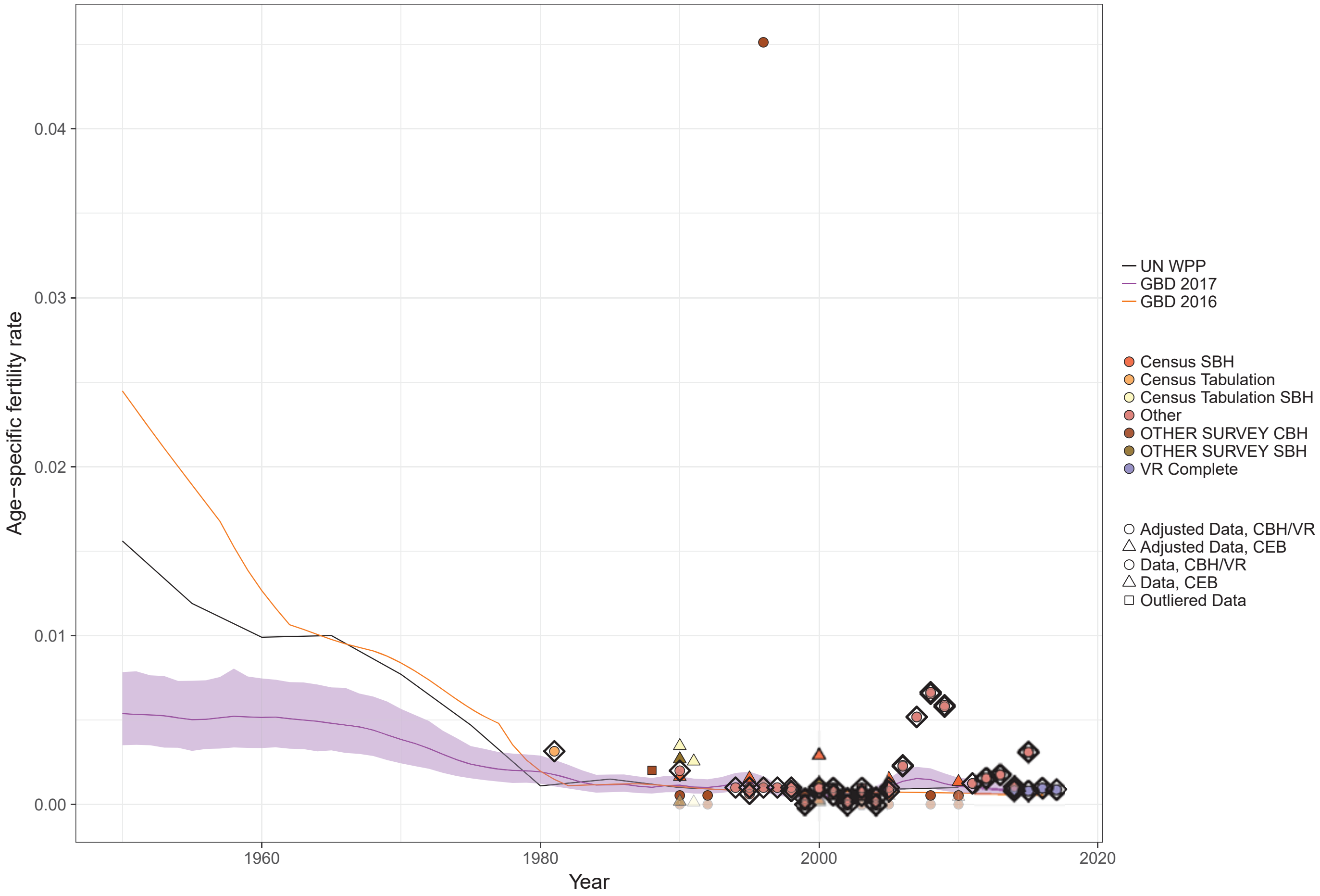

Age−specific fertility fitted trend lines, 1950−2017: China (without Hong Kong and Macao), Ages: 50−54
Data points enclosed with a diamond are reference sources

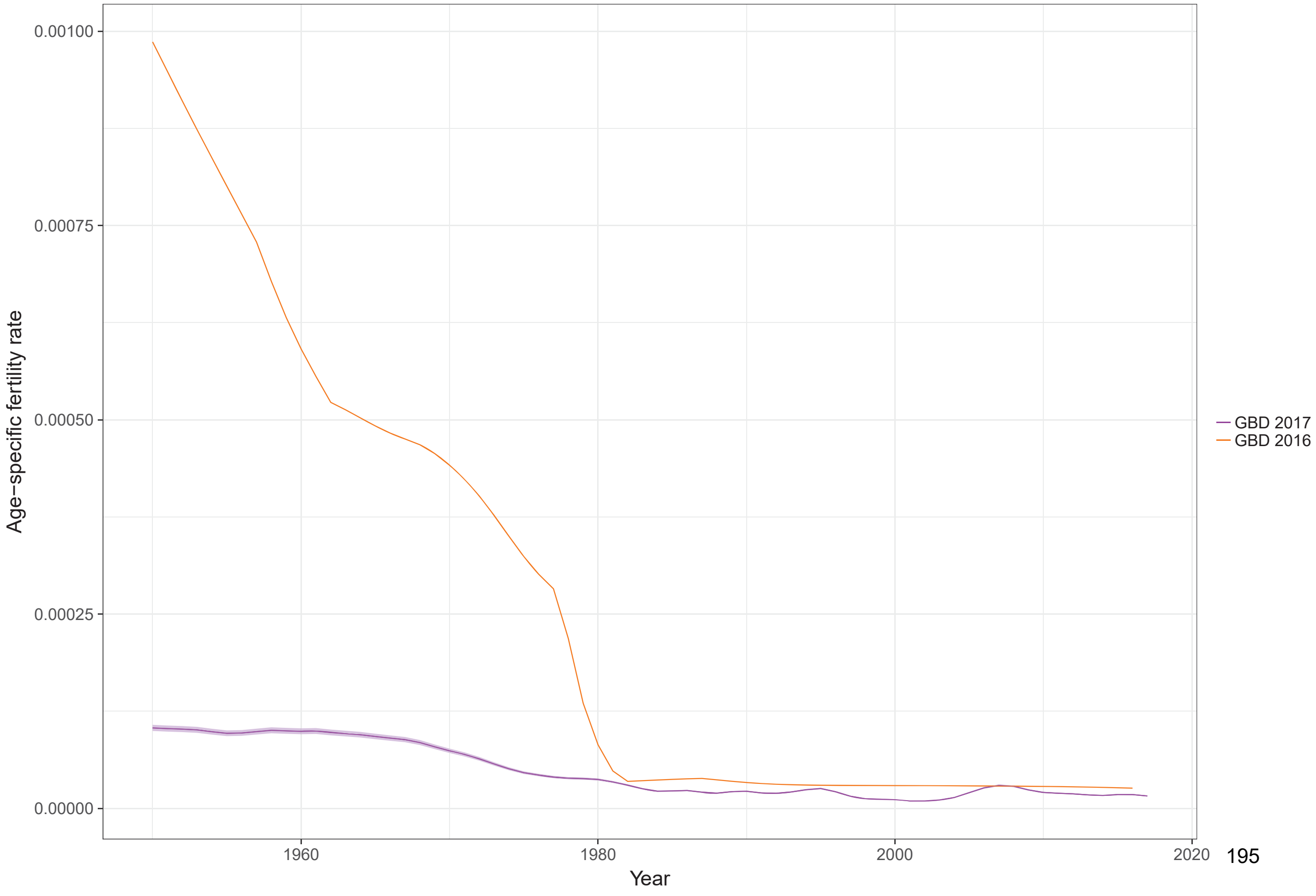

Age−specific fertility fitted trend lines, 1950−2017: Colombia, TFR
Data points enclosed with a diamond are reference sources

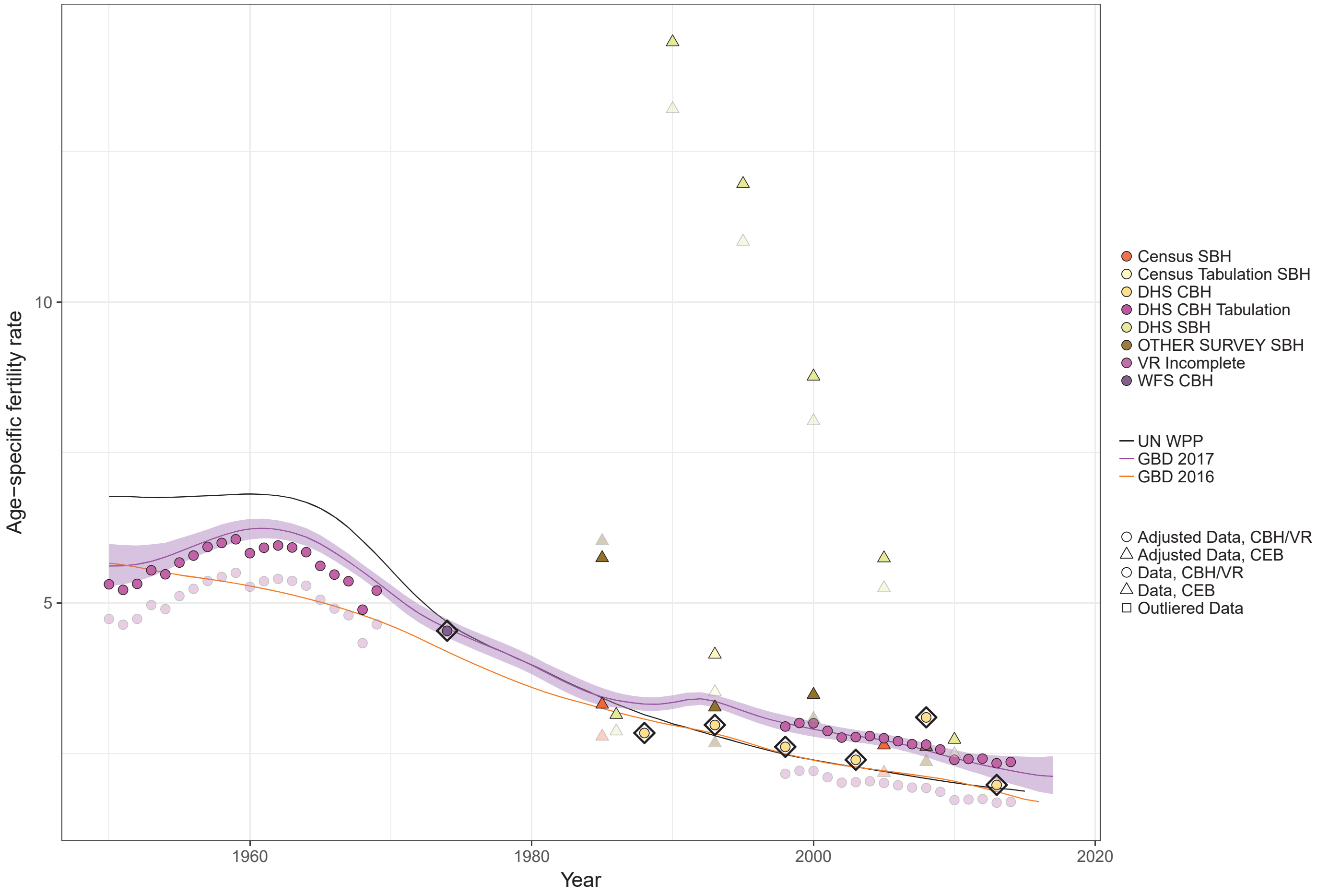

Age−specific fertility fitted trend lines, 1950−2017: Colombia, Ages: 10−14
Data points enclosed with a diamond are reference sources

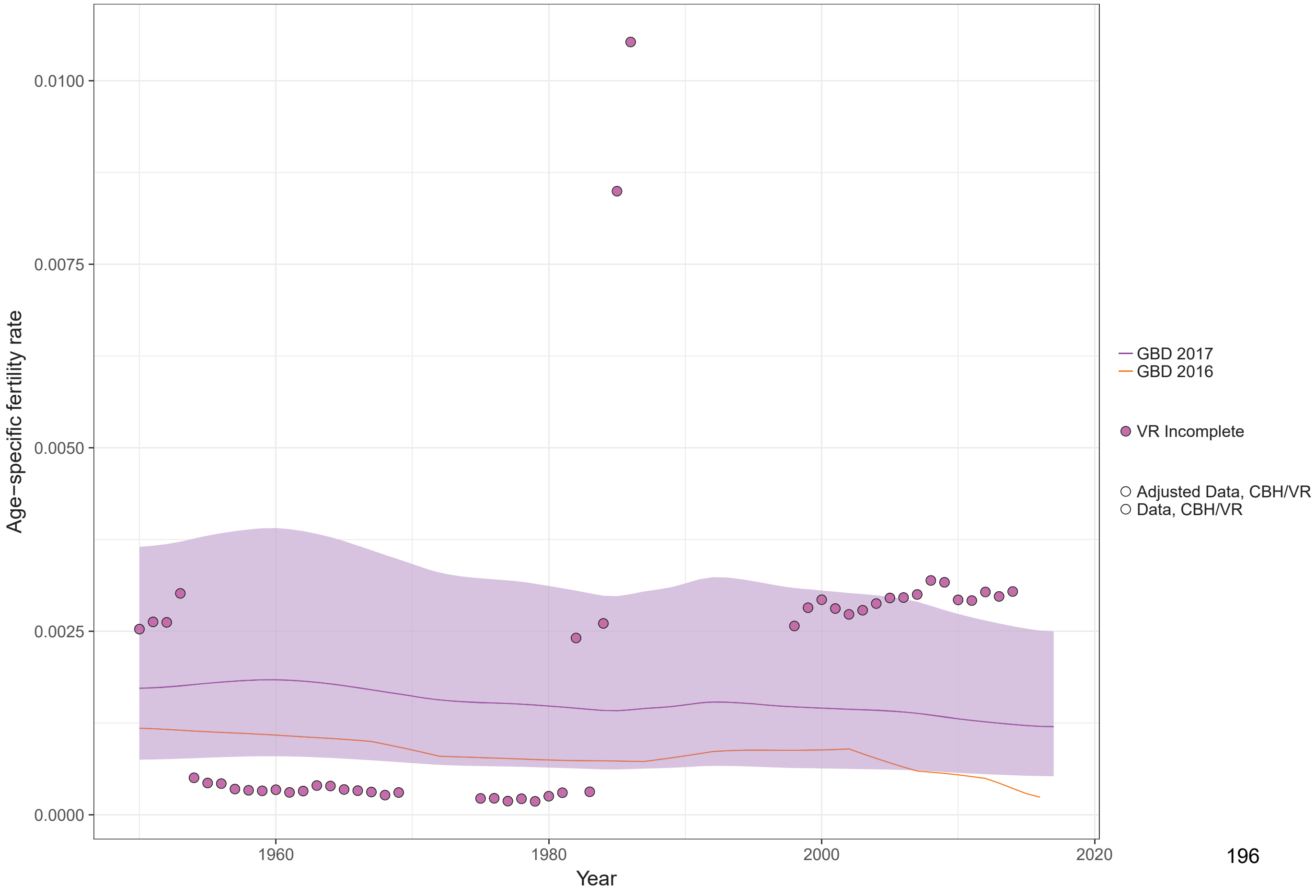

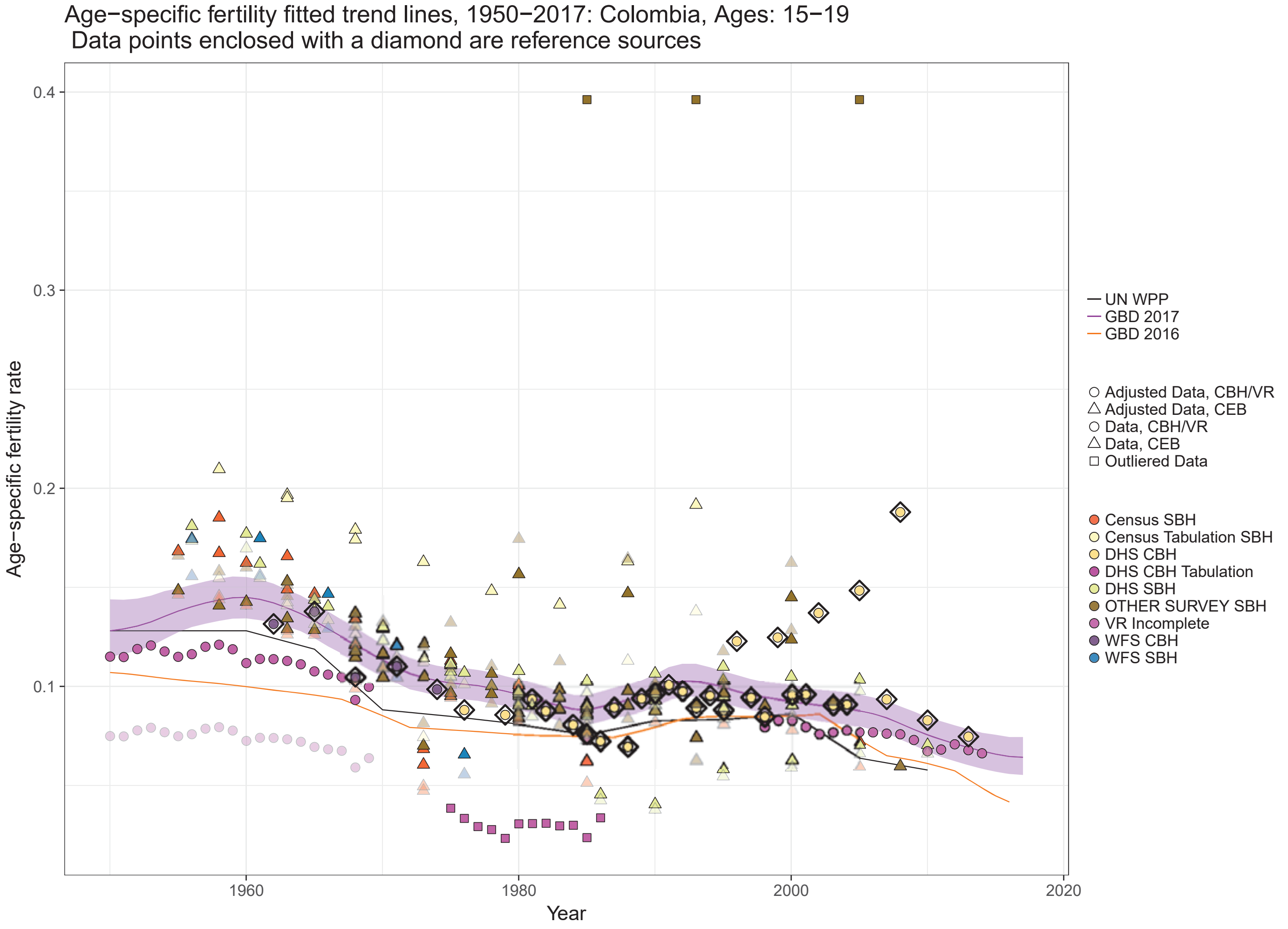
Age−specific fertility fitted trend lines, 1950−2017: Colombia, Ages: 15−19
Data points enclosed with a diamond are reference sources
Age−specific fertility rate
0.4
0.3
0.2
0.1
1960
1980
2000
2020
Year
UN WPP
GBD 2017
GBD 2016
Adjusted Data, CBH/VR
Adjusted Data, CEB
Data, CBH/VR
Data, CEB
Outliered Data
Census SBH
Census Tabulation SBH
DHS CBH
DHS CBH Tabulation
DHS SBH
OTHER SURVEY SBH
VR Incomplete
WFS CBH
WFS SBH

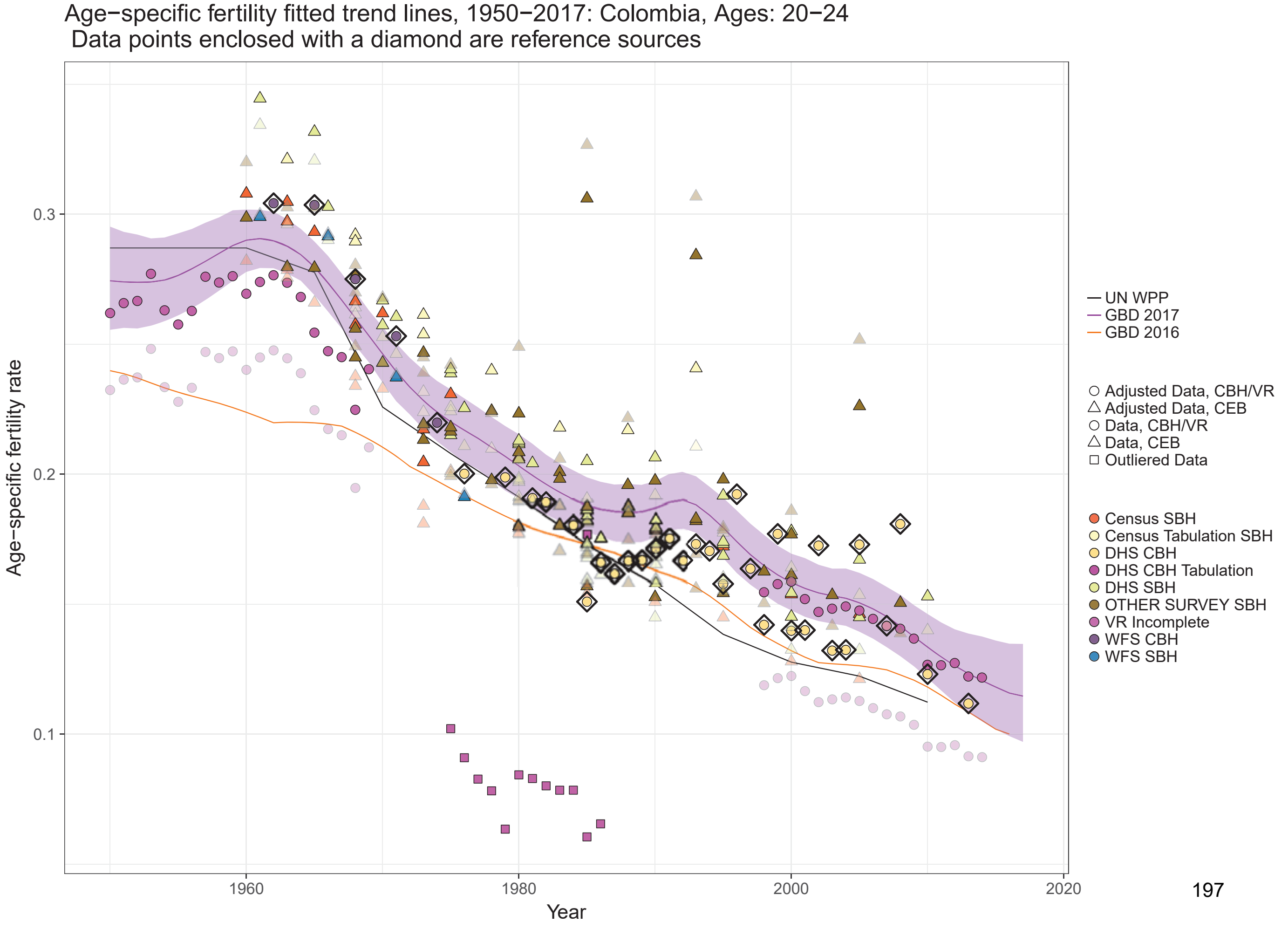
Age−specific fertility fitted trend lines, 1950−2017: Colombia, Ages: 20−24
Data points enclosed with a diamond are reference sources
Age−specific fertility rate
0.3
0.2
0.1
1960
1980
2000
2020
Year
UN WPP
GBD 2017
GBD 2016
Adjusted Data, CBH/VR
Adjusted Data, CEB
Data, CBH/VR
Data, CEB
Outliered Data
Census SBH
Census Tabulation SBH
DHS CBH
DHS CBH Tabulation
DHS SBH
OTHER SURVEY SBH
VR Incomplete
WFS CBH
WFS SBH

Age−specific fertility fitted trend lines, 1950−2017: Colombia, Ages: 25−29
Data points enclosed with a diamond are reference sources

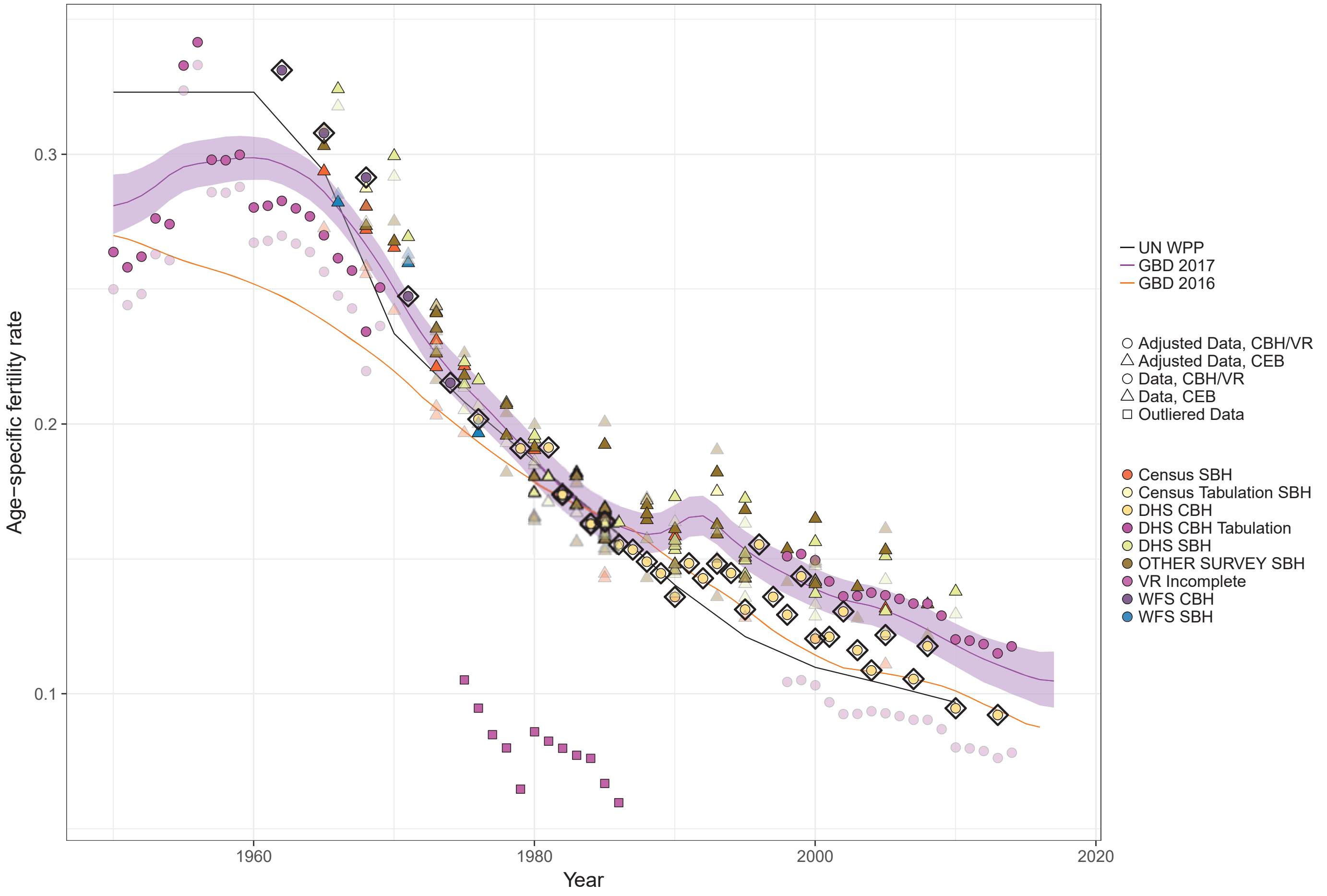

Age−specific fertility fitted trend lines, 1950−2017: Colombia, Ages: 30−34
Data points enclosed with a diamond are reference sources

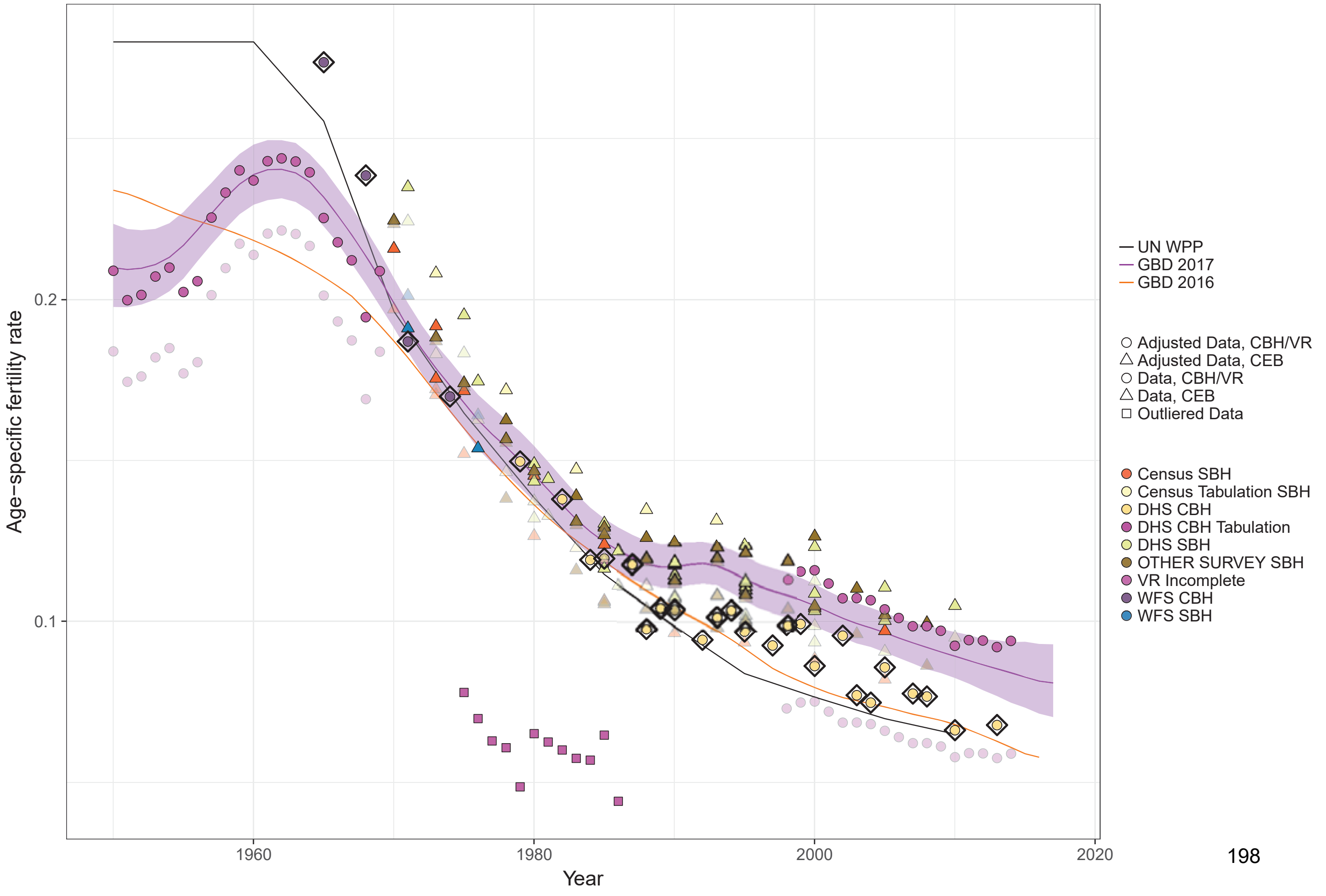

Age−specific fertility fitted trend lines, 1950−2017: Colombia, Ages: 35−39
Data points enclosed with a diamond are reference sources

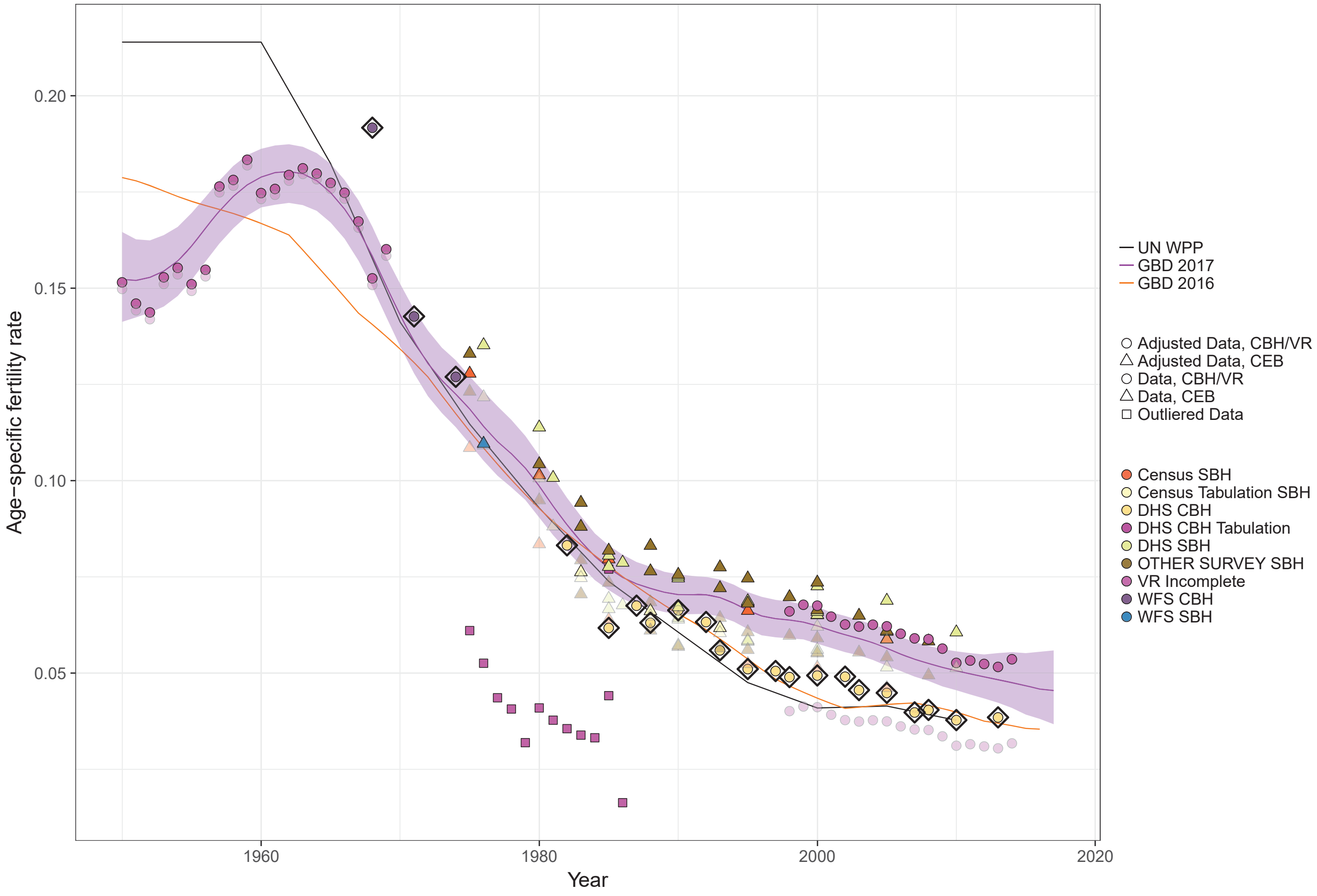

Age−specific fertility fitted trend lines, 1950−2017: Colombia, Ages: 40−44
Data points enclosed with a diamond are reference sources

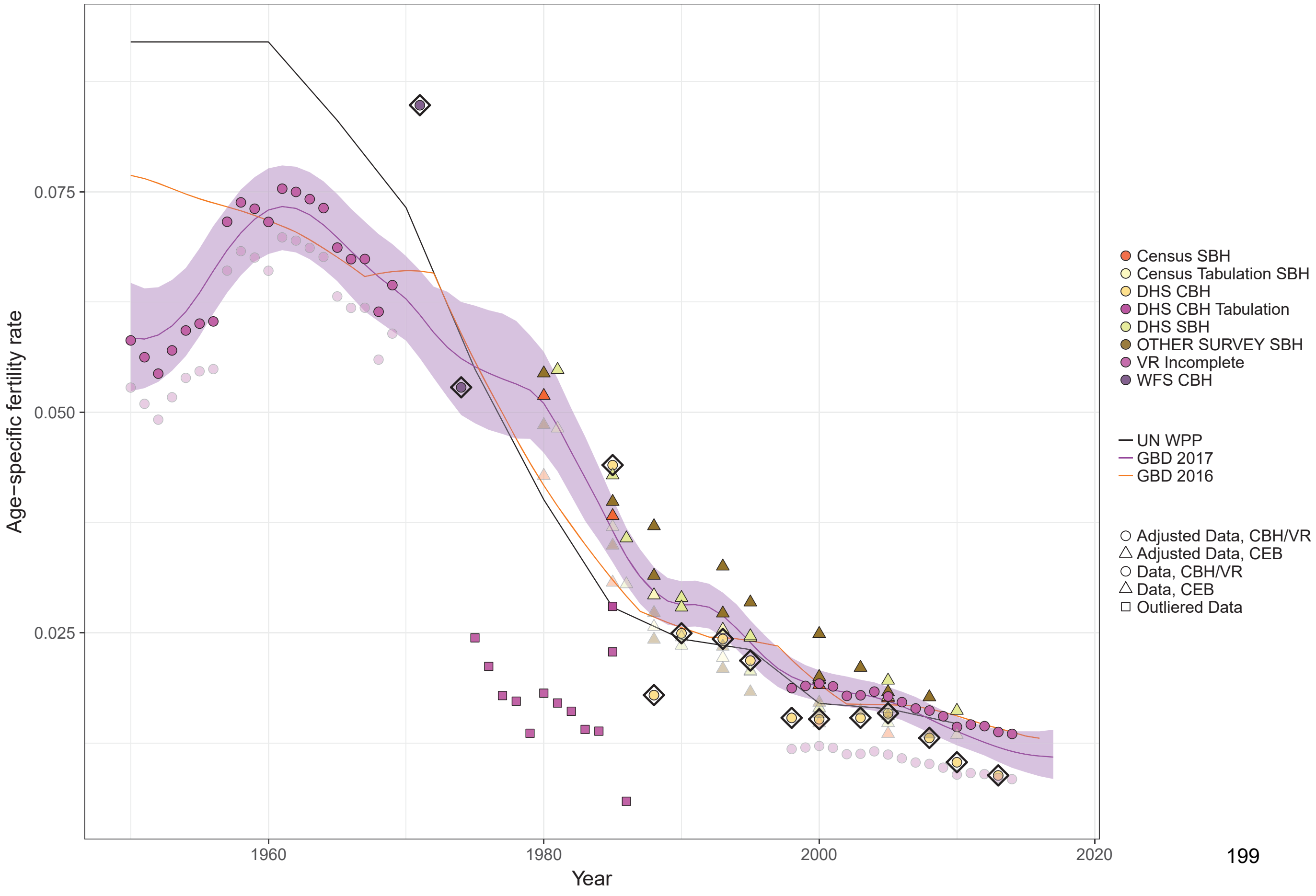

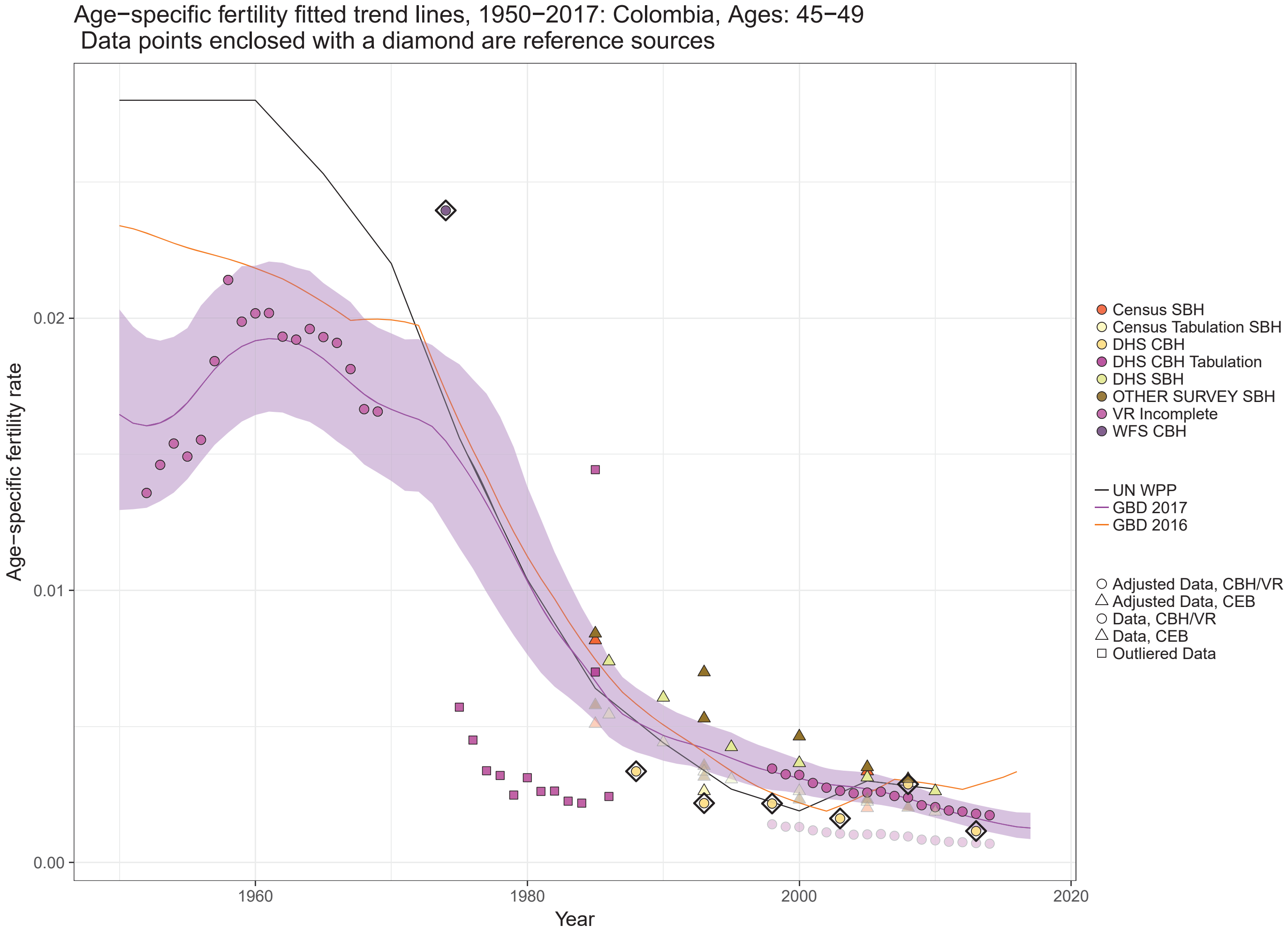
Age−specific fertility fitted trend lines, 1950−2017: Colombia, Ages: 45−49
Data points enclosed with a diamond are reference sources
Age−specific fertility rate
Year
0.00
0.01
0.02
1960
1980
2000
2020
Census SBH
Census Tabulation SBH
DHS CBH
DHS CBH Tabulation
DHS SBH
OTHER SURVEY SBH
VR Incomplete
WFS CBH
UN WPP
GBD 2017
GBD 2016
Adjusted Data, CBH/VR
Adjusted Data, CEB
Data, CBH/VR
Data, CEB
Outliered Data

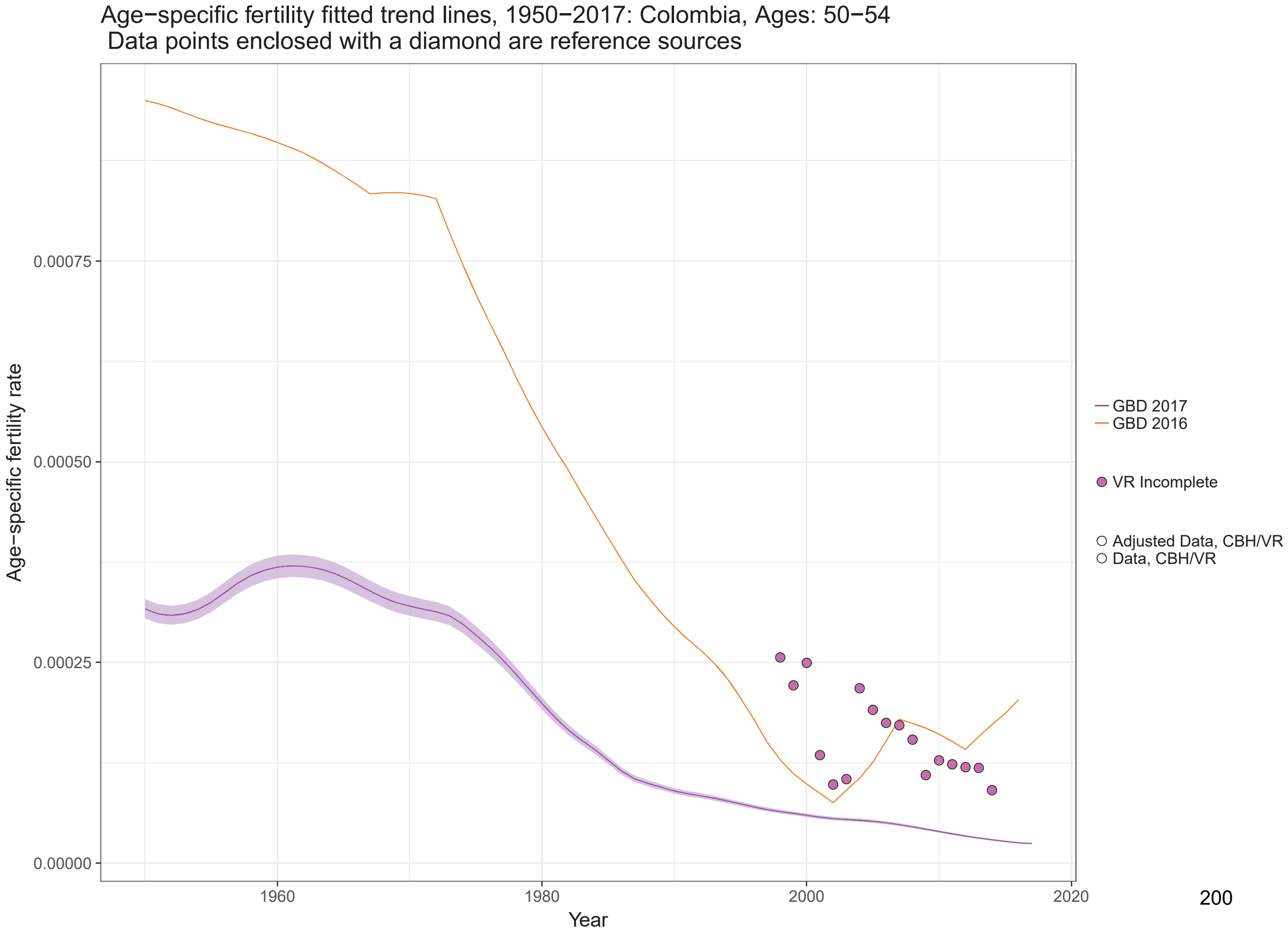
Age−specific fertility fitted trend lines, 1950−2017: Colombia, Ages: 50−54
Data points enclosed with a diamond are reference sources
Age−specific fertility rate
Year
0.00000
0.00025
0.00050
0.00075
1960
1980
2000
2020
GBD 2017
GBD 2016
VR Incomplete
Adjusted Data, CBH/VR
Data, CBH/VR

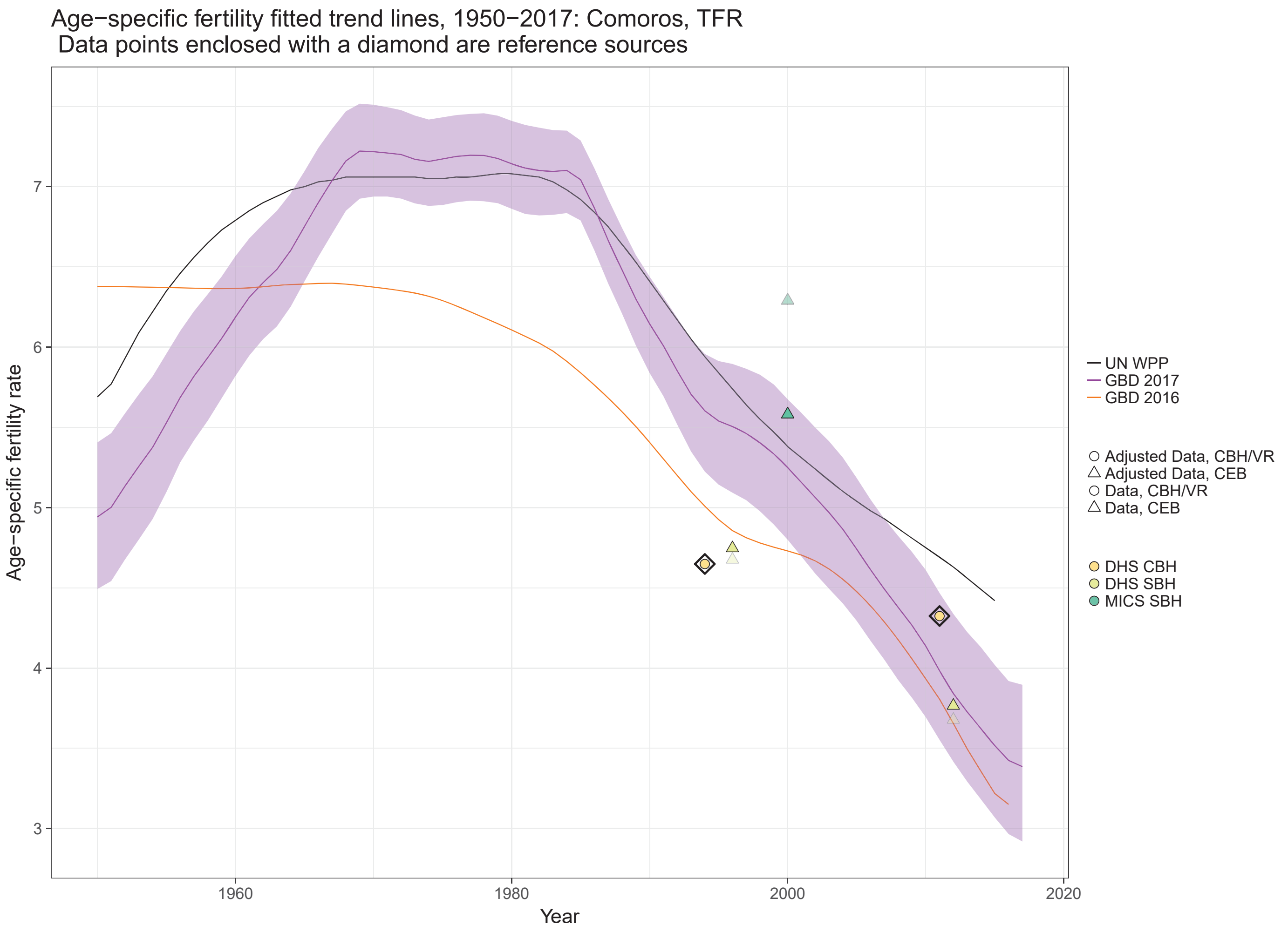

Age−specific fertility fitted trend lines, 1950−2017: Comoros, TFR
Data points enclosed with a diamond are reference sources
Age−specific fertility rate
Year
3
4
5
6
7
1960
1980
2000
2020
UN WPP
GBD 2017
GBD 2016
Adjusted Data, CBH/VR
Adjusted Data, CEB
Data, CBH/VR
Data, CEB
DHS CBH
DHS SBH
MICS SBH

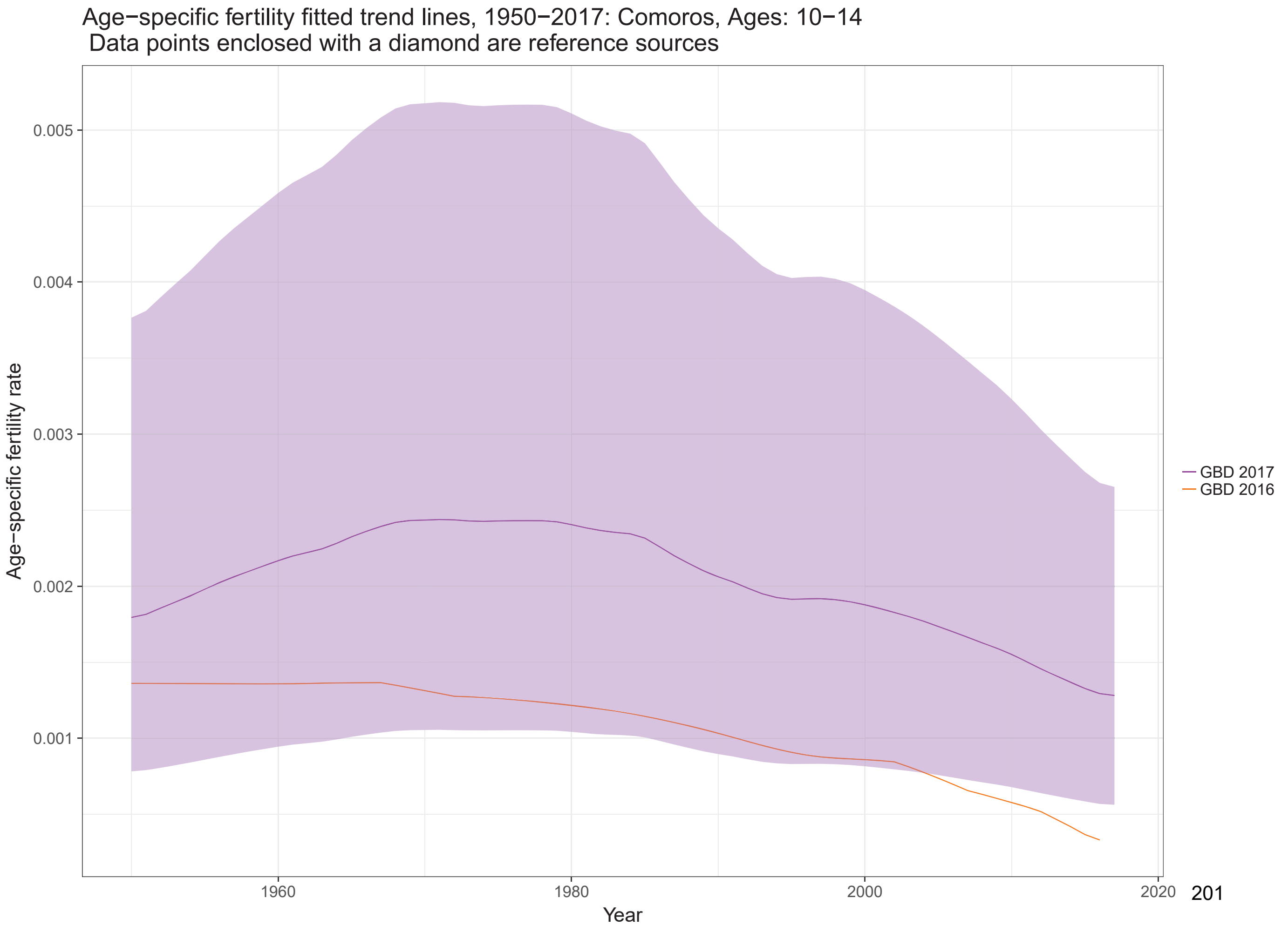

Age−specific fertility fitted trend lines, 1950−2017: Comoros, Ages: 10−14
Data points enclosed with a diamond are reference sources
Age−specific fertility rate
Year
0.001
0.002
0.003
0.004
0.005
1960
1980
2000
2020
GBD 2017
GBD 2016

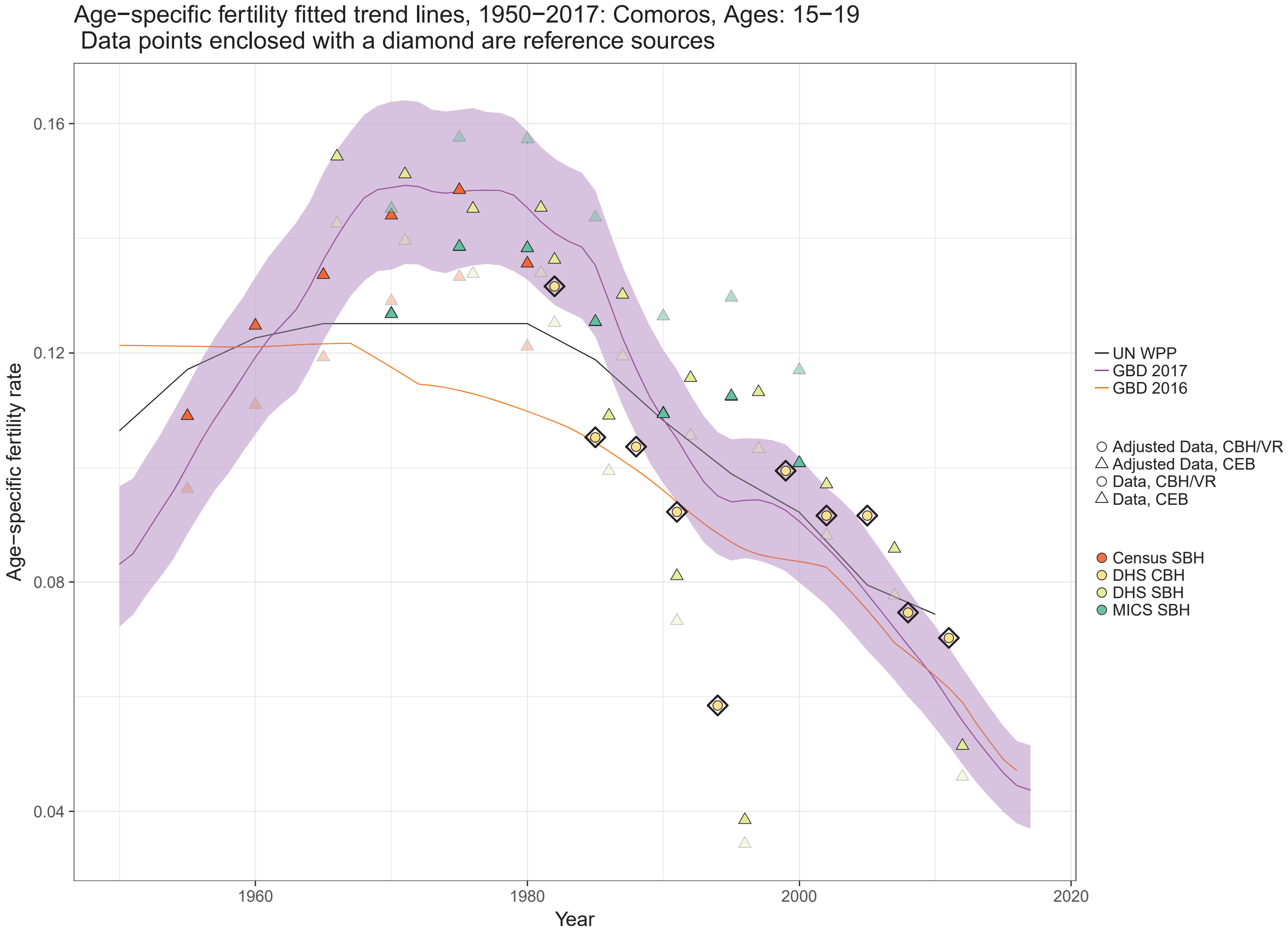
Age−specific fertility fitted trend lines, 1950−2017: Comoros, Ages: 15−19
Data points enclosed with a diamond are reference sources
Age−specific fertility rate
Year
0.04
0.08
0.12
0.16
1960
1980
2000
2020
UN WPP
GBD 2017
GBD 2016
Adjusted Data, CBH/VR
Adjusted Data, CEB
Data, CBH/VR
Data, CEB
Census SBH
DHS CBH
DHS SBH
MICS SBH

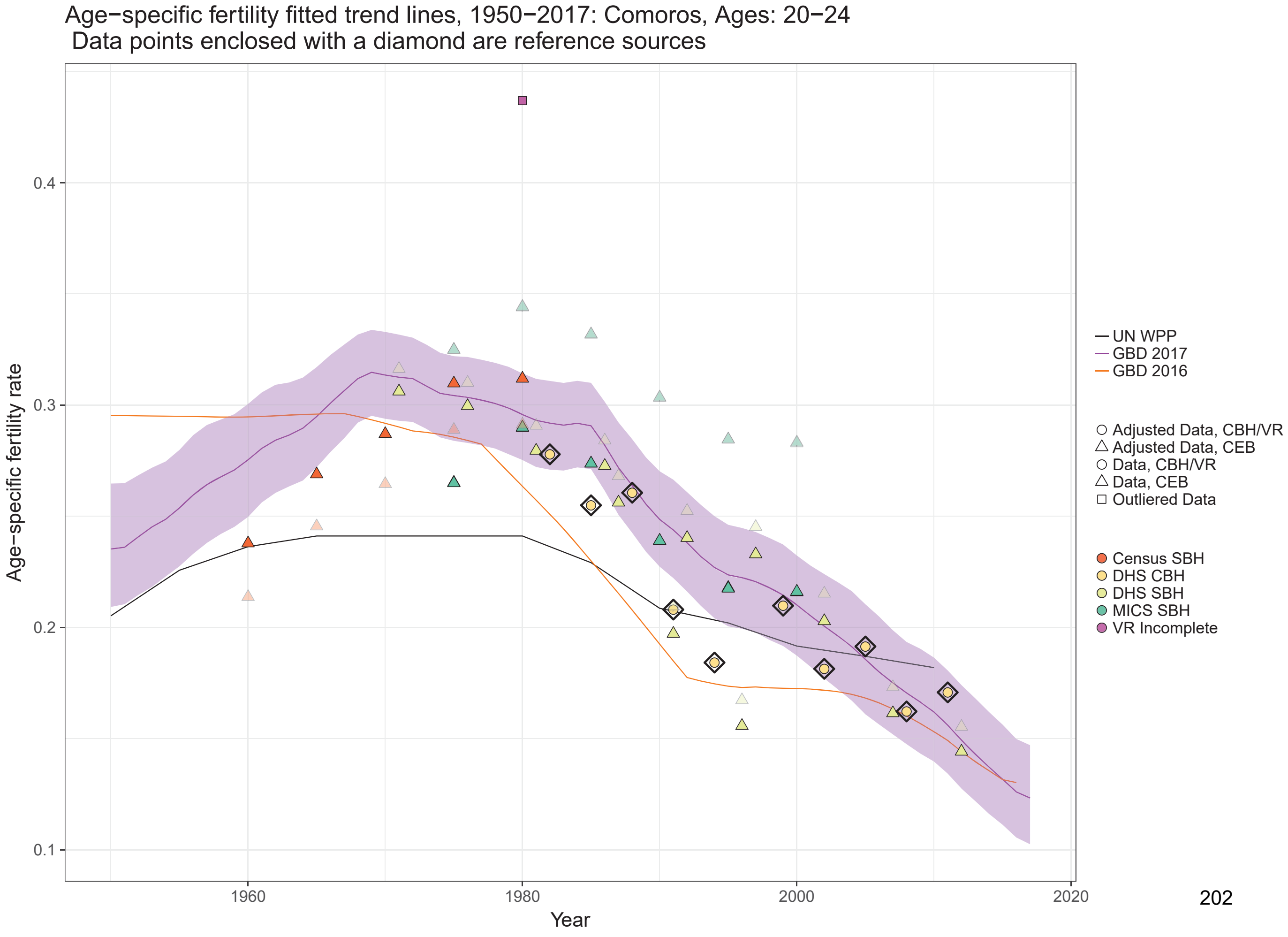
Age−specific fertility fitted trend lines, 1950−2017: Comoros, Ages: 20−24
Data points enclosed with a diamond are reference sources
Age−specific fertility rate
Year
0.1
0.2
0.3
0.4
1960
1980
2000
2020
UN WPP
GBD 2017
GBD 2016
Adjusted Data, CBH/VR
Adjusted Data, CEB
Data, CBH/VR
Data, CEB
Outliered Data
Census SBH
DHS CBH
DHS SBH
MICS SBH
VR Incomplete

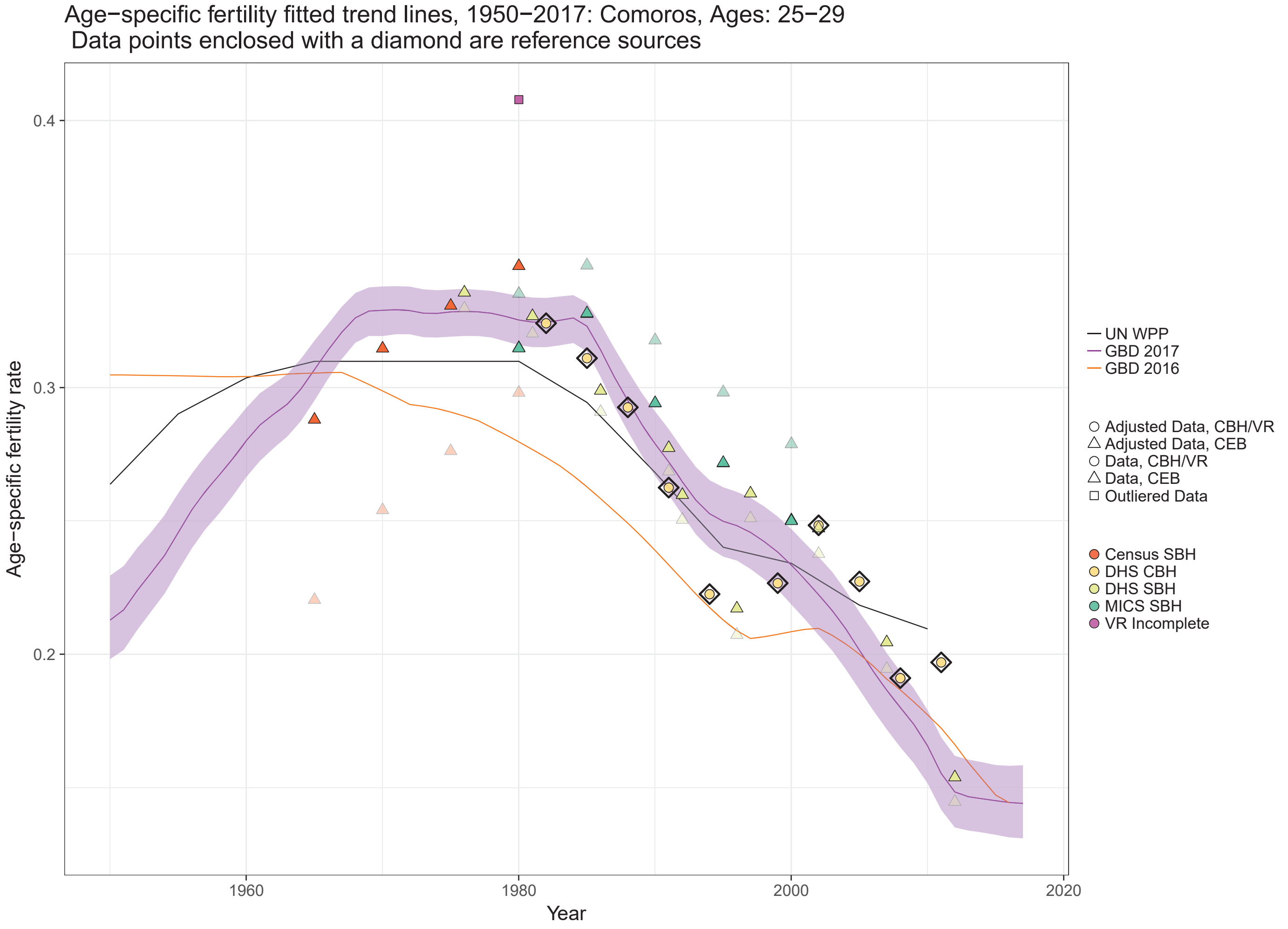
Age−specific fertility fitted trend lines, 1950−2017: Comoros, Ages: 25−29
Data points enclosed with a diamond are reference sources
Age−specific fertility rate
Year
0.4
0.3
0.2
1960
1980
2000
2020
UN WPP
GBD 2017
GBD 2016
Adjusted Data, CBH/VR
Adjusted Data, CEB
Data, CBH/VR
Data, CEB
Outliered Data
Census SBH
DHS CBH
DHS SBH
MICS SBH
VR Incomplete

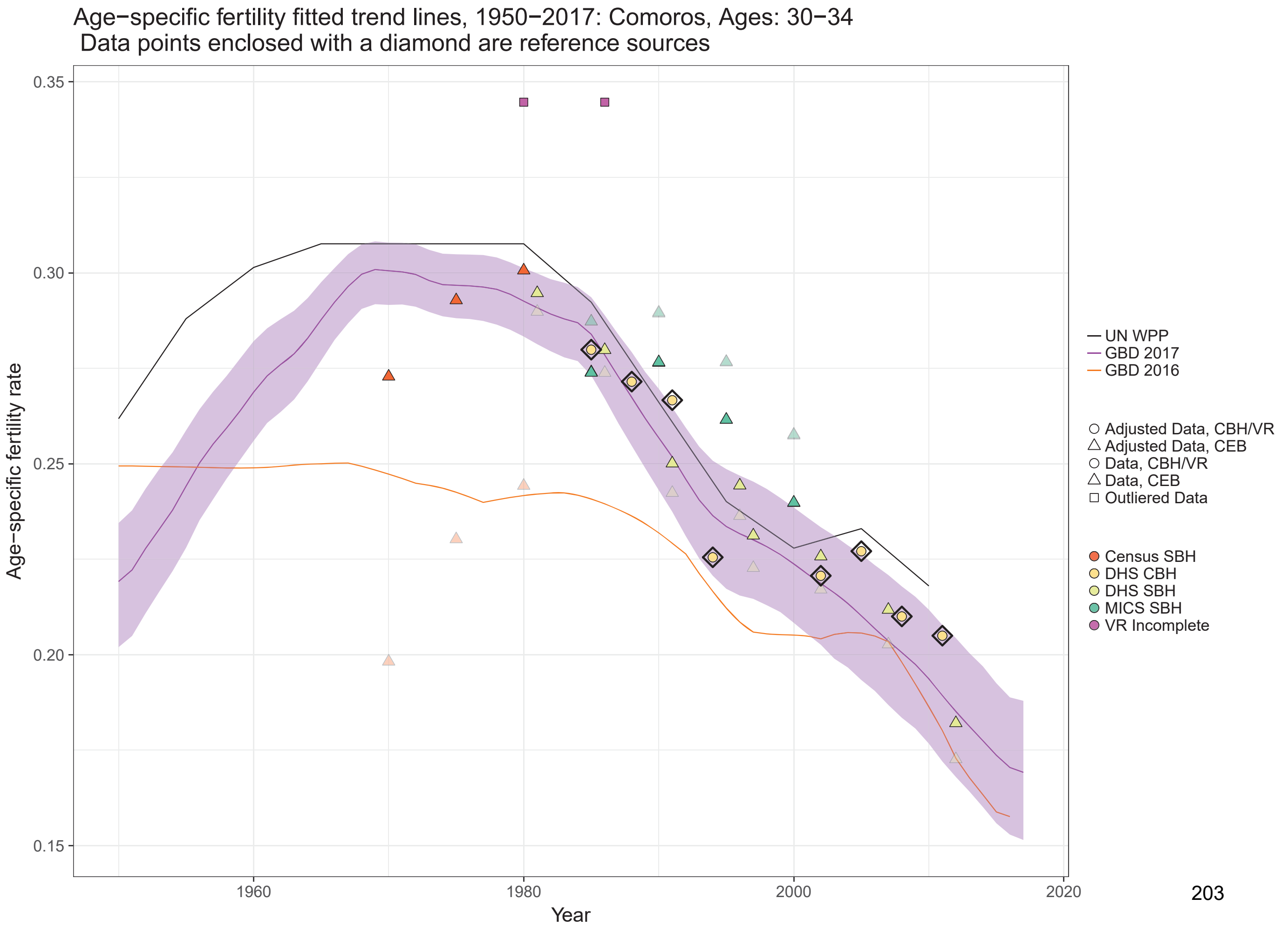
Age−specific fertility fitted trend lines, 1950−2017: Comoros, Ages: 30−34
Data points enclosed with a diamond are reference sources
Age−specific fertility rate
Year
0.35
0.30
0.25
0.20
0.15
1960
1980
2000
2020
UN WPP
GBD 2017
GBD 2016
Adjusted Data, CBH/VR
Adjusted Data, CEB
Data, CBH/VR
Data, CEB
Outliered Data
Census SBH
DHS CBH
DHS SBH
MICS SBH
VR Incomplete

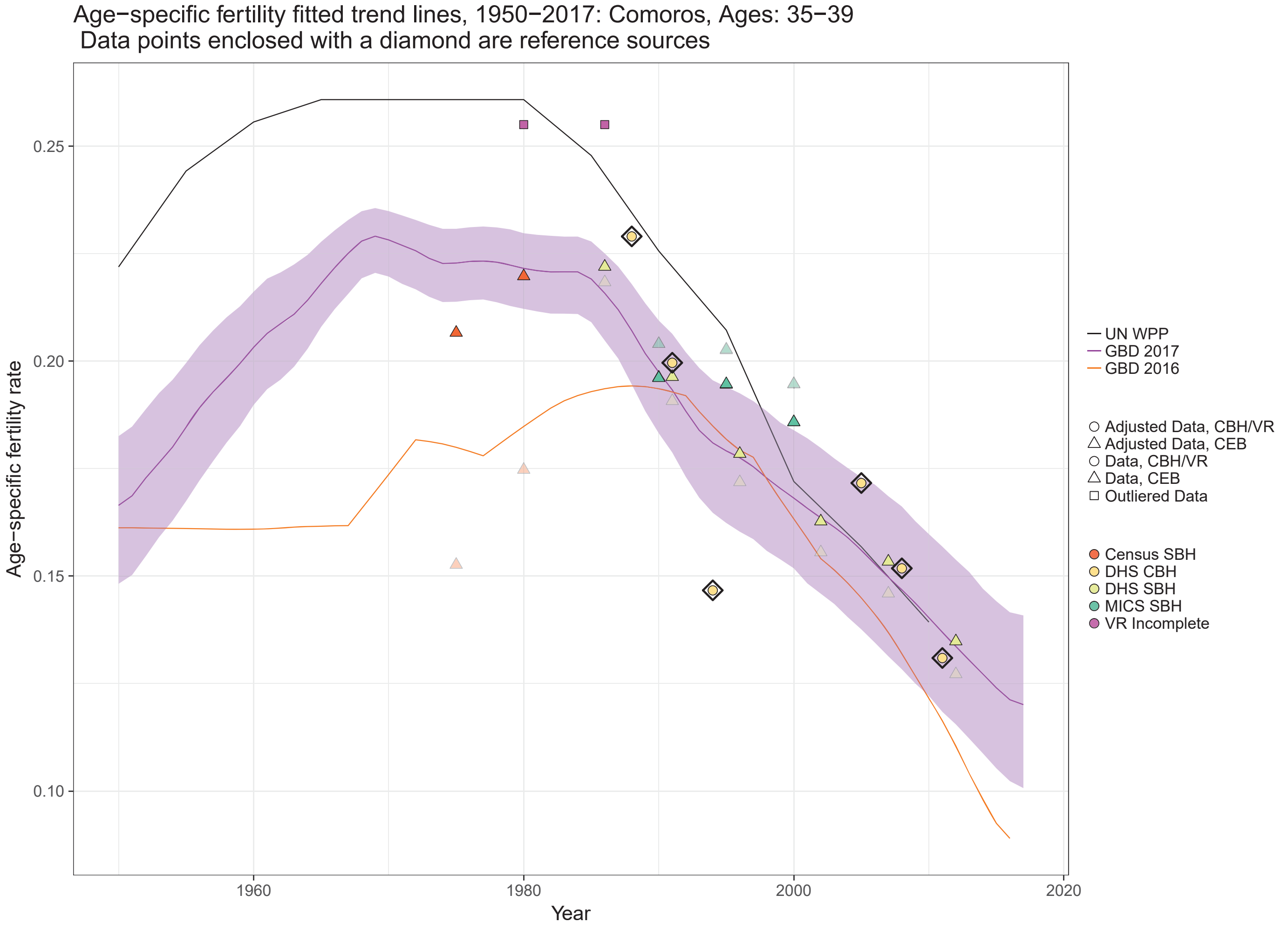

Age−specific fertility fitted trend lines, 1950−2017: Comoros, Ages: 35−39
Data points enclosed with a diamond are reference sources
Age−specific fertility rate
Year
0.10
0.15
0.20
0.25
1960
1980
2000
2020
UN WPP
GBD 2017
GBD 2016
Adjusted Data, CBH/VR
Adjusted Data, CEB
Data, CBH/VR
Data, CEB
Outliered Data
Census SBH
DHS CBH
DHS SBH
MICS SBH
VR Incomplete

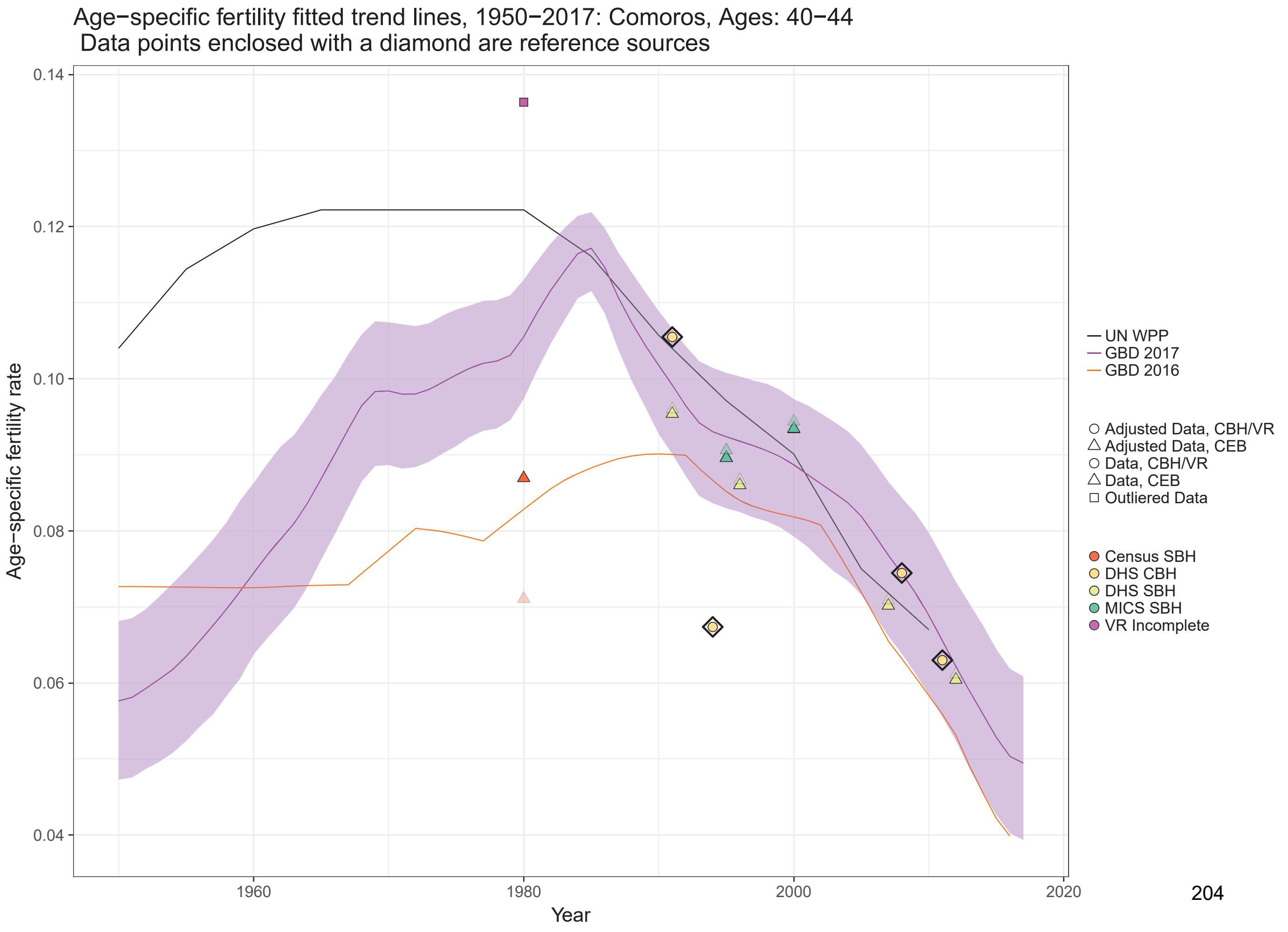

Age−specific fertility fitted trend lines, 1950−2017: Comoros, Ages: 40−44
Data points enclosed with a diamond are reference sources
Age−specific fertility rate
Year
0.04
0.06
0.08
0.10
0.12
0.14
1960
1980
2000
2020
UN WPP
GBD 2017
GBD 2016
Adjusted Data, CBH/VR
Adjusted Data, CEB
Data, CBH/VR
Data, CEB
Outliered Data
Census SBH
DHS CBH
DHS SBH
MICS SBH
VR Incomplete

Age−specific fertility fitted trend lines, 1950−2017: Comoros, Ages: 45−49
Data points enclosed with a diamond are reference sources

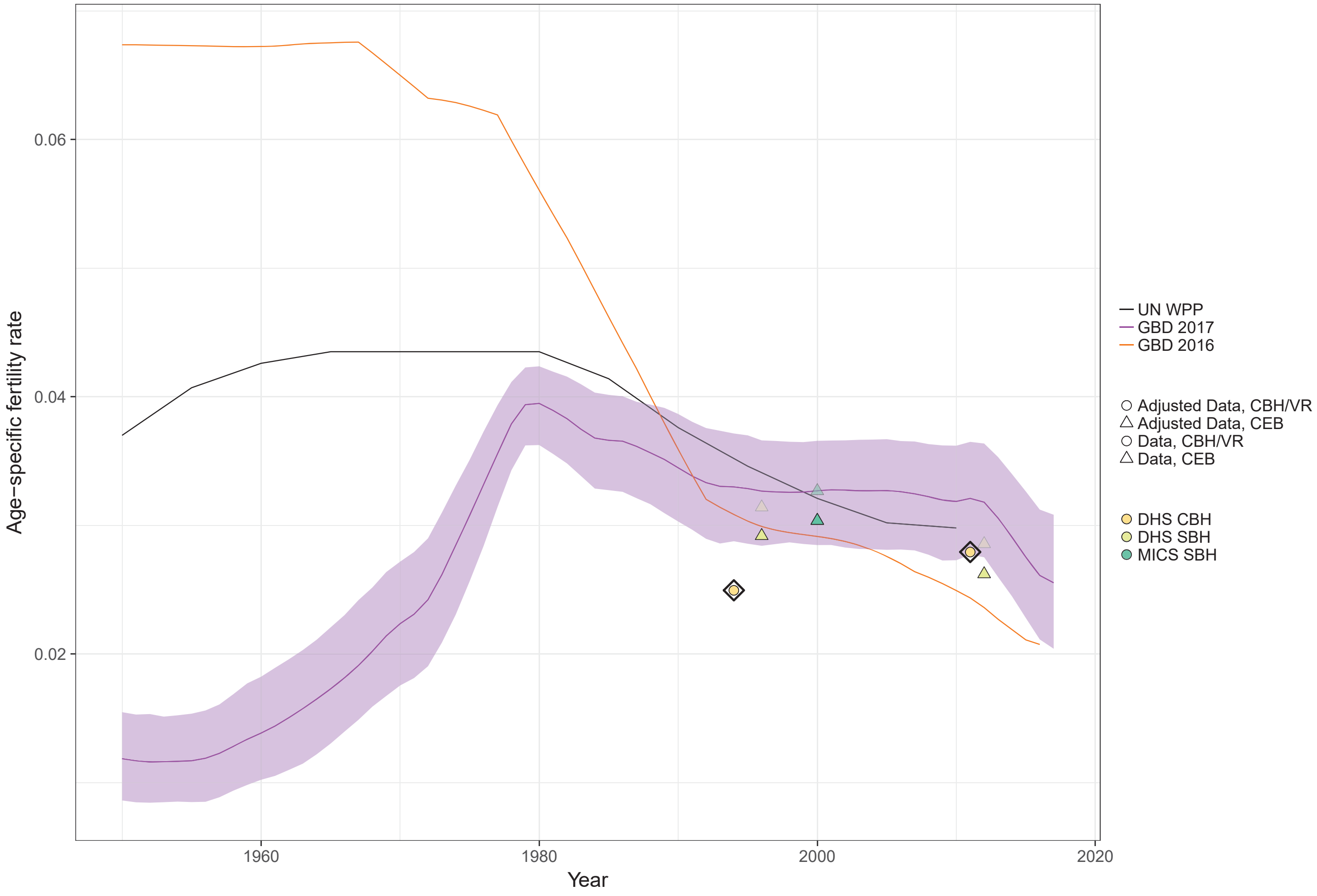

Age−specific fertility fitted trend lines, 1950−2017: Comoros, Ages: 50−54
Data points enclosed with a diamond are reference sources

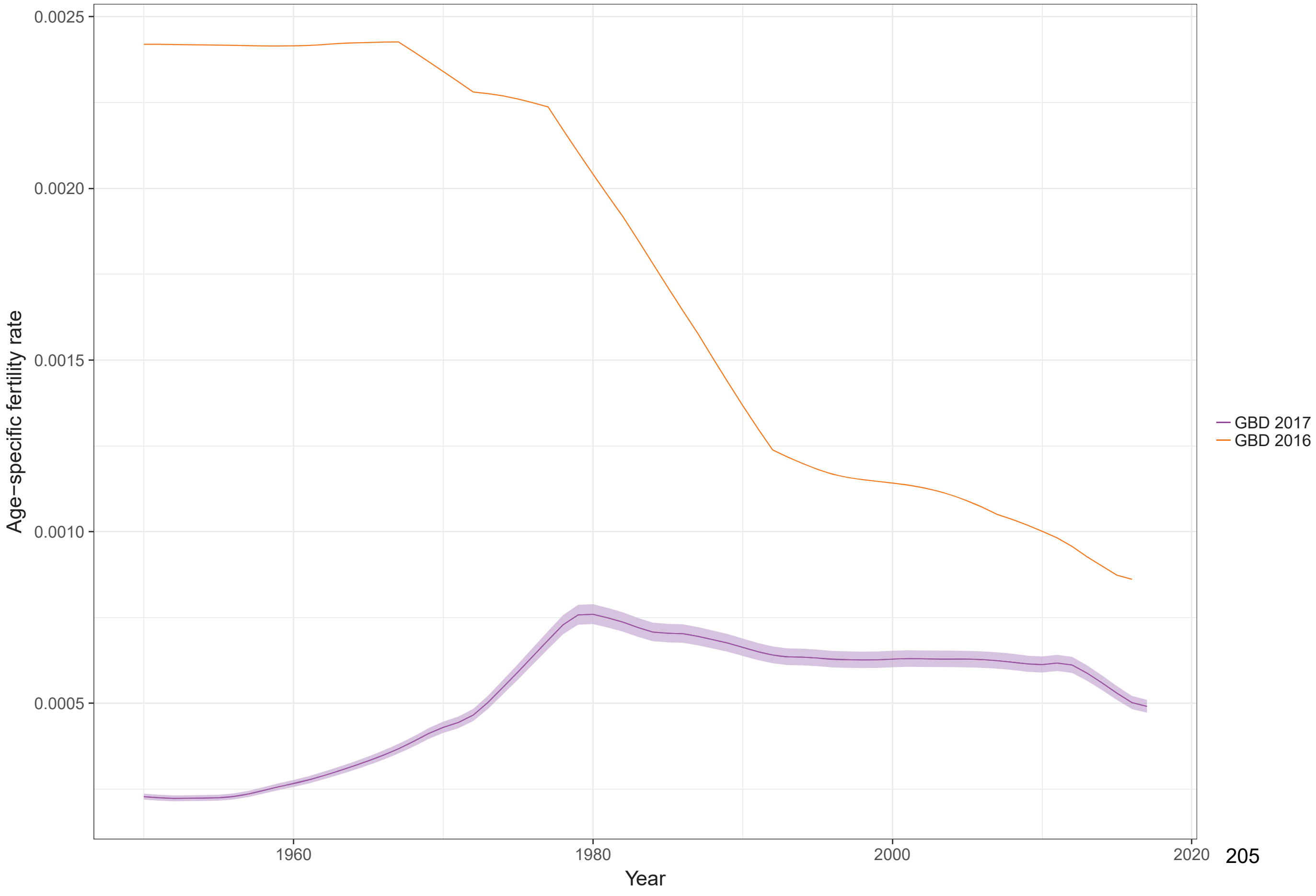

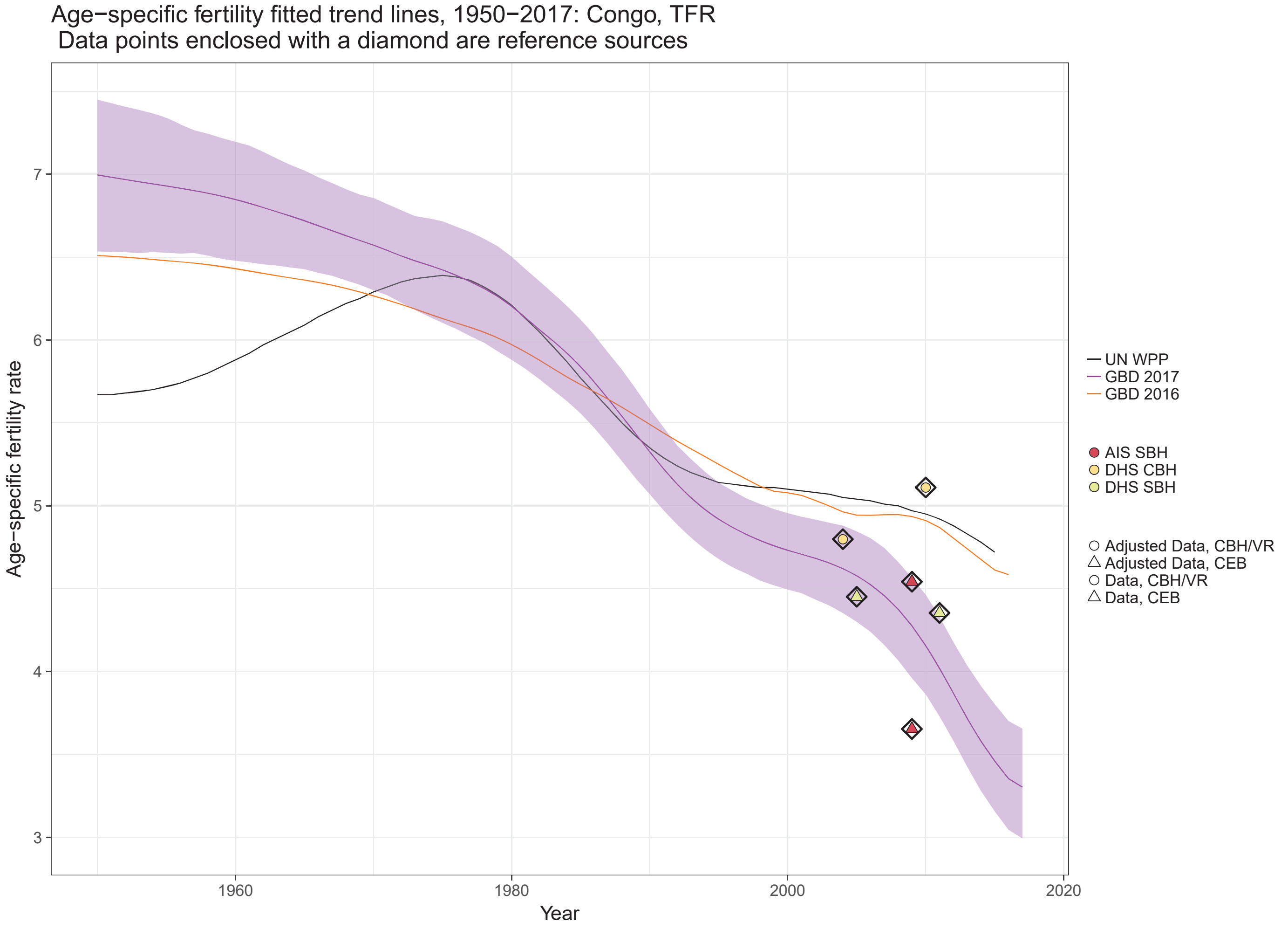
Age−specific fertility fitted trend lines, 1950−2017: Congo, TFR
Data points enclosed with a diamond are reference sources
Age−specific fertility rate
Year
3
4
5
6
7
1960
1980
2000
2020
UN WPP
GBD 2017
GBD 2016
AIS SBH
DHS CBH
DHS SBH
Adjusted Data, CBH/VR
Adjusted Data, CEB
Data, CBH/VR
Data, CEB

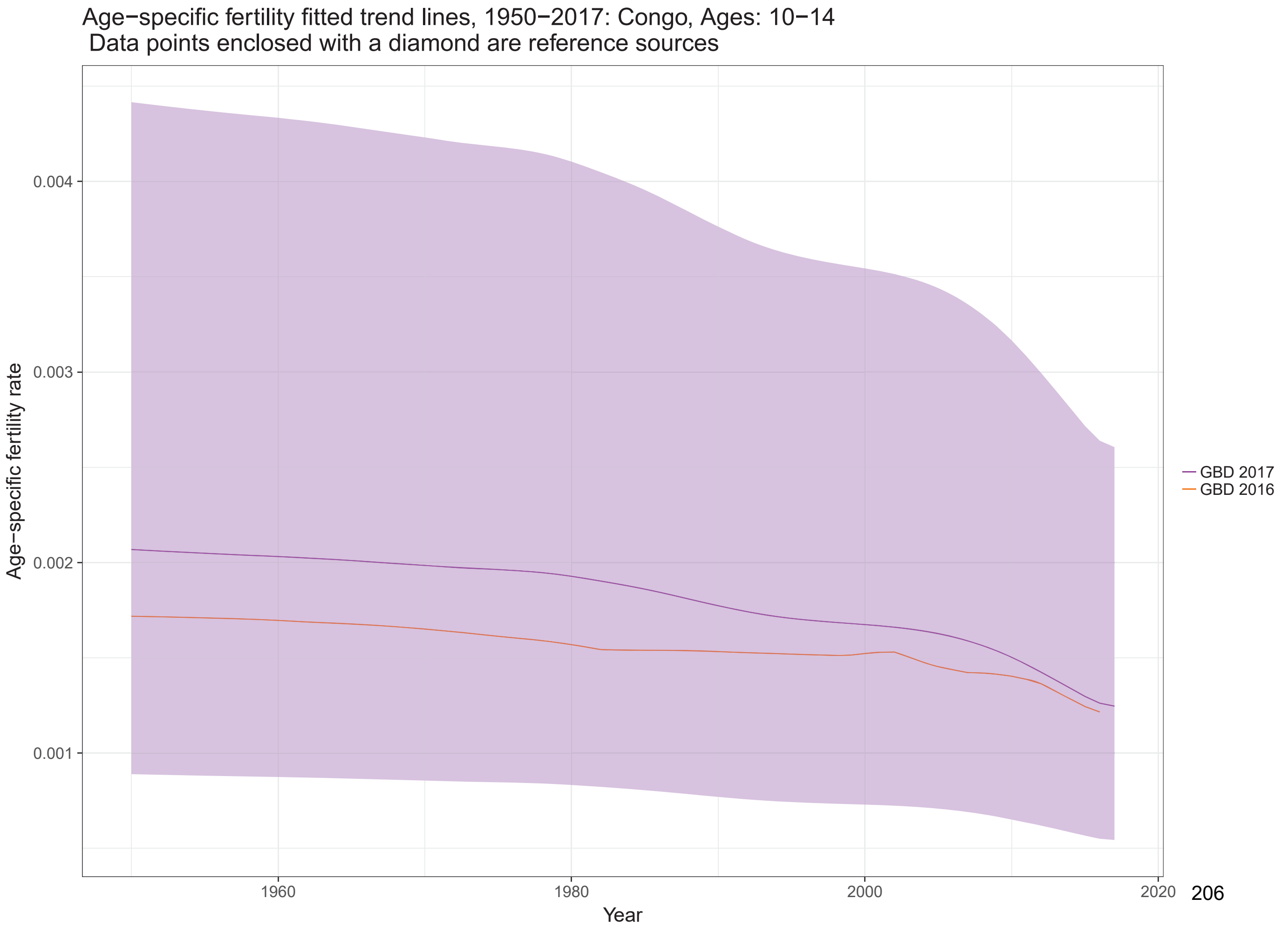
Age−specific fertility fitted trend lines, 1950−2017: Congo, Ages: 10−14
Data points enclosed with a diamond are reference sources
Age−specific fertility rate
Year
0.001
0.002
0.003
0.004
1960
1980
2000
2020
GBD 2017
GBD 2016

Age−specific fertility fitted trend lines, 1950−2017: Congo, Ages: 15−19
Data points enclosed with a diamond are reference sources

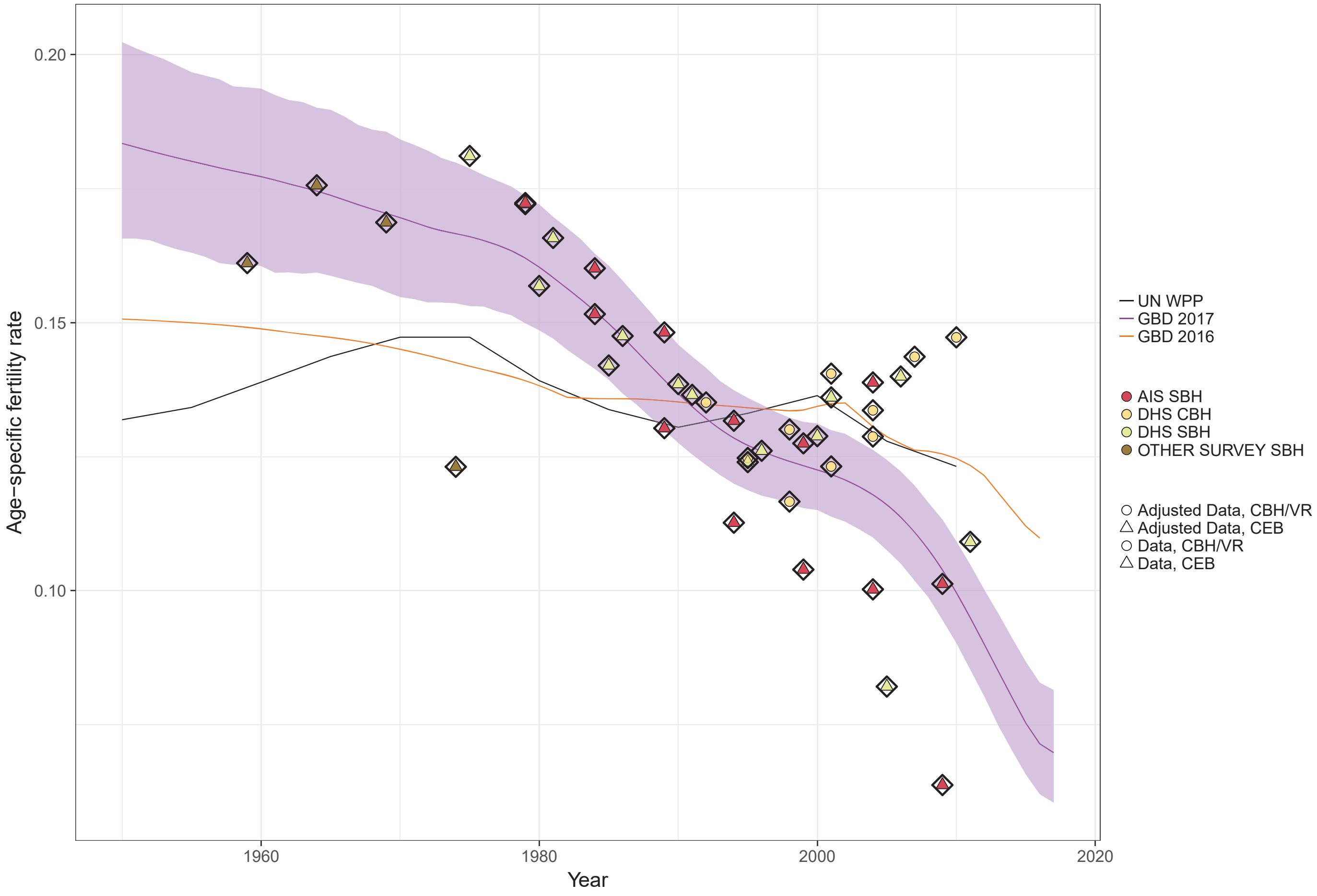

Age−specific fertility fitted trend lines, 1950−2017: Congo, Ages: 20−24
Data points enclosed with a diamond are reference sources

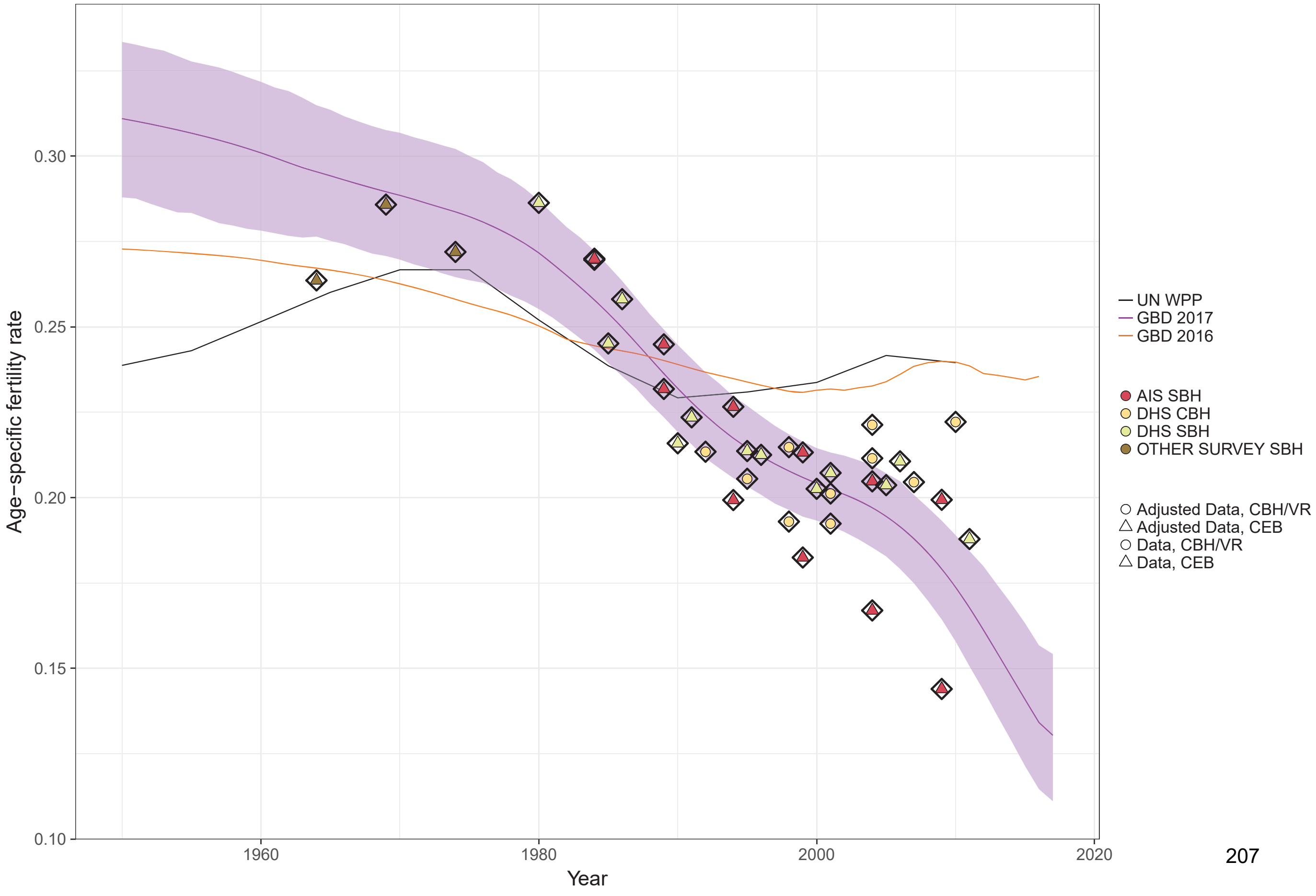

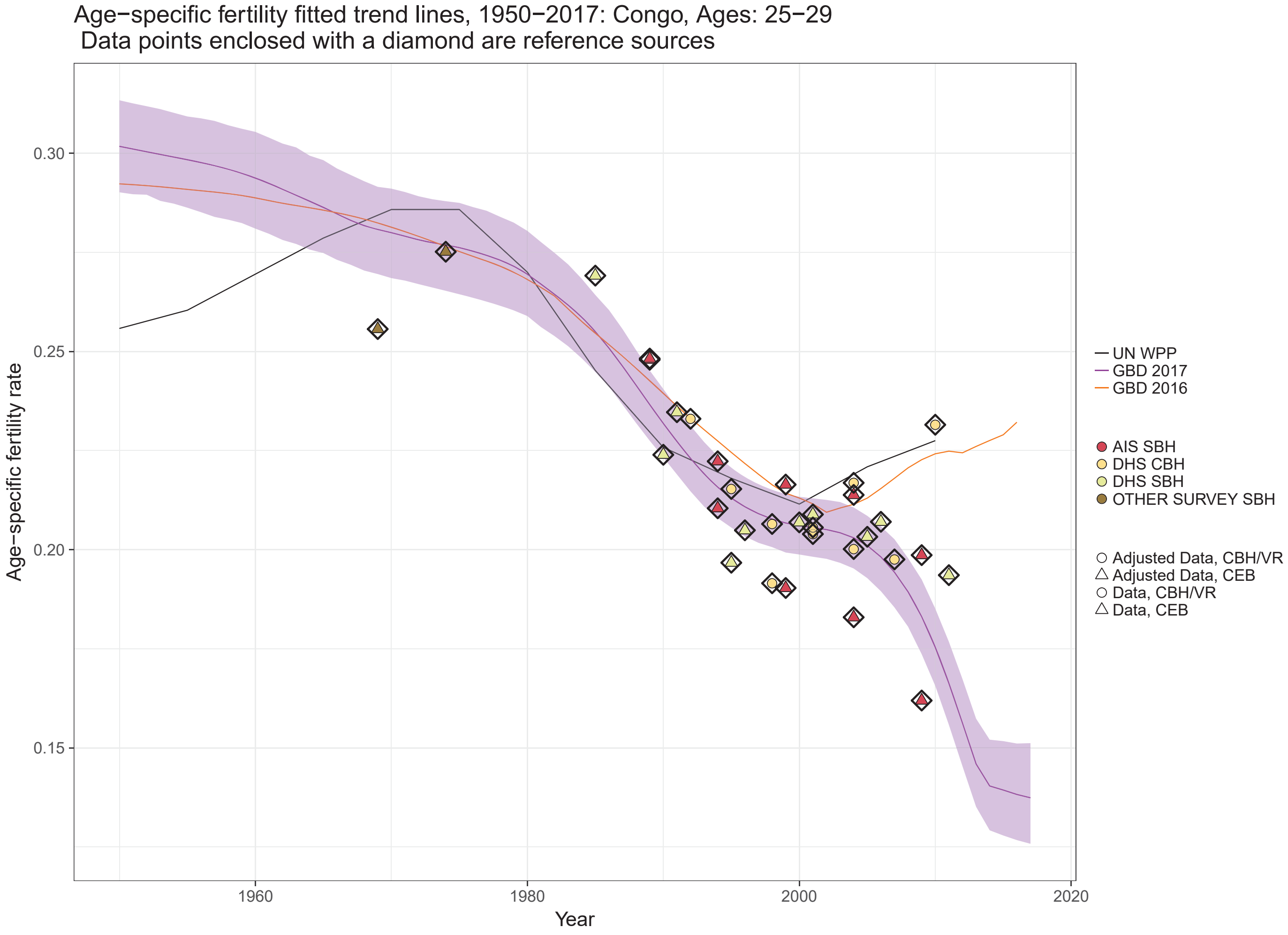
Age−specific fertility fitted trend lines, 1950−2017: Congo, Ages: 25−29
Data points enclosed with a diamond are reference sources
Age−specific fertility rate
0.30
0.25
0.20
0.15
1960
1980
2000
2020
Year
UN WPP
GBD 2017
GBD 2016
AIS SBH
DHS CBH
DHS SBH
OTHER SURVEY SBH
Adjusted Data, CBH/VR
Adjusted Data, CEB
Data, CBH/VR
Data, CEB

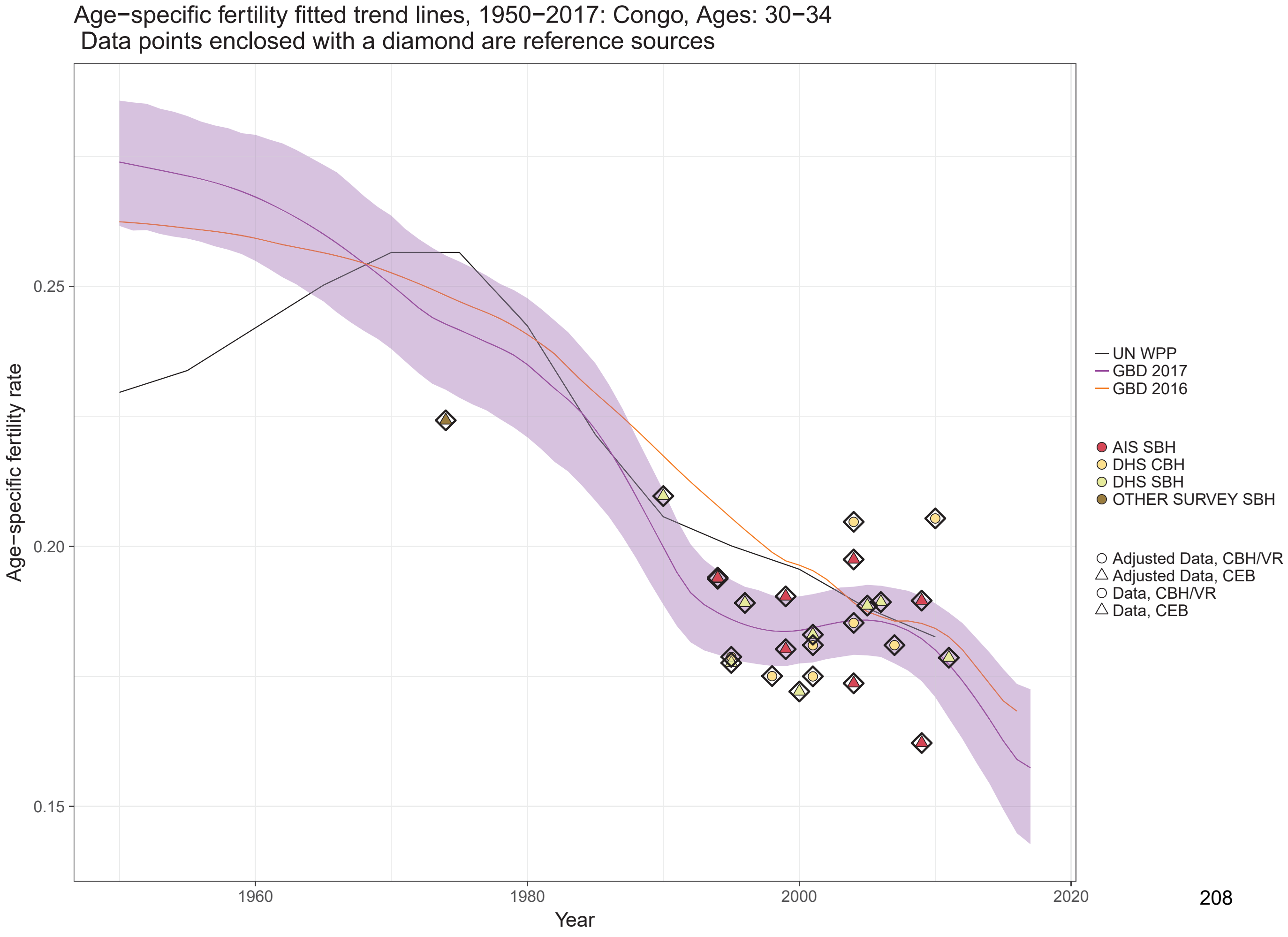
Age−specific fertility fitted trend lines, 1950−2017: Congo, Ages: 30−34
Data points enclosed with a diamond are reference sources
Age−specific fertility rate
0.25
0.20
0.15
1960
1980
2000
2020
Year
UN WPP
GBD 2017
GBD 2016
AIS SBH
DHS CBH
DHS SBH
OTHER SURVEY SBH
Adjusted Data, CBH/VR
Adjusted Data, CEB
Data, CBH/VR
Data, CEB

Age−specific fertility fitted trend lines, 1950−2017: Congo, Ages: 35−39
Data points enclosed with a diamond are reference sources

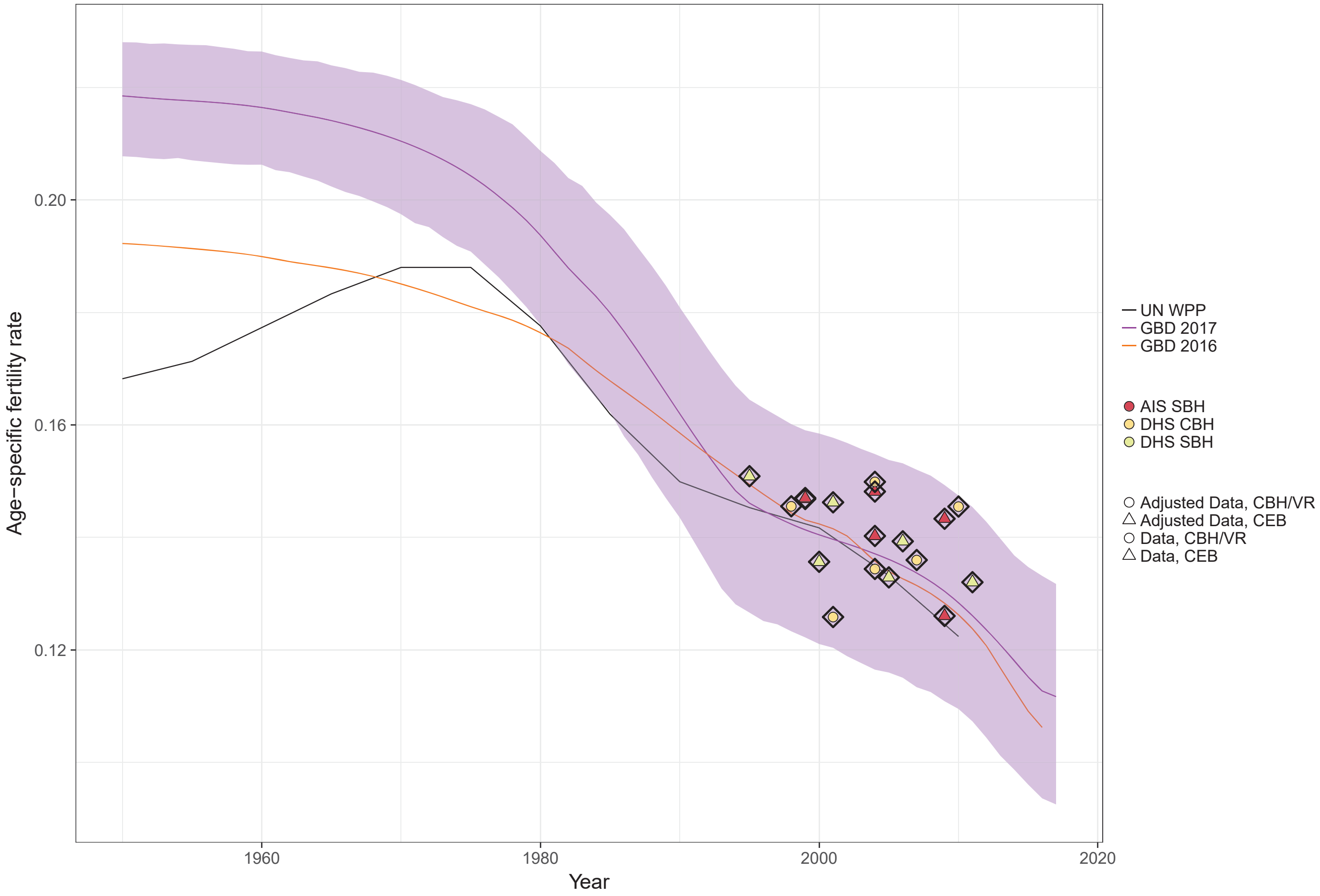

Age−specific fertility fitted trend lines, 1950−2017: Congo, Ages: 40−44
Data points enclosed with a diamond are reference sources

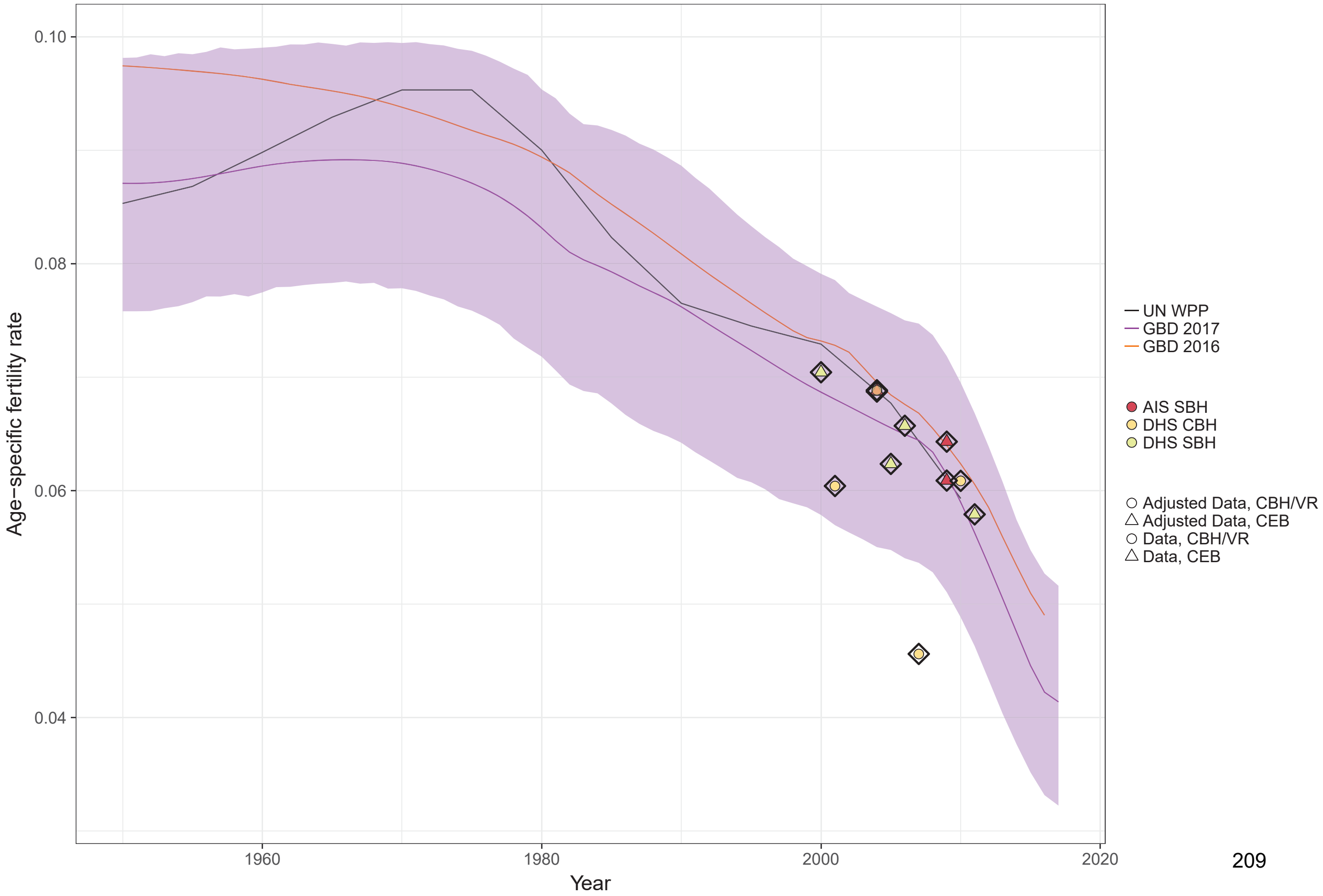

Age−specific fertility fitted trend lines, 1950−2017: Congo, Ages: 45−49
Data points enclosed with a diamond are reference sources

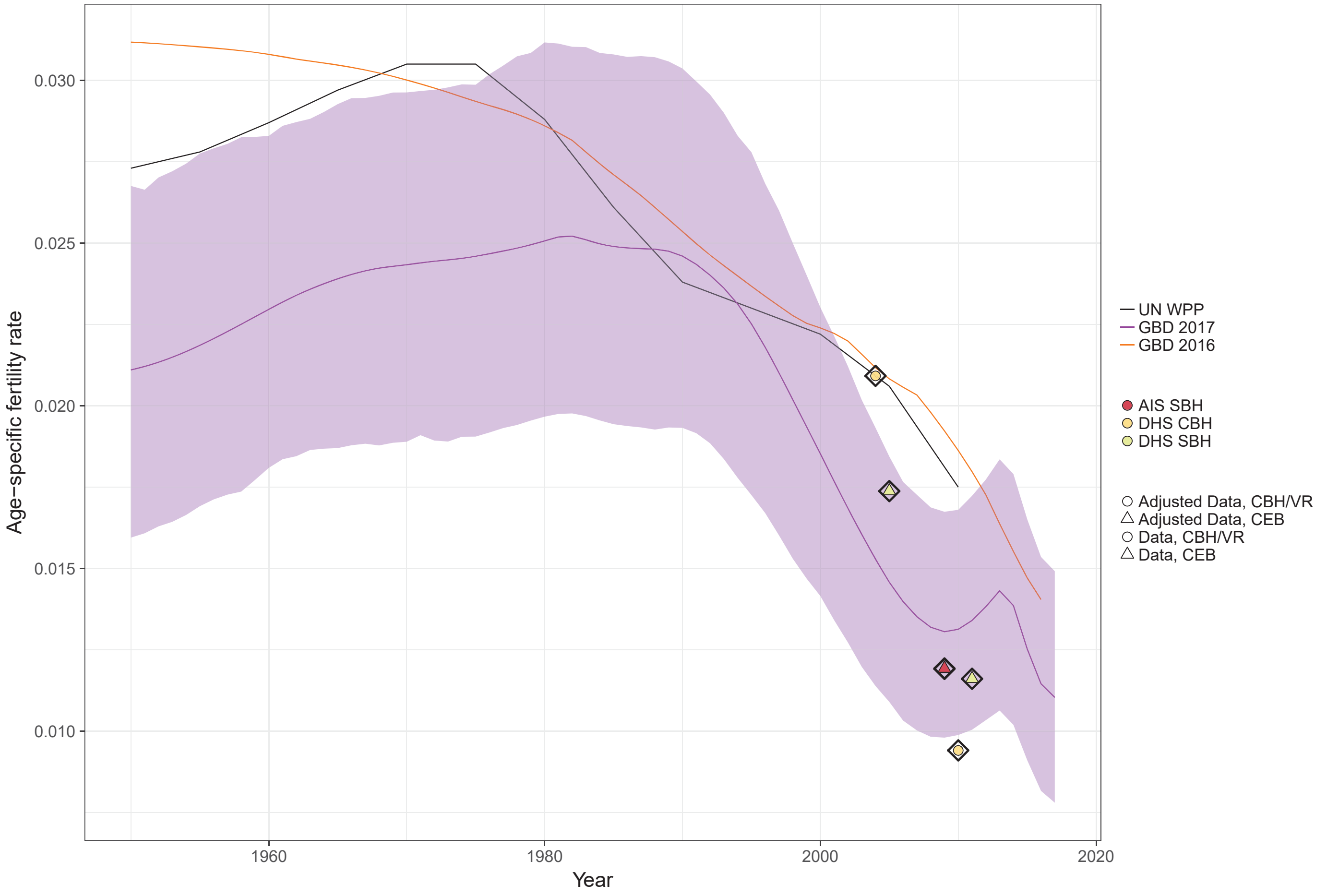

Age−specific fertility fitted trend lines, 1950−2017: Congo, Ages: 50−54
Data points enclosed with a diamond are reference sources

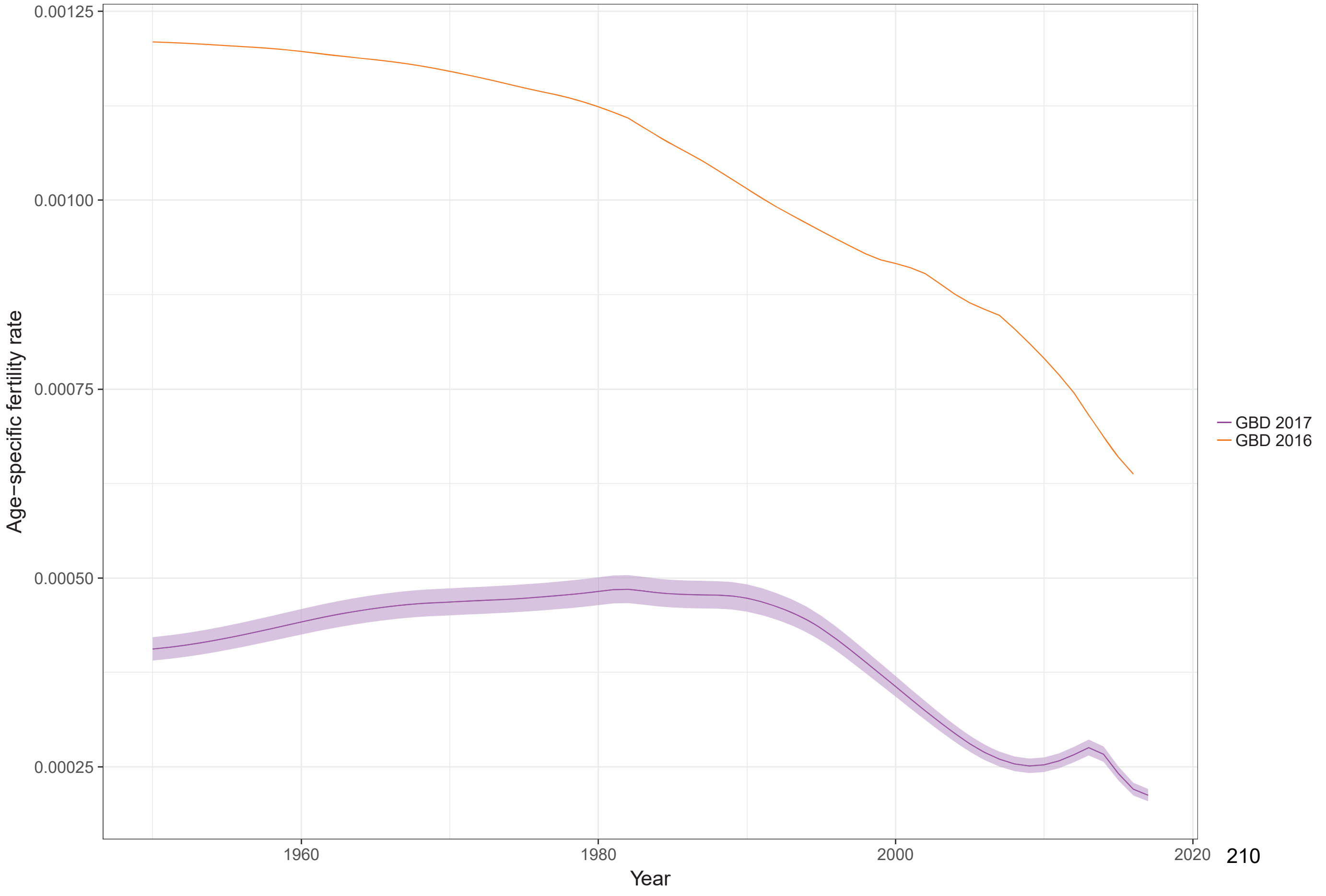

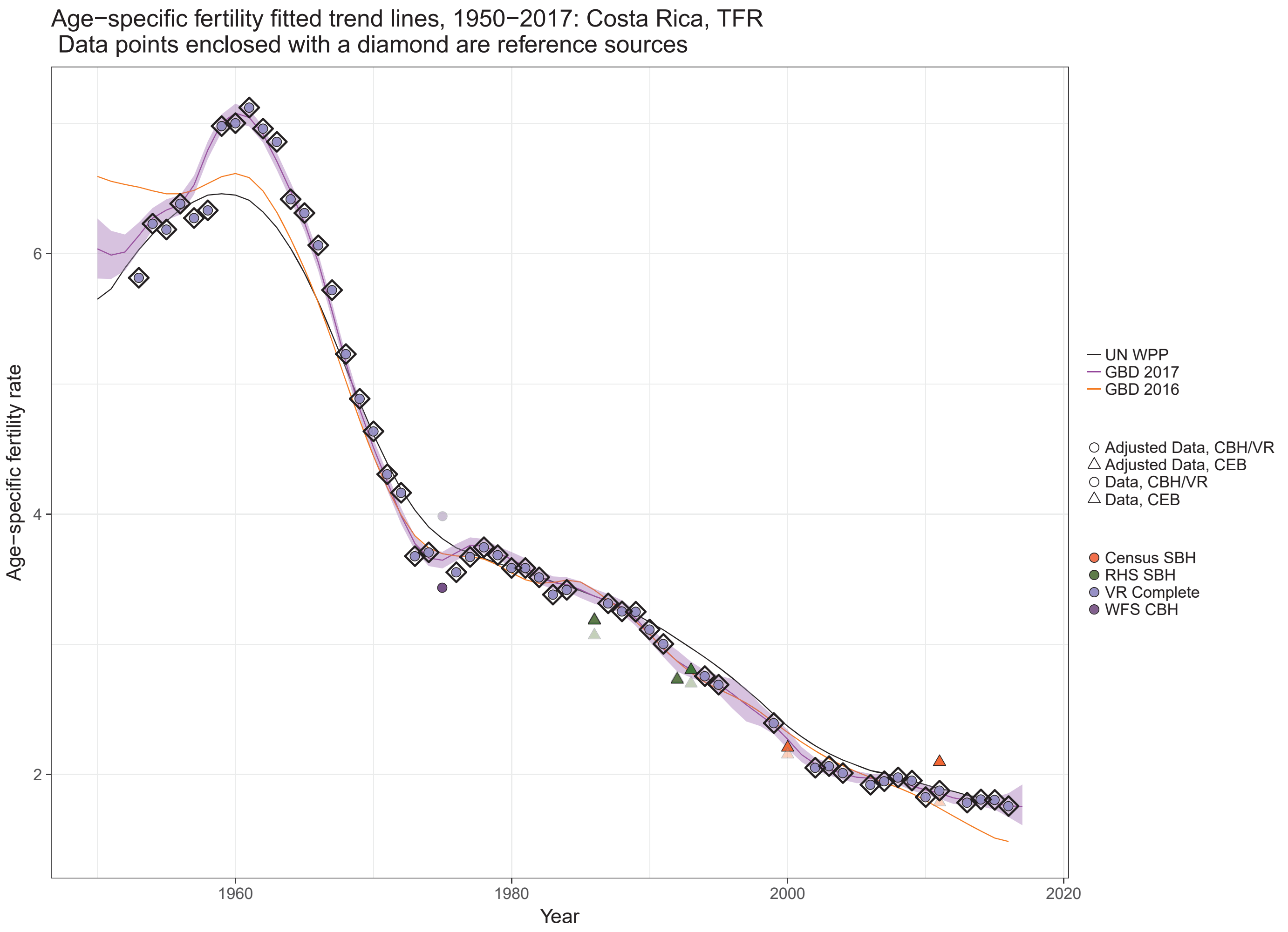

Age−specific fertility fitted trend lines, 1950−2017: Costa Rica, TFR
Data points enclosed with a diamond are reference sources
Age−specific fertility rate
Year
UN WPP
GBD 2017
GBD 2016
Adjusted Data, CBH/VR
Adjusted Data, CEB
Data, CBH/VR
Data, CEB
Census SBH
RHS SBH
VR Complete
WFS CBH

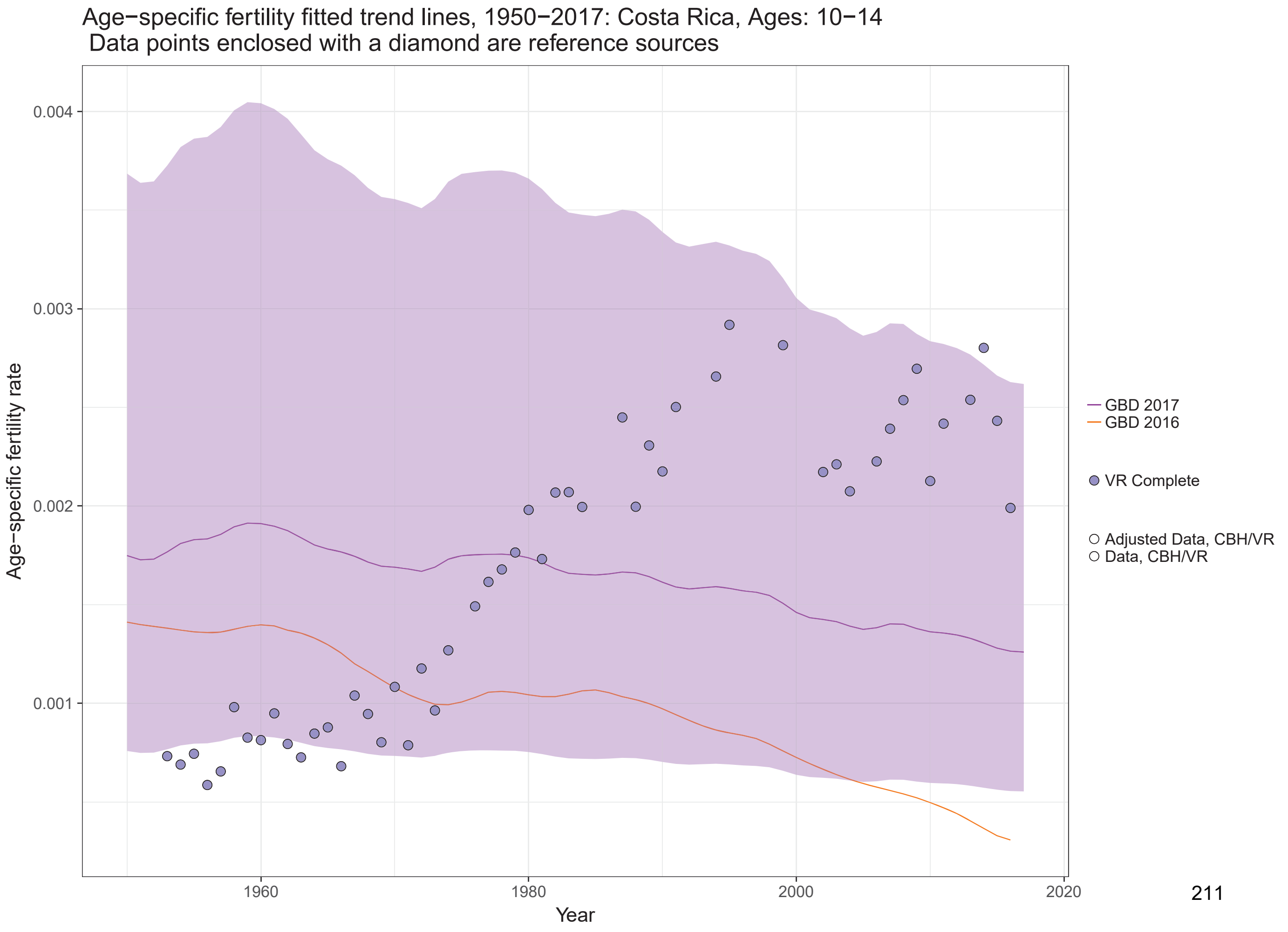

Age−specific fertility fitted trend lines, 1950−2017: Costa Rica, Ages: 10−14
Data points enclosed with a diamond are reference sources
Age−specific fertility rate
Year
GBD 2017
GBD 2016
VR Complete
Adjusted Data, CBH/VR
Data, CBH/VR

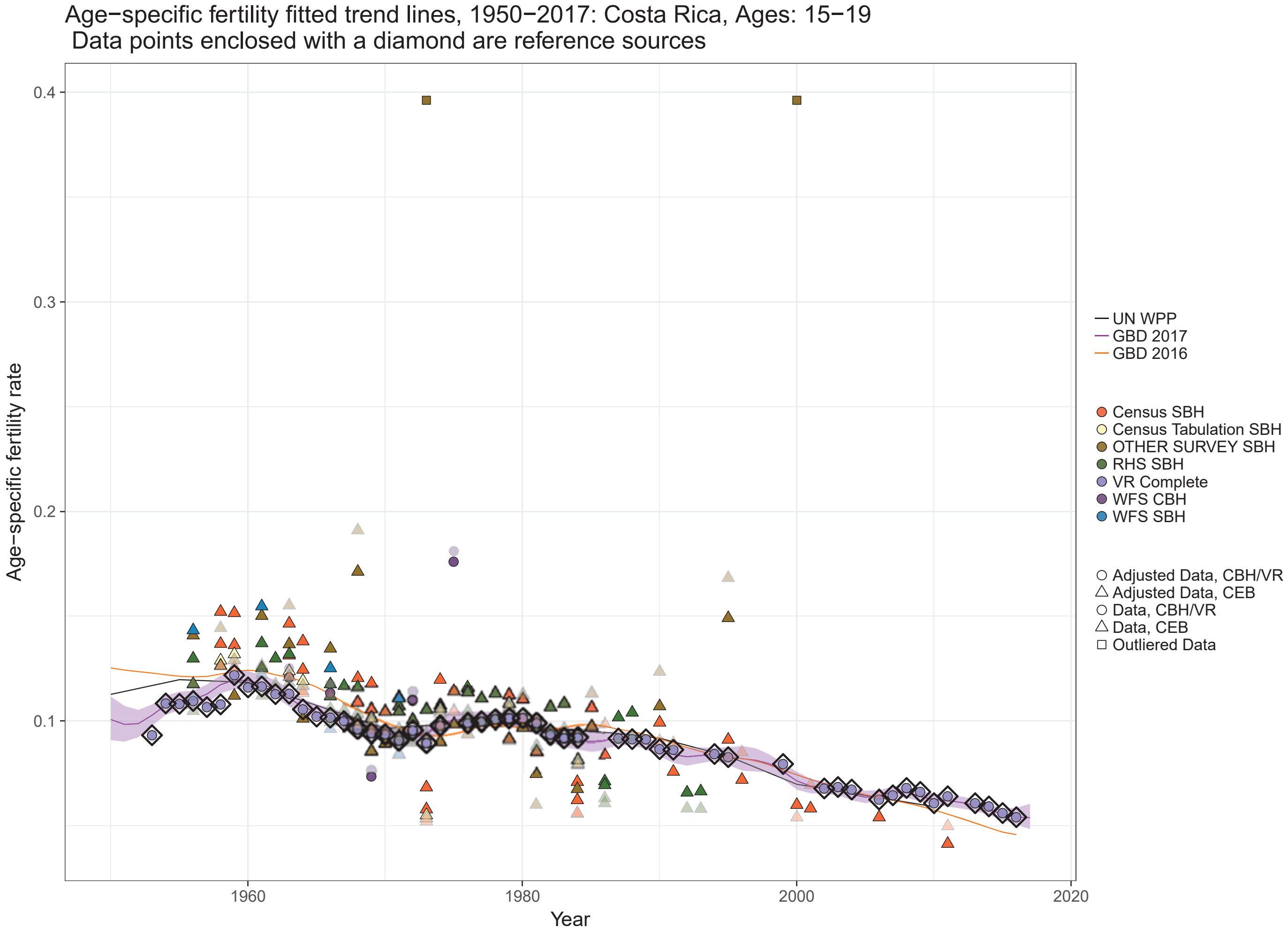

Age−specific fertility fitted trend lines, 1950−2017: Costa Rica, Ages: 15−19
Data points enclosed with a diamond are reference sources
Age−specific fertility rate
0.4
0.3
0.2
0.1
1960
1980
2000
2020
Year
UN WPP
GBD 2017
GBD 2016
Census SBH
Census Tabulation SBH
OTHER SURVEY SBH
RHS SBH
VR Complete
WFS CBH
WFS SBH
Adjusted Data, CBH/VR
Adjusted Data, CEB
Data, CBH/VR
Data, CEB
Outliered Data

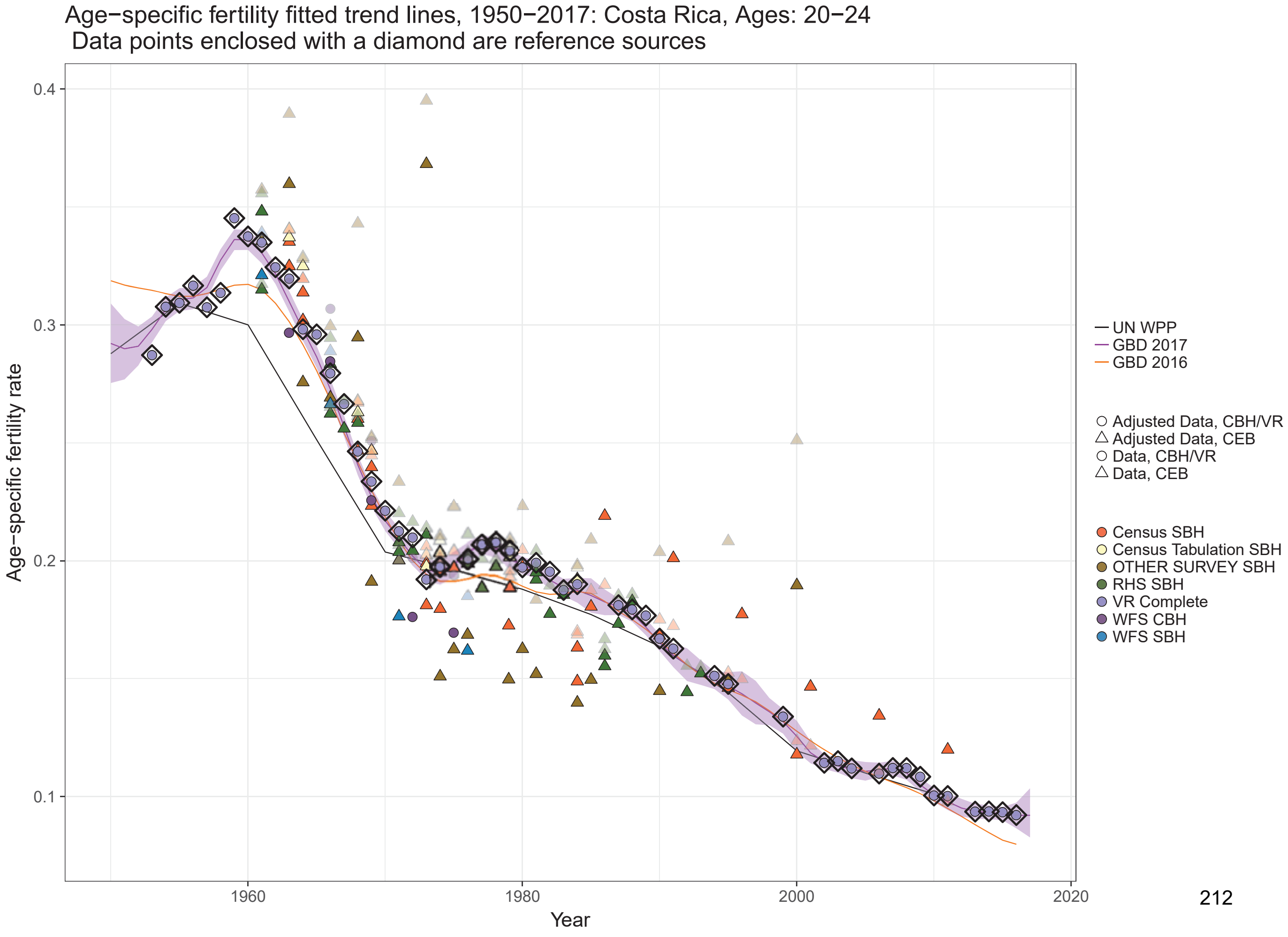

Age−specific fertility fitted trend lines, 1950−2017: Costa Rica, Ages: 20−24
Data points enclosed with a diamond are reference sources
Age−specific fertility rate
0.4
0.3
0.2
0.1
1960
1980
2000
2020
Year
UN WPP
GBD 2017
GBD 2016
Adjusted Data, CBH/VR
Adjusted Data, CEB
Data, CBH/VR
Data, CEB
Census SBH
Census Tabulation SBH
OTHER SURVEY SBH
RHS SBH
VR Complete
WFS CBH
WFS SBH

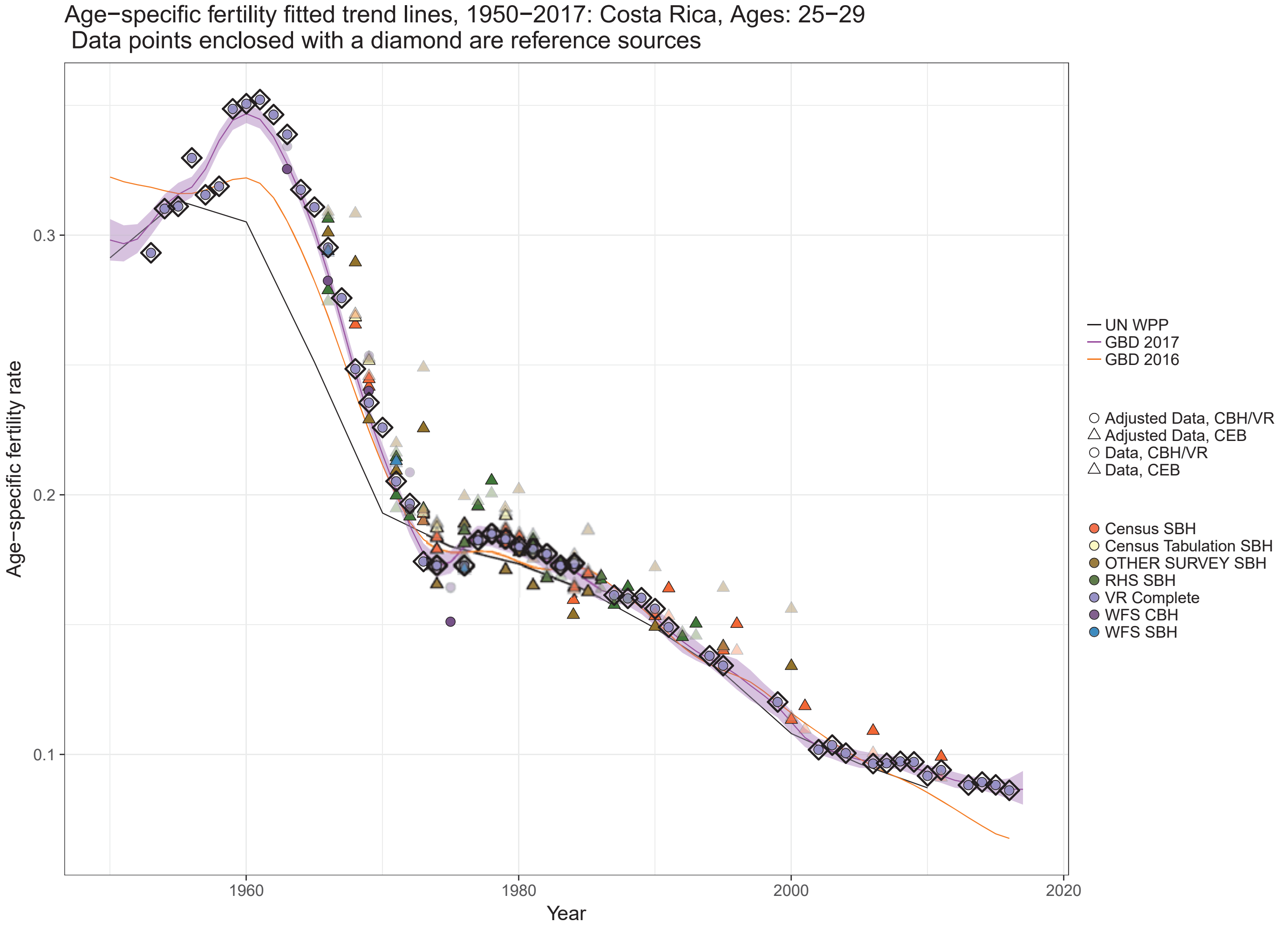
Age−specific fertility fitted trend lines, 1950−2017: Costa Rica, Ages: 25−29
Data points enclosed with a diamond are reference sources
Age-specific fertility rate
0.3
0.2
0.1
1960
1980
2000
2020
Year
UN WPP
GBD 2017
GBD 2016
Adjusted Data, CBH/VR
Adjusted Data, CEB
Data, CBH/VR
Data, CEB
Census SBH
Census Tabulation SBH
OTHER SURVEY SBH
RHS SBH
VR Complete
WFS CBH
WFS SBH

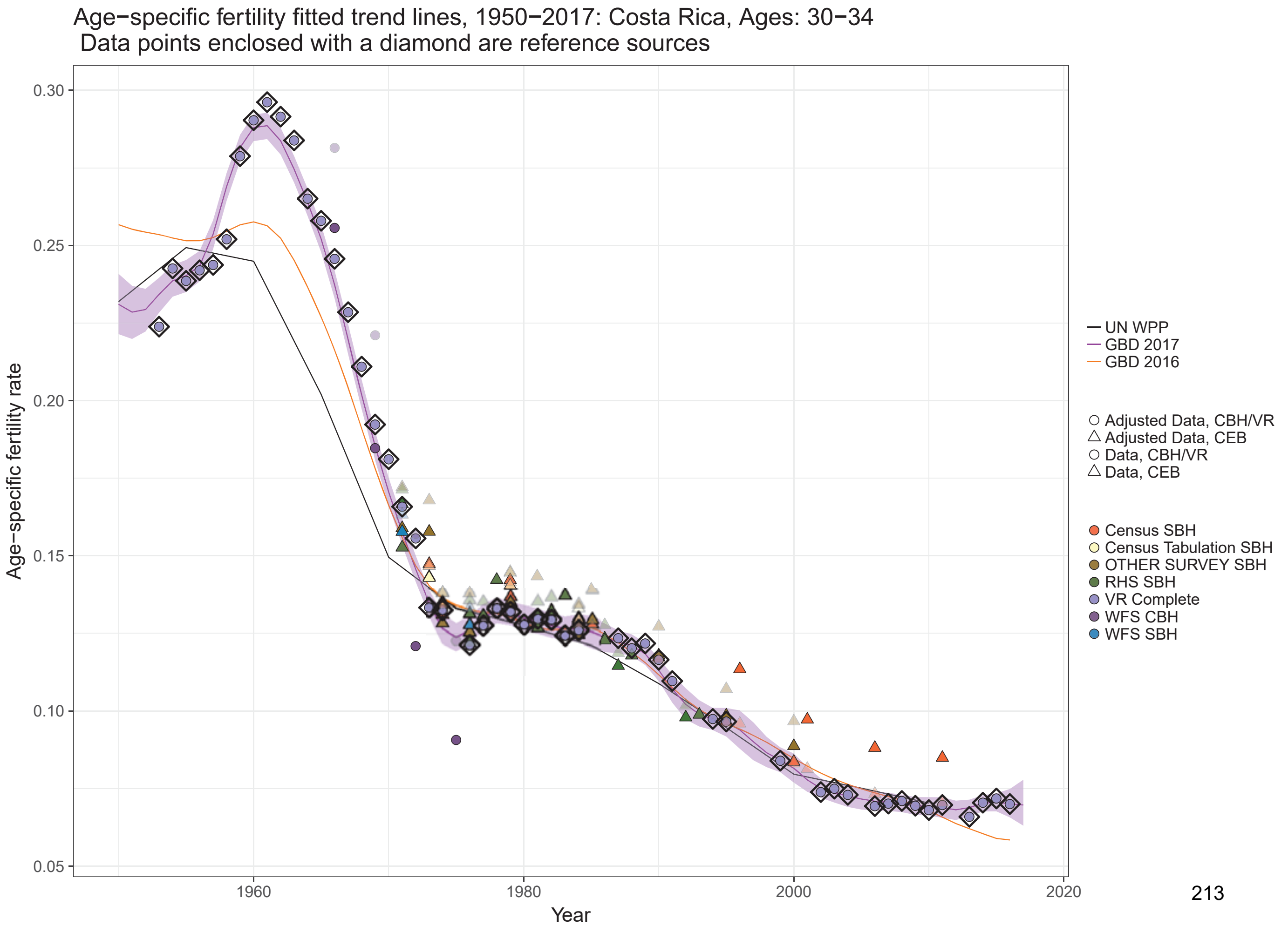
Age−specific fertility fitted trend lines, 1950−2017: Costa Rica, Ages: 30−34
Data points enclosed with a diamond are reference sources
Age-specific fertility rate
0.30
0.25
0.20
0.15
0.10
0.05
1960
1980
2000
2020
Year
UN WPP
GBD 2017
GBD 2016
Adjusted Data, CBH/VR
Adjusted Data, CEB
Data, CBH/VR
Data, CEB
Census SBH
Census Tabulation SBH
OTHER SURVEY SBH
RHS SBH
VR Complete
WFS CBH
WFS SBH

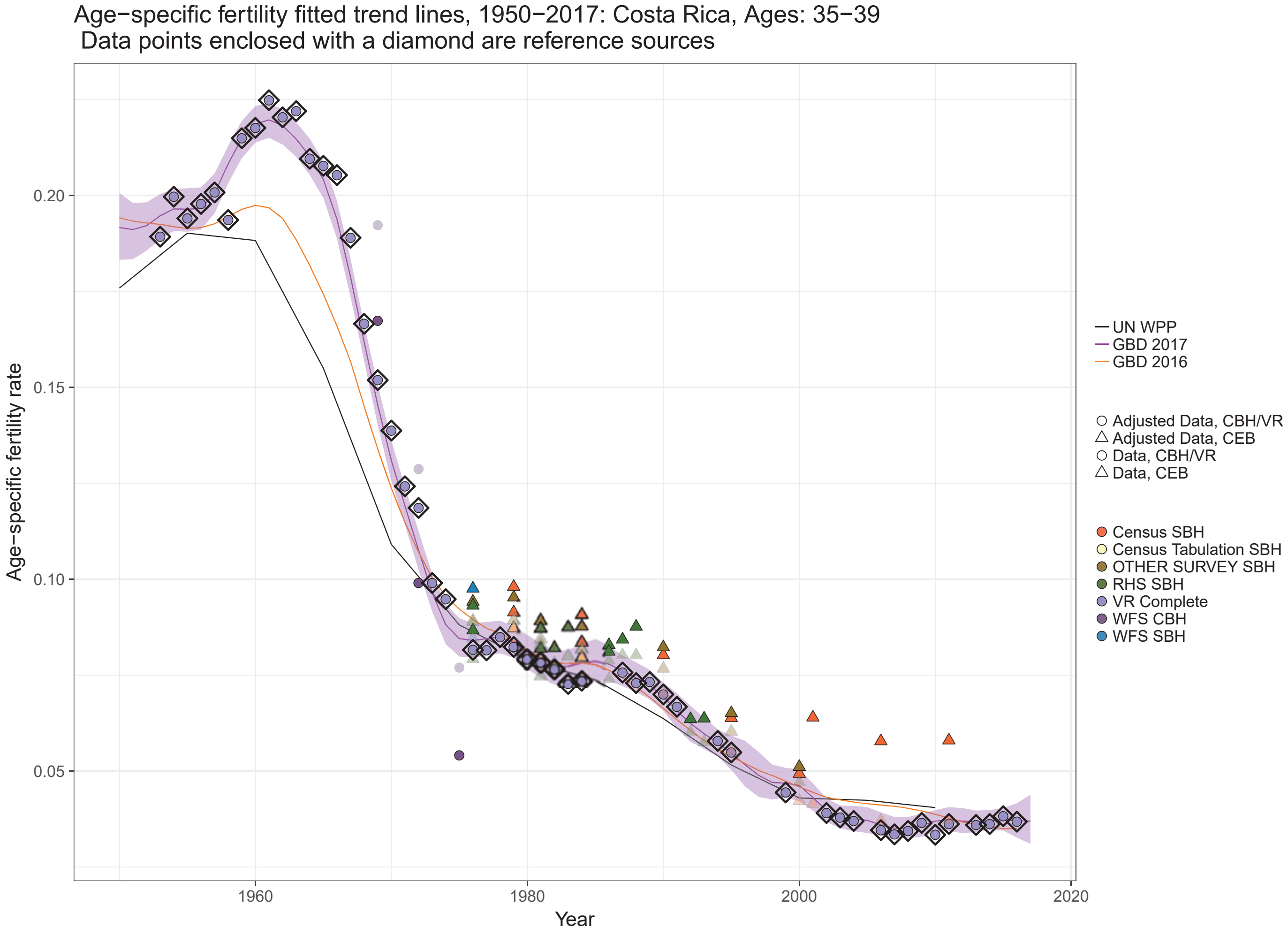

Age−specific fertility fitted trend lines, 1950−2017: Costa Rica, Ages: 35−39
Data points enclosed with a diamond are reference sources
Age-specific fertility rate
Year
UN WPP
GBD 2017
GBD 2016
Adjusted Data, CBH/VR
Adjusted Data, CEB
Data, CBH/VR
Data, CEB
Census SBH
Census Tabulation SBH
OTHER SURVEY SBH
RHS SBH
VR Complete
WFS CBH
WFS SBH

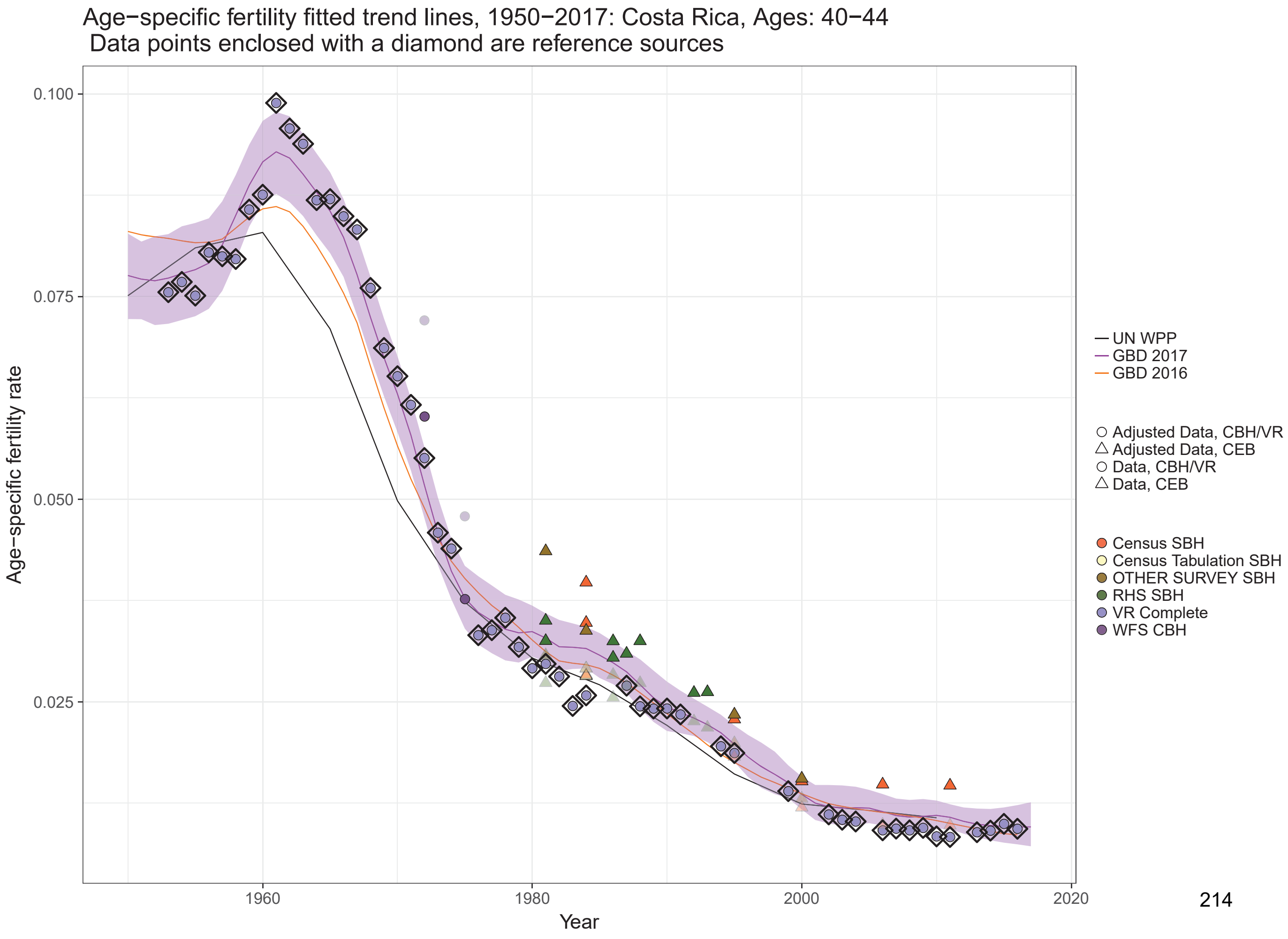

Age−specific fertility fitted trend lines, 1950−2017: Costa Rica, Ages: 40−44
Data points enclosed with a diamond are reference sources
Age-specific fertility rate
Year
UN WPP
GBD 2017
GBD 2016
Adjusted Data, CBH/VR
Adjusted Data, CEB
Data, CBH/VR
Data, CEB
Census SBH
Census Tabulation SBH
OTHER SURVEY SBH
RHS SBH
VR Complete
WFS CBH

Age−specific fertility fitted trend lines, 1950−2017: Costa Rica, Ages: 45−49
Data points enclosed with a diamond are reference sources

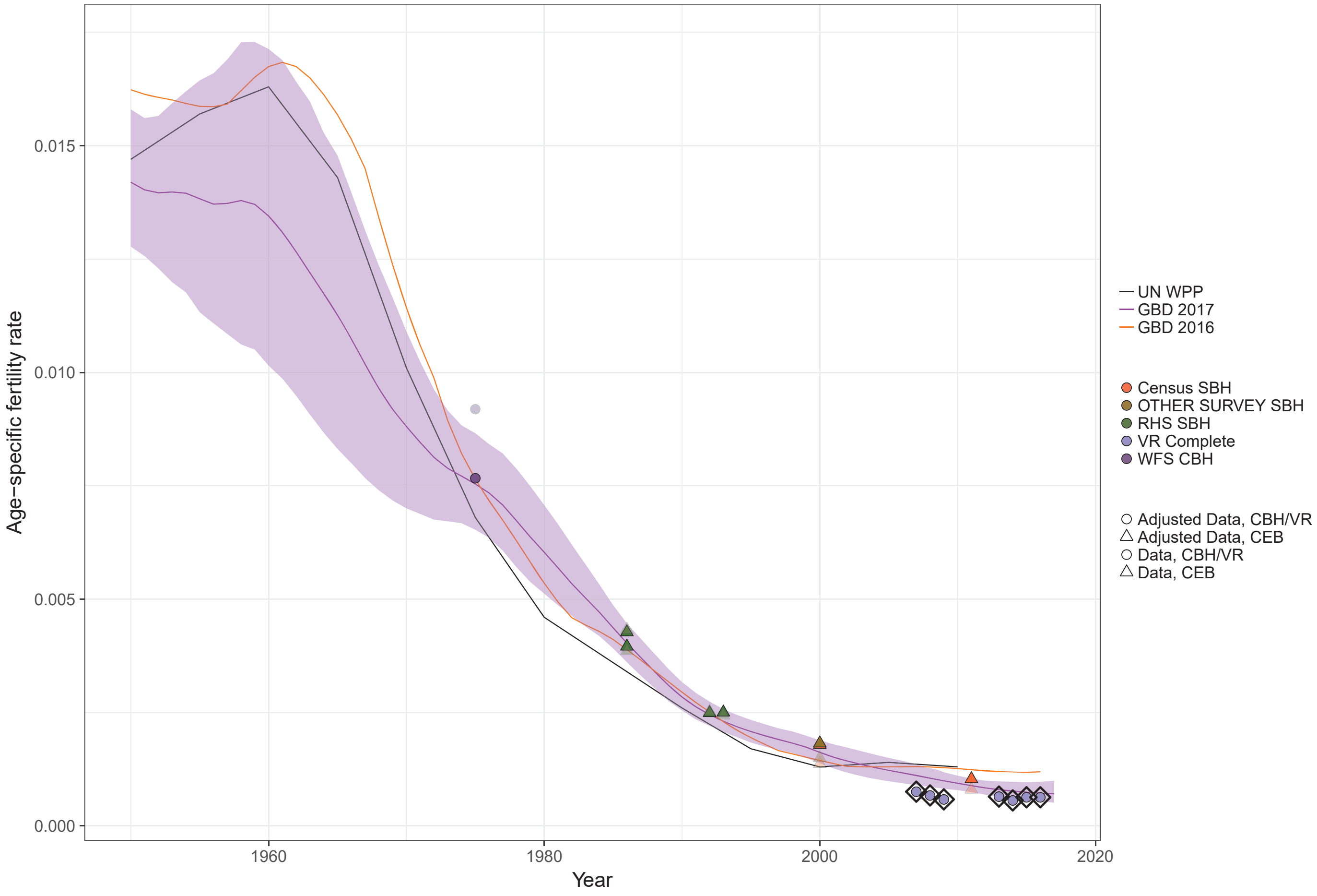

Age−specific fertility fitted trend lines, 1950−2017: Costa Rica, Ages: 50−54
Data points enclosed with a diamond are reference sources

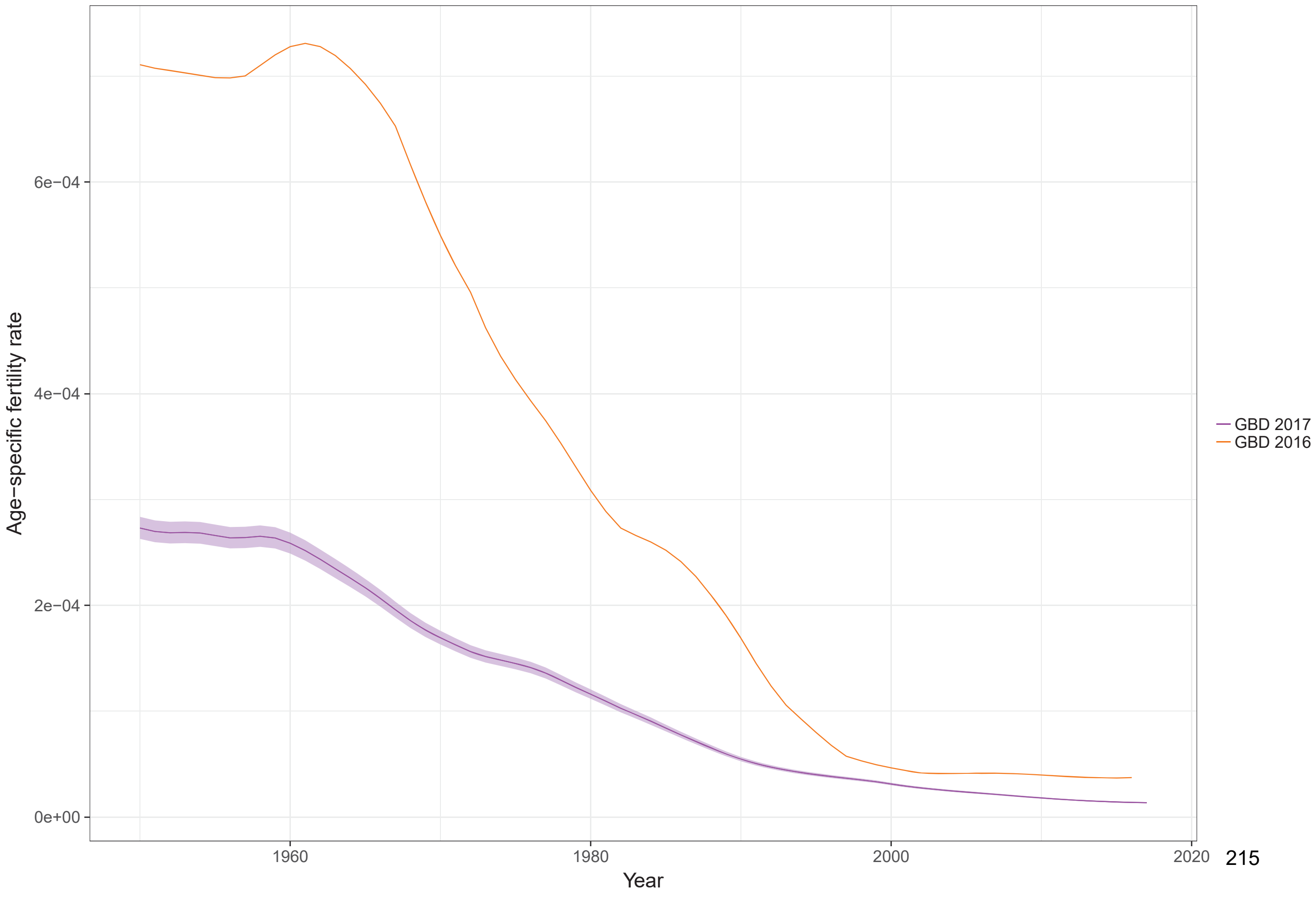

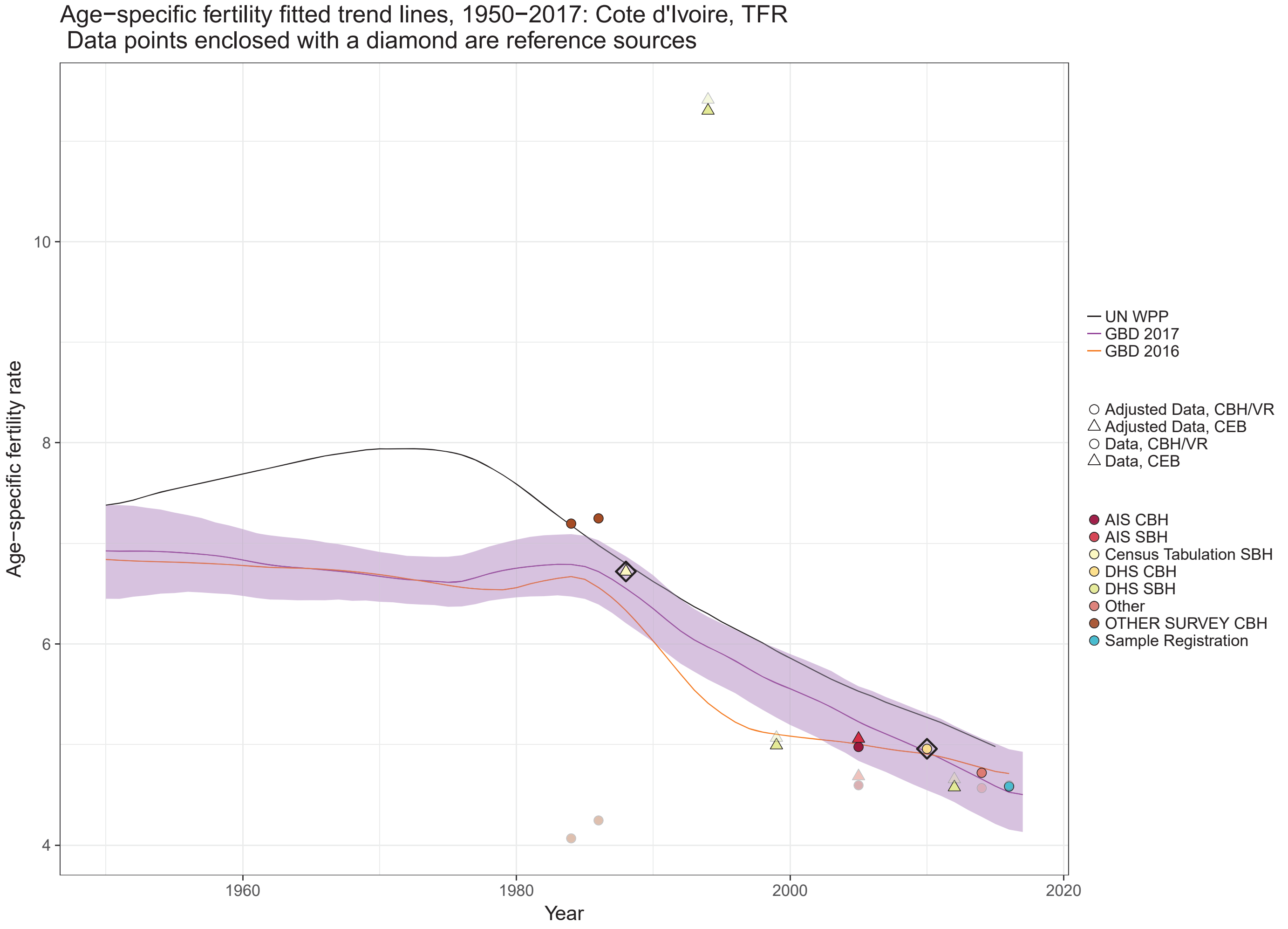
Age−specific fertility fitted trend lines, 1950−2017: Cote d'Ivoire, TFR
Data points enclosed with a diamond are reference sources
Age−specific fertility rate
Year
4
6
8
10
1960
1980
2000
2020
UN WPP
GBD 2017
GBD 2016
Adjusted Data, CBH/VR
Adjusted Data, CEB
Data, CBH/VR
Data, CEB
AIS CBH
AIS SBH
Census Tabulation SBH
DHS CBH
DHS SBH
Other
OTHER SURVEY CBH
Sample Registration

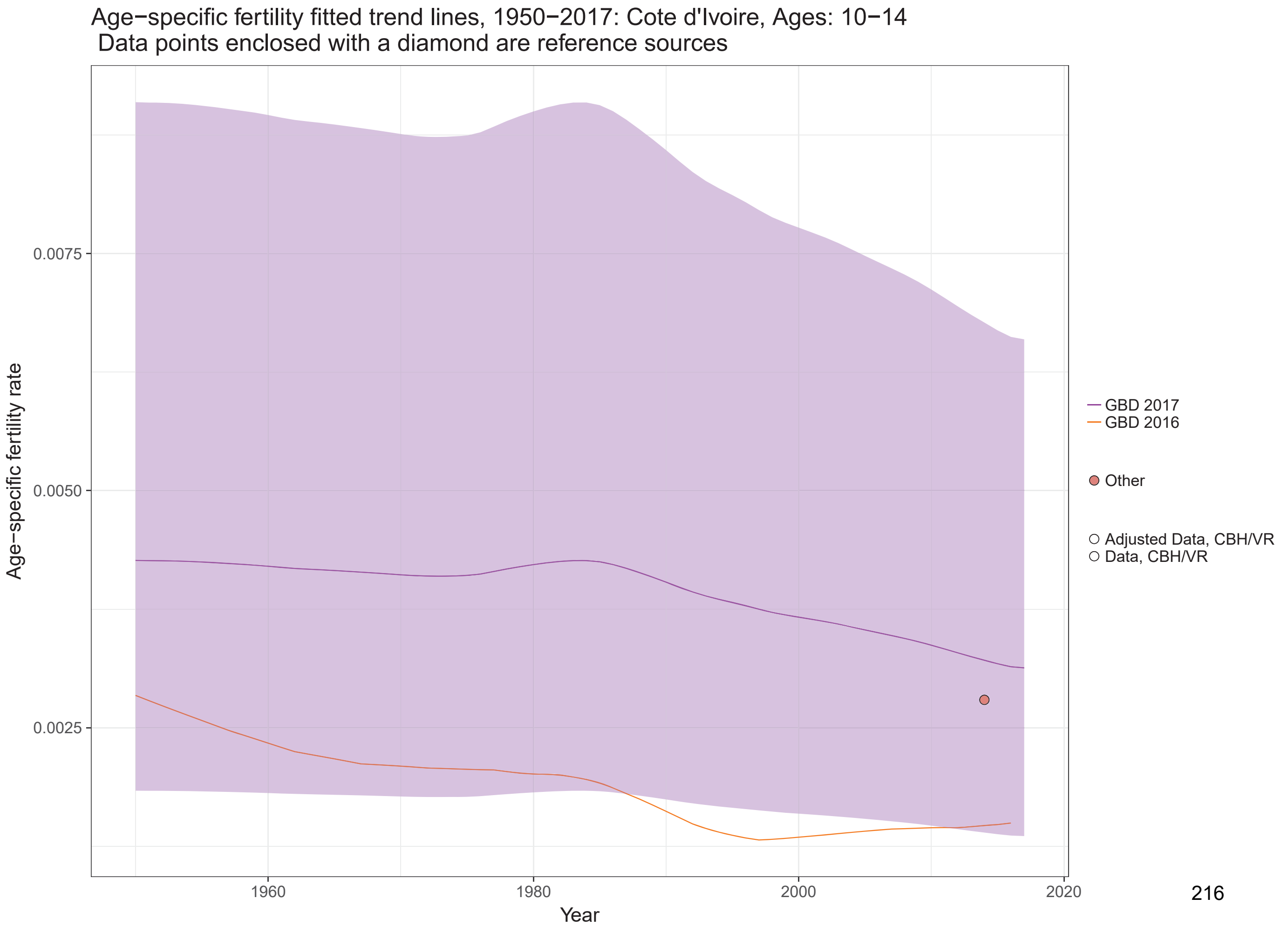
Age−specific fertility fitted trend lines, 1950−2017: Cote d'Ivoire, Ages: 10−14
Data points enclosed with a diamond are reference sources
Age−specific fertility rate
Year
0.0025
0.0050
0.0075
1960
1980
2000
2020
GBD 2017
GBD 2016
Other
Adjusted Data, CBH/VR
Data, CBH/VR

Age−specific fertility fitted trend lines, 1950−2017: Cote d'Ivoire, Ages: 15−19
Data points enclosed with a diamond are reference sources

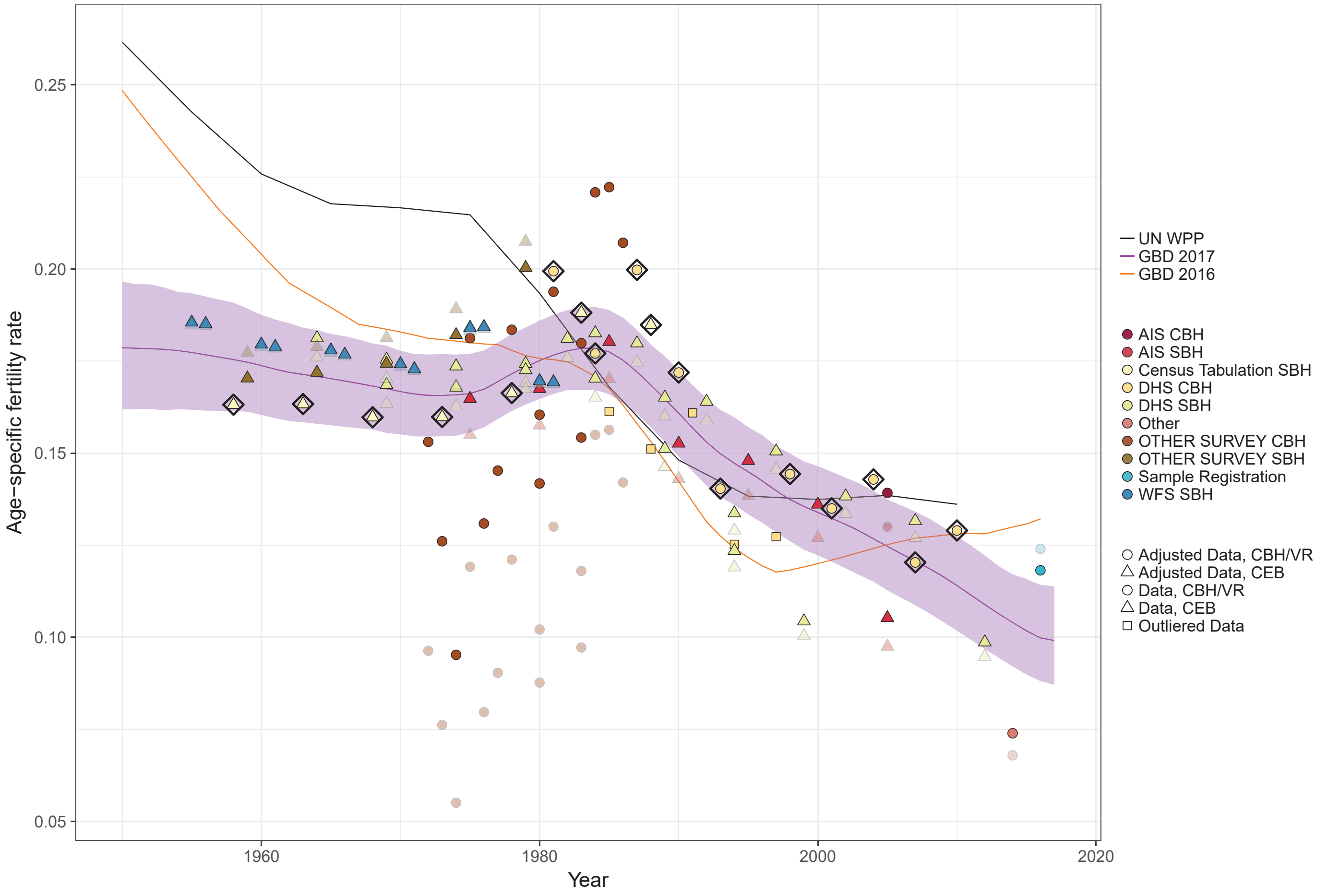

Age−specific fertility fitted trend lines, 1950−2017: Cote d'Ivoire, Ages: 20−24
Data points enclosed with a diamond are reference sources

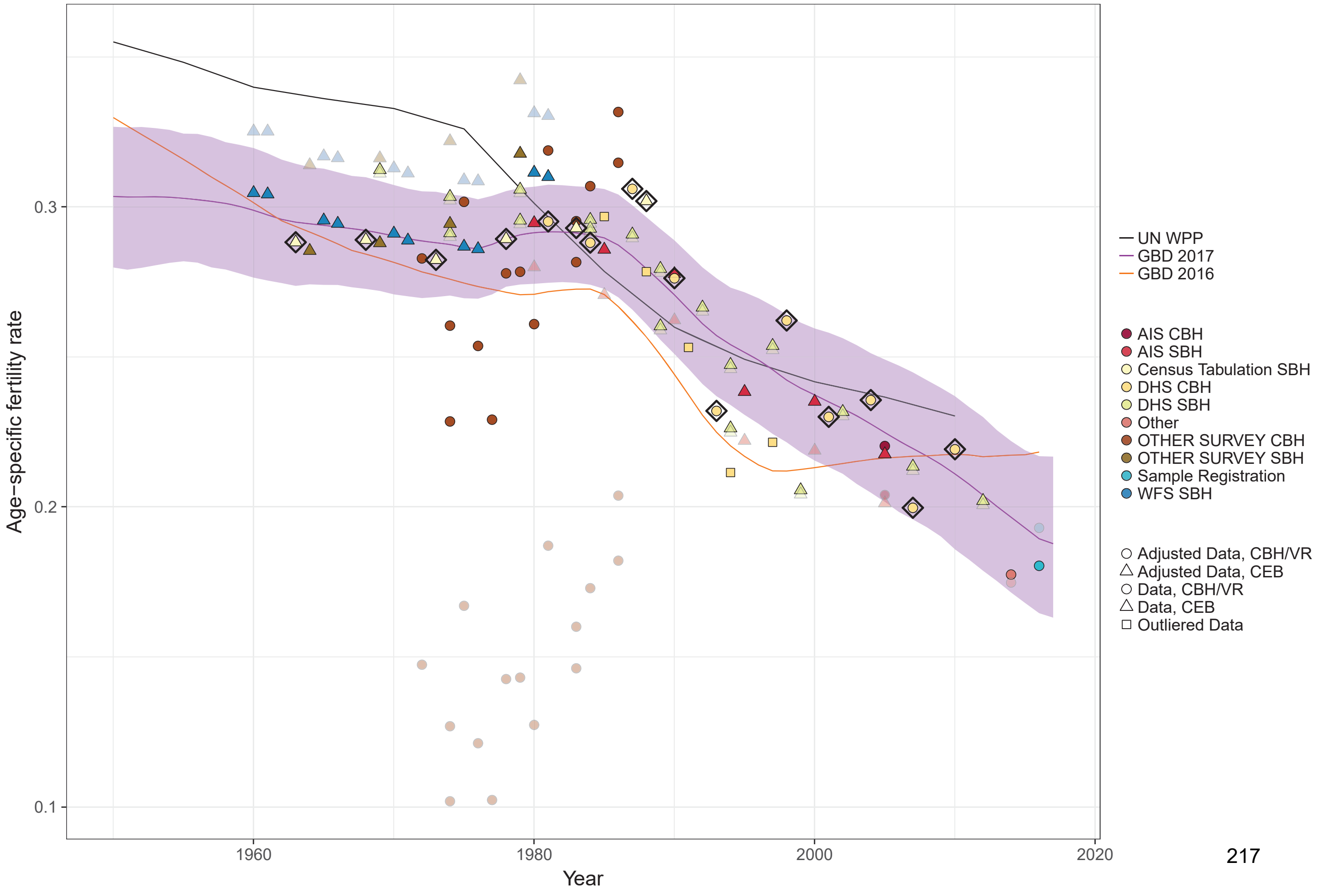

Age−specific fertility fitted trend lines, 1950−2017: Cote d'Ivoire, Ages: 25−29
Data points enclosed with a diamond are reference sources

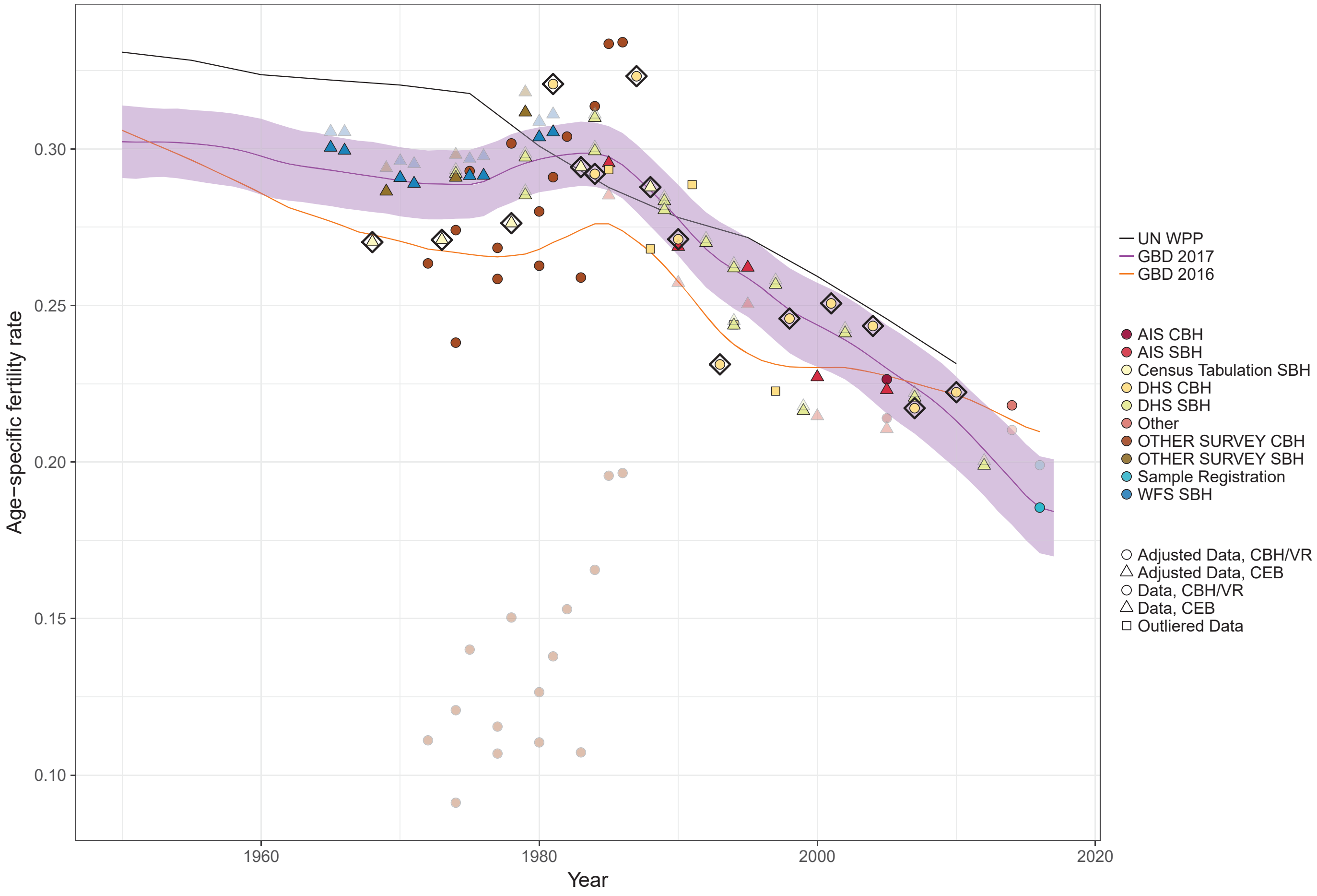

Age−specific fertility fitted trend lines, 1950−2017: Cote d'Ivoire, Ages: 30−34
Data points enclosed with a diamond are reference sources

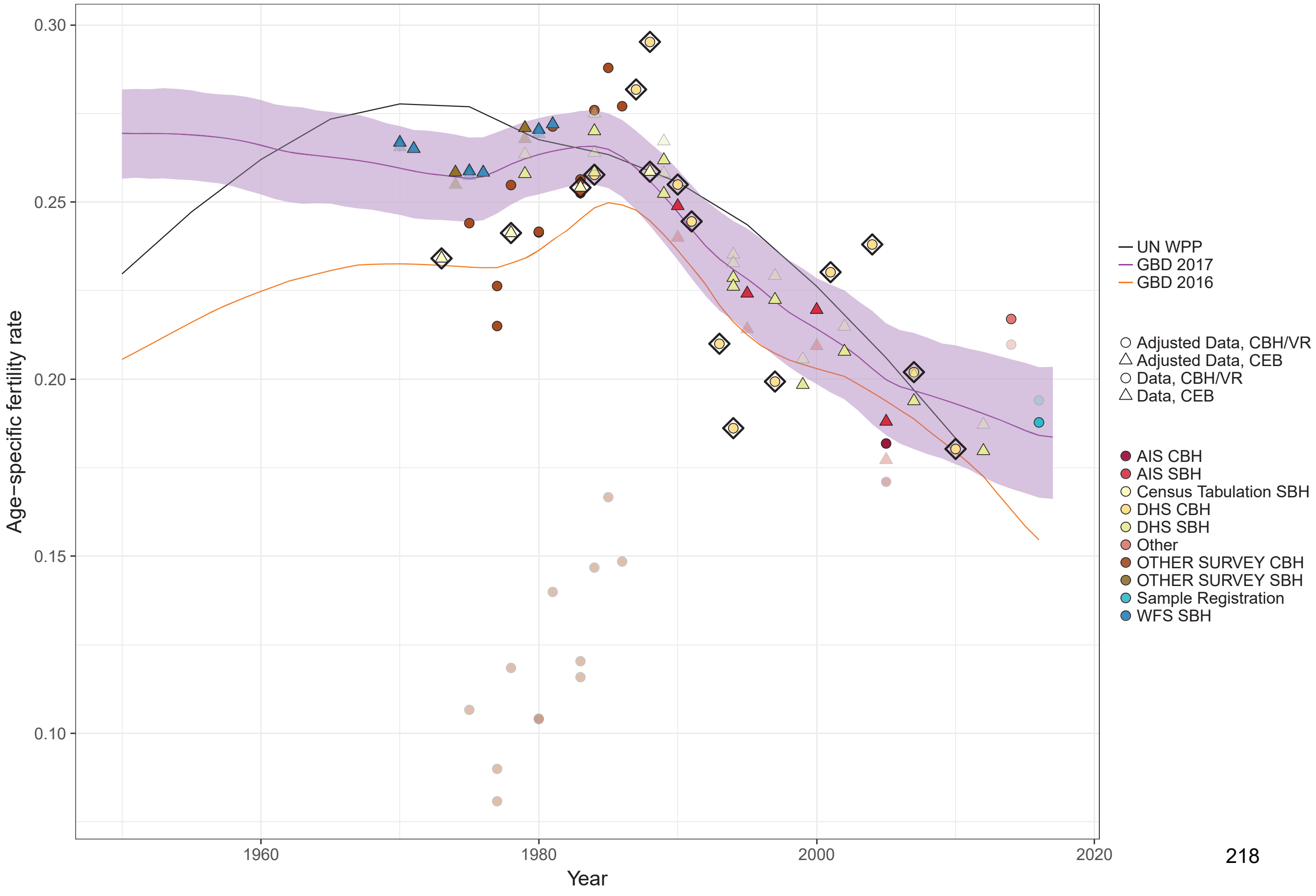

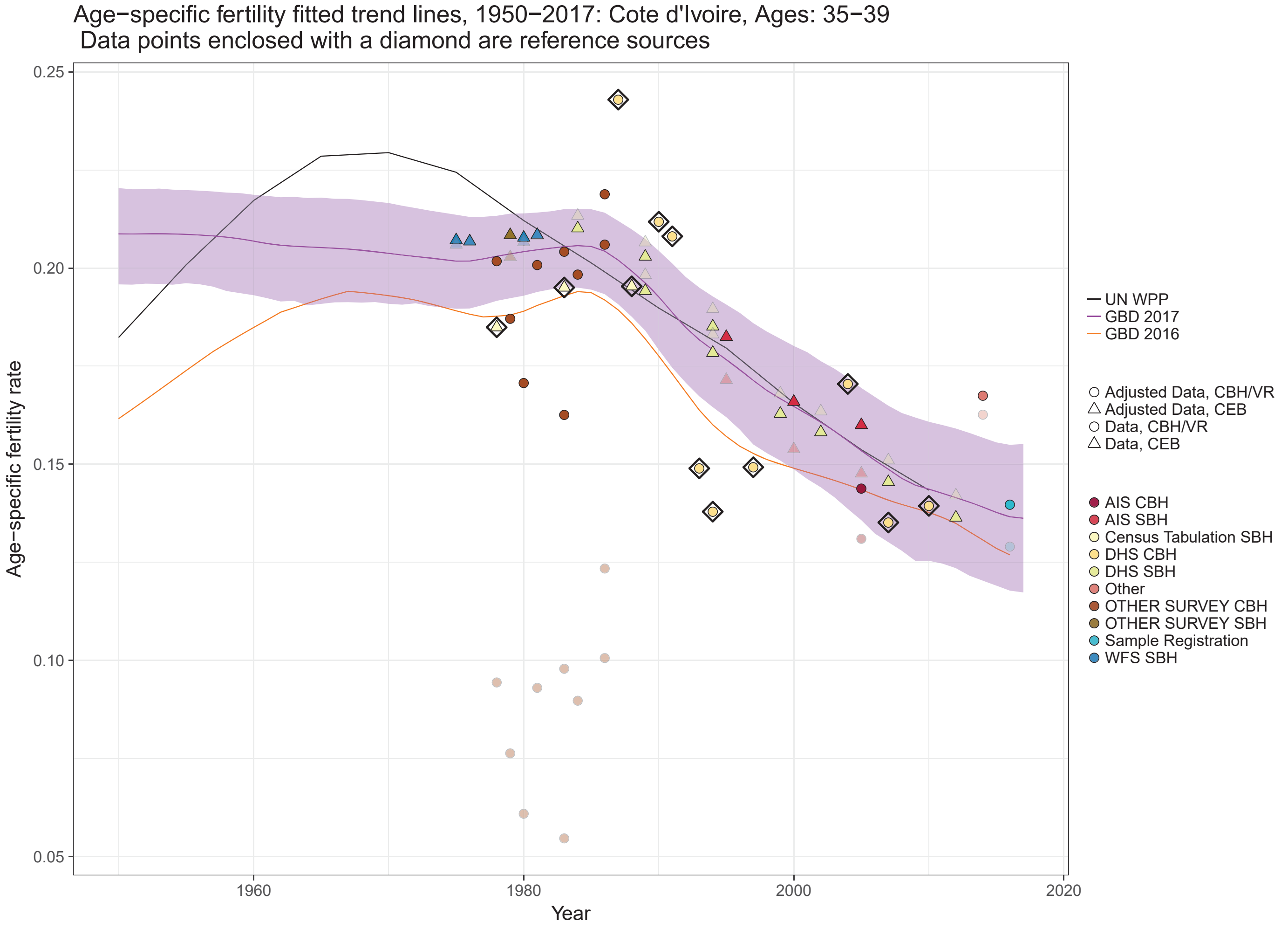

Age−specific fertility fitted trend lines, 1950−2017: Cote d'Ivoire, Ages: 35−39
Data points enclosed with a diamond are reference sources
Age−specific fertility rate
Year
0.25
0.20
0.15
0.10
0.05
1960
1980
2000
2020
UN WPP
GBD 2017
GBD 2016
Adjusted Data, CBH/VR
Adjusted Data, CEB
Data, CBH/VR
Data, CEB
AIS CBH
AIS SBH
Census Tabulation SBH
DHS CBH
DHS SBH
Other
OTHER SURVEY CBH
OTHER SURVEY SBH
Sample Registration
WFS SBH

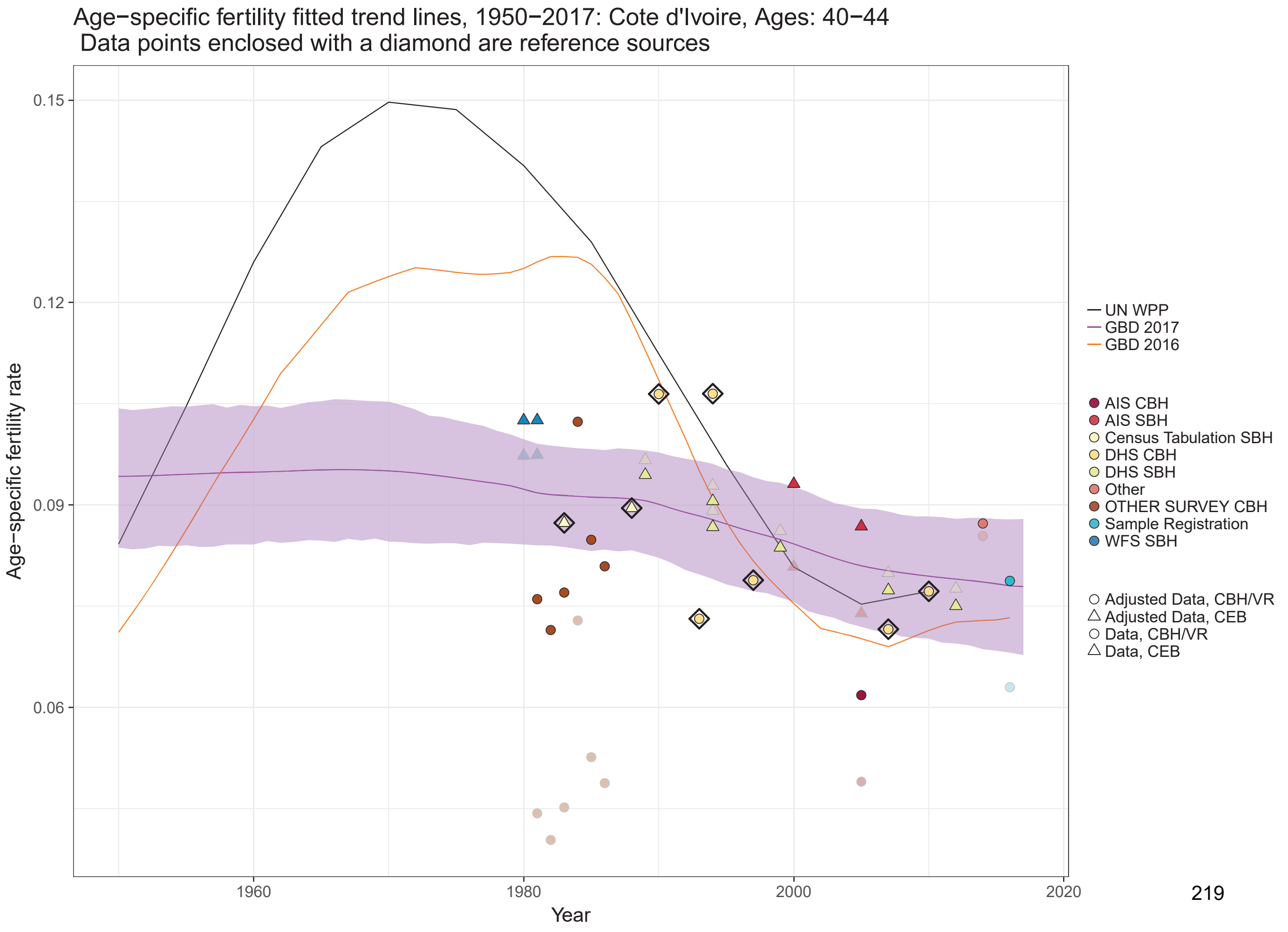

Age−specific fertility fitted trend lines, 1950−2017: Cote d'Ivoire, Ages: 40−44
Data points enclosed with a diamond are reference sources
Age−specific fertility rate
Year
0.15
0.12
0.09
0.06
1960
1980
2000
2020
UN WPP
GBD 2017
GBD 2016
AIS CBH
AIS SBH
Census Tabulation SBH
DHS CBH
DHS SBH
Other
OTHER SURVEY CBH
Sample Registration
WFS SBH
Adjusted Data, CBH/VR
Adjusted Data, CEB
Data, CBH/VR
Data, CEB

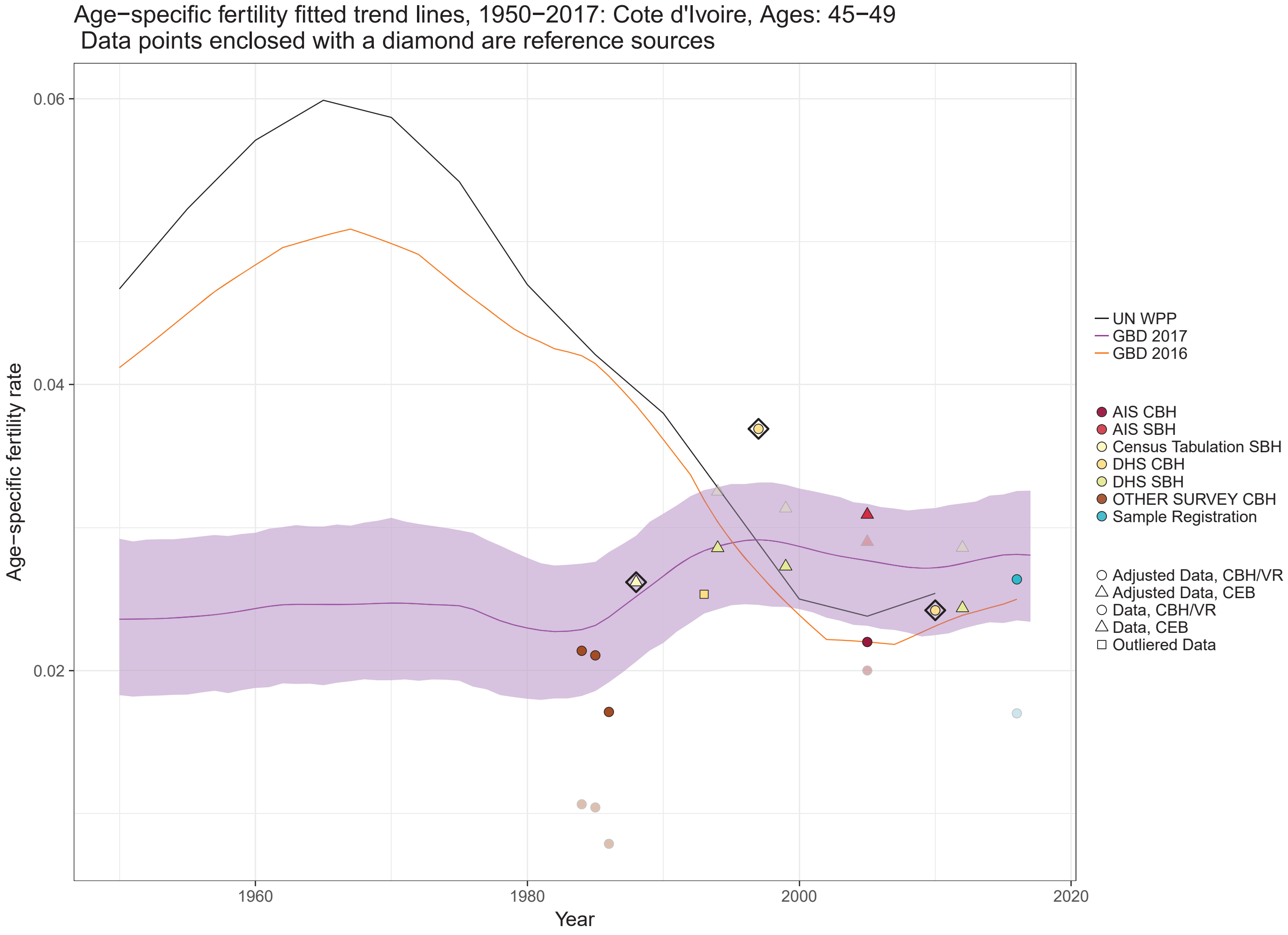
Age−specific fertility fitted trend lines, 1950−2017: Cote d'Ivoire, Ages: 45−49
Data points enclosed with a diamond are reference sources
Age−specific fertility rate
0.06
0.04
0.02
1960
1980
2000
2020
Year
UN WPP
GBD 2017
GBD 2016
AIS CBH
AIS SBH
Census Tabulation SBH
DHS CBH
DHS SBH
OTHER SURVEY CBH
Sample Registration
Adjusted Data, CBH/VR
Adjusted Data, CEB
Data, CBH/VR
Data, CEB
Outliered Data

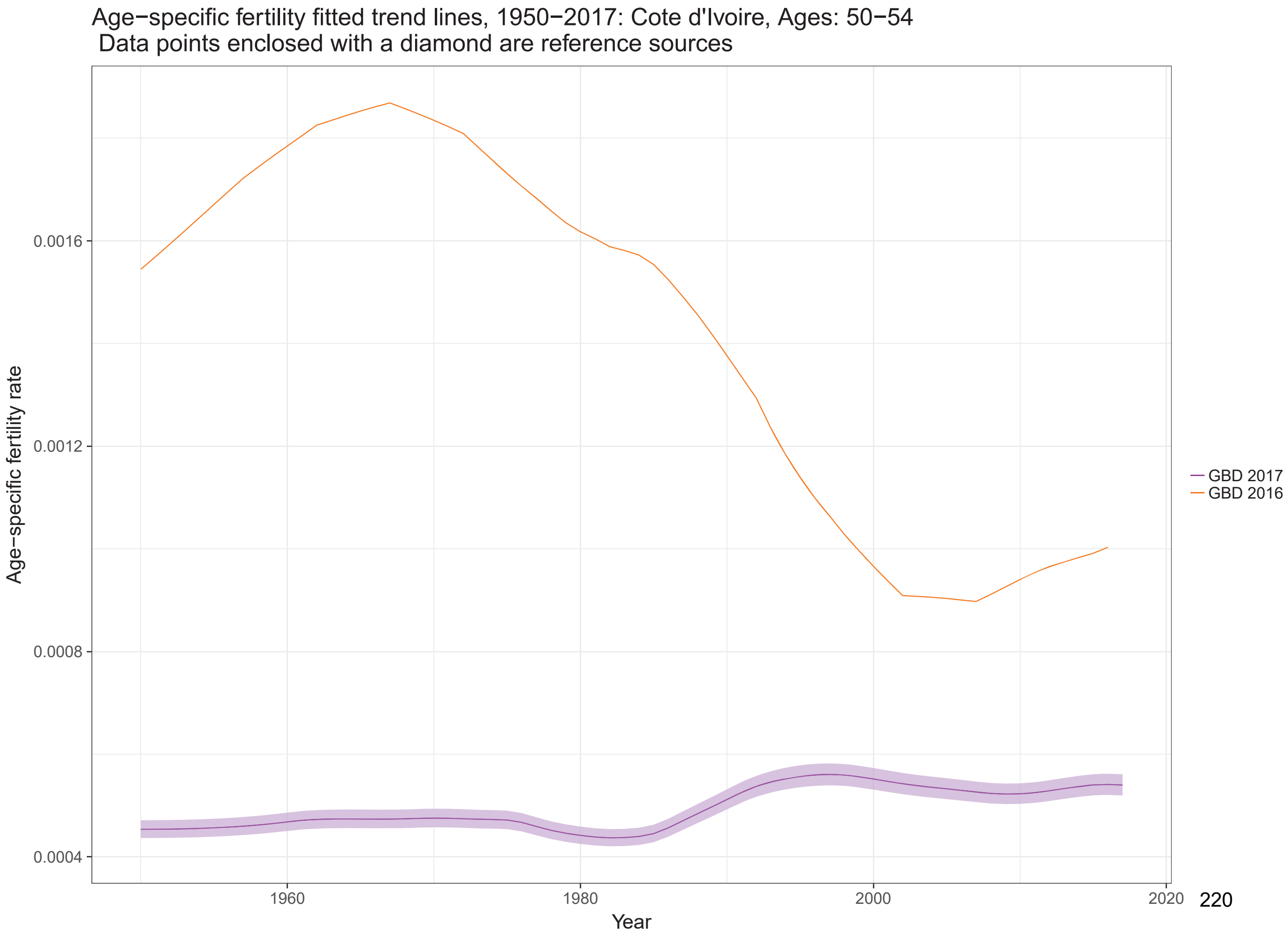
Age−specific fertility fitted trend lines, 1950−2017: Cote d'Ivoire, Ages: 50−54
Data points enclosed with a diamond are reference sources
Age−specific fertility rate
0.0016
0.0012
0.0008
0.0004
1960
1980
2000
2020
Year
GBD 2017
GBD 2016

Age−specific fertility fitted trend lines, 1950−2017: Croatia, TFR
Data points enclosed with a diamond are reference sources

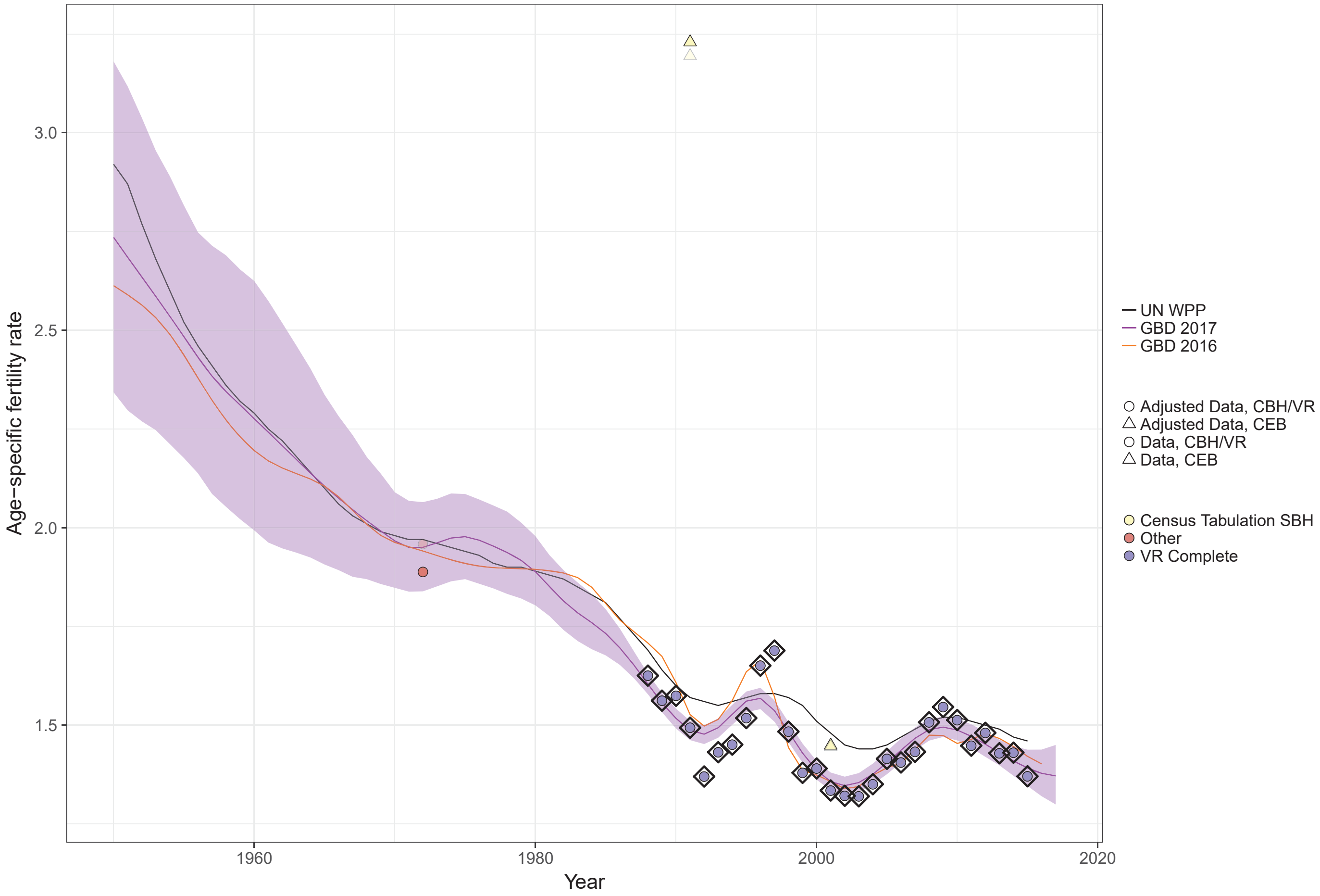

Age−specific fertility fitted trend lines, 1950−2017: Croatia, Ages: 10−14
Data points enclosed with a diamond are reference sources

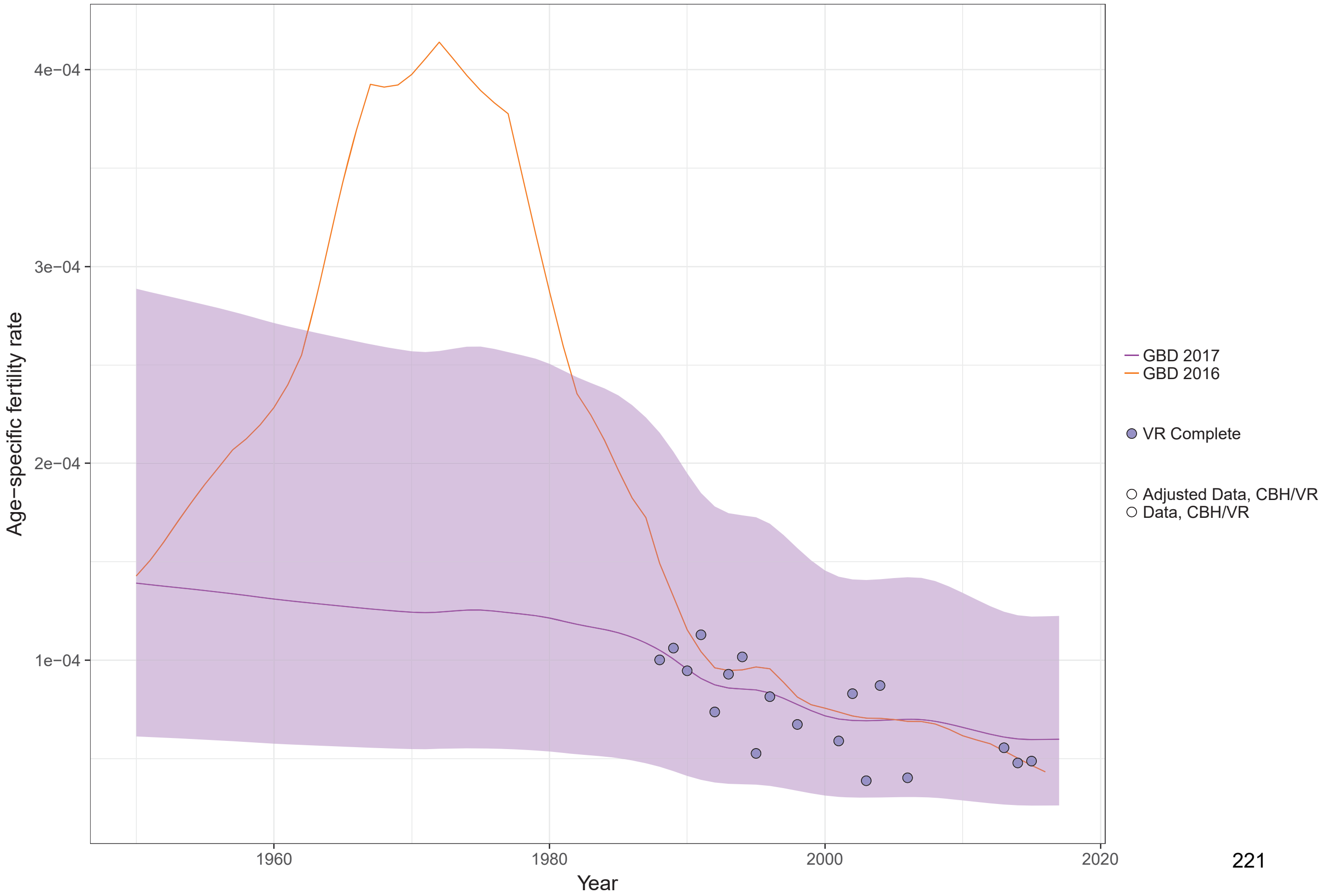

Age−specific fertility fitted trend lines, 1950−2017: Croatia, Ages: 15−19
Data points enclosed with a diamond are reference sources

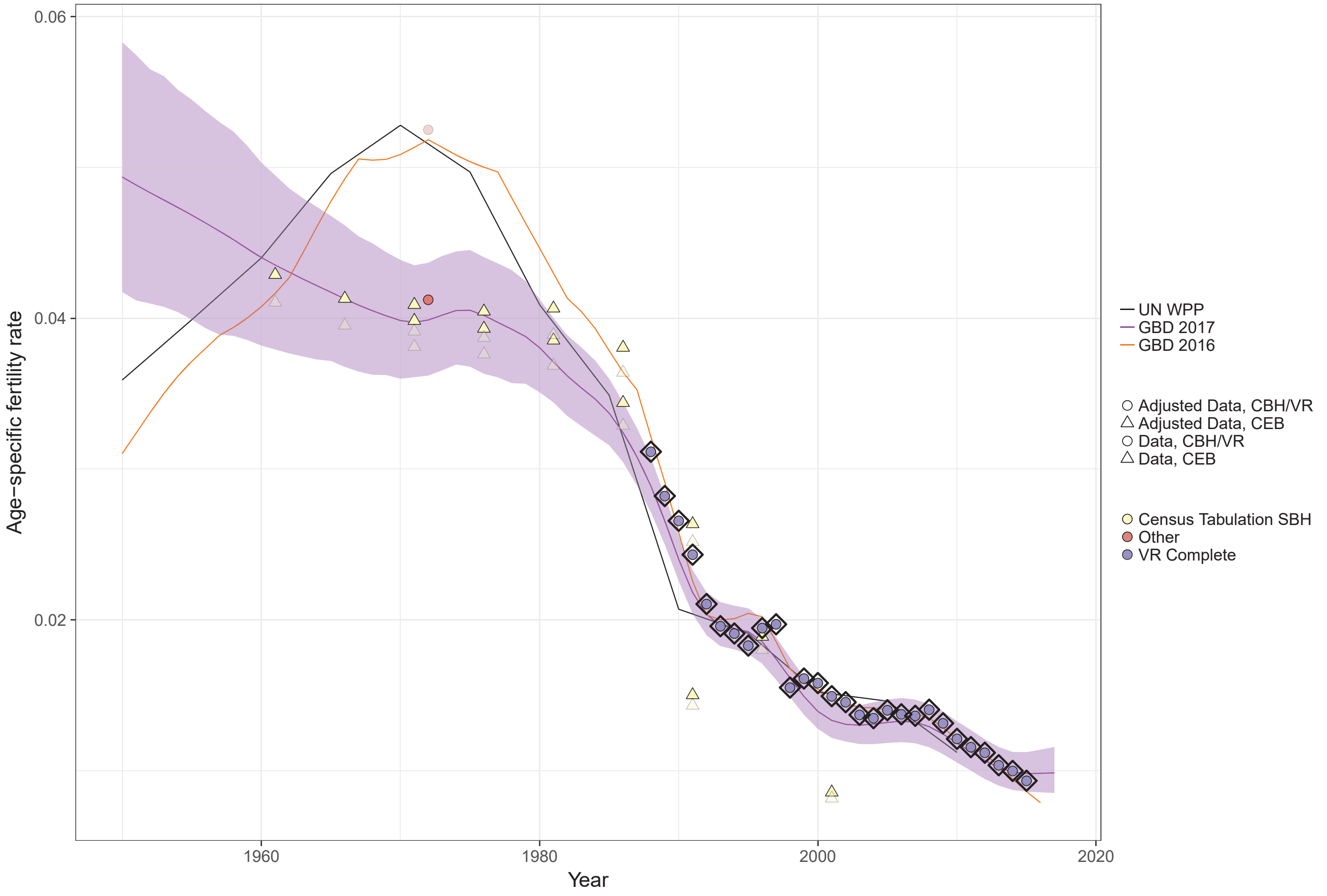

Age−specific fertility fitted trend lines, 1950−2017: Croatia, Ages: 20−24
Data points enclosed with a diamond are reference sources

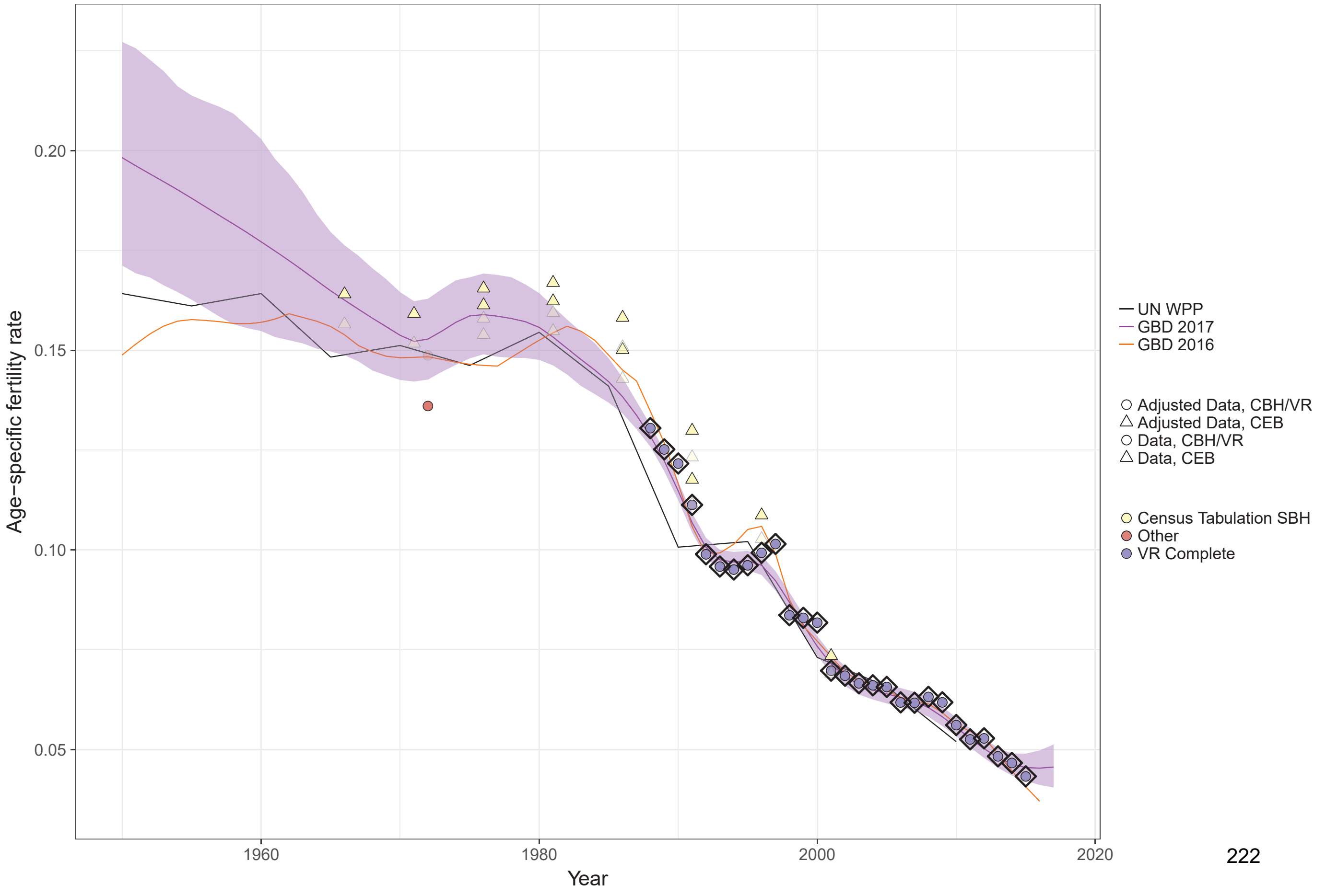

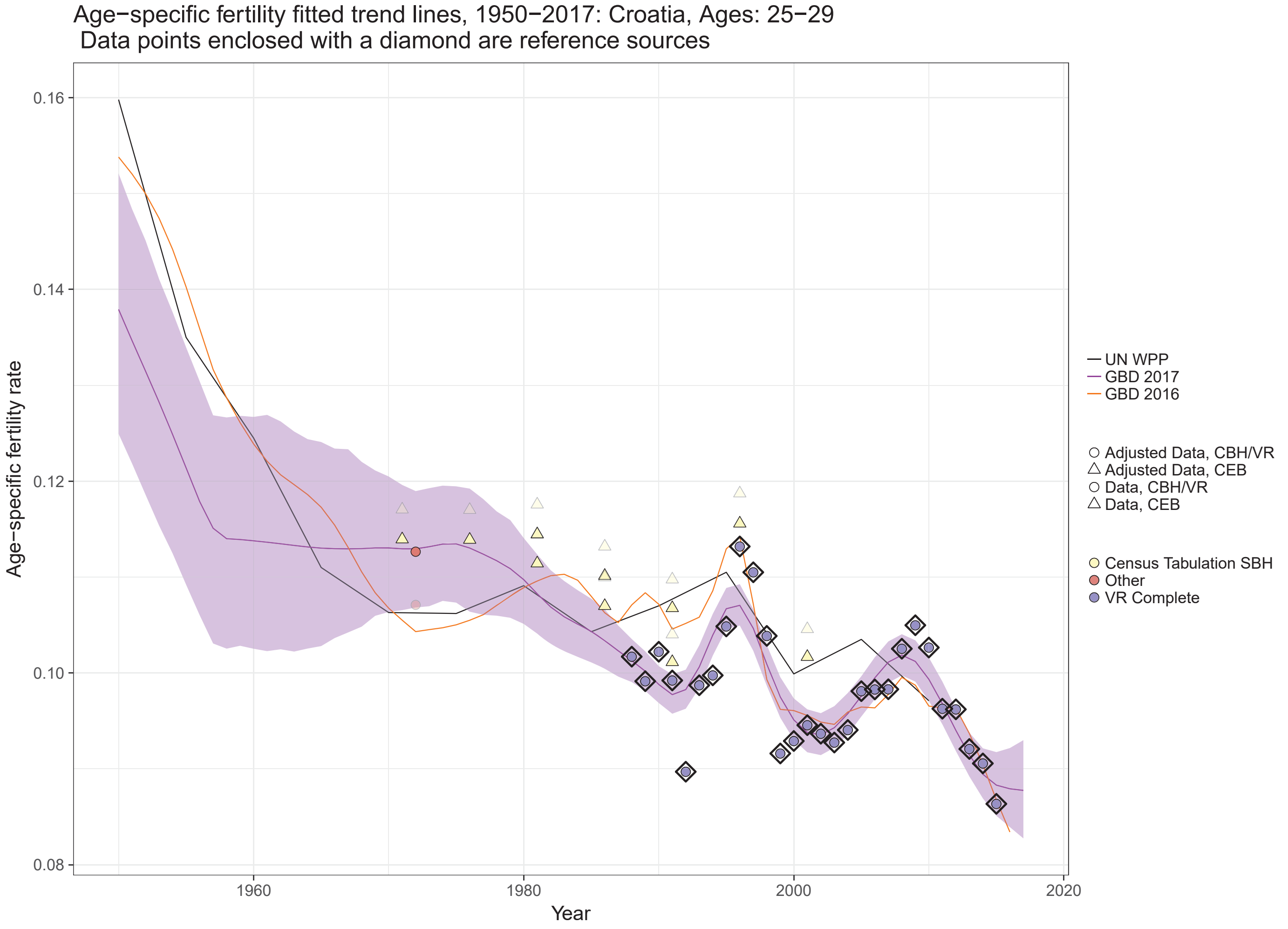
Age−specific fertility fitted trend lines, 1950−2017: Croatia, Ages: 25−29
Data points enclosed with a diamond are reference sources
Age−specific fertility rate
Year
0.16
0.14
0.12
0.10
0.08
1960
1980
2000
2020
UN WPP
GBD 2017
GBD 2016
Adjusted Data, CBH/VR
Adjusted Data, CEB
Data, CBH/VR
Data, CEB
Census Tabulation SBH
Other
VR Complete

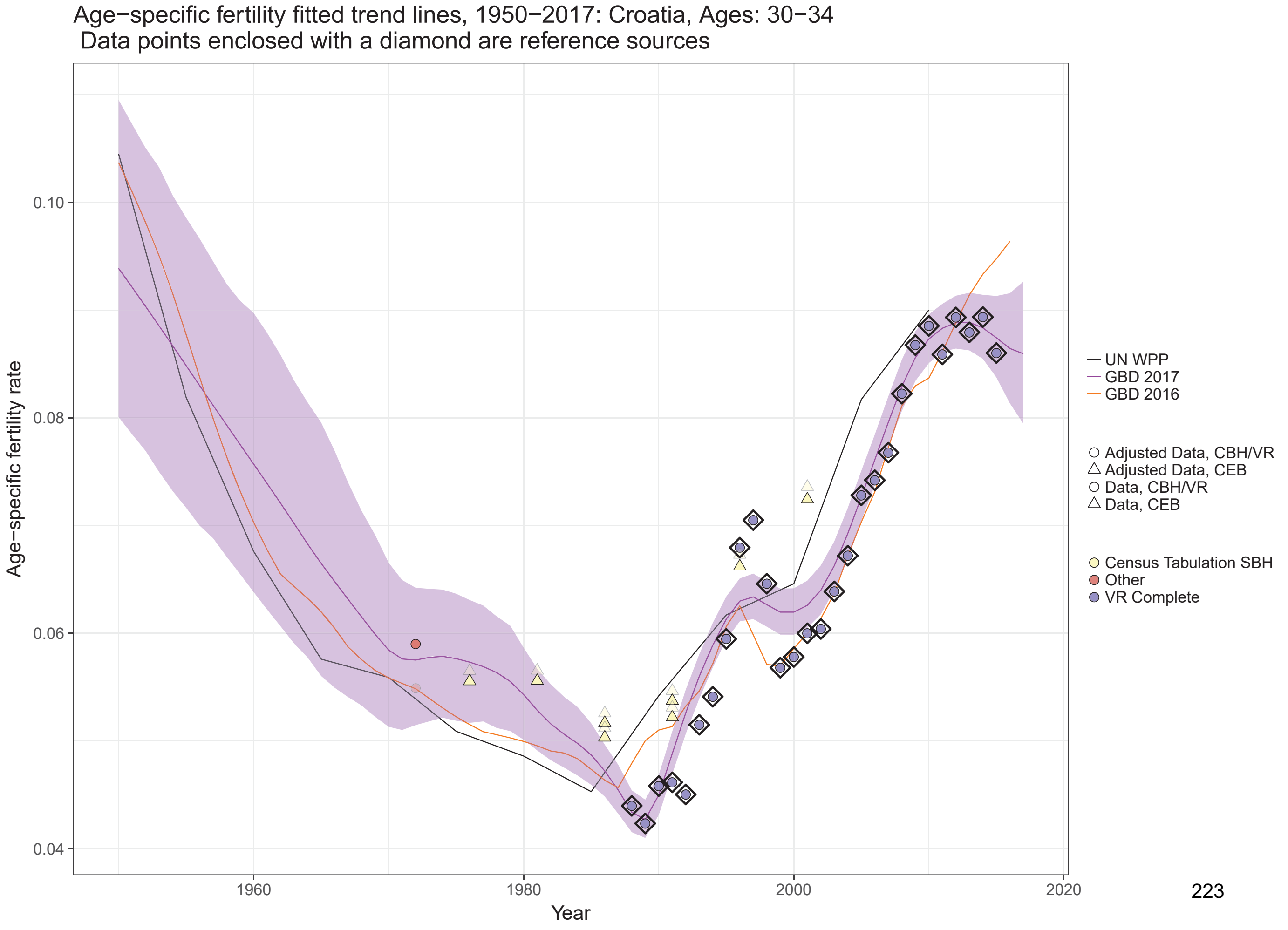
Age−specific fertility fitted trend lines, 1950−2017: Croatia, Ages: 30−34
Data points enclosed with a diamond are reference sources
Age−specific fertility rate
Year
0.10
0.08
0.06
0.04
1960
1980
2000
2020
UN WPP
GBD 2017
GBD 2016
Adjusted Data, CBH/VR
Adjusted Data, CEB
Data, CBH/VR
Data, CEB
Census Tabulation SBH
Other
VR Complete

Age−specific fertility fitted trend lines, 1950−2017: Croatia, Ages: 35−39
Data points enclosed with a diamond are reference sources

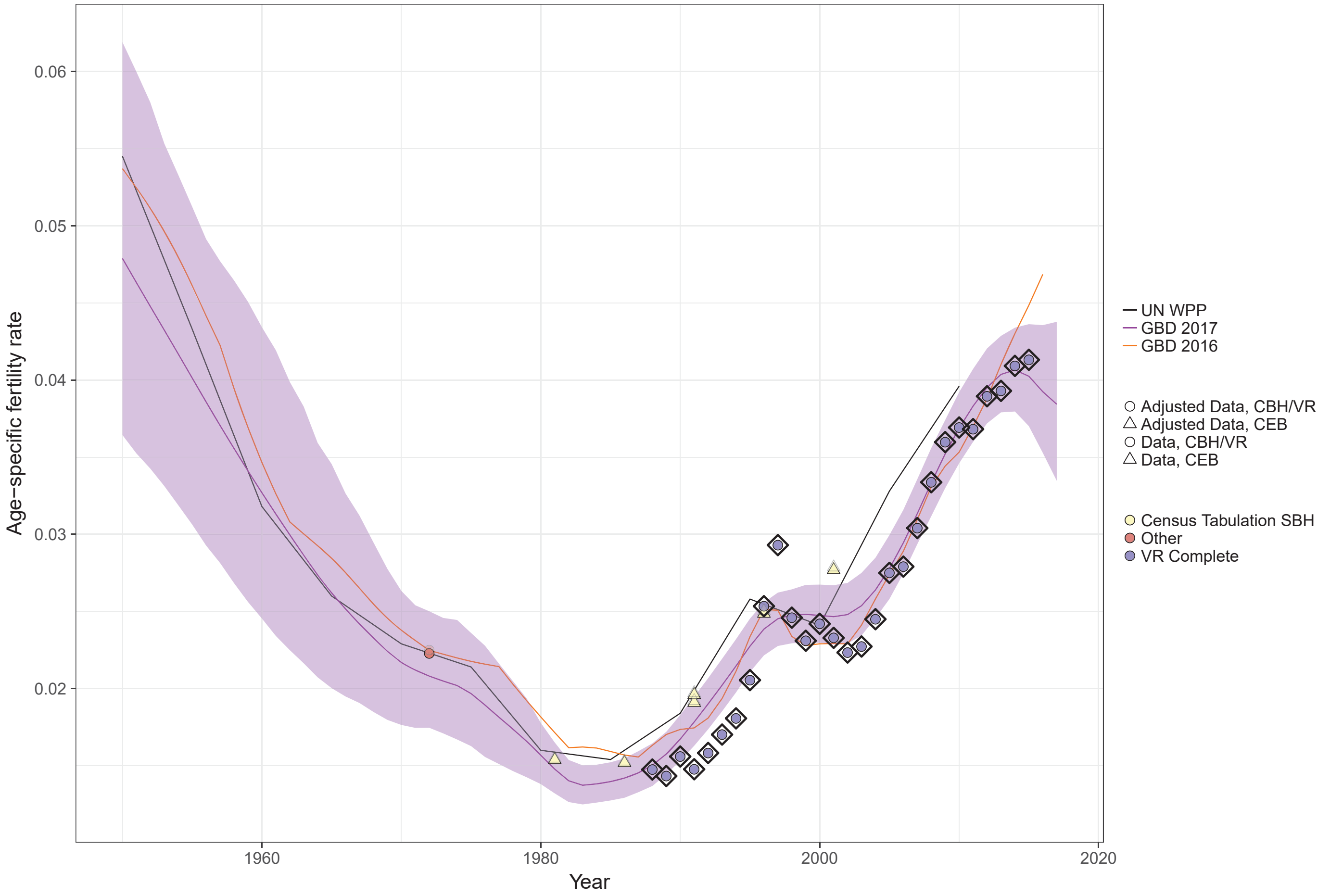

Age−specific fertility fitted trend lines, 1950−2017: Croatia, Ages: 40−44
Data points enclosed with a diamond are reference sources

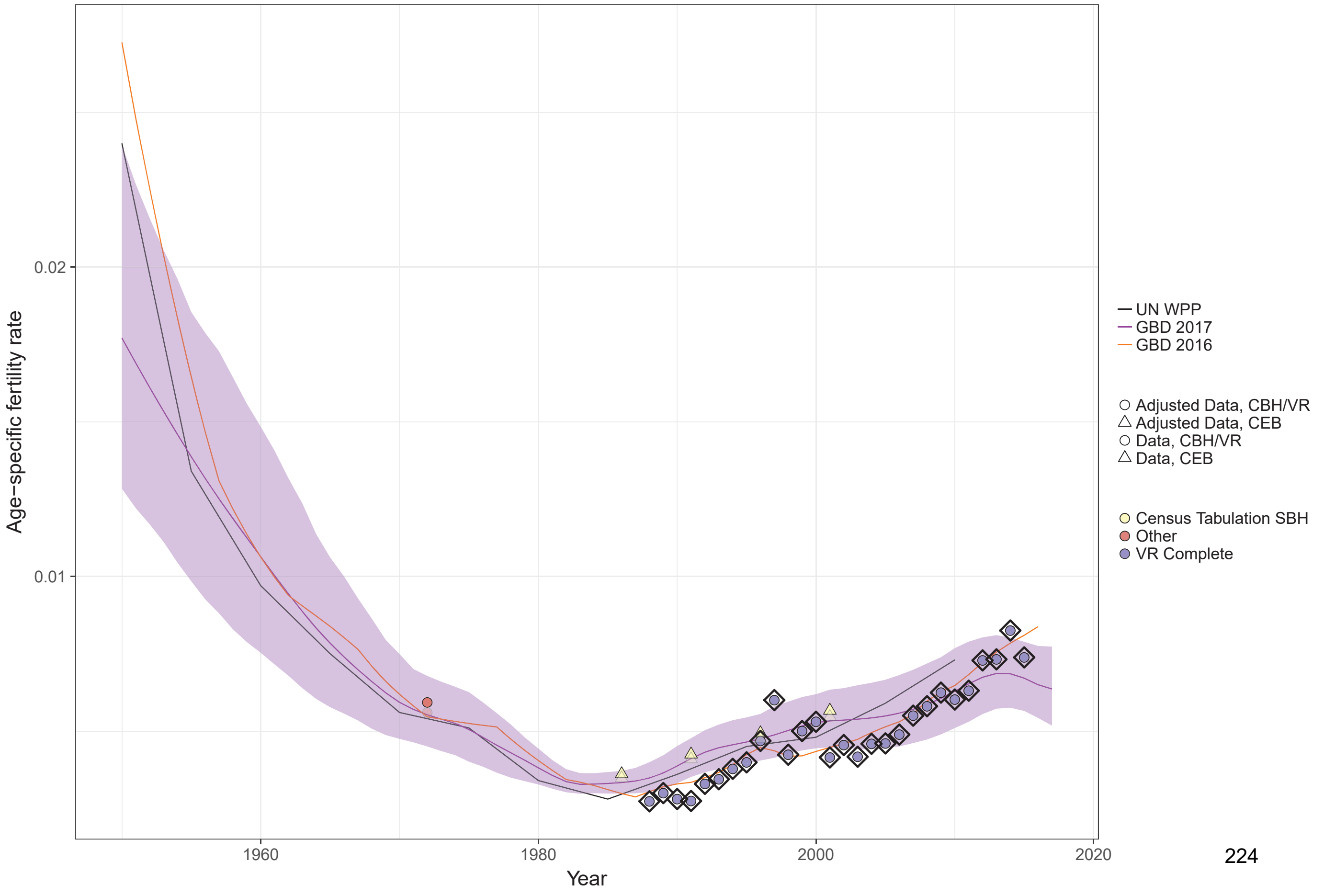

Age−specific fertility fitted trend lines, 1950−2017: Croatia, Ages: 45−49
Data points enclosed with a diamond are reference sources

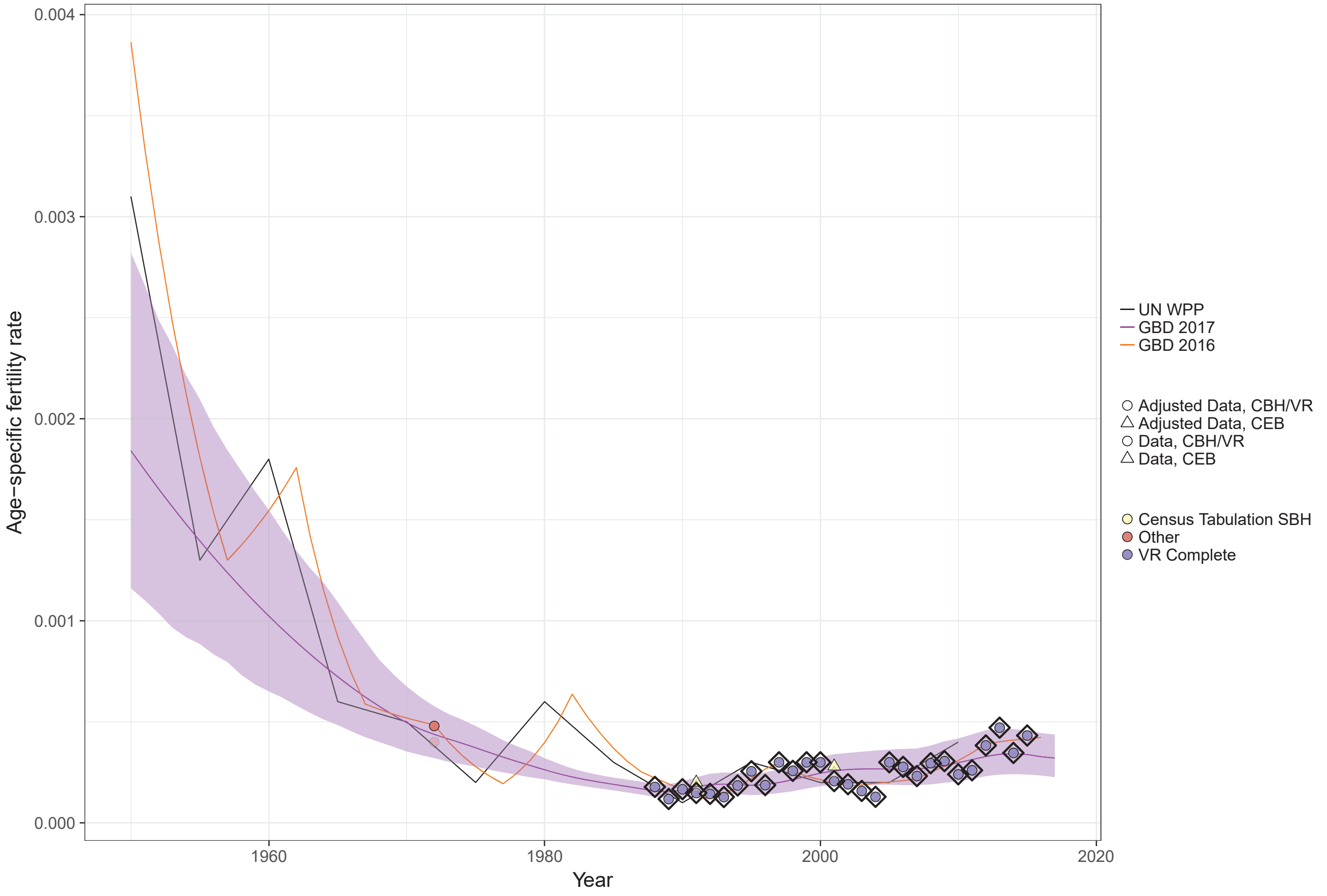

Age−specific fertility fitted trend lines, 1950−2017: Croatia, Ages: 50−54
Data points enclosed with a diamond are reference sources

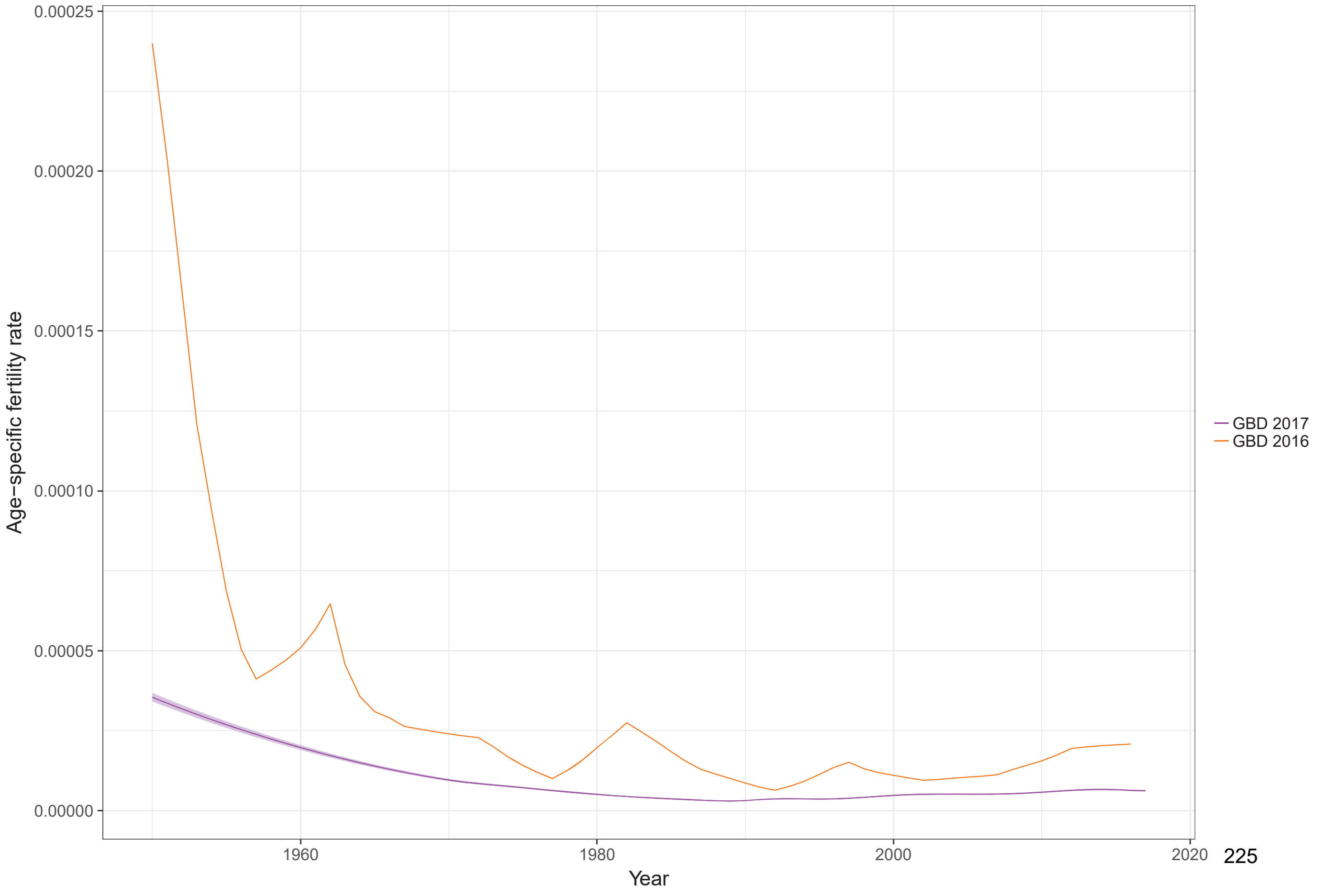

Age−specific fertility fitted trend lines, 1950−2017: Cuba, TFR
Data points enclosed with a diamond are reference sources

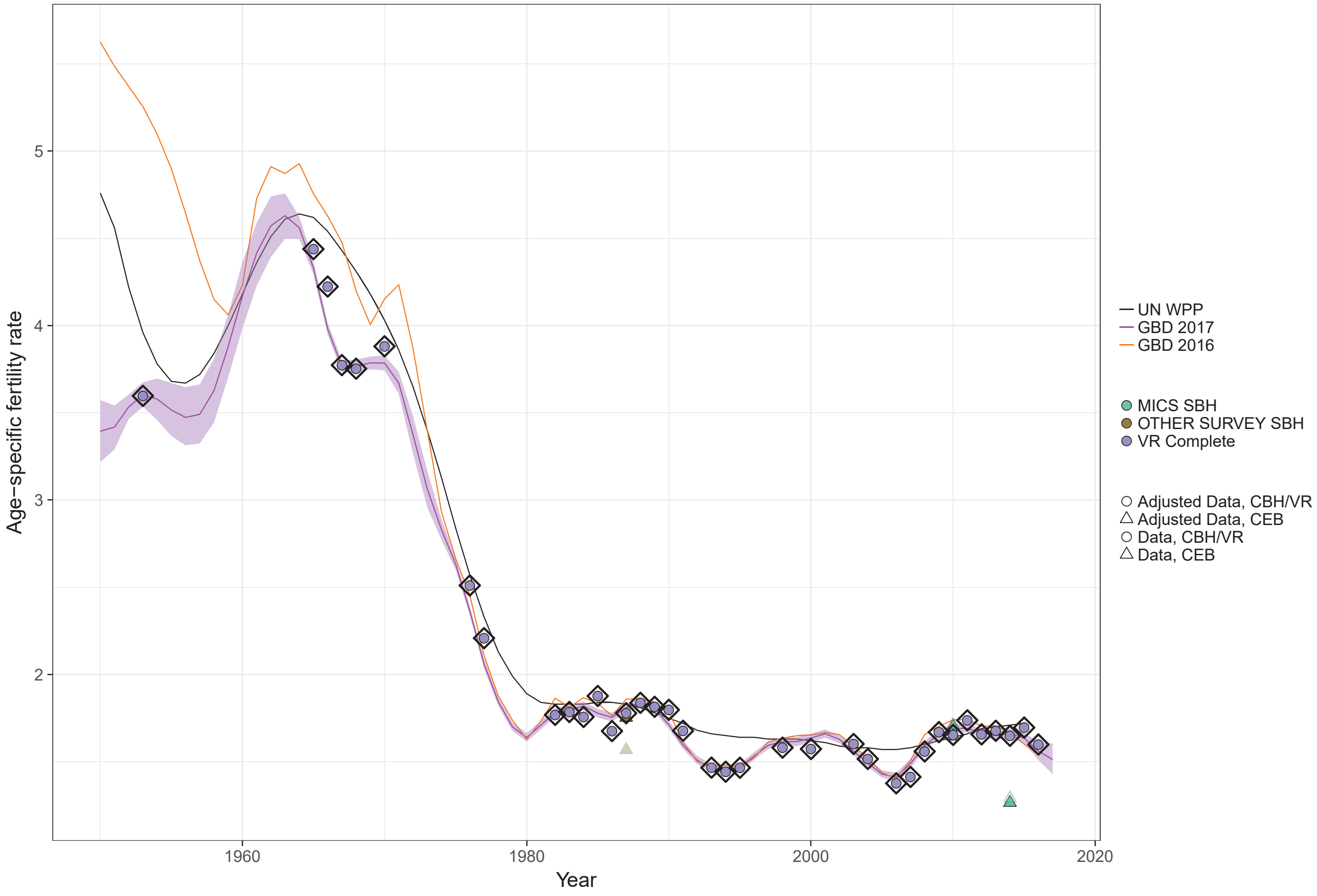

Age−specific fertility fitted trend lines, 1950−2017: Cuba, Ages: 10−14
Data points enclosed with a diamond are reference sources

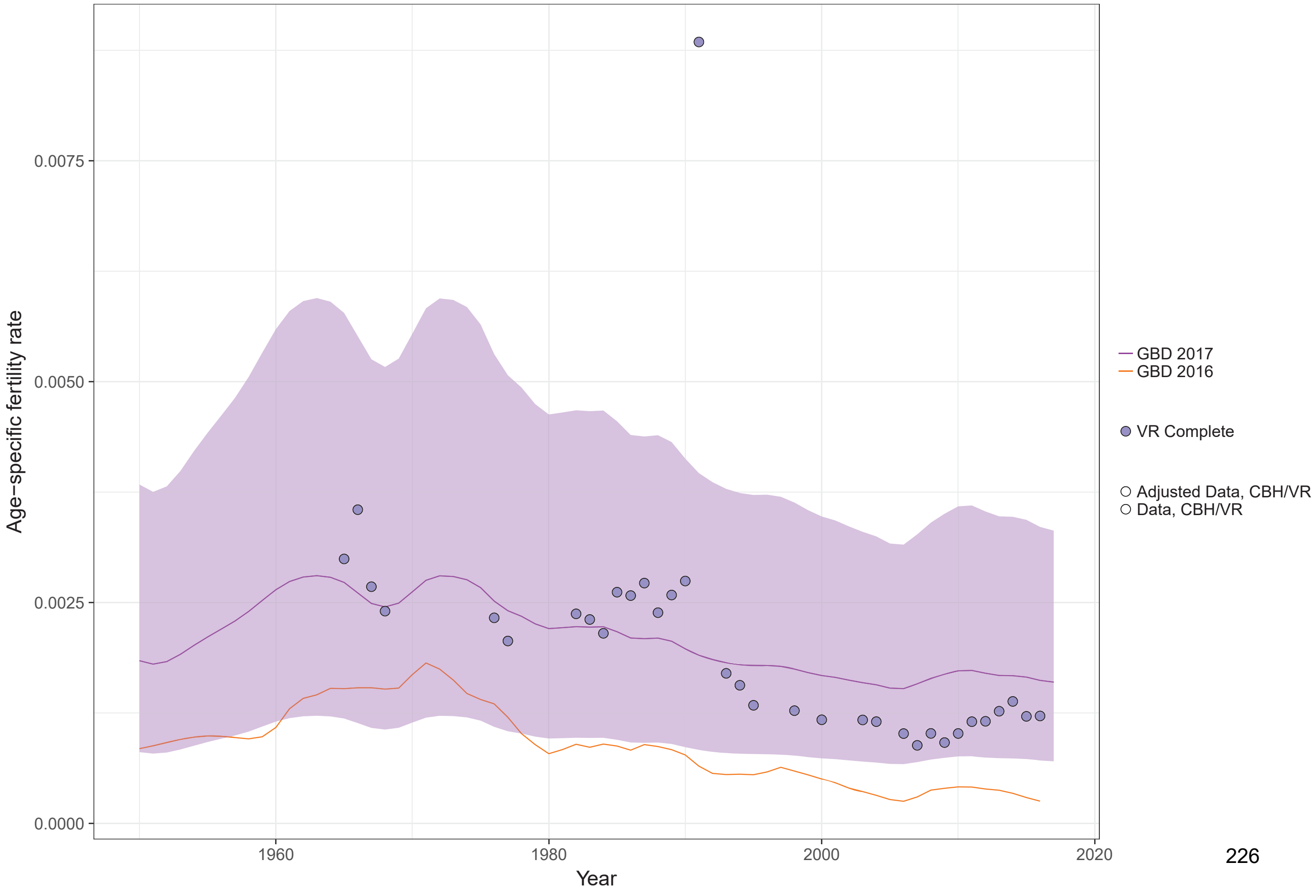

Age−specific fertility fitted trend lines, 1950−2017: Cuba, Ages: 15−19
Data points enclosed with a diamond are reference sources

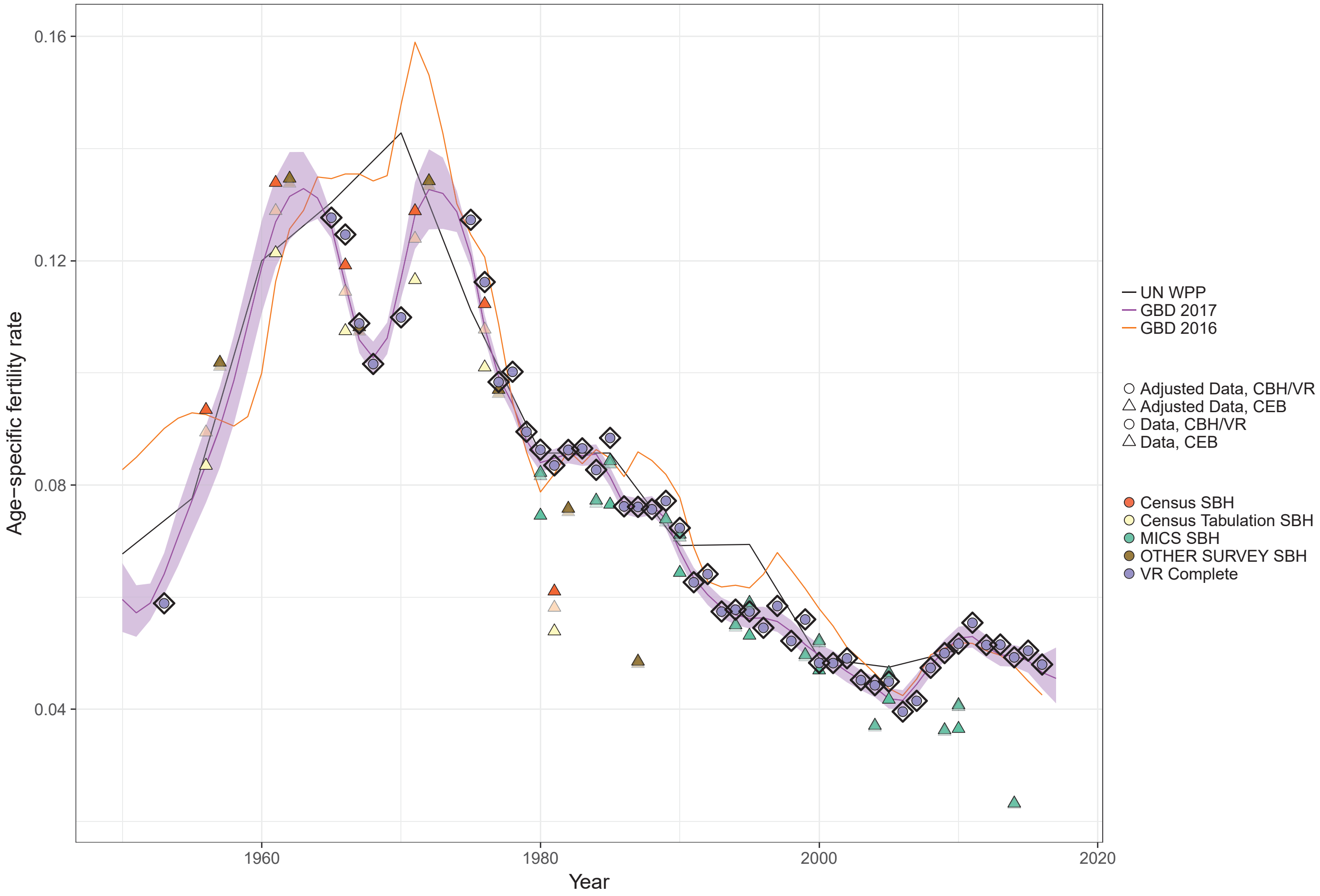

Age−specific fertility fitted trend lines, 1950−2017: Cuba, Ages: 20−24
Data points enclosed with a diamond are reference sources

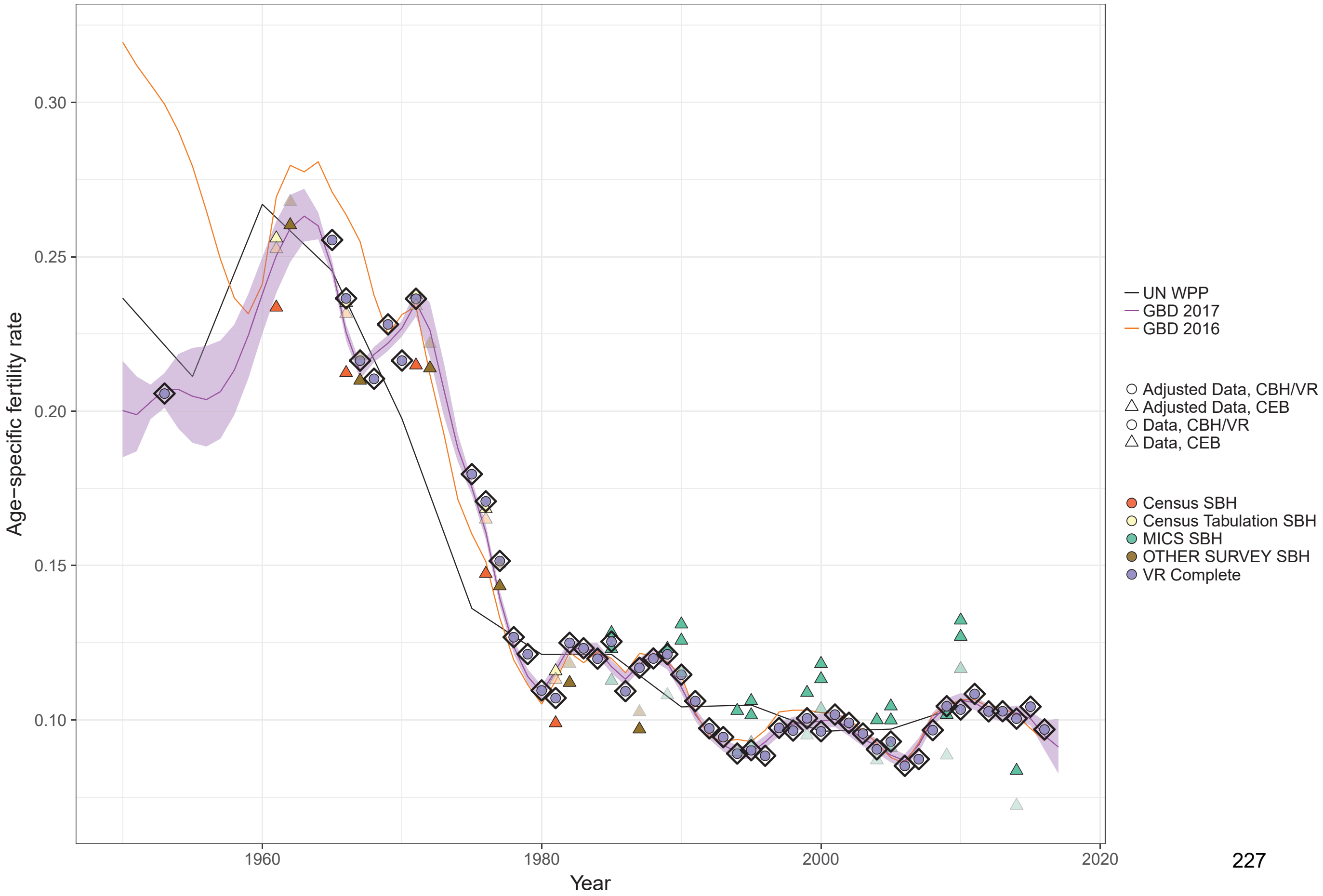

Age−specific fertility fitted trend lines, 1950−2017: Cuba, Ages: 25−29
Data points enclosed with a diamond are reference sources

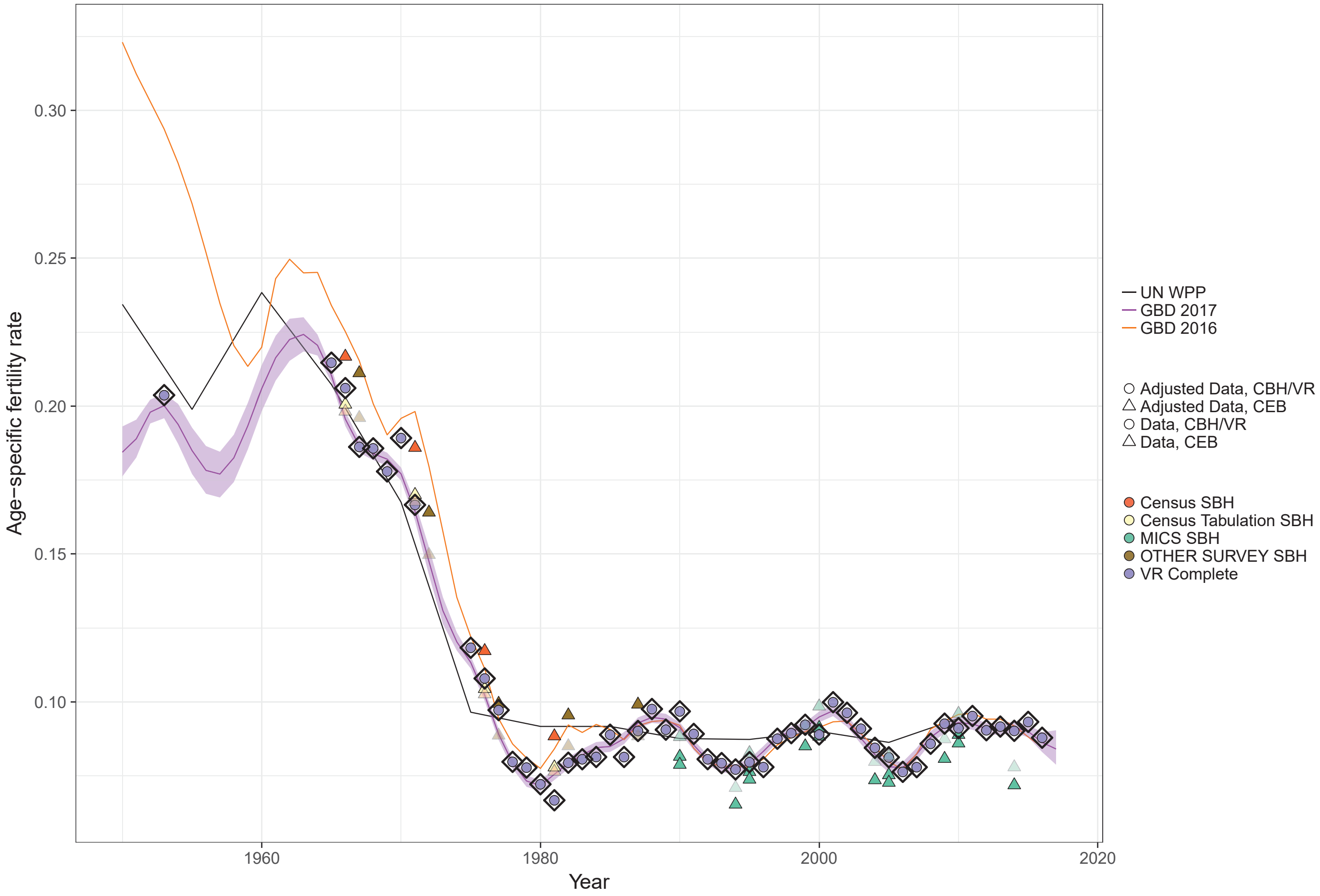

Age−specific fertility fitted trend lines, 1950−2017: Cuba, Ages: 30−34
Data points enclosed with a diamond are reference sources

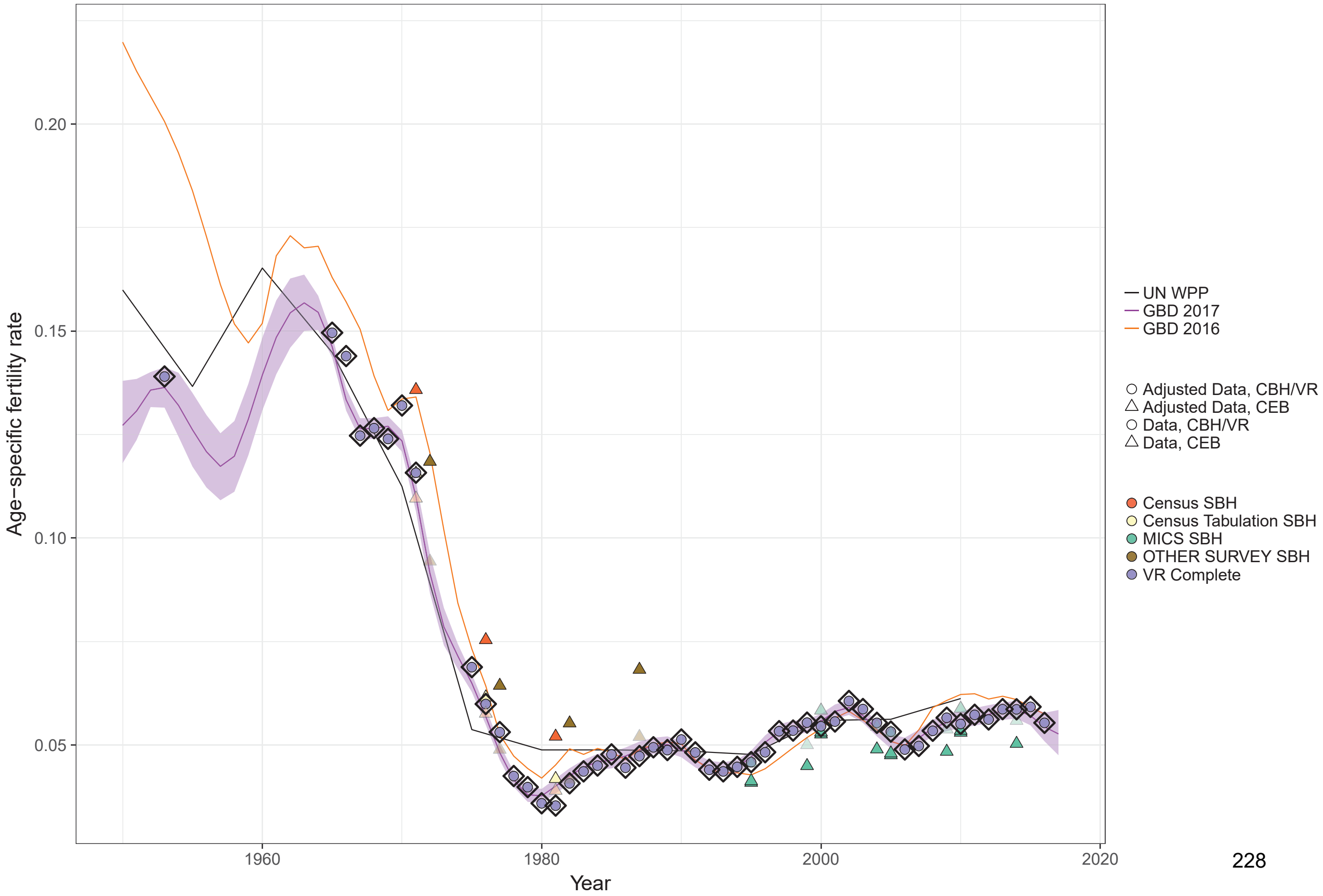

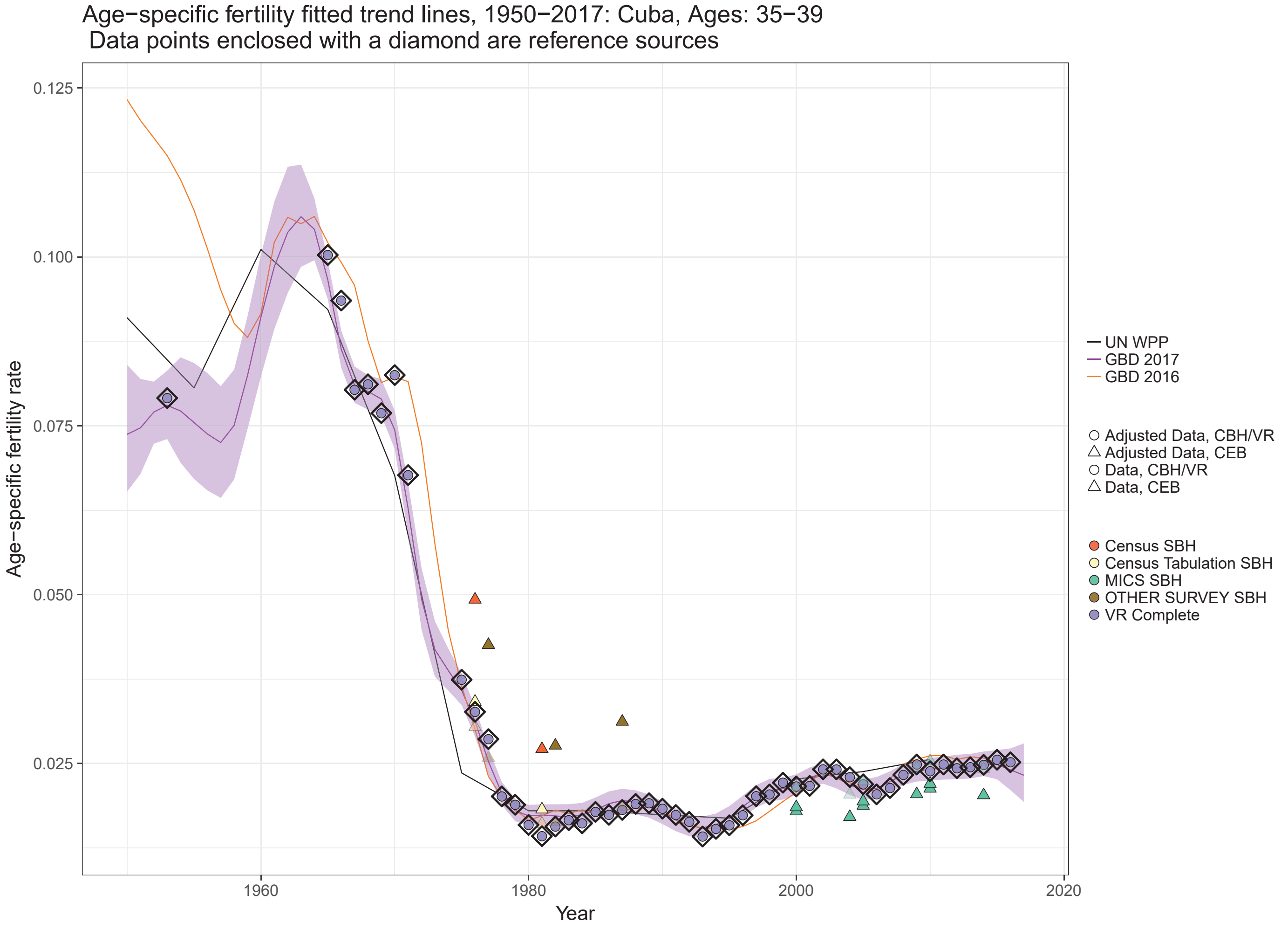

Age−specific fertility fitted trend lines, 1950−2017: Cuba, Ages: 35−39
Data points enclosed with a diamond are reference sources
Age−specific fertility rate
Year
UN WPP
GBD 2017
GBD 2016
Adjusted Data, CBH/VR
Adjusted Data, CEB
Data, CBH/VR
Data, CEB
Census SBH
Census Tabulation SBH
MICS SBH
OTHER SURVEY SBH
VR Complete

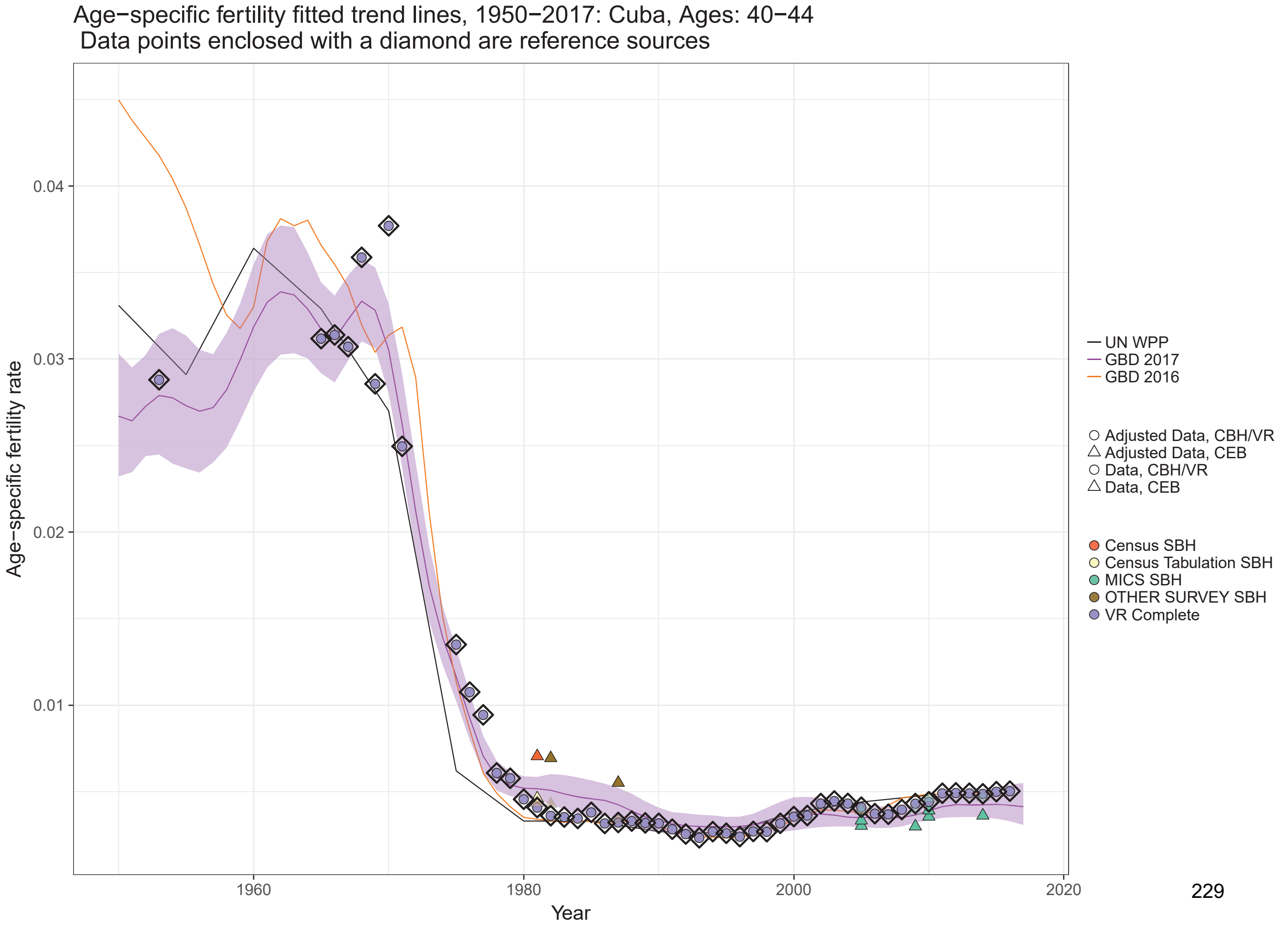

Age−specific fertility fitted trend lines, 1950−2017: Cuba, Ages: 40−44
Data points enclosed with a diamond are reference sources
Age−specific fertility rate
Year
UN WPP
GBD 2017
GBD 2016
Adjusted Data, CBH/VR
Adjusted Data, CEB
Data, CBH/VR
Data, CEB
Census SBH
Census Tabulation SBH
MICS SBH
OTHER SURVEY SBH
VR Complete

Age−specific fertility fitted trend lines, 1950−2017: Cuba, Ages: 45−49
Data points enclosed with a diamond are reference sources

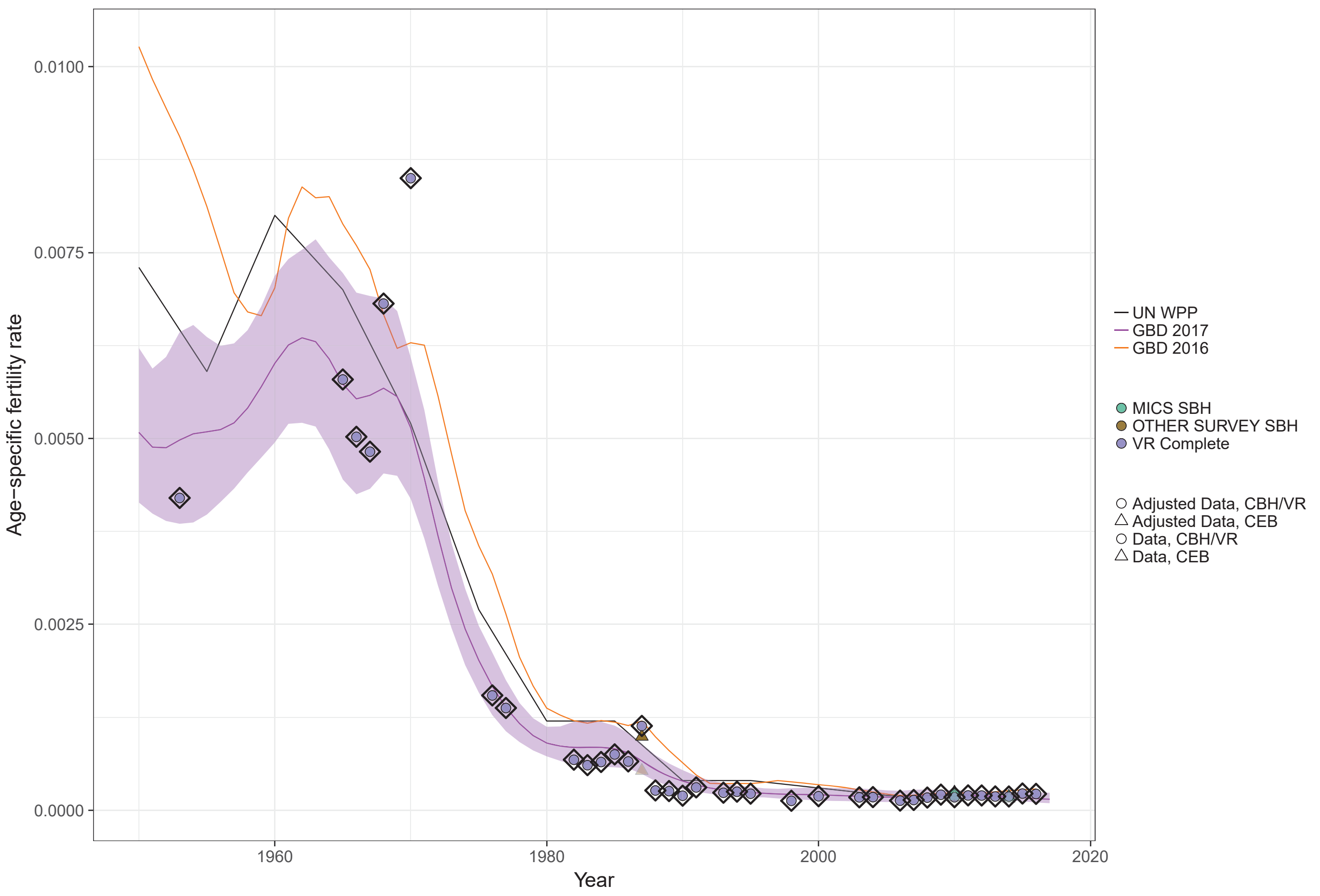

Age−specific fertility fitted trend lines, 1950−2017: Cuba, Ages: 50−54
Data points enclosed with a diamond are reference sources

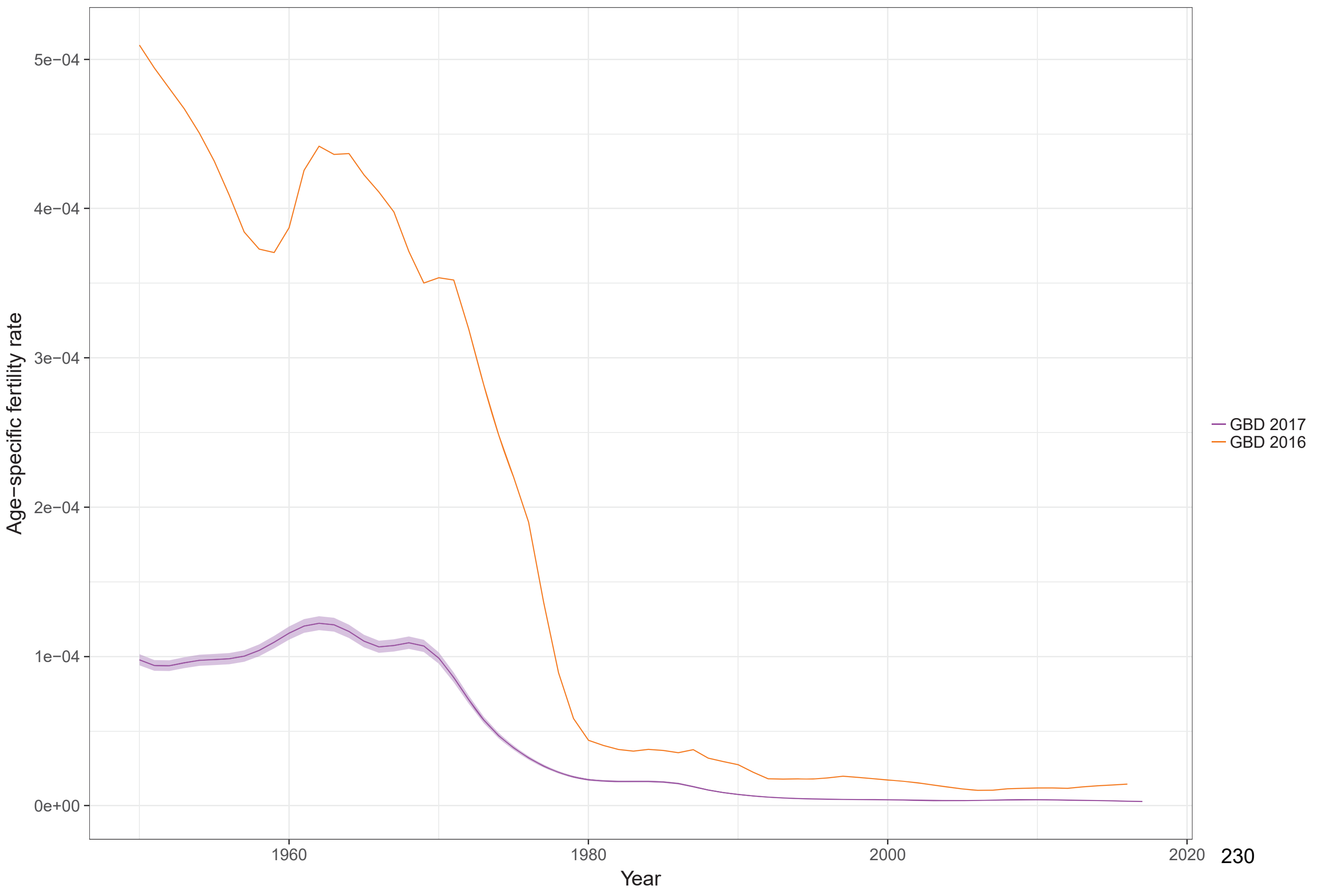

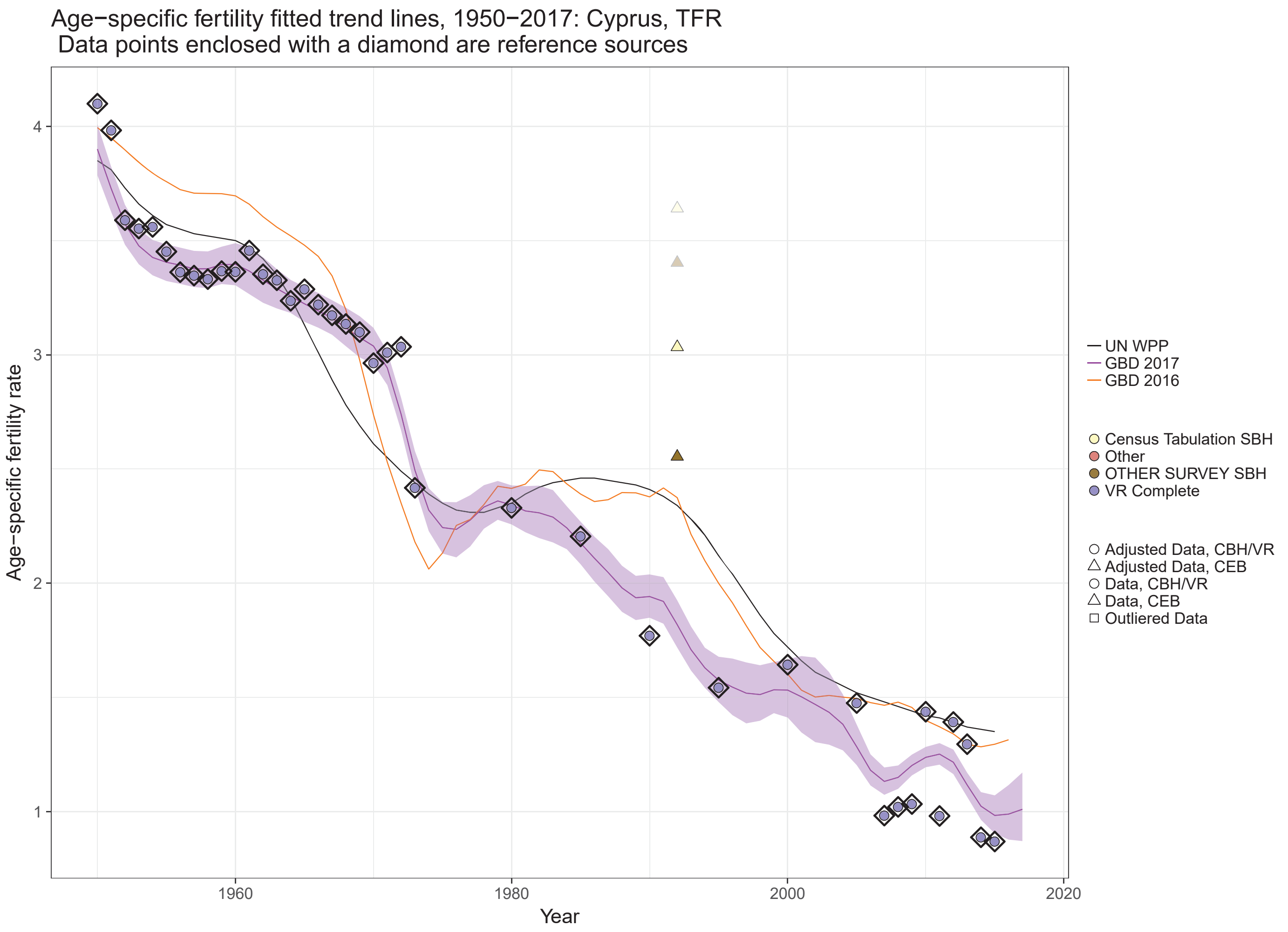

Age−specific fertility fitted trend lines, 1950−2017: Cyprus, TFR
Data points enclosed with a diamond are reference sources
Age-specific fertility rate
Year
1
2
3
4
1960
1980
2000
2020
UN WPP
GBD 2017
GBD 2016
Census Tabulation SBH
Other
OTHER SURVEY SBH
VR Complete
Adjusted Data, CBH/VR
Adjusted Data, CEB
Data, CBH/VR
Data, CEB
Outliered Data

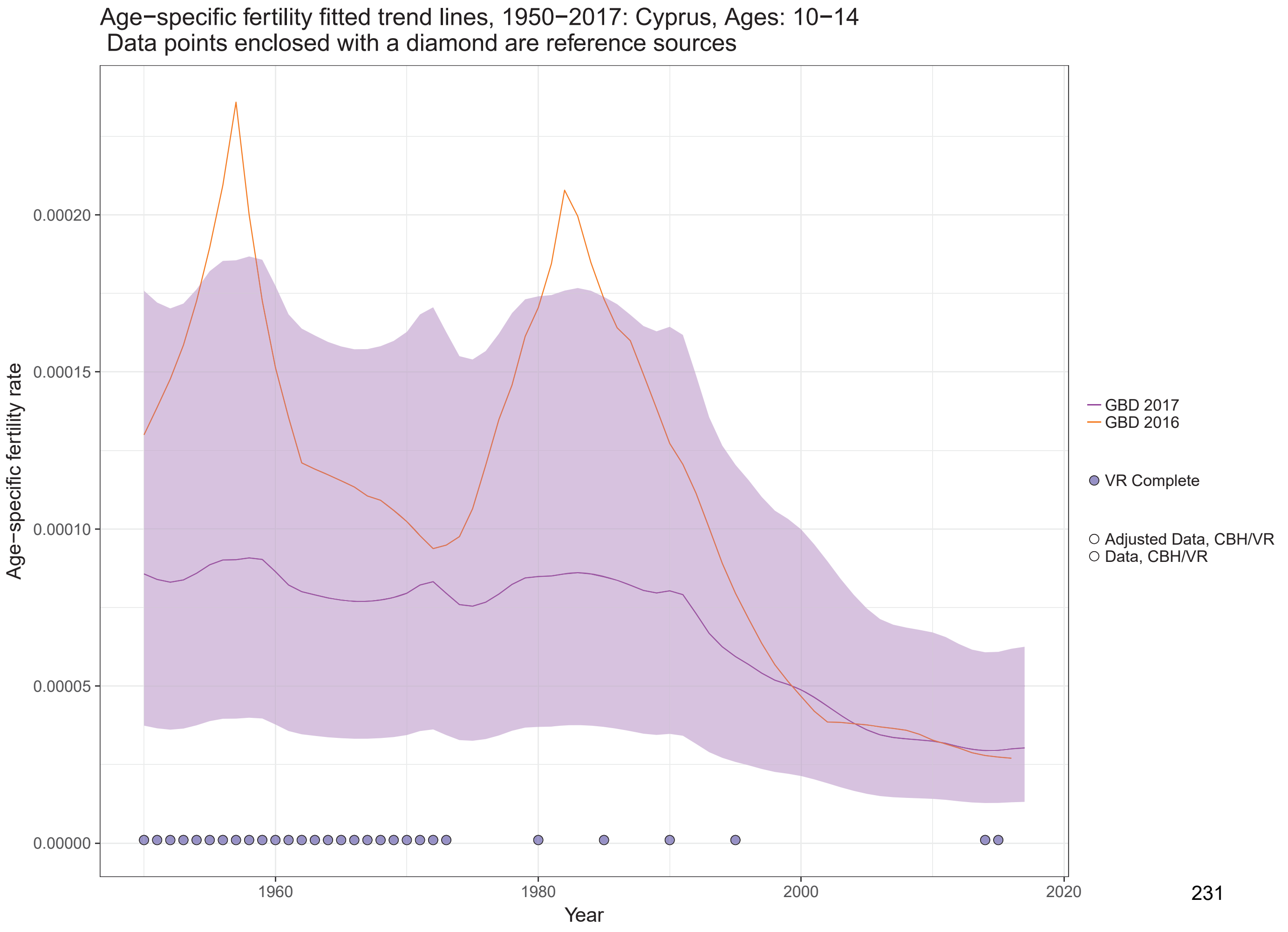

Age−specific fertility fitted trend lines, 1950−2017: Cyprus, Ages: 10−14
Data points enclosed with a diamond are reference sources
Age-specific fertility rate
Year
0.00000
0.00005
0.00010
0.00015
0.00020
1960
1980
2000
2020
GBD 2017
GBD 2016
VR Complete
Adjusted Data, CBH/VR
Data, CBH/VR

Age−specific fertility fitted trend lines, 1950−2017: Cyprus, Ages: 15−19
Data points enclosed with a diamond are reference sources

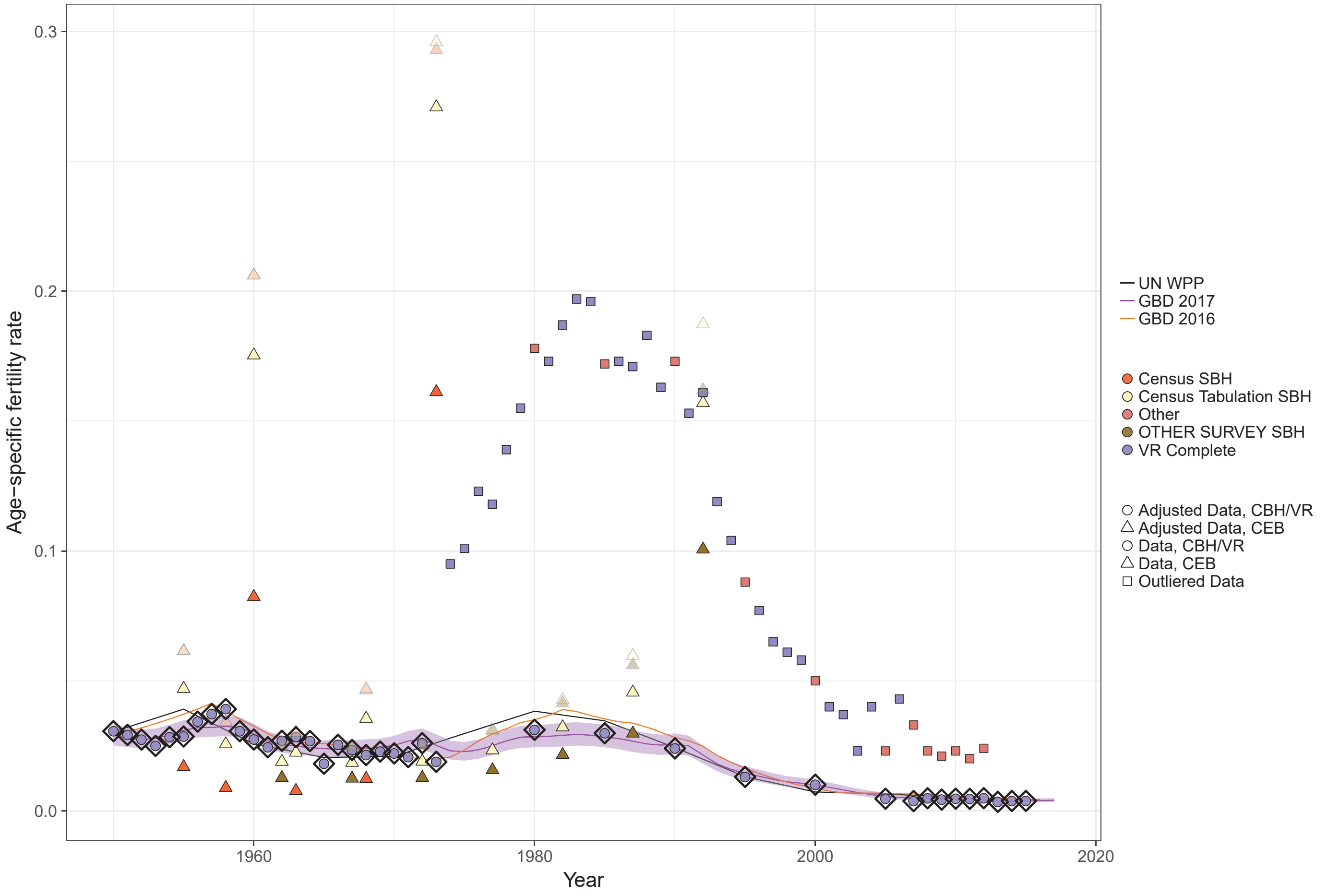

Age−specific fertility fitted trend lines, 1950−2017: Cyprus, Ages: 20−24
Data points enclosed with a diamond are reference sources

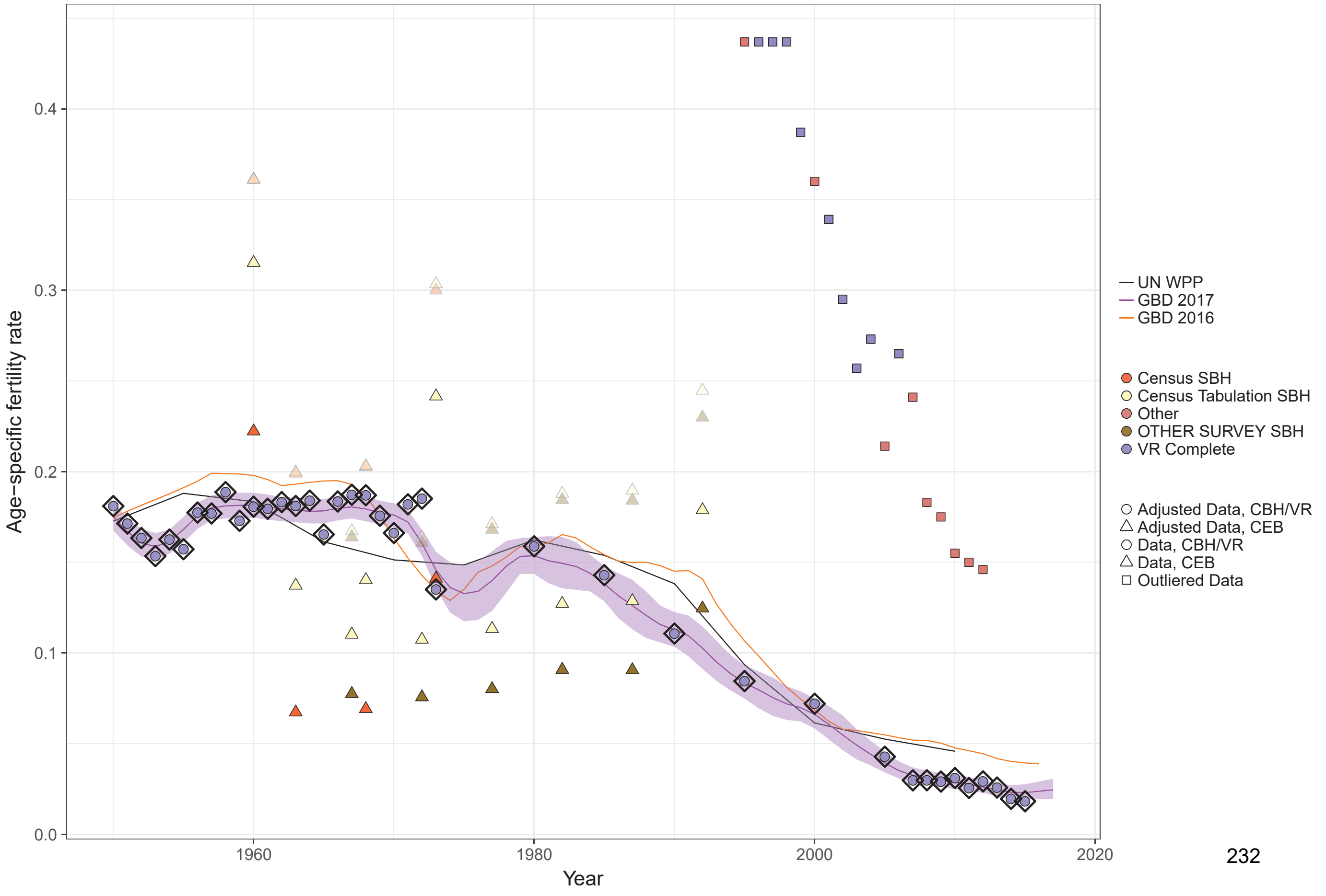

Age−specific fertility fitted trend lines, 1950−2017: Cyprus, Ages: 25−29
Data points enclosed with a diamond are reference sources

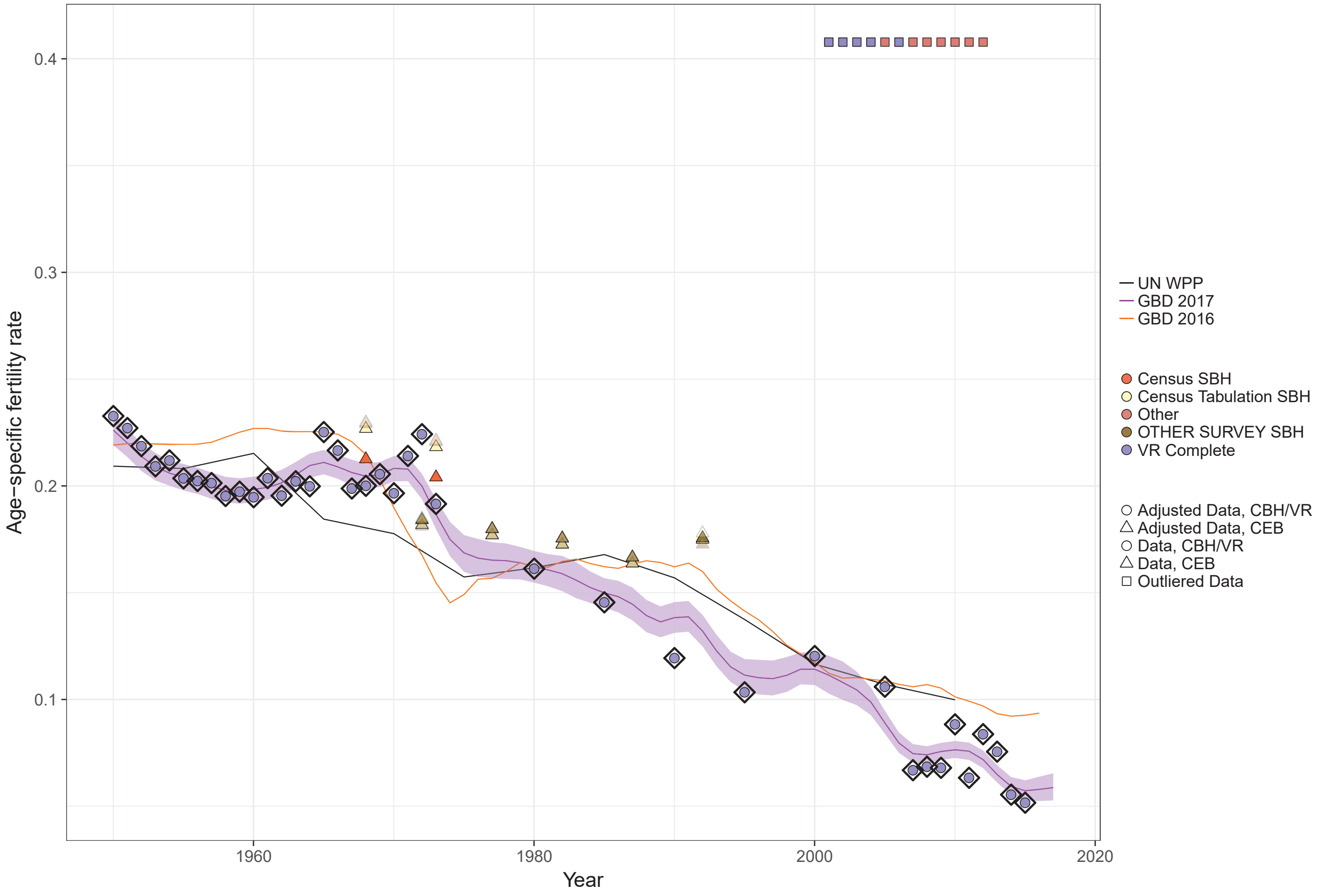

Age−specific fertility fitted trend lines, 1950−2017: Cyprus, Ages: 30−34
Data points enclosed with a diamond are reference sources

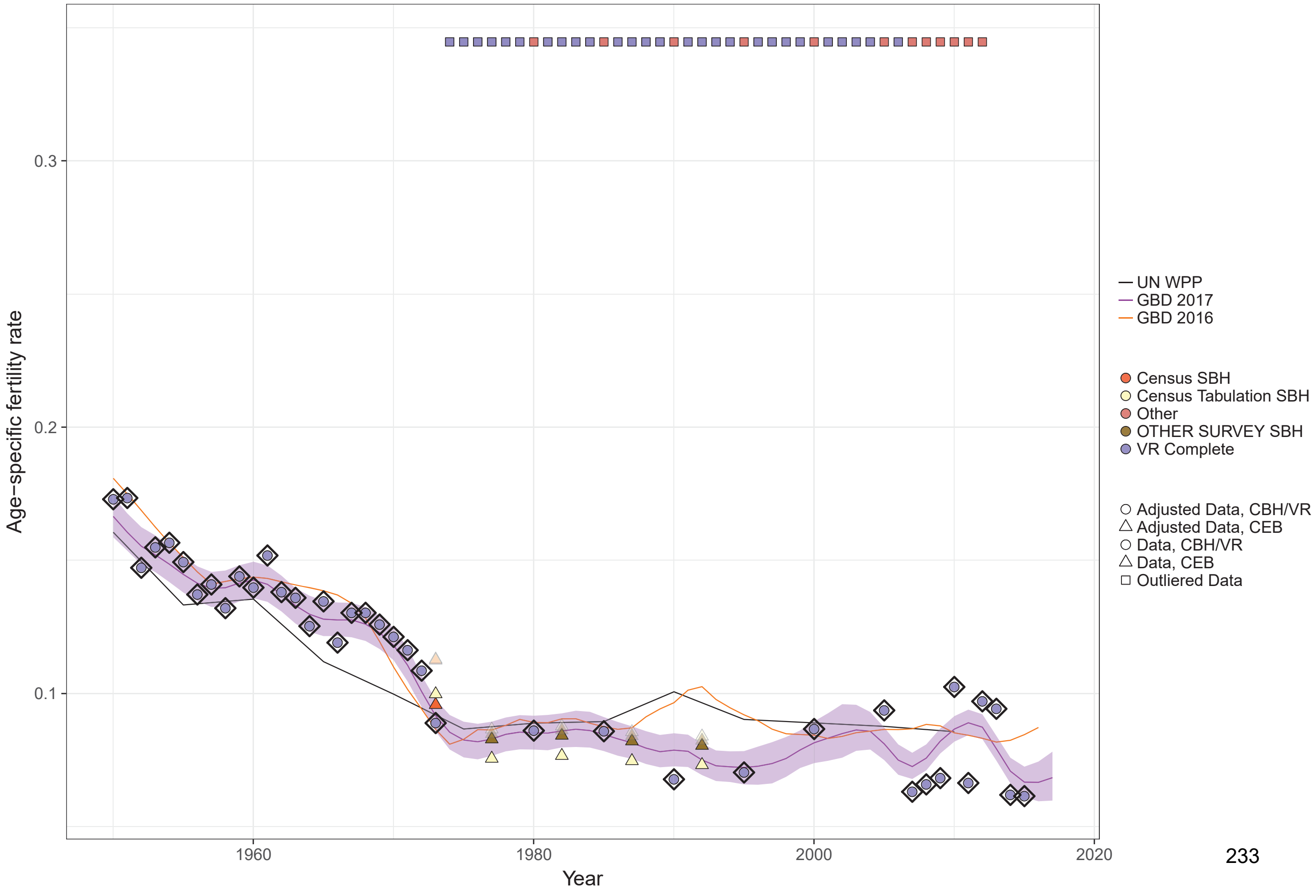

Age−specific fertility fitted trend lines, 1950−2017: Cyprus, Ages: 35−39
Data points enclosed with a diamond are reference sources

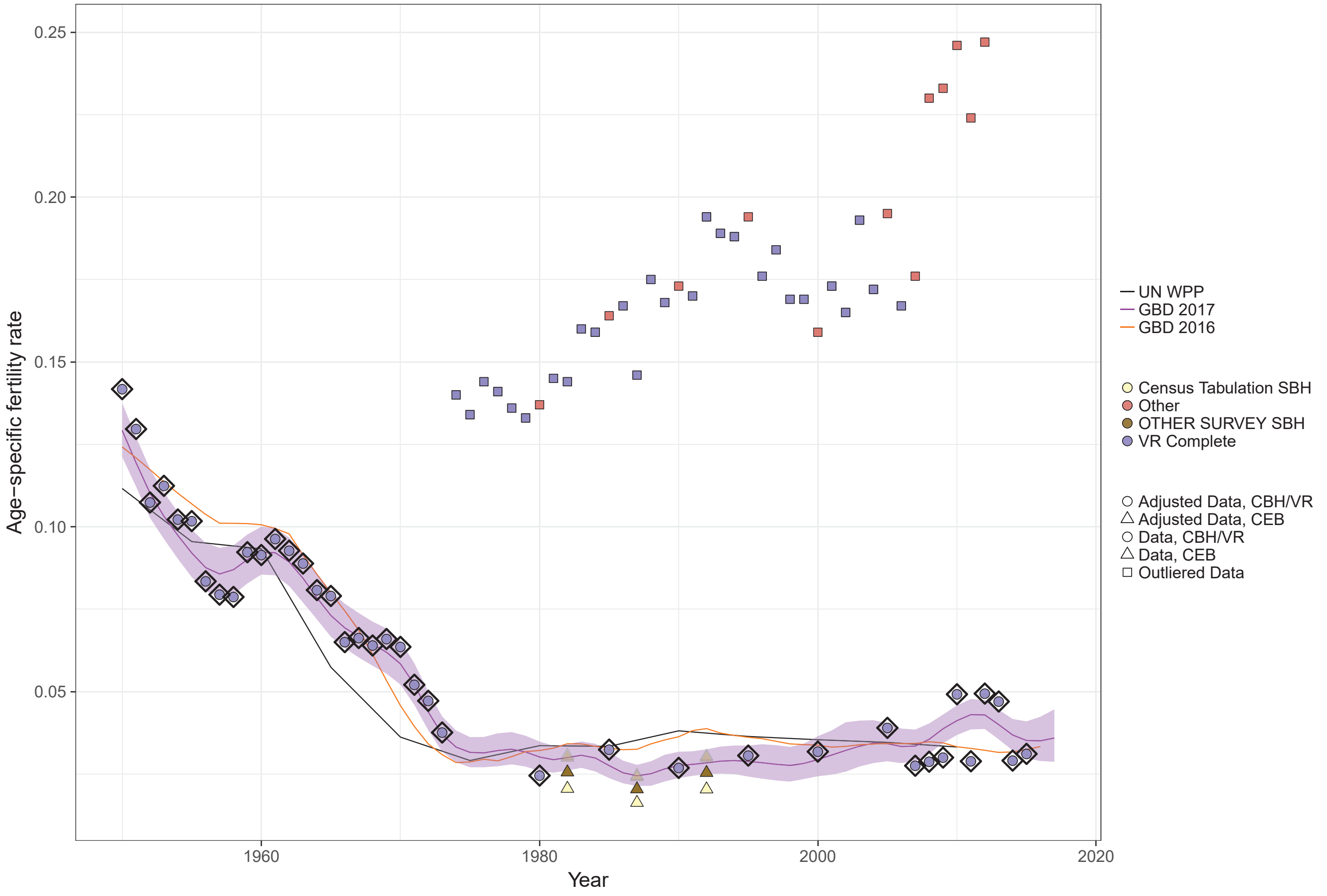

Age−specific fertility fitted trend lines, 1950−2017: Cyprus, Ages: 40−44
Data points enclosed with a diamond are reference sources

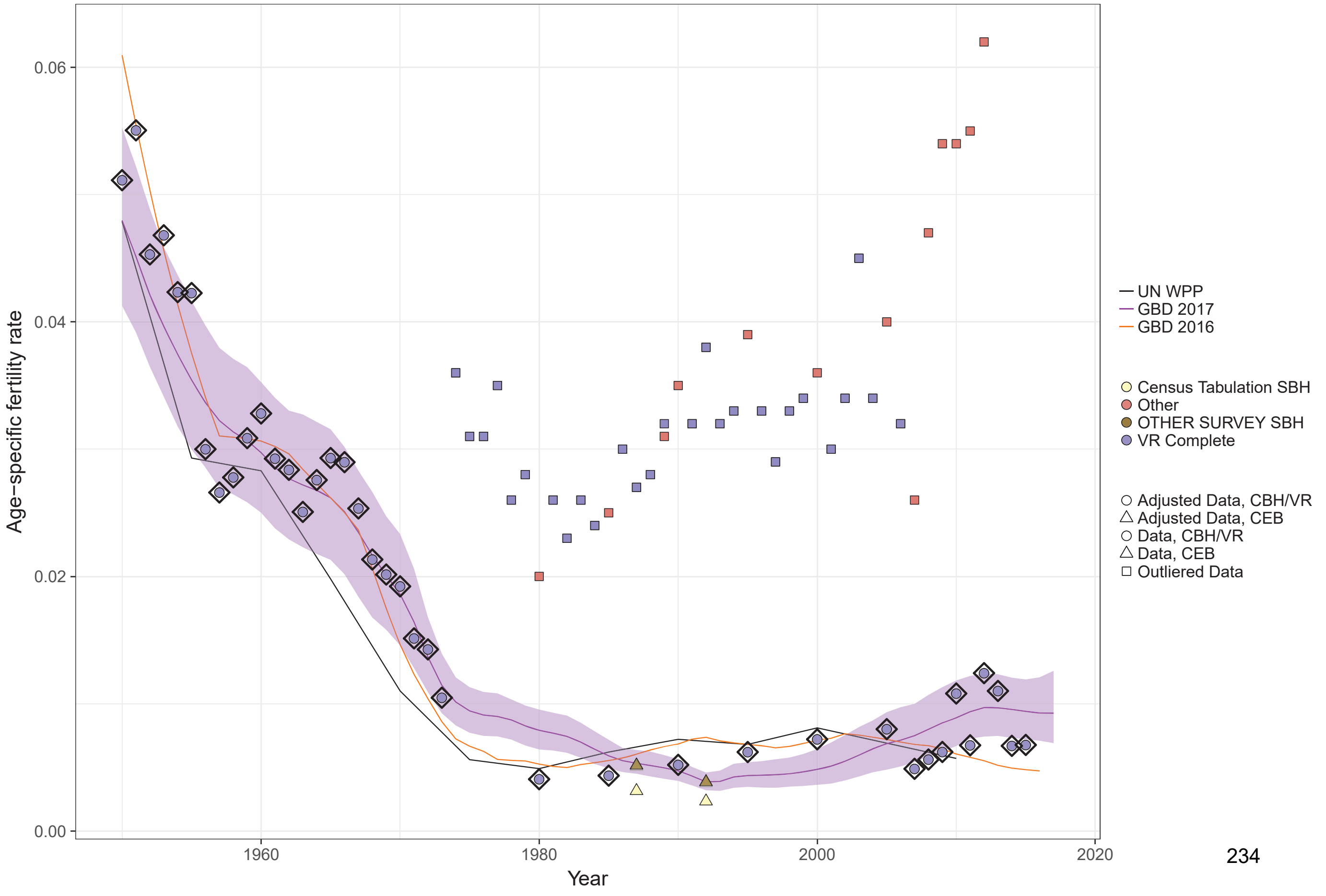

Age−specific fertility fitted trend lines, 1950−2017: Cyprus, Ages: 45−49
Data points enclosed with a diamond are reference sources

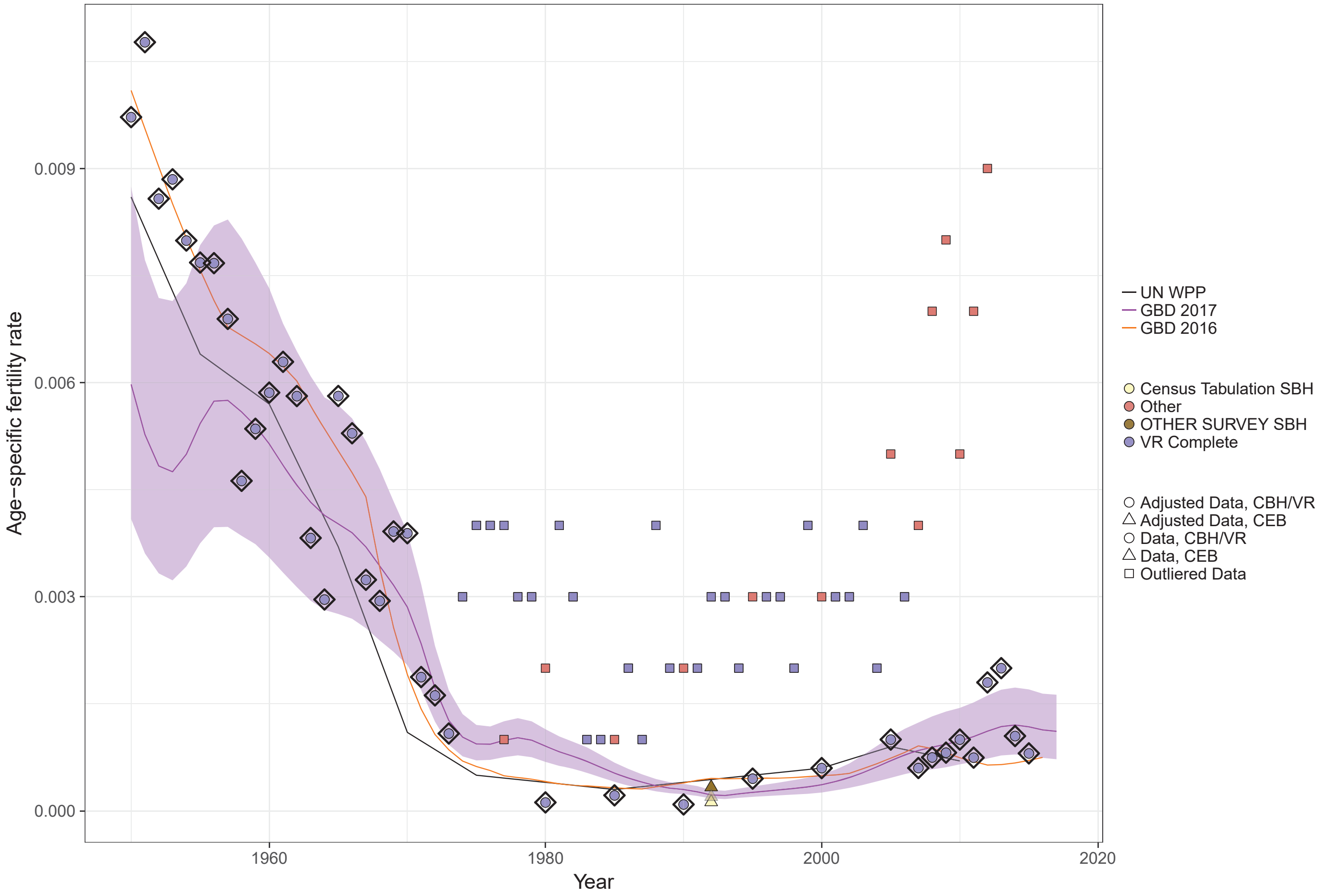

Age−specific fertility fitted trend lines, 1950−2017: Cyprus, Ages: 50−54
Data points enclosed with a diamond are reference sources

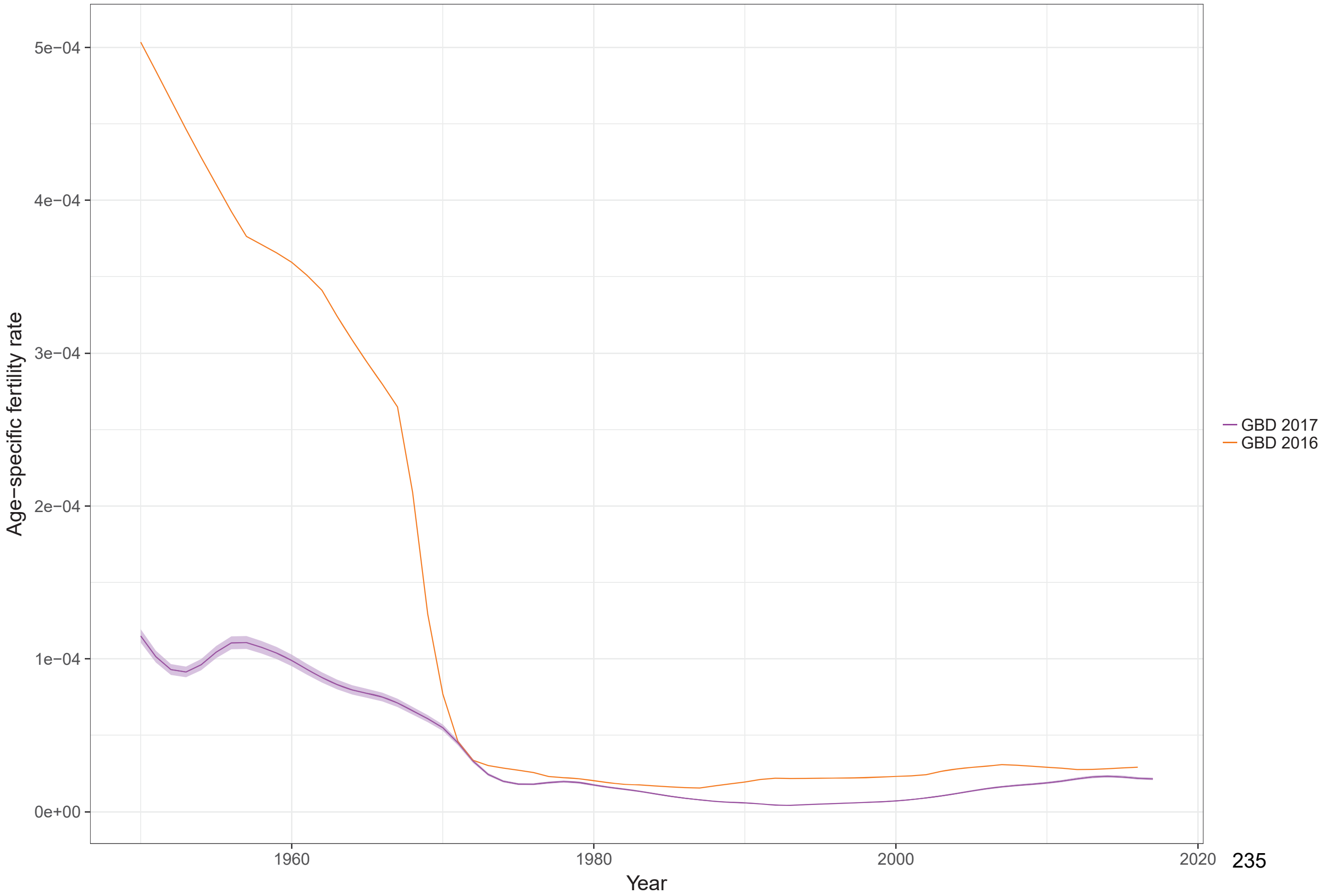

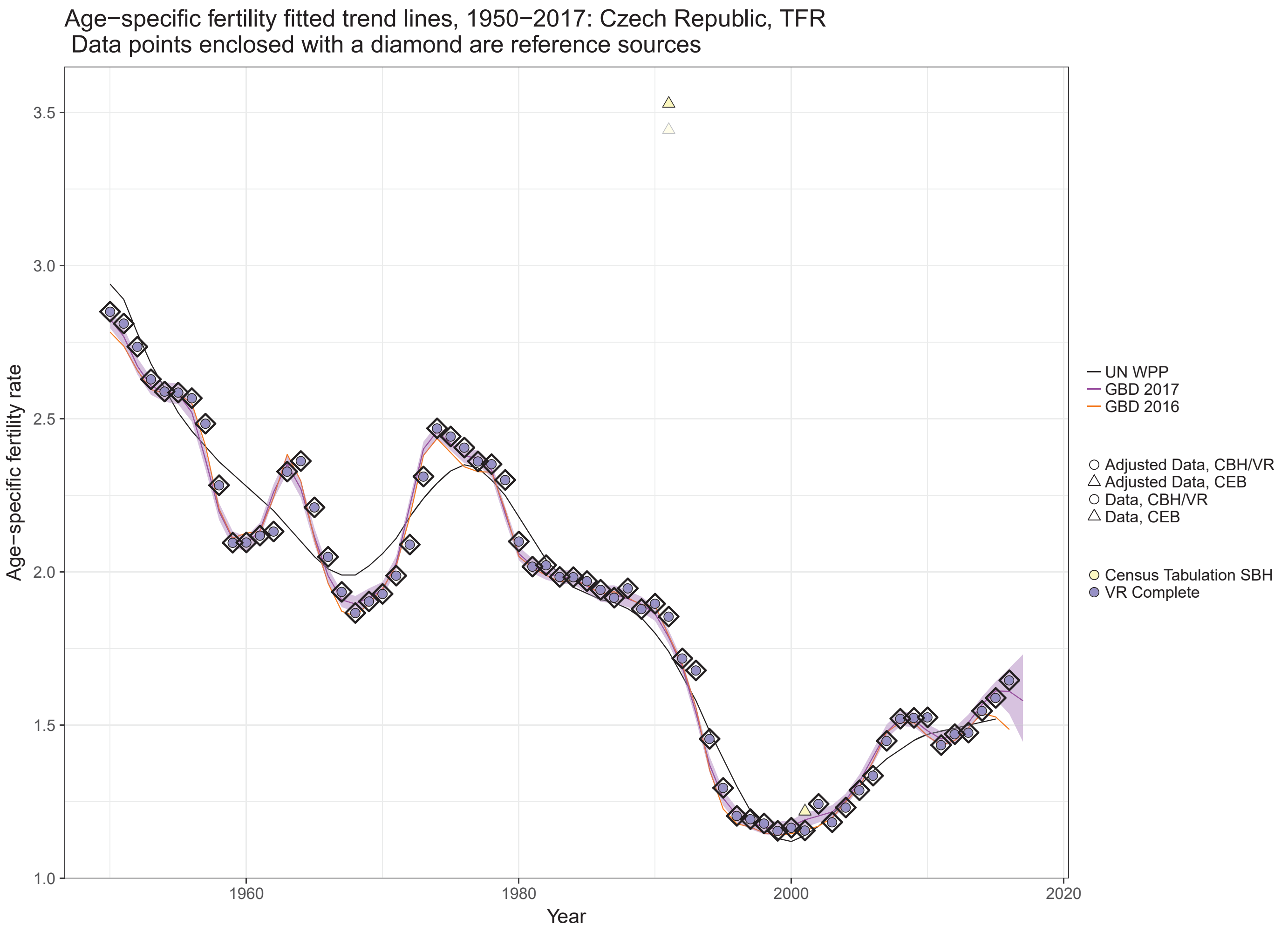
Age−specific fertility fitted trend lines, 1950−2017: Czech Republic, TFR
Data points enclosed with a diamond are reference sources
Age−specific fertility rate
Year
UN WPP
GBD 2017
GBD 2016
Adjusted Data, CBH/VR
Adjusted Data, CEB
Data, CBH/VR
Data, CEB
Census Tabulation SBH
VR Complete

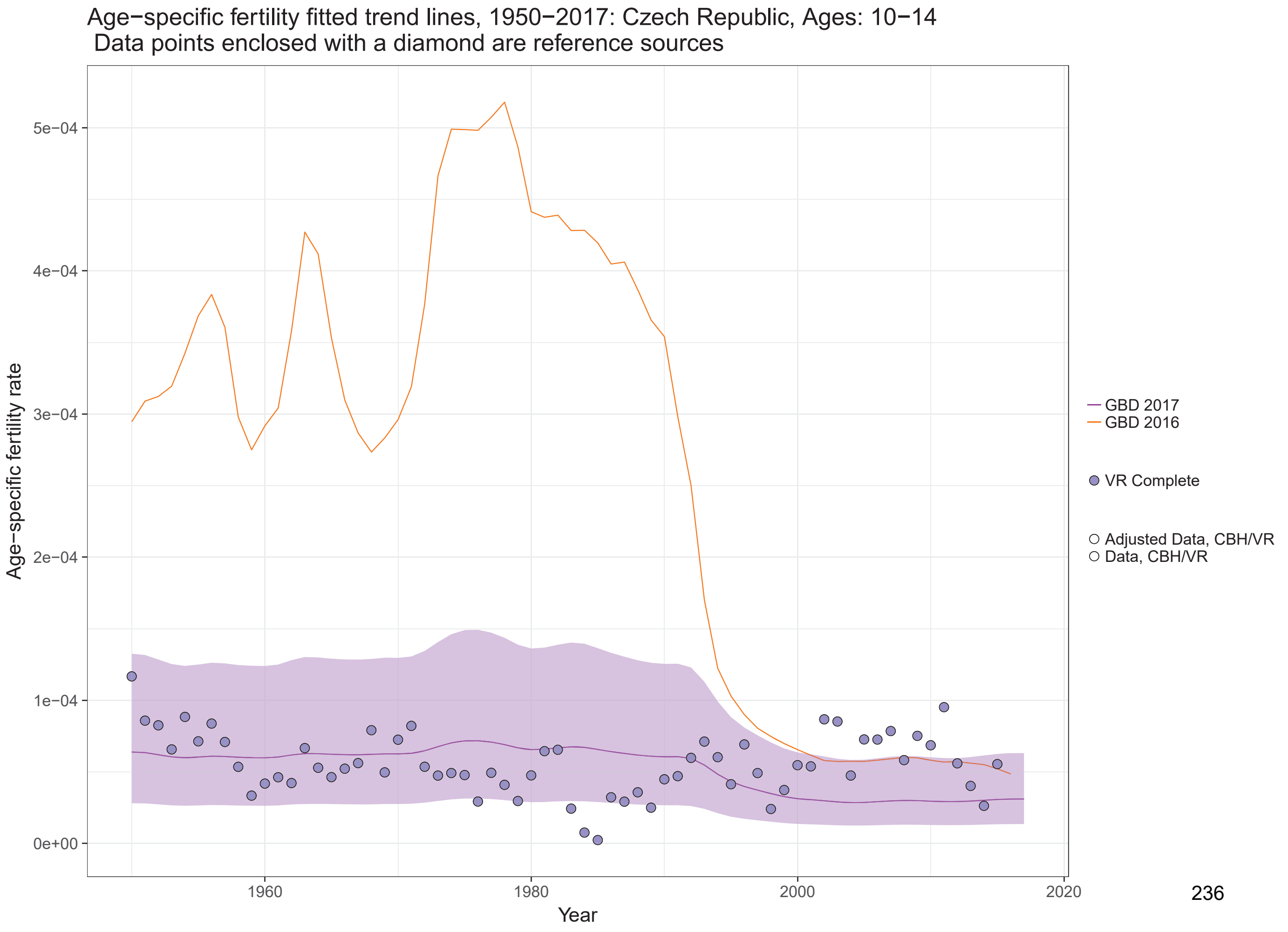
Age−specific fertility fitted trend lines, 1950−2017: Czech Republic, Ages: 10−14
Data points enclosed with a diamond are reference sources
Age−specific fertility rate
Year
GBD 2017
GBD 2016
VR Complete
Adjusted Data, CBH/VR
Data, CBH/VR

Age−specific fertility fitted trend lines, 1950−2017: Czech Republic, Ages: 15−19
Data points enclosed with a diamond are reference sources

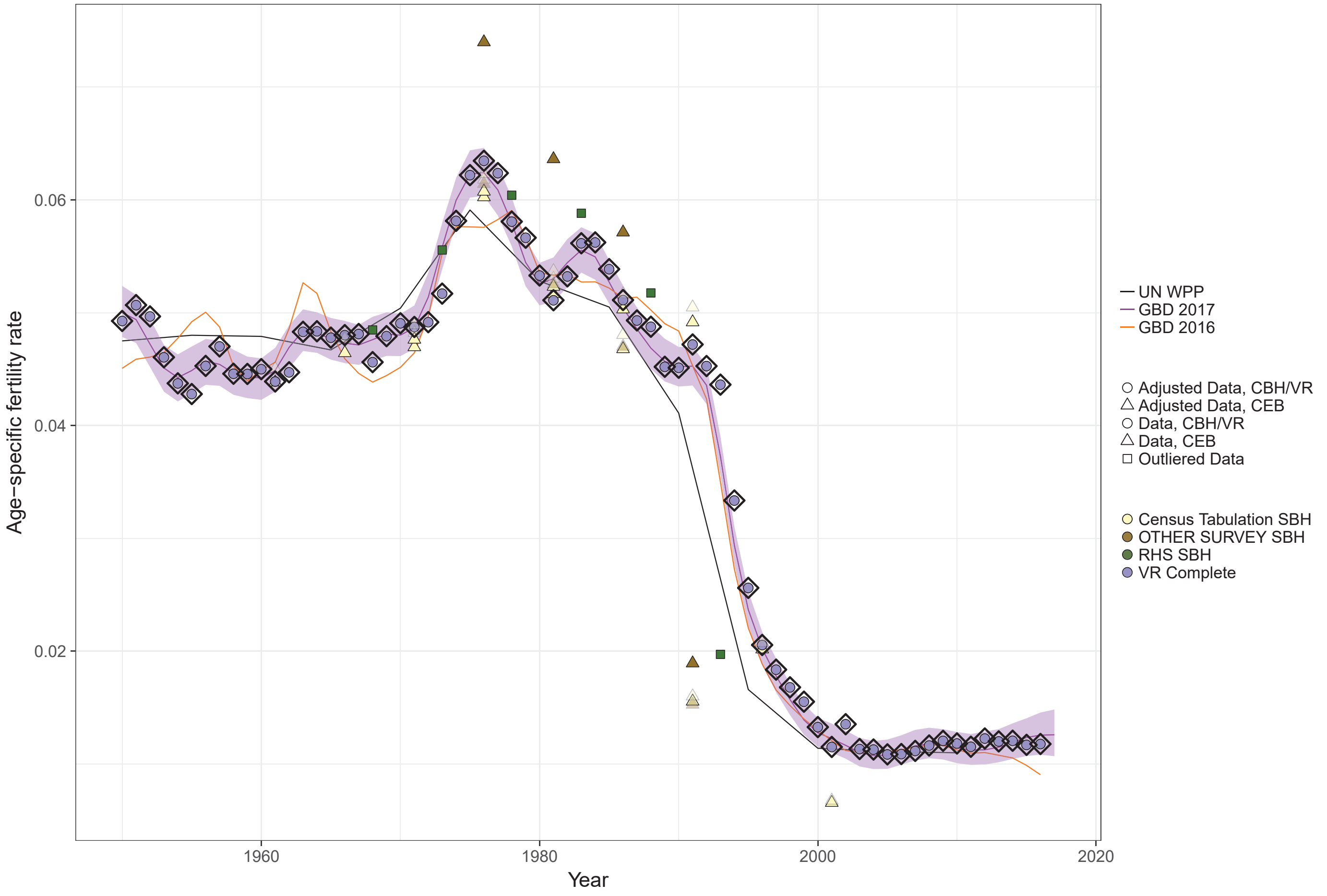

Age−specific fertility fitted trend lines, 1950−2017: Czech Republic, Ages: 20−24
Data points enclosed with a diamond are reference sources

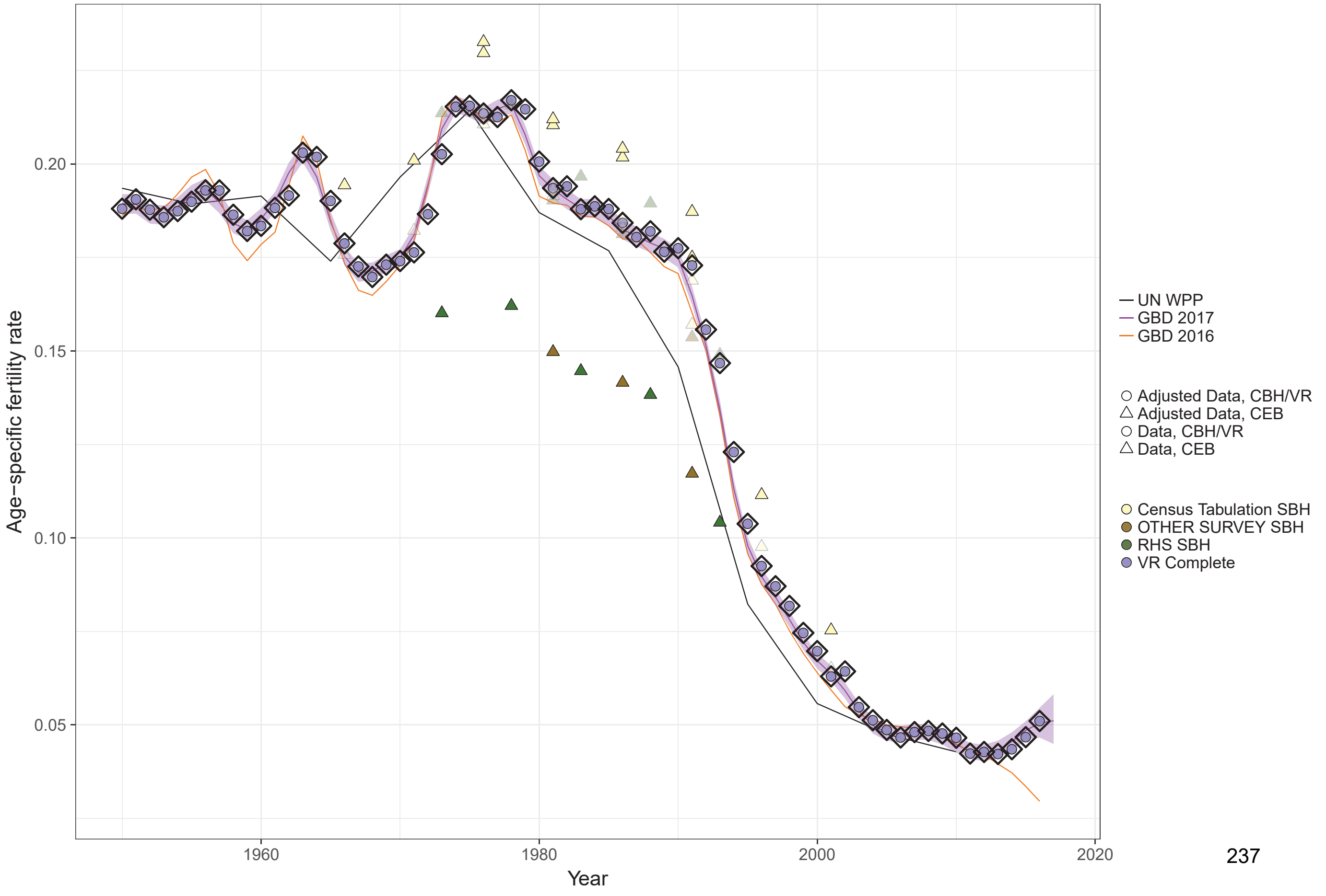

Age−specific fertility fitted trend lines, 1950−2017: Czech Republic, Ages: 25−29
Data points enclosed with a diamond are reference sources

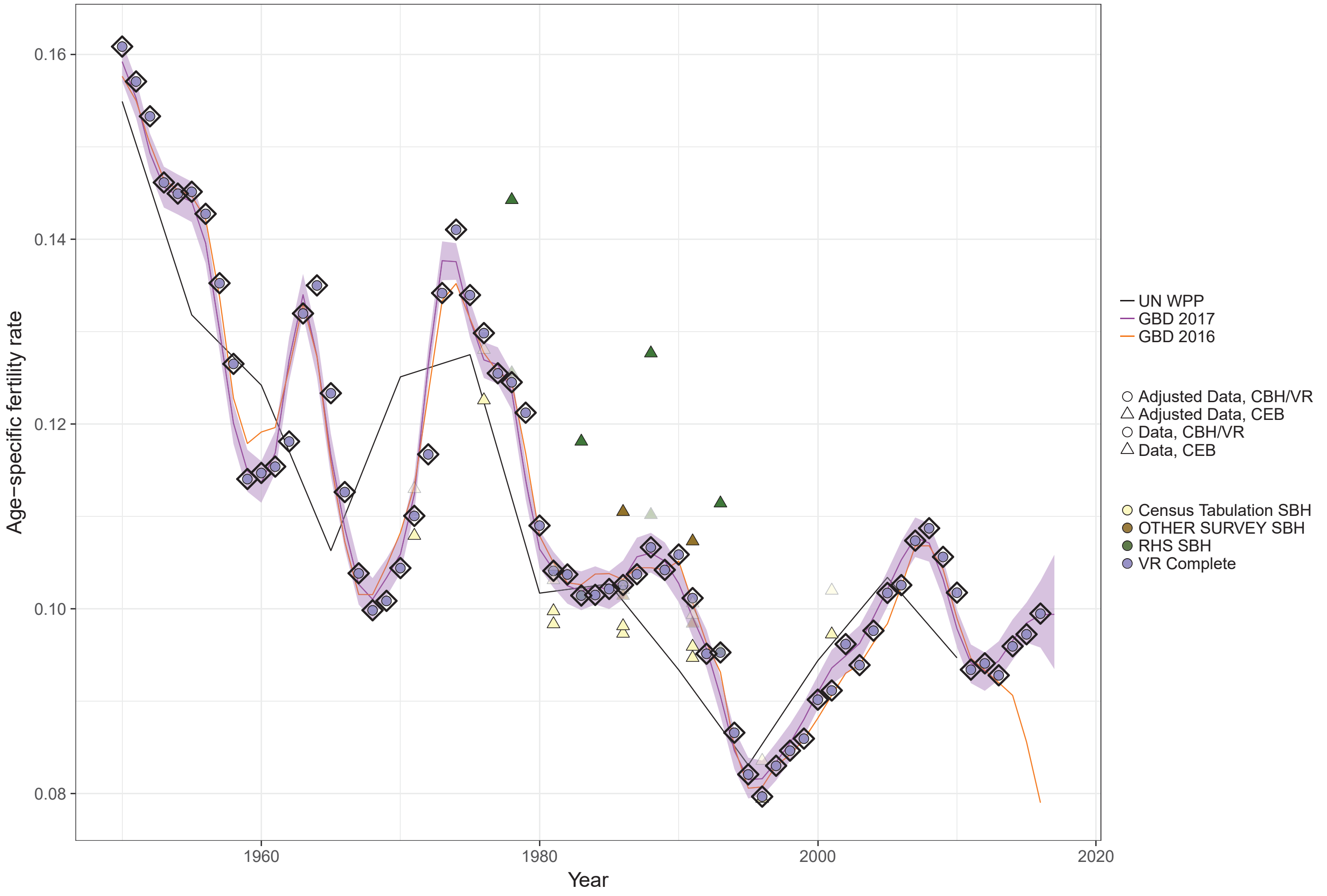

Age−specific fertility fitted trend lines, 1950−2017: Czech Republic, Ages: 30−34
Data points enclosed with a diamond are reference sources

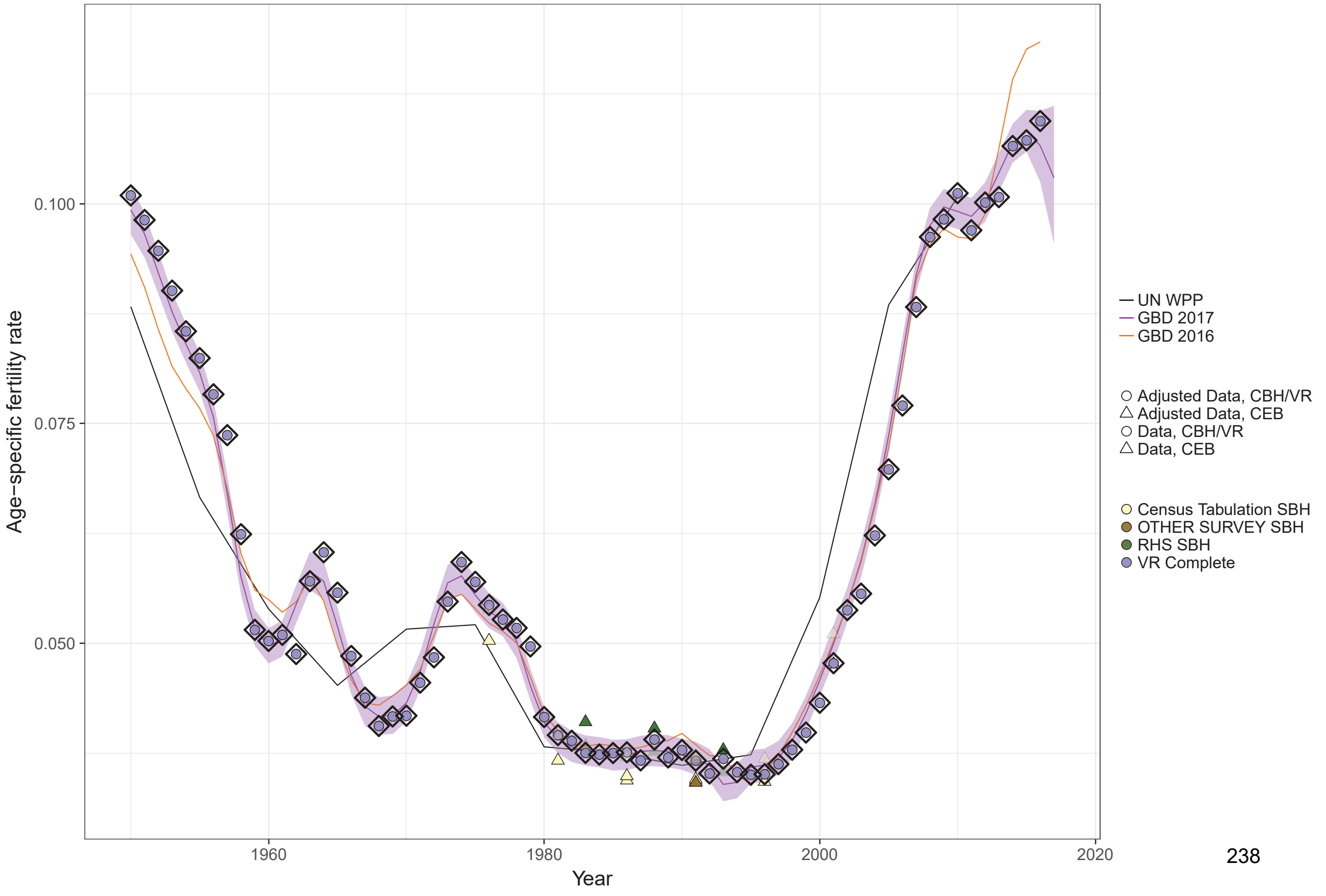

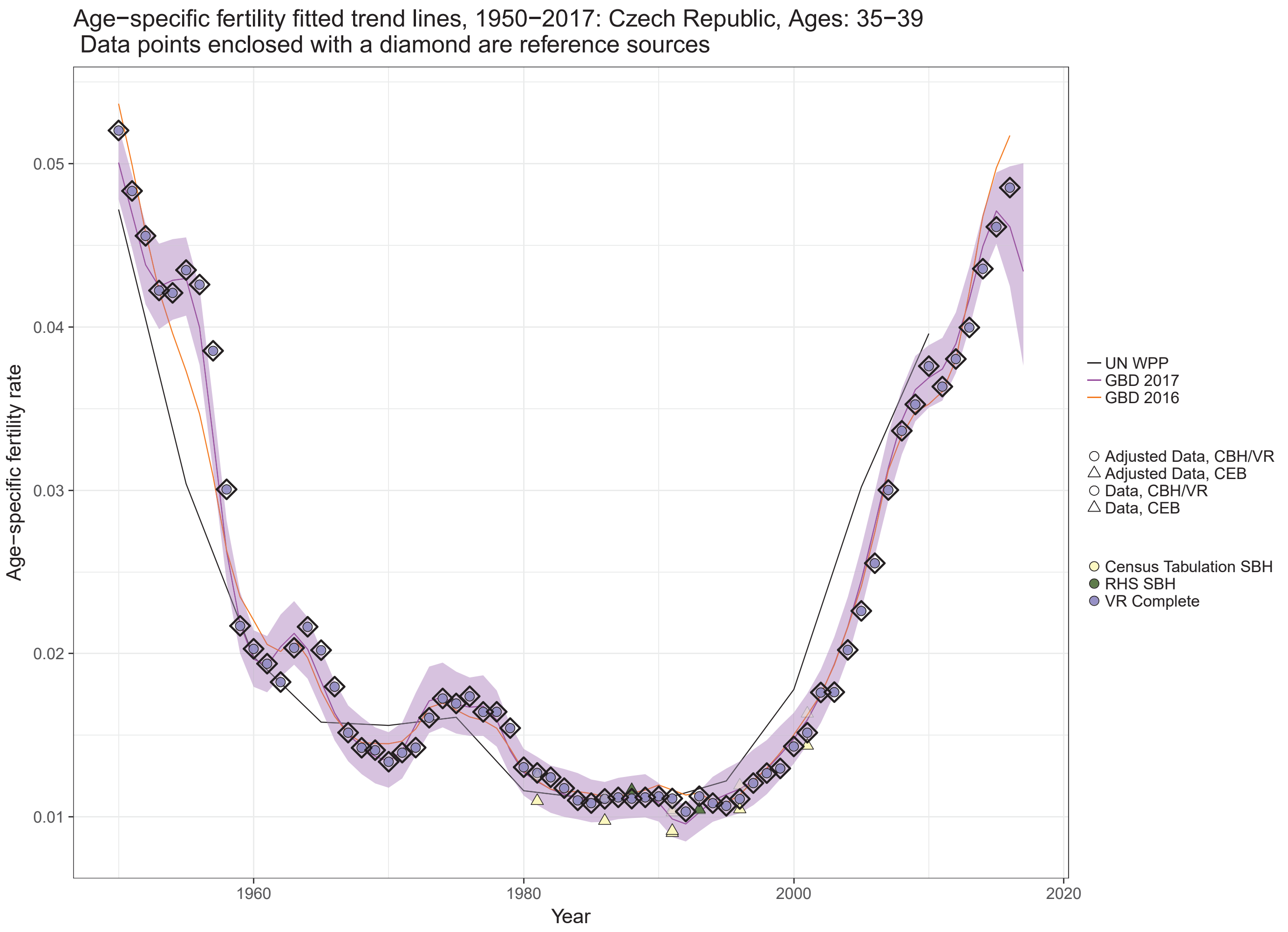
Age−specific fertility fitted trend lines, 1950−2017: Czech Republic, Ages: 35−39
Data points enclosed with a diamond are reference sources
Age−specific fertility rate
0.05
0.04
0.03
0.02
0.01
1960
1980
2000
2020
Year
UN WPP
GBD 2017
GBD 2016
Adjusted Data, CBH/VR
Adjusted Data, CEB
Data, CBH/VR
Data, CEB
Census Tabulation SBH
RHS SBH
VR Complete

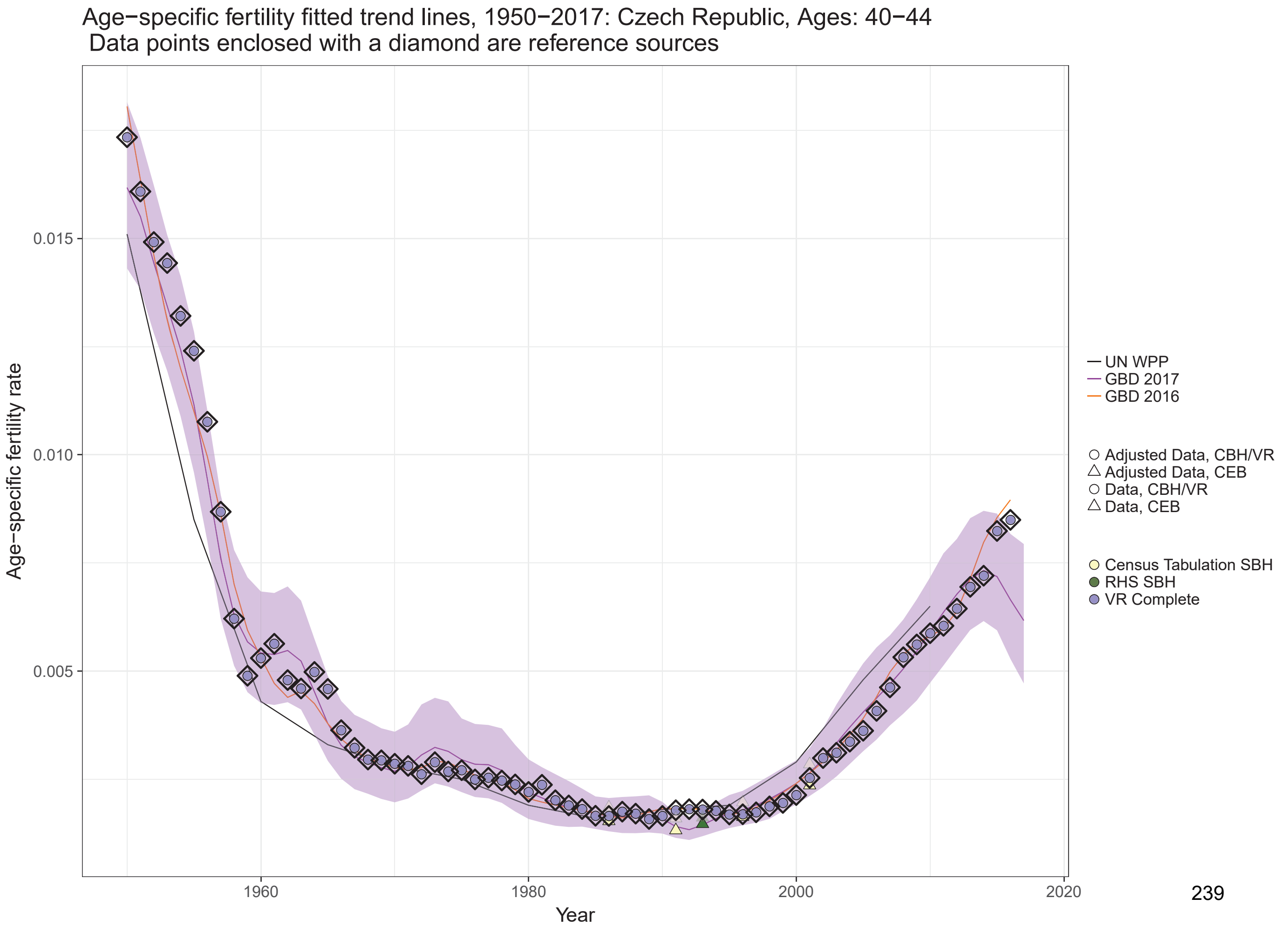
Age−specific fertility fitted trend lines, 1950−2017: Czech Republic, Ages: 40−44
Data points enclosed with a diamond are reference sources
Age−specific fertility rate
0.015
0.010
0.005
1960
1980
2000
2020
Year
UN WPP
GBD 2017
GBD 2016
Adjusted Data, CBH/VR
Adjusted Data, CEB
Data, CBH/VR
Data, CEB
Census Tabulation SBH
RHS SBH
VR Complete

Age−specific fertility fitted trend lines, 1950−2017: Czech Republic, Ages: 45−49
Data points enclosed with a diamond are reference sources

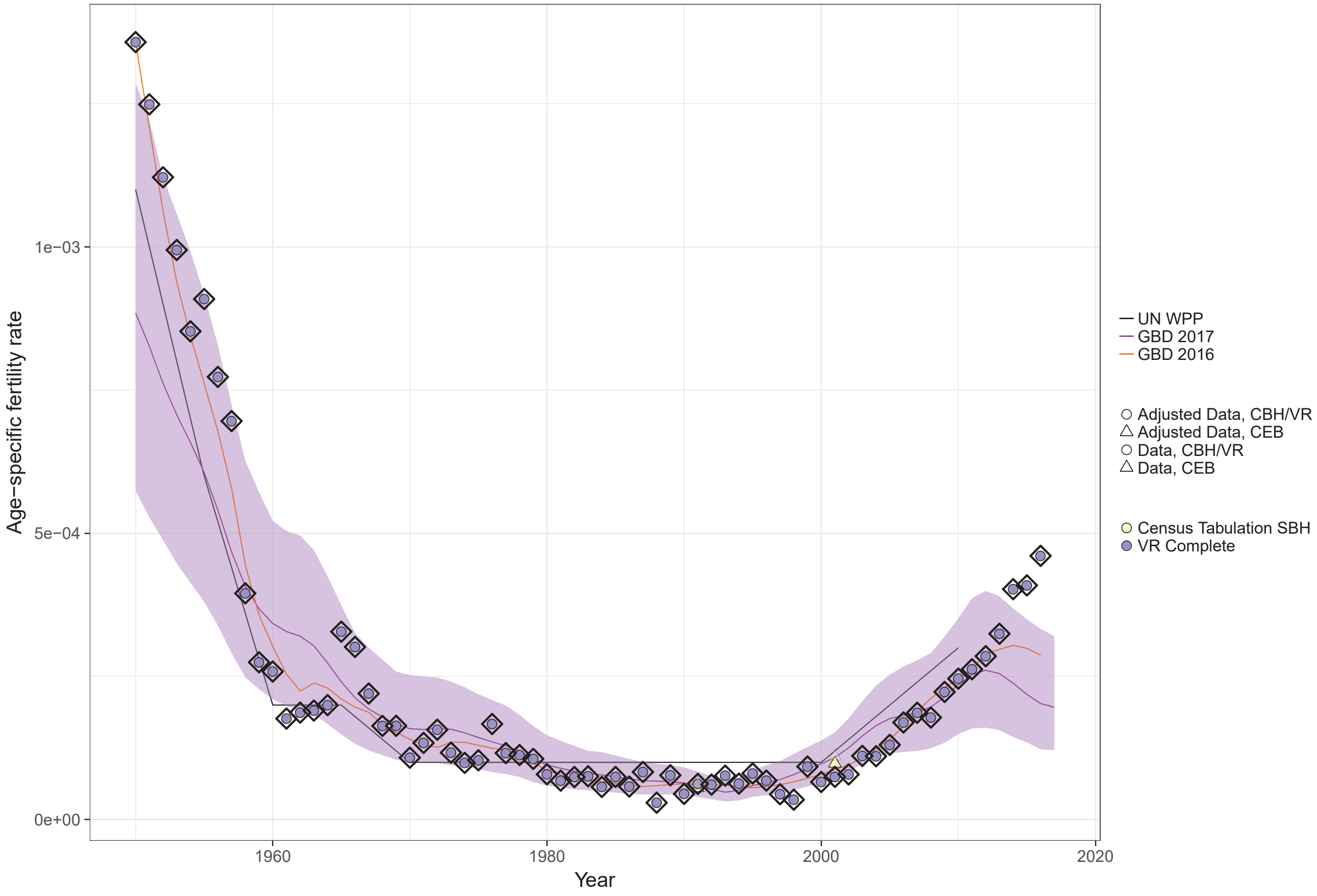

Age−specific fertility fitted trend lines, 1950−2017: Czech Republic, Ages: 50−54
Data points enclosed with a diamond are reference sources

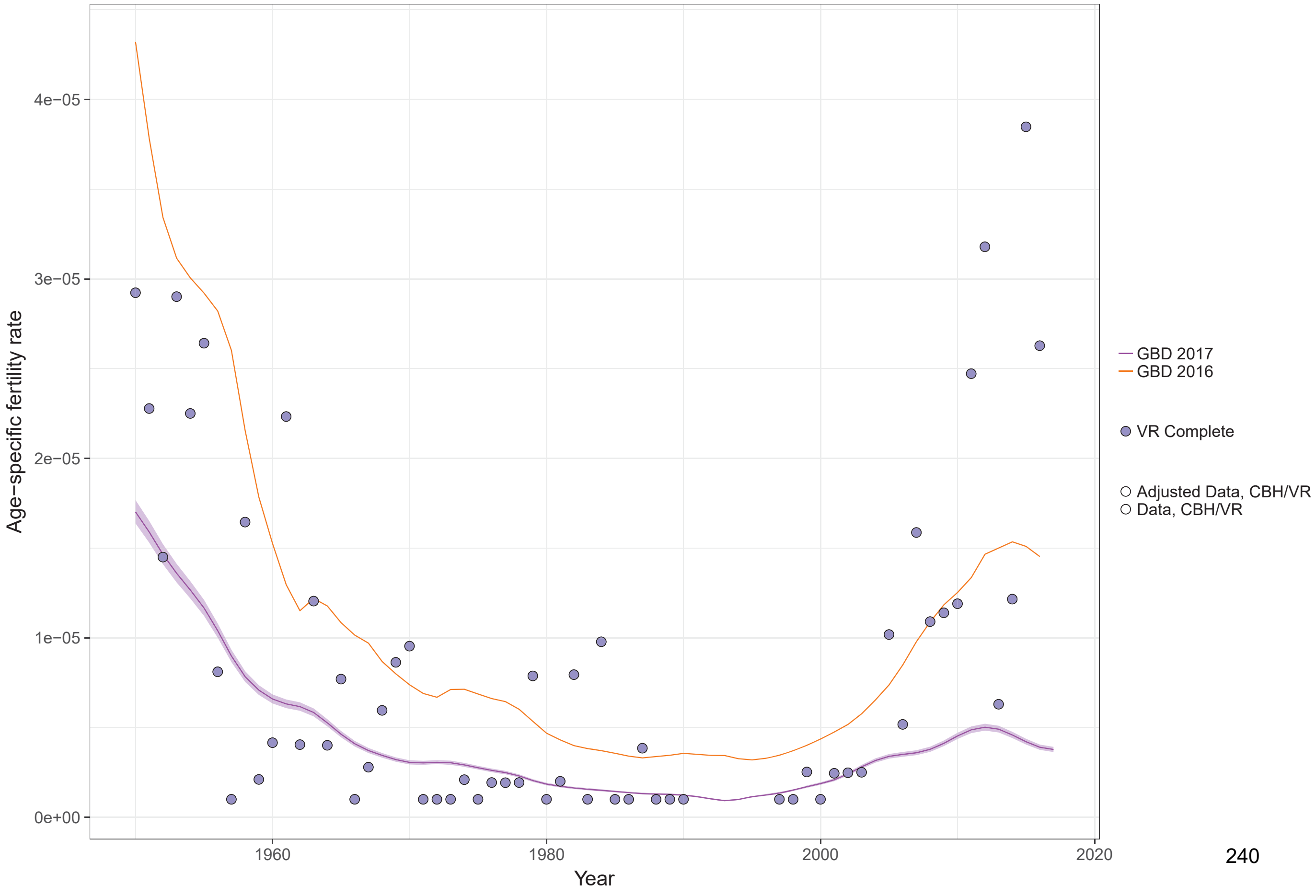

Age−specific fertility fitted trend lines, 1950−2017: Democratic Republic of the Congo, TFR
Data points enclosed with a diamond are reference sources

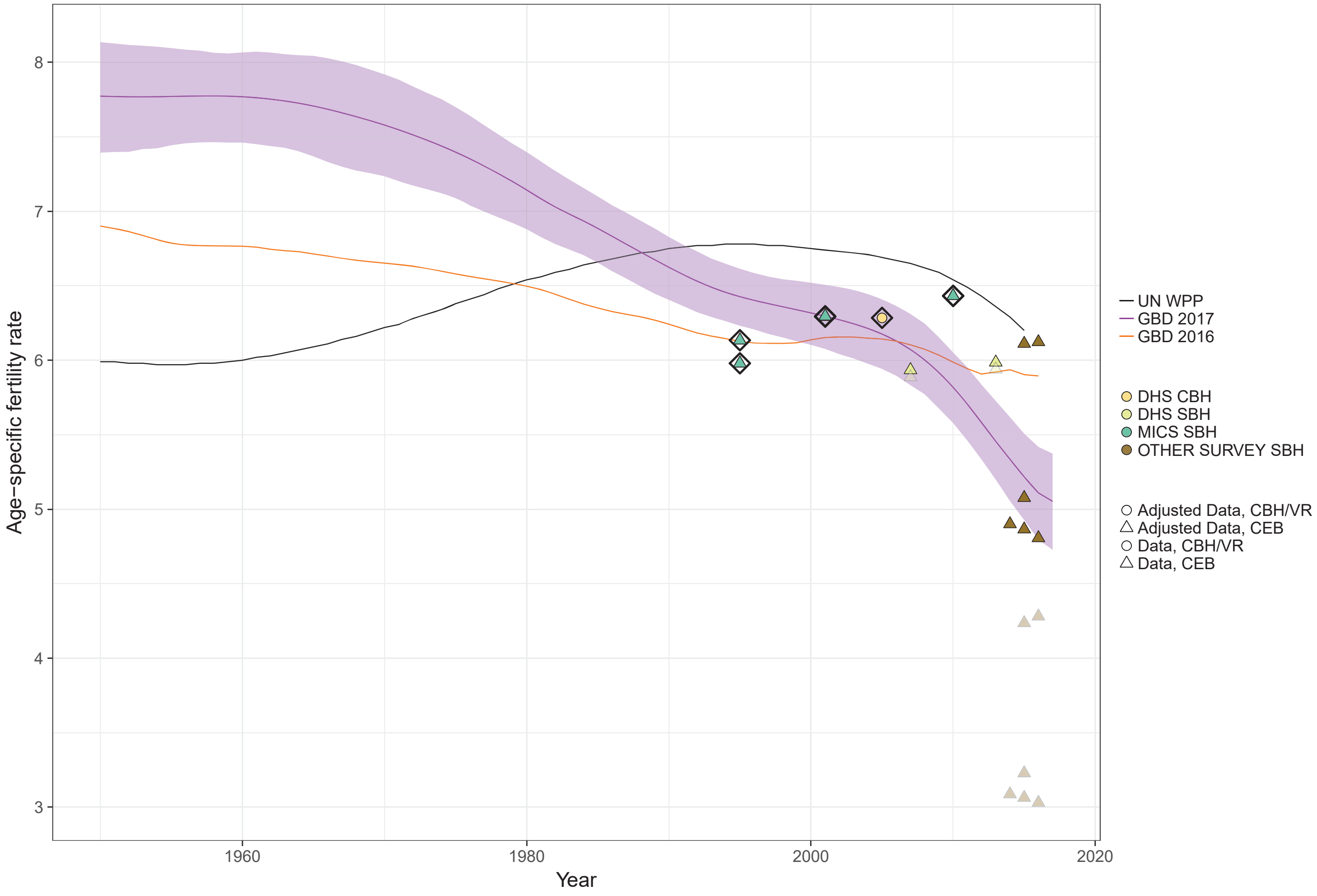

Age−specific fertility fitted trend lines, 1950−2017: Democratic Republic of the Congo, Ages: 10−14
Data points enclosed with a diamond are reference sources

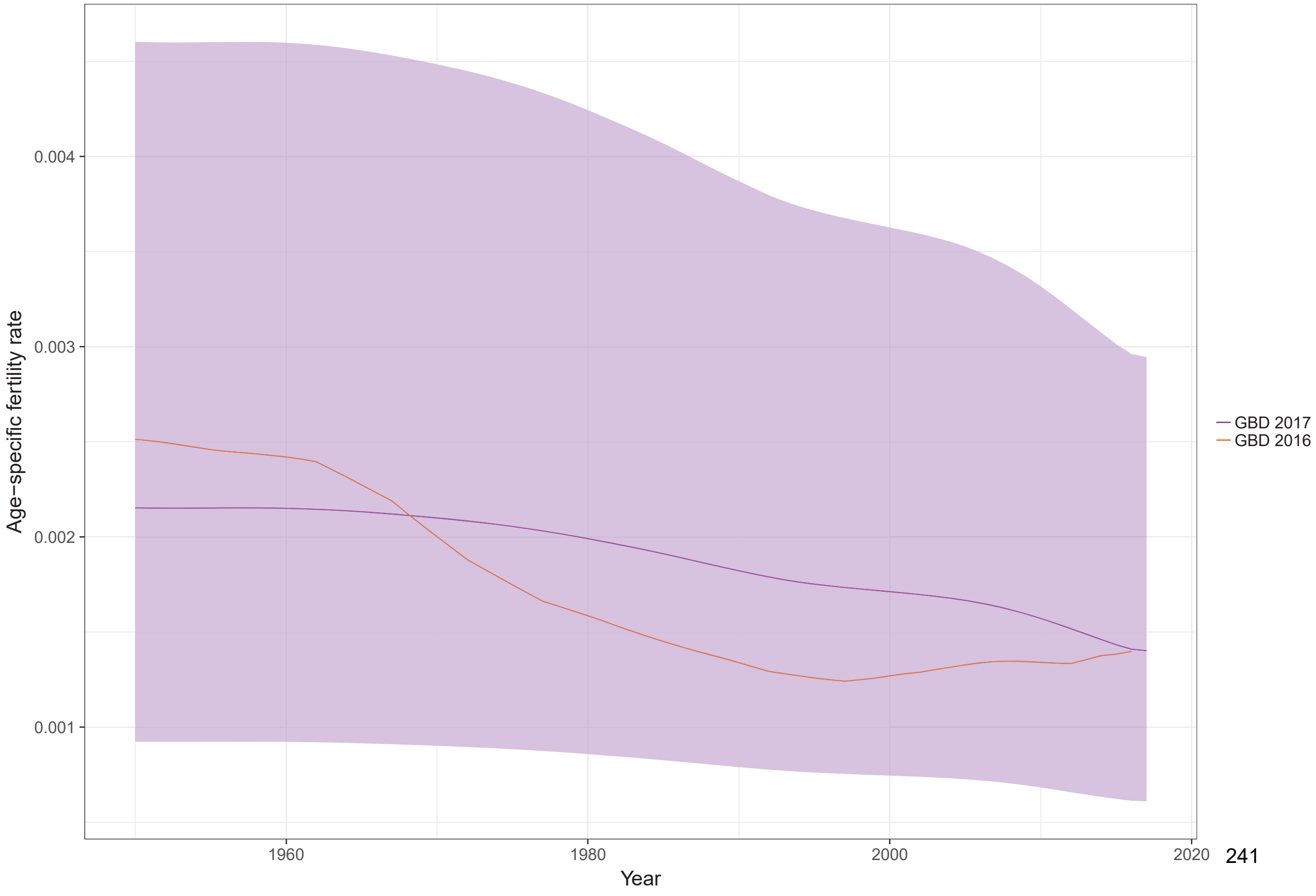

Age−specific fertility fitted trend lines, 1950−2017: Democratic Republic of the Congo, Ages: 15−19
Data points enclosed with a diamond are reference sources

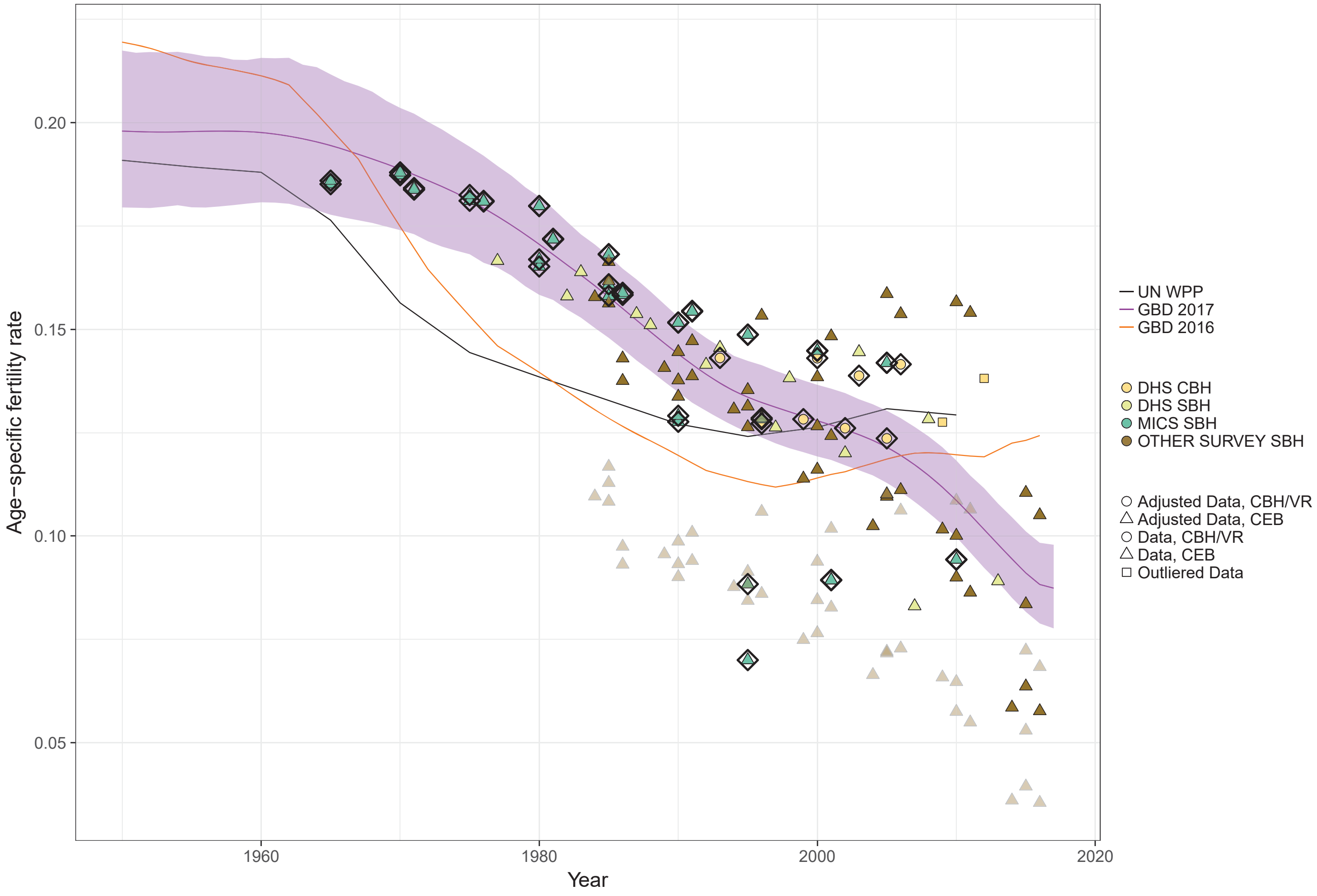

Age−specific fertility fitted trend lines, 1950−2017: Democratic Republic of the Congo, Ages: 20−24
Data points enclosed with a diamond are reference sources

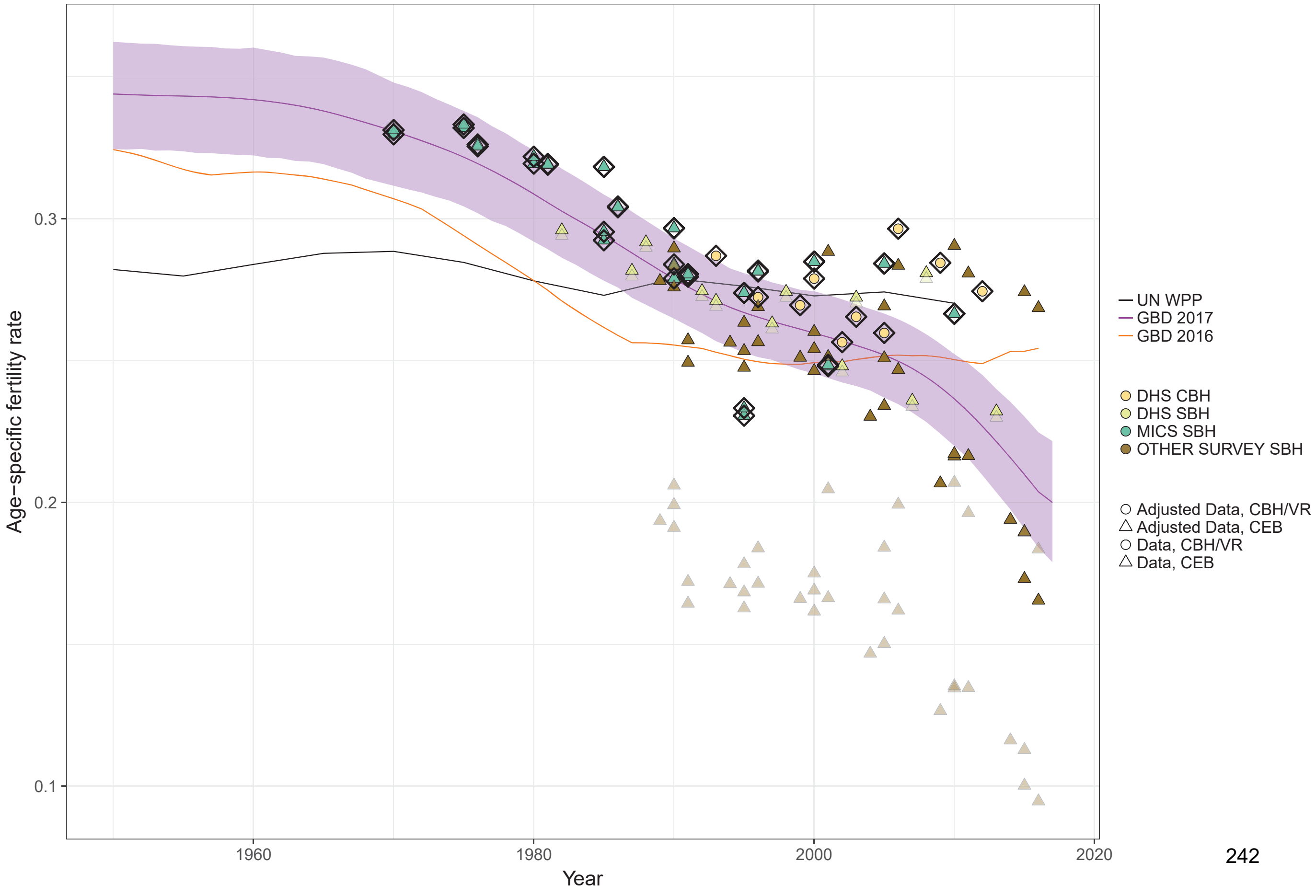

Age−specific fertility fitted trend lines, 1950−2017: Democratic Republic of the Congo, Ages: 25−29
Data points enclosed with a diamond are reference sources

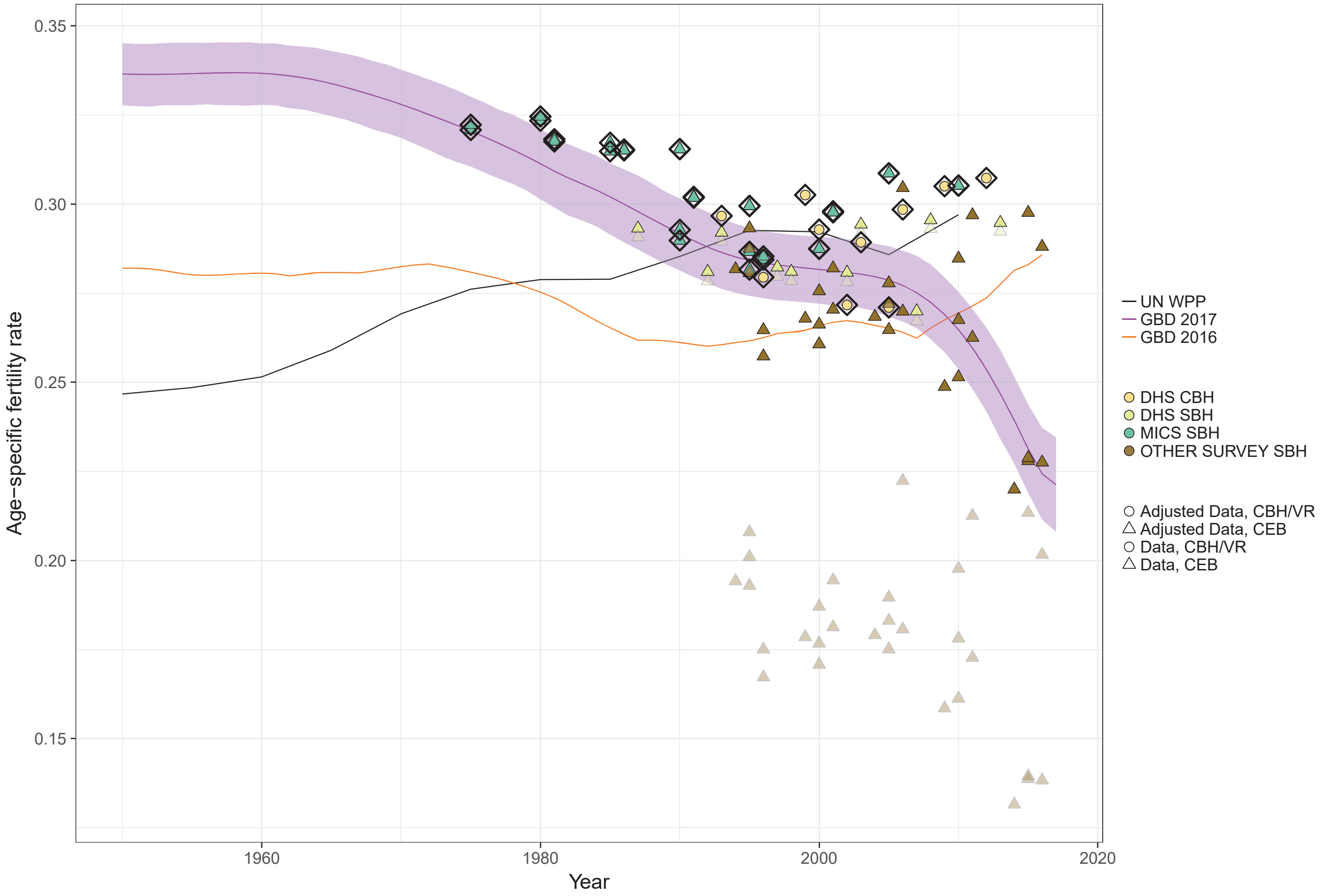

Age−specific fertility fitted trend lines, 1950−2017: Democratic Republic of the Congo, Ages: 30−34
Data points enclosed with a diamond are reference sources

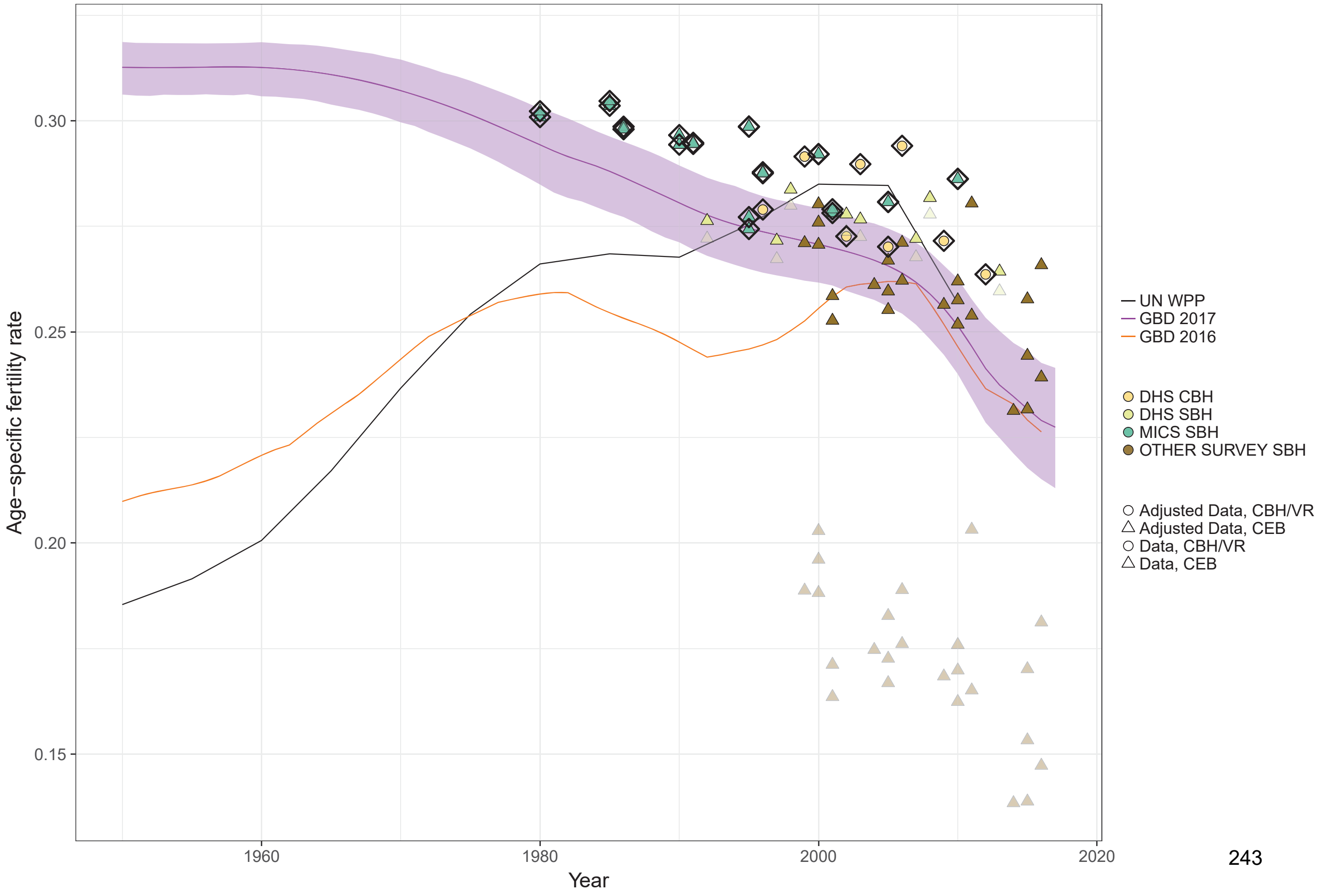

Age−specific fertility fitted trend lines, 1950−2017: Democratic Republic of the Congo, Ages: 35−39
Data points enclosed with a diamond are reference sources

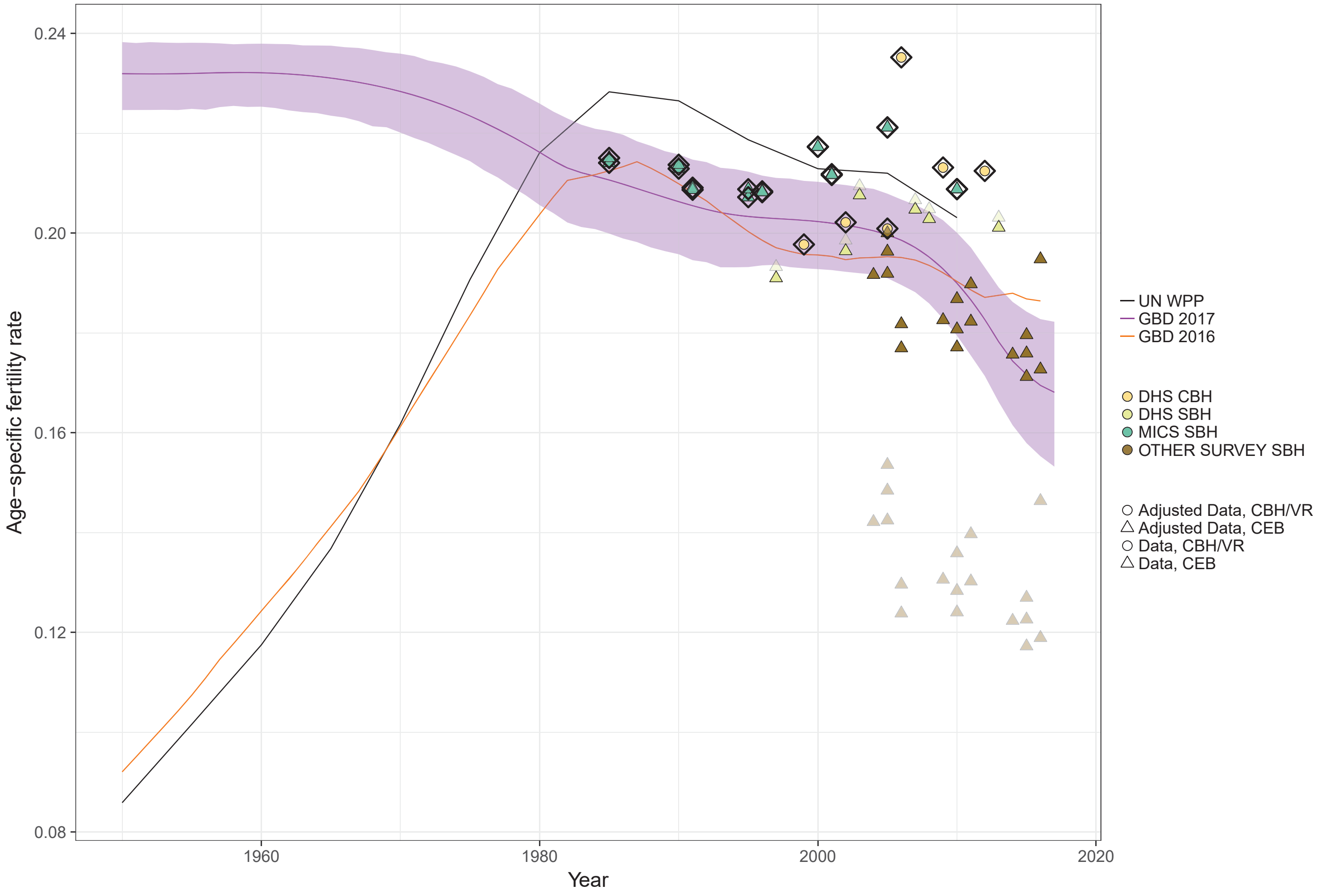

Age−specific fertility fitted trend lines, 1950−2017: Democratic Republic of the Congo, Ages: 40−44
Data points enclosed with a diamond are reference sources

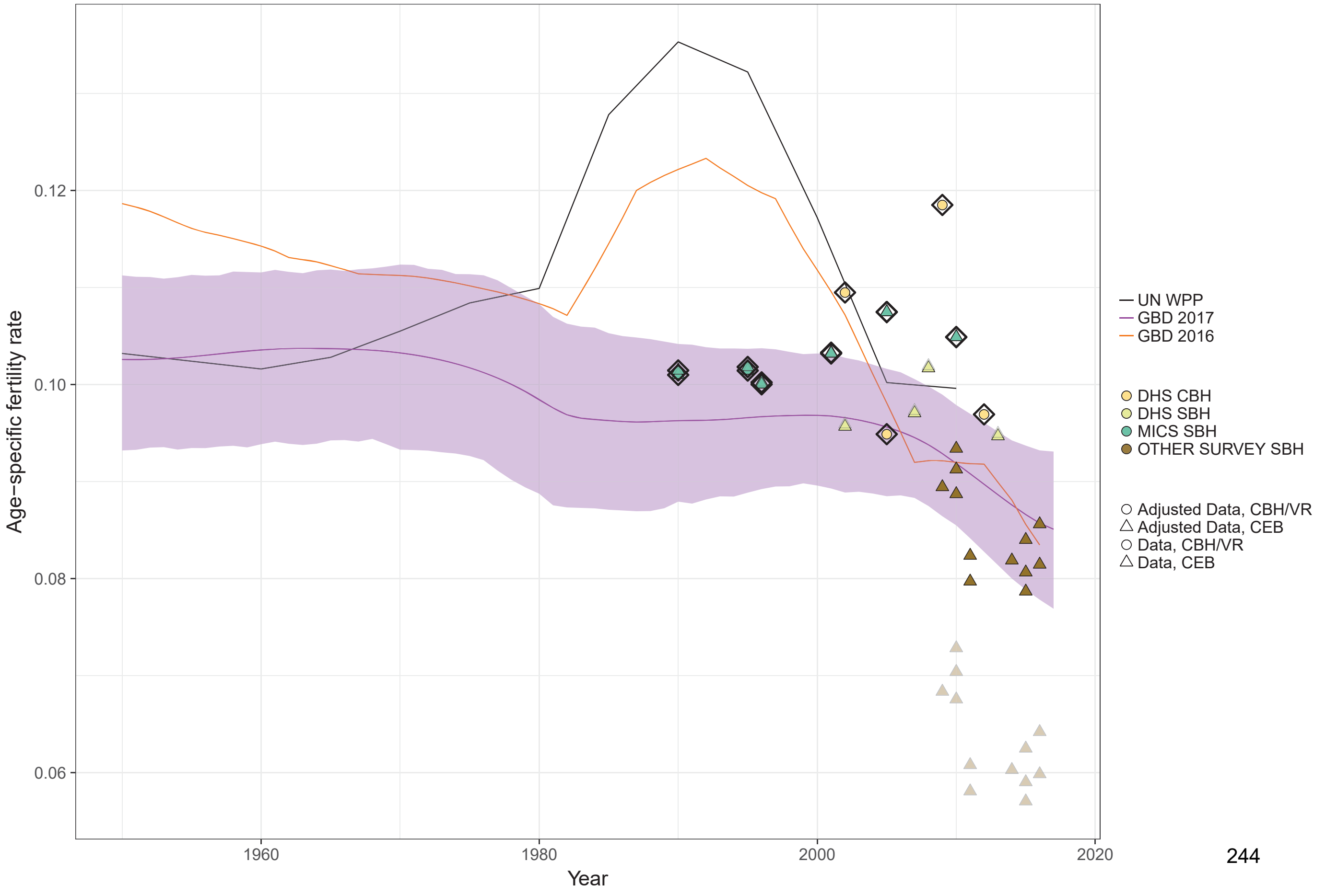

Age−specific fertility fitted trend lines, 1950−2017: Democratic Republic of the Congo, Ages: 45−49
Data points enclosed with a diamond are reference sources

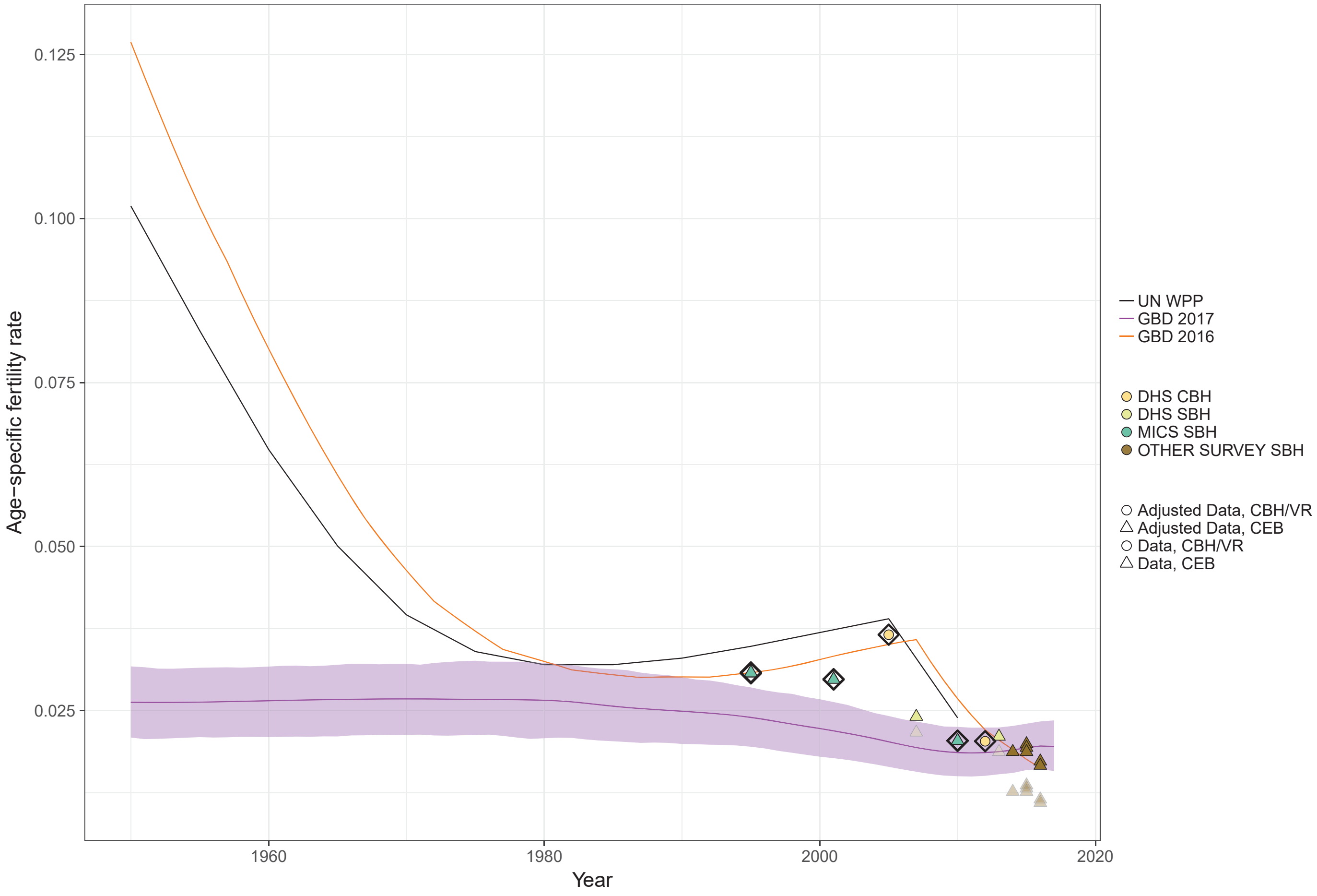

Age−specific fertility fitted trend lines, 1950−2017: Democratic Republic of the Congo, Ages: 50−54
Data points enclosed with a diamond are reference sources

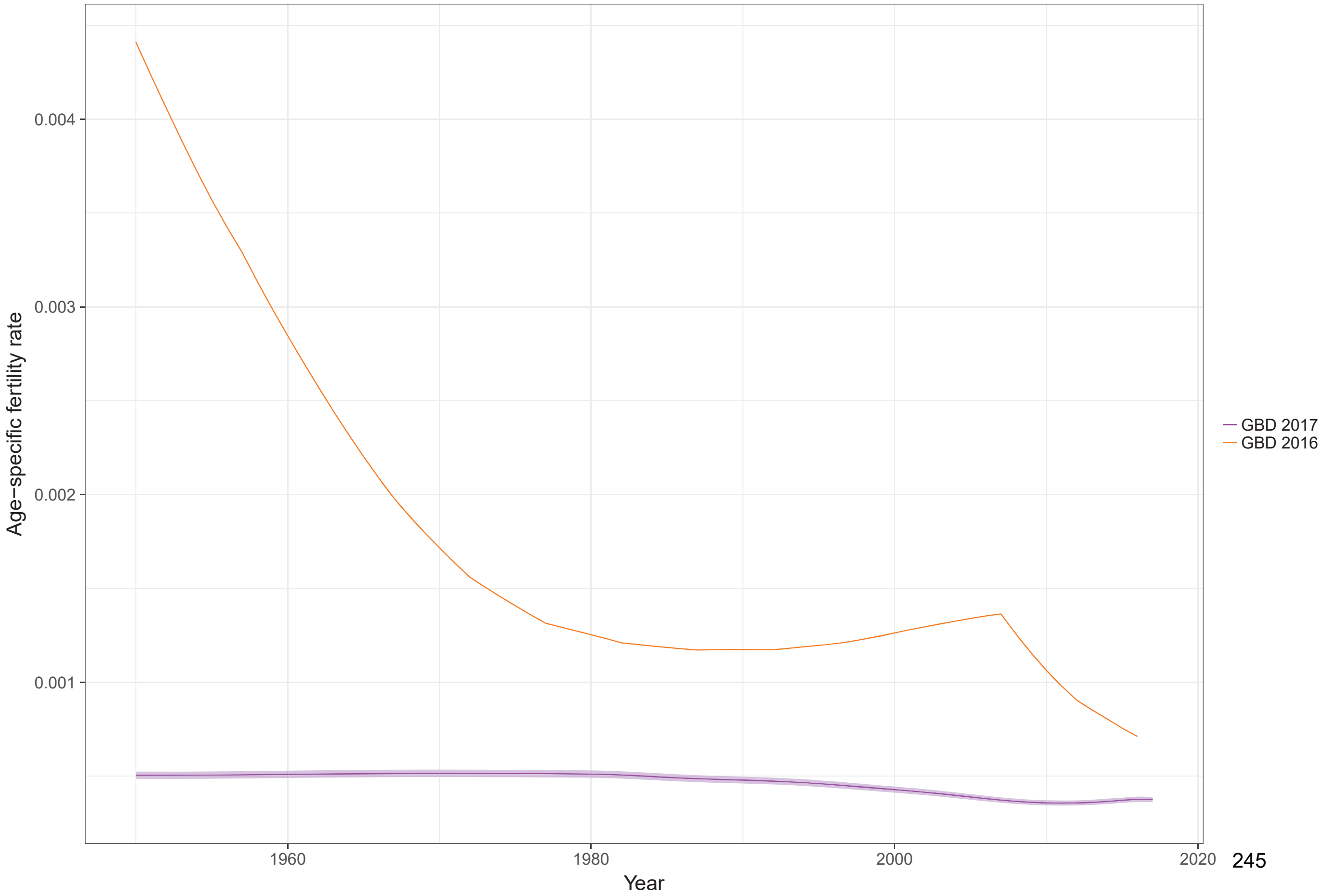

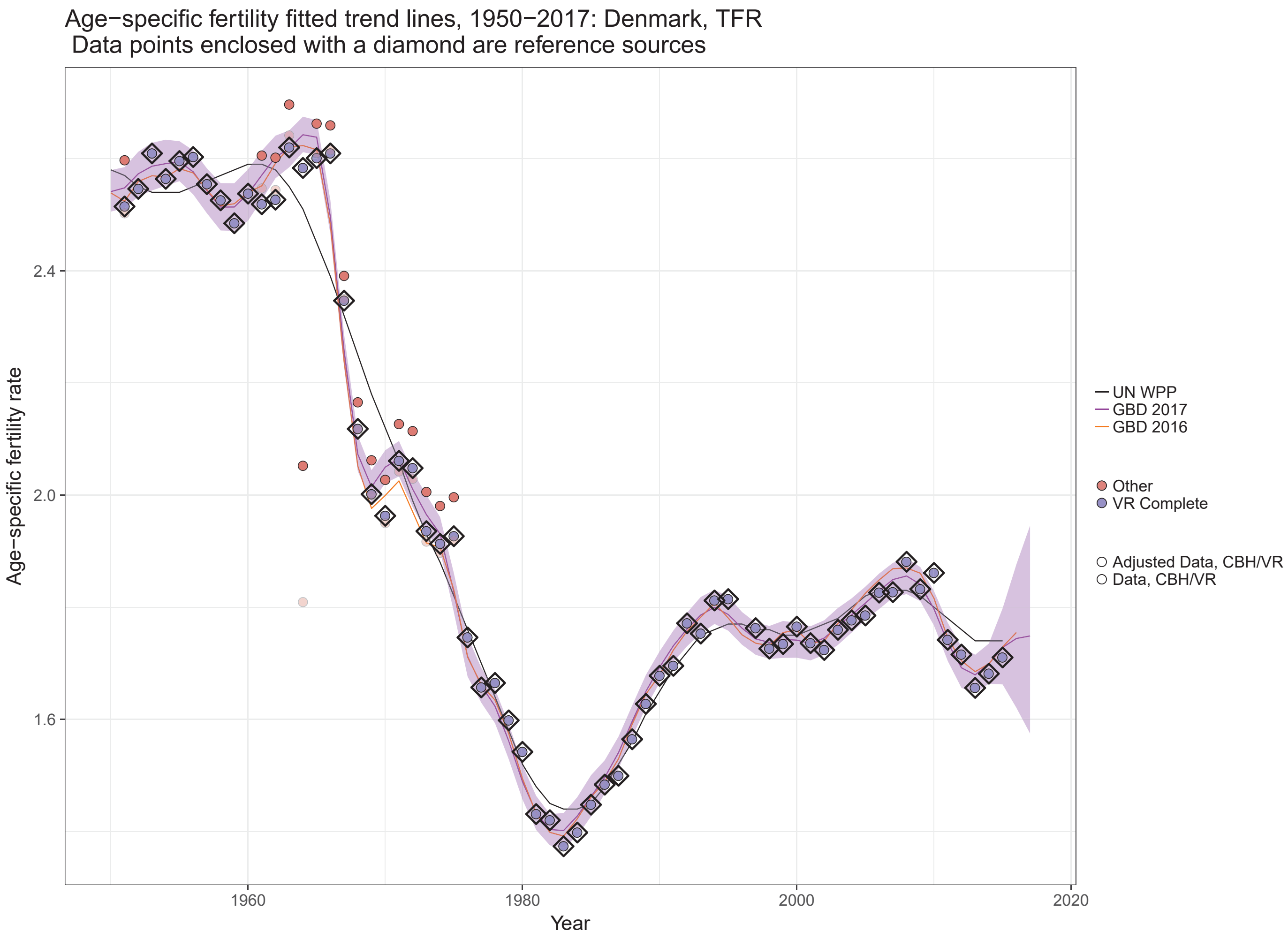
Age−specific fertility fitted trend lines, 1950−2017: Denmark, TFR
Data points enclosed with a diamond are reference sources
Age-specific fertility rate
2.4
2.0
1.6
1960
1980
2000
2020
Year
UN WPP
GBD 2017
GBD 2016
Other
VR Complete
Adjusted Data, CBH/VR
Data, CBH/VR

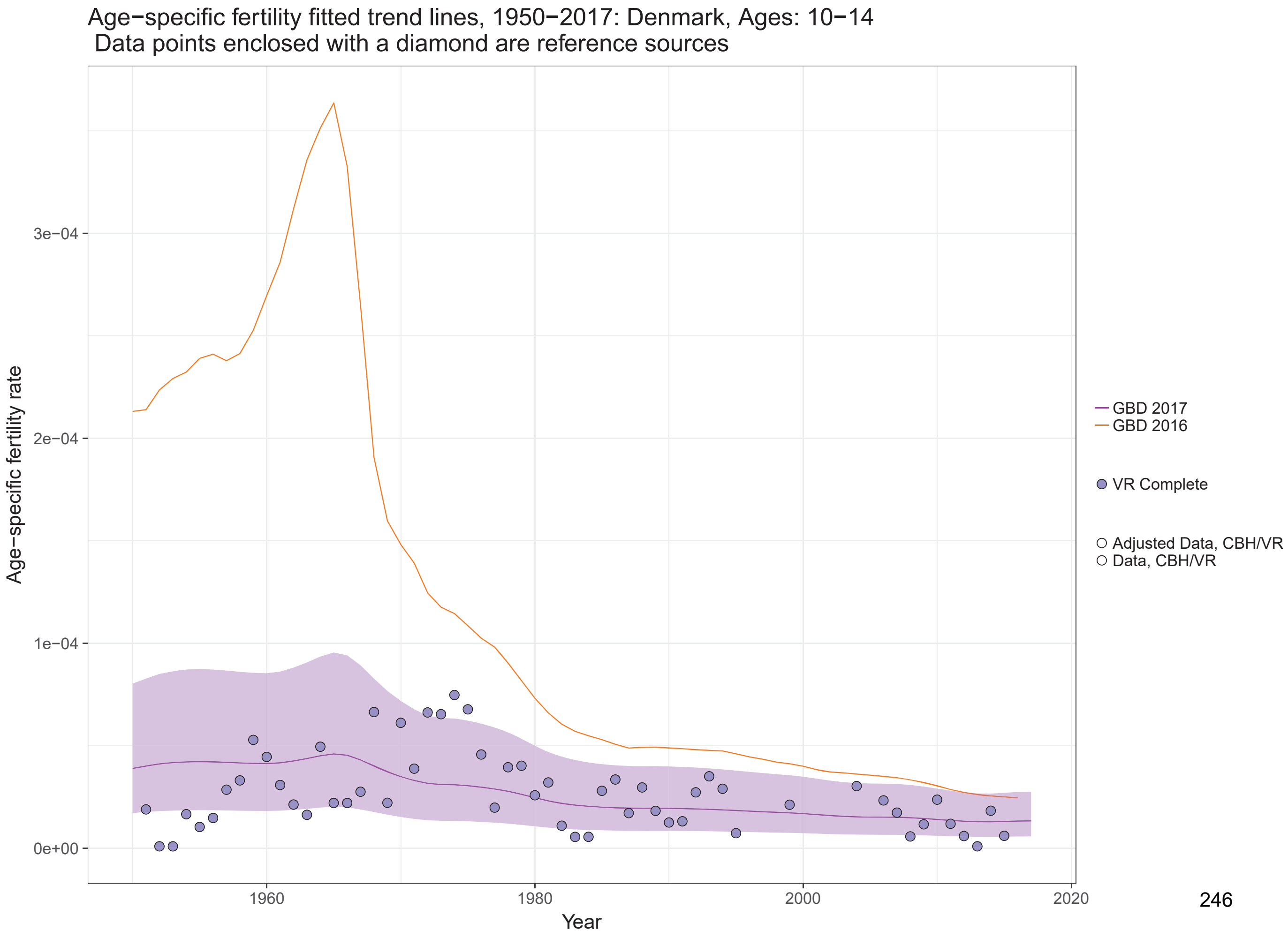
Age−specific fertility fitted trend lines, 1950−2017: Denmark, Ages: 10−14
Data points enclosed with a diamond are reference sources
Age-specific fertility rate
3e−04
2e−04
1e−04
0e+00
1960
1980
2000
2020
Year
GBD 2017
GBD 2016
VR Complete
Adjusted Data, CBH/VR
Data, CBH/VR

Age−specific fertility fitted trend lines, 1950−2017: Denmark, Ages: 15−19
Data points enclosed with a diamond are reference sources

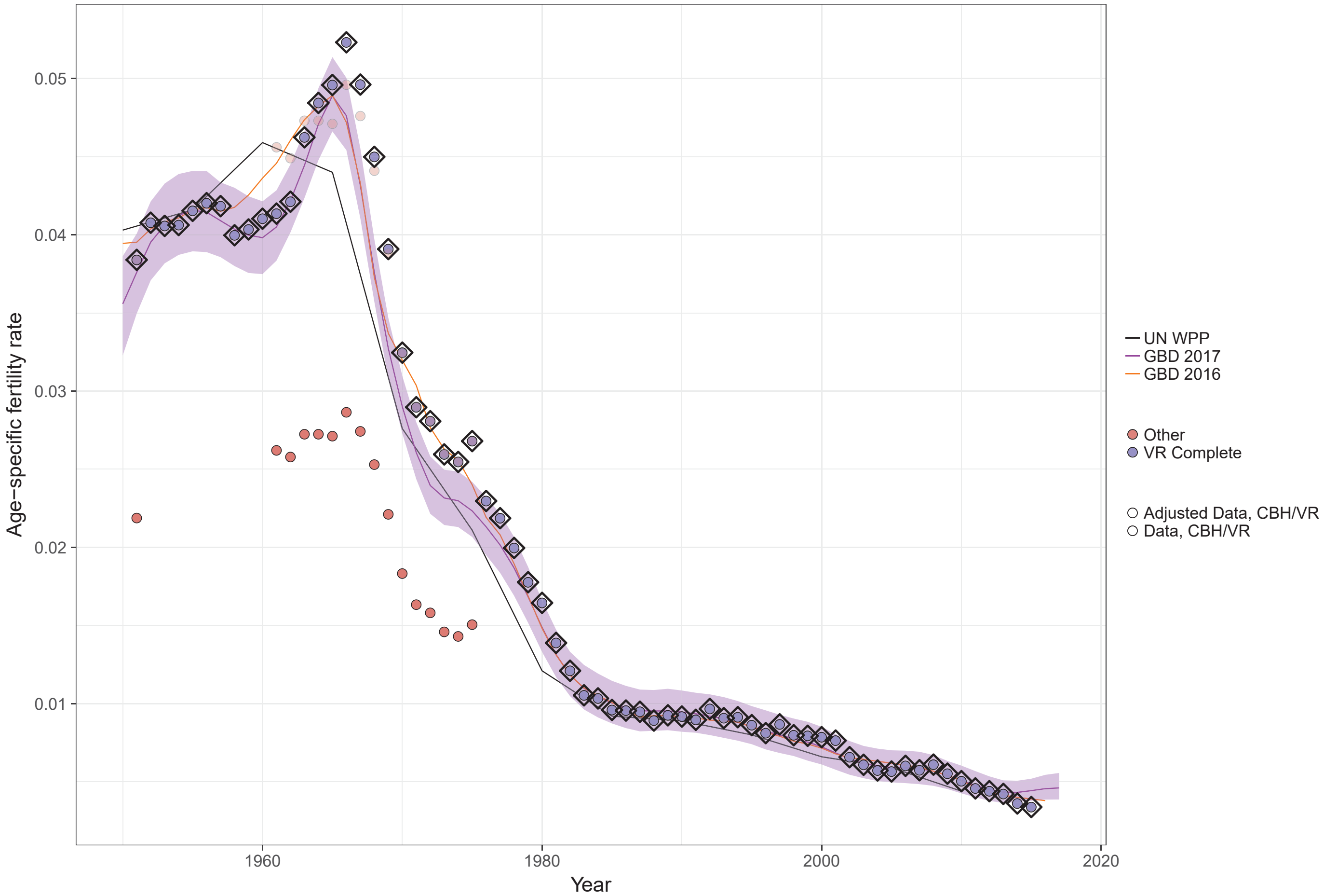

Age−specific fertility fitted trend lines, 1950−2017: Denmark, Ages: 20−24
Data points enclosed with a diamond are reference sources

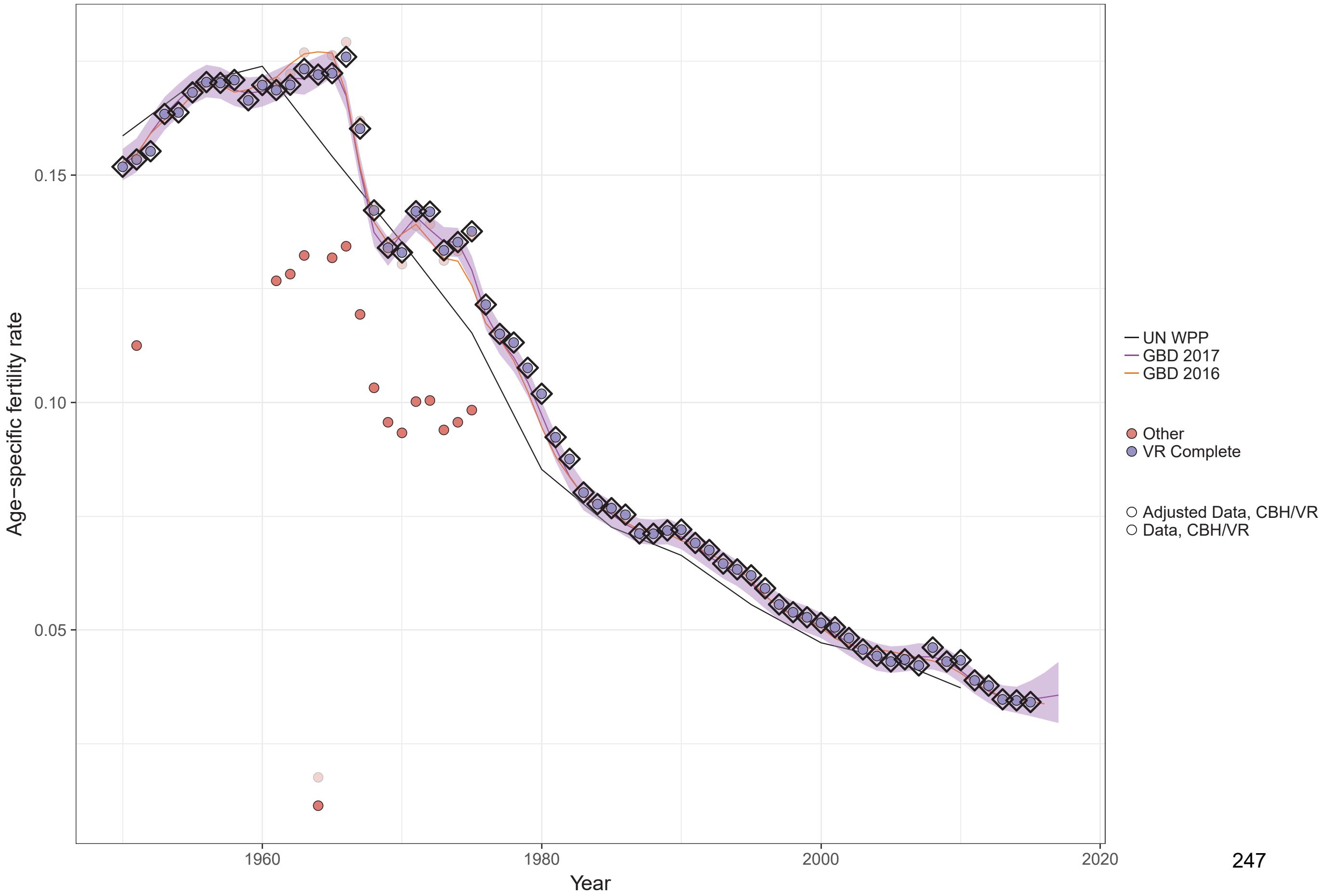

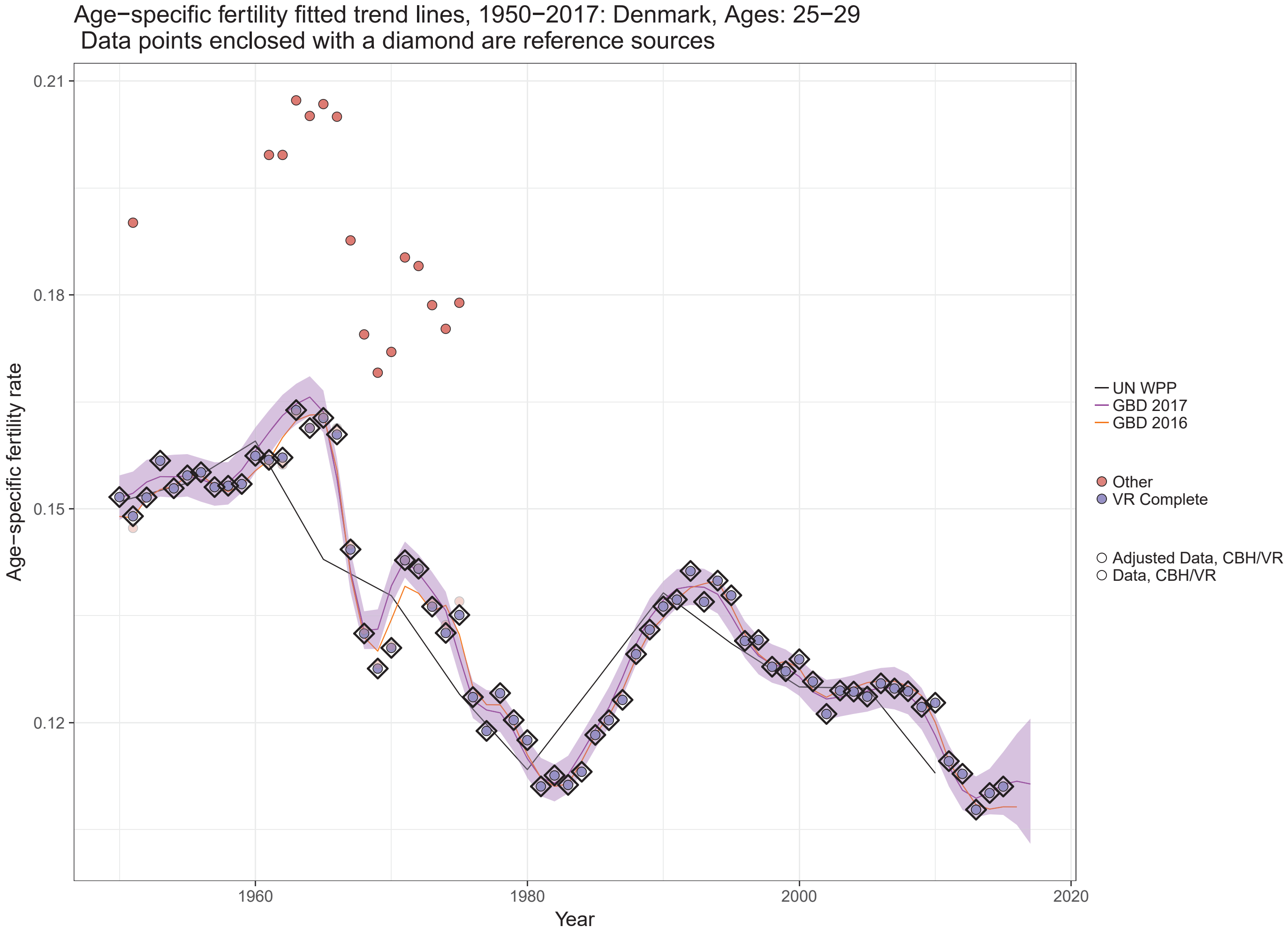

Age−specific fertility fitted trend lines, 1950−2017: Denmark, Ages: 25−29
Data points enclosed with a diamond are reference sources
Age−specific fertility rate
0.21
0.18
0.15
0.12
1960
1980
2000
2020
Year
UN WPP
GBD 2017
GBD 2016
Other
VR Complete
Adjusted Data, CBH/VR
Data, CBH/VR

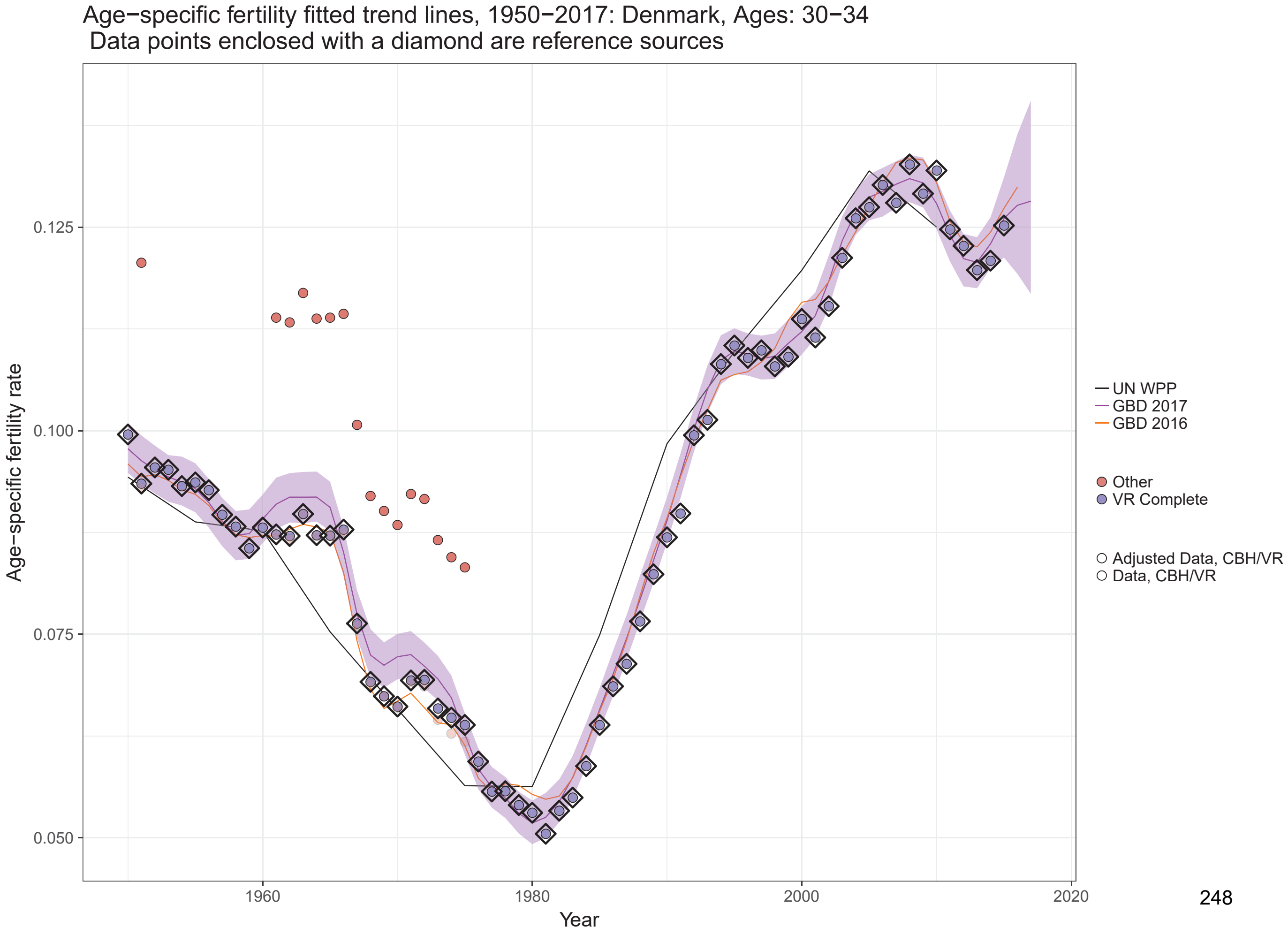

Age−specific fertility fitted trend lines, 1950−2017: Denmark, Ages: 30−34
Data points enclosed with a diamond are reference sources
Age−specific fertility rate
0.125
0.100
0.075
0.050
1960
1980
2000
2020
Year
UN WPP
GBD 2017
GBD 2016
Other
VR Complete
Adjusted Data, CBH/VR
Data, CBH/VR

Age−specific fertility fitted trend lines, 1950−2017: Denmark, Ages: 35−39
Data points enclosed with a diamond are reference sources

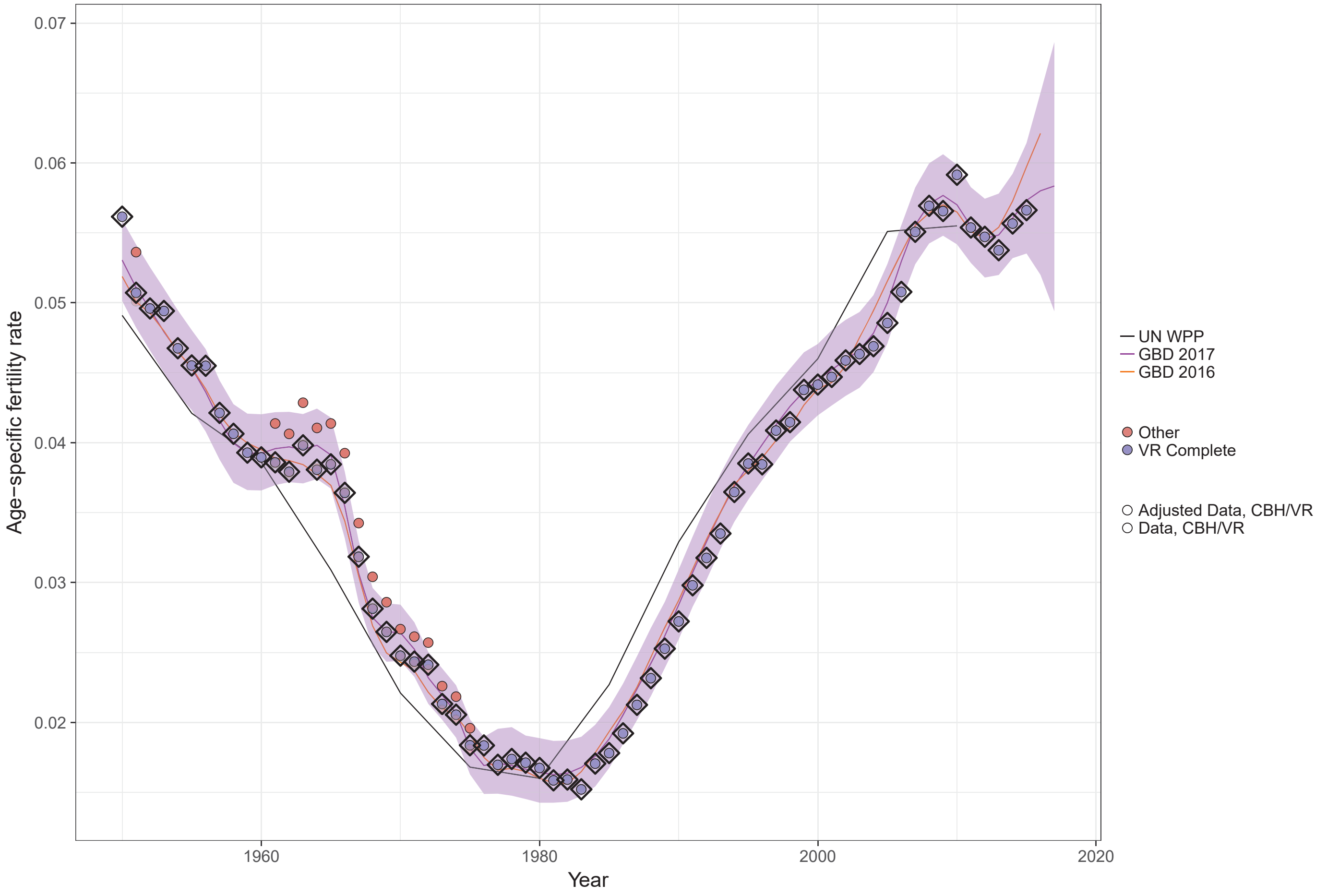

Age−specific fertility fitted trend lines, 1950−2017: Denmark, Ages: 40−44
Data points enclosed with a diamond are reference sources

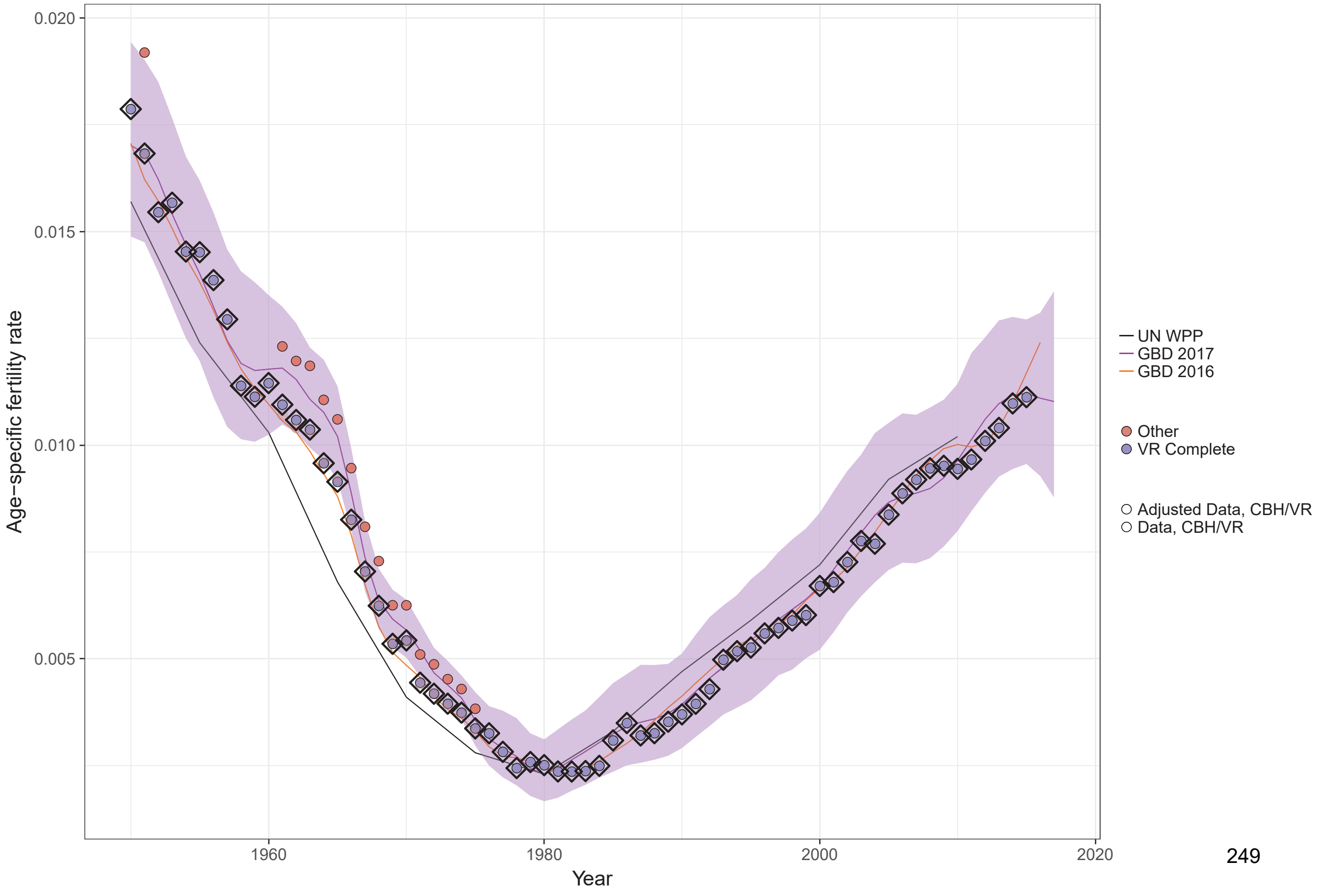

Age−specific fertility fitted trend lines, 1950−2017: Denmark, Ages: 45−49
Data points enclosed with a diamond are reference sources

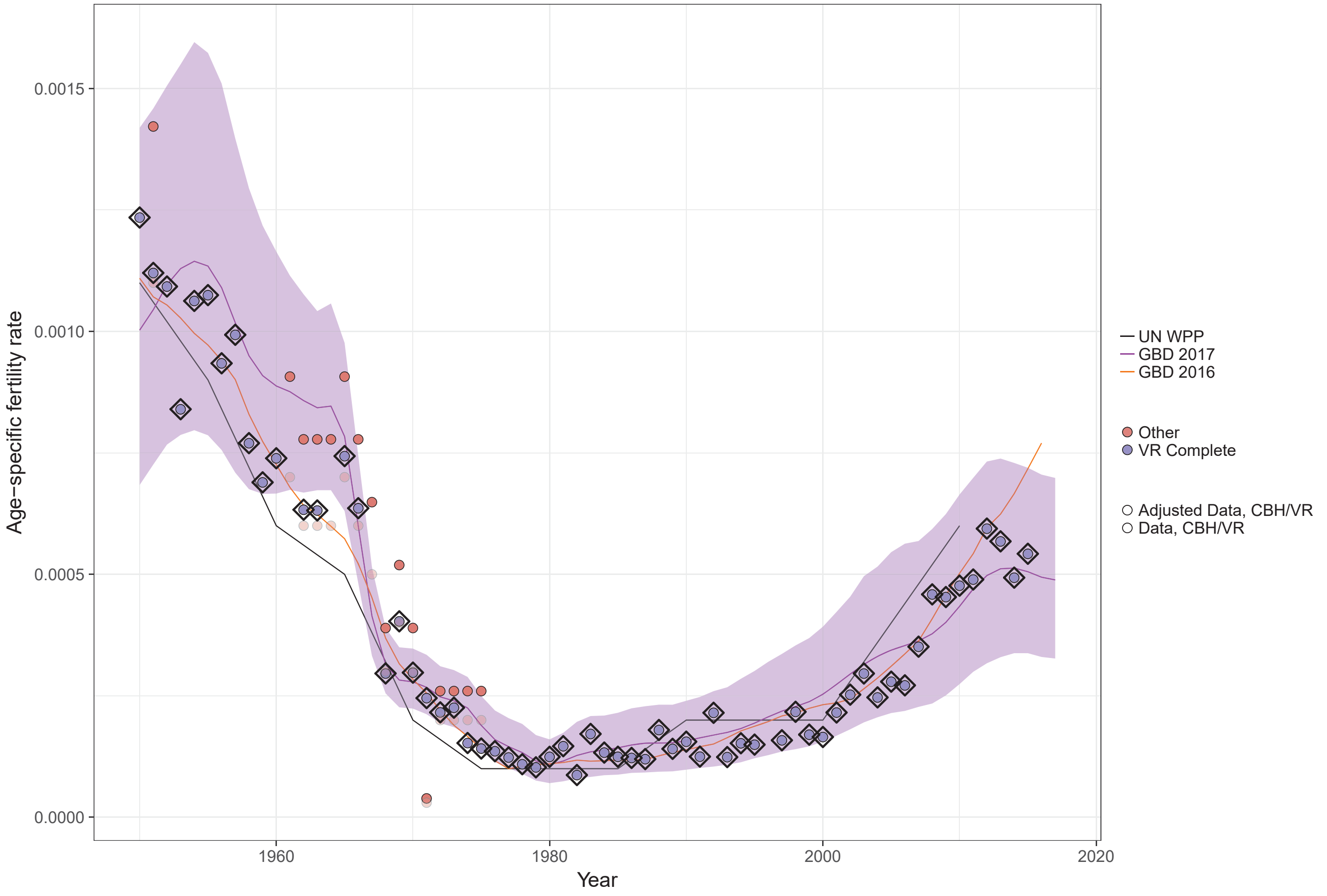

Age−specific fertility fitted trend lines, 1950−2017: Denmark, Ages: 50−54
Data points enclosed with a diamond are reference sources

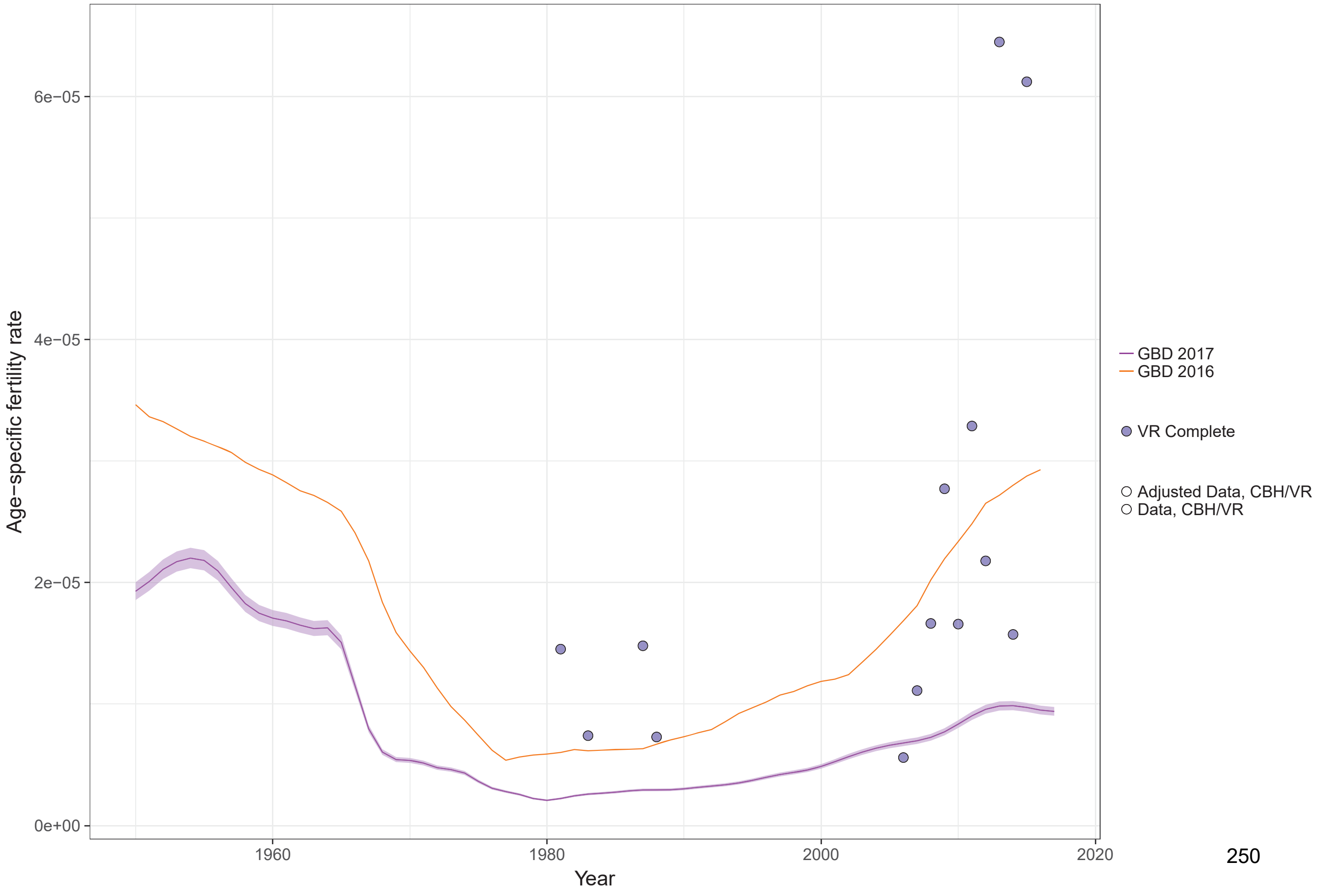

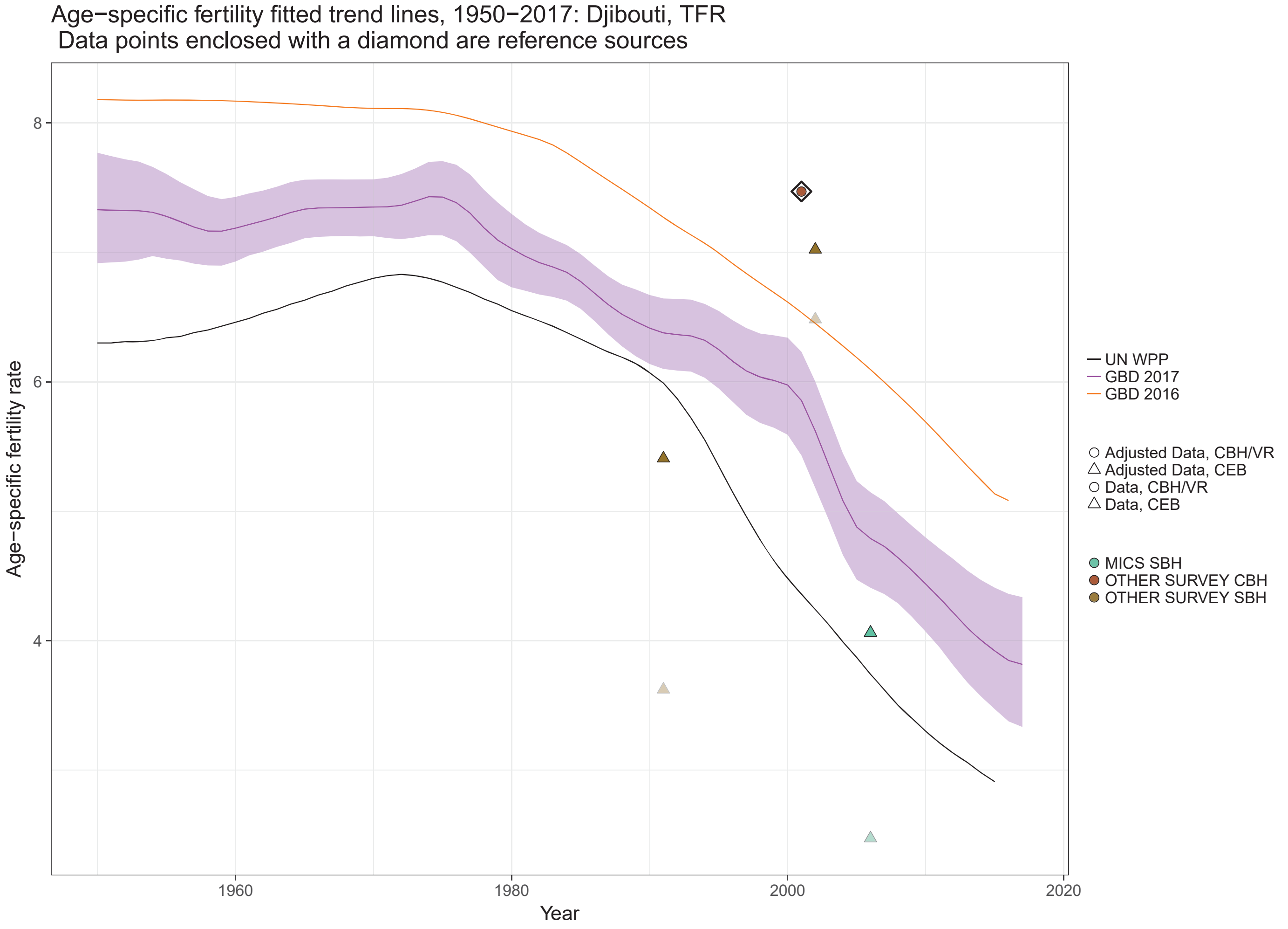
Age−specific fertility fitted trend lines, 1950−2017: Djibouti, TFR
Data points enclosed with a diamond are reference sources
Age−specific fertility rate
Year
8
6
4
1960
1980
2000
2020
UN WPP
GBD 2017
GBD 2016
Adjusted Data, CBH/VR
Adjusted Data, CEB
Data, CBH/VR
Data, CEB
MICS SBH
OTHER SURVEY CBH
OTHER SURVEY SBH

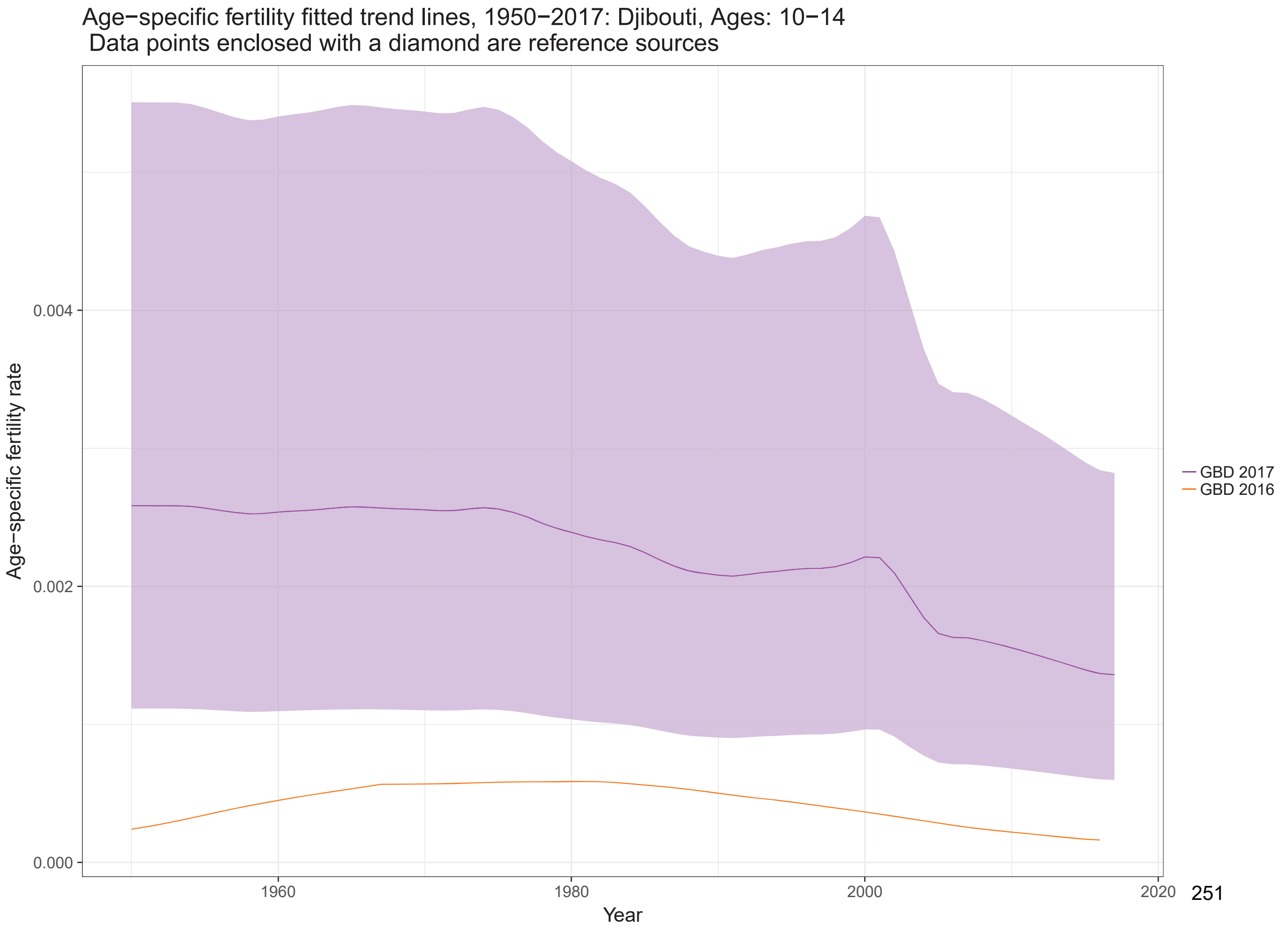
Age−specific fertility fitted trend lines, 1950−2017: Djibouti, Ages: 10−14
Data points enclosed with a diamond are reference sources
Age−specific fertility rate
Year
0.004
0.002
0.000
1960
1980
2000
2020
GBD 2017
GBD 2016

Age−specific fertility fitted trend lines, 1950−2017: Djibouti, Ages: 15−19
Data points enclosed with a diamond are reference sources

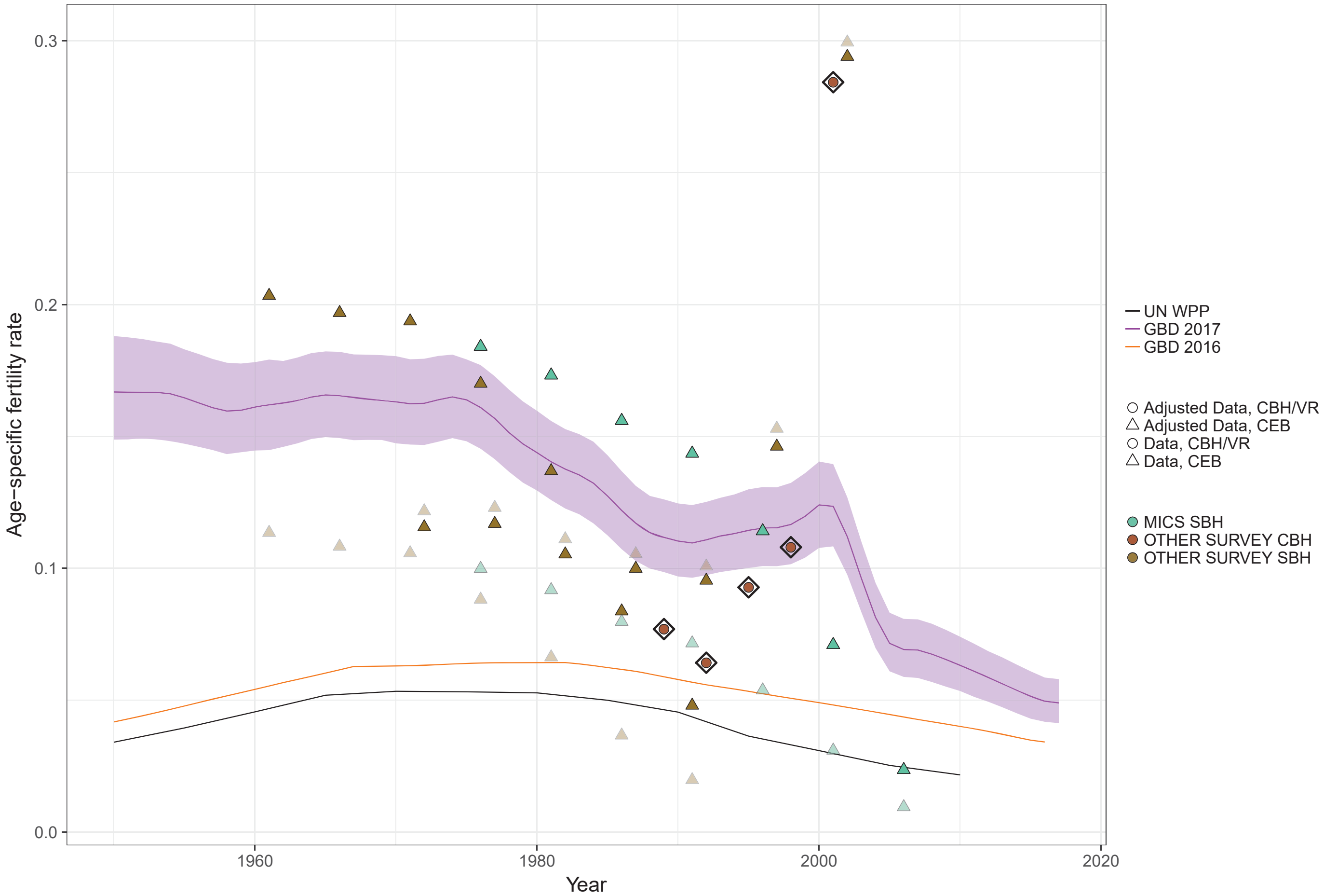

Age−specific fertility fitted trend lines, 1950−2017: Djibouti, Ages: 20−24
Data points enclosed with a diamond are reference sources

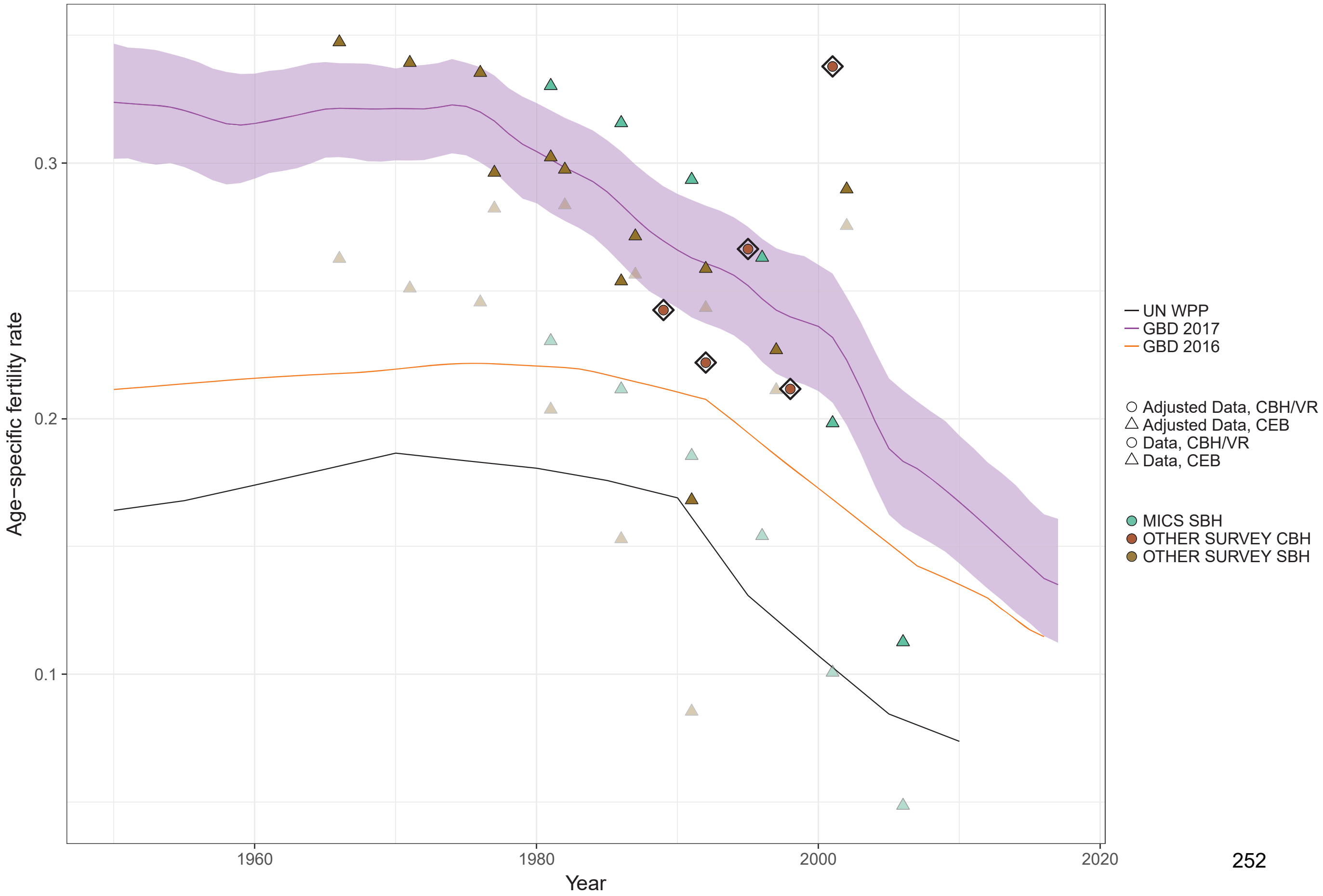

Age−specific fertility fitted trend lines, 1950−2017: Djibouti, Ages: 25−29
Data points enclosed with a diamond are reference sources

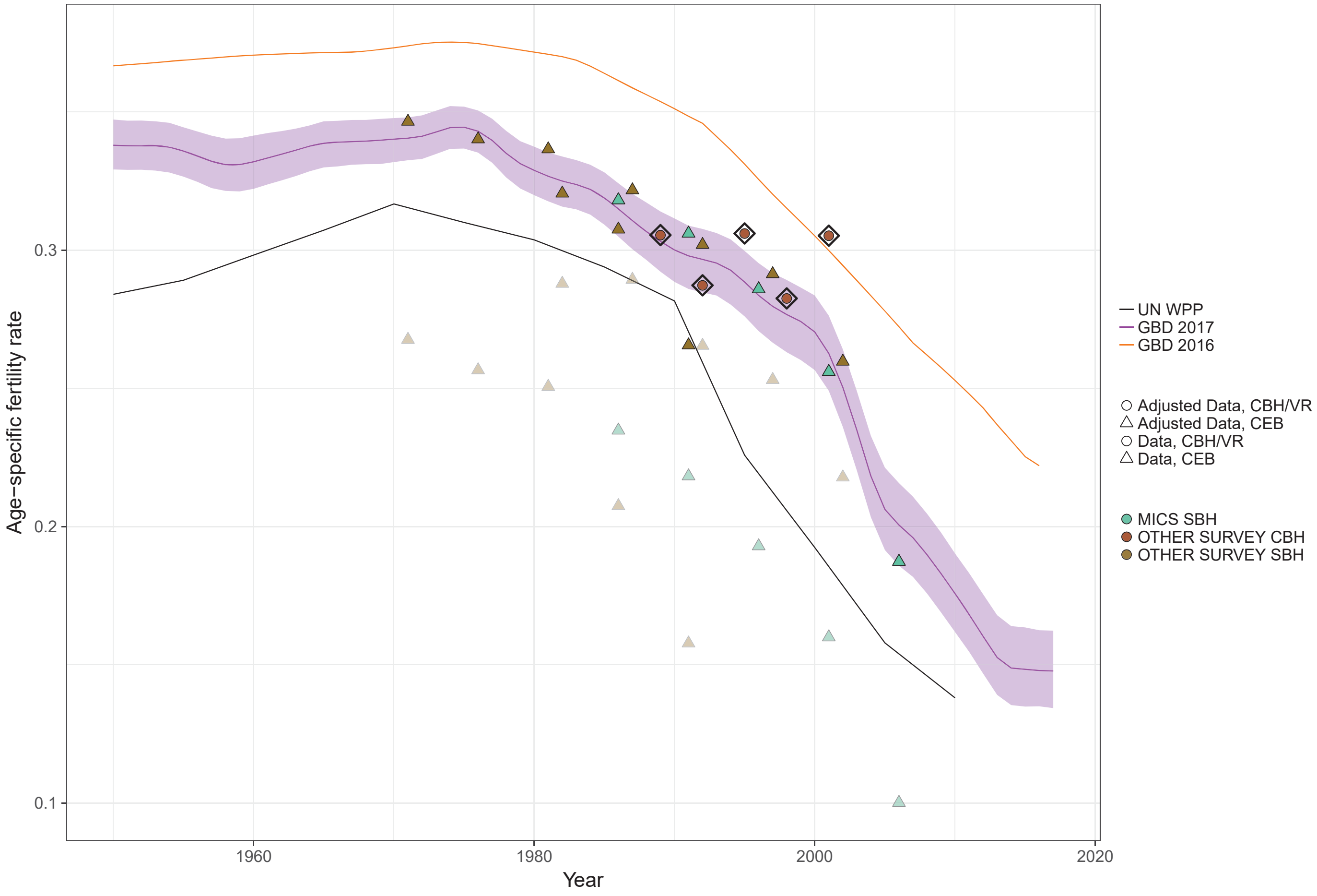

Age−specific fertility fitted trend lines, 1950−2017: Djibouti, Ages: 30−34
Data points enclosed with a diamond are reference sources

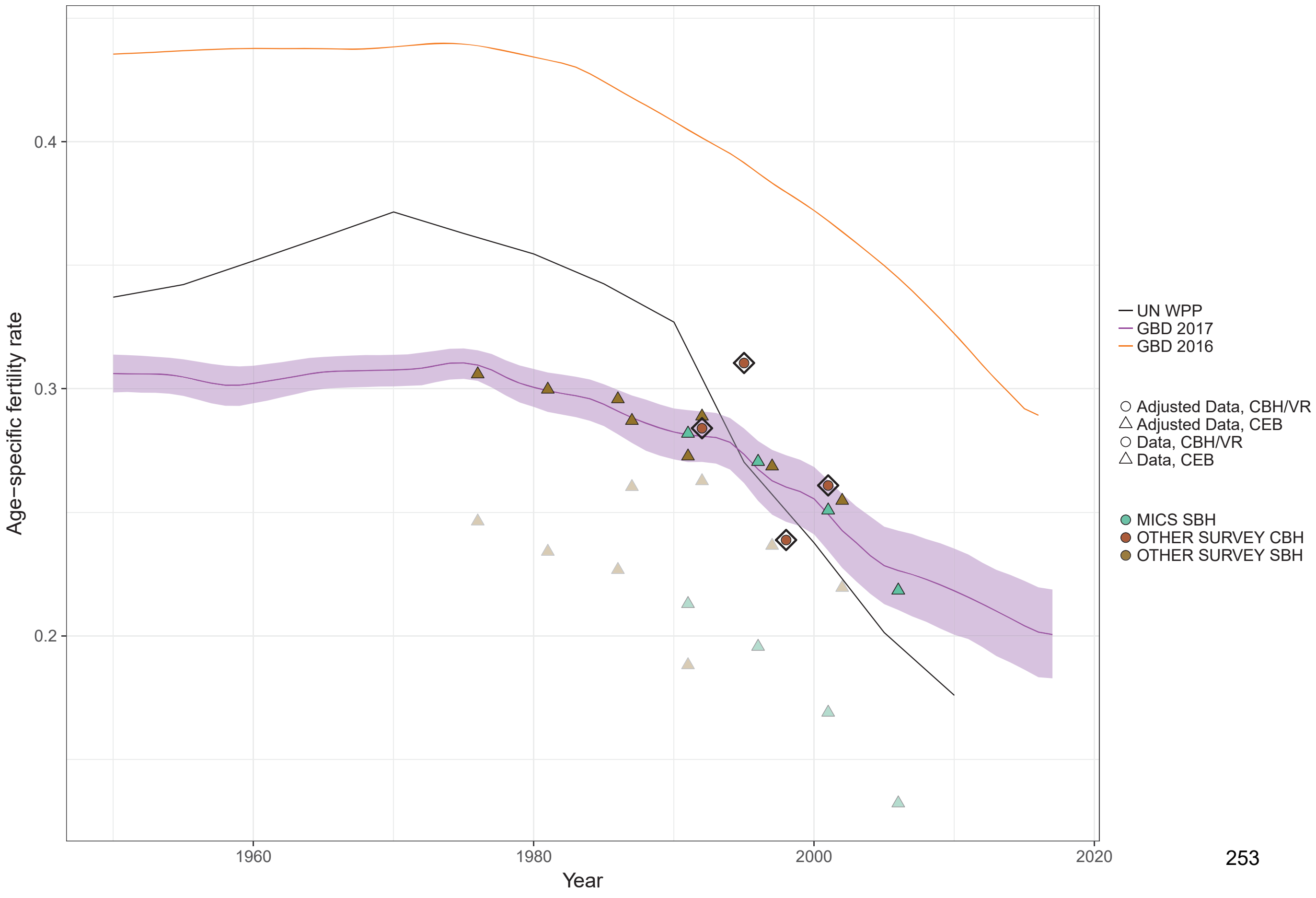

Age−specific fertility fitted trend lines, 1950−2017: Djibouti, Ages: 35−39
Data points enclosed with a diamond are reference sources

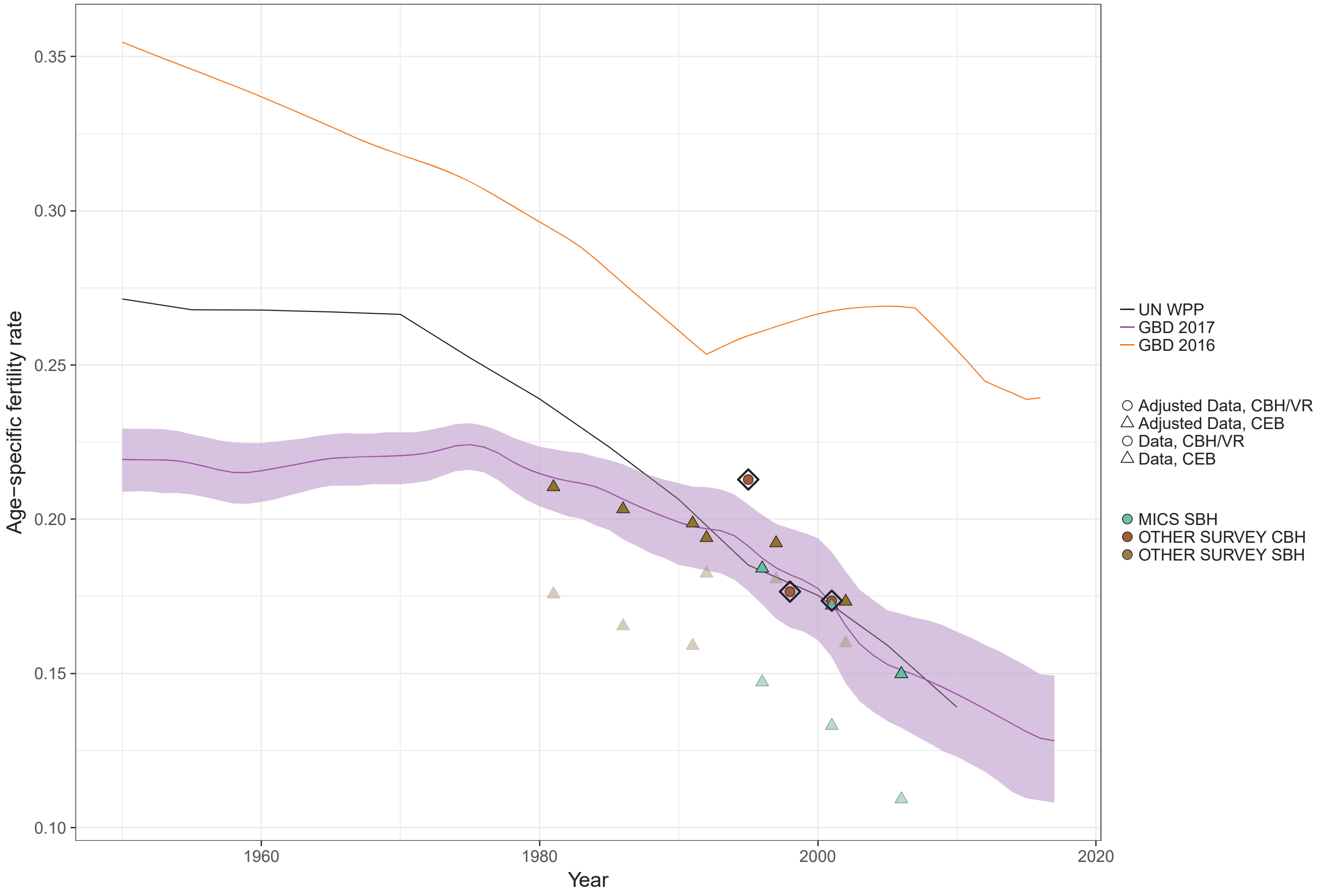

Age−specific fertility fitted trend lines, 1950−2017: Djibouti, Ages: 40−44
Data points enclosed with a diamond are reference sources

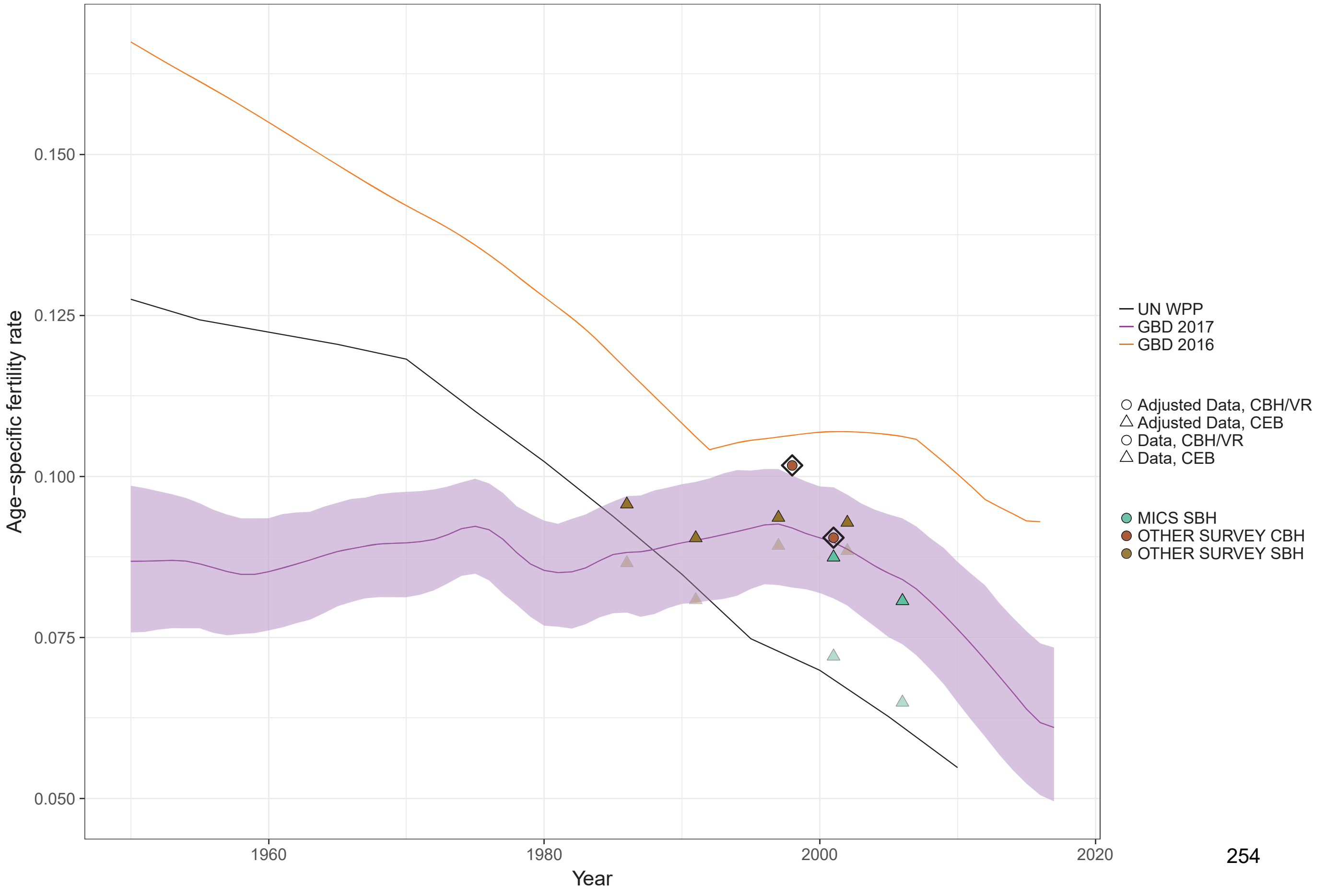

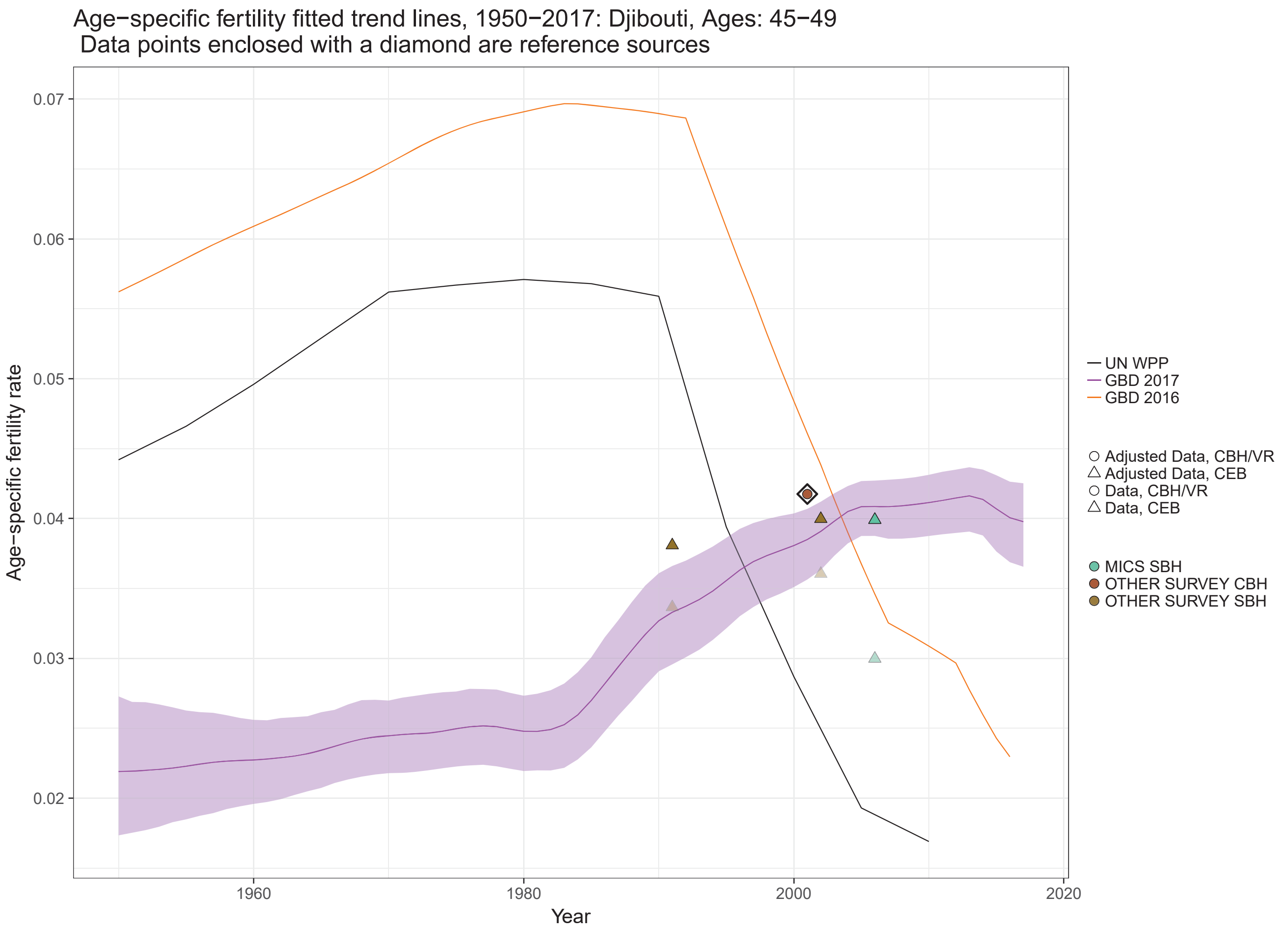
Age−specific fertility fitted trend lines, 1950−2017: Djibouti, Ages: 45−49
Data points enclosed with a diamond are reference sources
Age−specific fertility rate
0.07
0.06
0.05
0.04
0.03
0.02
1960
1980
2000
2020
Year
UN WPP
GBD 2017
GBD 2016
Adjusted Data, CBH/VR
Adjusted Data, CEB
Data, CBH/VR
Data, CEB
MICS SBH
OTHER SURVEY CBH
OTHER SURVEY SBH

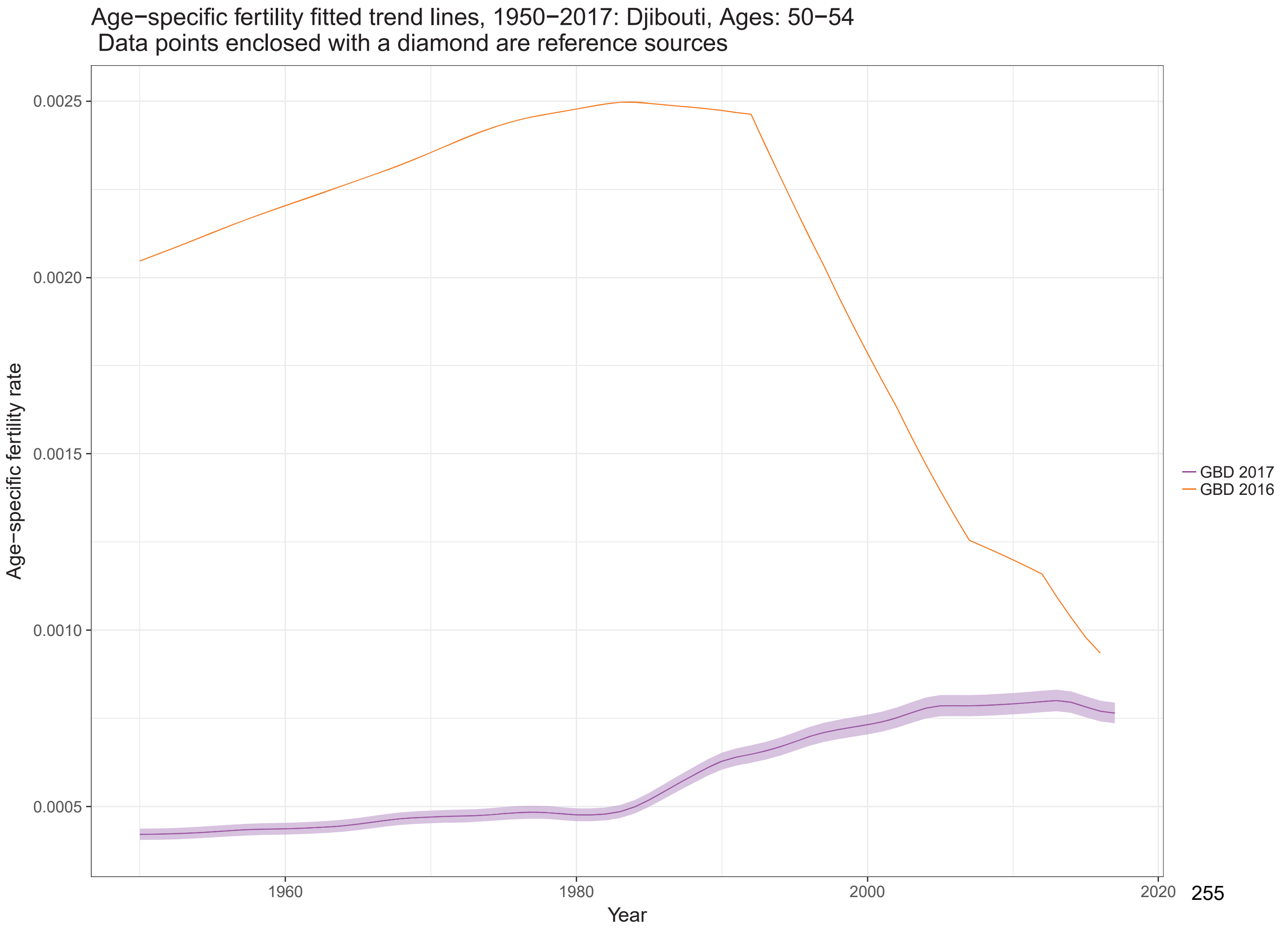
Age−specific fertility fitted trend lines, 1950−2017: Djibouti, Ages: 50−54
Data points enclosed with a diamond are reference sources
Age−specific fertility rate
0.0025
0.0020
0.0015
0.0010
0.0005
1960
1980
2000
2020
Year
GBD 2017
GBD 2016

Age−specific fertility fitted trend lines, 1950−2017: Dominica, TFR
Data points enclosed with a diamond are reference sources

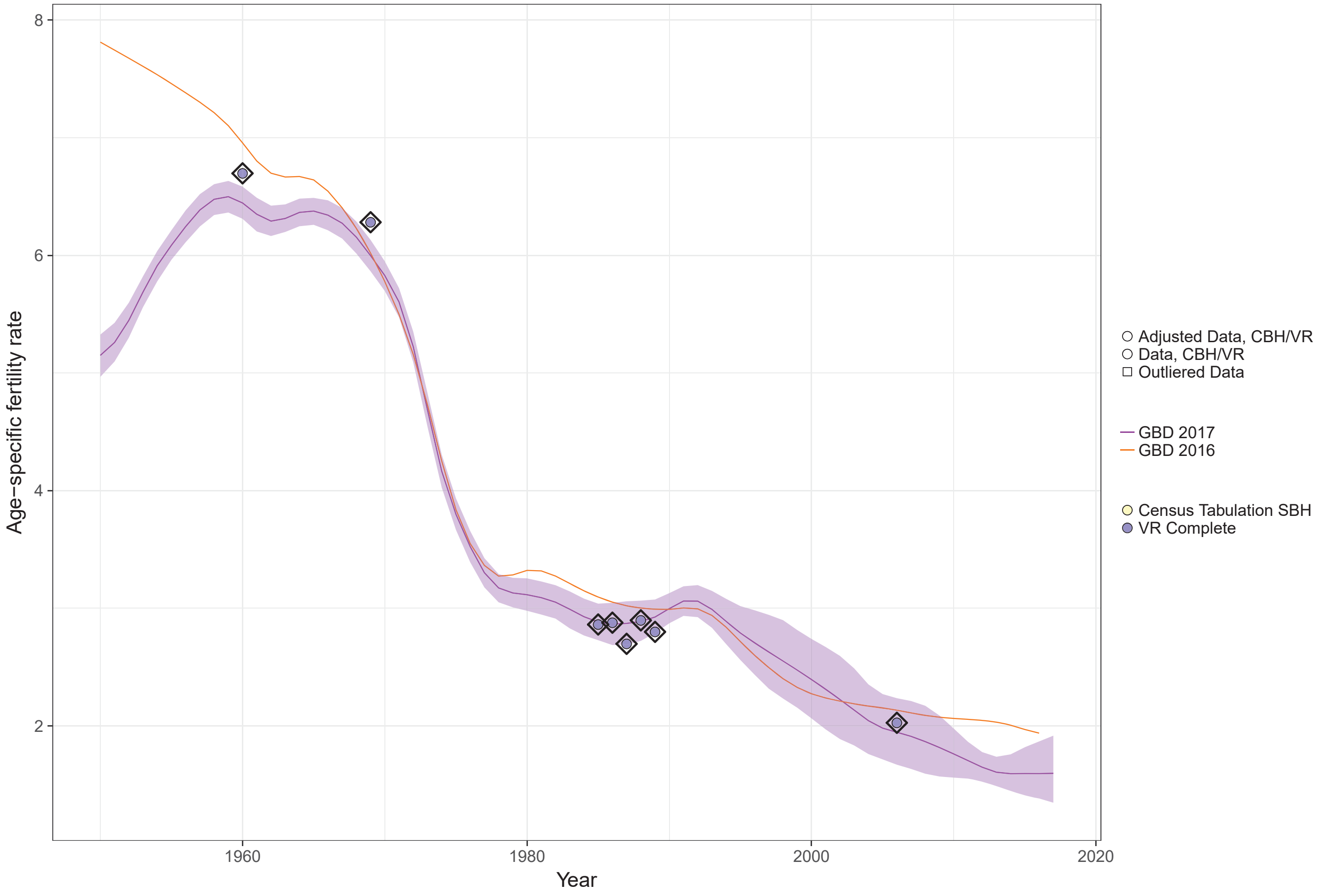

Age−specific fertility fitted trend lines, 1950−2017: Dominica, Ages: 10−14
Data points enclosed with a diamond are reference sources

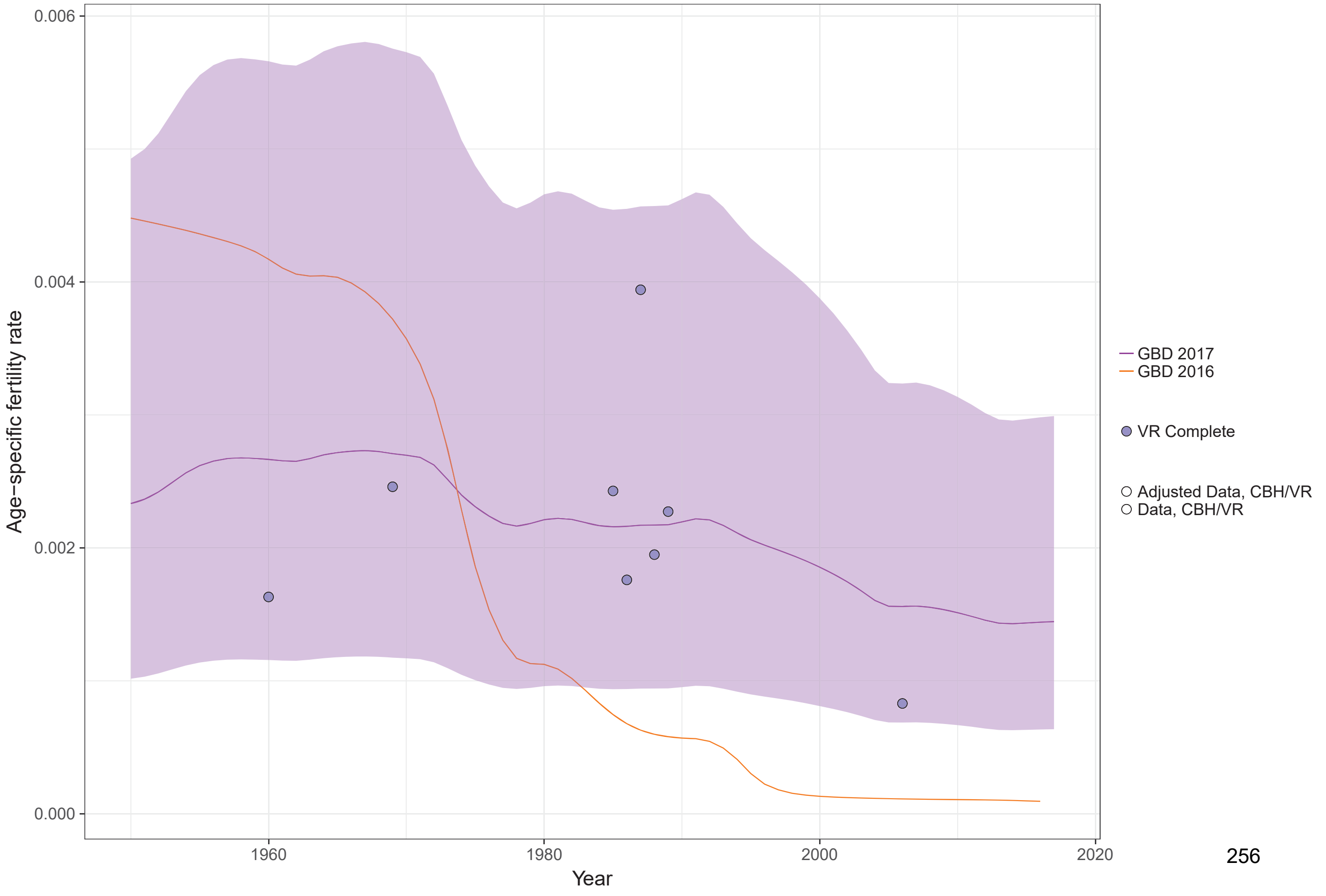

Age−specific fertility fitted trend lines, 1950−2017: Dominica, Ages: 15−19
Data points enclosed with a diamond are reference sources

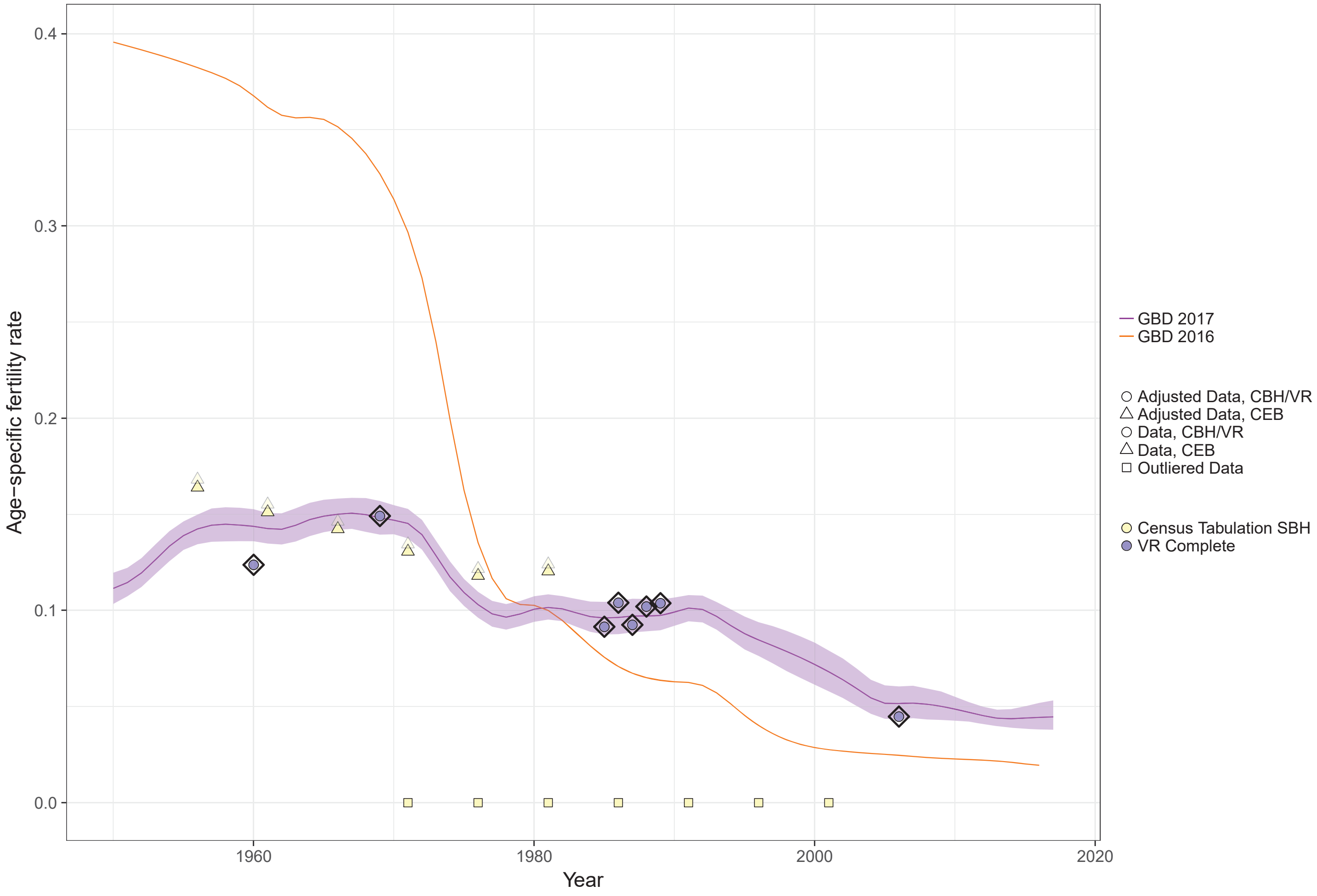

Age−specific fertility fitted trend lines, 1950−2017: Dominica, Ages: 20−24
Data points enclosed with a diamond are reference sources

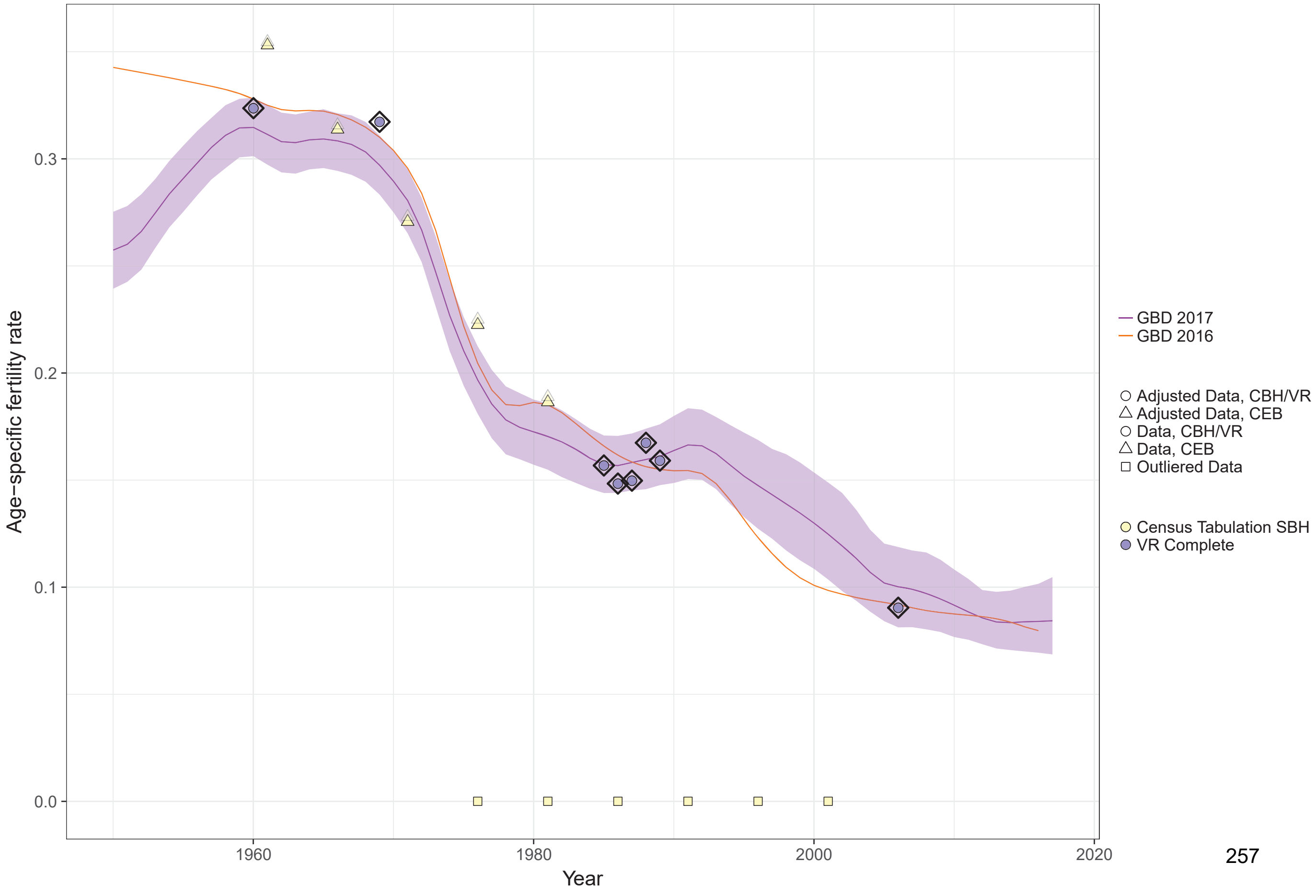

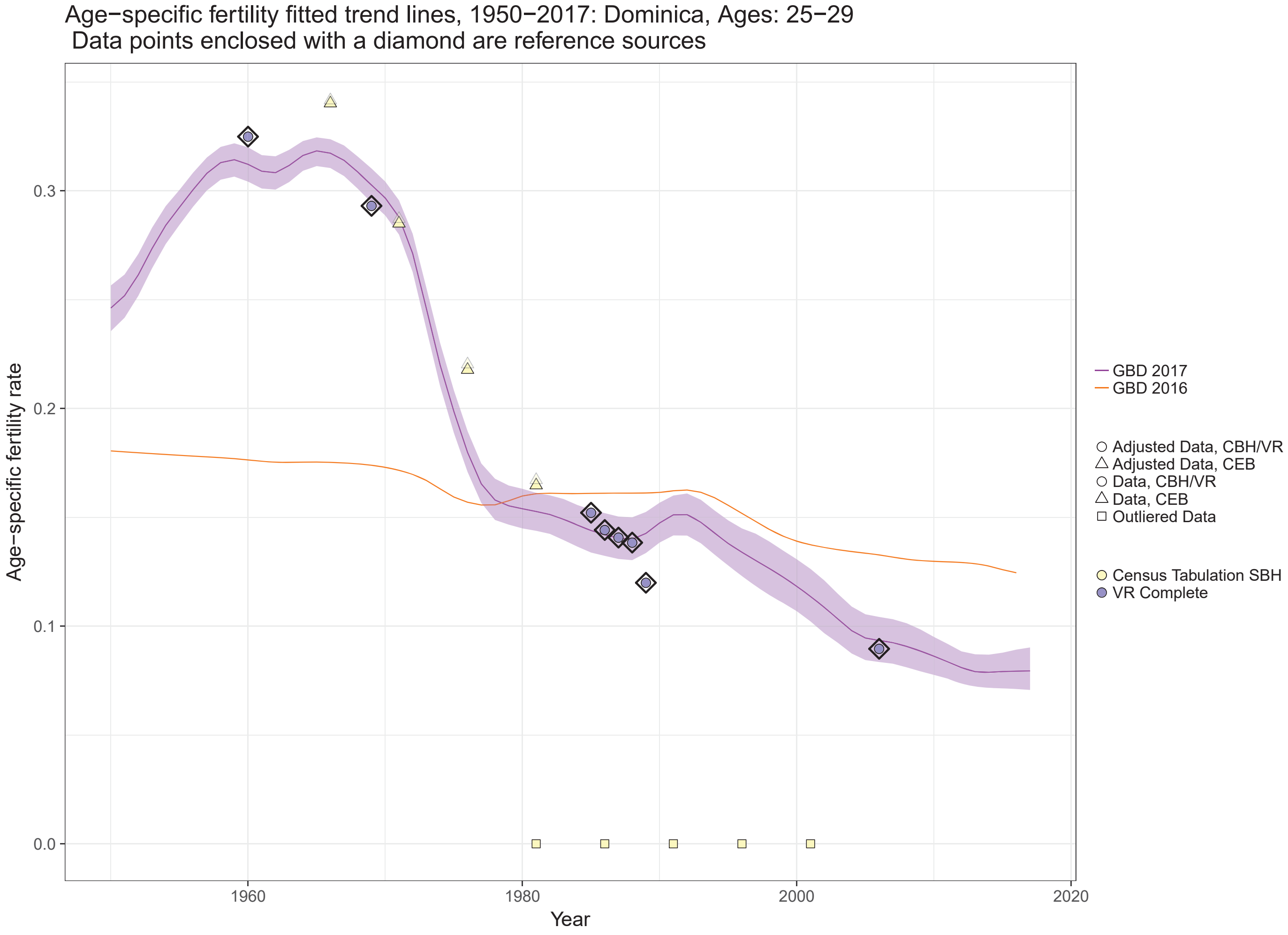
Age−specific fertility fitted trend lines, 1950−2017: Dominica, Ages: 25−29
Data points enclosed with a diamond are reference sources
Age−specific fertility rate
0.0
0.1
0.2
0.3
1960
1980
2000
2020
Year
GBD 2017
GBD 2016
Adjusted Data, CBH/VR
Adjusted Data, CEB
Data, CBH/VR
Data, CEB
Outliered Data
Census Tabulation SBH
VR Complete

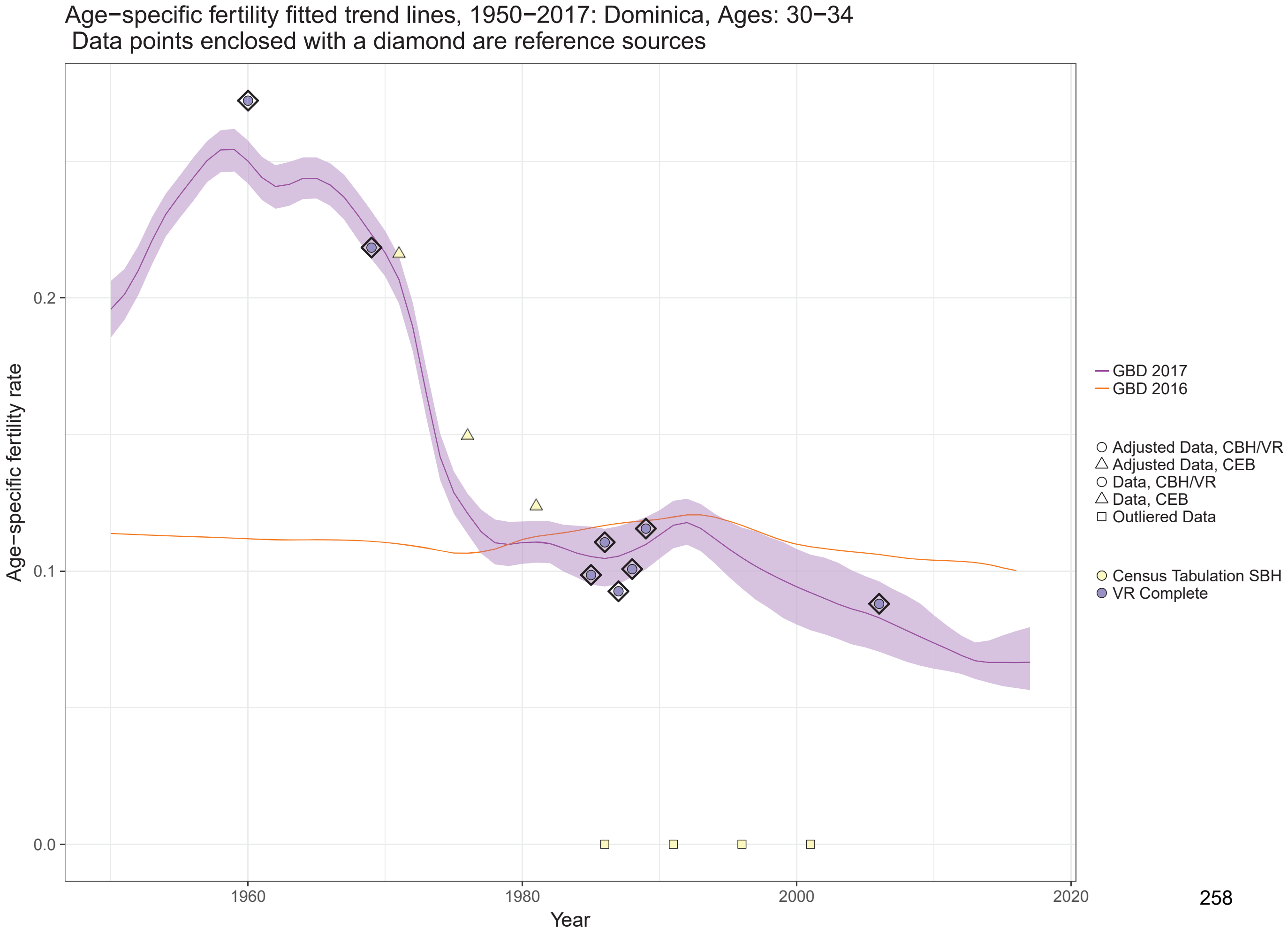
Age−specific fertility fitted trend lines, 1950−2017: Dominica, Ages: 30−34
Data points enclosed with a diamond are reference sources
Age−specific fertility rate
0.0
0.1
0.2
1960
1980
2000
2020
Year
GBD 2017
GBD 2016
Adjusted Data, CBH/VR
Adjusted Data, CEB
Data, CBH/VR
Data, CEB
Outliered Data
Census Tabulation SBH
VR Complete

Age−specific fertility fitted trend lines, 1950−2017: Dominica, Ages: 35−39
Data points enclosed with a diamond are reference sources

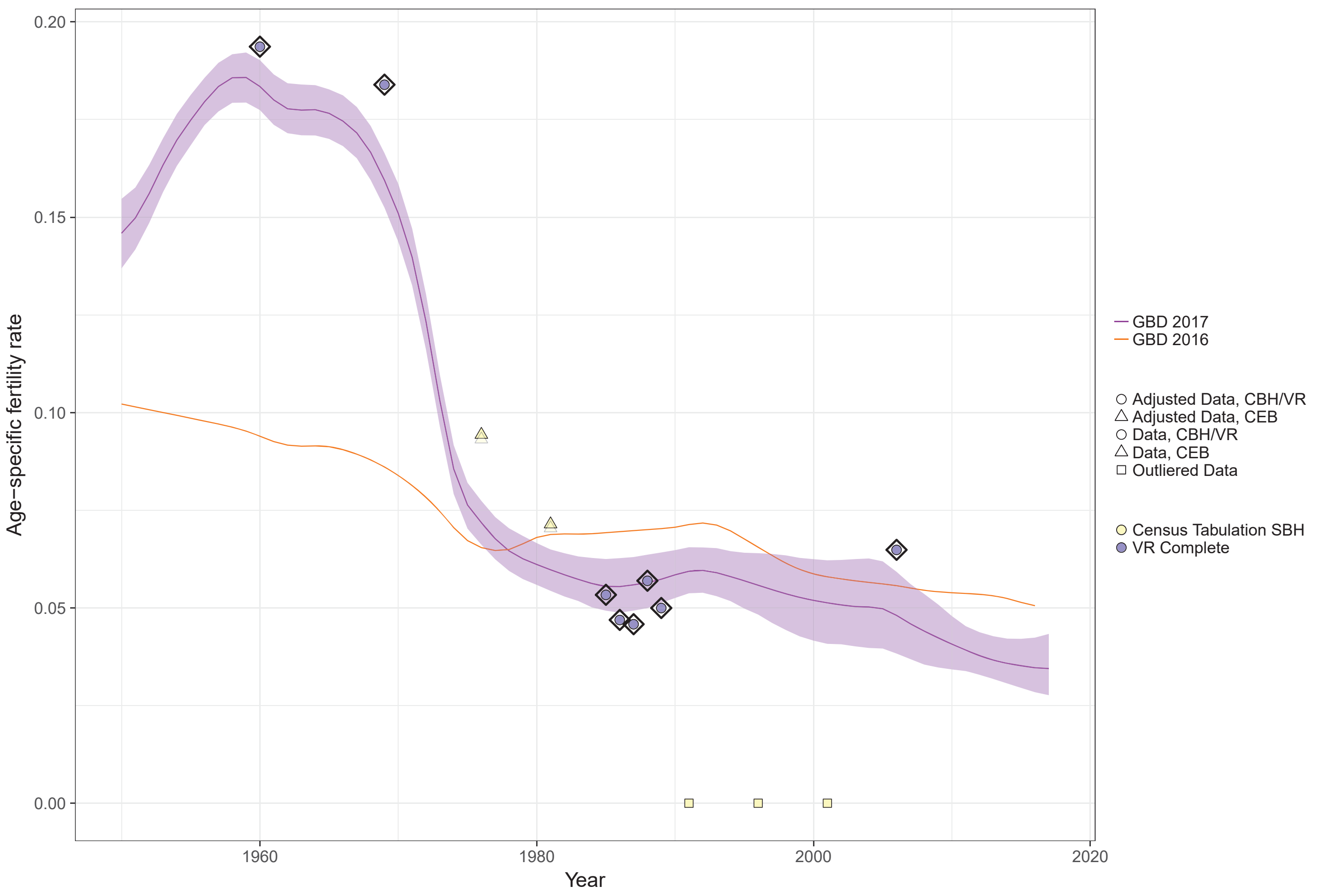

Age−specific fertility fitted trend lines, 1950−2017: Dominica, Ages: 40−44
Data points enclosed with a diamond are reference sources

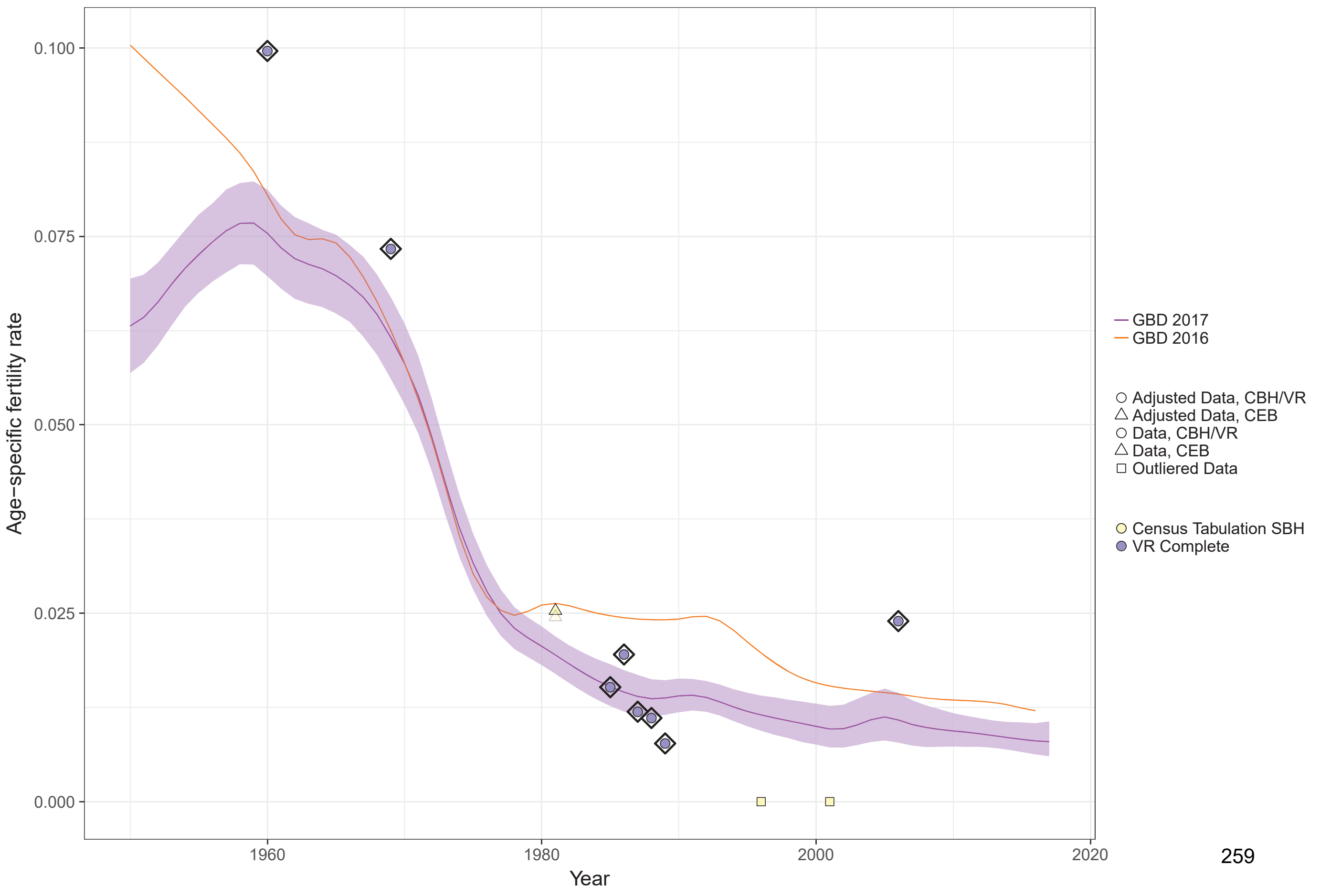

Age−specific fertility fitted trend lines, 1950−2017: Dominica, Ages: 45−49
Data points enclosed with a diamond are reference sources

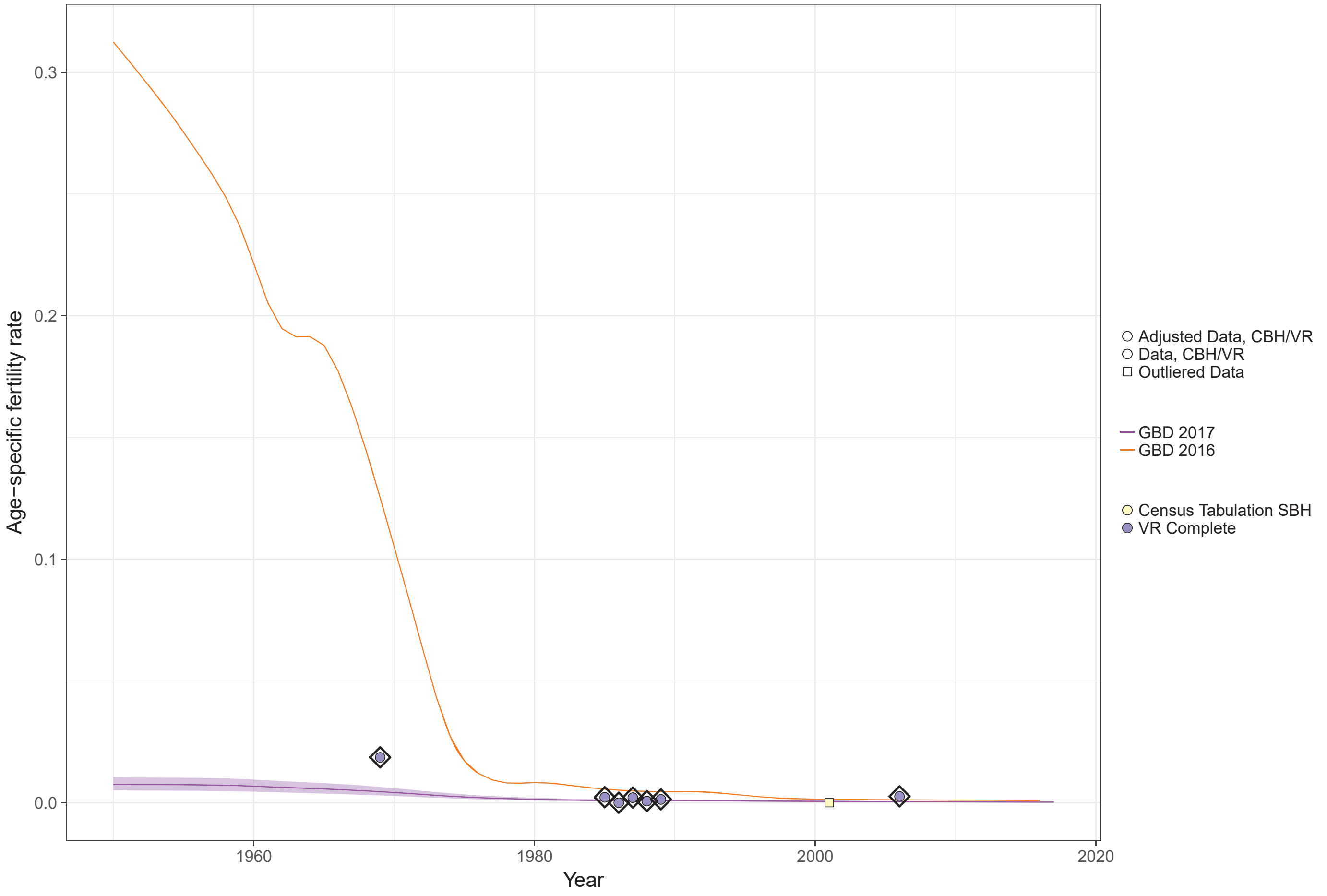

Age−specific fertility fitted trend lines, 1950−2017: Dominica, Ages: 50−54
Data points enclosed with a diamond are reference sources

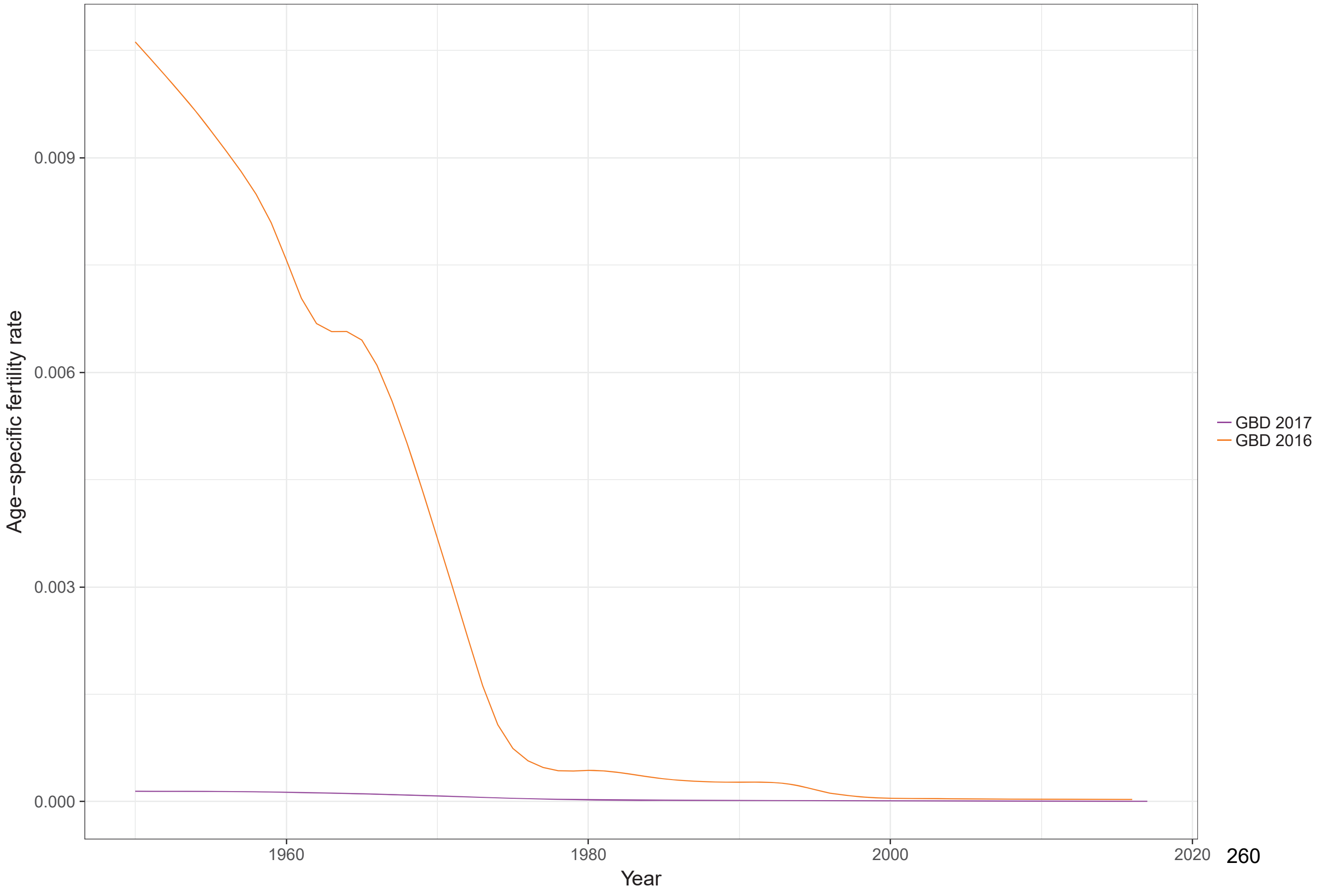

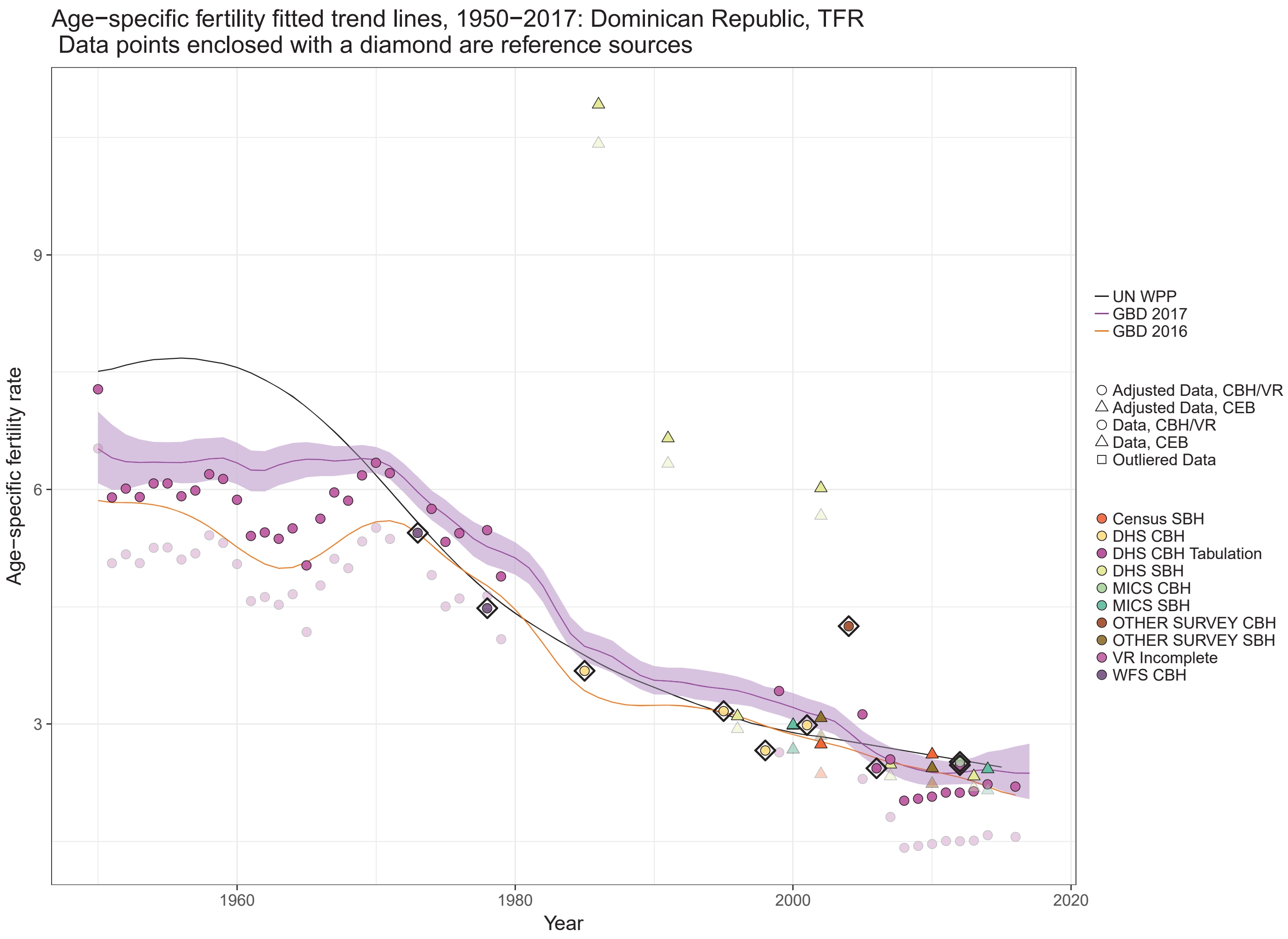

Age−specific fertility fitted trend lines, 1950−2017: Dominican Republic, TFR
Data points enclosed with a diamond are reference sources
Age−specific fertility rate
Year
3
6
9
1960
1980
2000
2020
UN WPP
GBD 2017
GBD 2016
Adjusted Data, CBH/VR
Adjusted Data, CEB
Data, CBH/VR
Data, CEB
Outliered Data
Census SBH
DHS CBH
DHS CBH Tabulation
DHS SBH
MICS CBH
MICS SBH
OTHER SURVEY CBH
OTHER SURVEY SBH
VR Incomplete
WFS CBH

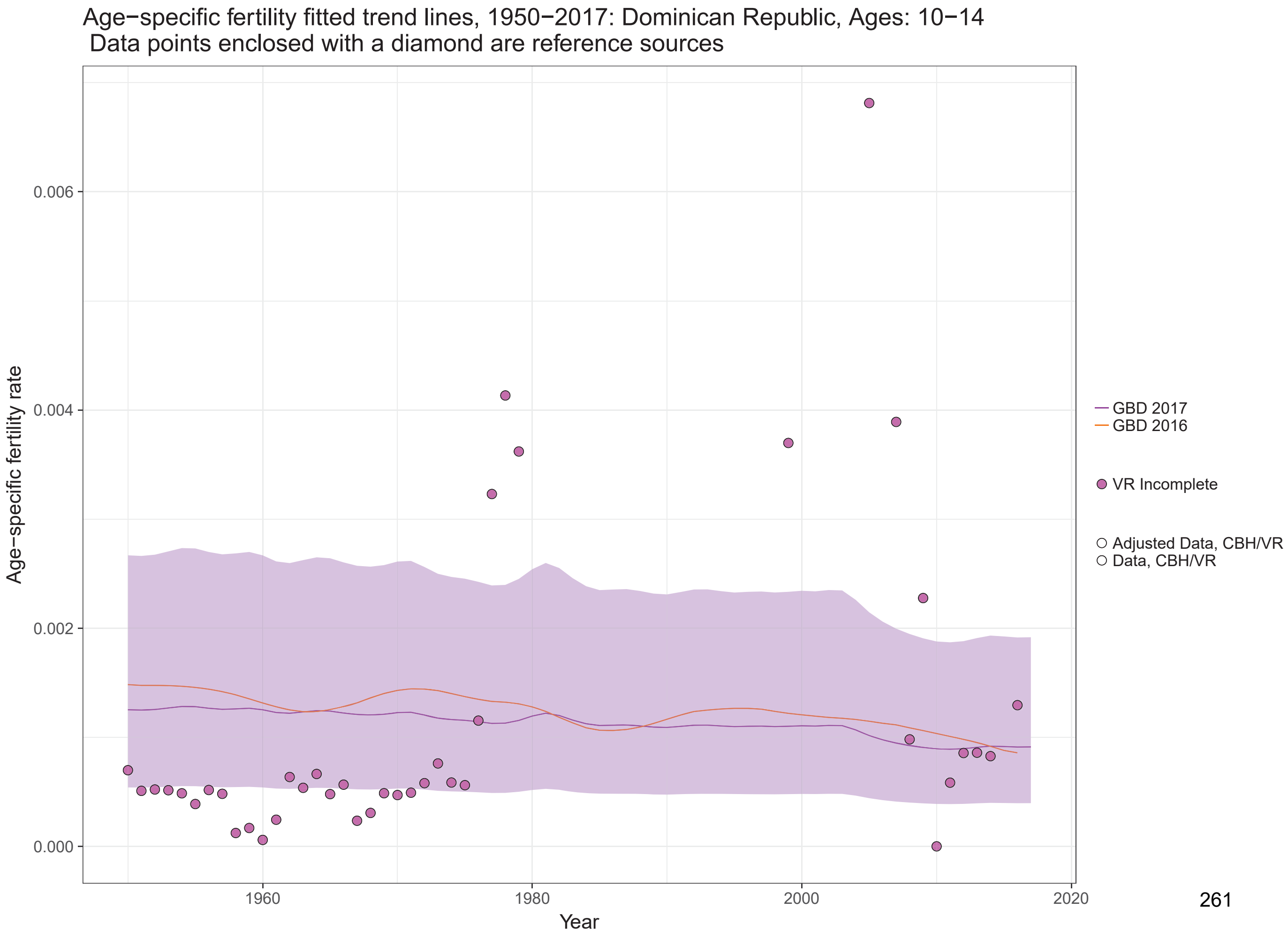

Age−specific fertility fitted trend lines, 1950−2017: Dominican Republic, Ages: 10−14
Data points enclosed with a diamond are reference sources
Age−specific fertility rate
Year
0.000
0.002
0.004
0.006
1960
1980
2000
2020
GBD 2017
GBD 2016
VR Incomplete
Adjusted Data, CBH/VR
Data, CBH/VR

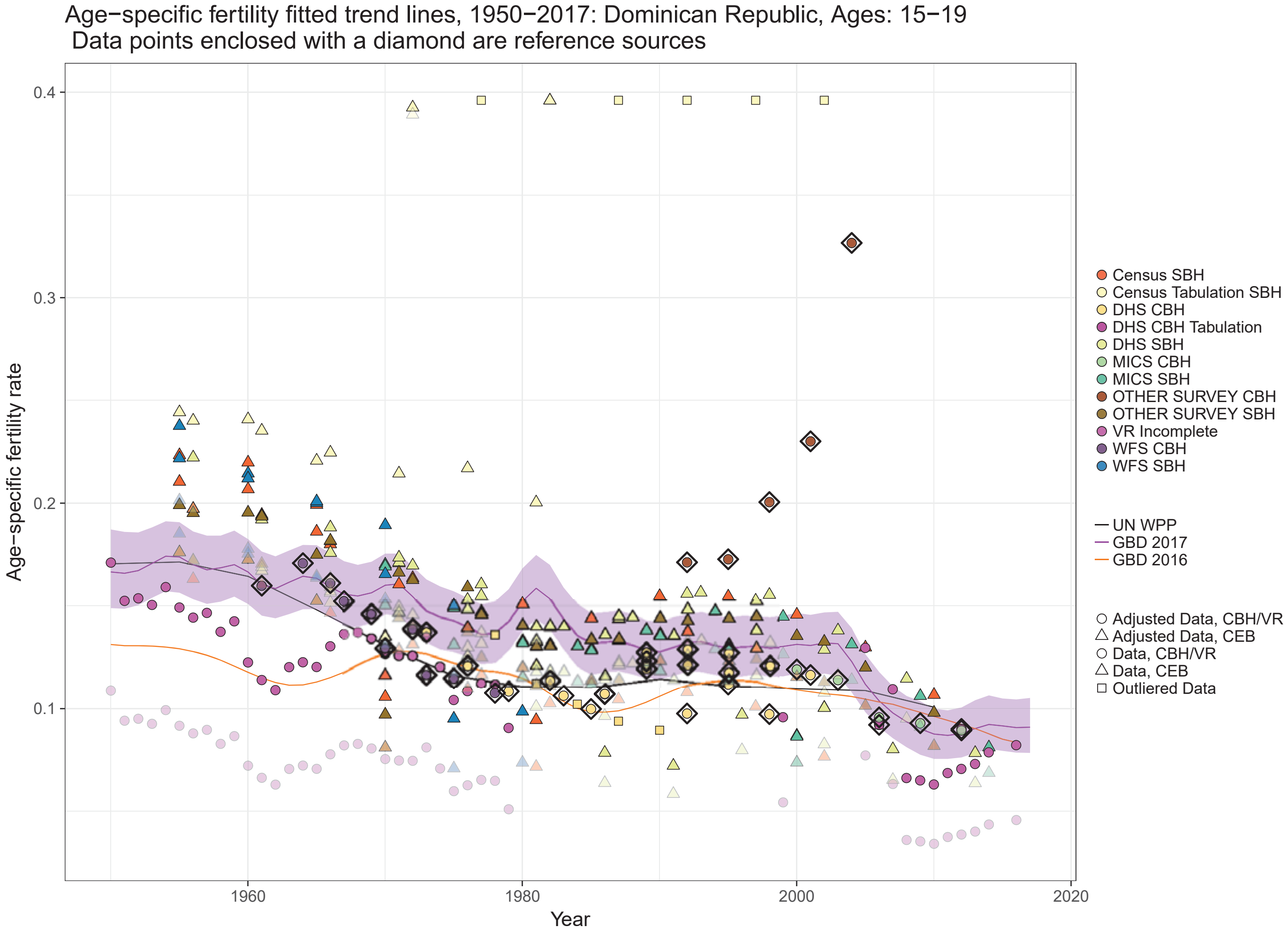

Age−specific fertility fitted trend lines, 1950−2017: Dominican Republic, Ages: 15−19
Data points enclosed with a diamond are reference sources
Age−specific fertility rate
Year
Census SBH
Census Tabulation SBH
DHS CBH
DHS CBH Tabulation
DHS SBH
MICS CBH
MICS SBH
OTHER SURVEY CBH
OTHER SURVEY SBH
VR Incomplete
WFS CBH
WFS SBH
UN WPP
GBD 2017
GBD 2016
Adjusted Data, CBH/VR
Adjusted Data, CEB
Data, CBH/VR
Data, CEB
Outliered Data

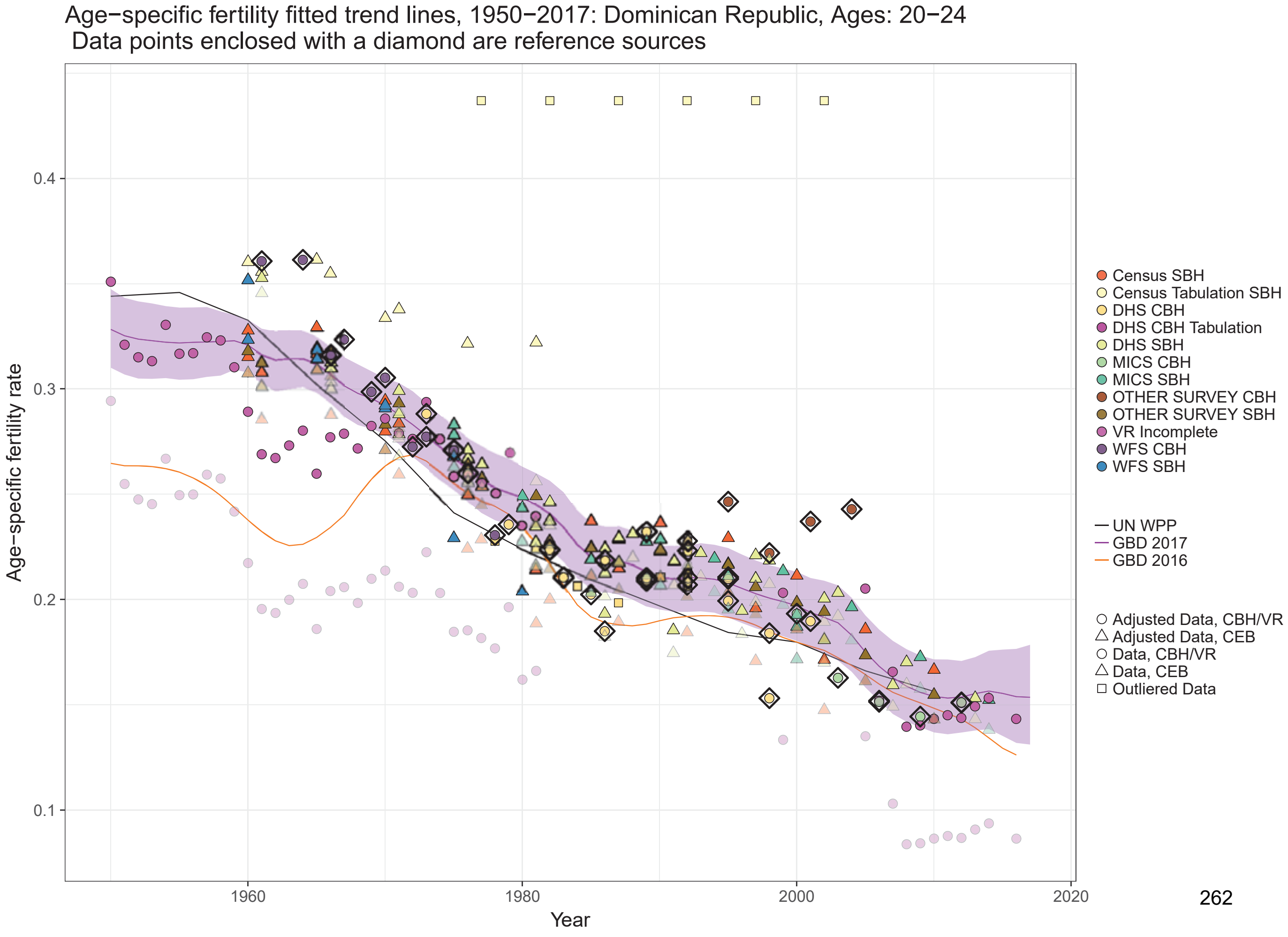

Age−specific fertility fitted trend lines, 1950−2017: Dominican Republic, Ages: 20−24
Data points enclosed with a diamond are reference sources
Age−specific fertility rate
Year
Census SBH
Census Tabulation SBH
DHS CBH
DHS CBH Tabulation
DHS SBH
MICS CBH
MICS SBH
OTHER SURVEY CBH
OTHER SURVEY SBH
VR Incomplete
WFS CBH
WFS SBH
UN WPP
GBD 2017
GBD 2016
Adjusted Data, CBH/VR
Adjusted Data, CEB
Data, CBH/VR
Data, CEB
Outliered Data

Age−specific fertility fitted trend lines, 1950−2017: Dominican Republic, Ages: 25−29
Data points enclosed with a diamond are reference sources

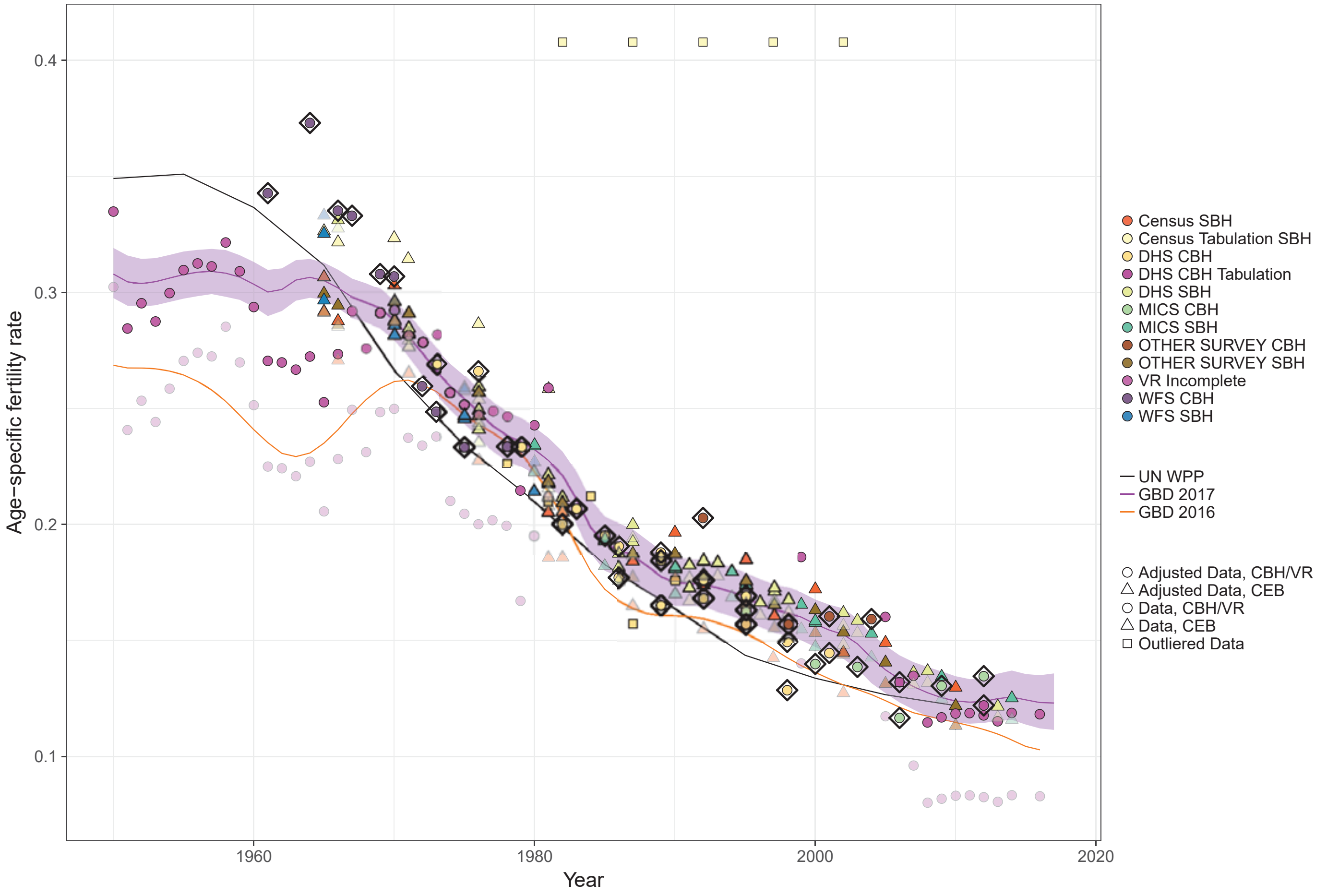

Age−specific fertility fitted trend lines, 1950−2017: Dominican Republic, Ages: 30−34
Data points enclosed with a diamond are reference sources

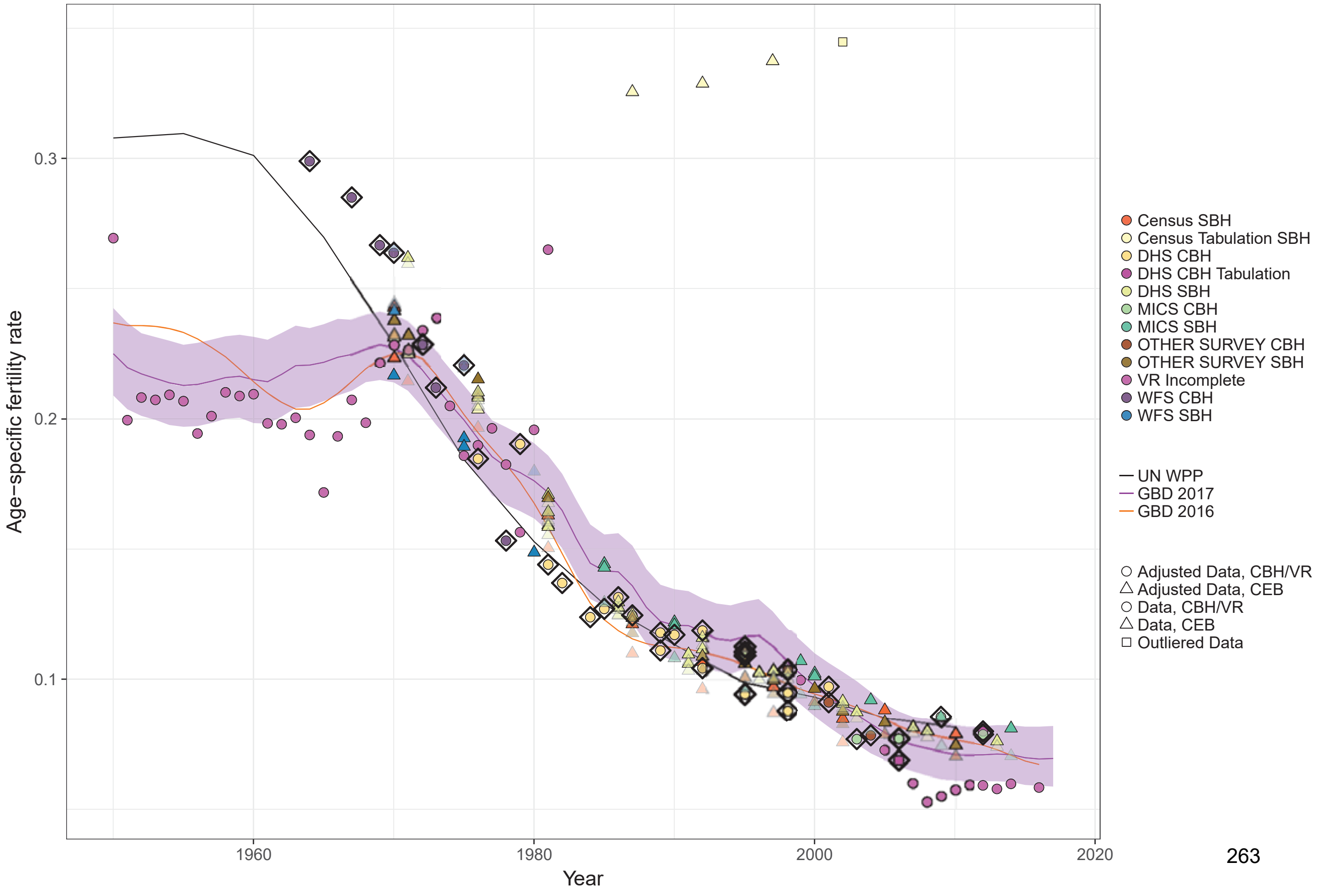

Age−specific fertility fitted trend lines, 1950−2017: Dominican Republic, Ages: 35−39
Data points enclosed with a diamond are reference sources

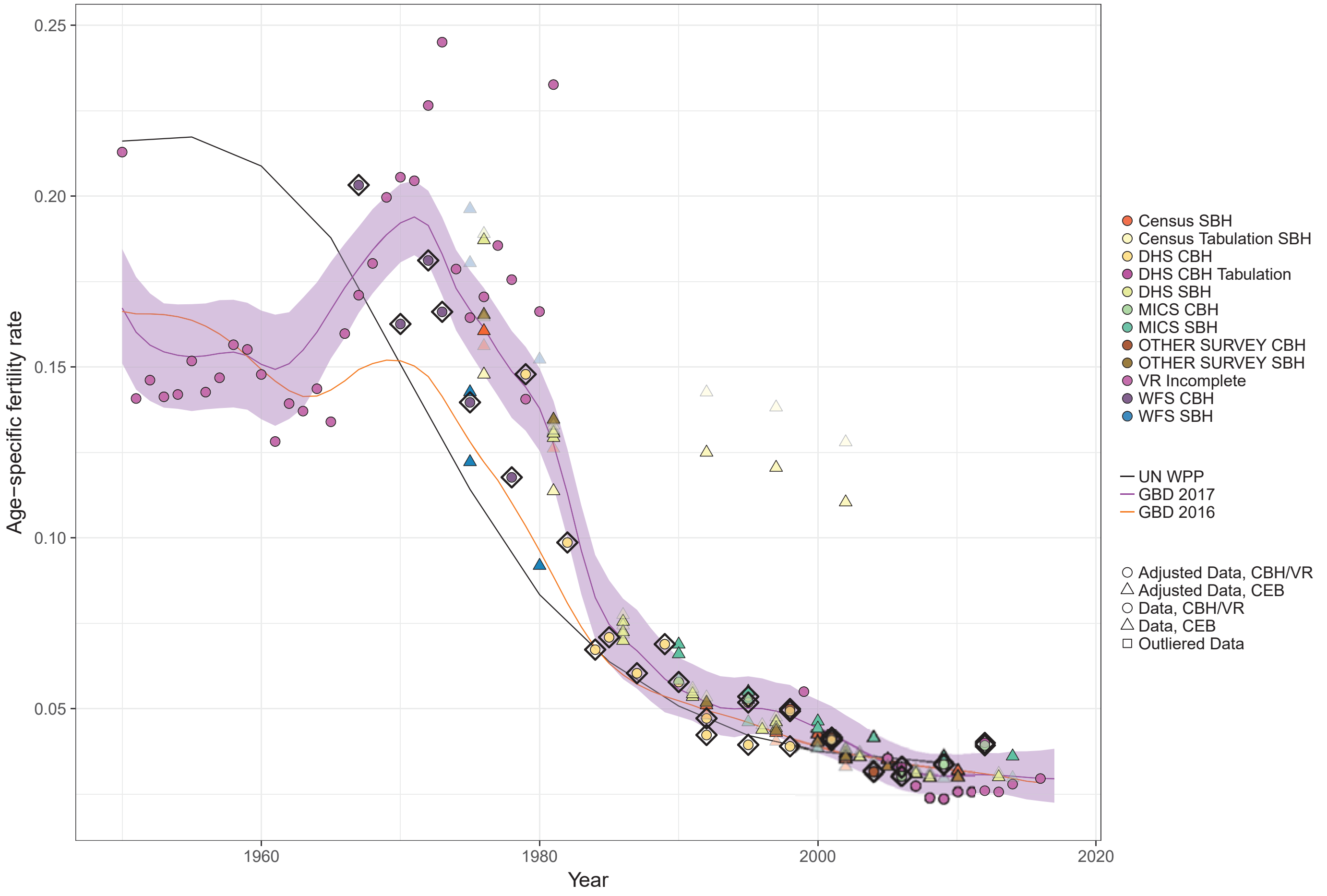

Age−specific fertility fitted trend lines, 1950−2017: Dominican Republic, Ages: 40−44
Data points enclosed with a diamond are reference sources

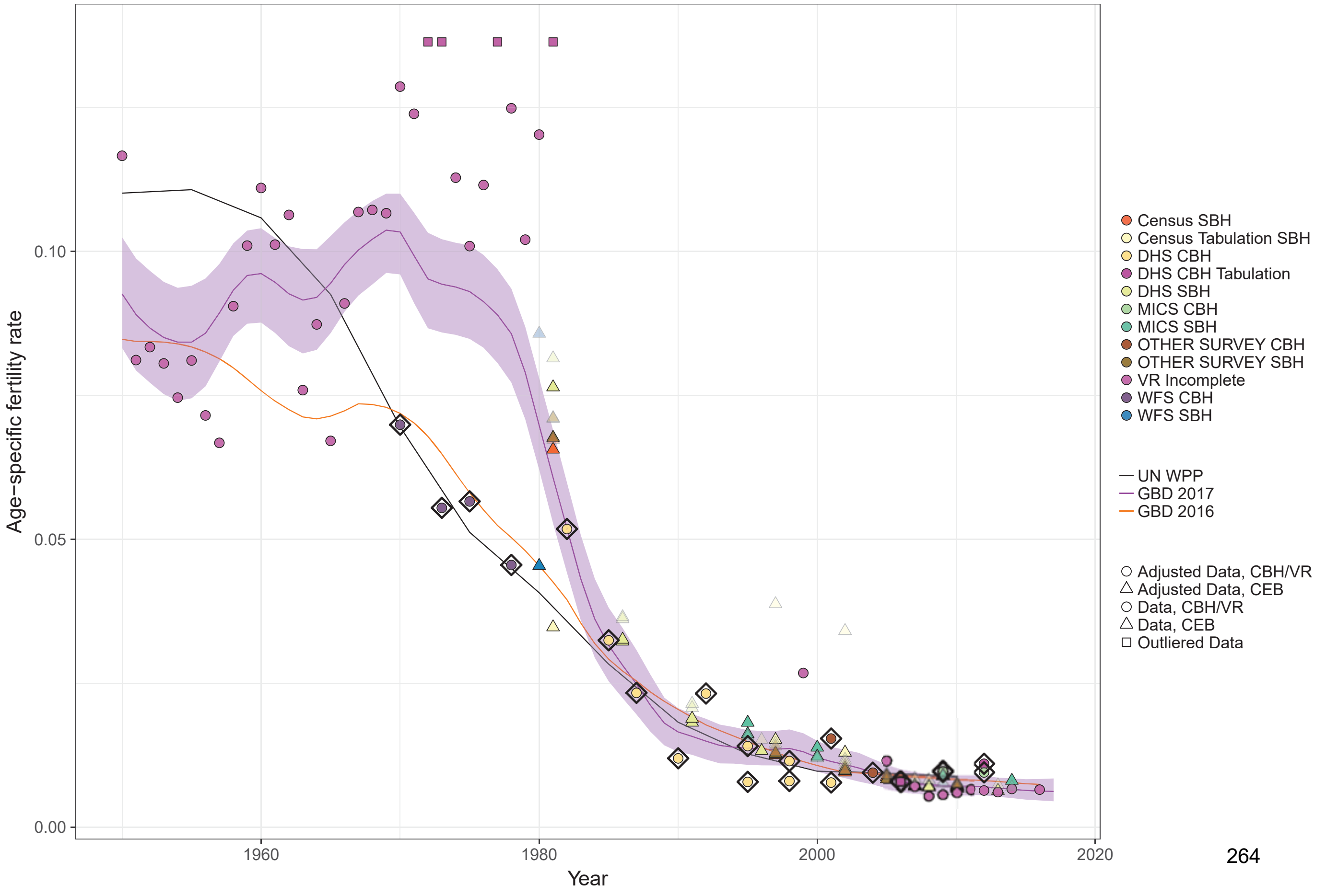

Age−specific fertility fitted trend lines, 1950−2017: Dominican Republic, Ages: 45−49
Data points enclosed with a diamond are reference sources

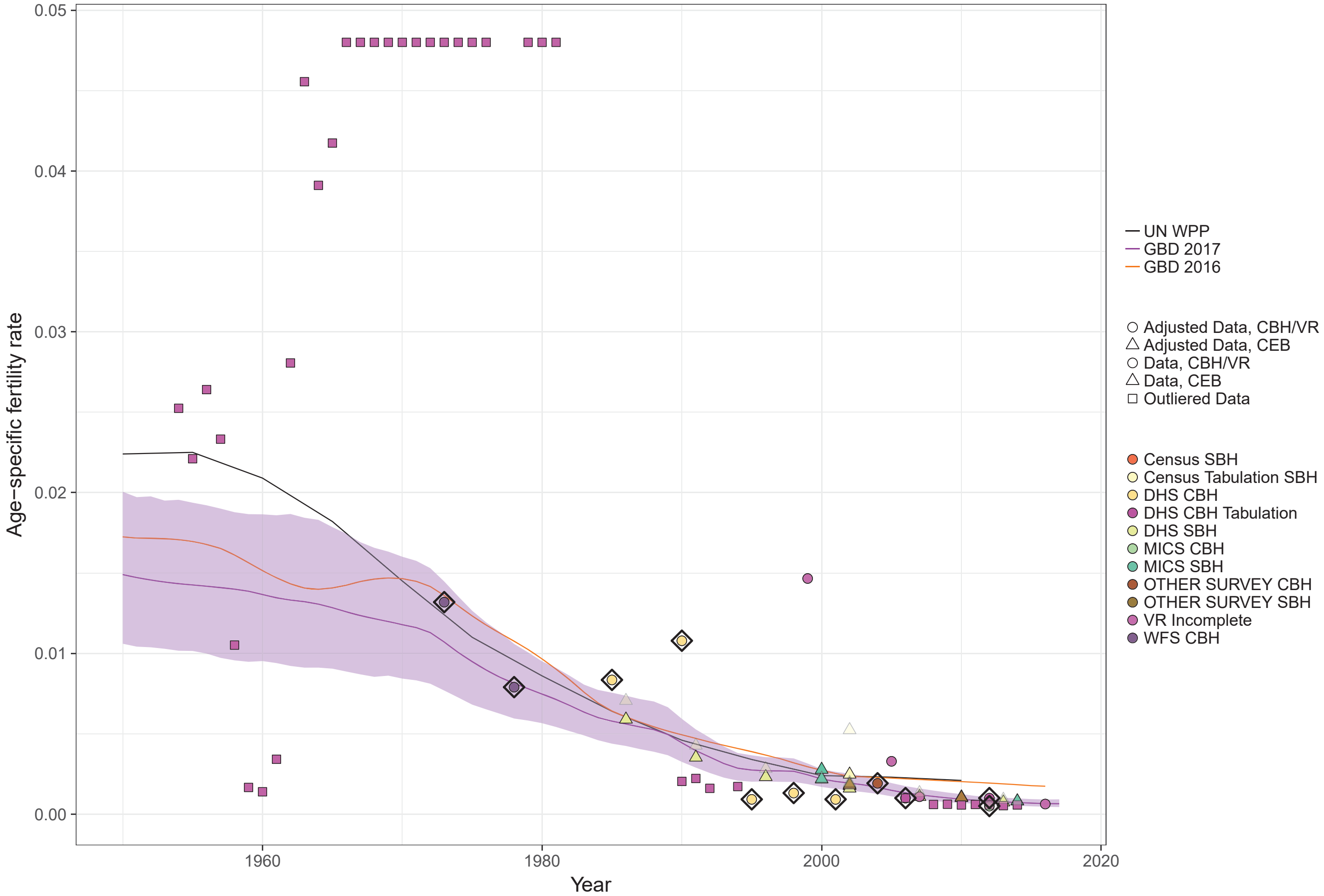

Age−specific fertility fitted trend lines, 1950−2017: Dominican Republic, Ages: 50−54
Data points enclosed with a diamond are reference sources

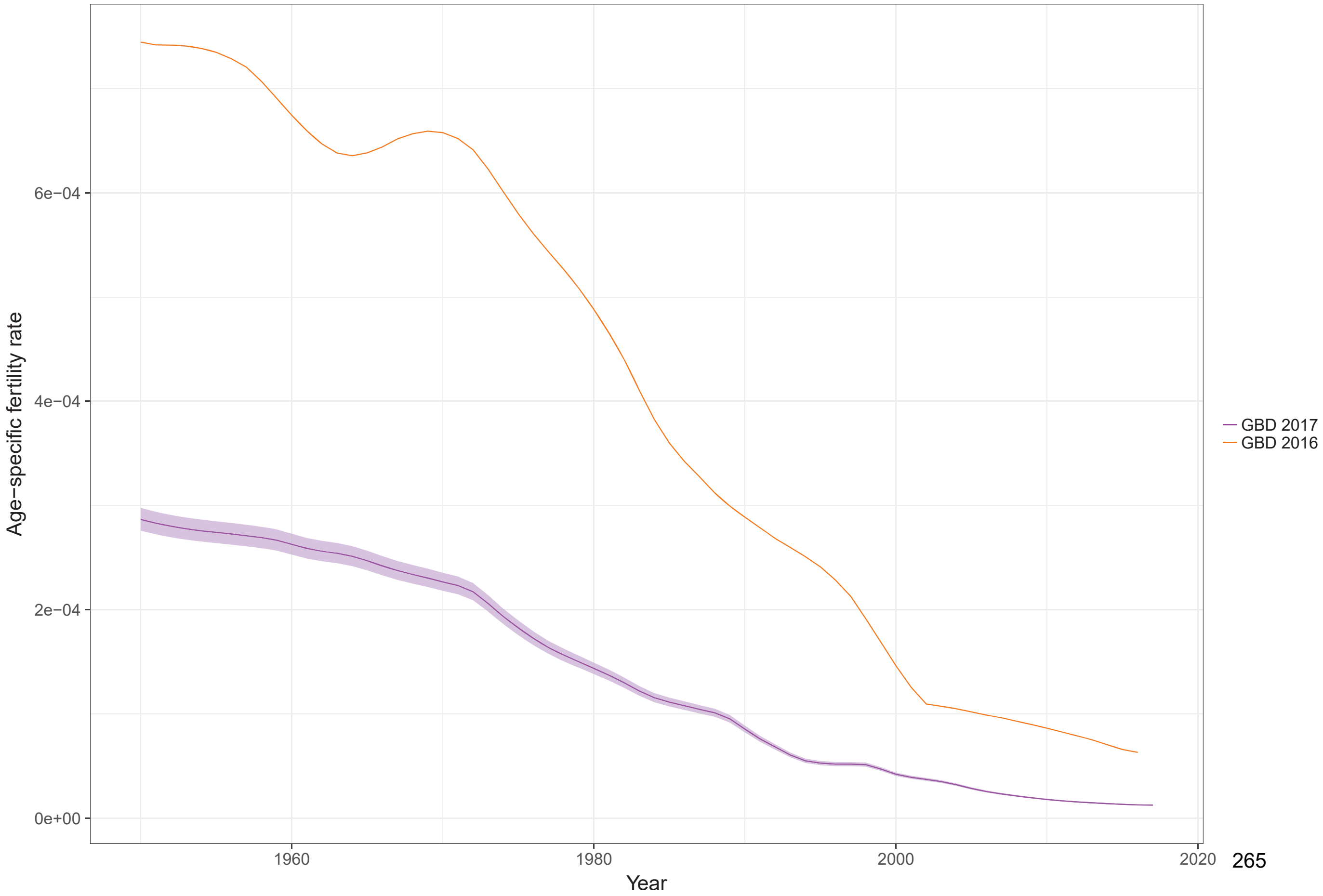

Age−specific fertility fitted trend lines, 1950−2017: Ecuador, TFR
Data points enclosed with a diamond are reference sources

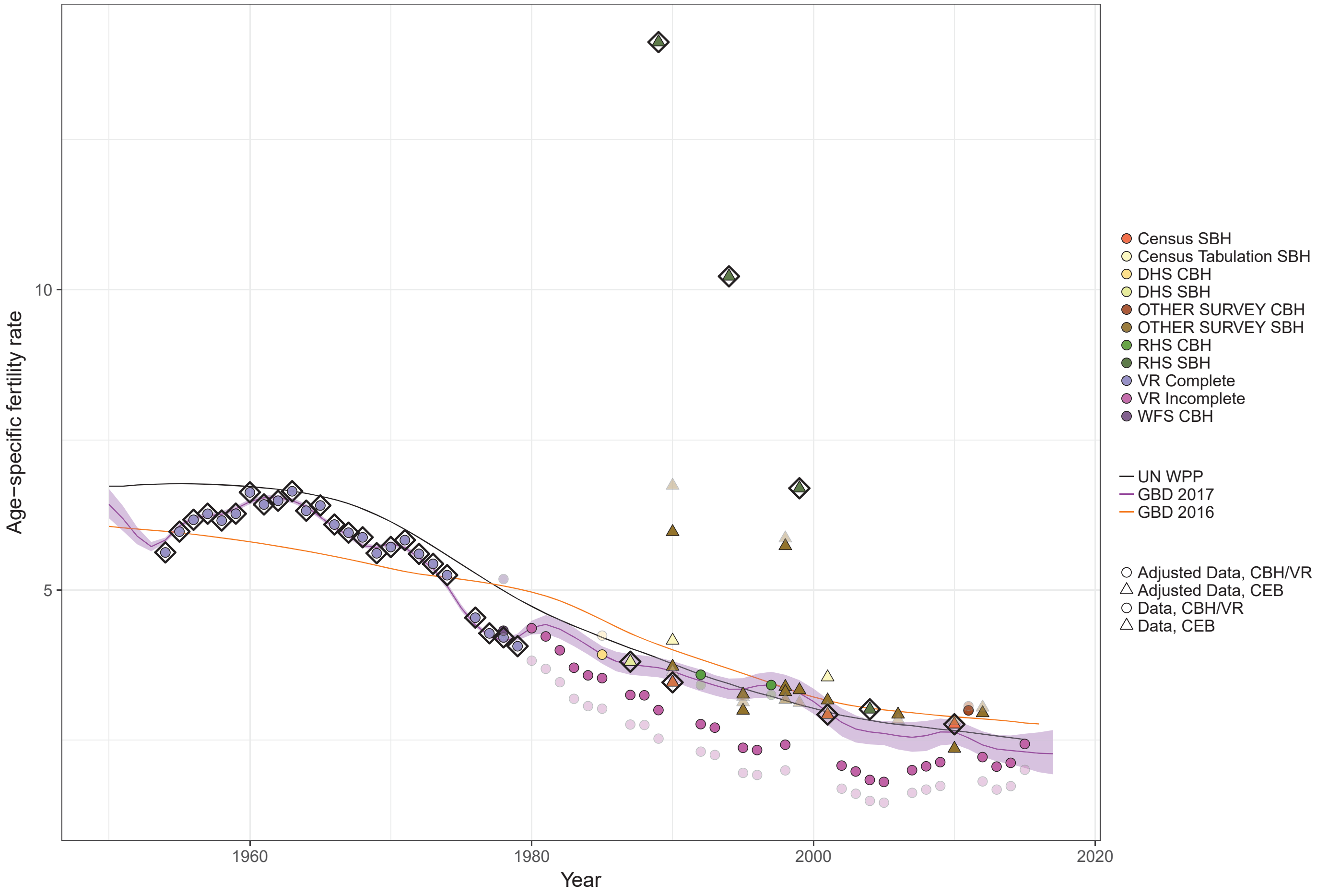

Age−specific fertility fitted trend lines, 1950−2017: Ecuador, Ages: 10−14
Data points enclosed with a diamond are reference sources

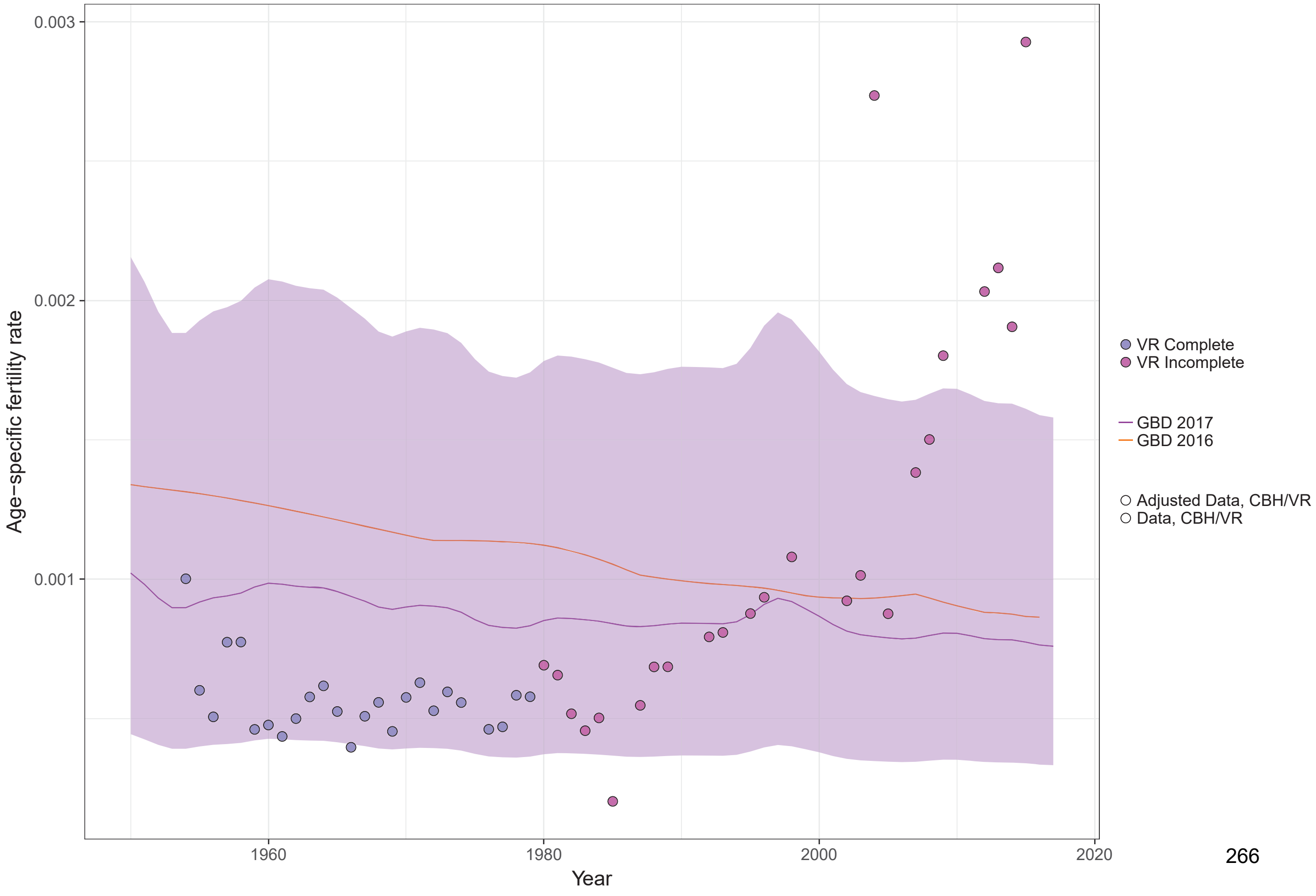

Age−specific fertility fitted trend lines, 1950−2017: Ecuador, Ages: 15−19
Data points enclosed with a diamond are reference sources

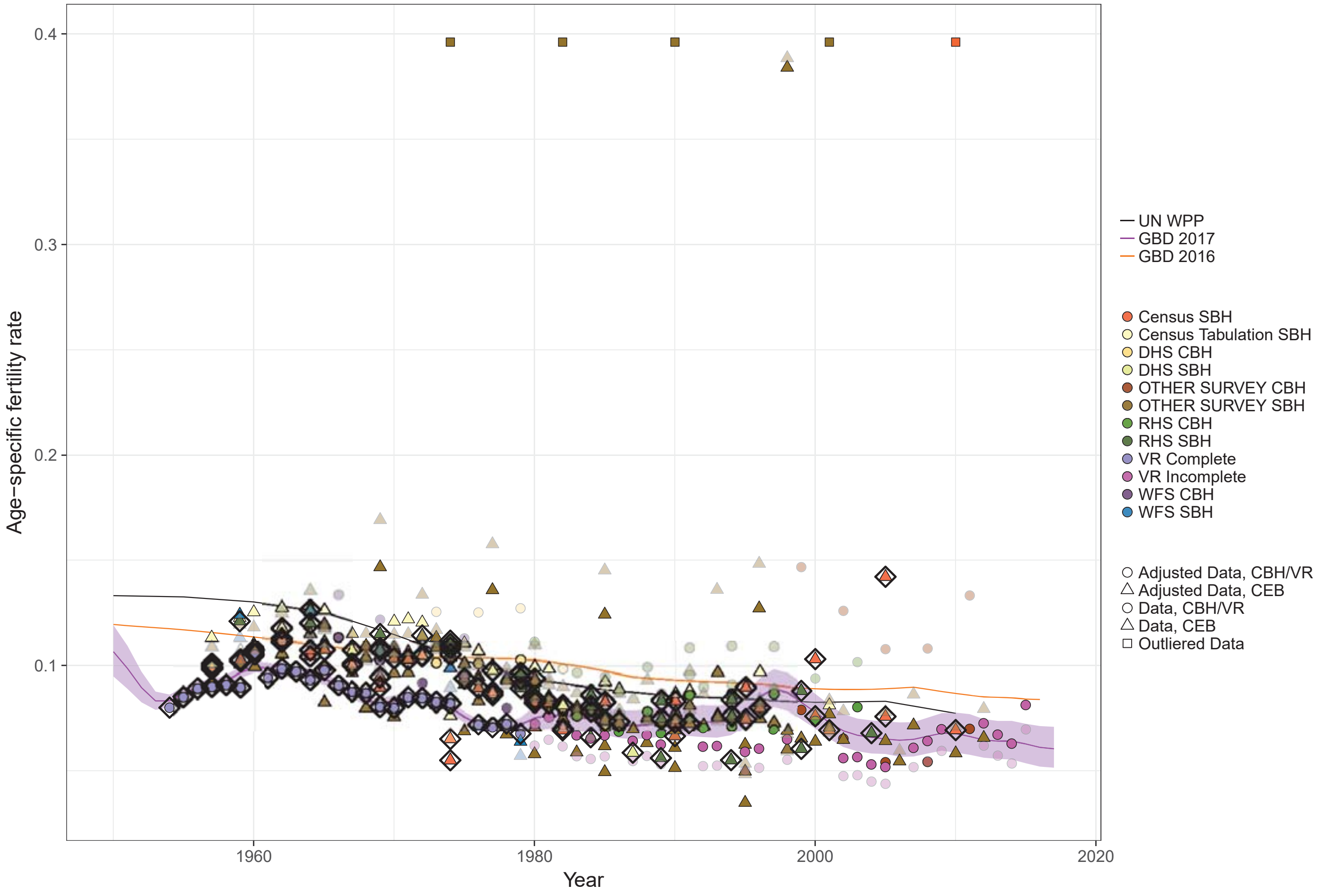

Age−specific fertility fitted trend lines, 1950−2017: Ecuador, Ages: 20−24
Data points enclosed with a diamond are reference sources

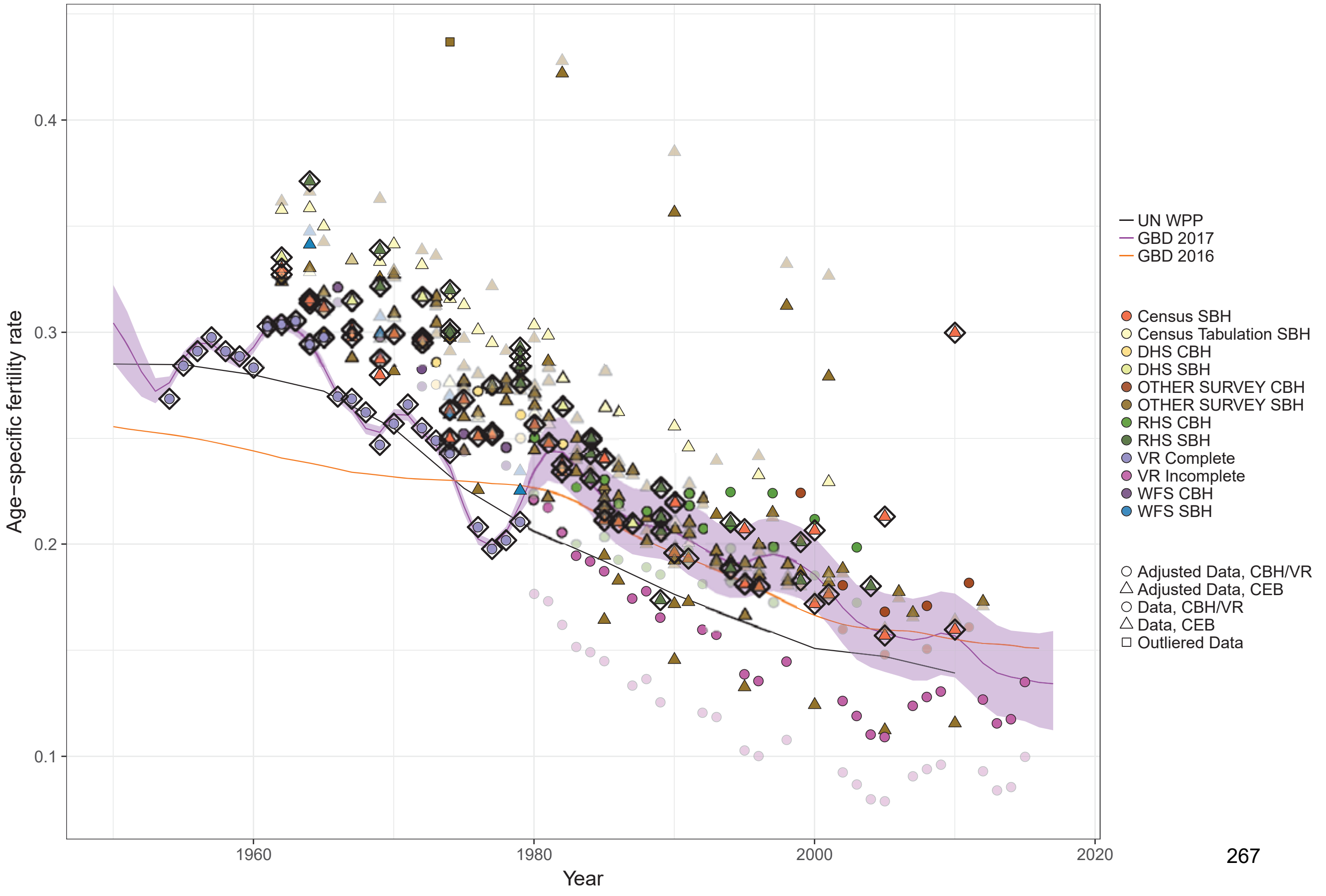

Age−specific fertility fitted trend lines, 1950−2017: Ecuador, Ages: 25−29
Data points enclosed with a diamond are reference sources

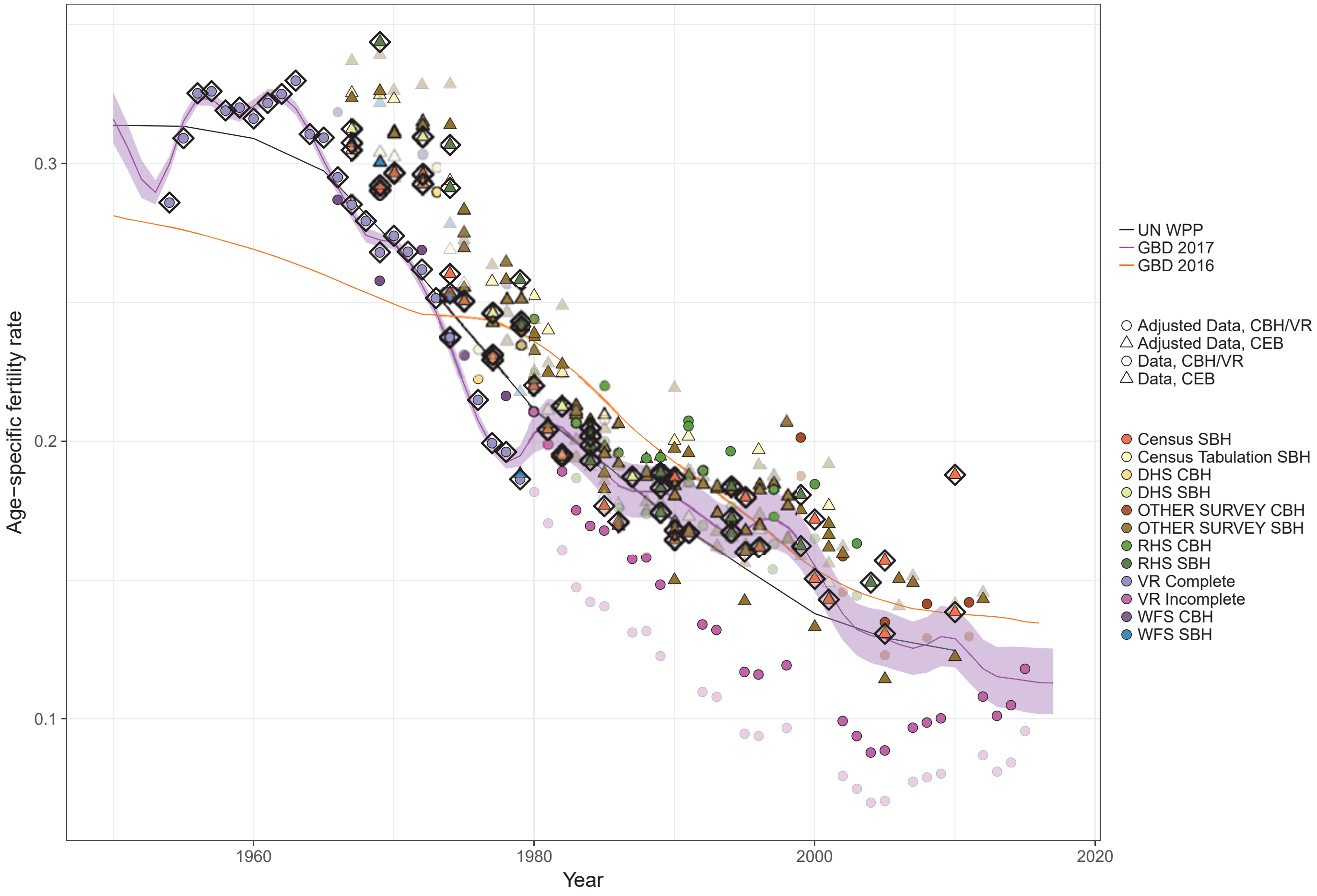

Age−specific fertility fitted trend lines, 1950−2017: Ecuador, Ages: 30−34
Data points enclosed with a diamond are reference sources

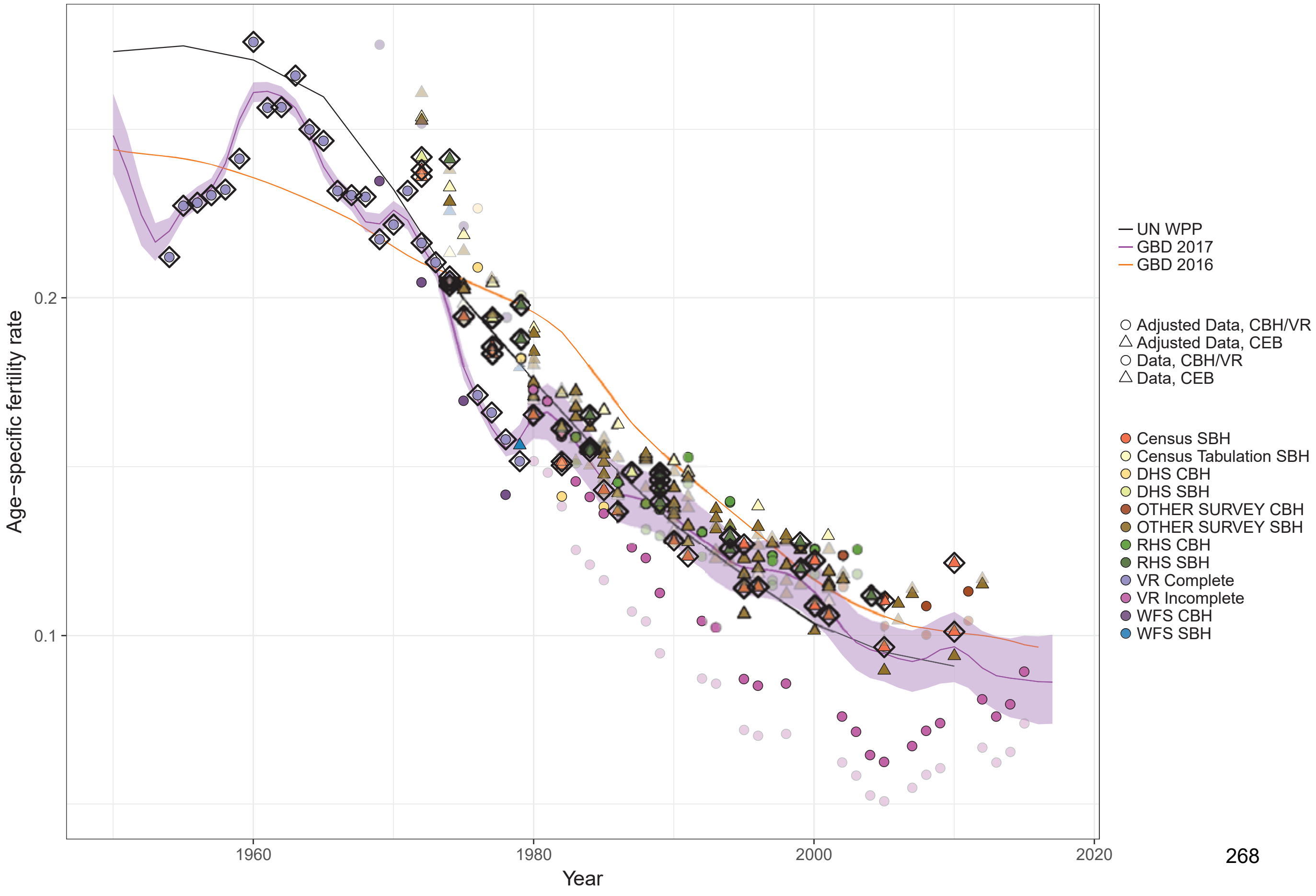

Age−specific fertility fitted trend lines, 1950−2017: Ecuador, Ages: 35−39
Data points enclosed with a diamond are reference sources

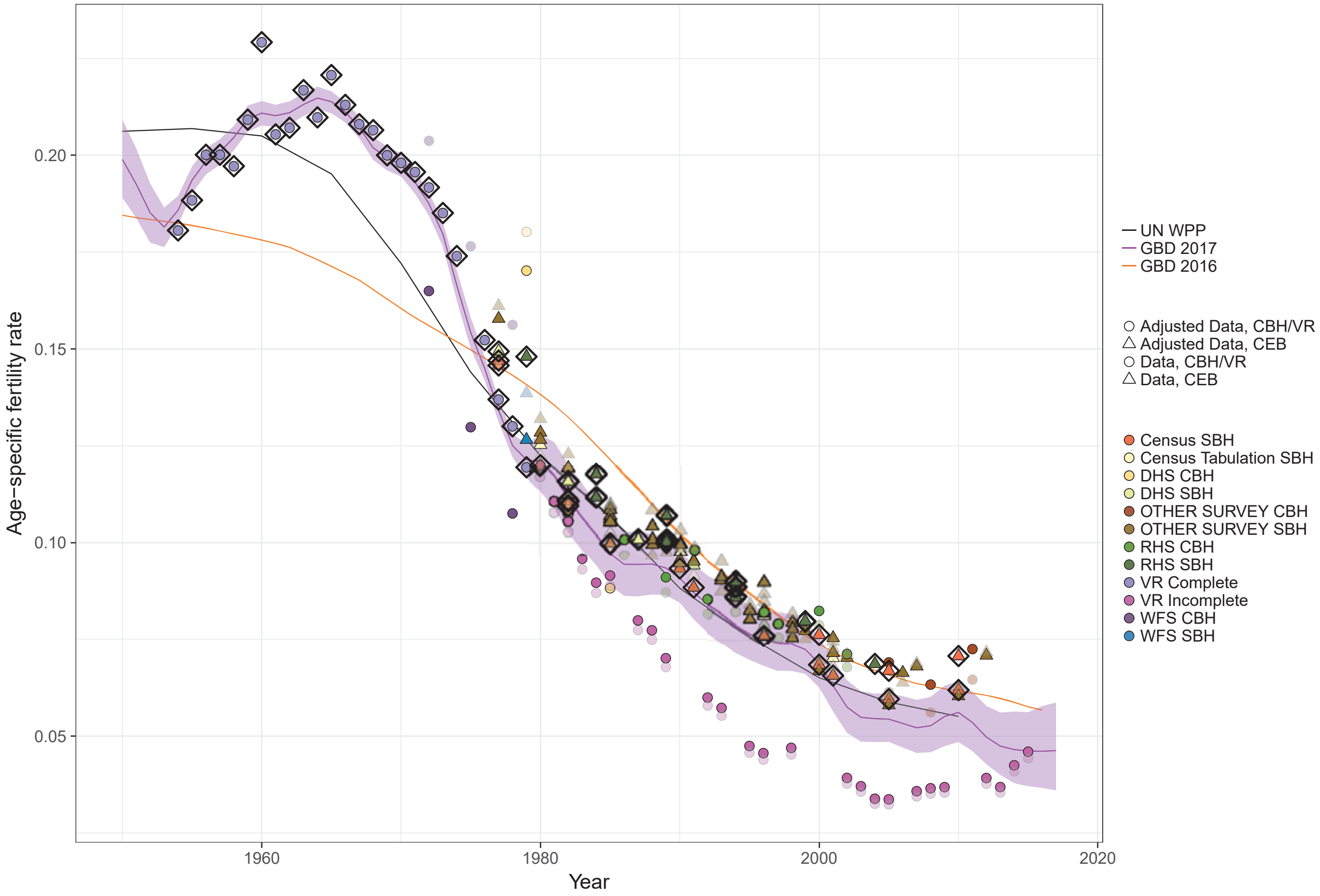

Age−specific fertility fitted trend lines, 1950−2017: Ecuador, Ages: 40−44
Data points enclosed with a diamond are reference sources

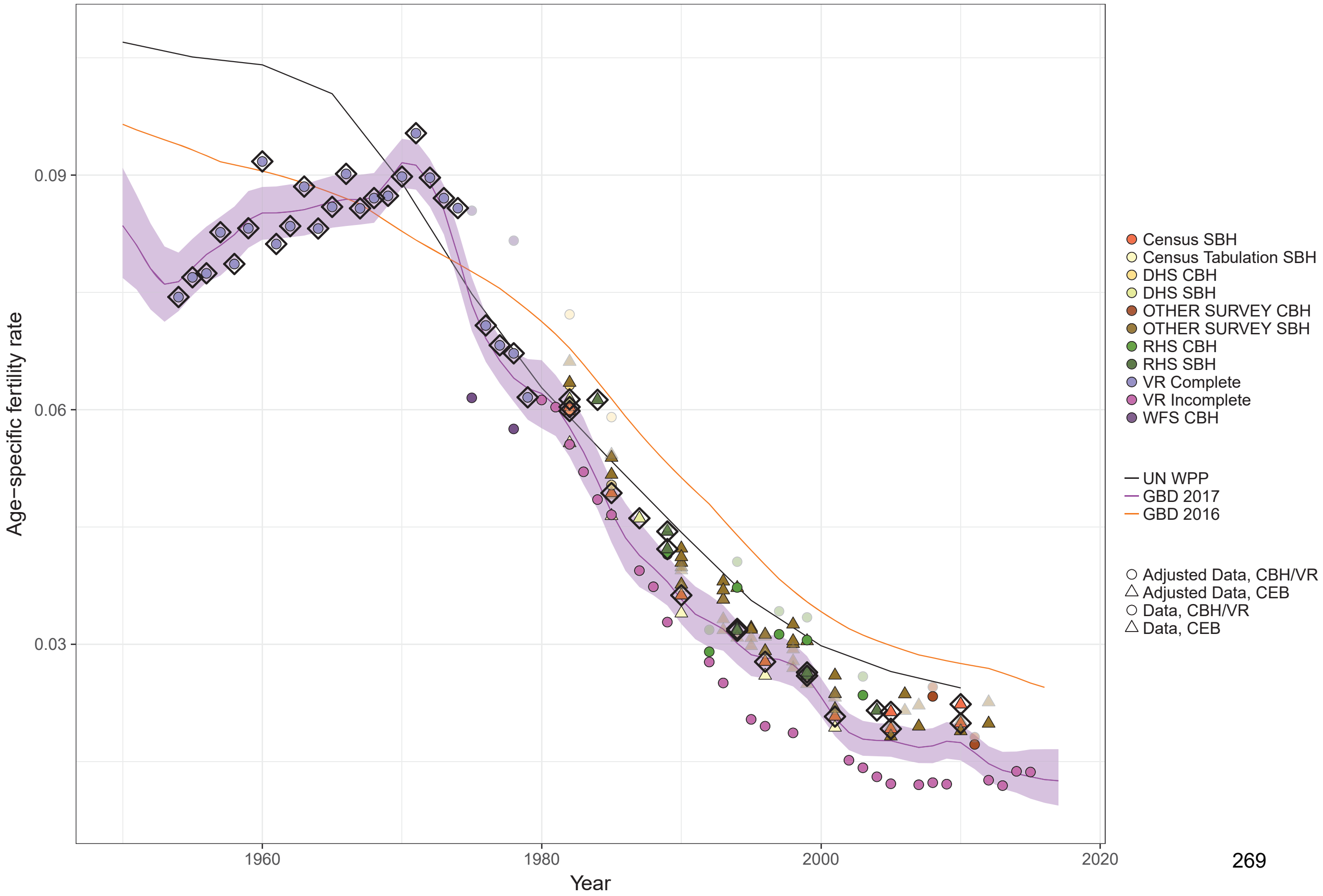

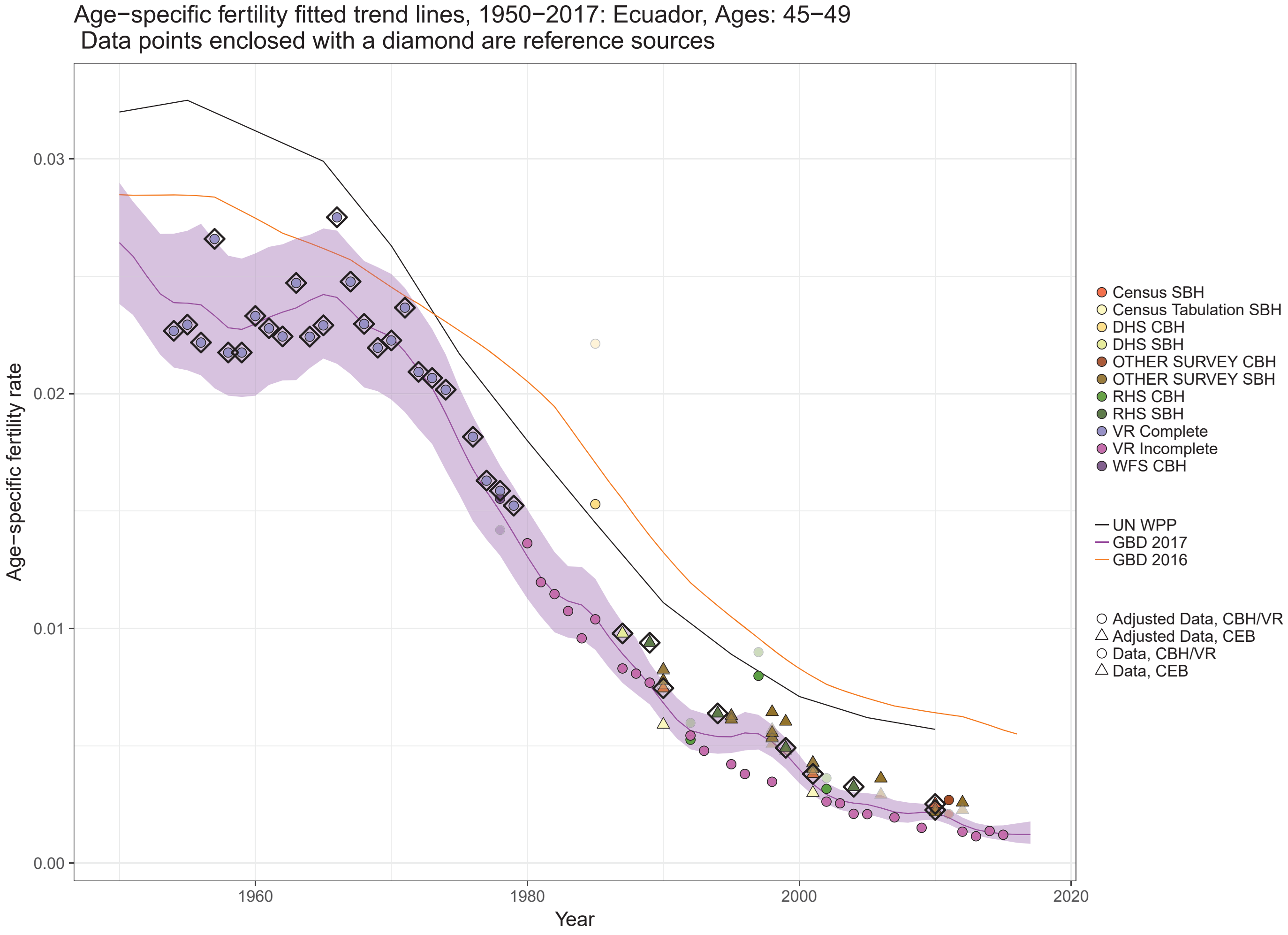

Age−specific fertility fitted trend lines, 1950−2017: Ecuador, Ages: 45−49
Data points enclosed with a diamond are reference sources
Age−specific fertility rate
0.00
0.01
0.02
0.03
Year
1960
1980
2000
2020
Census SBH
Census Tabulation SBH
DHS CBH
DHS SBH
OTHER SURVEY CBH
OTHER SURVEY SBH
RHS CBH
RHS SBH
VR Complete
VR Incomplete
WFS CBH
UN WPP
GBD 2017
GBD 2016
Adjusted Data, CBH/VR
Adjusted Data, CEB
Data, CBH/VR
Data, CEB

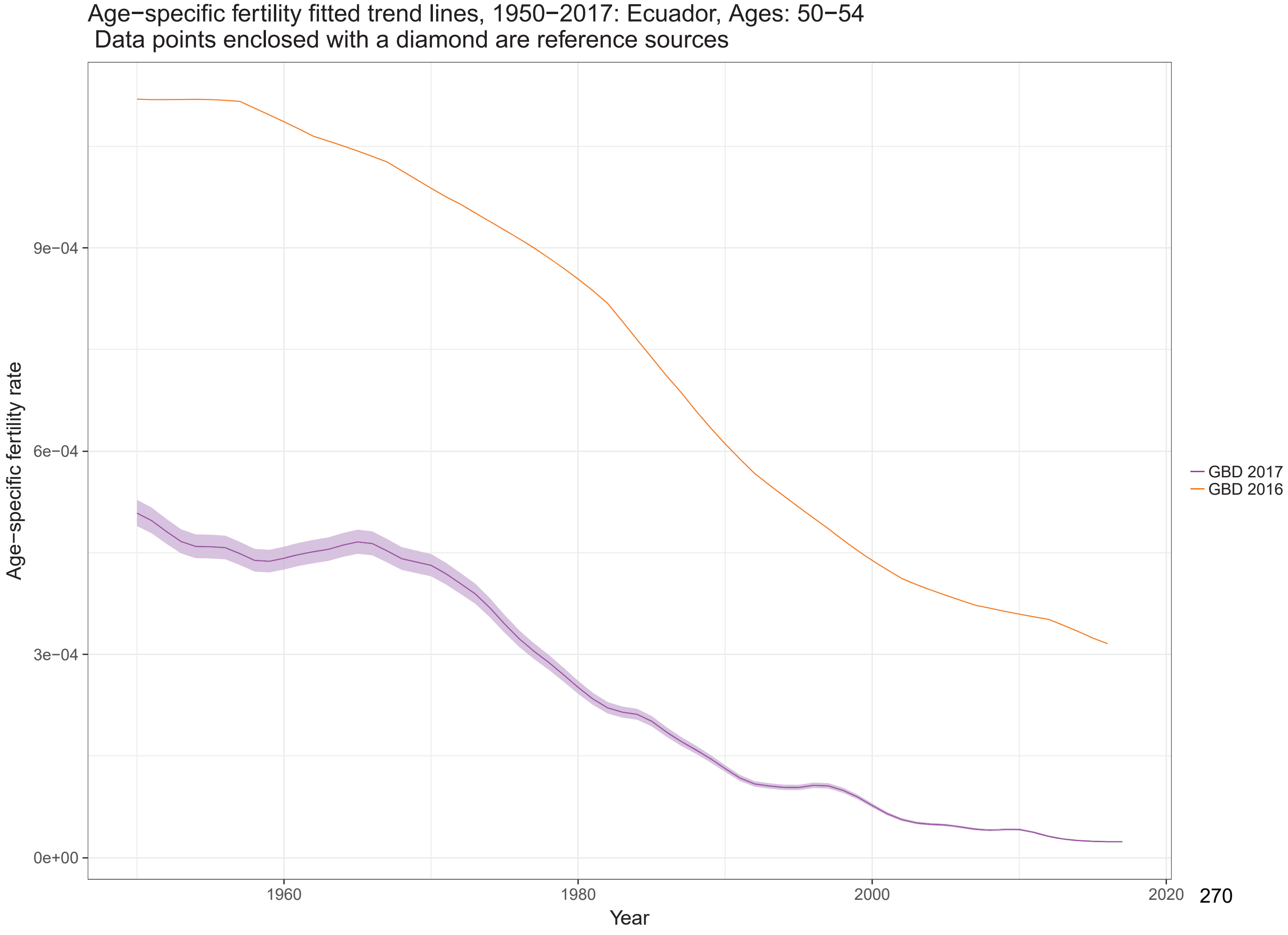

Age−specific fertility fitted trend lines, 1950−2017: Ecuador, Ages: 50−54
Data points enclosed with a diamond are reference sources
Age−specific fertility rate
0e+00
3e−04
6e−04
9e−04
Year
1960
1980
2000
2020
GBD 2017
GBD 2016

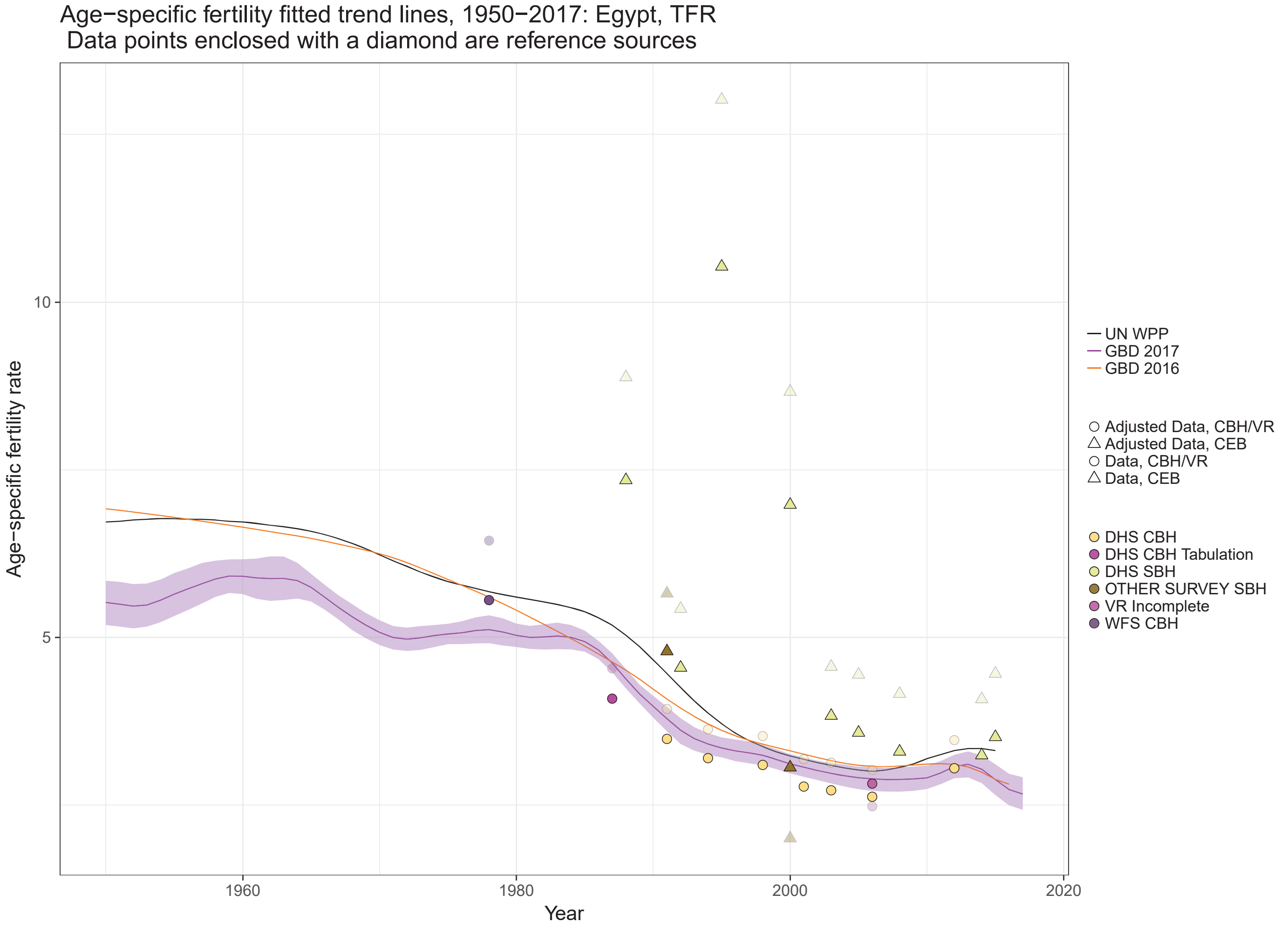

Age−specific fertility fitted trend lines, 1950−2017: Egypt, TFR
Data points enclosed with a diamond are reference sources
Age−specific fertility rate
Year
UN WPP
GBD 2017
GBD 2016
Adjusted Data, CBH/VR
Adjusted Data, CEB
Data, CBH/VR
Data, CEB
DHS CBH
DHS CBH Tabulation
DHS SBH
OTHER SURVEY SBH
VR Incomplete
WFS CBH

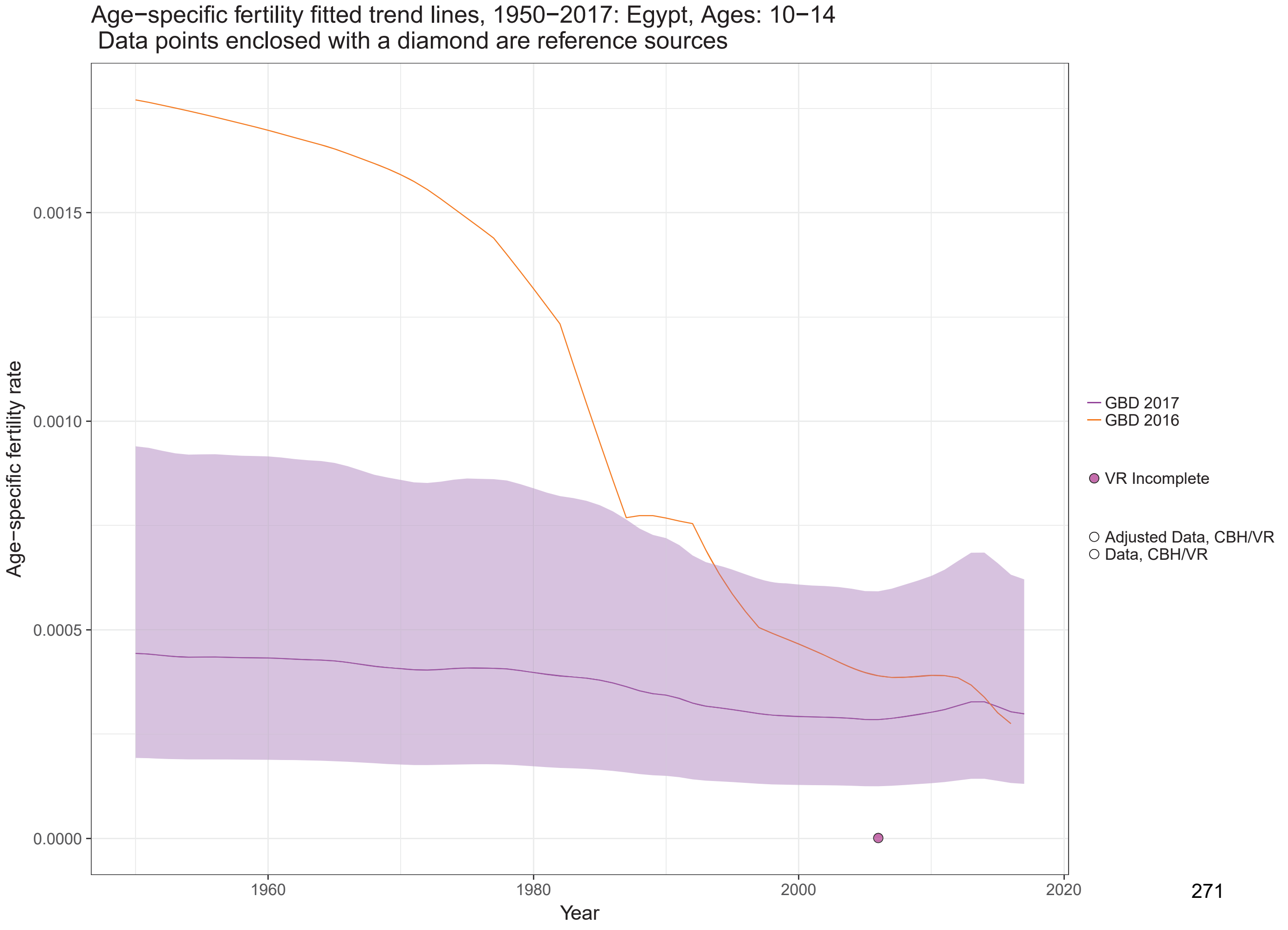

Age−specific fertility fitted trend lines, 1950−2017: Egypt, Ages: 10−14
Data points enclosed with a diamond are reference sources
Age−specific fertility rate
Year
GBD 2017
GBD 2016
VR Incomplete
Adjusted Data, CBH/VR
Data, CBH/VR

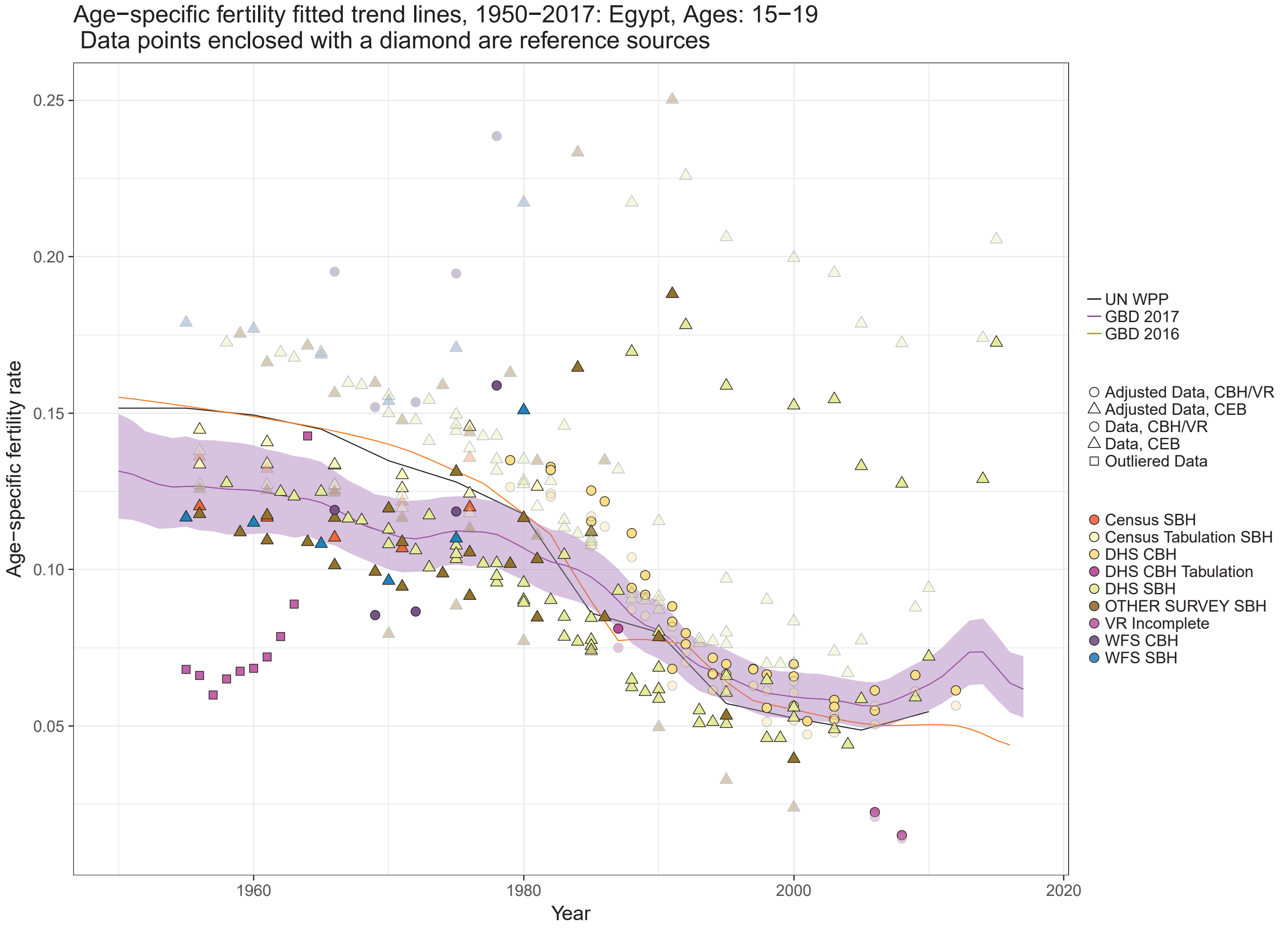

Age−specific fertility fitted trend lines, 1950−2017: Egypt, Ages: 15−19
Data points enclosed with a diamond are reference sources
Age-specific fertility rate
Year
0.25
0.20
0.15
0.10
0.05
1960
1980
2000
2020
UN WPP
GBD 2017
GBD 2016
Adjusted Data, CBH/VR
Adjusted Data, CEB
Data, CBH/VR
Data, CEB
Outliered Data
Census SBH
Census Tabulation SBH
DHS CBH
DHS CBH Tabulation
DHS SBH
OTHER SURVEY SBH
VR Incomplete
WFS CBH
WFS SBH

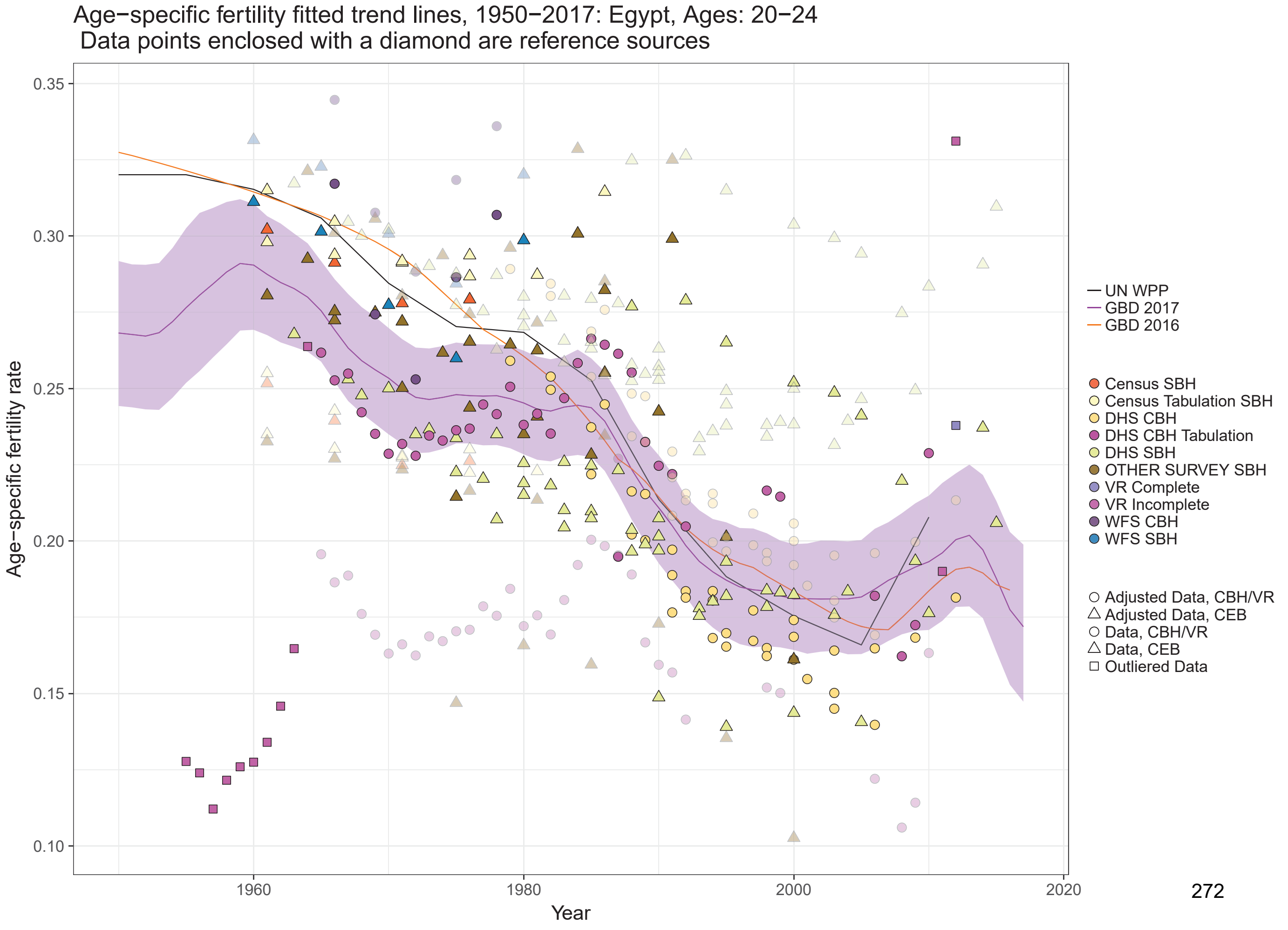

Age−specific fertility fitted trend lines, 1950−2017: Egypt, Ages: 20−24
Data points enclosed with a diamond are reference sources
Age-specific fertility rate
Year
0.35
0.30
0.25
0.20
0.15
0.10
1960
1980
2000
2020
UN WPP
GBD 2017
GBD 2016
Census SBH
Census Tabulation SBH
DHS CBH
DHS CBH Tabulation
DHS SBH
OTHER SURVEY SBH
VR Complete
VR Incomplete
WFS CBH
WFS SBH
Adjusted Data, CBH/VR
Adjusted Data, CEB
Data, CBH/VR
Data, CEB
Outliered Data

Age−specific fertility fitted trend lines, 1950−2017: Egypt, Ages: 25−29
Data points enclosed with a diamond are reference sources

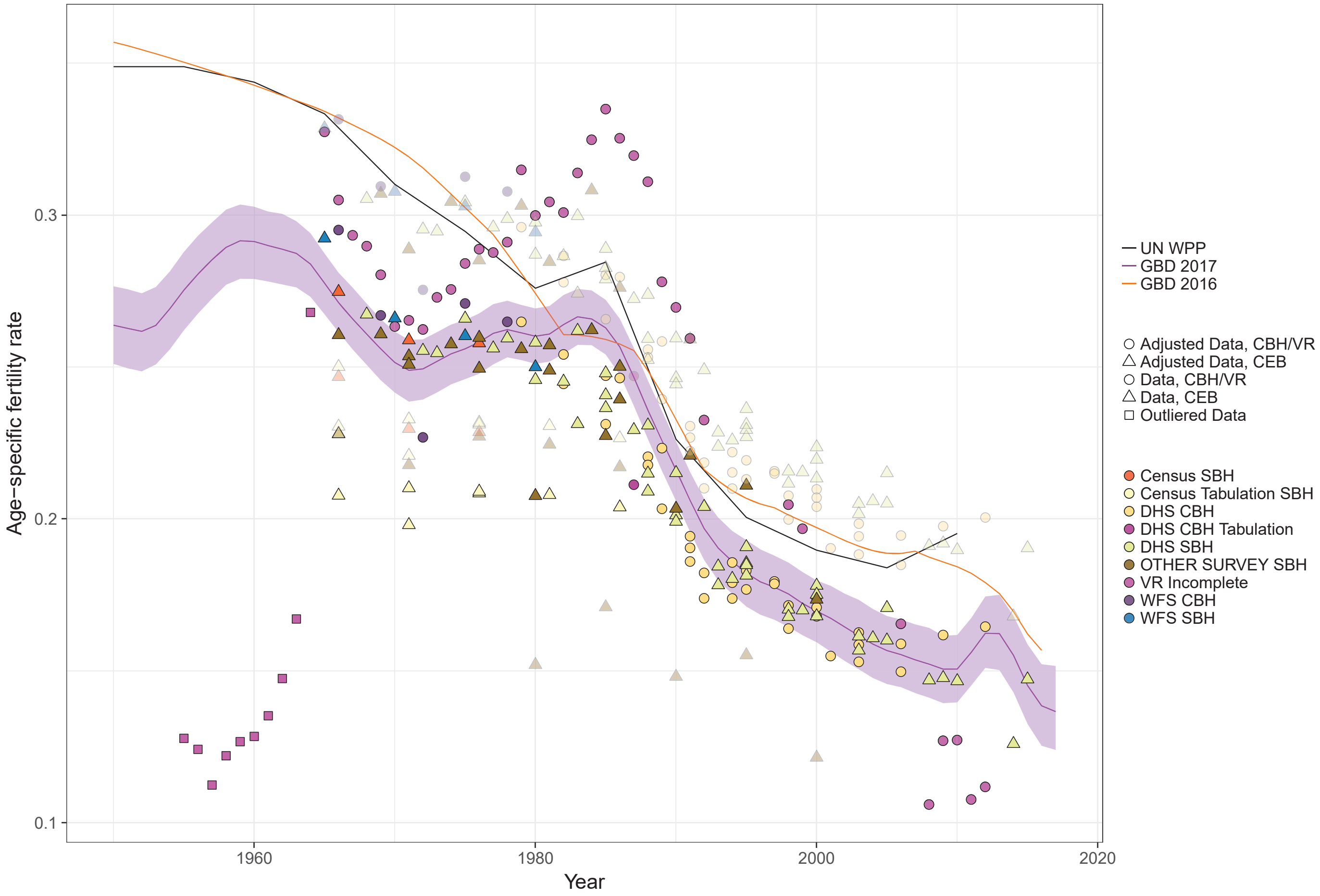

Age−specific fertility fitted trend lines, 1950−2017: Egypt, Ages: 30−34
Data points enclosed with a diamond are reference sources

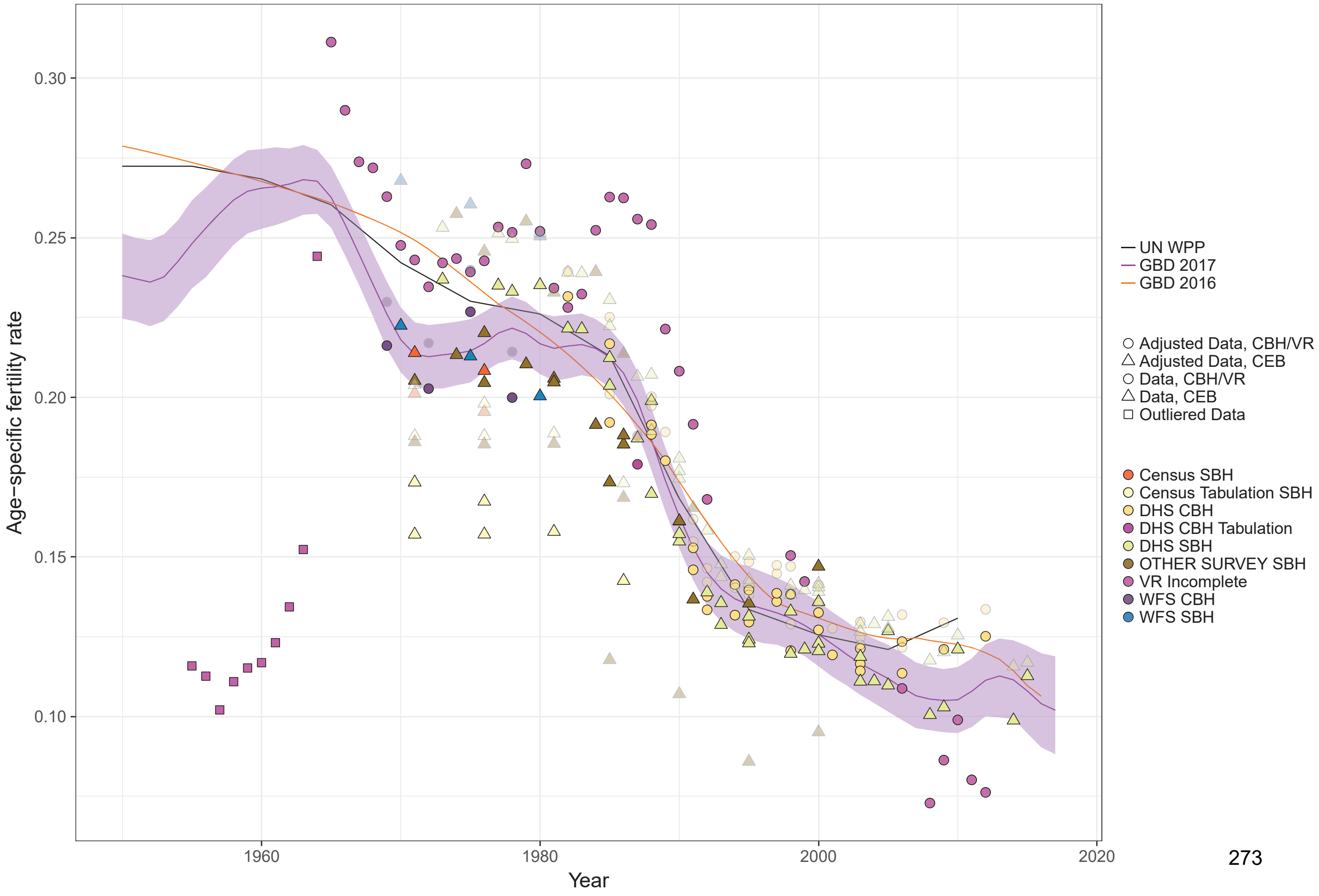

Age−specific fertility fitted trend lines, 1950−2017: Egypt, Ages: 35−39
Data points enclosed with a diamond are reference sources

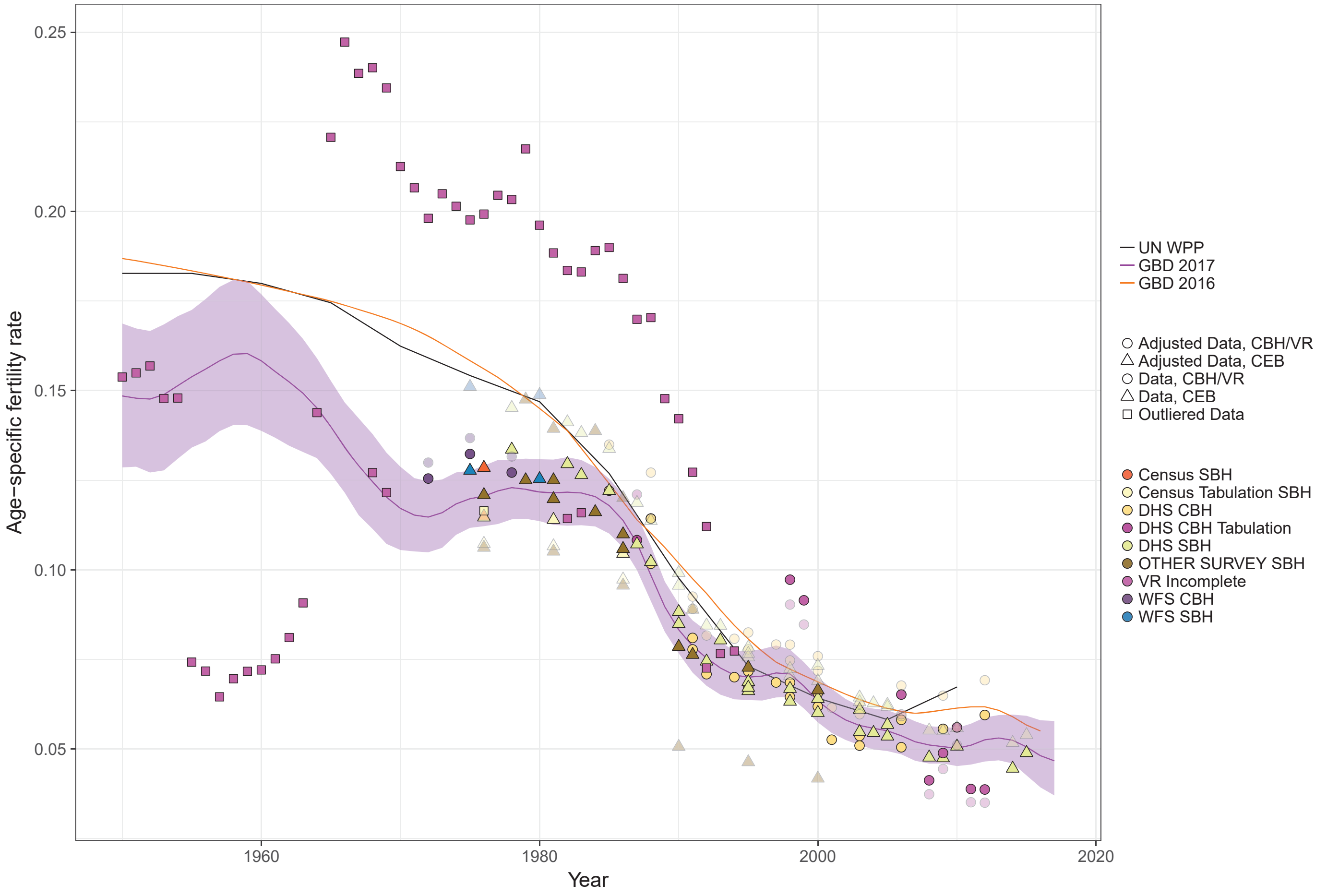

Age−specific fertility fitted trend lines, 1950−2017: Egypt, Ages: 40−44
Data points enclosed with a diamond are reference sources

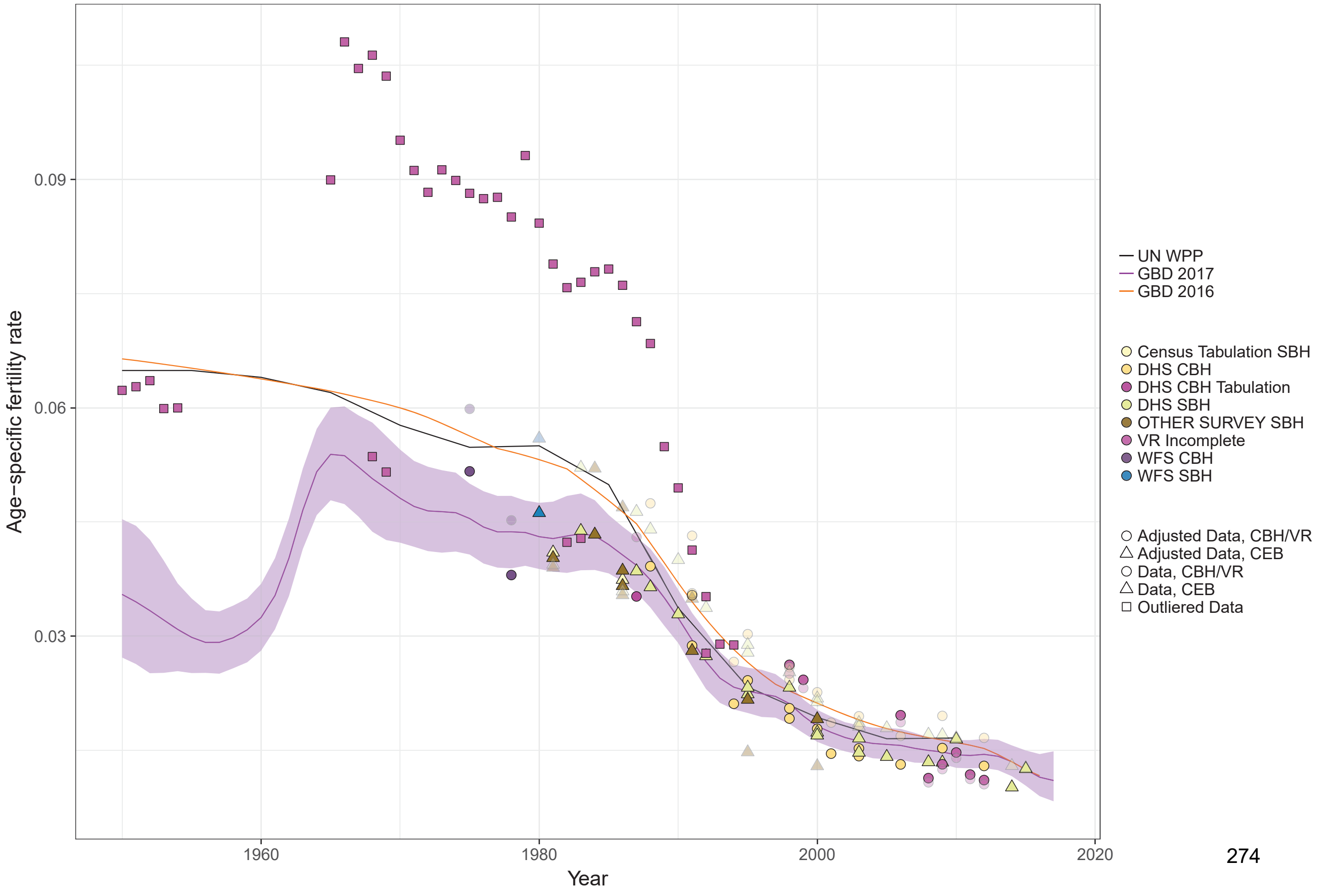

Age−specific fertility fitted trend lines, 1950−2017: Egypt, Ages: 45−49
Data points enclosed with a diamond are reference sources

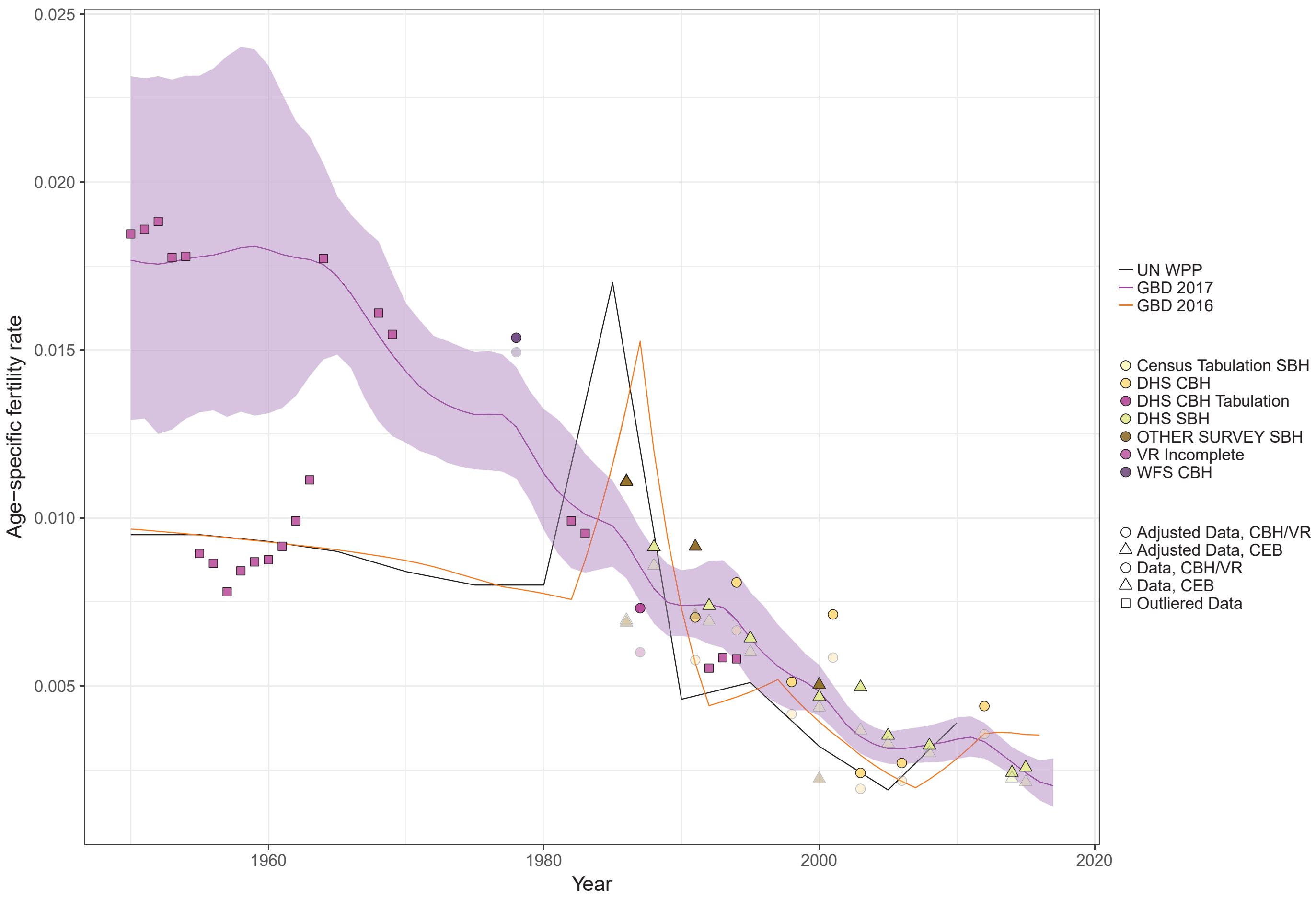

Age−specific fertility fitted trend lines, 1950−2017: Egypt, Ages: 50−54
Data points enclosed with a diamond are reference sources

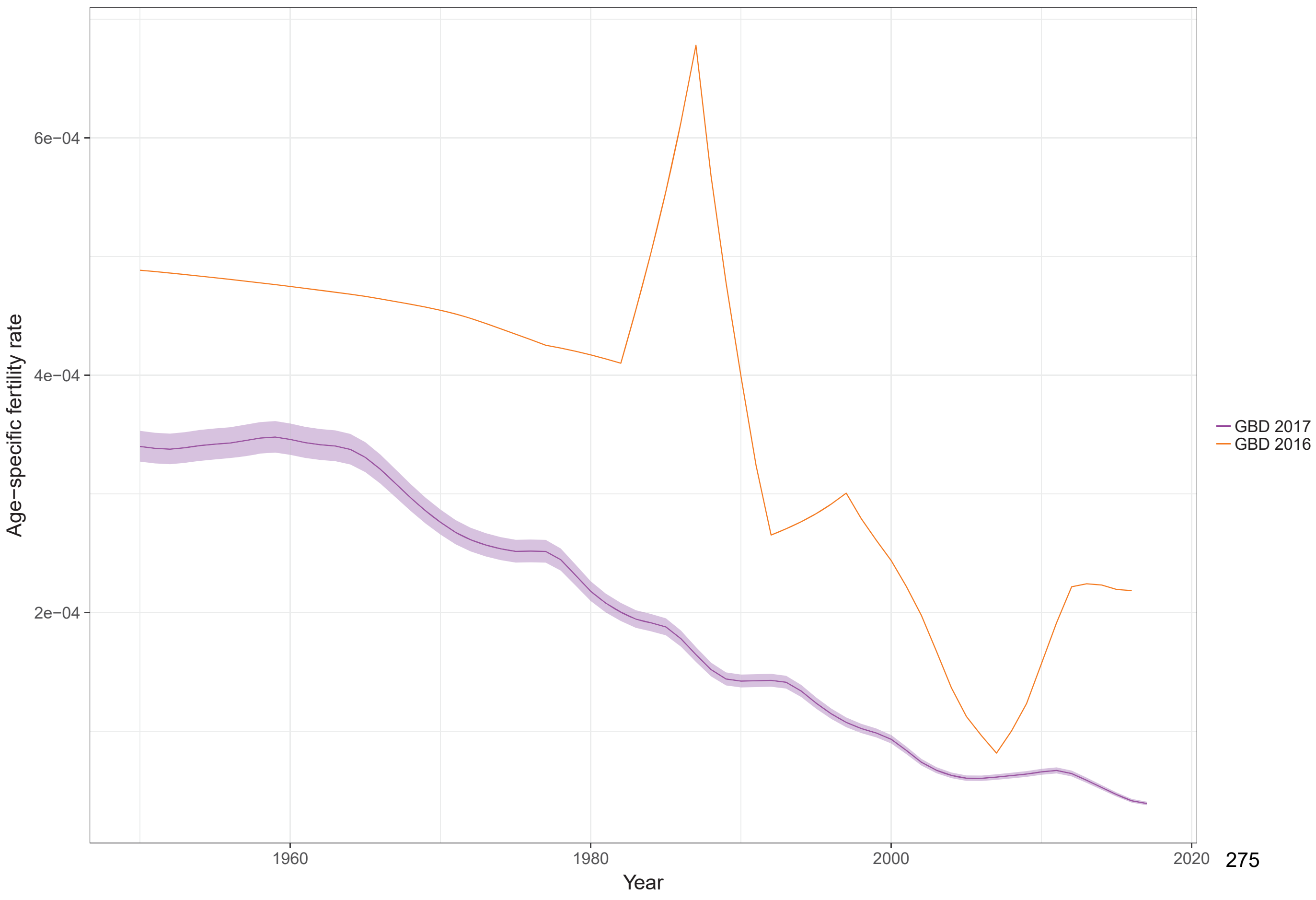

Age−specific fertility fitted trend lines, 1950−2017: El Salvador, TFR
Data points enclosed with a diamond are reference sources

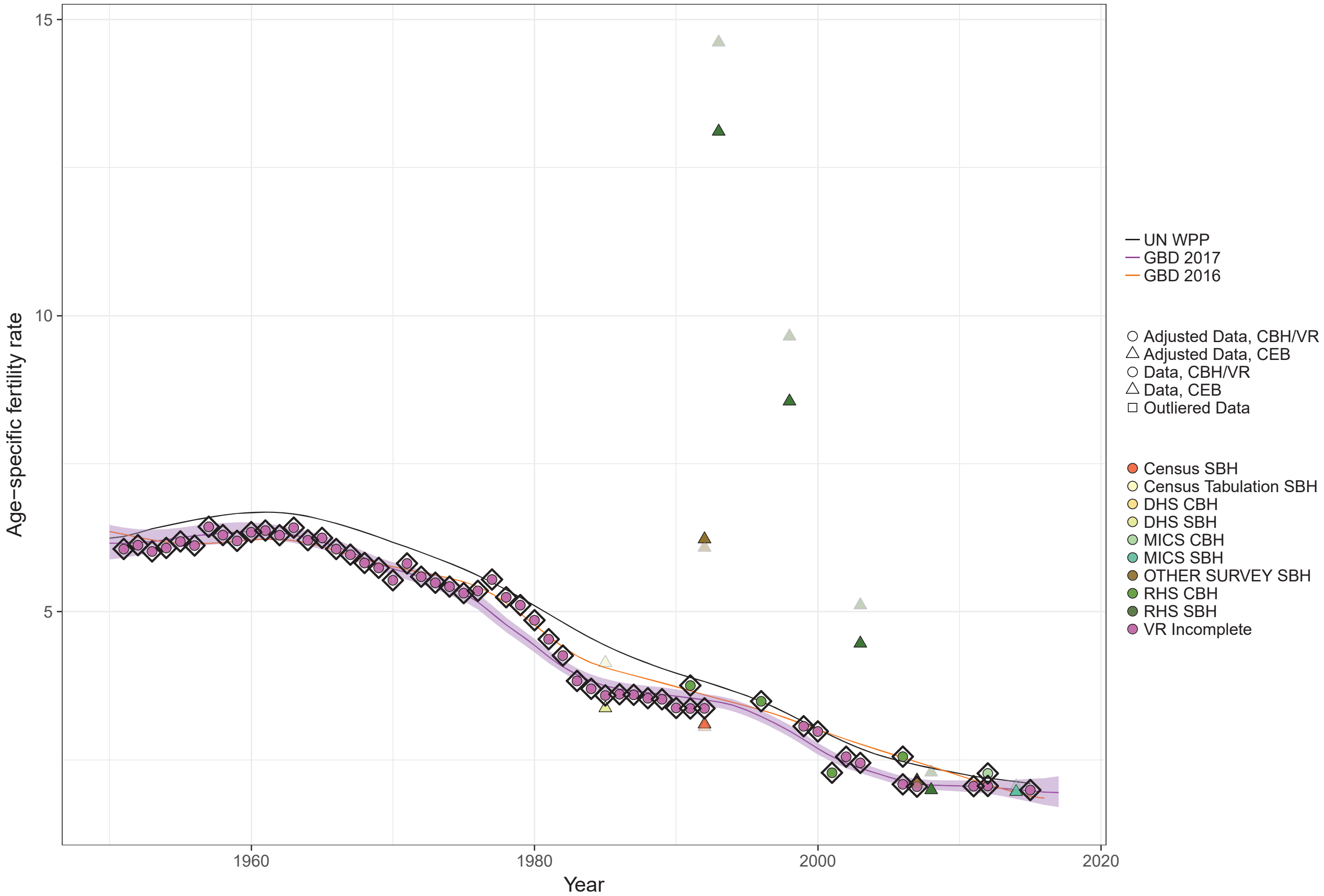

Age−specific fertility fitted trend lines, 1950−2017: El Salvador, Ages: 10−14
Data points enclosed with a diamond are reference sources

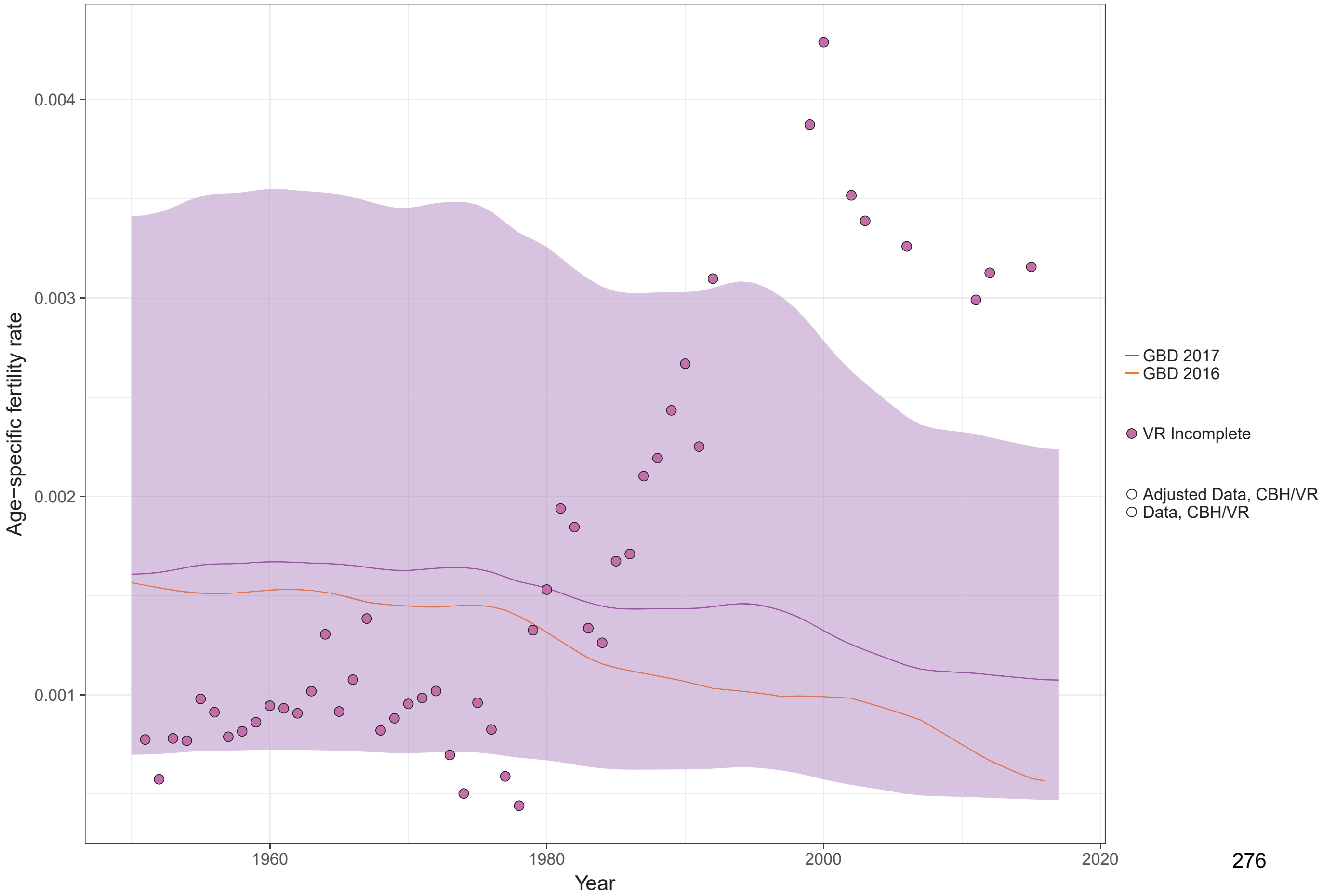

Age−specific fertility fitted trend lines, 1950−2017: El Salvador, Ages: 15−19
Data points enclosed with a diamond are reference sources

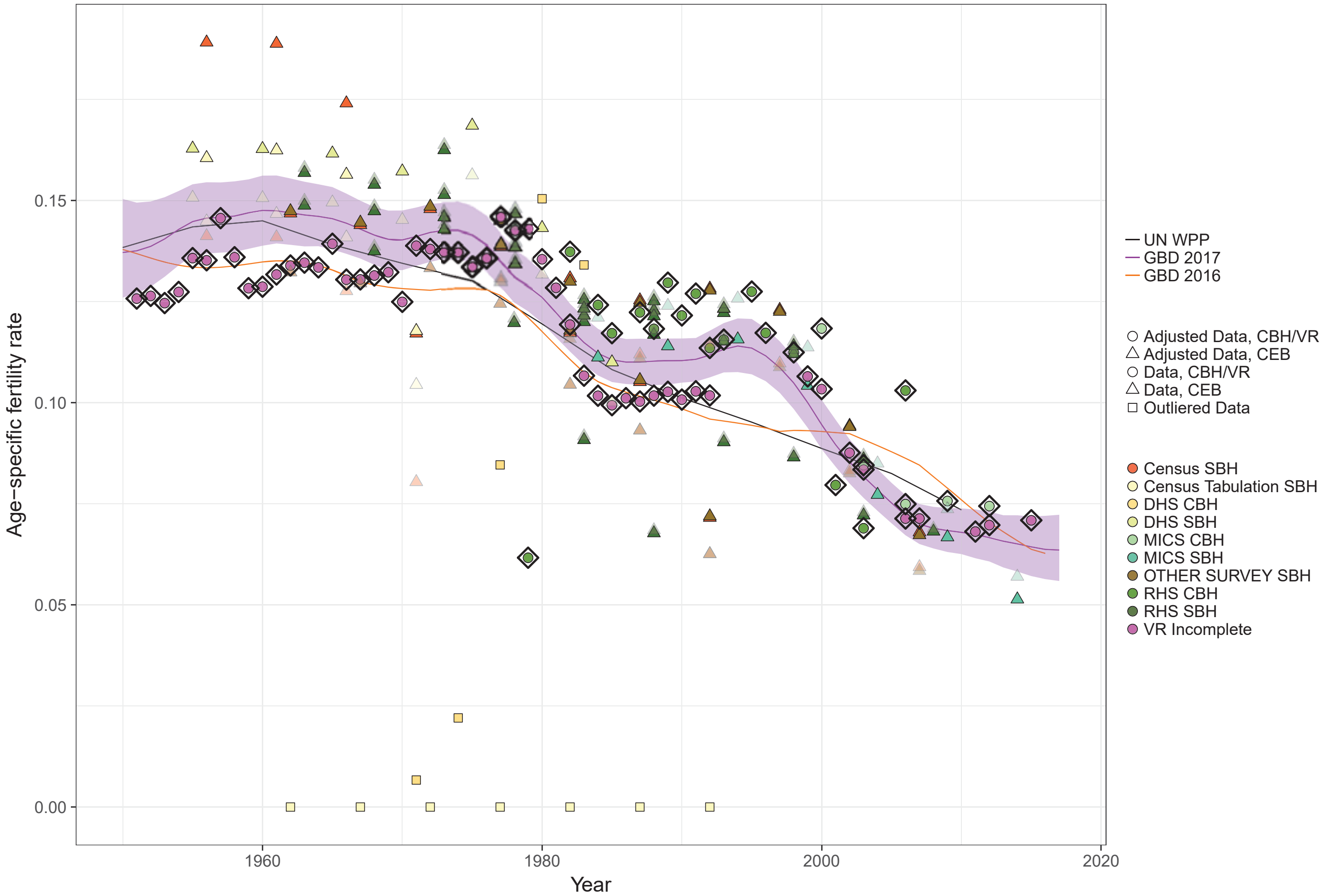

Age−specific fertility fitted trend lines, 1950−2017: El Salvador, Ages: 20−24
Data points enclosed with a diamond are reference sources

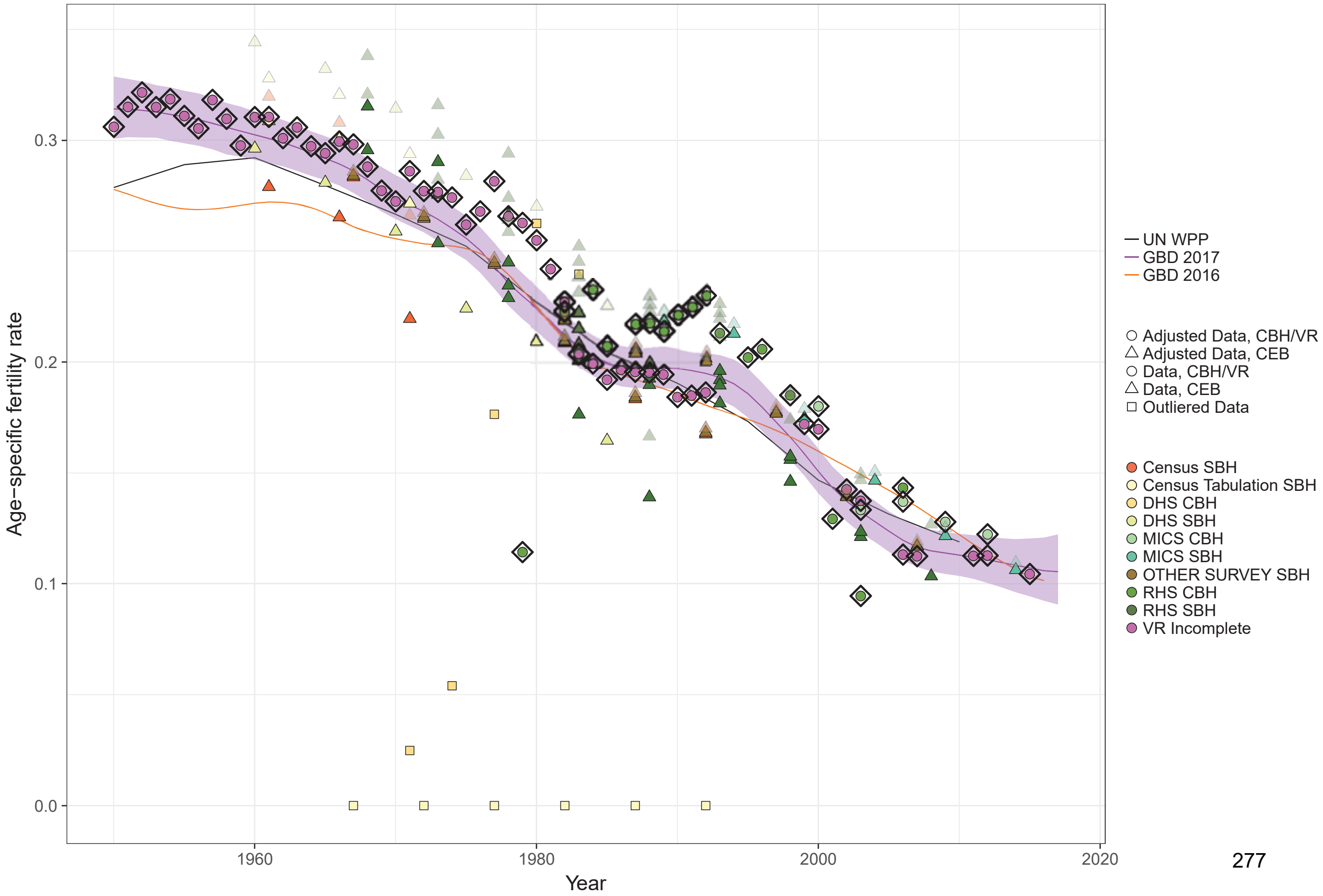

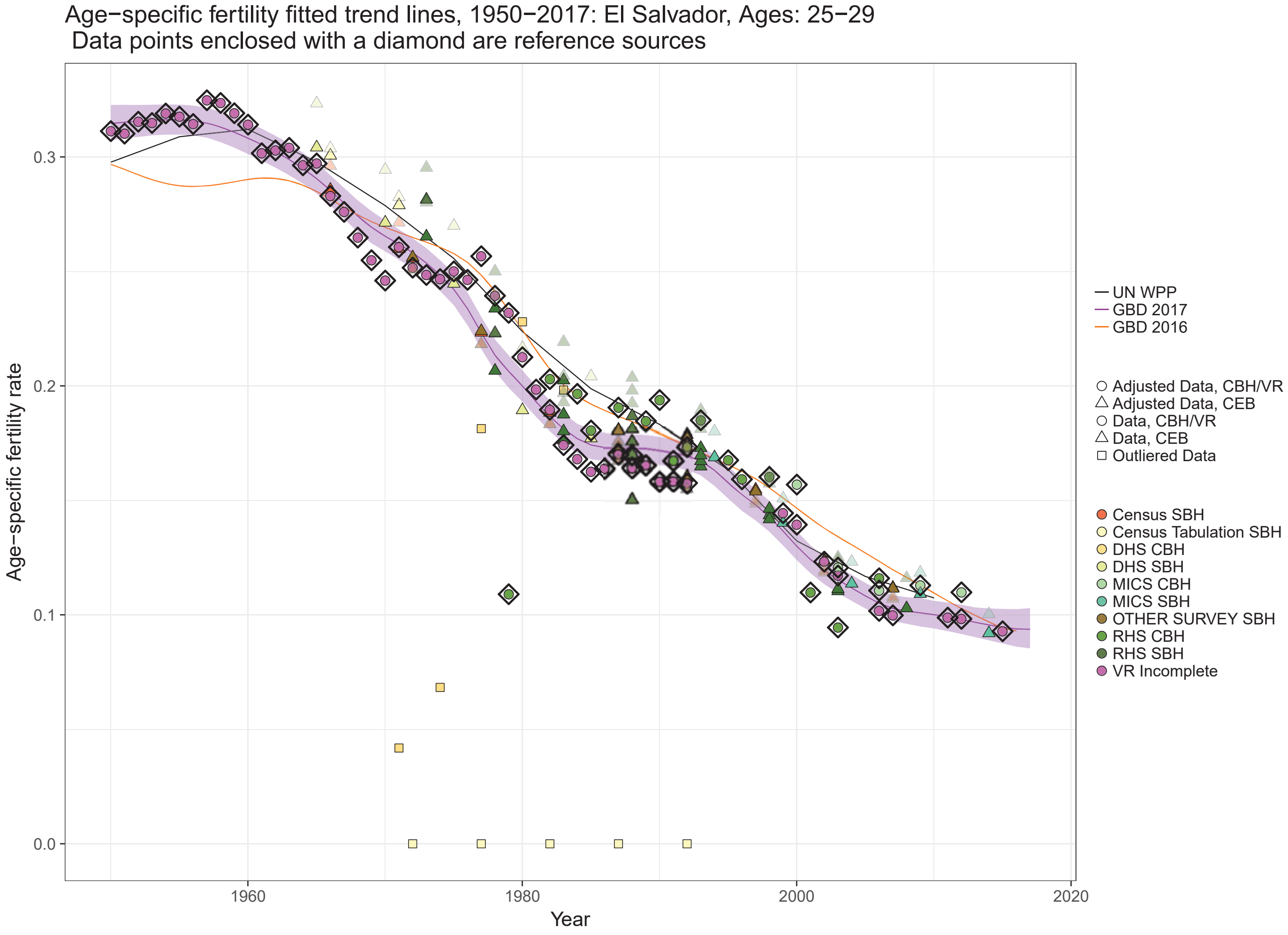
Age−specific fertility fitted trend lines, 1950−2017: El Salvador, Ages: 25−29
Data points enclosed with a diamond are reference sources
Age−specific fertility rate
0.0
0.1
0.2
0.3
1960
1980
2000
2020
Year
UN WPP
GBD 2017
GBD 2016
Adjusted Data, CBH/VR
Adjusted Data, CEB
Data, CBH/VR
Data, CEB
Outliered Data
Census SBH
Census Tabulation SBH
DHS CBH
DHS SBH
MICS CBH
MICS SBH
OTHER SURVEY SBH
RHS CBH
RHS SBH
VR Incomplete

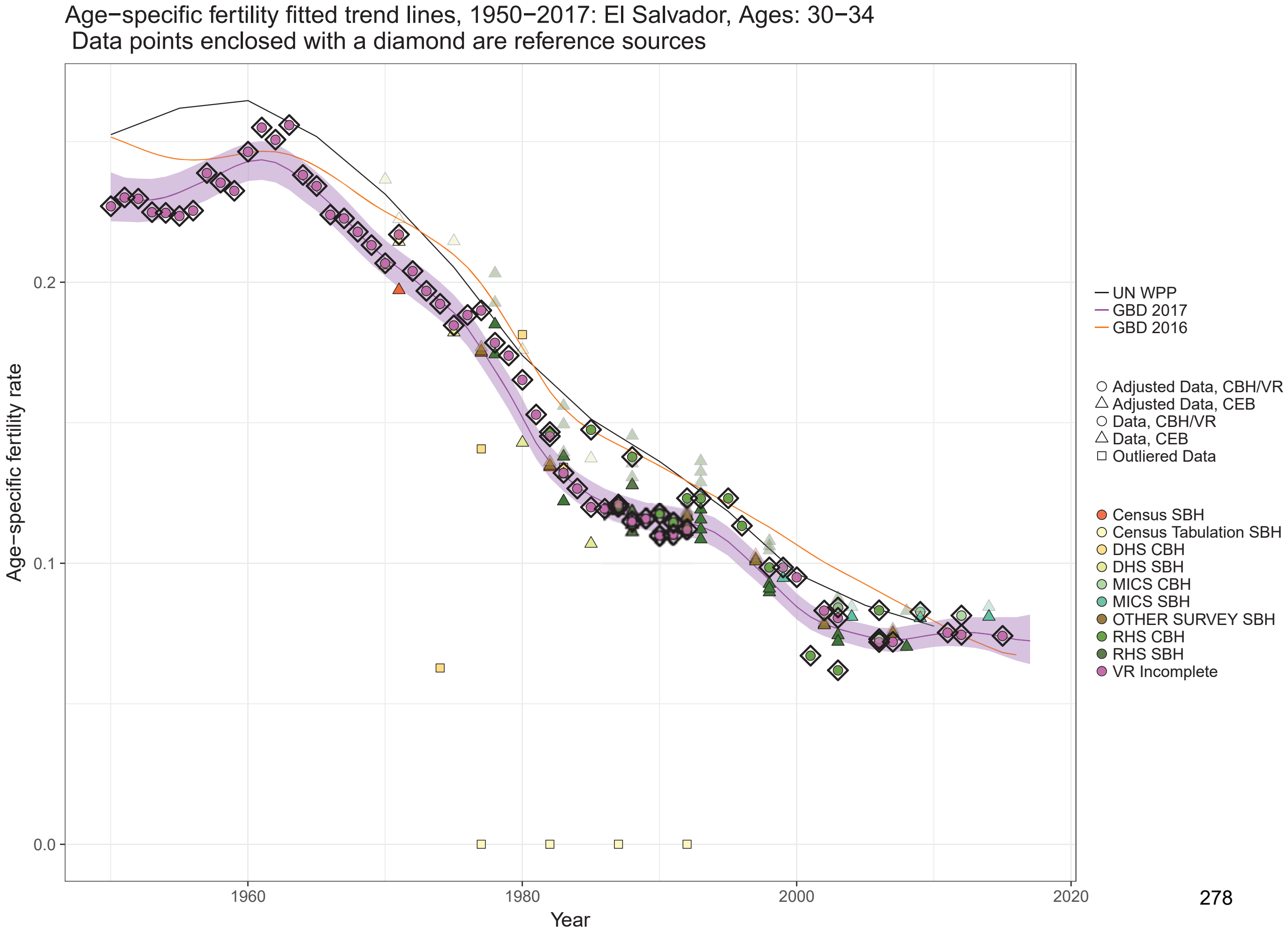
Age−specific fertility fitted trend lines, 1950−2017: El Salvador, Ages: 30−34
Data points enclosed with a diamond are reference sources
Age−specific fertility rate
0.0
0.1
0.2
1960
1980
2000
2020
Year
UN WPP
GBD 2017
GBD 2016
Adjusted Data, CBH/VR
Adjusted Data, CEB
Data, CBH/VR
Data, CEB
Outliered Data
Census SBH
Census Tabulation SBH
DHS CBH
DHS SBH
MICS CBH
MICS SBH
OTHER SURVEY SBH
RHS CBH
RHS SBH
VR Incomplete

Age−specific fertility fitted trend lines, 1950−2017: El Salvador, Ages: 35−39
Data points enclosed with a diamond are reference sources

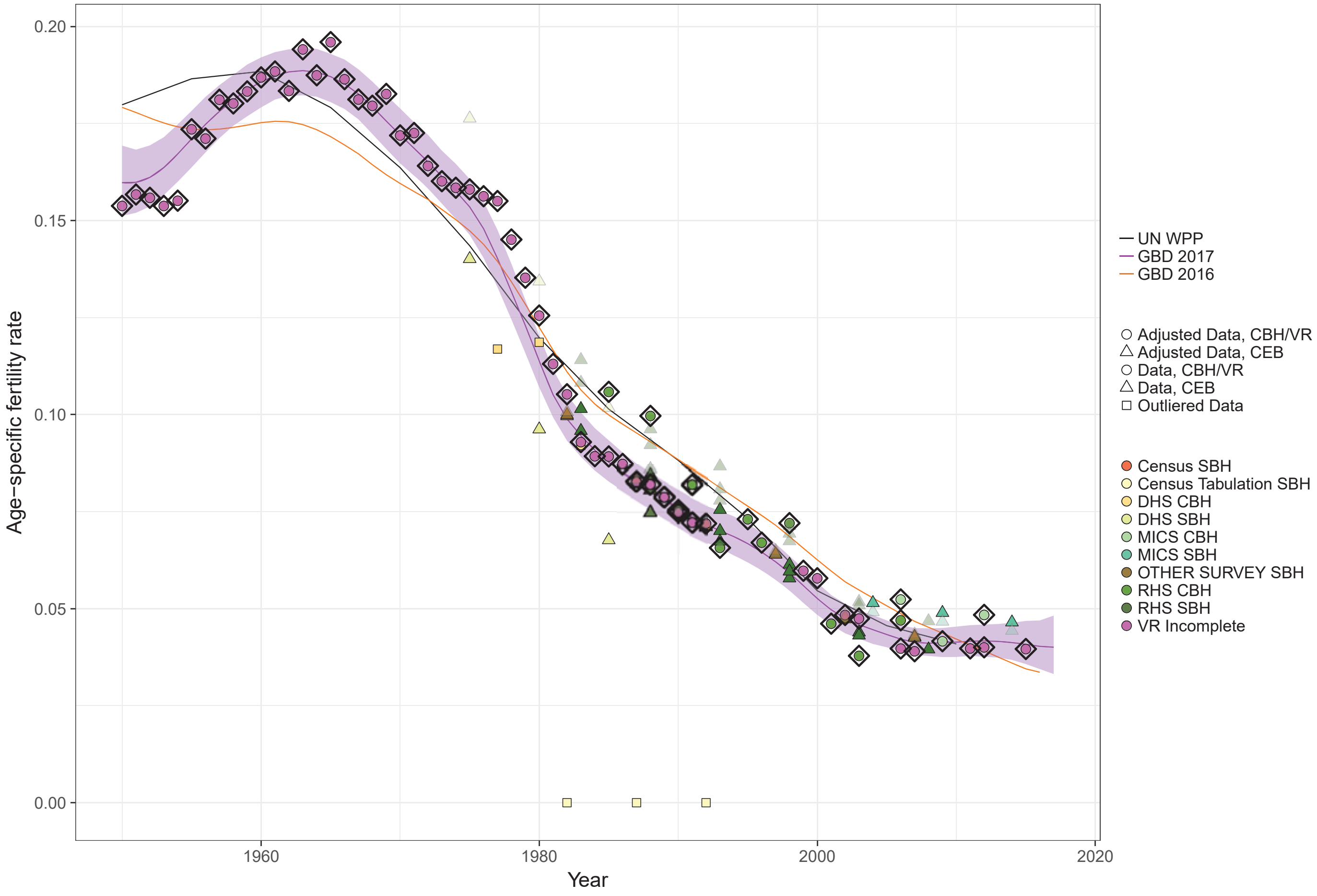

Age−specific fertility fitted trend lines, 1950−2017: El Salvador, Ages: 40−44
Data points enclosed with a diamond are reference sources

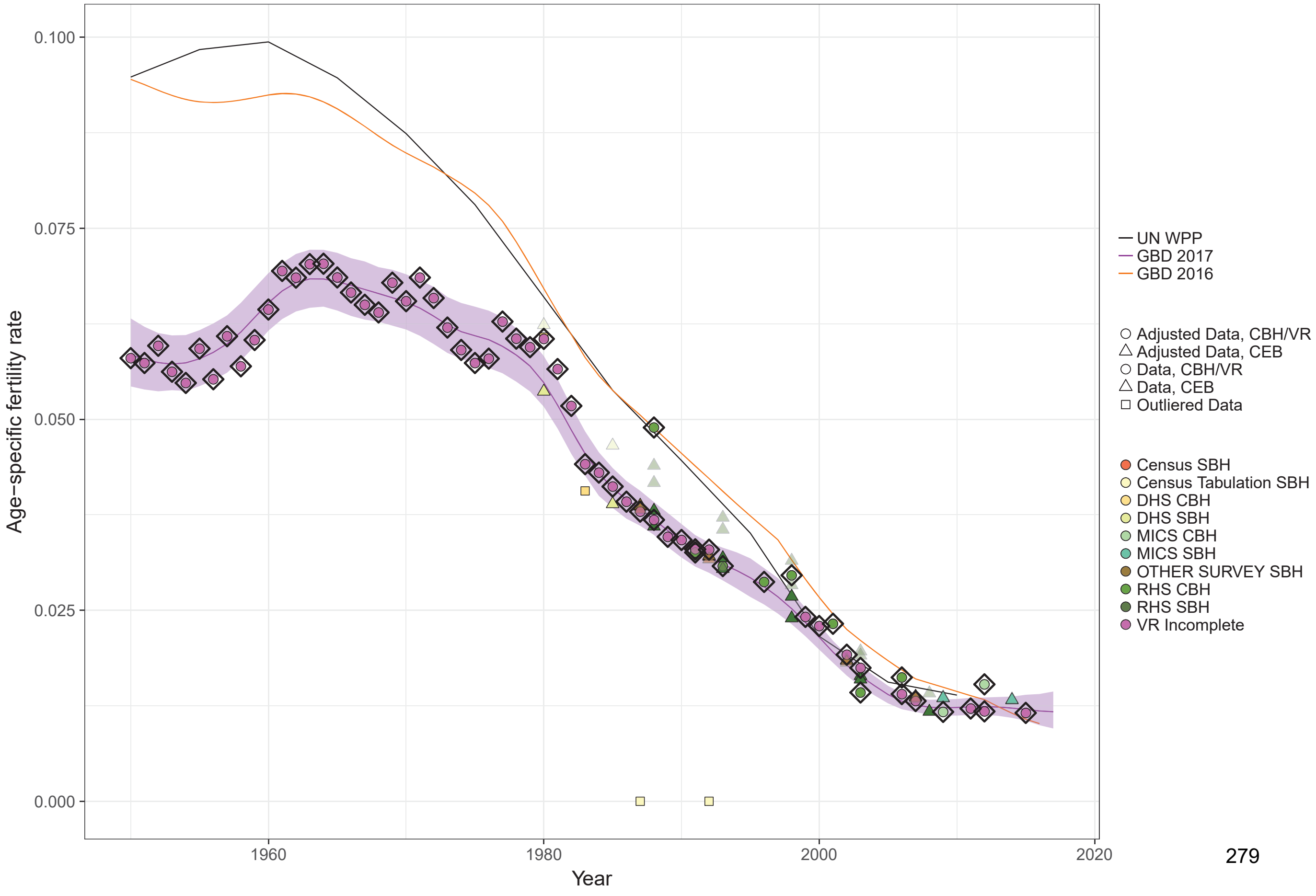

Age−specific fertility fitted trend lines, 1950−2017: El Salvador, Ages: 45−49
Data points enclosed with a diamond are reference sources

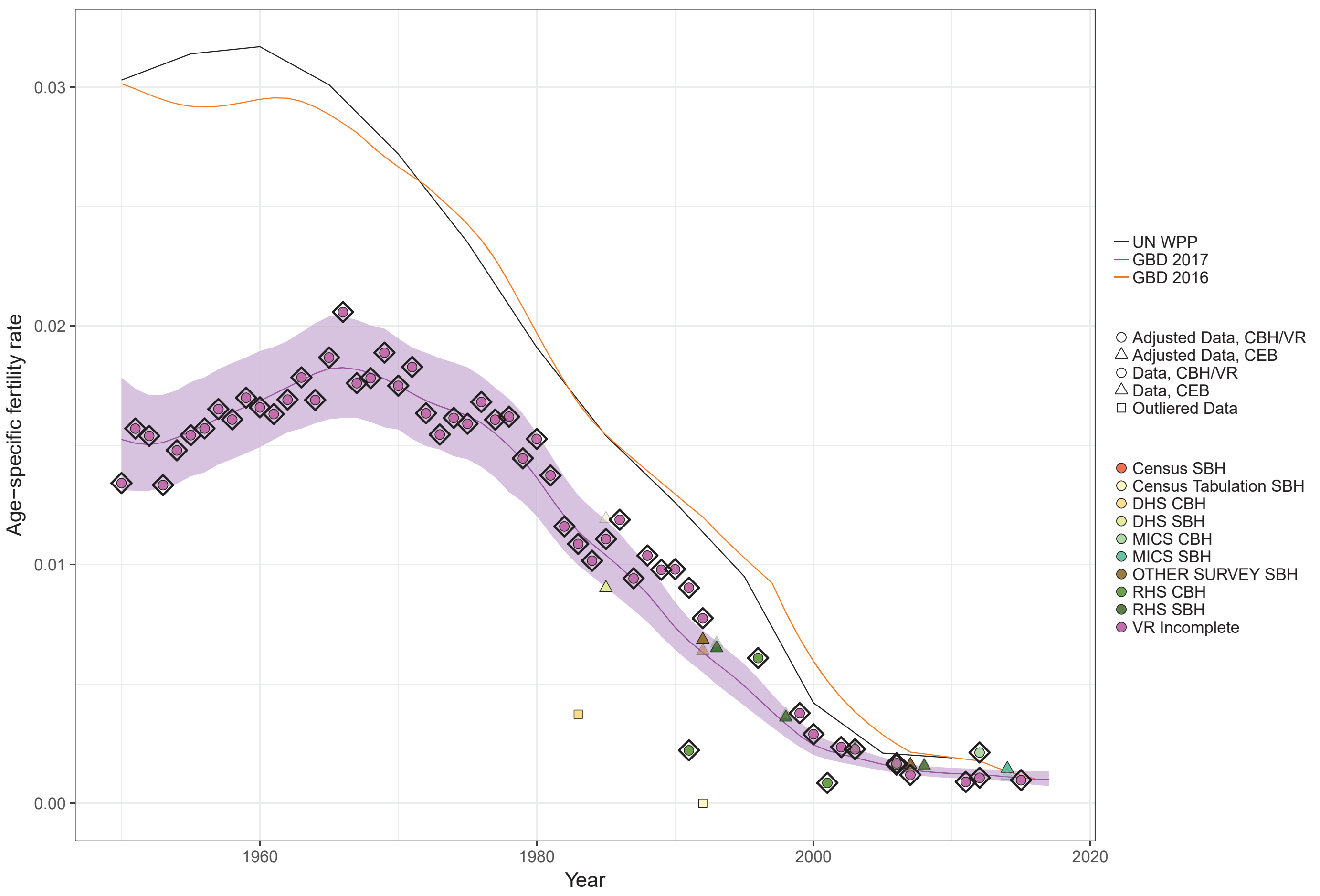

Age−specific fertility fitted trend lines, 1950−2017: El Salvador, Ages: 50−54
Data points enclosed with a diamond are reference sources

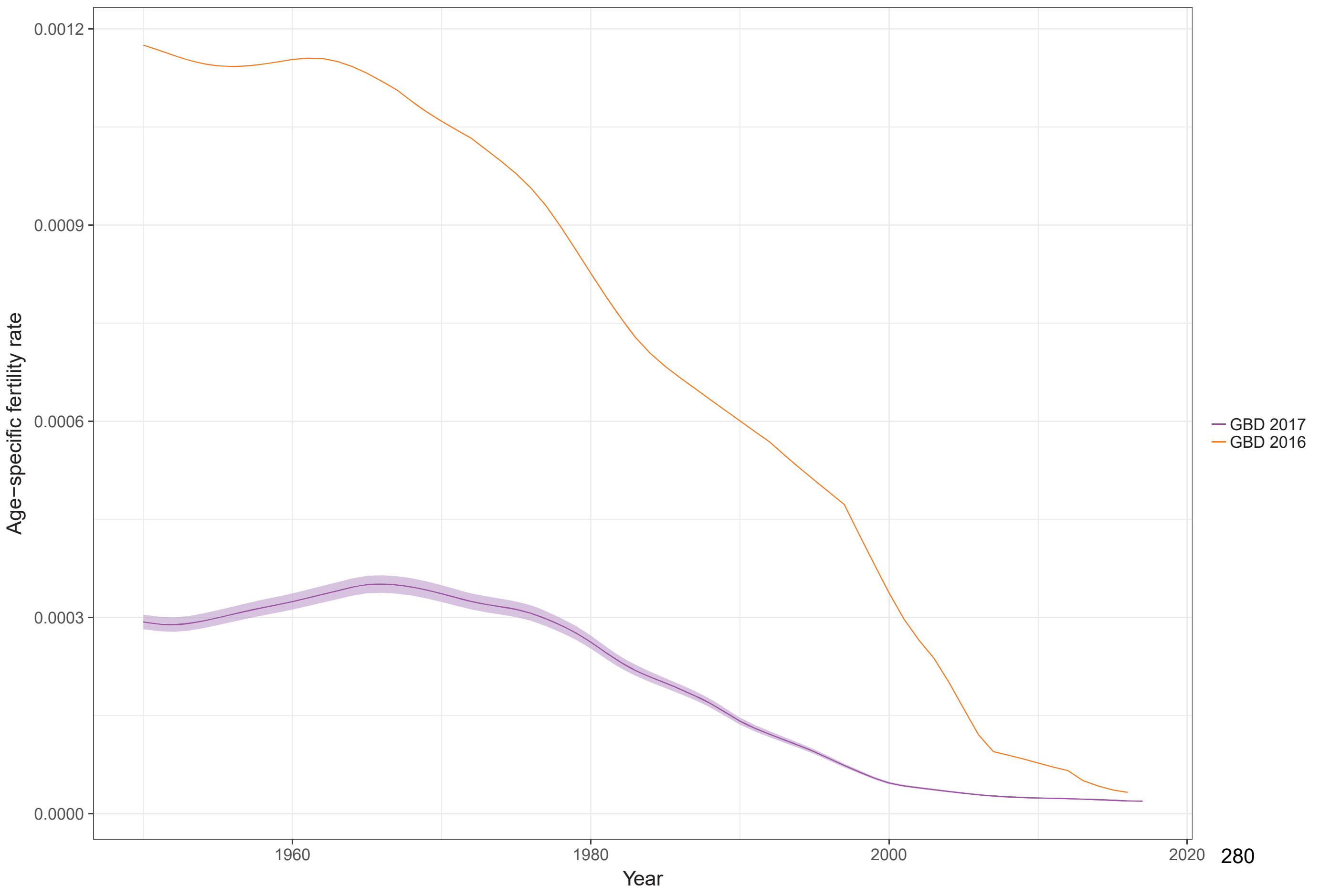

Age−specific fertility fitted trend lines, 1950−2017: England, TFR
Data points enclosed with a diamond are reference sources

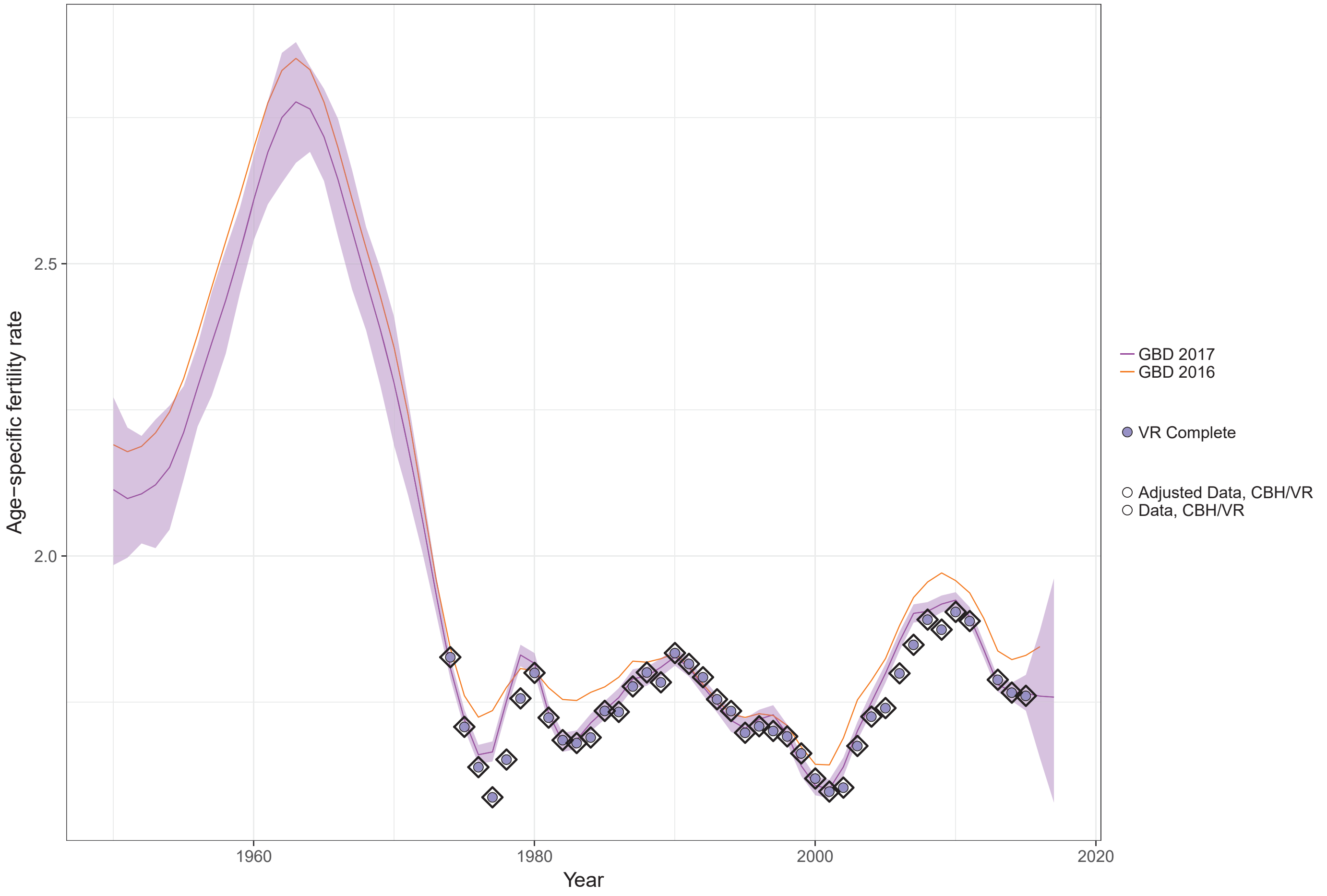

Age−specific fertility fitted trend lines, 1950−2017: England, Ages: 10−14
Data points enclosed with a diamond are reference sources

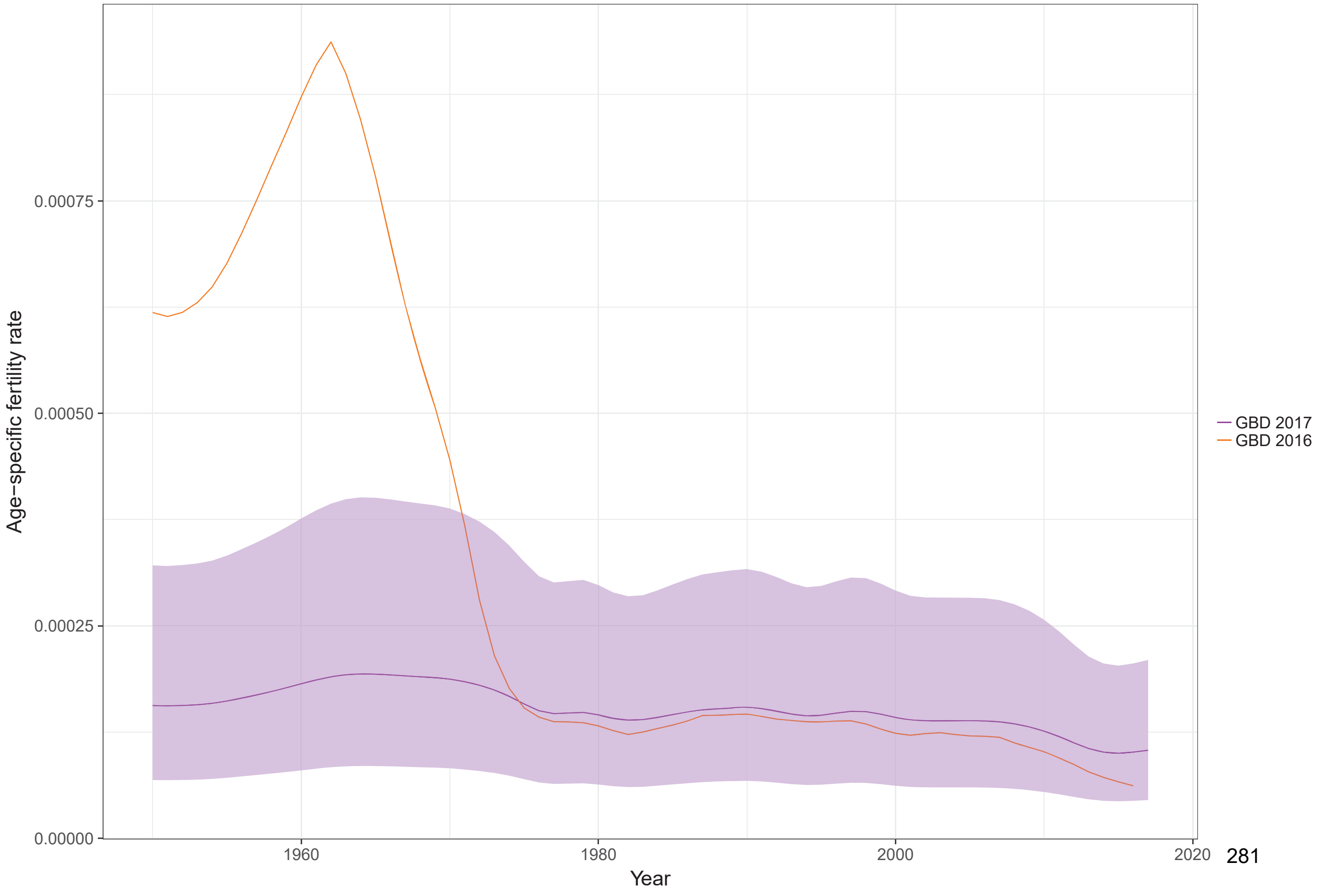

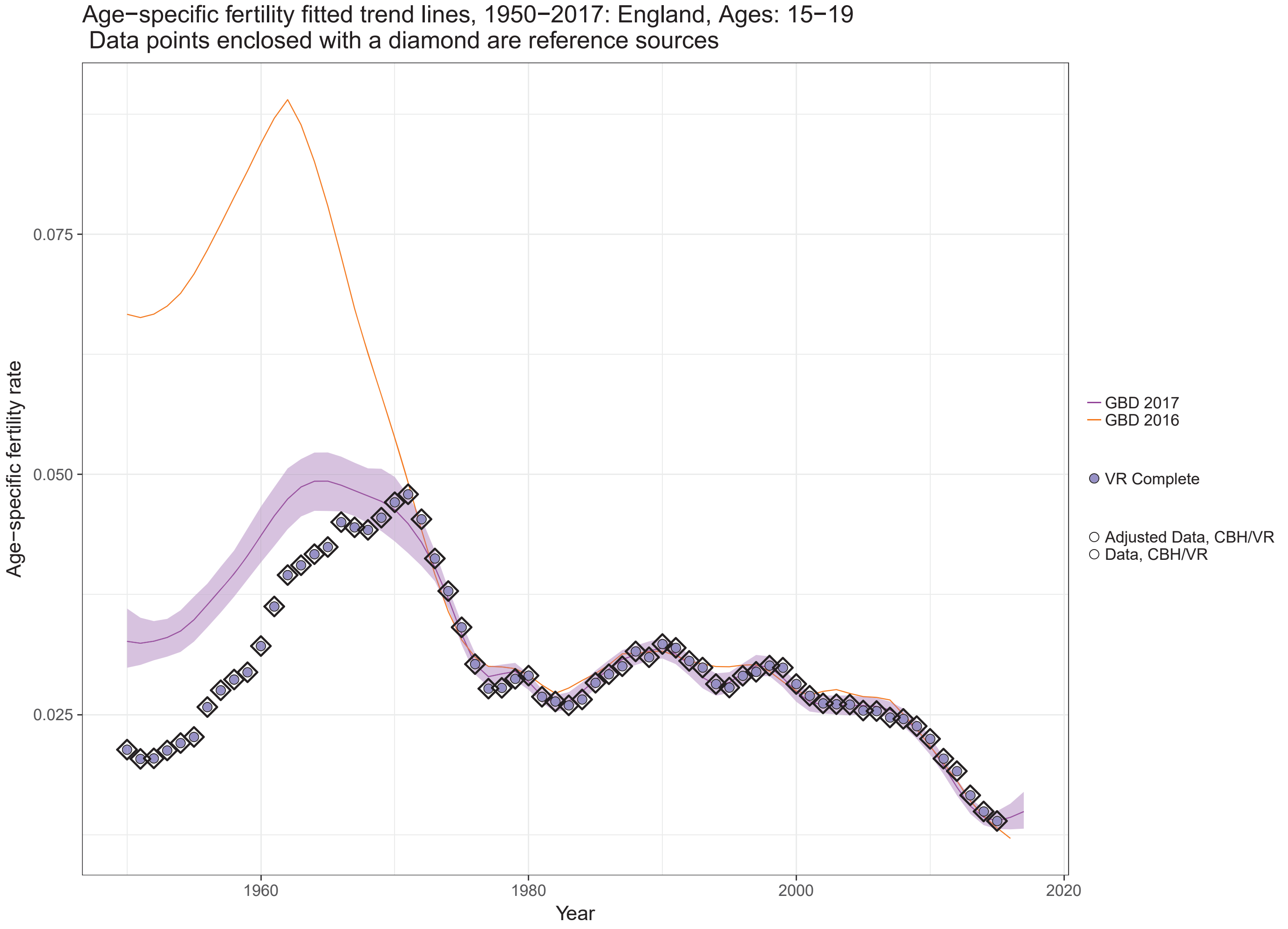

Age−specific fertility fitted trend lines, 1950−2017: England, Ages: 15−19
Data points enclosed with a diamond are reference sources
Age−specific fertility rate
0.075
0.050
0.025
1960
1980
2000
2020
Year
GBD 2017
GBD 2016
VR Complete
Adjusted Data, CBH/VR
Data, CBH/VR

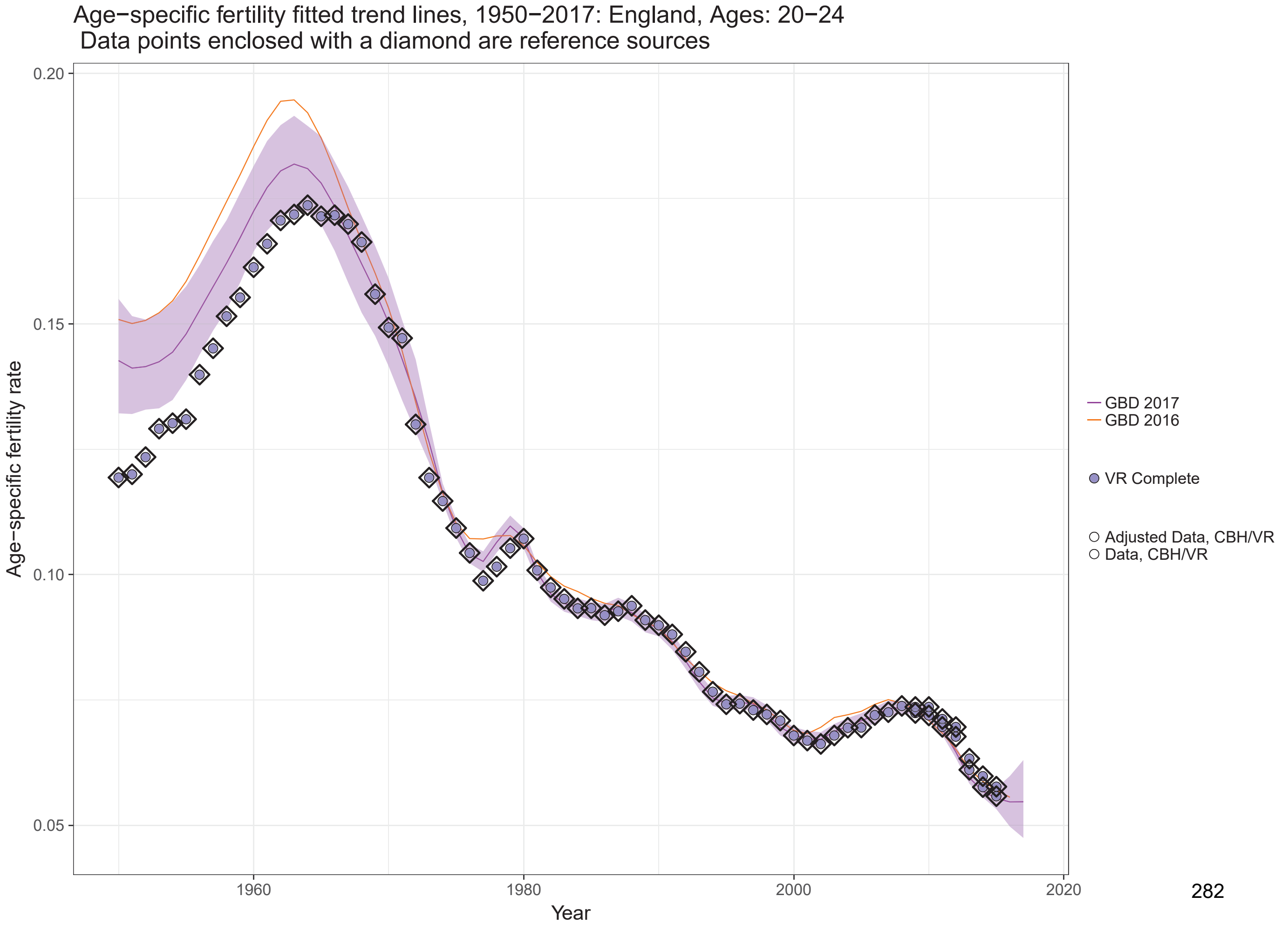

Age−specific fertility fitted trend lines, 1950−2017: England, Ages: 20−24
Data points enclosed with a diamond are reference sources
Age−specific fertility rate
0.20
0.15
0.10
0.05
1960
1980
2000
2020
Year
GBD 2017
GBD 2016
VR Complete
Adjusted Data, CBH/VR
Data, CBH/VR

Age−specific fertility fitted trend lines, 1950−2017: England, Ages: 25−29
Data points enclosed with a diamond are reference sources

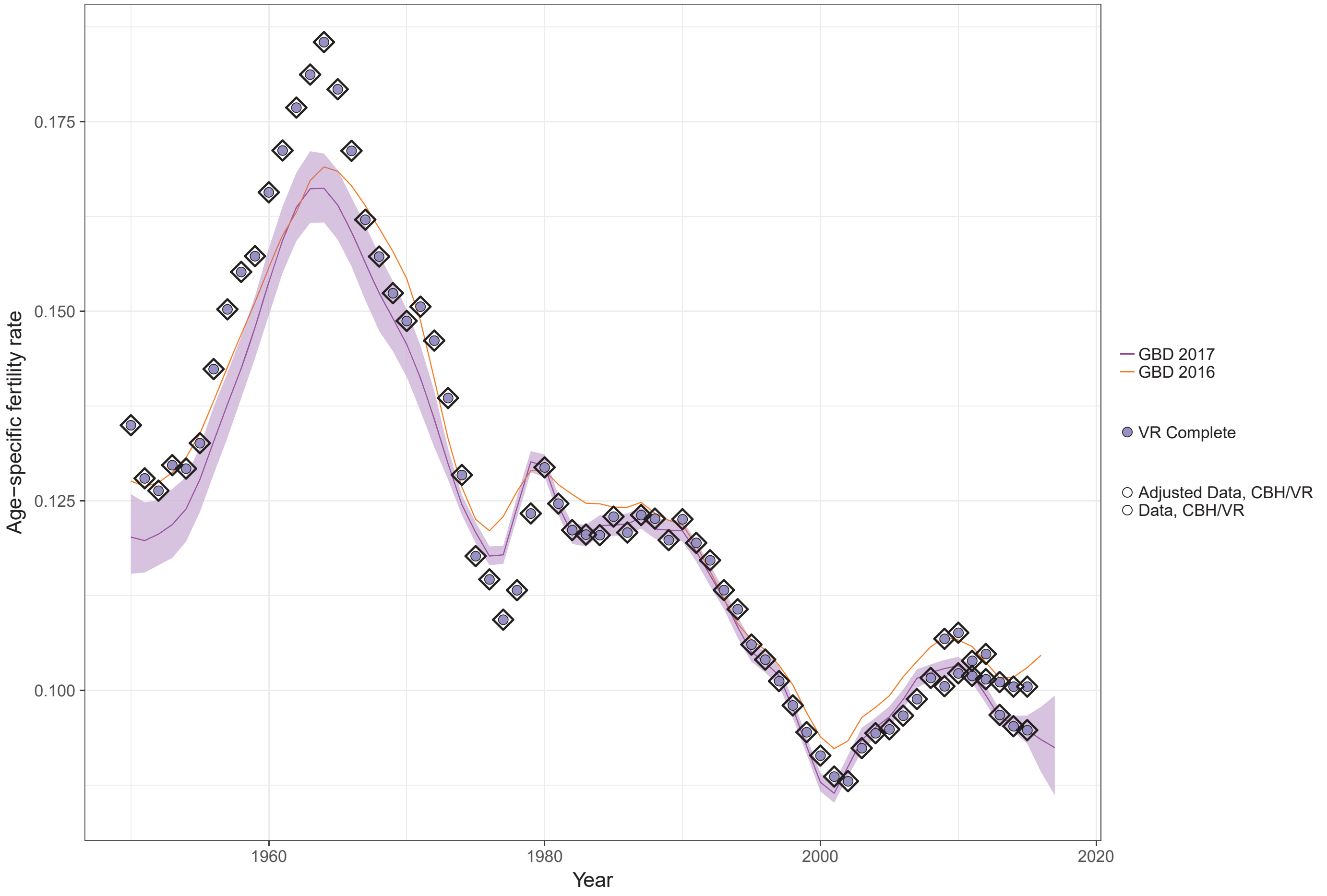

Age−specific fertility fitted trend lines, 1950−2017: England, Ages: 30−34
Data points enclosed with a diamond are reference sources

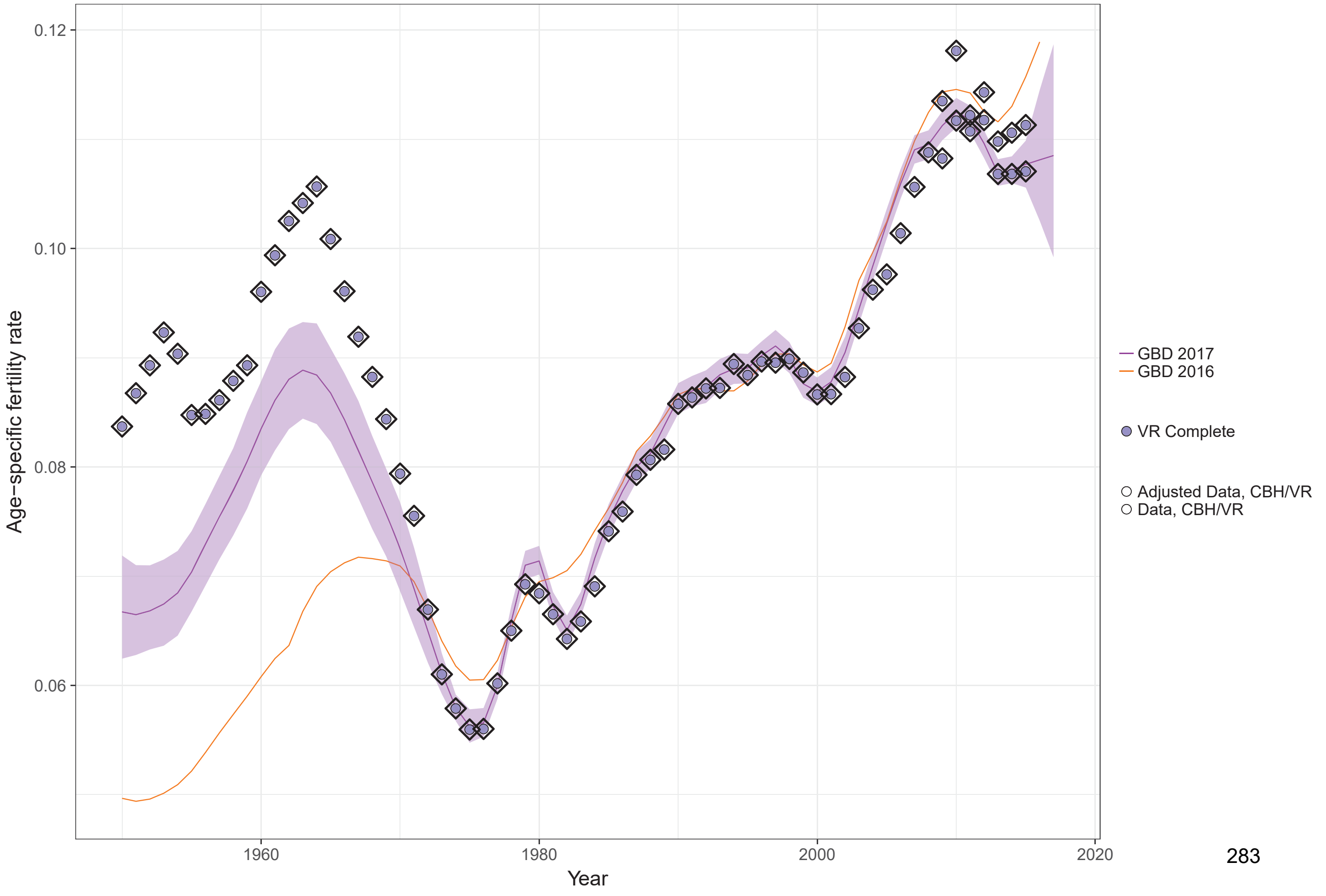

Age−specific fertility fitted trend lines, 1950−2017: England, Ages: 35−39
Data points enclosed with a diamond are reference sources

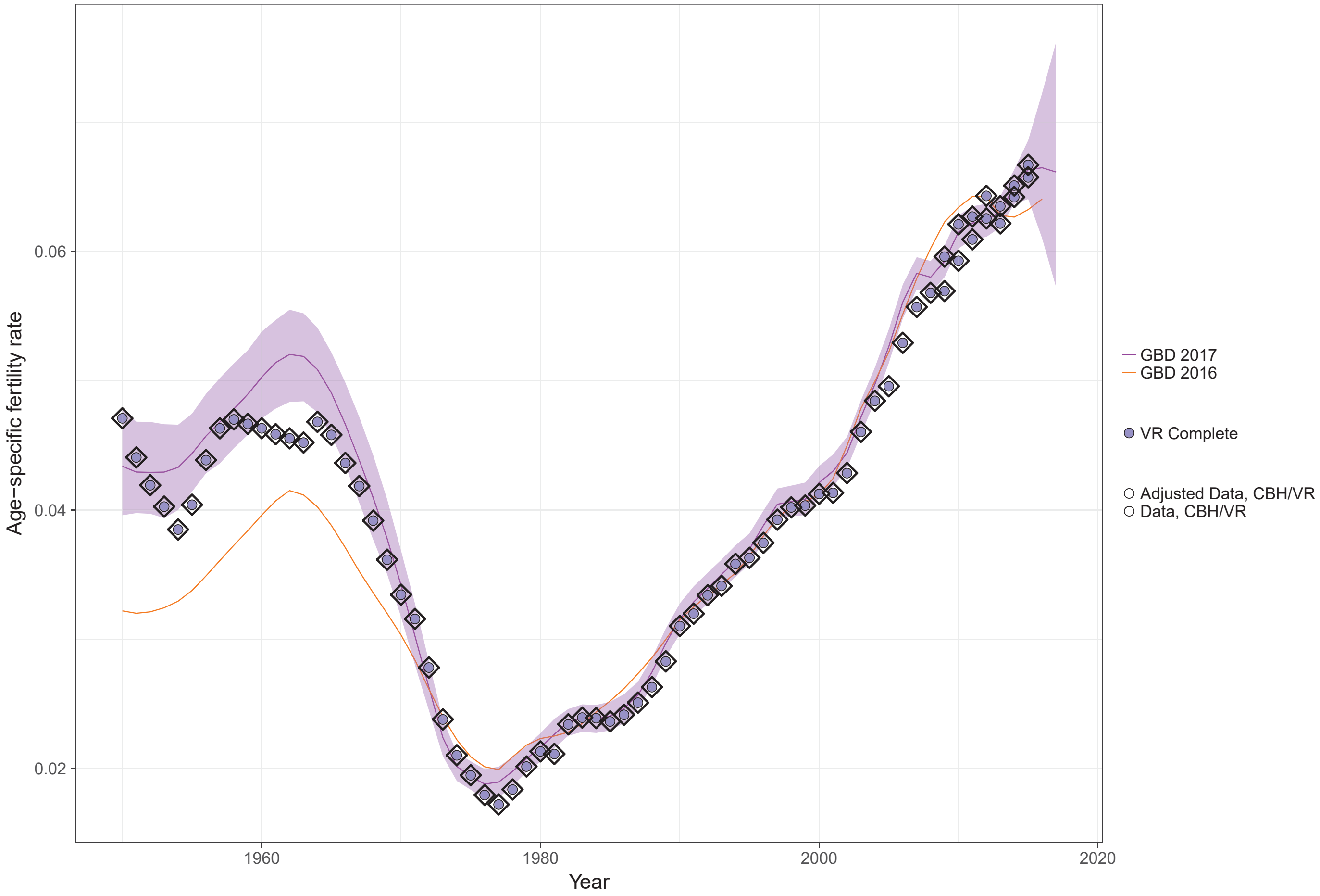

Age−specific fertility fitted trend lines, 1950−2017: England, Ages: 40−44
Data points enclosed with a diamond are reference sources

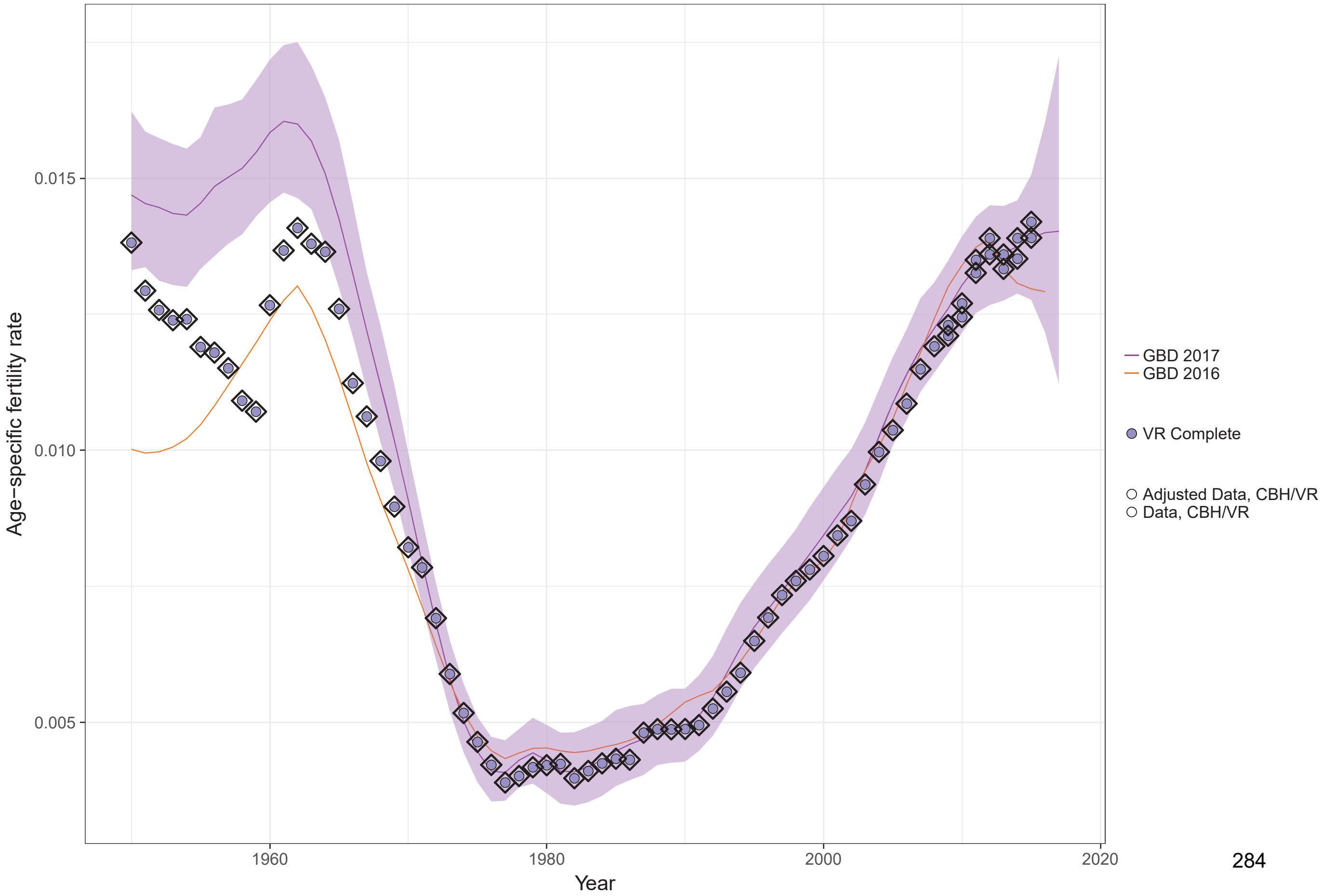

Age−specific fertility fitted trend lines, 1950−2017: England, Ages: 45−49
Data points enclosed with a diamond are reference sources

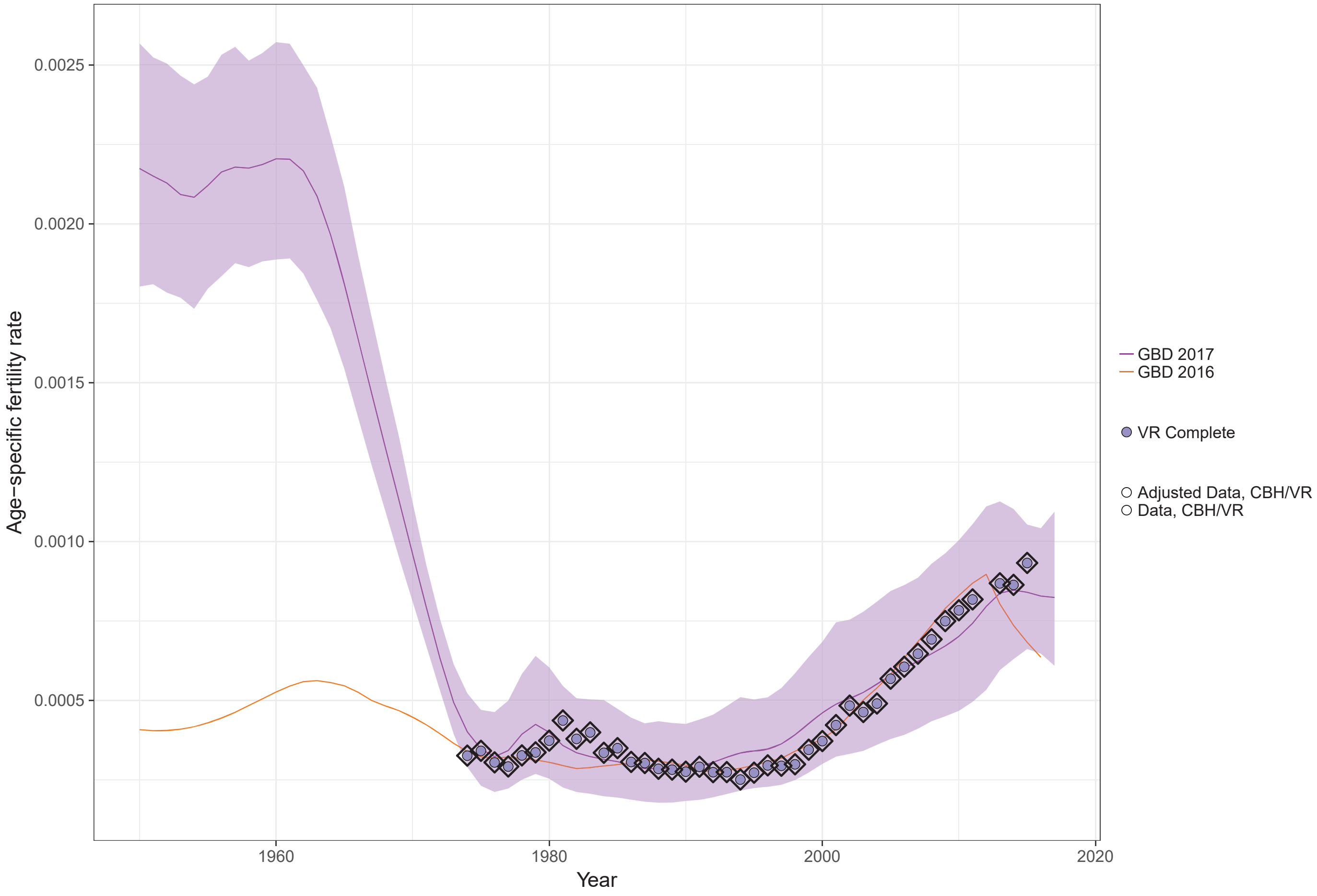

Age−specific fertility fitted trend lines, 1950−2017: England, Ages: 50−54
Data points enclosed with a diamond are reference sources

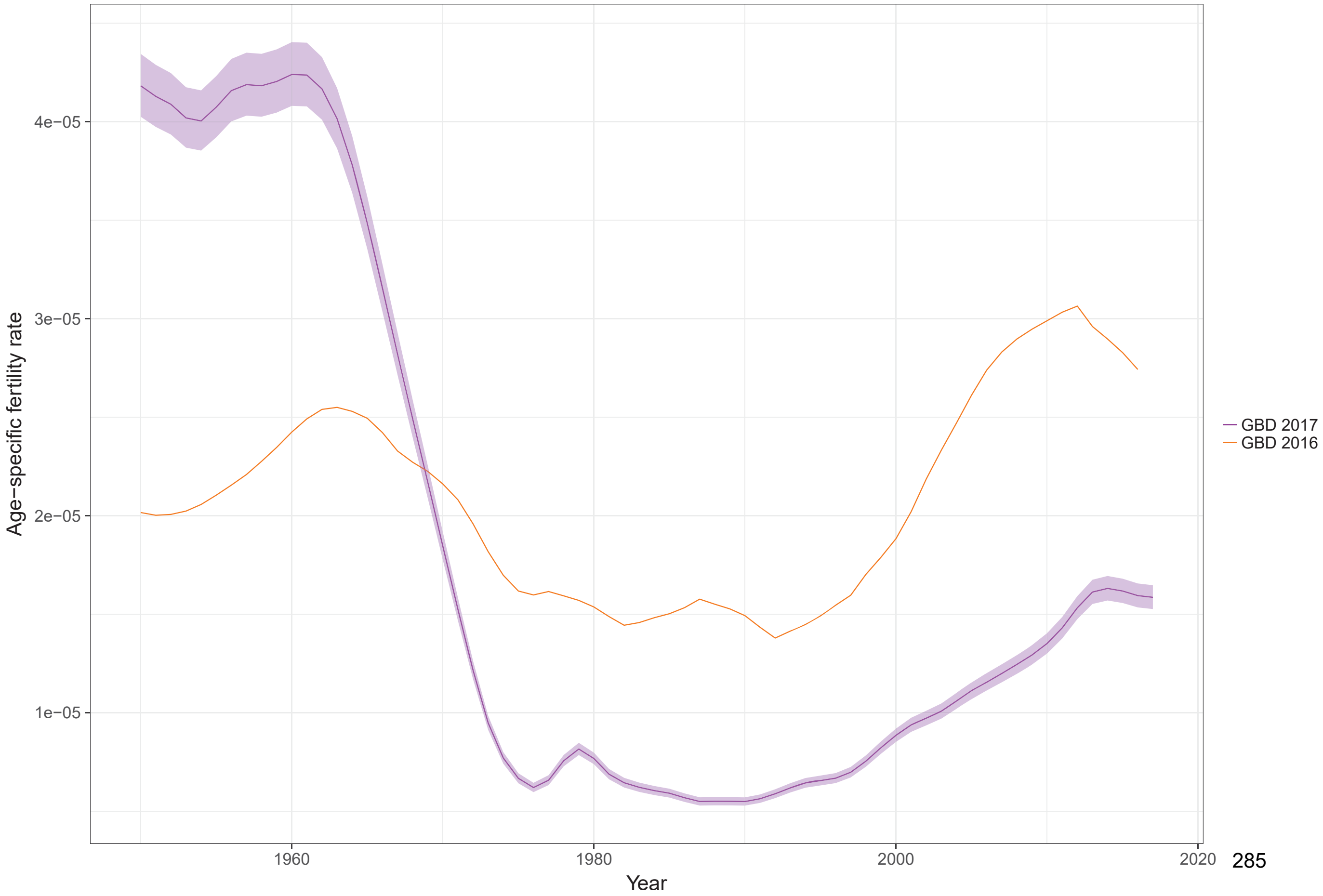

Age−specific fertility fitted trend lines, 1950−2017: Equatorial Guinea, TFR
Data points enclosed with a diamond are reference sources

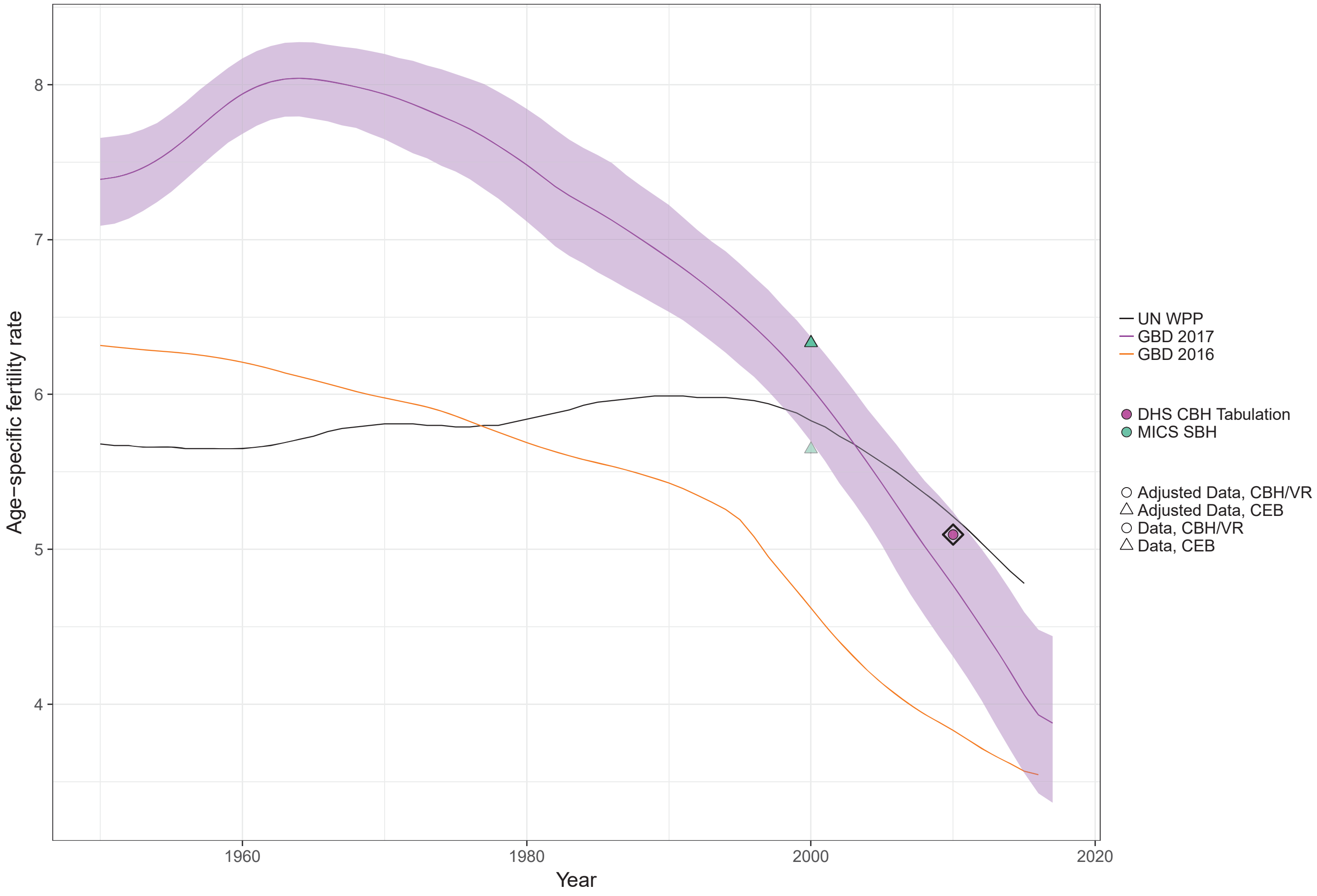

Age−specific fertility fitted trend lines, 1950−2017: Equatorial Guinea, Ages: 10−14
Data points enclosed with a diamond are reference sources

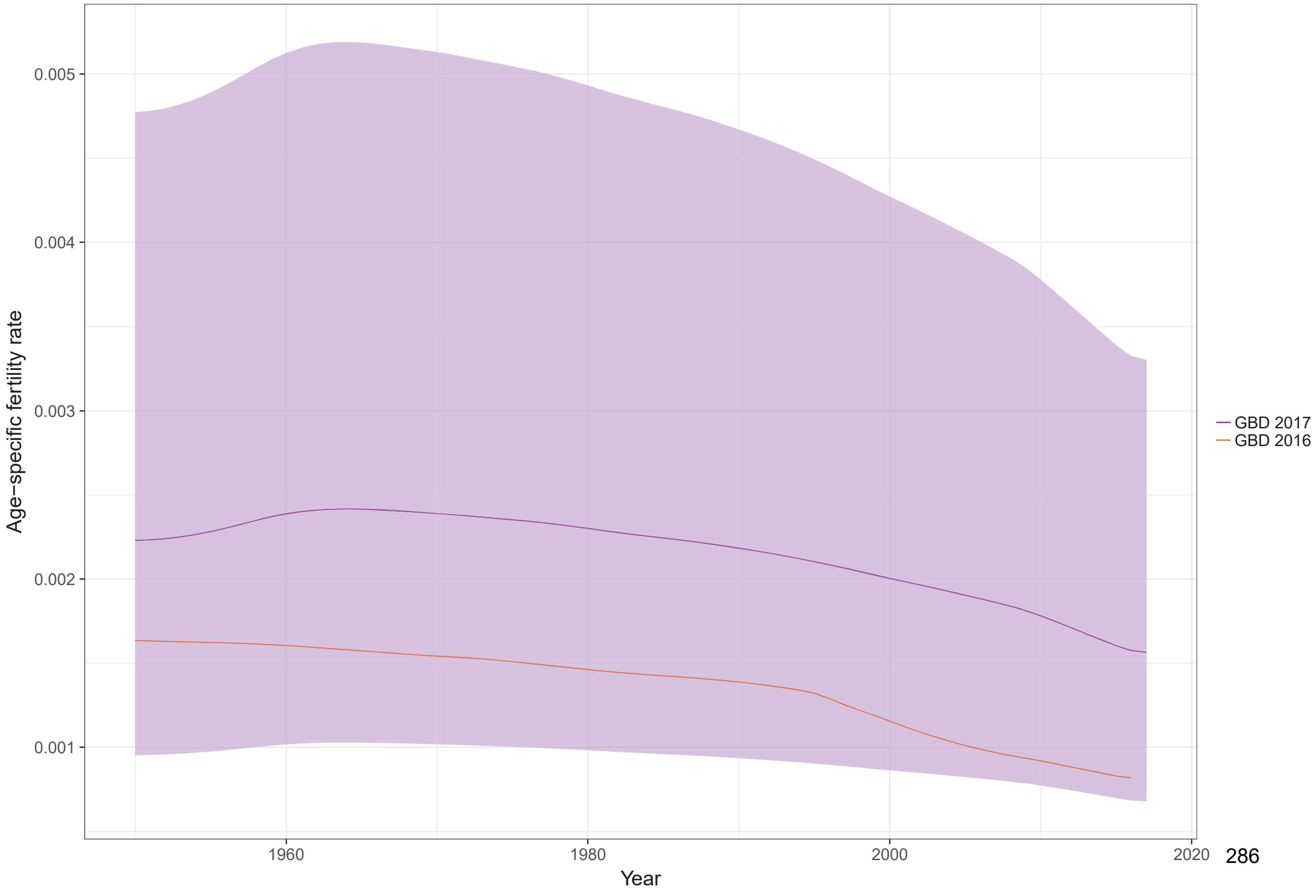

Age−specific fertility fitted trend lines, 1950−2017: Equatorial Guinea, Ages: 15−19
Data points enclosed with a diamond are reference sources

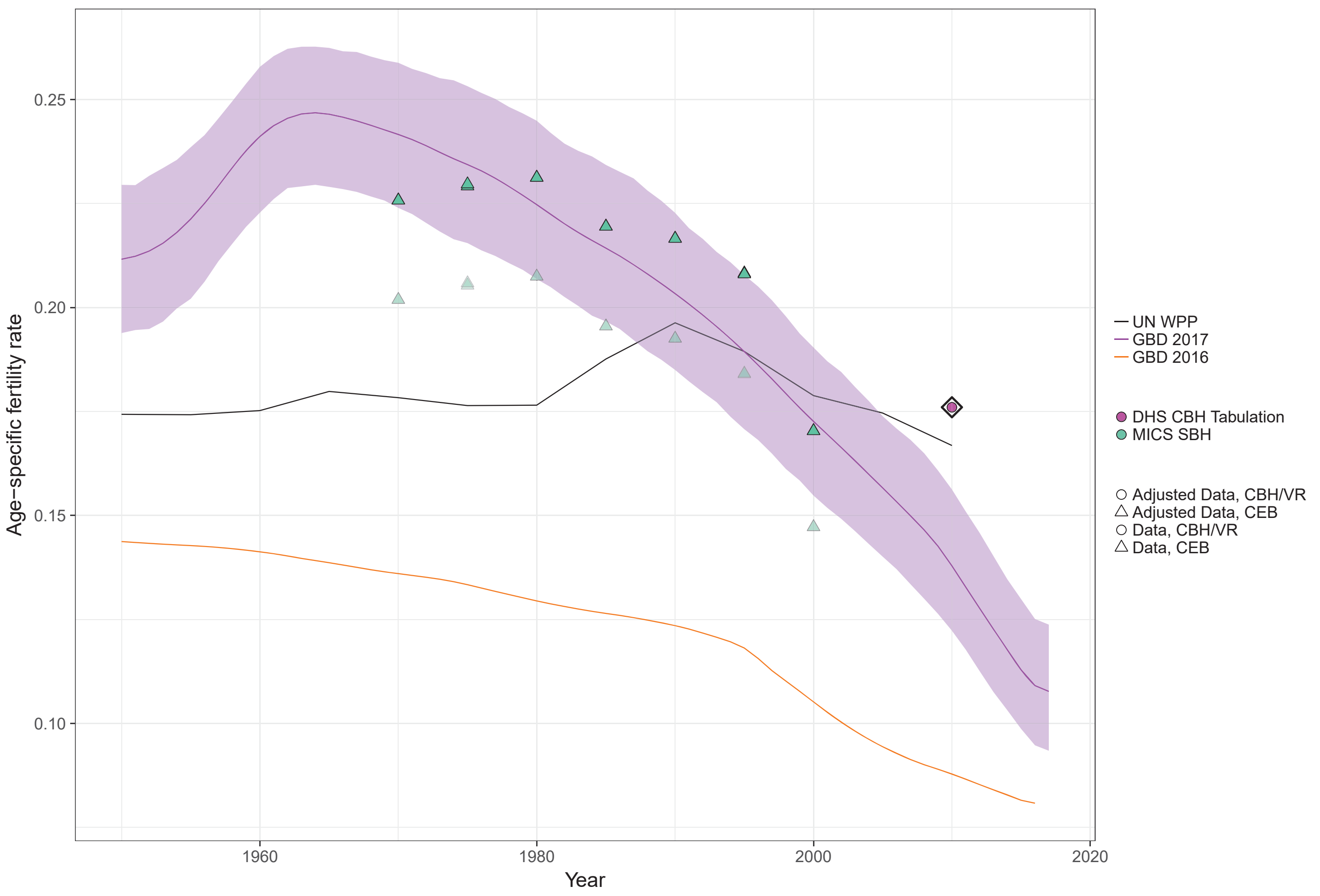

Age−specific fertility fitted trend lines, 1950−2017: Equatorial Guinea, Ages: 20−24
Data points enclosed with a diamond are reference sources

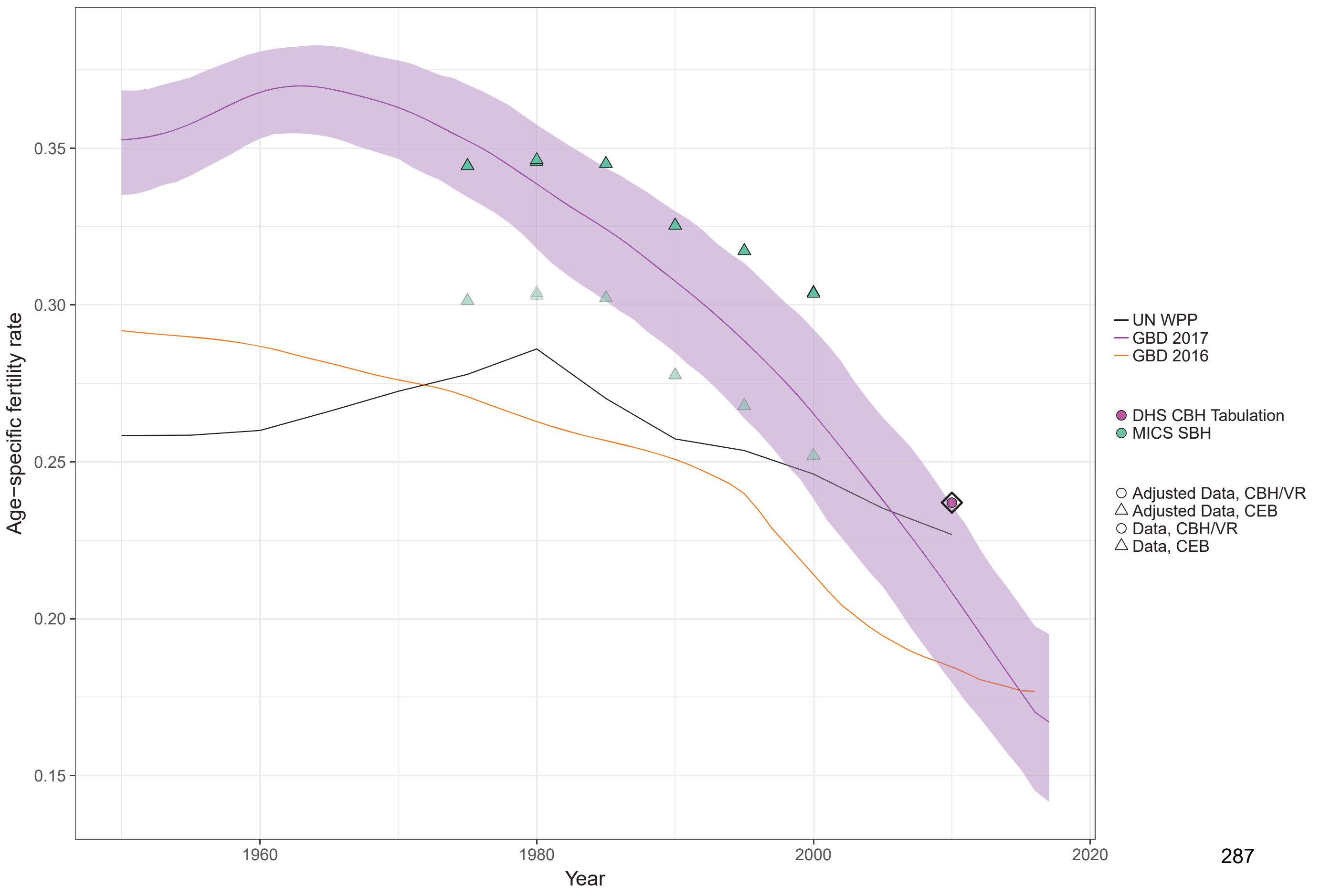

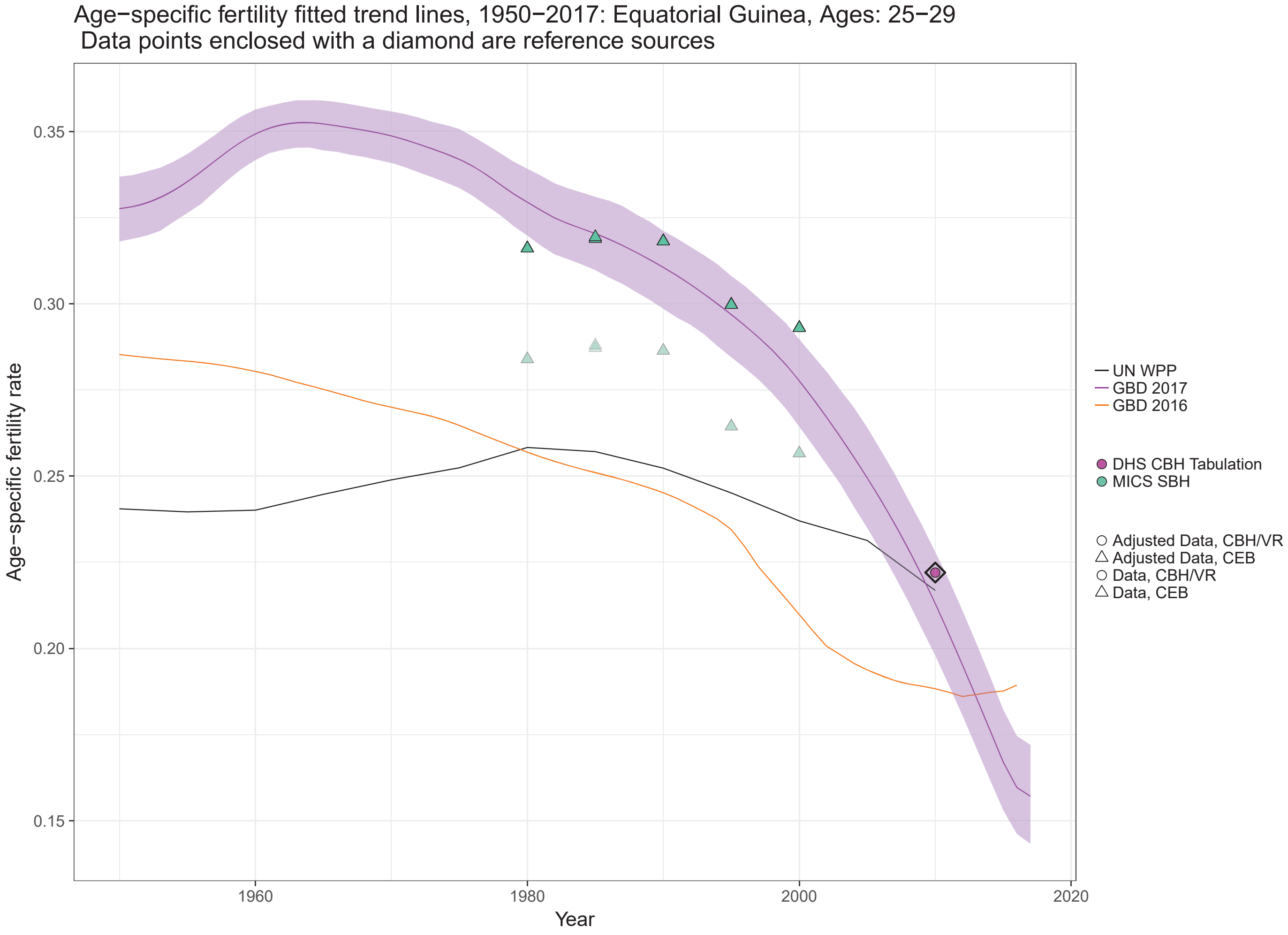

Age−specific fertility fitted trend lines, 1950−2017: Equatorial Guinea, Ages: 25−29
Data points enclosed with a diamond are reference sources
Age−specific fertility rate
0.35
0.30
0.25
0.20
0.15
1960
1980
2000
2020
Year
UN WPP
GBD 2017
GBD 2016
DHS CBH Tabulation
MICS SBH
Adjusted Data, CBH/VR
Adjusted Data, CEB
Data, CBH/VR
Data, CEB

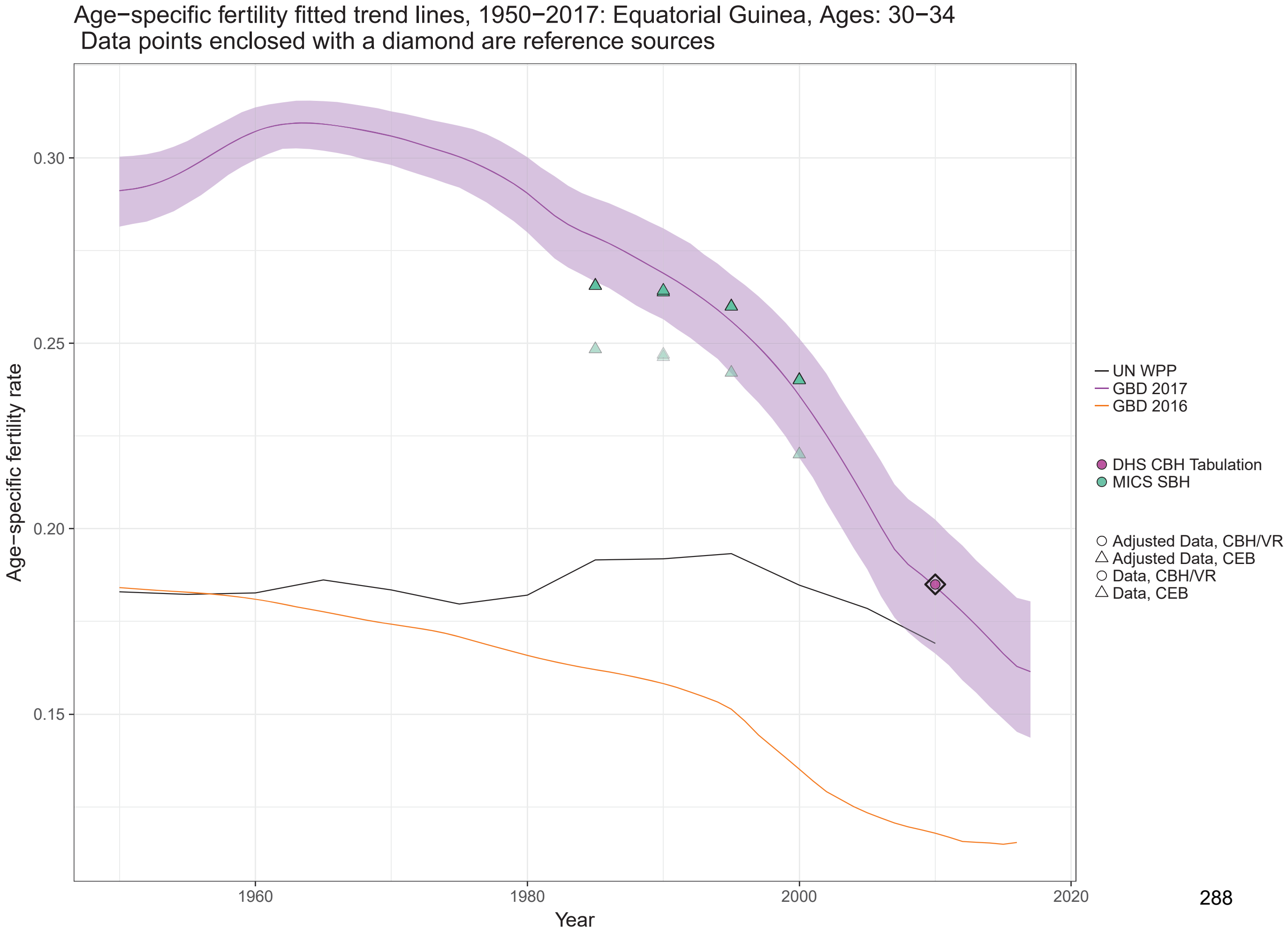

Age−specific fertility fitted trend lines, 1950−2017: Equatorial Guinea, Ages: 30−34
Data points enclosed with a diamond are reference sources
Age−specific fertility rate
0.30
0.25
0.20
0.15
1960
1980
2000
2020
Year
UN WPP
GBD 2017
GBD 2016
DHS CBH Tabulation
MICS SBH
Adjusted Data, CBH/VR
Adjusted Data, CEB
Data, CBH/VR
Data, CEB

Age−specific fertility fitted trend lines, 1950−2017: Equatorial Guinea, Ages: 35−39
Data points enclosed with a diamond are reference sources

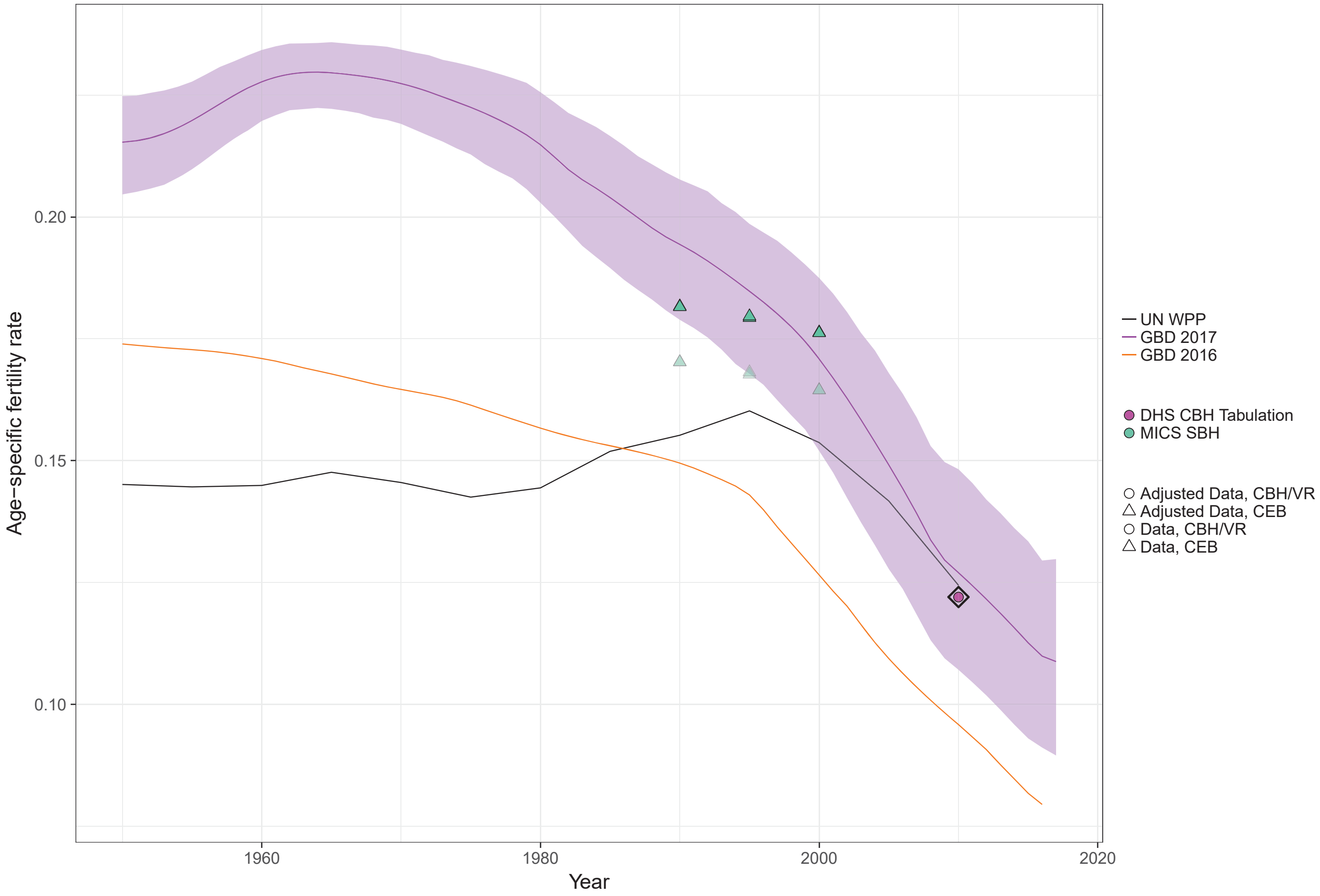

Age−specific fertility fitted trend lines, 1950−2017: Equatorial Guinea, Ages: 40−44
Data points enclosed with a diamond are reference sources

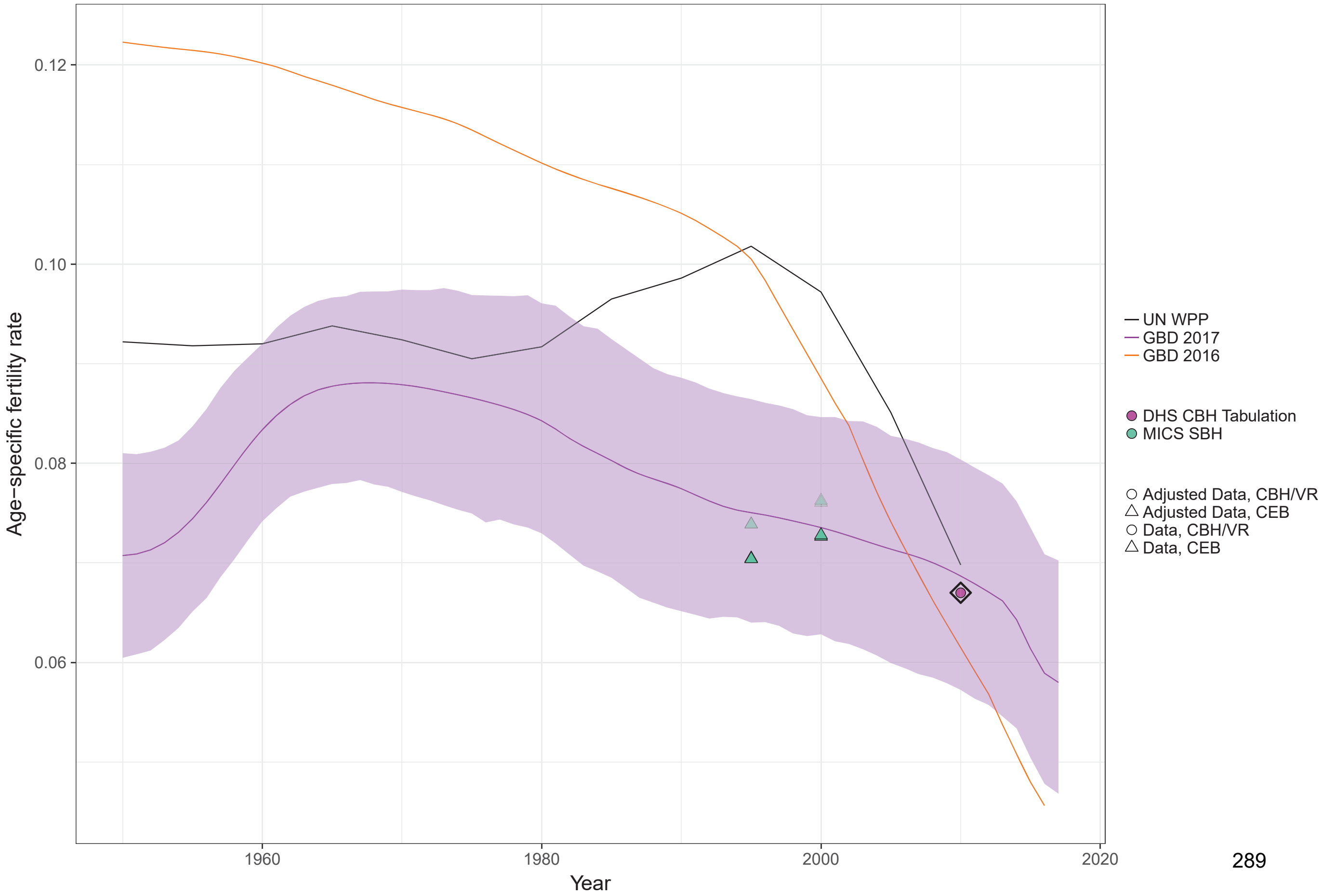

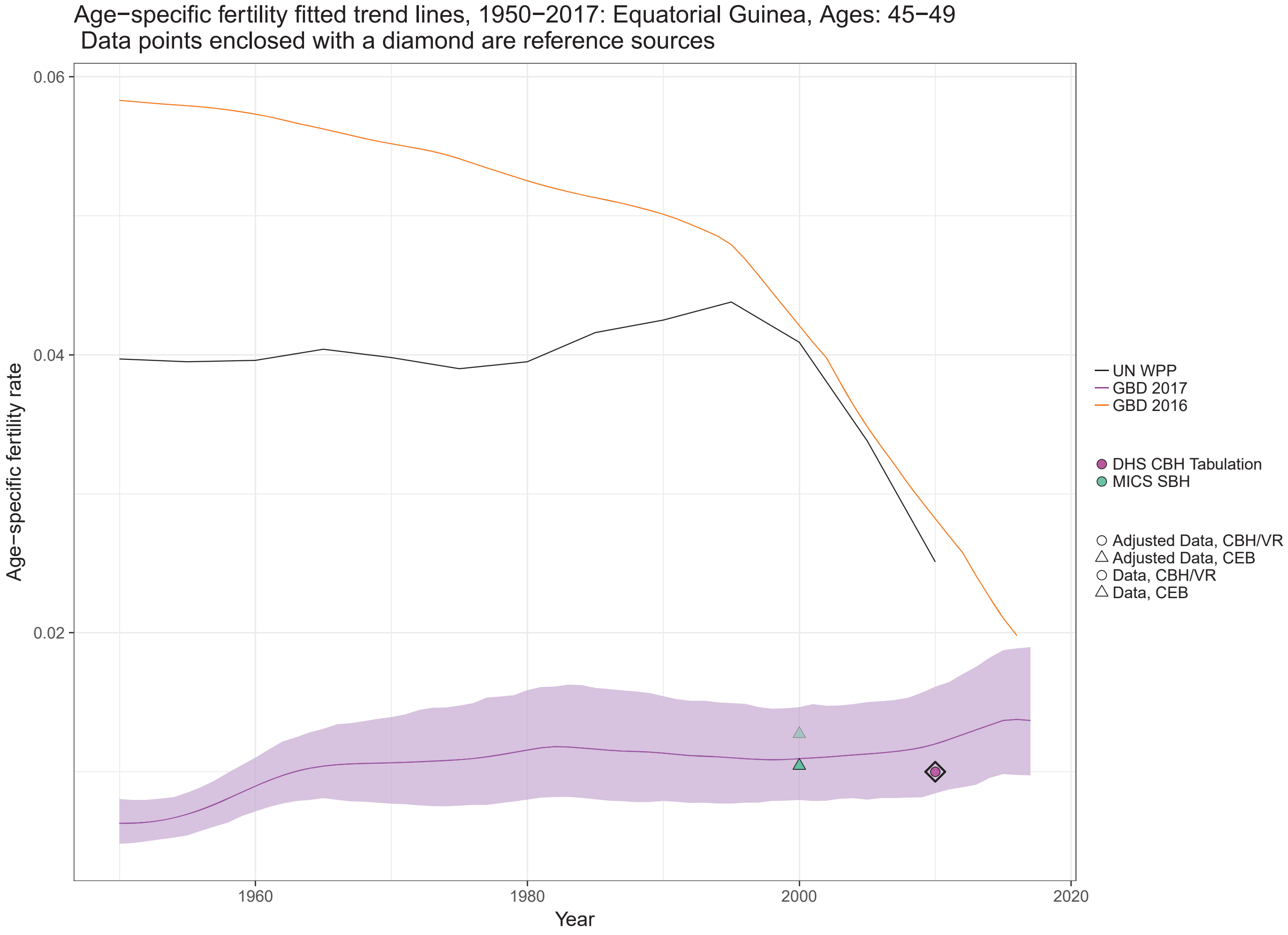

Age−specific fertility fitted trend lines, 1950−2017: Equatorial Guinea, Ages: 45−49
Data points enclosed with a diamond are reference sources
Age−specific fertility rate
0.06
0.04
0.02
1960
1980
2000
2020
Year
UN WPP
GBD 2017
GBD 2016
DHS CBH Tabulation
MICS SBH
Adjusted Data, CBH/VR
Adjusted Data, CEB
Data, CBH/VR
Data, CEB

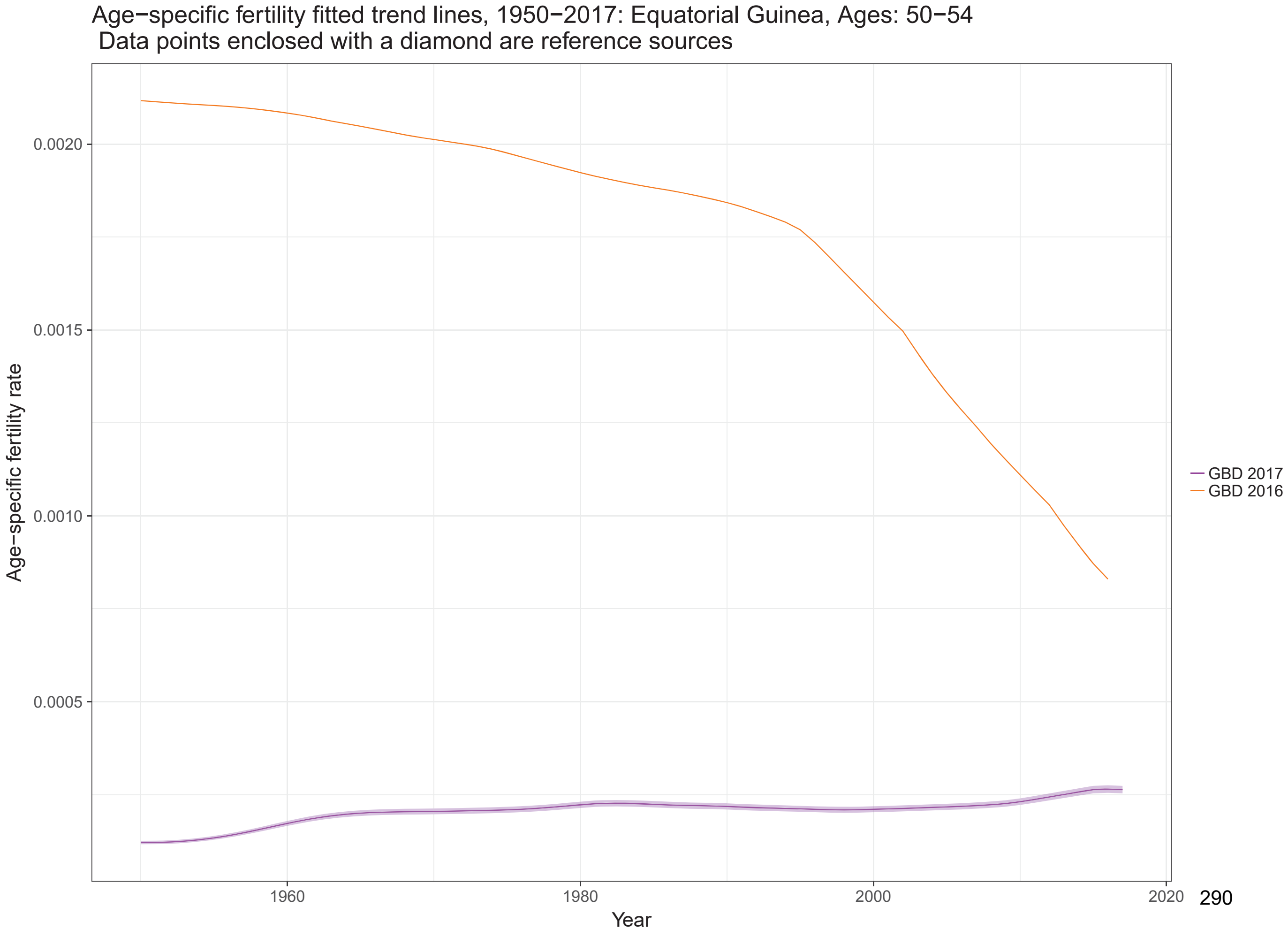

Age−specific fertility fitted trend lines, 1950−2017: Equatorial Guinea, Ages: 50−54
Data points enclosed with a diamond are reference sources
Age−specific fertility rate
0.0020
0.0015
0.0010
0.0005
1960
1980
2000
2020
Year
GBD 2017
GBD 2016

Age−specific fertility fitted trend lines, 1950−2017: Eritrea, TFR
Data points enclosed with a diamond are reference sources

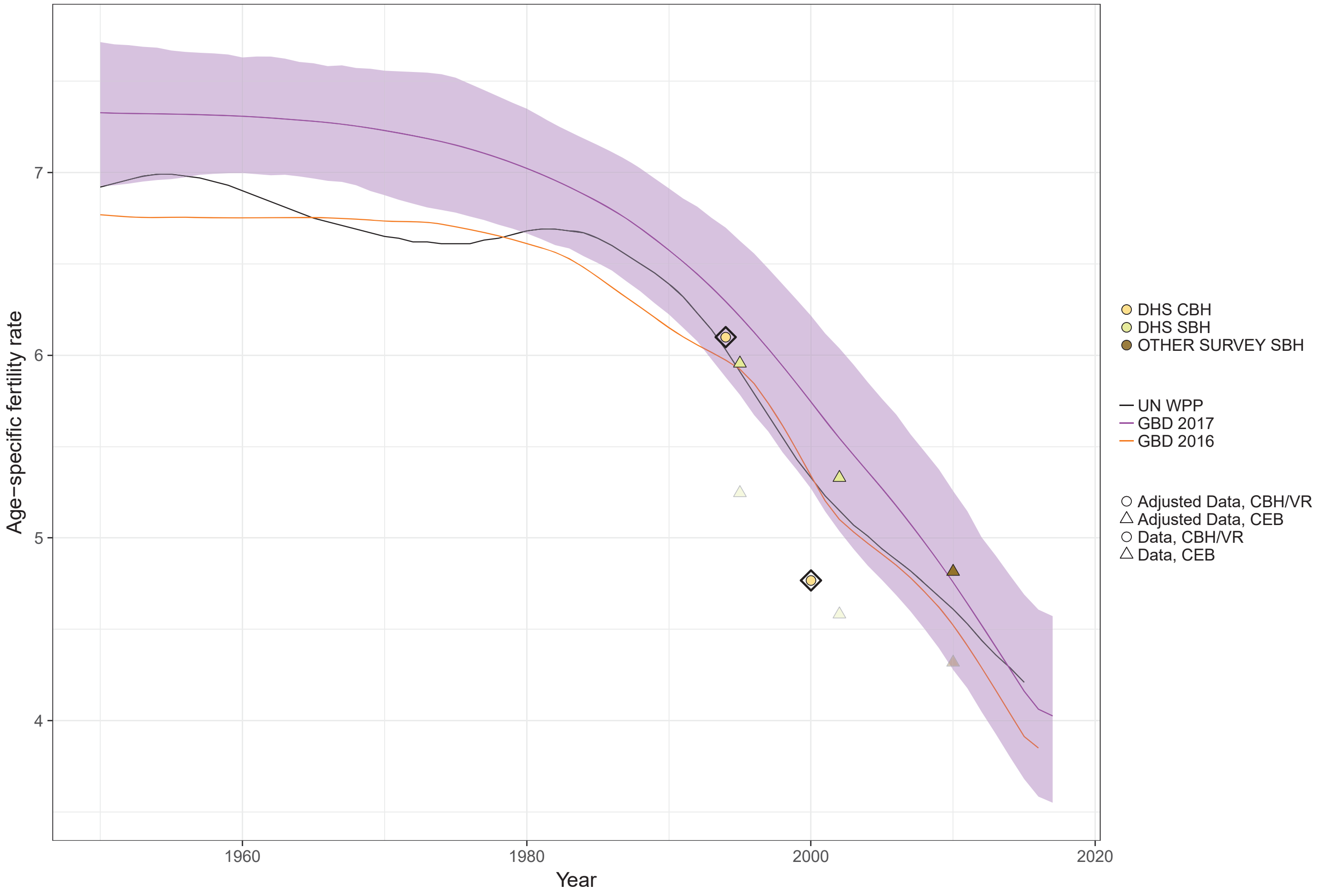

Age−specific fertility fitted trend lines, 1950−2017: Eritrea, Ages: 10−14
Data points enclosed with a diamond are reference sources

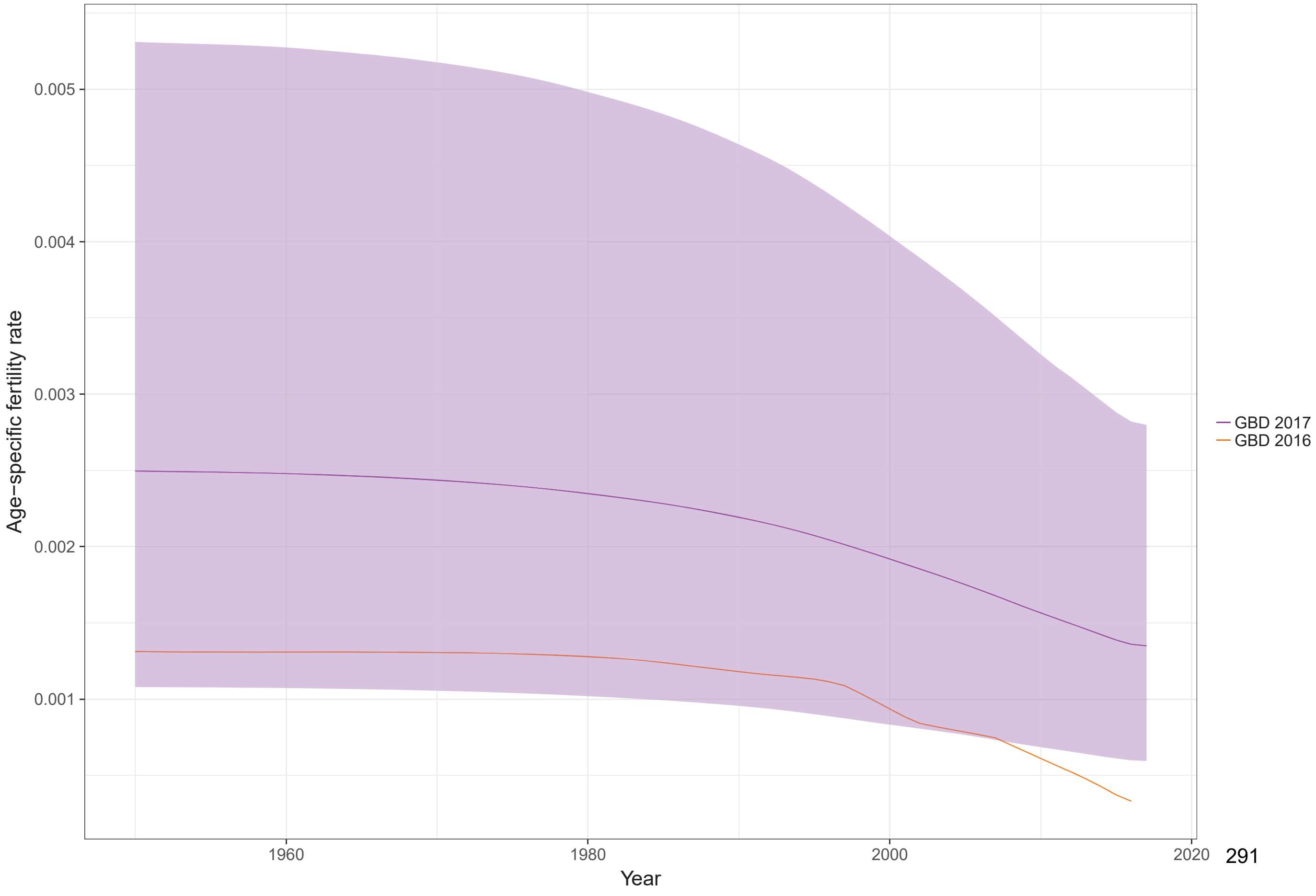

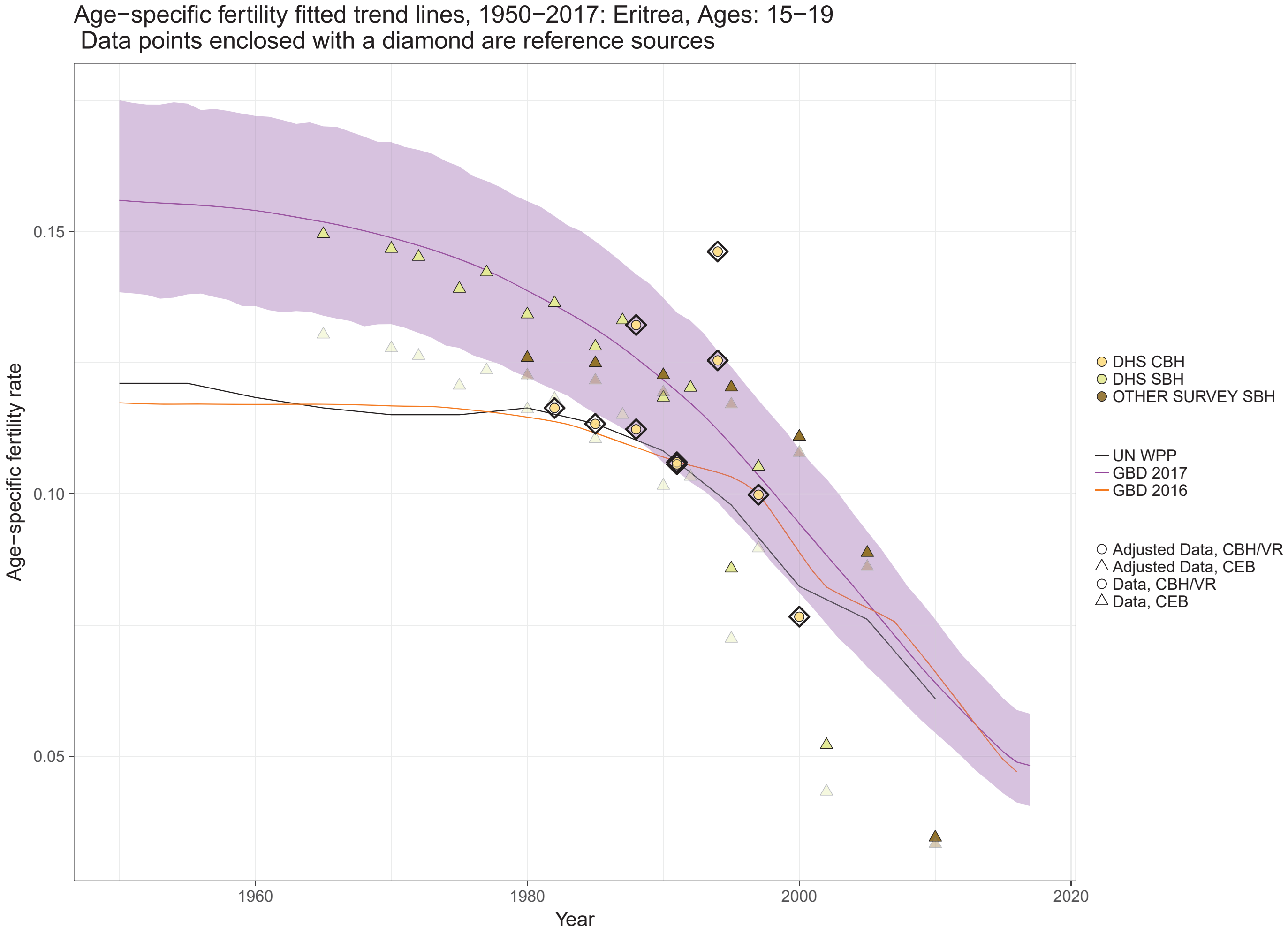
Age−specific fertility fitted trend lines, 1950−2017: Eritrea, Ages: 15−19
Data points enclosed with a diamond are reference sources
Age−specific fertility rate
0.15
0.10
0.05
1960
1980
2000
2020
Year
DHS CBH
DHS SBH
OTHER SURVEY SBH
UN WPP
GBD 2017
GBD 2016
Adjusted Data, CBH/VR
Adjusted Data, CEB
Data, CBH/VR
Data, CEB

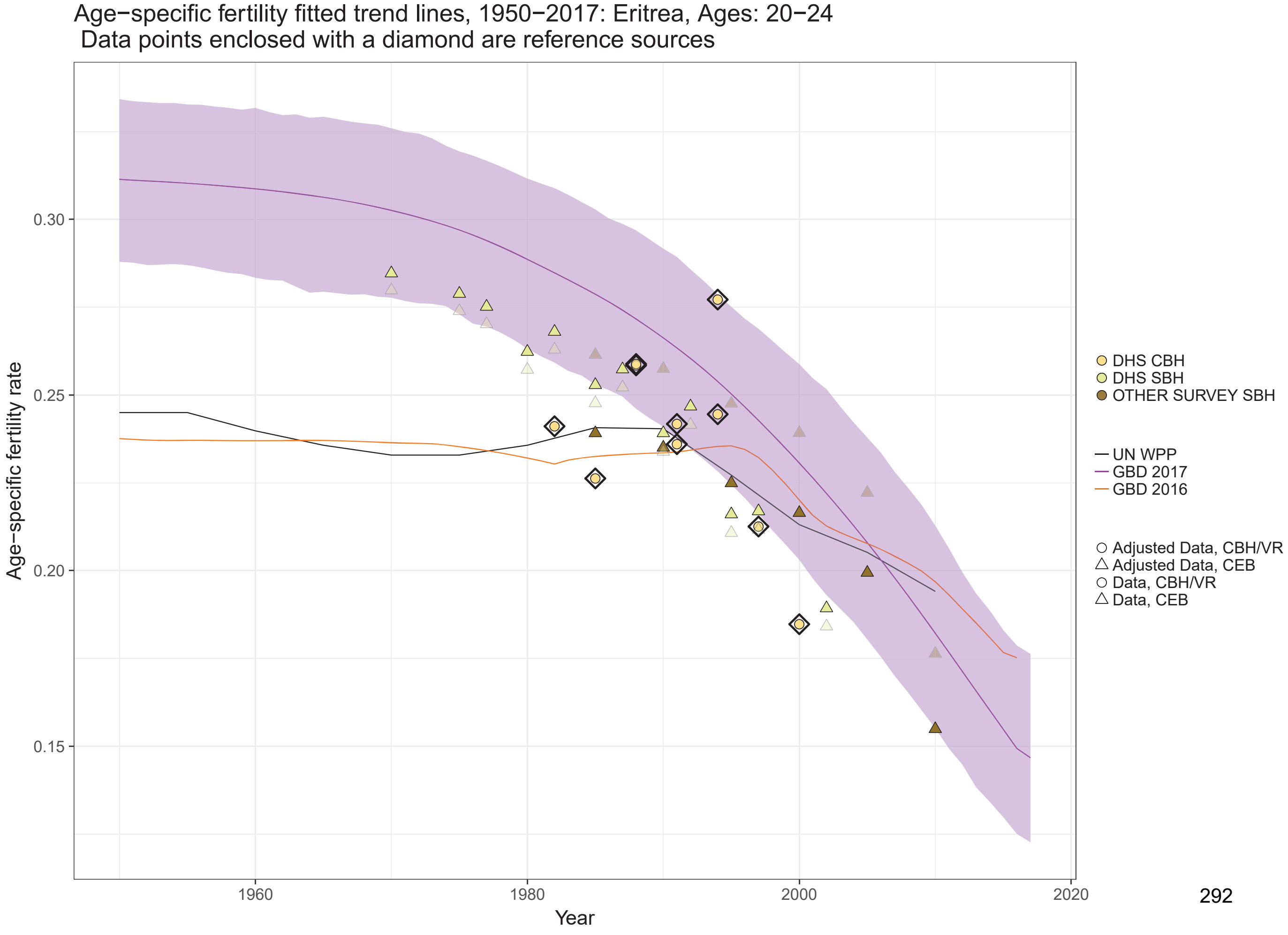
Age−specific fertility fitted trend lines, 1950−2017: Eritrea, Ages: 20−24
Data points enclosed with a diamond are reference sources
Age−specific fertility rate
0.30
0.25
0.20
0.15
1960
1980
2000
2020
Year
DHS CBH
DHS SBH
OTHER SURVEY SBH
UN WPP
GBD 2017
GBD 2016
Adjusted Data, CBH/VR
Adjusted Data, CEB
Data, CBH/VR
Data, CEB

Age−specific fertility fitted trend lines, 1950−2017: Eritrea, Ages: 25−29
Data points enclosed with a diamond are reference sources

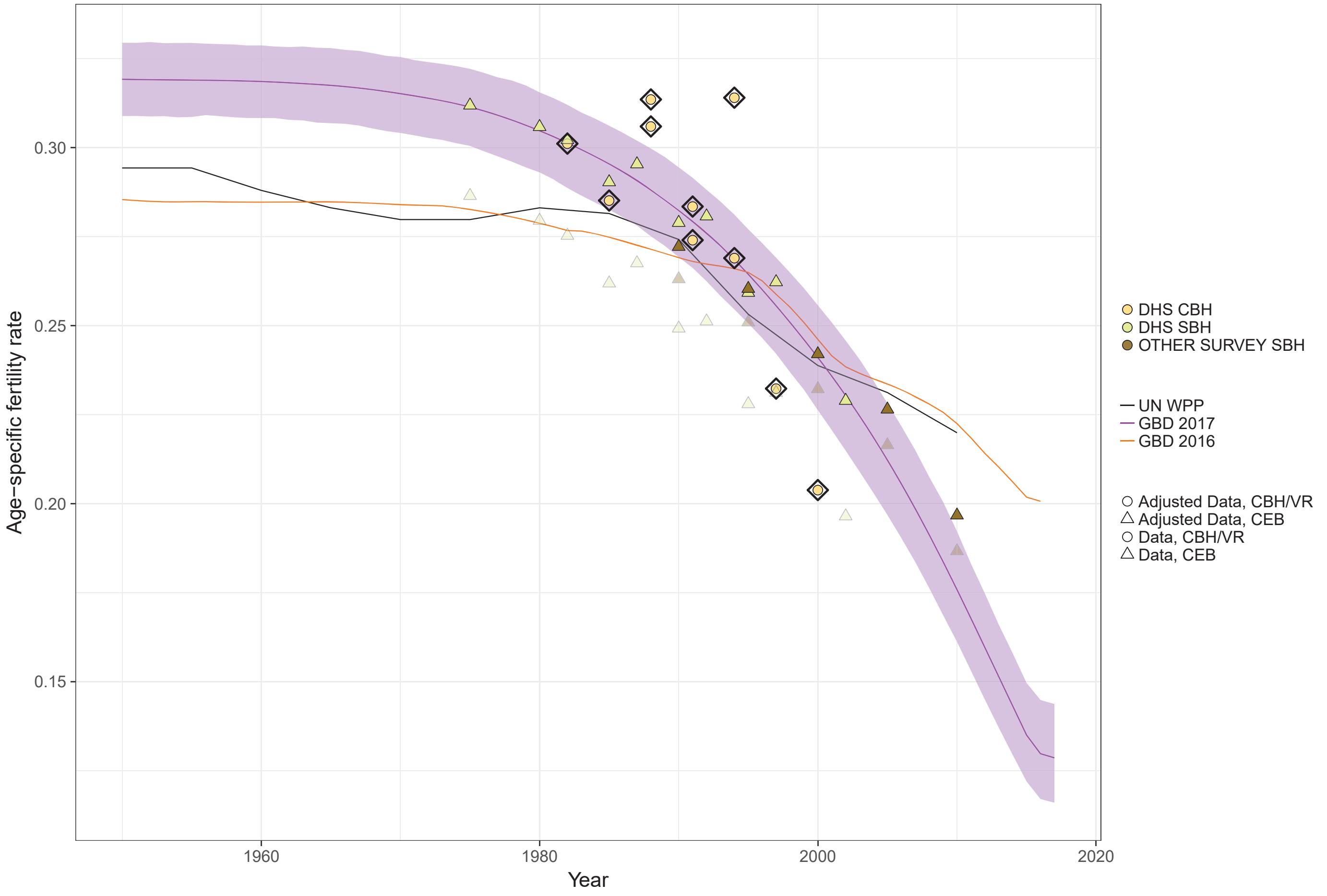

Age−specific fertility fitted trend lines, 1950−2017: Eritrea, Ages: 30−34
Data points enclosed with a diamond are reference sources

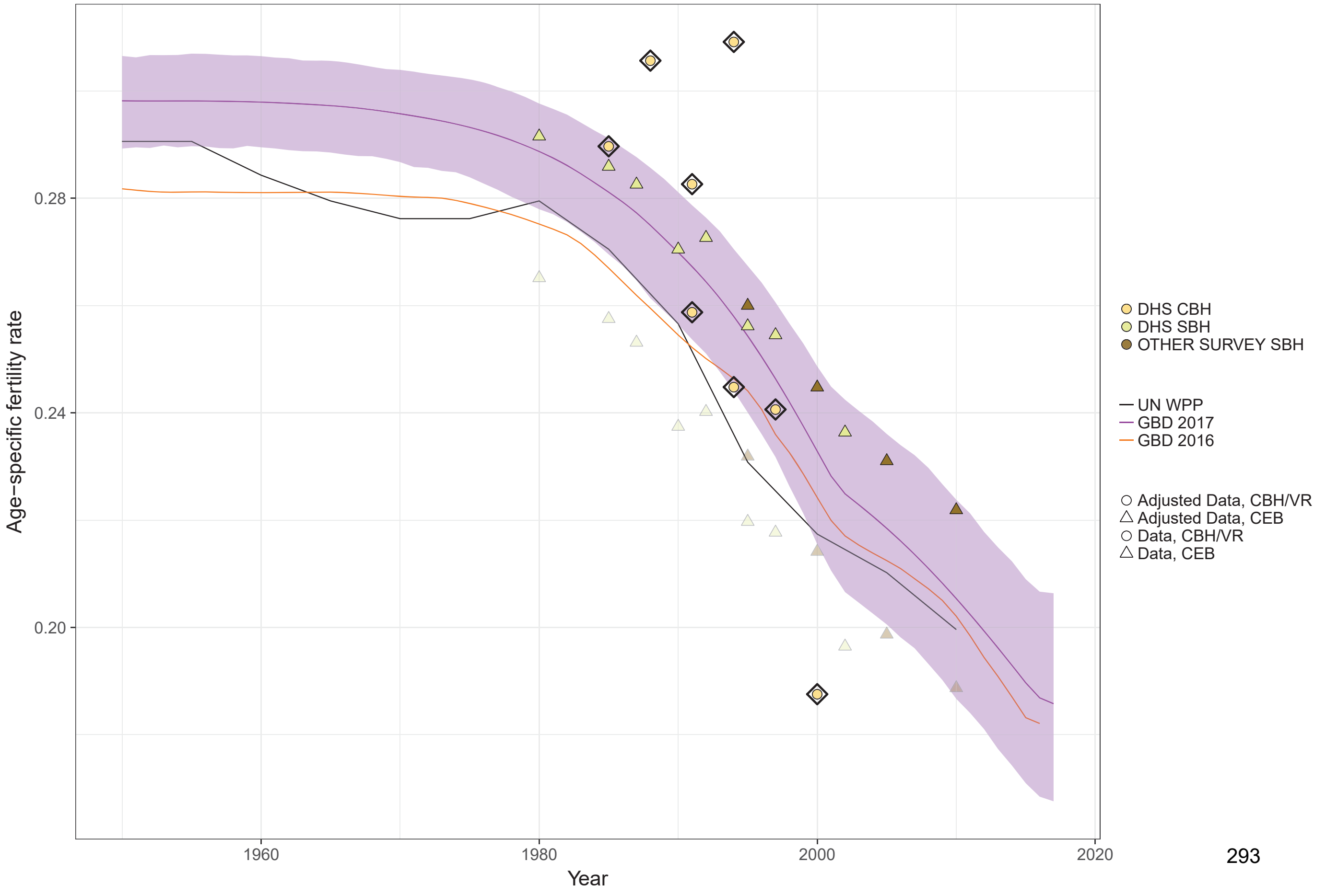

Age−specific fertility fitted trend lines, 1950−2017: Eritrea, Ages: 35−39
Data points enclosed with a diamond are reference sources

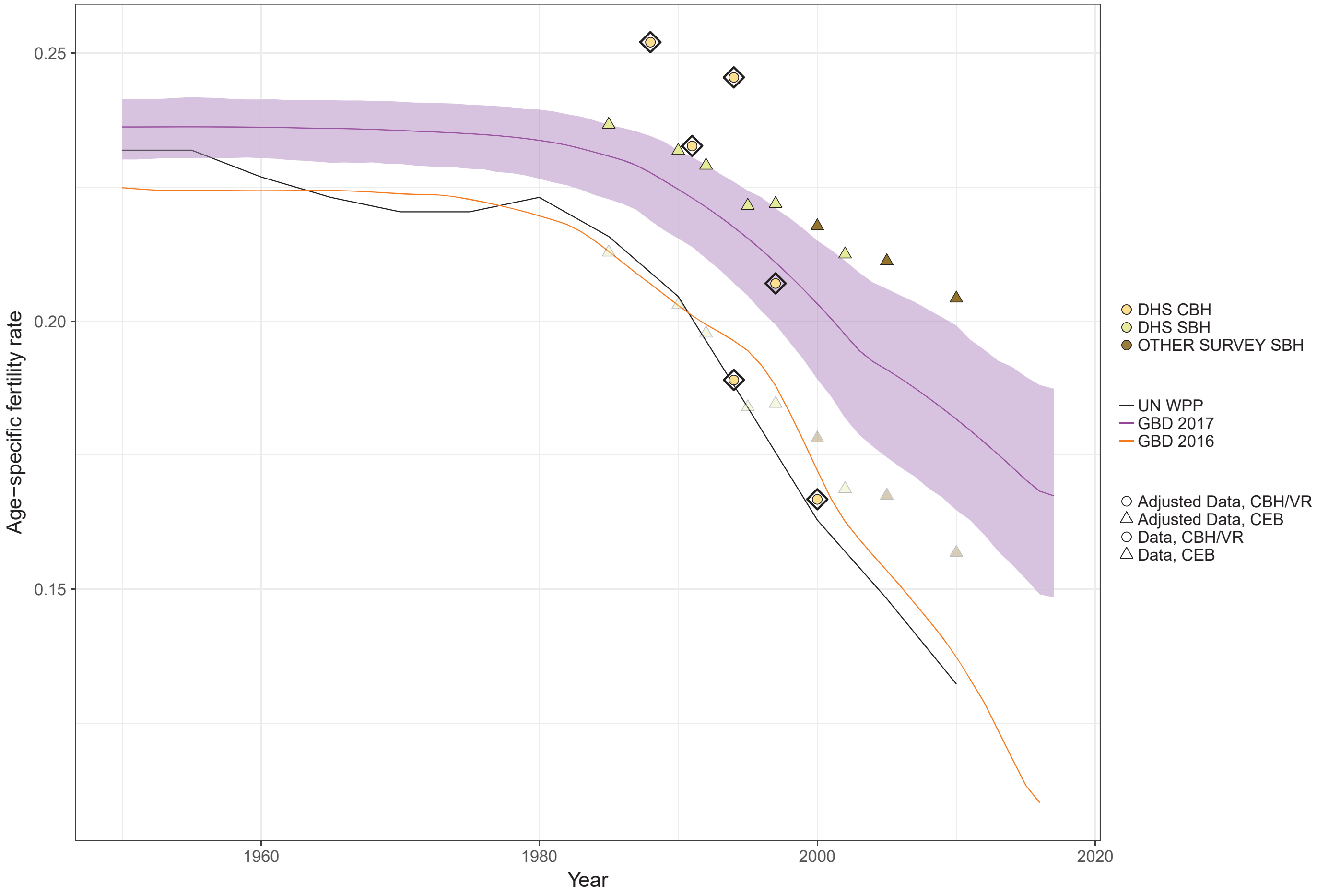

Age−specific fertility fitted trend lines, 1950−2017: Eritrea, Ages: 40−44
Data points enclosed with a diamond are reference sources

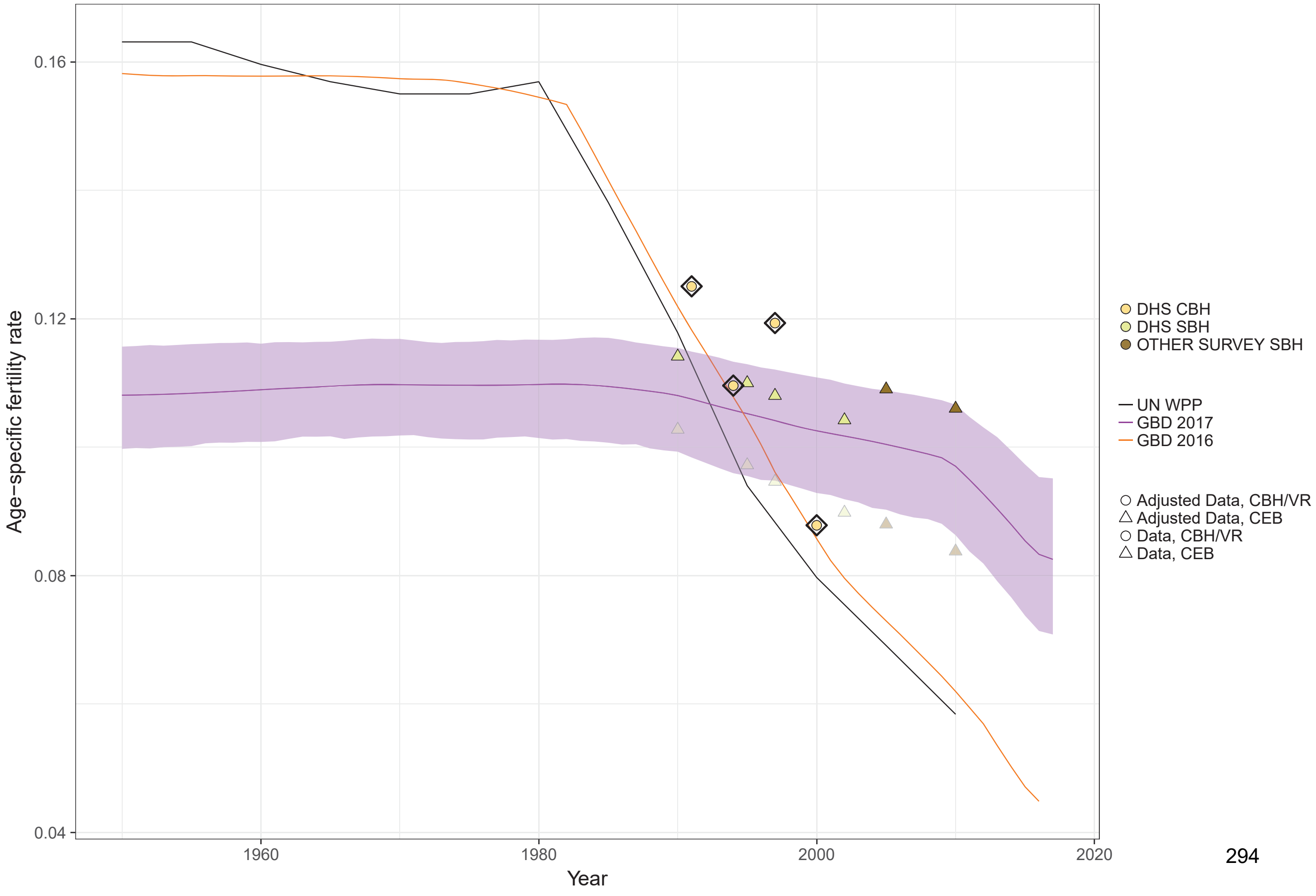

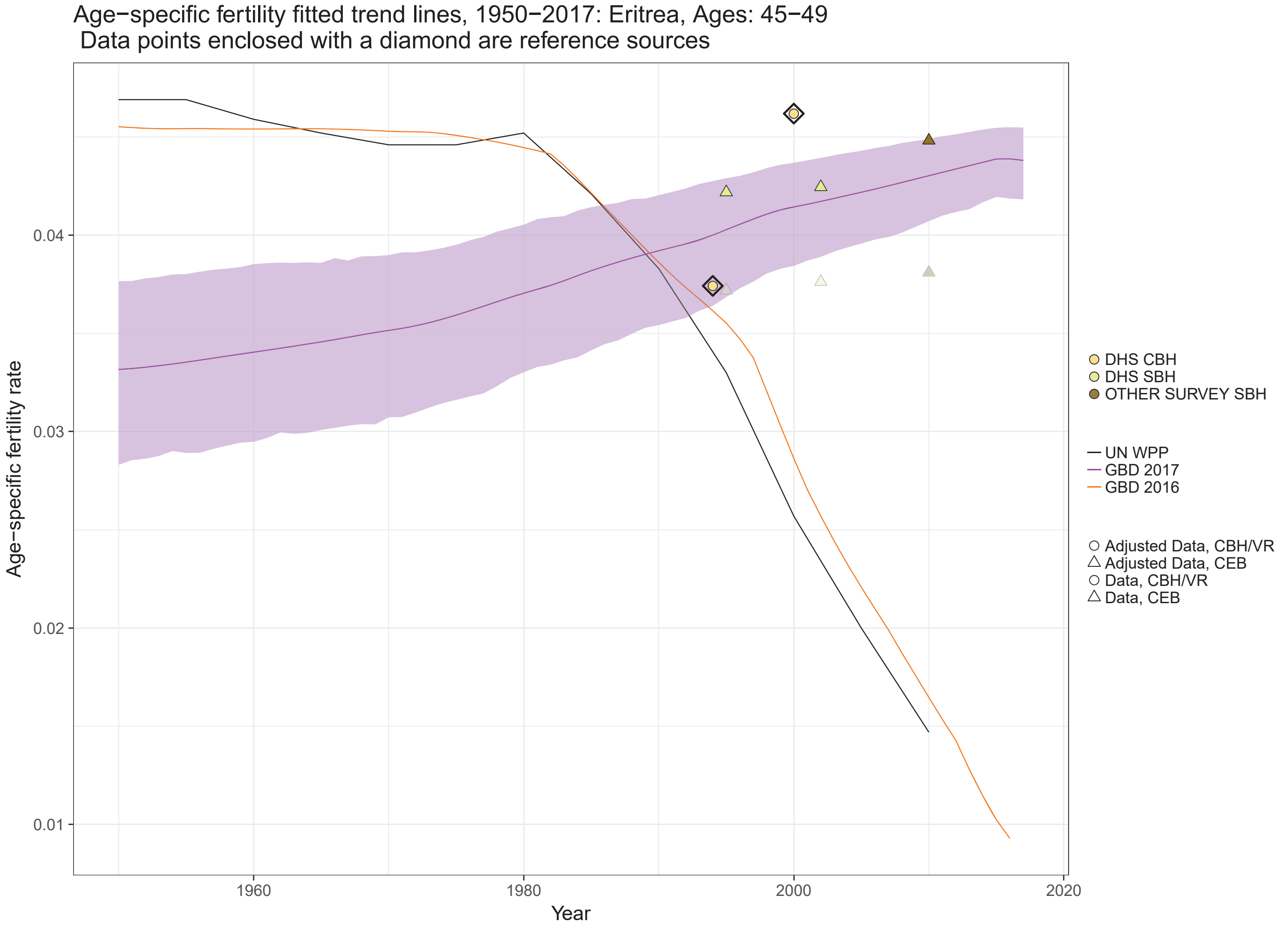

Age−specific fertility fitted trend lines, 1950−2017: Eritrea, Ages: 45−49
Data points enclosed with a diamond are reference sources
Age−specific fertility rate
0.04
0.03
0.02
0.01
1960
1980
2000
2020
Year
DHS CBH
DHS SBH
OTHER SURVEY SBH
UN WPP
GBD 2017
GBD 2016
Adjusted Data, CBH/VR
Adjusted Data, CEB
Data, CBH/VR
Data, CEB

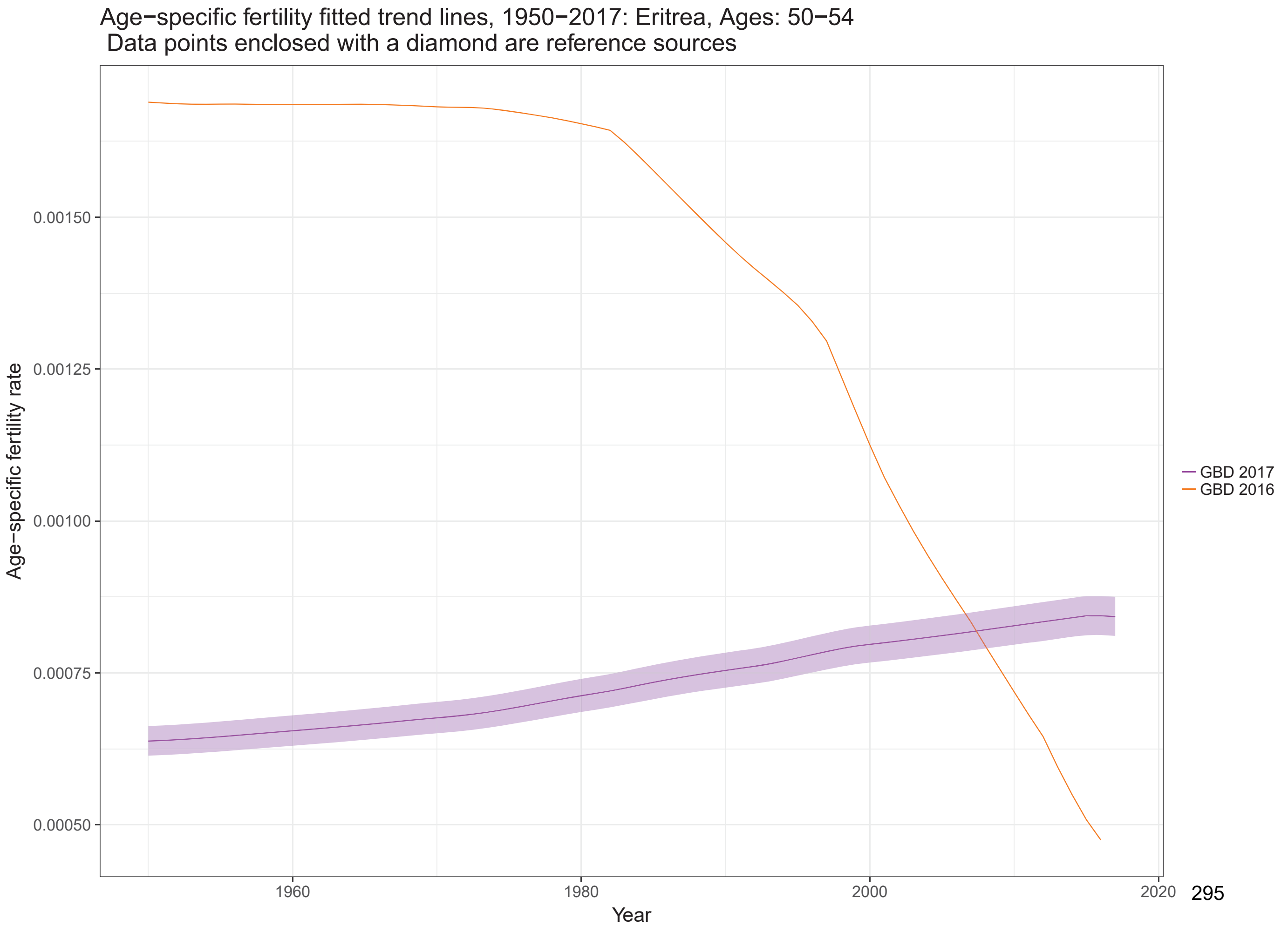

Age−specific fertility fitted trend lines, 1950−2017: Eritrea, Ages: 50−54
Data points enclosed with a diamond are reference sources
Age−specific fertility rate
0.00150
0.00125
0.00100
0.00075
0.00050
1960
1980
2000
2020
Year
GBD 2017
GBD 2016

Age−specific fertility fitted trend lines, 1950−2017: Estonia, TFR
Data points enclosed with a diamond are reference sources

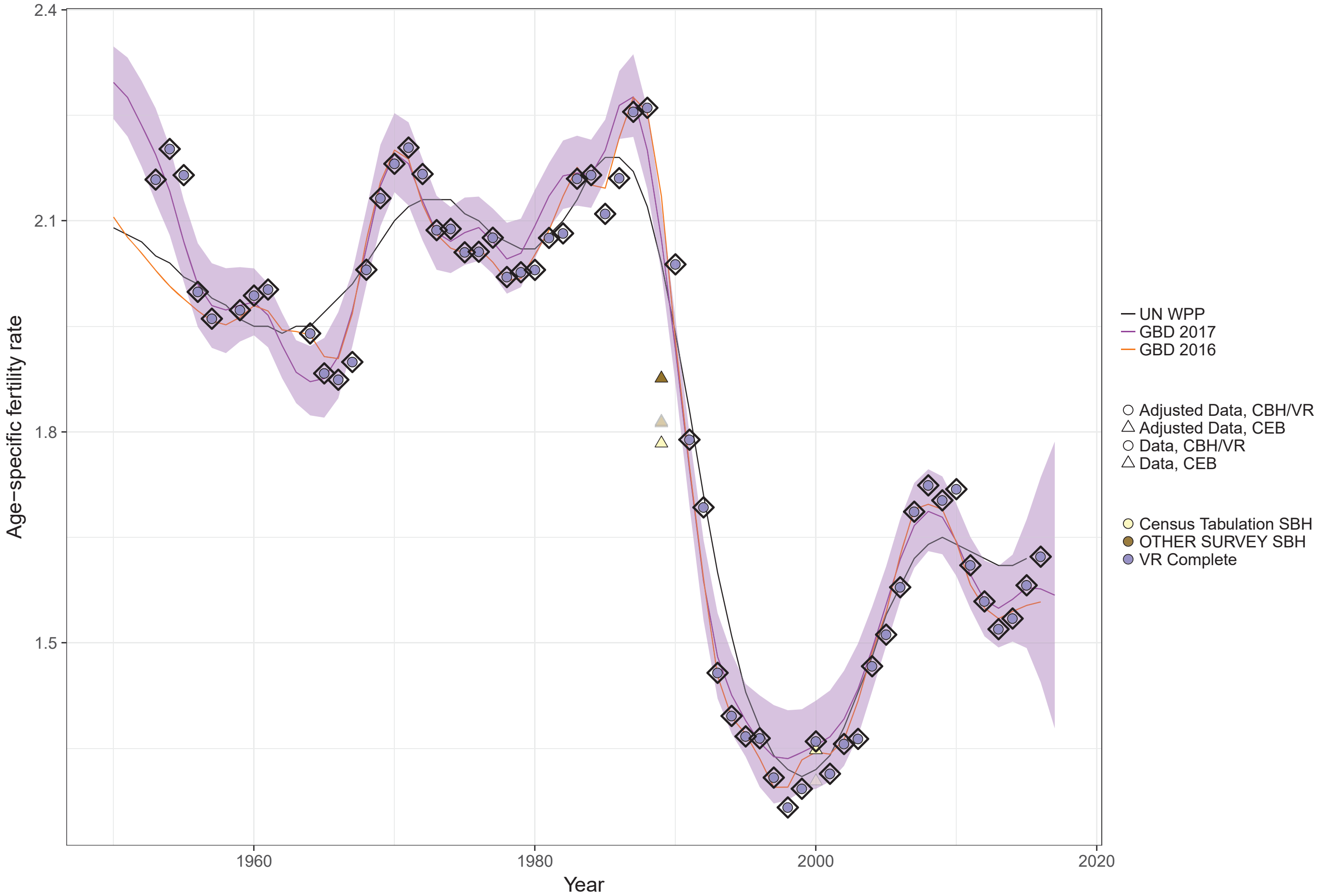

Age−specific fertility fitted trend lines, 1950−2017: Estonia, Ages: 10−14
Data points enclosed with a diamond are reference sources

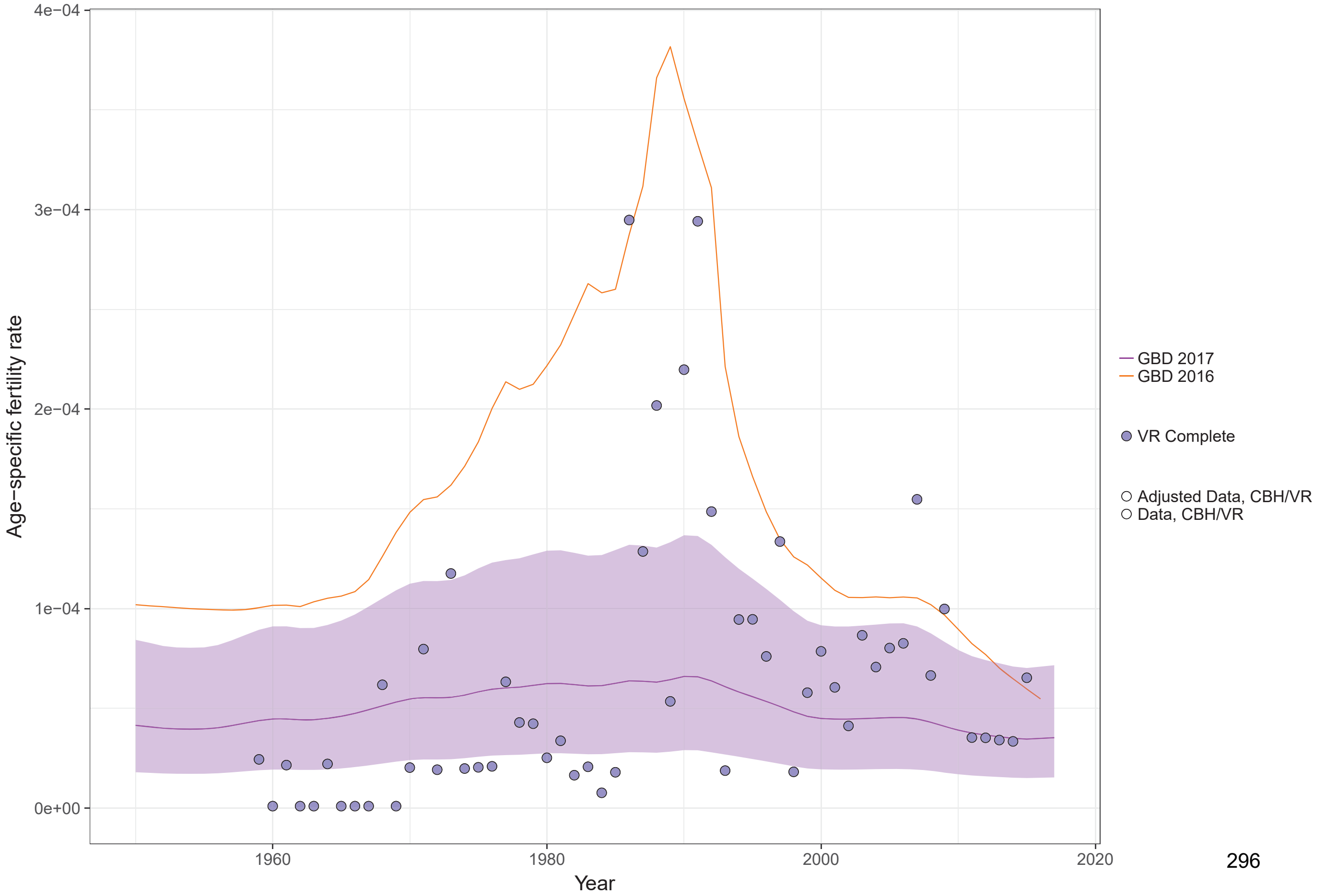

Age−specific fertility fitted trend lines, 1950−2017: Estonia, Ages: 15−19
Data points enclosed with a diamond are reference sources

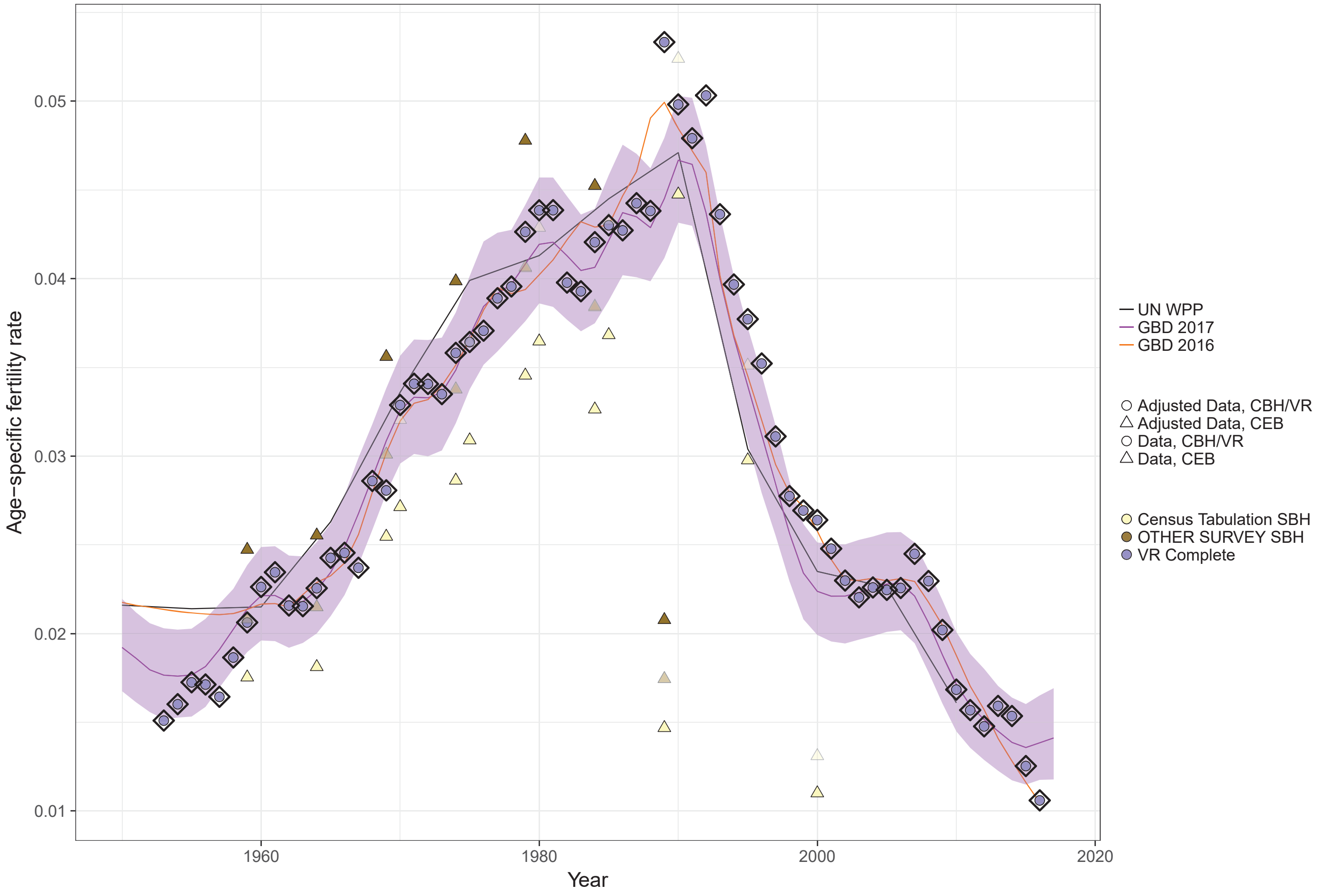

Age−specific fertility fitted trend lines, 1950−2017: Estonia, Ages: 20−24
Data points enclosed with a diamond are reference sources

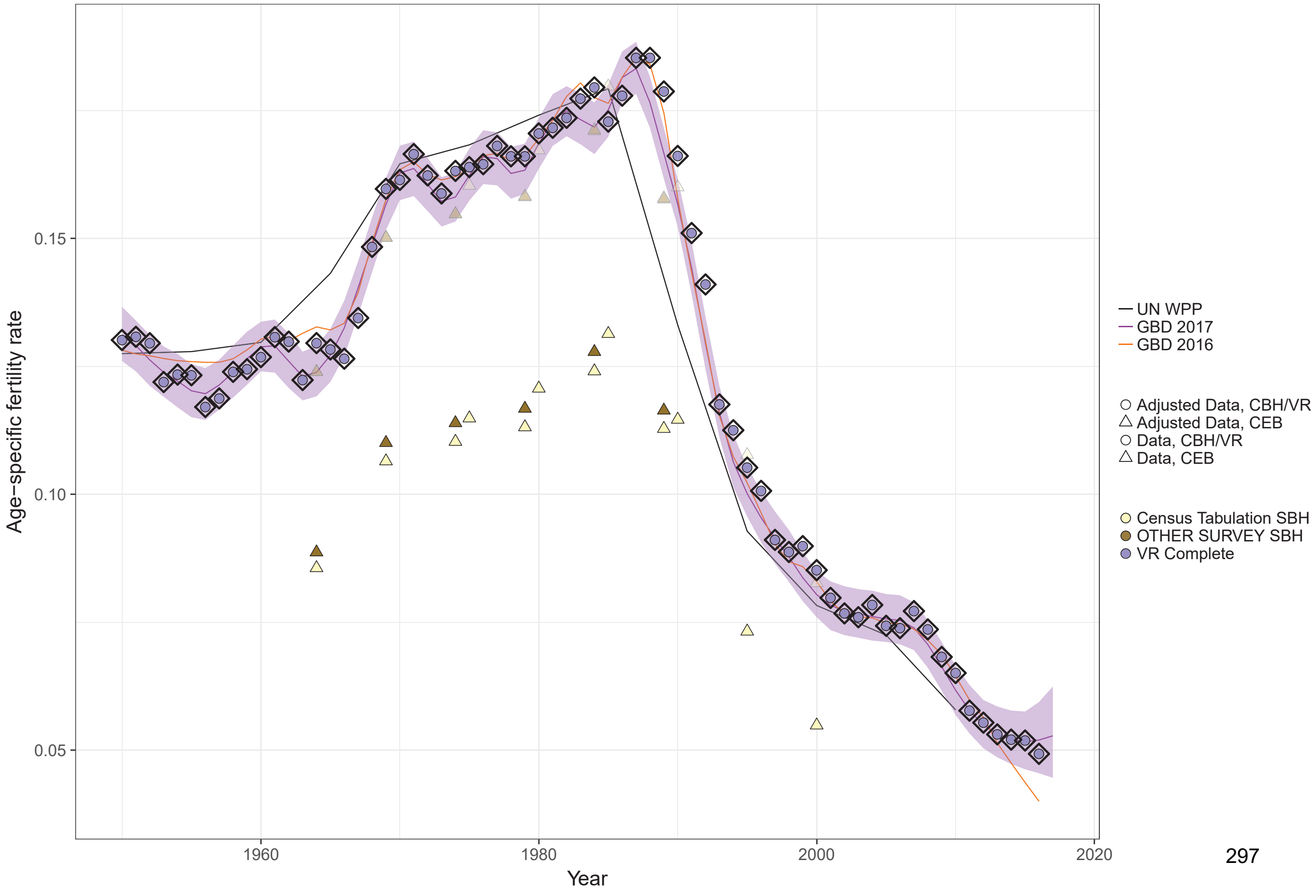

Age−specific fertility fitted trend lines, 1950−2017: Estonia, Ages: 25−29
Data points enclosed with a diamond are reference sources

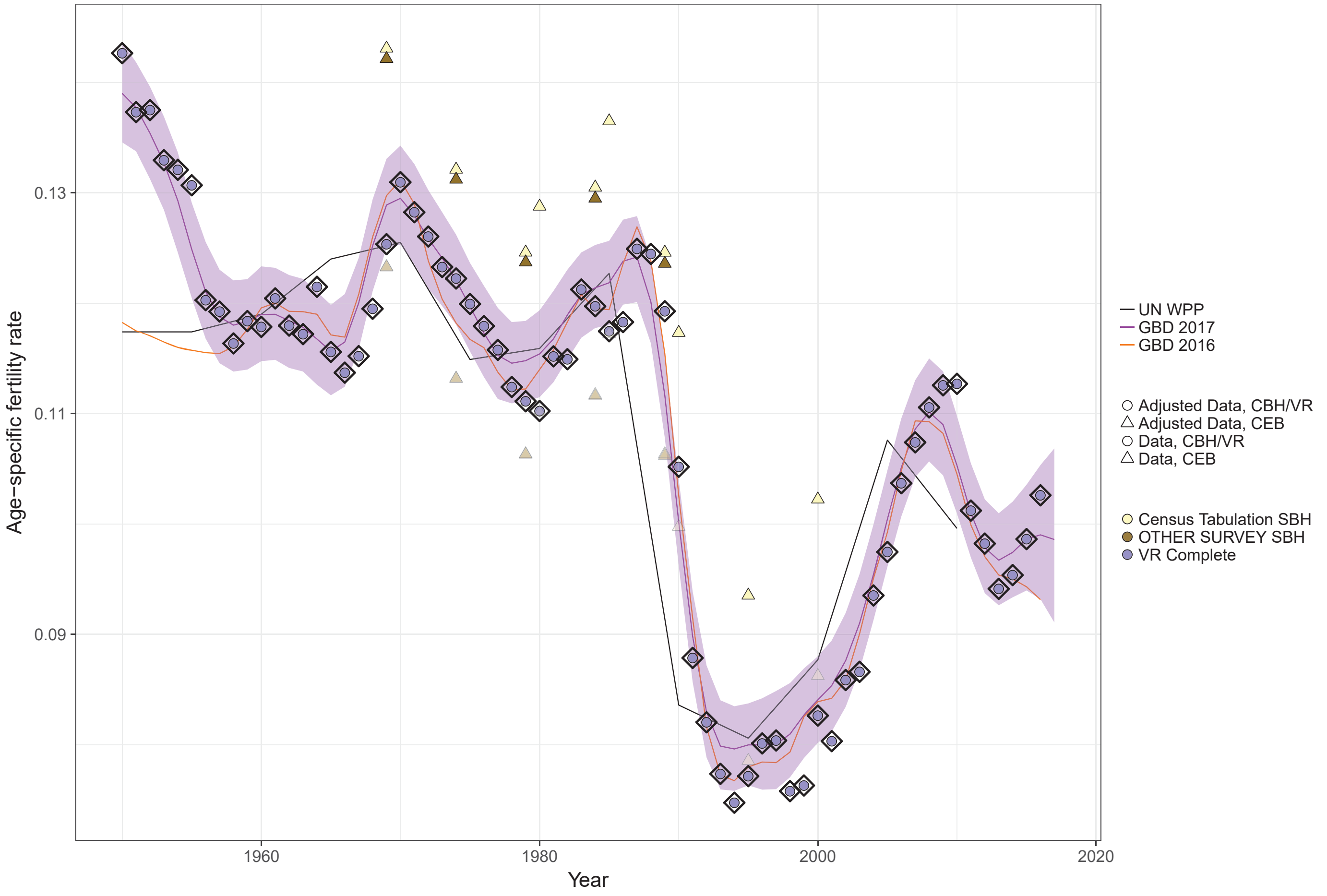

Age−specific fertility fitted trend lines, 1950−2017: Estonia, Ages: 30−34
Data points enclosed with a diamond are reference sources

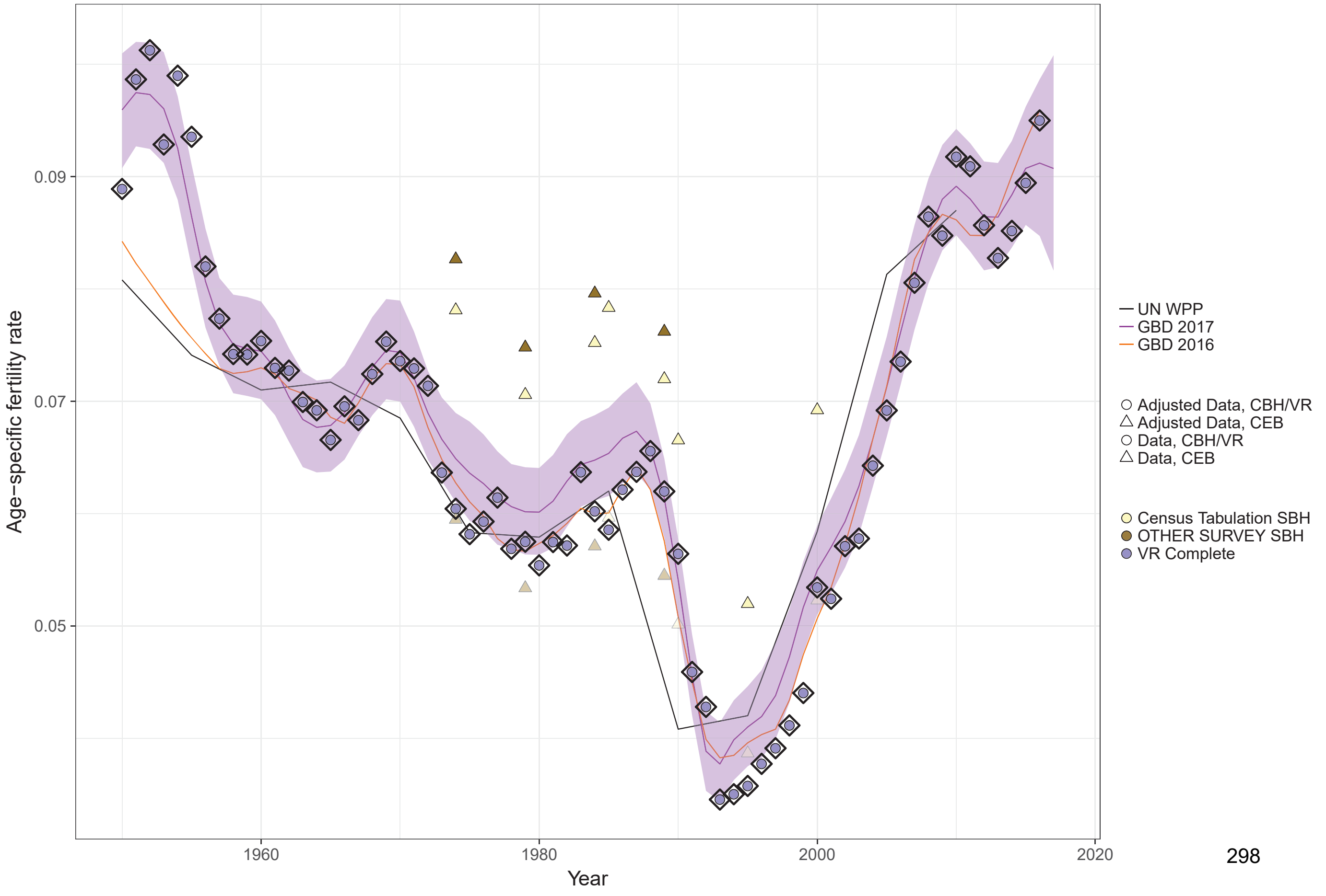

Age−specific fertility fitted trend lines, 1950−2017: Estonia, Ages: 35−39
Data points enclosed with a diamond are reference sources

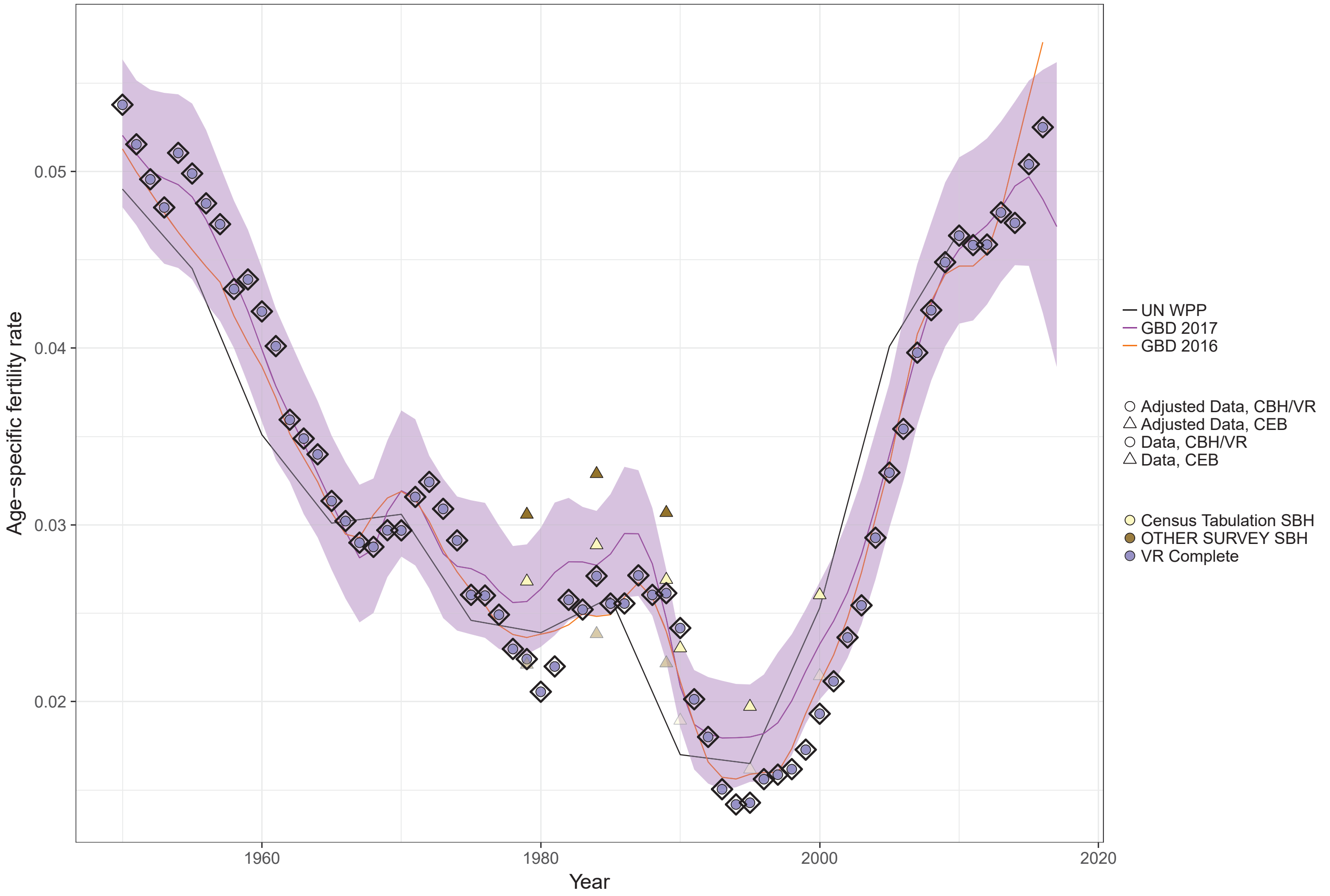

Age−specific fertility fitted trend lines, 1950−2017: Estonia, Ages: 40−44
Data points enclosed with a diamond are reference sources

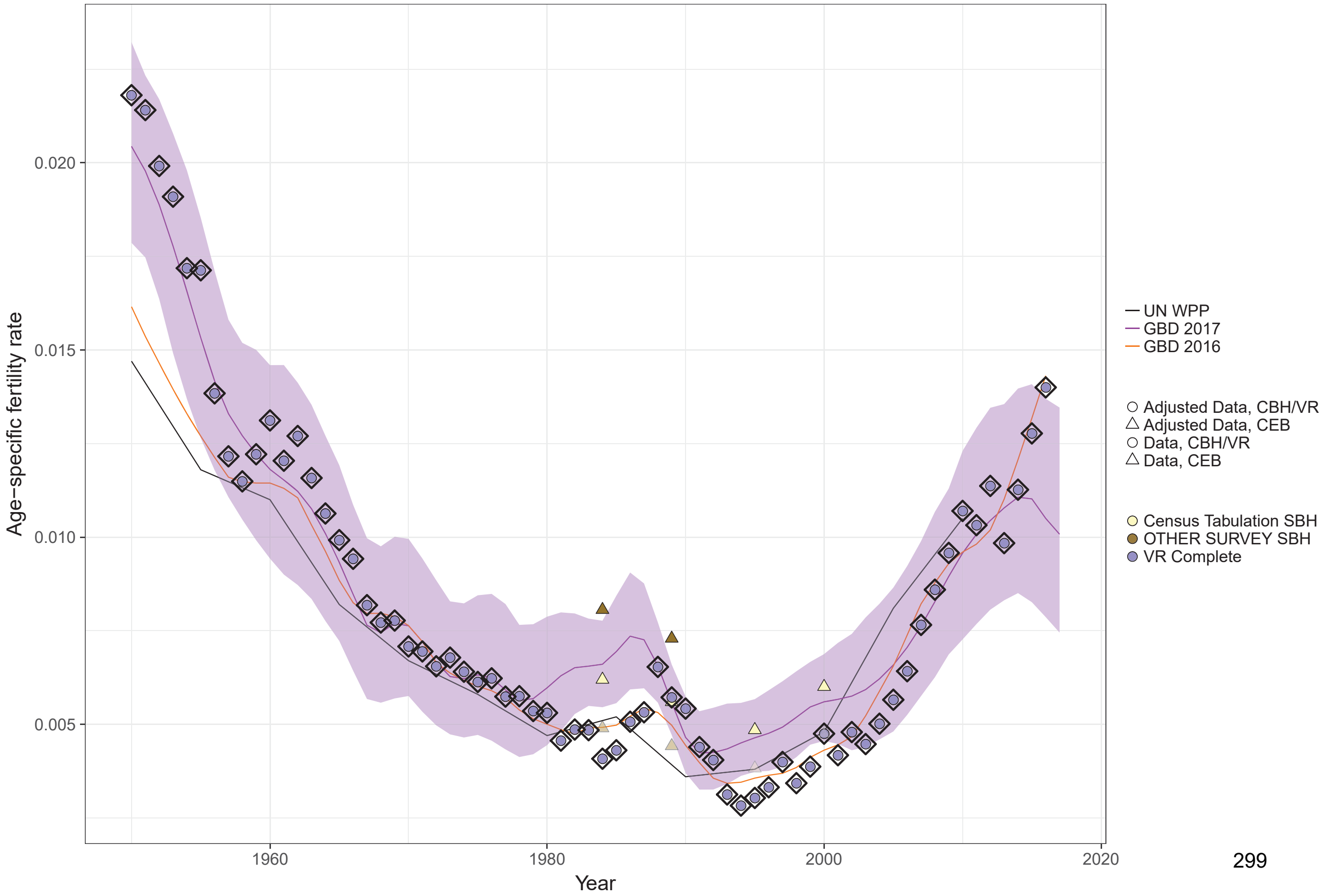

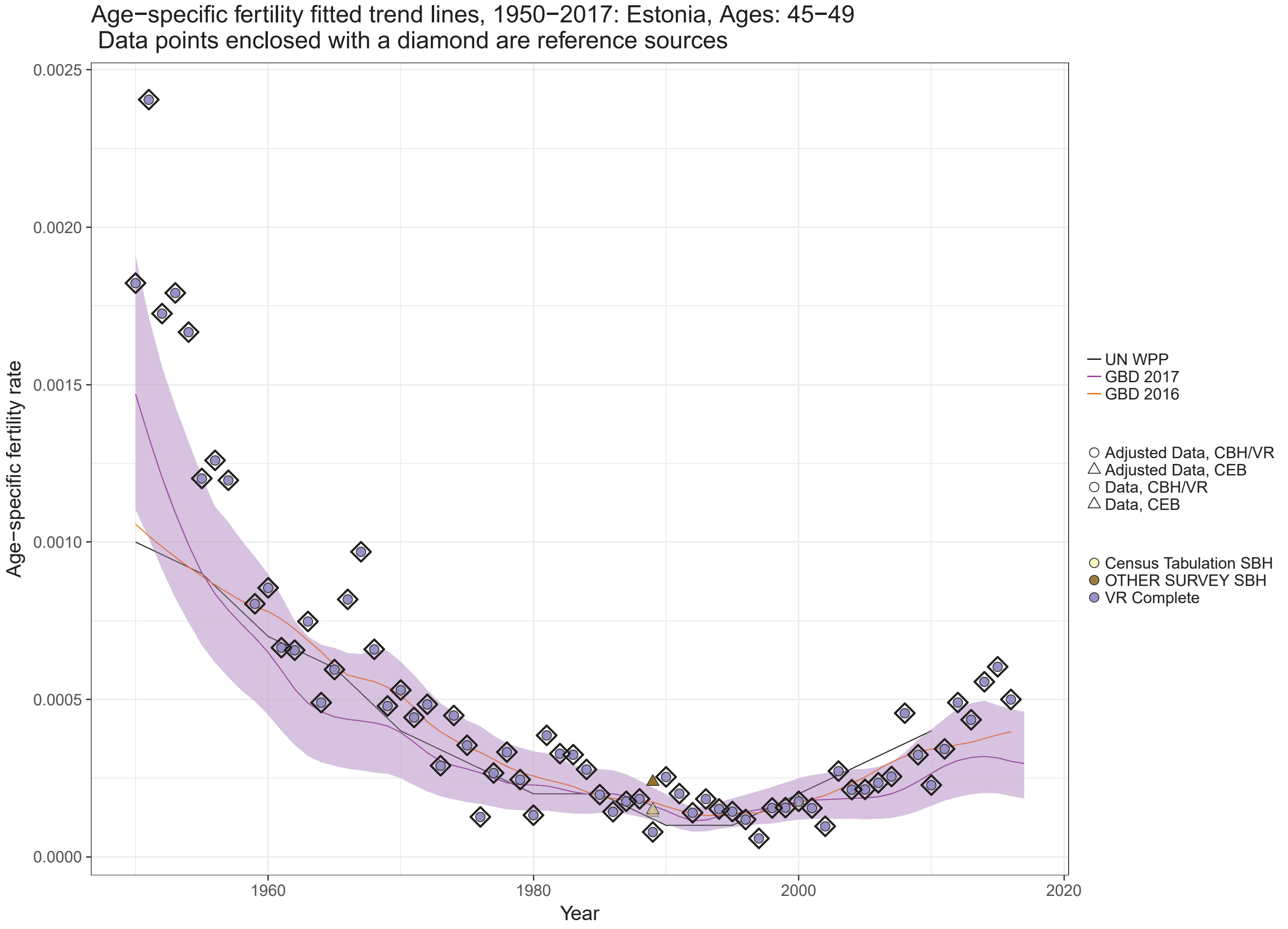
Age−specific fertility fitted trend lines, 1950−2017: Estonia, Ages: 45−49
Data points enclosed with a diamond are reference sources
Age−specific fertility rate
0.0025
0.0020
0.0015
0.0010
0.0005
0.0000
1960
1980
2000
2020
Year
UN WPP
GBD 2017
GBD 2016
Adjusted Data, CBH/VR
Adjusted Data, CEB
Data, CBH/VR
Data, CEB
Census Tabulation SBH
OTHER SURVEY SBH
VR Complete

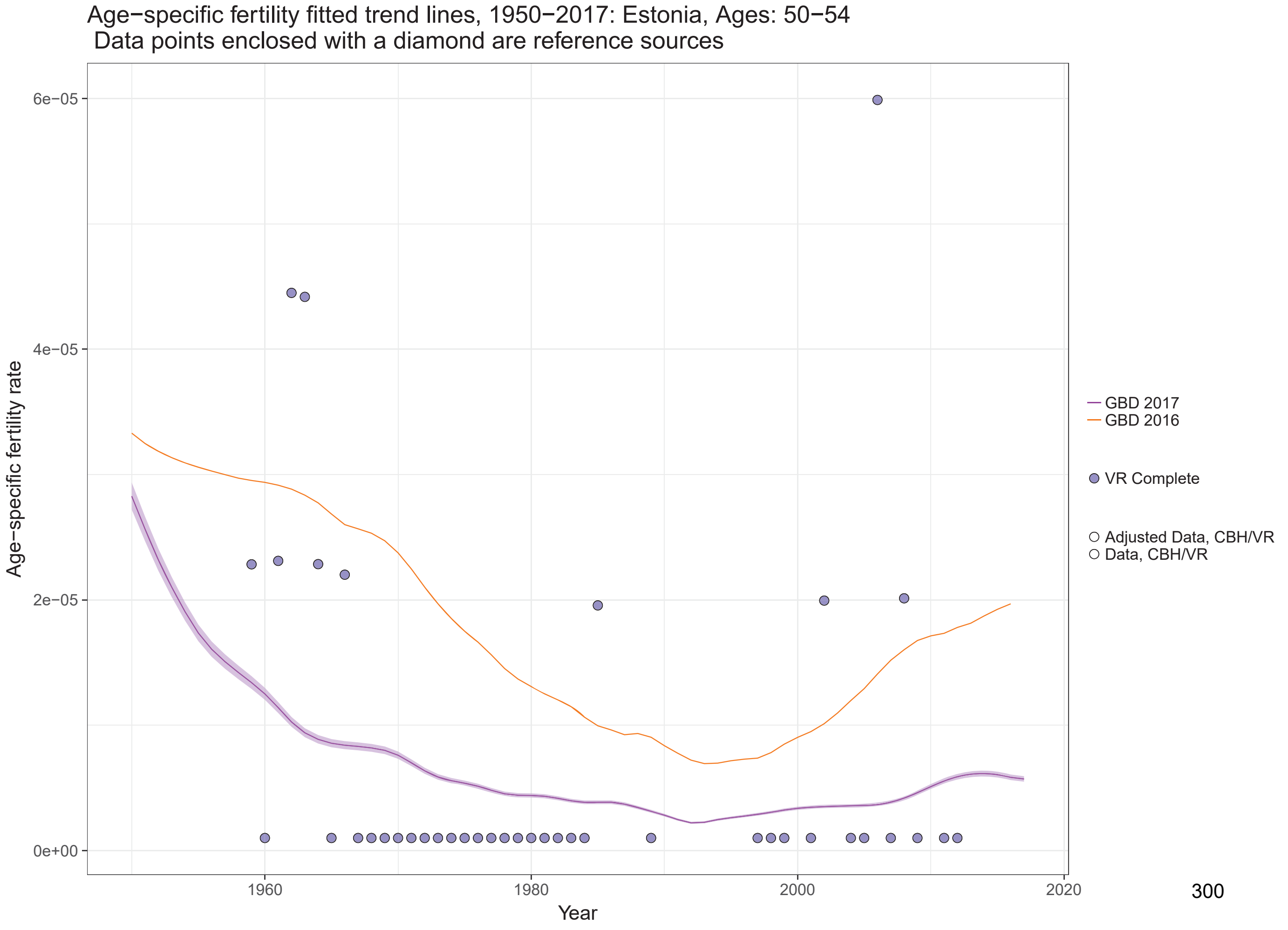
Age−specific fertility fitted trend lines, 1950−2017: Estonia, Ages: 50−54
Data points enclosed with a diamond are reference sources
Age−specific fertility rate
6e−05
4e−05
2e−05
0e+00
1960
1980
2000
2020
Year
GBD 2017
GBD 2016
VR Complete
Adjusted Data, CBH/VR
Data, CBH/VR

Age−specific fertility fitted trend lines, 1950−2017: Ethiopia, TFR
Data points enclosed with a diamond are reference sources

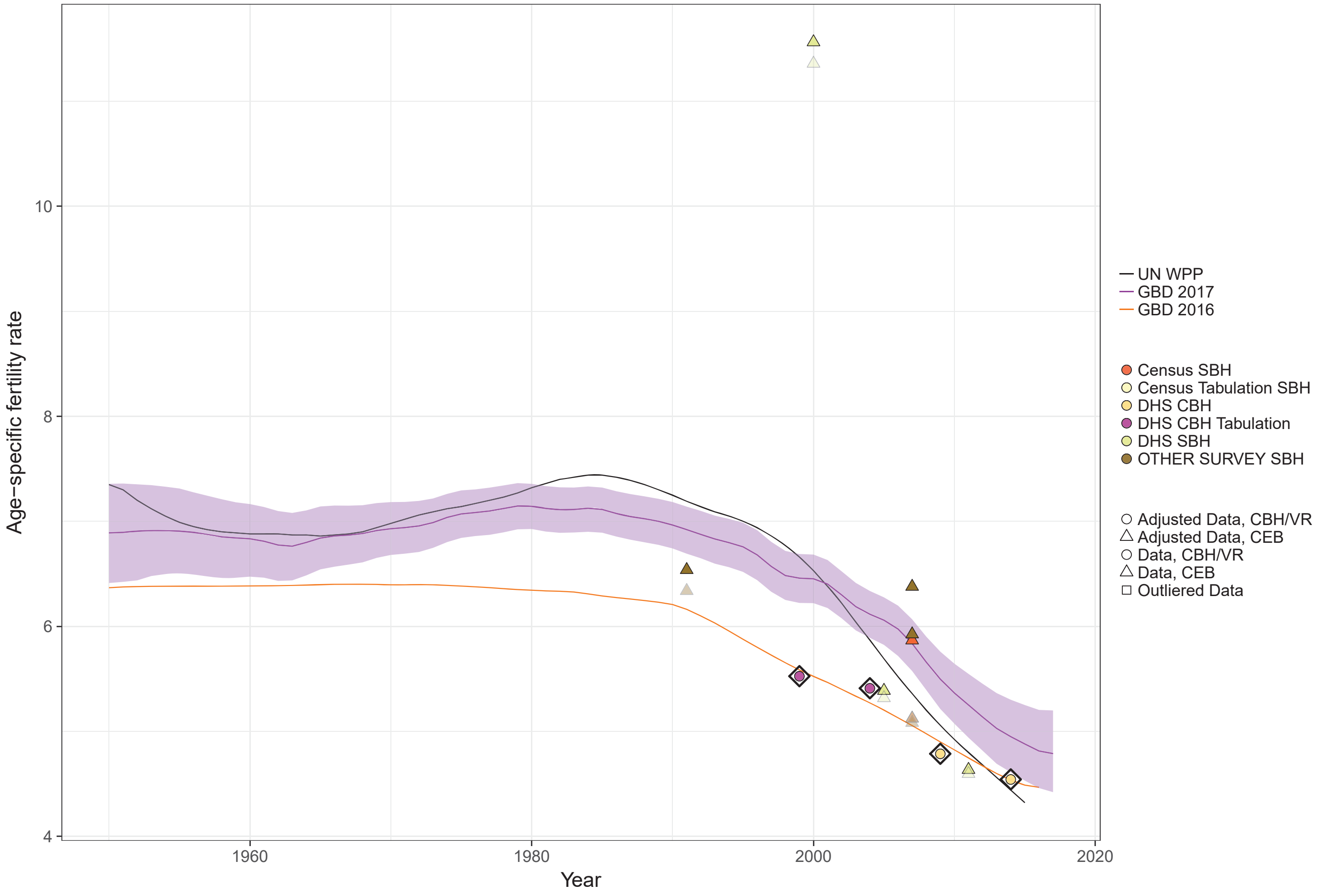

Age−specific fertility fitted trend lines, 1950−2017: Ethiopia, Ages: 10−14
Data points enclosed with a diamond are reference sources

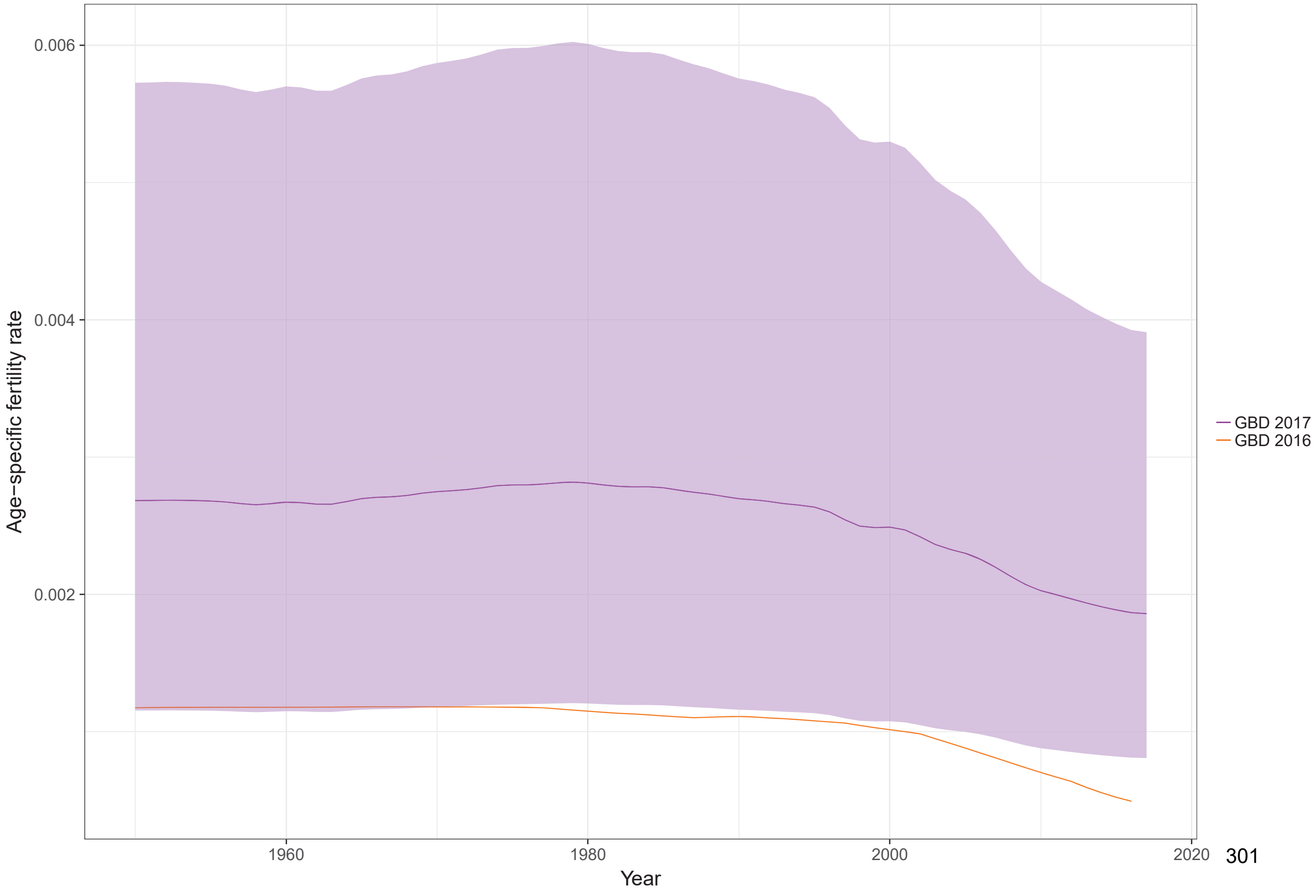

Age−specific fertility fitted trend lines, 1950−2017: Ethiopia, Ages: 15−19
Data points enclosed with a diamond are reference sources

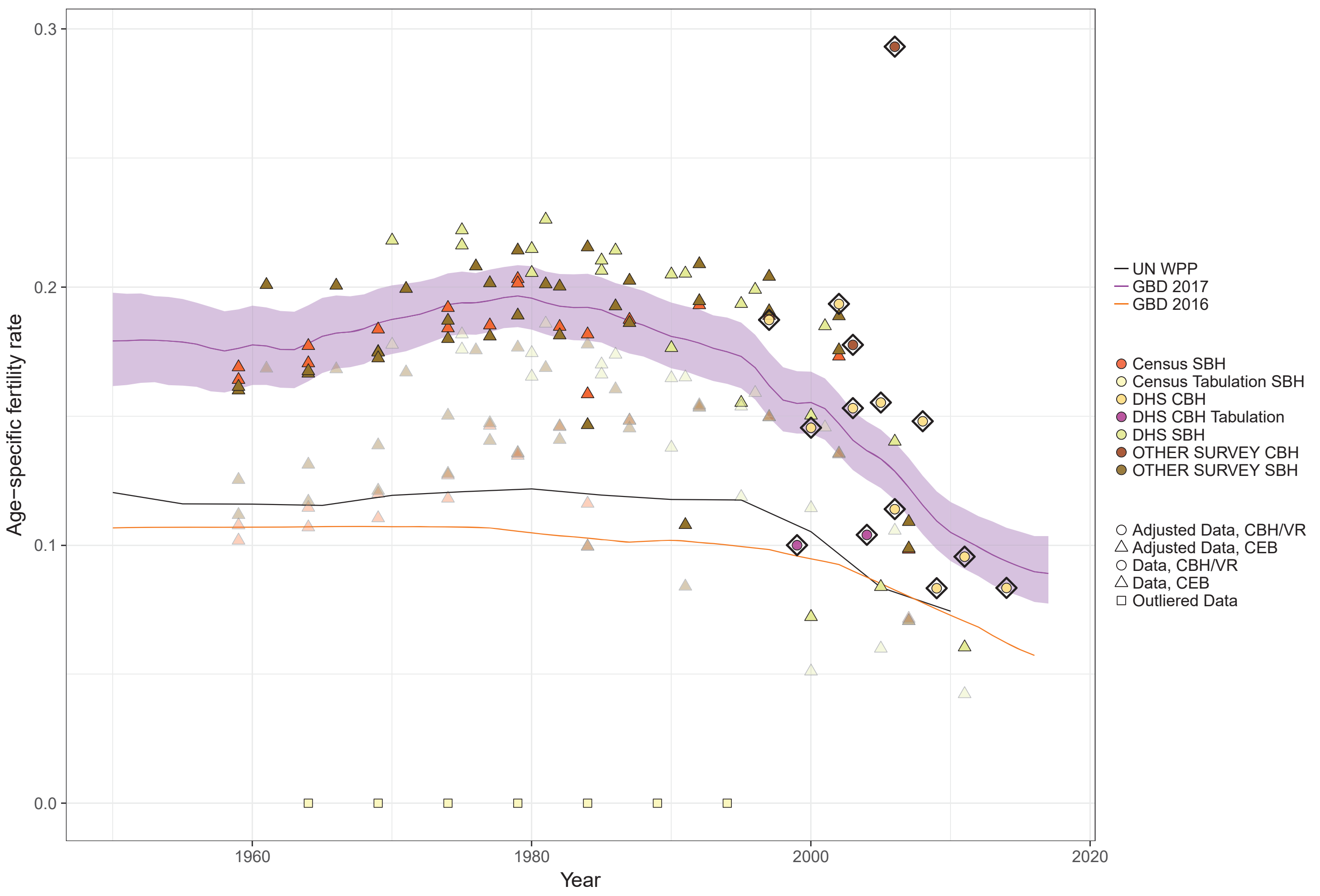

Age−specific fertility fitted trend lines, 1950−2017: Ethiopia, Ages: 20−24
Data points enclosed with a diamond are reference sources

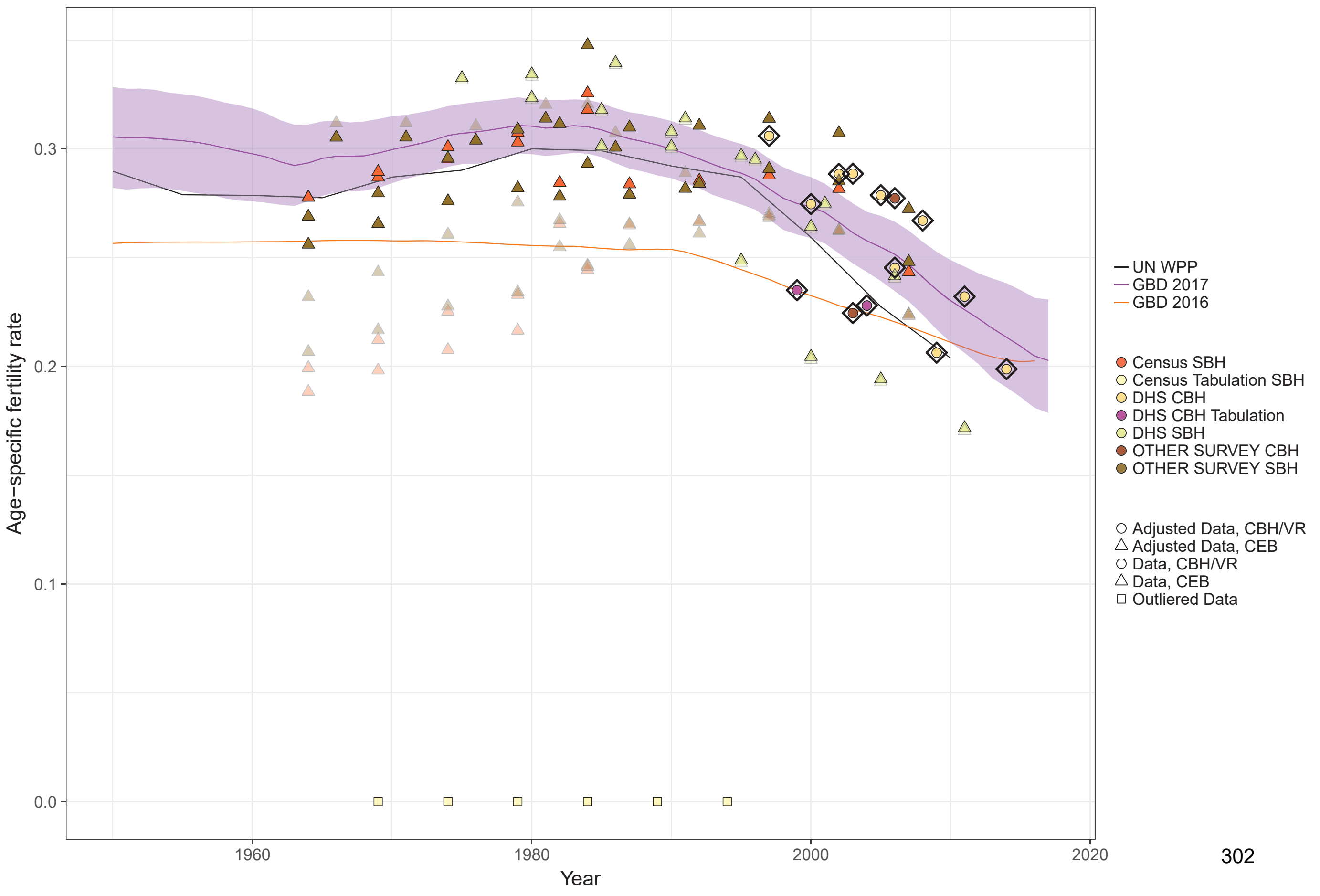

Age−specific fertility fitted trend lines, 1950−2017: Ethiopia, Ages: 25−29
Data points enclosed with a diamond are reference sources

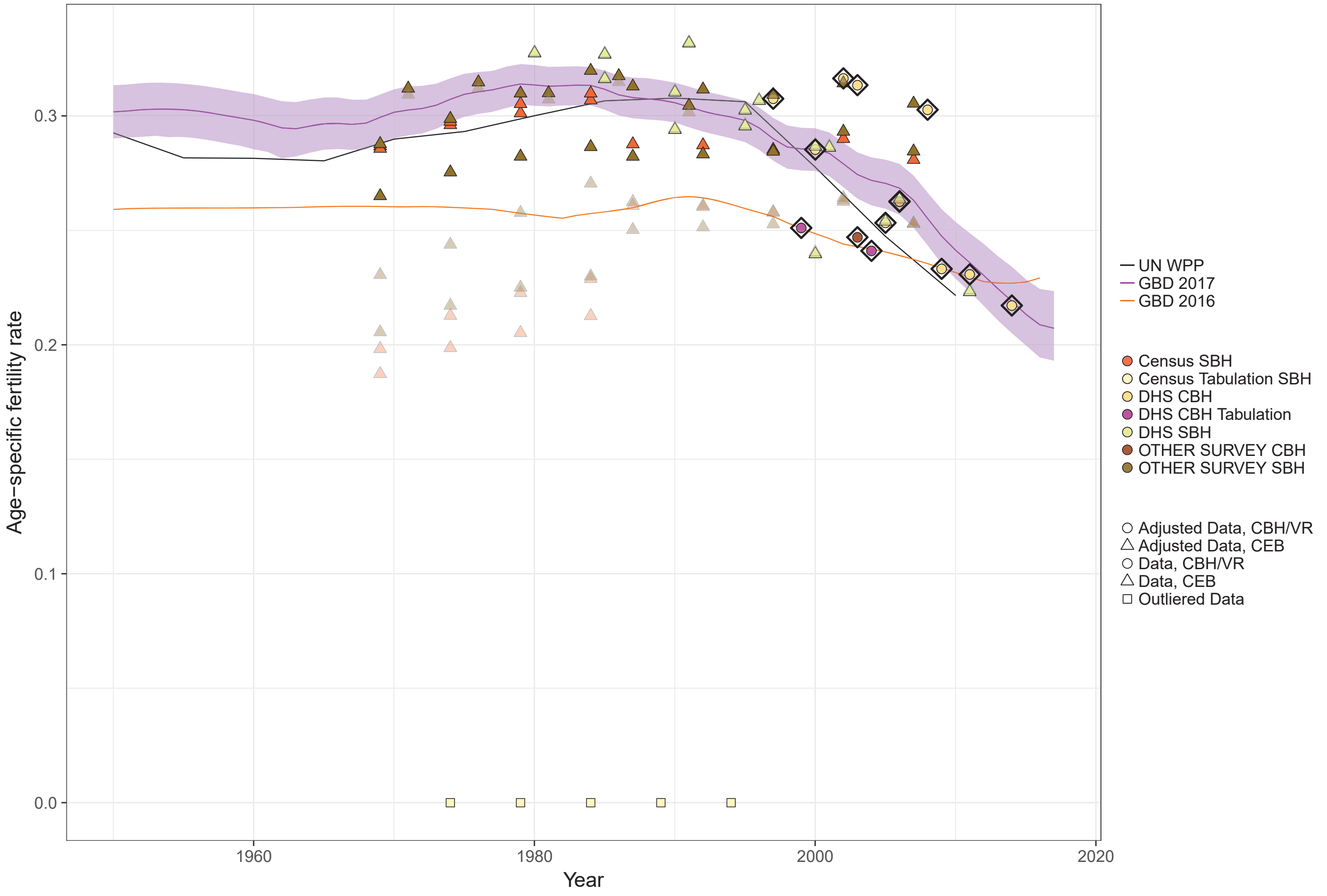

Age−specific fertility fitted trend lines, 1950−2017: Ethiopia, Ages: 30−34
Data points enclosed with a diamond are reference sources

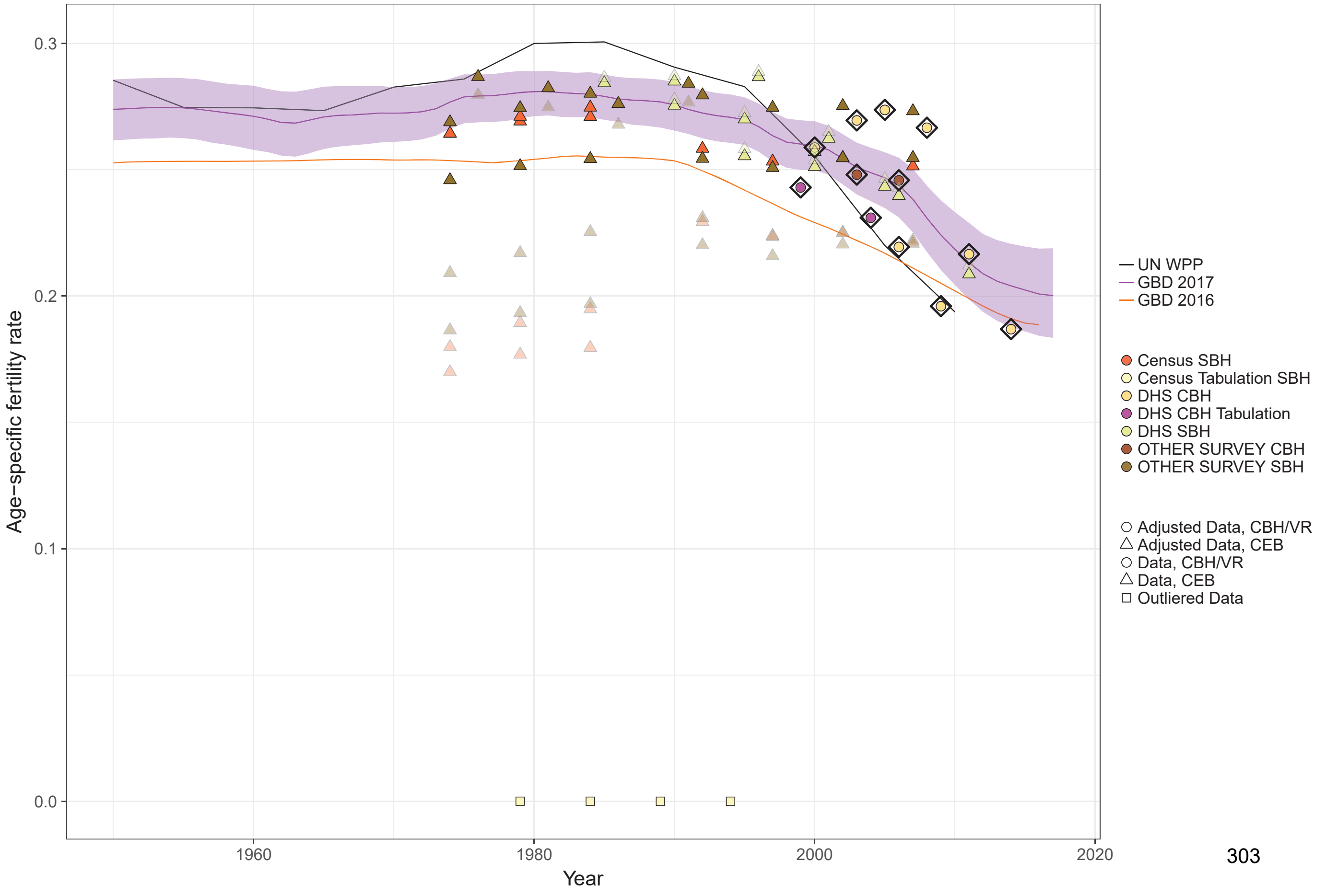

Age−specific fertility fitted trend lines, 1950−2017: Ethiopia, Ages: 35−39
Data points enclosed with a diamond are reference sources

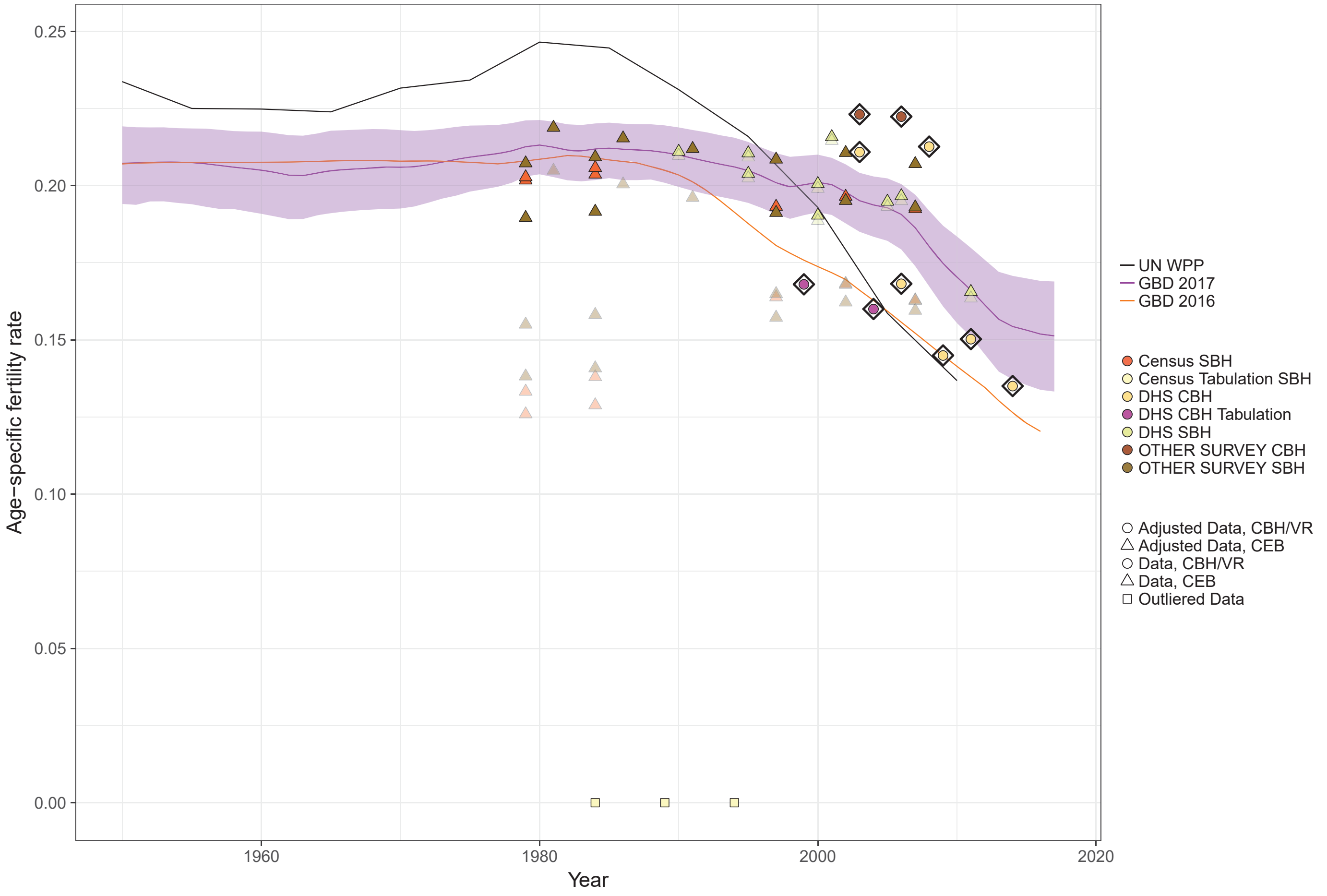

Age−specific fertility fitted trend lines, 1950−2017: Ethiopia, Ages: 40−44
Data points enclosed with a diamond are reference sources

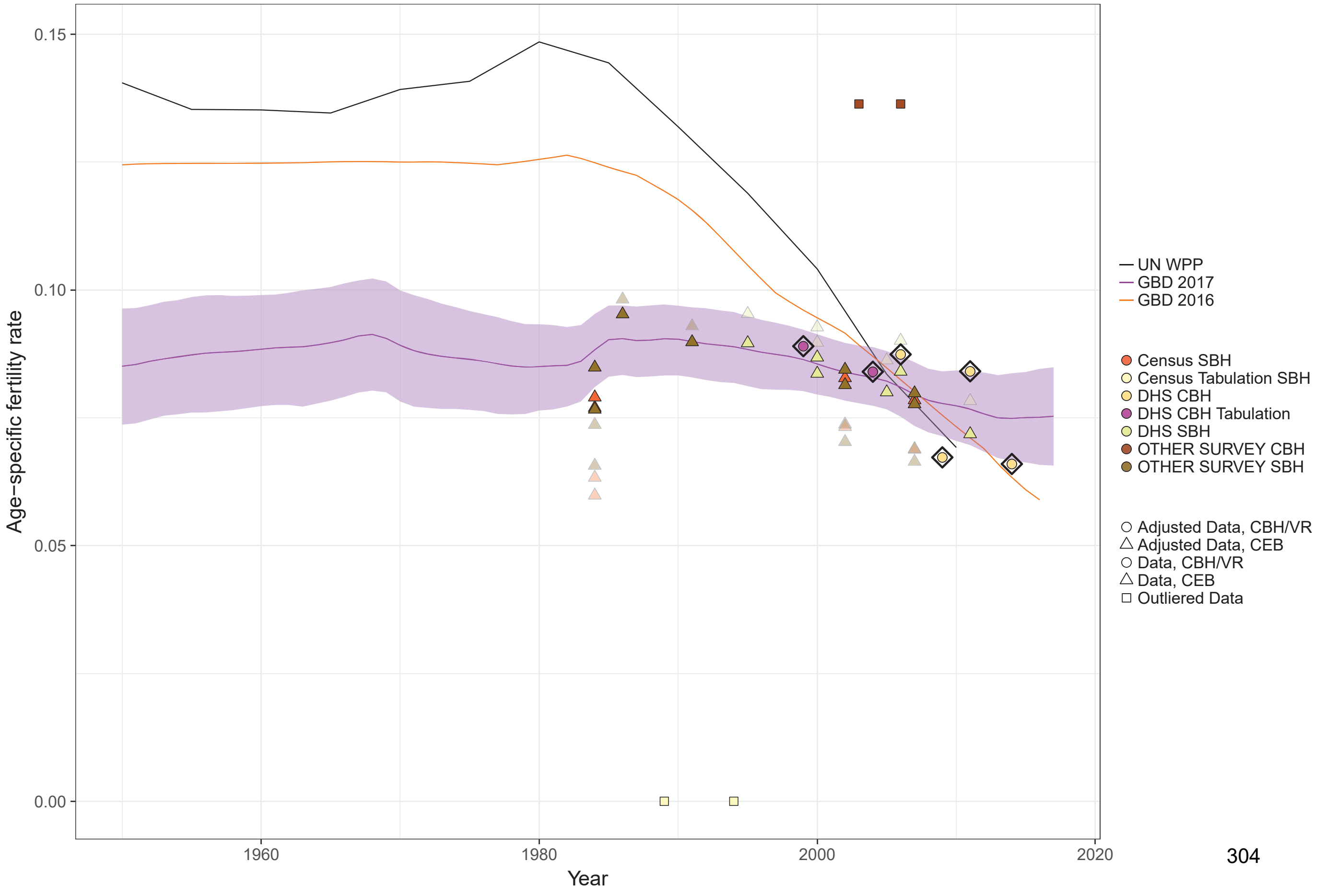

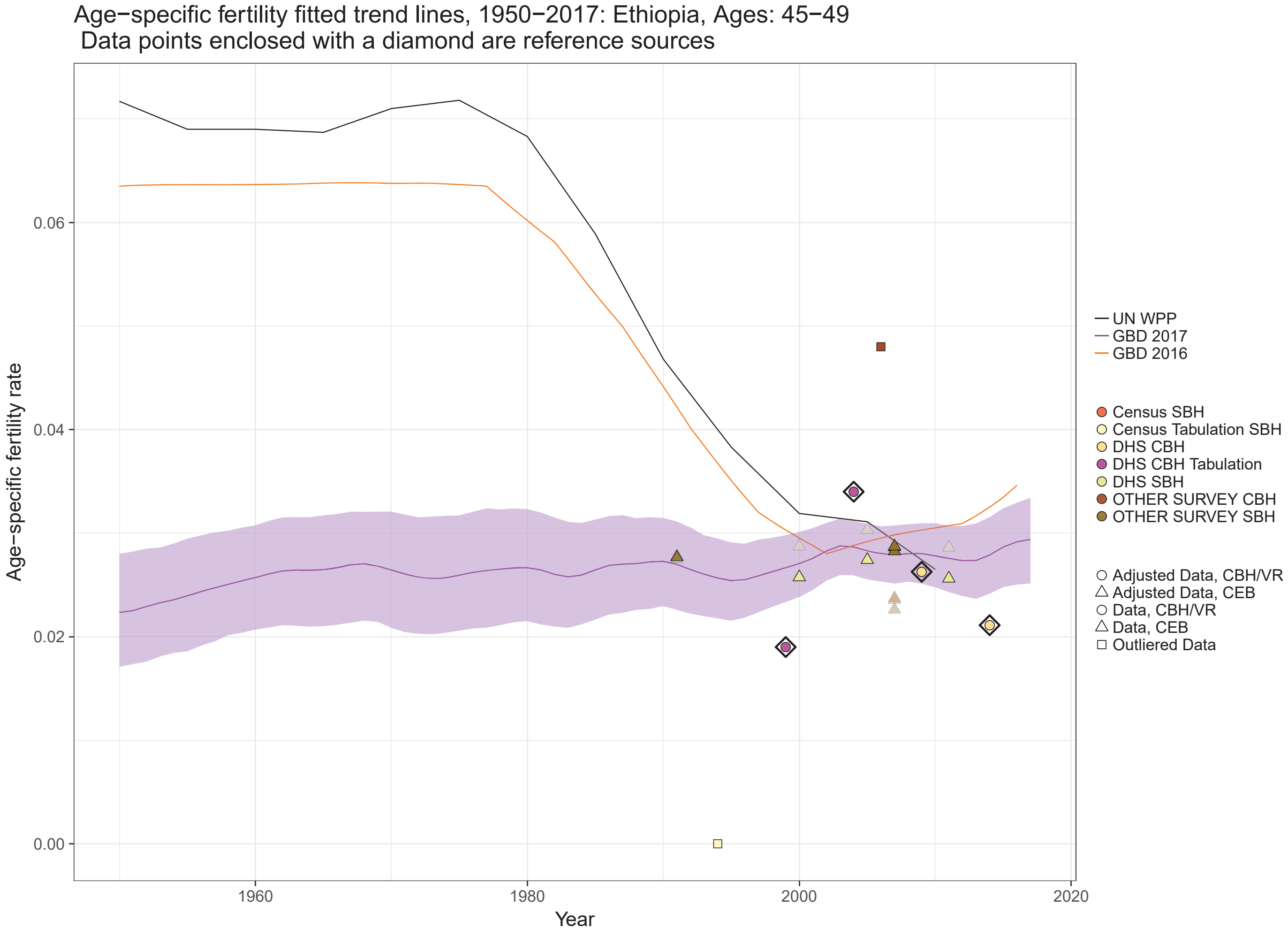
Age−specific fertility fitted trend lines, 1950−2017: Ethiopia, Ages: 45−49
Data points enclosed with a diamond are reference sources
Age−specific fertility rate
0.00
0.02
0.04
0.06
1960
1980
2000
2020
Year
UN WPP
GBD 2017
GBD 2016
Census SBH
Census Tabulation SBH
DHS CBH
DHS CBH Tabulation
DHS SBH
OTHER SURVEY CBH
OTHER SURVEY SBH
Adjusted Data, CBH/VR
Adjusted Data, CEB
Data, CBH/VR
Data, CEB
Outliered Data

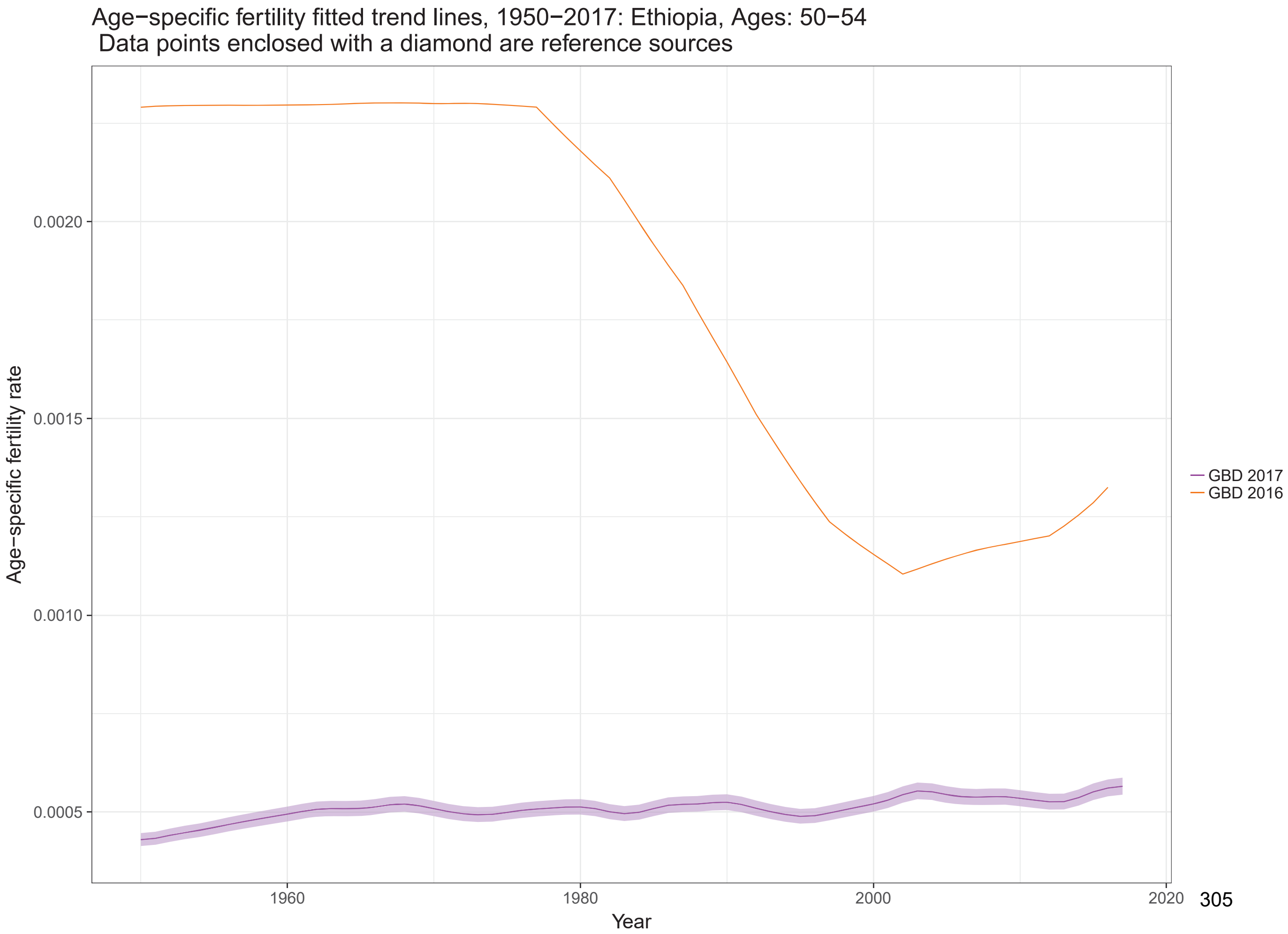
Age−specific fertility fitted trend lines, 1950−2017: Ethiopia, Ages: 50−54
Data points enclosed with a diamond are reference sources
Age−specific fertility rate
0.0005
0.0010
0.0015
0.0020
1960
1980
2000
2020
Year
GBD 2017
GBD 2016

Age−specific fertility fitted trend lines, 1950−2017: Federated States of Micronesia, TFR
Data points enclosed with a diamond are reference sources

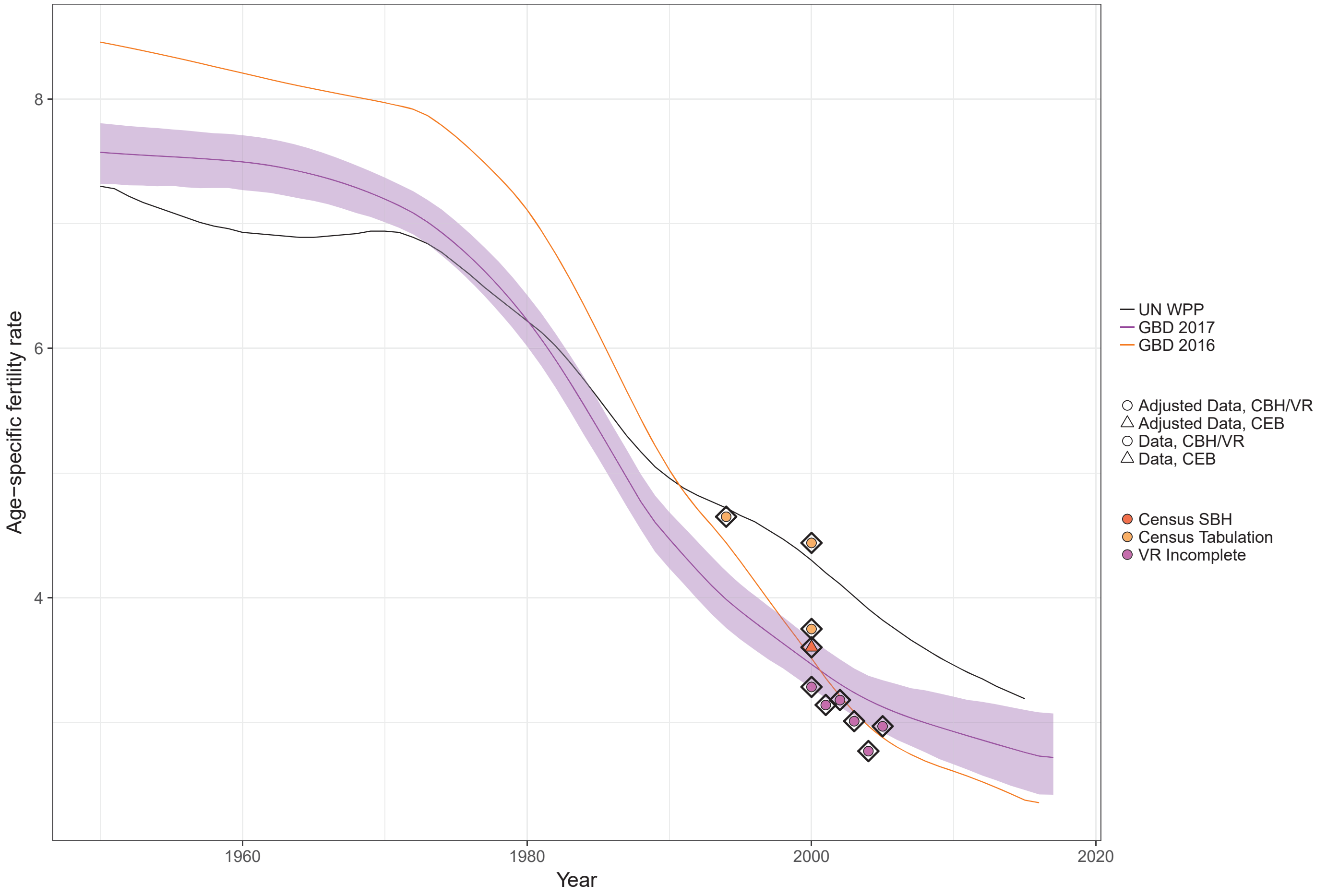

Age−specific fertility fitted trend lines, 1950−2017: Federated States of Micronesia, Ages: 10−14
Data points enclosed with a diamond are reference sources

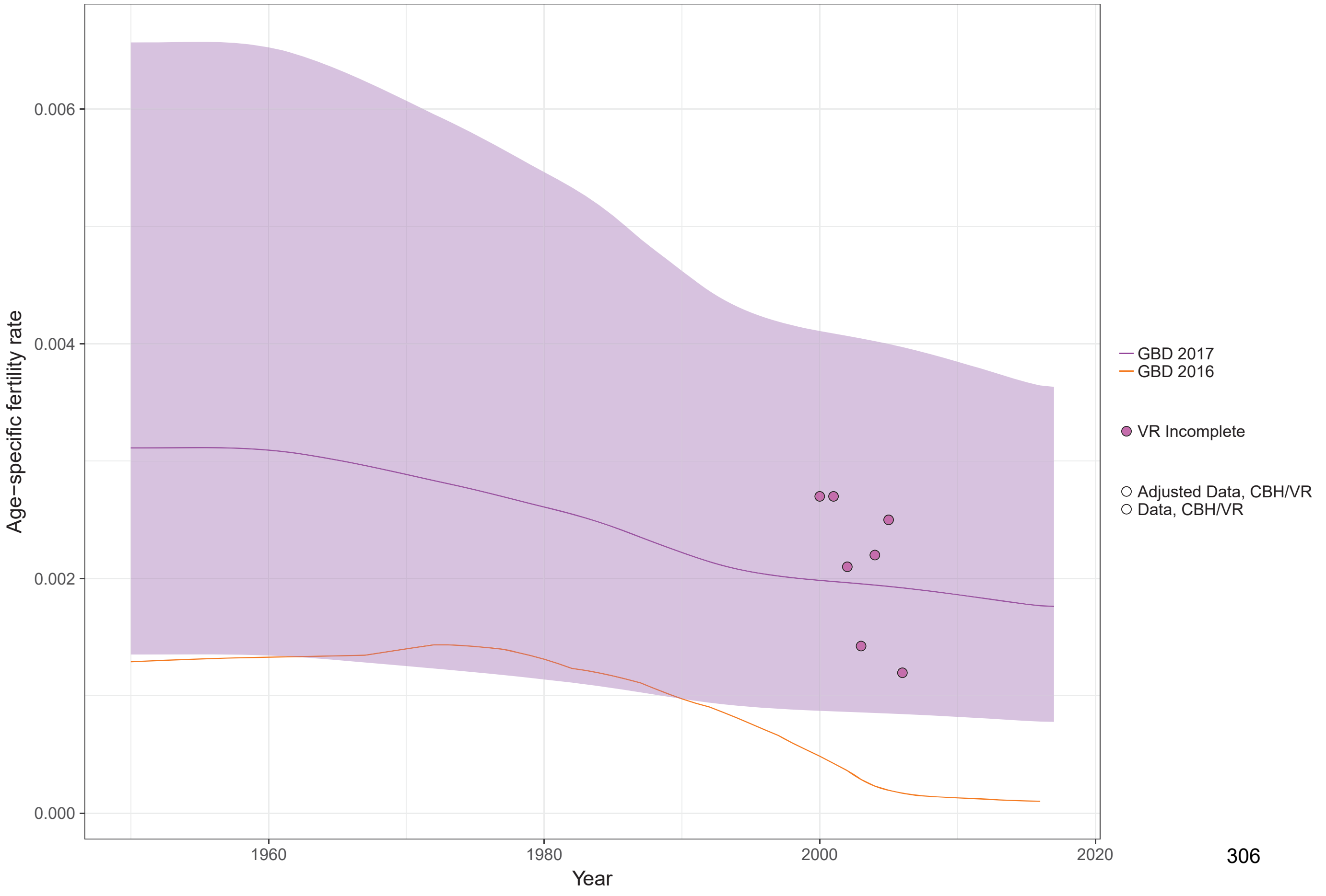

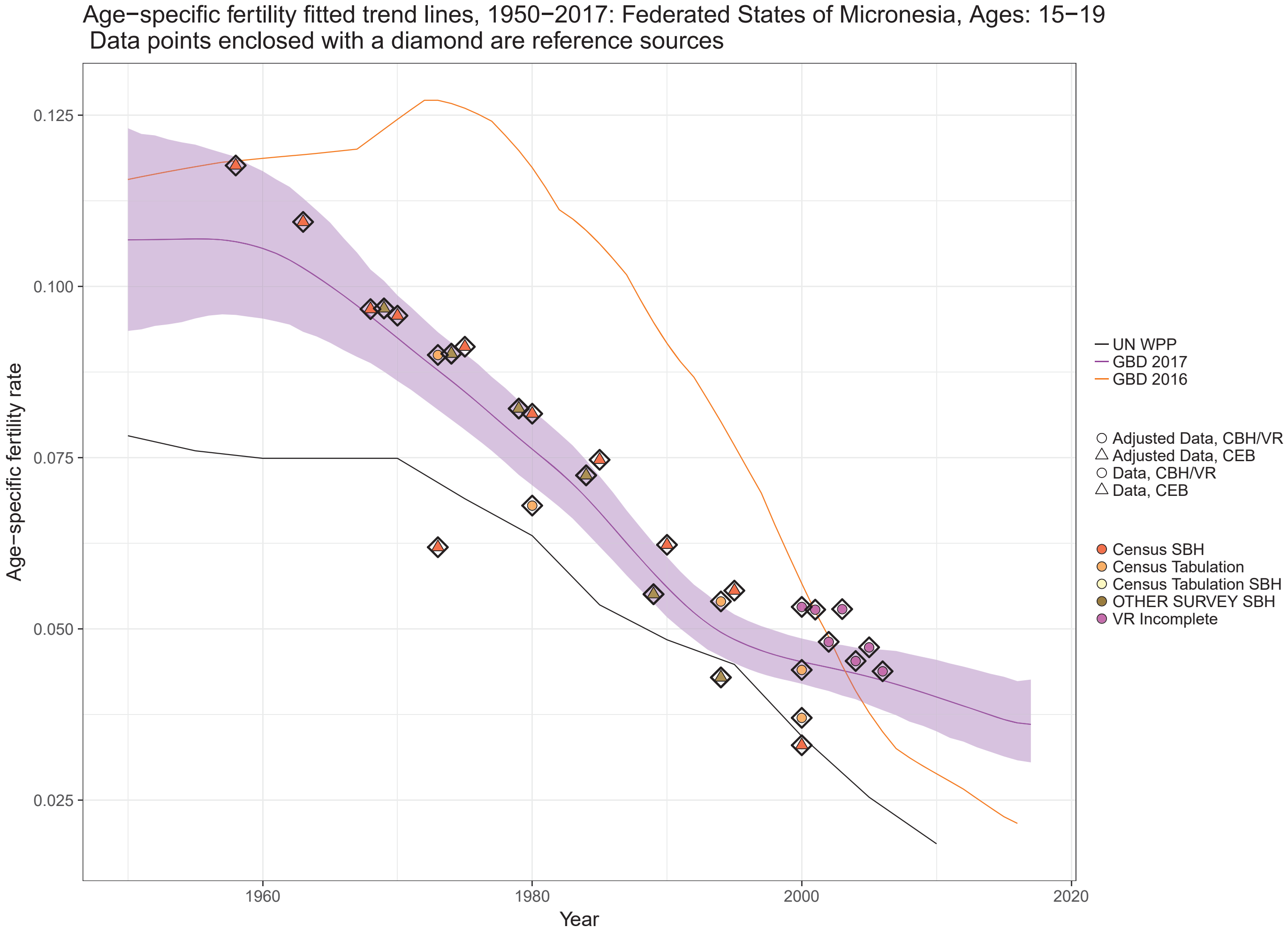

Age−specific fertility fitted trend lines, 1950−2017: Federated States of Micronesia, Ages: 15−19
Data points enclosed with a diamond are reference sources
Age−specific fertility rate
0.125
0.100
0.075
0.050
0.025
1960
1980
2000
2020
Year
UN WPP
GBD 2017
GBD 2016
Adjusted Data, CBH/VR
Adjusted Data, CEB
Data, CBH/VR
Data, CEB
Census SBH
Census Tabulation
Census Tabulation SBH
OTHER SURVEY SBH
VR Incomplete

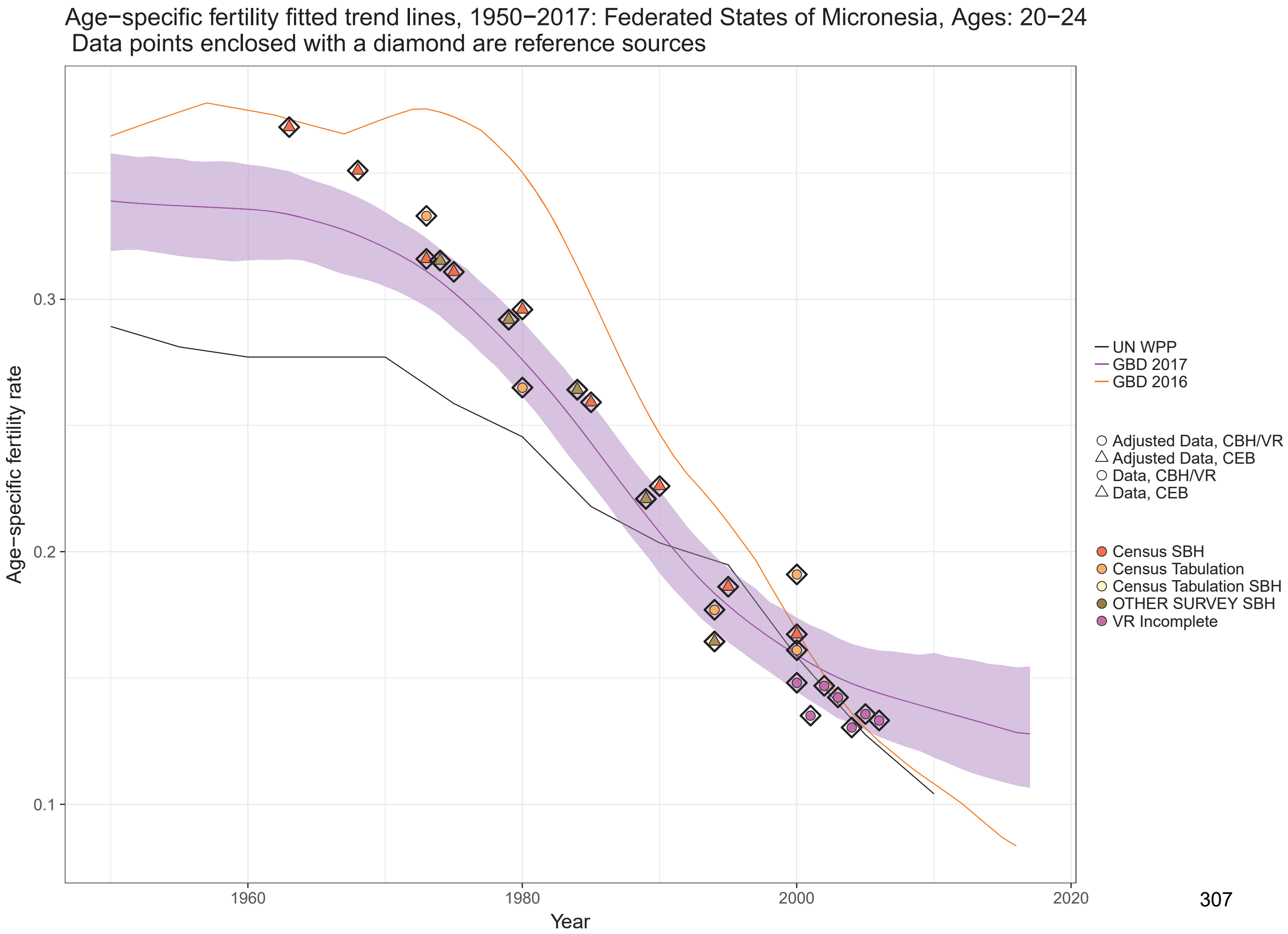

Age−specific fertility fitted trend lines, 1950−2017: Federated States of Micronesia, Ages: 20−24
Data points enclosed with a diamond are reference sources
Age−specific fertility rate
0.3
0.2
0.1
1960
1980
2000
2020
Year
UN WPP
GBD 2017
GBD 2016
Adjusted Data, CBH/VR
Adjusted Data, CEB
Data, CBH/VR
Data, CEB
Census SBH
Census Tabulation
Census Tabulation SBH
OTHER SURVEY SBH
VR Incomplete

Age−specific fertility fitted trend lines, 1950−2017: Federated States of Micronesia, Ages: 25−29
Data points enclosed with a diamond are reference sources

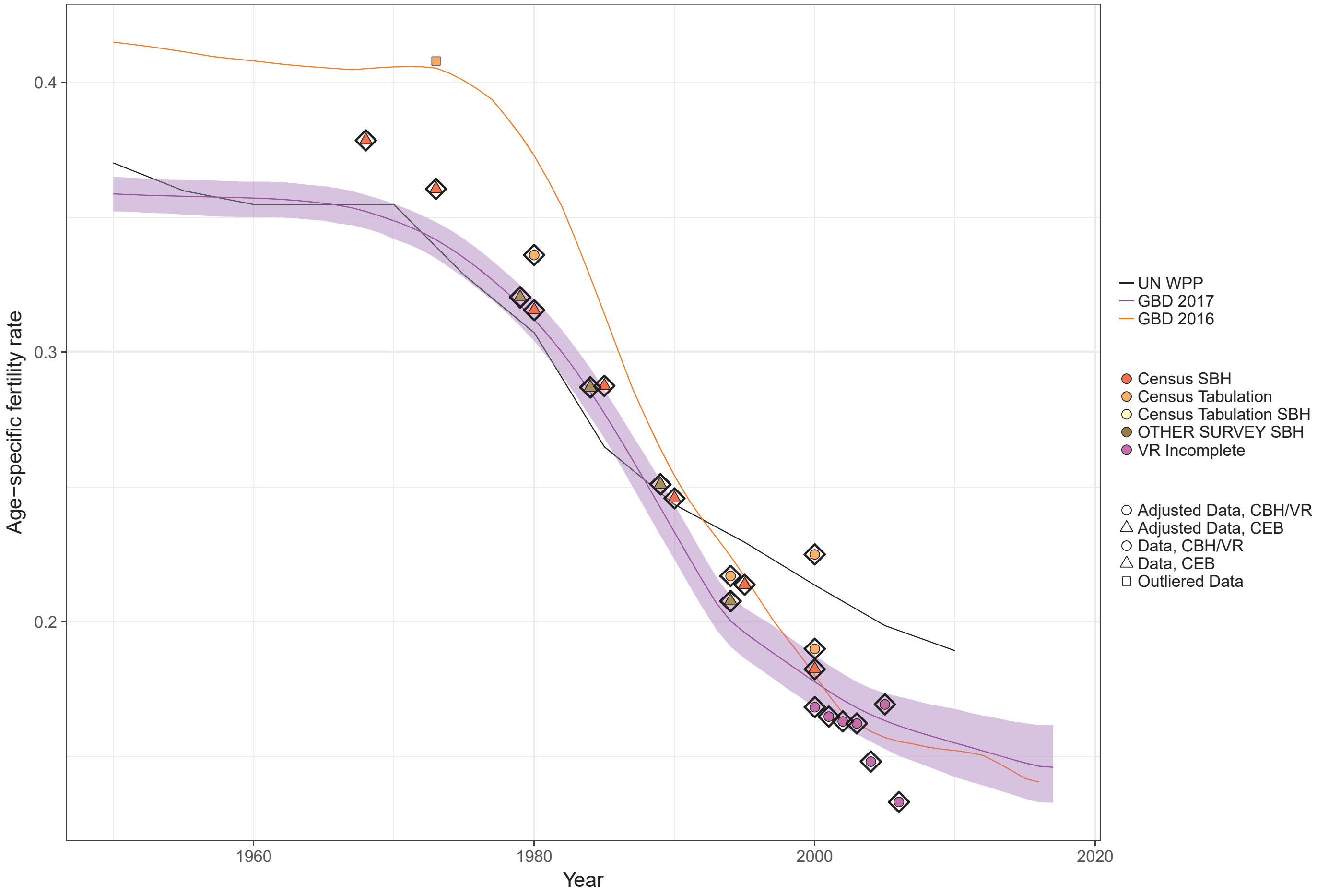

Age−specific fertility fitted trend lines, 1950−2017: Federated States of Micronesia, Ages: 30−34
Data points enclosed with a diamond are reference sources

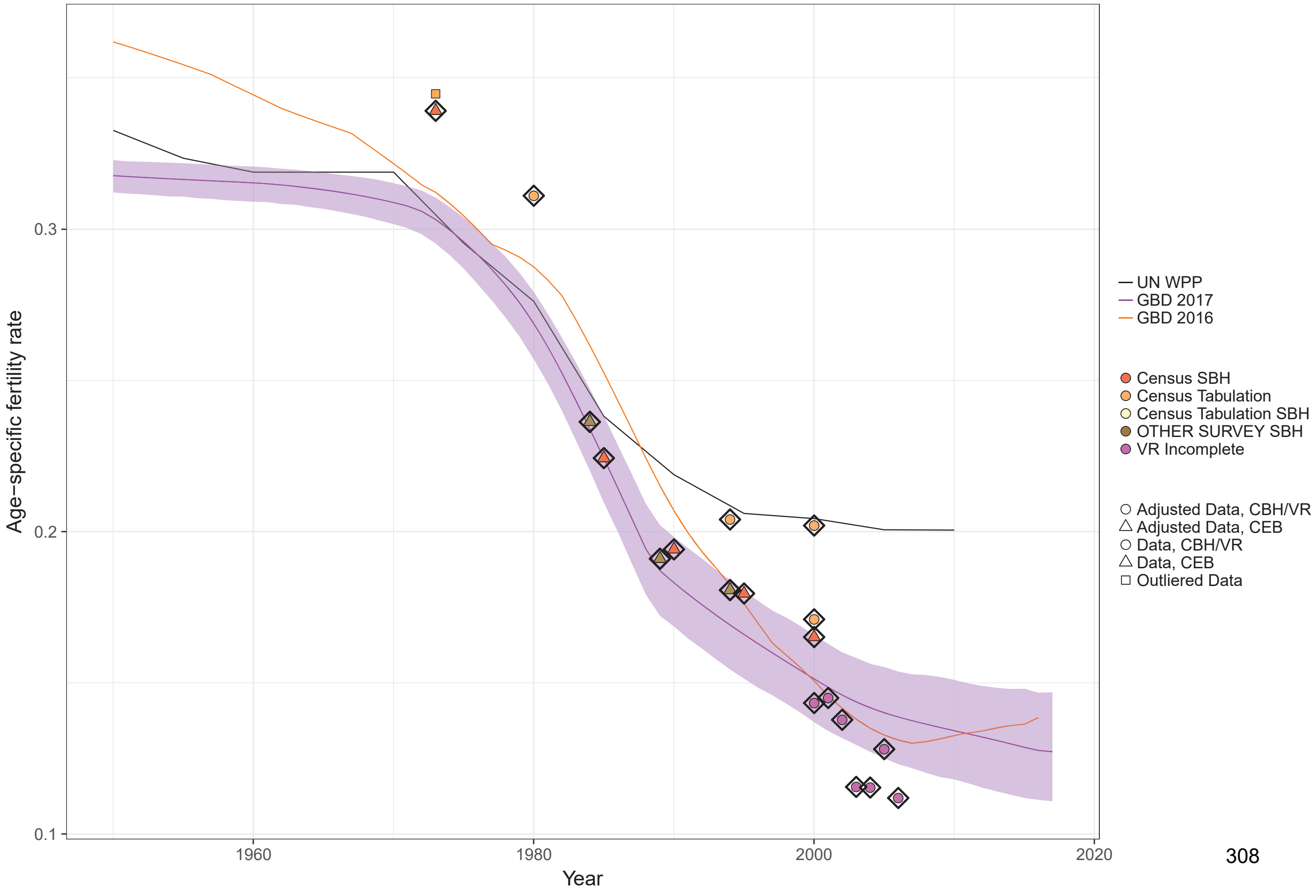

Age−specific fertility fitted trend lines, 1950−2017: Federated States of Micronesia, Ages: 35−39
Data points enclosed with a diamond are reference sources

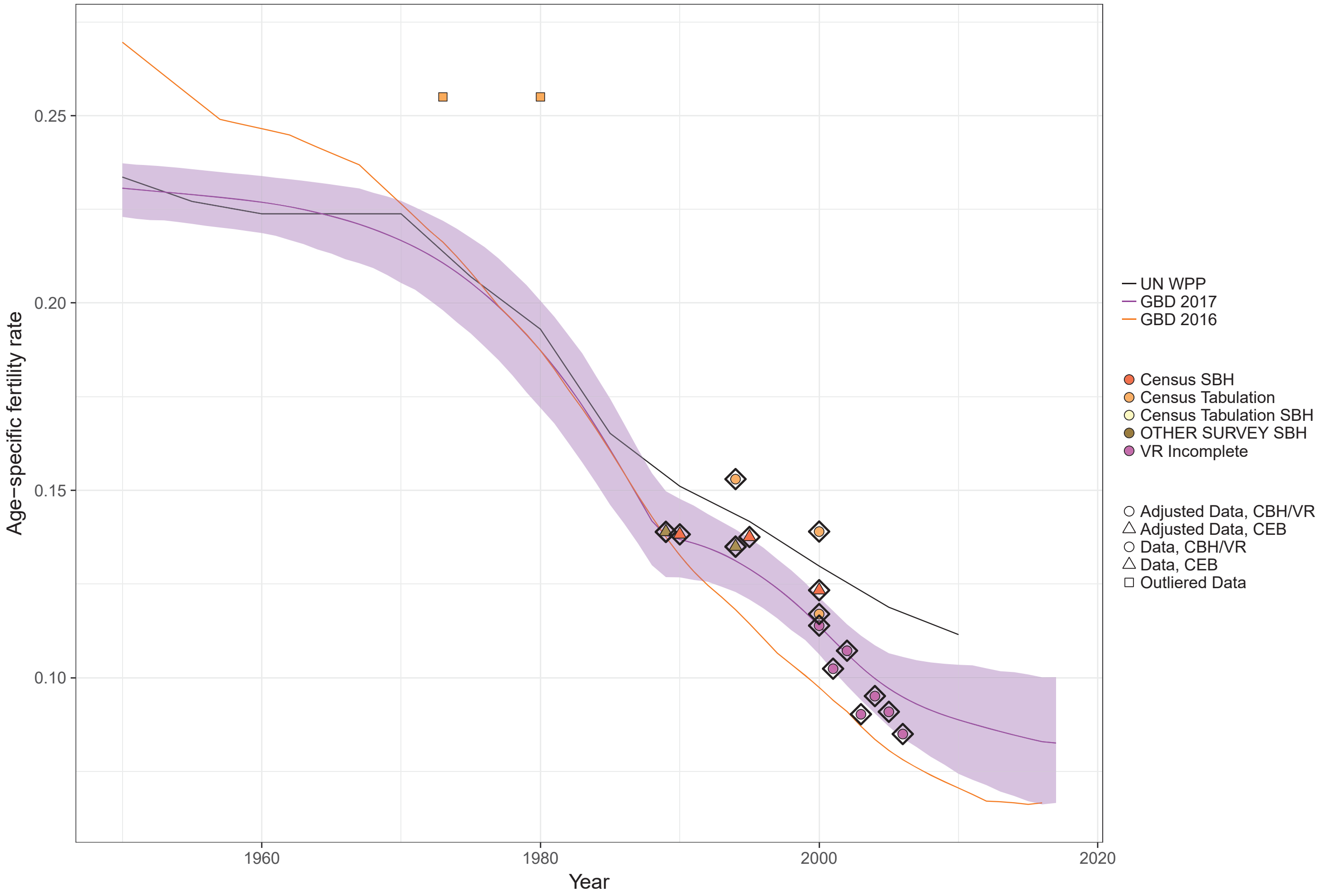

Age−specific fertility fitted trend lines, 1950−2017: Federated States of Micronesia, Ages: 40−44
Data points enclosed with a diamond are reference sources

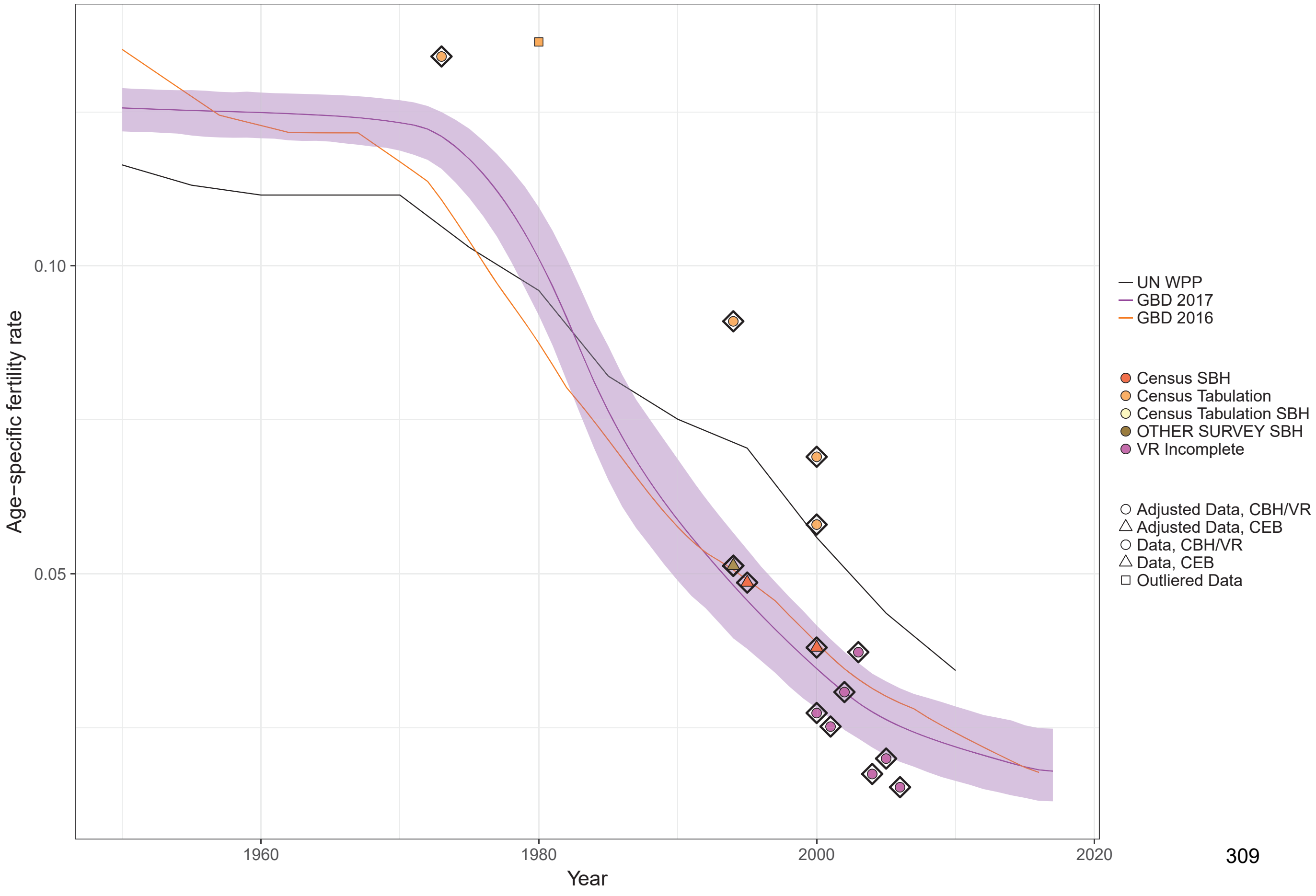

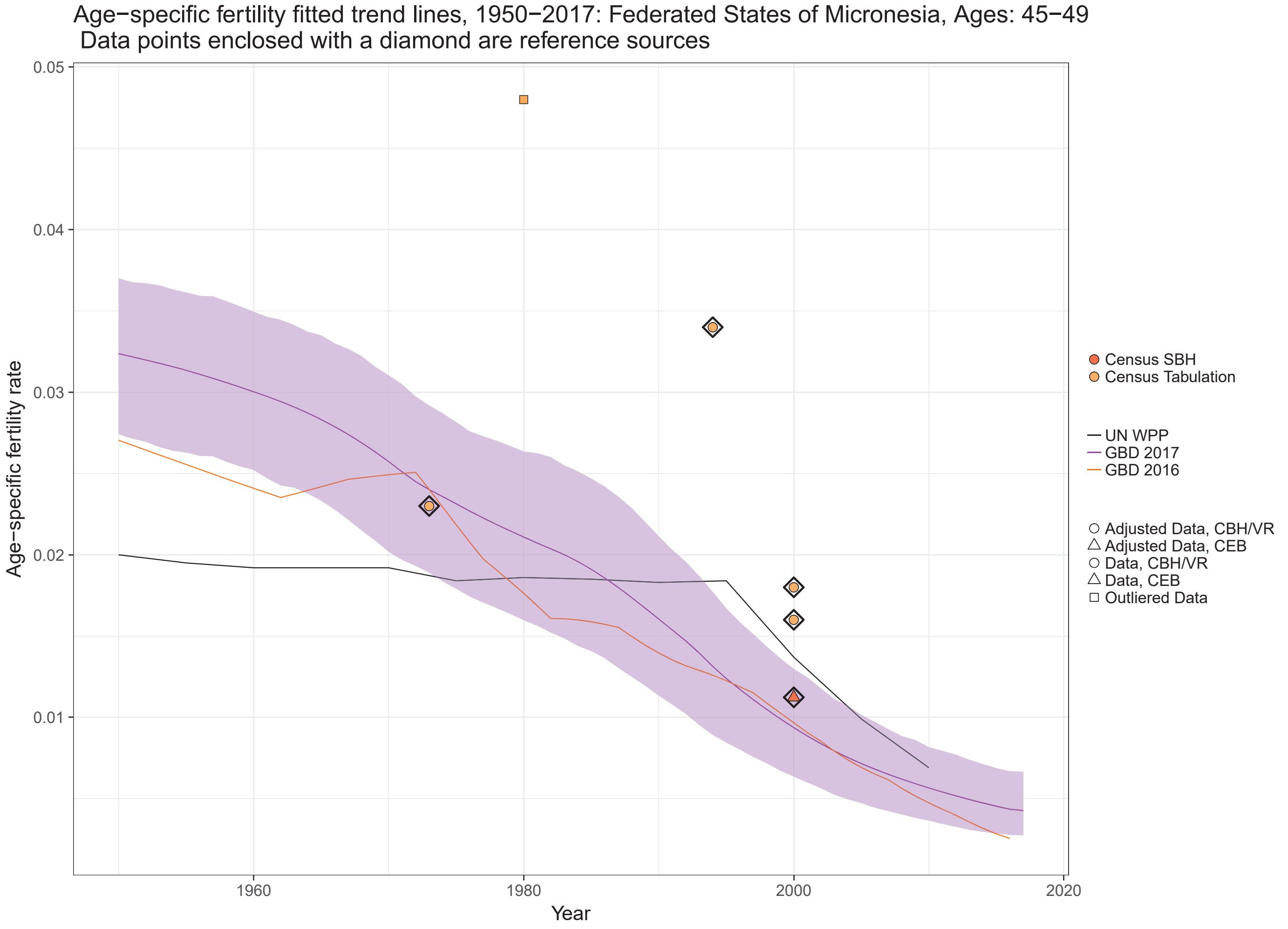

Age−specific fertility fitted trend lines, 1950−2017: Federated States of Micronesia, Ages: 45−49
Data points enclosed with a diamond are reference sources
Age−specific fertility rate
Year
0.01
0.02
0.03
0.04
0.05
1960
1980
2000
2020
Census SBH
Census Tabulation
UN WPP
GBD 2017
GBD 2016
Adjusted Data, CBH/VR
Adjusted Data, CEB
Data, CBH/VR
Data, CEB
Outliered Data

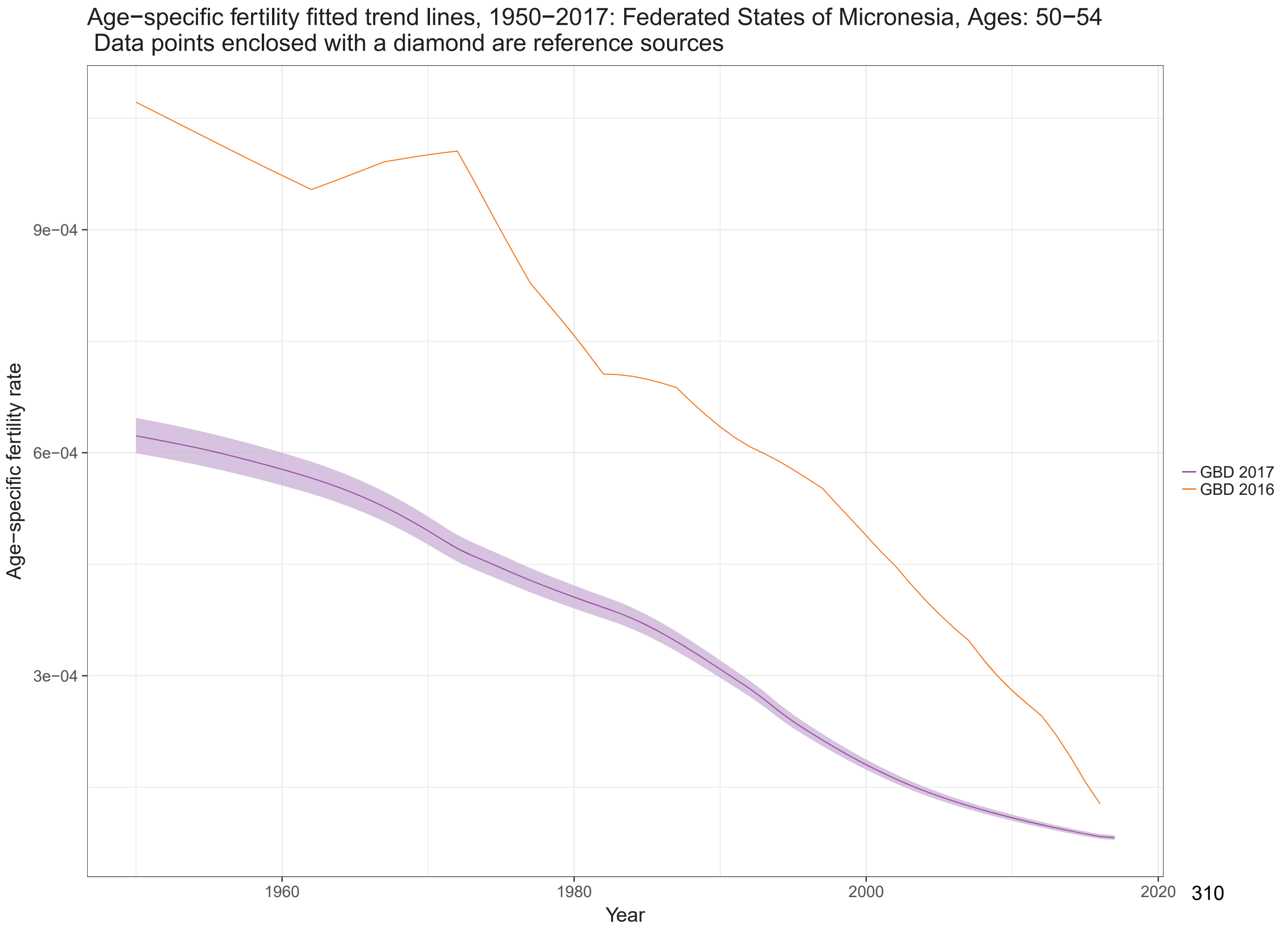

Age−specific fertility fitted trend lines, 1950−2017: Federated States of Micronesia, Ages: 50−54
Data points enclosed with a diamond are reference sources
Age−specific fertility rate
Year
3e−04
6e−04
9e−04
1960
1980
2000
2020
GBD 2017
GBD 2016

Age−specific fertility fitted trend lines, 1950−2017: Fiji, TFR
Data points enclosed with a diamond are reference sources

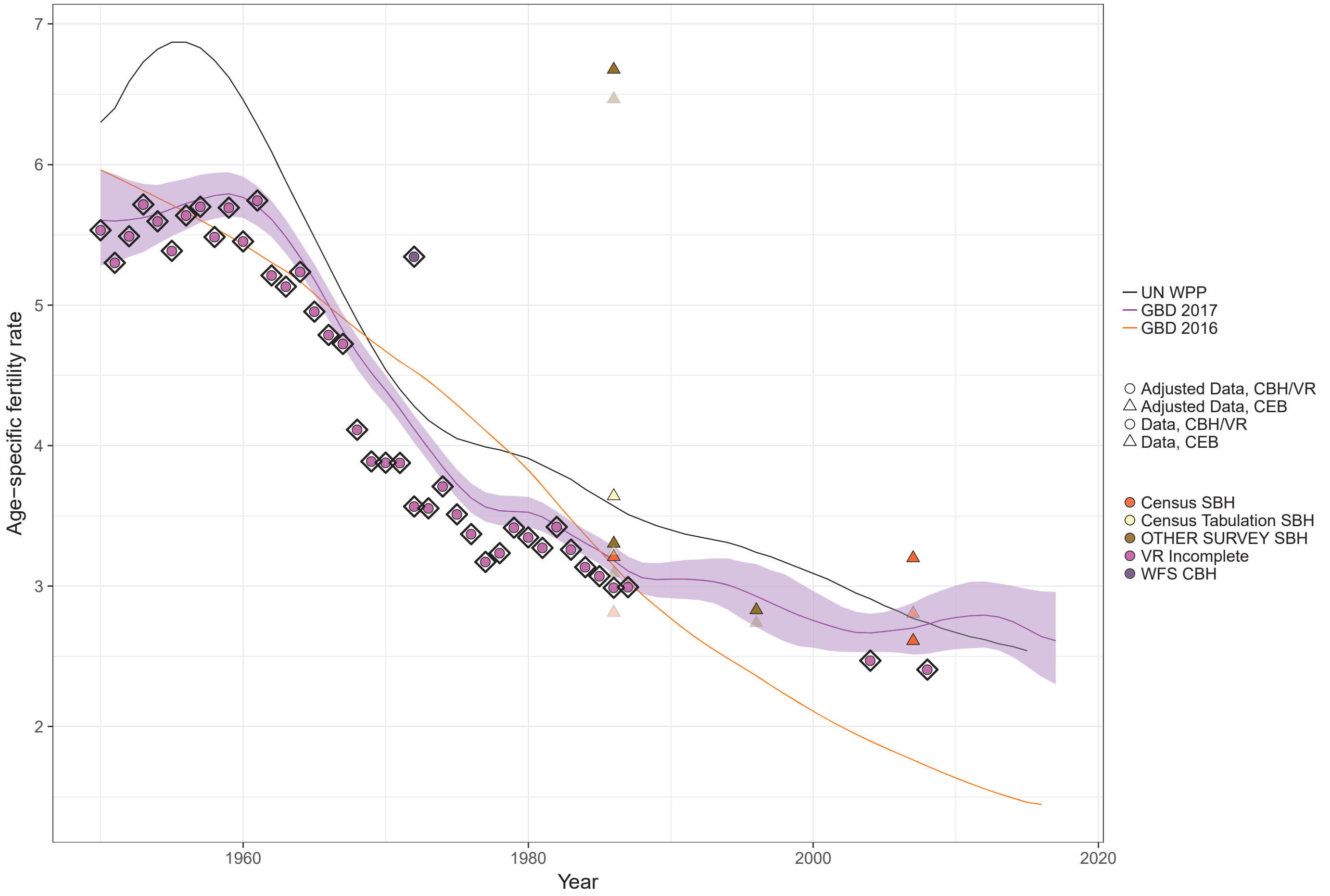

Age−specific fertility fitted trend lines, 1950−2017: Fiji, Ages: 10−14
Data points enclosed with a diamond are reference sources

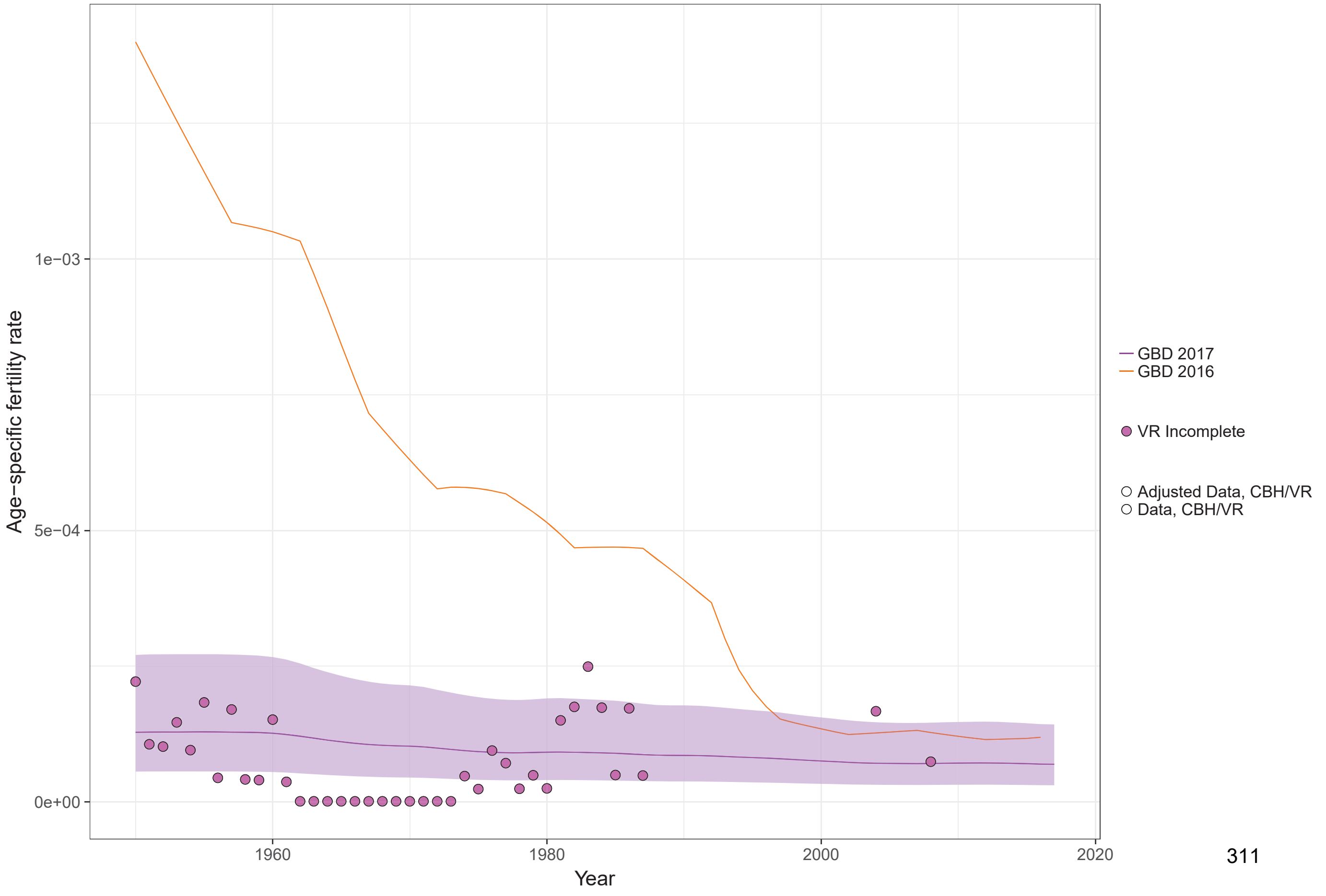

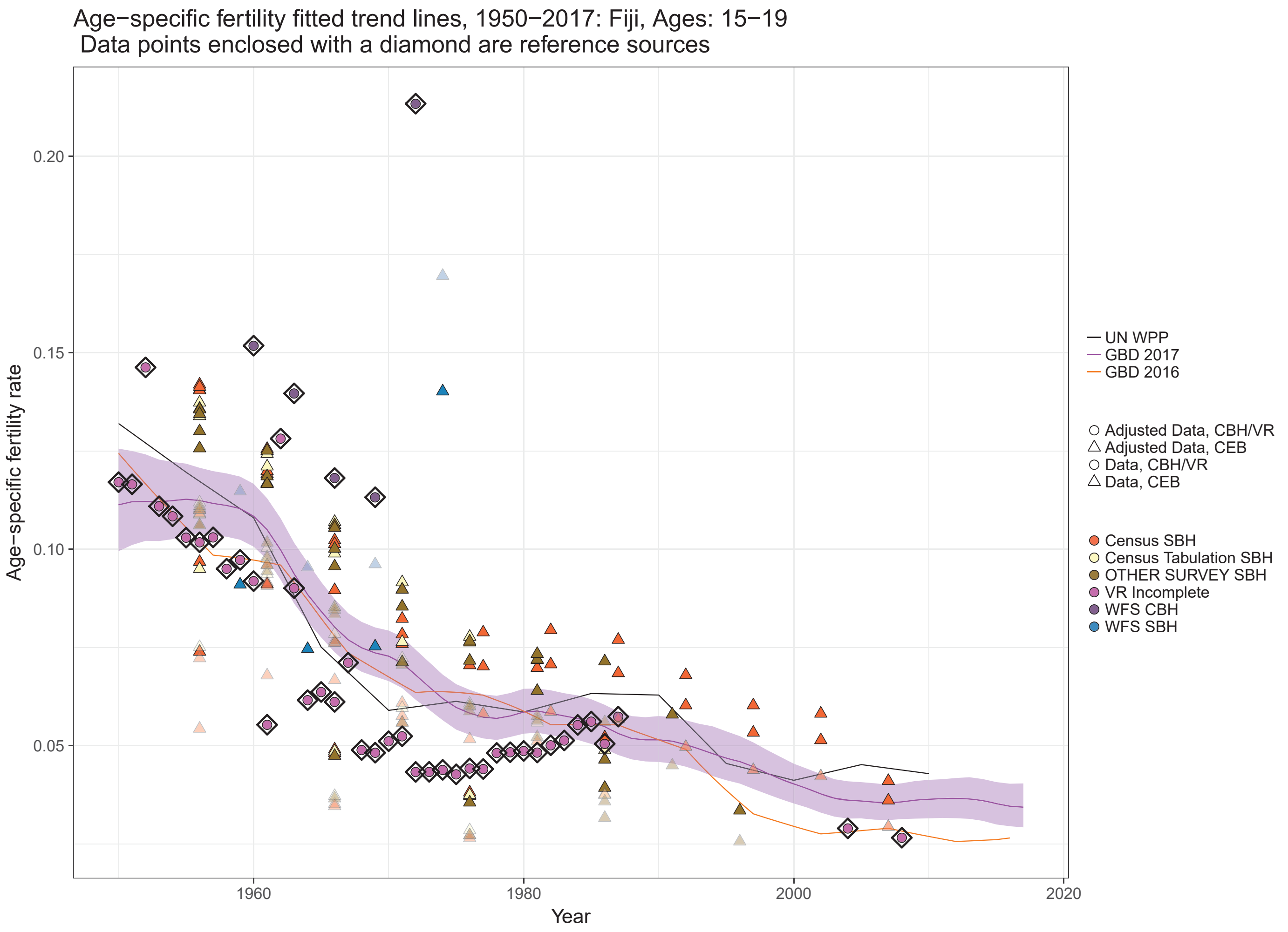
Age−specific fertility fitted trend lines, 1950−2017: Fiji, Ages: 15−19
Data points enclosed with a diamond are reference sources
Age−specific fertility rate
0.20
0.15
0.10
0.05
1960
1980
2000
2020
Year
UN WPP
GBD 2017
GBD 2016
Adjusted Data, CBH/VR
Adjusted Data, CEB
Data, CBH/VR
Data, CEB
Census SBH
Census Tabulation SBH
OTHER SURVEY SBH
VR Incomplete
WFS CBH
WFS SBH

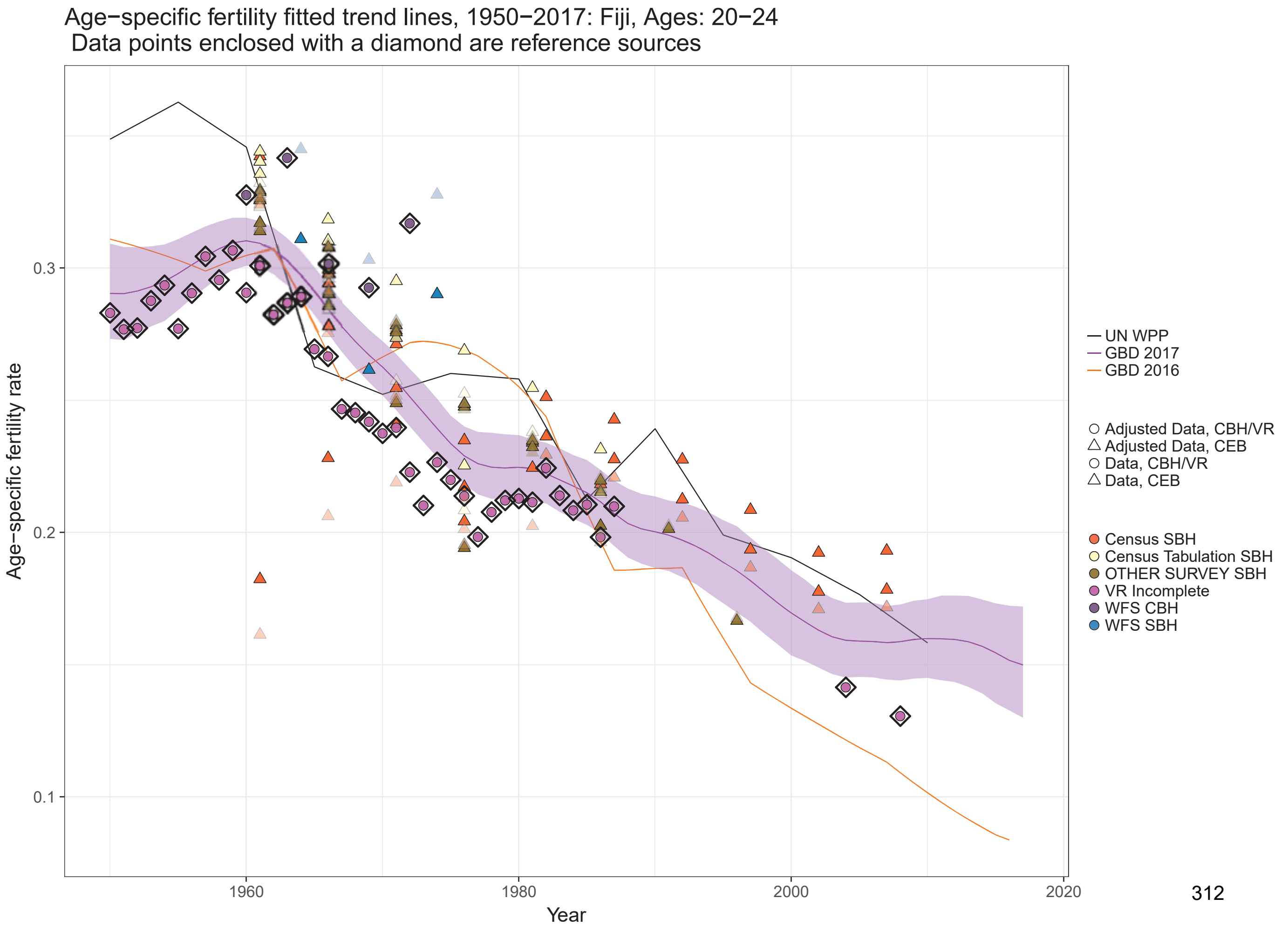
Age−specific fertility fitted trend lines, 1950−2017: Fiji, Ages: 20−24
Data points enclosed with a diamond are reference sources
Age−specific fertility rate
0.3
0.2
0.1
1960
1980
2000
2020
Year
UN WPP
GBD 2017
GBD 2016
Adjusted Data, CBH/VR
Adjusted Data, CEB
Data, CBH/VR
Data, CEB
Census SBH
Census Tabulation SBH
OTHER SURVEY SBH
VR Incomplete
WFS CBH
WFS SBH

Age−specific fertility fitted trend lines, 1950−2017: Fiji, Ages: 25−29
Data points enclosed with a diamond are reference sources

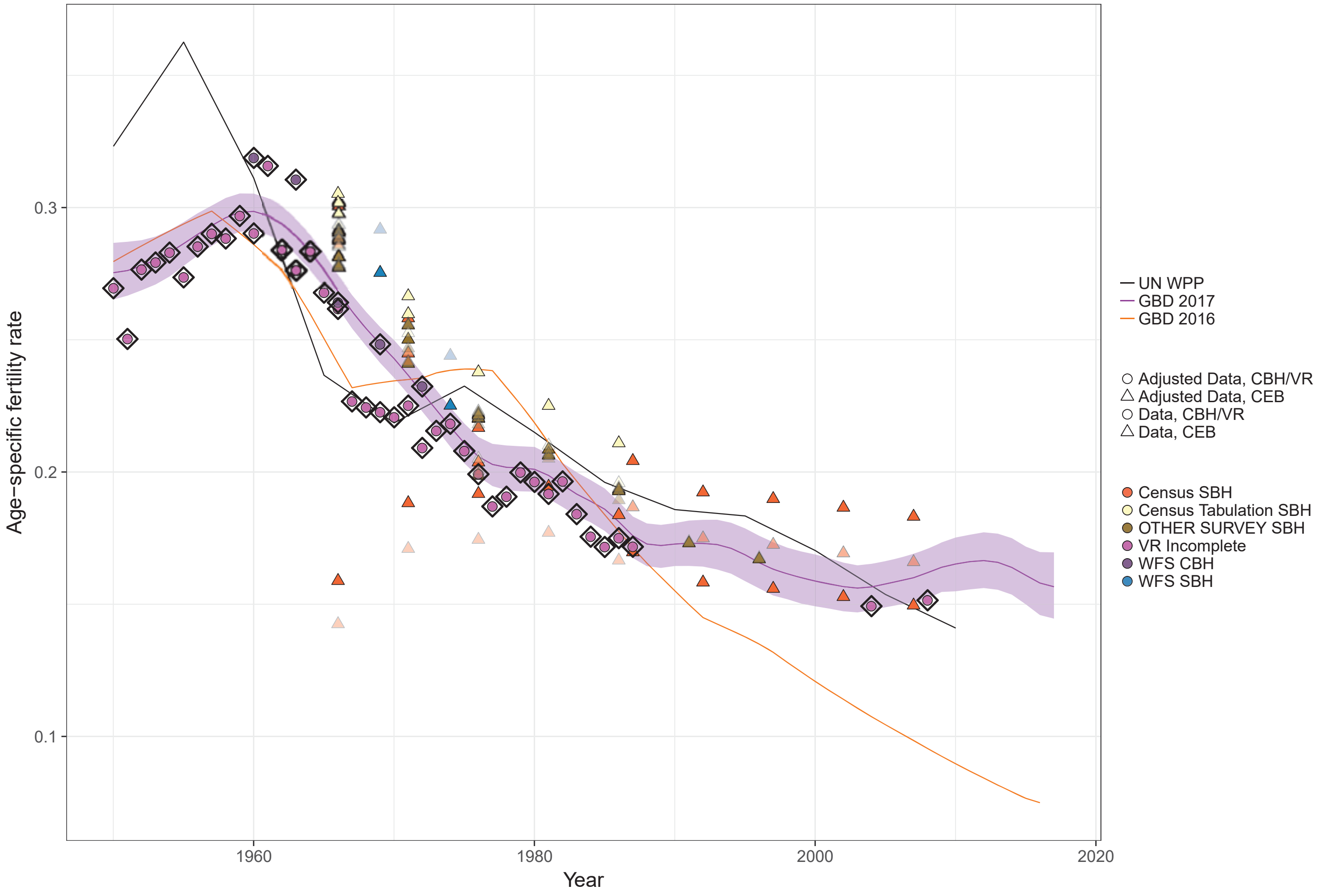

Age−specific fertility fitted trend lines, 1950−2017: Fiji, Ages: 30−34
Data points enclosed with a diamond are reference sources

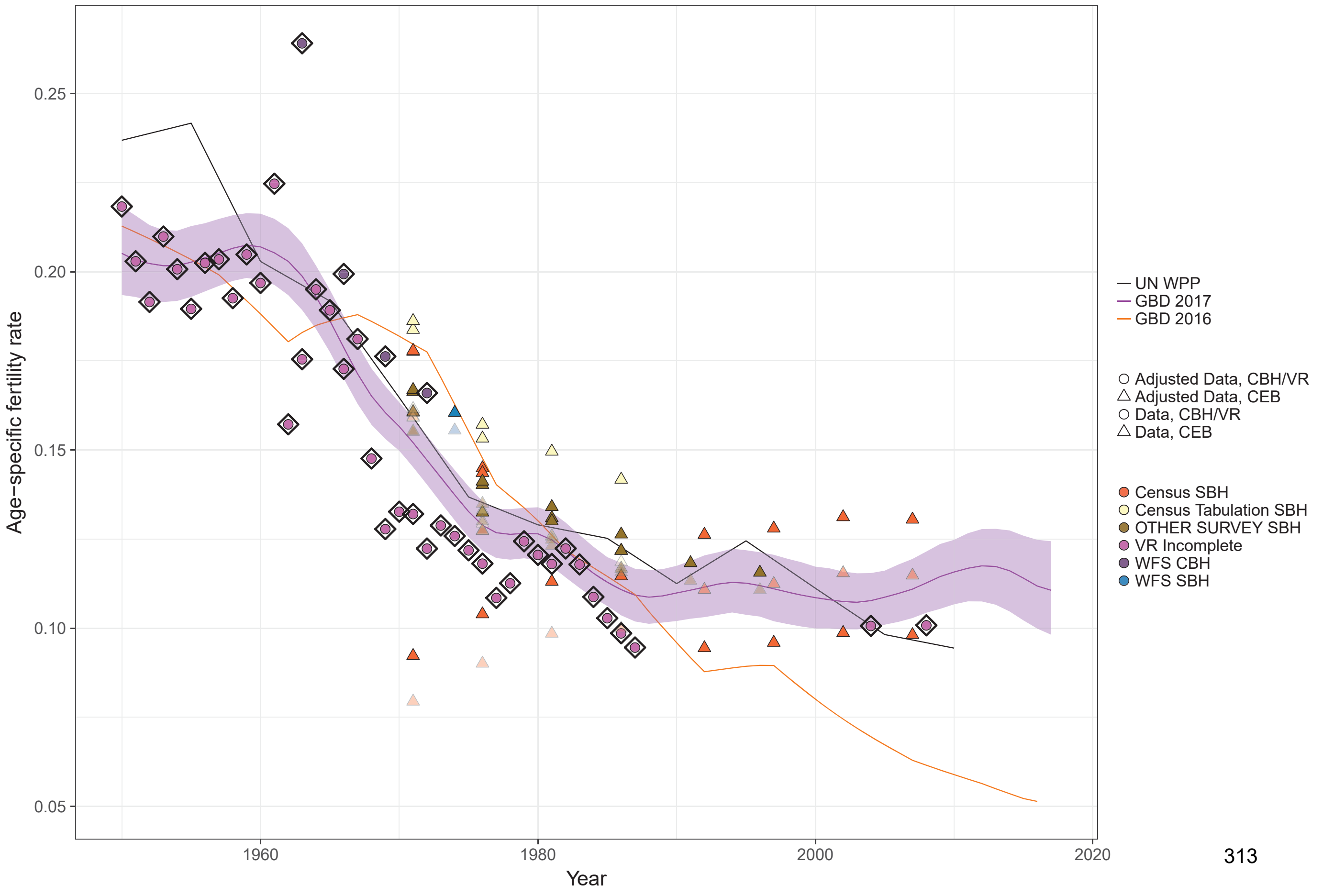

Age−specific fertility fitted trend lines, 1950−2017: Fiji, Ages: 35−39
Data points enclosed with a diamond are reference sources

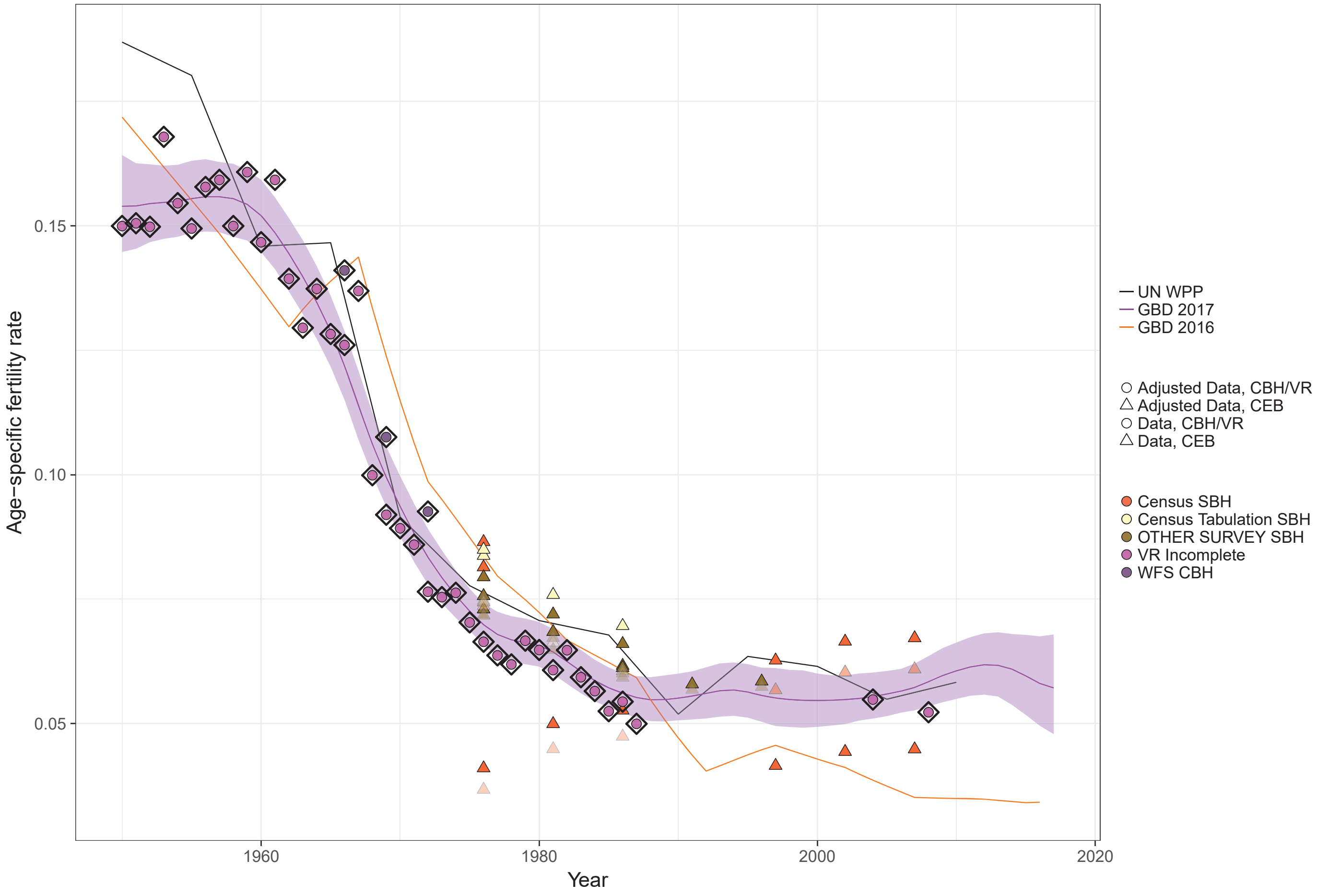

Age−specific fertility fitted trend lines, 1950−2017: Fiji, Ages: 40−44
Data points enclosed with a diamond are reference sources

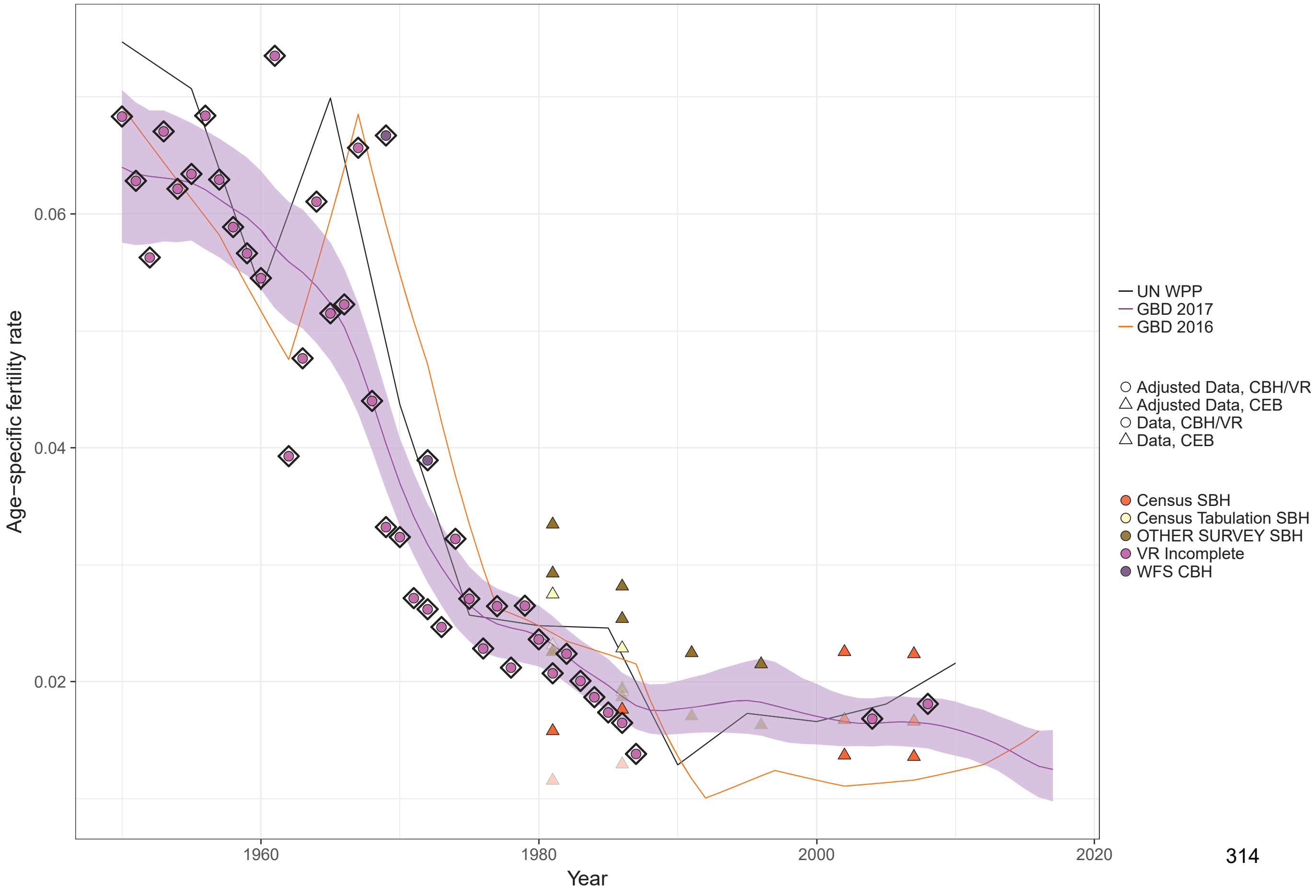

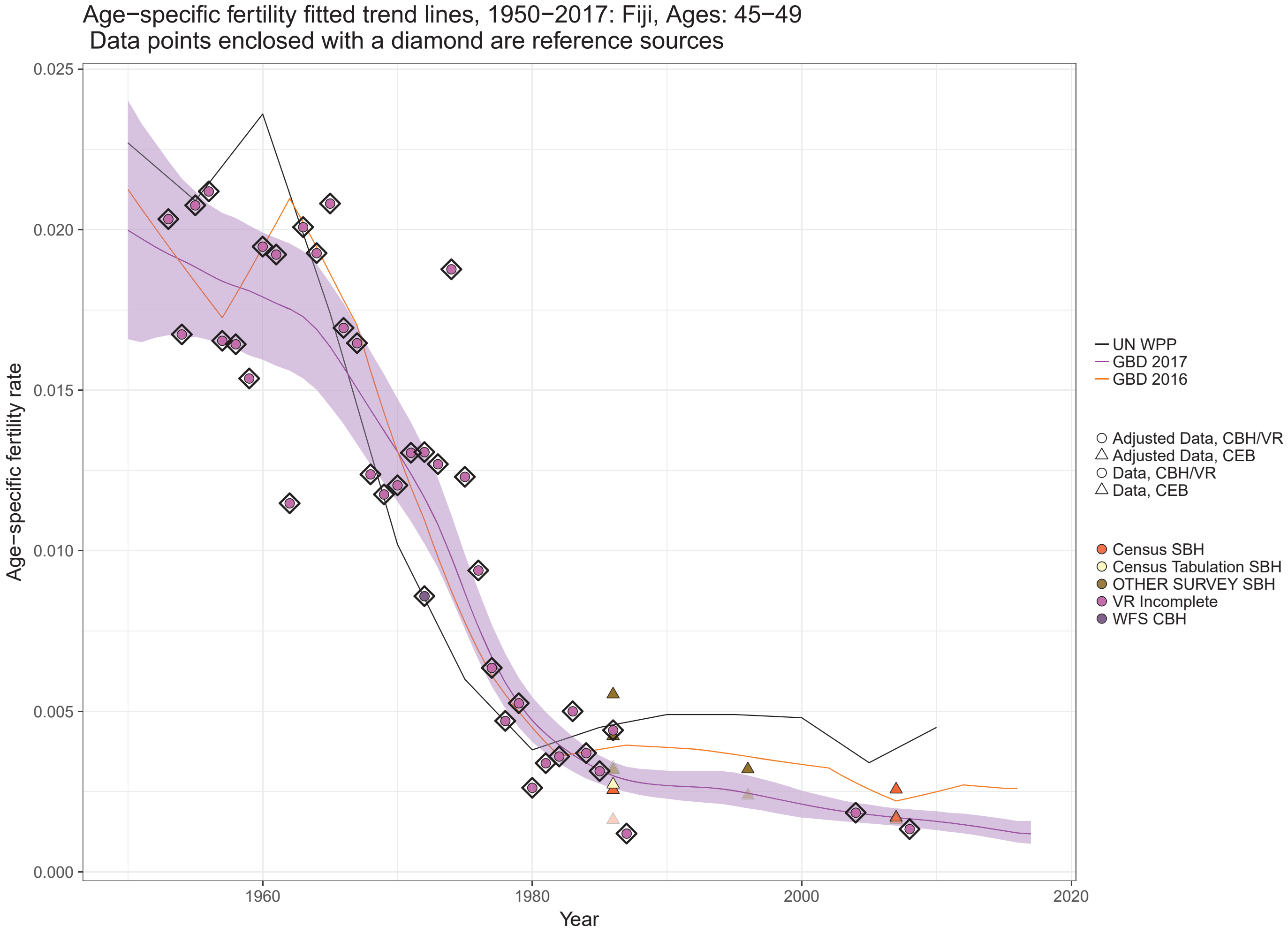

Age−specific fertility fitted trend lines, 1950−2017: Fiji, Ages: 45−49
Data points enclosed with a diamond are reference sources
Age−specific fertility rate
Year
0.000
0.005
0.010
0.015
0.020
0.025
1960
1980
2000
2020
UN WPP
GBD 2017
GBD 2016
Adjusted Data, CBH/VR
Adjusted Data, CEB
Data, CBH/VR
Data, CEB
Census SBH
Census Tabulation SBH
OTHER SURVEY SBH
VR Incomplete
WFS CBH

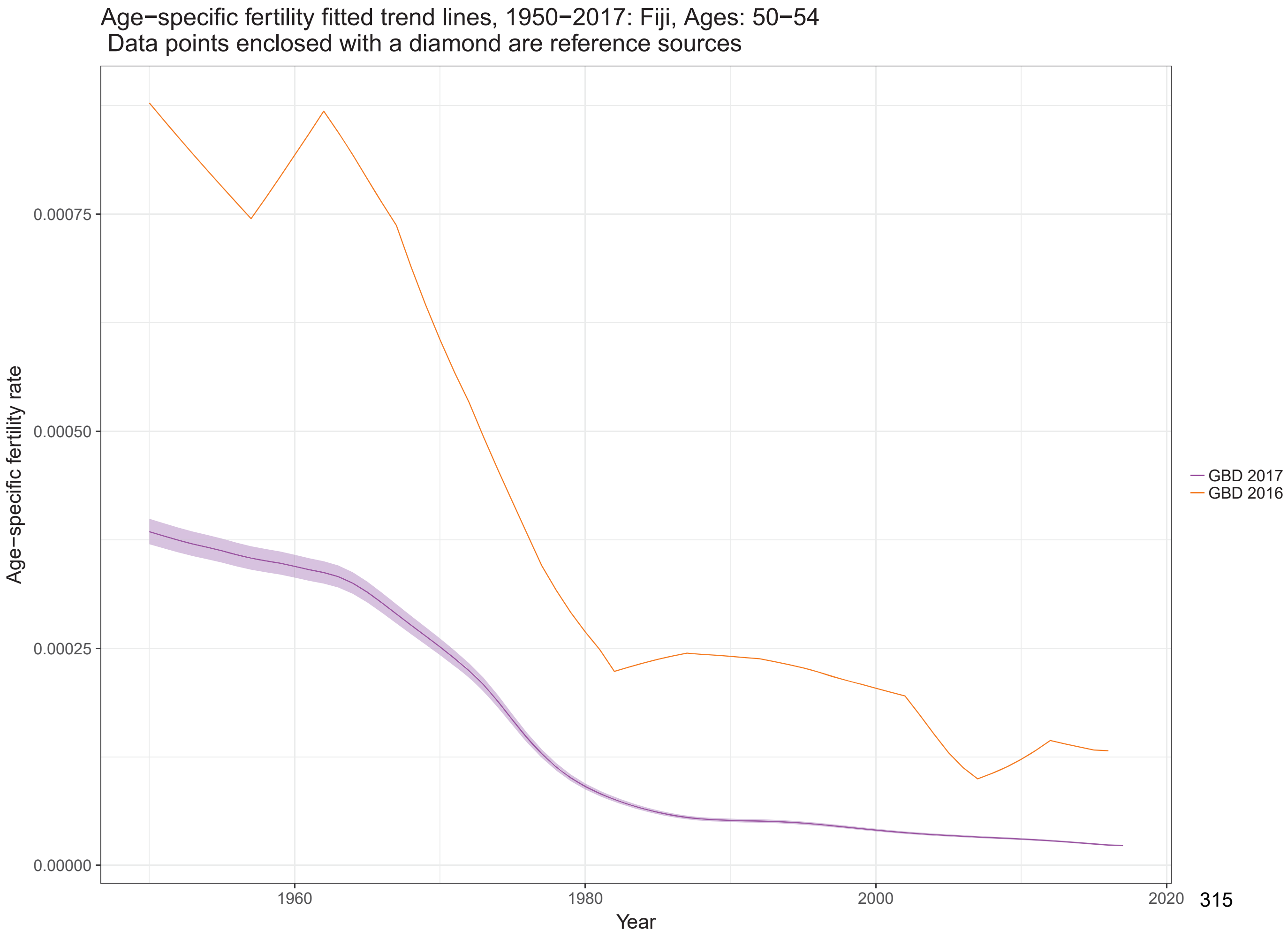

Age−specific fertility fitted trend lines, 1950−2017: Fiji, Ages: 50−54
Data points enclosed with a diamond are reference sources
Age−specific fertility rate
Year
0.00000
0.00025
0.00050
0.00075
1960
1980
2000
2020
GBD 2017
GBD 2016

Age−specific fertility fitted trend lines, 1950−2017: Finland, TFR
Data points enclosed with a diamond are reference sources

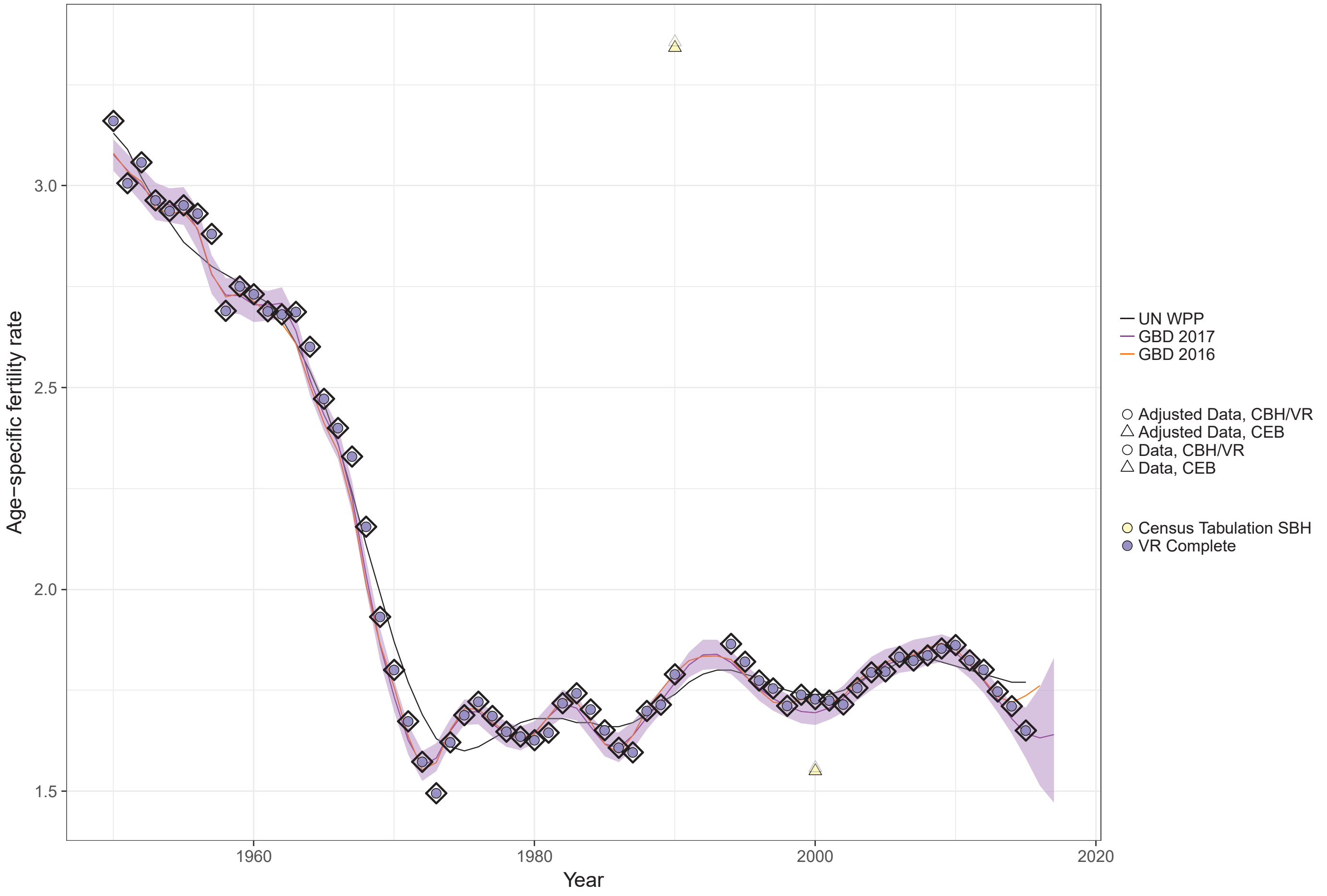

Age−specific fertility fitted trend lines, 1950−2017: Finland, Ages: 10−14
Data points enclosed with a diamond are reference sources

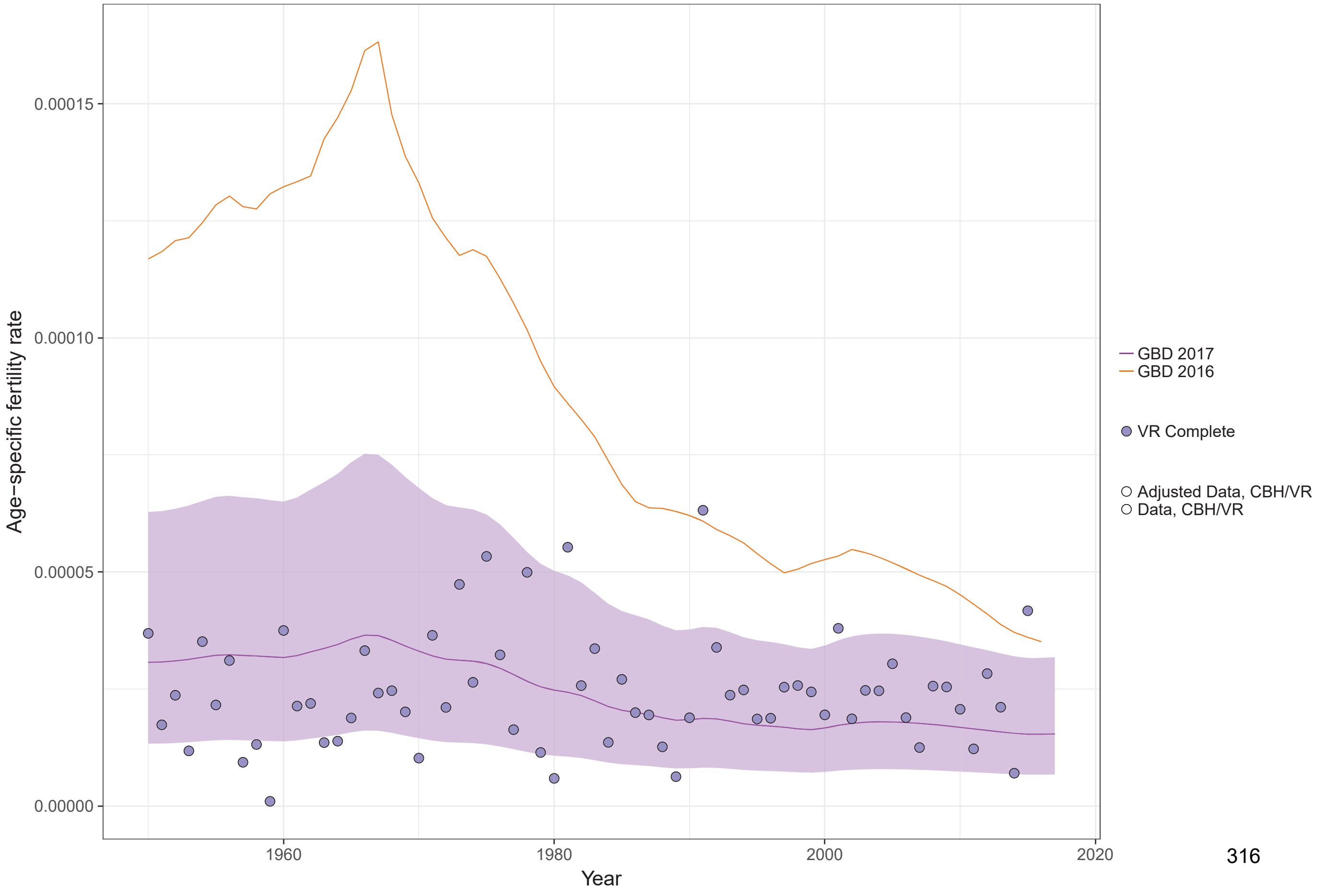

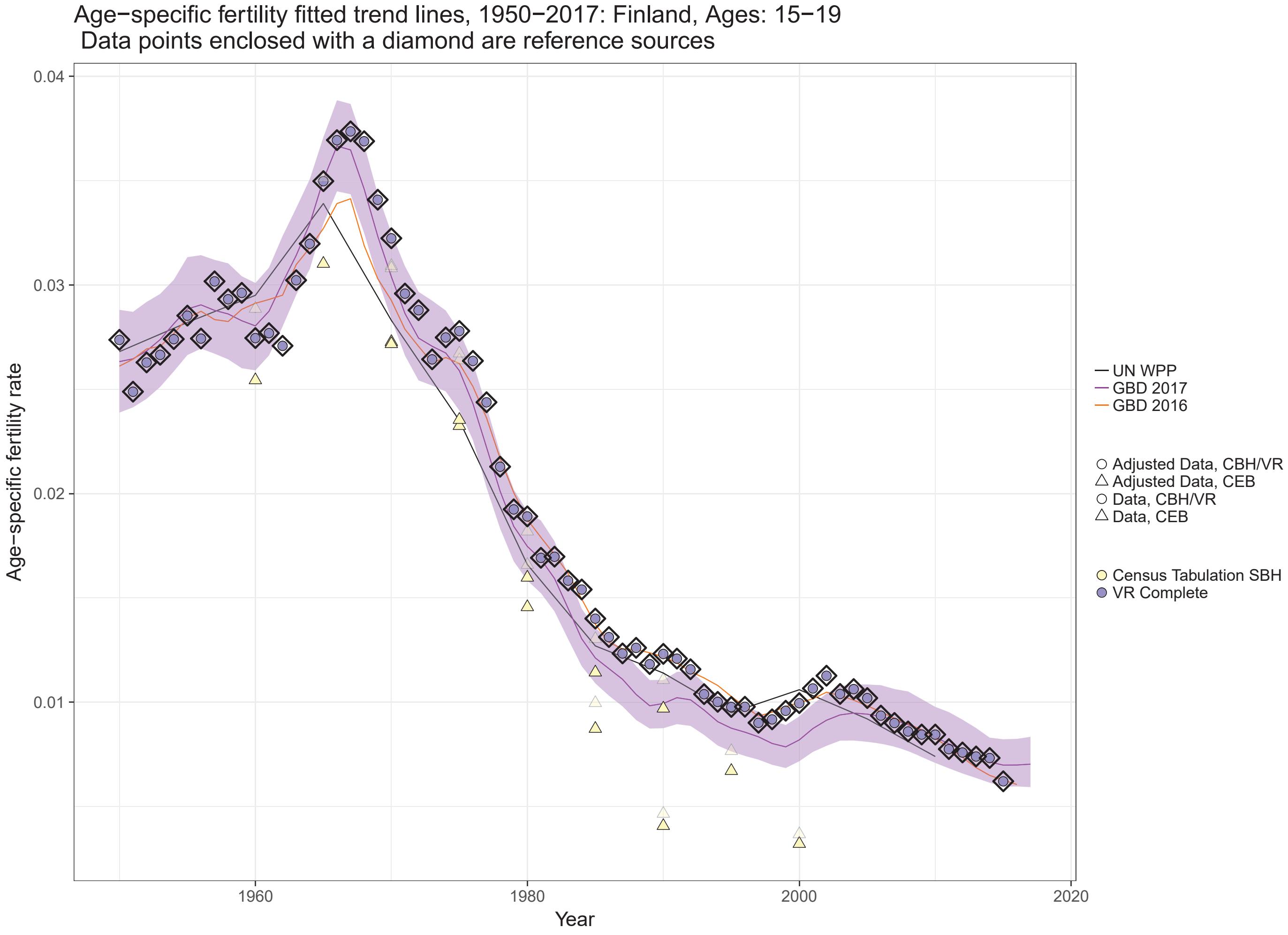

Age−specific fertility fitted trend lines, 1950−2017: Finland, Ages: 15−19
Data points enclosed with a diamond are reference sources
Age−specific fertility rate
0.04
0.03
0.02
0.01
1960
1980
2000
2020
Year
UN WPP
GBD 2017
GBD 2016
Adjusted Data, CBH/VR
Adjusted Data, CEB
Data, CBH/VR
Data, CEB
Census Tabulation SBH
VR Complete

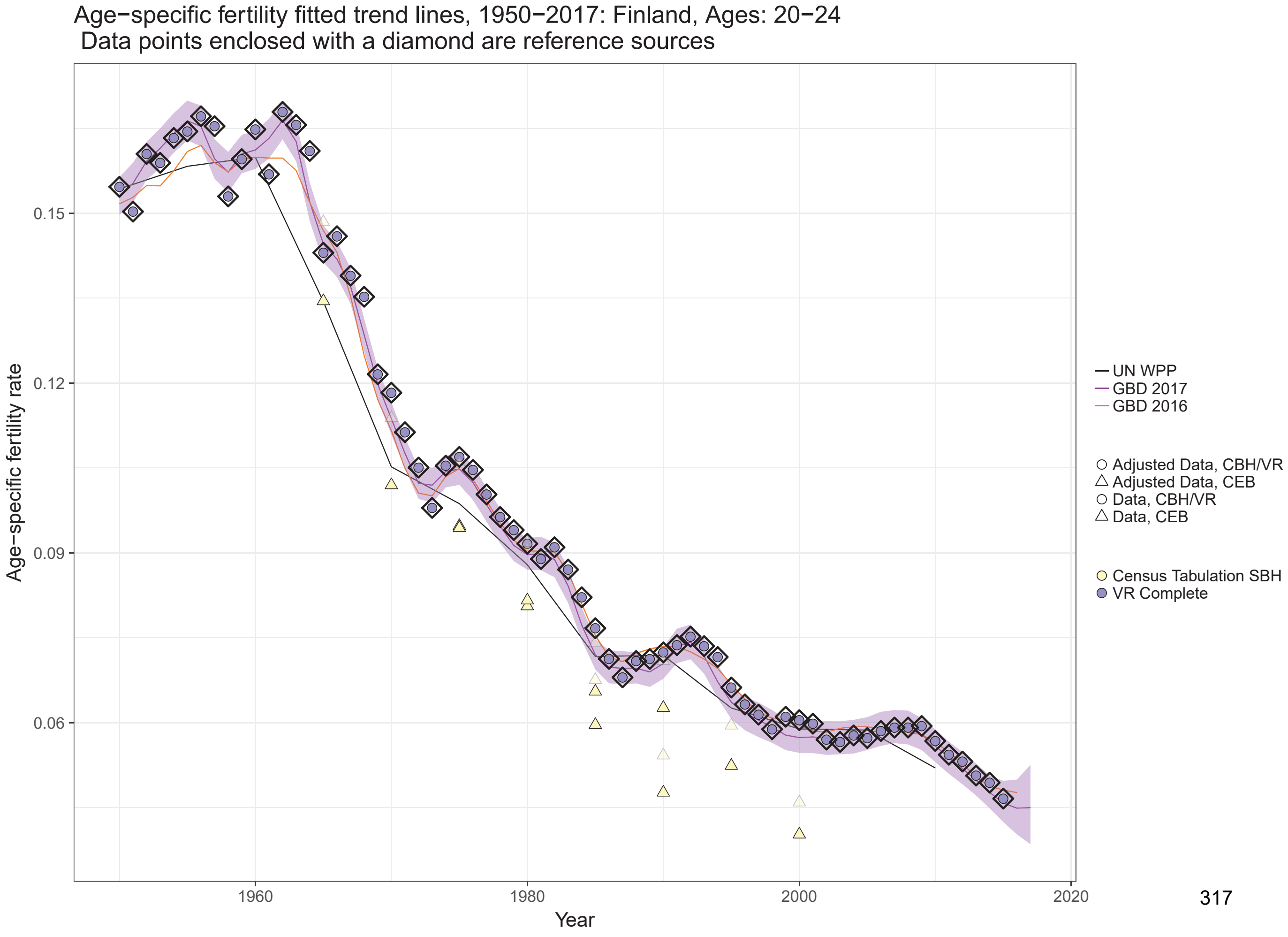

Age−specific fertility fitted trend lines, 1950−2017: Finland, Ages: 20−24
Data points enclosed with a diamond are reference sources
Age−specific fertility rate
0.15
0.12
0.09
0.06
1960
1980
2000
2020
Year
UN WPP
GBD 2017
GBD 2016
Adjusted Data, CBH/VR
Adjusted Data, CEB
Data, CBH/VR
Data, CEB
Census Tabulation SBH
VR Complete

Age−specific fertility fitted trend lines, 1950−2017: Finland, Ages: 25−29
Data points enclosed with a diamond are reference sources

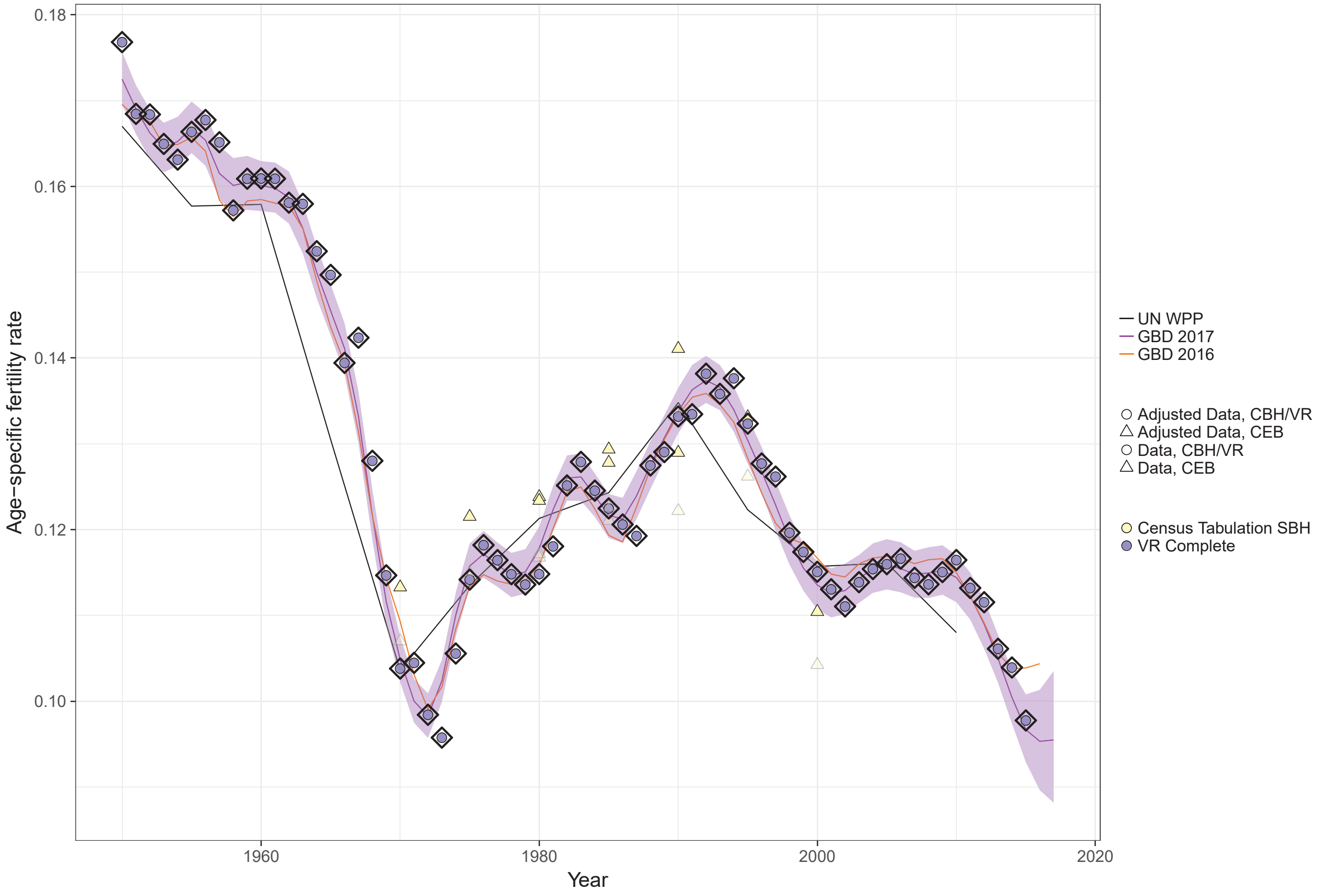

Age−specific fertility fitted trend lines, 1950−2017: Finland, Ages: 30−34
Data points enclosed with a diamond are reference sources

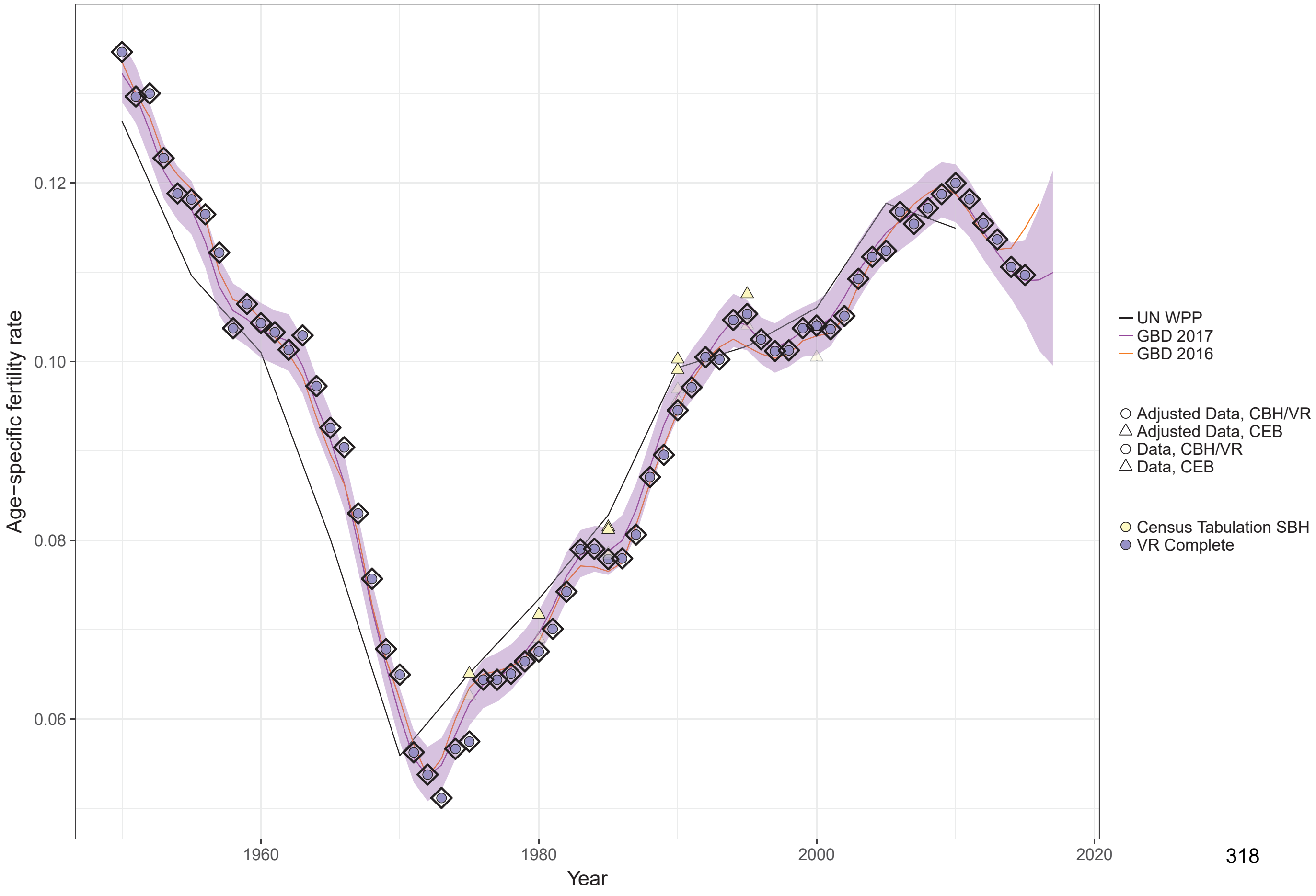

Age−specific fertility fitted trend lines, 1950−2017: Finland, Ages: 35−39
Data points enclosed with a diamond are reference sources

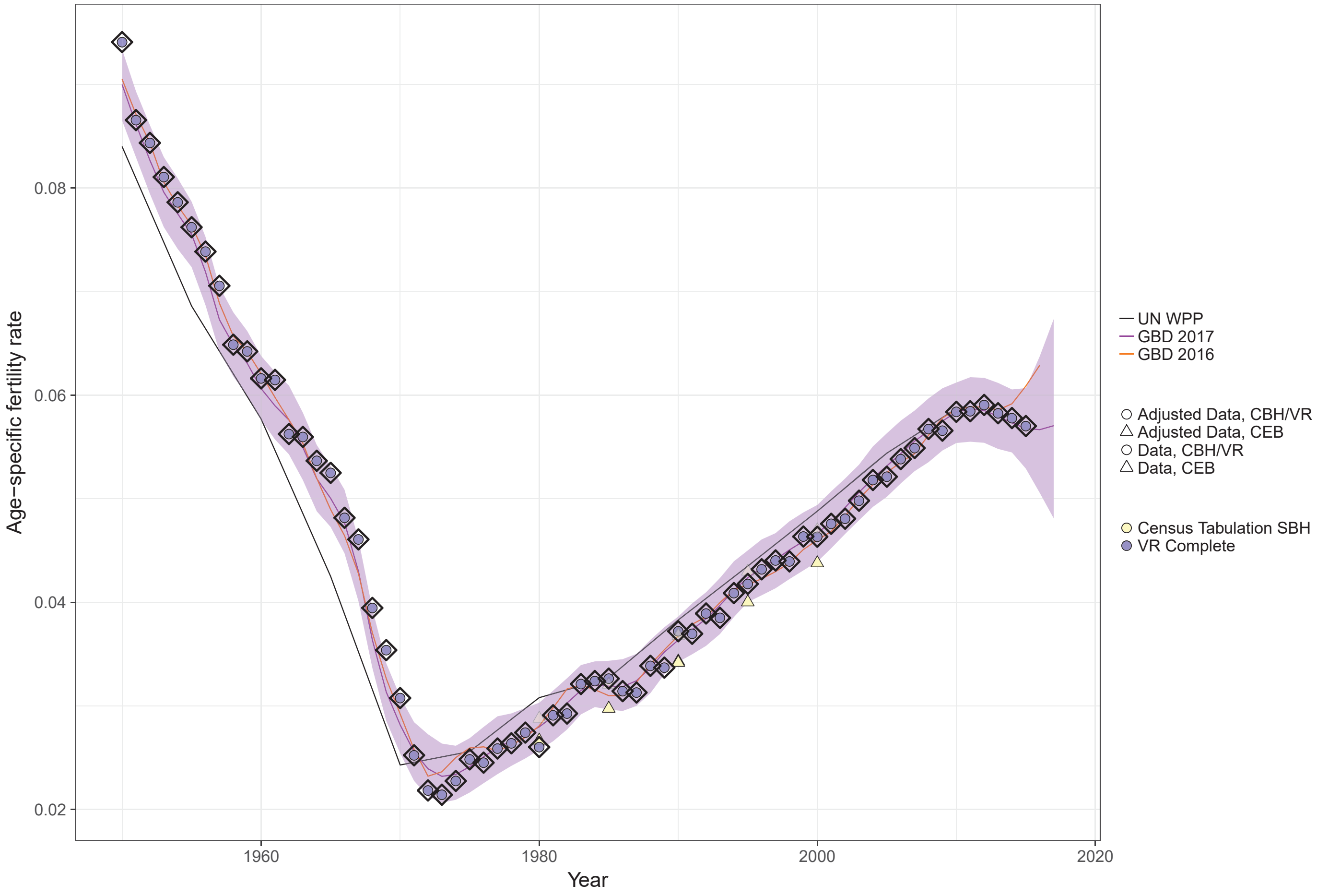

Age−specific fertility fitted trend lines, 1950−2017: Finland, Ages: 40−44
Data points enclosed with a diamond are reference sources

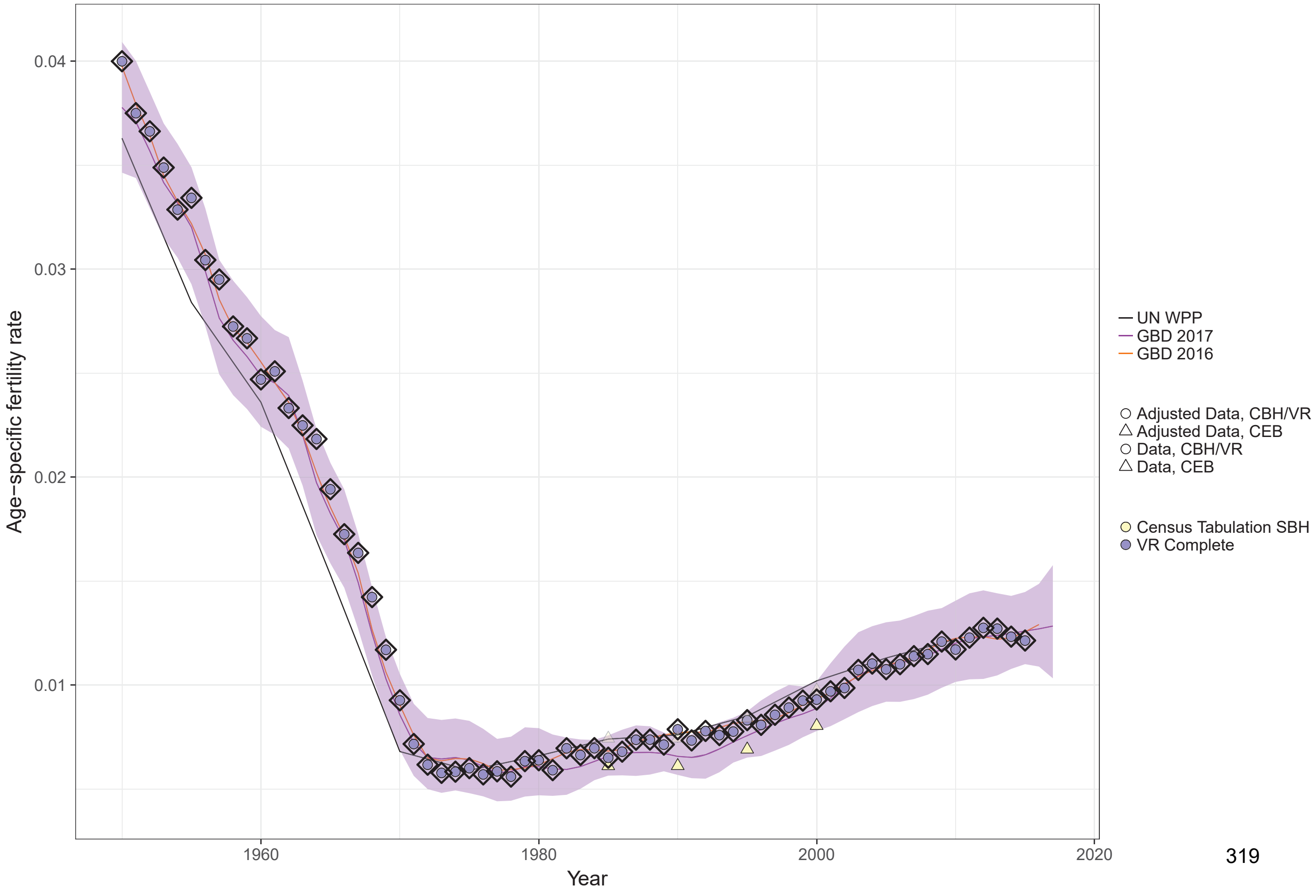

Age−specific fertility fitted trend lines, 1950−2017: Finland, Ages: 45−49
Data points enclosed with a diamond are reference sources

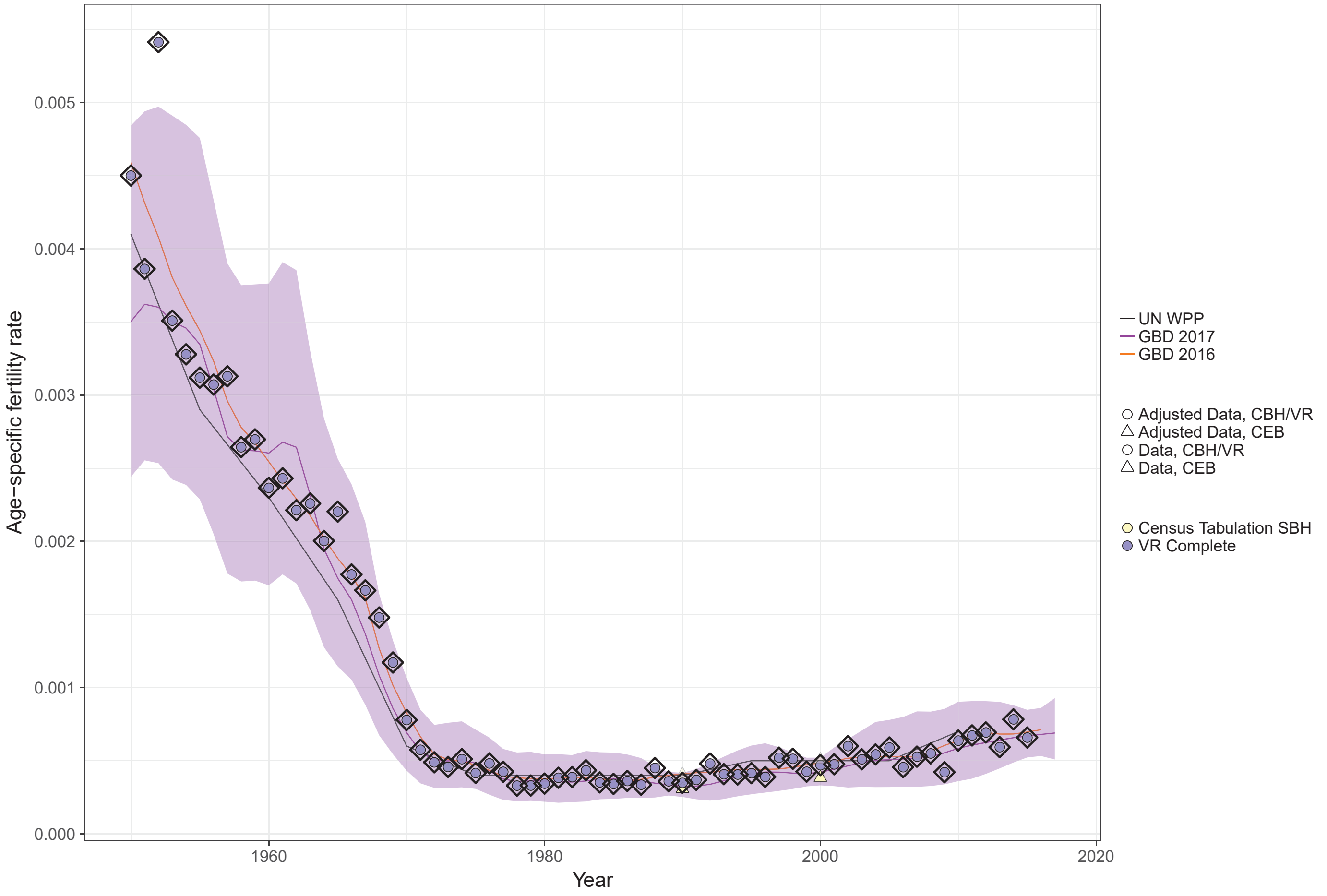

Age−specific fertility fitted trend lines, 1950−2017: Finland, Ages: 50−54
Data points enclosed with a diamond are reference sources

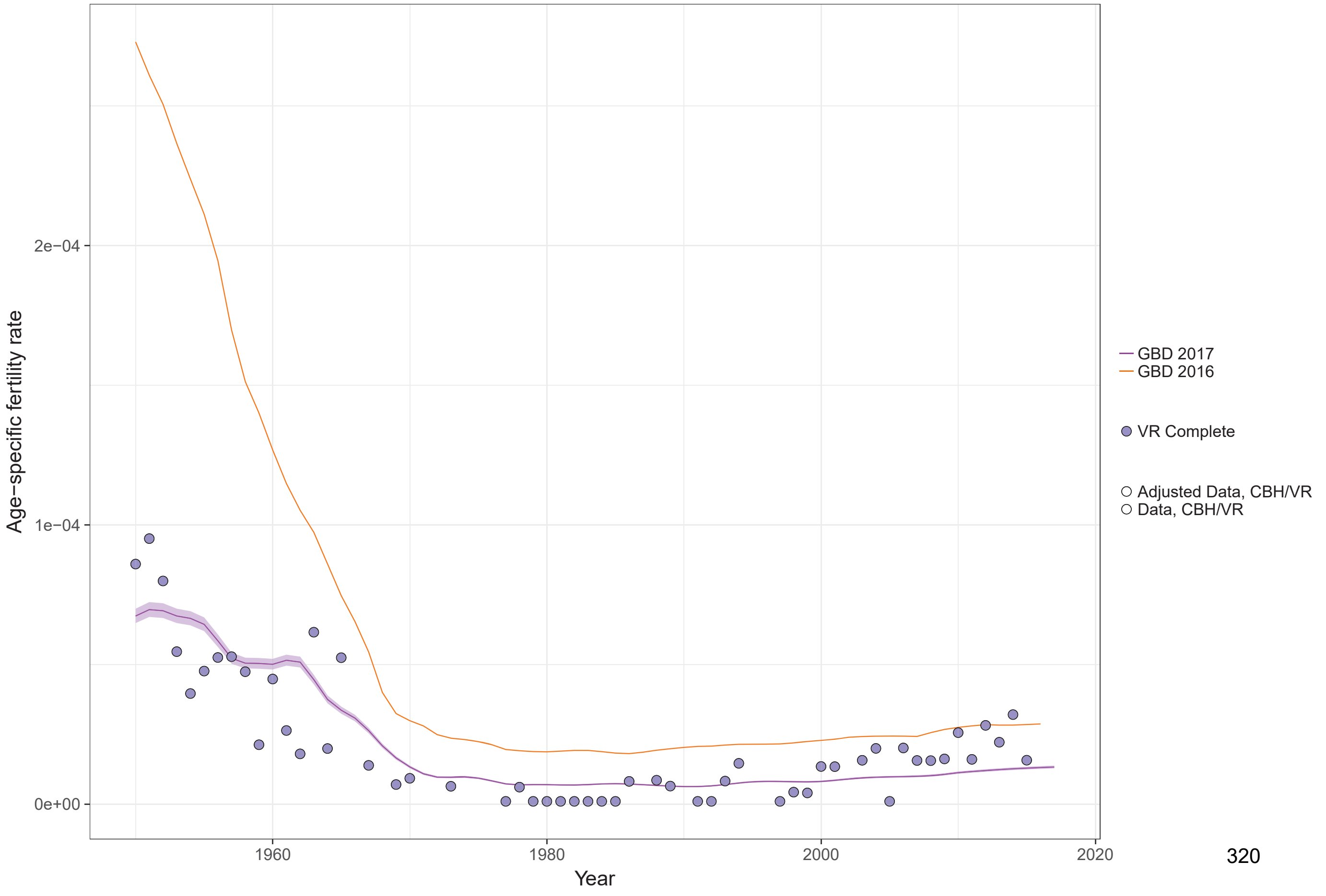

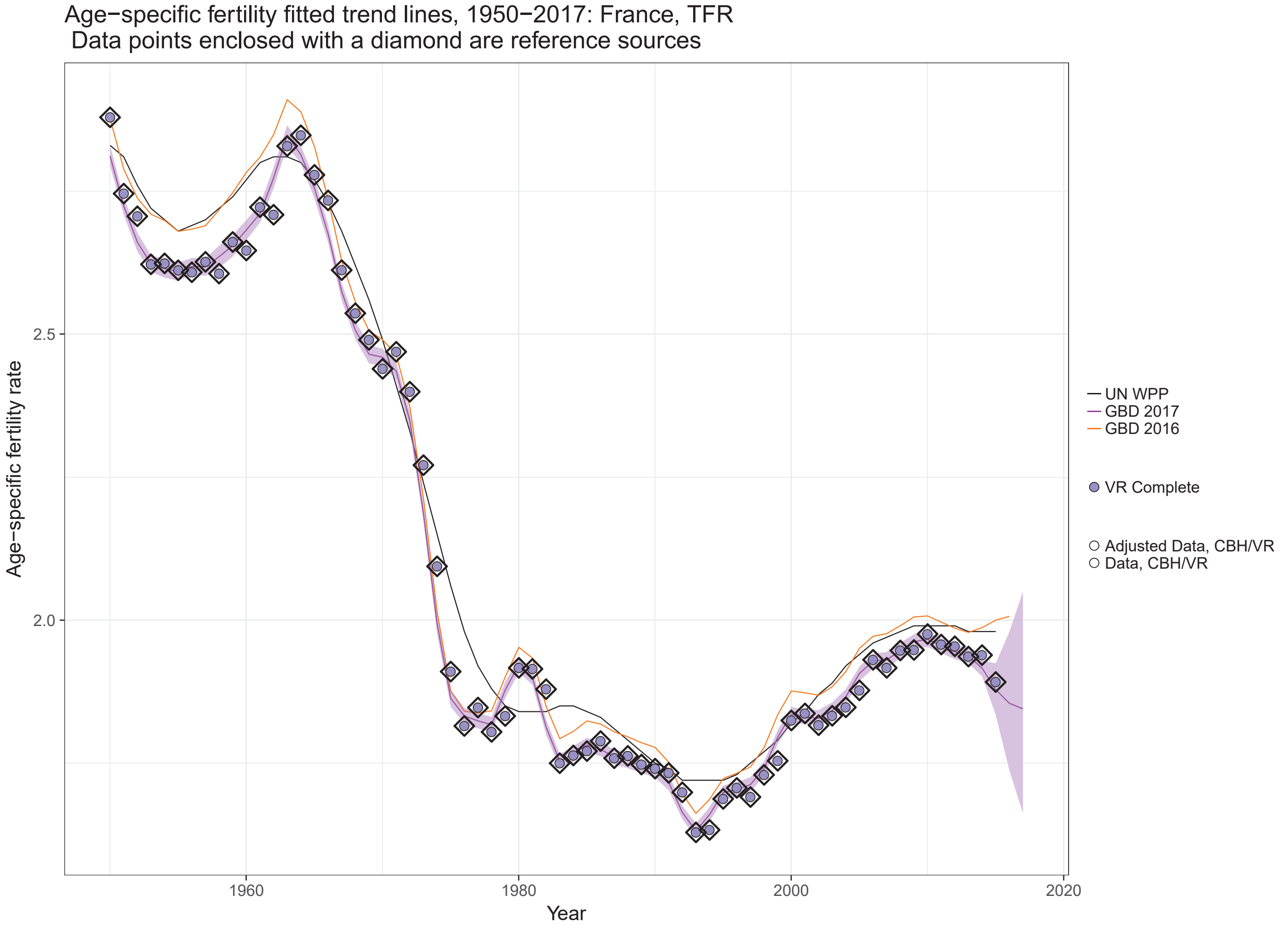
Age−specific fertility fitted trend lines, 1950−2017: France, TFR
Data points enclosed with a diamond are reference sources
Age−specific fertility rate
2.5
2.0
1960
1980
2000
2020
Year
UN WPP
GBD 2017
GBD 2016
VR Complete
Adjusted Data, CBH/VR
Data, CBH/VR

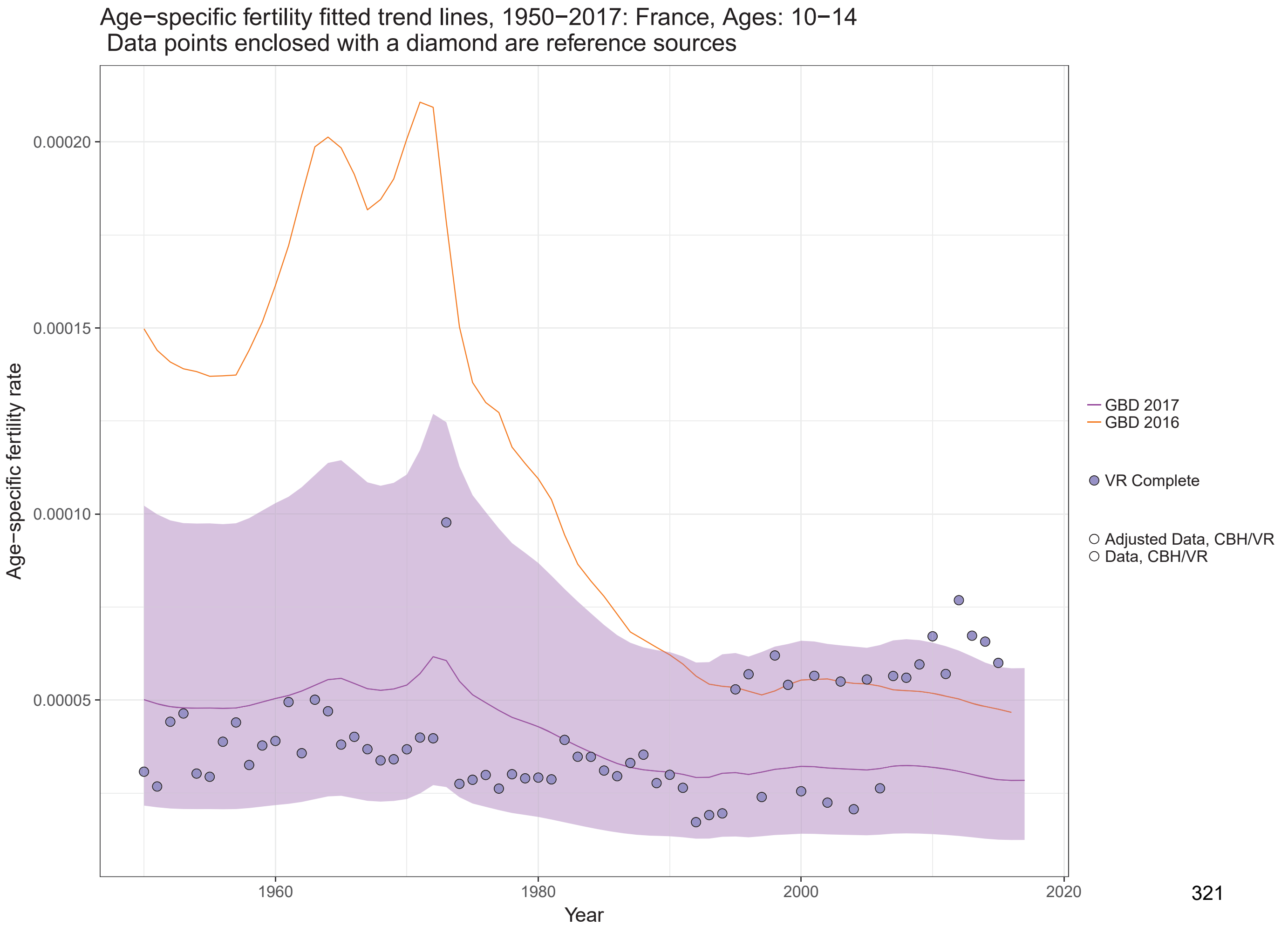
Age−specific fertility fitted trend lines, 1950−2017: France, Ages: 10−14
Data points enclosed with a diamond are reference sources
Age−specific fertility rate
0.00020
0.00015
0.00010
0.00005
1960
1980
2000
2020
Year
GBD 2017
GBD 2016
VR Complete
Adjusted Data, CBH/VR
Data, CBH/VR

Age−specific fertility fitted trend lines, 1950−2017: France, Ages: 15−19
Data points enclosed with a diamond are reference sources

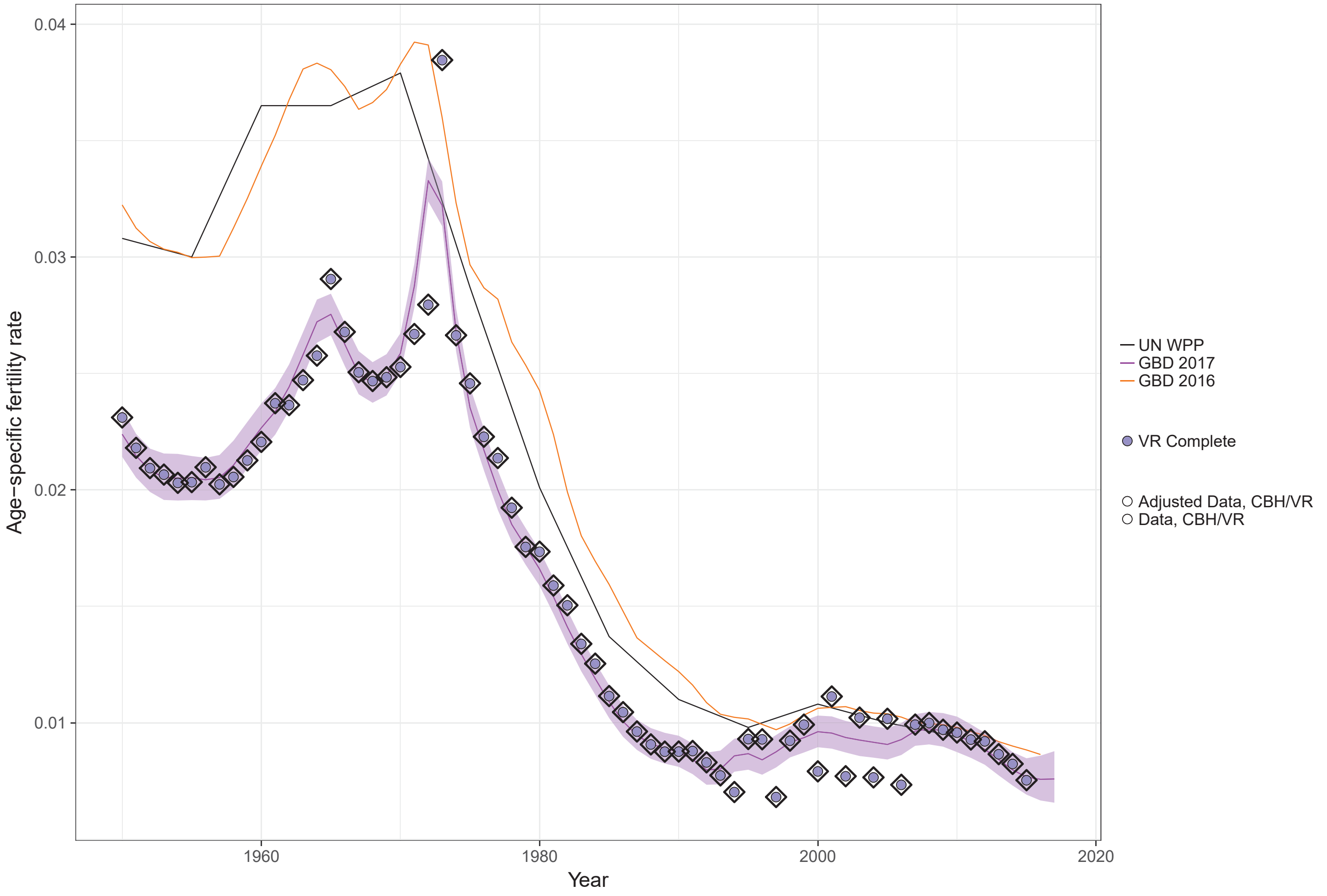

Age−specific fertility fitted trend lines, 1950−2017: France, Ages: 20−24
Data points enclosed with a diamond are reference sources

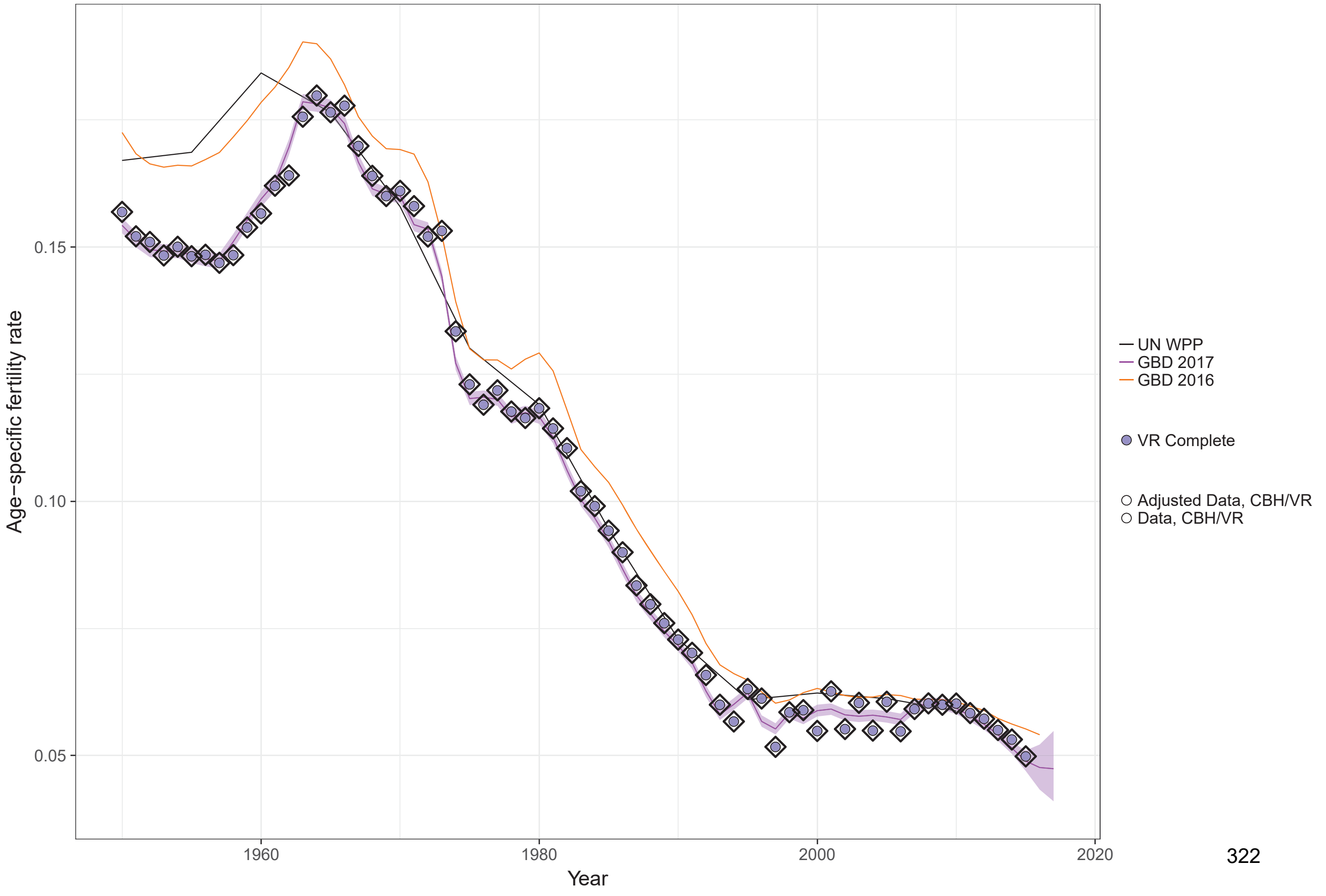

Age−specific fertility fitted trend lines, 1950−2017: France, Ages: 25−29
Data points enclosed with a diamond are reference sources

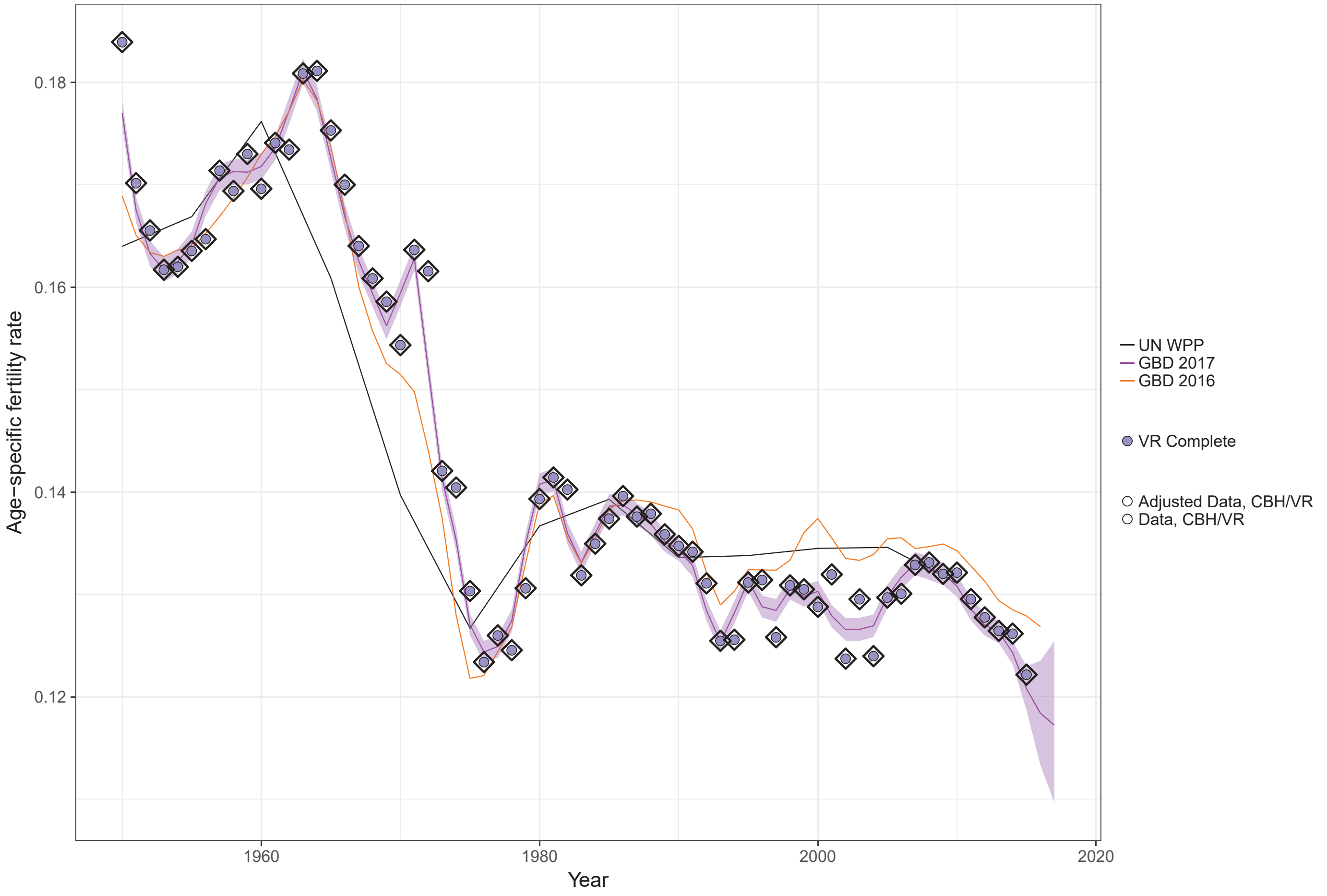

Age−specific fertility fitted trend lines, 1950−2017: France, Ages: 30−34
Data points enclosed with a diamond are reference sources

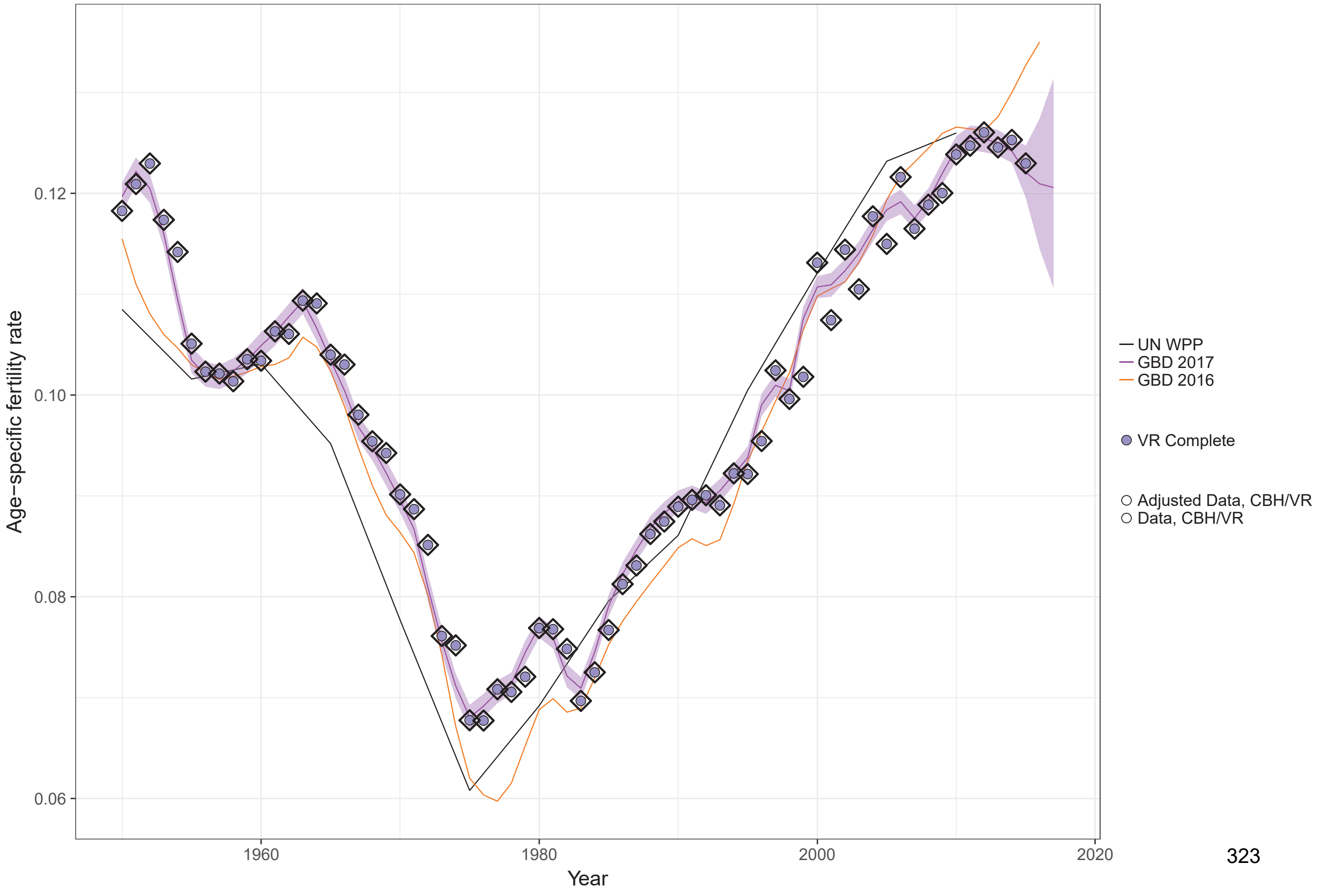

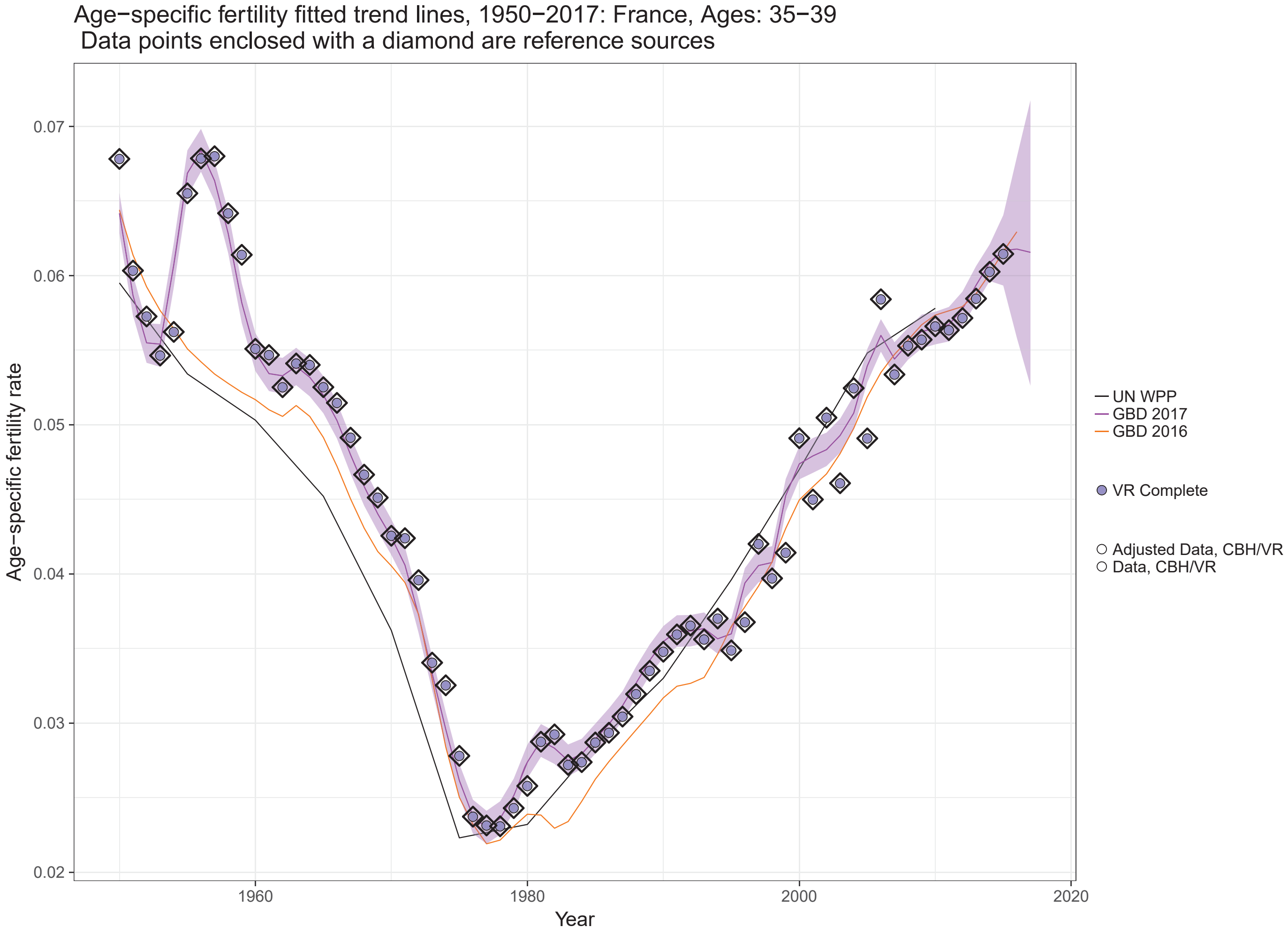

Age−specific fertility fitted trend lines, 1950−2017: France, Ages: 35−39
Data points enclosed with a diamond are reference sources
Age−specific fertility rate
0.07
0.06
0.05
0.04
0.03
0.02
1960
1980
2000
2020
Year
UN WPP
GBD 2017
GBD 2016
VR Complete
Adjusted Data, CBH/VR
Data, CBH/VR

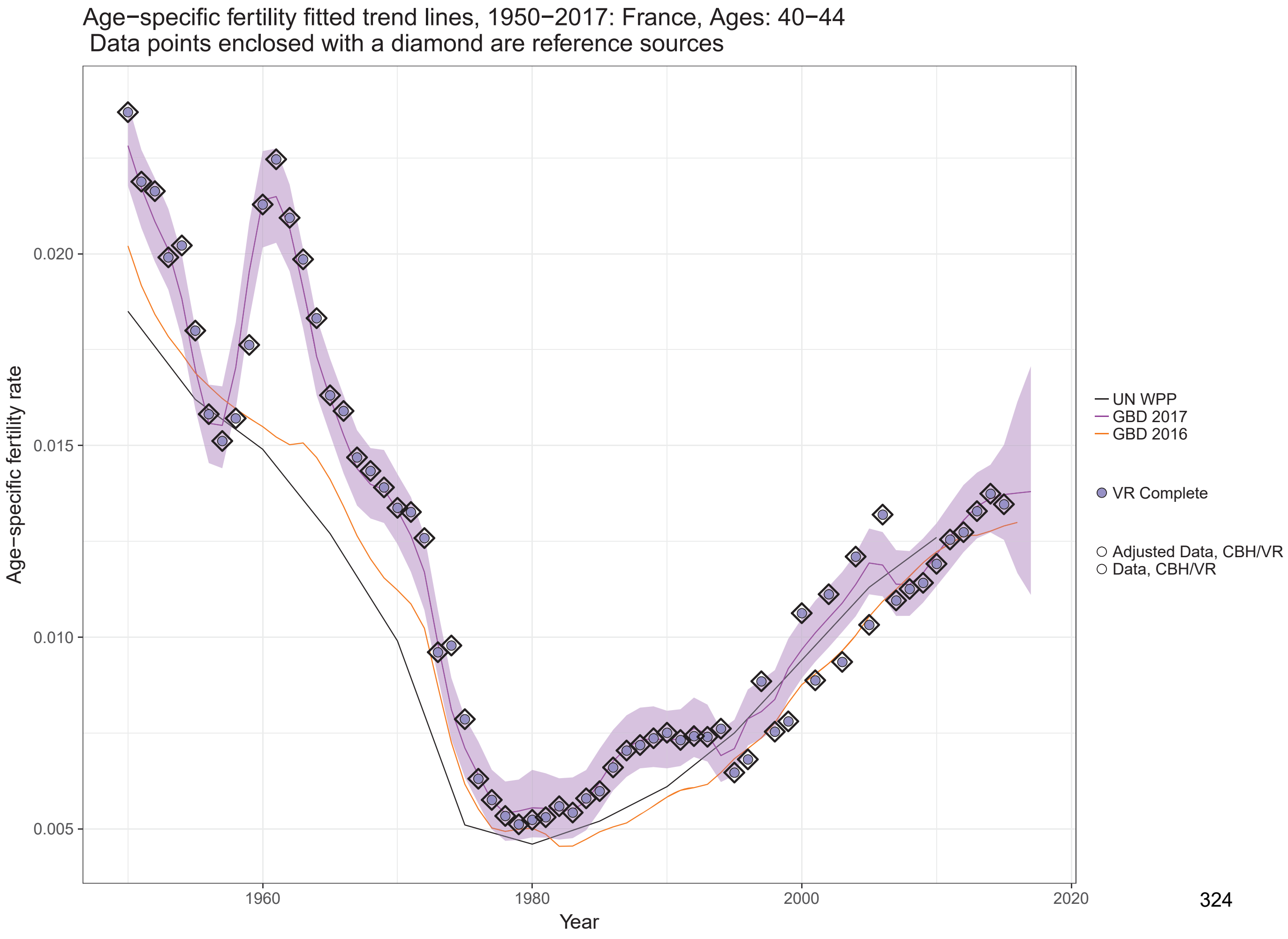

Age−specific fertility fitted trend lines, 1950−2017: France, Ages: 40−44
Data points enclosed with a diamond are reference sources
Age−specific fertility rate
0.020
0.015
0.010
0.005
1960
1980
2000
2020
Year
UN WPP
GBD 2017
GBD 2016
VR Complete
Adjusted Data, CBH/VR
Data, CBH/VR

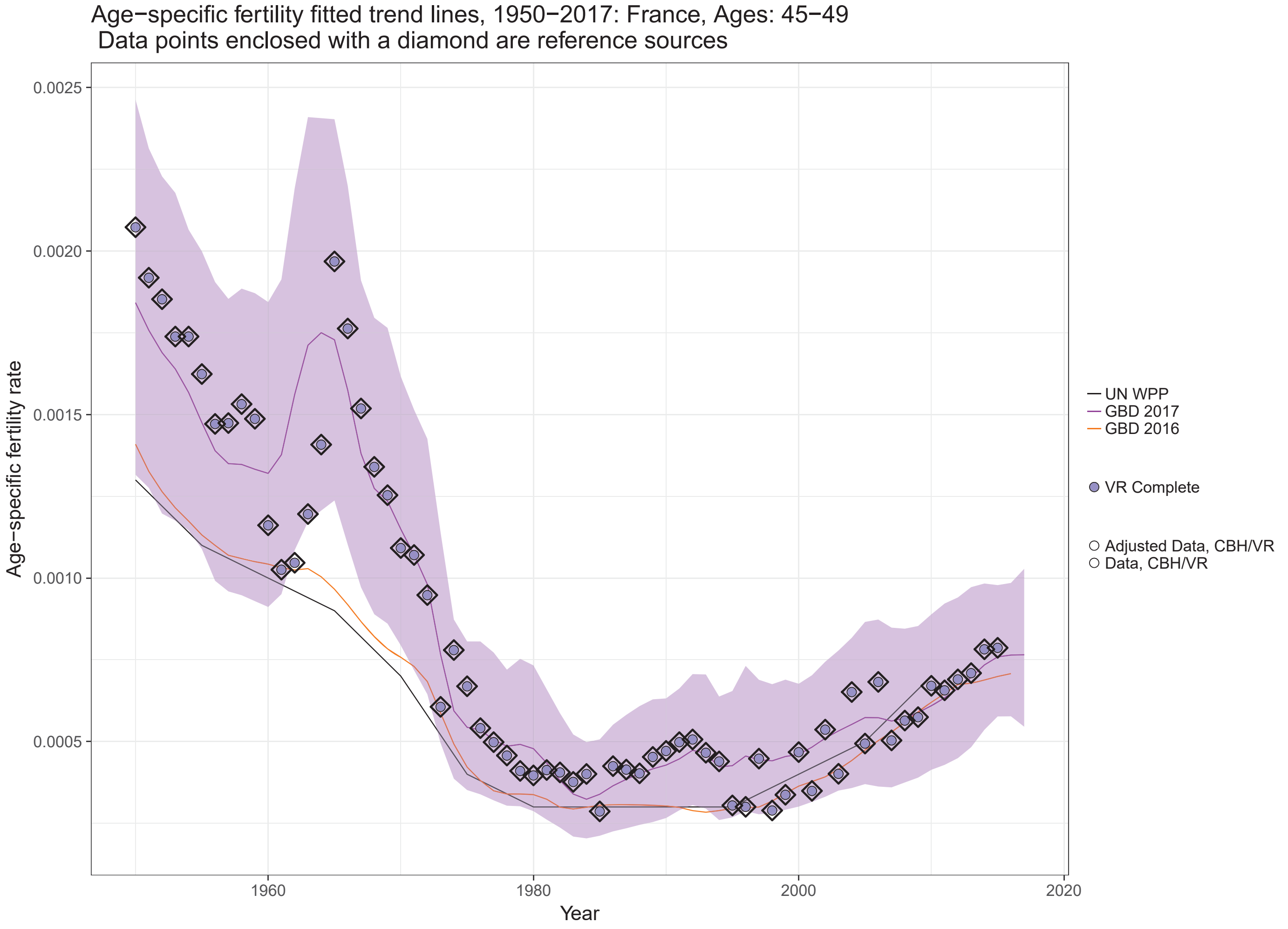

Age−specific fertility fitted trend lines, 1950−2017: France, Ages: 45−49
Data points enclosed with a diamond are reference sources
Age-specific fertility rate
Year
UN WPP
GBD 2017
GBD 2016
VR Complete
Adjusted Data, CBH/VR
Data, CBH/VR

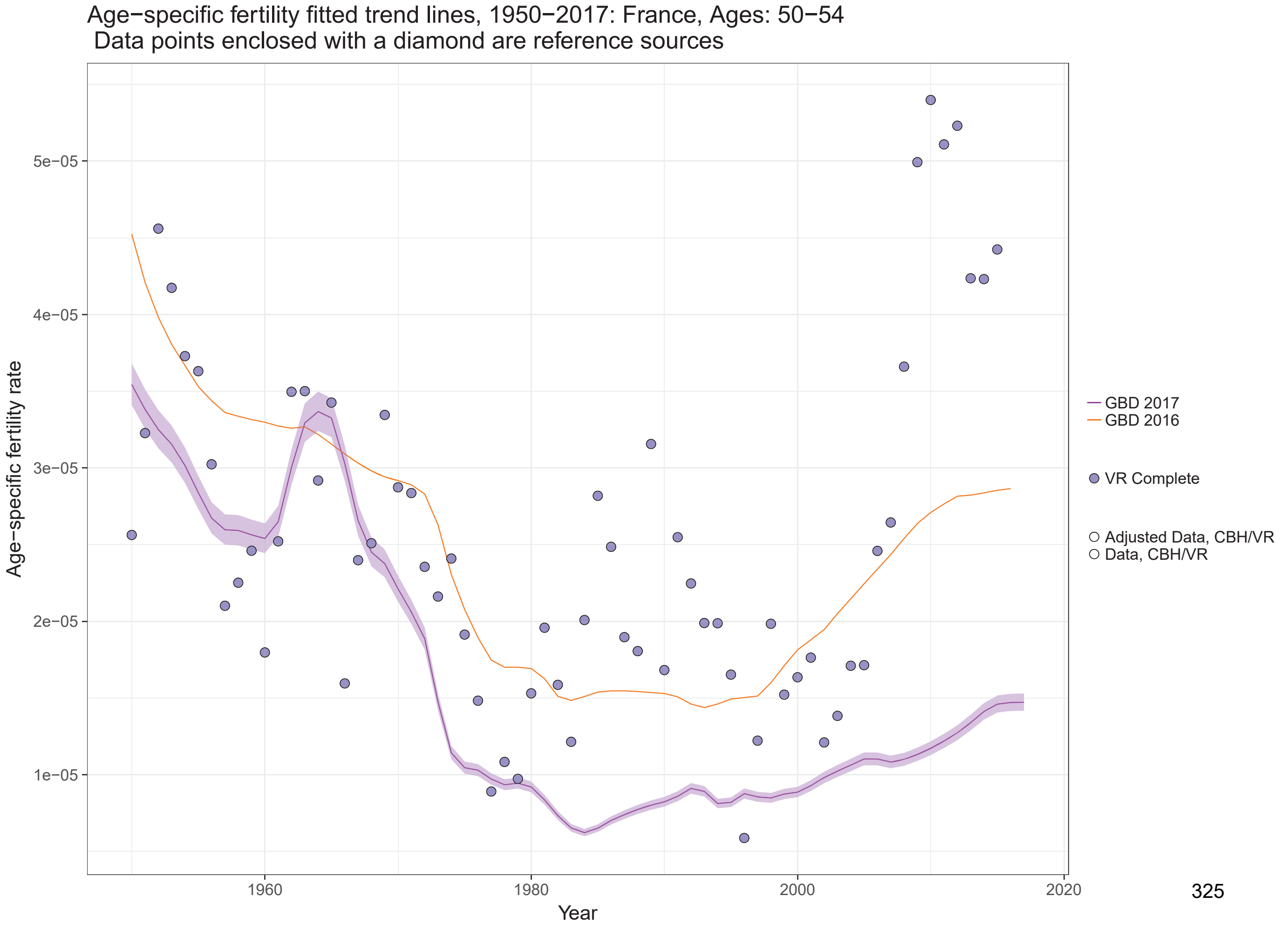

Age−specific fertility fitted trend lines, 1950−2017: France, Ages: 50−54
Data points enclosed with a diamond are reference sources
Age-specific fertility rate
Year
GBD 2017
GBD 2016
VR Complete
Adjusted Data, CBH/VR
Data, CBH/VR

Age−specific fertility fitted trend lines, 1950−2017: Gabon, TFR
Data points enclosed with a diamond are reference sources

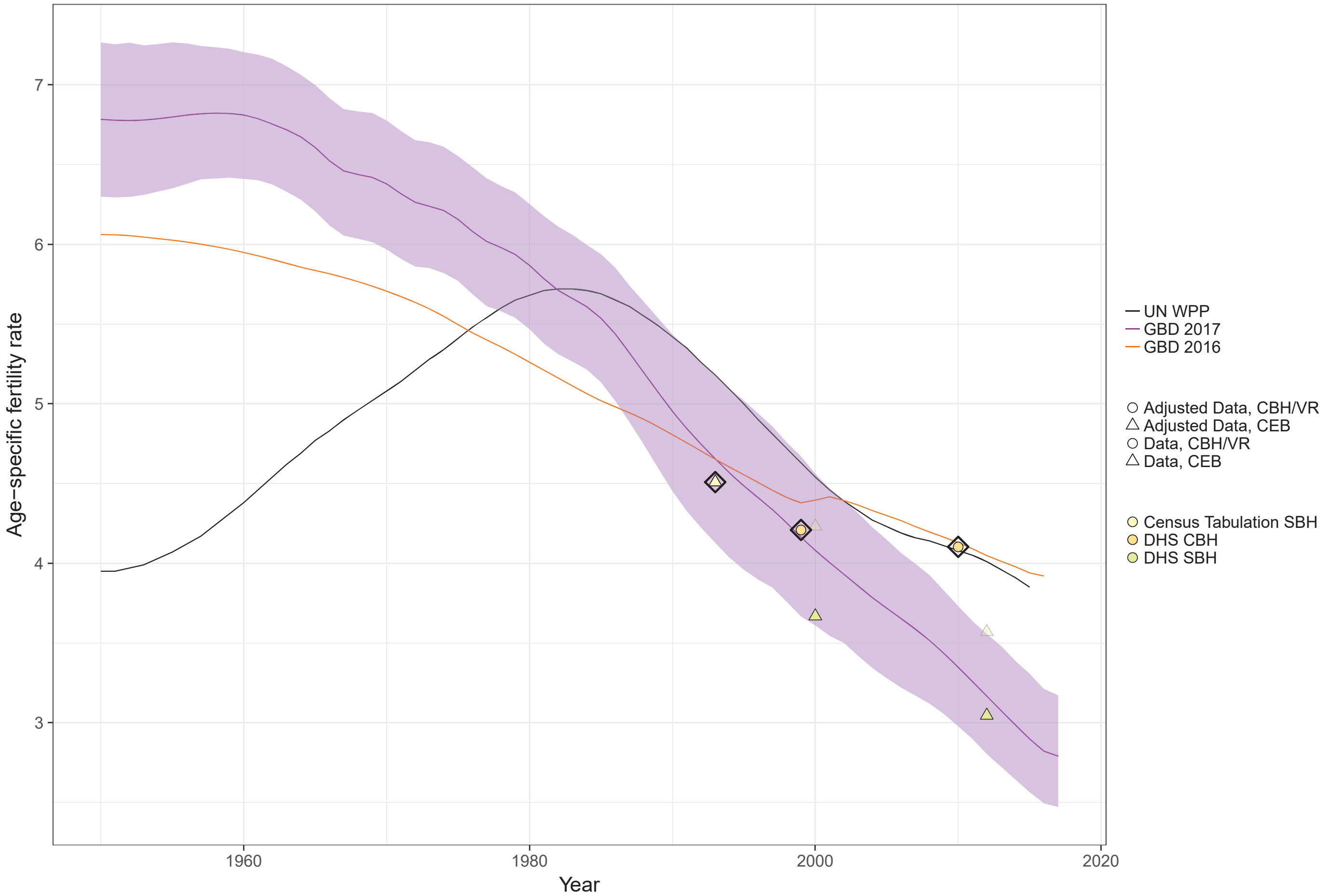

Age−specific fertility fitted trend lines, 1950−2017: Gabon, Ages: 10−14
Data points enclosed with a diamond are reference sources

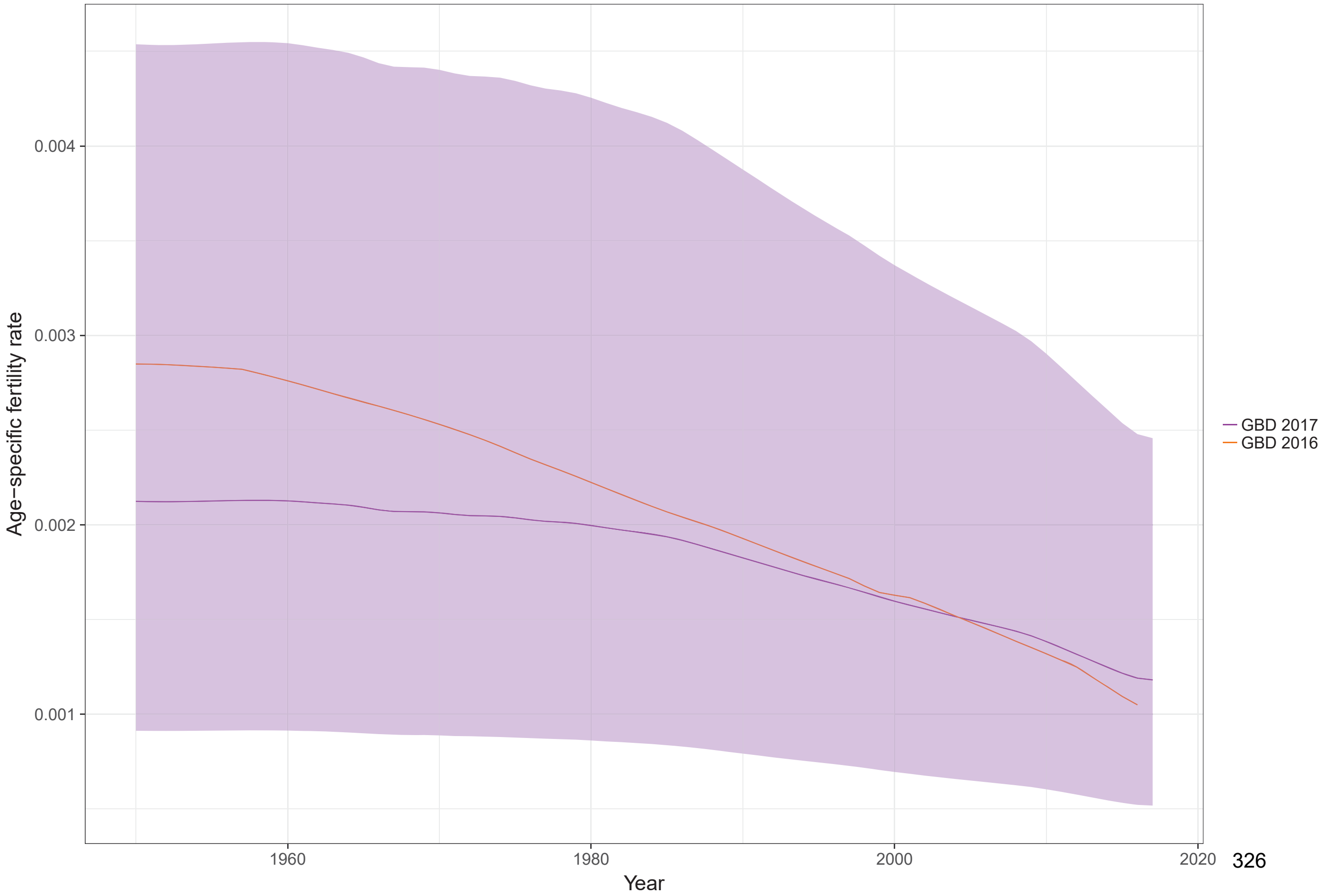

Age−specific fertility fitted trend lines, 1950−2017: Gabon, Ages: 15−19
Data points enclosed with a diamond are reference sources

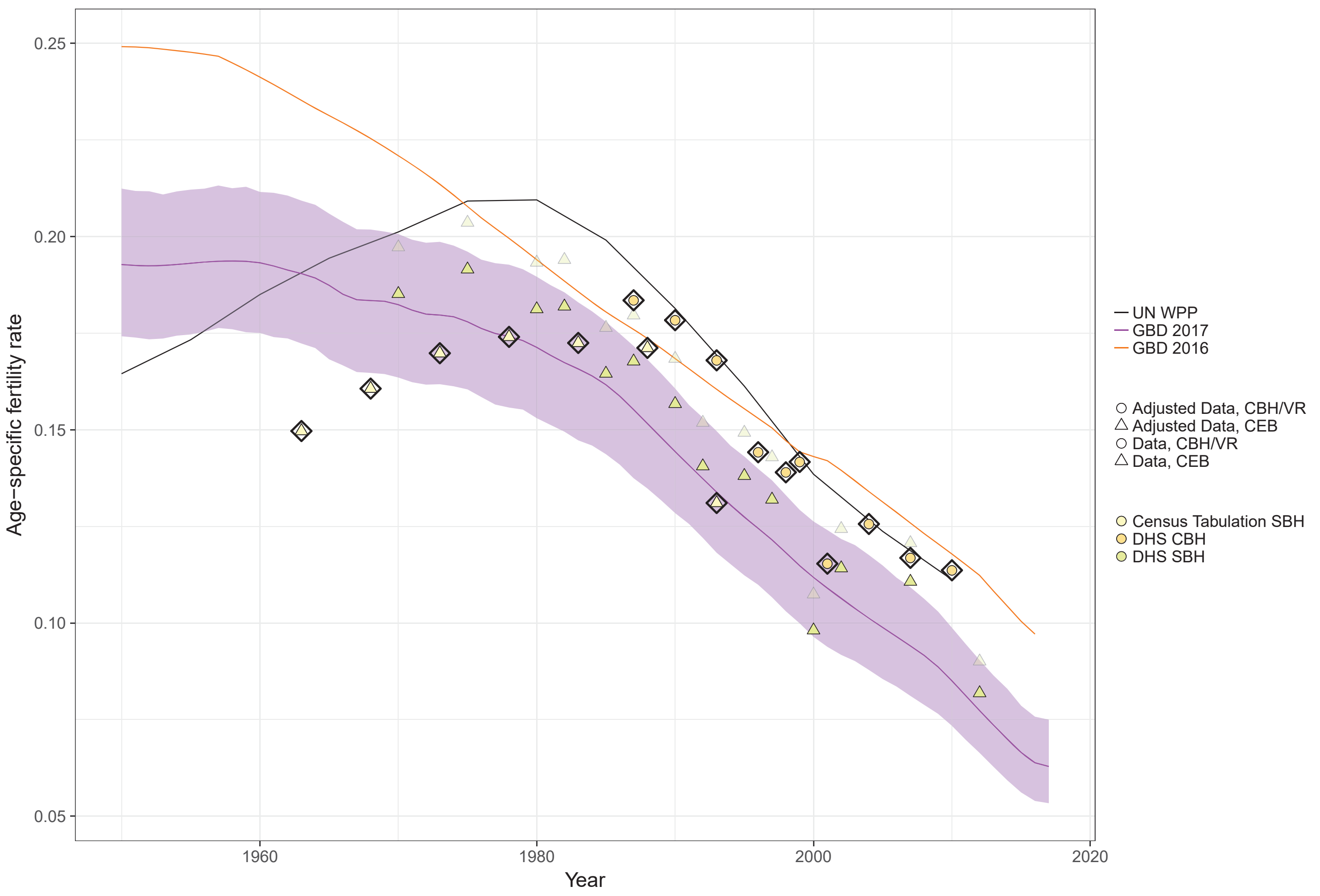

Age−specific fertility fitted trend lines, 1950−2017: Gabon, Ages: 20−24
Data points enclosed with a diamond are reference sources

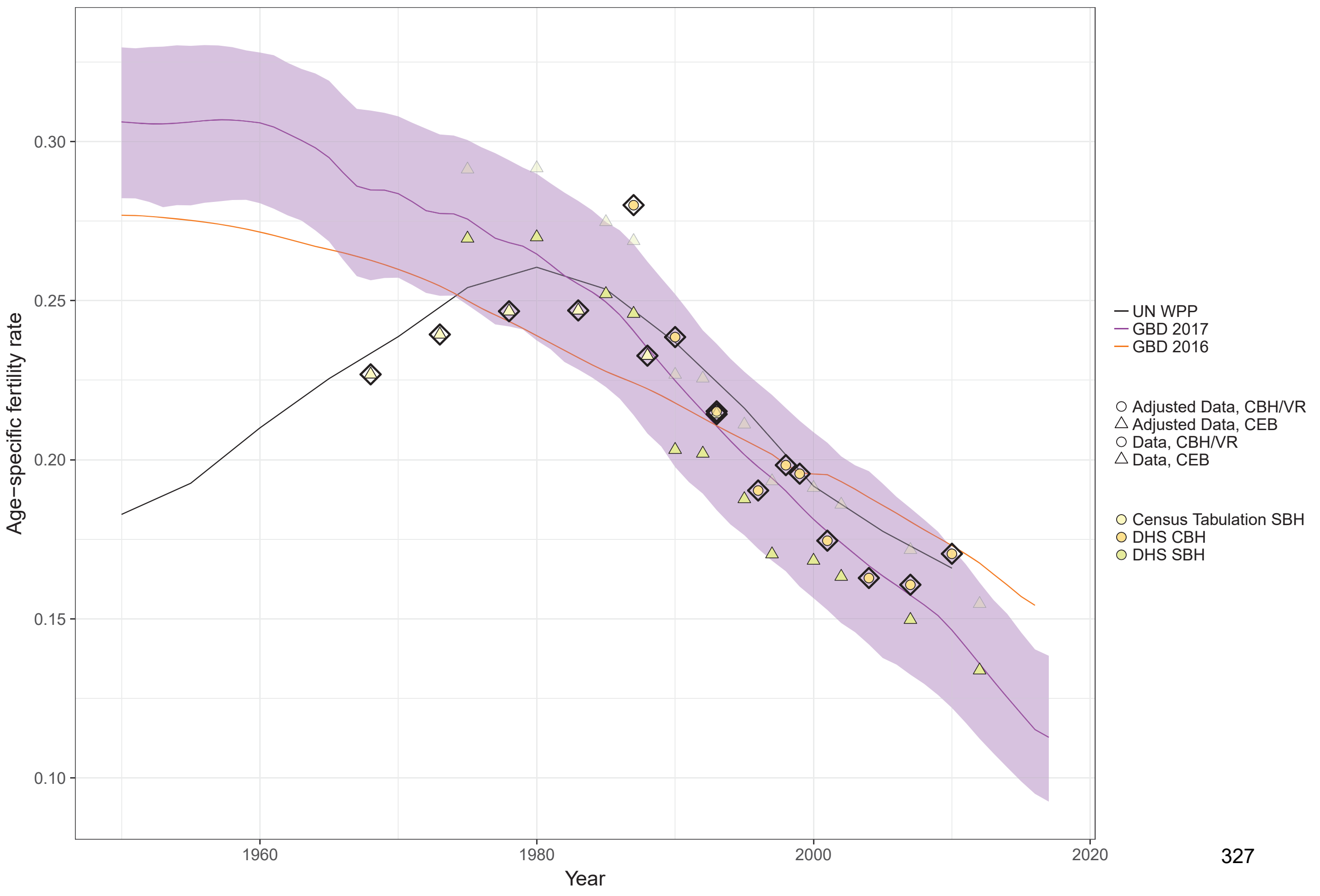

Age−specific fertility fitted trend lines, 1950−2017: Gabon, Ages: 25−29
Data points enclosed with a diamond are reference sources

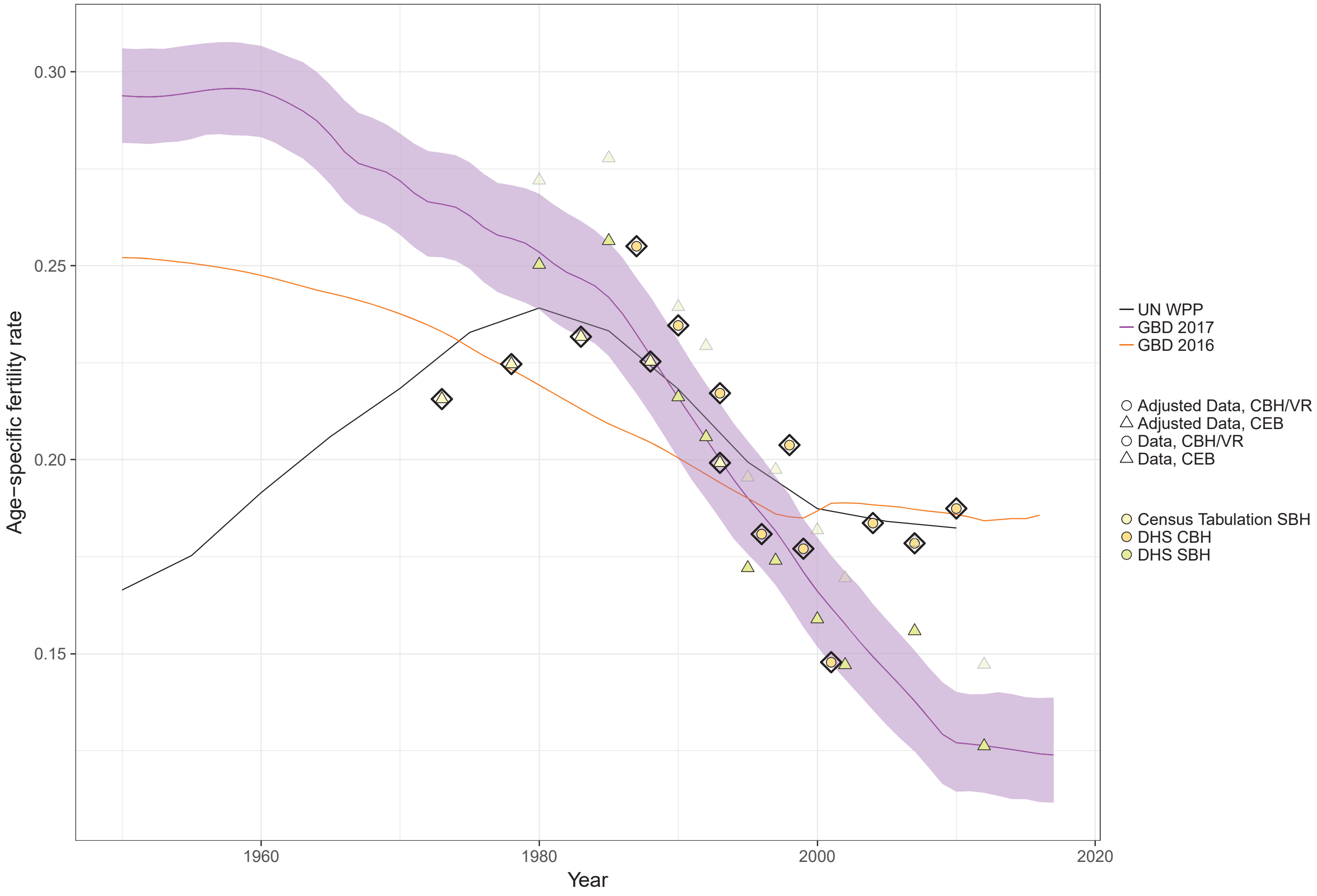

Age−specific fertility fitted trend lines, 1950−2017: Gabon, Ages: 30−34
Data points enclosed with a diamond are reference sources

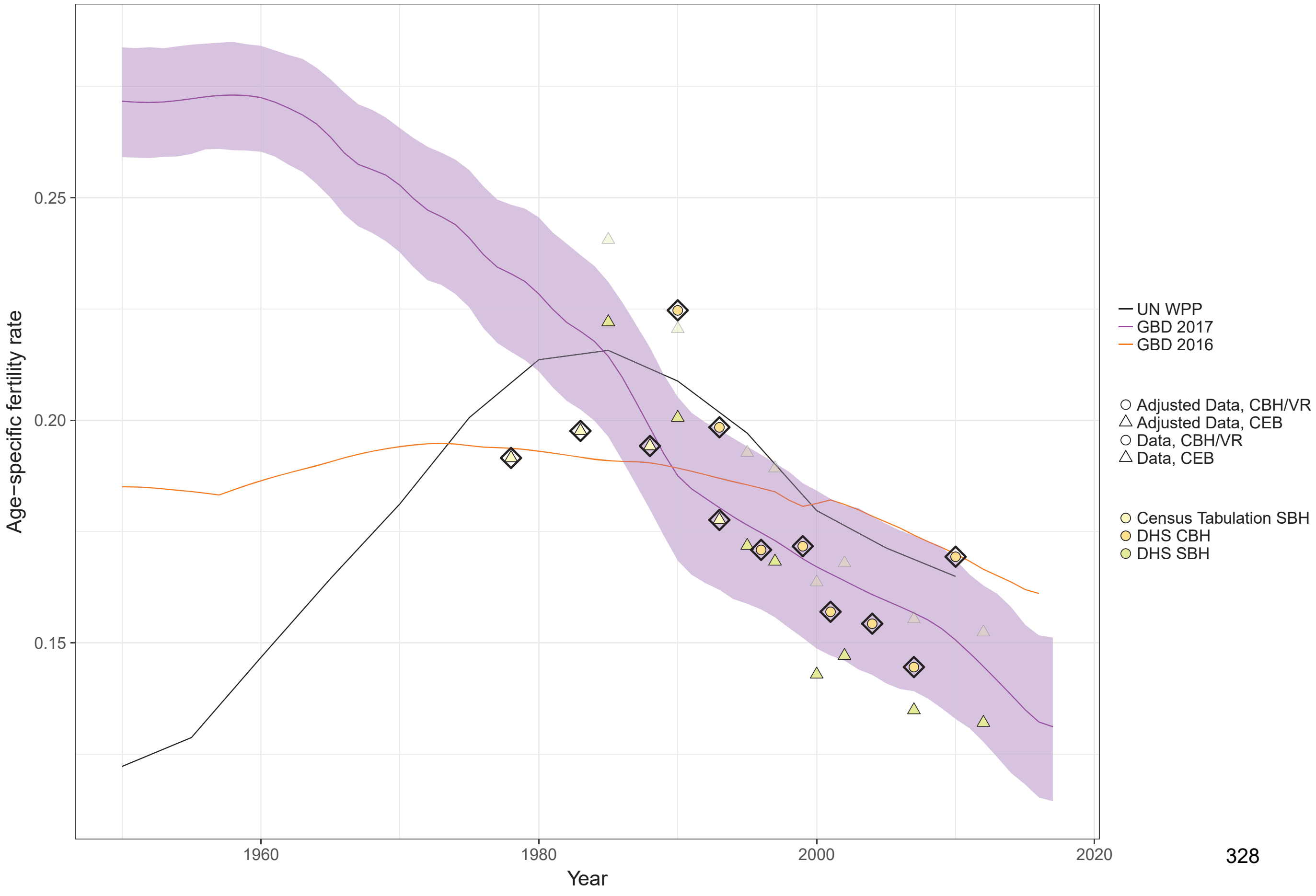

Age−specific fertility fitted trend lines, 1950−2017: Gabon, Ages: 35−39
Data points enclosed with a diamond are reference sources

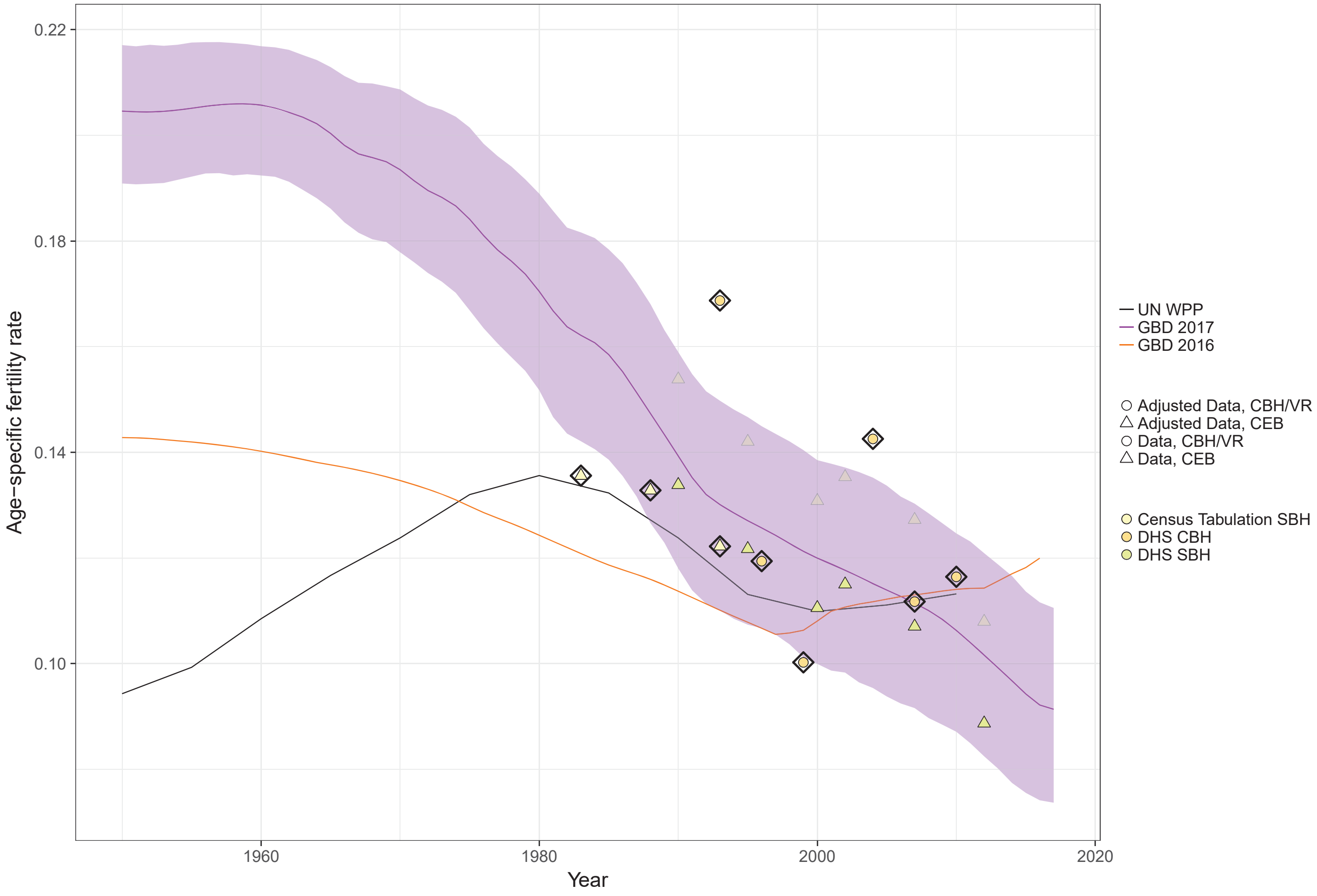

Age−specific fertility fitted trend lines, 1950−2017: Gabon, Ages: 40−44
Data points enclosed with a diamond are reference sources

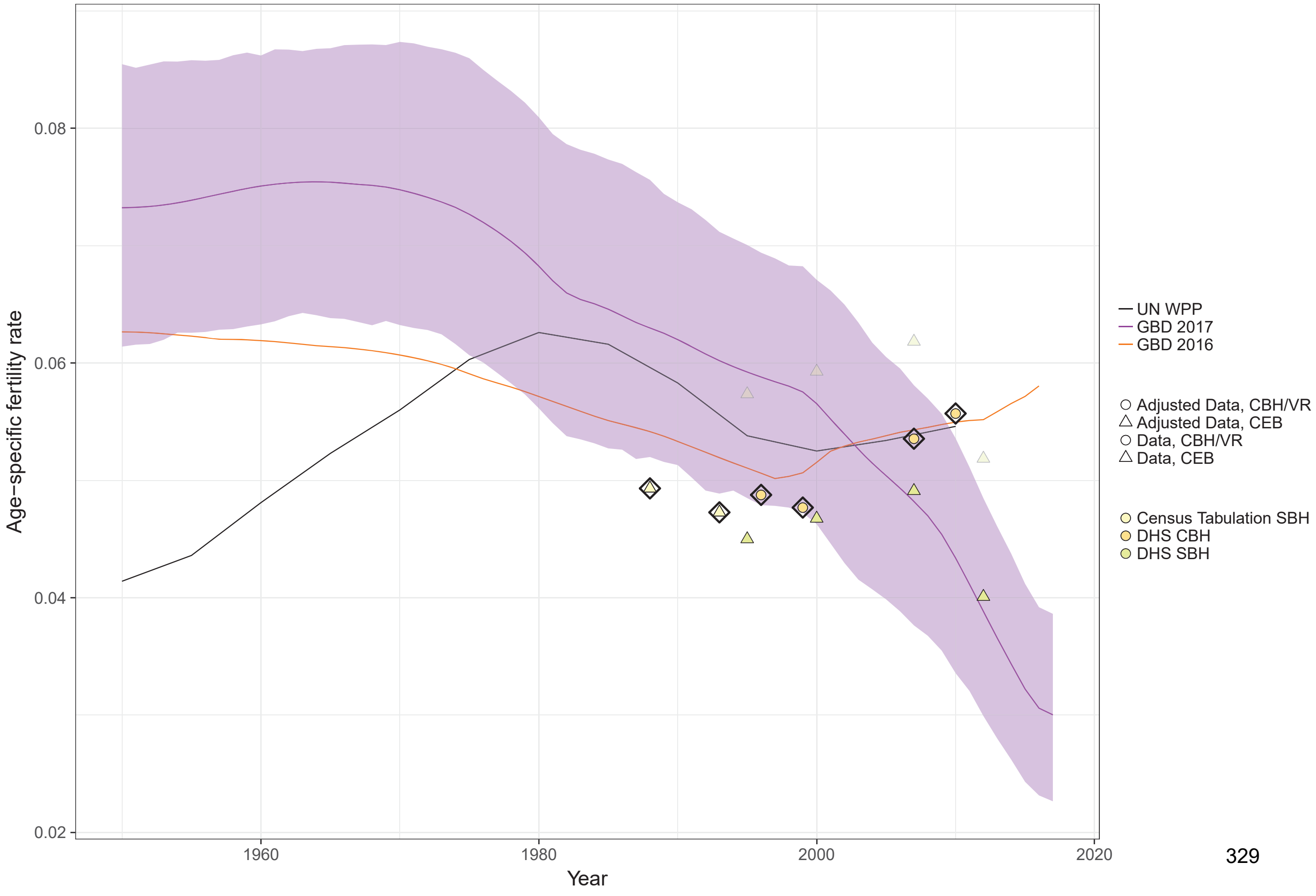

Age−specific fertility fitted trend lines, 1950−2017: Gabon, Ages: 45−49
Data points enclosed with a diamond are reference sources

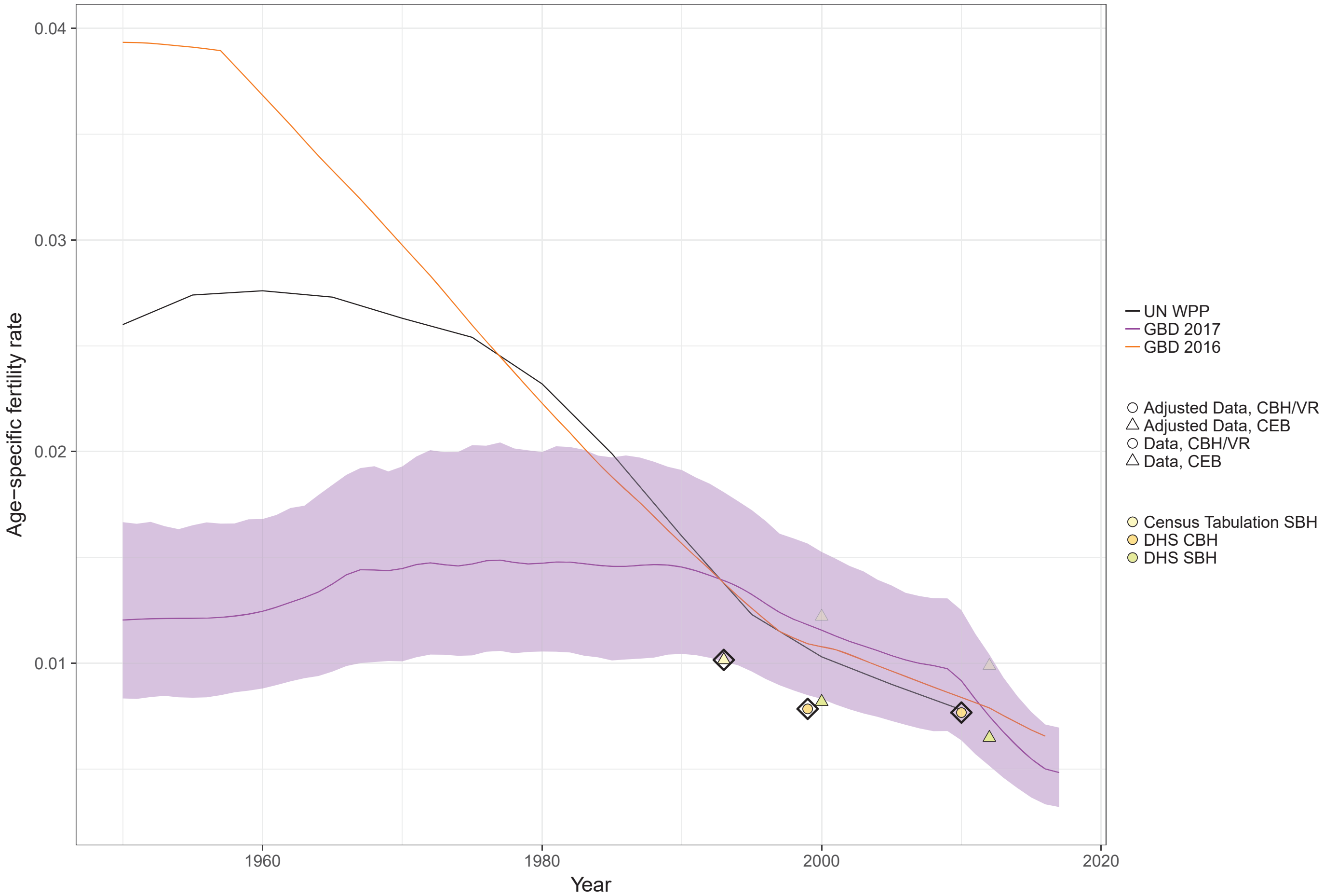

Age−specific fertility fitted trend lines, 1950−2017: Gabon, Ages: 50−54
Data points enclosed with a diamond are reference sources

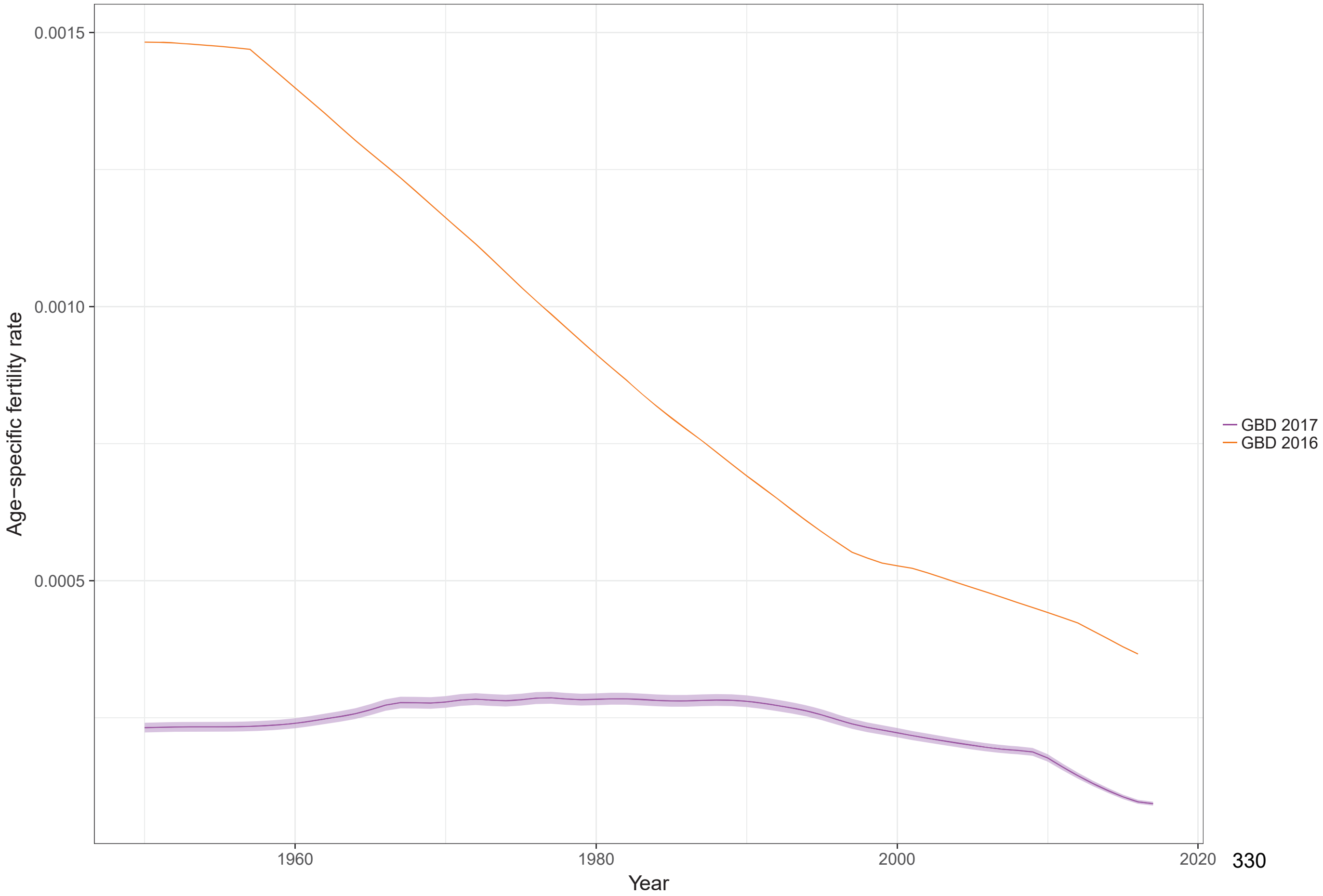

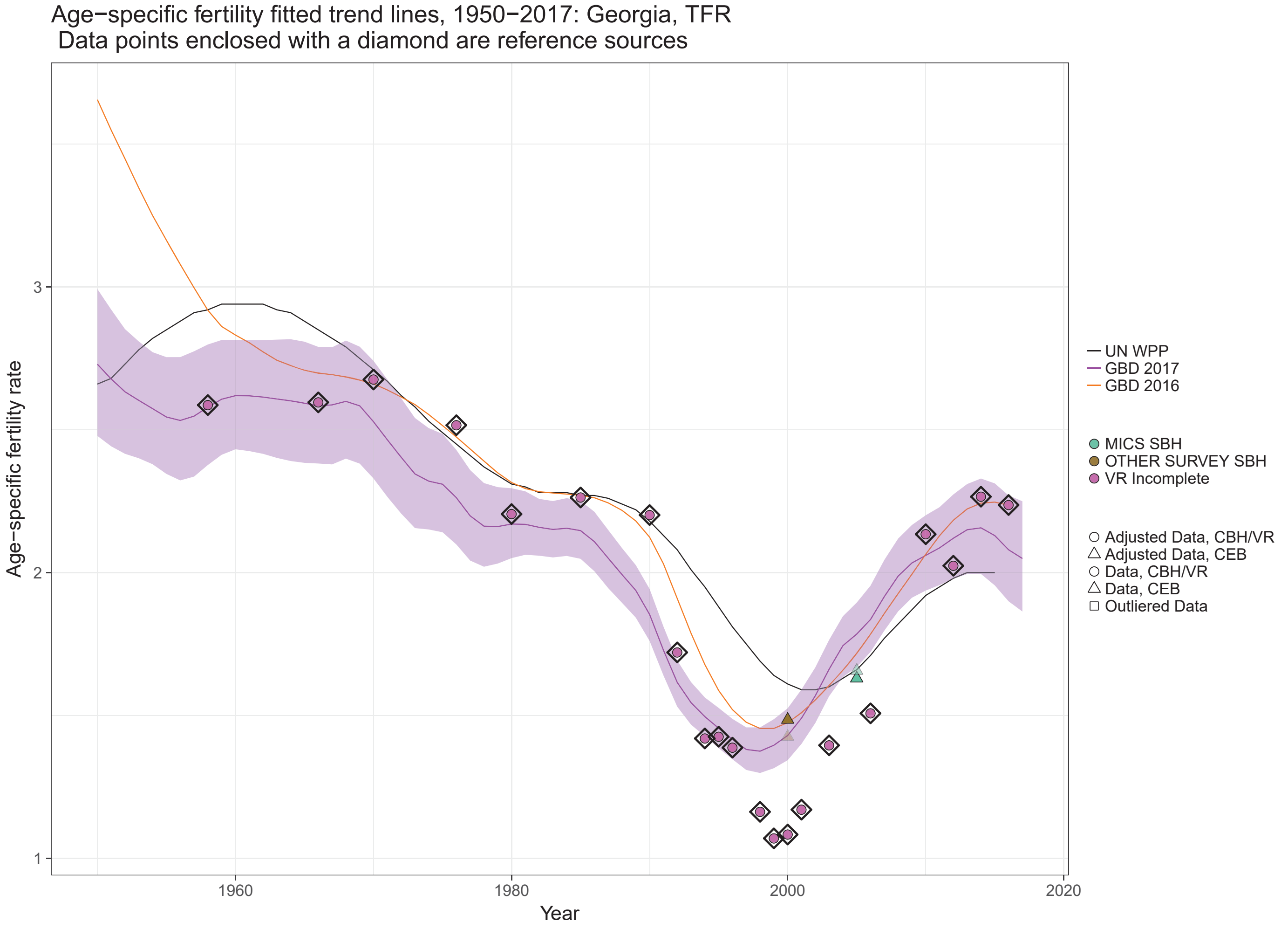

Age−specific fertility fitted trend lines, 1950−2017: Georgia, TFR
Data points enclosed with a diamond are reference sources
Age−specific fertility rate
Year
1
2
3
1960
1980
2000
2020
UN WPP
GBD 2017
GBD 2016
MICS SBH
OTHER SURVEY SBH
VR Incomplete
Adjusted Data, CBH/VR
Adjusted Data, CEB
Data, CBH/VR
Data, CEB
Outliered Data

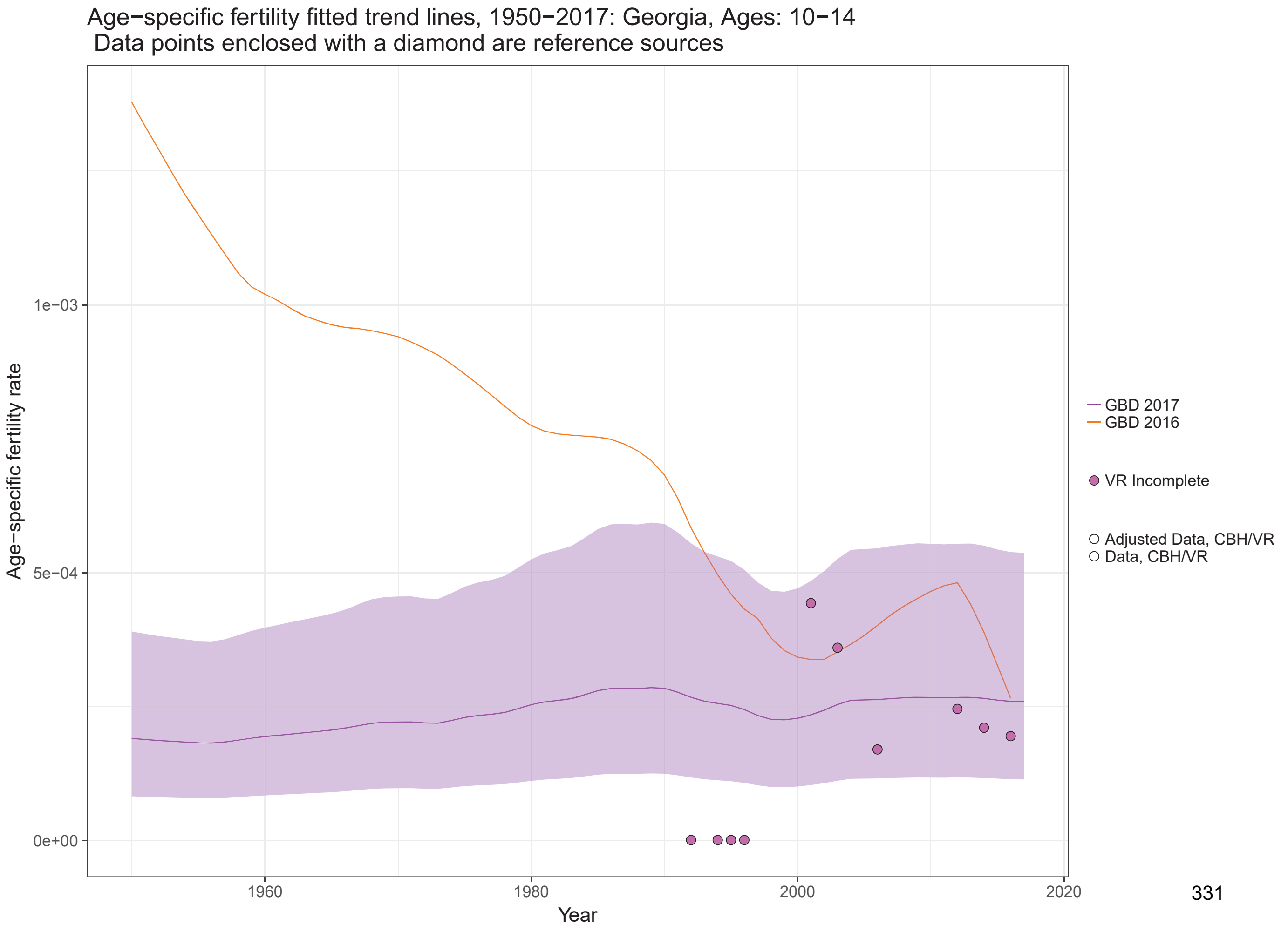

Age−specific fertility fitted trend lines, 1950−2017: Georgia, Ages: 10−14
Data points enclosed with a diamond are reference sources
Age−specific fertility rate
Year
0e+00
5e−04
1e−03
1960
1980
2000
2020
GBD 2017
GBD 2016
VR Incomplete
Adjusted Data, CBH/VR
Data, CBH/VR

Age−specific fertility fitted trend lines, 1950−2017: Georgia, Ages: 15−19
Data points enclosed with a diamond are reference sources

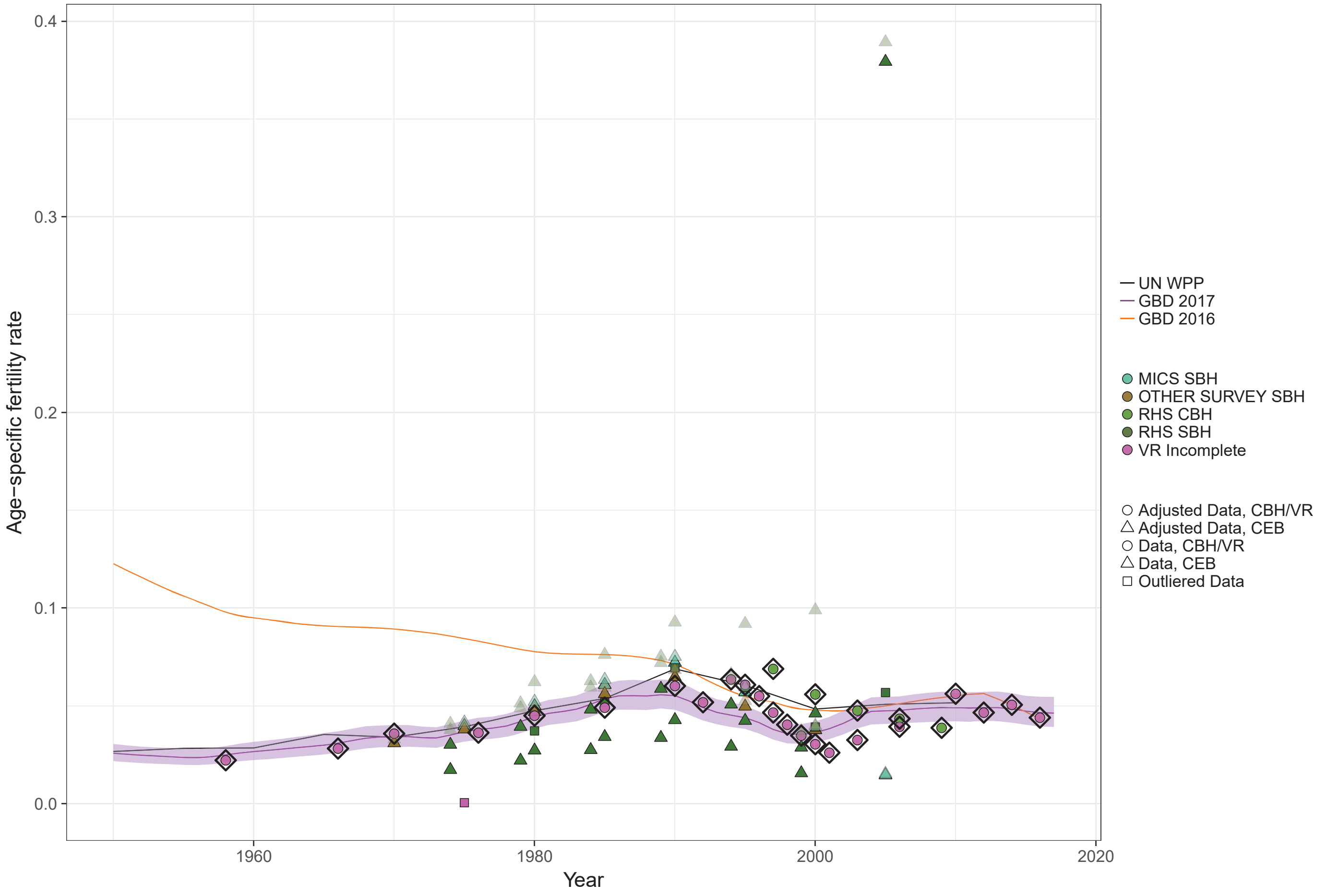

Age−specific fertility fitted trend lines, 1950−2017: Georgia, Ages: 20−24
Data points enclosed with a diamond are reference sources

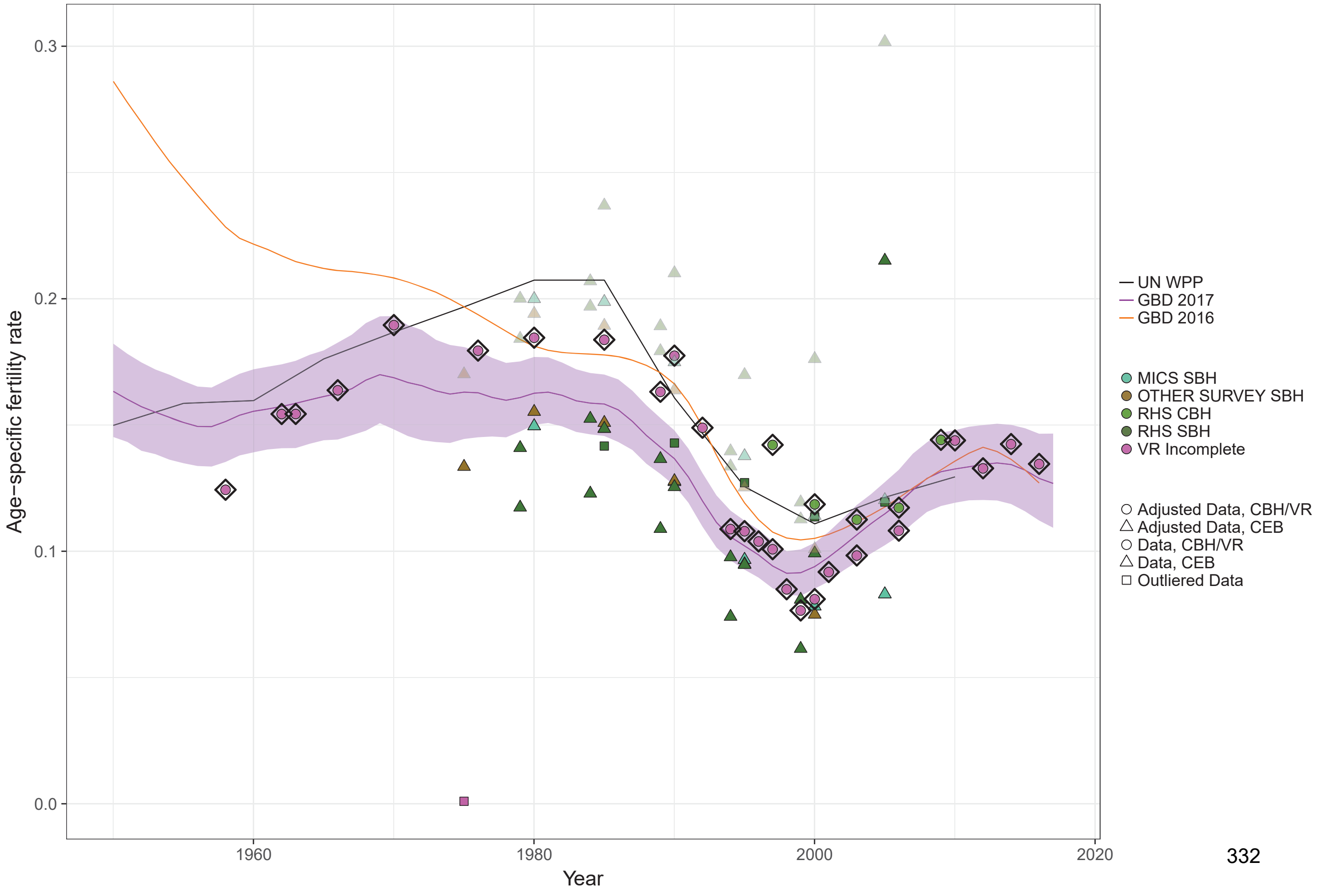

Age−specific fertility fitted trend lines, 1950−2017: Georgia, Ages: 25−29
Data points enclosed with a diamond are reference sources

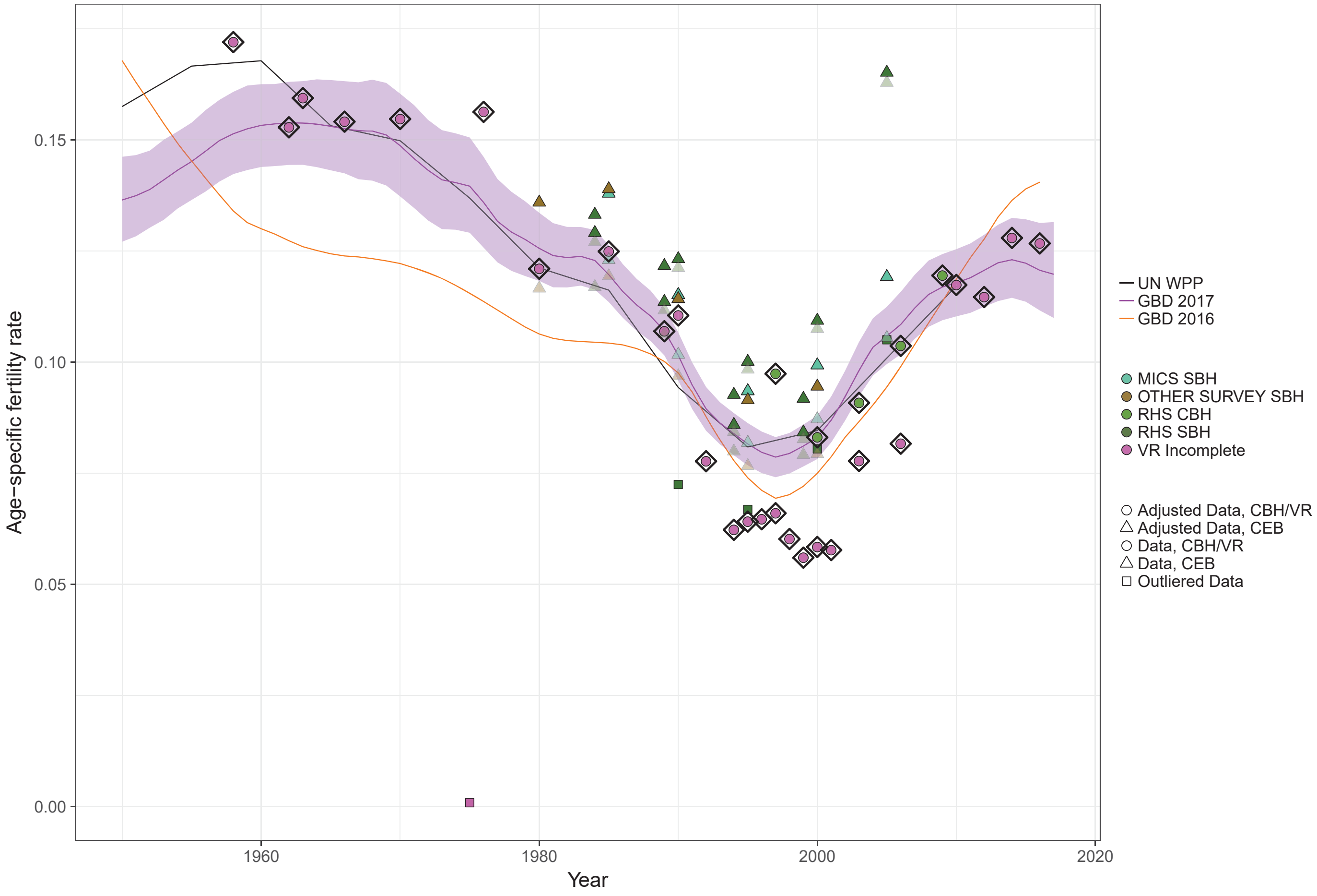

Age−specific fertility fitted trend lines, 1950−2017: Georgia, Ages: 30−34
Data points enclosed with a diamond are reference sources

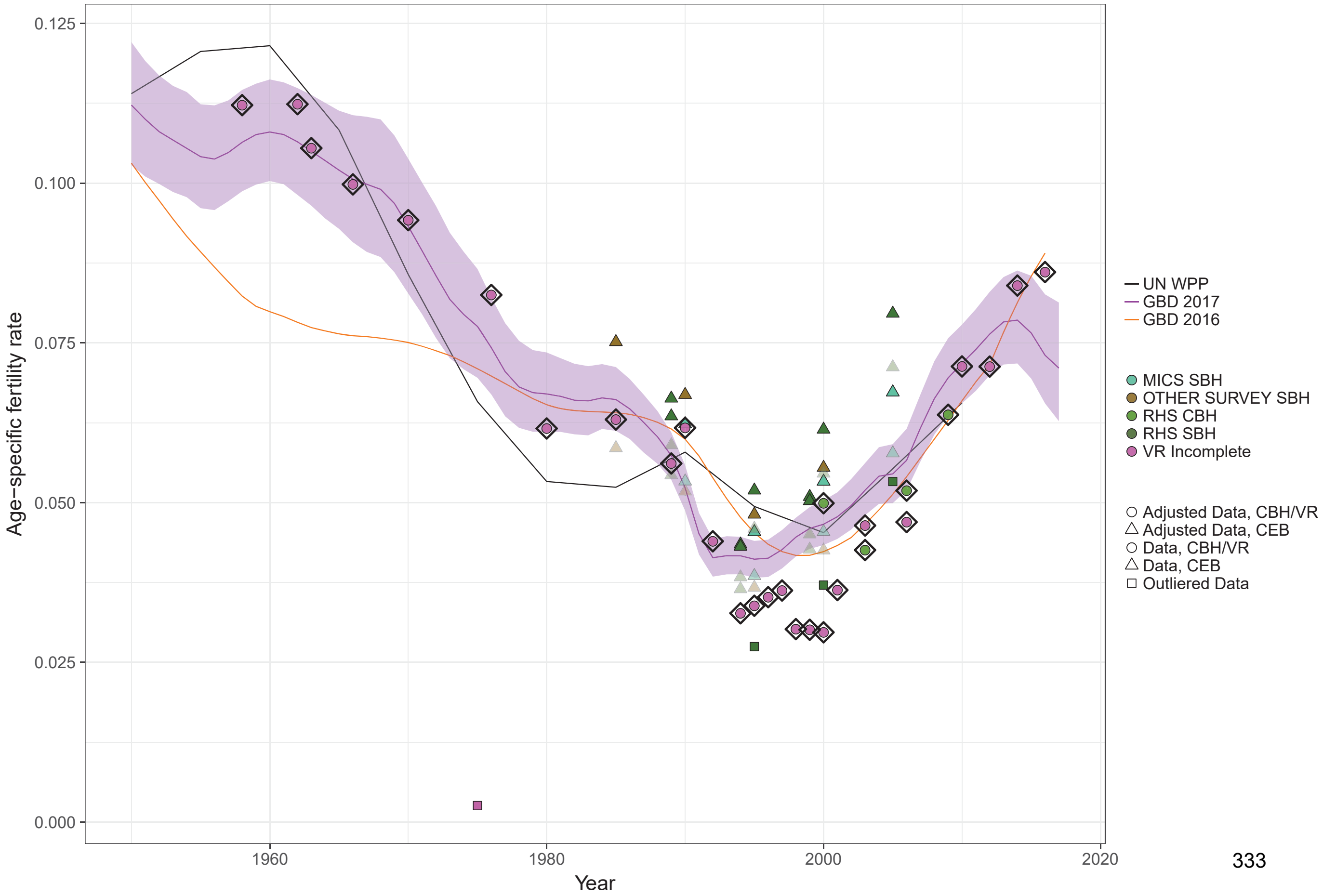

Age−specific fertility fitted trend lines, 1950−2017: Georgia, Ages: 35−39
Data points enclosed with a diamond are reference sources

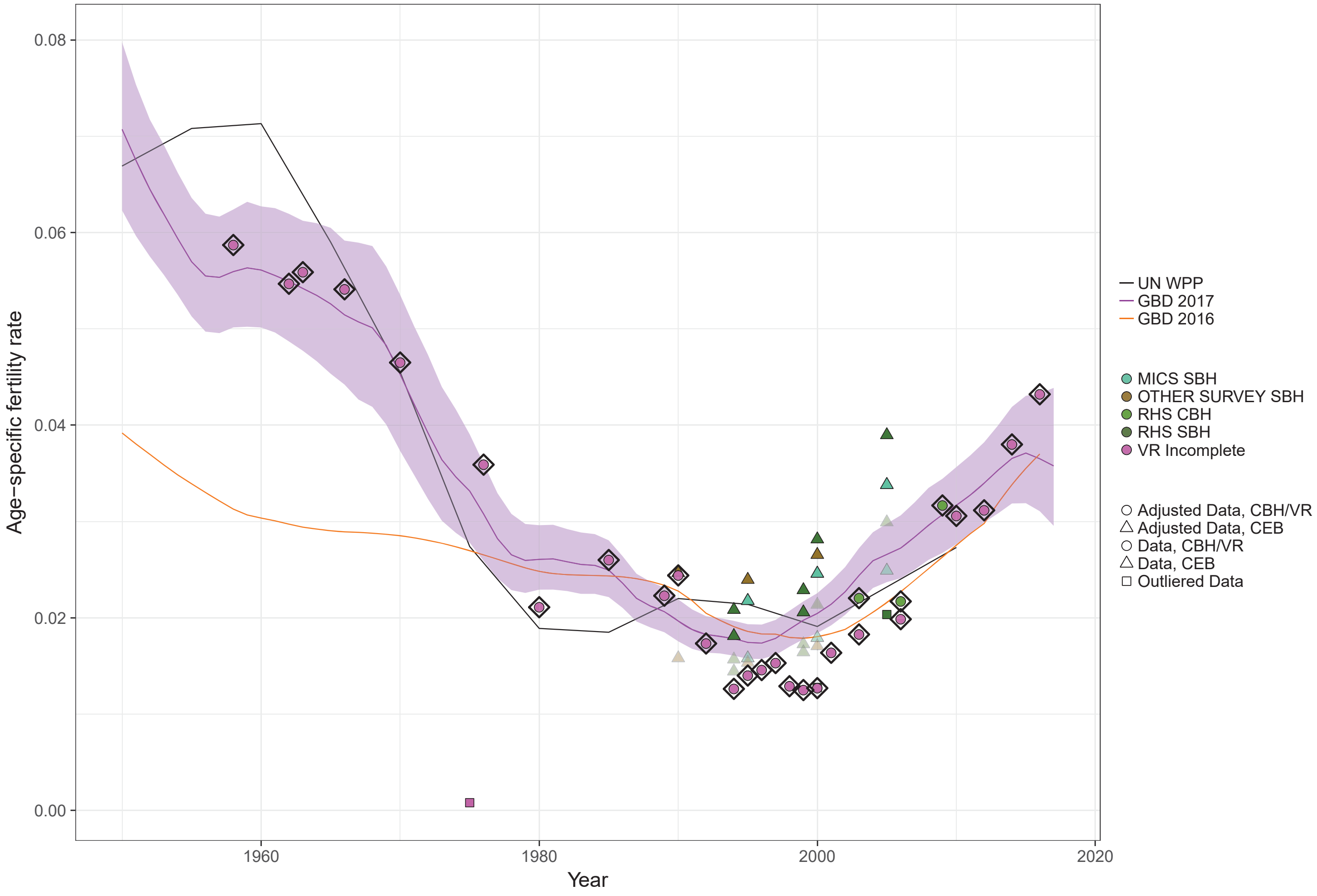

Age−specific fertility fitted trend lines, 1950−2017: Georgia, Ages: 40−44
Data points enclosed with a diamond are reference sources

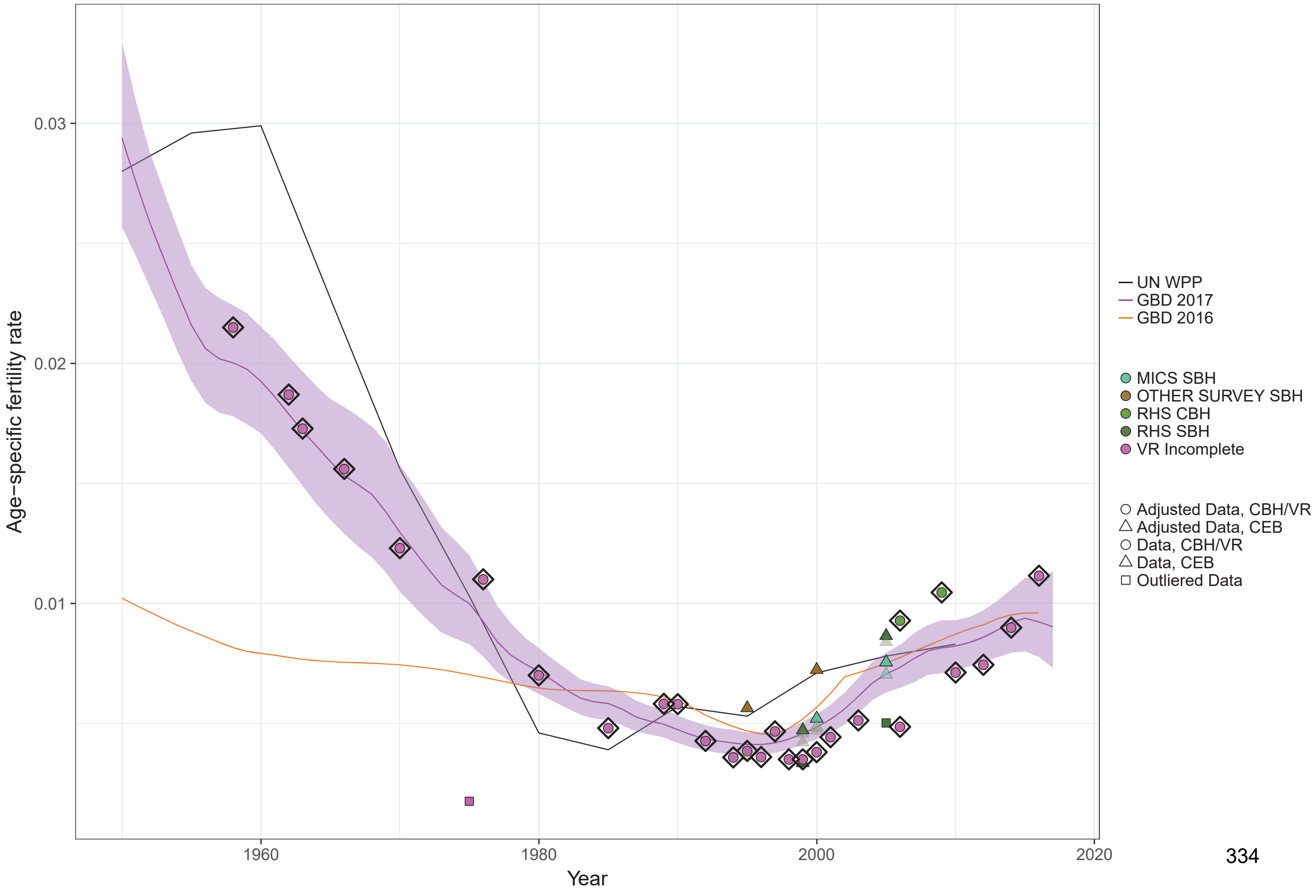

Age−specific fertility fitted trend lines, 1950−2017: Georgia, Ages: 45−49
Data points enclosed with a diamond are reference sources

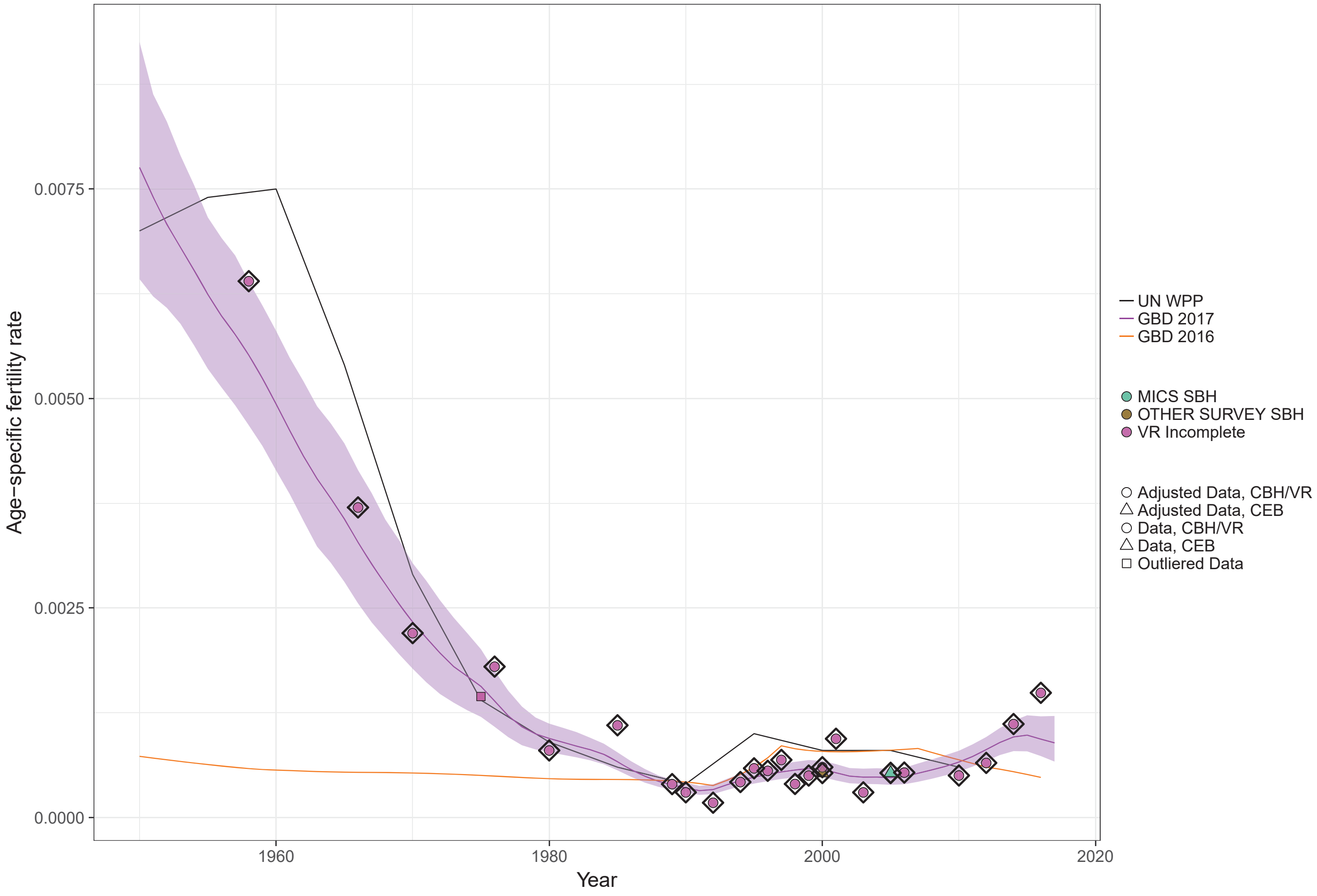

Age−specific fertility fitted trend lines, 1950−2017: Georgia, Ages: 50−54
Data points enclosed with a diamond are reference sources

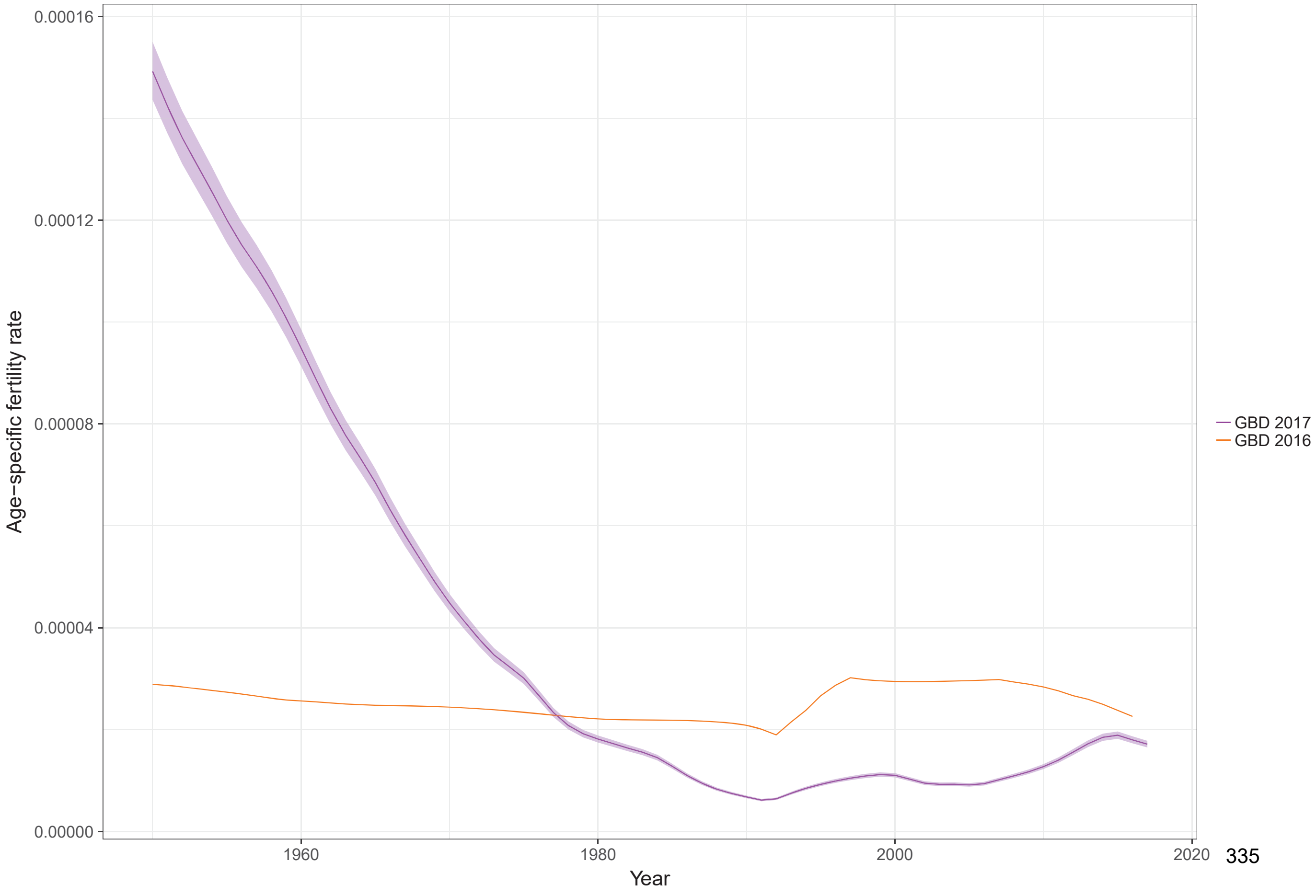

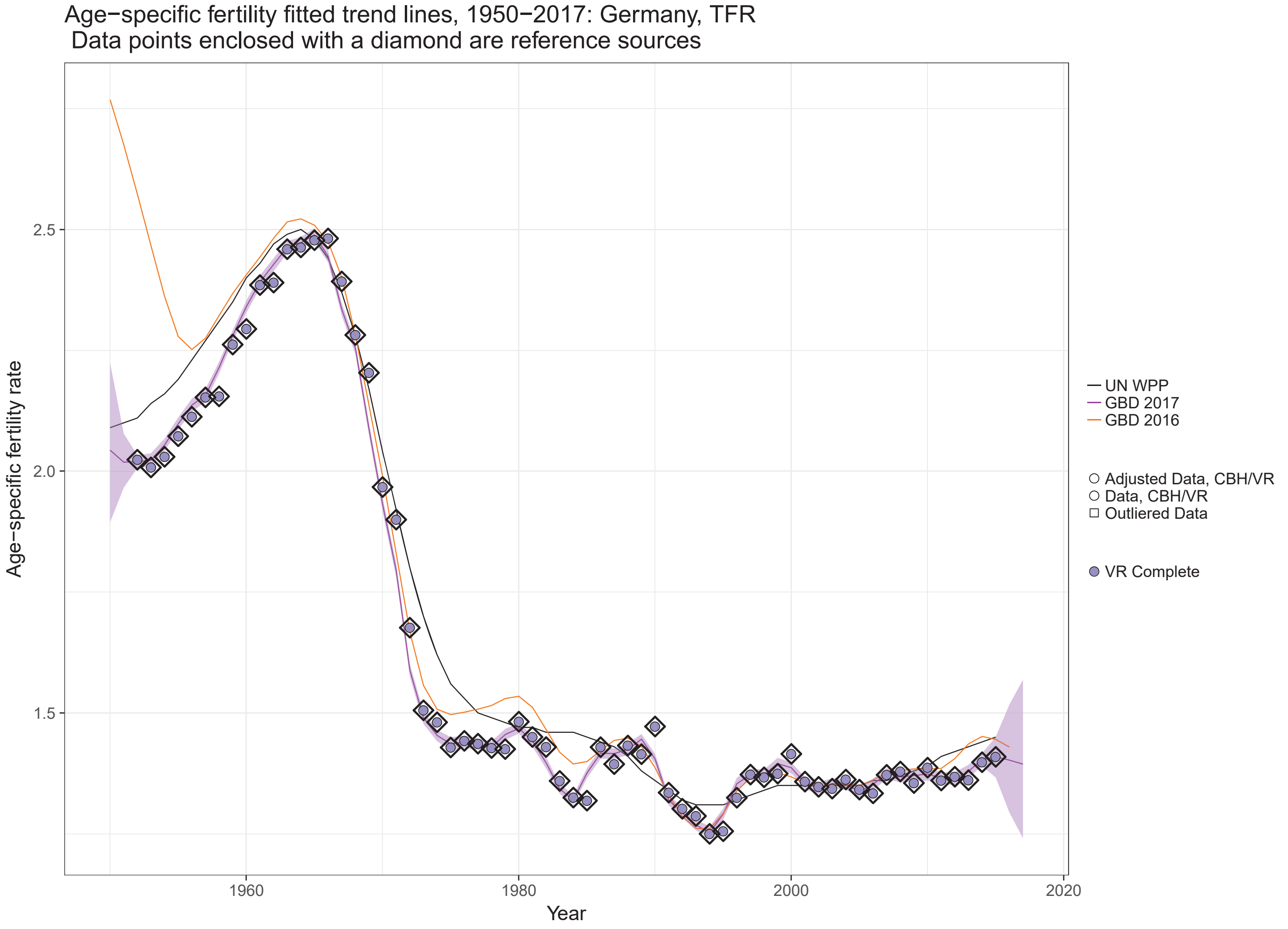

Age−specific fertility fitted trend lines, 1950−2017: Germany, TFR
Data points enclosed with a diamond are reference sources
Age−specific fertility rate
2.5
2.0
1.5
1960
1980
2000
2020
Year
UN WPP
GBD 2017
GBD 2016
Adjusted Data, CBH/VR
Data, CBH/VR
Outliered Data
VR Complete

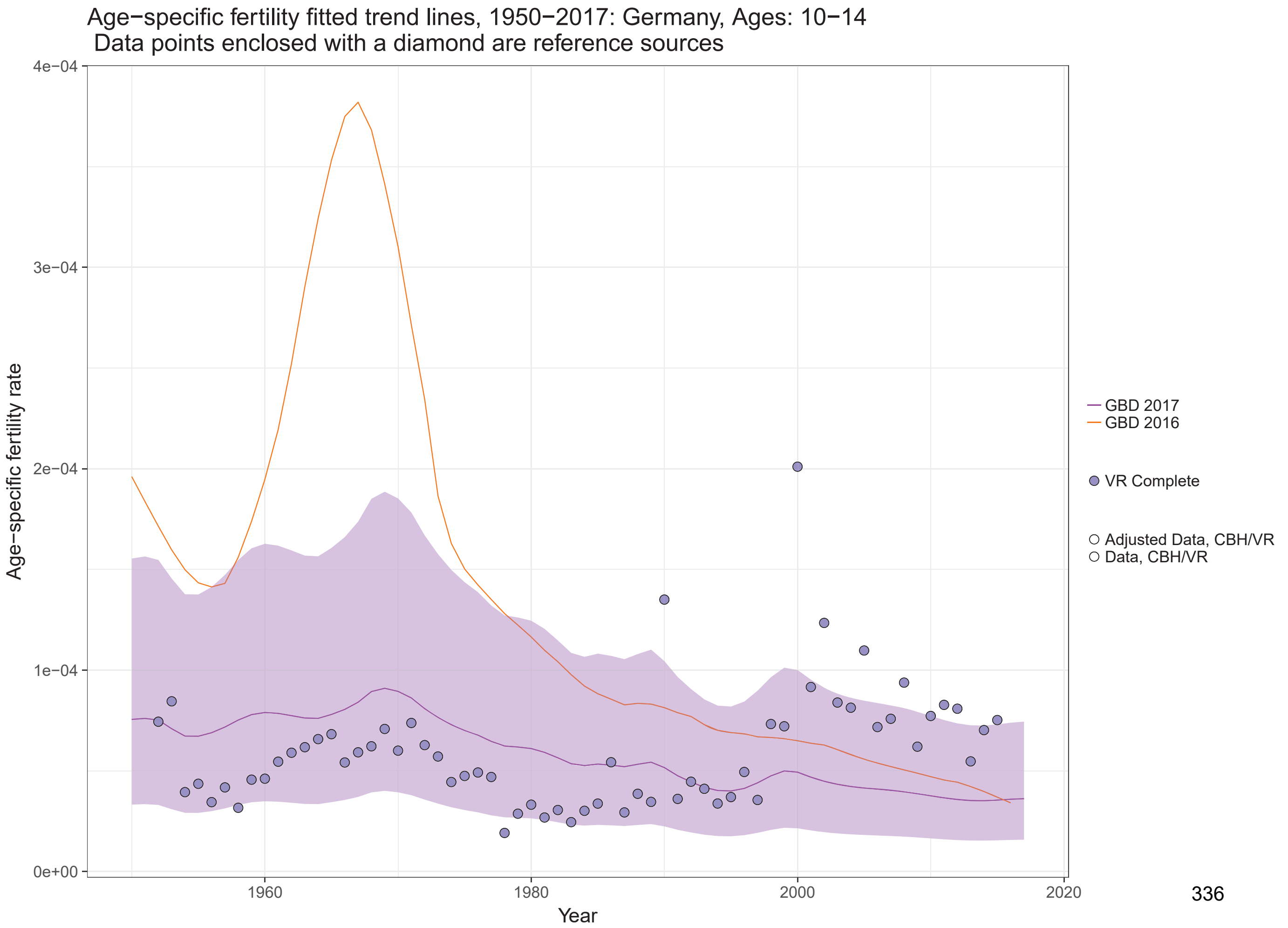

Age−specific fertility fitted trend lines, 1950−2017: Germany, Ages: 10−14
Data points enclosed with a diamond are reference sources
Age−specific fertility rate
4e−04
3e−04
2e−04
1e−04
0e+00
1960
1980
2000
2020
Year
GBD 2017
GBD 2016
VR Complete
Adjusted Data, CBH/VR
Data, CBH/VR

Age−specific fertility fitted trend lines, 1950−2017: Germany, Ages: 15−19
Data points enclosed with a diamond are reference sources

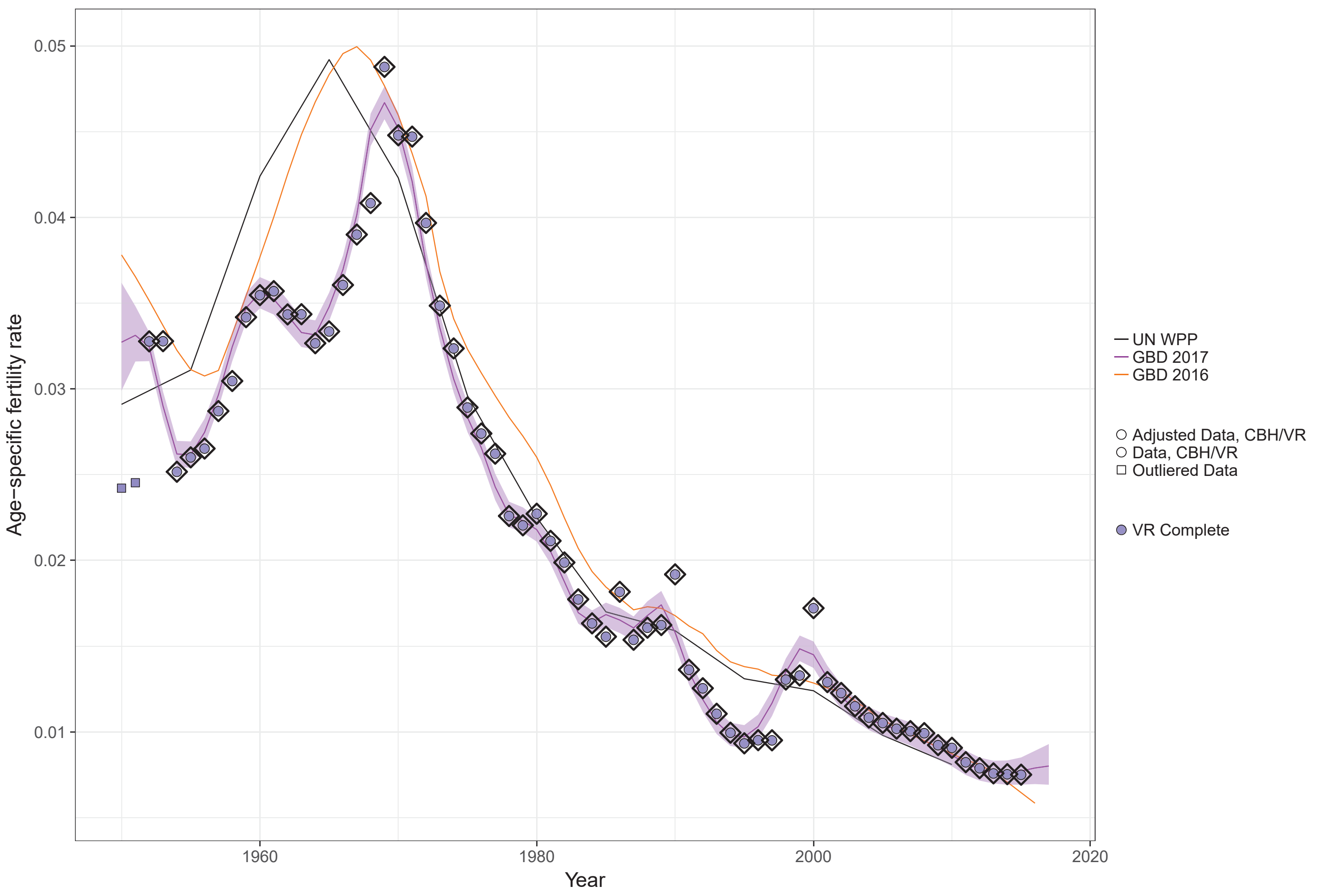

Age−specific fertility fitted trend lines, 1950−2017: Germany, Ages: 20−24
Data points enclosed with a diamond are reference sources

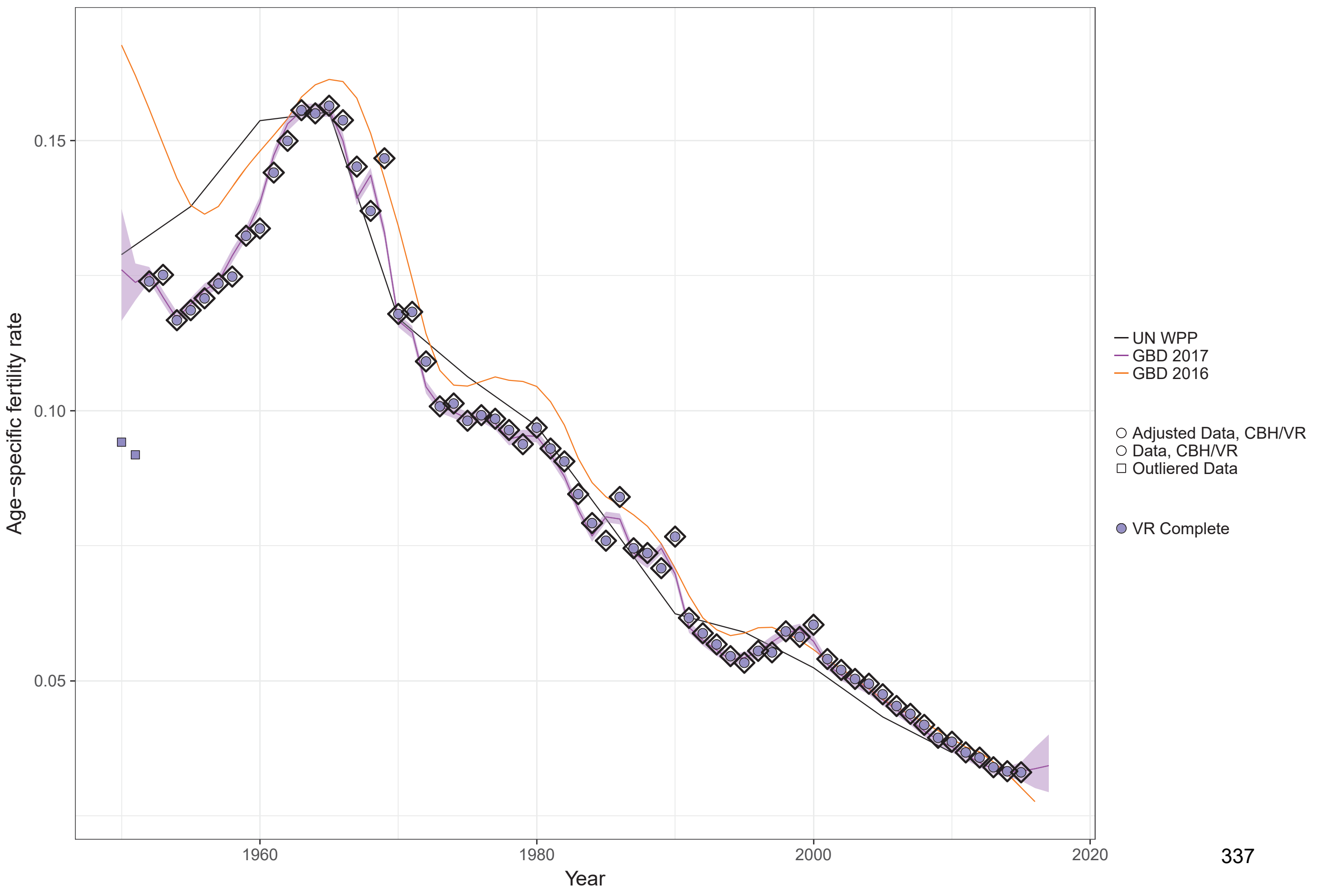

Age−specific fertility fitted trend lines, 1950−2017: Germany, Ages: 25−29
Data points enclosed with a diamond are reference sources

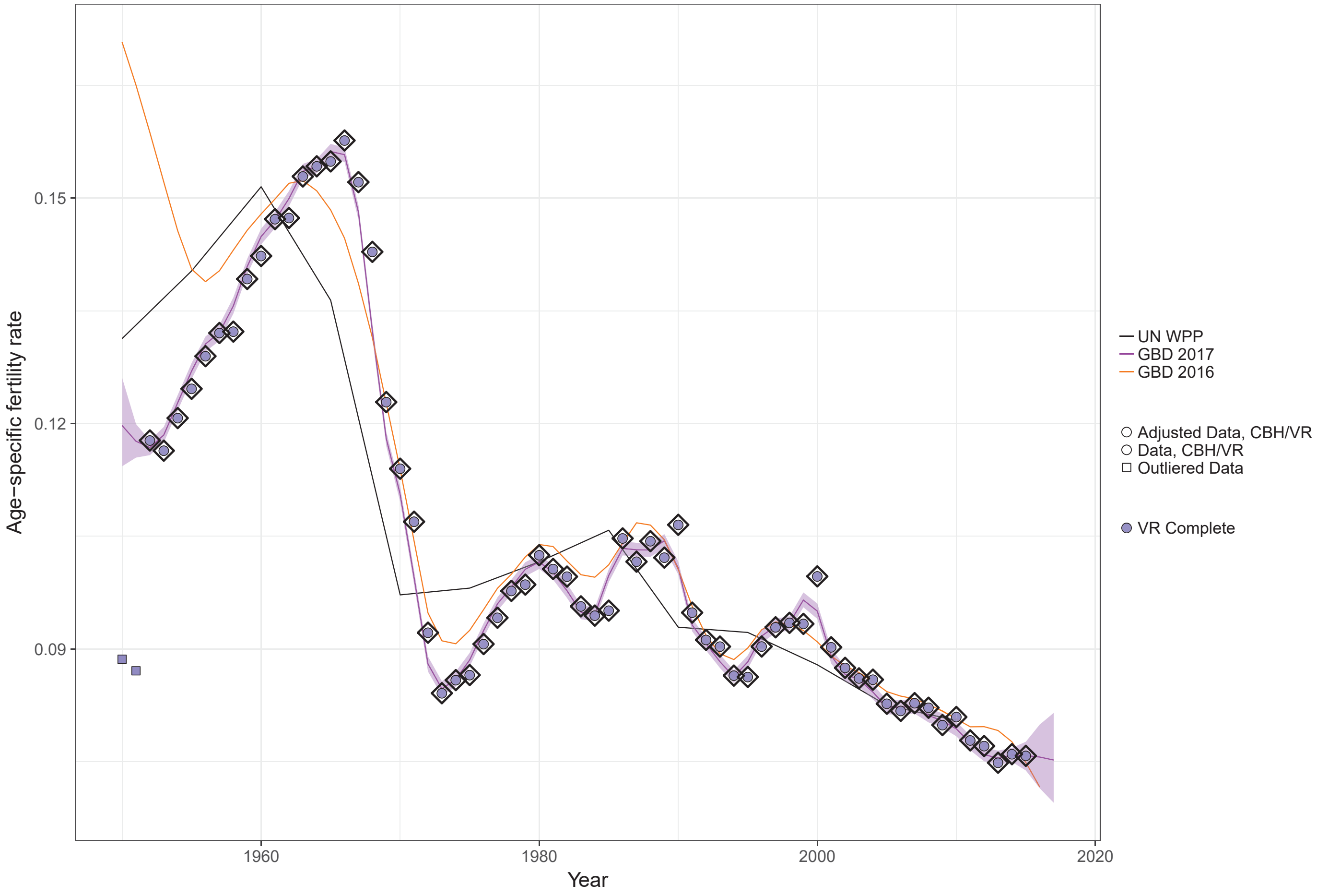

Age−specific fertility fitted trend lines, 1950−2017: Germany, Ages: 30−34
Data points enclosed with a diamond are reference sources

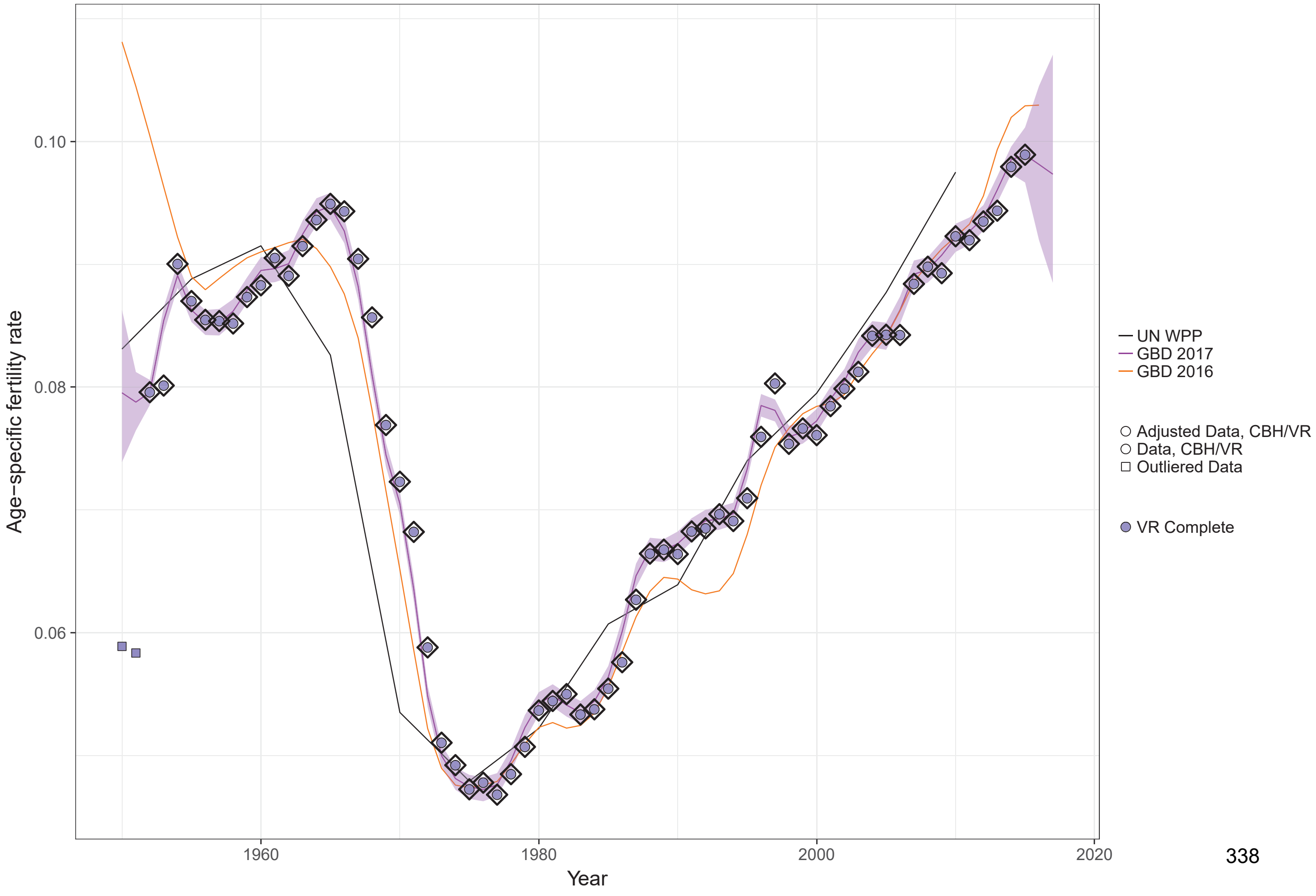

Age−specific fertility fitted trend lines, 1950−2017: Germany, Ages: 35−39
Data points enclosed with a diamond are reference sources

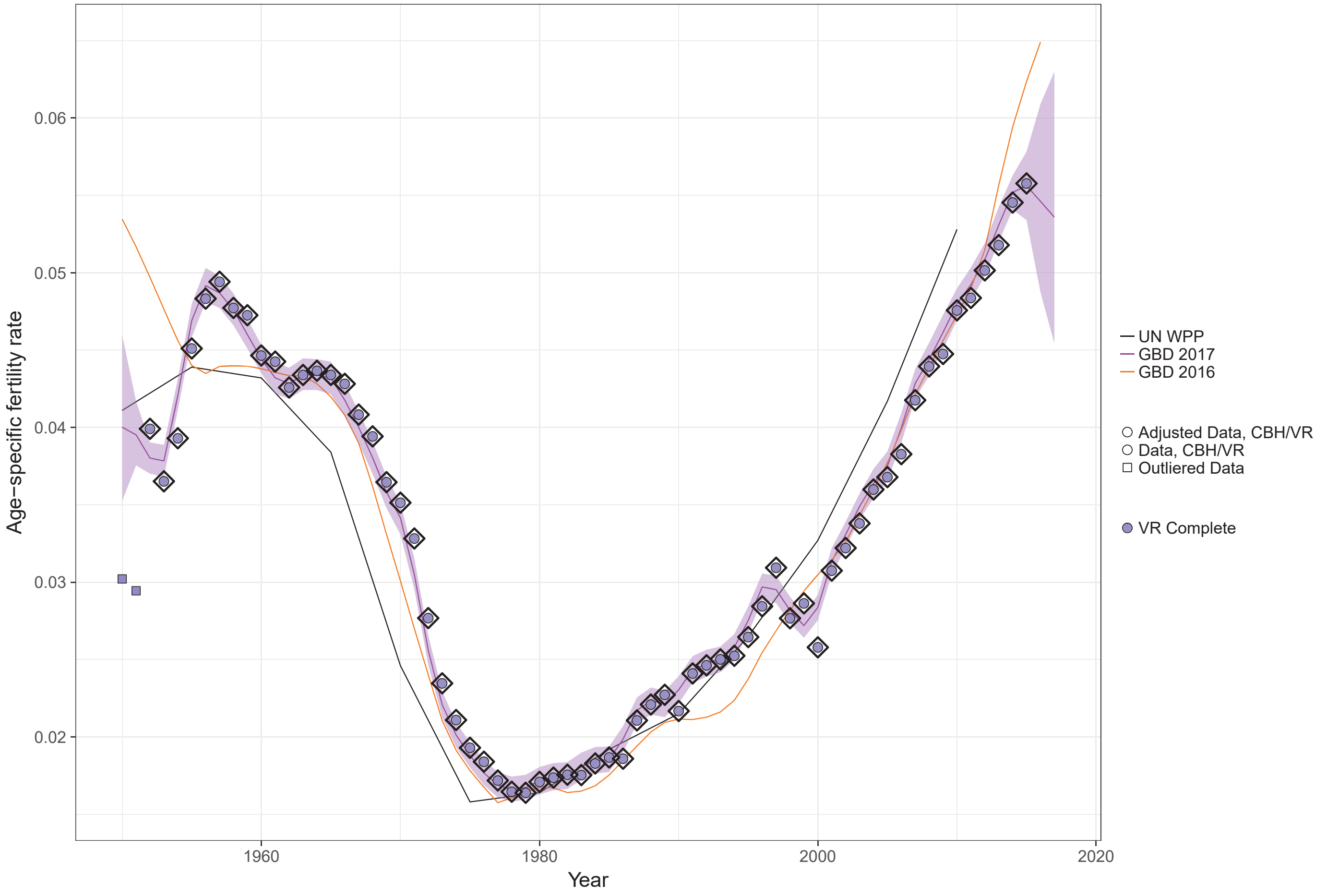

Age−specific fertility fitted trend lines, 1950−2017: Germany, Ages: 40−44
Data points enclosed with a diamond are reference sources

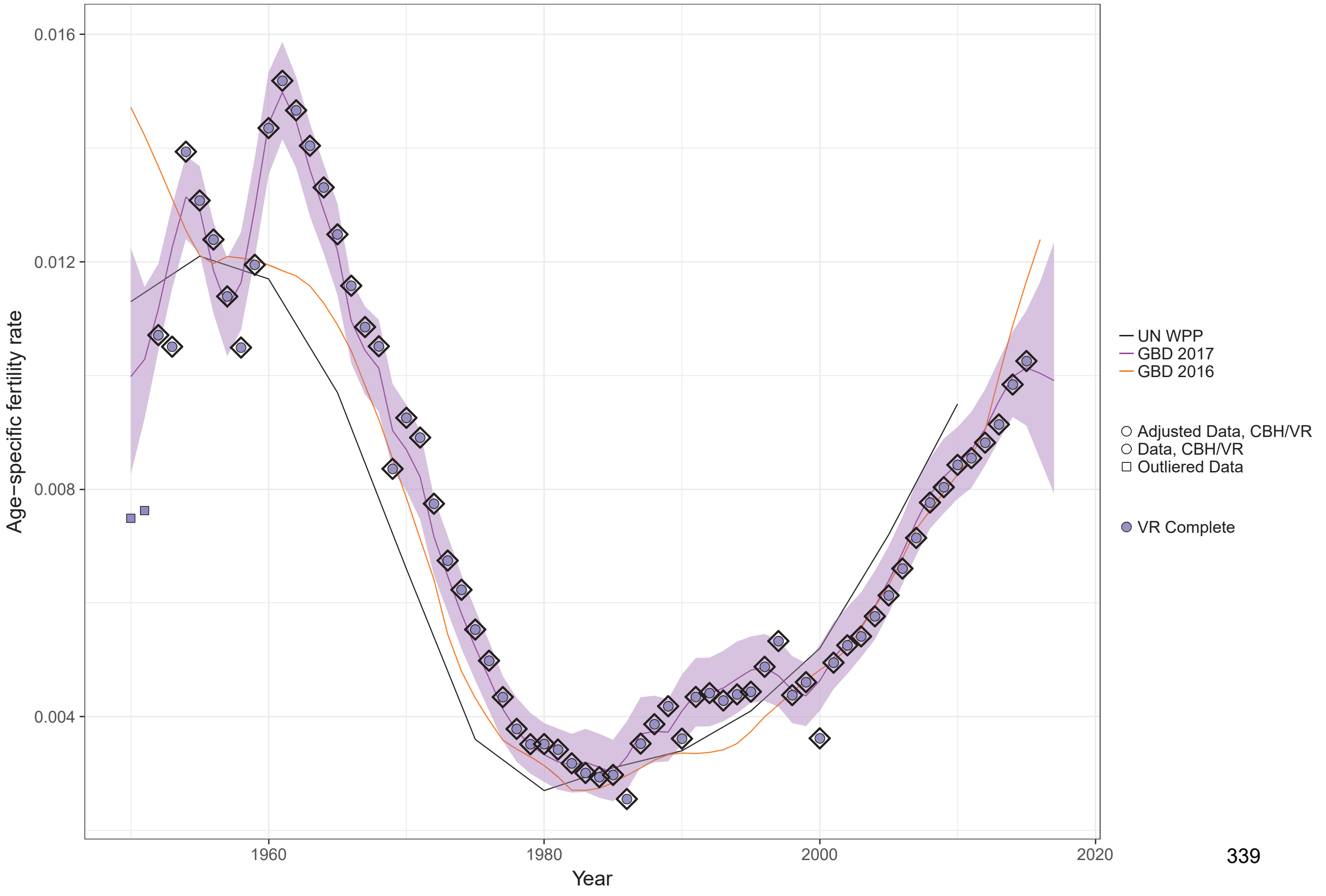

Age−specific fertility fitted trend lines, 1950−2017: Germany, Ages: 45−49
Data points enclosed with a diamond are reference sources

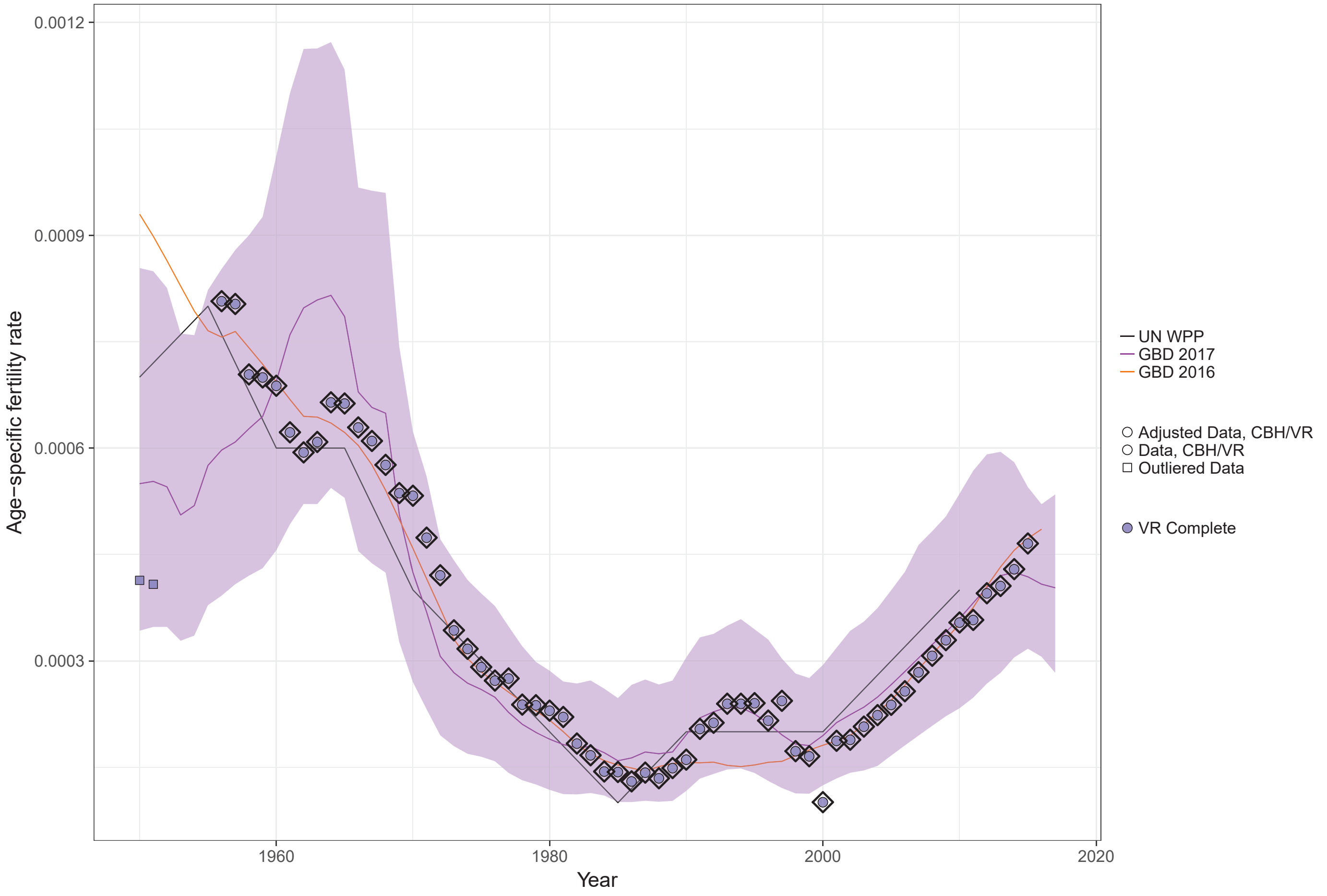

Age−specific fertility fitted trend lines, 1950−2017: Germany, Ages: 50−54
Data points enclosed with a diamond are reference sources

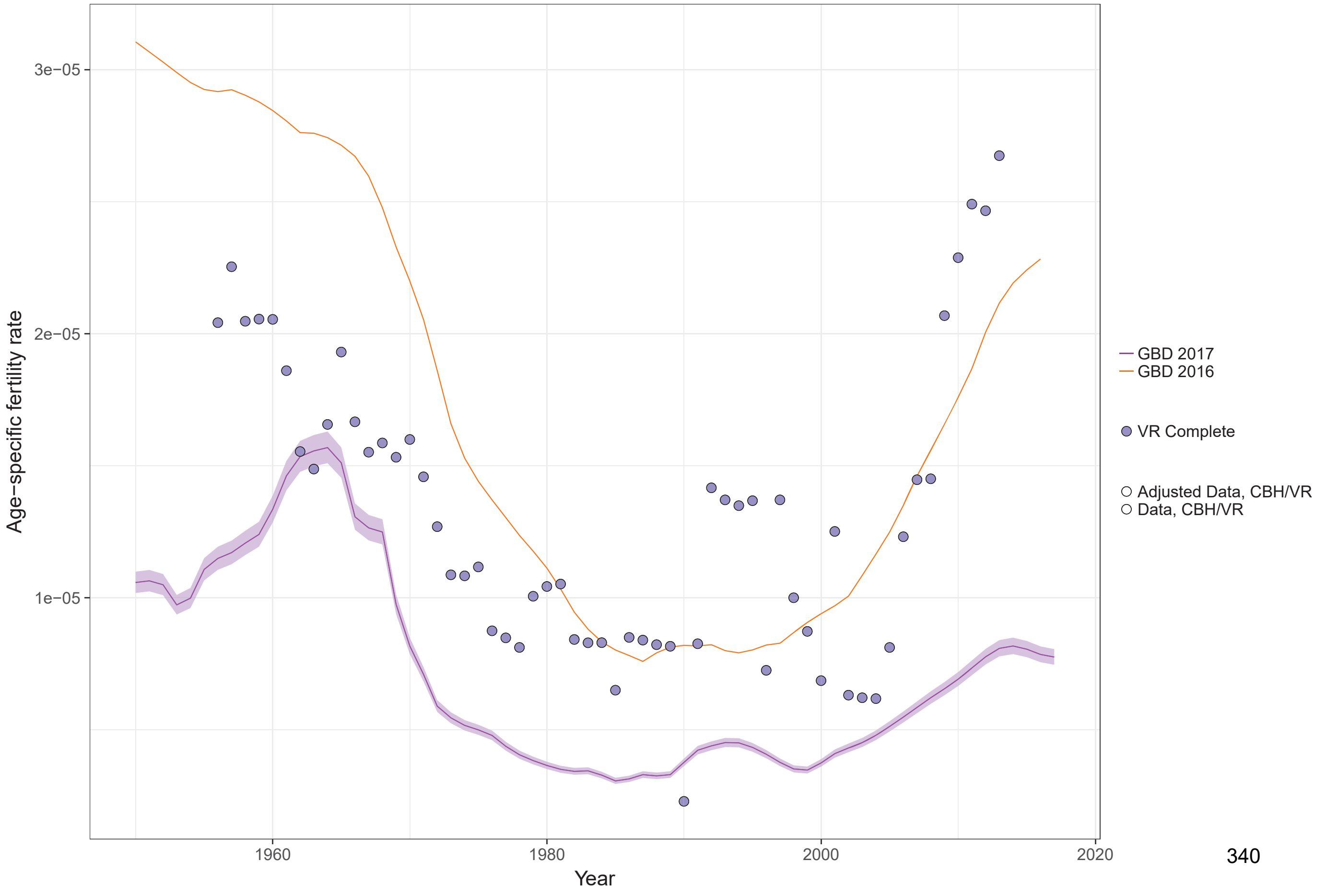

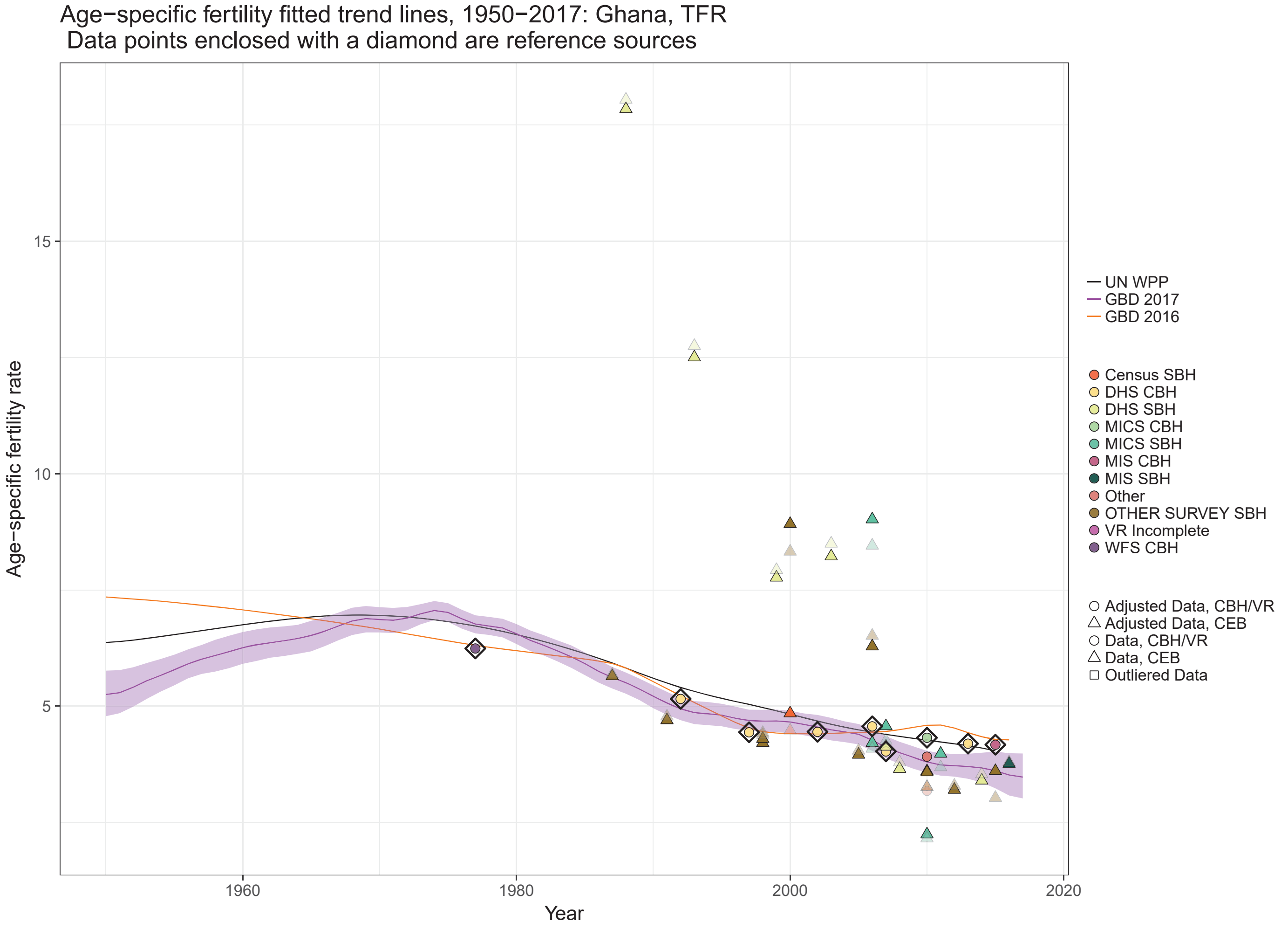

Age−specific fertility fitted trend lines, 1950−2017: Ghana, TFR
Data points enclosed with a diamond are reference sources
Age−specific fertility rate
15
10
5
1960
1980
2000
2020
Year
UN WPP
GBD 2017
GBD 2016
Census SBH
DHS CBH
DHS SBH
MICS CBH
MICS SBH
MIS CBH
MIS SBH
Other
OTHER SURVEY SBH
VR Incomplete
WFS CBH
Adjusted Data, CBH/VR
Adjusted Data, CEB
Data, CBH/VR
Data, CEB
Outliered Data

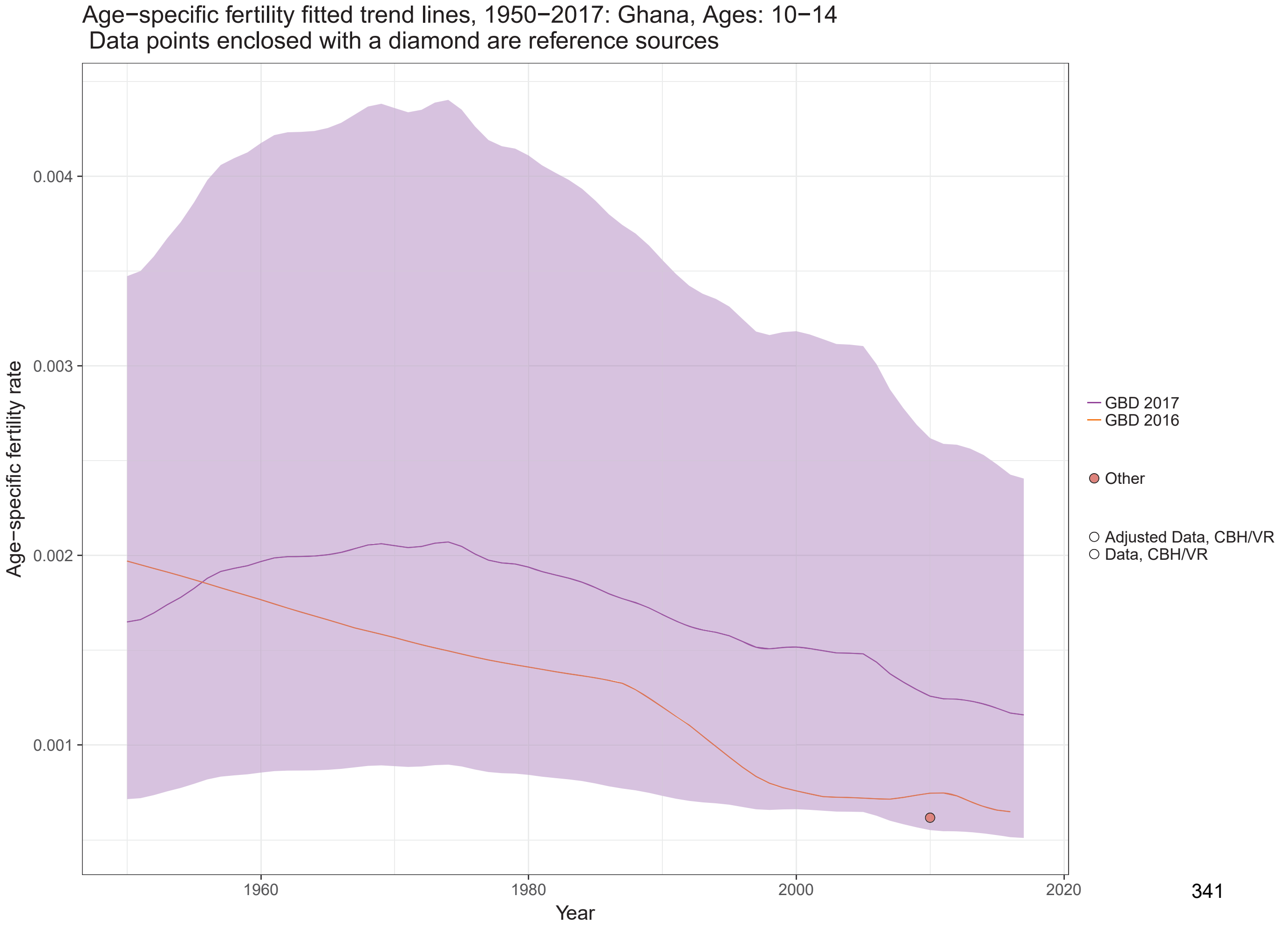

Age−specific fertility fitted trend lines, 1950−2017: Ghana, Ages: 10−14
Data points enclosed with a diamond are reference sources
Age−specific fertility rate
0.004
0.003
0.002
0.001
1960
1980
2000
2020
Year
GBD 2017
GBD 2016
Other
Adjusted Data, CBH/VR
Data, CBH/VR

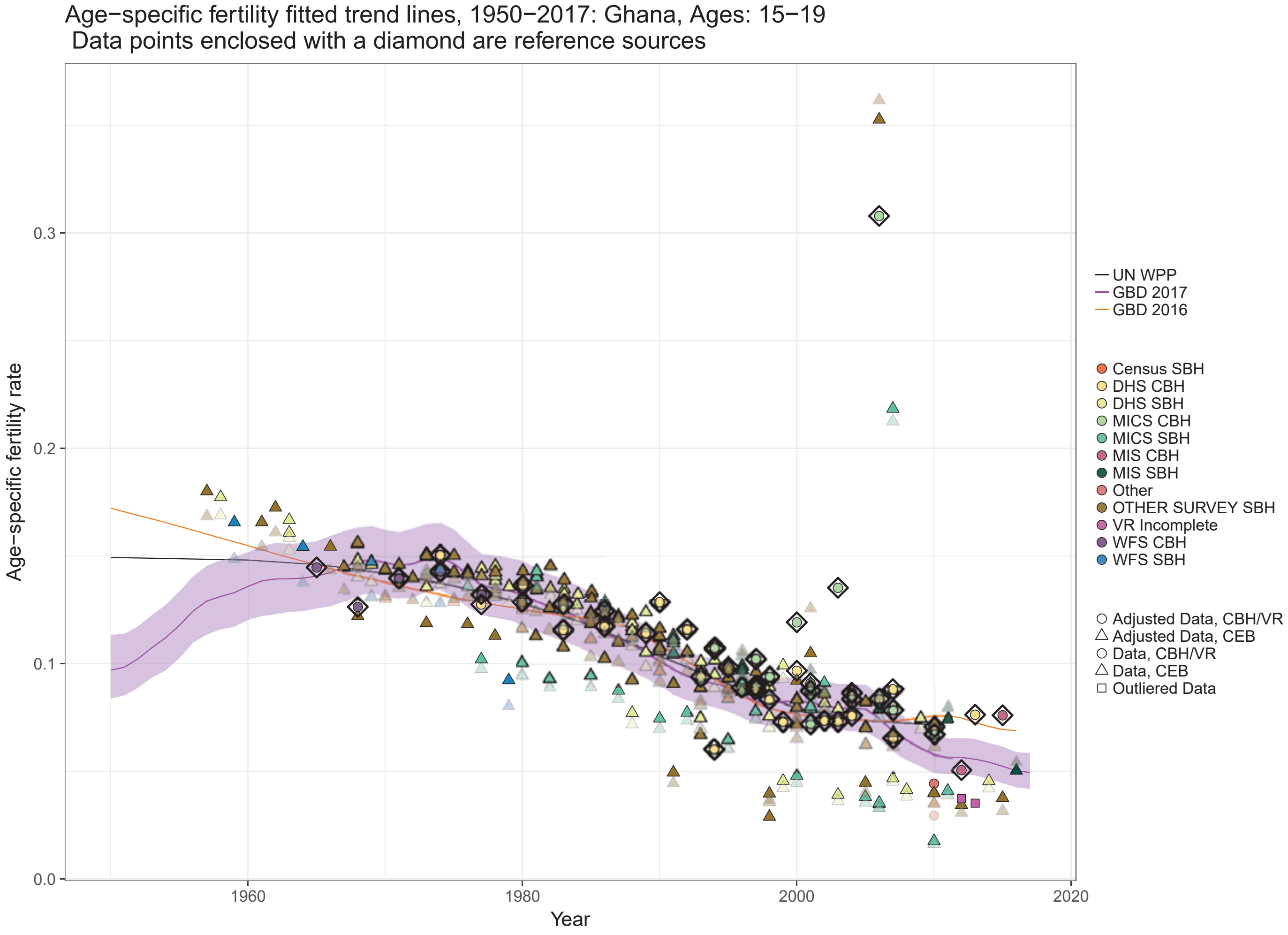

Age−specific fertility fitted trend lines, 1950−2017: Ghana, Ages: 15−19
Data points enclosed with a diamond are reference sources
Age−specific fertility rate
Year
0.0
0.1
0.2
0.3
1960
1980
2000
2020
UN WPP
GBD 2017
GBD 2016
Census SBH
DHS CBH
DHS SBH
MICS CBH
MICS SBH
MIS CBH
MIS SBH
Other
OTHER SURVEY SBH
VR Incomplete
WFS CBH
WFS SBH
Adjusted Data, CBH/VR
Adjusted Data, CEB
Data, CBH/VR
Data, CEB
Outliered Data

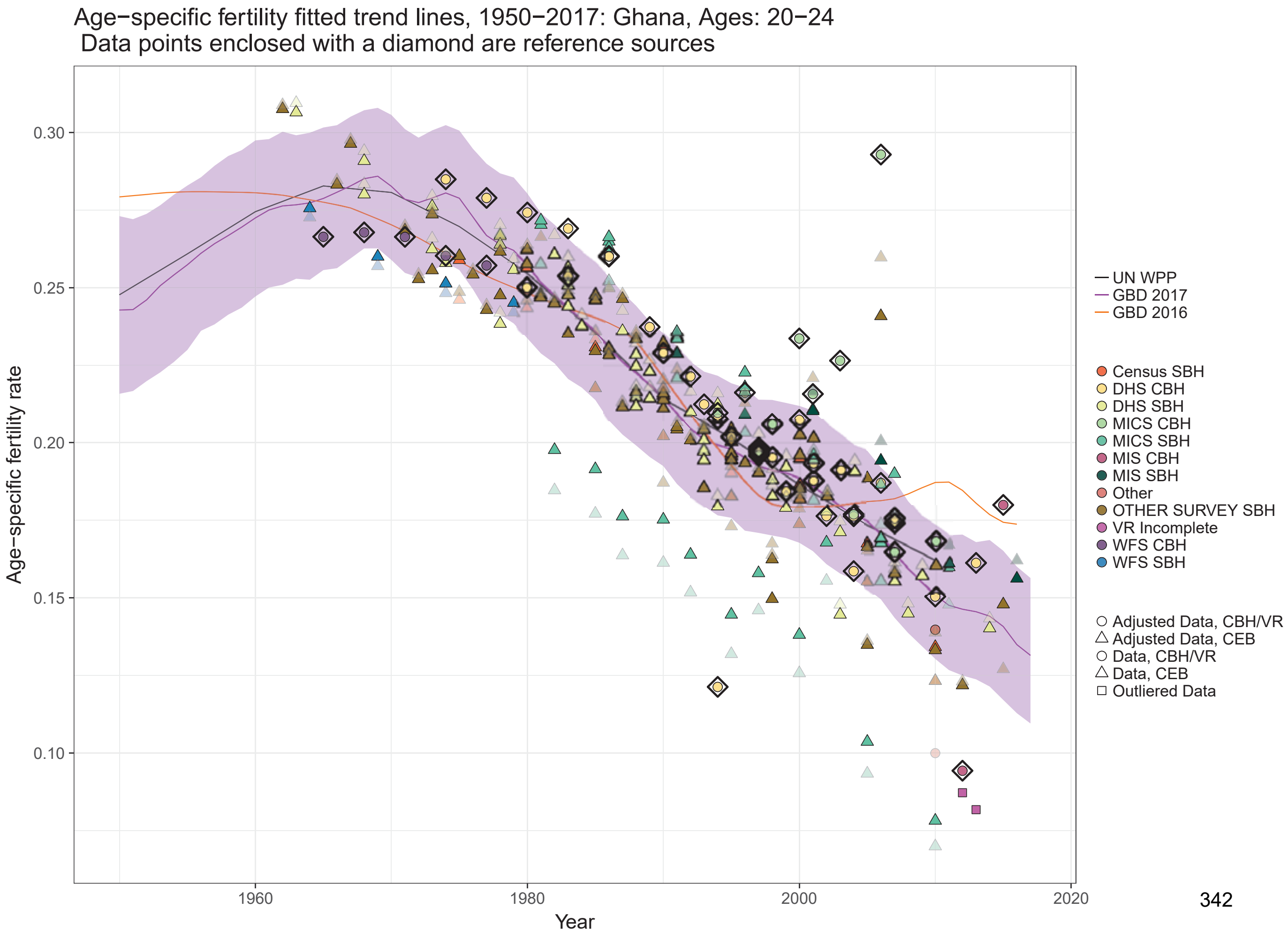

Age−specific fertility fitted trend lines, 1950−2017: Ghana, Ages: 20−24
Data points enclosed with a diamond are reference sources
Age−specific fertility rate
Year
0.10
0.15
0.20
0.25
0.30
1960
1980
2000
2020
UN WPP
GBD 2017
GBD 2016
Census SBH
DHS CBH
DHS SBH
MICS CBH
MICS SBH
MIS CBH
MIS SBH
Other
OTHER SURVEY SBH
VR Incomplete
WFS CBH
WFS SBH
Adjusted Data, CBH/VR
Adjusted Data, CEB
Data, CBH/VR
Data, CEB
Outliered Data

Age−specific fertility fitted trend lines, 1950−2017: Ghana, Ages: 25−29
Data points enclosed with a diamond are reference sources

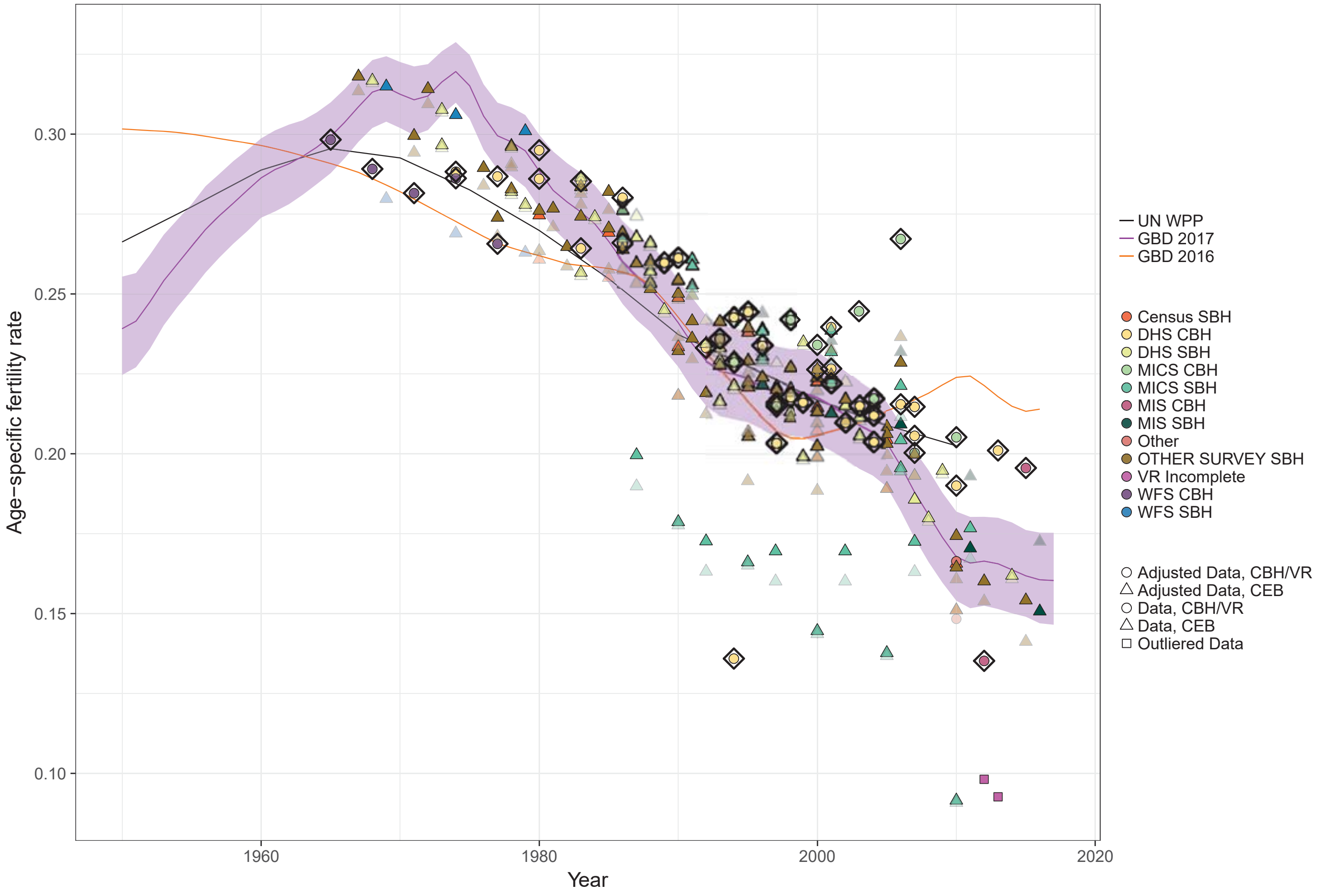

Age−specific fertility fitted trend lines, 1950−2017: Ghana, Ages: 30−34
Data points enclosed with a diamond are reference sources

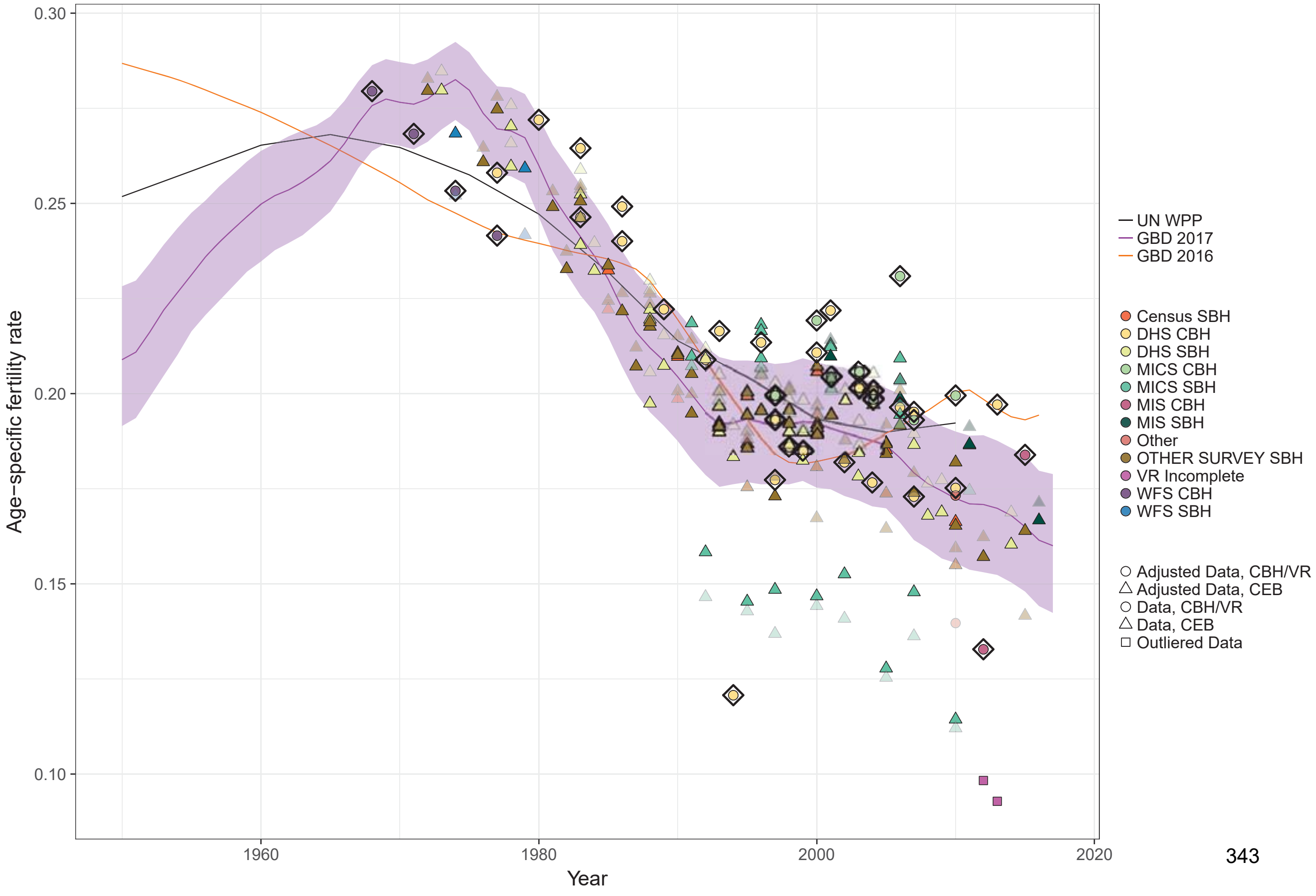

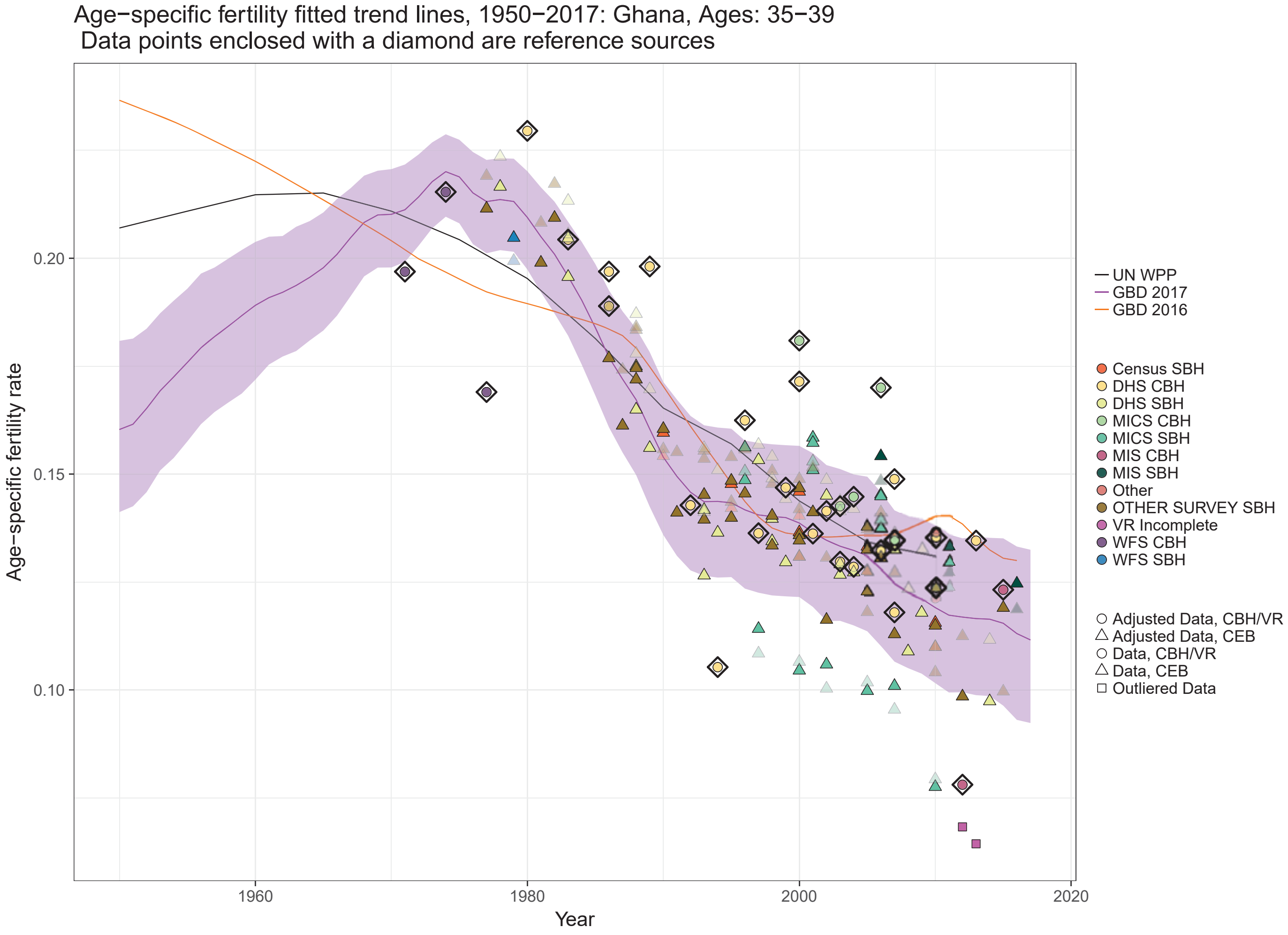
Age−specific fertility fitted trend lines, 1950−2017: Ghana, Ages: 35−39
Data points enclosed with a diamond are reference sources
Age−specific fertility rate
0.10
0.15
0.20
1960
1980
2000
2020
Year
UN WPP
GBD 2017
GBD 2016
Census SBH
DHS CBH
DHS SBH
MICS CBH
MICS SBH
MIS CBH
MIS SBH
Other
OTHER SURVEY SBH
VR Incomplete
WFS CBH
WFS SBH
Adjusted Data, CBH/VR
Adjusted Data, CEB
Data, CBH/VR
Data, CEB
Outliered Data

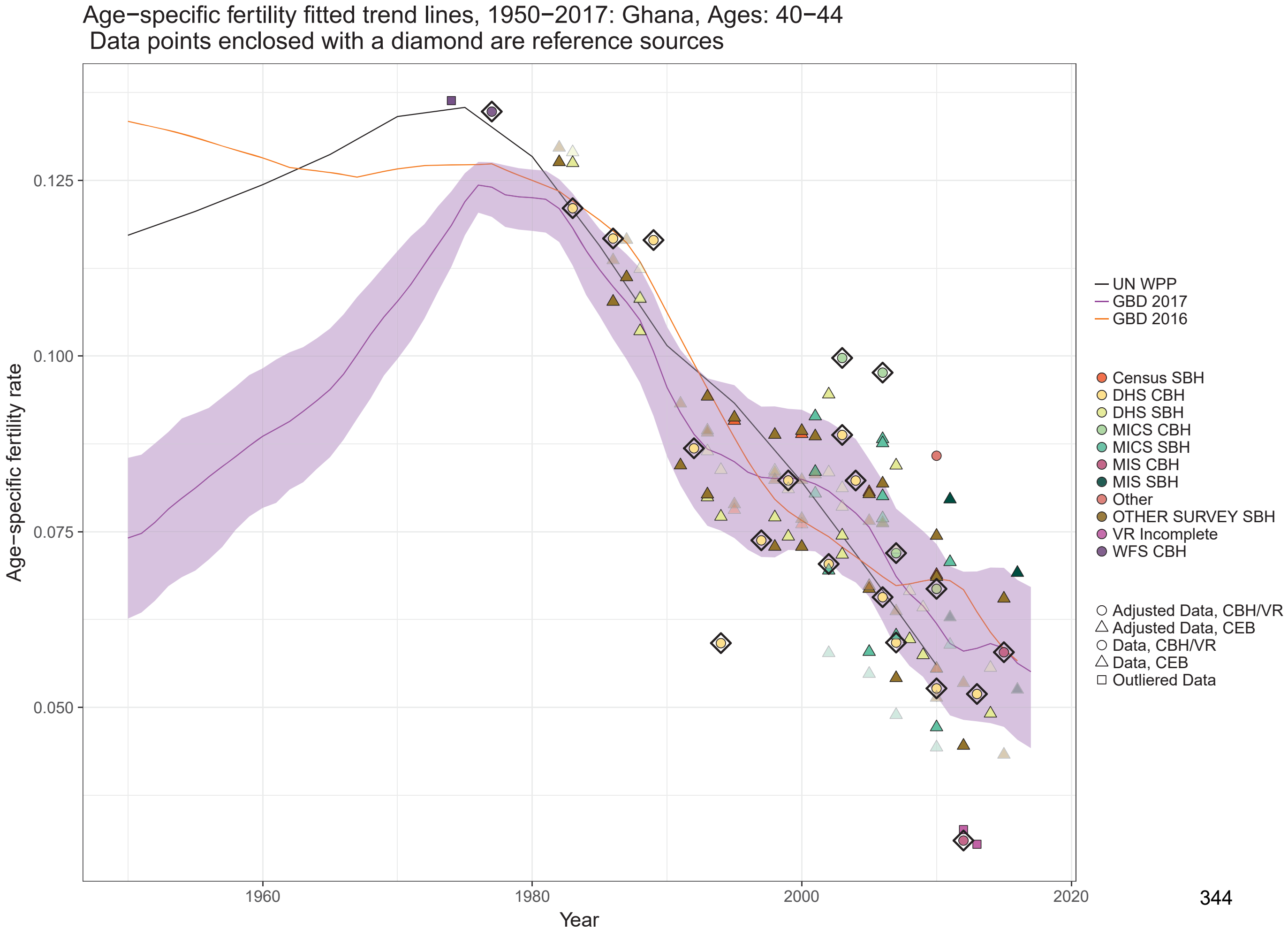
Age−specific fertility fitted trend lines, 1950−2017: Ghana, Ages: 40−44
Data points enclosed with a diamond are reference sources
Age−specific fertility rate
0.050
0.075
0.100
0.125
1960
1980
2000
2020
Year
UN WPP
GBD 2017
GBD 2016
Census SBH
DHS CBH
DHS SBH
MICS CBH
MICS SBH
MIS CBH
MIS SBH
Other
OTHER SURVEY SBH
VR Incomplete
WFS CBH
Adjusted Data, CBH/VR
Adjusted Data, CEB
Data, CBH/VR
Data, CEB
Outliered Data

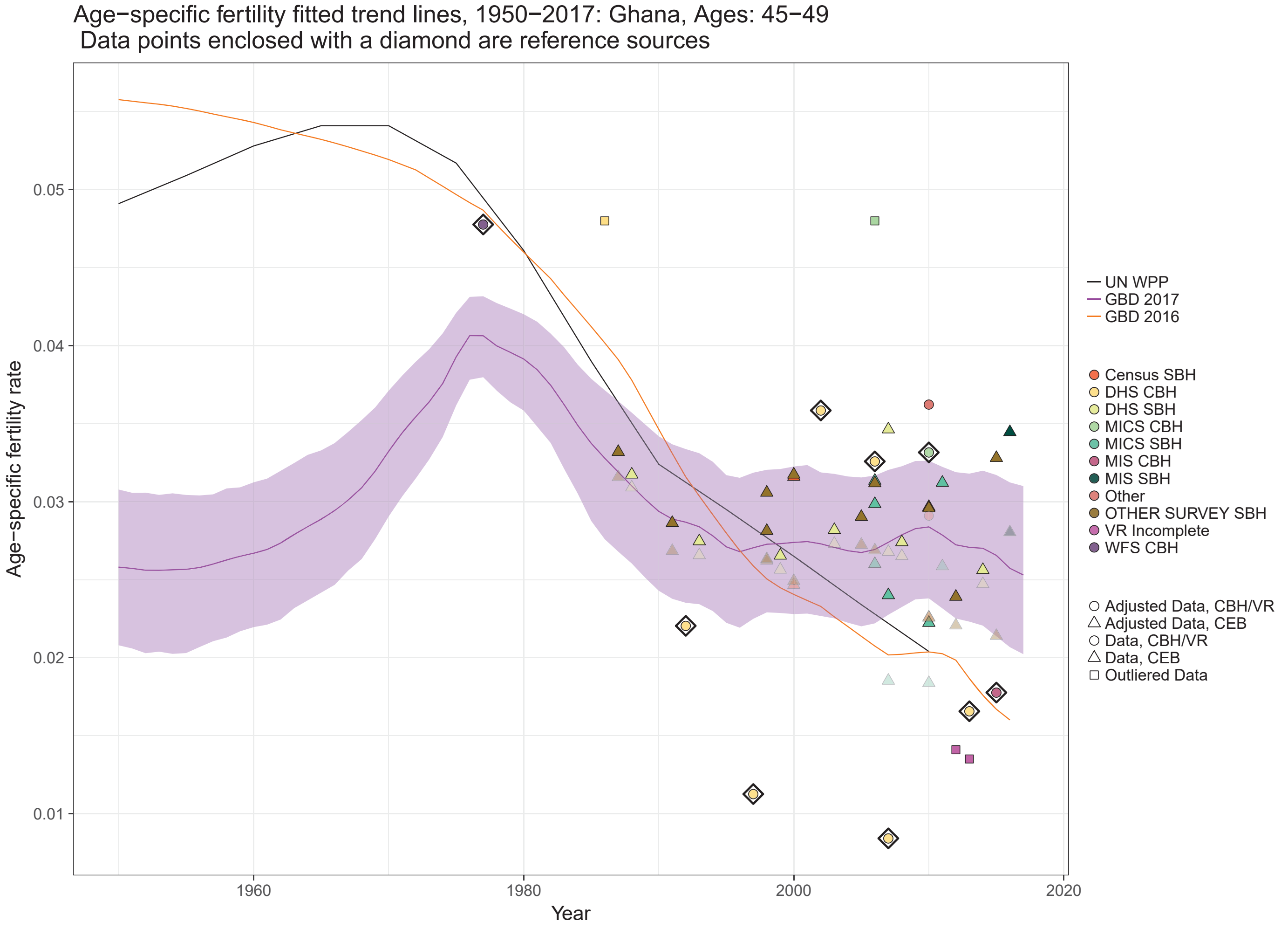

Age−specific fertility fitted trend lines, 1950−2017: Ghana, Ages: 45−49
Data points enclosed with a diamond are reference sources
Age-specific fertility rate
Year
0.01
0.02
0.03
0.04
0.05
1960
1980
2000
2020
UN WPP
GBD 2017
GBD 2016
Census SBH
DHS CBH
DHS SBH
MICS CBH
MICS SBH
MIS CBH
MIS SBH
Other
OTHER SURVEY SBH
VR Incomplete
WFS CBH
Adjusted Data, CBH/VR
Adjusted Data, CEB
Data, CBH/VR
Data, CEB
Outliered Data

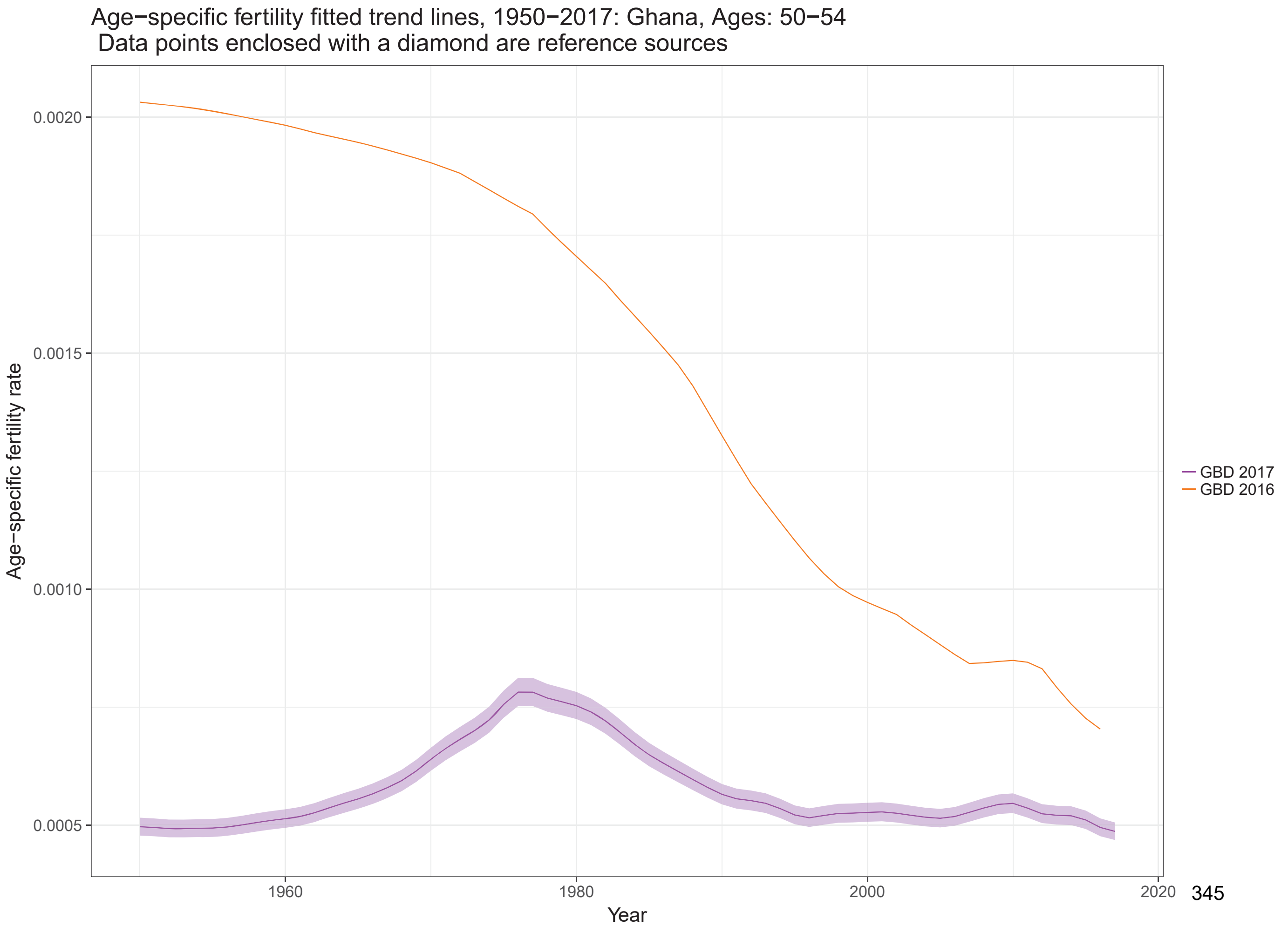

Age−specific fertility fitted trend lines, 1950−2017: Ghana, Ages: 50−54
Data points enclosed with a diamond are reference sources
Age-specific fertility rate
Year
0.0005
0.0010
0.0015
0.0020
1960
1980
2000
2020
GBD 2017
GBD 2016

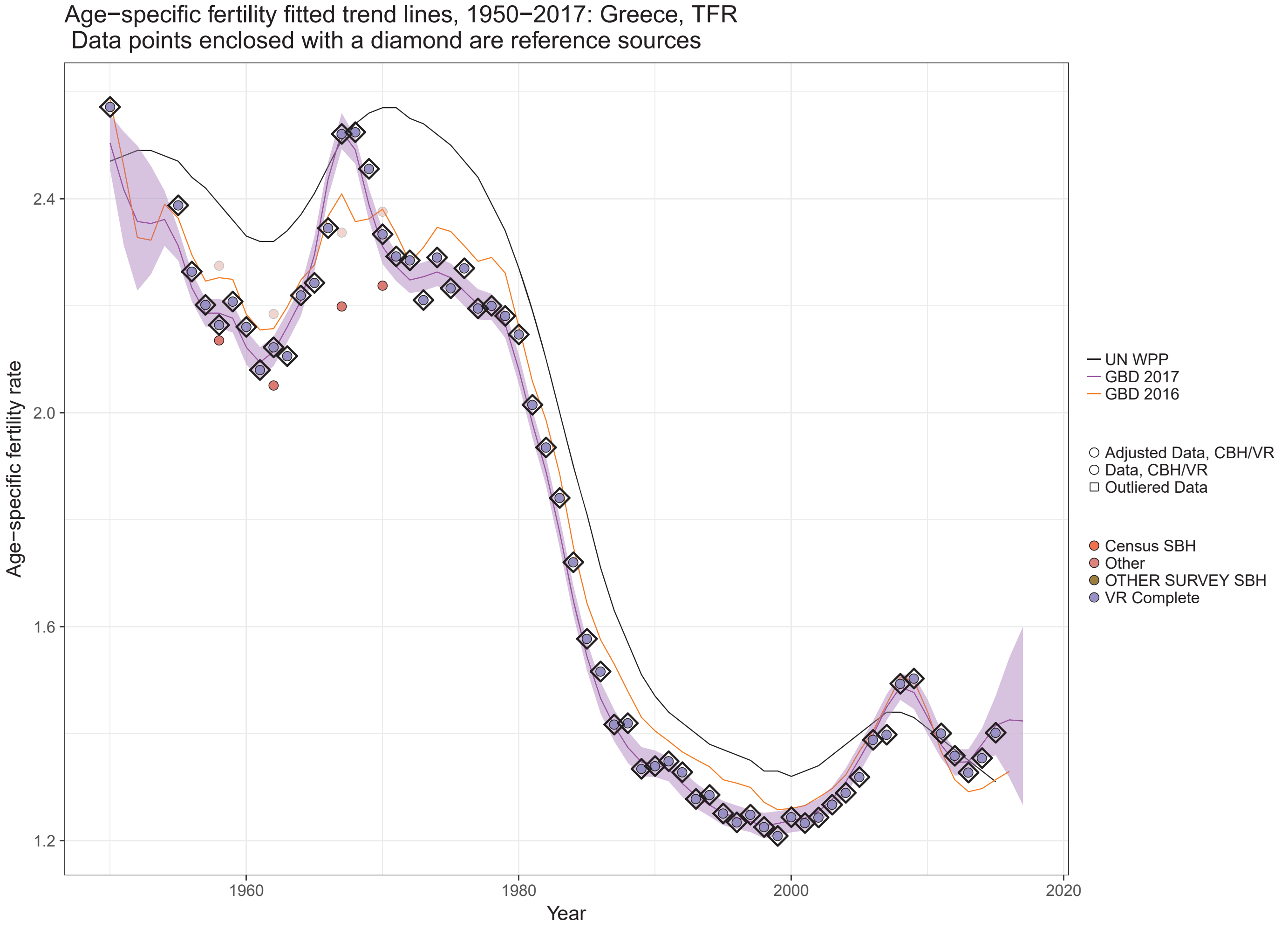

Age−specific fertility fitted trend lines, 1950−2017: Greece, TFR
Data points enclosed with a diamond are reference sources
Age−specific fertility rate
2.4
2.0
1.6
1.2
1960
1980
2000
2020
Year
UN WPP
GBD 2017
GBD 2016
Adjusted Data, CBH/VR
Data, CBH/VR
Outliered Data
Census SBH
Other
OTHER SURVEY SBH
VR Complete

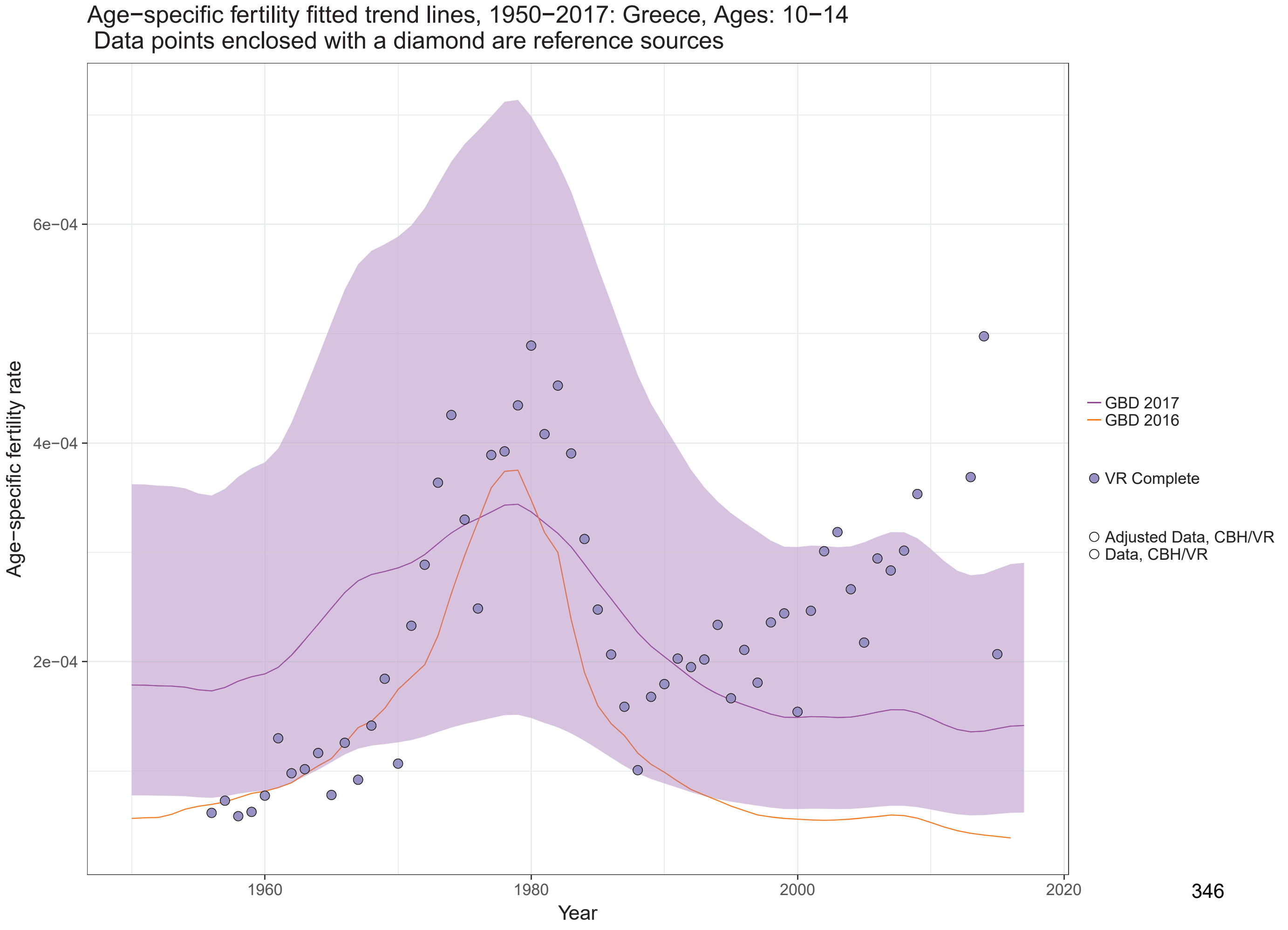

Age−specific fertility fitted trend lines, 1950−2017: Greece, Ages: 10−14
Data points enclosed with a diamond are reference sources
Age−specific fertility rate
6e−04
4e−04
2e−04
1960
1980
2000
2020
Year
GBD 2017
GBD 2016
VR Complete
Adjusted Data, CBH/VR
Data, CBH/VR

Age−specific fertility fitted trend lines, 1950−2017: Greece, Ages: 15−19
Data points enclosed with a diamond are reference sources

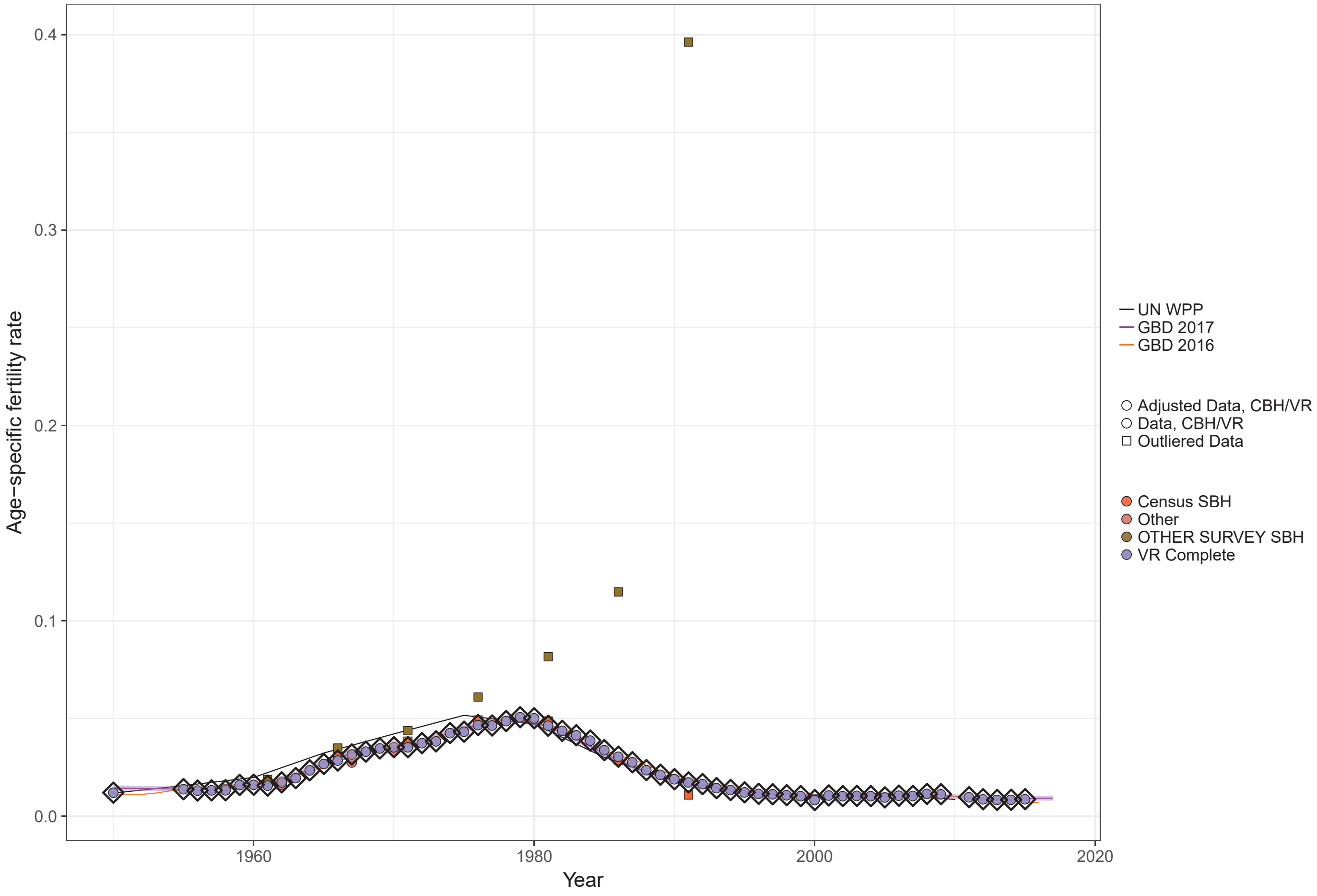

Age−specific fertility fitted trend lines, 1950−2017: Greece, Ages: 20−24
Data points enclosed with a diamond are reference sources

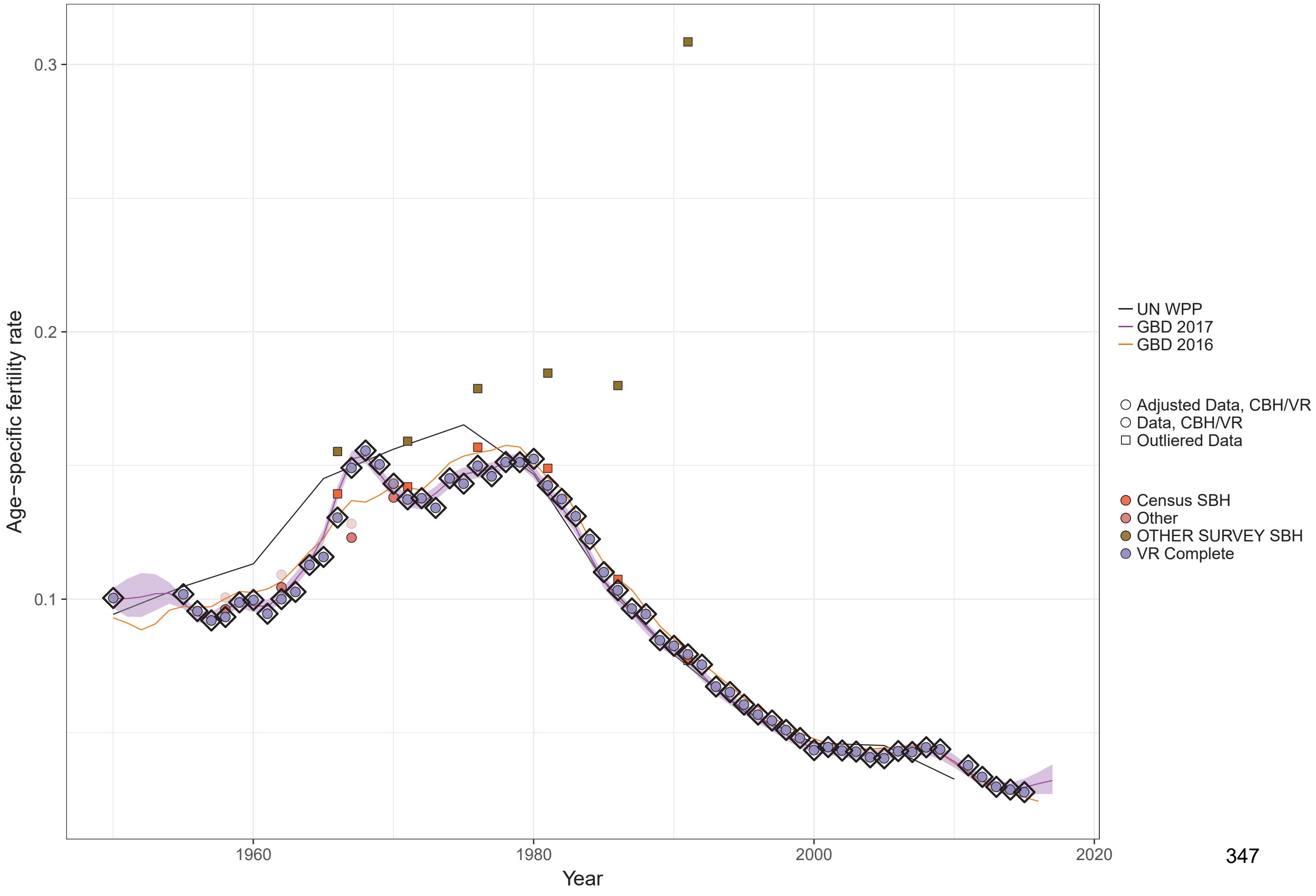

Age−specific fertility fitted trend lines, 1950−2017: Greece, Ages: 25−29
Data points enclosed with a diamond are reference sources

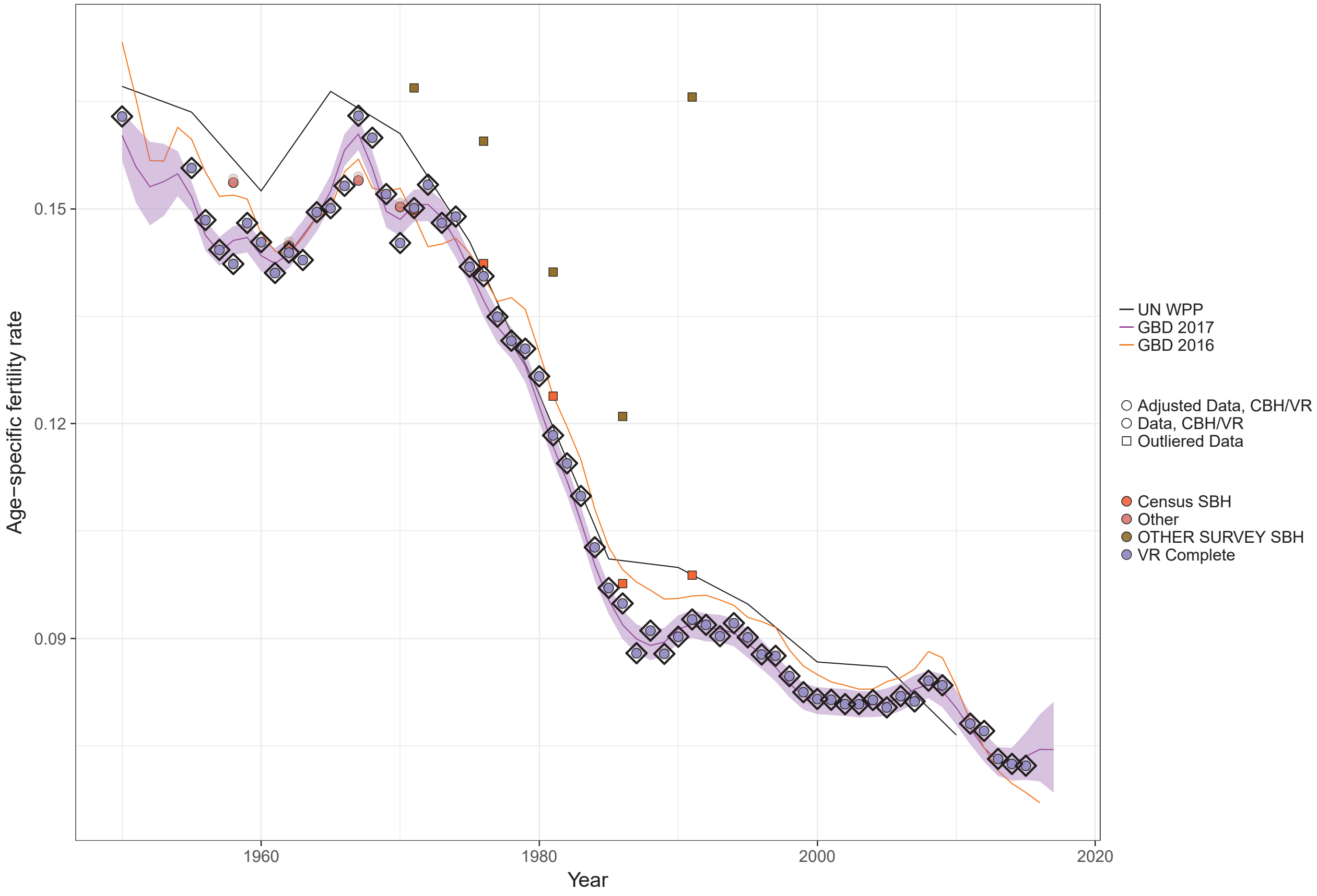

Age−specific fertility fitted trend lines, 1950−2017: Greece, Ages: 30−34
Data points enclosed with a diamond are reference sources

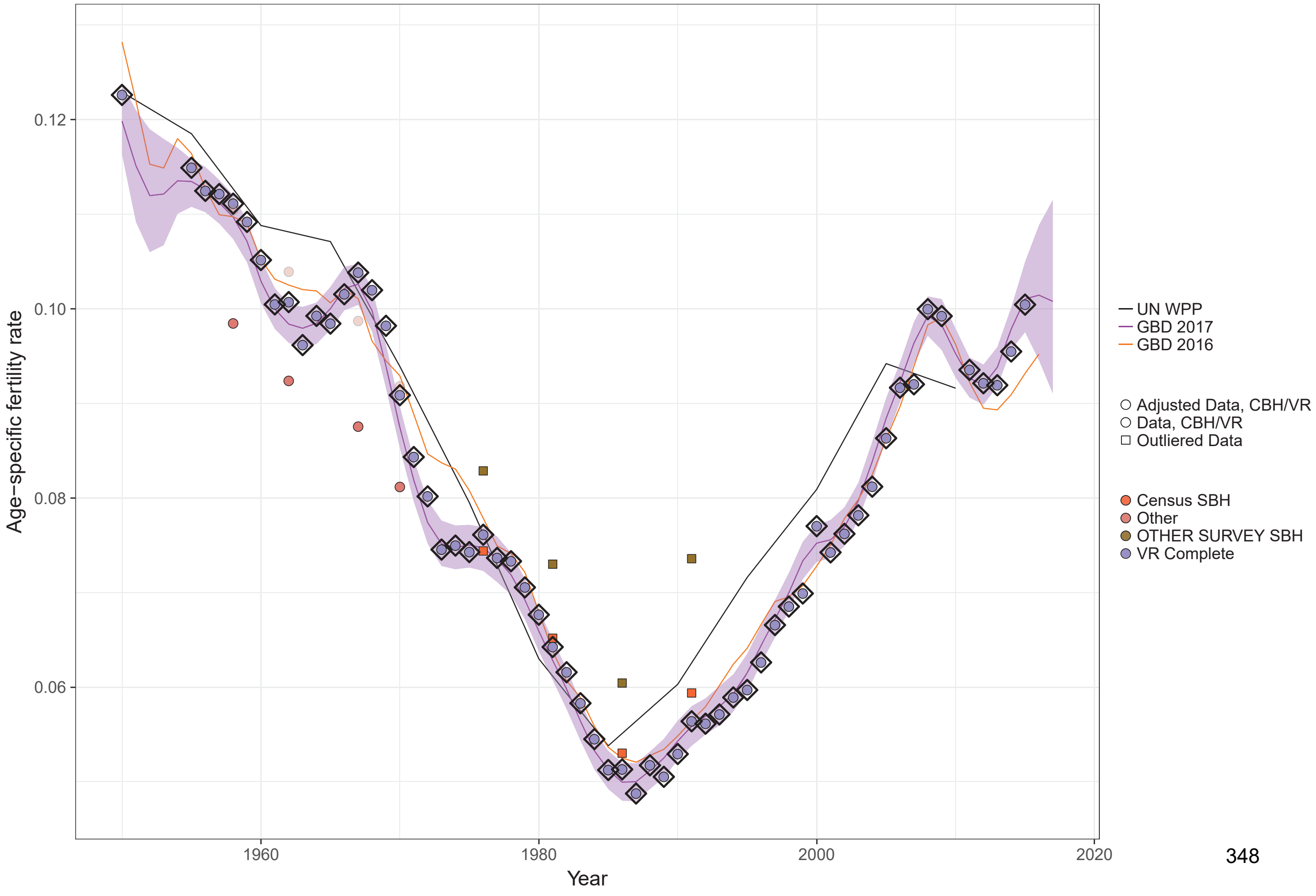

Age−specific fertility fitted trend lines, 1950−2017: Greece, Ages: 35−39
Data points enclosed with a diamond are reference sources

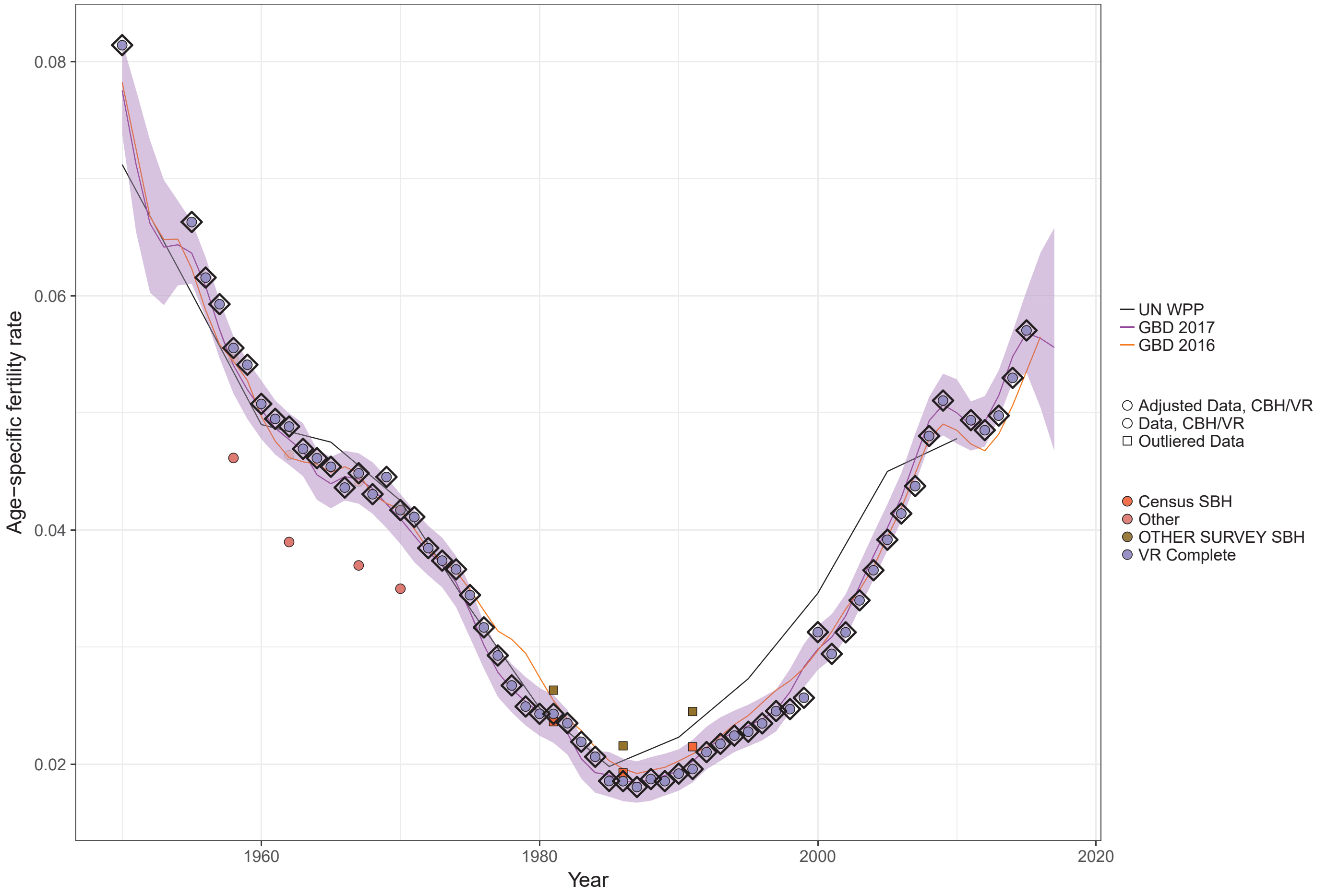

Age−specific fertility fitted trend lines, 1950−2017: Greece, Ages: 40−44
Data points enclosed with a diamond are reference sources

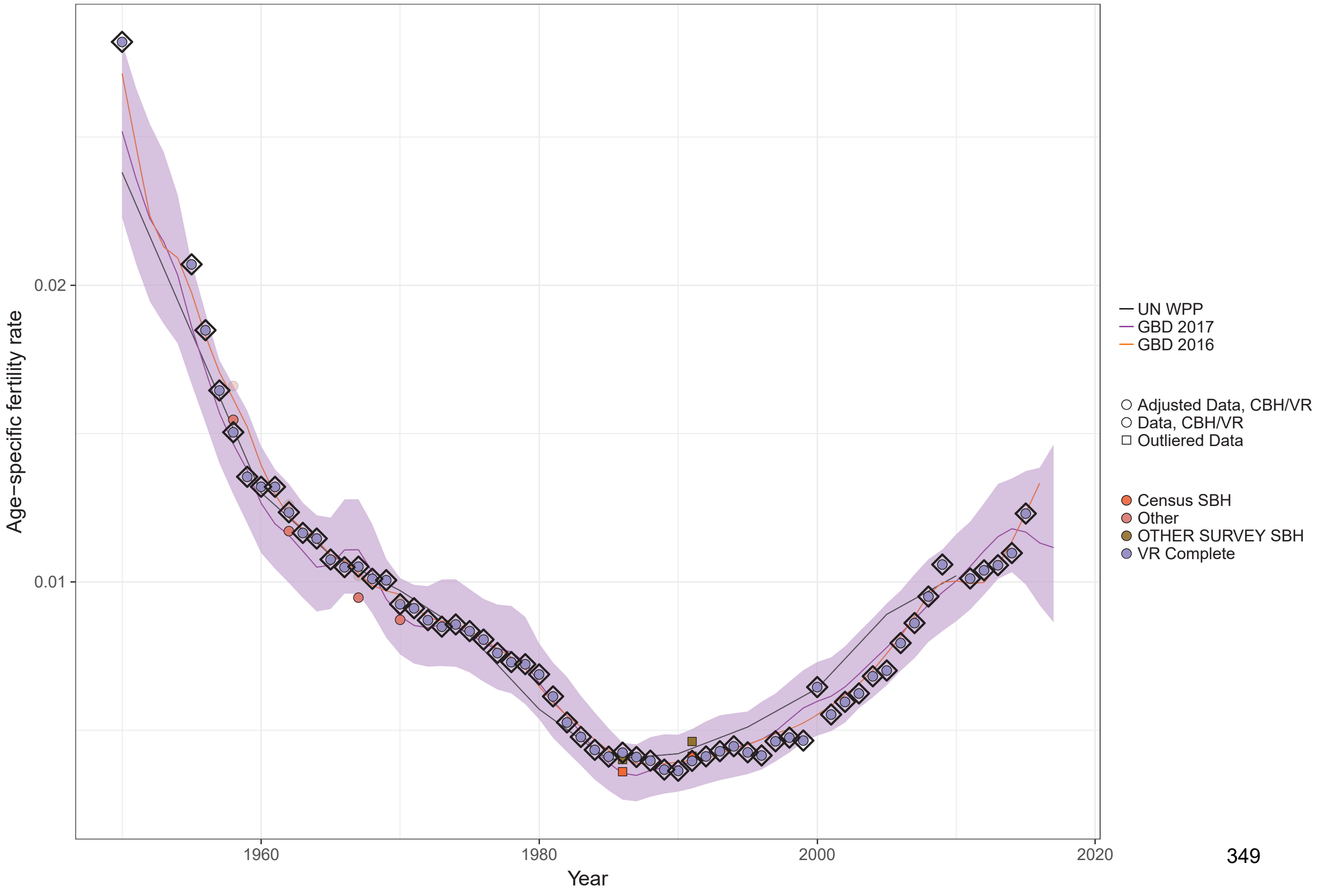

Age−specific fertility fitted trend lines, 1950−2017: Greece, Ages: 45−49
Data points enclosed with a diamond are reference sources

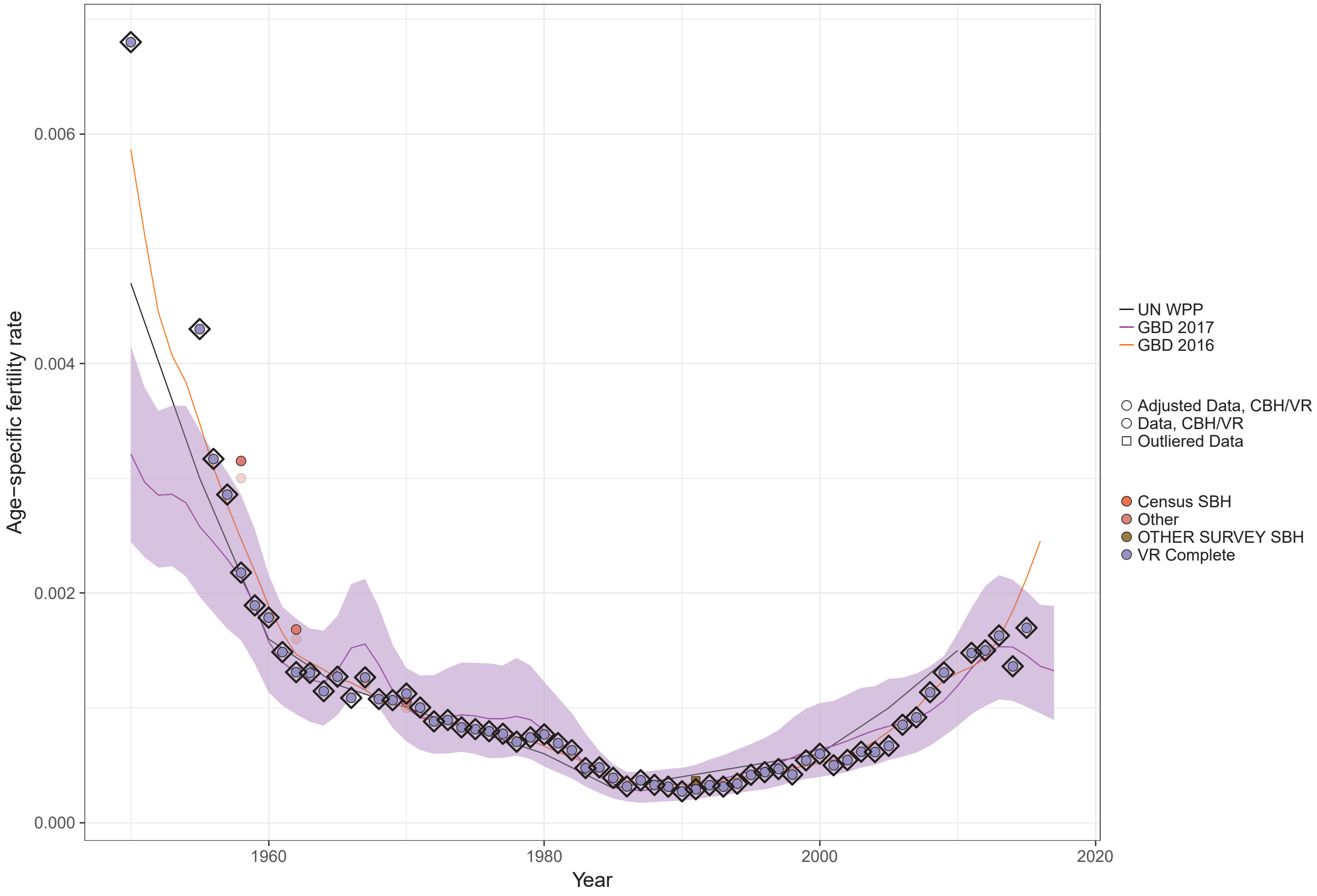

Age−specific fertility fitted trend lines, 1950−2017: Greece, Ages: 50−54
Data points enclosed with a diamond are reference sources

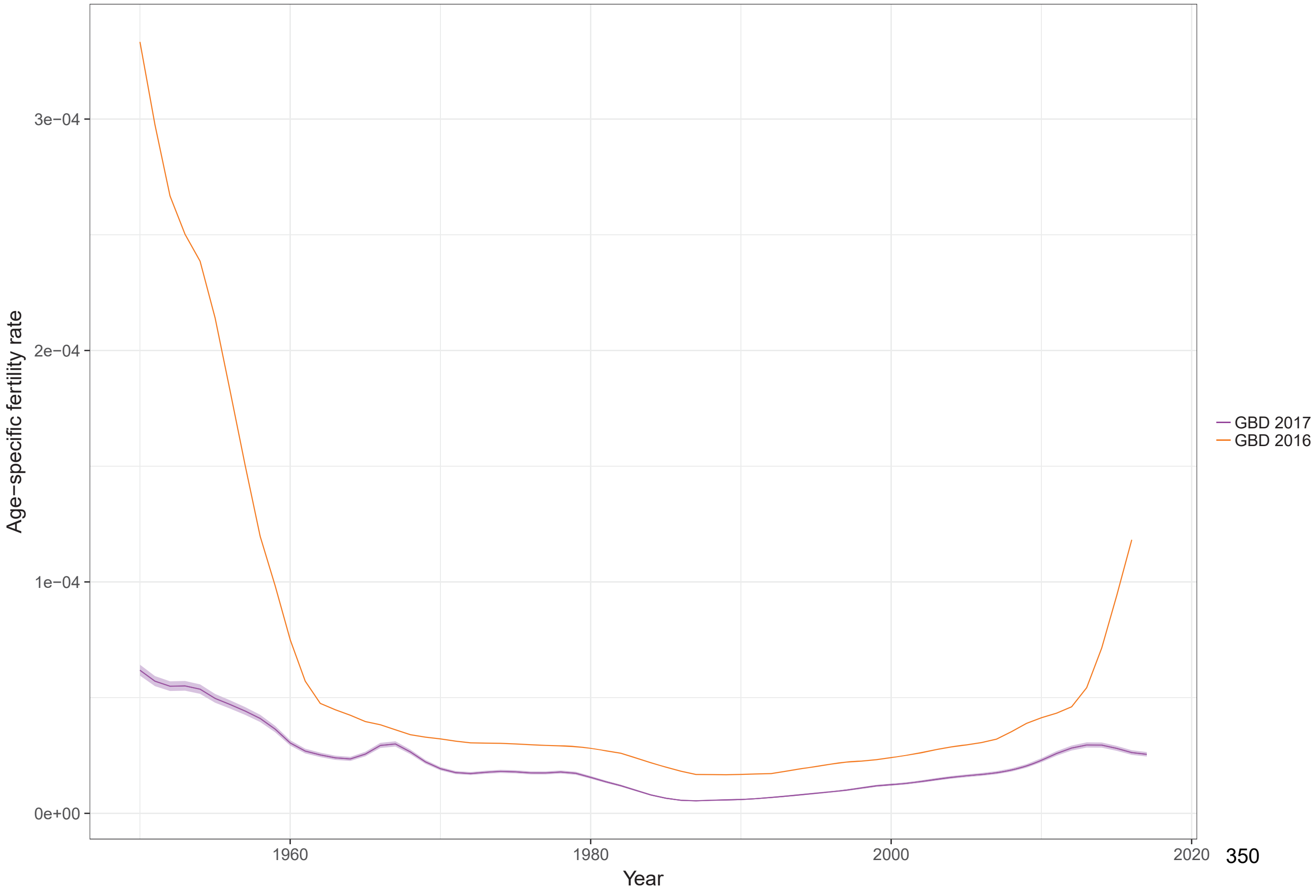

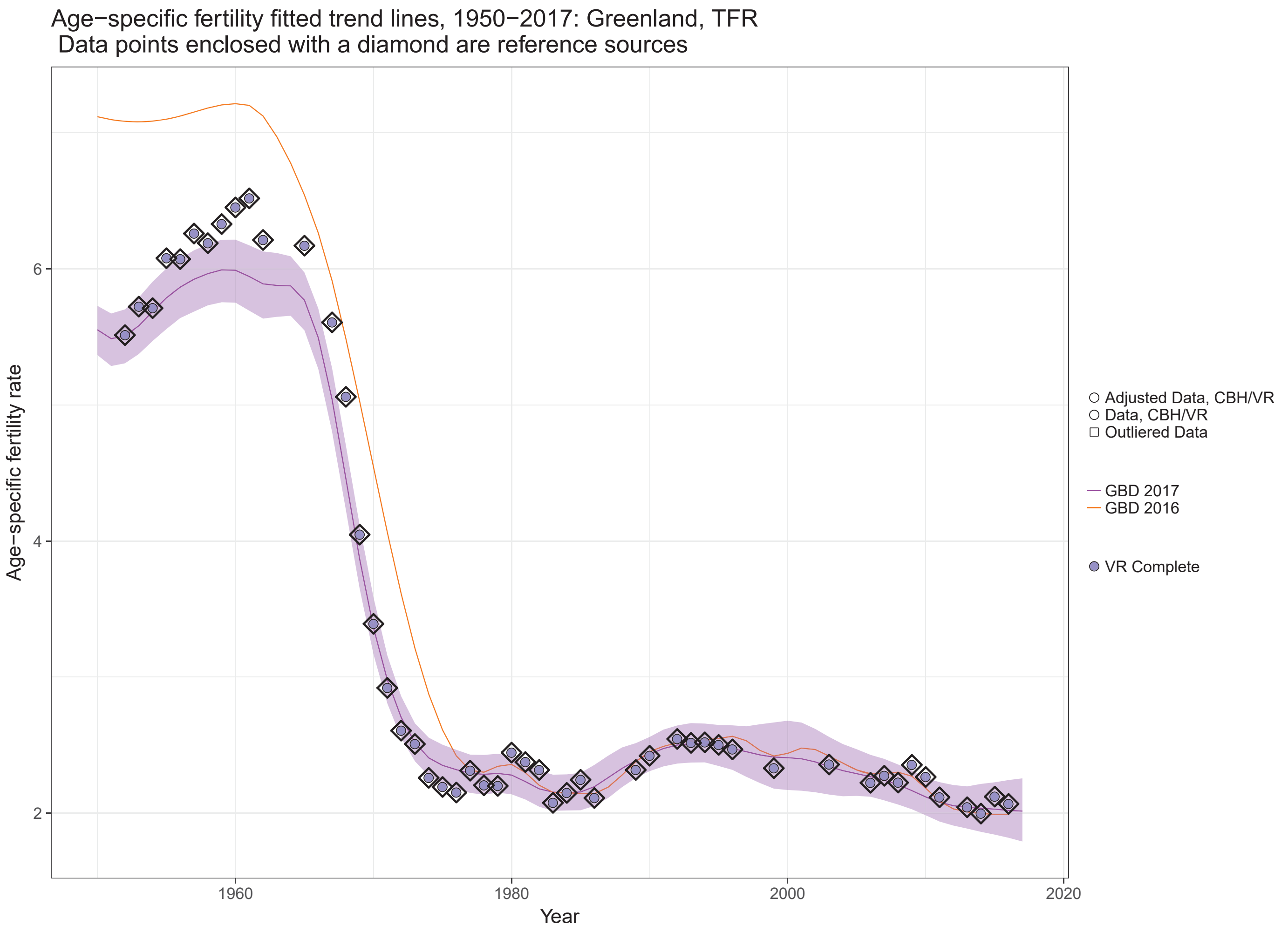

Age−specific fertility fitted trend lines, 1950−2017: Greenland, TFR
Data points enclosed with a diamond are reference sources
Age−specific fertility rate
Year
2
4
6
1960
1980
2000
2020
Adjusted Data, CBH/VR
Data, CBH/VR
Outliered Data
GBD 2017
GBD 2016
VR Complete

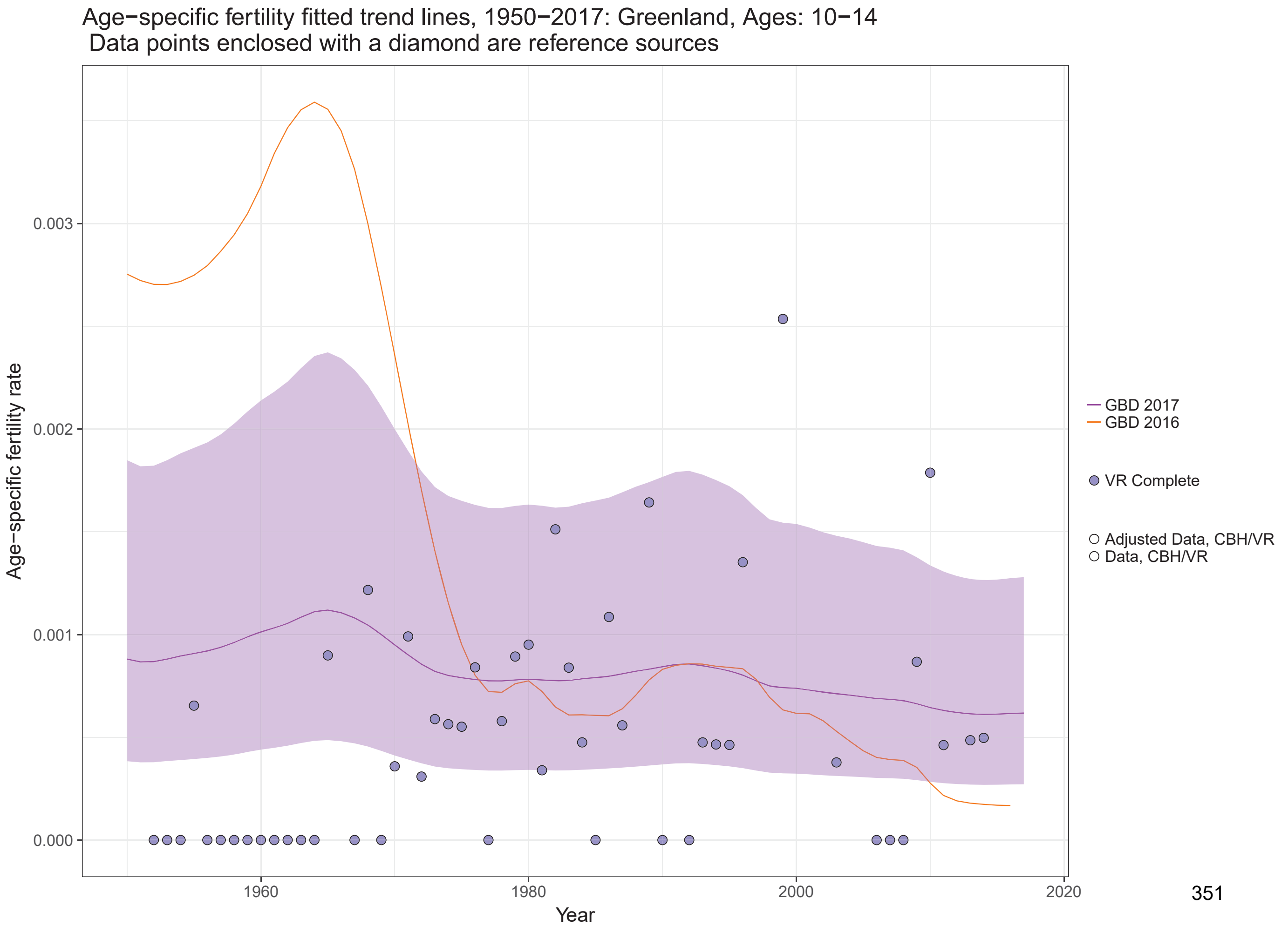

Age−specific fertility fitted trend lines, 1950−2017: Greenland, Ages: 10−14
Data points enclosed with a diamond are reference sources
Age−specific fertility rate
Year
0.000
0.001
0.002
0.003
1960
1980
2000
2020
GBD 2017
GBD 2016
VR Complete
Adjusted Data, CBH/VR
Data, CBH/VR

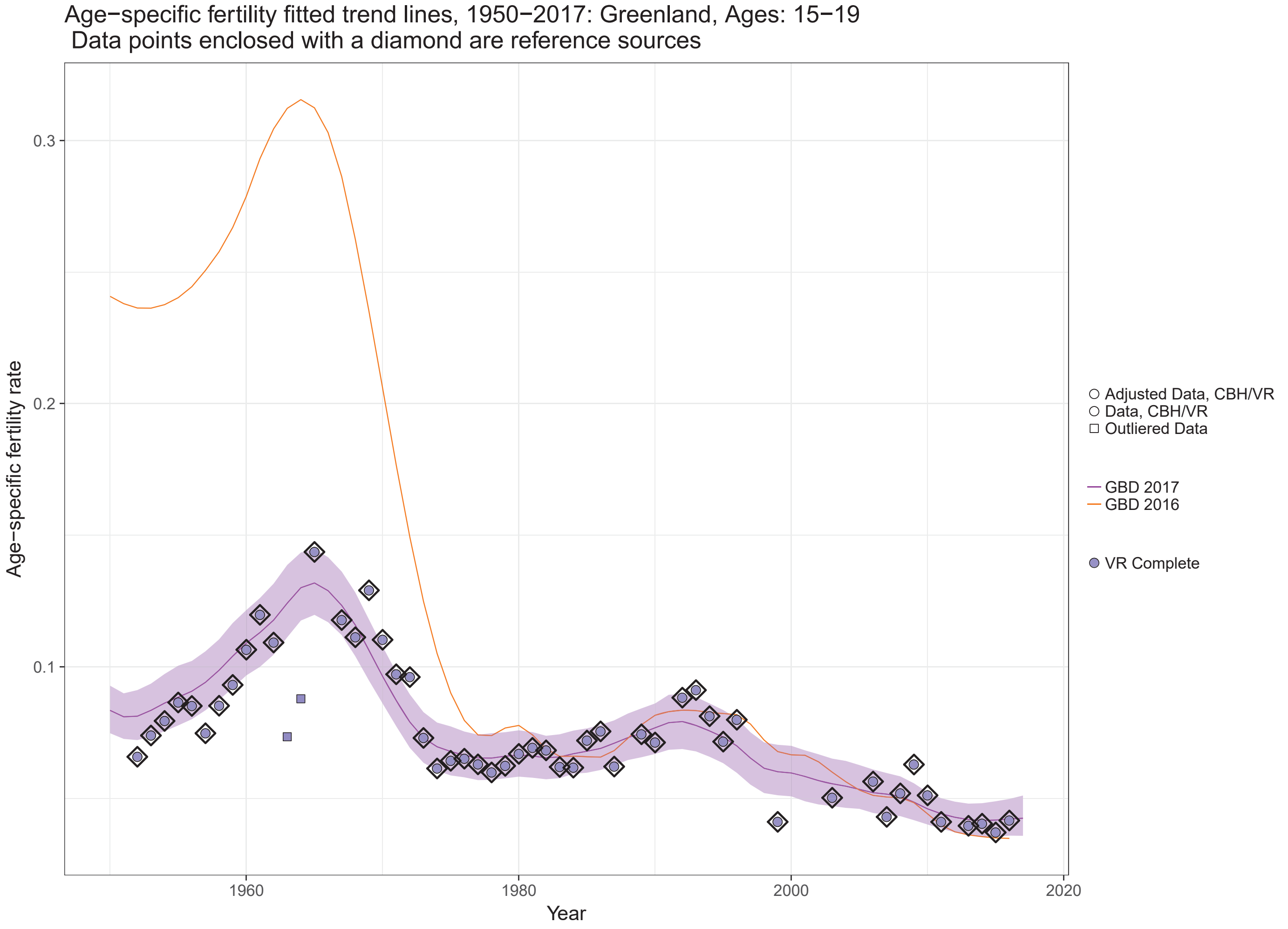
Age−specific fertility fitted trend lines, 1950−2017: Greenland, Ages: 15−19
Data points enclosed with a diamond are reference sources
Age-specific fertility rate
Year
0.1
0.2
0.3
1960
1980
2000
2020
Adjusted Data, CBH/VR
Data, CBH/VR
Outliered Data
GBD 2017
GBD 2016
VR Complete

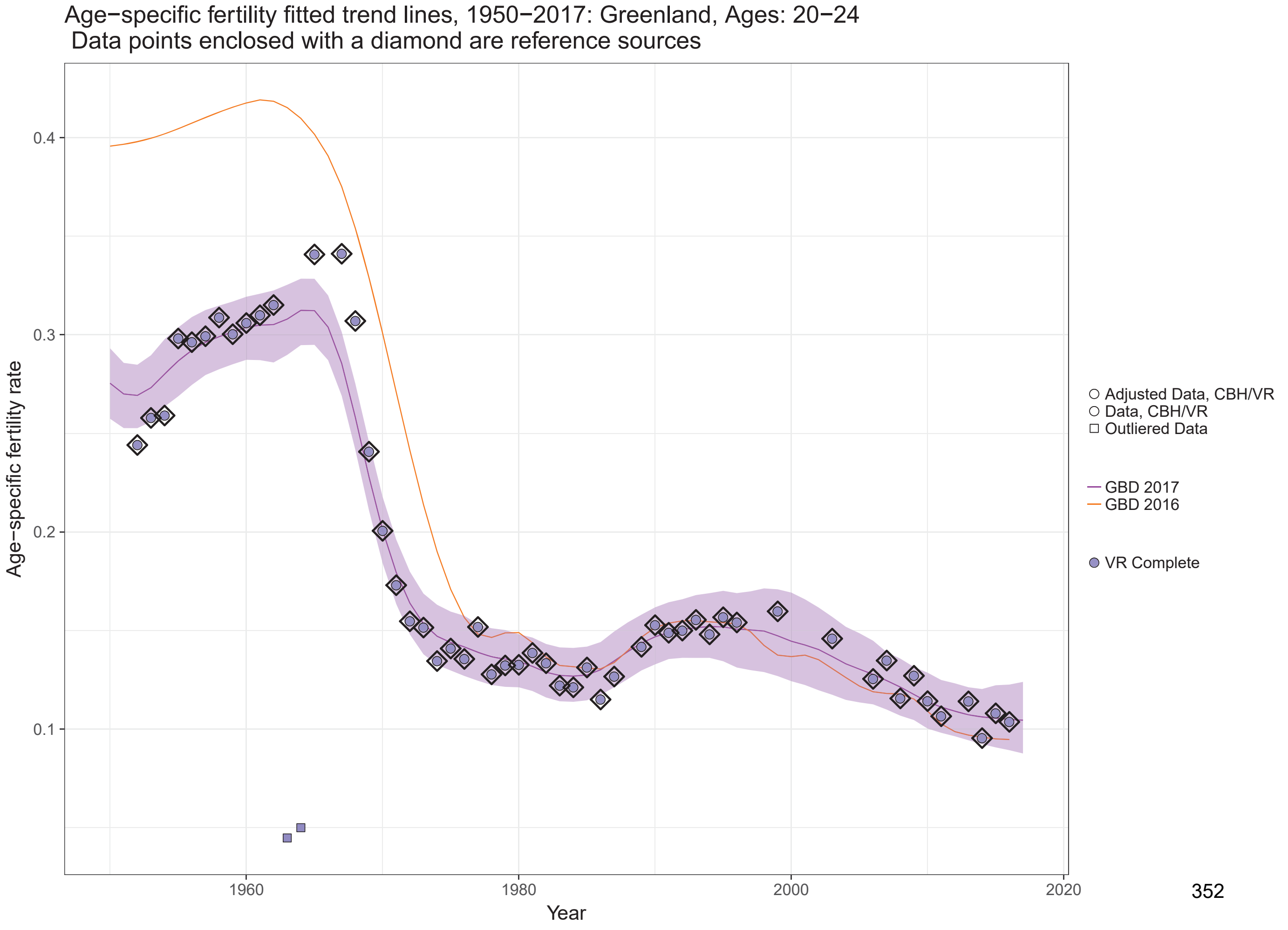
Age−specific fertility fitted trend lines, 1950−2017: Greenland, Ages: 20−24
Data points enclosed with a diamond are reference sources
Age-specific fertility rate
Year
0.1
0.2
0.3
0.4
1960
1980
2000
2020
Adjusted Data, CBH/VR
Data, CBH/VR
Outliered Data
GBD 2017
GBD 2016
VR Complete

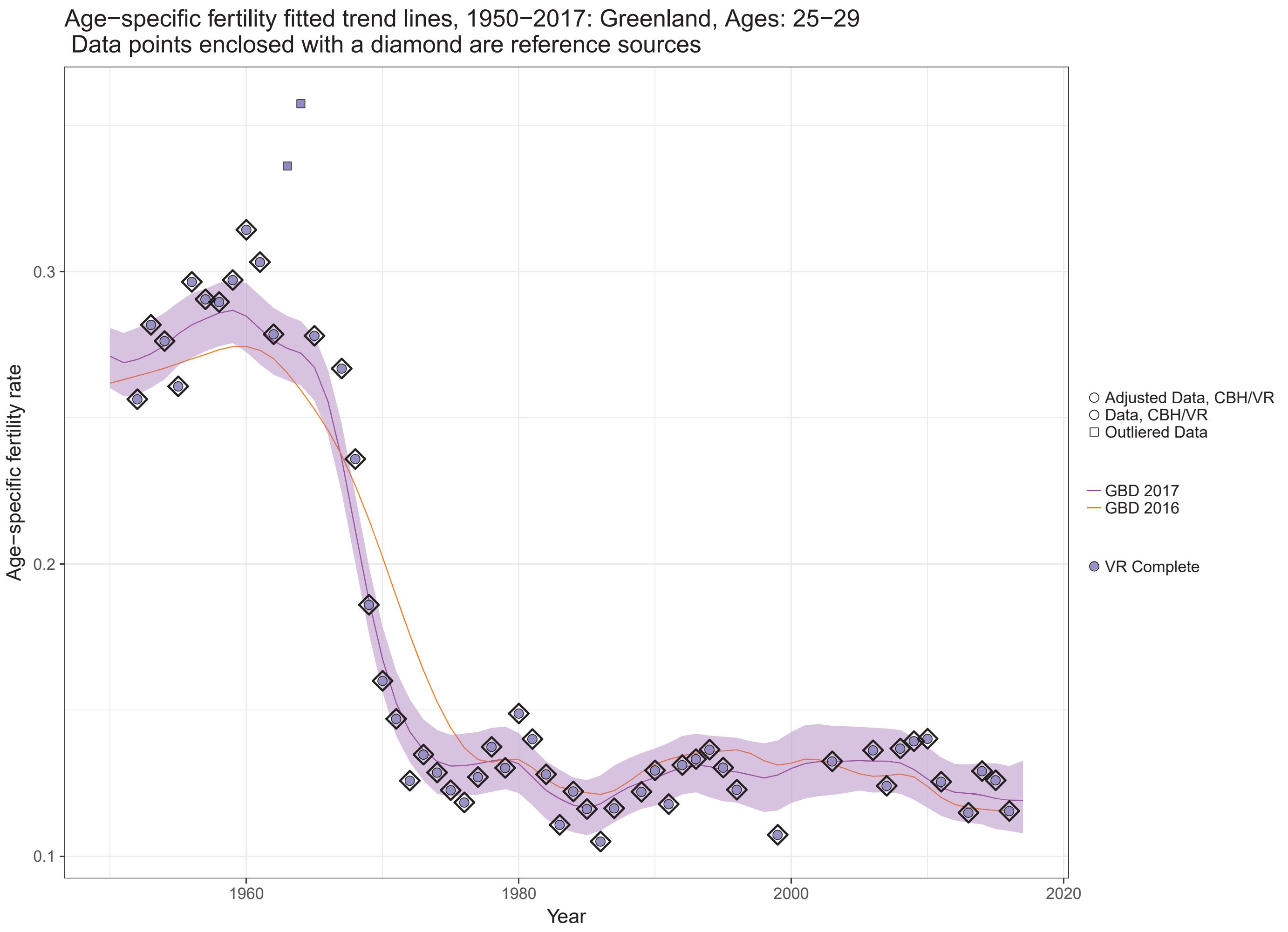

Age−specific fertility fitted trend lines, 1950−2017: Greenland, Ages: 25−29
Data points enclosed with a diamond are reference sources
Age−specific fertility rate
0.1
0.2
0.3
Year
1960
1980
2000
2020
Adjusted Data, CBH/VR
Data, CBH/VR
Outliered Data
GBD 2017
GBD 2016
VR Complete

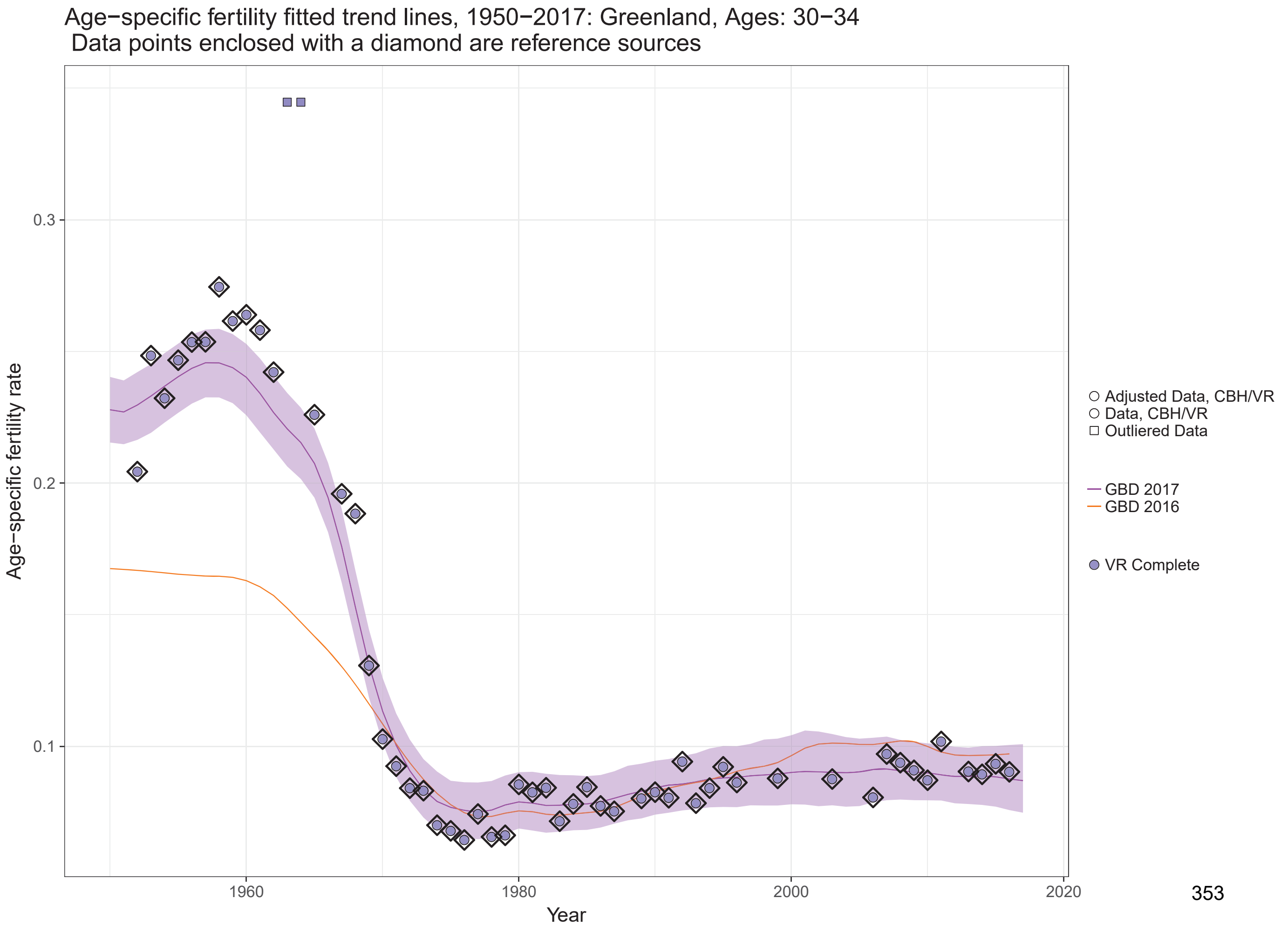

Age−specific fertility fitted trend lines, 1950−2017: Greenland, Ages: 30−34
Data points enclosed with a diamond are reference sources
Age−specific fertility rate
0.1
0.2
0.3
Year
1960
1980
2000
2020
Adjusted Data, CBH/VR
Data, CBH/VR
Outliered Data
GBD 2017
GBD 2016
VR Complete

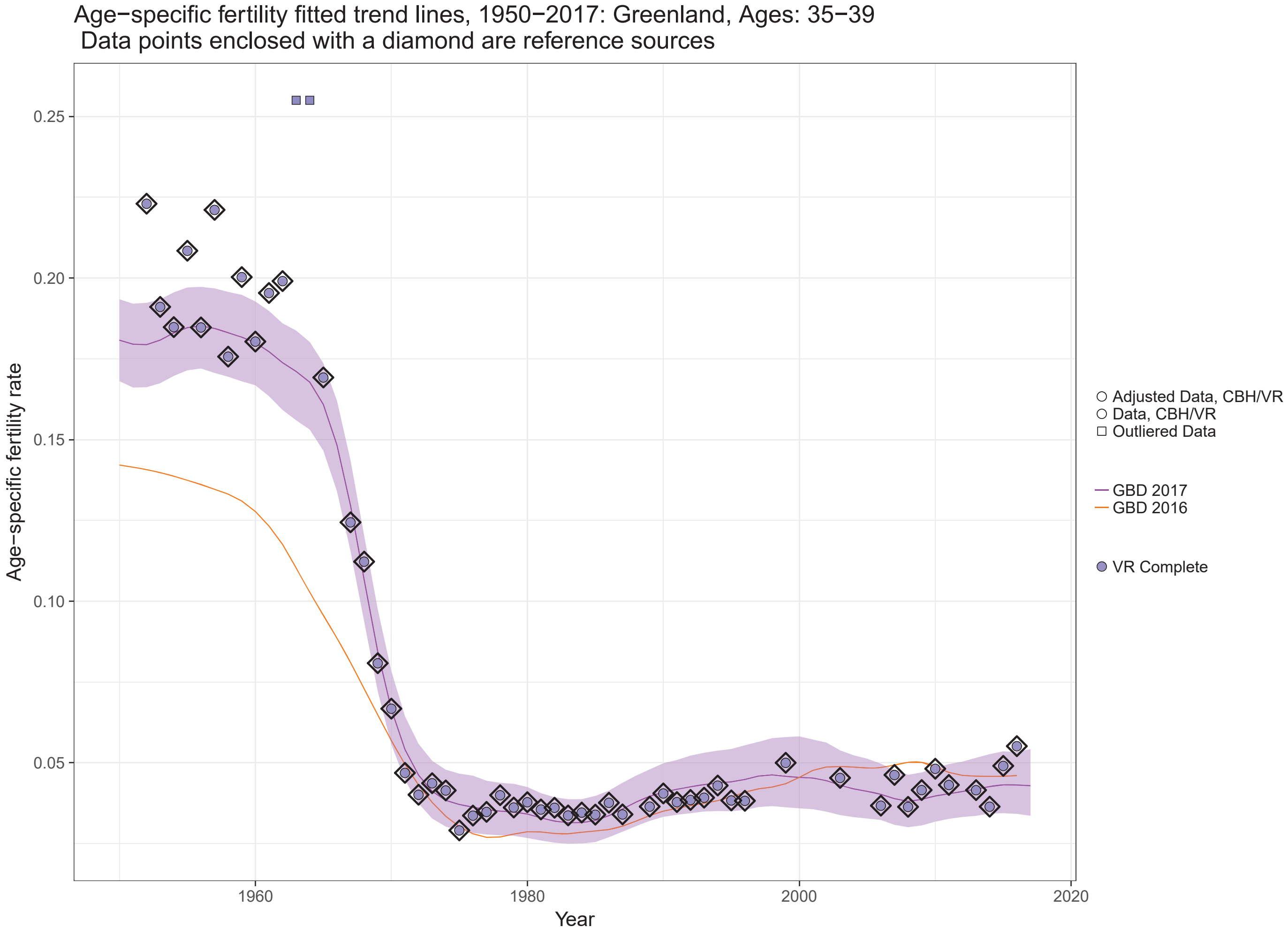

Age−specific fertility fitted trend lines, 1950−2017: Greenland, Ages: 35−39
Data points enclosed with a diamond are reference sources
Age−specific fertility rate
0.05
0.10
0.15
0.20
0.25
1960
1980
2000
2020
Year
Adjusted Data, CBH/VR
Data, CBH/VR
Outliered Data
GBD 2017
GBD 2016
VR Complete

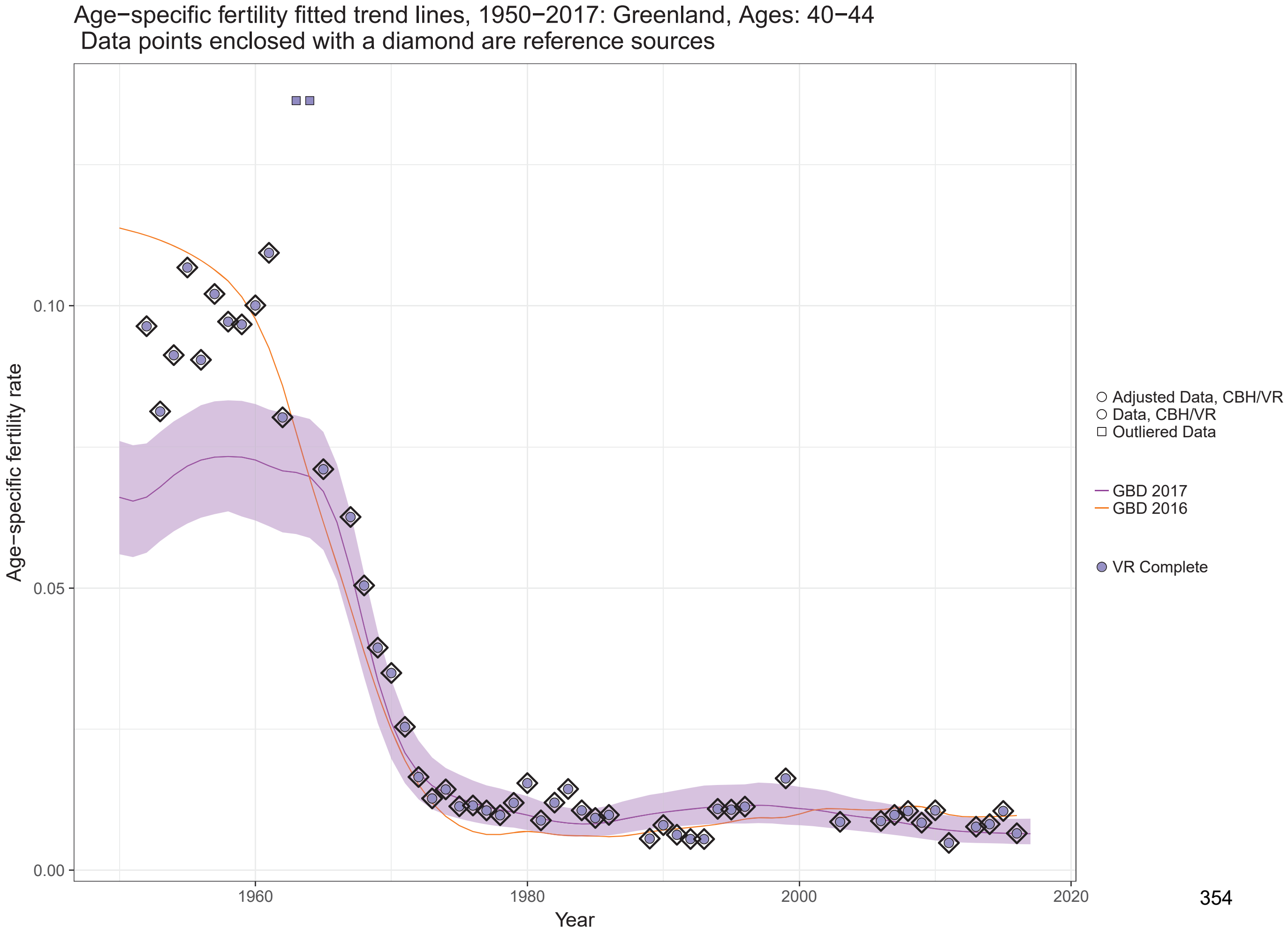

Age−specific fertility fitted trend lines, 1950−2017: Greenland, Ages: 40−44
Data points enclosed with a diamond are reference sources
Age−specific fertility rate
0.00
0.05
0.10
1960
1980
2000
2020
Year
Adjusted Data, CBH/VR
Data, CBH/VR
Outliered Data
GBD 2017
GBD 2016
VR Complete

Age−specific fertility fitted trend lines, 1950−2017: Greenland, Ages: 45−49
Data points enclosed with a diamond are reference sources

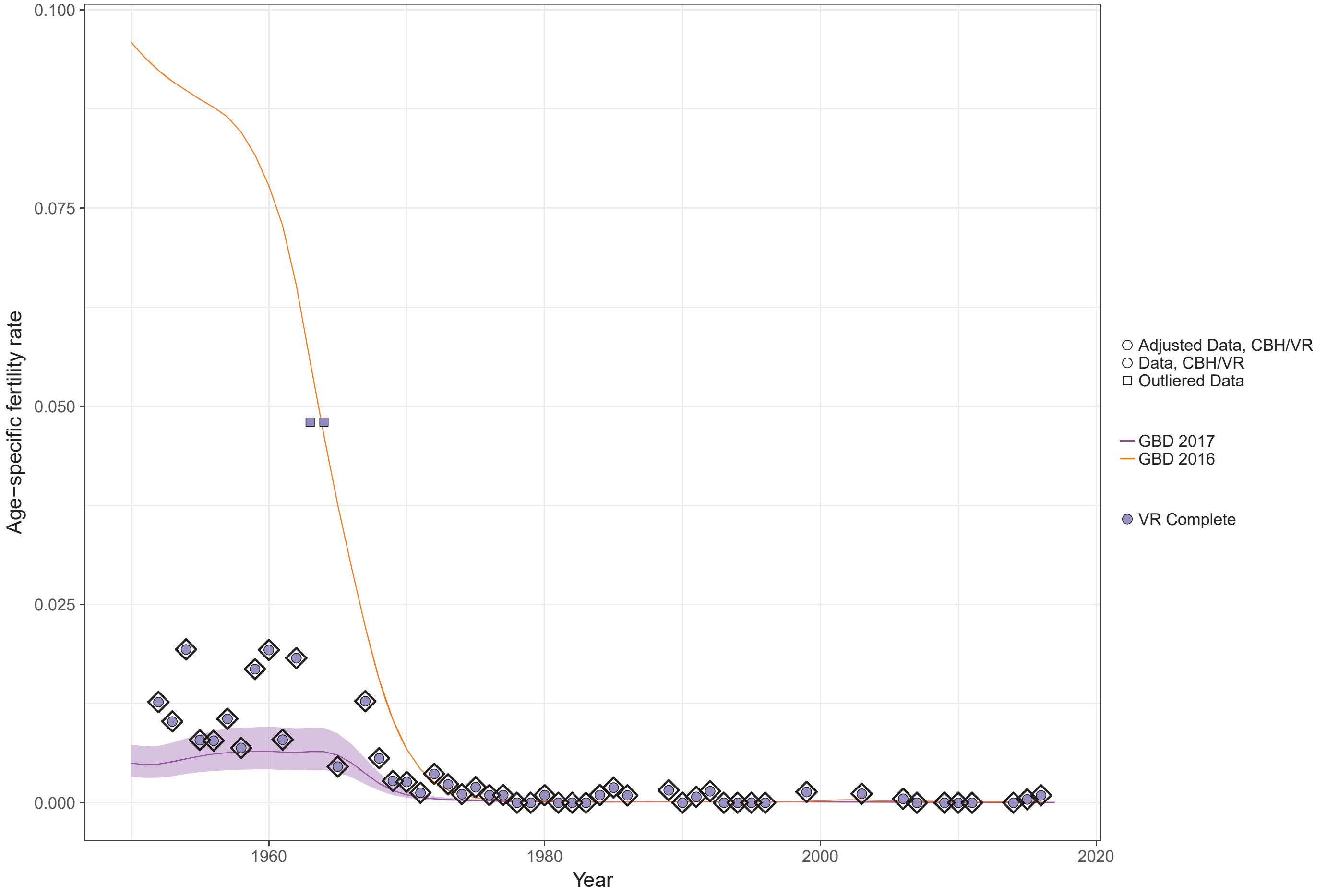

Age−specific fertility fitted trend lines, 1950−2017: Greenland, Ages: 50−54
Data points enclosed with a diamond are reference sources

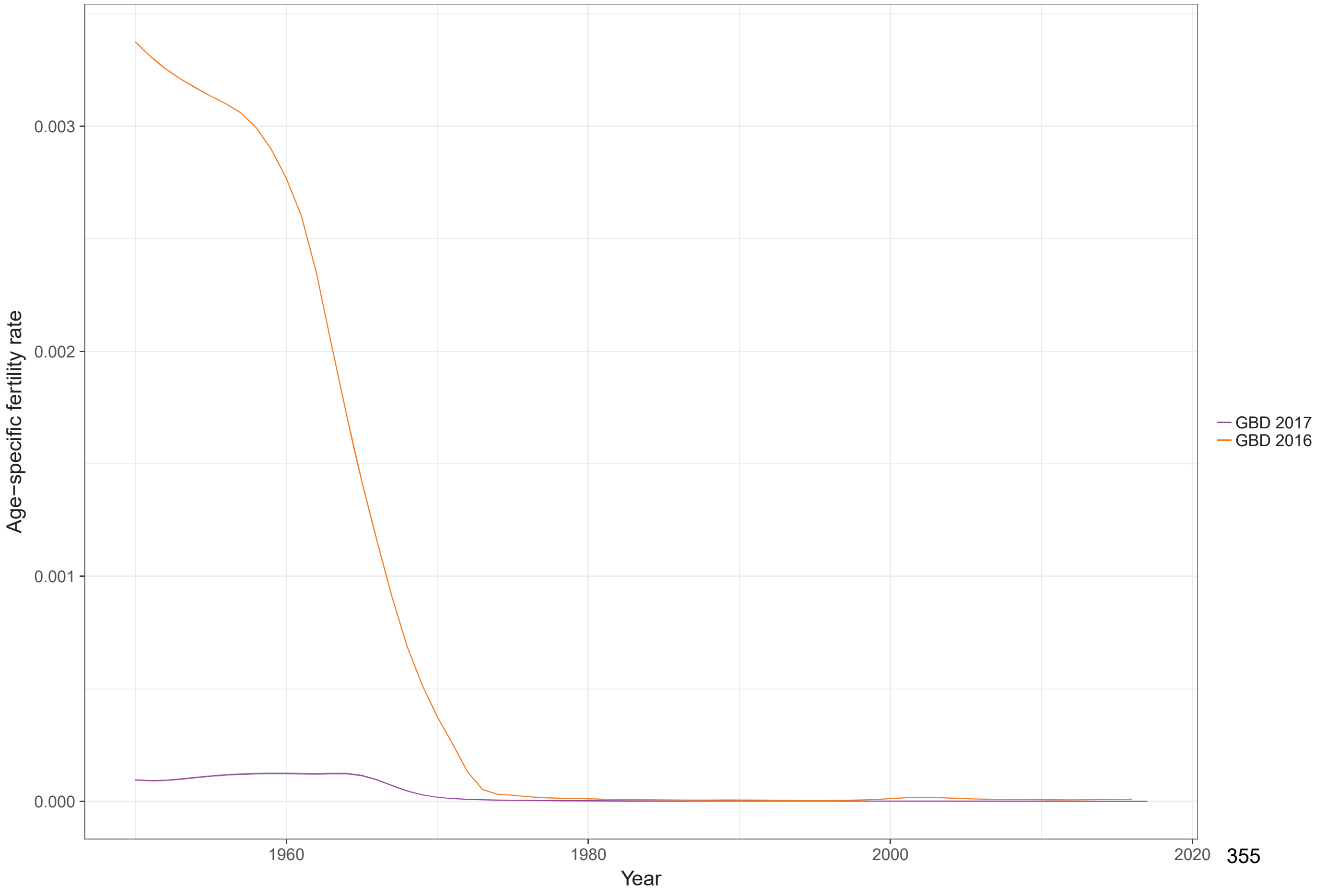

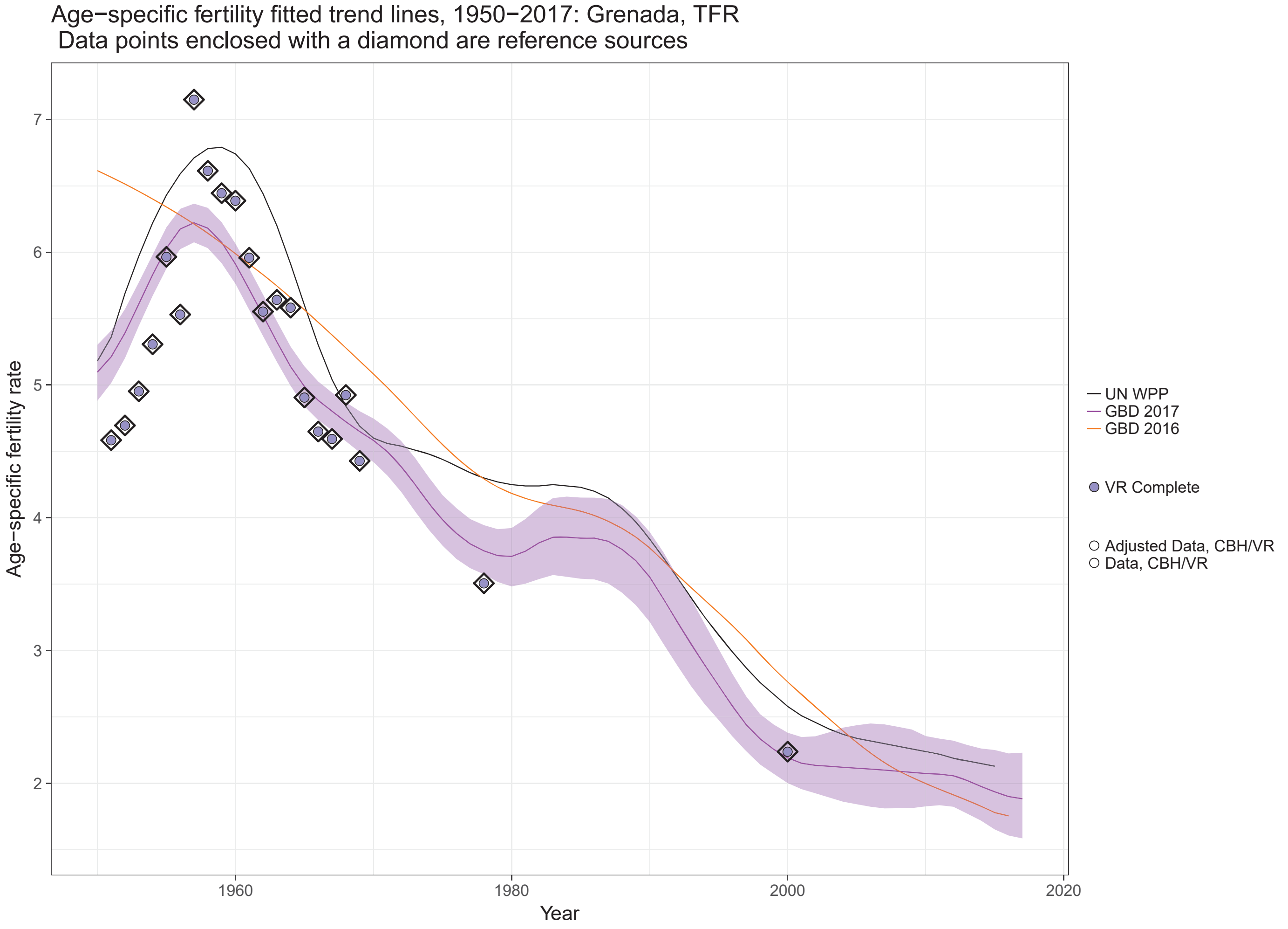

Age−specific fertility fitted trend lines, 1950−2017: Grenada, TFR
Data points enclosed with a diamond are reference sources
Age−specific fertility rate
Year
UN WPP
GBD 2017
GBD 2016
VR Complete
Adjusted Data, CBH/VR
Data, CBH/VR

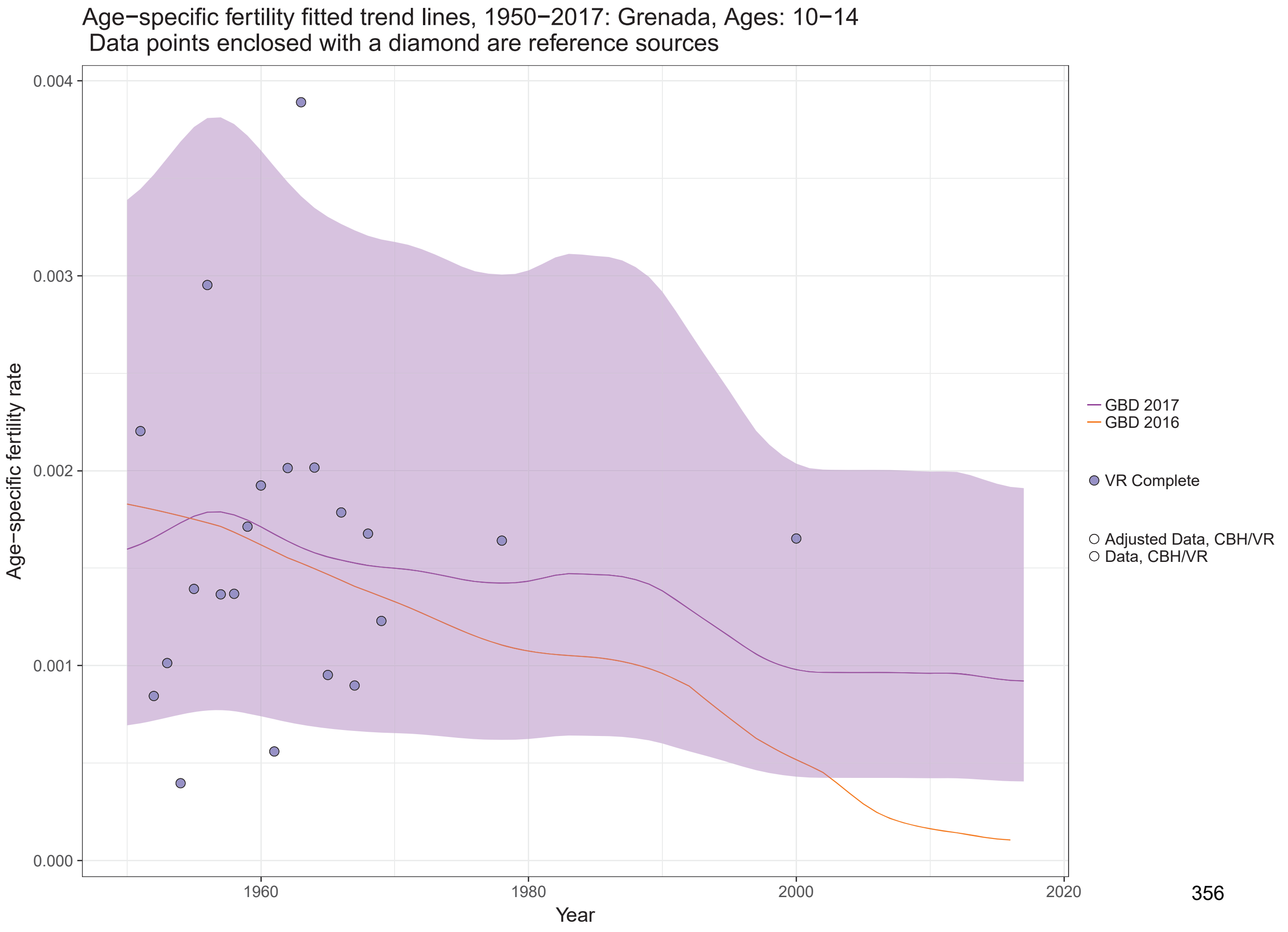

Age−specific fertility fitted trend lines, 1950−2017: Grenada, Ages: 10−14
Data points enclosed with a diamond are reference sources
Age−specific fertility rate
Year
GBD 2017
GBD 2016
VR Complete
Adjusted Data, CBH/VR
Data, CBH/VR

Age−specific fertility fitted trend lines, 1950−2017: Grenada, Ages: 15−19
Data points enclosed with a diamond are reference sources

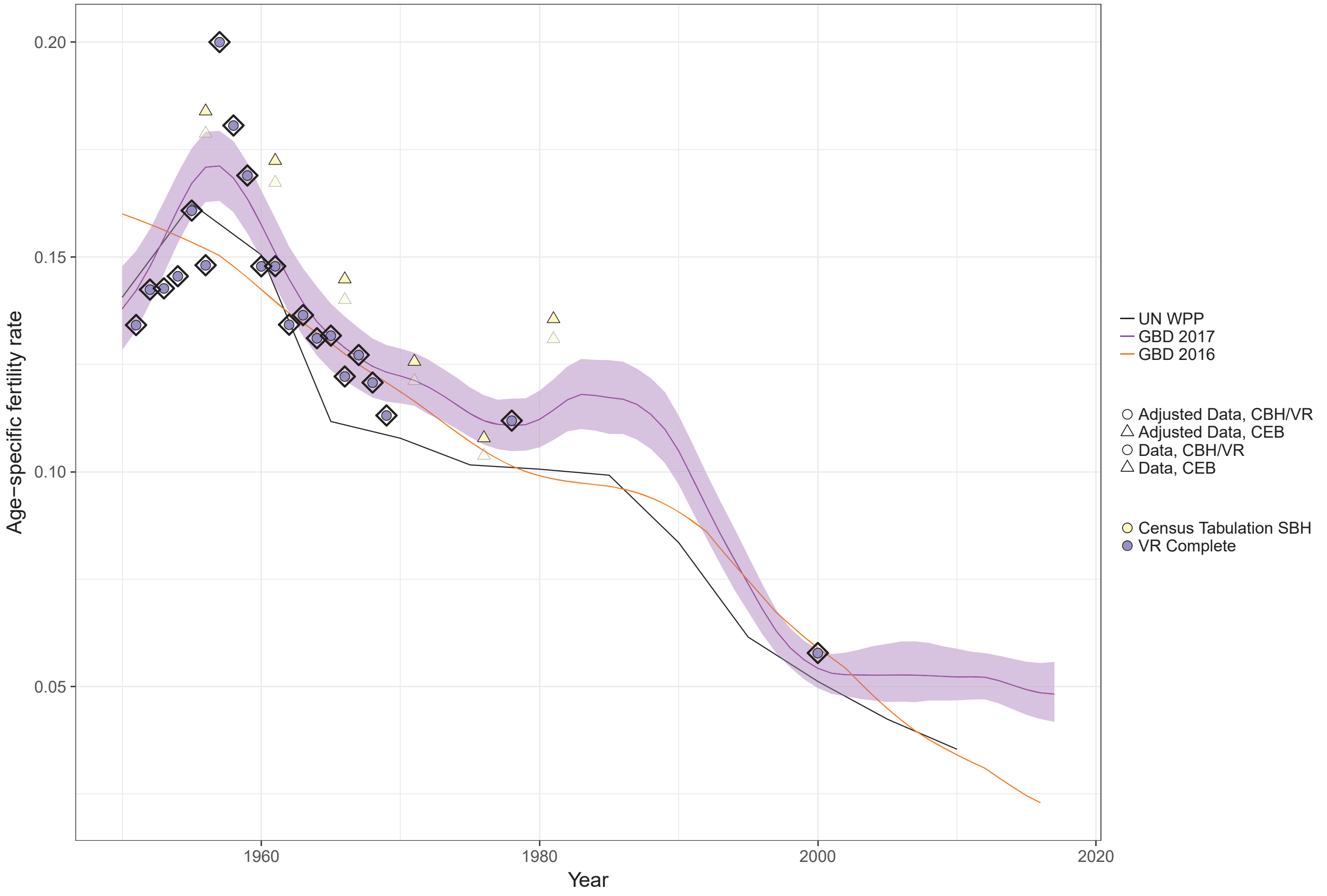

Age−specific fertility fitted trend lines, 1950−2017: Grenada, Ages: 20−24
Data points enclosed with a diamond are reference sources

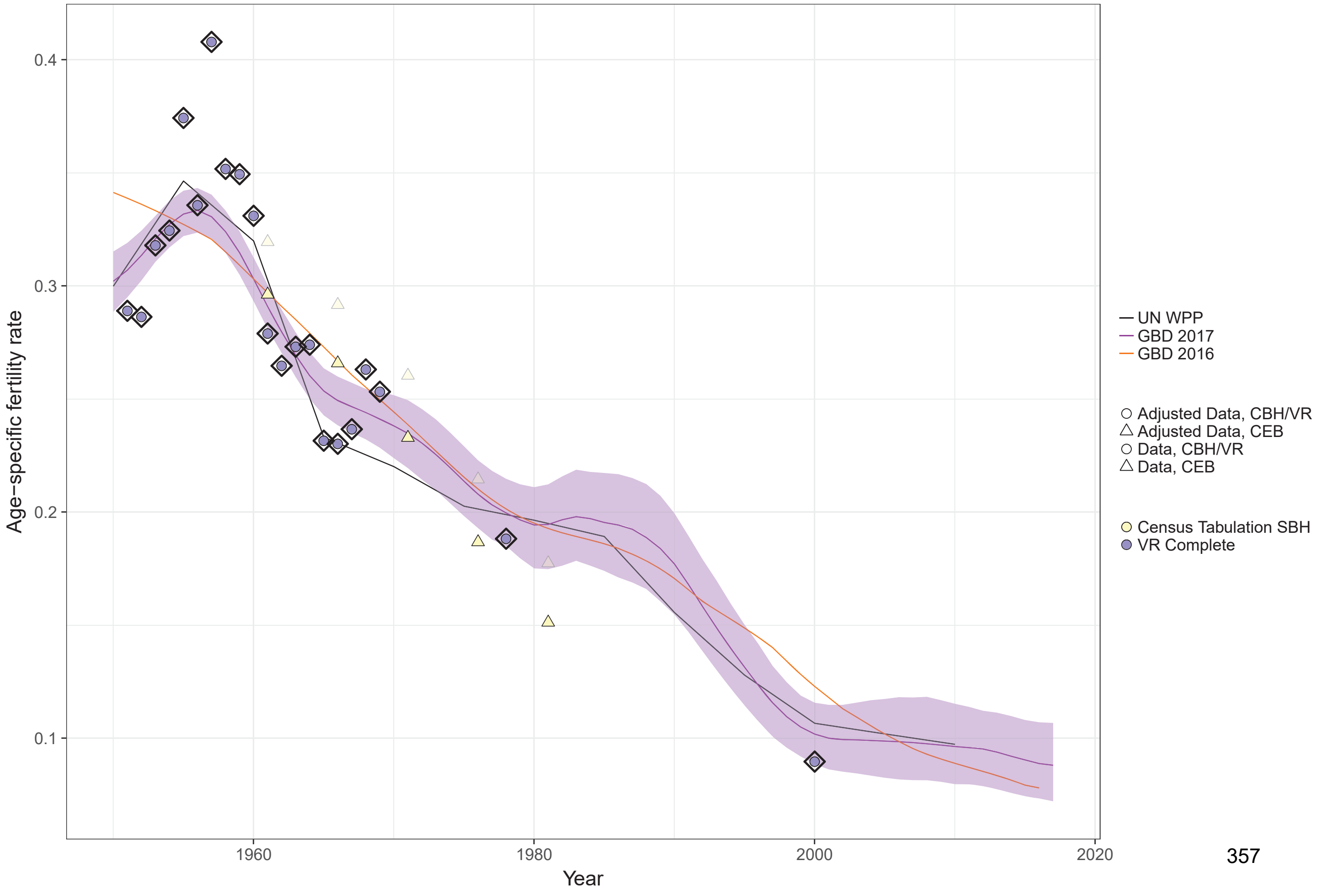

Age−specific fertility fitted trend lines, 1950−2017: Grenada, Ages: 25−29
Data points enclosed with a diamond are reference sources

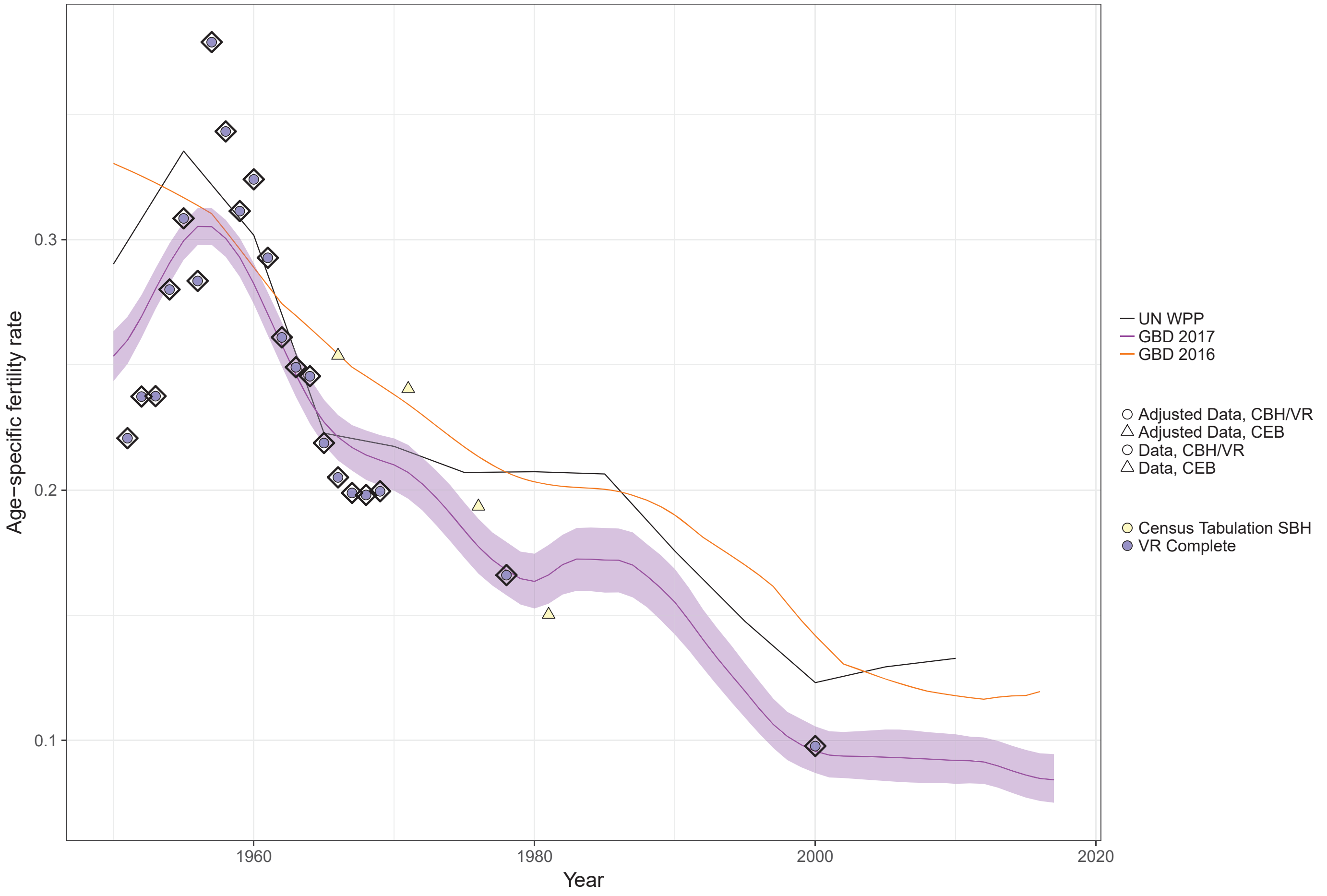

Age−specific fertility fitted trend lines, 1950−2017: Grenada, Ages: 30−34
Data points enclosed with a diamond are reference sources

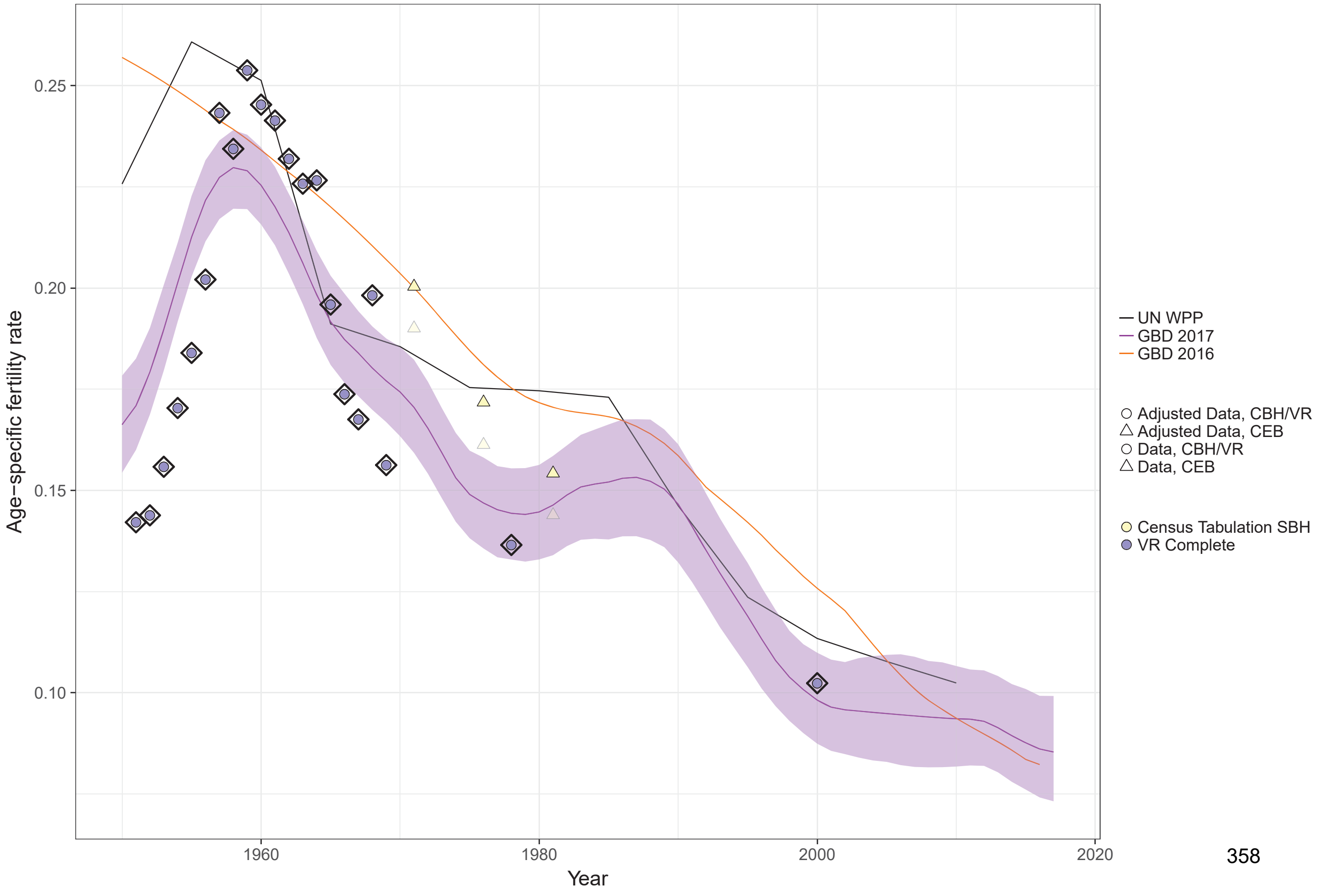

Age−specific fertility fitted trend lines, 1950−2017: Grenada, Ages: 35−39
Data points enclosed with a diamond are reference sources

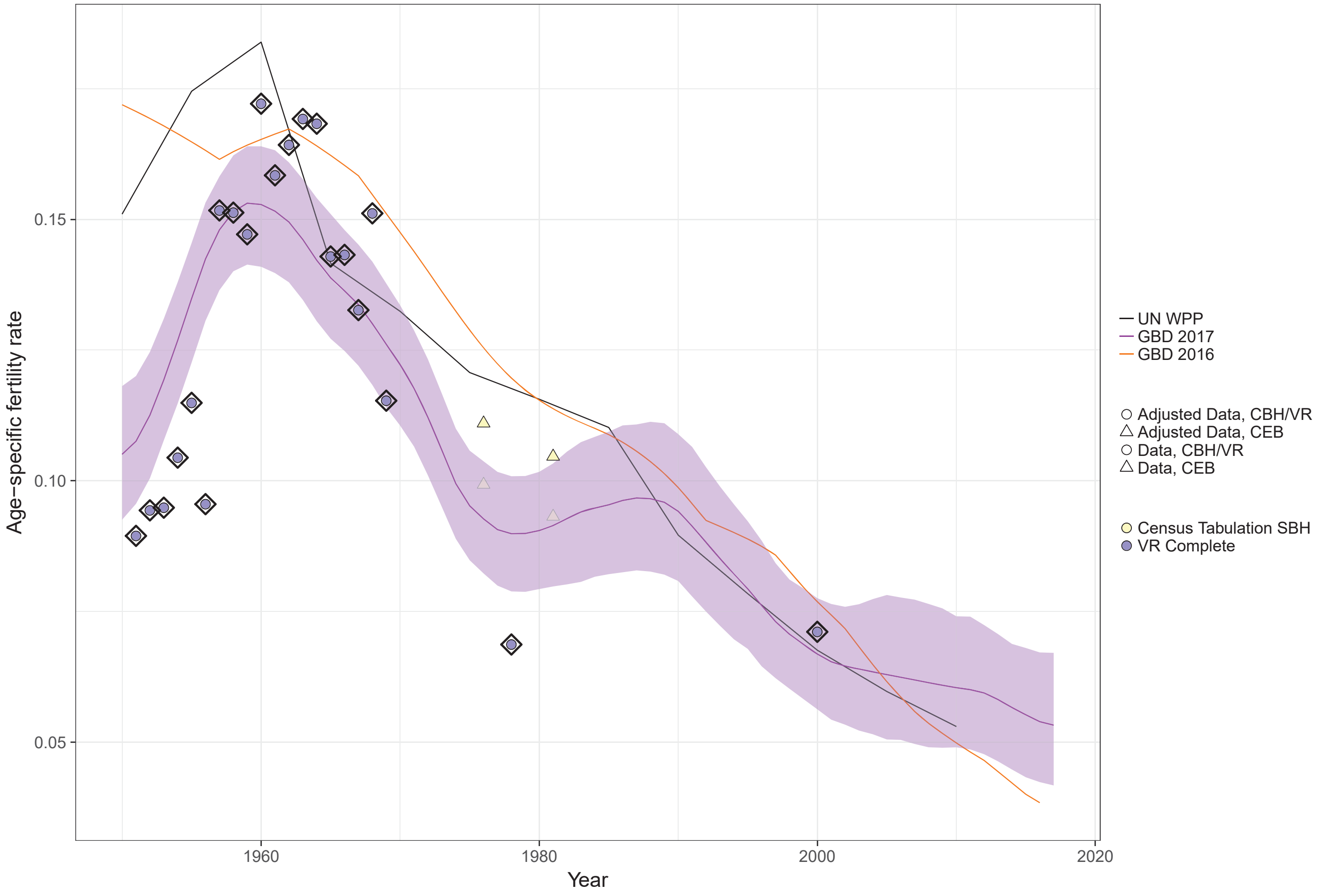

Age−specific fertility fitted trend lines, 1950−2017: Grenada, Ages: 40−44
Data points enclosed with a diamond are reference sources

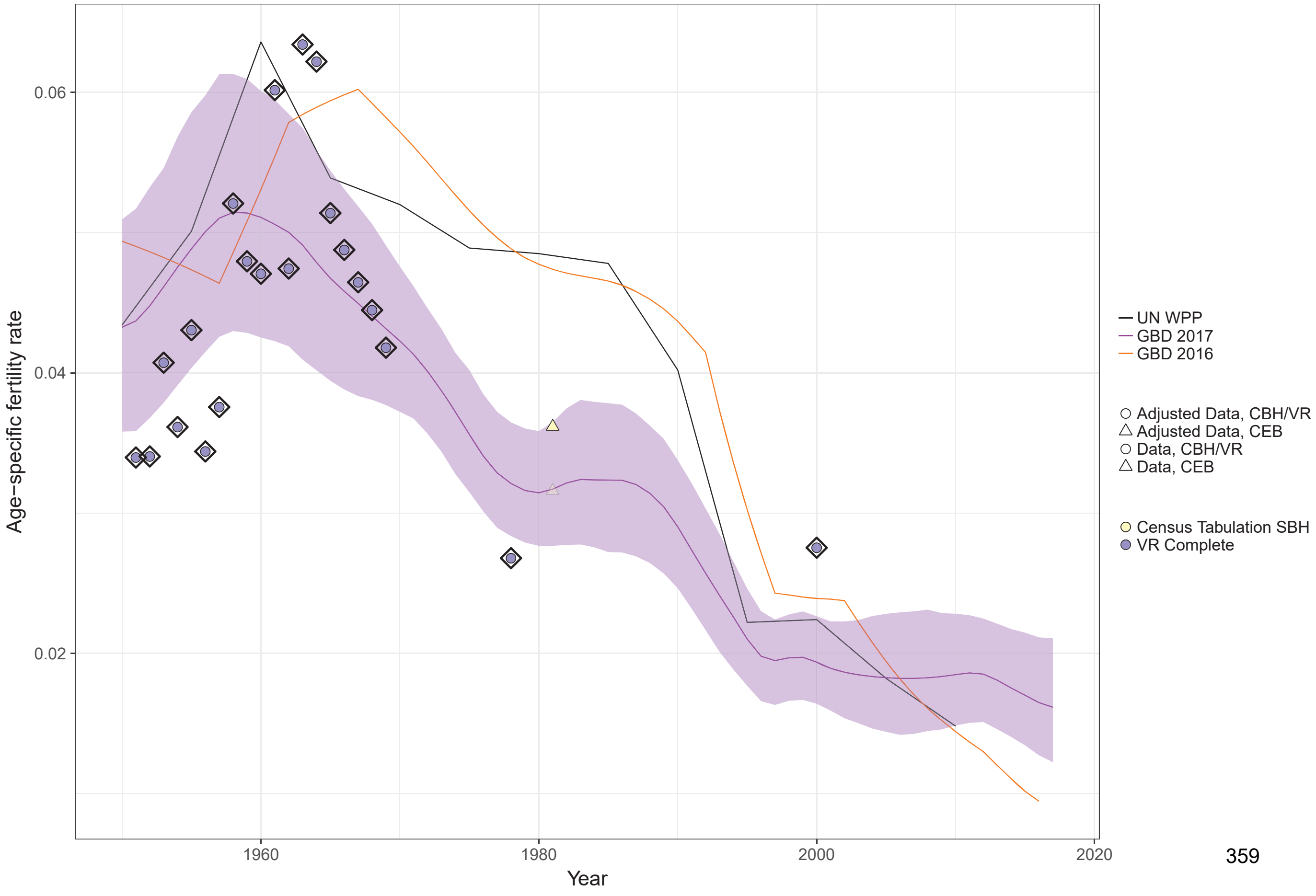

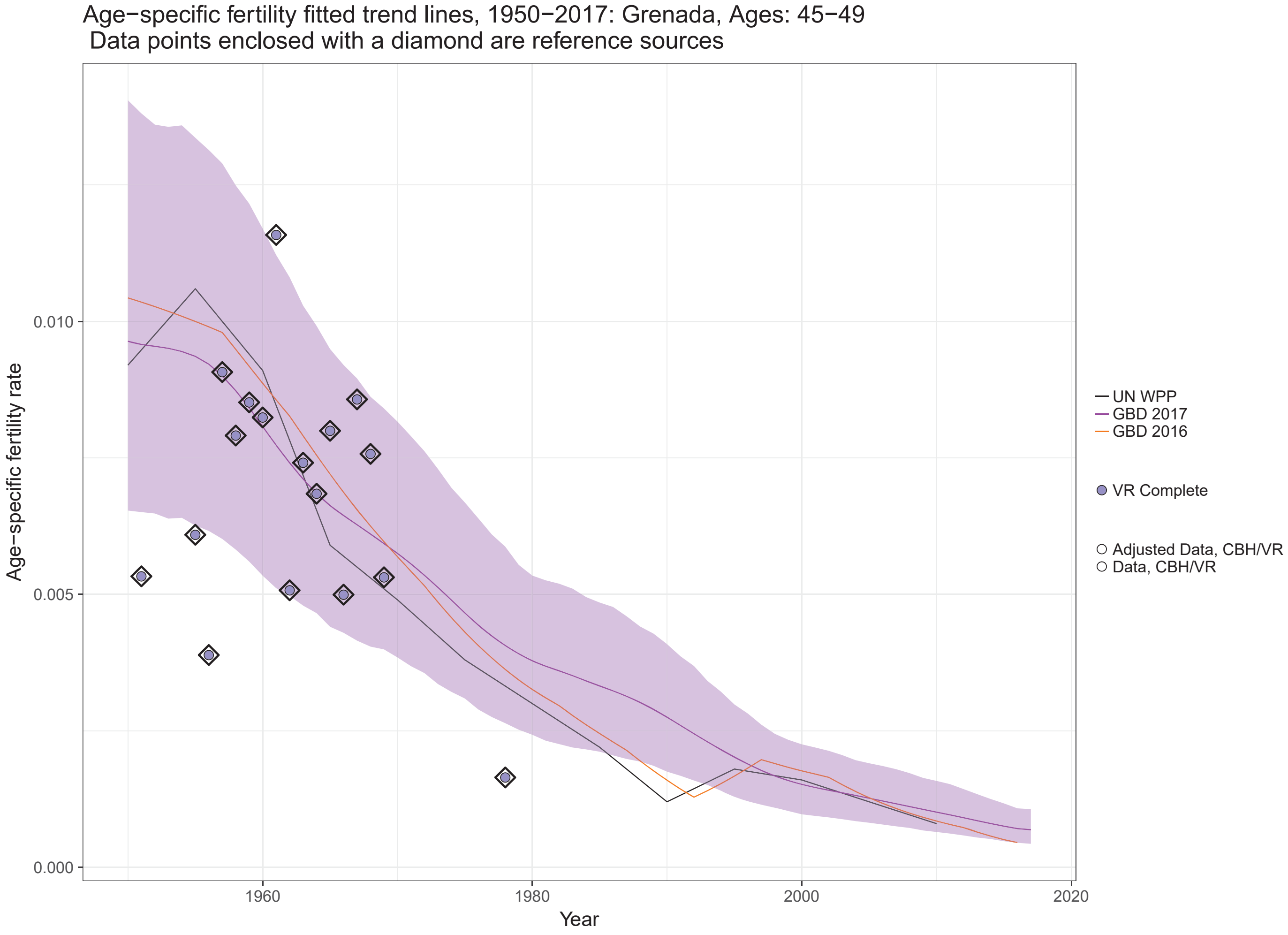
Age−specific fertility fitted trend lines, 1950−2017: Grenada, Ages: 45−49
Data points enclosed with a diamond are reference sources
Age−specific fertility rate
0.000
0.005
0.010
1960
1980
2000
2020
Year
UN WPP
GBD 2017
GBD 2016
VR Complete
Adjusted Data, CBH/VR
Data, CBH/VR

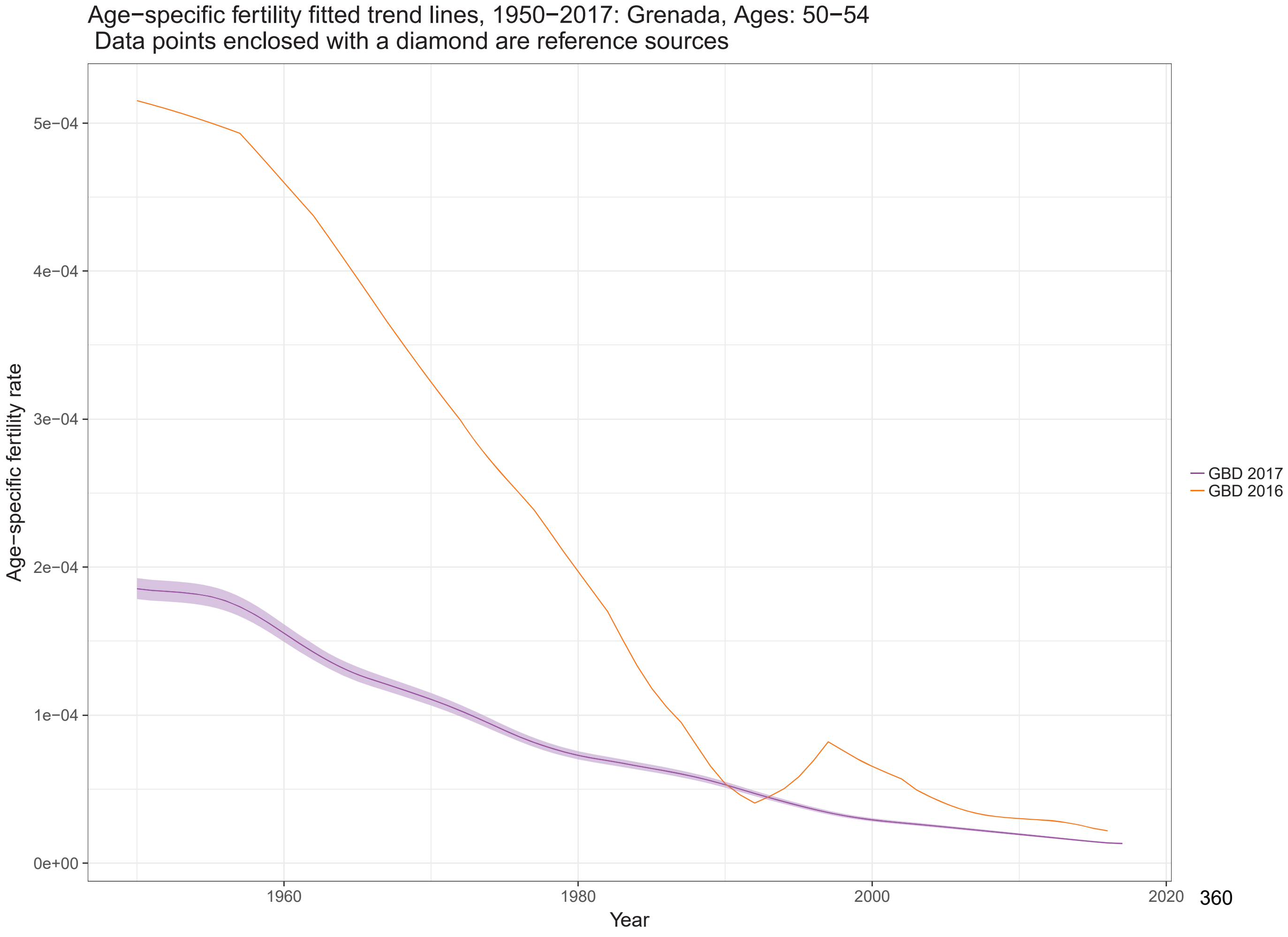
Age−specific fertility fitted trend lines, 1950−2017: Grenada, Ages: 50−54
Data points enclosed with a diamond are reference sources
Age−specific fertility rate
0e+00
1e−04
2e−04
3e−04
4e−04
5e−04
1960
1980
2000
2020
Year
GBD 2017
GBD 2016

Age−specific fertility fitted trend lines, 1950−2017: Guam, TFR
Data points enclosed with a diamond are reference sources

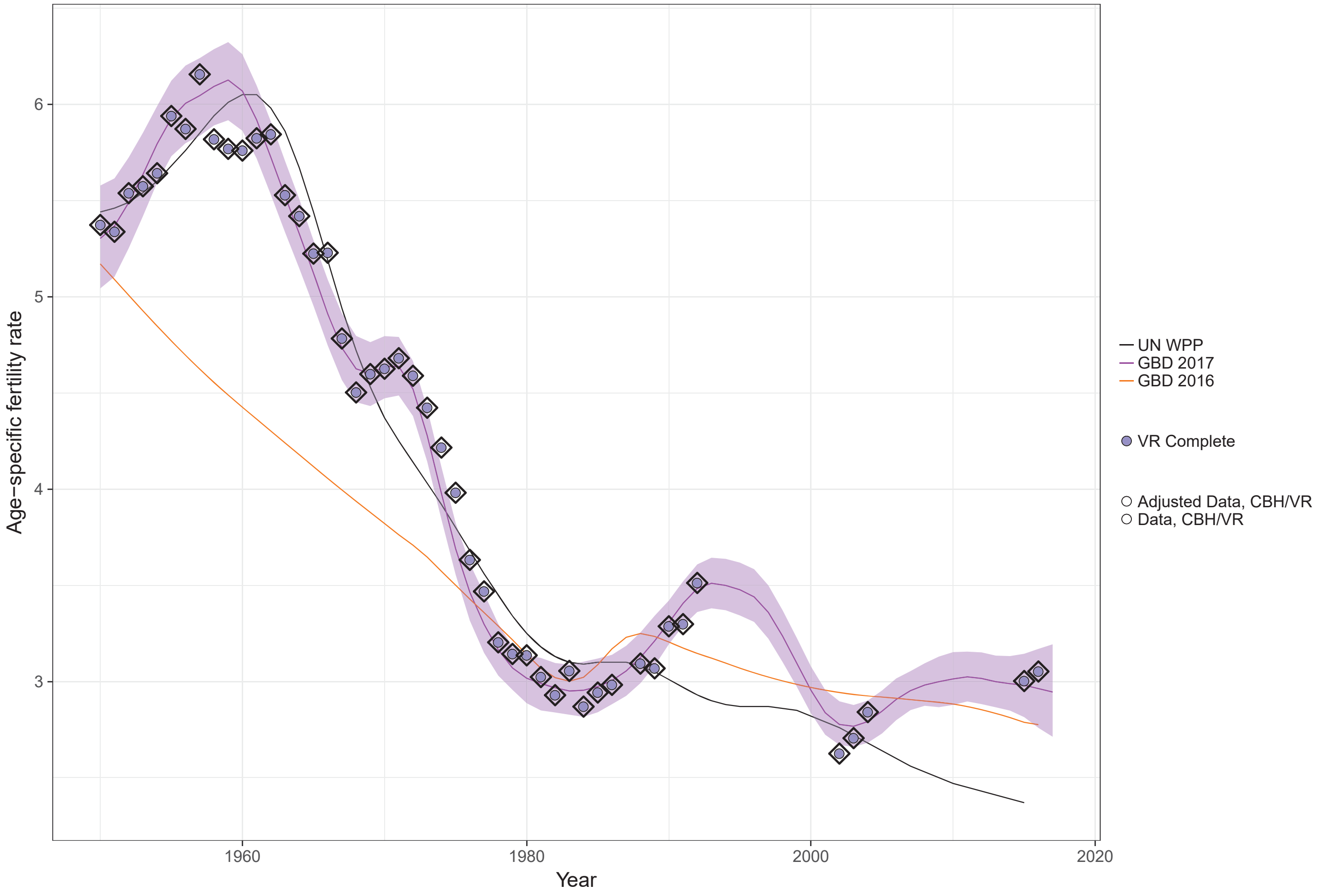

Age−specific fertility fitted trend lines, 1950−2017: Guam, Ages: 10−14
Data points enclosed with a diamond are reference sources

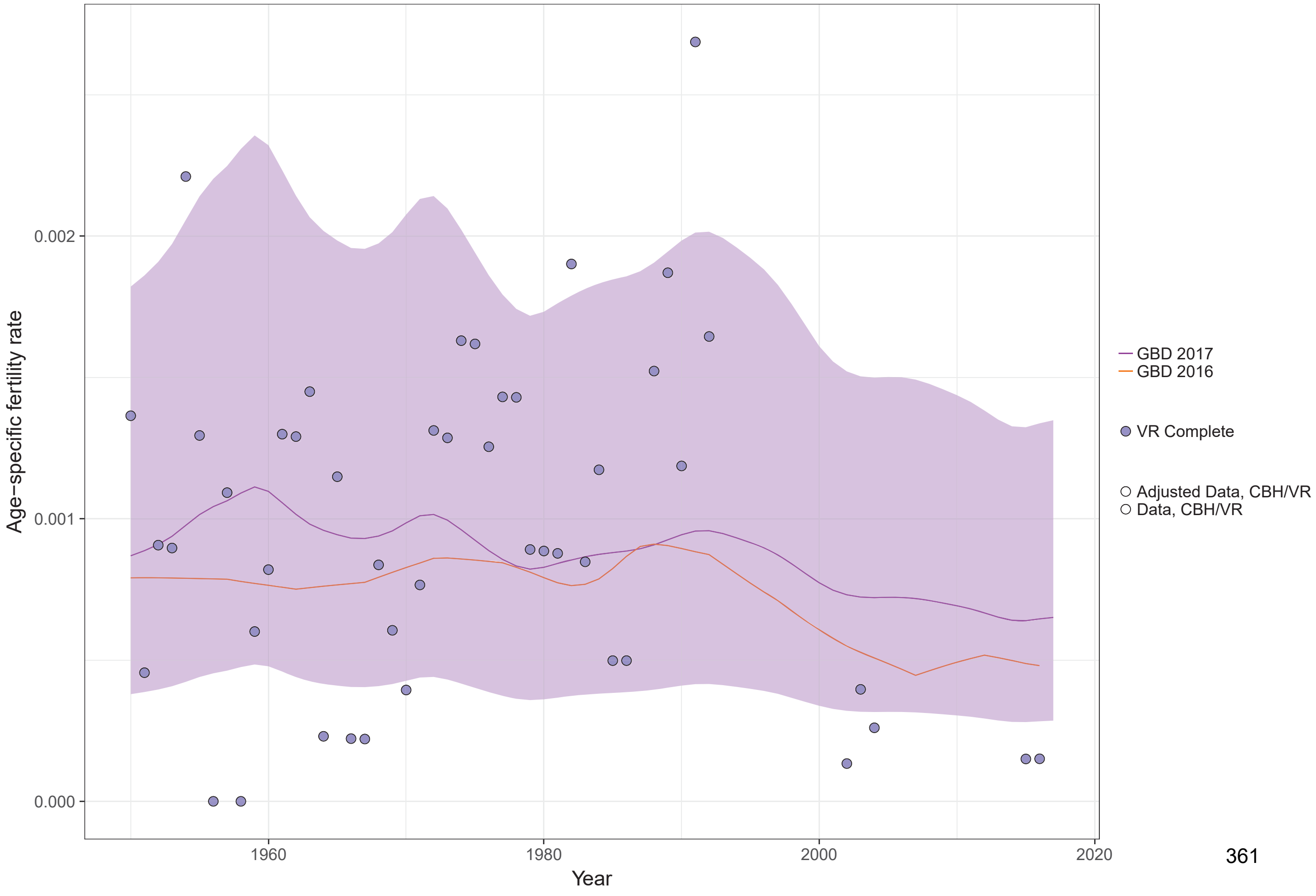

Age−specific fertility fitted trend lines, 1950−2017: Guam, Ages: 15−19
Data points enclosed with a diamond are reference sources

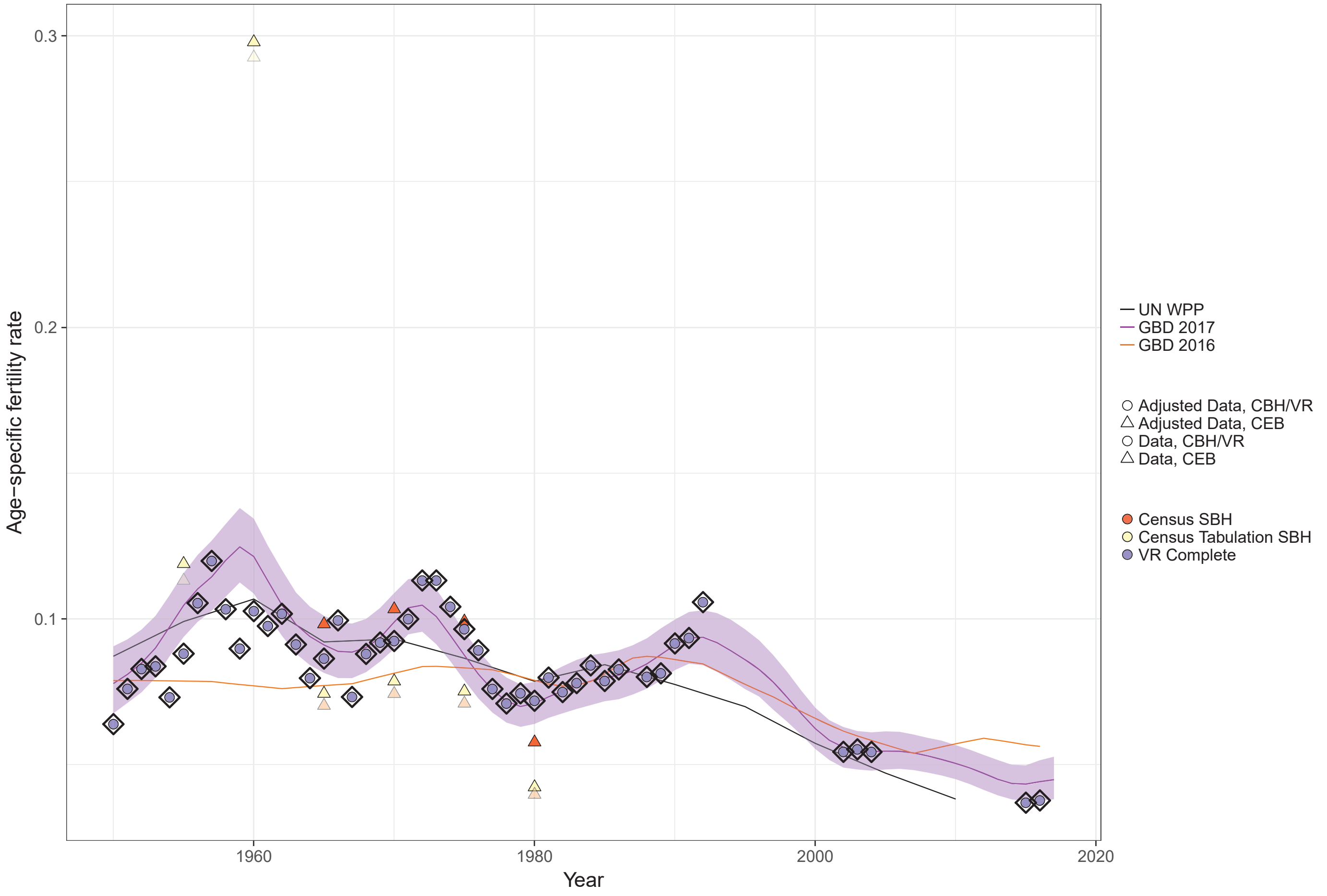

Age−specific fertility fitted trend lines, 1950−2017: Guam, Ages: 20−24
Data points enclosed with a diamond are reference sources

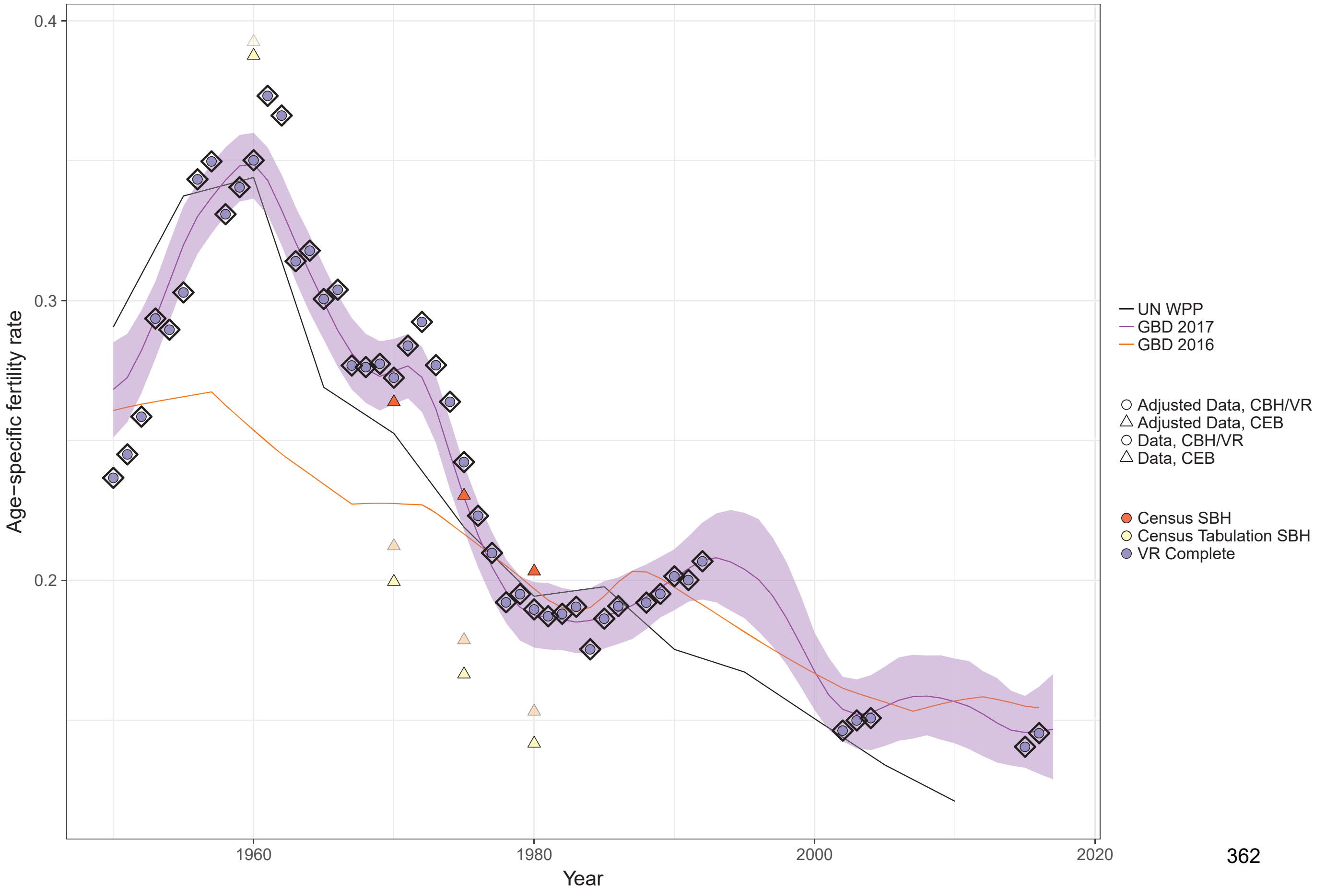

Age−specific fertility fitted trend lines, 1950−2017: Guam, Ages: 25−29
Data points enclosed with a diamond are reference sources

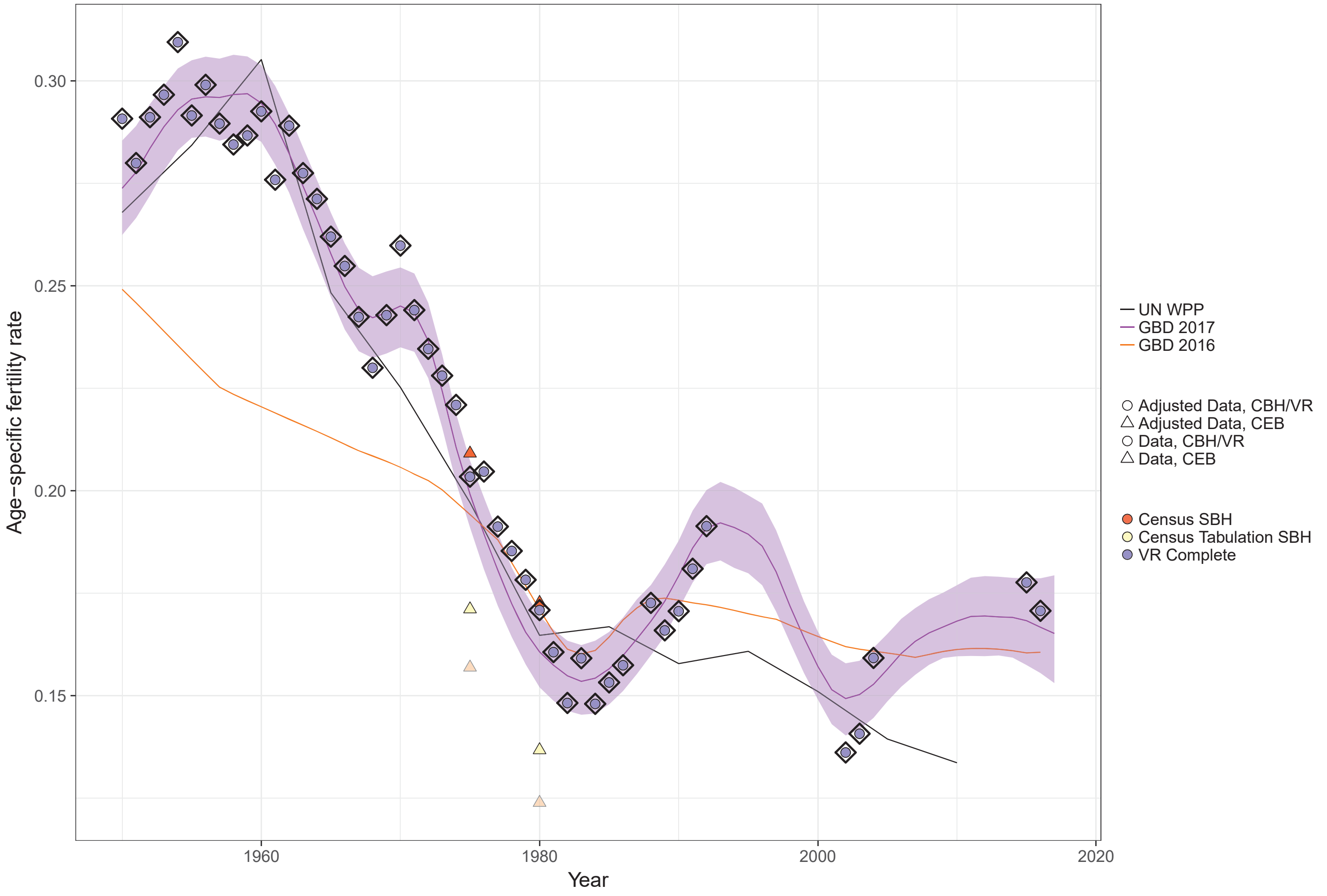

Age−specific fertility fitted trend lines, 1950−2017: Guam, Ages: 30−34
Data points enclosed with a diamond are reference sources

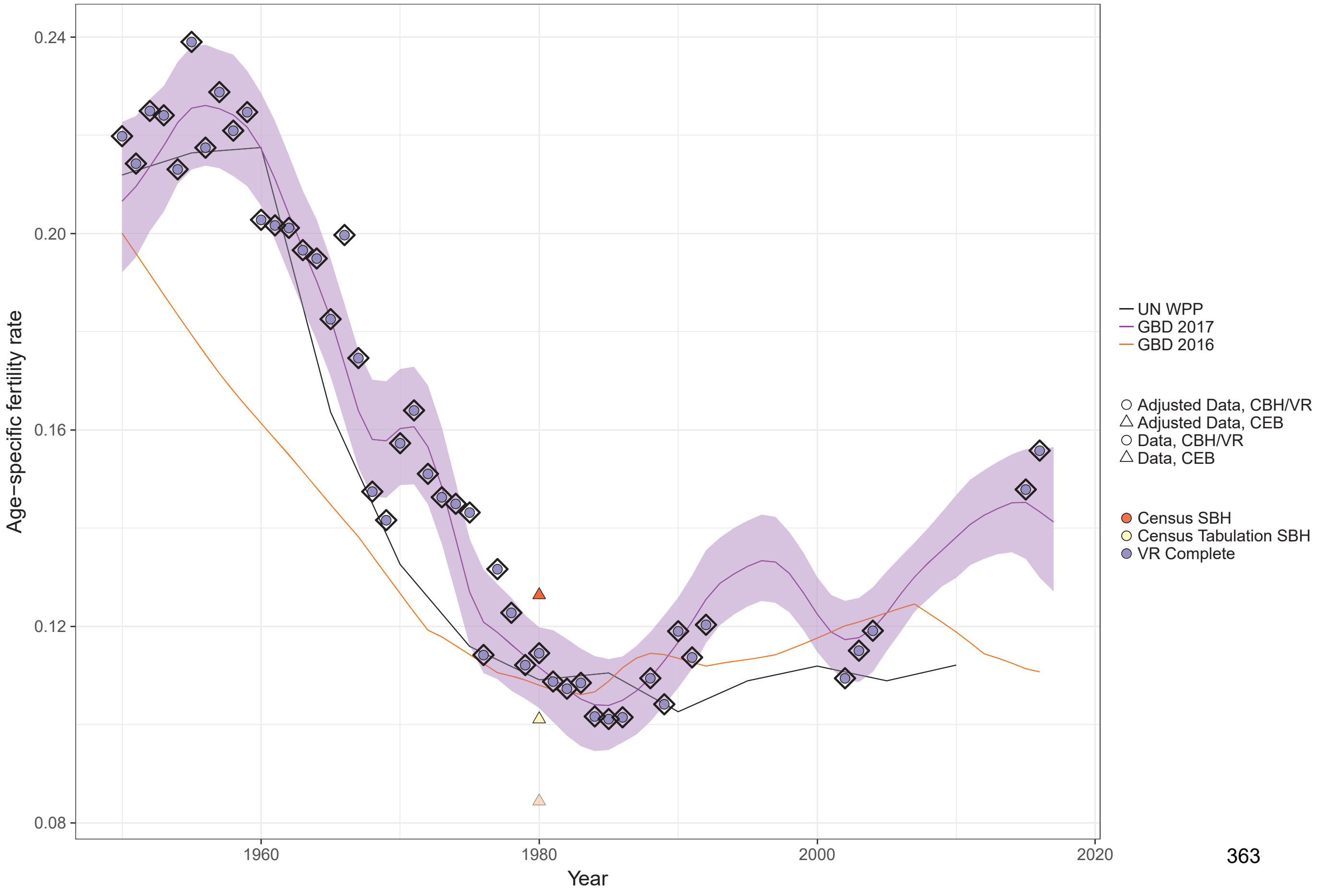

Age−specific fertility fitted trend lines, 1950−2017: Guam, Ages: 35−39
Data points enclosed with a diamond are reference sources

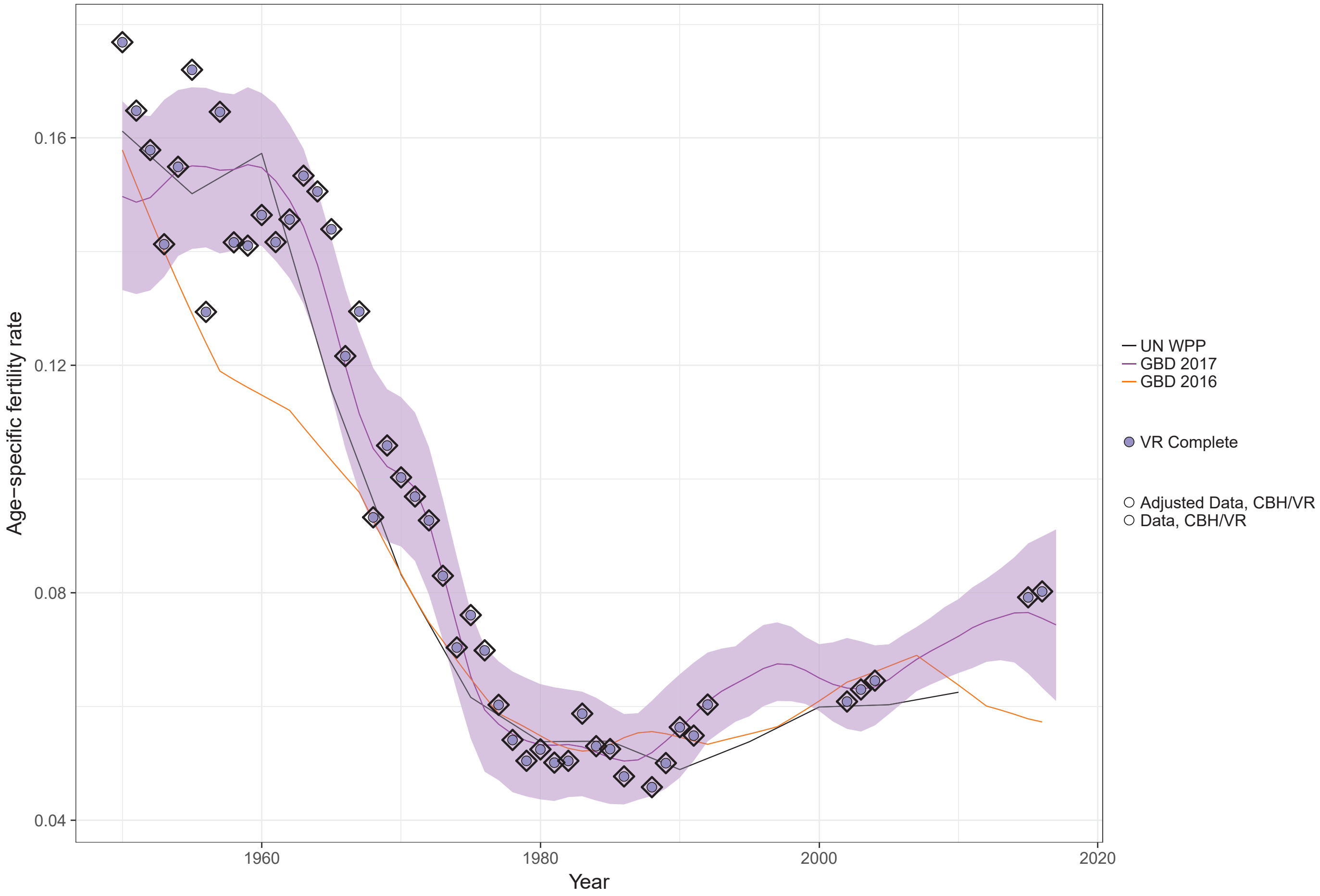

Age−specific fertility fitted trend lines, 1950−2017: Guam, Ages: 40−44
Data points enclosed with a diamond are reference sources

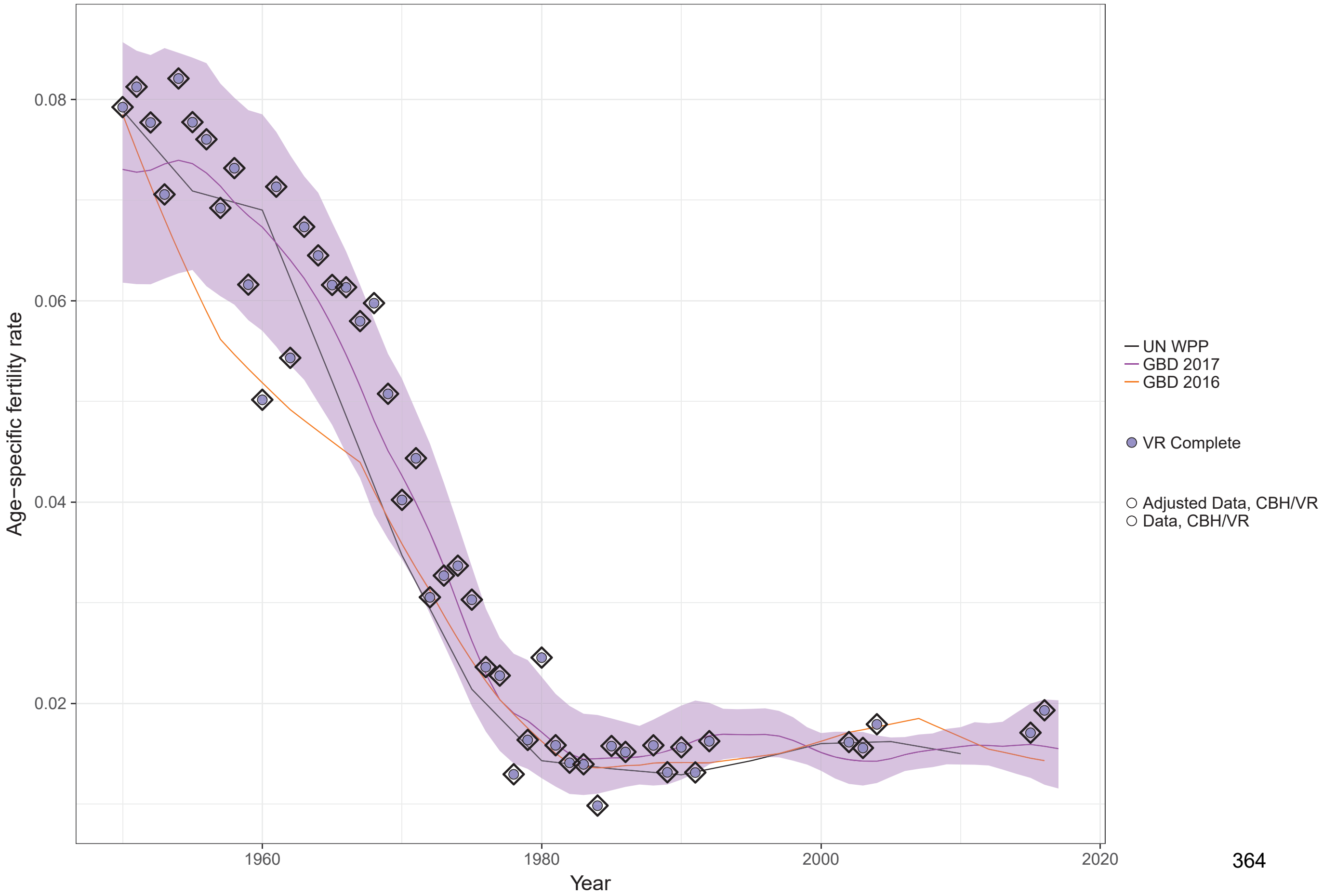

Age−specific fertility fitted trend lines, 1950−2017: Guam, Ages: 45−49
Data points enclosed with a diamond are reference sources

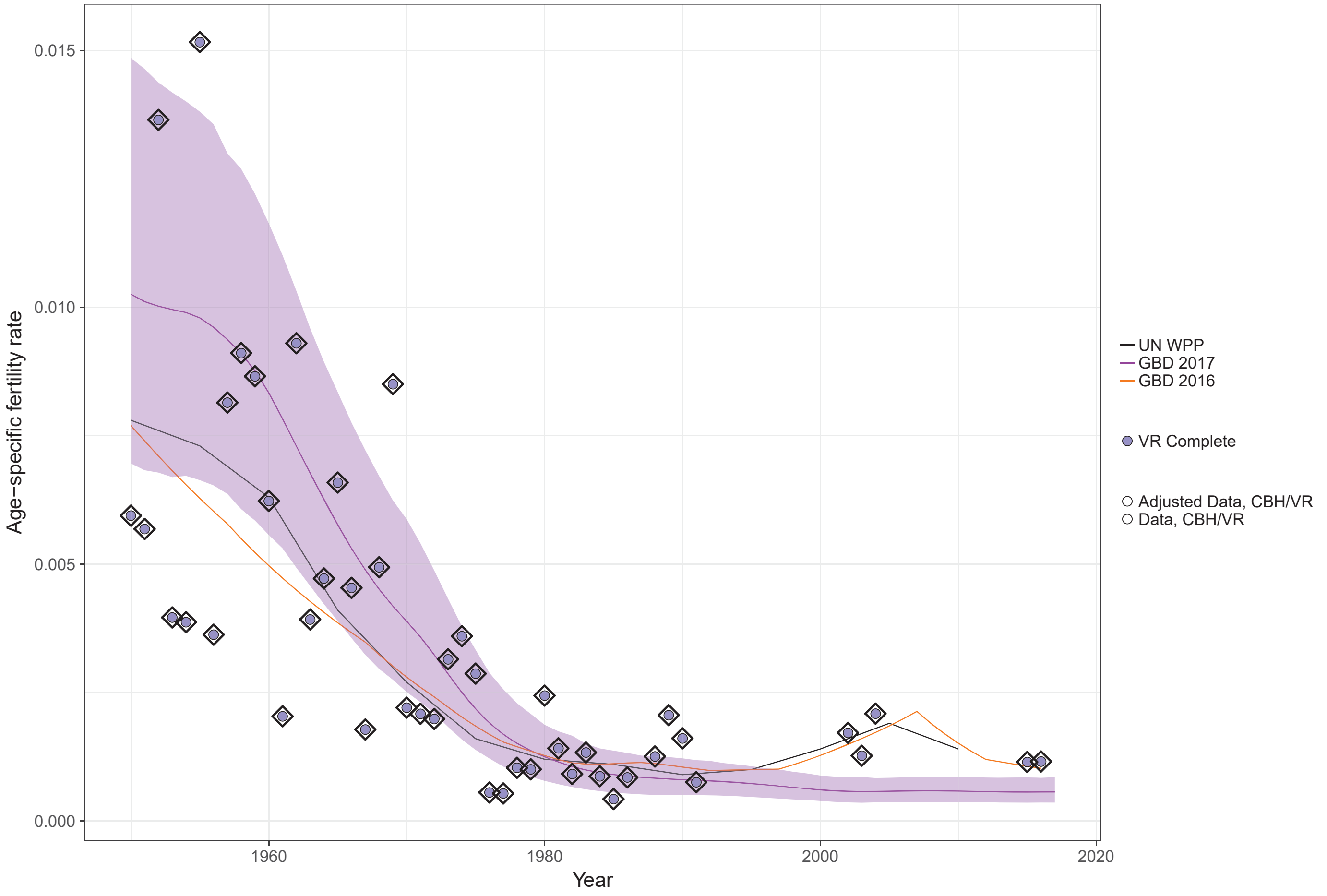

Age−specific fertility fitted trend lines, 1950−2017: Guam, Ages: 50−54
Data points enclosed with a diamond are reference sources

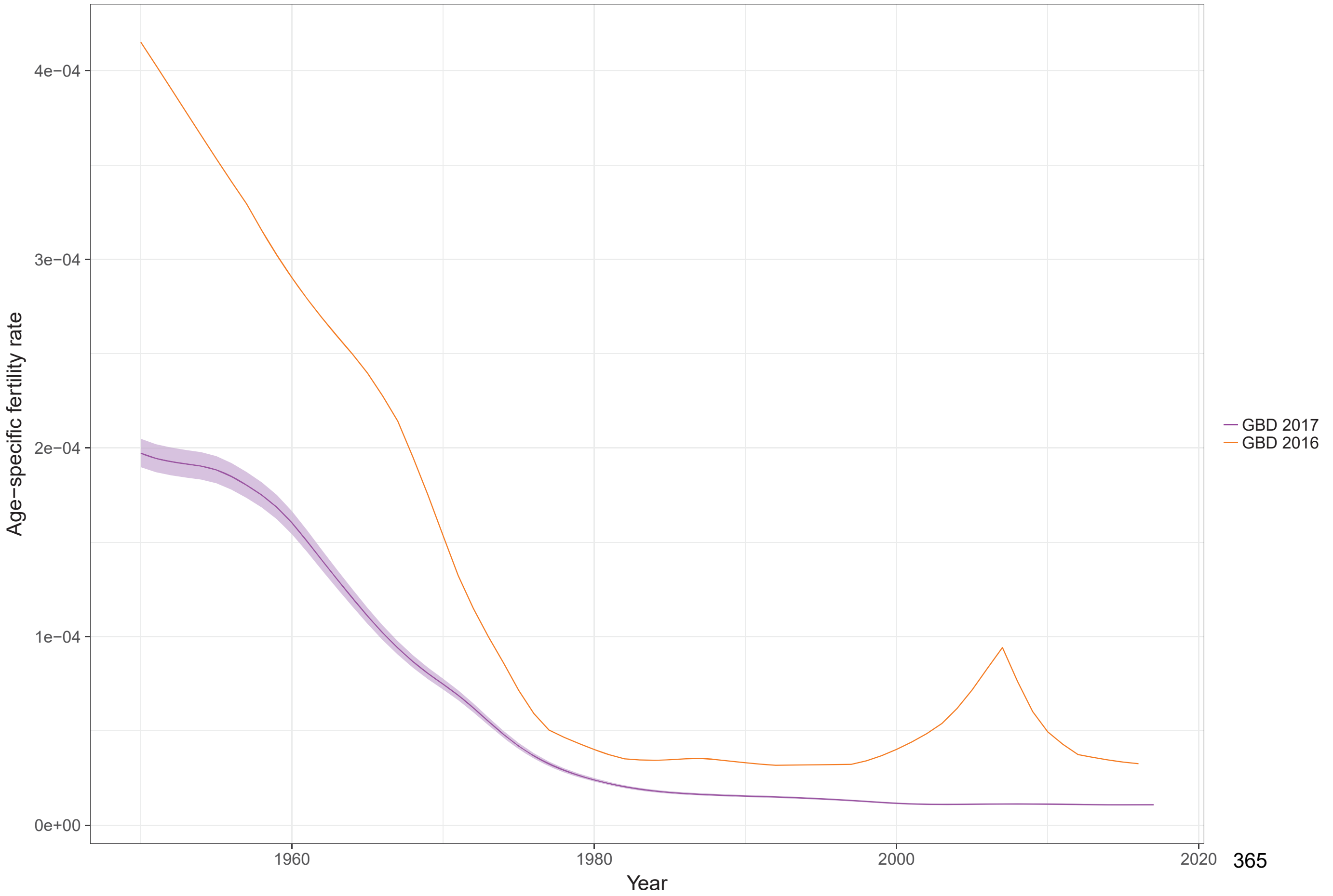

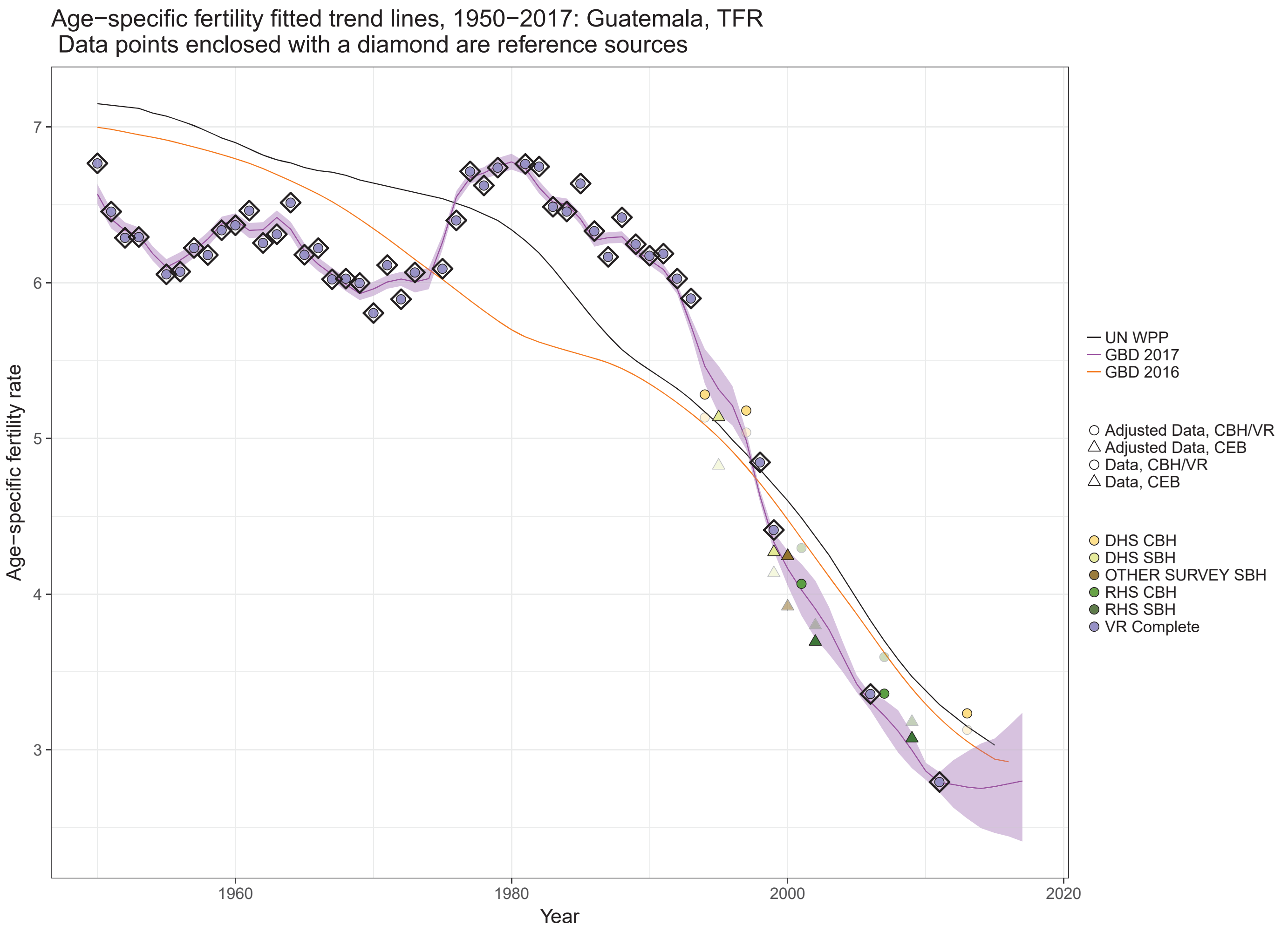
Age−specific fertility fitted trend lines, 1950−2017: Guatemala, TFR
Data points enclosed with a diamond are reference sources
Age−specific fertility rate
Year
UN WPP
GBD 2017
GBD 2016
Adjusted Data, CBH/VR
Adjusted Data, CEB
Data, CBH/VR
Data, CEB
DHS CBH
DHS SBH
OTHER SURVEY SBH
RHS CBH
RHS SBH
VR Complete

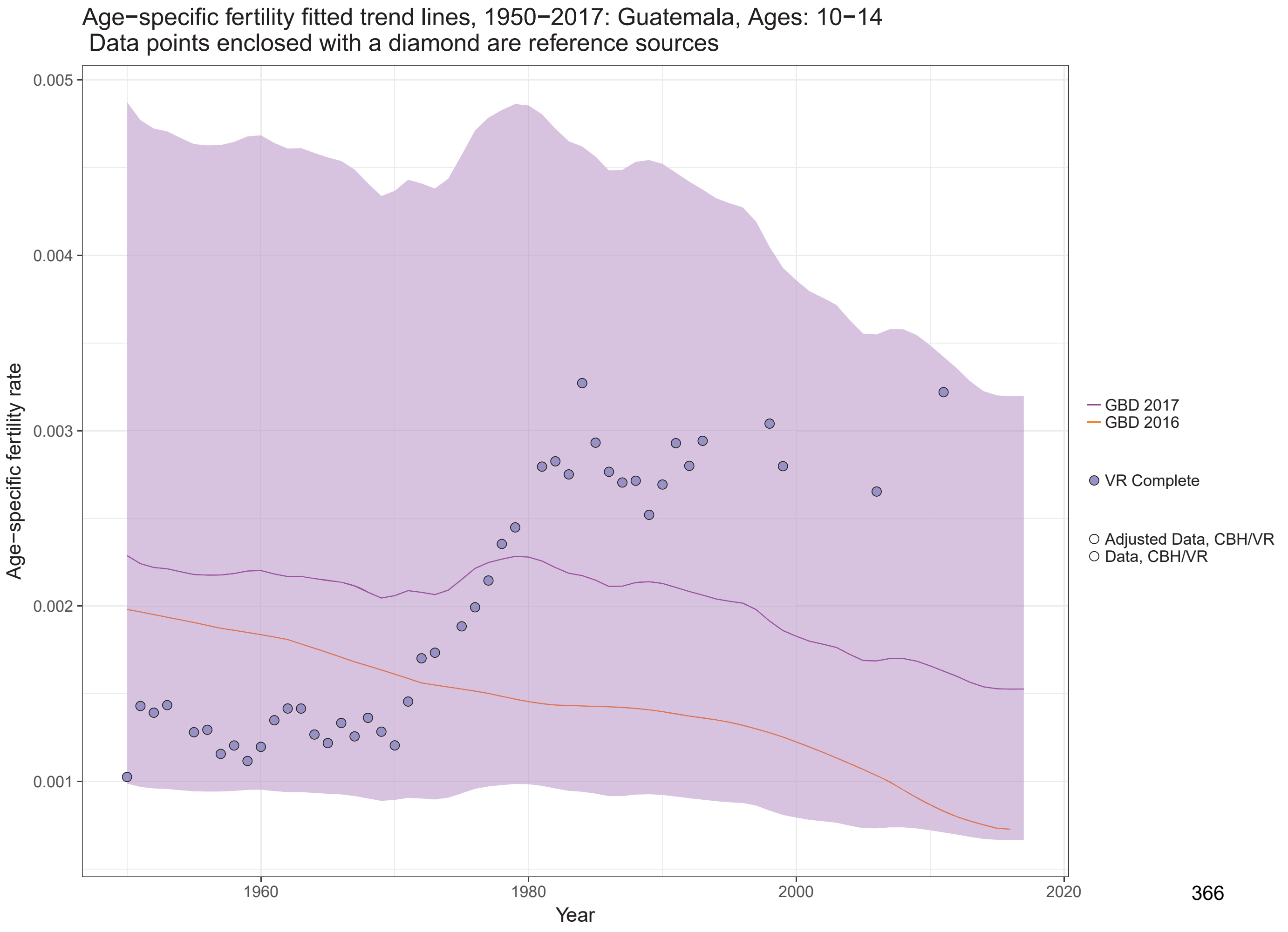
Age−specific fertility fitted trend lines, 1950−2017: Guatemala, Ages: 10−14
Data points enclosed with a diamond are reference sources
Age−specific fertility rate
Year
GBD 2017
GBD 2016
VR Complete
Adjusted Data, CBH/VR
Data, CBH/VR

Age−specific fertility fitted trend lines, 1950−2017: Guatemala, Ages: 15−19
Data points enclosed with a diamond are reference sources

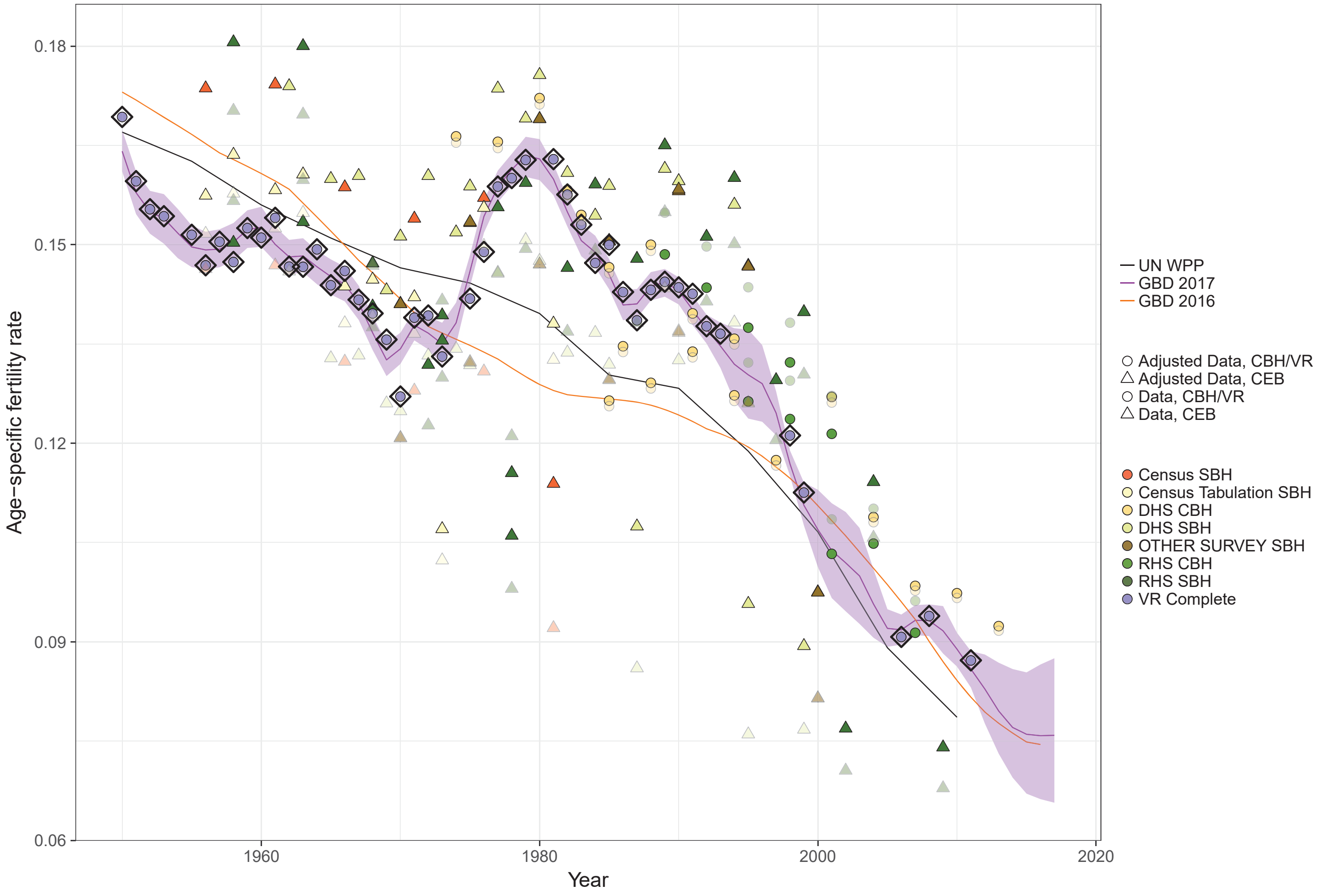

Age−specific fertility fitted trend lines, 1950−2017: Guatemala, Ages: 20−24
Data points enclosed with a diamond are reference sources

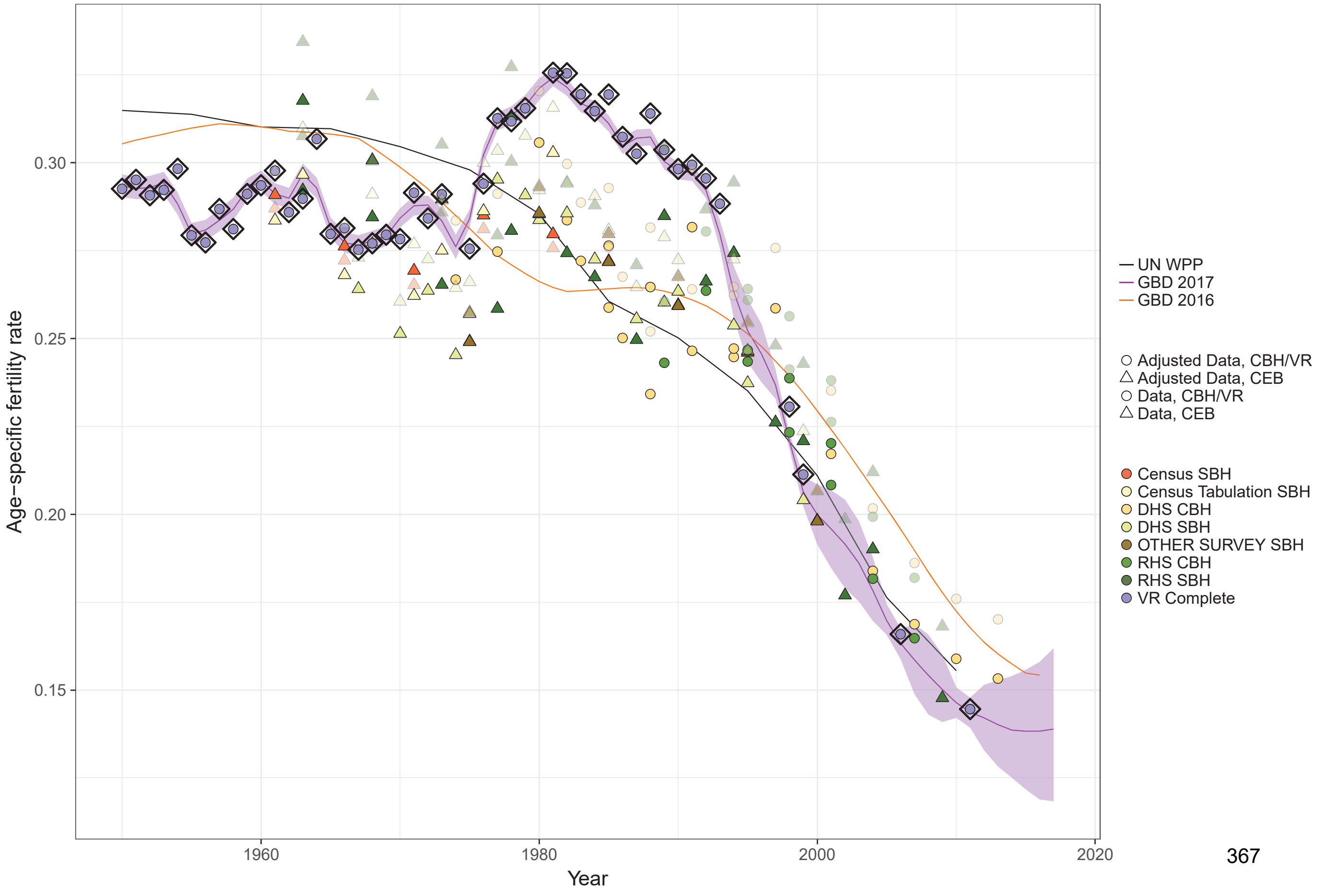

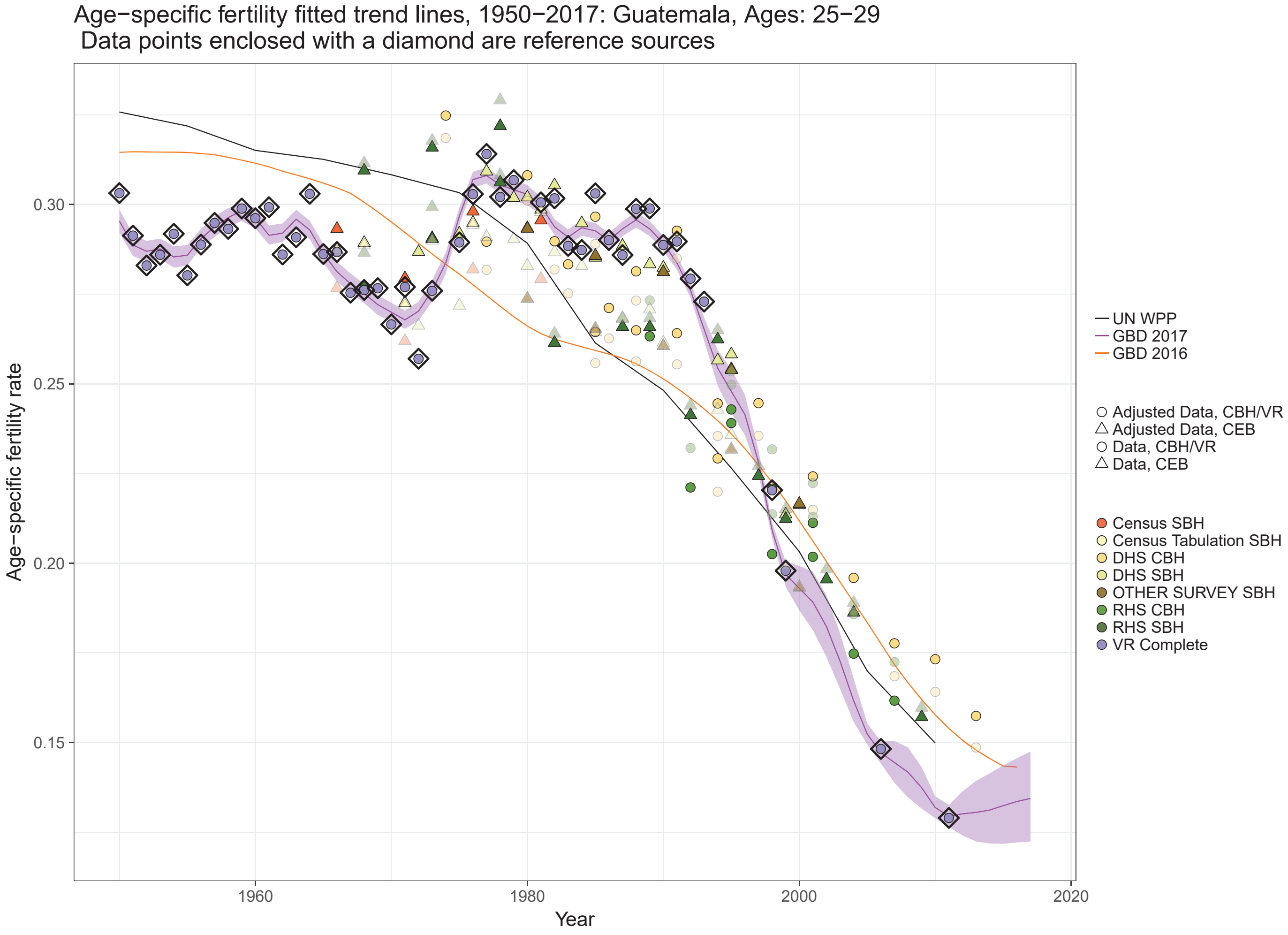

Age−specific fertility fitted trend lines, 1950−2017: Guatemala, Ages: 25−29
Data points enclosed with a diamond are reference sources
Age−specific fertility rate
0.30
0.25
0.20
0.15
1960
1980
2000
2020
Year
UN WPP
GBD 2017
GBD 2016
Adjusted Data, CBH/VR
Adjusted Data, CEB
Data, CBH/VR
Data, CEB
Census SBH
Census Tabulation SBH
DHS CBH
DHS SBH
OTHER SURVEY SBH
RHS CBH
RHS SBH
VR Complete

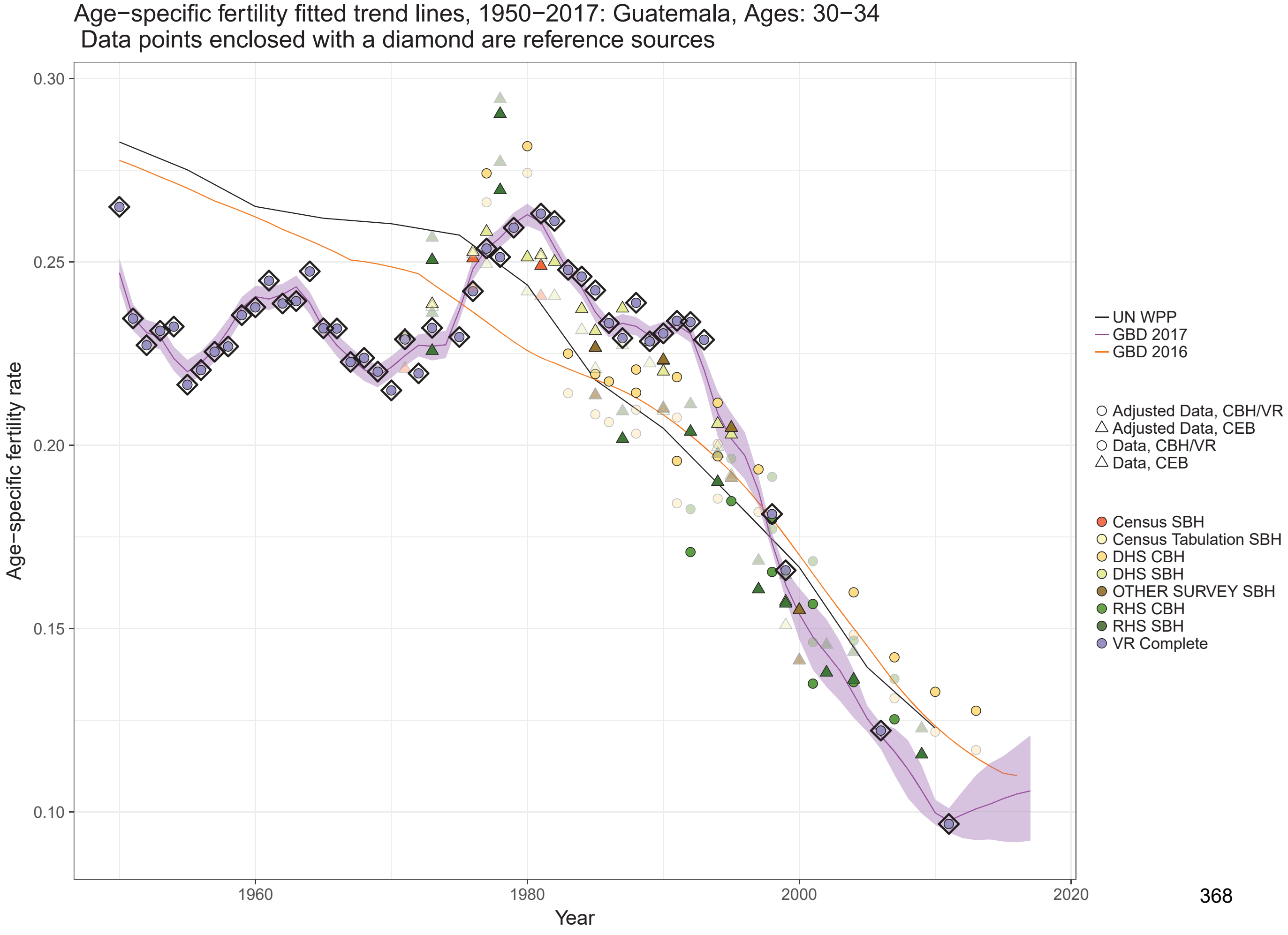

Age−specific fertility fitted trend lines, 1950−2017: Guatemala, Ages: 30−34
Data points enclosed with a diamond are reference sources
Age−specific fertility rate
0.30
0.25
0.20
0.15
0.10
1960
1980
2000
2020
Year
UN WPP
GBD 2017
GBD 2016
Adjusted Data, CBH/VR
Adjusted Data, CEB
Data, CBH/VR
Data, CEB
Census SBH
Census Tabulation SBH
DHS CBH
DHS SBH
OTHER SURVEY SBH
RHS CBH
RHS SBH
VR Complete

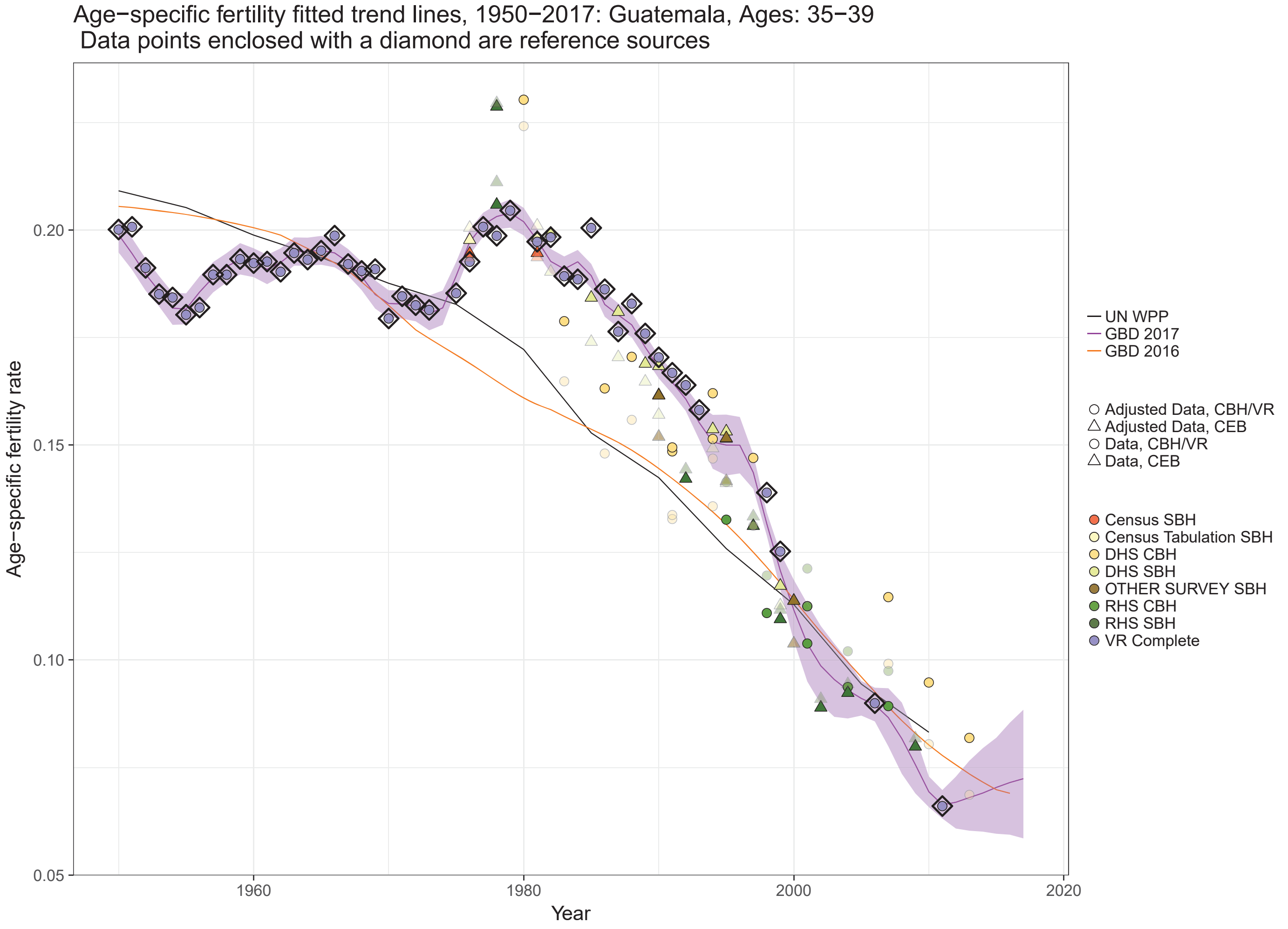

Age−specific fertility fitted trend lines, 1950−2017: Guatemala, Ages: 35−39
Data points enclosed with a diamond are reference sources
Age−specific fertility rate
Year
0.05
0.10
0.15
0.20
1960
1980
2000
2020
UN WPP
GBD 2017
GBD 2016
Adjusted Data, CBH/VR
Adjusted Data, CEB
Data, CBH/VR
Data, CEB
Census SBH
Census Tabulation SBH
DHS CBH
DHS SBH
OTHER SURVEY SBH
RHS CBH
RHS SBH
VR Complete

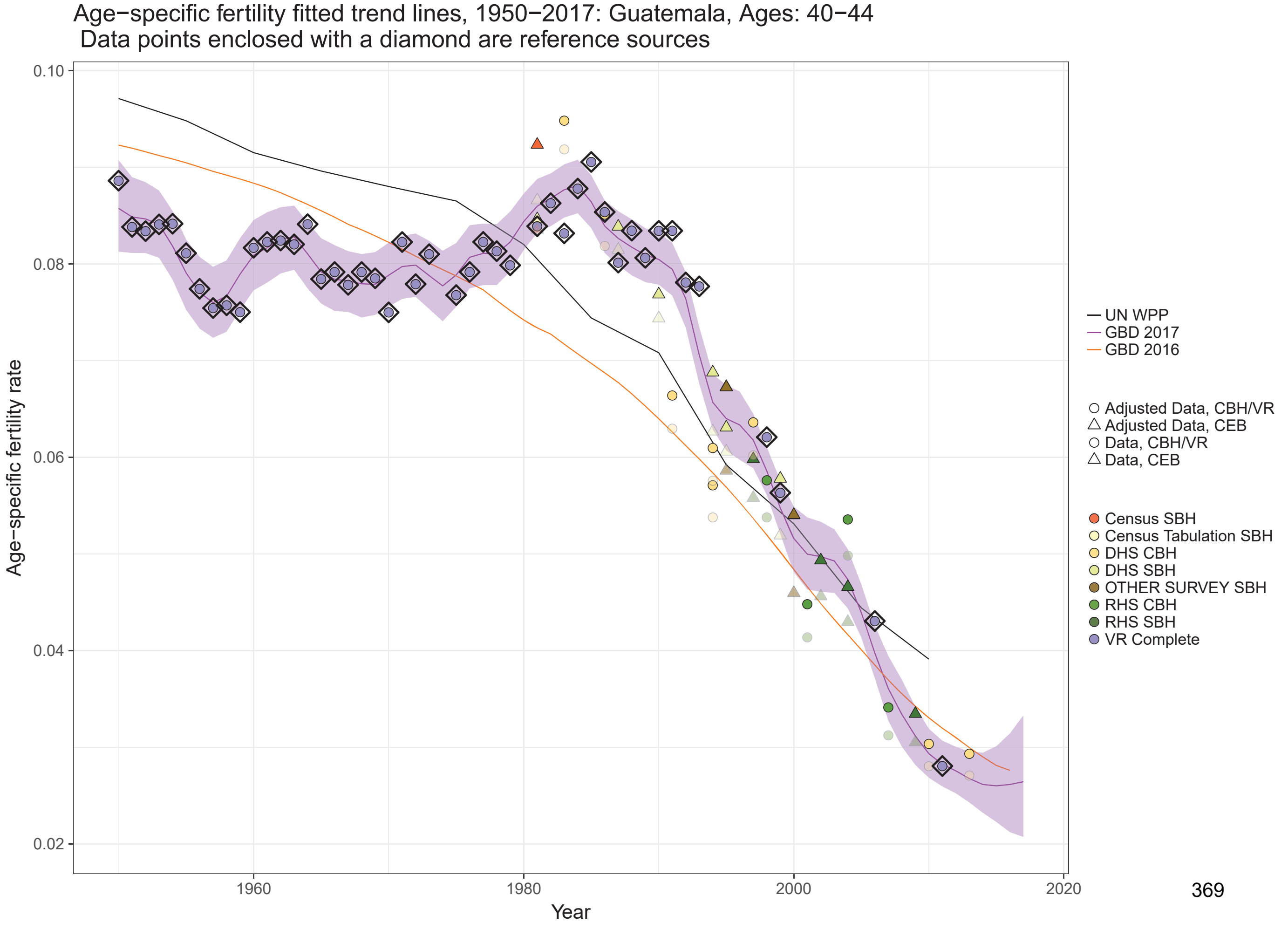

Age−specific fertility fitted trend lines, 1950−2017: Guatemala, Ages: 40−44
Data points enclosed with a diamond are reference sources
Age−specific fertility rate
Year
0.02
0.04
0.06
0.08
0.10
1960
1980
2000
2020
UN WPP
GBD 2017
GBD 2016
Adjusted Data, CBH/VR
Adjusted Data, CEB
Data, CBH/VR
Data, CEB
Census SBH
Census Tabulation SBH
DHS CBH
DHS SBH
OTHER SURVEY SBH
RHS CBH
RHS SBH
VR Complete

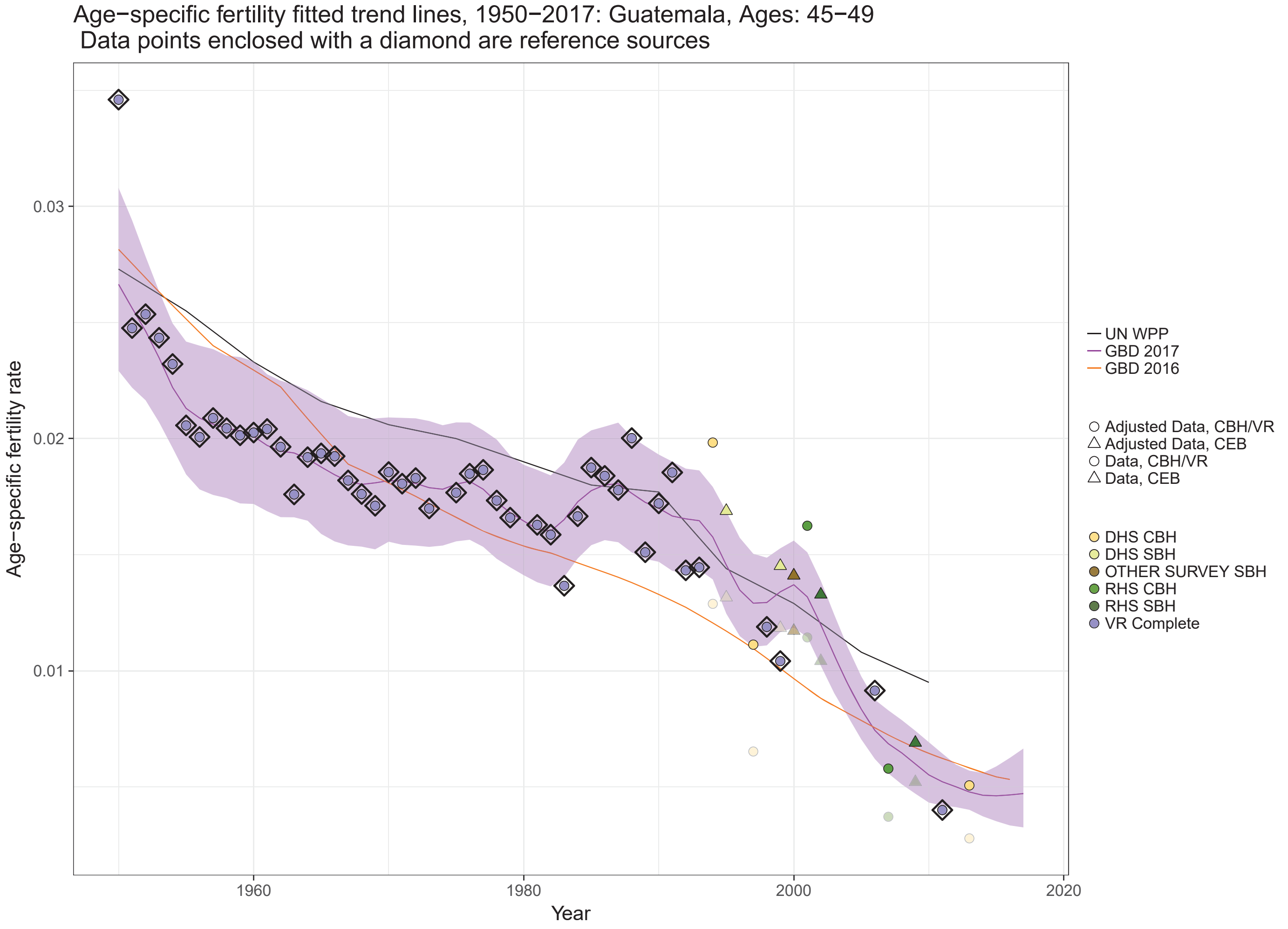
Age−specific fertility fitted trend lines, 1950−2017: Guatemala, Ages: 45−49
Data points enclosed with a diamond are reference sources
Age-specific fertility rate
0.03
0.02
0.01
1960
1980
2000
2020
Year
UN WPP
GBD 2017
GBD 2016
Adjusted Data, CBH/VR
Adjusted Data, CEB
Data, CBH/VR
Data, CEB
DHS CBH
DHS SBH
OTHER SURVEY SBH
RHS CBH
RHS SBH
VR Complete

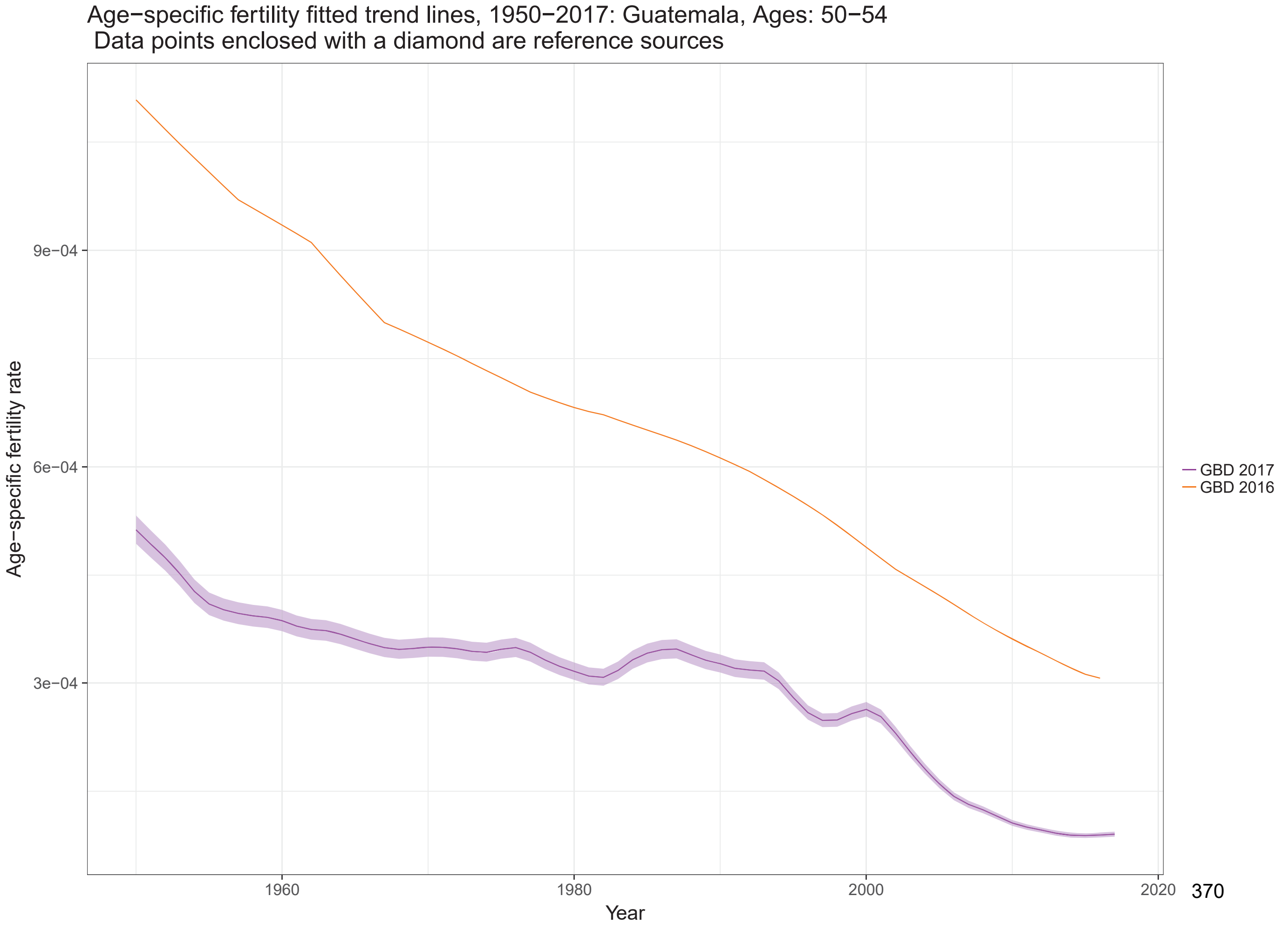
Age−specific fertility fitted trend lines, 1950−2017: Guatemala, Ages: 50−54
Data points enclosed with a diamond are reference sources
Age-specific fertility rate
9e−04
6e−04
3e−04
1960
1980
2000
2020
Year
GBD 2017
GBD 2016

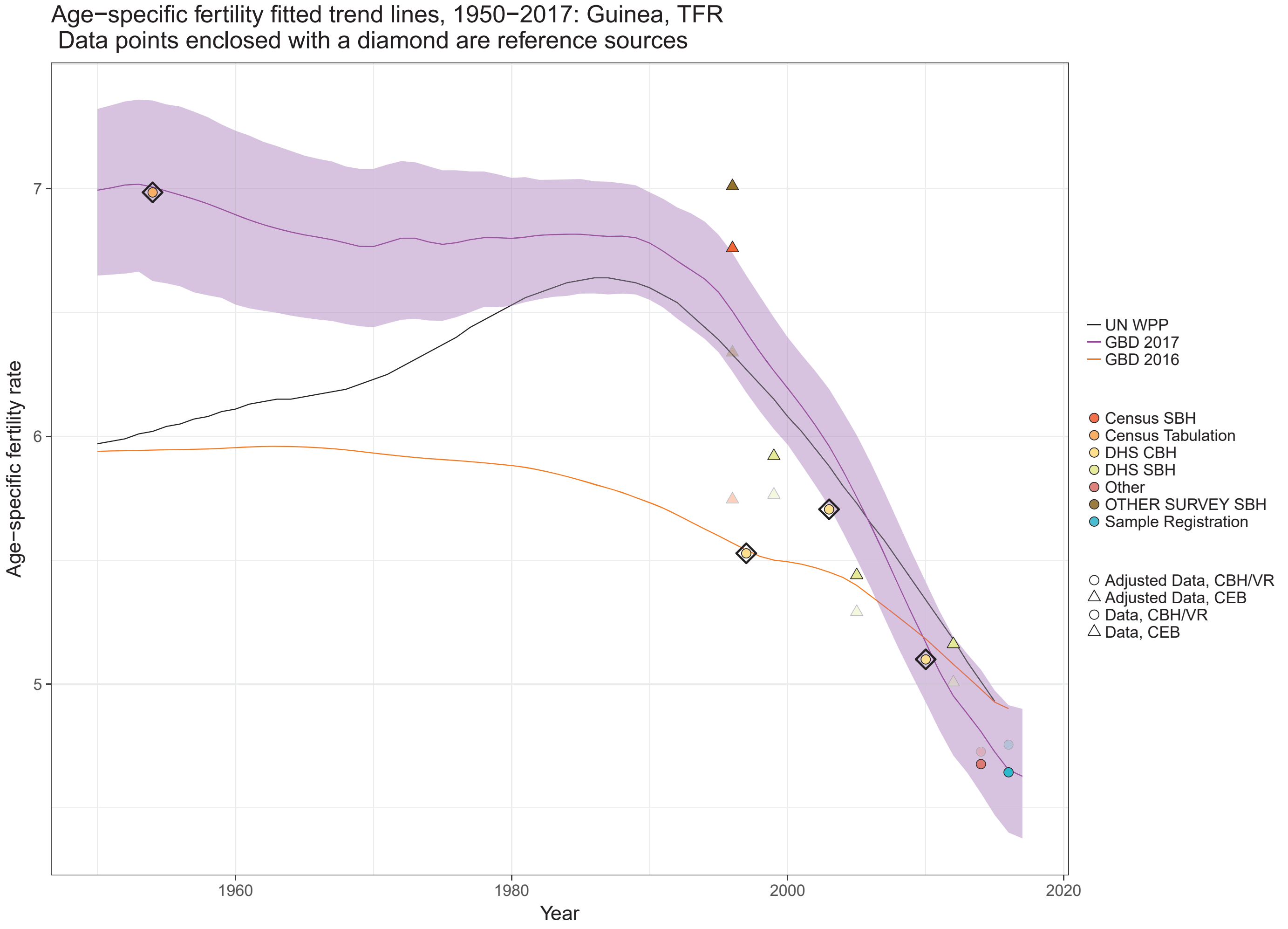

Age−specific fertility fitted trend lines, 1950−2017: Guinea, TFR
Data points enclosed with a diamond are reference sources
Age−specific fertility rate
Year
7
6
5
1960
1980
2000
2020
UN WPP
GBD 2017
GBD 2016
Census SBH
Census Tabulation
DHS CBH
DHS SBH
Other
OTHER SURVEY SBH
Sample Registration
Adjusted Data, CBH/VR
Adjusted Data, CEB
Data, CBH/VR
Data, CEB

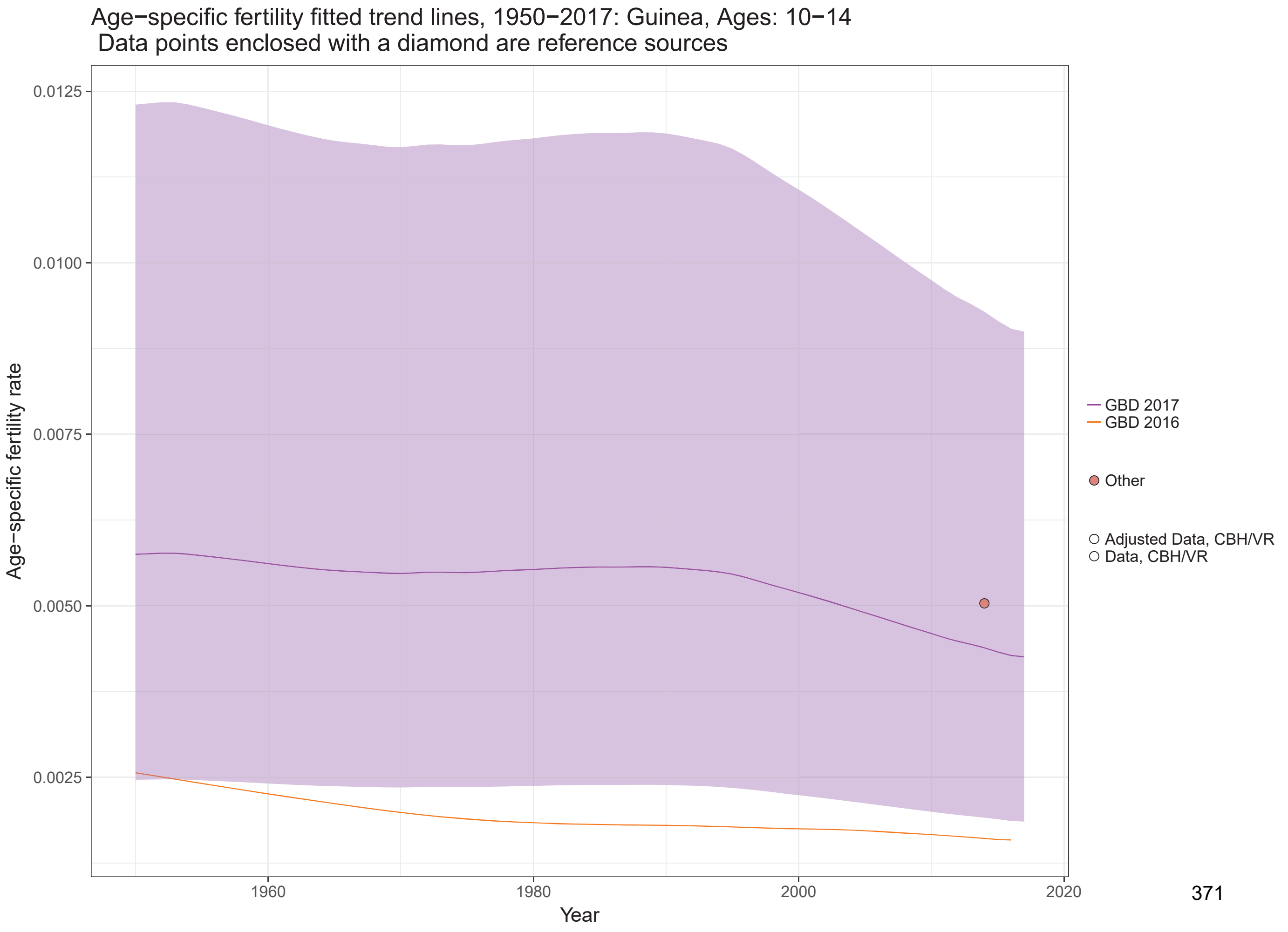

Age−specific fertility fitted trend lines, 1950−2017: Guinea, Ages: 10−14
Data points enclosed with a diamond are reference sources
Age−specific fertility rate
Year
0.0125
0.0100
0.0075
0.0050
0.0025
1960
1980
2000
2020
GBD 2017
GBD 2016
Other
Adjusted Data, CBH/VR
Data, CBH/VR

Age−specific fertility fitted trend lines, 1950−2017: Guinea, Ages: 15−19
Data points enclosed with a diamond are reference sources

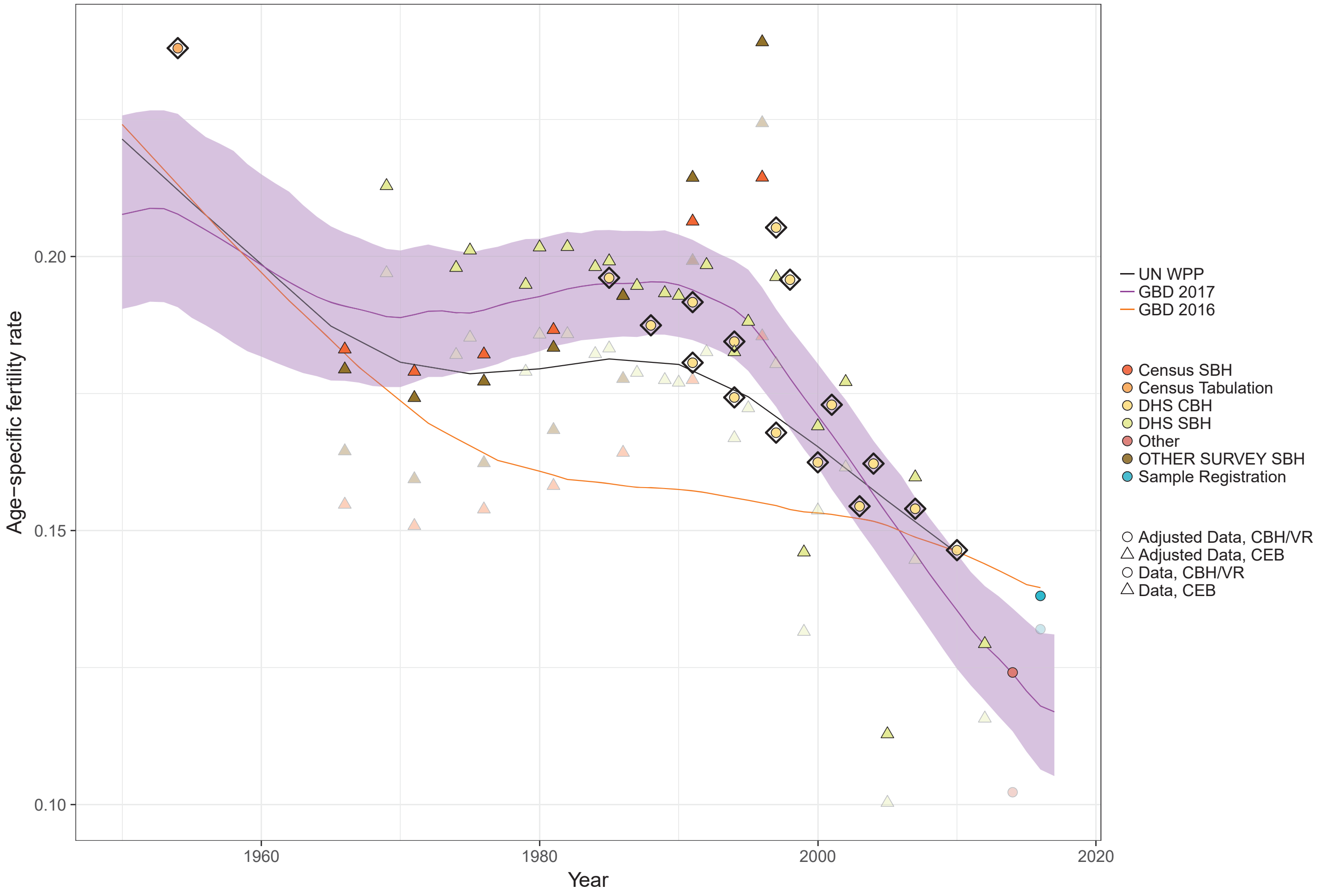

Age−specific fertility fitted trend lines, 1950−2017: Guinea, Ages: 20−24
Data points enclosed with a diamond are reference sources

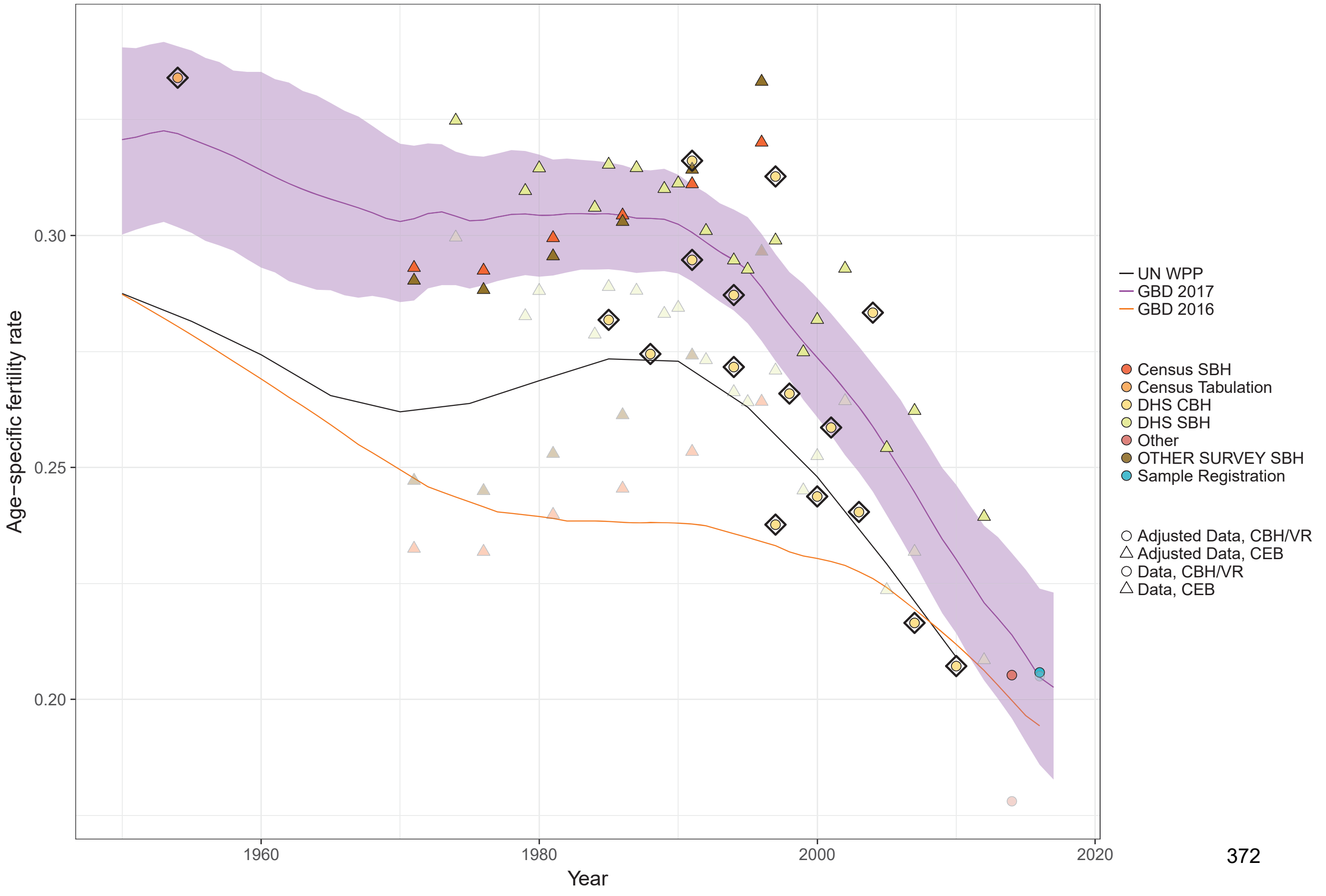

Age−specific fertility fitted trend lines, 1950−2017: Guinea, Ages: 25−29
Data points enclosed with a diamond are reference sources

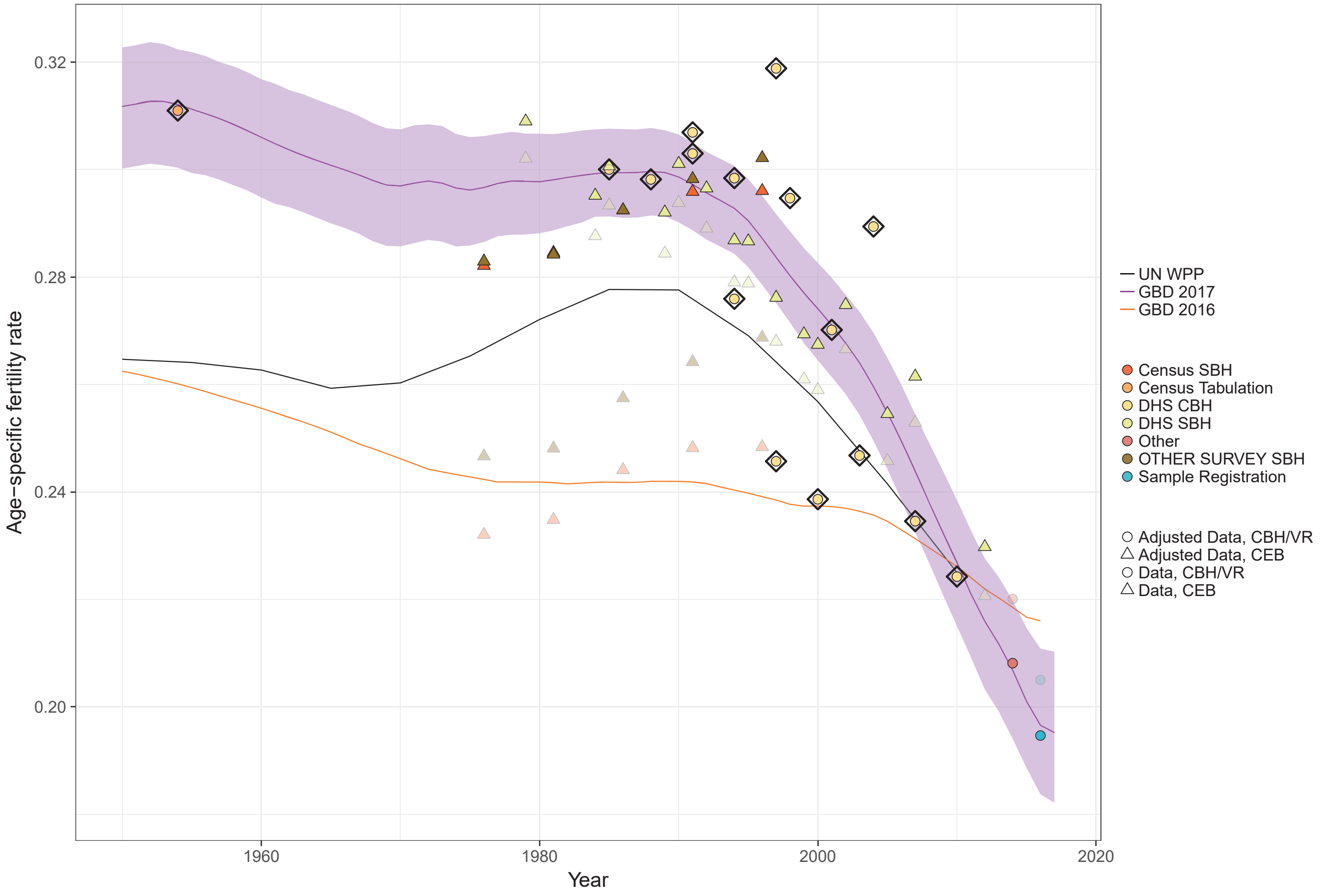

Age−specific fertility fitted trend lines, 1950−2017: Guinea, Ages: 30−34
Data points enclosed with a diamond are reference sources

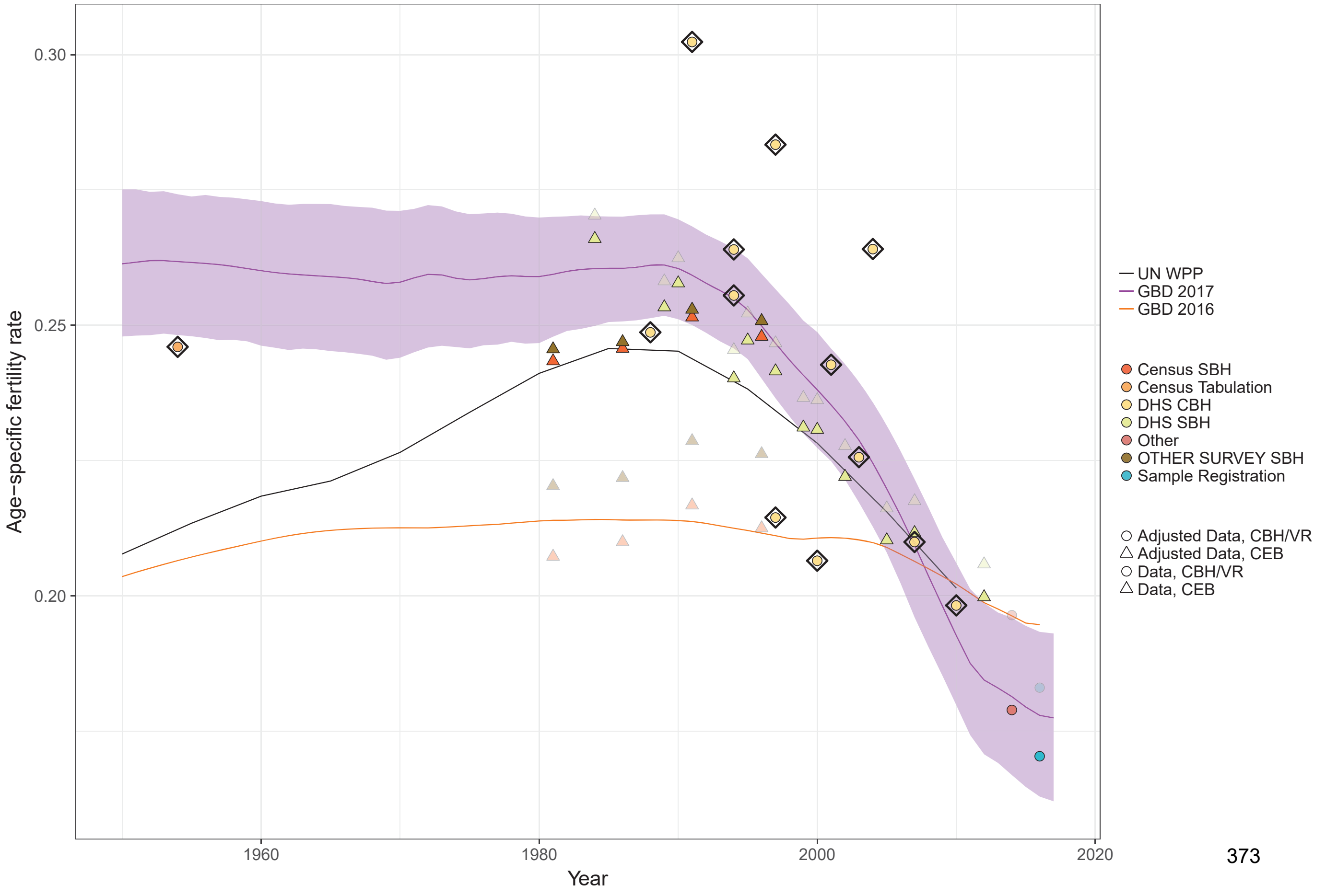

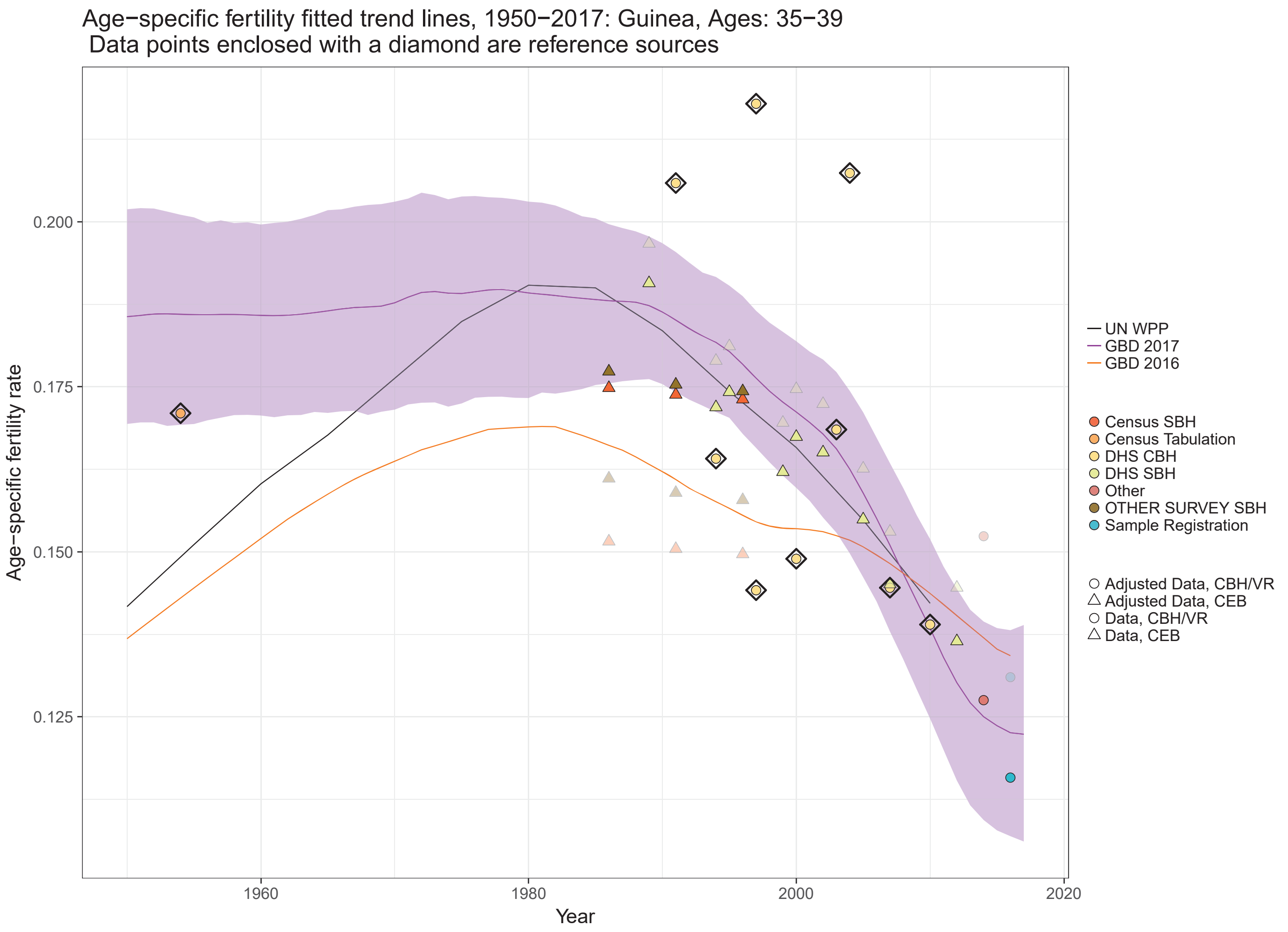

Age−specific fertility fitted trend lines, 1950−2017: Guinea, Ages: 35−39
Data points enclosed with a diamond are reference sources
Age−specific fertility rate
0.125
0.150
0.175
0.200
1960
1980
2000
2020
Year
UN WPP
GBD 2017
GBD 2016
Census SBH
Census Tabulation
DHS CBH
DHS SBH
Other
OTHER SURVEY SBH
Sample Registration
Adjusted Data, CBH/VR
Adjusted Data, CEB
Data, CBH/VR
Data, CEB

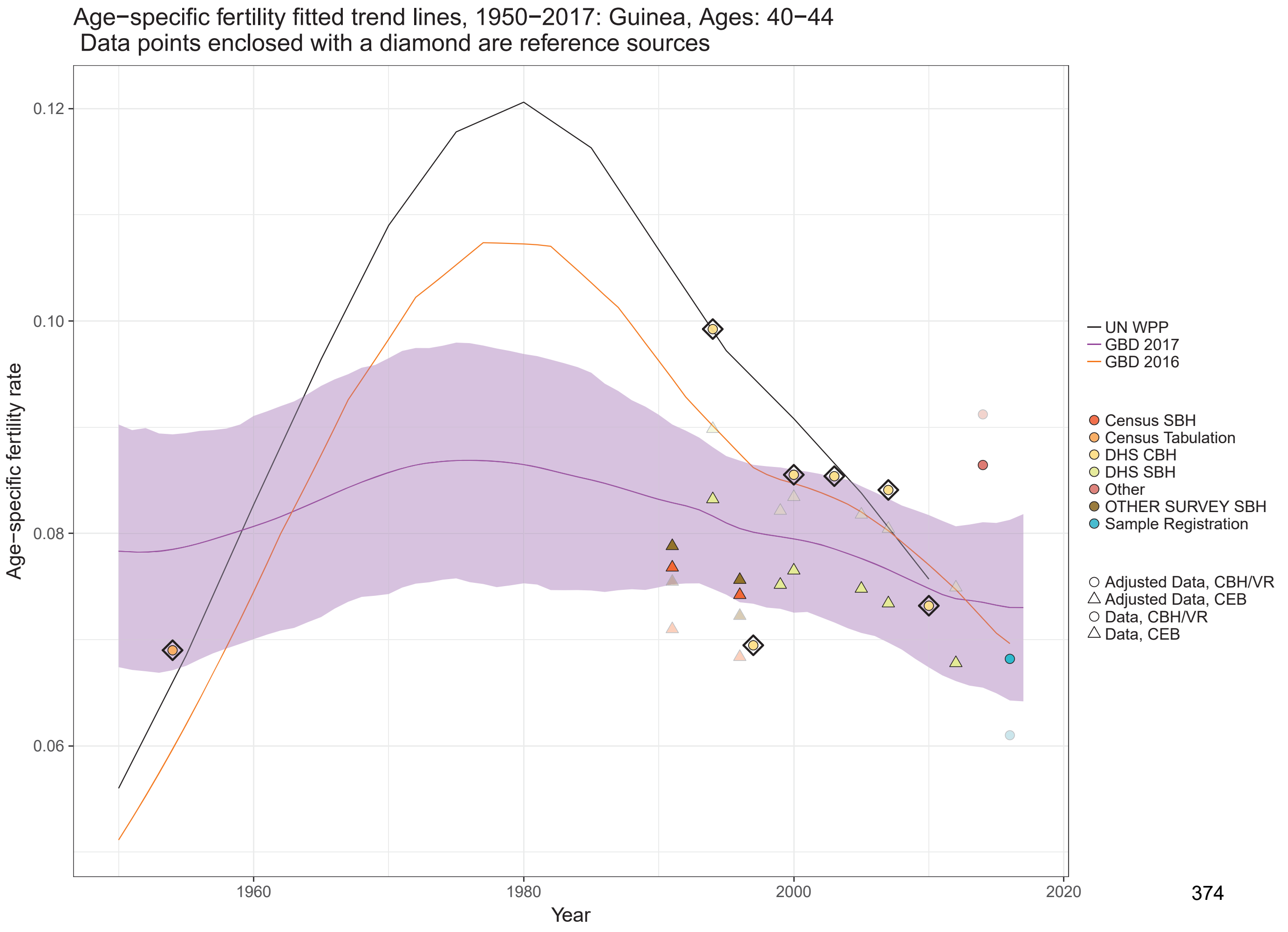

Age−specific fertility fitted trend lines, 1950−2017: Guinea, Ages: 40−44
Data points enclosed with a diamond are reference sources
Age−specific fertility rate
0.06
0.08
0.10
0.12
1960
1980
2000
2020
Year
UN WPP
GBD 2017
GBD 2016
Census SBH
Census Tabulation
DHS CBH
DHS SBH
Other
OTHER SURVEY SBH
Sample Registration
Adjusted Data, CBH/VR
Adjusted Data, CEB
Data, CBH/VR
Data, CEB

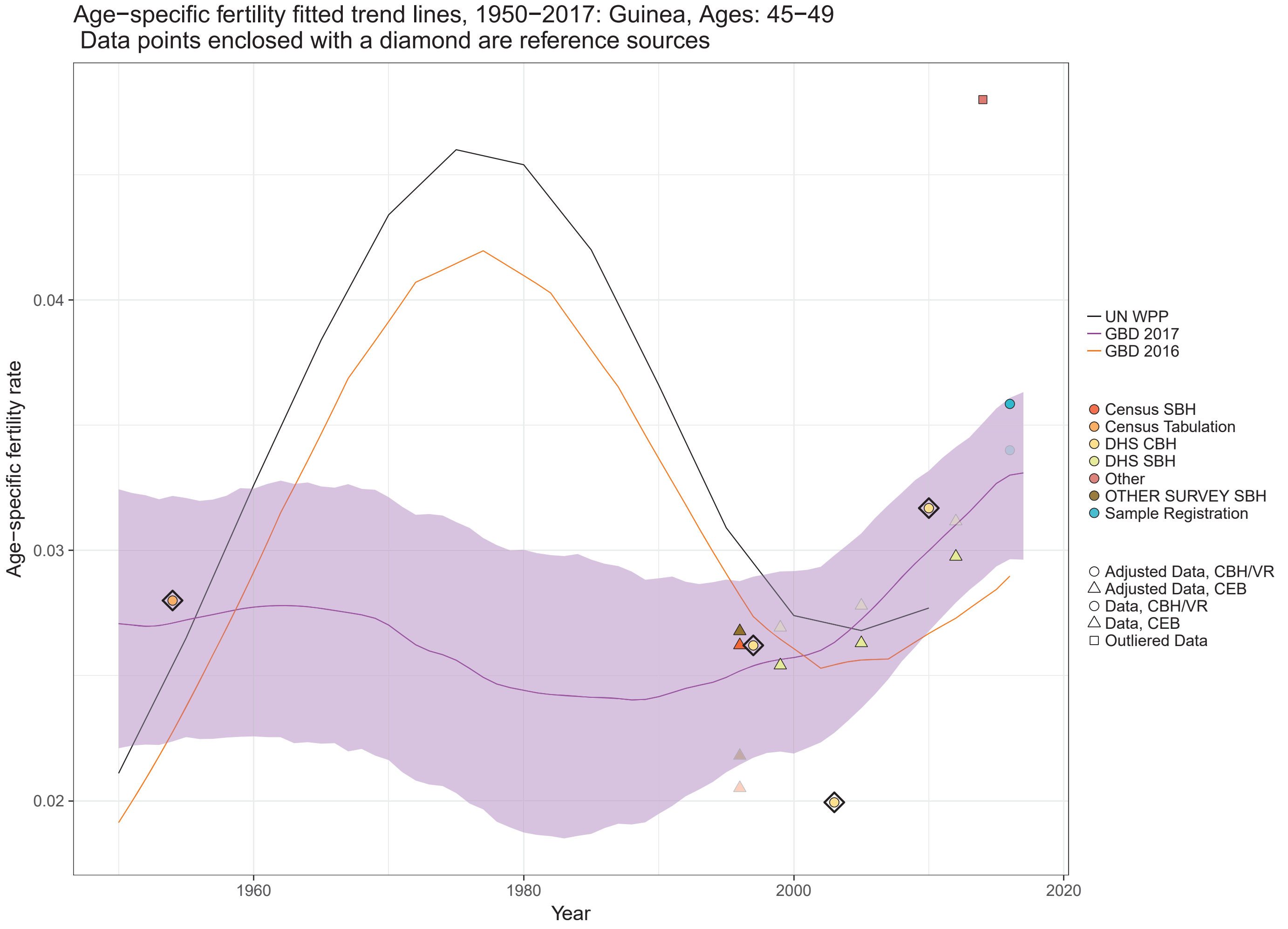
Age−specific fertility fitted trend lines, 1950−2017: Guinea, Ages: 45−49
Data points enclosed with a diamond are reference sources
Age−specific fertility rate
0.04
0.03
0.02
1960
1980
2000
2020
Year
UN WPP
GBD 2017
GBD 2016
Census SBH
Census Tabulation
DHS CBH
DHS SBH
Other
OTHER SURVEY SBH
Sample Registration
Adjusted Data, CBH/VR
Adjusted Data, CEB
Data, CBH/VR
Data, CEB
Outliered Data

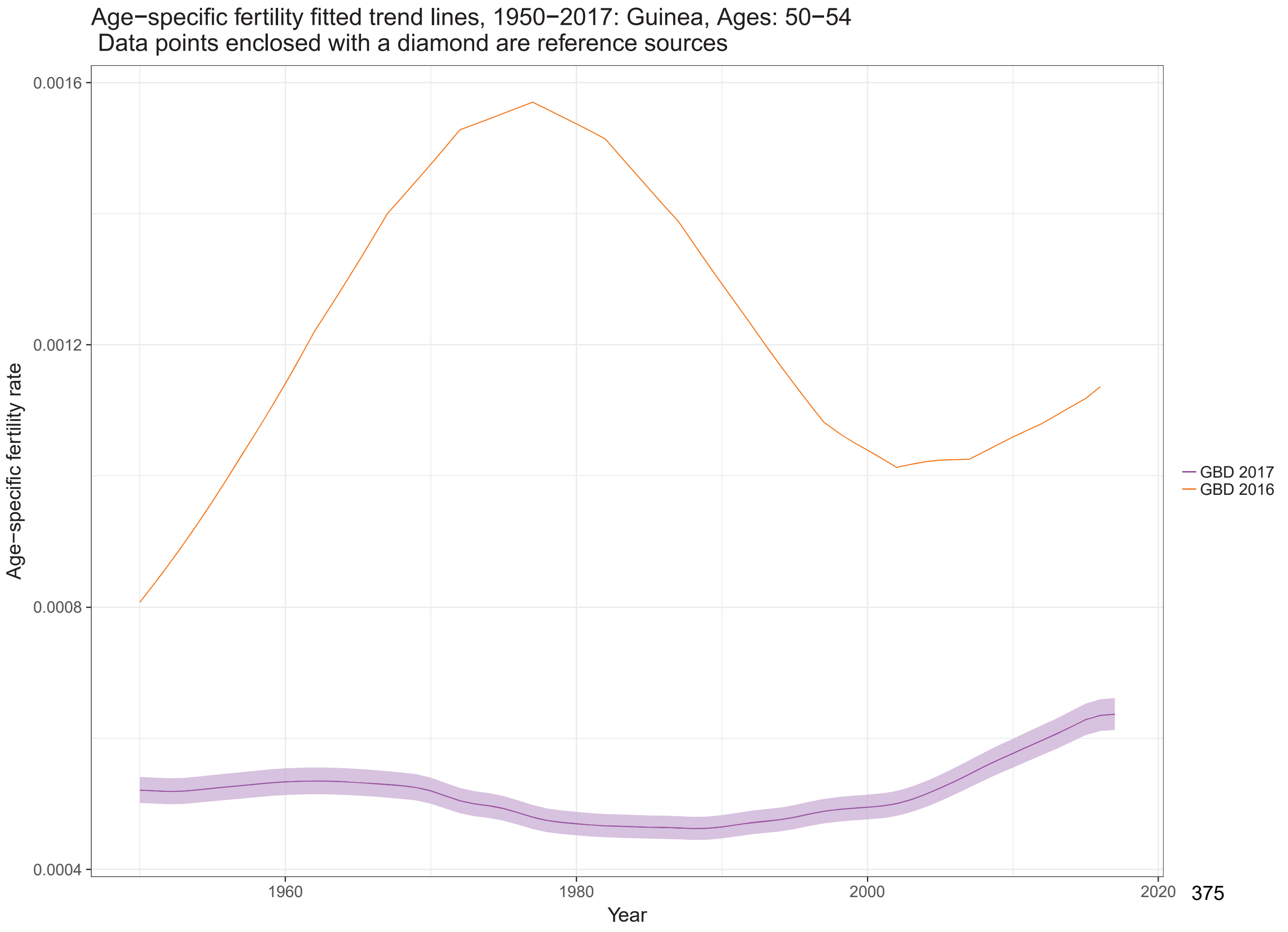
Age−specific fertility fitted trend lines, 1950−2017: Guinea, Ages: 50−54
Data points enclosed with a diamond are reference sources
Age−specific fertility rate
0.0016
0.0012
0.0008
0.0004
1960
1980
2000
2020
Year
GBD 2017
GBD 2016

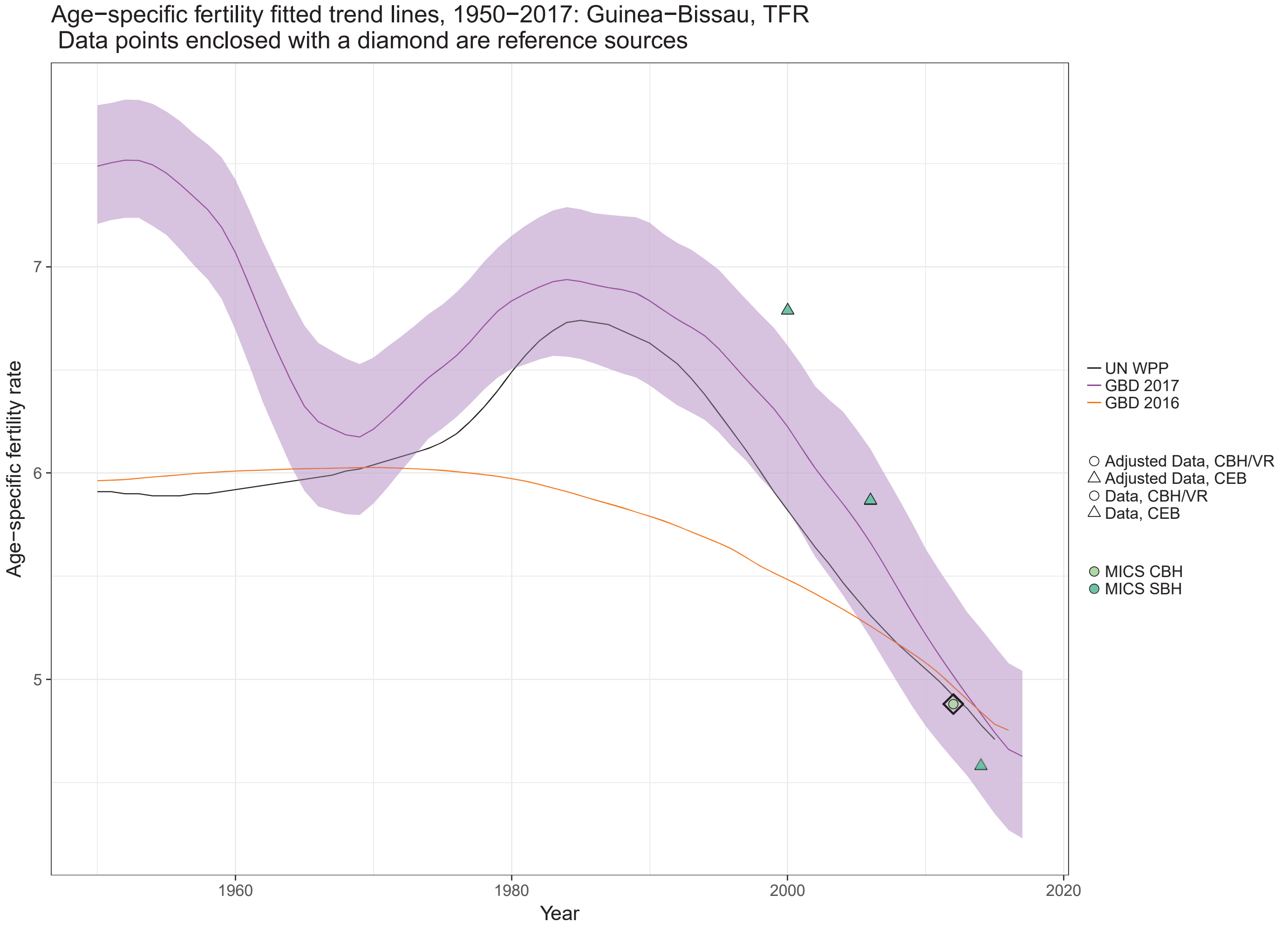
Age−specific fertility fitted trend lines, 1950−2017: Guinea−Bissau, TFR
Data points enclosed with a diamond are reference sources
Age−specific fertility rate
Year
7
6
5
1960
1980
2000
2020
UN WPP
GBD 2017
GBD 2016
Adjusted Data, CBH/VR
Adjusted Data, CEB
Data, CBH/VR
Data, CEB
MICS CBH
MICS SBH

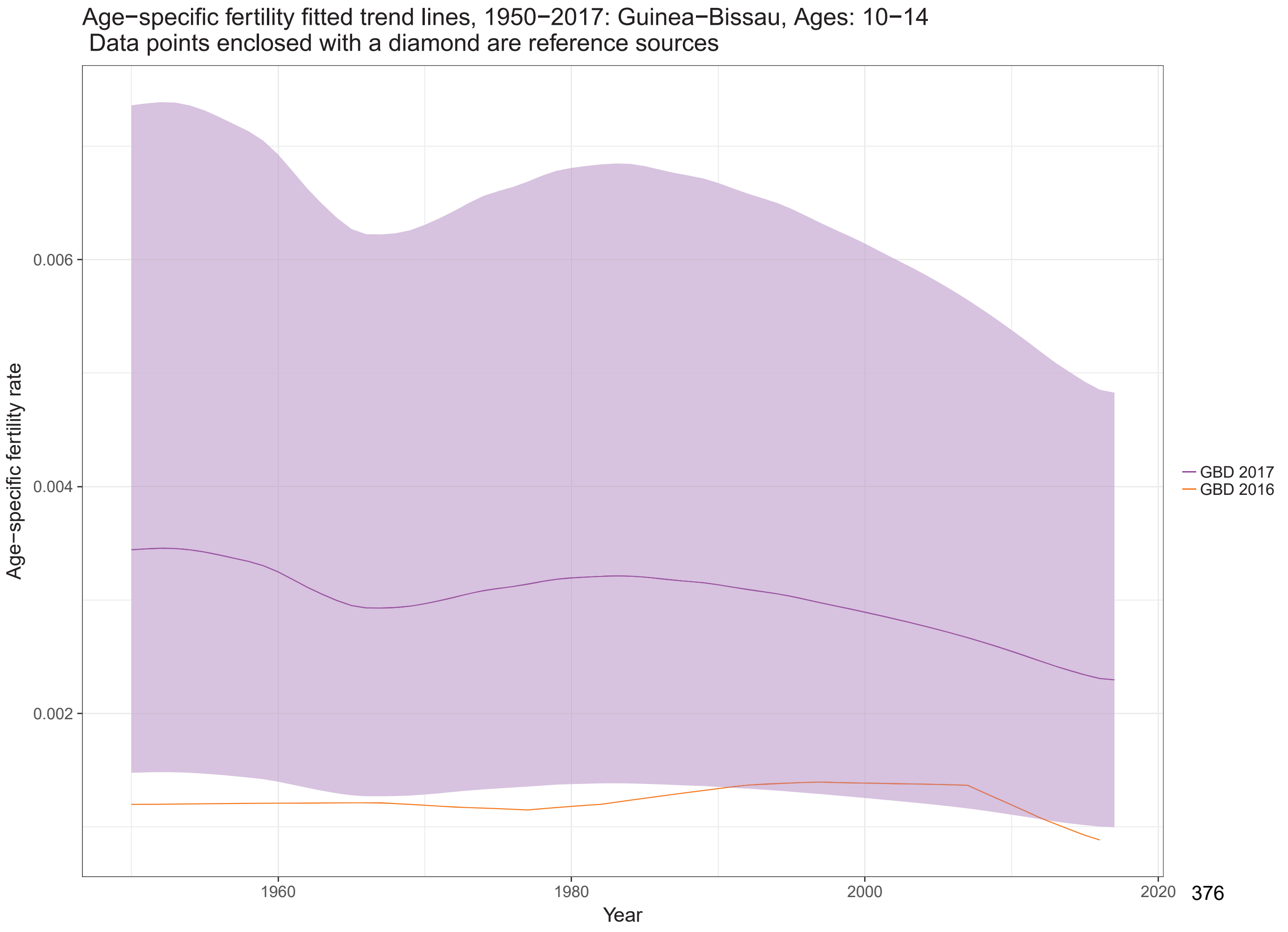
Age−specific fertility fitted trend lines, 1950−2017: Guinea−Bissau, Ages: 10−14
Data points enclosed with a diamond are reference sources
Age−specific fertility rate
Year
0.006
0.004
0.002
1960
1980
2000
2020
GBD 2017
GBD 2016

Age−specific fertility fitted trend lines, 1950−2017: Guinea−Bissau, Ages: 15−19
Data points enclosed with a diamond are reference sources

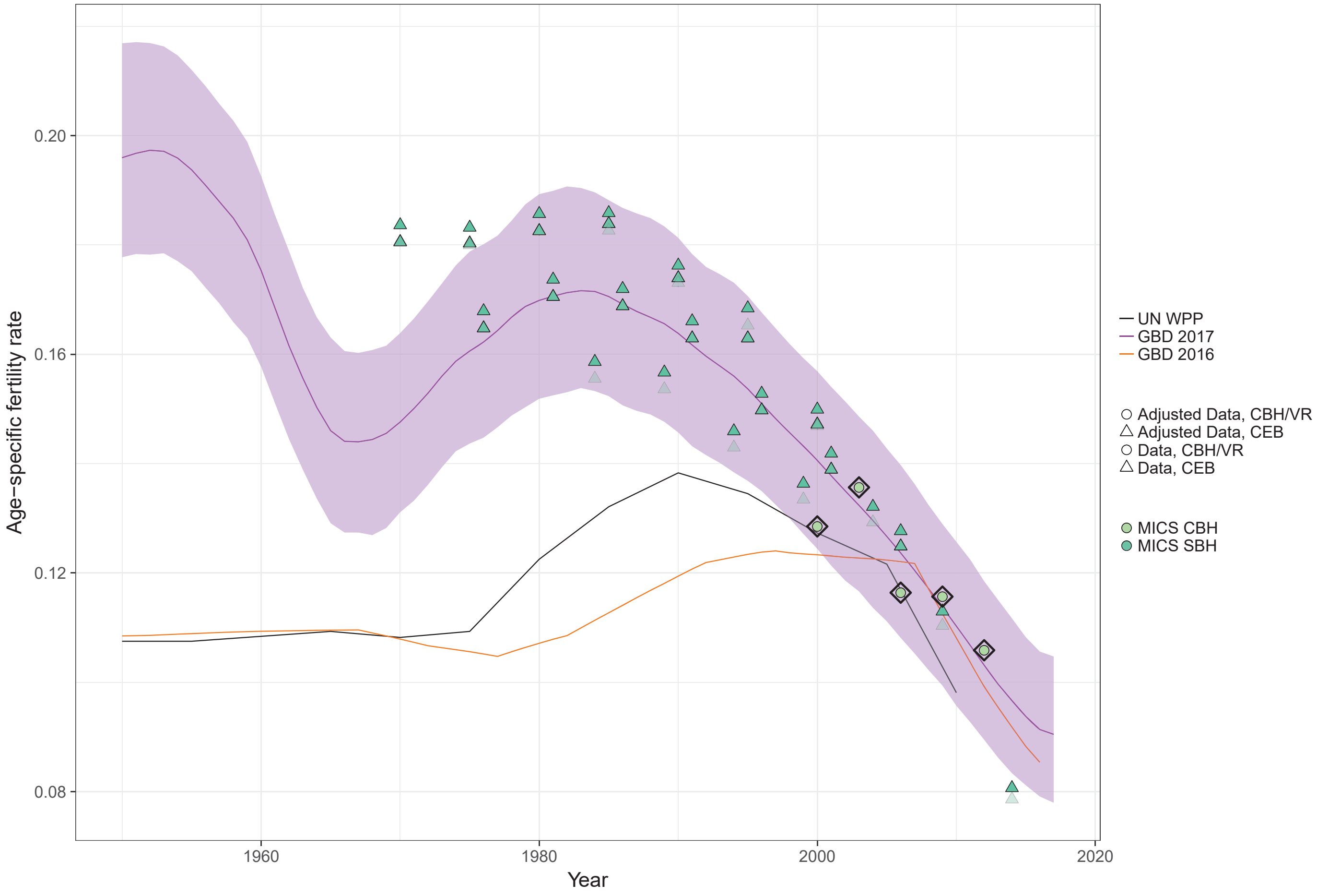

Age−specific fertility fitted trend lines, 1950−2017: Guinea−Bissau, Ages: 20−24
Data points enclosed with a diamond are reference sources

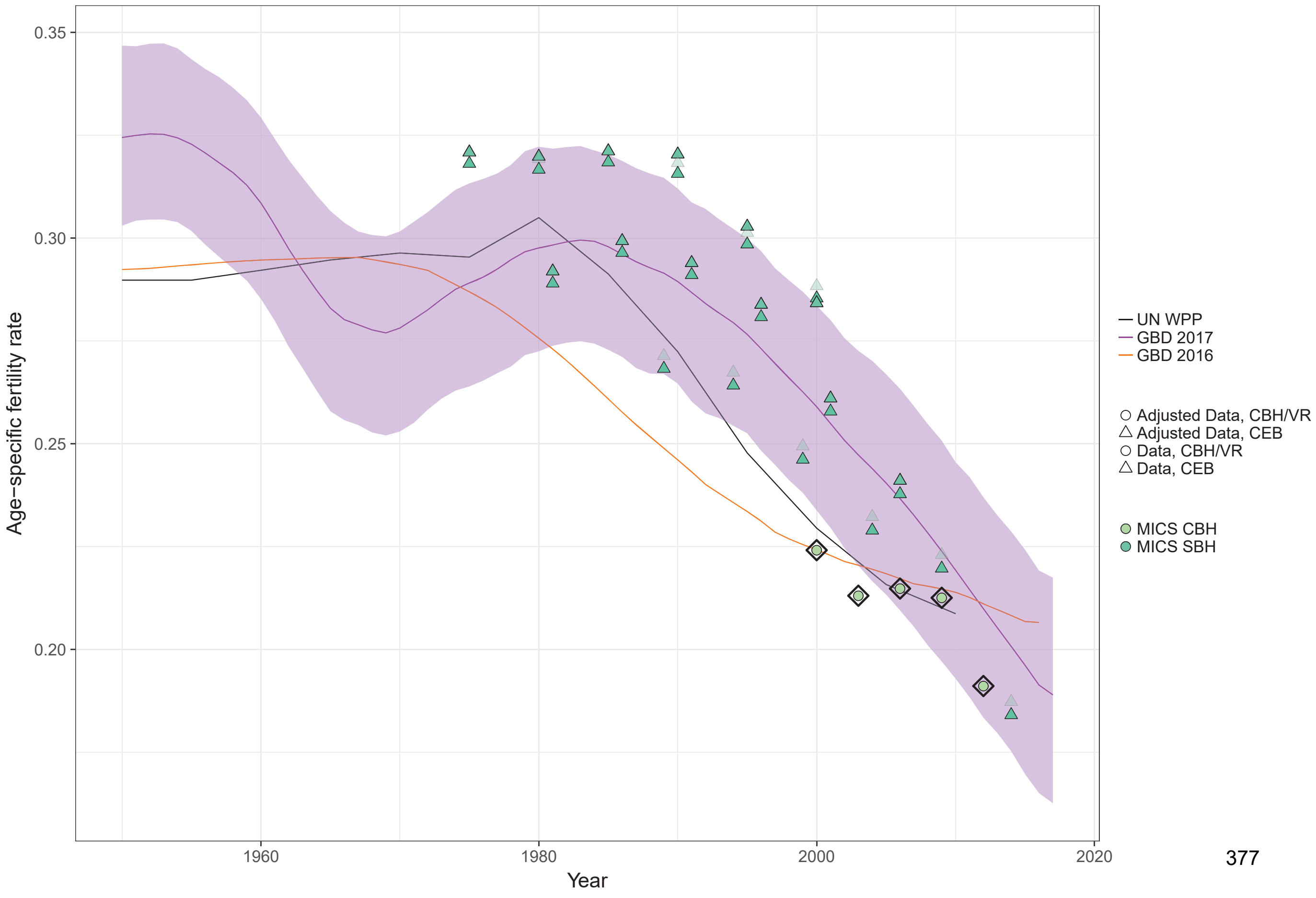

Age−specific fertility fitted trend lines, 1950−2017: Guinea−Bissau, Ages: 25−29
Data points enclosed with a diamond are reference sources

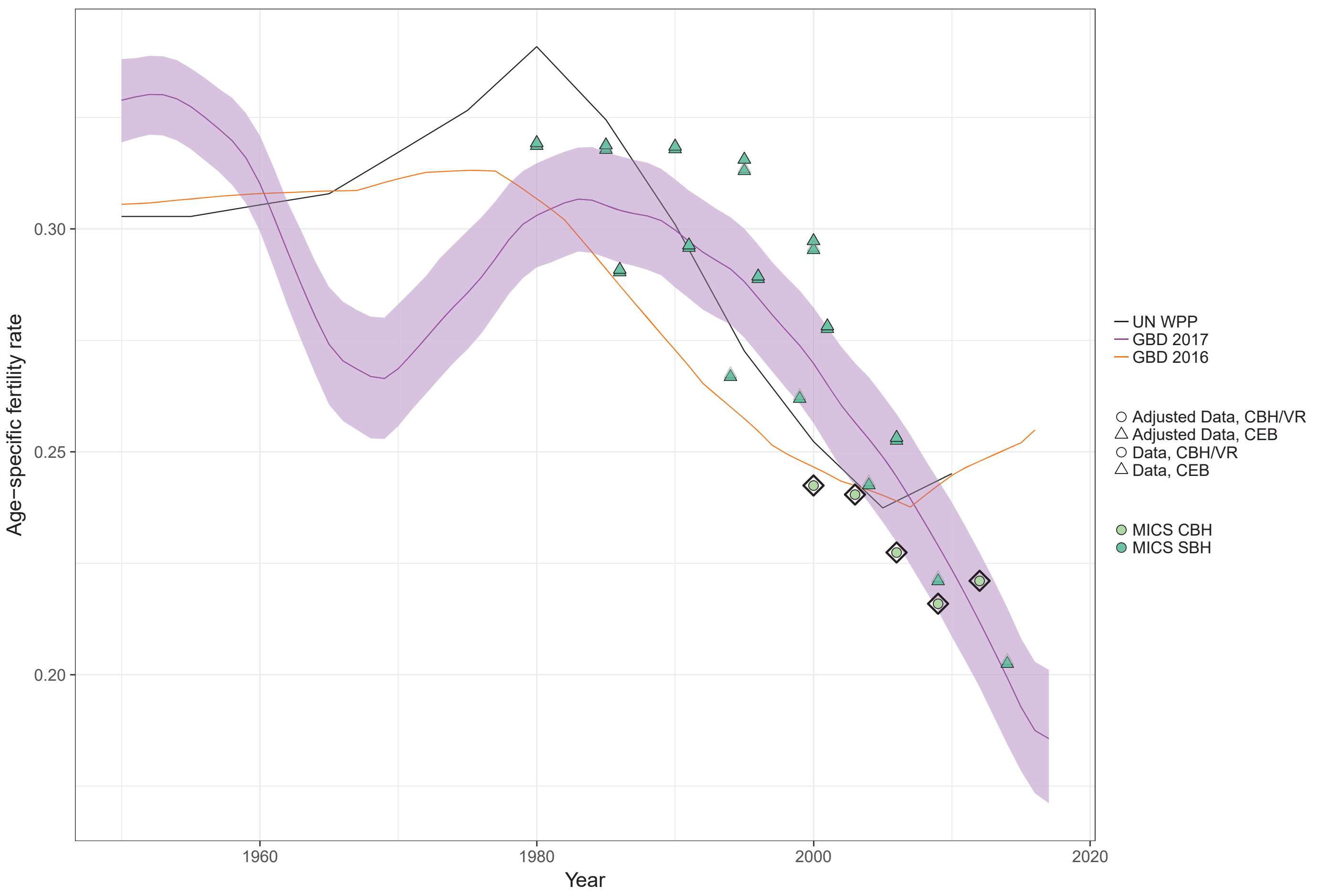

Age−specific fertility fitted trend lines, 1950−2017: Guinea−Bissau, Ages: 30−34
Data points enclosed with a diamond are reference sources

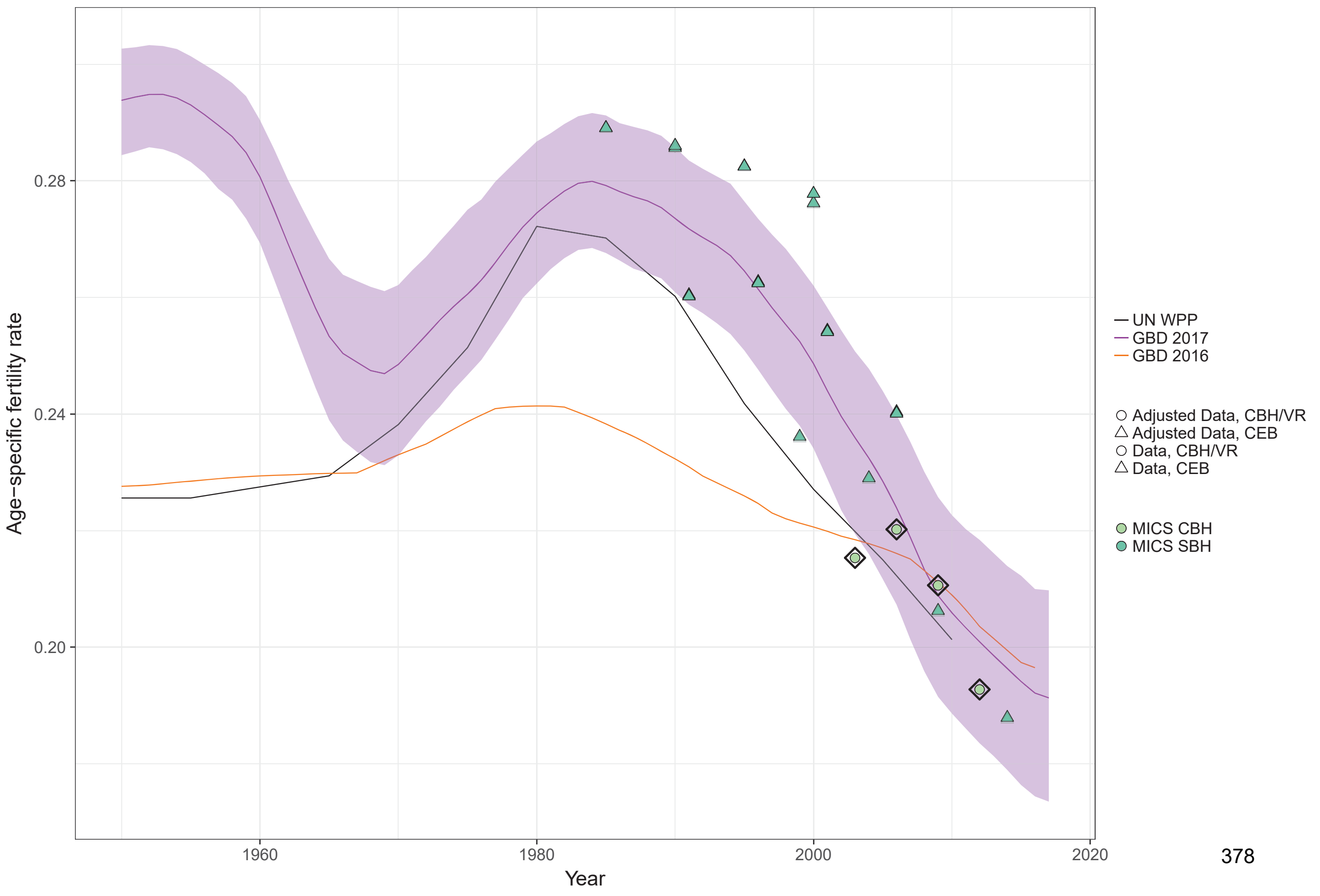

Age−specific fertility fitted trend lines, 1950−2017: Guinea−Bissau, Ages: 35−39
Data points enclosed with a diamond are reference sources

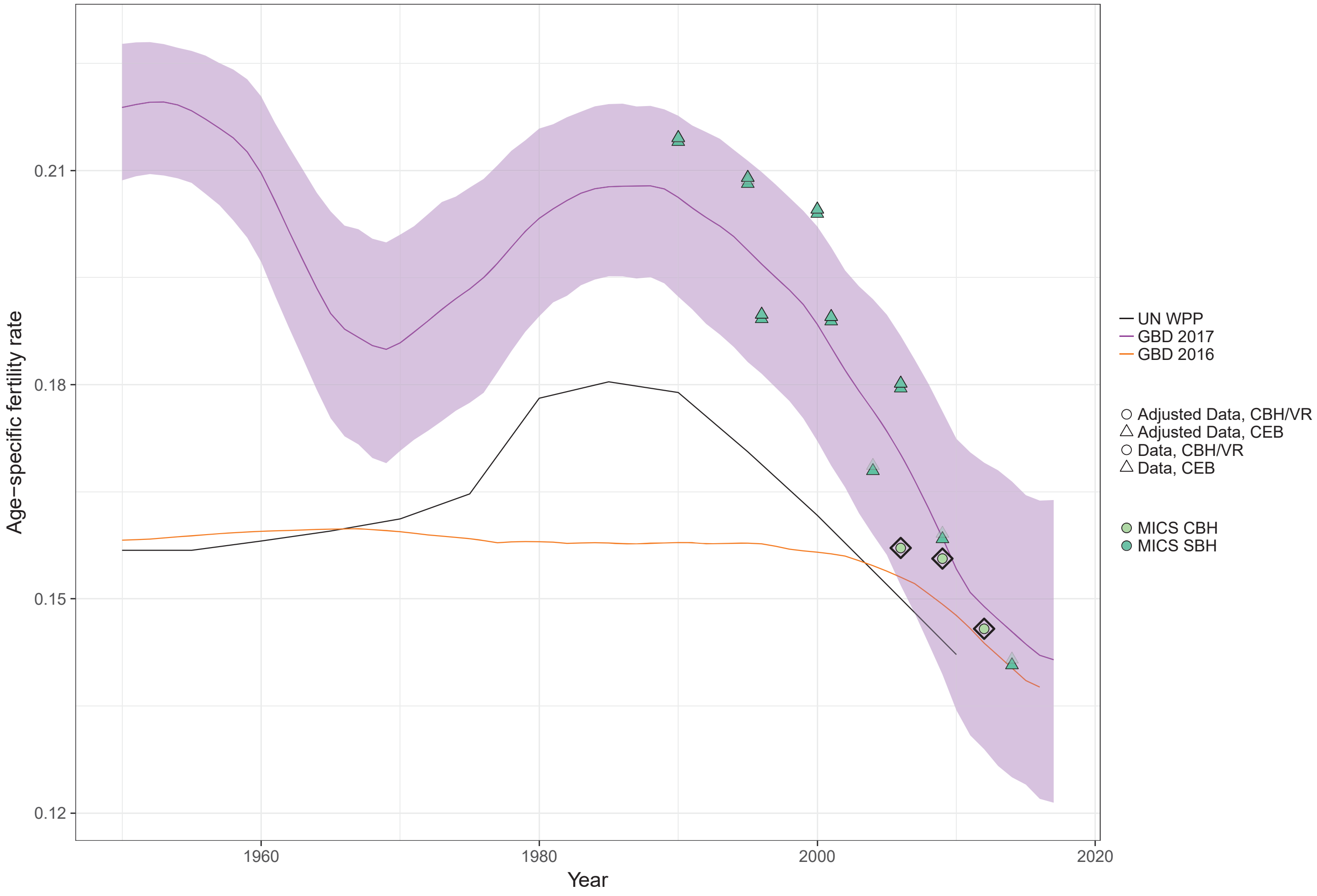

Age−specific fertility fitted trend lines, 1950−2017: Guinea−Bissau, Ages: 40−44
Data points enclosed with a diamond are reference sources

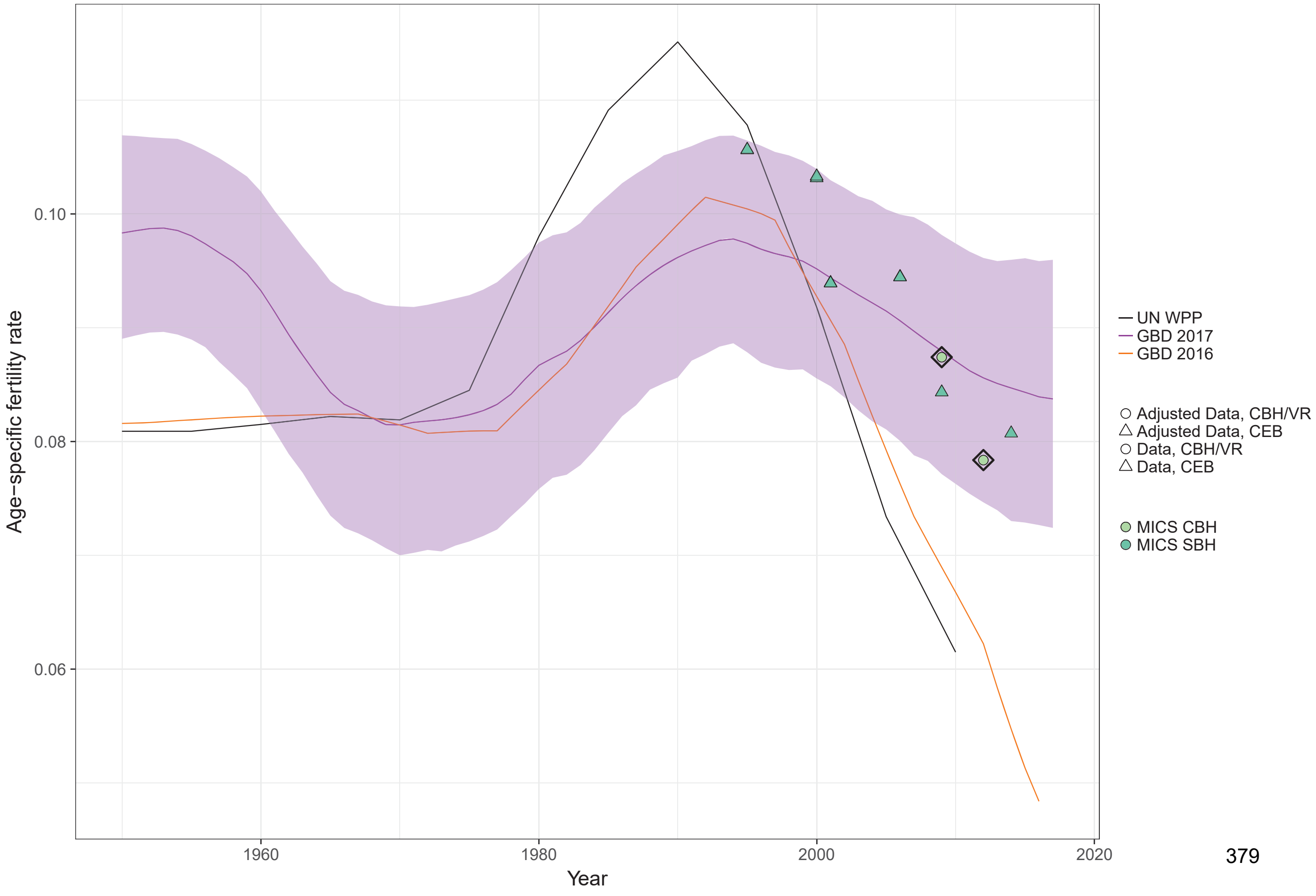

Age−specific fertility fitted trend lines, 1950−2017: Guinea−Bissau, Ages: 45−49
Data points enclosed with a diamond are reference sources

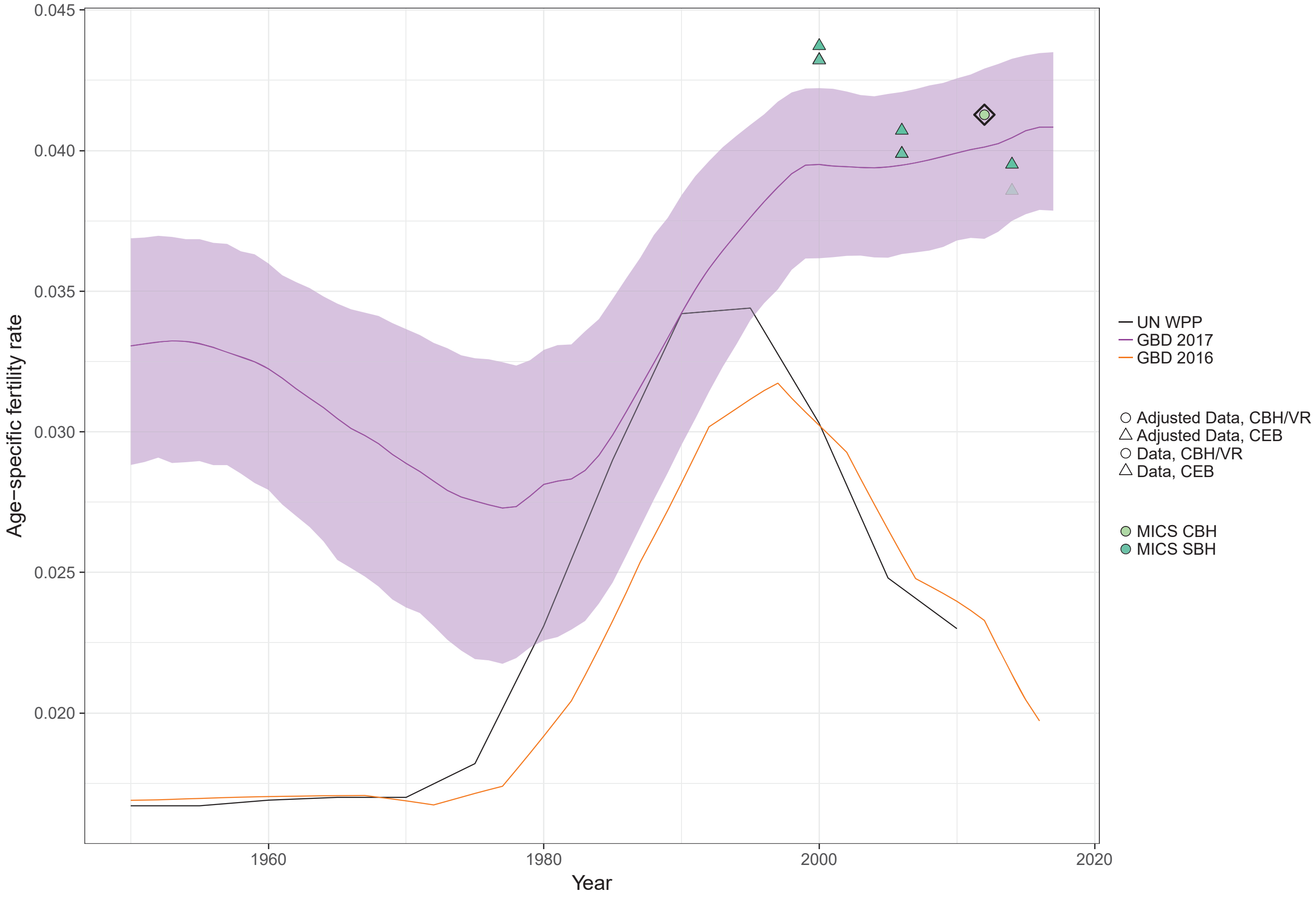

Age−specific fertility fitted trend lines, 1950−2017: Guinea−Bissau, Ages: 50−54
Data points enclosed with a diamond are reference sources

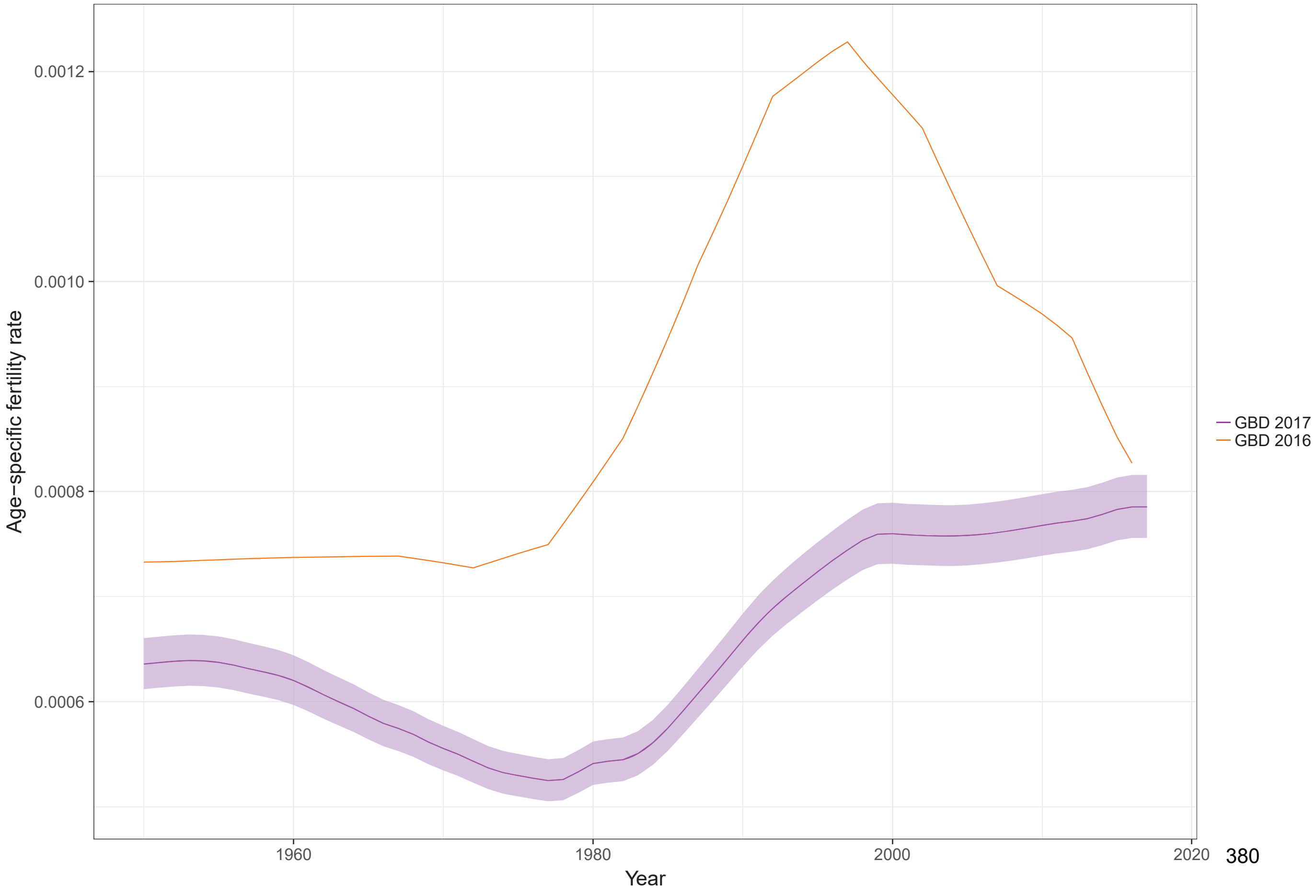

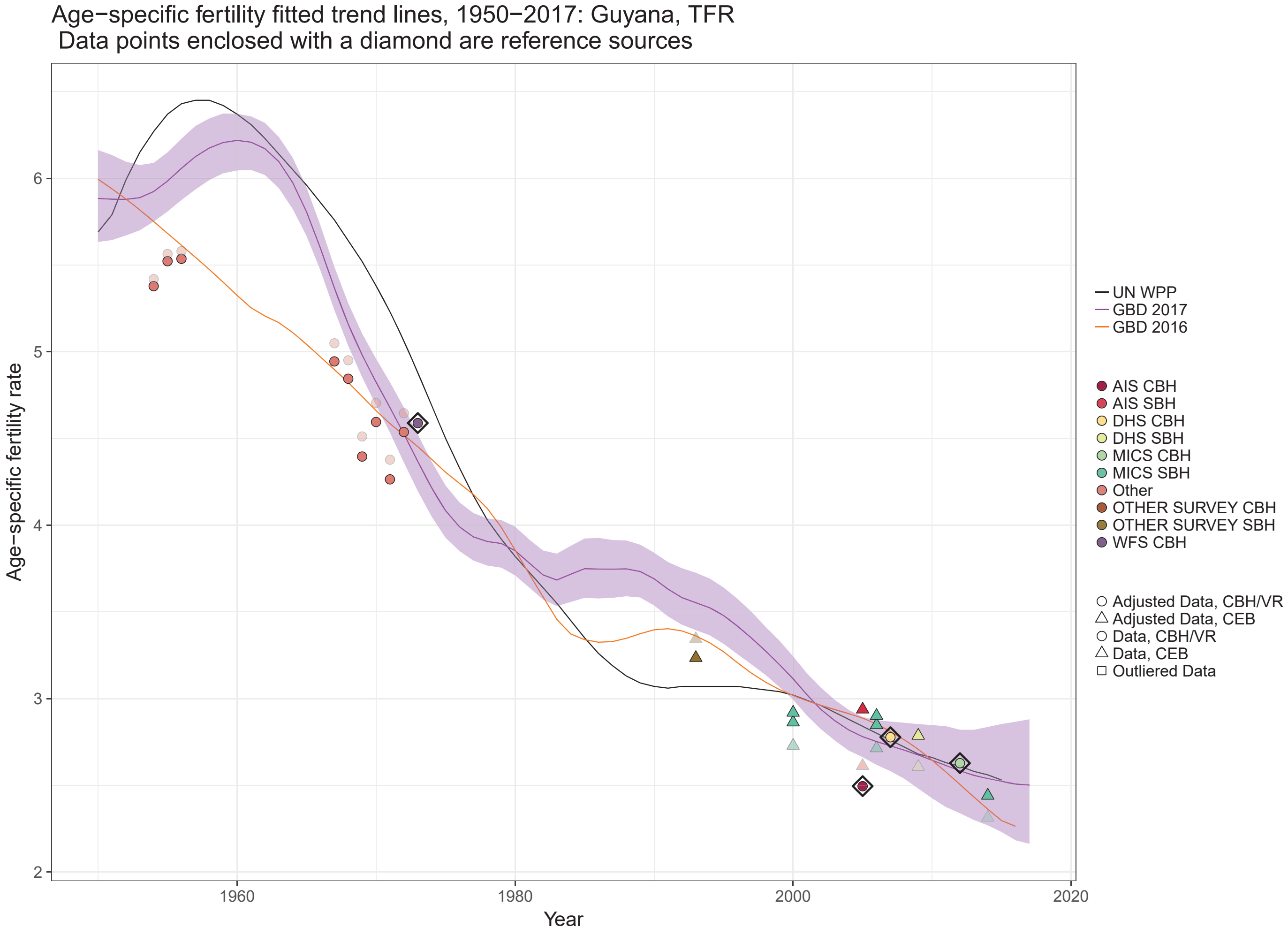
Age−specific fertility fitted trend lines, 1950−2017: Guyana, TFR
Data points enclosed with a diamond are reference sources
Age−specific fertility rate
Year
UN WPP
GBD 2017
GBD 2016
AIS CBH
AIS SBH
DHS CBH
DHS SBH
MICS CBH
MICS SBH
Other
OTHER SURVEY CBH
OTHER SURVEY SBH
WFS CBH
Adjusted Data, CBH/VR
Adjusted Data, CEB
Data, CBH/VR
Data, CEB
Outliered Data

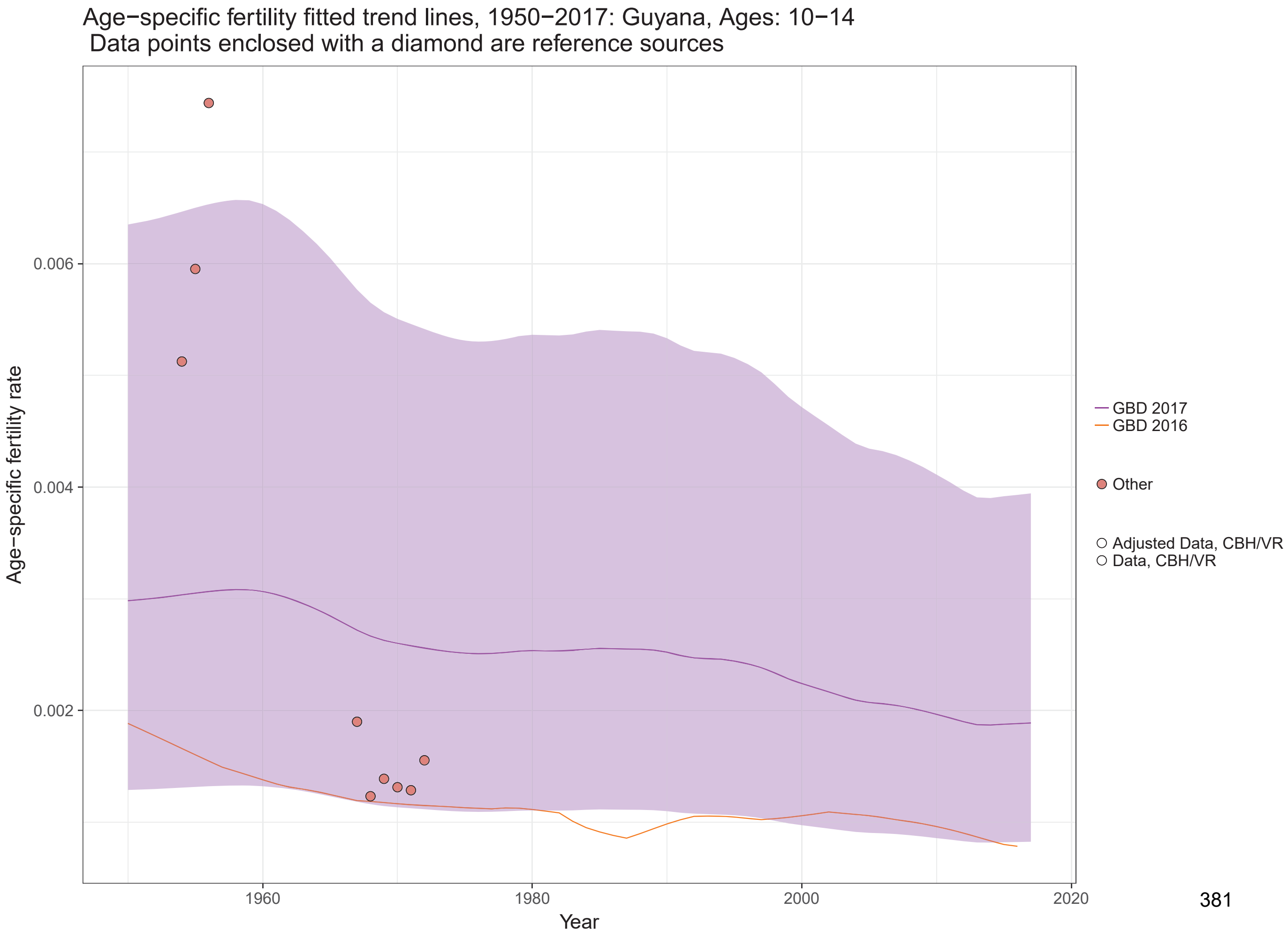
Age−specific fertility fitted trend lines, 1950−2017: Guyana, Ages: 10−14
Data points enclosed with a diamond are reference sources
Age−specific fertility rate
Year
GBD 2017
GBD 2016
Other
Adjusted Data, CBH/VR
Data, CBH/VR

Age−specific fertility fitted trend lines, 1950−2017: Guyana, Ages: 15−19
Data points enclosed with a diamond are reference sources

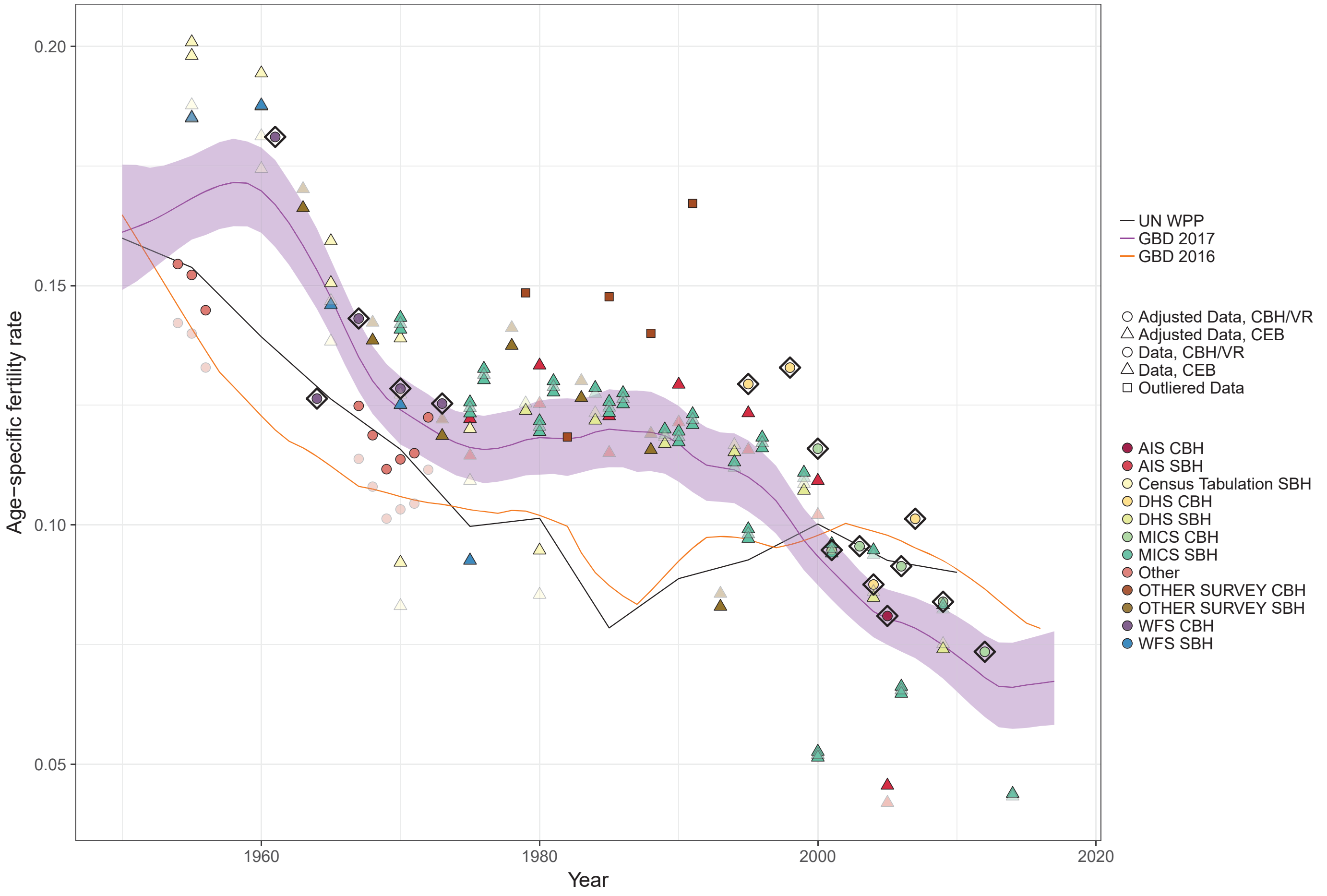

Age−specific fertility fitted trend lines, 1950−2017: Guyana, Ages: 20−24
Data points enclosed with a diamond are reference sources

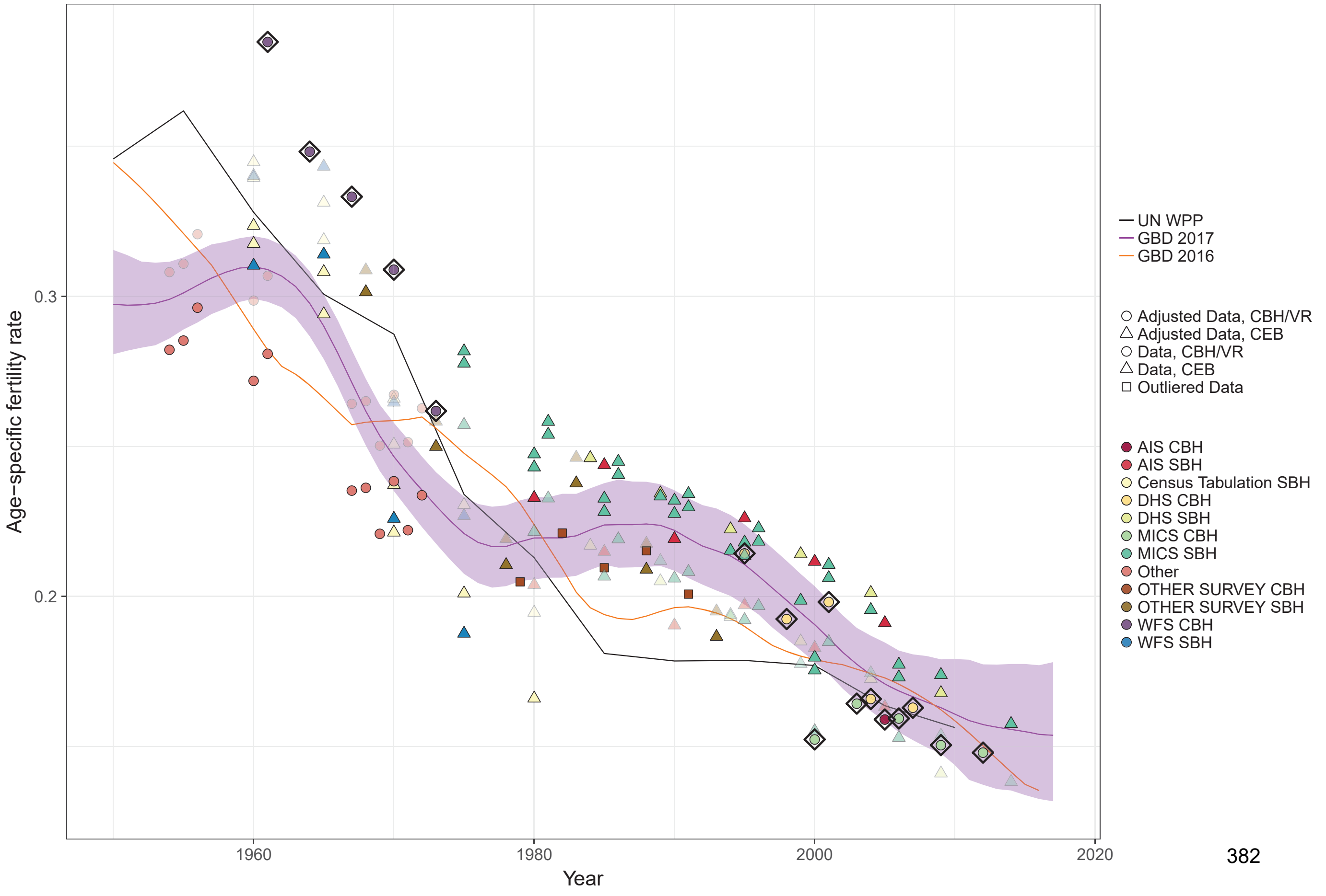

Age−specific fertility fitted trend lines, 1950−2017: Guyana, Ages: 25−29
Data points enclosed with a diamond are reference sources

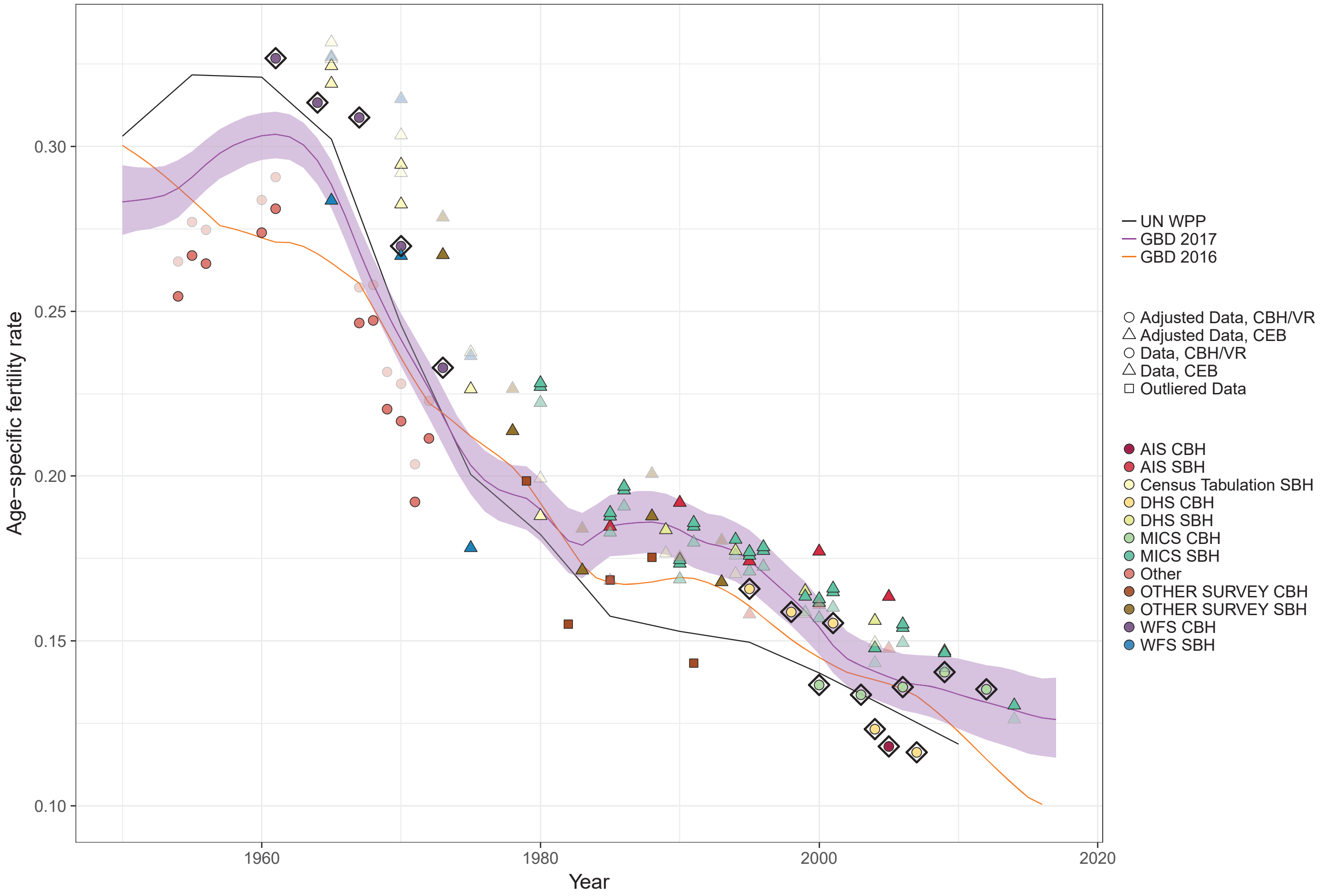

Age−specific fertility fitted trend lines, 1950−2017: Guyana, Ages: 30−34
Data points enclosed with a diamond are reference sources

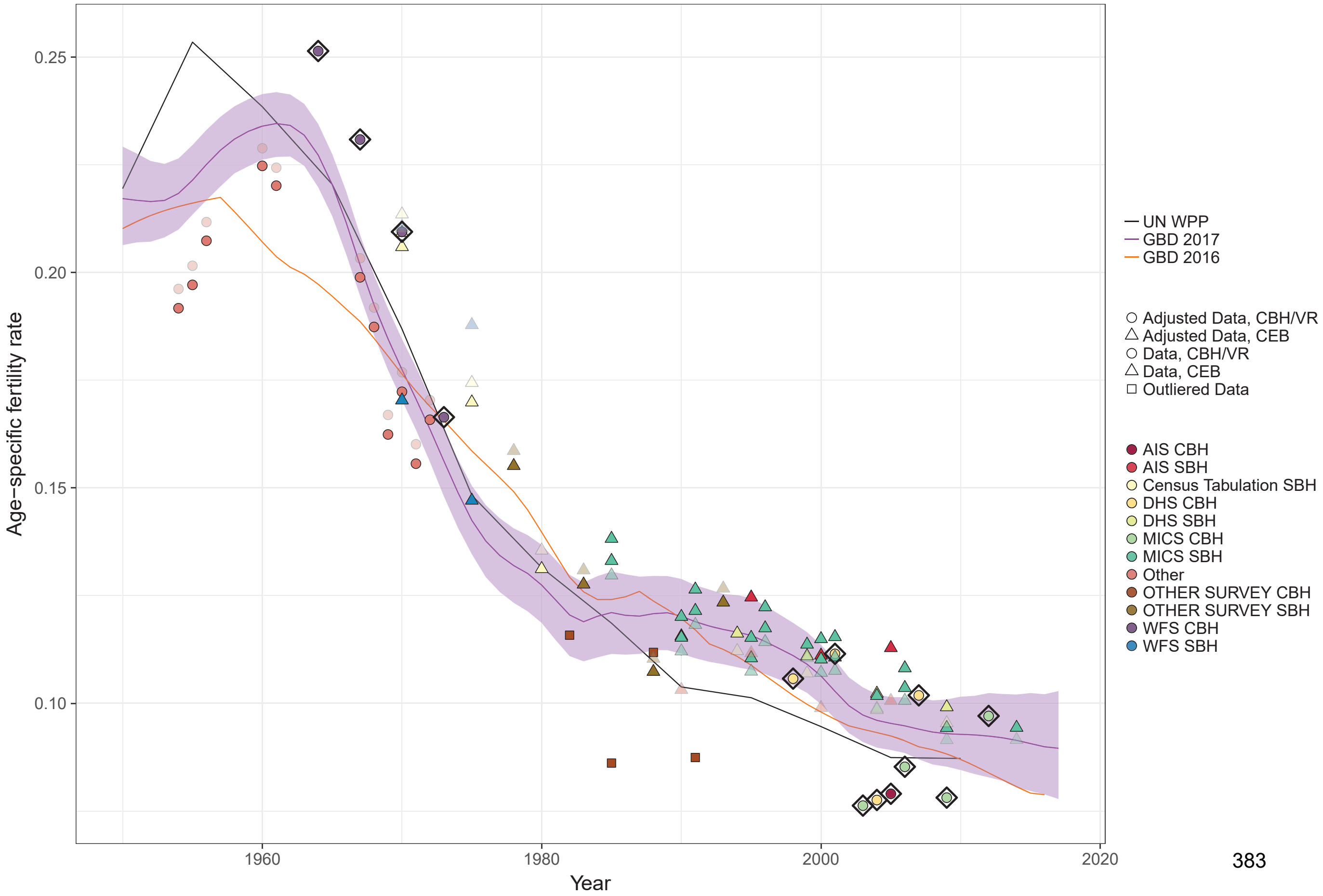

Age−specific fertility fitted trend lines, 1950−2017: Guyana, Ages: 35−39
Data points enclosed with a diamond are reference sources

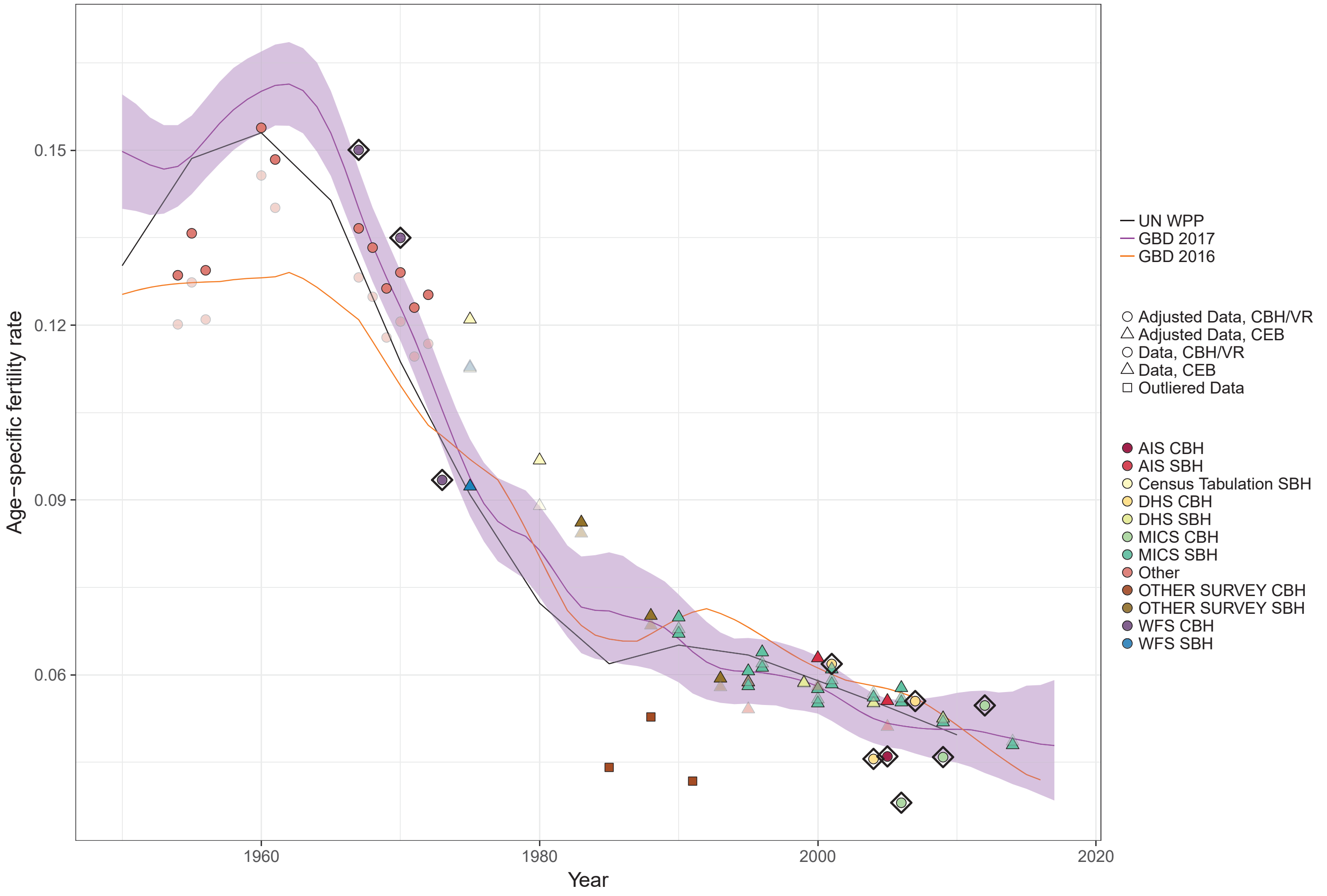

Age−specific fertility fitted trend lines, 1950−2017: Guyana, Ages: 40−44
Data points enclosed with a diamond are reference sources

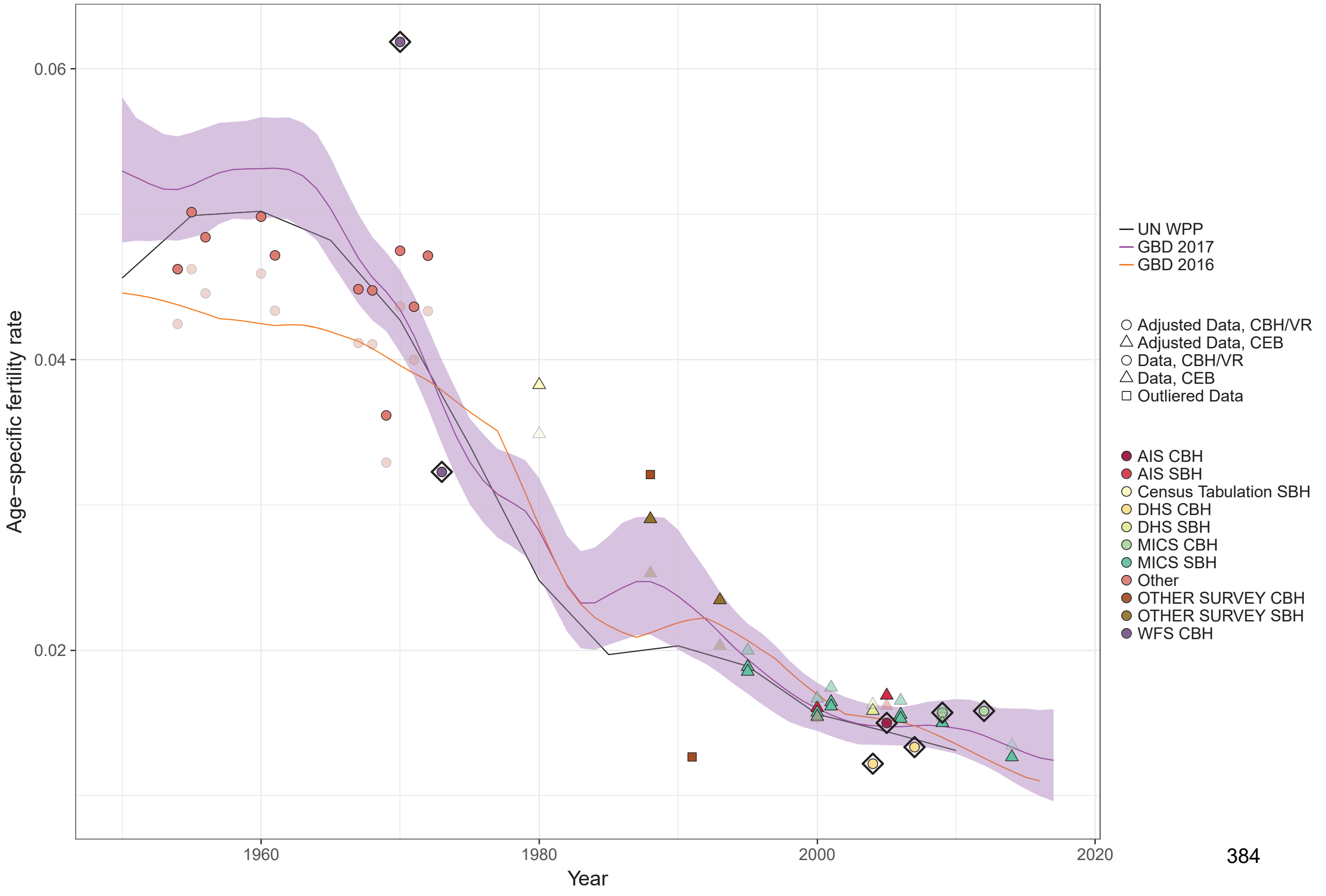

Age−specific fertility fitted trend lines, 1950−2017: Guyana, Ages: 45−49
Data points enclosed with a diamond are reference sources

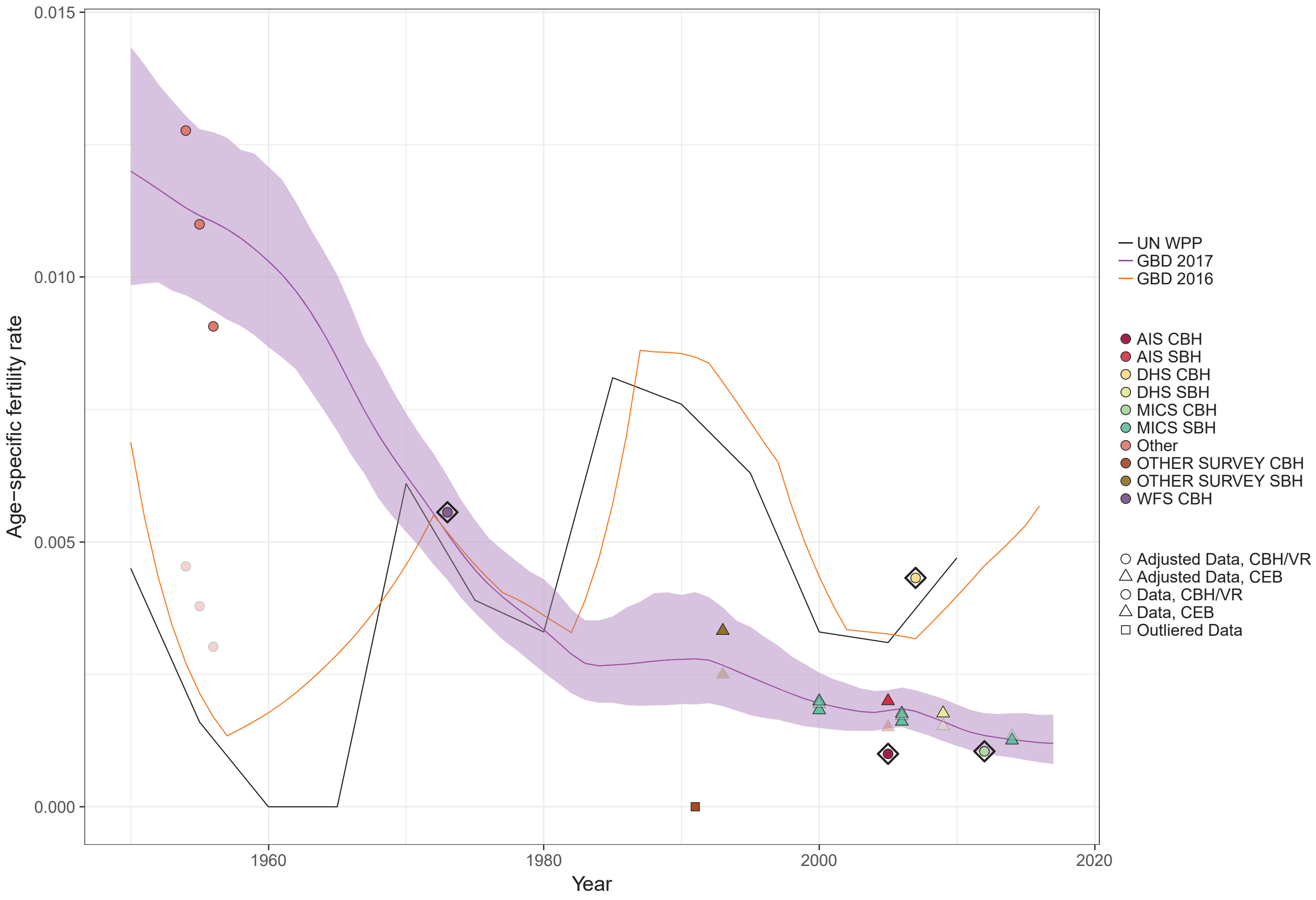

Age−specific fertility fitted trend lines, 1950−2017: Guyana, Ages: 50−54
Data points enclosed with a diamond are reference sources

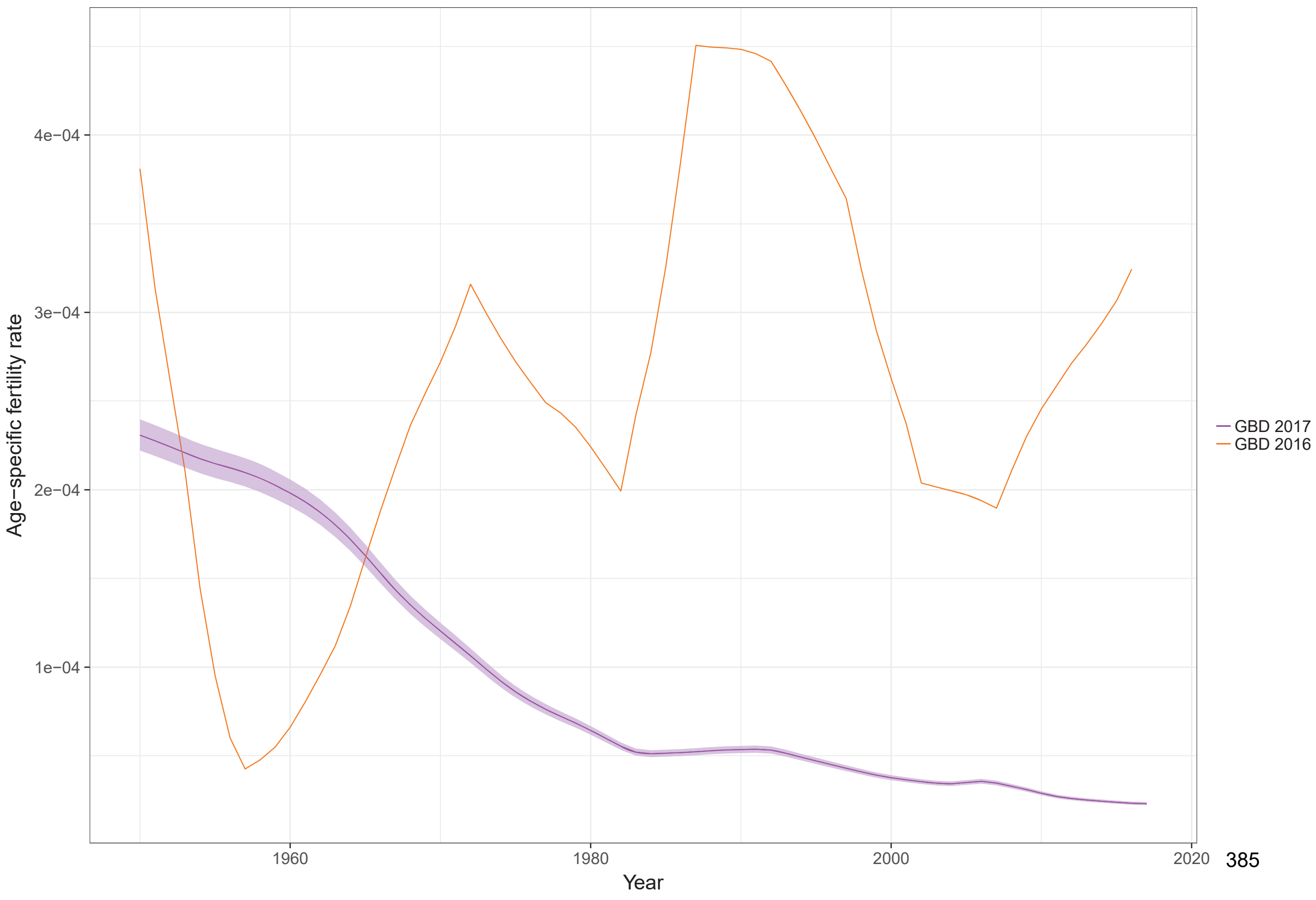

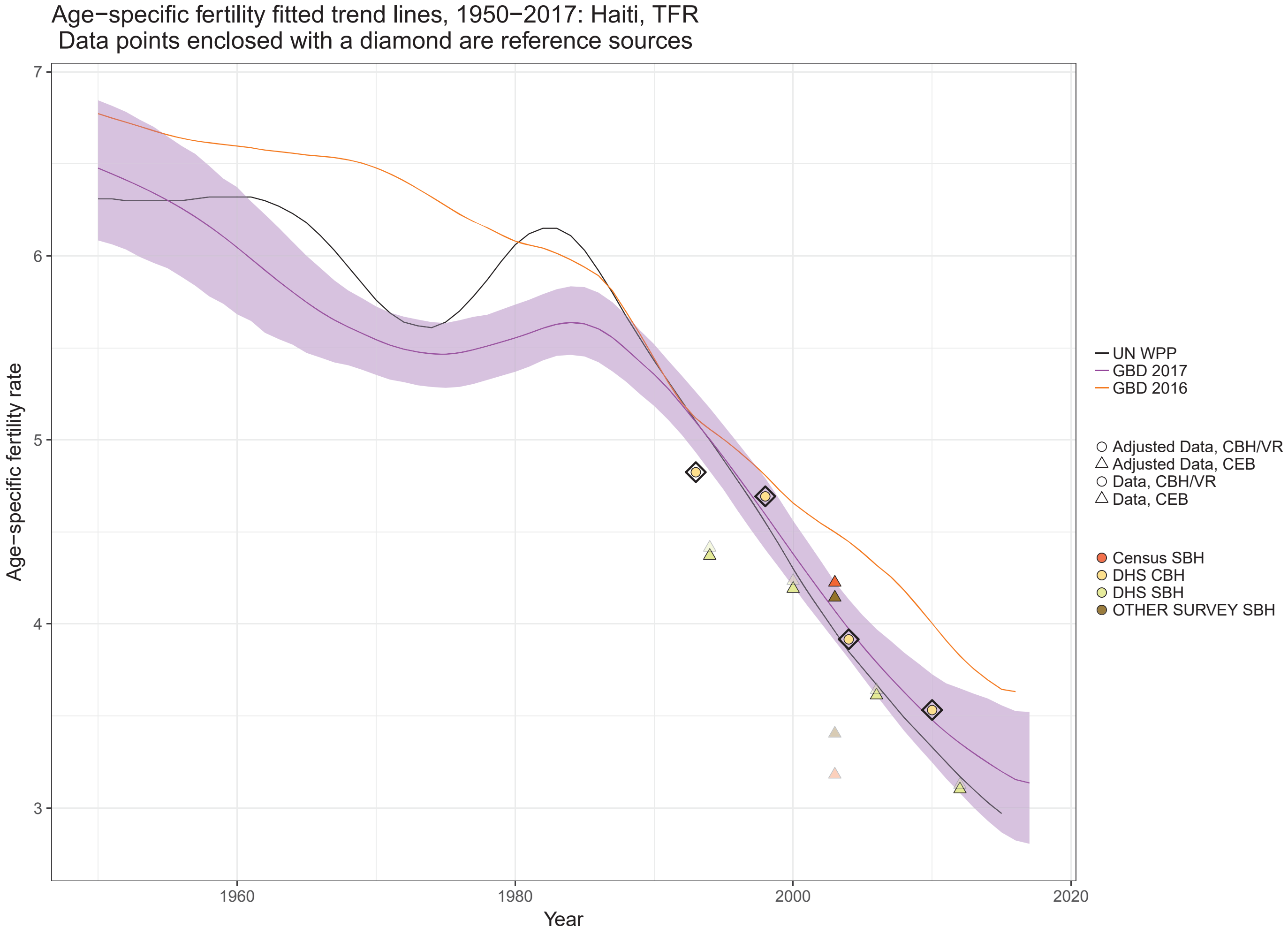

Age−specific fertility fitted trend lines, 1950−2017: Haiti, TFR
Data points enclosed with a diamond are reference sources
Age−specific fertility rate
Year
3
4
5
6
7
1960
1980
2000
2020
UN WPP
GBD 2017
GBD 2016
Adjusted Data, CBH/VR
Adjusted Data, CEB
Data, CBH/VR
Data, CEB
Census SBH
DHS CBH
DHS SBH
OTHER SURVEY SBH

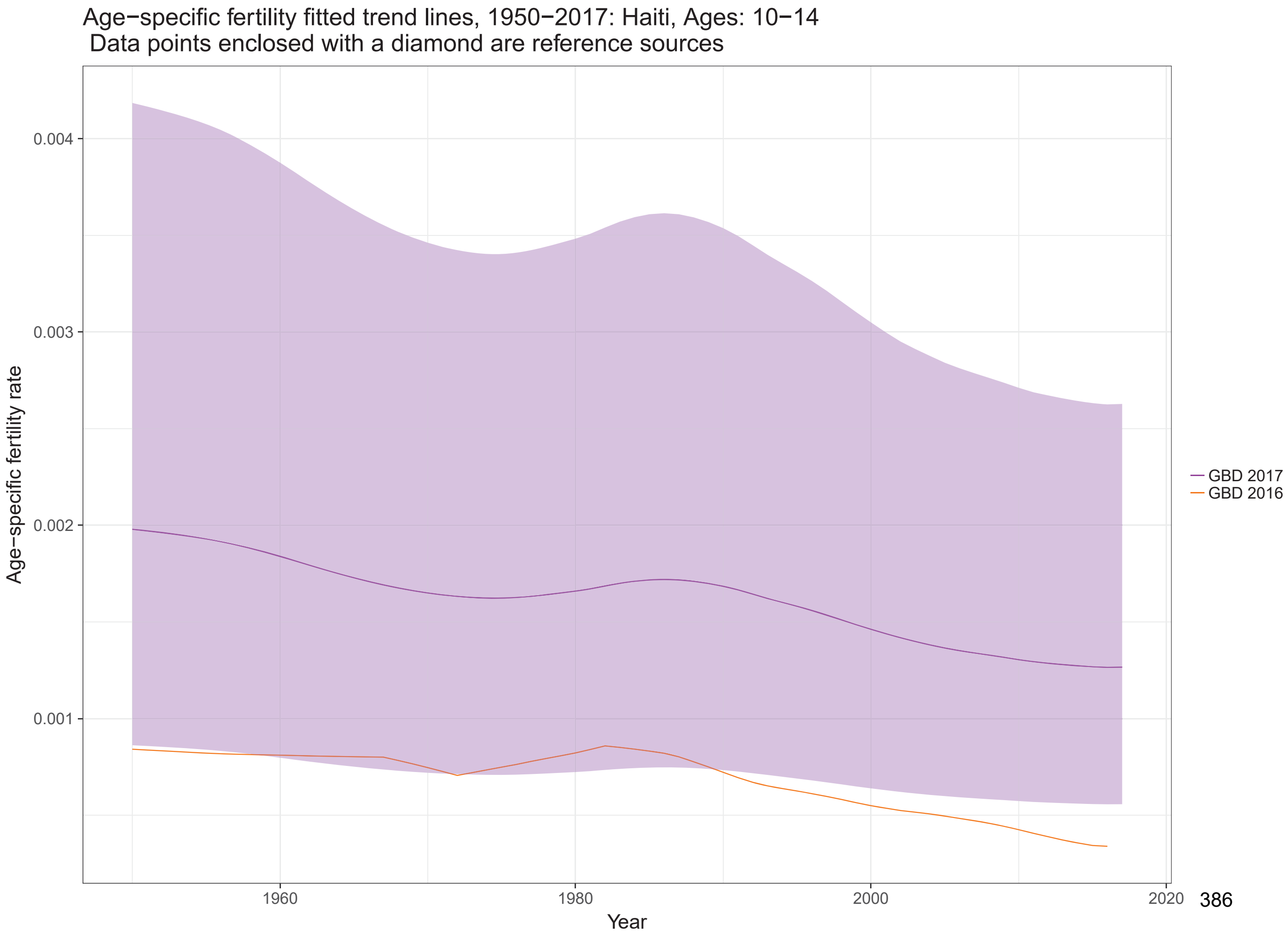

Age−specific fertility fitted trend lines, 1950−2017: Haiti, Ages: 10−14
Data points enclosed with a diamond are reference sources
Age−specific fertility rate
Year
0.001
0.002
0.003
0.004
1960
1980
2000
2020
GBD 2017
GBD 2016

Age−specific fertility fitted trend lines, 1950−2017: Haiti, Ages: 15−19
Data points enclosed with a diamond are reference sources

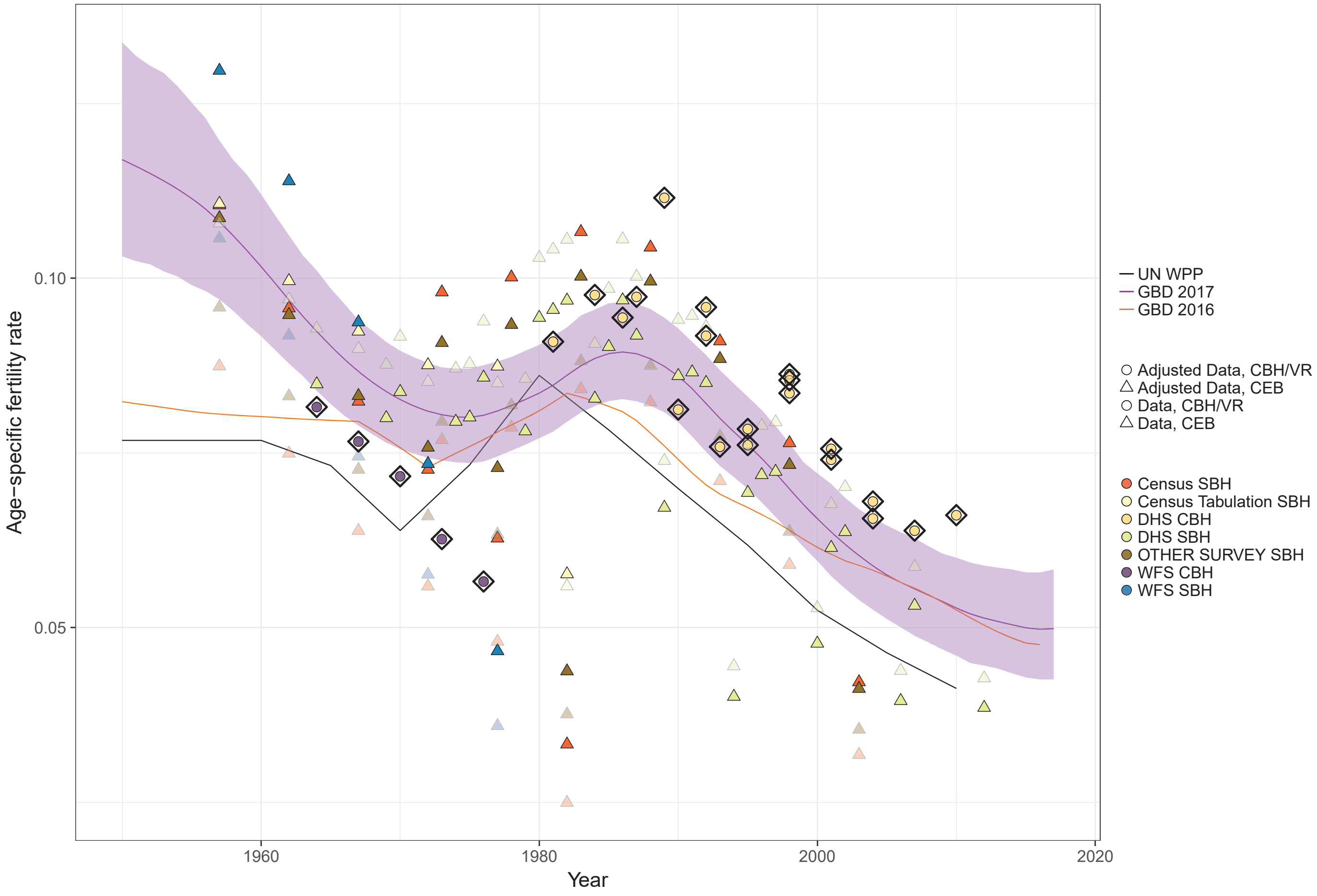

Age−specific fertility fitted trend lines, 1950−2017: Haiti, Ages: 20−24
Data points enclosed with a diamond are reference sources

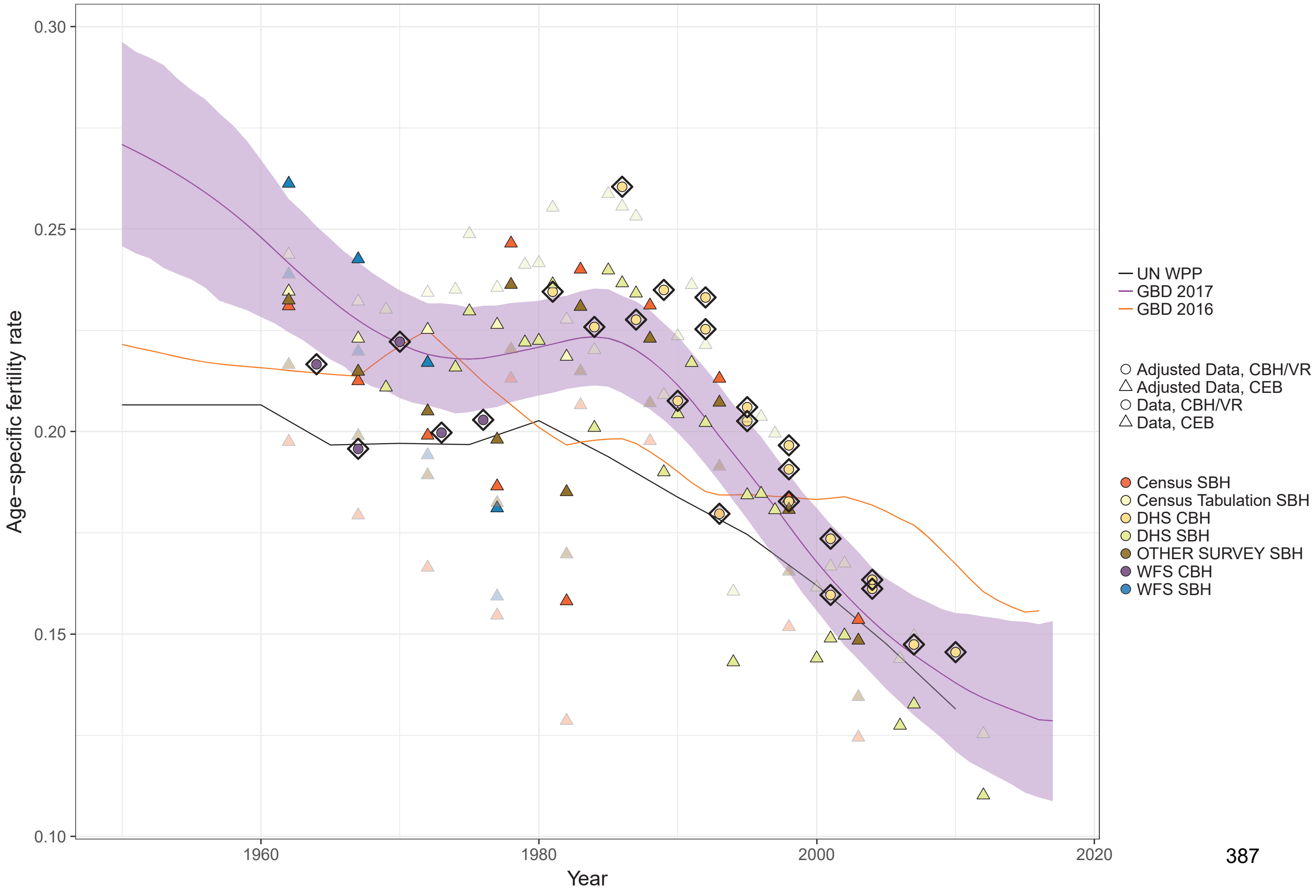

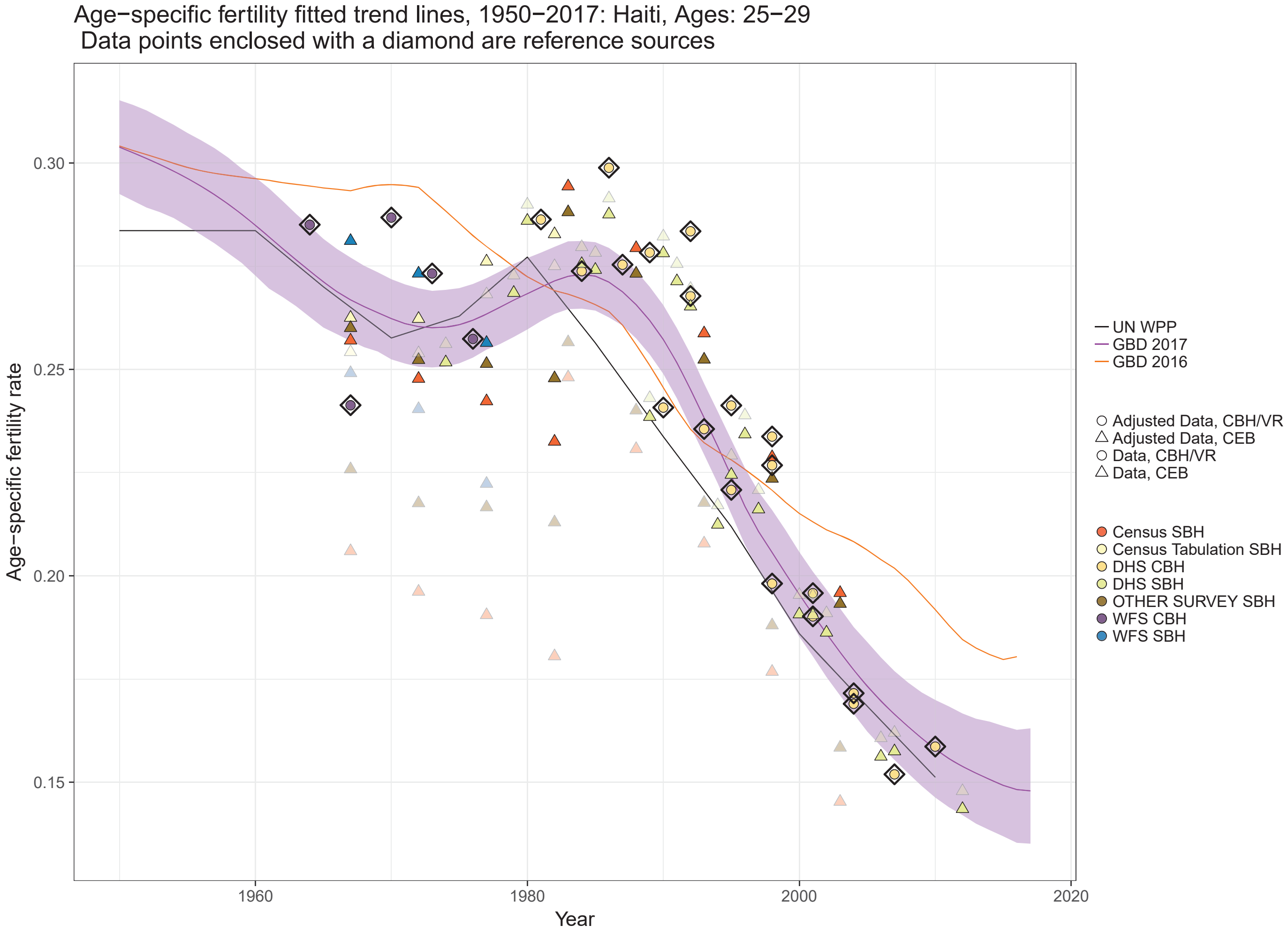
Age−specific fertility fitted trend lines, 1950−2017: Haiti, Ages: 25−29
Data points enclosed with a diamond are reference sources
Age−specific fertility rate
0.30
0.25
0.20
0.15
1960
1980
2000
2020
Year
UN WPP
GBD 2017
GBD 2016
Adjusted Data, CBH/VR
Adjusted Data, CEB
Data, CBH/VR
Data, CEB
Census SBH
Census Tabulation SBH
DHS CBH
DHS SBH
OTHER SURVEY SBH
WFS CBH
WFS SBH

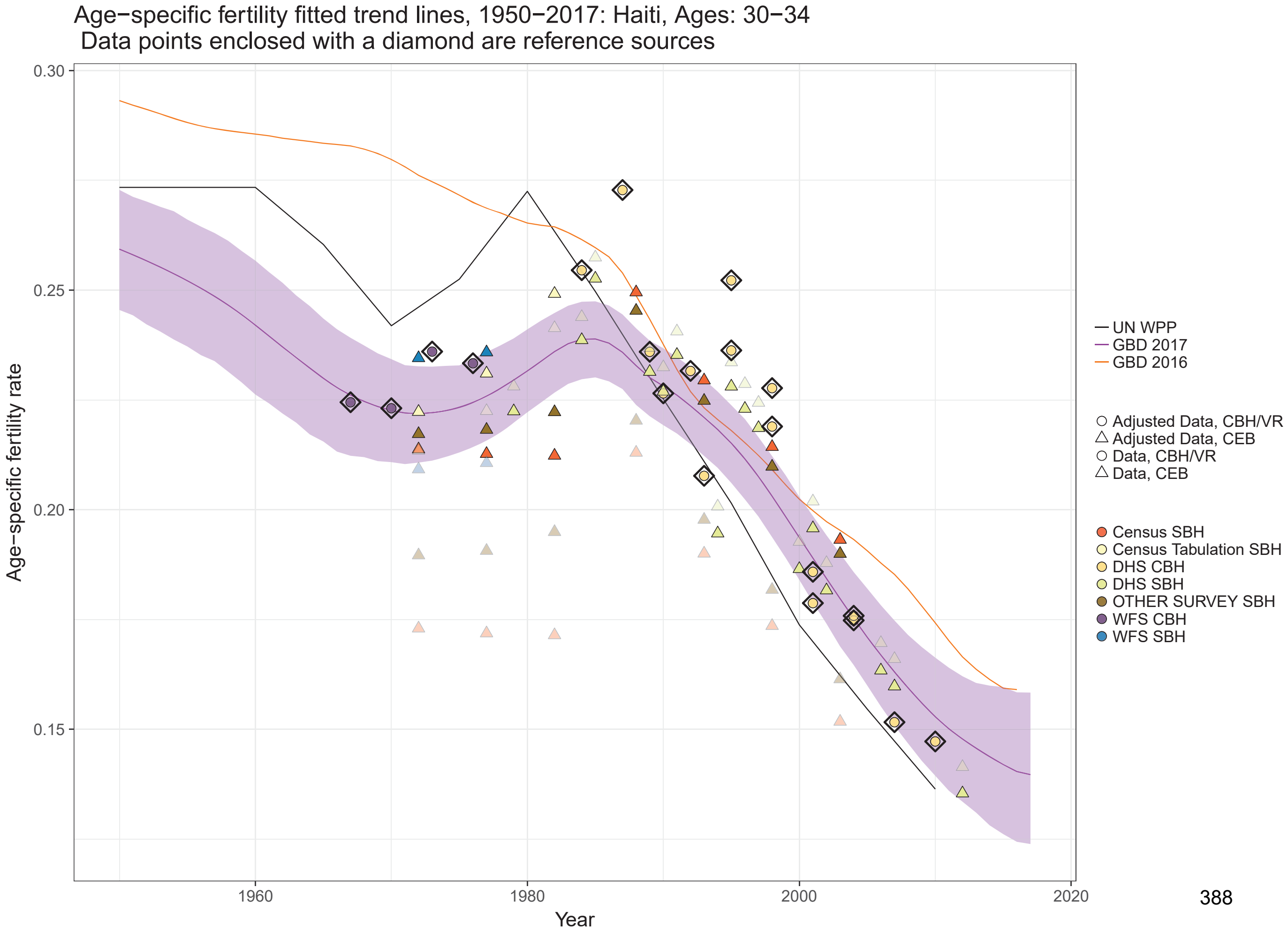
Age−specific fertility fitted trend lines, 1950−2017: Haiti, Ages: 30−34
Data points enclosed with a diamond are reference sources
Age−specific fertility rate
0.30
0.25
0.20
0.15
1960
1980
2000
2020
Year
UN WPP
GBD 2017
GBD 2016
Adjusted Data, CBH/VR
Adjusted Data, CEB
Data, CBH/VR
Data, CEB
Census SBH
Census Tabulation SBH
DHS CBH
DHS SBH
OTHER SURVEY SBH
WFS CBH
WFS SBH

Age−specific fertility fitted trend lines, 1950−2017: Haiti, Ages: 35−39
Data points enclosed with a diamond are reference sources

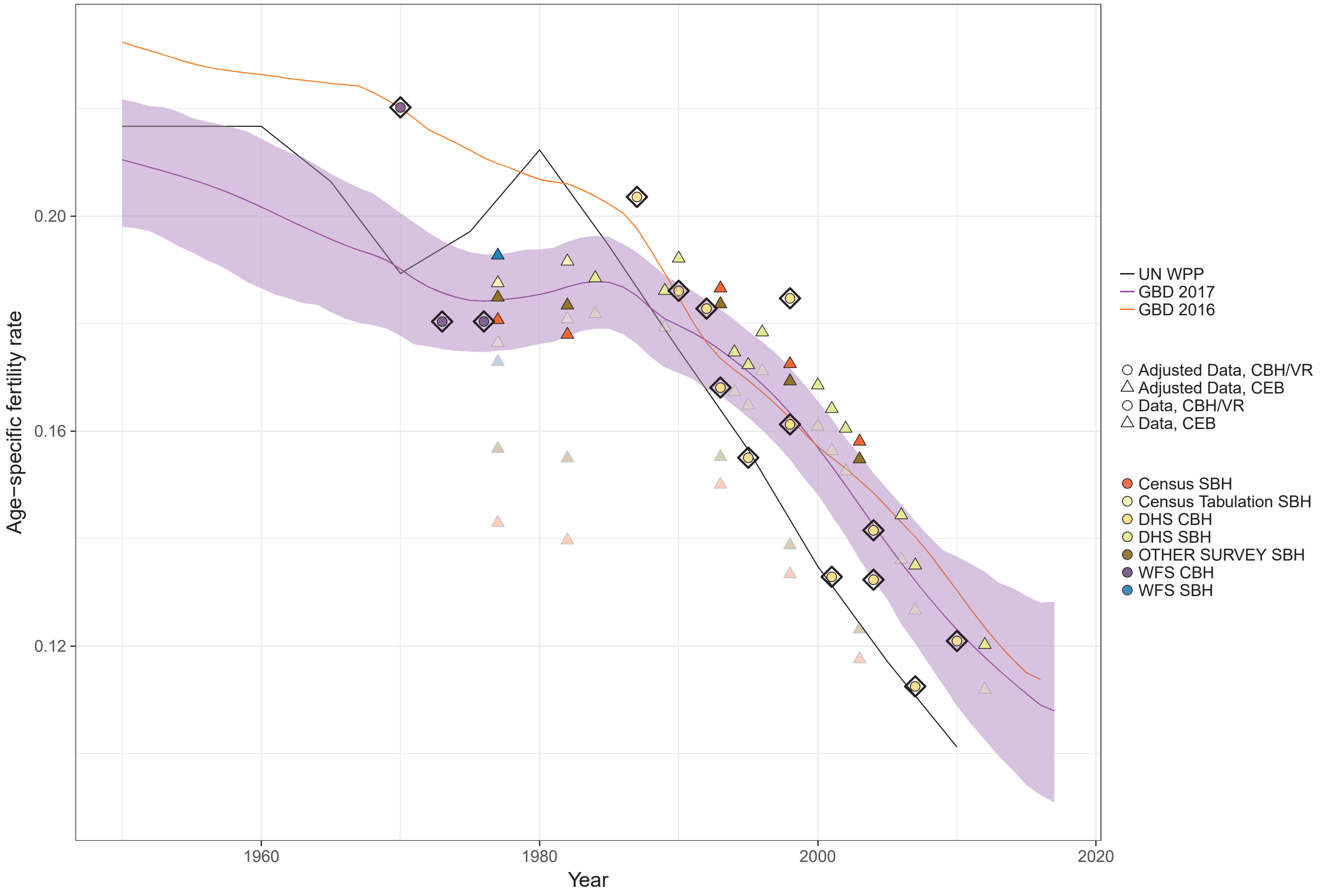

Age−specific fertility fitted trend lines, 1950−2017: Haiti, Ages: 40−44
Data points enclosed with a diamond are reference sources

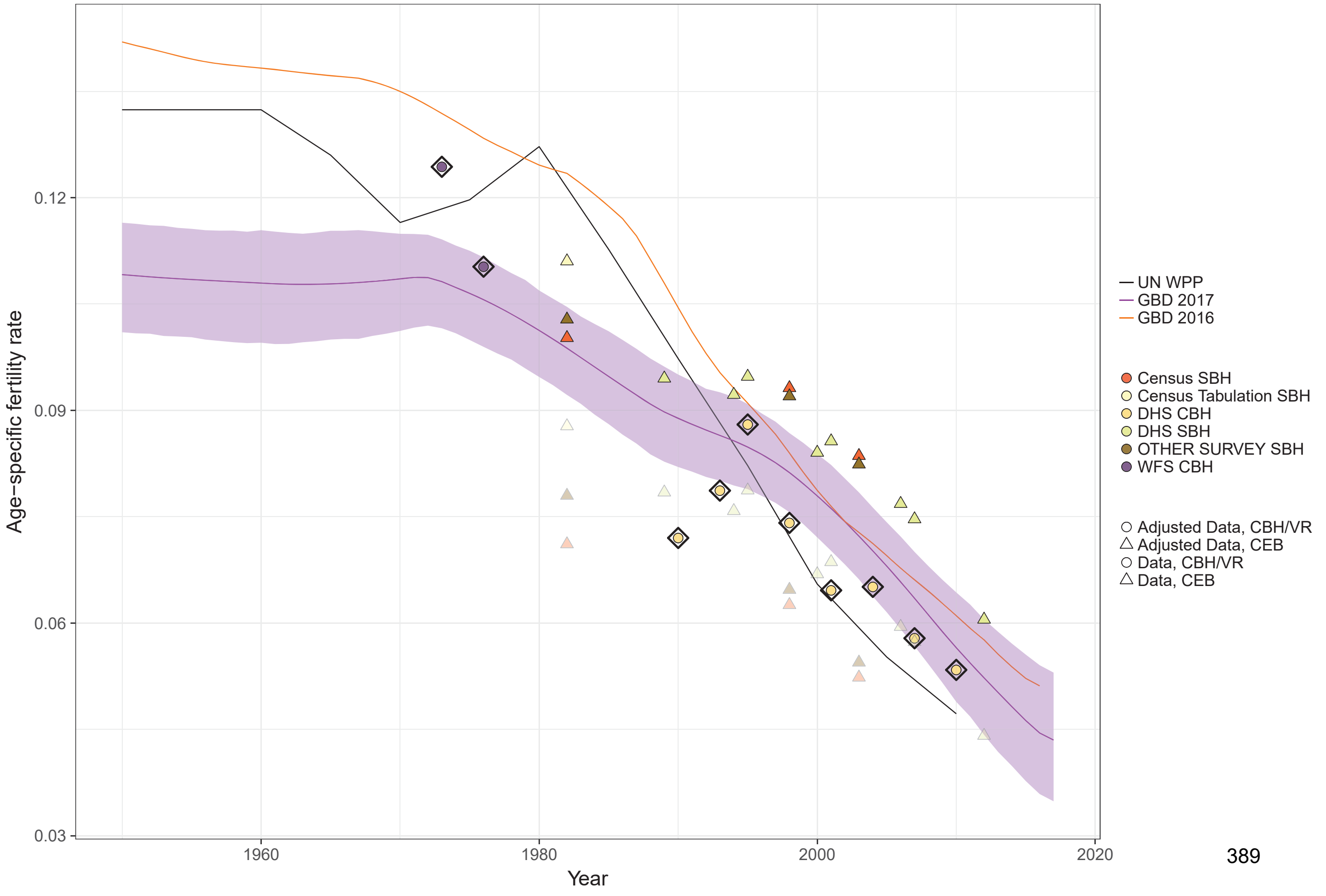

Age−specific fertility fitted trend lines, 1950−2017: Haiti, Ages: 45−49
Data points enclosed with a diamond are reference sources

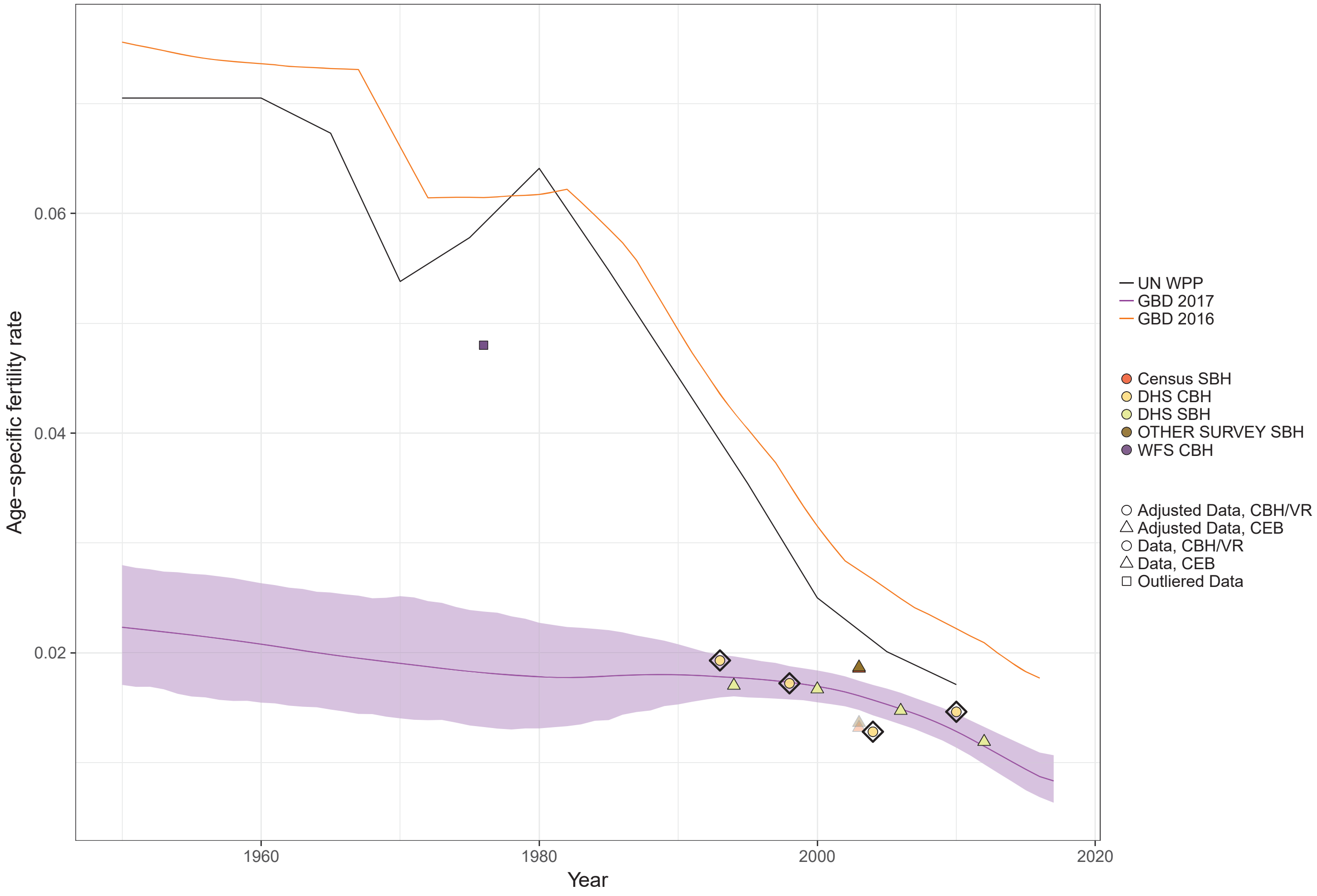

Age−specific fertility trend lines, 1950−2017: Haiti, Ages: 50−54
Data points enclosed with a diamond are reference sources

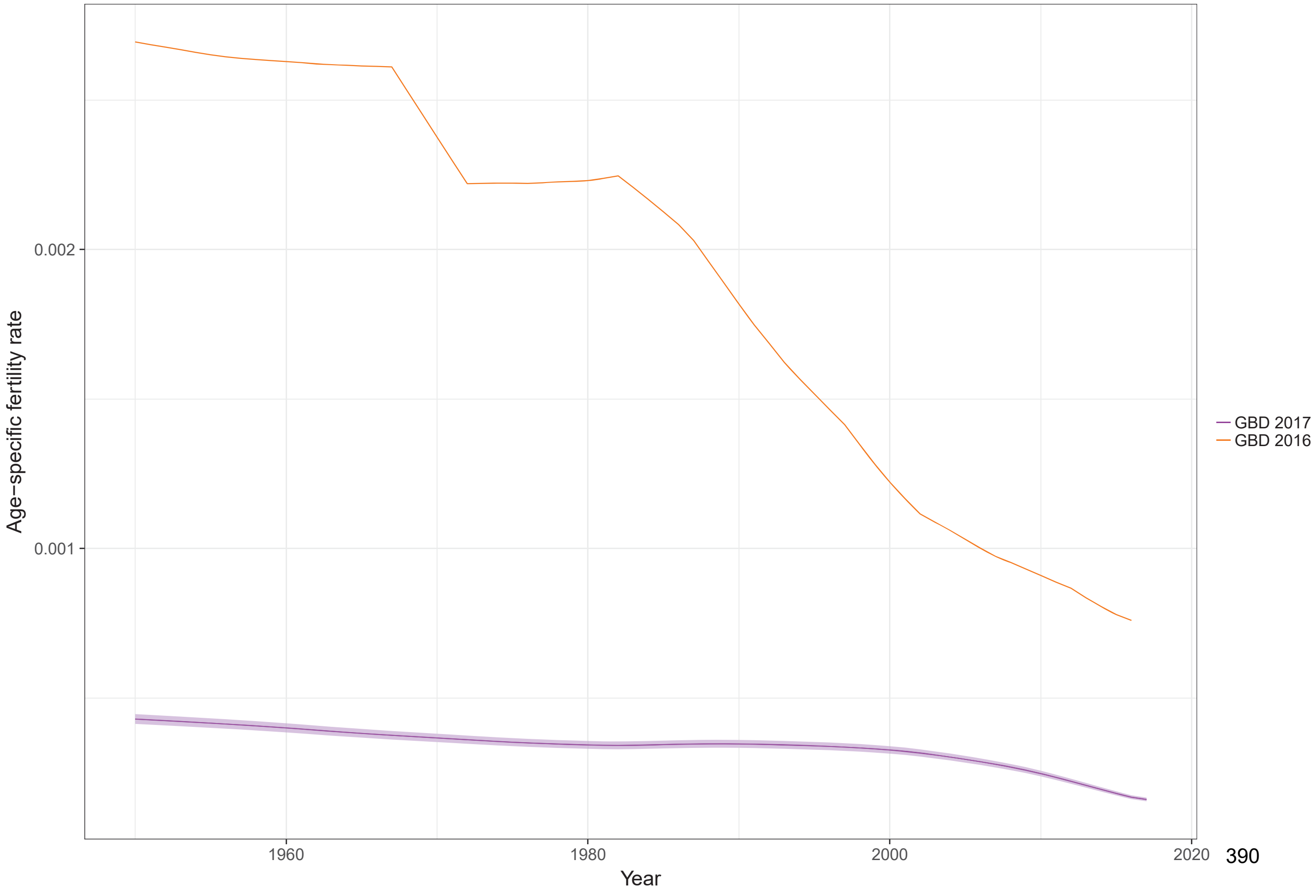

Age−specific fertility fitted trend lines, 1950−2017: Honduras, TFR
Data points enclosed with a diamond are reference sources

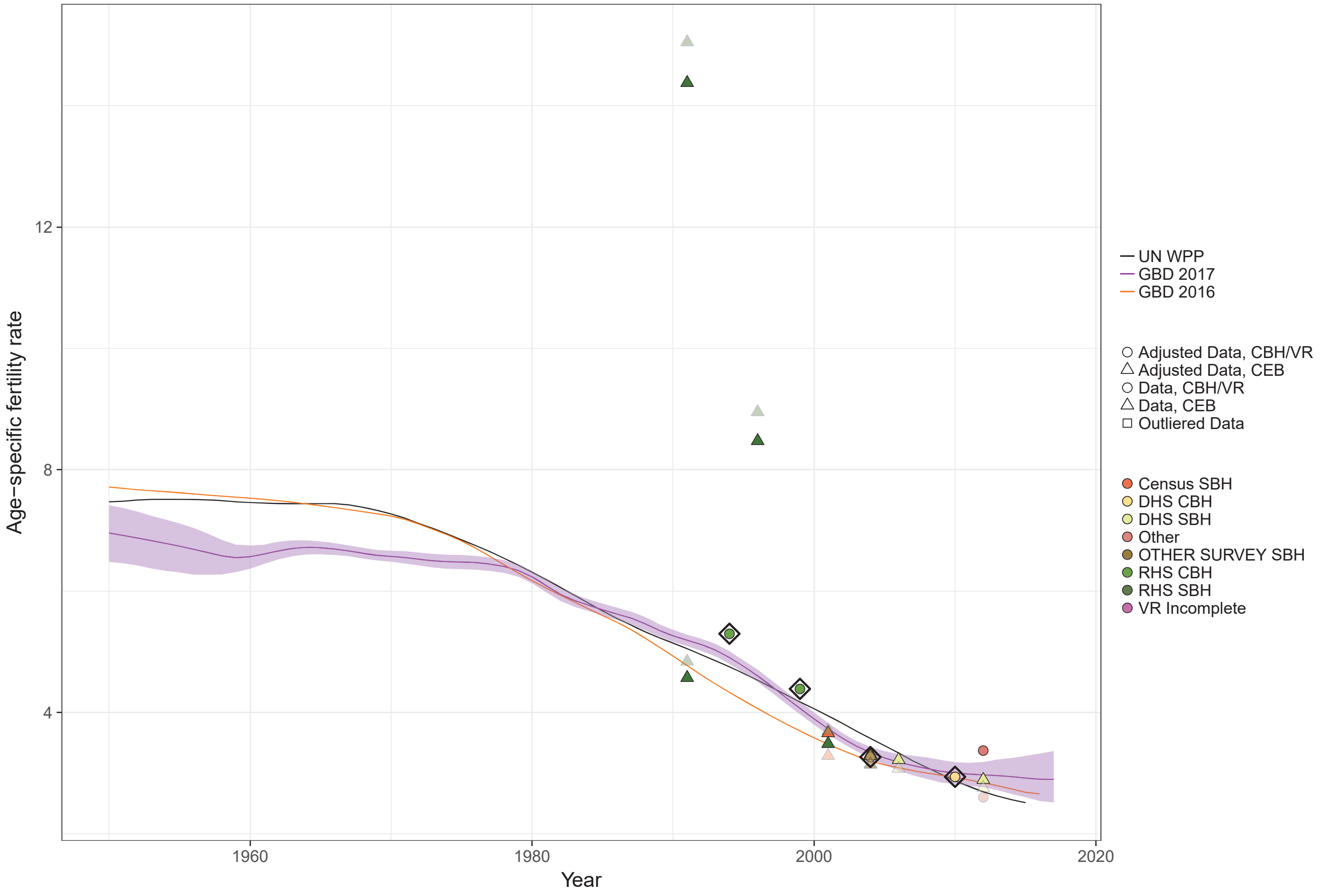

Age−specific fertility fitted trend lines, 1950−2017: Honduras, Ages: 10−14
Data points enclosed with a diamond are reference sources

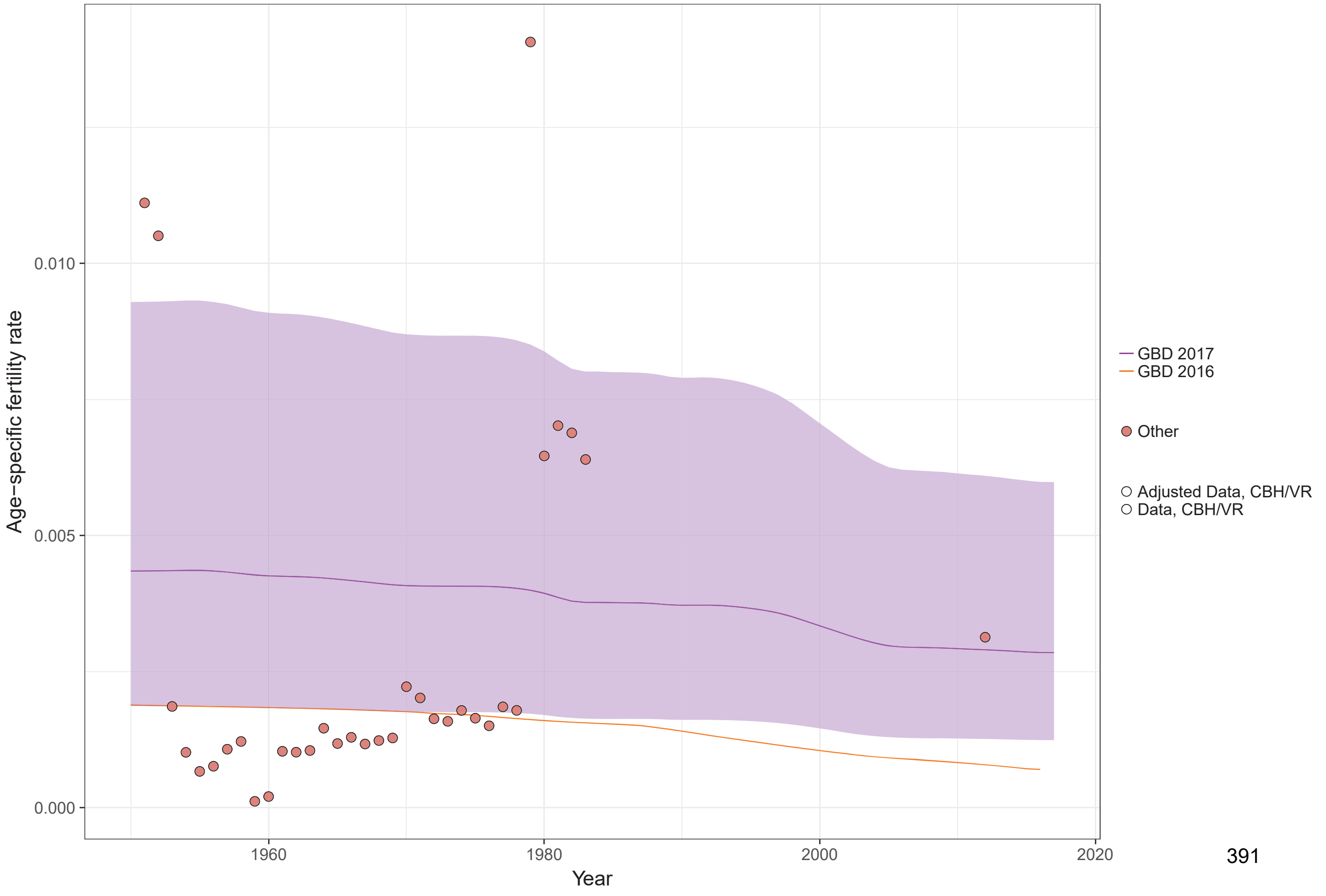

Age−specific fertility fitted trend lines, 1950−2017: Honduras, Ages: 15−19
Data points enclosed with a diamond are reference sources

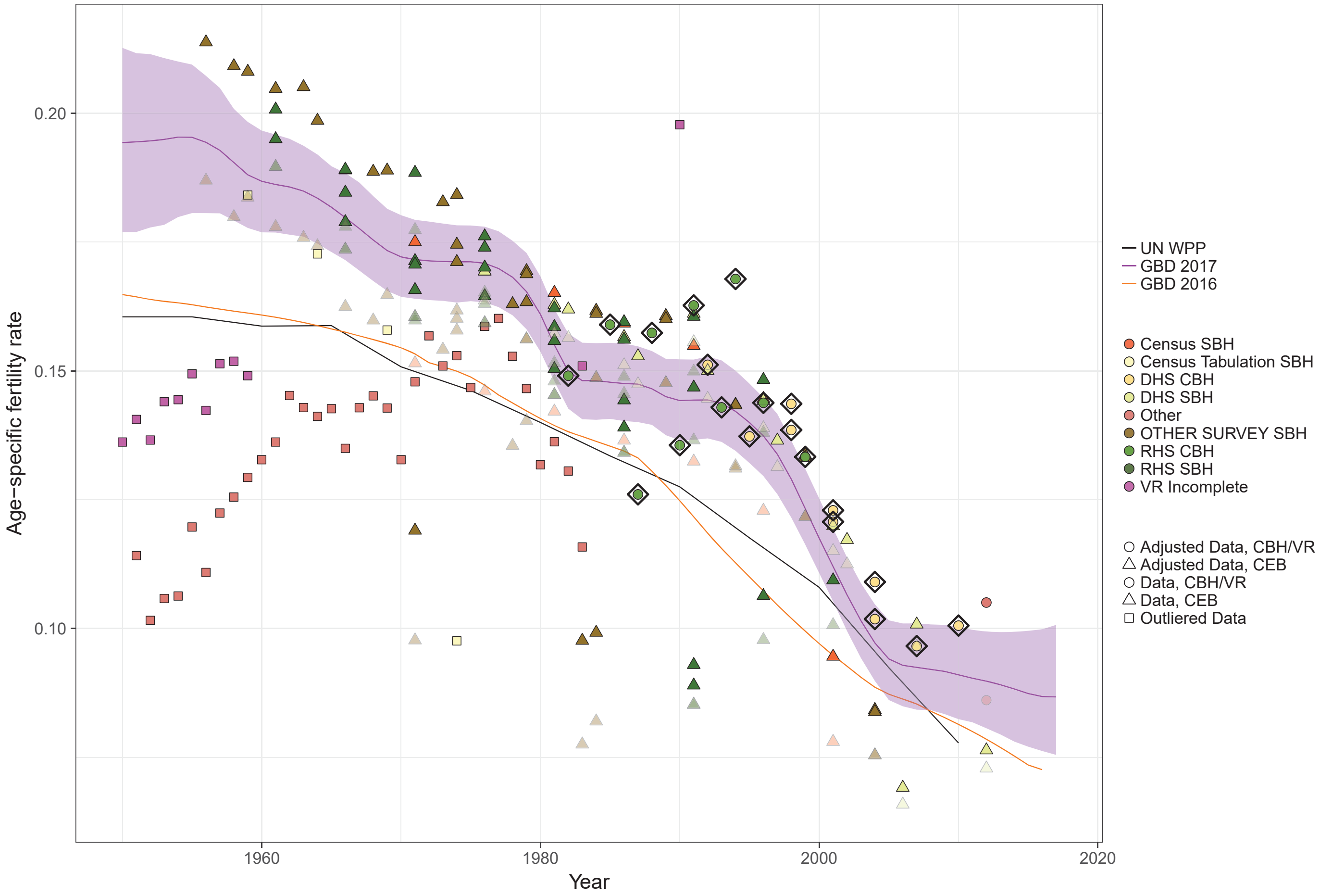

Age−specific fertility fitted trend lines, 1950−2017: Honduras, Ages: 20−24
Data points enclosed with a diamond are reference sources

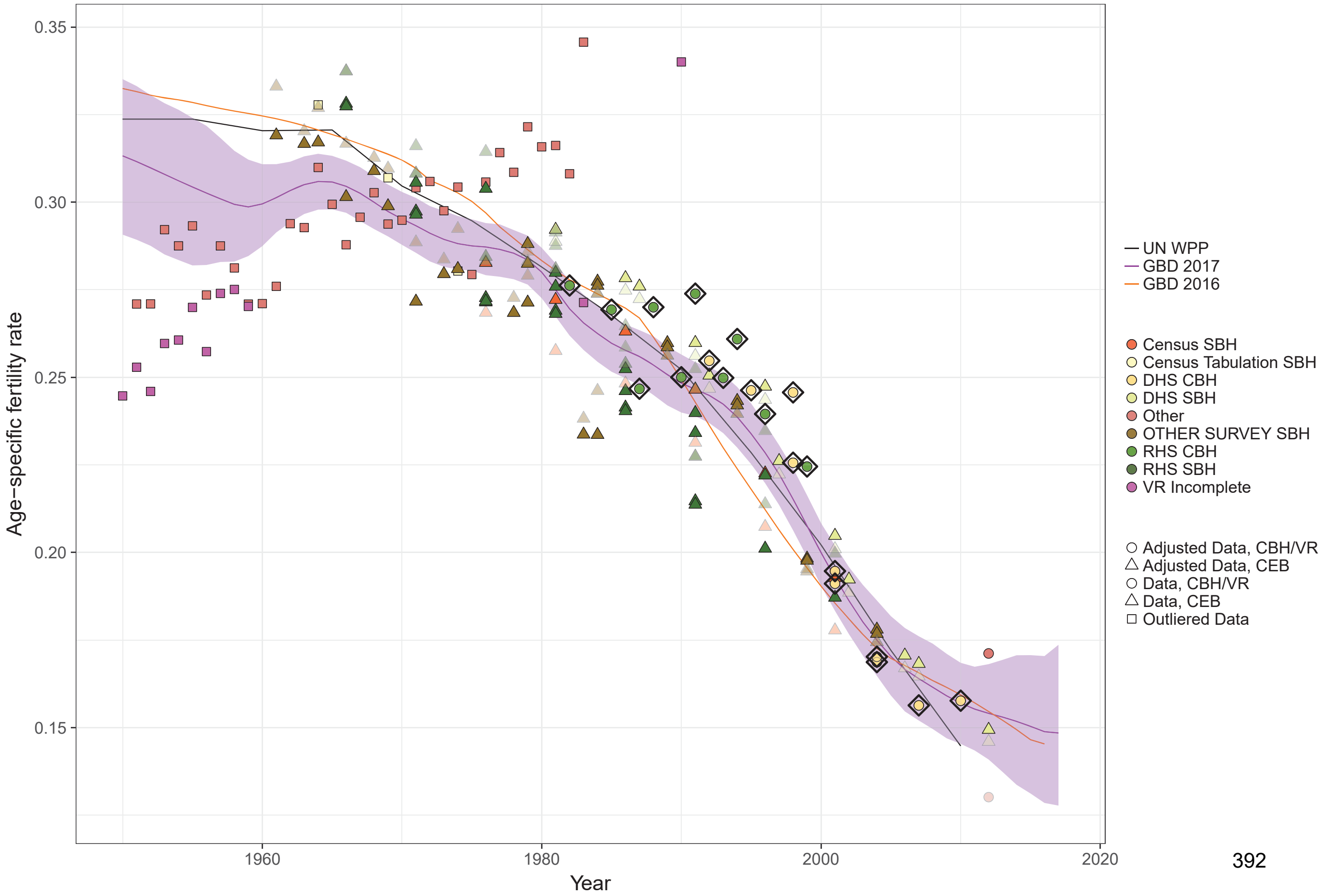

Age−specific fertility fitted trend lines, 1950−2017: Honduras, Ages: 25−29
Data points enclosed with a diamond are reference sources

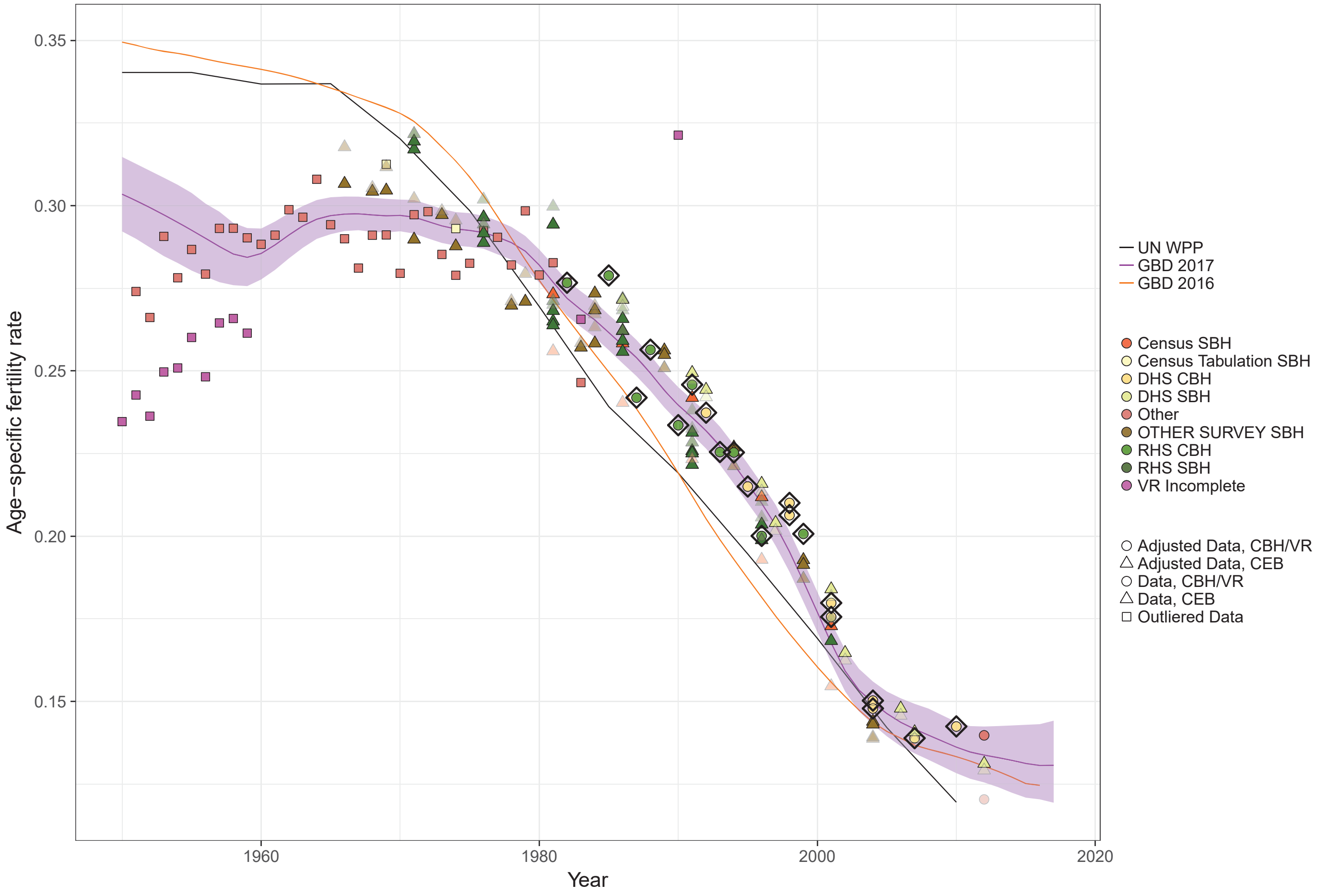

Age−specific fertility fitted trend lines, 1950−2017: Honduras, Ages: 30−34
Data points enclosed with a diamond are reference sources

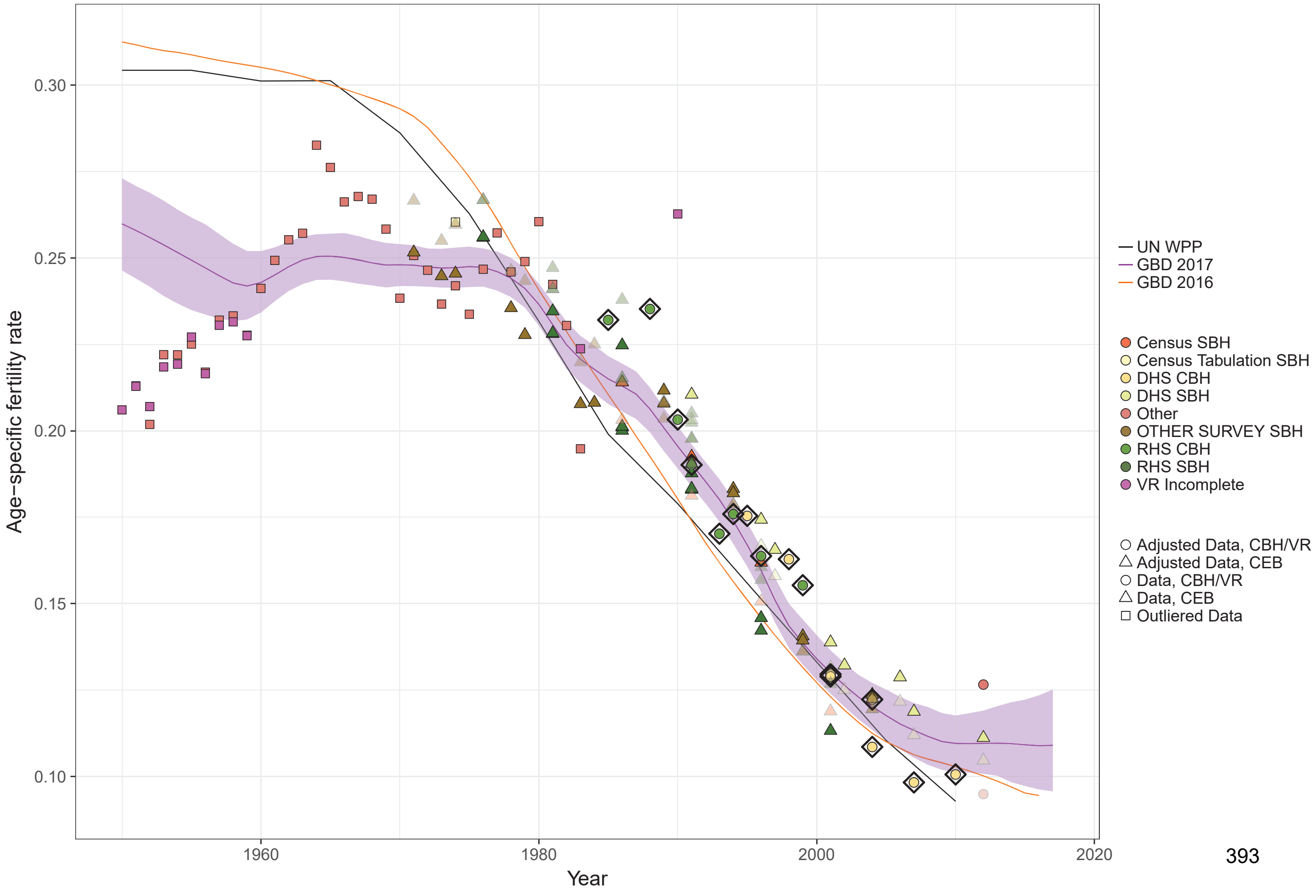

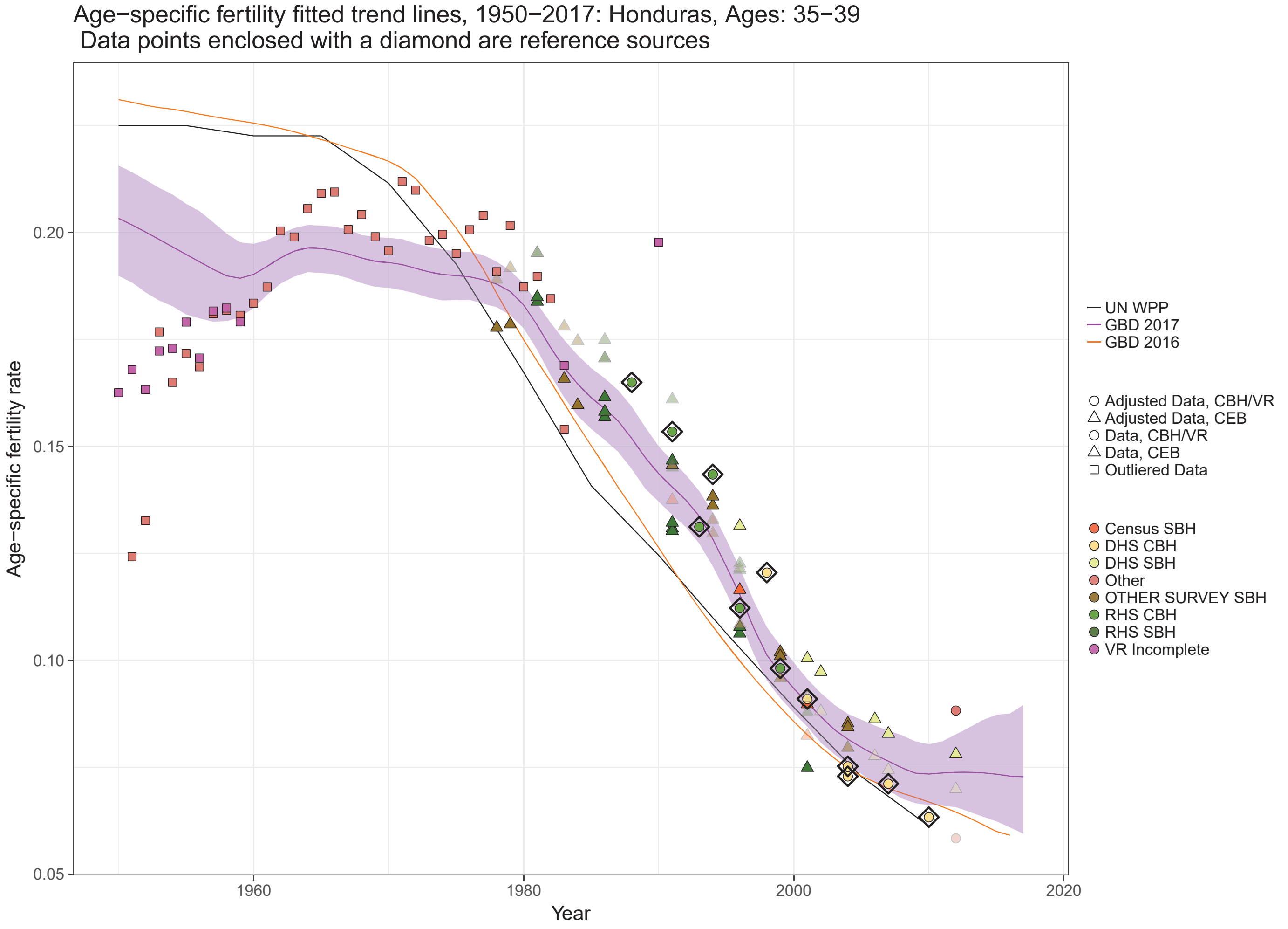
Age−specific fertility fitted trend lines, 1950−2017: Honduras, Ages: 35−39
Data points enclosed with a diamond are reference sources
Age-specific fertility rate
Year
UN WPP
GBD 2017
GBD 2016
Adjusted Data, CBH/VR
Adjusted Data, CEB
Data, CBH/VR
Data, CEB
Outliered Data
Census SBH
DHS CBH
DHS SBH
Other
OTHER SURVEY SBH
RHS CBH
RHS SBH
VR Incomplete

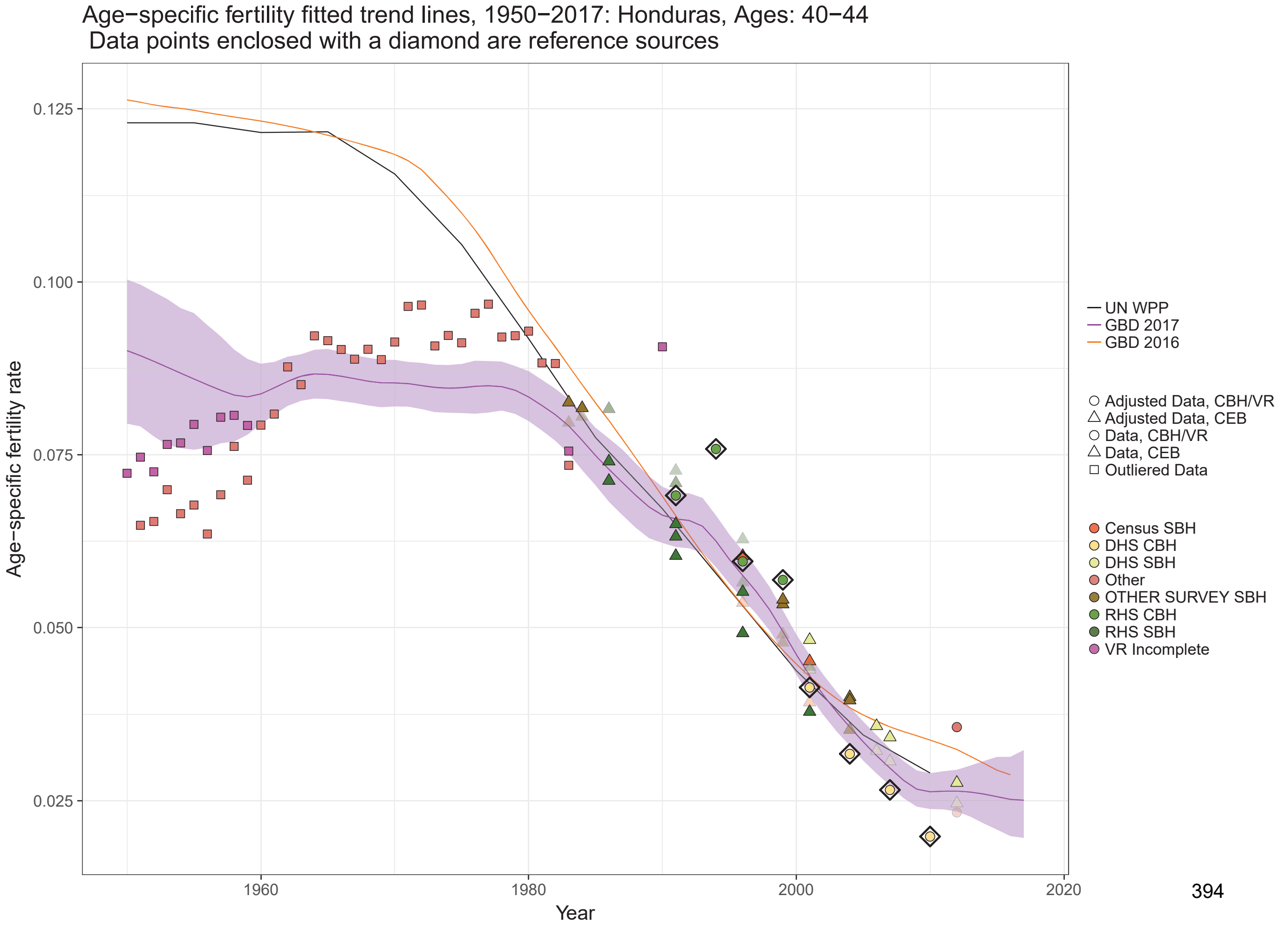
Age−specific fertility fitted trend lines, 1950−2017: Honduras, Ages: 40−44
Data points enclosed with a diamond are reference sources
Age-specific fertility rate
Year
UN WPP
GBD 2017
GBD 2016
Adjusted Data, CBH/VR
Adjusted Data, CEB
Data, CBH/VR
Data, CEB
Outliered Data
Census SBH
DHS CBH
DHS SBH
Other
OTHER SURVEY SBH
RHS CBH
RHS SBH
VR Incomplete

Age−specific fertility fitted trend lines, 1950−2017: Honduras, Ages: 45−49
Data points enclosed with a diamond are reference sources

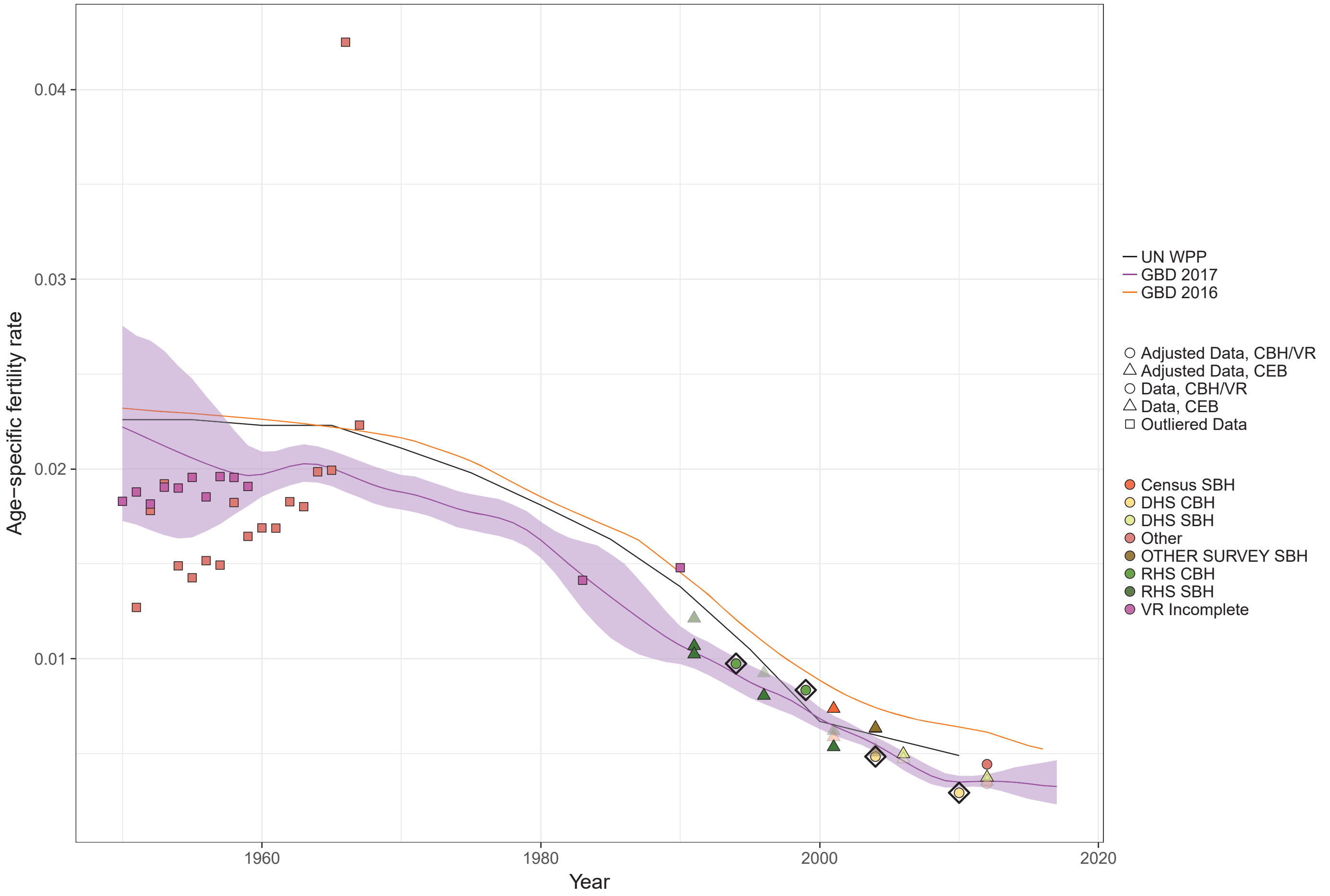

Age−specific fertility fitted trend lines, 1950−2017: Honduras, Ages: 50−54
Data points enclosed with a diamond are reference sources

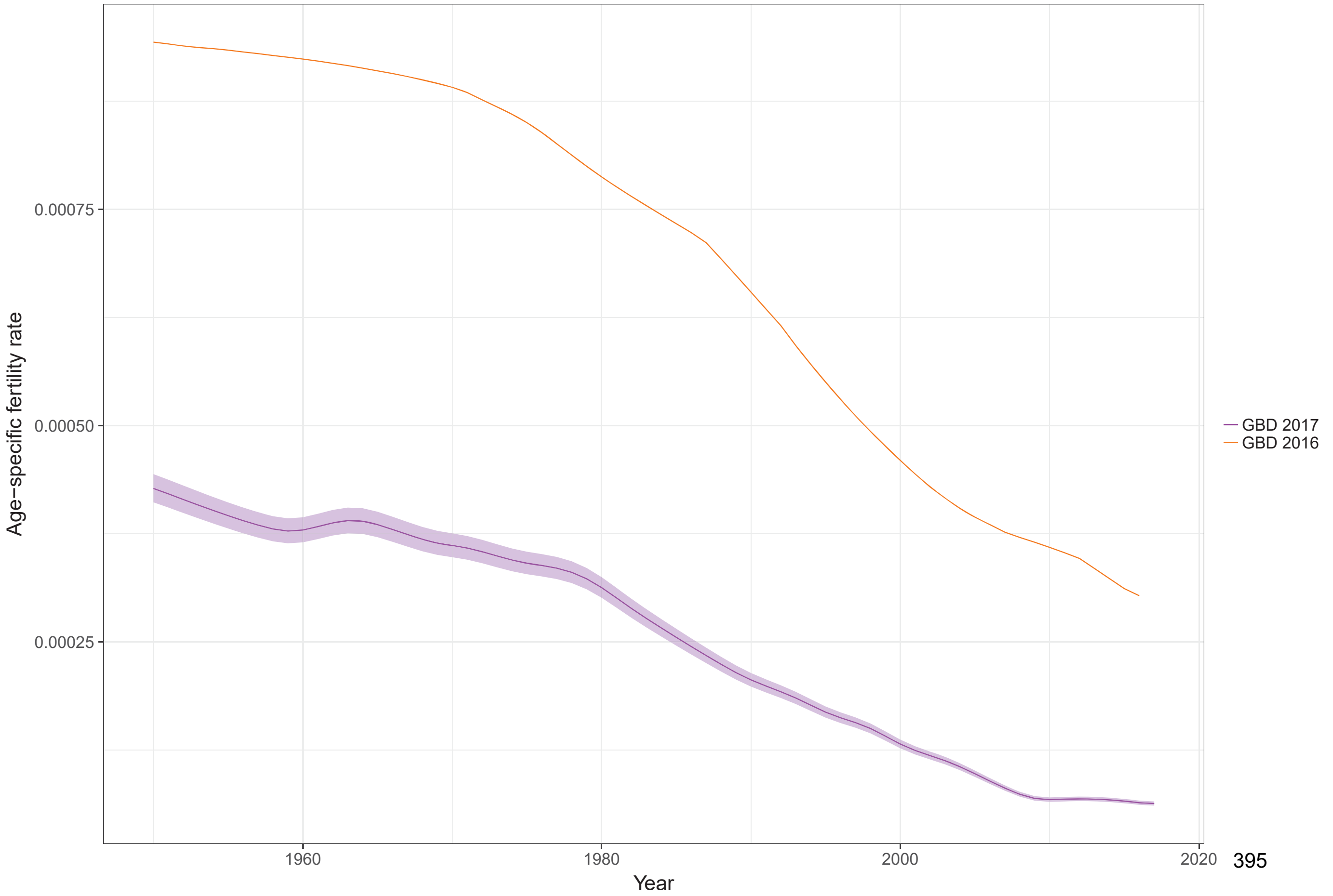

Age−specific fertility fitted trend lines, 1950−2017: Hong Kong Special Administrative Region of China, TFR
Data points enclosed with a diamond are reference sources

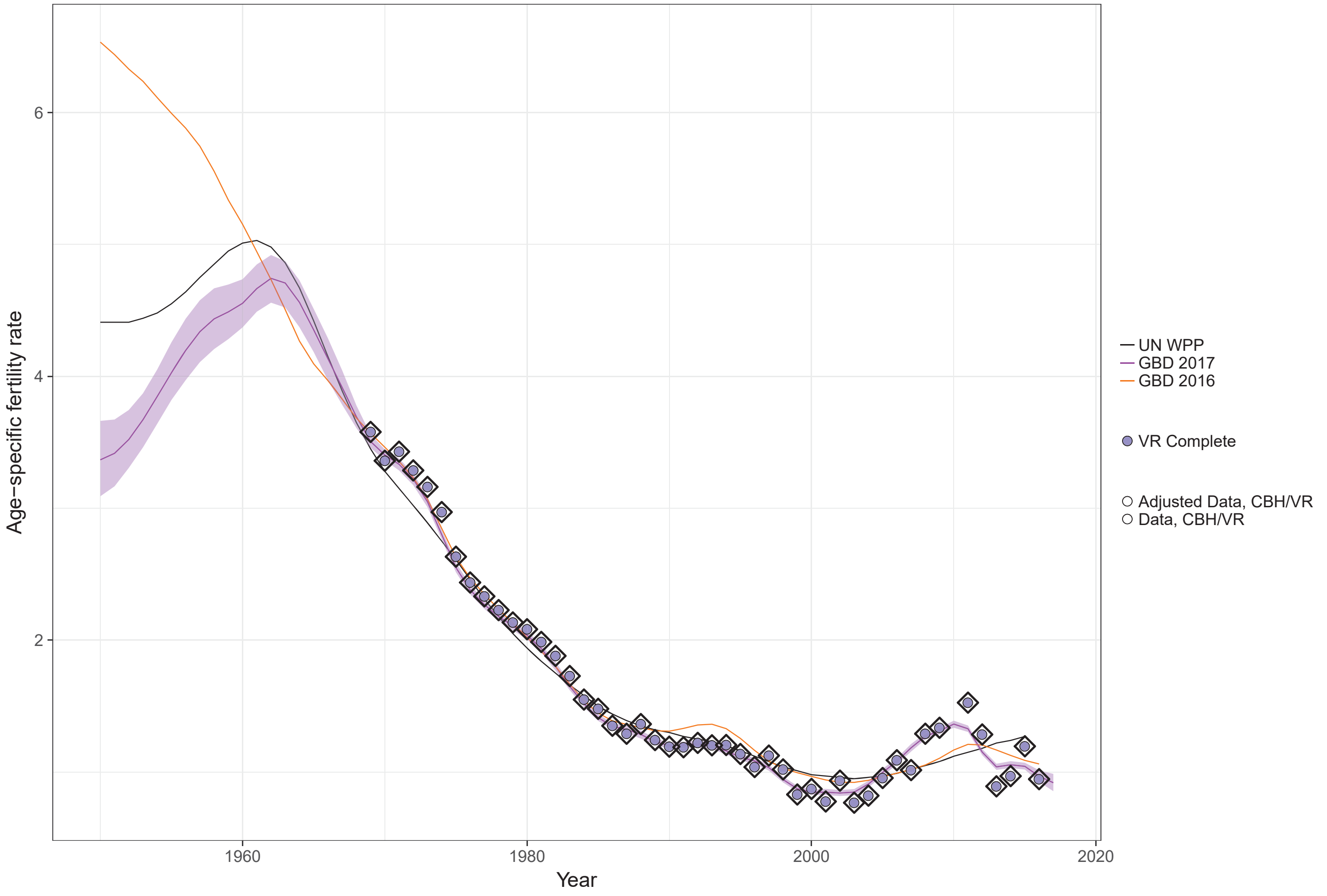

Age−specific fertility fitted trend lines, 1950−2017: Hong Kong Special Administrative Region of China, Ages: 10−1
Data points enclosed with a diamond are reference sources

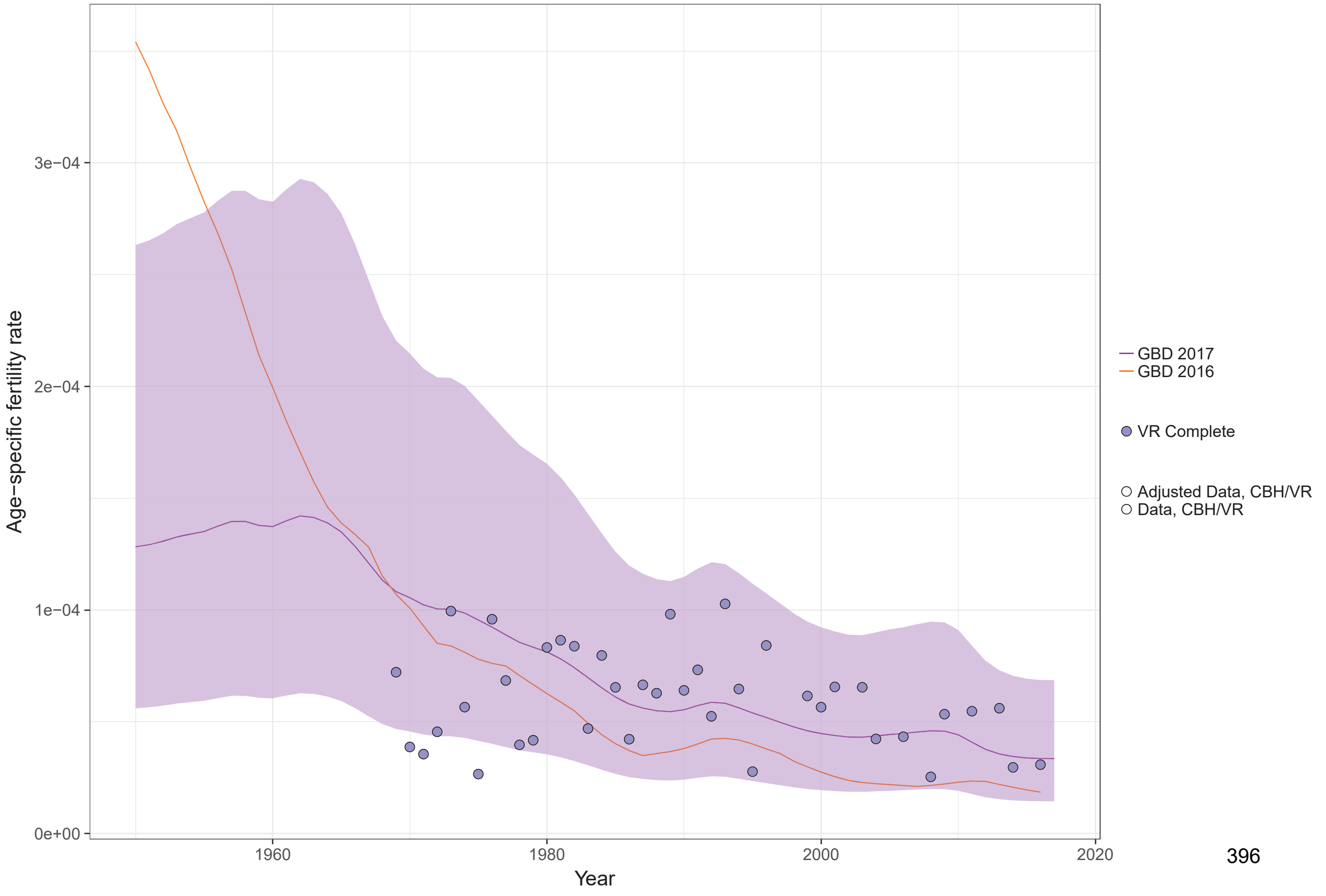

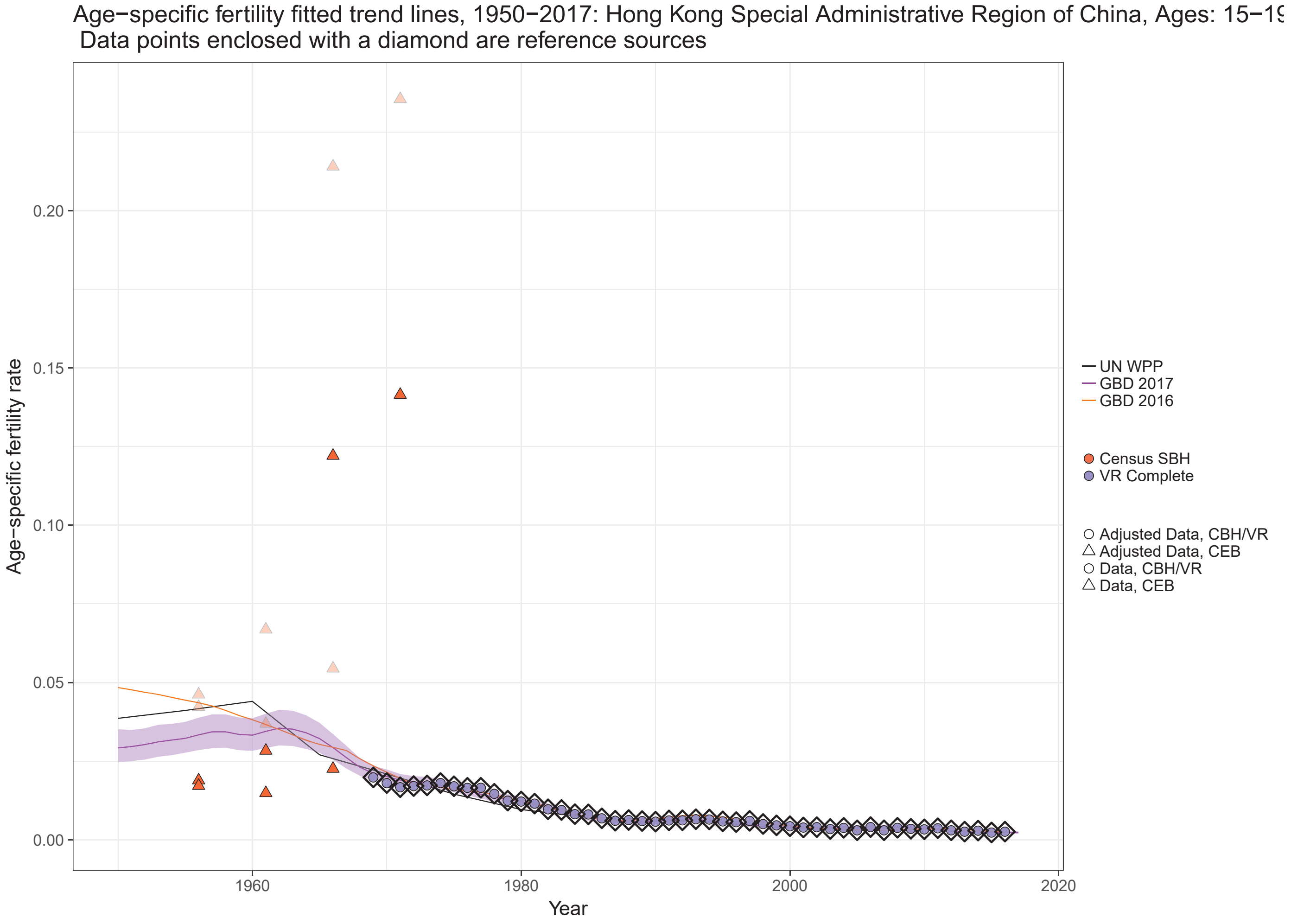

Age−specific fertility fitted trend lines, 1950−2017: Hong Kong Special Administrative Region of China, Ages: 15−19
Data points enclosed with a diamond are reference sources
Age−specific fertility rate
0.00
0.05
0.10
0.15
0.20
1960
1980
2000
2020
Year
UN WPP
GBD 2017
GBD 2016
Census SBH
VR Complete
Adjusted Data, CBH/VR
Adjusted Data, CEB
Data, CBH/VR
Data, CEB

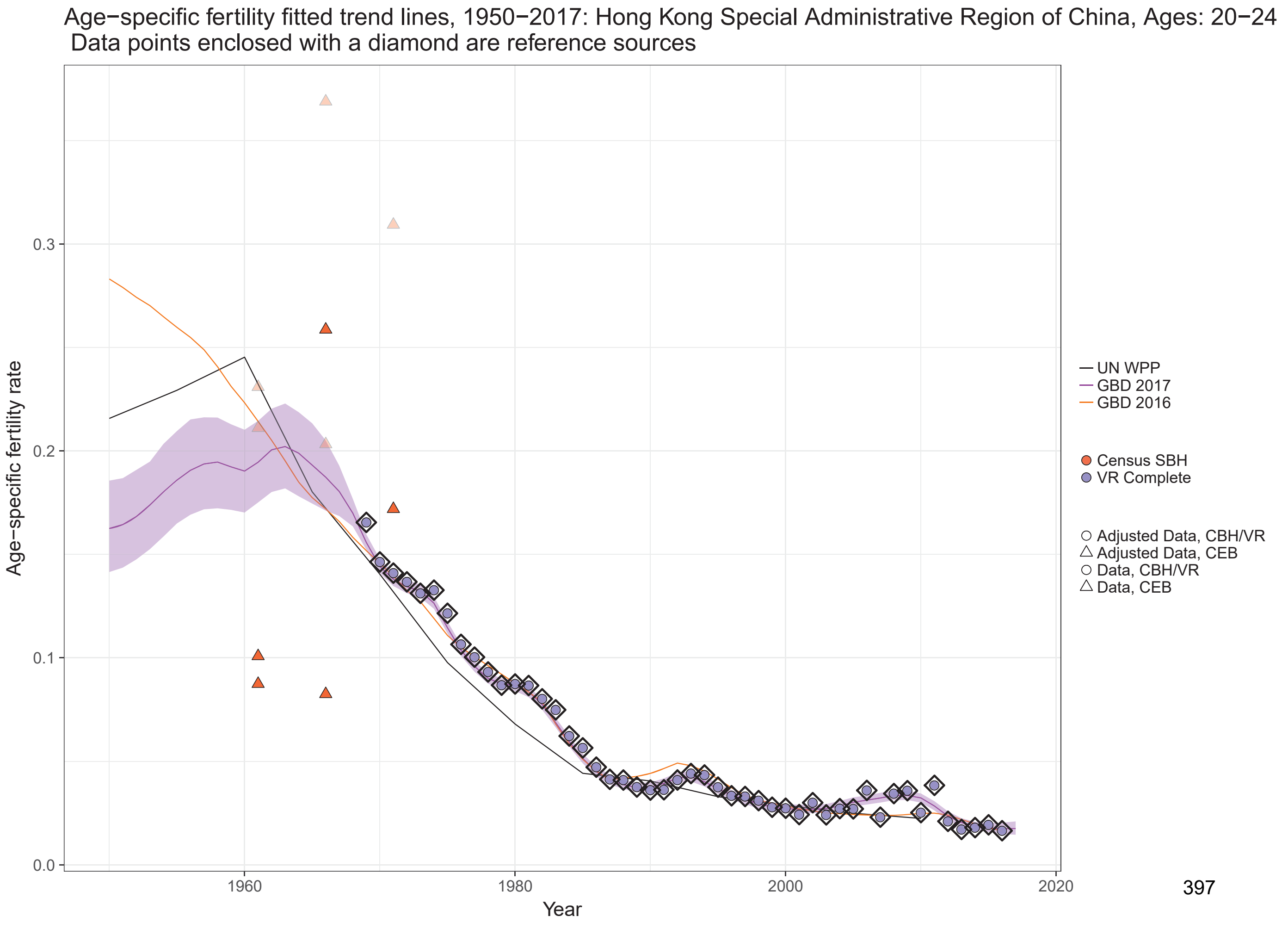

Age−specific fertility fitted trend lines, 1950−2017: Hong Kong Special Administrative Region of China, Ages: 20−24
Data points enclosed with a diamond are reference sources
Age−specific fertility rate
0.0
0.1
0.2
0.3
1960
1980
2000
2020
Year
UN WPP
GBD 2017
GBD 2016
Census SBH
VR Complete
Adjusted Data, CBH/VR
Adjusted Data, CEB
Data, CBH/VR
Data, CEB

Age−specific fertility fitted trend lines, 1950−2017: Hong Kong Special Administrative Region of China, Ages: 25−29
Data points enclosed with a diamond are reference sources

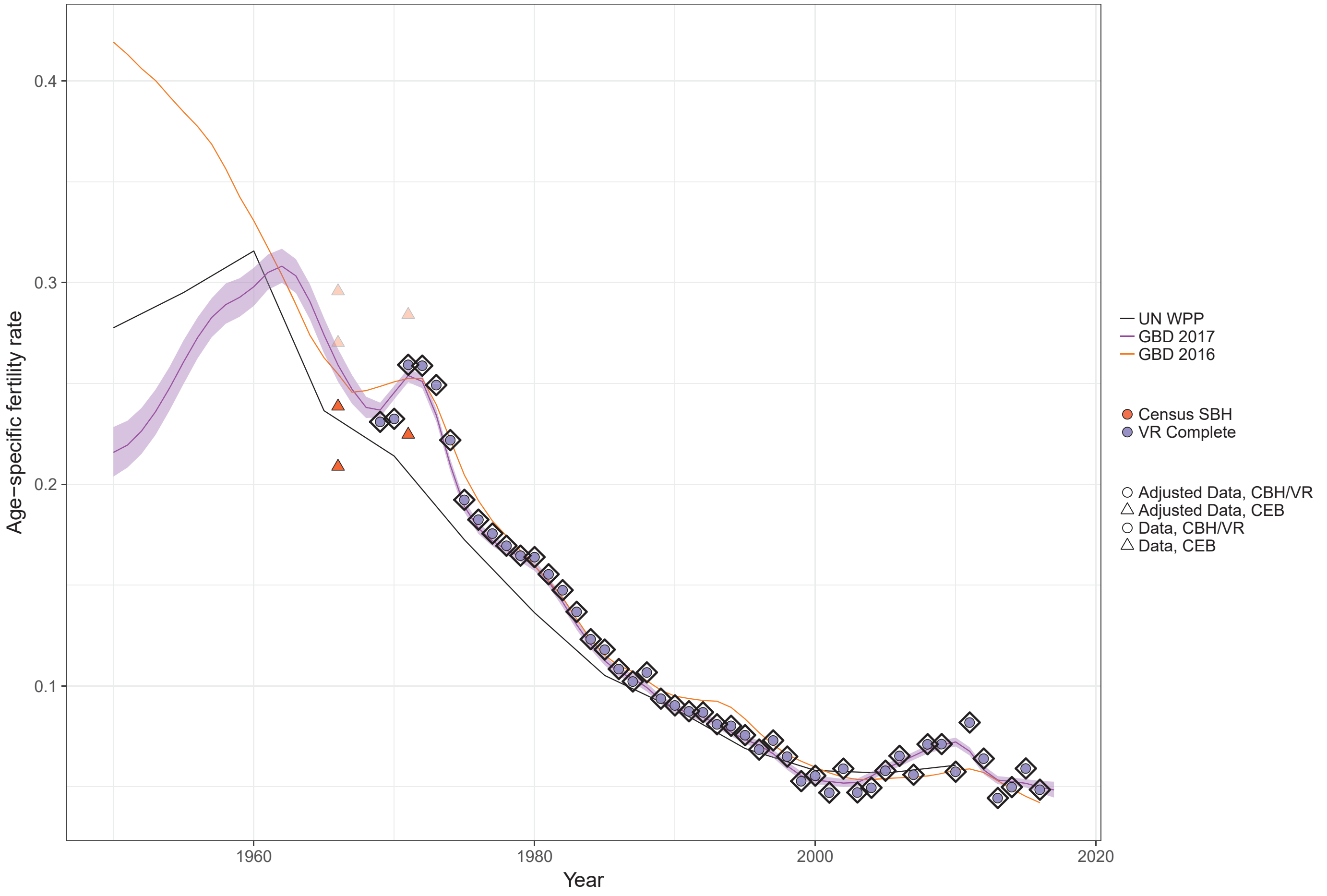

Age−specific fertility fitted trend lines, 1950−2017: Hong Kong Special Administrative Region of China, Ages: 30−34
Data points enclosed with a diamond are reference sources

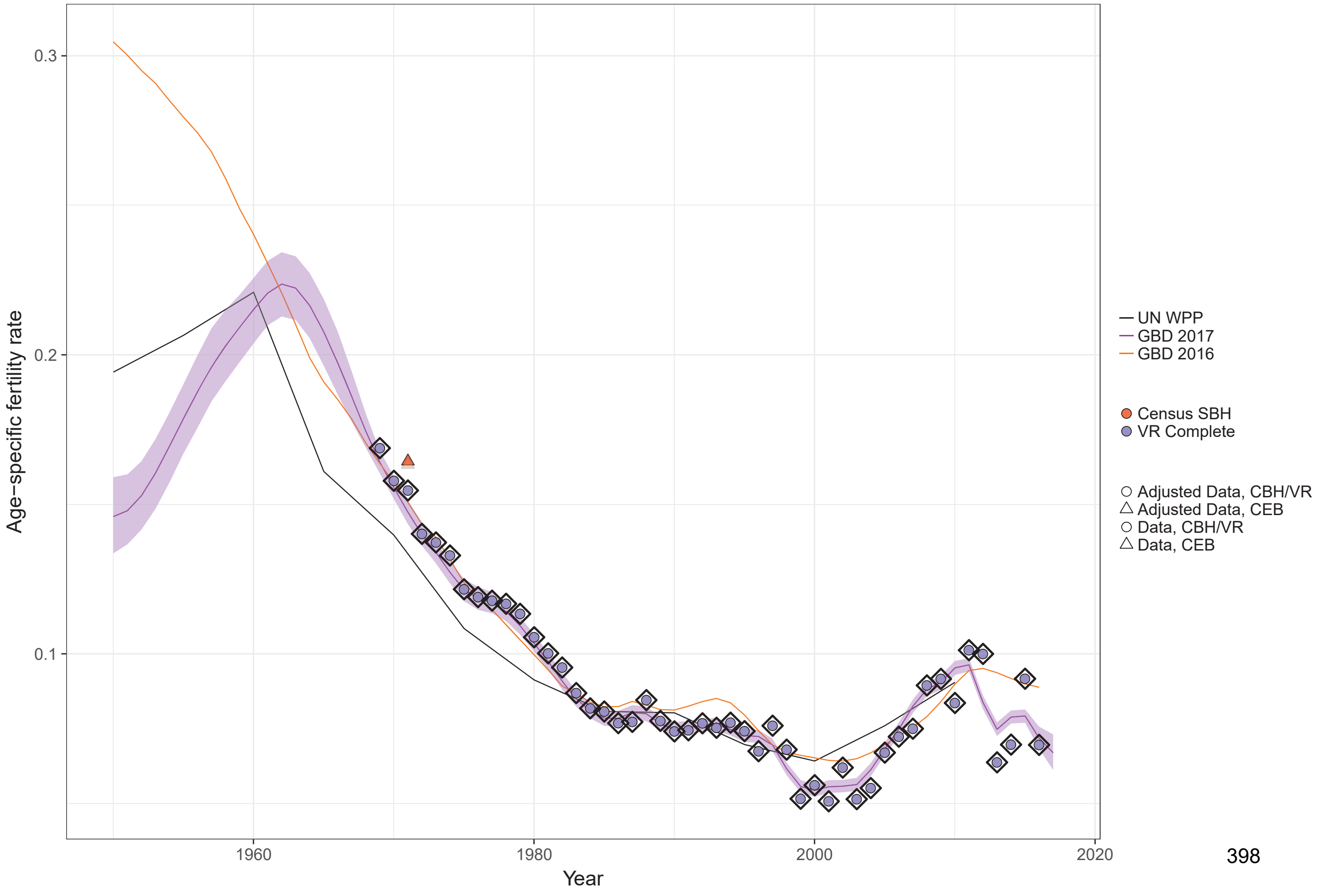

Age−specific fertility fitted trend lines, 1950−2017: Hong Kong Special Administrative Region of China, Ages: 35−39
Data points enclosed with a diamond are reference sources

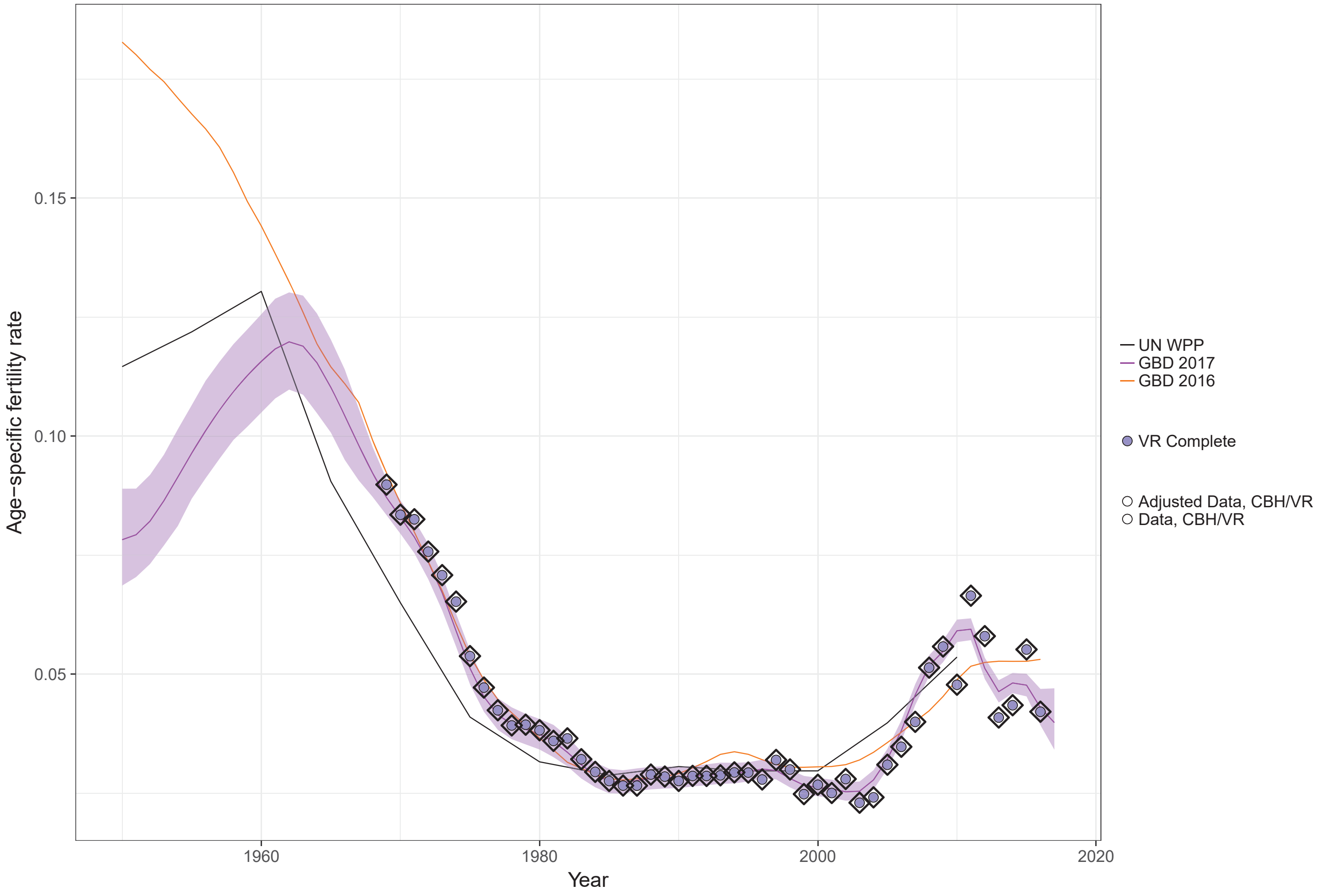

Age−specific fertility fitted trend lines, 1950−2017: Hong Kong Special Administrative Region of China, Ages: 40−44
Data points enclosed with a diamond are reference sources

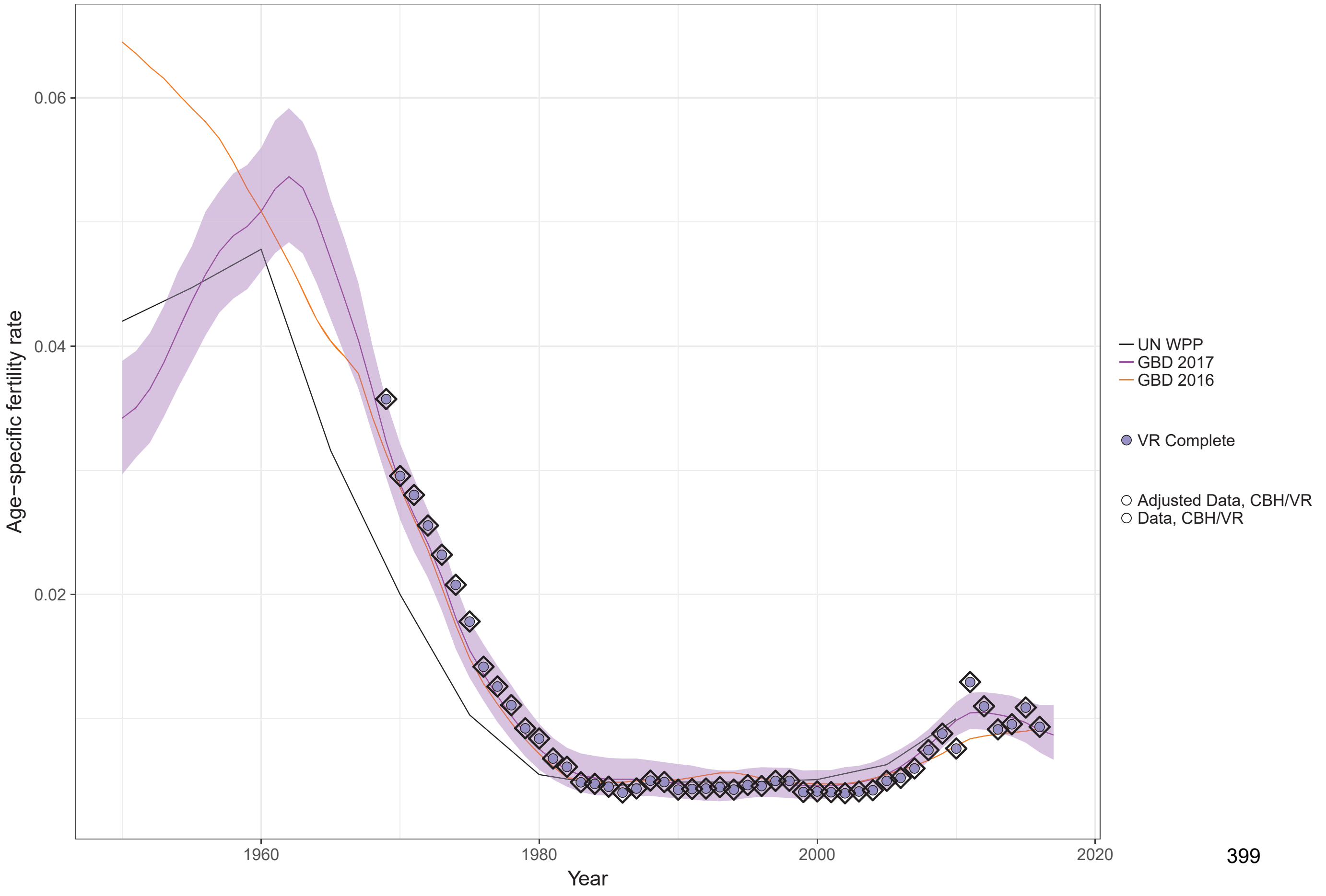

Age−specific fertility fitted trend lines, 1950−2017: Hong Kong Special Administrative Region of China, Ages: 45−4
Data points enclosed with a diamond are reference sources

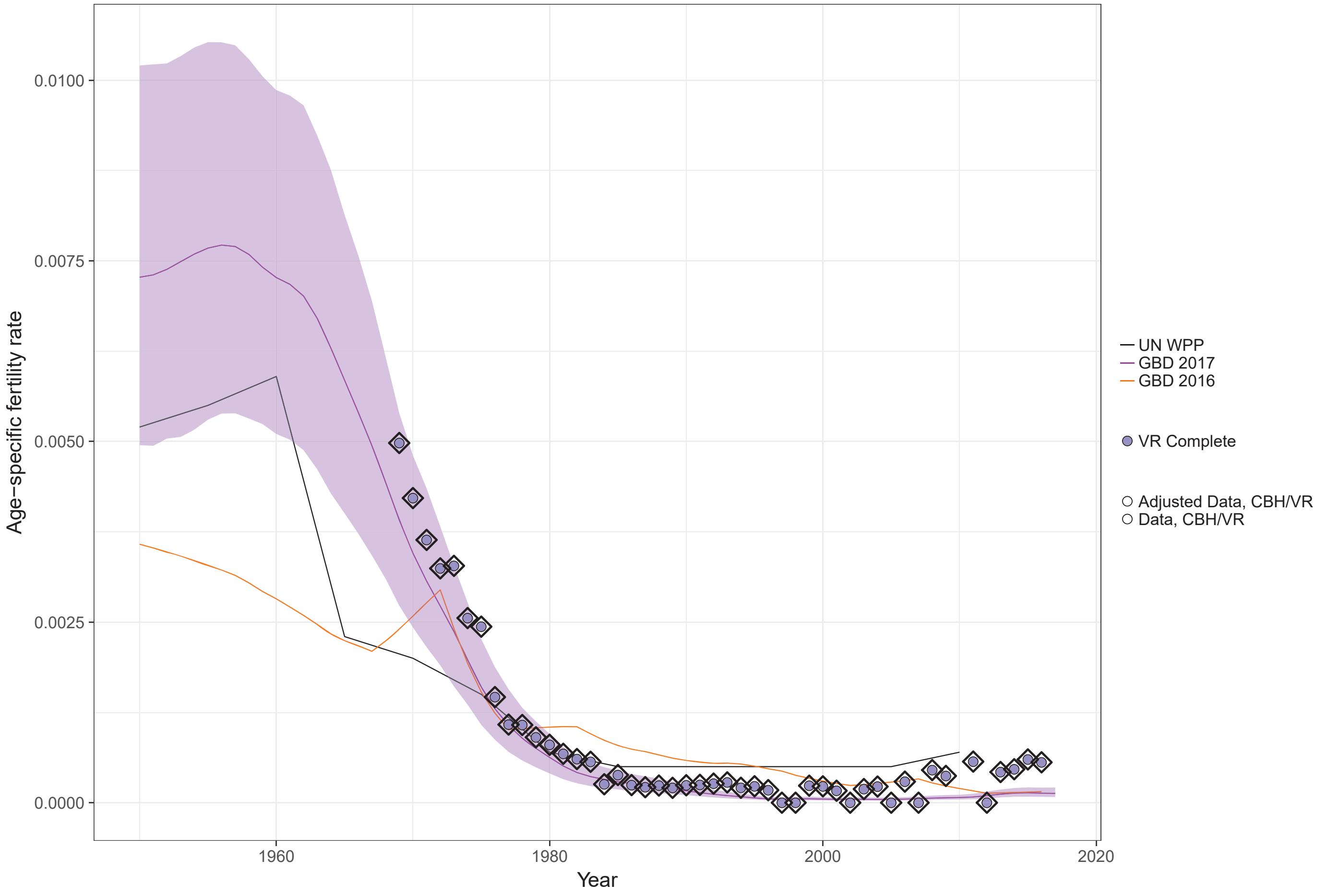

Age−specific fertility fitted trend lines, 1950−2017: Hong Kong Special Administrative Region of China, Ages: 50−
Data points enclosed with a diamond are reference sources

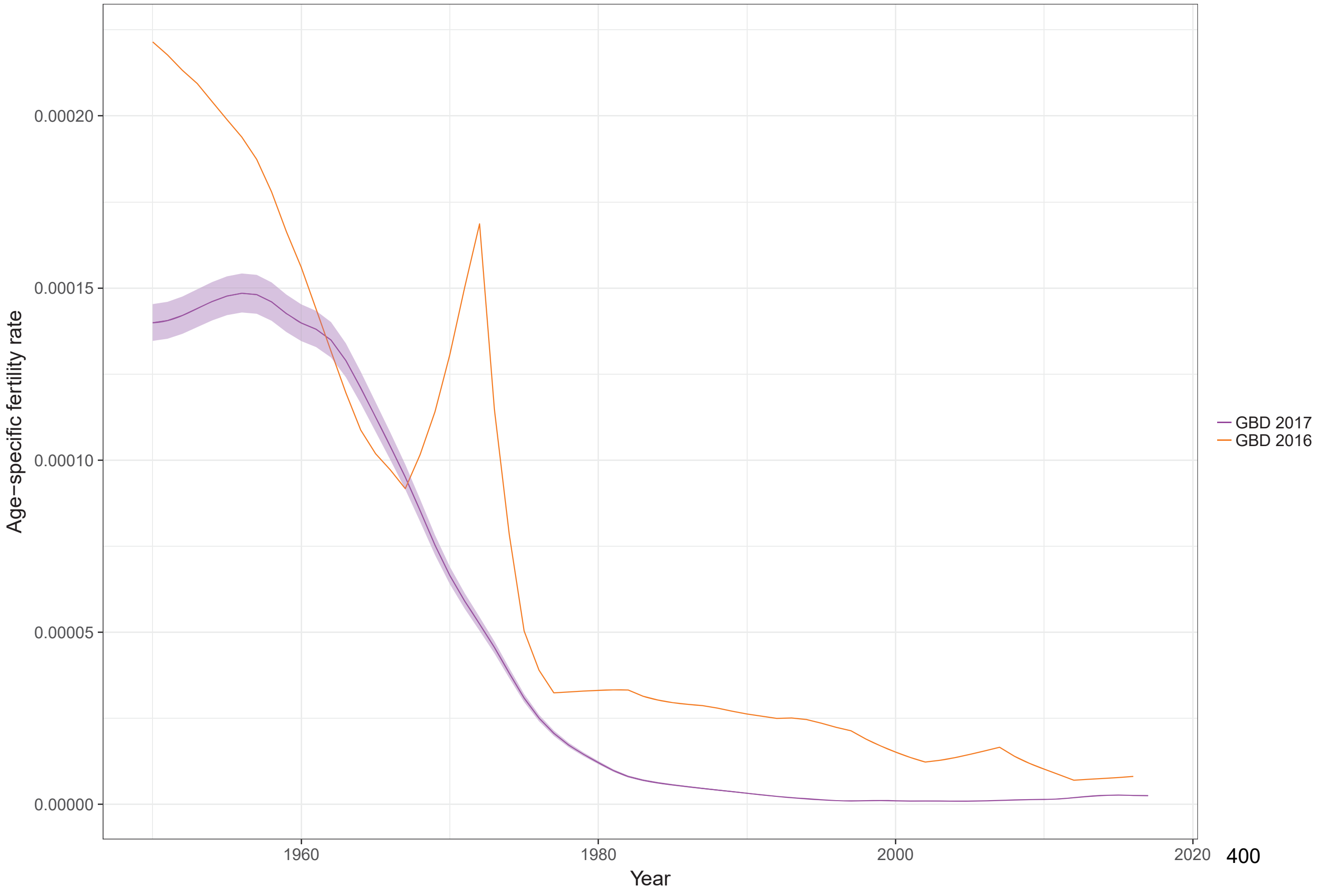

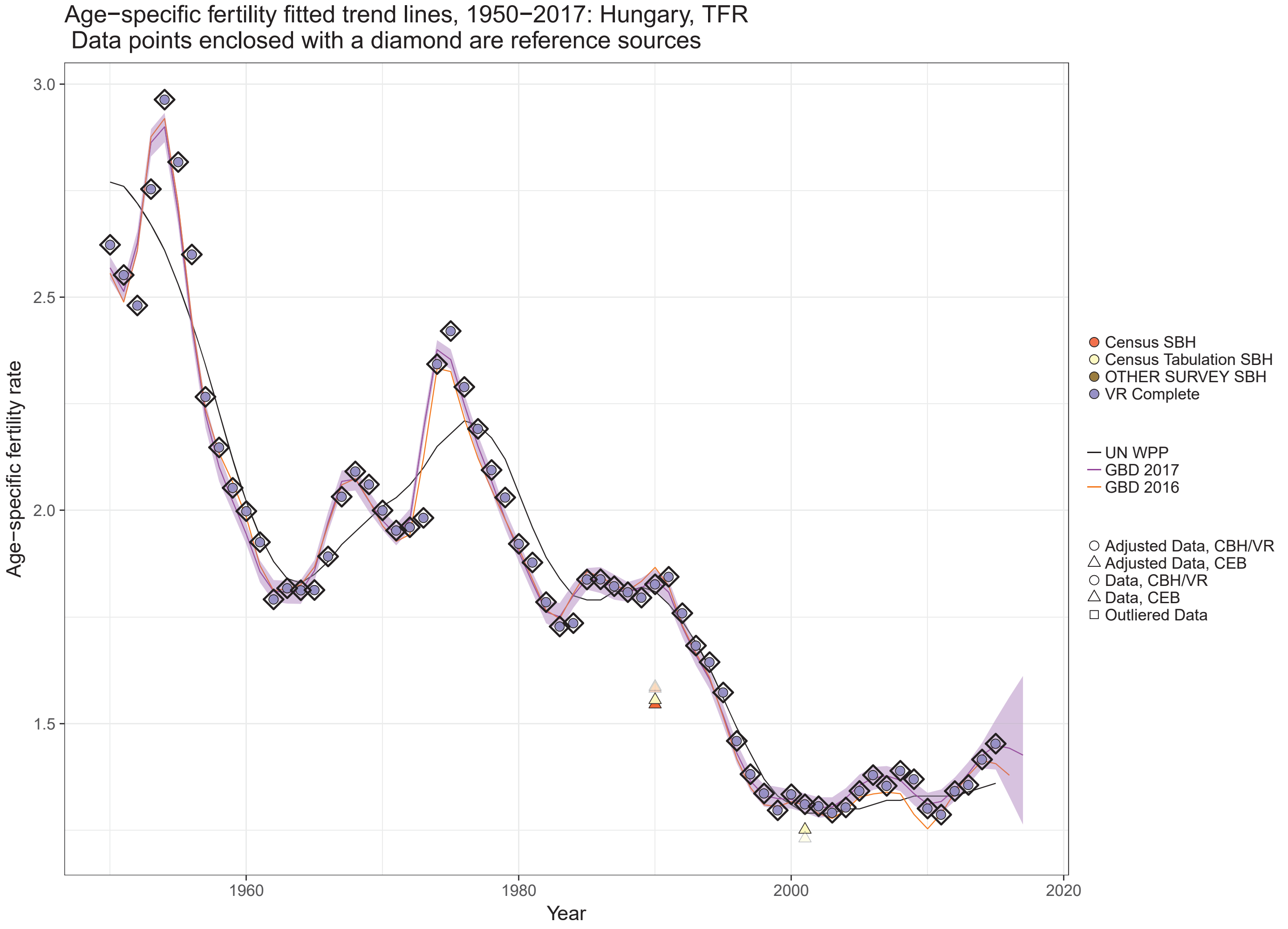
Age−specific fertility fitted trend lines, 1950−2017: Hungary, TFR
Data points enclosed with a diamond are reference sources
Age-specific fertility rate
Year
3.0
2.5
2.0
1.5
1960
1980
2000
2020
Census SBH
Census Tabulation SBH
OTHER SURVEY SBH
VR Complete
UN WPP
GBD 2017
GBD 2016
Adjusted Data, CBH/VR
Adjusted Data, CEB
Data, CBH/VR
Data, CEB
Outliered Data

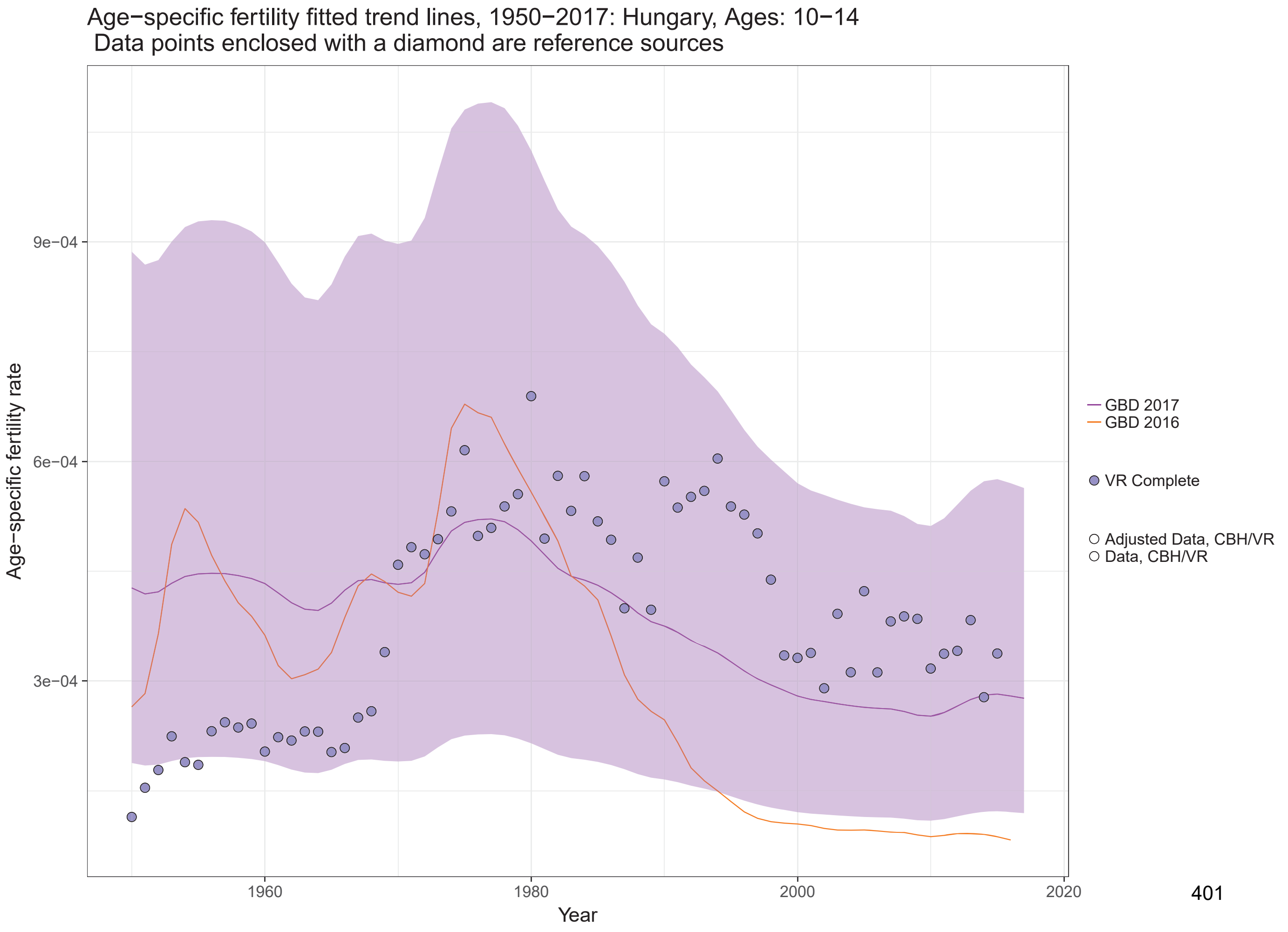
Age−specific fertility fitted trend lines, 1950−2017: Hungary, Ages: 10−14
Data points enclosed with a diamond are reference sources
Age-specific fertility rate
Year
9e−04
6e−04
3e−04
1960
1980
2000
2020
GBD 2017
GBD 2016
VR Complete
Adjusted Data, CBH/VR
Data, CBH/VR

Age−specific fertility fitted trend lines, 1950−2017: Hungary, Ages: 15−19
Data points enclosed with a diamond are reference sources

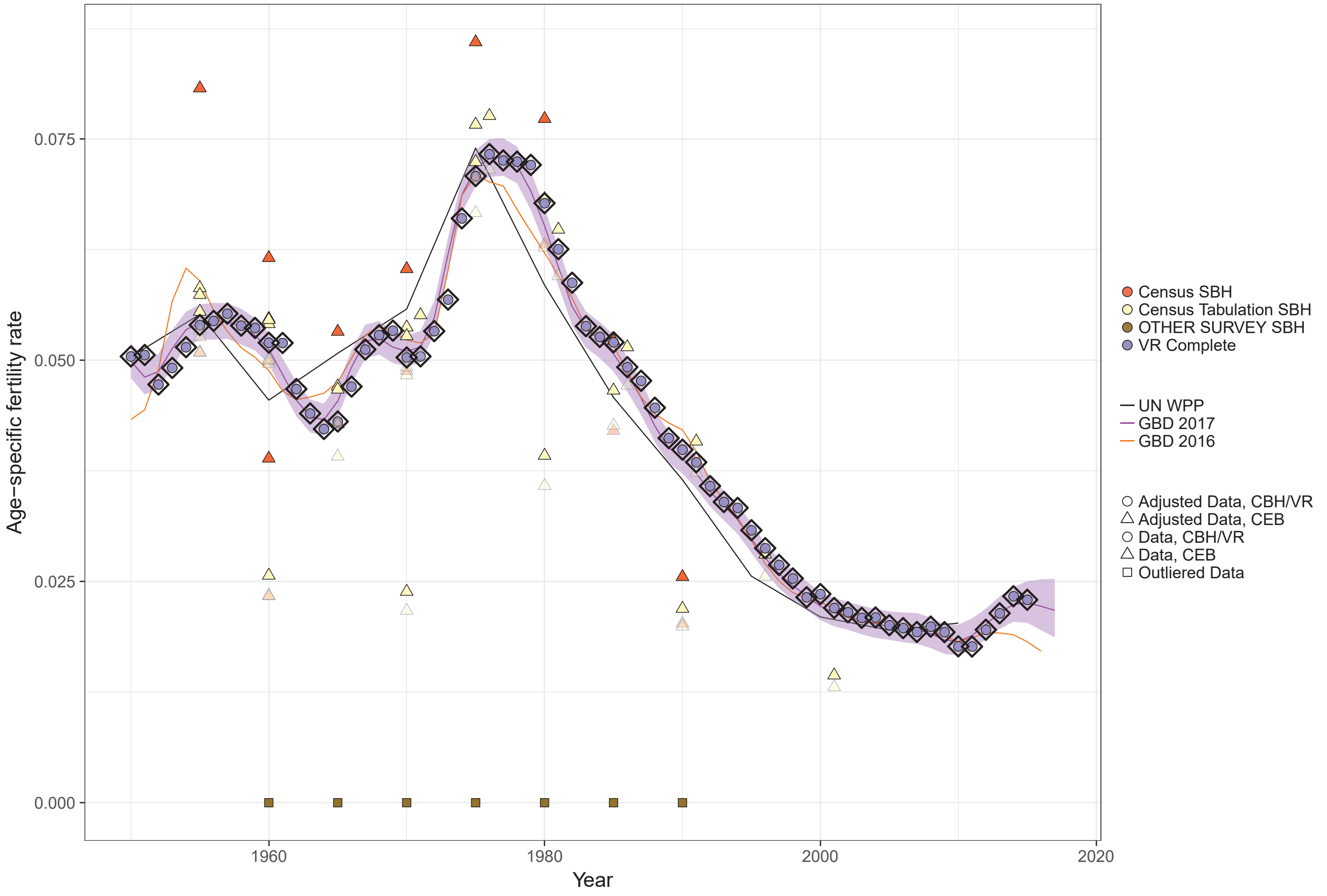

Age−specific fertility fitted trend lines, 1950−2017: Hungary, Ages: 20−24
Data points enclosed with a diamond are reference sources

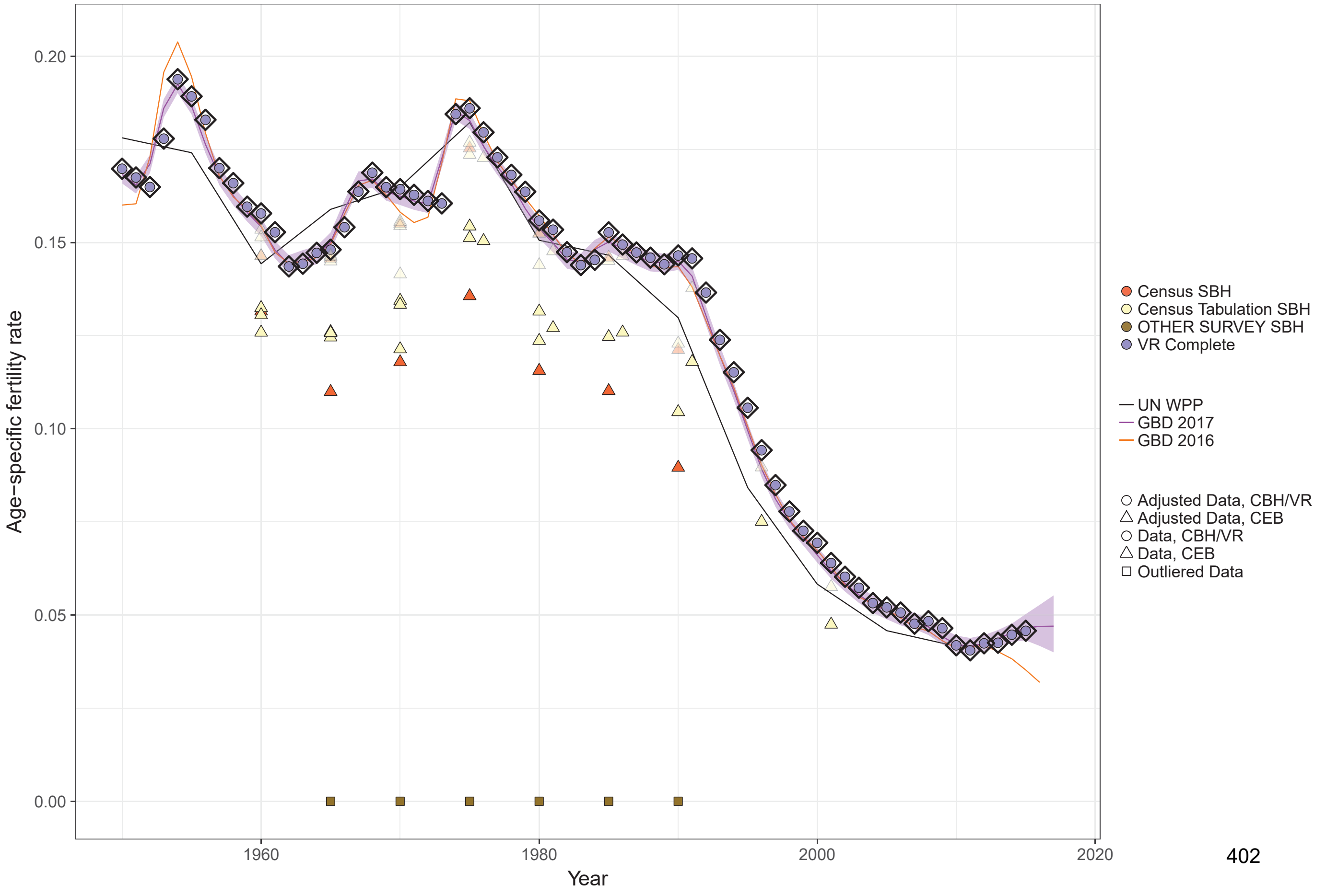

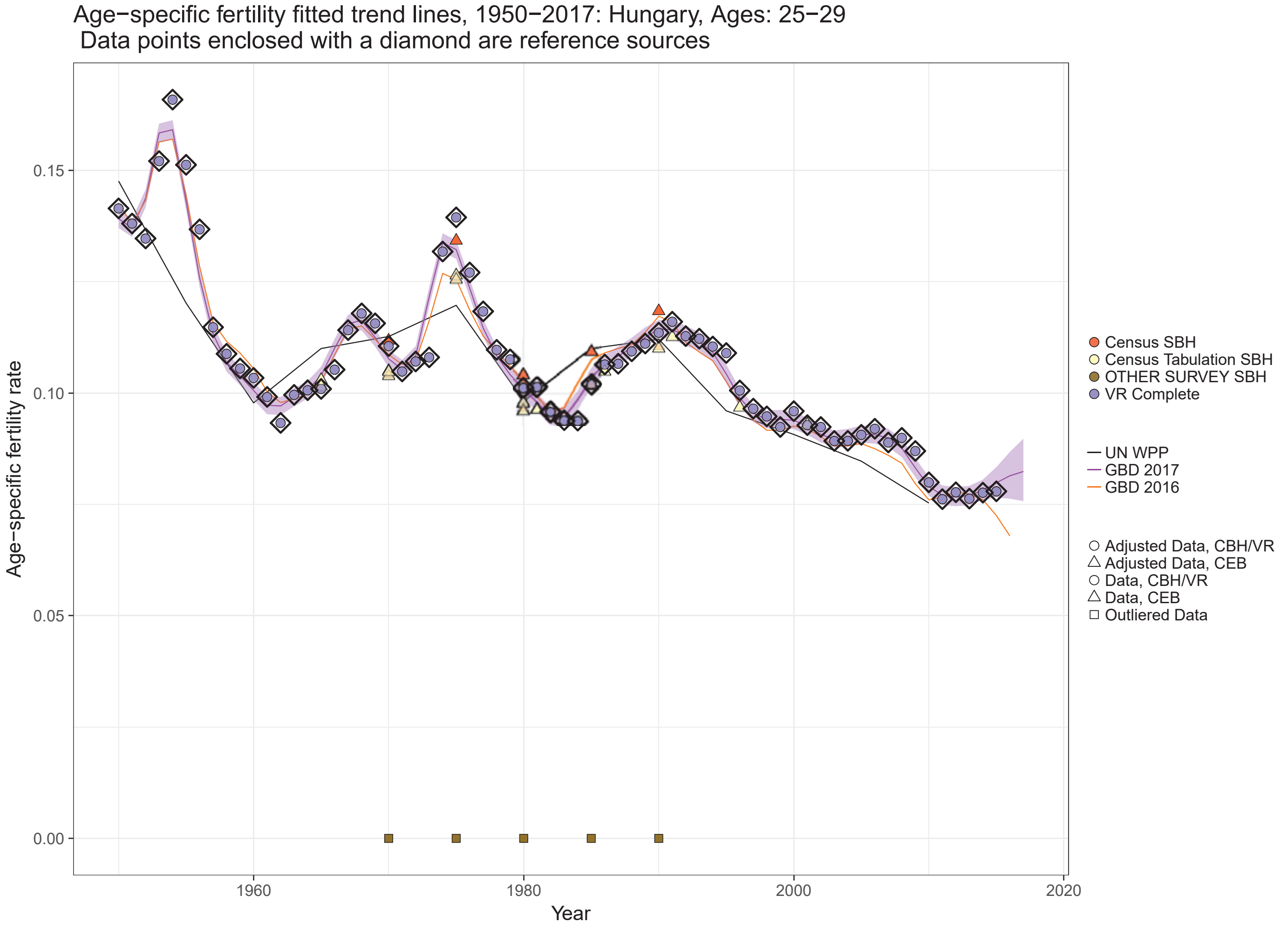

Age−specific fertility fitted trend lines, 1950−2017: Hungary, Ages: 25−29
Data points enclosed with a diamond are reference sources
Age−specific fertility rate
0.15
0.10
0.05
0.00
1960
1980
2000
2020
Year
Census SBH
Census Tabulation SBH
OTHER SURVEY SBH
VR Complete
UN WPP
GBD 2017
GBD 2016
Adjusted Data, CBH/VR
Adjusted Data, CEB
Data, CBH/VR
Data, CEB
Outliered Data

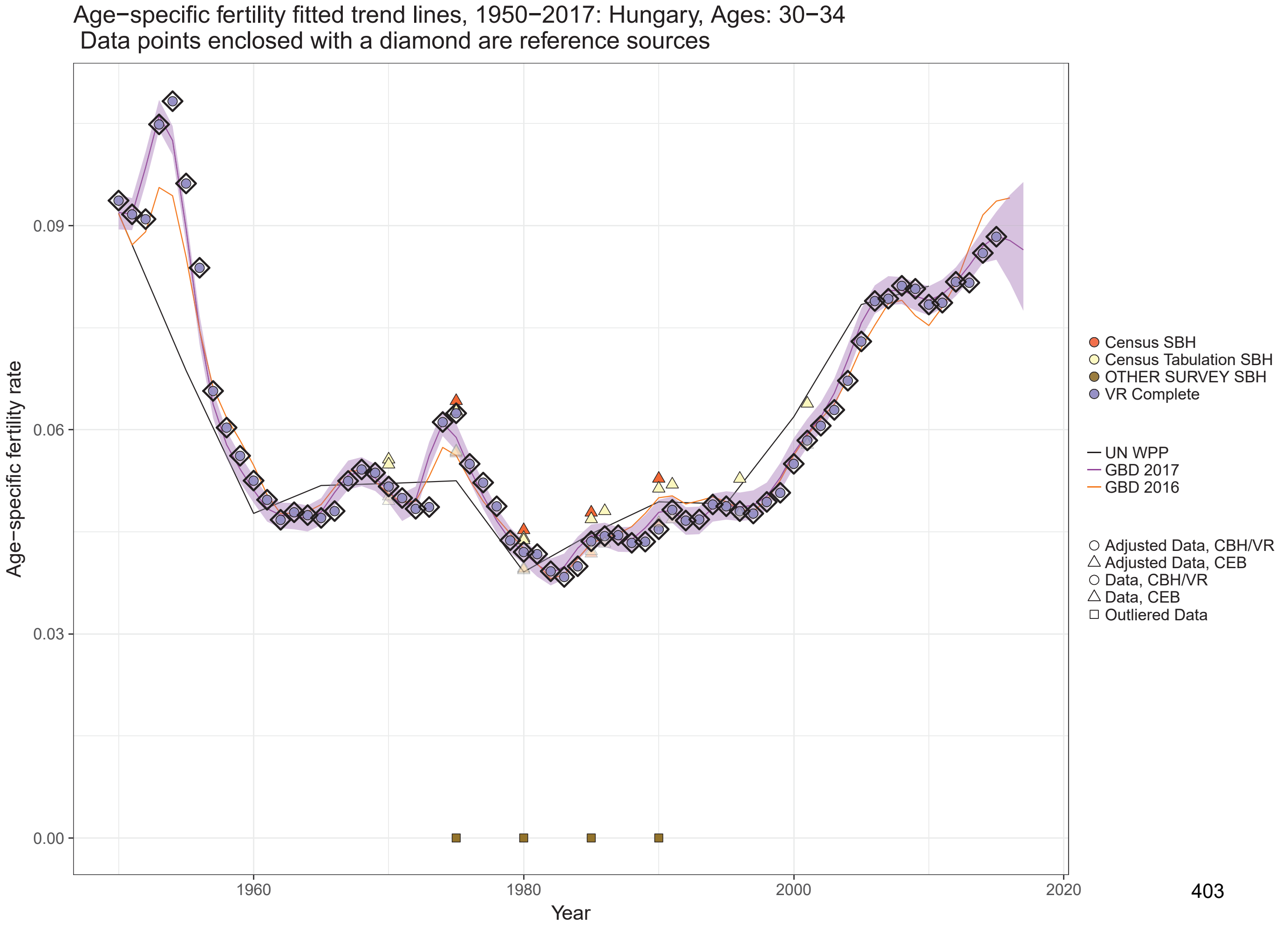

Age−specific fertility fitted trend lines, 1950−2017: Hungary, Ages: 30−34
Data points enclosed with a diamond are reference sources
Age−specific fertility rate
0.09
0.06
0.03
0.00
1960
1980
2000
2020
Year
Census SBH
Census Tabulation SBH
OTHER SURVEY SBH
VR Complete
UN WPP
GBD 2017
GBD 2016
Adjusted Data, CBH/VR
Adjusted Data, CEB
Data, CBH/VR
Data, CEB
Outliered Data

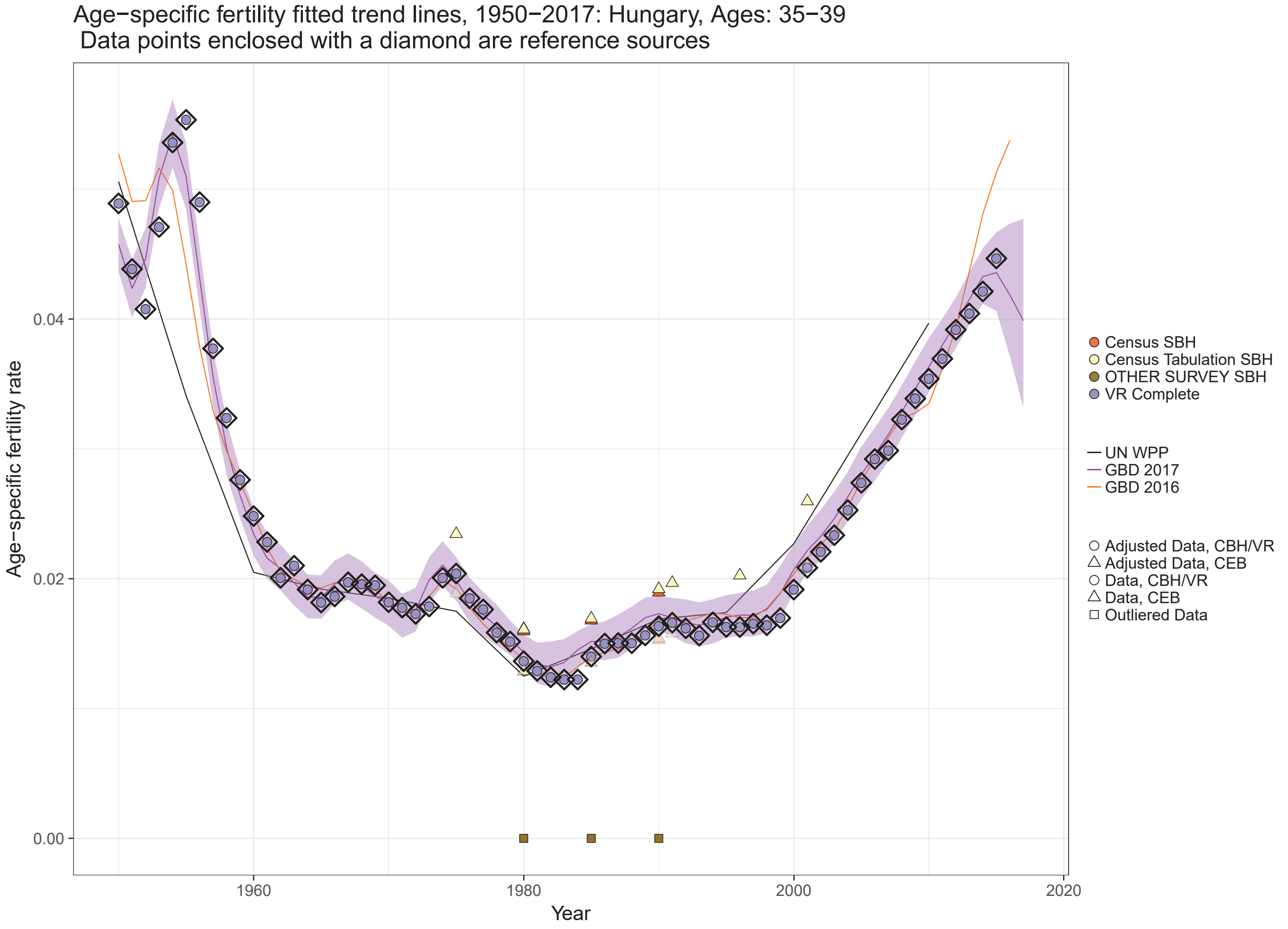
Age−specific fertility fitted trend lines, 1950−2017: Hungary, Ages: 35−39
Data points enclosed with a diamond are reference sources
Age-specific fertility rate
0.00
0.02
0.04
1960
1980
2000
2020
Year
Census SBH
Census Tabulation SBH
OTHER SURVEY SBH
VR Complete
UN WPP
GBD 2017
GBD 2016
Adjusted Data, CBH/VR
Adjusted Data, CEB
Data, CBH/VR
Data, CEB
Outliered Data

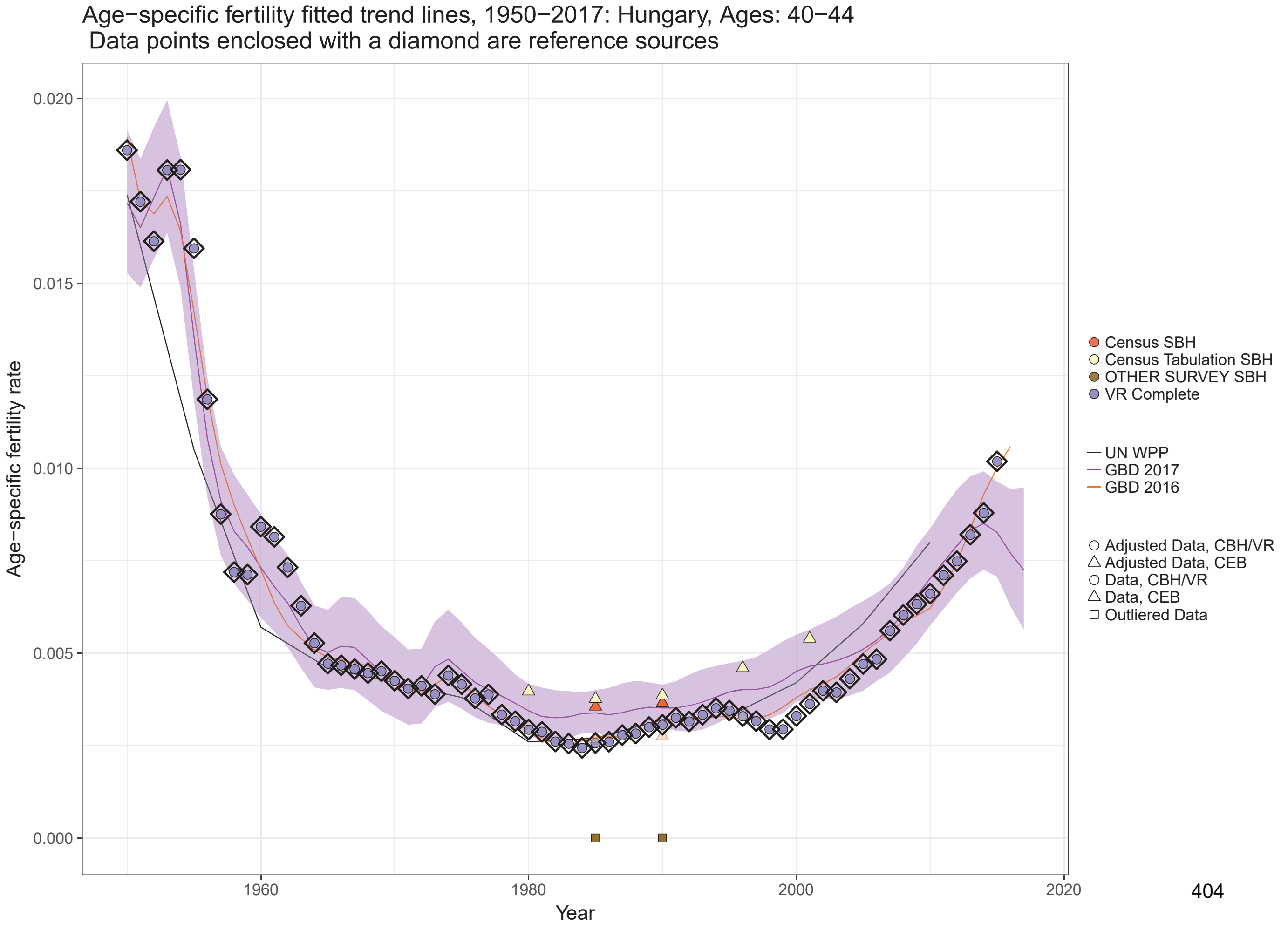
Age−specific fertility fitted trend lines, 1950−2017: Hungary, Ages: 40−44
Data points enclosed with a diamond are reference sources
Age-specific fertility rate
0.000
0.005
0.010
0.015
0.020
1960
1980
2000
2020
Year
Census SBH
Census Tabulation SBH
OTHER SURVEY SBH
VR Complete
UN WPP
GBD 2017
GBD 2016
Adjusted Data, CBH/VR
Adjusted Data, CEB
Data, CBH/VR
Data, CEB
Outliered Data

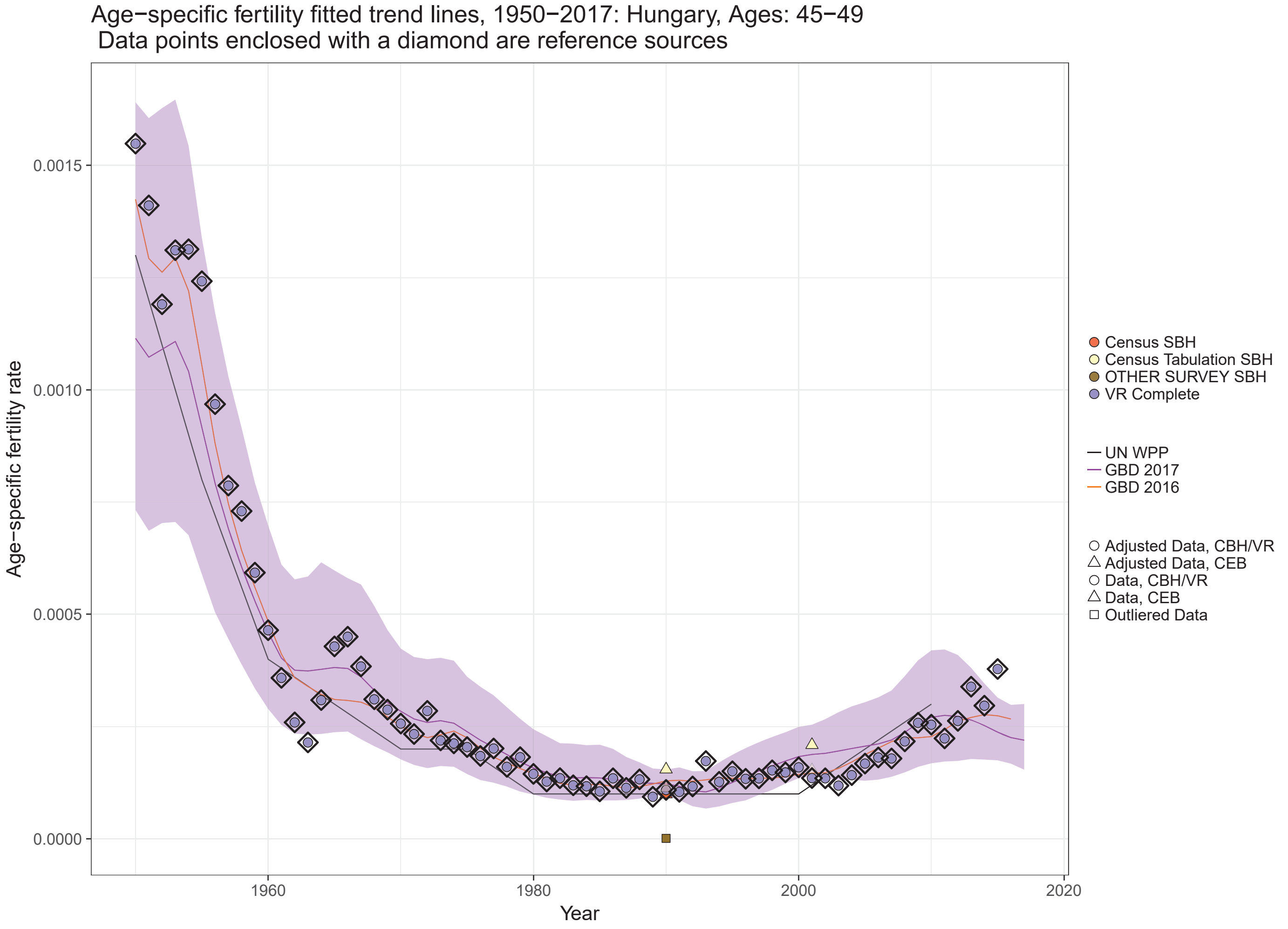

Age−specific fertility fitted trend lines, 1950−2017: Hungary, Ages: 45−49
Data points enclosed with a diamond are reference sources
Age−specific fertility rate
Year
Census SBH
Census Tabulation SBH
OTHER SURVEY SBH
VR Complete
UN WPP
GBD 2017
GBD 2016
Adjusted Data, CBH/VR
Adjusted Data, CEB
Data, CBH/VR
Data, CEB
Outliered Data

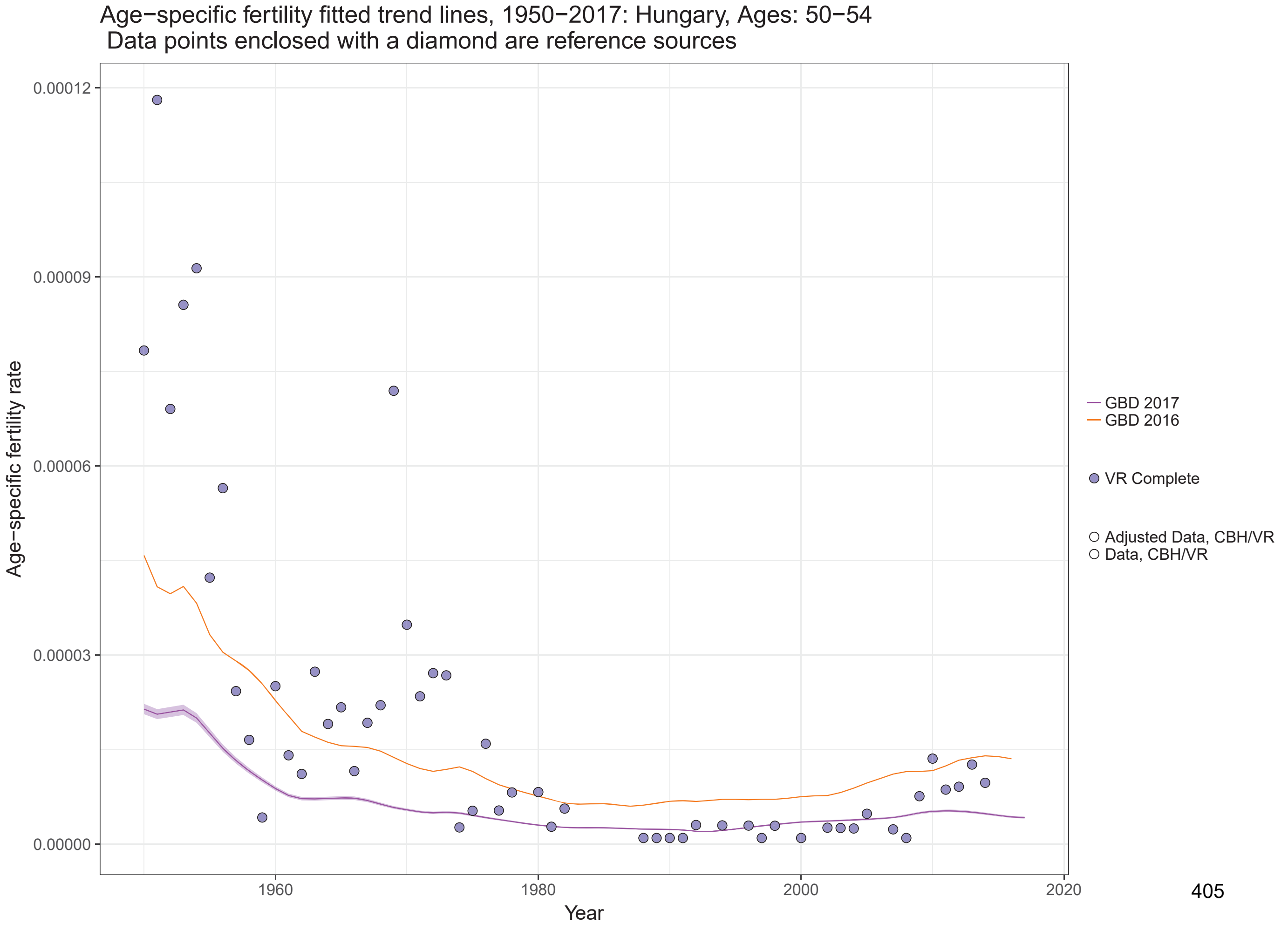

Age−specific fertility fitted trend lines, 1950−2017: Hungary, Ages: 50−54
Data points enclosed with a diamond are reference sources
Age−specific fertility rate
Year
GBD 2017
GBD 2016
VR Complete
Adjusted Data, CBH/VR
Data, CBH/VR

Age−specific fertility fitted trend lines, 1950−2017: Iceland, TFR
Data points enclosed with a diamond are reference sources

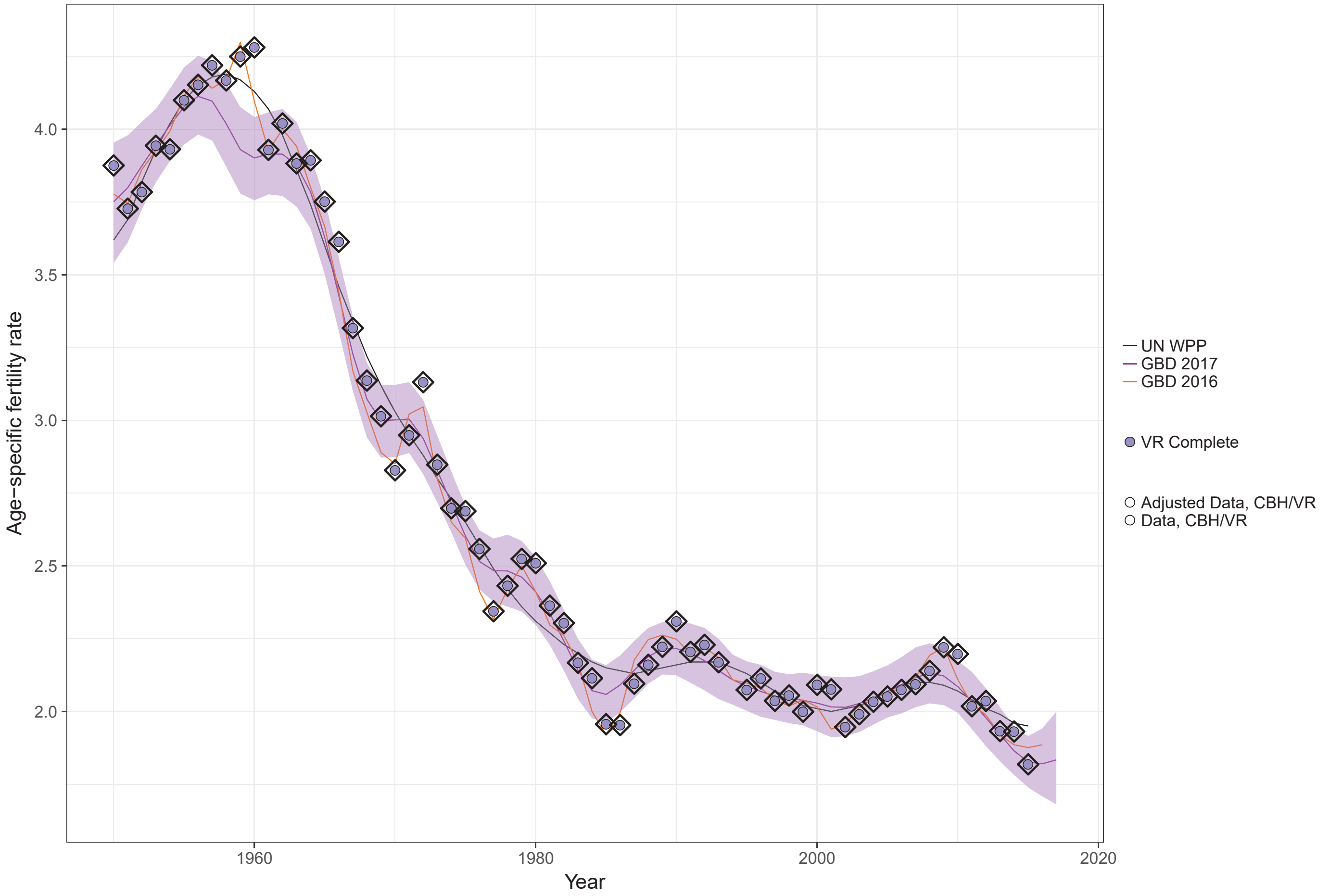

Age−specific fertility fitted trend lines, 1950−2017: Iceland, Ages: 10−14
Data points enclosed with a diamond are reference sources

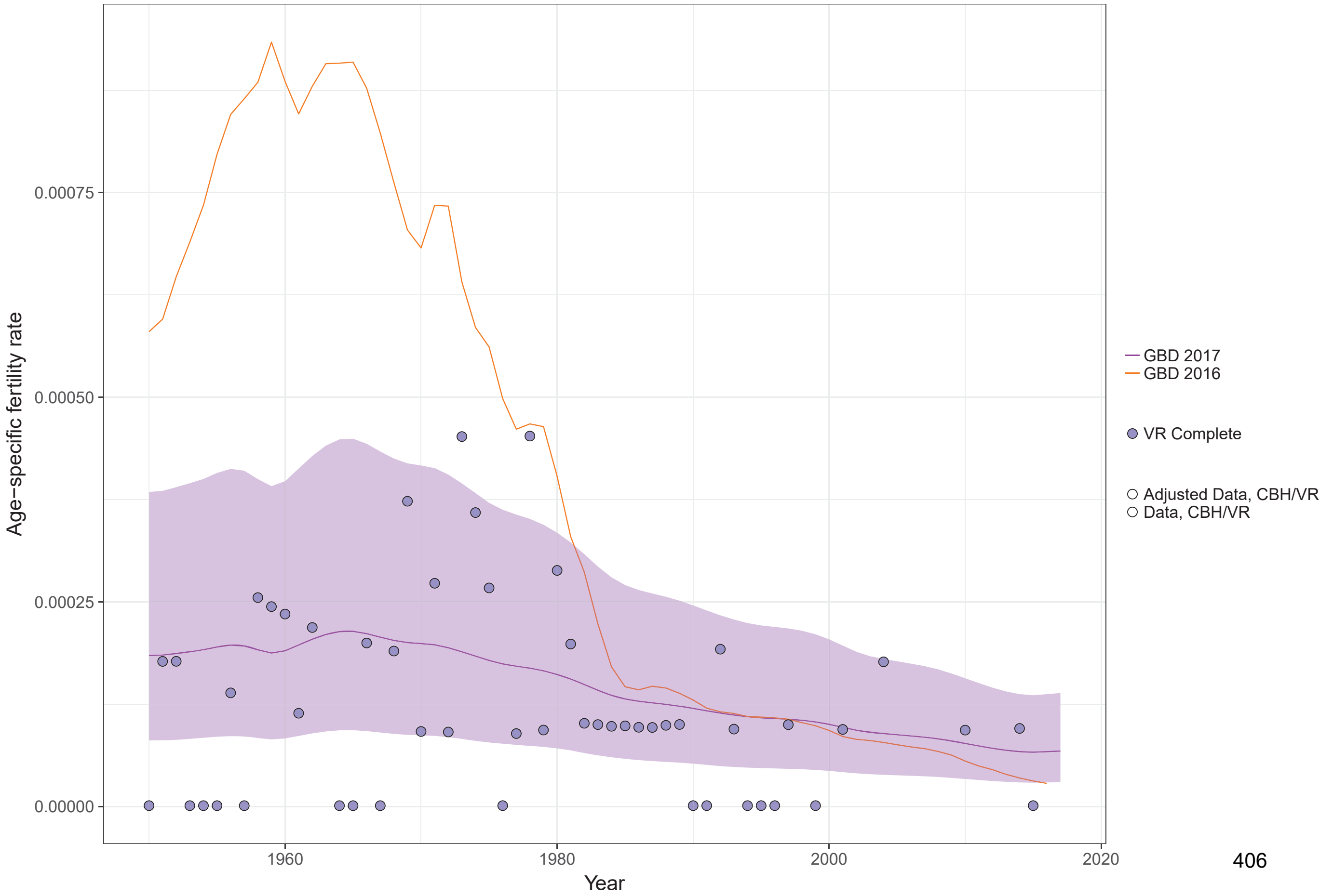

Age−specific fertility fitted trend lines, 1950−2017: Iceland, Ages: 15−19
Data points enclosed with a diamond are reference sources

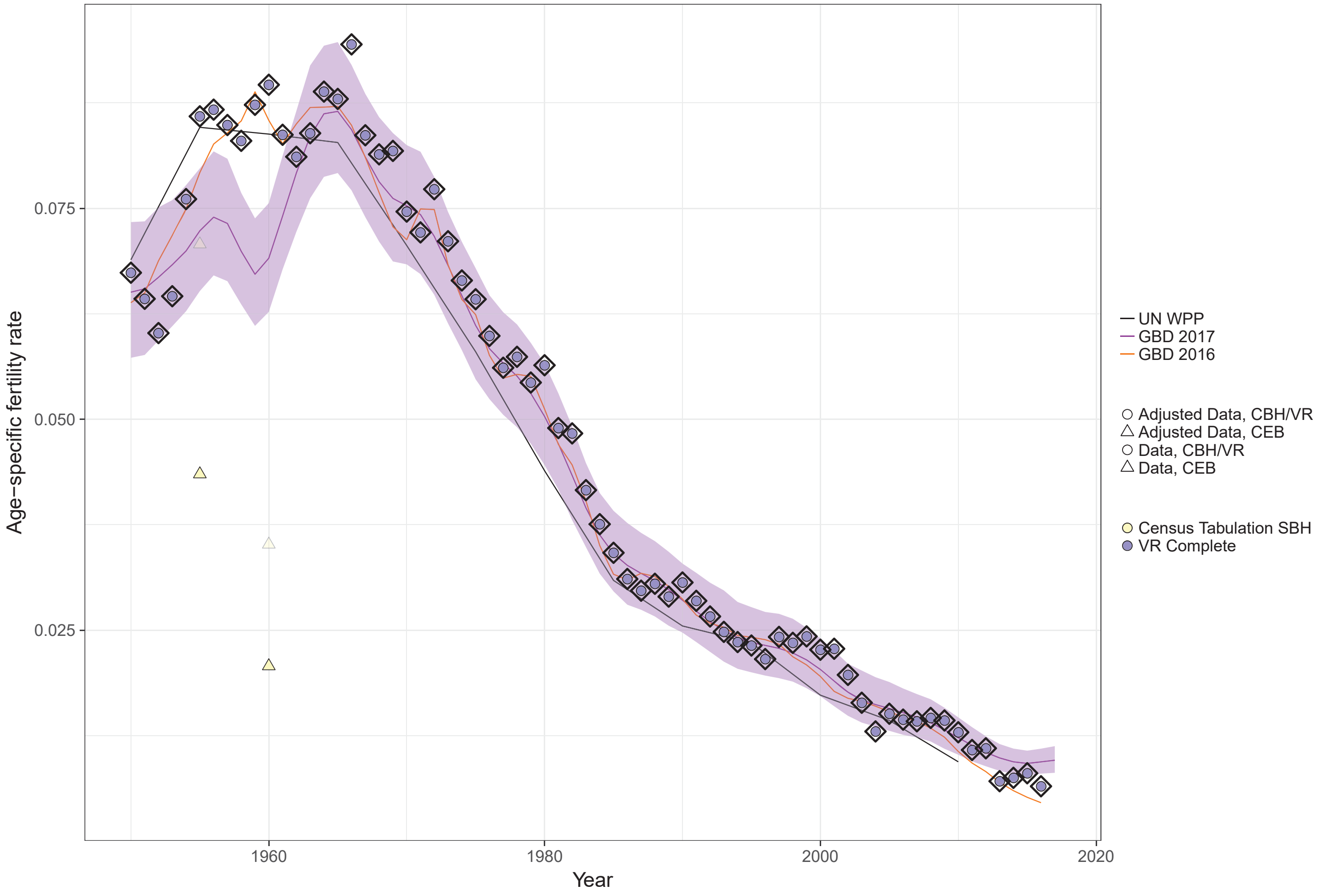

Age−specific fertility fitted trend lines, 1950−2017: Iceland, Ages: 20−24
Data points enclosed with a diamond are reference sources

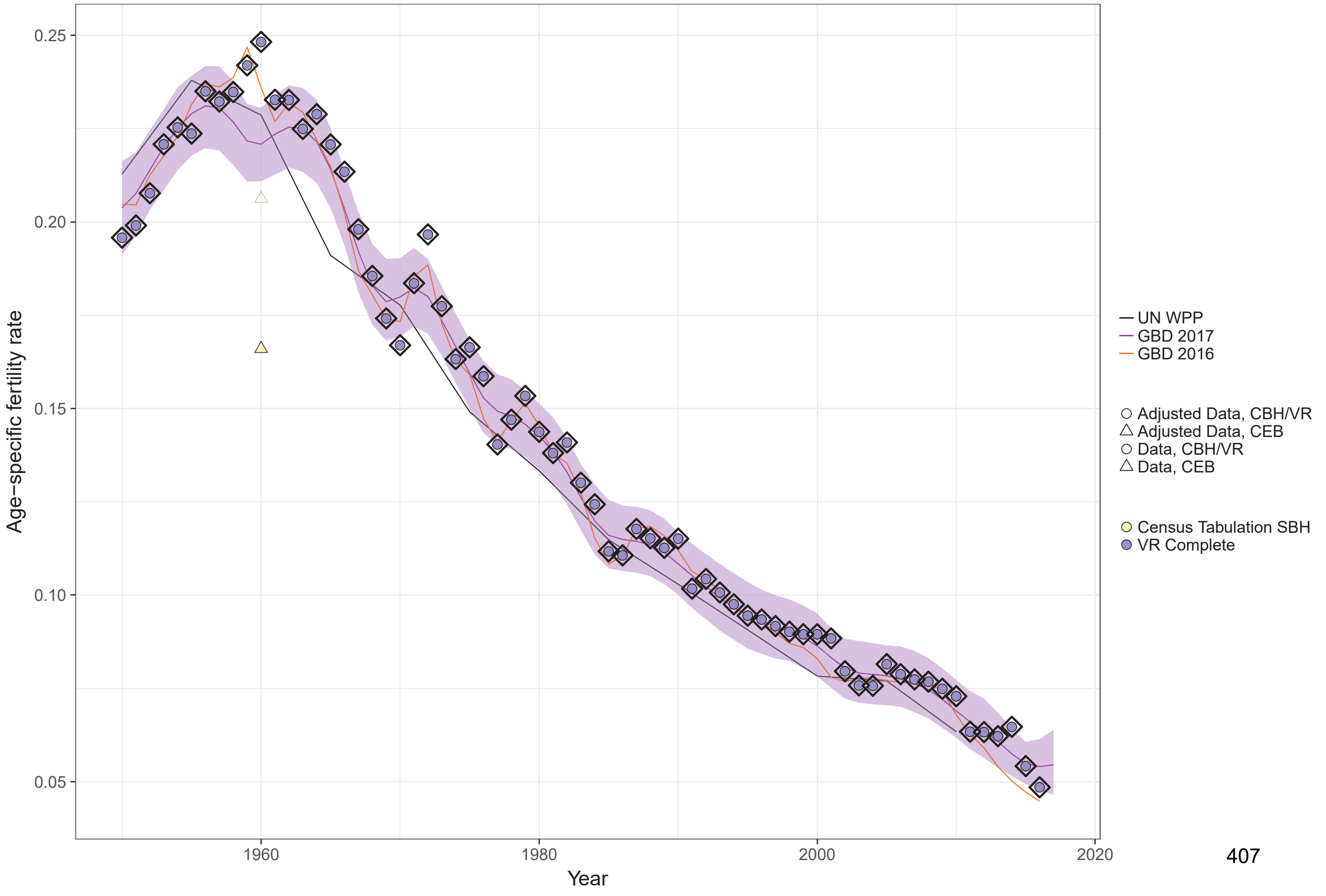

Age−specific fertility fitted trend lines, 1950−2017: Iceland, Ages: 25−29
Data points enclosed with a diamond are reference sources

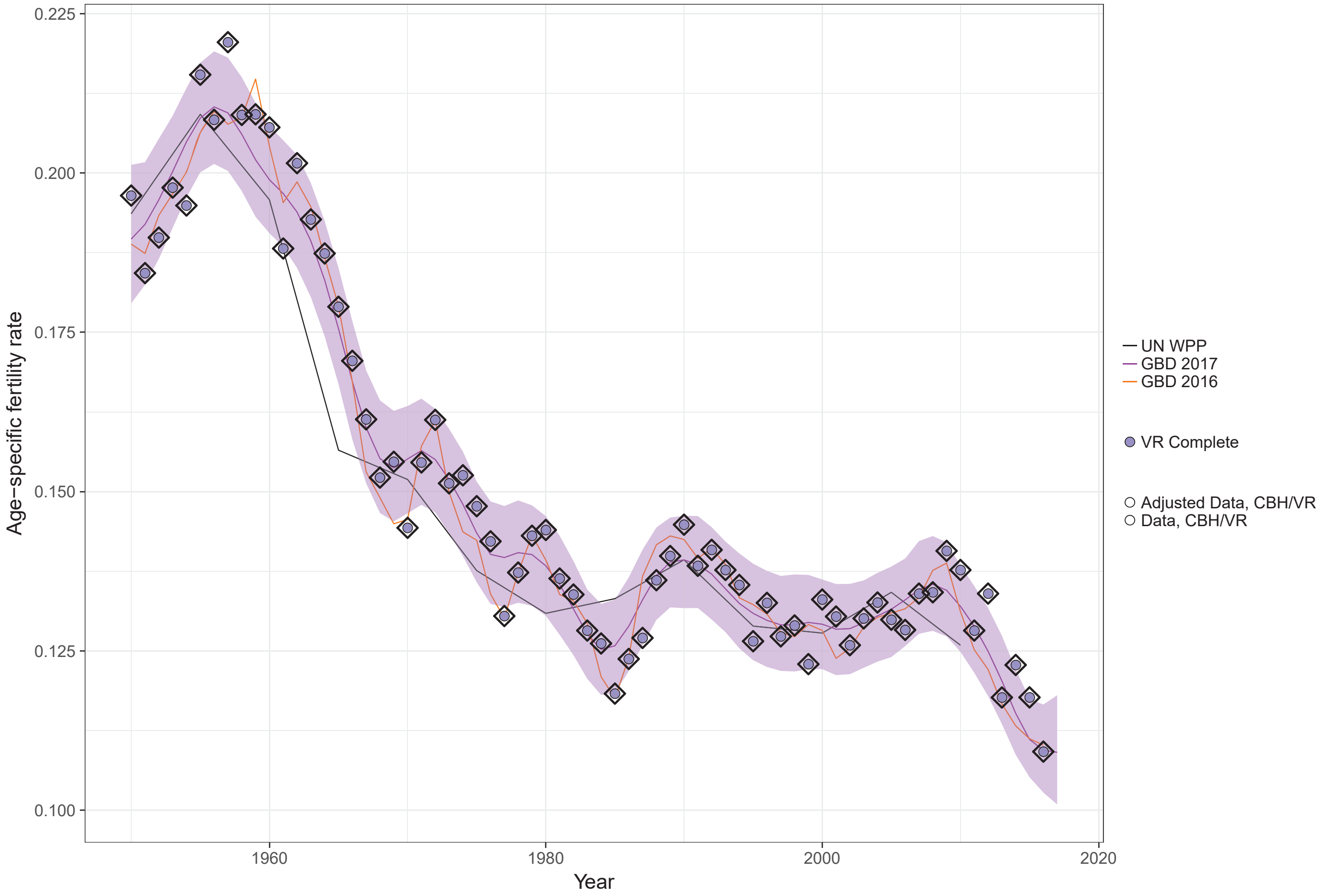

Age−specific fertility fitted trend lines, 1950−2017: Iceland, Ages: 30−34
Data points enclosed with a diamond are reference sources

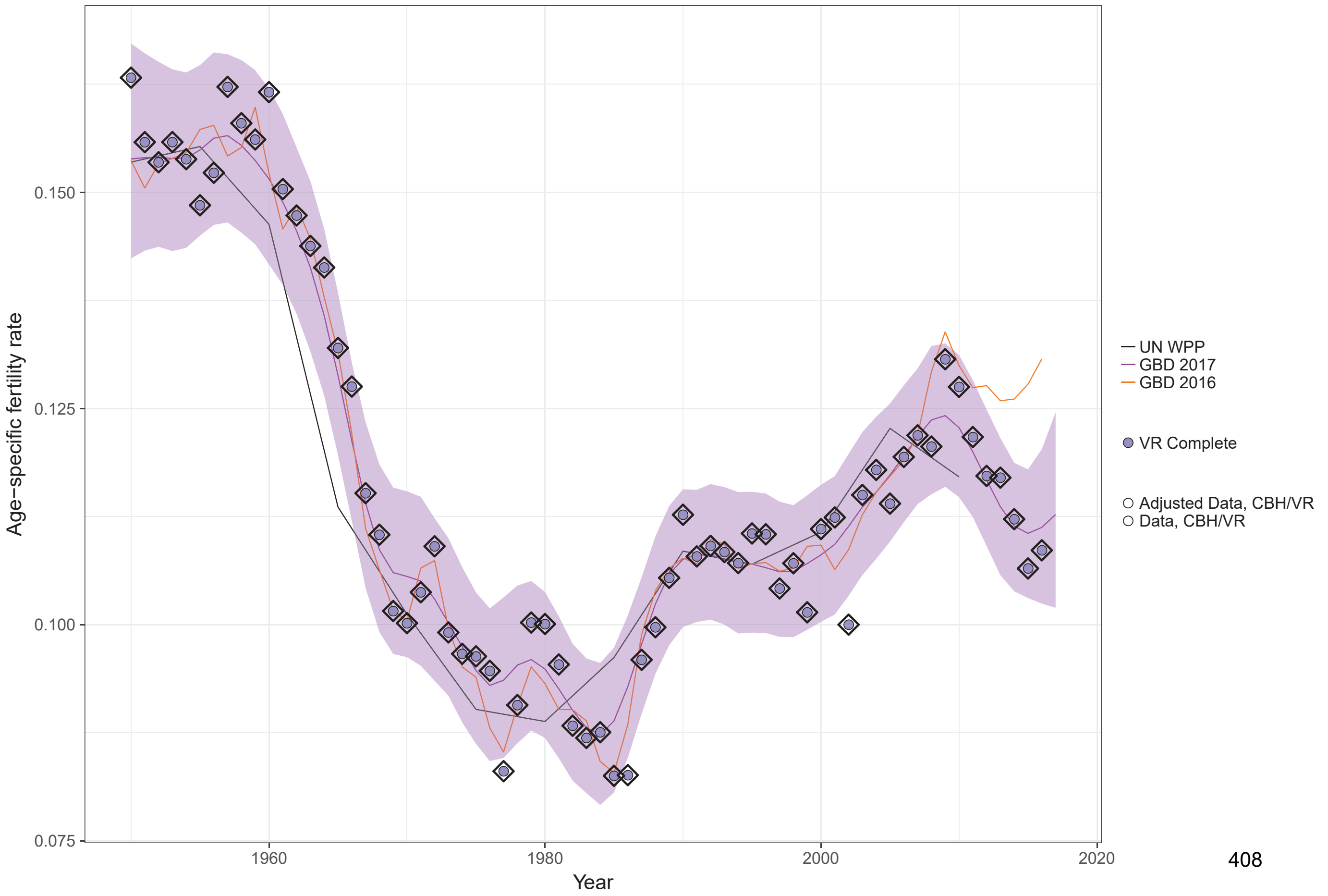

Age−specific fertility fitted trend lines, 1950−2017: Iceland, Ages: 35−39
Data points enclosed with a diamond are reference sources

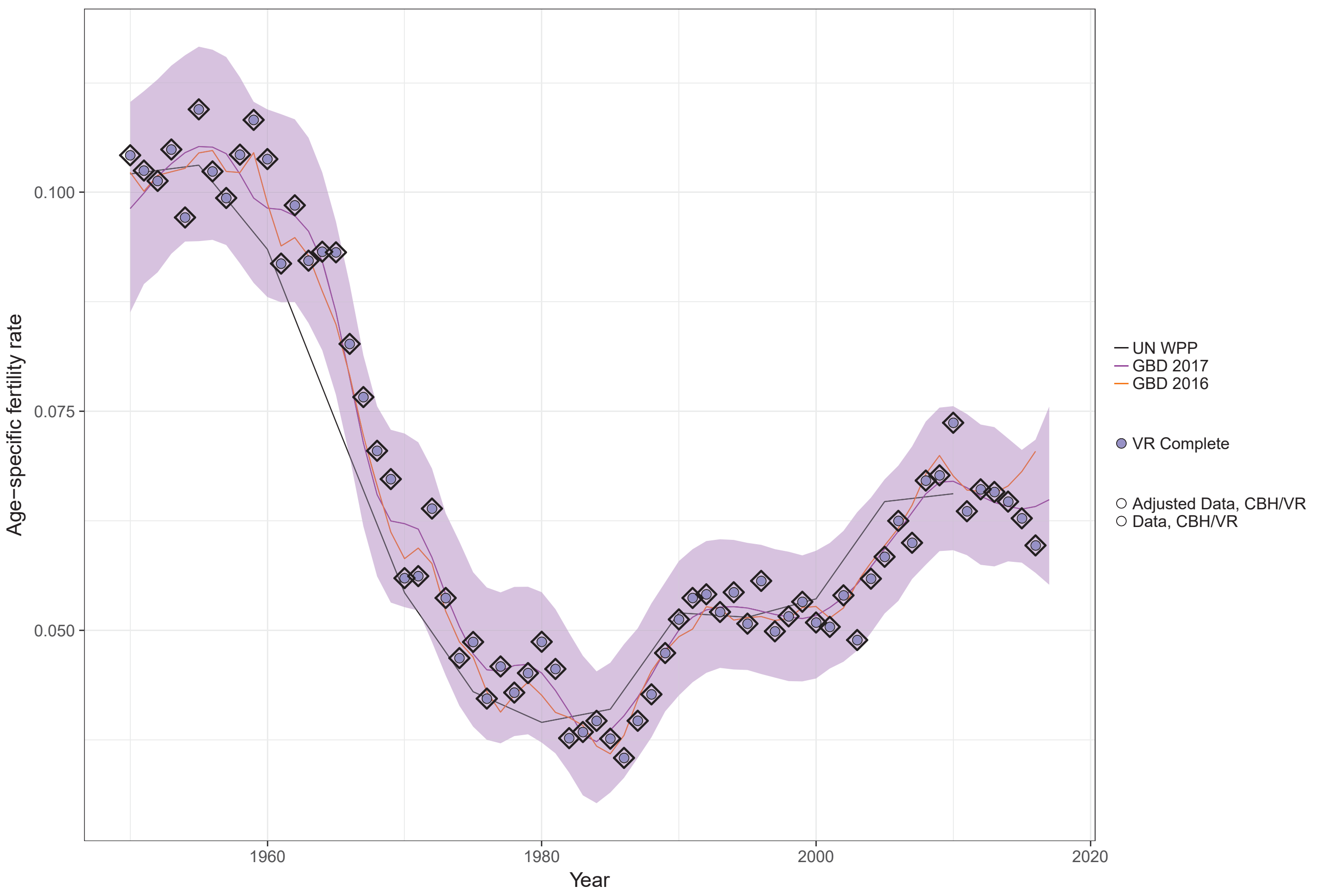

Age−specific fertility fitted trend lines, 1950−2017: Iceland, Ages: 40−44
Data points enclosed with a diamond are reference sources

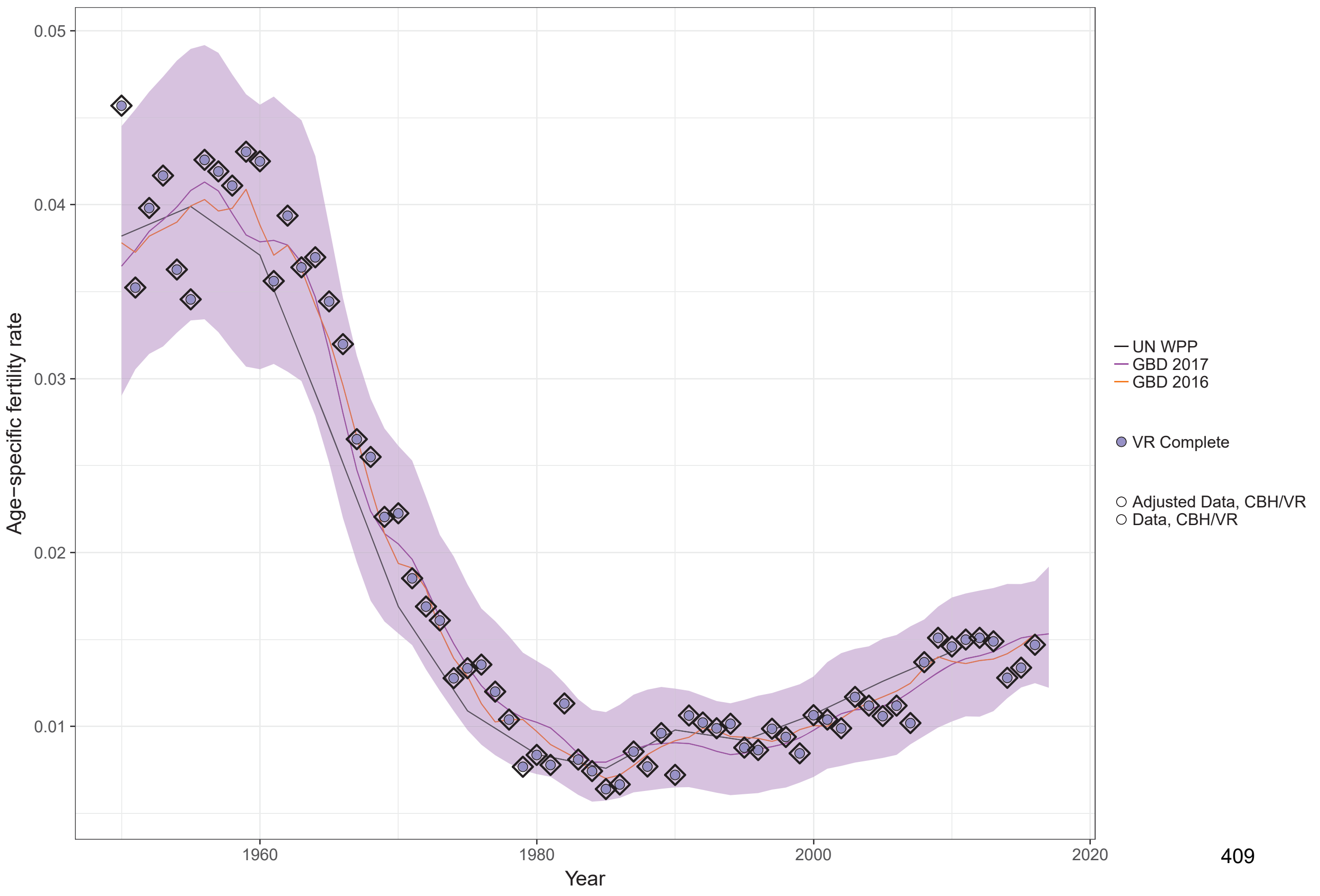

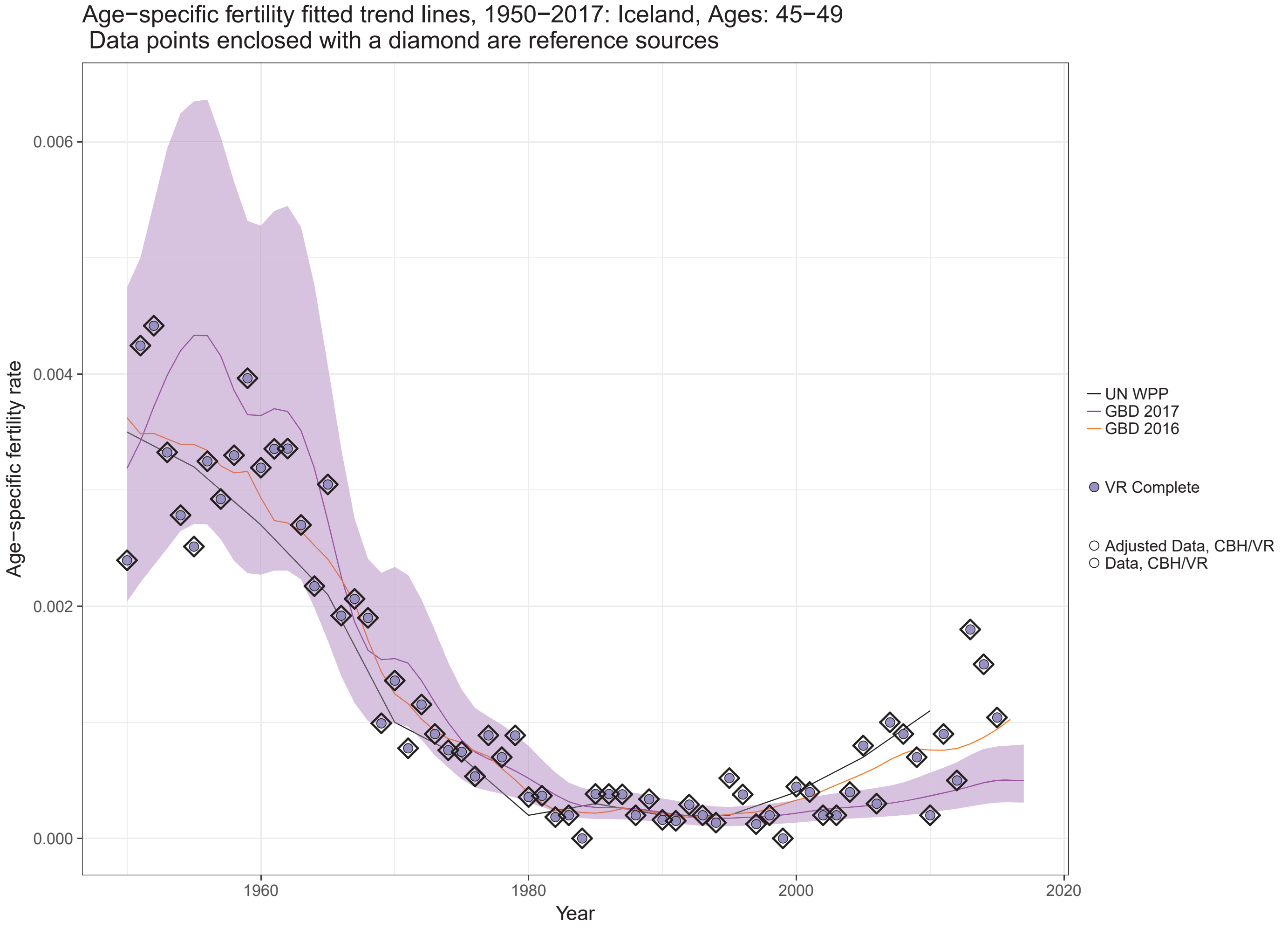

Age−specific fertility fitted trend lines, 1950−2017: Iceland, Ages: 45−49
Data points enclosed with a diamond are reference sources
Age−specific fertility rate
0.006
0.004
0.002
0.000
1960
1980
2000
2020
Year
UN WPP
GBD 2017
GBD 2016
VR Complete
Adjusted Data, CBH/VR
Data, CBH/VR

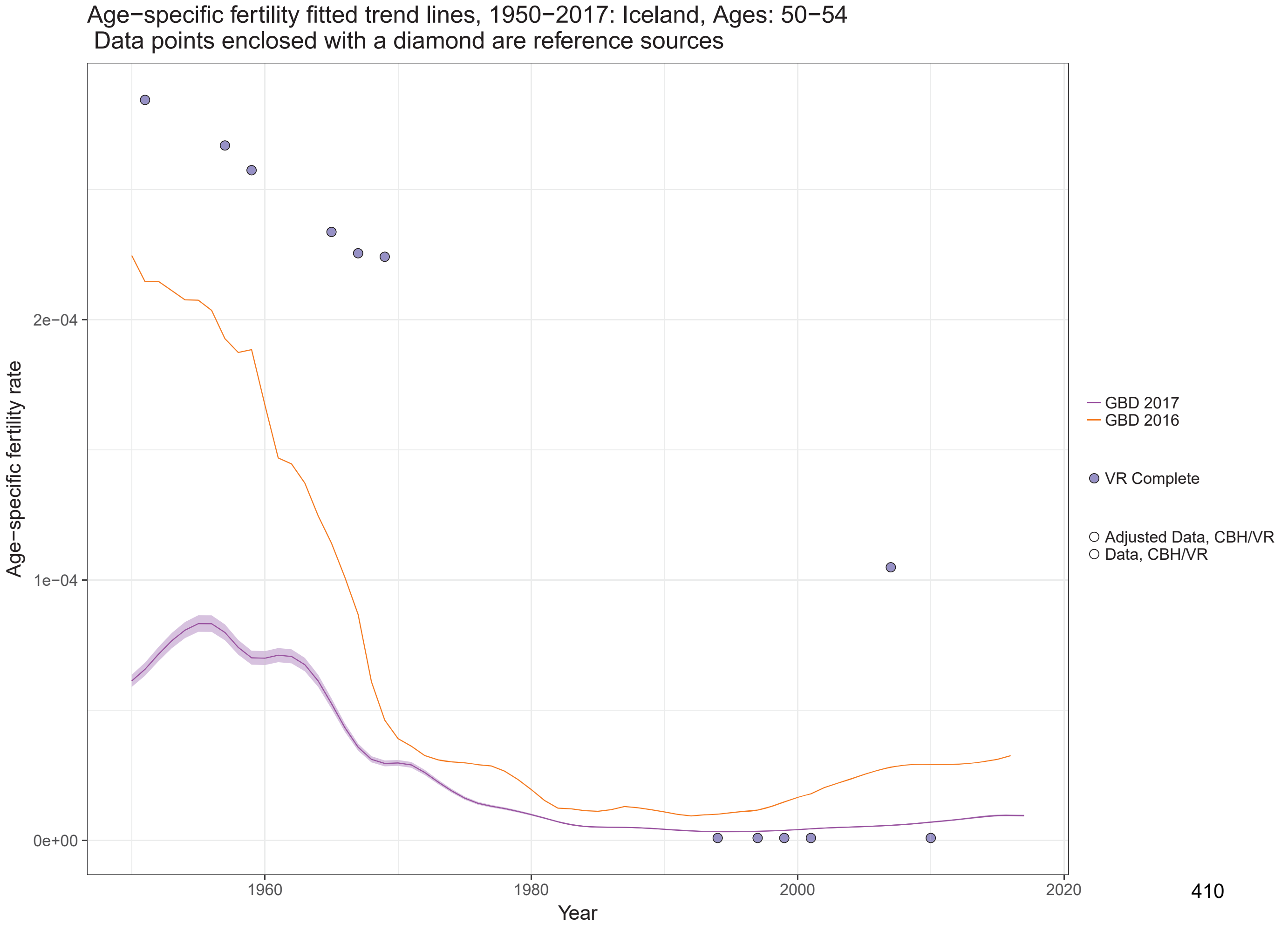

Age−specific fertility fitted trend lines, 1950−2017: Iceland, Ages: 50−54
Data points enclosed with a diamond are reference sources
Age−specific fertility rate
2e−04
1e−04
0e+00
1960
1980
2000
2020
Year
GBD 2017
GBD 2016
VR Complete
Adjusted Data, CBH/VR
Data, CBH/VR

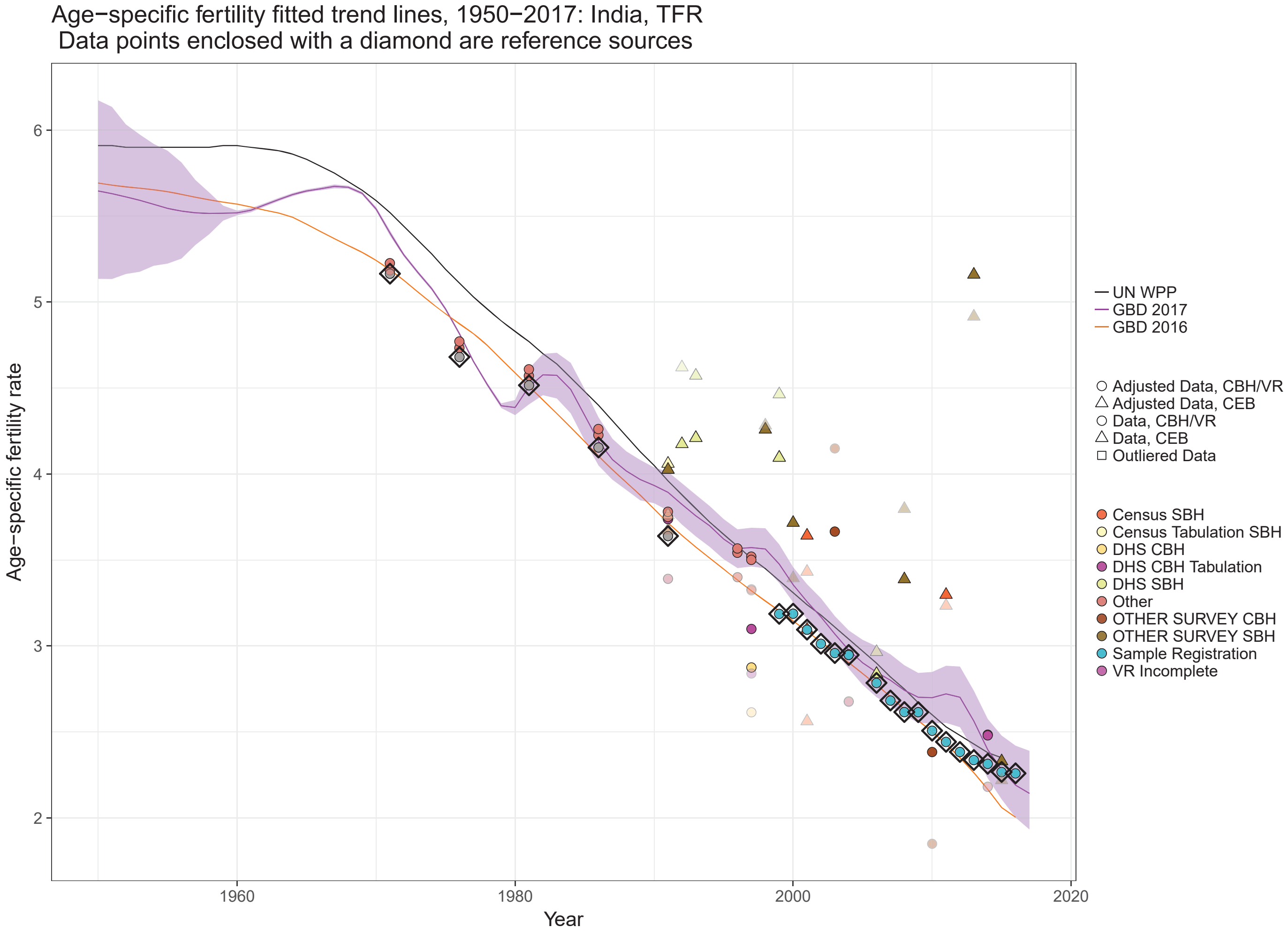
Age−specific fertility fitted trend lines, 1950−2017: India, TFR
Data points enclosed with a diamond are reference sources
Age−specific fertility rate
Year
UN WPP
GBD 2017
GBD 2016
Adjusted Data, CBH/VR
Adjusted Data, CEB
Data, CBH/VR
Data, CEB
Outliered Data
Census SBH
Census Tabulation SBH
DHS CBH
DHS CBH Tabulation
DHS SBH
Other
OTHER SURVEY CBH
OTHER SURVEY SBH
Sample Registration
VR Incomplete

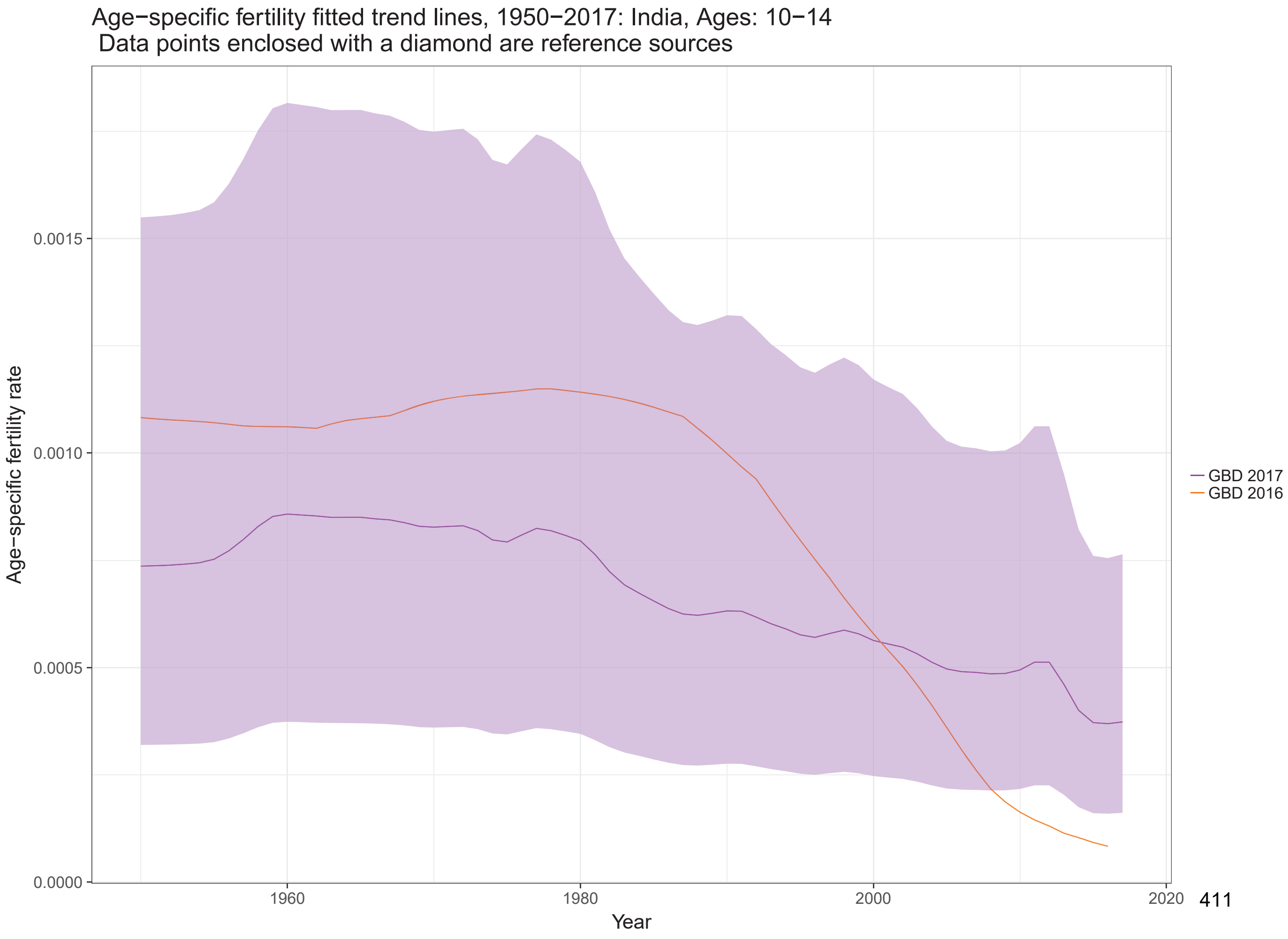
Age−specific fertility fitted trend lines, 1950−2017: India, Ages: 10−14
Data points enclosed with a diamond are reference sources
Age−specific fertility rate
Year
GBD 2017
GBD 2016

Age−specific fertility fitted trend lines, 1950−2017: India, Ages: 15−19
Data points enclosed with a diamond are reference sources

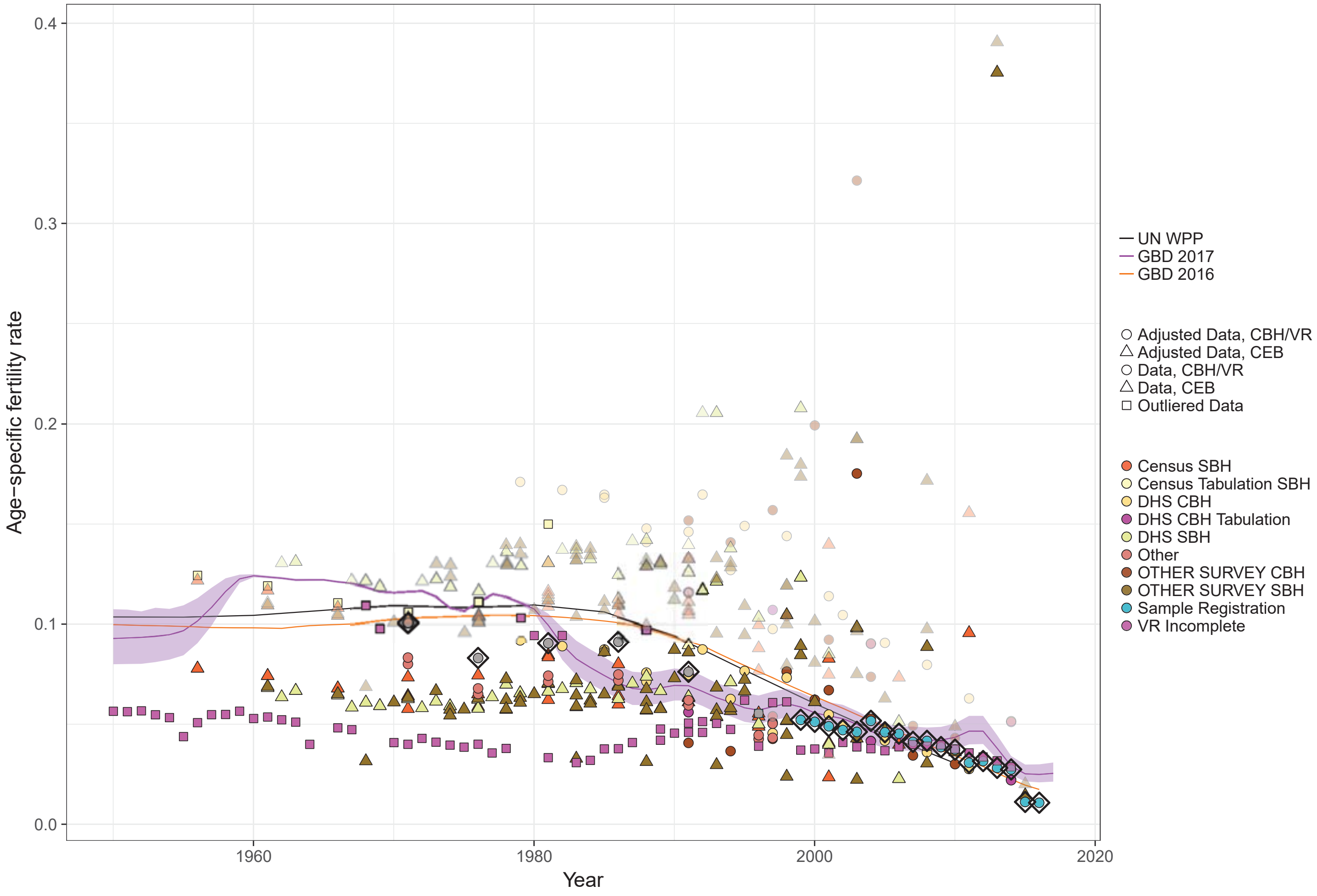

Age−specific fertility fitted trend lines, 1950−2017: India, Ages: 20−24
Data points enclosed with a diamond are reference sources

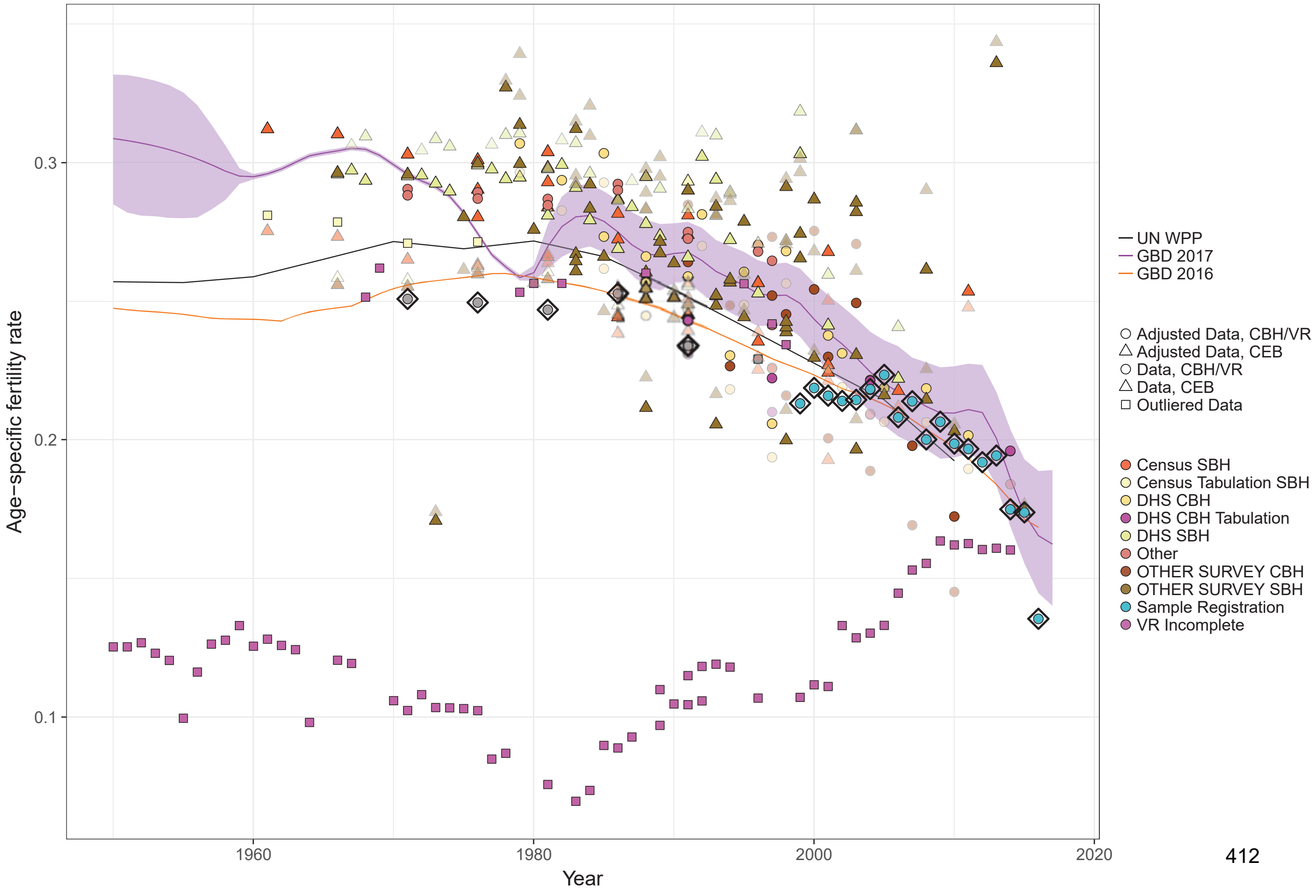

Age−specific fertility fitted trend lines, 1950−2017: India, Ages: 25−29
Data points enclosed with a diamond are reference sources

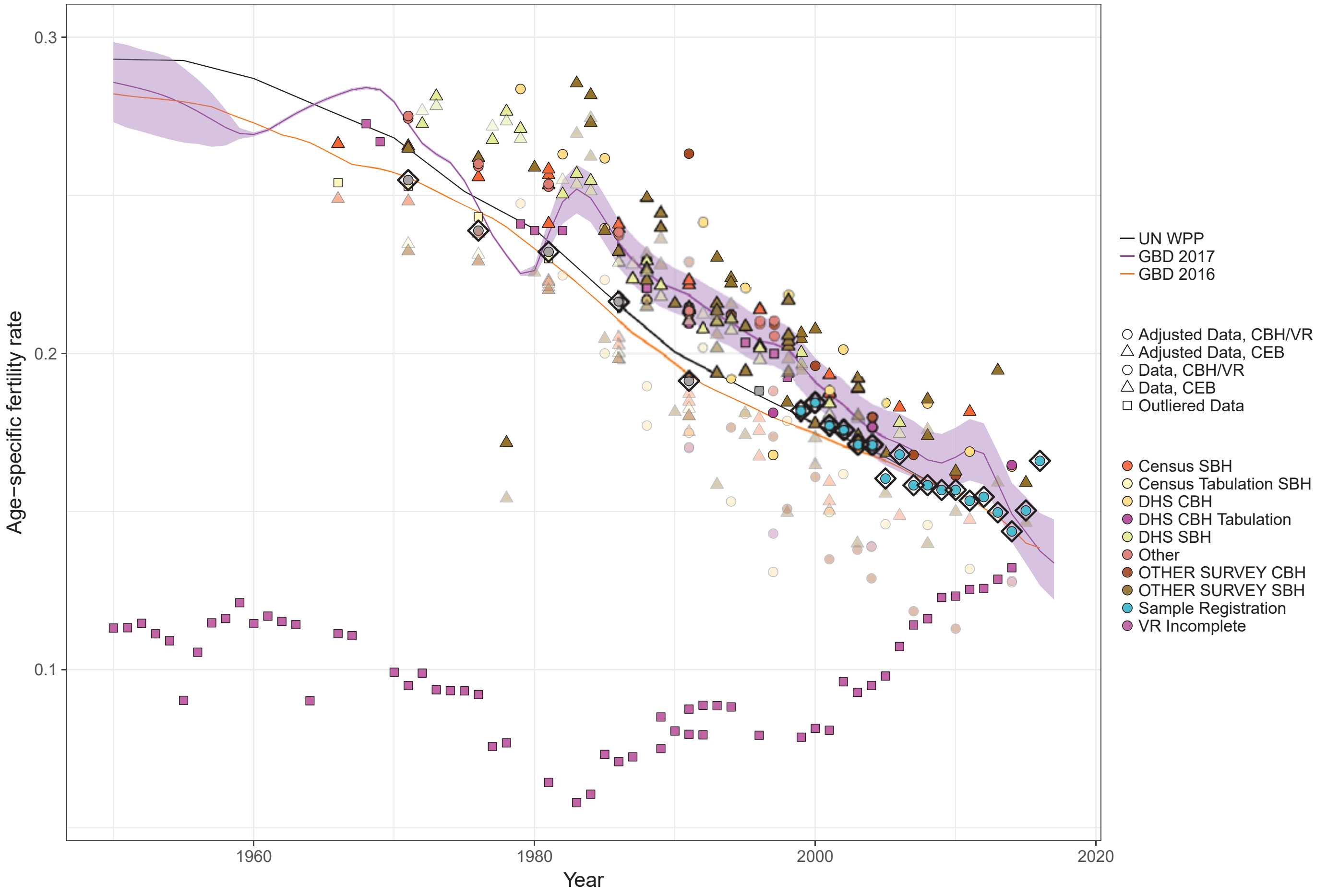

Age−specific fertility fitted trend lines, 1950−2017: India, Ages: 30−34
Data points enclosed with a diamond are reference sources

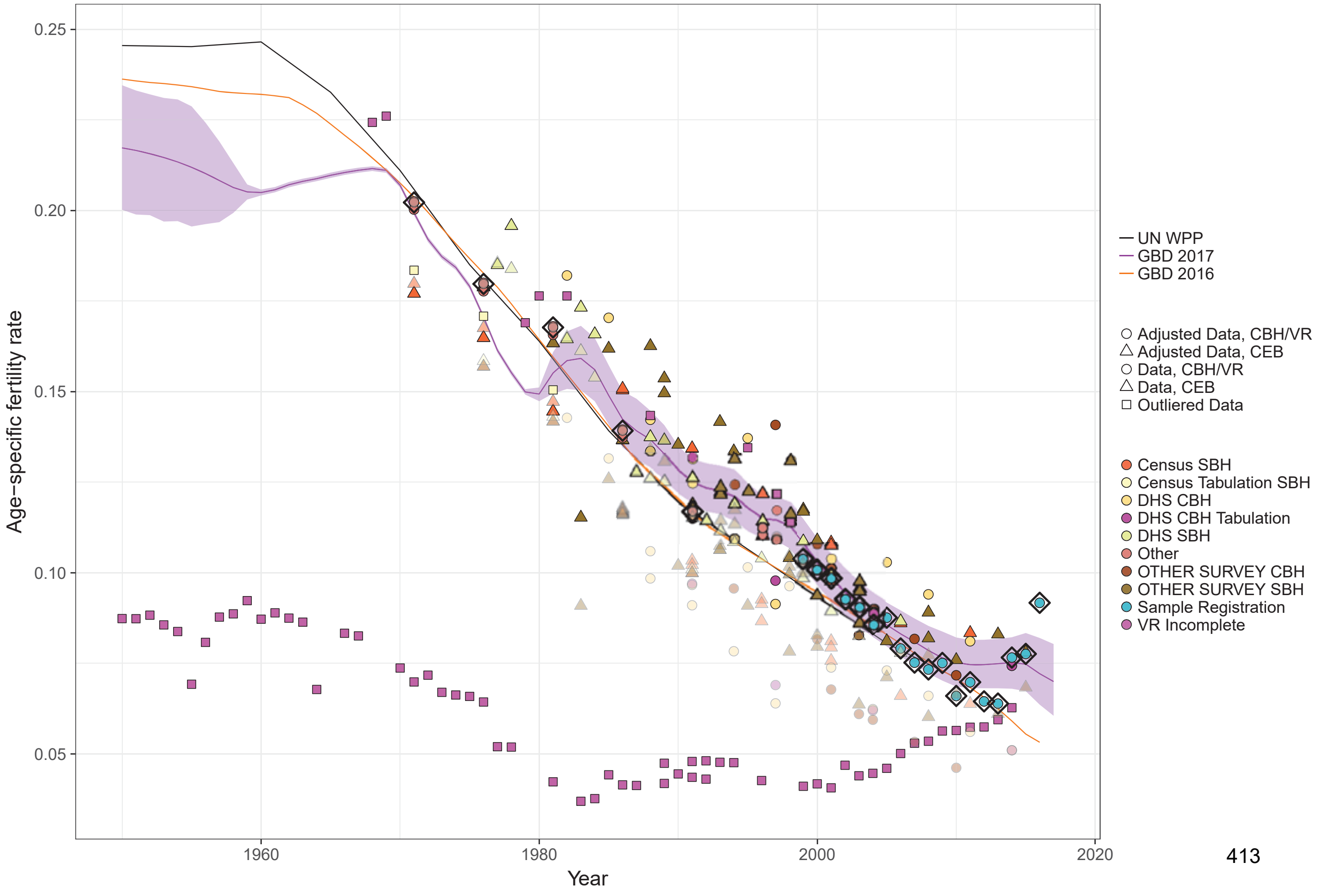

Age−specific fertility fitted trend lines, 1950−2017: India, Ages: 35−39
Data points enclosed with a diamond are reference sources

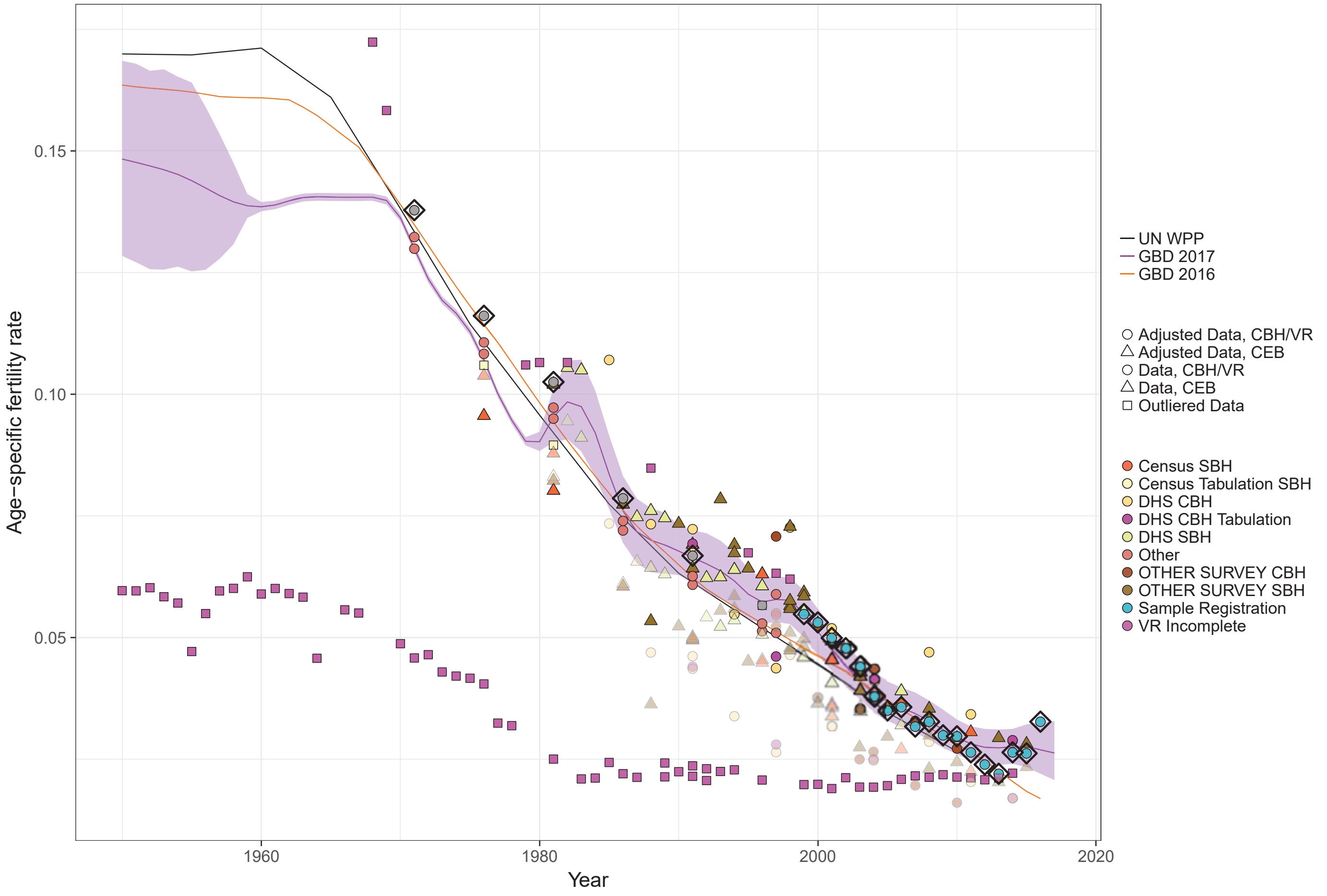

Age−specific fertility fitted trend lines, 1950−2017: India, Ages: 40−44
Data points enclosed with a diamond are reference sources

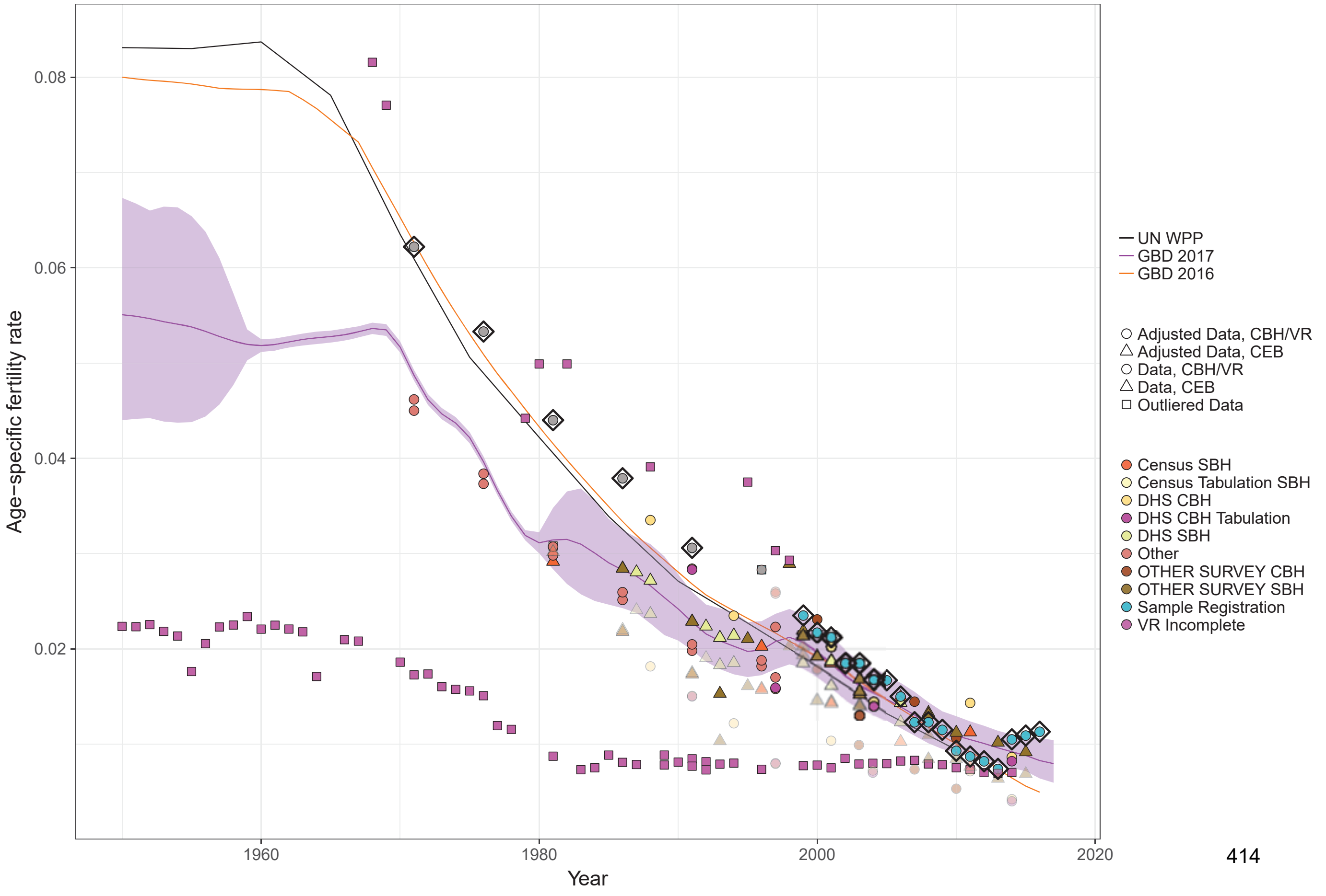

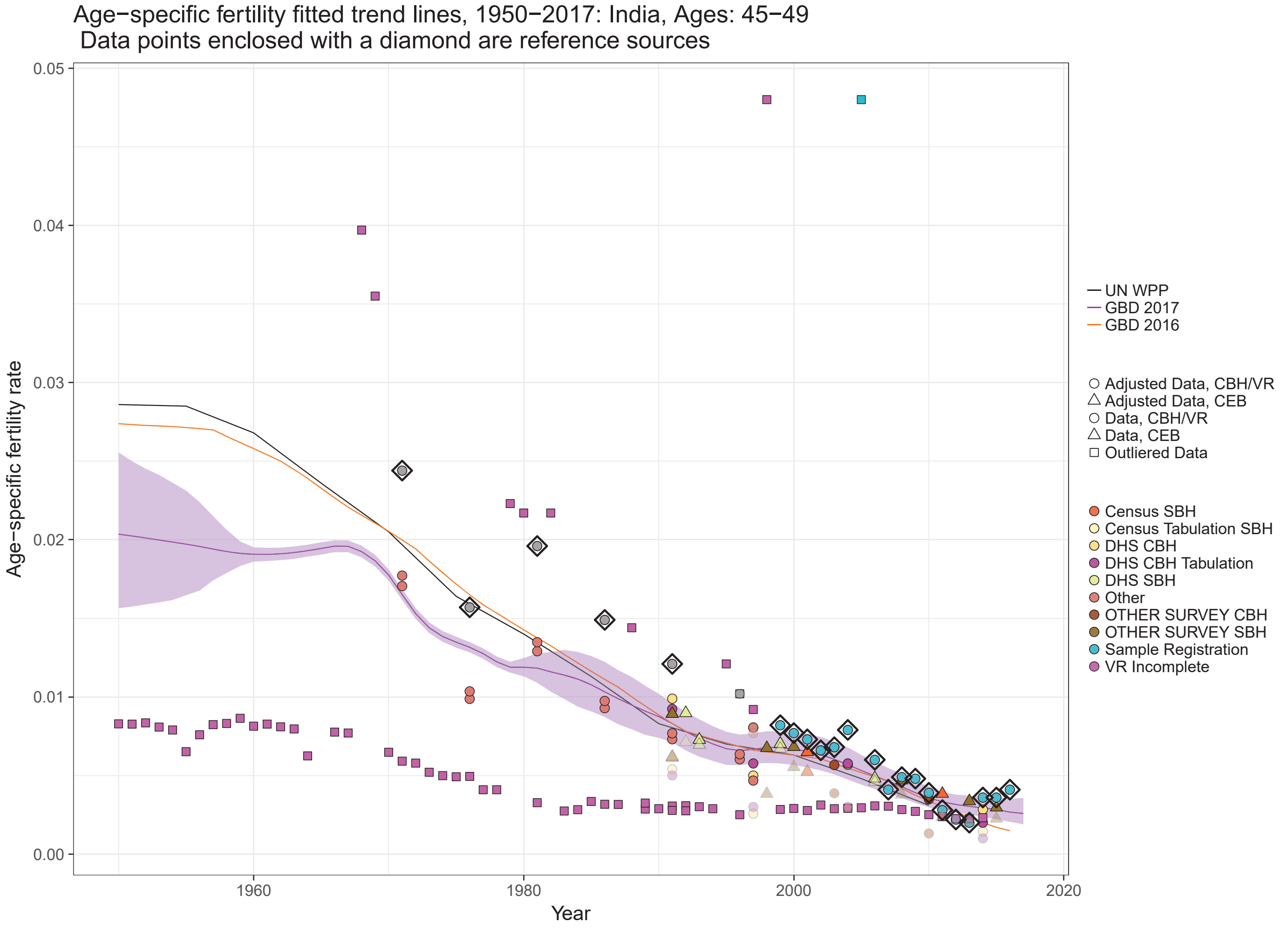

Age−specific fertility fitted trend lines, 1950−2017: India, Ages: 45−49
Data points enclosed with a diamond are reference sources
Age−specific fertility rate
0.05
0.04
0.03
0.02
0.01
0.00
1960
1980
2000
2020
Year
UN WPP
GBD 2017
GBD 2016
Adjusted Data, CBH/VR
Adjusted Data, CEB
Data, CBH/VR
Data, CEB
Outliered Data
Census SBH
Census Tabulation SBH
DHS CBH
DHS CBH Tabulation
DHS SBH
Other
OTHER SURVEY CBH
OTHER SURVEY SBH
Sample Registration
VR Incomplete

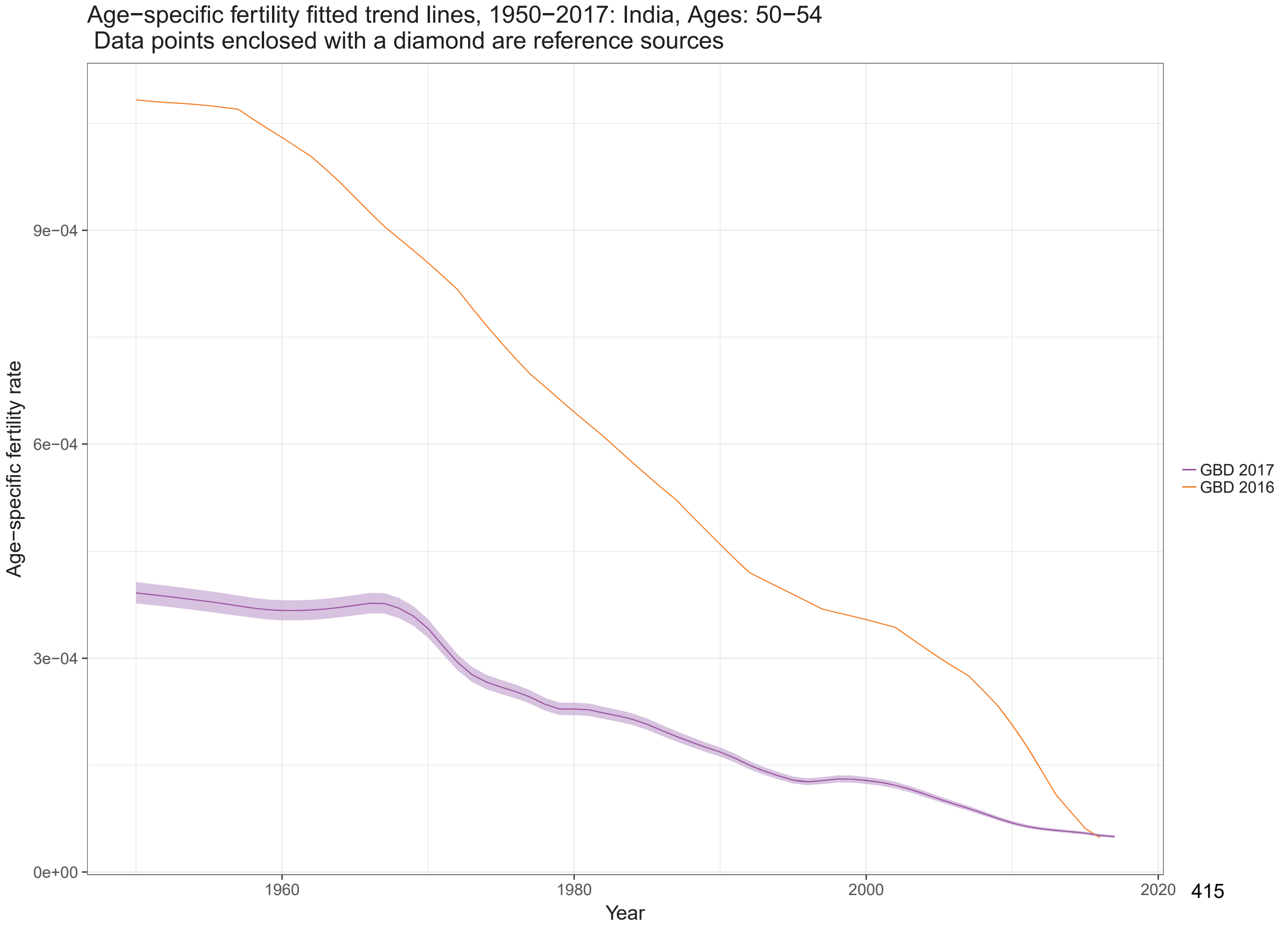

Age−specific fertility fitted trend lines, 1950−2017: India, Ages: 50−54
Data points enclosed with a diamond are reference sources
Age−specific fertility rate
9e−04
6e−04
3e−04
0e+00
1960
1980
2000
2020
Year
GBD 2017
GBD 2016

Age−specific fertility fitted trend lines, 1950−2017: Indonesia, TFR
Data points enclosed with a diamond are reference sources

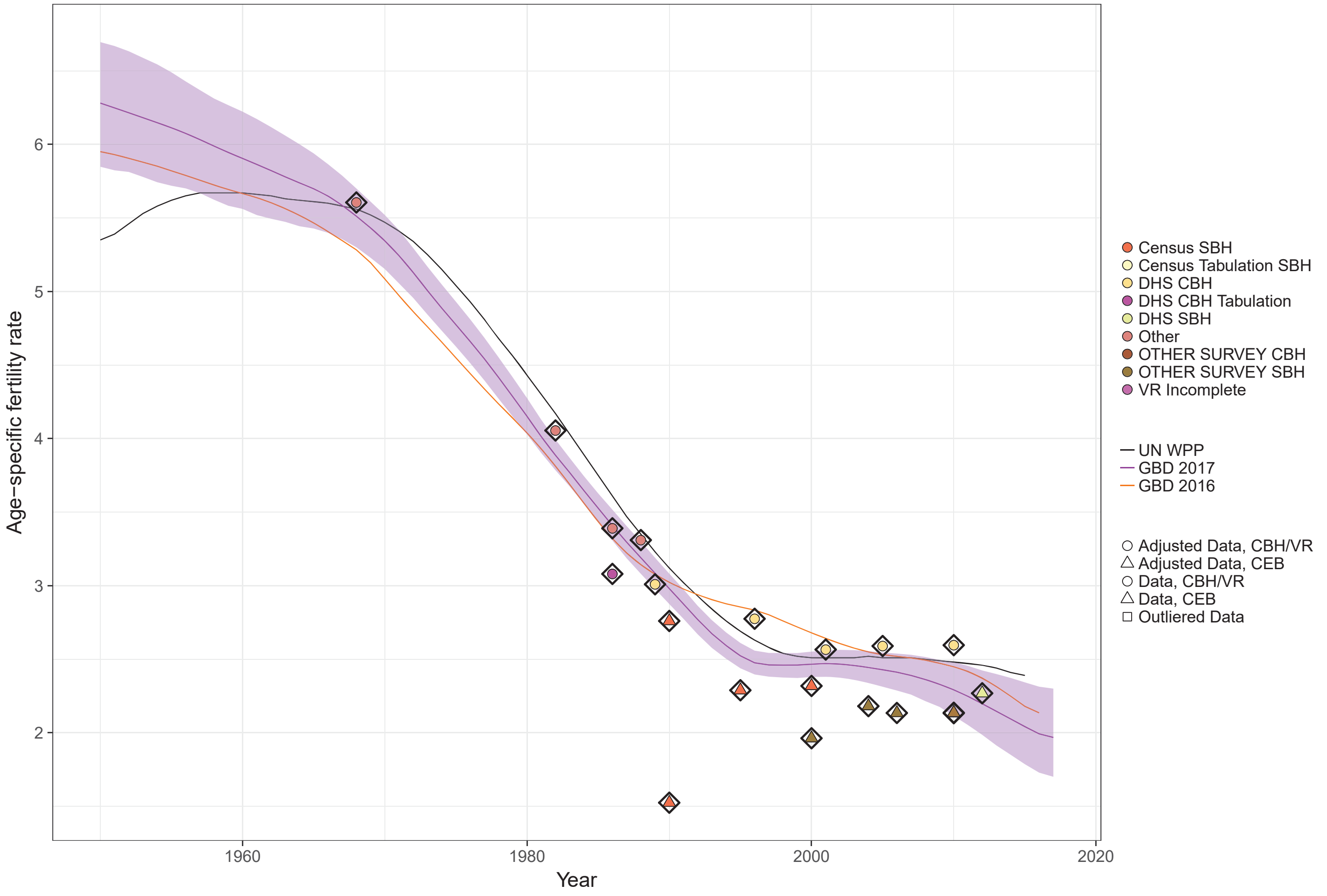

Age−specific fertility fitted trend lines, 1950−2017: Indonesia, Ages: 10−14
Data points enclosed with a diamond are reference sources

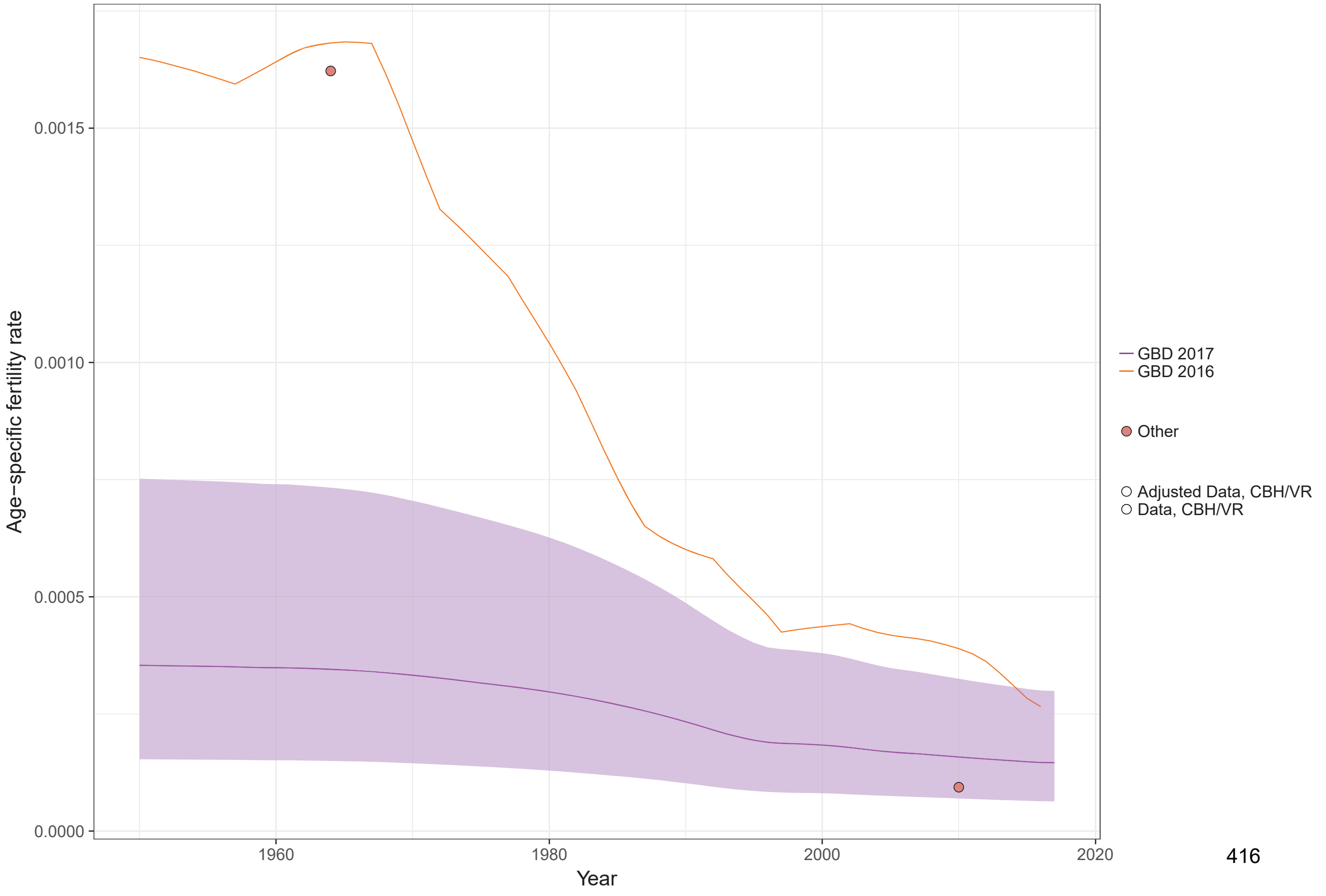

Age−specific fertility fitted trend lines, 1950−2017: Indonesia, Ages: 15−19
Data points enclosed with a diamond are reference sources

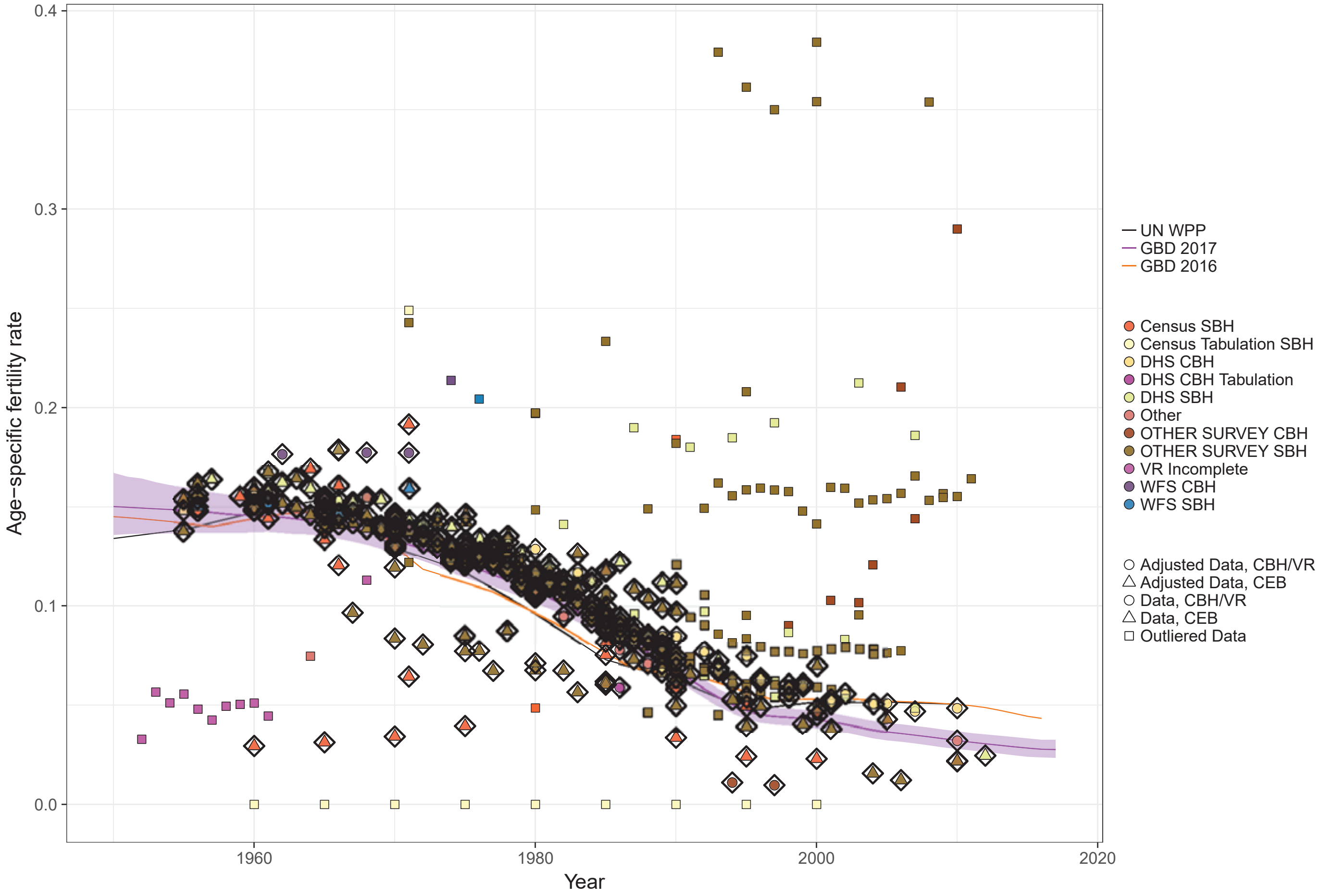

Age−specific fertility fitted trend lines, 1950−2017: Indonesia, Ages: 20−24
Data points enclosed with a diamond are reference sources

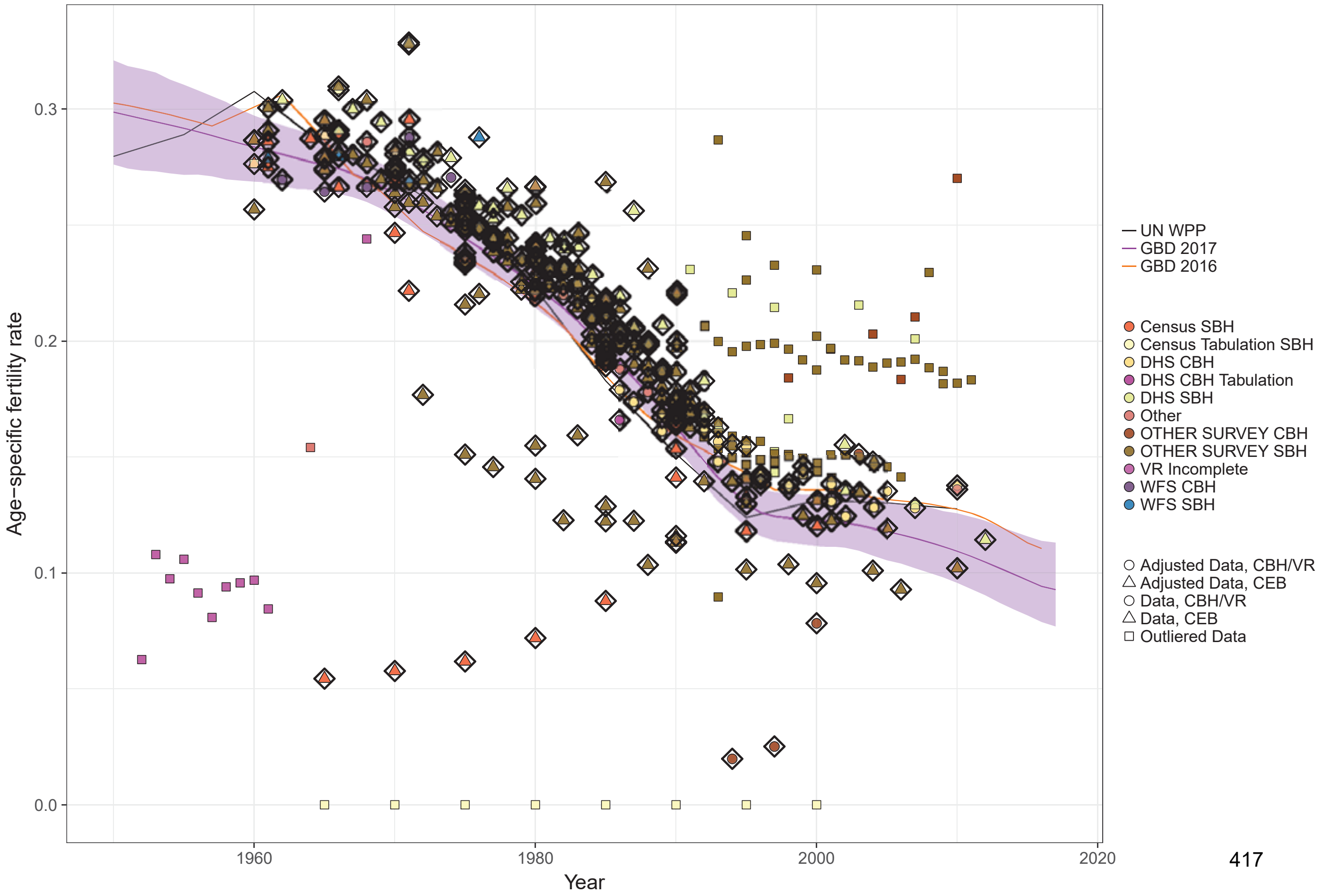

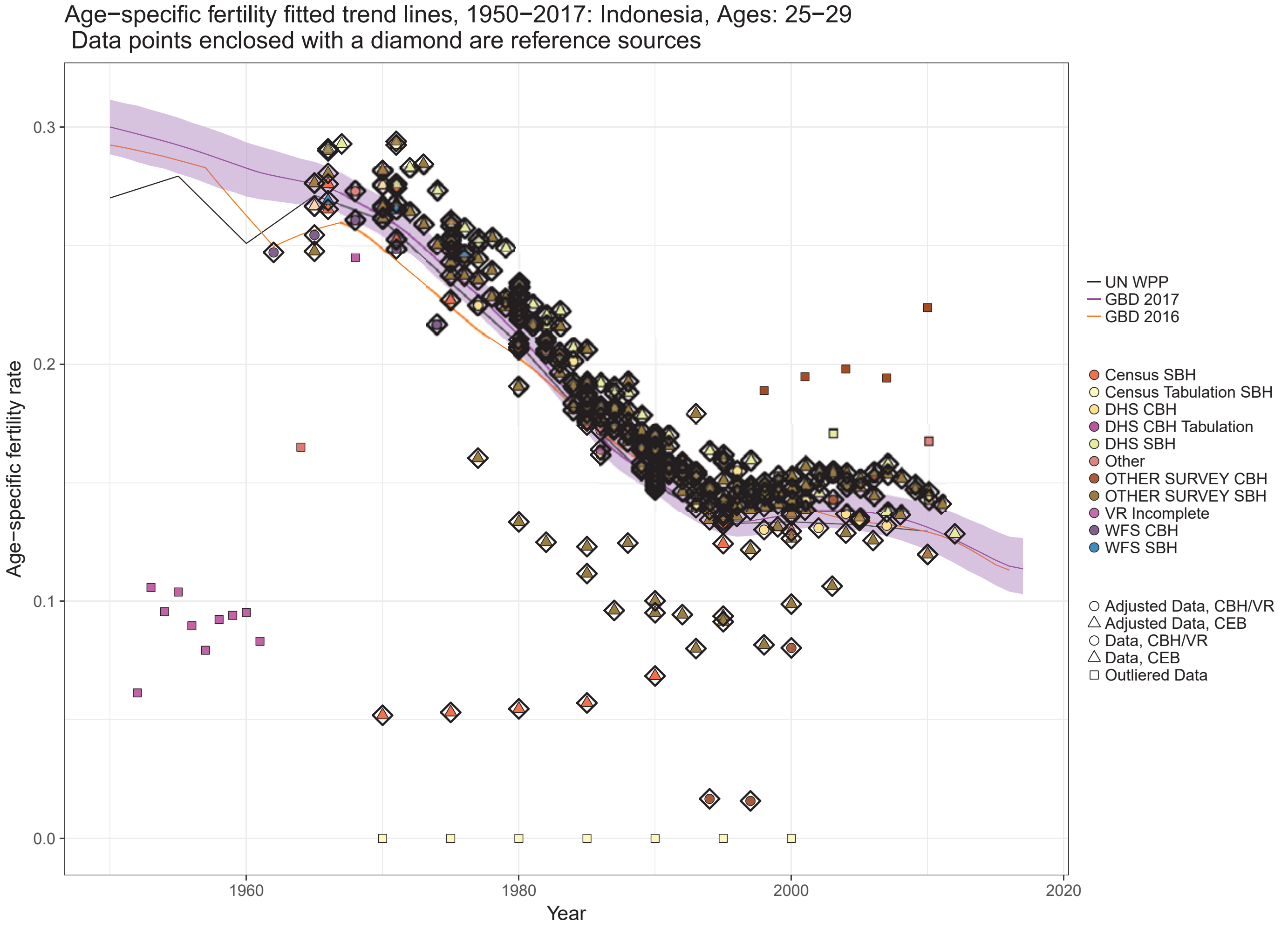

Age−specific fertility fitted trend lines, 1950−2017: Indonesia, Ages: 25−29
Data points enclosed with a diamond are reference sources
Age-specific fertility rate
Year
0.0
0.1
0.2
0.3
1960
1980
2000
2020
UN WPP
GBD 2017
GBD 2016
Census SBH
Census Tabulation SBH
DHS CBH
DHS CBH Tabulation
DHS SBH
Other
OTHER SURVEY CBH
OTHER SURVEY SBH
VR Incomplete
WFS CBH
WFS SBH
Adjusted Data, CBH/VR
Adjusted Data, CEB
Data, CBH/VR
Data, CEB
Outliered Data

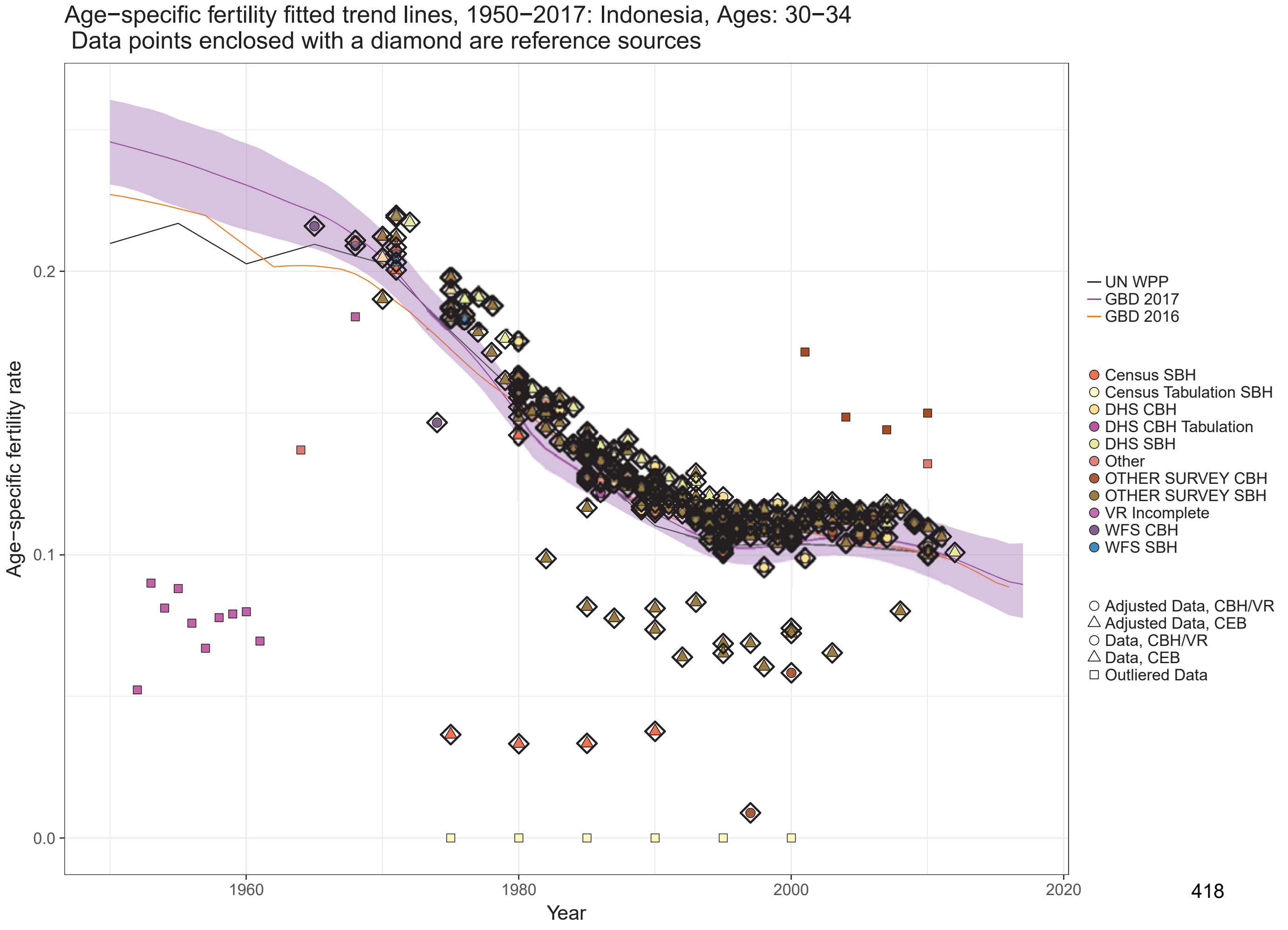

Age−specific fertility fitted trend lines, 1950−2017: Indonesia, Ages: 30−34
Data points enclosed with a diamond are reference sources
Age-specific fertility rate
Year
0.0
0.1
0.2
1960
1980
2000
2020
UN WPP
GBD 2017
GBD 2016
Census SBH
Census Tabulation SBH
DHS CBH
DHS CBH Tabulation
DHS SBH
Other
OTHER SURVEY CBH
OTHER SURVEY SBH
VR Incomplete
WFS CBH
WFS SBH
Adjusted Data, CBH/VR
Adjusted Data, CEB
Data, CBH/VR
Data, CEB
Outliered Data

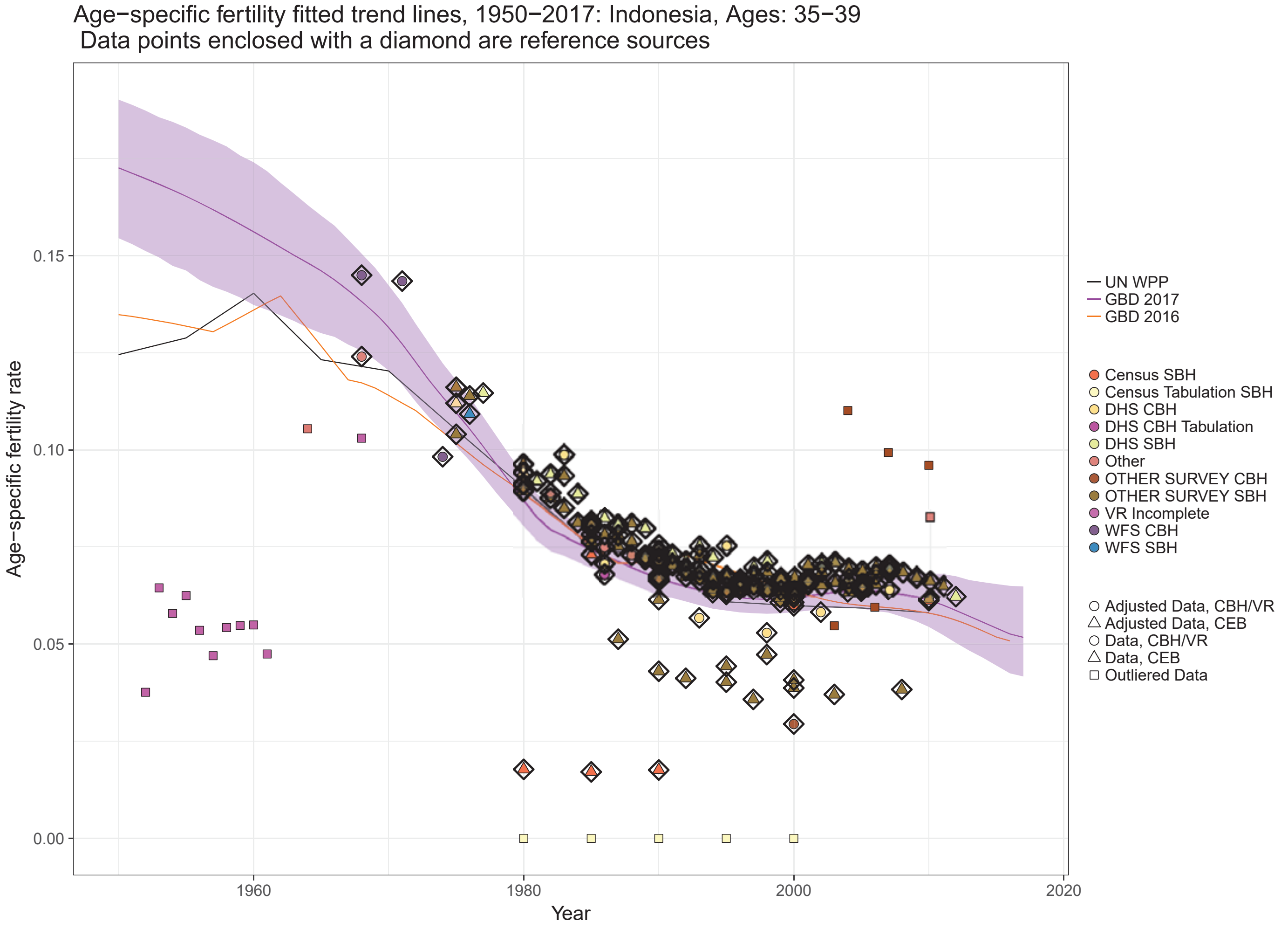

Age−specific fertility fitted trend lines, 1950−2017: Indonesia, Ages: 35−39
Data points enclosed with a diamond are reference sources
Age−specific fertility rate
0.15
0.10
0.05
0.00
1960
1980
2000
2020
Year
UN WPP
GBD 2017
GBD 2016
Census SBH
Census Tabulation SBH
DHS CBH
DHS CBH Tabulation
DHS SBH
Other
OTHER SURVEY CBH
OTHER SURVEY SBH
VR Incomplete
WFS CBH
WFS SBH
Adjusted Data, CBH/VR
Adjusted Data, CEB
Data, CBH/VR
Data, CEB
Outliered Data

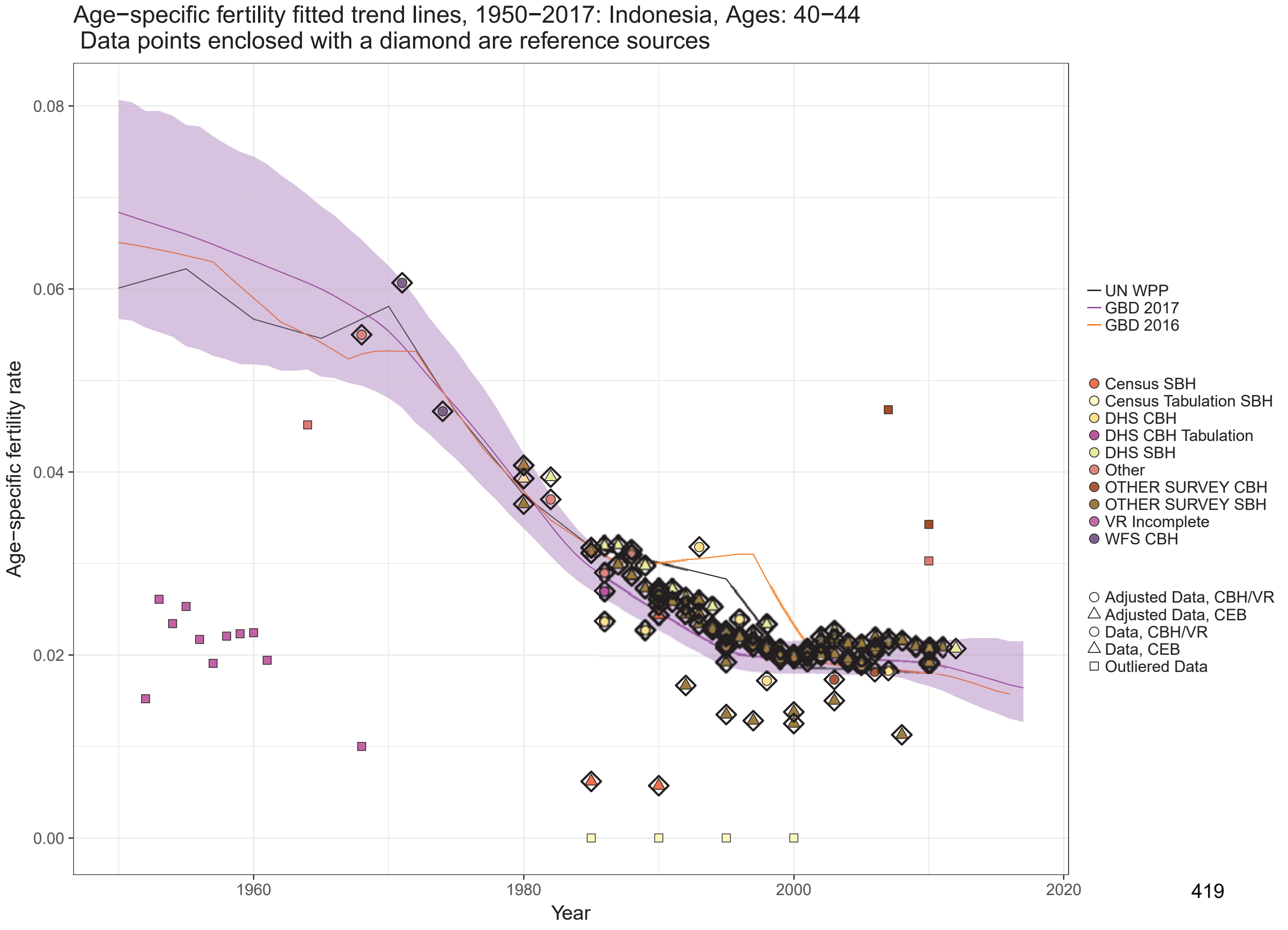

Age−specific fertility fitted trend lines, 1950−2017: Indonesia, Ages: 40−44
Data points enclosed with a diamond are reference sources
Age−specific fertility rate
0.08
0.06
0.04
0.02
0.00
1960
1980
2000
2020
Year
UN WPP
GBD 2017
GBD 2016
Census SBH
Census Tabulation SBH
DHS CBH
DHS CBH Tabulation
DHS SBH
Other
OTHER SURVEY CBH
OTHER SURVEY SBH
VR Incomplete
WFS CBH
Adjusted Data, CBH/VR
Adjusted Data, CEB
Data, CBH/VR
Data, CEB
Outliered Data

Age−specific fertility fitted trend lines, 1950−2017: Indonesia, Ages: 45−49
Data points enclosed with a diamond are reference sources

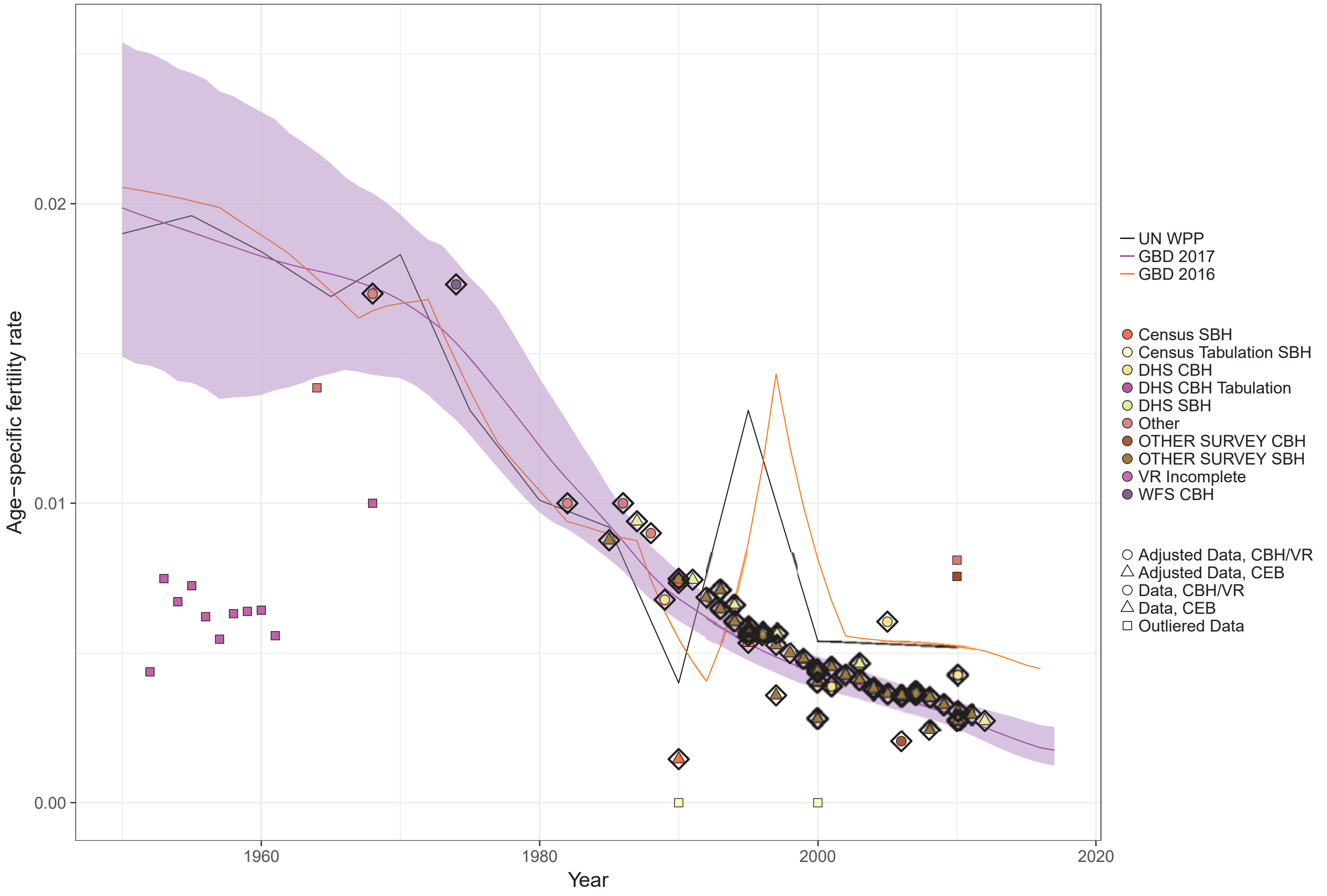

Age−specific fertility fitted trend lines, 1950−2017: Indonesia, Ages: 50−54
Data points enclosed with a diamond are reference sources

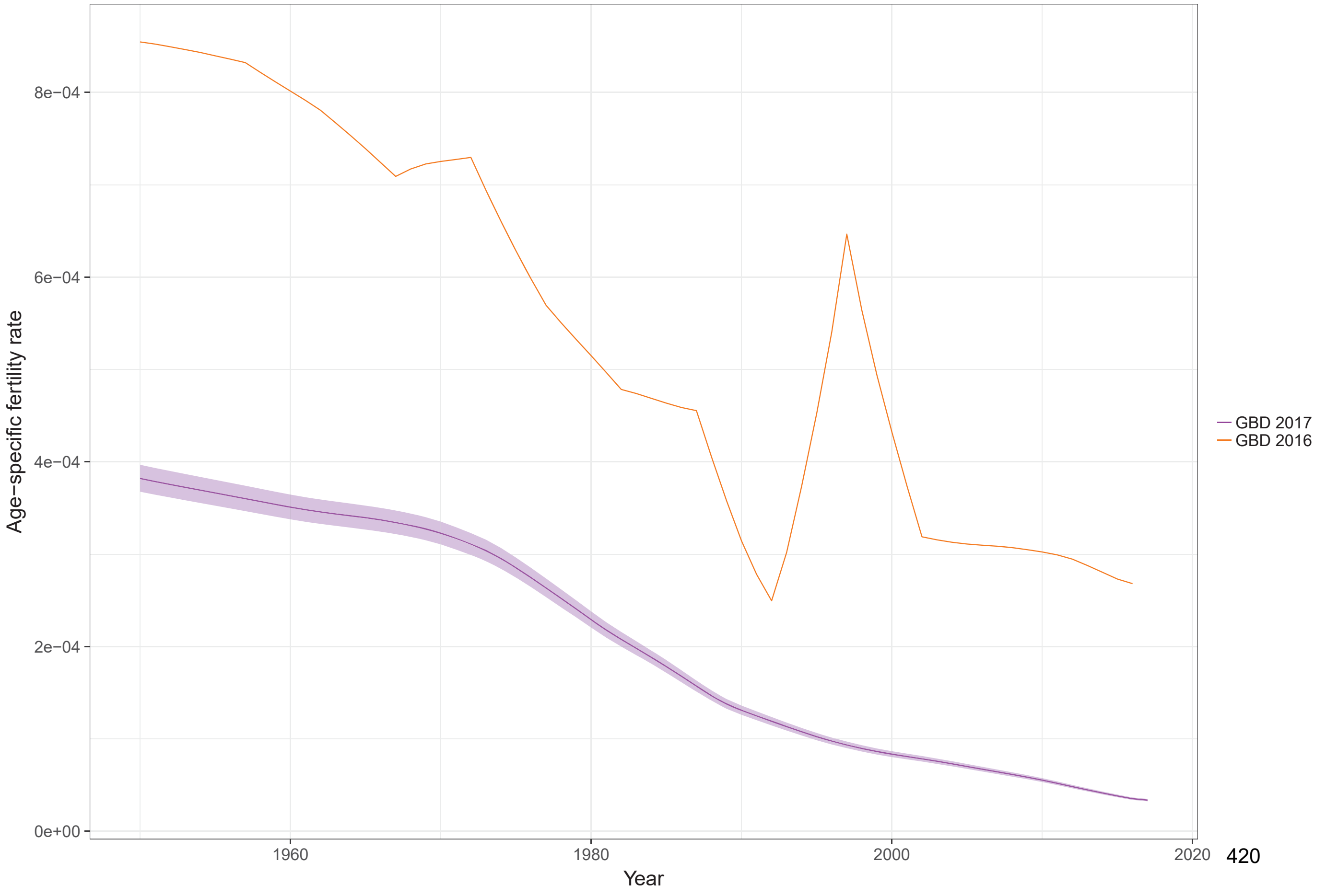

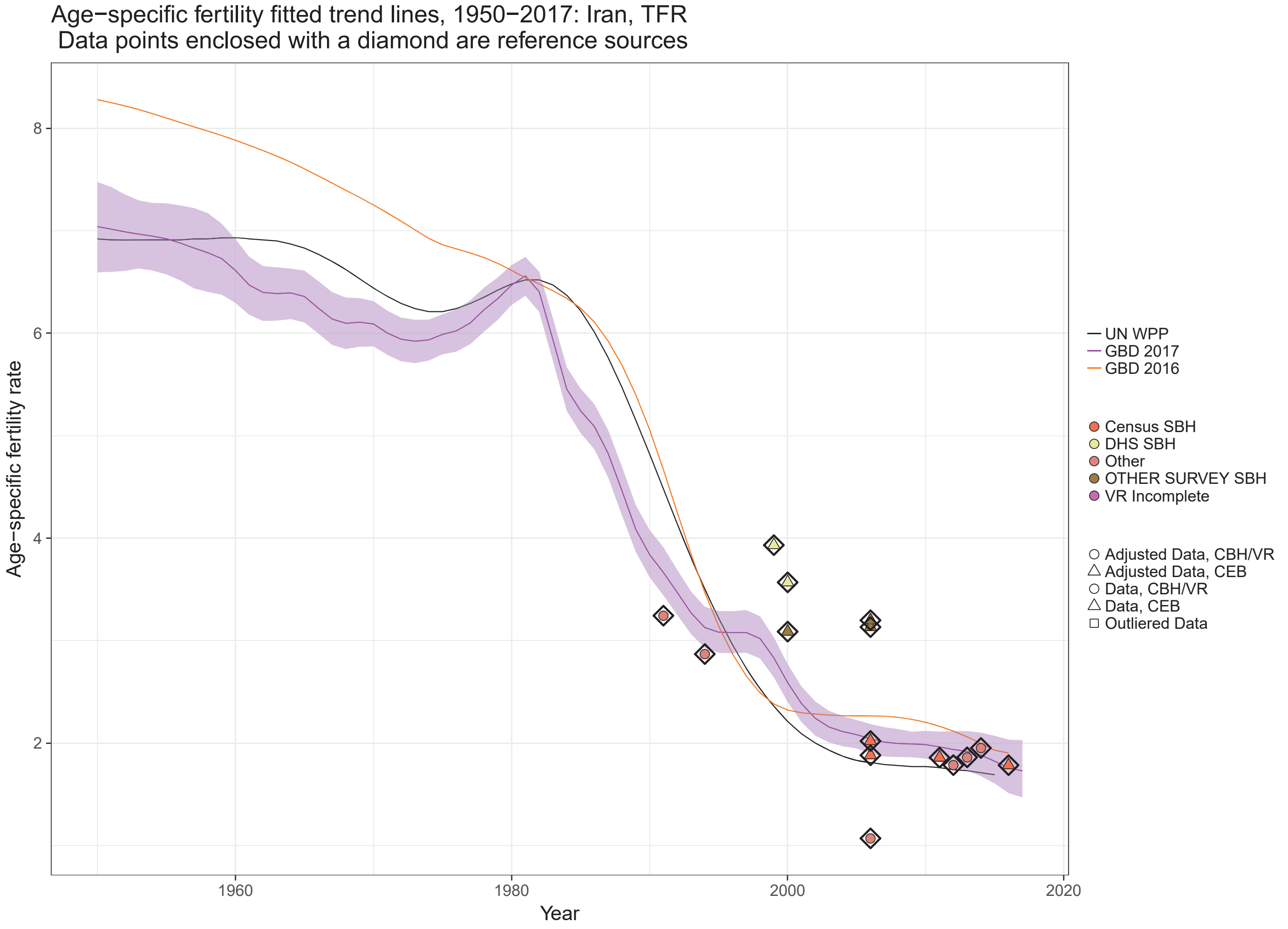

Age−specific fertility fitted trend lines, 1950−2017: Iran, TFR
Data points enclosed with a diamond are reference sources
Age−specific fertility rate
Year
8
6
4
2
1960
1980
2000
2020
UN WPP
GBD 2017
GBD 2016
Census SBH
DHS SBH
Other
OTHER SURVEY SBH
VR Incomplete
Adjusted Data, CBH/VR
Adjusted Data, CEB
Data, CBH/VR
Data, CEB
Outliered Data

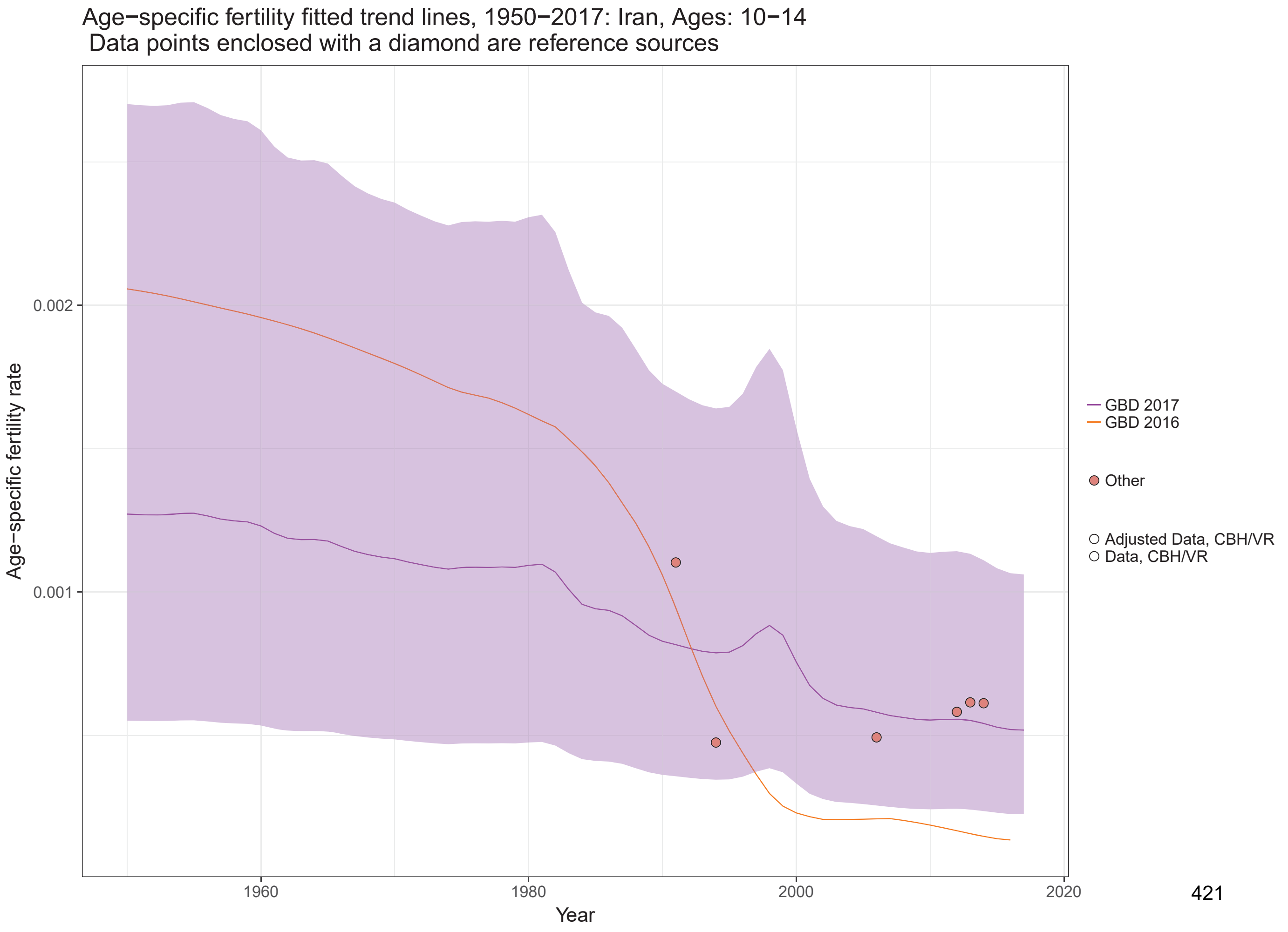

Age−specific fertility fitted trend lines, 1950−2017: Iran, Ages: 10−14
Data points enclosed with a diamond are reference sources
Age−specific fertility rate
Year
0.002
0.001
1960
1980
2000
2020
GBD 2017
GBD 2016
Other
Adjusted Data, CBH/VR
Data, CBH/VR

Age−specific fertility fitted trend lines, 1950−2017: Iran, Ages: 15−19
Data points enclosed with a diamond are reference sources

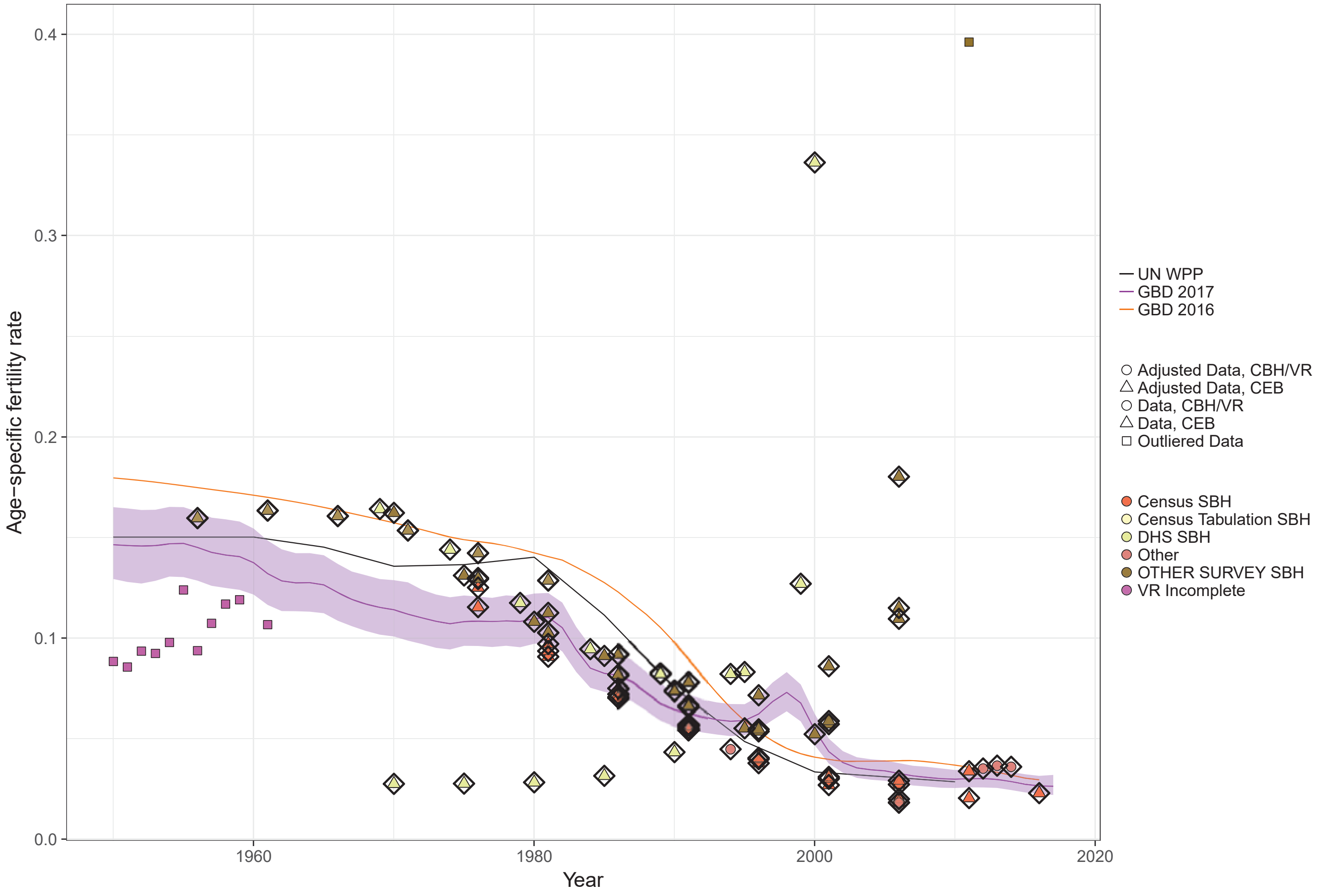

Age−specific fertility fitted trend lines, 1950−2017: Iran, Ages: 20−24
Data points enclosed with a diamond are reference sources

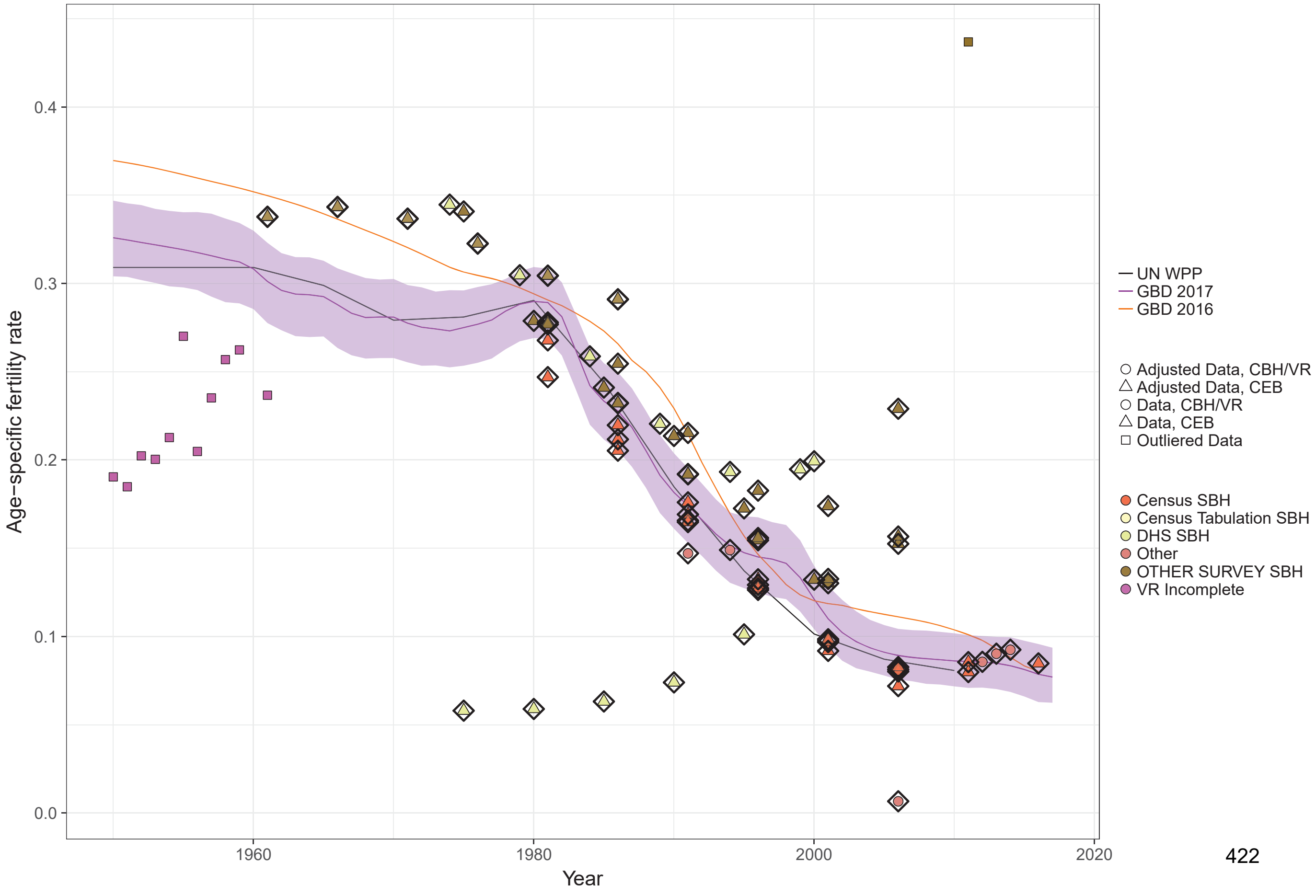

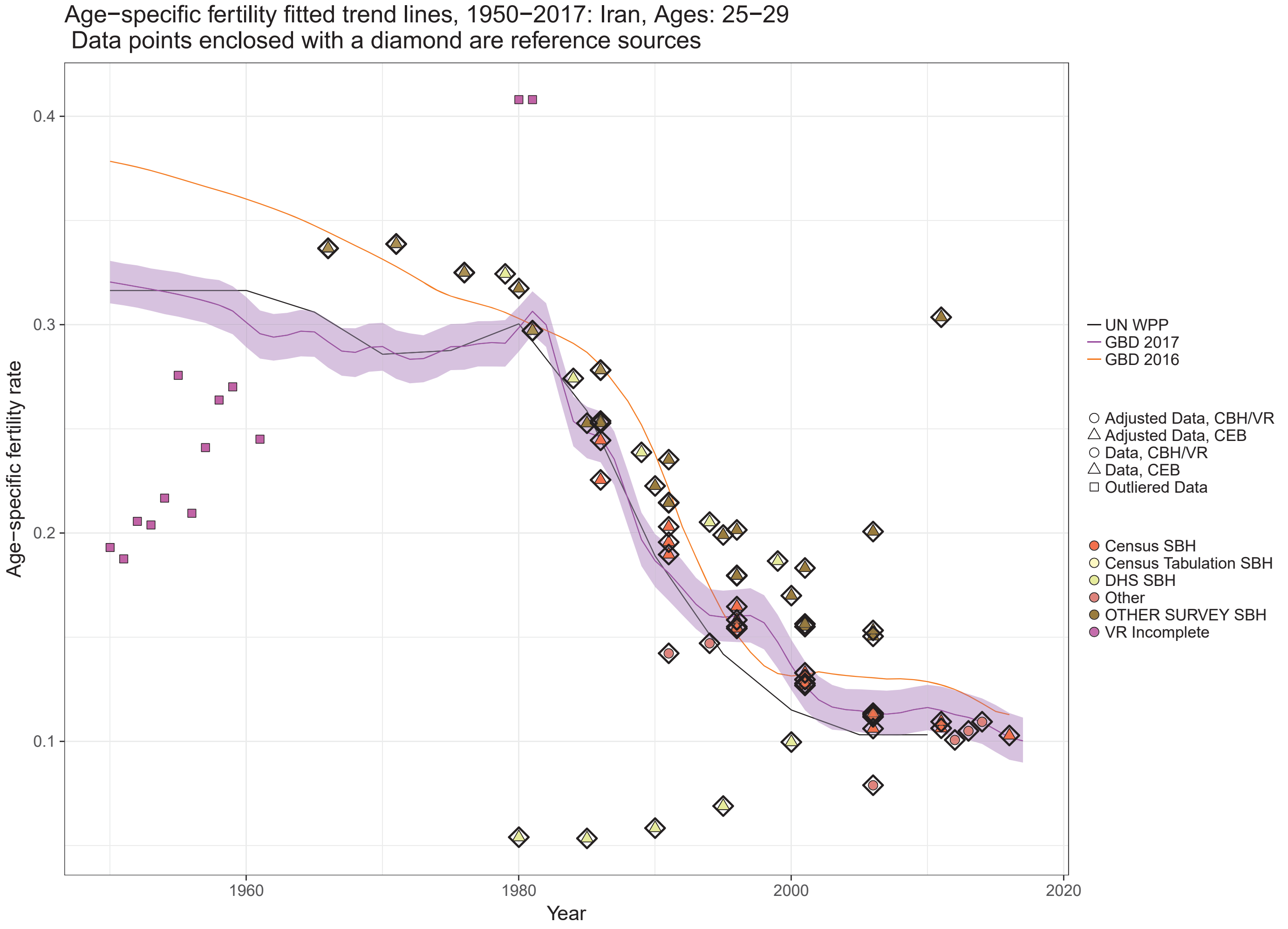

Age−specific fertility fitted trend lines, 1950−2017: Iran, Ages: 25−29
Data points enclosed with a diamond are reference sources
Age-specific fertility rate
0.4
0.3
0.2
0.1
1960
1980
2000
2020
Year
UN WPP
GBD 2017
GBD 2016
Adjusted Data, CBH/VR
Adjusted Data, CEB
Data, CBH/VR
Data, CEB
Outliered Data
Census SBH
Census Tabulation SBH
DHS SBH
Other
OTHER SURVEY SBH
VR Incomplete

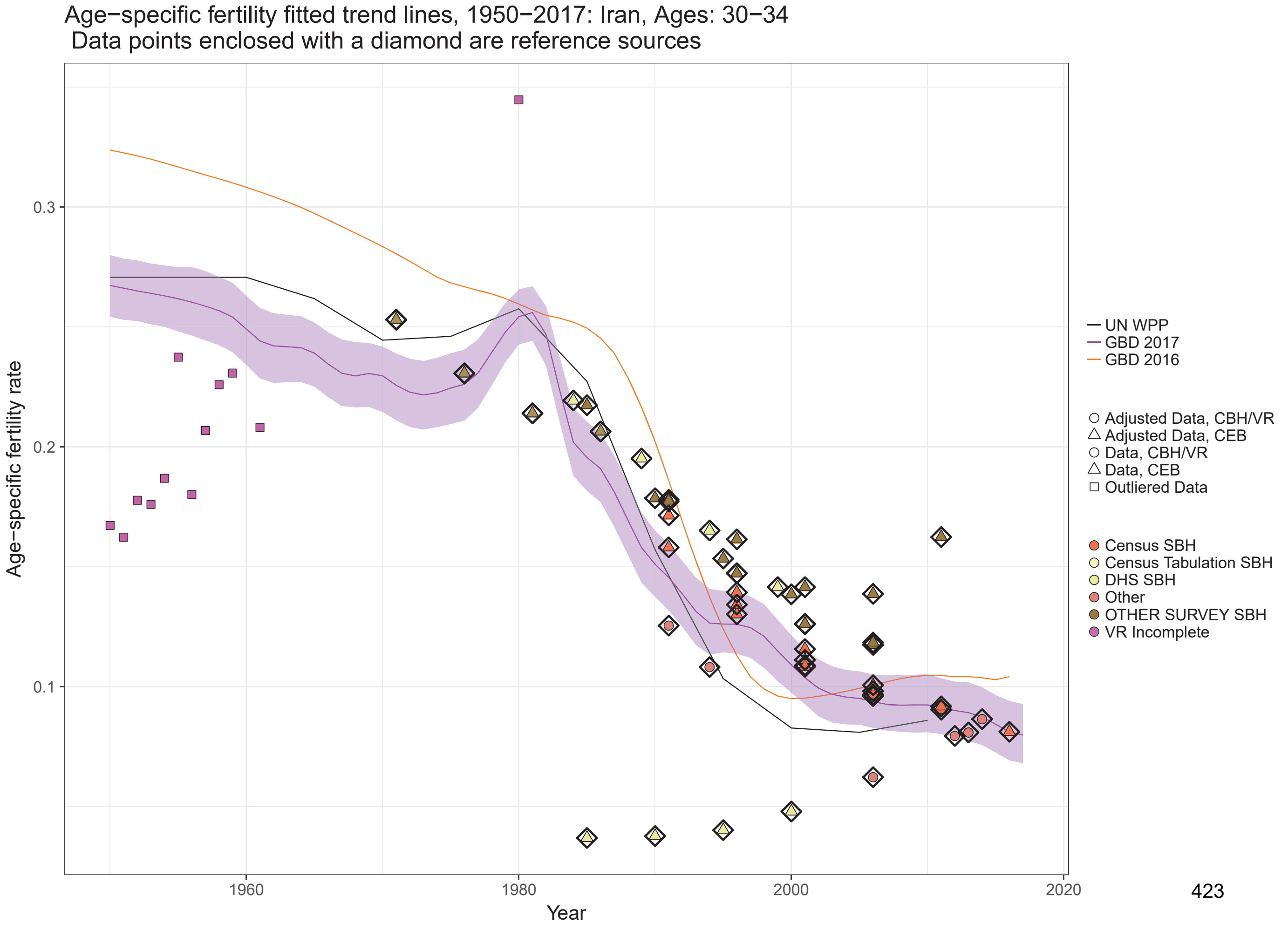

Age−specific fertility fitted trend lines, 1950−2017: Iran, Ages: 30−34
Data points enclosed with a diamond are reference sources
Age-specific fertility rate
0.3
0.2
0.1
1960
1980
2000
2020
Year
UN WPP
GBD 2017
GBD 2016
Adjusted Data, CBH/VR
Adjusted Data, CEB
Data, CBH/VR
Data, CEB
Outliered Data
Census SBH
Census Tabulation SBH
DHS SBH
Other
OTHER SURVEY SBH
VR Incomplete

Age−specific fertility fitted trend lines, 1950−2017: Iran, Ages: 35−39
Data points enclosed with a diamond are reference sources

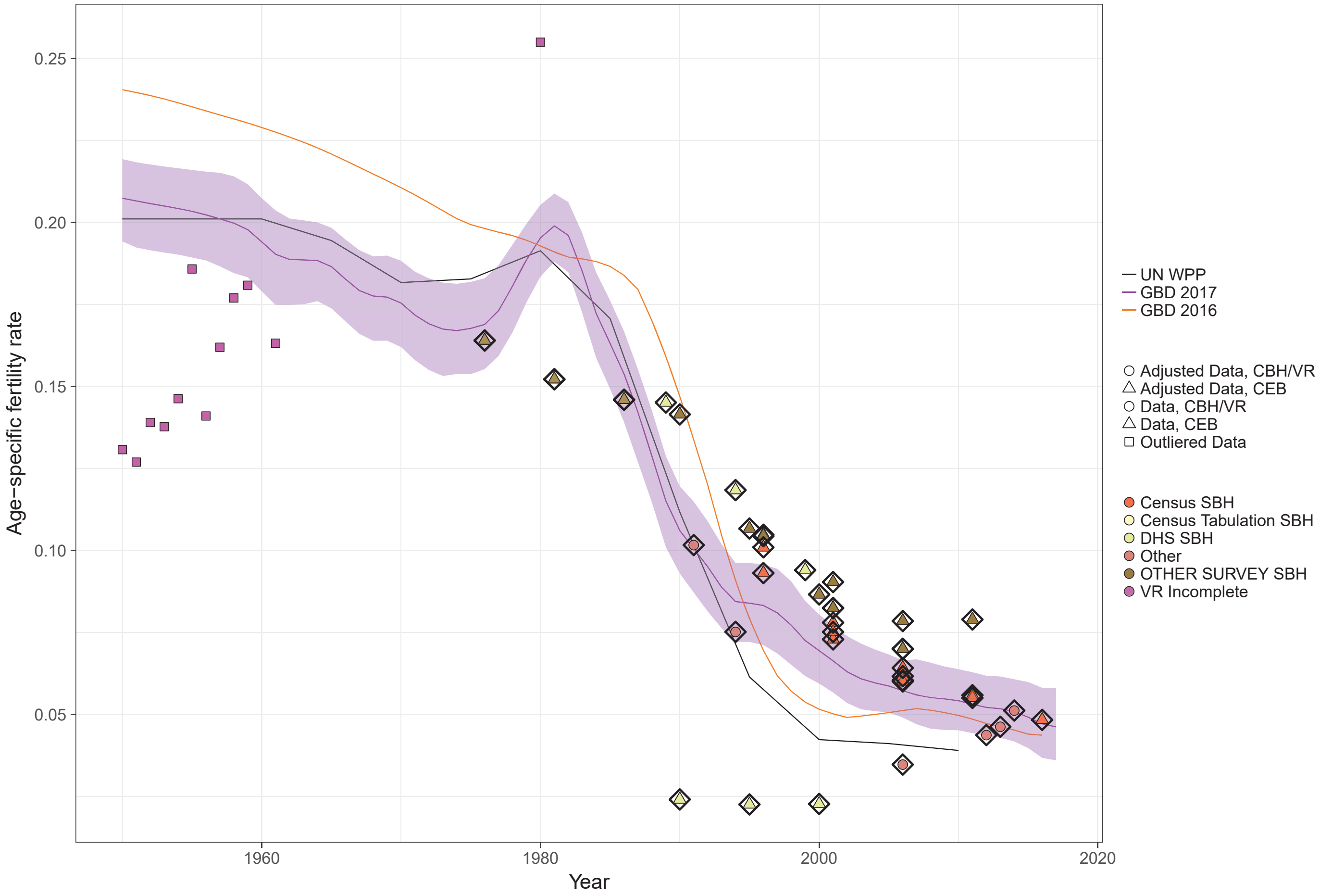

Age−specific fertility fitted trend lines, 1950−2017: Iran, Ages: 40−44
Data points enclosed with a diamond are reference sources

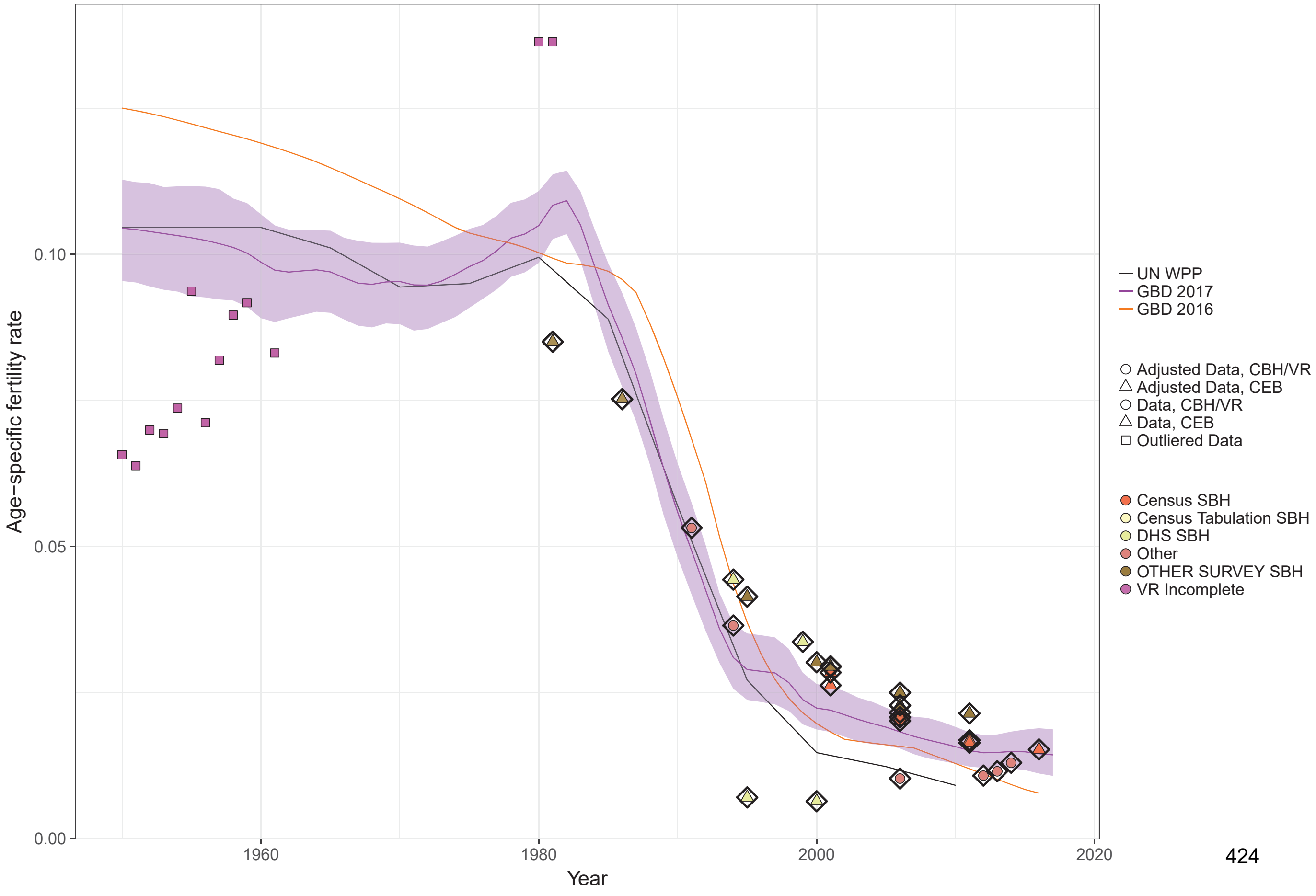

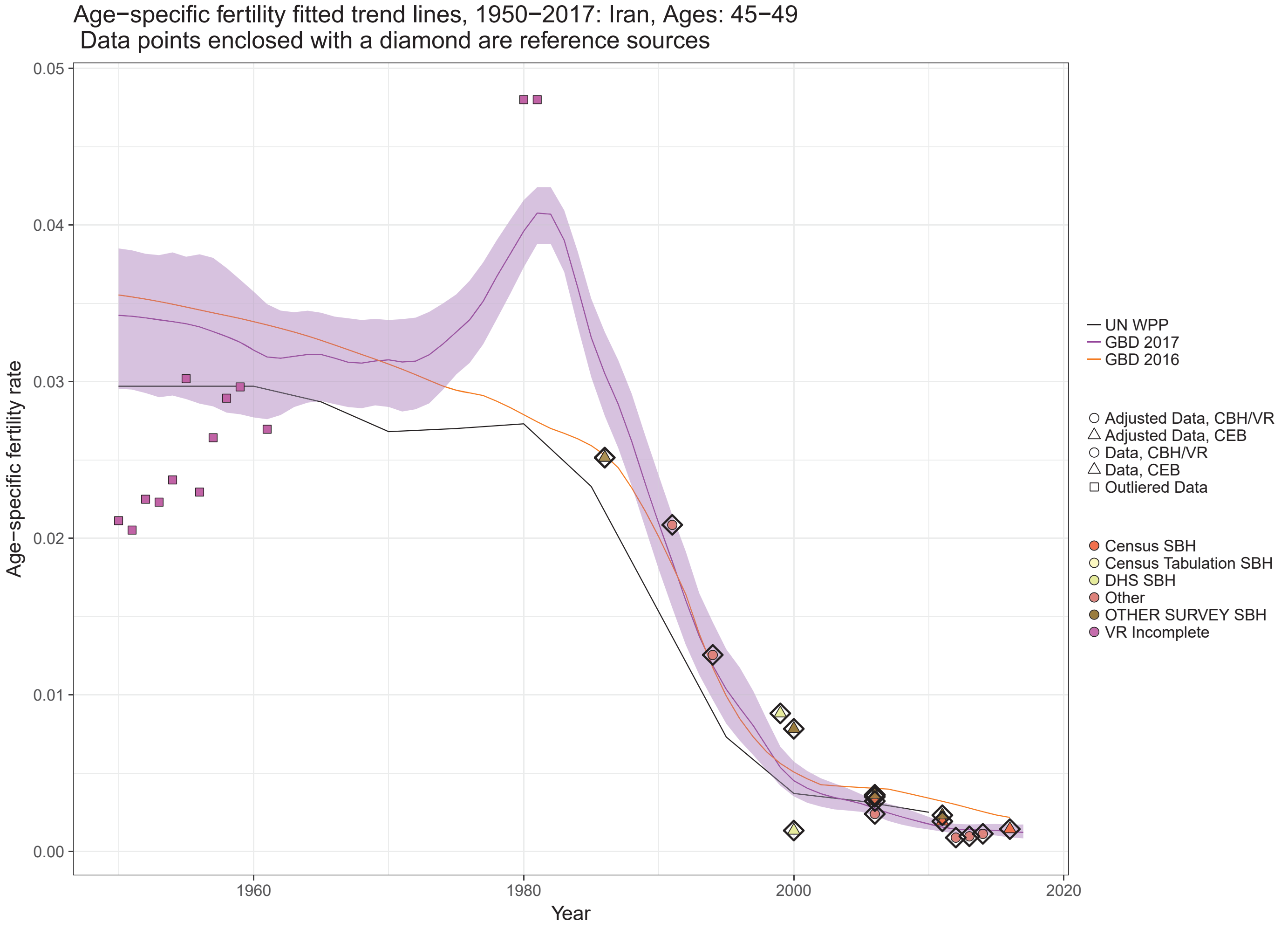

Age−specific fertility fitted trend lines, 1950−2017: Iran, Ages: 45−49
Data points enclosed with a diamond are reference sources
Age−specific fertility rate
Year
UN WPP
GBD 2017
GBD 2016
Adjusted Data, CBH/VR
Adjusted Data, CEB
Data, CBH/VR
Data, CEB
Outliered Data
Census SBH
Census Tabulation SBH
DHS SBH
Other
OTHER SURVEY SBH
VR Incomplete

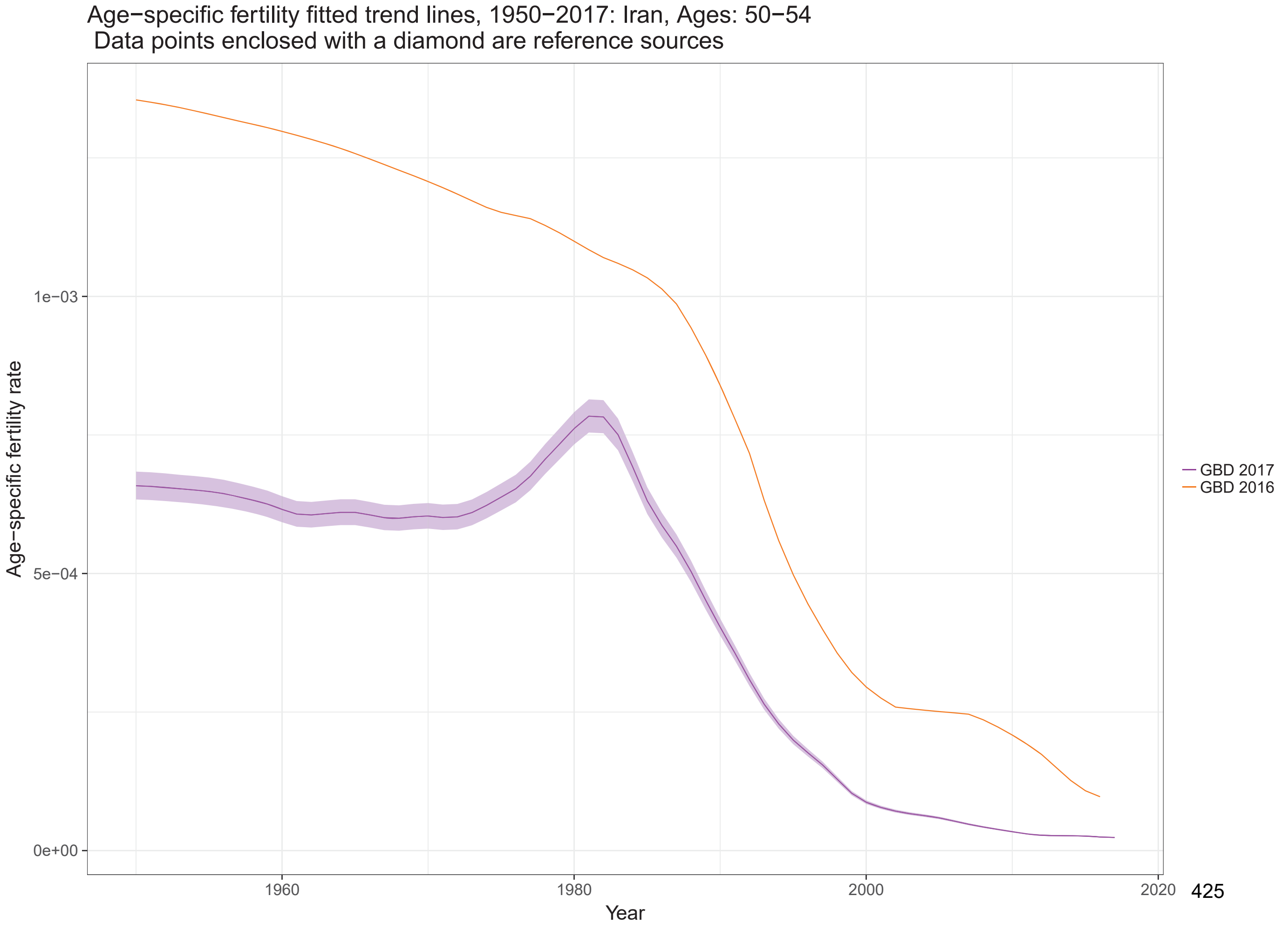

Age−specific fertility fitted trend lines, 1950−2017: Iran, Ages: 50−54
Data points enclosed with a diamond are reference sources
Age−specific fertility rate
Year
GBD 2017
GBD 2016

Age−specific fertility fitted trend lines, 1950−2017: Iraq, TFR
Data points enclosed with a diamond are reference sources

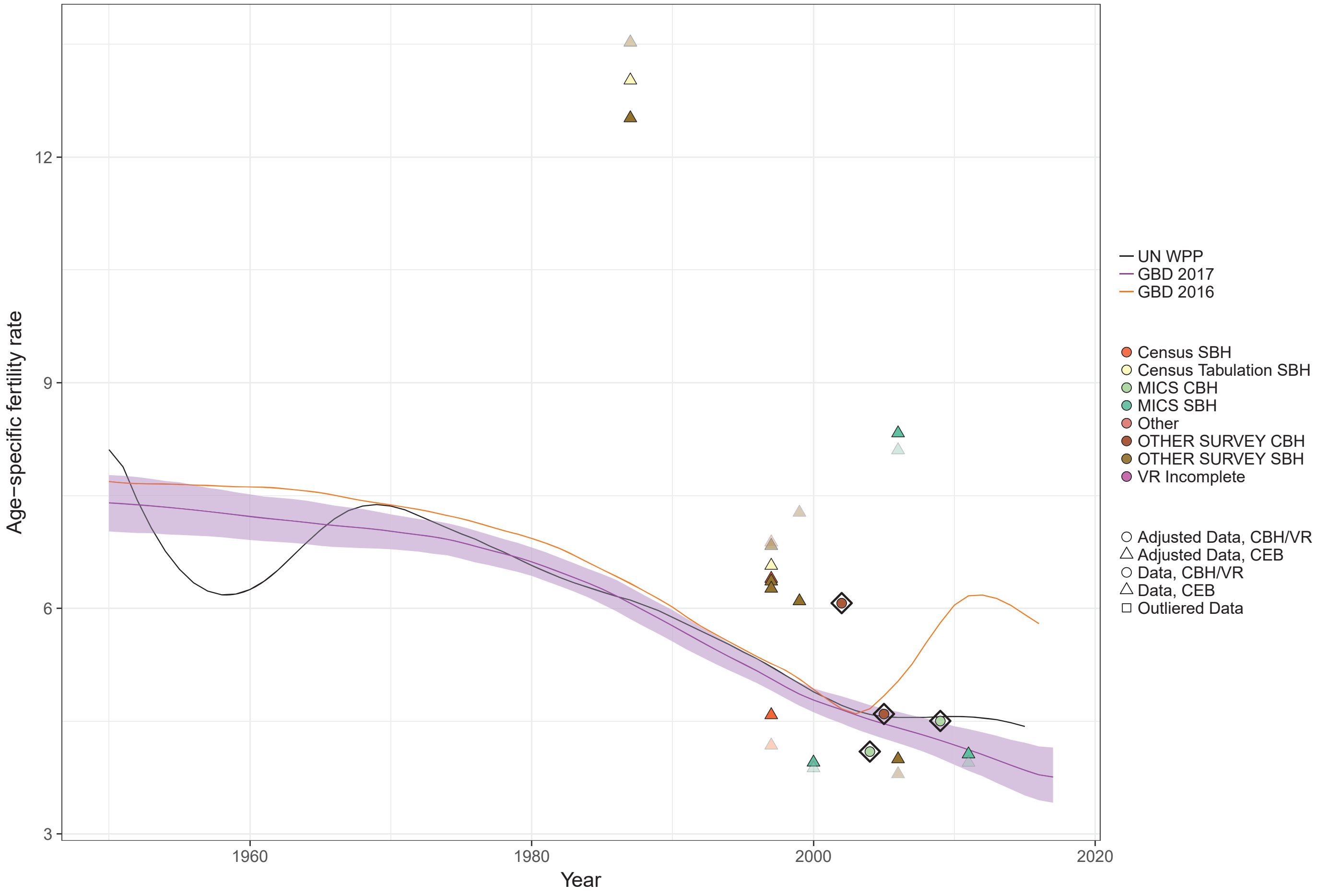

Age−specific fertility fitted trend lines, 1950−2017: Iraq, Ages: 10−14
Data points enclosed with a diamond are reference sources

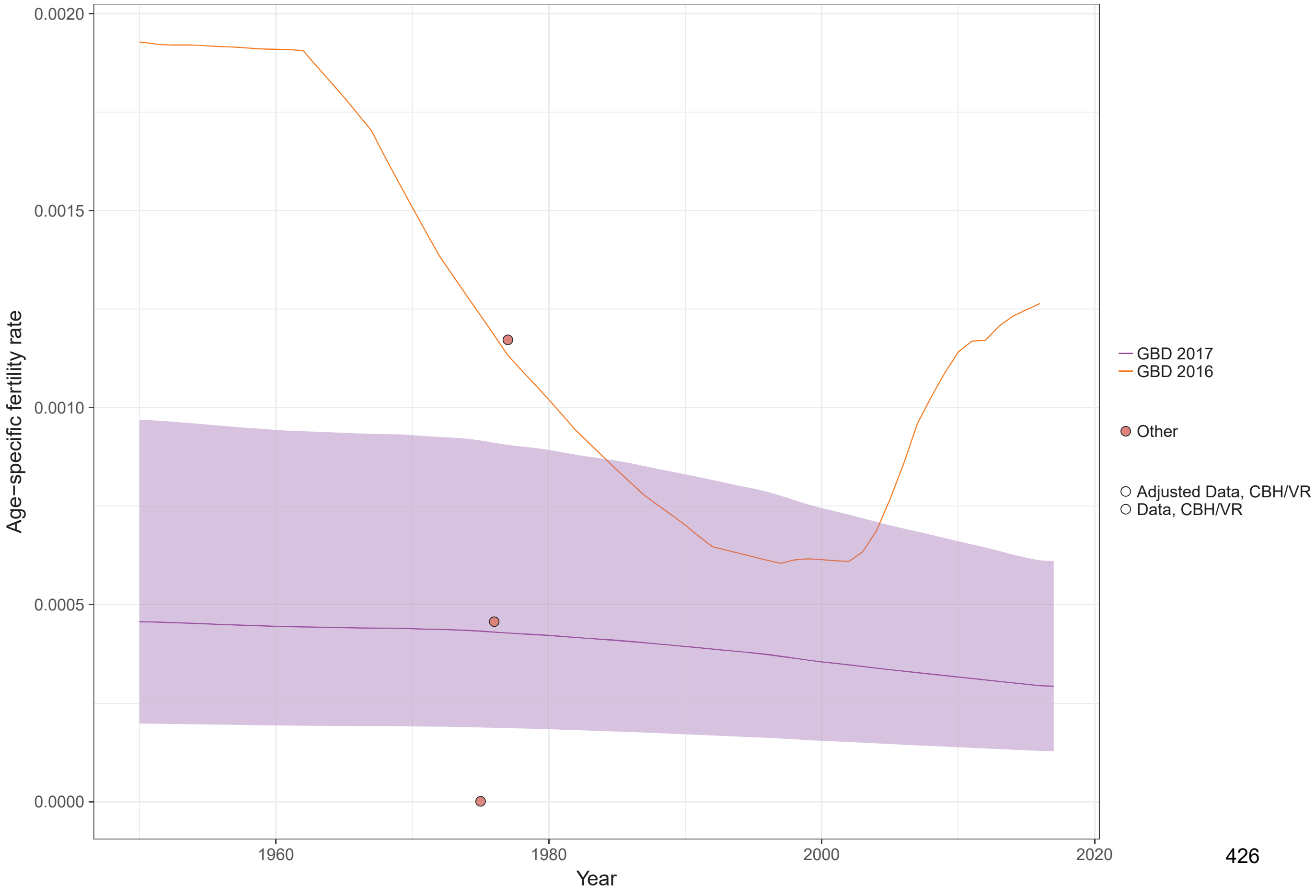

Age−specific fertility fitted trend lines, 1950−2017: Iraq, Ages: 15−19
Data points enclosed with a diamond are reference sources

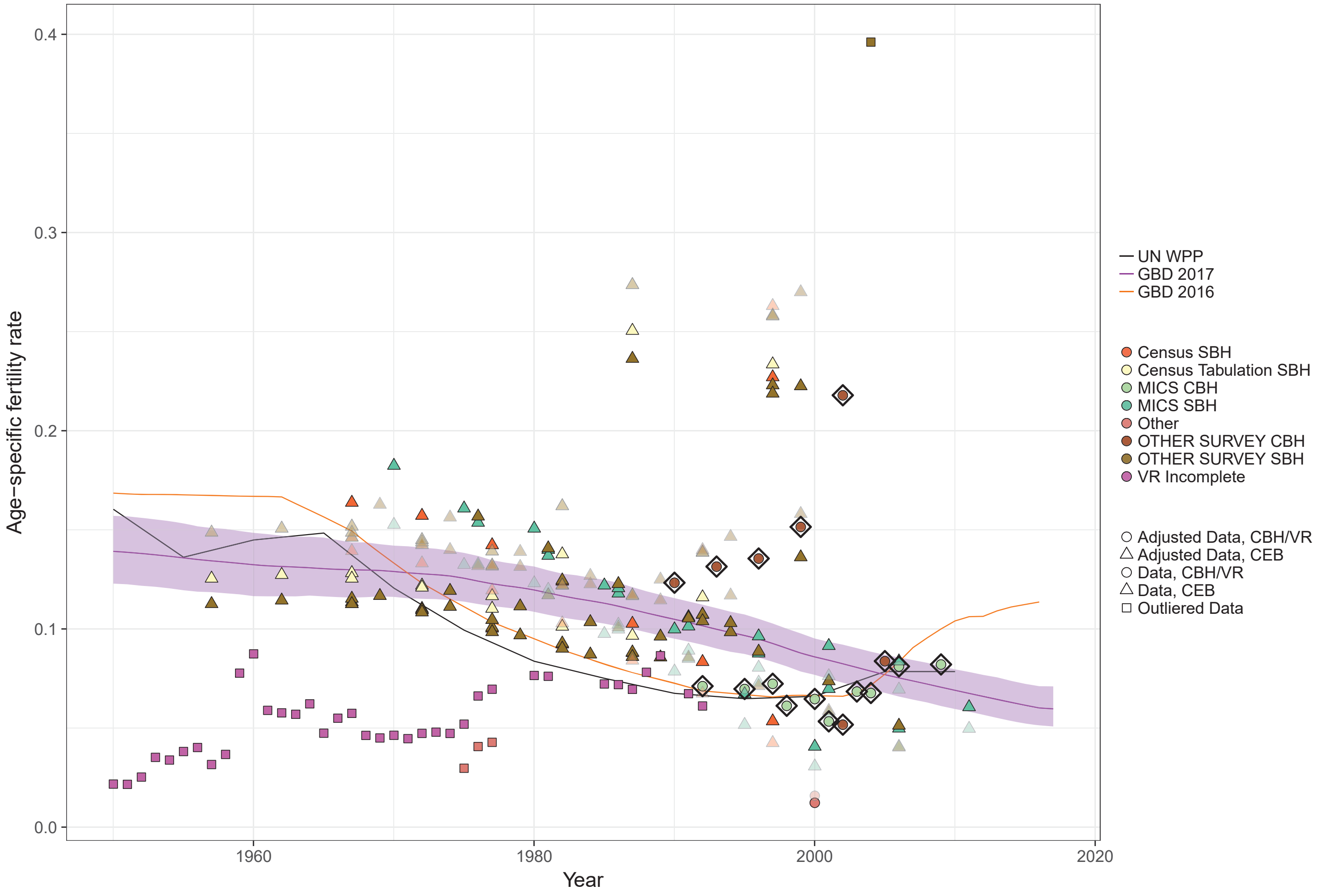

Age−specific fertility fitted trend lines, 1950−2017: Iraq, Ages: 20−24
Data points enclosed with a diamond are reference sources

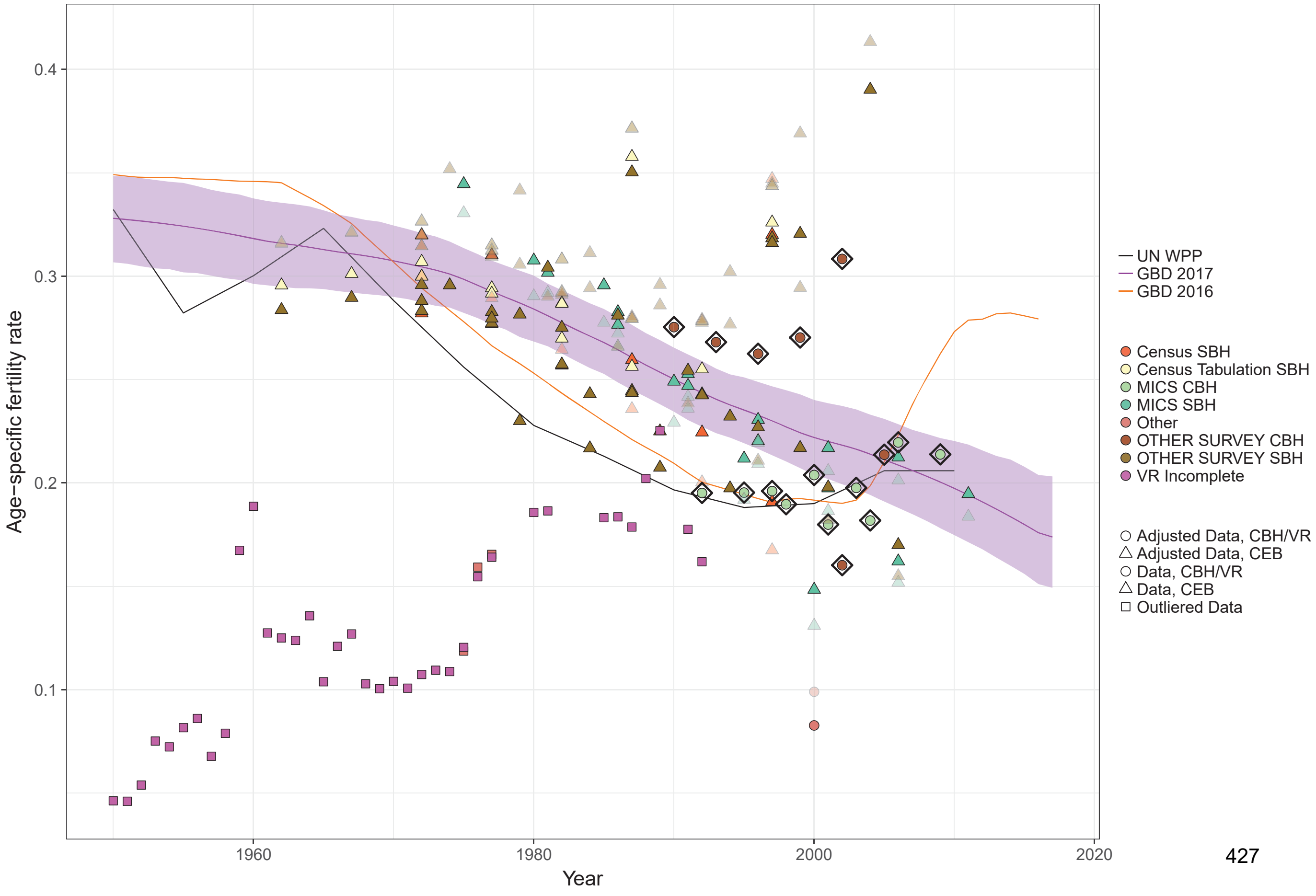

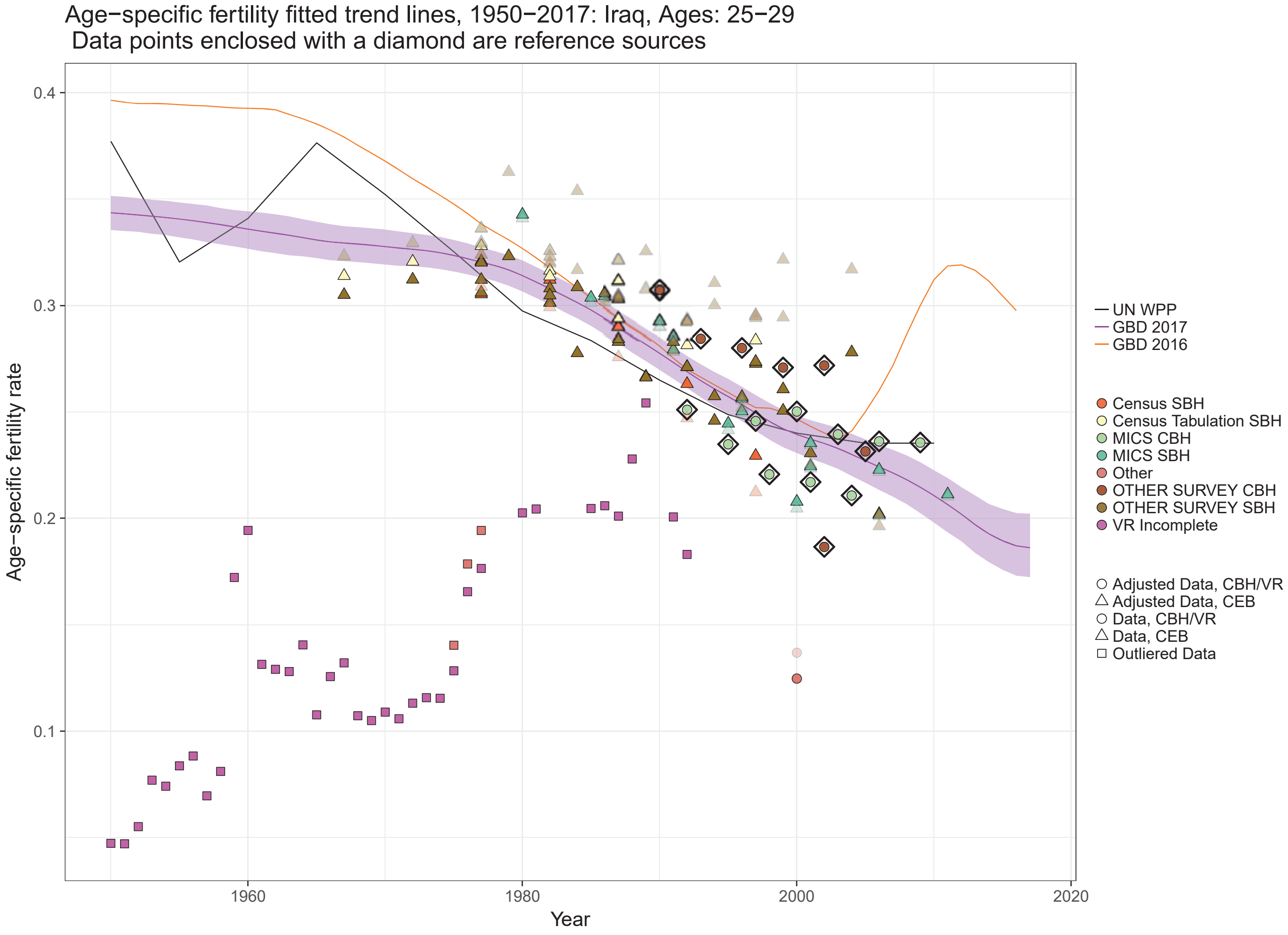

Age−specific fertility fitted trend lines, 1950−2017: Iraq, Ages: 25−29
Data points enclosed with a diamond are reference sources
Age−specific fertility rate
0.4
0.3
0.2
0.1
1960
1980
2000
2020
Year
UN WPP
GBD 2017
GBD 2016
Census SBH
Census Tabulation SBH
MICS CBH
MICS SBH
Other
OTHER SURVEY CBH
OTHER SURVEY SBH
VR Incomplete
Adjusted Data, CBH/VR
Adjusted Data, CEB
Data, CBH/VR
Data, CEB
Outliered Data

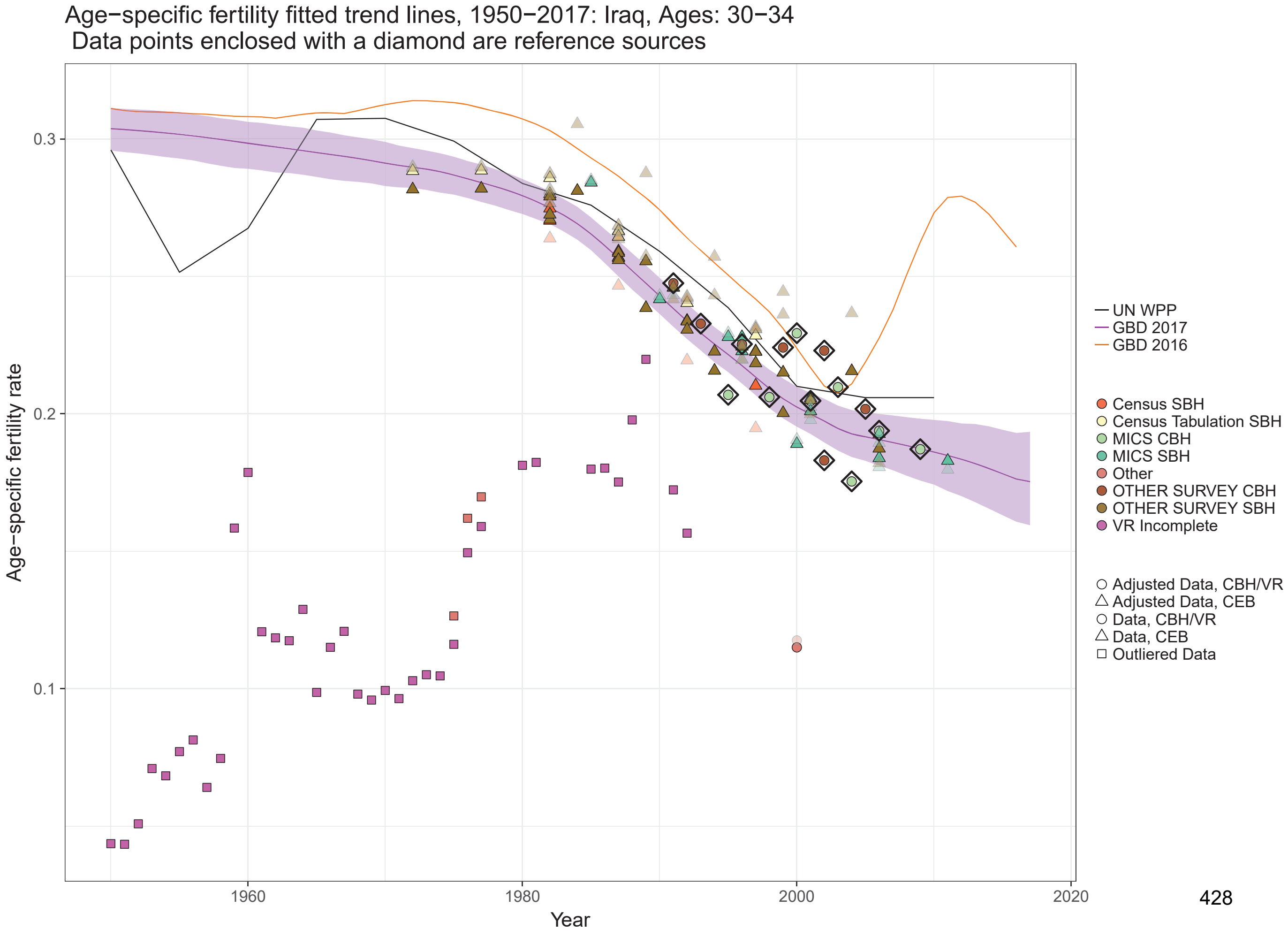

Age−specific fertility fitted trend lines, 1950−2017: Iraq, Ages: 30−34
Data points enclosed with a diamond are reference sources
Age−specific fertility rate
0.3
0.2
0.1
1960
1980
2000
2020
Year
UN WPP
GBD 2017
GBD 2016
Census SBH
Census Tabulation SBH
MICS CBH
MICS SBH
Other
OTHER SURVEY CBH
OTHER SURVEY SBH
VR Incomplete
Adjusted Data, CBH/VR
Adjusted Data, CEB
Data, CBH/VR
Data, CEB
Outliered Data

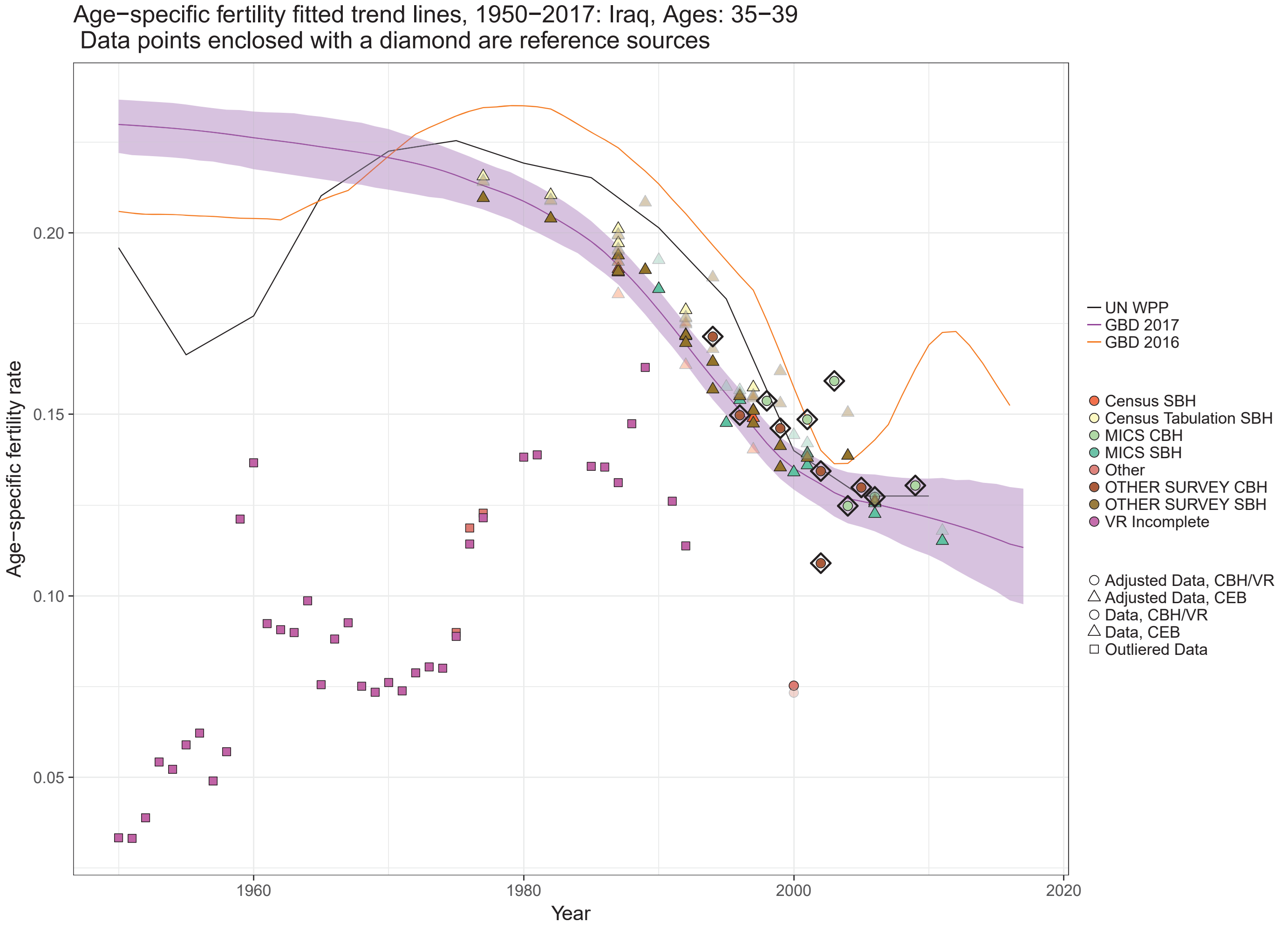

Age−specific fertility fitted trend lines, 1950−2017: Iraq, Ages: 35−39
Data points enclosed with a diamond are reference sources
Age-specific fertility rate
0.20
0.15
0.10
0.05
1960
1980
2000
2020
Year
UN WPP
GBD 2017
GBD 2016
Census SBH
Census Tabulation SBH
MICS CBH
MICS SBH
Other
OTHER SURVEY CBH
OTHER SURVEY SBH
VR Incomplete
Adjusted Data, CBH/VR
Adjusted Data, CEB
Data, CBH/VR
Data, CEB
Outliered Data

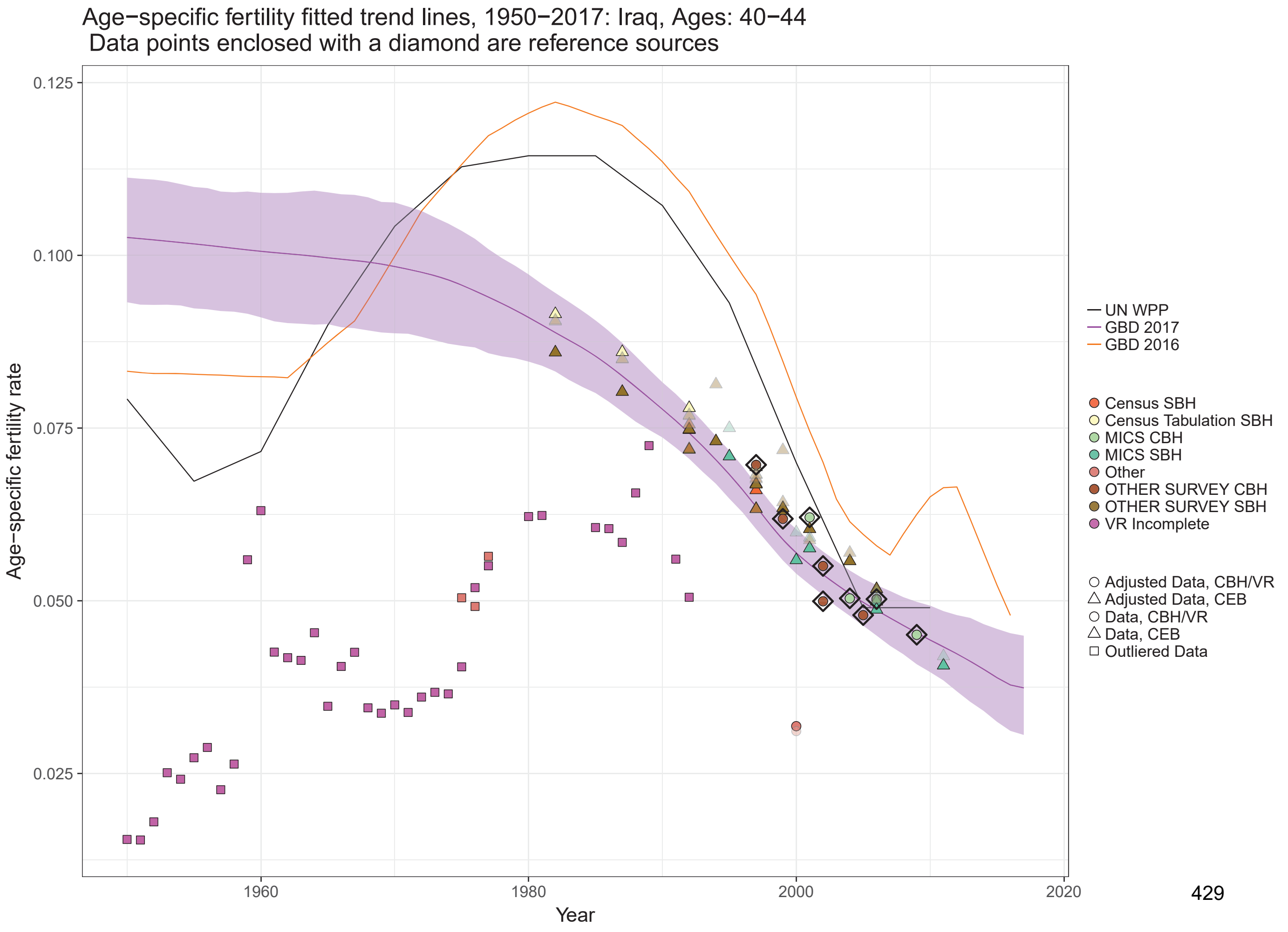

Age−specific fertility fitted trend lines, 1950−2017: Iraq, Ages: 40−44
Data points enclosed with a diamond are reference sources
Age-specific fertility rate
0.125
0.100
0.075
0.050
0.025
1960
1980
2000
2020
Year
UN WPP
GBD 2017
GBD 2016
Census SBH
Census Tabulation SBH
MICS CBH
MICS SBH
Other
OTHER SURVEY CBH
OTHER SURVEY SBH
VR Incomplete
Adjusted Data, CBH/VR
Adjusted Data, CEB
Data, CBH/VR
Data, CEB
Outliered Data

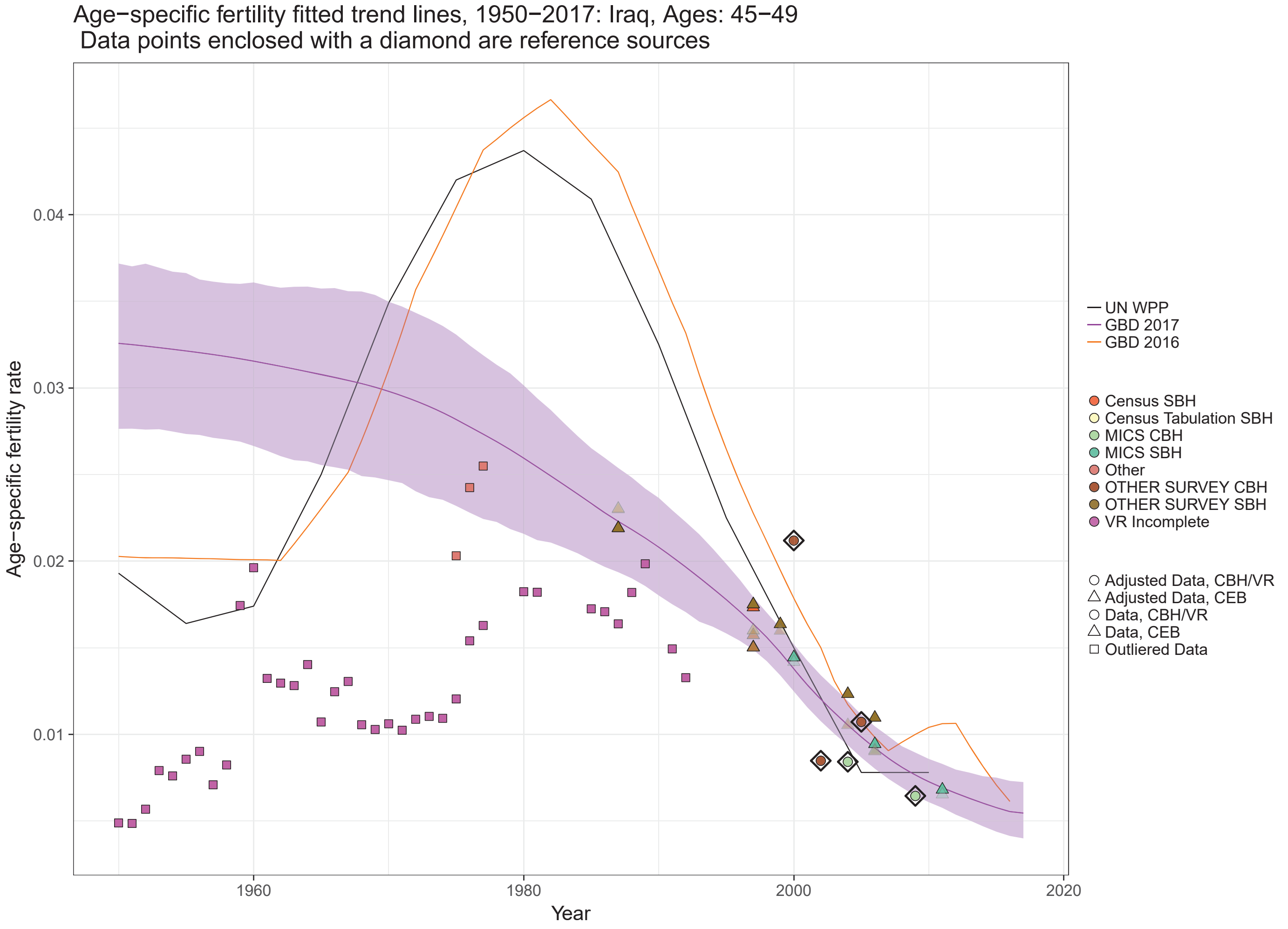

Age−specific fertility fitted trend lines, 1950−2017: Iraq, Ages: 45−49
Data points enclosed with a diamond are reference sources
Age−specific fertility rate
0.01
0.02
0.03
0.04
1960
1980
2000
2020
Year
UN WPP
GBD 2017
GBD 2016
Census SBH
Census Tabulation SBH
MICS CBH
MICS SBH
Other
OTHER SURVEY CBH
OTHER SURVEY SBH
VR Incomplete
Adjusted Data, CBH/VR
Adjusted Data, CEB
Data, CBH/VR
Data, CEB
Outliered Data

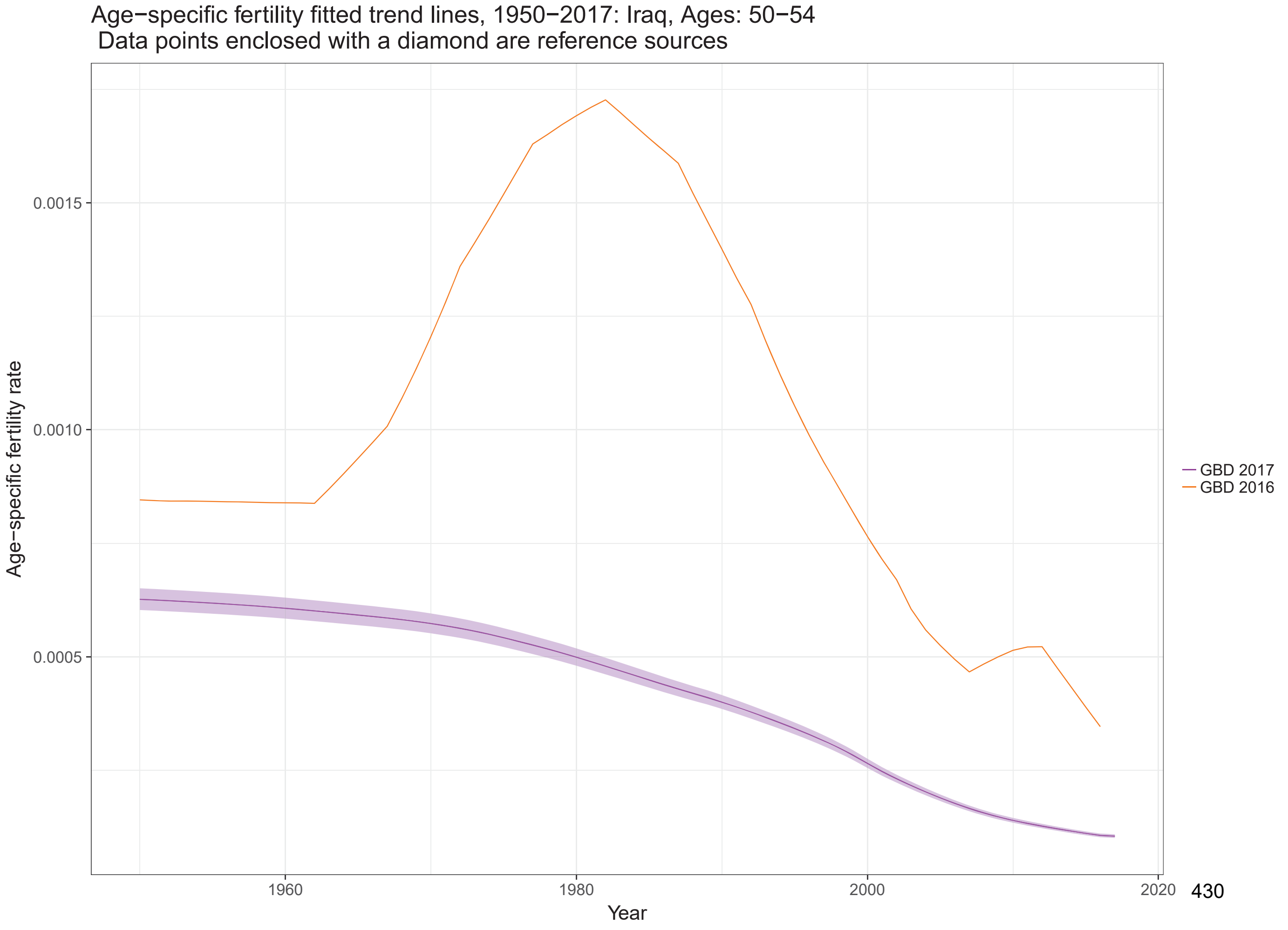

Age−specific fertility fitted trend lines, 1950−2017: Iraq, Ages: 50−54
Data points enclosed with a diamond are reference sources
Age−specific fertility rate
0.0005
0.0010
0.0015
1960
1980
2000
2020
Year
GBD 2017
GBD 2016

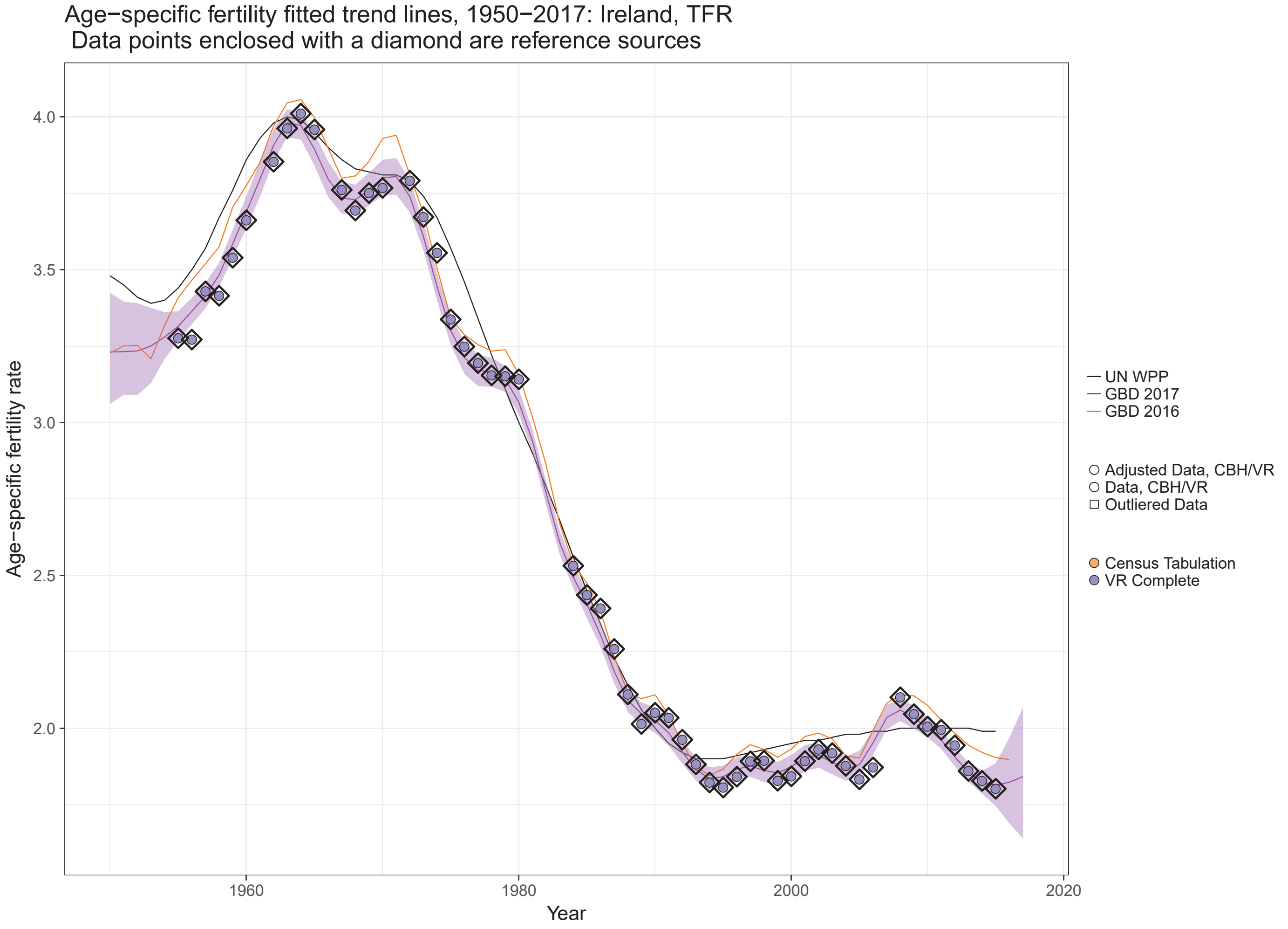
Age−specific fertility fitted trend lines, 1950−2017: Ireland, TFR
Data points enclosed with a diamond are reference sources
Age−specific fertility rate
Year
UN WPP
GBD 2017
GBD 2016
Adjusted Data, CBH/VR
Data, CBH/VR
Outliered Data
Census Tabulation
VR Complete

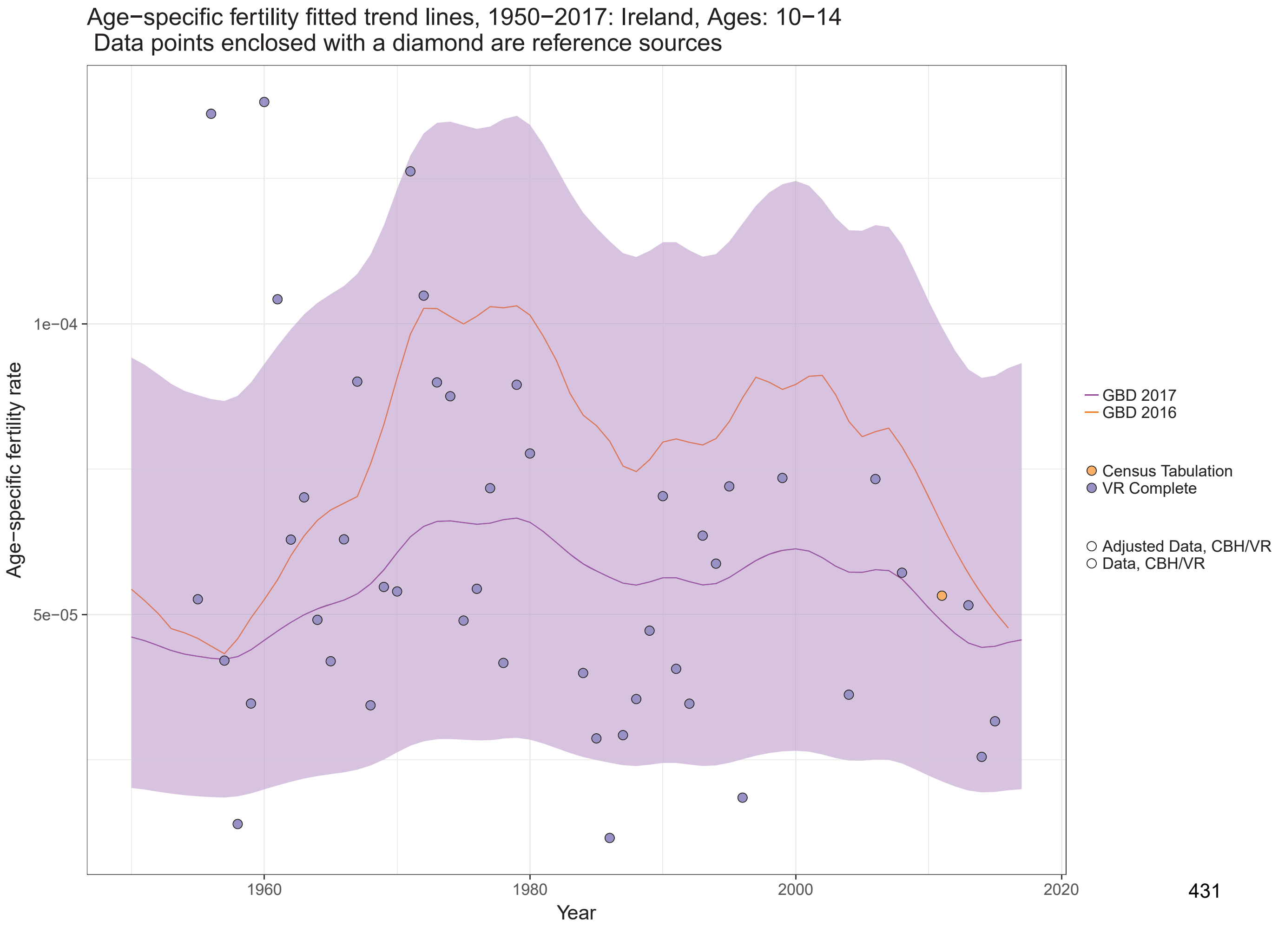
Age−specific fertility fitted trend lines, 1950−2017: Ireland, Ages: 10−14
Data points enclosed with a diamond are reference sources
Age−specific fertility rate
Year
GBD 2017
GBD 2016
Census Tabulation
VR Complete
Adjusted Data, CBH/VR
Data, CBH/VR

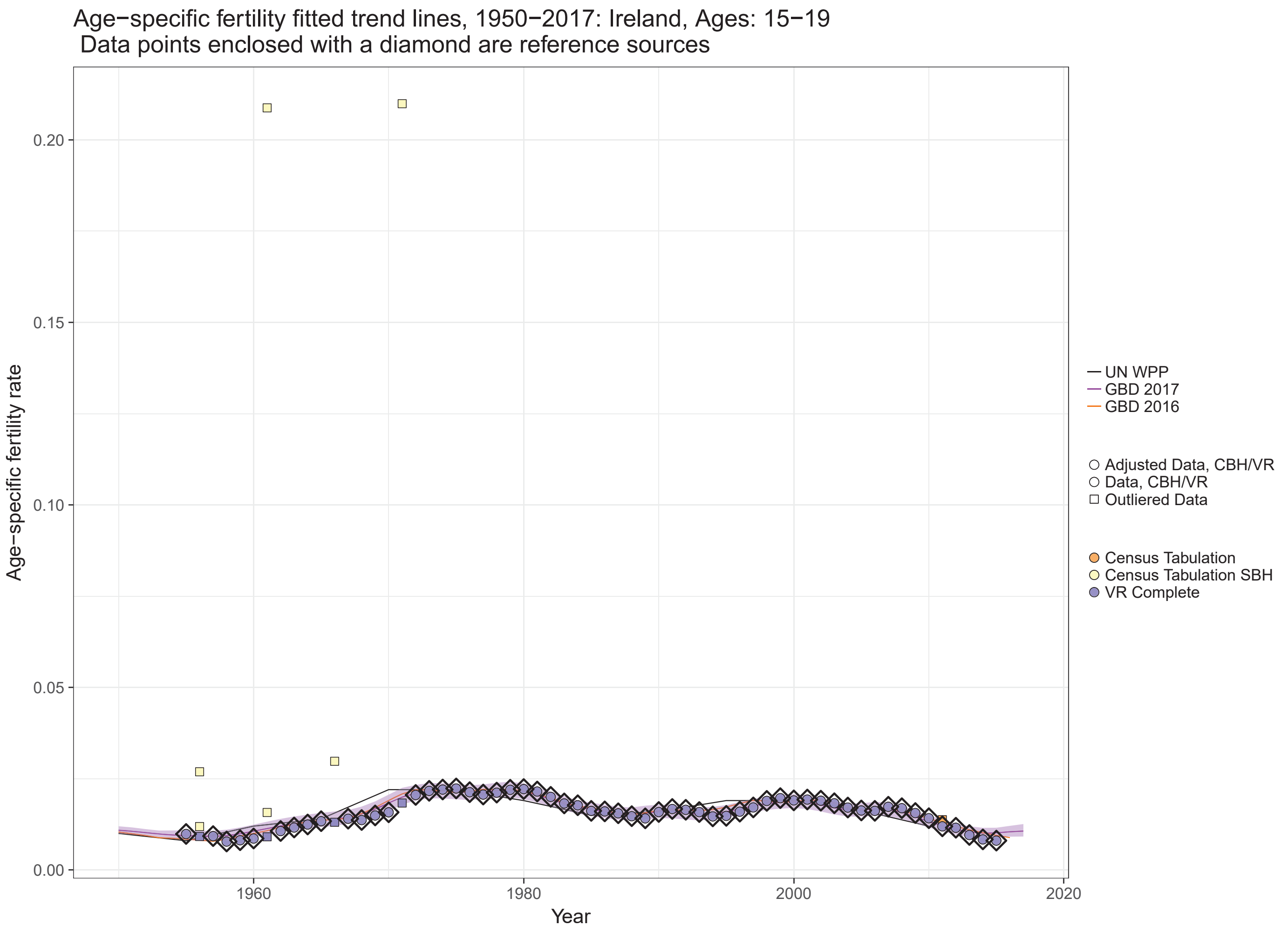

Age−specific fertility fitted trend lines, 1950−2017: Ireland, Ages: 15−19
Data points enclosed with a diamond are reference sources
Age−specific fertility rate
0.00
0.05
0.10
0.15
0.20
1960
1980
2000
2020
Year
UN WPP
GBD 2017
GBD 2016
Adjusted Data, CBH/VR
Data, CBH/VR
Outliered Data
Census Tabulation
Census Tabulation SBH
VR Complete

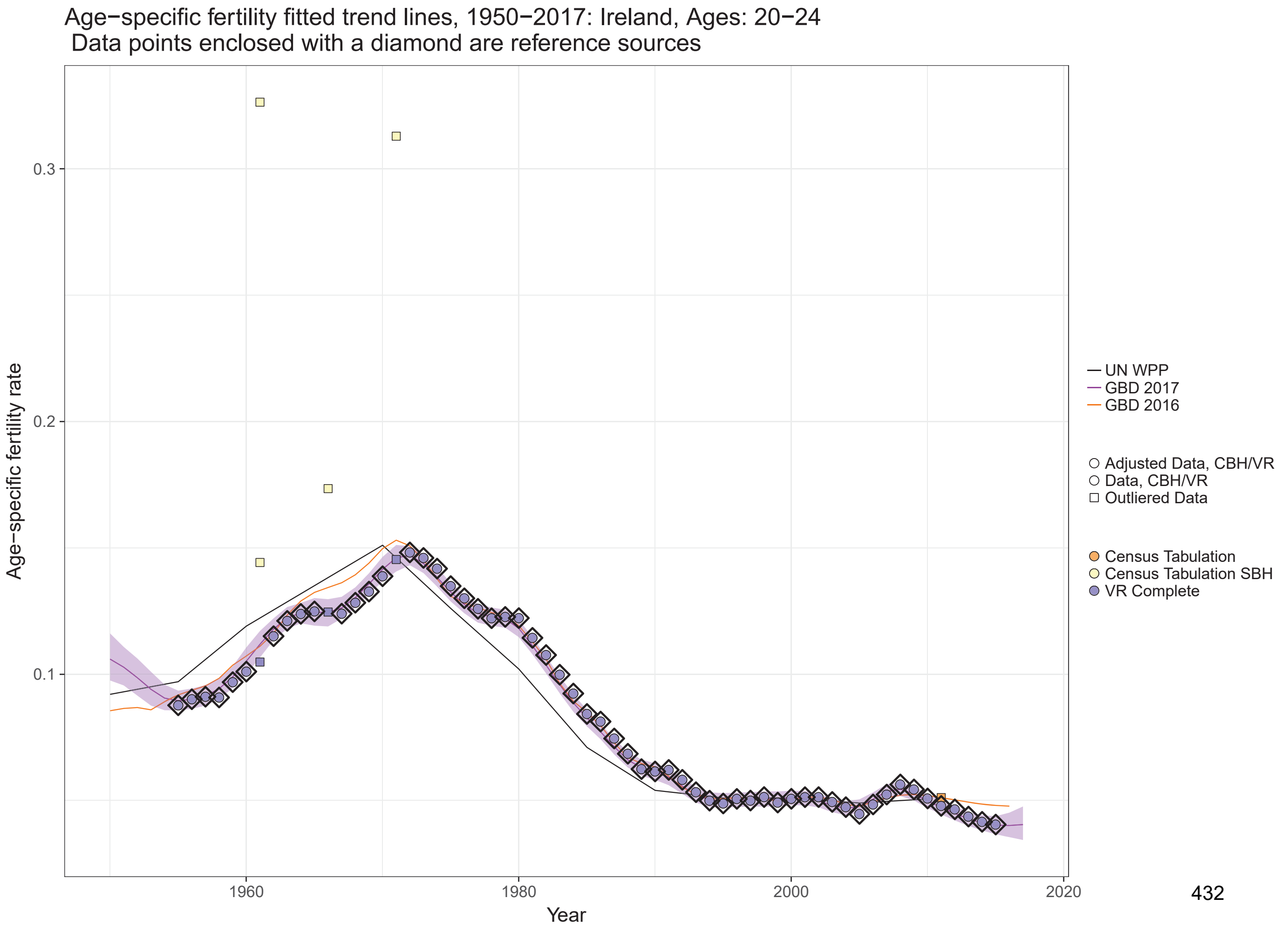

Age−specific fertility fitted trend lines, 1950−2017: Ireland, Ages: 20−24
Data points enclosed with a diamond are reference sources
Age−specific fertility rate
0.1
0.2
0.3
1960
1980
2000
2020
Year
UN WPP
GBD 2017
GBD 2016
Adjusted Data, CBH/VR
Data, CBH/VR
Outliered Data
Census Tabulation
Census Tabulation SBH
VR Complete

Age−specific fertility fitted trend lines, 1950−2017: Ireland, Ages: 25−29
Data points enclosed with a diamond are reference sources

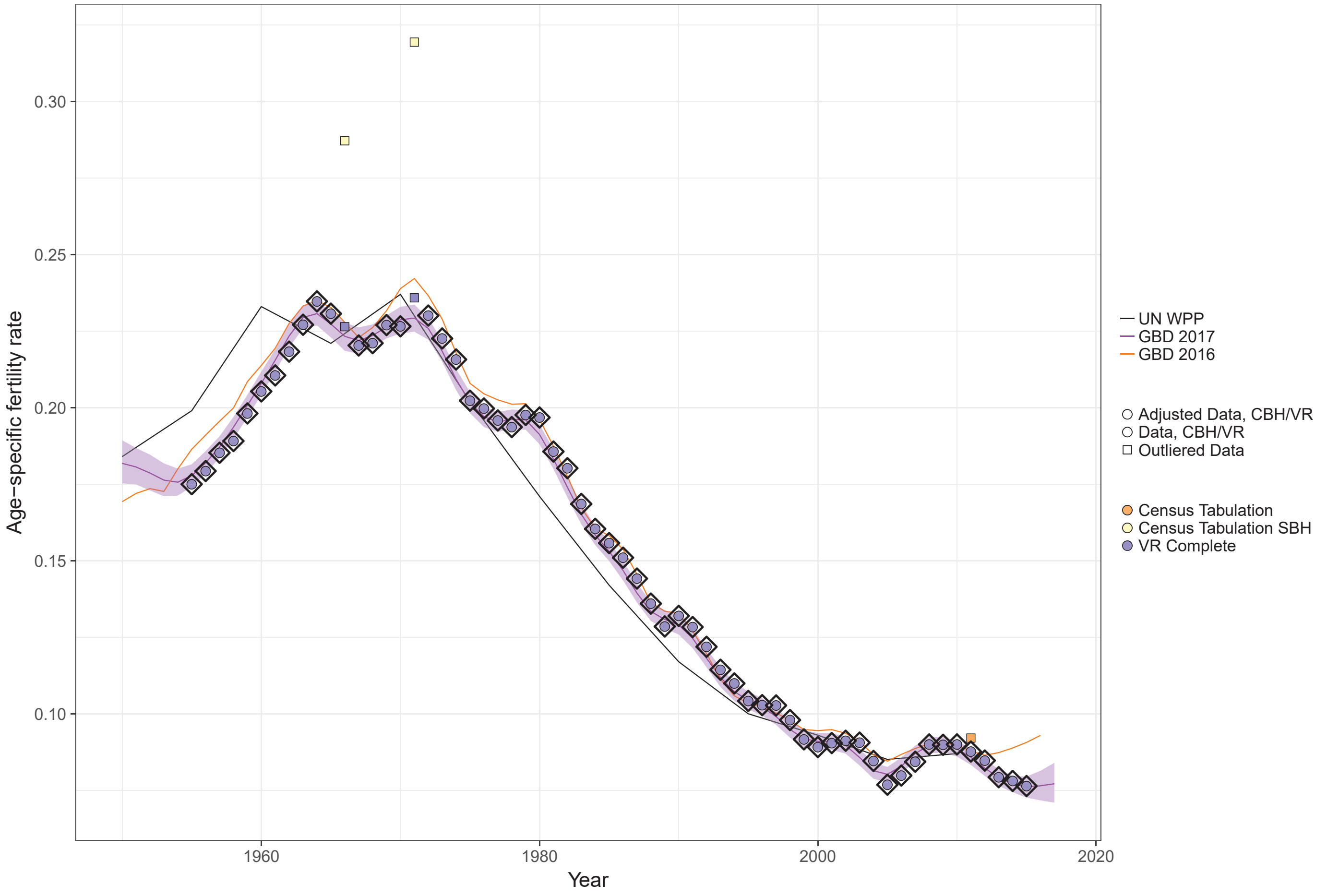

Age−specific fertility fitted trend lines, 1950−2017: Ireland, Ages: 30−34
Data points enclosed with a diamond are reference sources

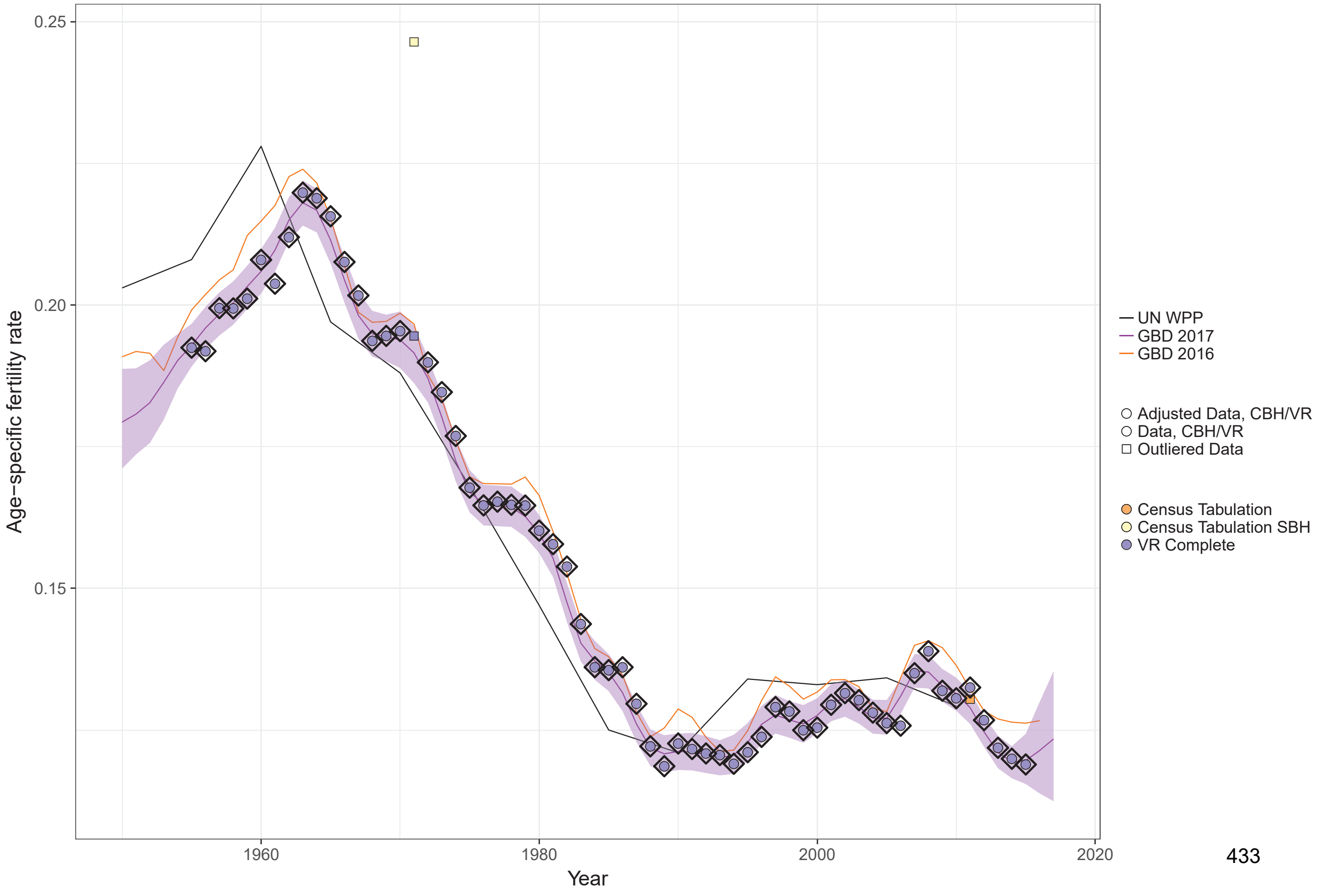

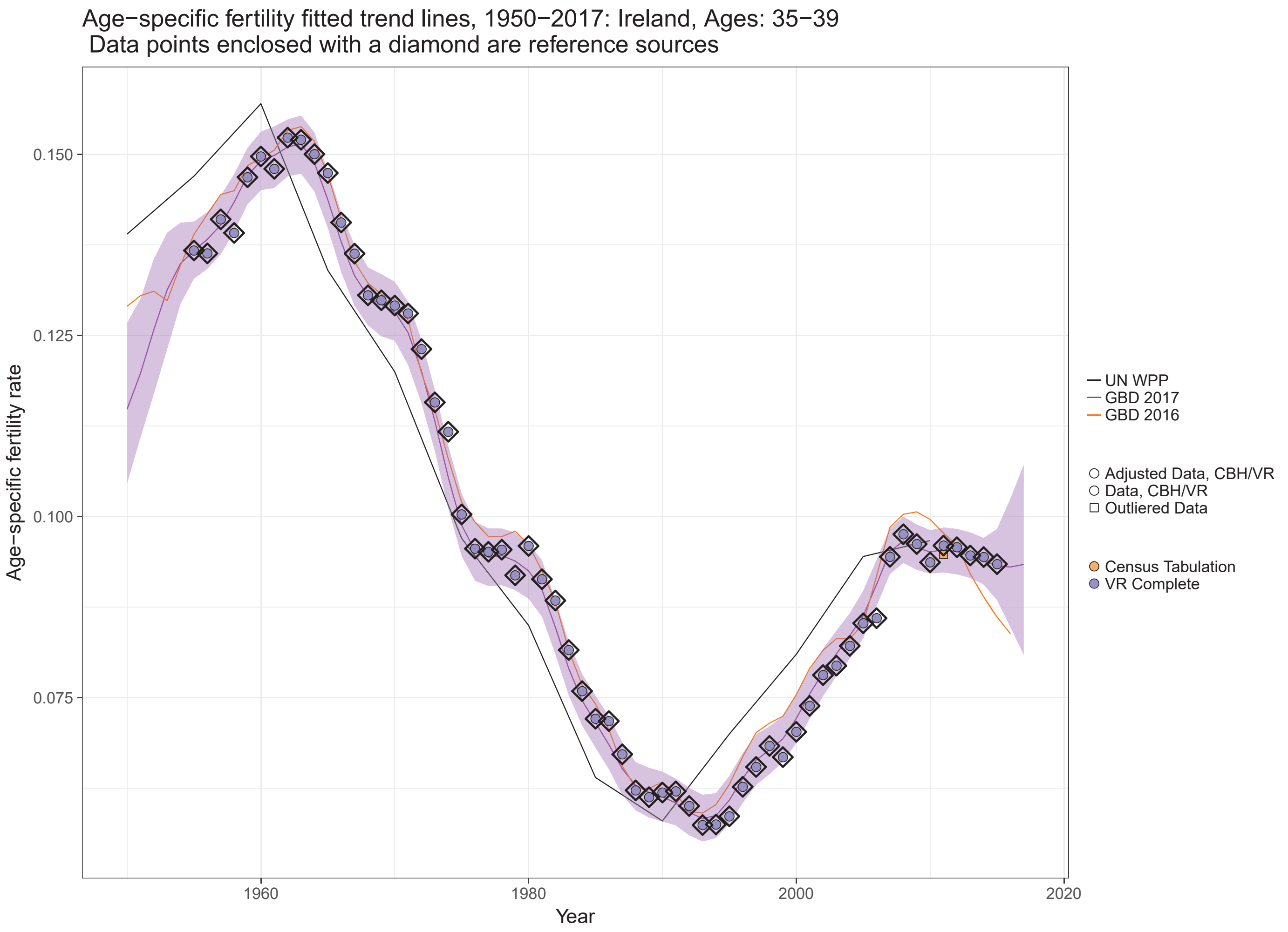
Age−specific fertility fitted trend lines, 1950−2017: Ireland, Ages: 35−39
Data points enclosed with a diamond are reference sources
Age−specific fertility rate
Year
UN WPP
GBD 2017
GBD 2016
Adjusted Data, CBH/VR
Data, CBH/VR
Outliered Data
Census Tabulation
VR Complete

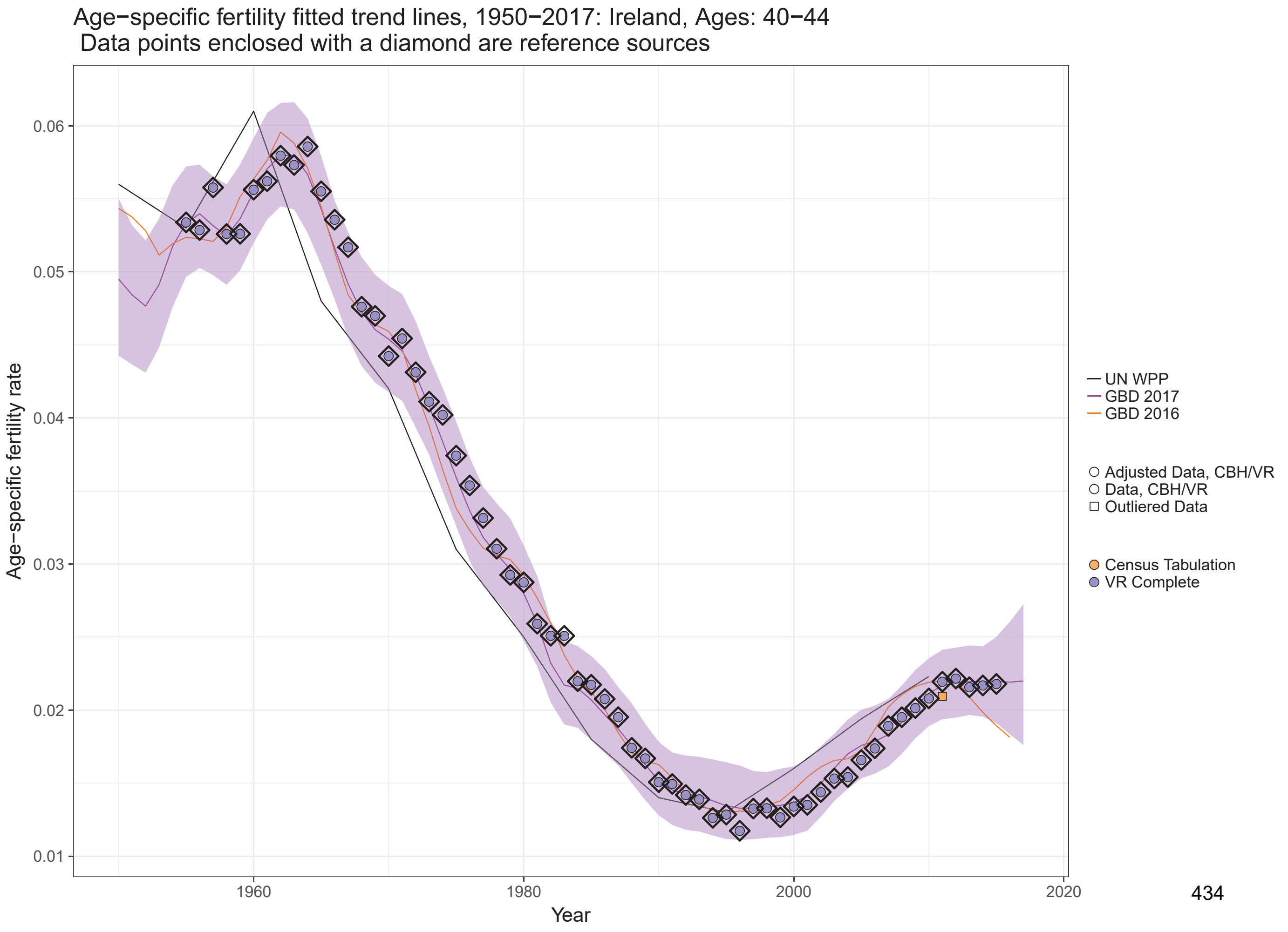
Age−specific fertility fitted trend lines, 1950−2017: Ireland, Ages: 40−44
Data points enclosed with a diamond are reference sources
Age−specific fertility rate
Year
UN WPP
GBD 2017
GBD 2016
Adjusted Data, CBH/VR
Data, CBH/VR
Outliered Data
Census Tabulation
VR Complete

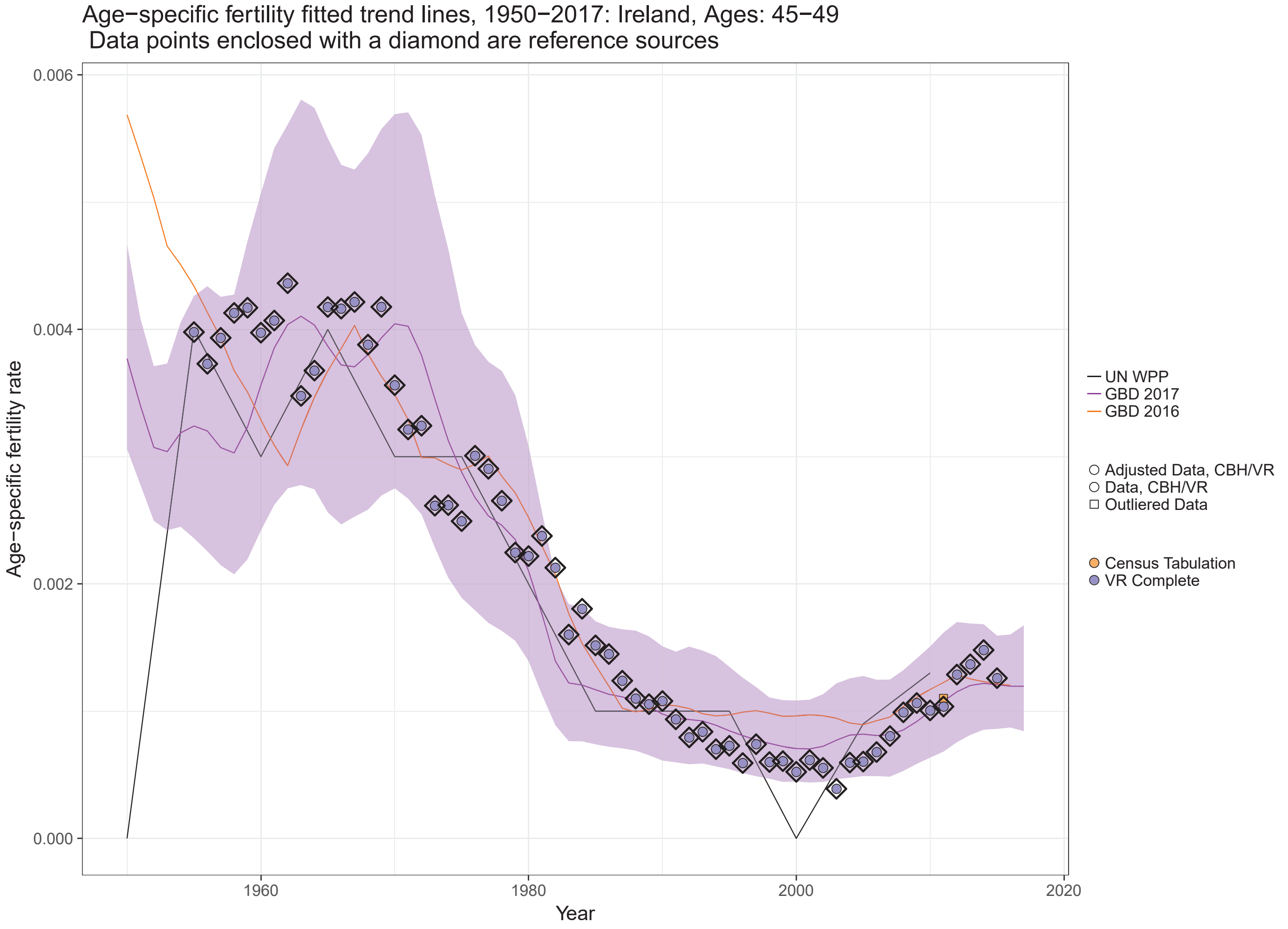
Age−specific fertility fitted trend lines, 1950−2017: Ireland, Ages: 45−49
Data points enclosed with a diamond are reference sources
Age−specific fertility rate
0.006
0.004
0.002
0.000
1960
1980
2000
2020
Year
UN WPP
GBD 2017
GBD 2016
Adjusted Data, CBH/VR
Data, CBH/VR
Outliered Data
Census Tabulation
VR Complete

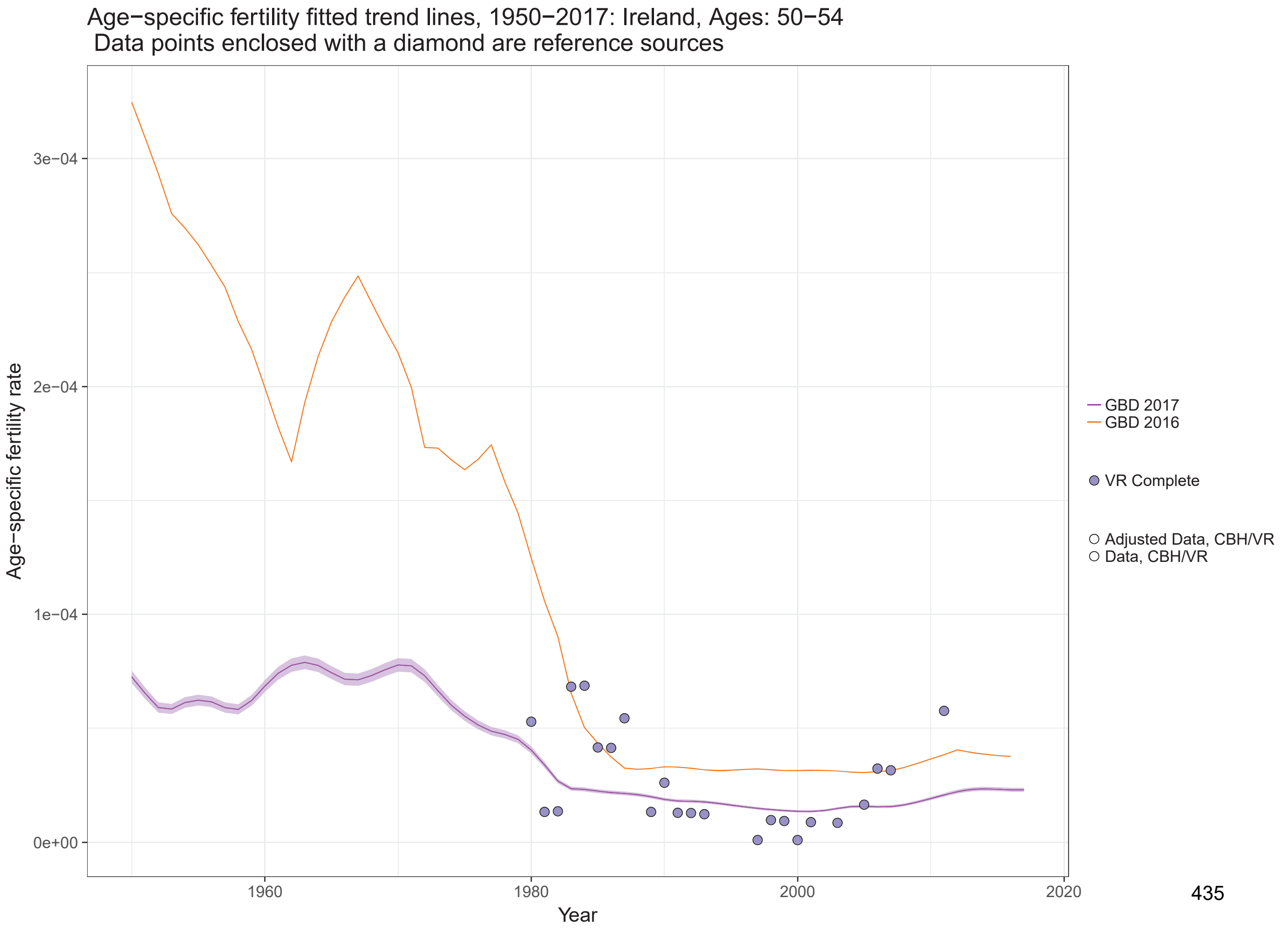
Age−specific fertility fitted trend lines, 1950−2017: Ireland, Ages: 50−54
Data points enclosed with a diamond are reference sources
Age−specific fertility rate
3e−04
2e−04
1e−04
0e+00
1960
1980
2000
2020
Year
GBD 2017
GBD 2016
VR Complete
Adjusted Data, CBH/VR
Data, CBH/VR

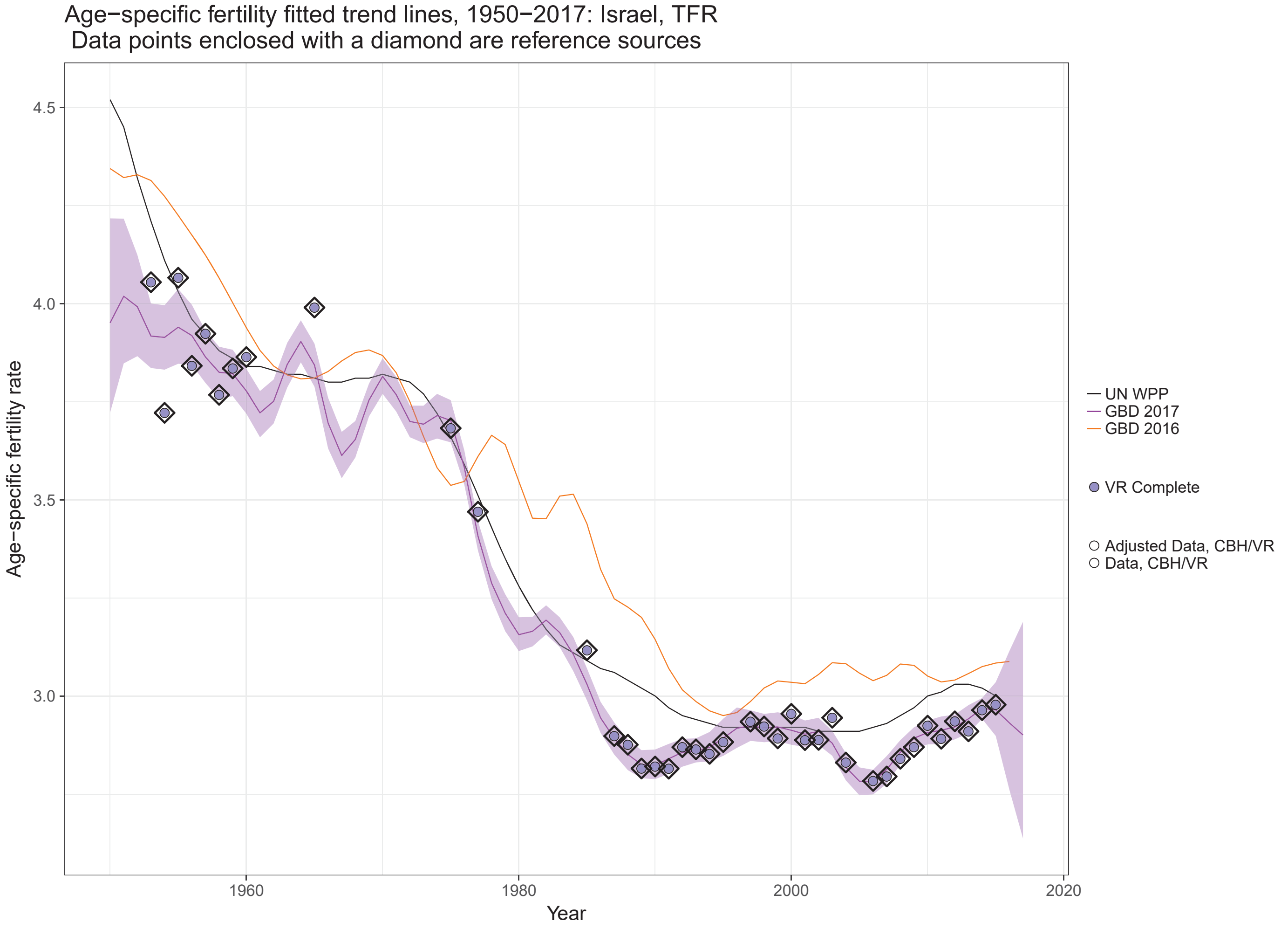
Age−specific fertility fitted trend lines, 1950−2017: Israel, TFR
Data points enclosed with a diamond are reference sources
Age−specific fertility rate
4.5
4.0
3.5
3.0
1960
1980
2000
2020
Year
UN WPP
GBD 2017
GBD 2016
VR Complete
Adjusted Data, CBH/VR
Data, CBH/VR

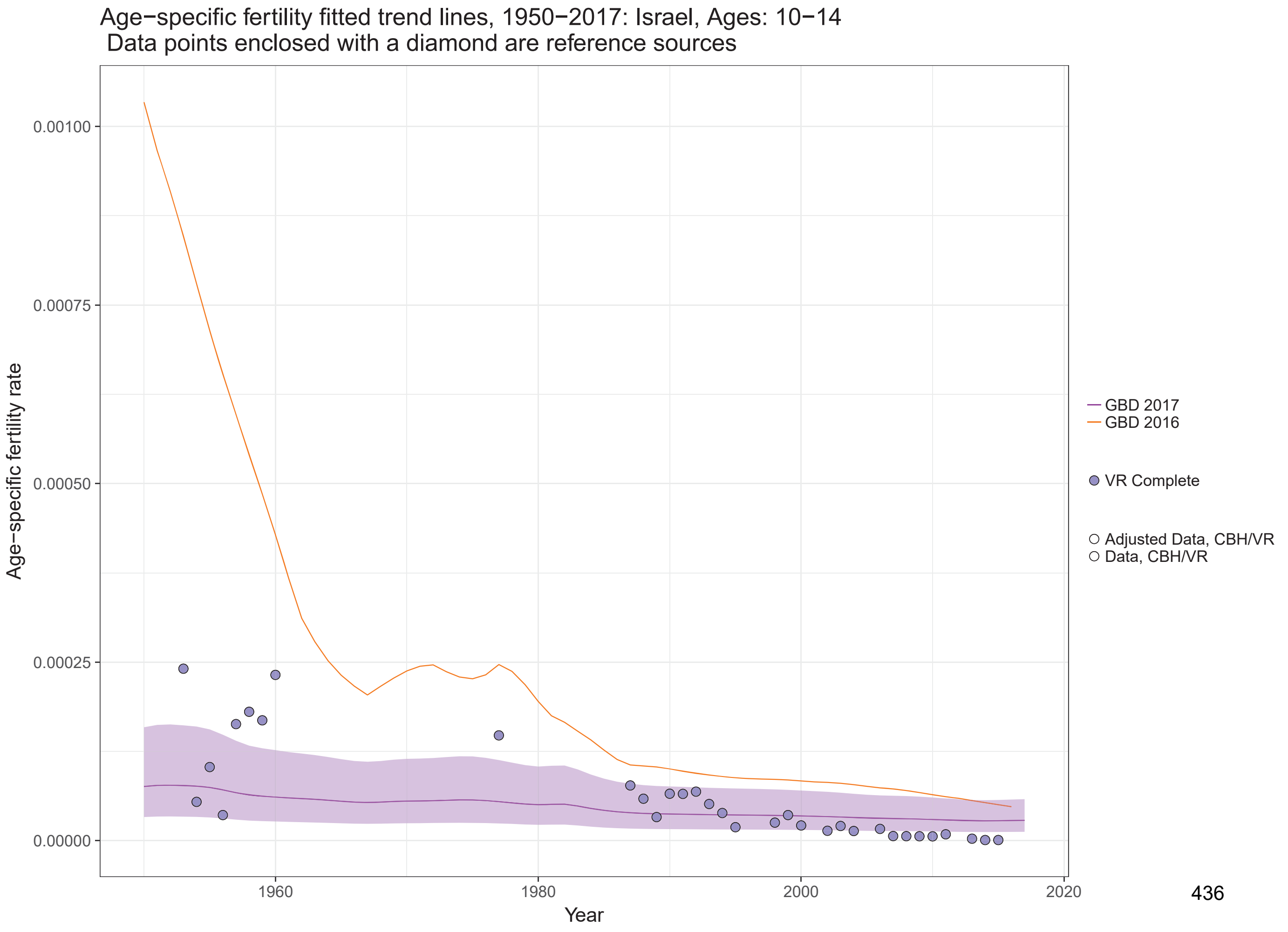
Age−specific fertility fitted trend lines, 1950−2017: Israel, Ages: 10−14
Data points enclosed with a diamond are reference sources
Age−specific fertility rate
0.00100
0.00075
0.00050
0.00025
0.00000
1960
1980
2000
2020
Year
GBD 2017
GBD 2016
VR Complete
Adjusted Data, CBH/VR
Data, CBH/VR

Age−specific fertility fitted trend lines, 1950−2017: Israel, Ages: 15−19
Data points enclosed with a diamond are reference sources

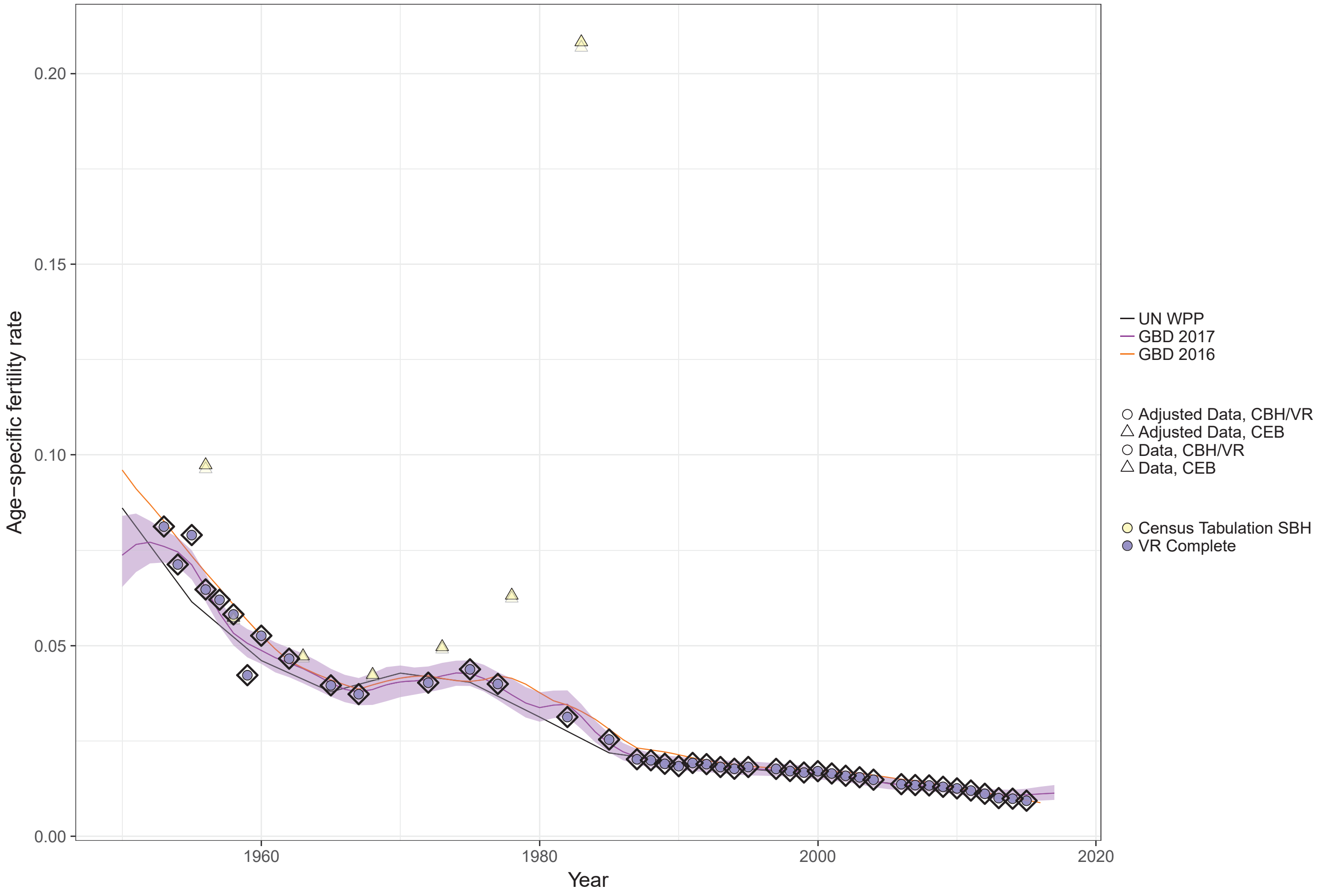

Age−specific fertility fitted trend lines, 1950−2017: Israel, Ages: 20−24
Data points enclosed with a diamond are reference sources

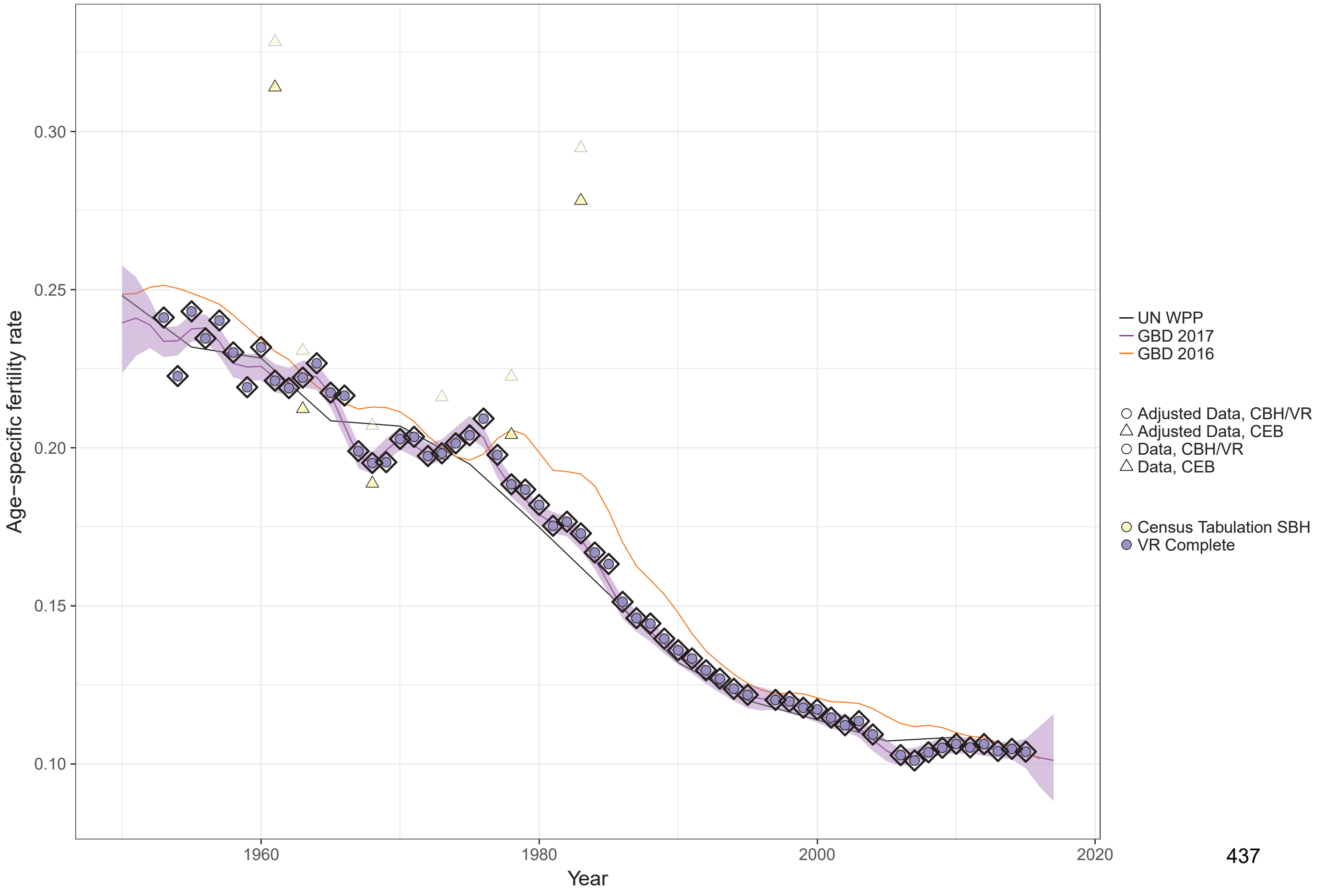

Age−specific fertility fitted trend lines, 1950−2017: Israel, Ages: 25−29
Data points enclosed with a diamond are reference sources

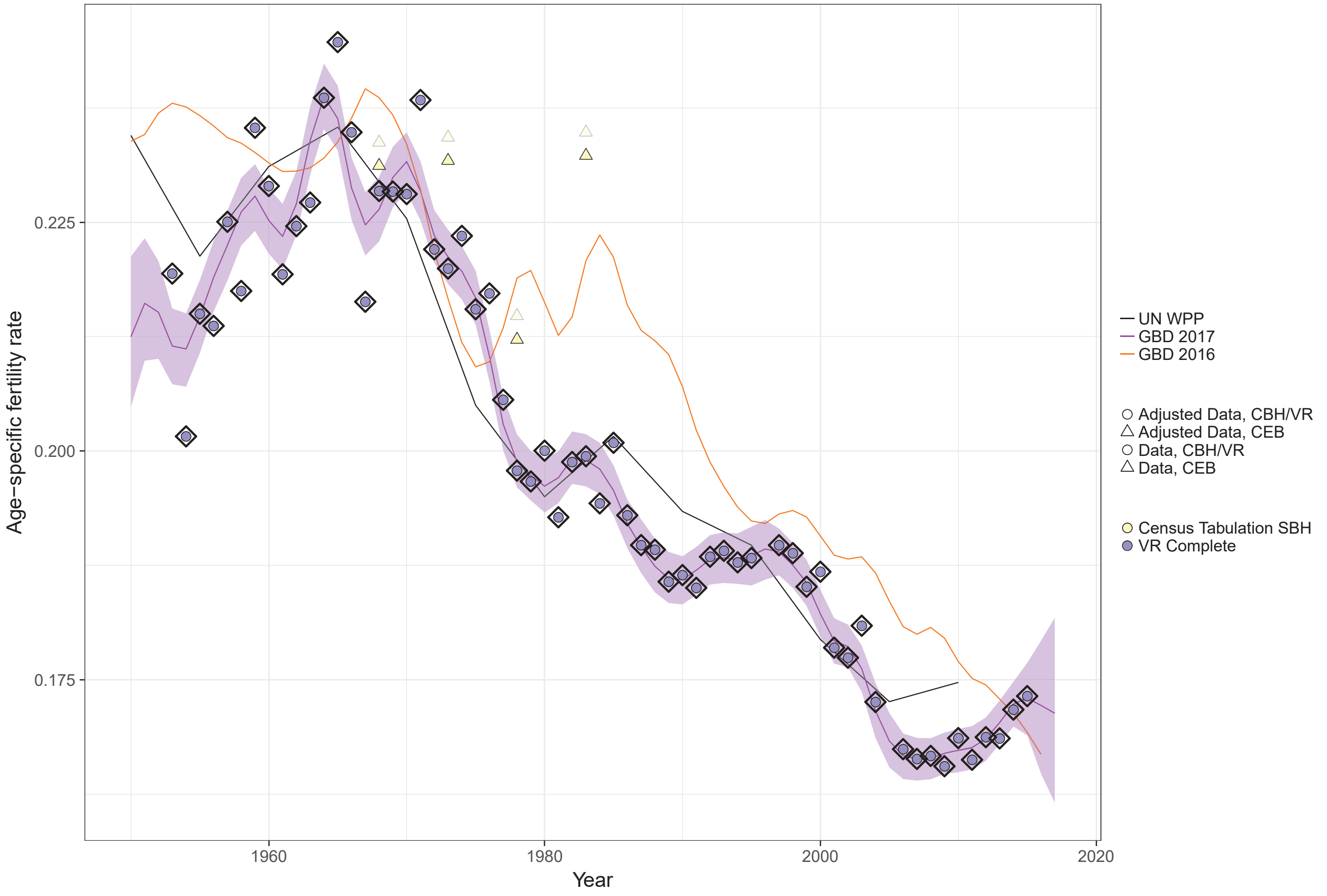

Age−specific fertility fitted trend lines, 1950−2017: Israel, Ages: 30−34
Data points enclosed with a diamond are reference sources

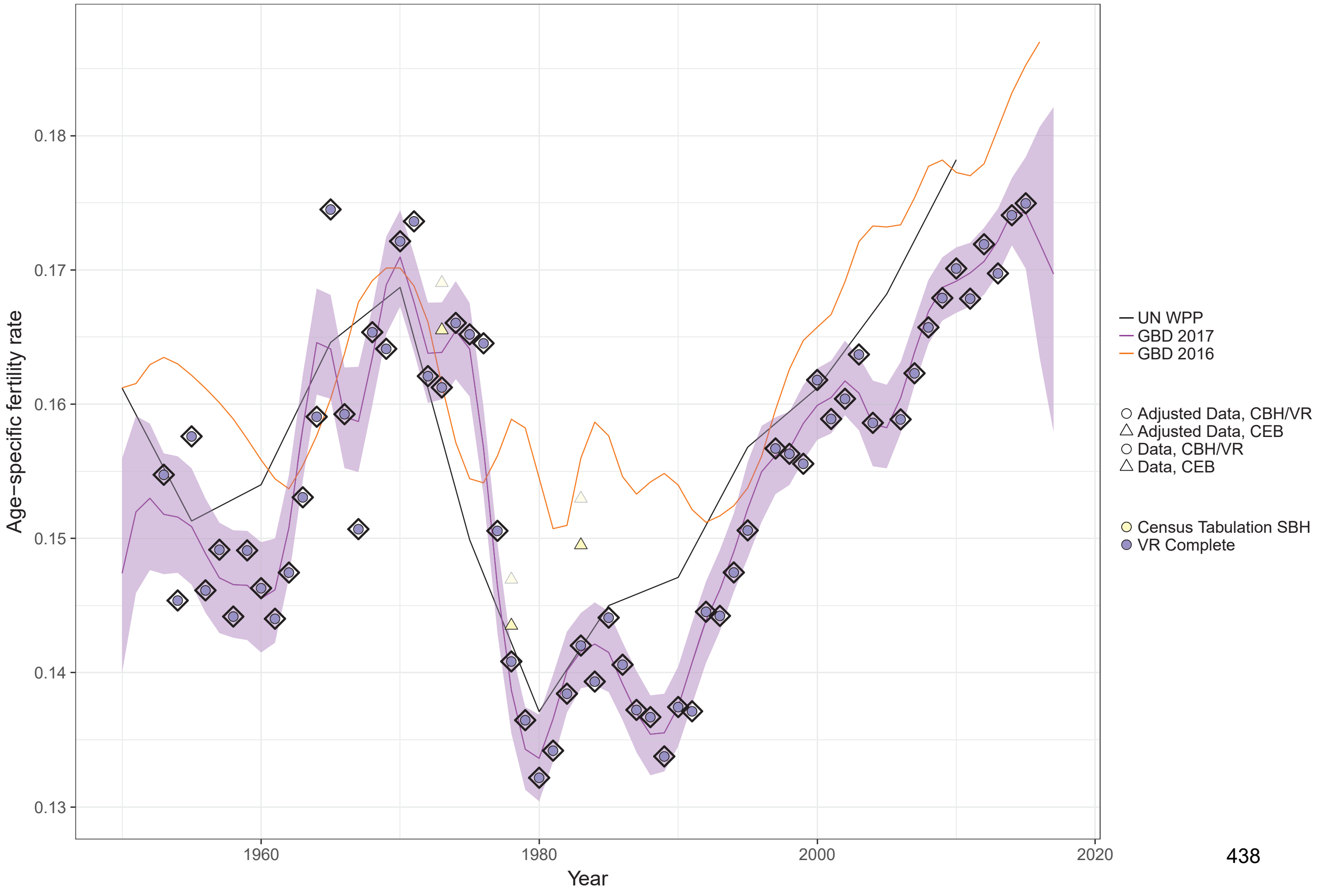

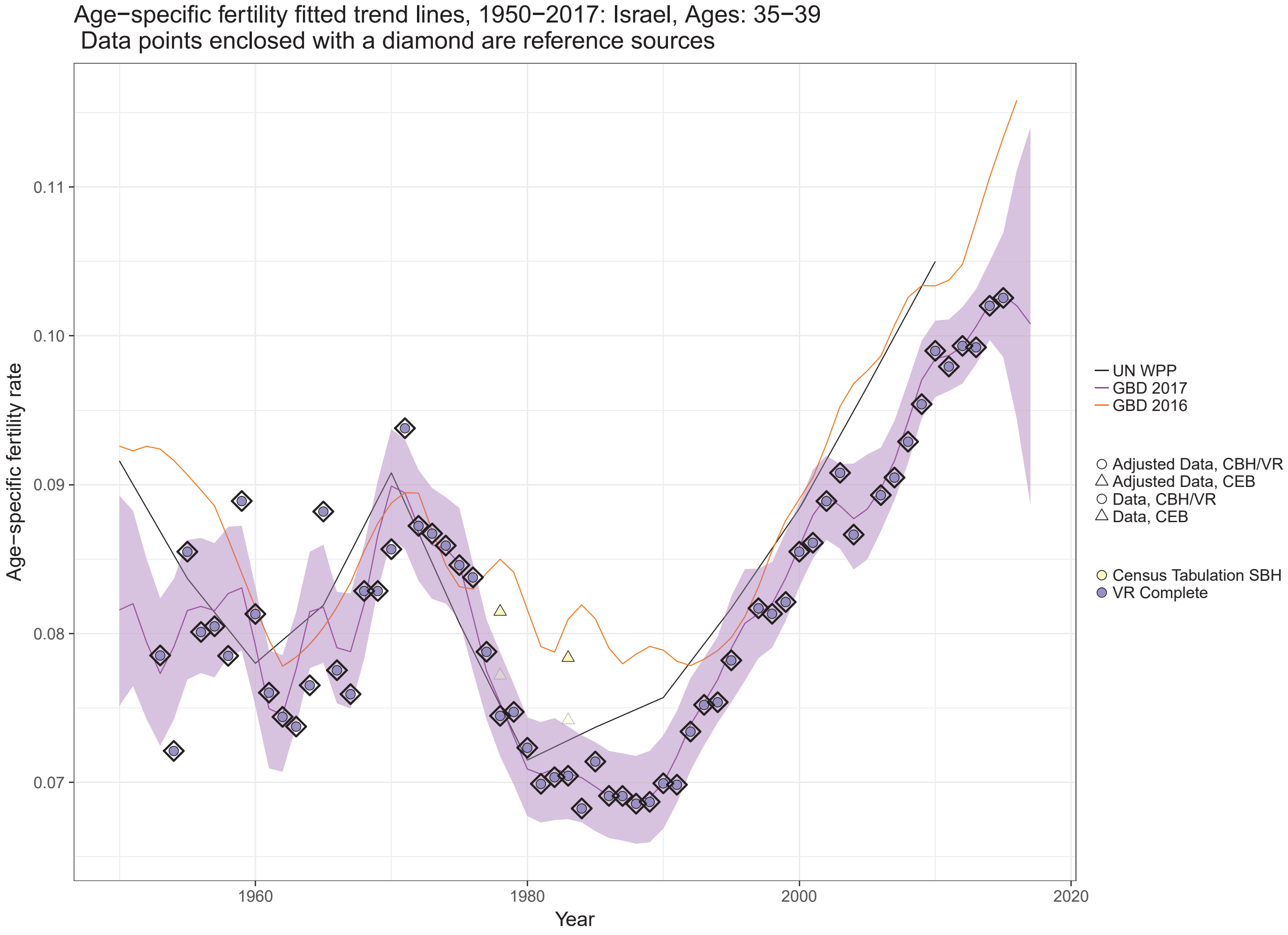

Age−specific fertility fitted trend lines, 1950−2017: Israel, Ages: 35−39
Data points enclosed with a diamond are reference sources
Age-specific fertility rate
0.11
0.10
0.09
0.08
0.07
1960
1980
2000
2020
Year
UN WPP
GBD 2017
GBD 2016
Adjusted Data, CBH/VR
Adjusted Data, CEB
Data, CBH/VR
Data, CEB
Census Tabulation SBH
VR Complete

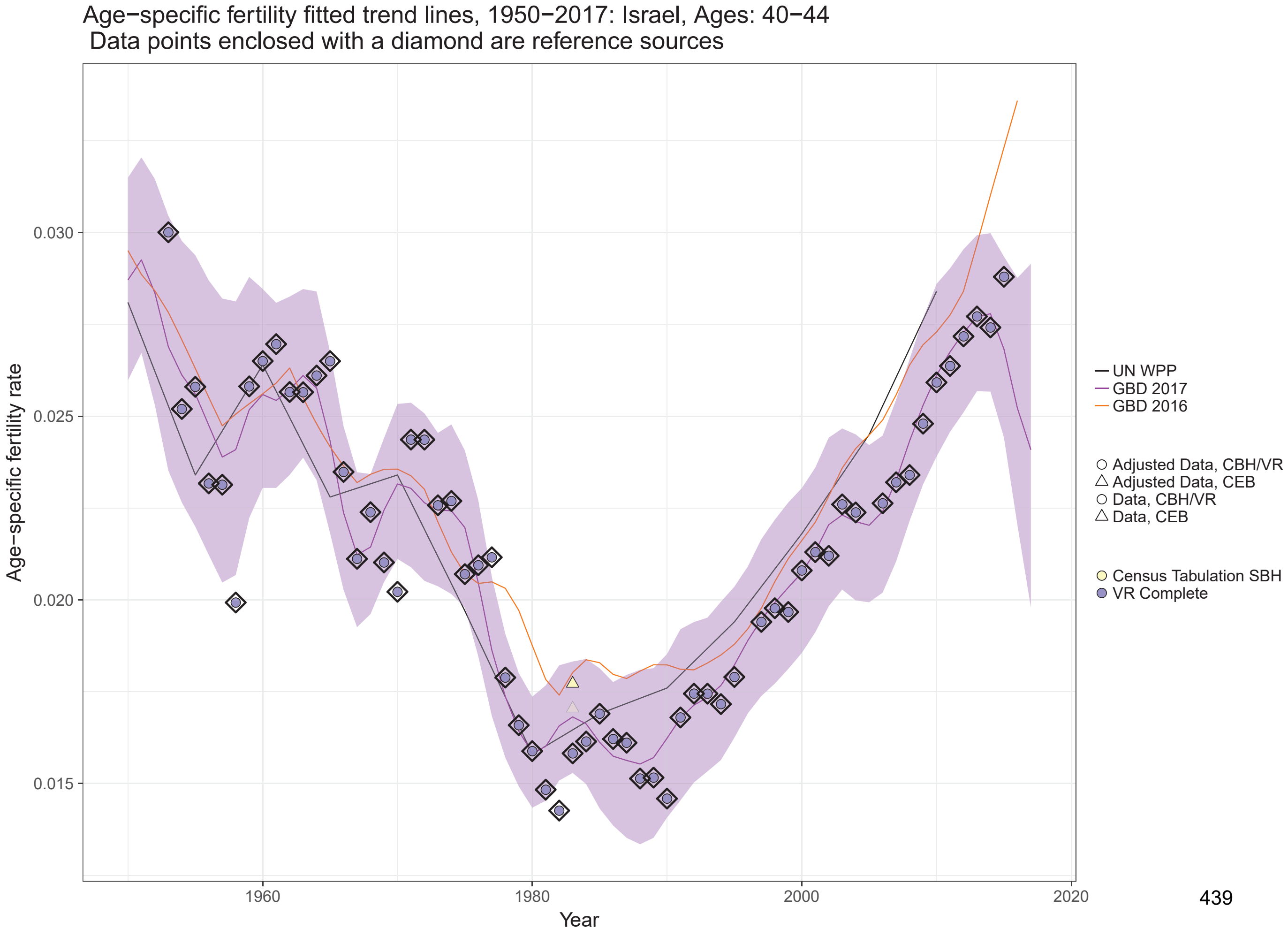

Age−specific fertility fitted trend lines, 1950−2017: Israel, Ages: 40−44
Data points enclosed with a diamond are reference sources
Age-specific fertility rate
0.030
0.025
0.020
0.015
1960
1980
2000
2020
Year
UN WPP
GBD 2017
GBD 2016
Adjusted Data, CBH/VR
Adjusted Data, CEB
Data, CBH/VR
Data, CEB
Census Tabulation SBH
VR Complete

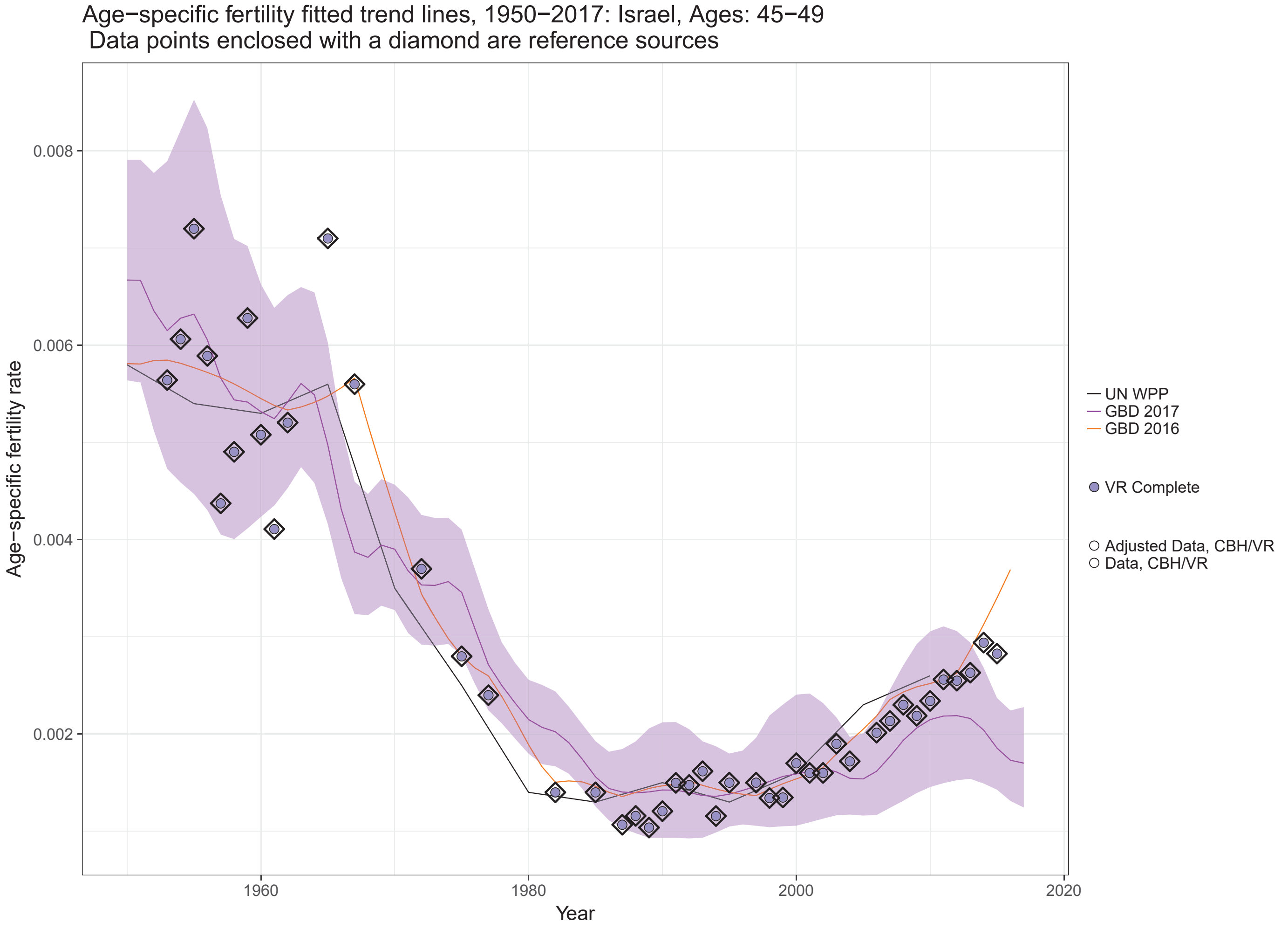

Age−specific fertility fitted trend lines, 1950−2017: Israel, Ages: 45−49
Data points enclosed with a diamond are reference sources
Age−specific fertility rate
0.008
0.006
0.004
0.002
1960
1980
2000
2020
Year
UN WPP
GBD 2017
GBD 2016
VR Complete
Adjusted Data, CBH/VR
Data, CBH/VR

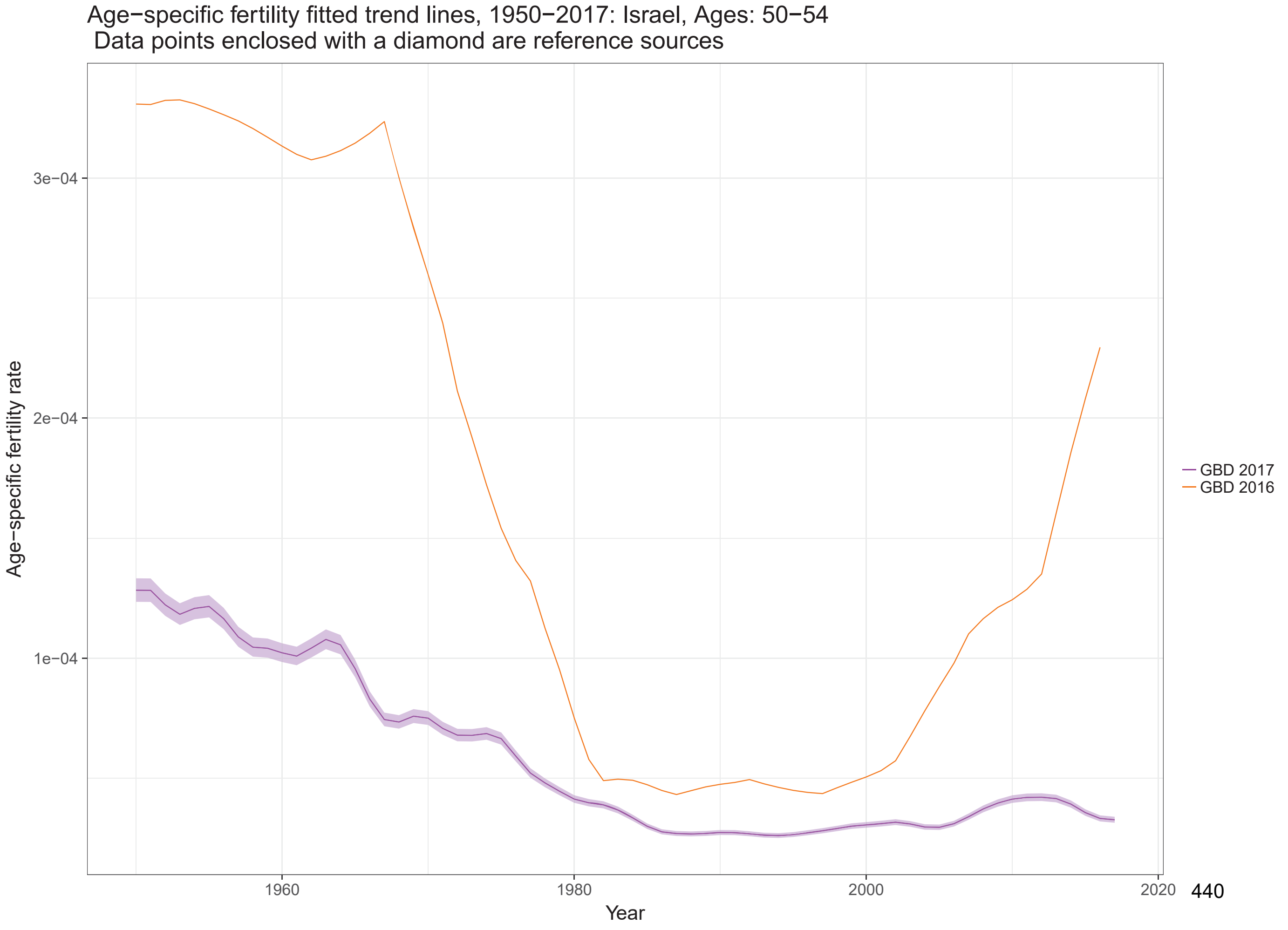

Age−specific fertility fitted trend lines, 1950−2017: Israel, Ages: 50−54
Data points enclosed with a diamond are reference sources
Age−specific fertility rate
3e−04
2e−04
1e−04
1960
1980
2000
2020
Year
GBD 2017
GBD 2016

Age−specific fertility fitted trend lines, 1950−2017: Italy, TFR
Data points enclosed with a diamond are reference sources

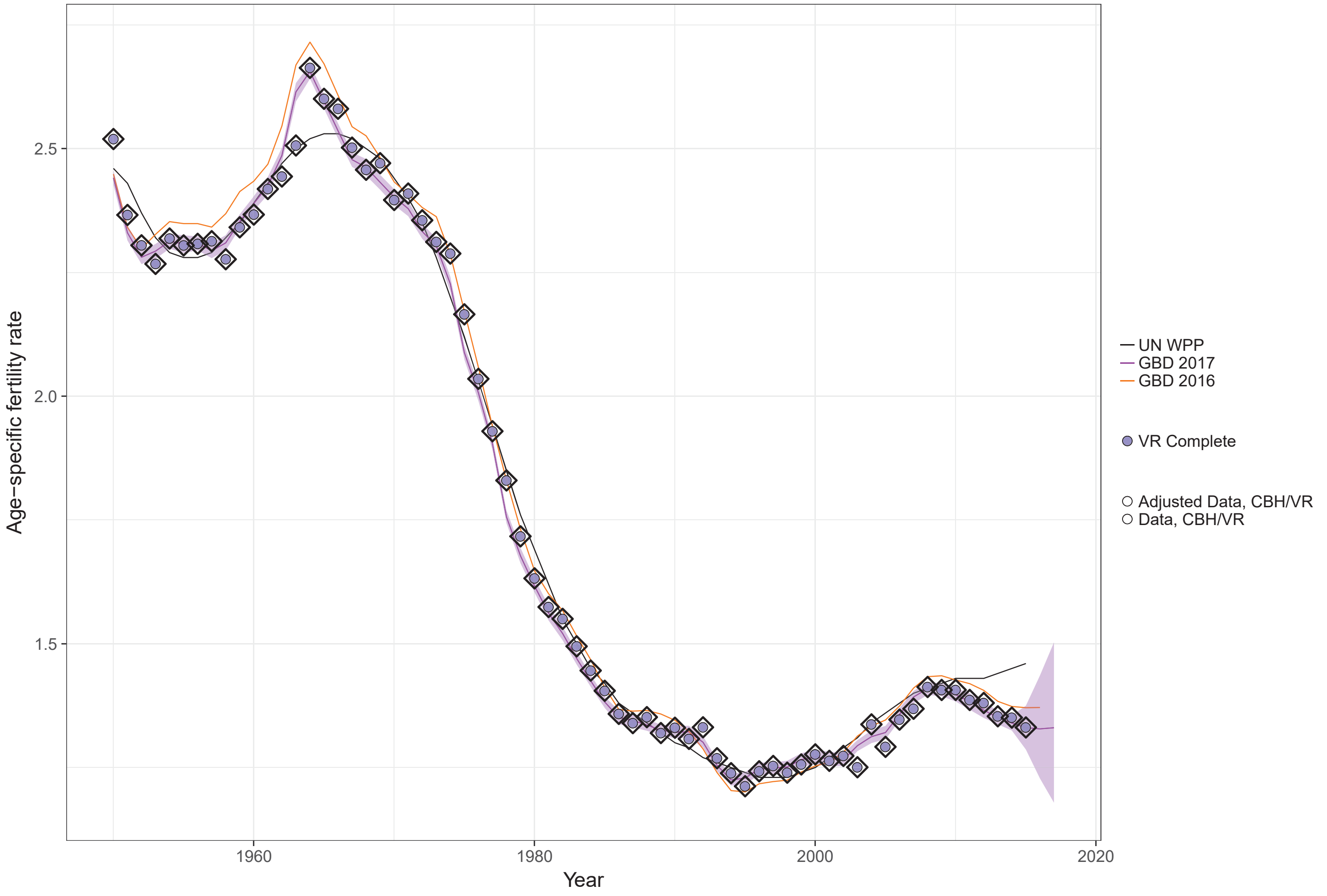

Age−specific fertility fitted trend lines, 1950−2017: Italy, Ages: 10−14
Data points enclosed with a diamond are reference sources

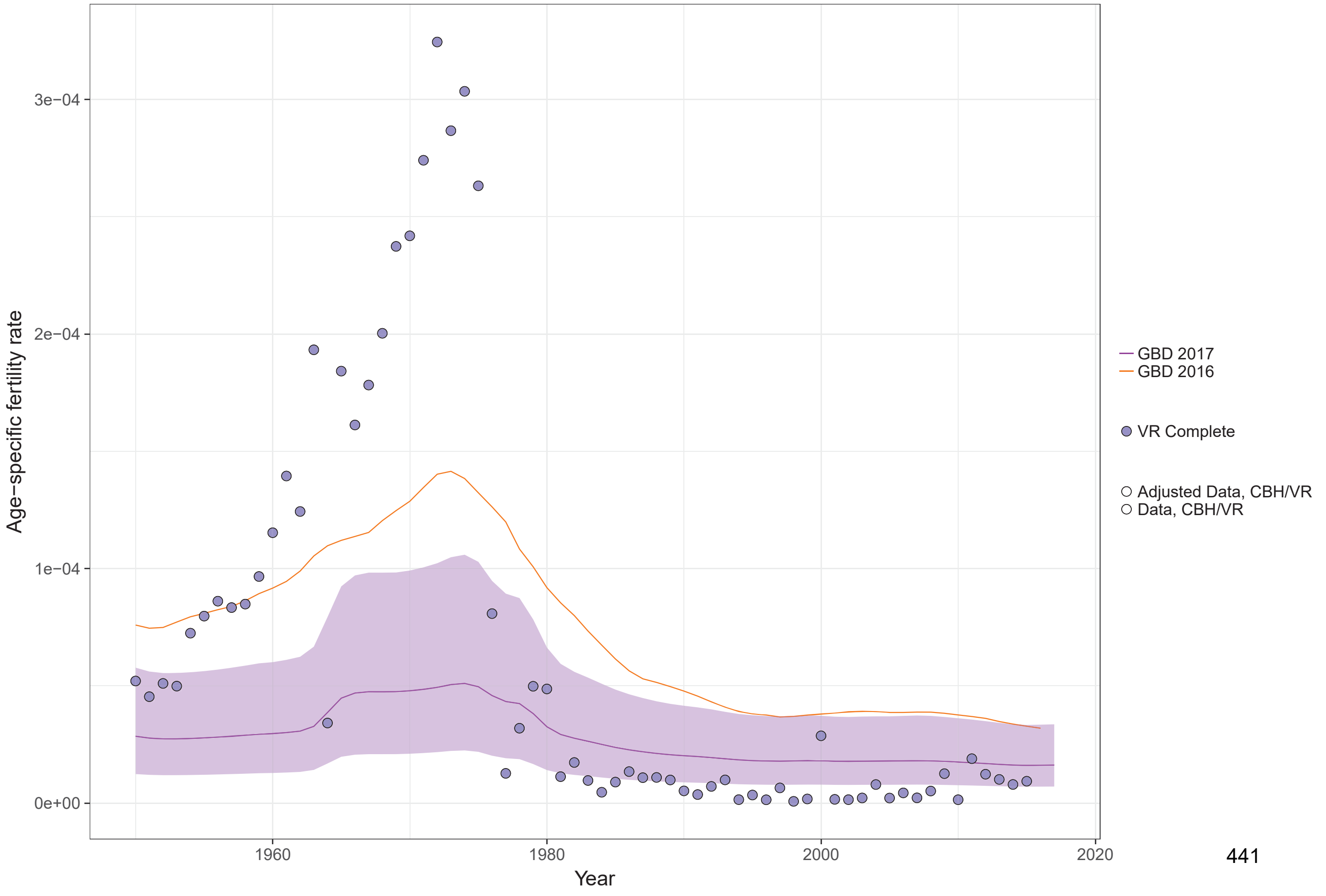

Age−specific fertility fitted trend lines, 1950−2017: Italy, Ages: 15−19
Data points enclosed with a diamond are reference sources

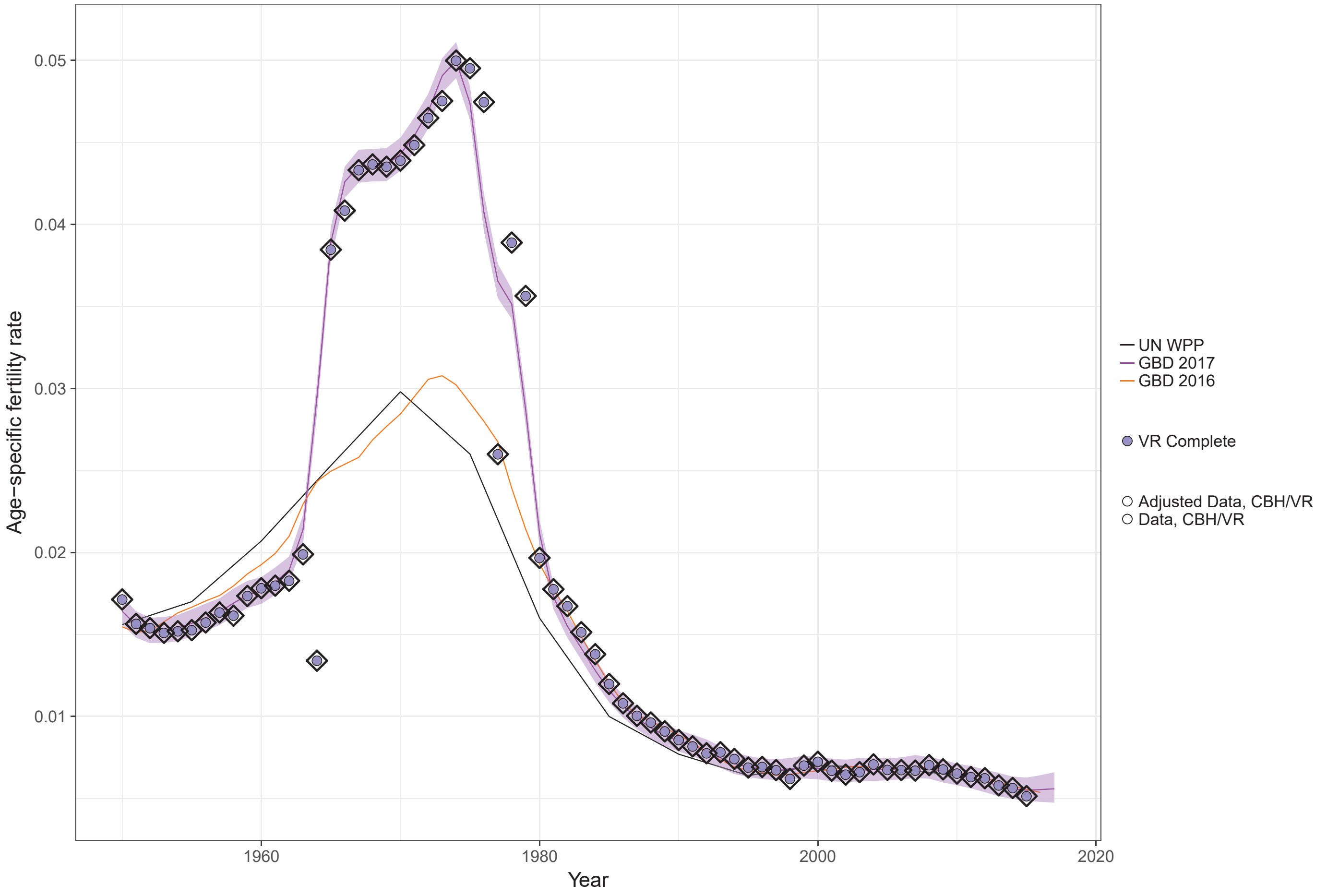

Age−specific fertility fitted trend lines, 1950−2017: Italy, Ages: 20−24
Data points enclosed with a diamond are reference sources

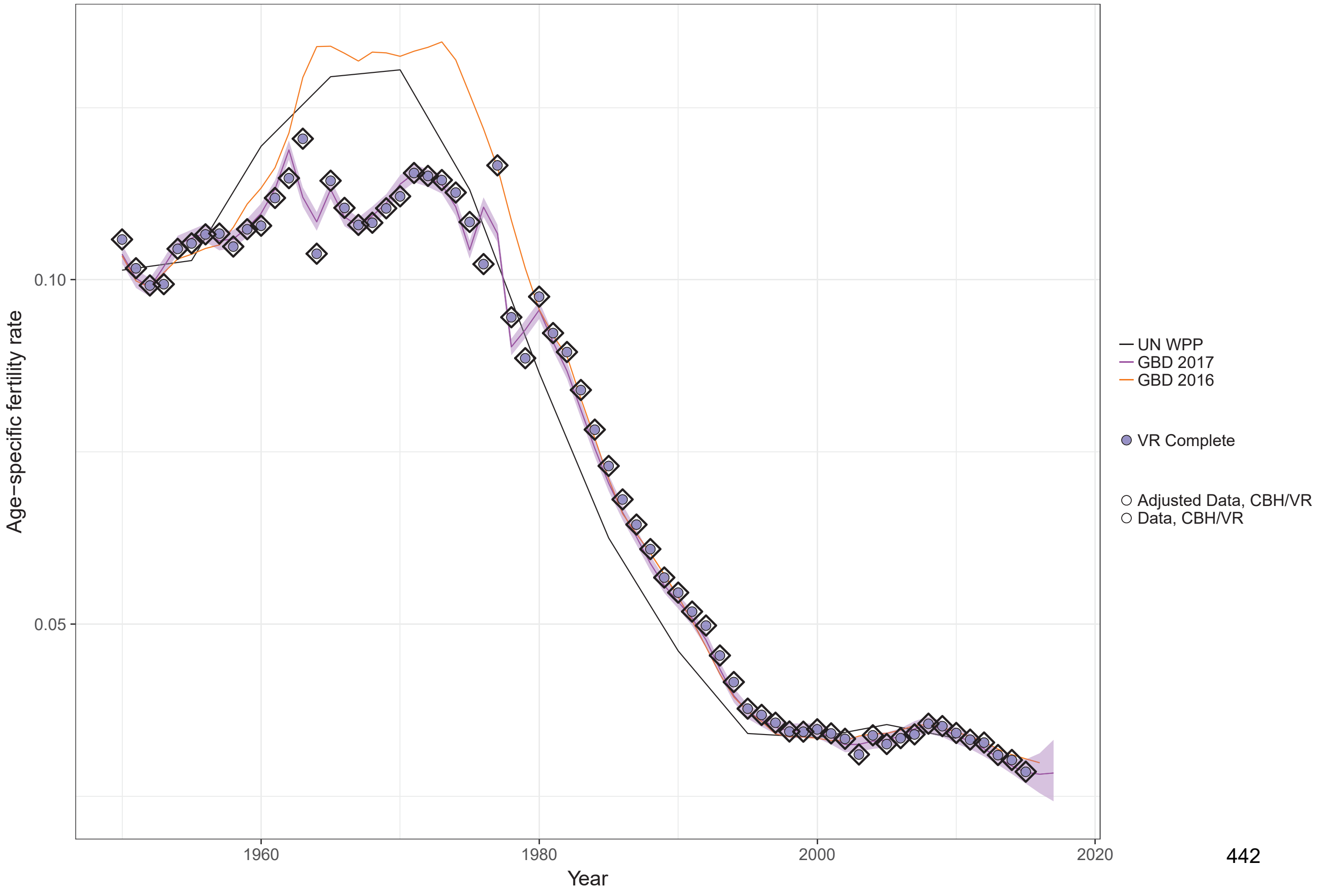

Age−specific fertility fitted trend lines, 1950−2017: Italy, Ages: 25−29
Data points enclosed with a diamond are reference sources

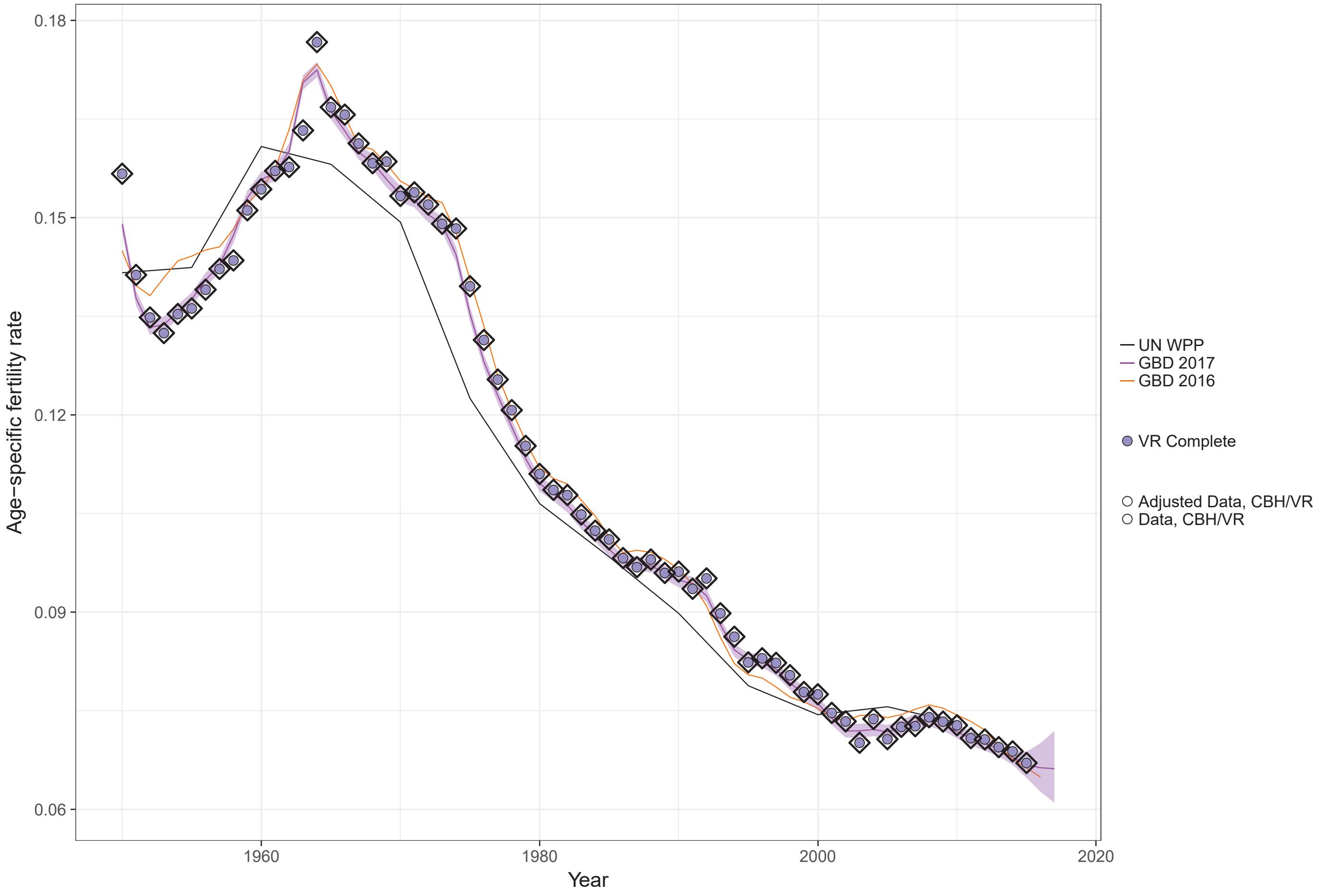

Age−specific fertility fitted trend lines, 1950−2017: Italy, Ages: 30−34
Data points enclosed with a diamond are reference sources

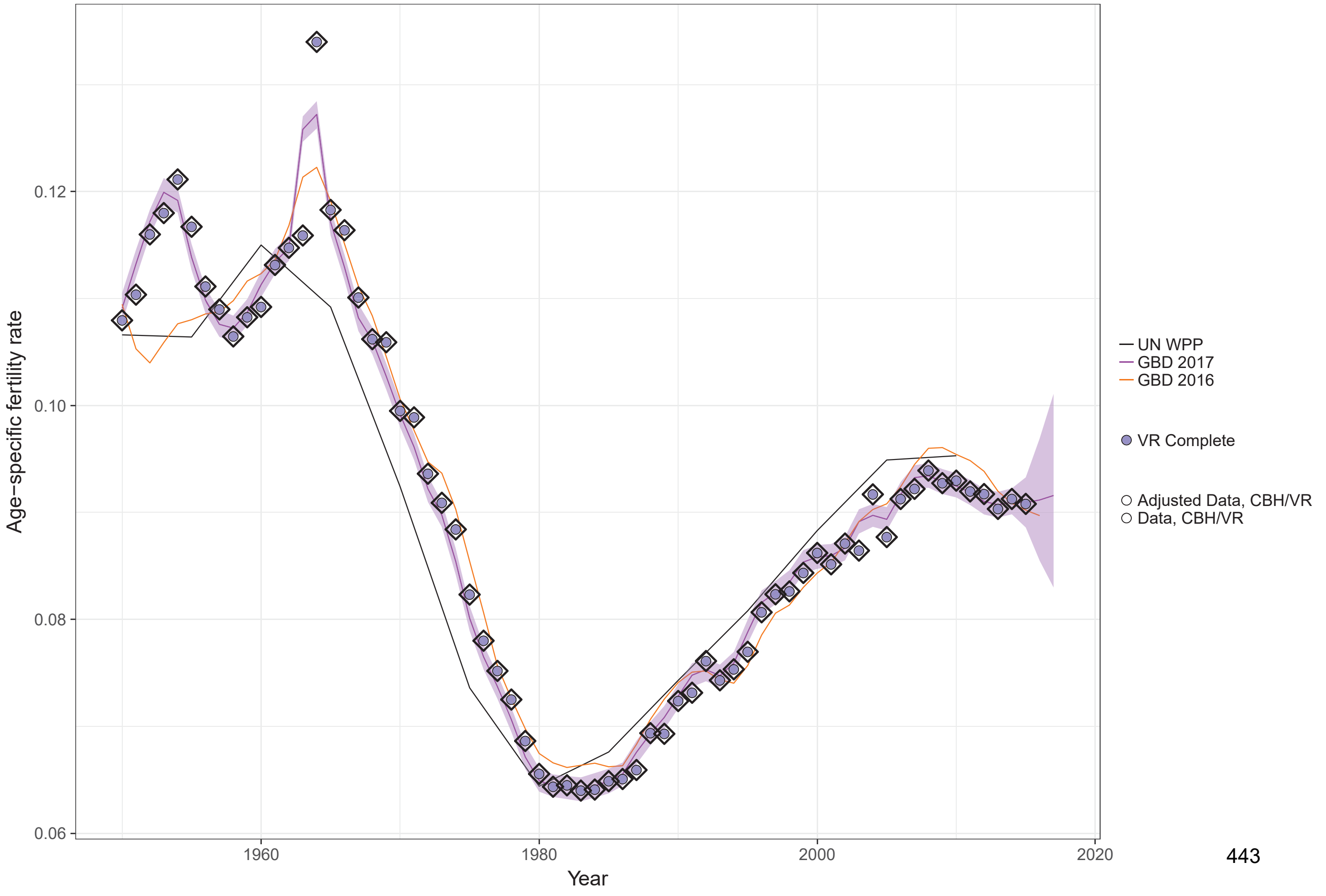

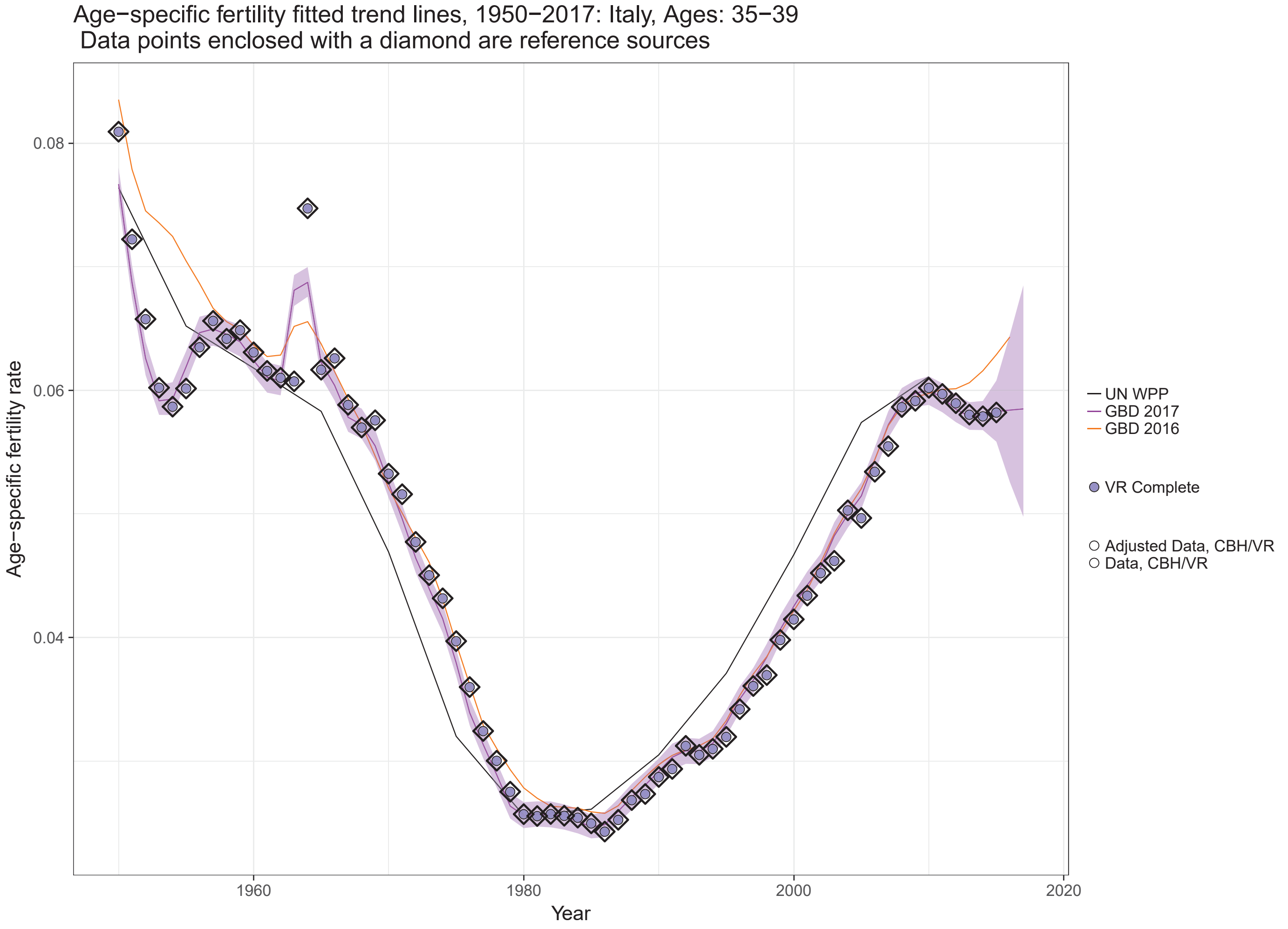

Age−specific fertility fitted trend lines, 1950−2017: Italy, Ages: 35−39
Data points enclosed with a diamond are reference sources
Age−specific fertility rate
0.08
0.06
0.04
1960
1980
2000
2020
Year
UN WPP
GBD 2017
GBD 2016
VR Complete
Adjusted Data, CBH/VR
Data, CBH/VR

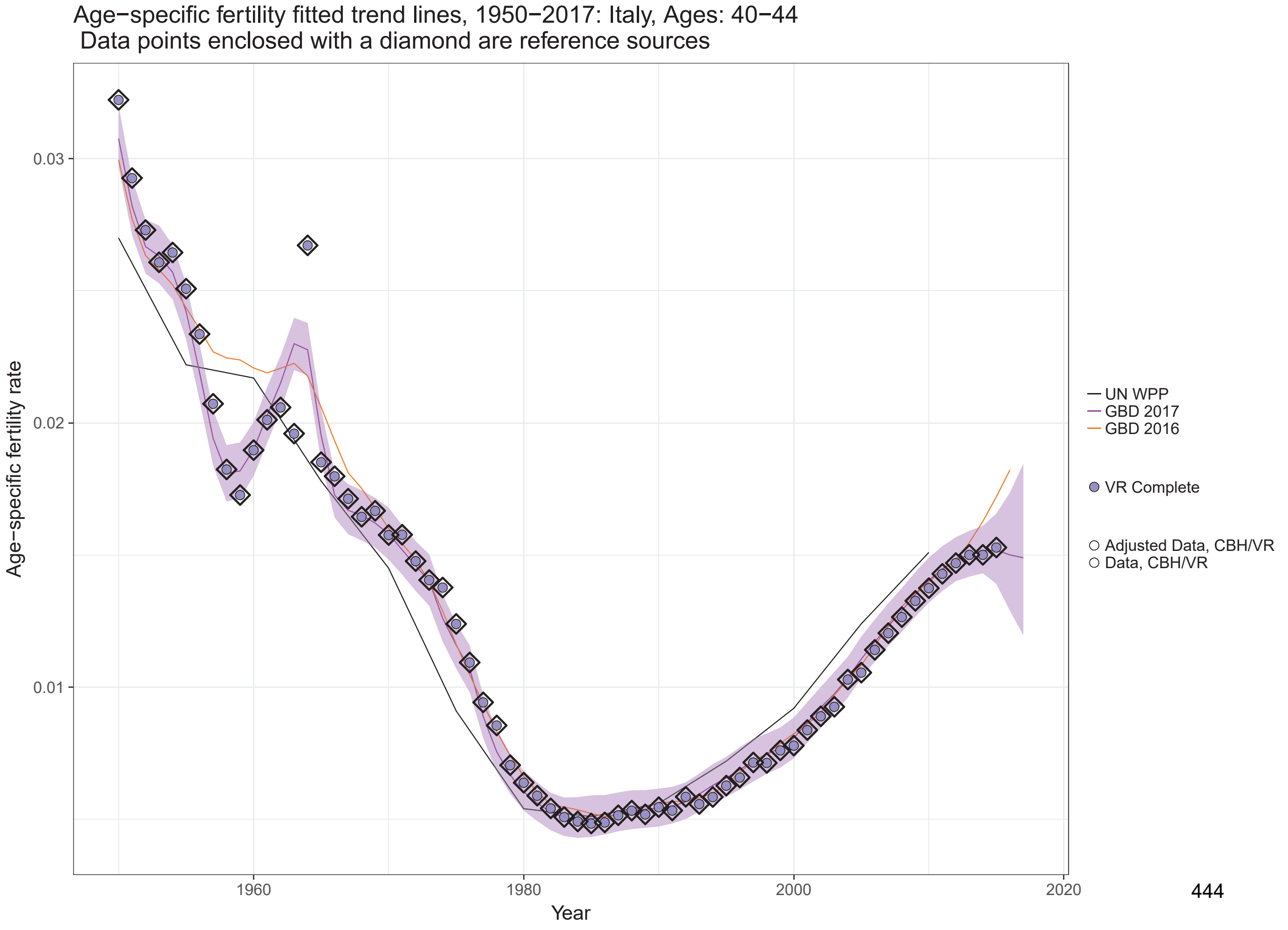

Age−specific fertility fitted trend lines, 1950−2017: Italy, Ages: 40−44
Data points enclosed with a diamond are reference sources
Age−specific fertility rate
0.03
0.02
0.01
1960
1980
2000
2020
Year
UN WPP
GBD 2017
GBD 2016
VR Complete
Adjusted Data, CBH/VR
Data, CBH/VR

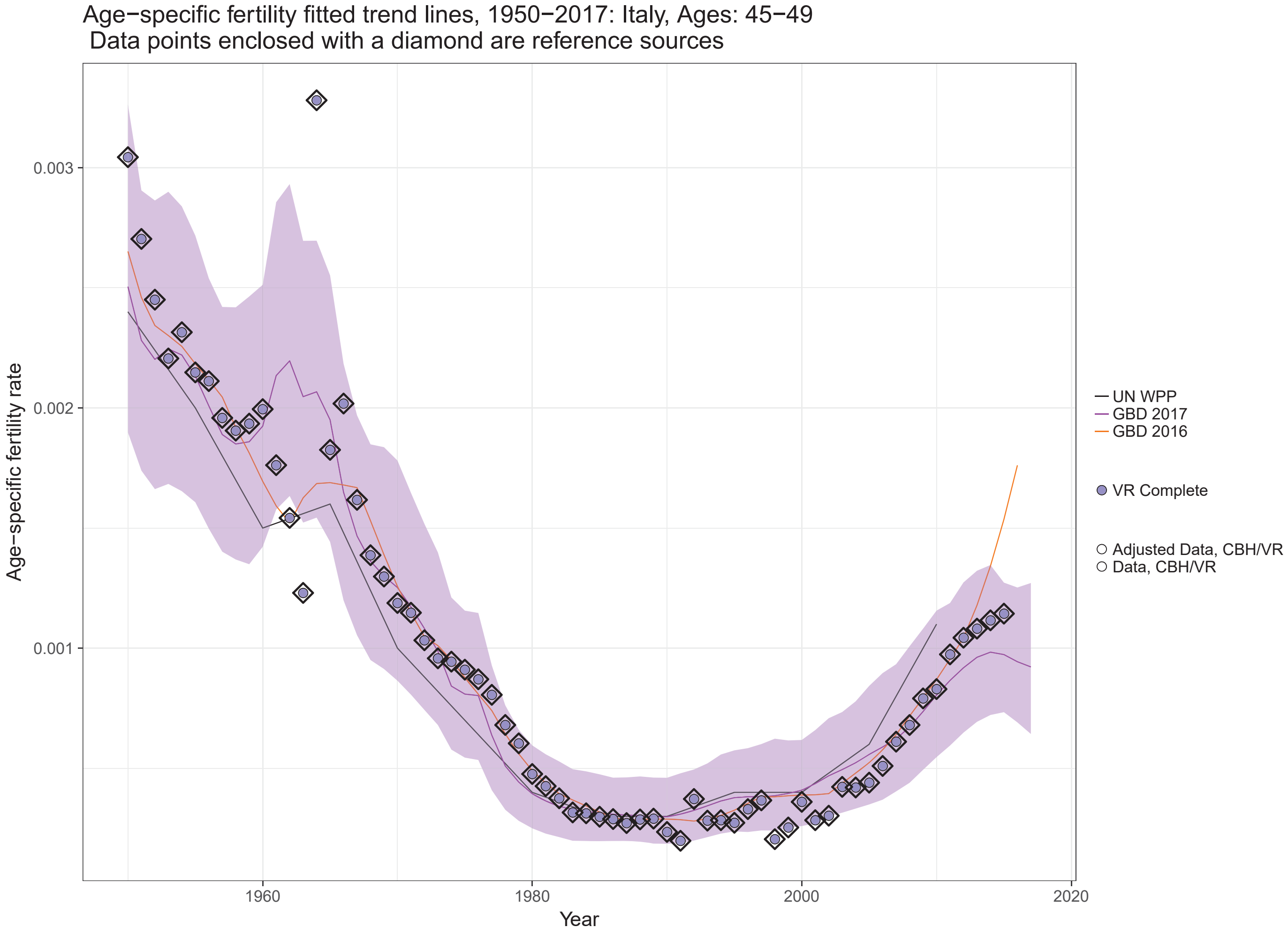

Age−specific fertility fitted trend lines, 1950−2017: Italy, Ages: 45−49
Data points enclosed with a diamond are reference sources
Age−specific fertility rate
0.003
0.002
0.001
1960
1980
2000
2020
Year
UN WPP
GBD 2017
GBD 2016
VR Complete
Adjusted Data, CBH/VR
Data, CBH/VR

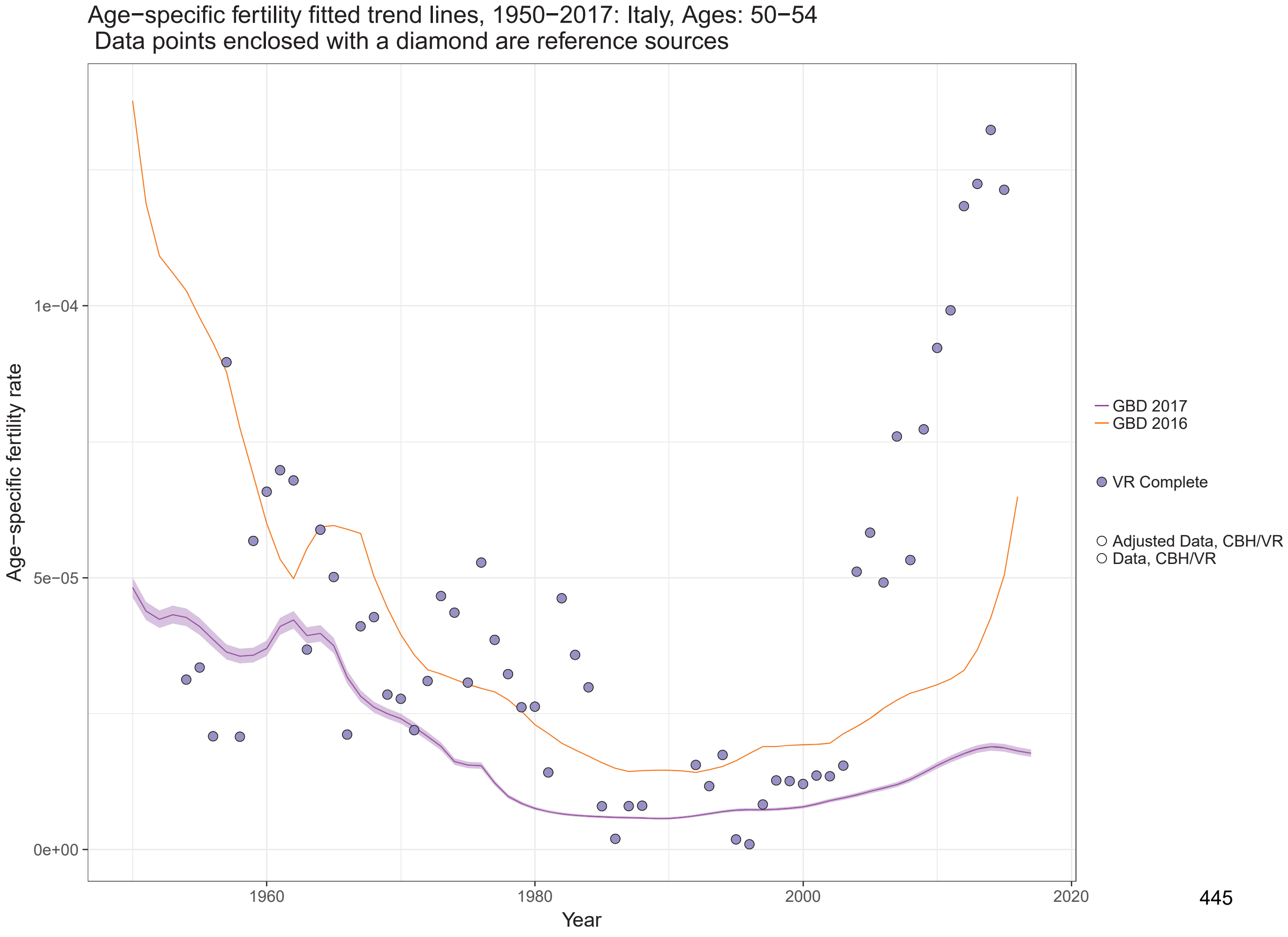

Age−specific fertility fitted trend lines, 1950−2017: Italy, Ages: 50−54
Data points enclosed with a diamond are reference sources
Age−specific fertility rate
1e−04
5e−05
0e+00
1960
1980
2000
2020
Year
GBD 2017
GBD 2016
VR Complete
Adjusted Data, CBH/VR
Data, CBH/VR

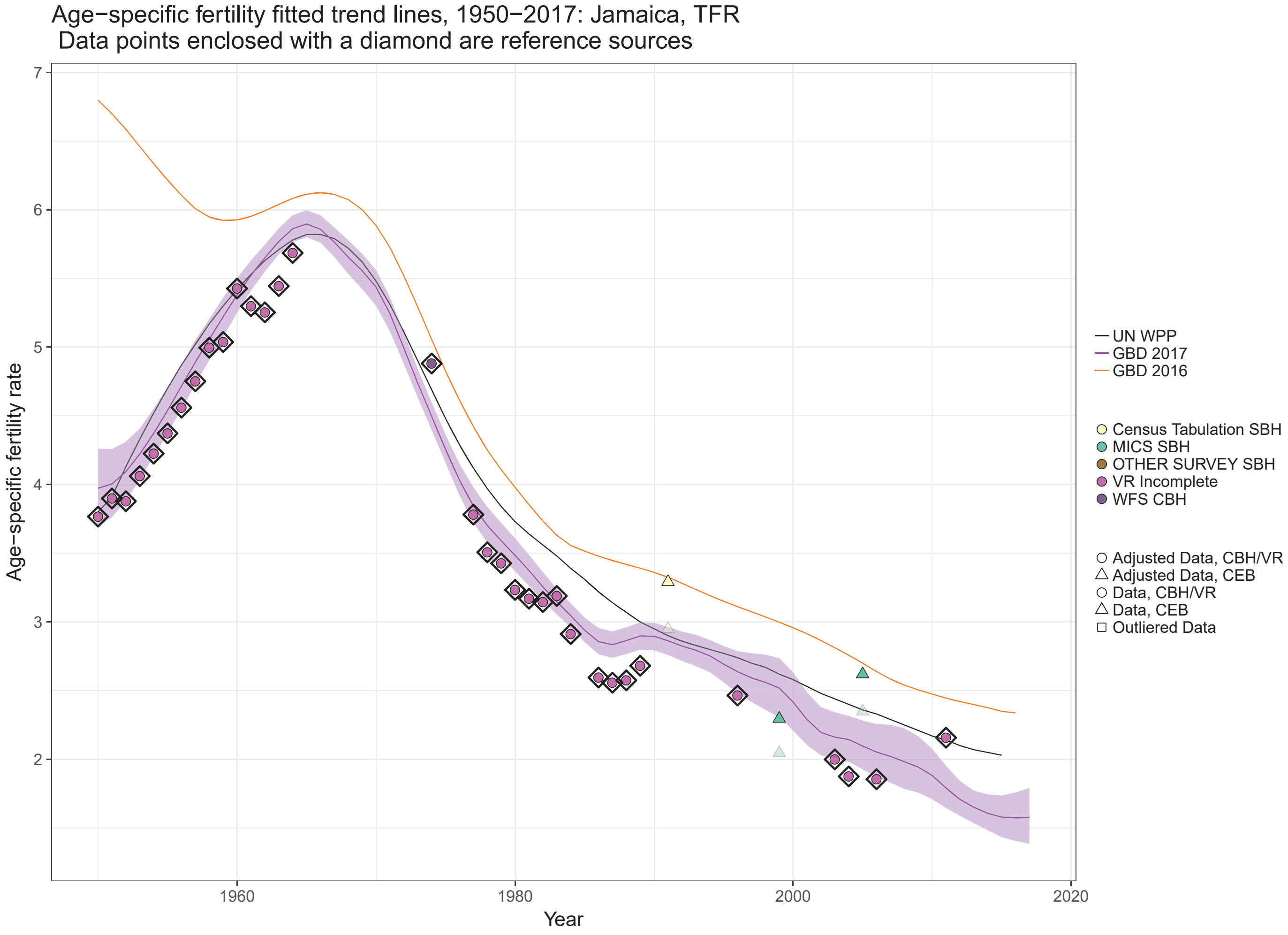

Age−specific fertility fitted trend lines, 1950−2017: Jamaica, TFR
Data points enclosed with a diamond are reference sources
Age−specific fertility rate
Year
UN WPP
GBD 2017
GBD 2016
Census Tabulation SBH
MICS SBH
OTHER SURVEY SBH
VR Incomplete
WFS CBH
Adjusted Data, CBH/VR
Adjusted Data, CEB
Data, CBH/VR
Data, CEB
Outliered Data

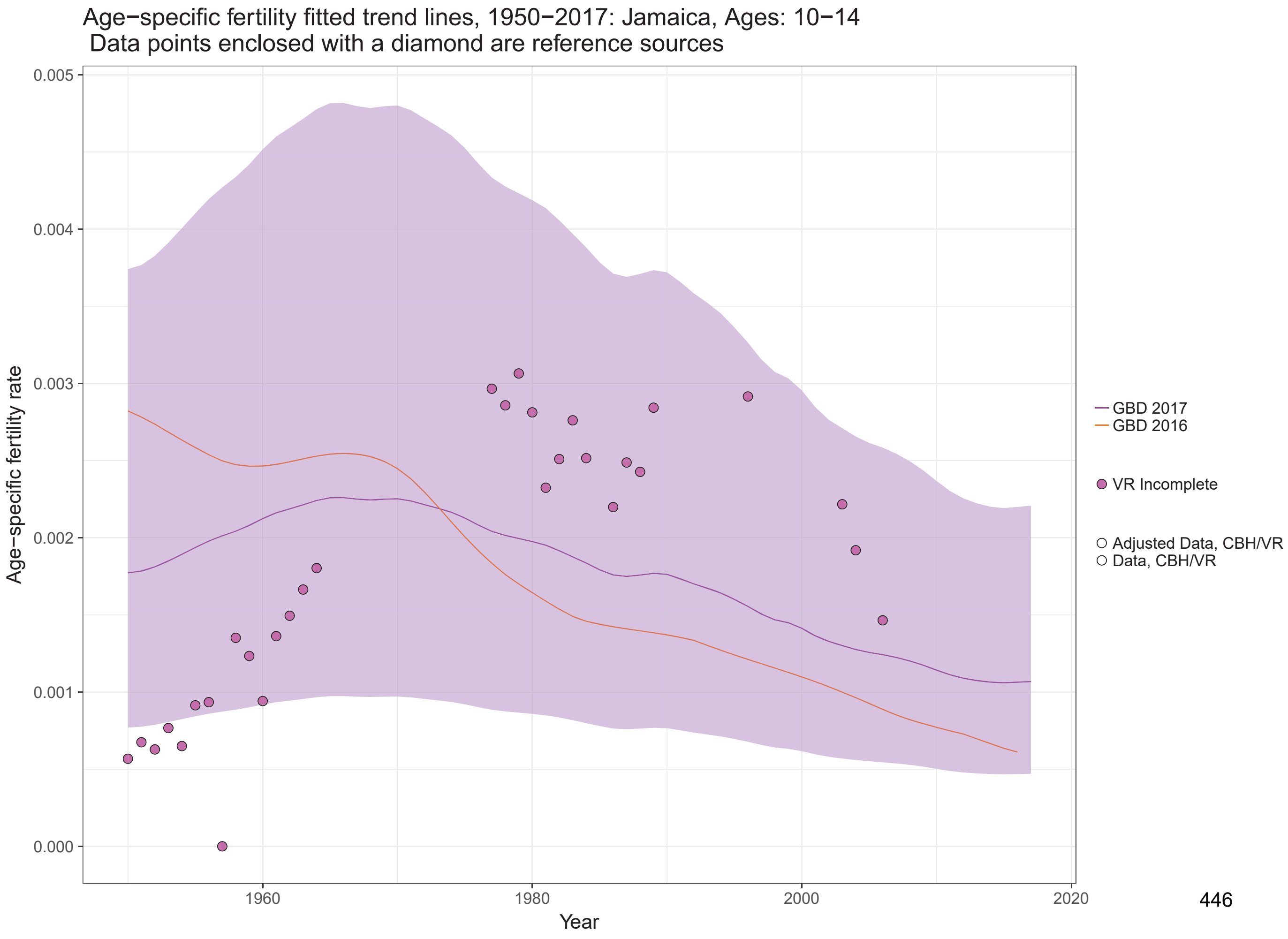

Age−specific fertility fitted trend lines, 1950−2017: Jamaica, Ages: 10−14
Data points enclosed with a diamond are reference sources
Age−specific fertility rate
Year
GBD 2017
GBD 2016
VR Incomplete
Adjusted Data, CBH/VR
Data, CBH/VR

Age−specific fertility fitted trend lines, 1950−2017: Jamaica, Ages: 15−19
Data points enclosed with a diamond are reference sources

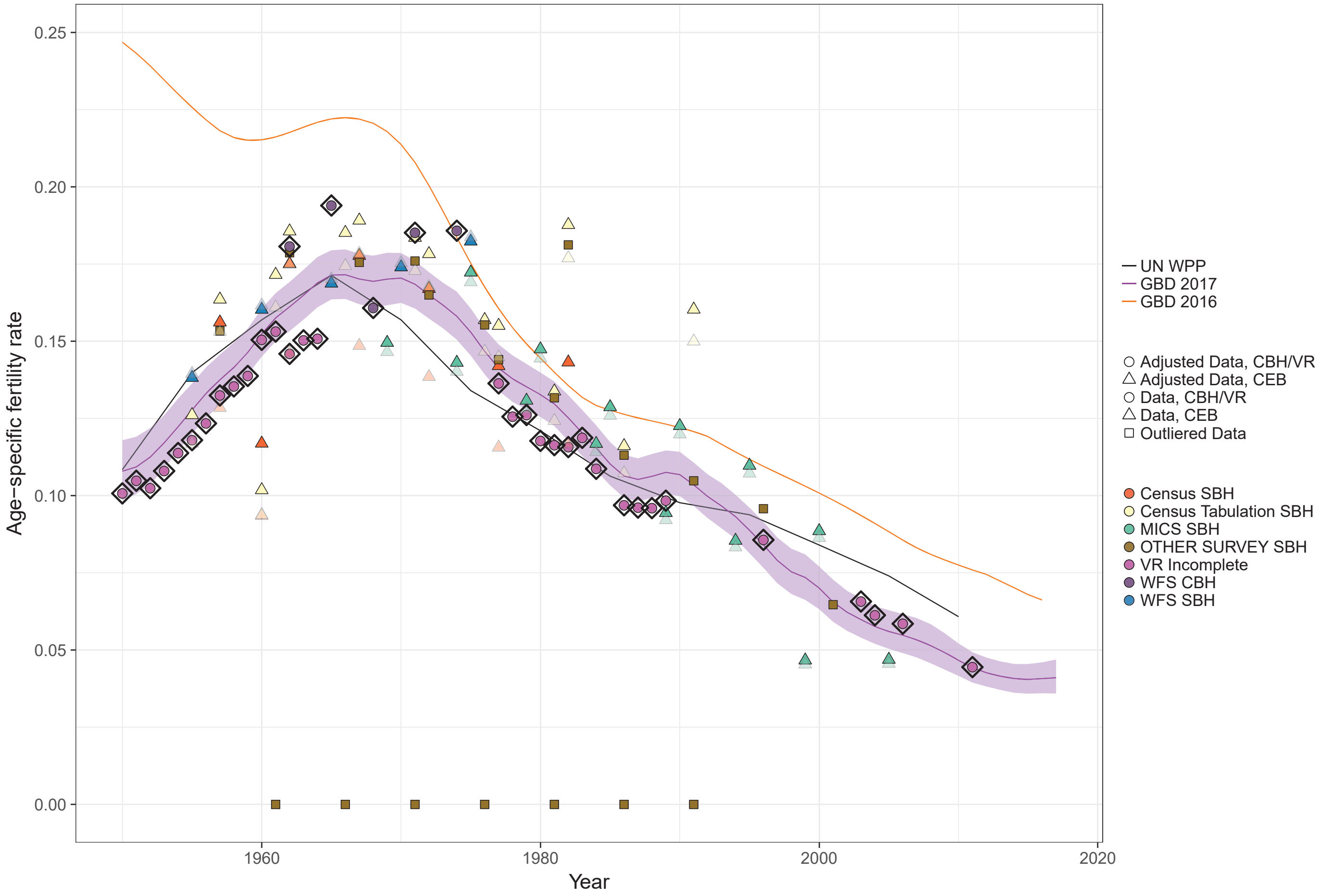

Age−specific fertility fitted trend lines, 1950−2017: Jamaica, Ages: 20−24
Data points enclosed with a diamond are reference sources

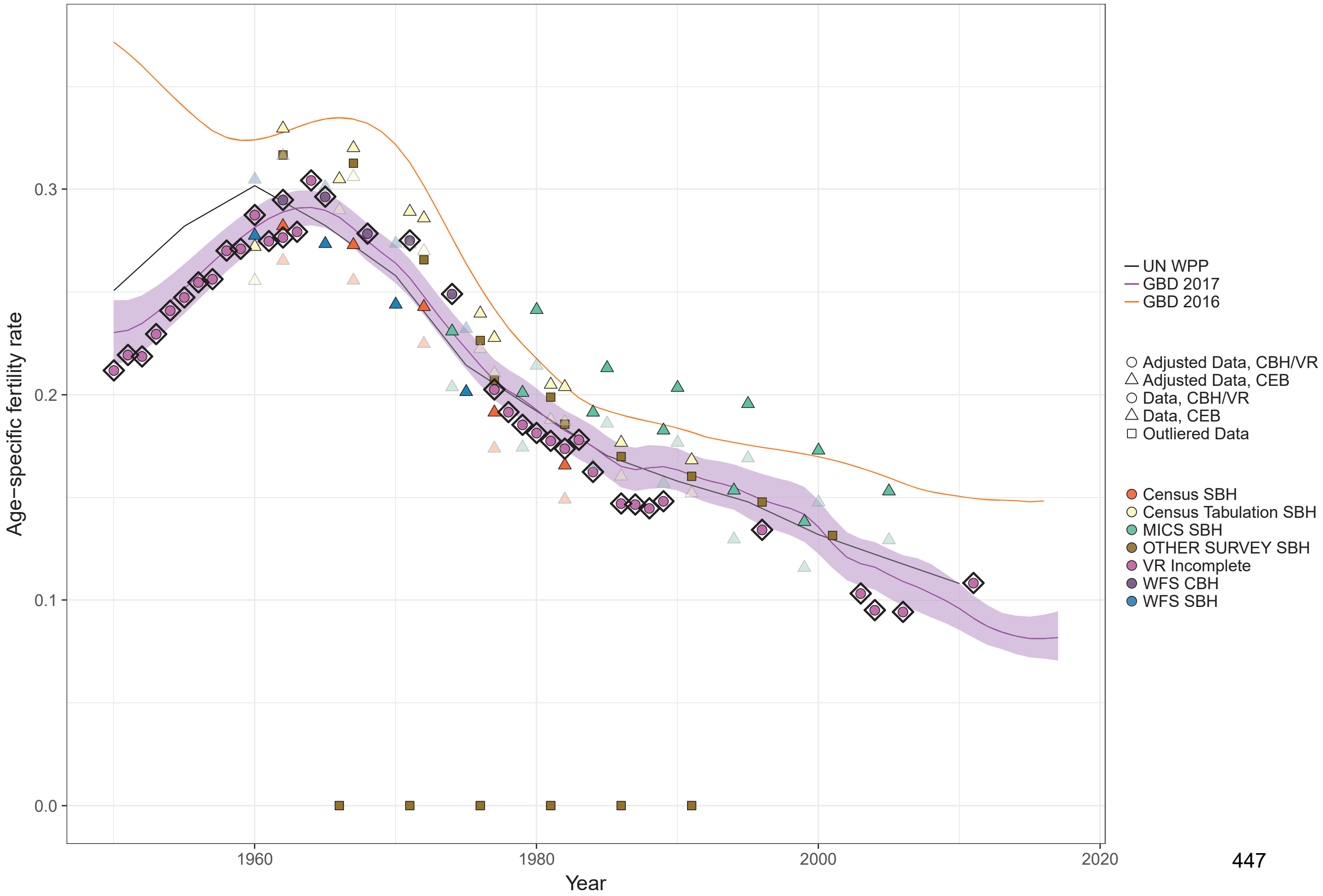

Age−specific fertility fitted trend lines, 1950−2017: Jamaica, Ages: 25−29
Data points enclosed with a diamond are reference sources

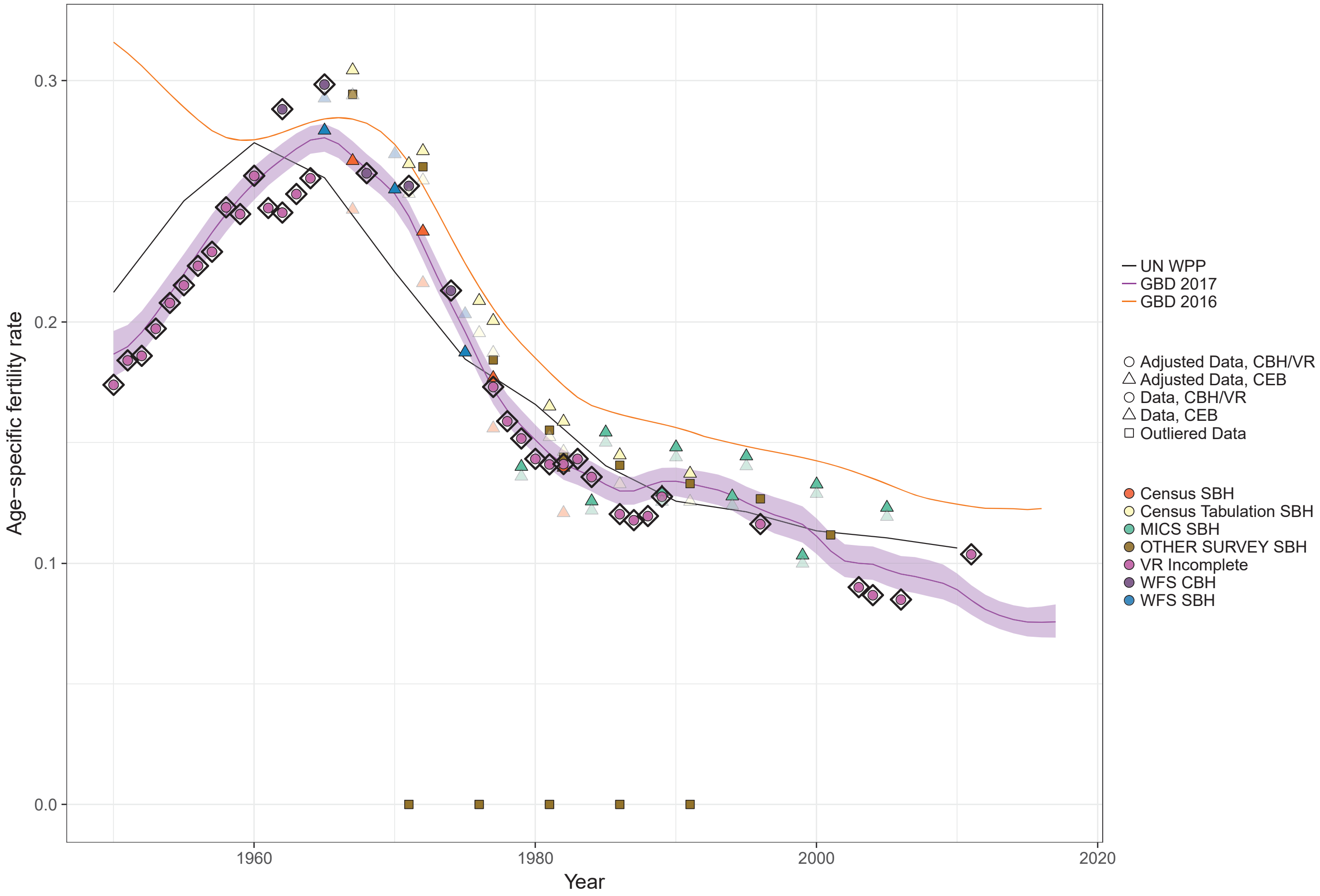

Age−specific fertility fitted trend lines, 1950−2017: Jamaica, Ages: 30−34
Data points enclosed with a diamond are reference sources

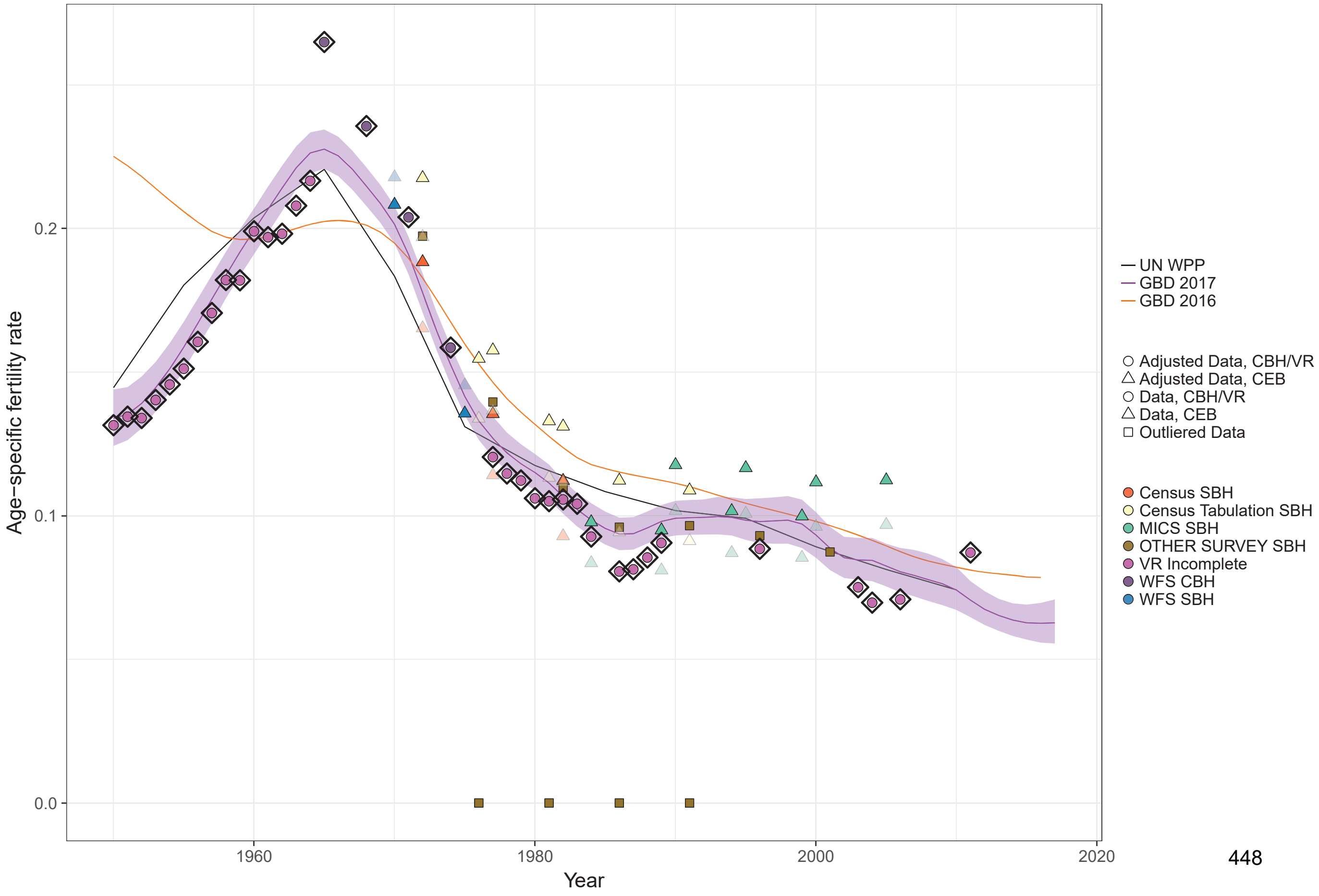

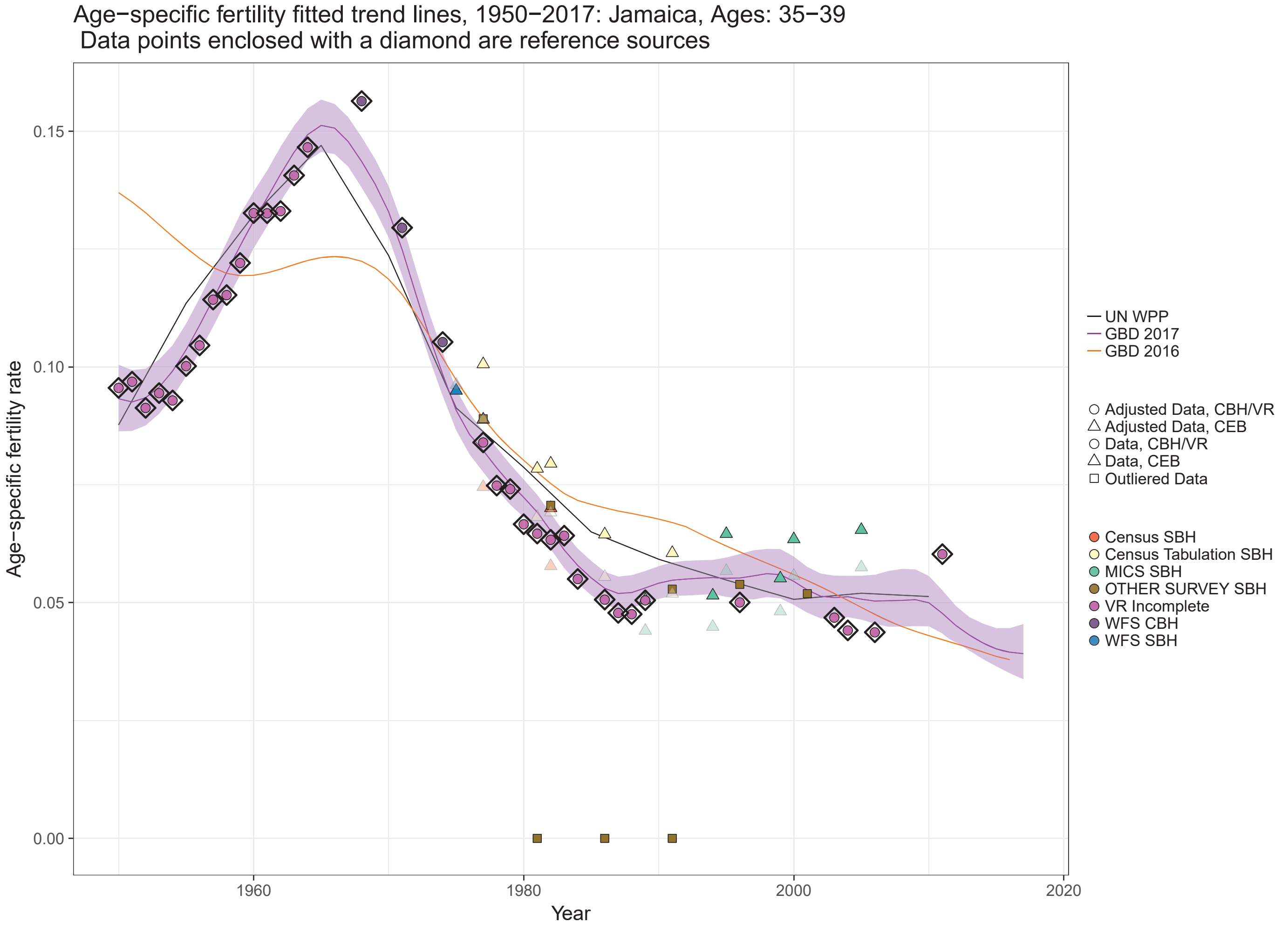

Age−specific fertility fitted trend lines, 1950−2017: Jamaica, Ages: 35−39
Data points enclosed with a diamond are reference sources
Age−specific fertility rate
0.15
0.10
0.05
0.00
1960
1980
2000
2020
Year
UN WPP
GBD 2017
GBD 2016
Adjusted Data, CBH/VR
Adjusted Data, CEB
Data, CBH/VR
Data, CEB
Outliered Data
Census SBH
Census Tabulation SBH
MICS SBH
OTHER SURVEY SBH
VR Incomplete
WFS CBH
WFS SBH

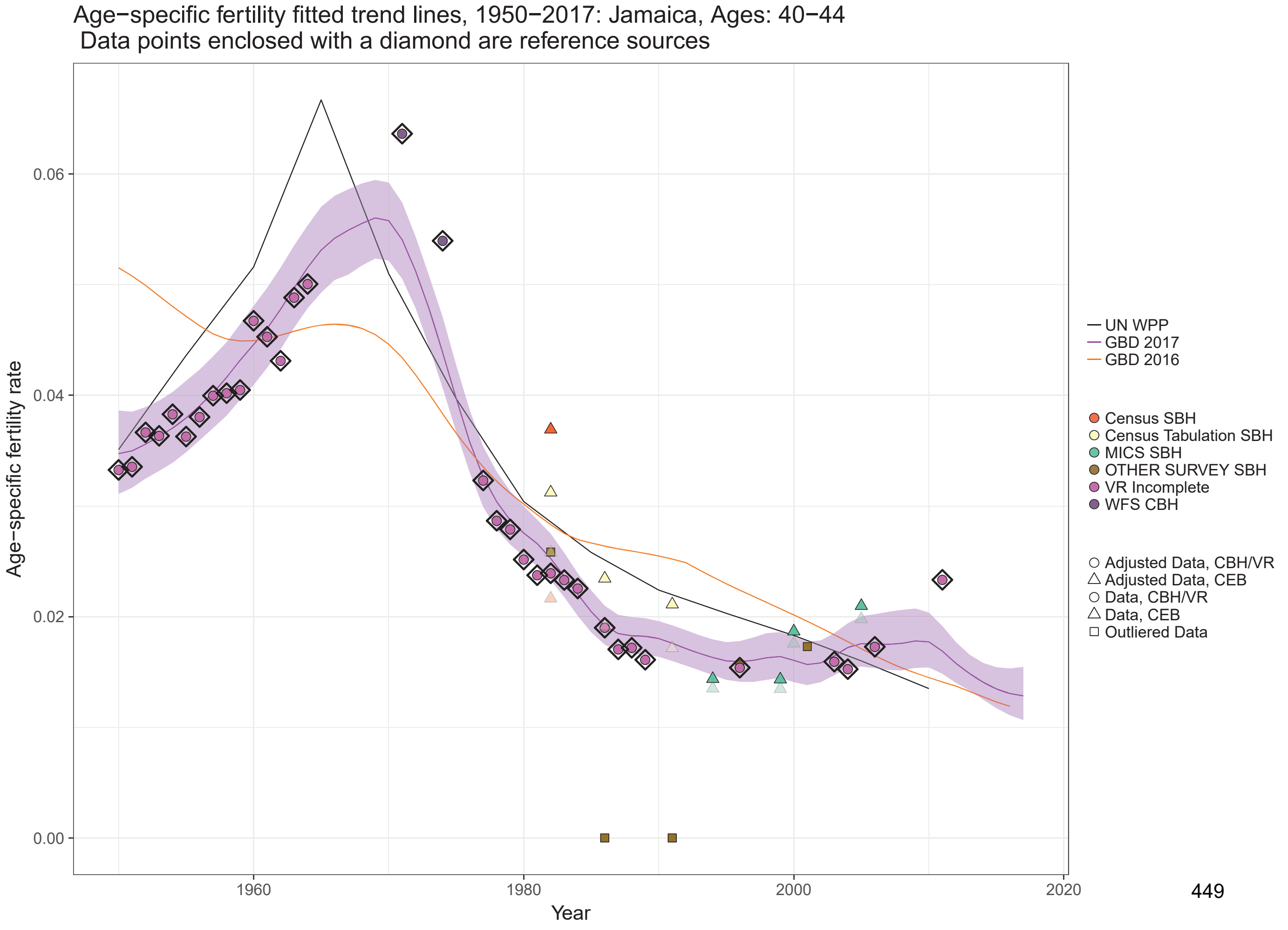

Age−specific fertility fitted trend lines, 1950−2017: Jamaica, Ages: 40−44
Data points enclosed with a diamond are reference sources
Age−specific fertility rate
0.06
0.04
0.02
0.00
1960
1980
2000
2020
Year
UN WPP
GBD 2017
GBD 2016
Census SBH
Census Tabulation SBH
MICS SBH
OTHER SURVEY SBH
VR Incomplete
WFS CBH
Adjusted Data, CBH/VR
Adjusted Data, CEB
Data, CBH/VR
Data, CEB
Outliered Data

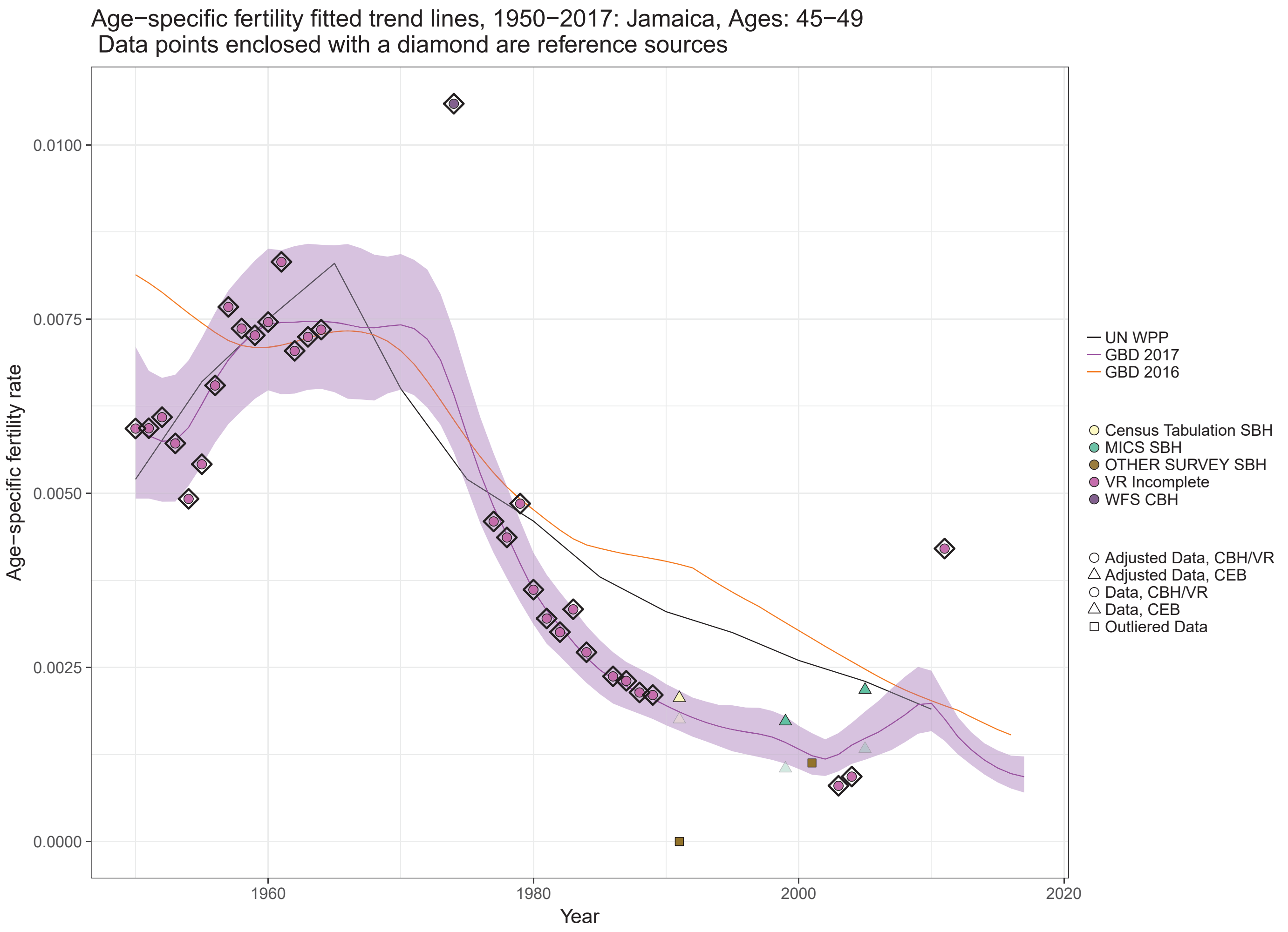

Age−specific fertility fitted trend lines, 1950−2017: Jamaica, Ages: 45−49
Data points enclosed with a diamond are reference sources
Age−specific fertility rate
0.0100
0.0075
0.0050
0.0025
0.0000
1960
1980
2000
2020
Year
UN WPP
GBD 2017
GBD 2016
Census Tabulation SBH
MICS SBH
OTHER SURVEY SBH
VR Incomplete
WFS CBH
Adjusted Data, CBH/VR
Adjusted Data, CEB
Data, CBH/VR
Data, CEB
Outliered Data

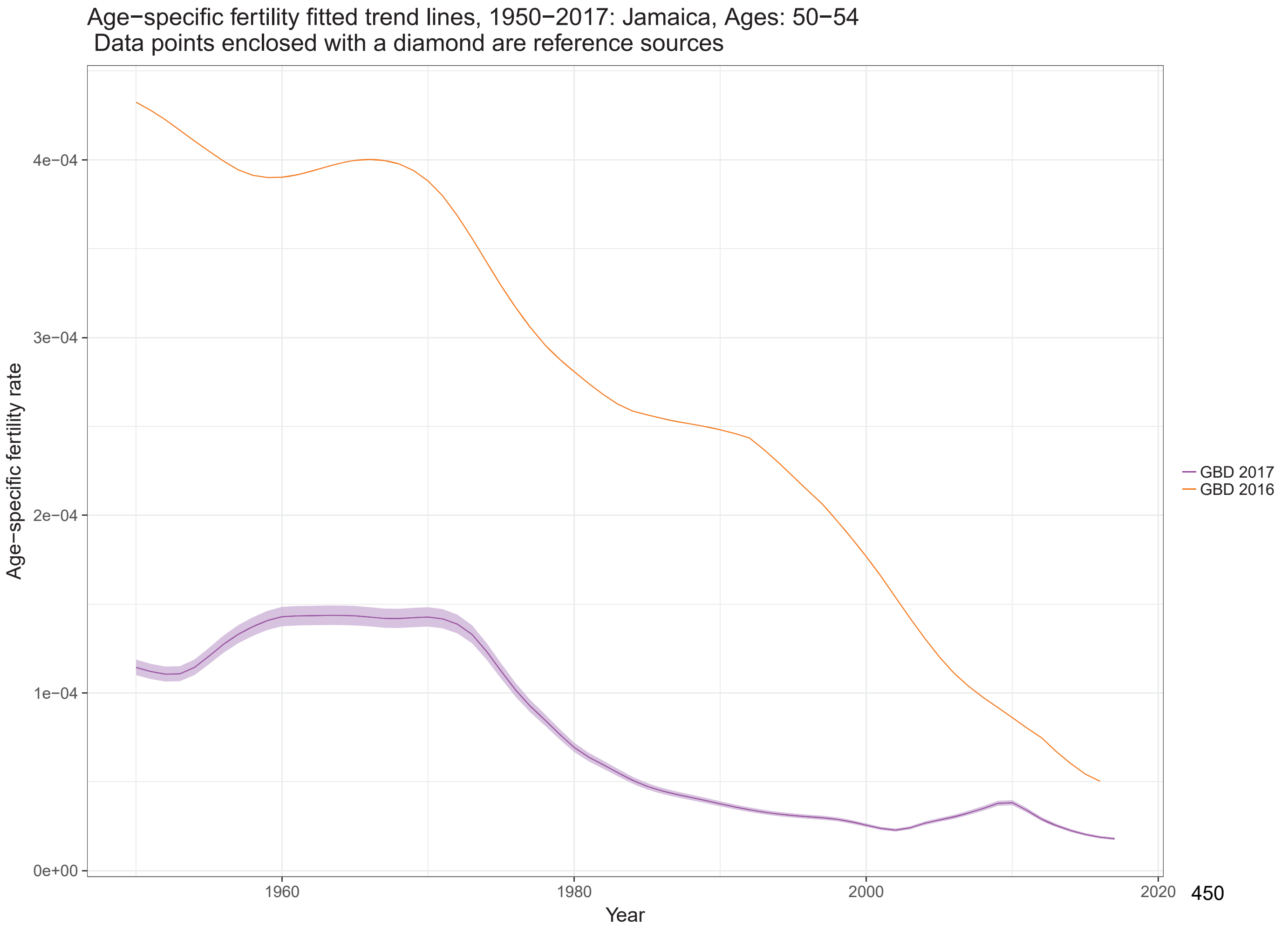

Age−specific fertility fitted trend lines, 1950−2017: Jamaica, Ages: 50−54
Data points enclosed with a diamond are reference sources
Age−specific fertility rate
4e−04
3e−04
2e−04
1e−04
0e+00
1960
1980
2000
2020
Year
GBD 2017
GBD 2016

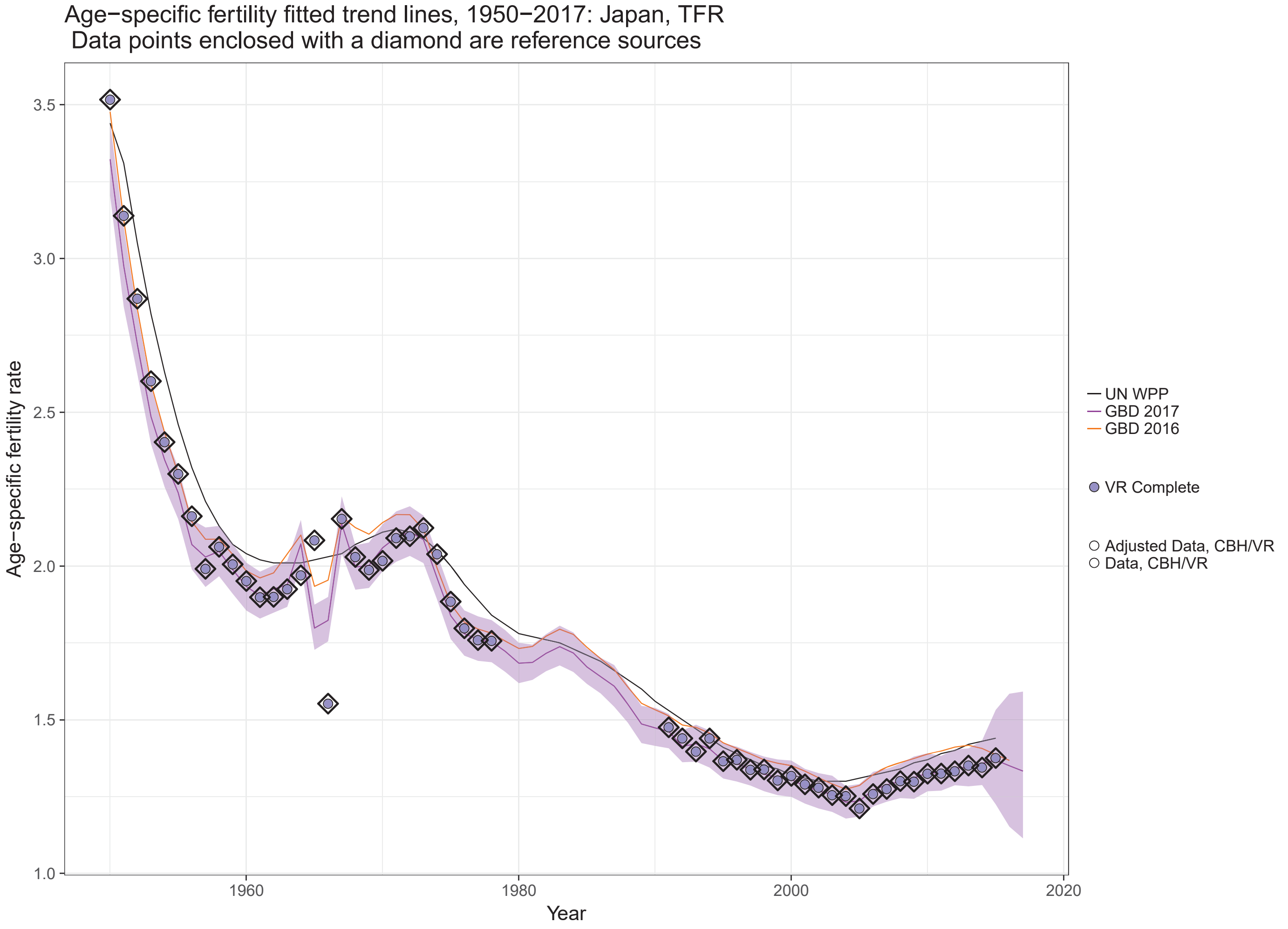
Age−specific fertility fitted trend lines, 1950−2017: Japan, TFR
Data points enclosed with a diamond are reference sources
Age-specific fertility rate
Year
UN WPP
GBD 2017
GBD 2016
VR Complete
Adjusted Data, CBH/VR
Data, CBH/VR

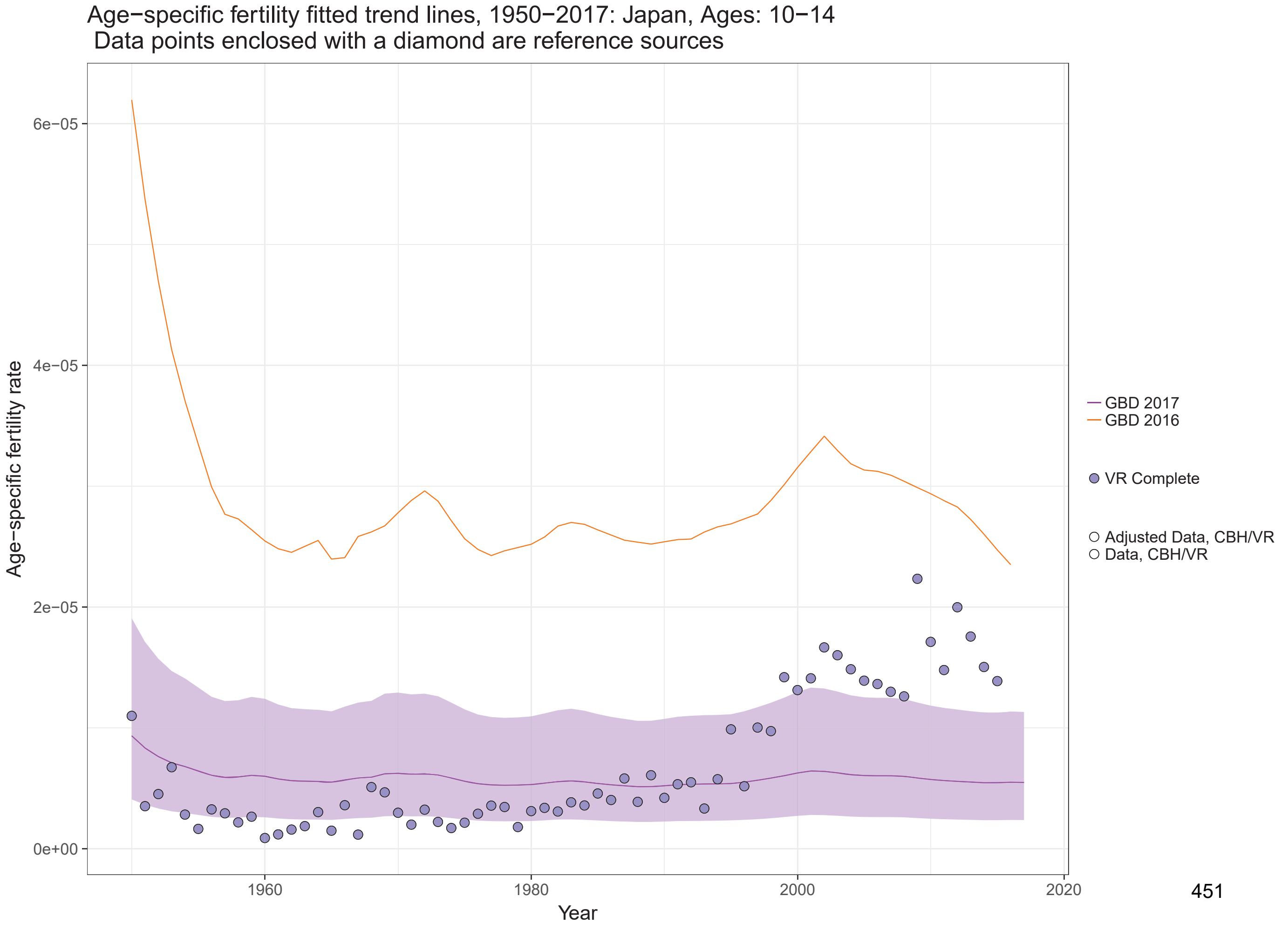
Age−specific fertility fitted trend lines, 1950−2017: Japan, Ages: 10−14
Data points enclosed with a diamond are reference sources
Age-specific fertility rate
Year
GBD 2017
GBD 2016
VR Complete
Adjusted Data, CBH/VR
Data, CBH/VR

Age−specific fertility fitted trend lines, 1950−2017: Japan, Ages: 15−19
Data points enclosed with a diamond are reference sources

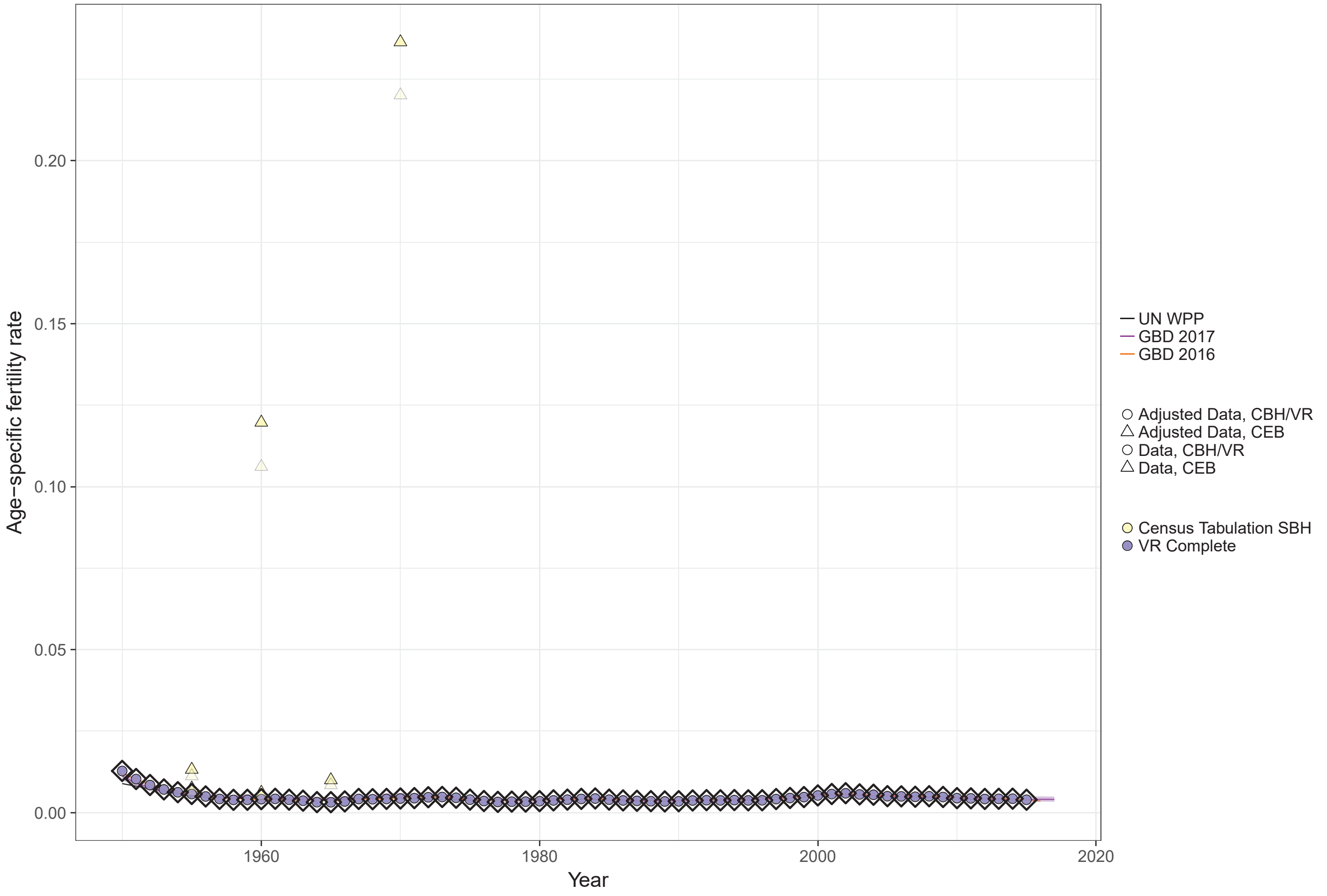

Age−specific fertility fitted trend lines, 1950−2017: Japan, Ages: 20−24
Data points enclosed with a diamond are reference sources

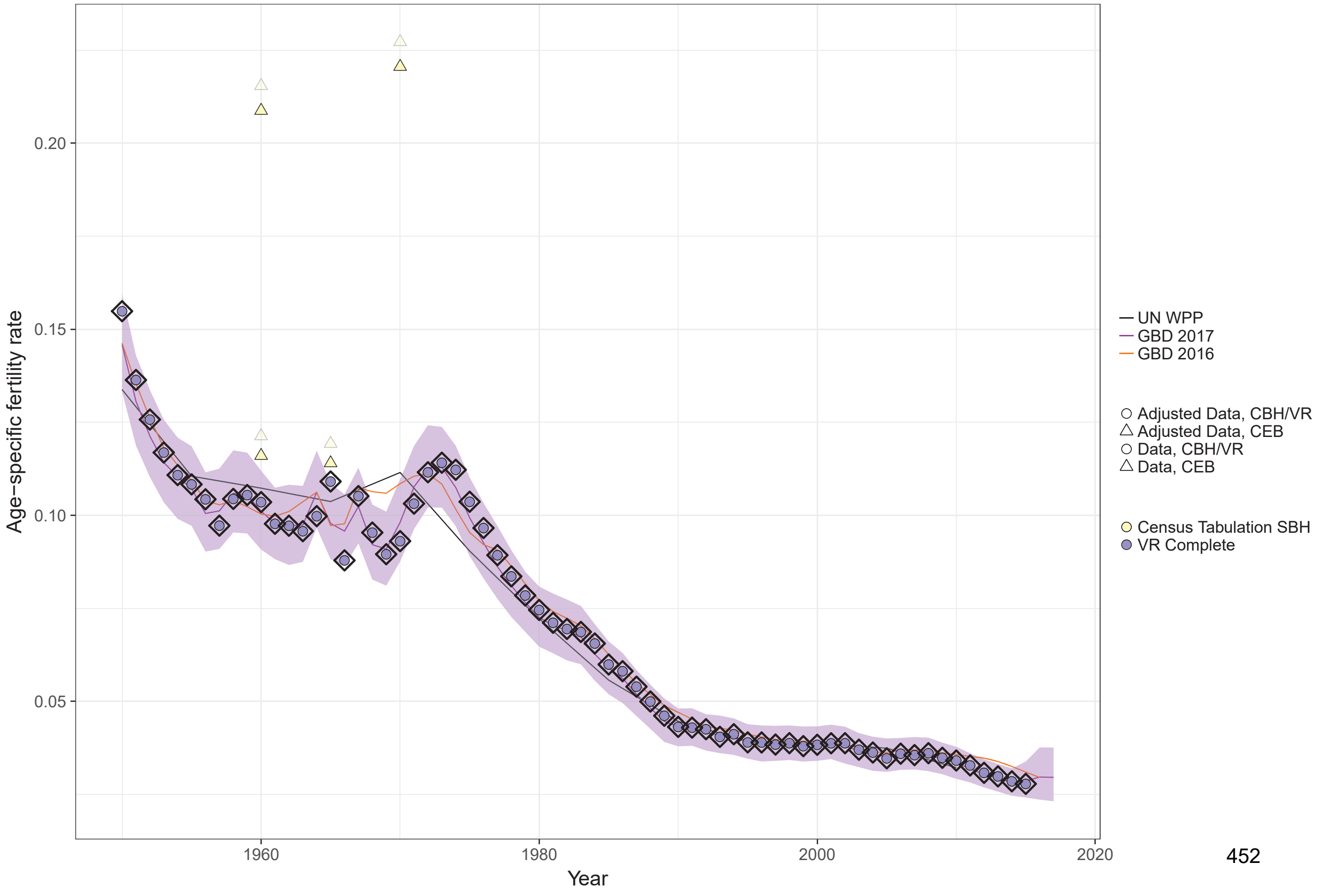

Age−specific fertility fitted trend lines, 1950−2017: Japan, Ages: 25−29
Data points enclosed with a diamond are reference sources

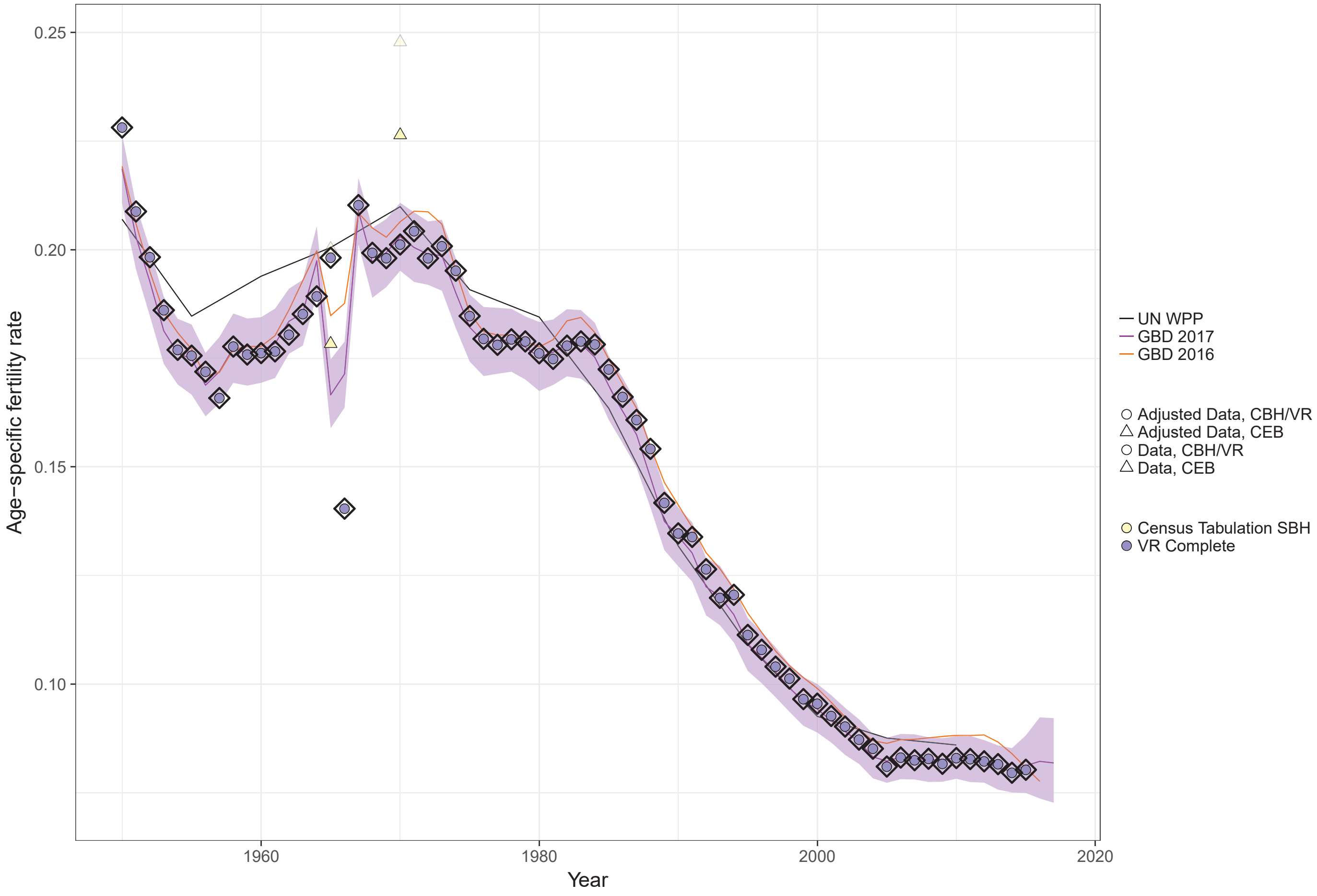

Age−specific fertility fitted trend lines, 1950−2017: Japan, Ages: 30−34
Data points enclosed with a diamond are reference sources

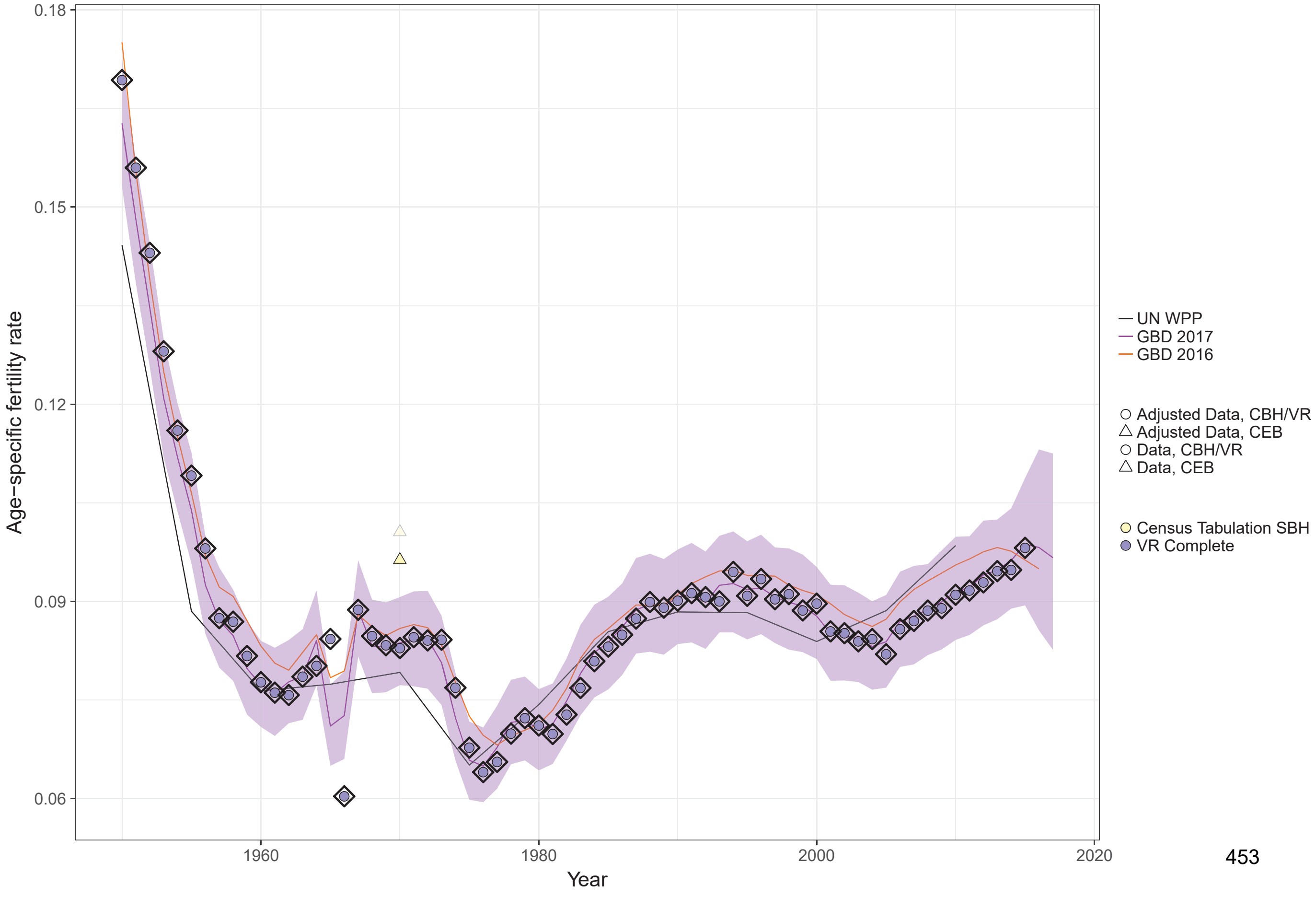

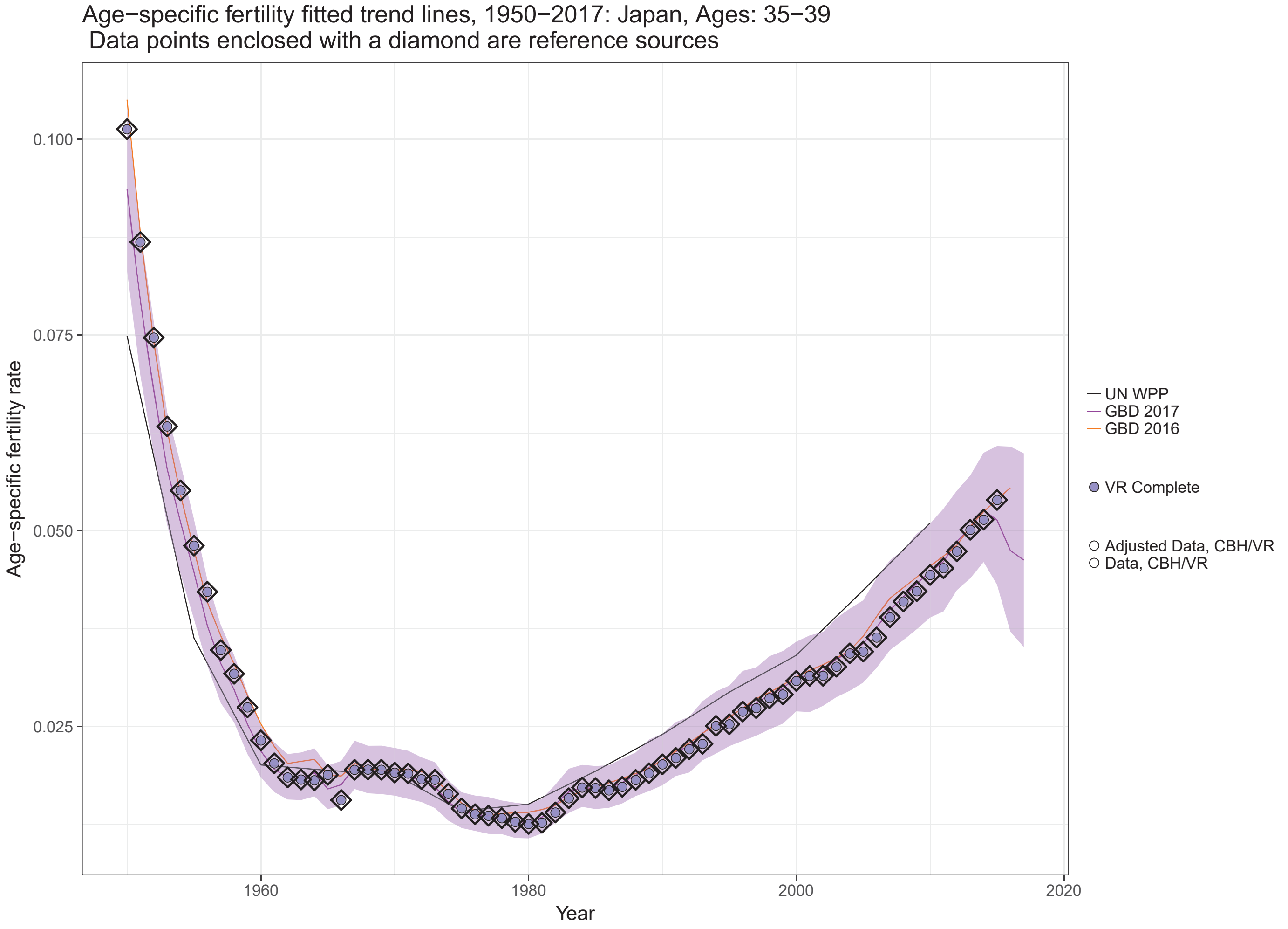
Age−specific fertility fitted trend lines, 1950−2017: Japan, Ages: 35−39
Data points enclosed with a diamond are reference sources
Age−specific fertility rate
0.100
0.075
0.050
0.025
1960
1980
2000
2020
Year
UN WPP
GBD 2017
GBD 2016
VR Complete
Adjusted Data, CBH/VR
Data, CBH/VR

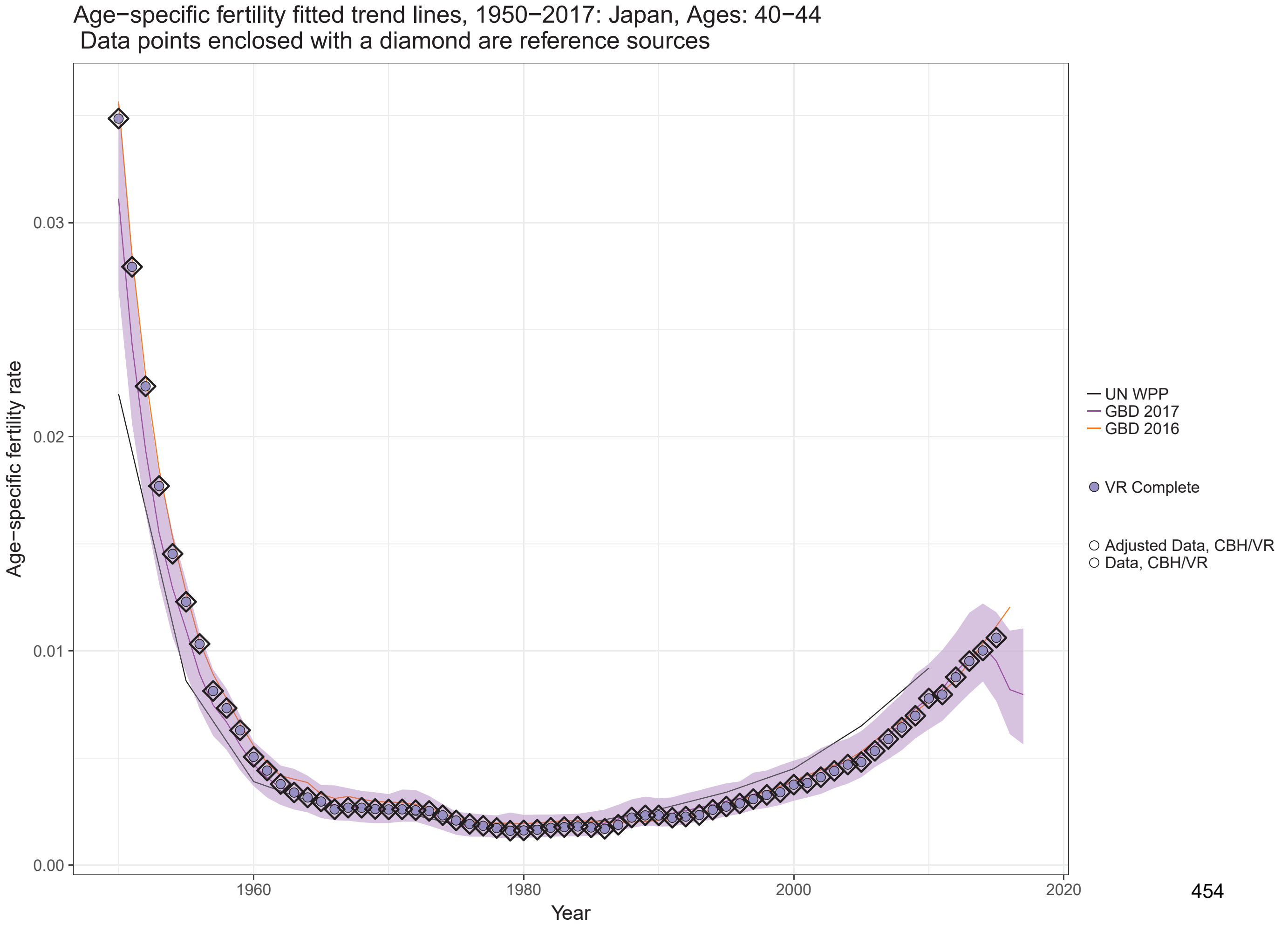
Age−specific fertility fitted trend lines, 1950−2017: Japan, Ages: 40−44
Data points enclosed with a diamond are reference sources
Age−specific fertility rate
0.03
0.02
0.01
0.00
1960
1980
2000
2020
Year
UN WPP
GBD 2017
GBD 2016
VR Complete
Adjusted Data, CBH/VR
Data, CBH/VR

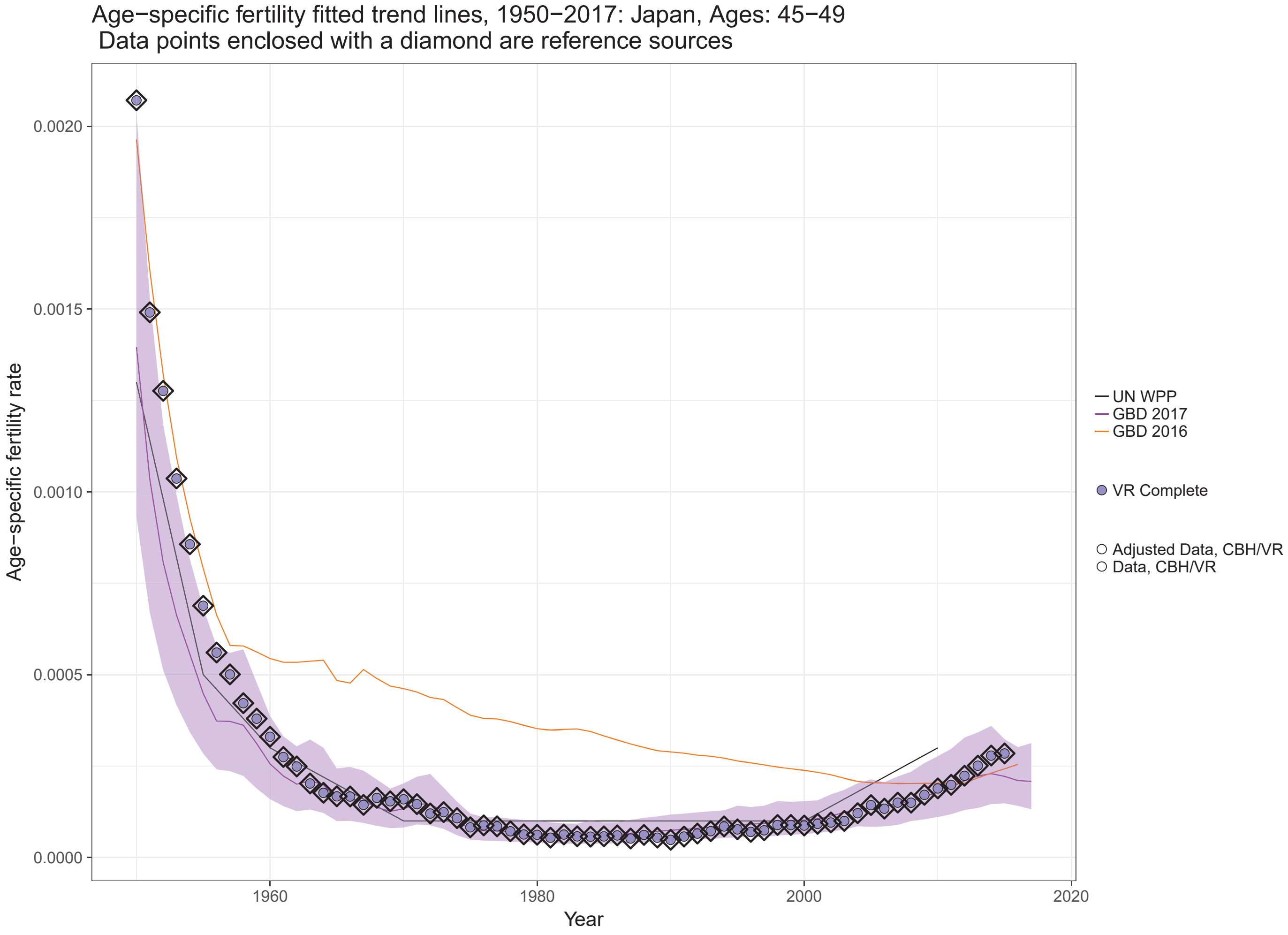

Age−specific fertility fitted trend lines, 1950−2017: Japan, Ages: 45−49
Data points enclosed with a diamond are reference sources
Age-specific fertility rate
0.0020
0.0015
0.0010
0.0005
0.0000
1960
1980
2000
2020
Year
UN WPP
GBD 2017
GBD 2016
VR Complete
Adjusted Data, CBH/VR
Data, CBH/VR

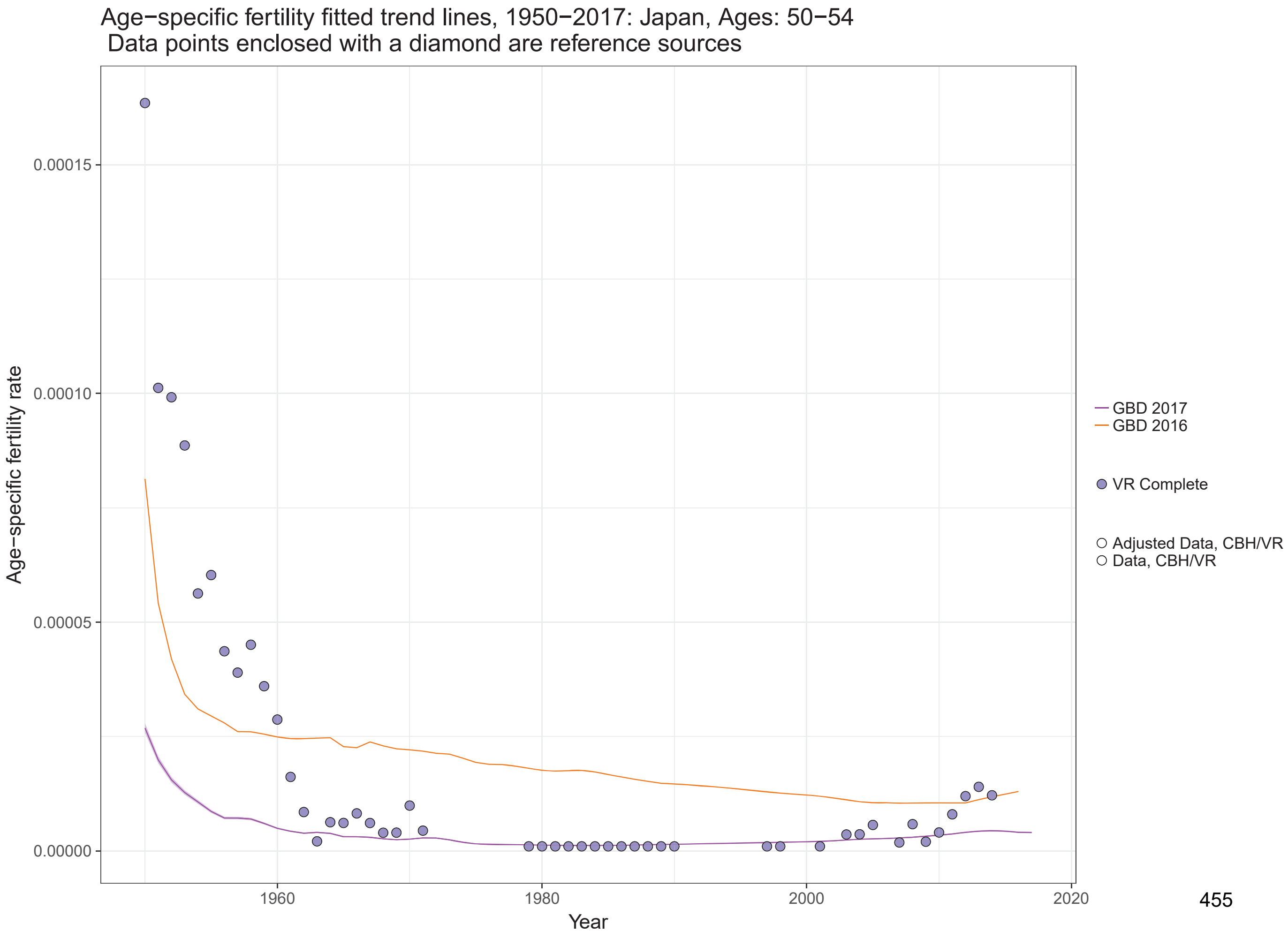

Age−specific fertility fitted trend lines, 1950−2017: Japan, Ages: 50−54
Data points enclosed with a diamond are reference sources
Age-specific fertility rate
0.00015
0.00010
0.00005
0.00000
1960
1980
2000
2020
Year
GBD 2017
GBD 2016
VR Complete
Adjusted Data, CBH/VR
Data, CBH/VR

Age−specific fertility fitted trend lines, 1950−2017: Jordan, TFR
Data points enclosed with a diamond are reference sources

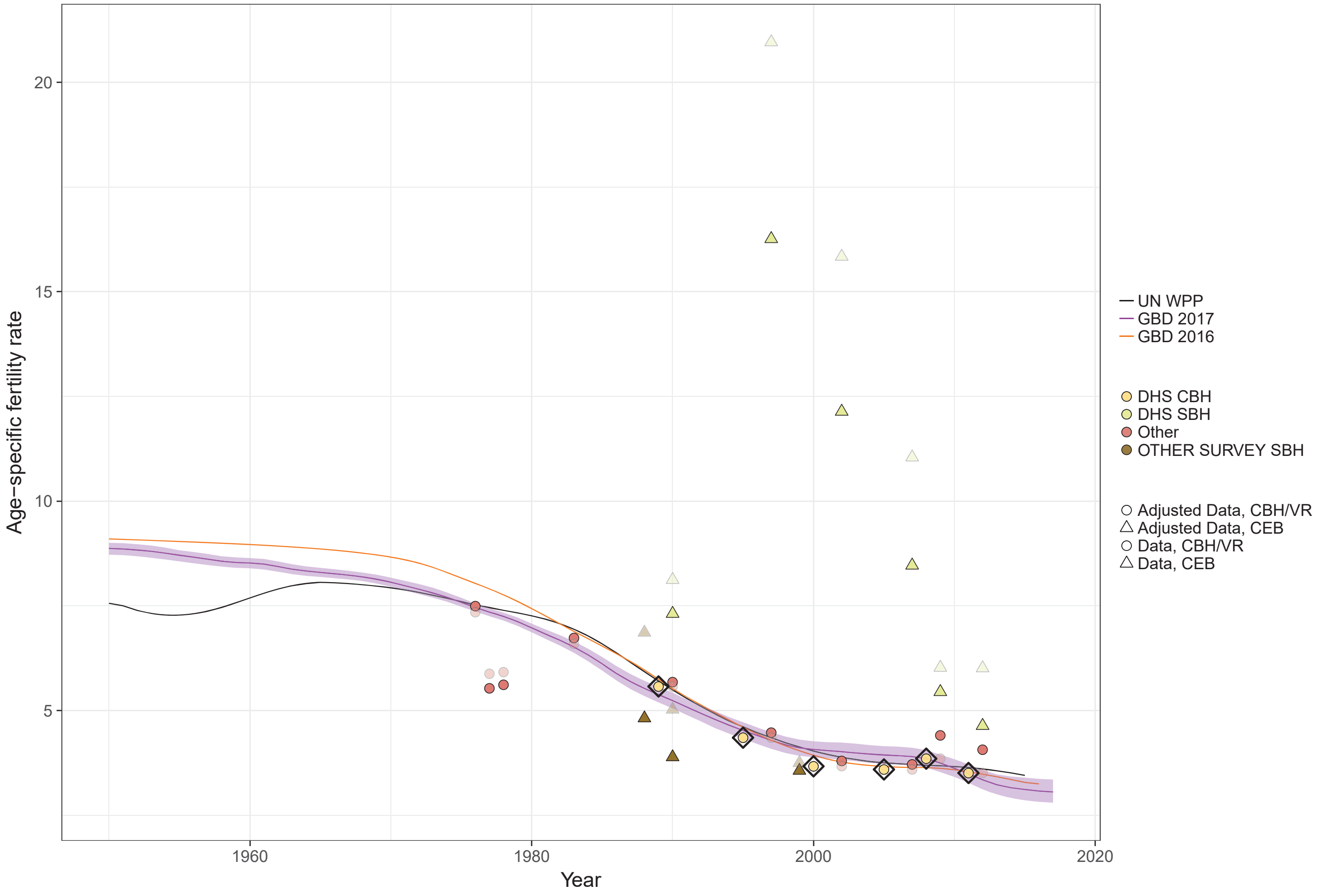

Age−specific fertility fitted trend lines, 1950−2017: Jordan, Ages: 10−14
Data points enclosed with a diamond are reference sources

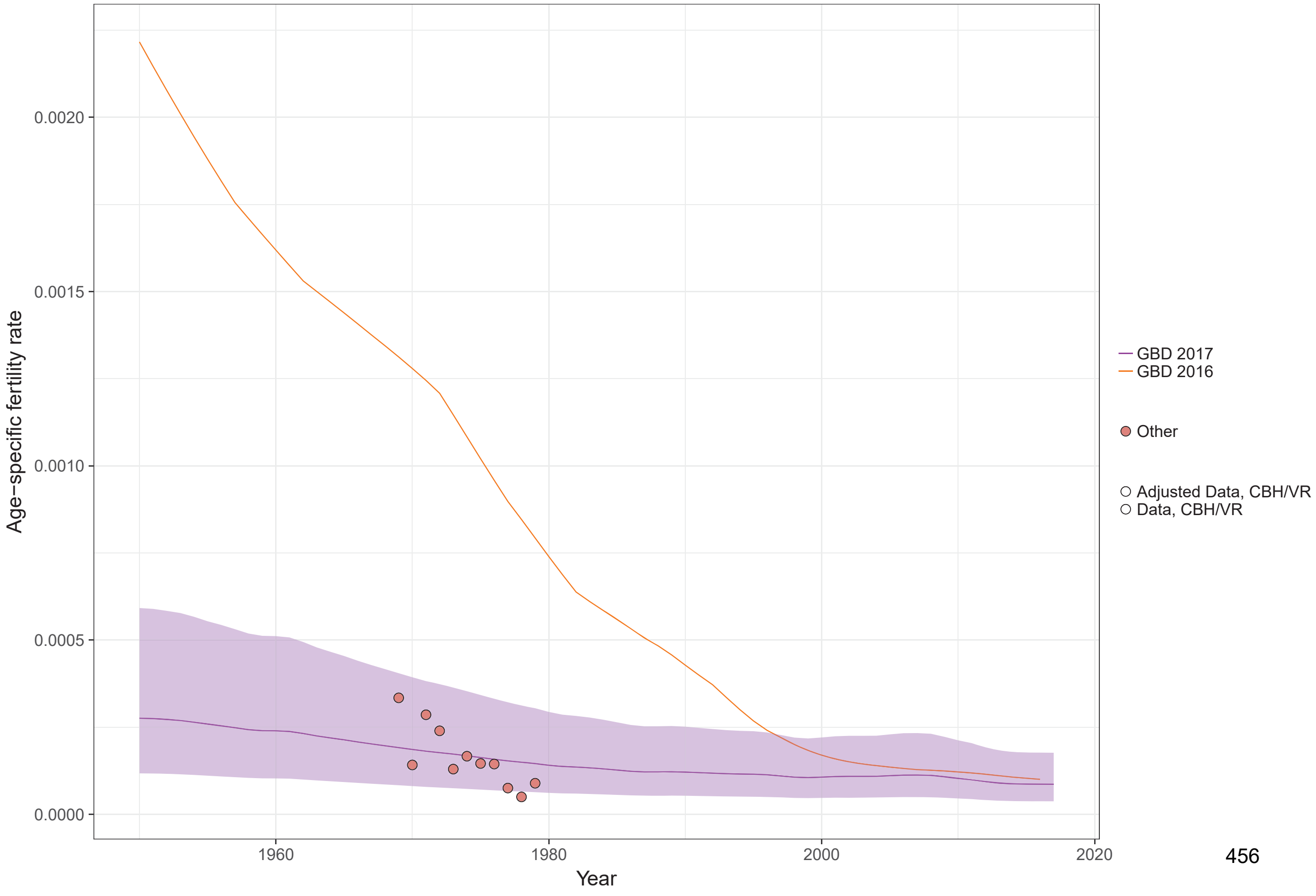

Age−specific fertility fitted trend lines, 1950−2017: Jordan, Ages: 15−19
Data points enclosed with a diamond are reference sources

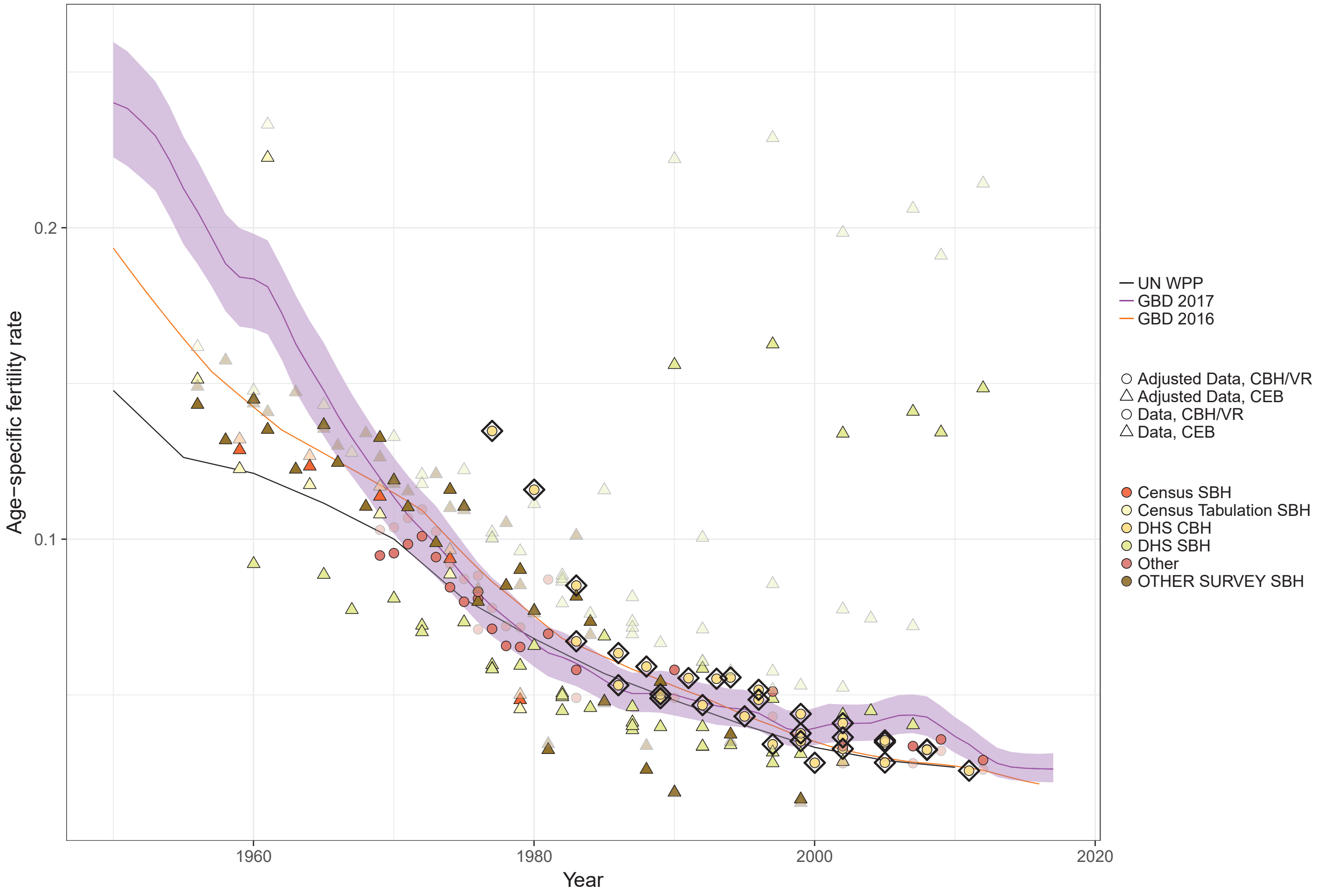

Age−specific fertility fitted trend lines, 1950−2017: Jordan, Ages: 20−24
Data points enclosed with a diamond are reference sources

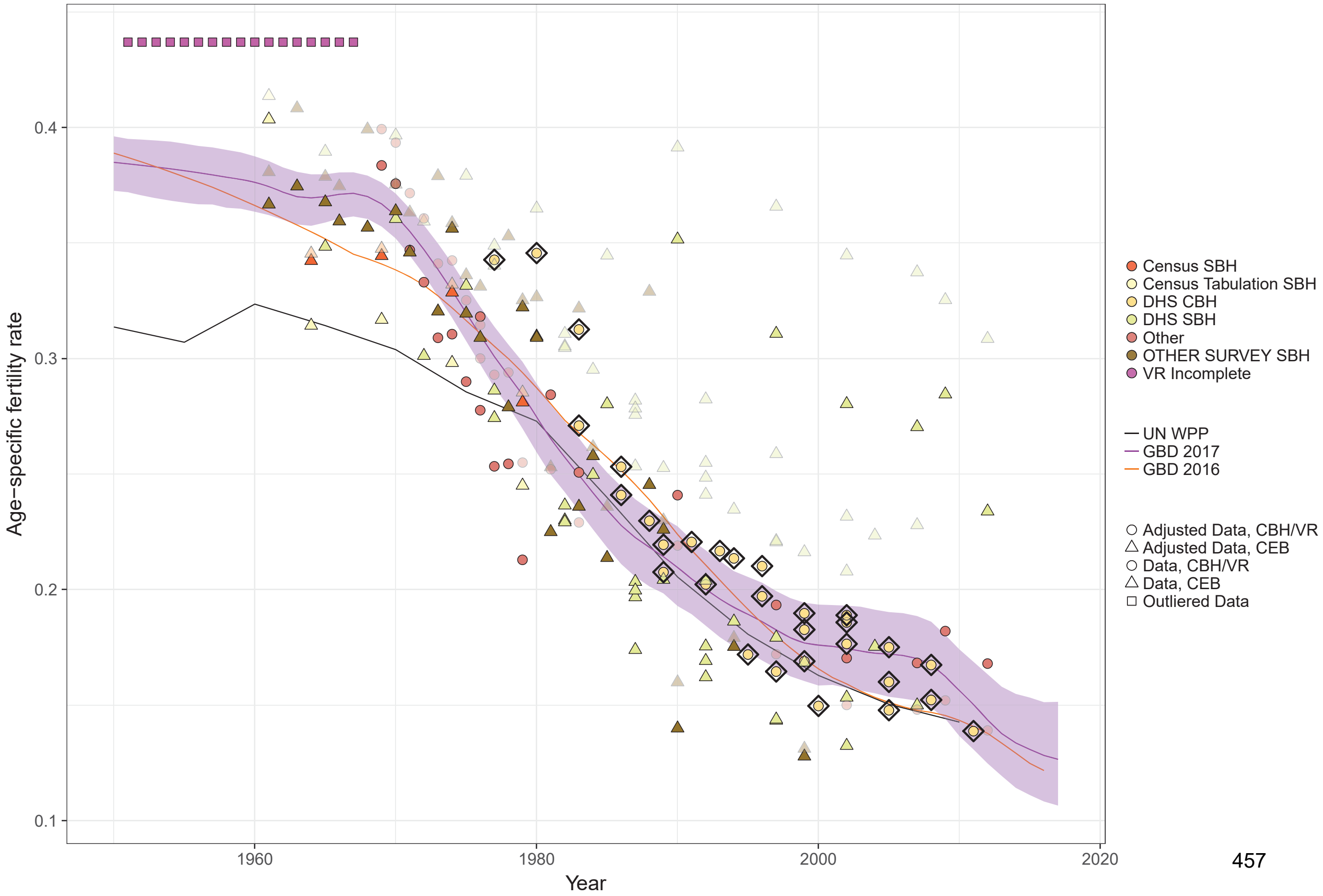

Age−specific fertility fitted trend lines, 1950−2017: Jordan, Ages: 25−29
Data points enclosed with a diamond are reference sources

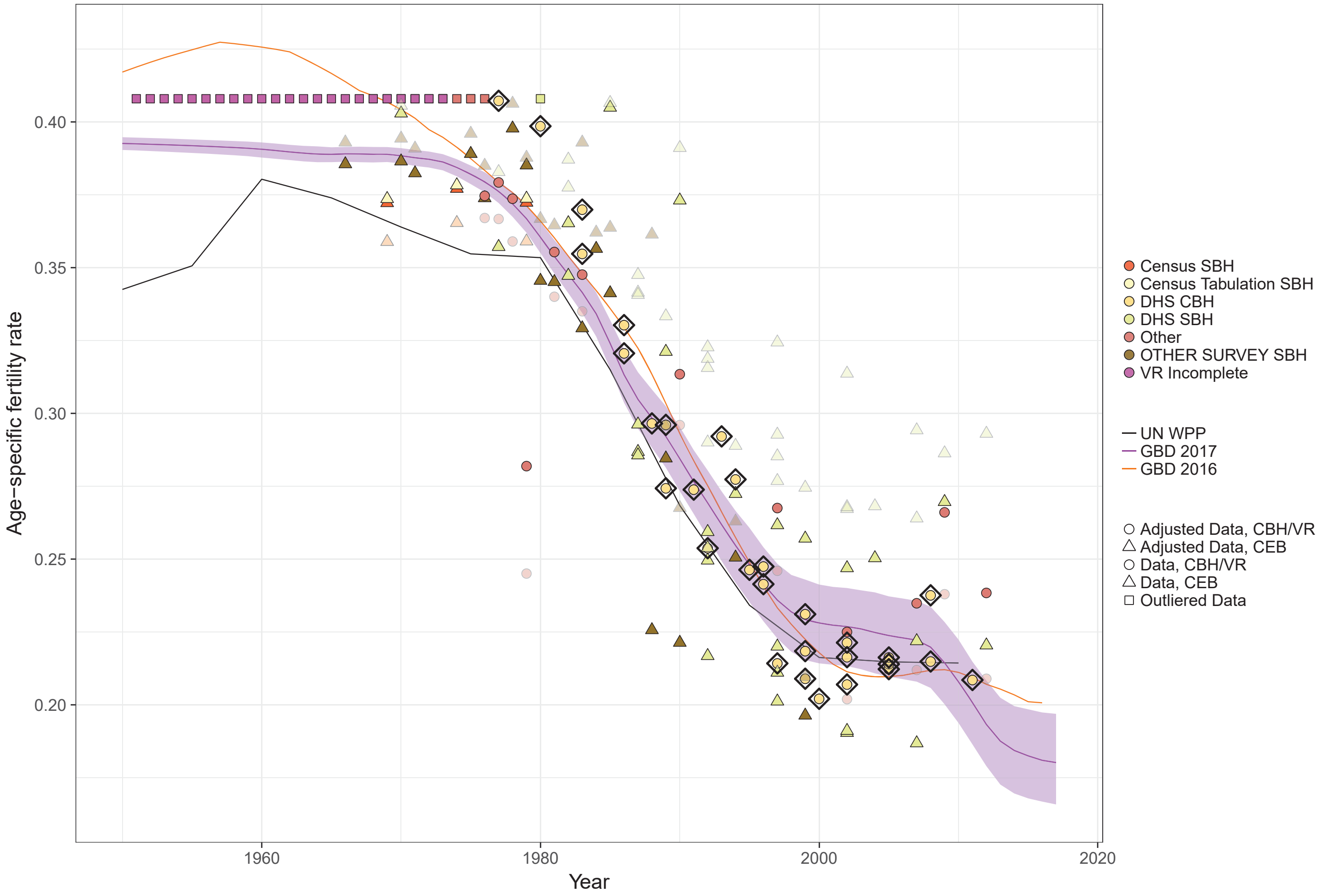

Age−specific fertility fitted trend lines, 1950−2017: Jordan, Ages: 30−34
Data points enclosed with a diamond are reference sources

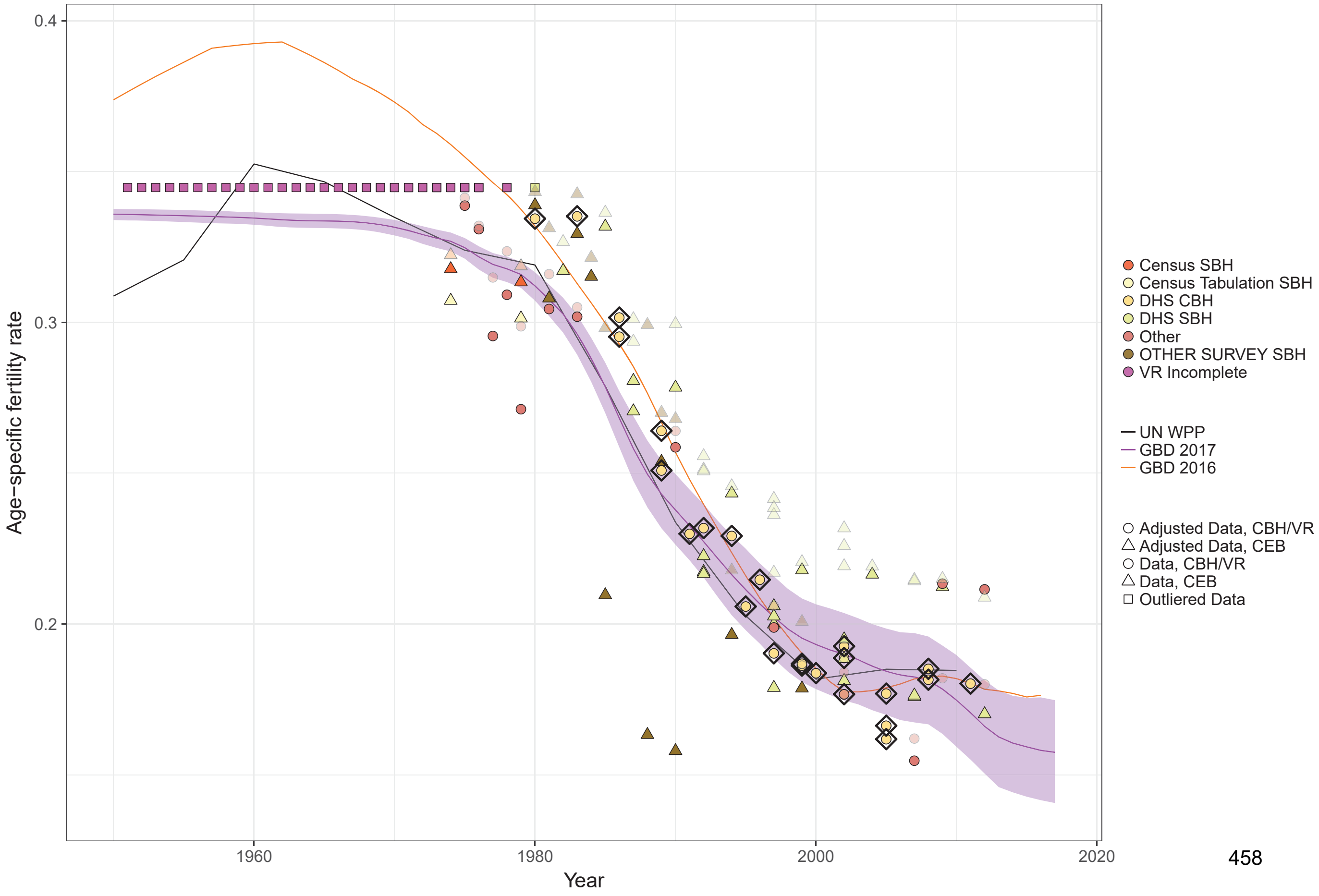

Age−specific fertility fitted trend lines, 1950−2017: Jordan, Ages: 35−39
Data points enclosed with a diamond are reference sources

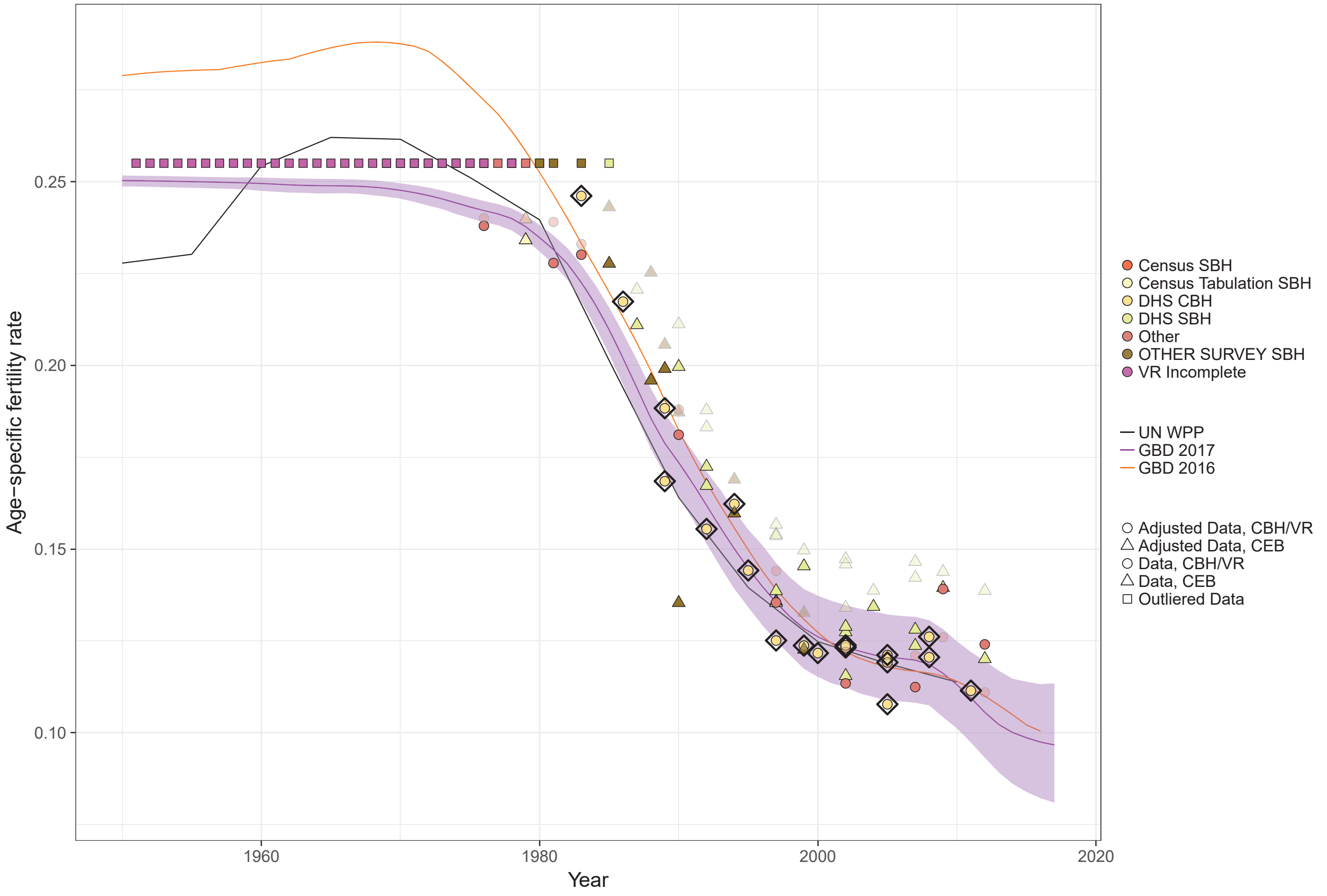

Age−specific fertility fitted trend lines, 1950−2017: Jordan, Ages: 40−44
Data points enclosed with a diamond are reference sources

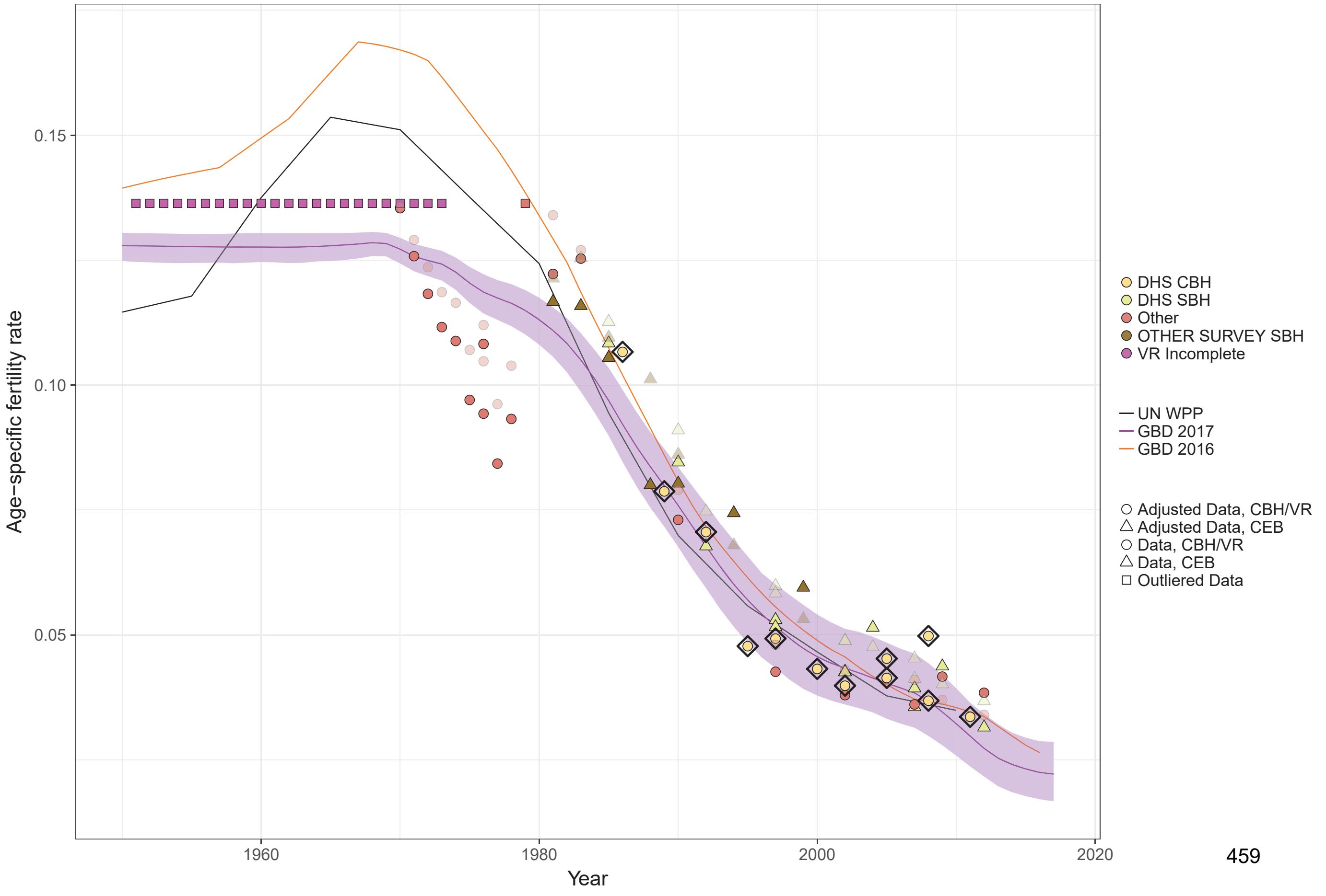

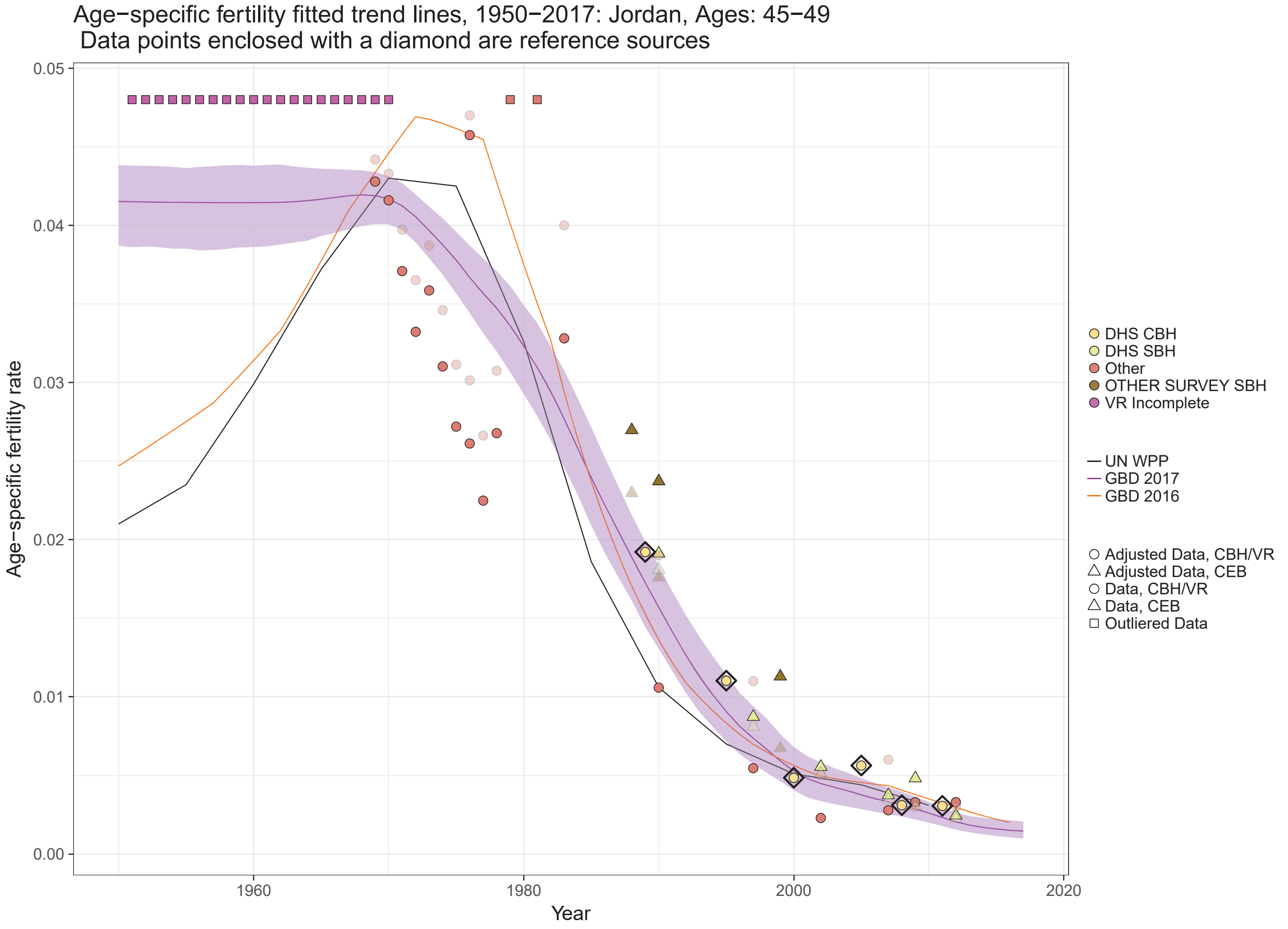
Age−specific fertility fitted trend lines, 1950−2017: Jordan, Ages: 45−49
Data points enclosed with a diamond are reference sources
Age−specific fertility rate
Year
DHS CBH
DHS SBH
Other
OTHER SURVEY SBH
VR Incomplete
UN WPP
GBD 2017
GBD 2016
Adjusted Data, CBH/VR
Adjusted Data, CEB
Data, CBH/VR
Data, CEB
Outliered Data

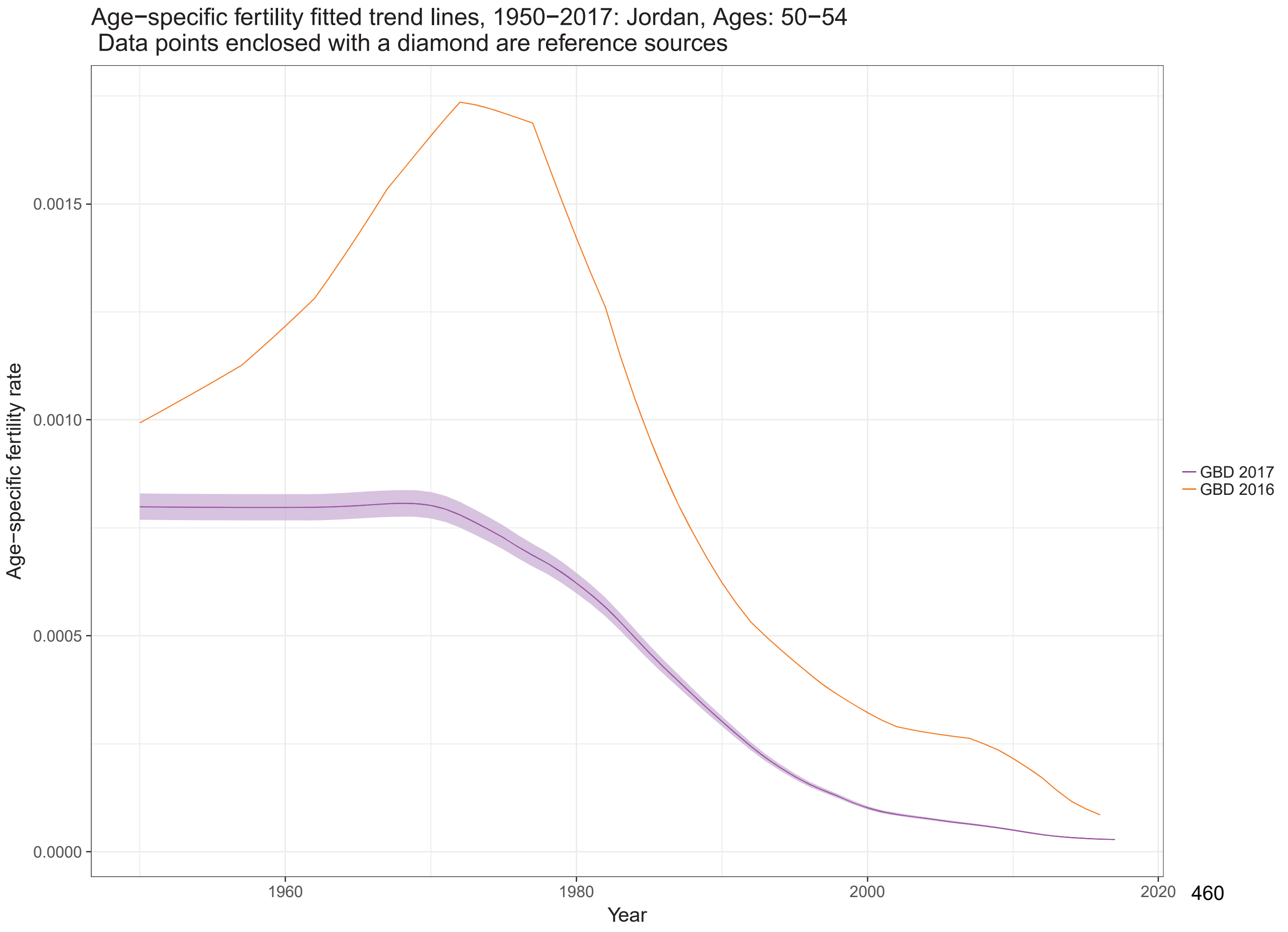
Age−specific fertility fitted trend lines, 1950−2017: Jordan, Ages: 50−54
Data points enclosed with a diamond are reference sources
Age−specific fertility rate
Year
GBD 2017
GBD 2016

Age−specific fertility fitted trend lines, 1950−2017: Kazakhstan, TFR
Data points enclosed with a diamond are reference sources

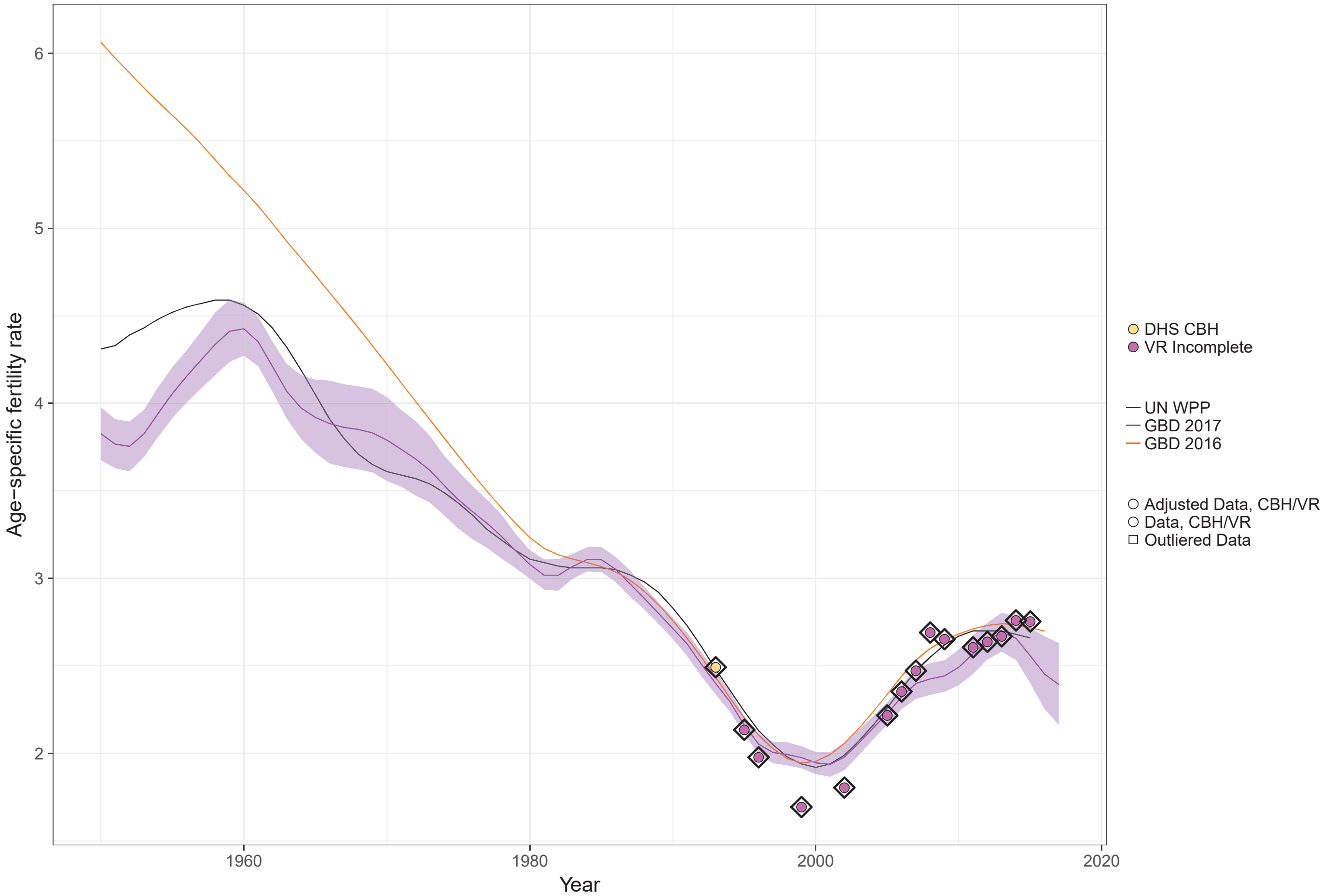

Age−specific fertility fitted trend lines, 1950−2017: Kazakhstan, Ages: 10−14
Data points enclosed with a diamond are reference sources

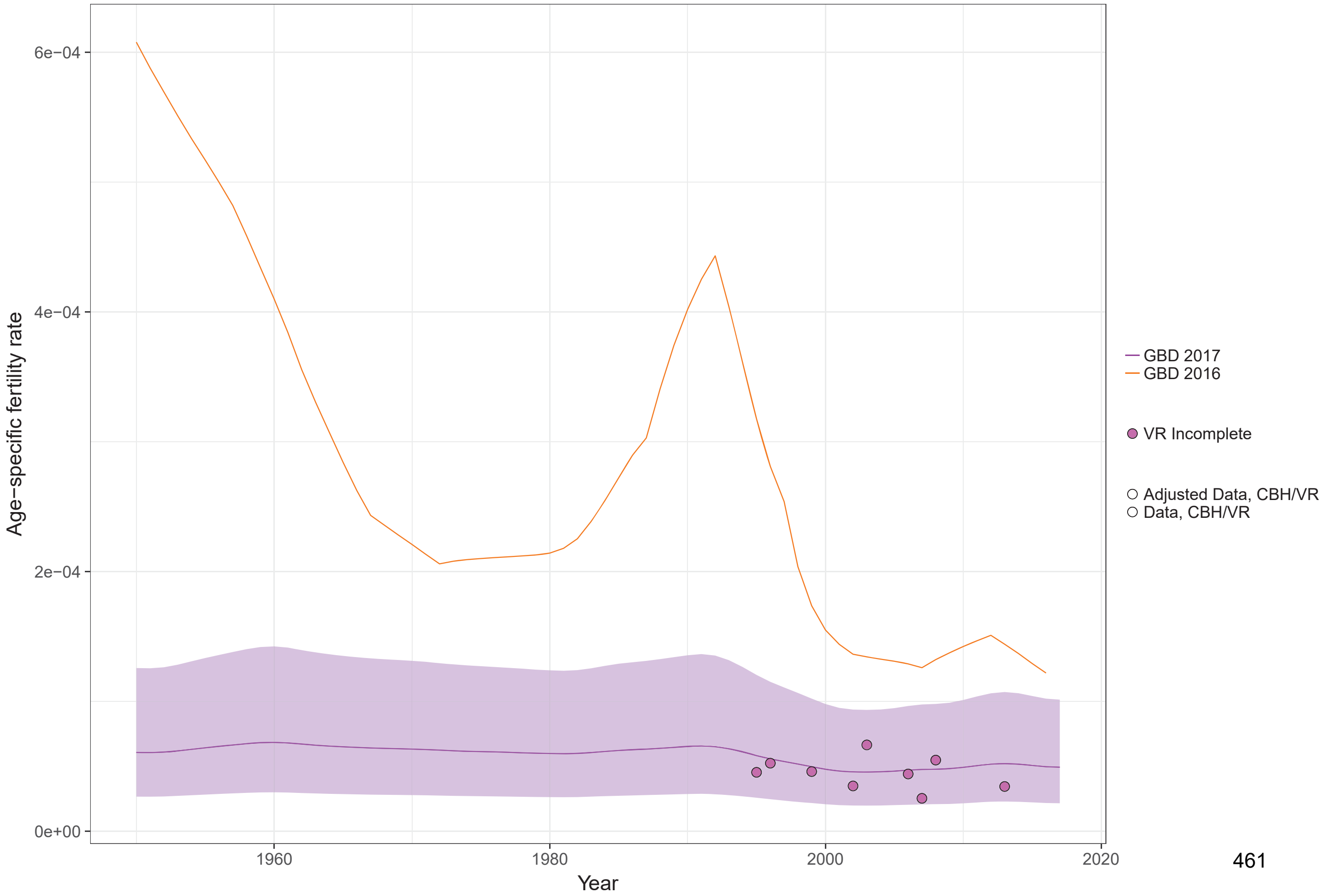

Age−specific fertility fitted trend lines, 1950−2017: Kazakhstan, Ages: 15−19
Data points enclosed with a diamond are reference sources

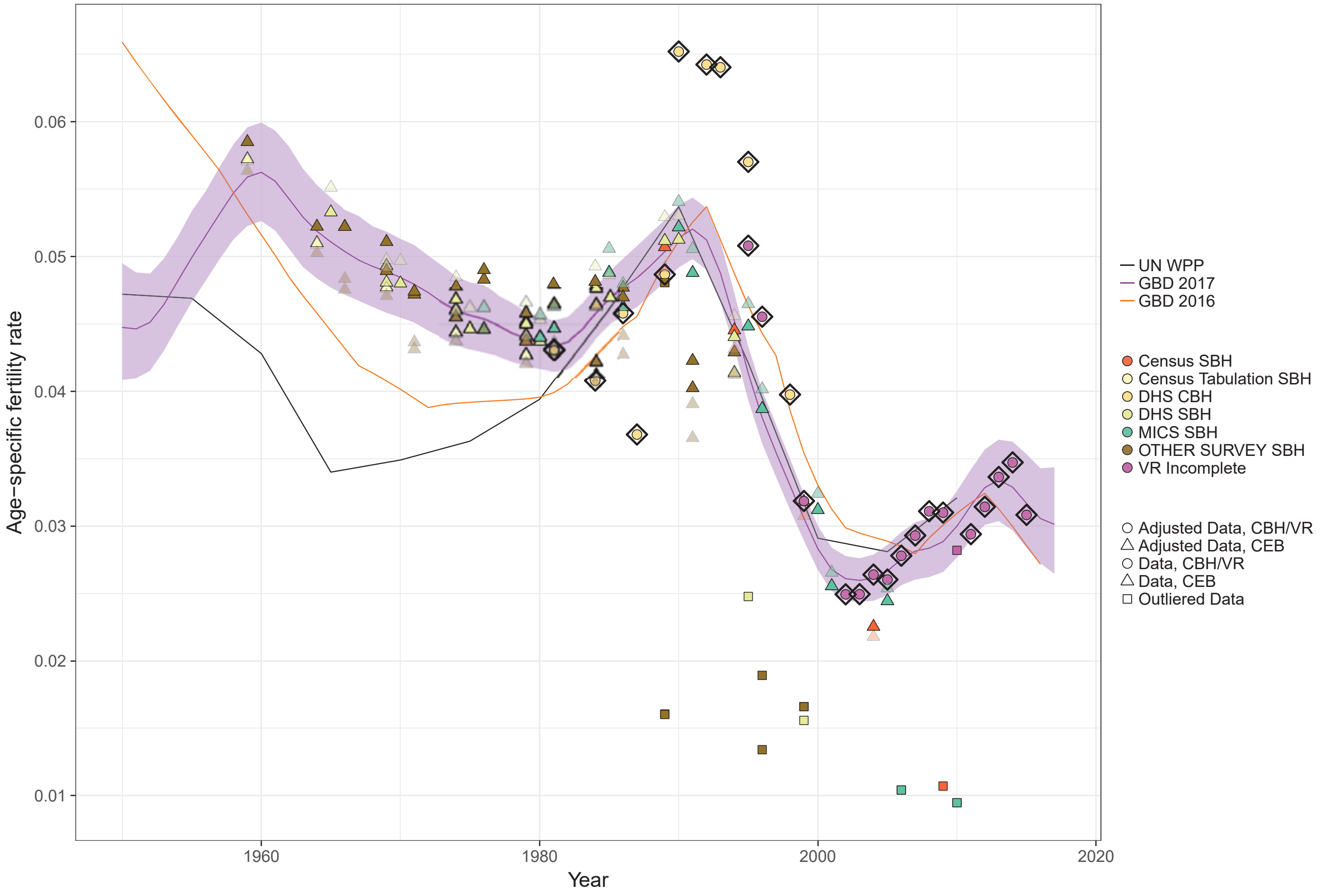

Age−specific fertility fitted trend lines, 1950−2017: Kazakhstan, Ages: 20−24
Data points enclosed with a diamond are reference sources

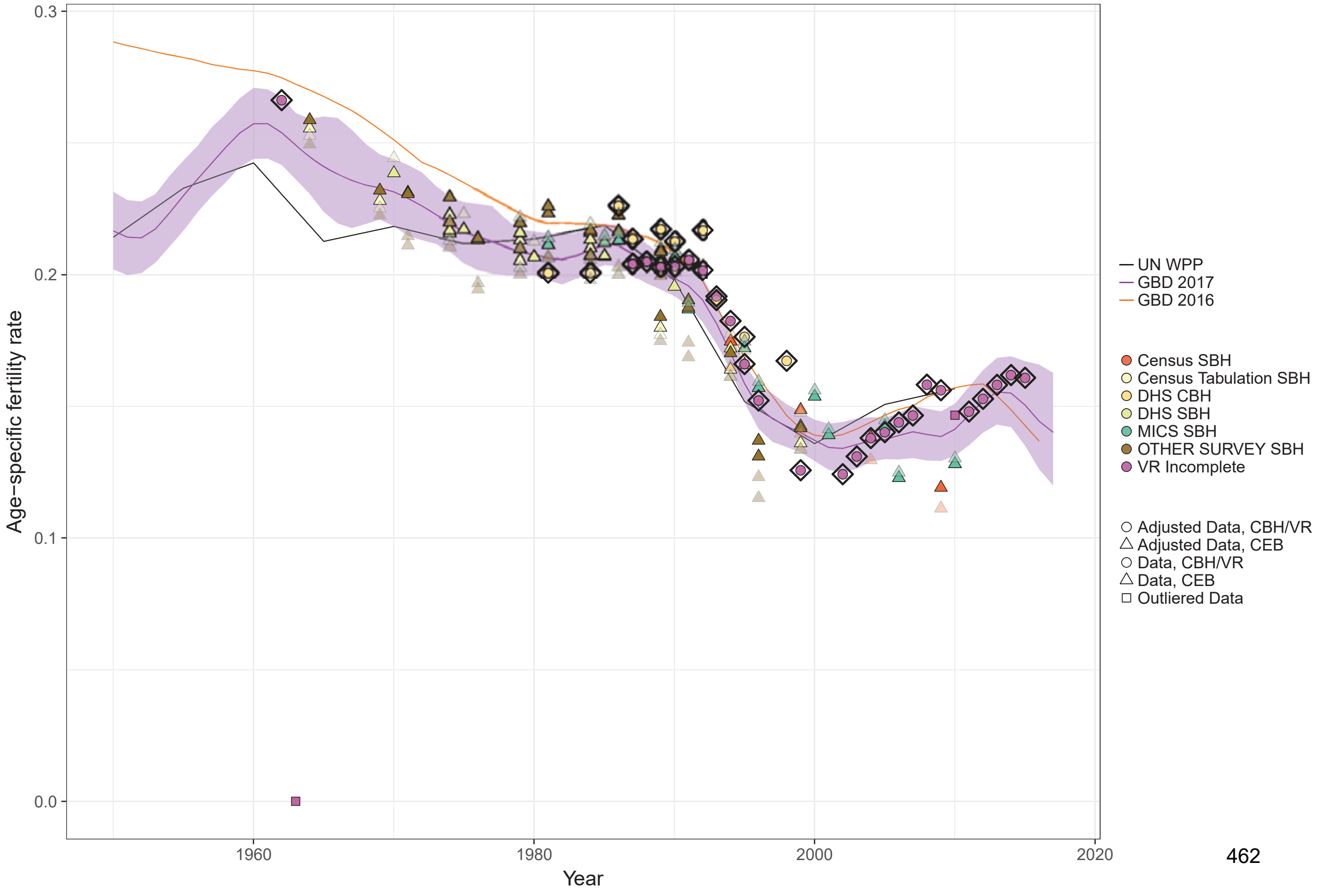

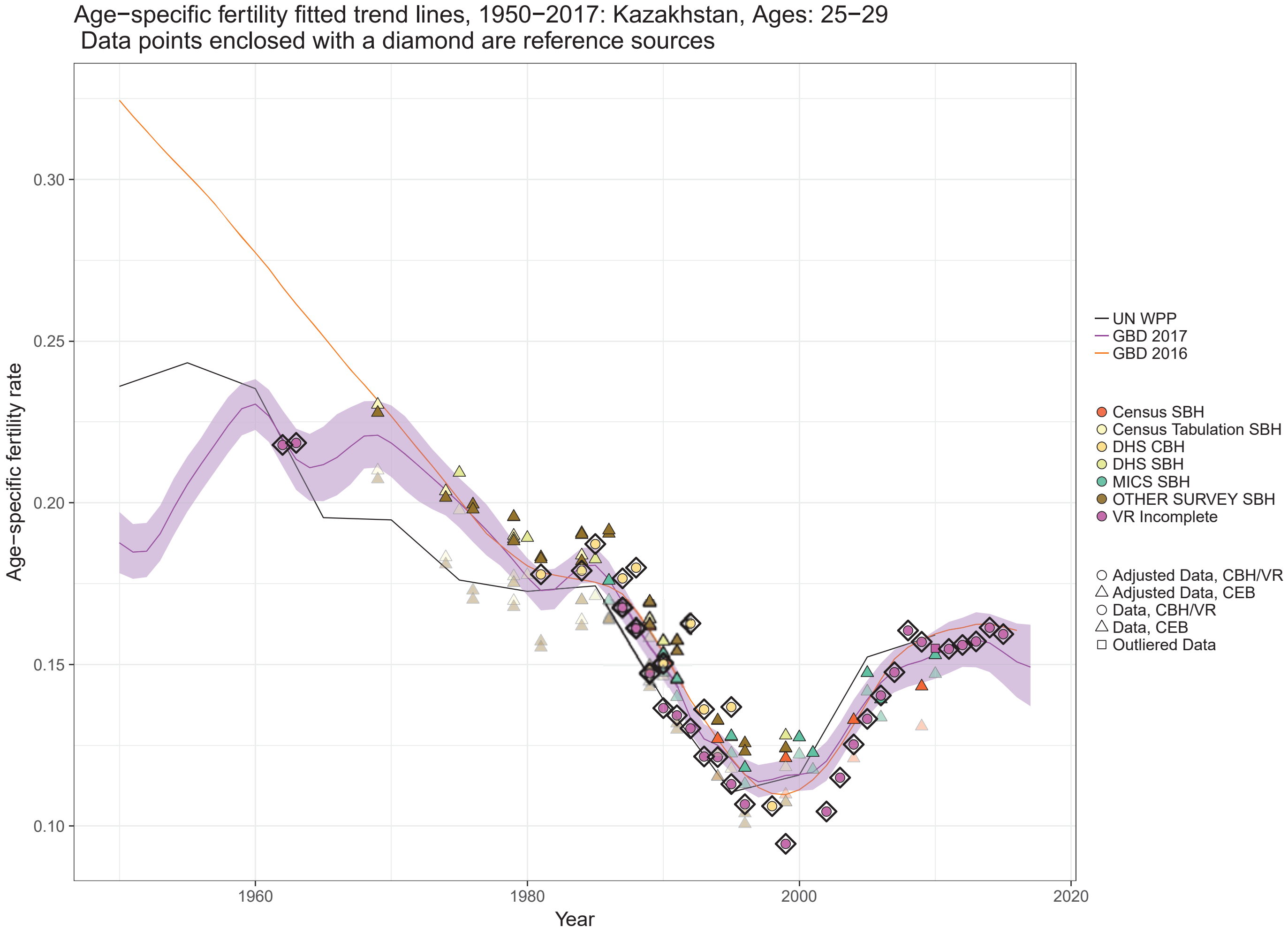

Age−specific fertility fitted trend lines, 1950−2017: Kazakhstan, Ages: 25−29
Data points enclosed with a diamond are reference sources
Age-specific fertility rate
0.30
0.25
0.20
0.15
0.10
1960
1980
2000
2020
Year
UN WPP
GBD 2017
GBD 2016
Census SBH
Census Tabulation SBH
DHS CBH
DHS SBH
MICS SBH
OTHER SURVEY SBH
VR Incomplete
Adjusted Data, CBH/VR
Adjusted Data, CEB
Data, CBH/VR
Data, CEB
Outliered Data

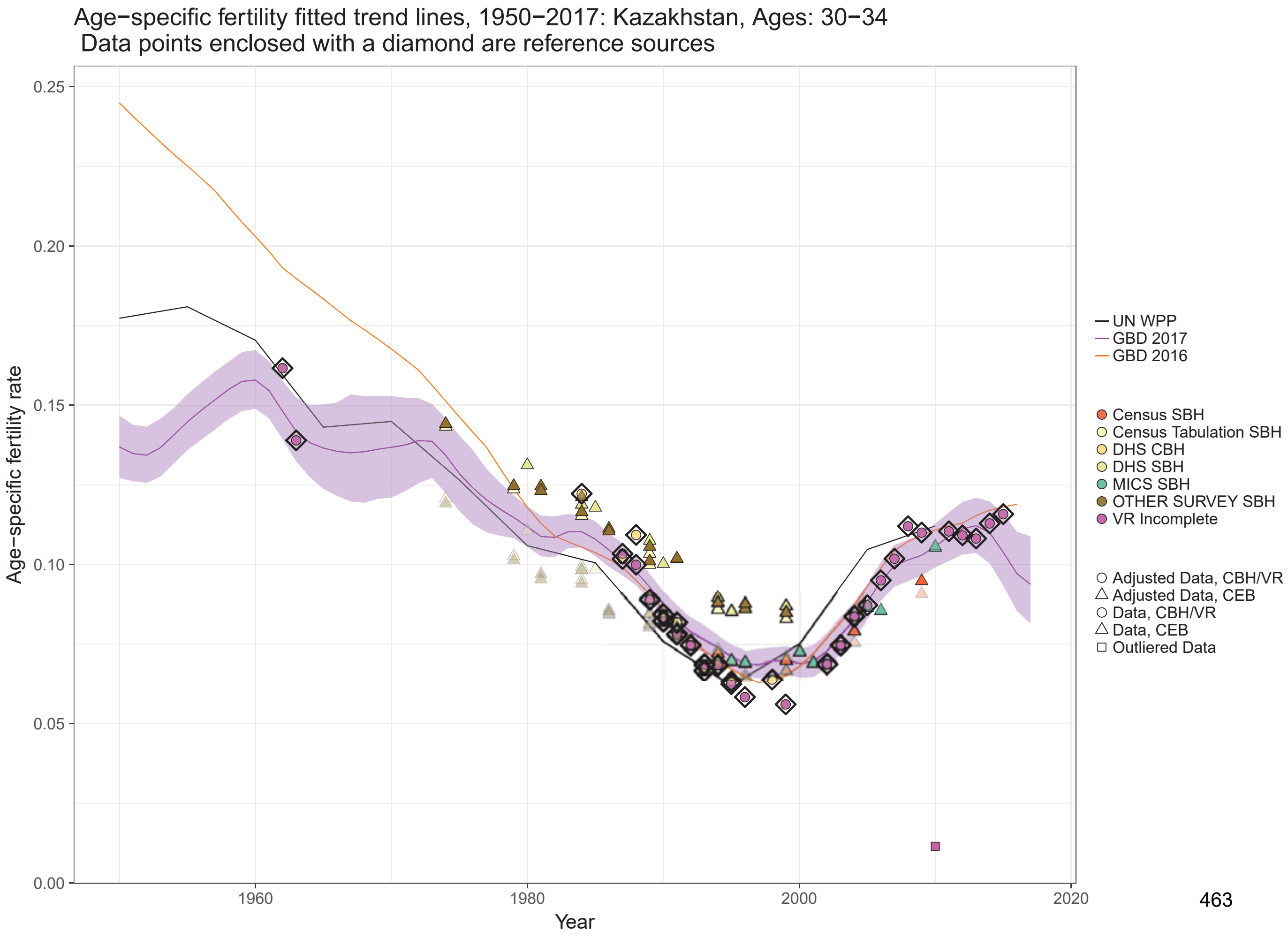

Age−specific fertility fitted trend lines, 1950−2017: Kazakhstan, Ages: 30−34
Data points enclosed with a diamond are reference sources
Age-specific fertility rate
0.25
0.20
0.15
0.10
0.05
0.00
1960
1980
2000
2020
Year
UN WPP
GBD 2017
GBD 2016
Census SBH
Census Tabulation SBH
DHS CBH
DHS SBH
MICS SBH
OTHER SURVEY SBH
VR Incomplete
Adjusted Data, CBH/VR
Adjusted Data, CEB
Data, CBH/VR
Data, CEB
Outliered Data

Age−specific fertility fitted trend lines, 1950−2017: Kazakhstan, Ages: 35−39
Data points enclosed with a diamond are reference sources

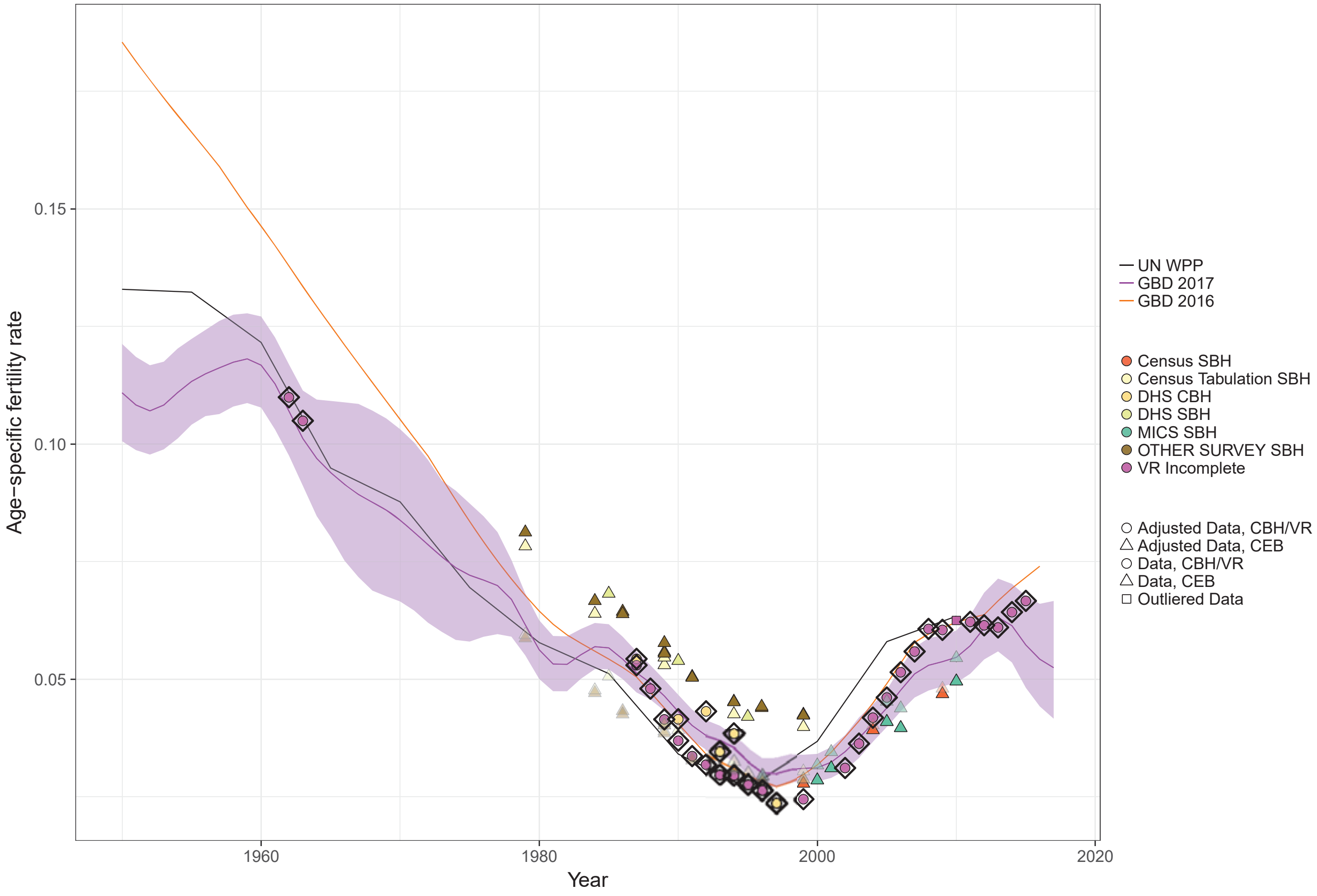

Age−specific fertility fitted trend lines, 1950−2017: Kazakhstan, Ages: 40−44
Data points enclosed with a diamond are reference sources

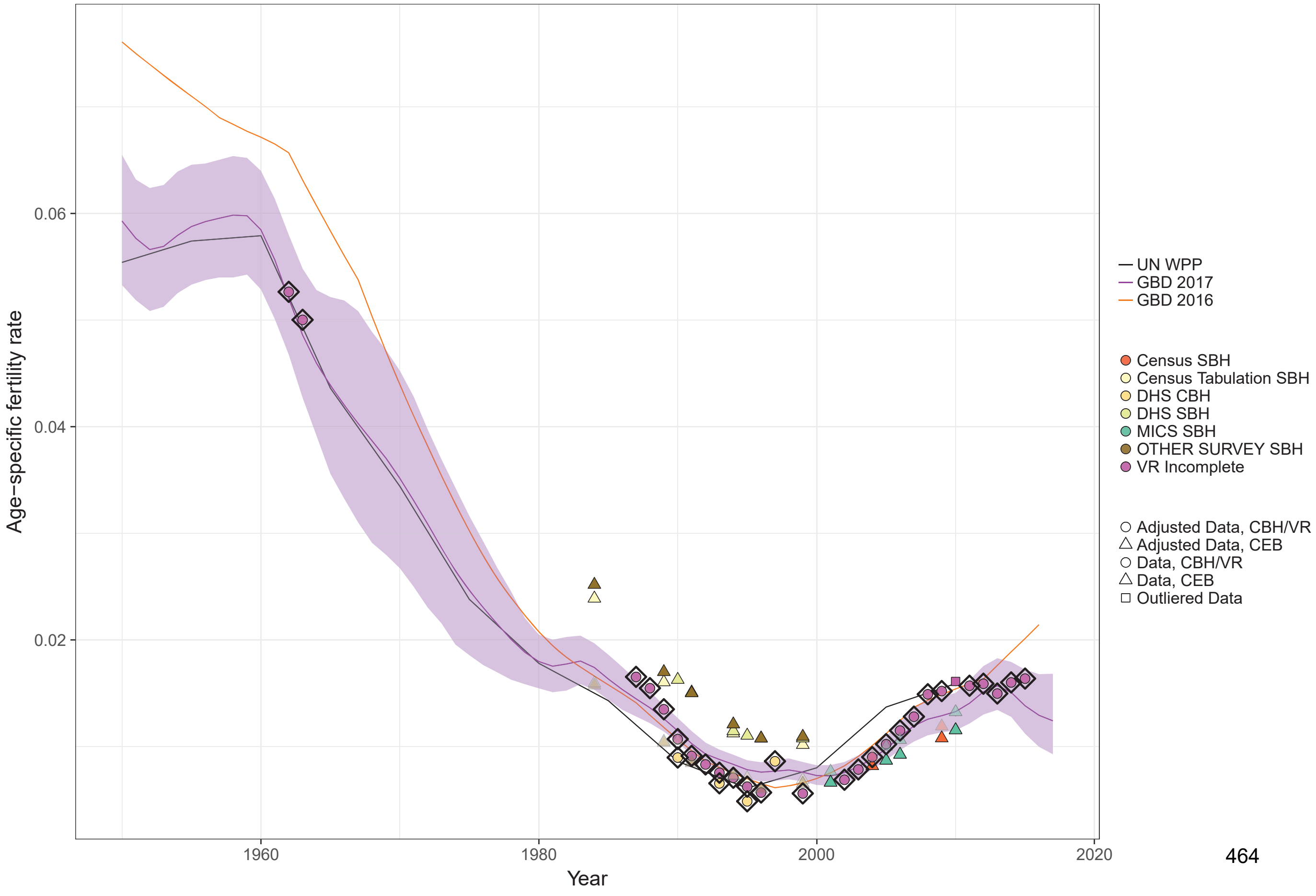

Age−specific fertility fitted trend lines, 1950−2017: Kazakhstan, Ages: 45−49
Data points enclosed with a diamond are reference sources

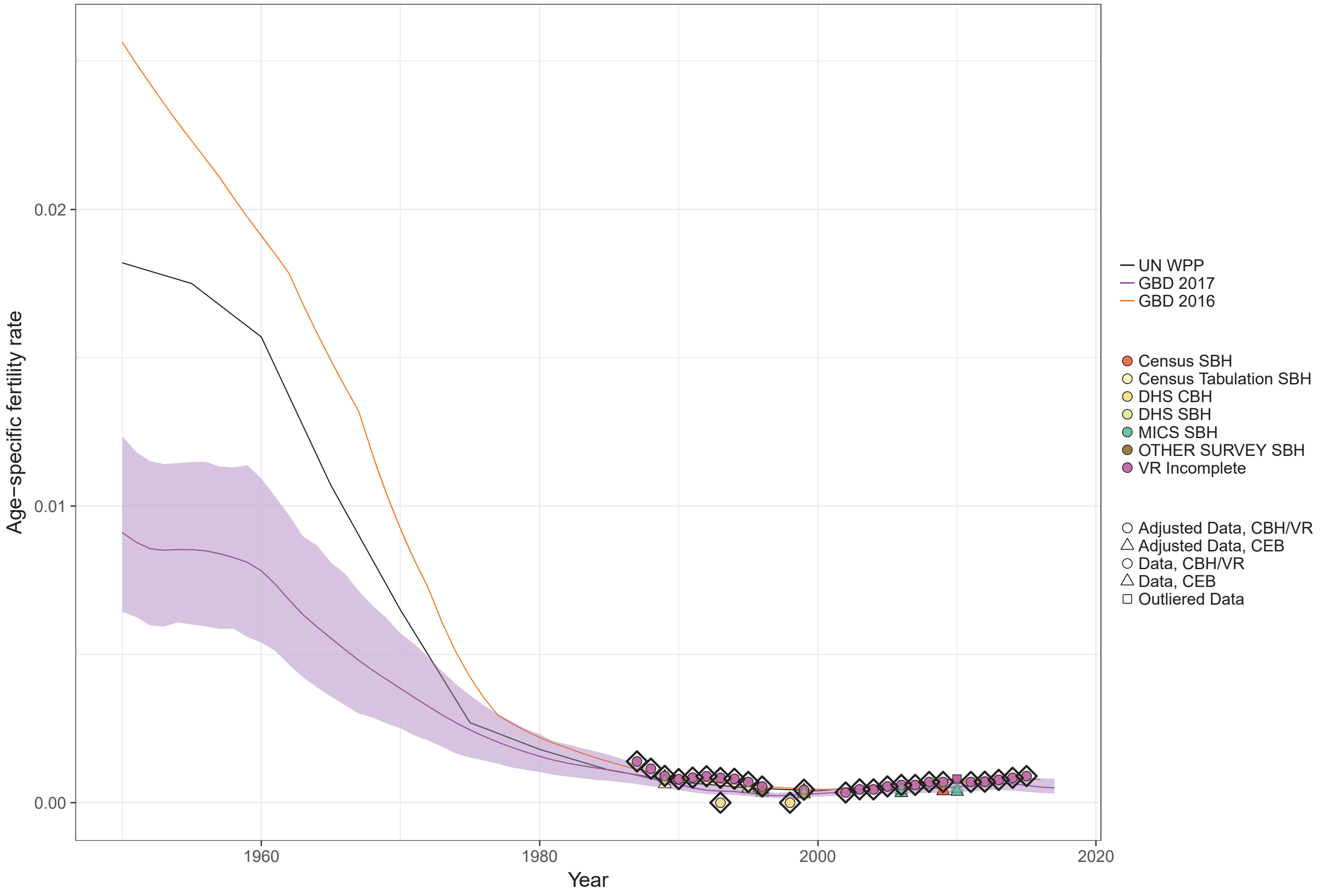

Age−specific fertility fitted trend lines, 1950−2017: Kazakhstan, Ages: 50−54
Data points enclosed with a diamond are reference sources

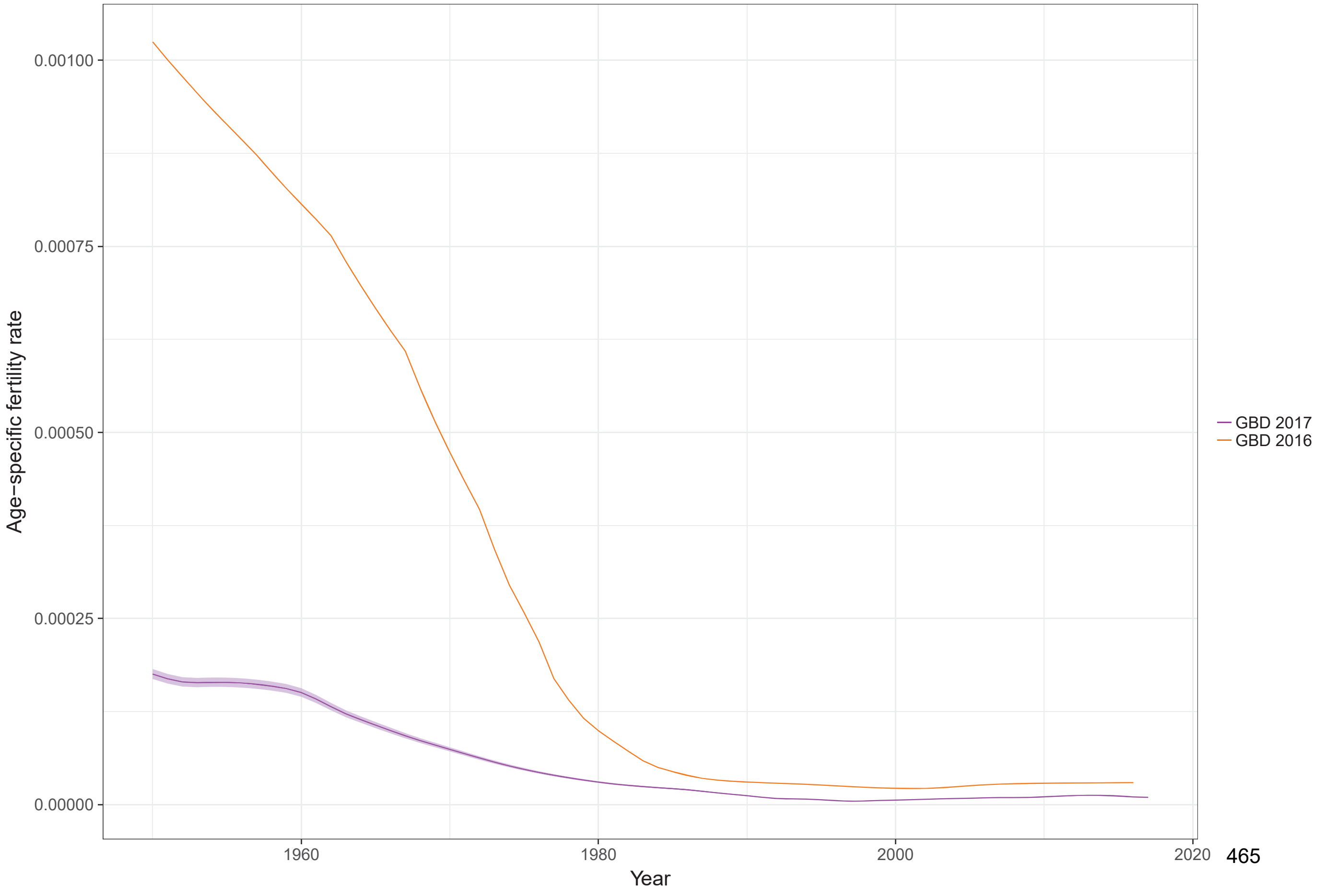

Age−specific fertility fitted trend lines, 1950−2017: Kenya, TFR
Data points enclosed with a diamond are reference sources

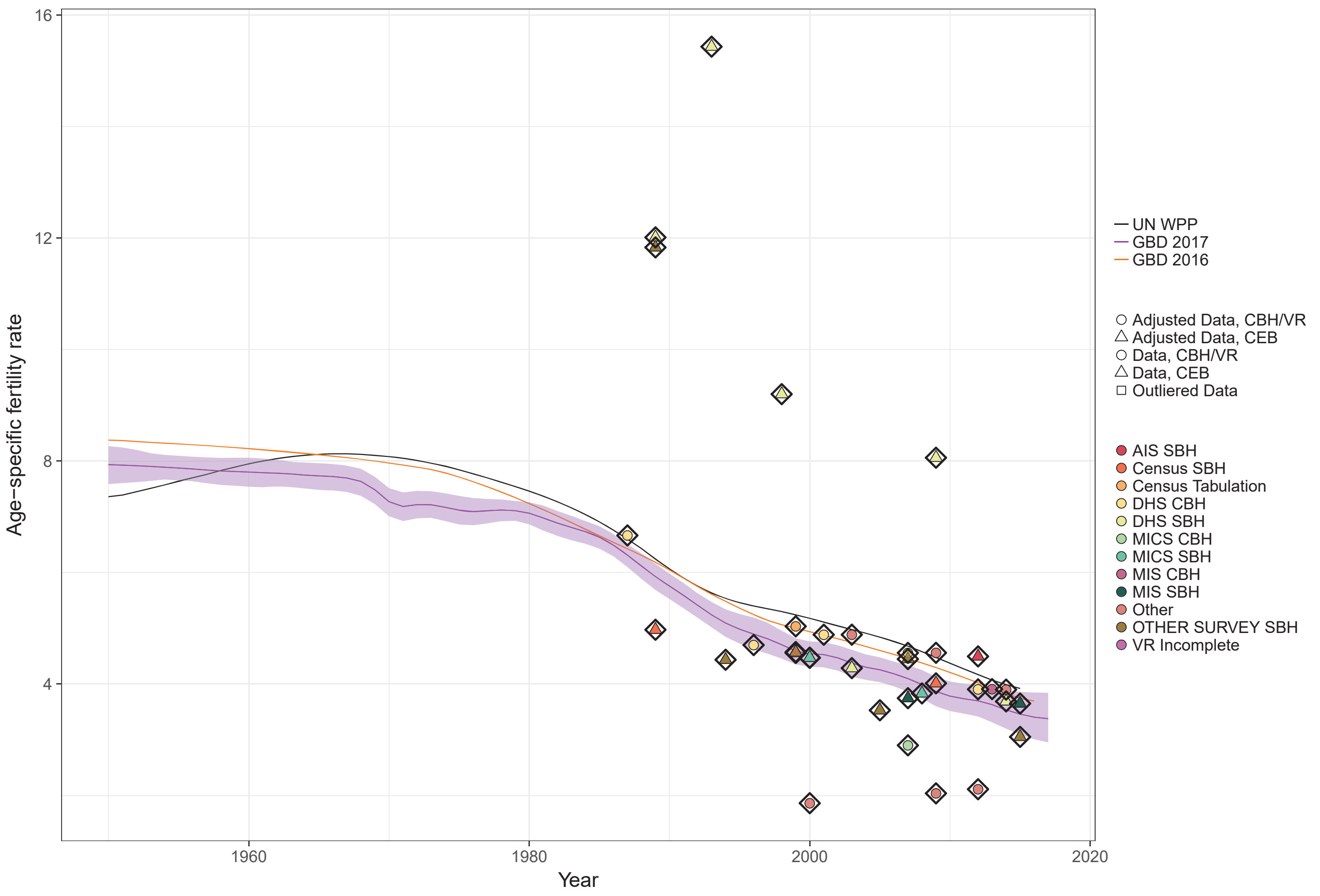

Age−specific fertility fitted trend lines, 1950−2017: Kenya, Ages: 10−14
Data points enclosed with a diamond are reference sources

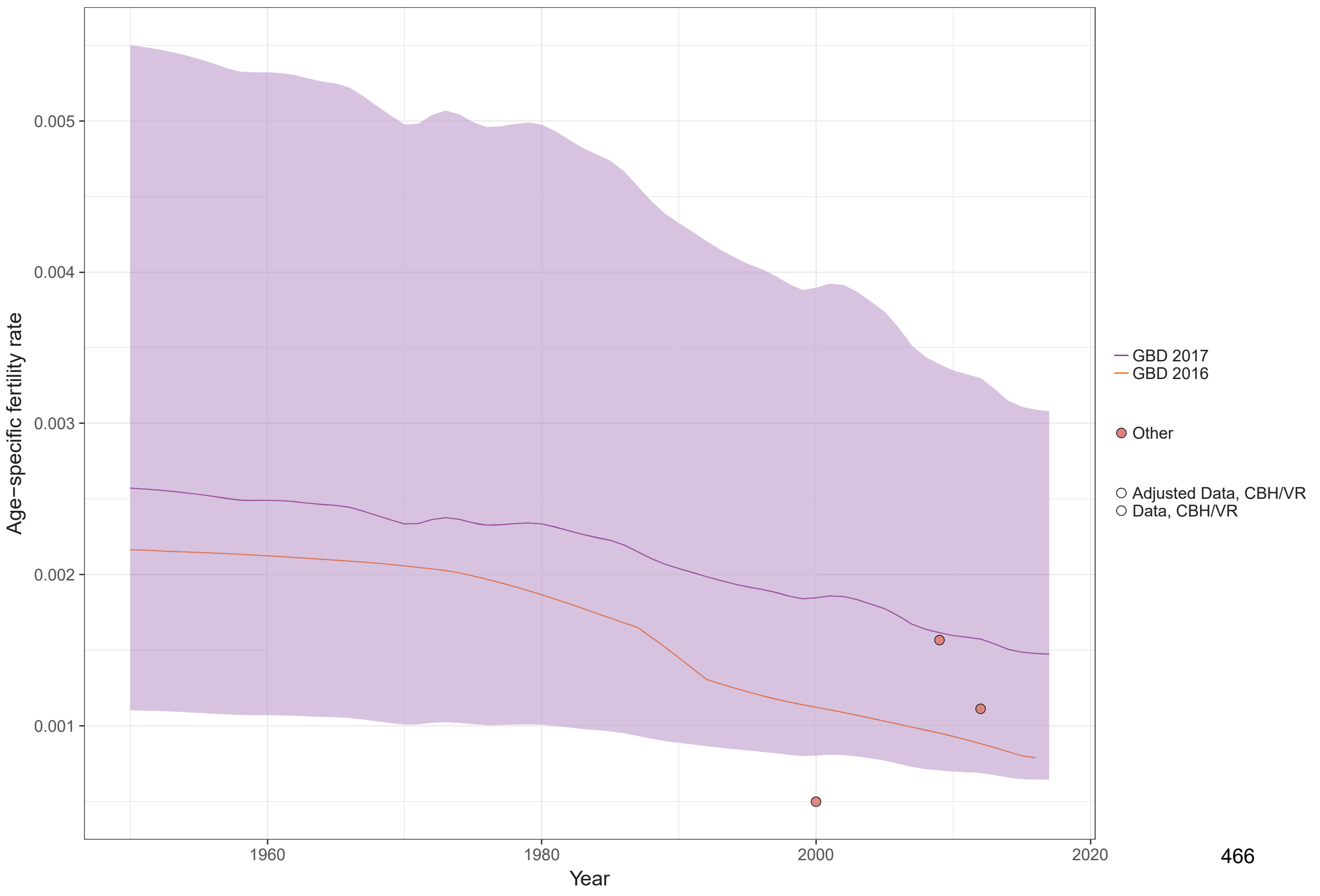

Age−specific fertility fitted trend lines, 1950−2017: Kenya, Ages: 15−19
Data points enclosed with a diamond are reference sources

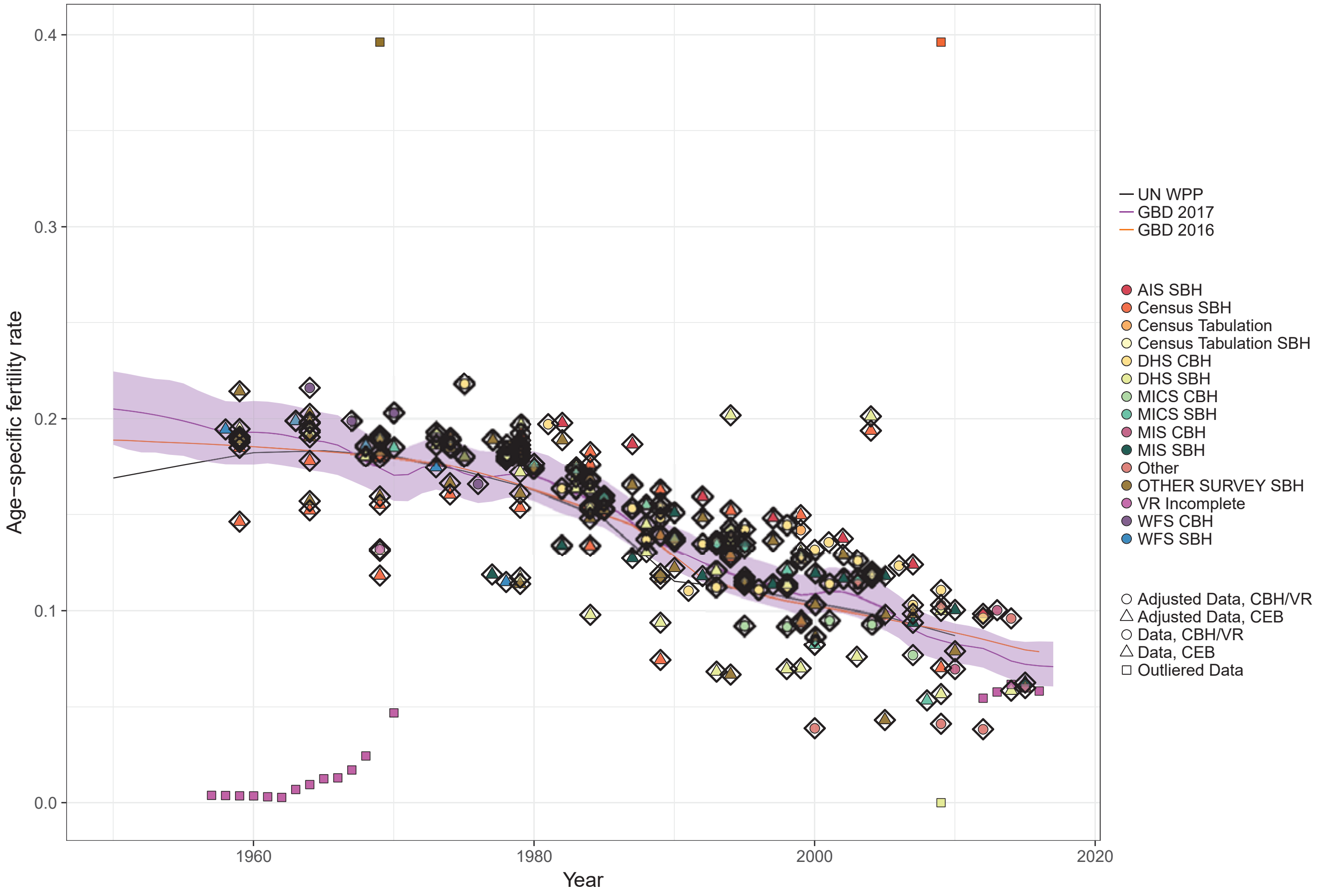

Age−specific fertility fitted trend lines, 1950−2017: Kenya, Ages: 20−24
Data points enclosed with a diamond are reference sources

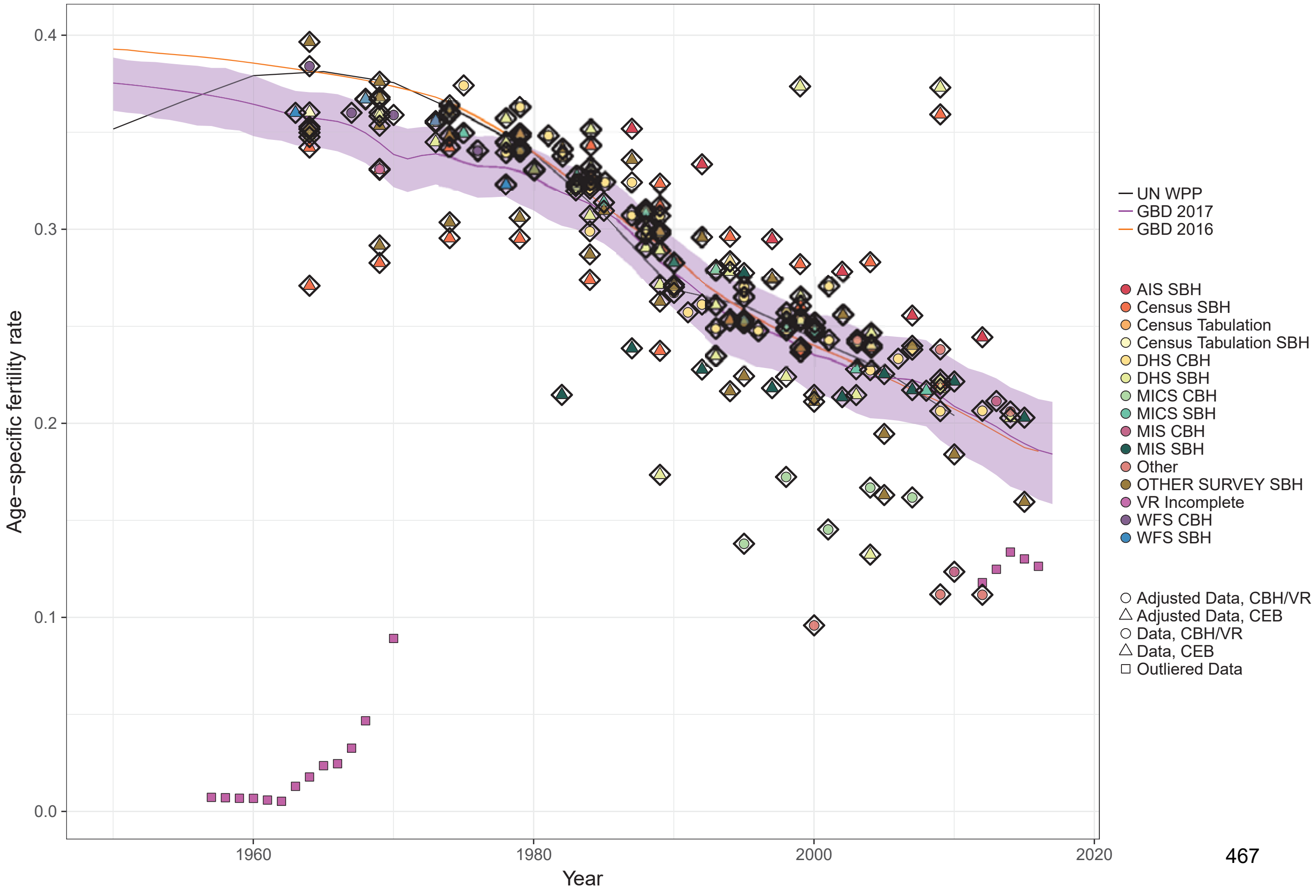

Age−specific fertility fitted trend lines, 1950−2017: Kenya, Ages: 25−29
Data points enclosed with a diamond are reference sources

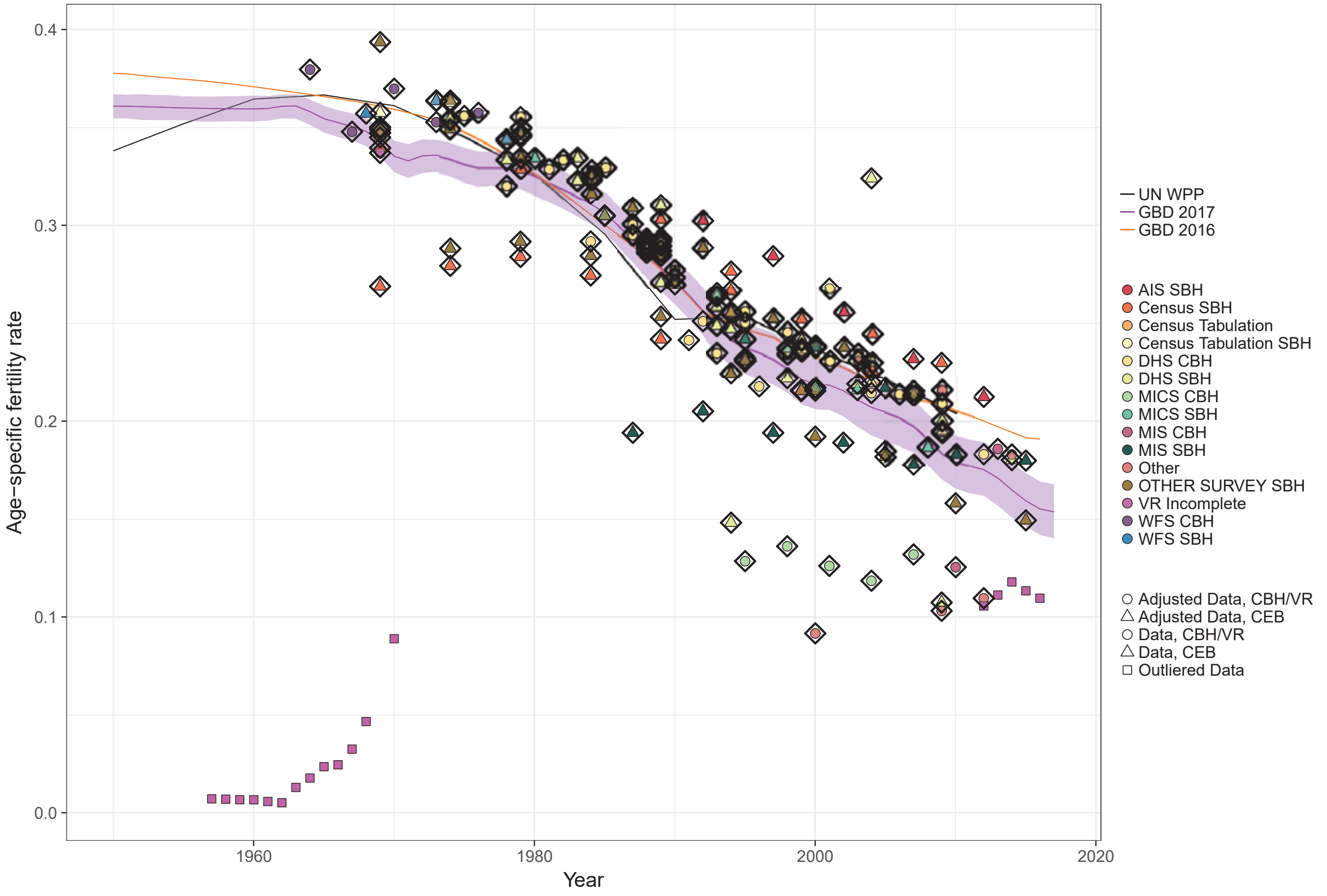

Age−specific fertility fitted trend lines, 1950−2017: Kenya, Ages: 30−34
Data points enclosed with a diamond are reference sources

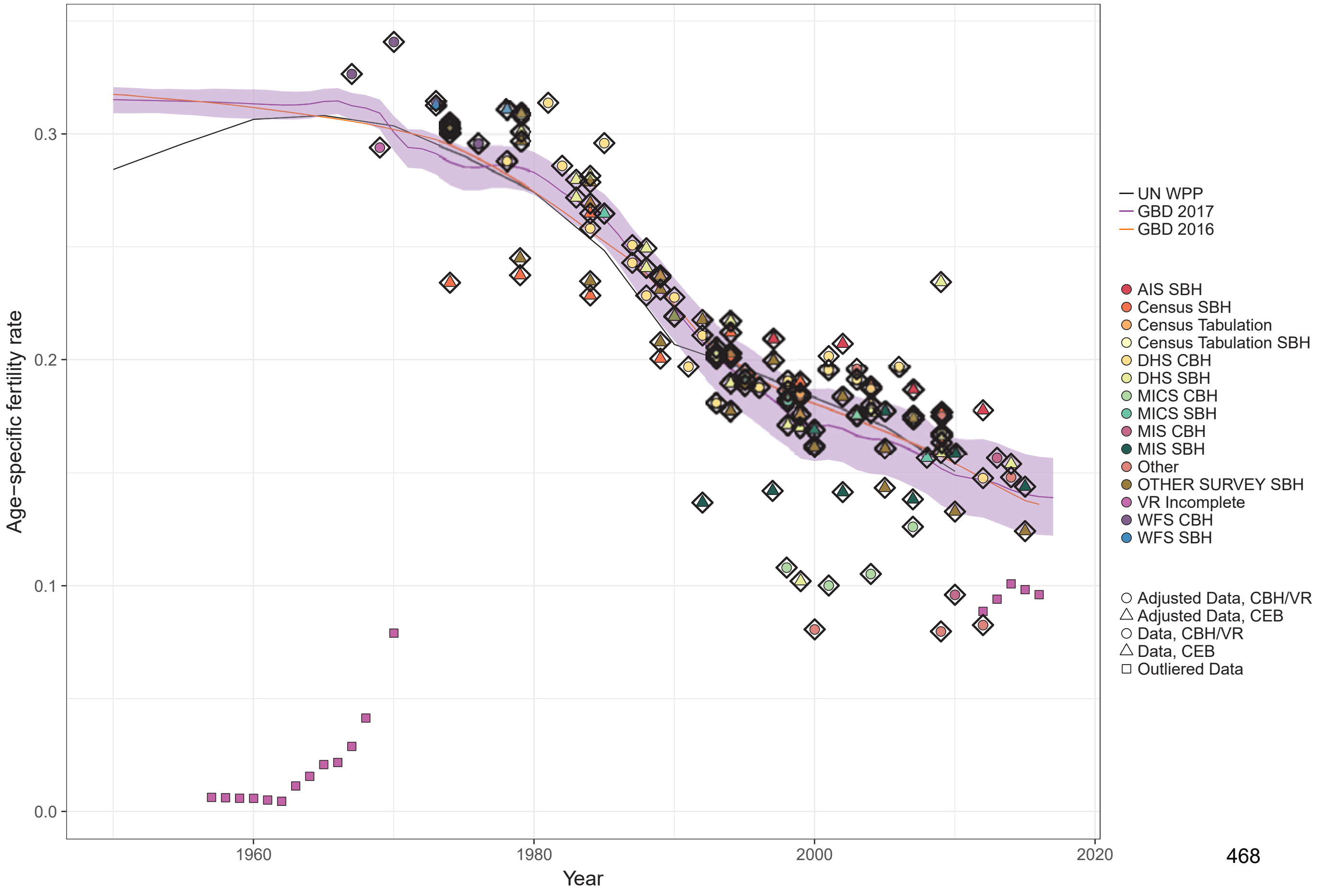

Age−specific fertility fitted trend lines, 1950−2017: Kenya, Ages: 35−39
Data points enclosed with a diamond are reference sources

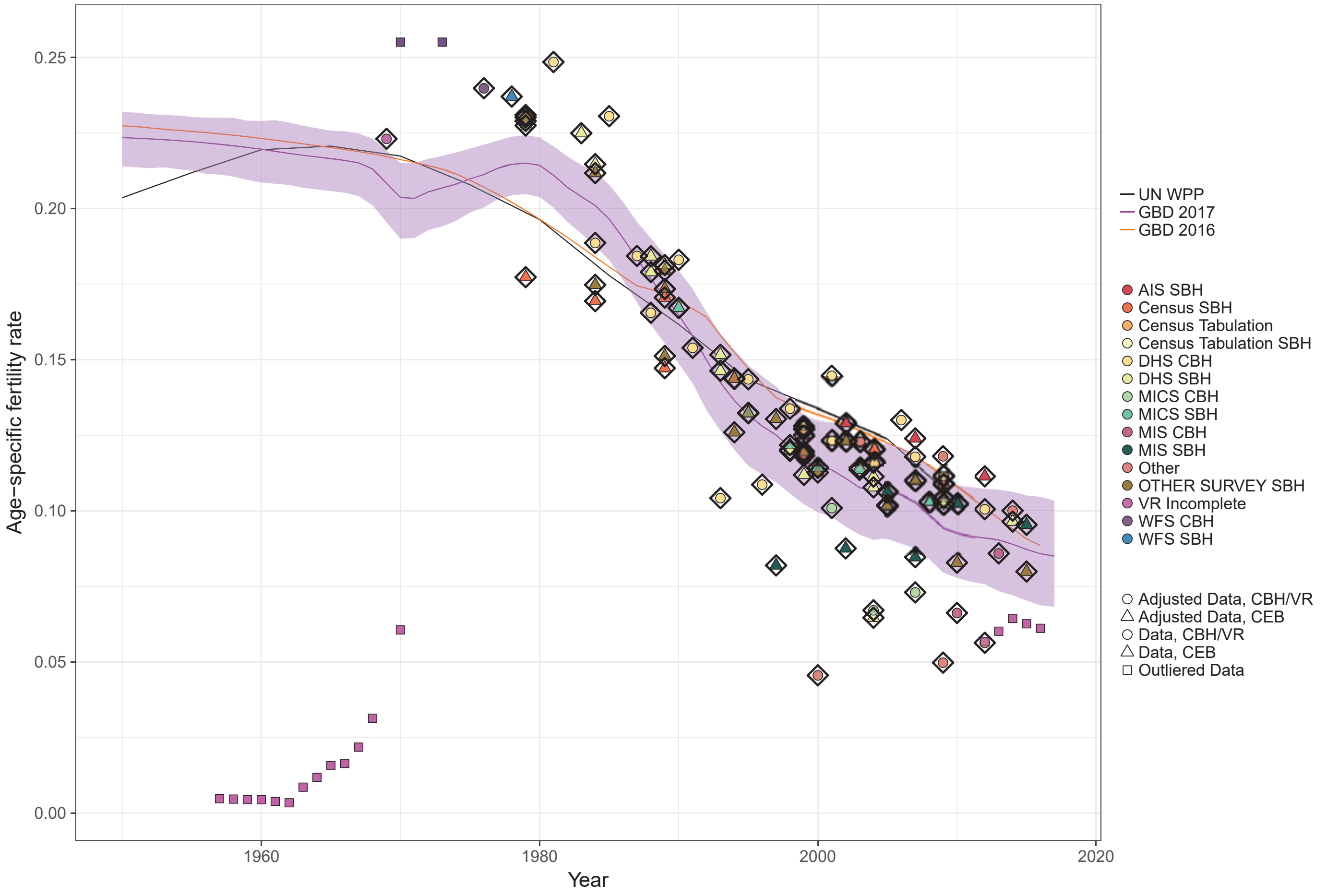

Age−specific fertility fitted trend lines, 1950−2017: Kenya, Ages: 40−44
Data points enclosed with a diamond are reference sources

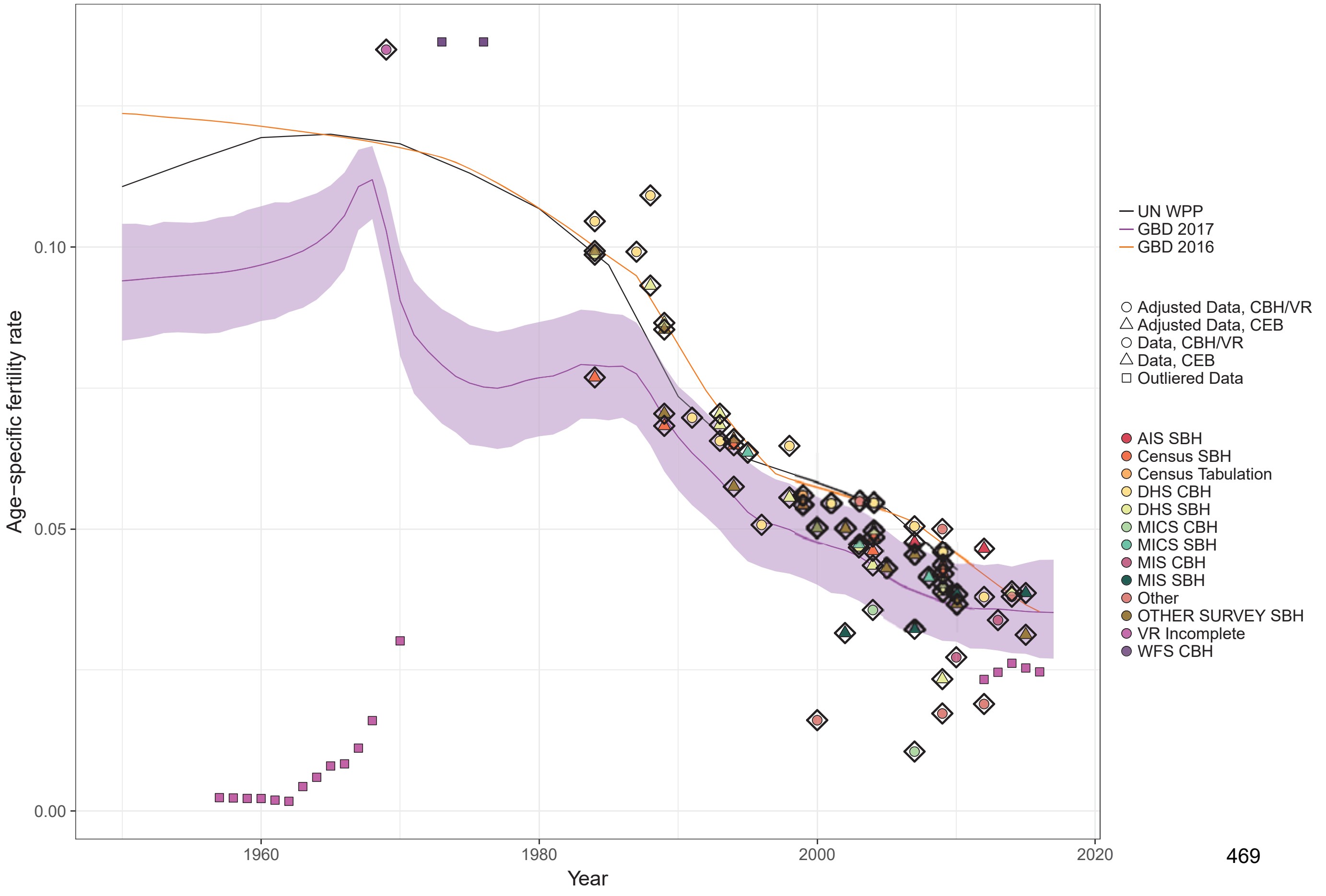

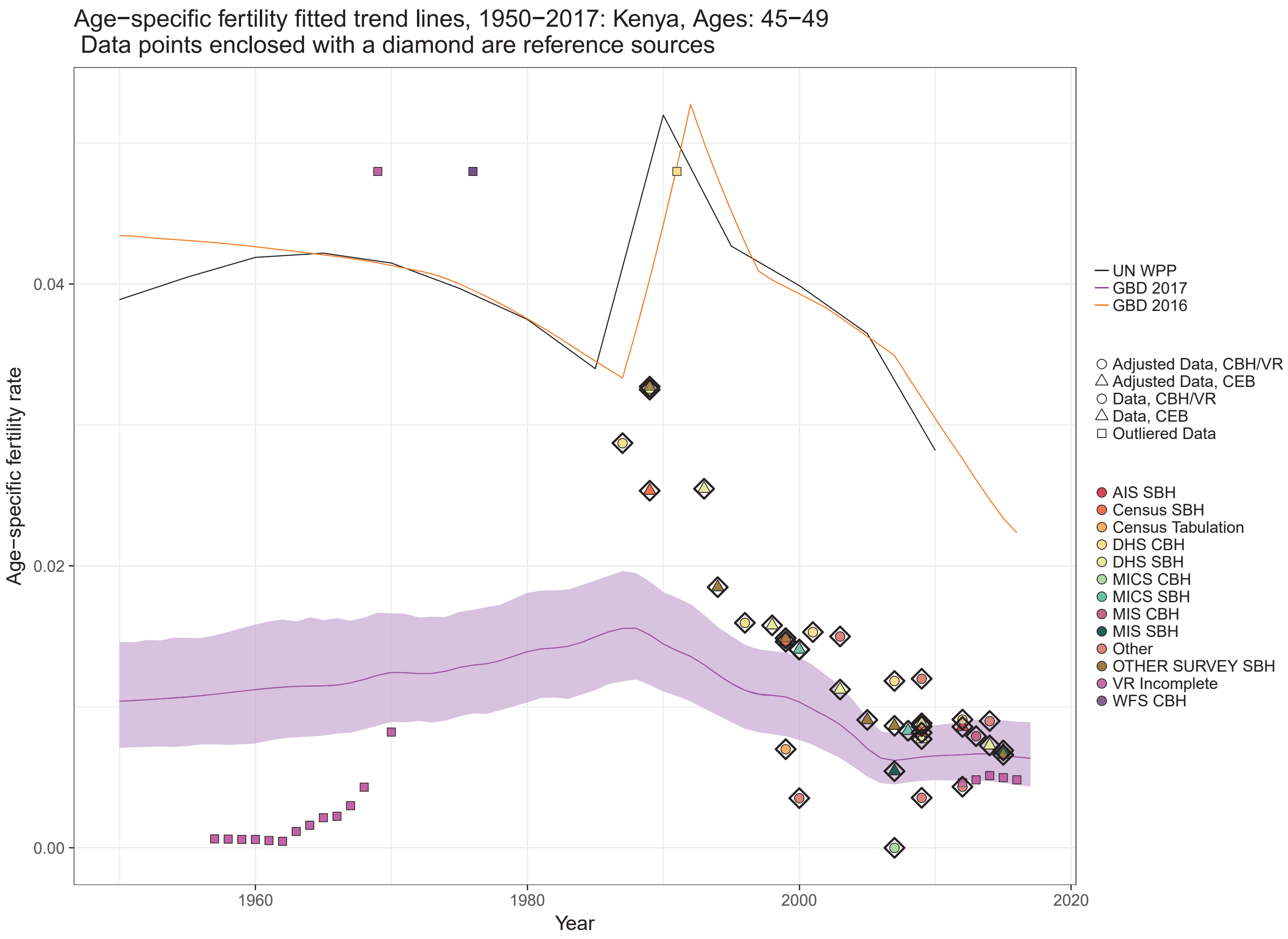

Age−specific fertility fitted trend lines, 1950−2017: Kenya, Ages: 45−49
Data points enclosed with a diamond are reference sources
Age−specific fertility rate
0.04
0.02
0.00
1960
1980
2000
2020
Year
UN WPP
GBD 2017
GBD 2016
Adjusted Data, CBH/VR
Adjusted Data, CEB
Data, CBH/VR
Data, CEB
Outliered Data
AIS SBH
Census SBH
Census Tabulation
DHS CBH
DHS SBH
MICS CBH
MICS SBH
MIS CBH
MIS SBH
Other
OTHER SURVEY SBH
VR Incomplete
WFS CBH

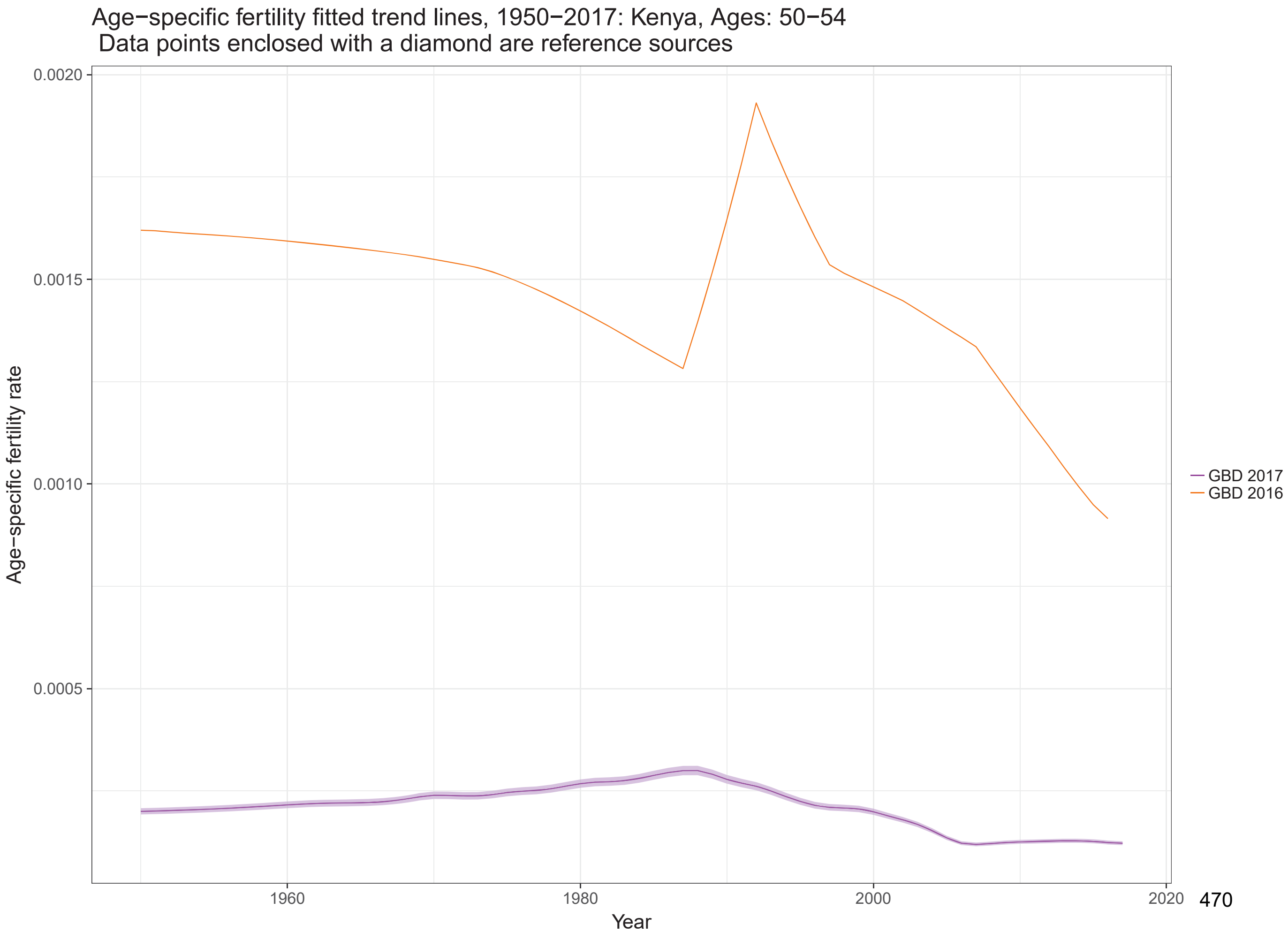

Age−specific fertility fitted trend lines, 1950−2017: Kenya, Ages: 50−54
Data points enclosed with a diamond are reference sources
Age−specific fertility rate
0.0020
0.0015
0.0010
0.0005
1960
1980
2000
2020
Year
GBD 2017
GBD 2016

Age−specific fertility fitted trend lines, 1950−2017: Kiribati, TFR
Data points enclosed with a diamond are reference sources

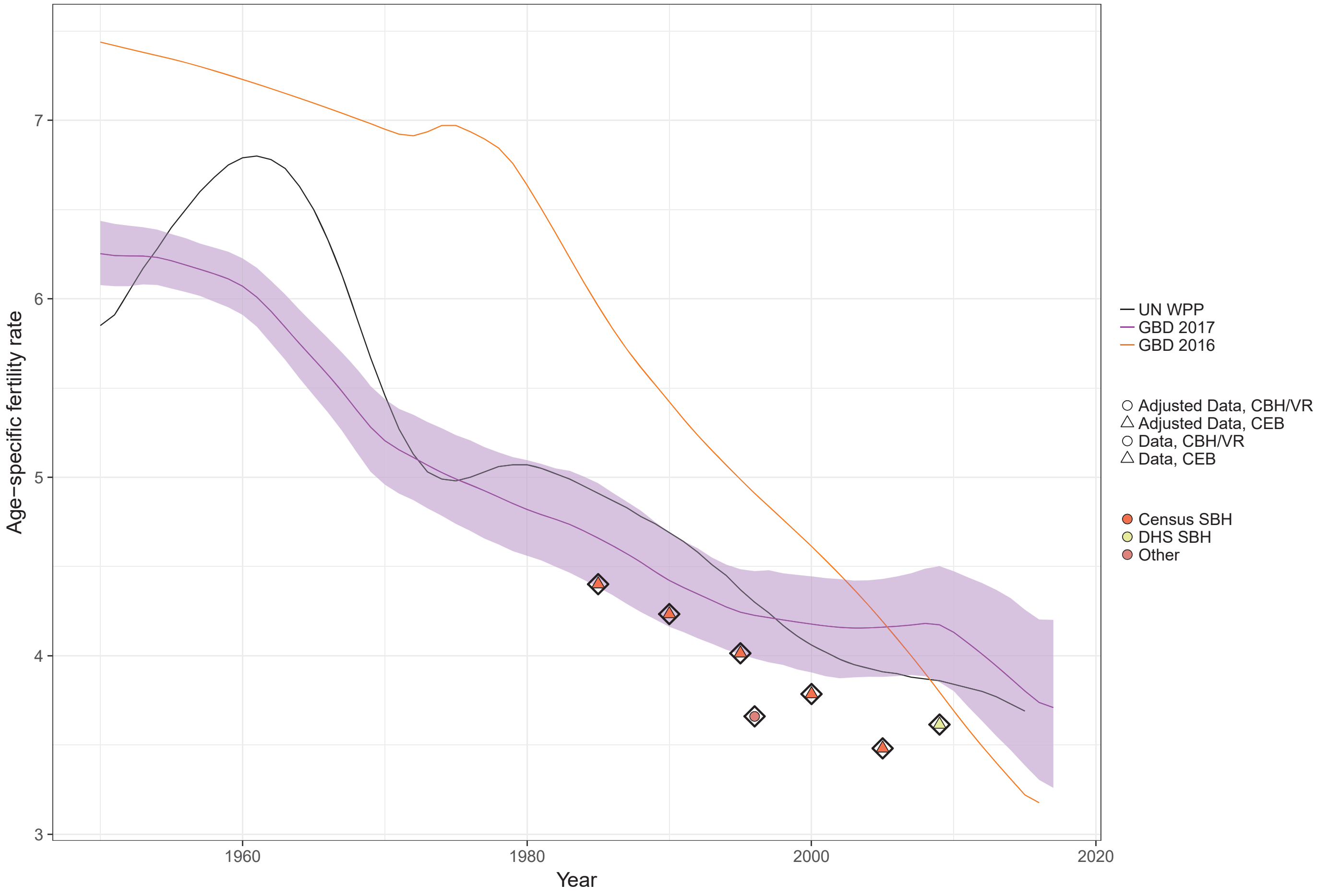

Age−specific fertility fitted trend lines, 1950−2017: Kiribati, Ages: 10−14
Data points enclosed with a diamond are reference sources

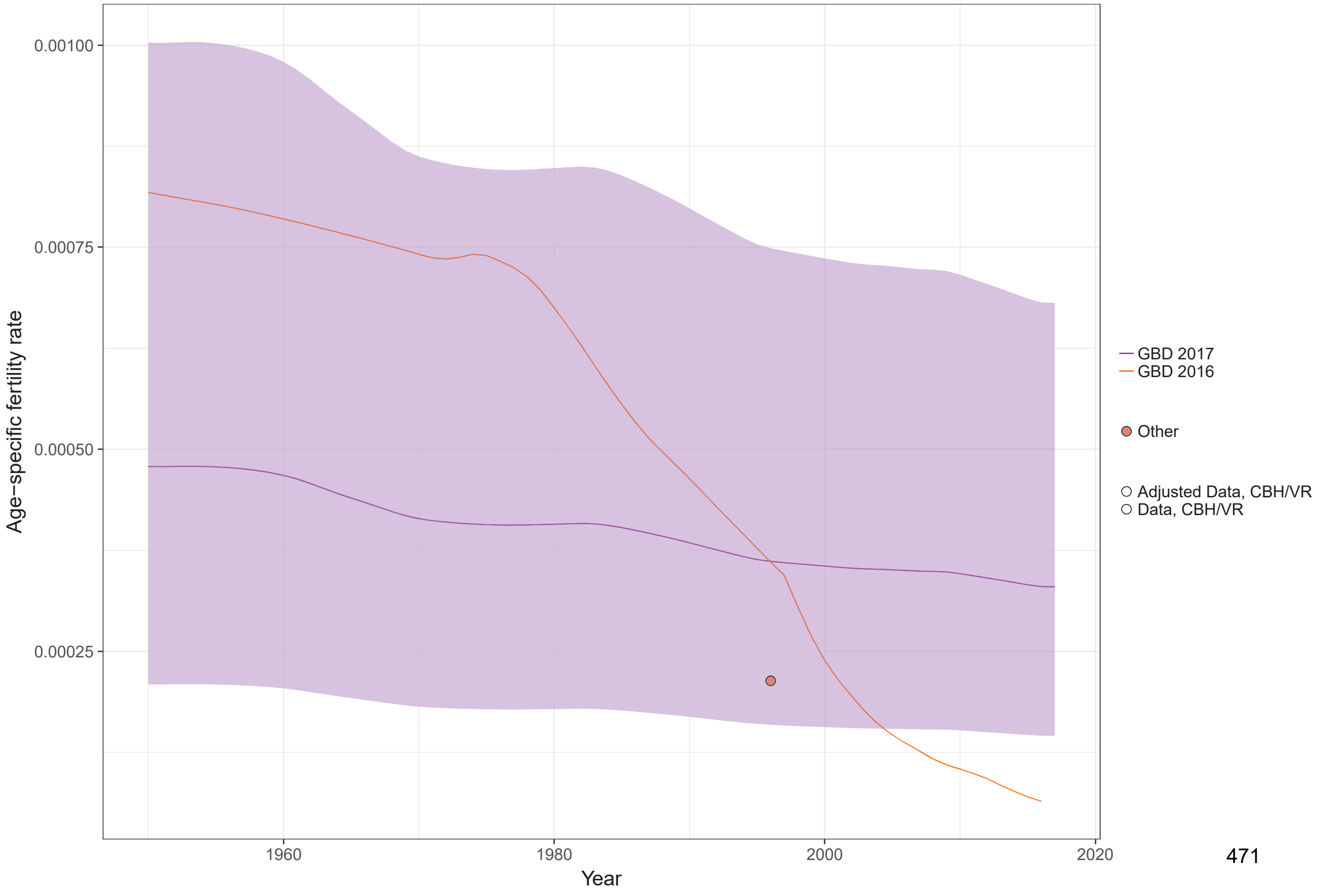

Age−specific fertility fitted trend lines, 1950−2017: Kiribati, Ages: 15−19
Data points enclosed with a diamond are reference sources

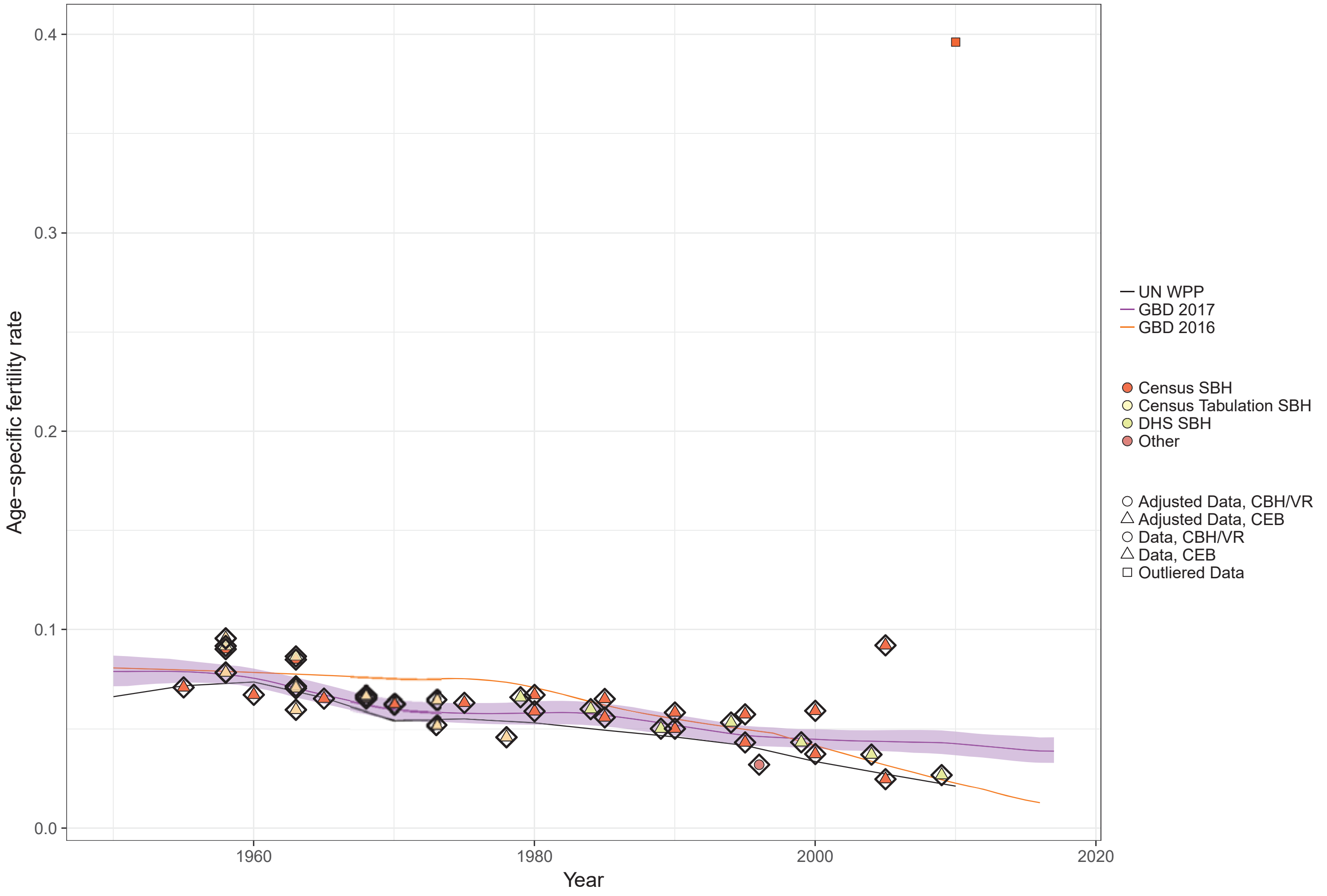

Age−specific fertility fitted trend lines, 1950−2017: Kiribati, Ages: 20−24
Data points enclosed with a diamond are reference sources

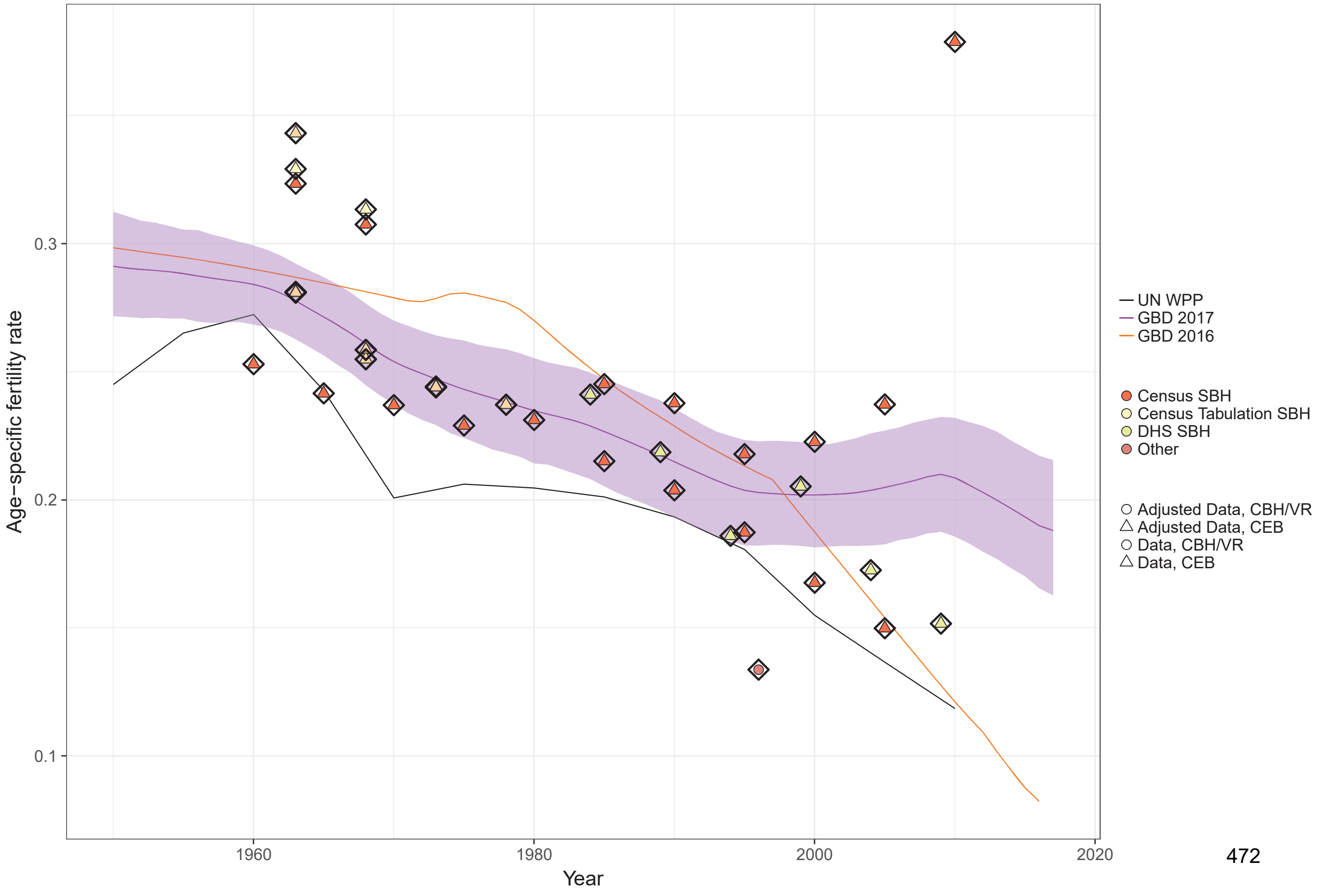

Age−specific fertility fitted trend lines, 1950−2017: Kiribati, Ages: 25−29
Data points enclosed with a diamond are reference sources

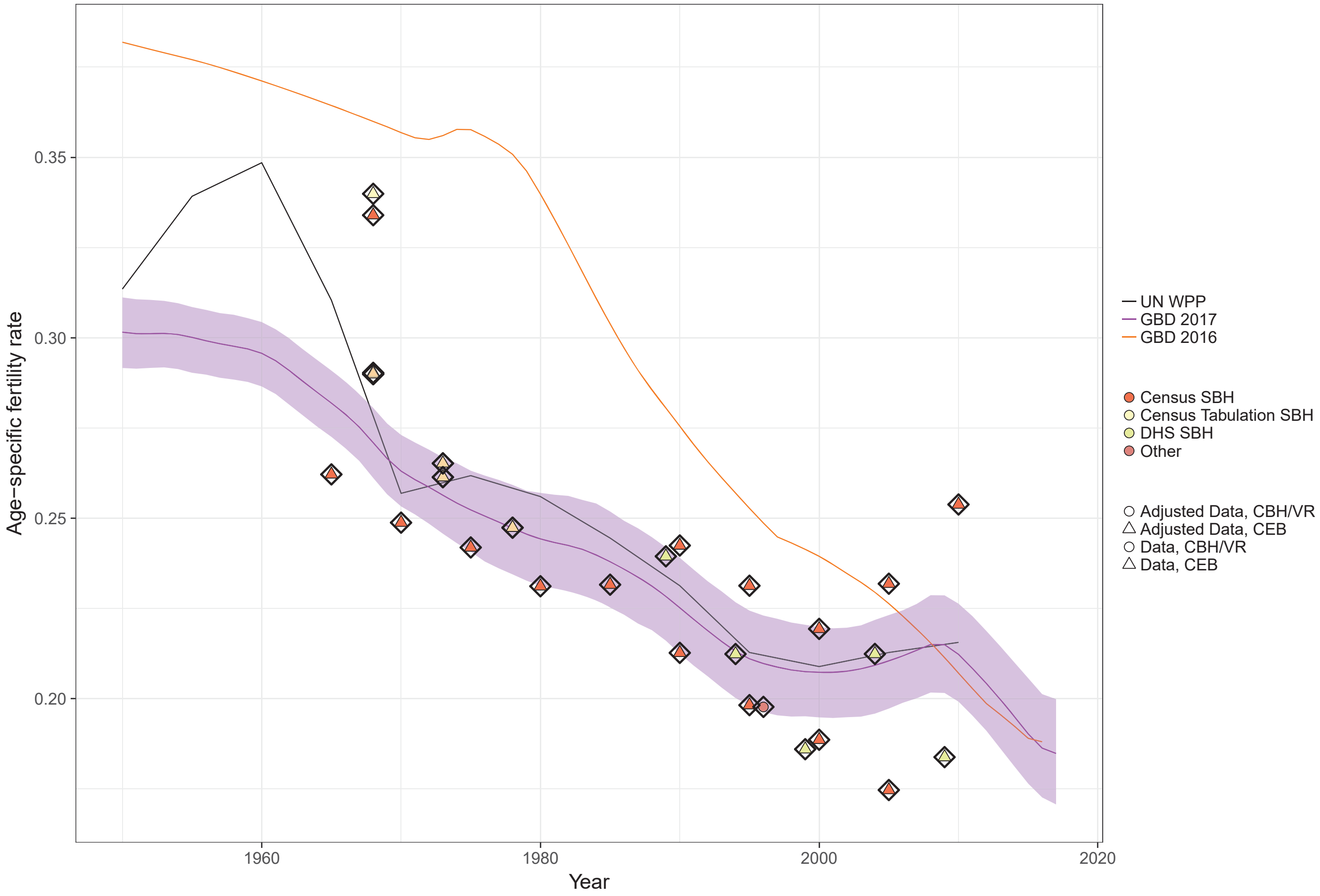

Age−specific fertility fitted trend lines, 1950−2017: Kiribati, Ages: 30−34
Data points enclosed with a diamond are reference sources

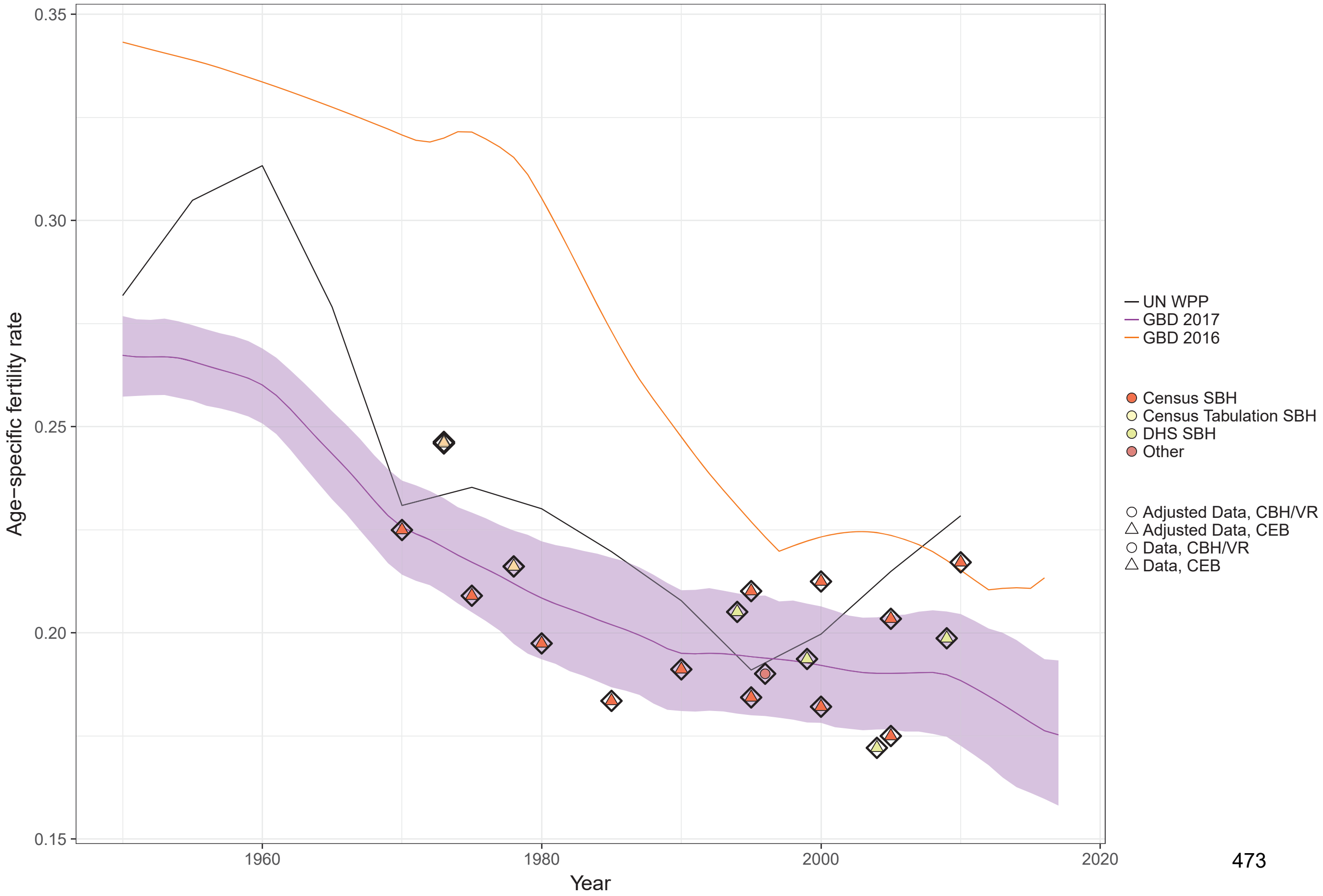

Age−specific fertility fitted trend lines, 1950−2017: Kiribati, Ages: 35−39
Data points enclosed with a diamond are reference sources

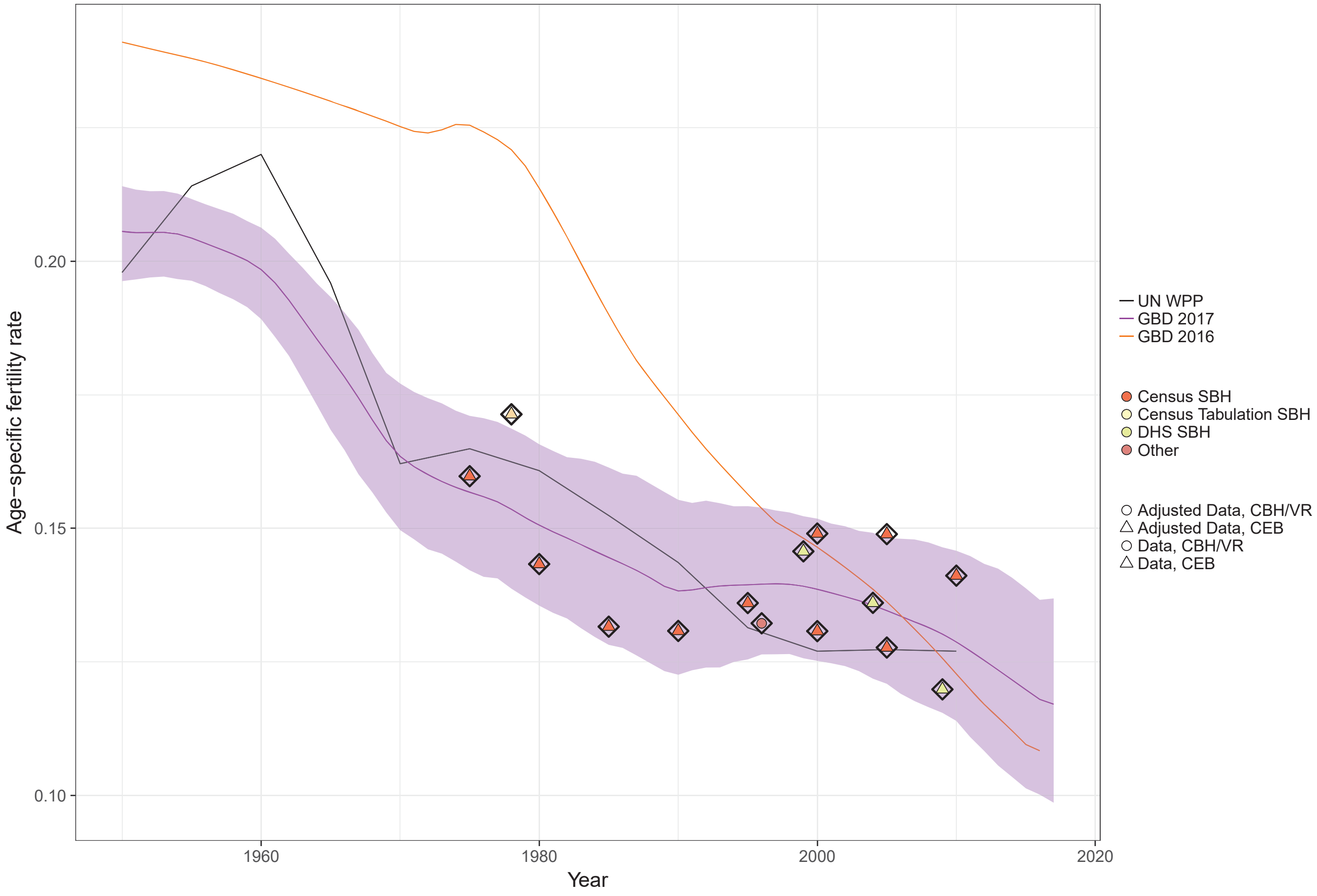

Age−specific fertility fitted trend lines, 1950−2017: Kiribati, Ages: 40−44
Data points enclosed with a diamond are reference sources

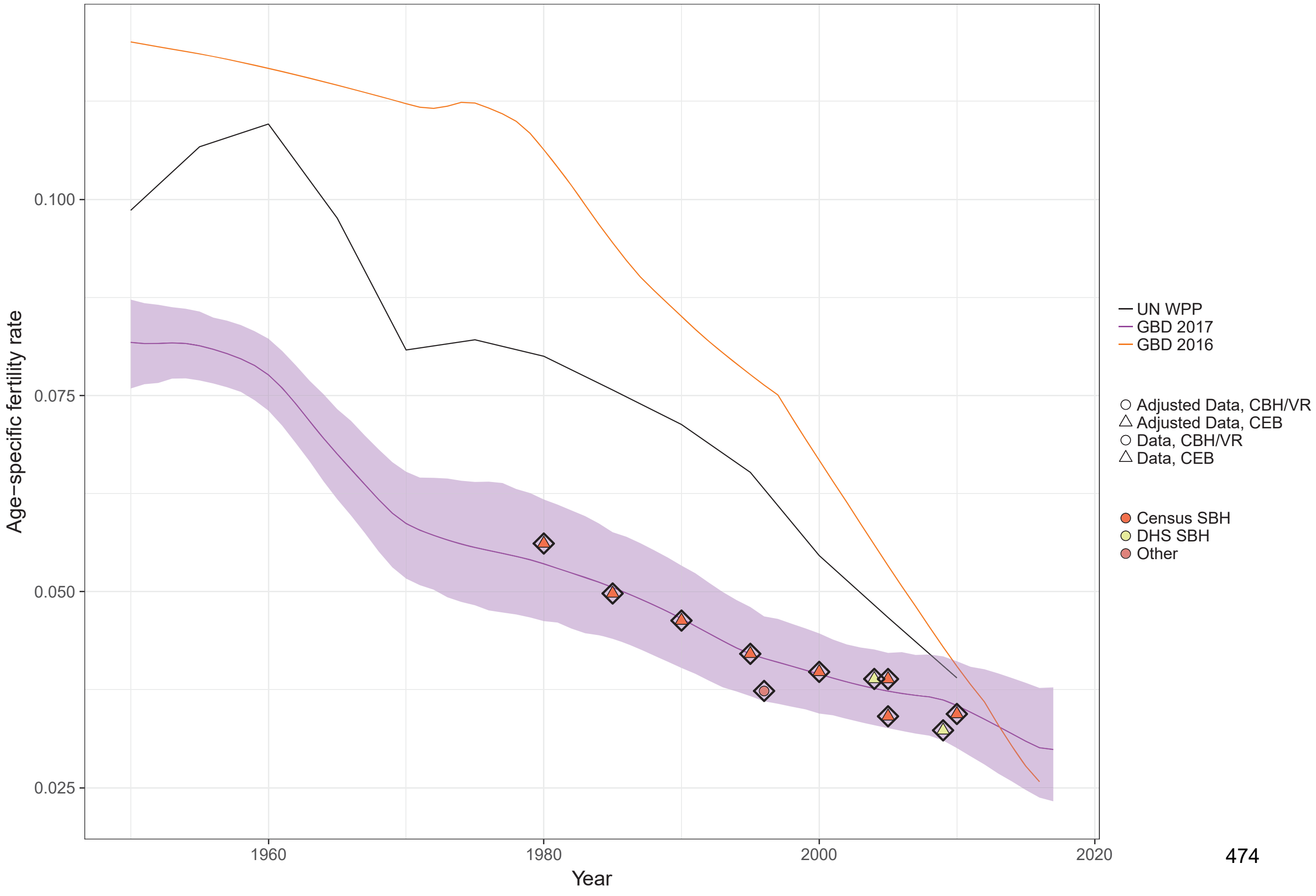

Age−specific fertility fitted trend lines, 1950−2017: Kiribati, Ages: 45−49
Data points enclosed with a diamond are reference sources

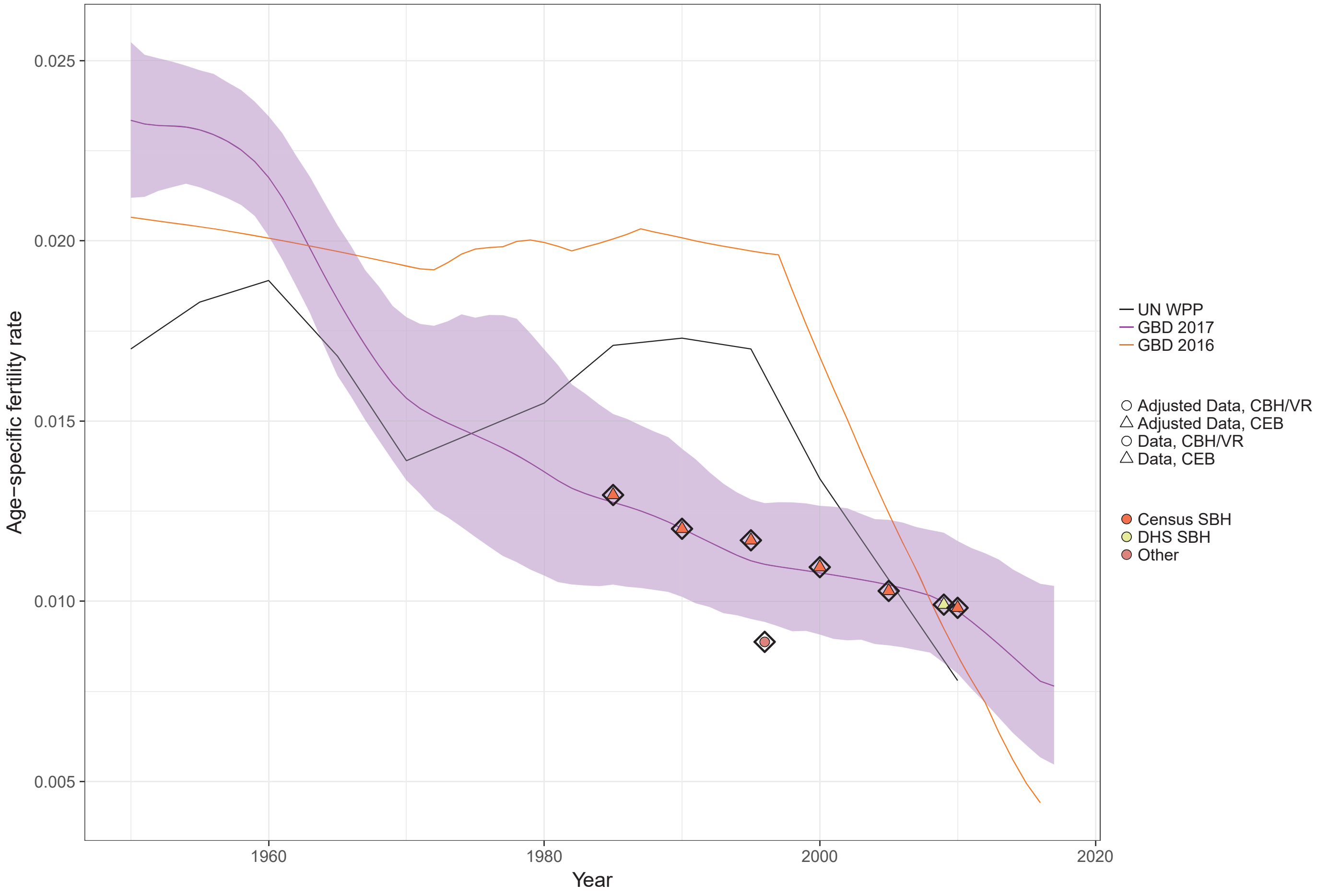

Age−specific fertility fitted trend lines, 1950−2017: Kiribati, Ages: 50−54
Data points enclosed with a diamond are reference sources

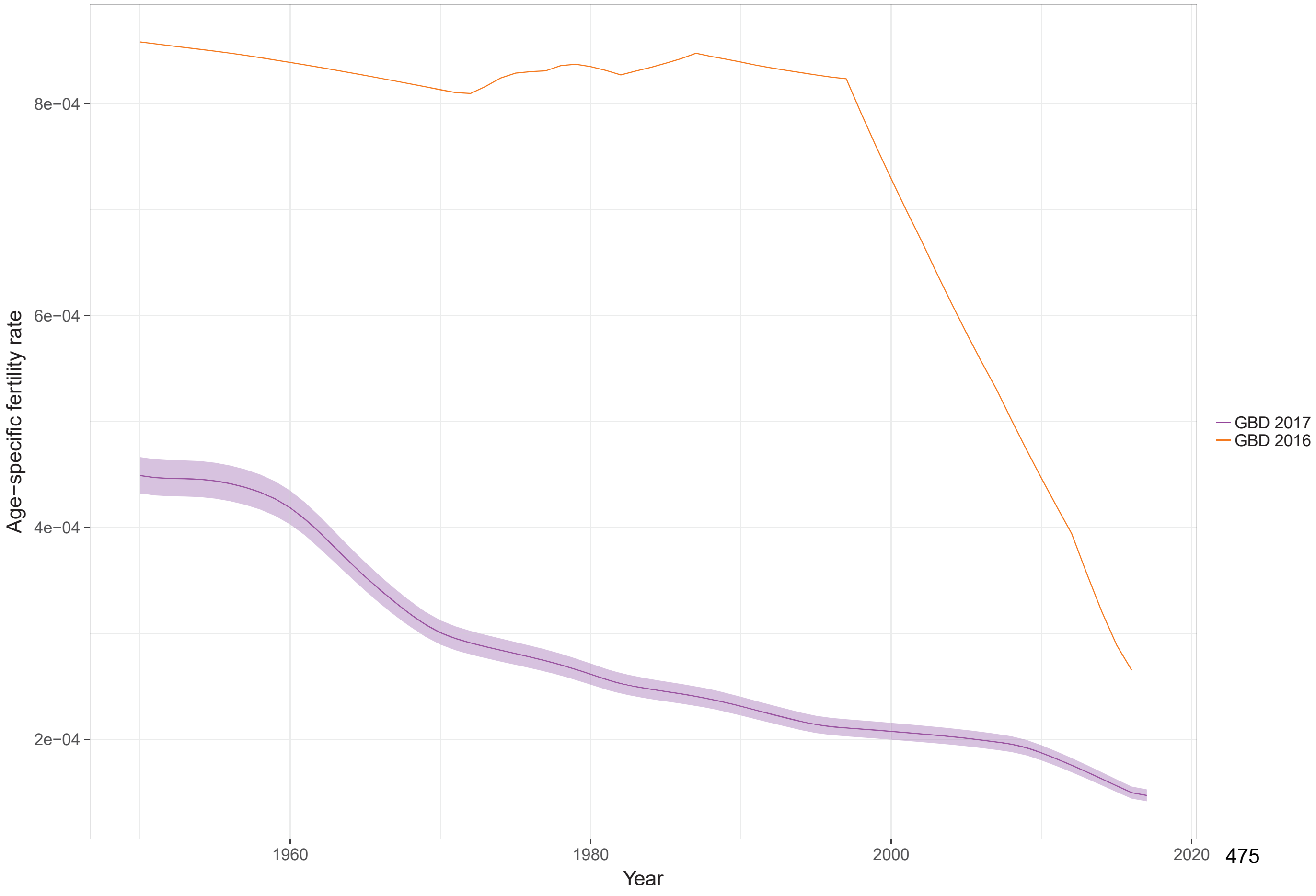

Age−specific fertility fitted trend lines, 1950−2017: Kuwait, TFR
Data points enclosed with a diamond are reference sources

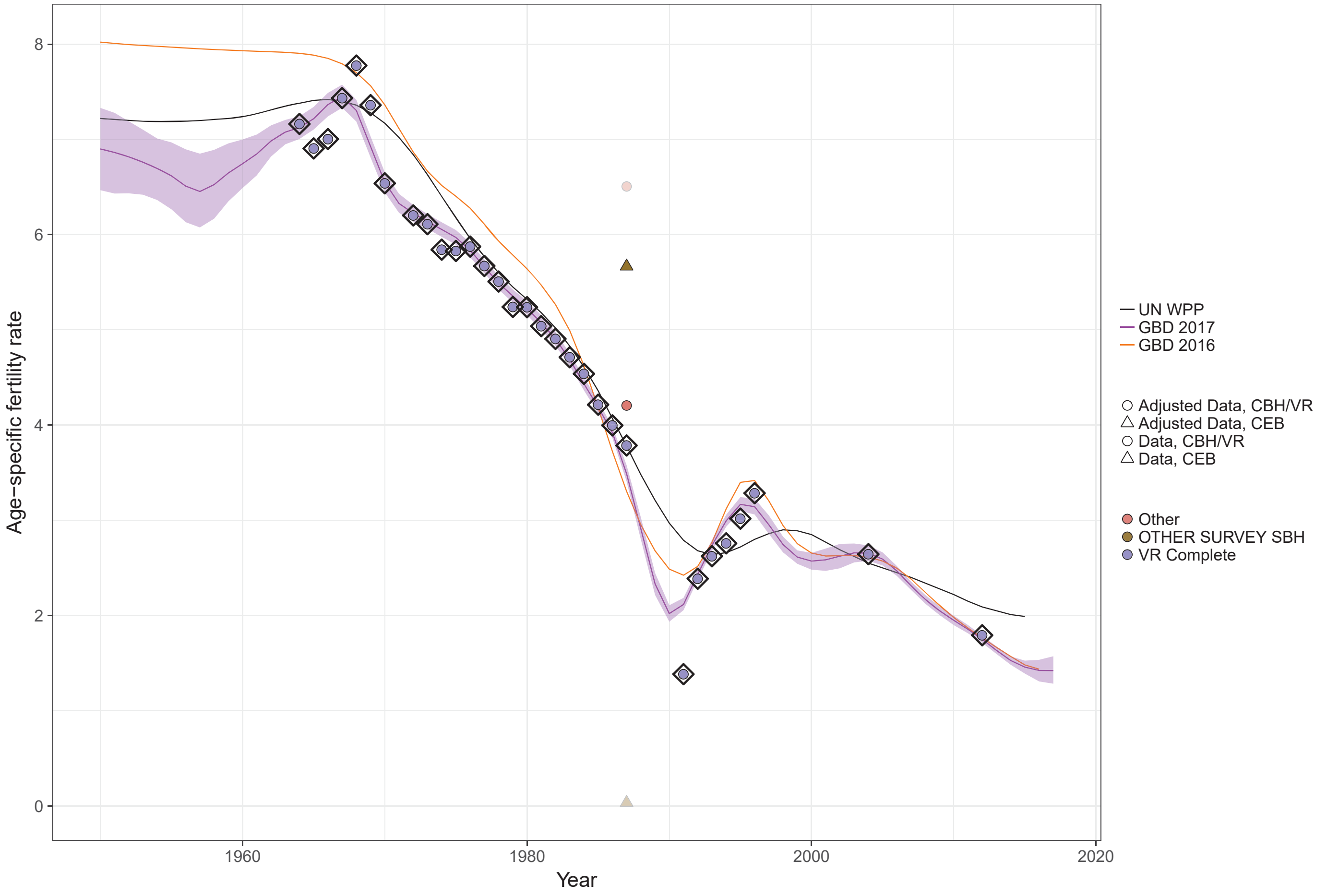

Age−specific fertility fitted trend lines, 1950−2017: Kuwait, Ages: 10−14
Data points enclosed with a diamond are reference sources

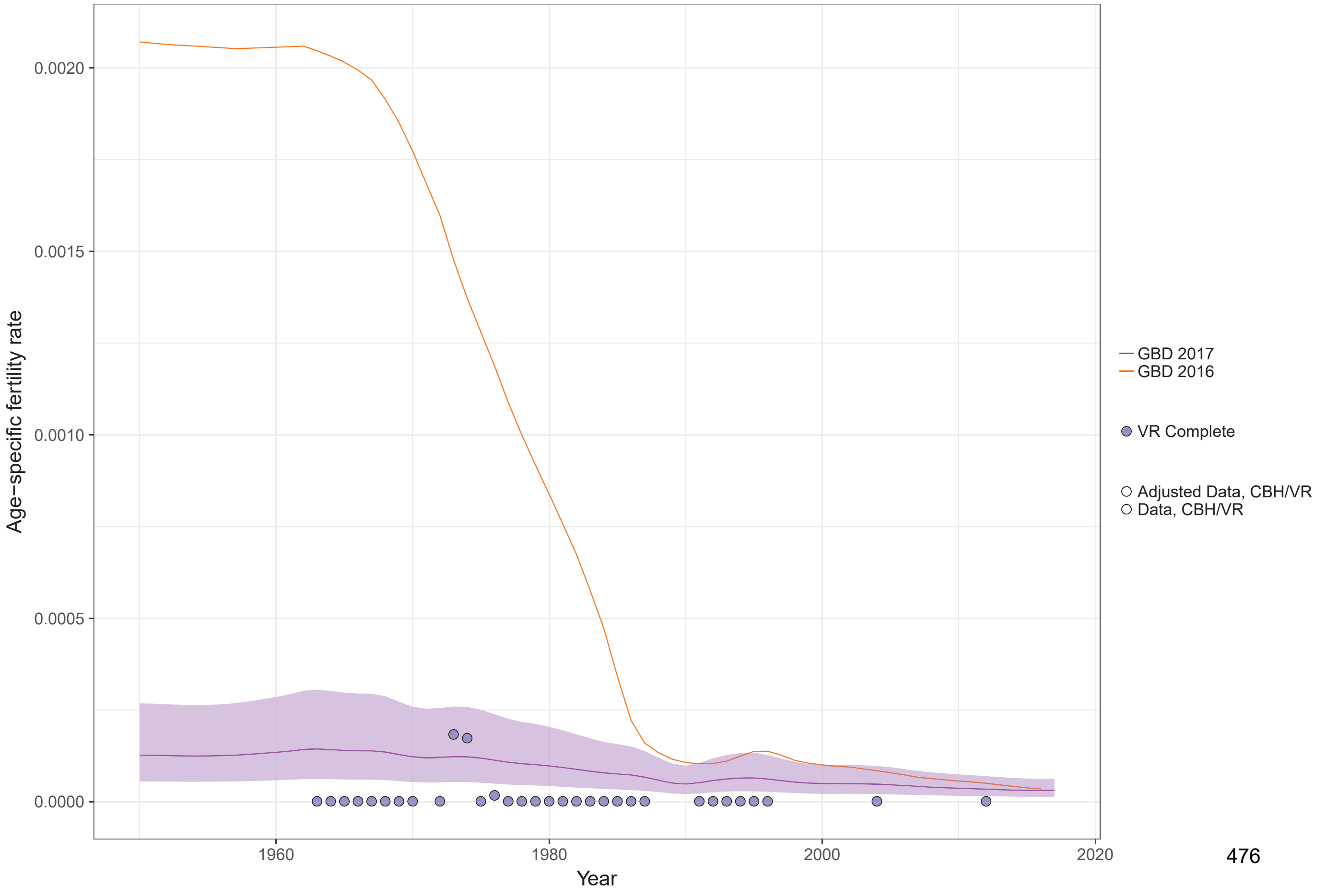

Age−specific fertility fitted trend lines, 1950−2017: Kuwait, Ages: 15−19
Data points enclosed with a diamond are reference sources

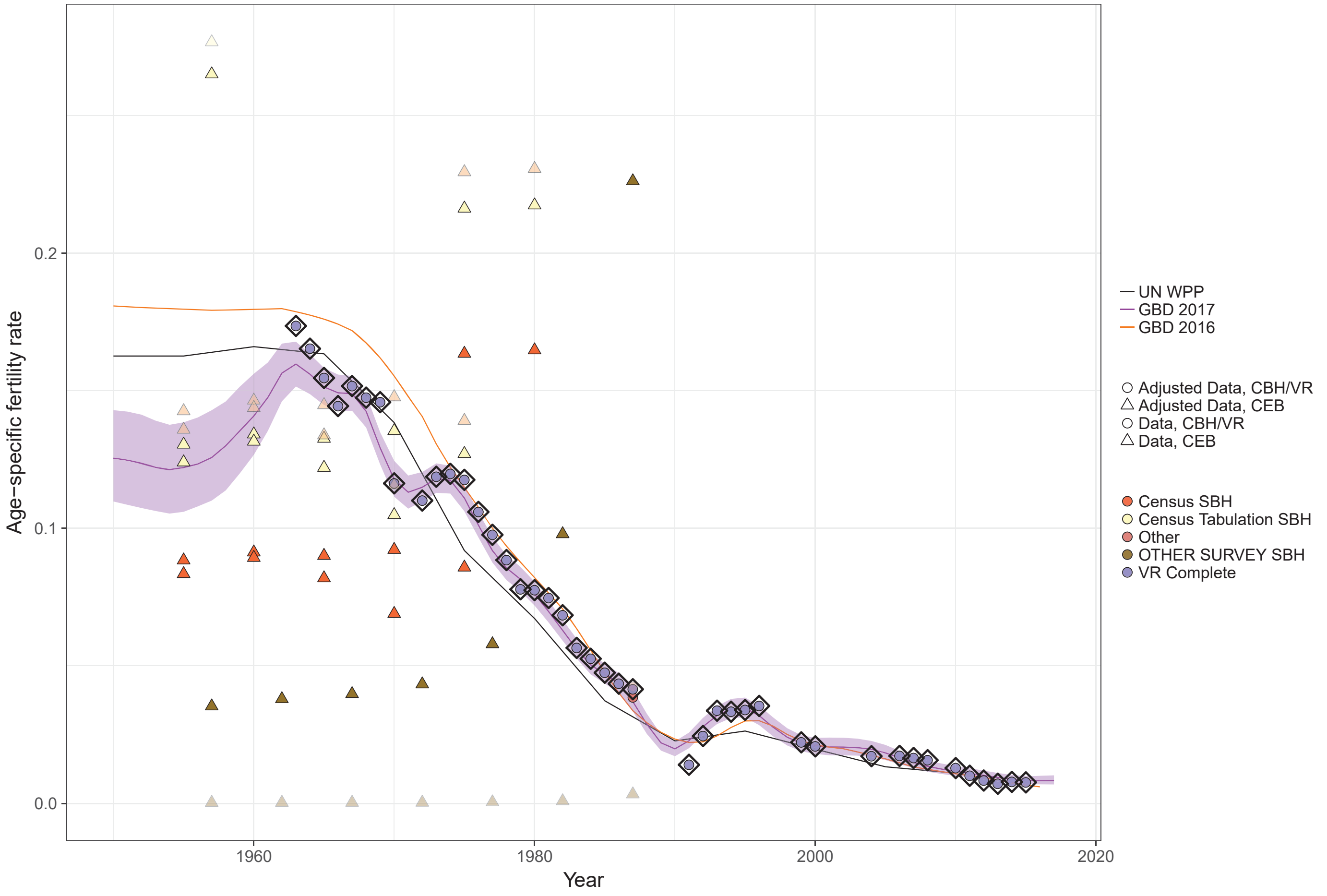

Age−specific fertility fitted trend lines, 1950−2017: Kuwait, Ages: 20−24
Data points enclosed with a diamond are reference sources

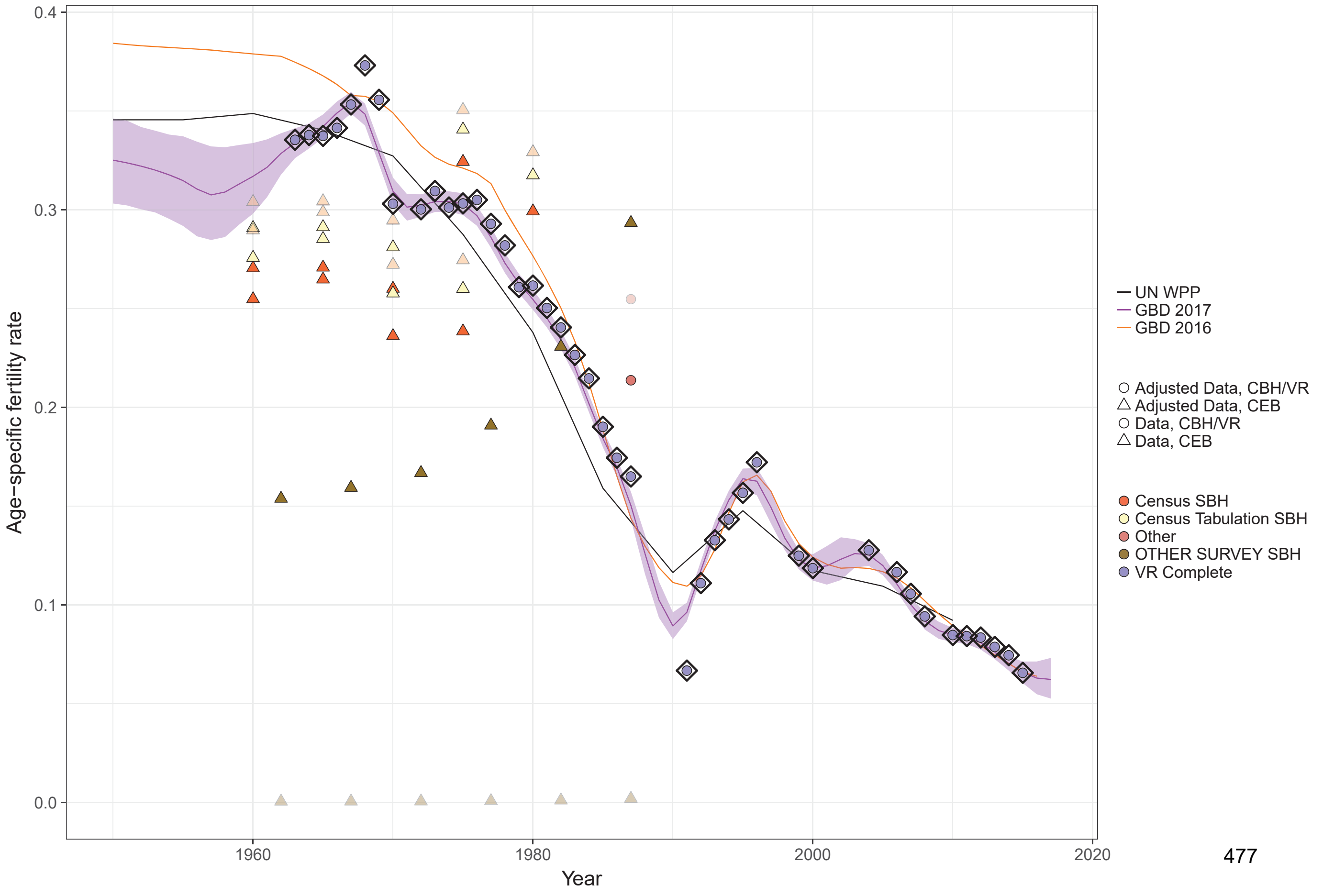

Age−specific fertility fitted trend lines, 1950−2017: Kuwait, Ages: 25−29
Data points enclosed with a diamond are reference sources

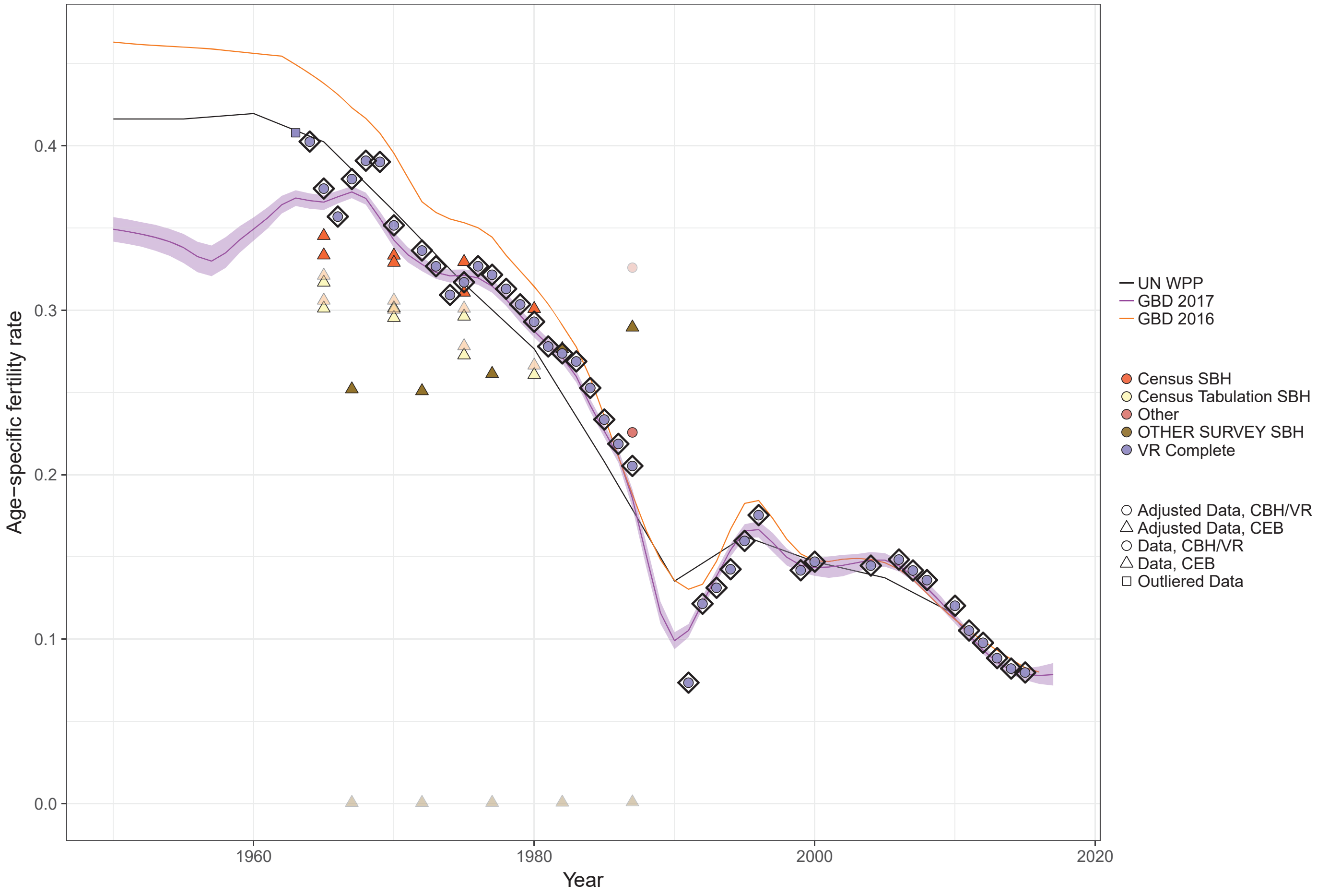

Age−specific fertility fitted trend lines, 1950−2017: Kuwait, Ages: 30−34
Data points enclosed with a diamond are reference sources

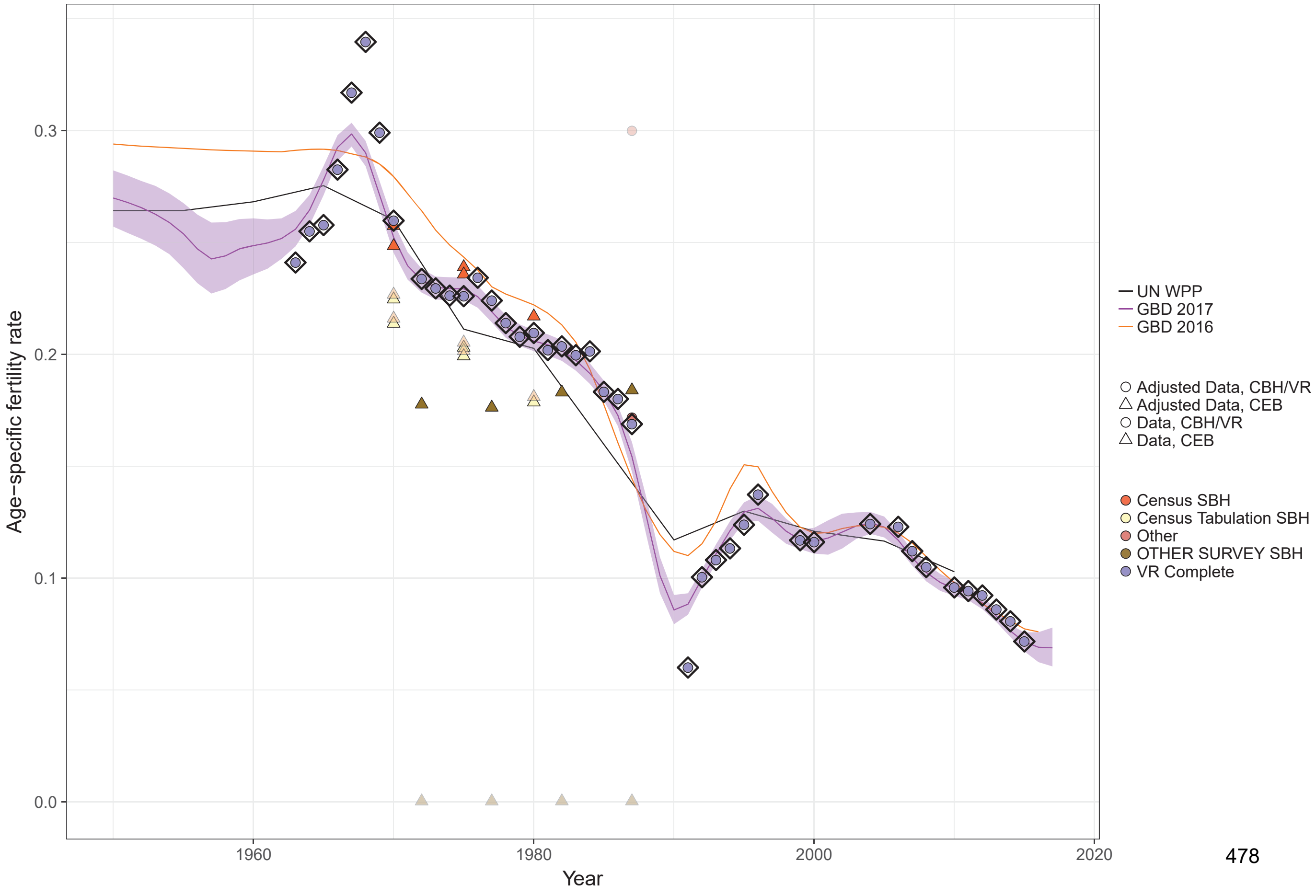

Age−specific fertility fitted trend lines, 1950−2017: Kuwait, Ages: 35−39
Data points enclosed with a diamond are reference sources

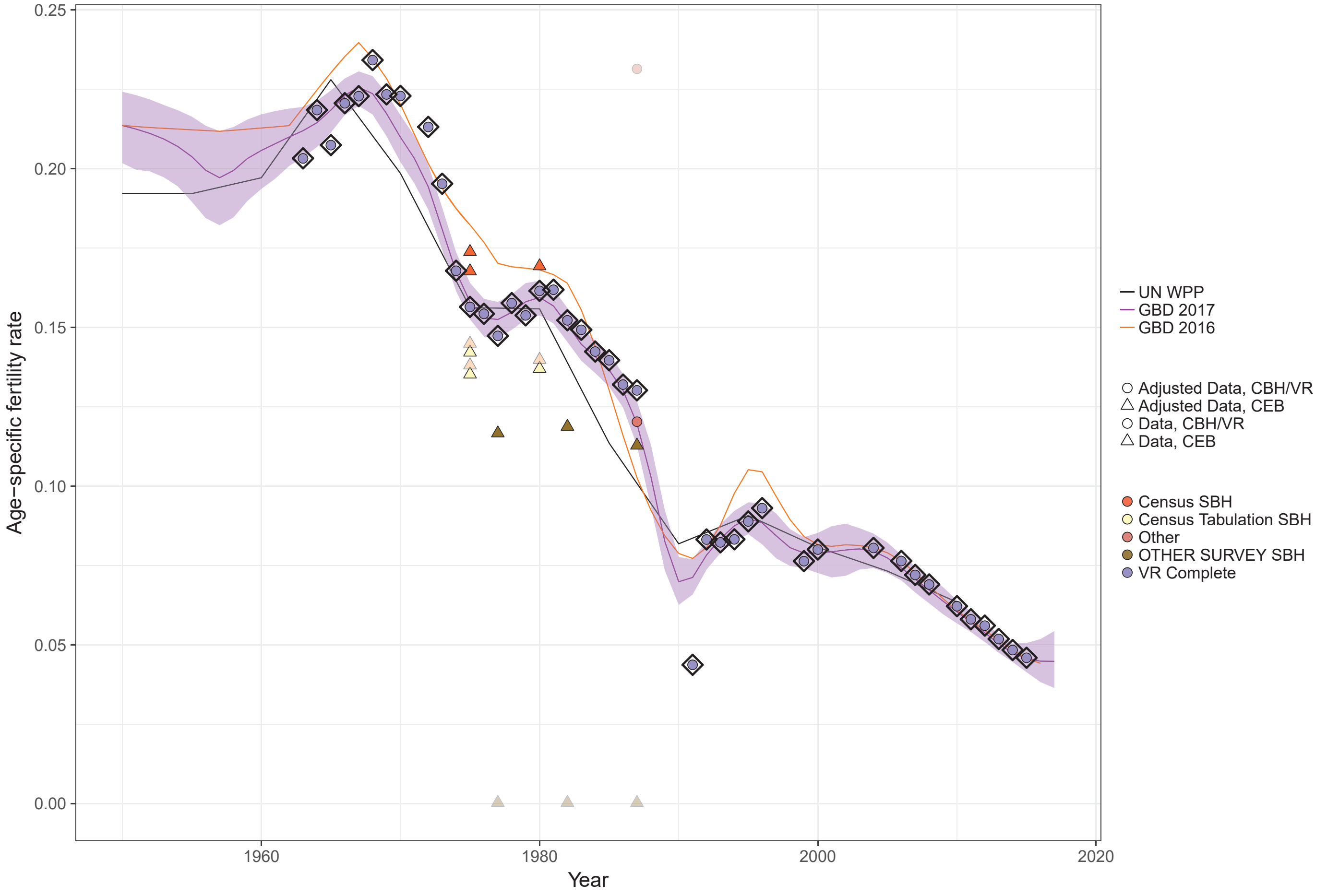

Age−specific fertility fitted trend lines, 1950−2017: Kuwait, Ages: 40−44
Data points enclosed with a diamond are reference sources

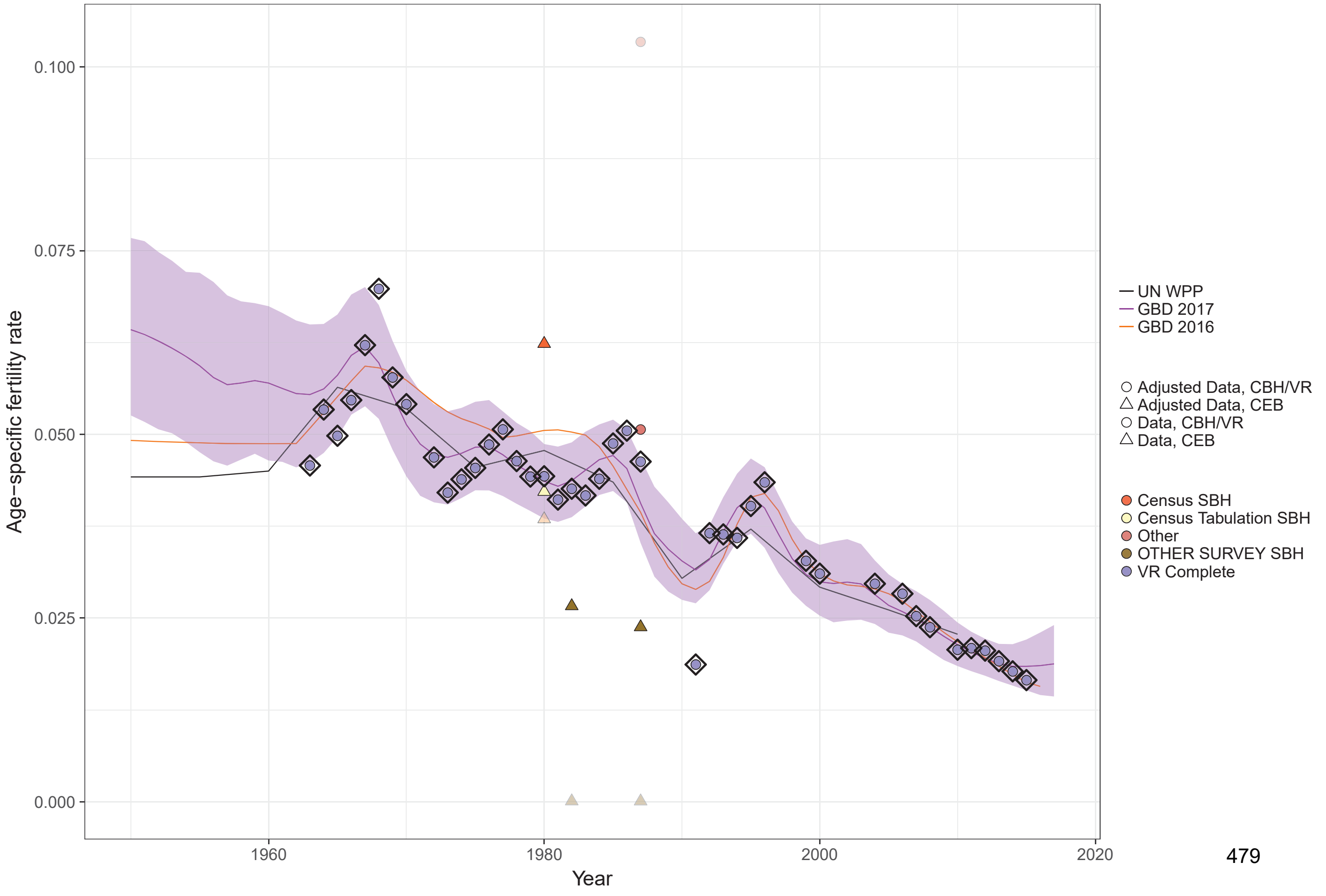

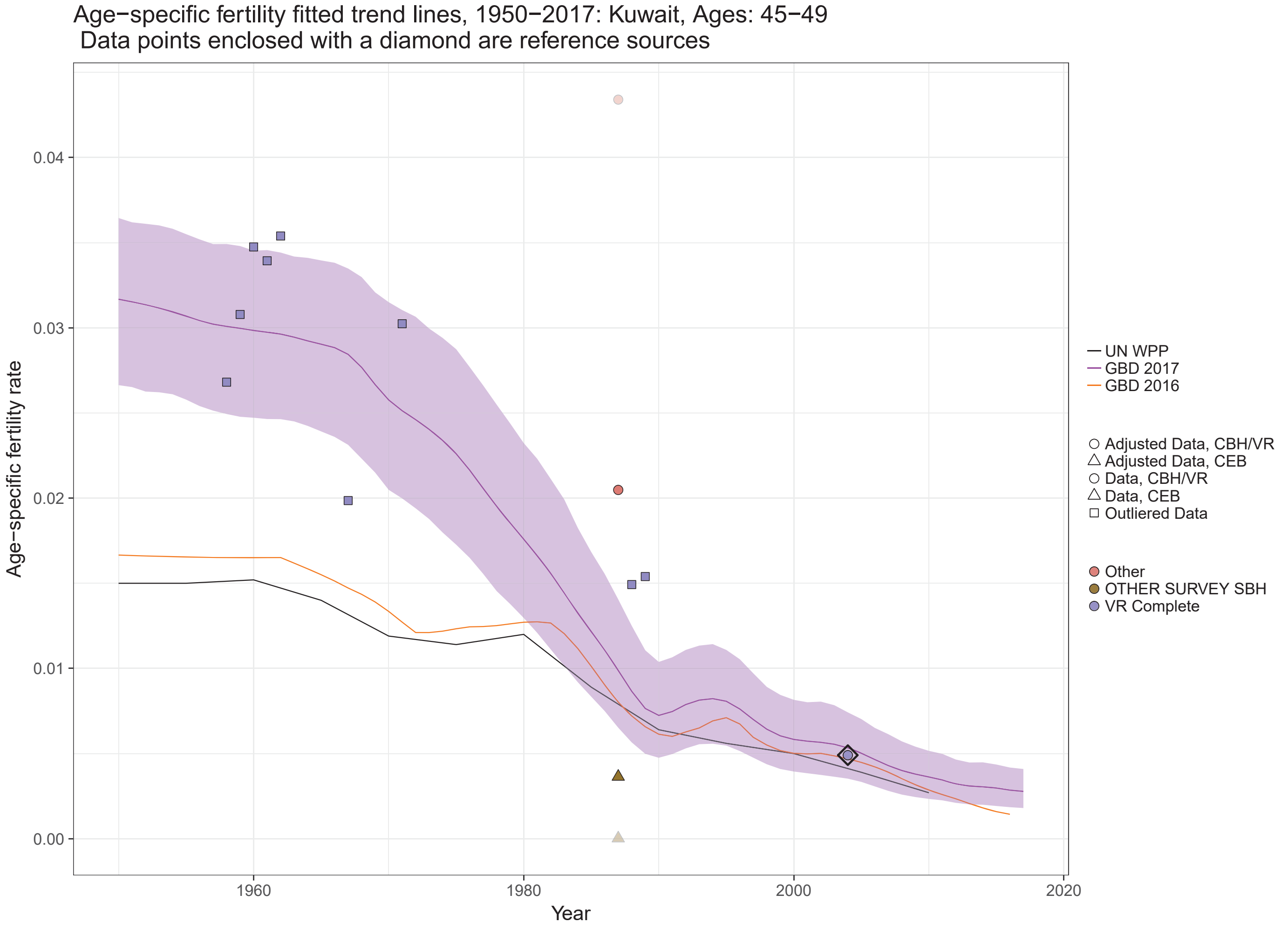

Age−specific fertility fitted trend lines, 1950−2017: Kuwait, Ages: 45−49
Data points enclosed with a diamond are reference sources
Age−specific fertility rate
0.00
0.01
0.02
0.03
0.04
1960
1980
2000
2020
Year
UN WPP
GBD 2017
GBD 2016
Adjusted Data, CBH/VR
Adjusted Data, CEB
Data, CBH/VR
Data, CEB
Outliered Data
Other
OTHER SURVEY SBH
VR Complete

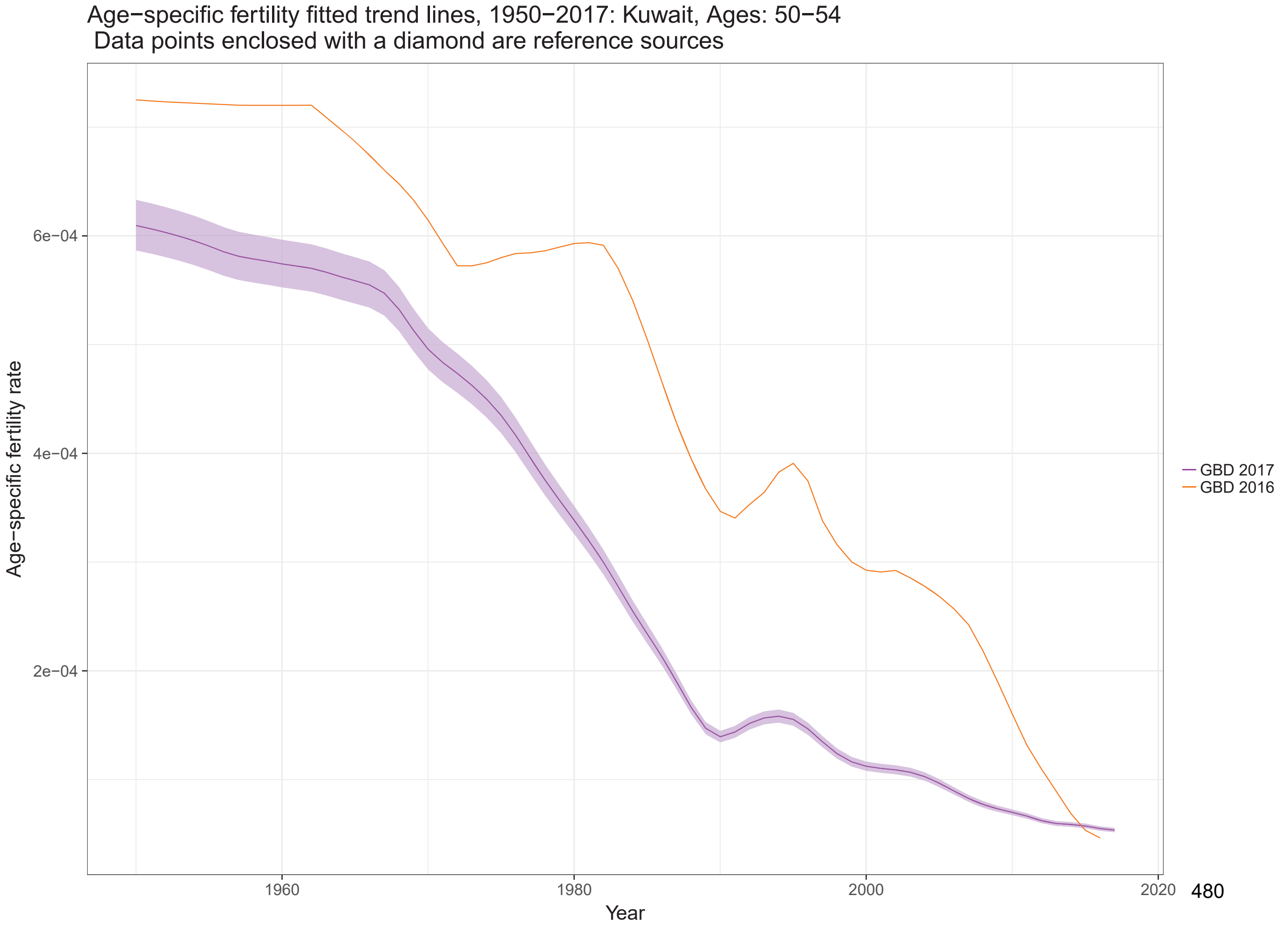

Age−specific fertility fitted trend lines, 1950−2017: Kuwait, Ages: 50−54
Data points enclosed with a diamond are reference sources
Age−specific fertility rate
2e−04
4e−04
6e−04
1960
1980
2000
2020
Year
GBD 2017
GBD 2016

Age−specific fertility fitted trend lines, 1950−2017: Kyrgyzstan, TFR
Data points enclosed with a diamond are reference sources

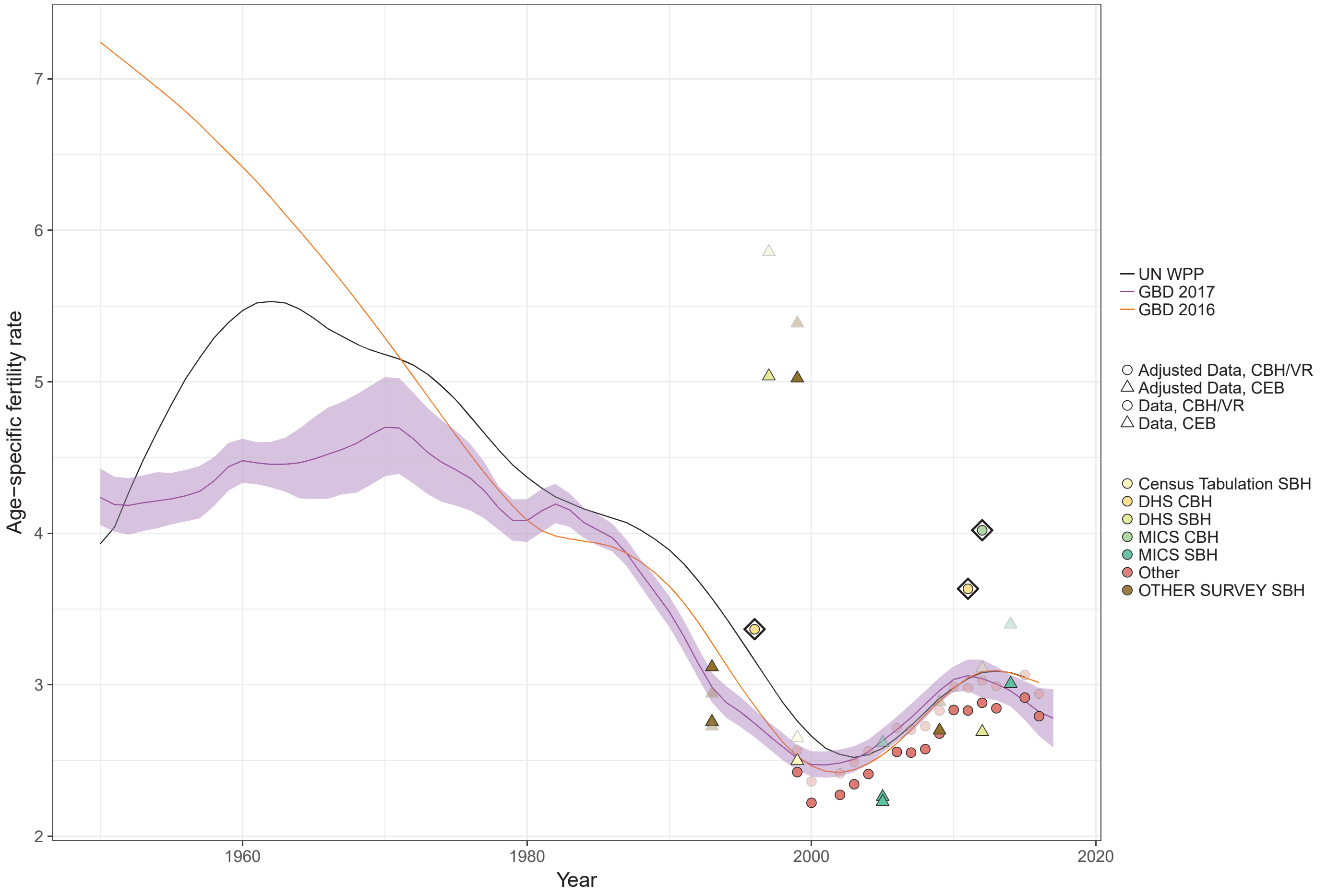

Age−specific fertility fitted trend lines, 1950−2017: Kyrgyzstan, Ages: 10−14
Data points enclosed with a diamond are reference sources

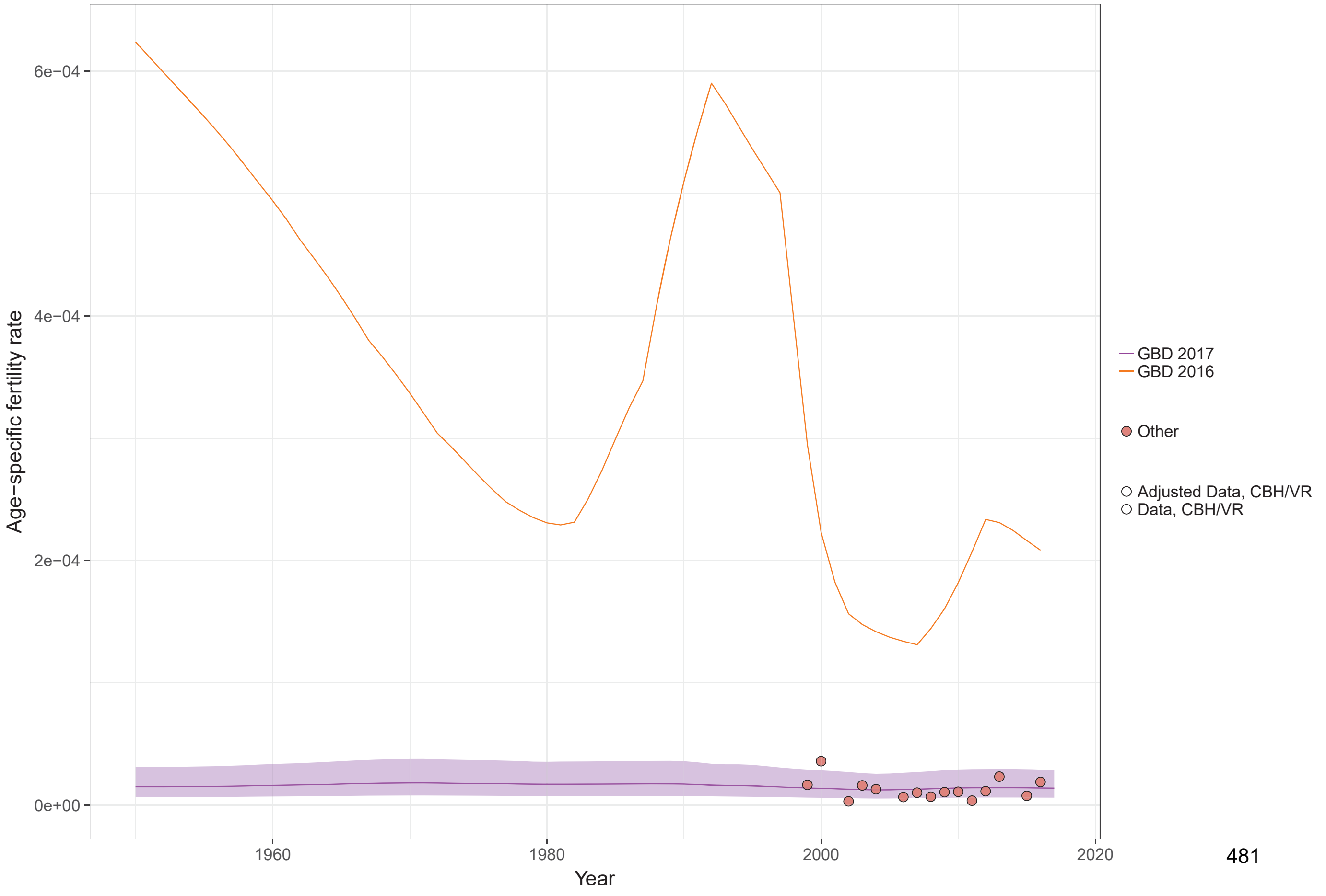

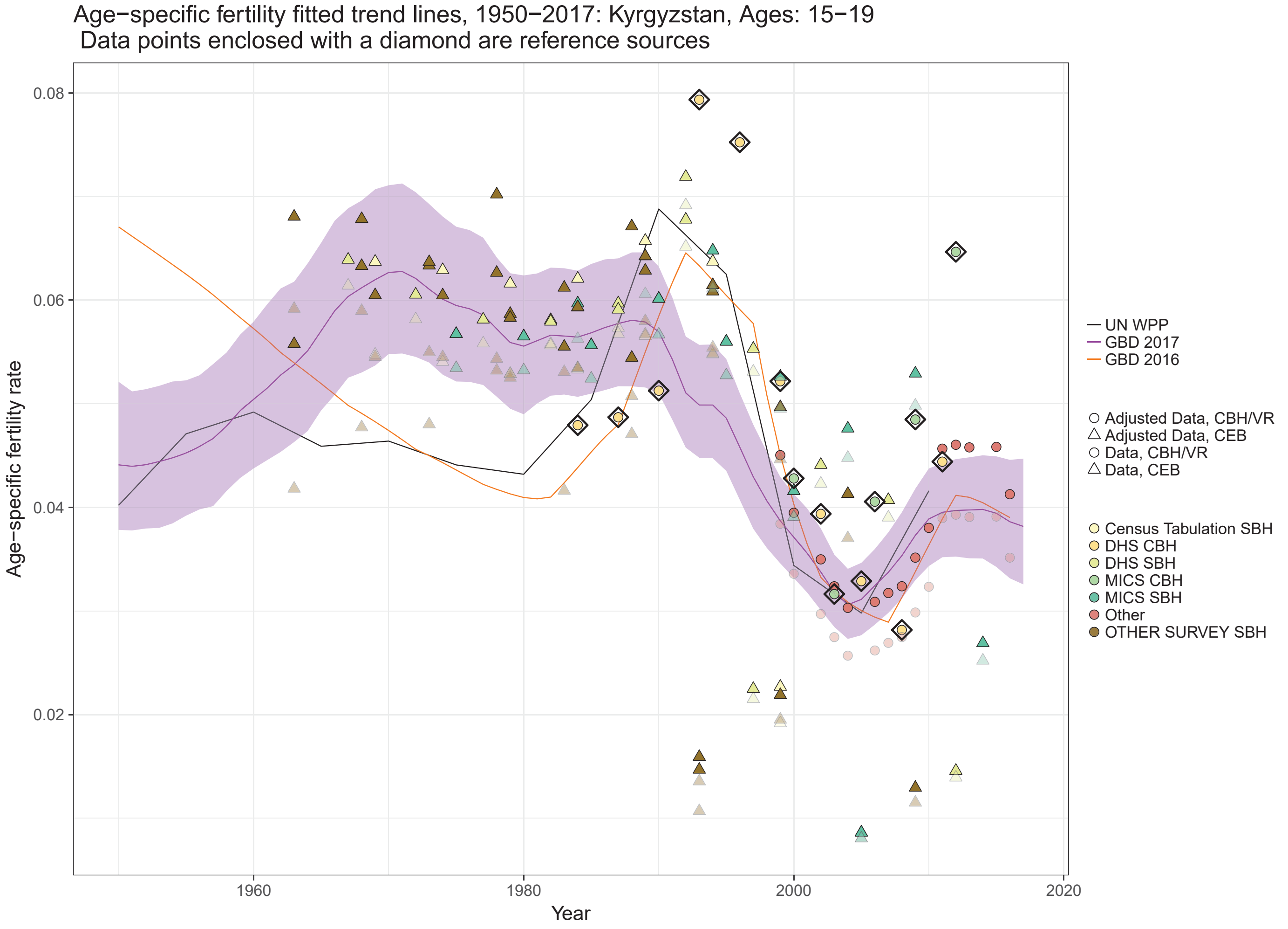
Age−specific fertility fitted trend lines, 1950−2017: Kyrgyzstan, Ages: 15−19
Data points enclosed with a diamond are reference sources
Age-specific fertility rate
Year
0.08
0.06
0.04
0.02
1960
1980
2000
2020
UN WPP
GBD 2017
GBD 2016
Adjusted Data, CBH/VR
Adjusted Data, CEB
Data, CBH/VR
Data, CEB
Census Tabulation SBH
DHS CBH
DHS SBH
MICS CBH
MICS SBH
Other
OTHER SURVEY SBH

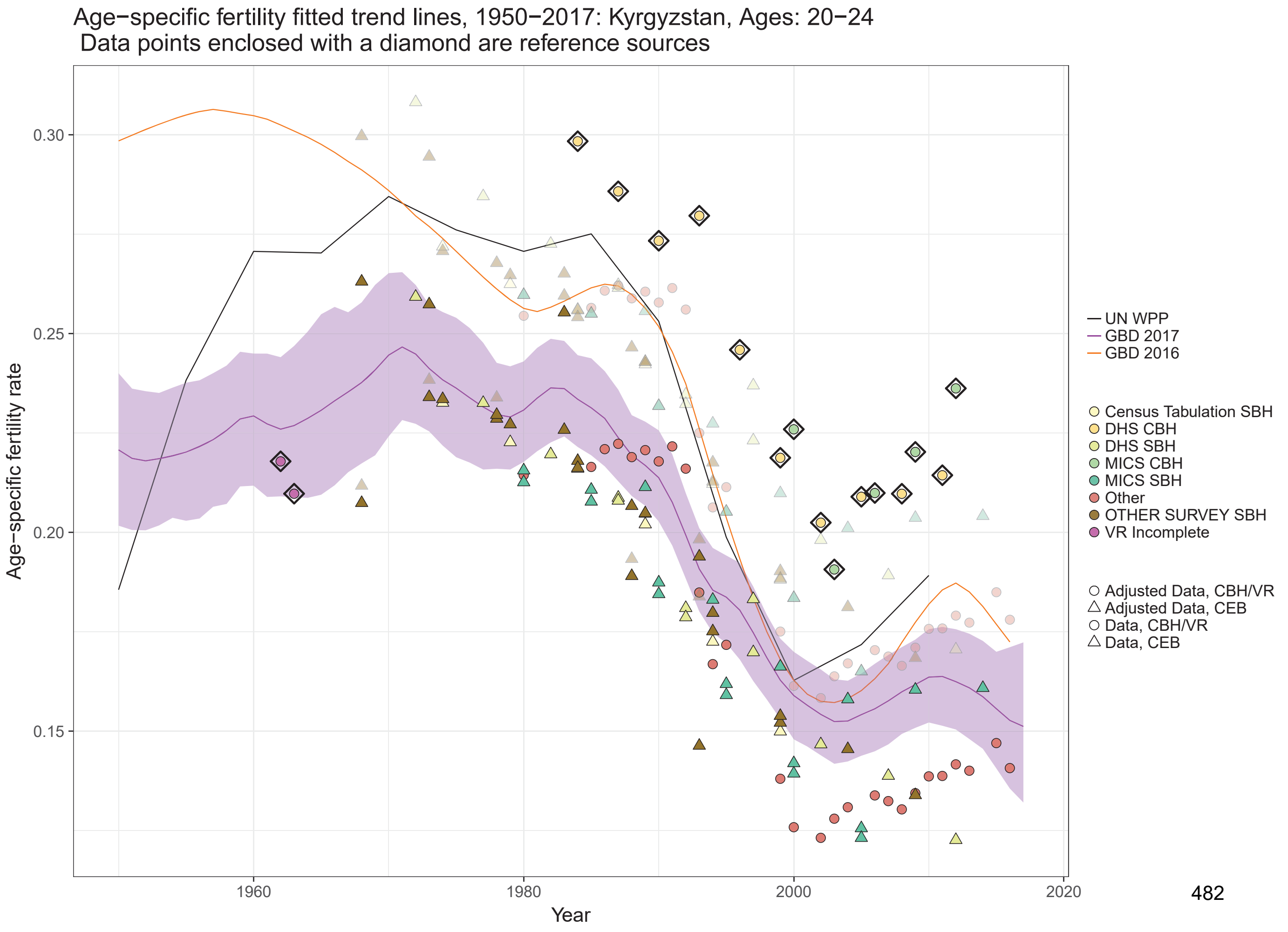
Age−specific fertility fitted trend lines, 1950−2017: Kyrgyzstan, Ages: 20−24
Data points enclosed with a diamond are reference sources
Age-specific fertility rate
Year
0.30
0.25
0.20
0.15
1960
1980
2000
2020
UN WPP
GBD 2017
GBD 2016
Census Tabulation SBH
DHS CBH
DHS SBH
MICS CBH
MICS SBH
Other
OTHER SURVEY SBH
VR Incomplete
Adjusted Data, CBH/VR
Adjusted Data, CEB
Data, CBH/VR
Data, CEB

Age−specific fertility fitted trend lines, 1950−2017: Kyrgyzstan, Ages: 25−29
Data points enclosed with a diamond are reference sources

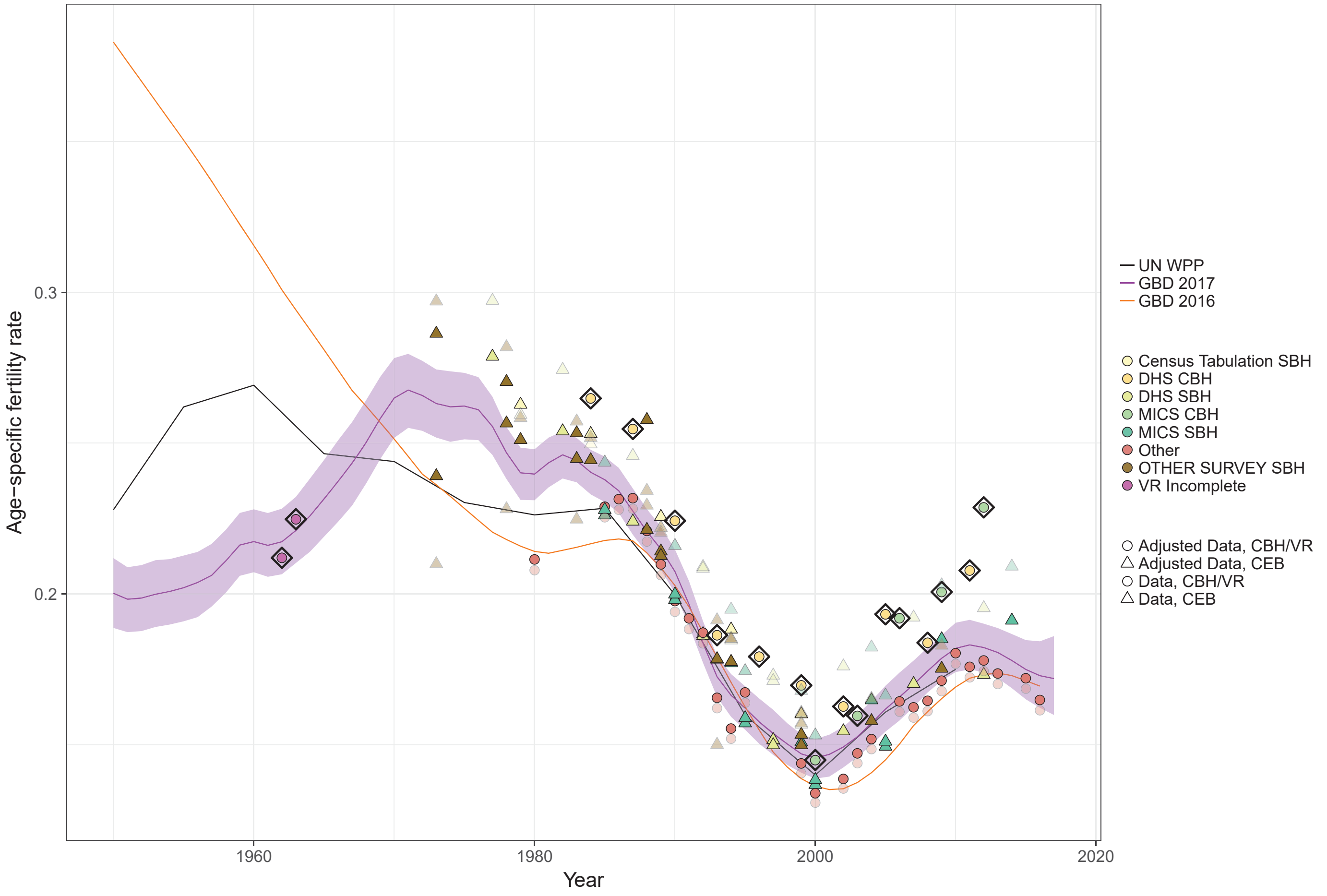

Age−specific fertility fitted trend lines, 1950−2017: Kyrgyzstan, Ages: 30−34
Data points enclosed with a diamond are reference sources

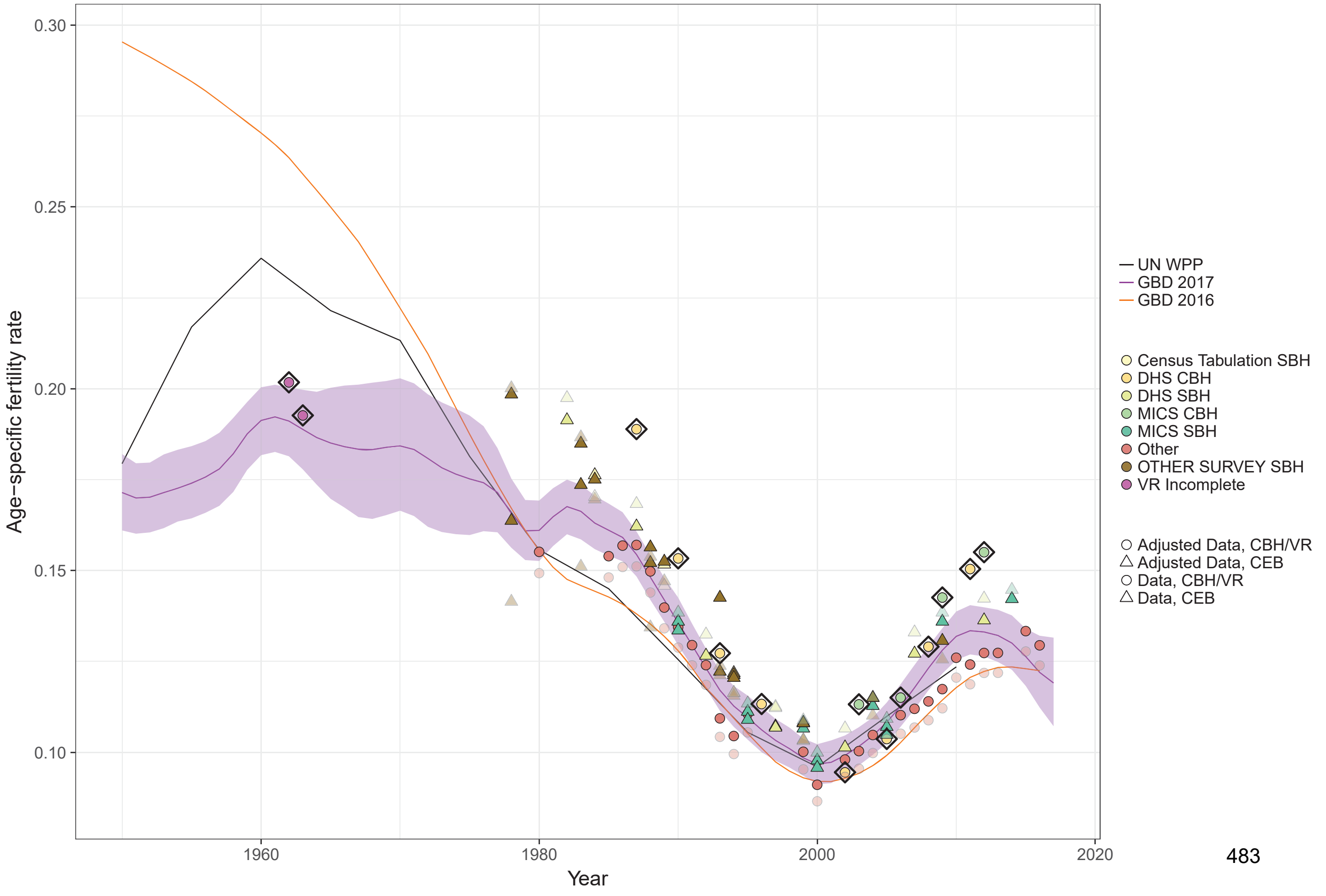

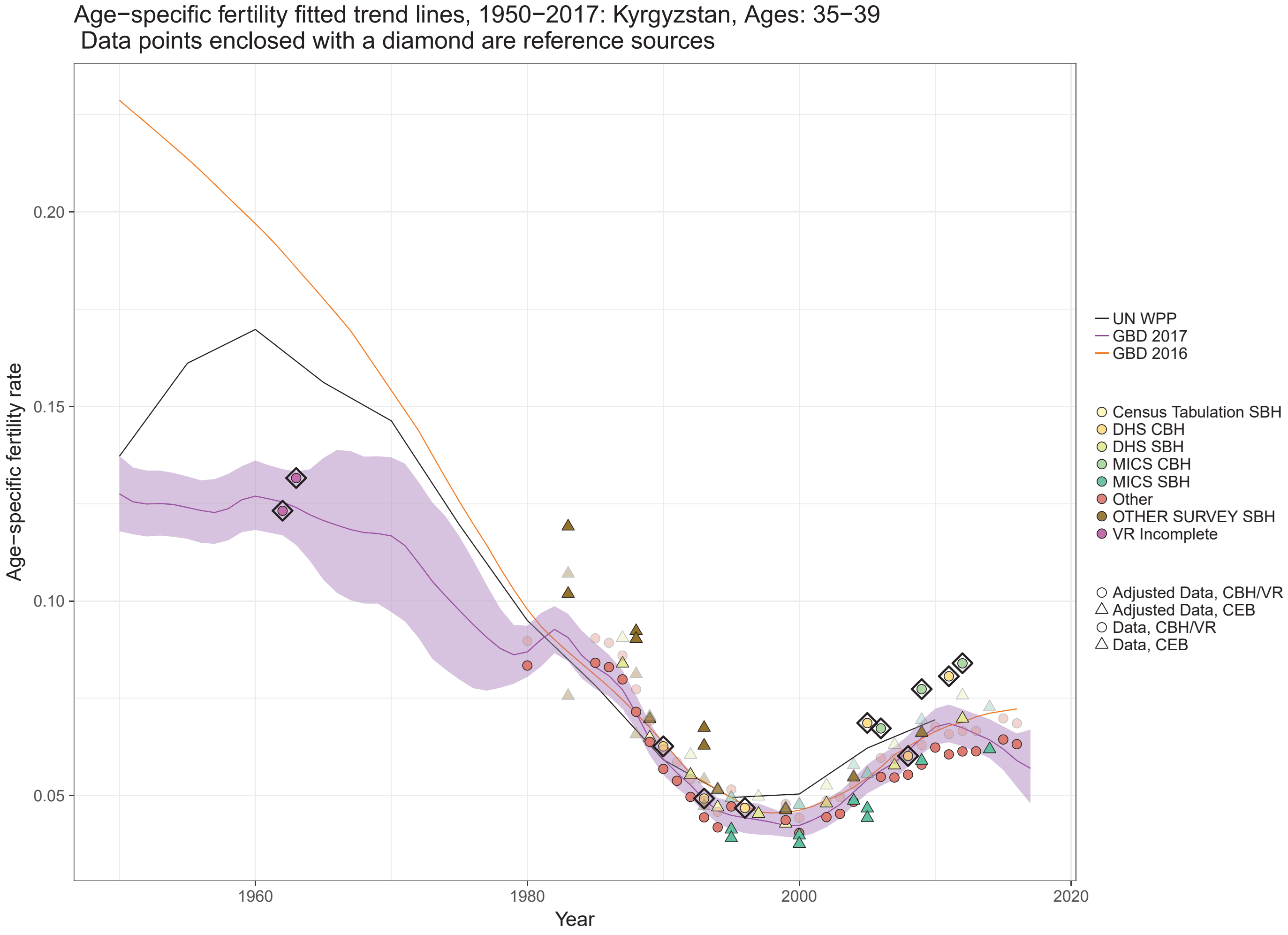

Age−specific fertility fitted trend lines, 1950−2017: Kyrgyzstan, Ages: 35−39
Data points enclosed with a diamond are reference sources
Age−specific fertility rate
0.20
0.15
0.10
0.05
1960
1980
2000
2020
Year
UN WPP
GBD 2017
GBD 2016
Census Tabulation SBH
DHS CBH
DHS SBH
MICS CBH
MICS SBH
Other
OTHER SURVEY SBH
VR Incomplete
Adjusted Data, CBH/VR
Adjusted Data, CEB
Data, CBH/VR
Data, CEB

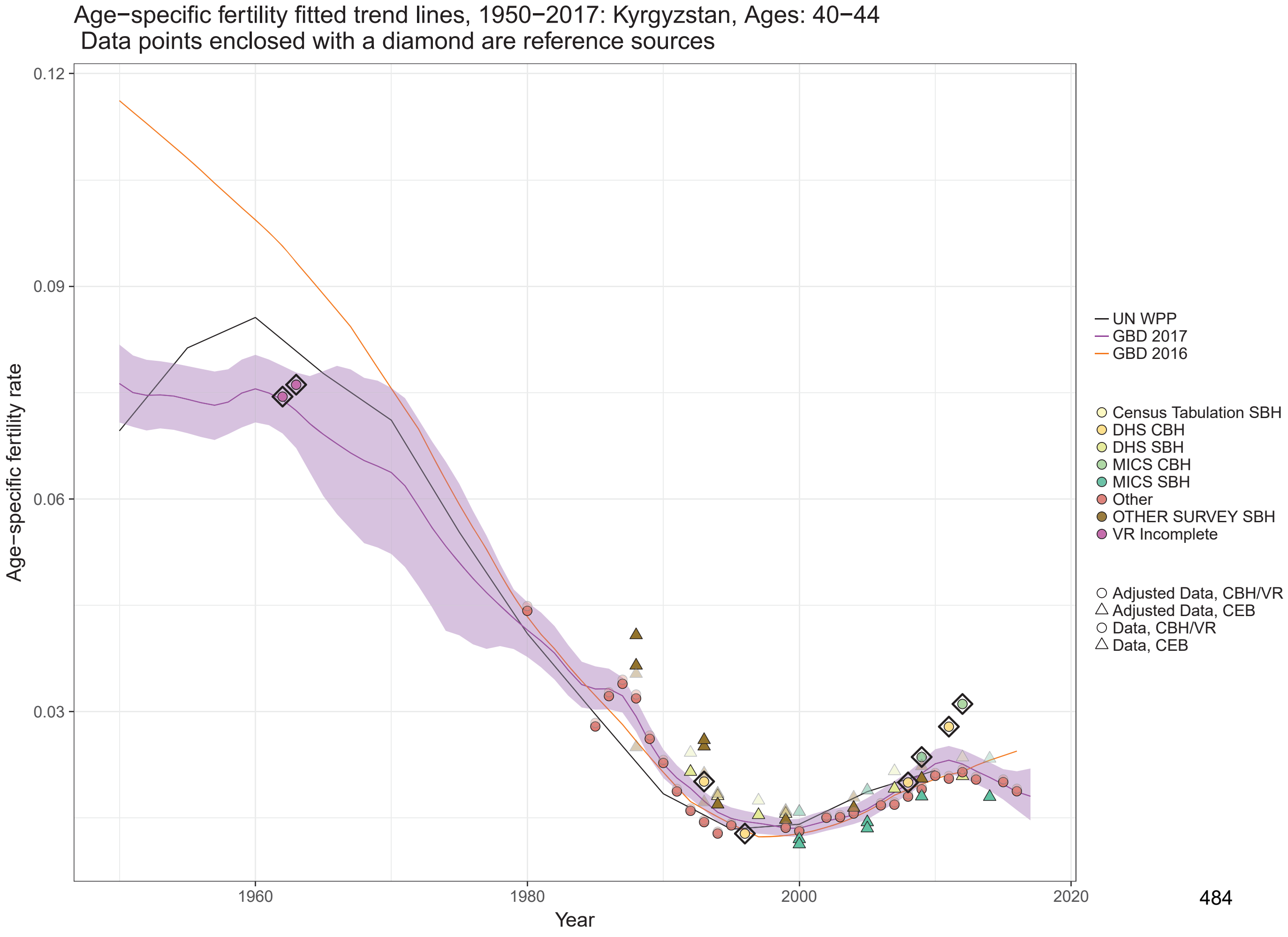

Age−specific fertility fitted trend lines, 1950−2017: Kyrgyzstan, Ages: 40−44
Data points enclosed with a diamond are reference sources
Age−specific fertility rate
0.12
0.09
0.06
0.03
1960
1980
2000
2020
Year
UN WPP
GBD 2017
GBD 2016
Census Tabulation SBH
DHS CBH
DHS SBH
MICS CBH
MICS SBH
Other
OTHER SURVEY SBH
VR Incomplete
Adjusted Data, CBH/VR
Adjusted Data, CEB
Data, CBH/VR
Data, CEB

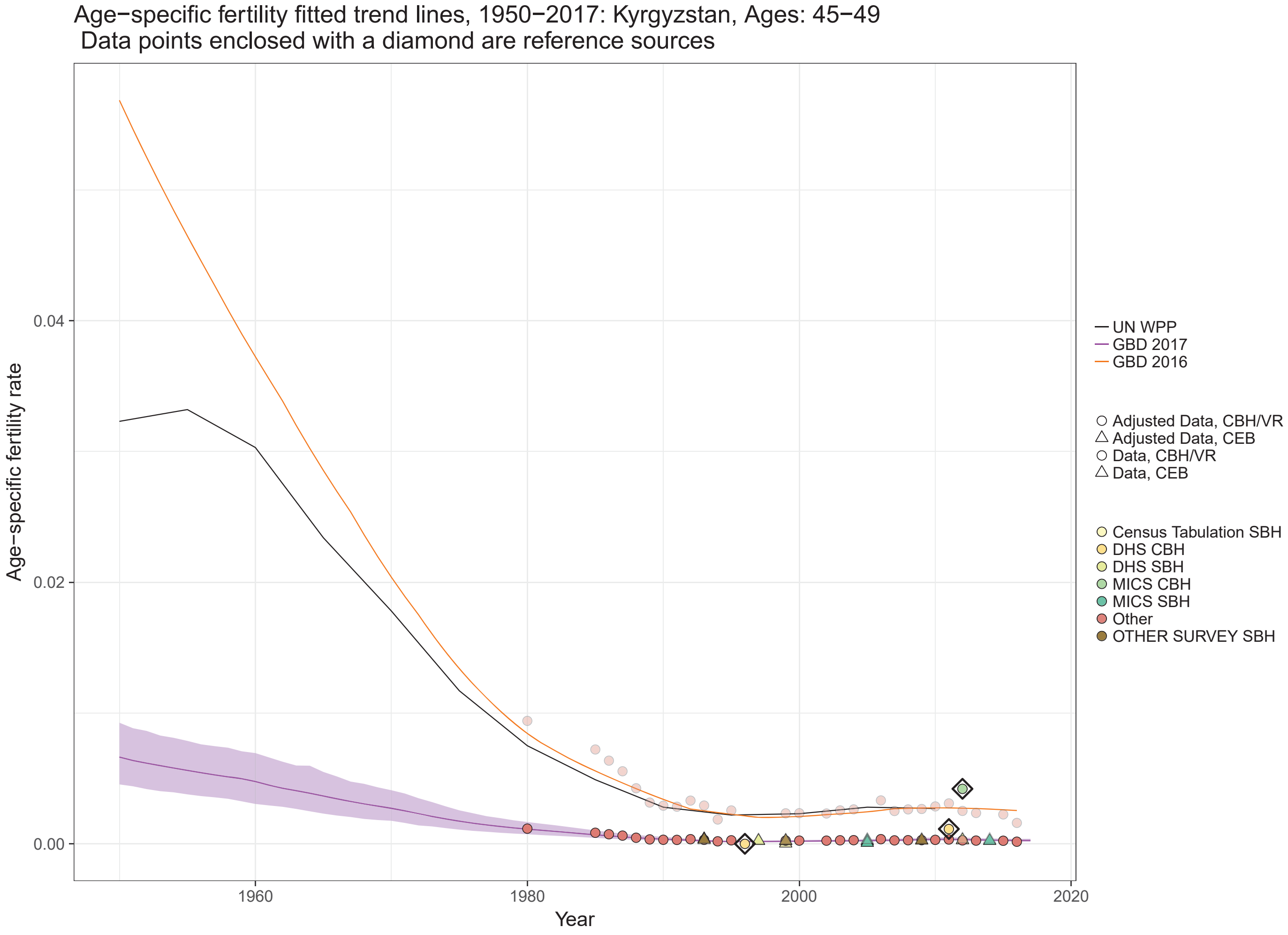

Age−specific fertility fitted trend lines, 1950−2017: Kyrgyzstan, Ages: 45−49
Data points enclosed with a diamond are reference sources
Age−specific fertility rate
0.00
0.02
0.04
Year
1960
1980
2000
2020
UN WPP
GBD 2017
GBD 2016
Adjusted Data, CBH/VR
Adjusted Data, CEB
Data, CBH/VR
Data, CEB
Census Tabulation SBH
DHS CBH
DHS SBH
MICS CBH
MICS SBH
Other
OTHER SURVEY SBH

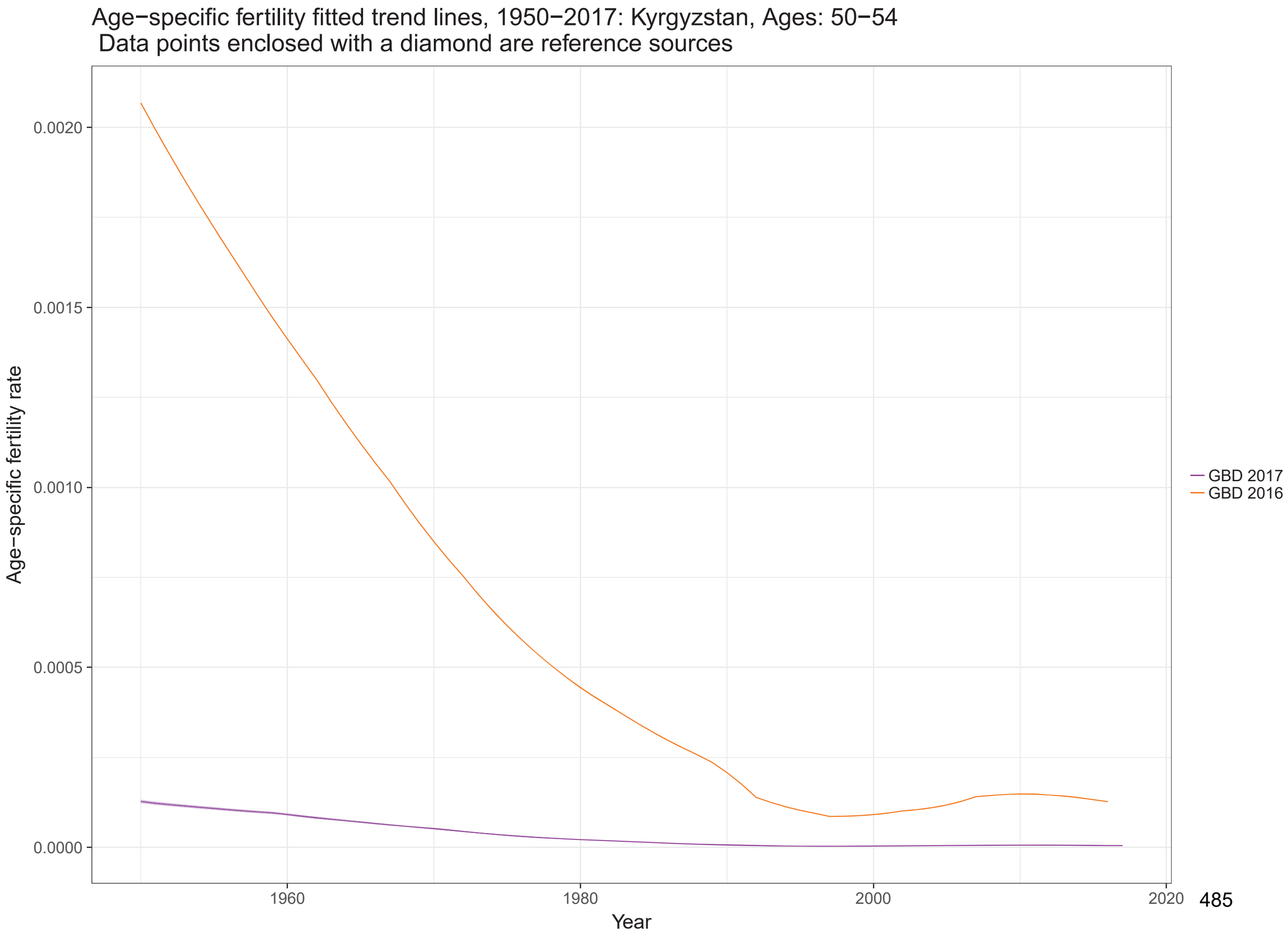

Age−specific fertility fitted trend lines, 1950−2017: Kyrgyzstan, Ages: 50−54
Data points enclosed with a diamond are reference sources
Age−specific fertility rate
0.0000
0.0005
0.0010
0.0015
0.0020
Year
1960
1980
2000
2020
GBD 2017
GBD 2016

Age−specific fertility fitted trend lines, 1950−2017: Laos, TFR
Data points enclosed with a diamond are reference sources

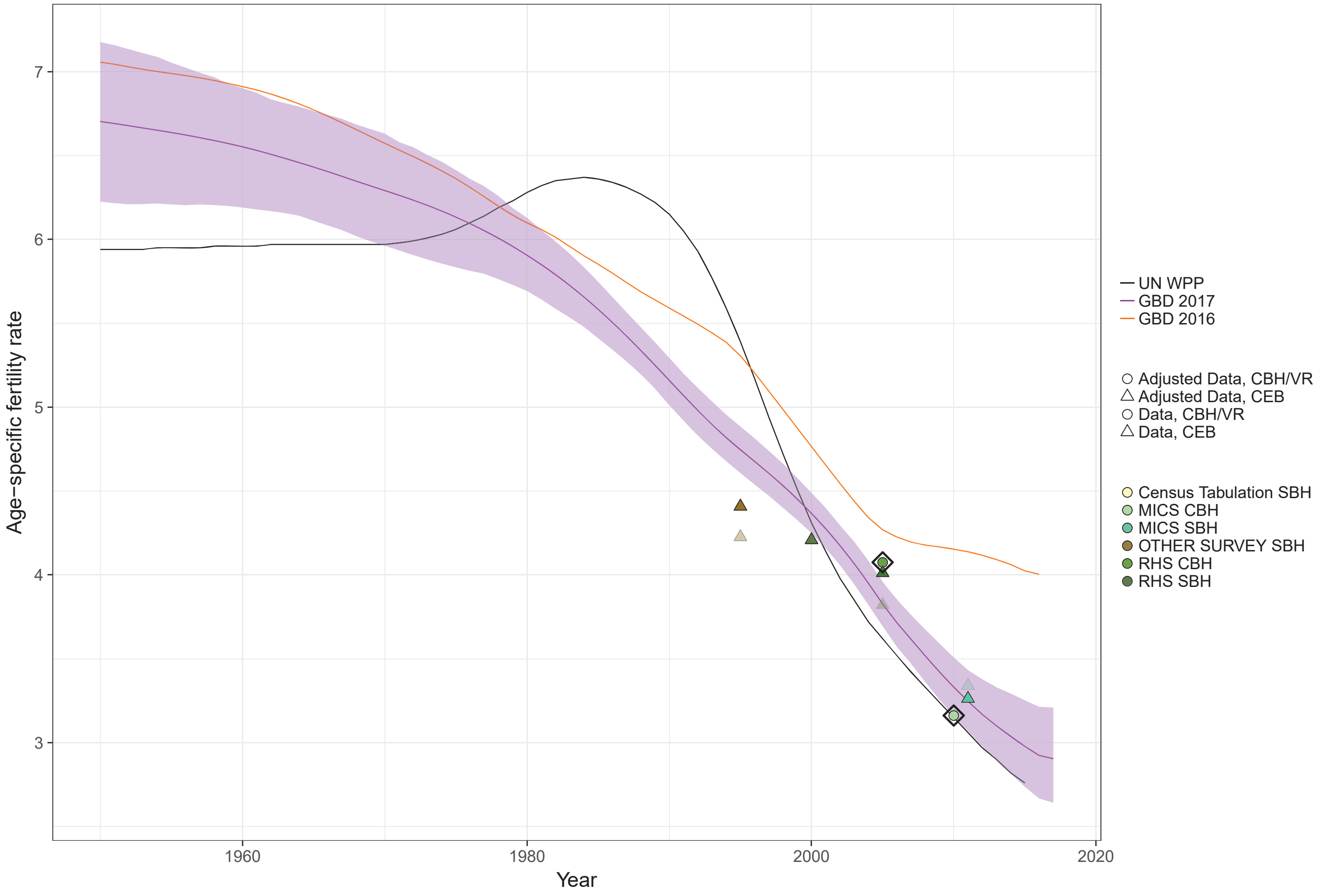

Age−specific fertility fitted trend lines, 1950−2017: Laos, Ages: 10−14
Data points enclosed with a diamond are reference sources

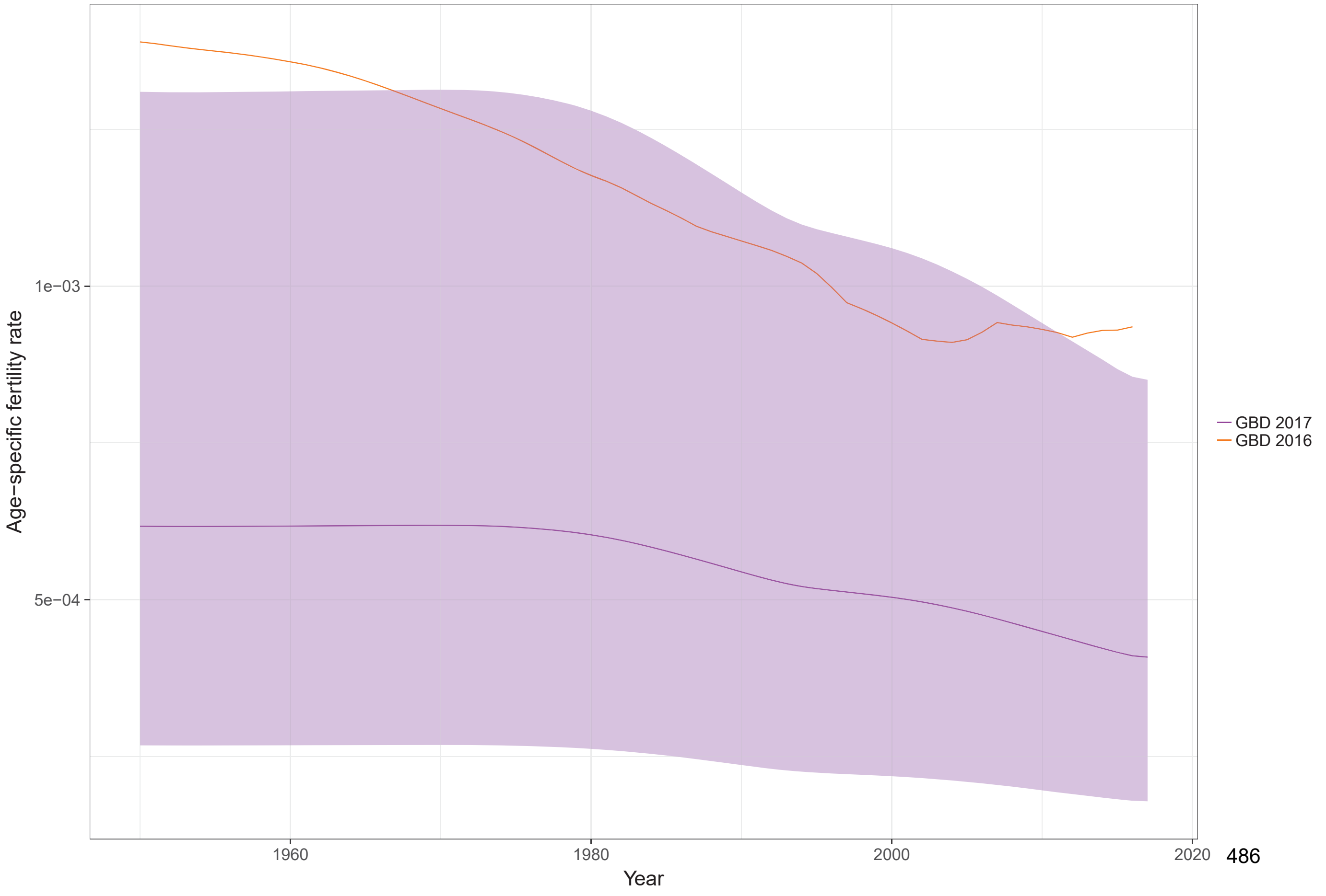

Age−specific fertility fitted trend lines, 1950−2017: Laos, Ages: 15−19
Data points enclosed with a diamond are reference sources

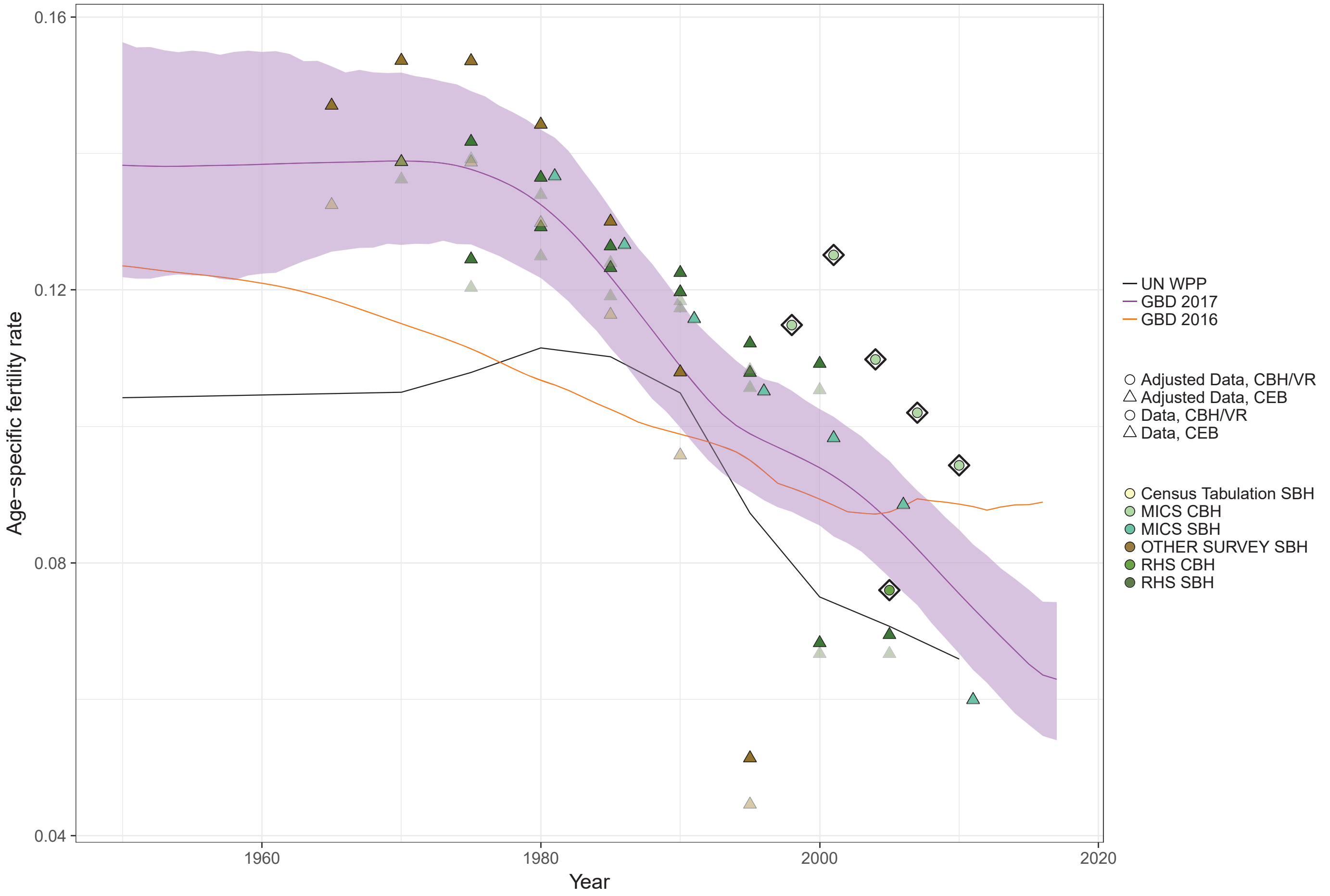

Age−specific fertility fitted trend lines, 1950−2017: Laos, Ages: 20−24
Data points enclosed with a diamond are reference sources

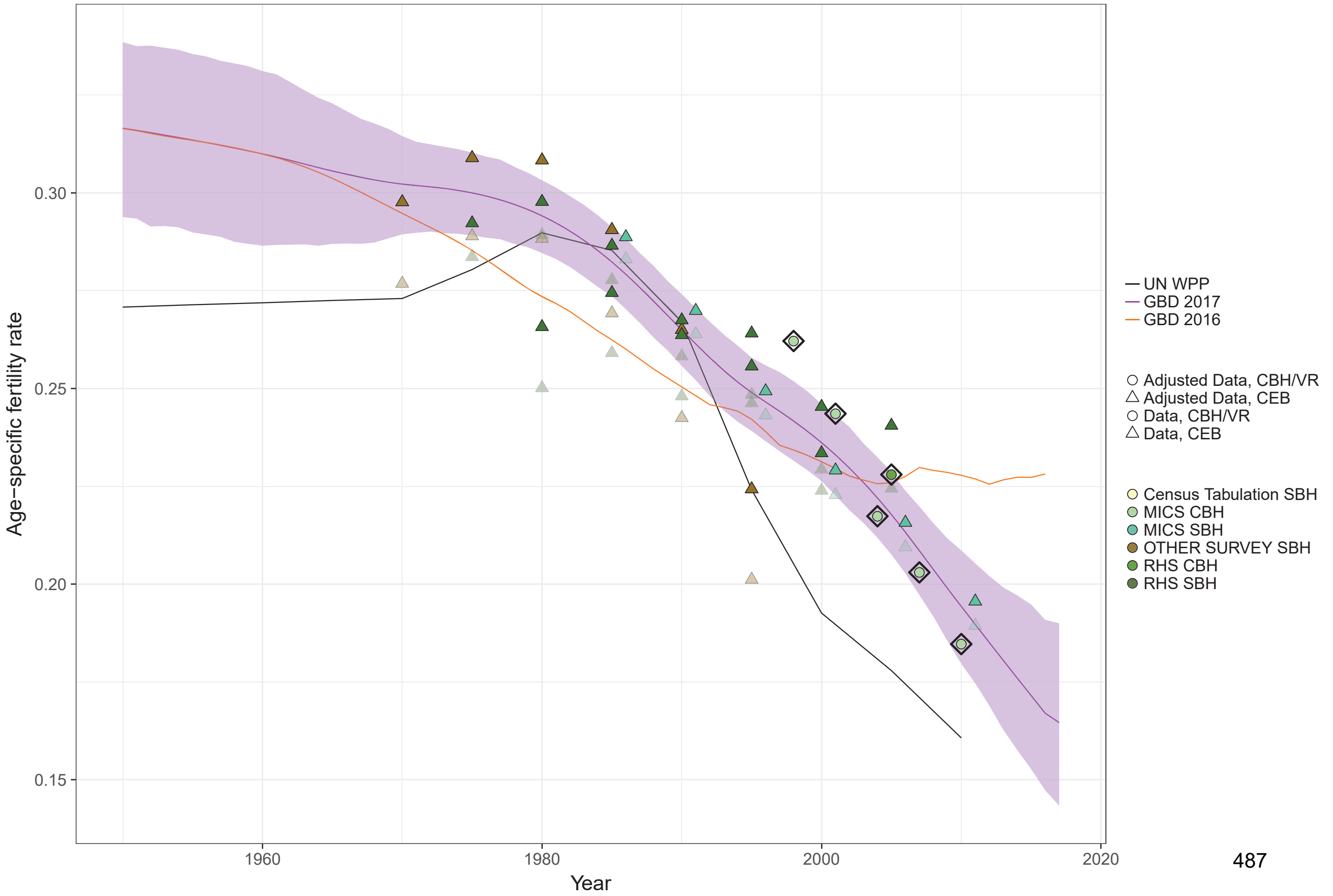

Age−specific fertility fitted trend lines, 1950−2017: Laos, Ages: 25−29
Data points enclosed with a diamond are reference sources

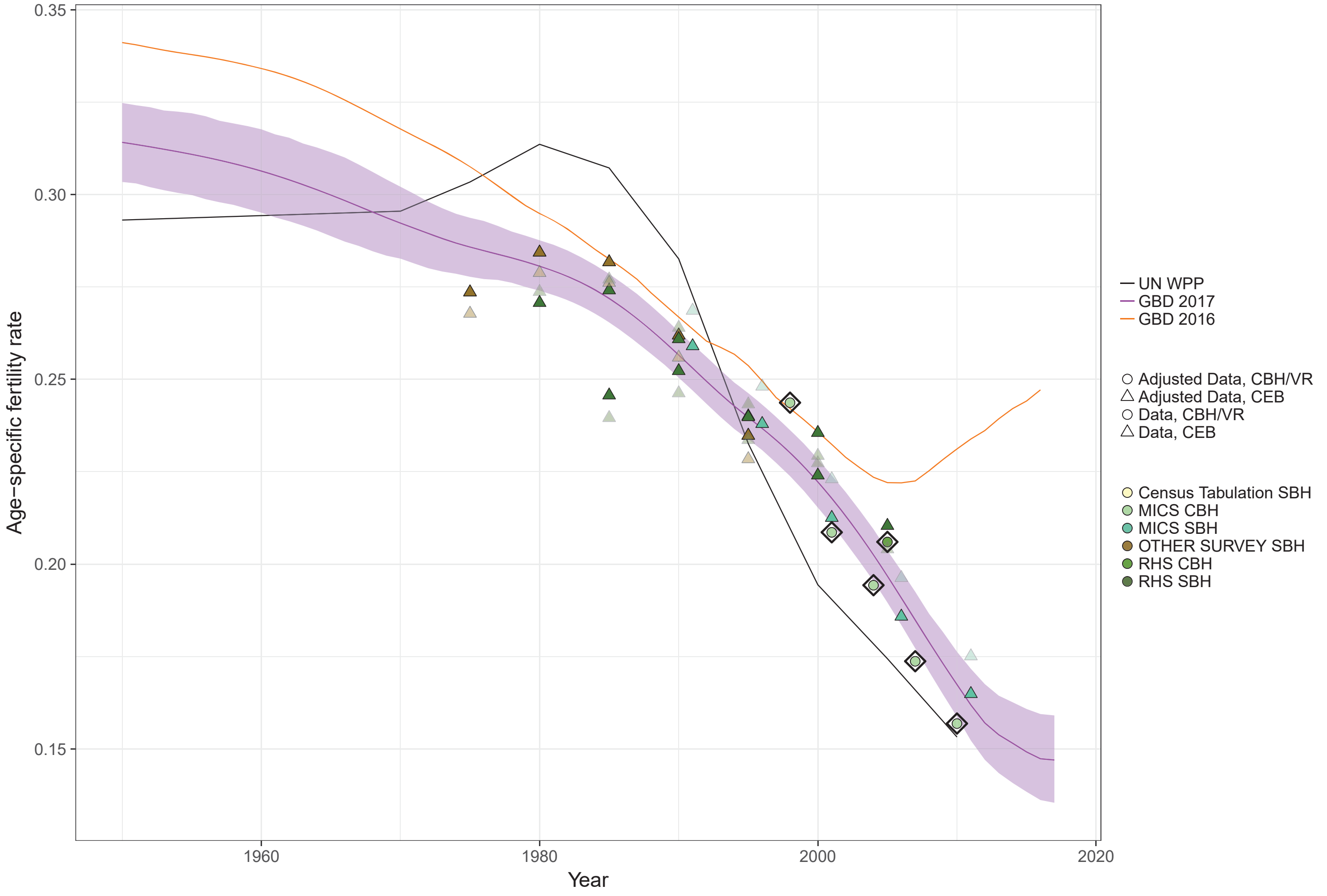

Age−specific fertility fitted trend lines, 1950−2017: Laos, Ages: 30−34
Data points enclosed with a diamond are reference sources

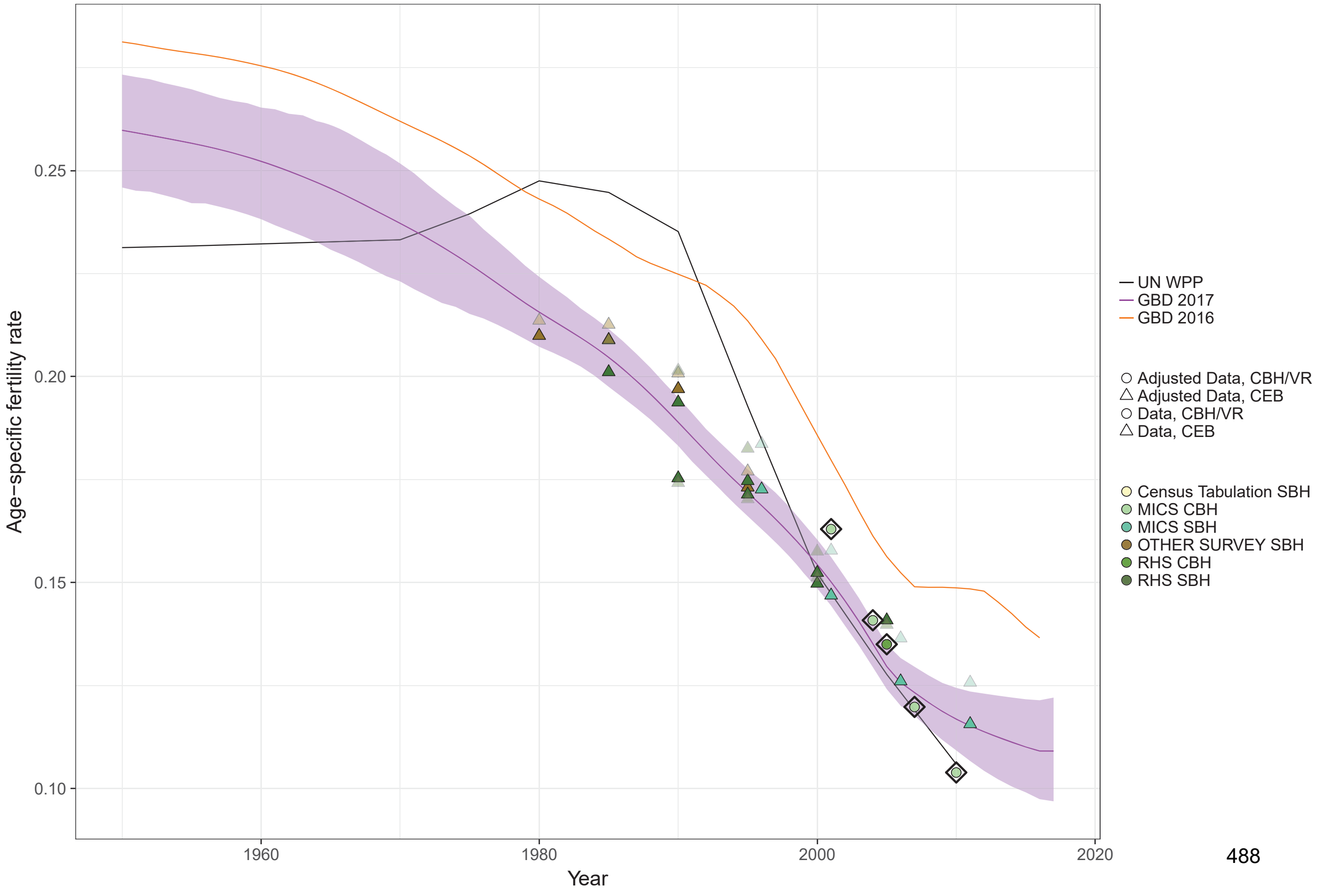

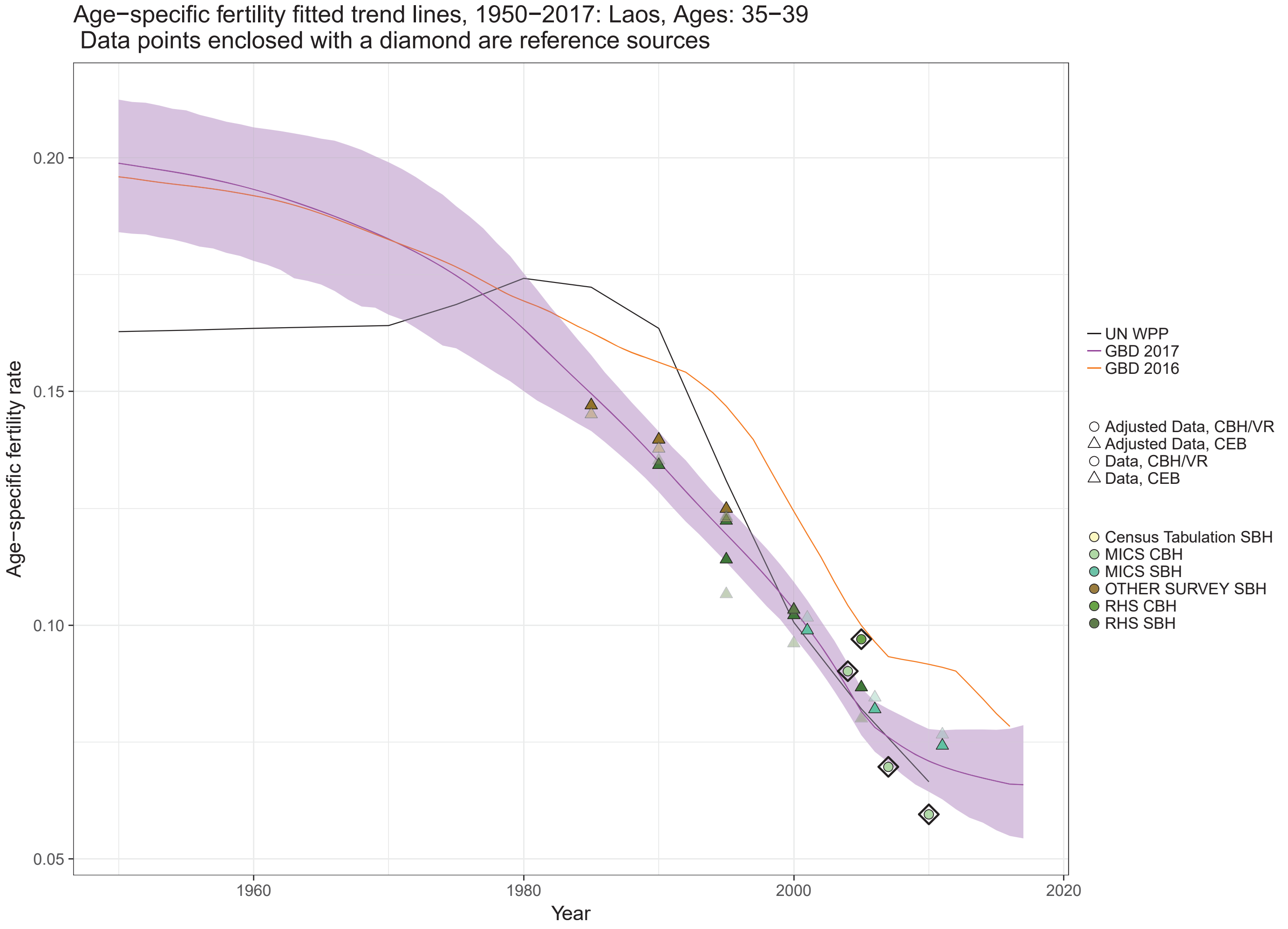
Age−specific fertility fitted trend lines, 1950−2017: Laos, Ages: 35−39
Data points enclosed with a diamond are reference sources
Age−specific fertility rate
0.20
0.15
0.10
0.05
1960
1980
2000
2020
Year
UN WPP
GBD 2017
GBD 2016
Adjusted Data, CBH/VR
Adjusted Data, CEB
Data, CBH/VR
Data, CEB
Census Tabulation SBH
MICS CBH
MICS SBH
OTHER SURVEY SBH
RHS CBH
RHS SBH

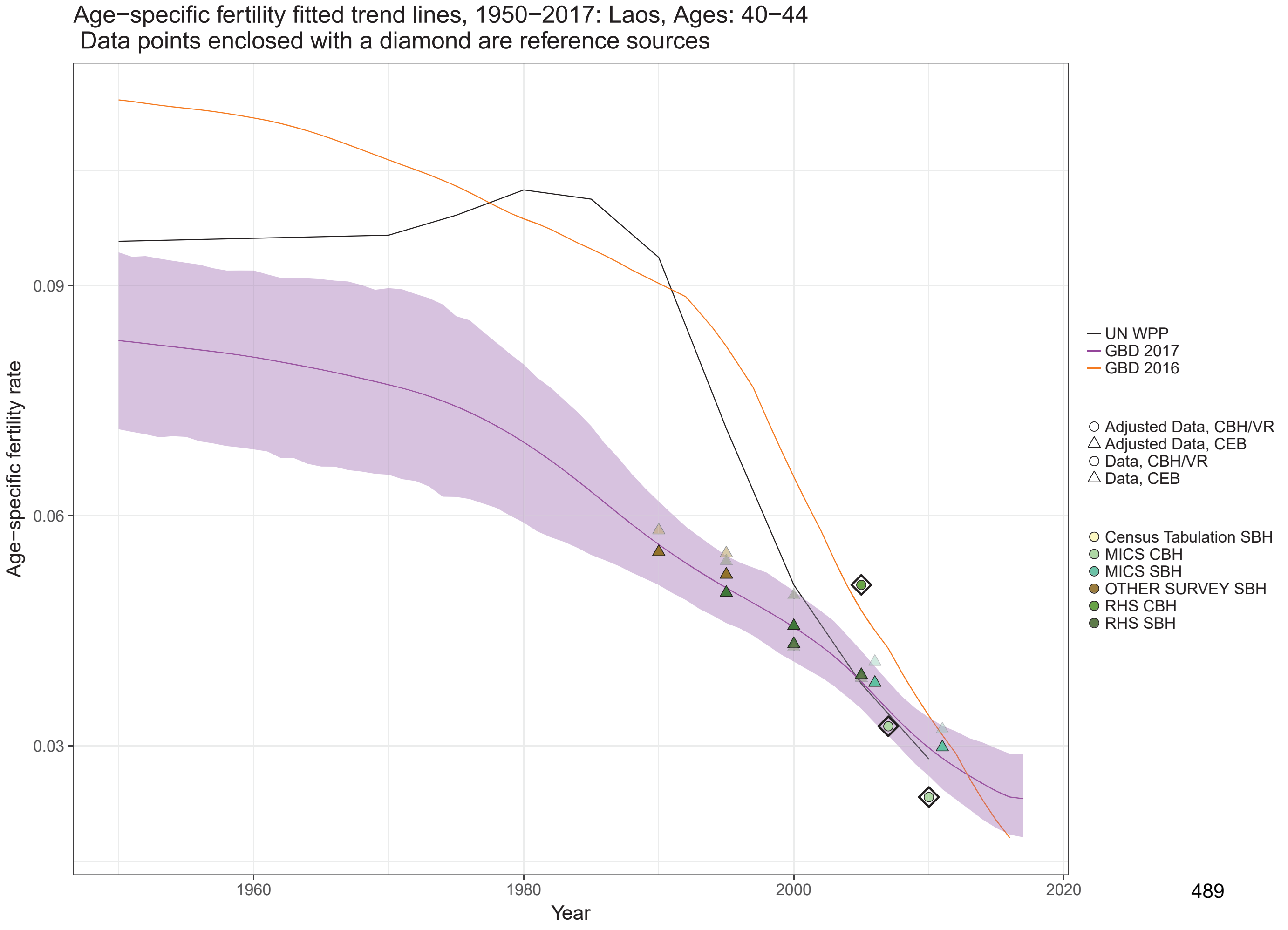
Age−specific fertility fitted trend lines, 1950−2017: Laos, Ages: 40−44
Data points enclosed with a diamond are reference sources
Age−specific fertility rate
0.09
0.06
0.03
1960
1980
2000
2020
Year
UN WPP
GBD 2017
GBD 2016
Adjusted Data, CBH/VR
Adjusted Data, CEB
Data, CBH/VR
Data, CEB
Census Tabulation SBH
MICS CBH
MICS SBH
OTHER SURVEY SBH
RHS CBH
RHS SBH

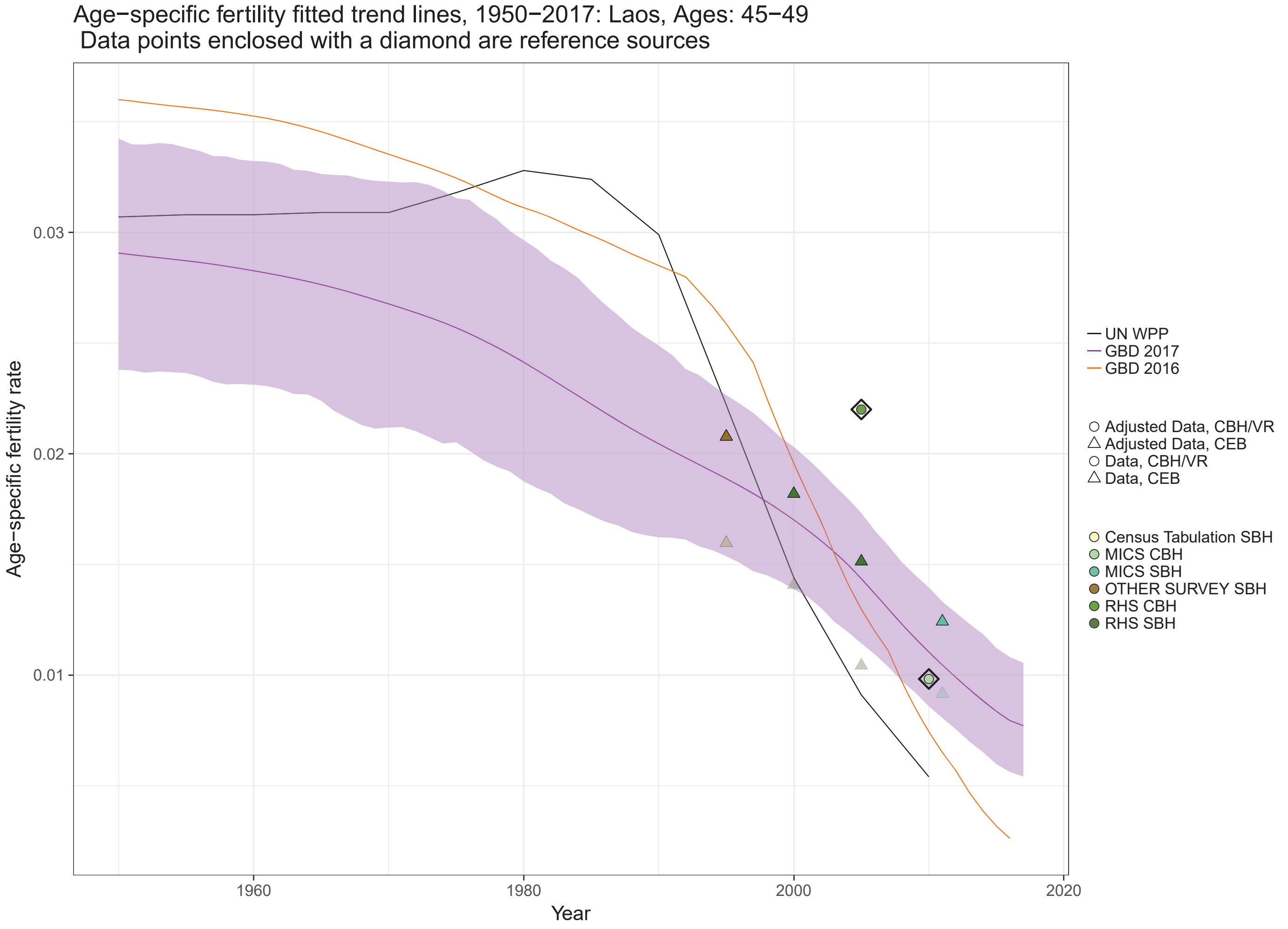

Age−specific fertility fitted trend lines, 1950−2017: Laos, Ages: 45−49
Data points enclosed with a diamond are reference sources
Age−specific fertility rate
0.03
0.02
0.01
1960
1980
2000
2020
Year
UN WPP
GBD 2017
GBD 2016
Adjusted Data, CBH/VR
Adjusted Data, CEB
Data, CBH/VR
Data, CEB
Census Tabulation SBH
MICS CBH
MICS SBH
OTHER SURVEY SBH
RHS CBH
RHS SBH

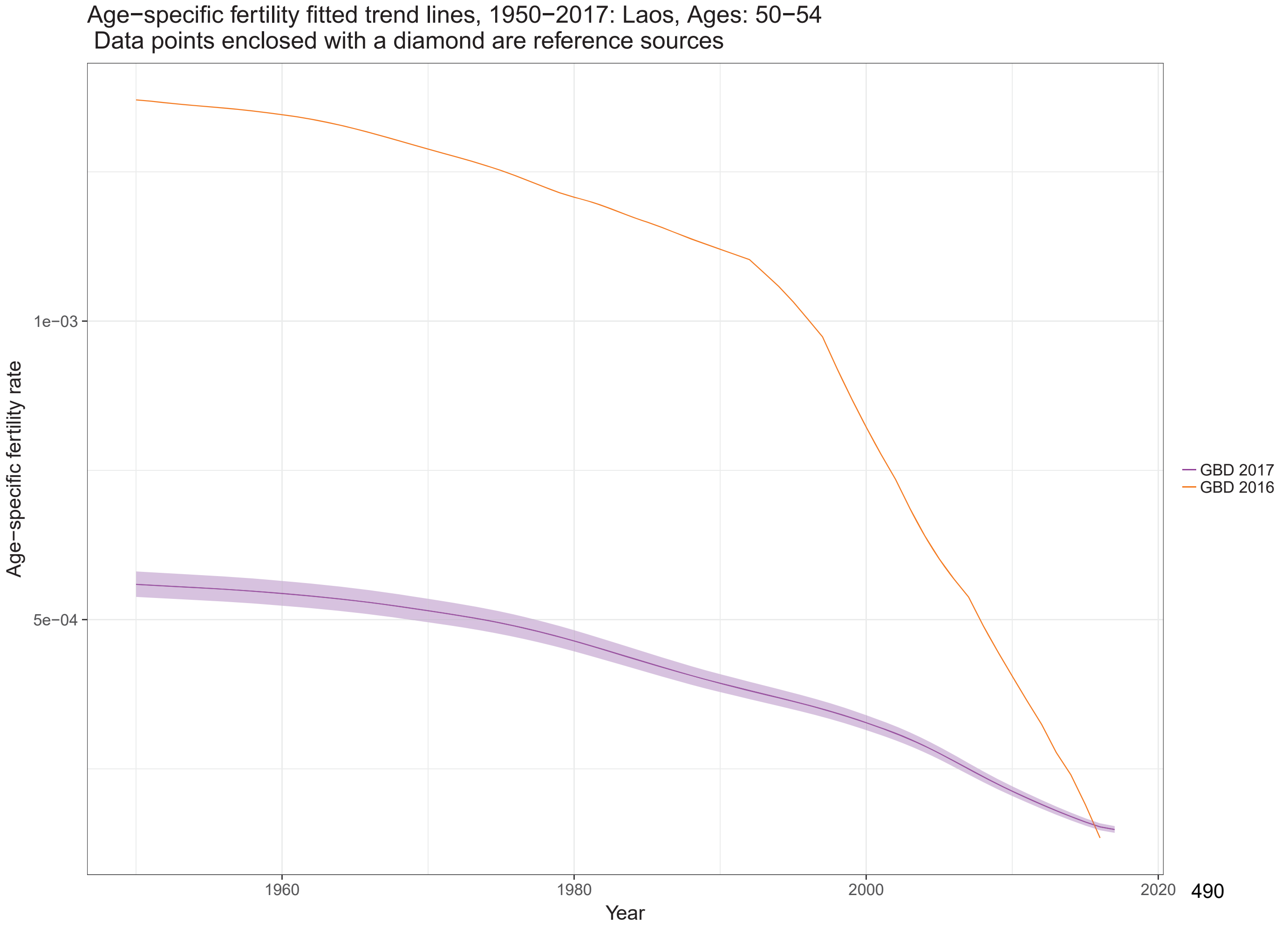

Age−specific fertility fitted trend lines, 1950−2017: Laos, Ages: 50−54
Data points enclosed with a diamond are reference sources
Age−specific fertility rate
1e−03
5e−04
1960
1980
2000
2020
Year
GBD 2017
GBD 2016

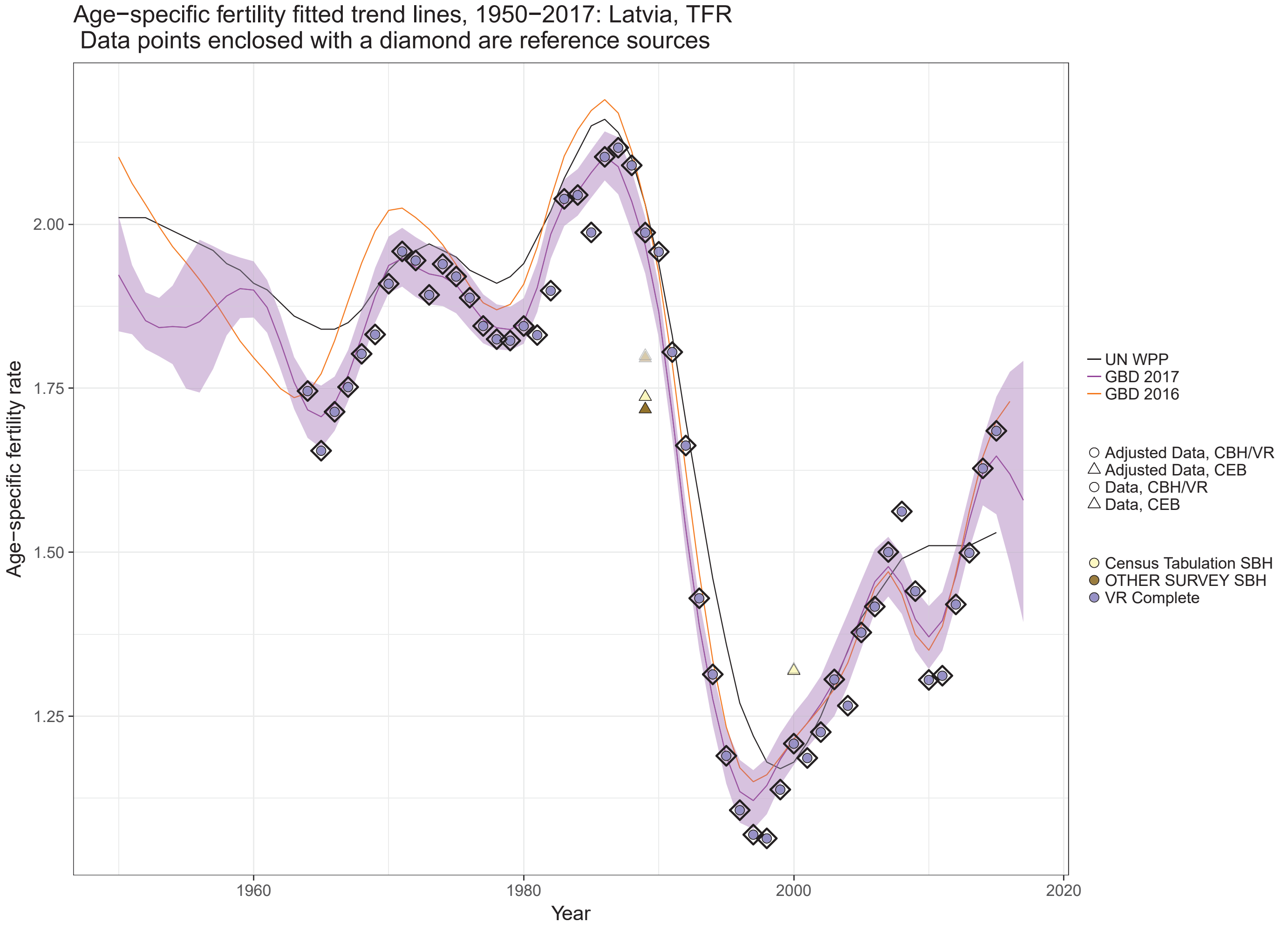
Age−specific fertility fitted trend lines, 1950−2017: Latvia, TFR
Data points enclosed with a diamond are reference sources
Age−specific fertility rate
Year
UN WPP
GBD 2017
GBD 2016
Adjusted Data, CBH/VR
Adjusted Data, CEB
Data, CBH/VR
Data, CEB
Census Tabulation SBH
OTHER SURVEY SBH
VR Complete

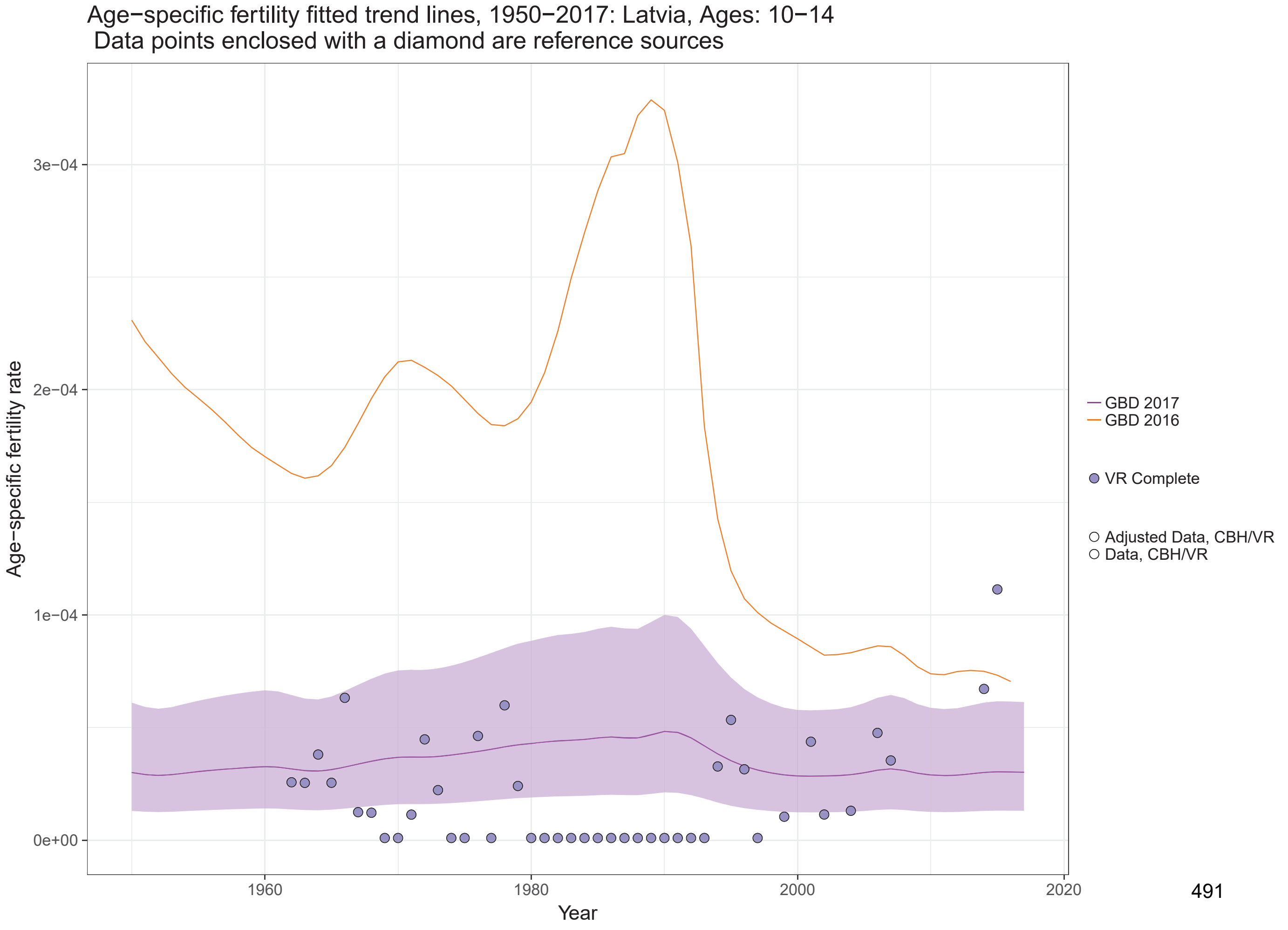
Age−specific fertility fitted trend lines, 1950−2017: Latvia, Ages: 10−14
Data points enclosed with a diamond are reference sources
Age−specific fertility rate
Year
GBD 2017
GBD 2016
VR Complete
Adjusted Data, CBH/VR
Data, CBH/VR

Age−specific fertility fitted trend lines, 1950−2017: Latvia, Ages: 15−19
Data points enclosed with a diamond are reference sources

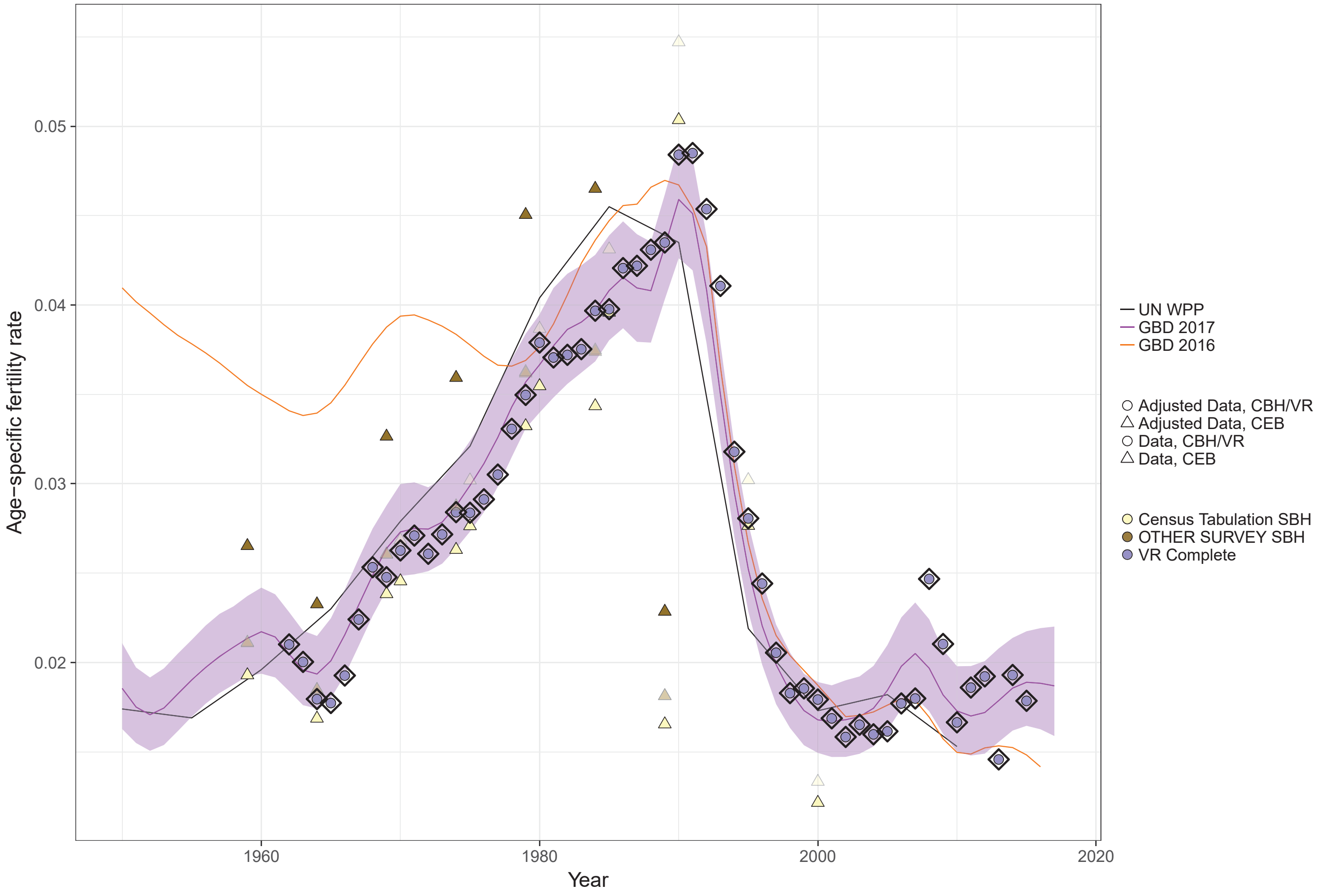

Age−specific fertility fitted trend lines, 1950−2017: Latvia, Ages: 20−24
Data points enclosed with a diamond are reference sources

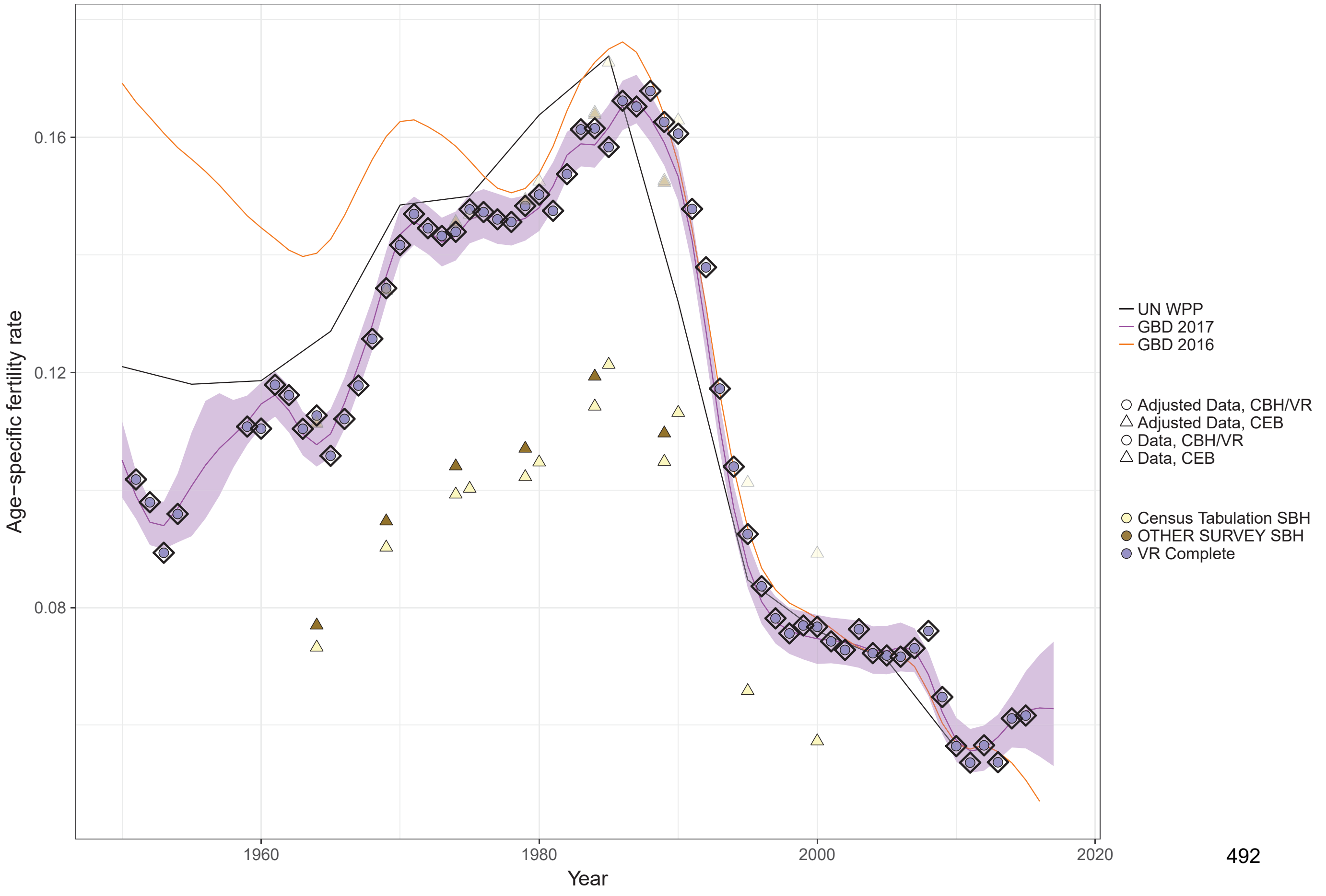

Age−specific fertility fitted trend lines, 1950−2017: Latvia, Ages: 25−29
Data points enclosed with a diamond are reference sources

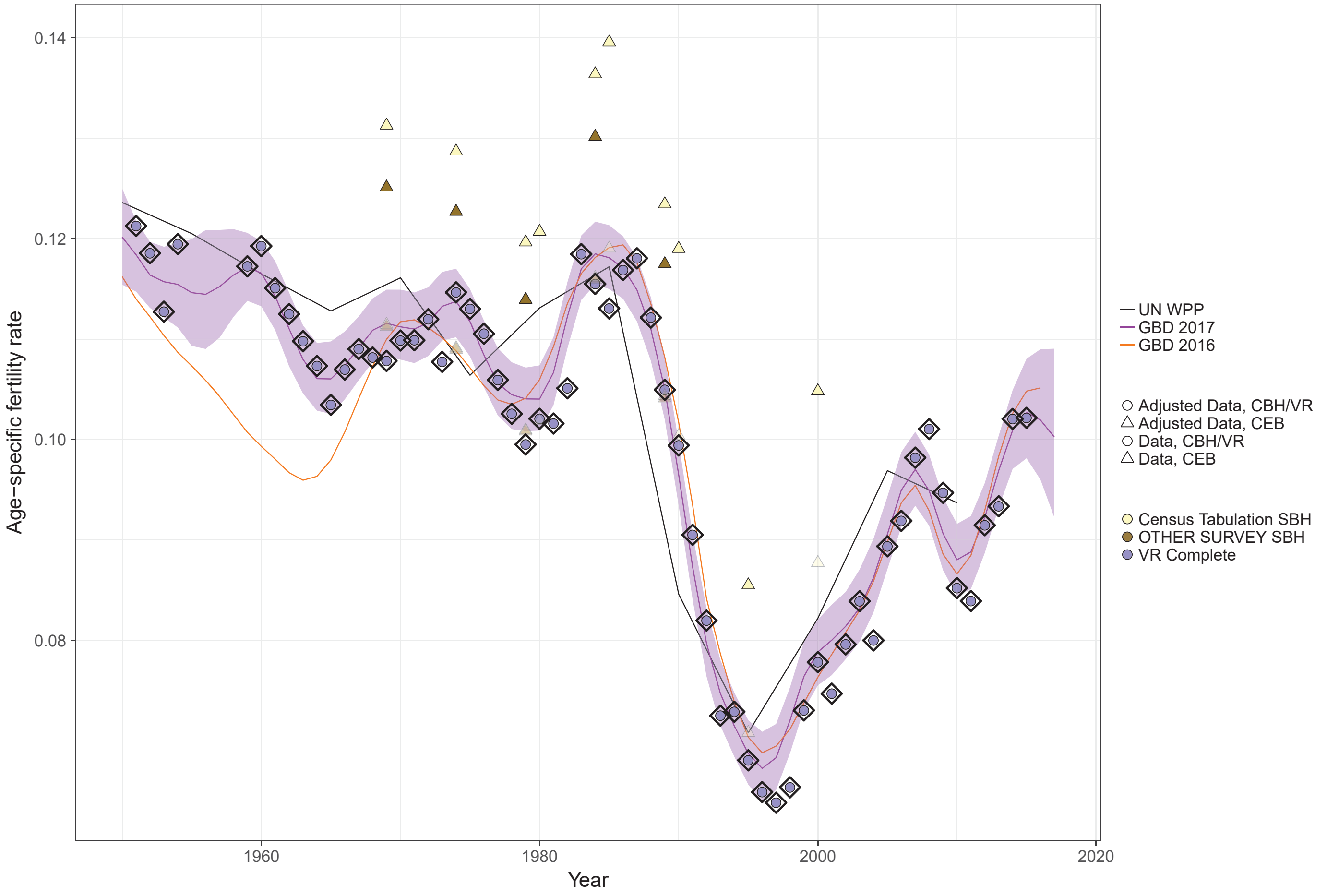

Age−specific fertility fitted trend lines, 1950−2017: Latvia, Ages: 30−34
Data points enclosed with a diamond are reference sources

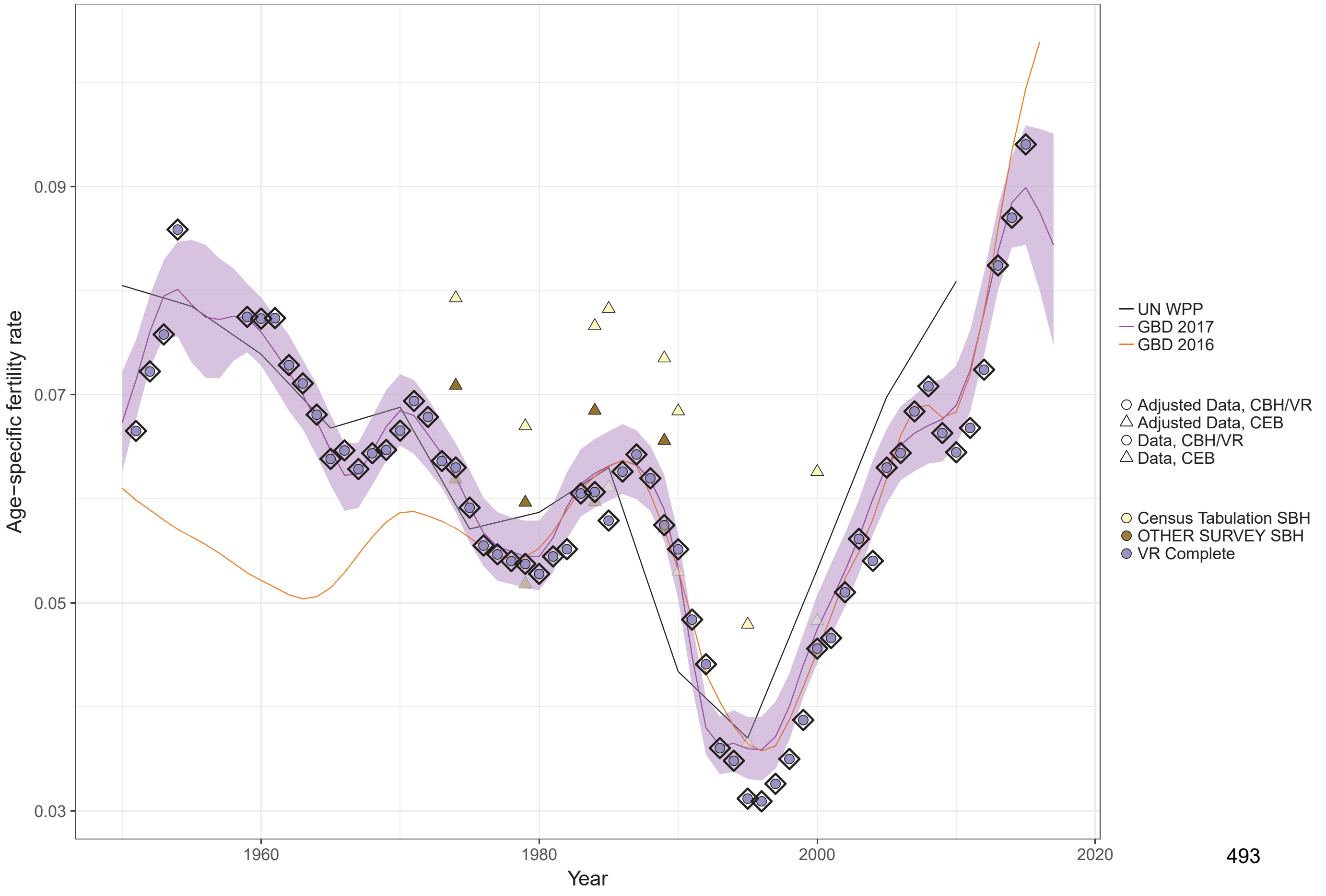

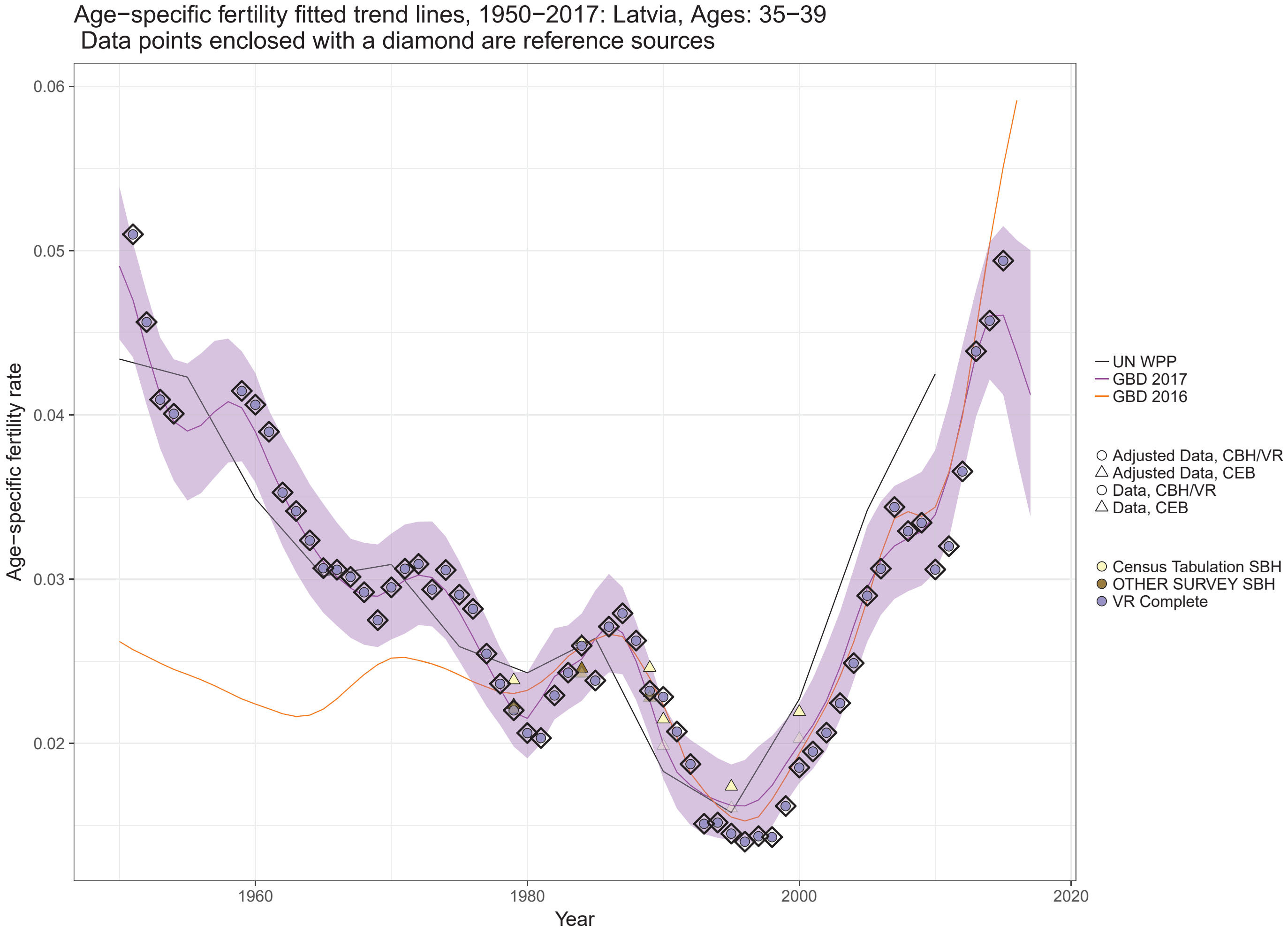

Age−specific fertility fitted trend lines, 1950−2017: Latvia, Ages: 35−39
Data points enclosed with a diamond are reference sources
Age−specific fertility rate
Year
UN WPP
GBD 2017
GBD 2016
Adjusted Data, CBH/VR
Adjusted Data, CEB
Data, CBH/VR
Data, CEB
Census Tabulation SBH
OTHER SURVEY SBH
VR Complete

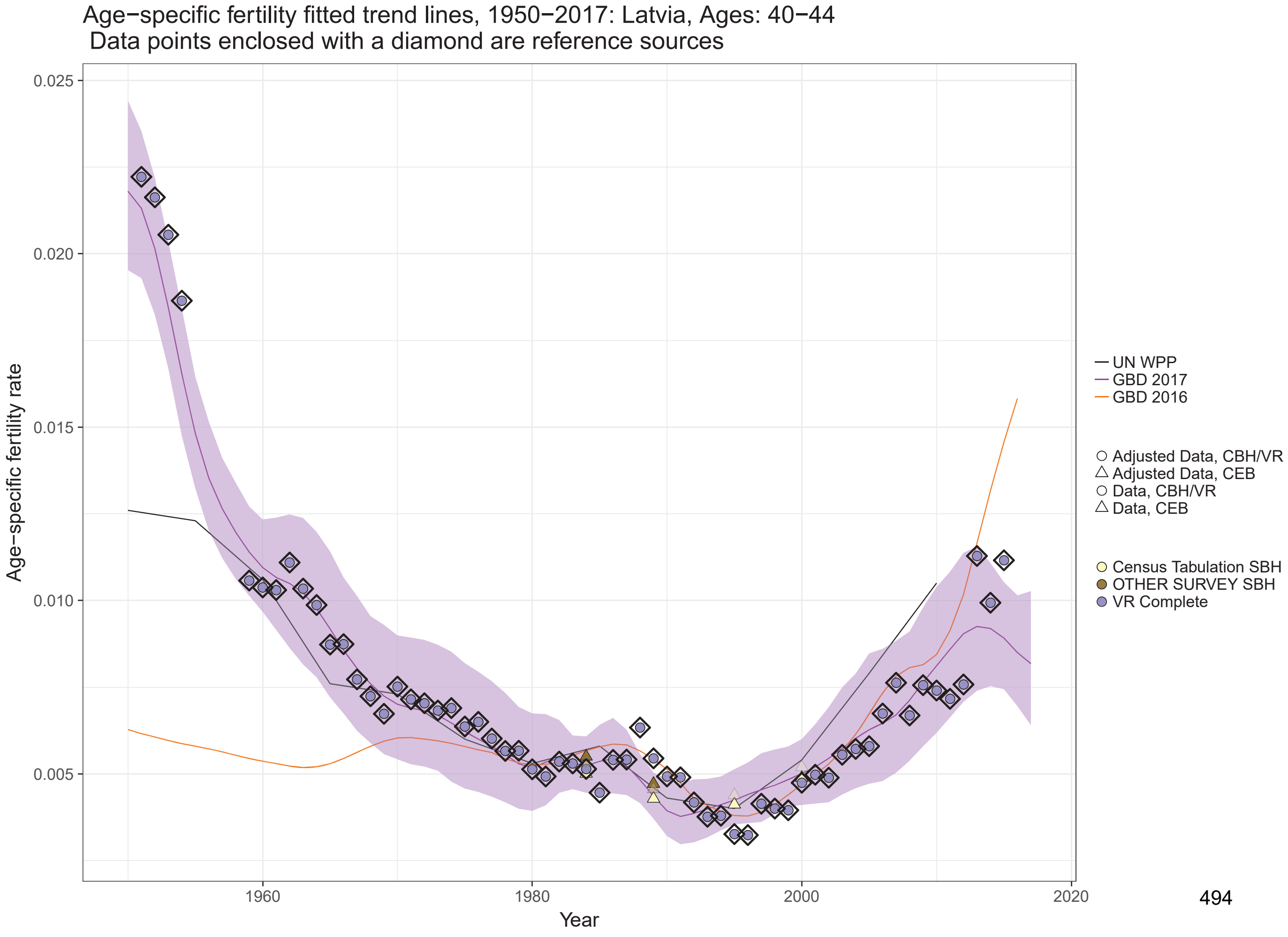

Age−specific fertility fitted trend lines, 1950−2017: Latvia, Ages: 40−44
Data points enclosed with a diamond are reference sources
Age−specific fertility rate
Year
UN WPP
GBD 2017
GBD 2016
Adjusted Data, CBH/VR
Adjusted Data, CEB
Data, CBH/VR
Data, CEB
Census Tabulation SBH
OTHER SURVEY SBH
VR Complete

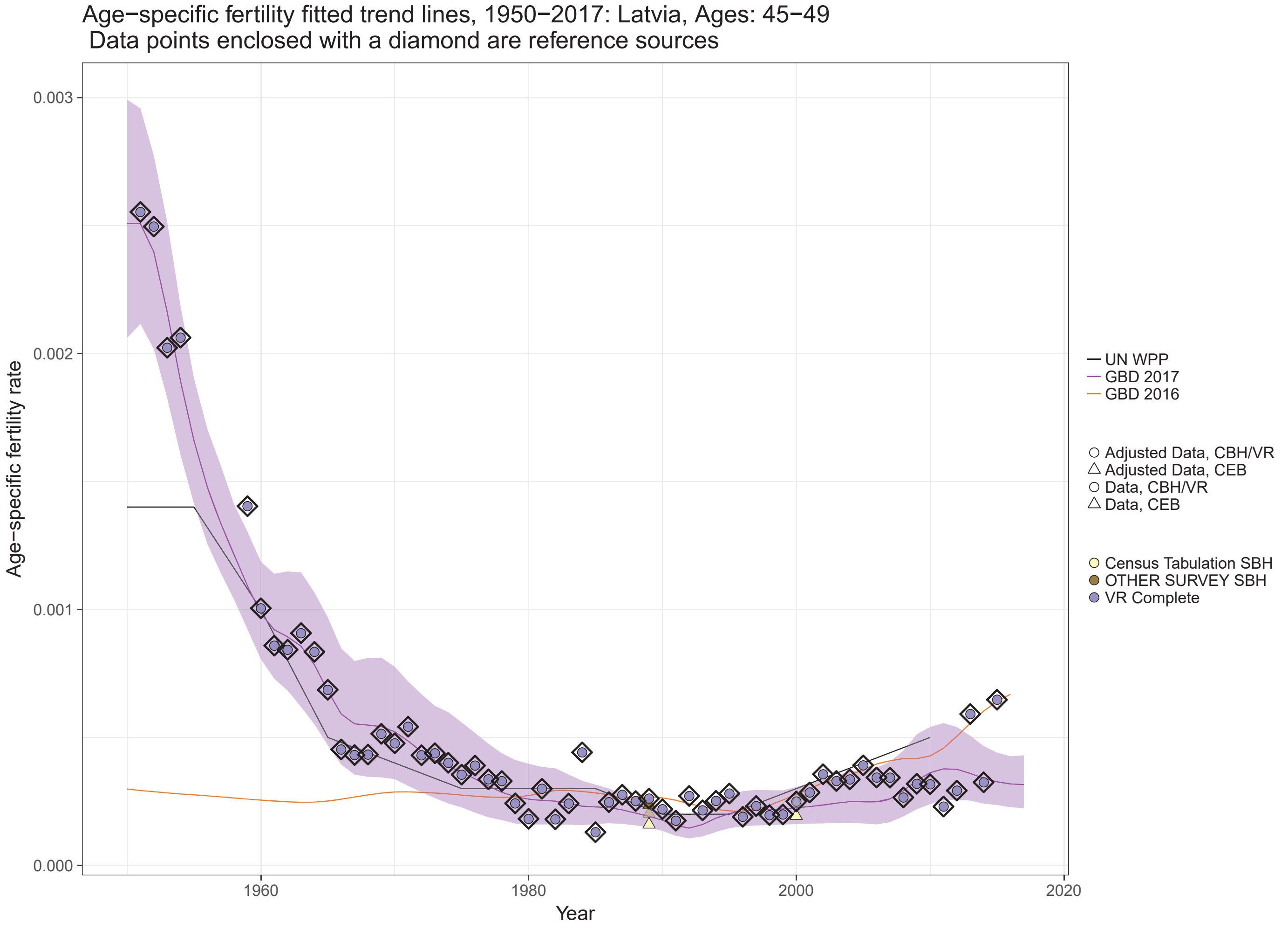
Age−specific fertility fitted trend lines, 1950−2017: Latvia, Ages: 45−49
Data points enclosed with a diamond are reference sources
Age−specific fertility rate
0.003
0.002
0.001
0.000
1960
1980
2000
2020
Year
UN WPP
GBD 2017
GBD 2016
Adjusted Data, CBH/VR
Adjusted Data, CEB
Data, CBH/VR
Data, CEB
Census Tabulation SBH
OTHER SURVEY SBH
VR Complete

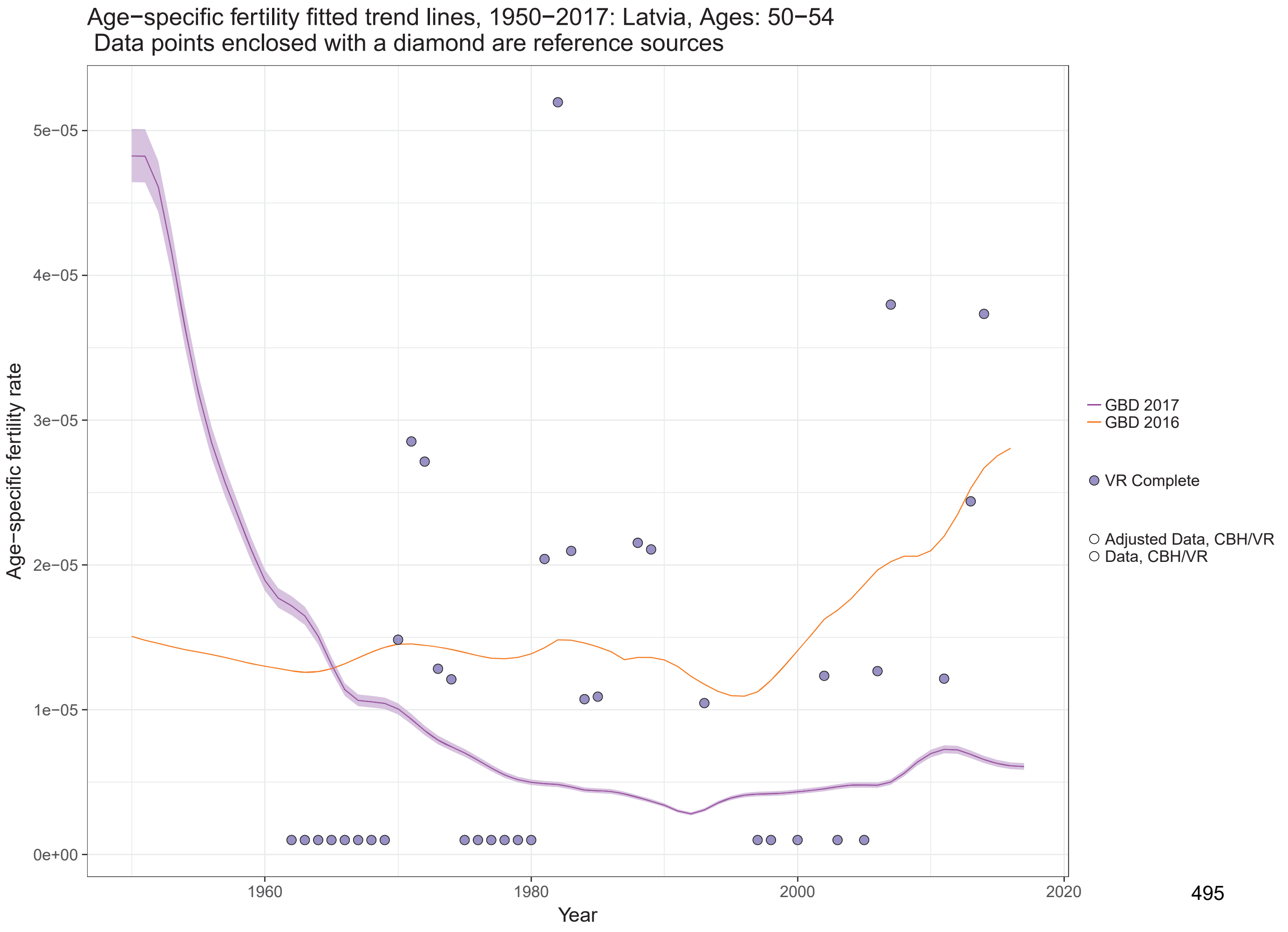
Age−specific fertility fitted trend lines, 1950−2017: Latvia, Ages: 50−54
Data points enclosed with a diamond are reference sources
Age−specific fertility rate
5e−05
4e−05
3e−05
2e−05
1e−05
0e+00
1960
1980
2000
2020
Year
GBD 2017
GBD 2016
VR Complete
Adjusted Data, CBH/VR
Data, CBH/VR

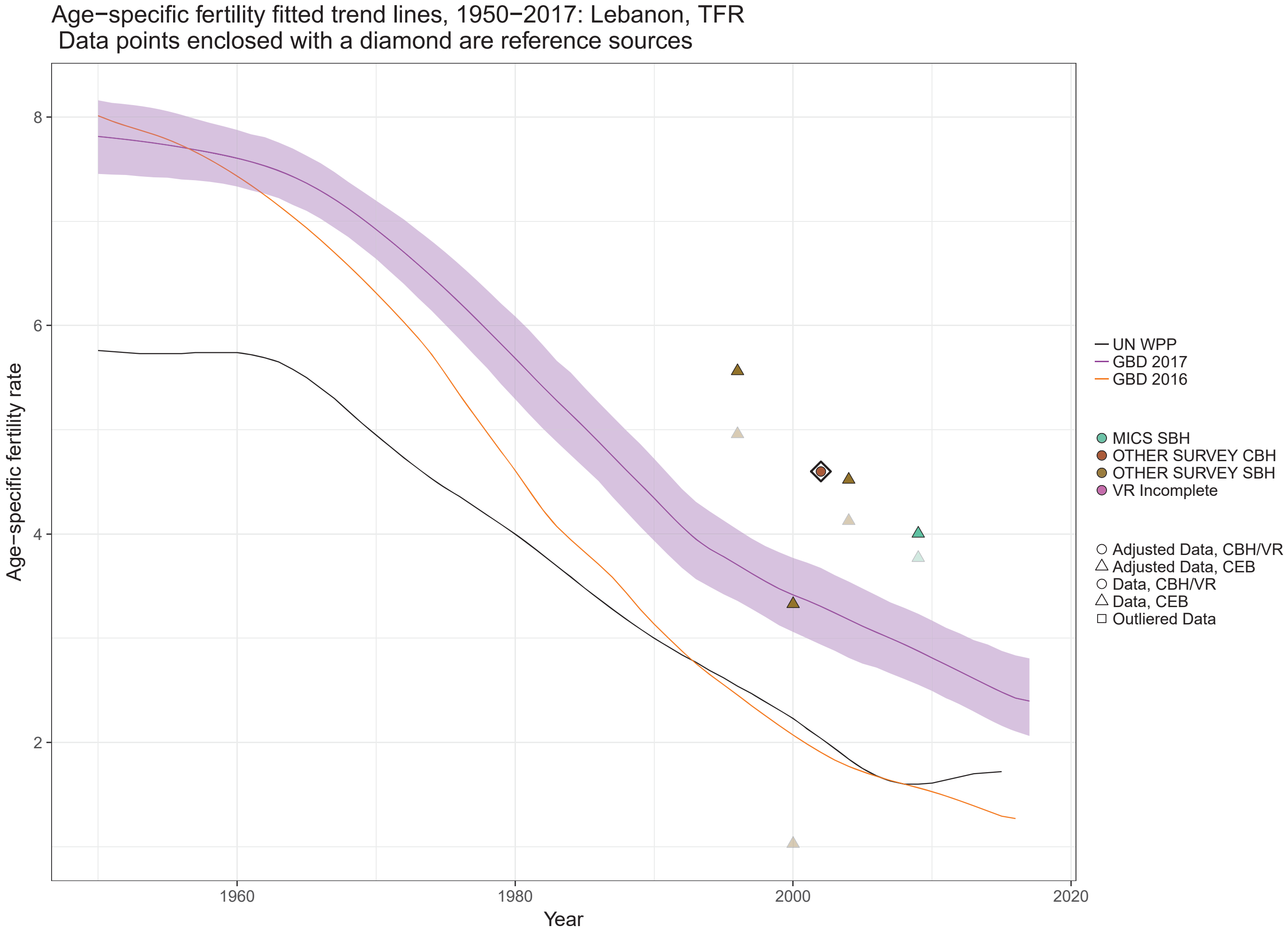

Age−specific fertility fitted trend lines, 1950−2017: Lebanon, TFR
Data points enclosed with a diamond are reference sources
Age−specific fertility rate
Year
UN WPP
GBD 2017
GBD 2016
MICS SBH
OTHER SURVEY CBH
OTHER SURVEY SBH
VR Incomplete
Adjusted Data, CBH/VR
Adjusted Data, CEB
Data, CBH/VR
Data, CEB
Outliered Data

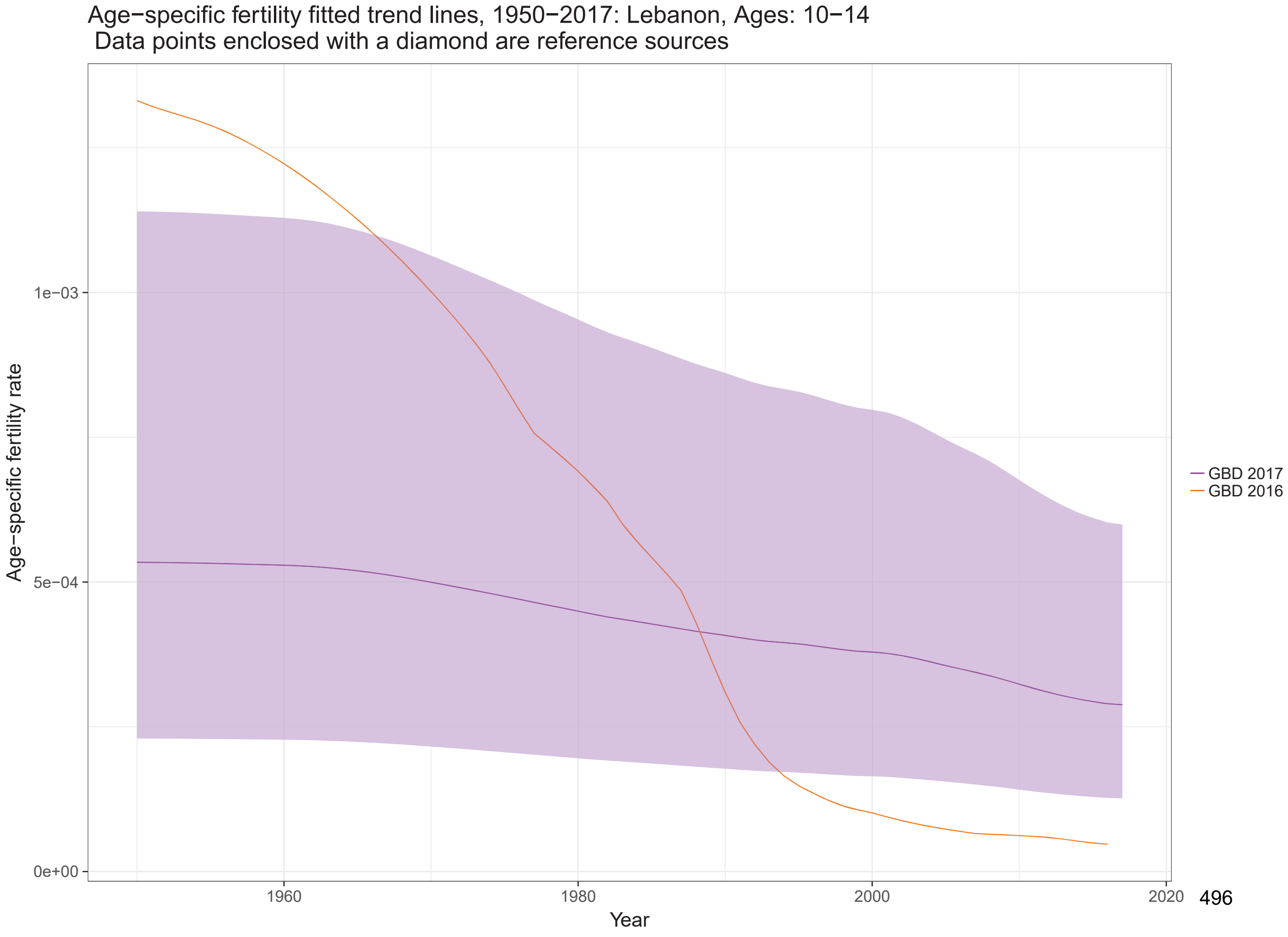

Age−specific fertility fitted trend lines, 1950−2017: Lebanon, Ages: 10−14
Data points enclosed with a diamond are reference sources
Age−specific fertility rate
Year
GBD 2017
GBD 2016

Age−specific fertility fitted trend lines, 1950−2017: Lebanon, Ages: 15−19
Data points enclosed with a diamond are reference sources

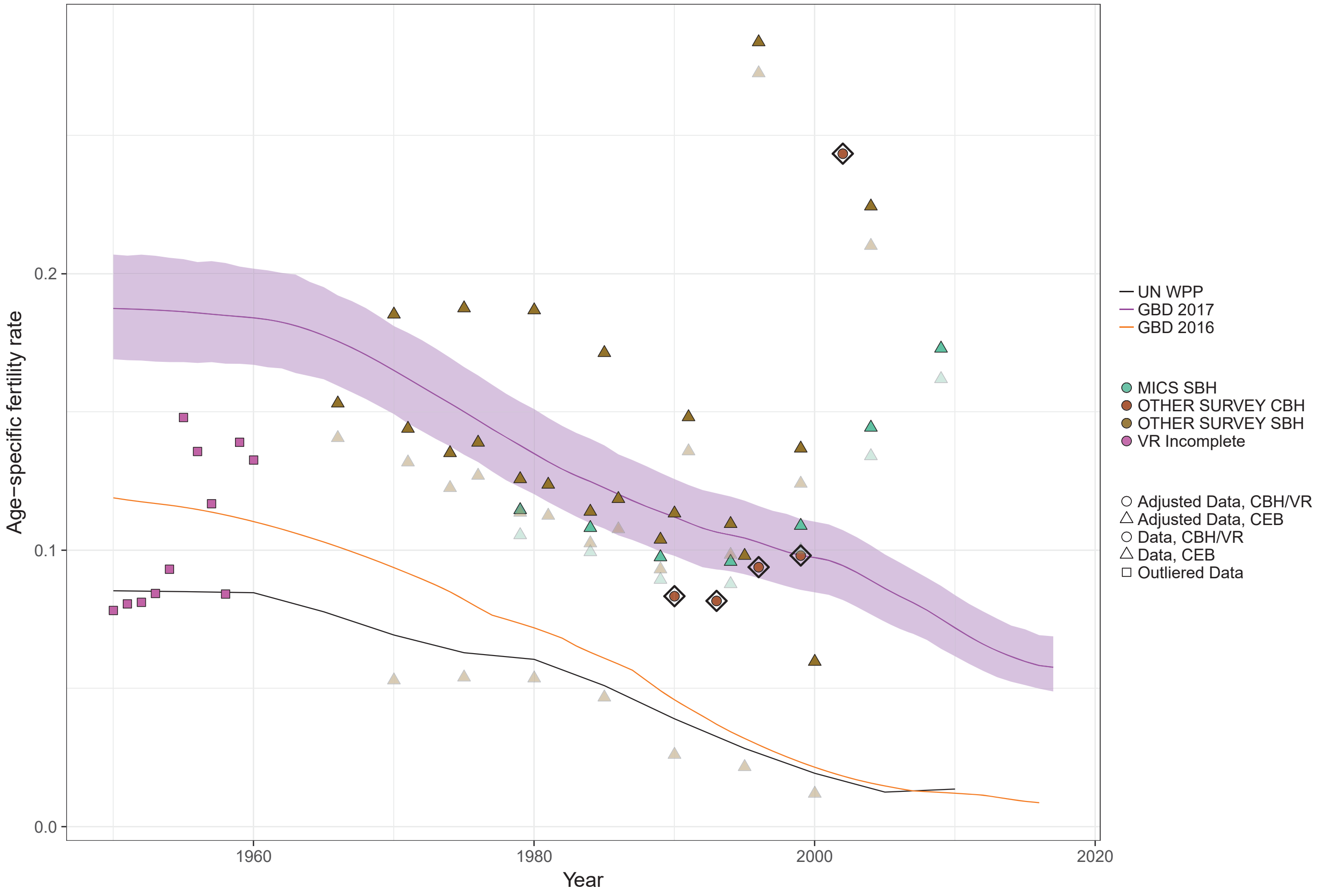

Age−specific fertility fitted trend lines, 1950−2017: Lebanon, Ages: 20−24
Data points enclosed with a diamond are reference sources

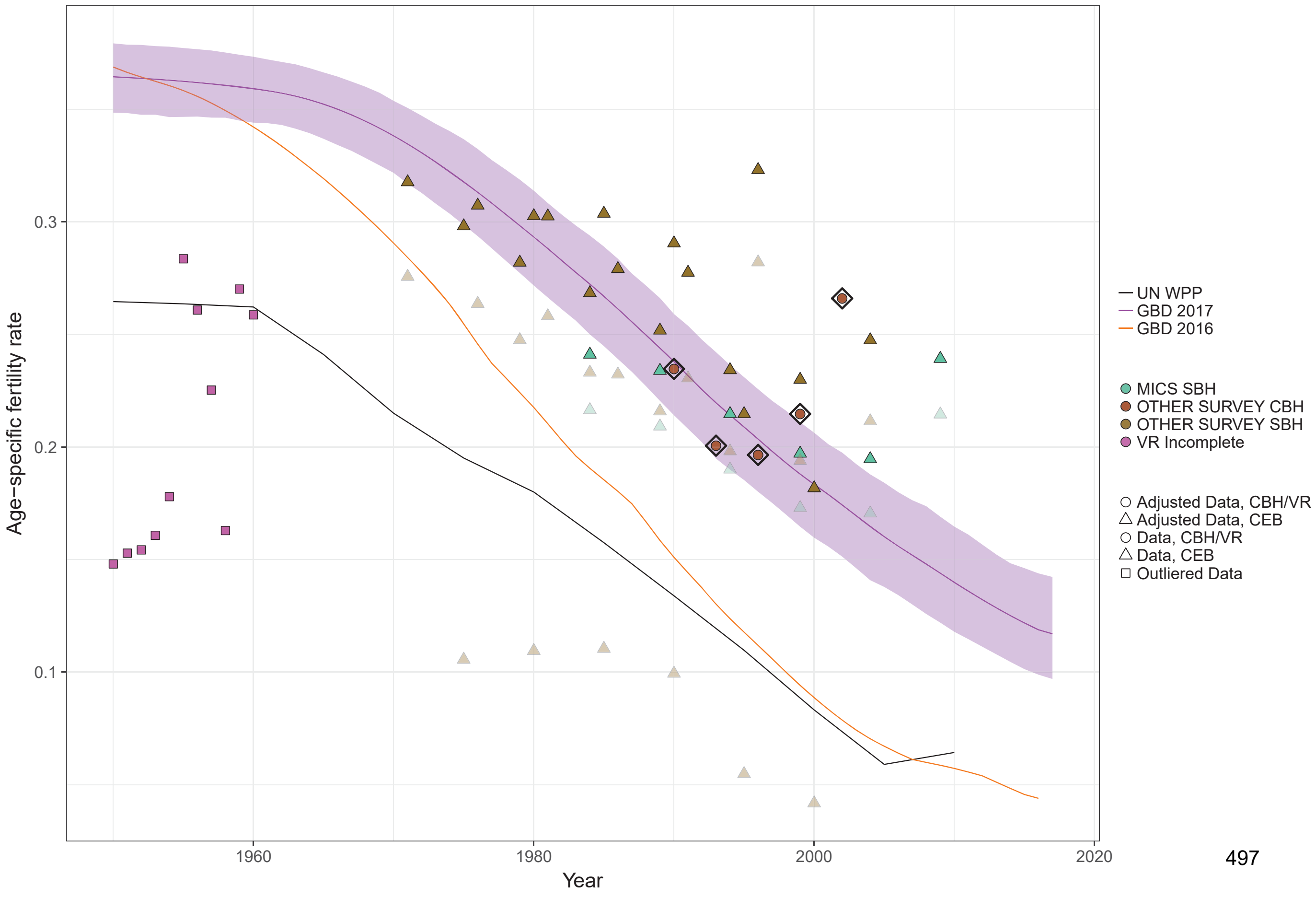

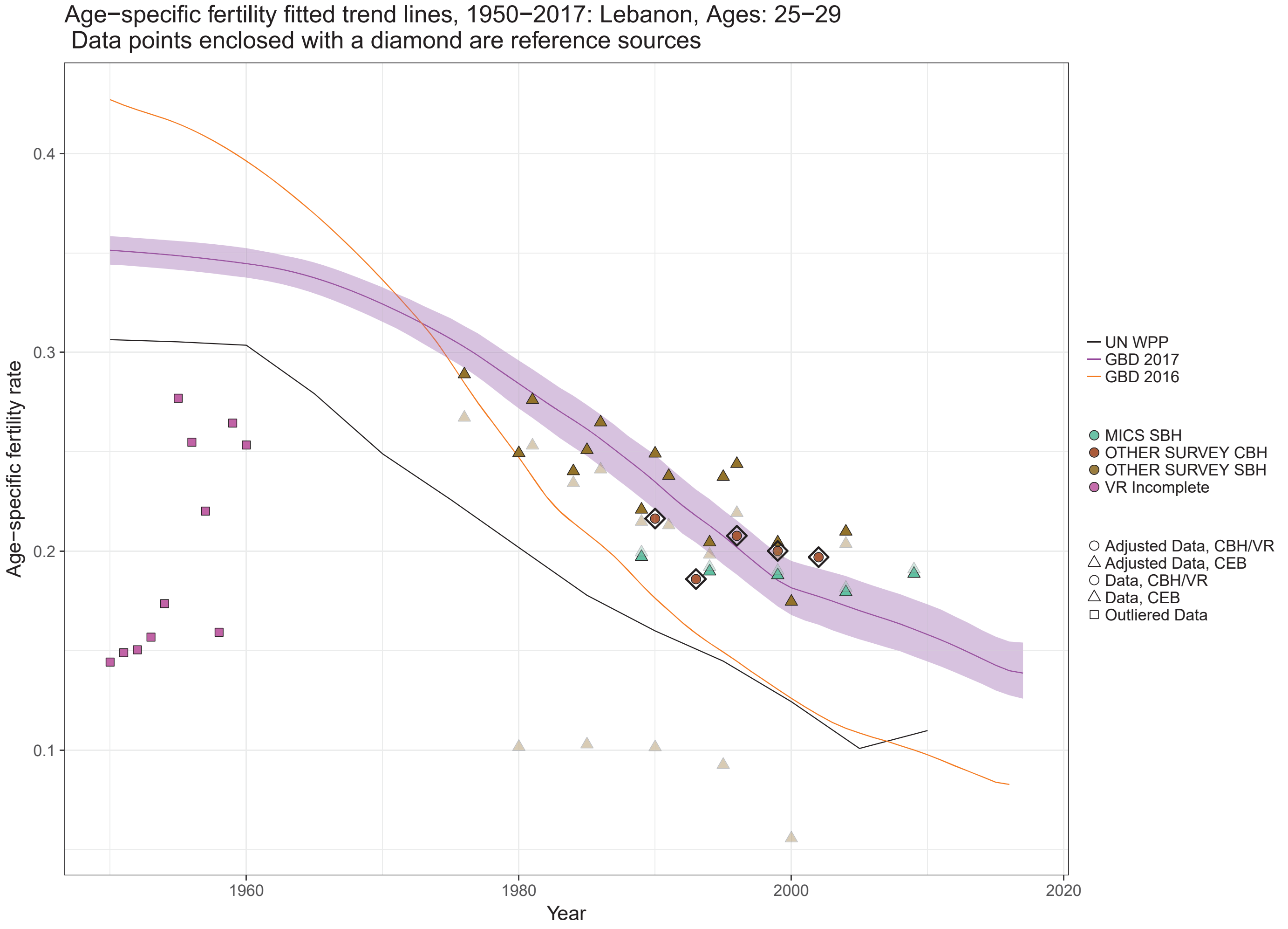
Age−specific fertility fitted trend lines, 1950−2017: Lebanon, Ages: 25−29
Data points enclosed with a diamond are reference sources
Age−specific fertility rate
Year
UN WPP
GBD 2017
GBD 2016
MICS SBH
OTHER SURVEY CBH
OTHER SURVEY SBH
VR Incomplete
Adjusted Data, CBH/VR
Adjusted Data, CEB
Data, CBH/VR
Data, CEB
Outliered Data

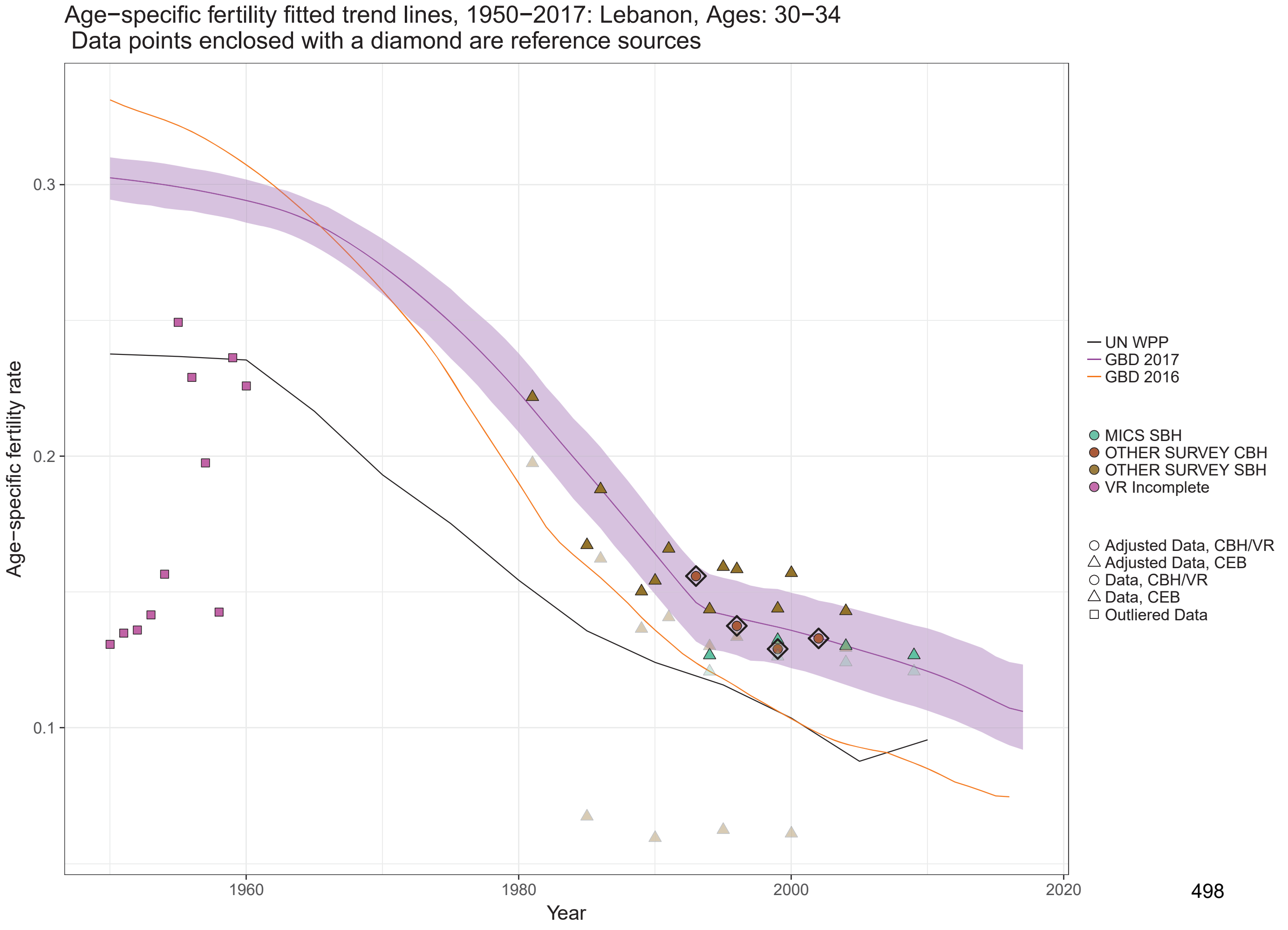
Age−specific fertility fitted trend lines, 1950−2017: Lebanon, Ages: 30−34
Data points enclosed with a diamond are reference sources
Age−specific fertility rate
Year
UN WPP
GBD 2017
GBD 2016
MICS SBH
OTHER SURVEY CBH
OTHER SURVEY SBH
VR Incomplete
Adjusted Data, CBH/VR
Adjusted Data, CEB
Data, CBH/VR
Data, CEB
Outliered Data

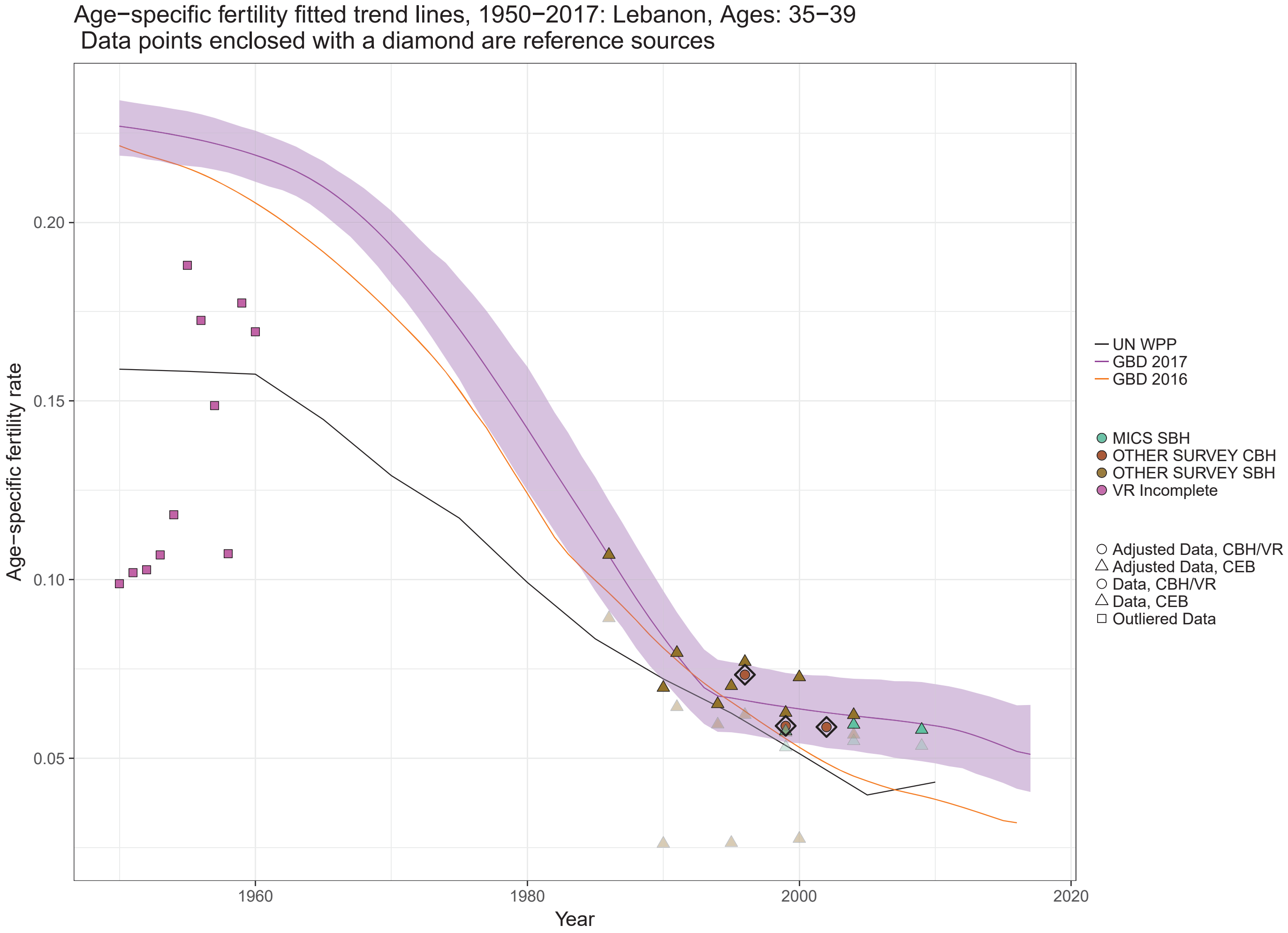
Age−specific fertility fitted trend lines, 1950−2017: Lebanon, Ages: 35−39
Data points enclosed with a diamond are reference sources
Age−specific fertility rate
0.20
0.15
0.10
0.05
1960
1980
2000
2020
Year
UN WPP
GBD 2017
GBD 2016
MICS SBH
OTHER SURVEY CBH
OTHER SURVEY SBH
VR Incomplete
Adjusted Data, CBH/VR
Adjusted Data, CEB
Data, CBH/VR
Data, CEB
Outliered Data

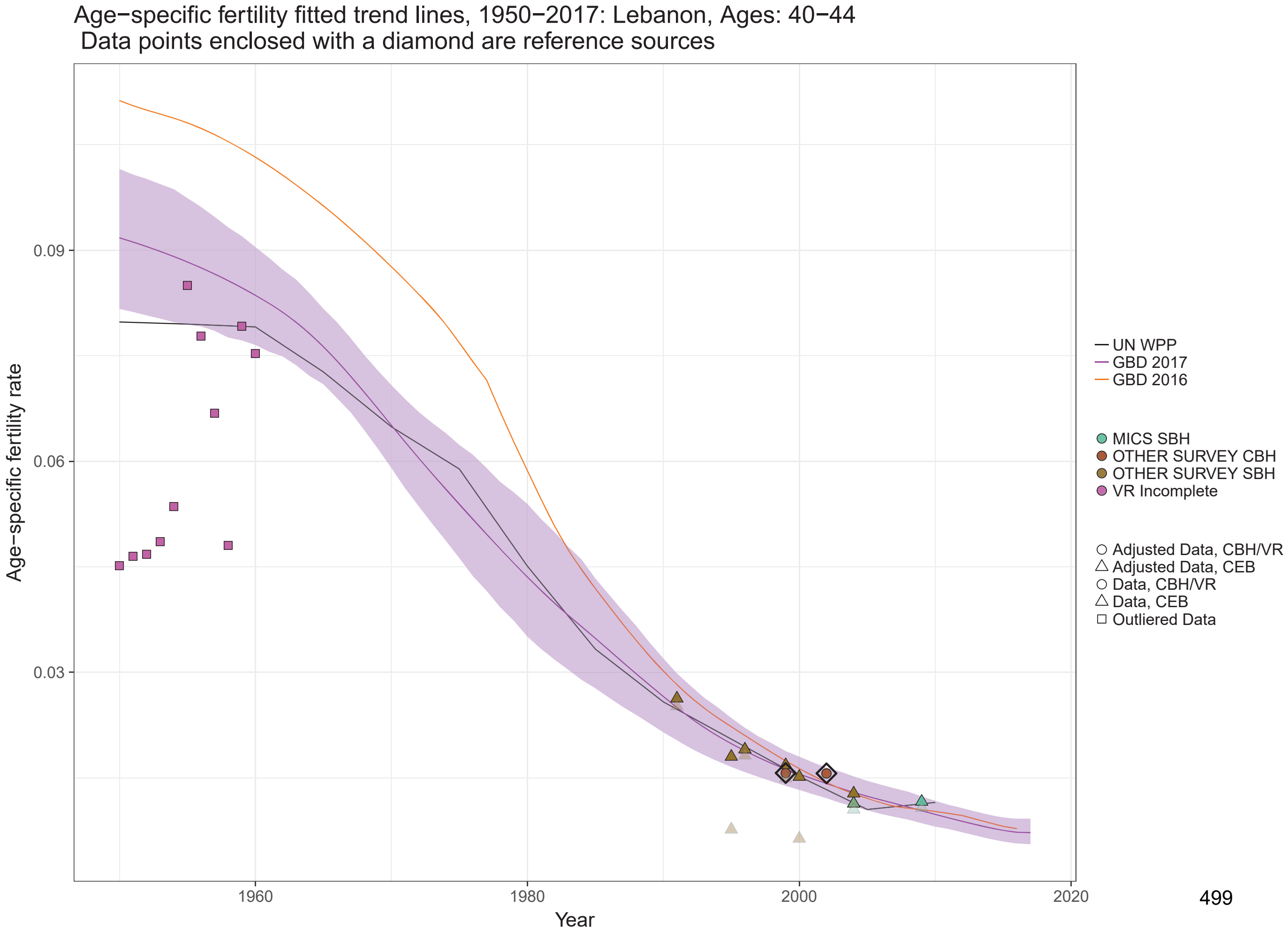
Age−specific fertility fitted trend lines, 1950−2017: Lebanon, Ages: 40−44
Data points enclosed with a diamond are reference sources
Age−specific fertility rate
0.09
0.06
0.03
1960
1980
2000
2020
Year
UN WPP
GBD 2017
GBD 2016
MICS SBH
OTHER SURVEY CBH
OTHER SURVEY SBH
VR Incomplete
Adjusted Data, CBH/VR
Adjusted Data, CEB
Data, CBH/VR
Data, CEB
Outliered Data

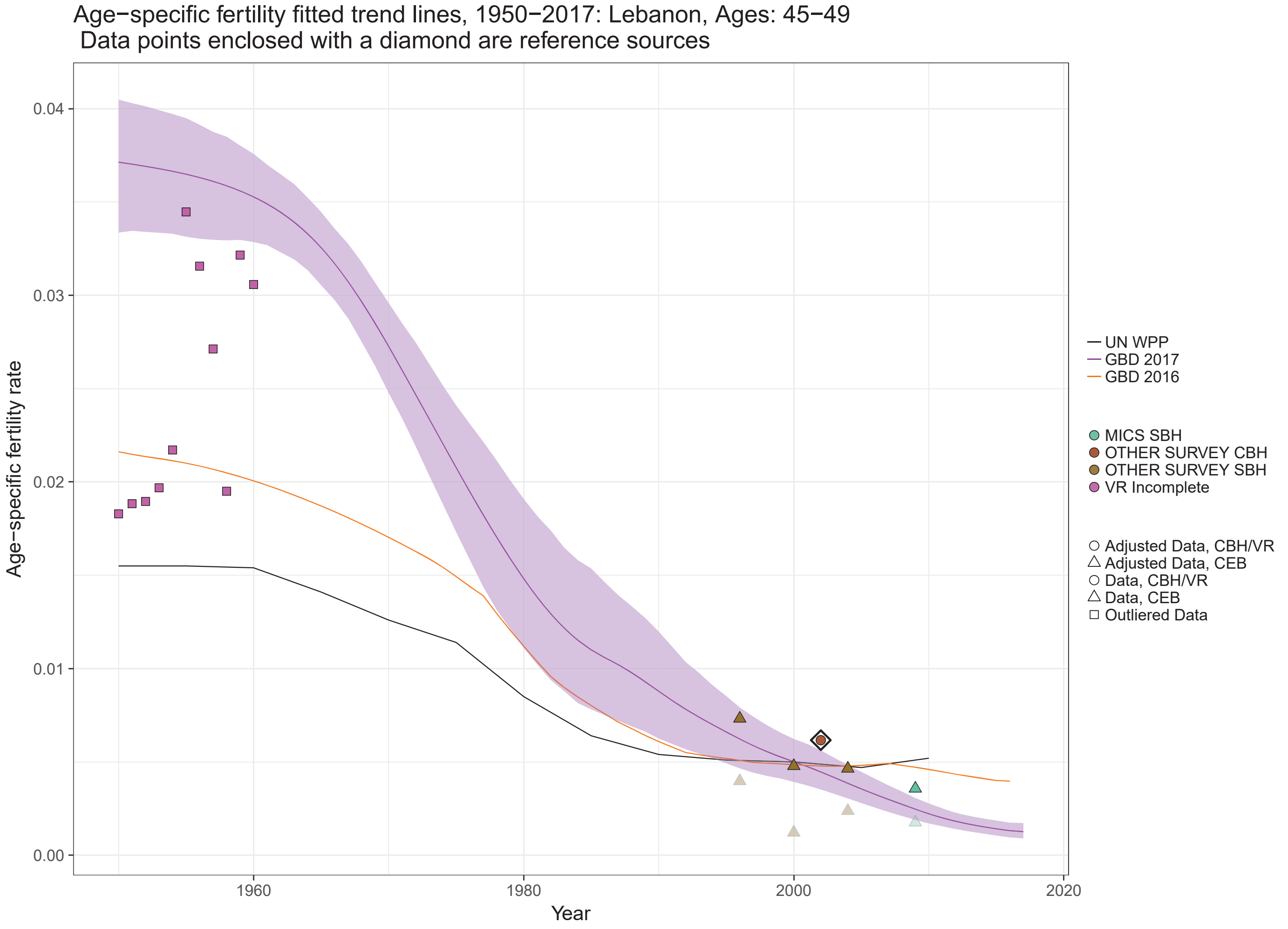

Age−specific fertility fitted trend lines, 1950−2017: Lebanon, Ages: 45−49
Data points enclosed with a diamond are reference sources
Age−specific fertility rate
Year
0.00
0.01
0.02
0.03
0.04
1960
1980
2000
2020
UN WPP
GBD 2017
GBD 2016
MICS SBH
OTHER SURVEY CBH
OTHER SURVEY SBH
VR Incomplete
Adjusted Data, CBH/VR
Adjusted Data, CEB
Data, CBH/VR
Data, CEB
Outliered Data

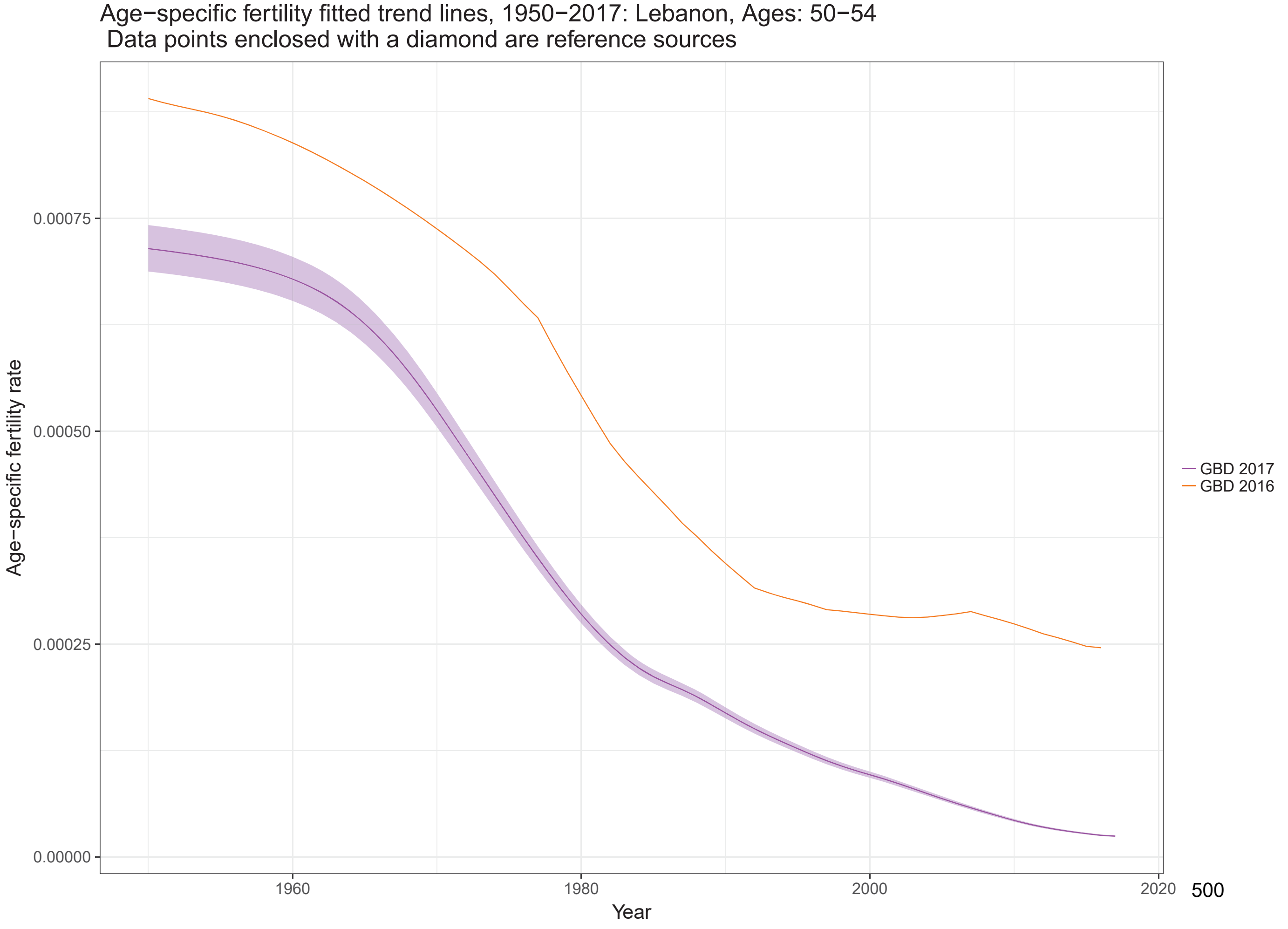

Age−specific fertility fitted trend lines, 1950−2017: Lebanon, Ages: 50−54
Data points enclosed with a diamond are reference sources
Age−specific fertility rate
Year
0.00000
0.00025
0.00050
0.00075
1960
1980
2000
2020
GBD 2017
GBD 2016

Age−specific fertility fitted trend lines, 1950−2017: Lesotho, TFR
Data points enclosed with a diamond are reference sources

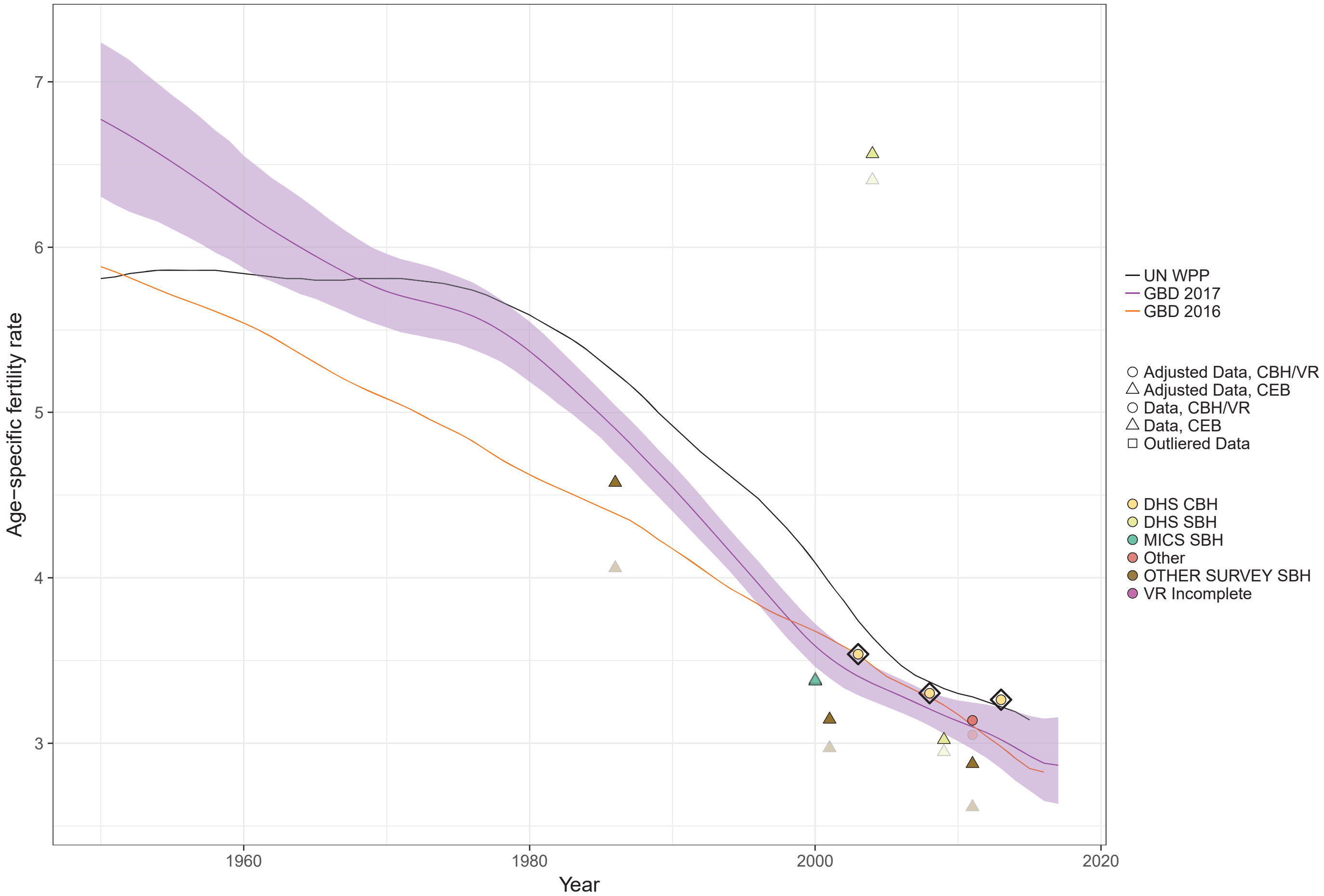

Age−specific fertility fitted trend lines, 1950−2017: Lesotho, Ages: 10−14
Data points enclosed with a diamond are reference sources

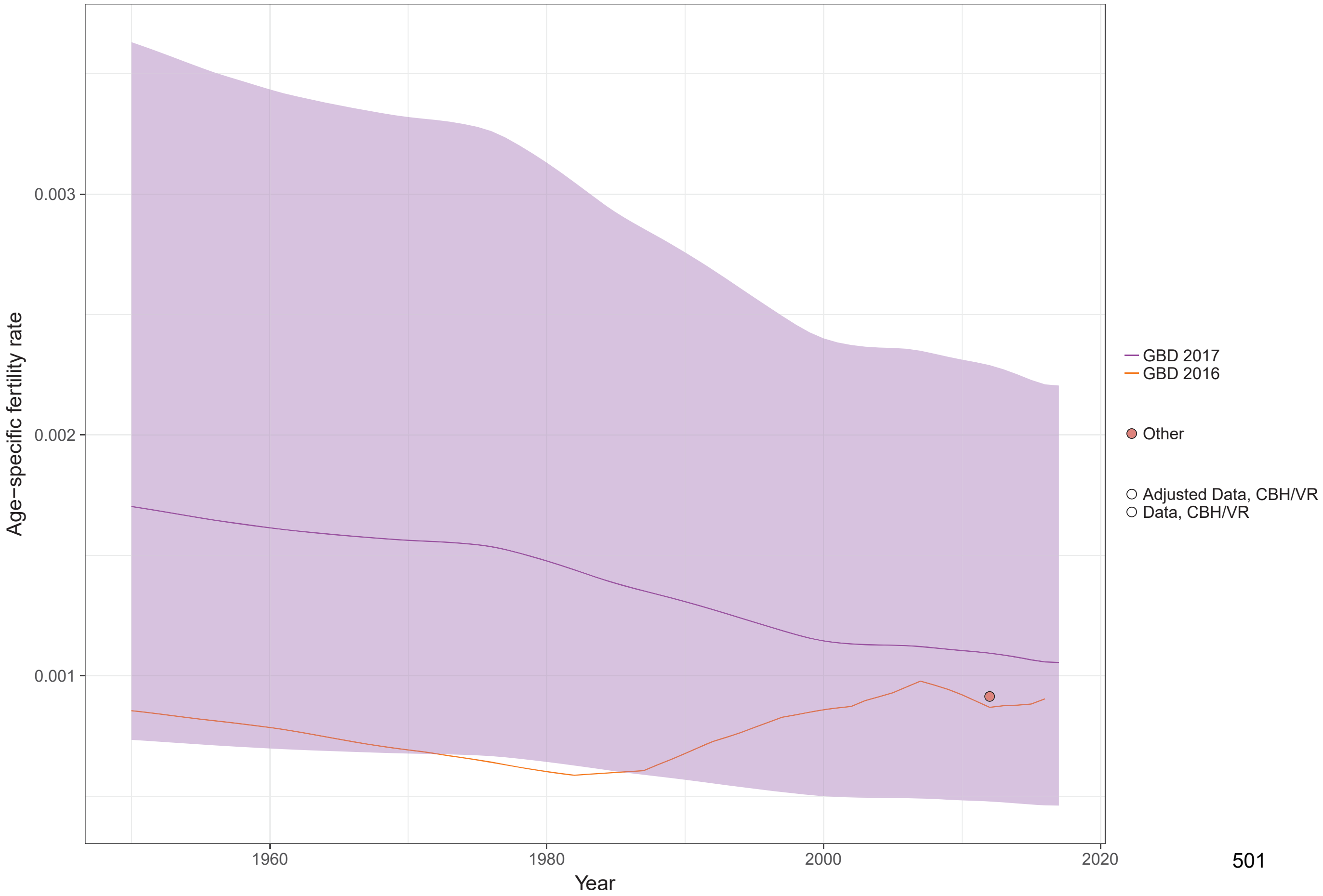

Age−specific fertility fitted trend lines, 1950−2017: Lesotho, Ages: 15−19
Data points enclosed with a diamond are reference sources

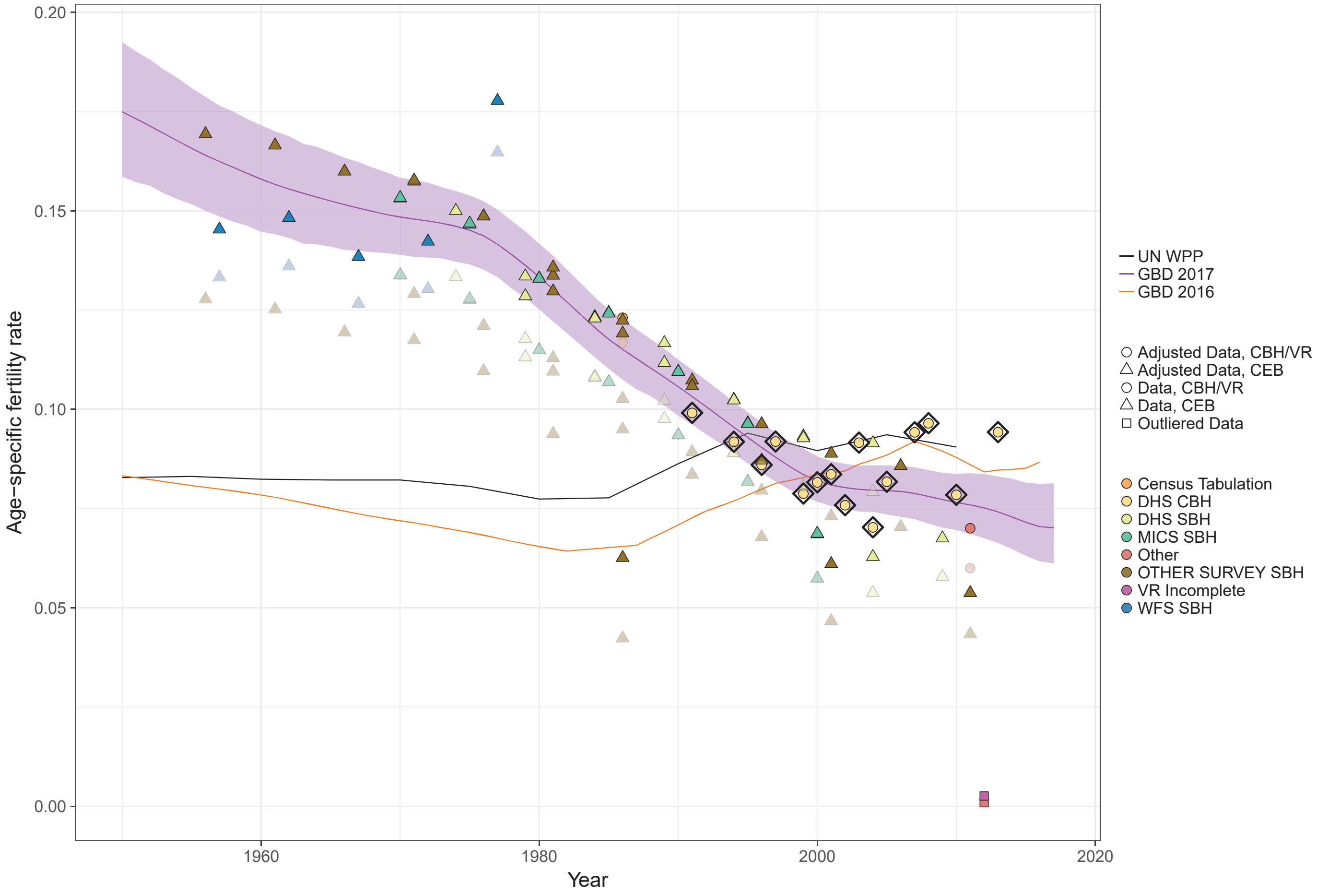

Age−specific fertility fitted trend lines, 1950−2017: Lesotho, Ages: 20−24
Data points enclosed with a diamond are reference sources

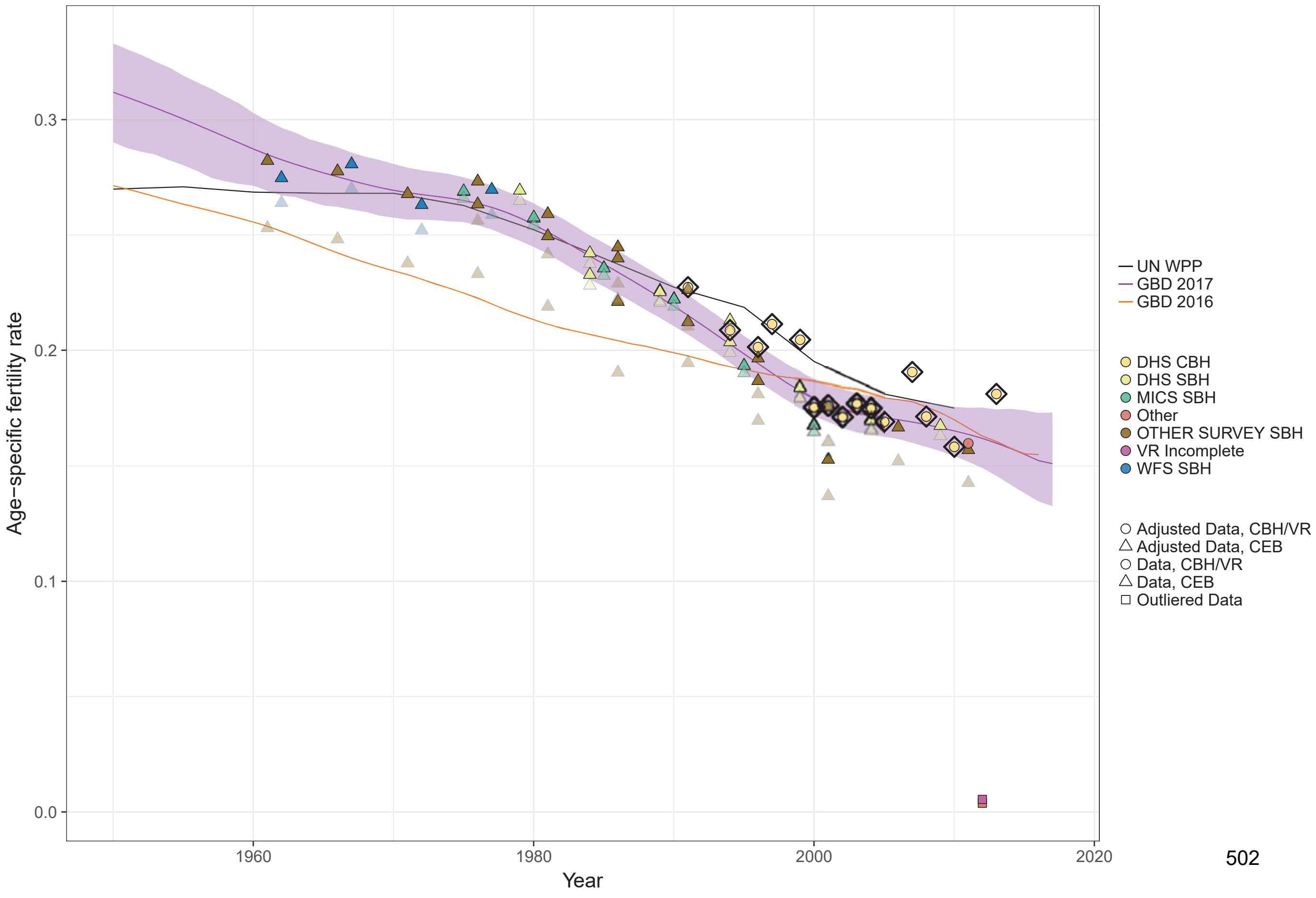

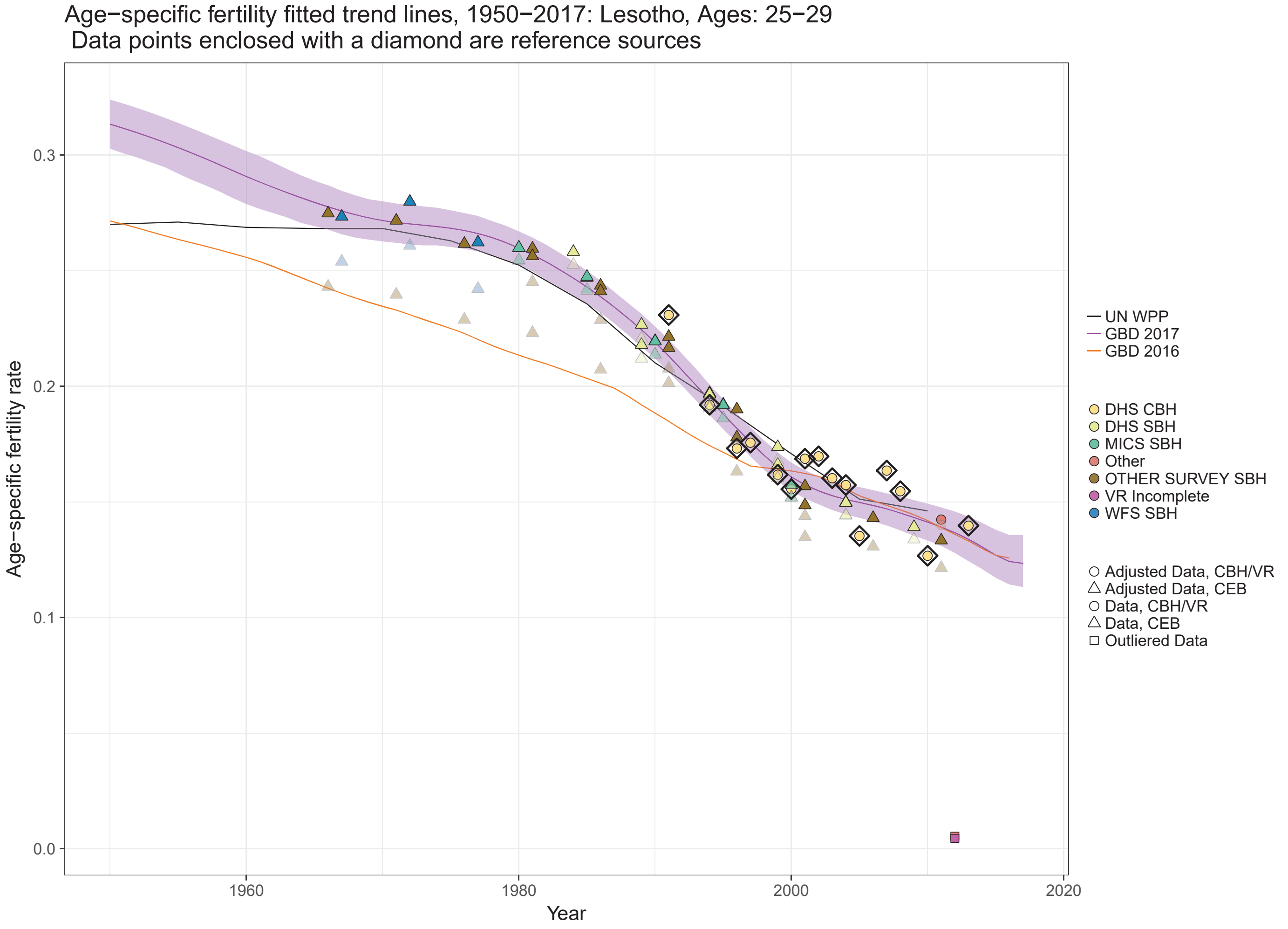

Age−specific fertility fitted trend lines, 1950−2017: Lesotho, Ages: 25−29
Data points enclosed with a diamond are reference sources
Age−specific fertility rate
0.0
0.1
0.2
0.3
Year
1960
1980
2000
2020
UN WPP
GBD 2017
GBD 2016
DHS CBH
DHS SBH
MICS SBH
Other
OTHER SURVEY SBH
VR Incomplete
WFS SBH
Adjusted Data, CBH/VR
Adjusted Data, CEB
Data, CBH/VR
Data, CEB
Outliered Data

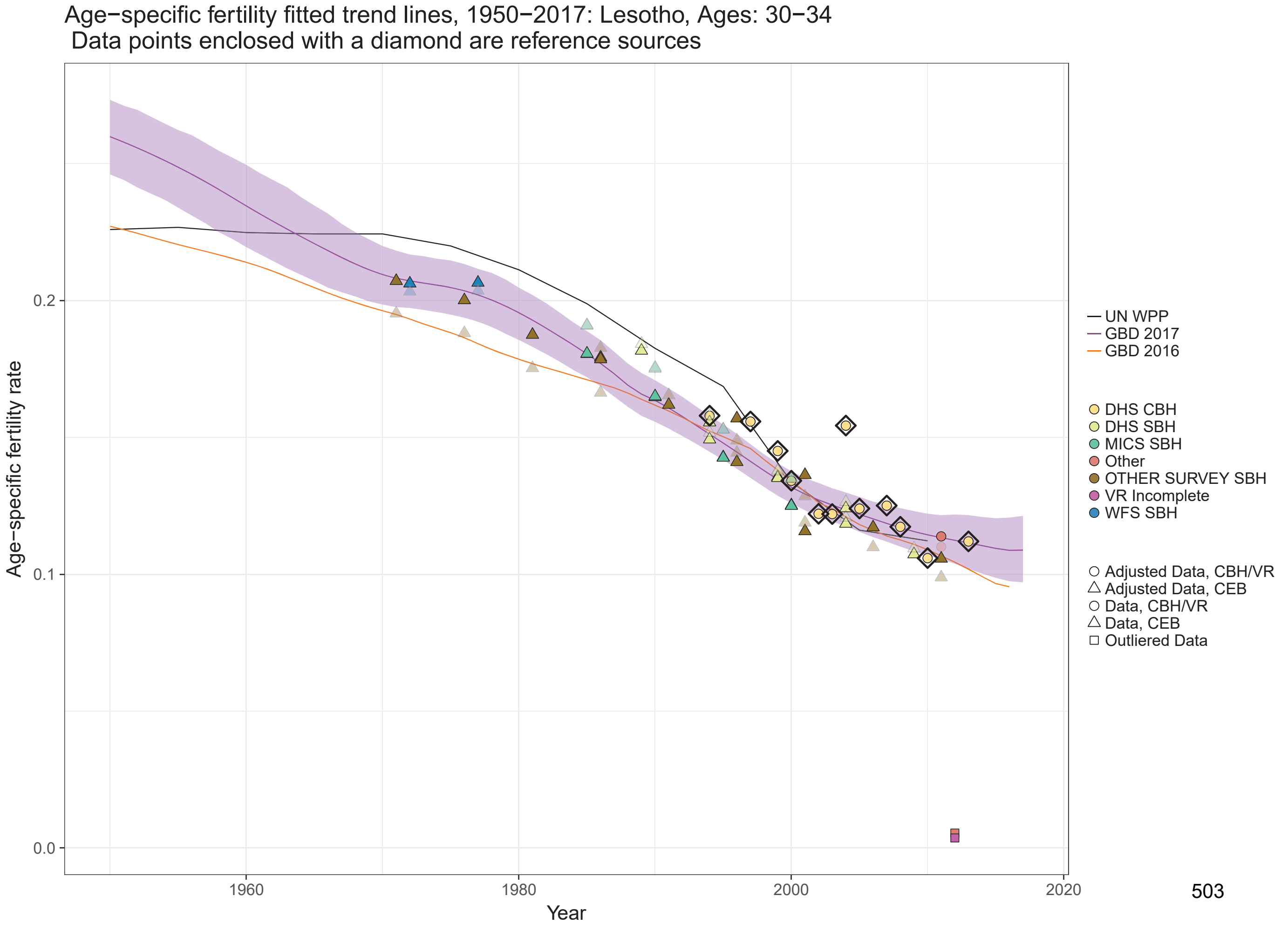

Age−specific fertility fitted trend lines, 1950−2017: Lesotho, Ages: 30−34
Data points enclosed with a diamond are reference sources
Age−specific fertility rate
0.0
0.1
0.2
Year
1960
1980
2000
2020
UN WPP
GBD 2017
GBD 2016
DHS CBH
DHS SBH
MICS SBH
Other
OTHER SURVEY SBH
VR Incomplete
WFS SBH
Adjusted Data, CBH/VR
Adjusted Data, CEB
Data, CBH/VR
Data, CEB
Outliered Data

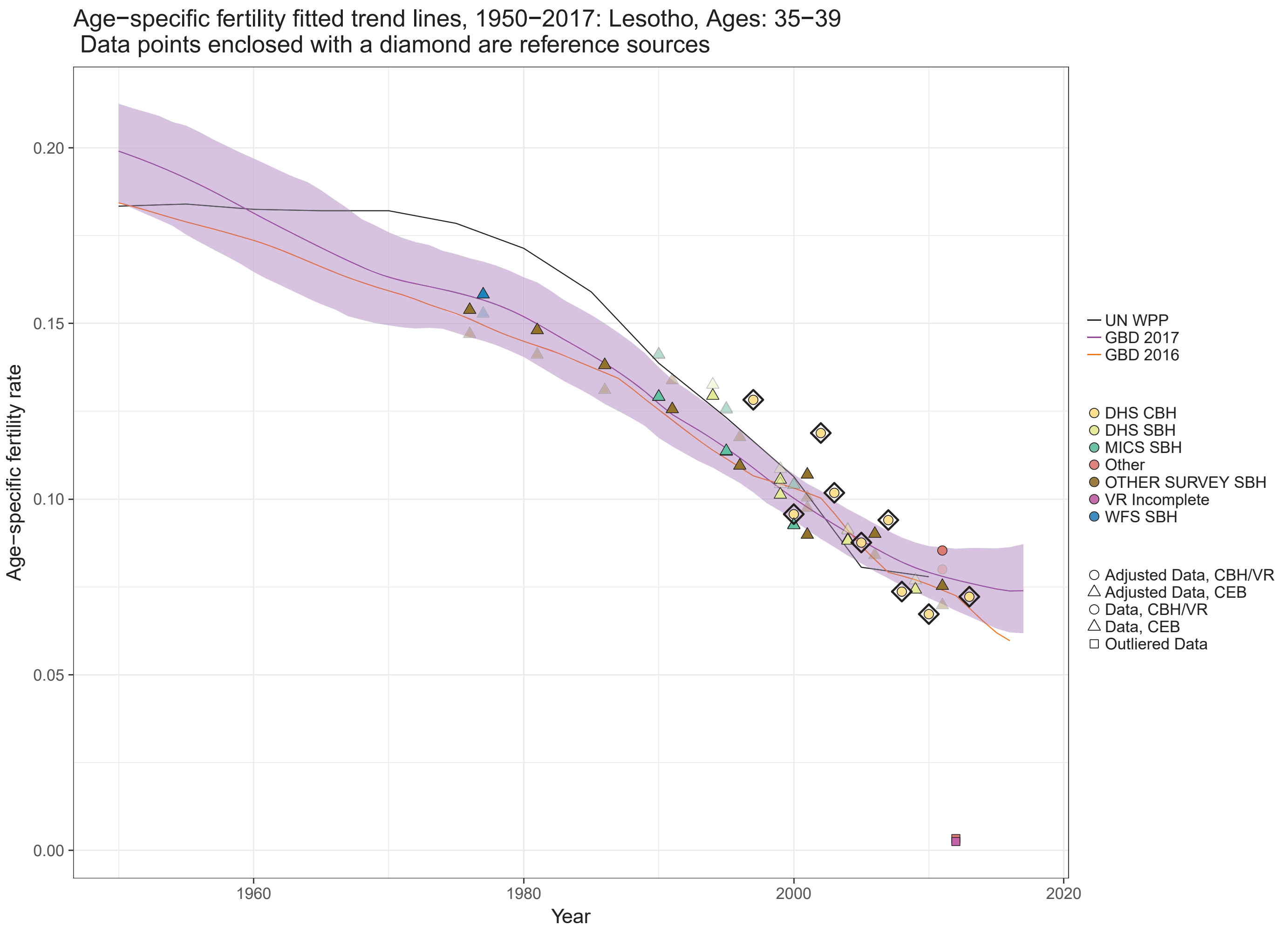

Age−specific fertility fitted trend lines, 1950−2017: Lesotho, Ages: 35−39
Data points enclosed with a diamond are reference sources
Age−specific fertility rate
Year
UN WPP
GBD 2017
GBD 2016
DHS CBH
DHS SBH
MICS SBH
Other
OTHER SURVEY SBH
VR Incomplete
WFS SBH
Adjusted Data, CBH/VR
Adjusted Data, CEB
Data, CBH/VR
Data, CEB
Outliered Data

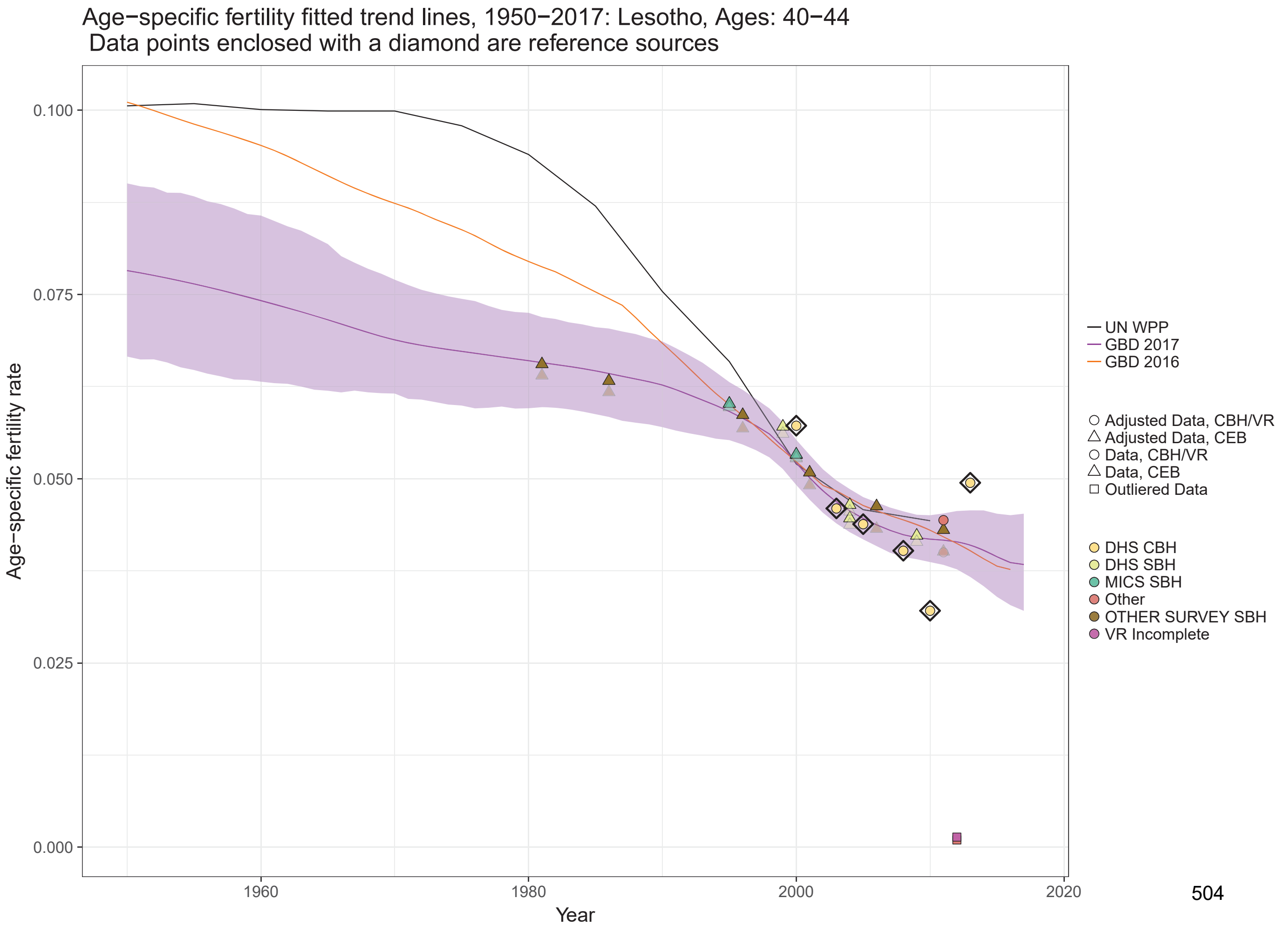

Age−specific fertility fitted trend lines, 1950−2017: Lesotho, Ages: 40−44
Data points enclosed with a diamond are reference sources
Age−specific fertility rate
Year
UN WPP
GBD 2017
GBD 2016
Adjusted Data, CBH/VR
Adjusted Data, CEB
Data, CBH/VR
Data, CEB
Outliered Data
DHS CBH
DHS SBH
MICS SBH
Other
OTHER SURVEY SBH
VR Incomplete

Age−specific fertility fitted trend lines, 1950−2017: Lesotho, Ages: 45−49
Data points enclosed with a diamond are reference sources

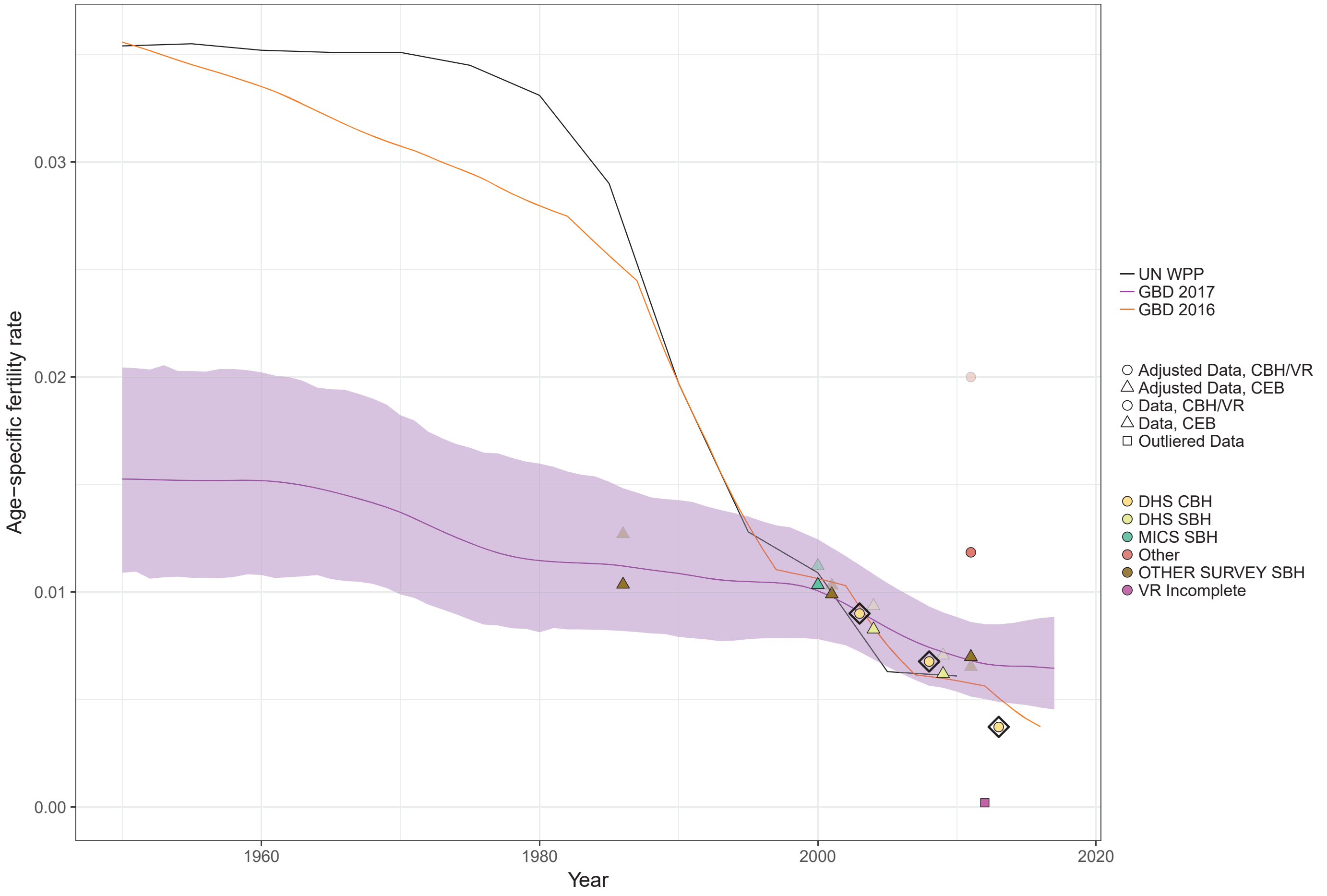

Age−specific fertility fitted trend lines, 1950−2017: Lesotho, Ages: 50−54
Data points enclosed with a diamond are reference sources

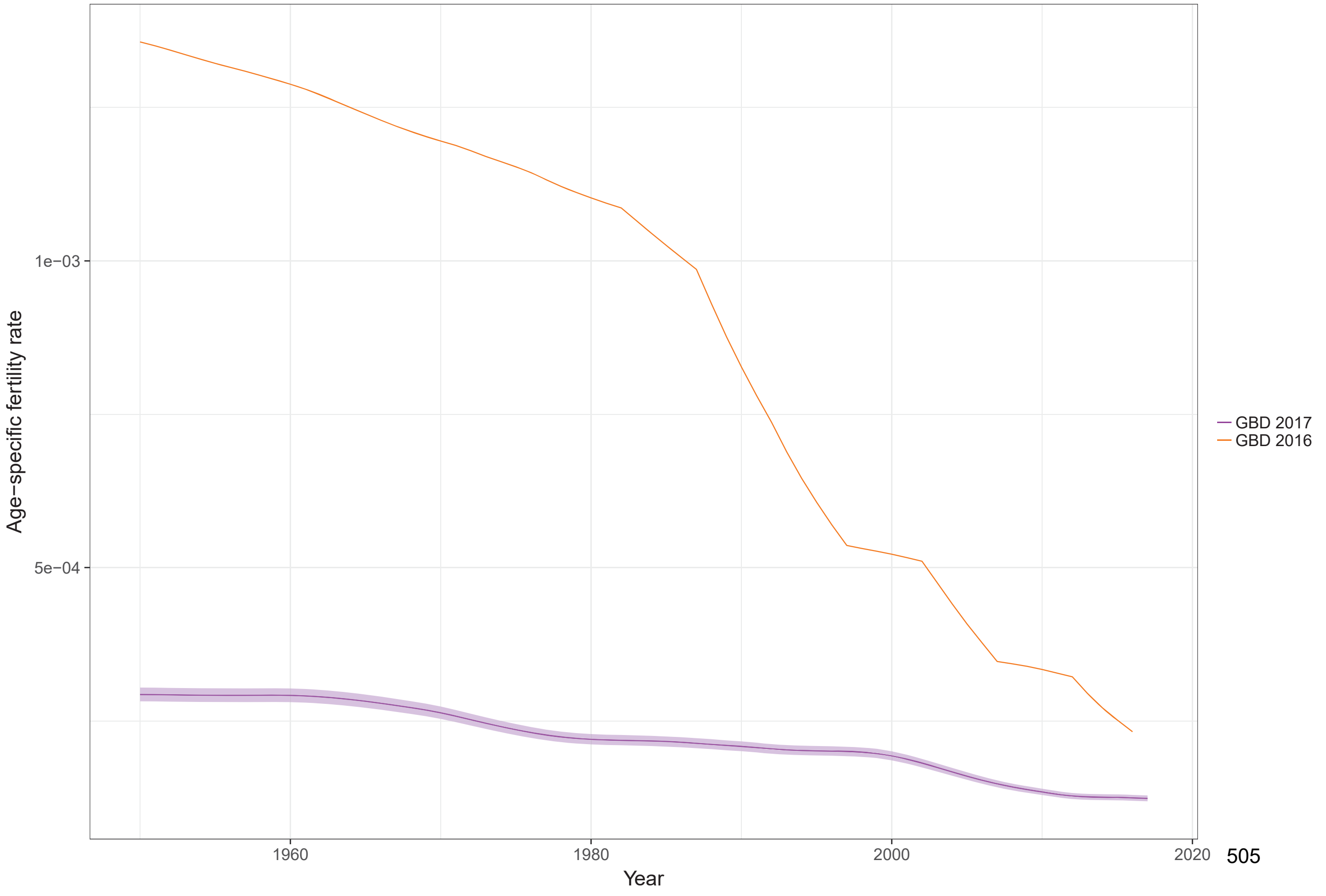

Age−specific fertility fitted trend lines, 1950−2017: Liberia, TFR
Data points enclosed with a diamond are reference sources

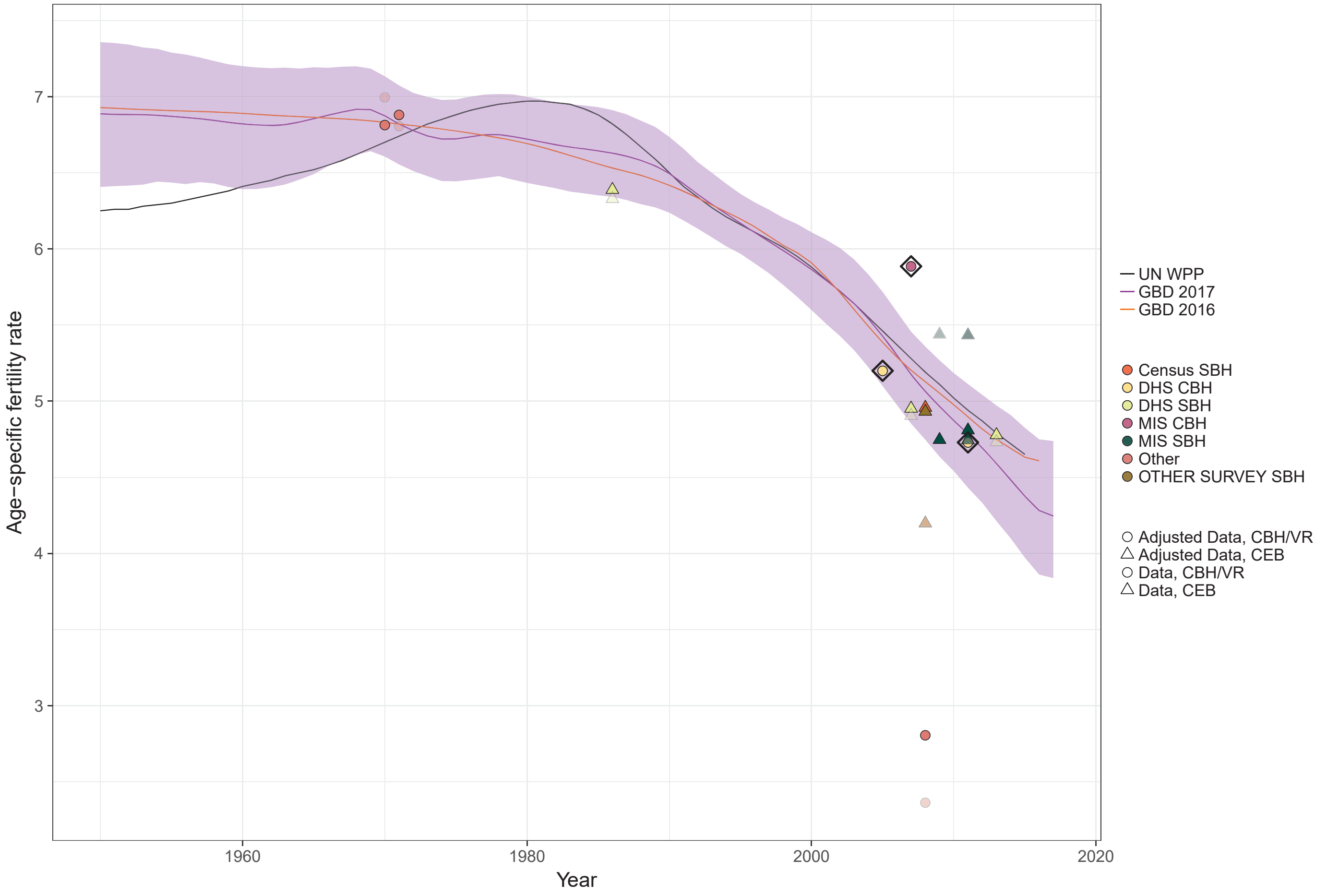

Age−specific fertility fitted trend lines, 1950−2017: Liberia, Ages: 10−14
Data points enclosed with a diamond are reference sources

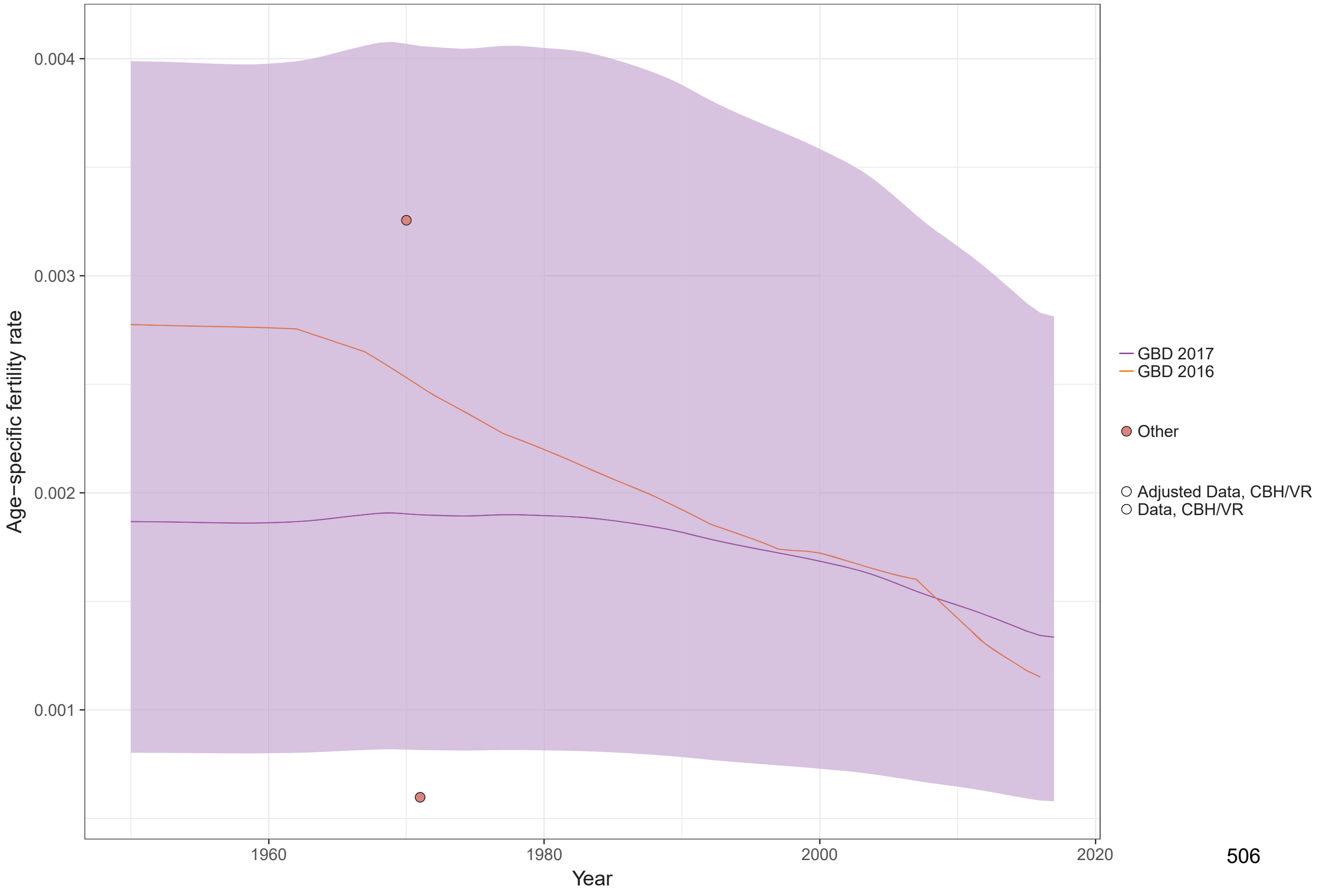

Age−specific fertility fitted trend lines, 1950−2017: Liberia, Ages: 15−19
Data points enclosed with a diamond are reference sources

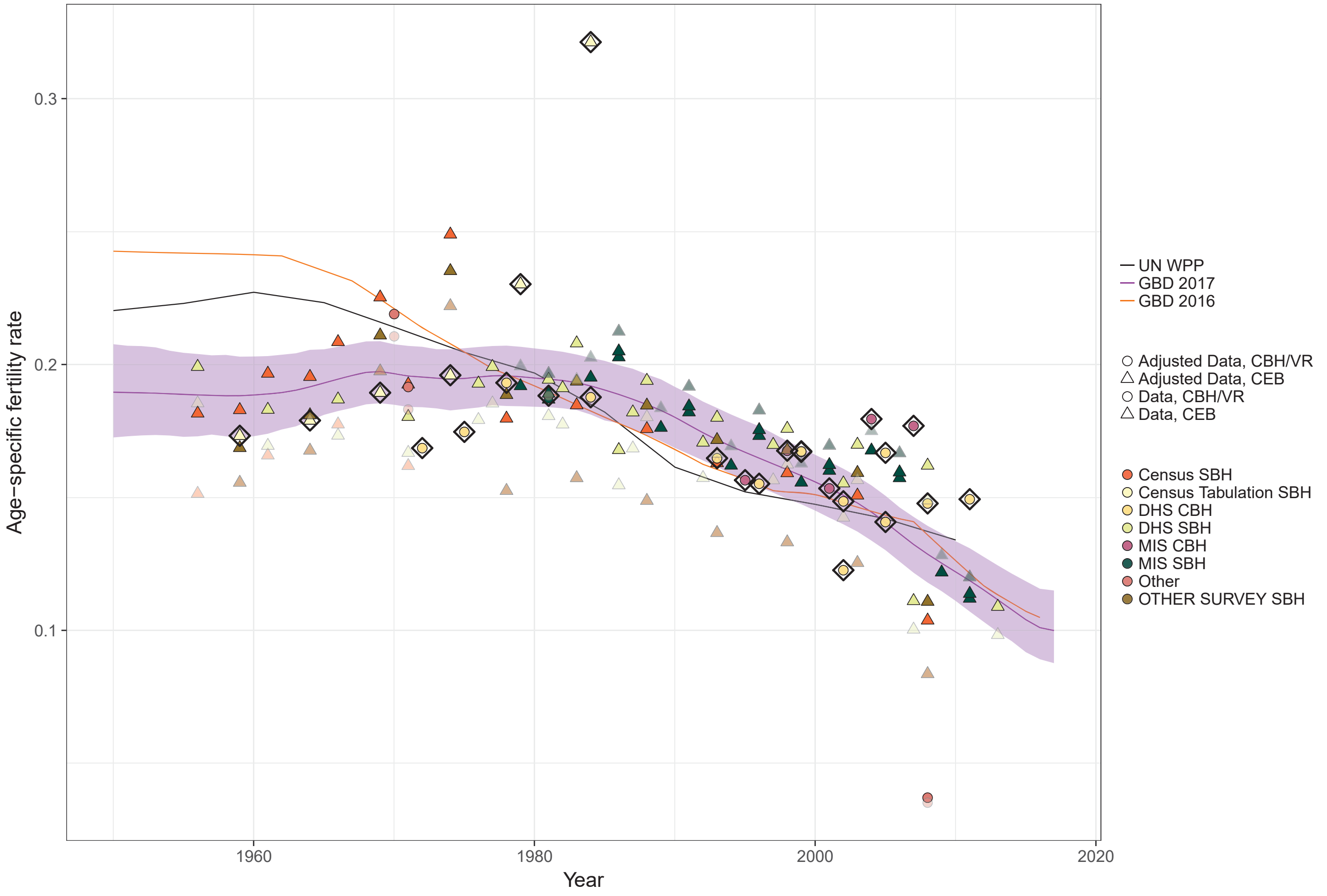

Age−specific fertility fitted trend lines, 1950−2017: Liberia, Ages: 20−24
Data points enclosed with a diamond are reference sources

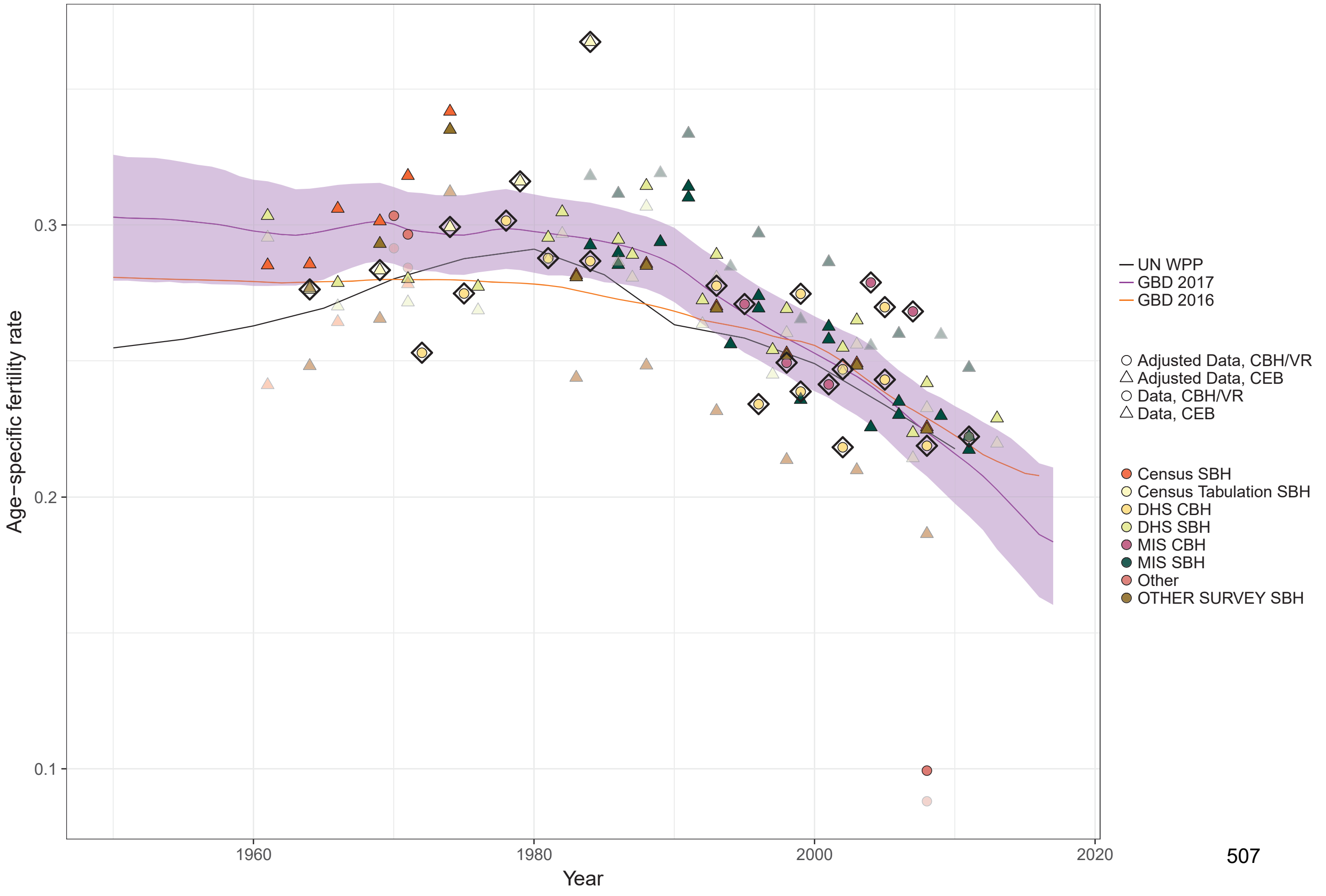

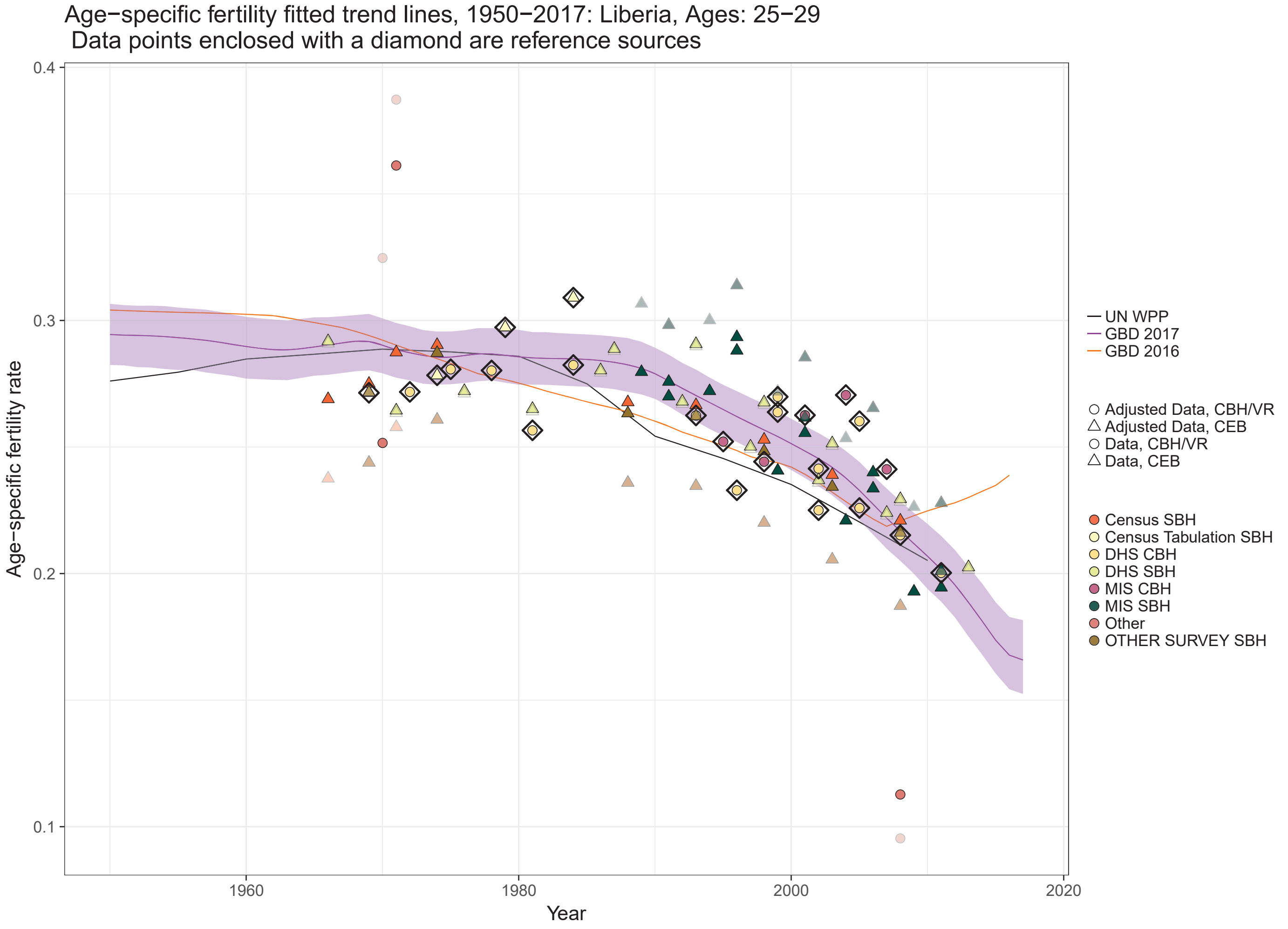
Age−specific fertility fitted trend lines, 1950−2017: Liberia, Ages: 25−29
Data points enclosed with a diamond are reference sources
Age−specific fertility rate
Year
0.1
0.2
0.3
0.4
1960
1980
2000
2020
UN WPP
GBD 2017
GBD 2016
Adjusted Data, CBH/VR
Adjusted Data, CEB
Data, CBH/VR
Data, CEB
Census SBH
Census Tabulation SBH
DHS CBH
DHS SBH
MIS CBH
MIS SBH
Other
OTHER SURVEY SBH

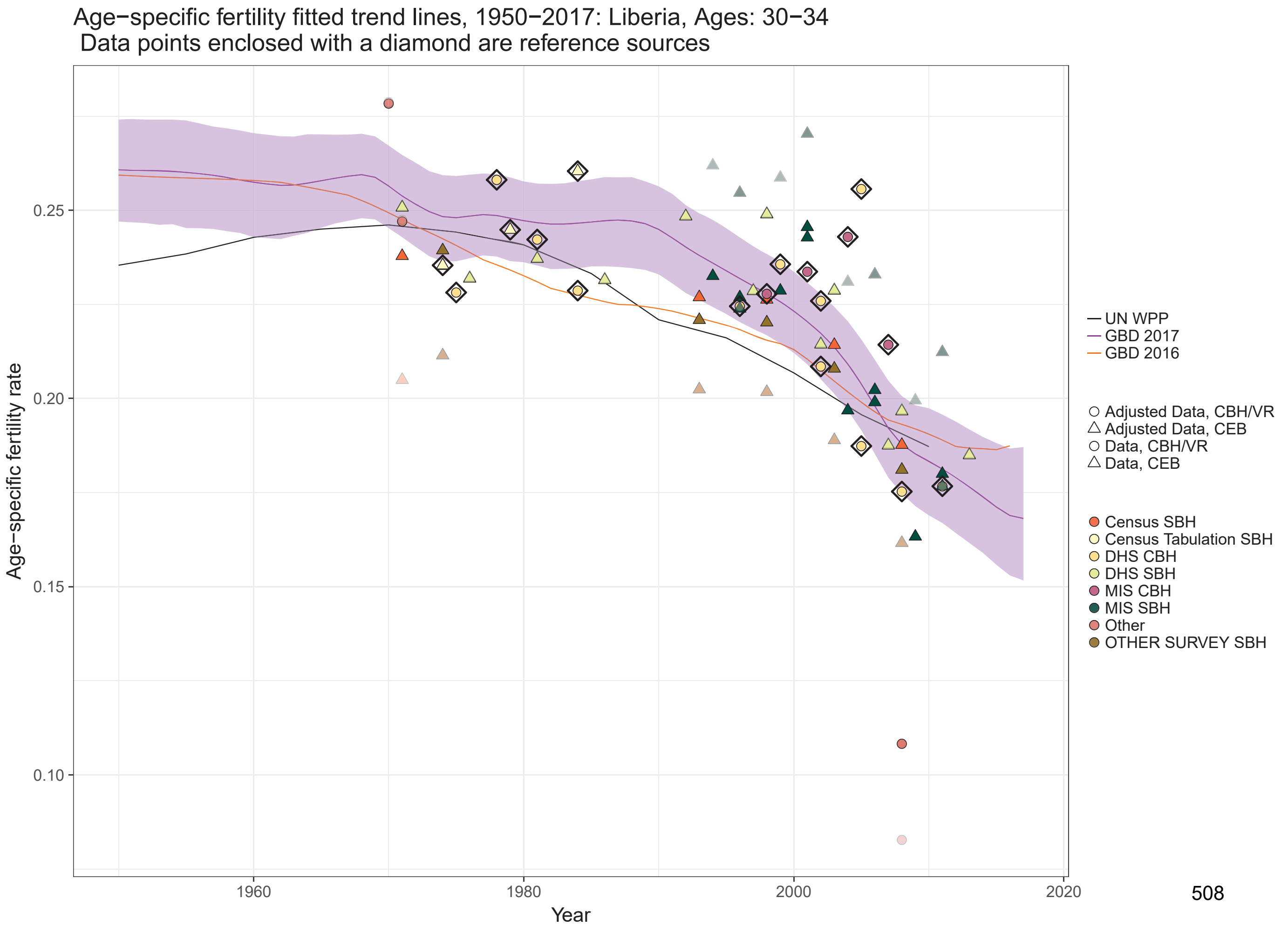
Age−specific fertility fitted trend lines, 1950−2017: Liberia, Ages: 30−34
Data points enclosed with a diamond are reference sources
Age−specific fertility rate
Year
0.10
0.15
0.20
0.25
1960
1980
2000
2020
UN WPP
GBD 2017
GBD 2016
Adjusted Data, CBH/VR
Adjusted Data, CEB
Data, CBH/VR
Data, CEB
Census SBH
Census Tabulation SBH
DHS CBH
DHS SBH
MIS CBH
MIS SBH
Other
OTHER SURVEY SBH

Age−specific fertility fitted trend lines, 1950−2017: Liberia, Ages: 35−39
Data points enclosed with a diamond are reference sources

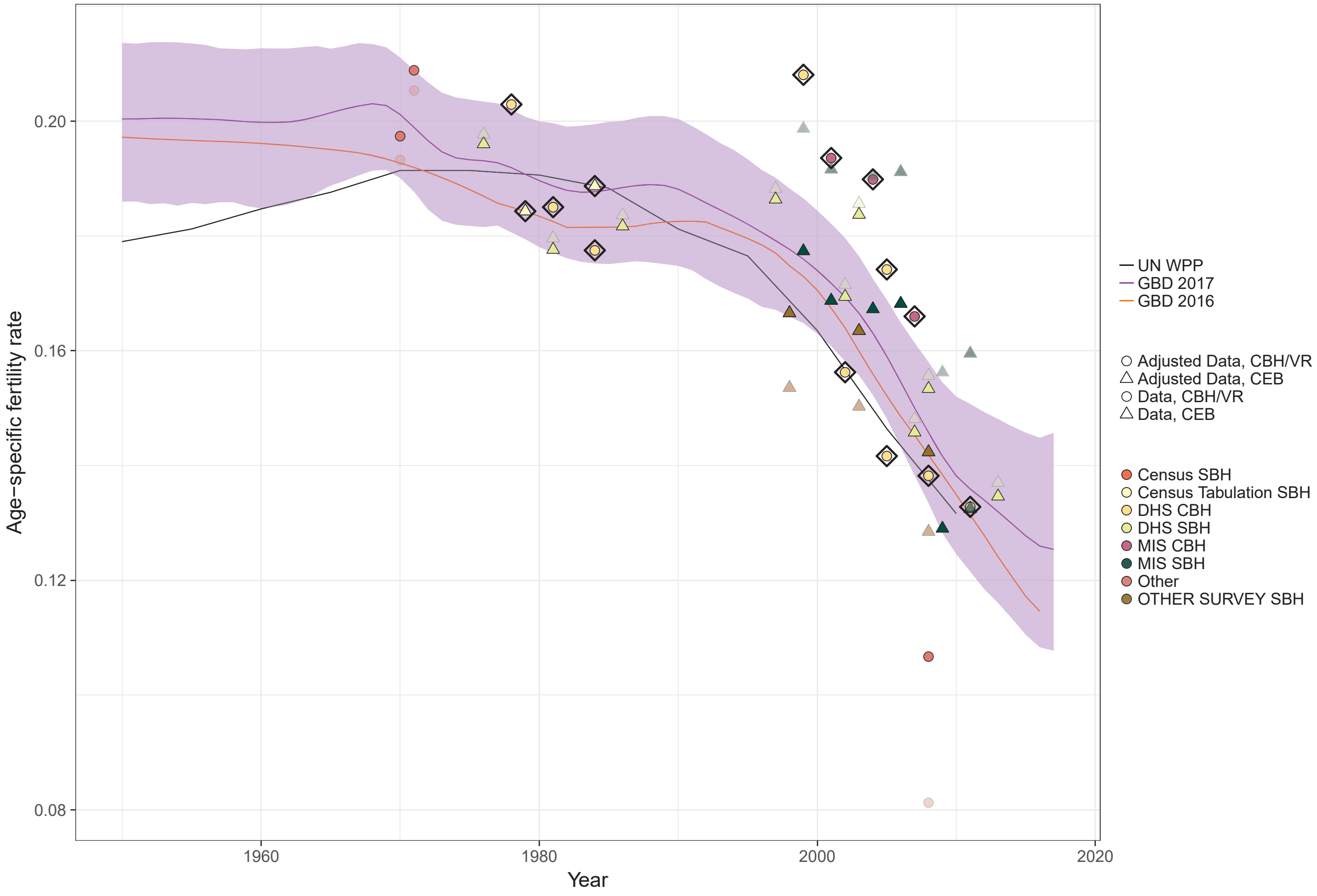

Age−specific fertility fitted trend lines, 1950−2017: Liberia, Ages: 40−44
Data points enclosed with a diamond are reference sources

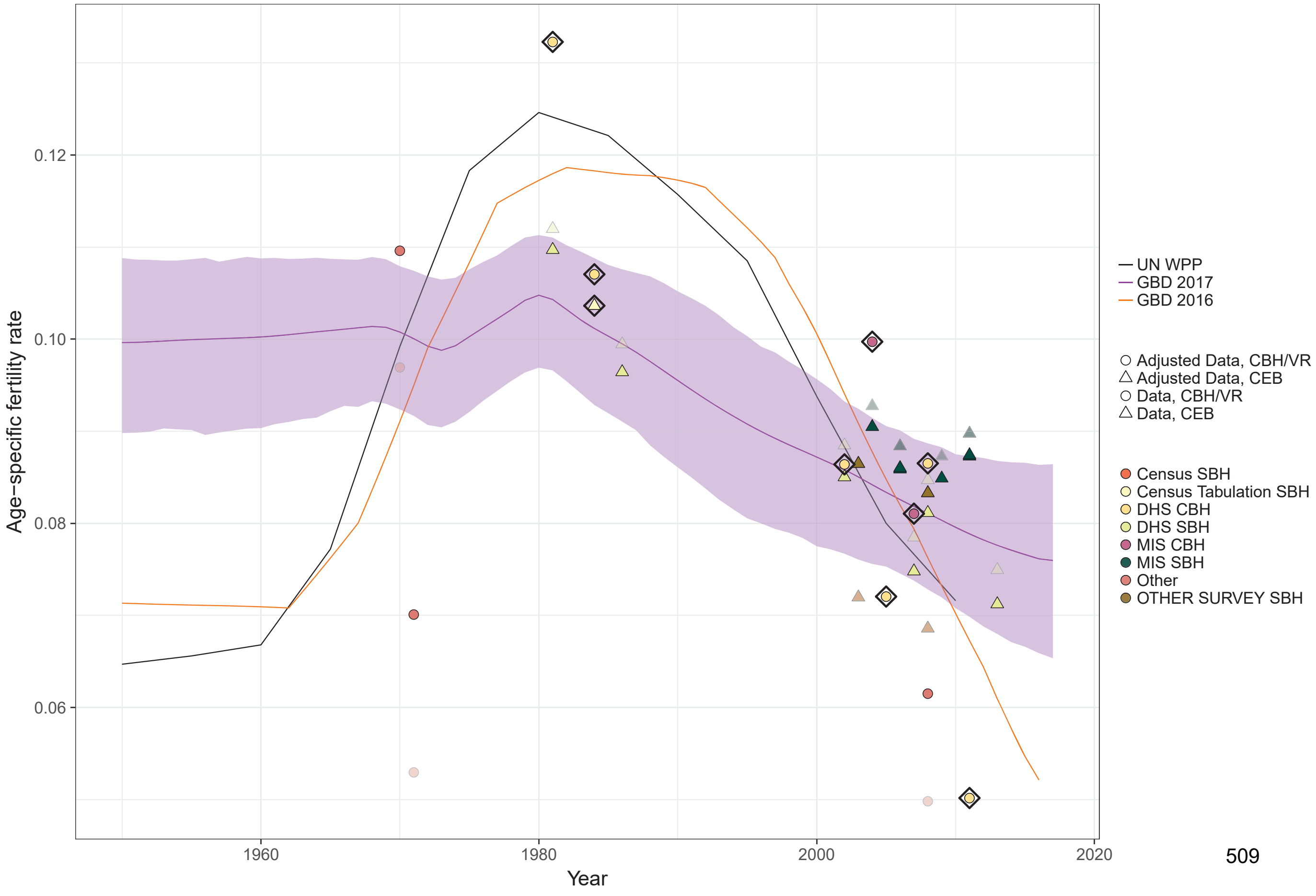

Age−specific fertility fitted trend lines, 1950−2017: Liberia, Ages: 45−49
Data points enclosed with a diamond are reference sources

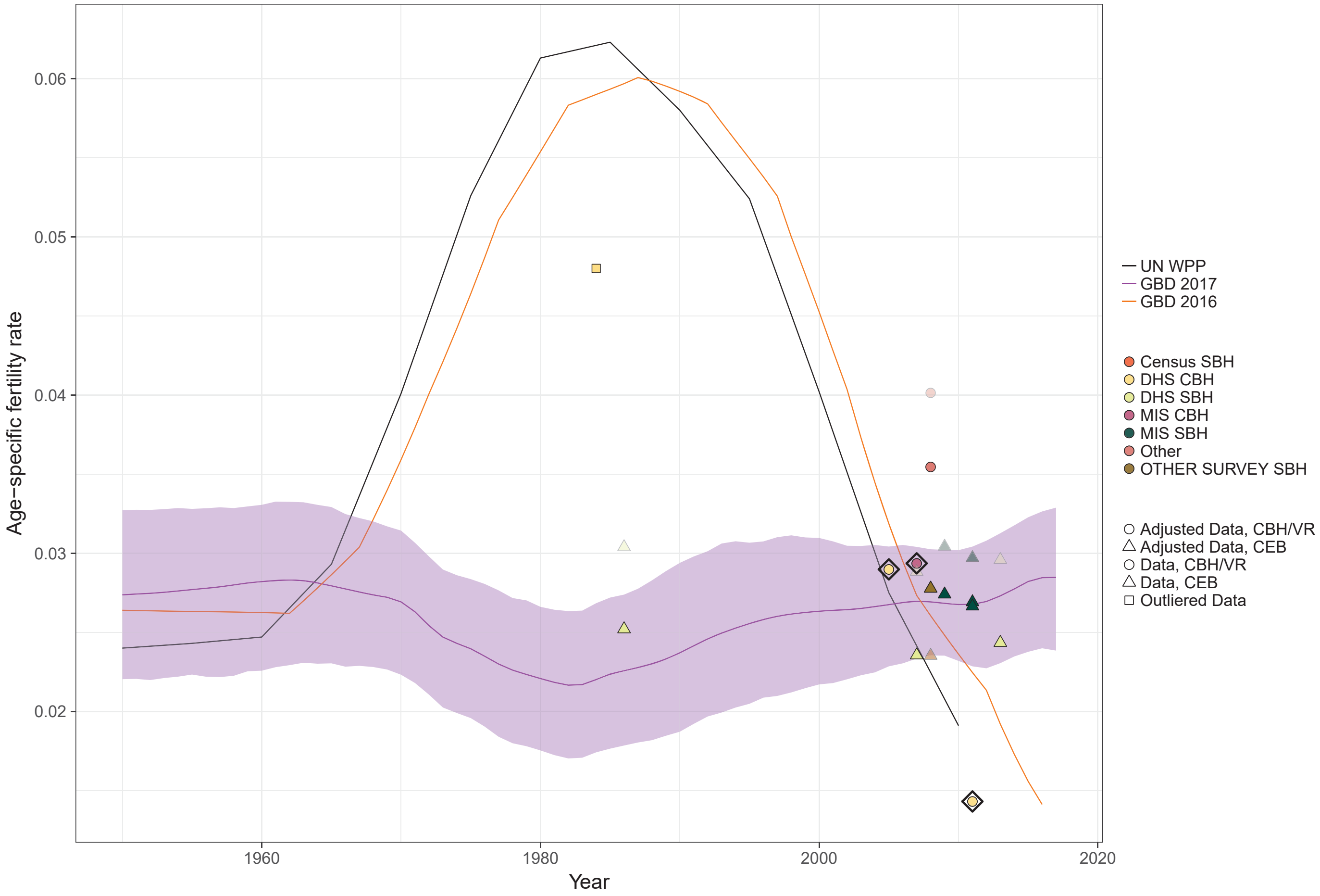

Age−specific fertility fitted trend lines, 1950−2017: Liberia, Ages: 50−54
Data points enclosed with a diamond are reference sources

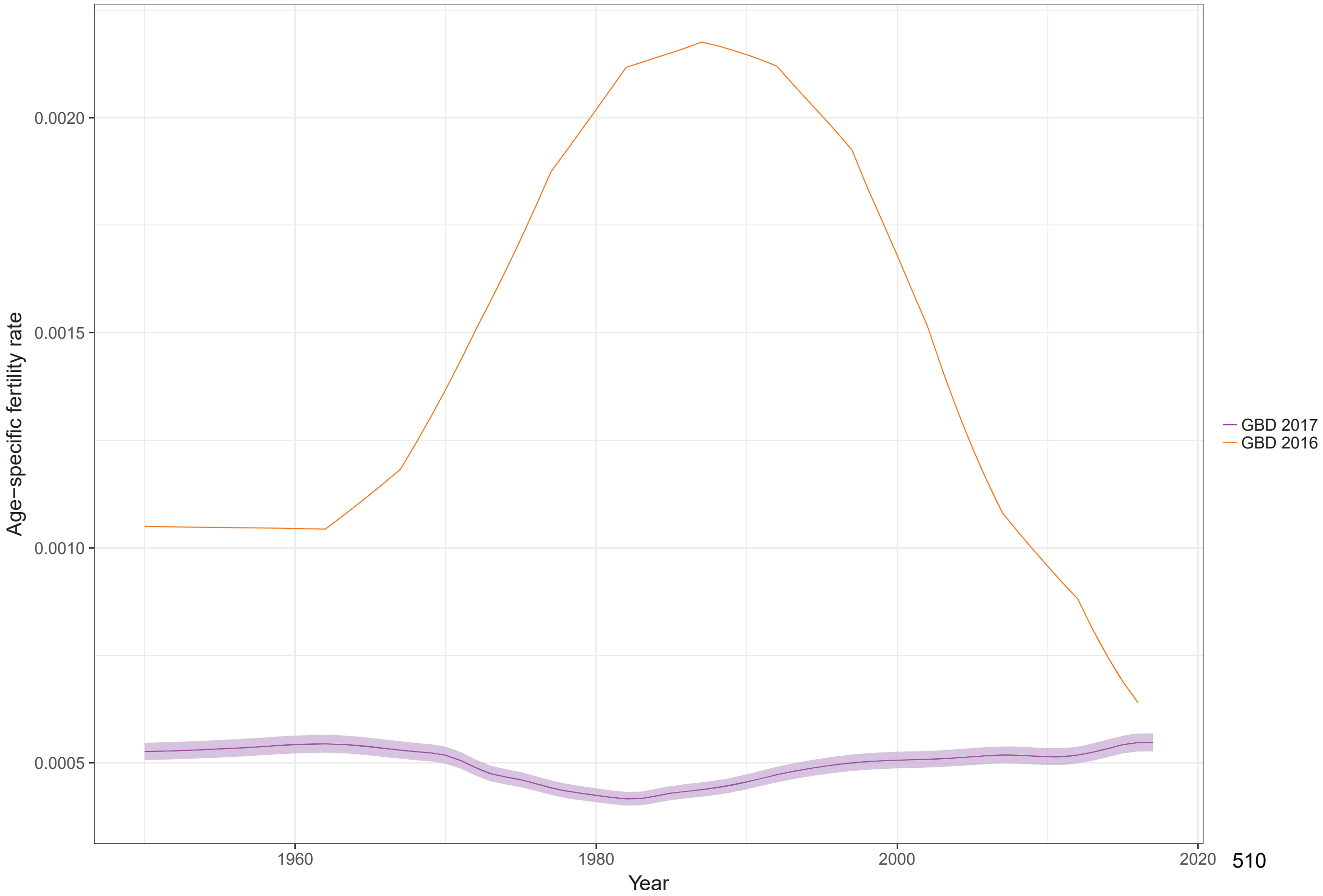

Age−specific fertility fitted trend lines, 1950−2017: Libya, TFR
Data points enclosed with a diamond are reference sources

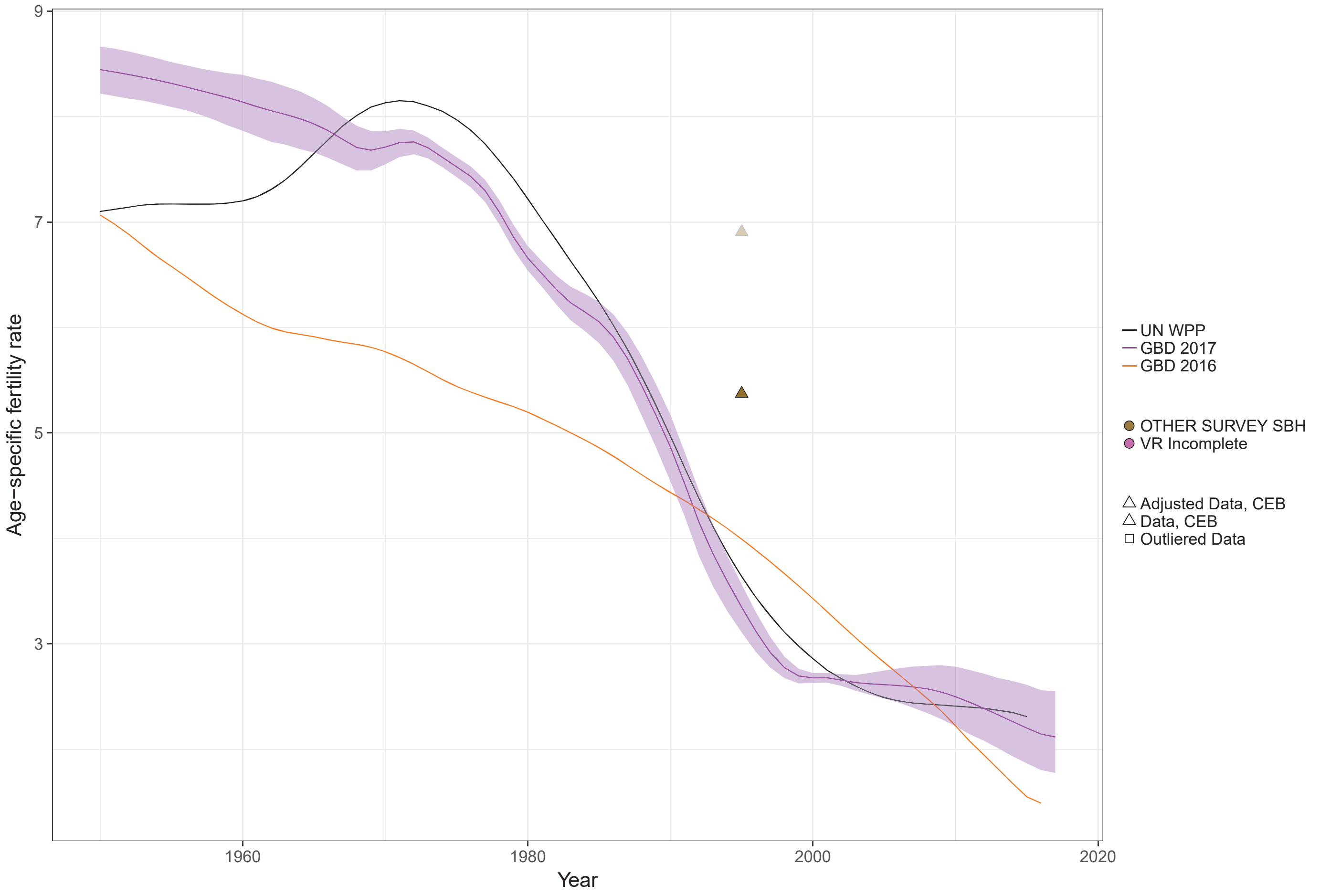

Age−specific fertility fitted trend lines, 1950−2017: Libya, Ages: 10−14
Data points enclosed with a diamond are reference sources

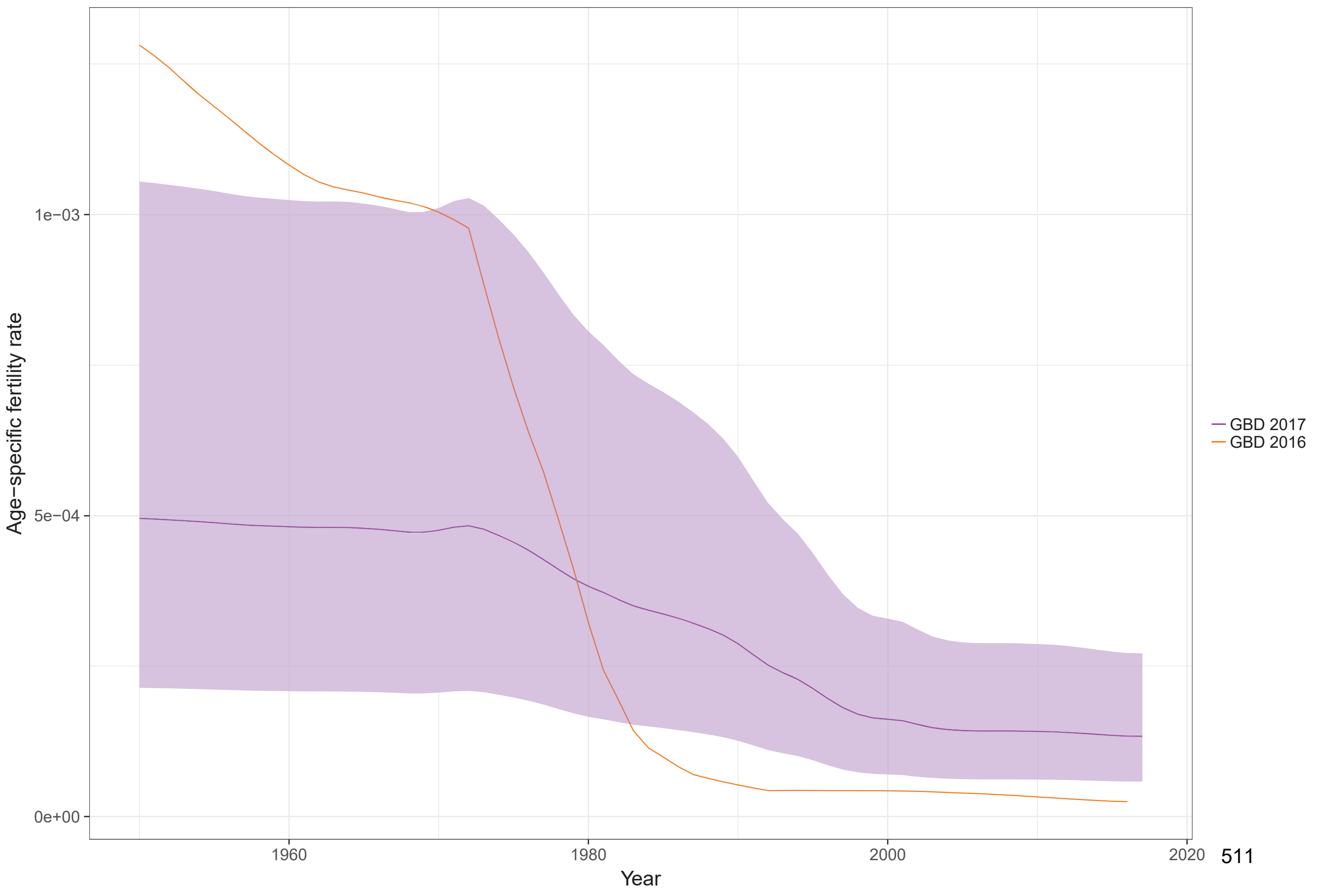

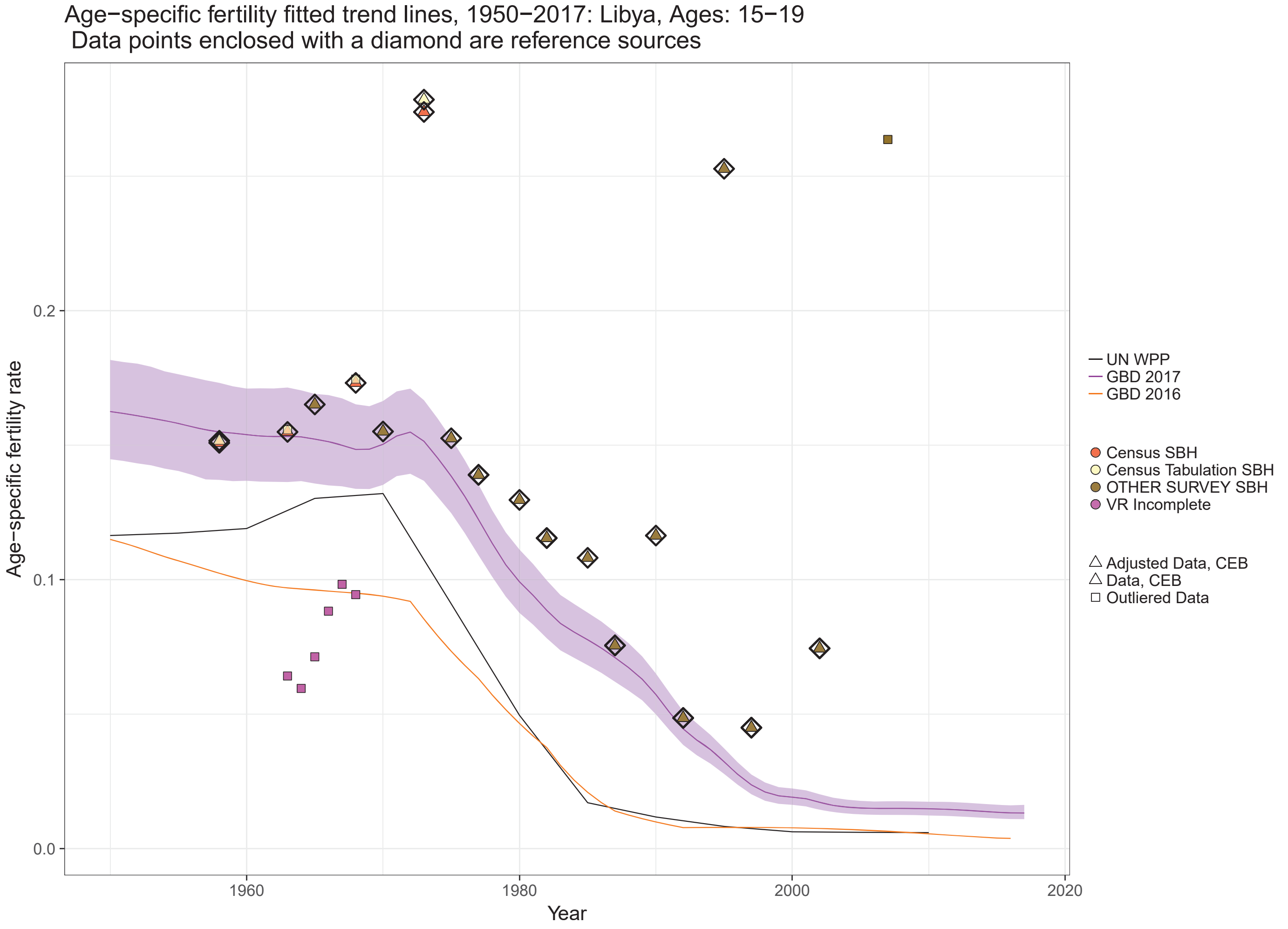

Age−specific fertility fitted trend lines, 1950−2017: Libya, Ages: 15−19
Data points enclosed with a diamond are reference sources
Age−specific fertility rate
Year
0.0
0.1
0.2
1960
1980
2000
2020
UN WPP
GBD 2017
GBD 2016
Census SBH
Census Tabulation SBH
OTHER SURVEY SBH
VR Incomplete
Adjusted Data, CEB
Data, CEB
Outliered Data

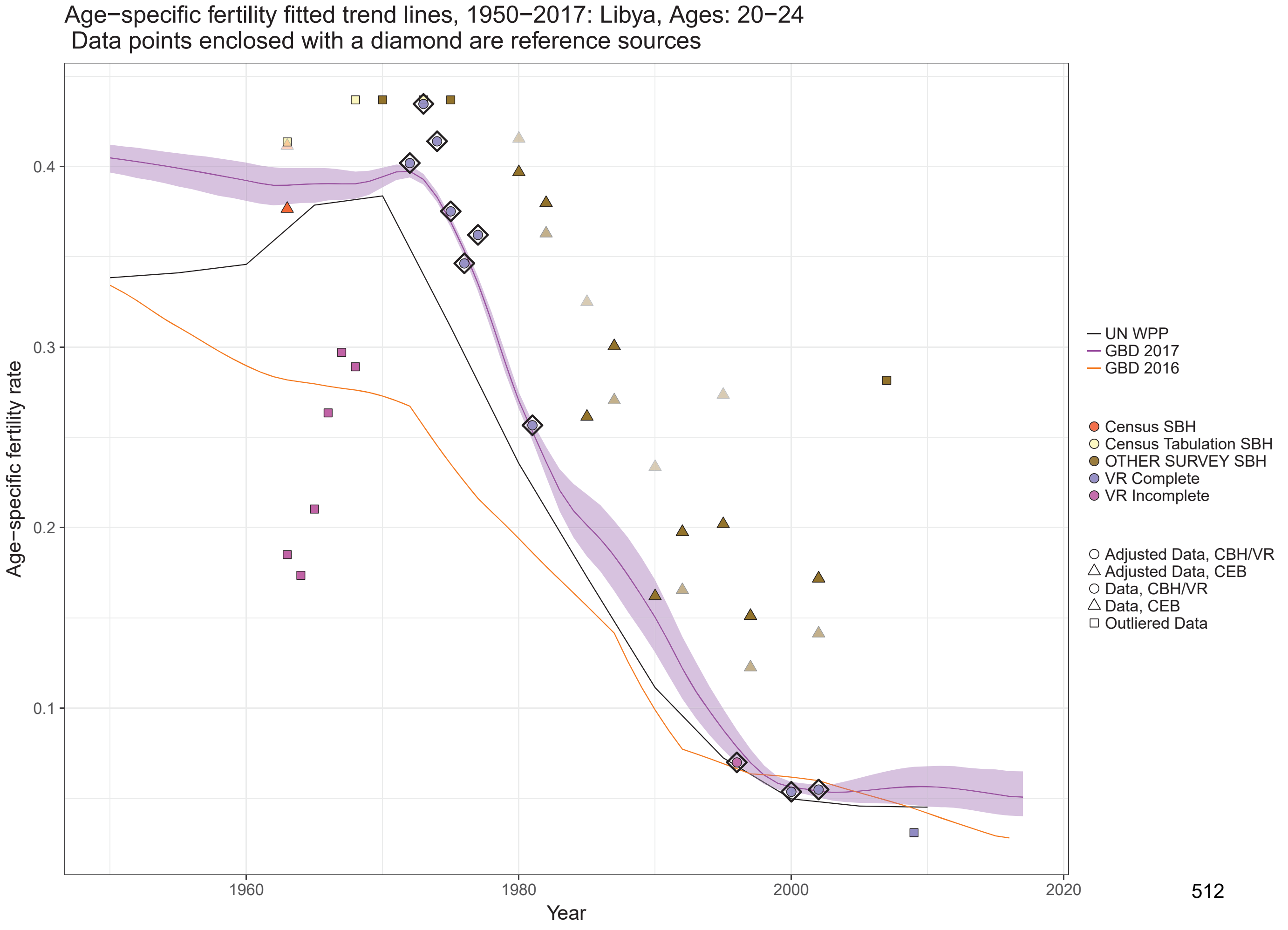

Age−specific fertility fitted trend lines, 1950−2017: Libya, Ages: 20−24
Data points enclosed with a diamond are reference sources
Age−specific fertility rate
Year
0.1
0.2
0.3
0.4
1960
1980
2000
2020
UN WPP
GBD 2017
GBD 2016
Census SBH
Census Tabulation SBH
OTHER SURVEY SBH
VR Complete
VR Incomplete
Adjusted Data, CBH/VR
Adjusted Data, CEB
Data, CBH/VR
Data, CEB
Outliered Data

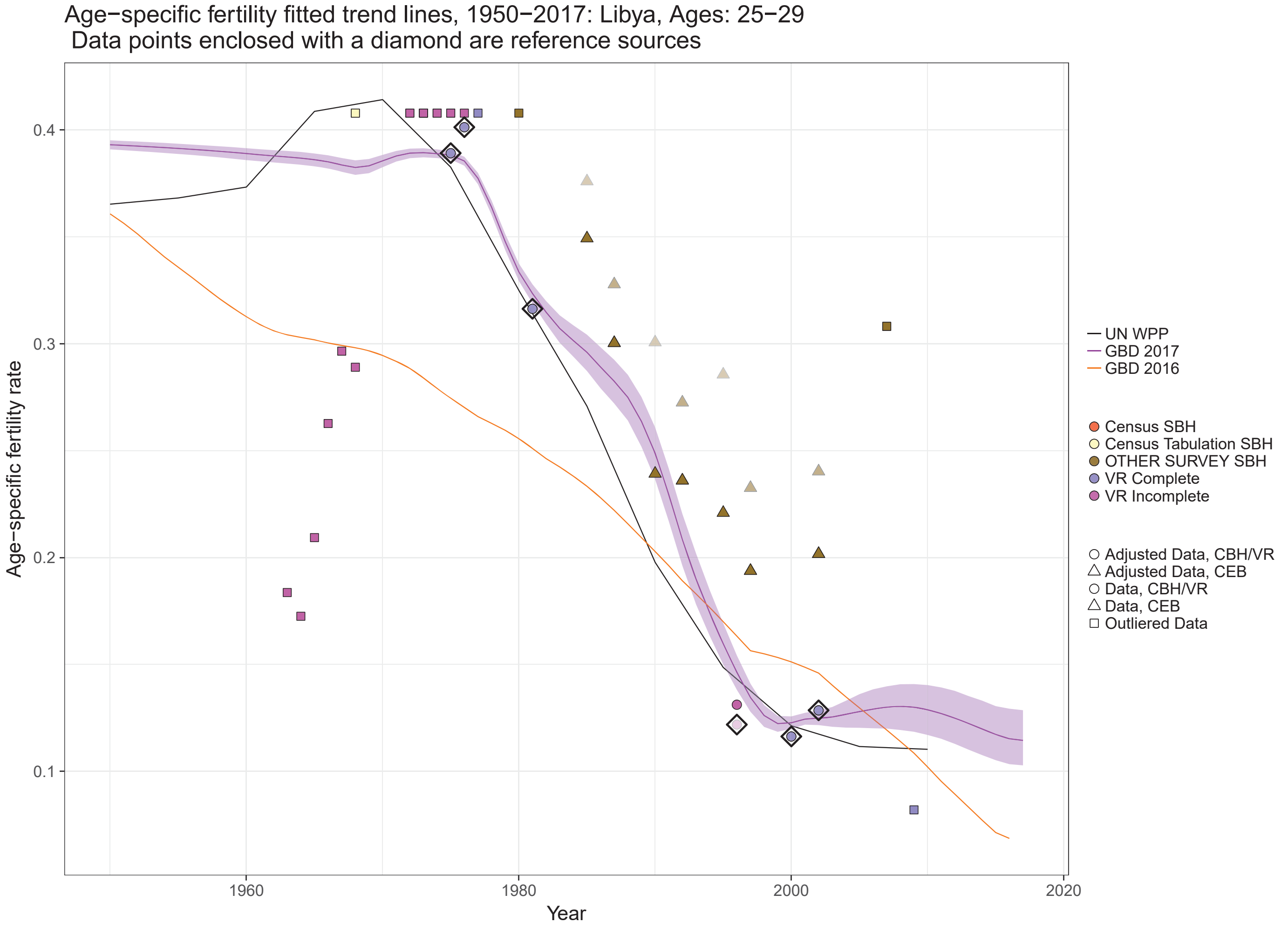

Age−specific fertility fitted trend lines, 1950−2017: Libya, Ages: 25−29
Data points enclosed with a diamond are reference sources
Age−specific fertility rate
Year
0.1
0.2
0.3
0.4
1960
1980
2000
2020
UN WPP
GBD 2017
GBD 2016
Census SBH
Census Tabulation SBH
OTHER SURVEY SBH
VR Complete
VR Incomplete
Adjusted Data, CBH/VR
Adjusted Data, CEB
Data, CBH/VR
Data, CEB
Outliered Data

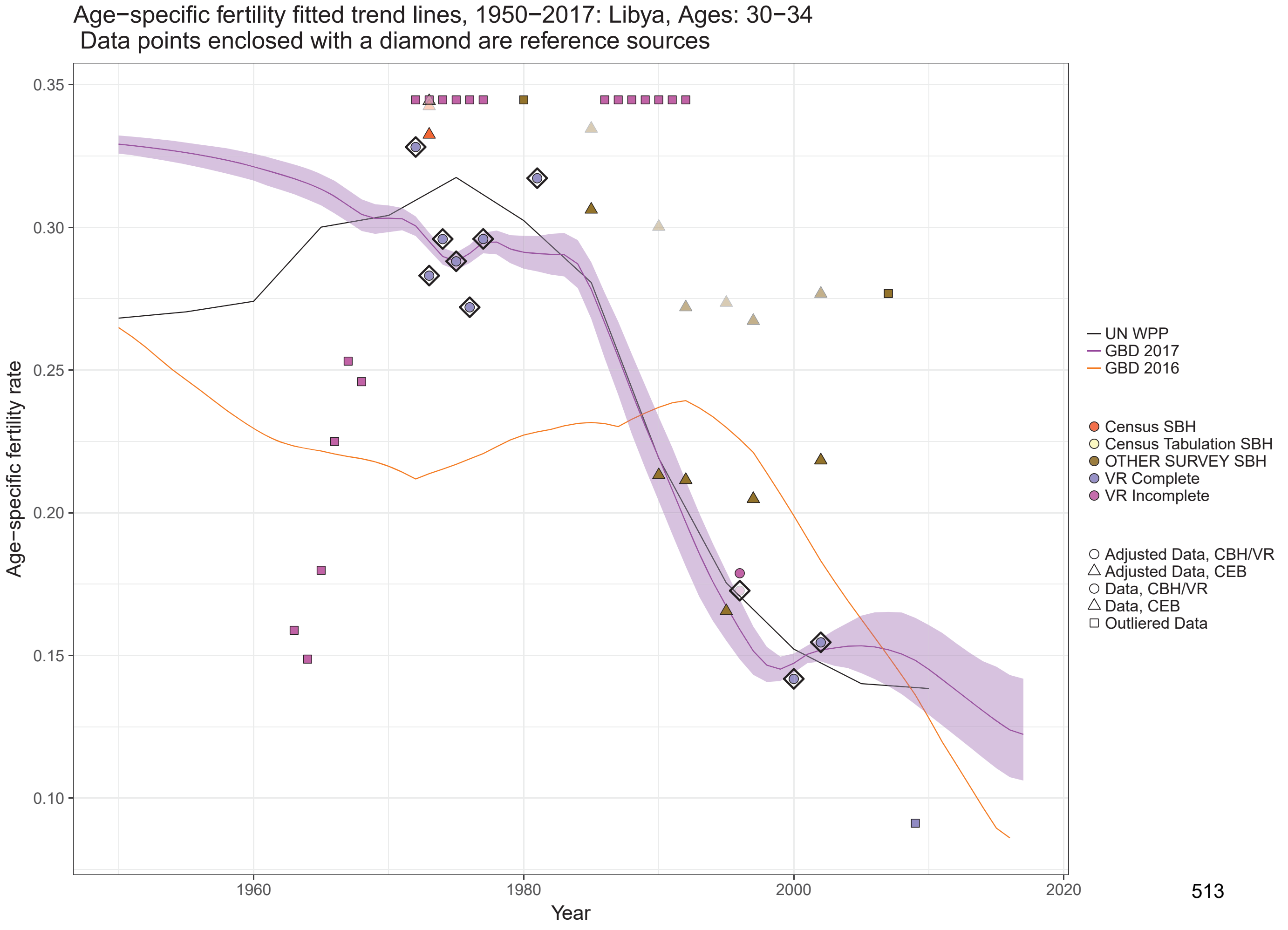

Age−specific fertility fitted trend lines, 1950−2017: Libya, Ages: 30−34
Data points enclosed with a diamond are reference sources
Age−specific fertility rate
Year
0.10
0.15
0.20
0.25
0.30
0.35
1960
1980
2000
2020
UN WPP
GBD 2017
GBD 2016
Census SBH
Census Tabulation SBH
OTHER SURVEY SBH
VR Complete
VR Incomplete
Adjusted Data, CBH/VR
Adjusted Data, CEB
Data, CBH/VR
Data, CEB
Outliered Data

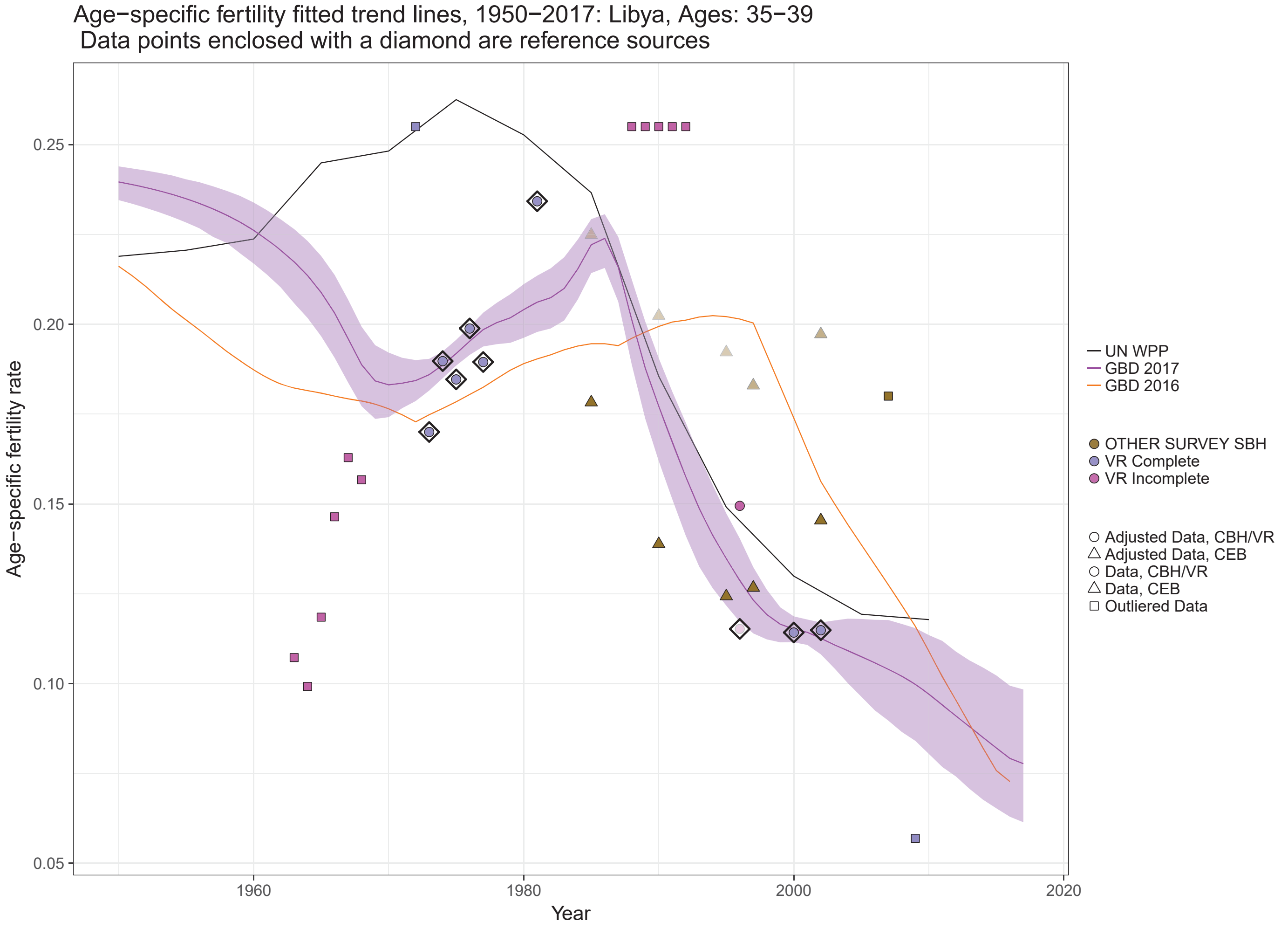

Age−specific fertility fitted trend lines, 1950−2017: Libya, Ages: 35−39
Data points enclosed with a diamond are reference sources
Age−specific fertility rate
Year
0.25
0.20
0.15
0.10
0.05
1960
1980
2000
2020
UN WPP
GBD 2017
GBD 2016
OTHER SURVEY SBH
VR Complete
VR Incomplete
Adjusted Data, CBH/VR
Adjusted Data, CEB
Data, CBH/VR
Data, CEB
Outliered Data

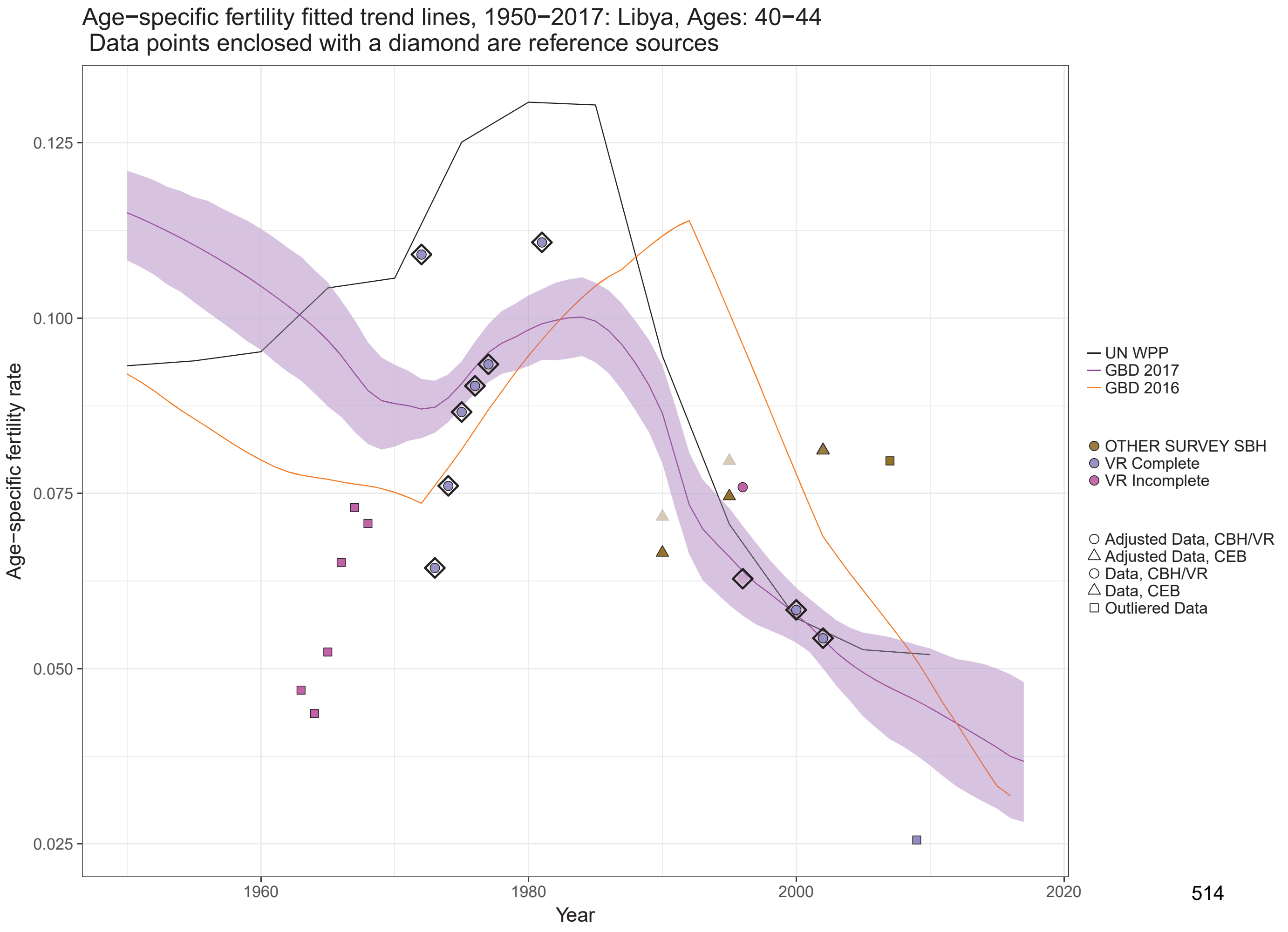

Age−specific fertility fitted trend lines, 1950−2017: Libya, Ages: 40−44
Data points enclosed with a diamond are reference sources
Age−specific fertility rate
Year
0.125
0.100
0.075
0.050
0.025
1960
1980
2000
2020
UN WPP
GBD 2017
GBD 2016
OTHER SURVEY SBH
VR Complete
VR Incomplete
Adjusted Data, CBH/VR
Adjusted Data, CEB
Data, CBH/VR
Data, CEB
Outliered Data

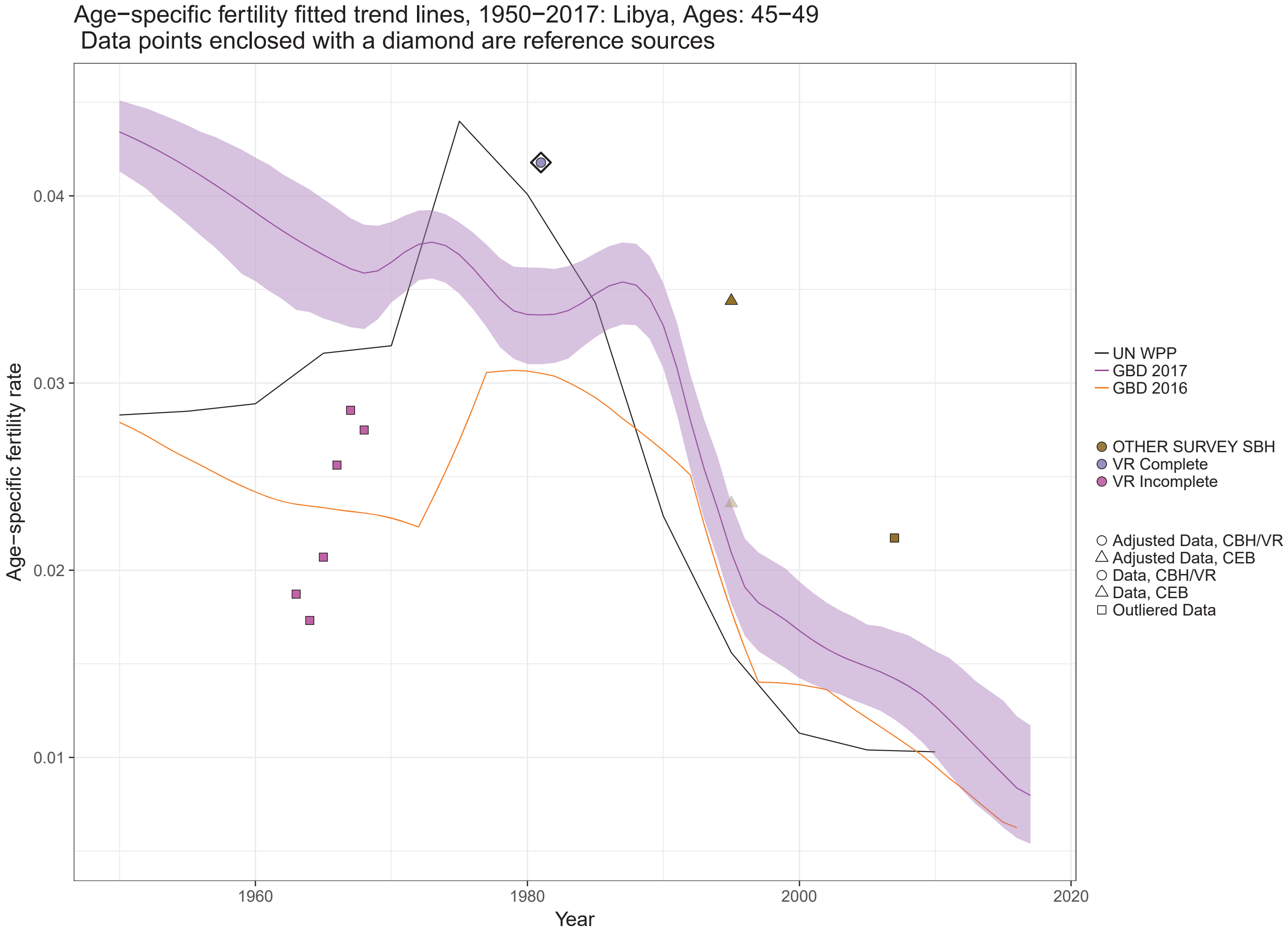

Age−specific fertility fitted trend lines, 1950−2017: Libya, Ages: 45−49
Data points enclosed with a diamond are reference sources
Age−specific fertility rate
0.01
0.02
0.03
0.04
1960
1980
2000
2020
Year
UN WPP
GBD 2017
GBD 2016
OTHER SURVEY SBH
VR Complete
VR Incomplete
Adjusted Data, CBH/VR
Adjusted Data, CEB
Data, CBH/VR
Data, CEB
Outliered Data

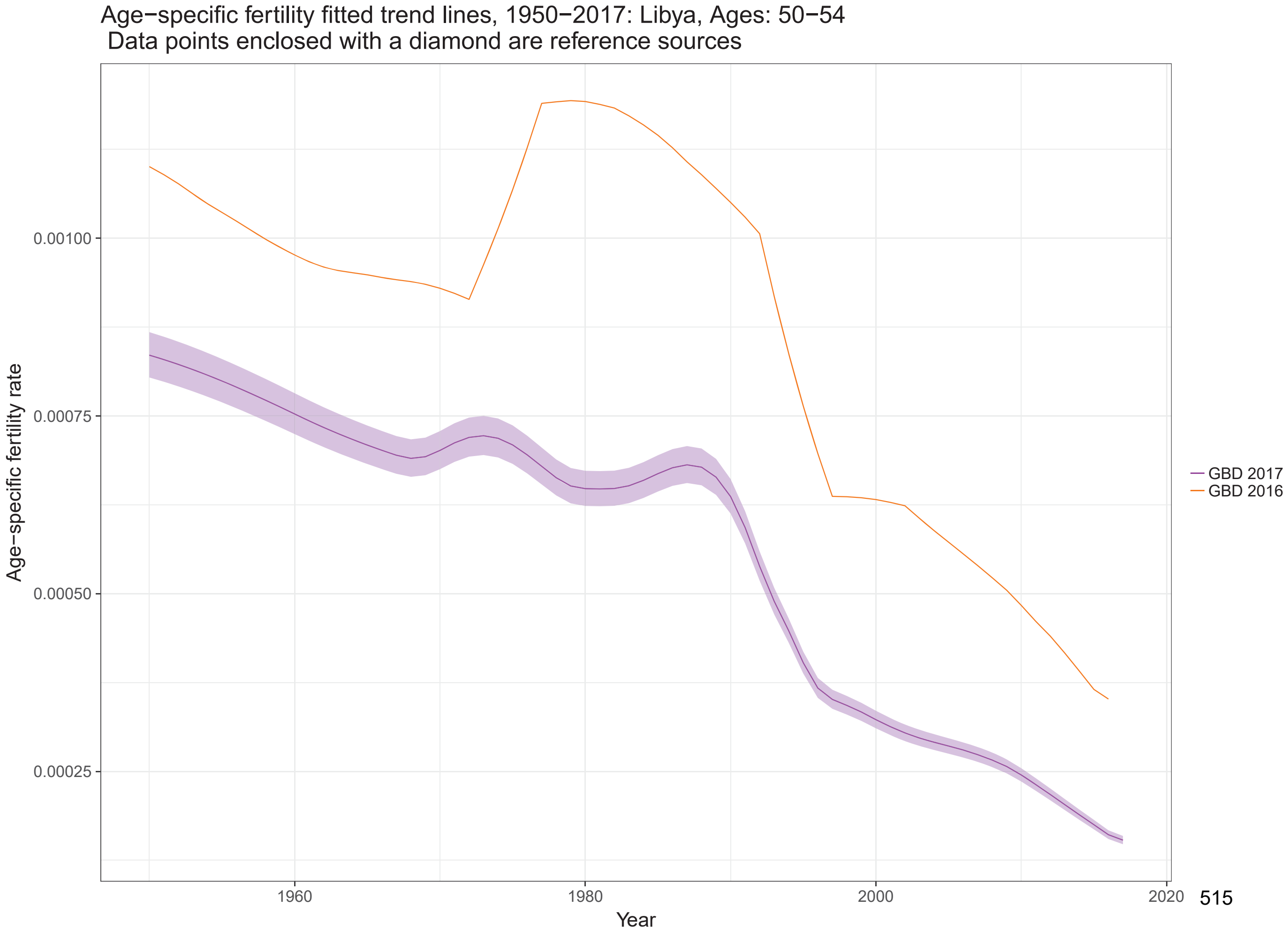

Age−specific fertility fitted trend lines, 1950−2017: Libya, Ages: 50−54
Data points enclosed with a diamond are reference sources
Age−specific fertility rate
0.00025
0.00050
0.00075
0.00100
1960
1980
2000
2020
Year
GBD 2017
GBD 2016

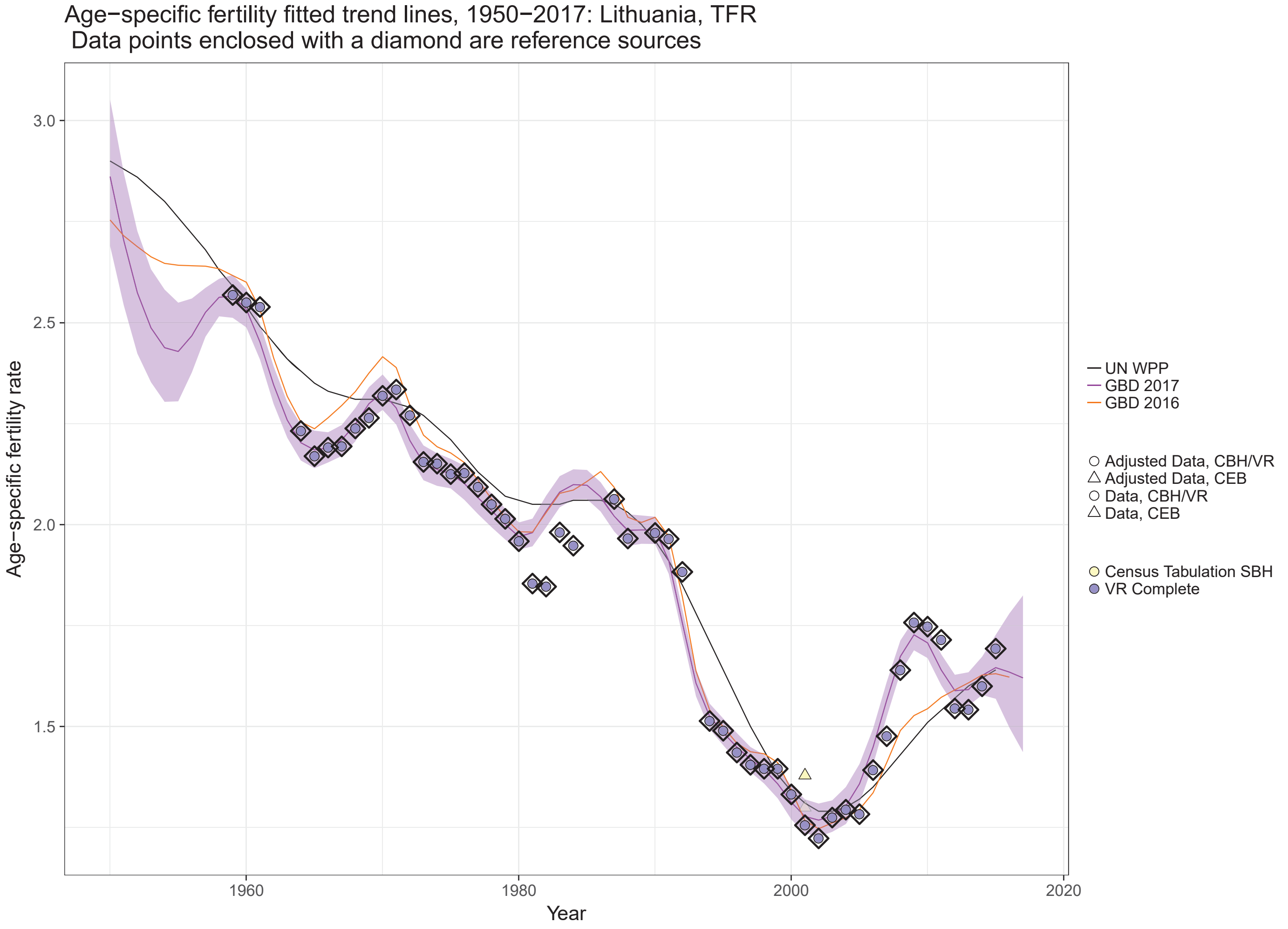
Age−specific fertility fitted trend lines, 1950−2017: Lithuania, TFR
Data points enclosed with a diamond are reference sources
Age−specific fertility rate
Year
3.0
2.5
2.0
1.5
1960
1980
2000
2020
UN WPP
GBD 2017
GBD 2016
Adjusted Data, CBH/VR
Adjusted Data, CEB
Data, CBH/VR
Data, CEB
Census Tabulation SBH
VR Complete

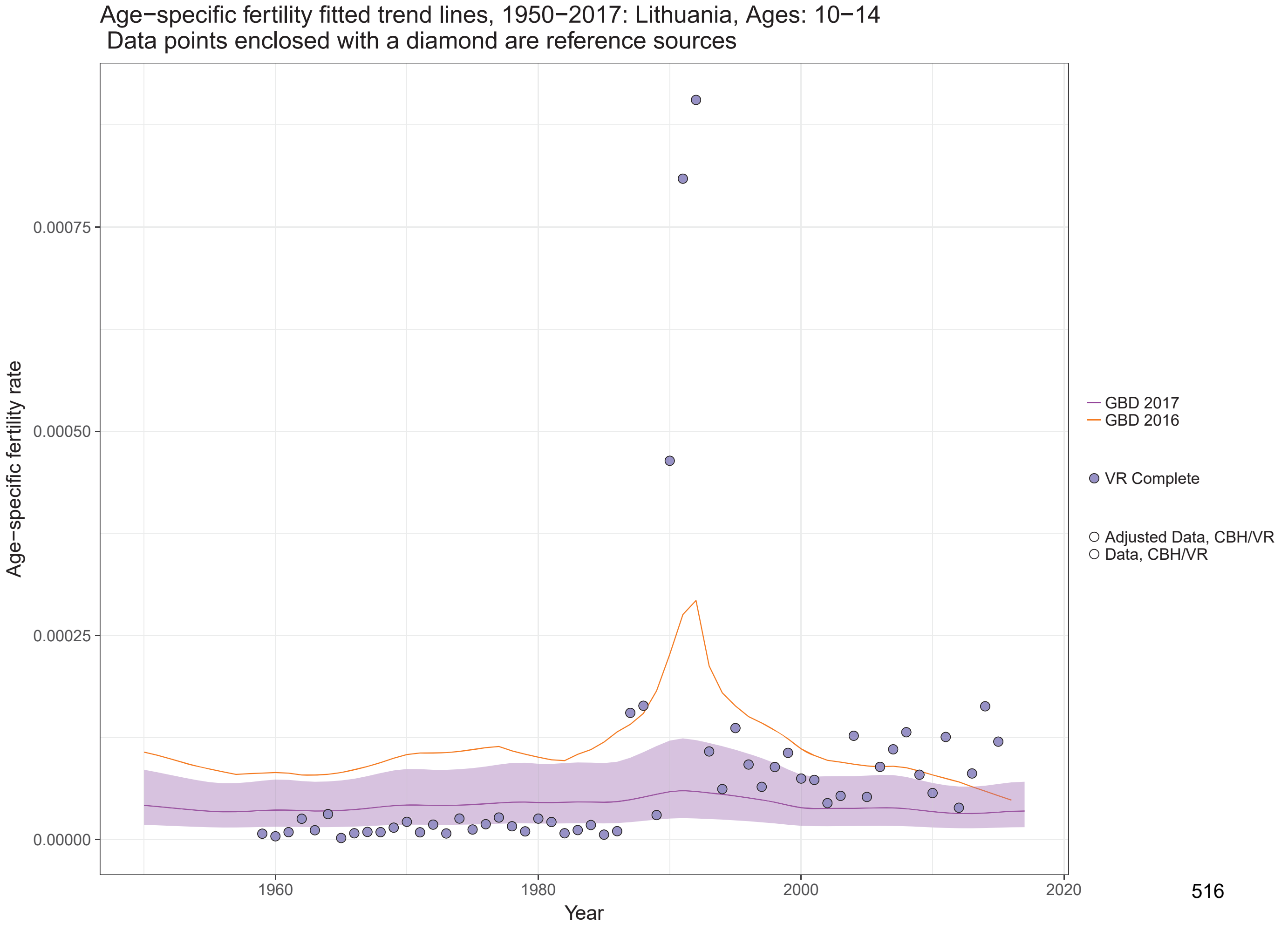
Age−specific fertility fitted trend lines, 1950−2017: Lithuania, Ages: 10−14
Data points enclosed with a diamond are reference sources
Age−specific fertility rate
Year
0.00075
0.00050
0.00025
0.00000
1960
1980
2000
2020
GBD 2017
GBD 2016
VR Complete
Adjusted Data, CBH/VR
Data, CBH/VR

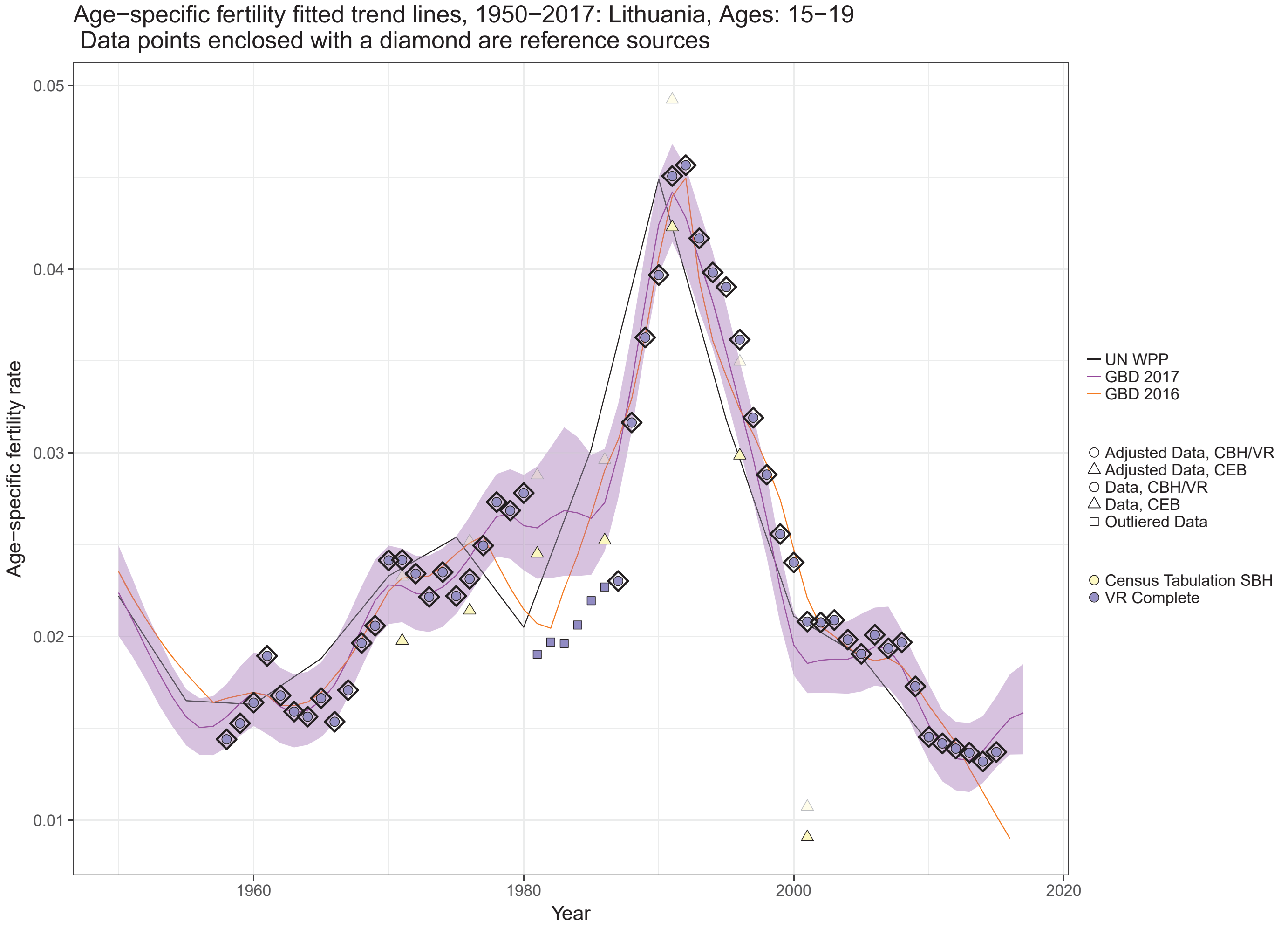
Age−specific fertility fitted trend lines, 1950−2017: Lithuania, Ages: 15−19
Data points enclosed with a diamond are reference sources
Age−specific fertility rate
0.05
0.04
0.03
0.02
0.01
1960
1980
2000
2020
Year
UN WPP
GBD 2017
GBD 2016
Adjusted Data, CBH/VR
Adjusted Data, CEB
Data, CBH/VR
Data, CEB
Outliered Data
Census Tabulation SBH
VR Complete

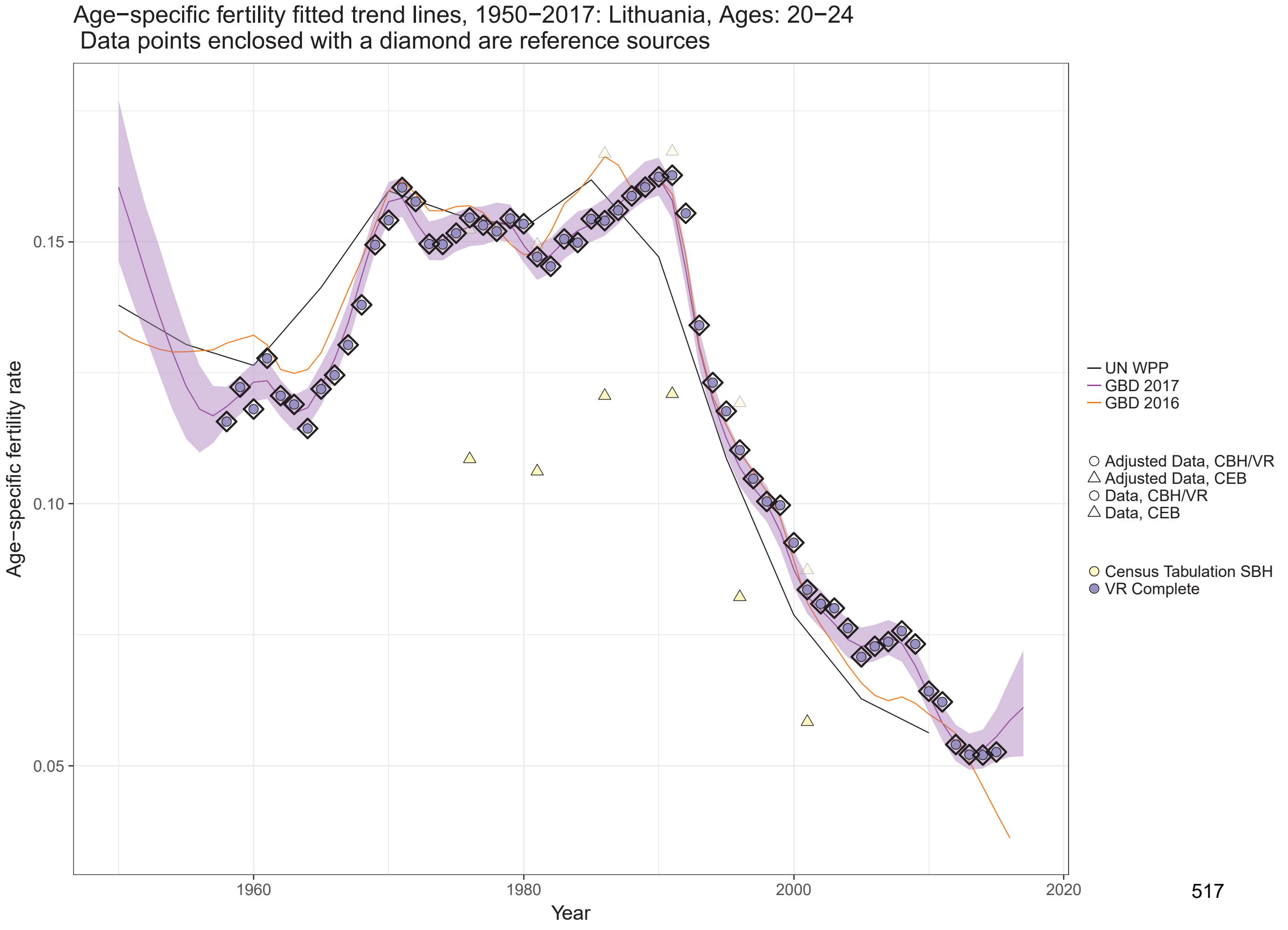
Age−specific fertility fitted trend lines, 1950−2017: Lithuania, Ages: 20−24
Data points enclosed with a diamond are reference sources
Age−specific fertility rate
0.15
0.10
0.05
1960
1980
2000
2020
Year
UN WPP
GBD 2017
GBD 2016
Adjusted Data, CBH/VR
Adjusted Data, CEB
Data, CBH/VR
Data, CEB
Census Tabulation SBH
VR Complete

Age−specific fertility fitted trend lines, 1950−2017: Lithuania, Ages: 25−29
Data points enclosed with a diamond are reference sources

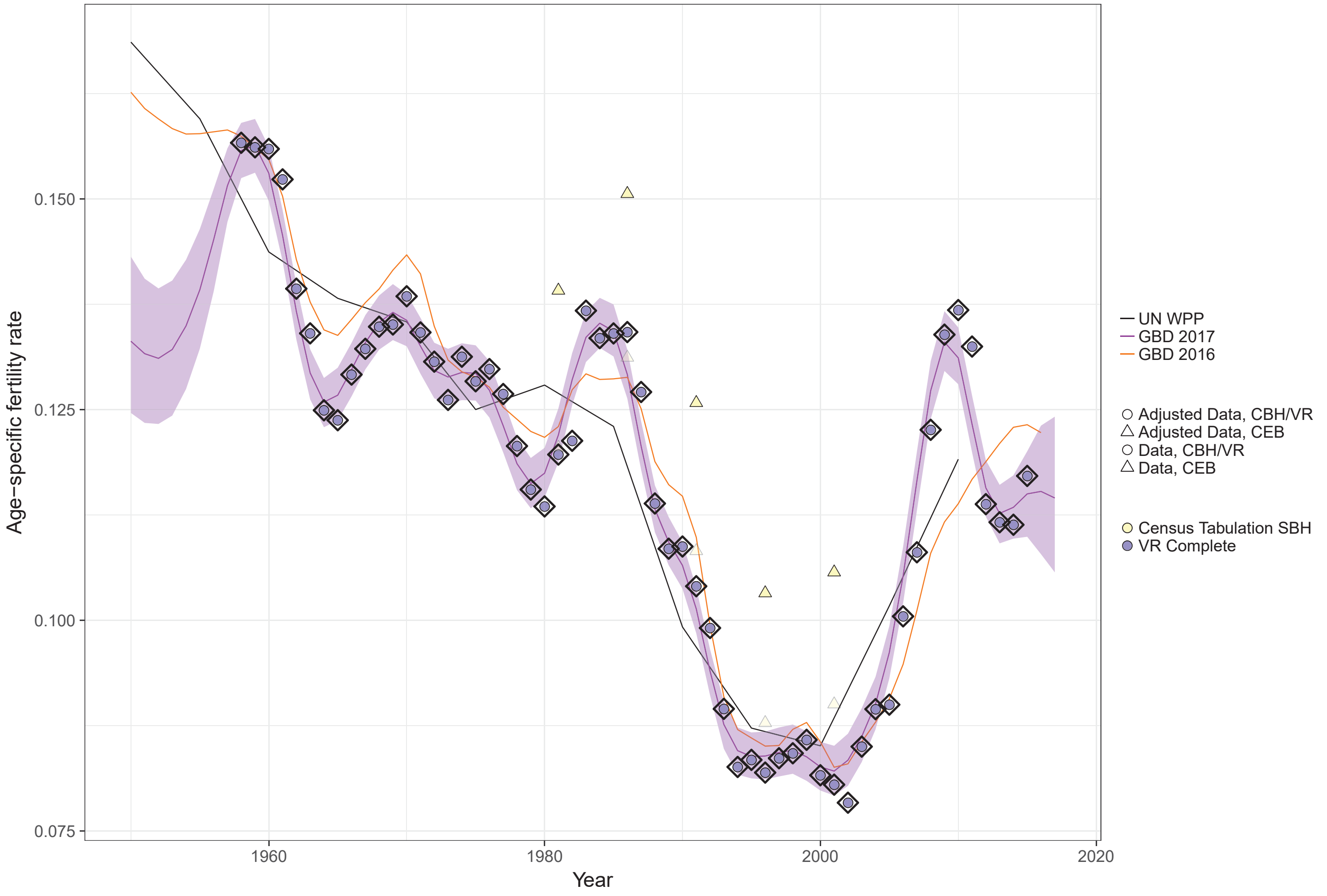

Age−specific fertility fitted trend lines, 1950−2017: Lithuania, Ages: 30−34
Data points enclosed with a diamond are reference sources

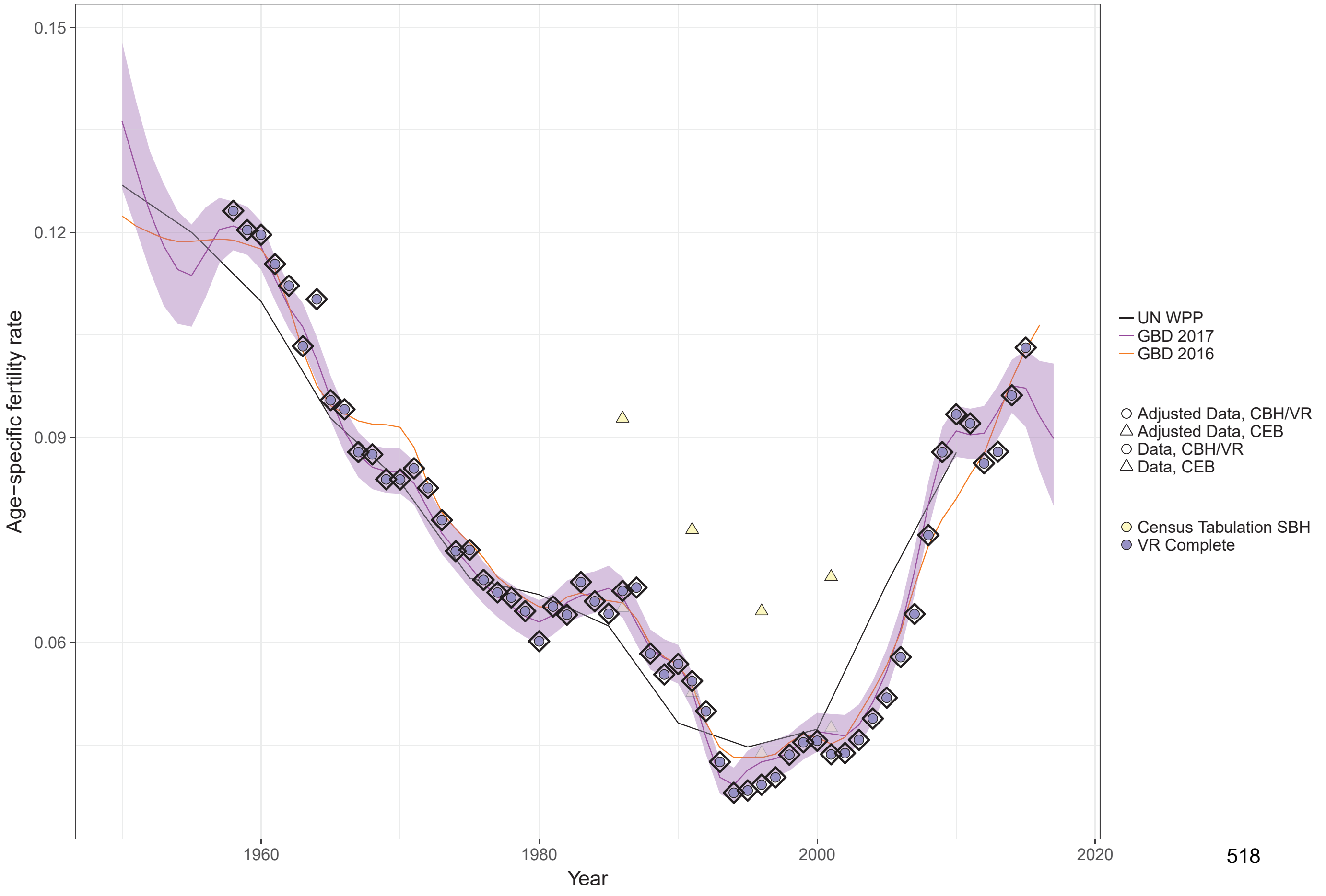

Age−specific fertility fitted trend lines, 1950−2017: Lithuania, Ages: 35−39
Data points enclosed with a diamond are reference sources

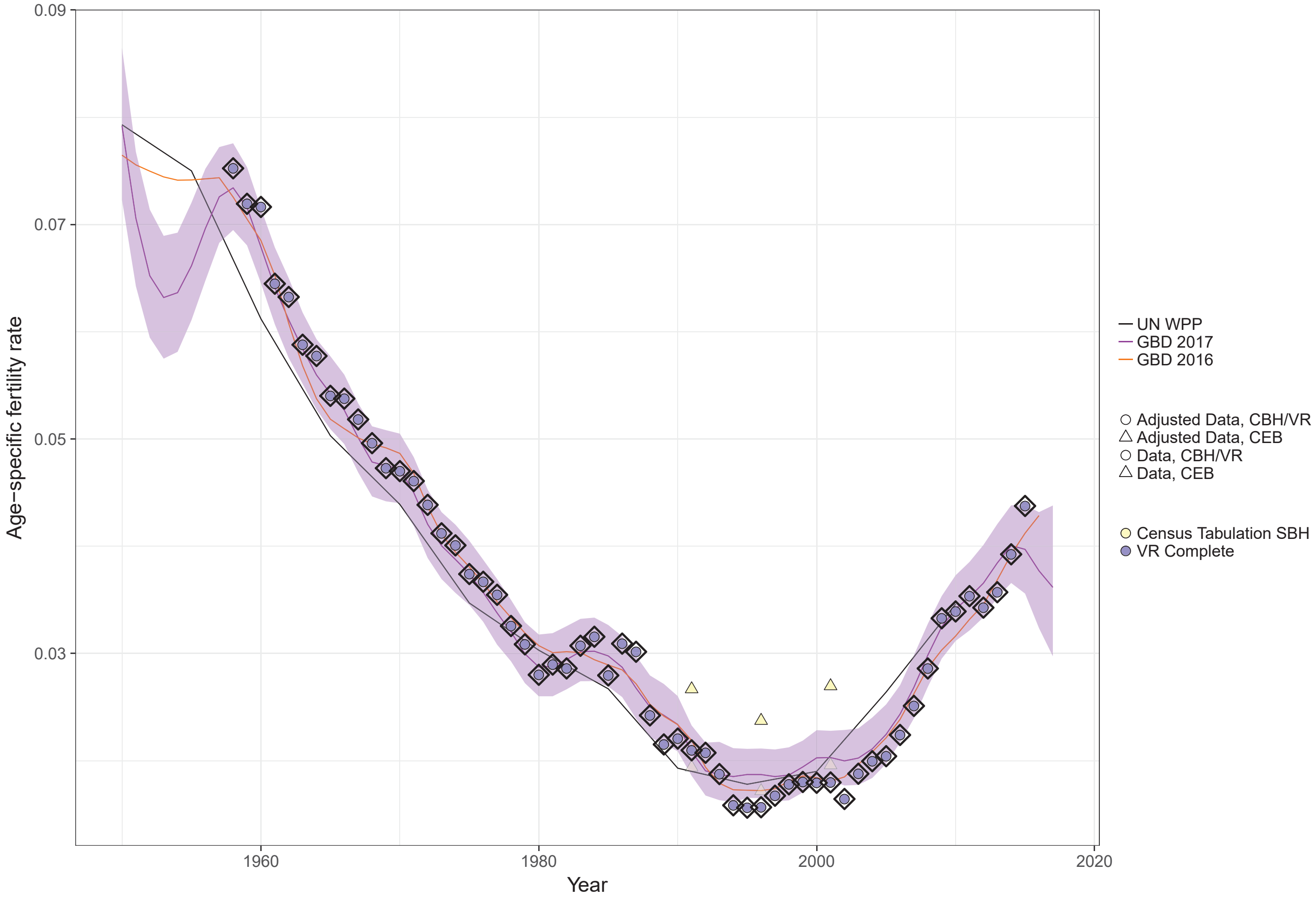

Age−specific fertility fitted trend lines, 1950−2017: Lithuania, Ages: 40−44
Data points enclosed with a diamond are reference sources

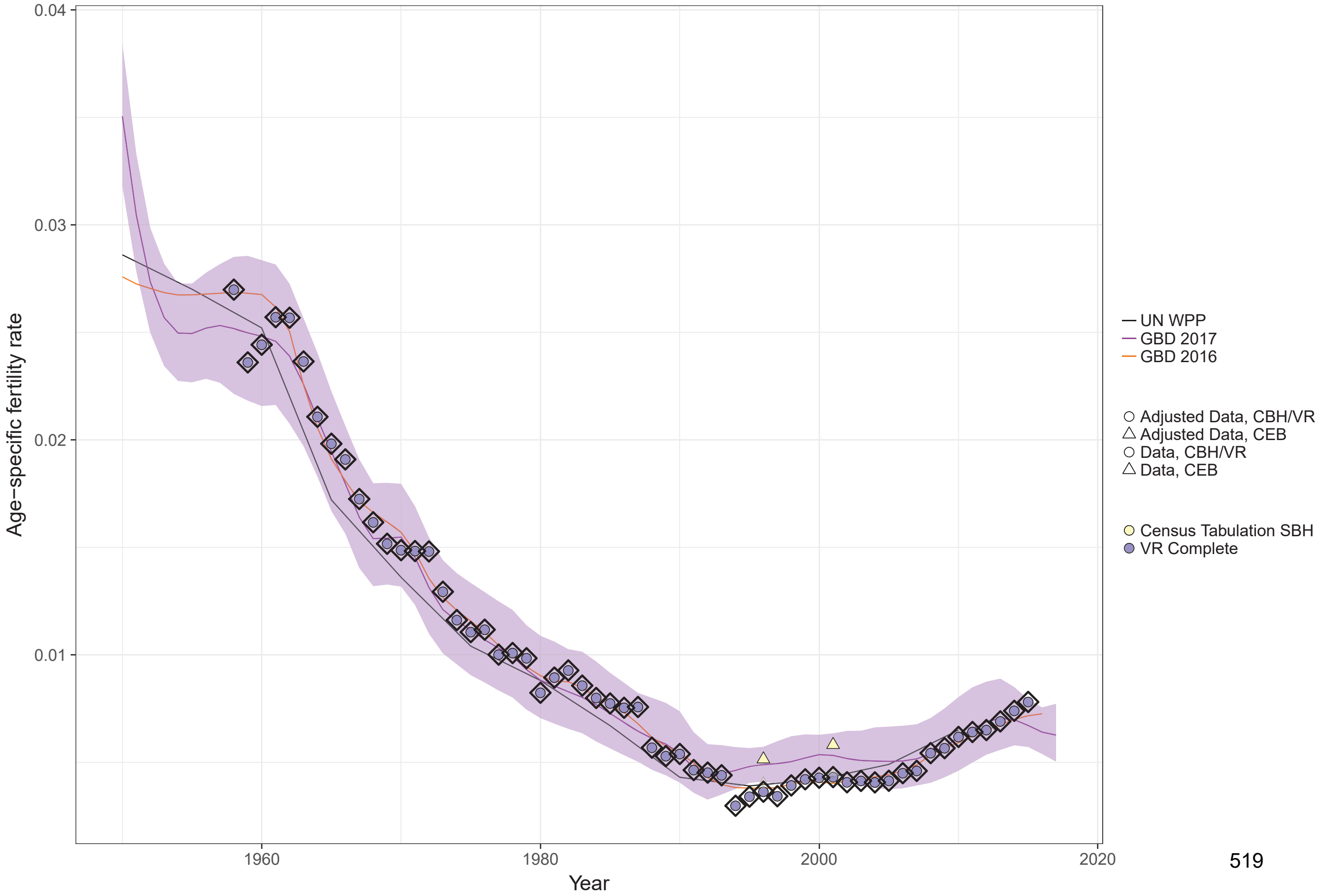

Age−specific fertility fitted trend lines, 1950−2017: Lithuania, Ages: 45−49
Data points enclosed with a diamond are reference sources

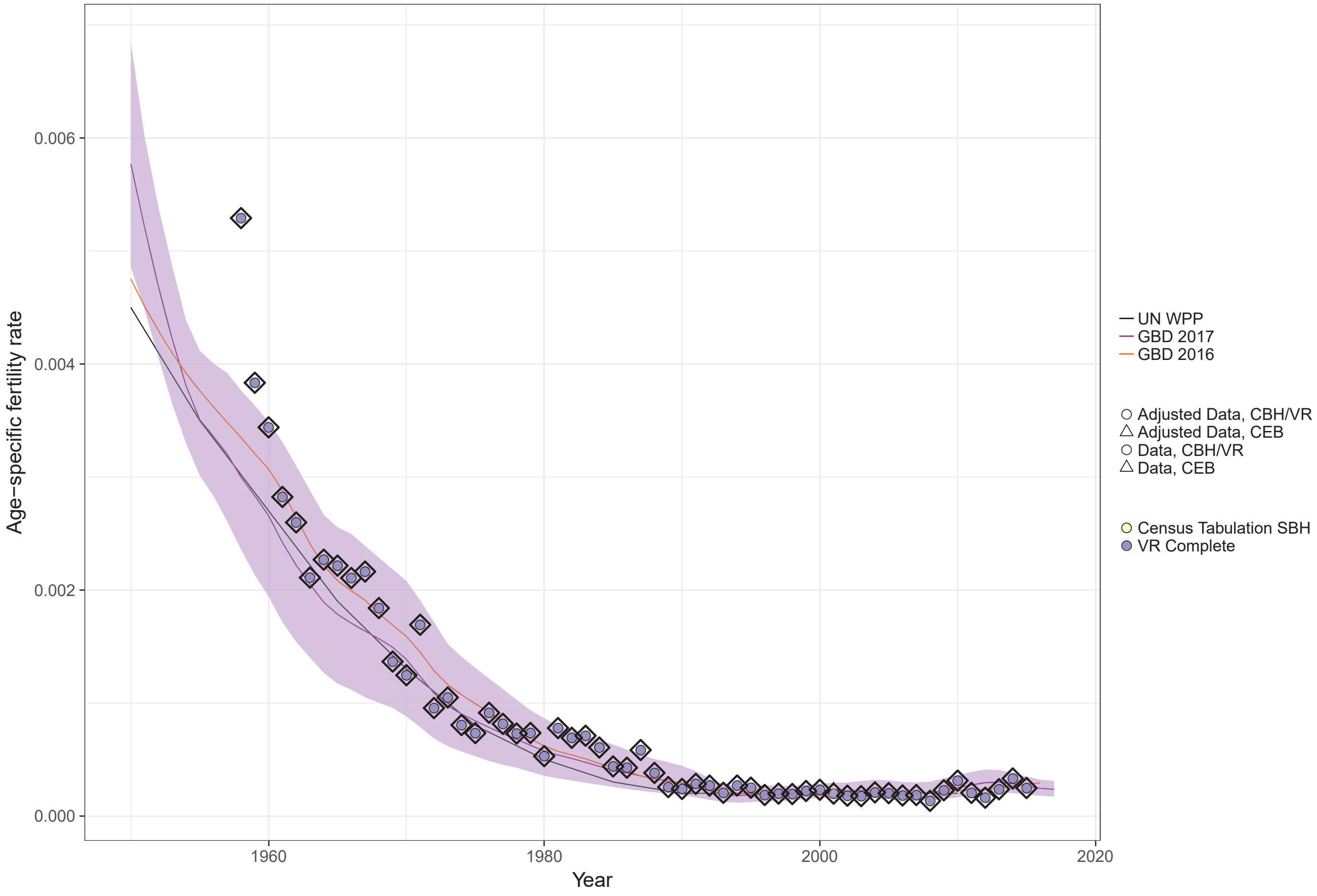

Age−specific fertility fitted trend lines, 1950−2017: Lithuania, Ages: 50−54
Data points enclosed with a diamond are reference sources

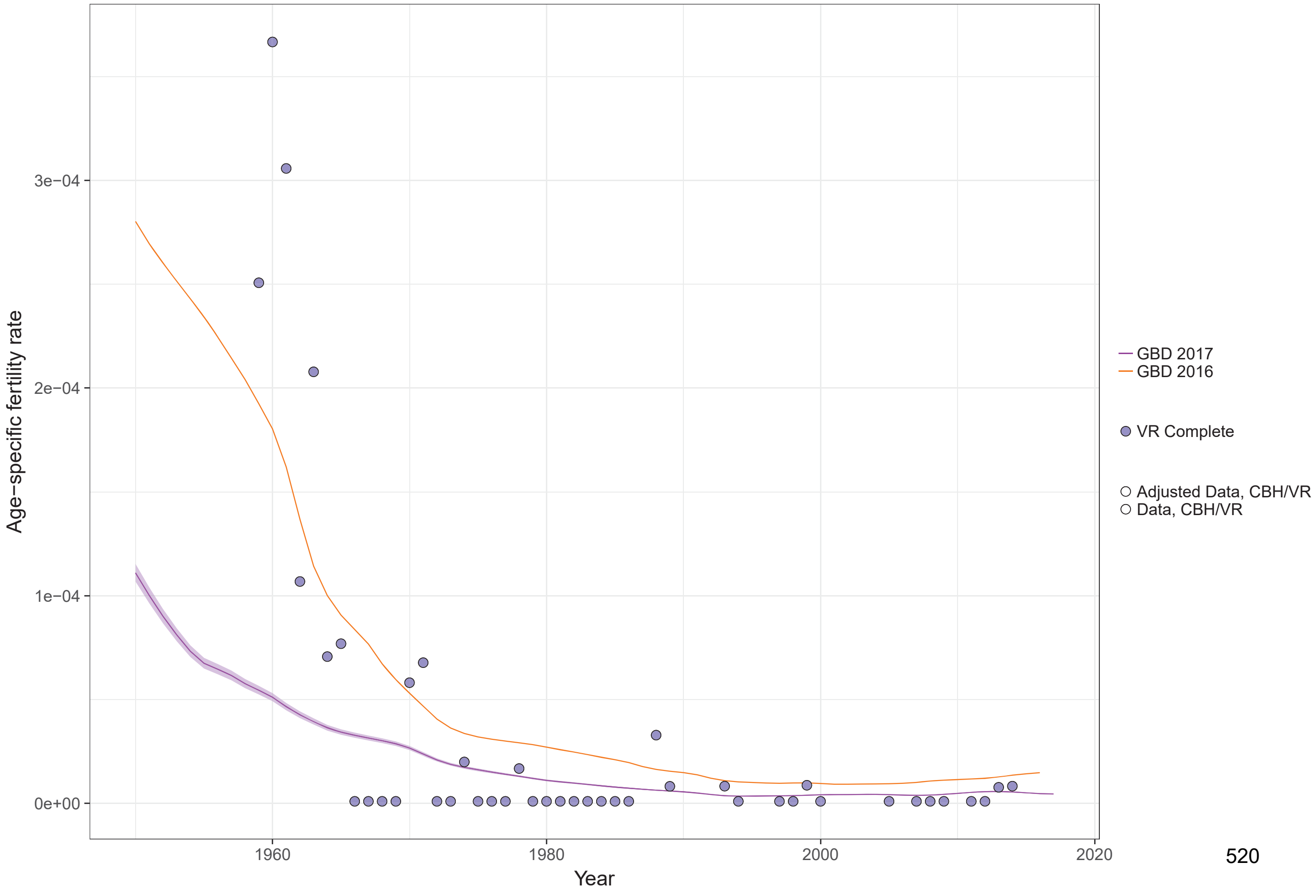

Age−specific fertility fitted trend lines, 1950−2017: Luxembourg, TFR
Data points enclosed with a diamond are reference sources

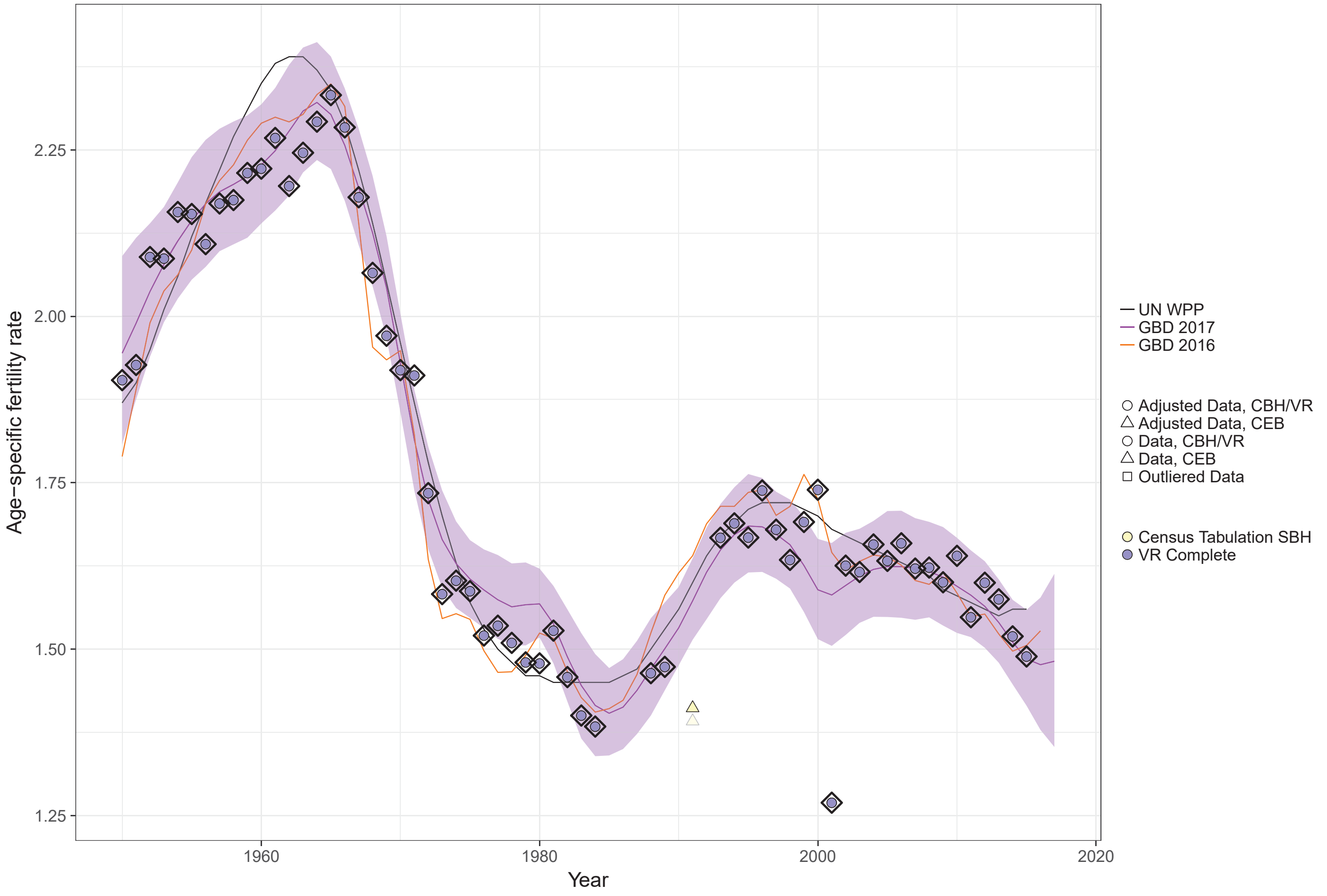

Age−specific fertility fitted trend lines, 1950−2017: Luxembourg, Ages: 10−14
Data points enclosed with a diamond are reference sources

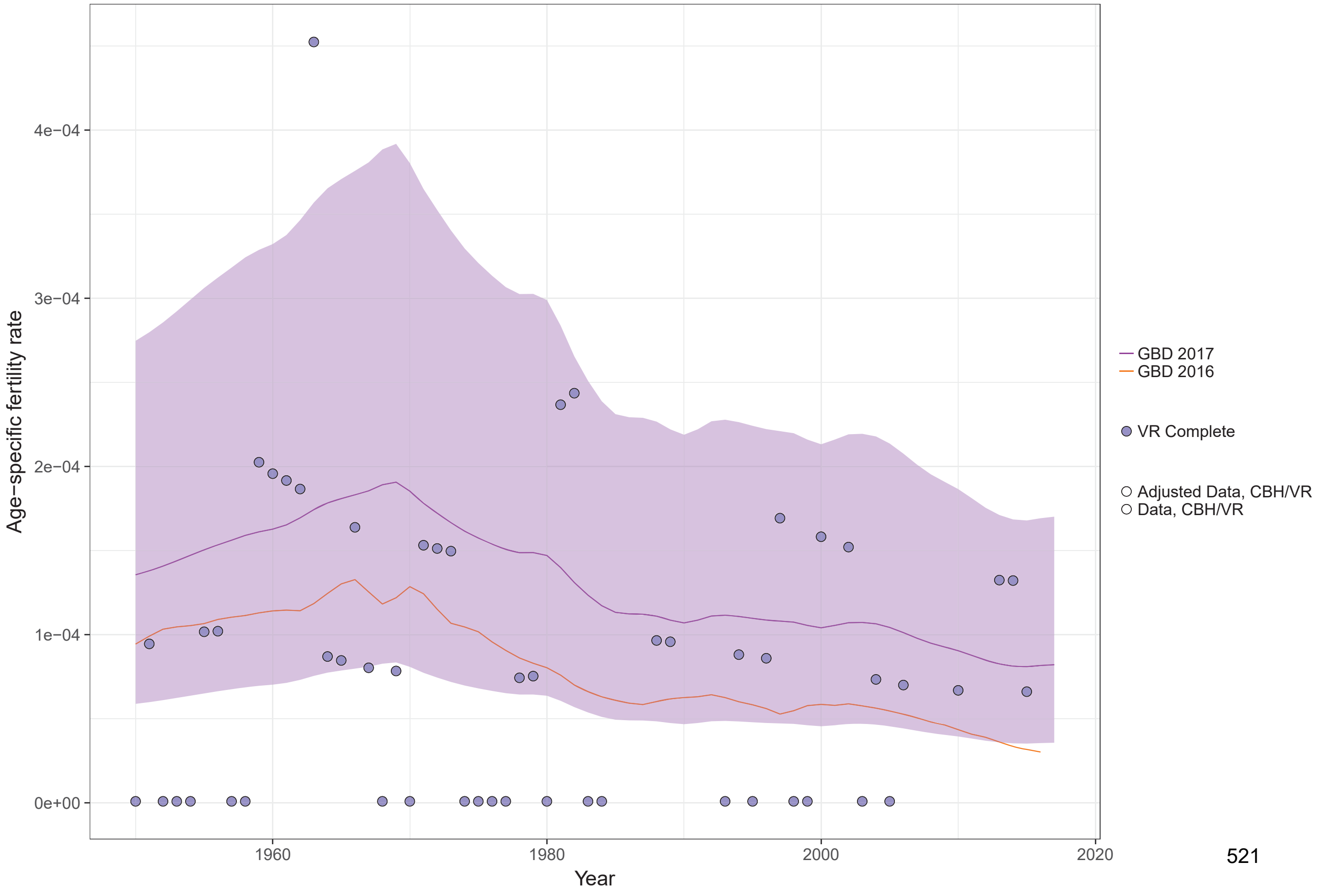

Age−specific fertility fitted trend lines, 1950−2017: Luxembourg, Ages: 15−19
Data points enclosed with a diamond are reference sources

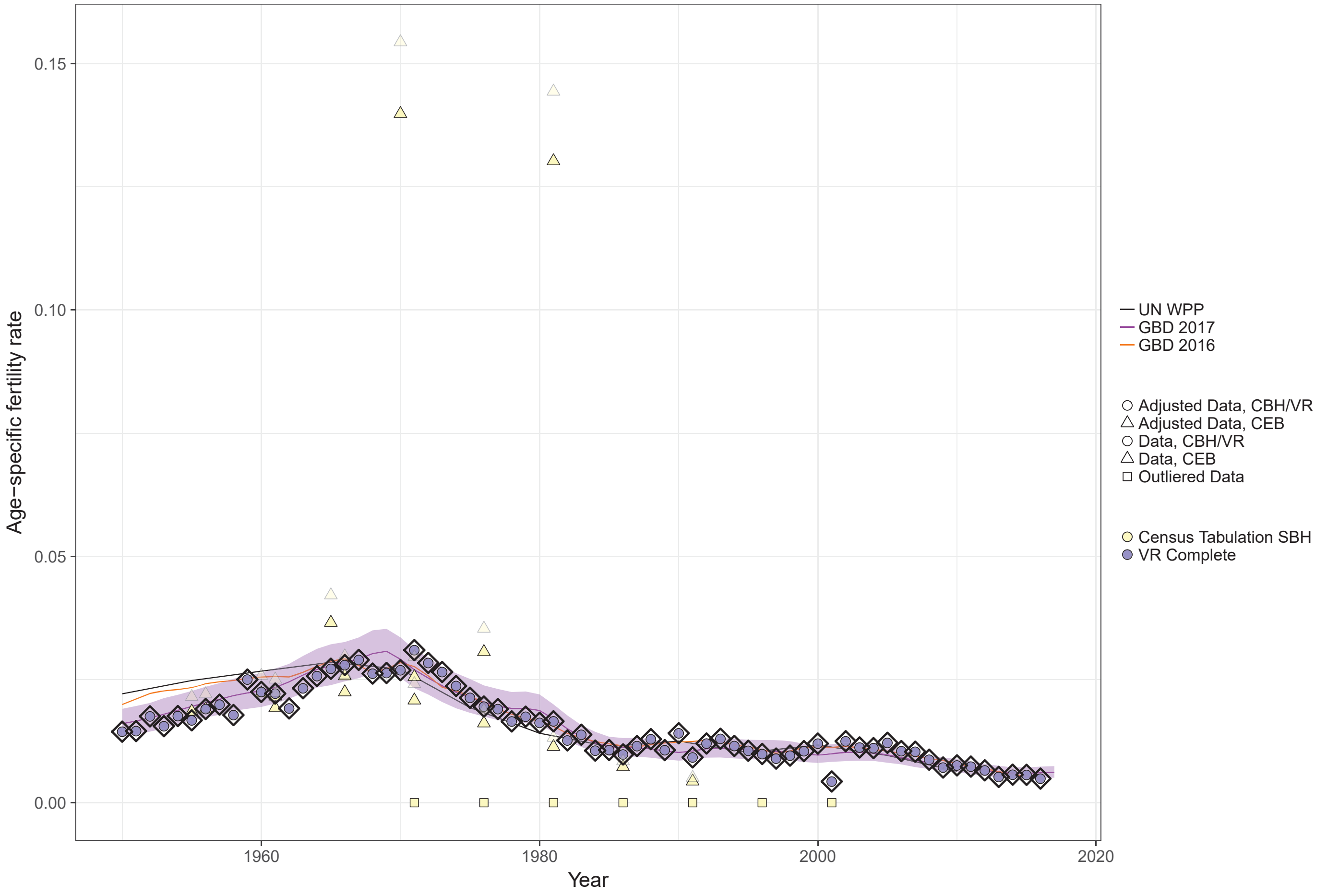

Age−specific fertility fitted trend lines, 1950−2017: Luxembourg, Ages: 20−24
Data points enclosed with a diamond are reference sources

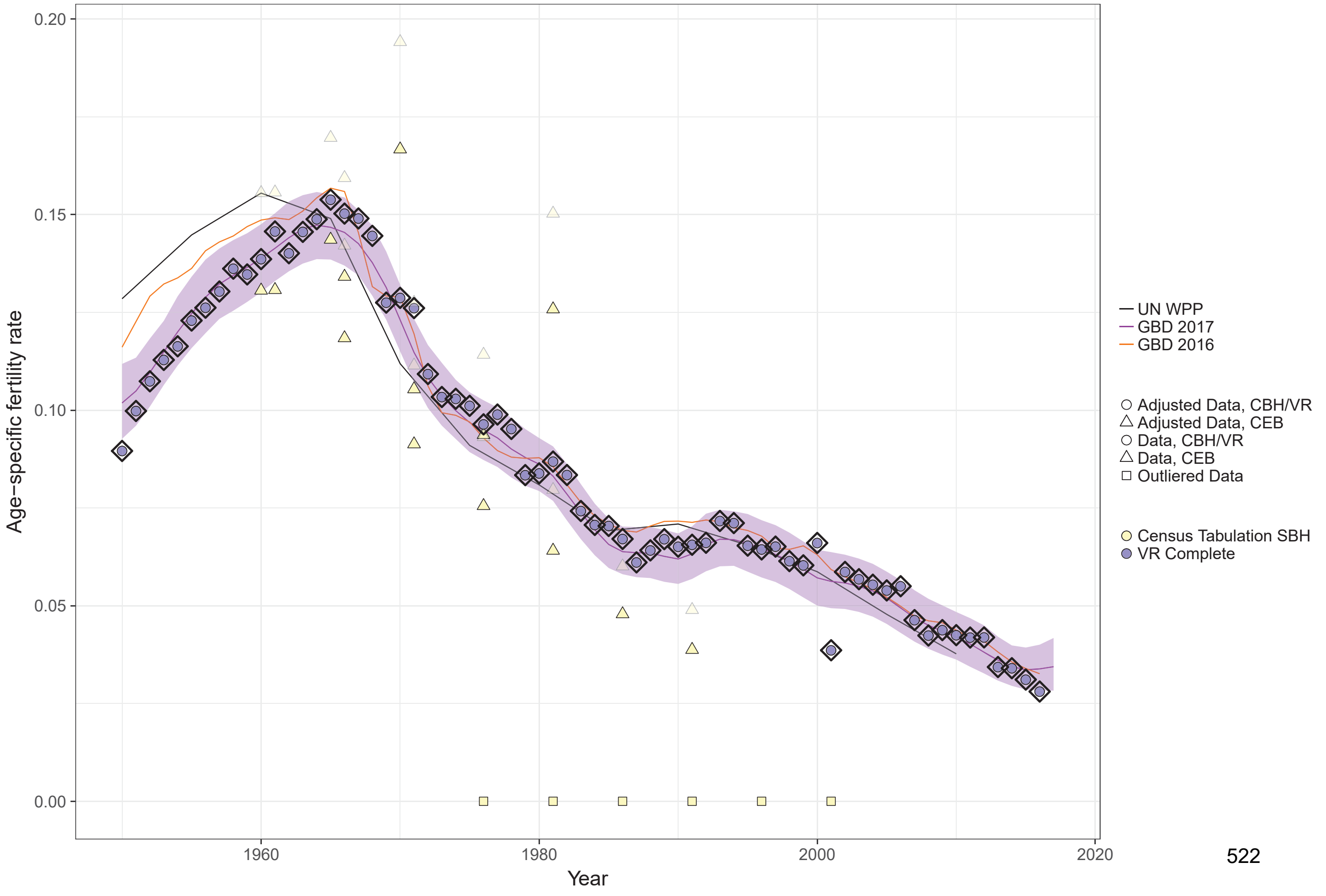

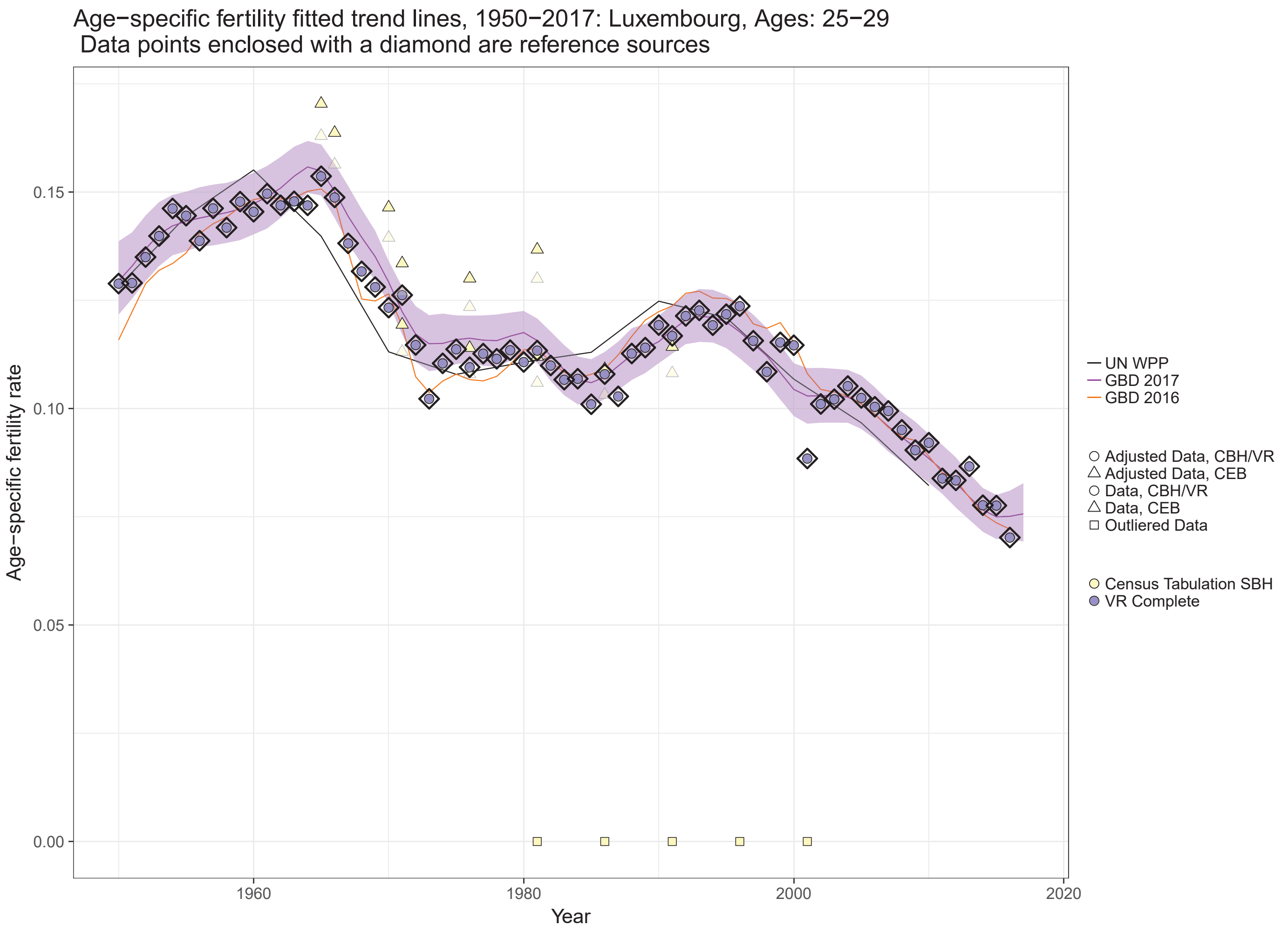

Age−specific fertility fitted trend lines, 1950−2017: Luxembourg, Ages: 25−29
Data points enclosed with a diamond are reference sources
Age−specific fertility rate
0.00
0.05
0.10
0.15
1960
1980
2000
2020
Year
UN WPP
GBD 2017
GBD 2016
Adjusted Data, CBH/VR
Adjusted Data, CEB
Data, CBH/VR
Data, CEB
Outliered Data
Census Tabulation SBH
VR Complete

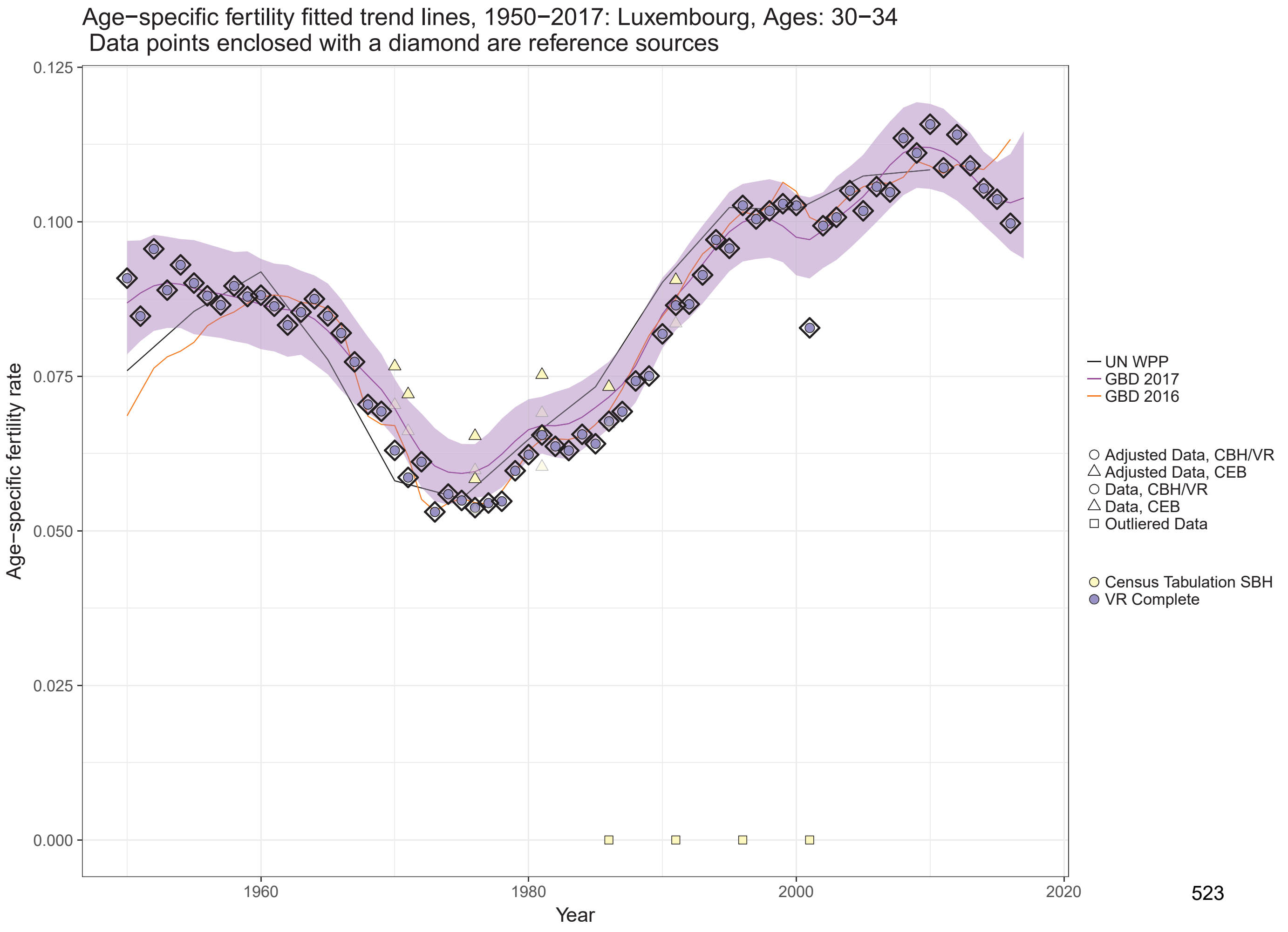

Age−specific fertility fitted trend lines, 1950−2017: Luxembourg, Ages: 30−34
Data points enclosed with a diamond are reference sources
Age−specific fertility rate
0.000
0.025
0.050
0.075
0.100
0.125
1960
1980
2000
2020
Year
UN WPP
GBD 2017
GBD 2016
Adjusted Data, CBH/VR
Adjusted Data, CEB
Data, CBH/VR
Data, CEB
Outliered Data
Census Tabulation SBH
VR Complete

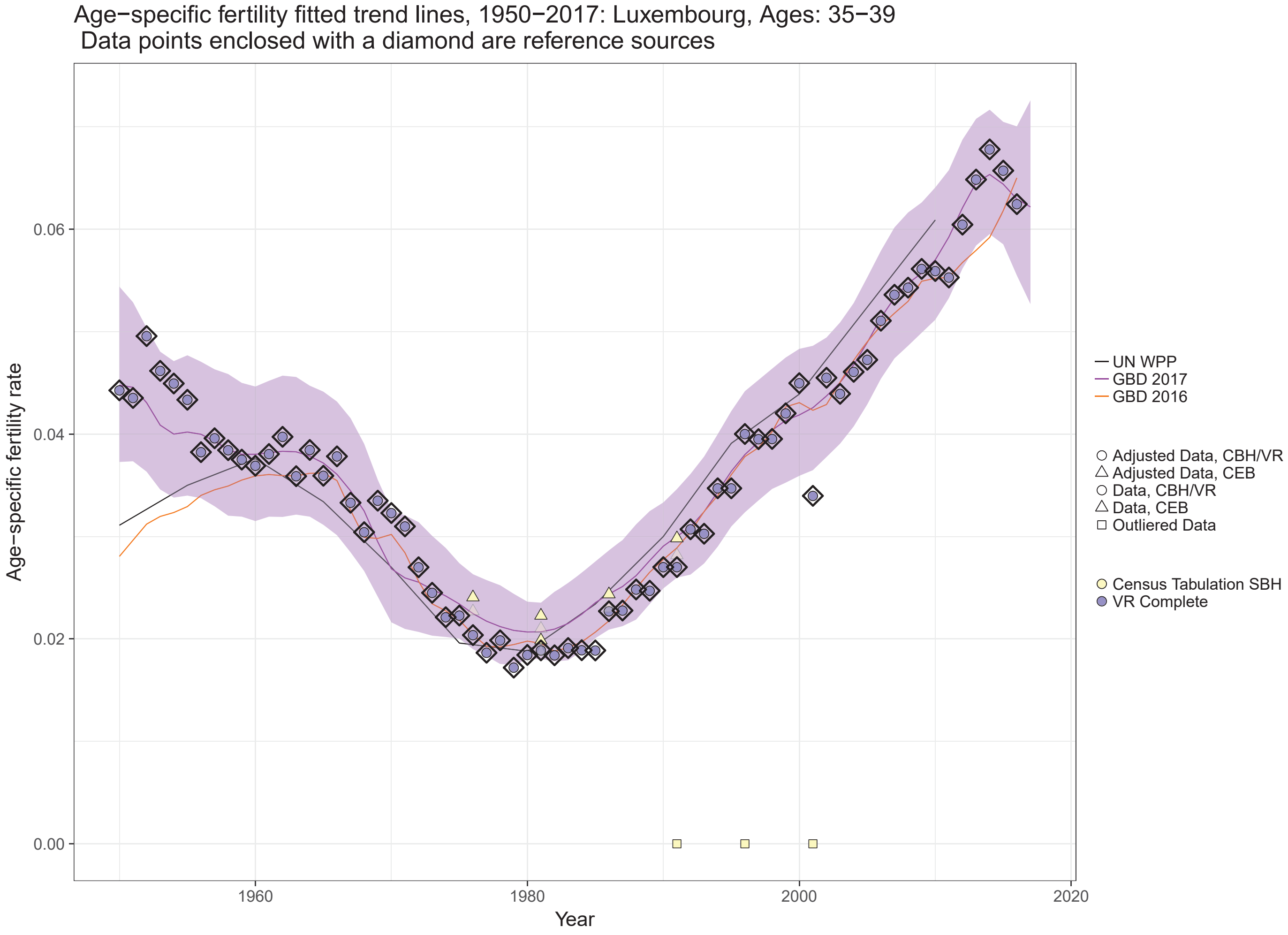
Age−specific fertility fitted trend lines, 1950−2017: Luxembourg, Ages: 35−39
Data points enclosed with a diamond are reference sources
Age−specific fertility rate
0.06
0.04
0.02
0.00
1960
1980
2000
2020
Year
UN WPP
GBD 2017
GBD 2016
Adjusted Data, CBH/VR
Adjusted Data, CEB
Data, CBH/VR
Data, CEB
Outliered Data
Census Tabulation SBH
VR Complete

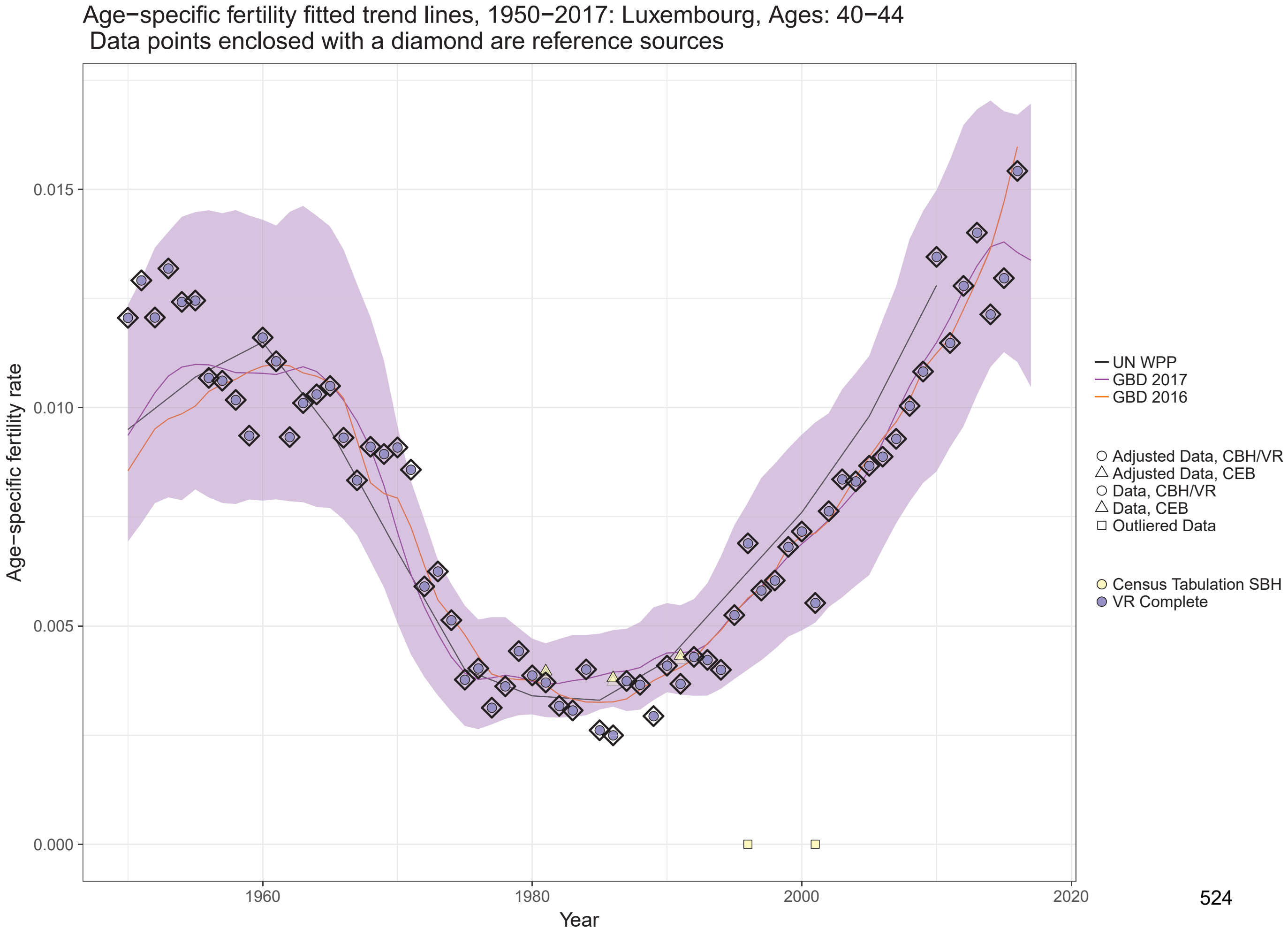
Age−specific fertility fitted trend lines, 1950−2017: Luxembourg, Ages: 40−44
Data points enclosed with a diamond are reference sources
Age−specific fertility rate
0.015
0.010
0.005
0.000
1960
1980
2000
2020
Year
UN WPP
GBD 2017
GBD 2016
Adjusted Data, CBH/VR
Adjusted Data, CEB
Data, CBH/VR
Data, CEB
Outliered Data
Census Tabulation SBH
VR Complete

Age−specific fertility fitted trend lines, 1950−2017: Luxembourg, Ages: 45−49
Data points enclosed with a diamond are reference sources

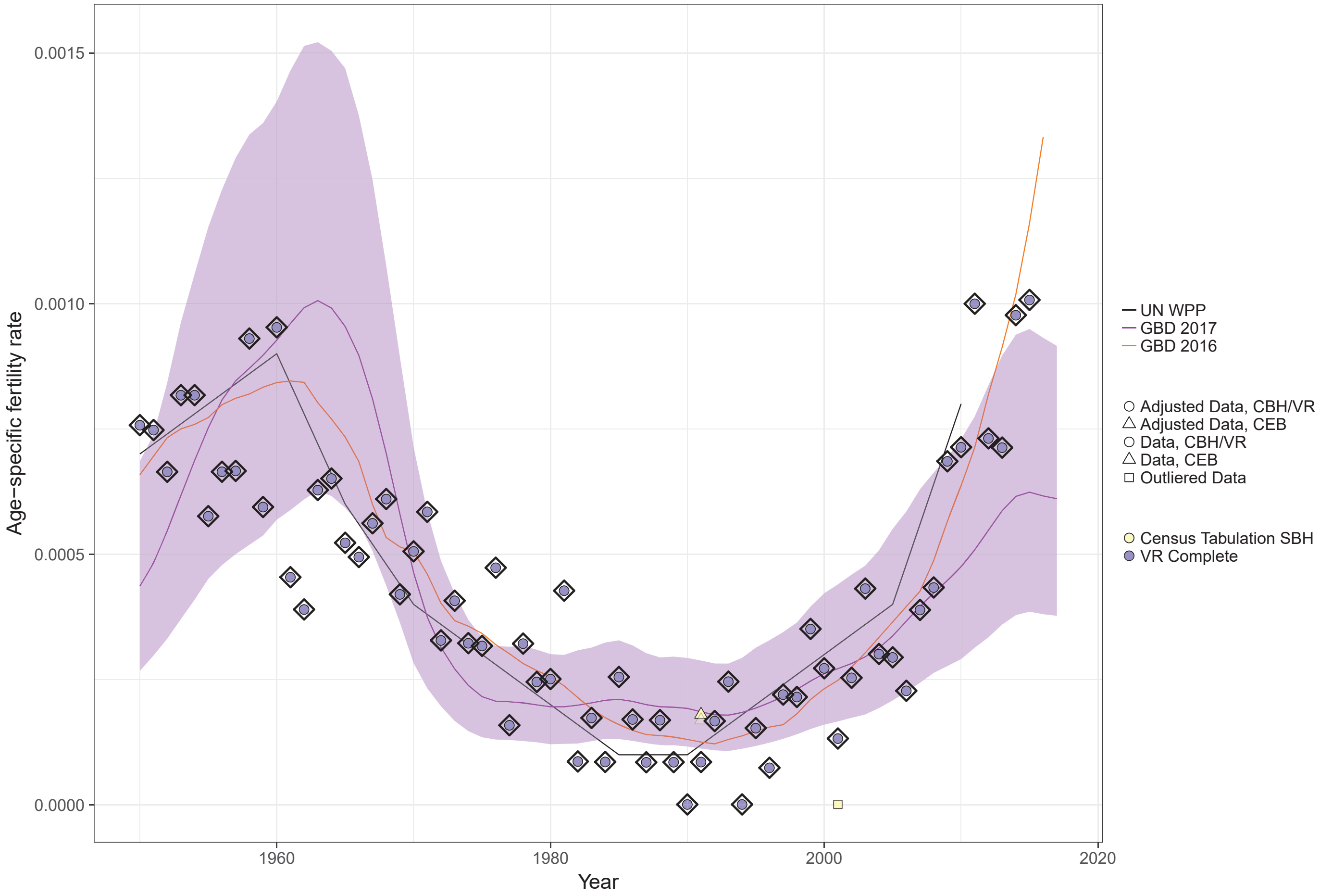

Age−specific fertility fitted trend lines, 1950−2017: Luxembourg, Ages: 50−54
Data points enclosed with a diamond are reference sources

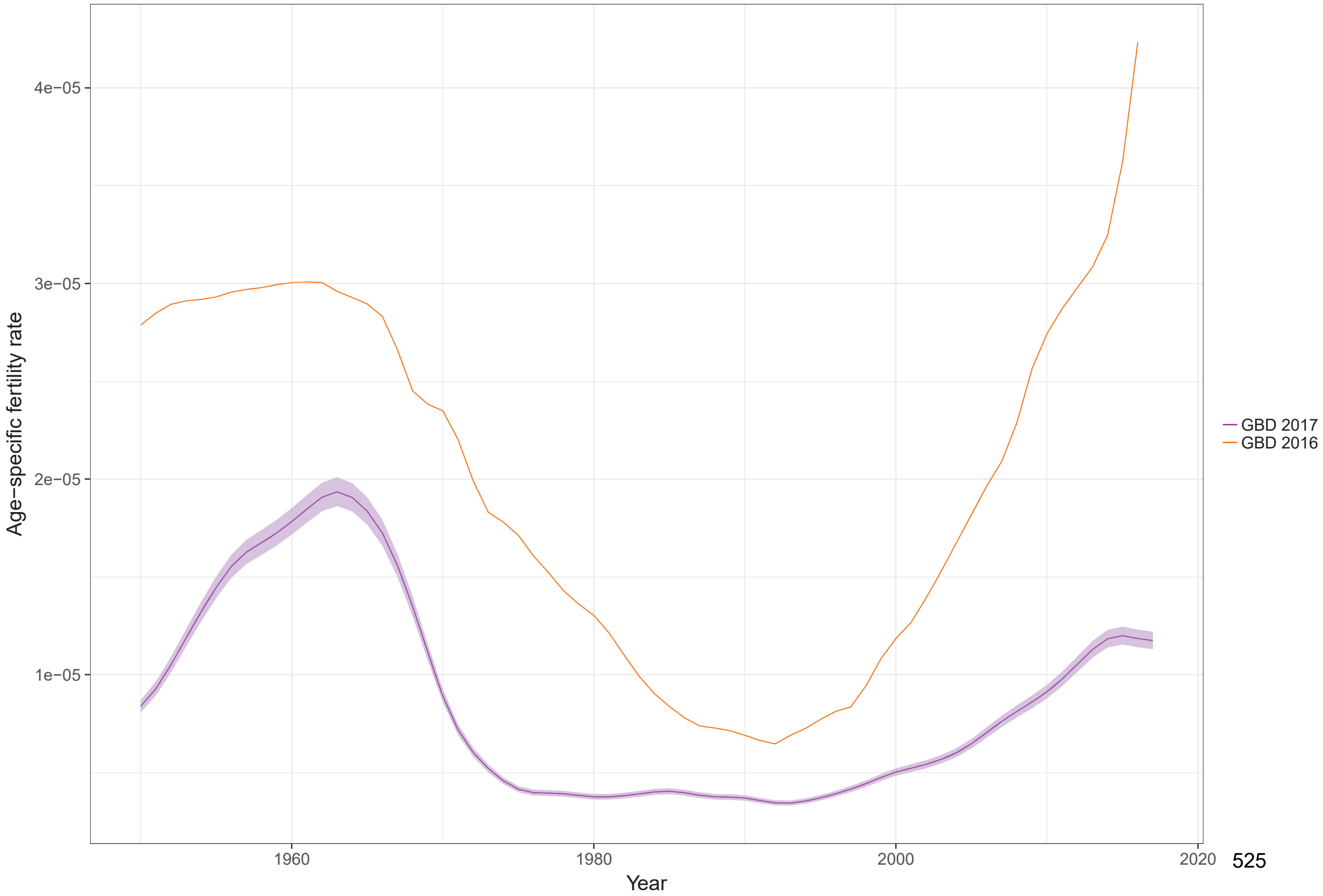

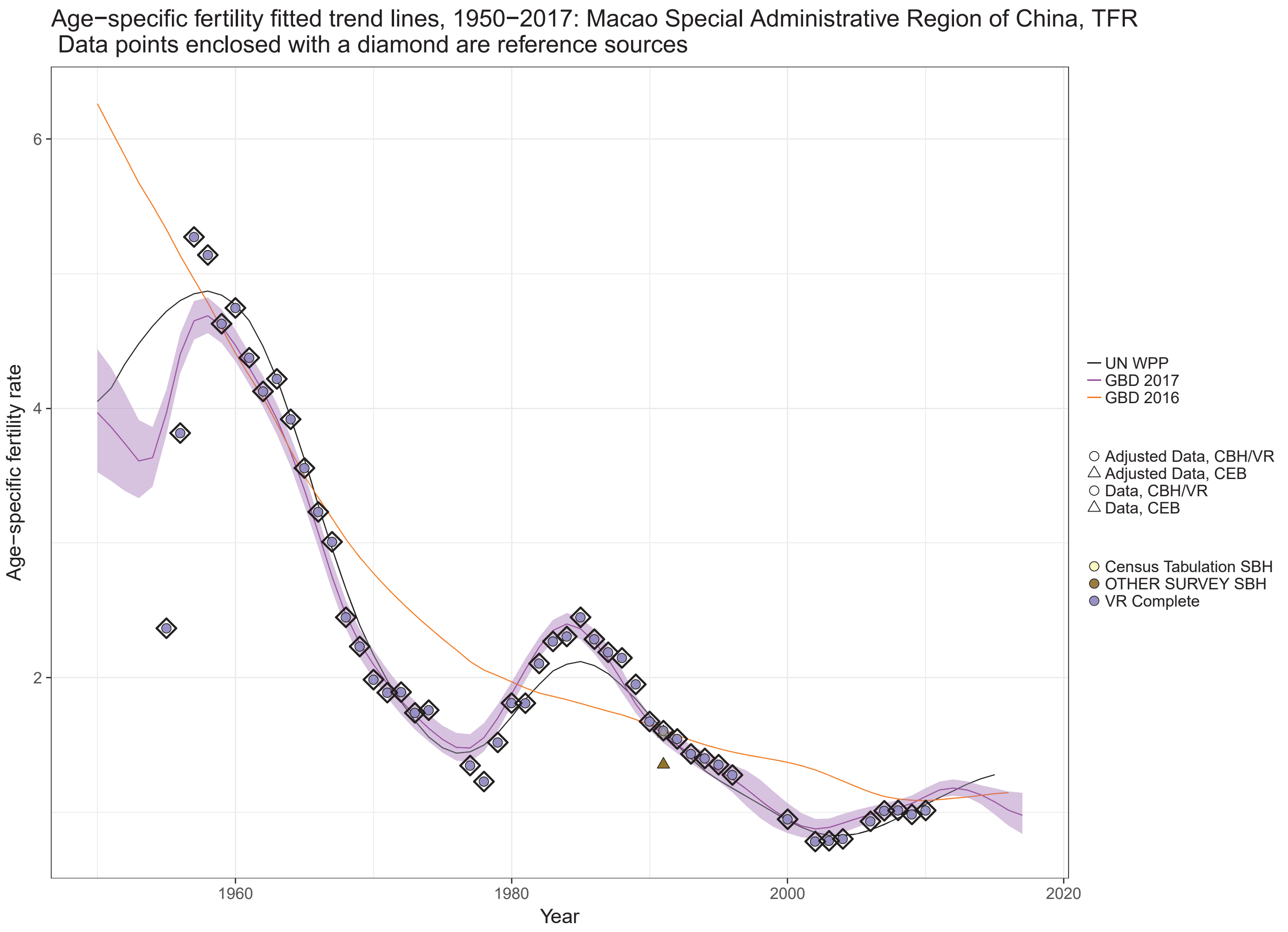

Age−specific fertility fitted trend lines, 1950−2017: Macao Special Administrative Region of China, TFR
Data points enclosed with a diamond are reference sources
Age−specific fertility rate
Year
UN WPP
GBD 2017
GBD 2016
Adjusted Data, CBH/VR
Adjusted Data, CEB
Data, CBH/VR
Data, CEB
Census Tabulation SBH
OTHER SURVEY SBH
VR Complete

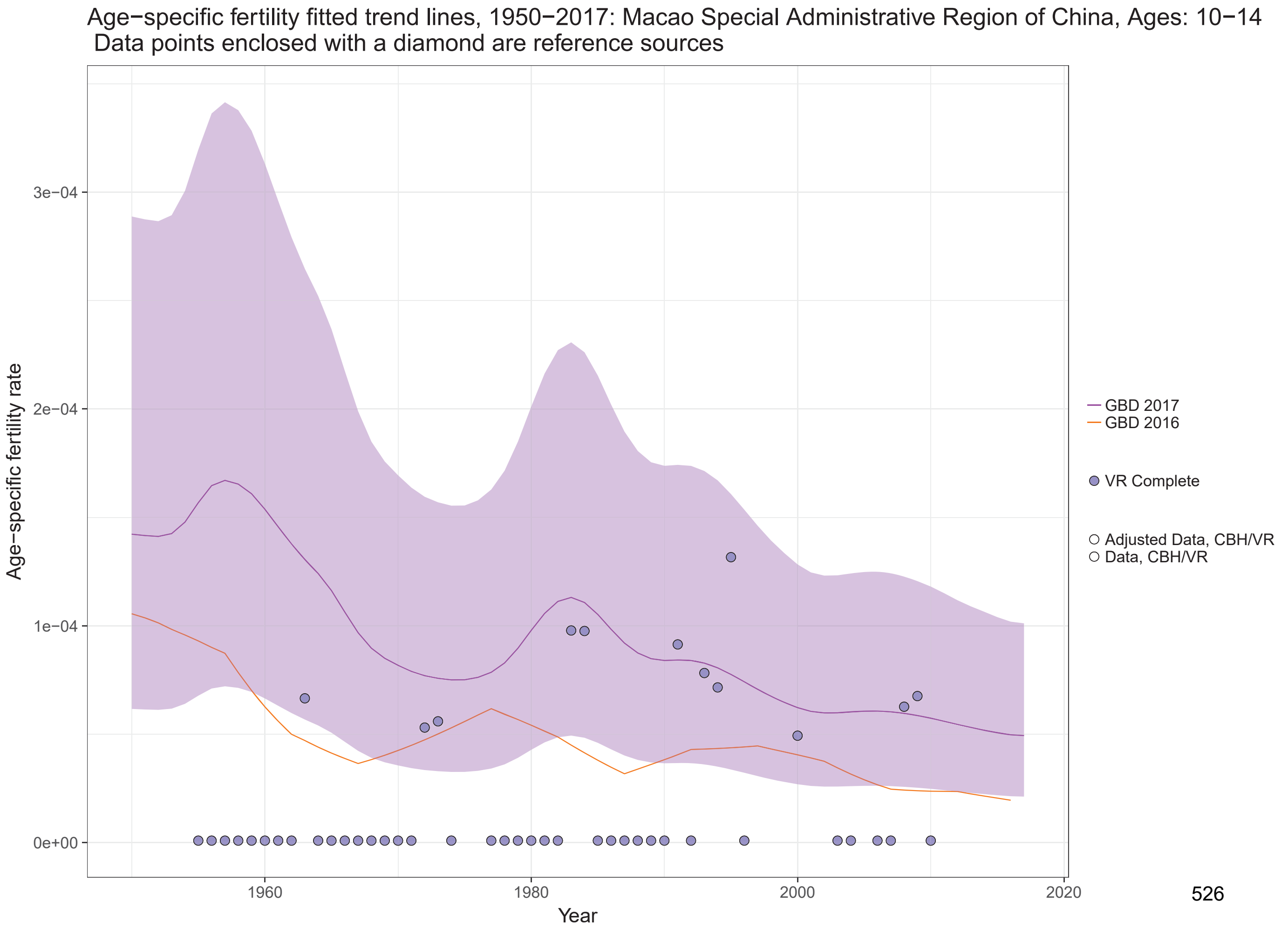

Age−specific fertility fitted trend lines, 1950−2017: Macao Special Administrative Region of China, Ages: 10−14
Data points enclosed with a diamond are reference sources
Age−specific fertility rate
Year
GBD 2017
GBD 2016
VR Complete
Adjusted Data, CBH/VR
Data, CBH/VR

Age−specific fertility fitted trend lines, 1950−2017: Macao Special Administrative Region of China, Ages: 15−19
Data points enclosed with a diamond are reference sources

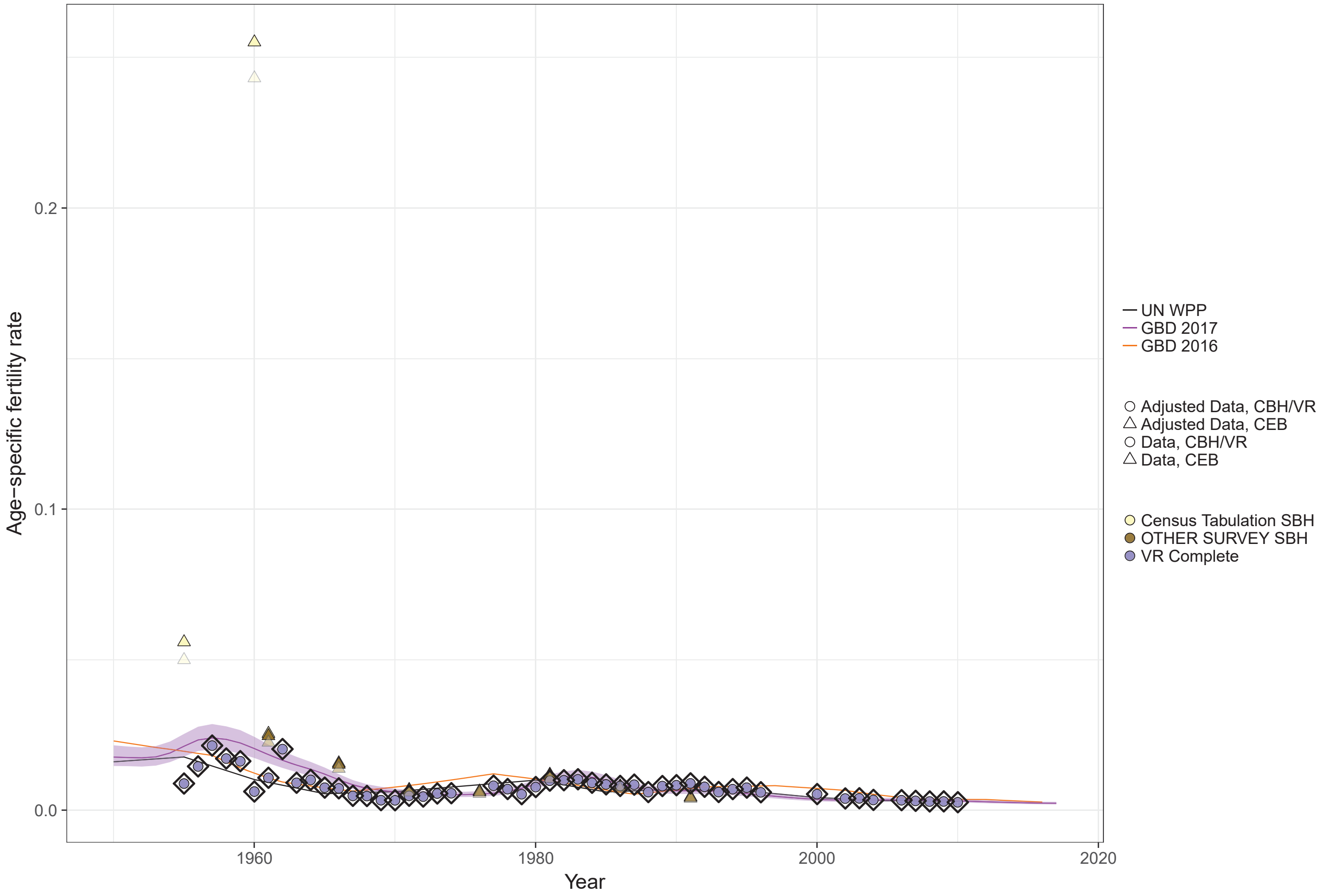

Age−specific fertility fitted trend lines, 1950−2017: Macao Special Administrative Region of China, Ages: 20−24
Data points enclosed with a diamond are reference sources

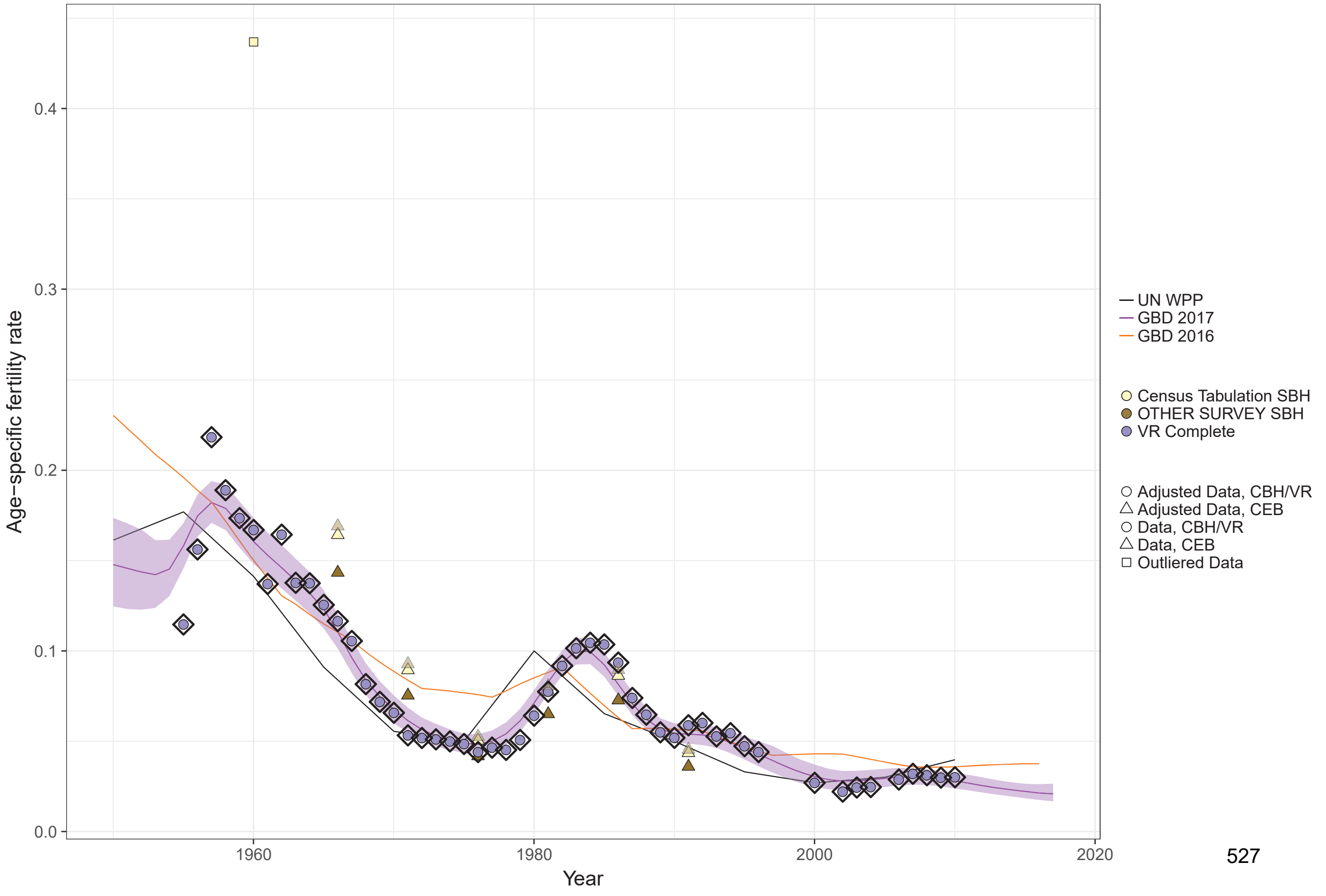

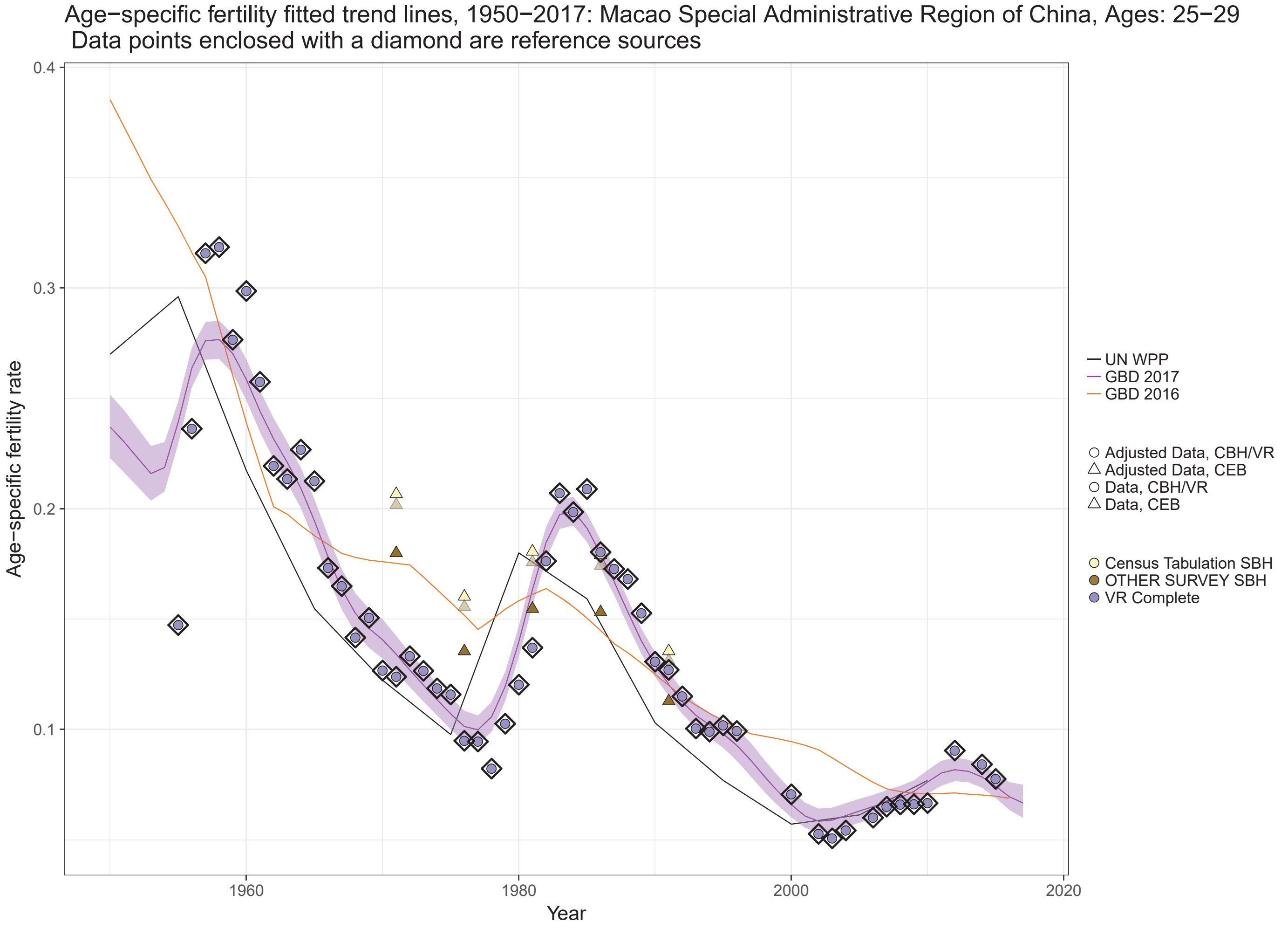
Age−specific fertility fitted trend lines, 1950−2017: Macao Special Administrative Region of China, Ages: 25−29
Data points enclosed with a diamond are reference sources
Age−specific fertility rate
Year
UN WPP
GBD 2017
GBD 2016
Adjusted Data, CBH/VR
Adjusted Data, CEB
Data, CBH/VR
Data, CEB
Census Tabulation SBH
OTHER SURVEY SBH
VR Complete

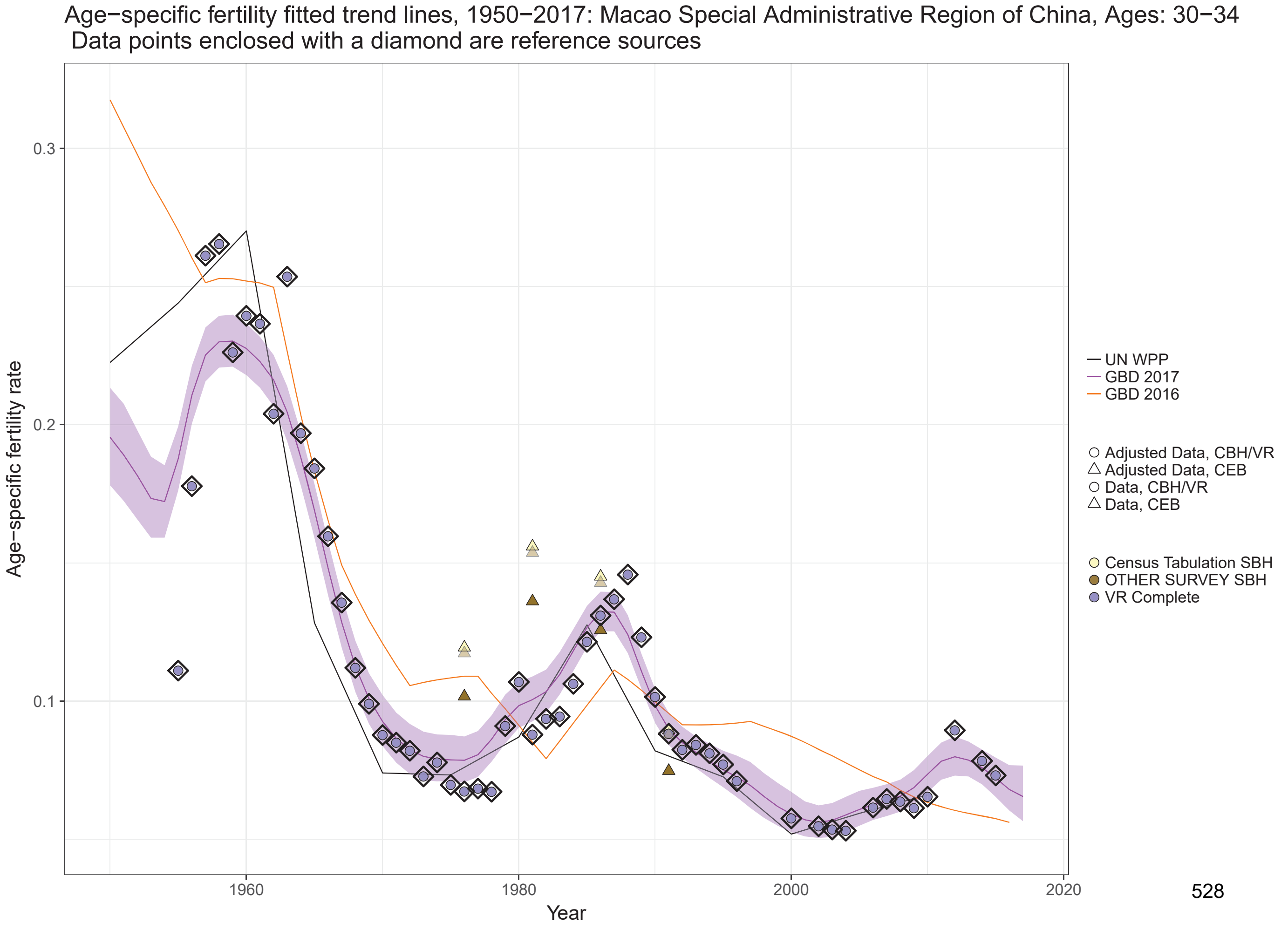
Age−specific fertility fitted trend lines, 1950−2017: Macao Special Administrative Region of China, Ages: 30−34
Data points enclosed with a diamond are reference sources
Age−specific fertility rate
Year
UN WPP
GBD 2017
GBD 2016
Adjusted Data, CBH/VR
Adjusted Data, CEB
Data, CBH/VR
Data, CEB
Census Tabulation SBH
OTHER SURVEY SBH
VR Complete

Age−specific fertility fitted trend lines, 1950−2017: Macao Special Administrative Region of China, Ages: 35−39
Data points enclosed with a diamond are reference sources

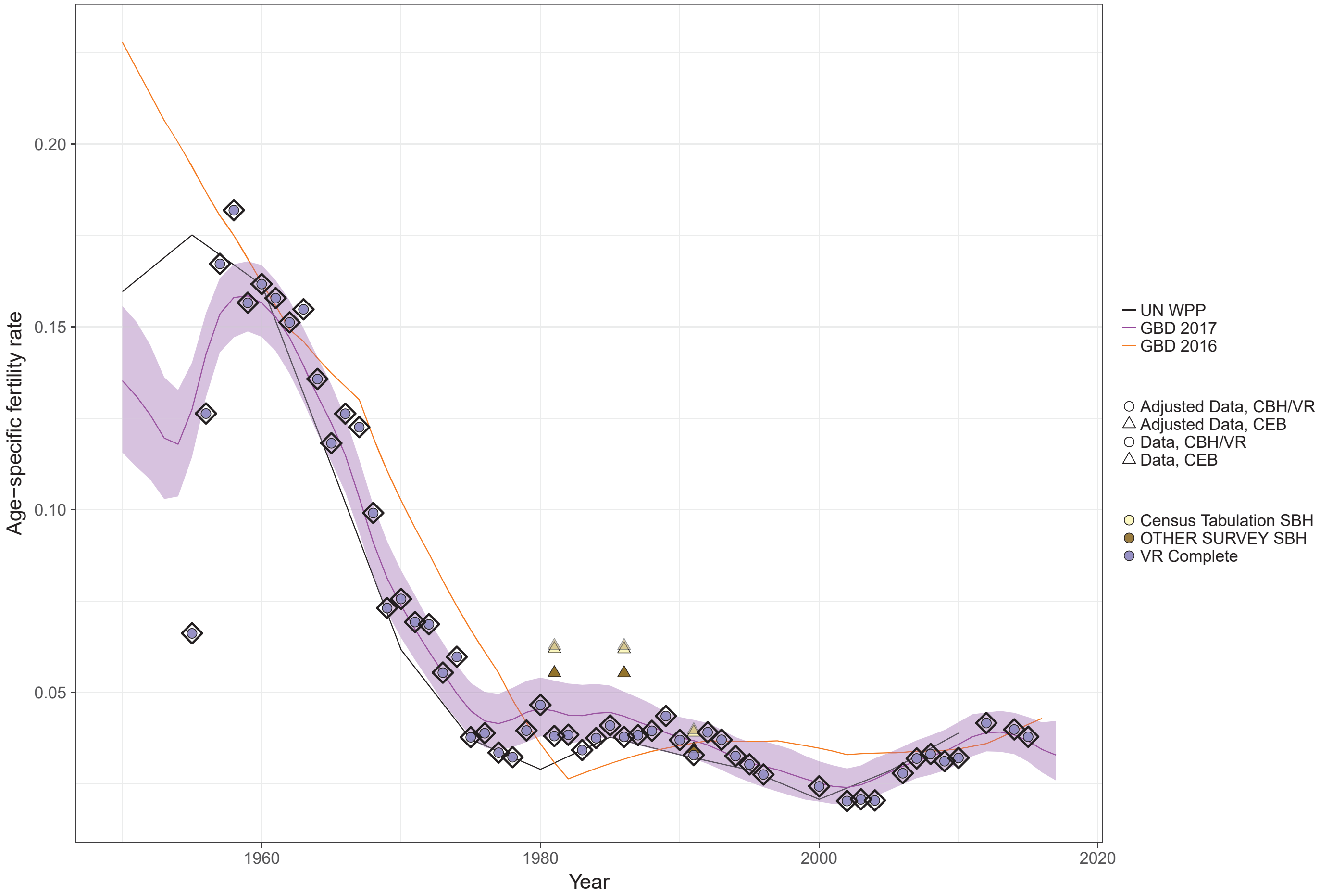

Age−specific fertility fitted trend lines, 1950−2017: Macao Special Administrative Region of China, Ages: 40−44
Data points enclosed with a diamond are reference sources

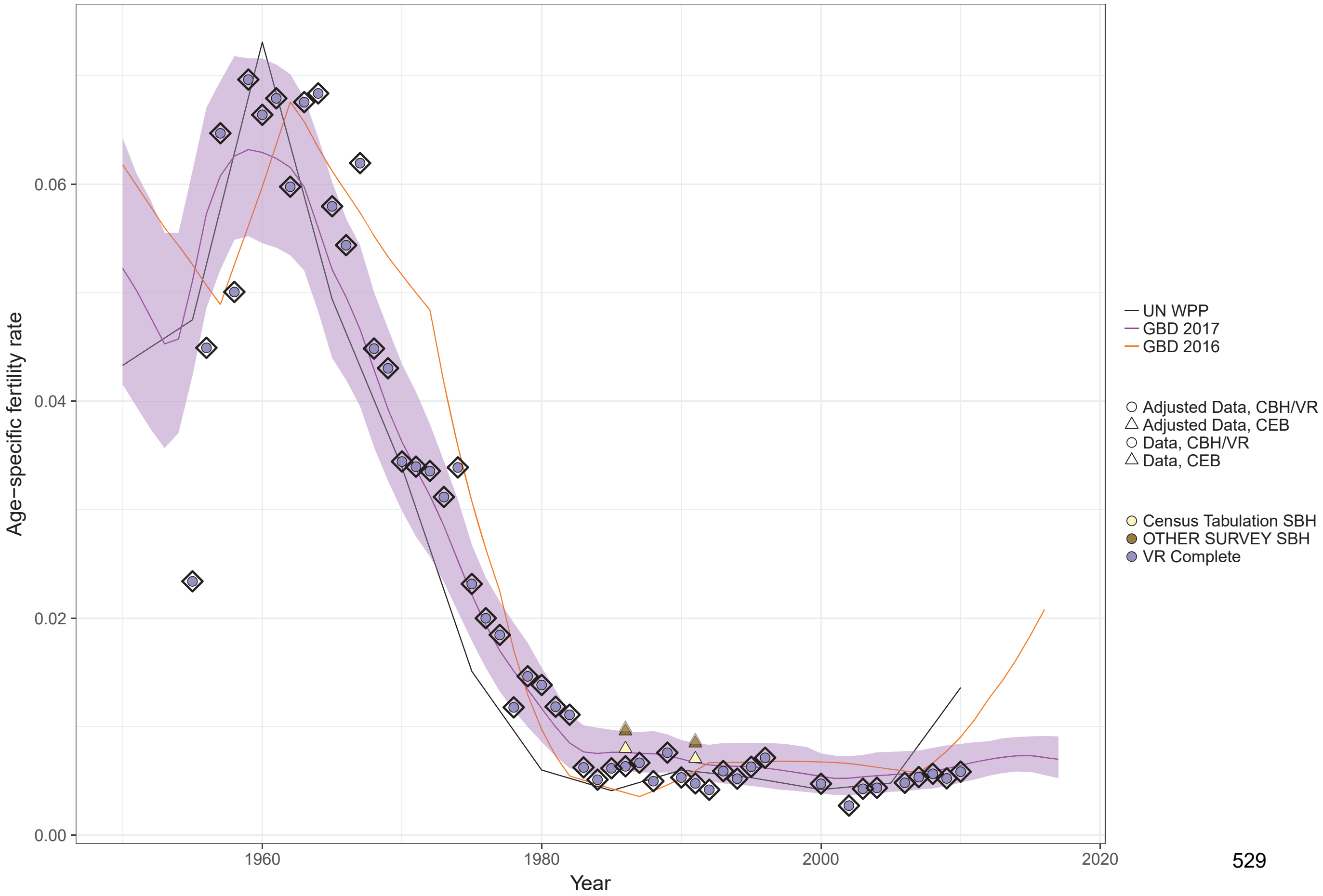

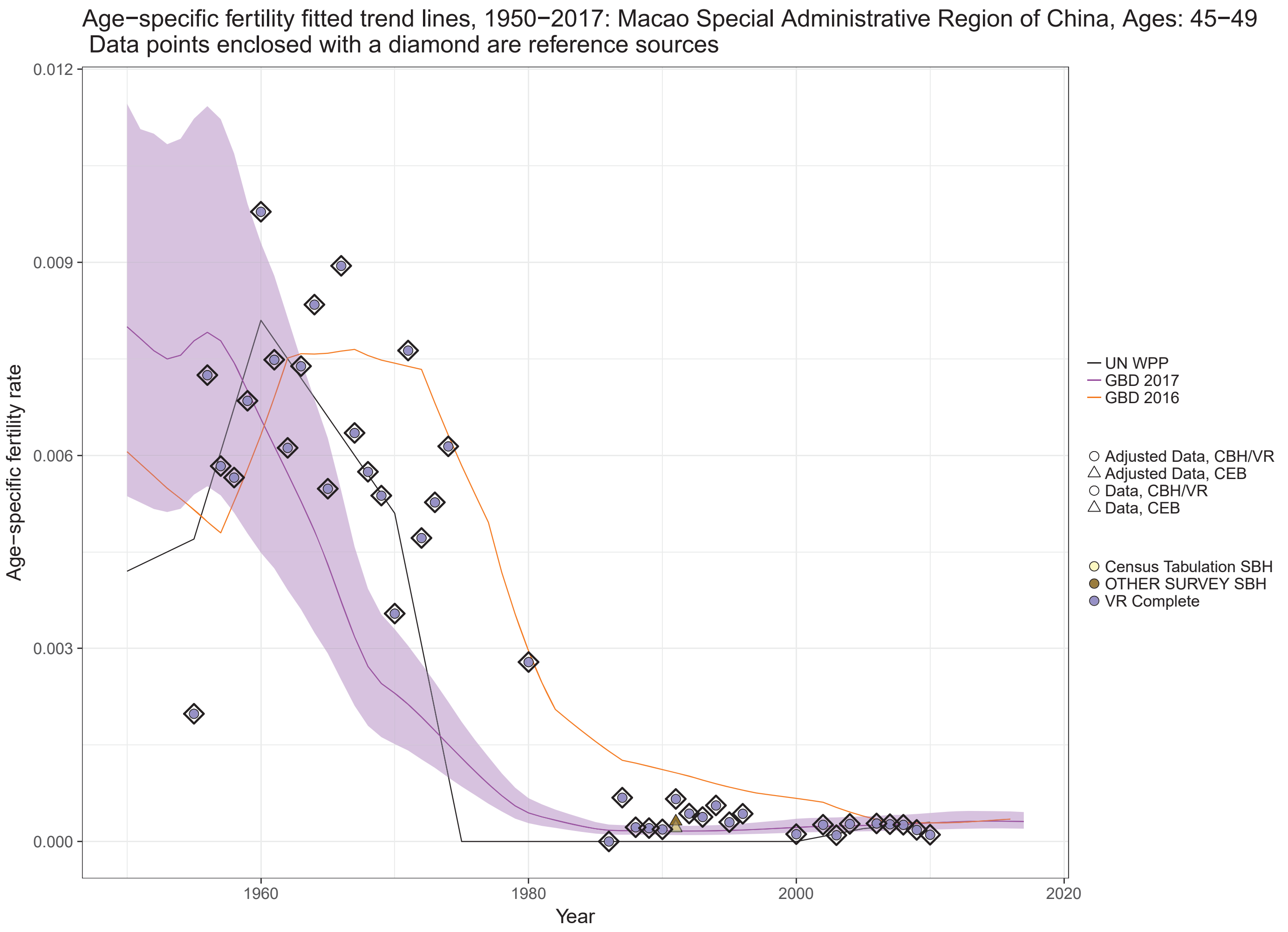

Age−specific fertility fitted trend lines, 1950−2017: Macao Special Administrative Region of China, Ages: 45−49
Data points enclosed with a diamond are reference sources
Age−specific fertility rate
0.012
0.009
0.006
0.003
0.000
1960
1980
2000
2020
Year
UN WPP
GBD 2017
GBD 2016
Adjusted Data, CBH/VR
Adjusted Data, CEB
Data, CBH/VR
Data, CEB
Census Tabulation SBH
OTHER SURVEY SBH
VR Complete

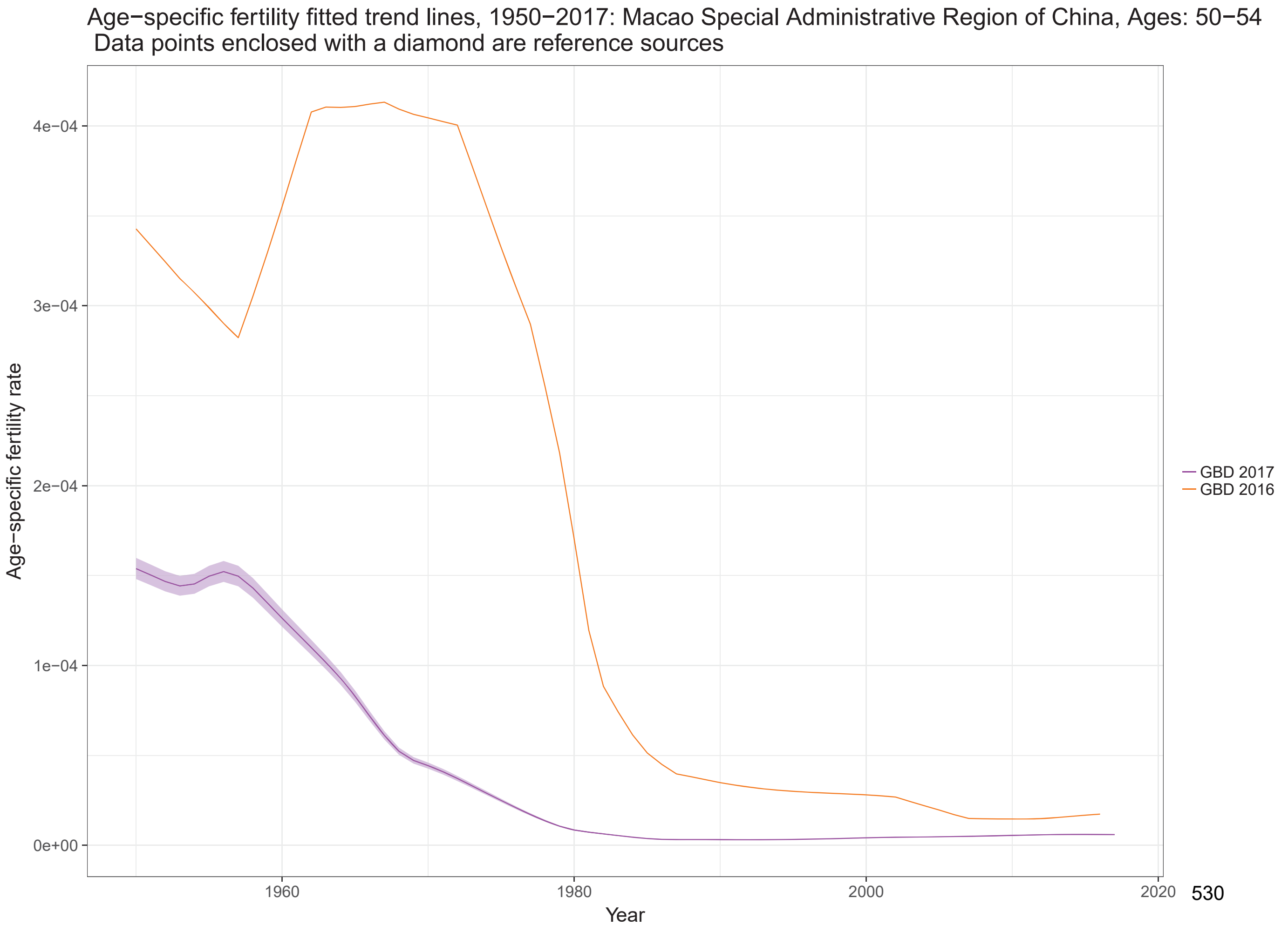

Age−specific fertility fitted trend lines, 1950−2017: Macao Special Administrative Region of China, Ages: 50−54
Data points enclosed with a diamond are reference sources
Age−specific fertility rate
4e−04
3e−04
2e−04
1e−04
0e+00
1960
1980
2000
2020
Year
GBD 2017
GBD 2016

Age−specific fertility fitted trend lines, 1950−2017: Macedonia, TFR
Data points enclosed with a diamond are reference sources

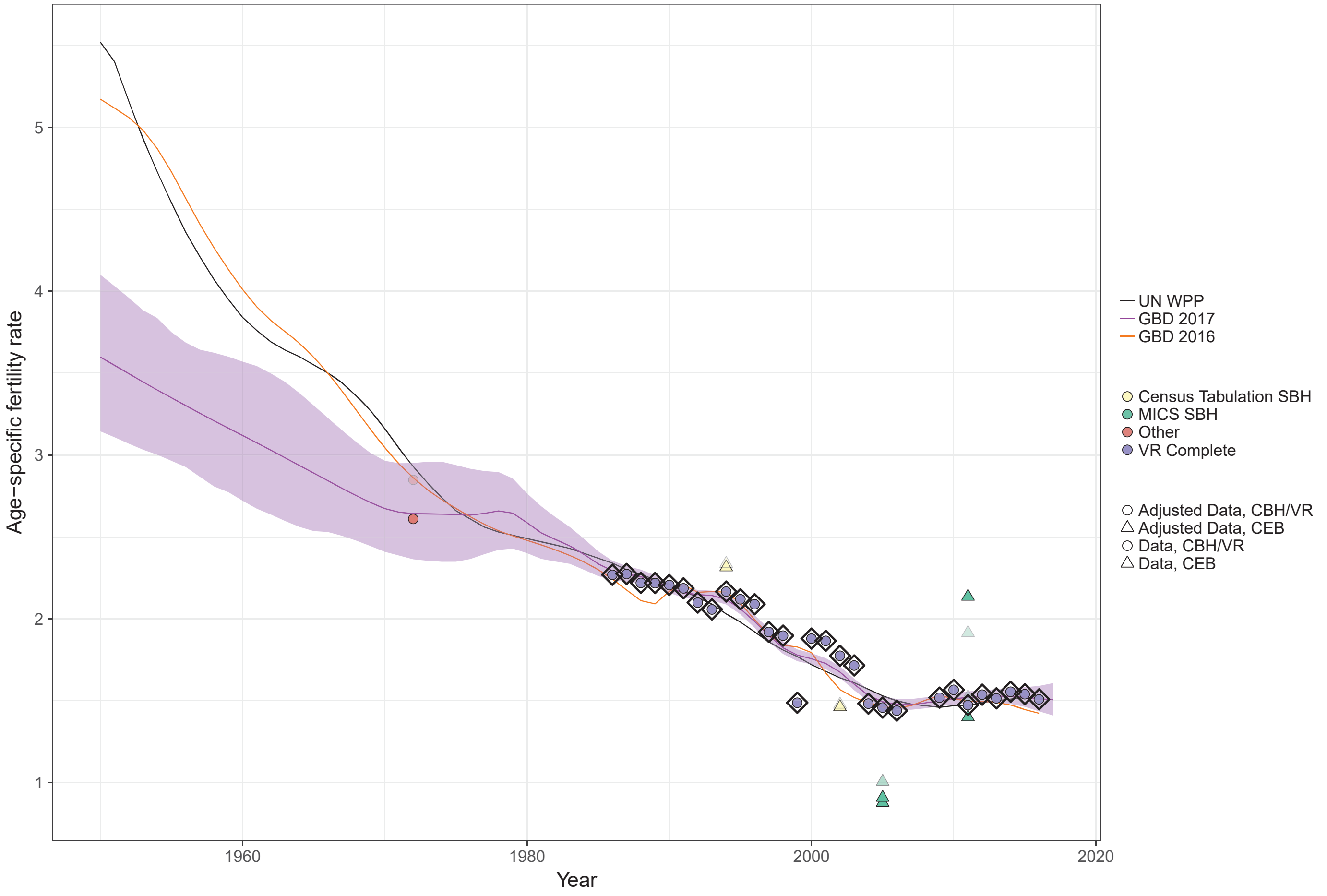

Age−specific fertility fitted trend lines, 1950−2017: Macedonia, Ages: 10−14
Data points enclosed with a diamond are reference sources

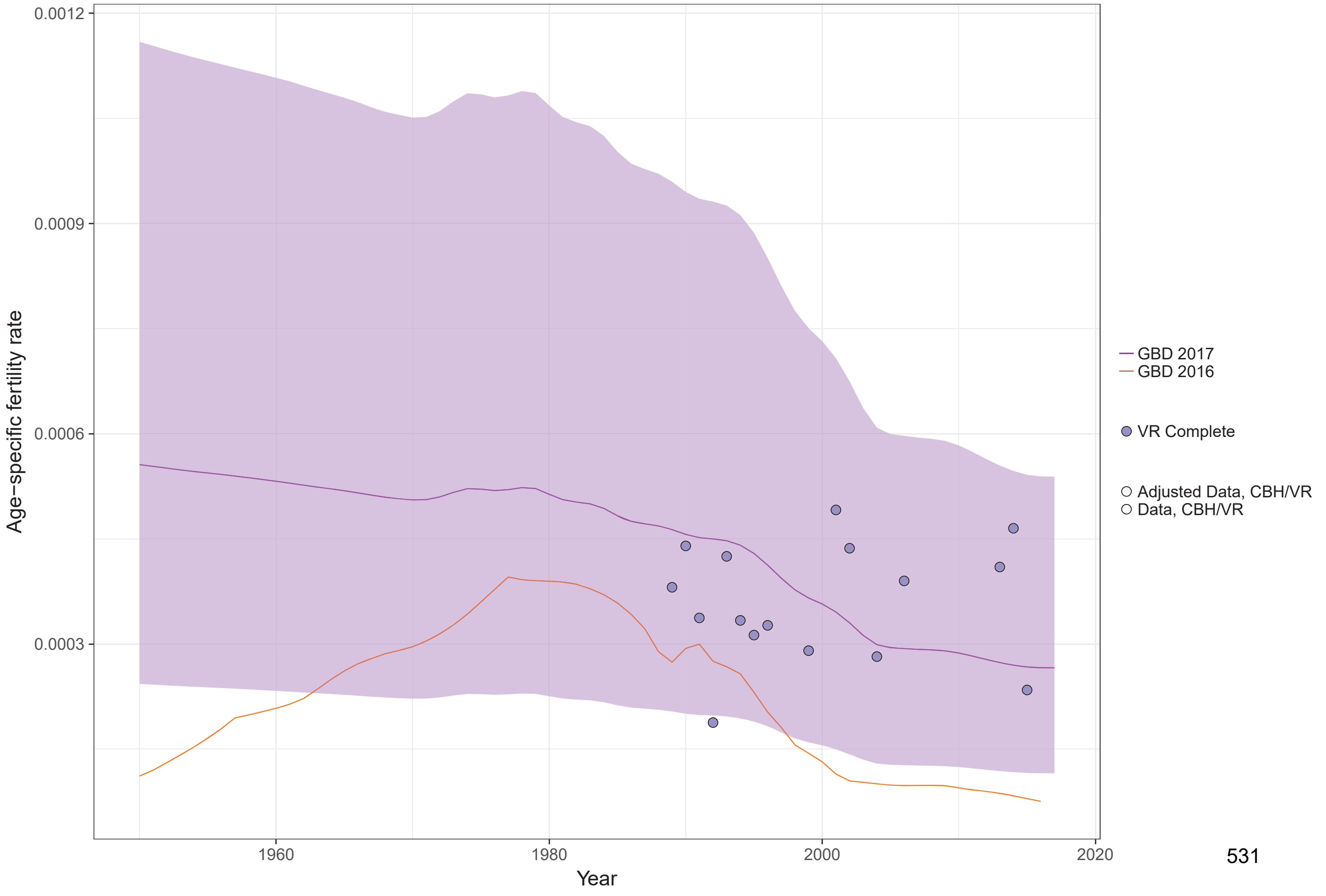

Age−specific fertility fitted trend lines, 1950−2017: Macedonia, Ages: 15−19
Data points enclosed with a diamond are reference sources

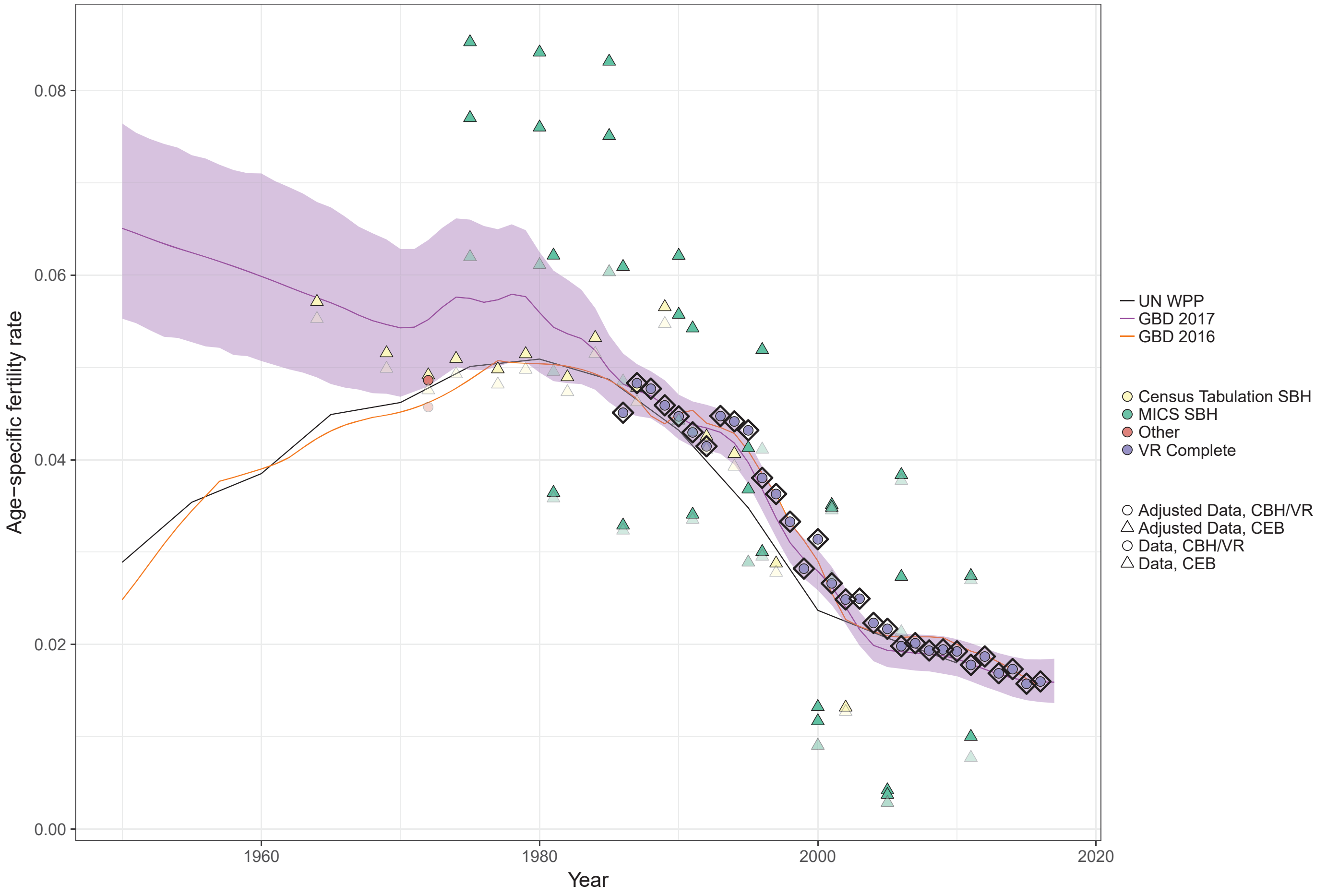

Age−specific fertility fitted trend lines, 1950−2017: Macedonia, Ages: 20−24
Data points enclosed with a diamond are reference sources

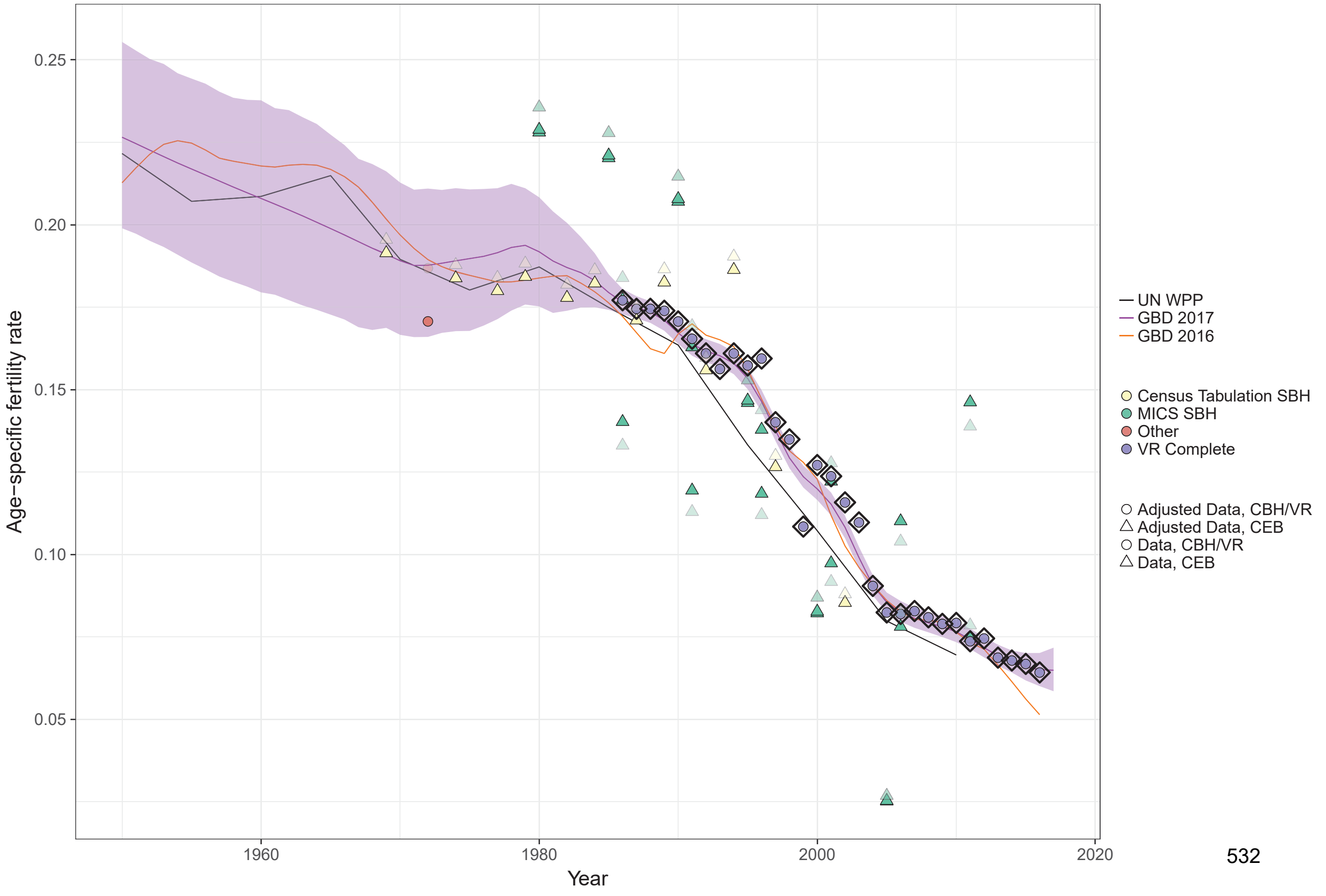

Age−specific fertility fitted trend lines, 1950−2017: Macedonia, Ages: 25−29
Data points enclosed with a diamond are reference sources

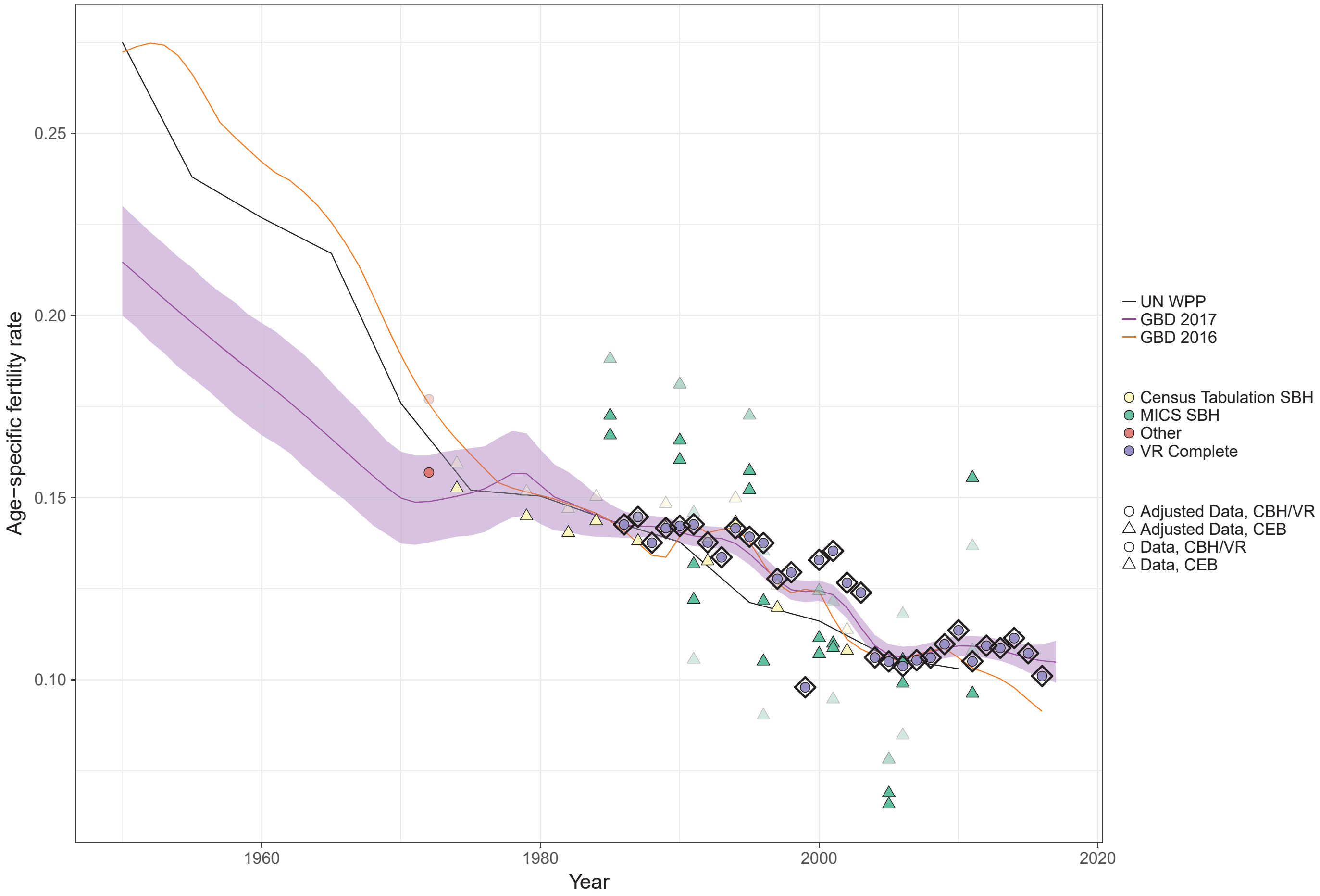

Age−specific fertility fitted trend lines, 1950−2017: Macedonia, Ages: 30−34
Data points enclosed with a diamond are reference sources

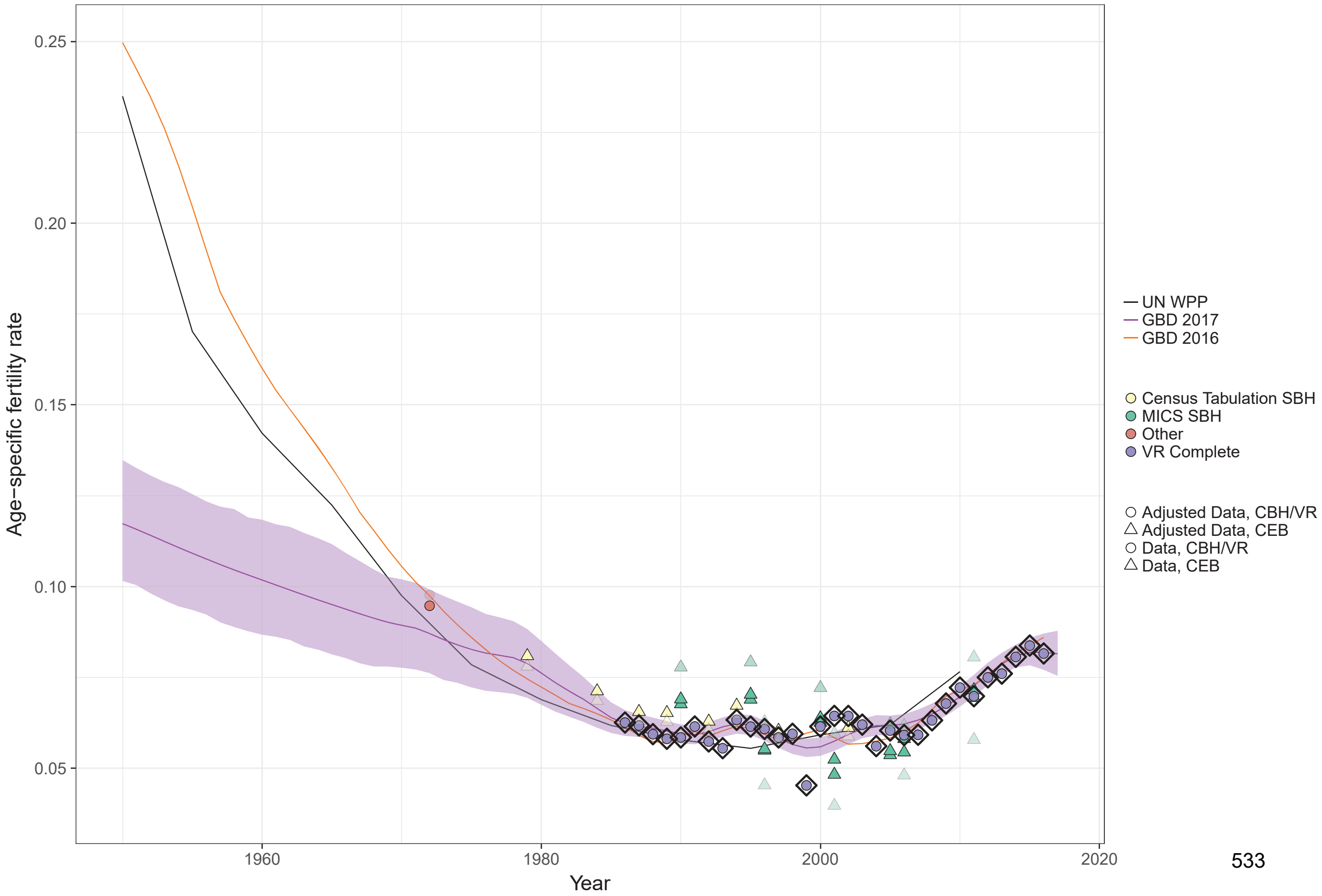

Age−specific fertility fitted trend lines, 1950−2017: Macedonia, Ages: 35−39
Data points enclosed with a diamond are reference sources

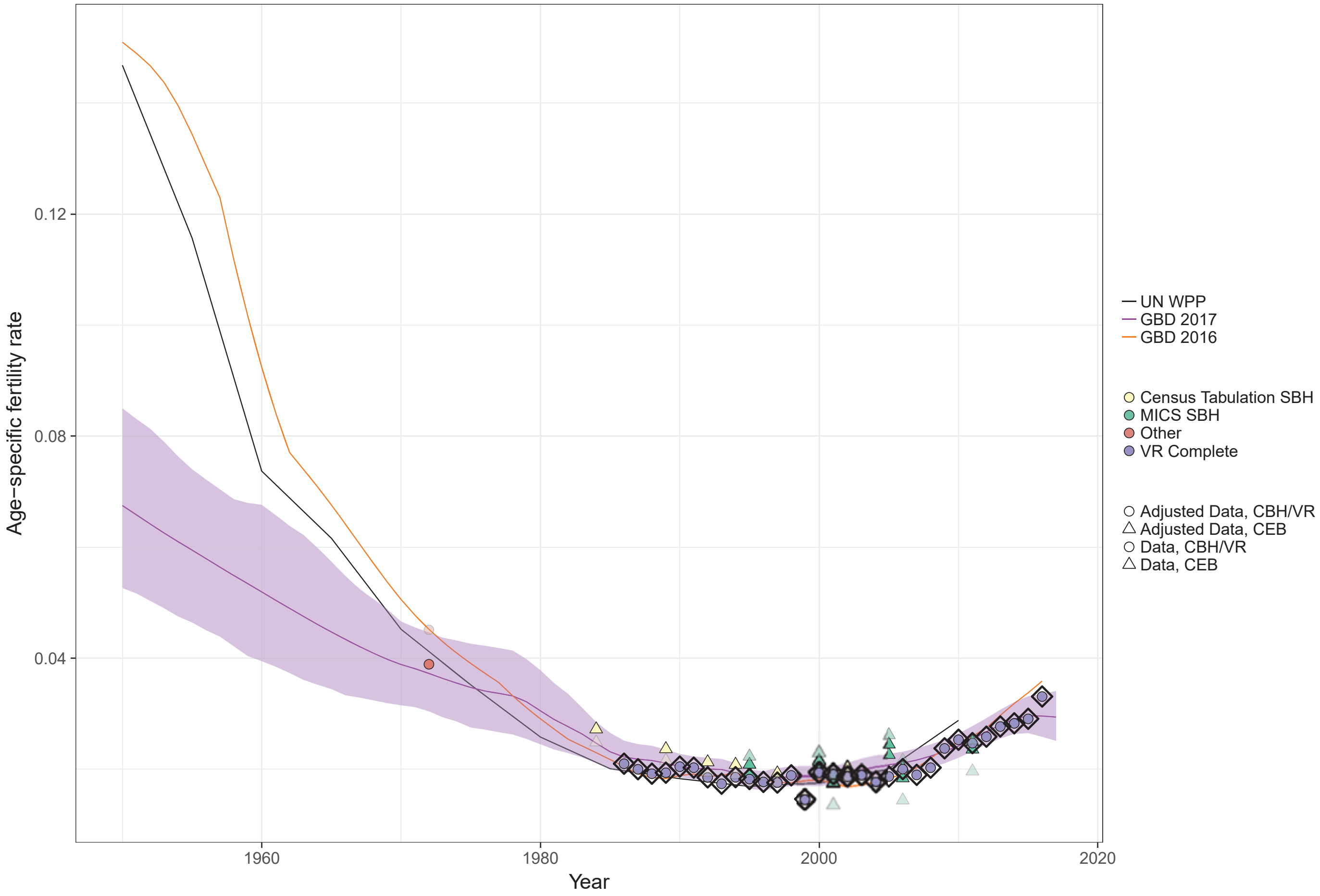

Age−specific fertility fitted trend lines, 1950−2017: Macedonia, Ages: 40−44
Data points enclosed with a diamond are reference sources

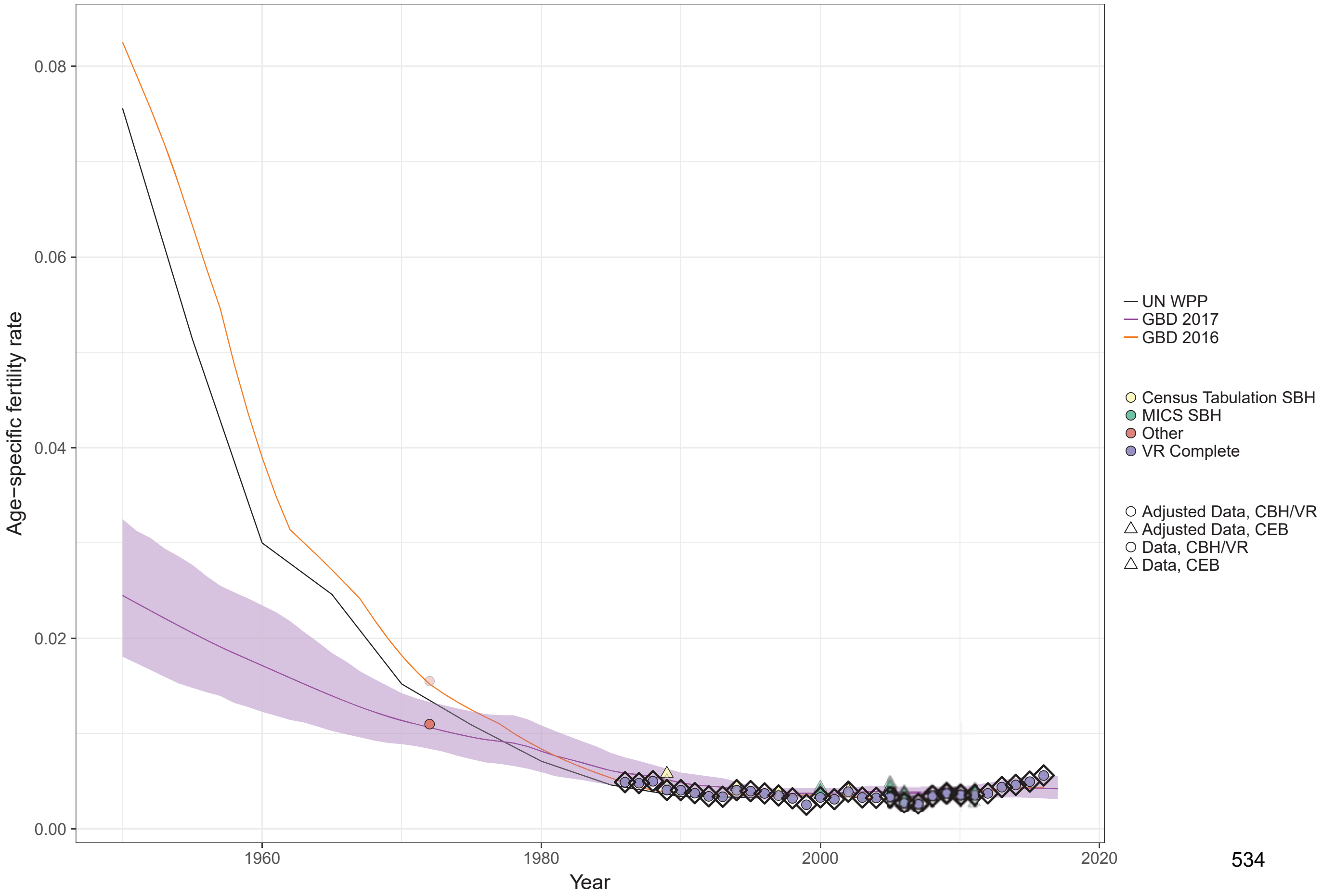

Age−specific fertility fitted trend lines, 1950−2017: Macedonia, Ages: 45−49
Data points enclosed with a diamond are reference sources

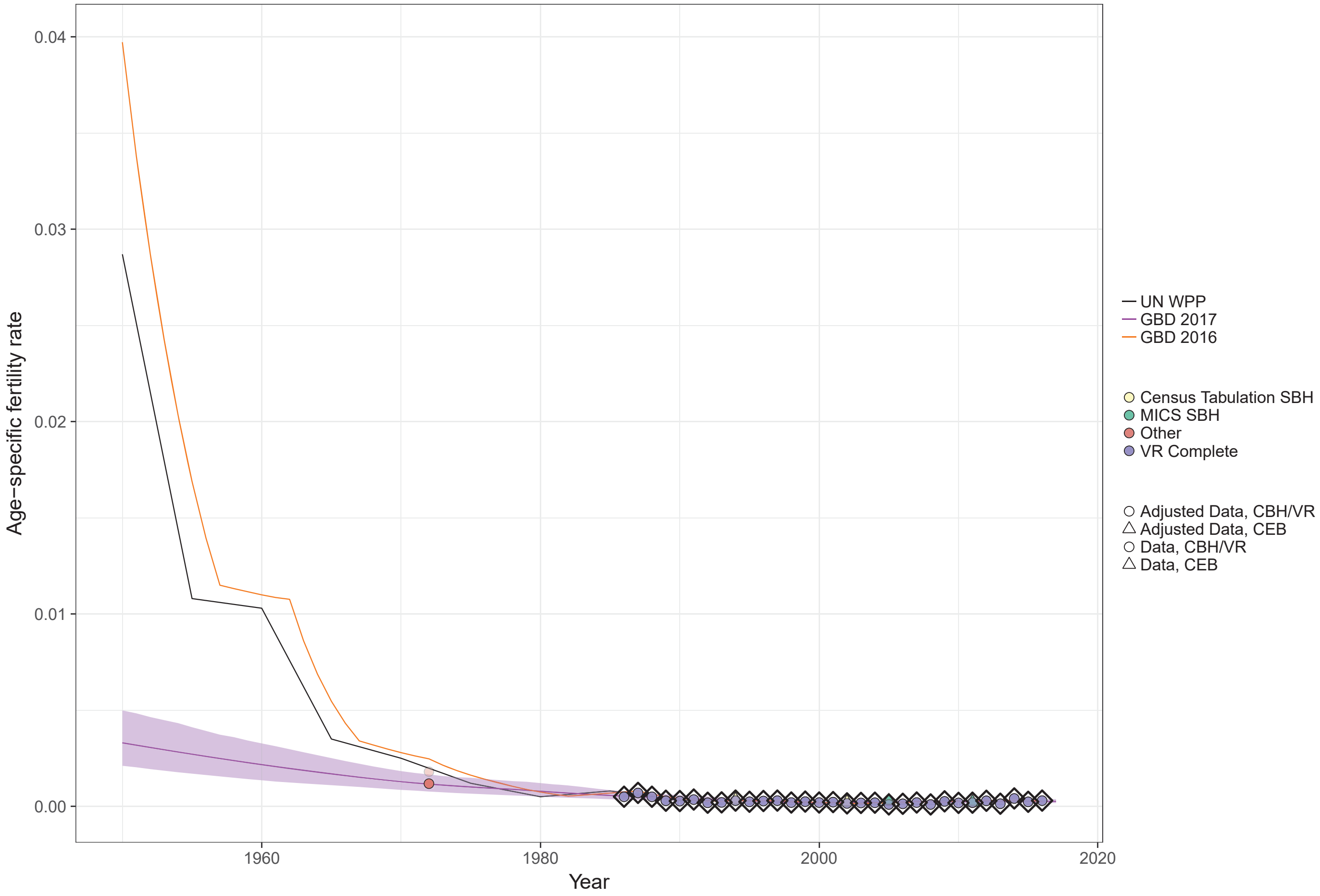

Age−specific fertility fitted trend lines, 1950−2017: Macedonia, Ages: 50−54
Data points enclosed with a diamond are reference sources

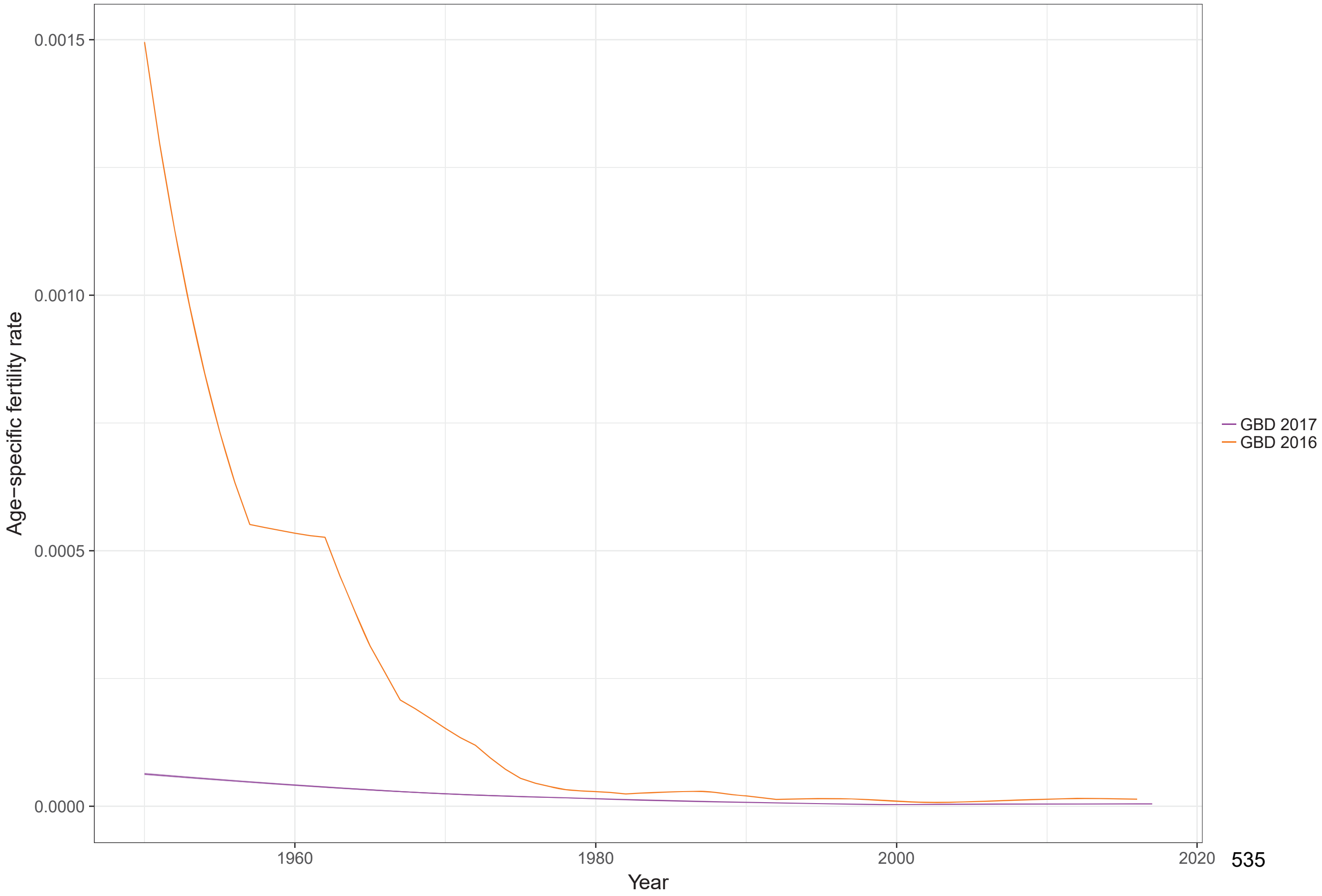

Age−specific fertility fitted trend lines, 1950−2017: Madagascar, TFR
Data points enclosed with a diamond are reference sources

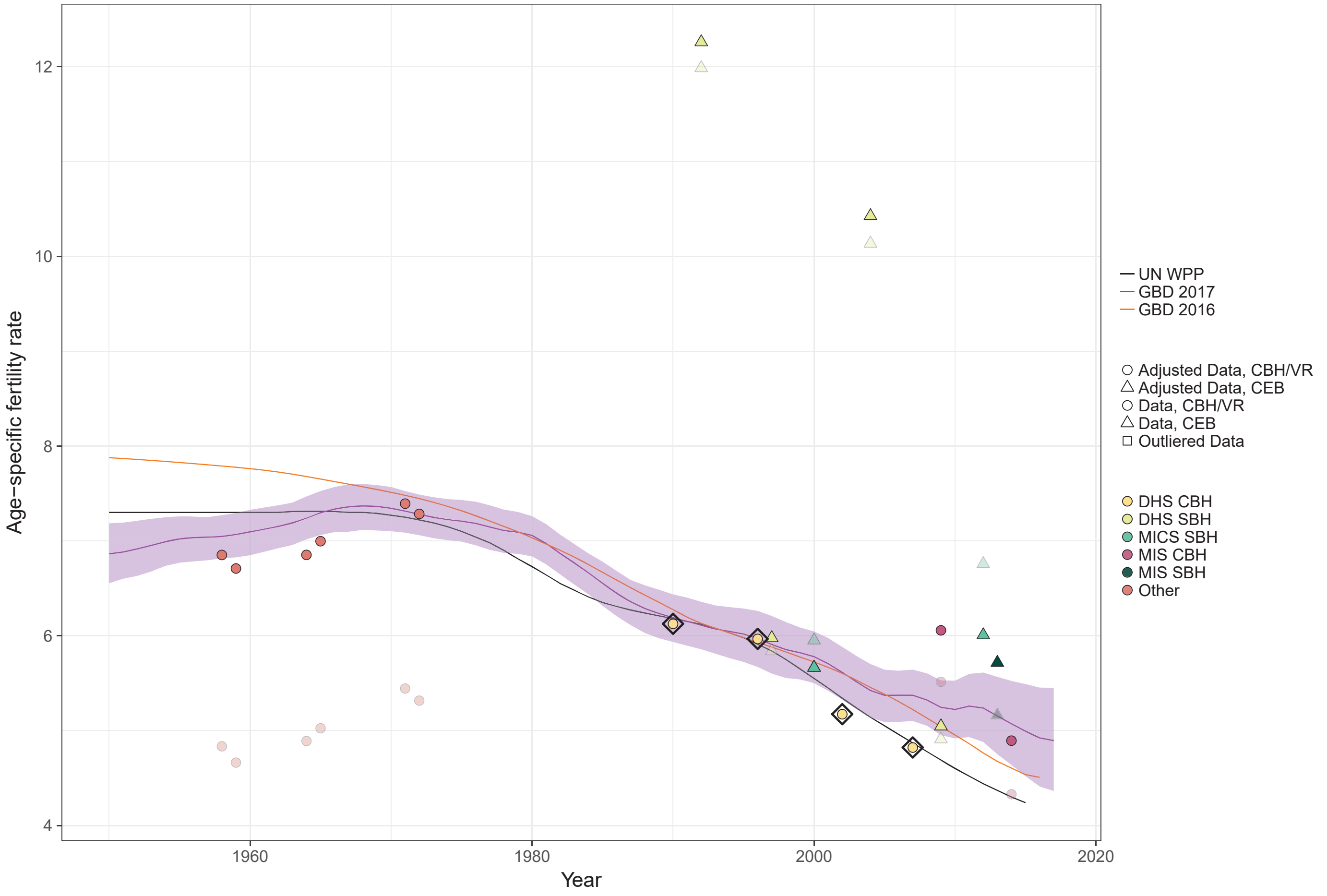

Age−specific fertility fitted trend lines, 1950−2017: Madagascar, Ages: 10−14
Data points enclosed with a diamond are reference sources

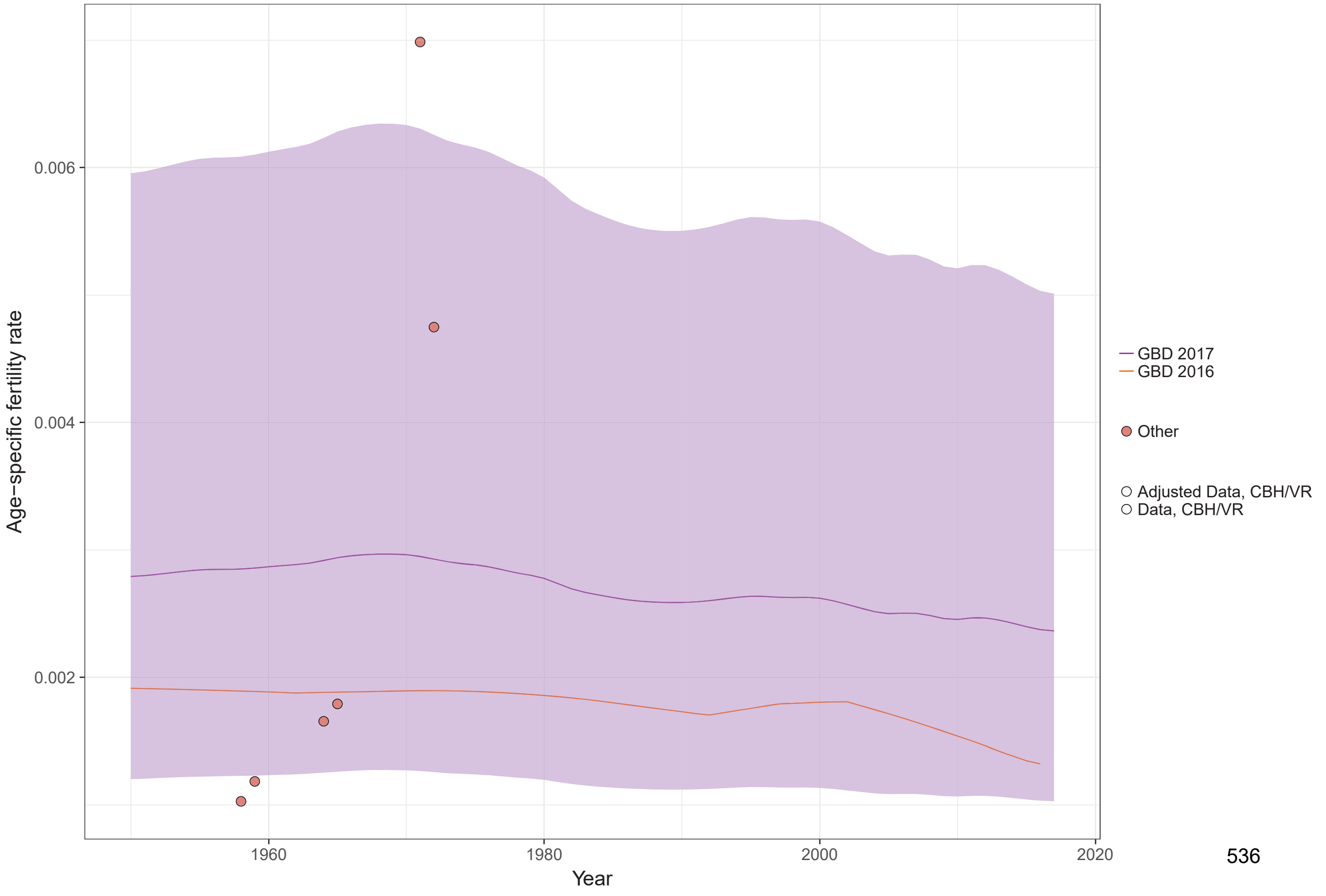

Age−specific fertility fitted trend lines, 1950−2017: Madagascar, Ages: 15−19
Data points enclosed with a diamond are reference sources

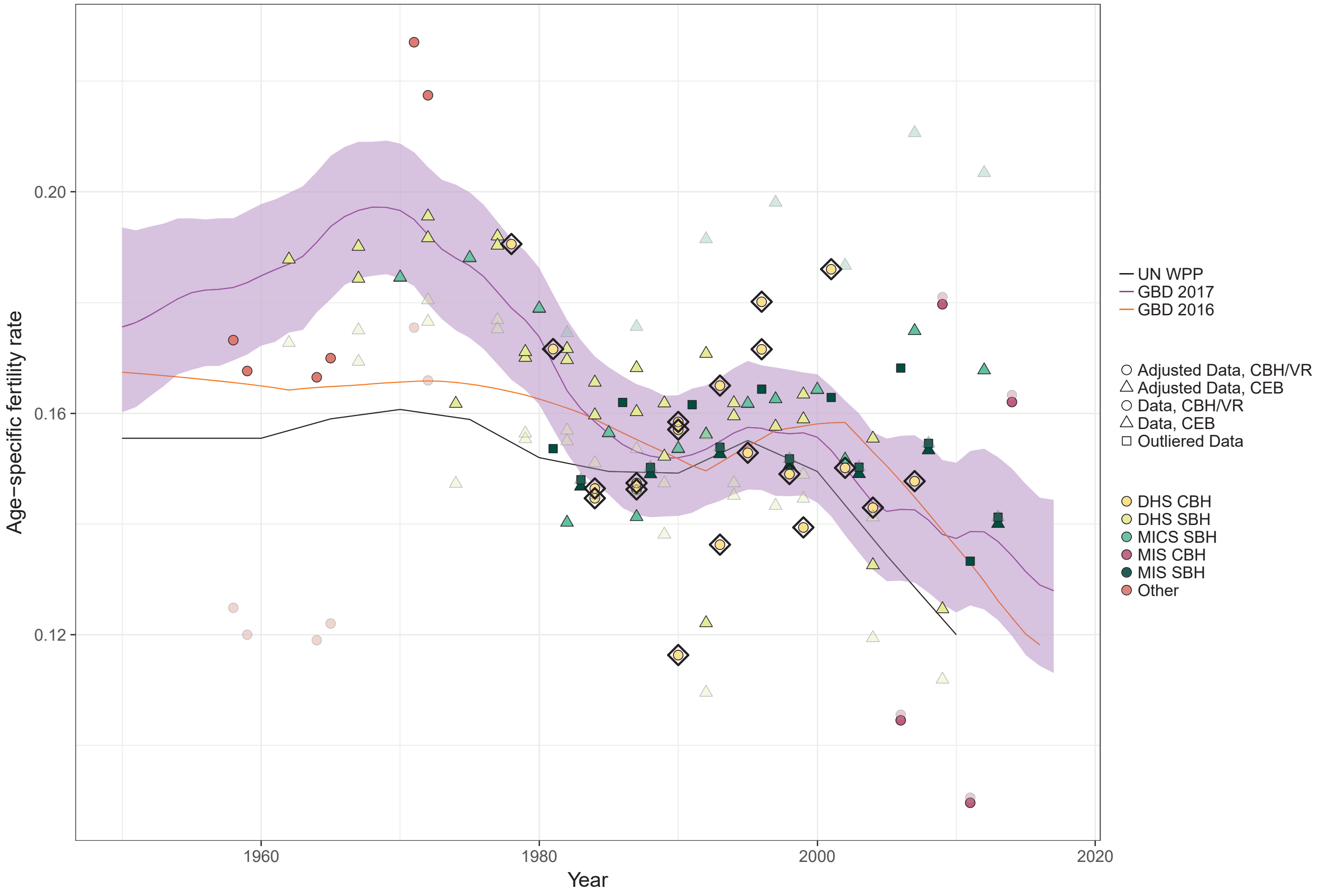

Age−specific fertility fitted trend lines, 1950−2017: Madagascar, Ages: 20−24
Data points enclosed with a diamond are reference sources

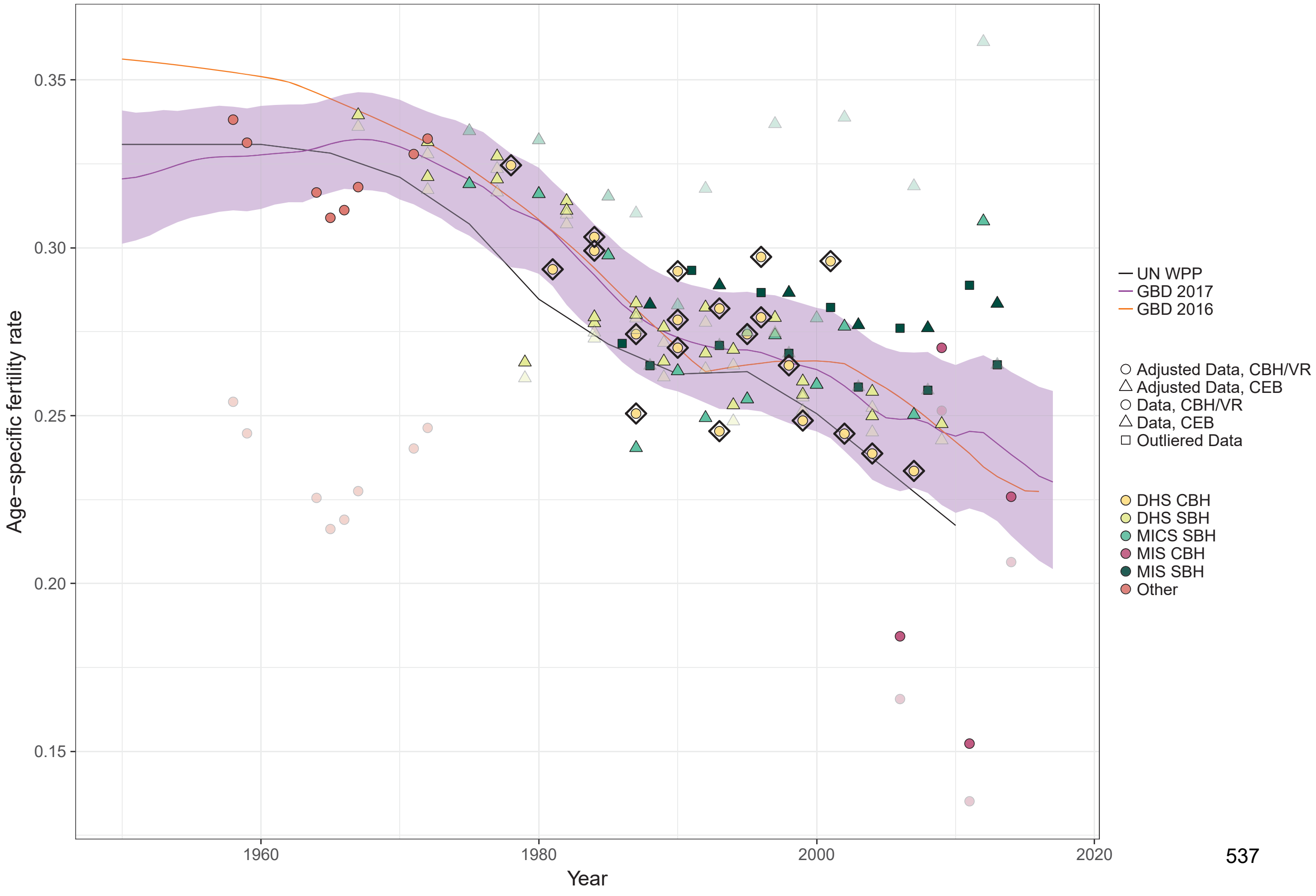

Age−specific fertility fitted trend lines, 1950−2017: Madagascar, Ages: 25−29
Data points enclosed with a diamond are reference sources

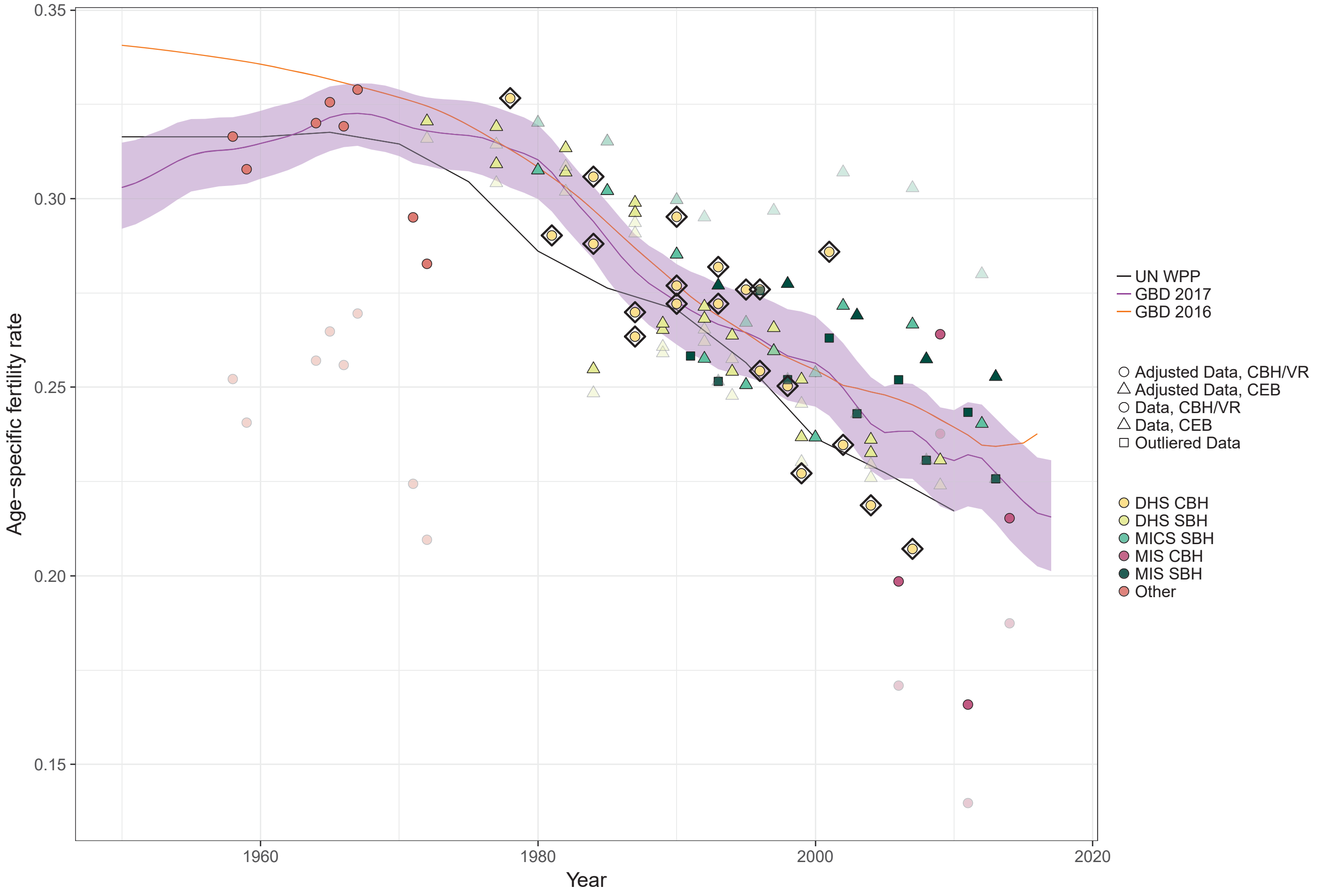

Age−specific fertility fitted trend lines, 1950−2017: Madagascar, Ages: 30−34
Data points enclosed with a diamond are reference sources

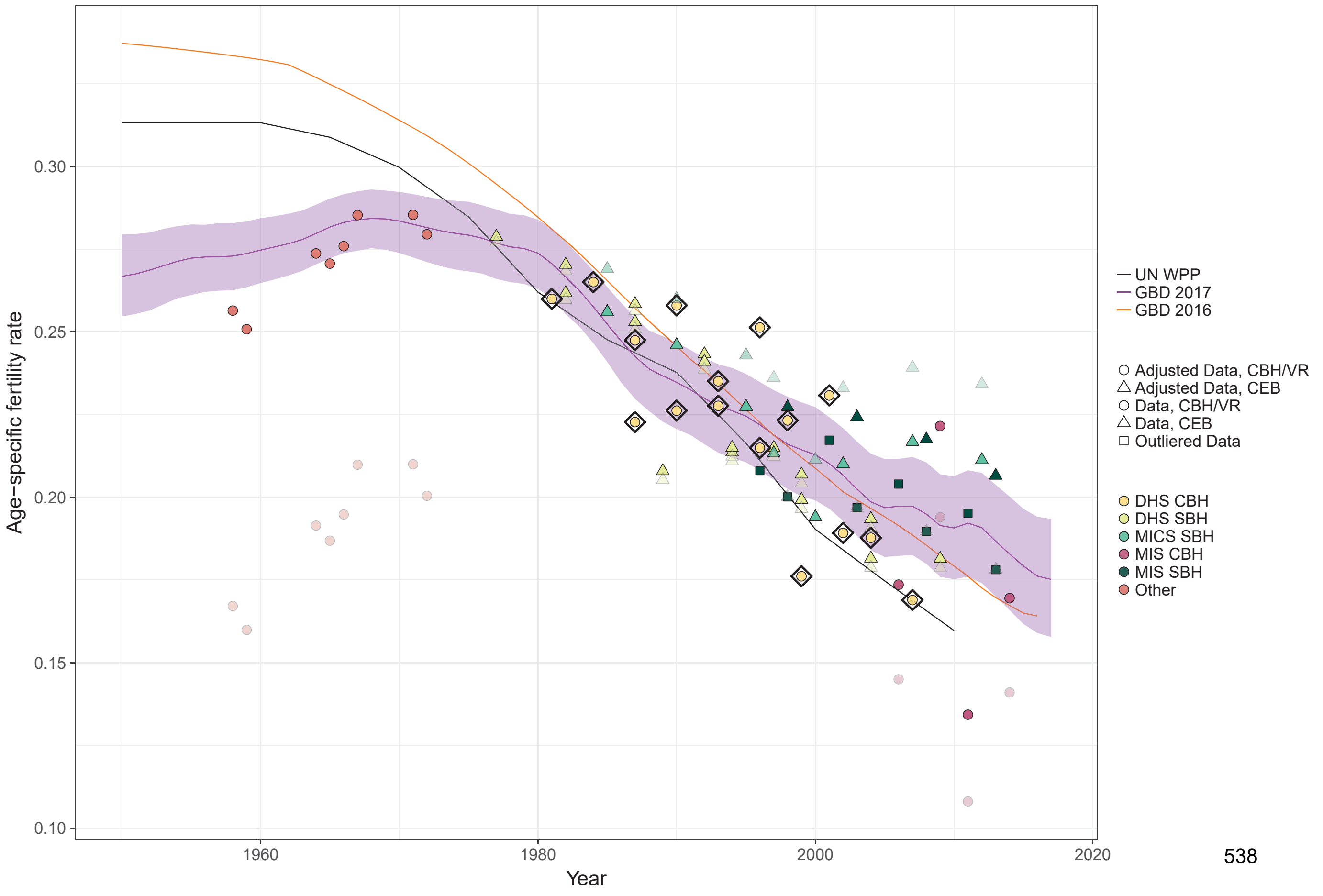

Age−specific fertility fitted trend lines, 1950−2017: Madagascar, Ages: 35−39
Data points enclosed with a diamond are reference sources

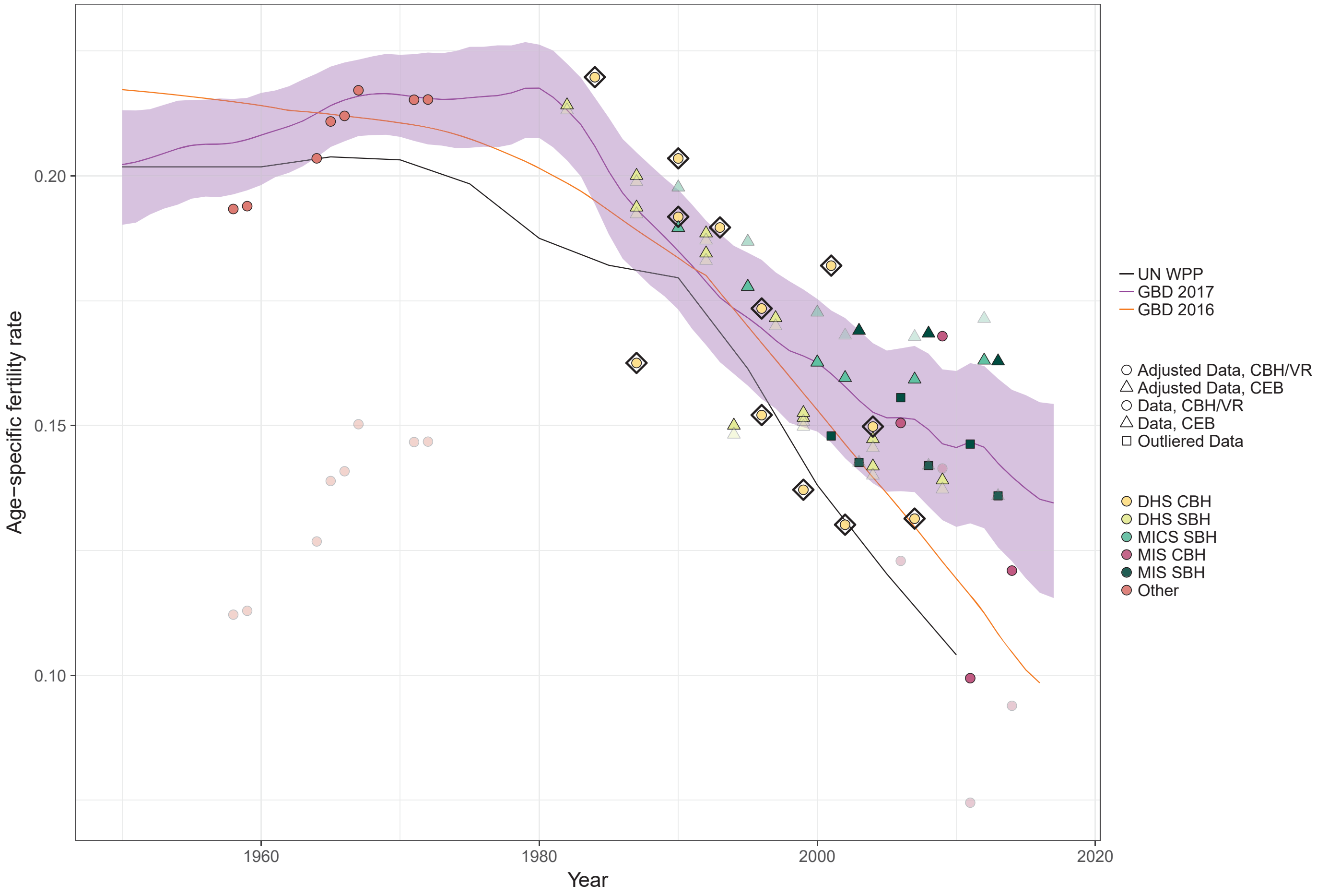

Age−specific fertility fitted trend lines, 1950−2017: Madagascar, Ages: 40−44
Data points enclosed with a diamond are reference sources

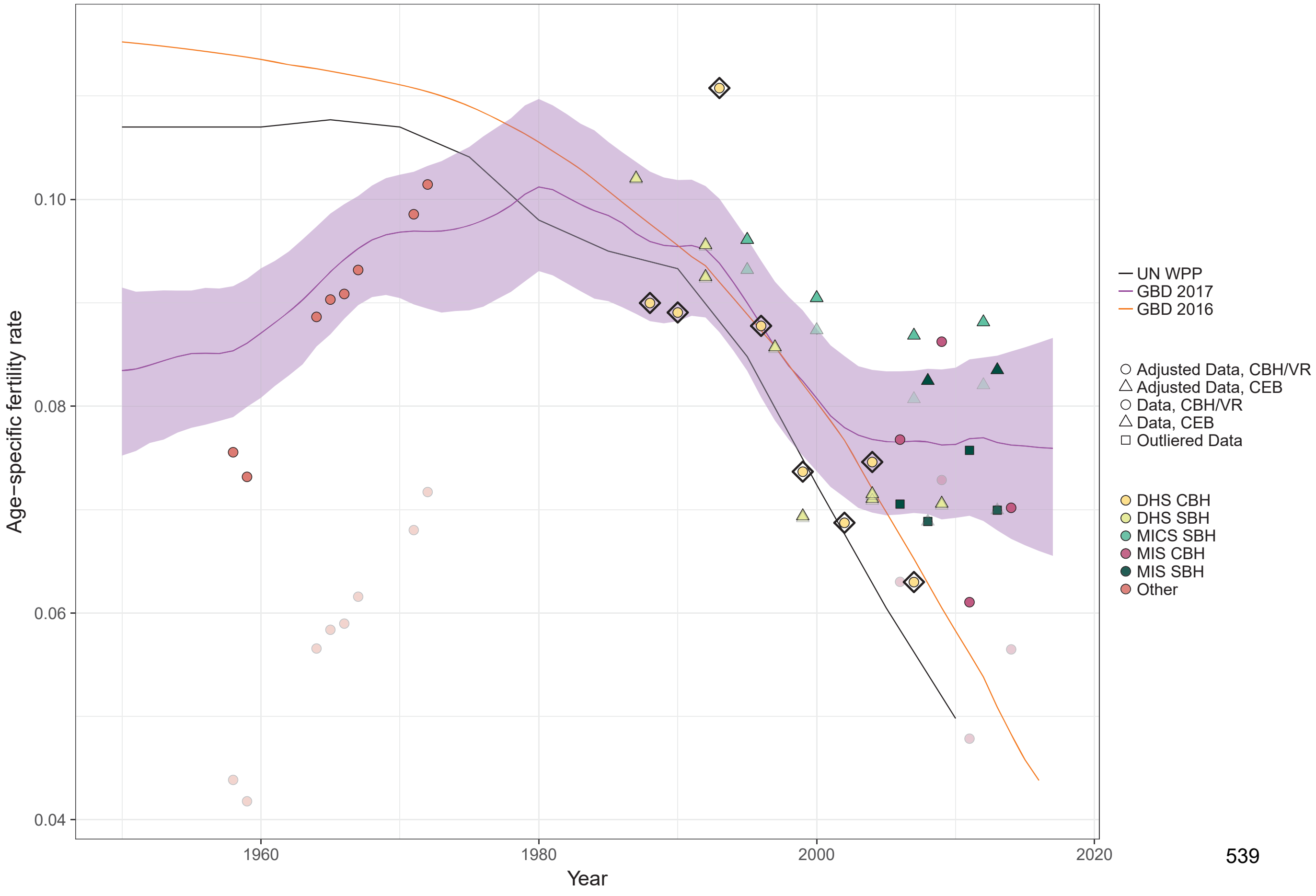

Age−specific fertility fitted trend lines, 1950−2017: Madagascar, Ages: 45−49
Data points enclosed with a diamond are reference sources

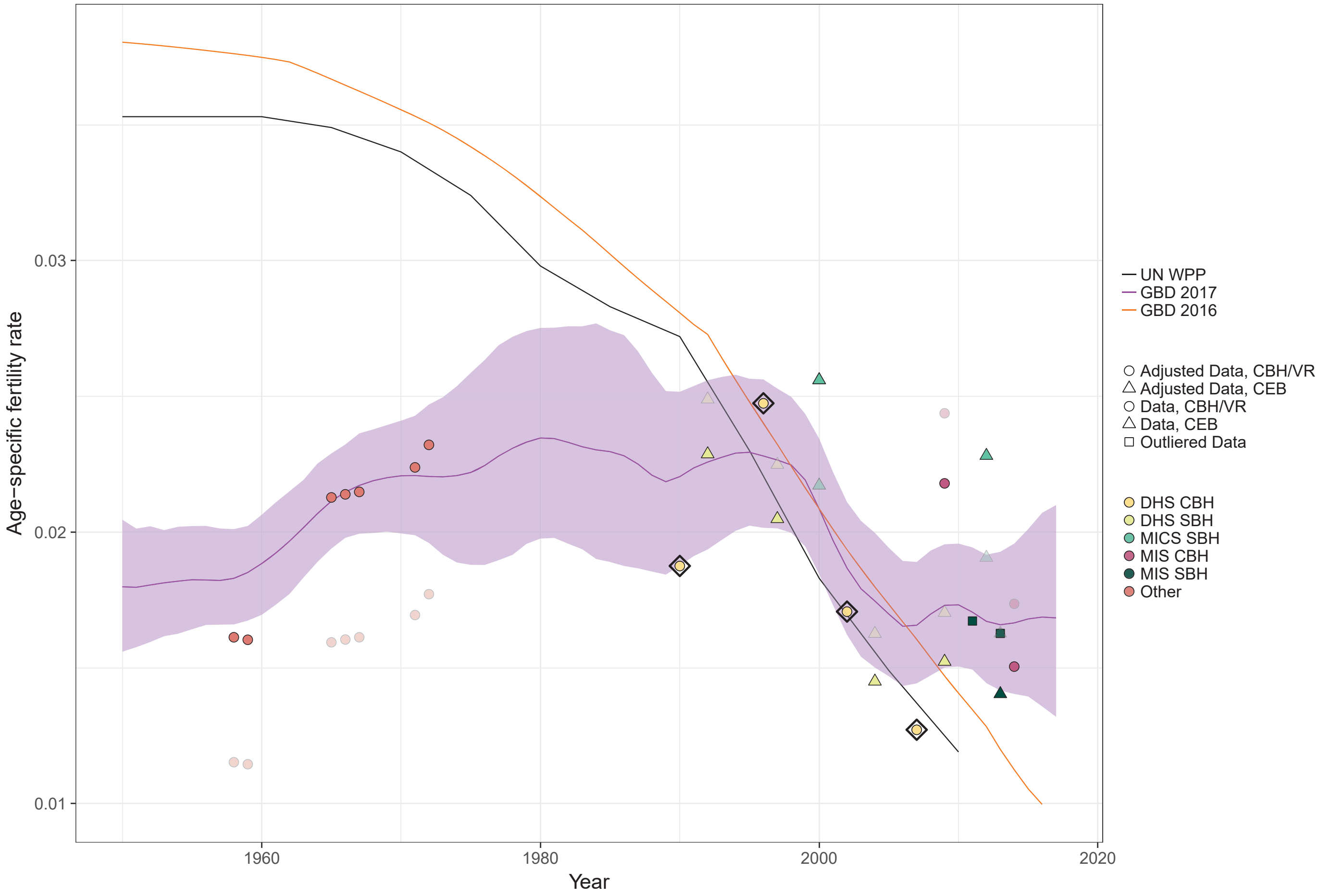

Age−specific fertility fitted trend lines, 1950−2017: Madagascar, Ages: 50−54
Data points enclosed with a diamond are reference sources

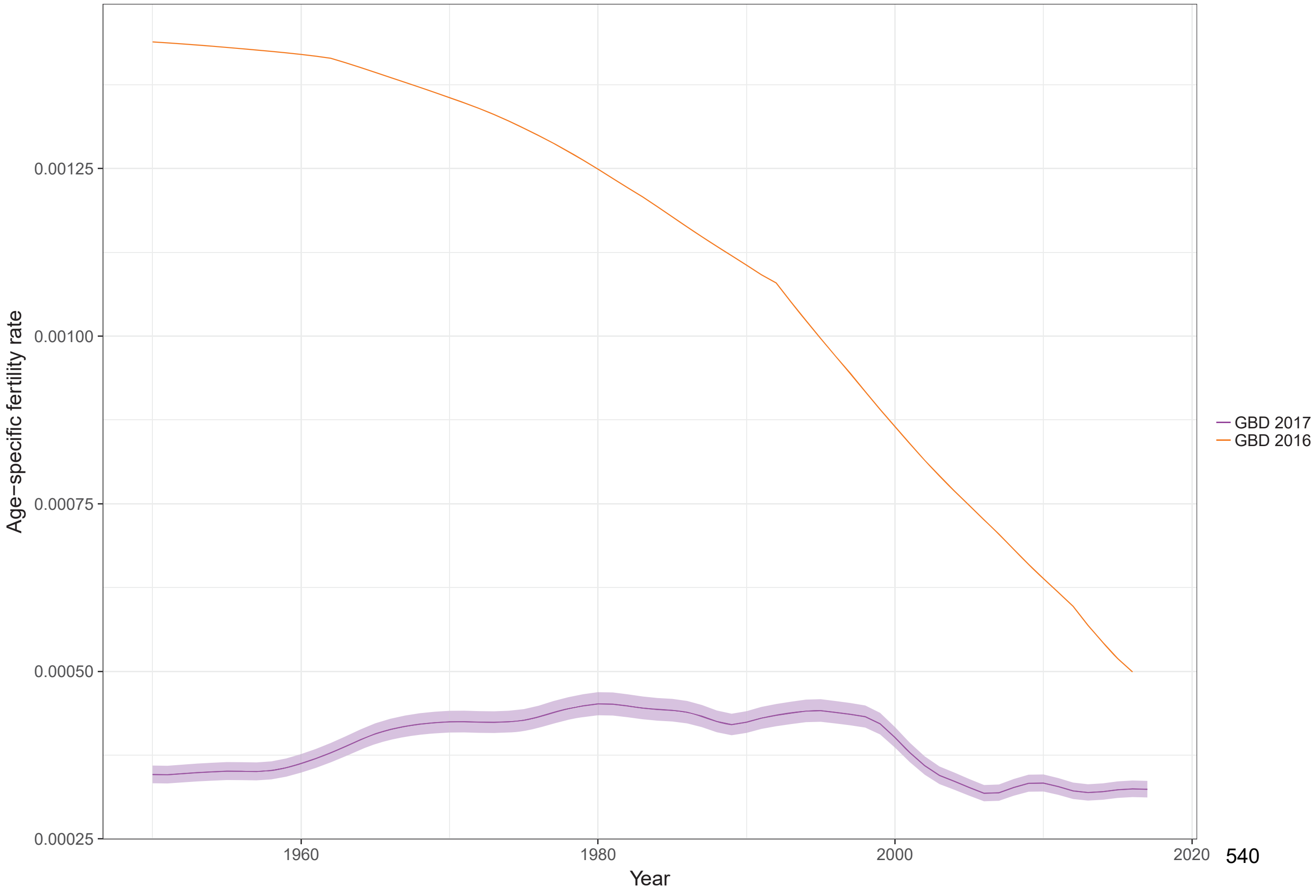

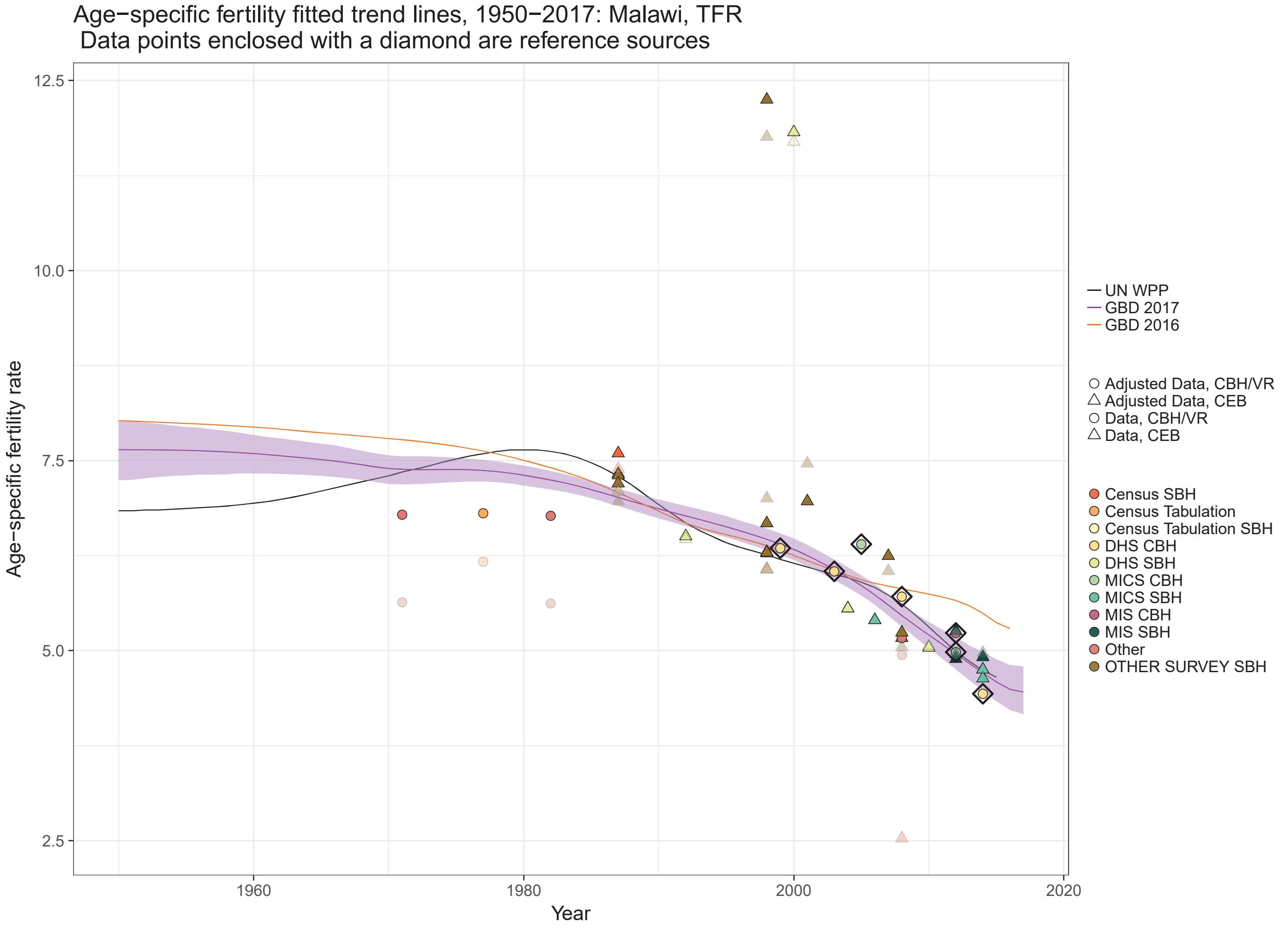

Age−specific fertility fitted trend lines, 1950−2017: Malawi, TFR
Data points enclosed with a diamond are reference sources
Age−specific fertility rate
Year
12.5
10.0
7.5
5.0
2.5
1960
1980
2000
2020
UN WPP
GBD 2017
GBD 2016
Adjusted Data, CBH/VR
Adjusted Data, CEB
Data, CBH/VR
Data, CEB
Census SBH
Census Tabulation
Census Tabulation SBH
DHS CBH
DHS SBH
MICS CBH
MICS SBH
MIS CBH
MIS SBH
Other
OTHER SURVEY SBH

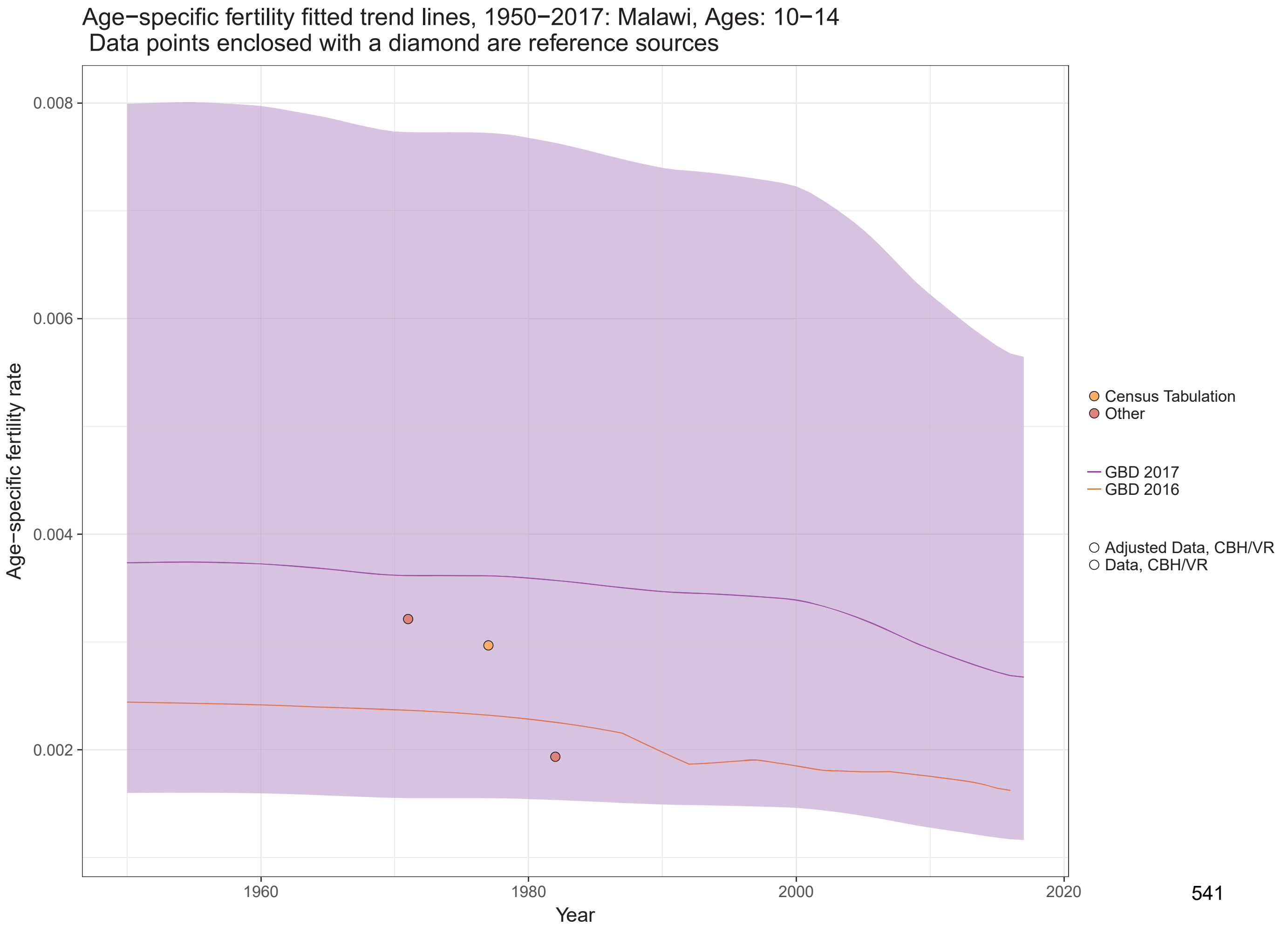

Age−specific fertility fitted trend lines, 1950−2017: Malawi, Ages: 10−14
Data points enclosed with a diamond are reference sources
Age−specific fertility rate
Year
0.008
0.006
0.004
0.002
1960
1980
2000
2020
Census Tabulation
Other
GBD 2017
GBD 2016
Adjusted Data, CBH/VR
Data, CBH/VR

Age−specific fertility fitted trend lines, 1950−2017: Malawi, Ages: 15−19
Data points enclosed with a diamond are reference sources

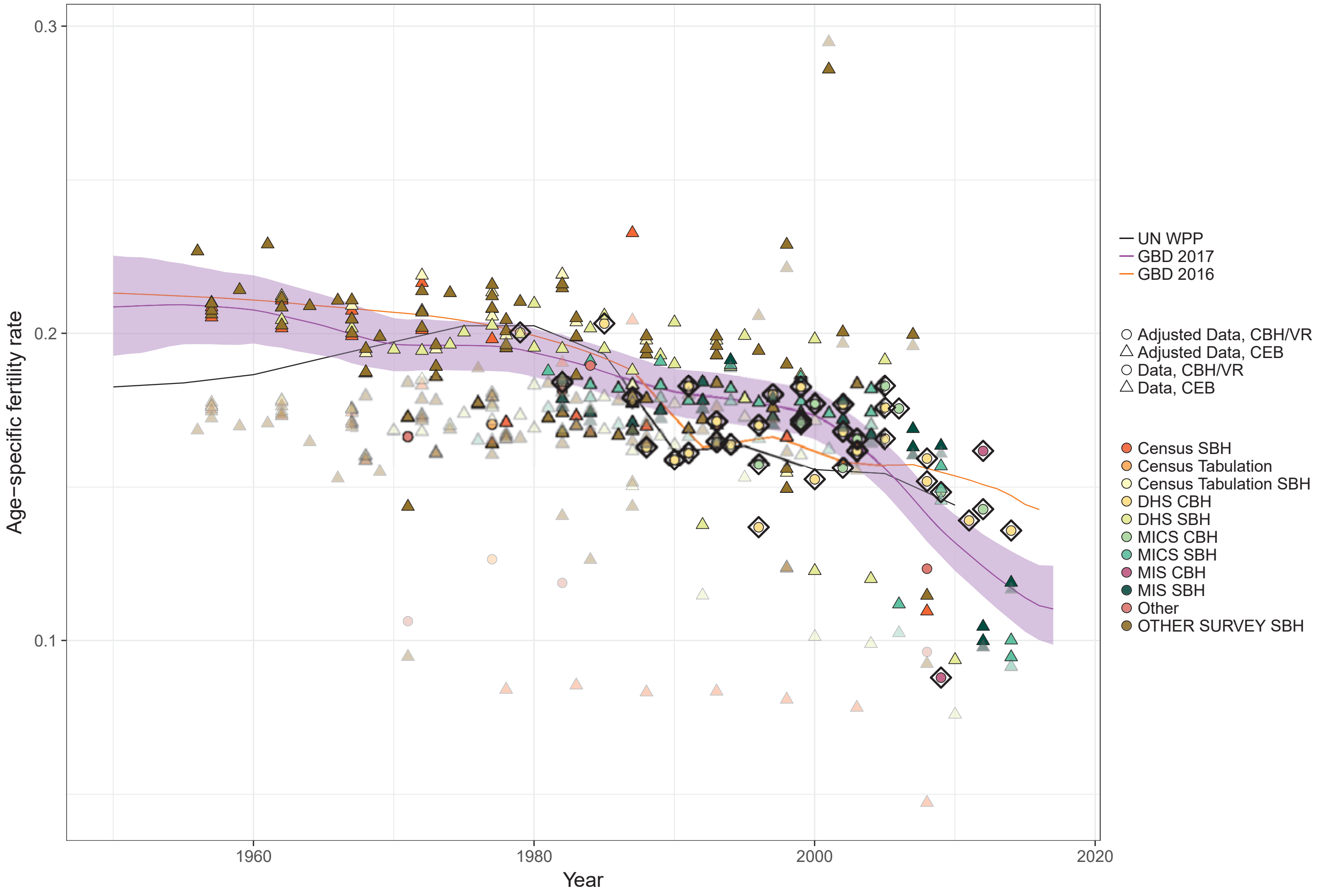

Age−specific fertility fitted trend lines, 1950−2017: Malawi, Ages: 20−24
Data points enclosed with a diamond are reference sources

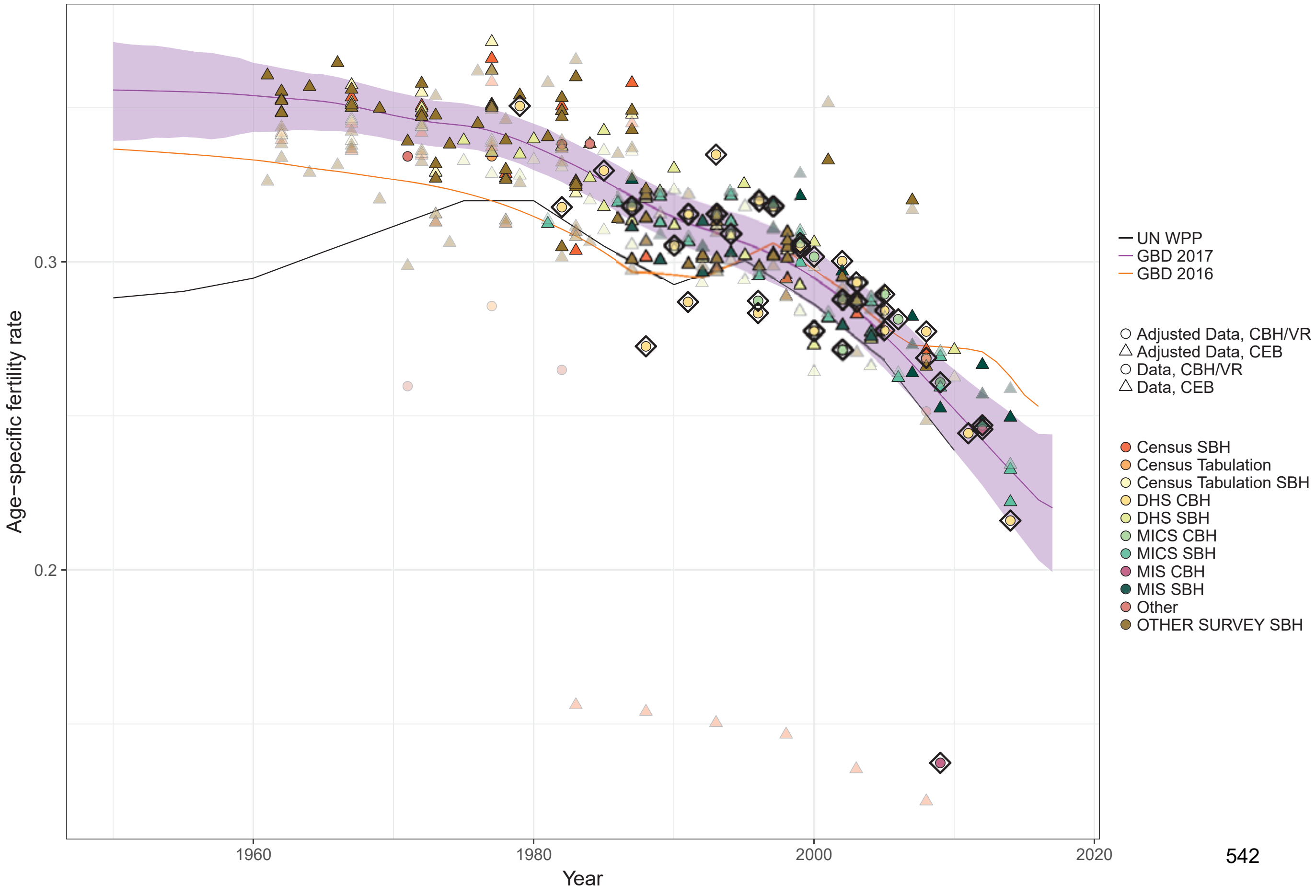

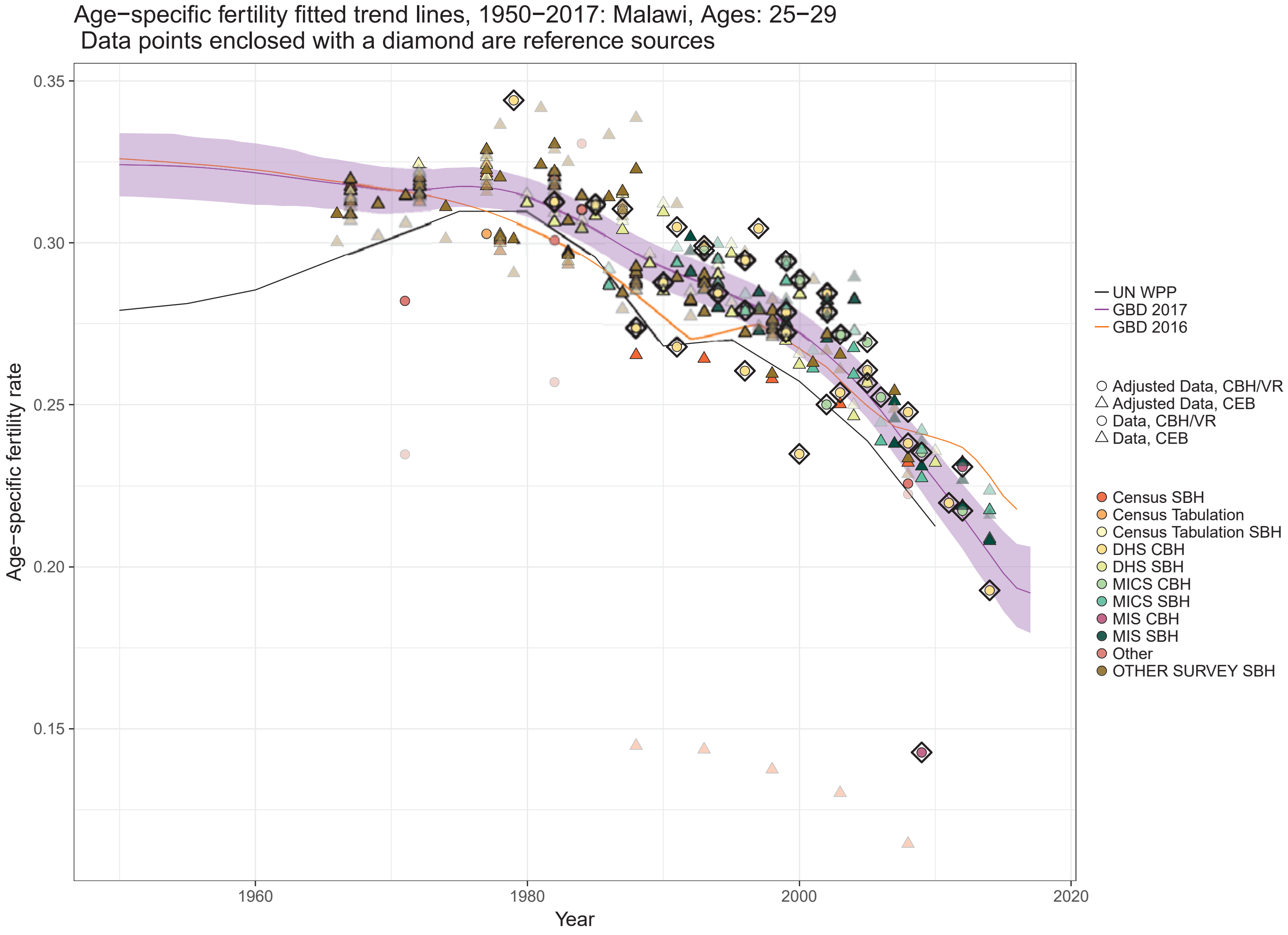

Age−specific fertility fitted trend lines, 1950−2017: Malawi, Ages: 25−29
Data points enclosed with a diamond are reference sources
Age−specific fertility rate
Year
0.35
0.30
0.25
0.20
0.15
1960
1980
2000
2020
UN WPP
GBD 2017
GBD 2016
Adjusted Data, CBH/VR
Adjusted Data, CEB
Data, CBH/VR
Data, CEB
Census SBH
Census Tabulation
Census Tabulation SBH
DHS CBH
DHS SBH
MICS CBH
MICS SBH
MIS CBH
MIS SBH
Other
OTHER SURVEY SBH

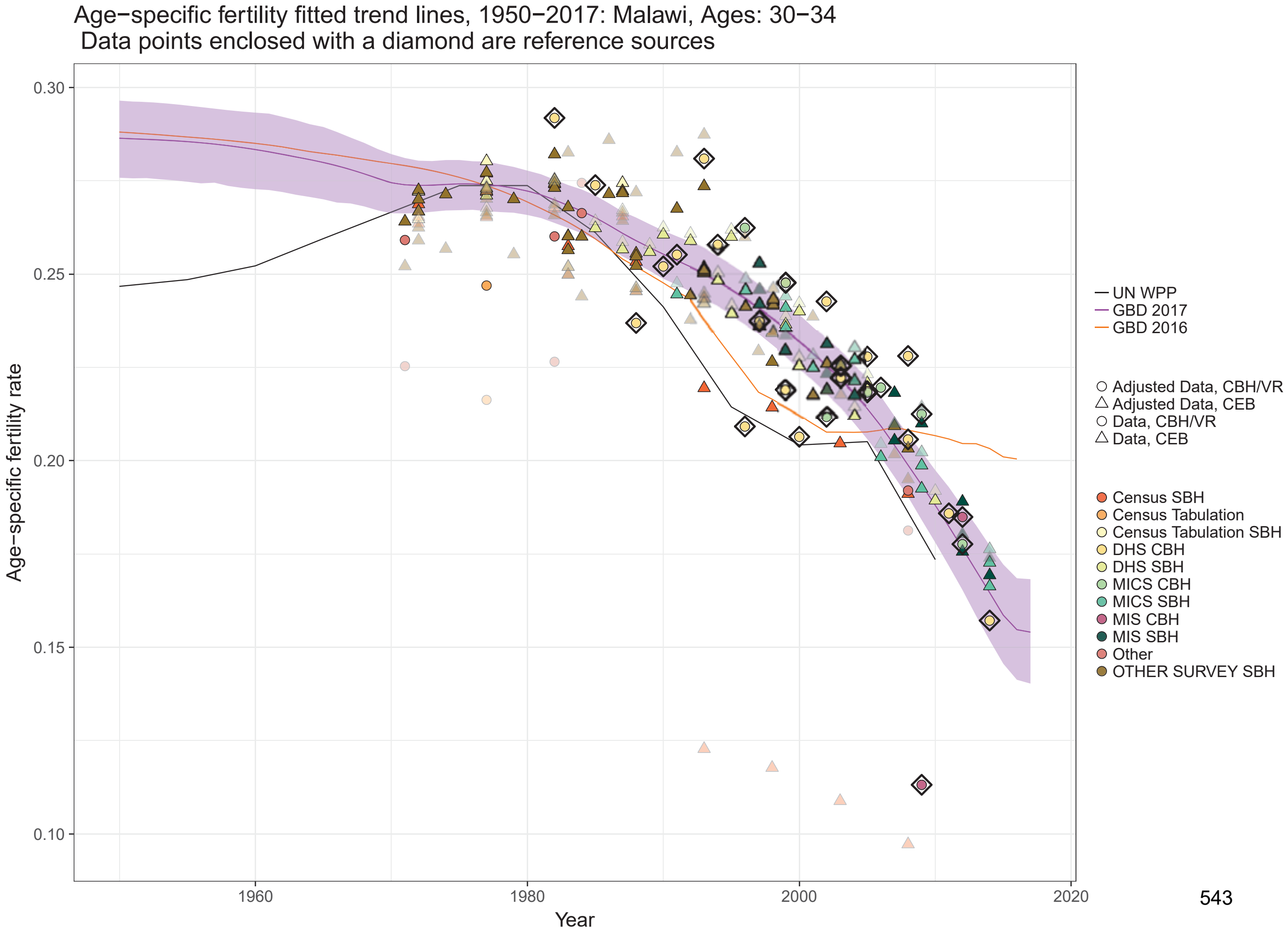

Age−specific fertility fitted trend lines, 1950−2017: Malawi, Ages: 30−34
Data points enclosed with a diamond are reference sources
Age−specific fertility rate
Year
0.30
0.25
0.20
0.15
0.10
1960
1980
2000
2020
UN WPP
GBD 2017
GBD 2016
Adjusted Data, CBH/VR
Adjusted Data, CEB
Data, CBH/VR
Data, CEB
Census SBH
Census Tabulation
Census Tabulation SBH
DHS CBH
DHS SBH
MICS CBH
MICS SBH
MIS CBH
MIS SBH
Other
OTHER SURVEY SBH

Age−specific fertility fitted trend lines, 1950−2017: Malawi, Ages: 35−39
Data points enclosed with a diamond are reference sources

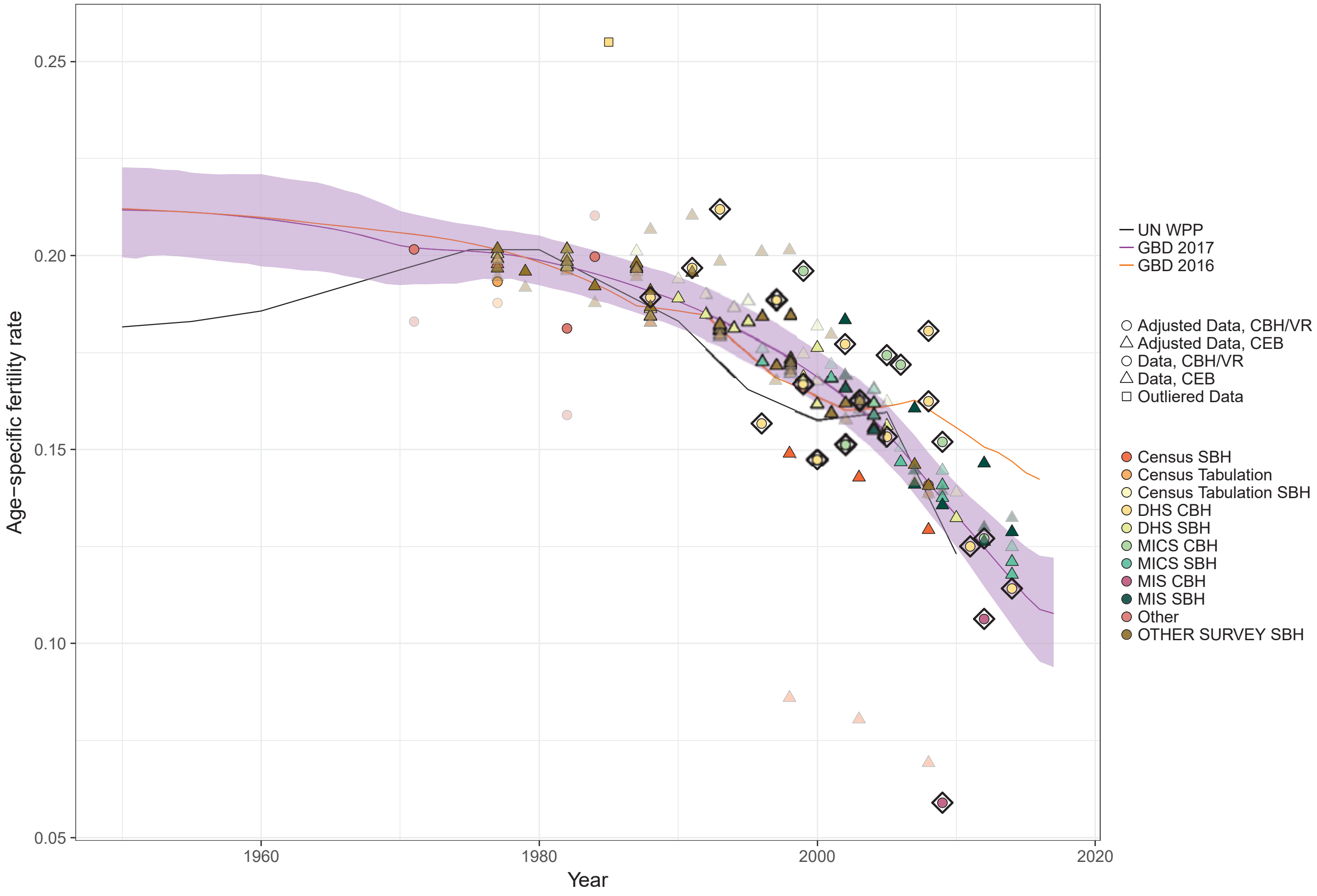

Age−specific fertility fitted trend lines, 1950−2017: Malawi, Ages: 40−44
Data points enclosed with a diamond are reference sources

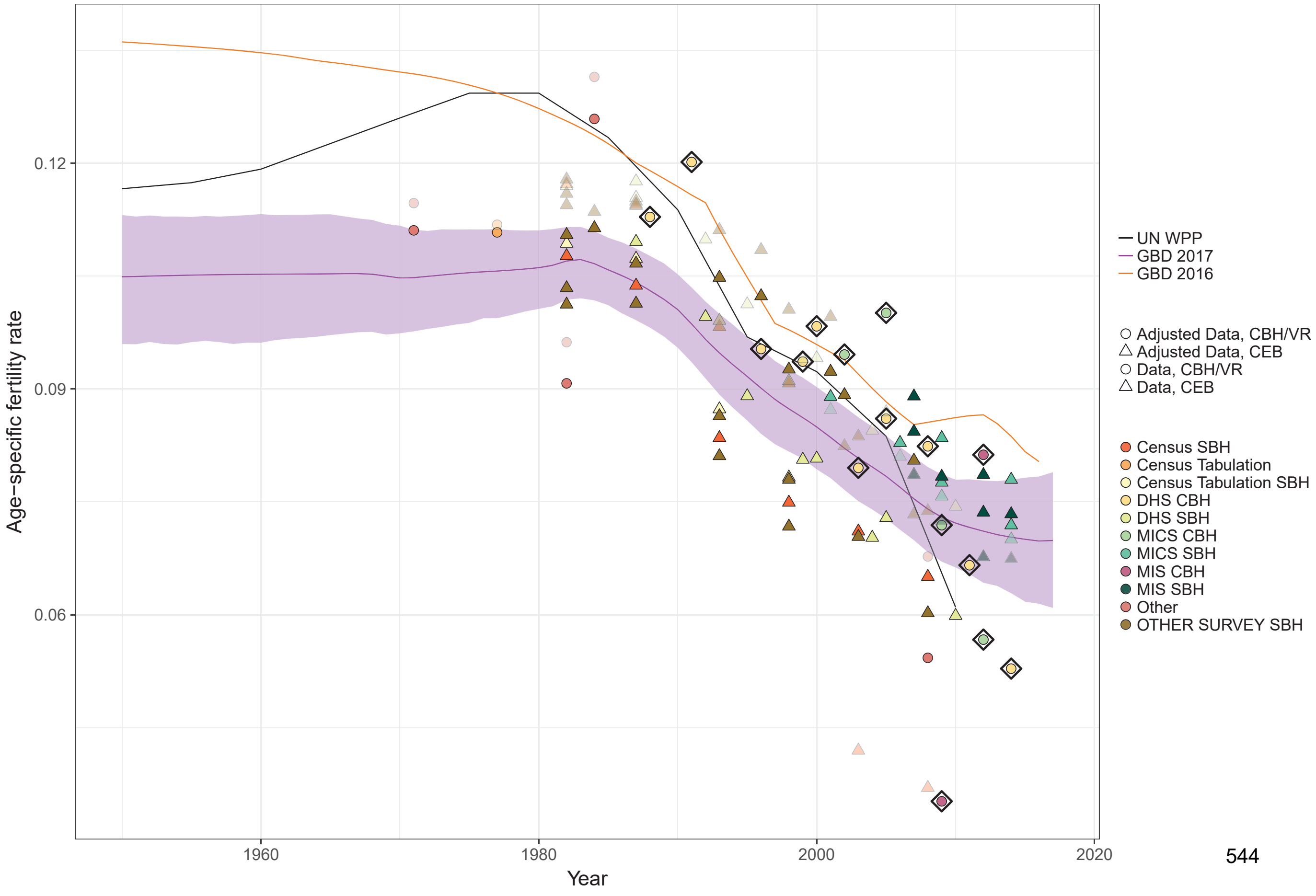

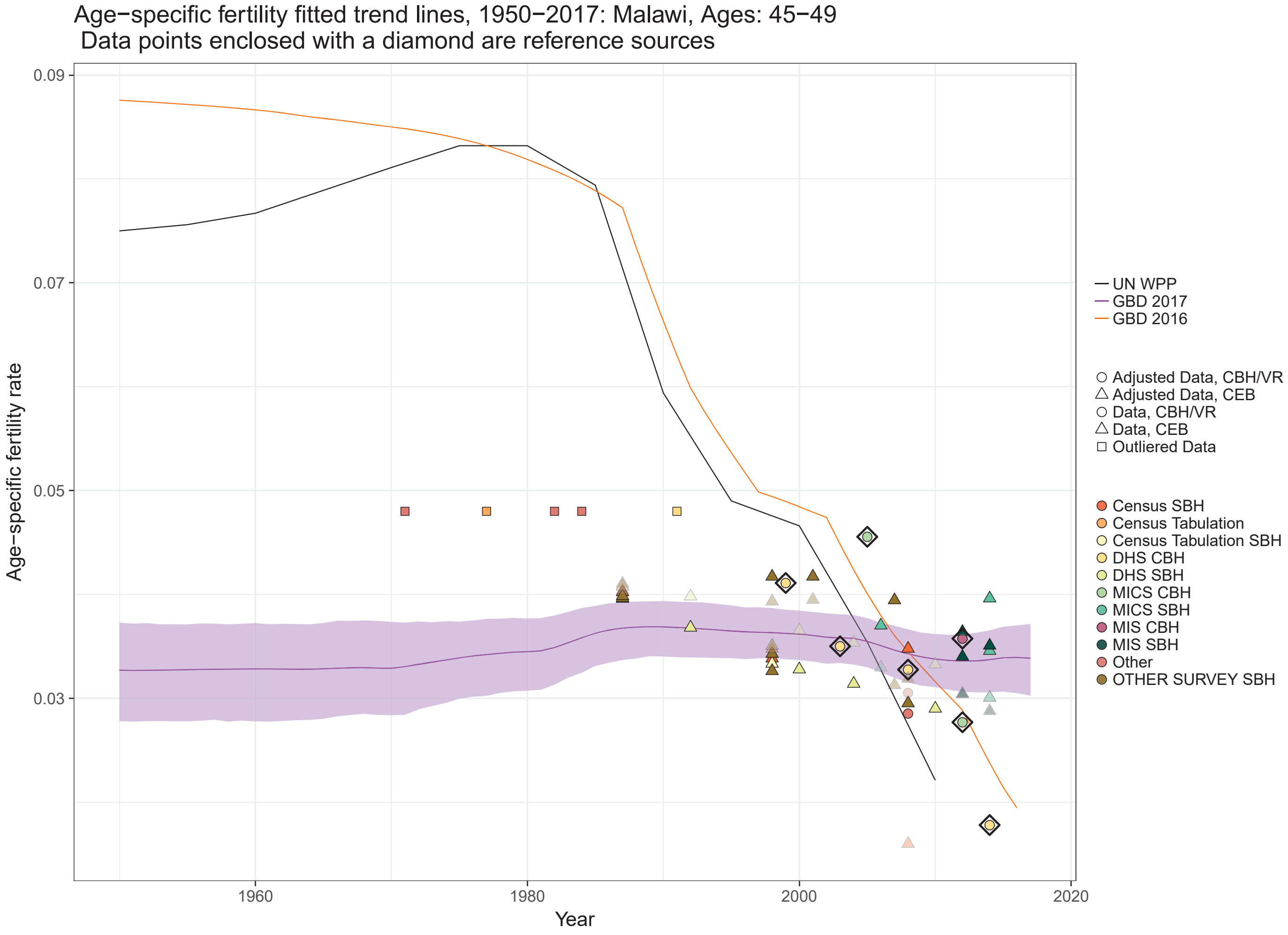

Age−specific fertility fitted trend lines, 1950−2017: Malawi, Ages: 45−49
Data points enclosed with a diamond are reference sources
0.09
0.07
0.05
0.03
Age−specific fertility rate
1960
1980
2000
2020
Year
UN WPP
GBD 2017
GBD 2016
Adjusted Data, CBH/VR
Adjusted Data, CEB
Data, CBH/VR
Data, CEB
Outliered Data
Census SBH
Census Tabulation
Census Tabulation SBH
DHS CBH
DHS SBH
MICS CBH
MICS SBH
MIS CBH
MIS SBH
Other
OTHER SURVEY SBH

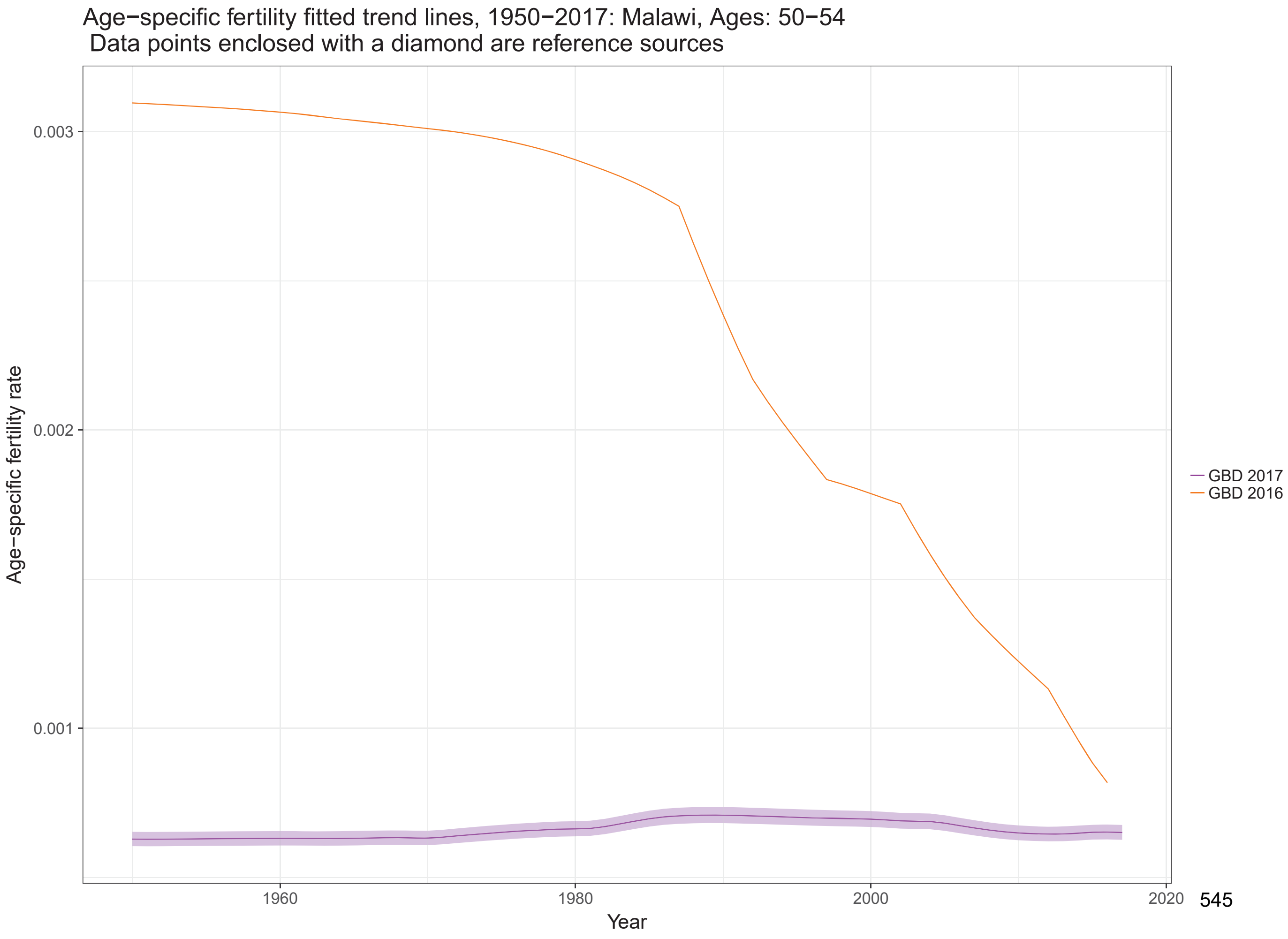

Age−specific fertility fitted trend lines, 1950−2017: Malawi, Ages: 50−54
Data points enclosed with a diamond are reference sources
0.003
0.002
0.001
Age−specific fertility rate
1960
1980
2000
2020
Year
GBD 2017
GBD 2016

Age−specific fertility fitted trend lines, 1950−2017: Malaysia, TFR
Data points enclosed with a diamond are reference sources

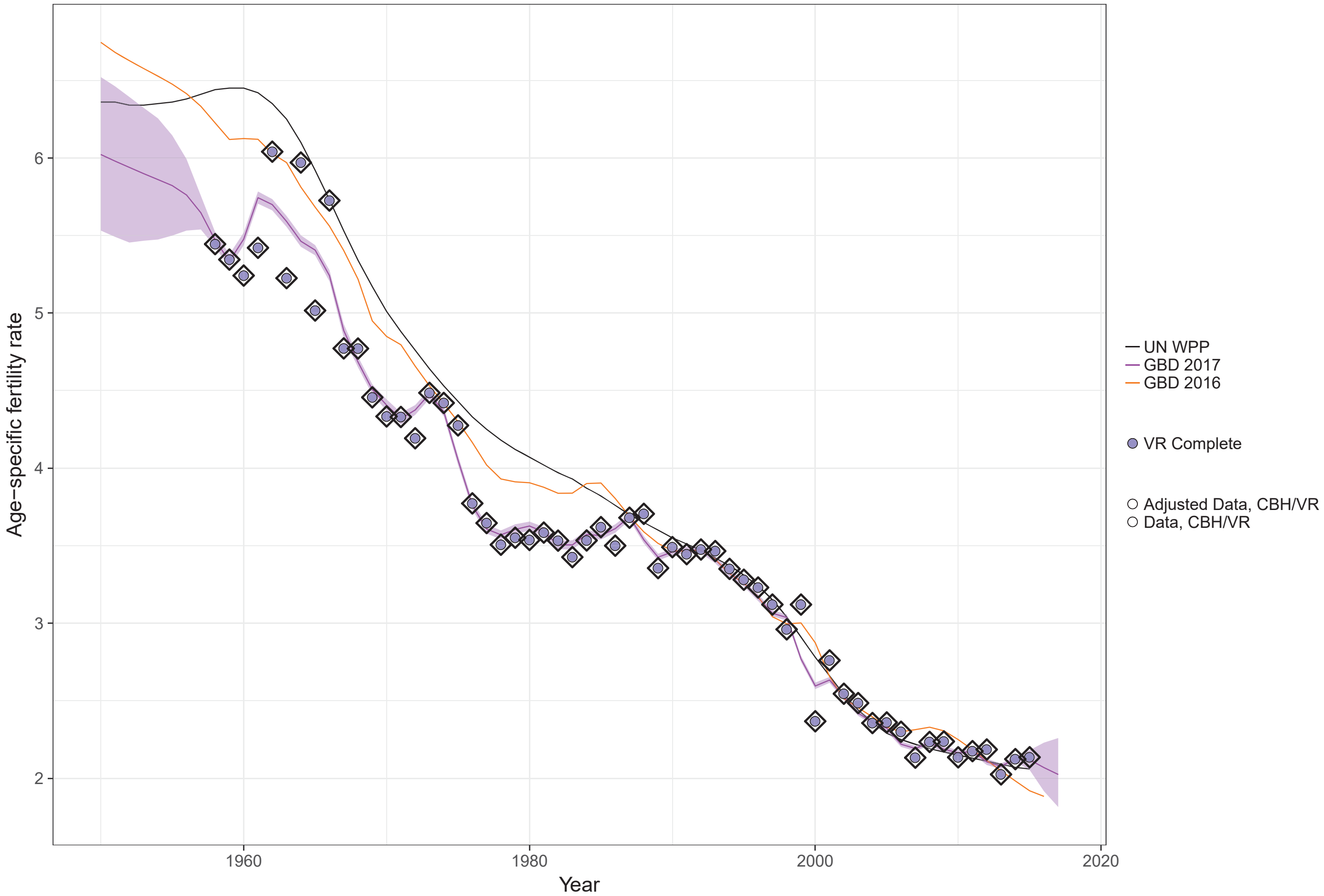

Age−specific fertility fitted trend lines, 1950−2017: Malaysia, Ages: 10−14
Data points enclosed with a diamond are reference sources

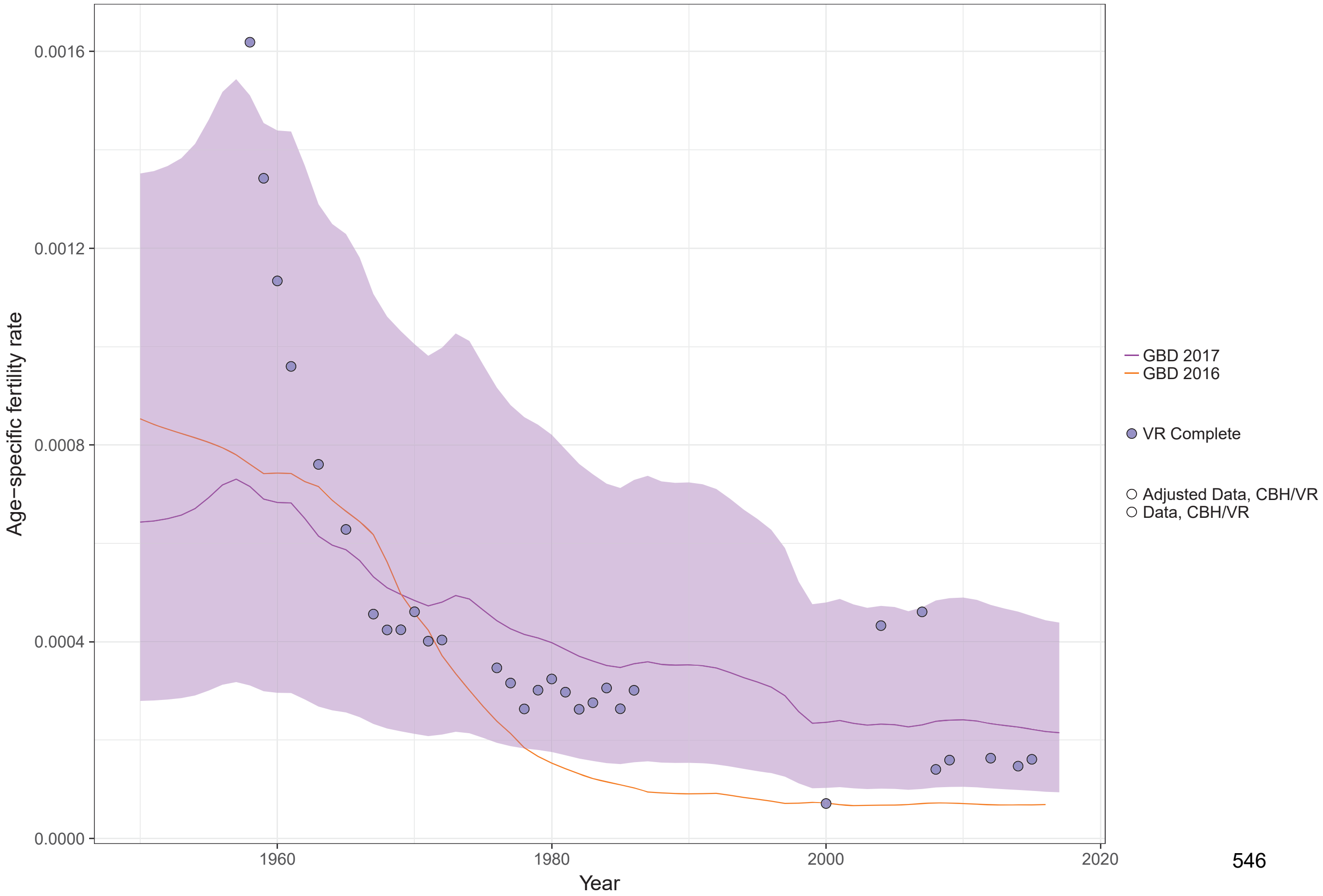

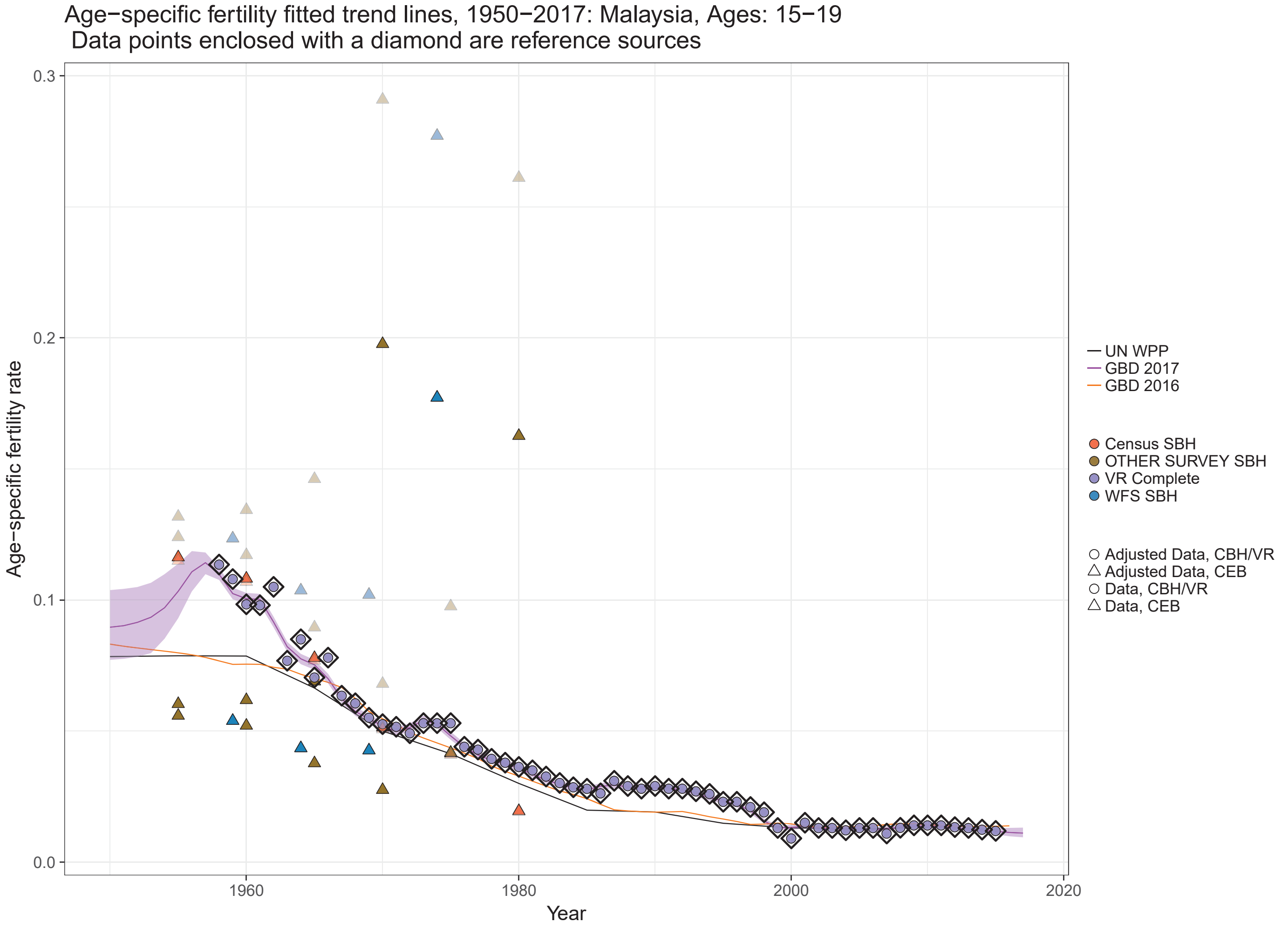
Age−specific fertility fitted trend lines, 1950−2017: Malaysia, Ages: 15−19
Data points enclosed with a diamond are reference sources
Age−specific fertility rate
Year
UN WPP
GBD 2017
GBD 2016
Census SBH
OTHER SURVEY SBH
VR Complete
WFS SBH
Adjusted Data, CBH/VR
Adjusted Data, CEB
Data, CBH/VR
Data, CEB

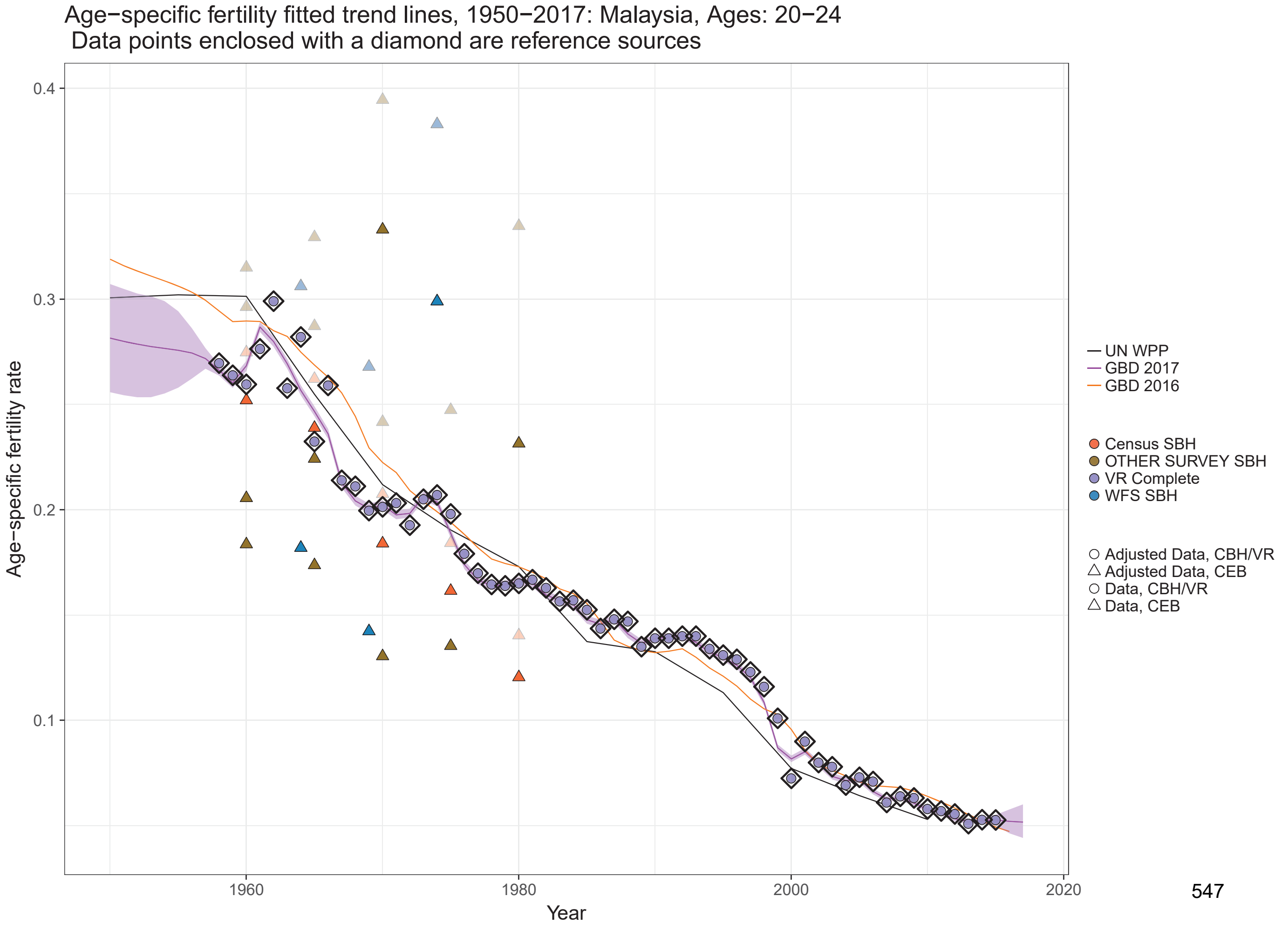
Age−specific fertility fitted trend lines, 1950−2017: Malaysia, Ages: 20−24
Data points enclosed with a diamond are reference sources
Age−specific fertility rate
Year
UN WPP
GBD 2017
GBD 2016
Census SBH
OTHER SURVEY SBH
VR Complete
WFS SBH
Adjusted Data, CBH/VR
Adjusted Data, CEB
Data, CBH/VR
Data, CEB

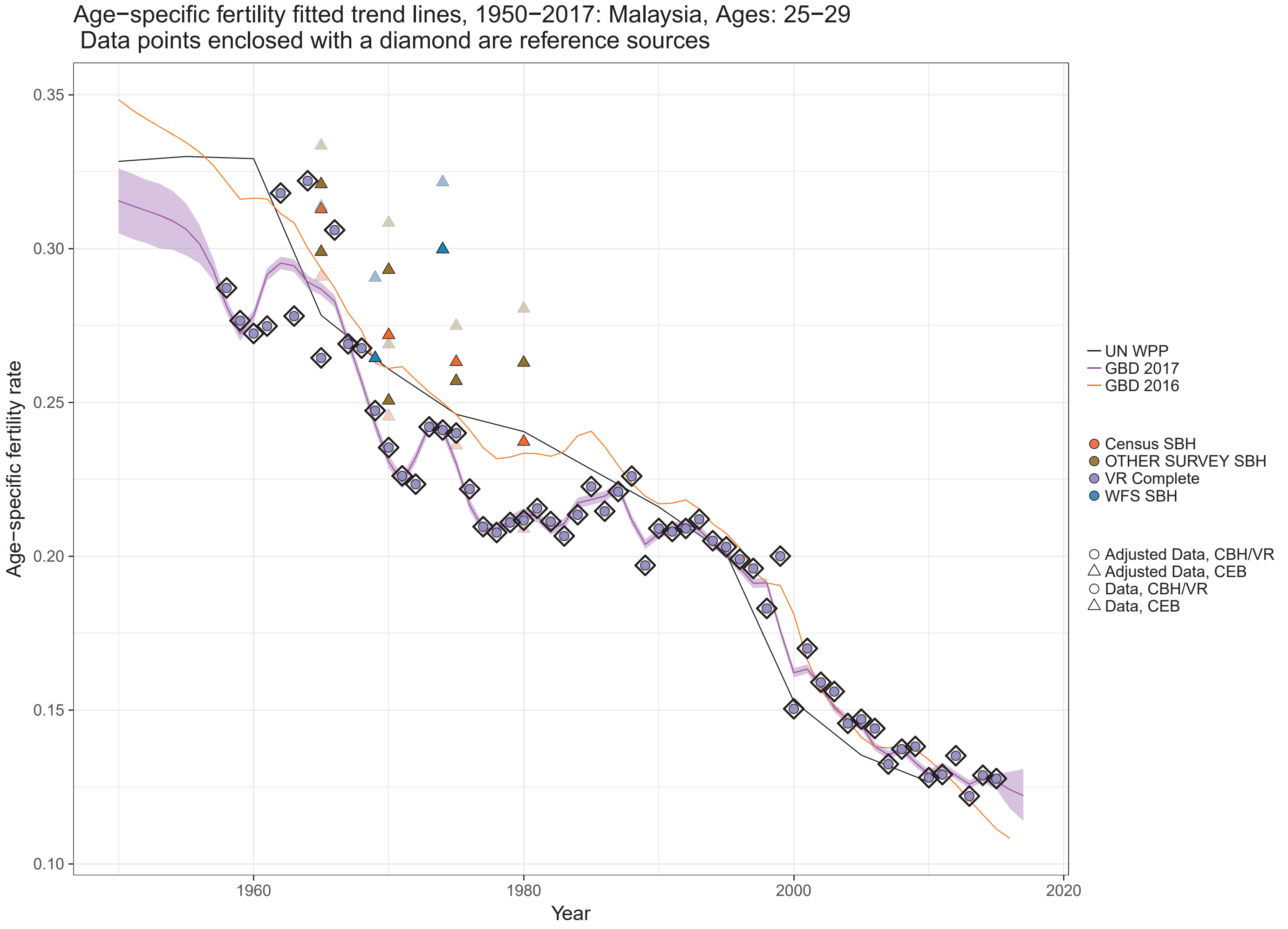

Age−specific fertility fitted trend lines, 1950−2017: Malaysia, Ages: 25−29
Data points enclosed with a diamond are reference sources
Age-specific fertility rate
Year
UN WPP
GBD 2017
GBD 2016
Census SBH
OTHER SURVEY SBH
VR Complete
WFS SBH
Adjusted Data, CBH/VR
Adjusted Data, CEB
Data, CBH/VR
Data, CEB

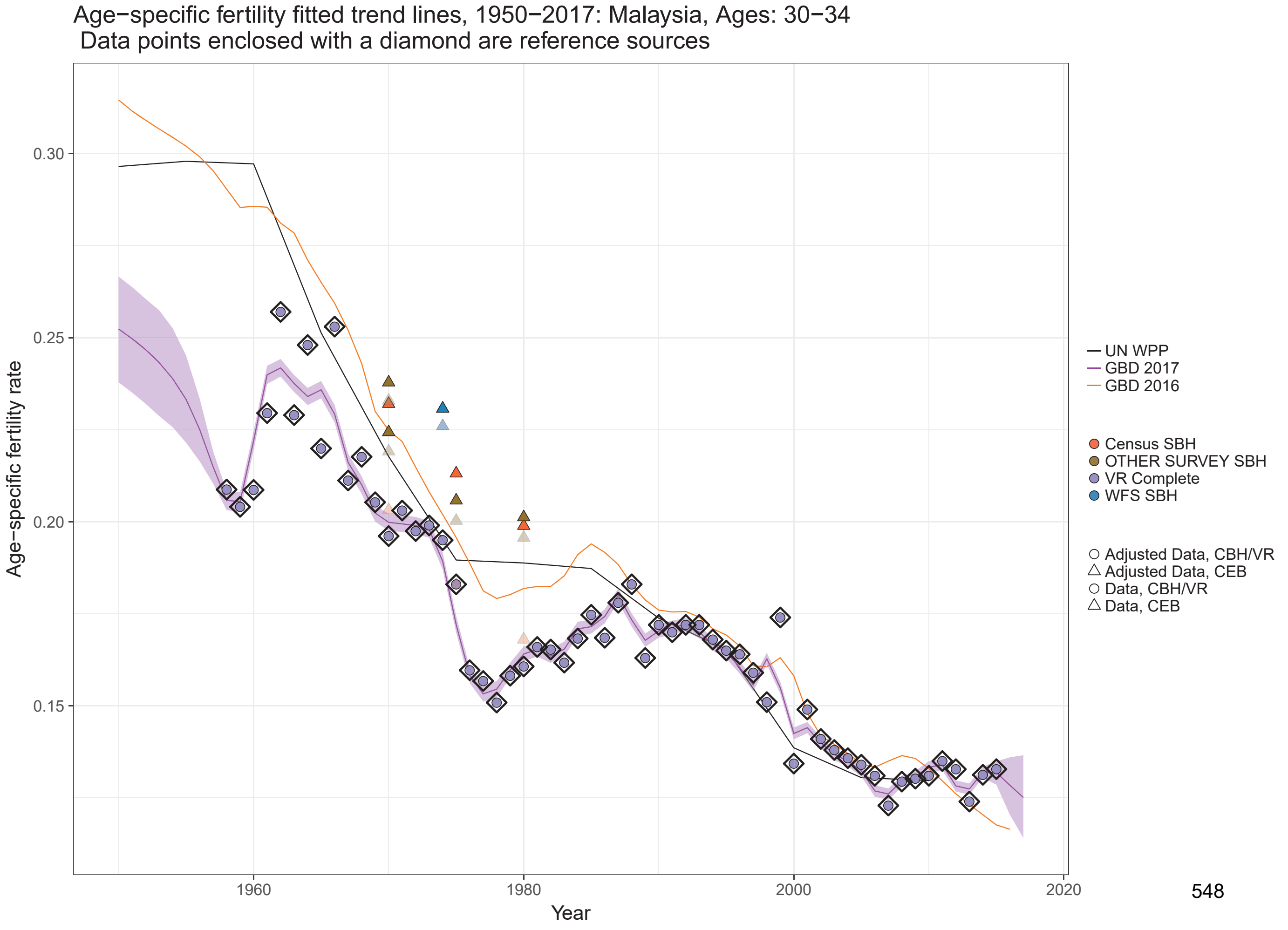

Age−specific fertility fitted trend lines, 1950−2017: Malaysia, Ages: 30−34
Data points enclosed with a diamond are reference sources
Age-specific fertility rate
Year
UN WPP
GBD 2017
GBD 2016
Census SBH
OTHER SURVEY SBH
VR Complete
WFS SBH
Adjusted Data, CBH/VR
Adjusted Data, CEB
Data, CBH/VR
Data, CEB

Age−specific fertility fitted trend lines, 1950−2017: Malaysia, Ages: 35−39
Data points enclosed with a diamond are reference sources

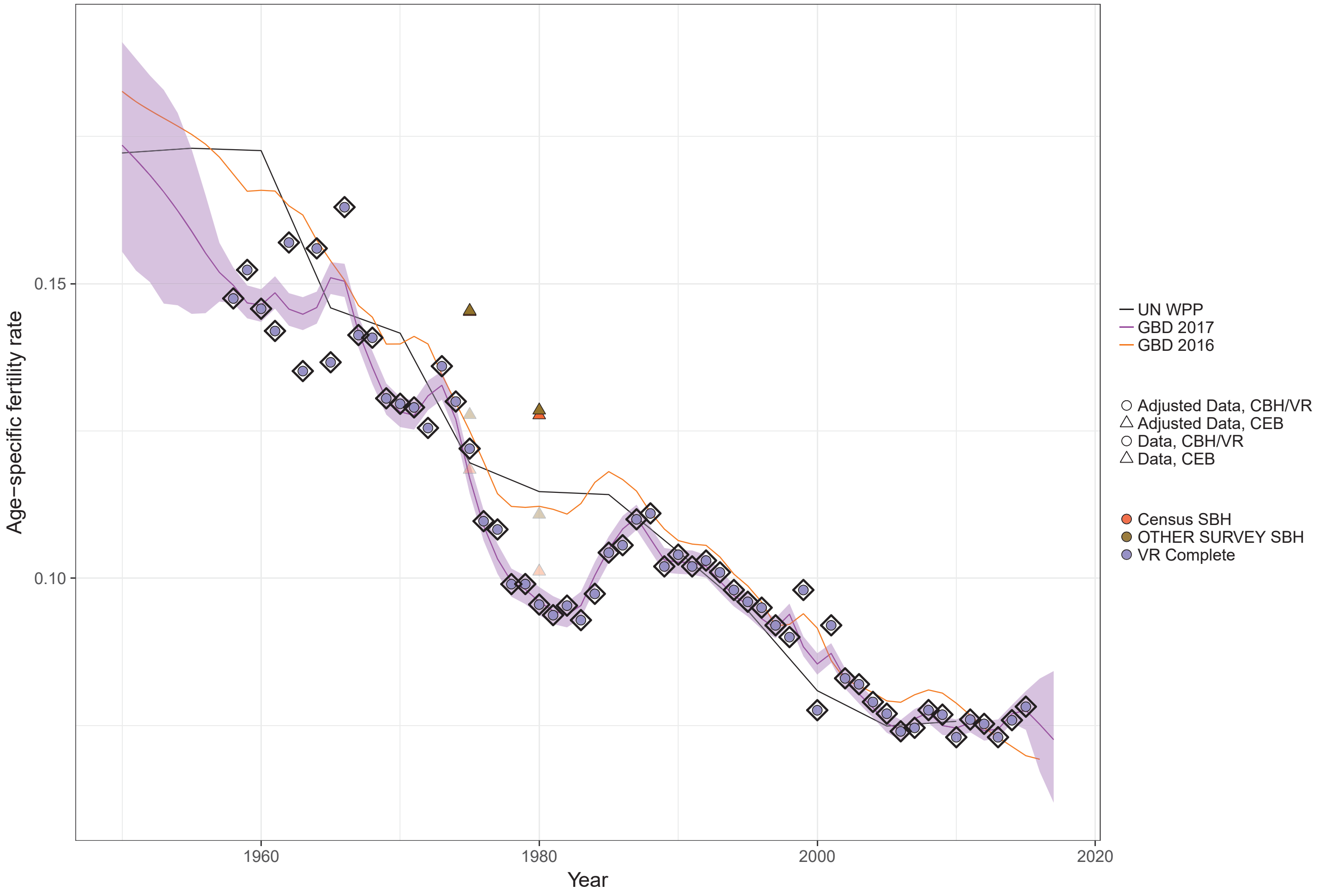

Age−specific fertility fitted trend lines, 1950−2017: Malaysia, Ages: 40−44
Data points enclosed with a diamond are reference sources

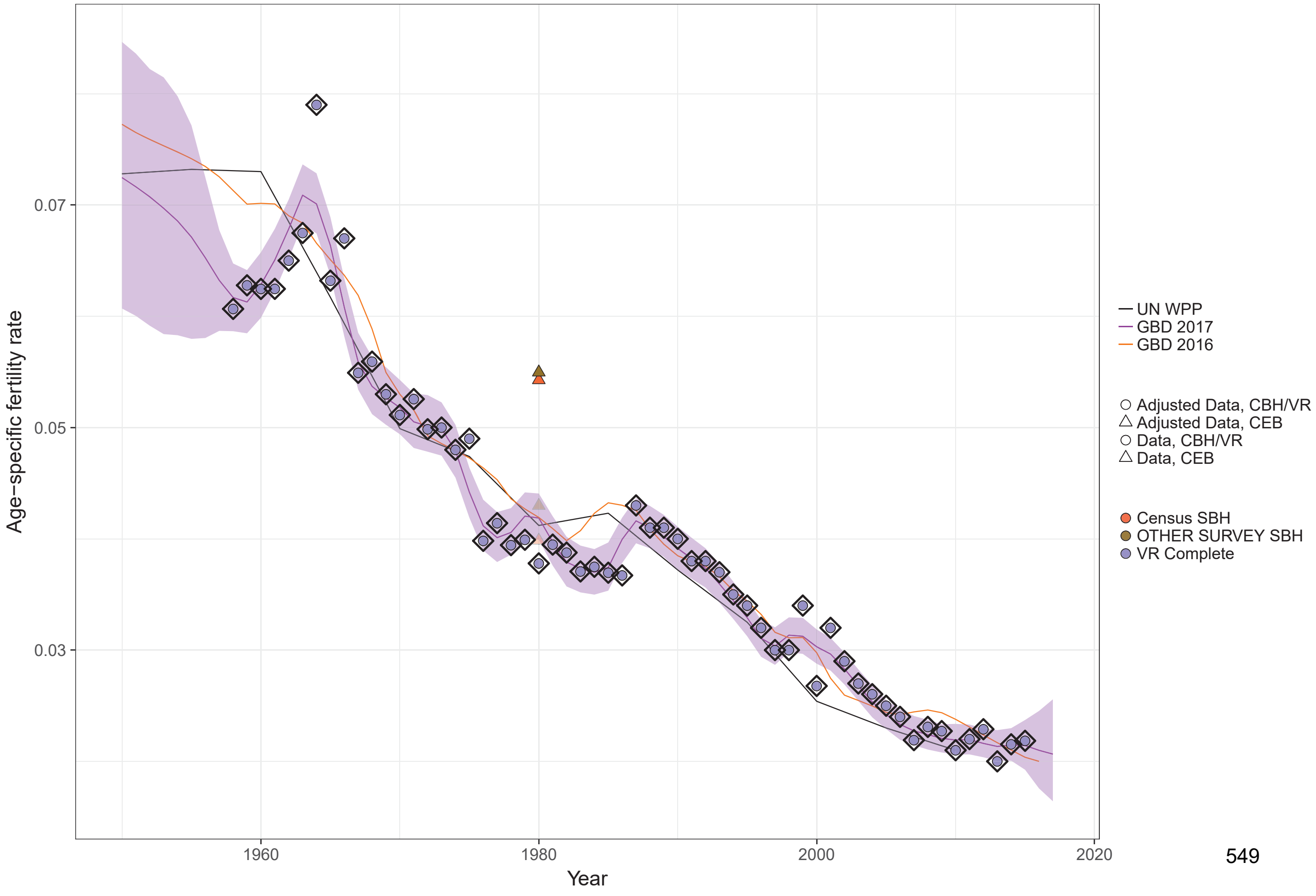

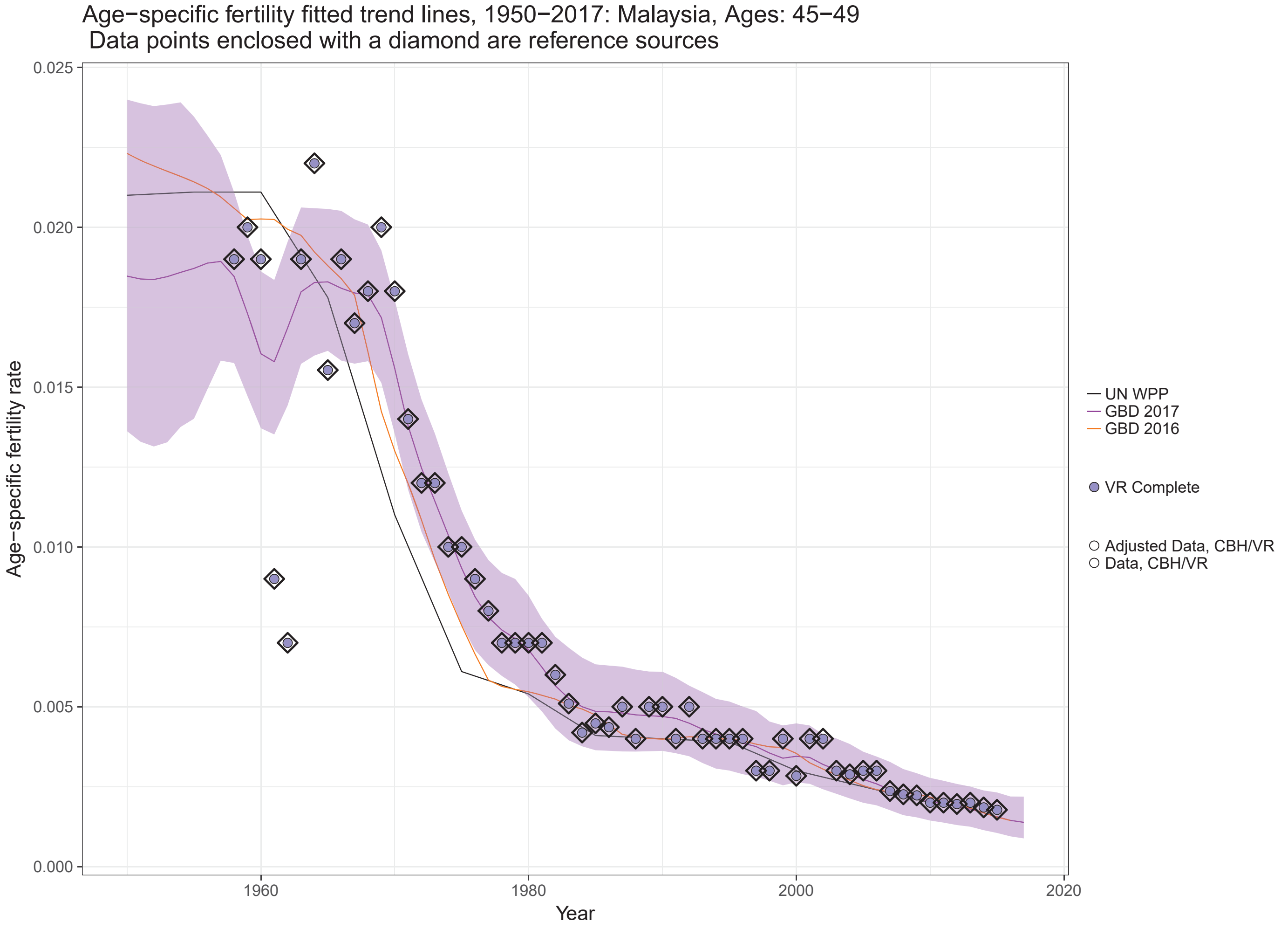

Age−specific fertility fitted trend lines, 1950−2017: Malaysia, Ages: 45−49
Data points enclosed with a diamond are reference sources
Age−specific fertility rate
0.025
0.020
0.015
0.010
0.005
0.000
1960
1980
2000
2020
Year
UN WPP
GBD 2017
GBD 2016
VR Complete
Adjusted Data, CBH/VR
Data, CBH/VR

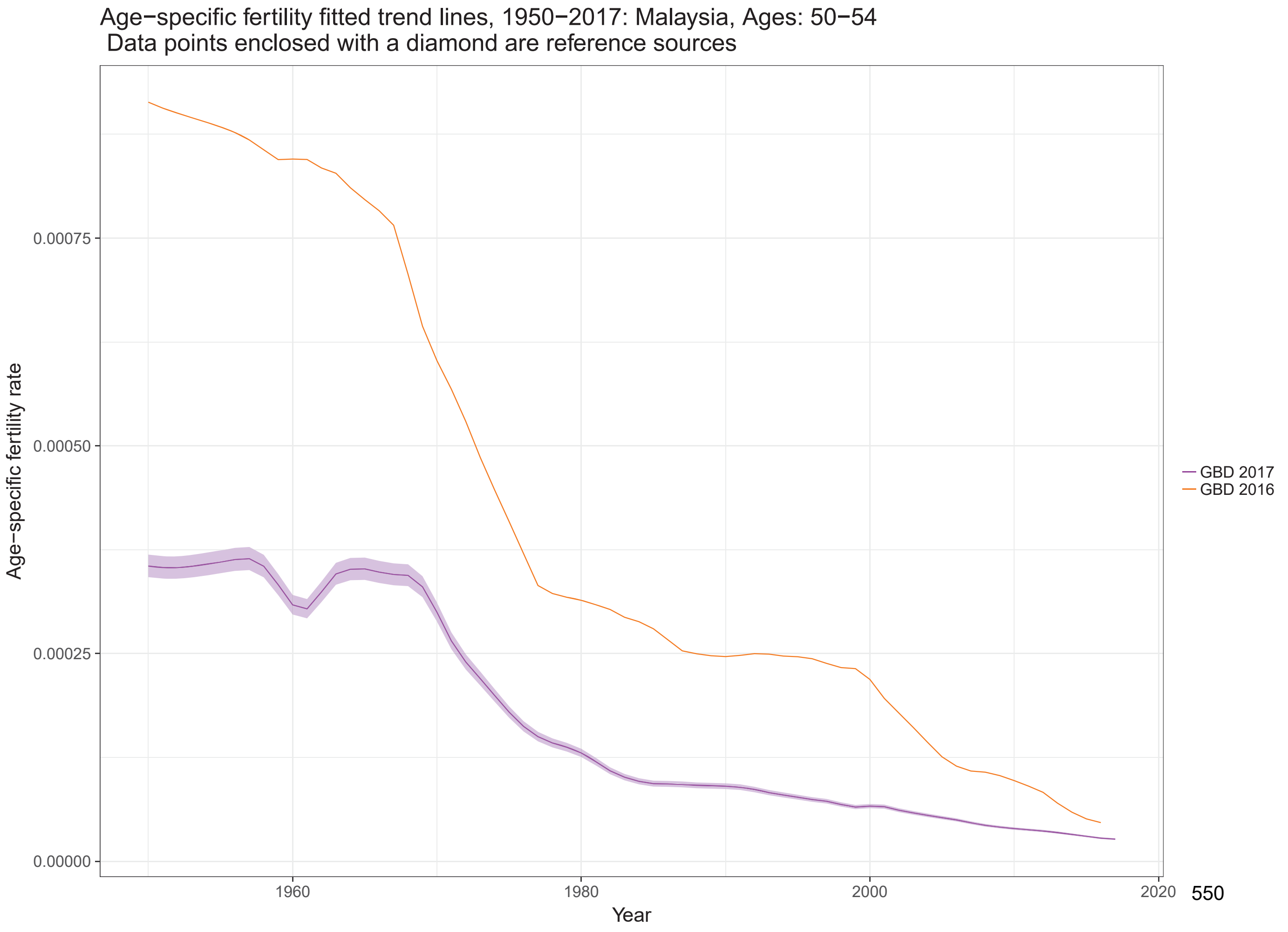

Age−specific fertility fitted trend lines, 1950−2017: Malaysia, Ages: 50−54
Data points enclosed with a diamond are reference sources
Age−specific fertility rate
0.00075
0.00050
0.00025
0.00000
1960
1980
2000
2020
Year
GBD 2017
GBD 2016

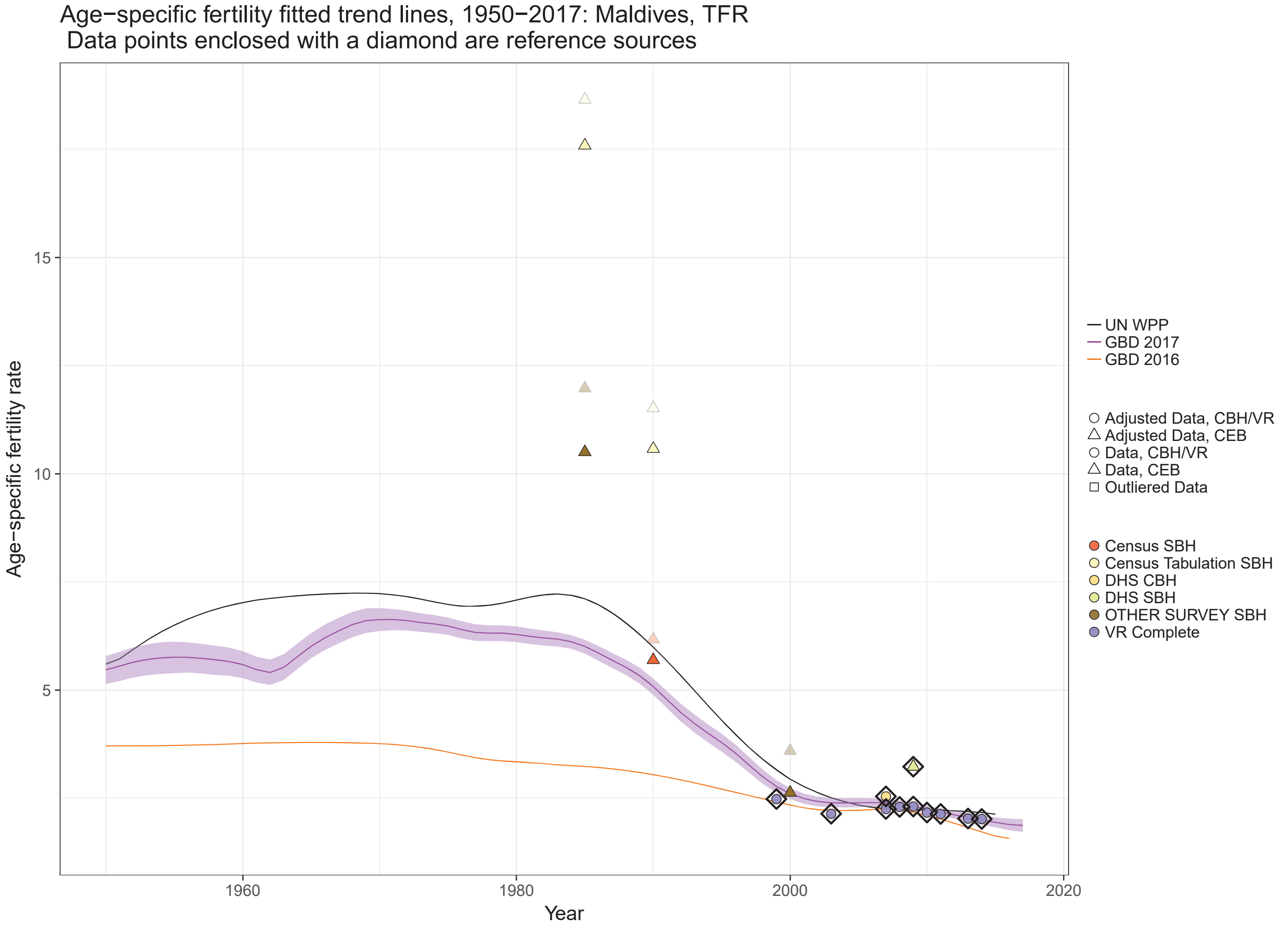

Age−specific fertility fitted trend lines, 1950−2017: Maldives, TFR
Data points enclosed with a diamond are reference sources
Age−specific fertility rate
15
10
5
1960
1980
2000
2020
Year
UN WPP
GBD 2017
GBD 2016
Adjusted Data, CBH/VR
Adjusted Data, CEB
Data, CBH/VR
Data, CEB
Outliered Data
Census SBH
Census Tabulation SBH
DHS CBH
DHS SBH
OTHER SURVEY SBH
VR Complete

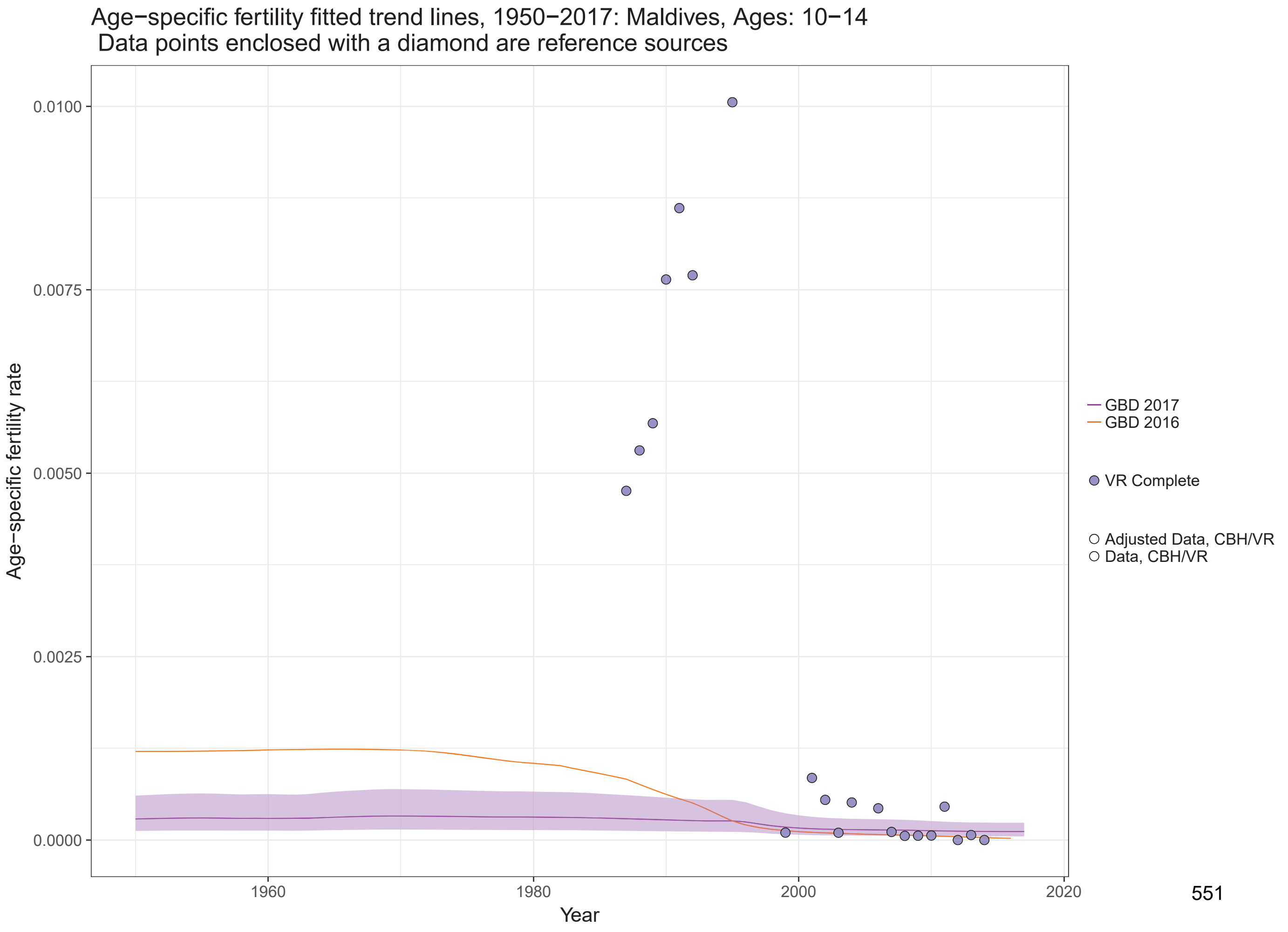

Age−specific fertility fitted trend lines, 1950−2017: Maldives, Ages: 10−14
Data points enclosed with a diamond are reference sources
Age−specific fertility rate
0.0100
0.0075
0.0050
0.0025
0.0000
1960
1980
2000
2020
Year
GBD 2017
GBD 2016
VR Complete
Adjusted Data, CBH/VR
Data, CBH/VR

Age−specific fertility fitted trend lines, 1950−2017: Maldives, Ages: 15−19
Data points enclosed with a diamond are reference sources

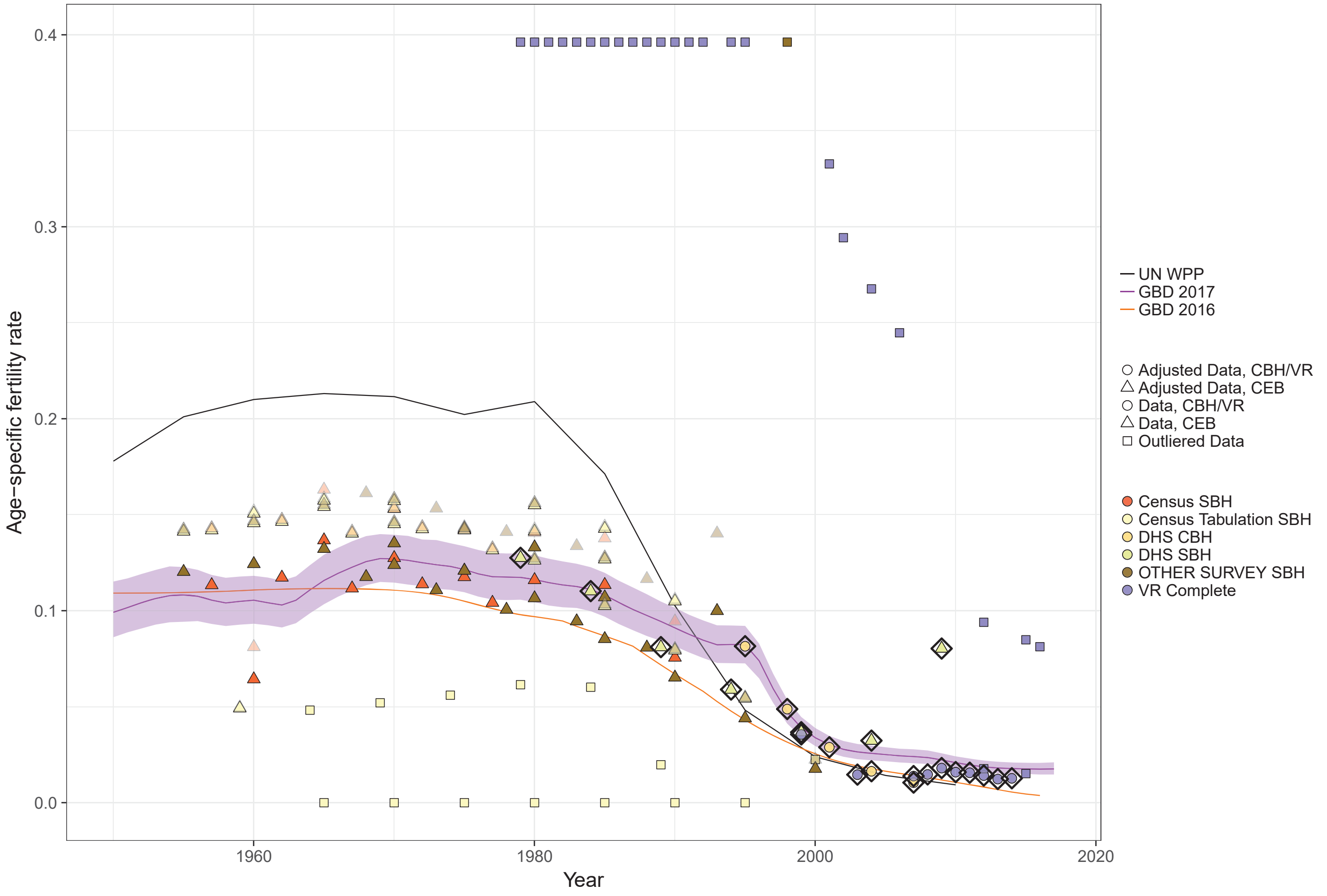

Age−specific fertility fitted trend lines, 1950−2017: Maldives, Ages: 20−24
Data points enclosed with a diamond are reference sources

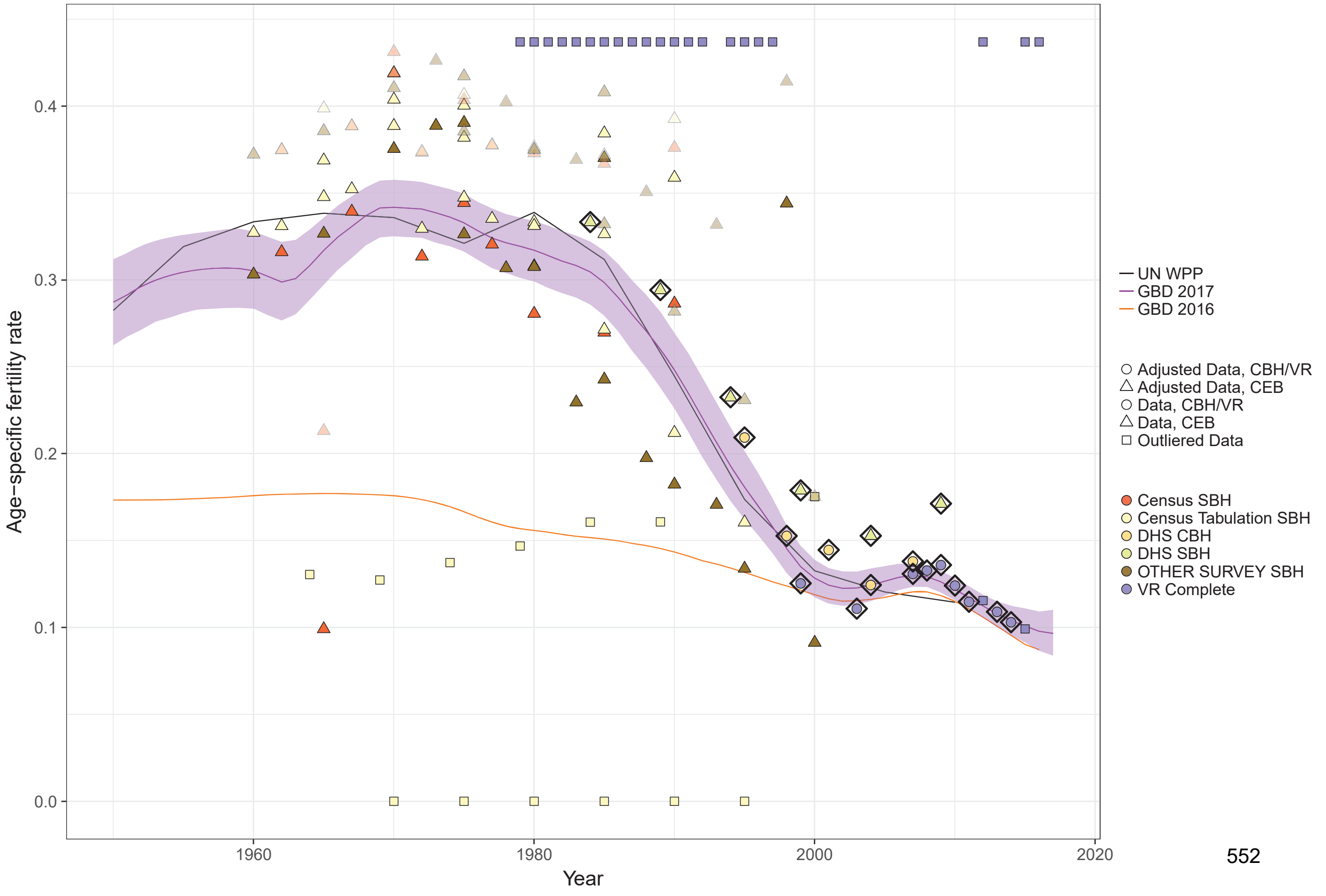

Age−specific fertility fitted trend lines, 1950−2017: Maldives, Ages: 25−29
Data points enclosed with a diamond are reference sources

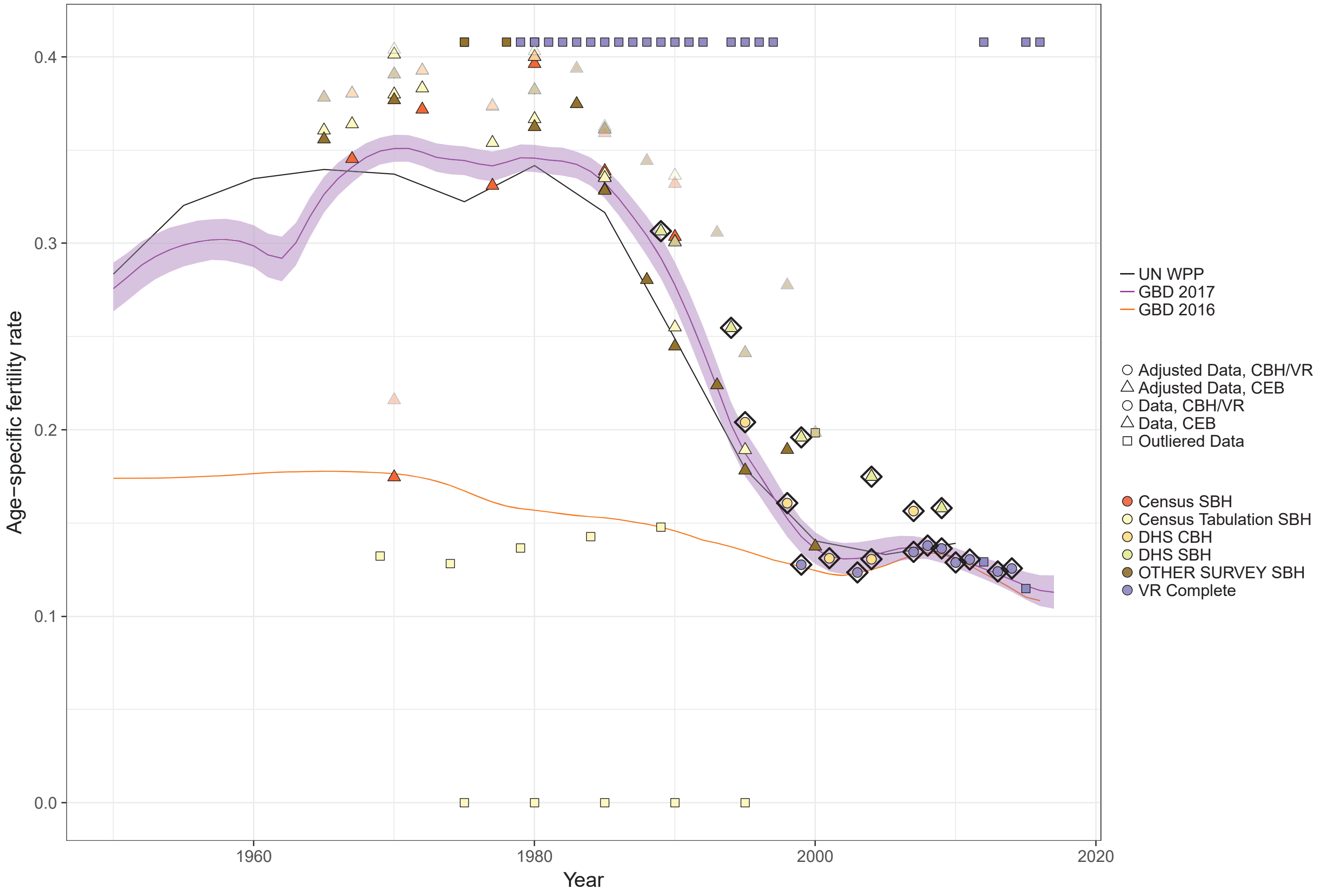

Age−specific fertility fitted trend lines, 1950−2017: Maldives, Ages: 30−34
Data points enclosed with a diamond are reference sources

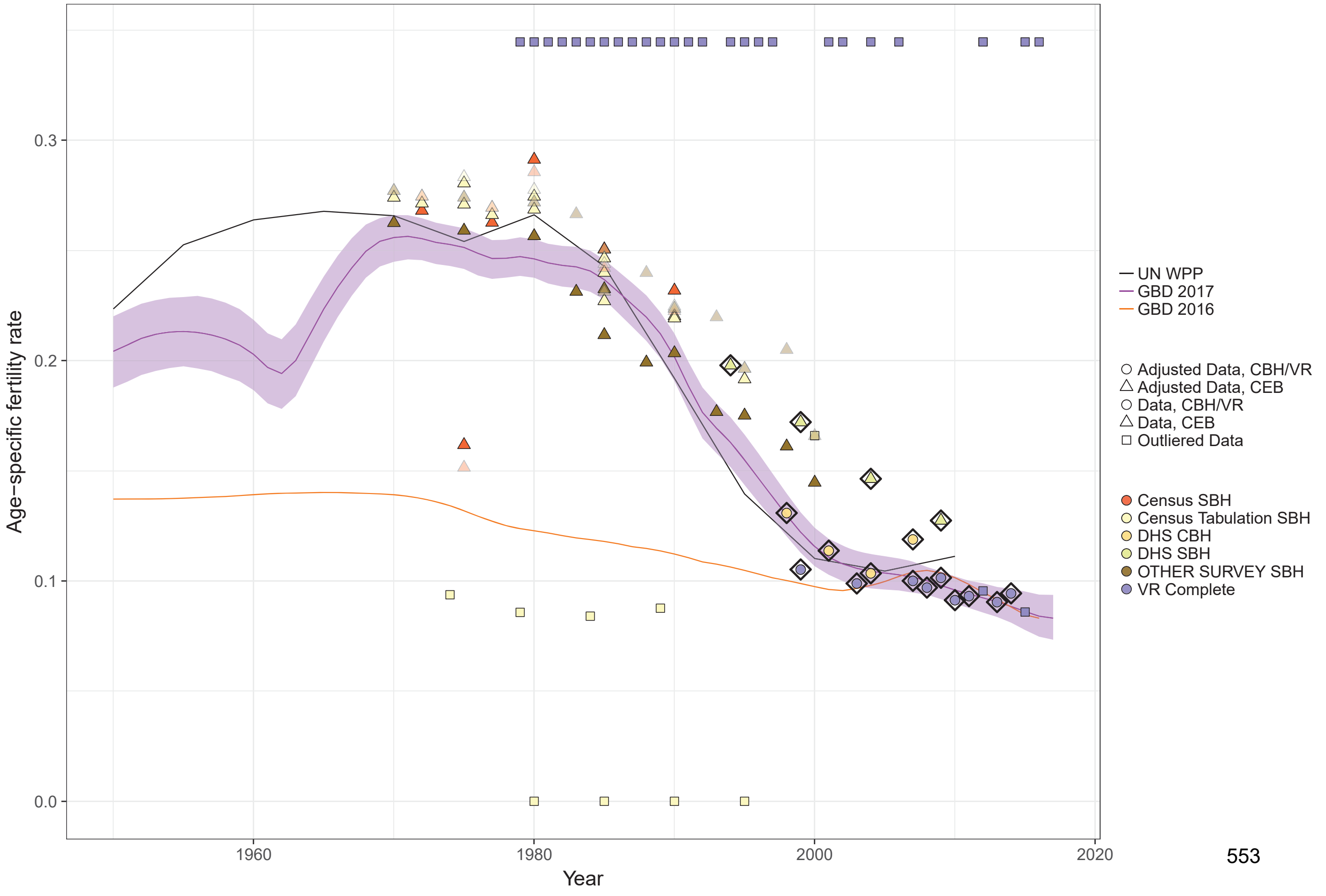

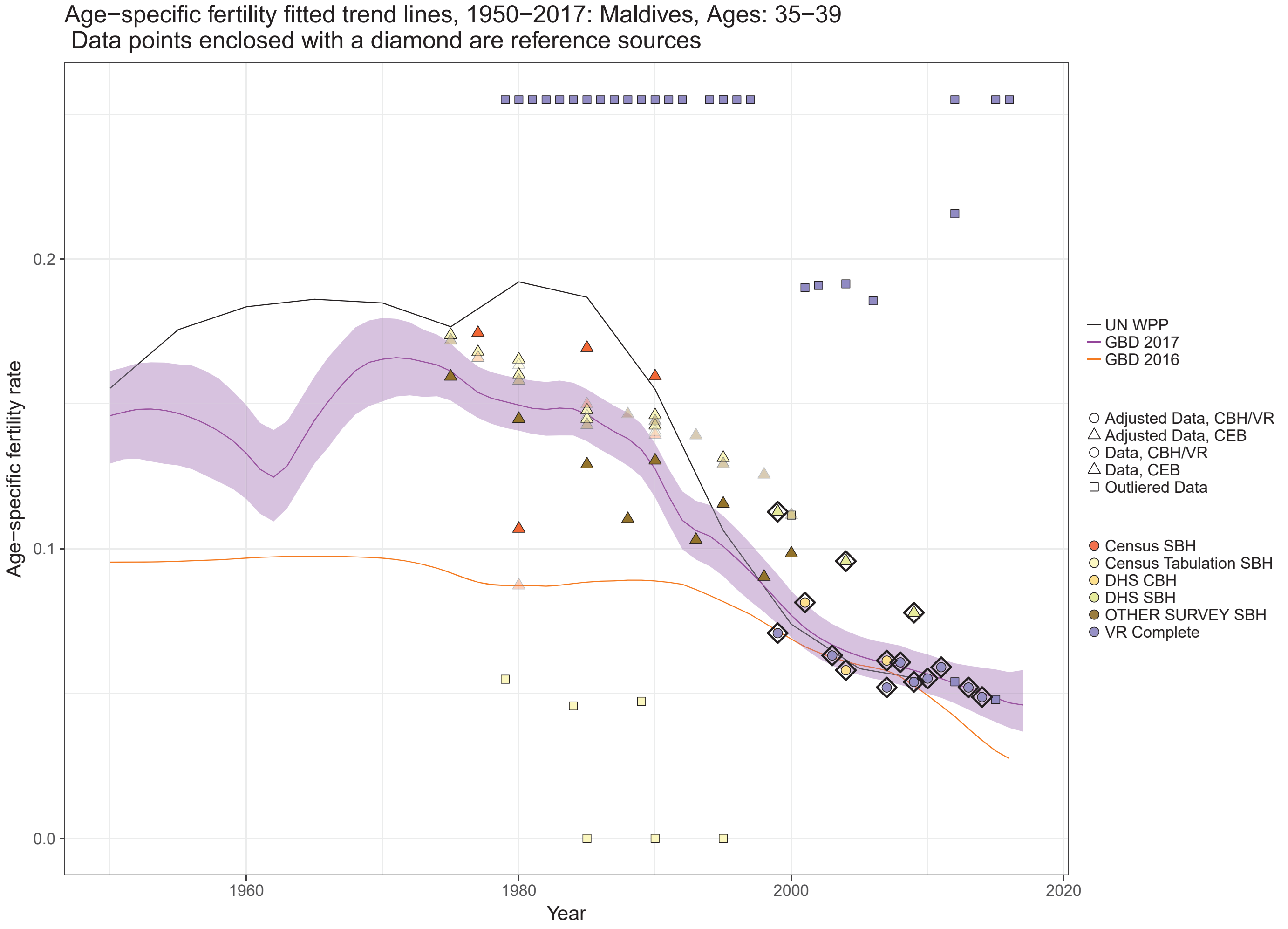

Age−specific fertility fitted trend lines, 1950−2017: Maldives, Ages: 35−39
Data points enclosed with a diamond are reference sources
Age−specific fertility rate
0.0
0.1
0.2
1960
1980
2000
2020
Year
UN WPP
GBD 2017
GBD 2016
Adjusted Data, CBH/VR
Adjusted Data, CEB
Data, CBH/VR
Data, CEB
Outliered Data
Census SBH
Census Tabulation SBH
DHS CBH
DHS SBH
OTHER SURVEY SBH
VR Complete

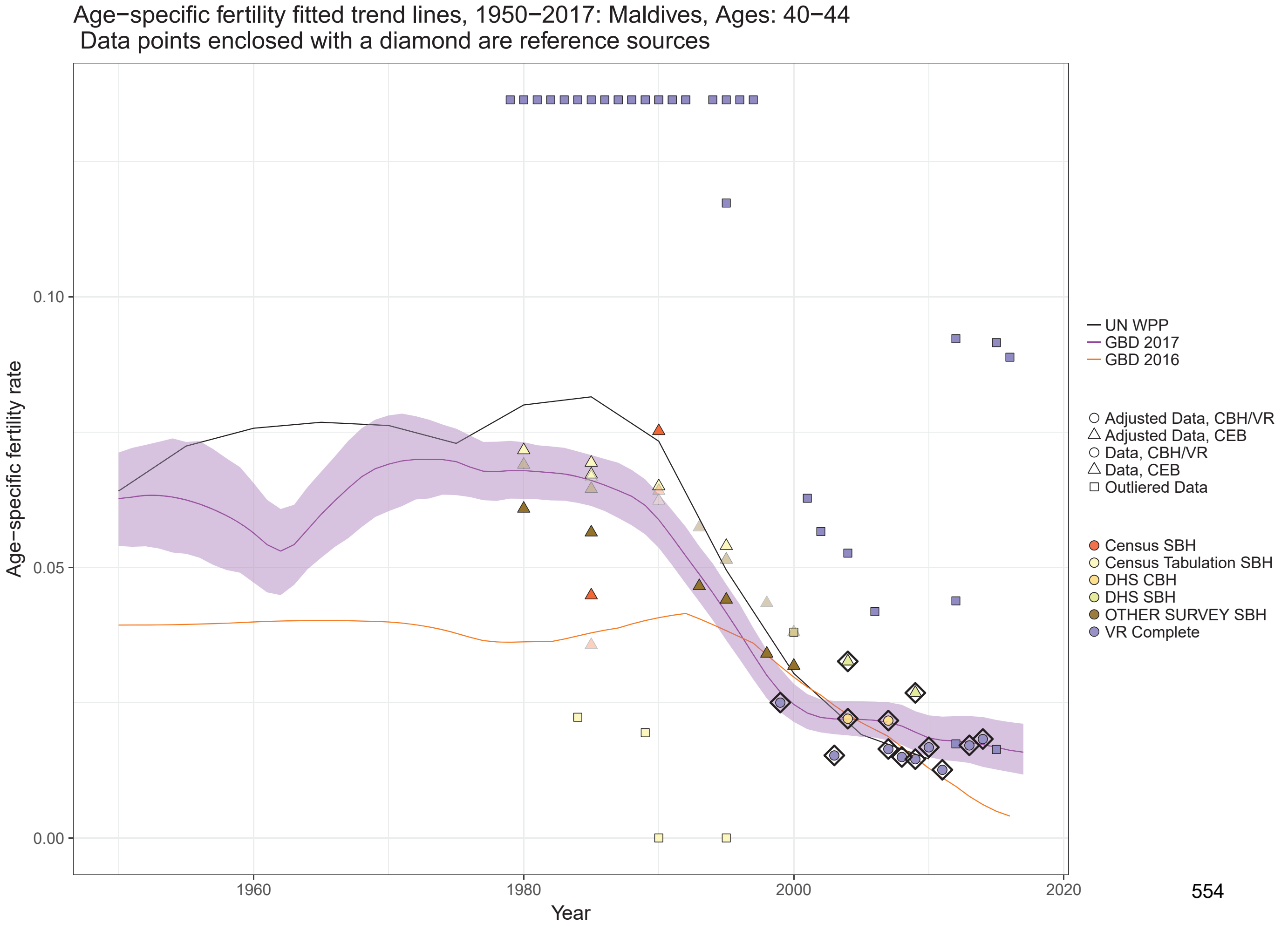

Age−specific fertility fitted trend lines, 1950−2017: Maldives, Ages: 40−44
Data points enclosed with a diamond are reference sources
Age−specific fertility rate
0.00
0.05
0.10
1960
1980
2000
2020
Year
UN WPP
GBD 2017
GBD 2016
Adjusted Data, CBH/VR
Adjusted Data, CEB
Data, CBH/VR
Data, CEB
Outliered Data
Census SBH
Census Tabulation SBH
DHS CBH
DHS SBH
OTHER SURVEY SBH
VR Complete

Age−specific fertility fitted trend lines, 1950−2017: Maldives, Ages: 45−49
Data points enclosed with a diamond are reference sources

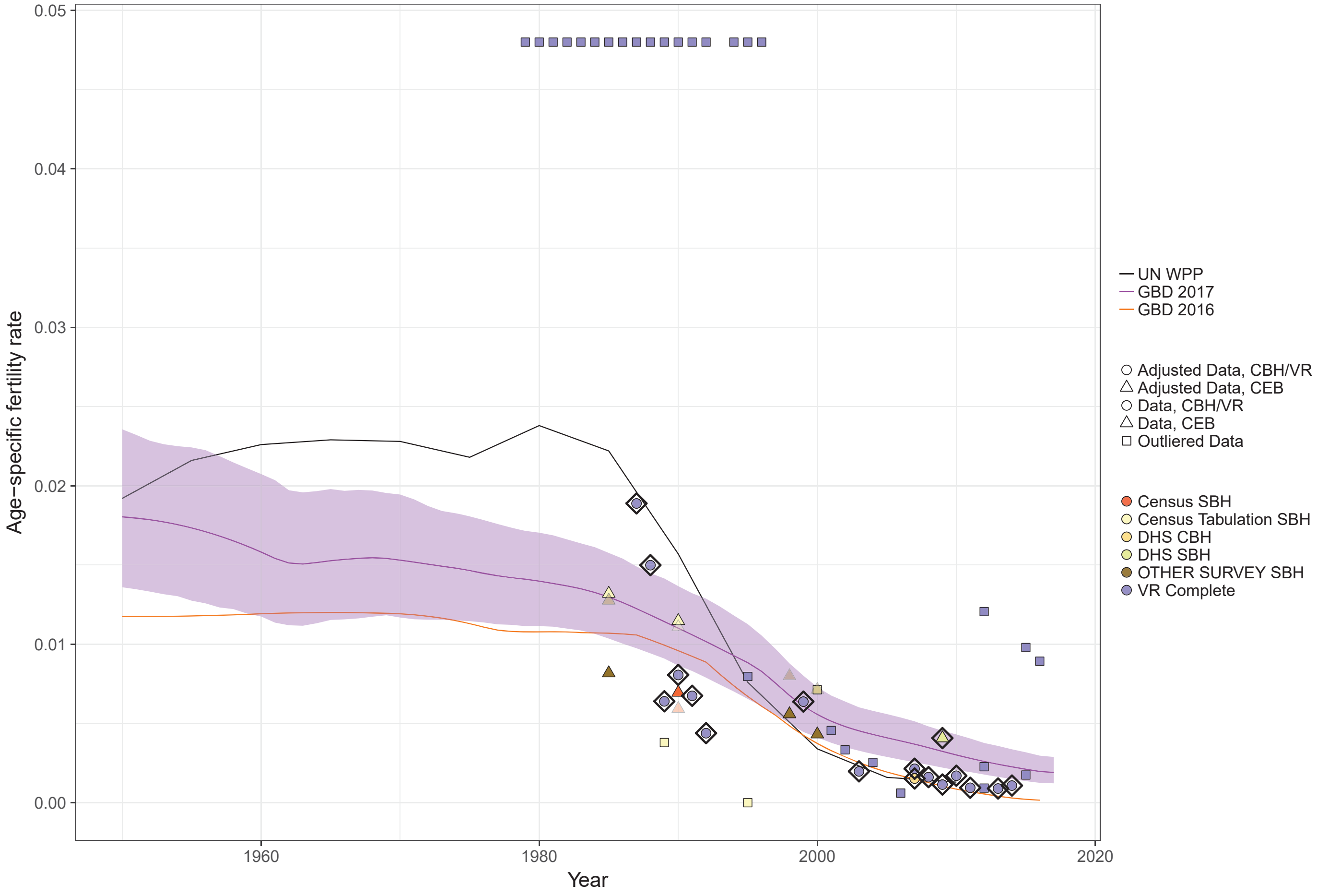

Age−specific fertility fitted trend lines, 1950−2017: Maldives, Ages: 50−54
Data points enclosed with a diamond are reference sources

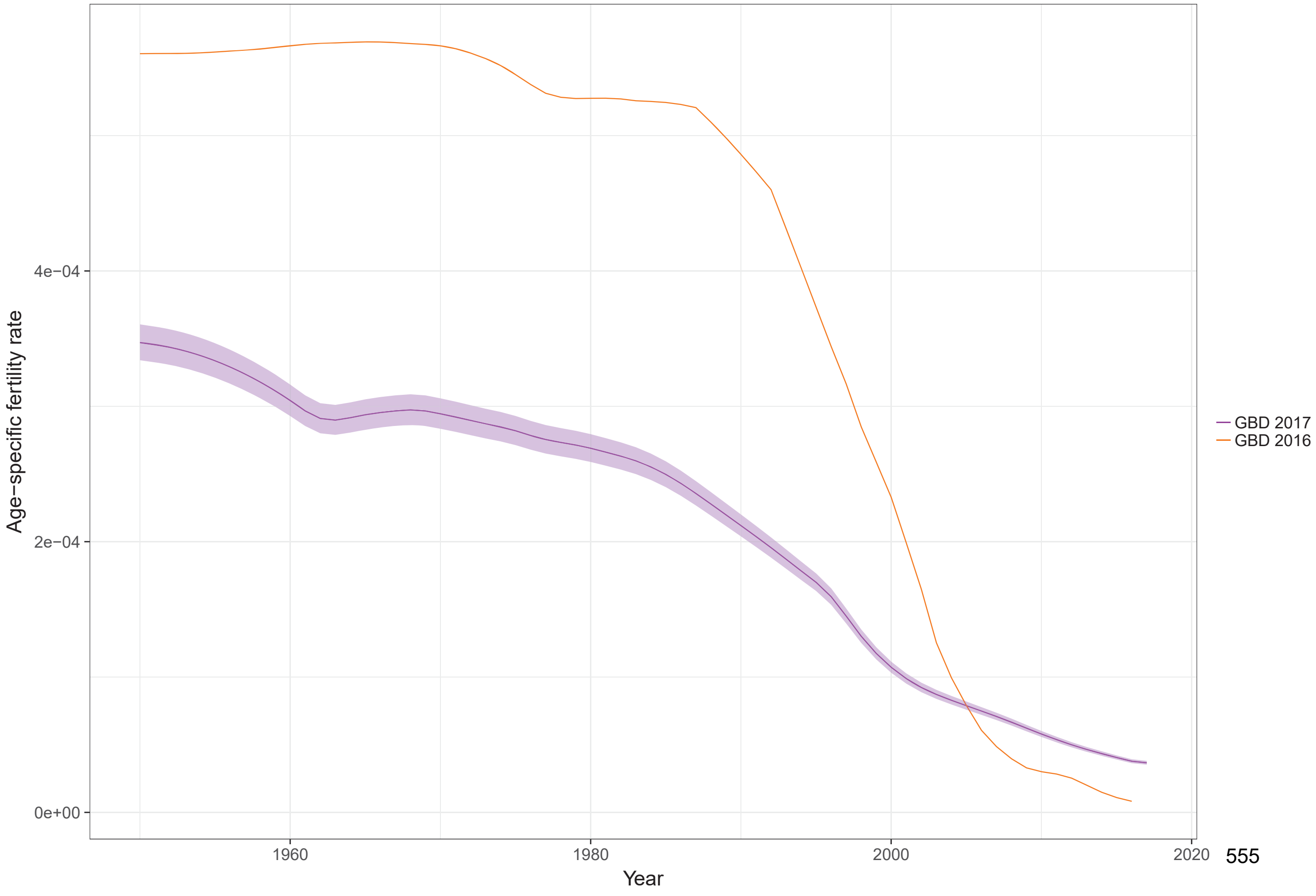

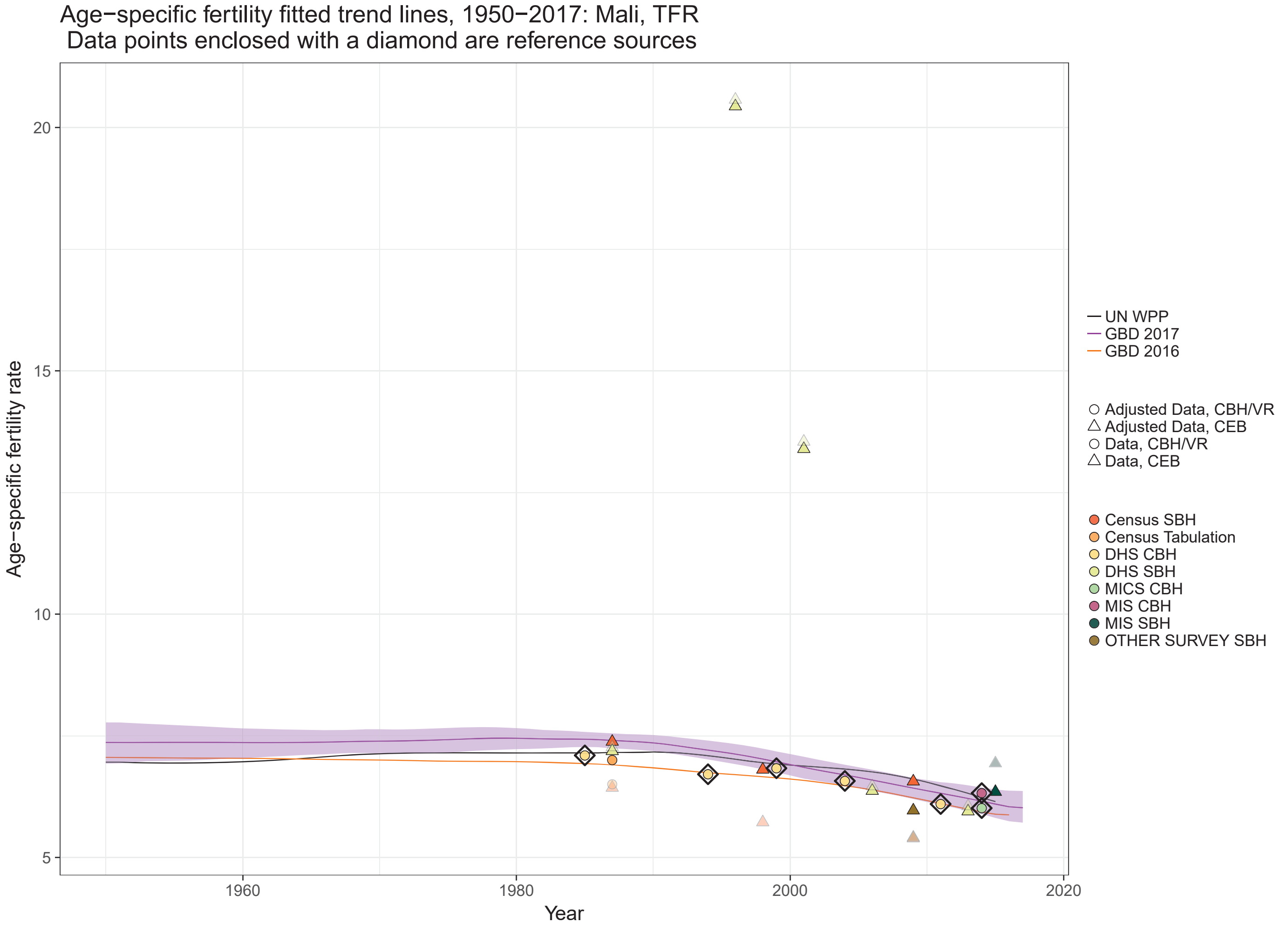

Age−specific fertility fitted trend lines, 1950−2017: Mali, TFR
Data points enclosed with a diamond are reference sources
Age−specific fertility rate
Year
20
15
10
5
1960
1980
2000
2020
UN WPP
GBD 2017
GBD 2016
Adjusted Data, CBH/VR
Adjusted Data, CEB
Data, CBH/VR
Data, CEB
Census SBH
Census Tabulation
DHS CBH
DHS SBH
MICS CBH
MIS CBH
MIS SBH
OTHER SURVEY SBH

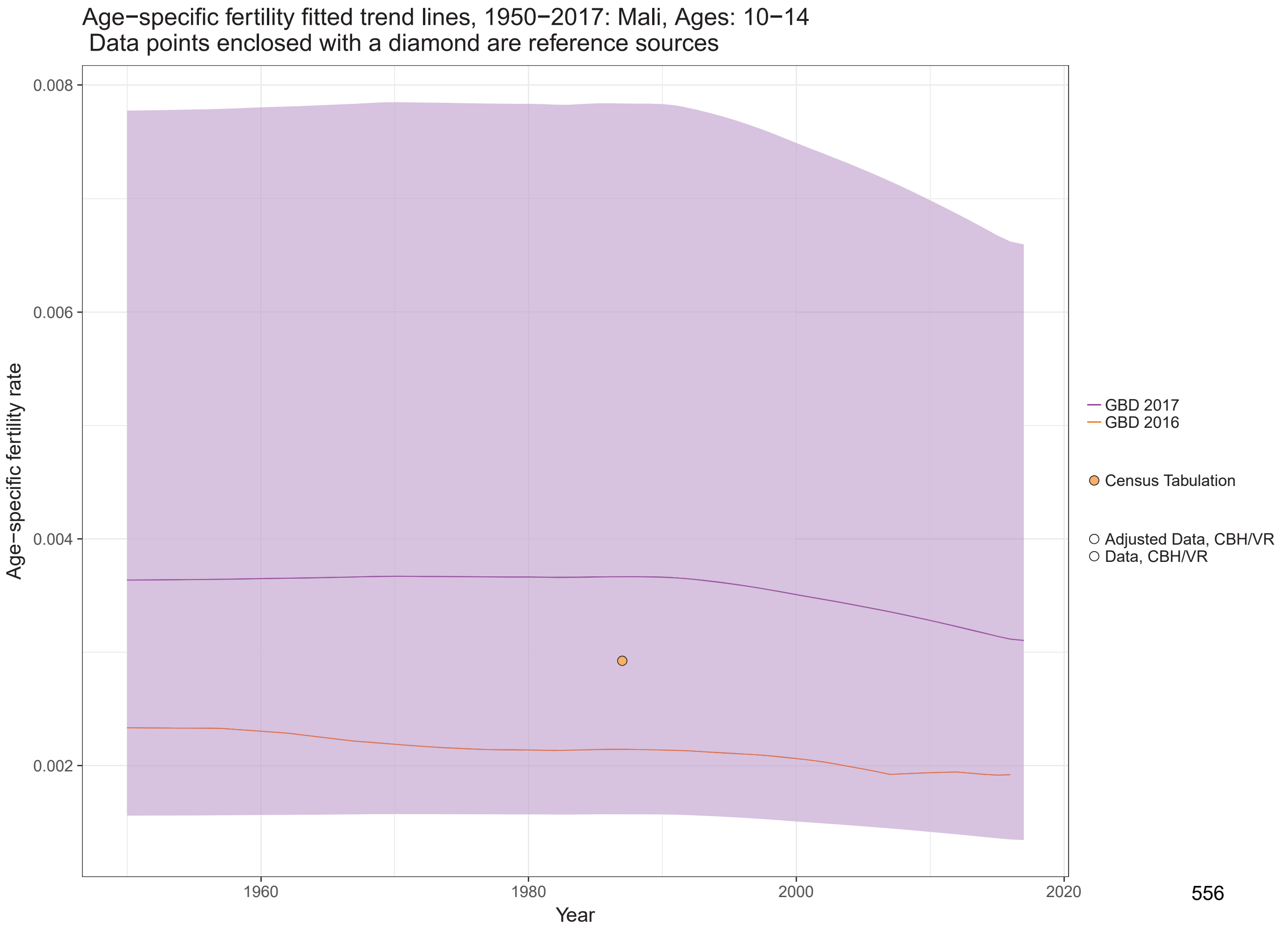

Age−specific fertility fitted trend lines, 1950−2017: Mali, Ages: 10−14
Data points enclosed with a diamond are reference sources
Age−specific fertility rate
Year
0.008
0.006
0.004
0.002
1960
1980
2000
2020
GBD 2017
GBD 2016
Census Tabulation
Adjusted Data, CBH/VR
Data, CBH/VR

Age−specific fertility fitted trend lines, 1950−2017: Mali, Ages: 15−19
Data points enclosed with a diamond are reference sources

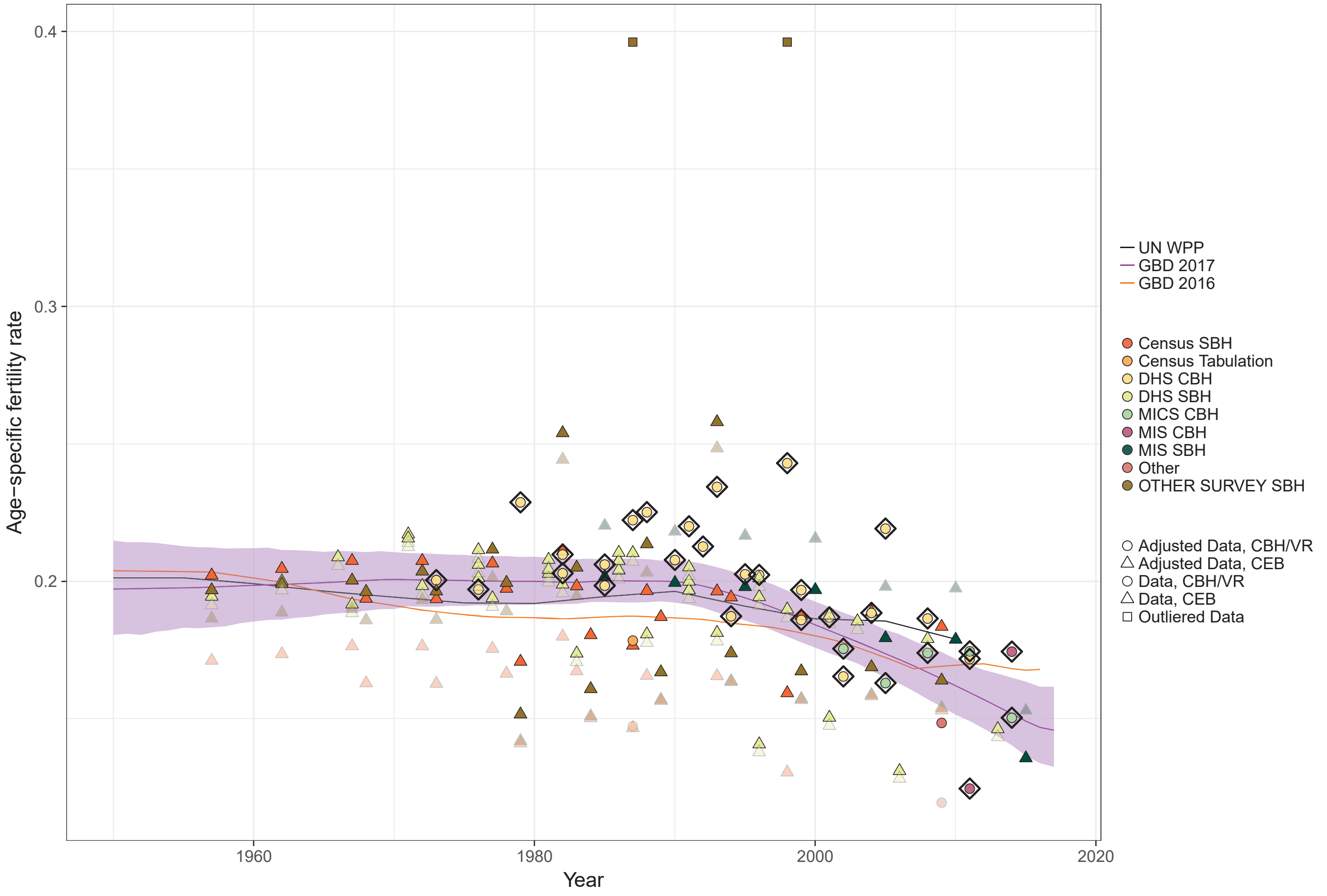

Age−specific fertility fitted trend lines, 1950−2017: Mali, Ages: 20−24
Data points enclosed with a diamond are reference sources

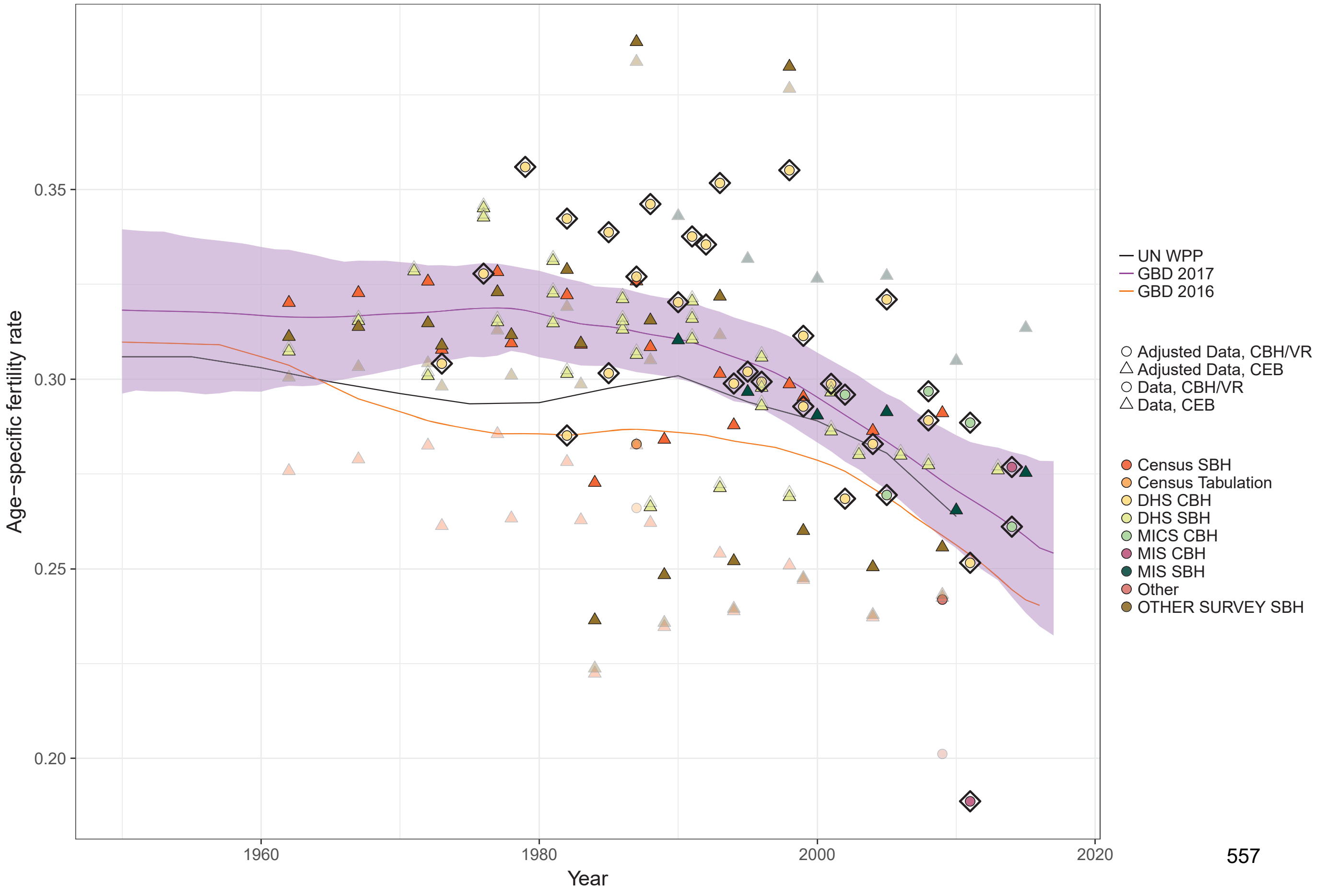

Age−specific fertility fitted trend lines, 1950−2017: Mali, Ages: 25−29
Data points enclosed with a diamond are reference sources

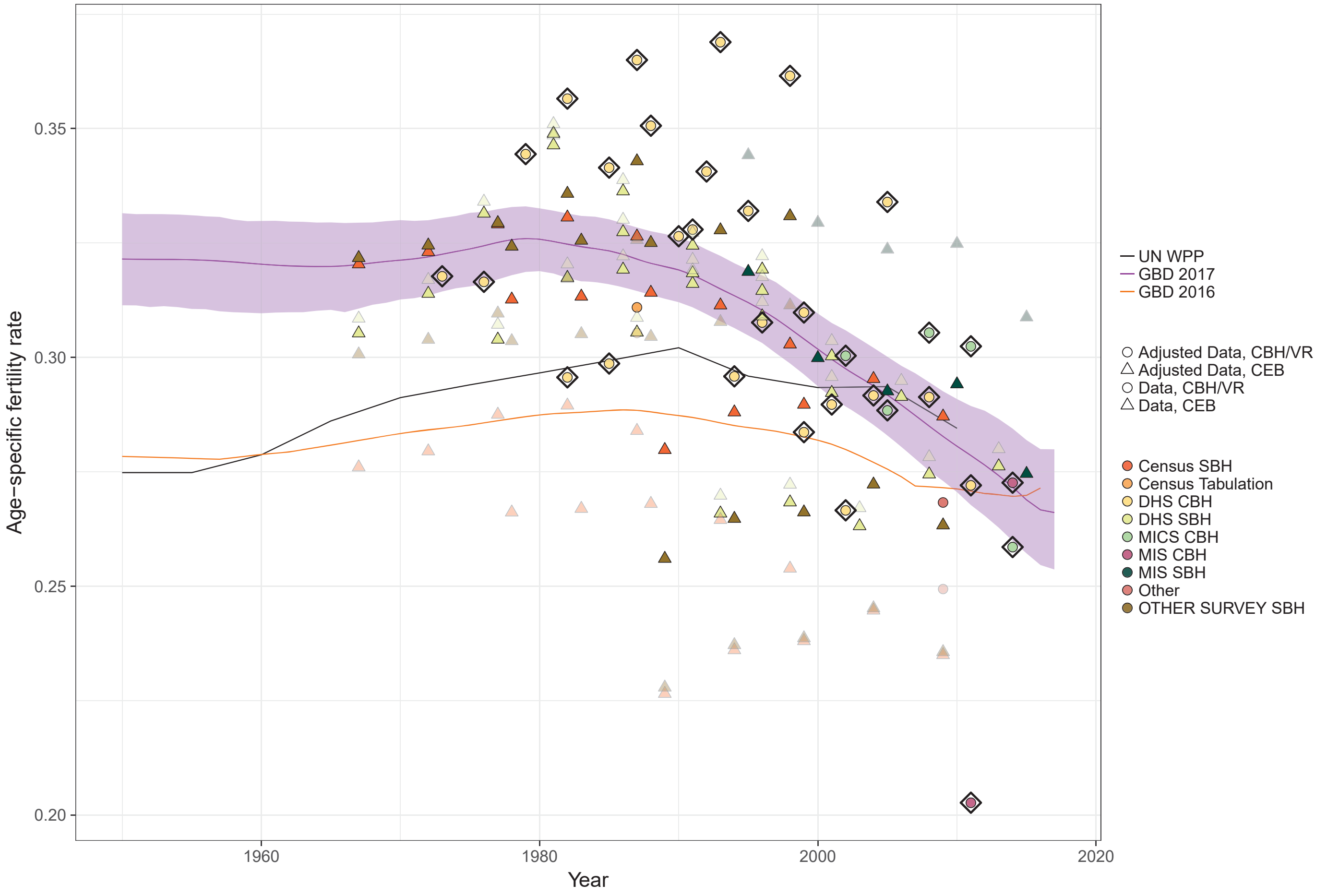

Age−specific fertility fitted trend lines, 1950−2017: Mali, Ages: 30−34
Data points enclosed with a diamond are reference sources

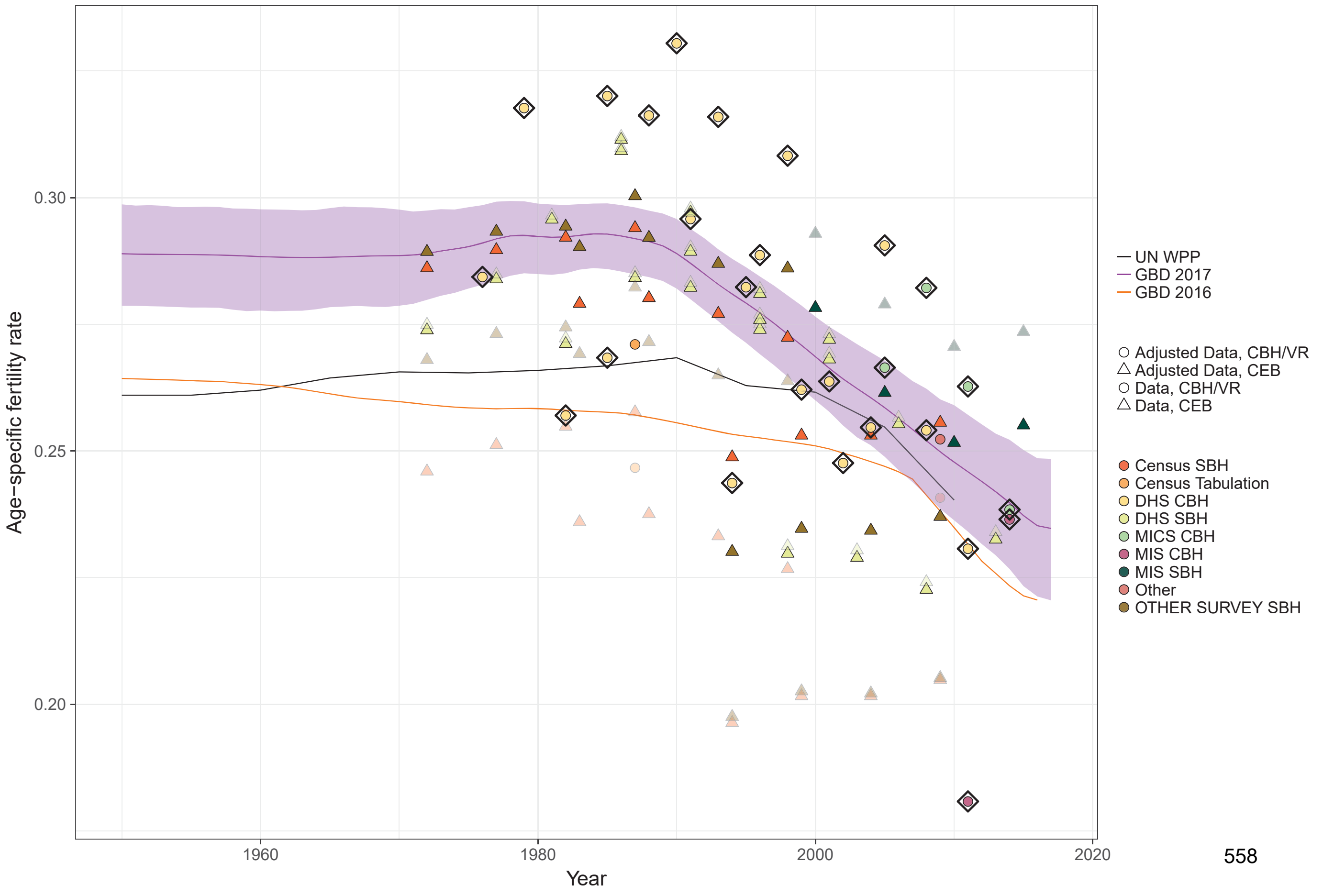

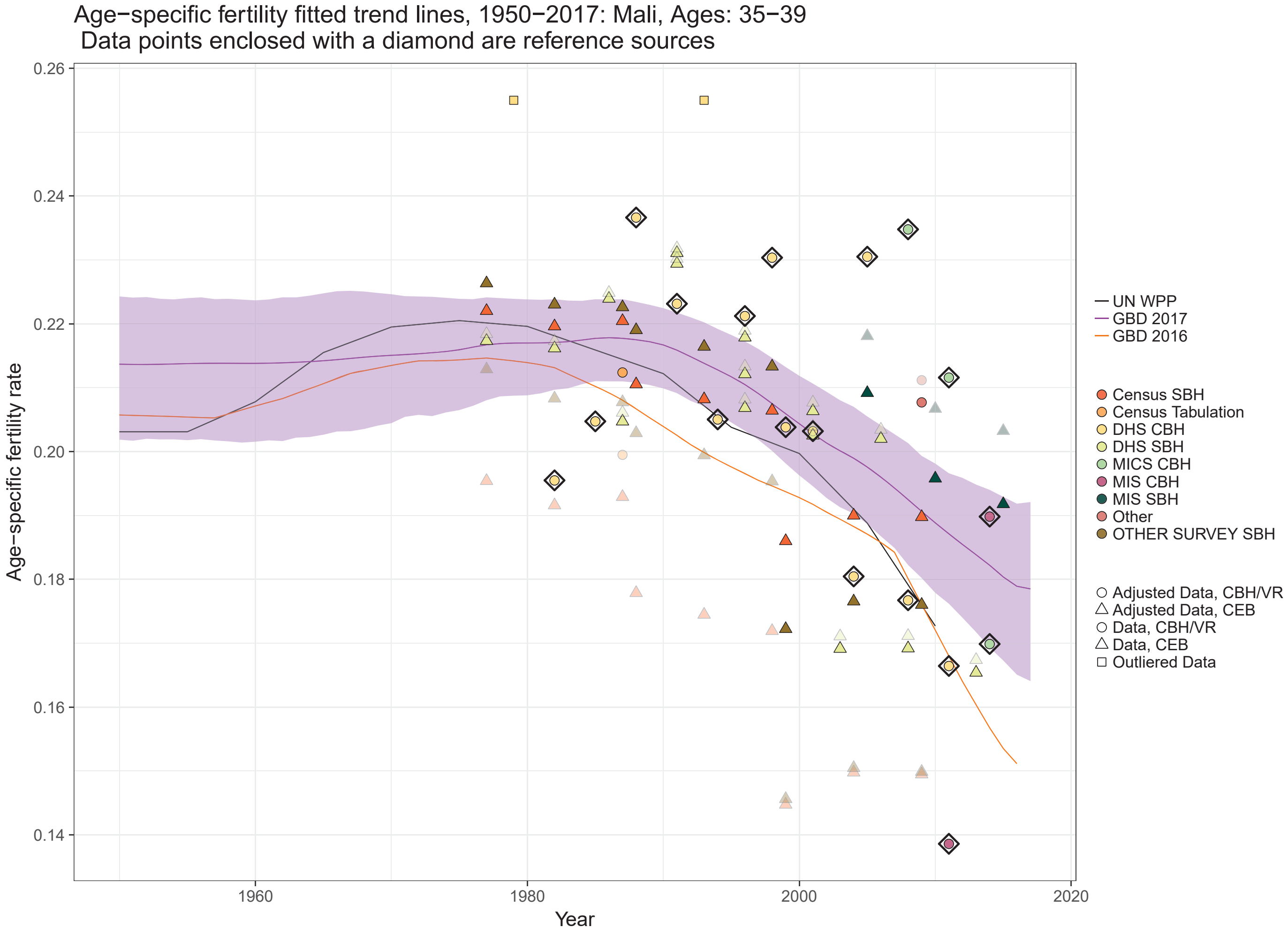
Age−specific fertility fitted trend lines, 1950−2017: Mali, Ages: 35−39
Data points enclosed with a diamond are reference sources
Age−specific fertility rate
Year
UN WPP
GBD 2017
GBD 2016
Census SBH
Census Tabulation
DHS CBH
DHS SBH
MICS CBH
MIS CBH
MIS SBH
Other
OTHER SURVEY SBH
Adjusted Data, CBH/VR
Adjusted Data, CEB
Data, CBH/VR
Data, CEB
Outliered Data

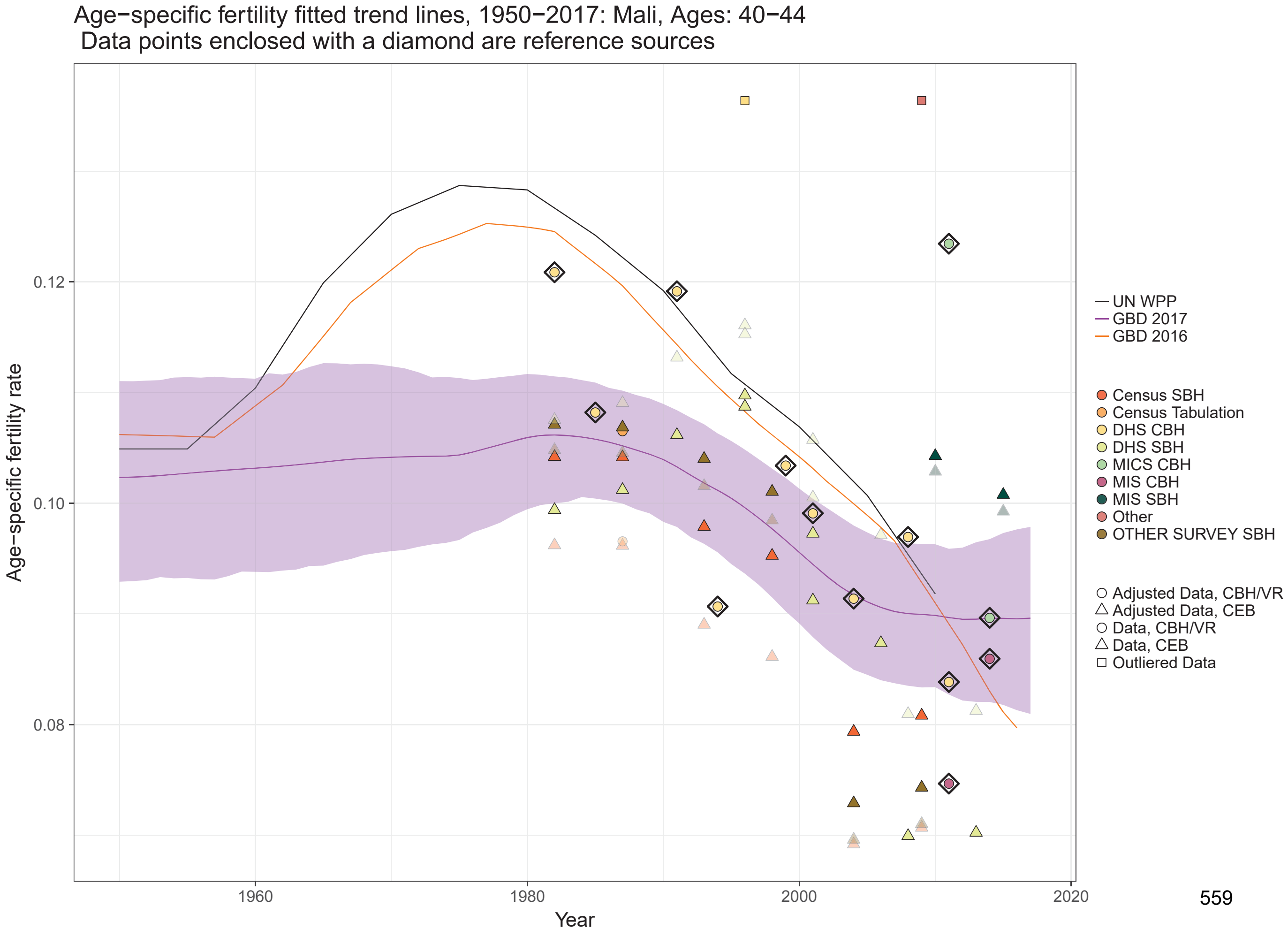
Age−specific fertility fitted trend lines, 1950−2017: Mali, Ages: 40−44
Data points enclosed with a diamond are reference sources
Age−specific fertility rate
Year
UN WPP
GBD 2017
GBD 2016
Census SBH
Census Tabulation
DHS CBH
DHS SBH
MICS CBH
MIS CBH
MIS SBH
Other
OTHER SURVEY SBH
Adjusted Data, CBH/VR
Adjusted Data, CEB
Data, CBH/VR
Data, CEB
Outliered Data

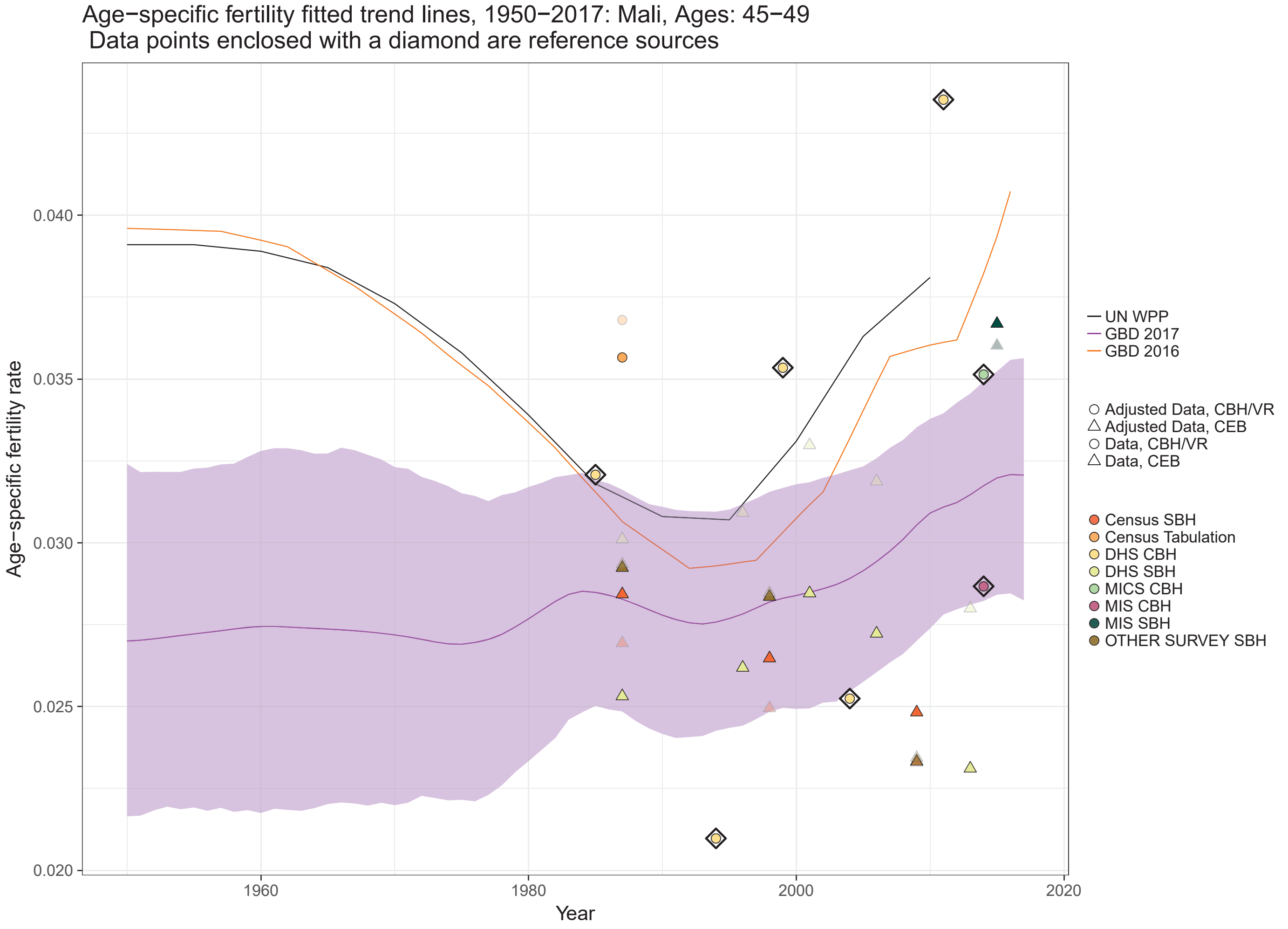

Age−specific fertility fitted trend lines, 1950−2017: Mali, Ages: 45−49
Data points enclosed with a diamond are reference sources
Age−specific fertility rate
0.020
0.025
0.030
0.035
0.040
Year
1960
1980
2000
2020
UN WPP
GBD 2017
GBD 2016
Adjusted Data, CBH/VR
Adjusted Data, CEB
Data, CBH/VR
Data, CEB
Census SBH
Census Tabulation
DHS CBH
DHS SBH
MICS CBH
MIS CBH
MIS SBH
OTHER SURVEY SBH

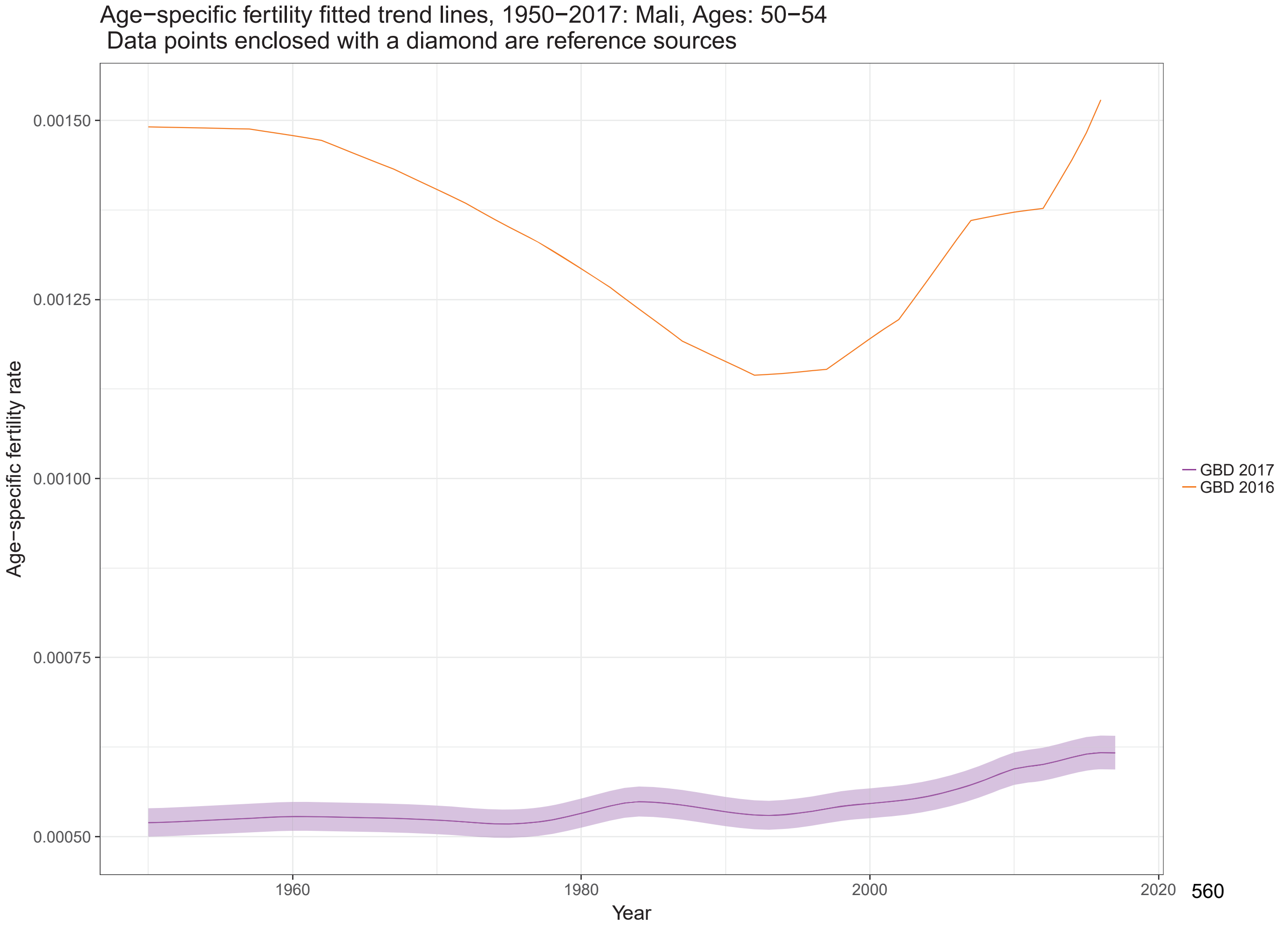

Age−specific fertility fitted trend lines, 1950−2017: Mali, Ages: 50−54
Data points enclosed with a diamond are reference sources
Age−specific fertility rate
0.00050
0.00075
0.00100
0.00125
0.00150
Year
1960
1980
2000
2020
GBD 2017
GBD 2016

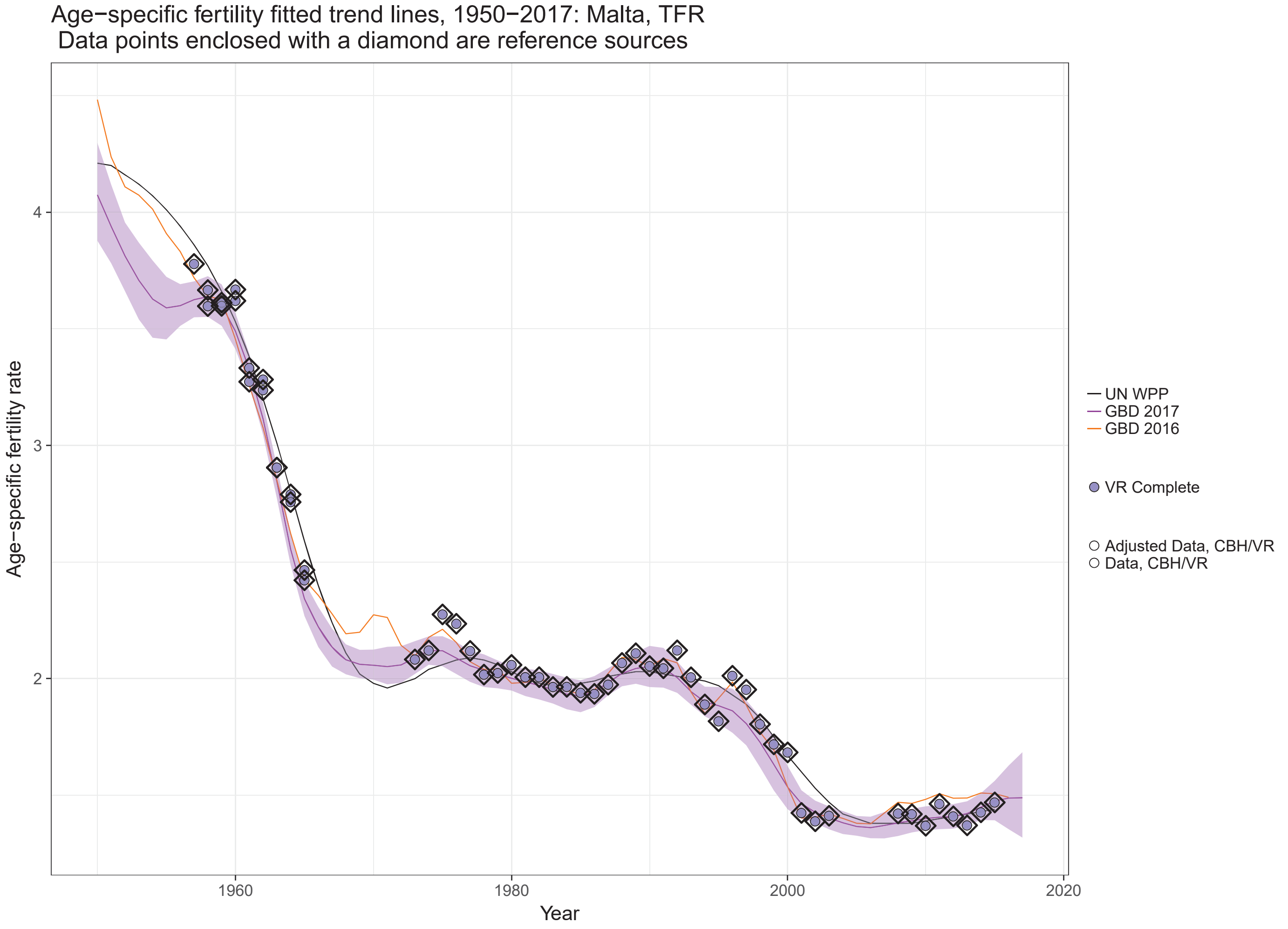

Age−specific fertility fitted trend lines, 1950−2017: Malta, TFR
Data points enclosed with a diamond are reference sources
Age−specific fertility rate
Year
4
3
2
1960
1980
2000
2020
UN WPP
GBD 2017
GBD 2016
VR Complete
Adjusted Data, CBH/VR
Data, CBH/VR

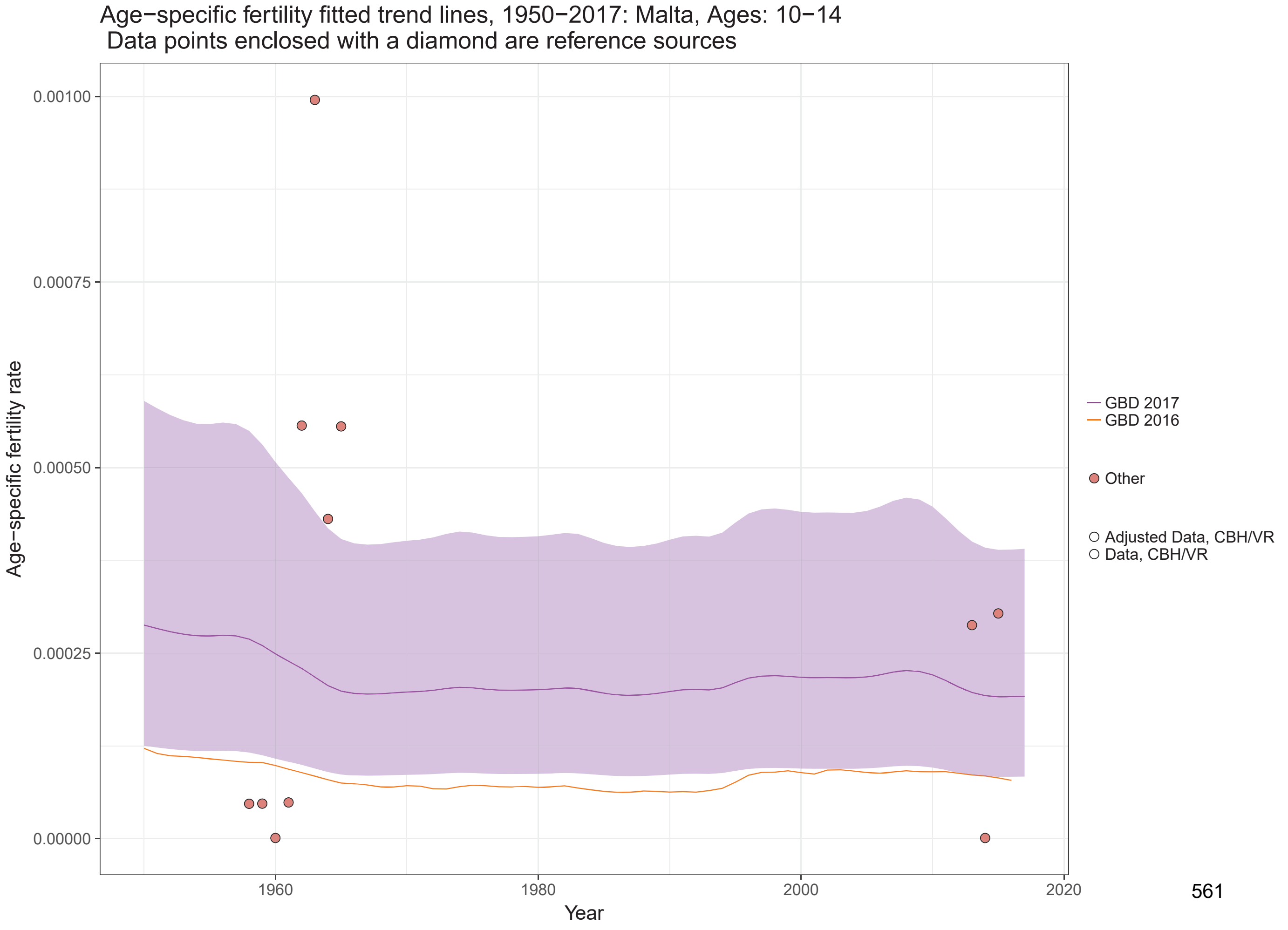

Age−specific fertility fitted trend lines, 1950−2017: Malta, Ages: 10−14
Data points enclosed with a diamond are reference sources
Age−specific fertility rate
Year
0.00100
0.00075
0.00050
0.00025
0.00000
1960
1980
2000
2020
GBD 2017
GBD 2016
Other
Adjusted Data, CBH/VR
Data, CBH/VR

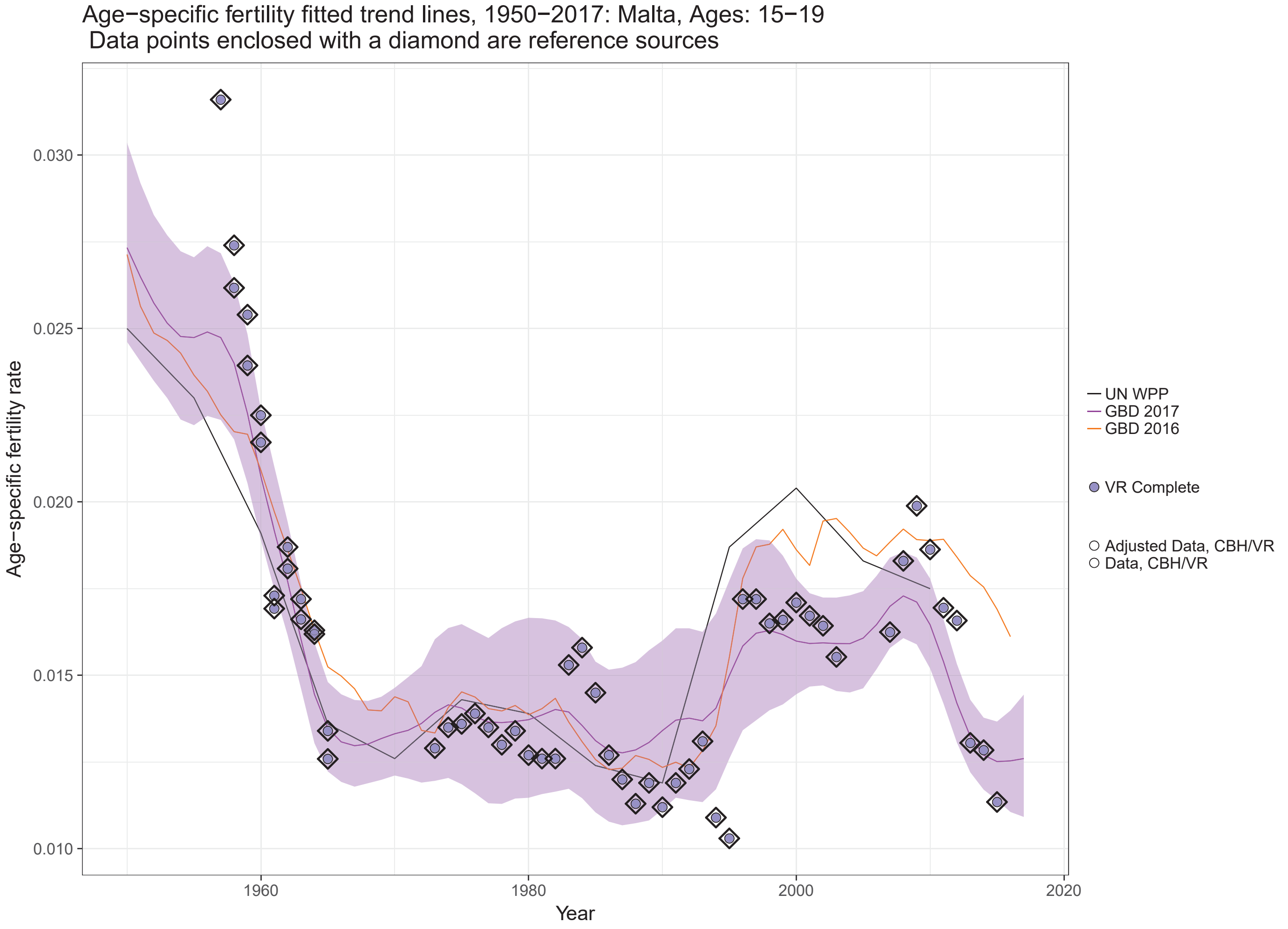

Age−specific fertility fitted trend lines, 1950−2017: Malta, Ages: 15−19
Data points enclosed with a diamond are reference sources
Age−specific fertility rate
0.030
0.025
0.020
0.015
0.010
1960
1980
2000
2020
Year
UN WPP
GBD 2017
GBD 2016
VR Complete
Adjusted Data, CBH/VR
Data, CBH/VR

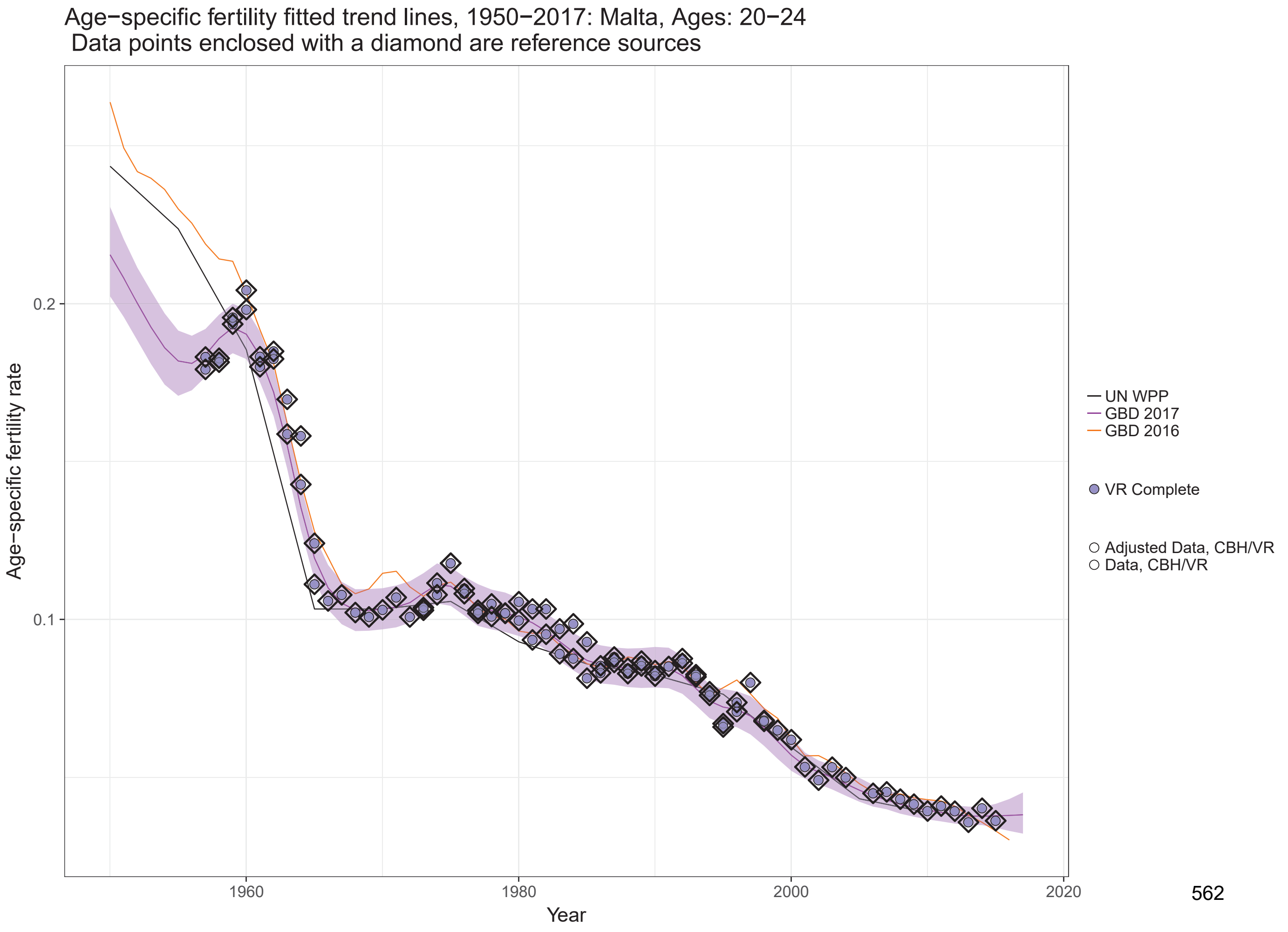

Age−specific fertility fitted trend lines, 1950−2017: Malta, Ages: 20−24
Data points enclosed with a diamond are reference sources
Age−specific fertility rate
0.2
0.1
1960
1980
2000
2020
Year
UN WPP
GBD 2017
GBD 2016
VR Complete
Adjusted Data, CBH/VR
Data, CBH/VR

Age−specific fertility fitted trend lines, 1950−2017: Malta, Ages: 25−29
Data points enclosed with a diamond are reference sources

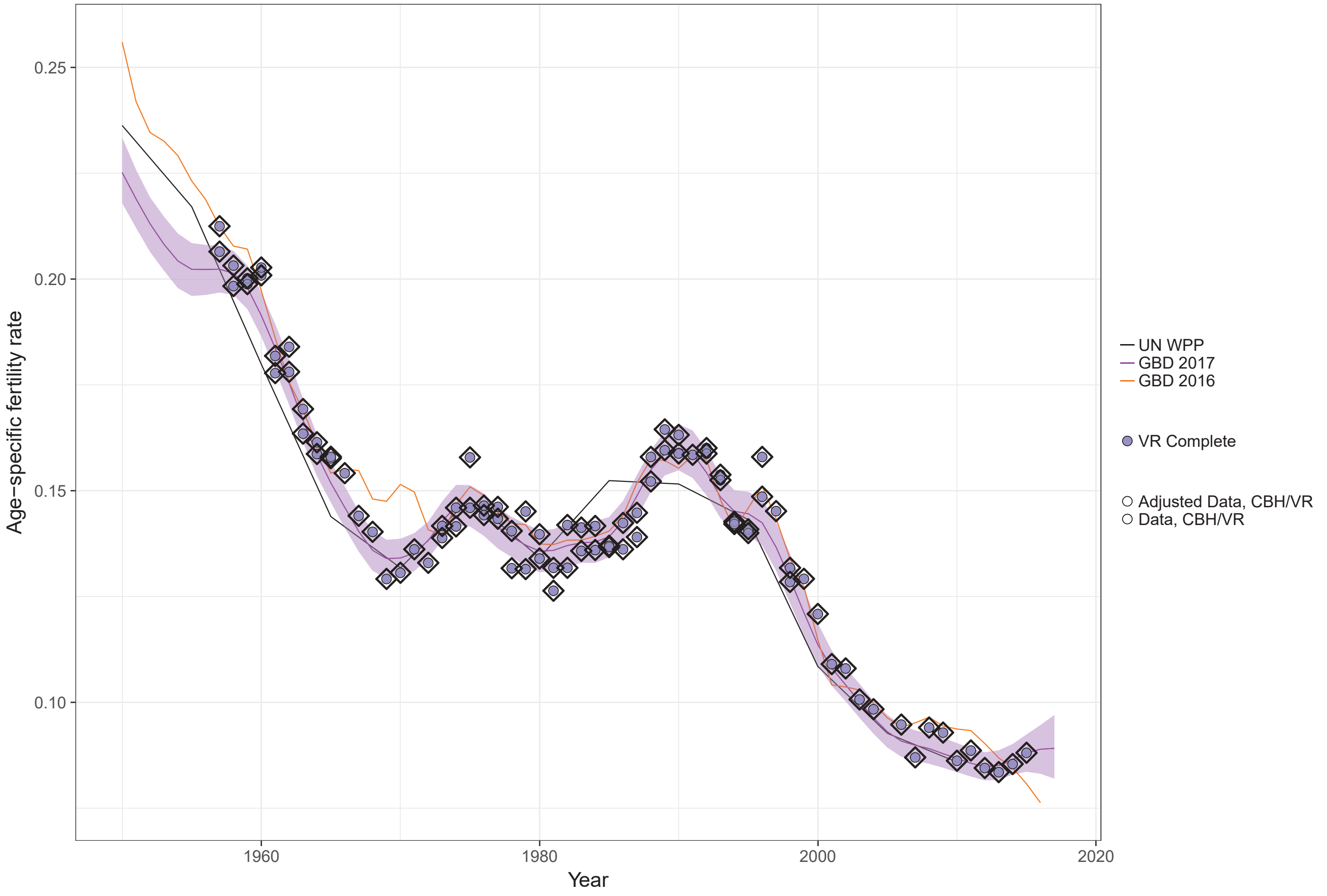

Age−specific fertility fitted trend lines, 1950−2017: Malta, Ages: 30−34
Data points enclosed with a diamond are reference sources

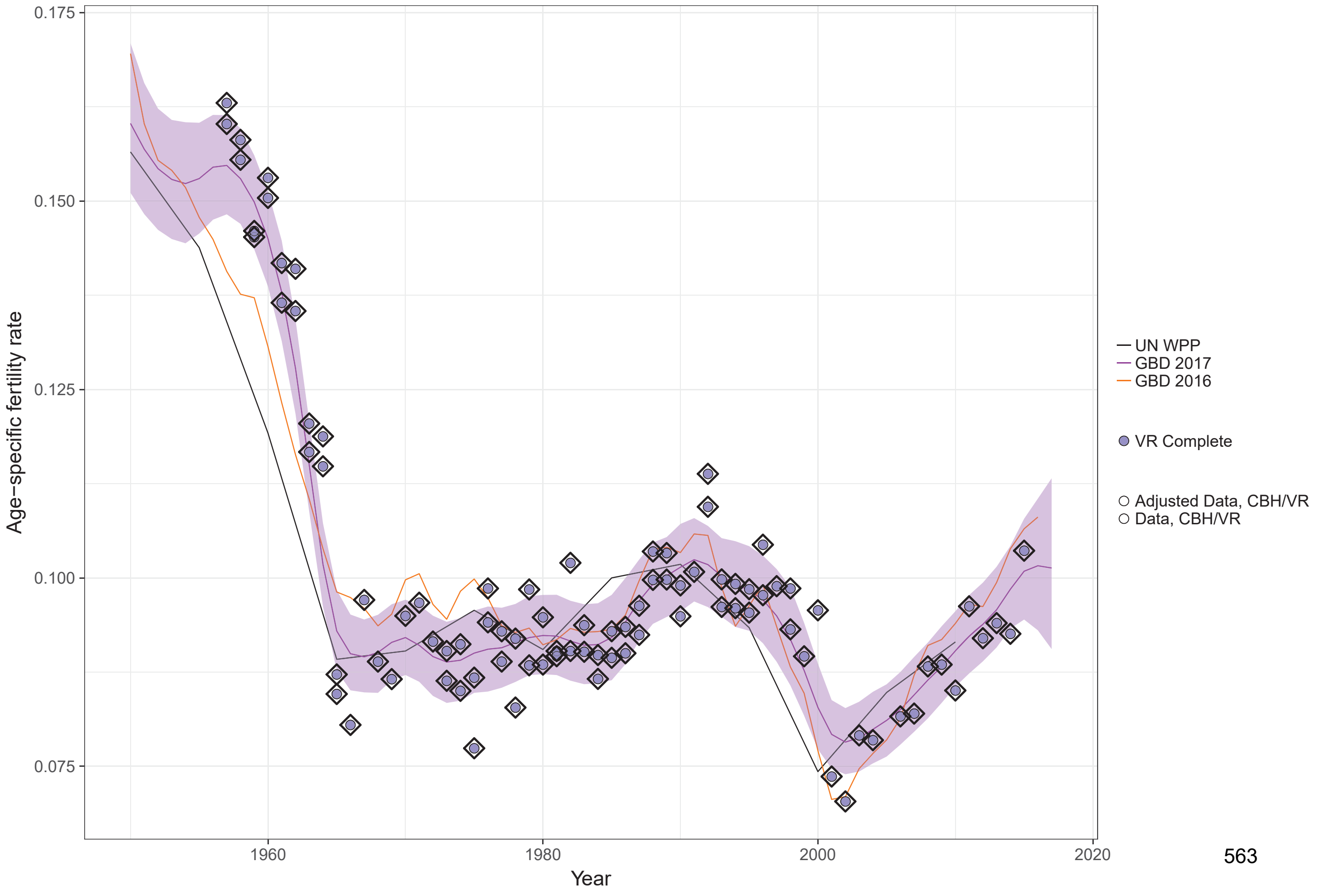

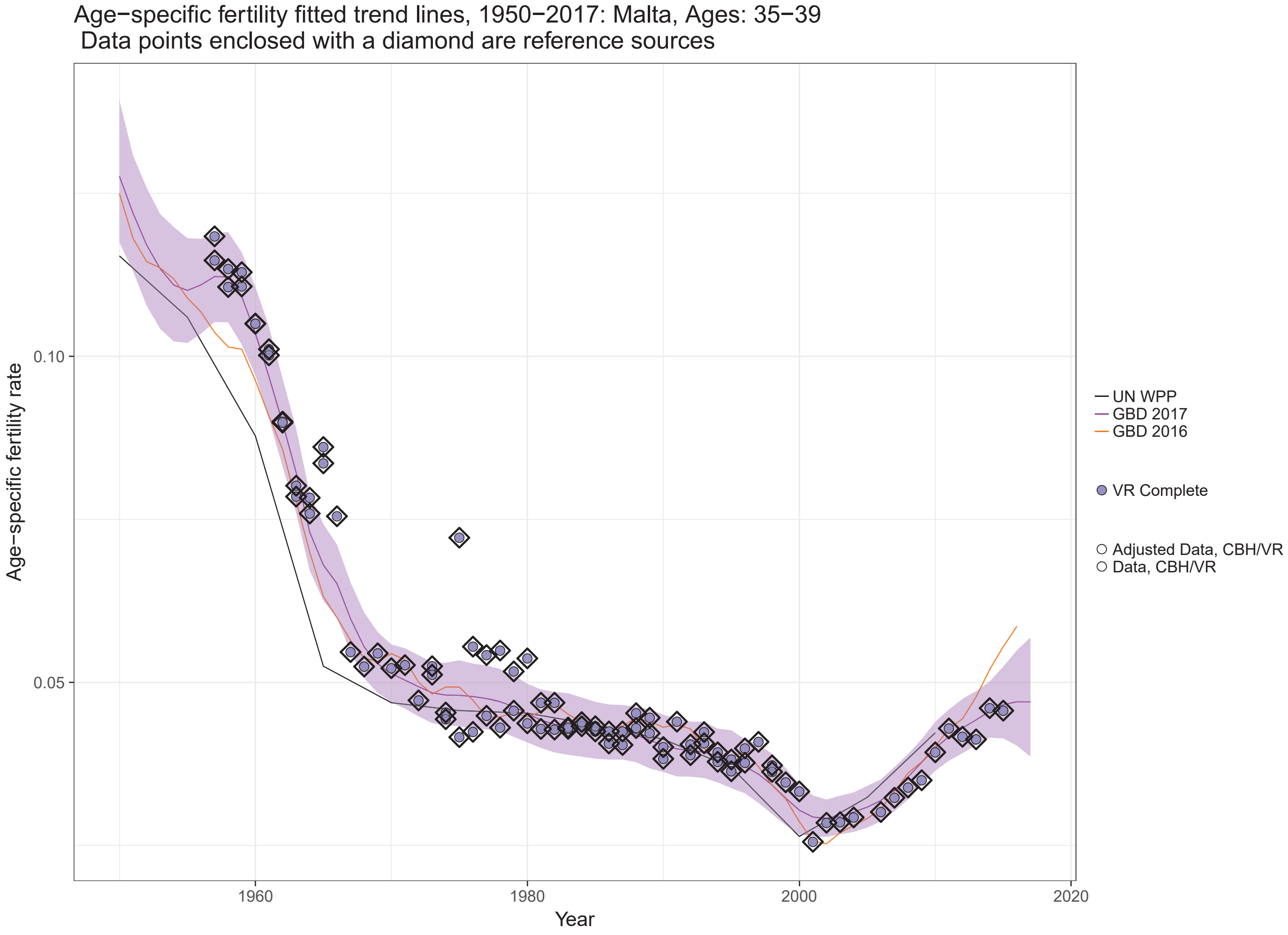
Age−specific fertility fitted trend lines, 1950−2017: Malta, Ages: 35−39
Data points enclosed with a diamond are reference sources
Age−specific fertility rate
0.10
0.05
1960
1980
2000
2020
Year
UN WPP
GBD 2017
GBD 2016
VR Complete
Adjusted Data, CBH/VR
Data, CBH/VR

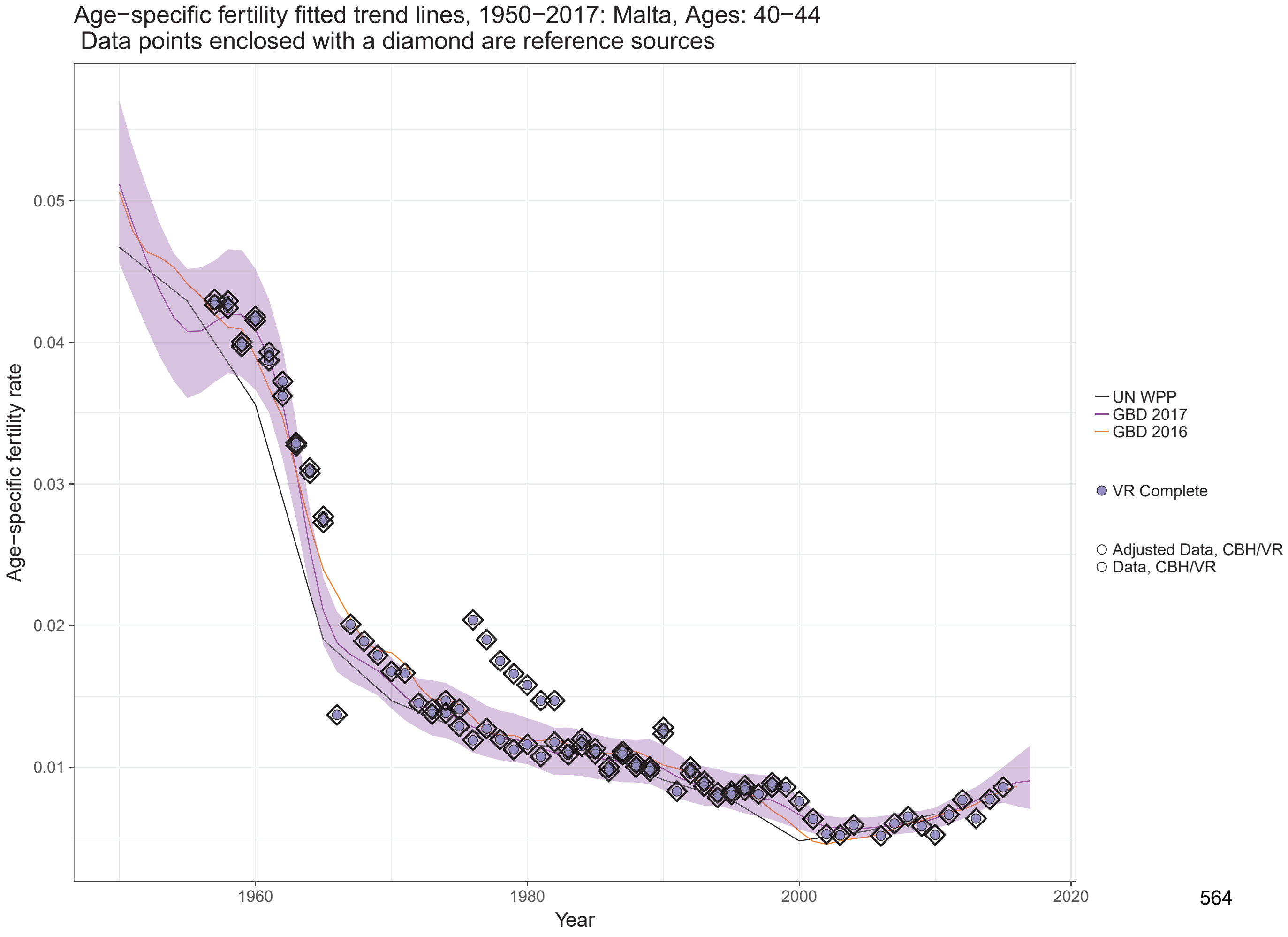
Age−specific fertility fitted trend lines, 1950−2017: Malta, Ages: 40−44
Data points enclosed with a diamond are reference sources
Age−specific fertility rate
0.05
0.04
0.03
0.02
0.01
1960
1980
2000
2020
Year
UN WPP
GBD 2017
GBD 2016
VR Complete
Adjusted Data, CBH/VR
Data, CBH/VR

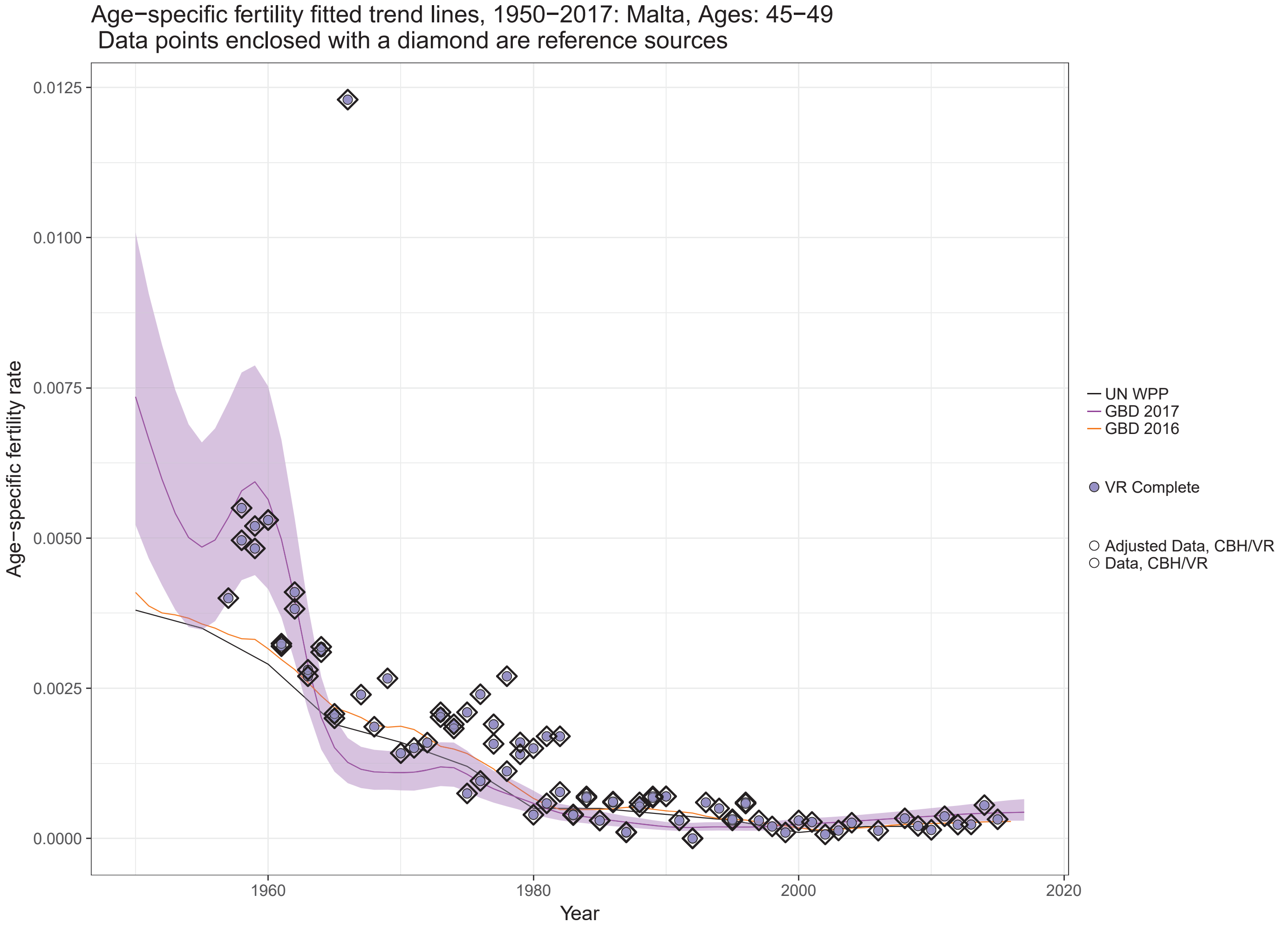
Age−specific fertility fitted trend lines, 1950−2017: Malta, Ages: 45−49
Data points enclosed with a diamond are reference sources
0.0125
0.0100
0.0075
0.0050
0.0025
0.0000
Age−specific fertility rate
1960
1980
2000
2020
Year
UN WPP
GBD 2017
GBD 2016
VR Complete
Adjusted Data, CBH/VR
Data, CBH/VR

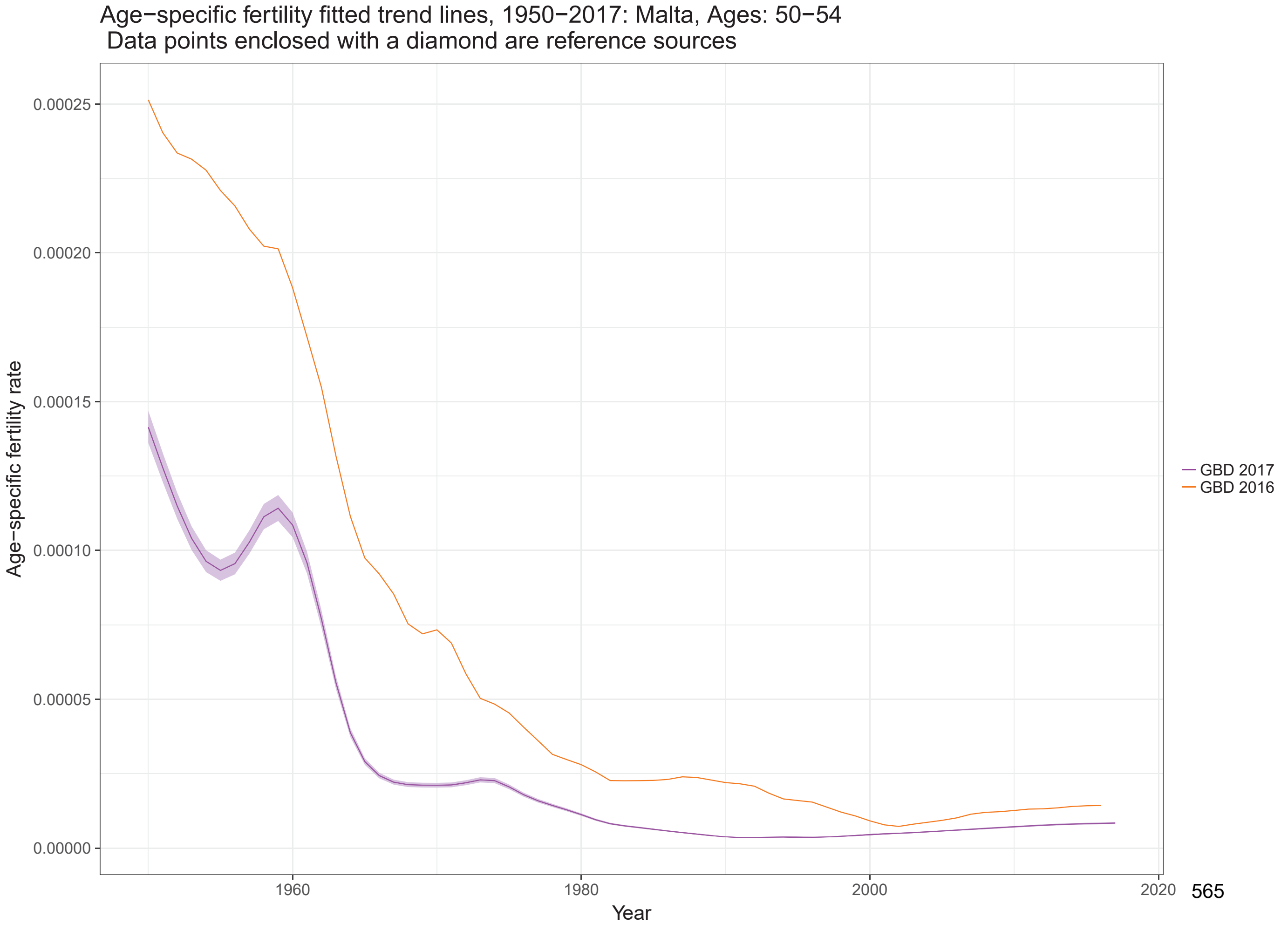
Age−specific fertility fitted trend lines, 1950−2017: Malta, Ages: 50−54
Data points enclosed with a diamond are reference sources
0.00025
0.00020
0.00015
0.00010
0.00005
0.00000
Age−specific fertility rate
1960
1980
2000
2020
Year
GBD 2017
GBD 2016

Age−specific fertility fitted trend lines, 1950−2017: Marshall Islands, TFR
Data points enclosed with a diamond are reference sources

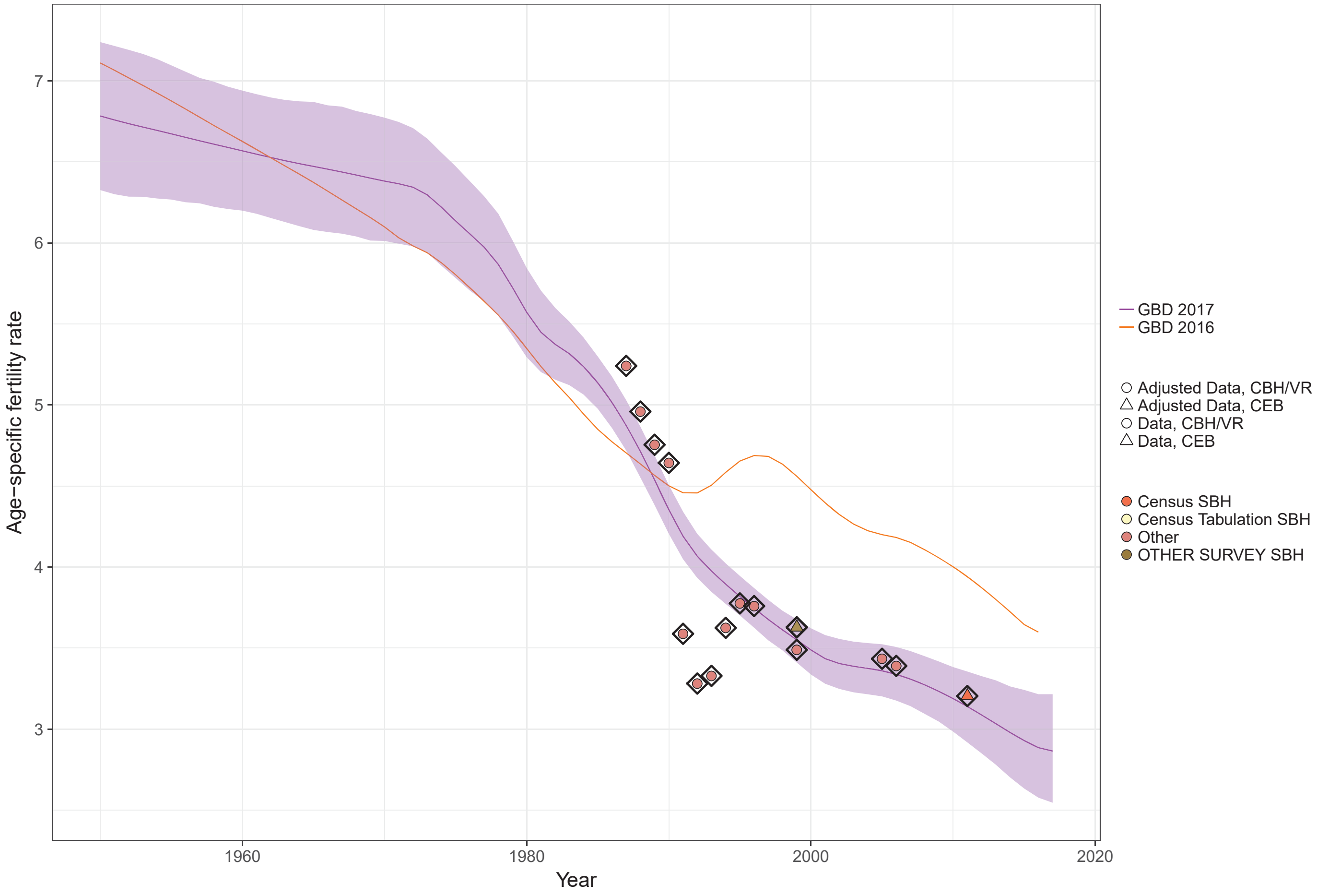

Age−specific fertility fitted trend lines, 1950−2017: Marshall Islands, Ages: 10−14
Data points enclosed with a diamond are reference sources

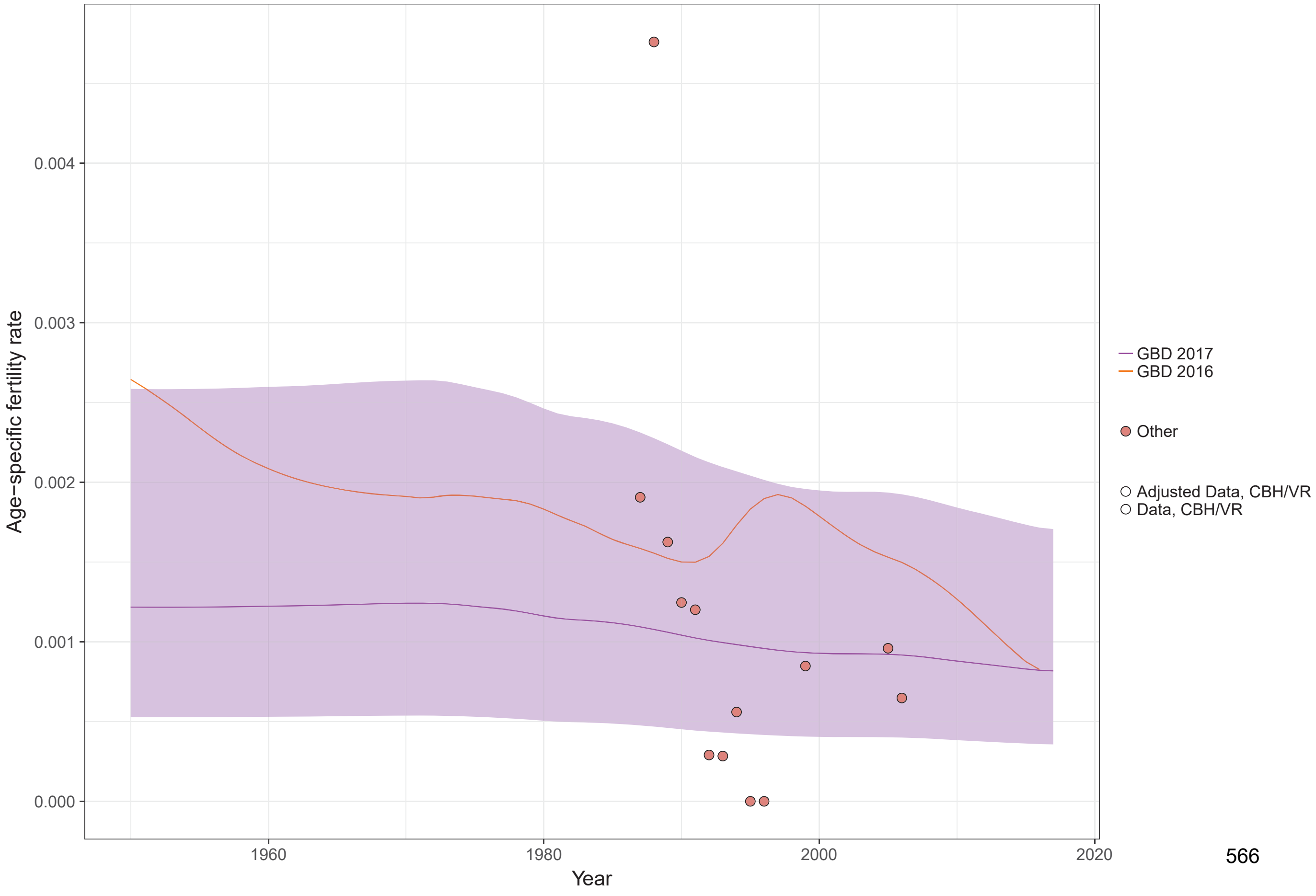

Age−specific fertility fitted trend lines, 1950−2017: Marshall Islands, Ages: 15−19
Data points enclosed with a diamond are reference sources

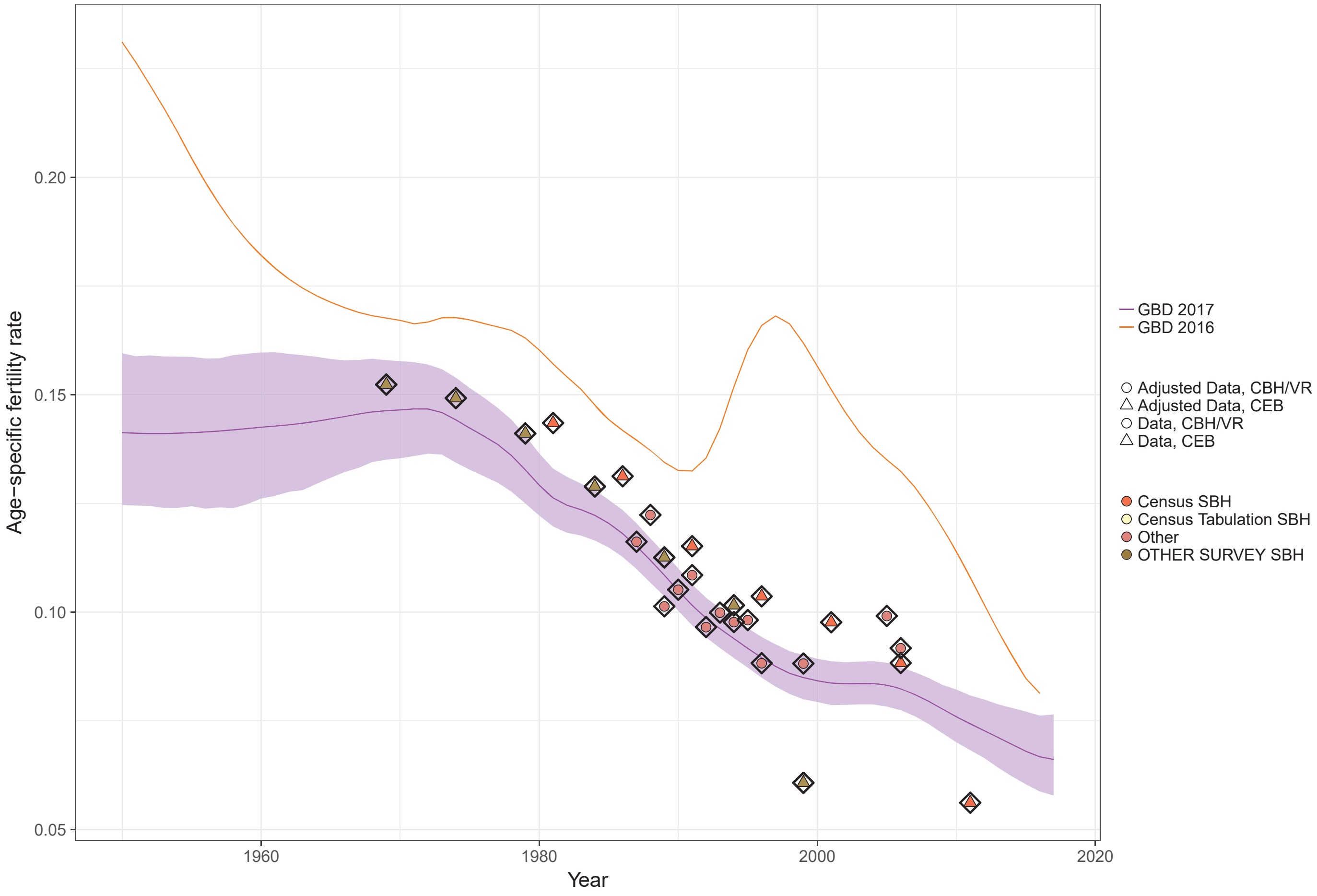

Age−specific fertility fitted trend lines, 1950−2017: Marshall Islands, Ages: 20−24
Data points enclosed with a diamond are reference sources

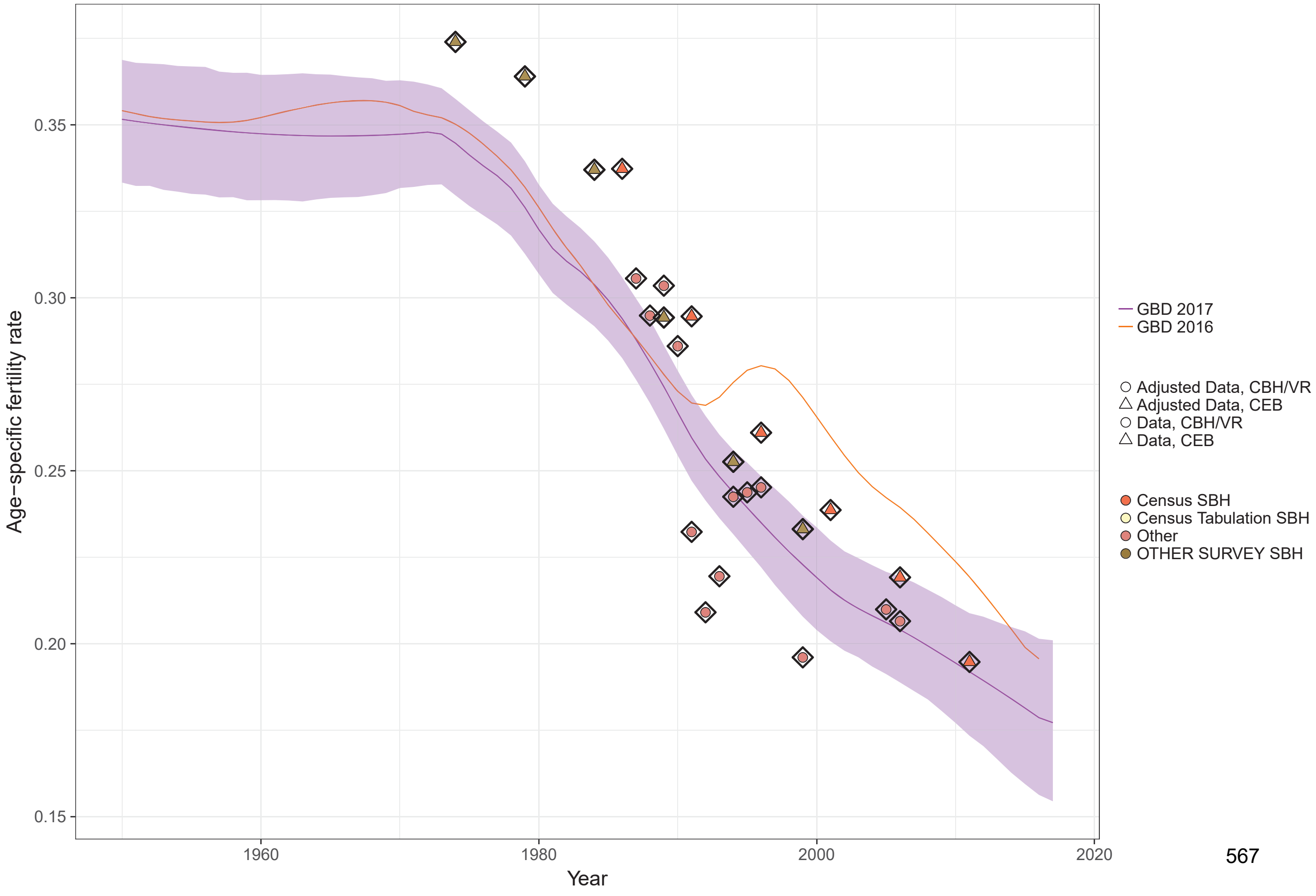

Age−specific fertility fitted trend lines, 1950−2017: Marshall Islands, Ages: 25−29
Data points enclosed with a diamond are reference sources

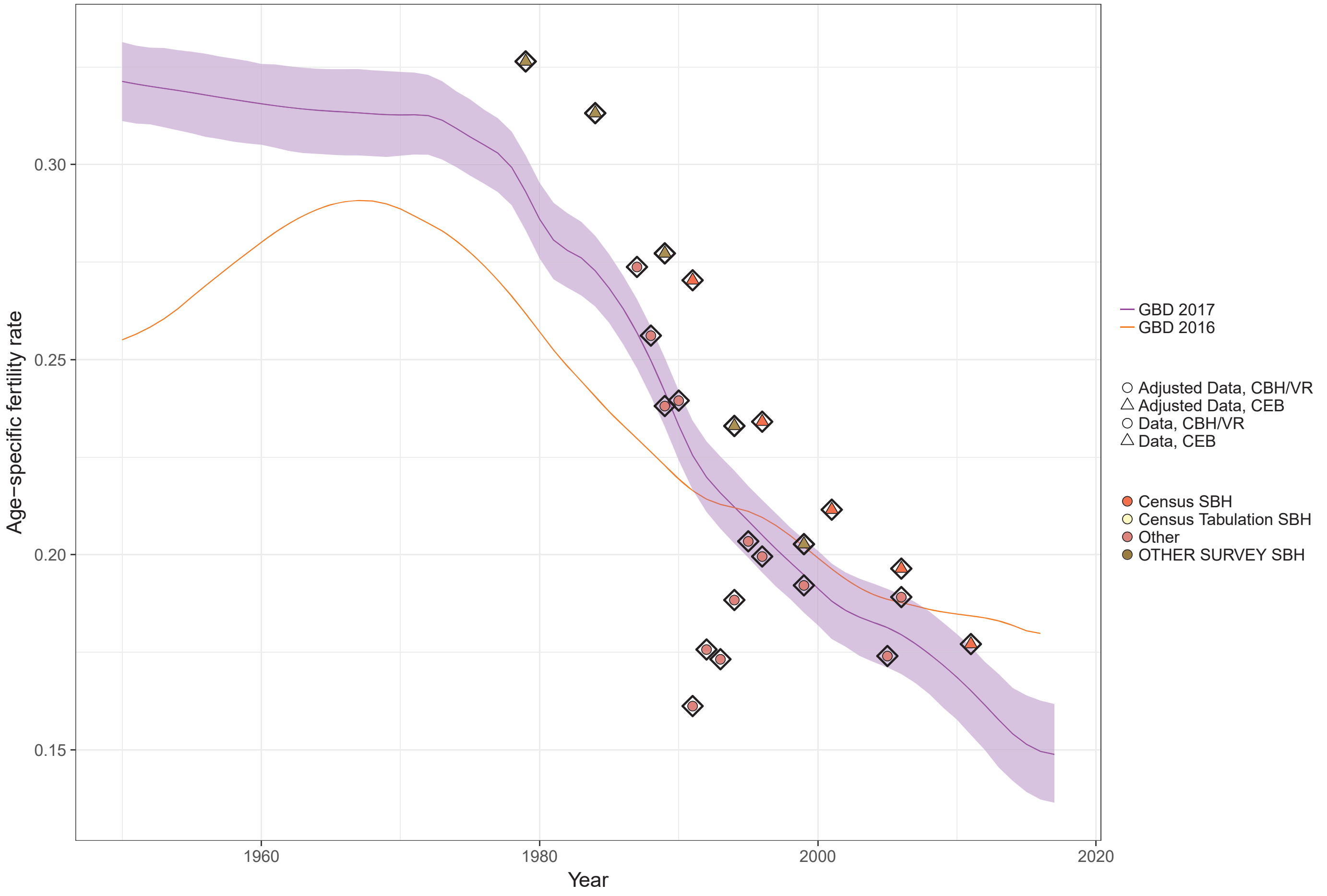

Age−specific fertility fitted trend lines, 1950−2017: Marshall Islands, Ages: 30−34
Data points enclosed with a diamond are reference sources

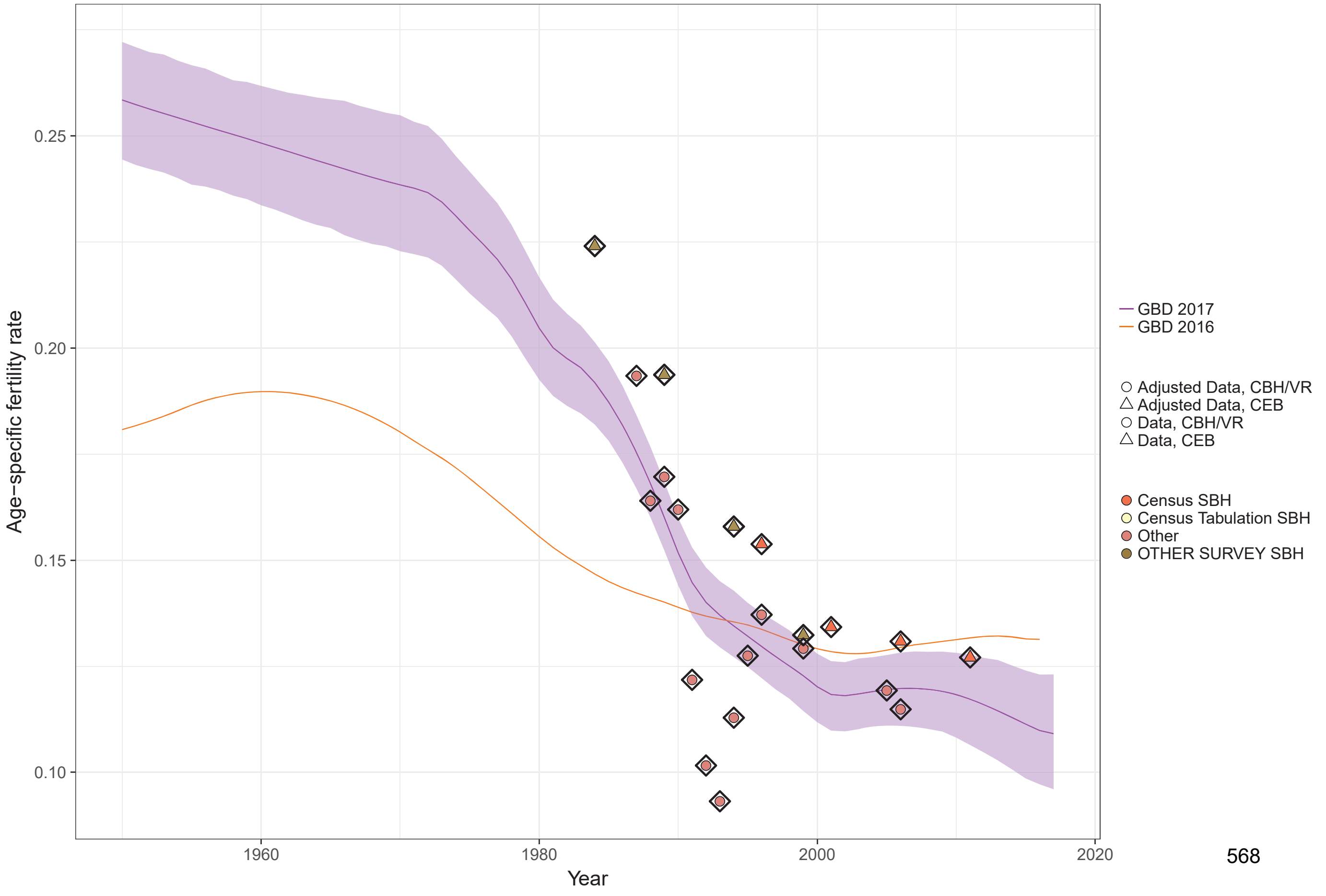

Age−specific fertility fitted trend lines, 1950−2017: Marshall Islands, Ages: 35−39
Data points enclosed with a diamond are reference sources

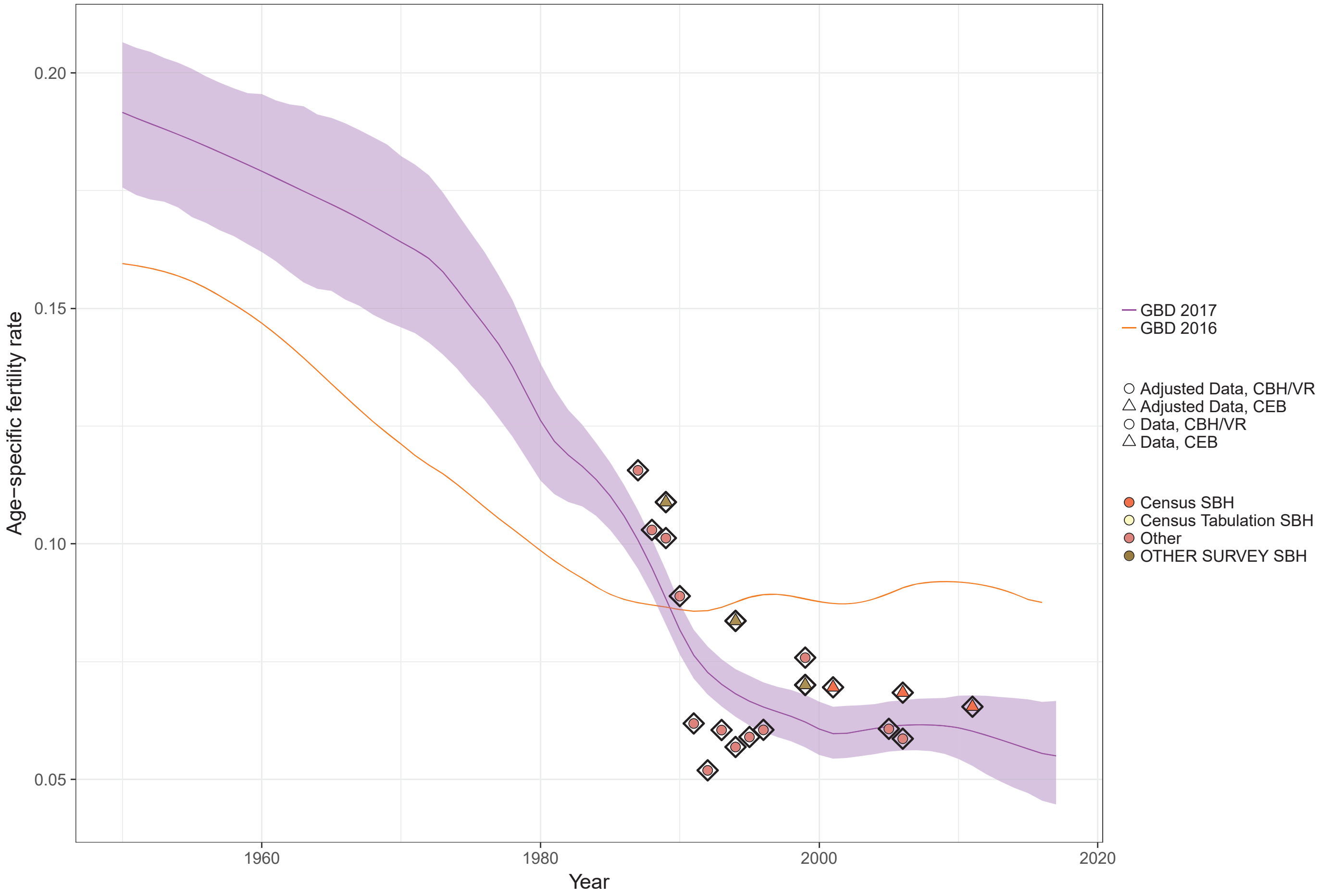

Age−specific fertility fitted trend lines, 1950−2017: Marshall Islands, Ages: 40−44
Data points enclosed with a diamond are reference sources

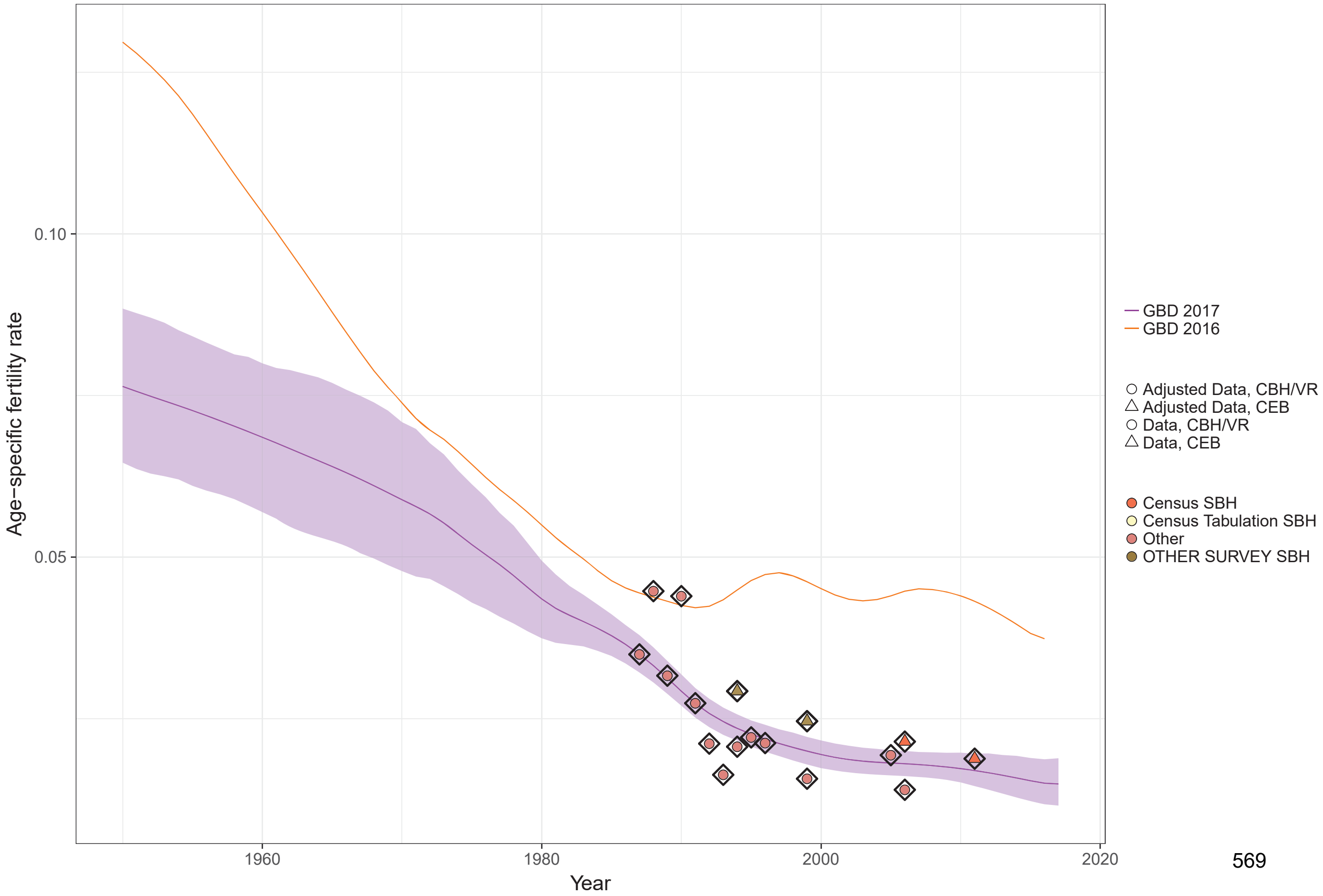

Age−specific fertility fitted trend lines, 1950−2017: Marshall Islands, Ages: 45−49
Data points enclosed with a diamond are reference sources

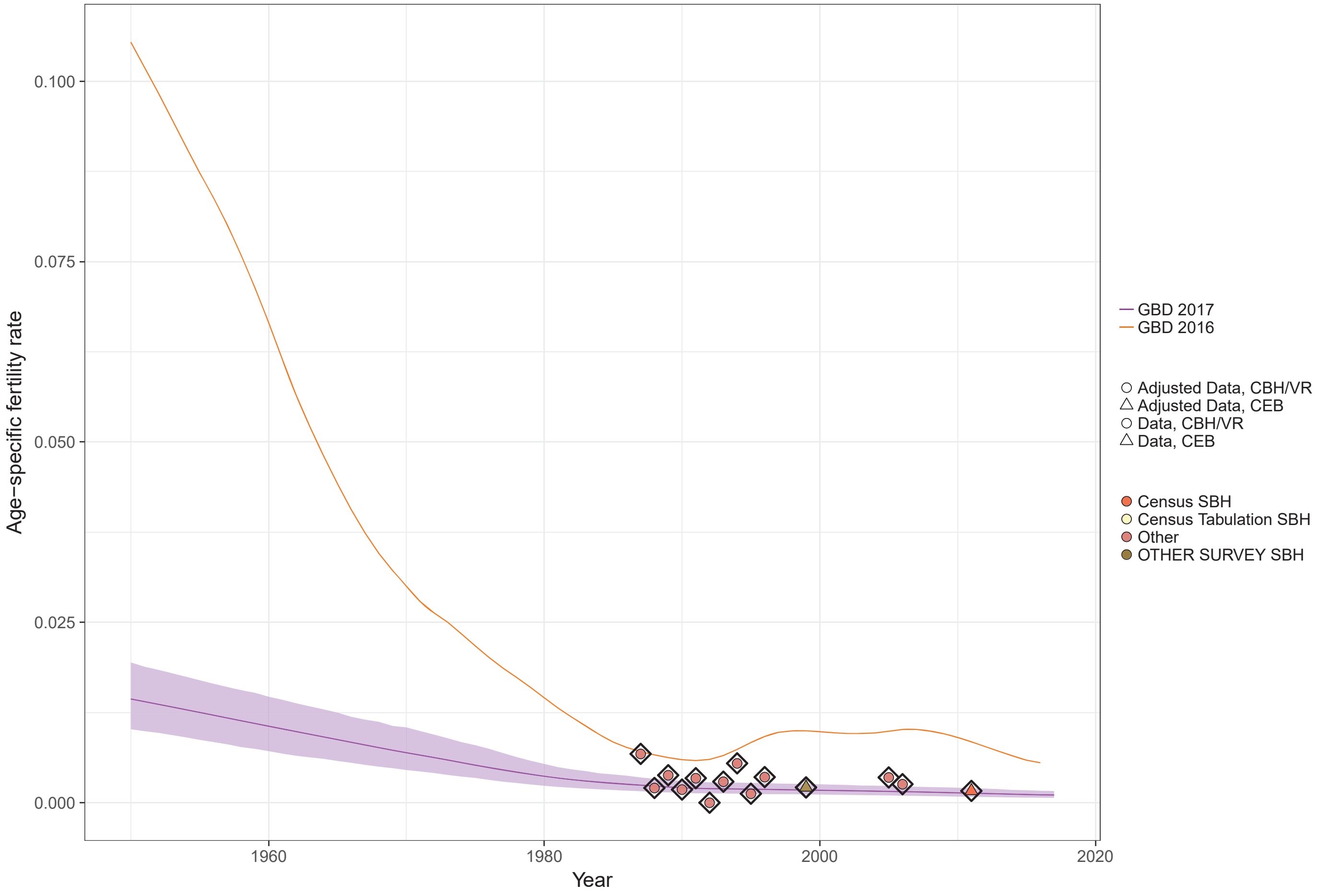

Age−specific fertility fitted trend lines, 1950−2017: Marshall Islands, Ages: 50−54
Data points enclosed with a diamond are reference sources

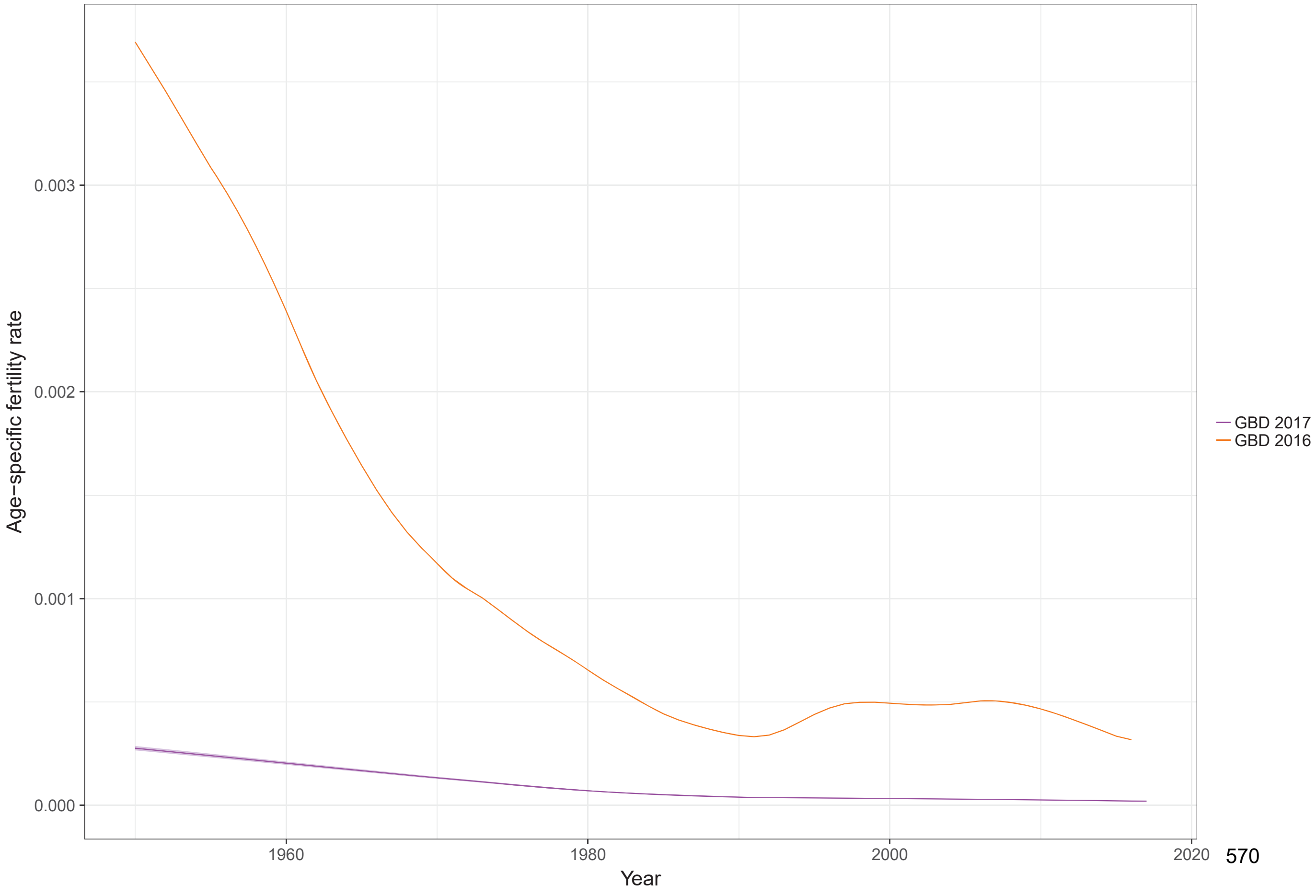

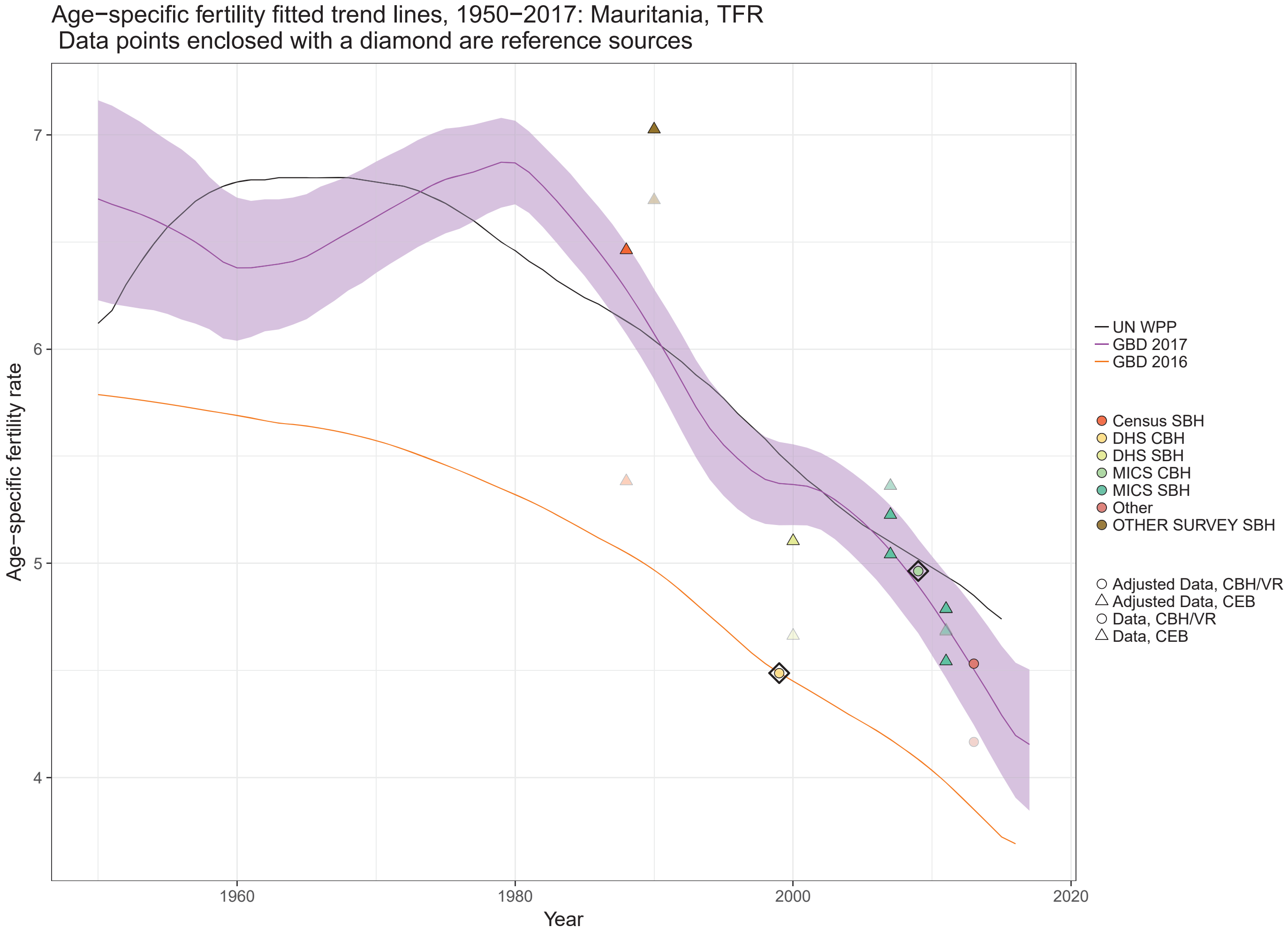

Age−specific fertility fitted trend lines, 1950−2017: Mauritania, TFR
Data points enclosed with a diamond are reference sources
Age−specific fertility rate
Year
UN WPP
GBD 2017
GBD 2016
Census SBH
DHS CBH
DHS SBH
MICS CBH
MICS SBH
Other
OTHER SURVEY SBH
Adjusted Data, CBH/VR
Adjusted Data, CEB
Data, CBH/VR
Data, CEB

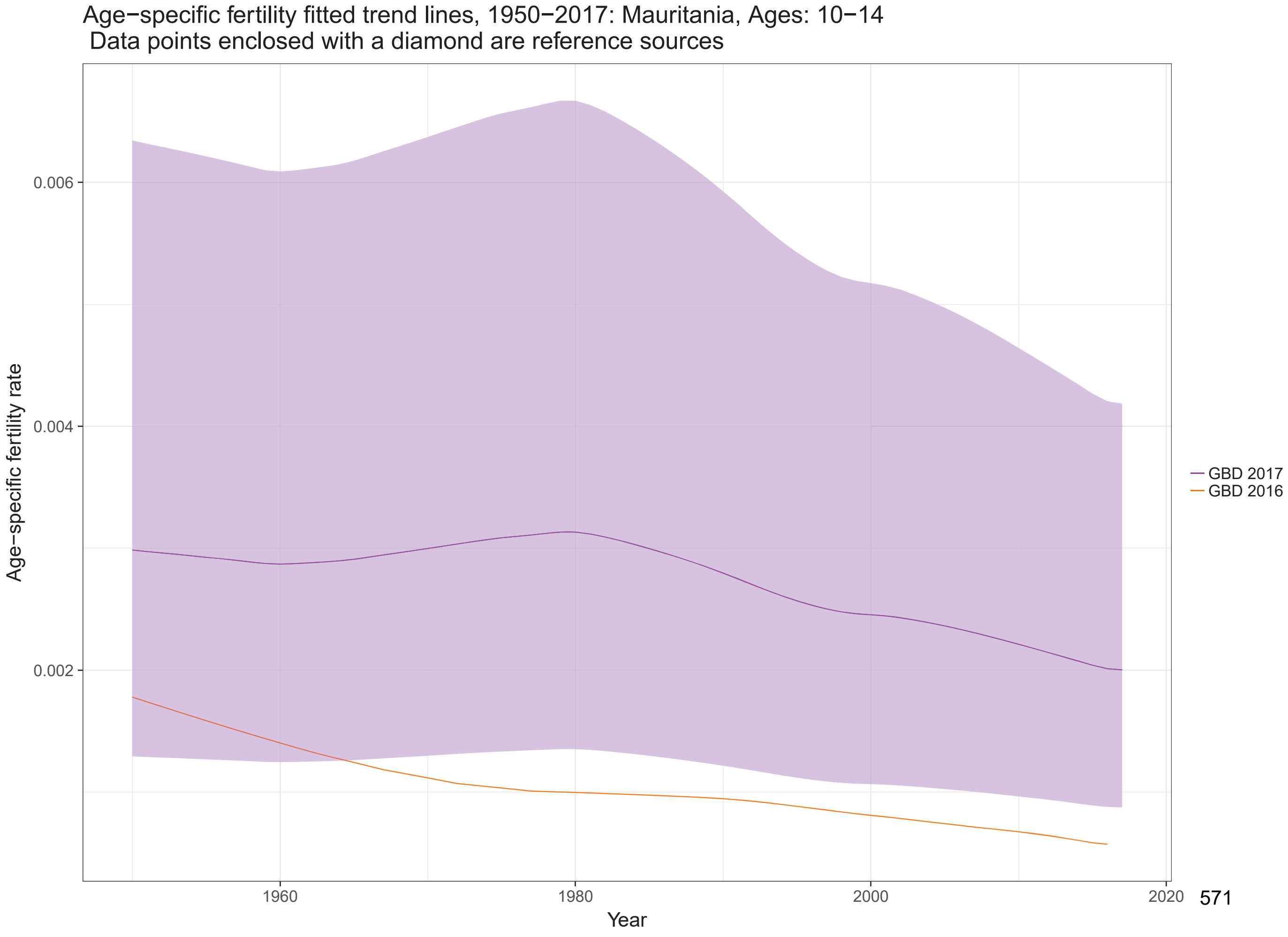

Age−specific fertility fitted trend lines, 1950−2017: Mauritania, Ages: 10−14
Data points enclosed with a diamond are reference sources
Age−specific fertility rate
Year
GBD 2017
GBD 2016

Age−specific fertility fitted trend lines, 1950−2017: Mauritania, Ages: 15−19
Data points enclosed with a diamond are reference sources

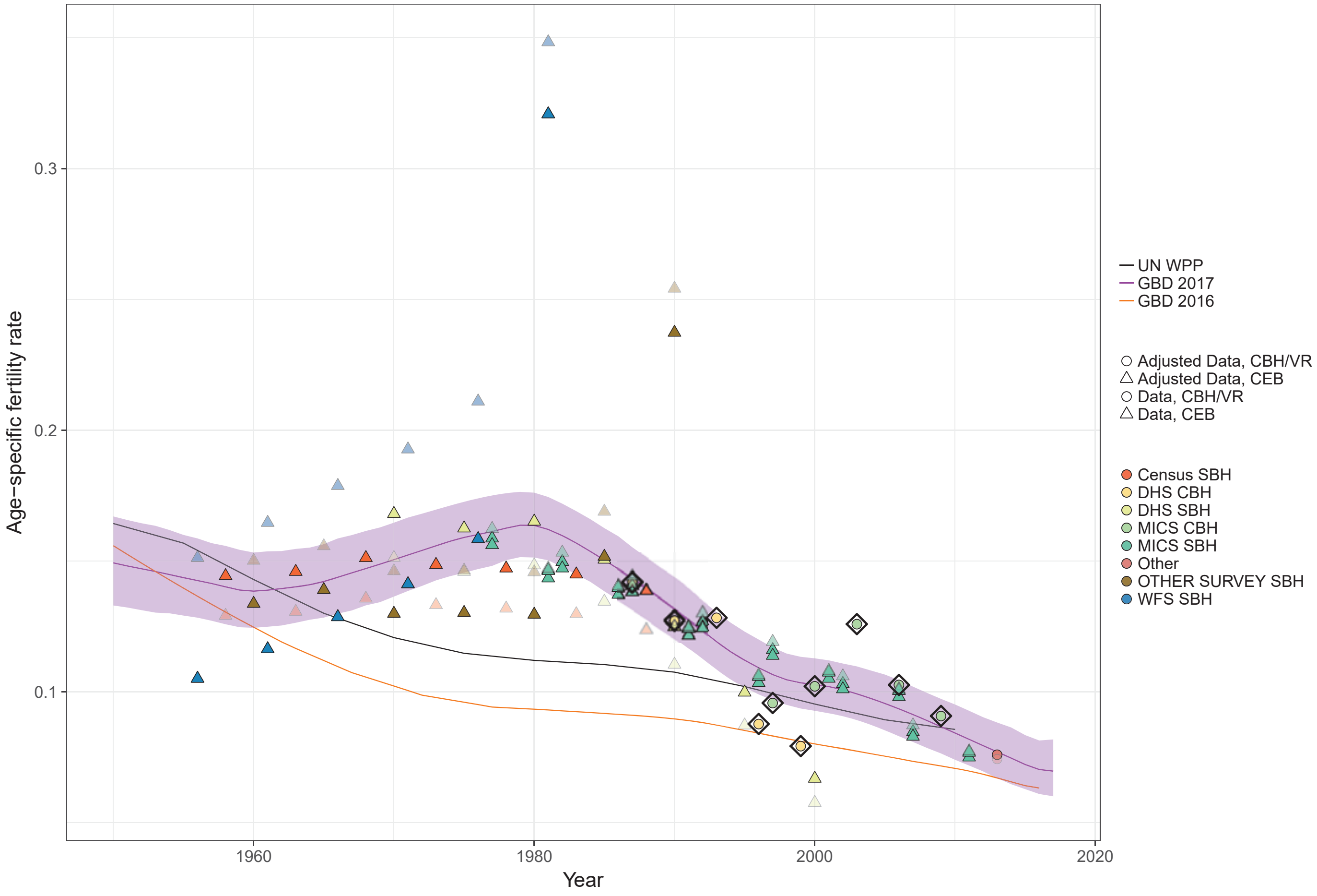

Age−specific fertility fitted trend lines, 1950−2017: Mauritania, Ages: 20−24
Data points enclosed with a diamond are reference sources

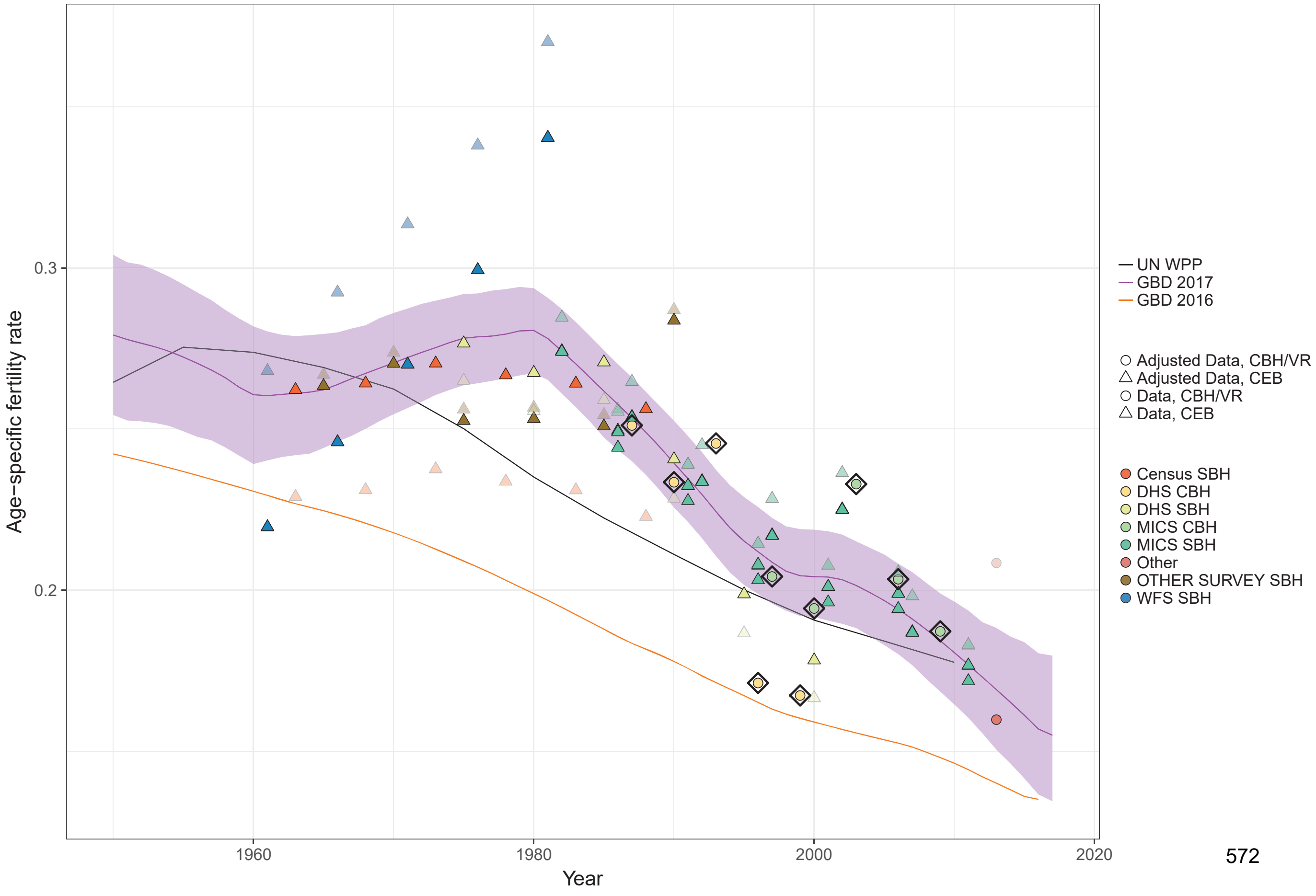

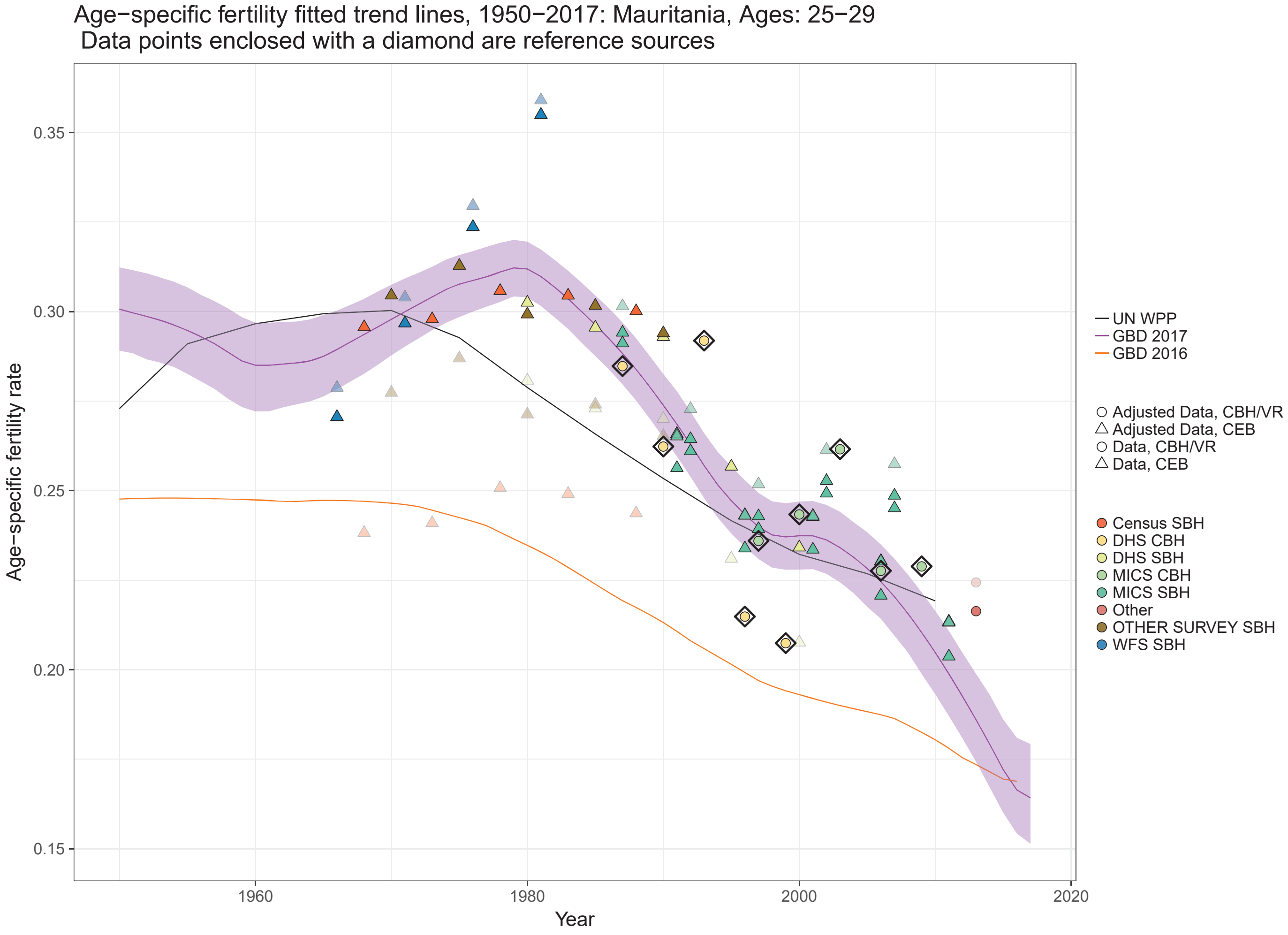

Age−specific fertility fitted trend lines, 1950−2017: Mauritania, Ages: 25−29
Data points enclosed with a diamond are reference sources
Age-specific fertility rate
Year
0.15
0.20
0.25
0.30
0.35
1960
1980
2000
2020
UN WPP
GBD 2017
GBD 2016
Adjusted Data, CBH/VR
Adjusted Data, CEB
Data, CBH/VR
Data, CEB
Census SBH
DHS CBH
DHS SBH
MICS CBH
MICS SBH
Other
OTHER SURVEY SBH
WFS SBH

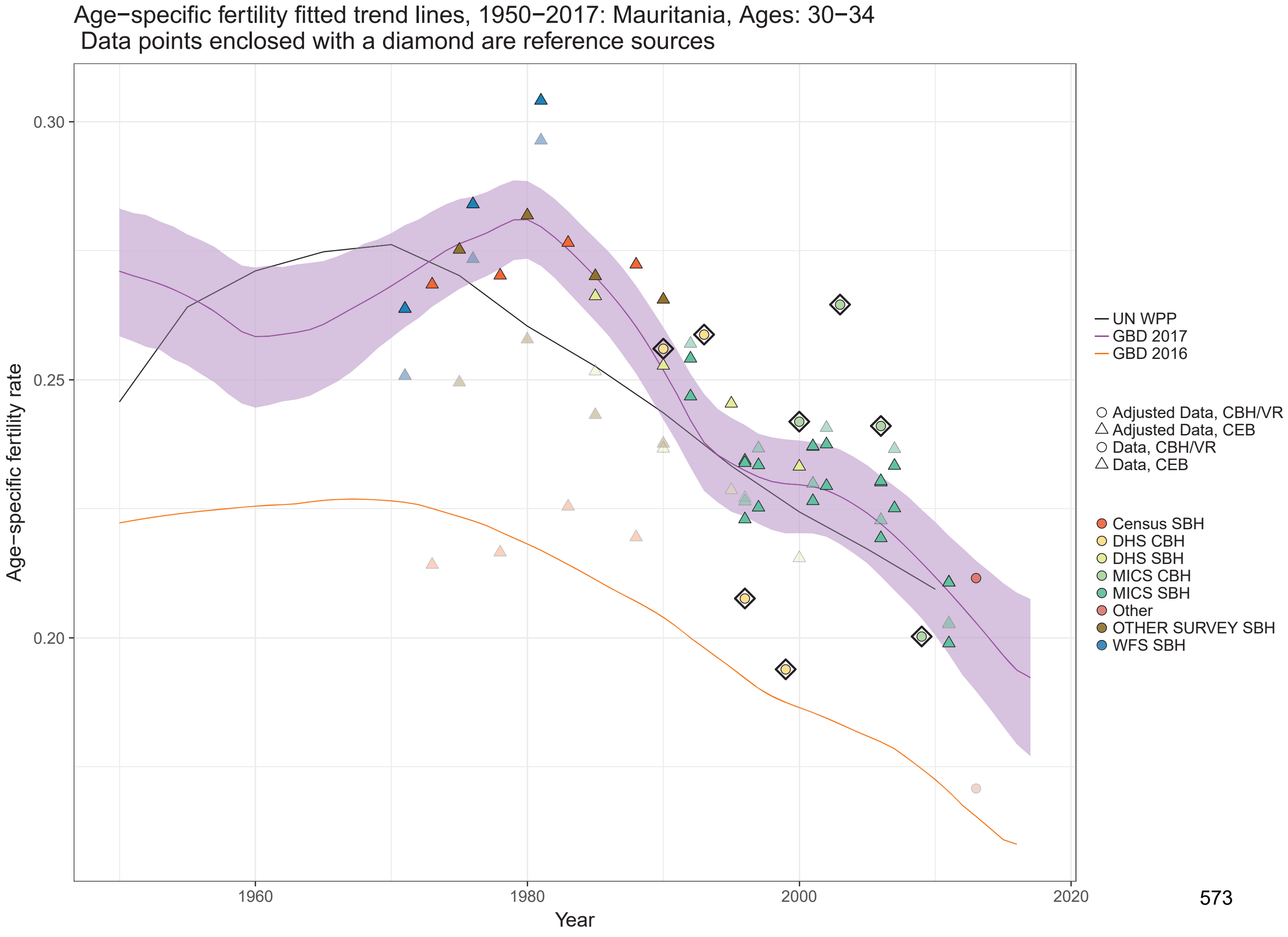

Age−specific fertility fitted trend lines, 1950−2017: Mauritania, Ages: 30−34
Data points enclosed with a diamond are reference sources
Age-specific fertility rate
Year
0.20
0.25
0.30
1960
1980
2000
2020
UN WPP
GBD 2017
GBD 2016
Adjusted Data, CBH/VR
Adjusted Data, CEB
Data, CBH/VR
Data, CEB
Census SBH
DHS CBH
DHS SBH
MICS CBH
MICS SBH
Other
OTHER SURVEY SBH
WFS SBH

Age−specific fertility fitted trend lines, 1950−2017: Mauritania, Ages: 35−39
Data points enclosed with a diamond are reference sources

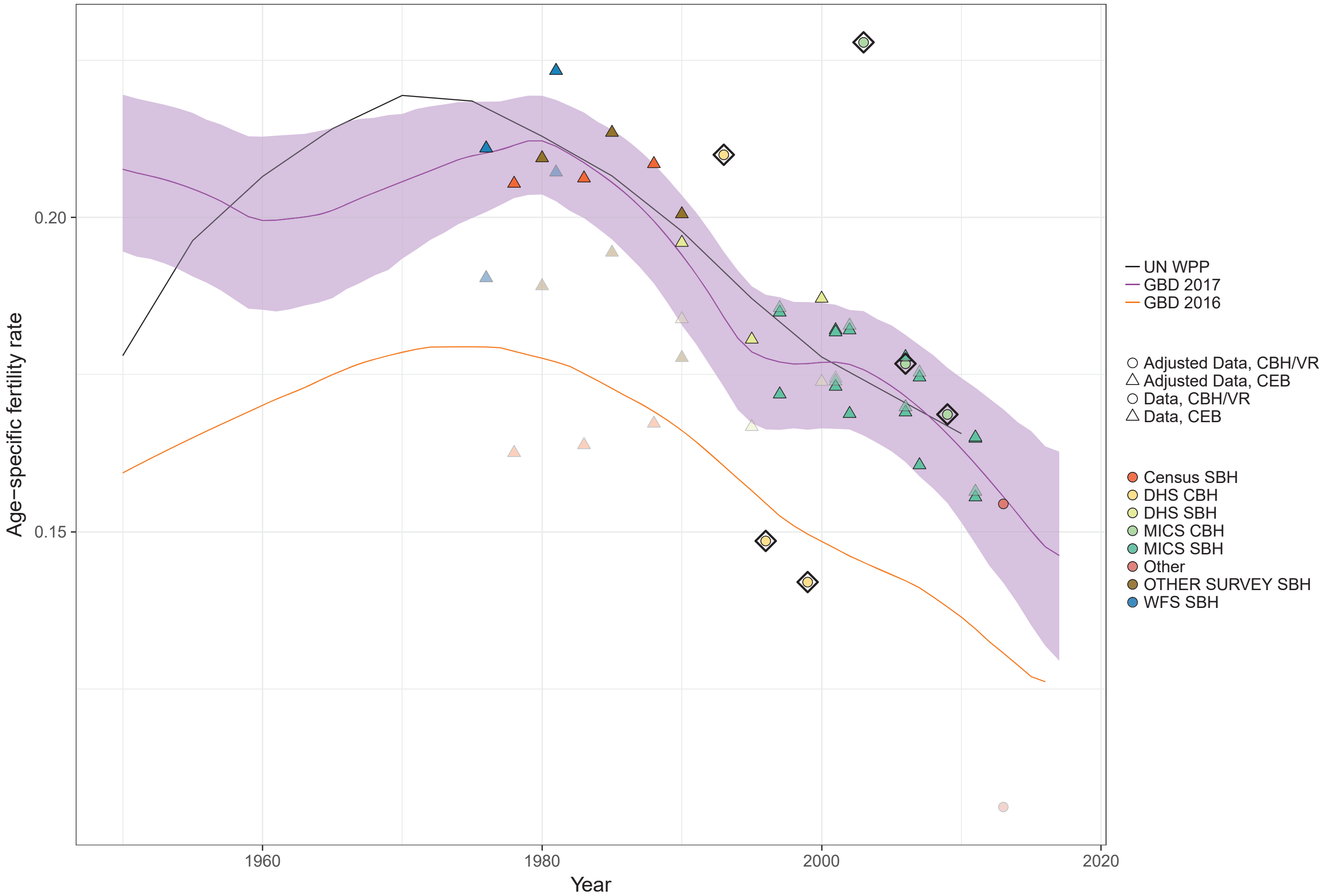

Age−specific fertility fitted trend lines, 1950−2017: Mauritania, Ages: 40−44
Data points enclosed with a diamond are reference sources

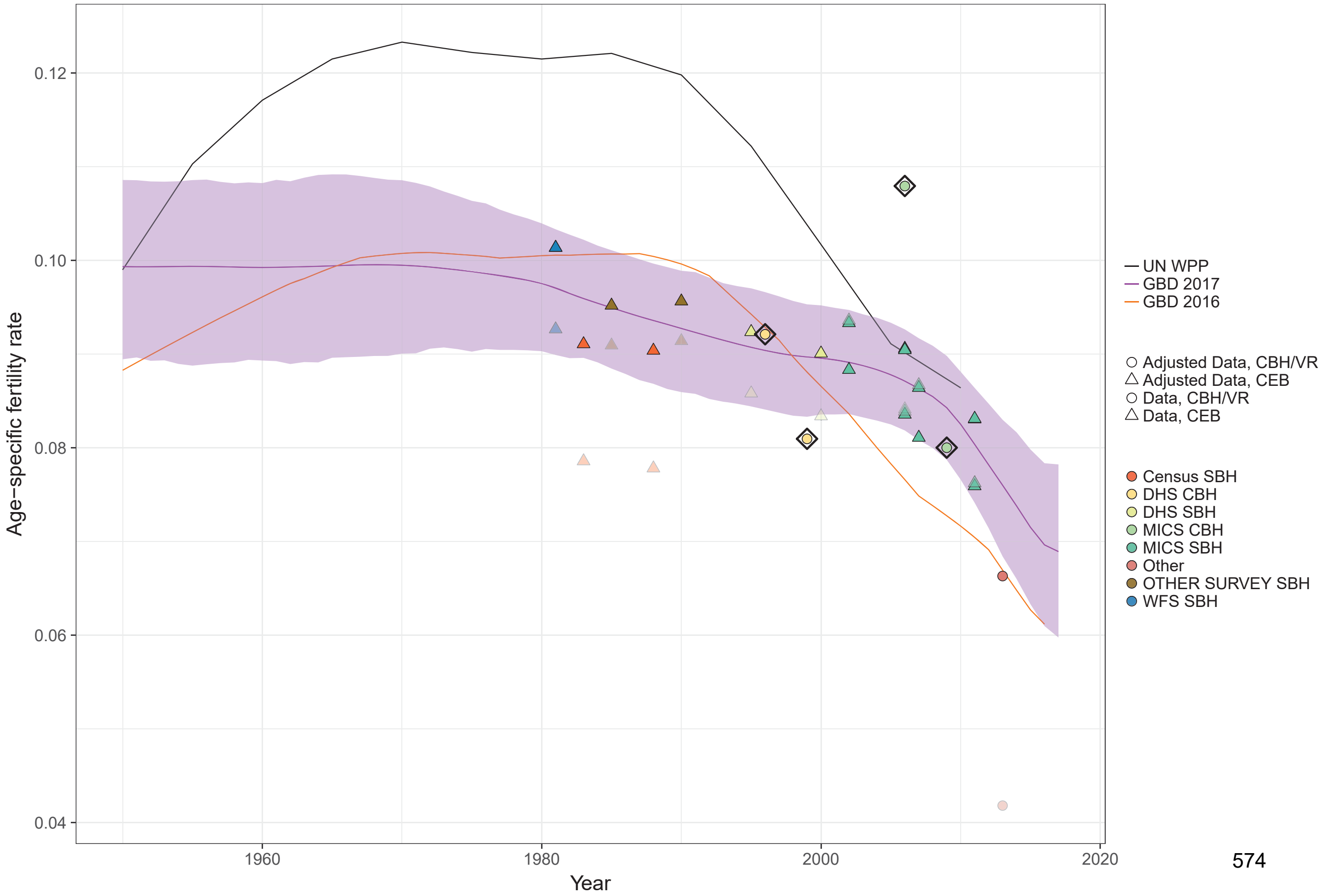

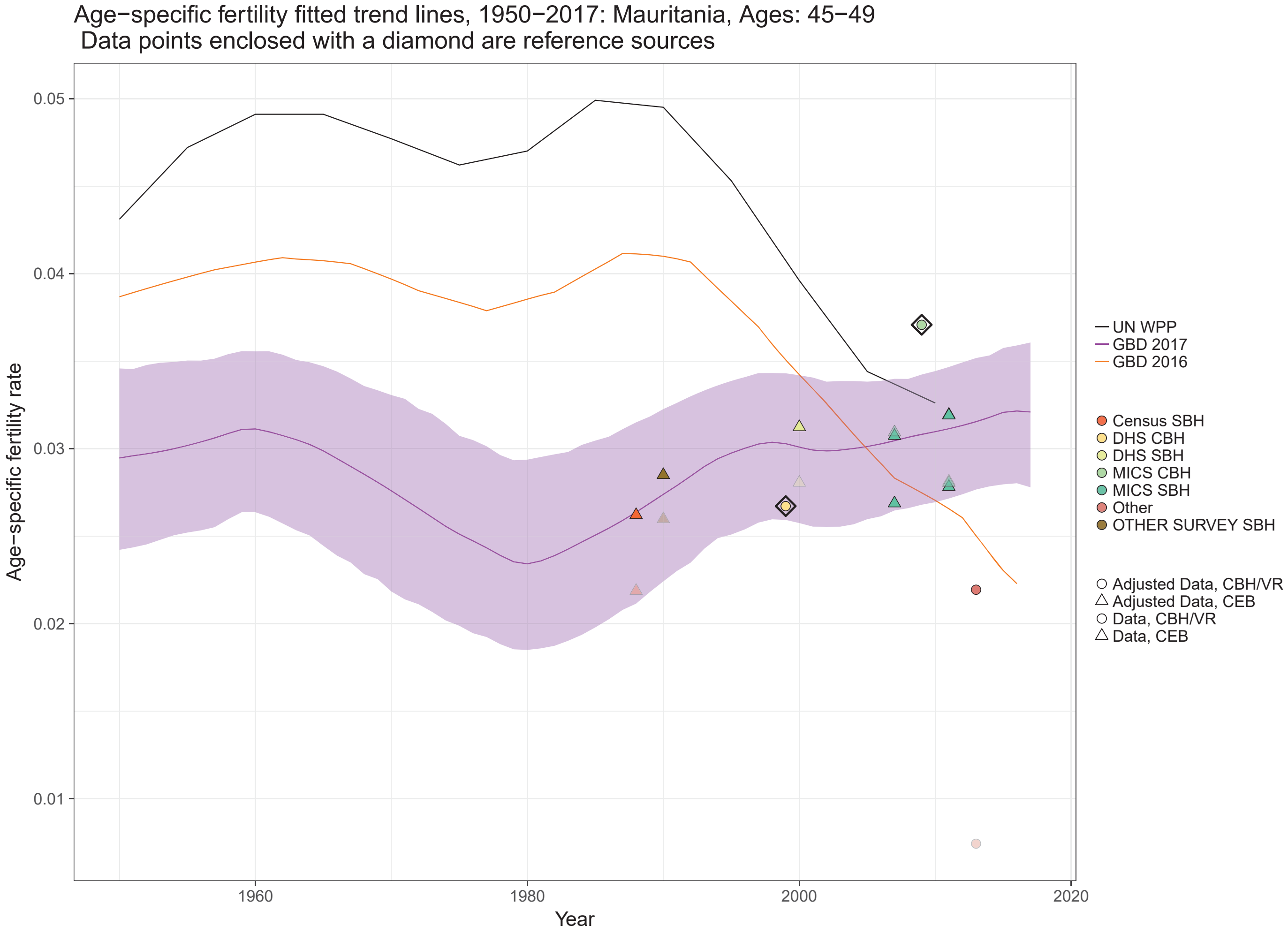

Age−specific fertility fitted trend lines, 1950−2017: Mauritania, Ages: 45−49
Data points enclosed with a diamond are reference sources
Age−specific fertility rate
0.05
0.04
0.03
0.02
0.01
1960
1980
2000
2020
Year
UN WPP
GBD 2017
GBD 2016
Census SBH
DHS CBH
DHS SBH
MICS CBH
MICS SBH
Other
OTHER SURVEY SBH
Adjusted Data, CBH/VR
Adjusted Data, CEB
Data, CBH/VR
Data, CEB

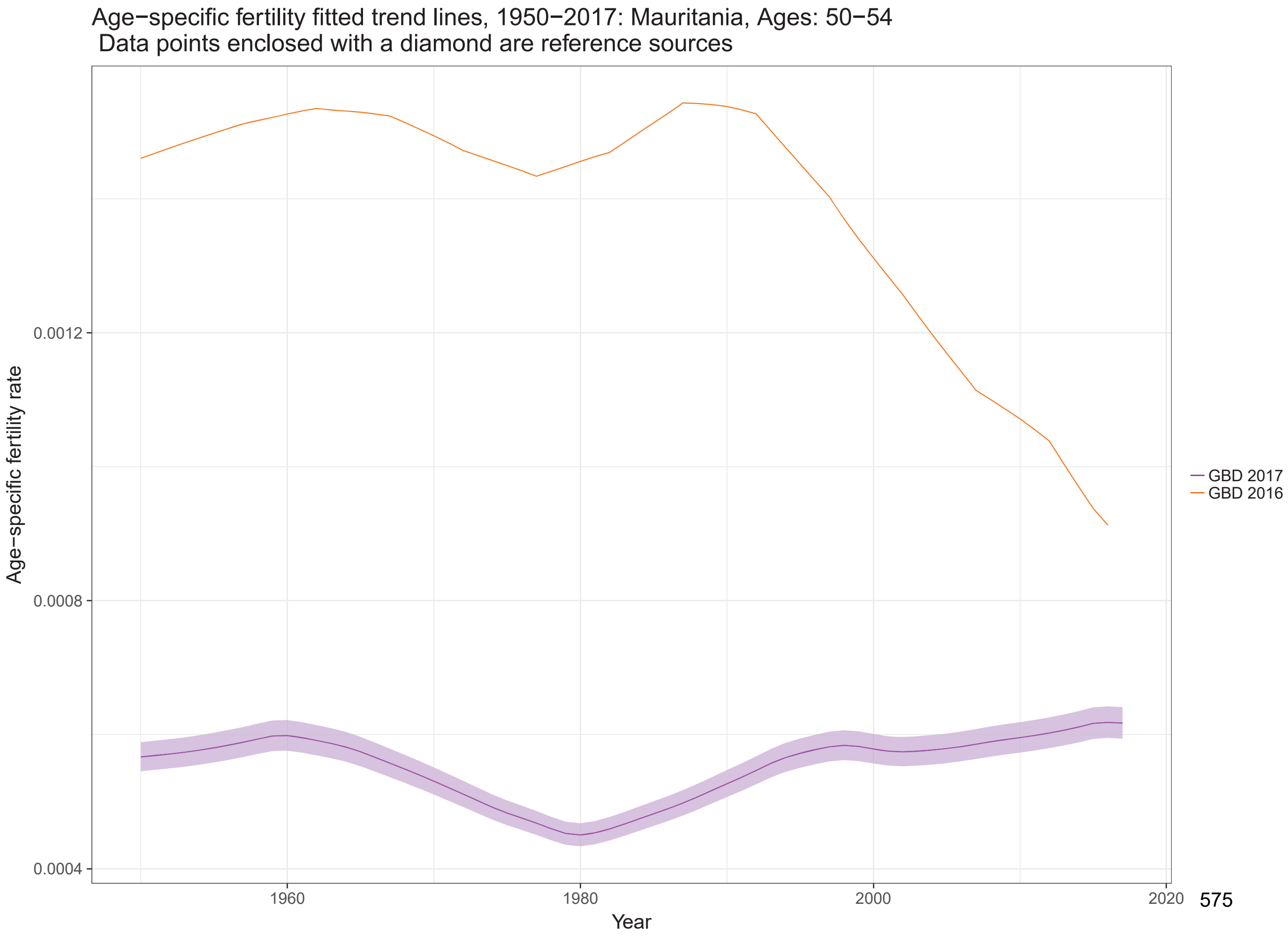

Age−specific fertility fitted trend lines, 1950−2017: Mauritania, Ages: 50−54
Data points enclosed with a diamond are reference sources
Age−specific fertility rate
0.0012
0.0008
0.0004
1960
1980
2000
2020
Year
GBD 2017
GBD 2016

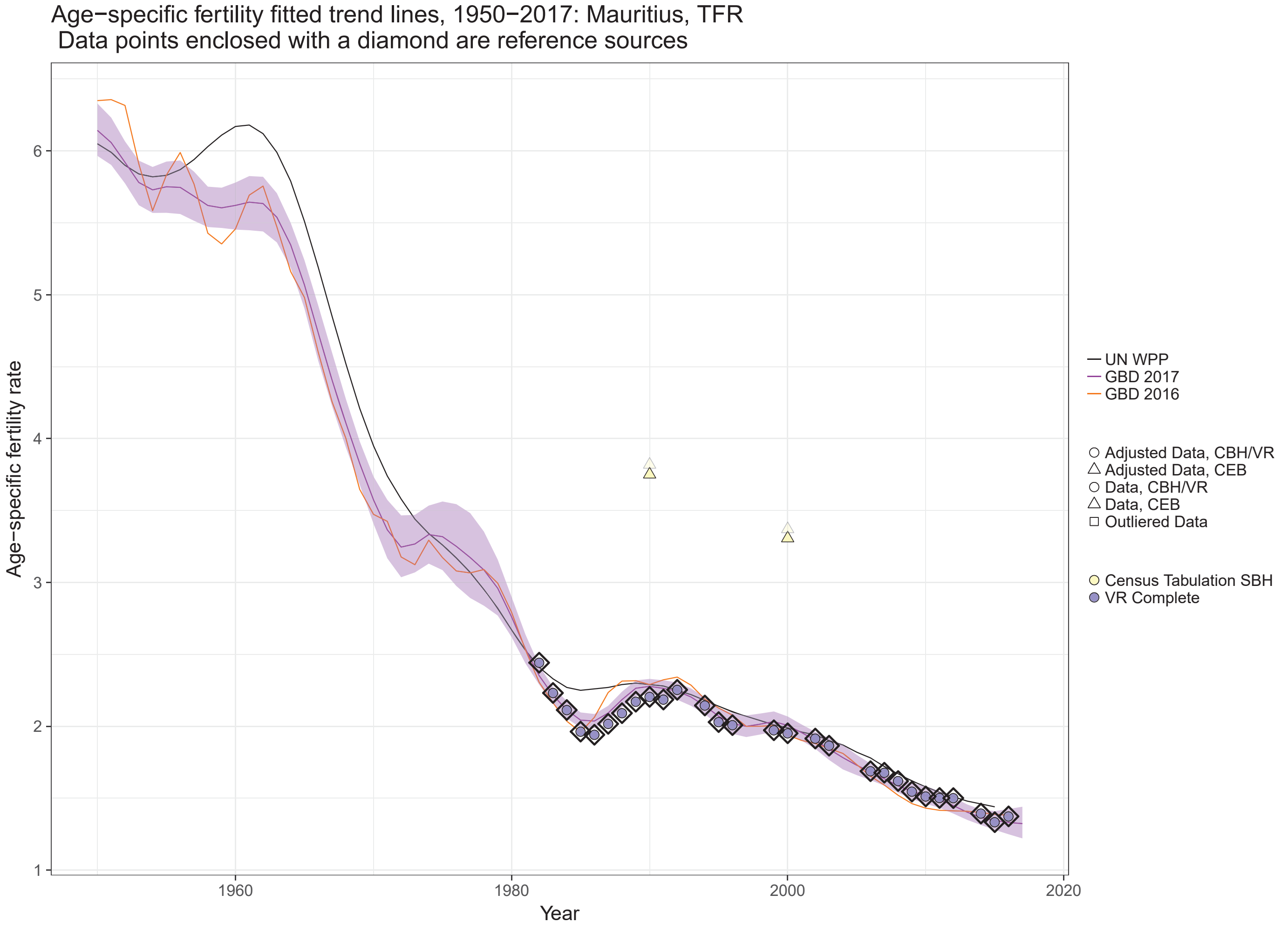

Age−specific fertility fitted trend lines, 1950−2017: Mauritius, TFR
Data points enclosed with a diamond are reference sources
Age−specific fertility rate
Year
UN WPP
GBD 2017
GBD 2016
Adjusted Data, CBH/VR
Adjusted Data, CEB
Data, CBH/VR
Data, CEB
Outliered Data
Census Tabulation SBH
VR Complete

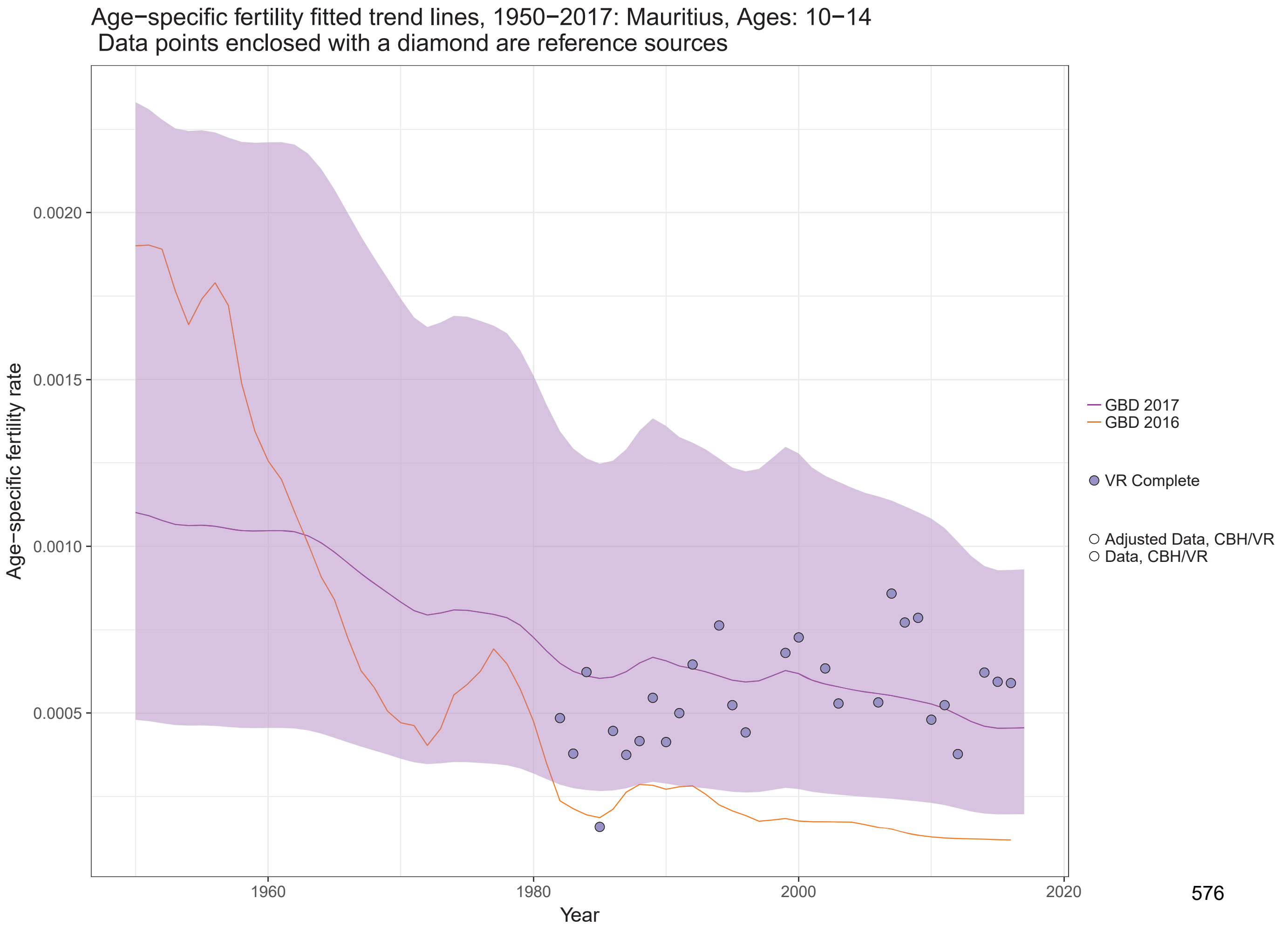

Age−specific fertility fitted trend lines, 1950−2017: Mauritius, Ages: 10−14
Data points enclosed with a diamond are reference sources
Age−specific fertility rate
Year
GBD 2017
GBD 2016
VR Complete
Adjusted Data, CBH/VR
Data, CBH/VR

Age−specific fertility fitted trend lines, 1950−2017: Mauritius, Ages: 15−19
Data points enclosed with a diamond are reference sources

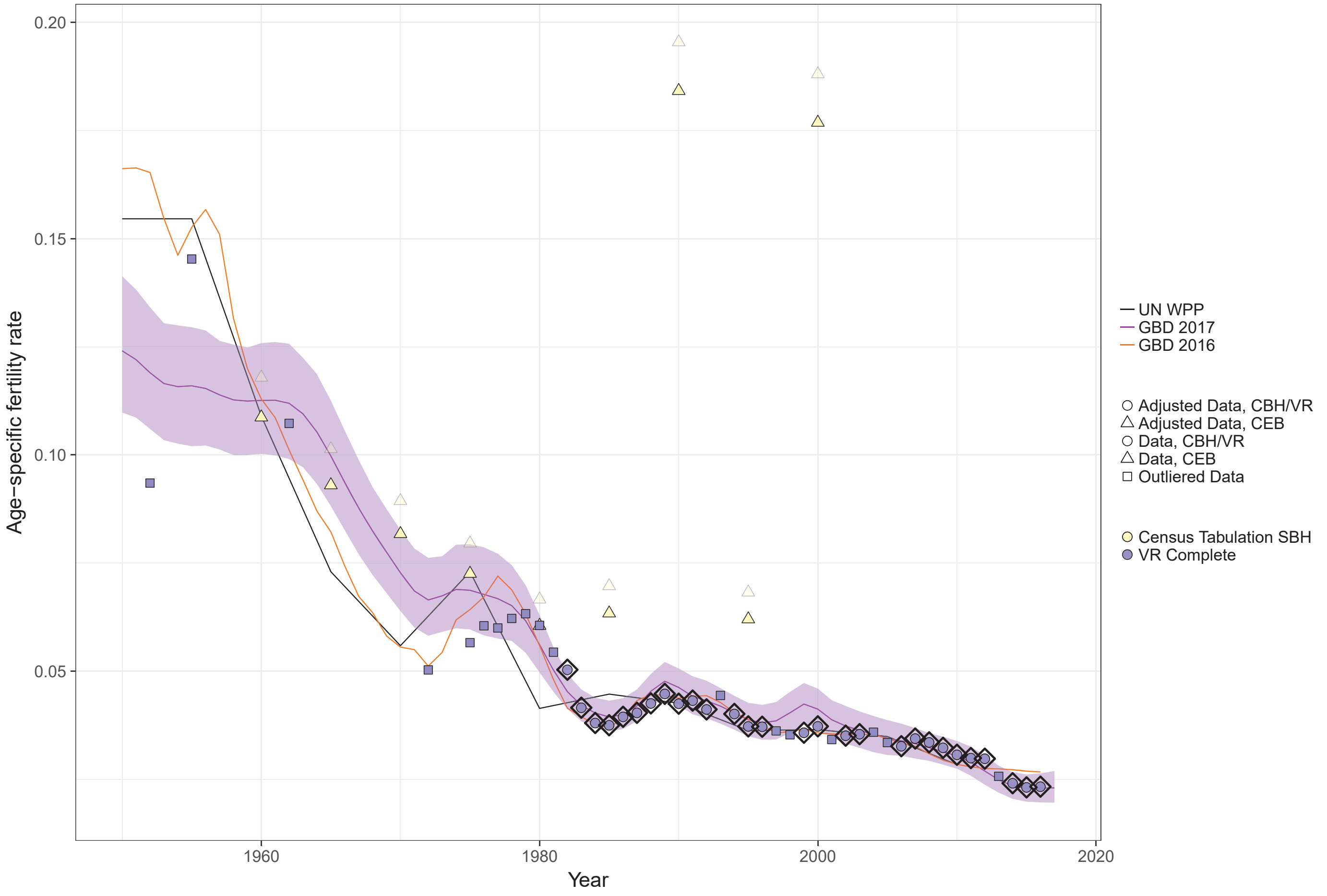

Age−specific fertility fitted trend lines, 1950−2017: Mauritius, Ages: 20−24
Data points enclosed with a diamond are reference sources

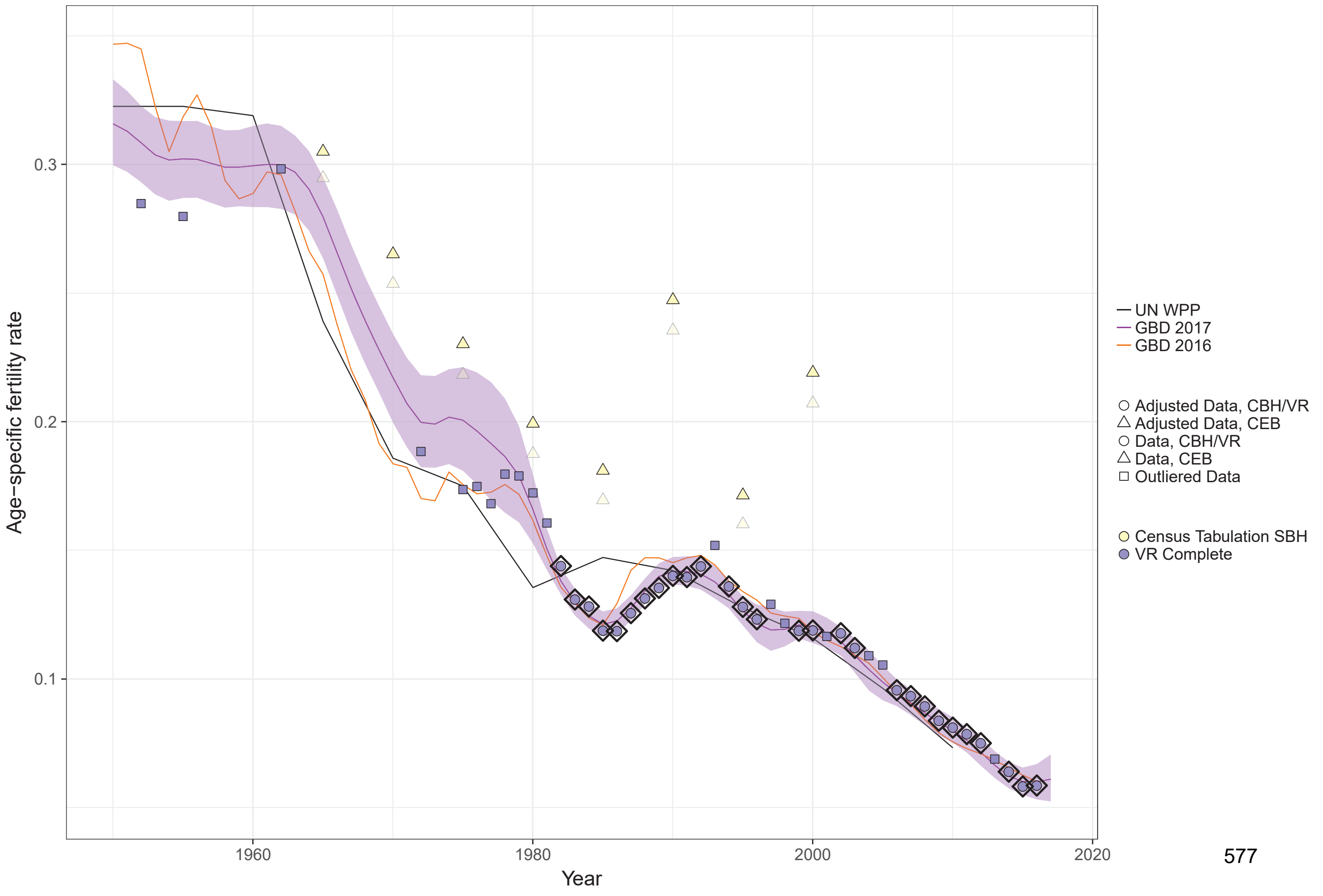

Age−specific fertility fitted trend lines, 1950−2017: Mauritius, Ages: 25−29
Data points enclosed with a diamond are reference sources

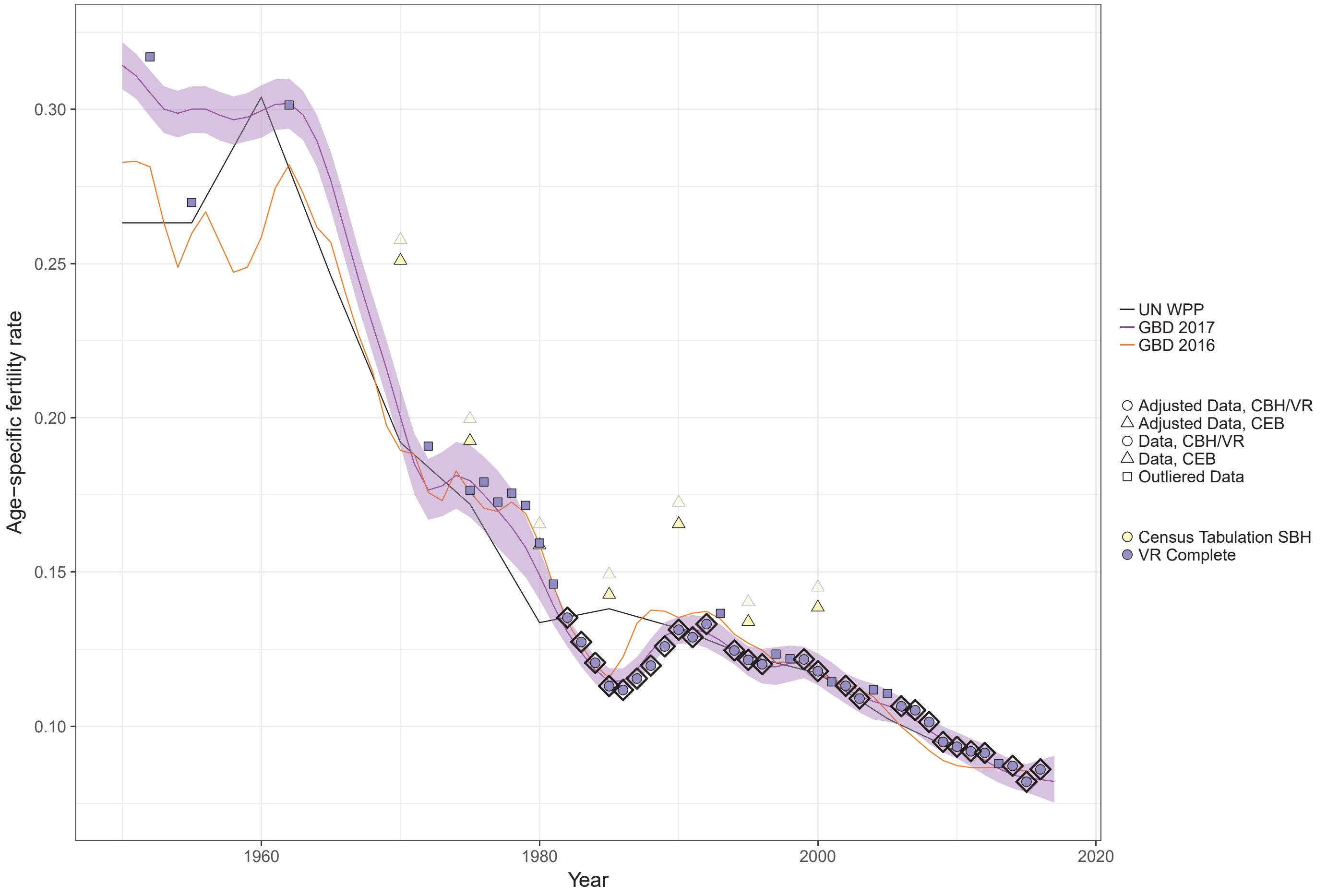

Age−specific fertility fitted trend lines, 1950−2017: Mauritius, Ages: 30−34
Data points enclosed with a diamond are reference sources

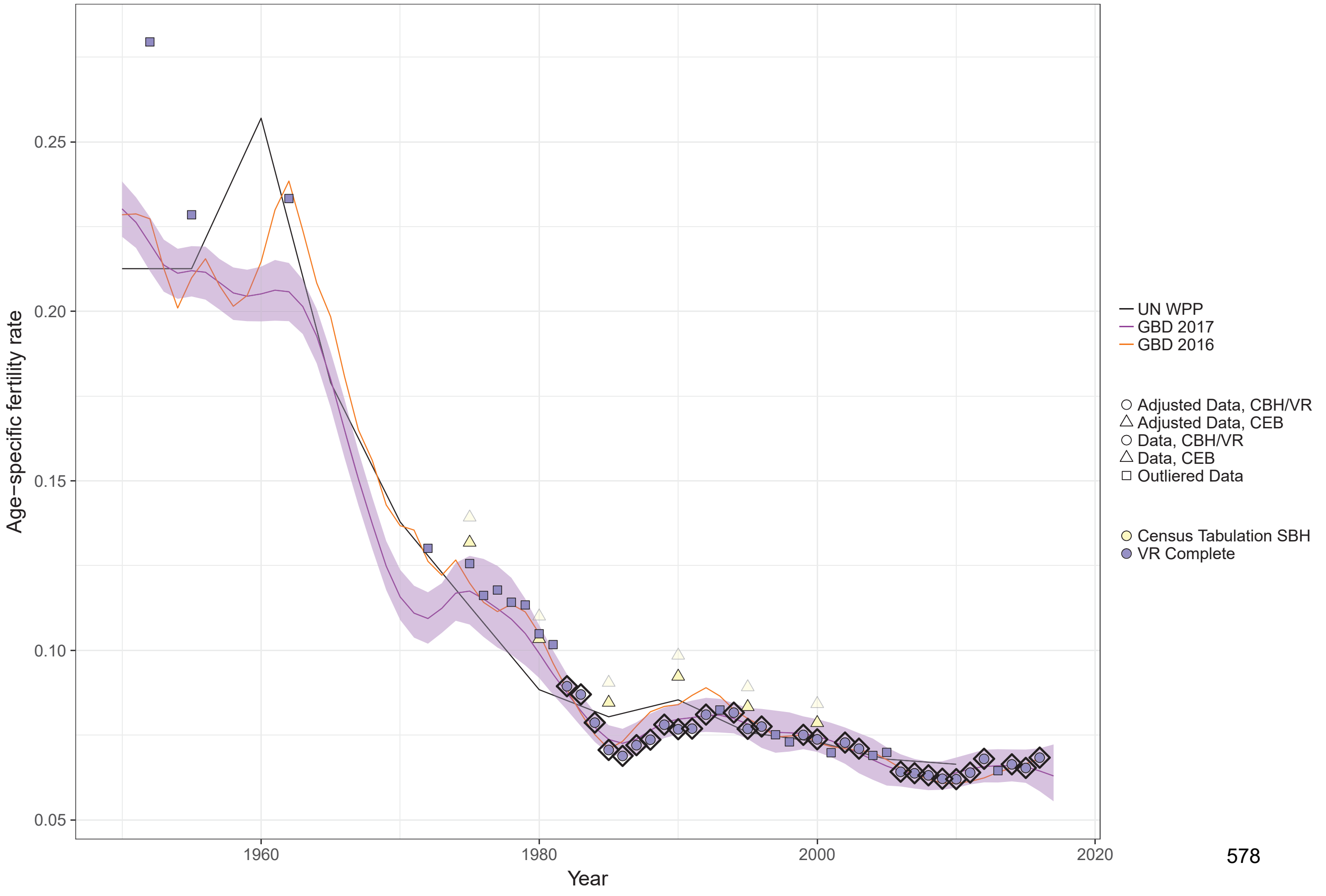

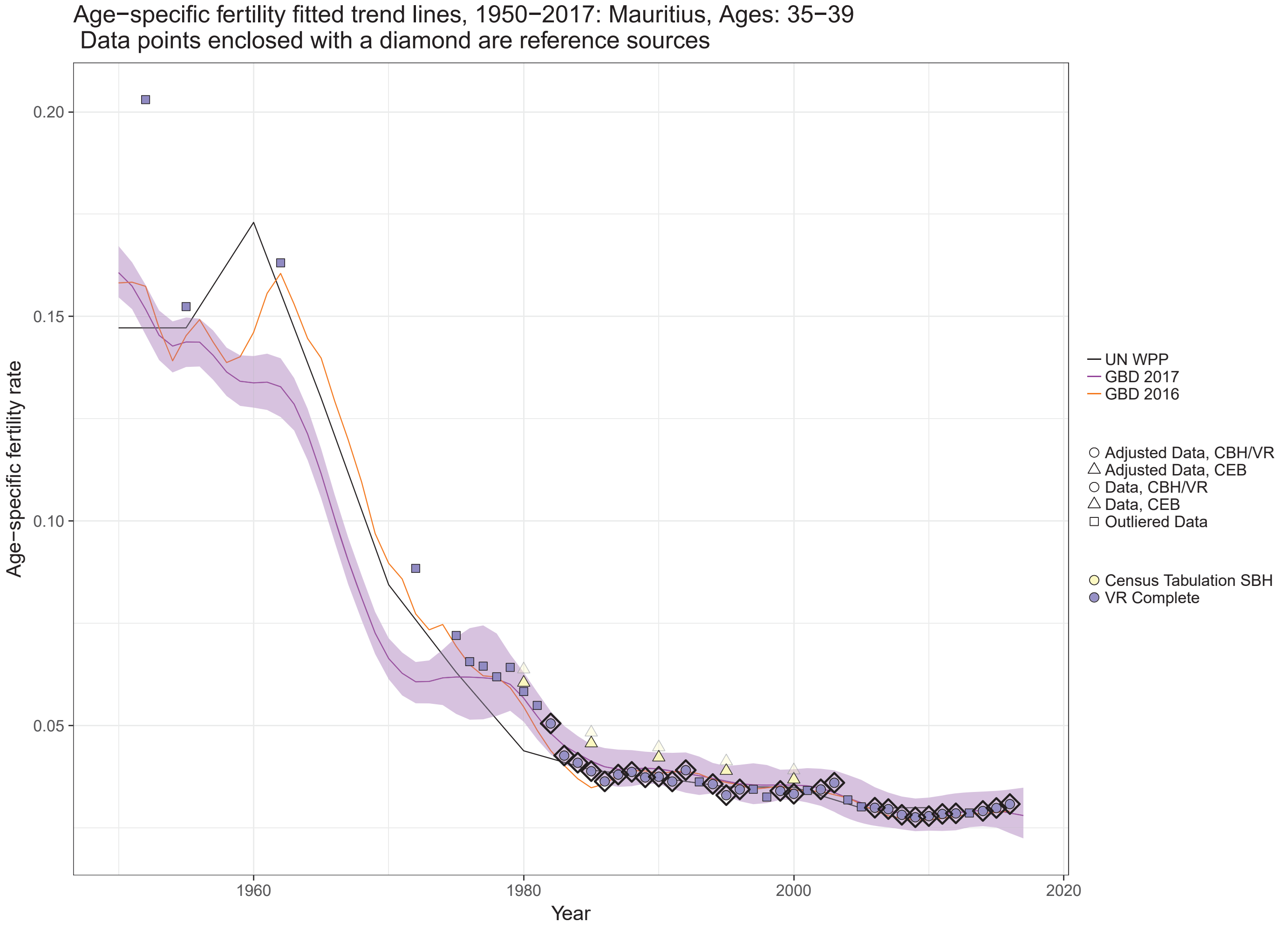
Age−specific fertility fitted trend lines, 1950−2017: Mauritius, Ages: 35−39
Data points enclosed with a diamond are reference sources
Age−specific fertility rate
0.20
0.15
0.10
0.05
1960
1980
2000
2020
Year
UN WPP
GBD 2017
GBD 2016
Adjusted Data, CBH/VR
Adjusted Data, CEB
Data, CBH/VR
Data, CEB
Outliered Data
Census Tabulation SBH
VR Complete

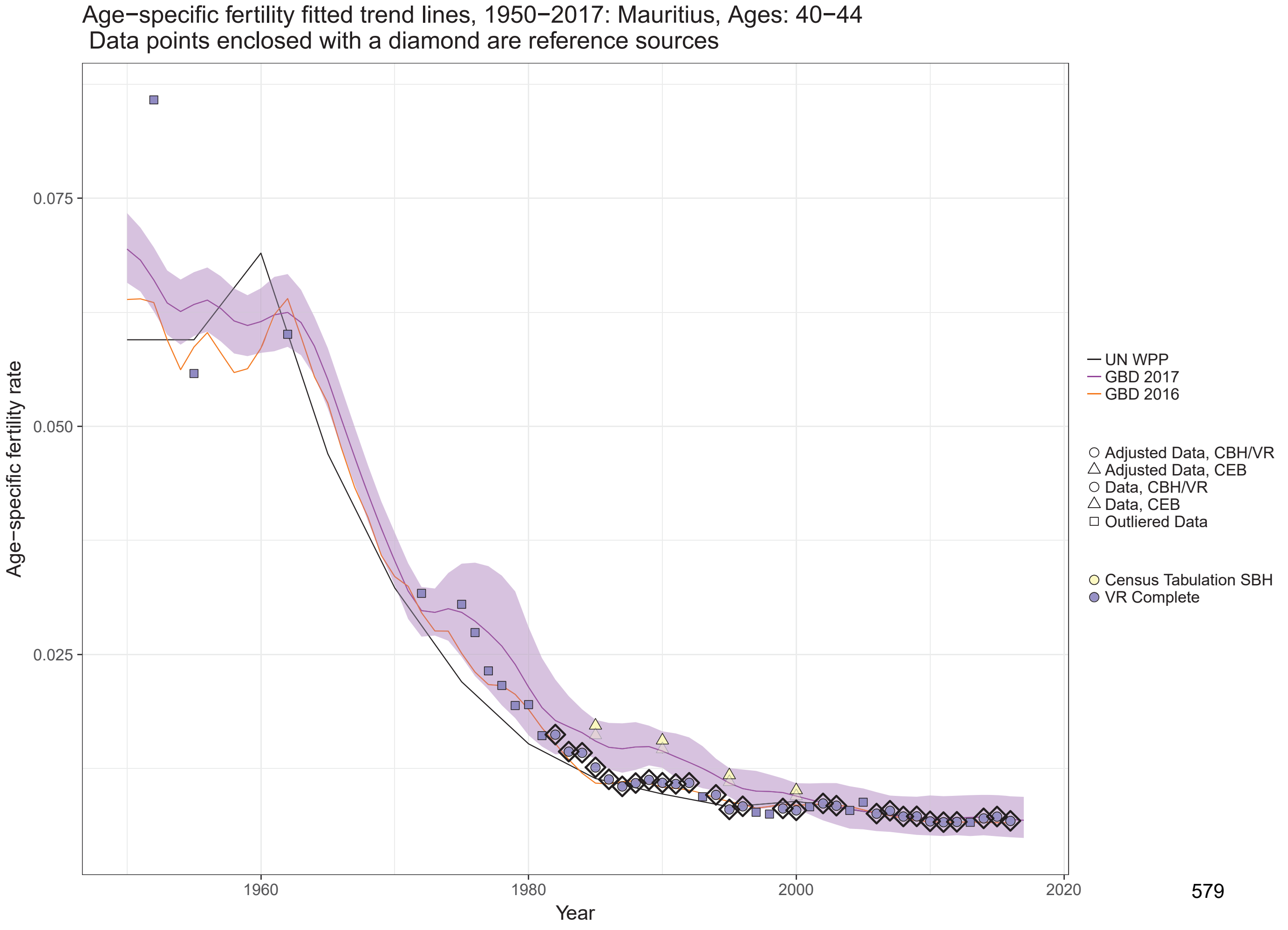
Age−specific fertility fitted trend lines, 1950−2017: Mauritius, Ages: 40−44
Data points enclosed with a diamond are reference sources
Age−specific fertility rate
0.075
0.050
0.025
1960
1980
2000
2020
Year
UN WPP
GBD 2017
GBD 2016
Adjusted Data, CBH/VR
Adjusted Data, CEB
Data, CBH/VR
Data, CEB
Outliered Data
Census Tabulation SBH
VR Complete

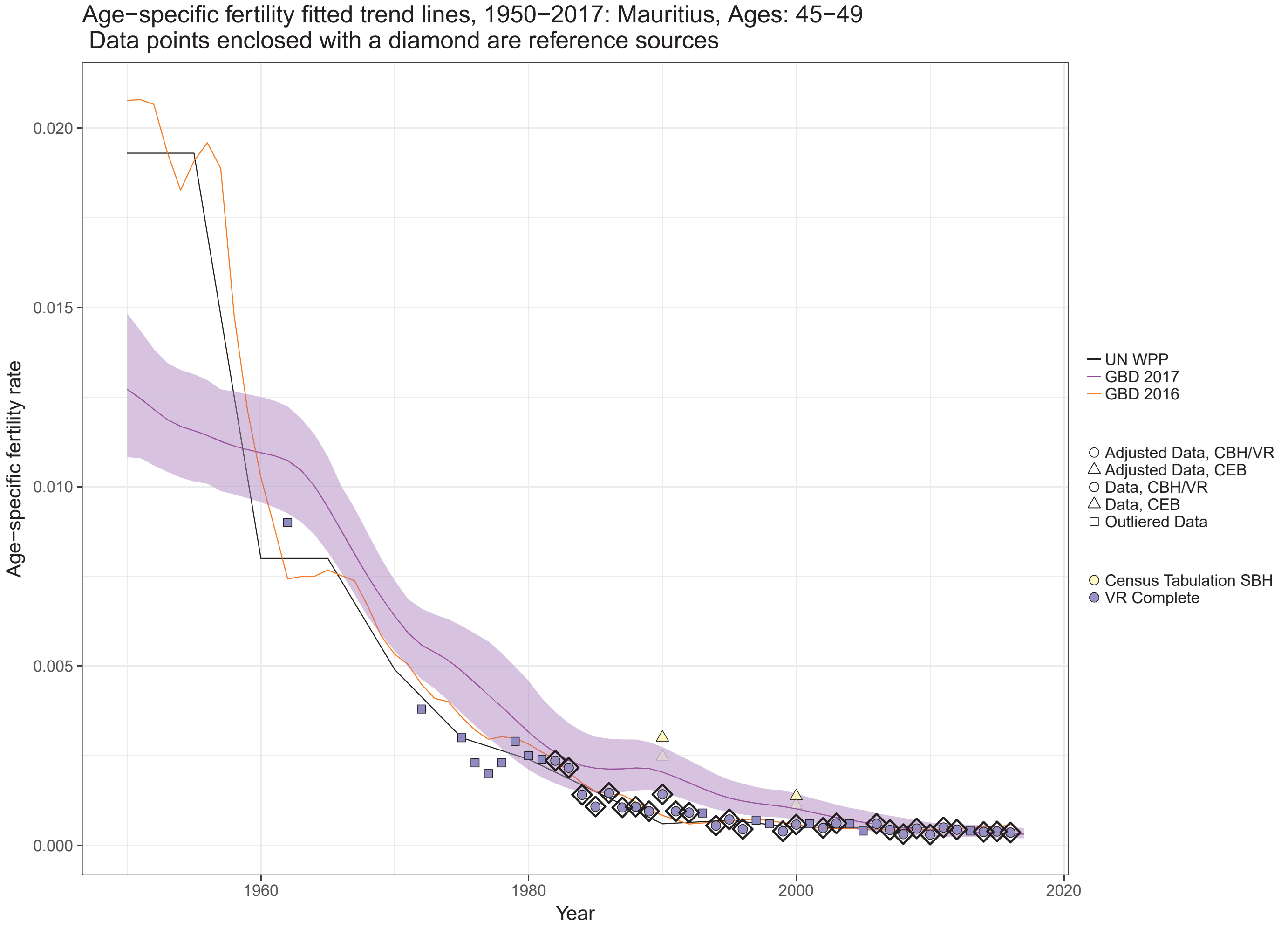
Age−specific fertility fitted trend lines, 1950−2017: Mauritius, Ages: 45−49
Data points enclosed with a diamond are reference sources
Age−specific fertility rate
Year
0.000
0.005
0.010
0.015
0.020
1960
1980
2000
2020
UN WPP
GBD 2017
GBD 2016
Adjusted Data, CBH/VR
Adjusted Data, CEB
Data, CBH/VR
Data, CEB
Outliered Data
Census Tabulation SBH
VR Complete

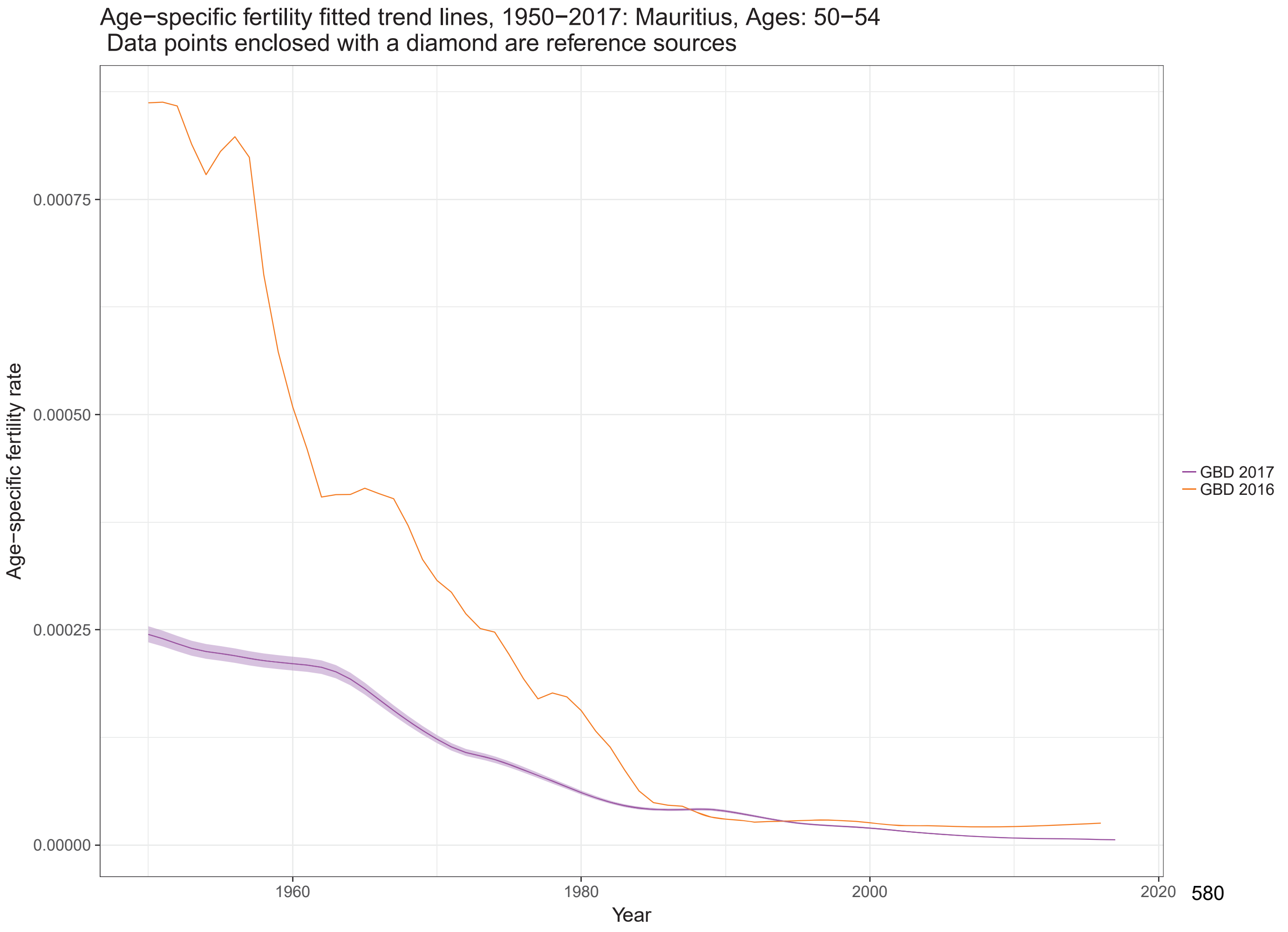
Age−specific fertility fitted trend lines, 1950−2017: Mauritius, Ages: 50−54
Data points enclosed with a diamond are reference sources
Age−specific fertility rate
Year
0.00000
0.00025
0.00050
0.00075
1960
1980
2000
2020
GBD 2017
GBD 2016

Age−specific fertility fitted trend lines, 1950−2017: Mexico, TFR
Data points enclosed with a diamond are reference sources

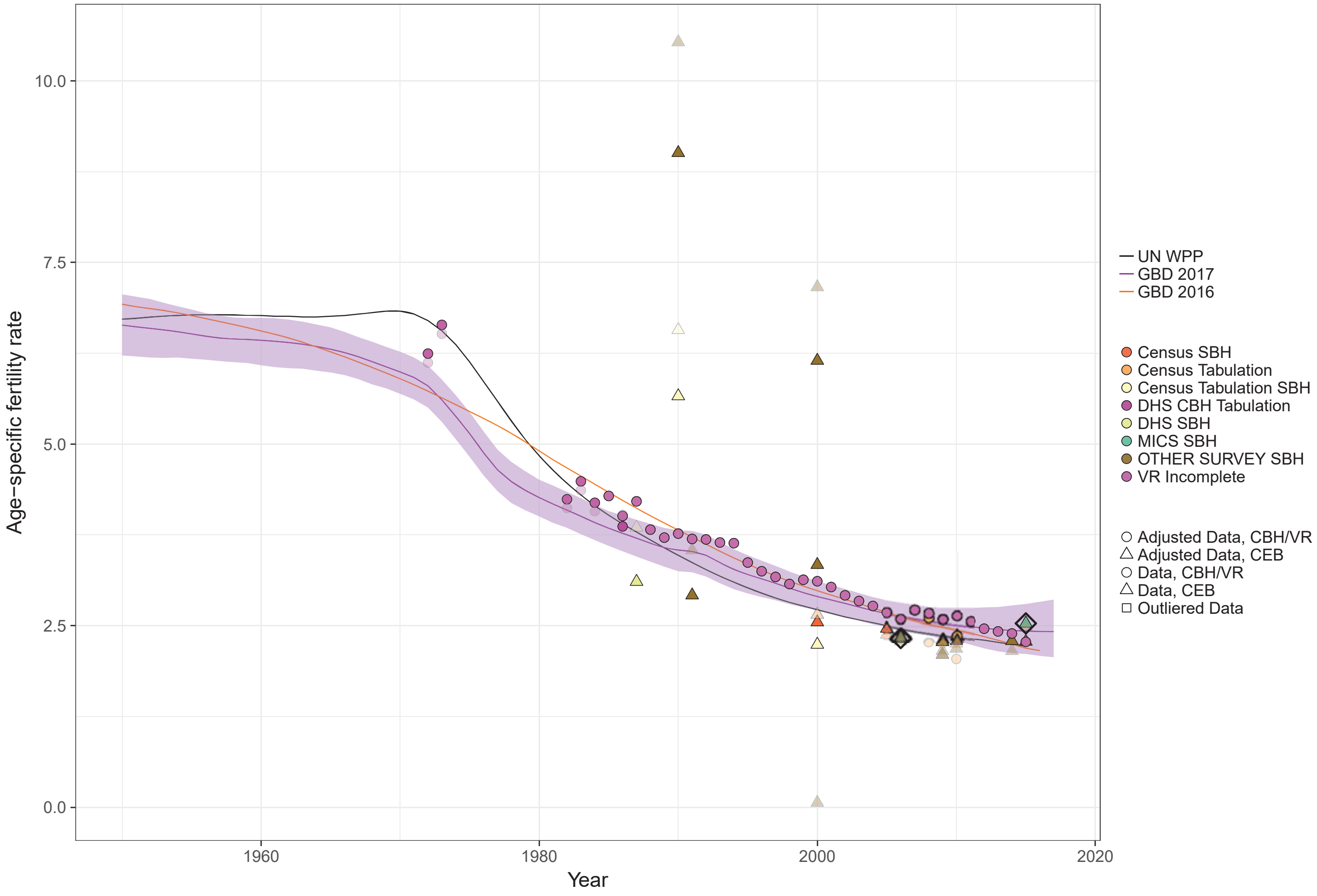

Age−specific fertility fitted trend lines, 1950−2017: Mexico, Ages: 10−14
Data points enclosed with a diamond are reference sources

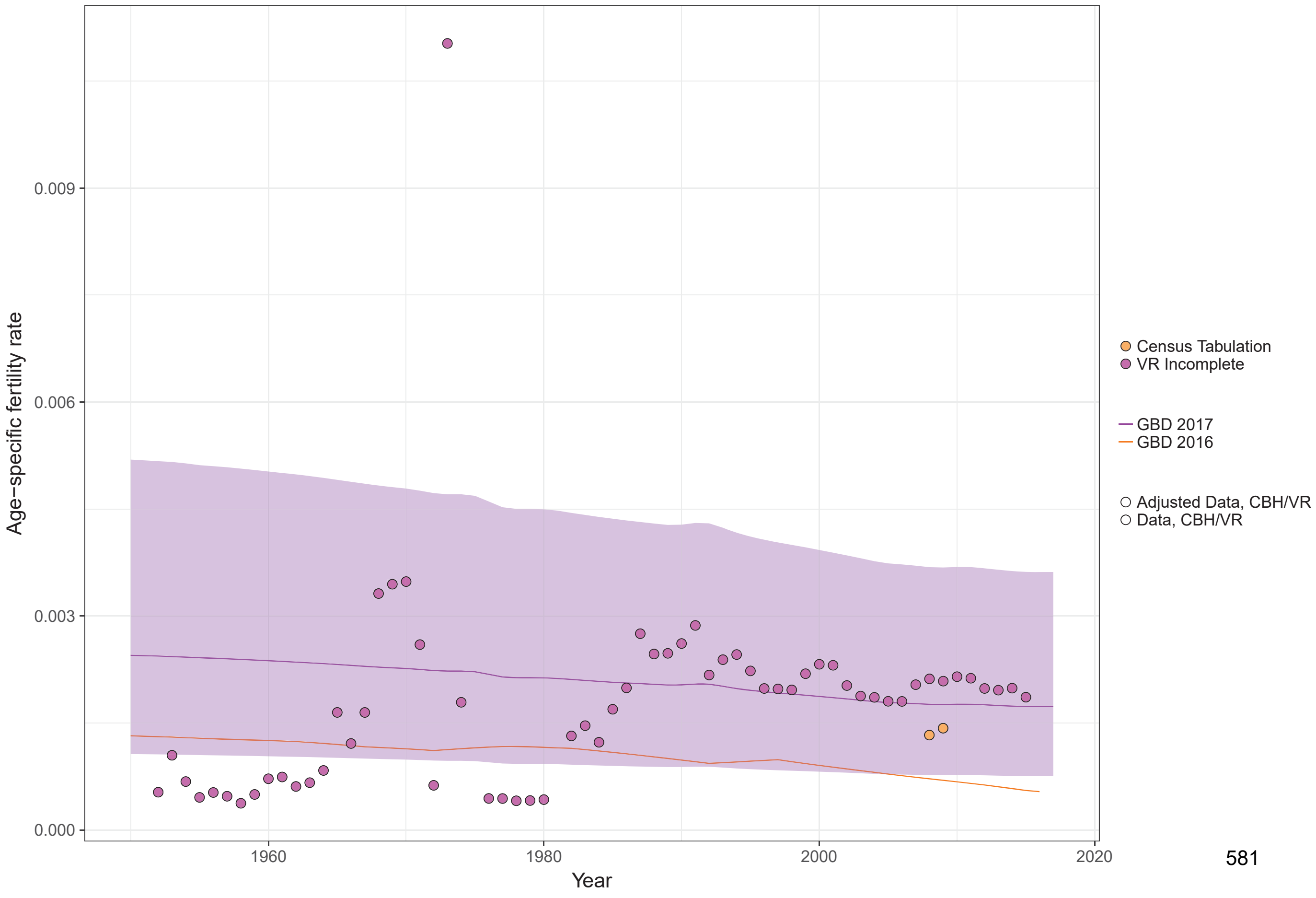

Age−specific fertility fitted trend lines, 1950−2017: Mexico, Ages: 15−19
Data points enclosed with a diamond are reference sources

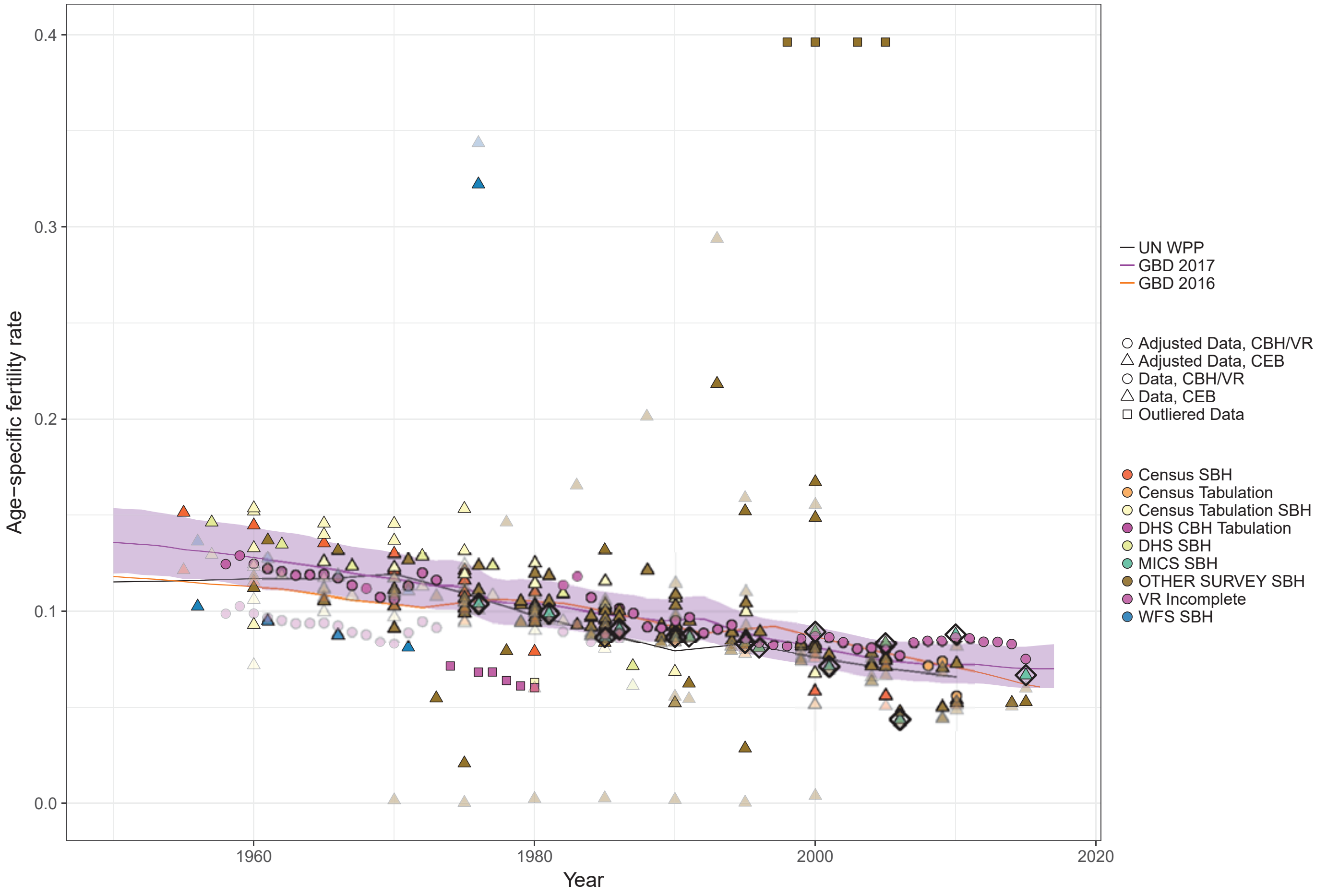

Age−specific fertility fitted trend lines, 1950−2017: Mexico, Ages: 20−24
Data points enclosed with a diamond are reference sources

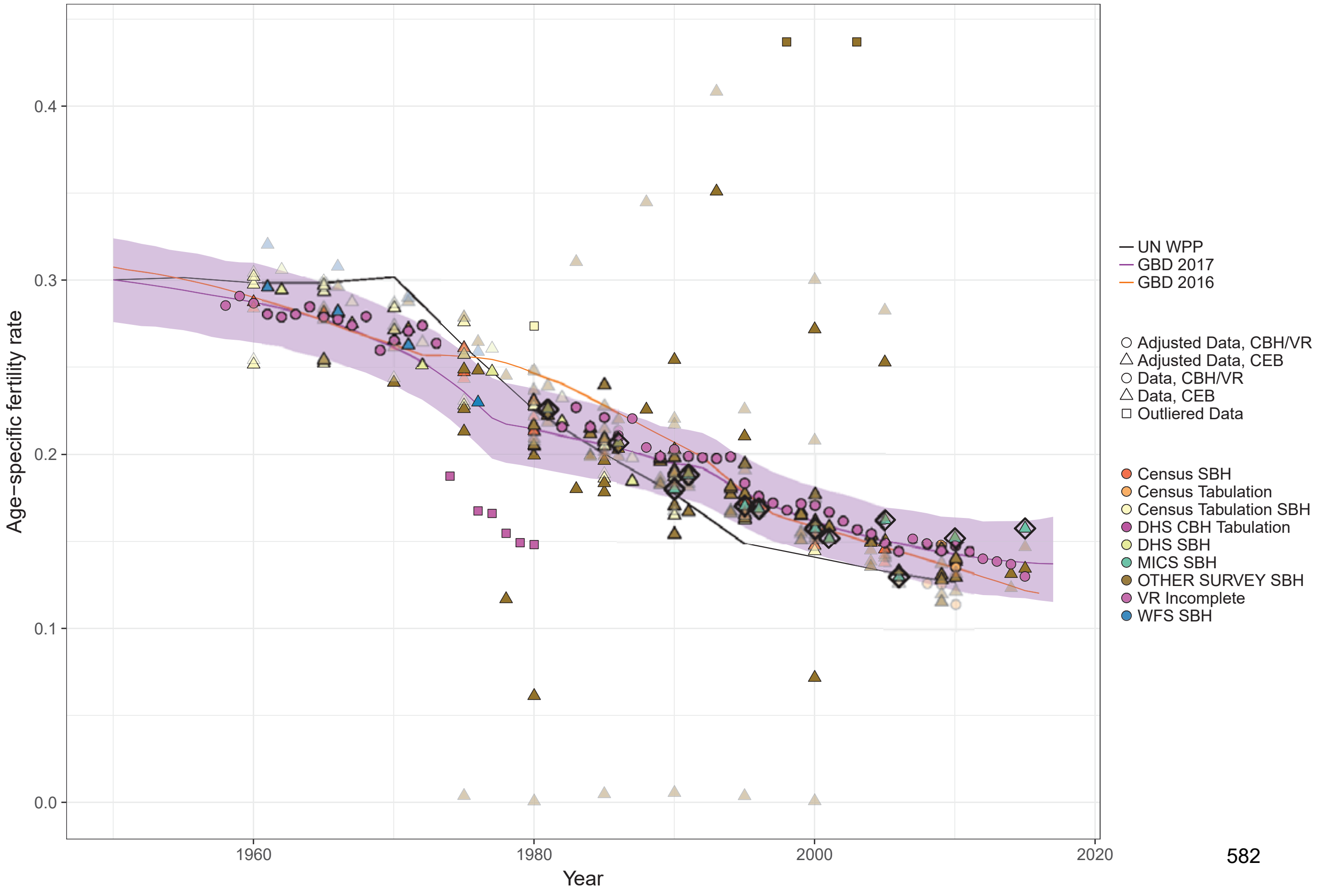

Age−specific fertility fitted trend lines, 1950−2017: Mexico, Ages: 25−29
Data points enclosed with a diamond are reference sources

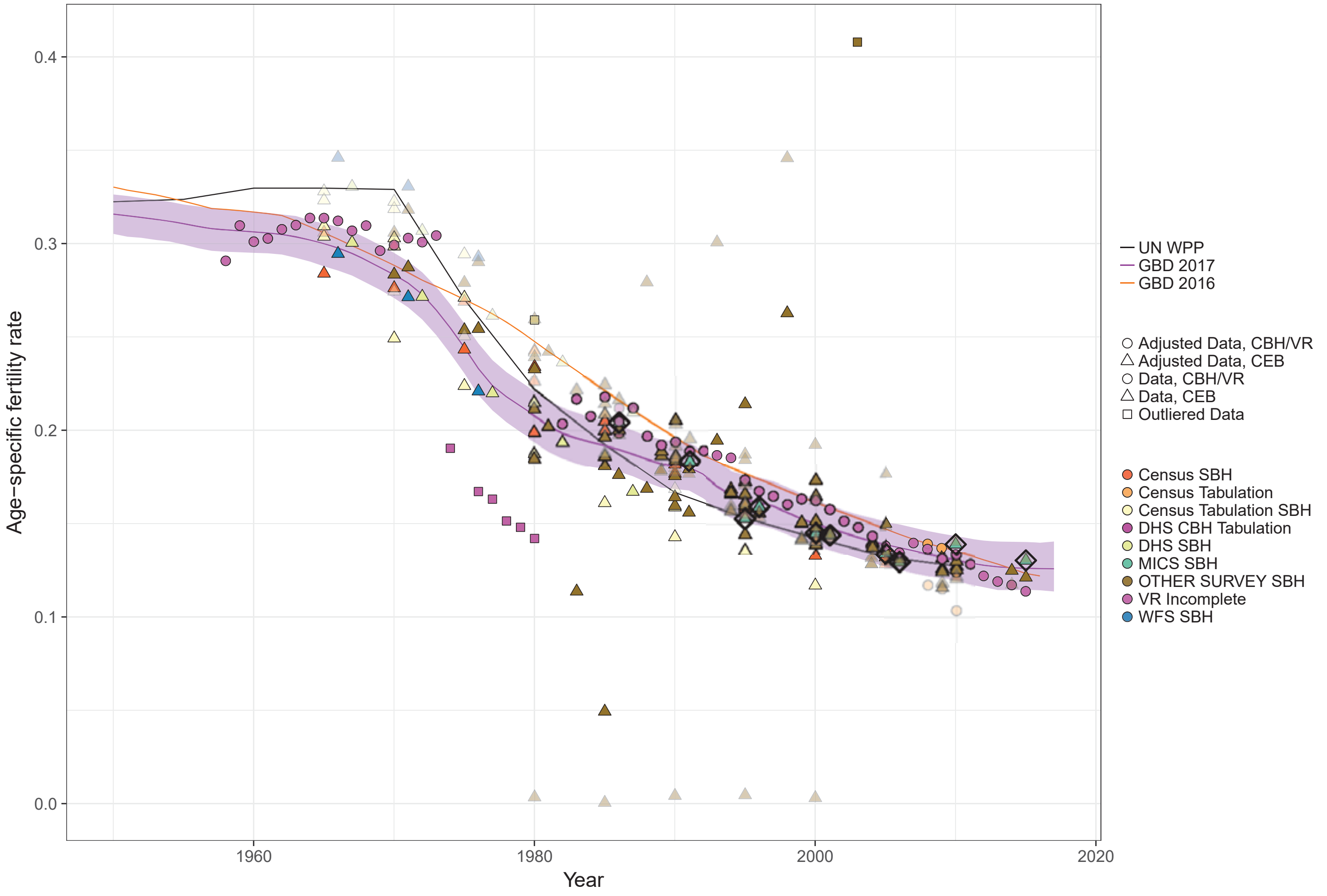

Age−specific fertility fitted trend lines, 1950−2017: Mexico, Ages: 30−34
Data points enclosed with a diamond are reference sources

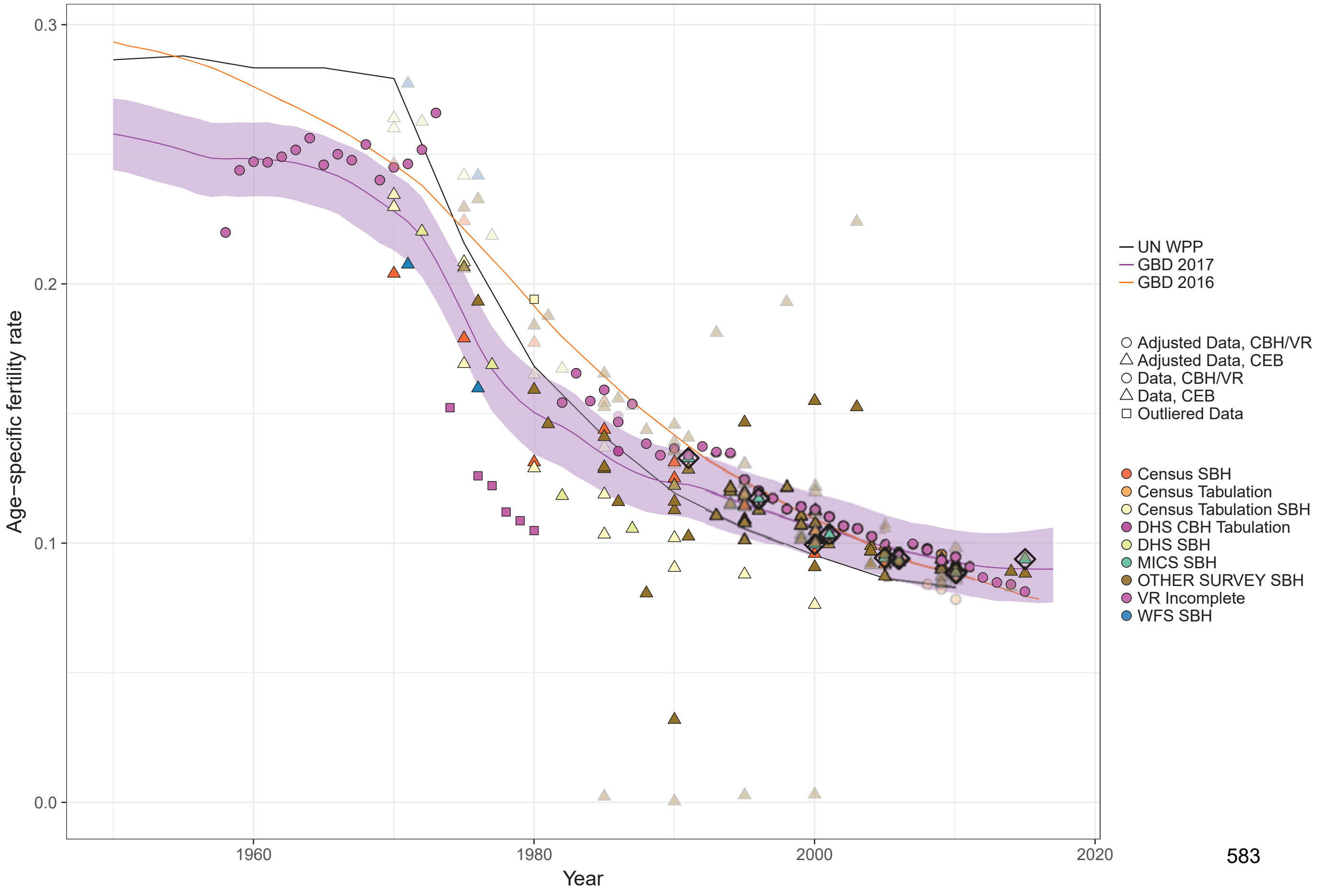

Age−specific fertility fitted trend lines, 1950−2017: Mexico, Ages: 35−39
Data points enclosed with a diamond are reference sources

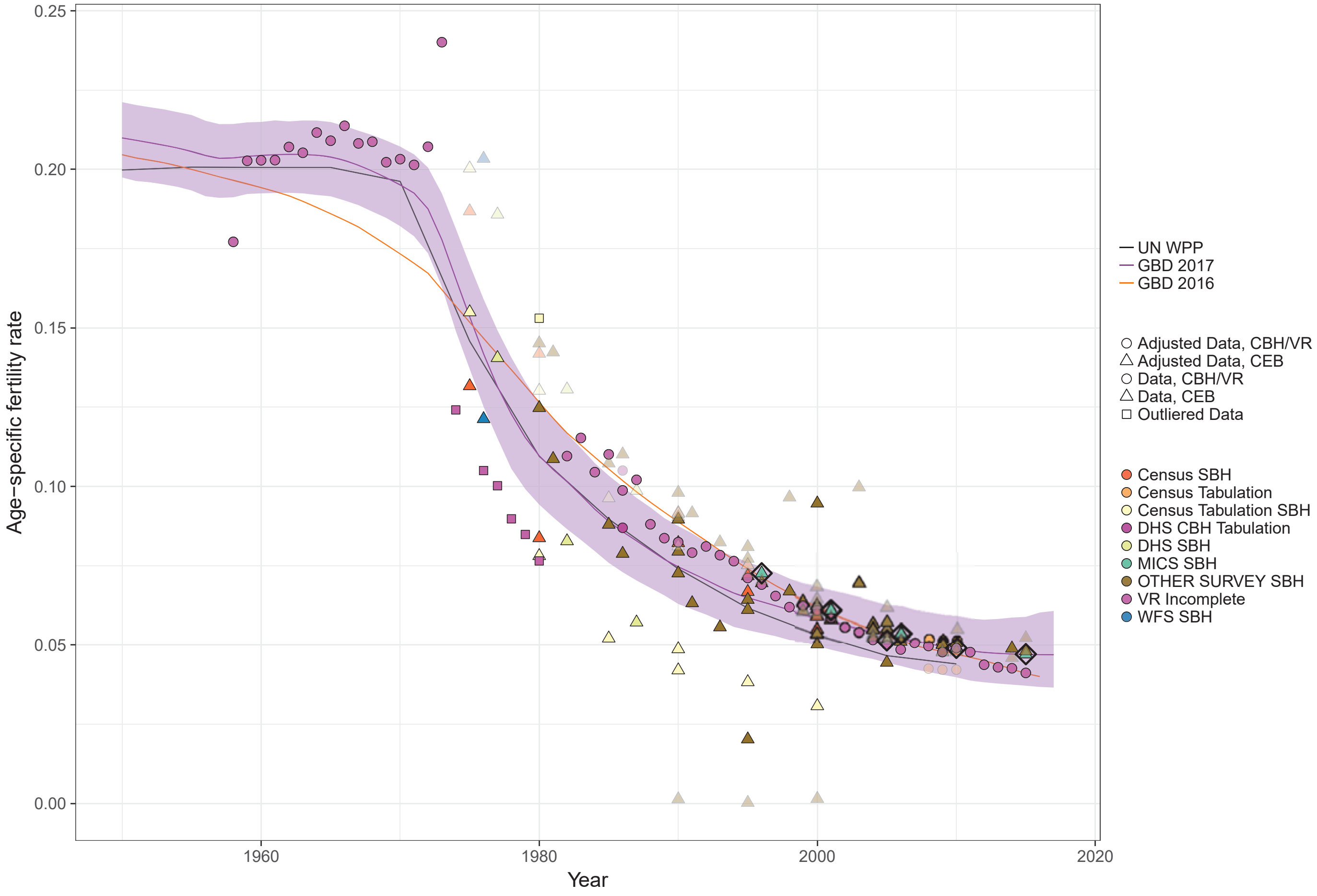

Age−specific fertility fitted trend lines, 1950−2017: Mexico, Ages: 40−44
Data points enclosed with a diamond are reference sources

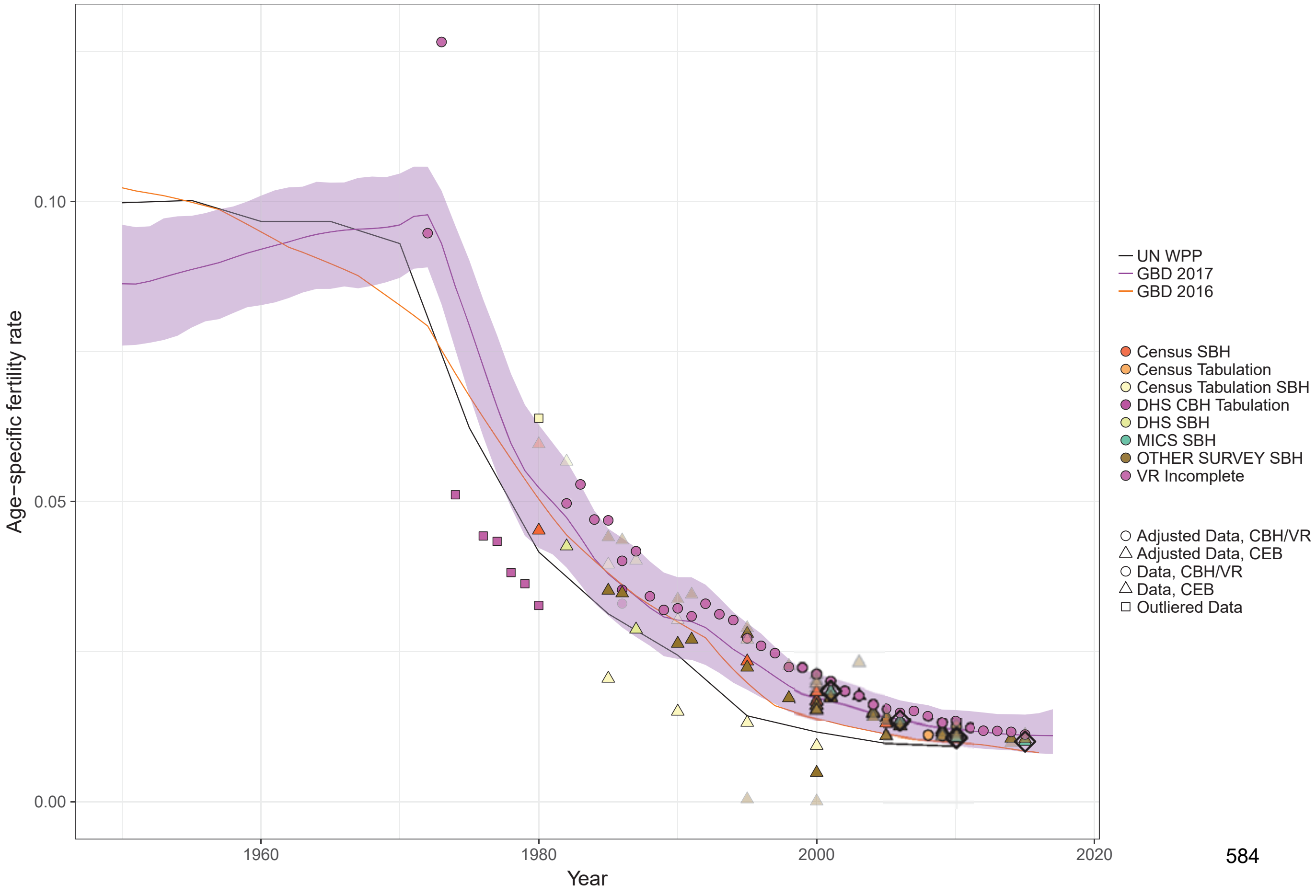

Age−specific fertility fitted trend lines, 1950−2017: Mexico, Ages: 45−49
Data points enclosed with a diamond are reference sources

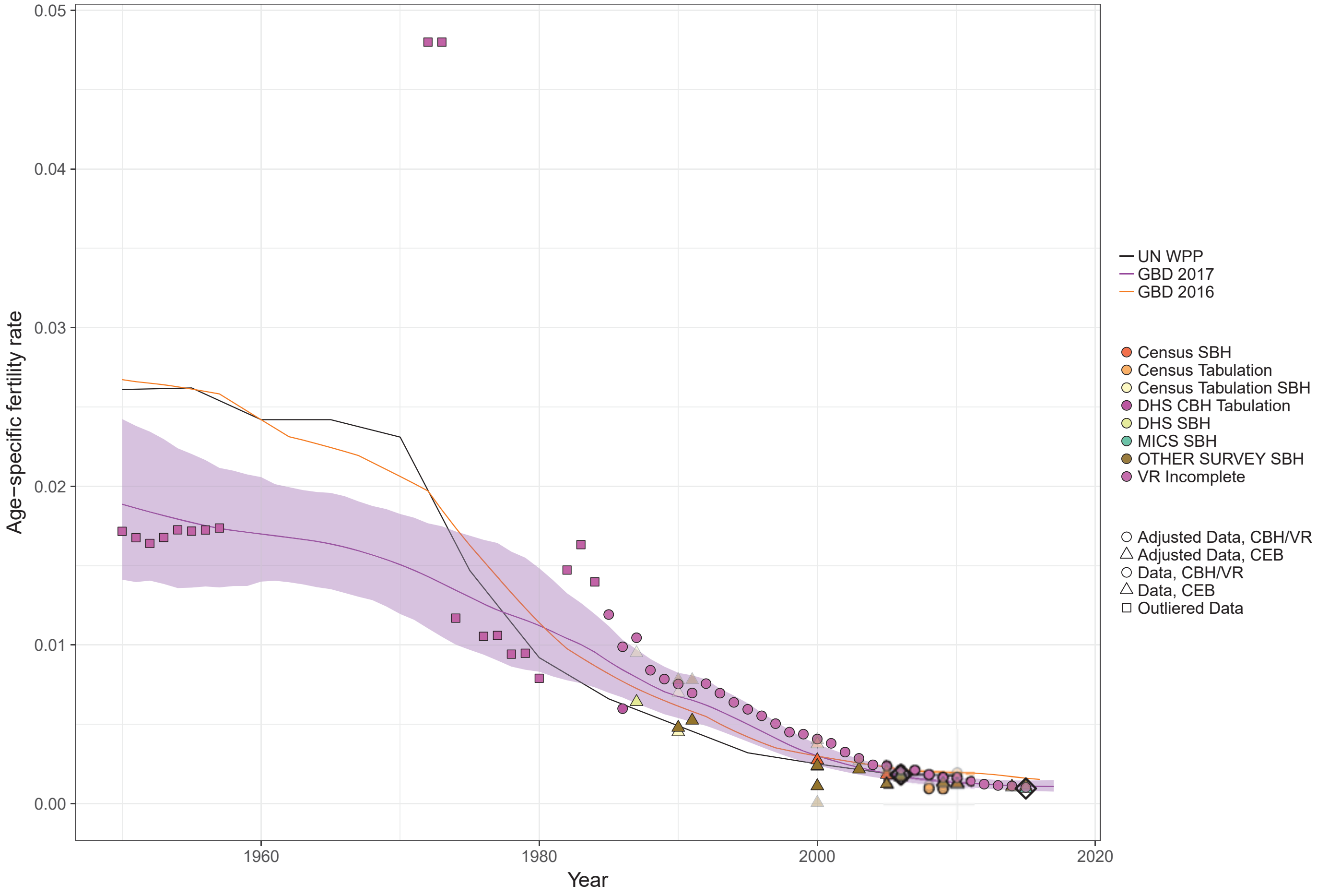

Age−specific fertility fitted trend lines, 1950−2017: Mexico, Ages: 50−54
Data points enclosed with a diamond are reference sources

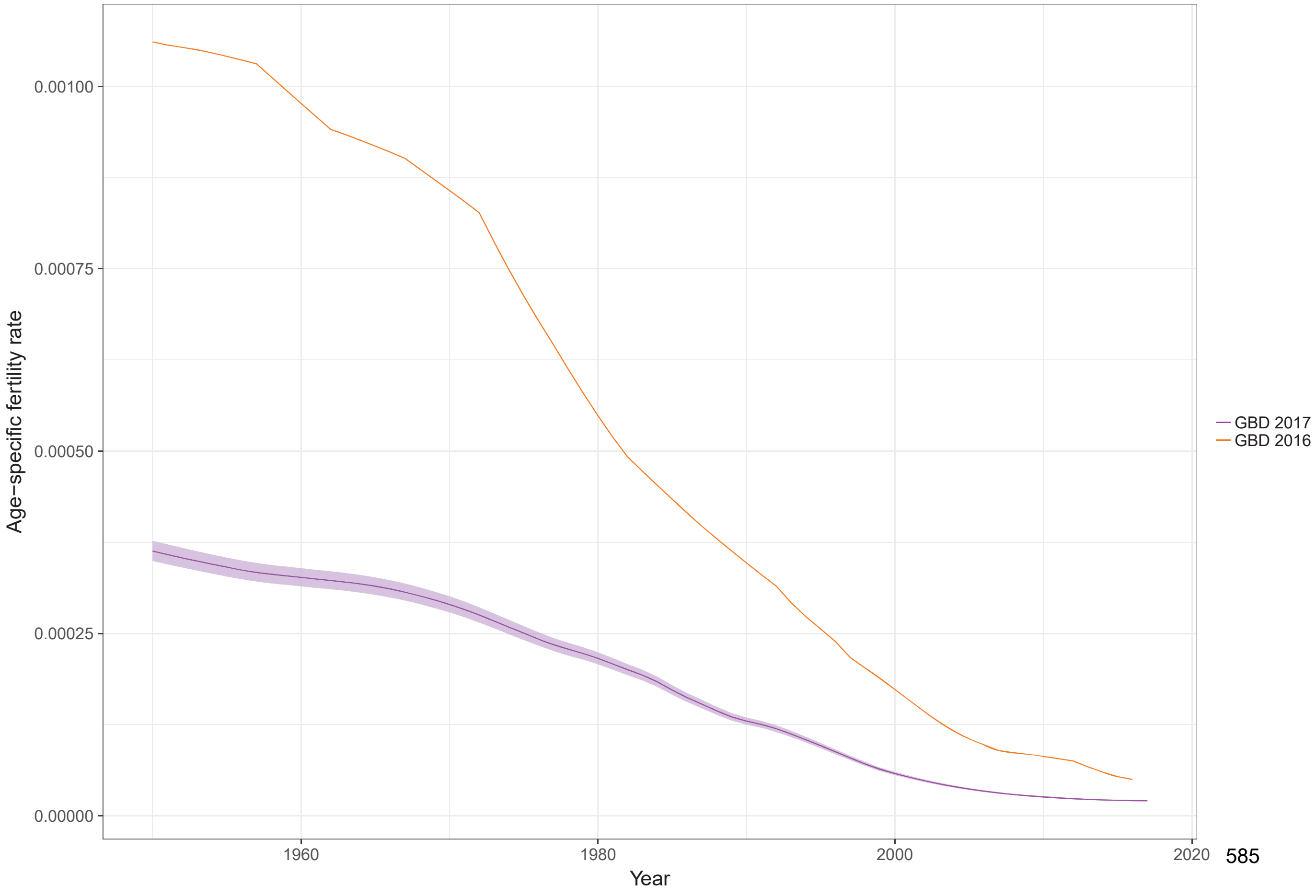

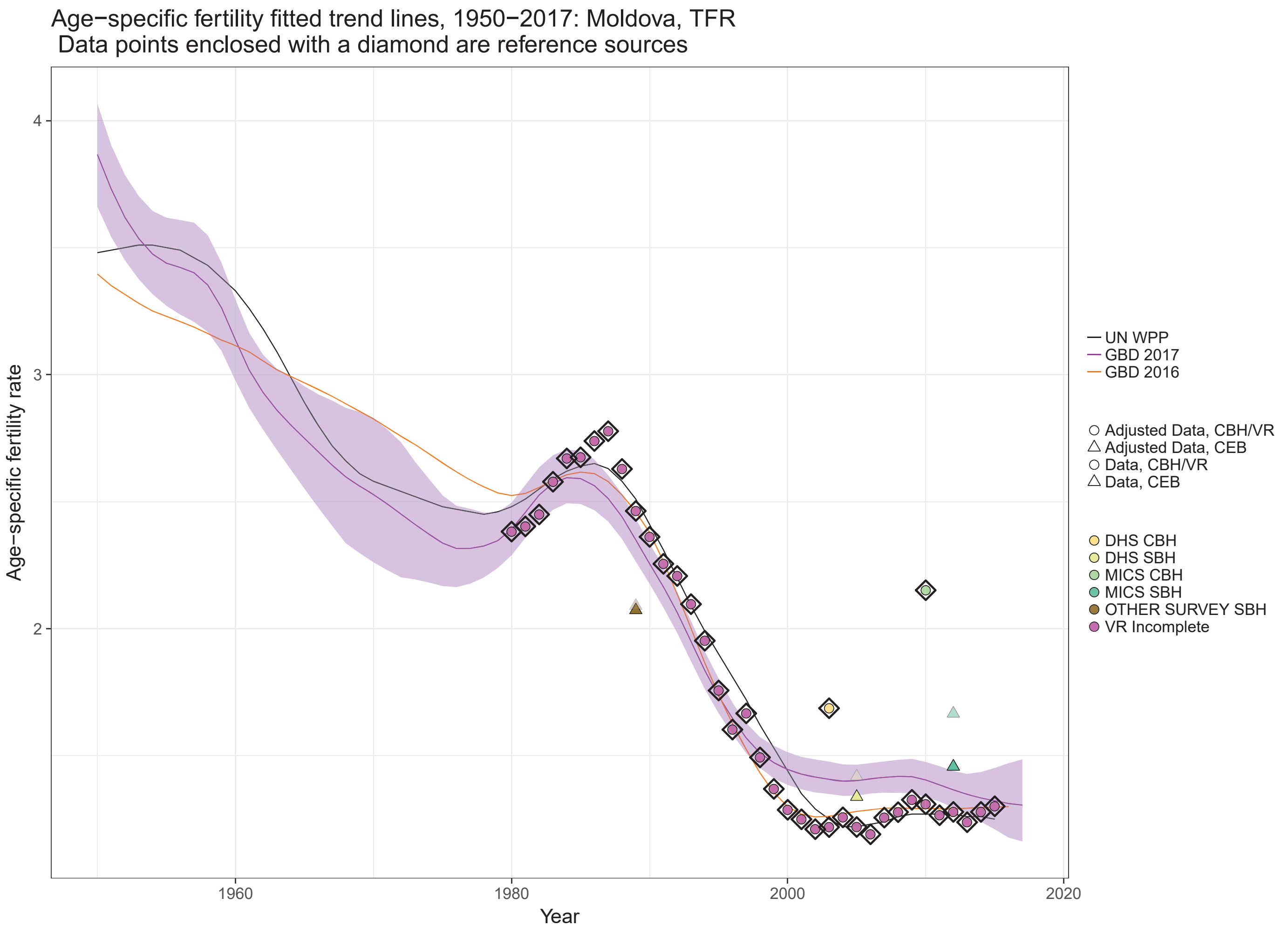

Age−specific fertility fitted trend lines, 1950−2017: Moldova, TFR
Data points enclosed with a diamond are reference sources
Age−specific fertility rate
Year
4
3
2
1960
1980
2000
2020
UN WPP
GBD 2017
GBD 2016
Adjusted Data, CBH/VR
Adjusted Data, CEB
Data, CBH/VR
Data, CEB
DHS CBH
DHS SBH
MICS CBH
MICS SBH
OTHER SURVEY SBH
VR Incomplete

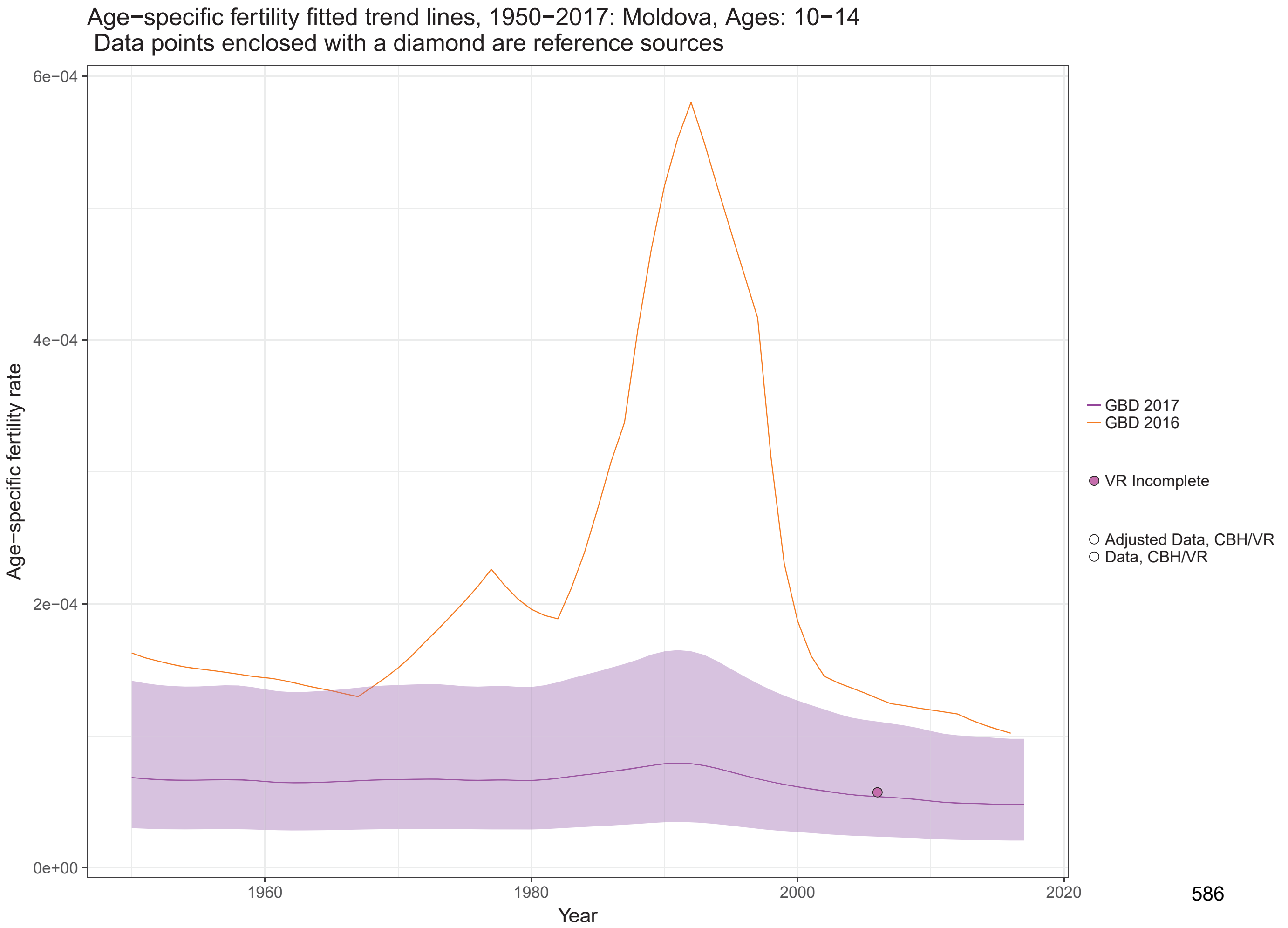

Age−specific fertility fitted trend lines, 1950−2017: Moldova, Ages: 10−14
Data points enclosed with a diamond are reference sources
Age−specific fertility rate
Year
6e−04
4e−04
2e−04
0e+00
1960
1980
2000
2020
GBD 2017
GBD 2016
VR Incomplete
Adjusted Data, CBH/VR
Data, CBH/VR

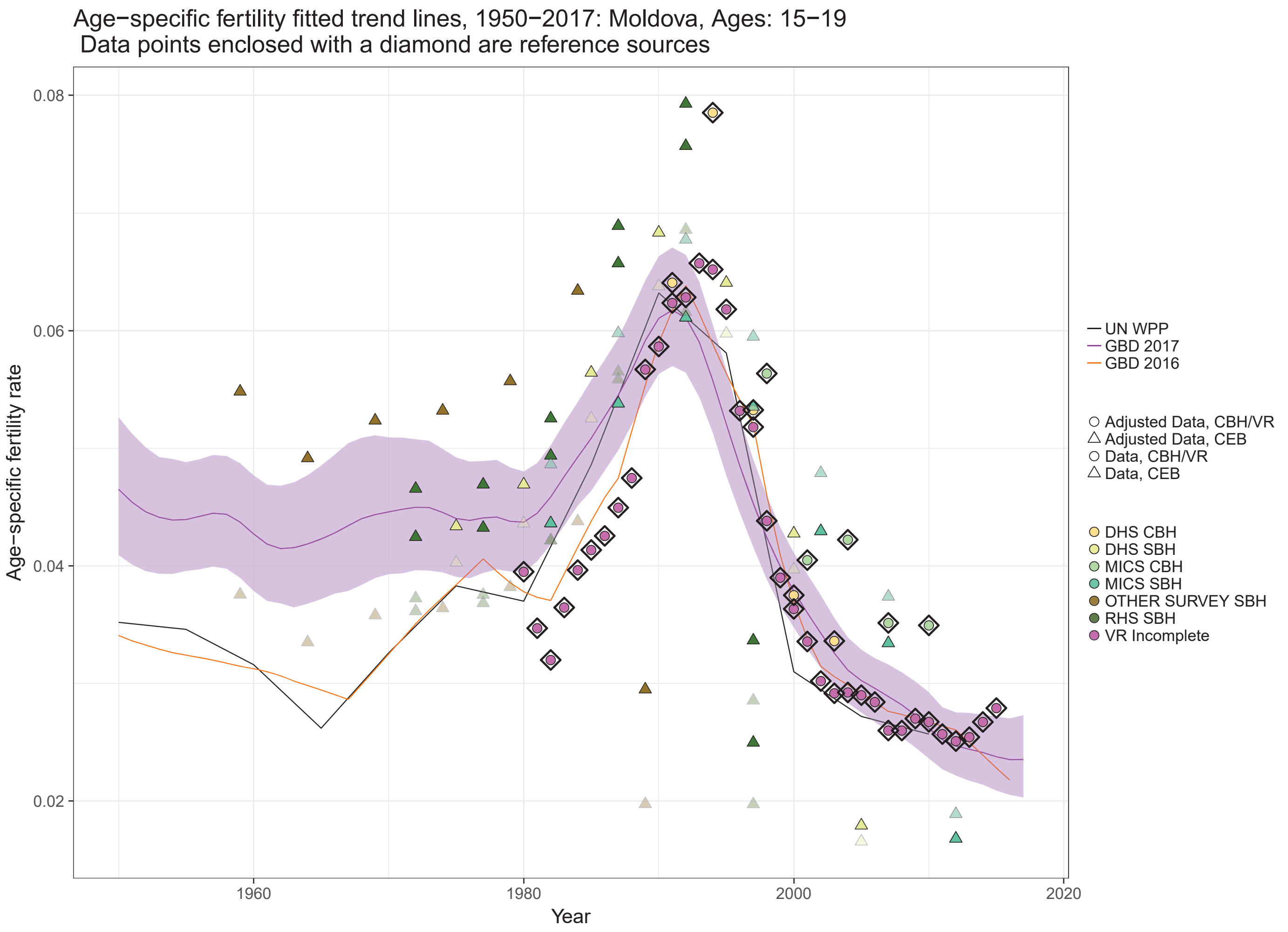

Age−specific fertility fitted trend lines, 1950−2017: Moldova, Ages: 15−19
Data points enclosed with a diamond are reference sources
Age−specific fertility rate
Year
0.02
0.04
0.06
0.08
1960
1980
2000
2020
UN WPP
GBD 2017
GBD 2016
Adjusted Data, CBH/VR
Adjusted Data, CEB
Data, CBH/VR
Data, CEB
DHS CBH
DHS SBH
MICS CBH
MICS SBH
OTHER SURVEY SBH
RHS SBH
VR Incomplete

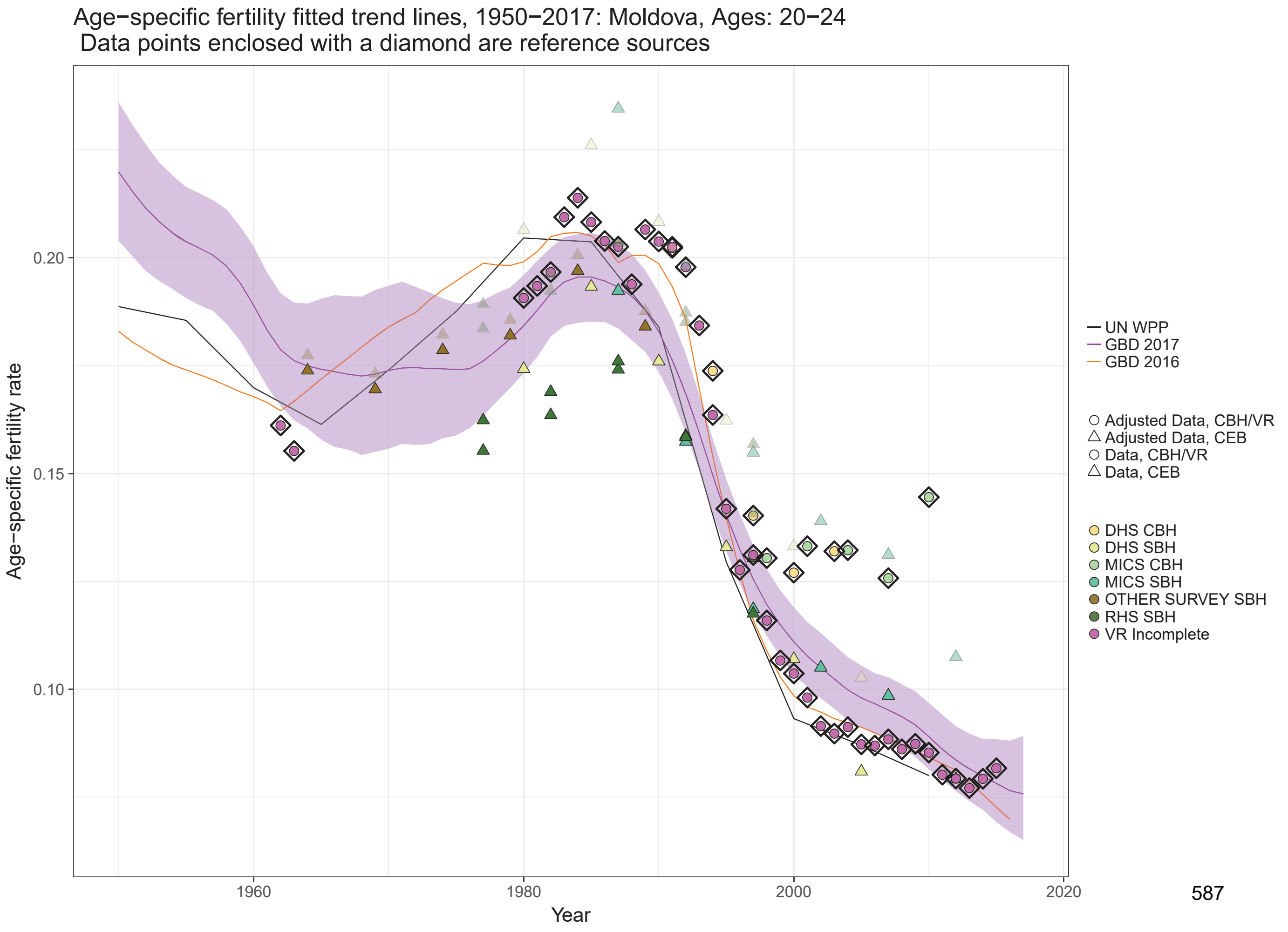

Age−specific fertility fitted trend lines, 1950−2017: Moldova, Ages: 20−24
Data points enclosed with a diamond are reference sources
Age−specific fertility rate
Year
0.10
0.15
0.20
1960
1980
2000
2020
UN WPP
GBD 2017
GBD 2016
Adjusted Data, CBH/VR
Adjusted Data, CEB
Data, CBH/VR
Data, CEB
DHS CBH
DHS SBH
MICS CBH
MICS SBH
OTHER SURVEY SBH
RHS SBH
VR Incomplete

Age−specific fertility fitted trend lines, 1950−2017: Moldova, Ages: 25−29
Data points enclosed with a diamond are reference sources

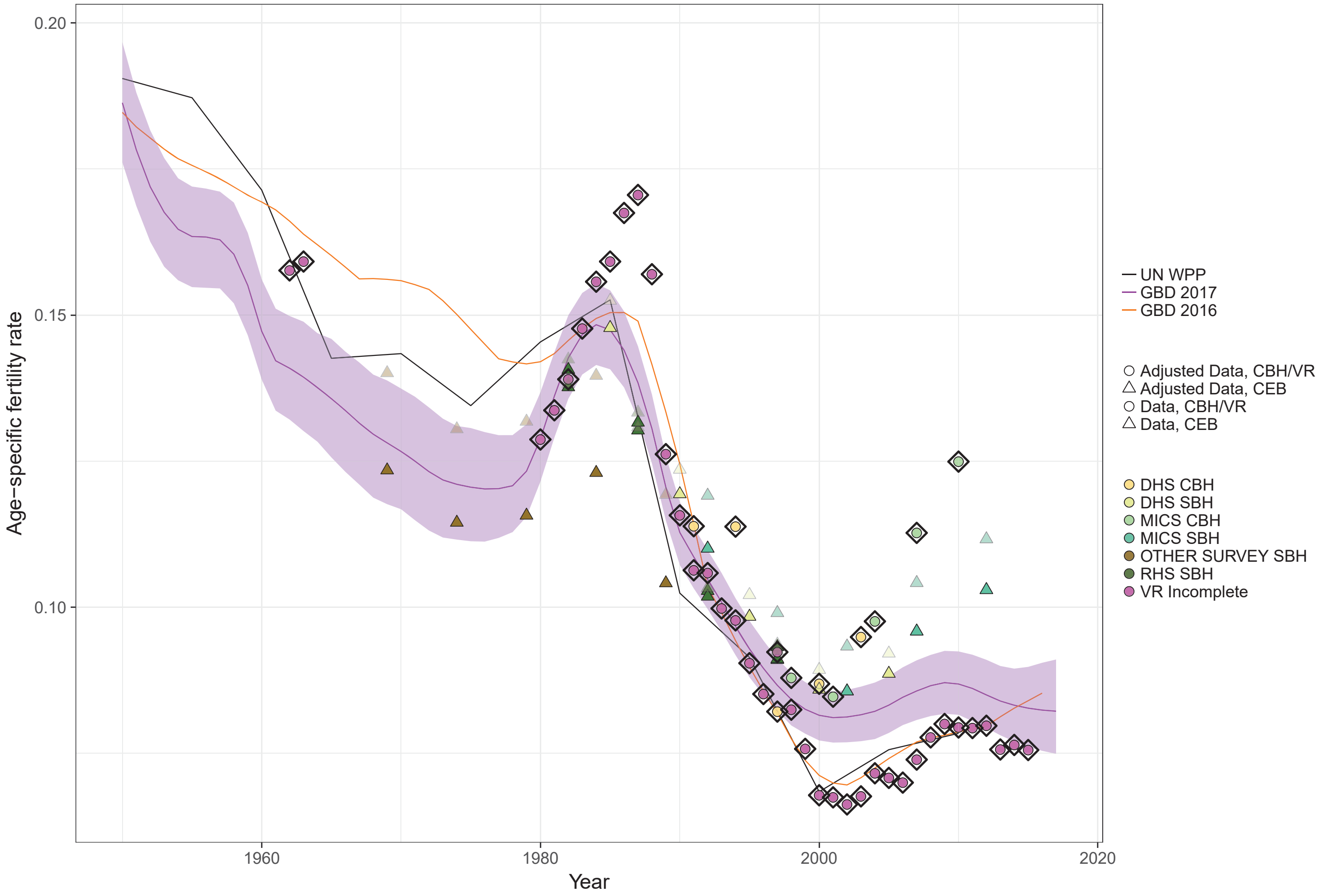

Age−specific fertility fitted trend lines, 1950−2017: Moldova, Ages: 30−34
Data points enclosed with a diamond are reference sources

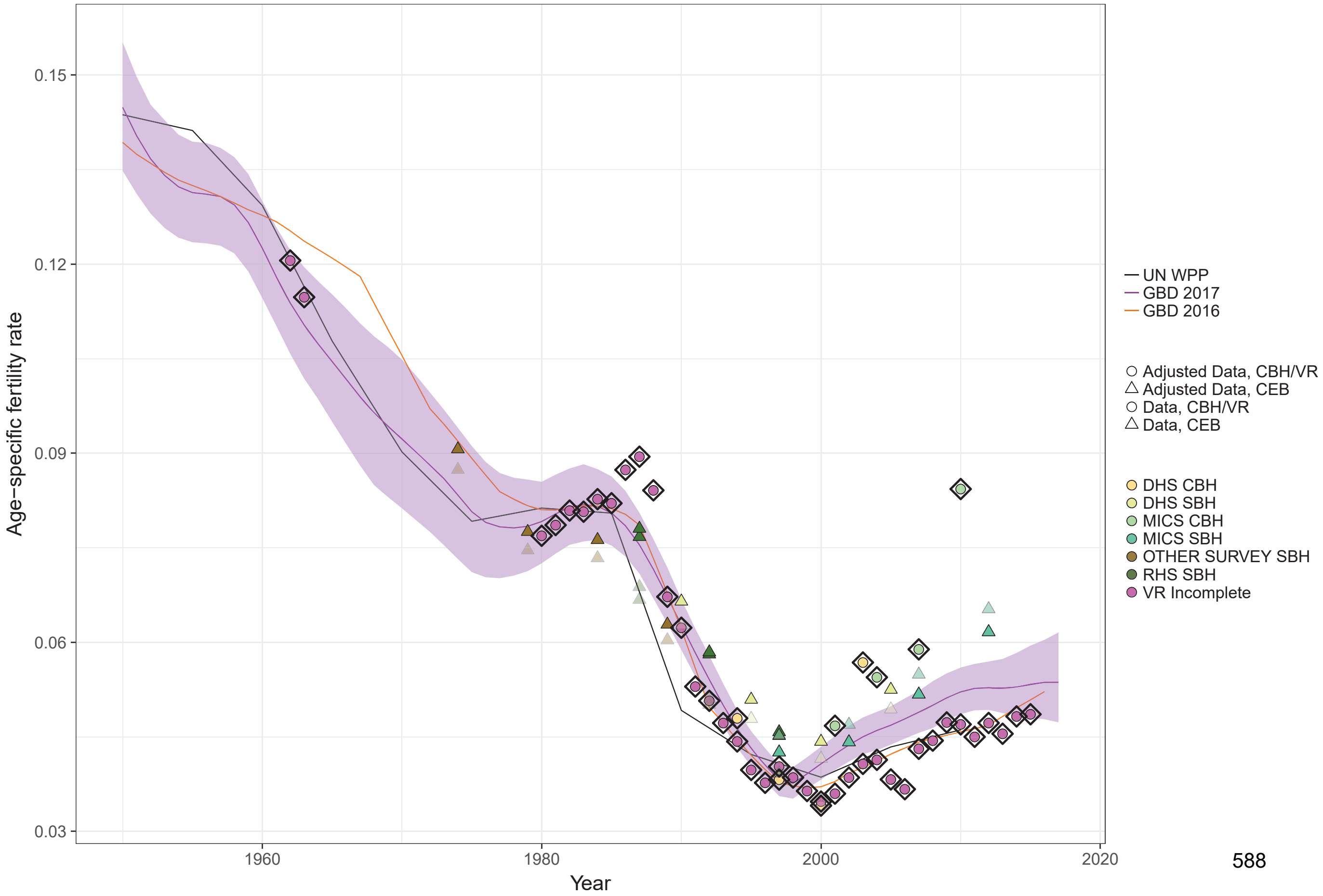

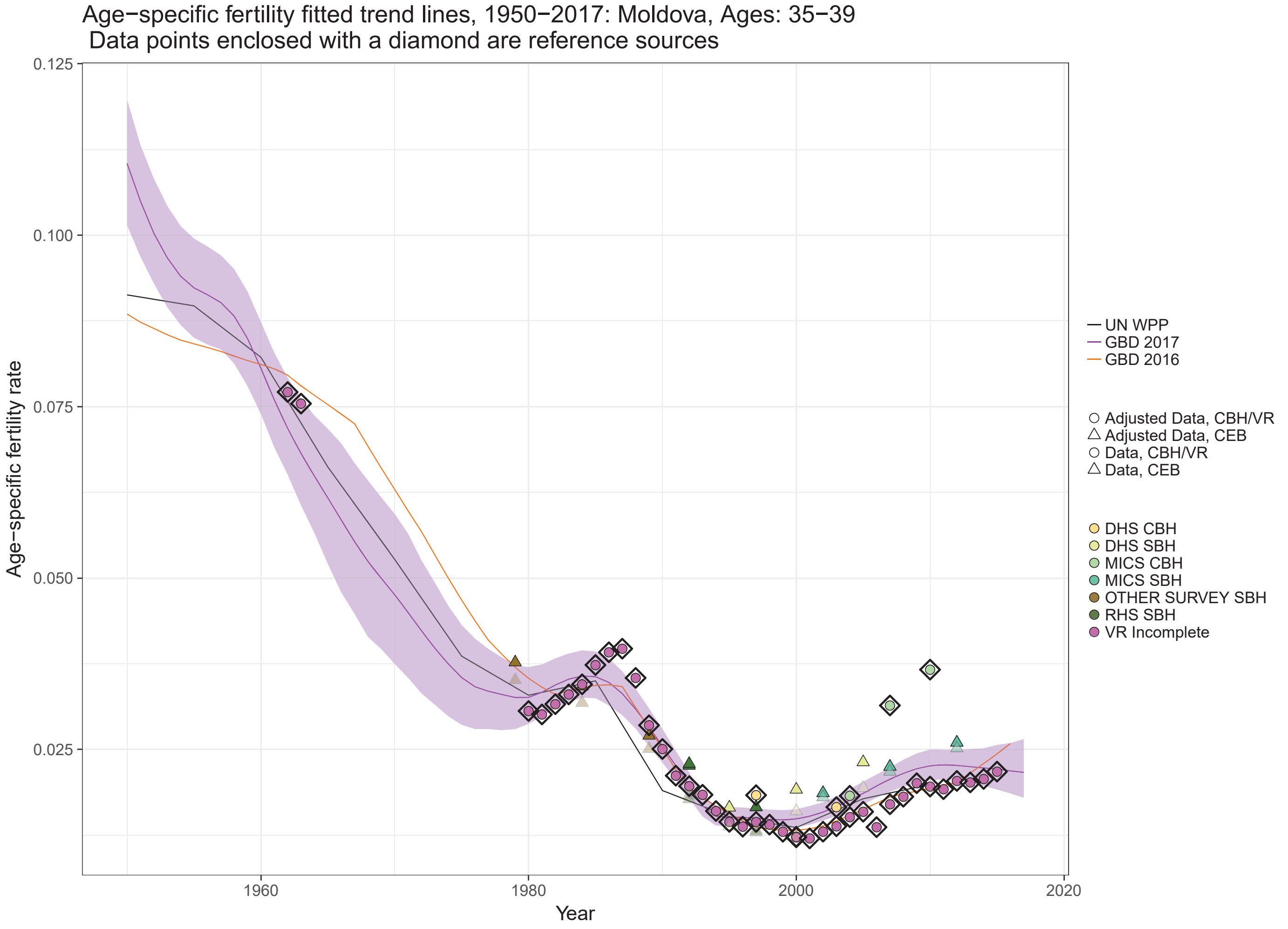

Age−specific fertility fitted trend lines, 1950−2017: Moldova, Ages: 35−39
Data points enclosed with a diamond are reference sources
Age−specific fertility rate
0.125
0.100
0.075
0.050
0.025
1960
1980
2000
2020
Year
UN WPP
GBD 2017
GBD 2016
Adjusted Data, CBH/VR
Adjusted Data, CEB
Data, CBH/VR
Data, CEB
DHS CBH
DHS SBH
MICS CBH
MICS SBH
OTHER SURVEY SBH
RHS SBH
VR Incomplete

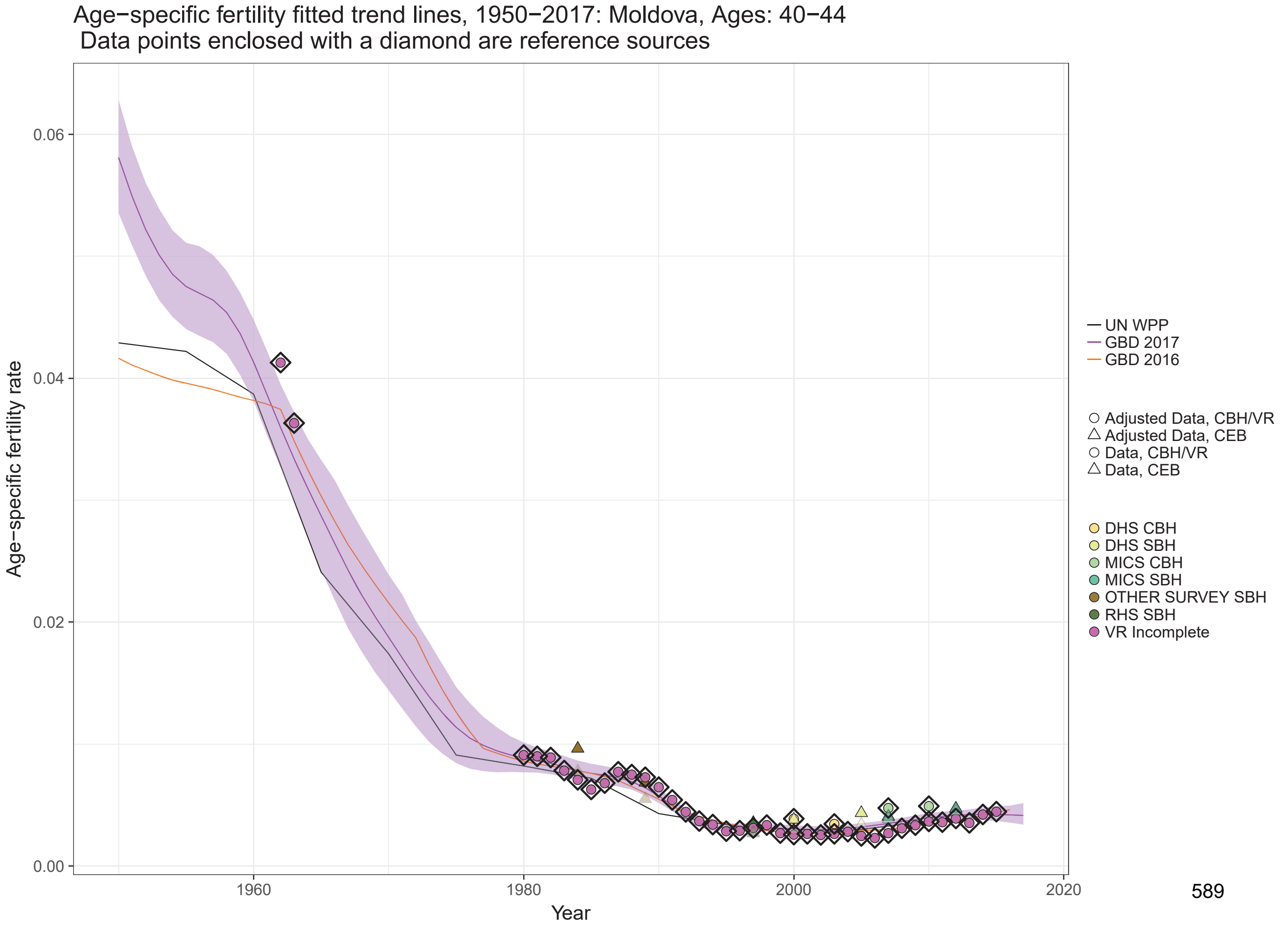

Age−specific fertility fitted trend lines, 1950−2017: Moldova, Ages: 40−44
Data points enclosed with a diamond are reference sources
Age−specific fertility rate
0.06
0.04
0.02
0.00
1960
1980
2000
2020
Year
UN WPP
GBD 2017
GBD 2016
Adjusted Data, CBH/VR
Adjusted Data, CEB
Data, CBH/VR
Data, CEB
DHS CBH
DHS SBH
MICS CBH
MICS SBH
OTHER SURVEY SBH
RHS SBH
VR Incomplete

Age−specific fertility fitted trend lines, 1950−2017: Moldova, Ages: 45−49
Data points enclosed with a diamond are reference sources

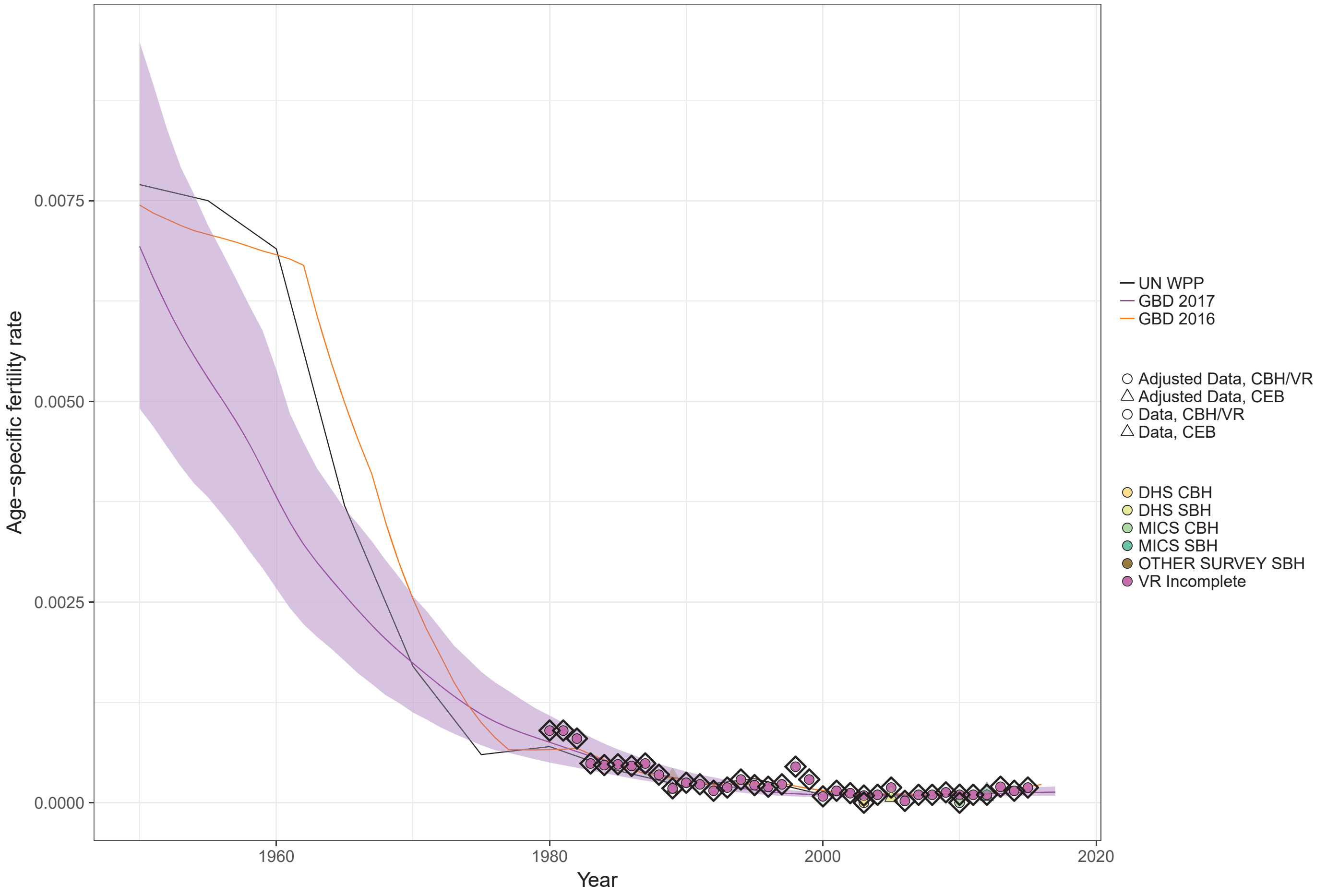

Age−specific fertility fitted trend lines, 1950−2017: Moldova, Ages: 50−54
Data points enclosed with a diamond are reference sources

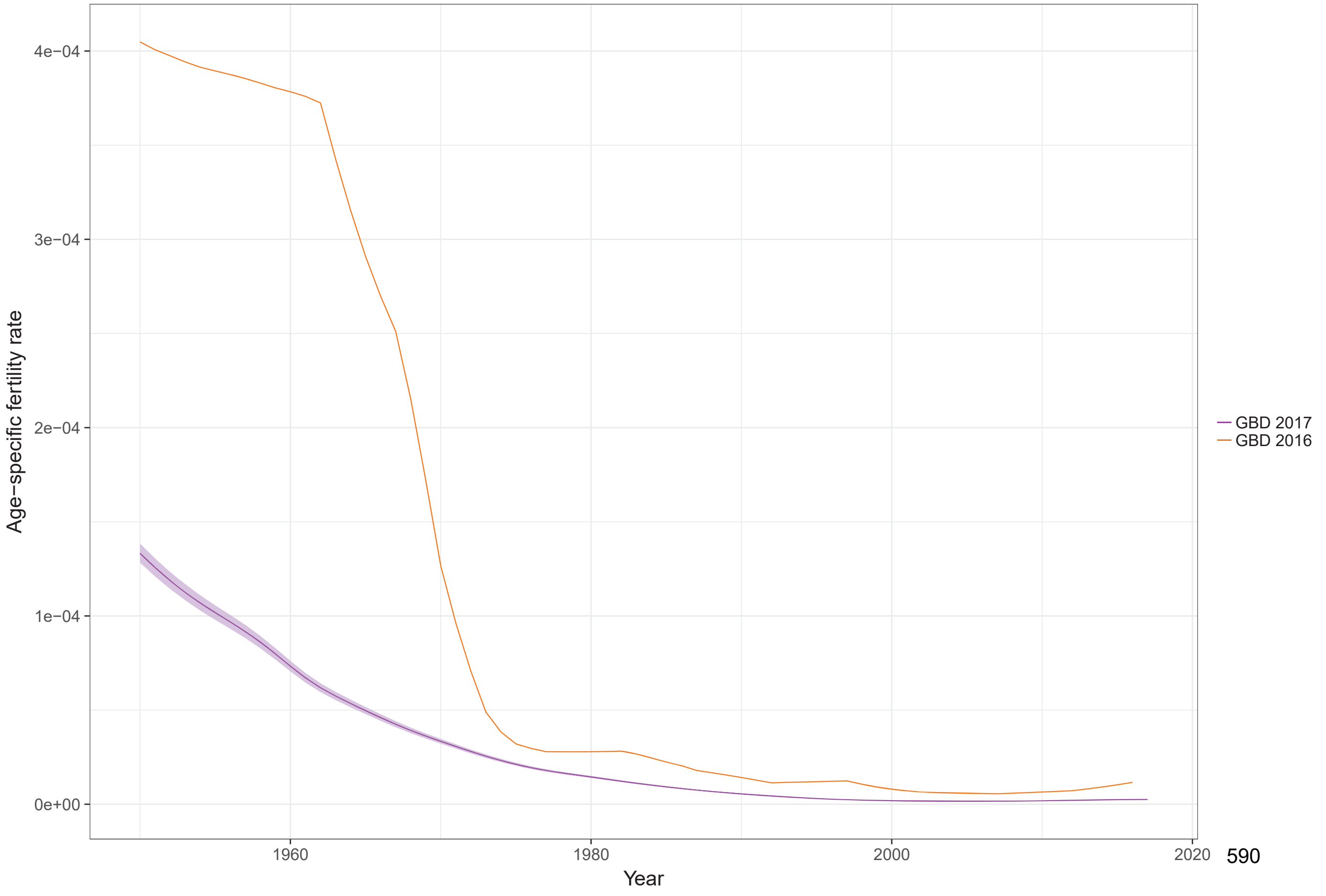

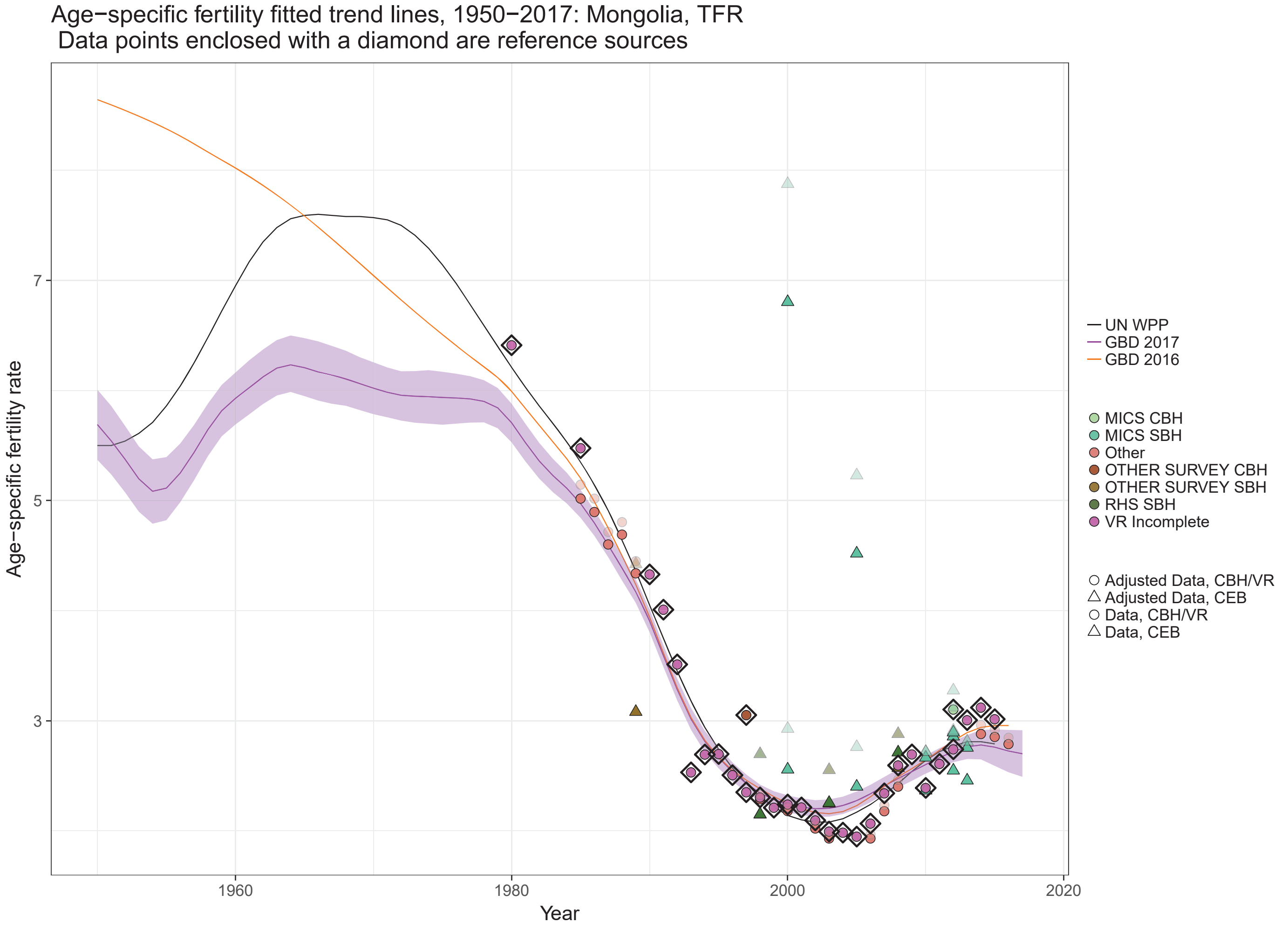

Age−specific fertility fitted trend lines, 1950−2017: Mongolia, TFR
Data points enclosed with a diamond are reference sources
Age−specific fertility rate
Year
UN WPP
GBD 2017
GBD 2016
MICS CBH
MICS SBH
Other
OTHER SURVEY CBH
OTHER SURVEY SBH
RHS SBH
VR Incomplete
Adjusted Data, CBH/VR
Adjusted Data, CEB
Data, CBH/VR
Data, CEB

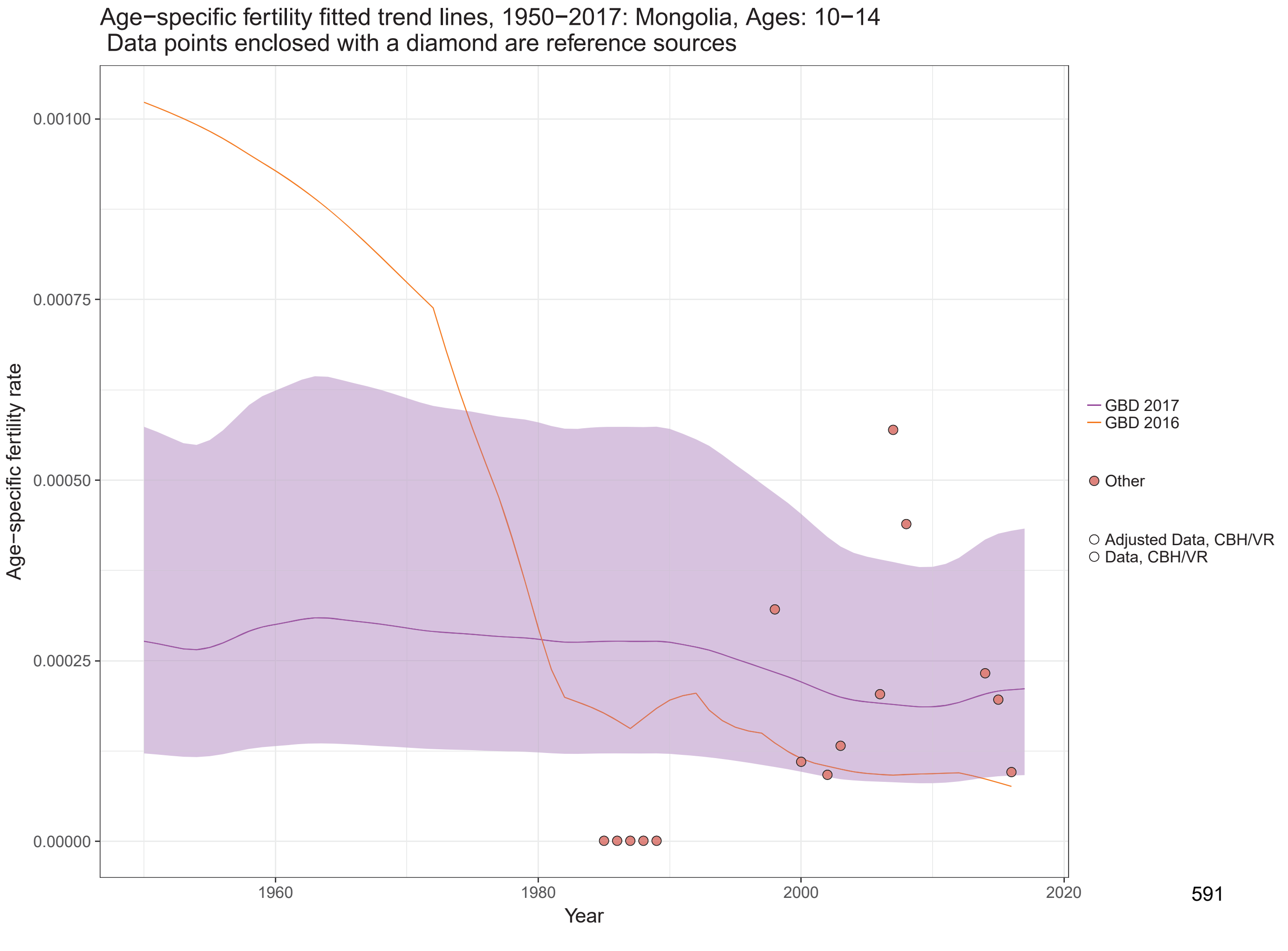

Age−specific fertility fitted trend lines, 1950−2017: Mongolia, Ages: 10−14
Data points enclosed with a diamond are reference sources
Age−specific fertility rate
Year
GBD 2017
GBD 2016
Other
Adjusted Data, CBH/VR
Data, CBH/VR

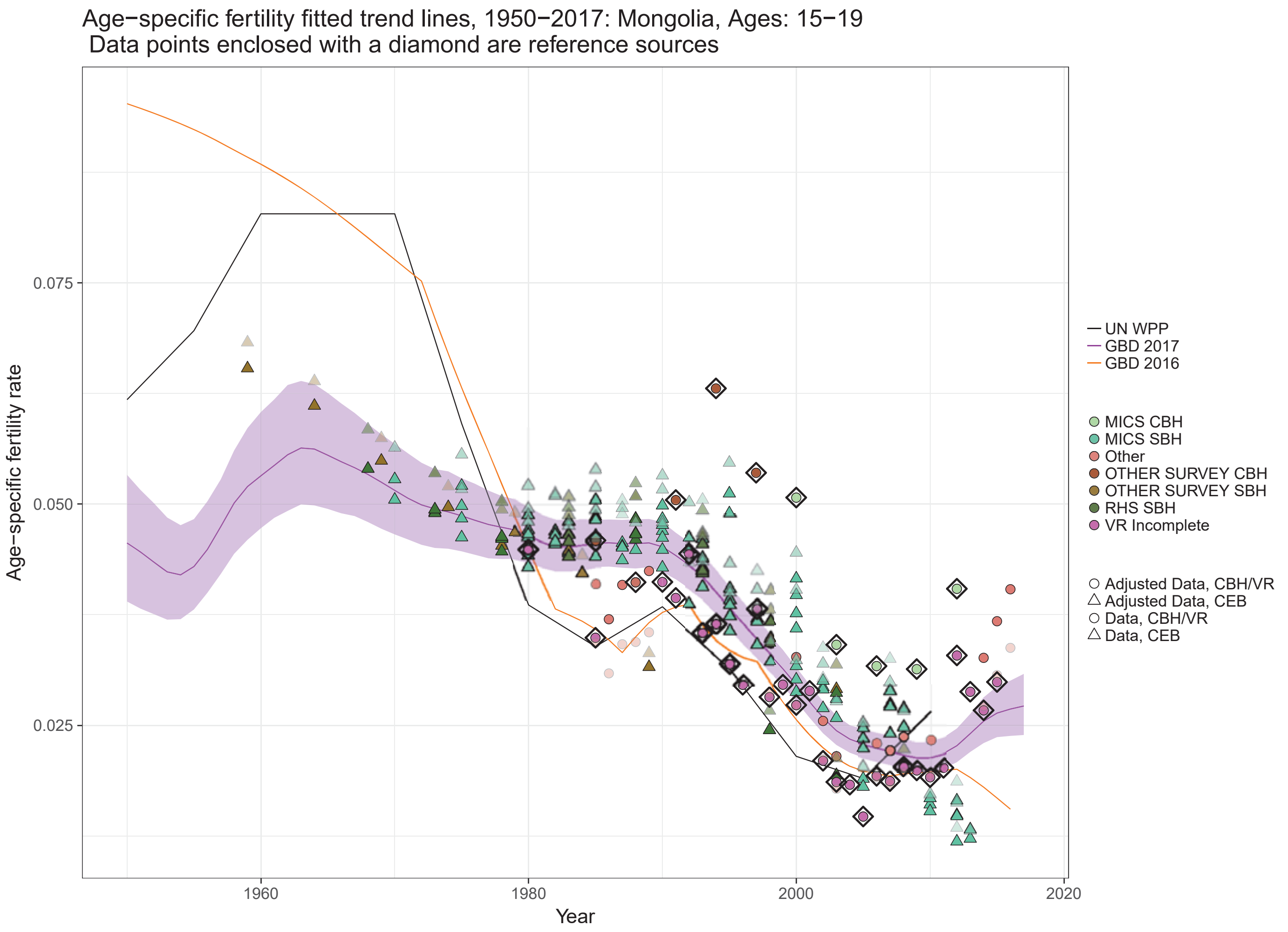

Age−specific fertility fitted trend lines, 1950−2017: Mongolia, Ages: 15−19
Data points enclosed with a diamond are reference sources
Age−specific fertility rate
0.075
0.050
0.025
1960
1980
2000
2020
Year
UN WPP
GBD 2017
GBD 2016
MICS CBH
MICS SBH
Other
OTHER SURVEY CBH
OTHER SURVEY SBH
RHS SBH
VR Incomplete
Adjusted Data, CBH/VR
Adjusted Data, CEB
Data, CBH/VR
Data, CEB

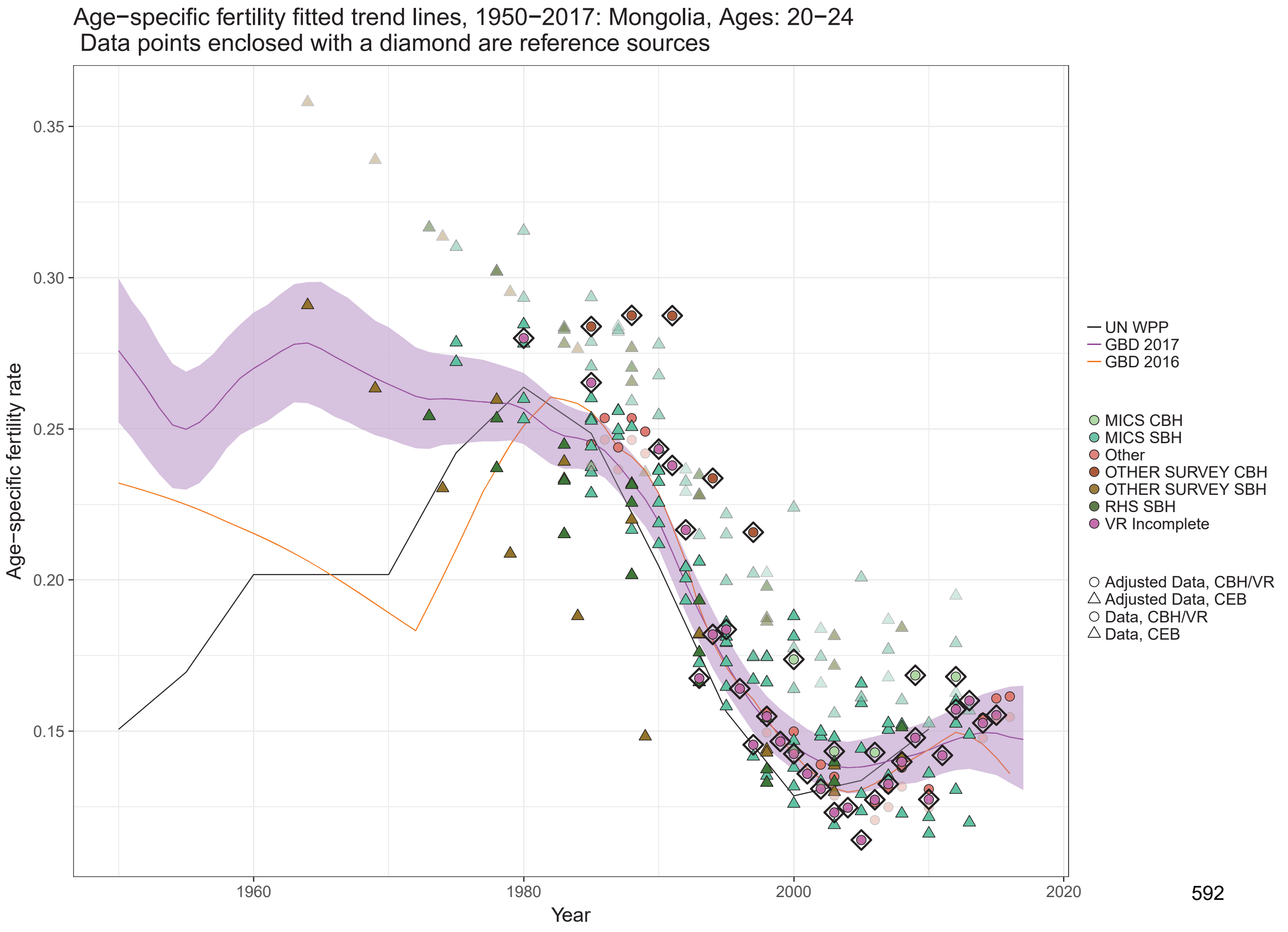

Age−specific fertility fitted trend lines, 1950−2017: Mongolia, Ages: 20−24
Data points enclosed with a diamond are reference sources
Age−specific fertility rate
0.35
0.30
0.25
0.20
0.15
1960
1980
2000
2020
Year
UN WPP
GBD 2017
GBD 2016
MICS CBH
MICS SBH
Other
OTHER SURVEY CBH
OTHER SURVEY SBH
RHS SBH
VR Incomplete
Adjusted Data, CBH/VR
Adjusted Data, CEB
Data, CBH/VR
Data, CEB

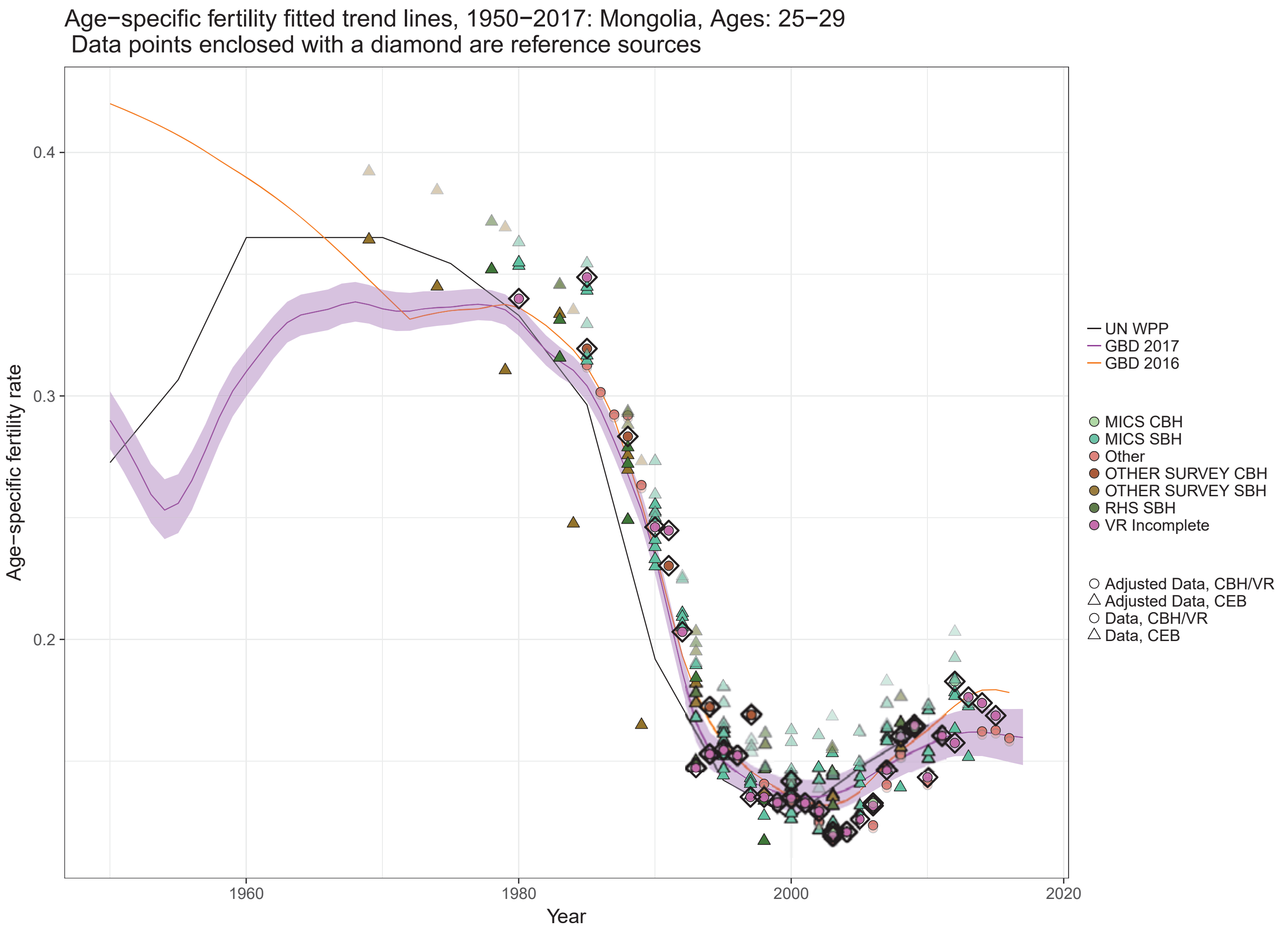
Age−specific fertility fitted trend lines, 1950−2017: Mongolia, Ages: 25−29
Data points enclosed with a diamond are reference sources
Age-specific fertility rate
Year
UN WPP
GBD 2017
GBD 2016
MICS CBH
MICS SBH
Other
OTHER SURVEY CBH
OTHER SURVEY SBH
RHS SBH
VR Incomplete
Adjusted Data, CBH/VR
Adjusted Data, CEB
Data, CBH/VR
Data, CEB

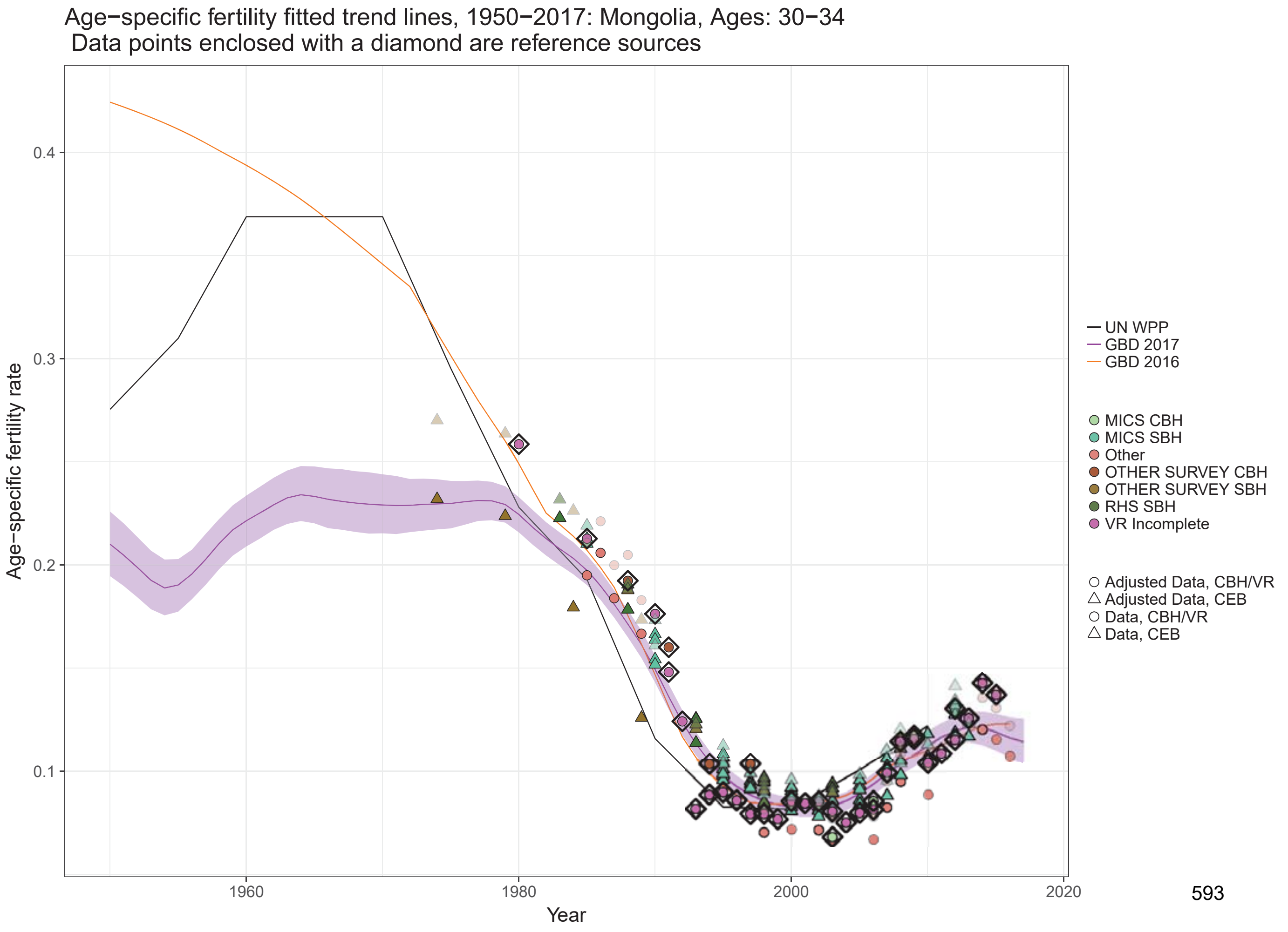
Age−specific fertility fitted trend lines, 1950−2017: Mongolia, Ages: 30−34
Data points enclosed with a diamond are reference sources
Age-specific fertility rate
Year
UN WPP
GBD 2017
GBD 2016
MICS CBH
MICS SBH
Other
OTHER SURVEY CBH
OTHER SURVEY SBH
RHS SBH
VR Incomplete
Adjusted Data, CBH/VR
Adjusted Data, CEB
Data, CBH/VR
Data, CEB

Age−specific fertility fitted trend lines, 1950−2017: Mongolia, Ages: 35−39
Data points enclosed with a diamond are reference sources

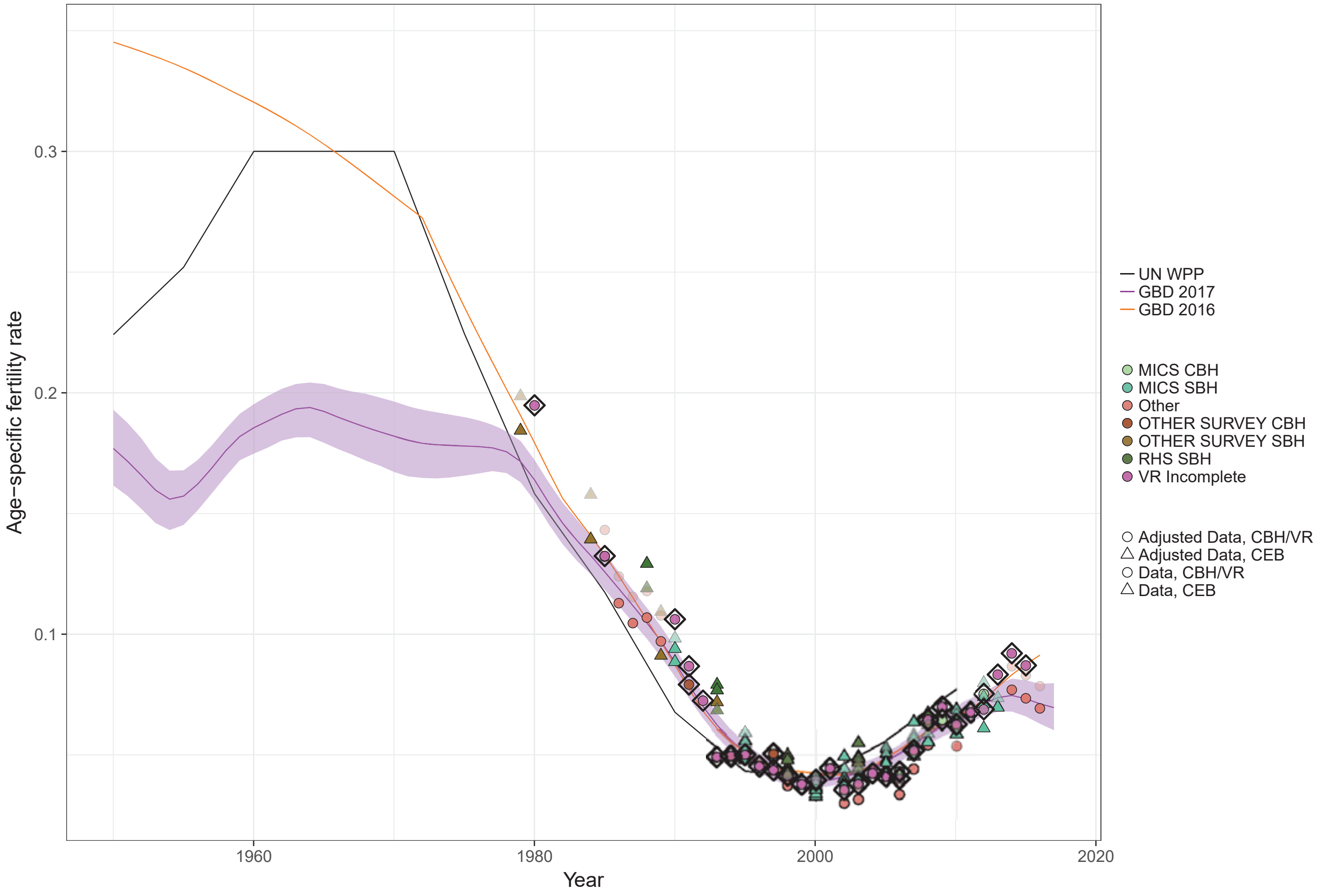

Age−specific fertility fitted trend lines, 1950−2017: Mongolia, Ages: 40−44
Data points enclosed with a diamond are reference sources

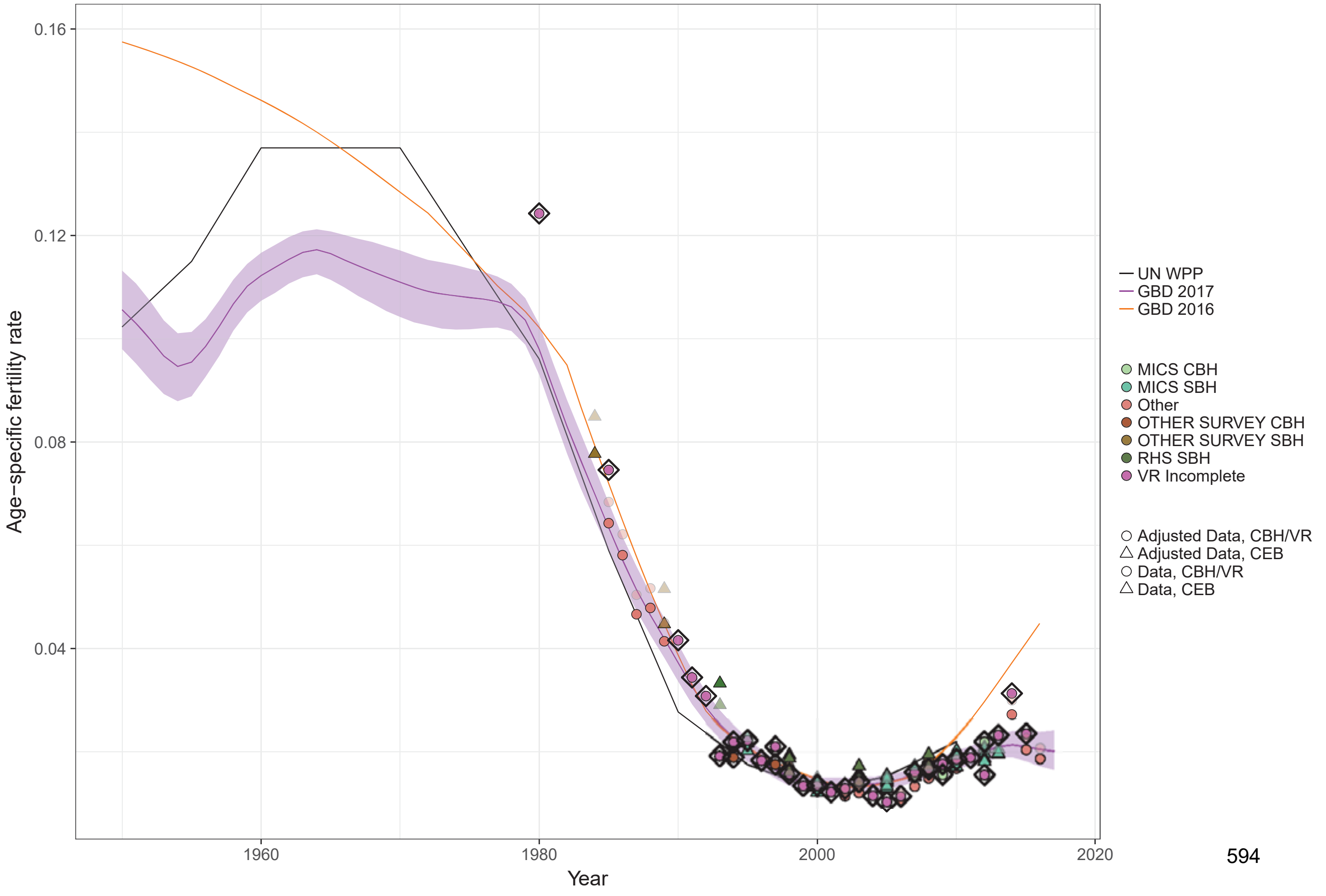

Age−specific fertility fitted trend lines, 1950−2017: Mongolia, Ages: 45−49
Data points enclosed with a diamond are reference sources

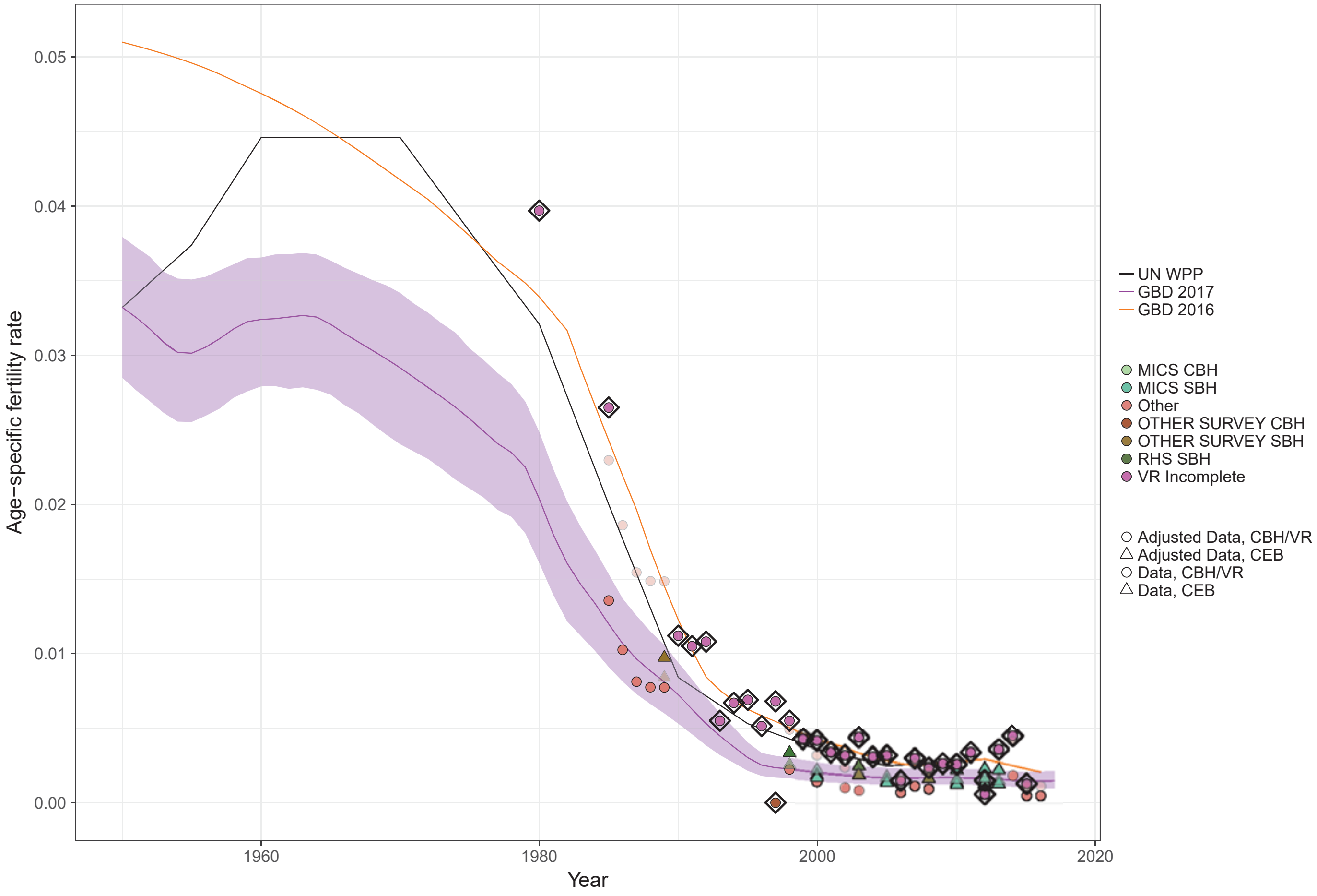

Age−specific fertility fitted trend lines, 1950−2017: Mongolia, Ages: 50−54
Data points enclosed with a diamond are reference sources

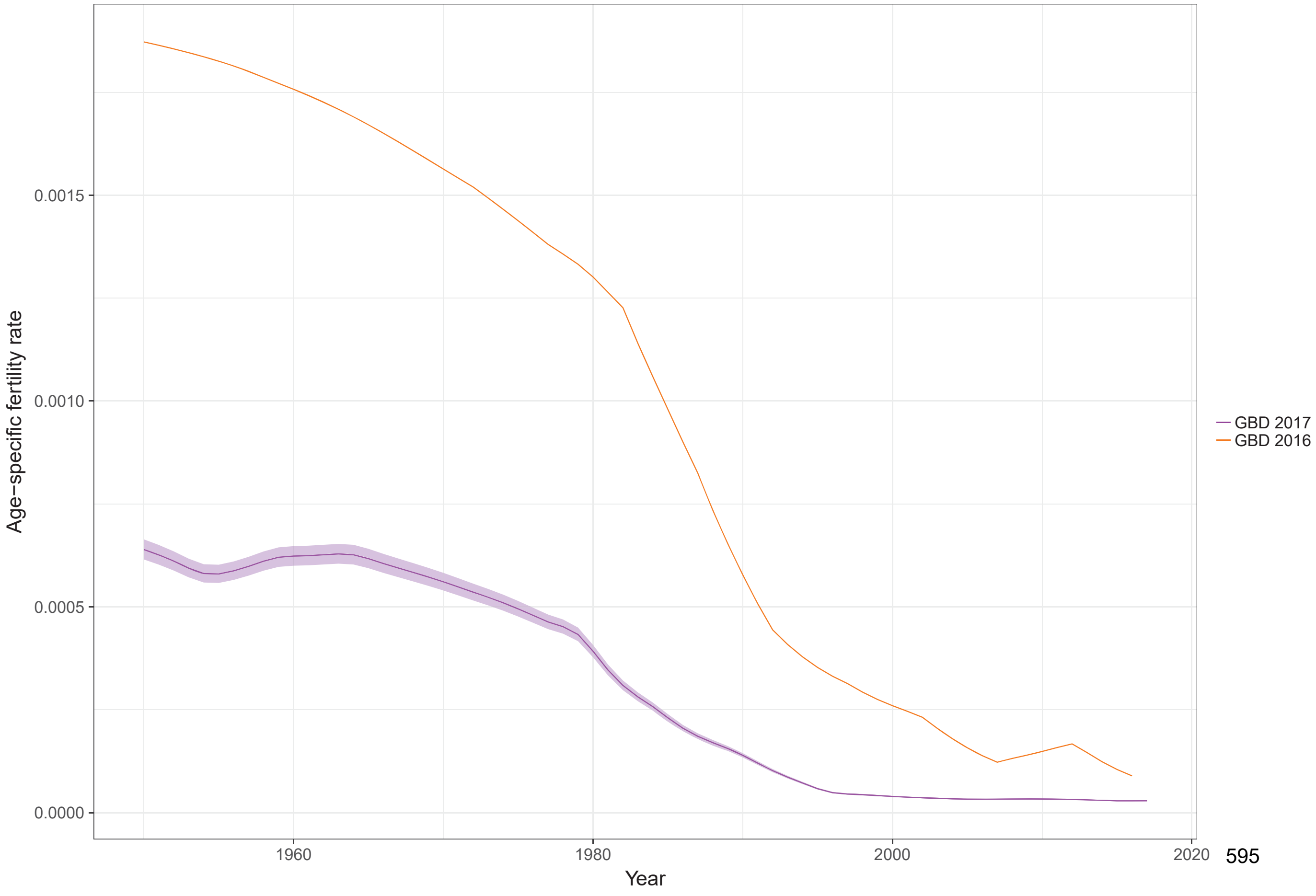

Age−specific fertility fitted trend lines, 1950−2017: Montenegro, TFR
Data points enclosed with a diamond are reference sources

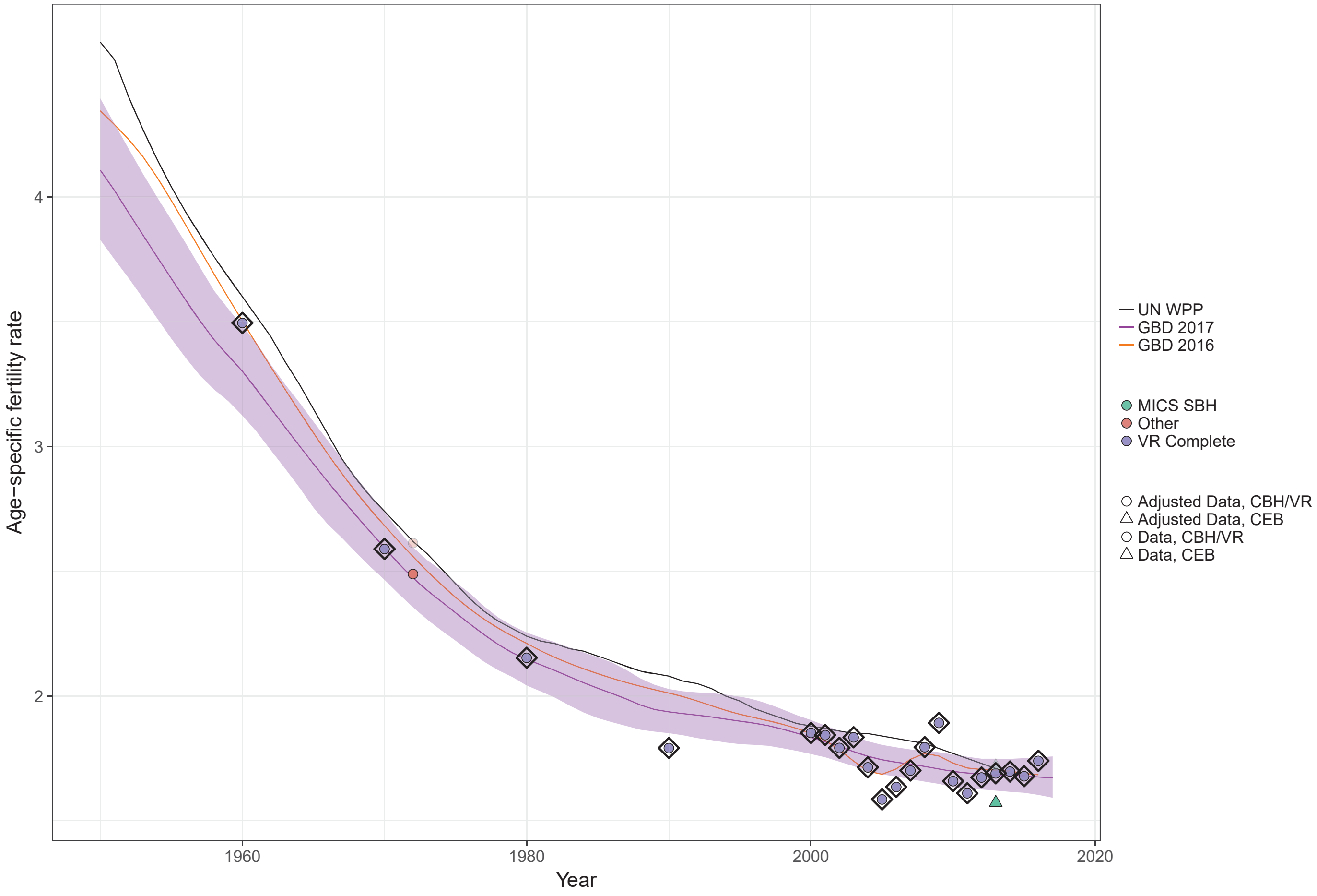

Age−specific fertility fitted trend lines, 1950−2017: Montenegro, Ages: 10−14
Data points enclosed with a diamond are reference sources

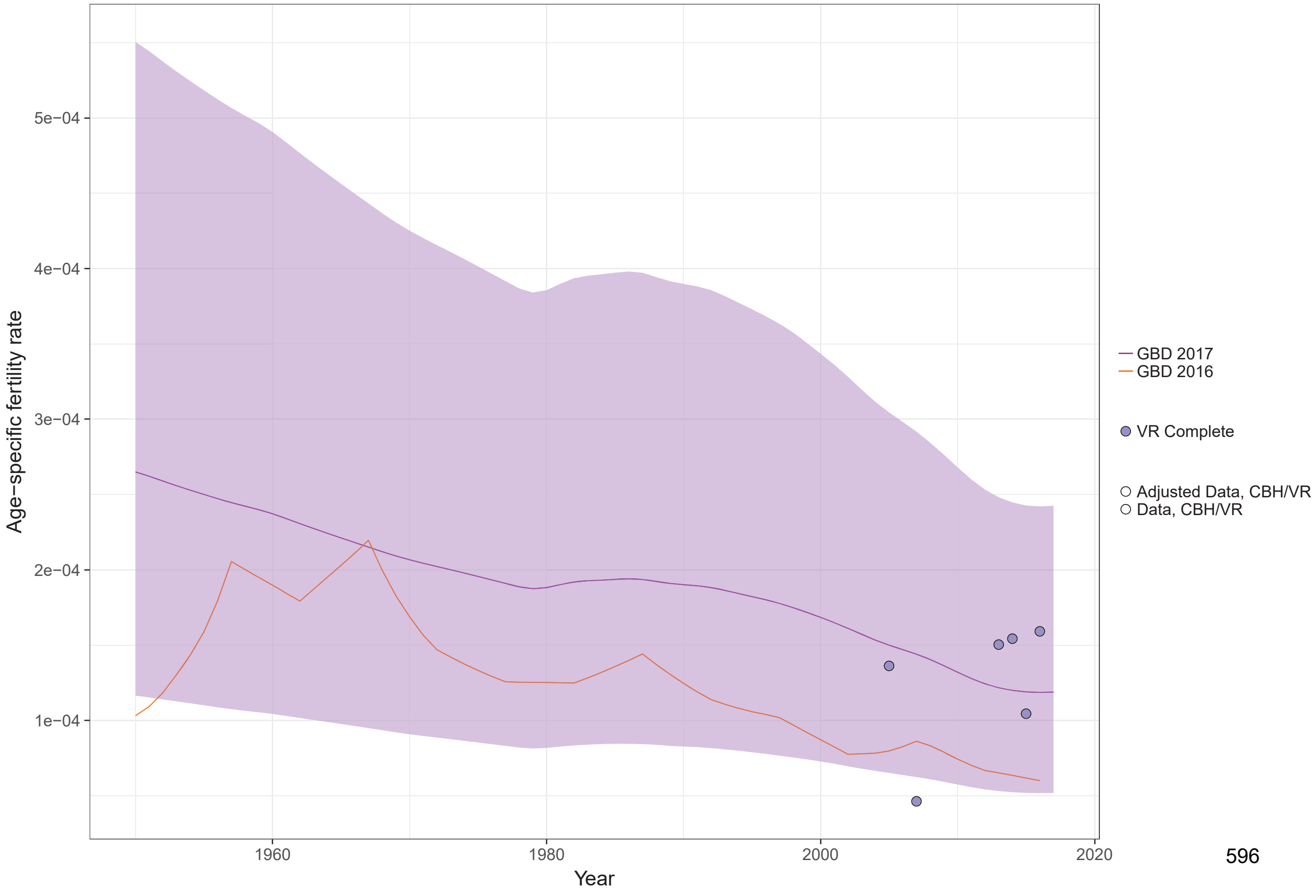

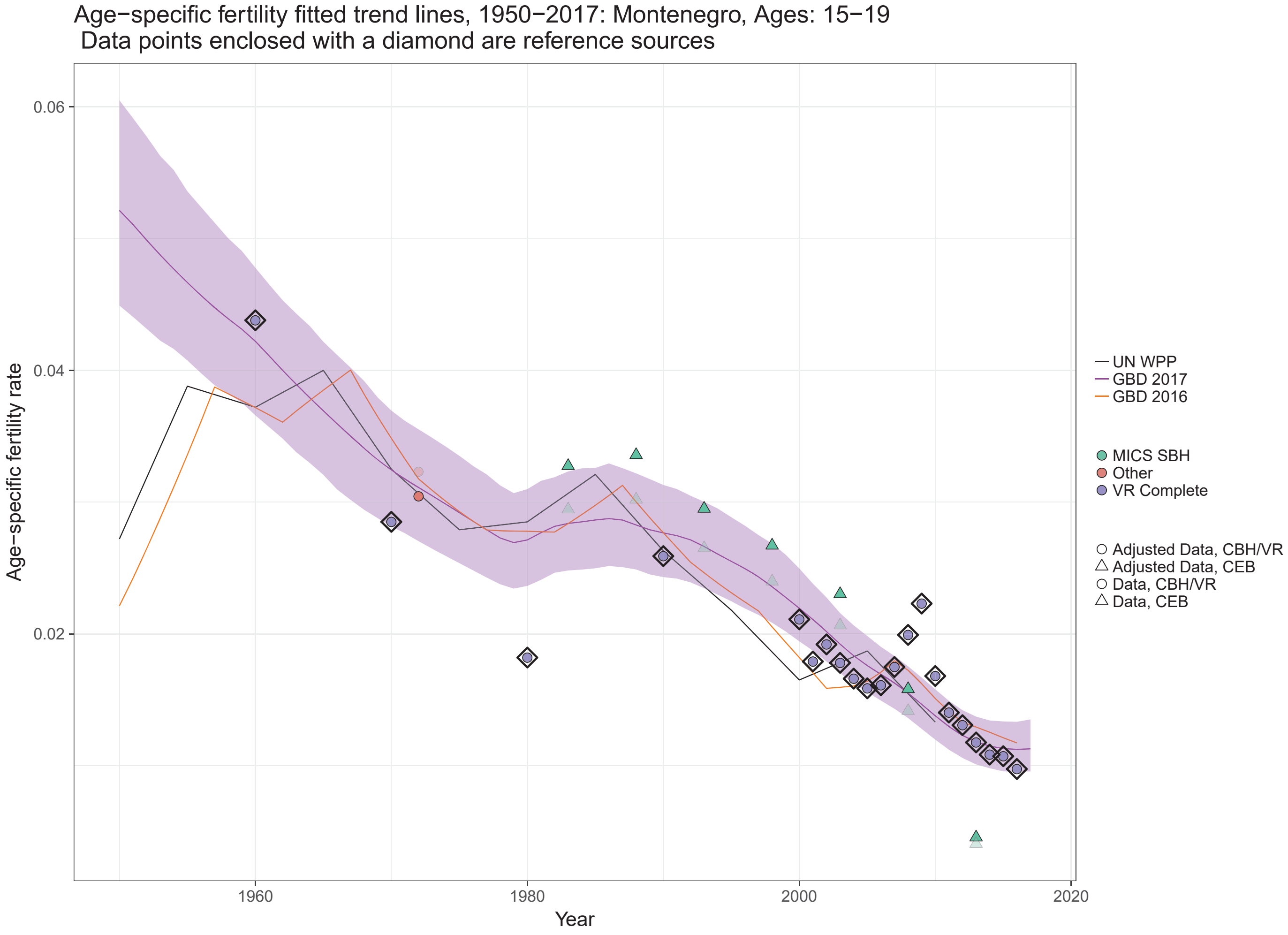
Age−specific fertility fitted trend lines, 1950−2017: Montenegro, Ages: 15−19
Data points enclosed with a diamond are reference sources
Age−specific fertility rate
0.06
0.04
0.02
1960
1980
2000
2020
Year
UN WPP
GBD 2017
GBD 2016
MICS SBH
Other
VR Complete
Adjusted Data, CBH/VR
Adjusted Data, CEB
Data, CBH/VR
Data, CEB

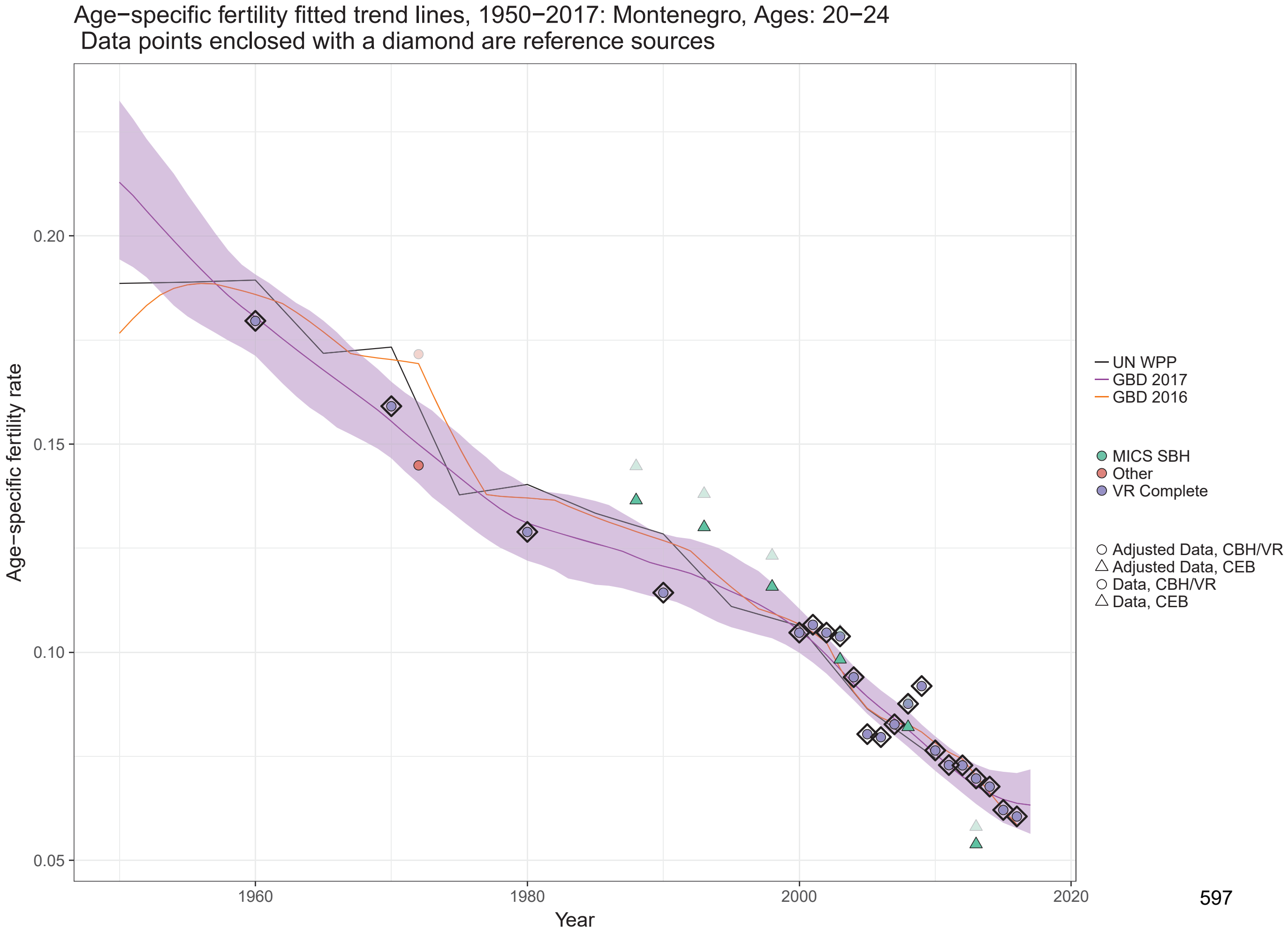
Age−specific fertility fitted trend lines, 1950−2017: Montenegro, Ages: 20−24
Data points enclosed with a diamond are reference sources
Age−specific fertility rate
0.20
0.15
0.10
0.05
1960
1980
2000
2020
Year
UN WPP
GBD 2017
GBD 2016
MICS SBH
Other
VR Complete
Adjusted Data, CBH/VR
Adjusted Data, CEB
Data, CBH/VR
Data, CEB

Age−specific fertility fitted trend lines, 1950−2017: Montenegro, Ages: 25−29
Data points enclosed with a diamond are reference sources

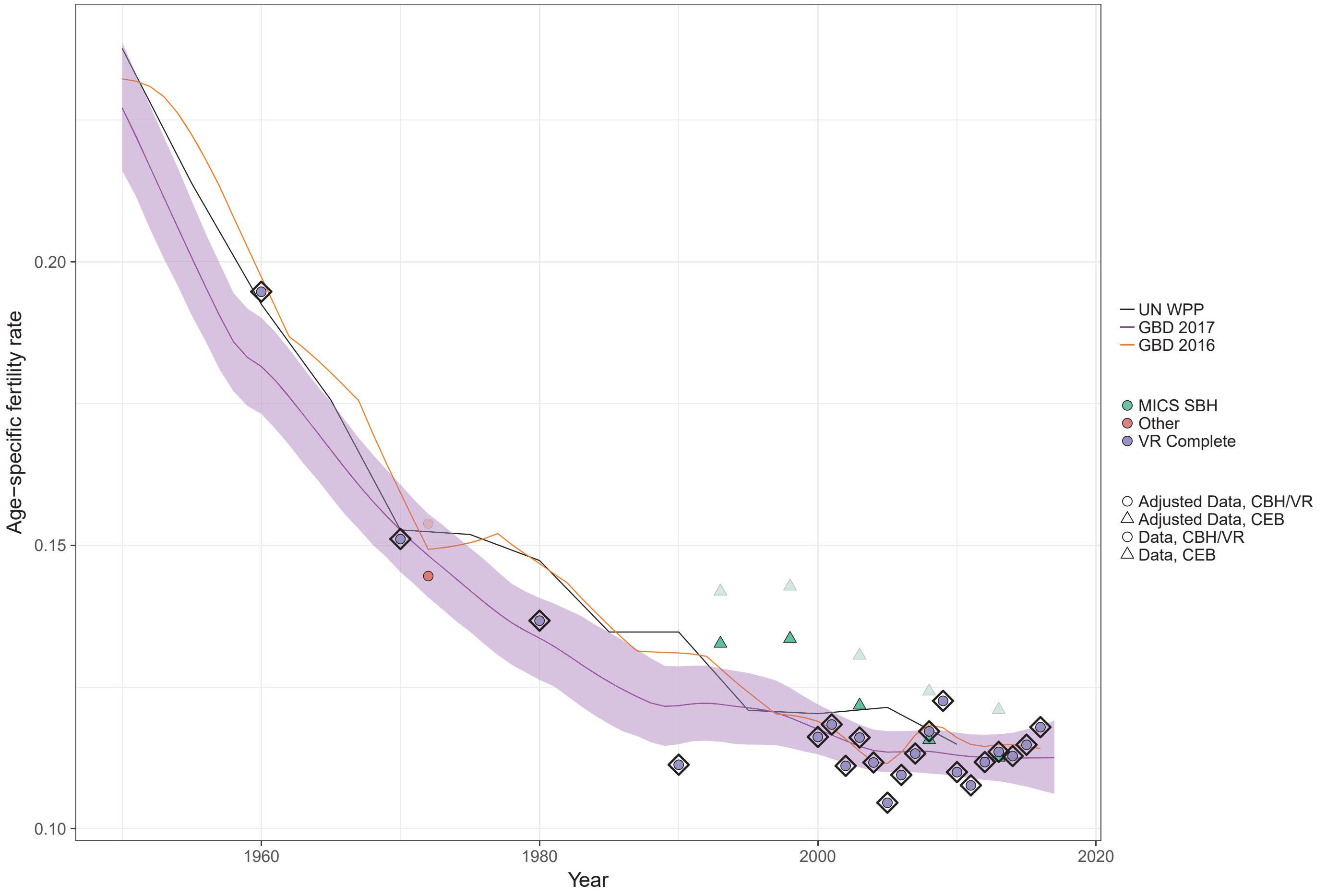

Age−specific fertility fitted trend lines, 1950−2017: Montenegro, Ages: 30−34
Data points enclosed with a diamond are reference sources

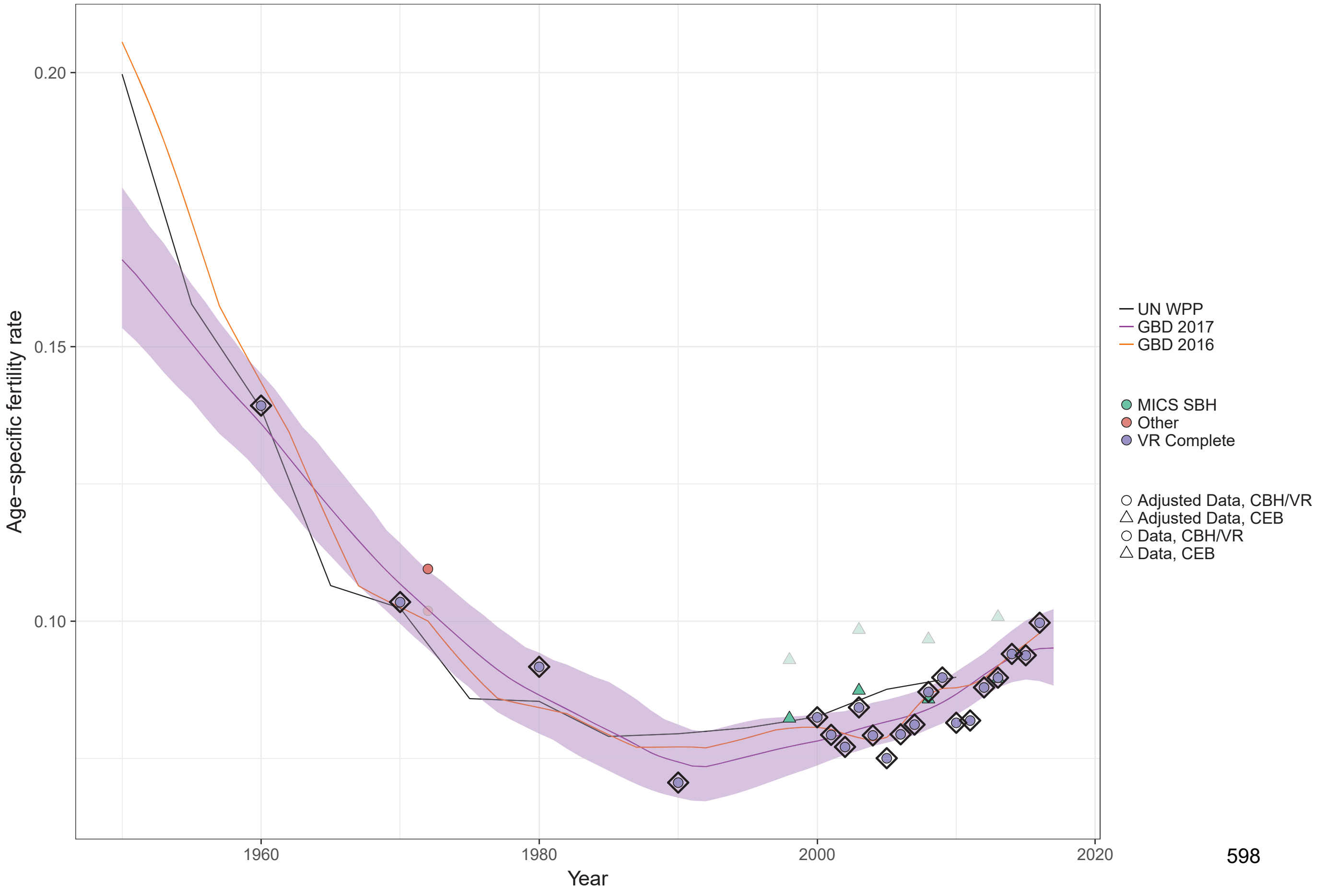

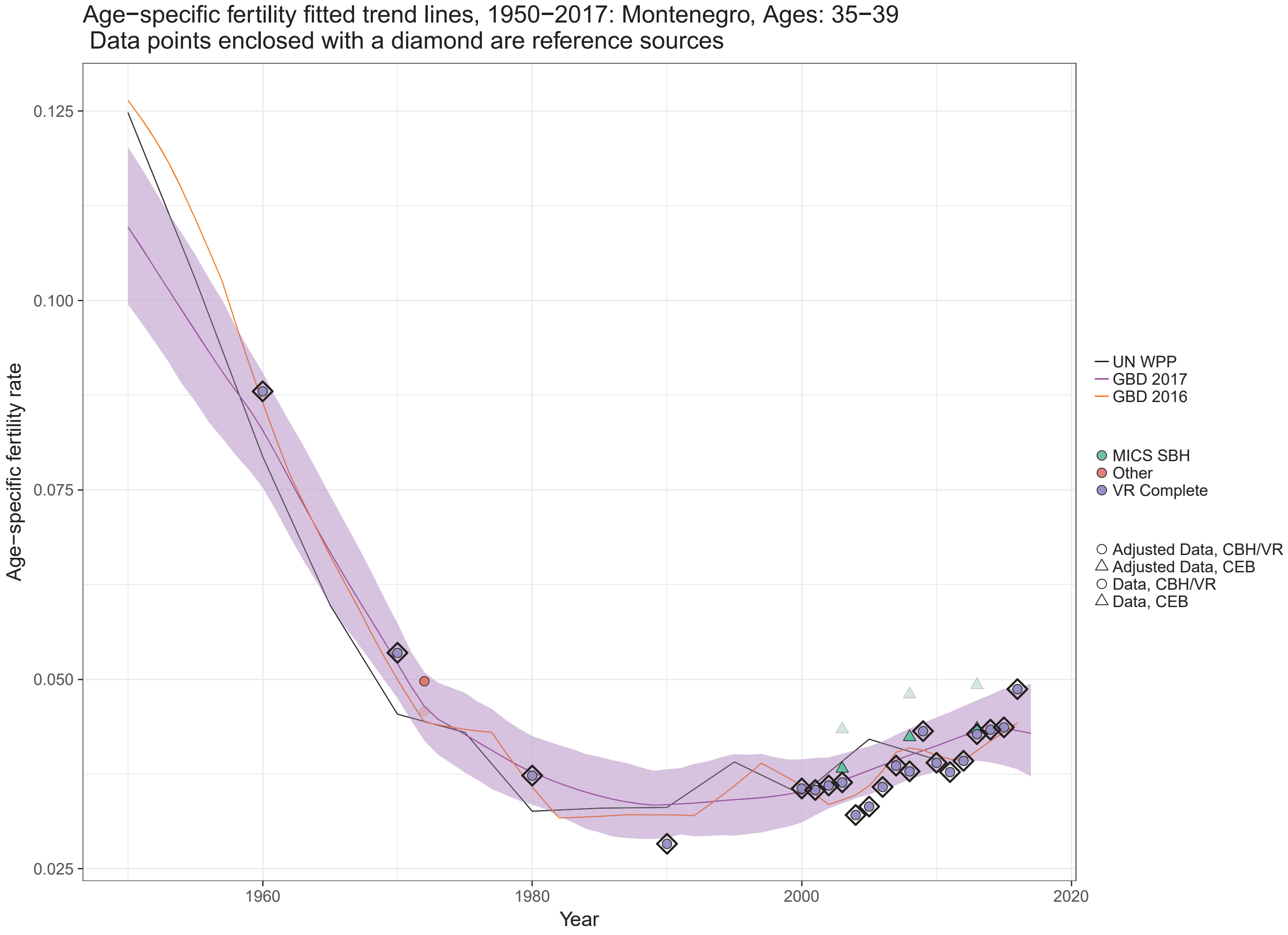

Age−specific fertility fitted trend lines, 1950−2017: Montenegro, Ages: 35−39
Data points enclosed with a diamond are reference sources
Age−specific fertility rate
0.125
0.100
0.075
0.050
0.025
1960
1980
2000
2020
Year
UN WPP
GBD 2017
GBD 2016
MICS SBH
Other
VR Complete
Adjusted Data, CBH/VR
Adjusted Data, CEB
Data, CBH/VR
Data, CEB

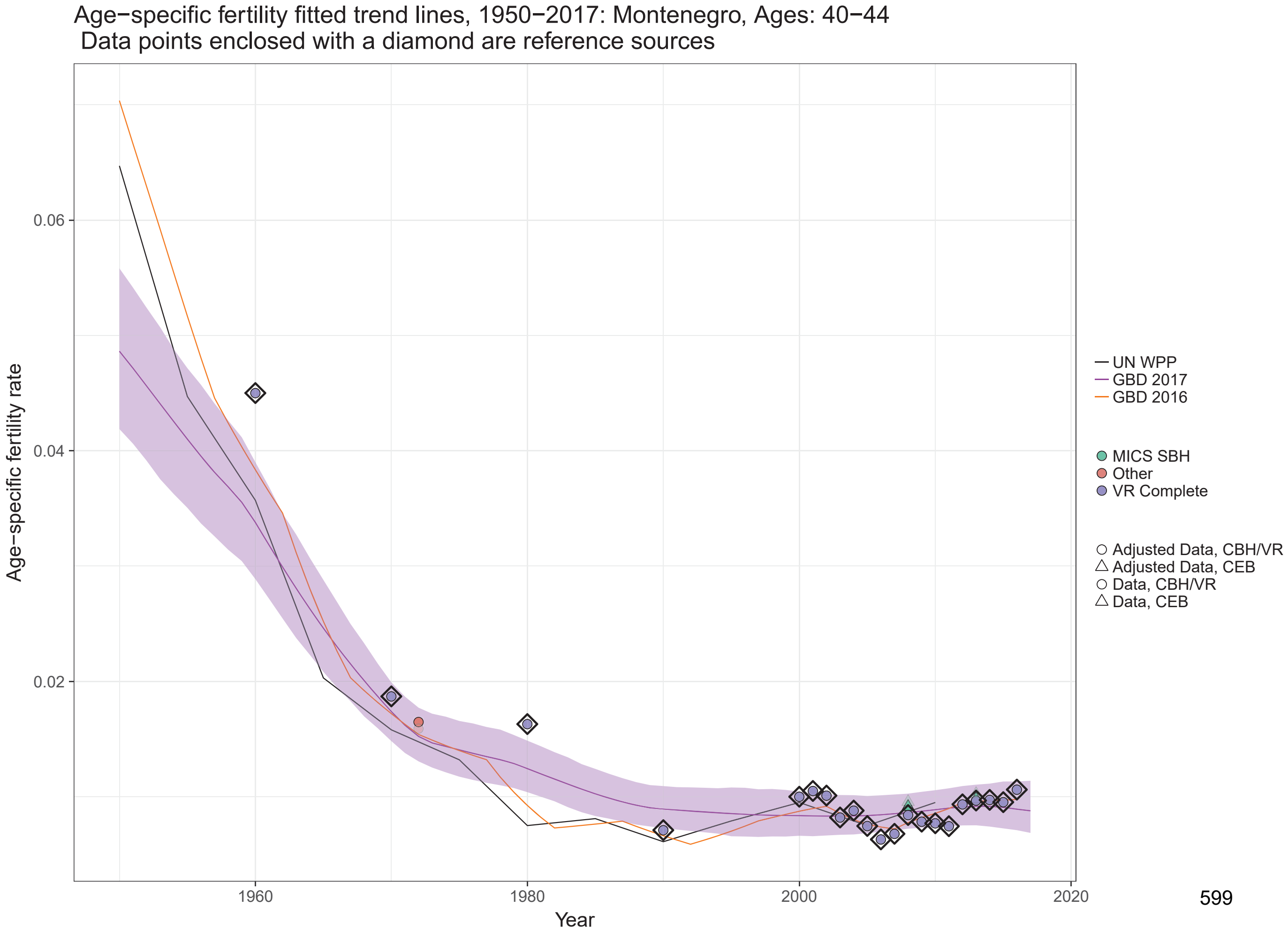

Age−specific fertility fitted trend lines, 1950−2017: Montenegro, Ages: 40−44
Data points enclosed with a diamond are reference sources
Age−specific fertility rate
0.06
0.04
0.02
1960
1980
2000
2020
Year
UN WPP
GBD 2017
GBD 2016
MICS SBH
Other
VR Complete
Adjusted Data, CBH/VR
Adjusted Data, CEB
Data, CBH/VR
Data, CEB

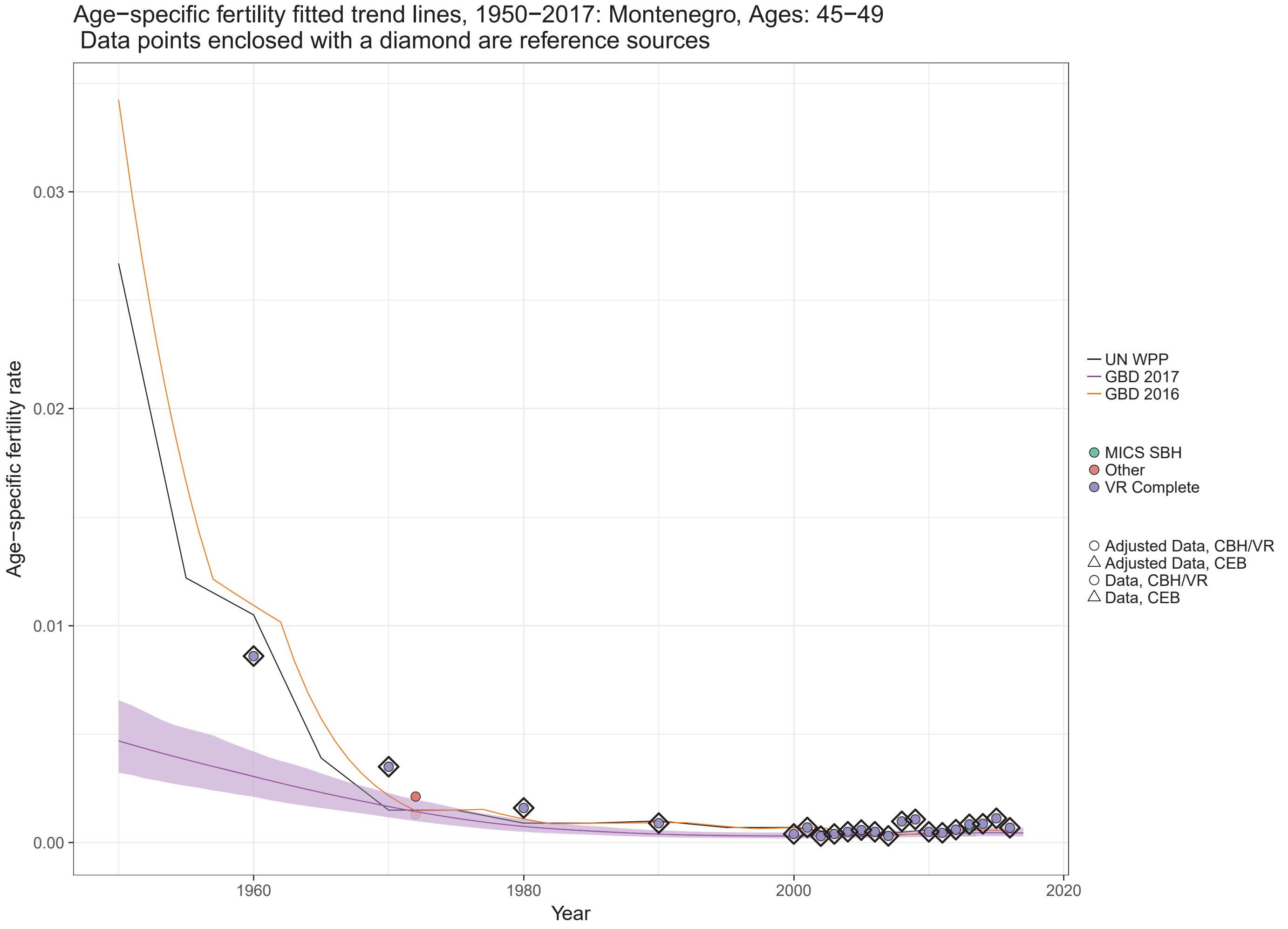
Age−specific fertility fitted trend lines, 1950−2017: Montenegro, Ages: 45−49
Data points enclosed with a diamond are reference sources
Age−specific fertility rate
0.03
0.02
0.01
0.00
1960
1980
2000
2020
Year
UN WPP
GBD 2017
GBD 2016
MICS SBH
Other
VR Complete
Adjusted Data, CBH/VR
Adjusted Data, CEB
Data, CBH/VR
Data, CEB

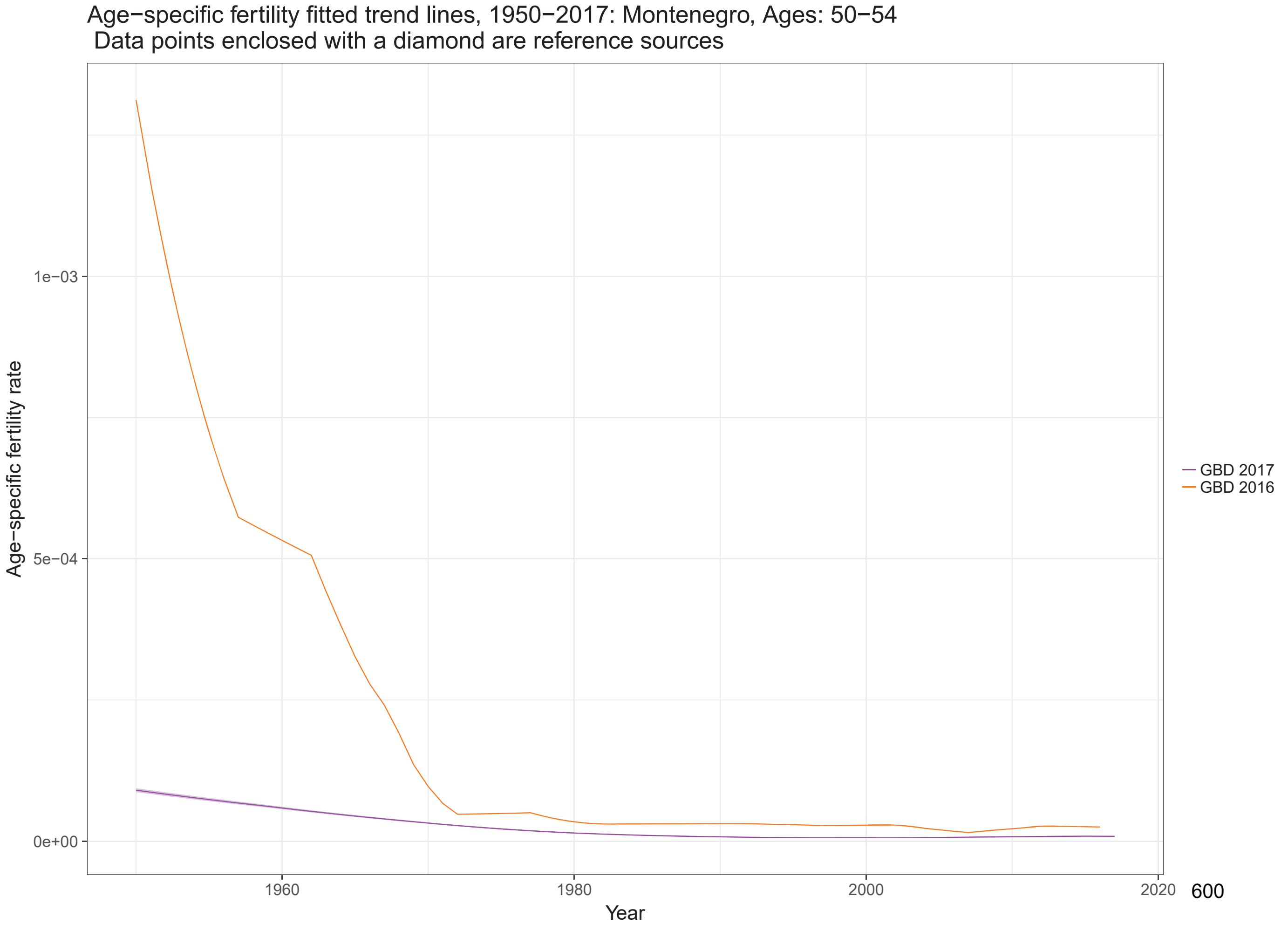
Age−specific fertility fitted trend lines, 1950−2017: Montenegro, Ages: 50−54
Data points enclosed with a diamond are reference sources
Age−specific fertility rate
1e−03
5e−04
0e+00
1960
1980
2000
2020
Year
GBD 2017
GBD 2016

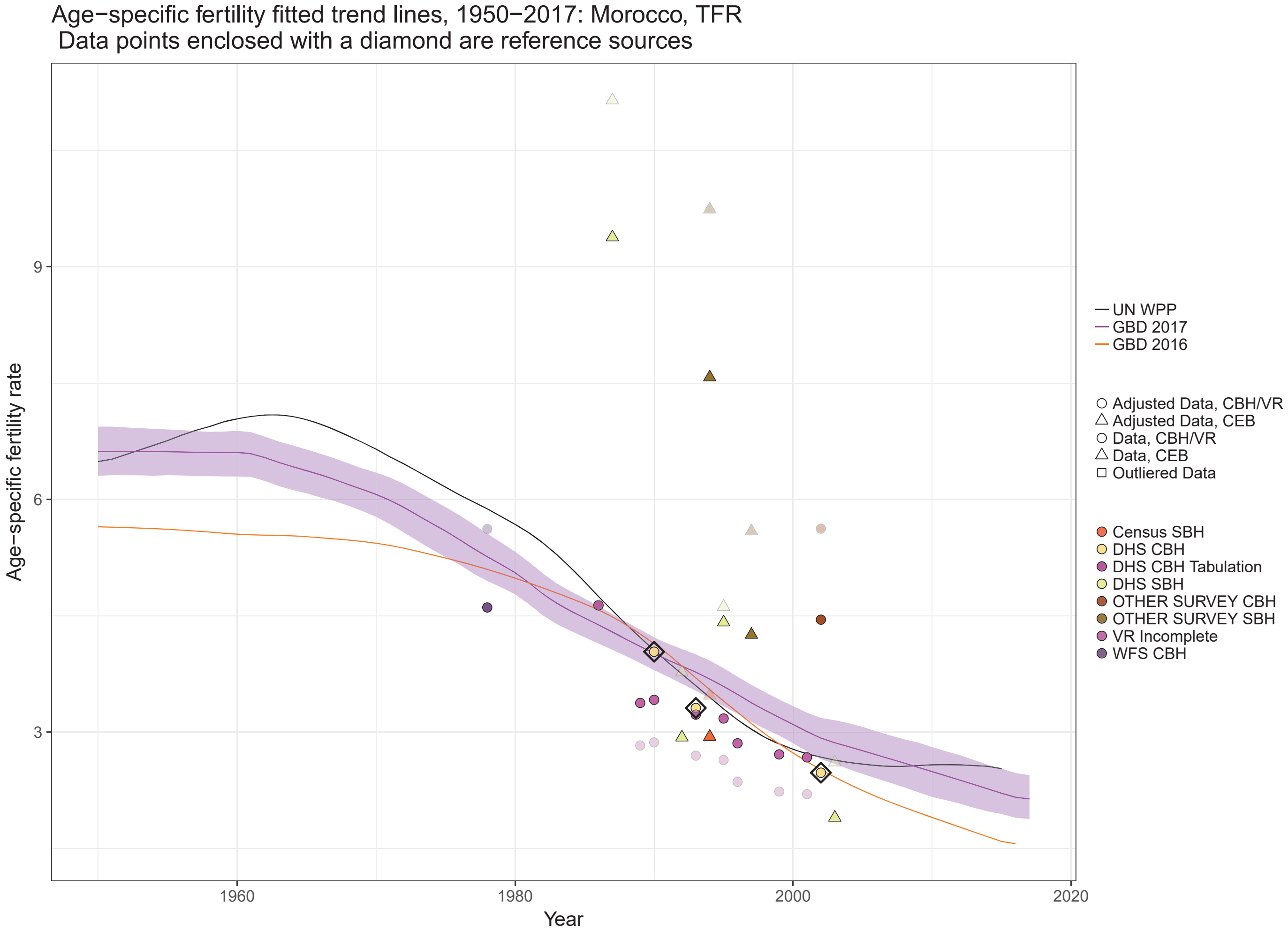
Age−specific fertility fitted trend lines, 1950−2017: Morocco, TFR
Data points enclosed with a diamond are reference sources
Age-specific fertility rate
Year
UN WPP
GBD 2017
GBD 2016
Adjusted Data, CBH/VR
Adjusted Data, CEB
Data, CBH/VR
Data, CEB
Outliered Data
Census SBH
DHS CBH
DHS CBH Tabulation
DHS SBH
OTHER SURVEY CBH
OTHER SURVEY SBH
VR Incomplete
WFS CBH

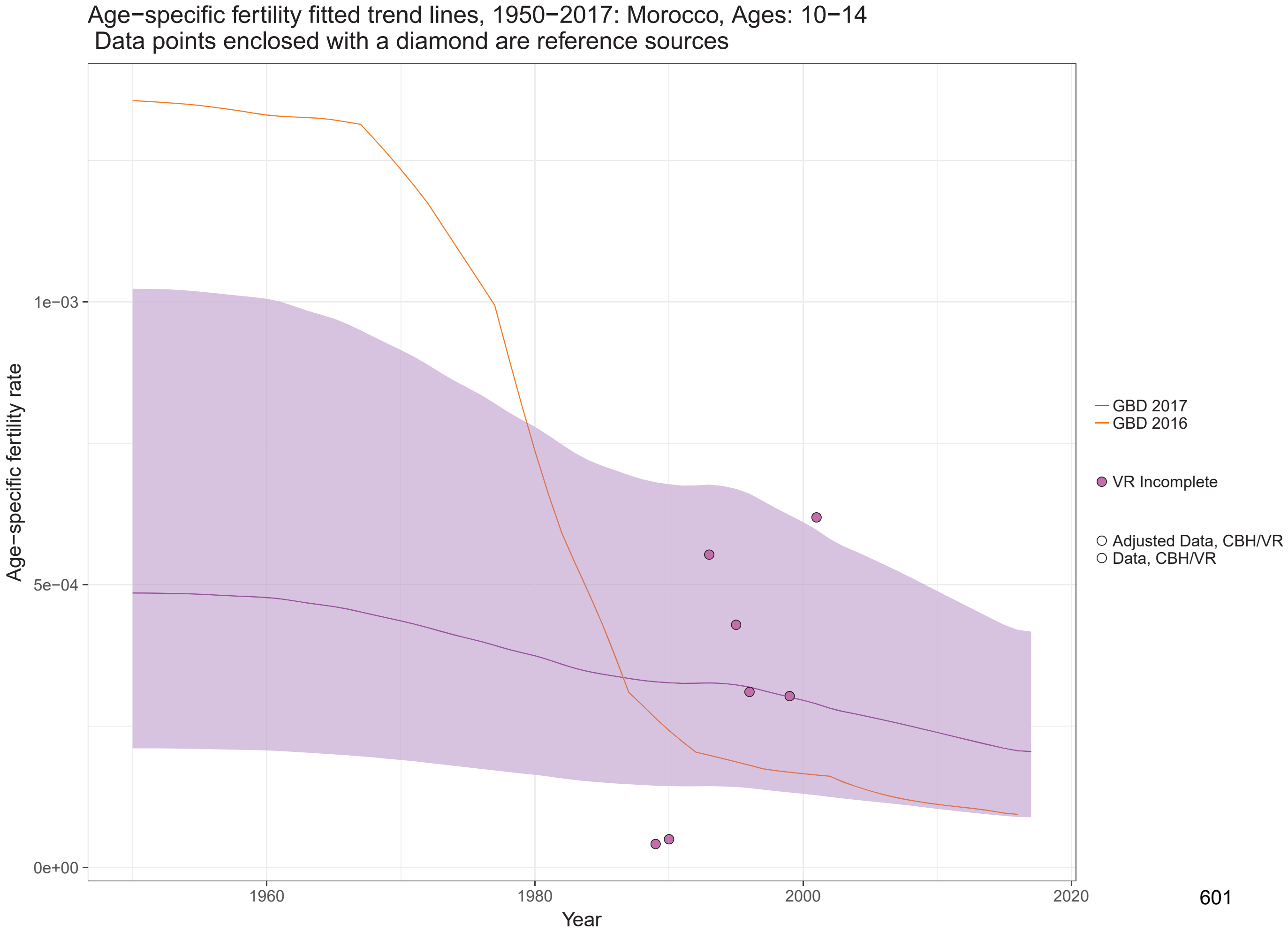
Age−specific fertility fitted trend lines, 1950−2017: Morocco, Ages: 10−14
Data points enclosed with a diamond are reference sources
Age-specific fertility rate
Year
GBD 2017
GBD 2016
VR Incomplete
Adjusted Data, CBH/VR
Data, CBH/VR

Age−specific fertility fitted trend lines, 1950−2017: Morocco, Ages: 15−19
Data points enclosed with a diamond are reference sources

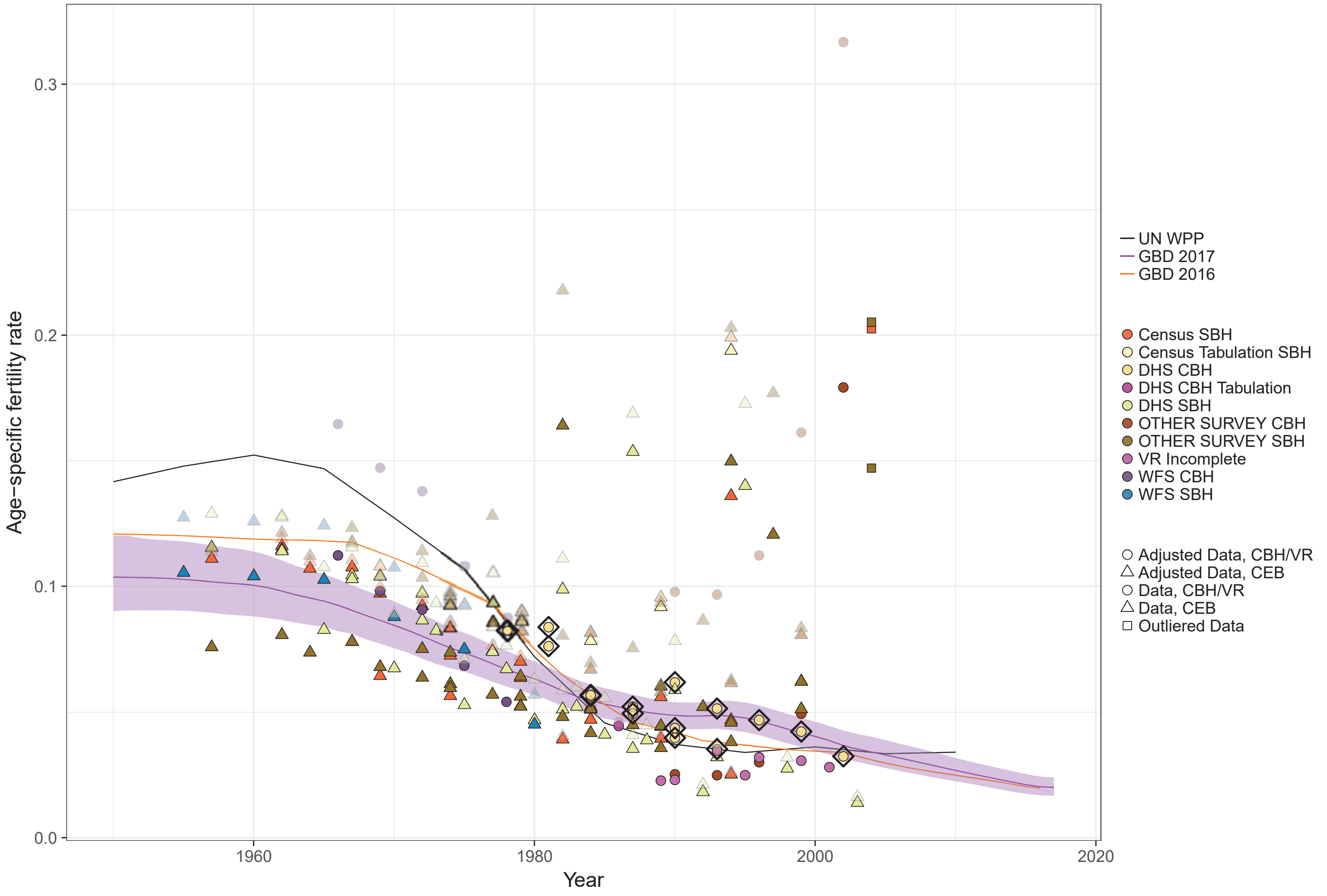

Age−specific fertility fitted trend lines, 1950−2017: Morocco, Ages: 20−24
Data points enclosed with a diamond are reference sources

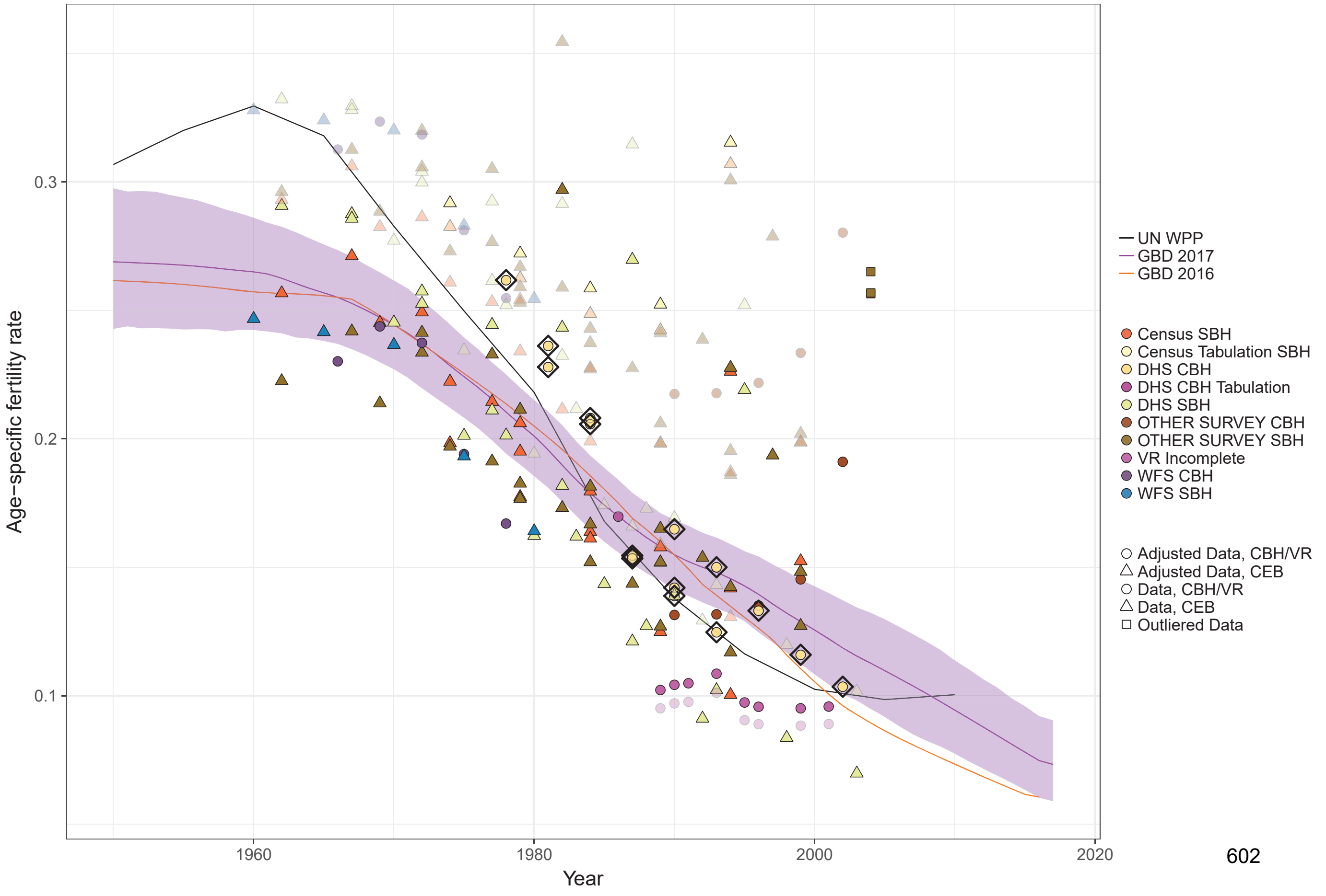

Age−specific fertility fitted trend lines, 1950−2017: Morocco, Ages: 25−29
Data points enclosed with a diamond are reference sources

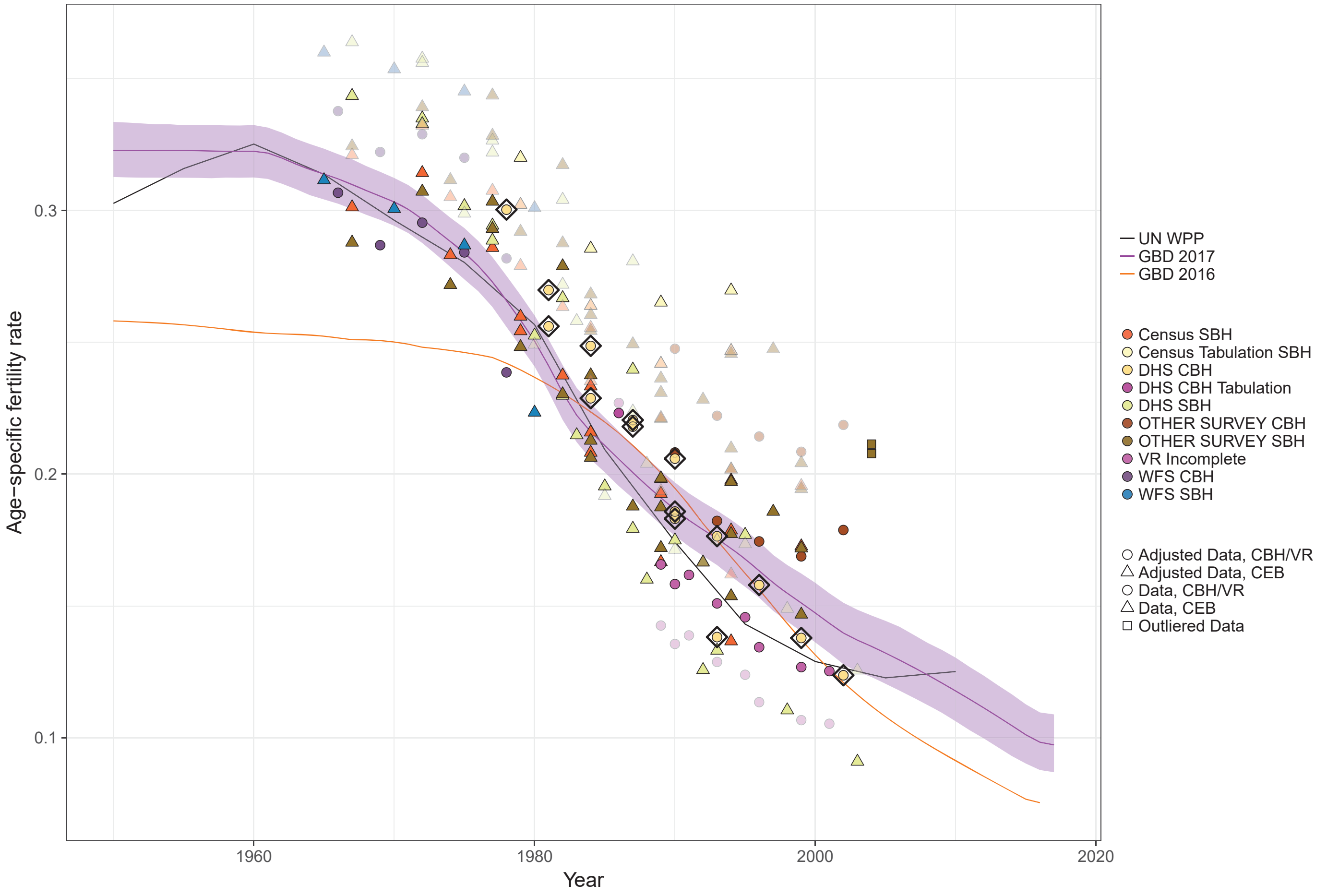

Age−specific fertility fitted trend lines, 1950−2017: Morocco, Ages: 30−34
Data points enclosed with a diamond are reference sources

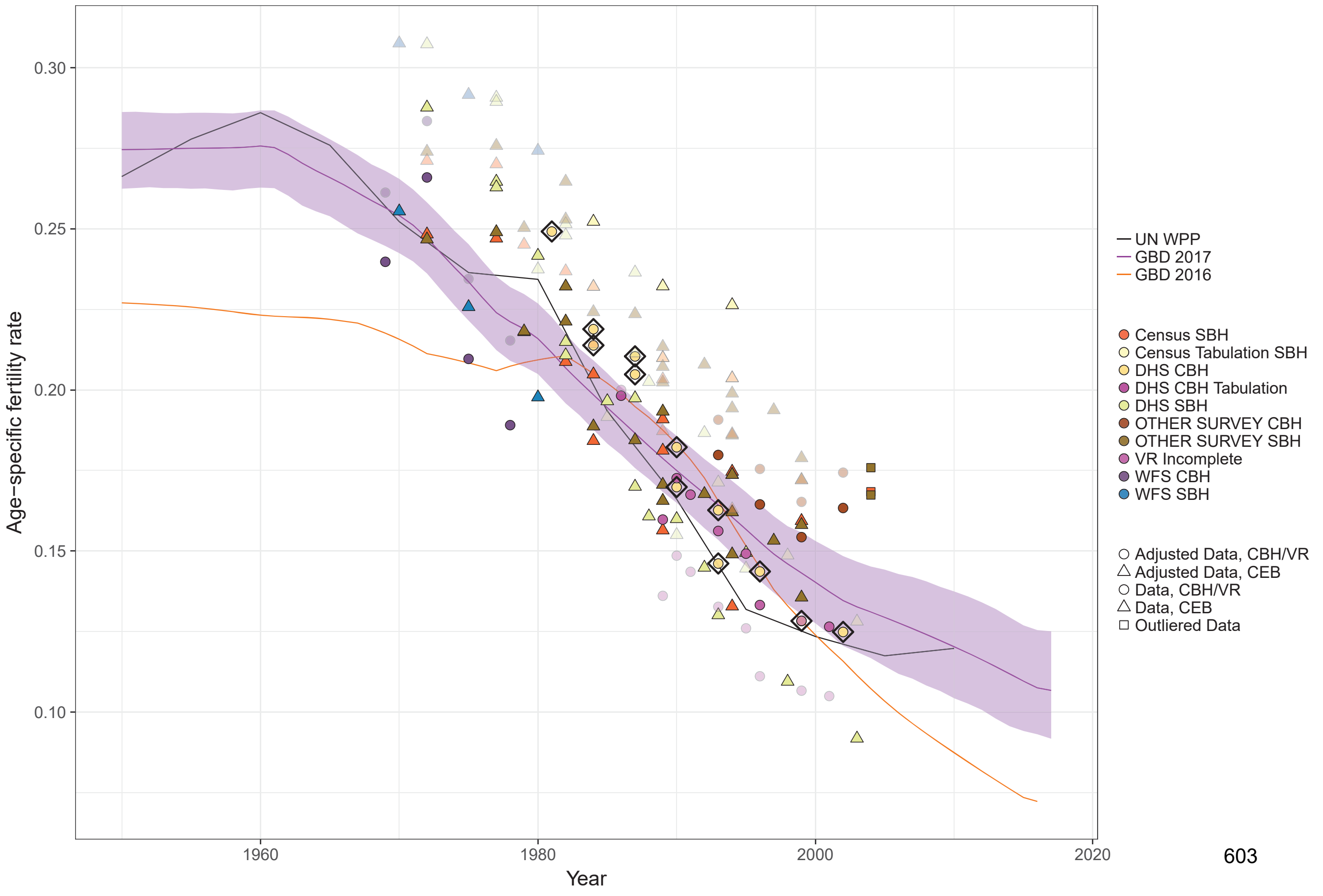

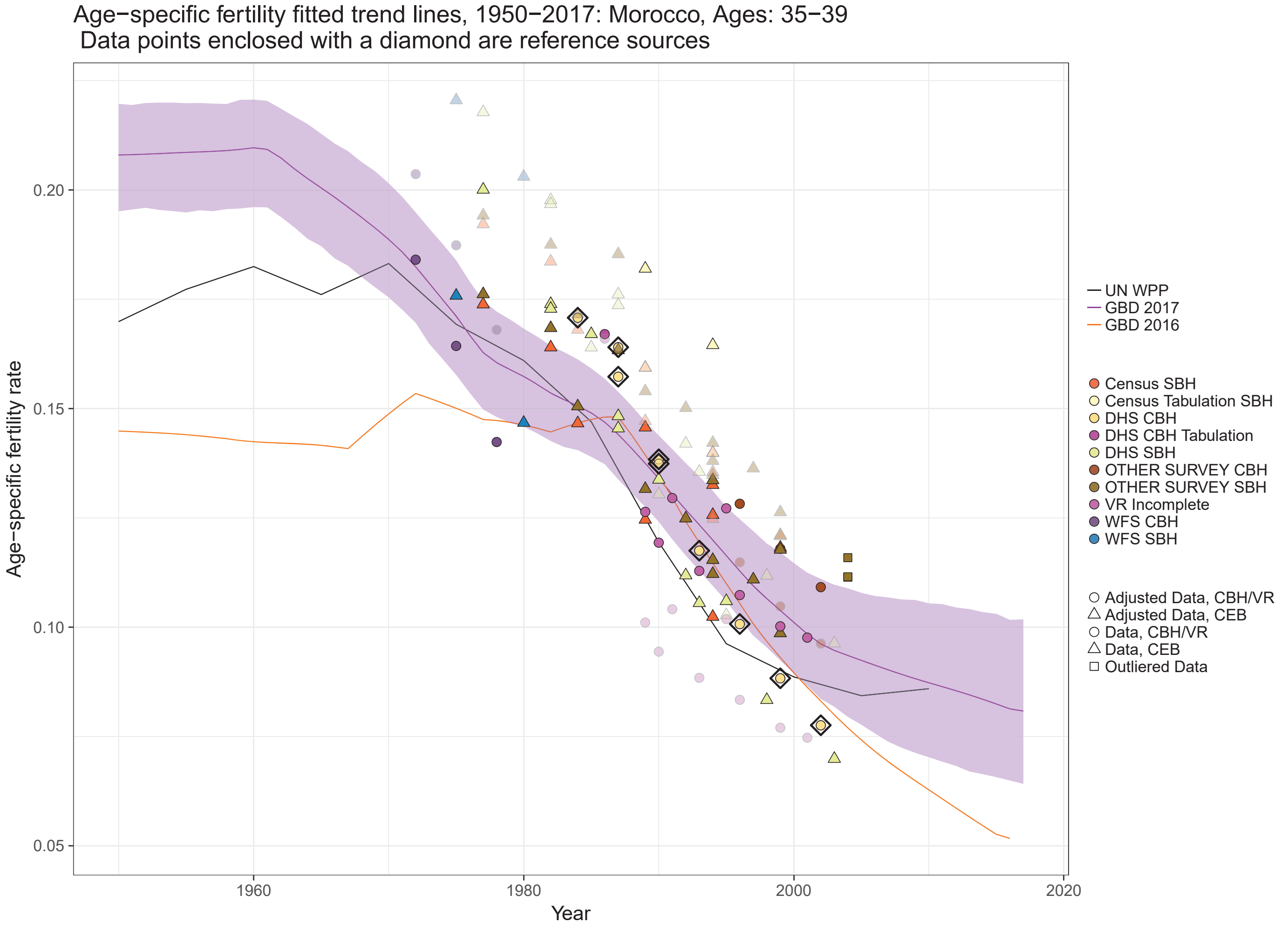
Age−specific fertility fitted trend lines, 1950−2017: Morocco, Ages: 35−39
Data points enclosed with a diamond are reference sources
Age−specific fertility rate
0.20
0.15
0.10
0.05
1960
1980
2000
2020
Year
UN WPP
GBD 2017
GBD 2016
Census SBH
Census Tabulation SBH
DHS CBH
DHS CBH Tabulation
DHS SBH
OTHER SURVEY CBH
OTHER SURVEY SBH
VR Incomplete
WFS CBH
WFS SBH
Adjusted Data, CBH/VR
Adjusted Data, CEB
Data, CBH/VR
Data, CEB
Outliered Data

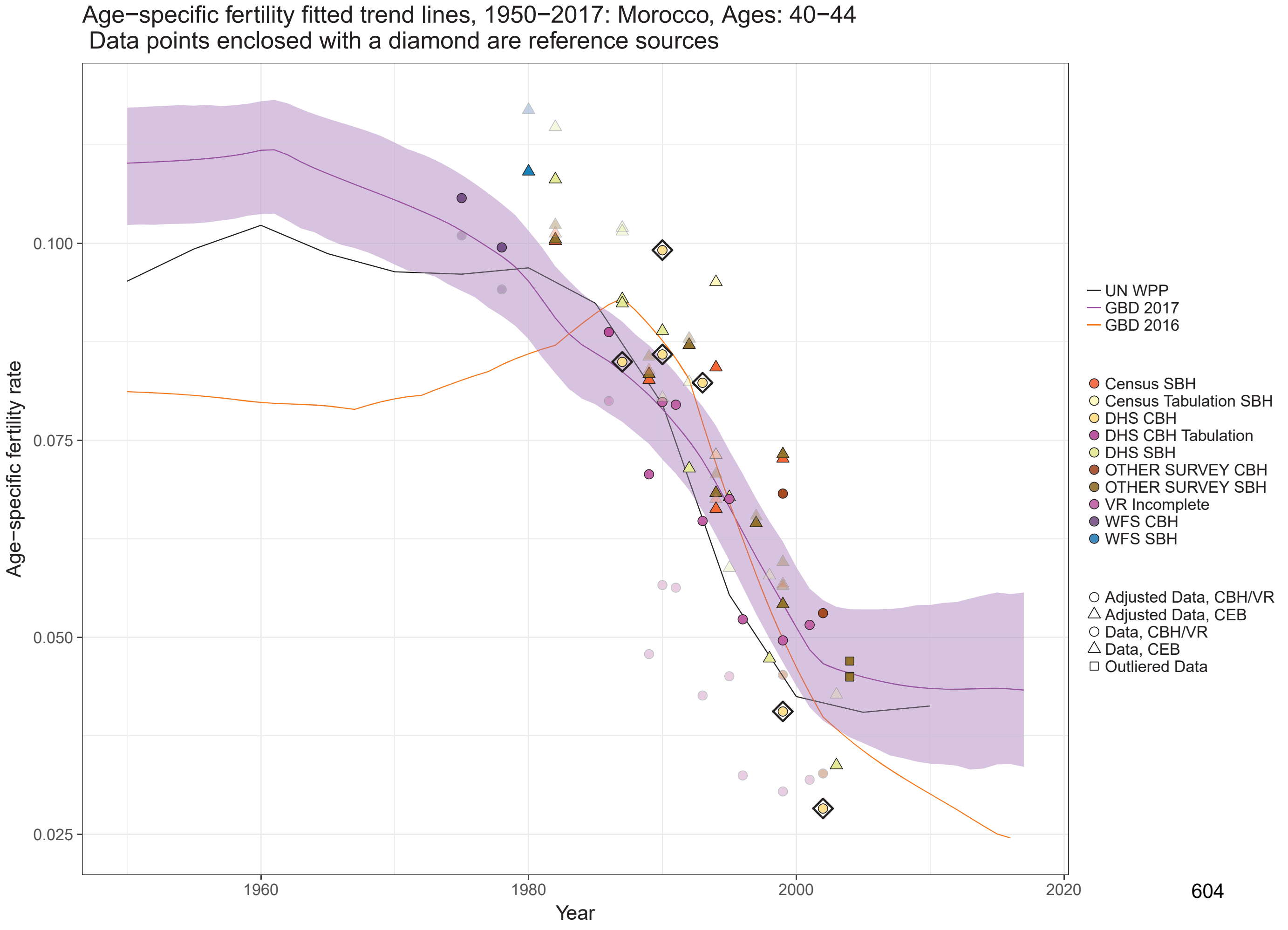
Age−specific fertility fitted trend lines, 1950−2017: Morocco, Ages: 40−44
Data points enclosed with a diamond are reference sources
Age−specific fertility rate
0.100
0.075
0.050
0.025
1960
1980
2000
2020
Year
UN WPP
GBD 2017
GBD 2016
Census SBH
Census Tabulation SBH
DHS CBH
DHS CBH Tabulation
DHS SBH
OTHER SURVEY CBH
OTHER SURVEY SBH
VR Incomplete
WFS CBH
WFS SBH
Adjusted Data, CBH/VR
Adjusted Data, CEB
Data, CBH/VR
Data, CEB
Outliered Data

Age−specific fertility fitted trend lines, 1950−2017: Morocco, Ages: 45−49
Data points enclosed with a diamond are reference sources

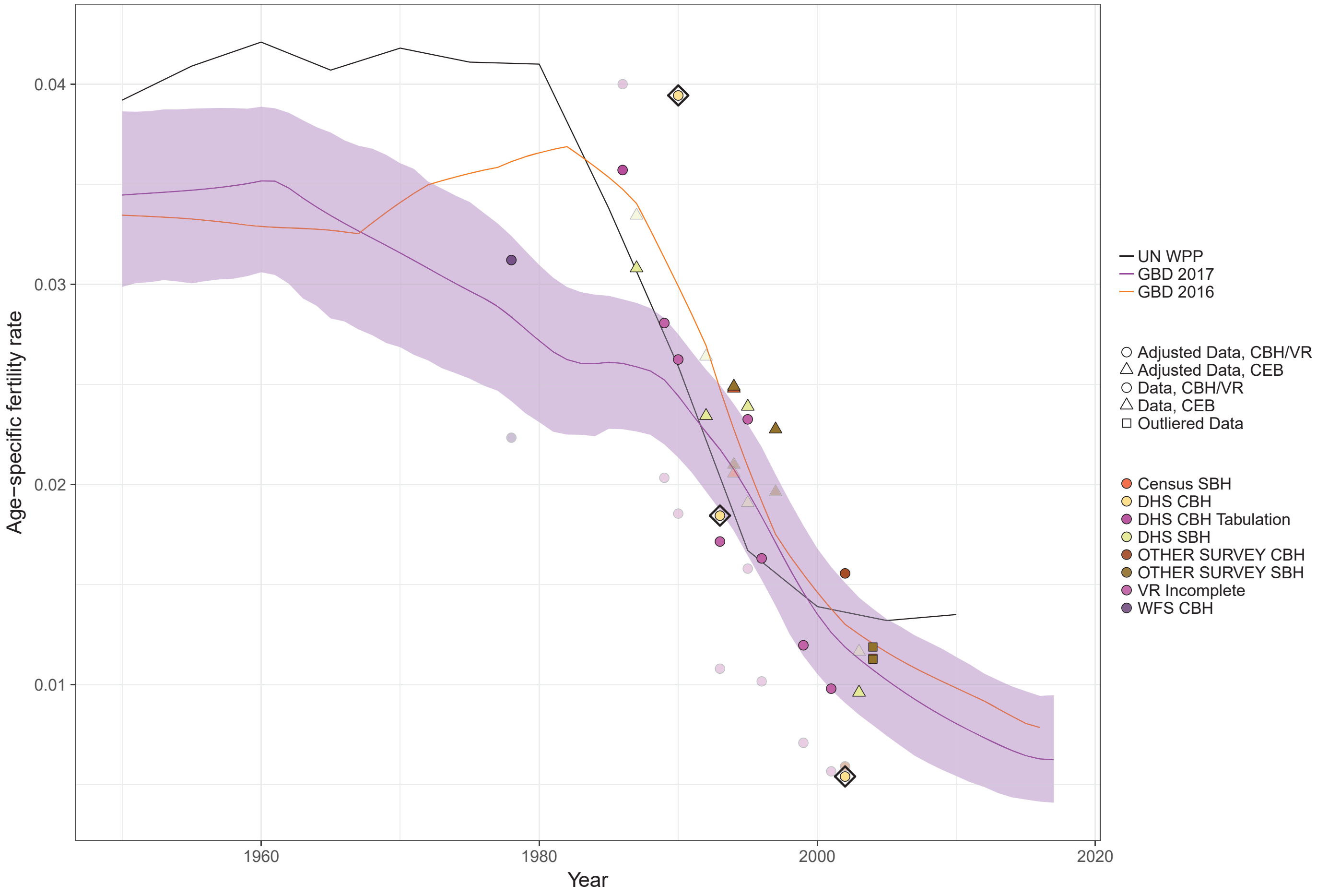

Age−specific fertility fitted trend lines, 1950−2017: Morocco, Ages: 50−54
Data points enclosed with a diamond are reference sources

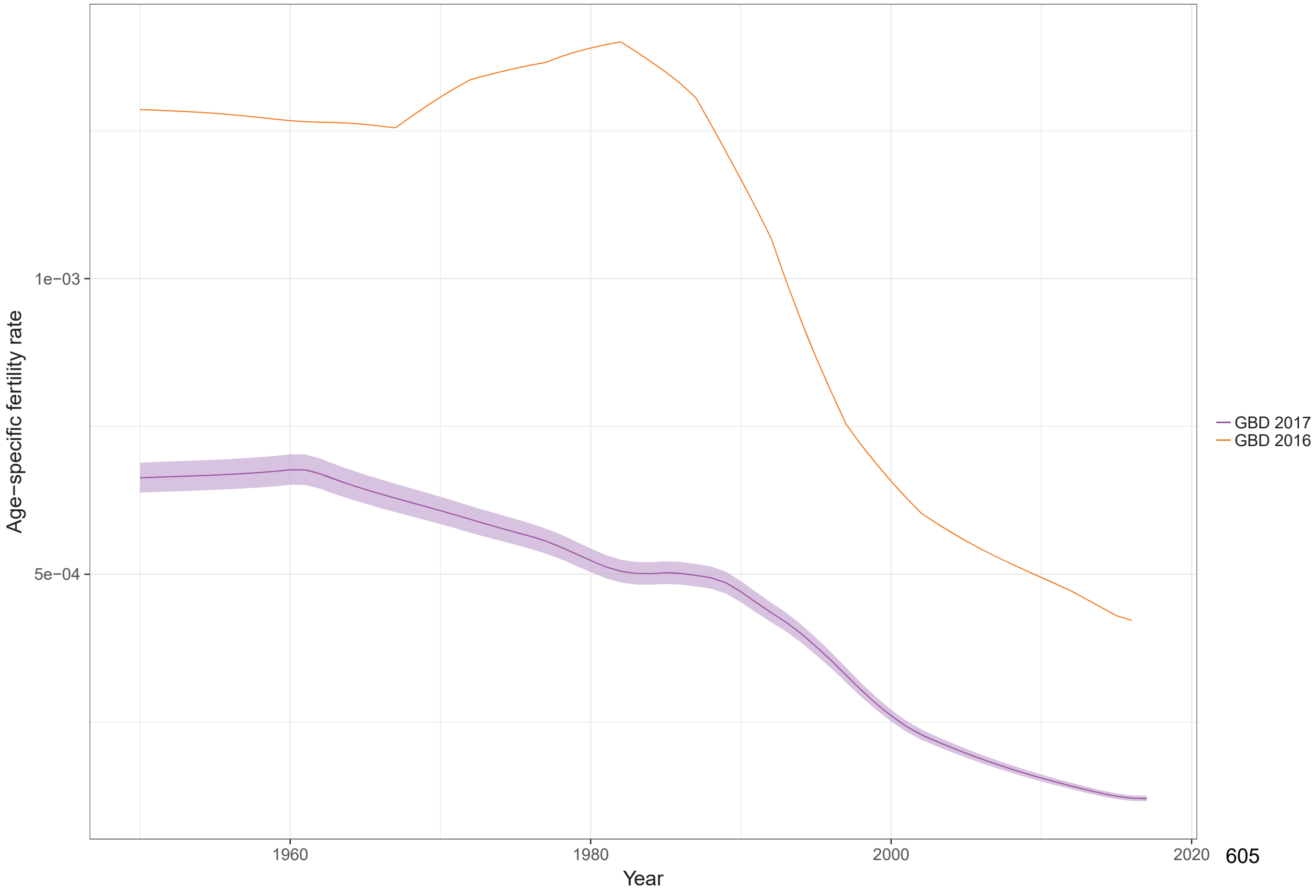

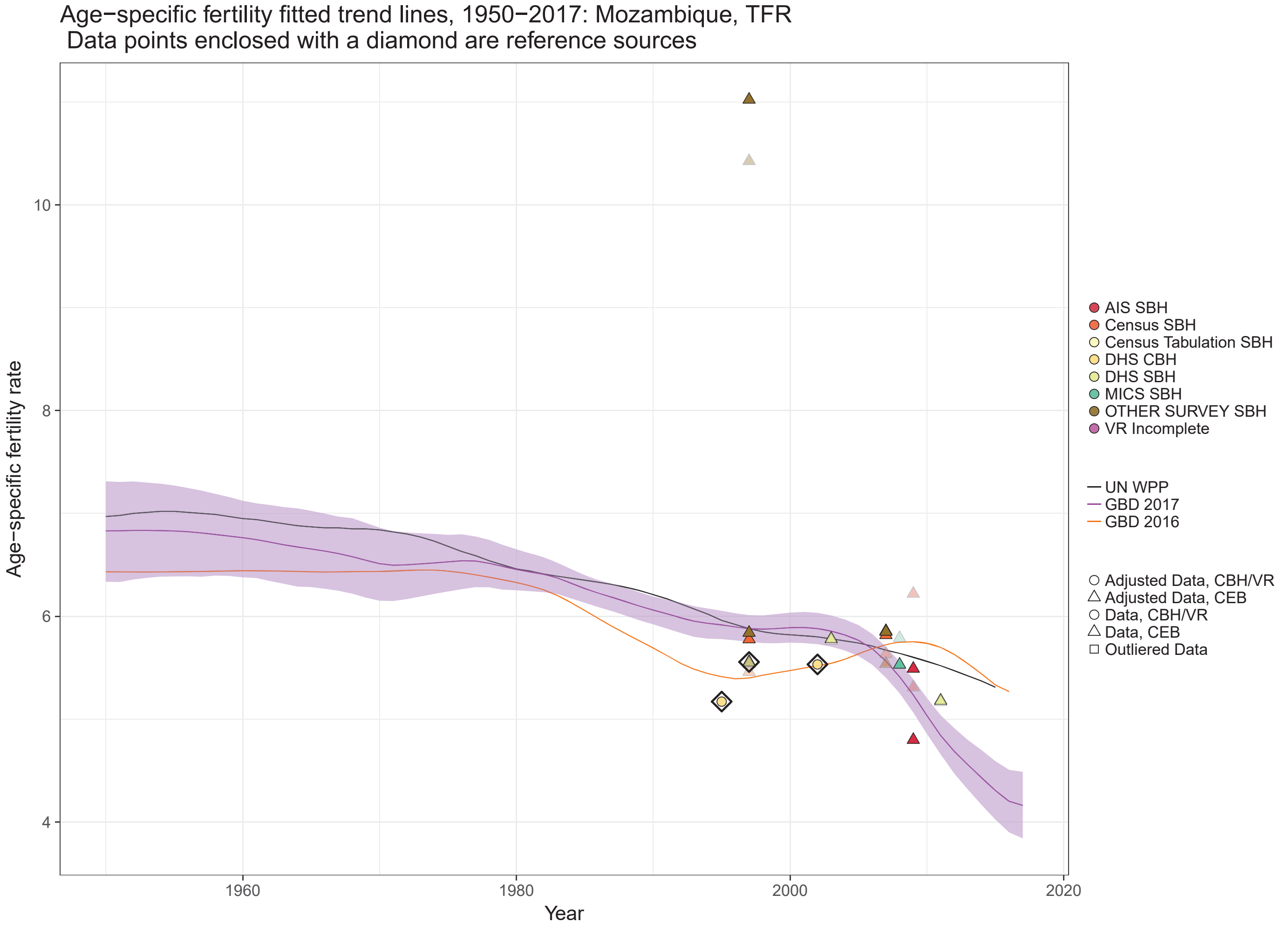

Age−specific fertility fitted trend lines, 1950−2017: Mozambique, TFR
Data points enclosed with a diamond are reference sources
Age−specific fertility rate
Year
10
8
6
4
1960
1980
2000
2020
AIS SBH
Census SBH
Census Tabulation SBH
DHS CBH
DHS SBH
MICS SBH
OTHER SURVEY SBH
VR Incomplete
UN WPP
GBD 2017
GBD 2016
Adjusted Data, CBH/VR
Adjusted Data, CEB
Data, CBH/VR
Data, CEB
Outliered Data

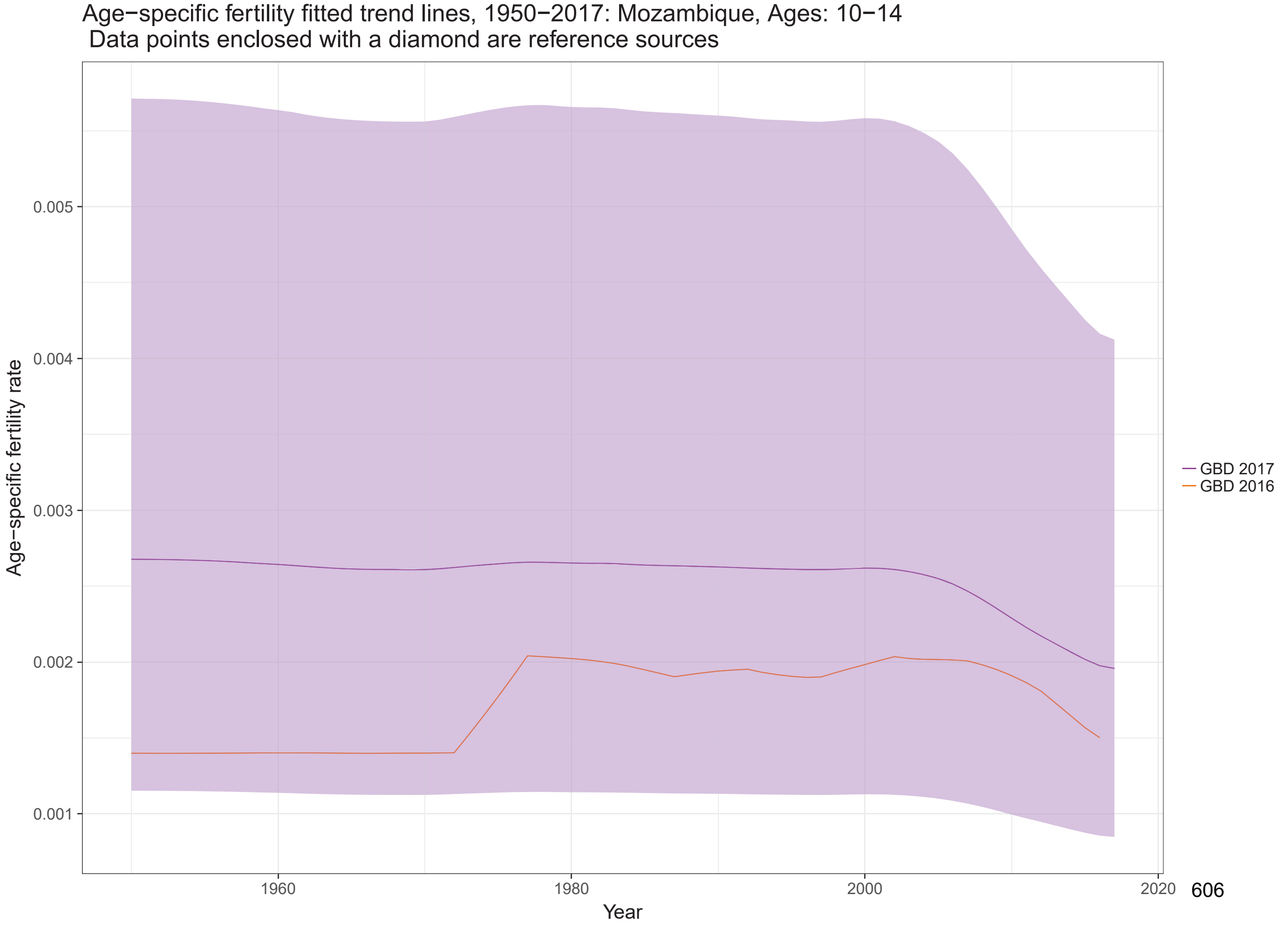

Age−specific fertility fitted trend lines, 1950−2017: Mozambique, Ages: 10−14
Data points enclosed with a diamond are reference sources
Age−specific fertility rate
Year
0.005
0.004
0.003
0.002
0.001
1960
1980
2000
2020
GBD 2017
GBD 2016

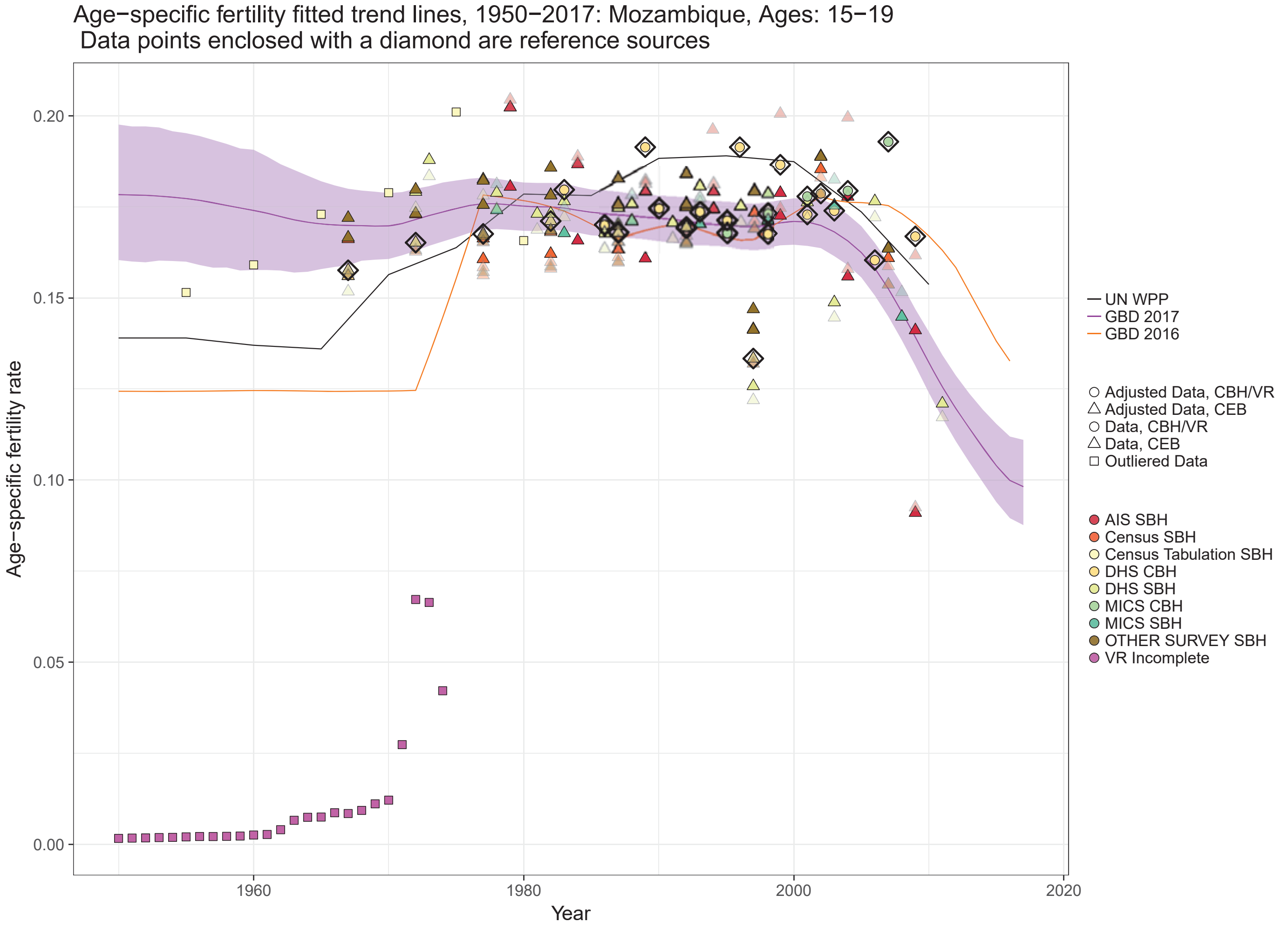
Age−specific fertility fitted trend lines, 1950−2017: Mozambique, Ages: 15−19
Data points enclosed with a diamond are reference sources
Age−specific fertility rate
Year
UN WPP
GBD 2017
GBD 2016
Adjusted Data, CBH/VR
Adjusted Data, CEB
Data, CBH/VR
Data, CEB
Outliered Data
AIS SBH
Census SBH
Census Tabulation SBH
DHS CBH
DHS SBH
MICS CBH
MICS SBH
OTHER SURVEY SBH
VR Incomplete

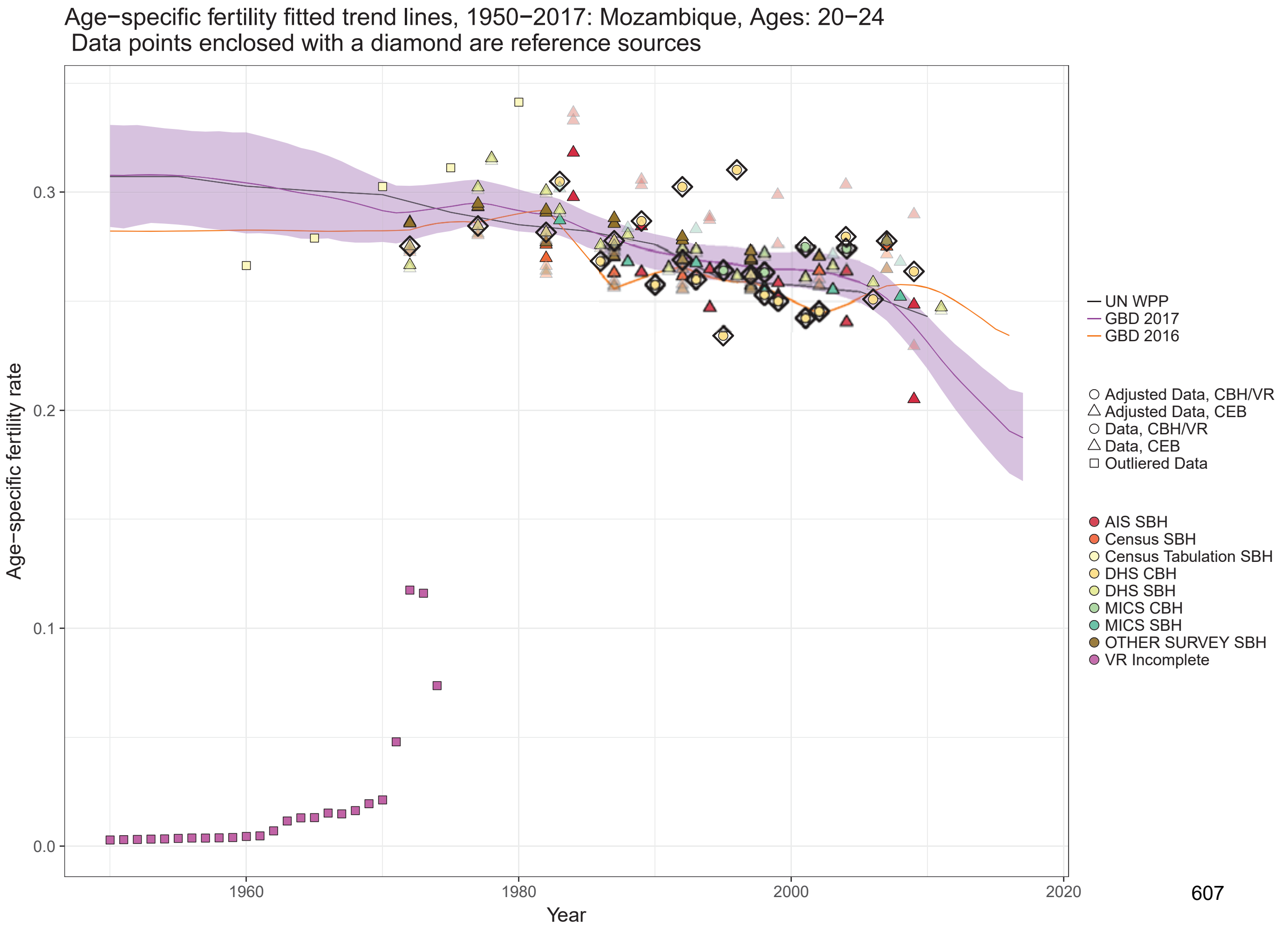
Age−specific fertility fitted trend lines, 1950−2017: Mozambique, Ages: 20−24
Data points enclosed with a diamond are reference sources
Age−specific fertility rate
Year
UN WPP
GBD 2017
GBD 2016
Adjusted Data, CBH/VR
Adjusted Data, CEB
Data, CBH/VR
Data, CEB
Outliered Data
AIS SBH
Census SBH
Census Tabulation SBH
DHS CBH
DHS SBH
MICS CBH
MICS SBH
OTHER SURVEY SBH
VR Incomplete

Age−specific fertility fitted trend lines, 1950−2017: Mozambique, Ages: 25−29
Data points enclosed with a diamond are reference sources

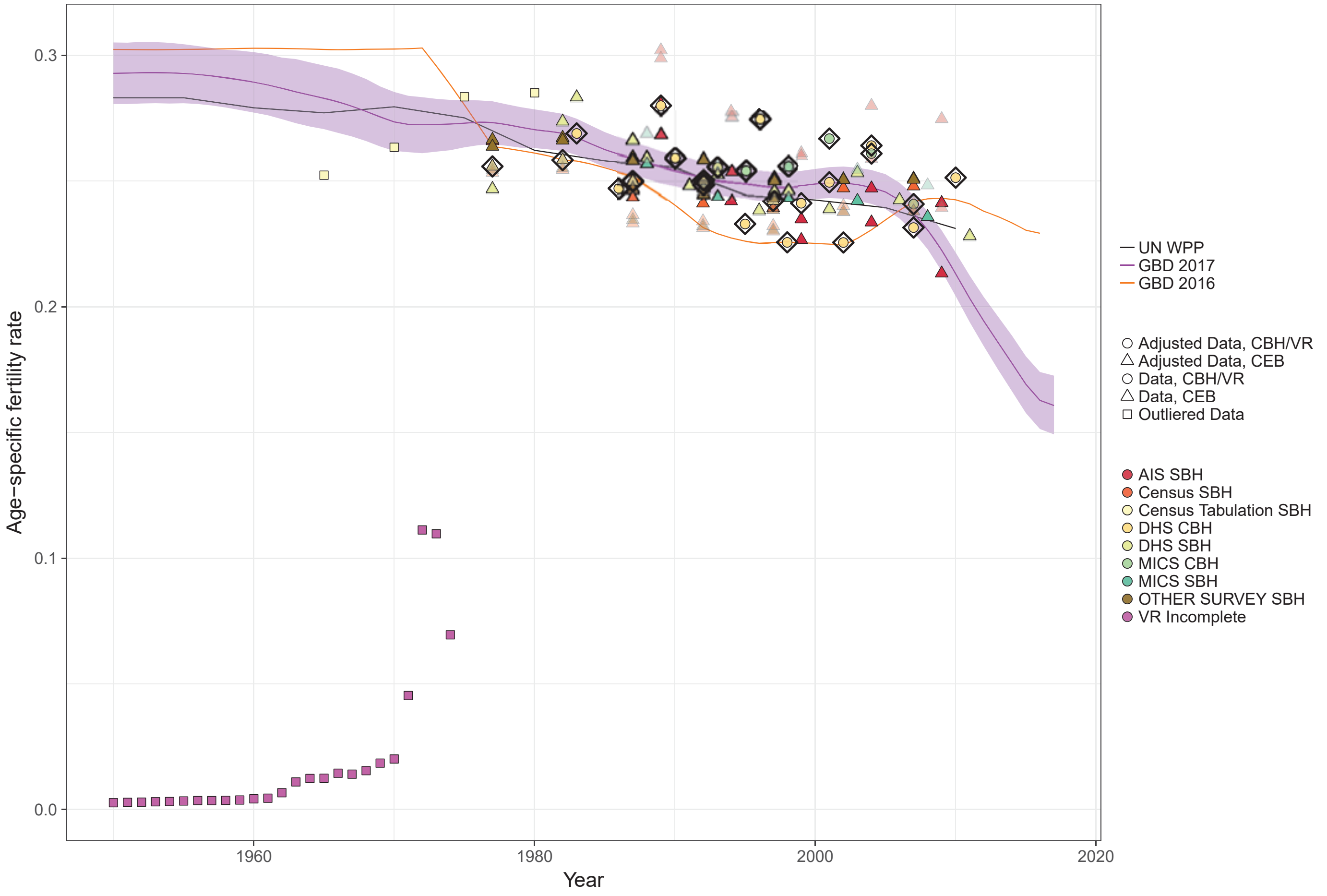

Age−specific fertility fitted trend lines, 1950−2017: Mozambique, Ages: 30−34
Data points enclosed with a diamond are reference sources

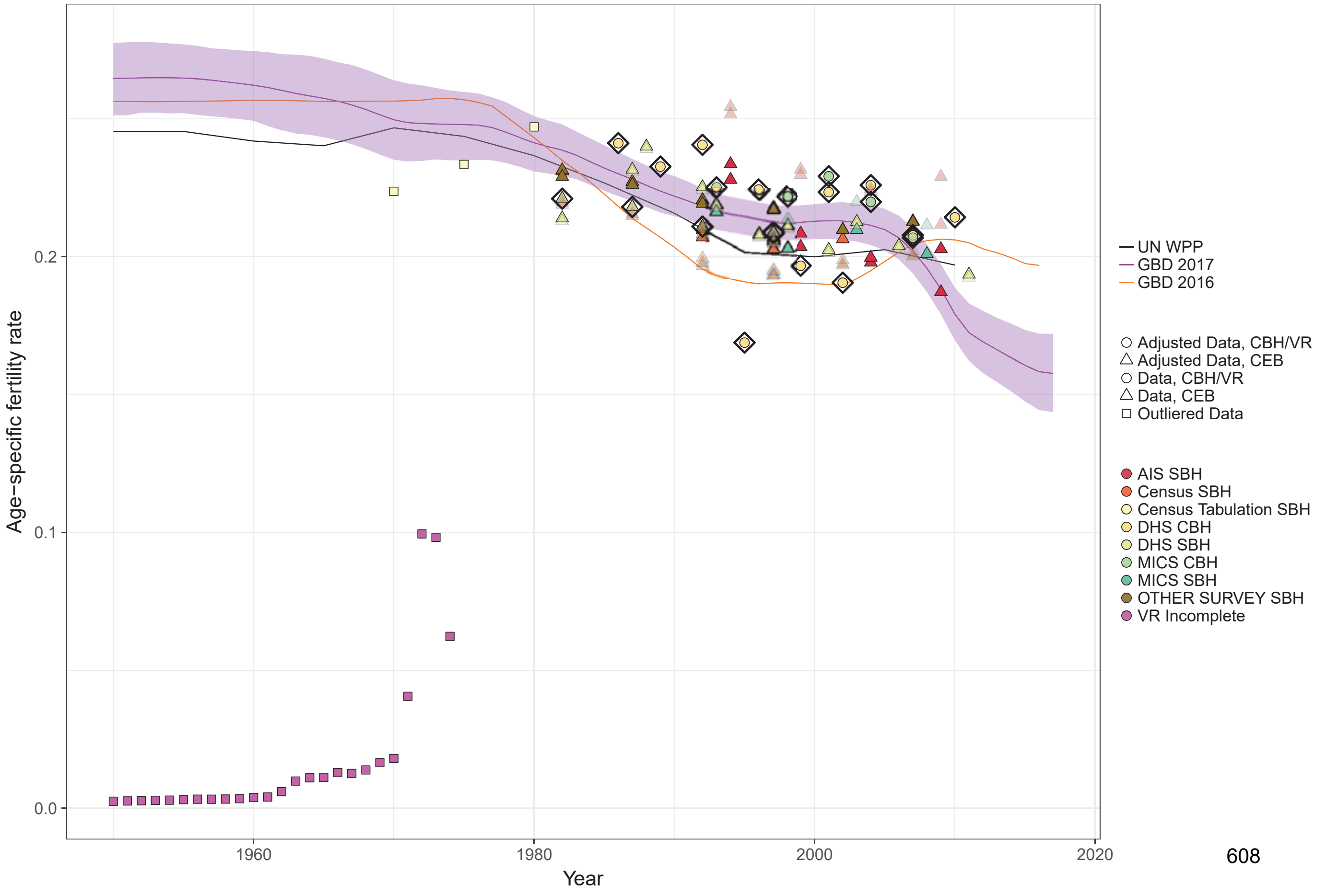

Age−specific fertility fitted trend lines, 1950−2017: Mozambique, Ages: 35−39
Data points enclosed with a diamond are reference sources

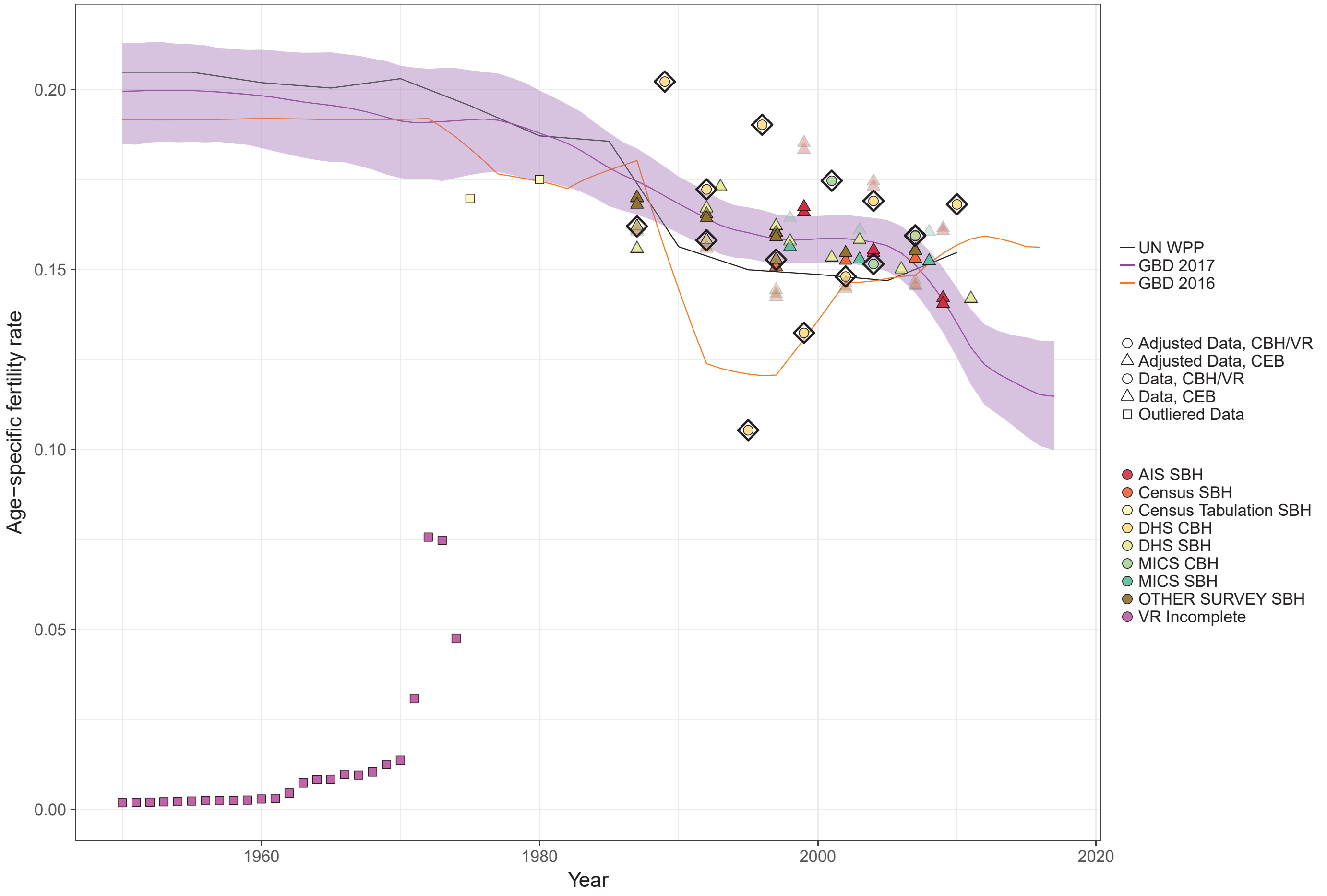

Age−specific fertility fitted trend lines, 1950−2017: Mozambique, Ages: 40−44
Data points enclosed with a diamond are reference sources

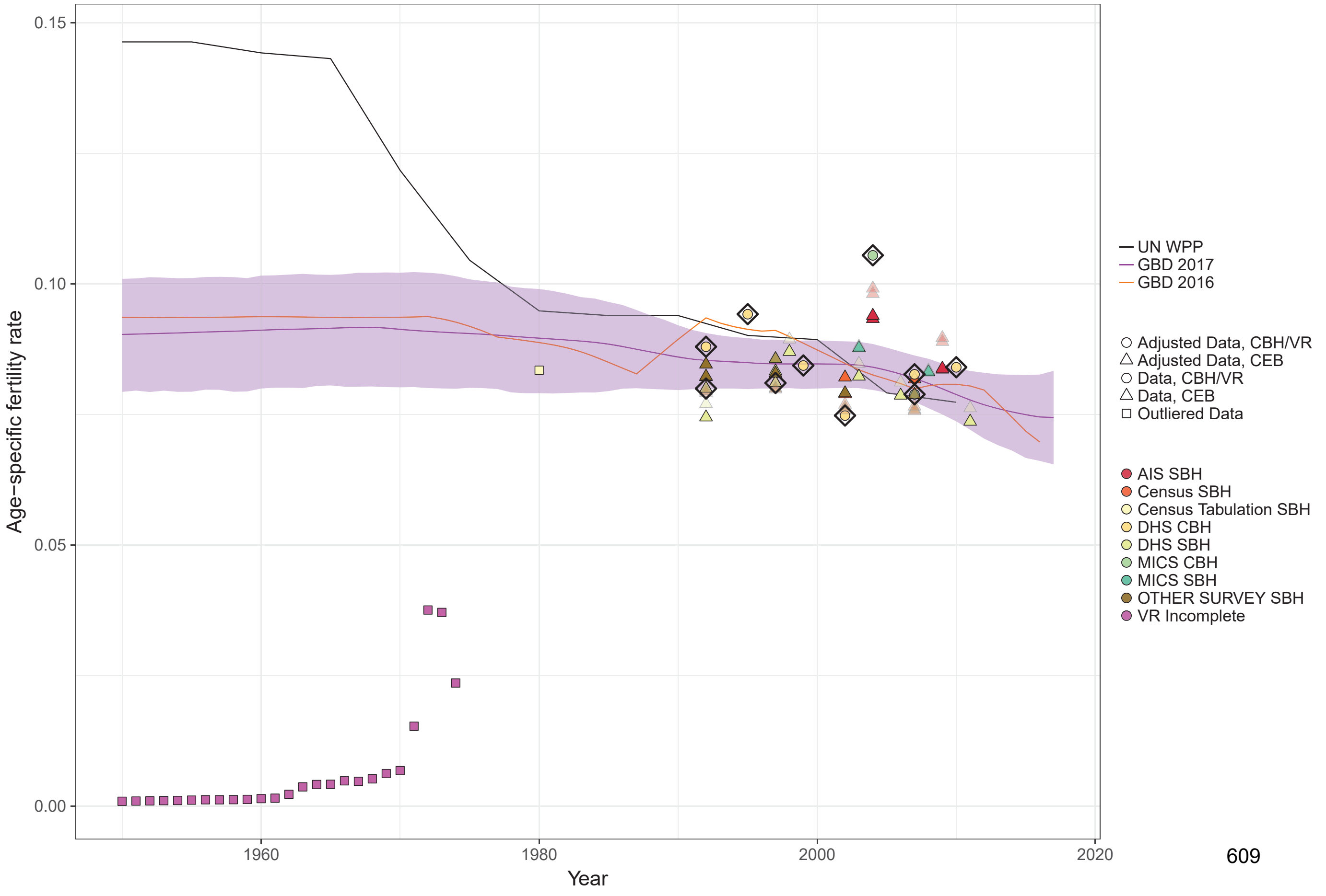

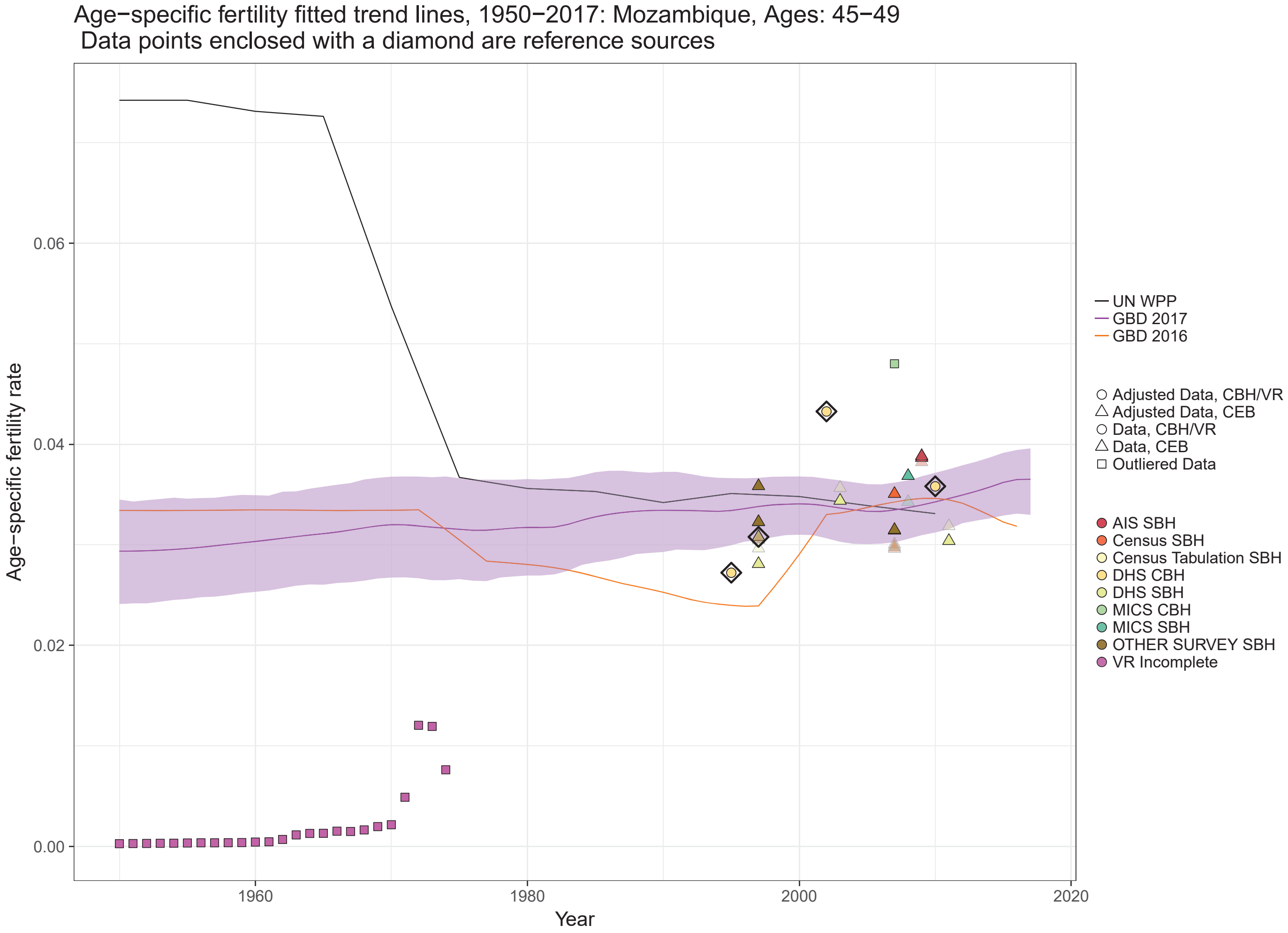

Age−specific fertility fitted trend lines, 1950−2017: Mozambique, Ages: 45−49
Data points enclosed with a diamond are reference sources
Age−specific fertility rate
Year
0.00
0.02
0.04
0.06
1960
1980
2000
2020
UN WPP
GBD 2017
GBD 2016
Adjusted Data, CBH/VR
Adjusted Data, CEB
Data, CBH/VR
Data, CEB
Outliered Data
AIS SBH
Census SBH
Census Tabulation SBH
DHS CBH
DHS SBH
MICS CBH
MICS SBH
OTHER SURVEY SBH
VR Incomplete

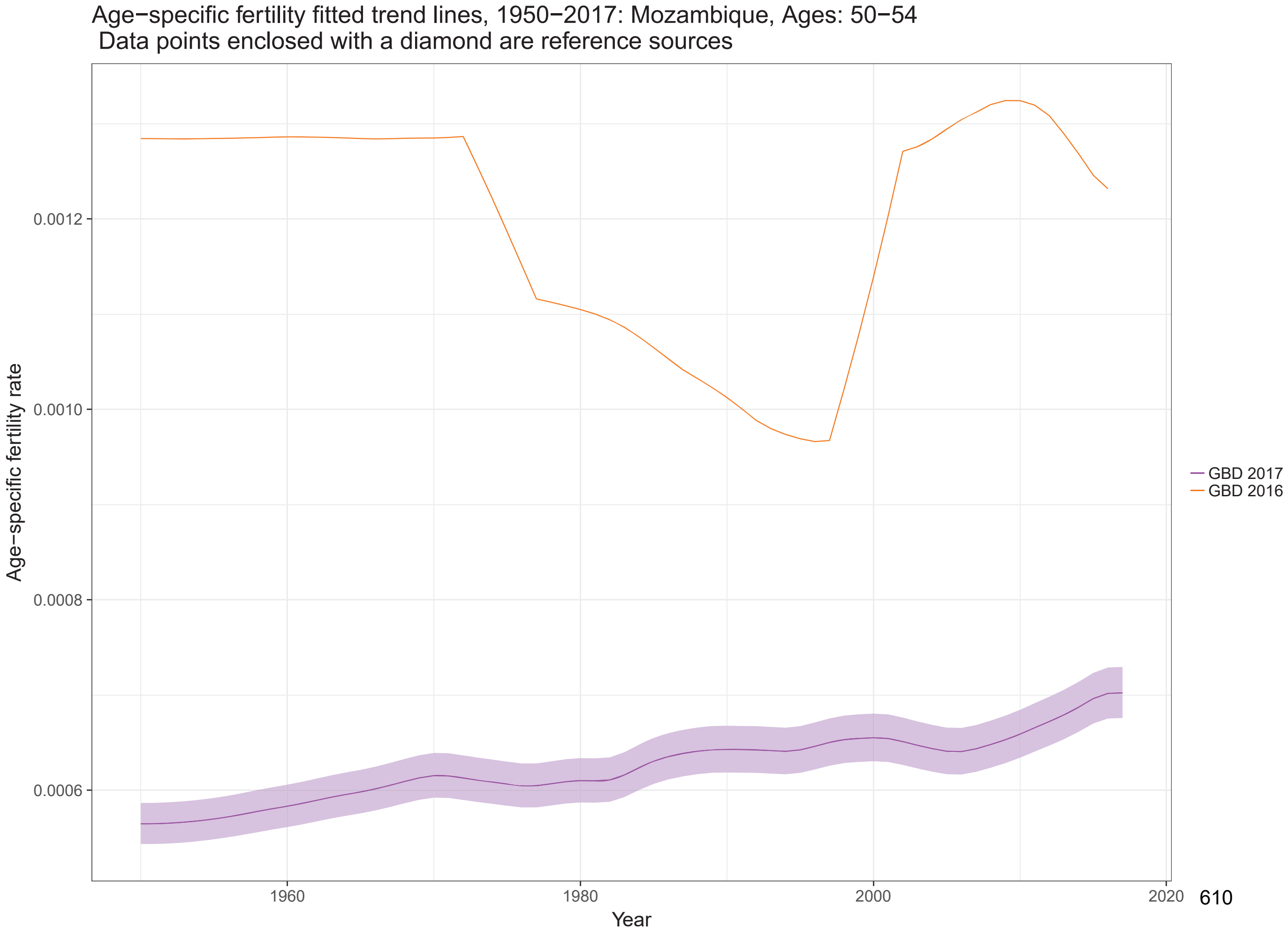

Age−specific fertility fitted trend lines, 1950−2017: Mozambique, Ages: 50−54
Data points enclosed with a diamond are reference sources
Age−specific fertility rate
Year
0.0006
0.0008
0.0010
0.0012
1960
1980
2000
2020
GBD 2017
GBD 2016

Age−specific fertility fitted trend lines, 1950−2017: Myanmar, TFR
Data points enclosed with a diamond are reference sources

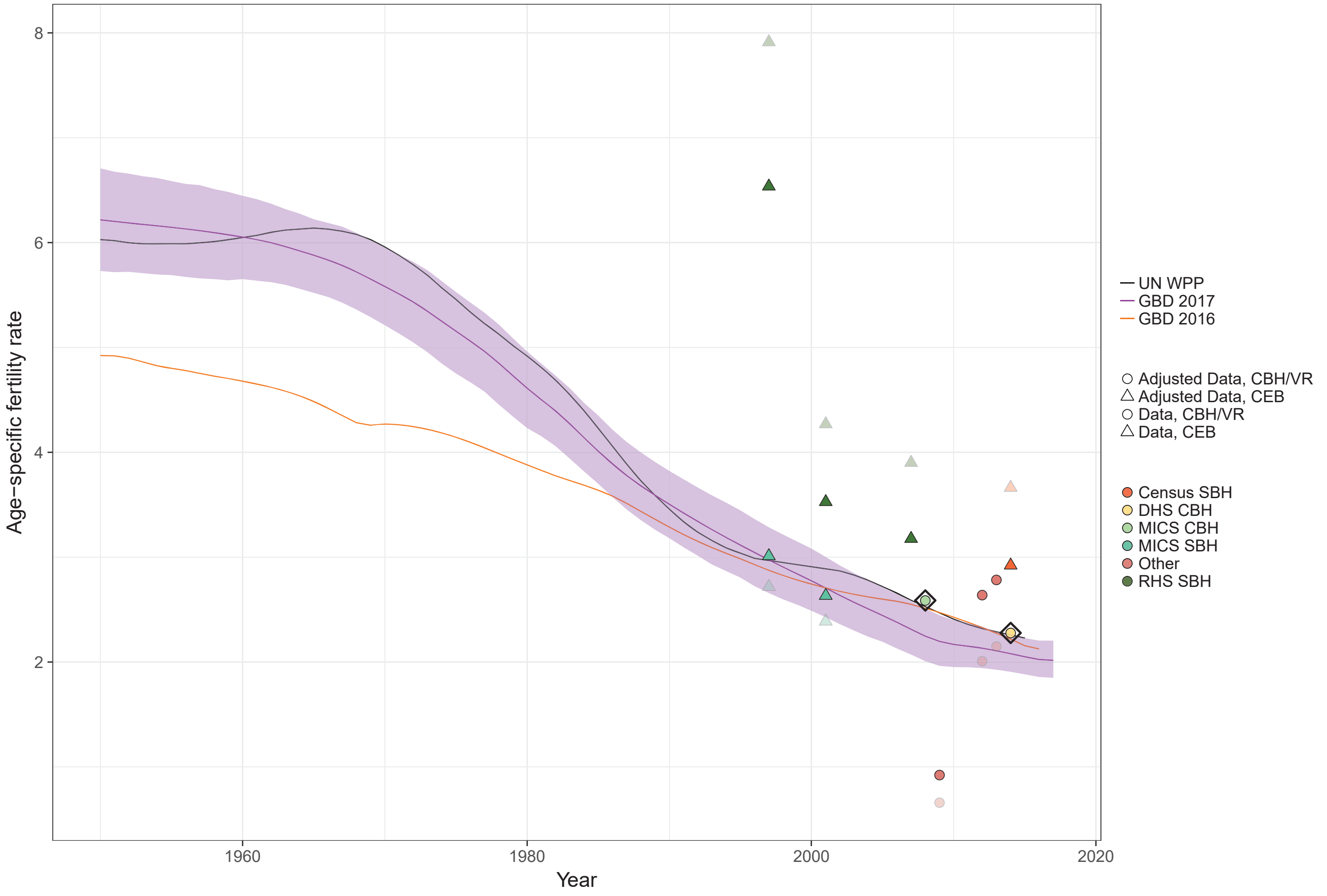

Age−specific fertility fitted trend lines, 1950−2017: Myanmar, Ages: 10−14
Data points enclosed with a diamond are reference sources

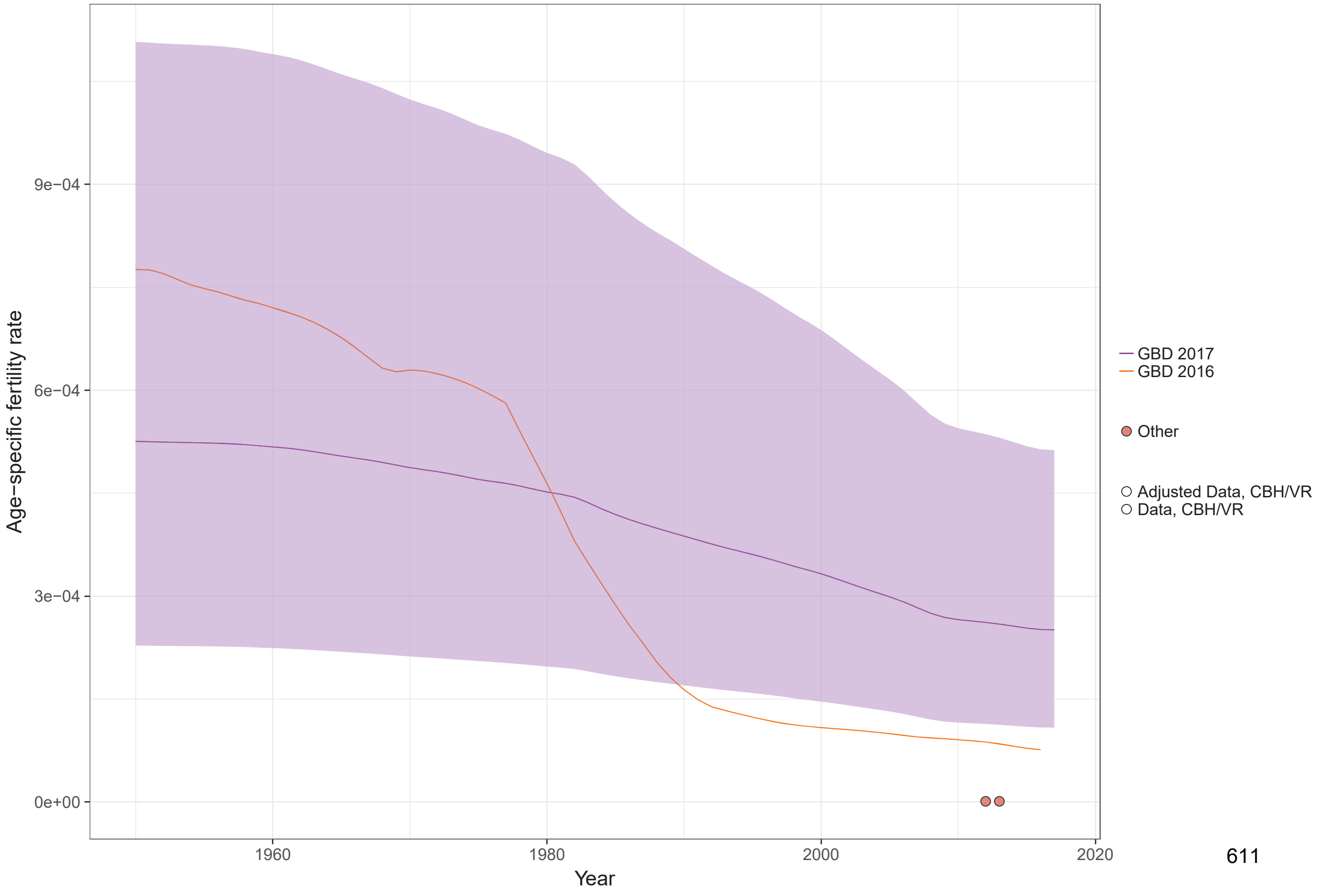

Age−specific fertility fitted trend lines, 1950−2017: Myanmar, Ages: 15−19
Data points enclosed with a diamond are reference sources

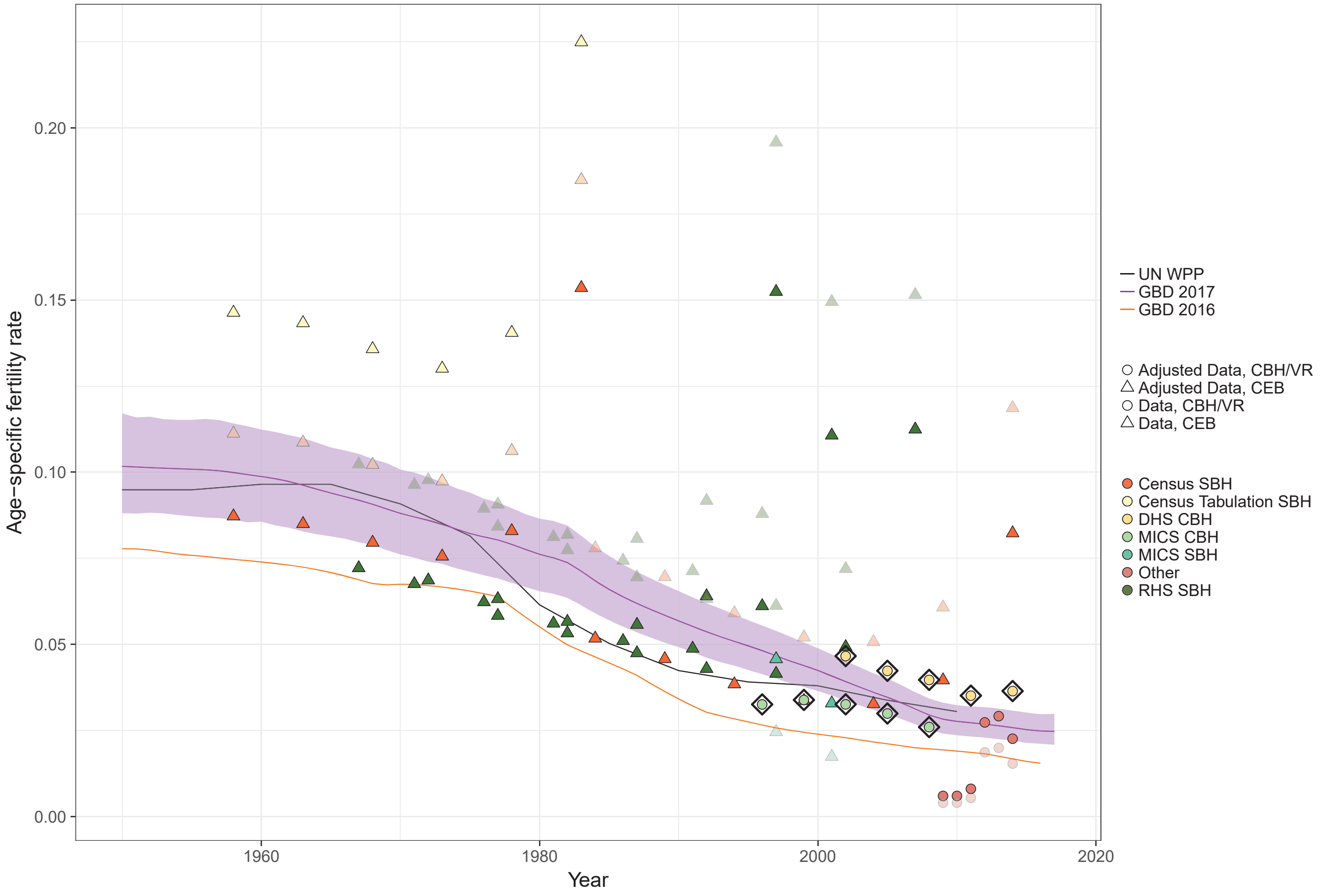

Age−specific fertility fitted trend lines, 1950−2017: Myanmar, Ages: 20−24
Data points enclosed with a diamond are reference sources

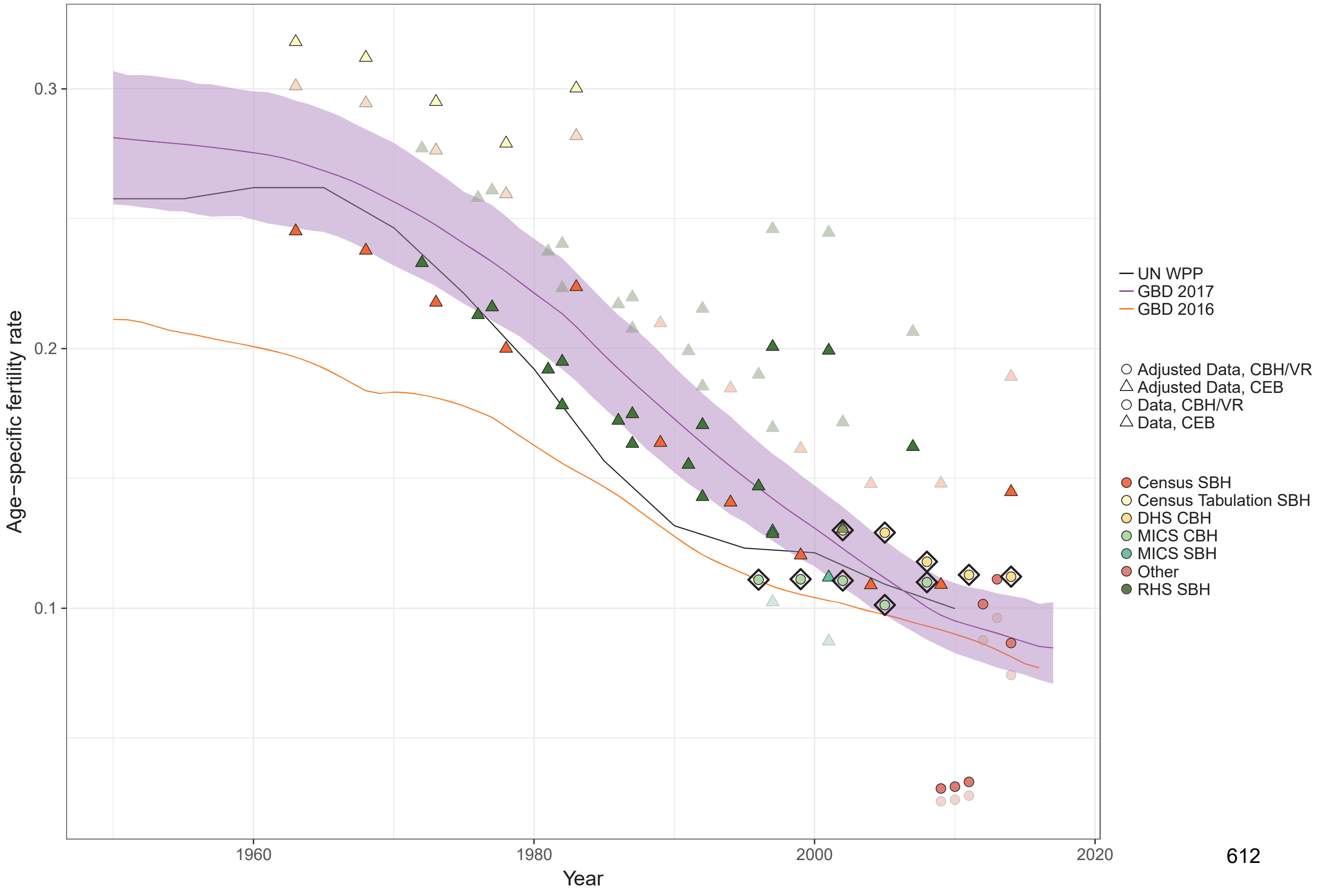

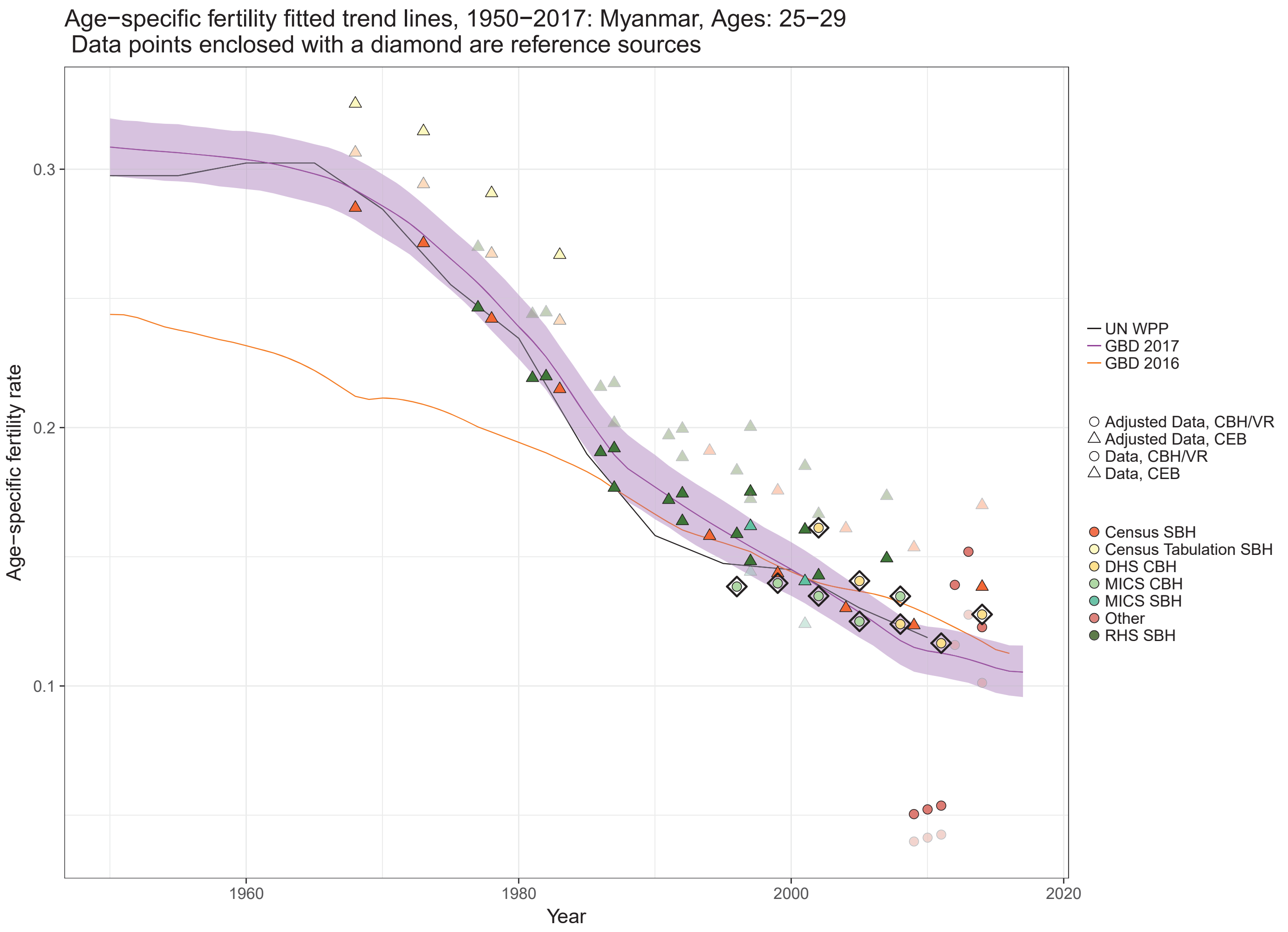
Age−specific fertility fitted trend lines, 1950−2017: Myanmar, Ages: 25−29
Data points enclosed with a diamond are reference sources
Age-specific fertility rate
Year
0.1
0.2
0.3
1960
1980
2000
2020
UN WPP
GBD 2017
GBD 2016
Adjusted Data, CBH/VR
Adjusted Data, CEB
Data, CBH/VR
Data, CEB
Census SBH
Census Tabulation SBH
DHS CBH
MICS CBH
MICS SBH
Other
RHS SBH

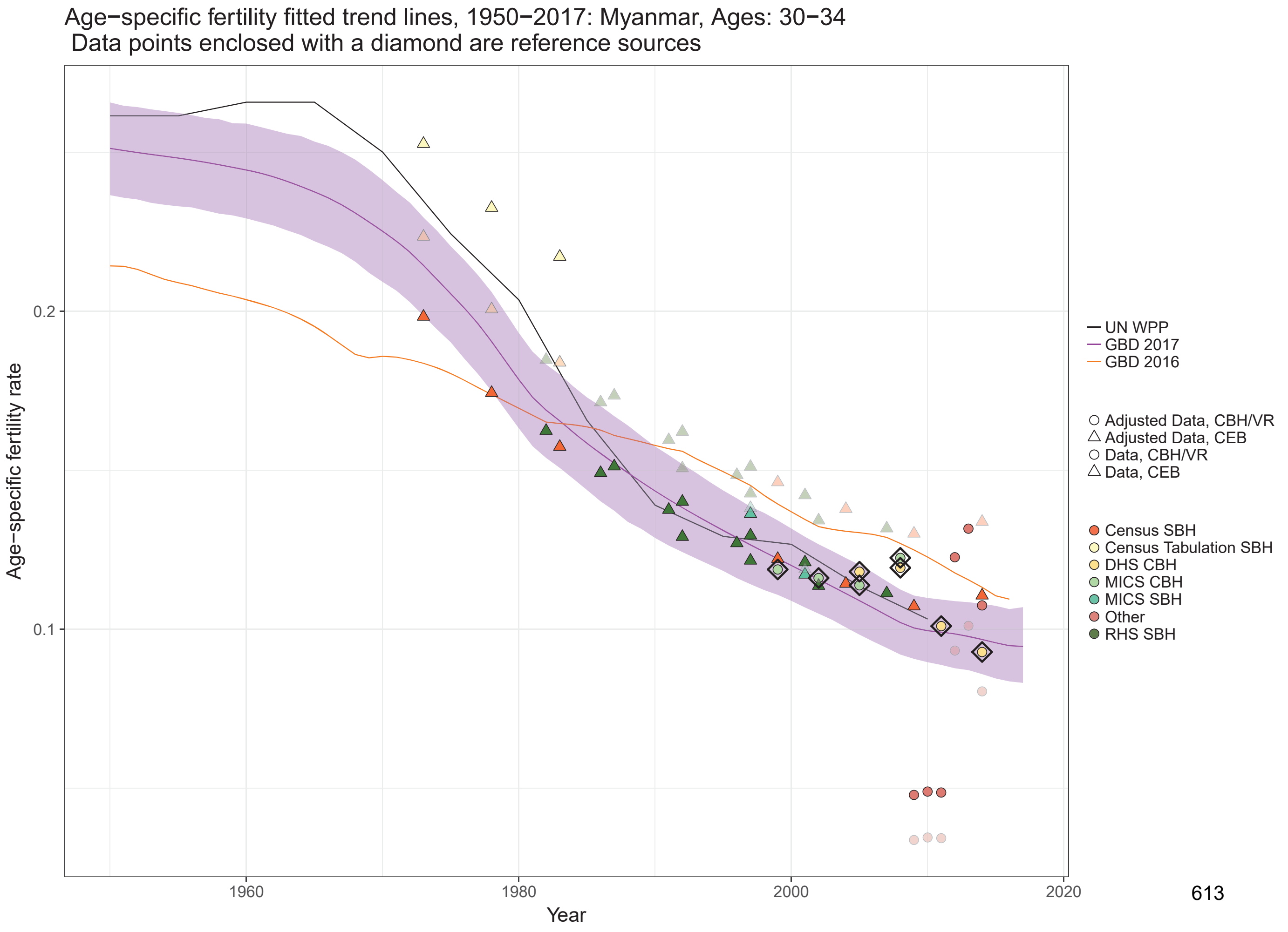
Age−specific fertility fitted trend lines, 1950−2017: Myanmar, Ages: 30−34
Data points enclosed with a diamond are reference sources
Age-specific fertility rate
Year
0.1
0.2
1960
1980
2000
2020
UN WPP
GBD 2017
GBD 2016
Adjusted Data, CBH/VR
Adjusted Data, CEB
Data, CBH/VR
Data, CEB
Census SBH
Census Tabulation SBH
DHS CBH
MICS CBH
MICS SBH
Other
RHS SBH

Age−specific fertility fitted trend lines, 1950−2017: Myanmar, Ages: 35−39
Data points enclosed with a diamond are reference sources

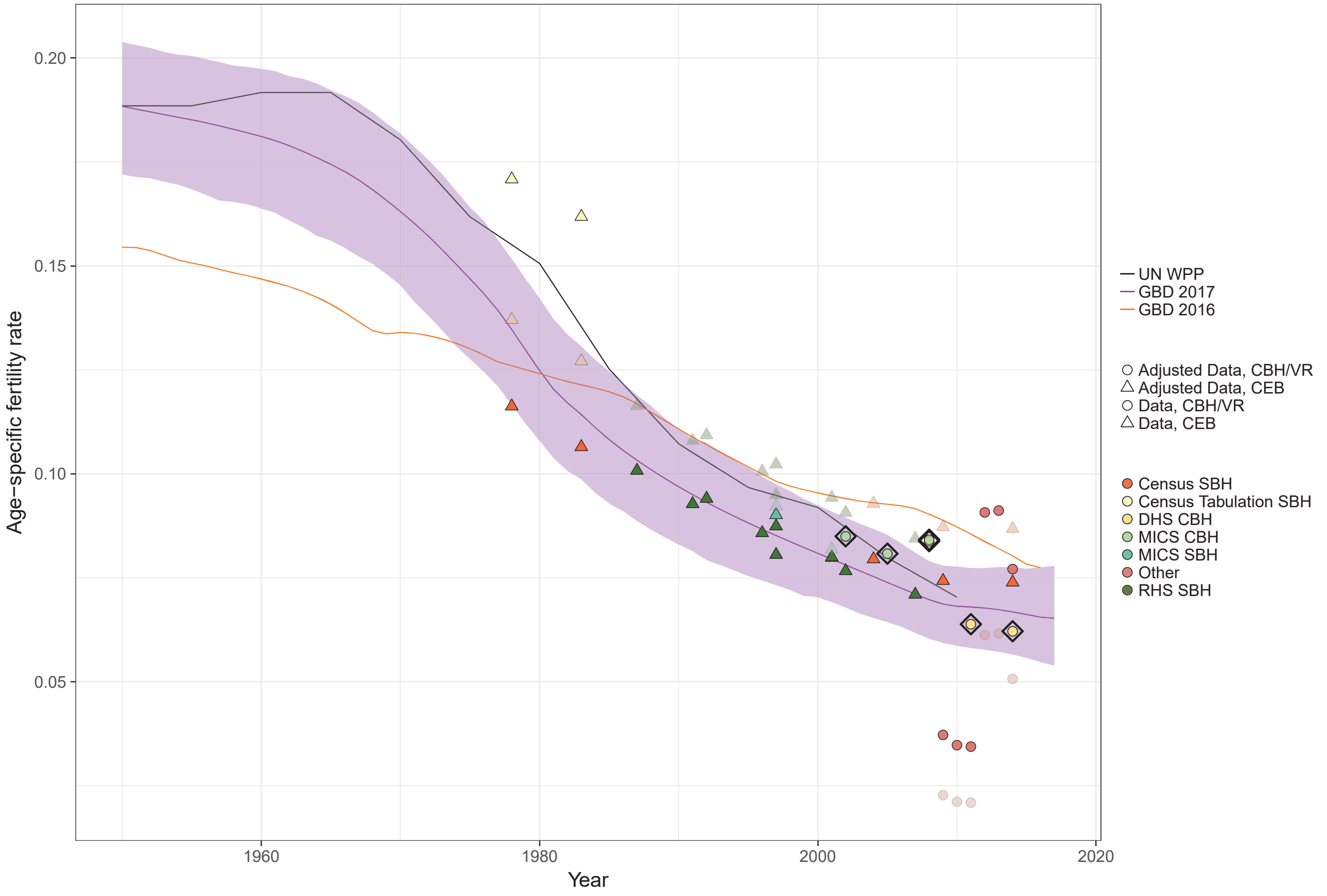

Age−specific fertility fitted trend lines, 1950−2017: Myanmar, Ages: 40−44
Data points enclosed with a diamond are reference sources

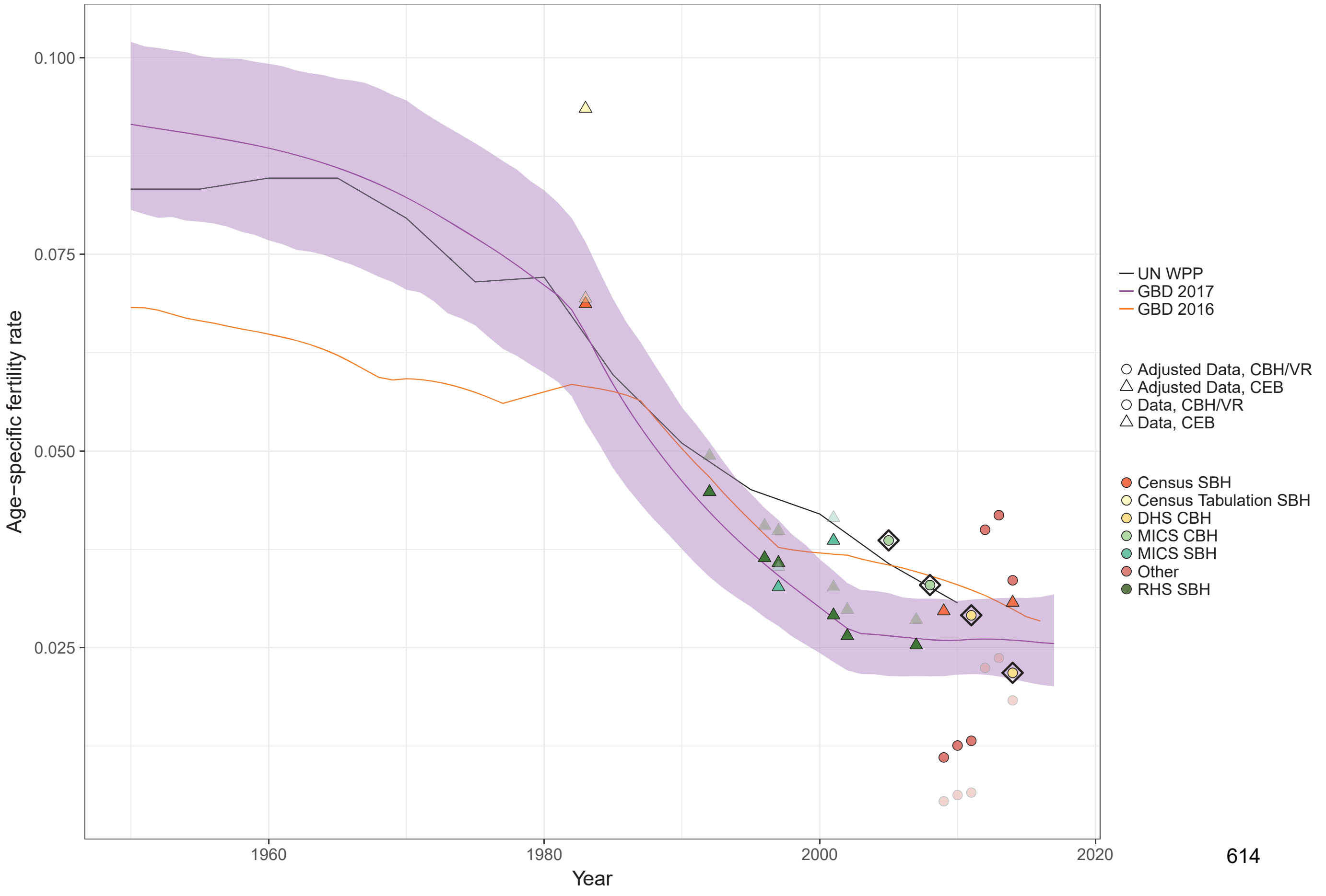

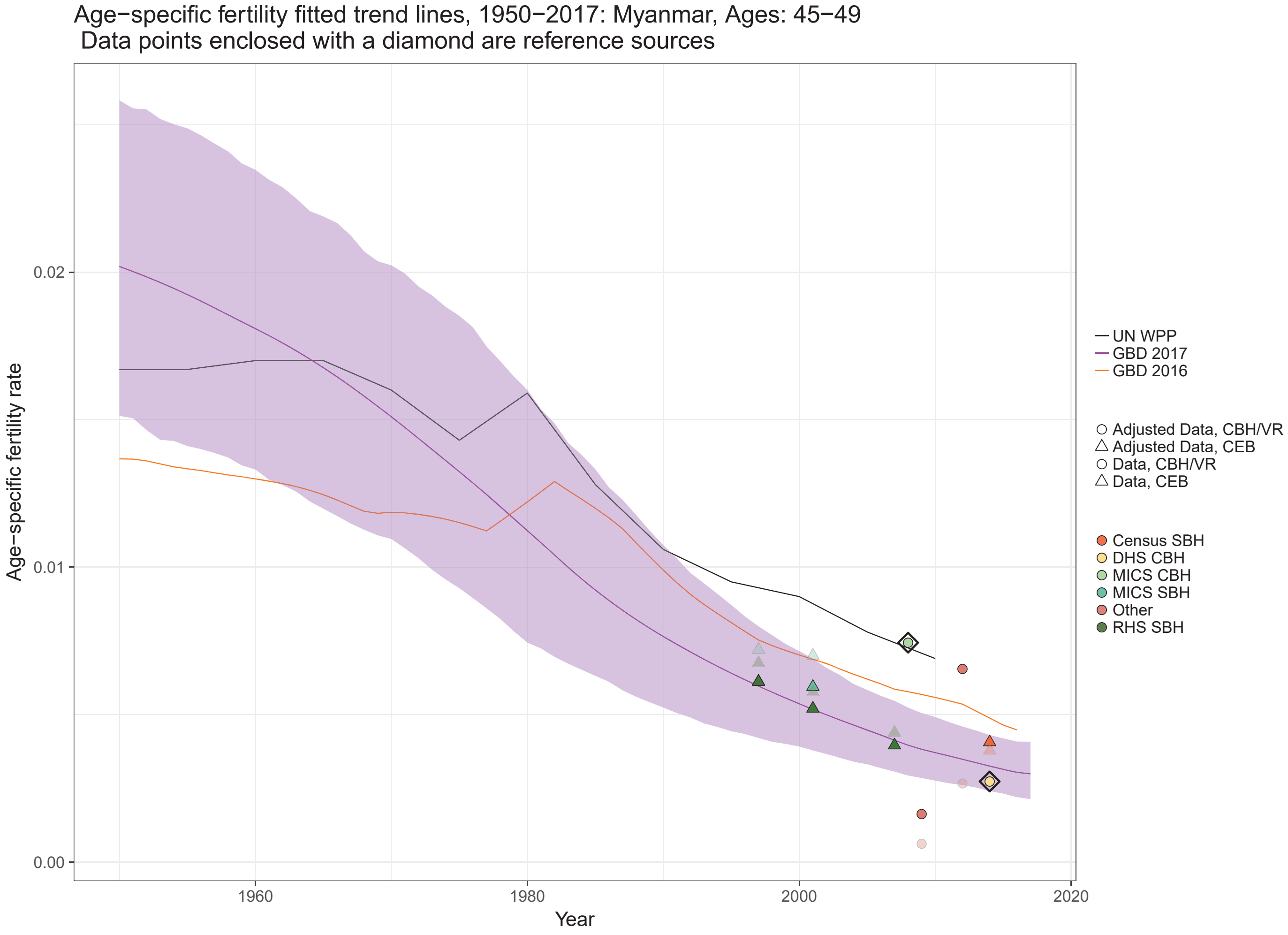

Age−specific fertility fitted trend lines, 1950−2017: Myanmar, Ages: 45−49
Data points enclosed with a diamond are reference sources
Age−specific fertility rate
0.00
0.01
0.02
1960
1980
2000
2020
Year
UN WPP
GBD 2017
GBD 2016
Adjusted Data, CBH/VR
Adjusted Data, CEB
Data, CBH/VR
Data, CEB
Census SBH
DHS CBH
MICS CBH
MICS SBH
Other
RHS SBH

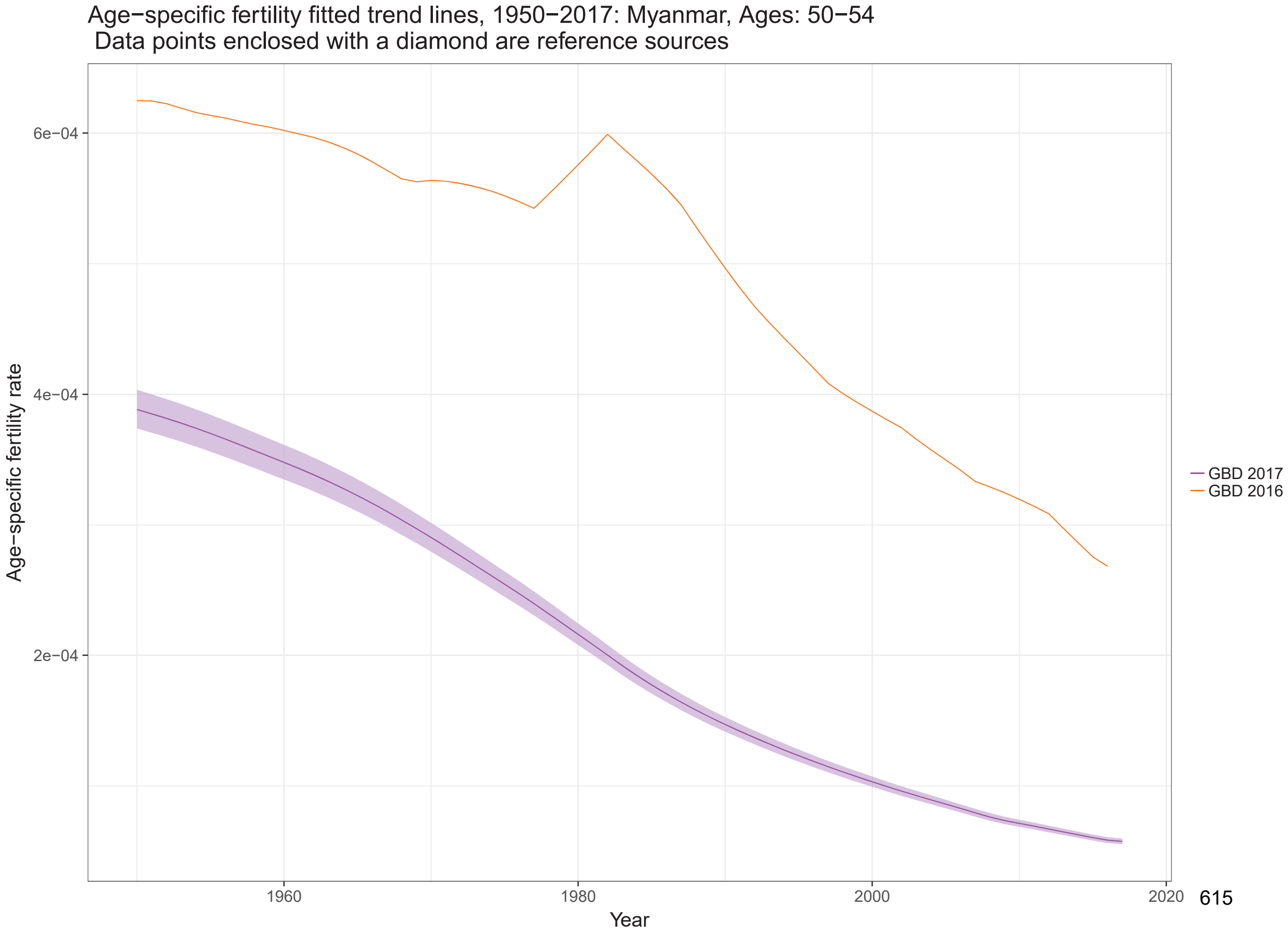

Age−specific fertility fitted trend lines, 1950−2017: Myanmar, Ages: 50−54
Data points enclosed with a diamond are reference sources
Age−specific fertility rate
2e−04
4e−04
6e−04
1960
1980
2000
2020
Year
GBD 2017
GBD 2016

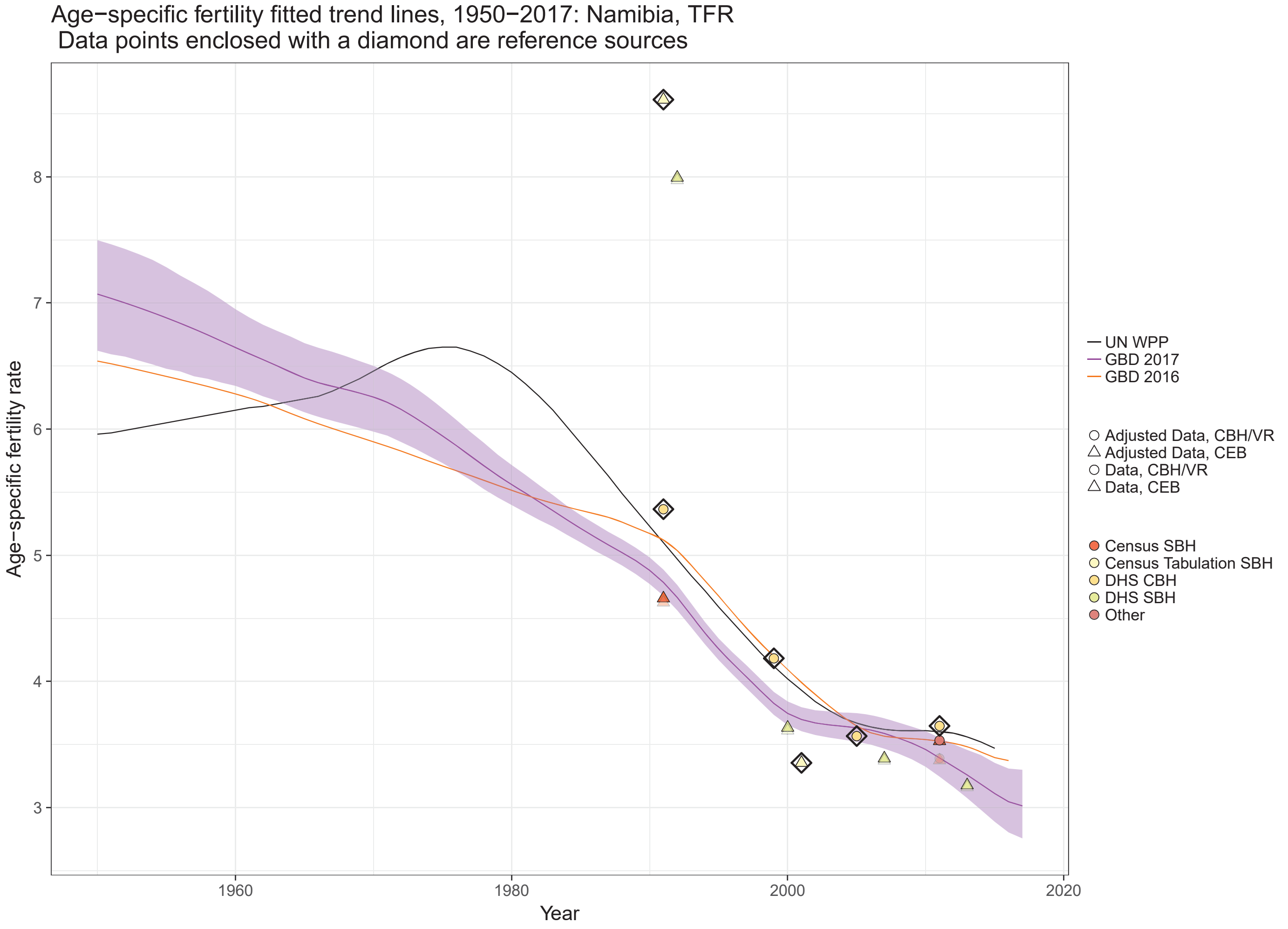

Age−specific fertility fitted trend lines, 1950−2017: Namibia, TFR
Data points enclosed with a diamond are reference sources
Age−specific fertility rate
Year
UN WPP
GBD 2017
GBD 2016
Adjusted Data, CBH/VR
Adjusted Data, CEB
Data, CBH/VR
Data, CEB
Census SBH
Census Tabulation SBH
DHS CBH
DHS SBH
Other

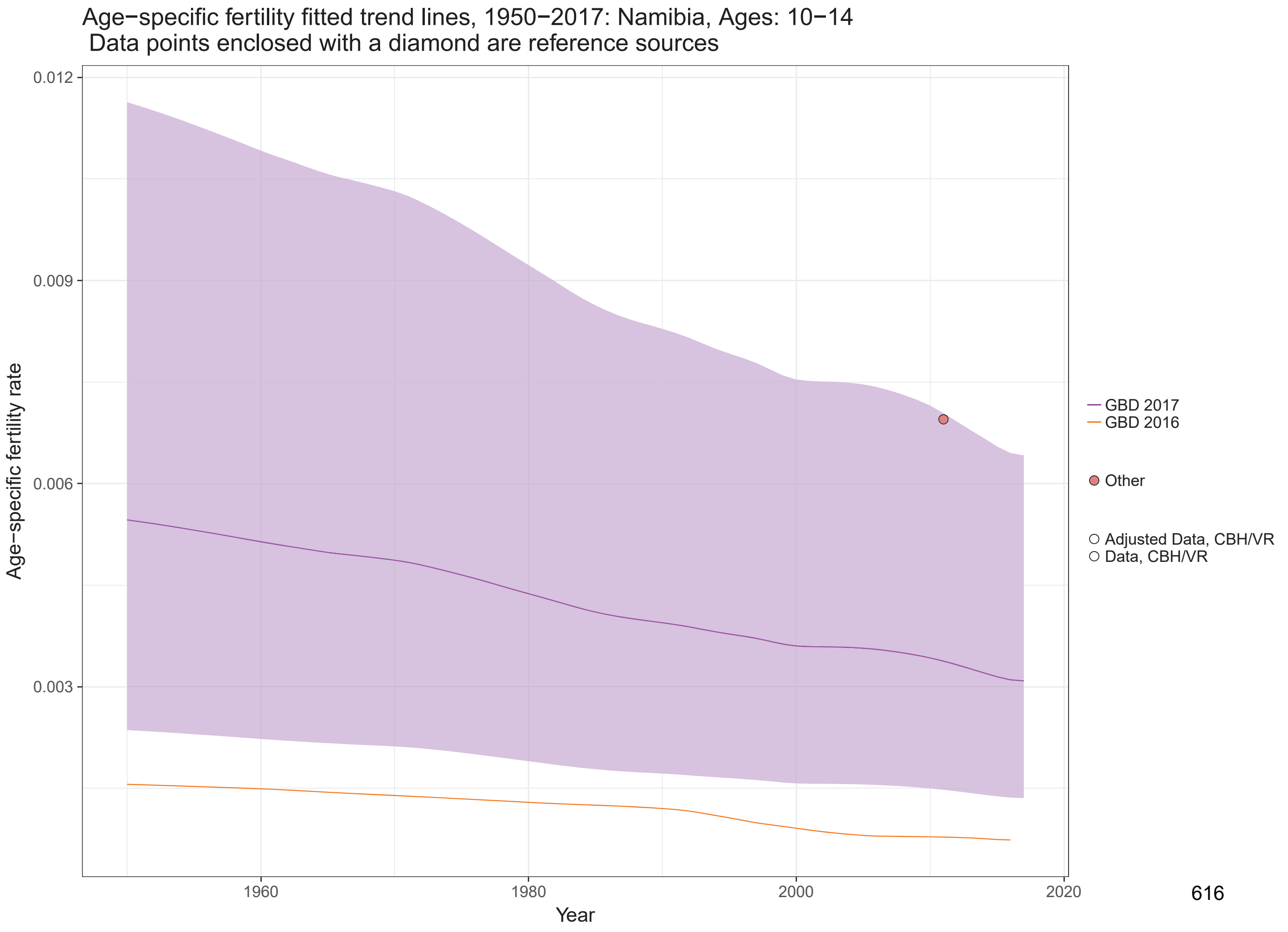

Age−specific fertility fitted trend lines, 1950−2017: Namibia, Ages: 10−14
Data points enclosed with a diamond are reference sources
Age−specific fertility rate
Year
GBD 2017
GBD 2016
Other
Adjusted Data, CBH/VR
Data, CBH/VR

Age−specific fertility fitted trend lines, 1950−2017: Namibia, Ages: 15−19
Data points enclosed with a diamond are reference sources

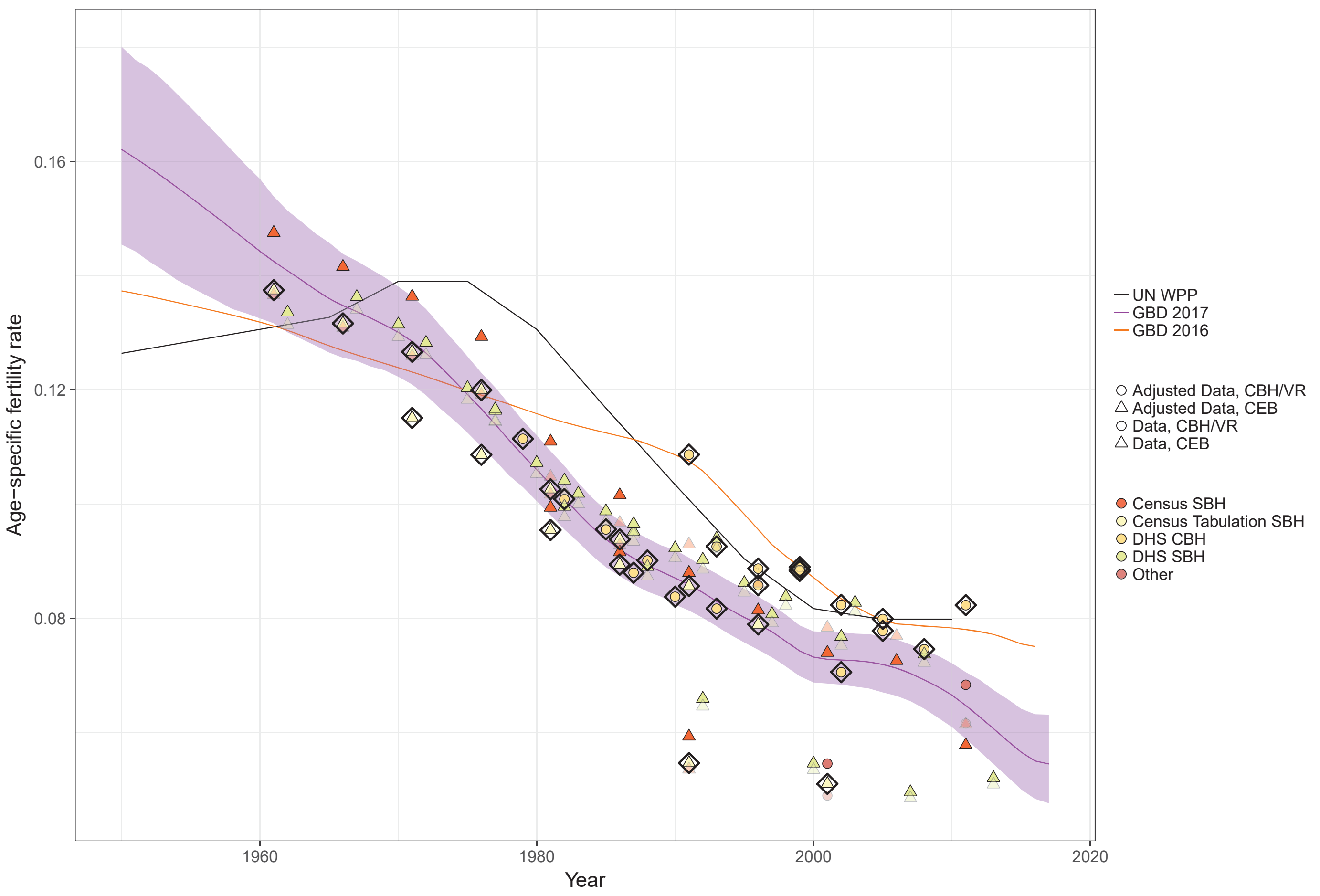

Age−specific fertility fitted trend lines, 1950−2017: Namibia, Ages: 20−24
Data points enclosed with a diamond are reference sources

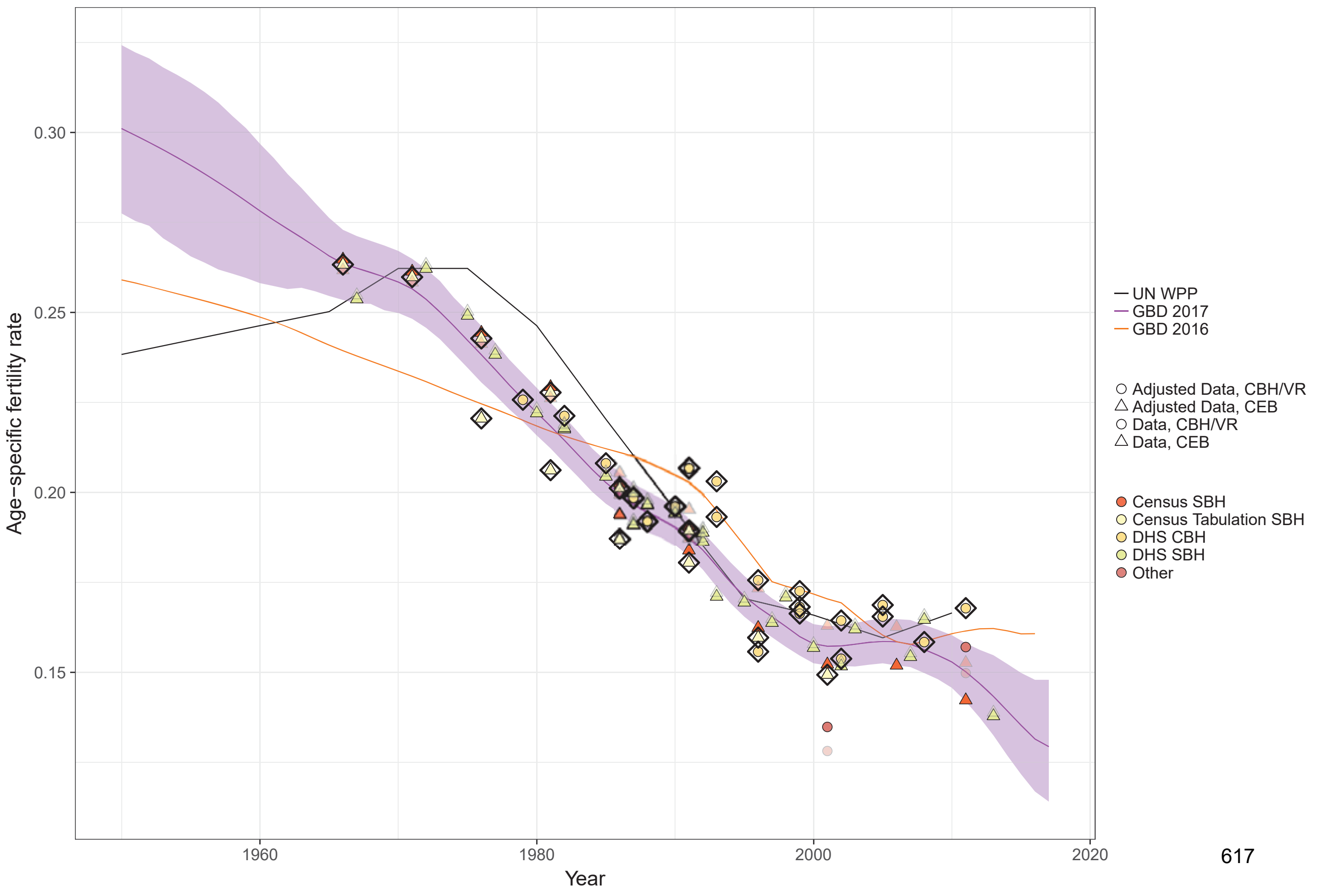

Age−specific fertility fitted trend lines, 1950−2017: Namibia, Ages: 25−29
Data points enclosed with a diamond are reference sources

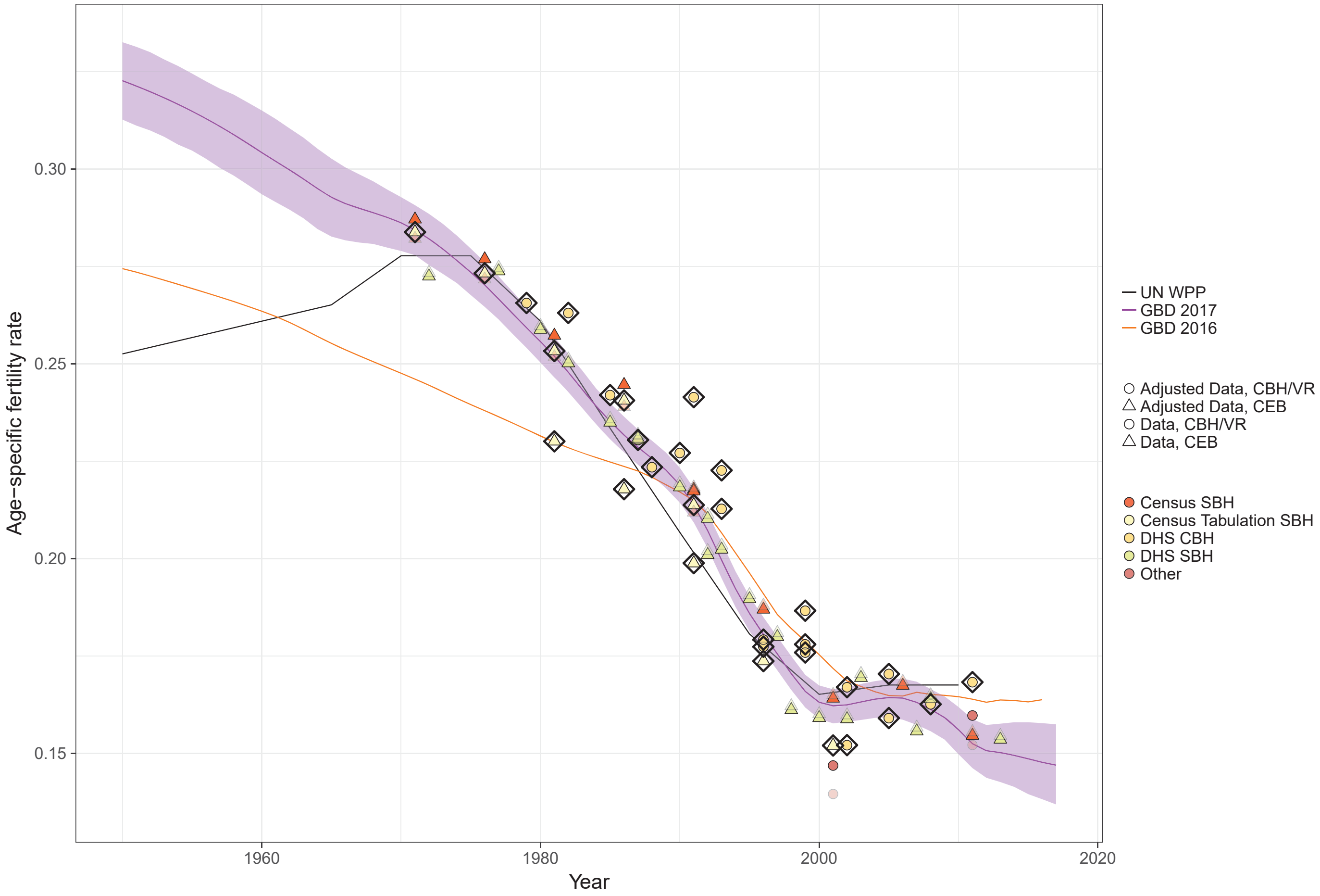

Age−specific fertility fitted trend lines, 1950−2017: Namibia, Ages: 30−34
Data points enclosed with a diamond are reference sources

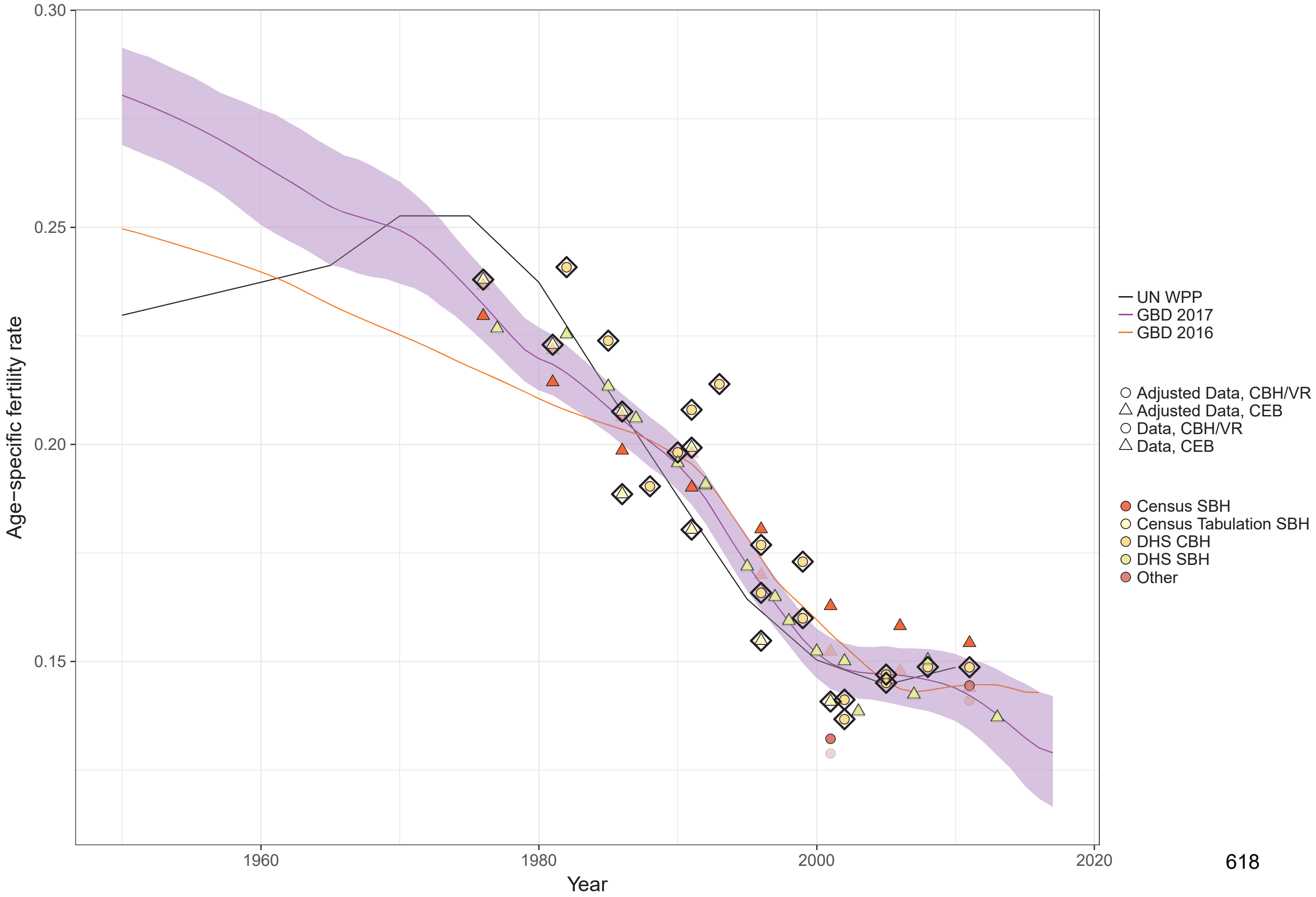

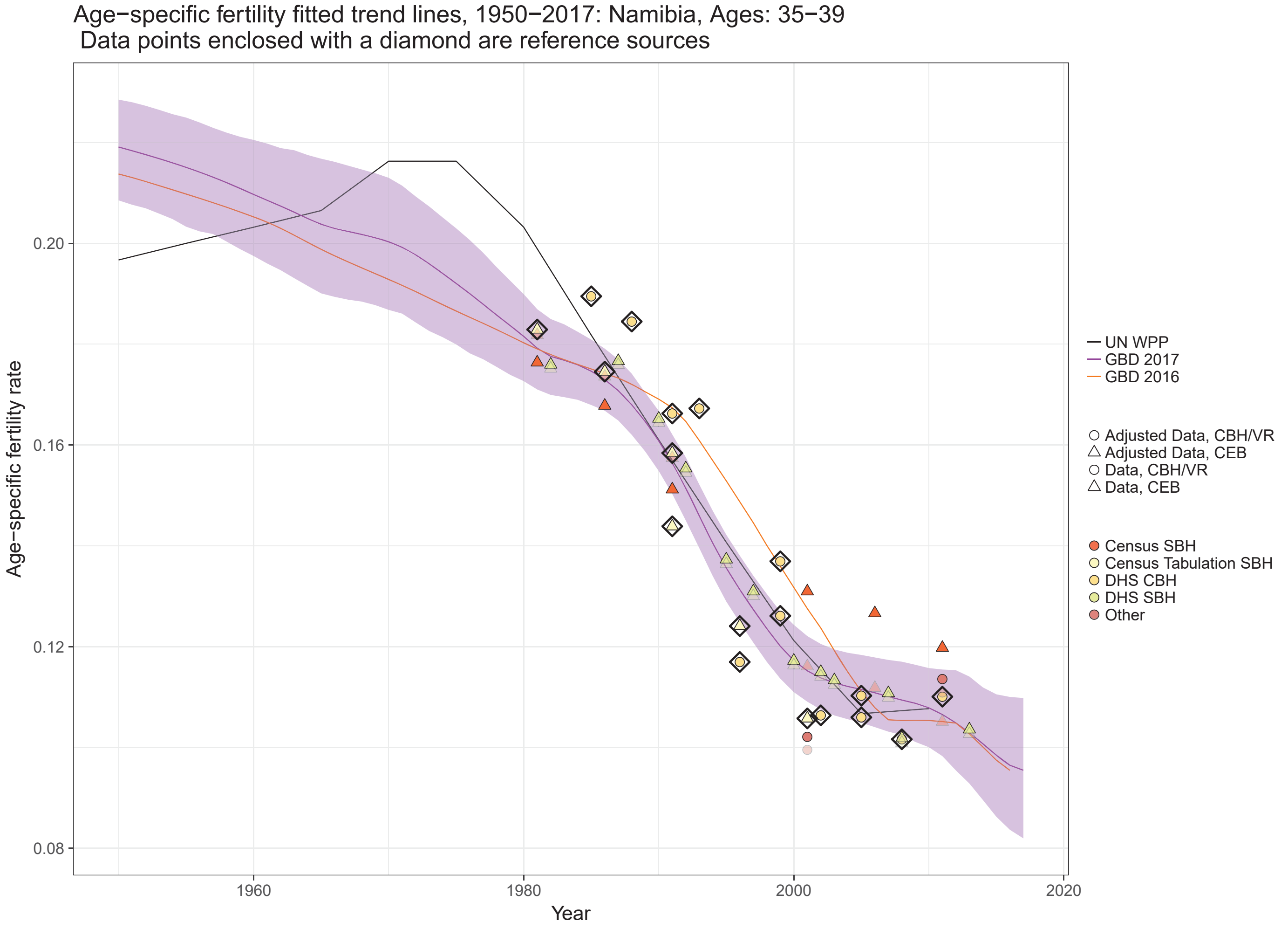
Age−specific fertility fitted trend lines, 1950−2017: Namibia, Ages: 35−39
Data points enclosed with a diamond are reference sources
Age−specific fertility rate
Year
UN WPP
GBD 2017
GBD 2016
Adjusted Data, CBH/VR
Adjusted Data, CEB
Data, CBH/VR
Data, CEB
Census SBH
Census Tabulation SBH
DHS CBH
DHS SBH
Other

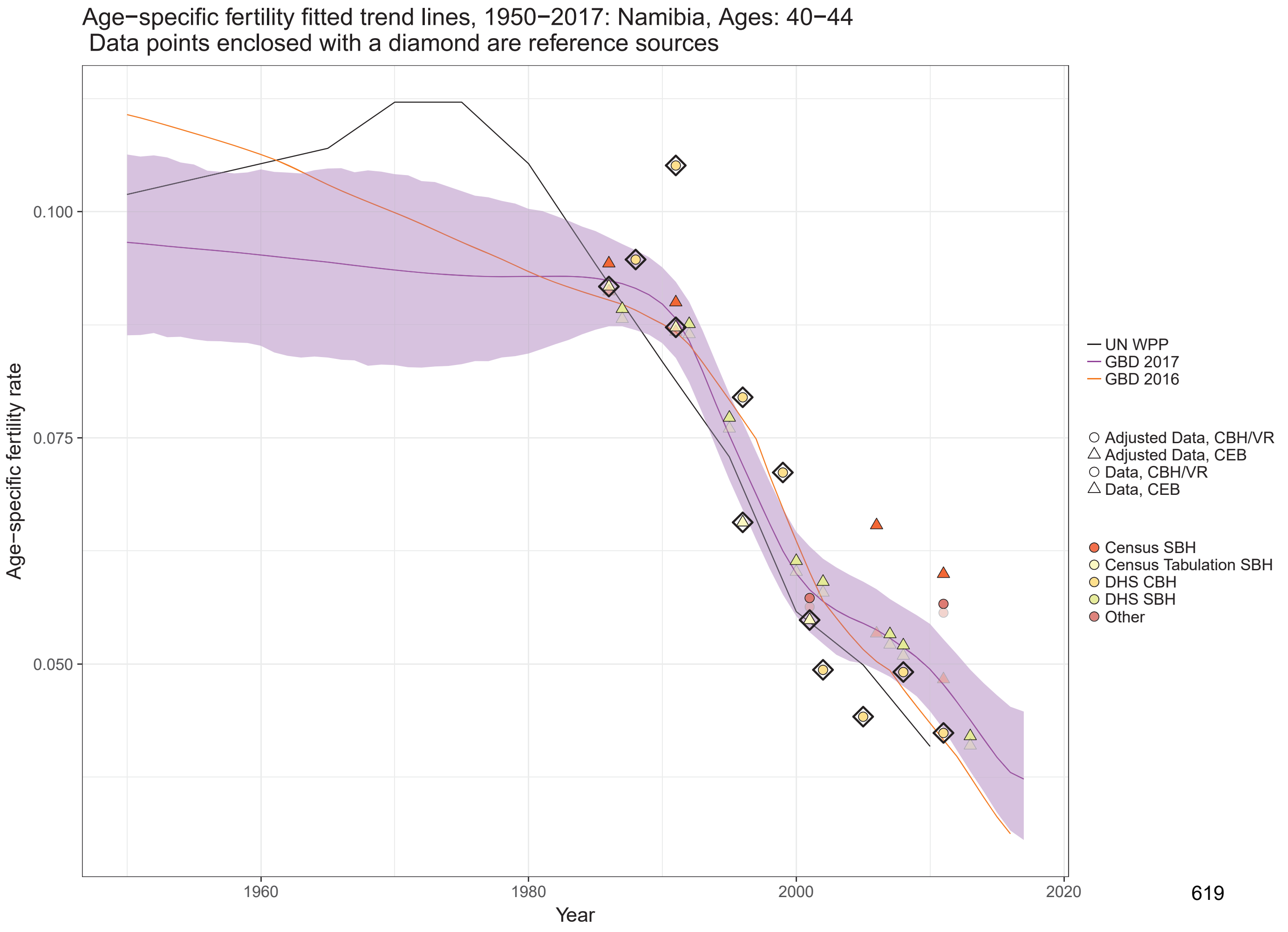
Age−specific fertility fitted trend lines, 1950−2017: Namibia, Ages: 40−44
Data points enclosed with a diamond are reference sources
Age−specific fertility rate
Year
UN WPP
GBD 2017
GBD 2016
Adjusted Data, CBH/VR
Adjusted Data, CEB
Data, CBH/VR
Data, CEB
Census SBH
Census Tabulation SBH
DHS CBH
DHS SBH
Other

Age−specific fertility fitted trend lines, 1950−2017: Namibia, Ages: 45−49
Data points enclosed with a diamond are reference sources

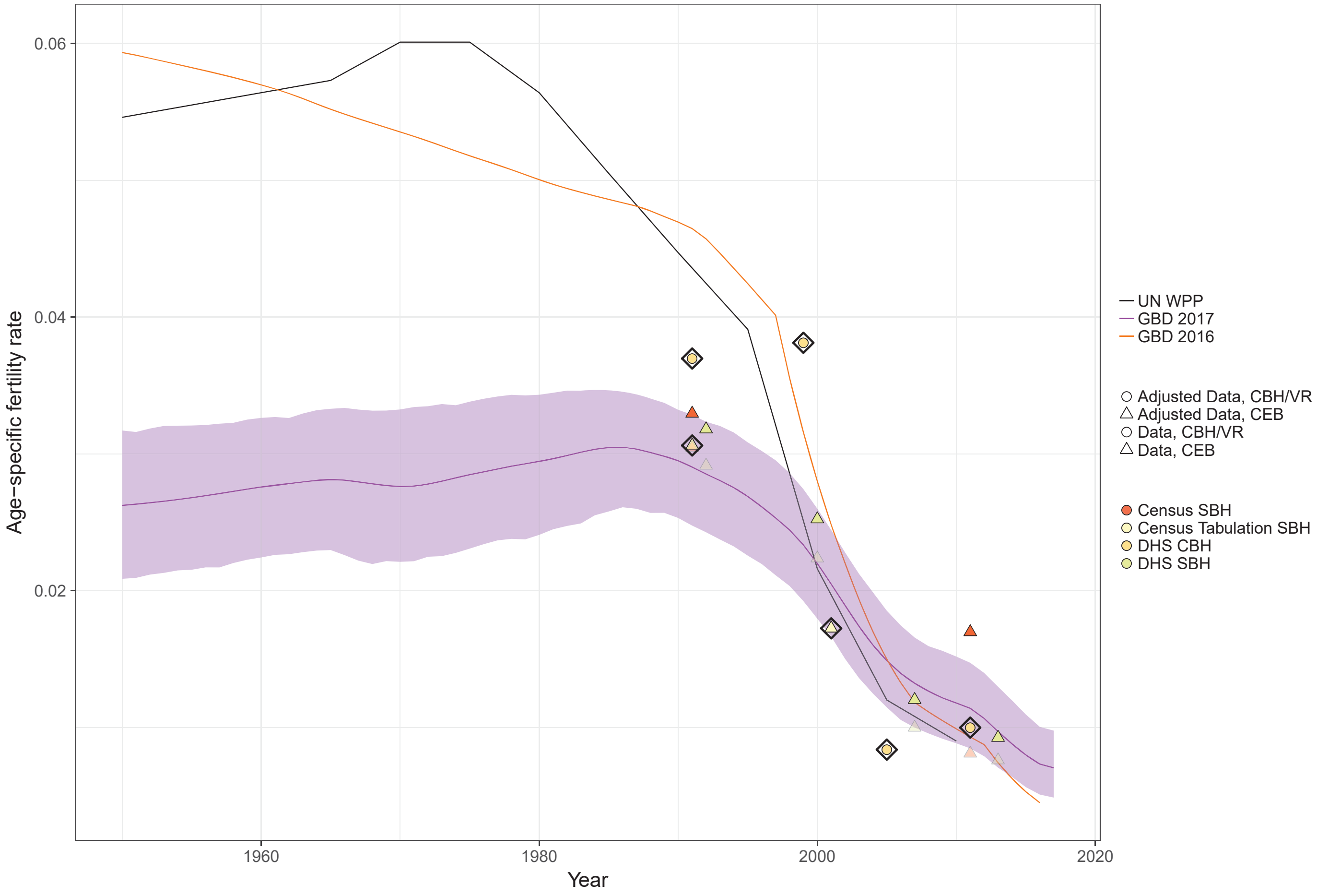

Age−specific fertility fitted trend lines, 1950−2017: Namibia, Ages: 50−54
Data points enclosed with a diamond are reference sources

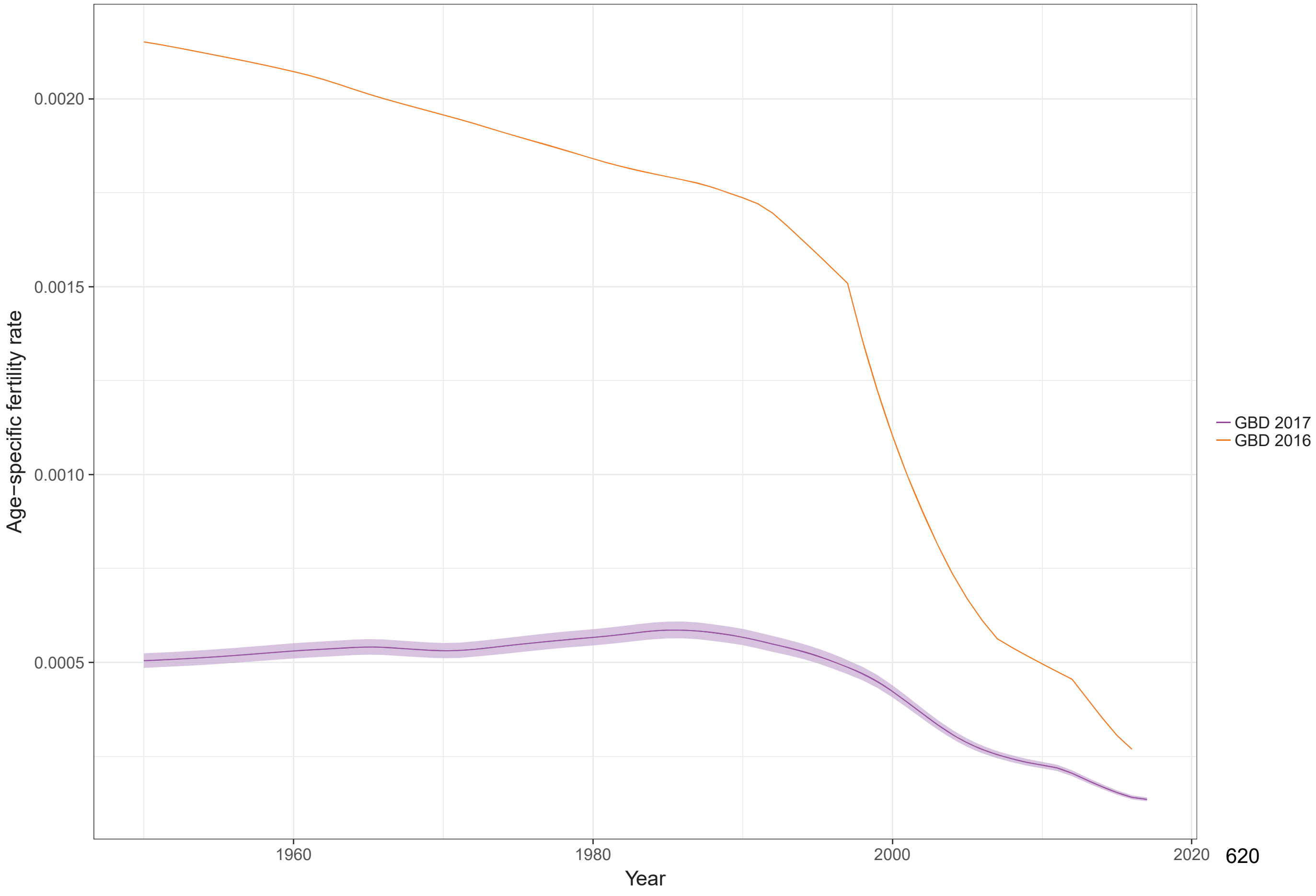

Age−specific fertility fitted trend lines, 1950−2017: Nepal, TFR
Data points enclosed with a diamond are reference sources

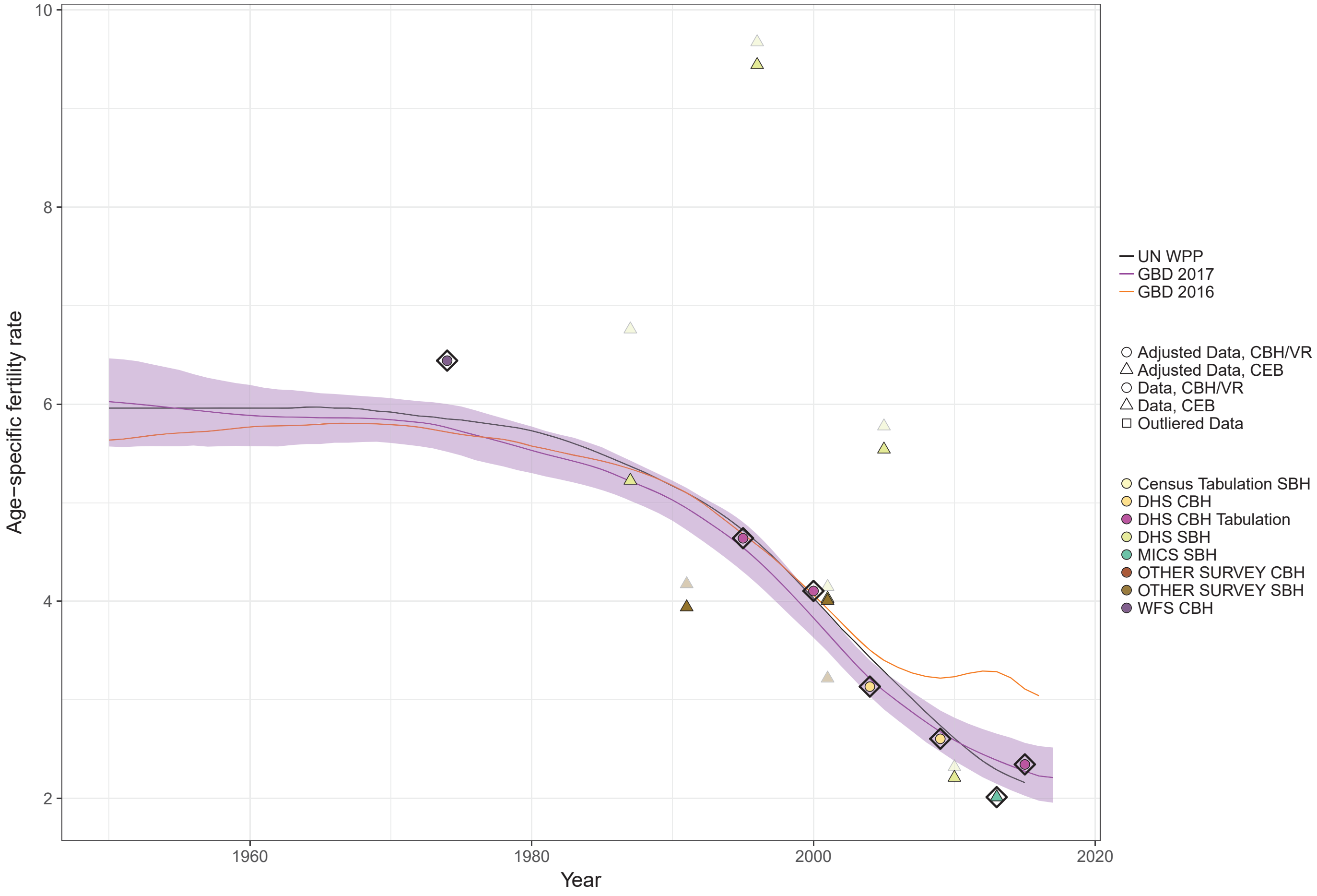

Age−specific fertility fitted trend lines, 1950−2017: Nepal, Ages: 10−14
Data points enclosed with a diamond are reference sources

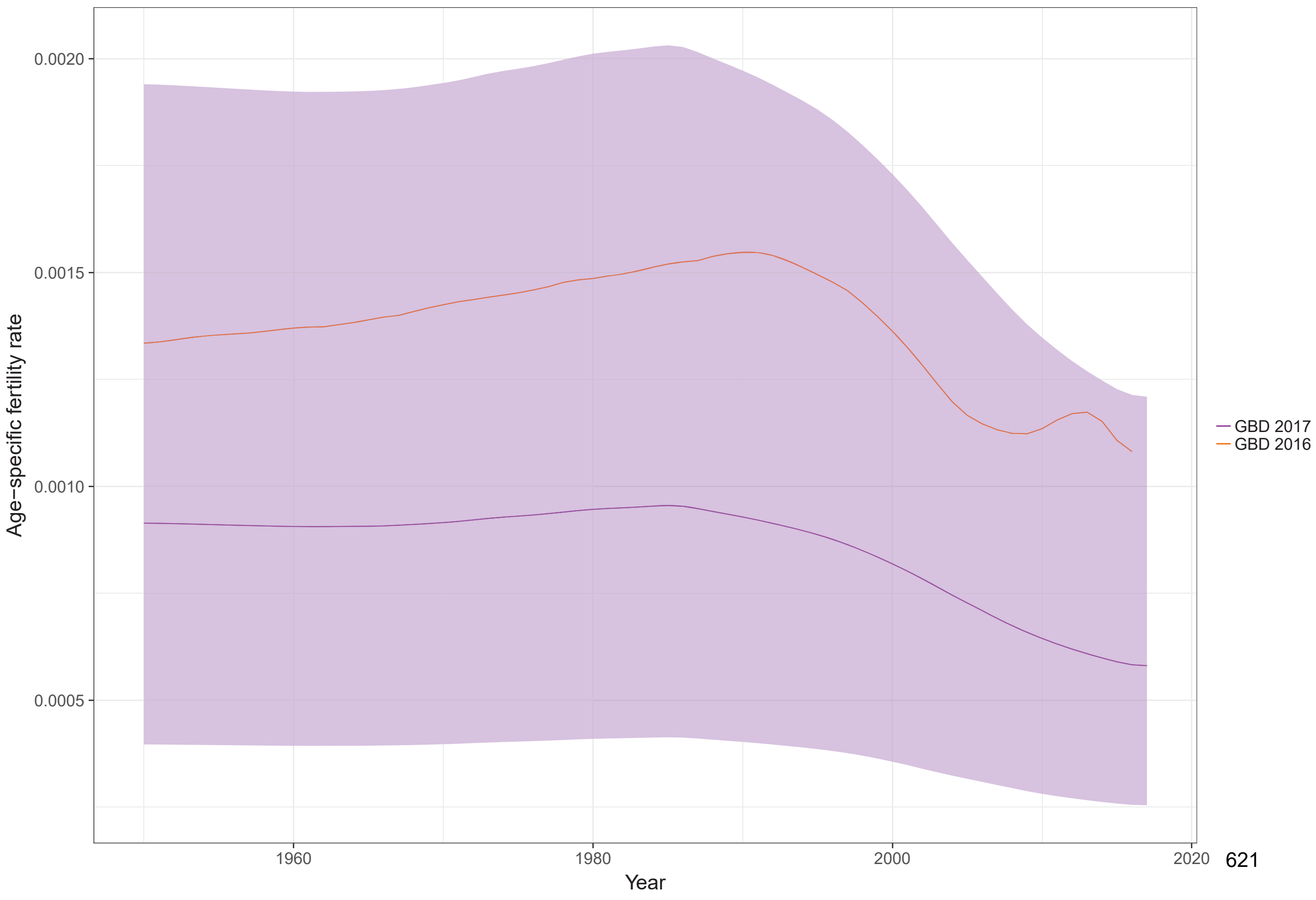

Age−specific fertility fitted trend lines, 1950−2017: Nepal, Ages: 15−19
Data points enclosed with a diamond are reference sources

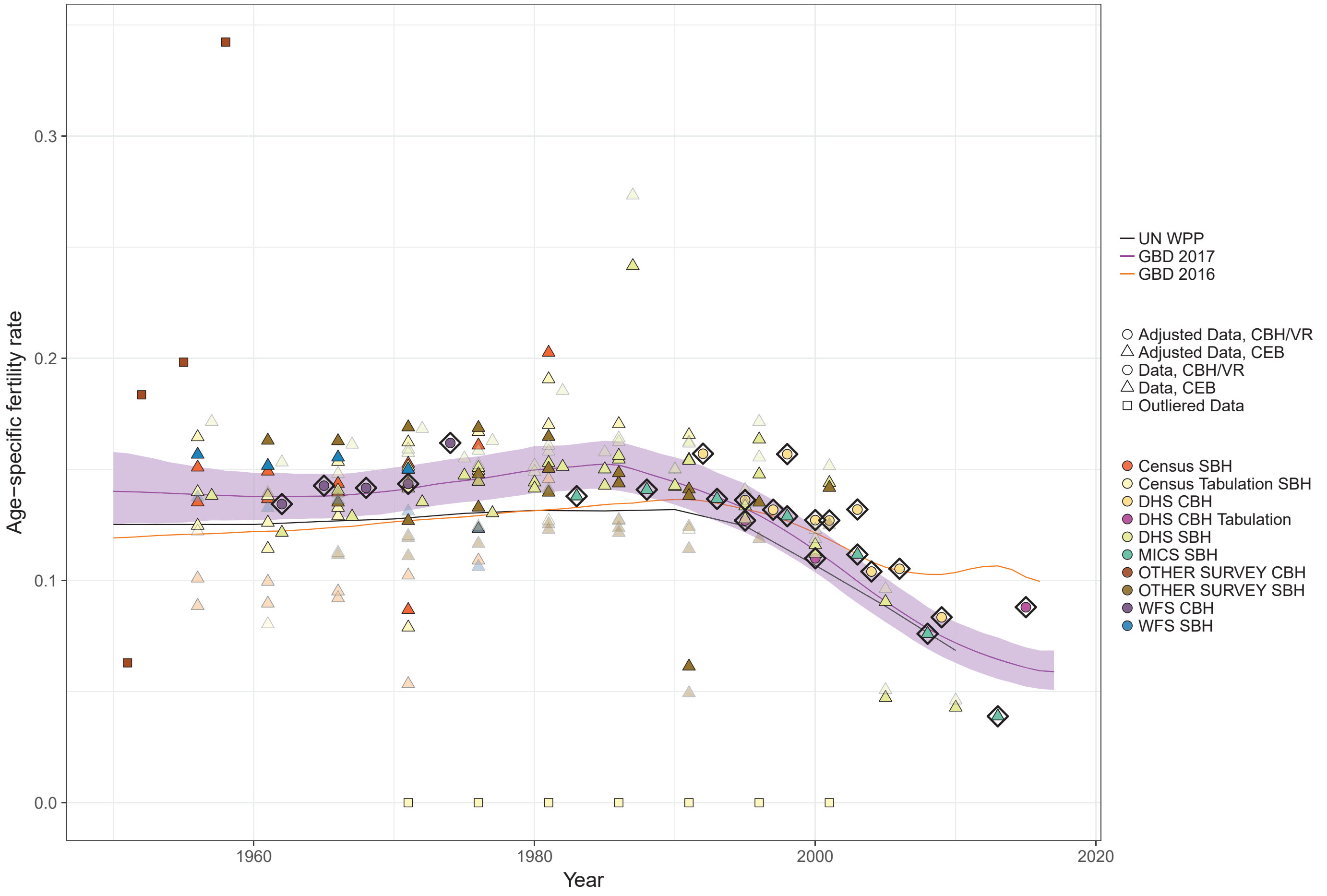

Age−specific fertility fitted trend lines, 1950−2017: Nepal, Ages: 20−24
Data points enclosed with a diamond are reference sources

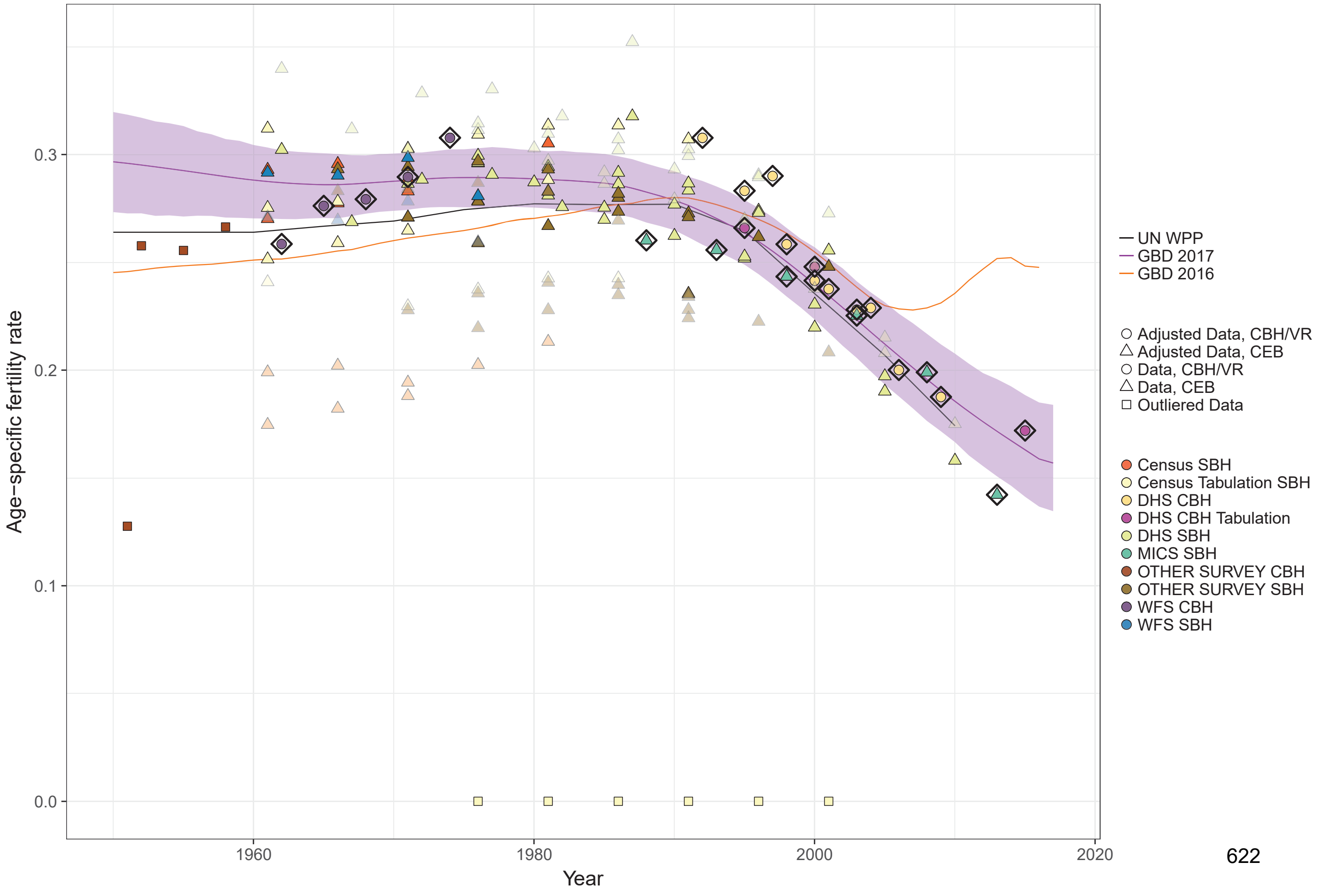

Age−specific fertility fitted trend lines, 1950−2017: Nepal, Ages: 25−29
Data points enclosed with a diamond are reference sources

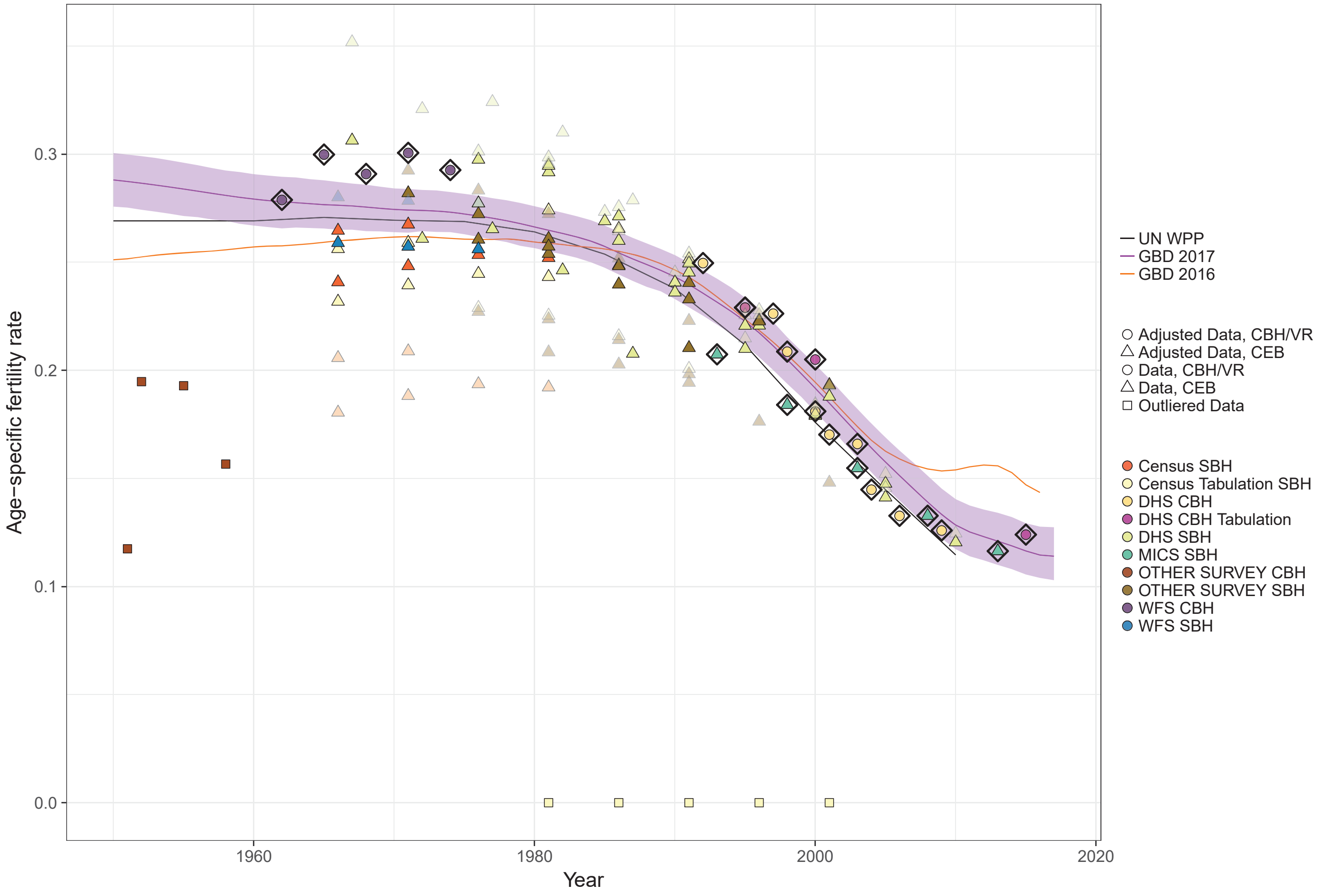

Age−specific fertility fitted trend lines, 1950−2017: Nepal, Ages: 30−34
Data points enclosed with a diamond are reference sources

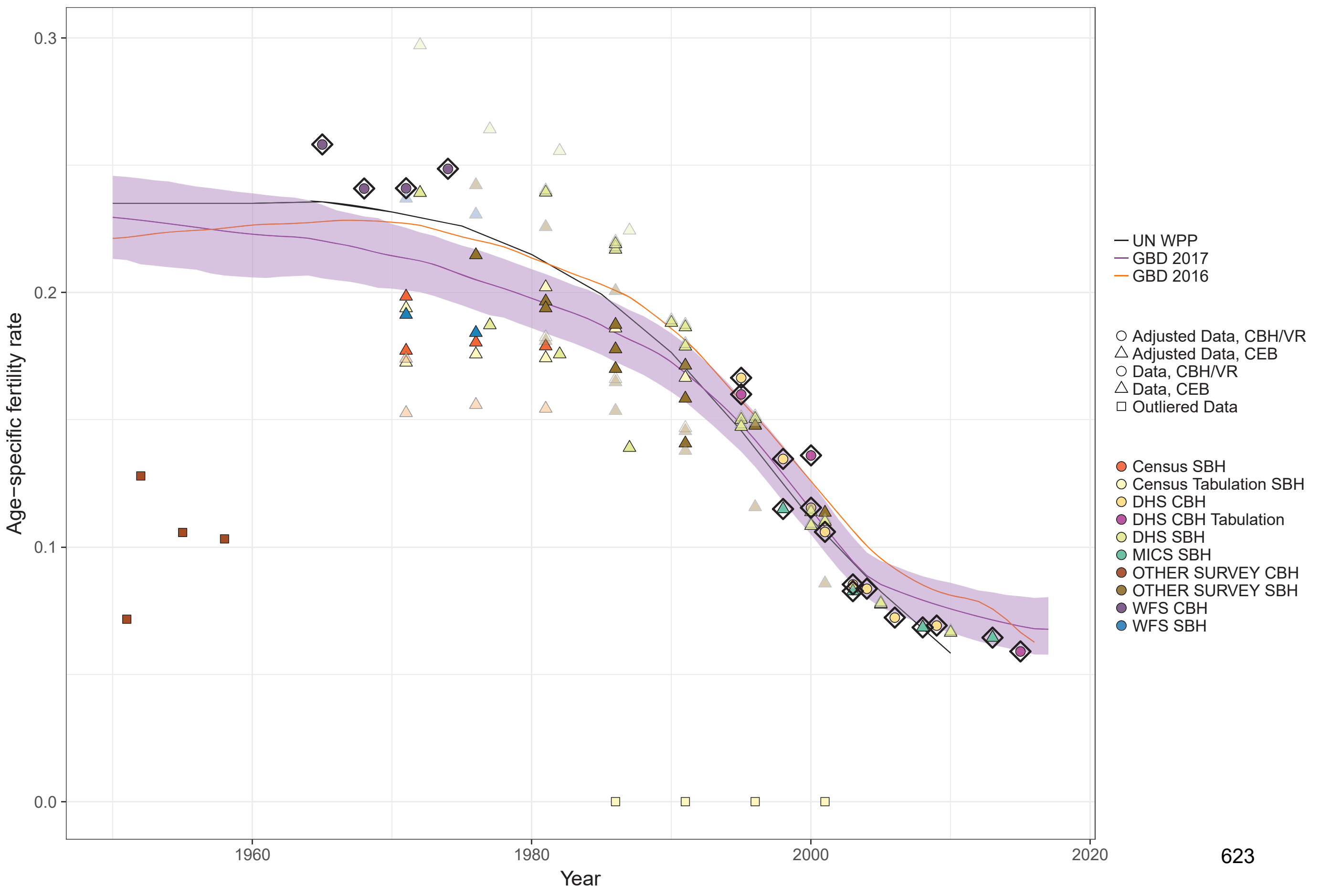

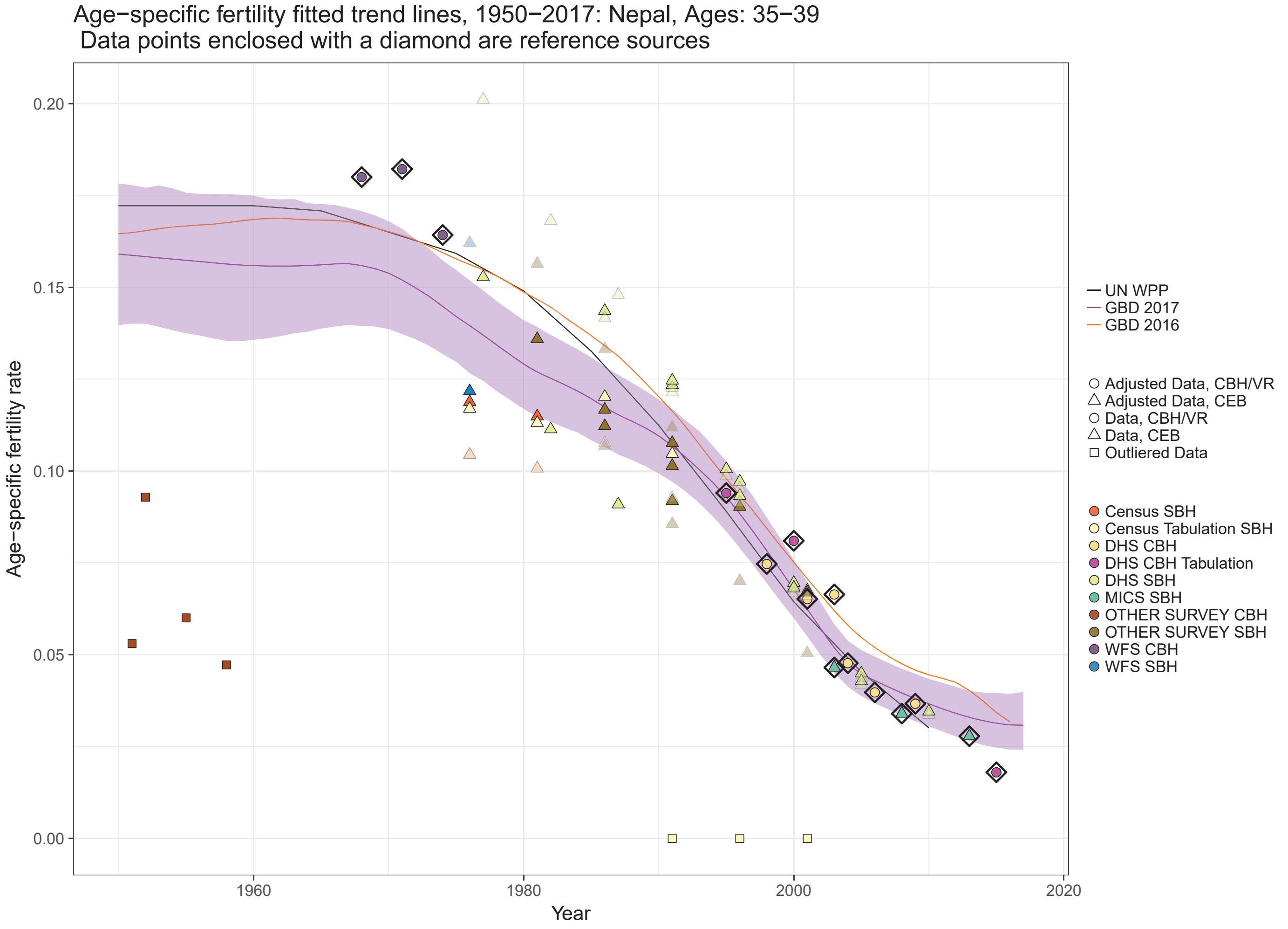
Age−specific fertility fitted trend lines, 1950−2017: Nepal, Ages: 35−39
Data points enclosed with a diamond are reference sources
Age−specific fertility rate
Year
UN WPP
GBD 2017
GBD 2016
Adjusted Data, CBH/VR
Adjusted Data, CEB
Data, CBH/VR
Data, CEB
Outliered Data
Census SBH
Census Tabulation SBH
DHS CBH
DHS CBH Tabulation
DHS SBH
MICS SBH
OTHER SURVEY CBH
OTHER SURVEY SBH
WFS CBH
WFS SBH

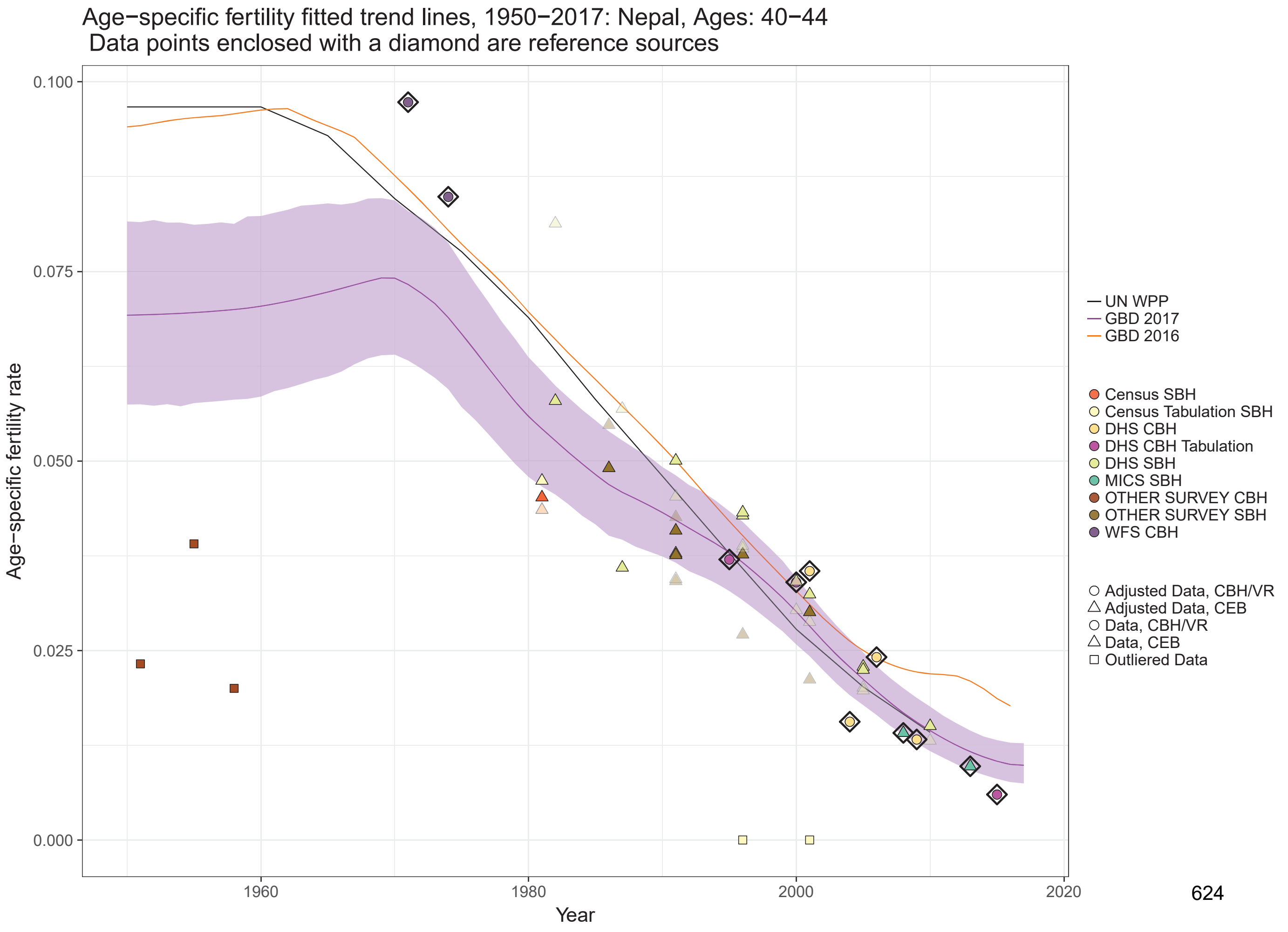
Age−specific fertility fitted trend lines, 1950−2017: Nepal, Ages: 40−44
Data points enclosed with a diamond are reference sources
Age−specific fertility rate
Year
UN WPP
GBD 2017
GBD 2016
Census SBH
Census Tabulation SBH
DHS CBH
DHS CBH Tabulation
DHS SBH
MICS SBH
OTHER SURVEY CBH
OTHER SURVEY SBH
WFS CBH
Adjusted Data, CBH/VR
Adjusted Data, CEB
Data, CBH/VR
Data, CEB
Outliered Data

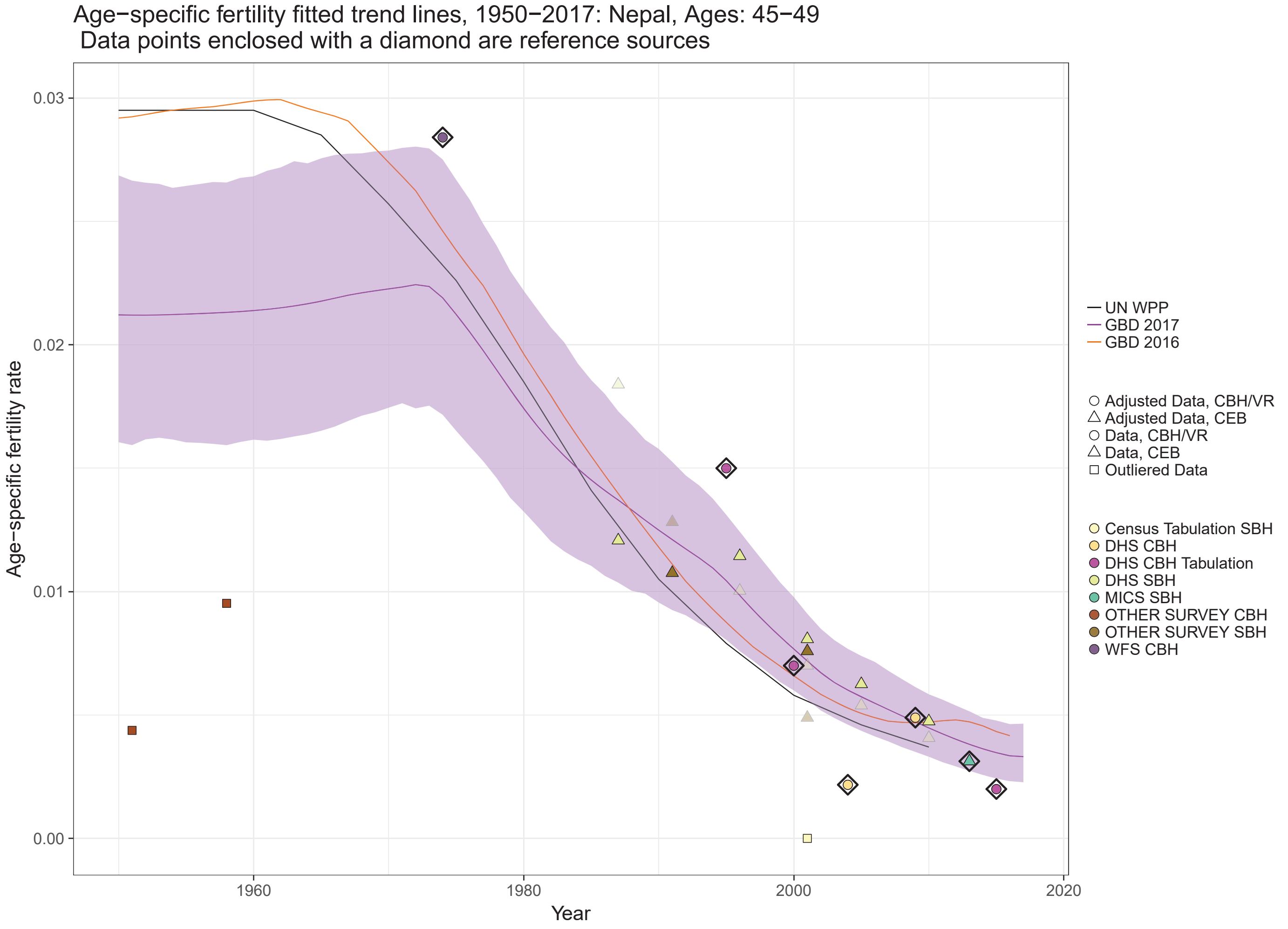

Age−specific fertility fitted trend lines, 1950−2017: Nepal, Ages: 45−49
Data points enclosed with a diamond are reference sources
Age−specific fertility rate
0.00
0.01
0.02
0.03
Year
1960
1980
2000
2020
UN WPP
GBD 2017
GBD 2016
Adjusted Data, CBH/VR
Adjusted Data, CEB
Data, CBH/VR
Data, CEB
Outliered Data
Census Tabulation SBH
DHS CBH
DHS CBH Tabulation
DHS SBH
MICS SBH
OTHER SURVEY CBH
OTHER SURVEY SBH
WFS CBH

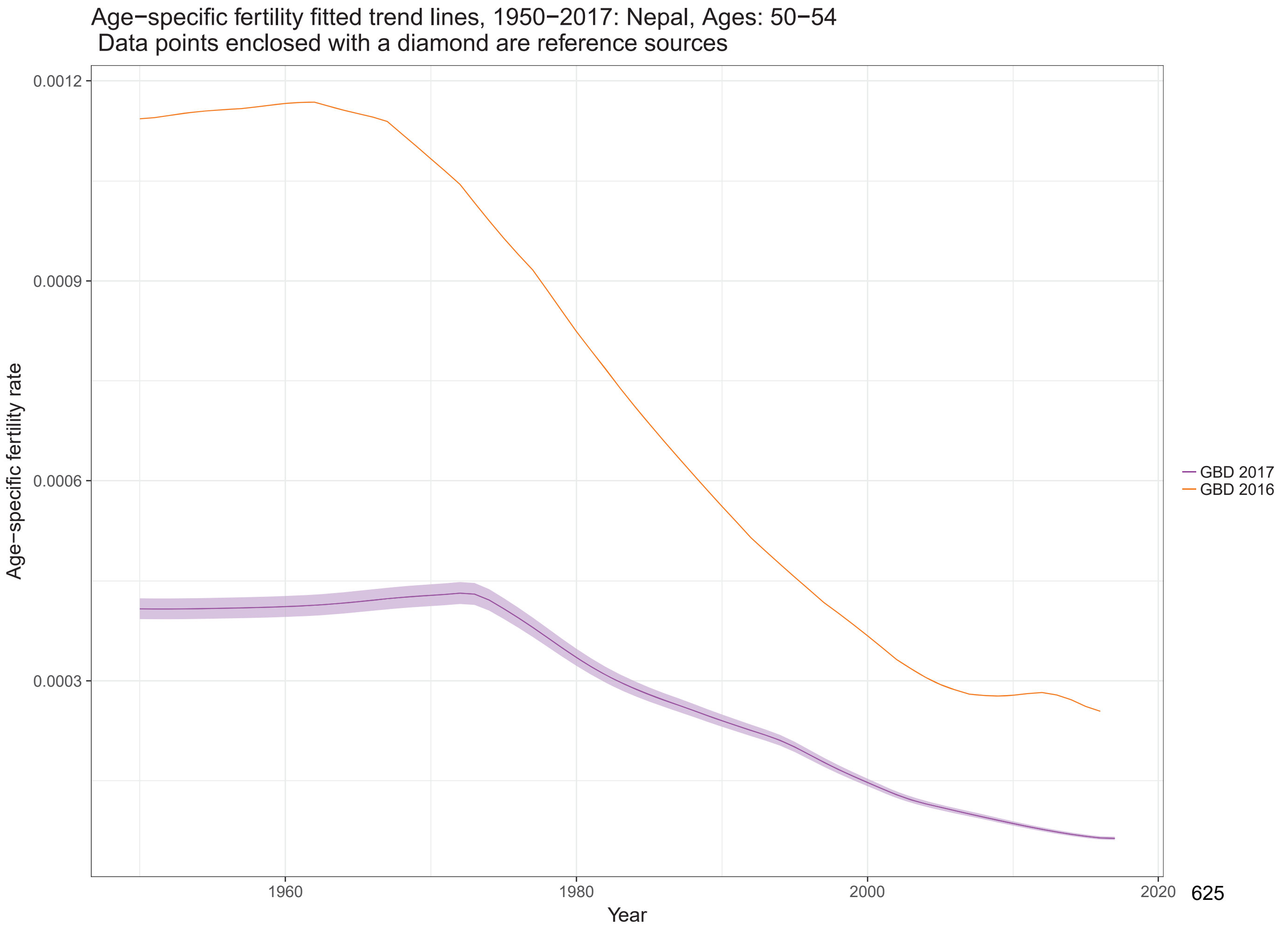

Age−specific fertility fitted trend lines, 1950−2017: Nepal, Ages: 50−54
Data points enclosed with a diamond are reference sources
Age−specific fertility rate
0.0003
0.0006
0.0009
0.0012
Year
1960
1980
2000
2020
GBD 2017
GBD 2016

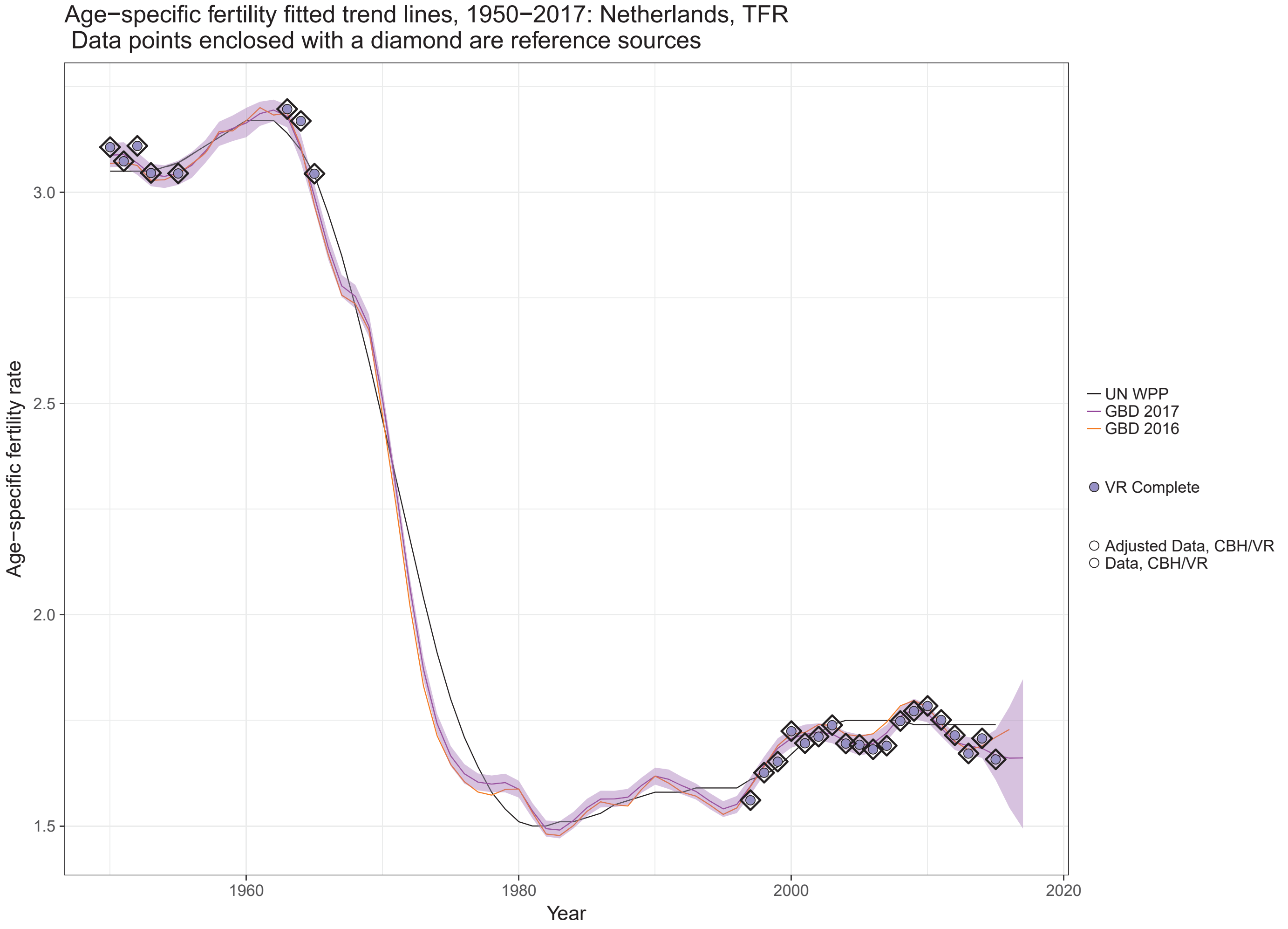

Age−specific fertility fitted trend lines, 1950−2017: Netherlands, TFR
Data points enclosed with a diamond are reference sources
Age−specific fertility rate
3.0
2.5
2.0
1.5
1960
1980
2000
2020
Year
UN WPP
GBD 2017
GBD 2016
VR Complete
Adjusted Data, CBH/VR
Data, CBH/VR

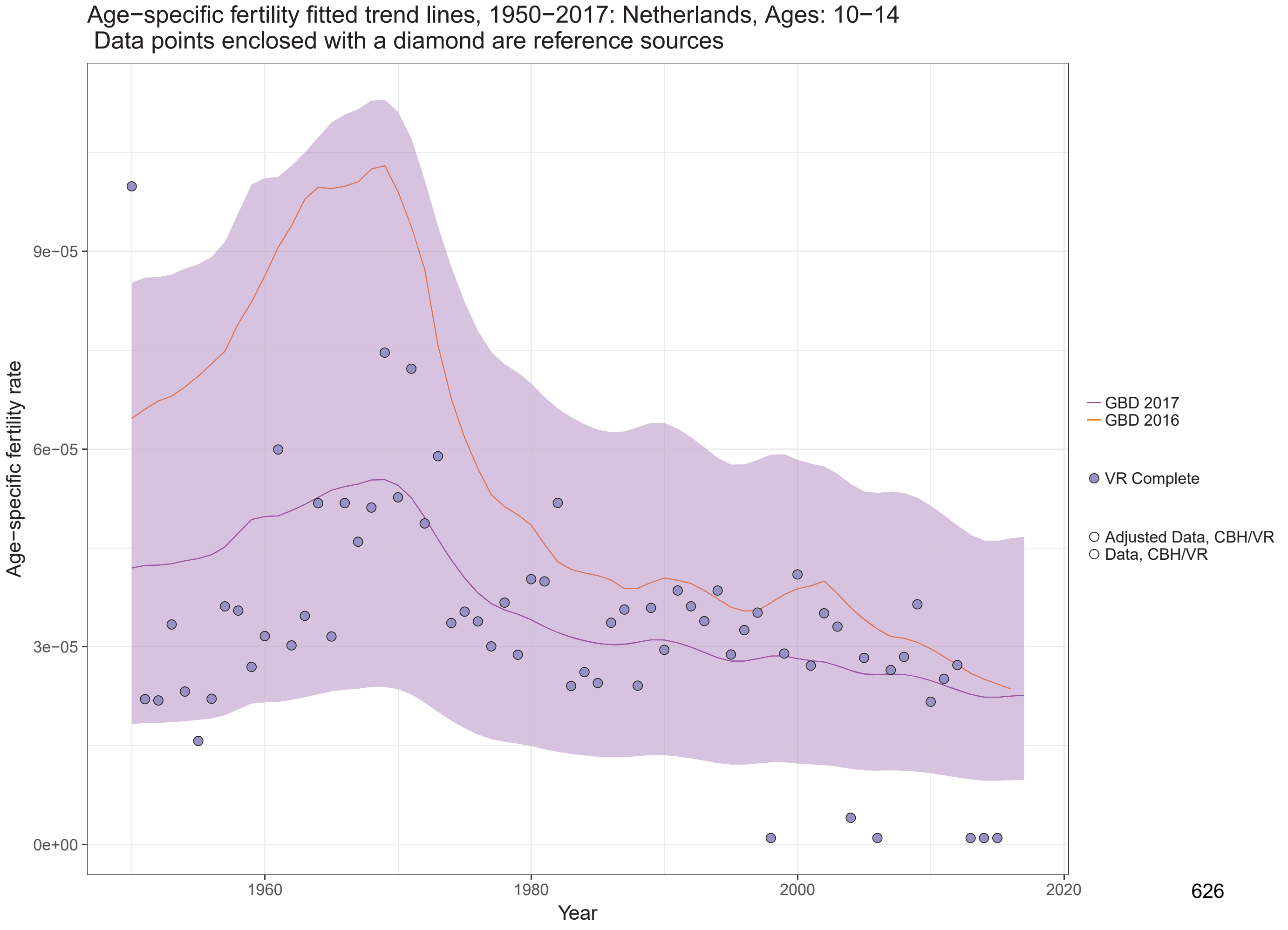

Age−specific fertility fitted trend lines, 1950−2017: Netherlands, Ages: 10−14
Data points enclosed with a diamond are reference sources
Age−specific fertility rate
9e−05
6e−05
3e−05
0e+00
1960
1980
2000
2020
Year
GBD 2017
GBD 2016
VR Complete
Adjusted Data, CBH/VR
Data, CBH/VR

Age−specific fertility fitted trend lines, 1950−2017: Netherlands, Ages: 15−19
Data points enclosed with a diamond are reference sources

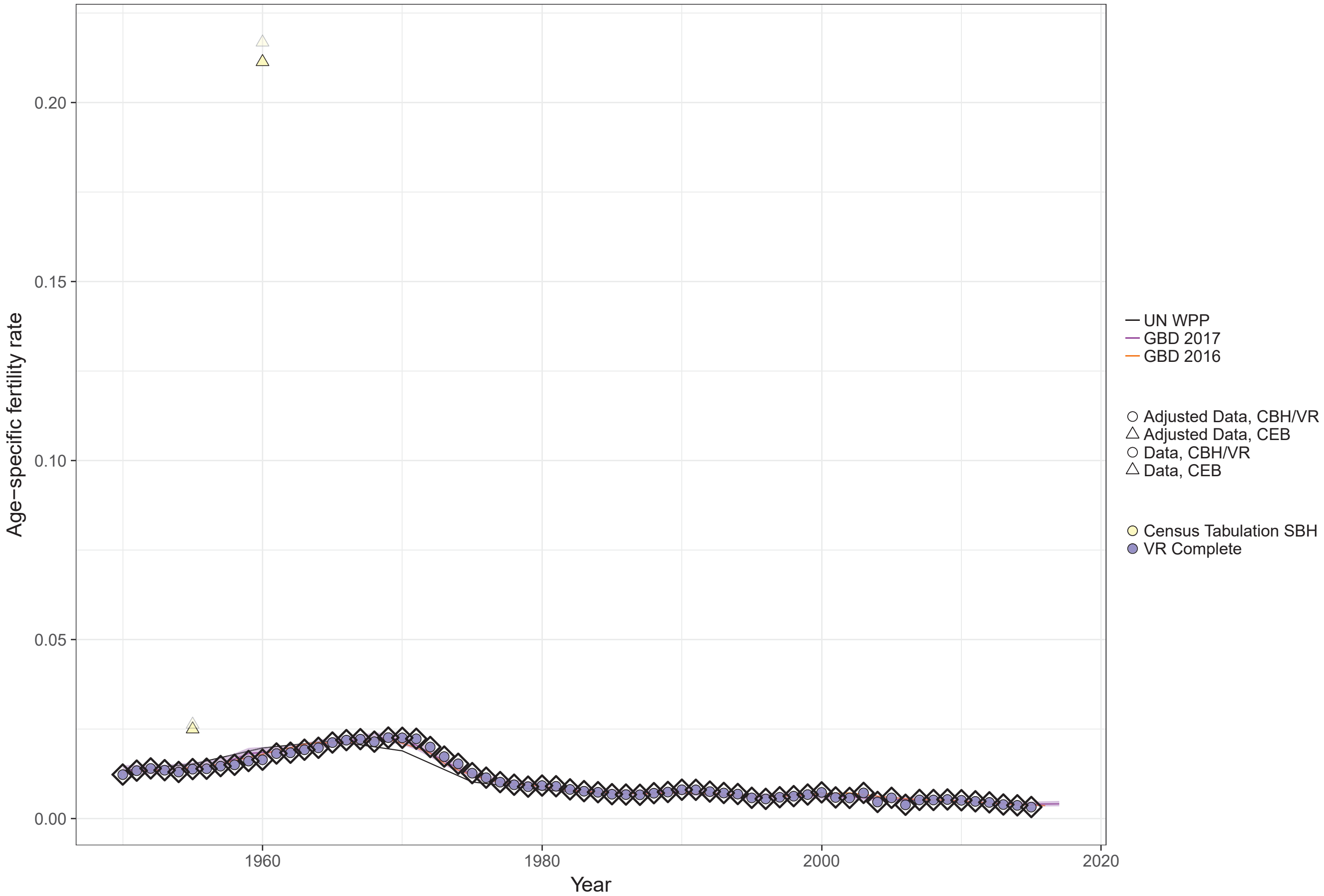

Age−specific fertility fitted trend lines, 1950−2017: Netherlands, Ages: 20−24
Data points enclosed with a diamond are reference sources

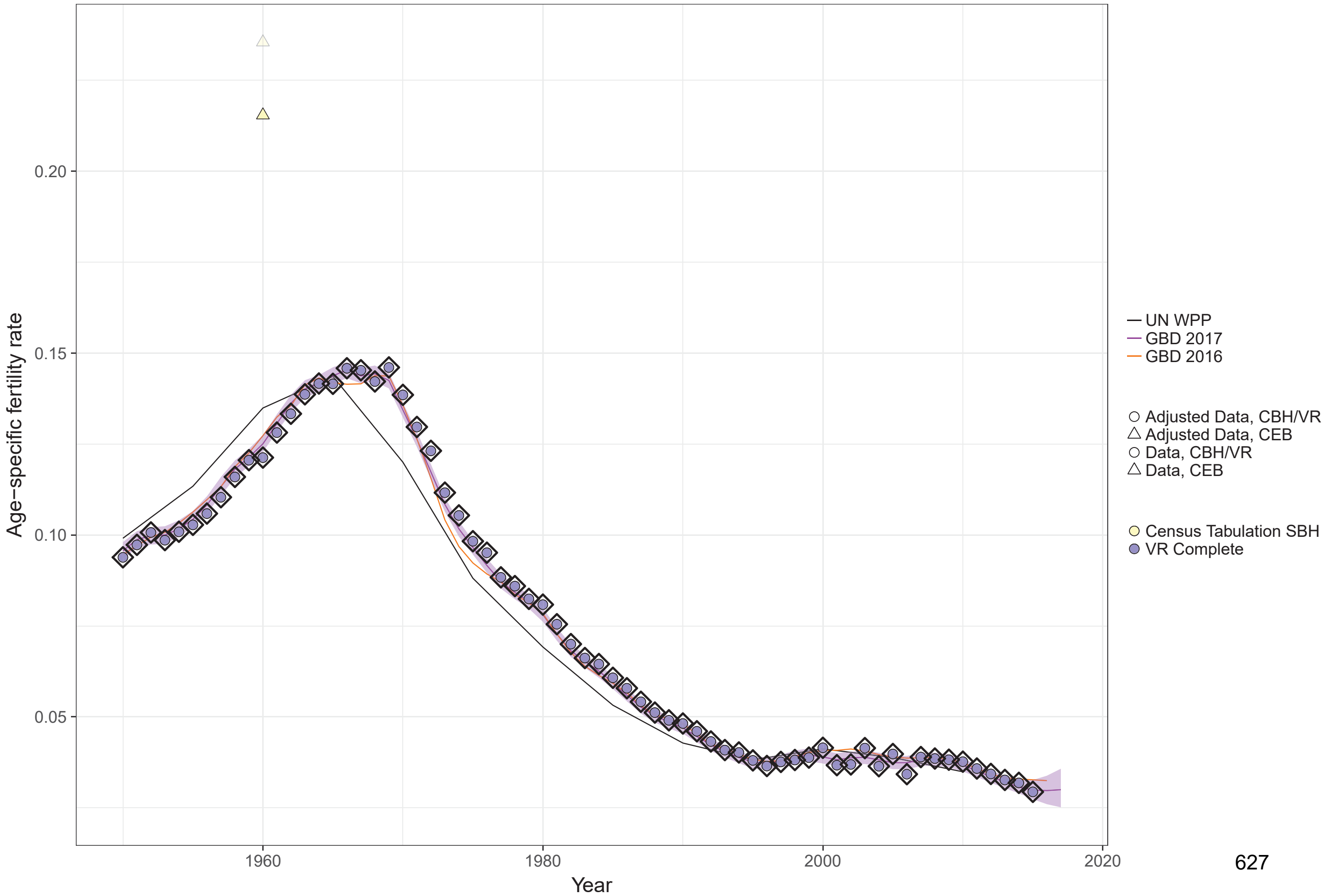

Age−specific fertility fitted trend lines, 1950−2017: Netherlands, Ages: 25−29
Data points enclosed with a diamond are reference sources

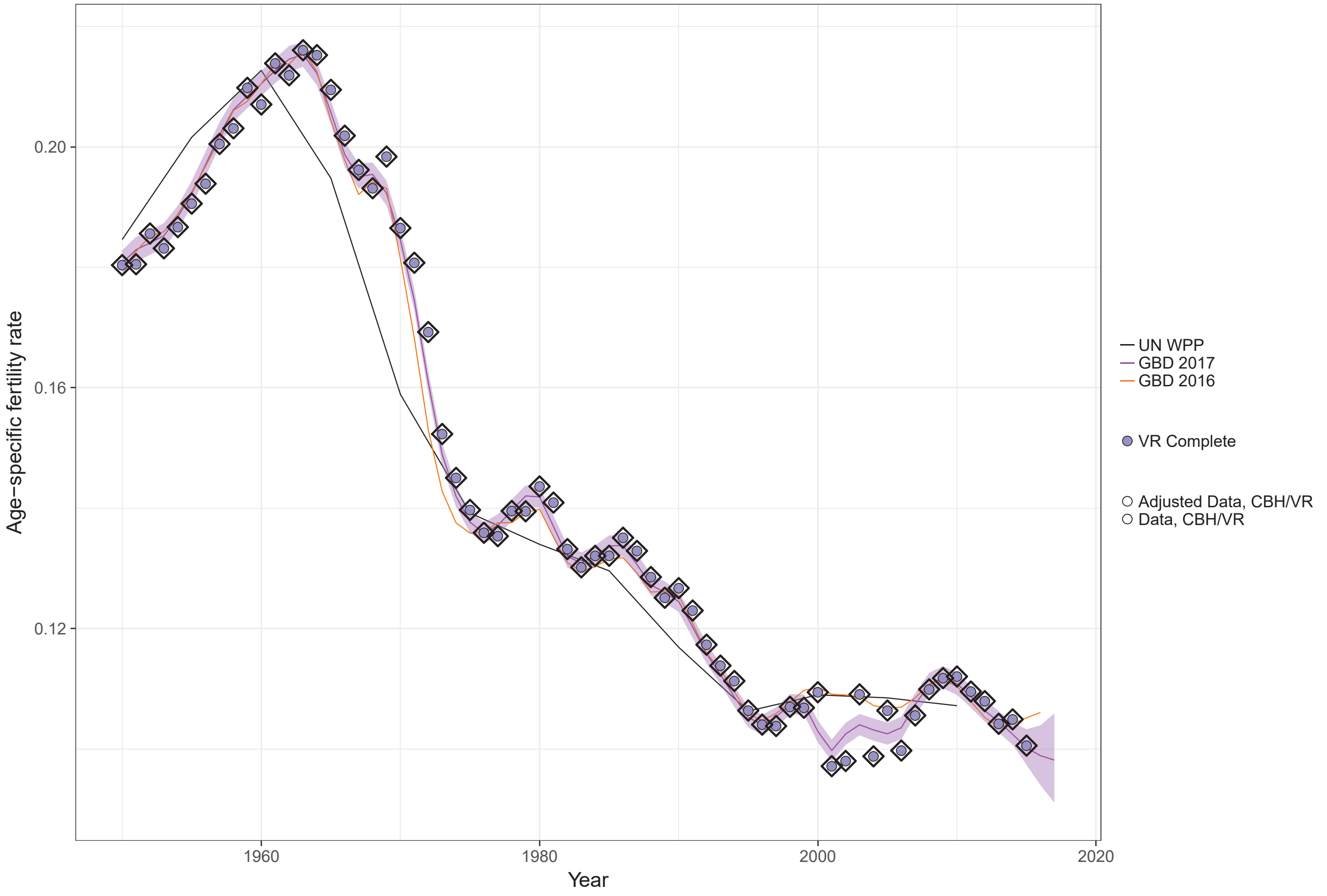

Age−specific fertility fitted trend lines, 1950−2017: Netherlands, Ages: 30−34
Data points enclosed with a diamond are reference sources

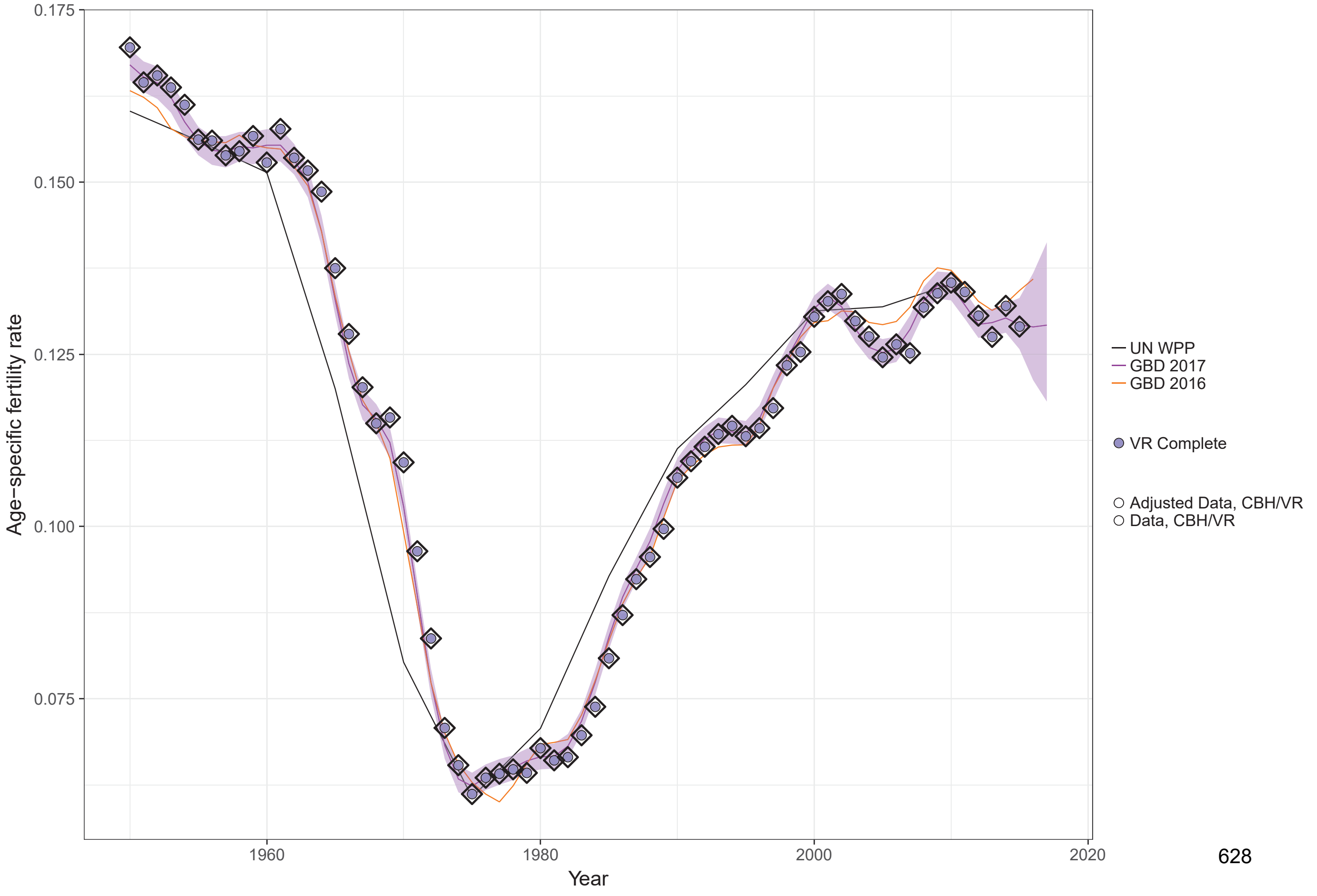

Age−specific fertility fitted trend lines, 1950−2017: Netherlands, Ages: 35−39
Data points enclosed with a diamond are reference sources

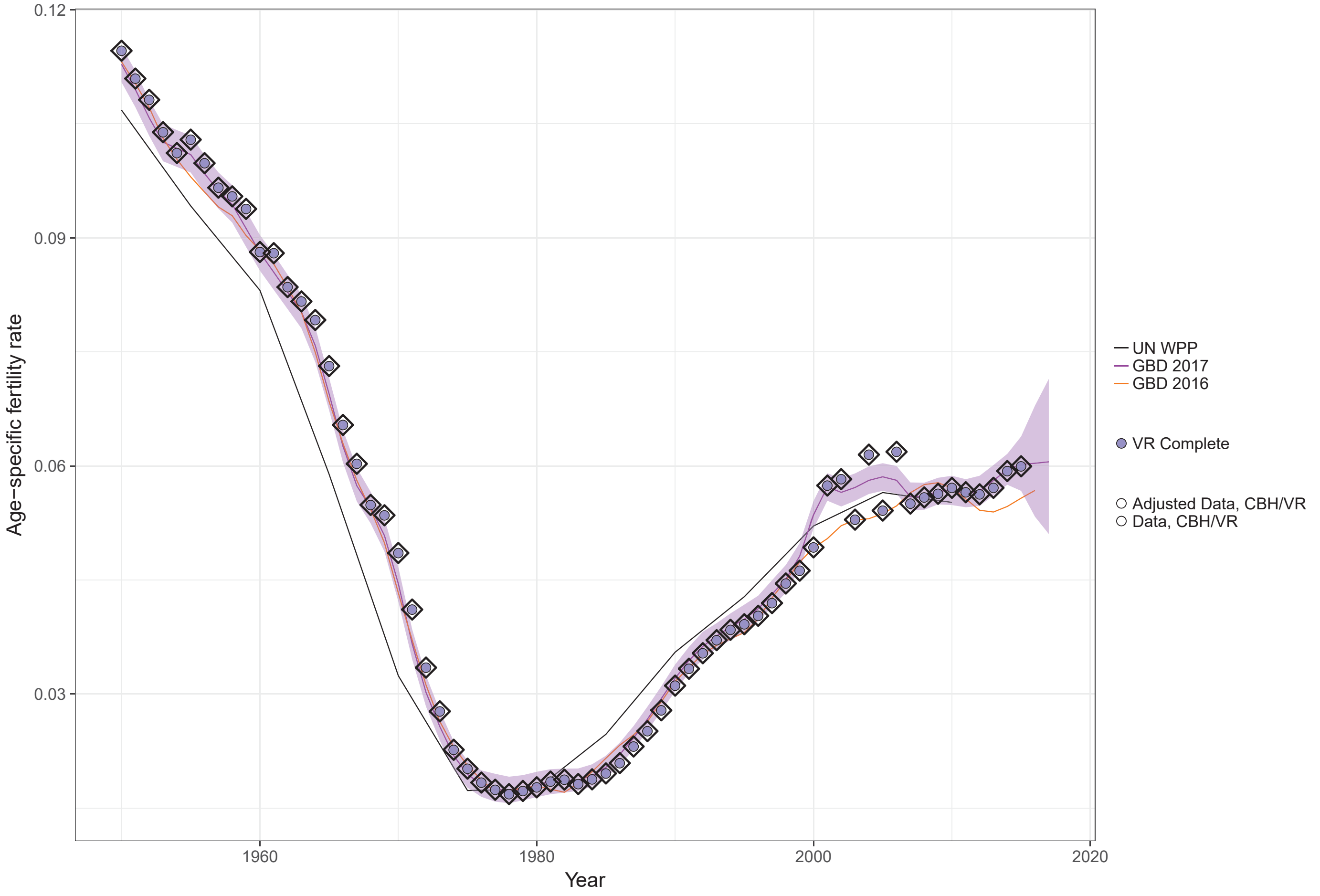

Age−specific fertility fitted trend lines, 1950−2017: Netherlands, Ages: 40−44
Data points enclosed with a diamond are reference sources

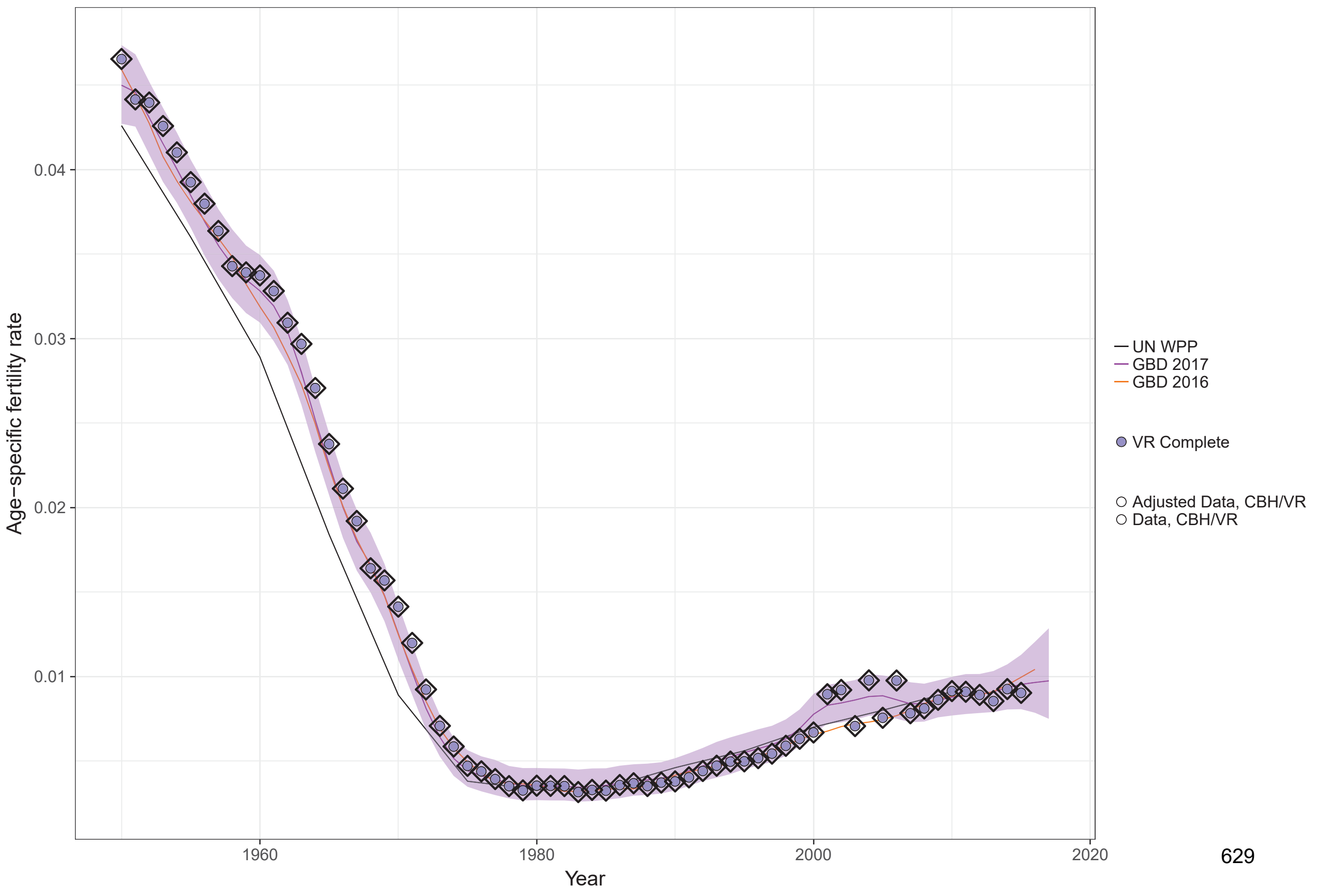

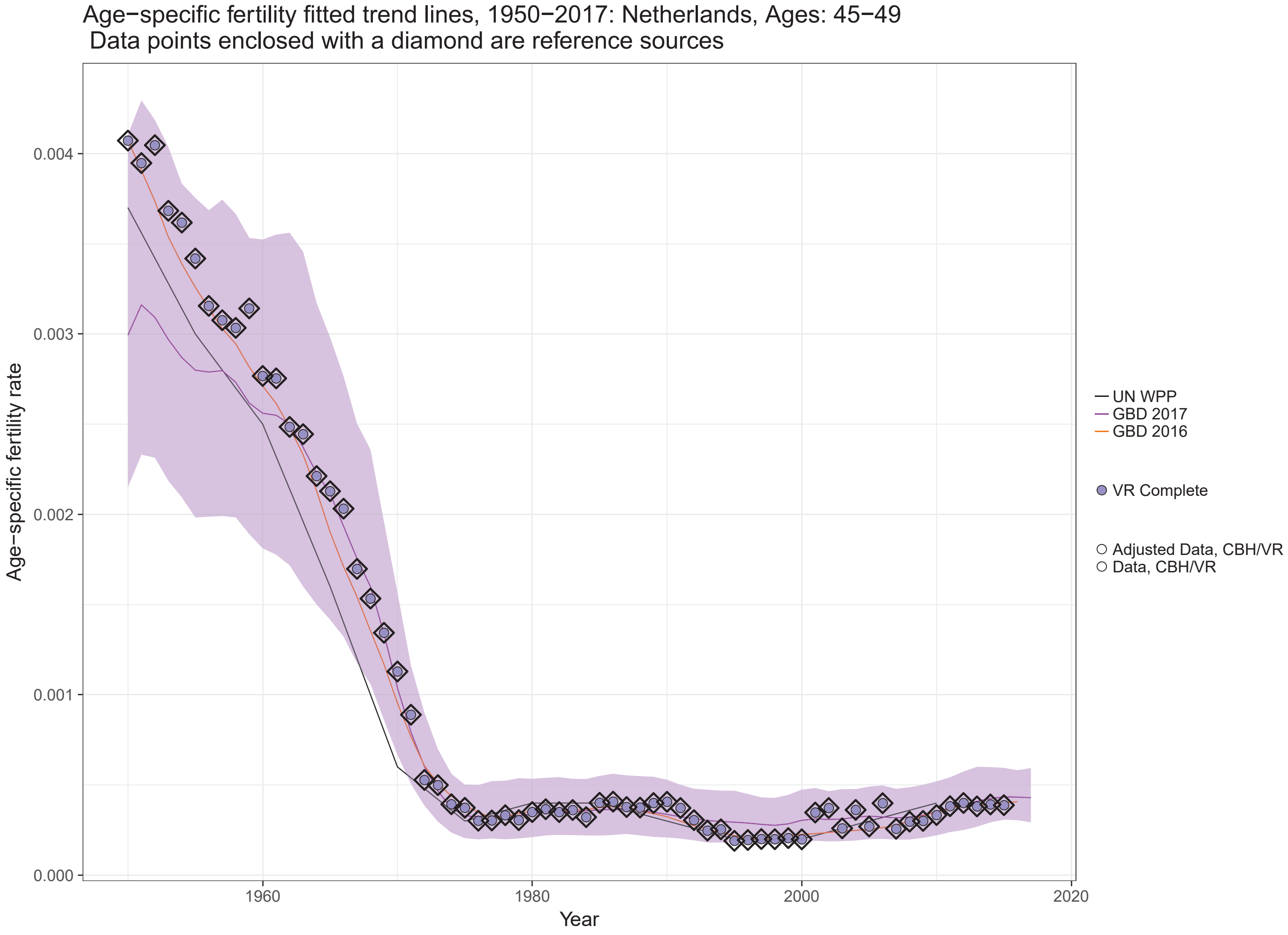

Age−specific fertility fitted trend lines, 1950−2017: Netherlands, Ages: 45−49
Data points enclosed with a diamond are reference sources
Age−specific fertility rate
0.000
0.001
0.002
0.003
0.004
1960
1980
2000
2020
Year
UN WPP
GBD 2017
GBD 2016
VR Complete
Adjusted Data, CBH/VR
Data, CBH/VR

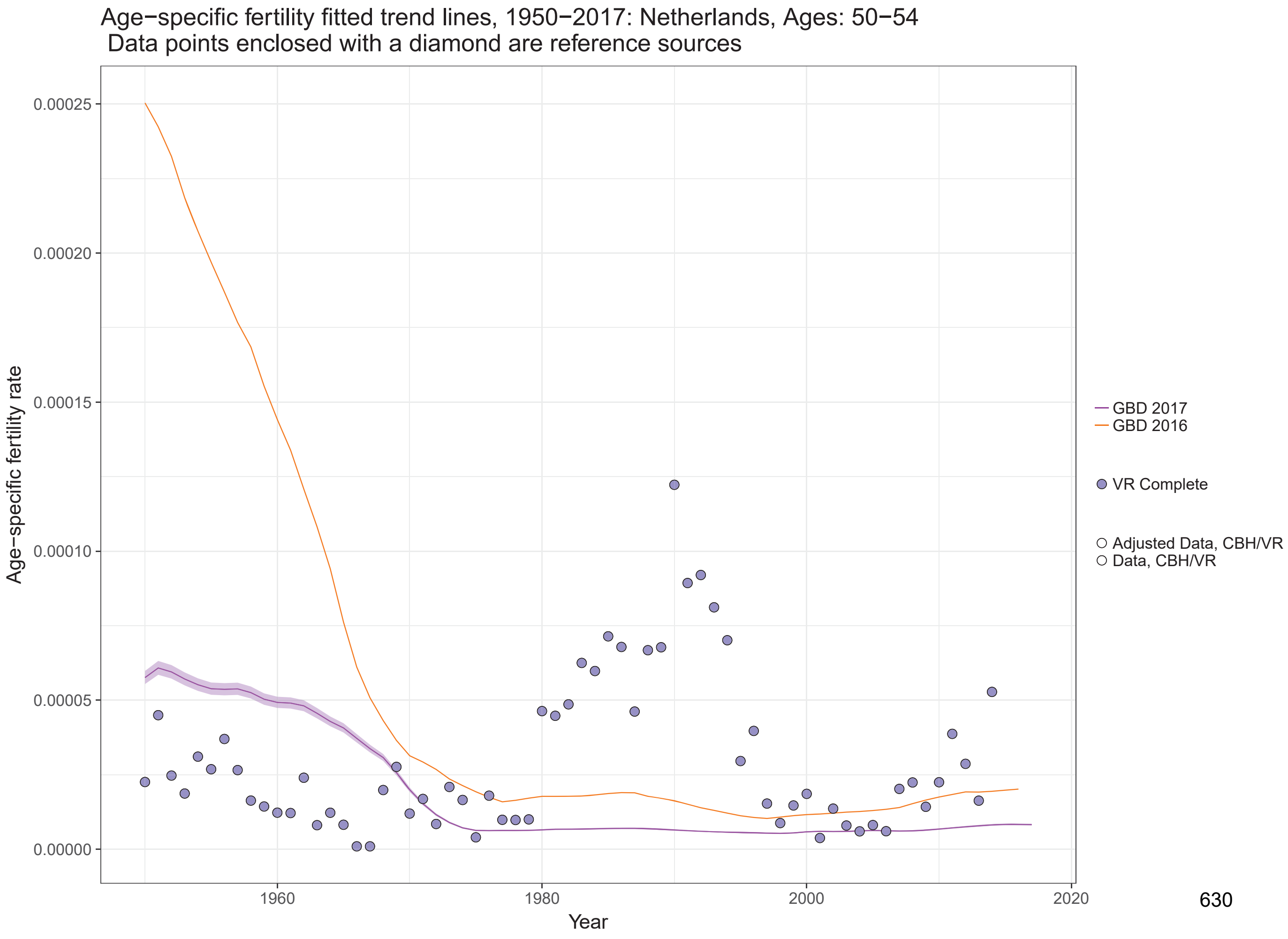

Age−specific fertility fitted trend lines, 1950−2017: Netherlands, Ages: 50−54
Data points enclosed with a diamond are reference sources
Age−specific fertility rate
0.00000
0.00005
0.00010
0.00015
0.00020
0.00025
1960
1980
2000
2020
Year
GBD 2017
GBD 2016
VR Complete
Adjusted Data, CBH/VR
Data, CBH/VR

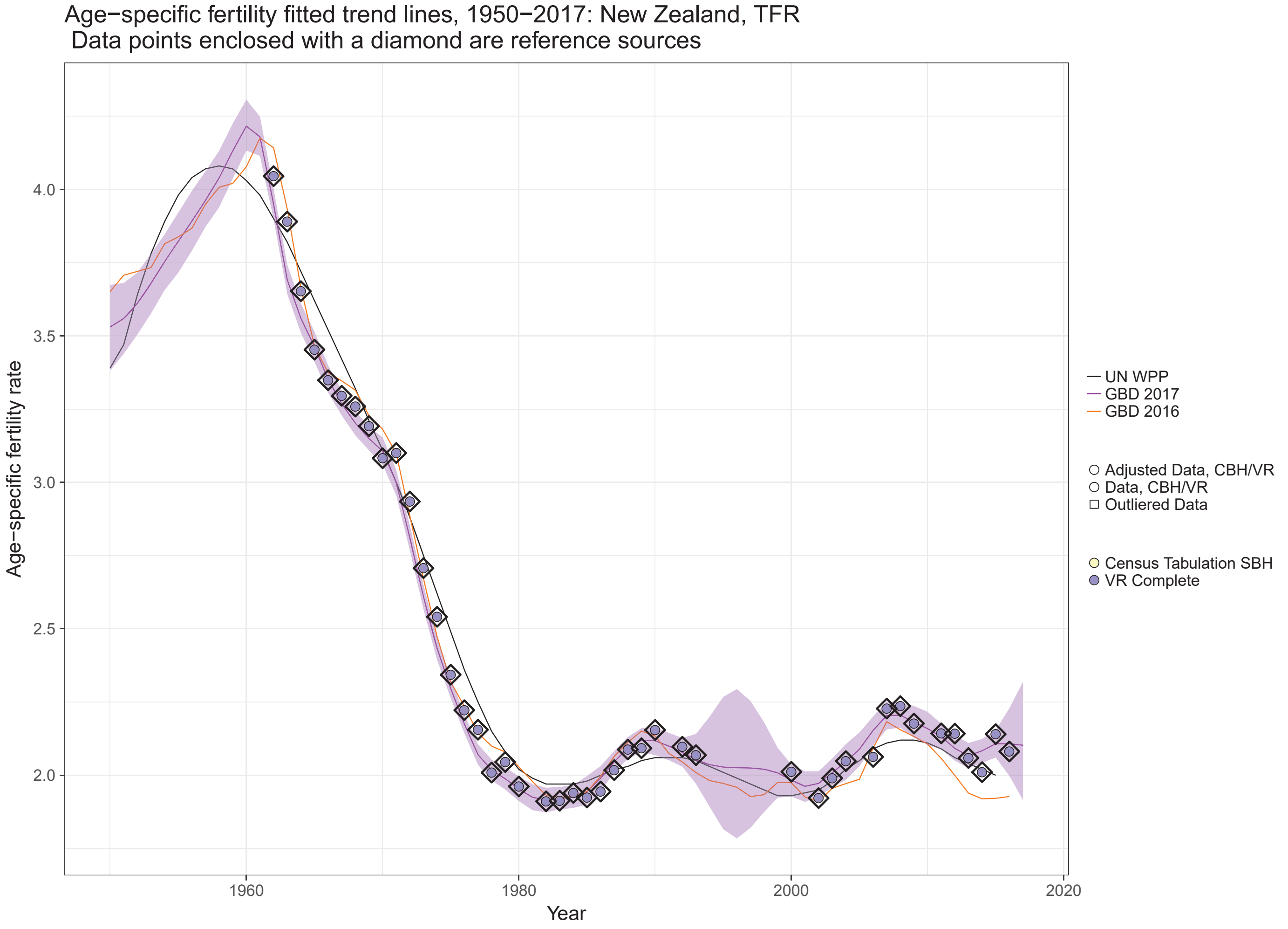

Age−specific fertility fitted trend lines, 1950−2017: New Zealand, TFR
Data points enclosed with a diamond are reference sources
Age−specific fertility rate
Year
UN WPP
GBD 2017
GBD 2016
Adjusted Data, CBH/VR
Data, CBH/VR
Outliered Data
Census Tabulation SBH
VR Complete

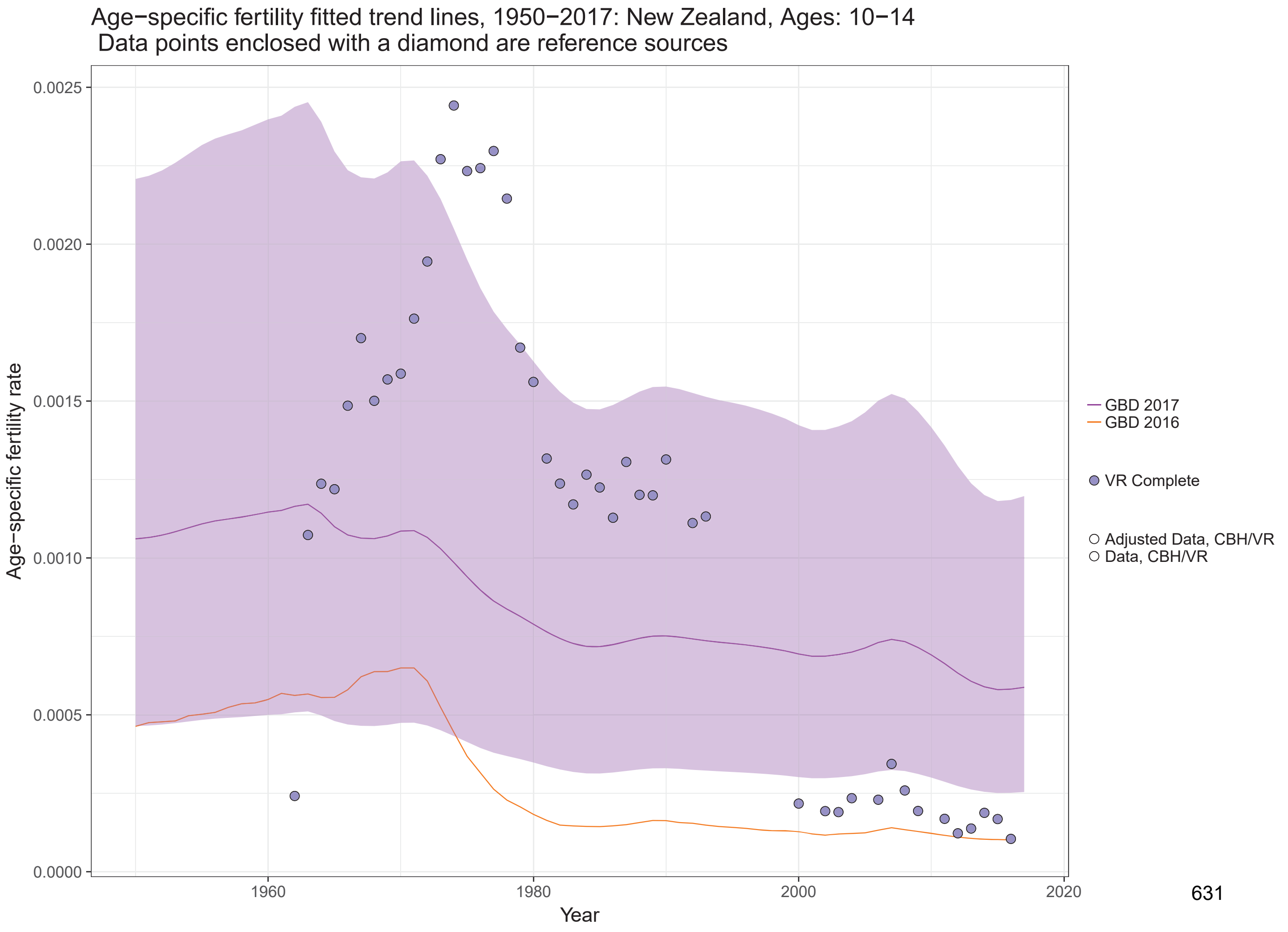

Age−specific fertility fitted trend lines, 1950−2017: New Zealand, Ages: 10−14
Data points enclosed with a diamond are reference sources
Age−specific fertility rate
Year
GBD 2017
GBD 2016
VR Complete
Adjusted Data, CBH/VR
Data, CBH/VR

Age−specific fertility fitted trend lines, 1950−2017: New Zealand, Ages: 15−19
Data points enclosed with a diamond are reference sources

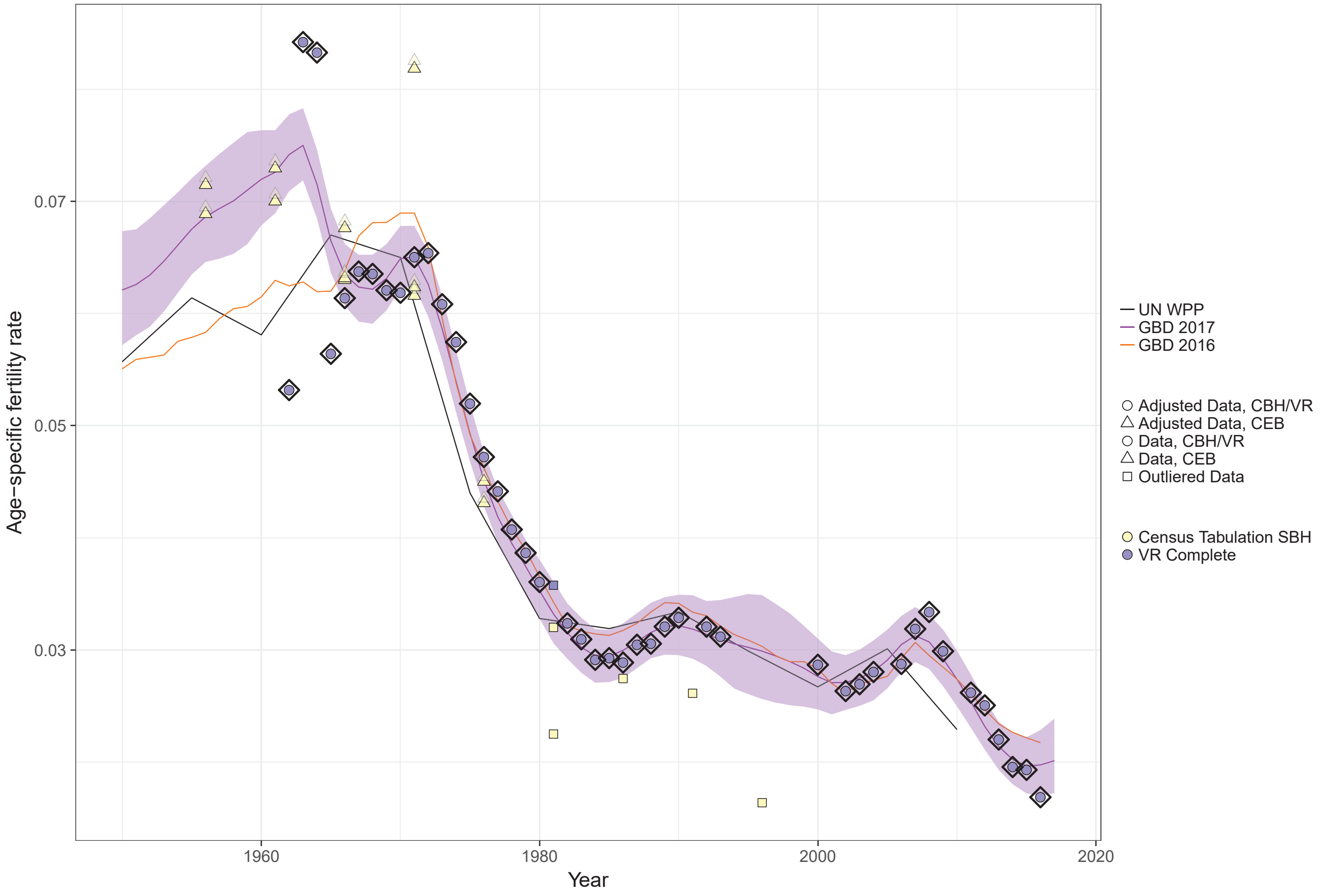

Age−specific fertility fitted trend lines, 1950−2017: New Zealand, Ages: 20−24
Data points enclosed with a diamond are reference sources

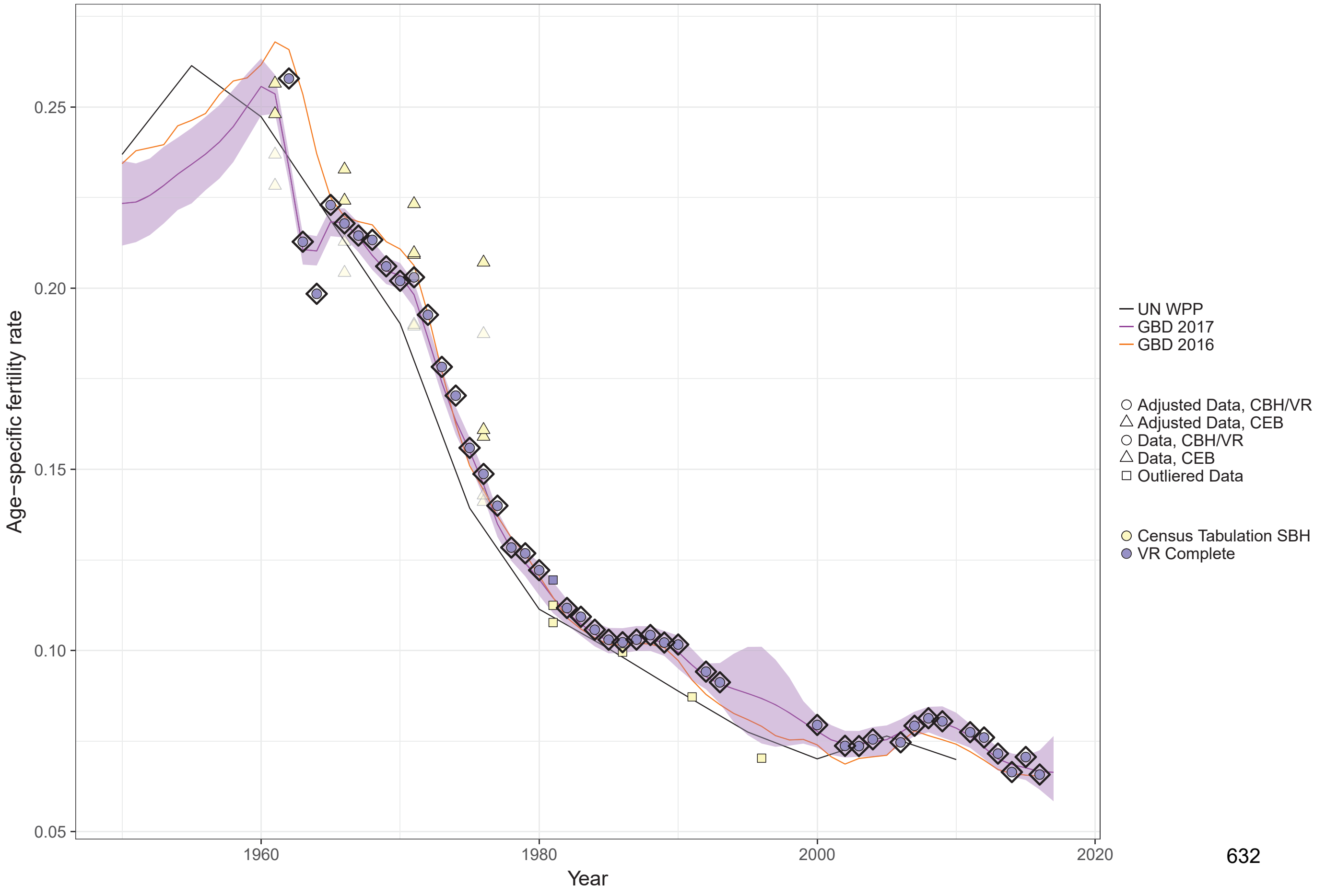

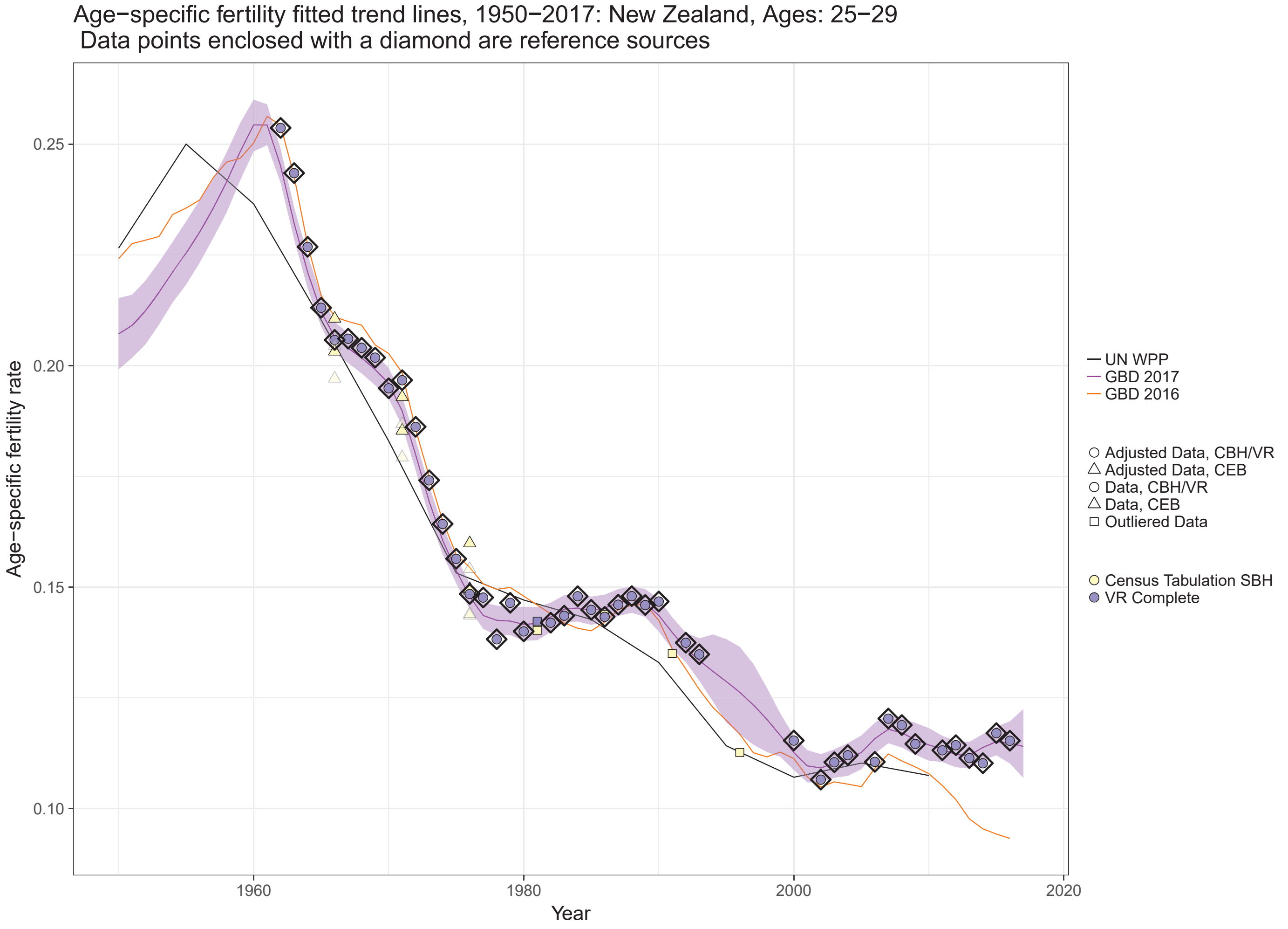

Age−specific fertility fitted trend lines, 1950−2017: New Zealand, Ages: 25−29
Data points enclosed with a diamond are reference sources
Age−specific fertility rate
0.25
0.20
0.15
0.10
Year
1960
1980
2000
2020
UN WPP
GBD 2017
GBD 2016
Adjusted Data, CBH/VR
Adjusted Data, CEB
Data, CBH/VR
Data, CEB
Outliered Data
Census Tabulation SBH
VR Complete

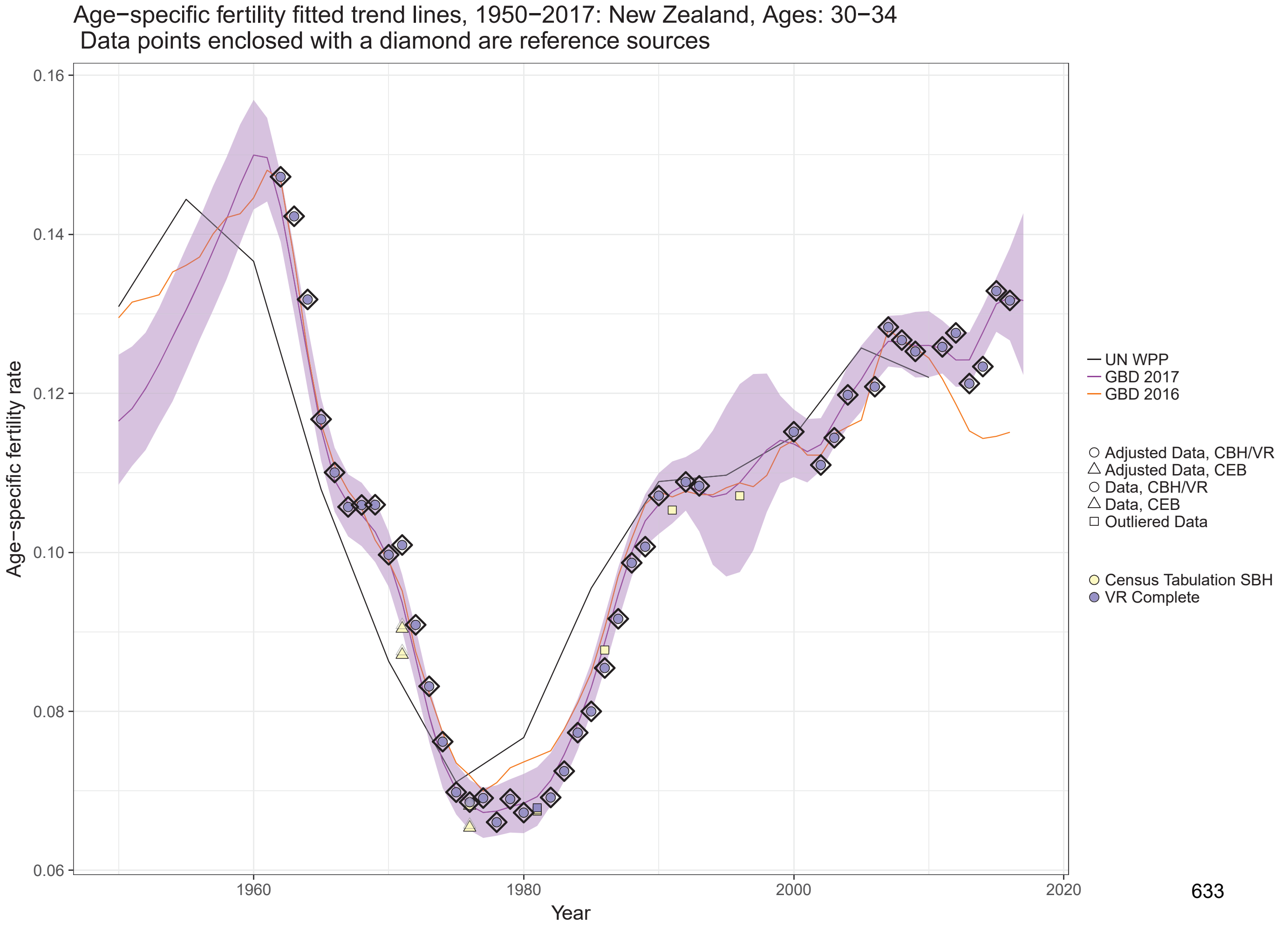

Age−specific fertility fitted trend lines, 1950−2017: New Zealand, Ages: 30−34
Data points enclosed with a diamond are reference sources
Age−specific fertility rate
0.16
0.14
0.12
0.10
0.08
0.06
Year
1960
1980
2000
2020
UN WPP
GBD 2017
GBD 2016
Adjusted Data, CBH/VR
Adjusted Data, CEB
Data, CBH/VR
Data, CEB
Outliered Data
Census Tabulation SBH
VR Complete

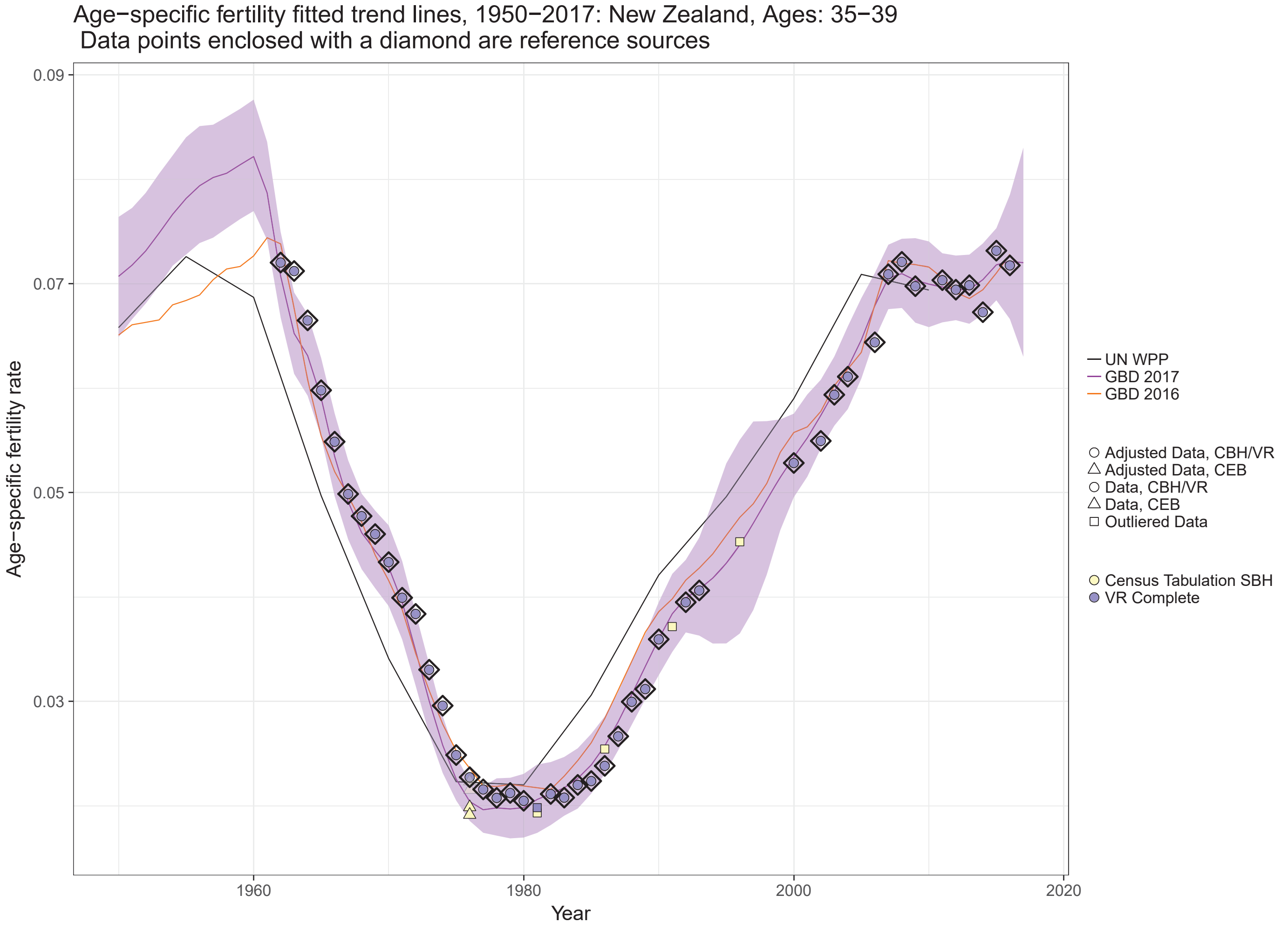
Age−specific fertility fitted trend lines, 1950−2017: New Zealand, Ages: 35−39
Data points enclosed with a diamond are reference sources
Age−specific fertility rate
0.09
0.07
0.05
0.03
1960
1980
2000
2020
Year
UN WPP
GBD 2017
GBD 2016
Adjusted Data, CBH/VR
Adjusted Data, CEB
Data, CBH/VR
Data, CEB
Outliered Data
Census Tabulation SBH
VR Complete

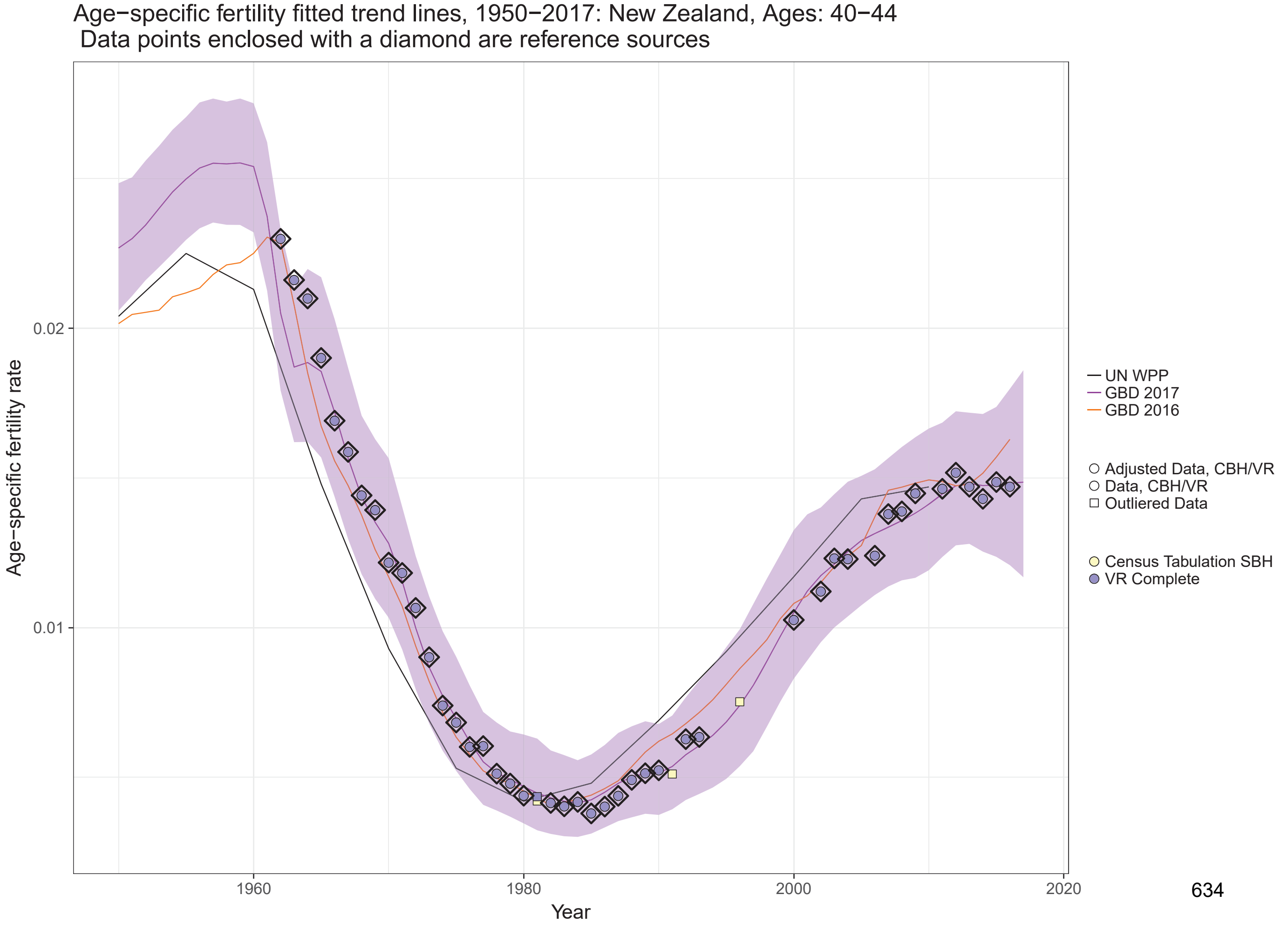
Age−specific fertility fitted trend lines, 1950−2017: New Zealand, Ages: 40−44
Data points enclosed with a diamond are reference sources
Age−specific fertility rate
0.02
0.01
1960
1980
2000
2020
Year
UN WPP
GBD 2017
GBD 2016
Adjusted Data, CBH/VR
Data, CBH/VR
Outliered Data
Census Tabulation SBH
VR Complete

Age−specific fertility fitted trend lines, 1950−2017: New Zealand, Ages: 45−49
Data points enclosed with a diamond are reference sources

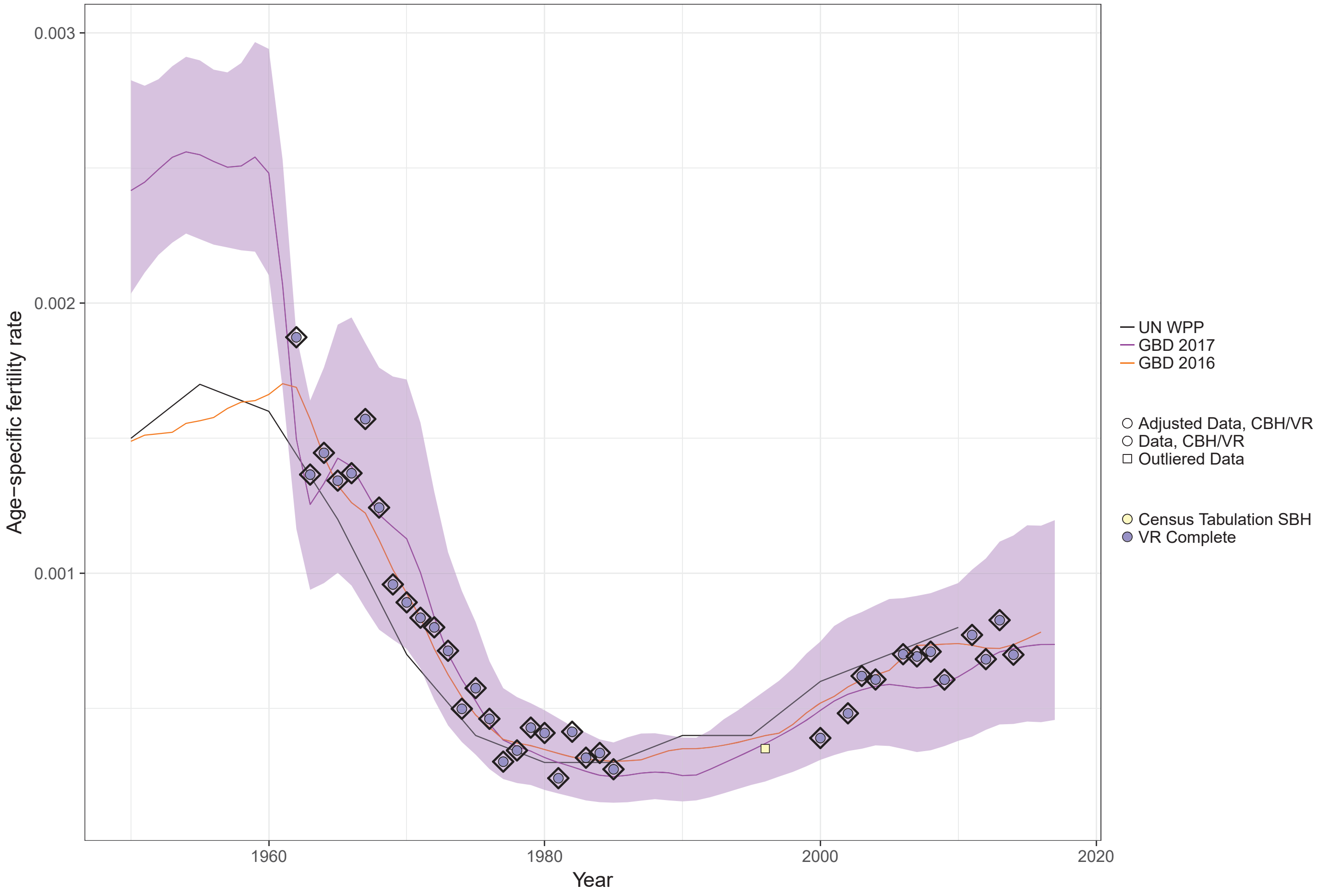

Age−specific fertility fitted trend lines, 1950−2017: New Zealand, Ages: 50−54
Data points enclosed with a diamond are reference sources

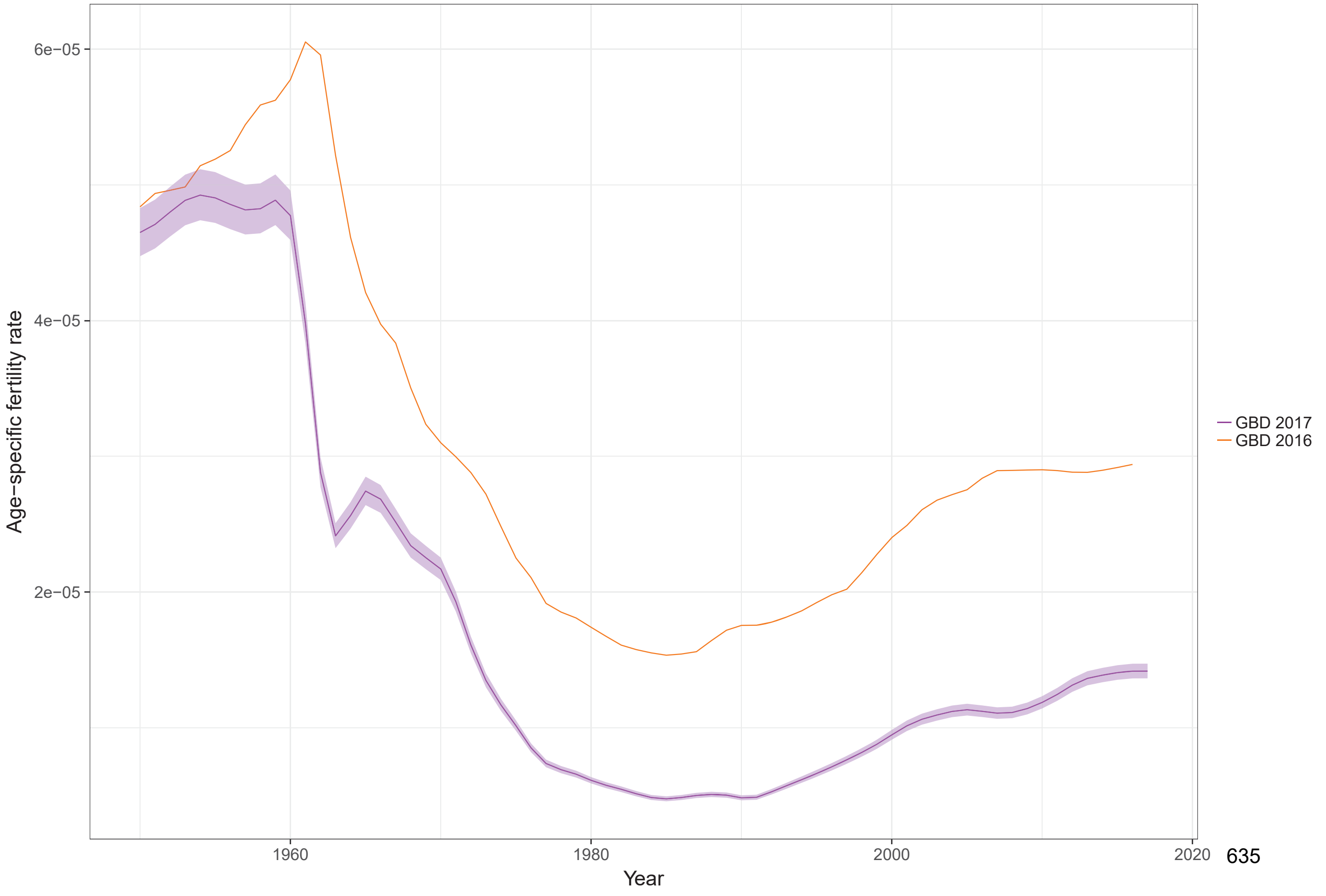

Age−specific fertility fitted trend lines, 1950−2017: Nicaragua, TFR
Data points enclosed with a diamond are reference sources

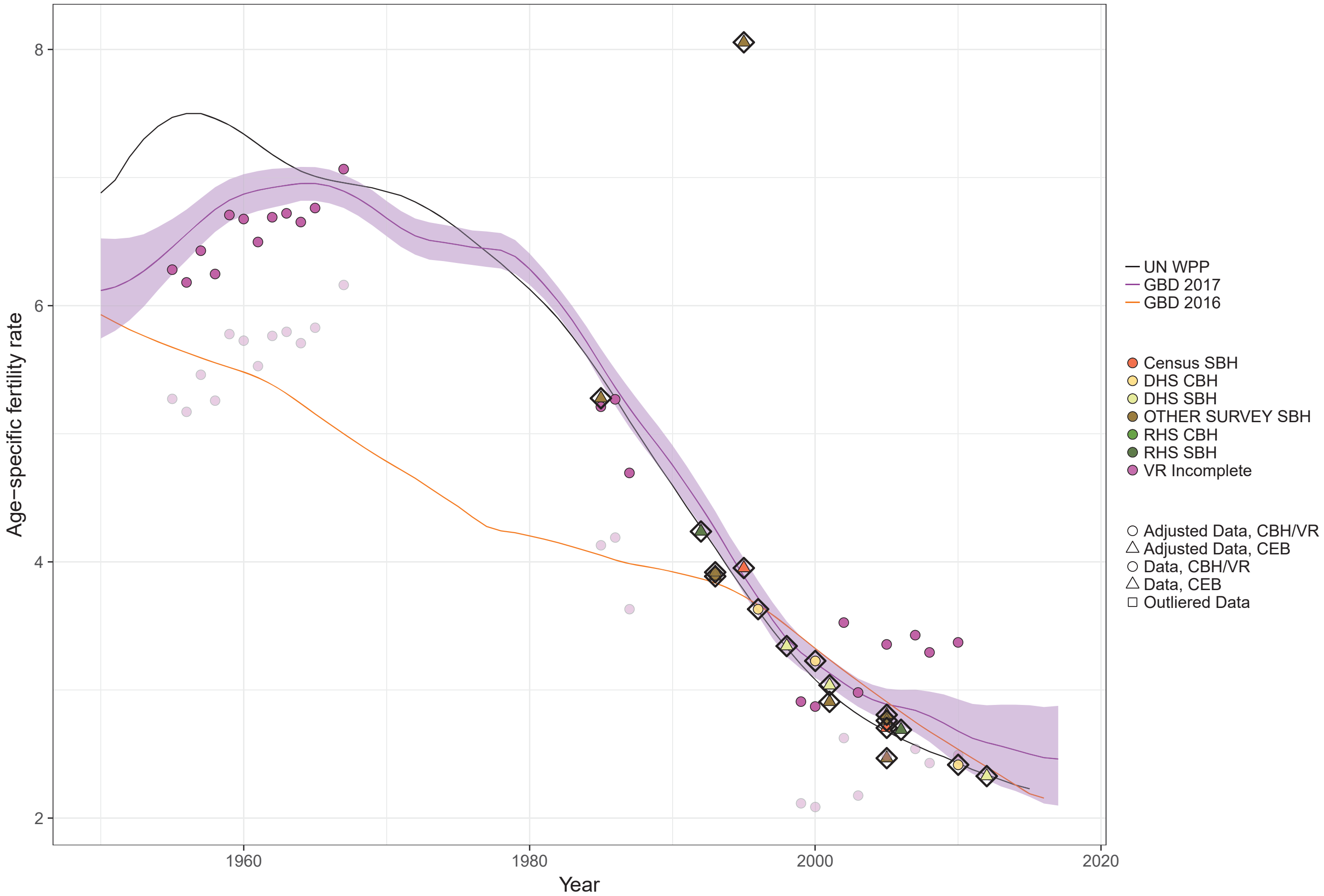

Age−specific fertility fitted trend lines, 1950−2017: Nicaragua, Ages: 10−14
Data points enclosed with a diamond are reference sources

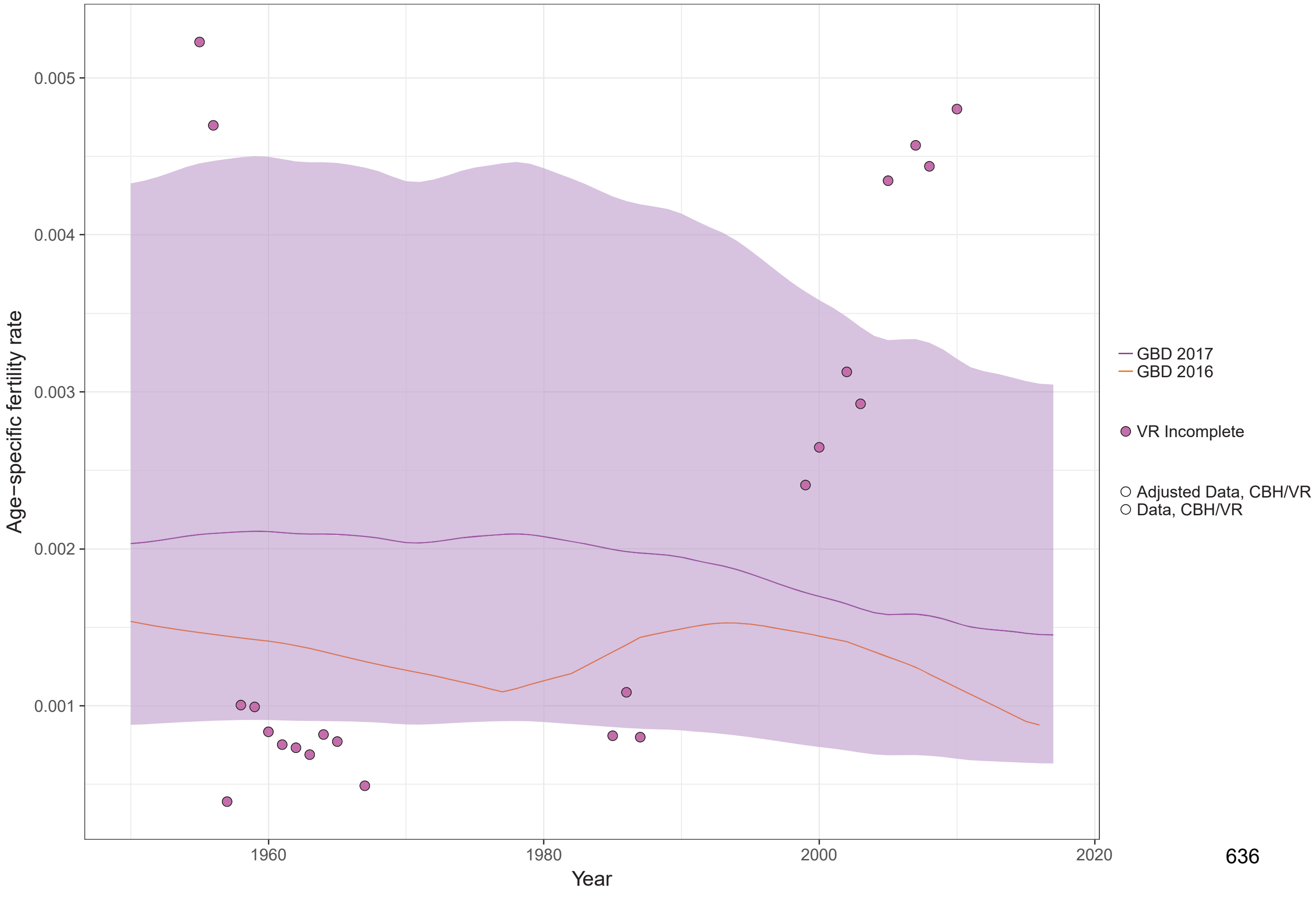

Age−specific fertility fitted trend lines, 1950−2017: Nicaragua, Ages: 15−19
Data points enclosed with a diamond are reference sources

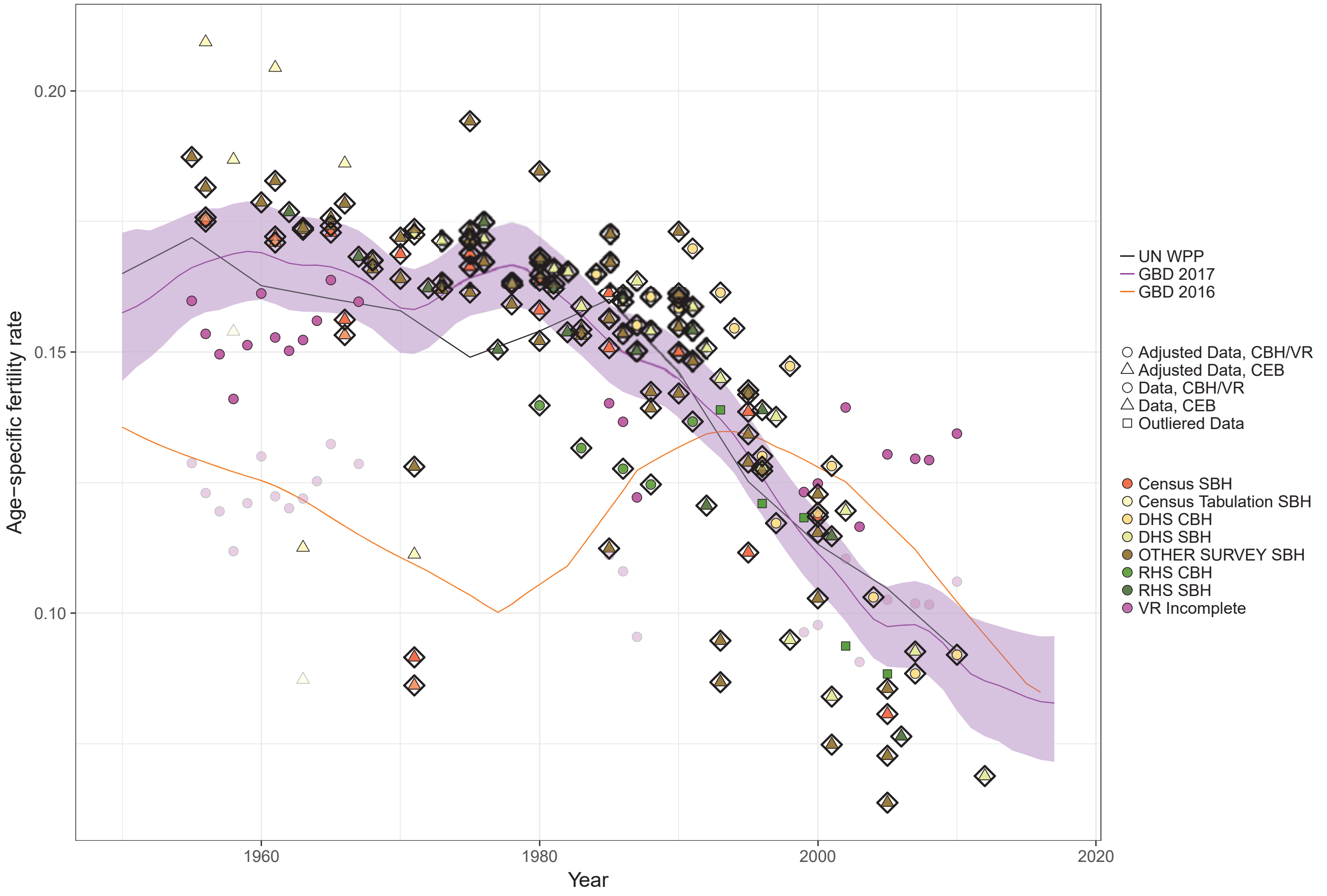

Age−specific fertility fitted trend lines, 1950−2017: Nicaragua, Ages: 20−24
Data points enclosed with a diamond are reference sources

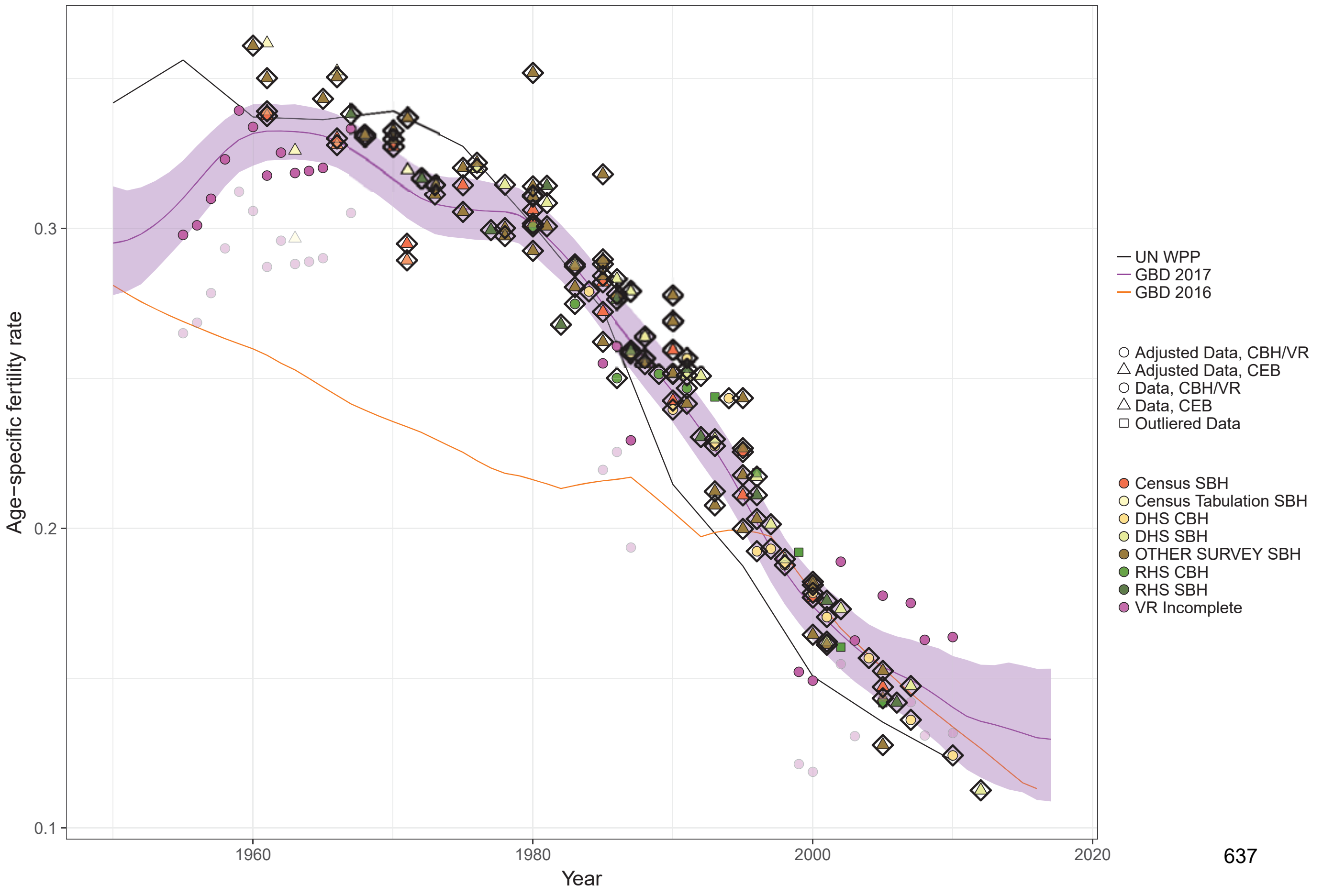

Age−specific fertility fitted trend lines, 1950−2017: Nicaragua, Ages: 25−29
Data points enclosed with a diamond are reference sources

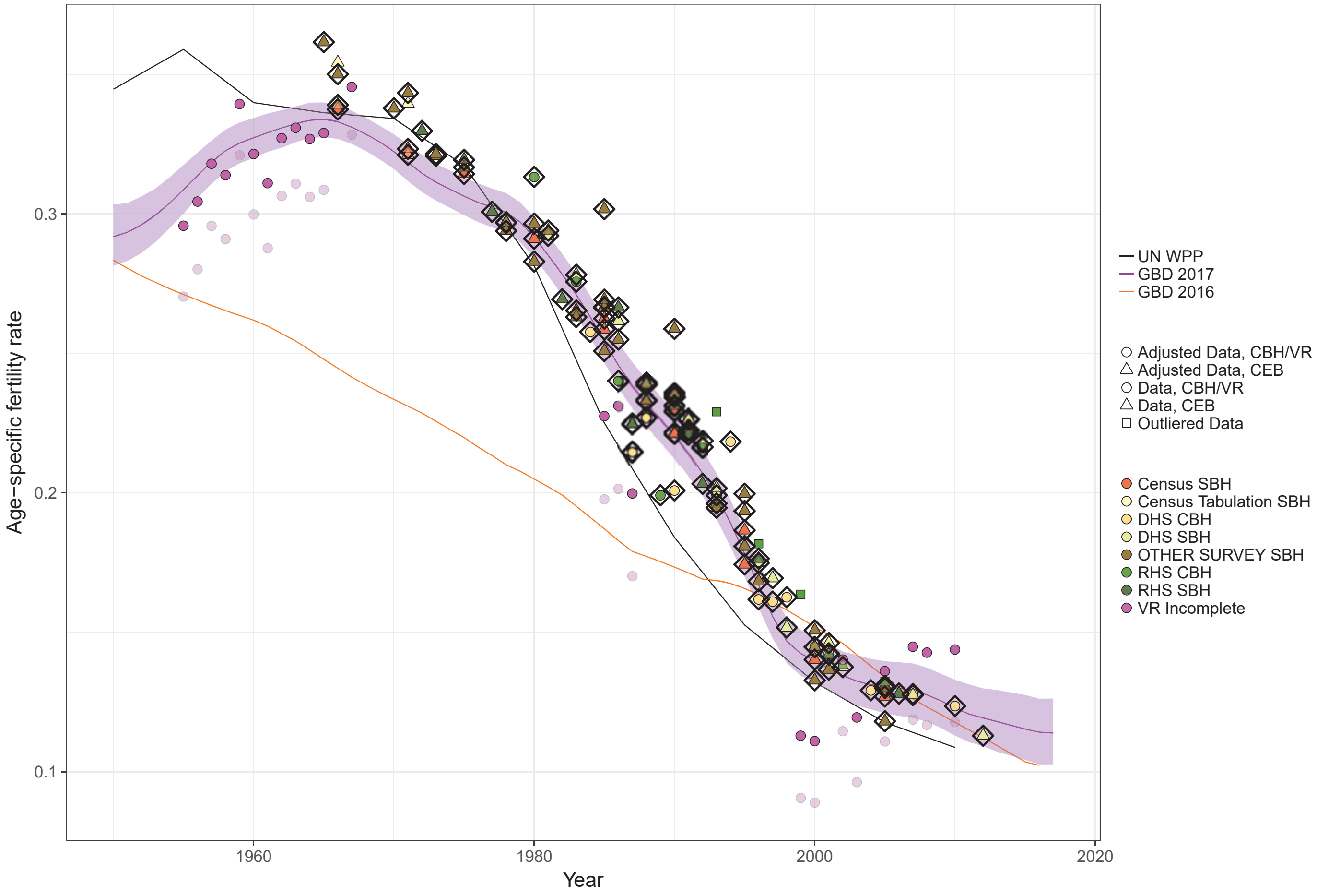

Age−specific fertility fitted trend lines, 1950−2017: Nicaragua, Ages: 30−34
Data points enclosed with a diamond are reference sources

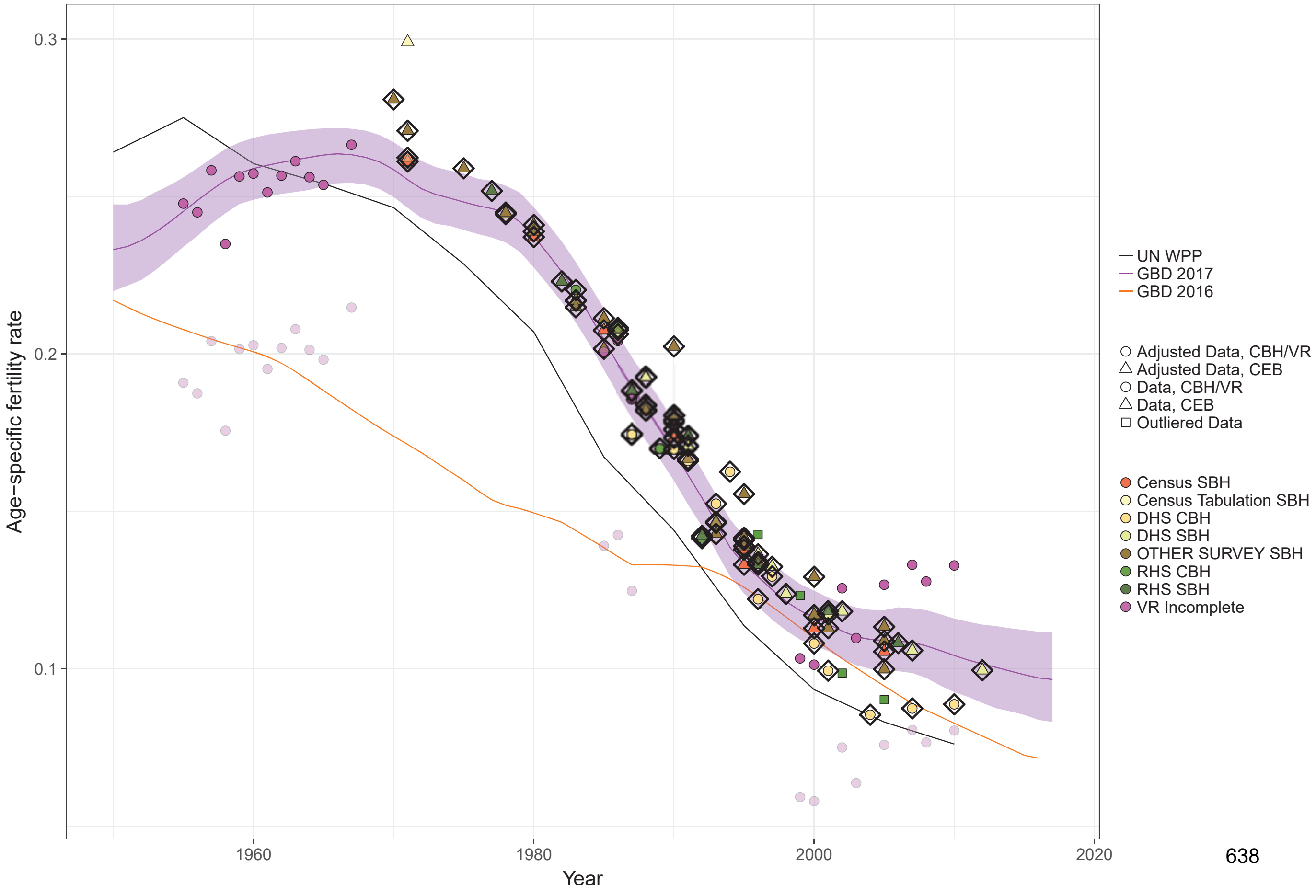

Age−specific fertility fitted trend lines, 1950−2017: Nicaragua, Ages: 35−39
Data points enclosed with a diamond are reference sources

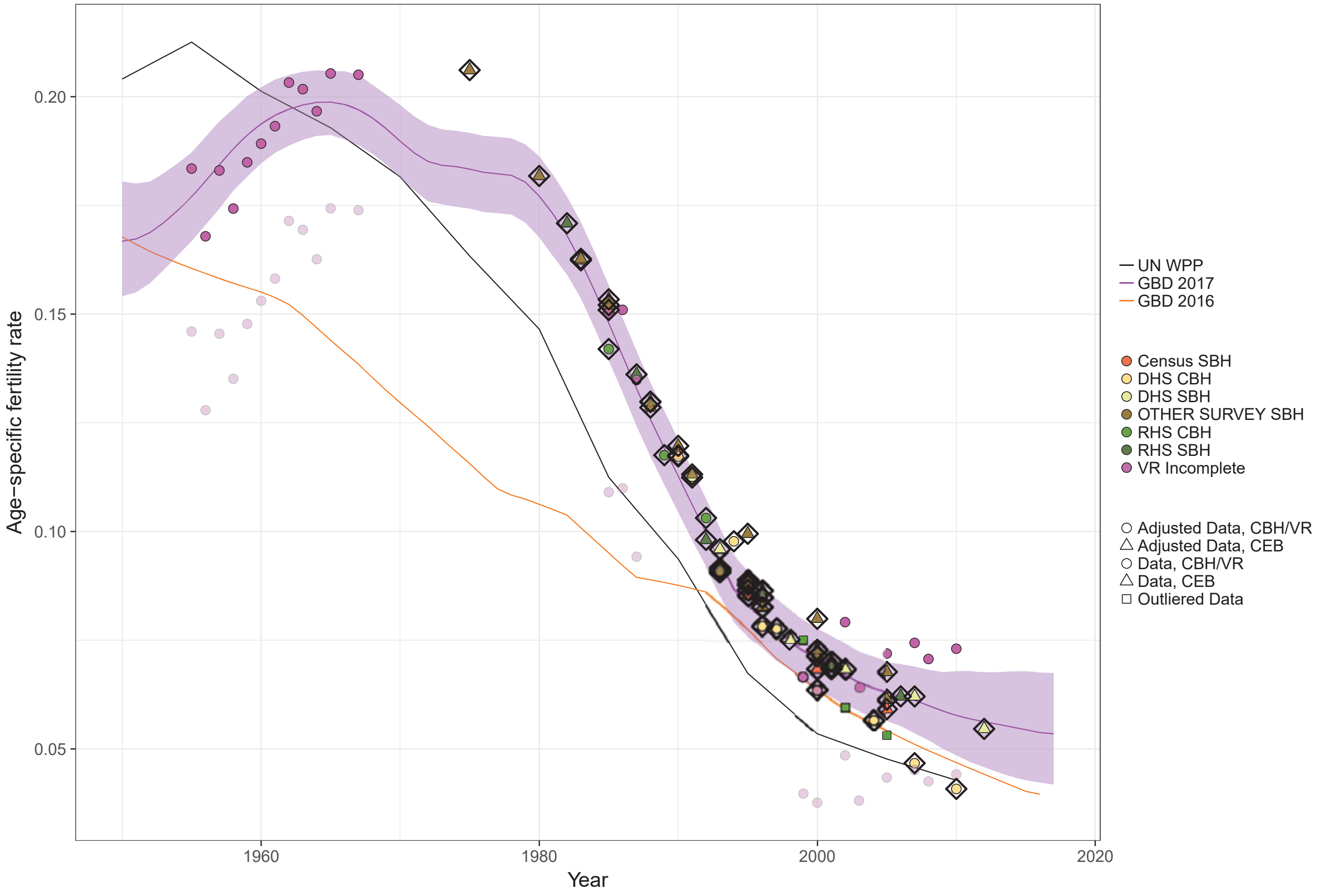

Age−specific fertility fitted trend lines, 1950−2017: Nicaragua, Ages: 40−44
Data points enclosed with a diamond are reference sources

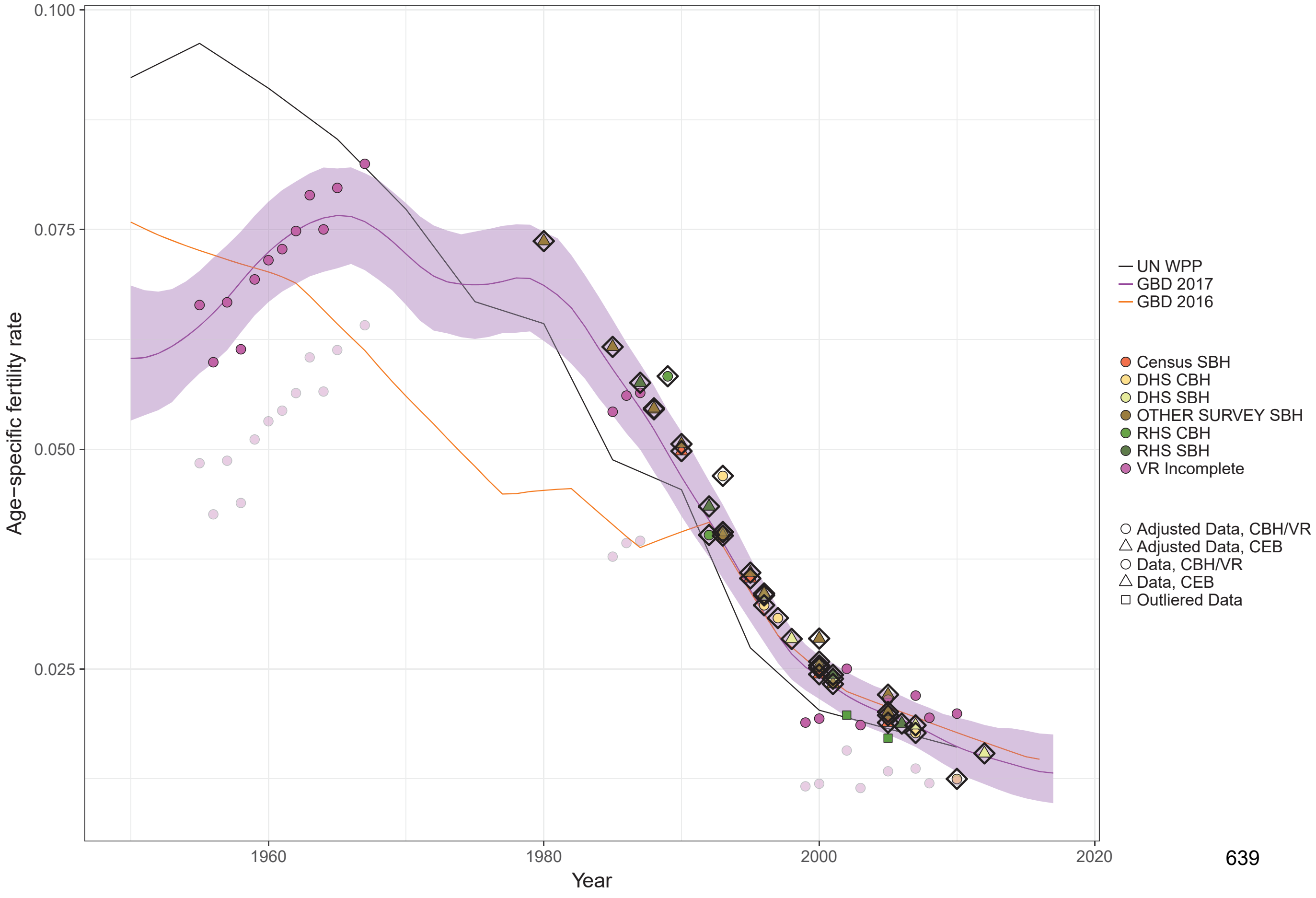

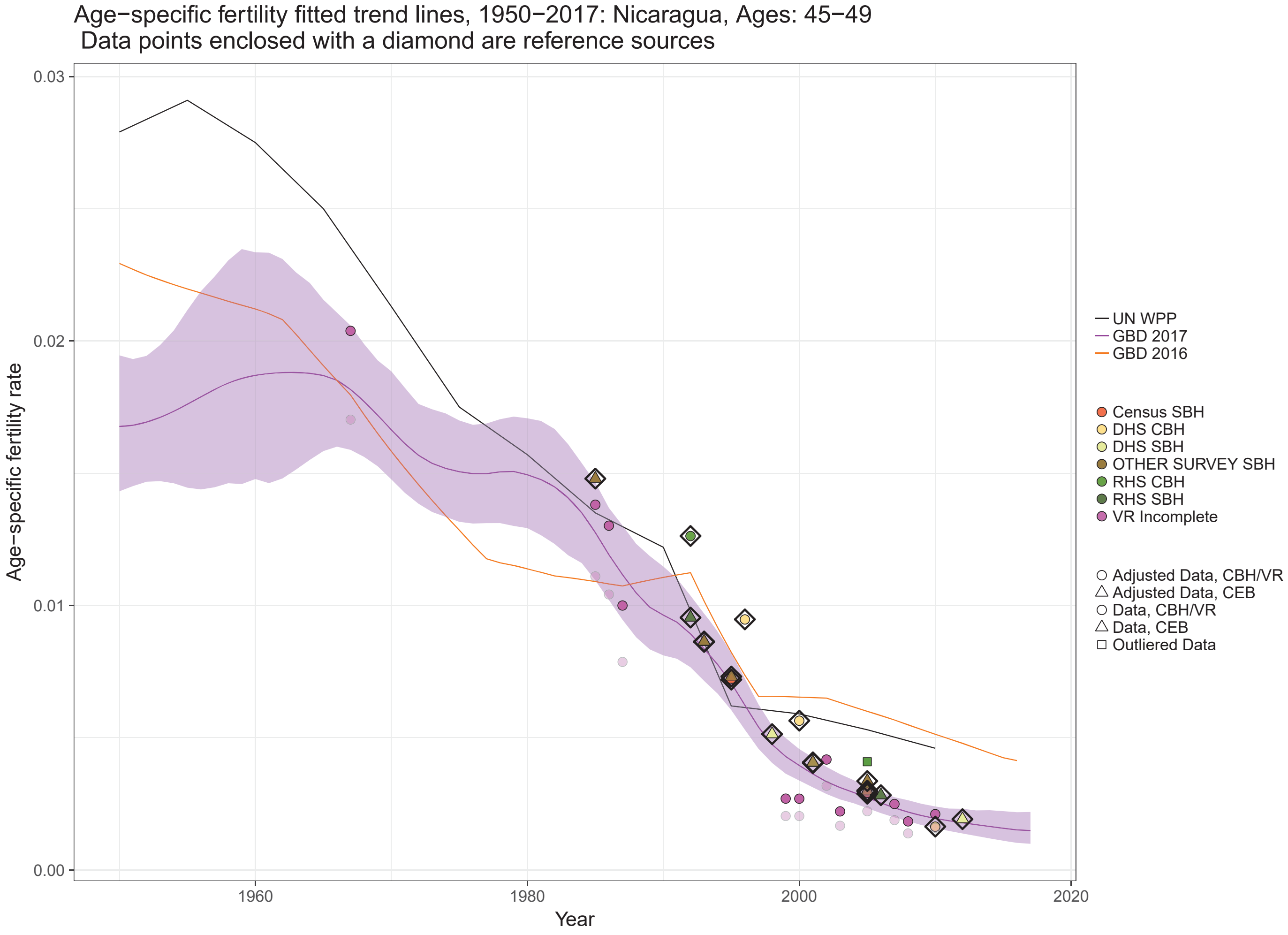
Age−specific fertility fitted trend lines, 1950−2017: Nicaragua, Ages: 45−49
Data points enclosed with a diamond are reference sources
Age−specific fertility rate
Year
0.00
0.01
0.02
0.03
1960
1980
2000
2020
UN WPP
GBD 2017
GBD 2016
Census SBH
DHS CBH
DHS SBH
OTHER SURVEY SBH
RHS CBH
RHS SBH
VR Incomplete
Adjusted Data, CBH/VR
Adjusted Data, CEB
Data, CBH/VR
Data, CEB
Outliered Data

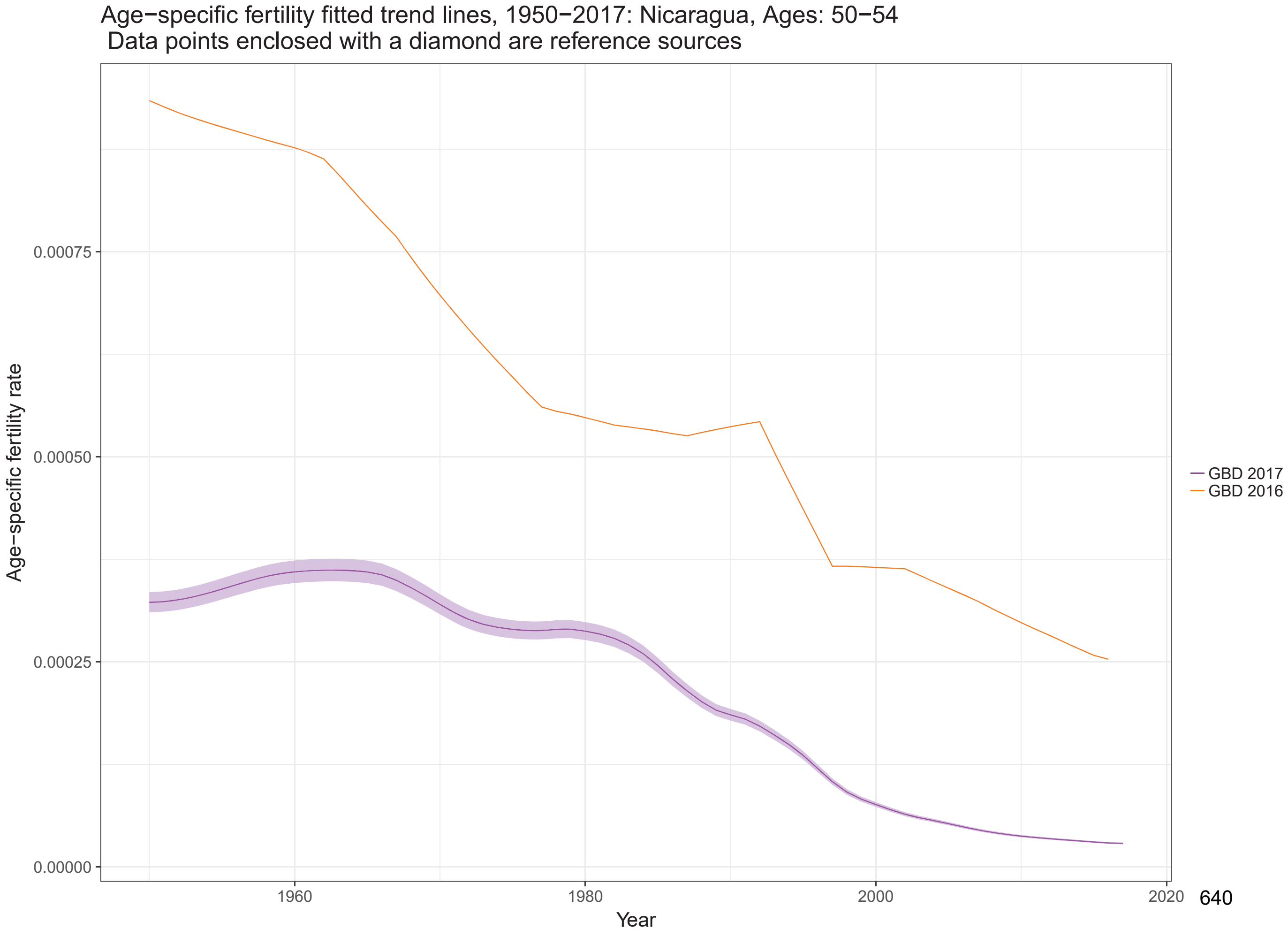
Age−specific fertility fitted trend lines, 1950−2017: Nicaragua, Ages: 50−54
Data points enclosed with a diamond are reference sources
Age−specific fertility rate
Year
0.00000
0.00025
0.00050
0.00075
1960
1980
2000
2020
GBD 2017
GBD 2016

Age−specific fertility fitted trend lines, 1950−2017: Niger, TFR
Data points enclosed with a diamond are reference sources

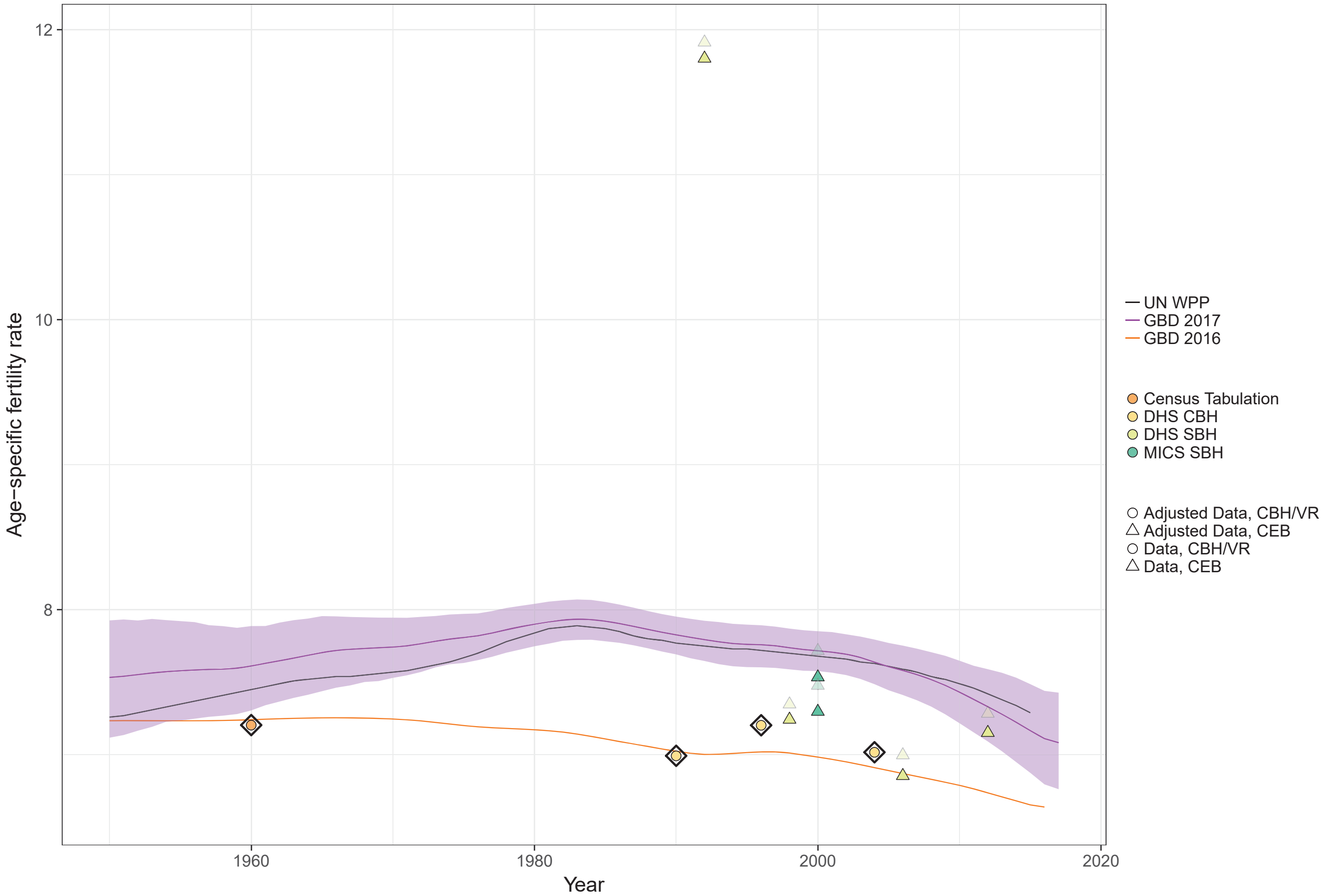

Age−specific fertility fitted trend lines, 1950−2017: Niger, Ages: 10−14
Data points enclosed with a diamond are reference sources

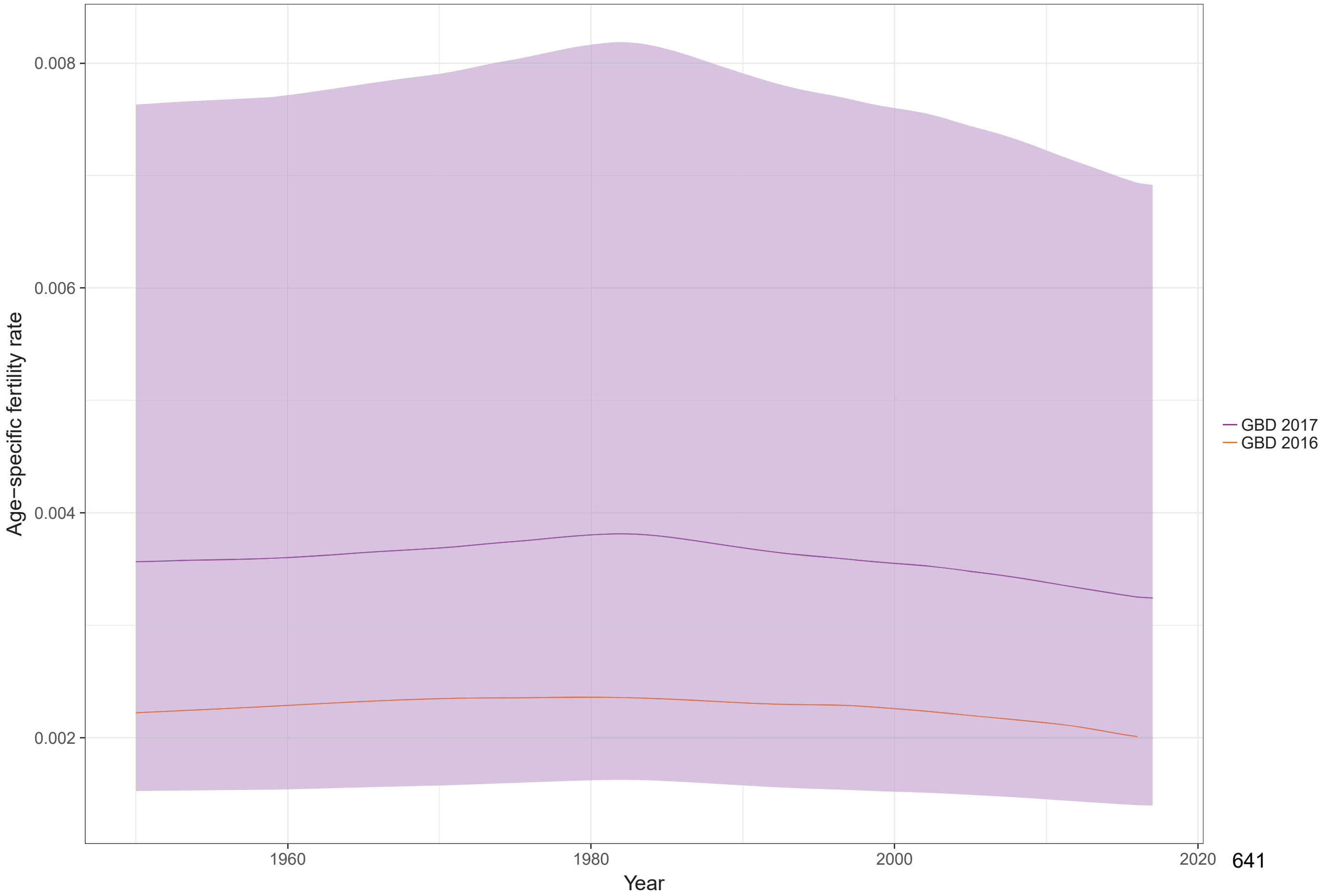

Age−specific fertility fitted trend lines, 1950−2017: Niger, Ages: 15−19
Data points enclosed with a diamond are reference sources

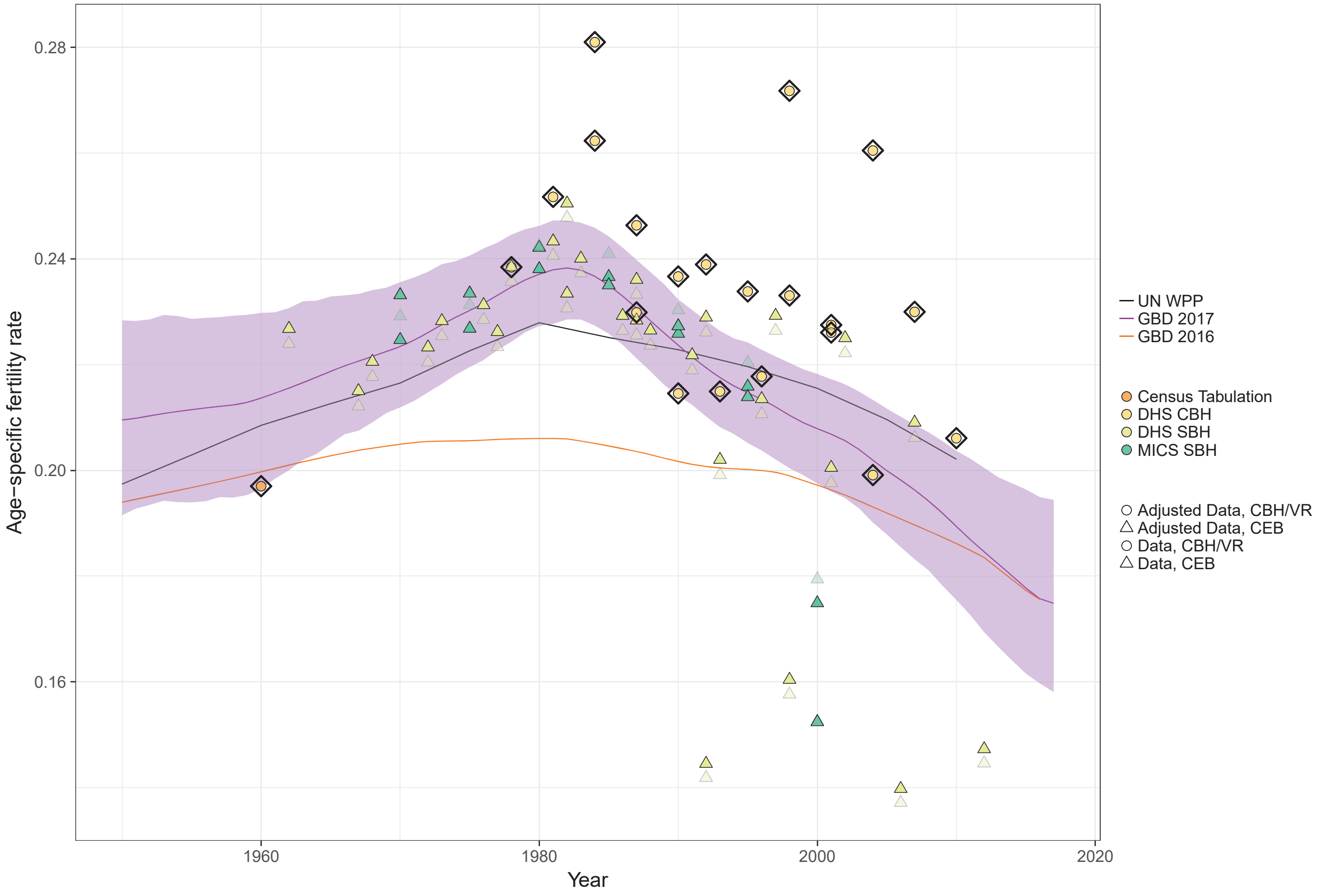

Age−specific fertility fitted trend lines, 1950−2017: Niger, Ages: 20−24
Data points enclosed with a diamond are reference sources

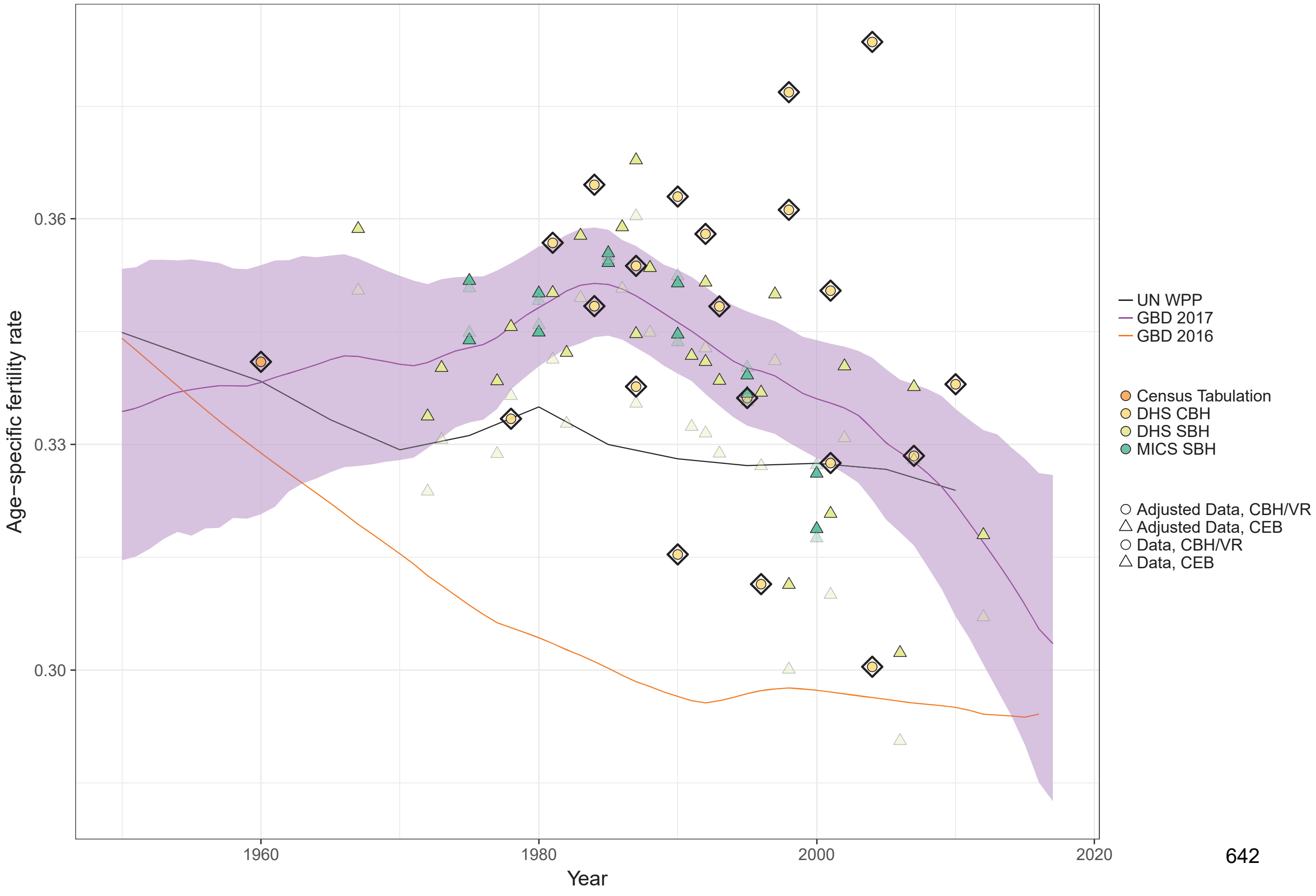

Age−specific fertility fitted trend lines, 1950−2017: Niger, Ages: 25−29
Data points enclosed with a diamond are reference sources

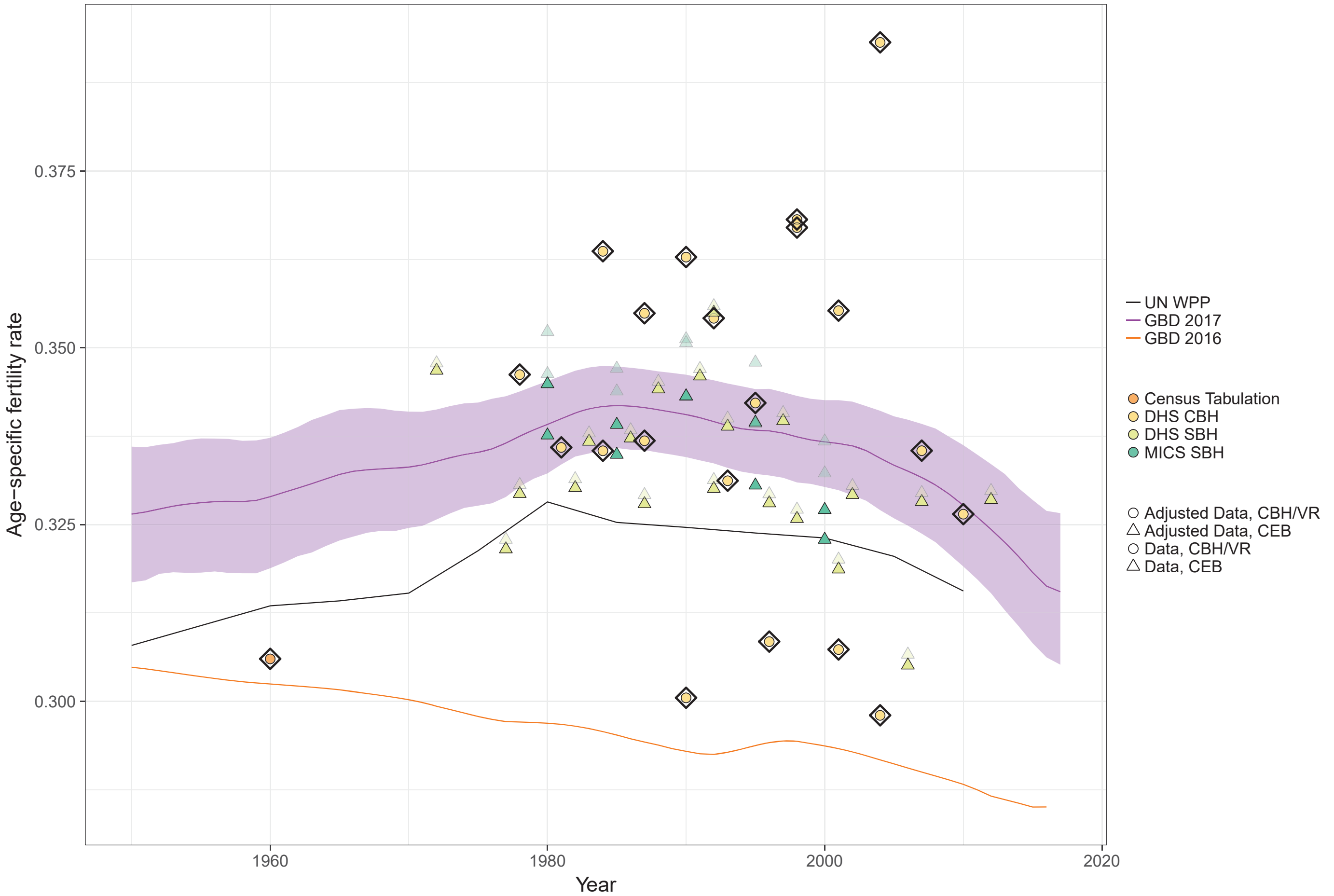

Age−specific fertility fitted trend lines, 1950−2017: Niger, Ages: 30−34
Data points enclosed with a diamond are reference sources

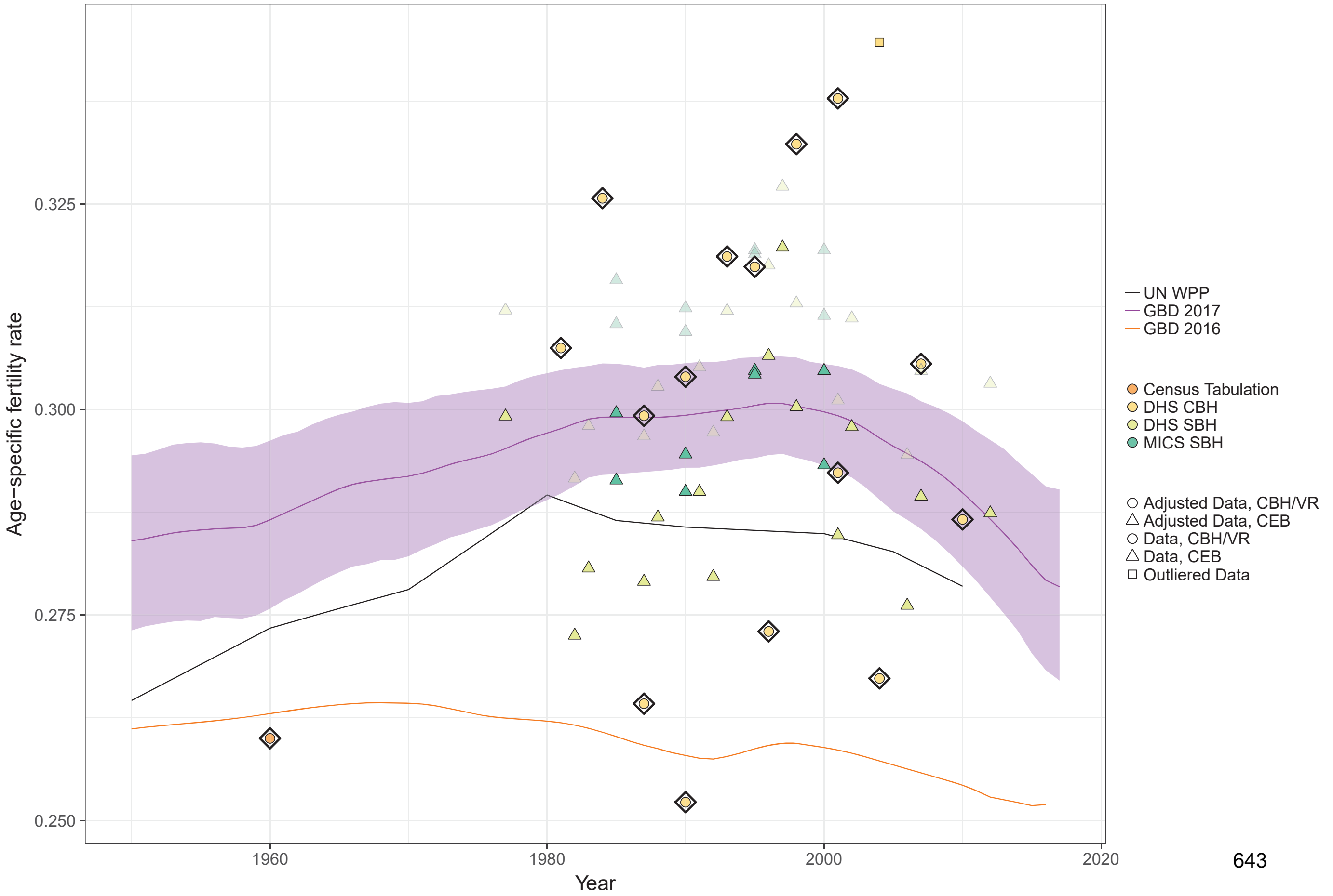

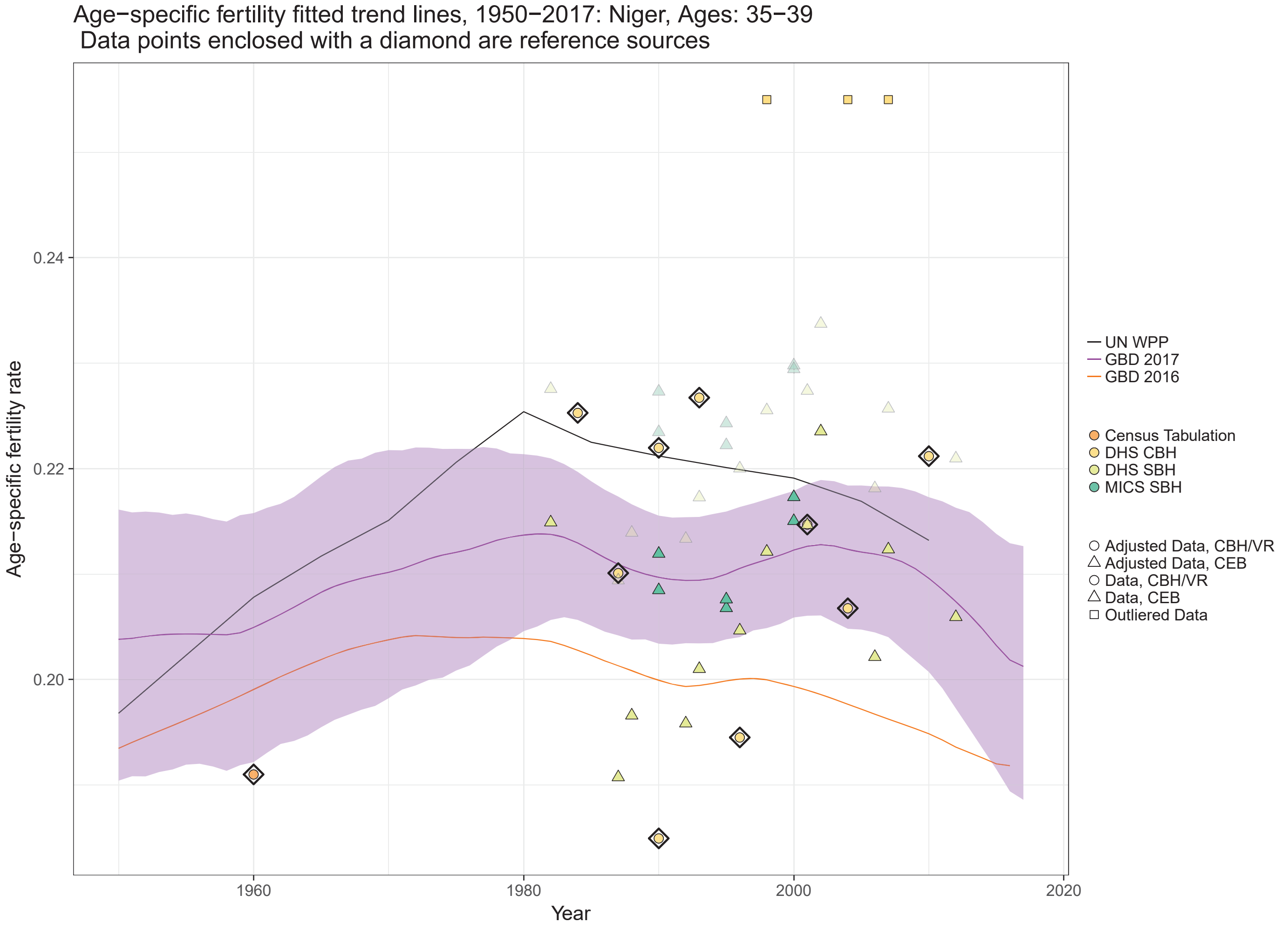
Age−specific fertility fitted trend lines, 1950−2017: Niger, Ages: 35−39
Data points enclosed with a diamond are reference sources
Age−specific fertility rate
0.20
0.22
0.24
1960
1980
2000
2020
Year
UN WPP
GBD 2017
GBD 2016
Census Tabulation
DHS CBH
DHS SBH
MICS SBH
Adjusted Data, CBH/VR
Adjusted Data, CEB
Data, CBH/VR
Data, CEB
Outliered Data

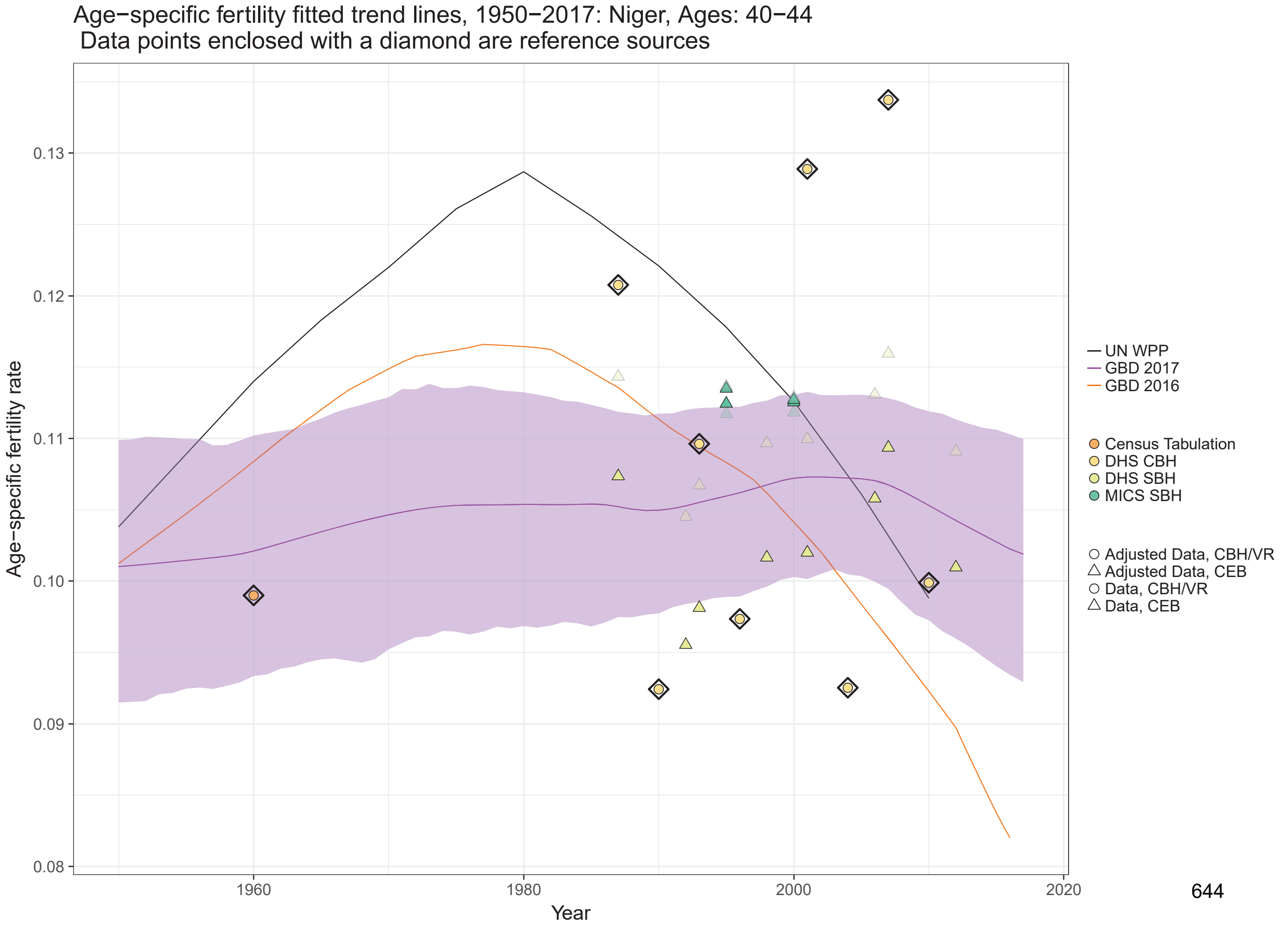
Age−specific fertility fitted trend lines, 1950−2017: Niger, Ages: 40−44
Data points enclosed with a diamond are reference sources
Age−specific fertility rate
0.08
0.09
0.10
0.11
0.12
0.13
1960
1980
2000
2020
Year
UN WPP
GBD 2017
GBD 2016
Census Tabulation
DHS CBH
DHS SBH
MICS SBH
Adjusted Data, CBH/VR
Adjusted Data, CEB
Data, CBH/VR
Data, CEB

Age−specific fertility fitted trend lines, 1950−2017: Niger, Ages: 45−49
Data points enclosed with a diamond are reference sources

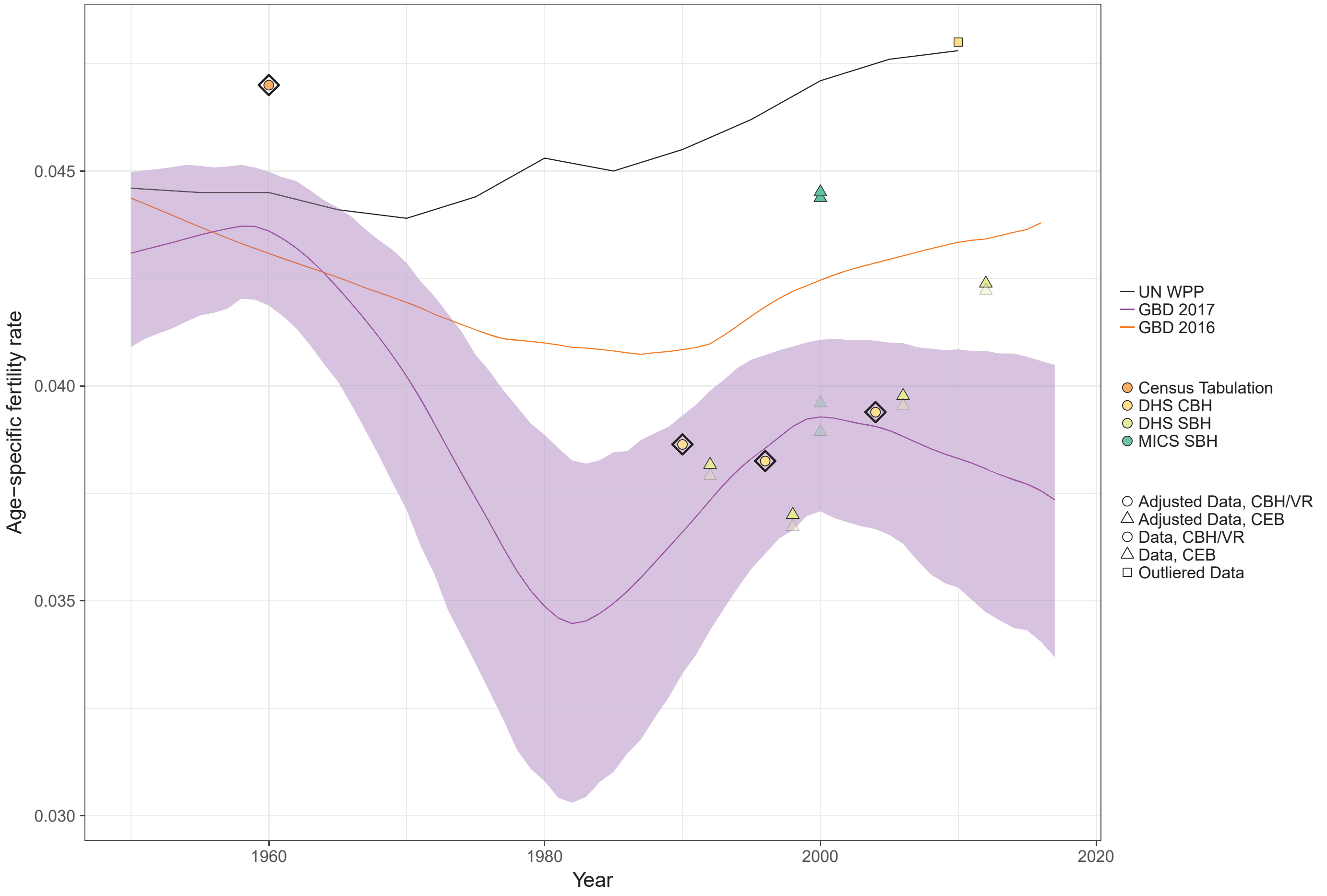

Age−specific fertility fitted trend lines, 1950−2017: Niger, Ages: 50−54
Data points enclosed with a diamond are reference sources

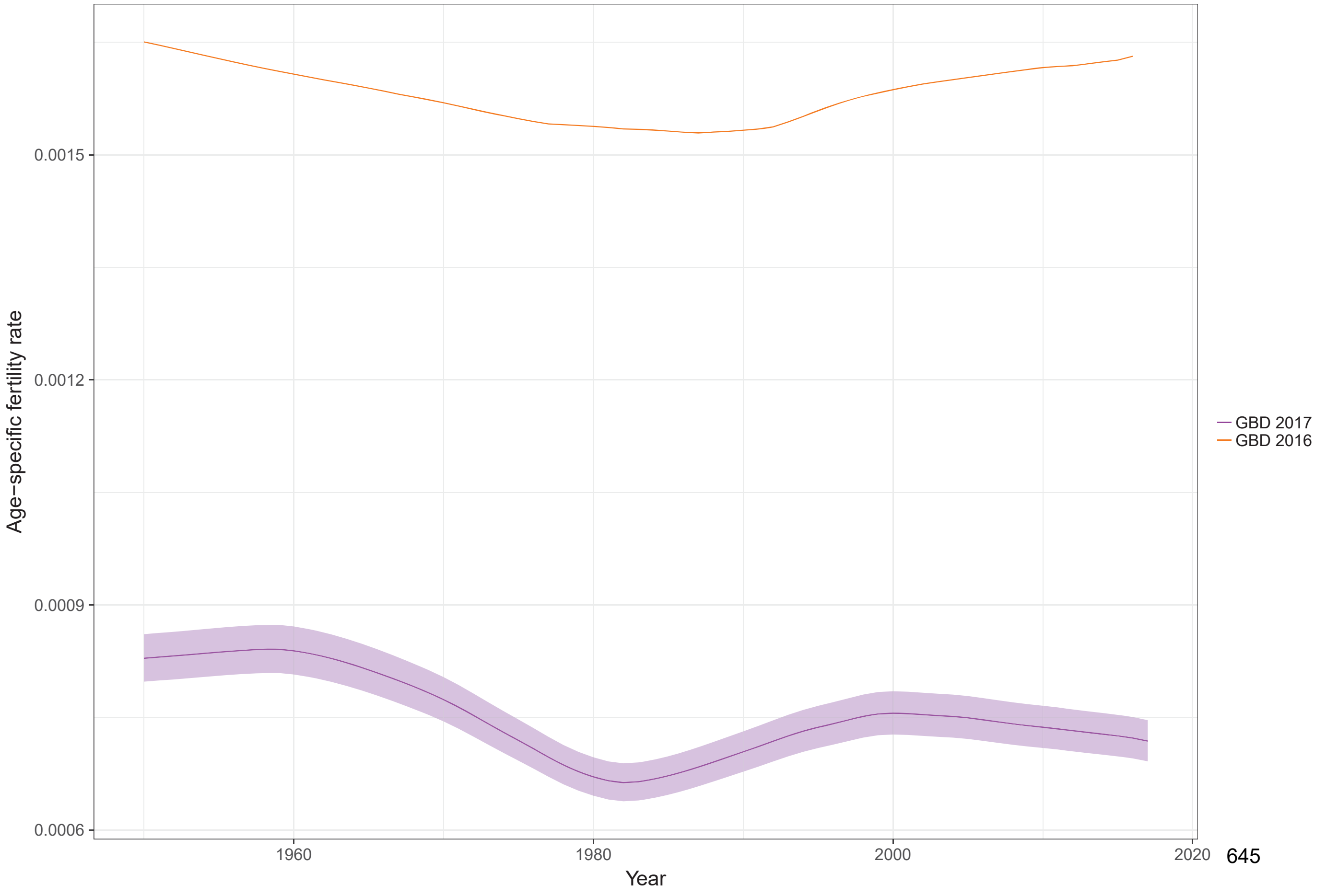

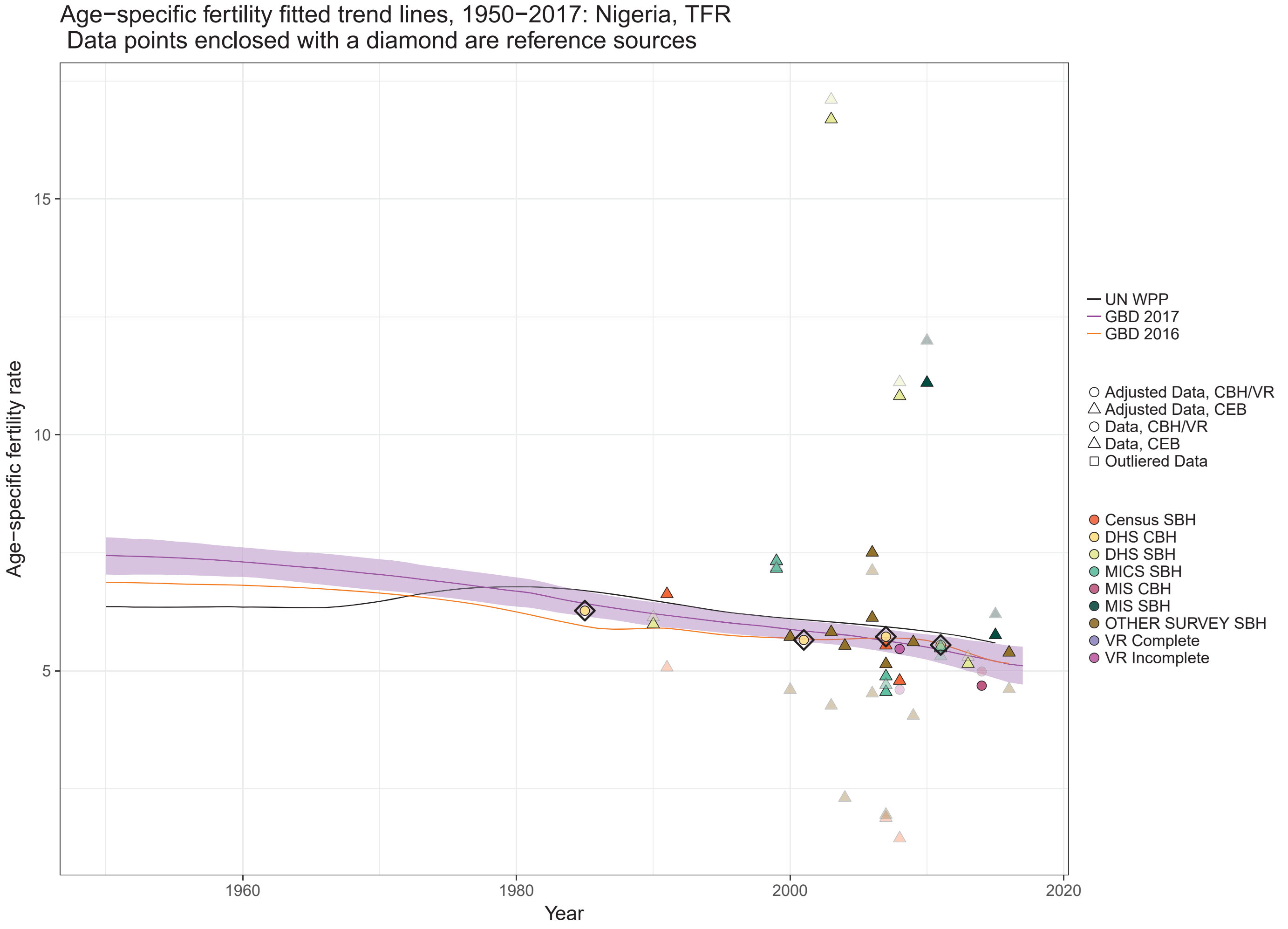

Age−specific fertility fitted trend lines, 1950−2017: Nigeria, TFR
Data points enclosed with a diamond are reference sources
Age-specific fertility rate
Year
UN WPP
GBD 2017
GBD 2016
Adjusted Data, CBH/VR
Adjusted Data, CEB
Data, CBH/VR
Data, CEB
Outliered Data
Census SBH
DHS CBH
DHS SBH
MICS SBH
MIS CBH
MIS SBH
OTHER SURVEY SBH
VR Complete
VR Incomplete

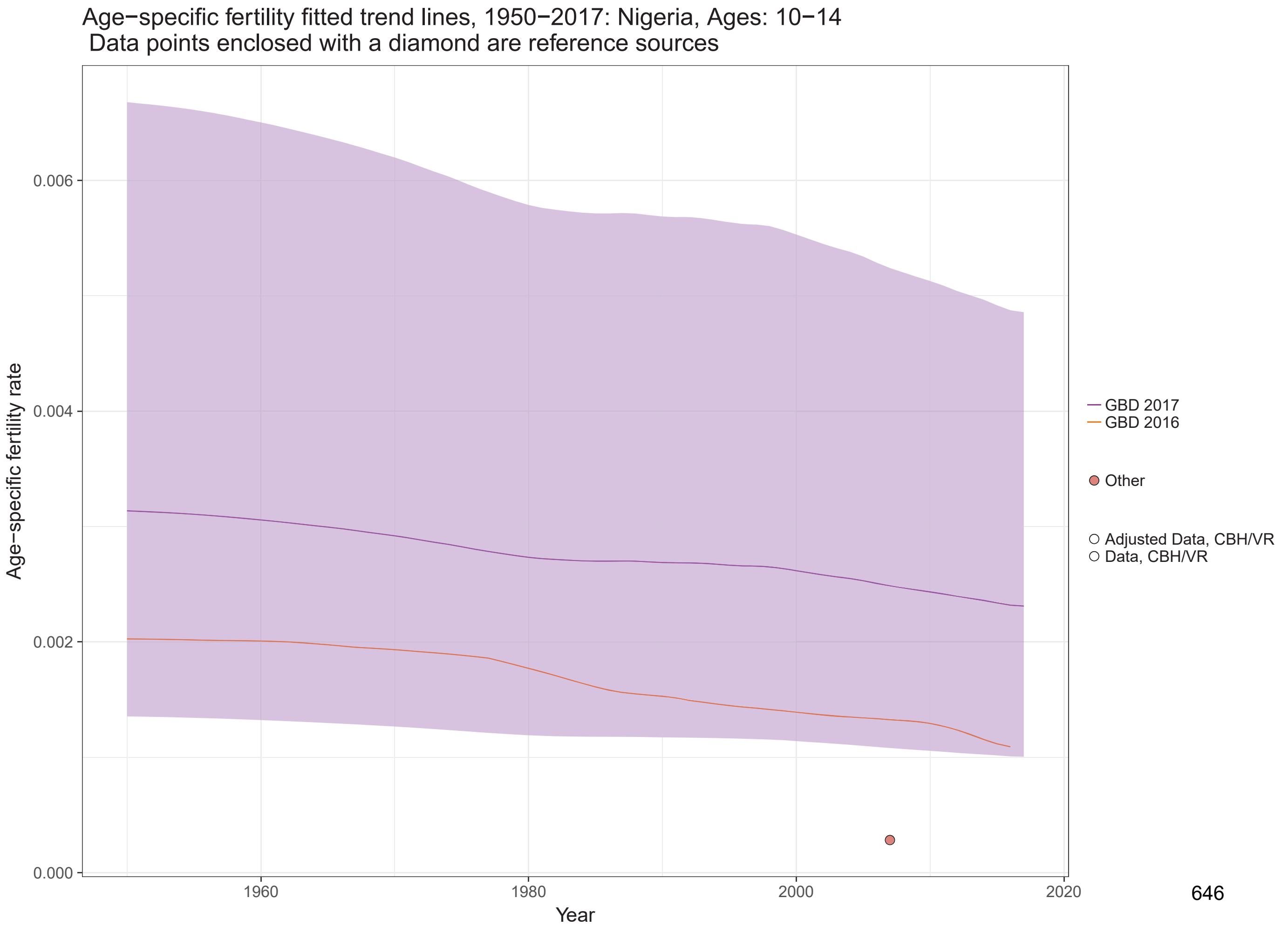

Age−specific fertility fitted trend lines, 1950−2017: Nigeria, Ages: 10−14
Data points enclosed with a diamond are reference sources
Age-specific fertility rate
Year
GBD 2017
GBD 2016
Other
Adjusted Data, CBH/VR
Data, CBH/VR

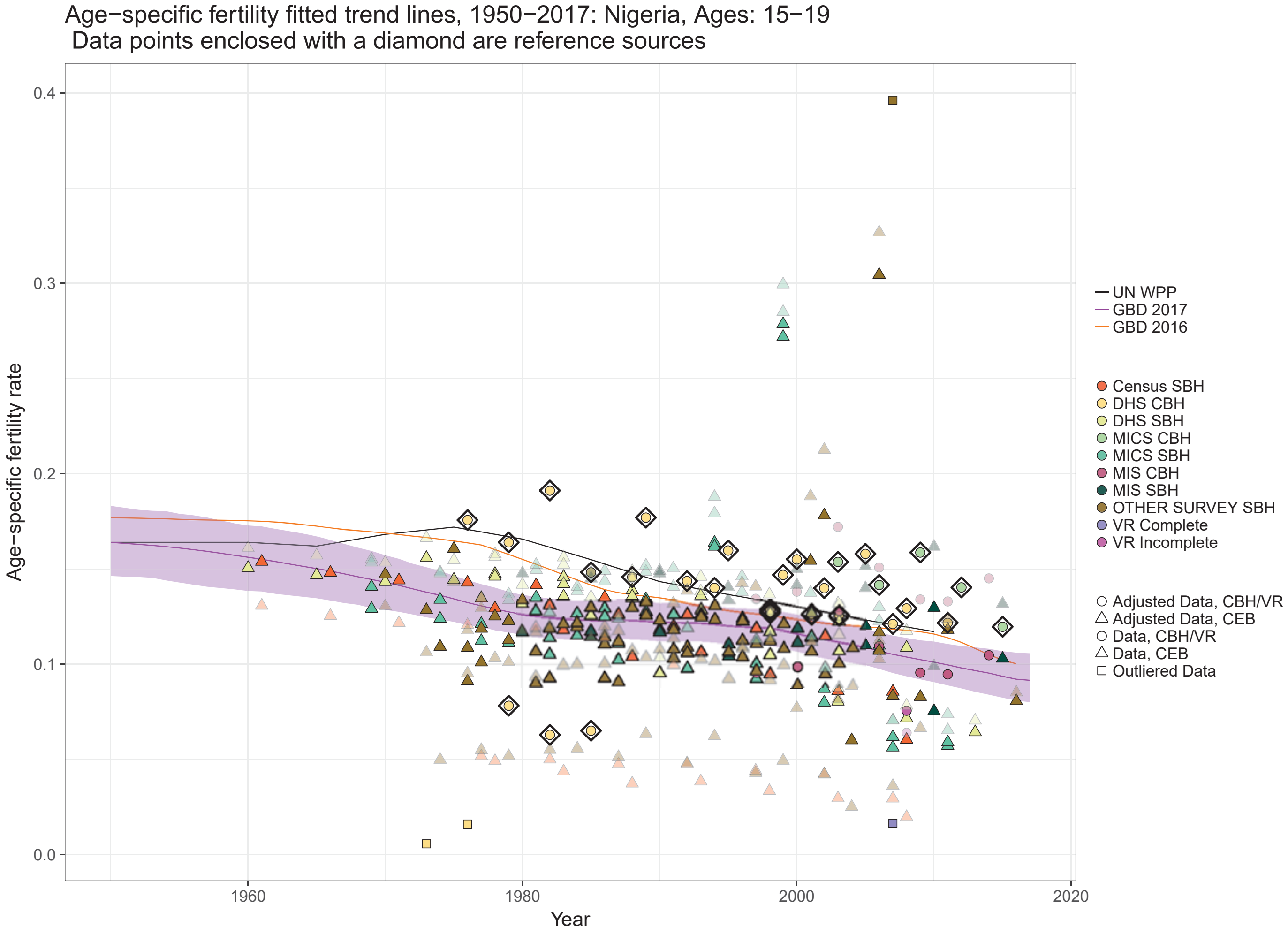

Age−specific fertility fitted trend lines, 1950−2017: Nigeria, Ages: 15−19
Data points enclosed with a diamond are reference sources
Age-specific fertility rate
Year
UN WPP
GBD 2017
GBD 2016
Census SBH
DHS CBH
DHS SBH
MICS CBH
MICS SBH
MIS CBH
MIS SBH
OTHER SURVEY SBH
VR Complete
VR Incomplete
Adjusted Data, CBH/VR
Adjusted Data, CEB
Data, CBH/VR
Data, CEB
Outliered Data

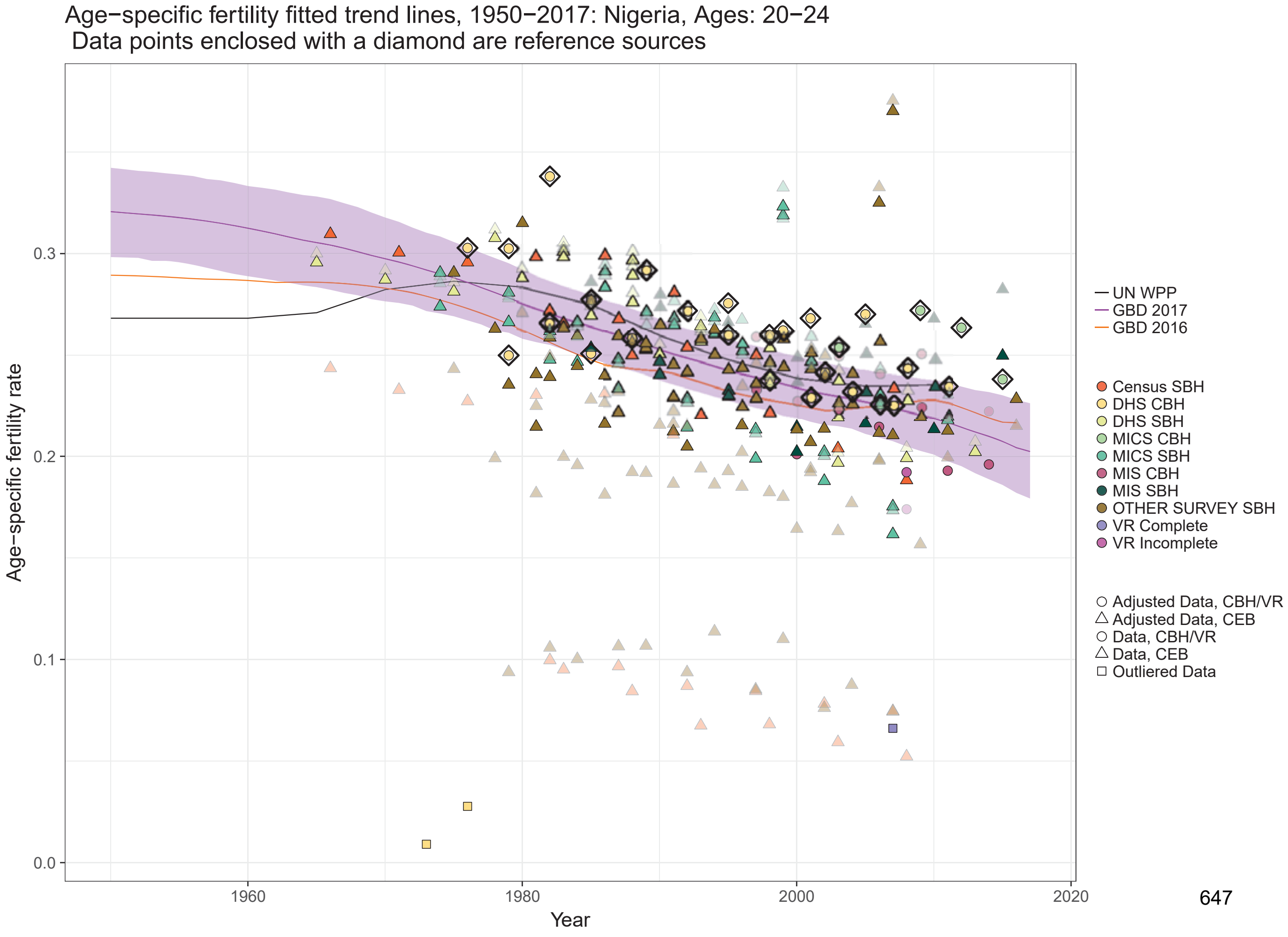

Age−specific fertility fitted trend lines, 1950−2017: Nigeria, Ages: 20−24
Data points enclosed with a diamond are reference sources
Age-specific fertility rate
Year
UN WPP
GBD 2017
GBD 2016
Census SBH
DHS CBH
DHS SBH
MICS CBH
MICS SBH
MIS CBH
MIS SBH
OTHER SURVEY SBH
VR Complete
VR Incomplete
Adjusted Data, CBH/VR
Adjusted Data, CEB
Data, CBH/VR
Data, CEB
Outliered Data

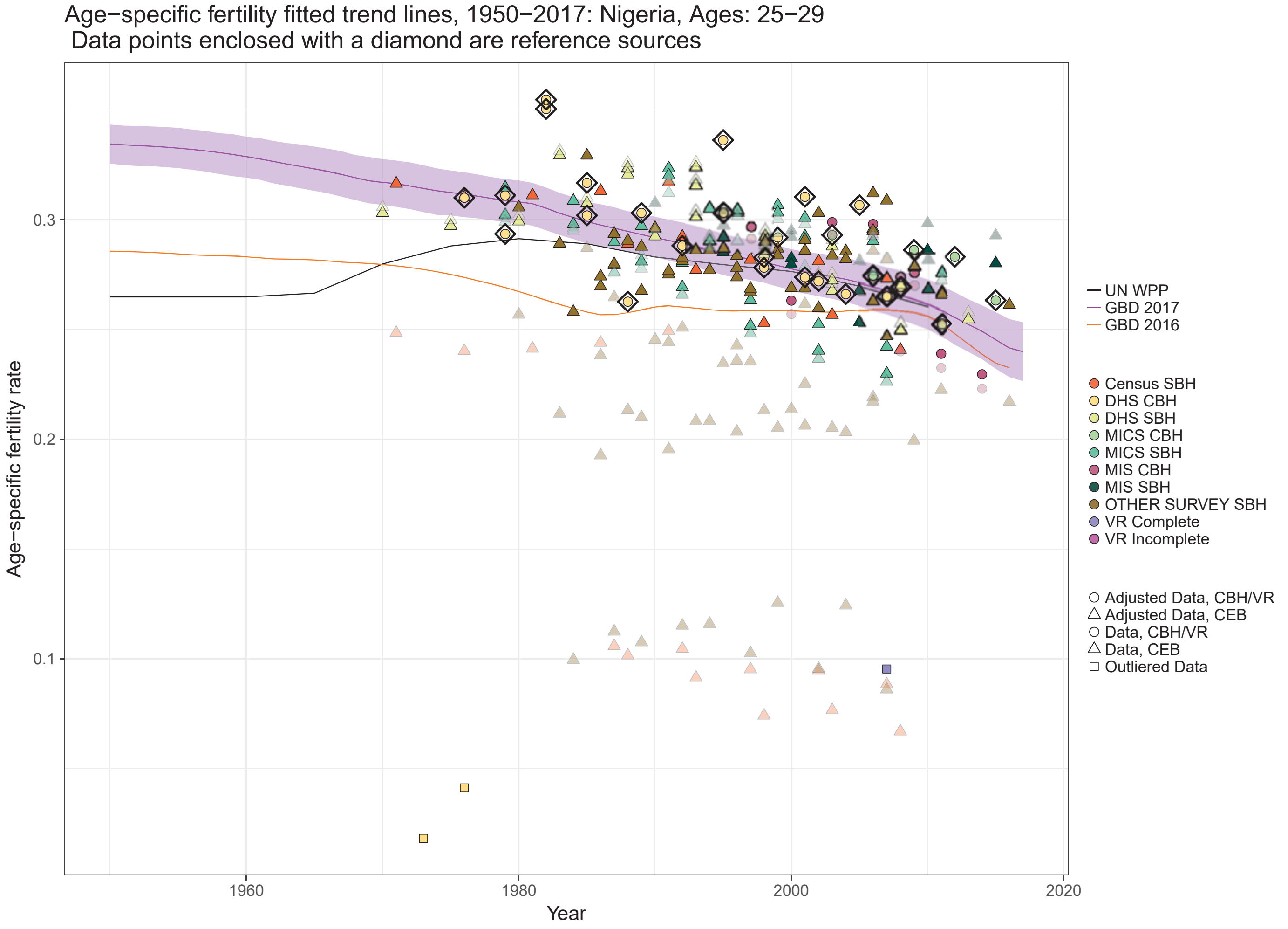
Age−specific fertility fitted trend lines, 1950−2017: Nigeria, Ages: 25−29
Data points enclosed with a diamond are reference sources
Age−specific fertility rate
0.1
0.2
0.3
1960
1980
2000
2020
Year
UN WPP
GBD 2017
GBD 2016
Census SBH
DHS CBH
DHS SBH
MICS CBH
MICS SBH
MIS CBH
MIS SBH
OTHER SURVEY SBH
VR Complete
VR Incomplete
Adjusted Data, CBH/VR
Adjusted Data, CEB
Data, CBH/VR
Data, CEB
Outliered Data

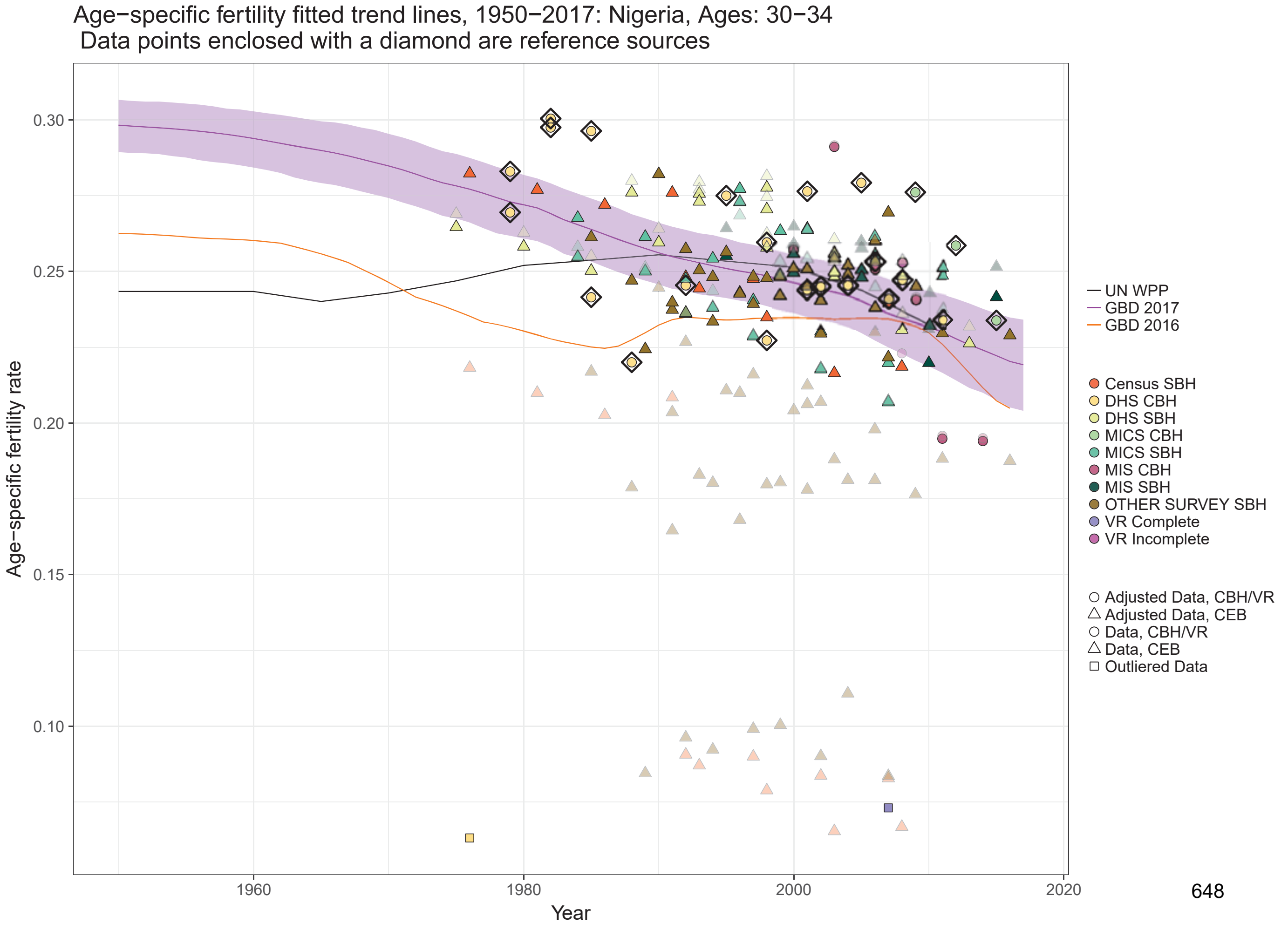
Age−specific fertility fitted trend lines, 1950−2017: Nigeria, Ages: 30−34
Data points enclosed with a diamond are reference sources
Age−specific fertility rate
0.10
0.15
0.20
0.25
0.30
1960
1980
2000
2020
Year
UN WPP
GBD 2017
GBD 2016
Census SBH
DHS CBH
DHS SBH
MICS CBH
MICS SBH
MIS CBH
MIS SBH
OTHER SURVEY SBH
VR Complete
VR Incomplete
Adjusted Data, CBH/VR
Adjusted Data, CEB
Data, CBH/VR
Data, CEB
Outliered Data

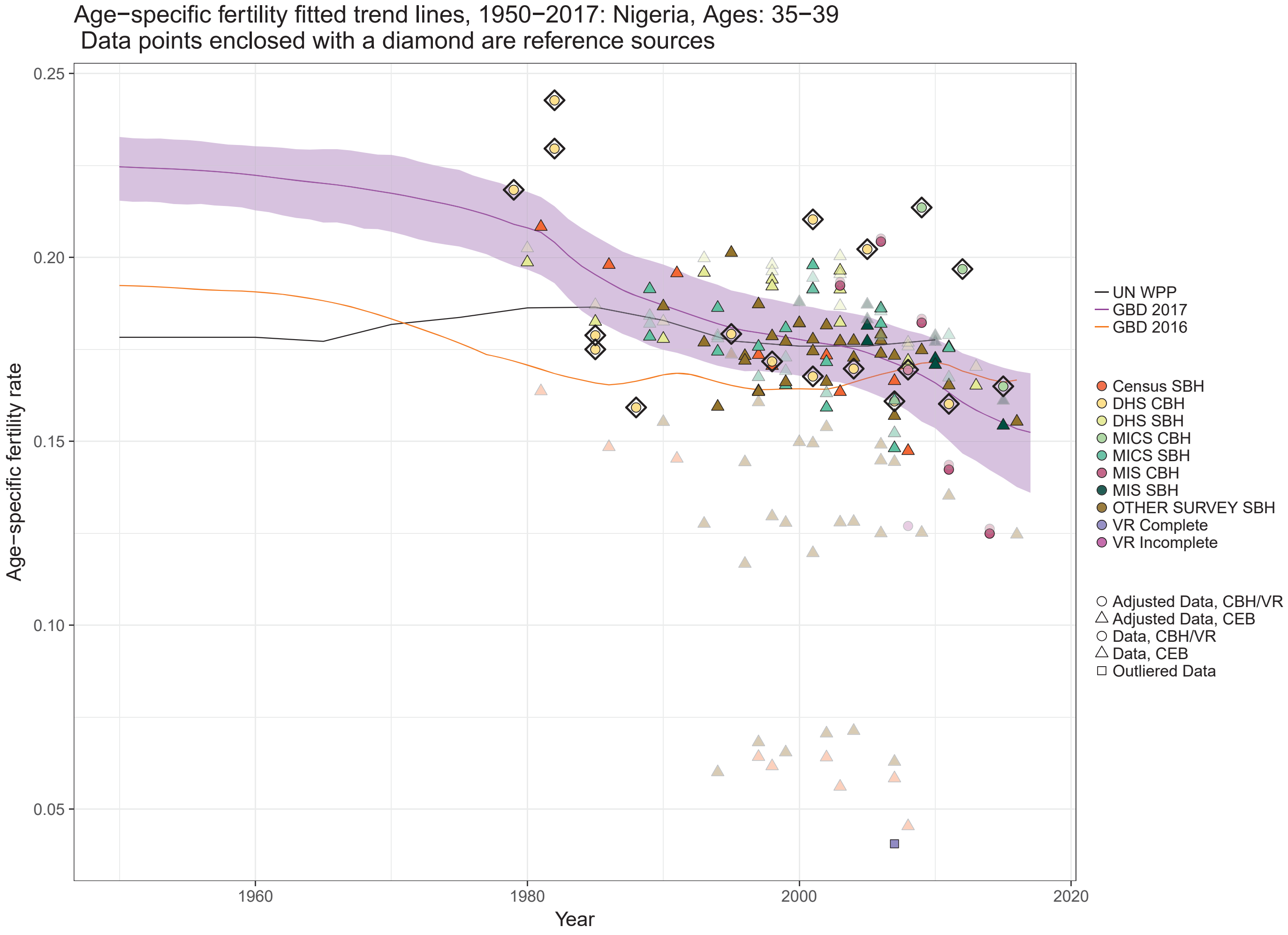

Age−specific fertility fitted trend lines, 1950−2017: Nigeria, Ages: 35−39
Data points enclosed with a diamond are reference sources
Age−specific fertility rate
0.25
0.20
0.15
0.10
0.05
1960
1980
2000
2020
Year
UN WPP
GBD 2017
GBD 2016
Census SBH
DHS CBH
DHS SBH
MICS CBH
MICS SBH
MIS CBH
MIS SBH
OTHER SURVEY SBH
VR Complete
VR Incomplete
Adjusted Data, CBH/VR
Adjusted Data, CEB
Data, CBH/VR
Data, CEB
Outliered Data

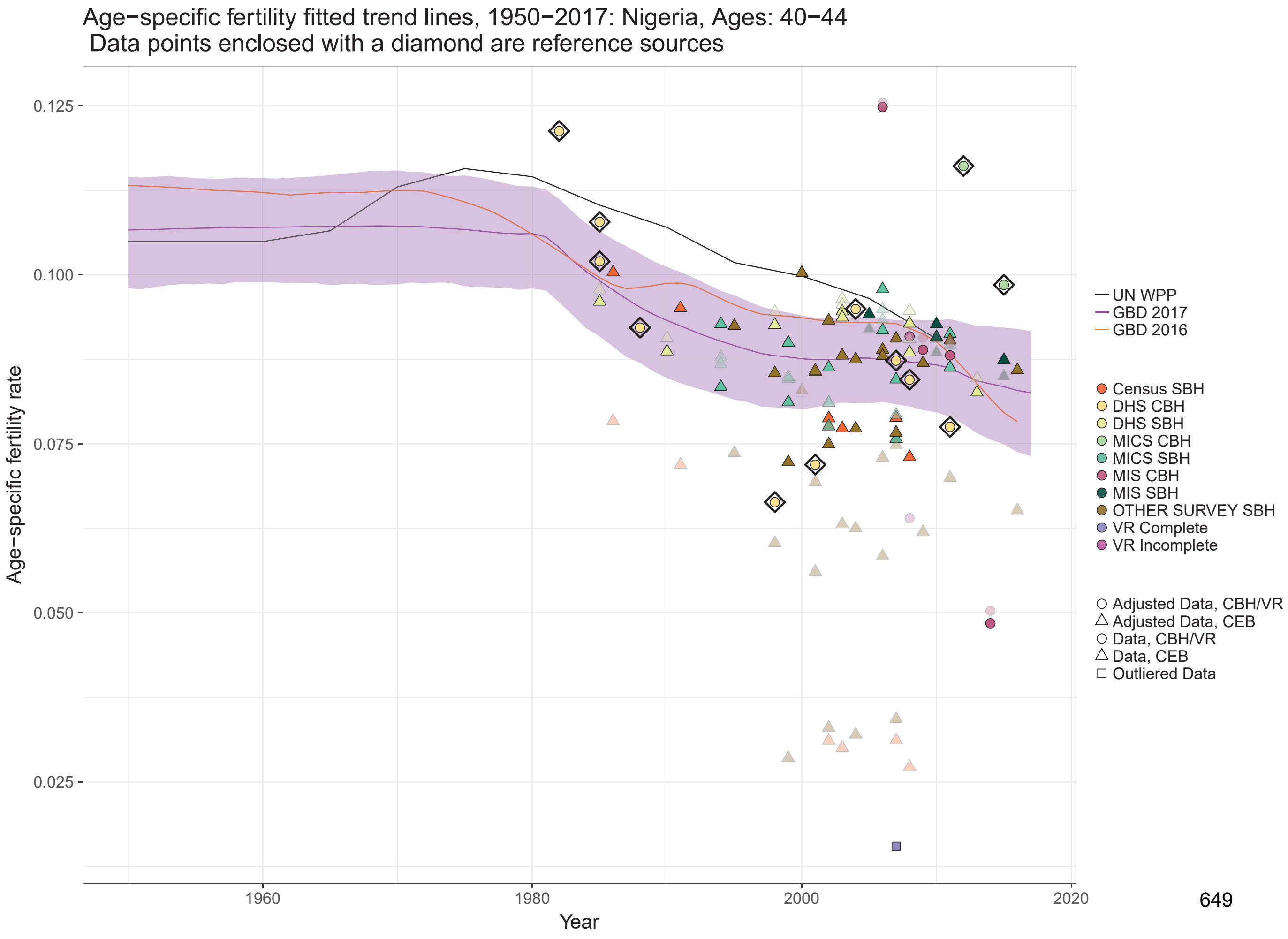

Age−specific fertility fitted trend lines, 1950−2017: Nigeria, Ages: 40−44
Data points enclosed with a diamond are reference sources
Age−specific fertility rate
0.125
0.100
0.075
0.050
0.025
1960
1980
2000
2020
Year
UN WPP
GBD 2017
GBD 2016
Census SBH
DHS CBH
DHS SBH
MICS CBH
MICS SBH
MIS CBH
MIS SBH
OTHER SURVEY SBH
VR Complete
VR Incomplete
Adjusted Data, CBH/VR
Adjusted Data, CEB
Data, CBH/VR
Data, CEB
Outliered Data

Age−specific fertility fitted trend lines, 1950−2017: Nigeria, Ages: 45−49
Data points enclosed with a diamond are reference sources

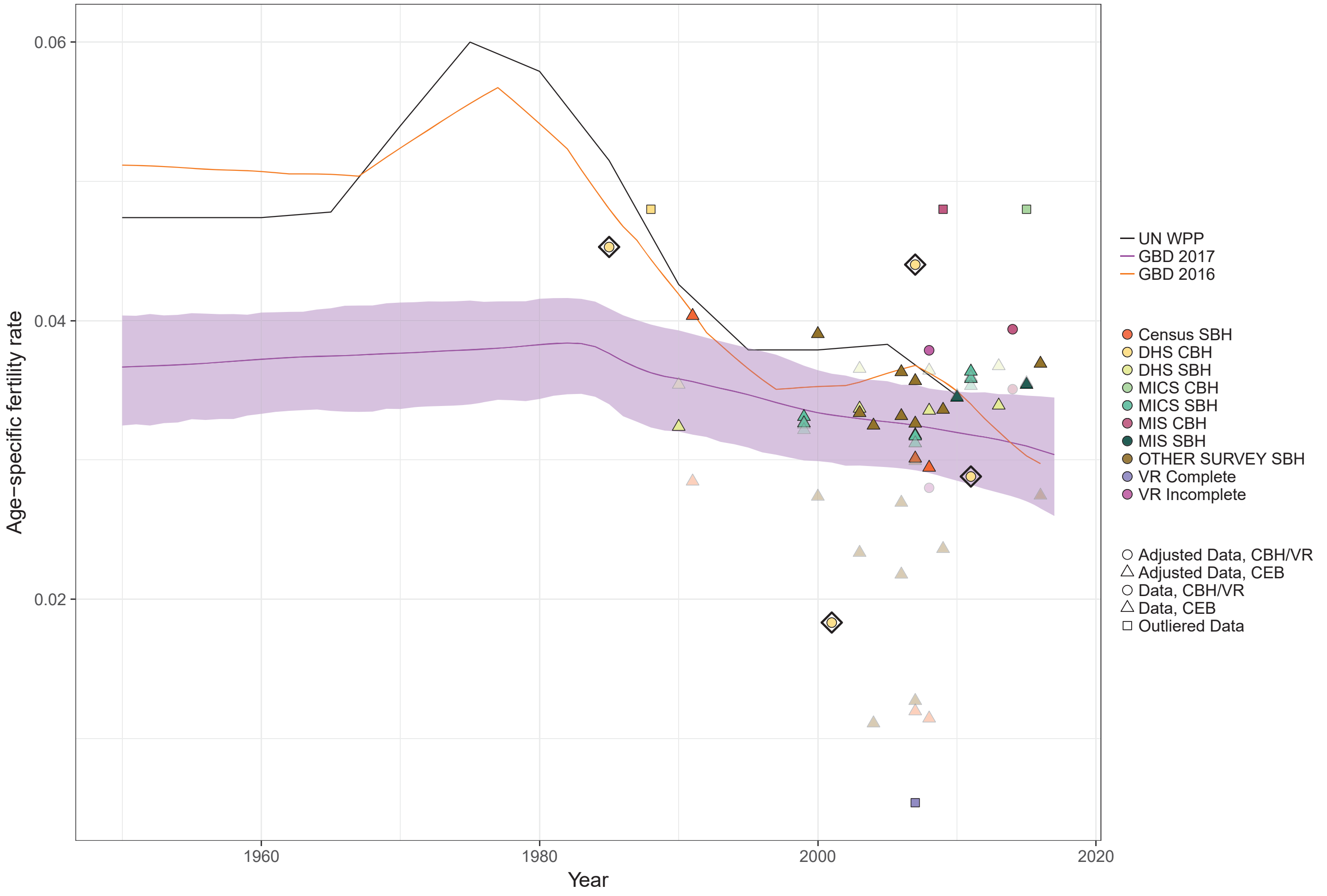

Age−specific fertility fitted trend lines, 1950−2017: Nigeria, Ages: 50−54
Data points enclosed with a diamond are reference sources

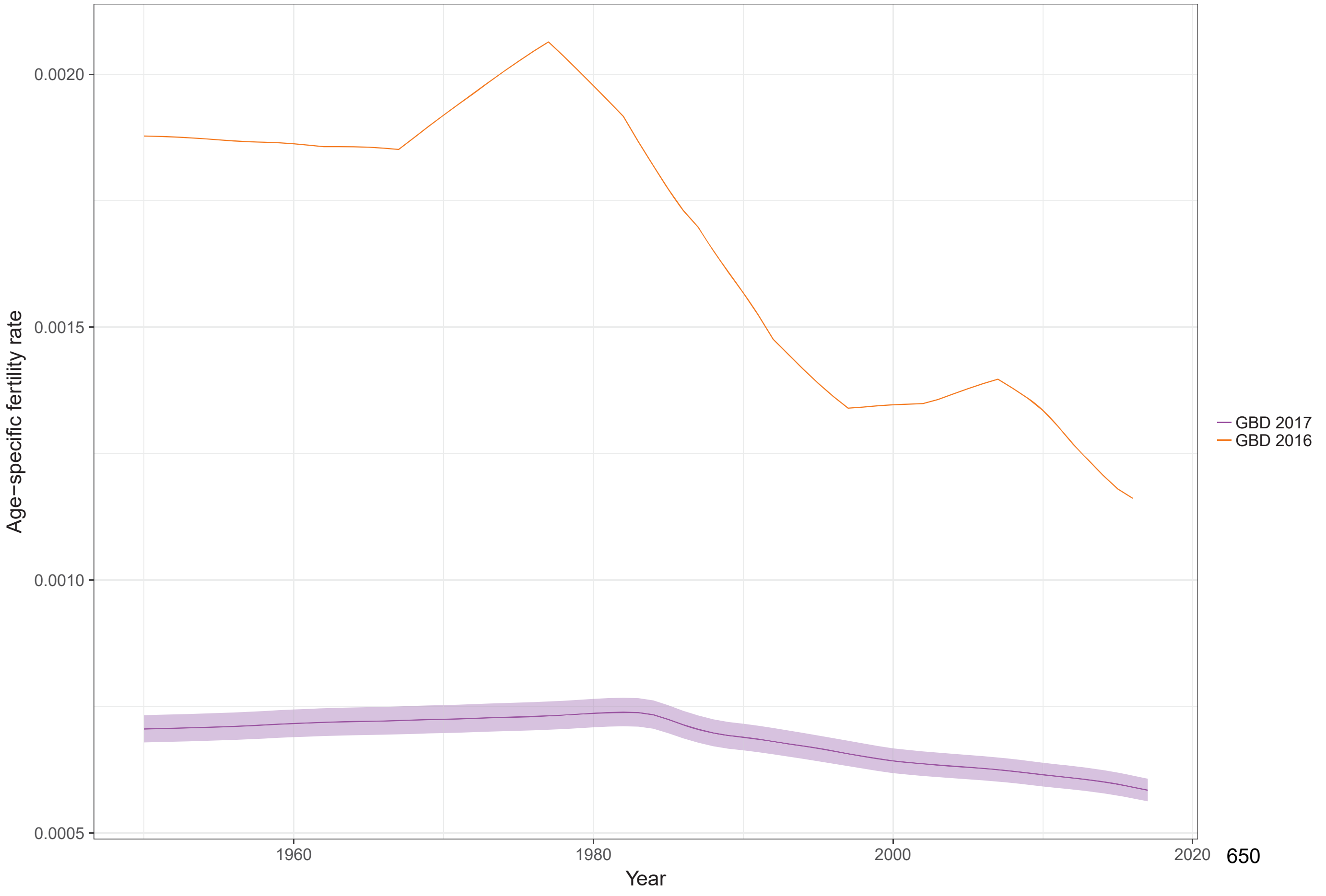

Age−specific fertility fitted trend lines, 1950−2017: North Korea, TFR
Data points enclosed with a diamond are reference sources

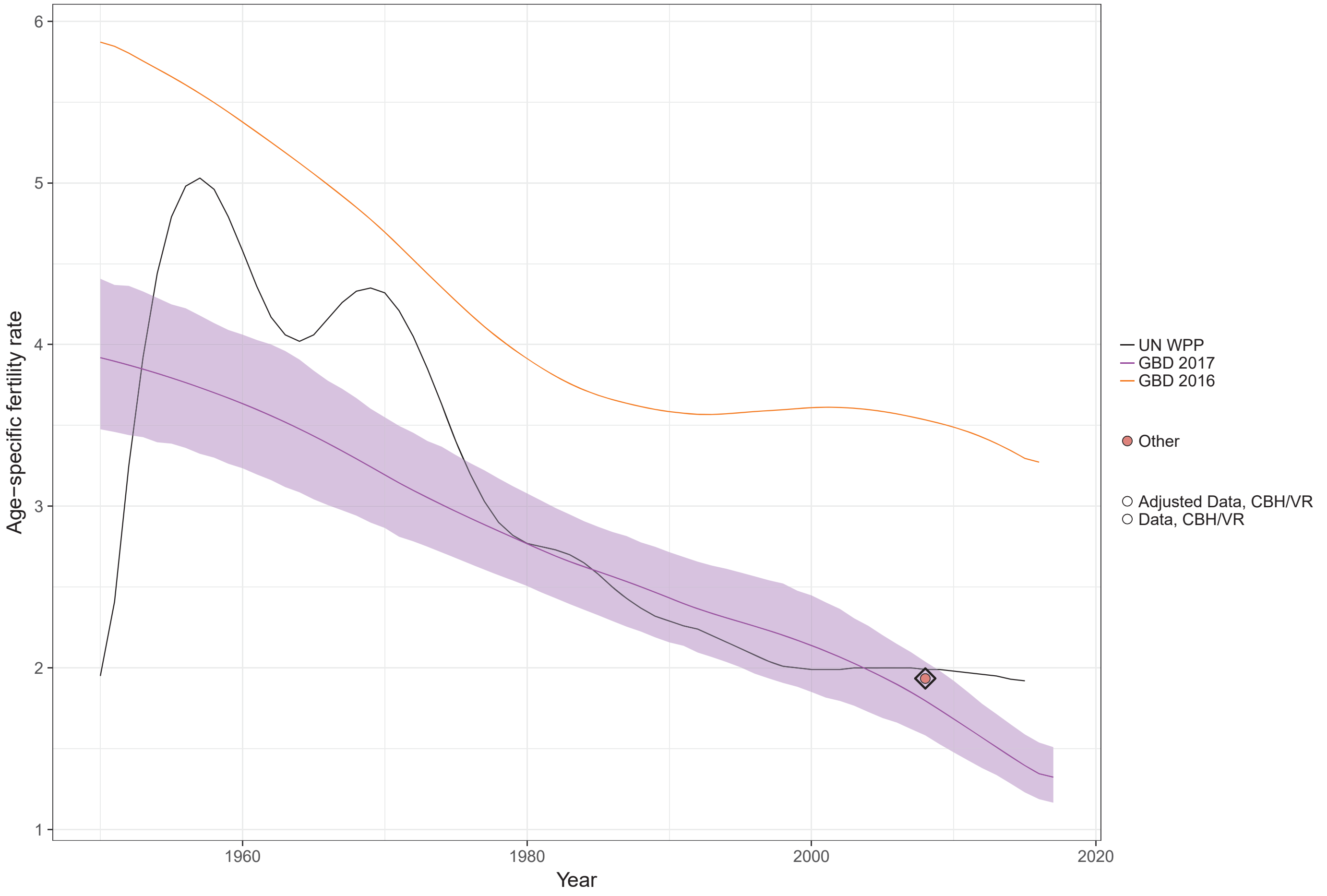

Age−specific fertility fitted trend lines, 1950−2017: North Korea, Ages: 10−14
Data points enclosed with a diamond are reference sources

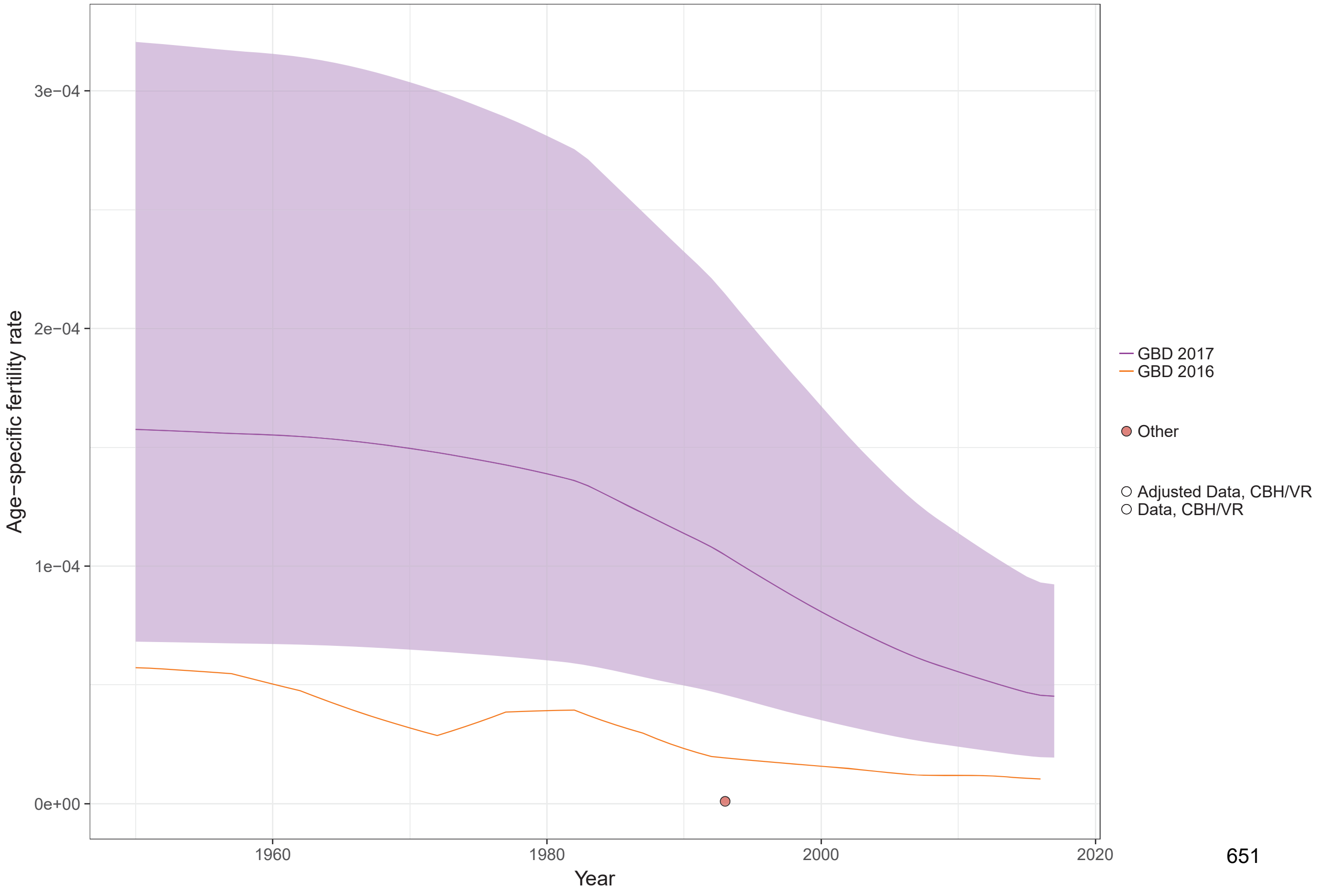

Age−specific fertility fitted trend lines, 1950−2017: North Korea, Ages: 15−19
Data points enclosed with a diamond are reference sources

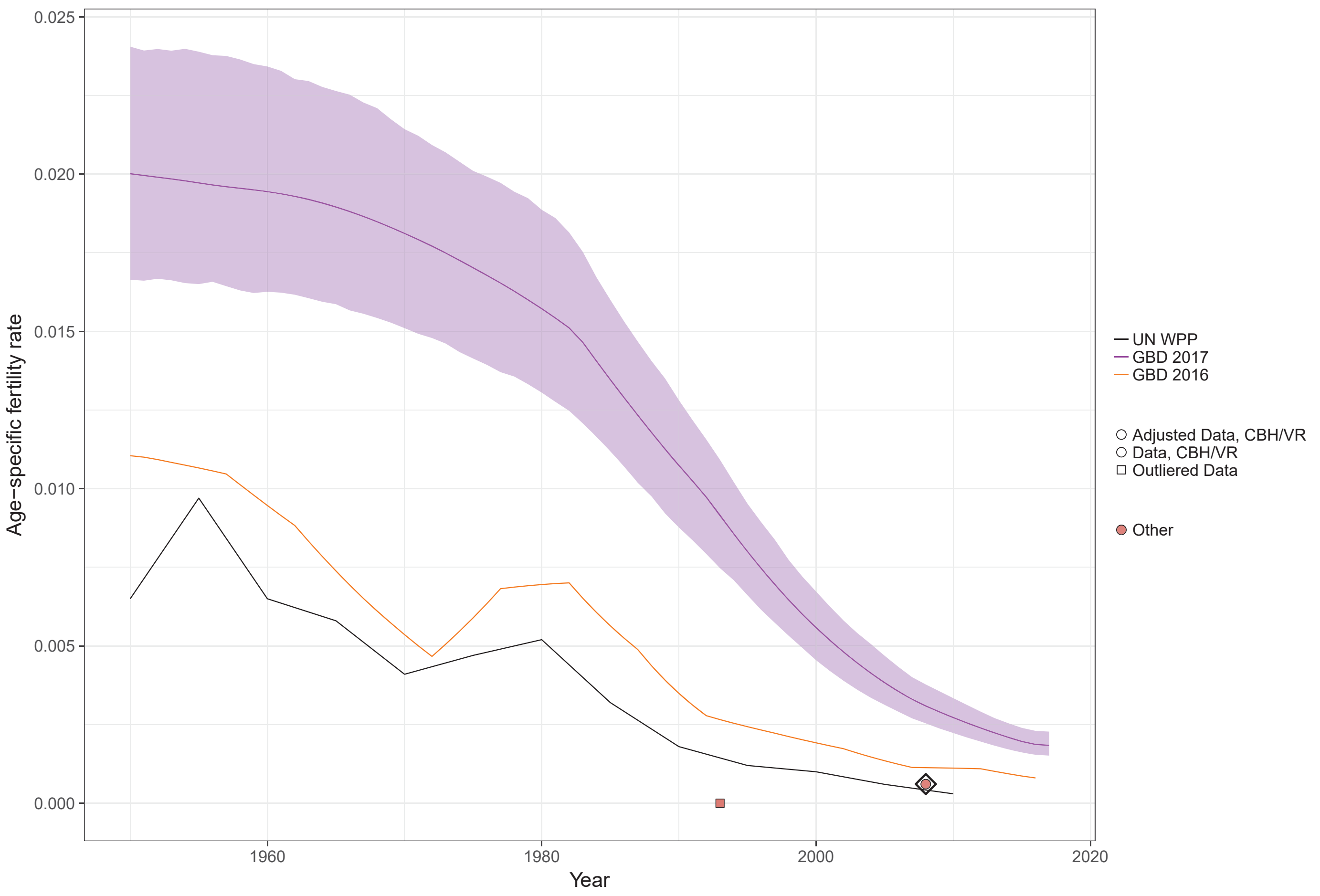

Age−specific fertility fitted trend lines, 1950−2017: North Korea, Ages: 20−24
Data points enclosed with a diamond are reference sources

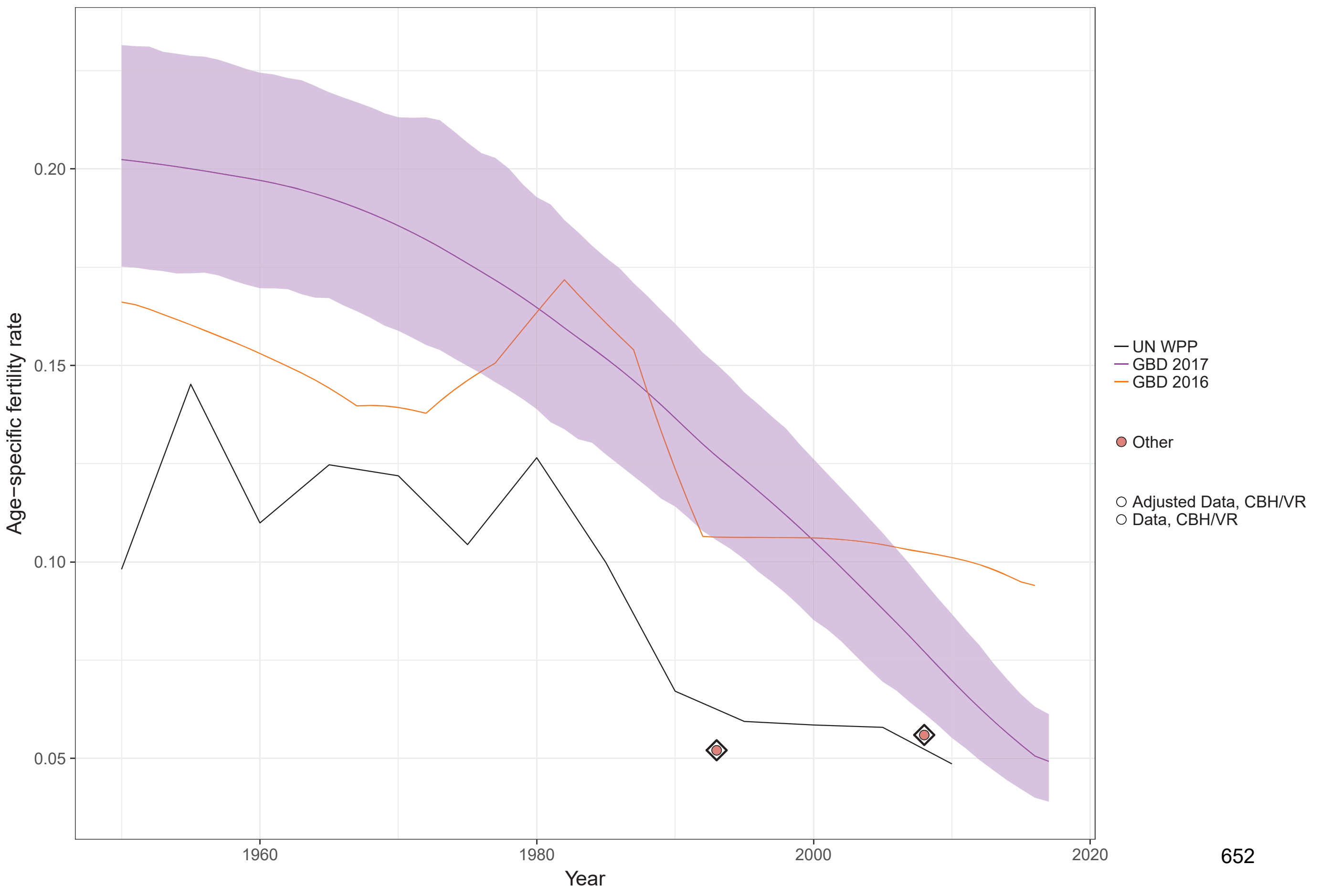

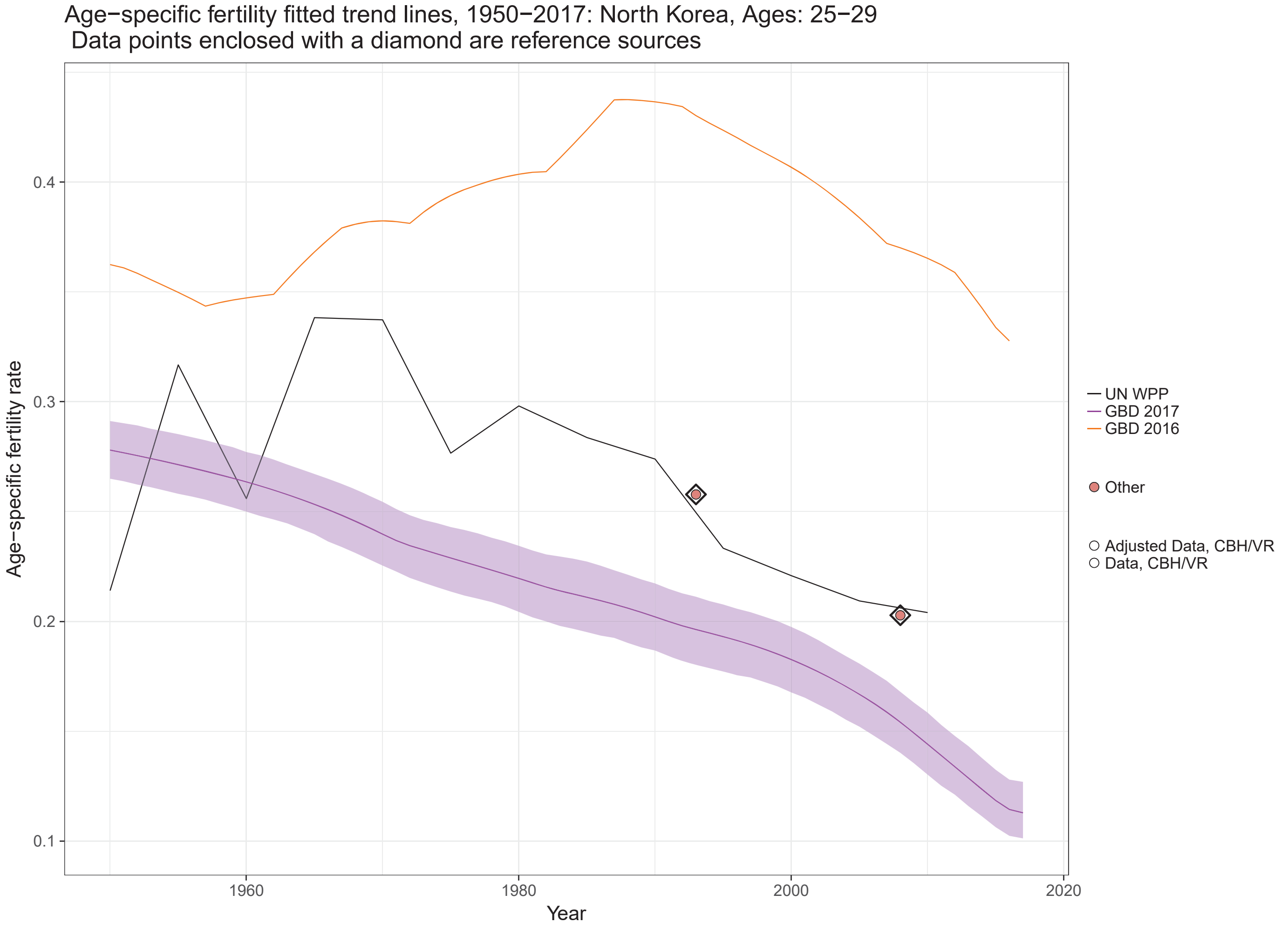

Age−specific fertility fitted trend lines, 1950−2017: North Korea, Ages: 25−29
Data points enclosed with a diamond are reference sources
Age−specific fertility rate
0.1
0.2
0.3
0.4
1960
1980
2000
2020
Year
UN WPP
GBD 2017
GBD 2016
Other
Adjusted Data, CBH/VR
Data, CBH/VR

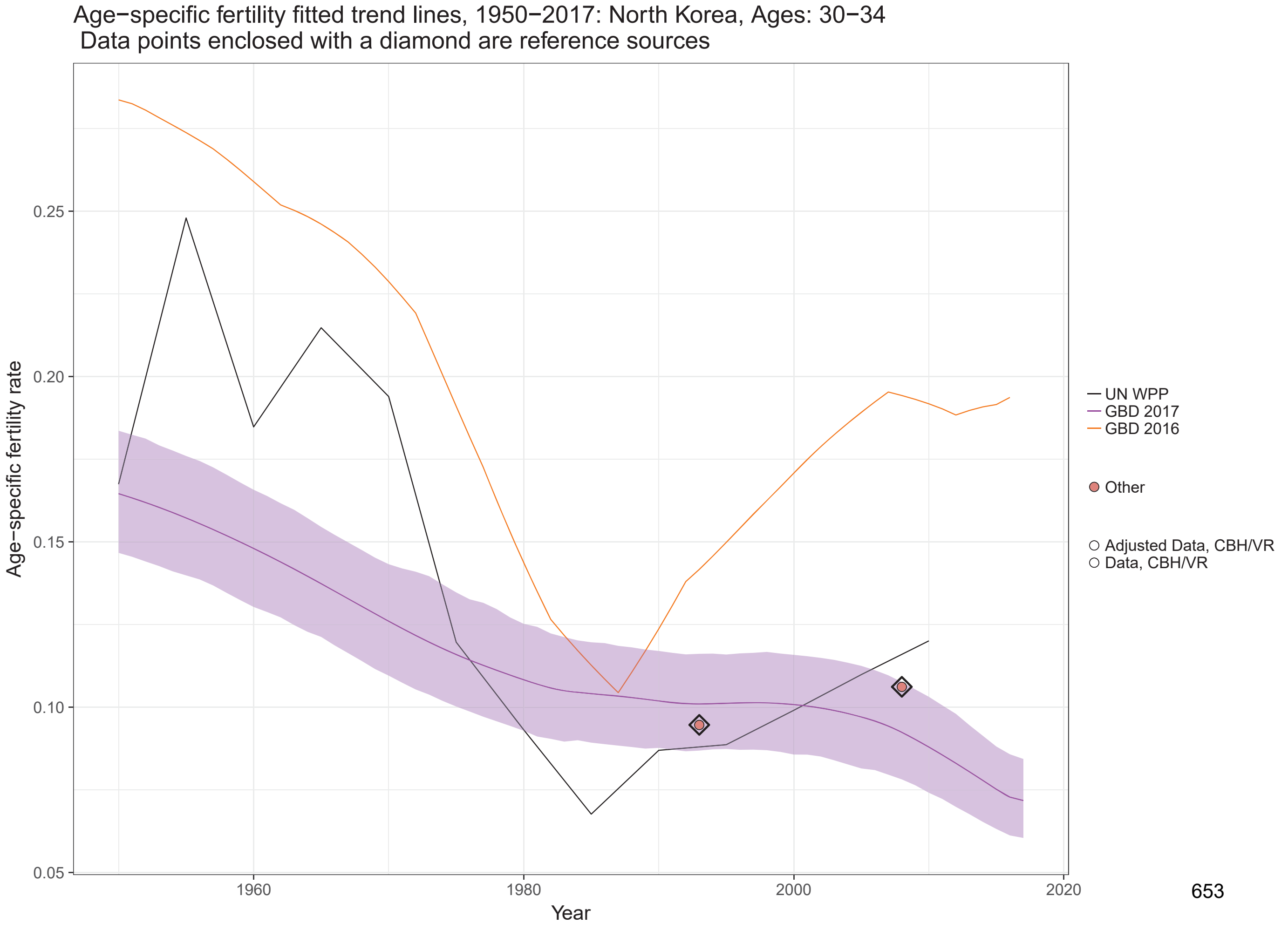

Age−specific fertility fitted trend lines, 1950−2017: North Korea, Ages: 30−34
Data points enclosed with a diamond are reference sources
Age−specific fertility rate
0.05
0.10
0.15
0.20
0.25
1960
1980
2000
2020
Year
UN WPP
GBD 2017
GBD 2016
Other
Adjusted Data, CBH/VR
Data, CBH/VR

Age−specific fertility fitted trend lines, 1950−2017: North Korea, Ages: 35−39
Data points enclosed with a diamond are reference sources

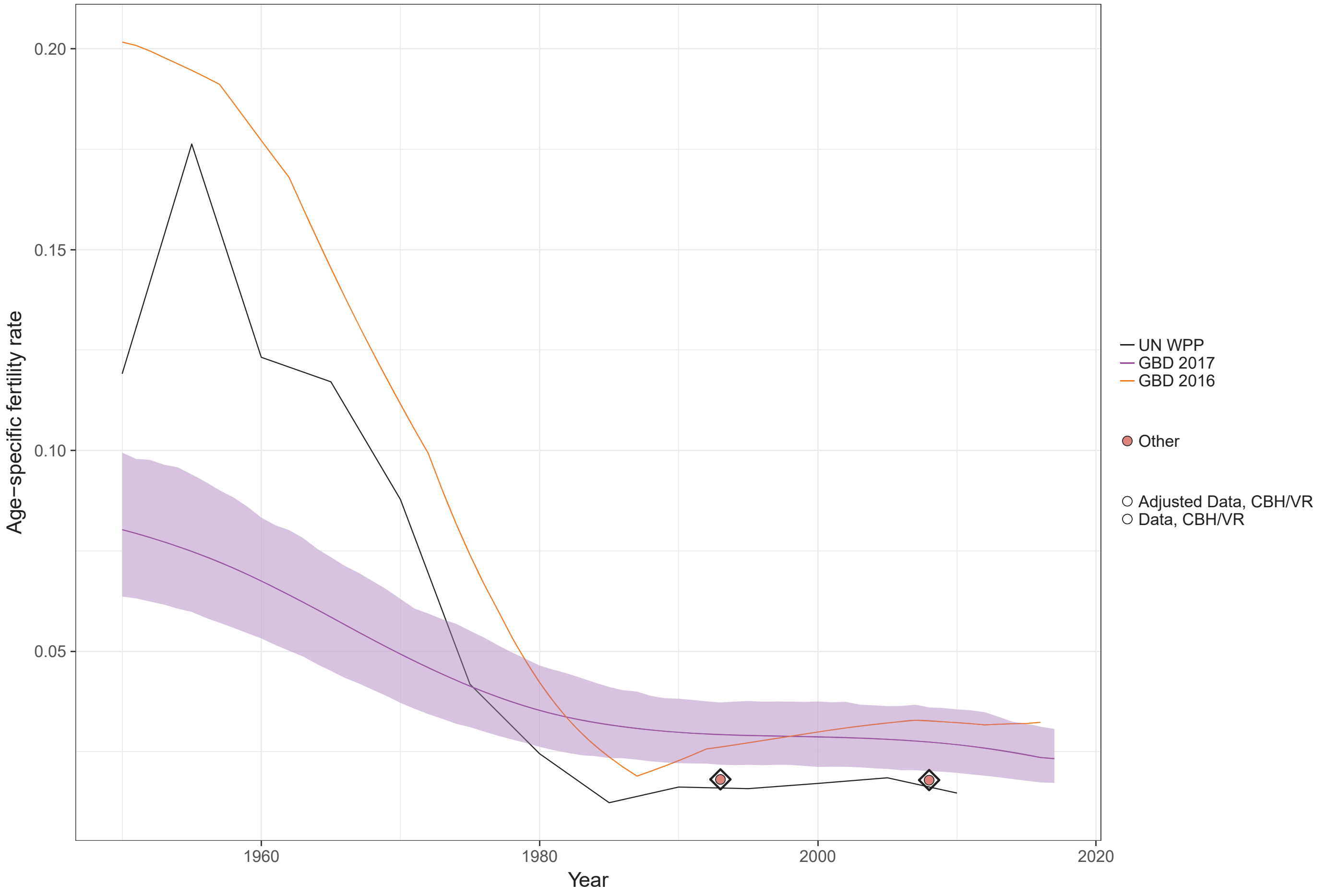

Age−specific fertility fitted trend lines, 1950−2017: North Korea, Ages: 40−44
Data points enclosed with a diamond are reference sources

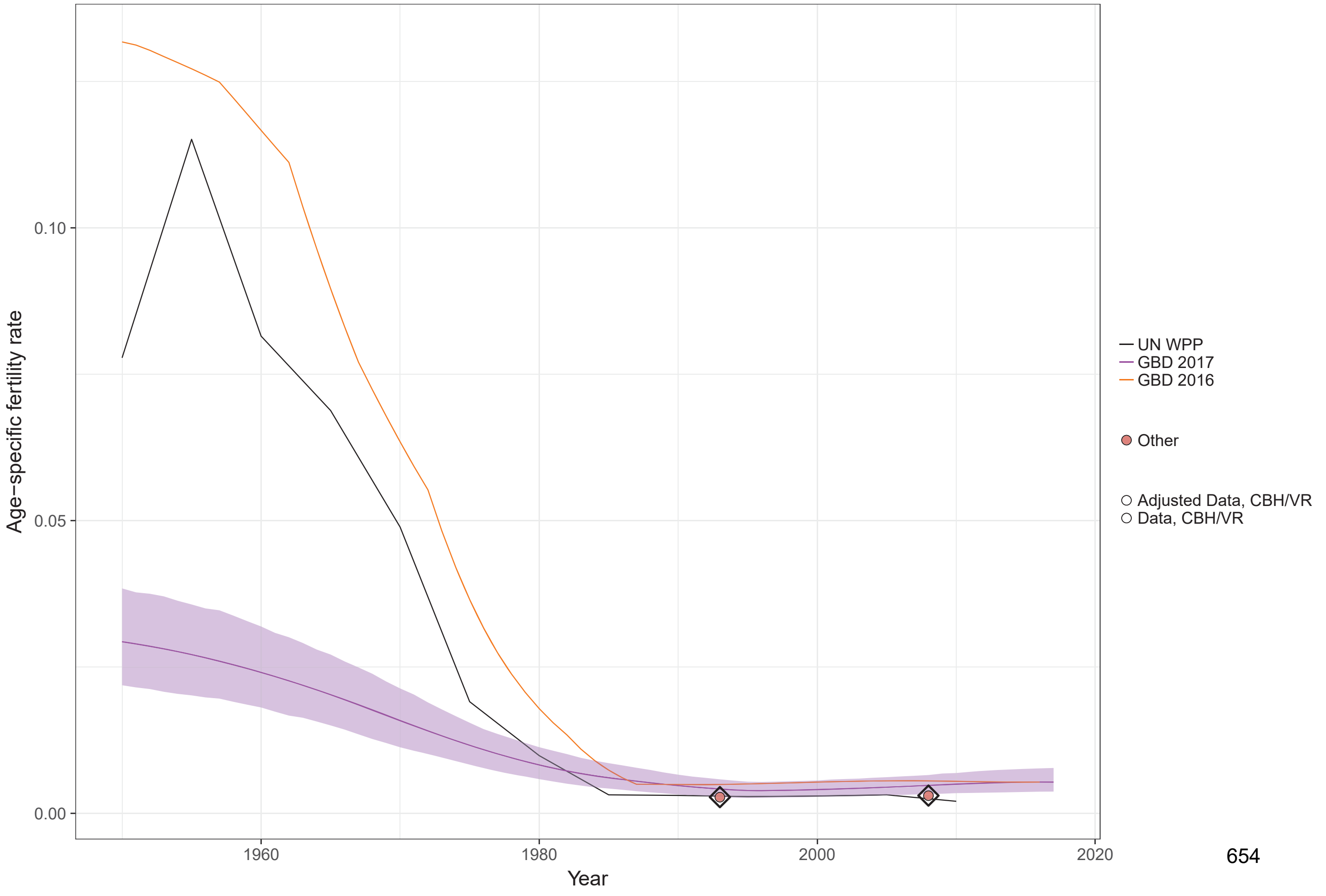

Age−specific fertility fitted trend lines, 1950−2017: North Korea, Ages: 45−49
Data points enclosed with a diamond are reference sources

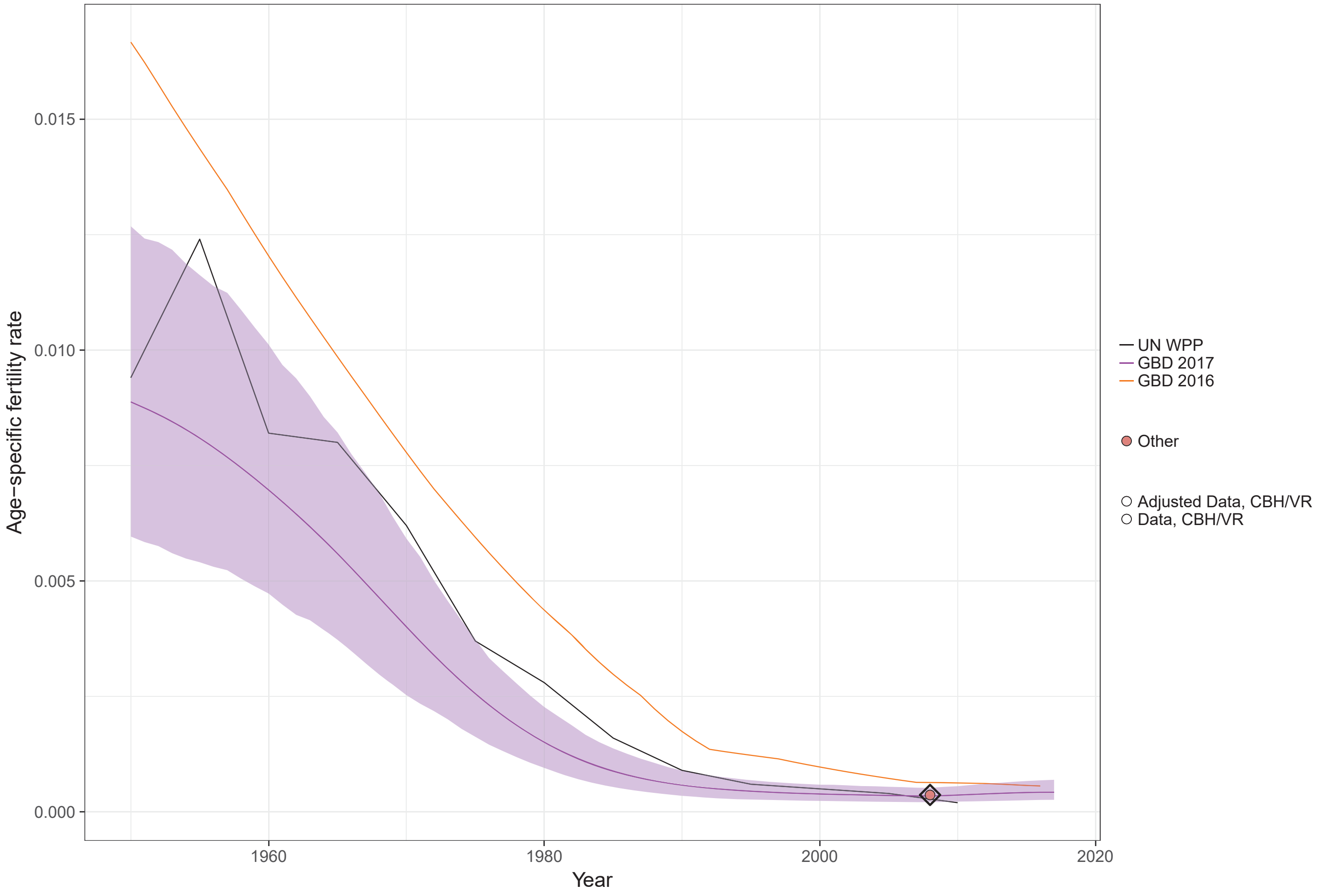

Age−specific fertility fitted trend lines, 1950−2017: North Korea, Ages: 50−54
Data points enclosed with a diamond are reference sources

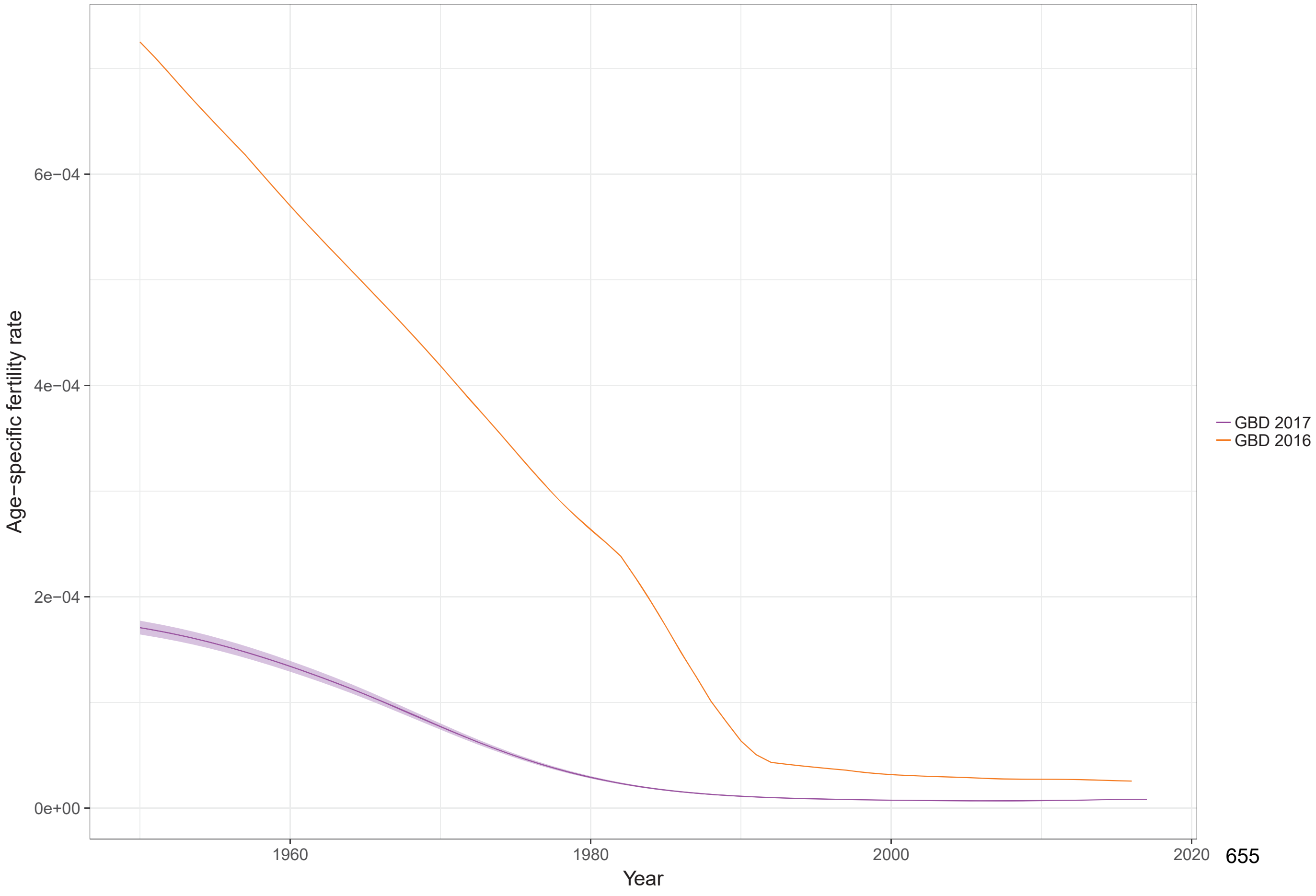

Age−specific fertility fitted trend lines, 1950−2017: Northern Ireland, TFR
Data points enclosed with a diamond are reference sources

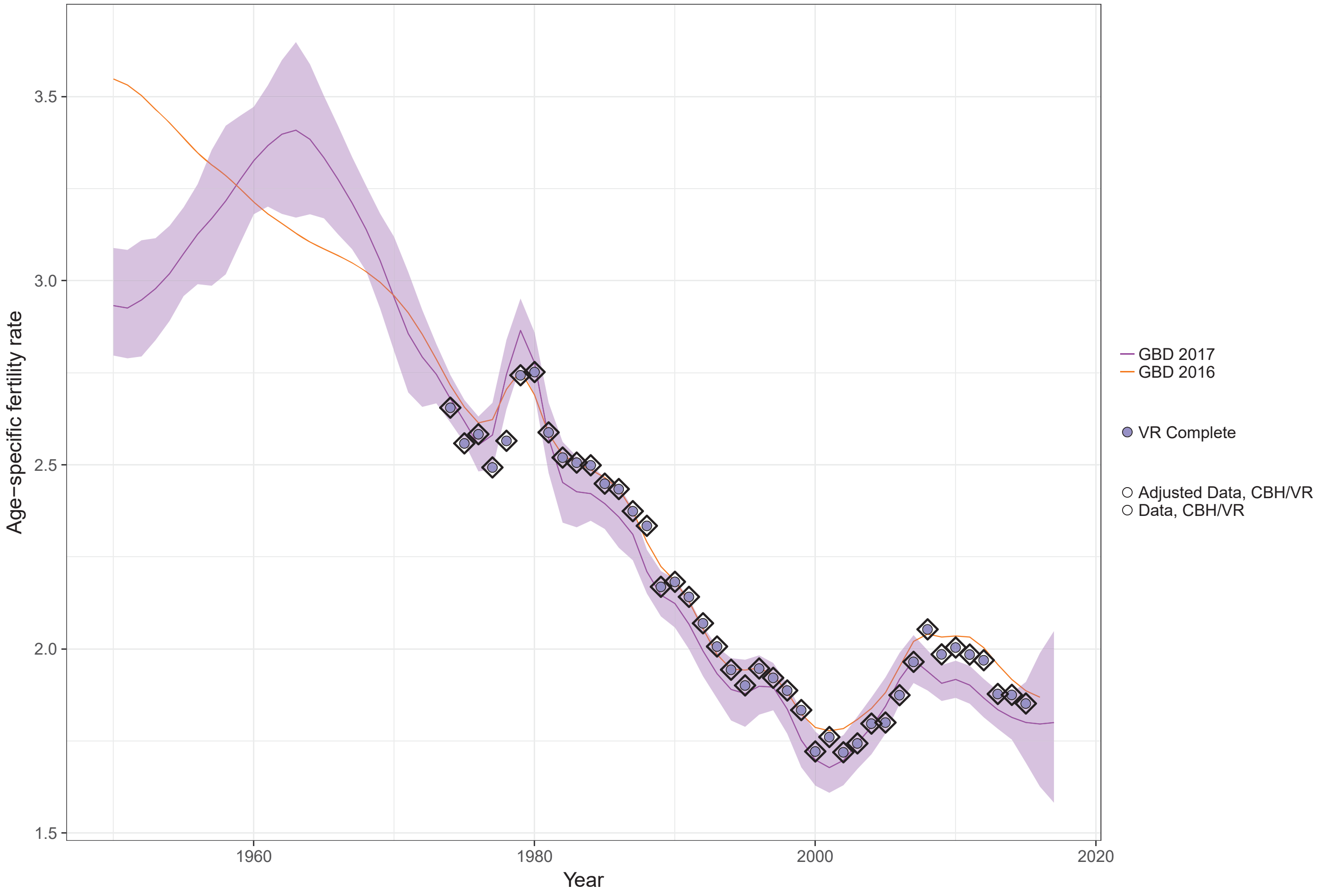

Age−specific fertility fitted trend lines, 1950−2017: Northern Ireland, Ages: 10−14
Data points enclosed with a diamond are reference sources

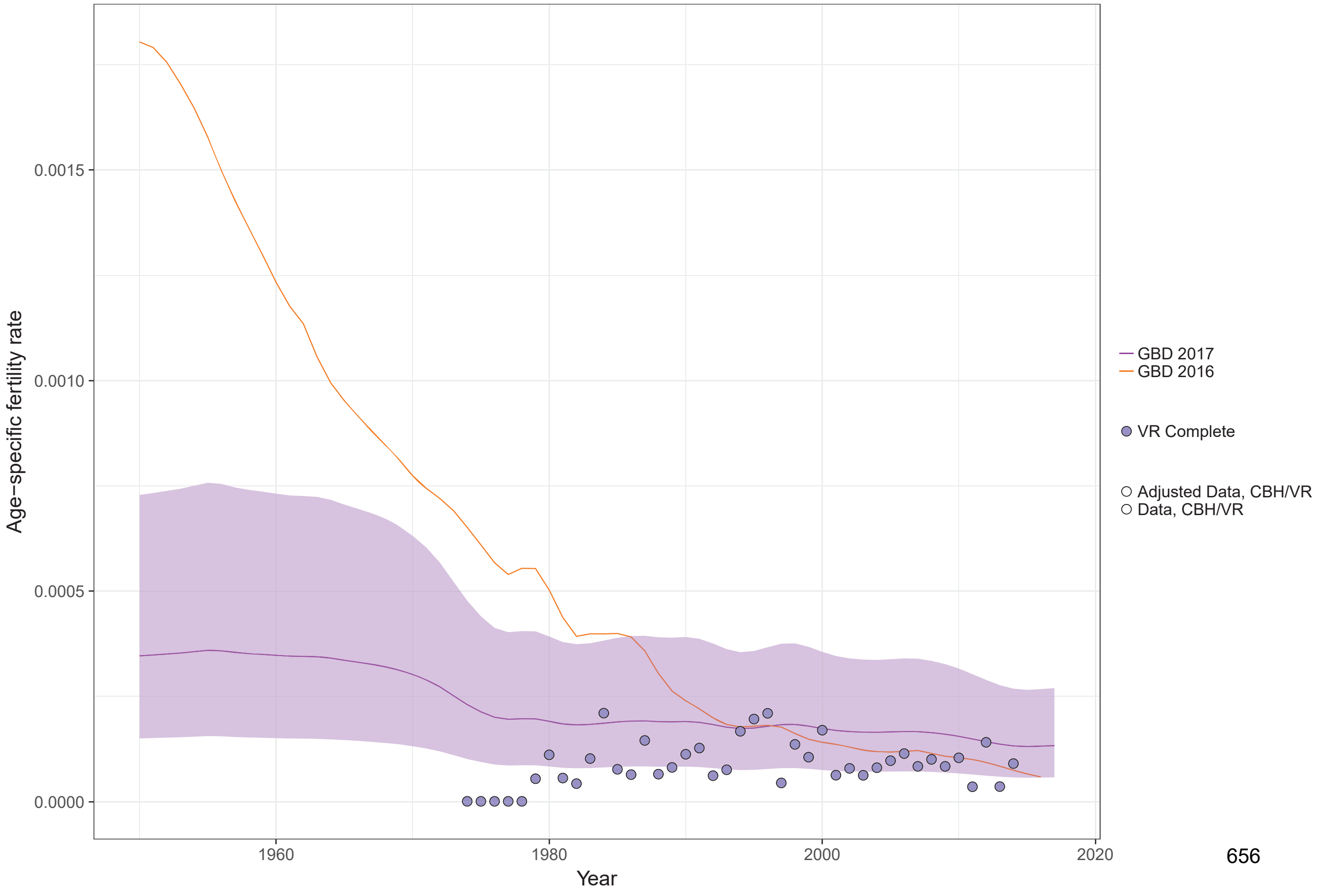

Age−specific fertility fitted trend lines, 1950−2017: Northern Ireland, Ages: 15−19
Data points enclosed with a diamond are reference sources

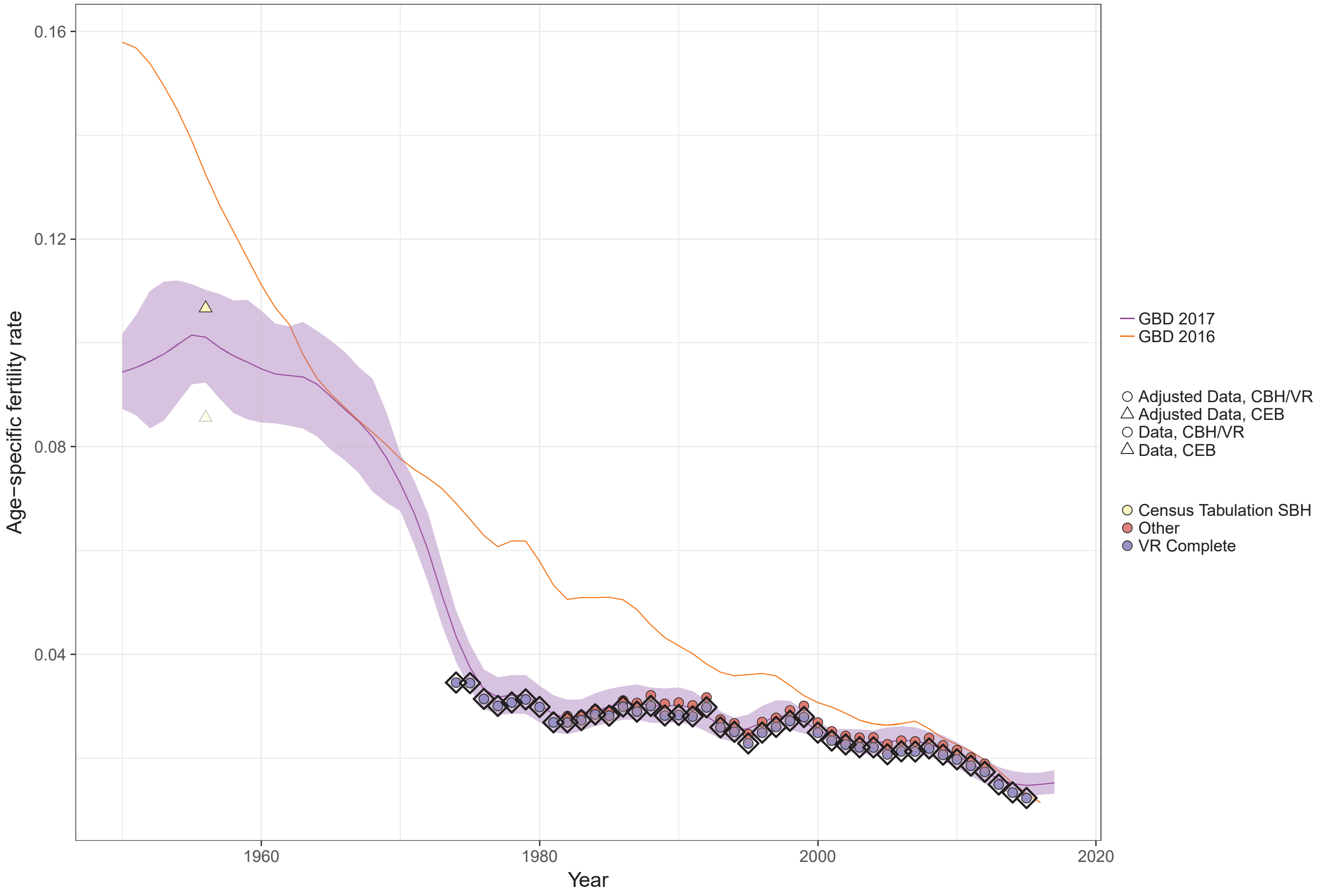

Age−specific fertility fitted trend lines, 1950−2017: Northern Ireland, Ages: 20−24
Data points enclosed with a diamond are reference sources

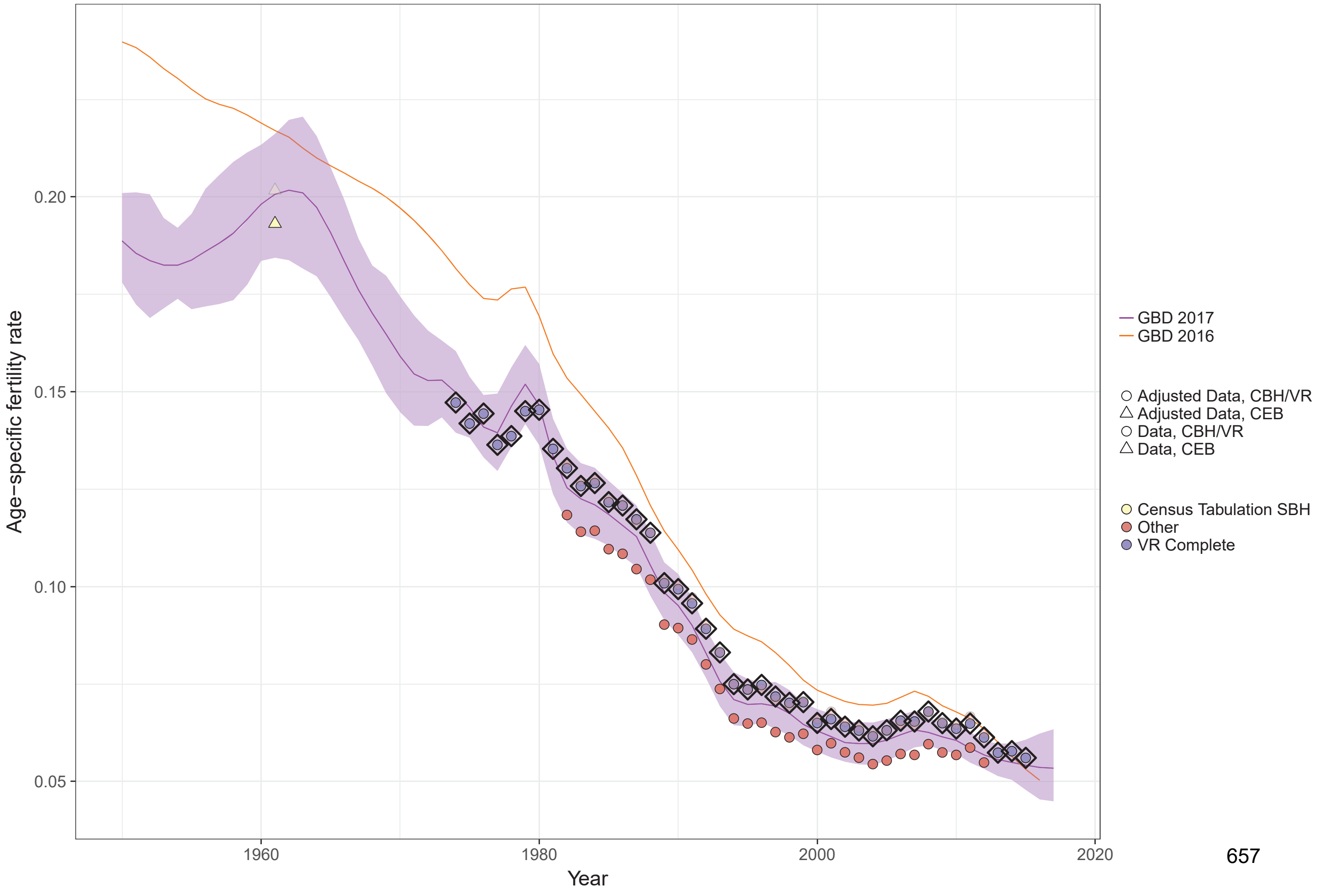

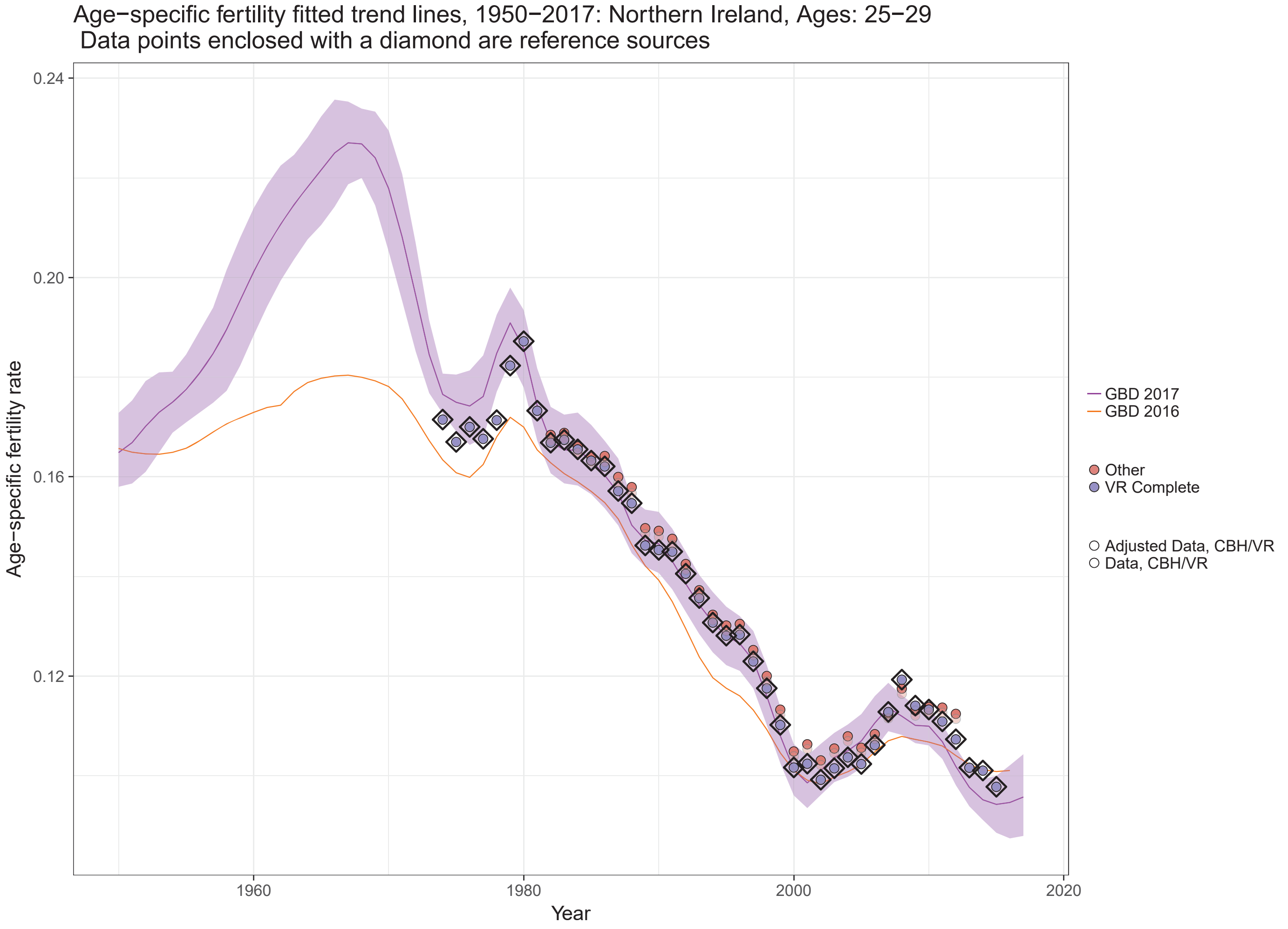

Age−specific fertility fitted trend lines, 1950−2017: Northern Ireland, Ages: 25−29
Data points enclosed with a diamond are reference sources
Age−specific fertility rate
0.24
0.20
0.16
0.12
1960
1980
2000
2020
Year
GBD 2017
GBD 2016
Other
VR Complete
Adjusted Data, CBH/VR
Data, CBH/VR

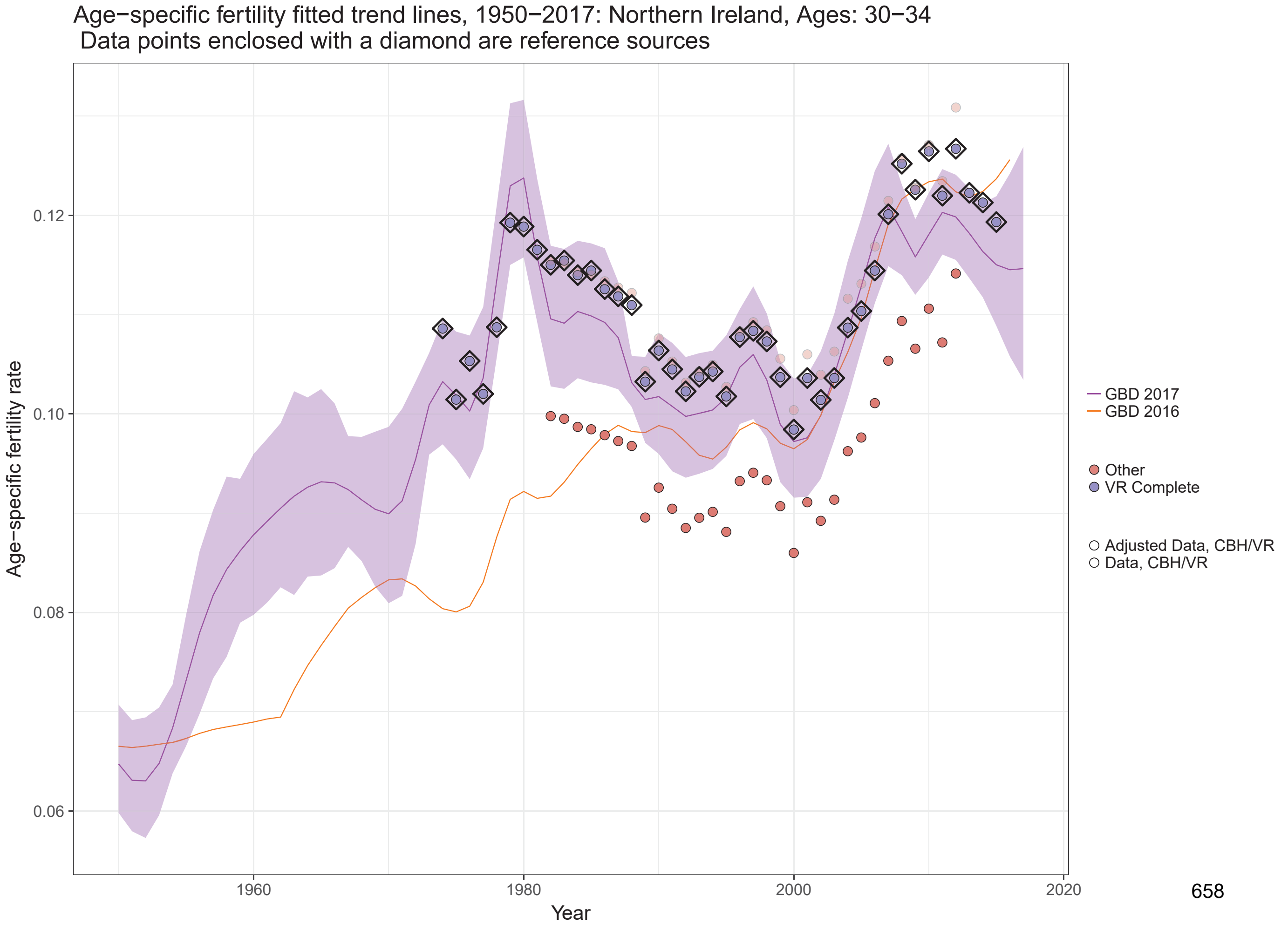

Age−specific fertility fitted trend lines, 1950−2017: Northern Ireland, Ages: 30−34
Data points enclosed with a diamond are reference sources
Age−specific fertility rate
0.12
0.10
0.08
0.06
1960
1980
2000
2020
Year
GBD 2017
GBD 2016
Other
VR Complete
Adjusted Data, CBH/VR
Data, CBH/VR

Age−specific fertility fitted trend lines, 1950−2017: Northern Ireland, Ages: 35−39
Data points enclosed with a diamond are reference sources

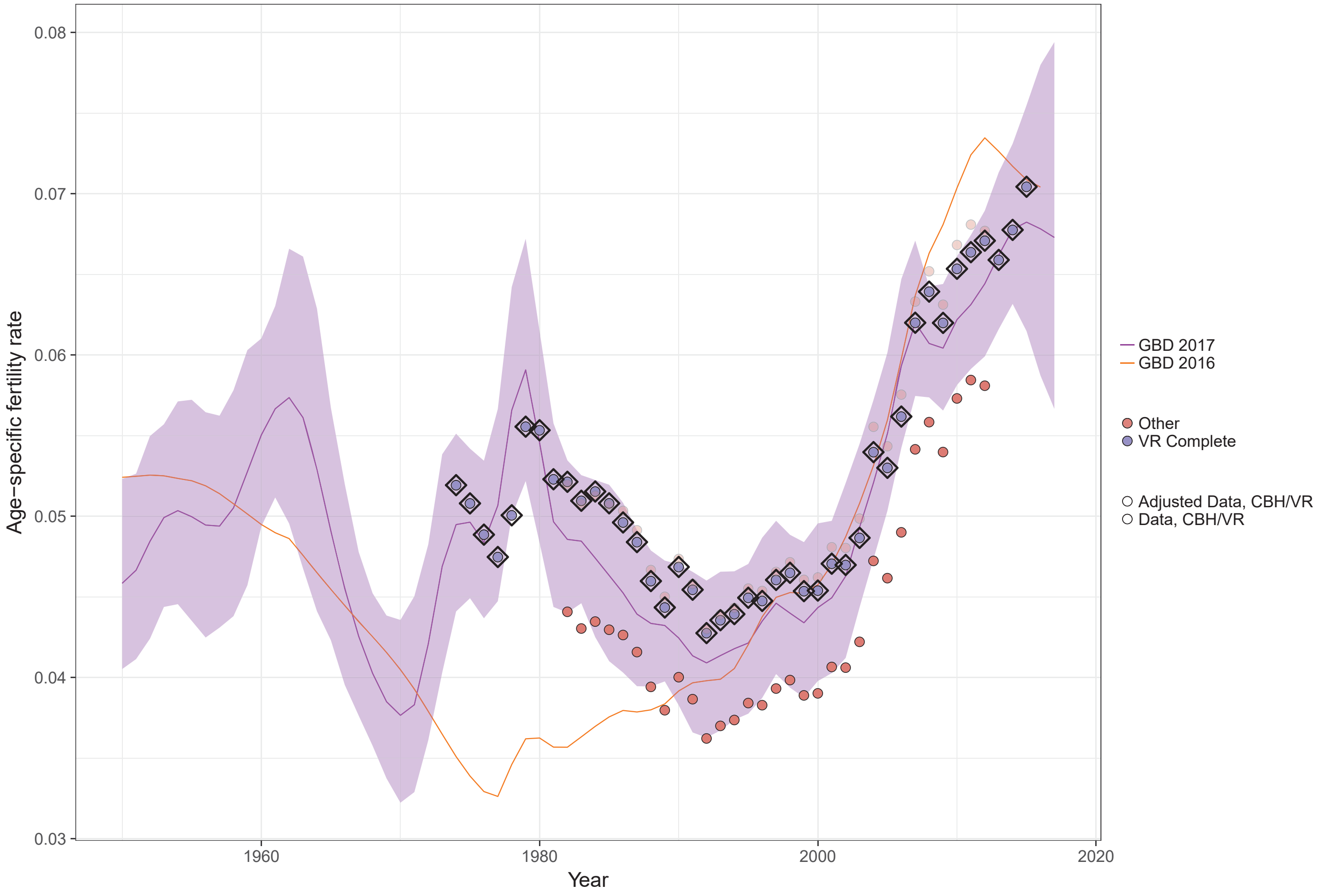

Age−specific fertility fitted trend lines, 1950−2017: Northern Ireland, Ages: 40−44
Data points enclosed with a diamond are reference sources

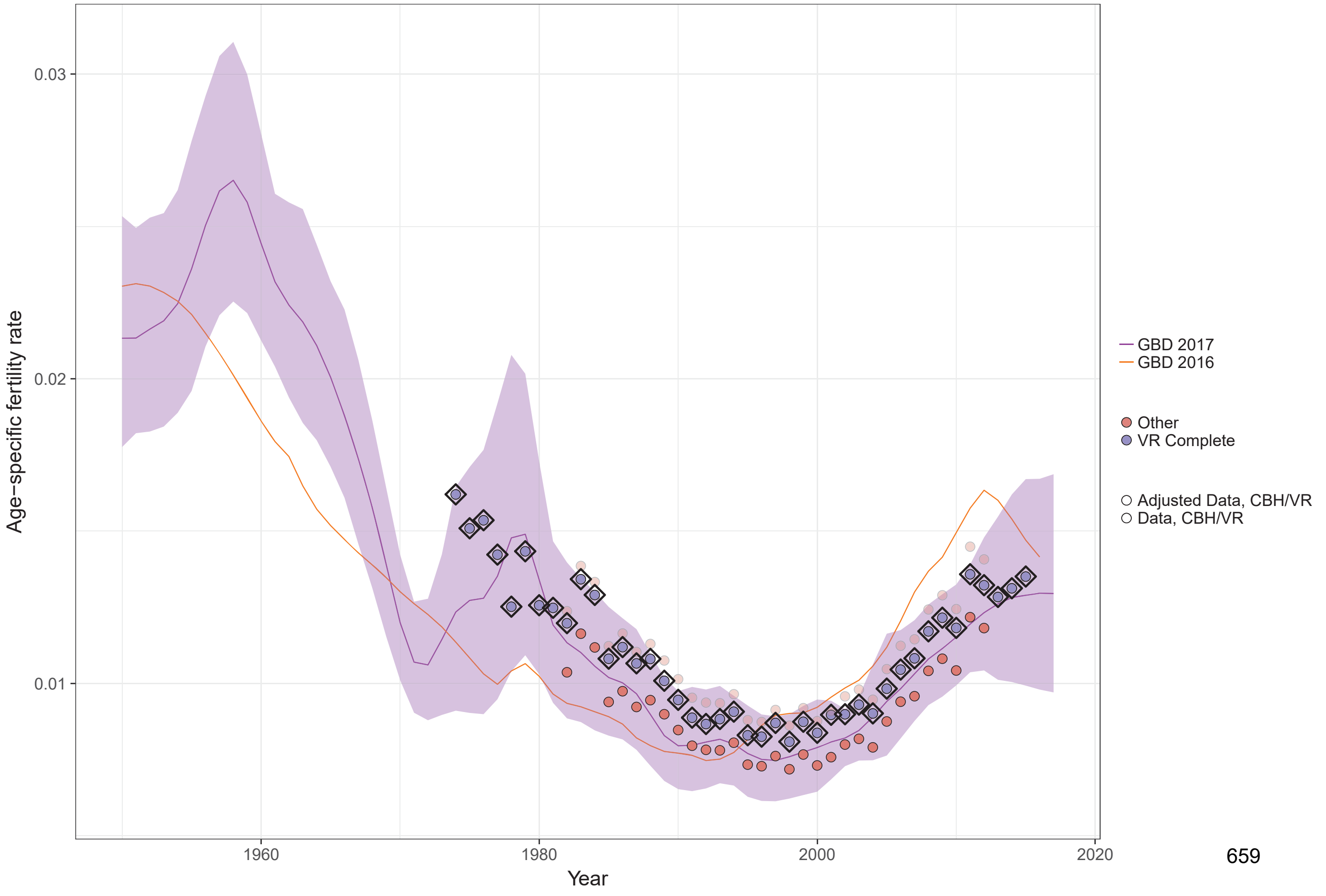

Age−specific fertility fitted trend lines, 1950−2017: Northern Ireland, Ages: 45−49
Data points enclosed with a diamond are reference sources

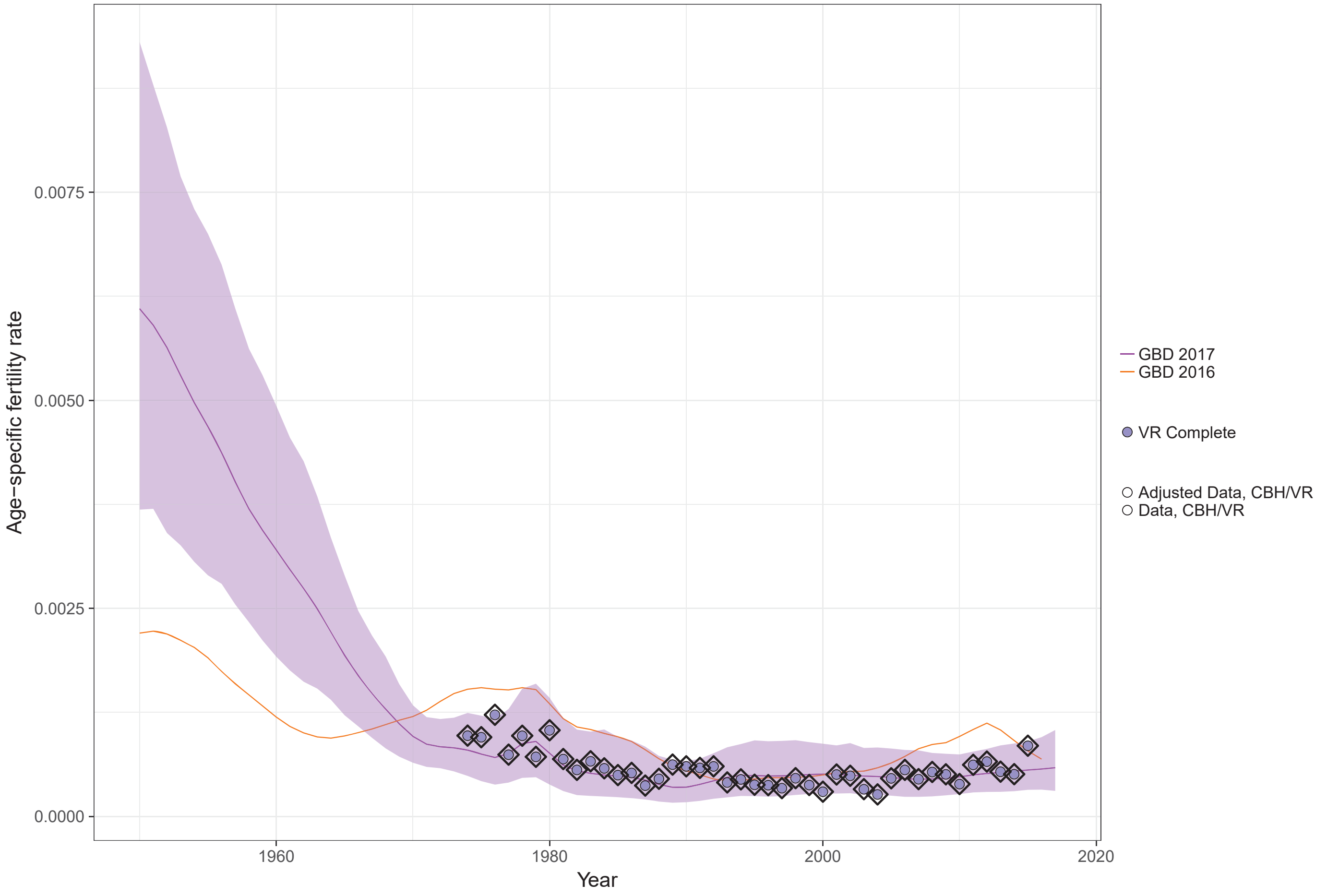

Age−specific fertility fitted trend lines, 1950−2017: Northern Ireland, Ages: 50−54
Data points enclosed with a diamond are reference sources

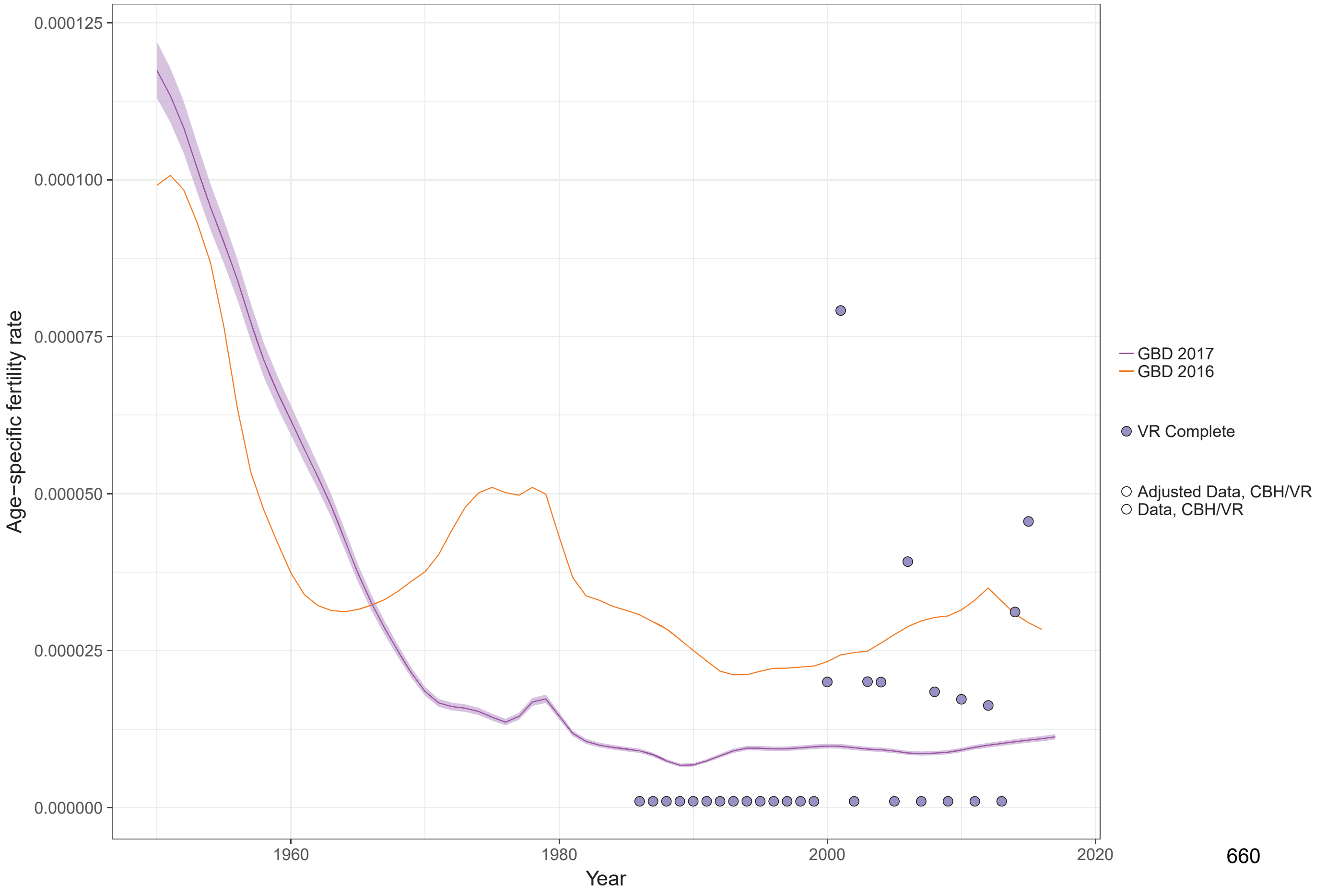

Age−specific fertility fitted trend lines, 1950−2017: Northern Mariana Islands, TFR
Data points enclosed with a diamond are reference sources

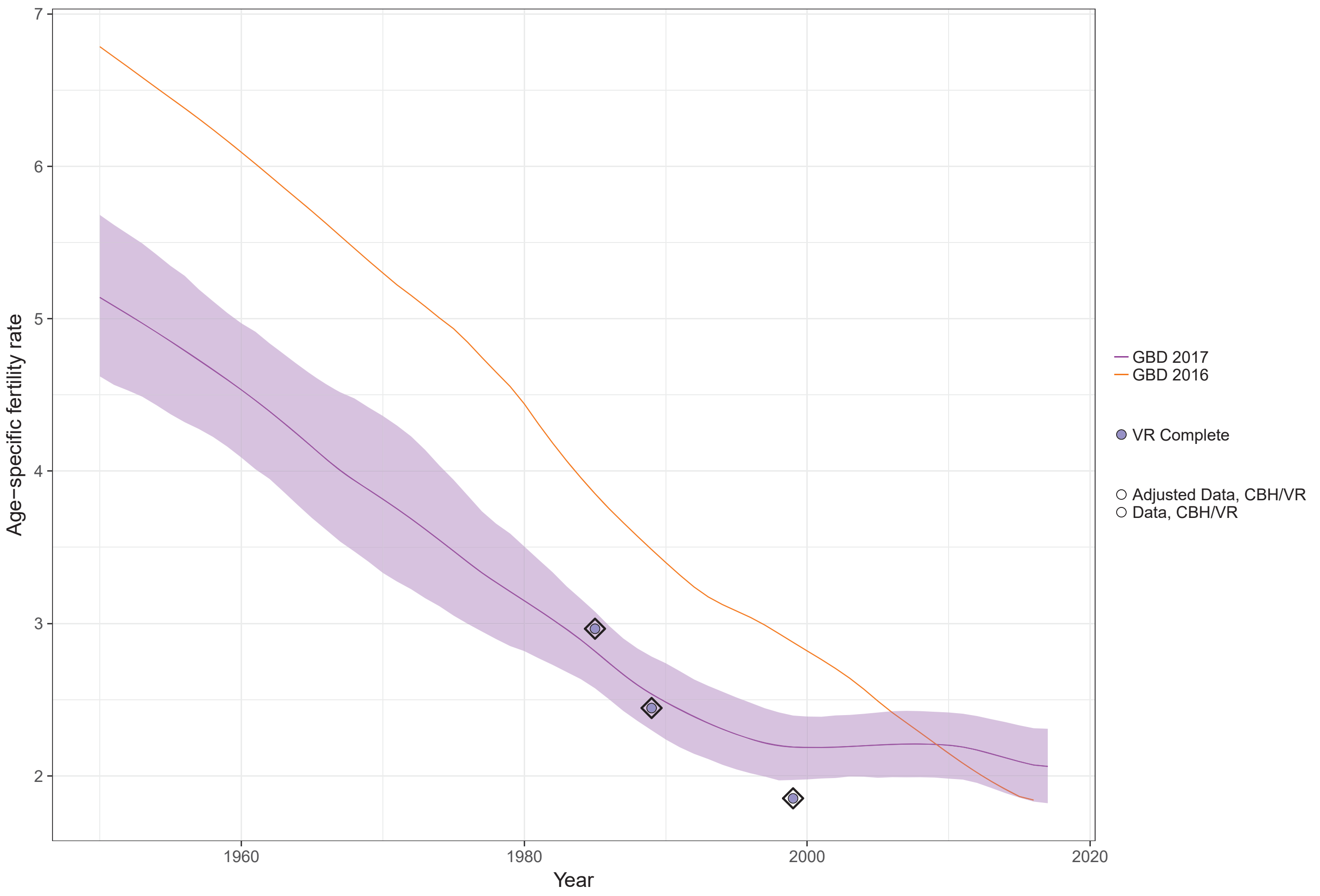

Age−specific fertility fitted trend lines, 1950−2017: Northern Mariana Islands, Ages: 10−14
Data points enclosed with a diamond are reference sources

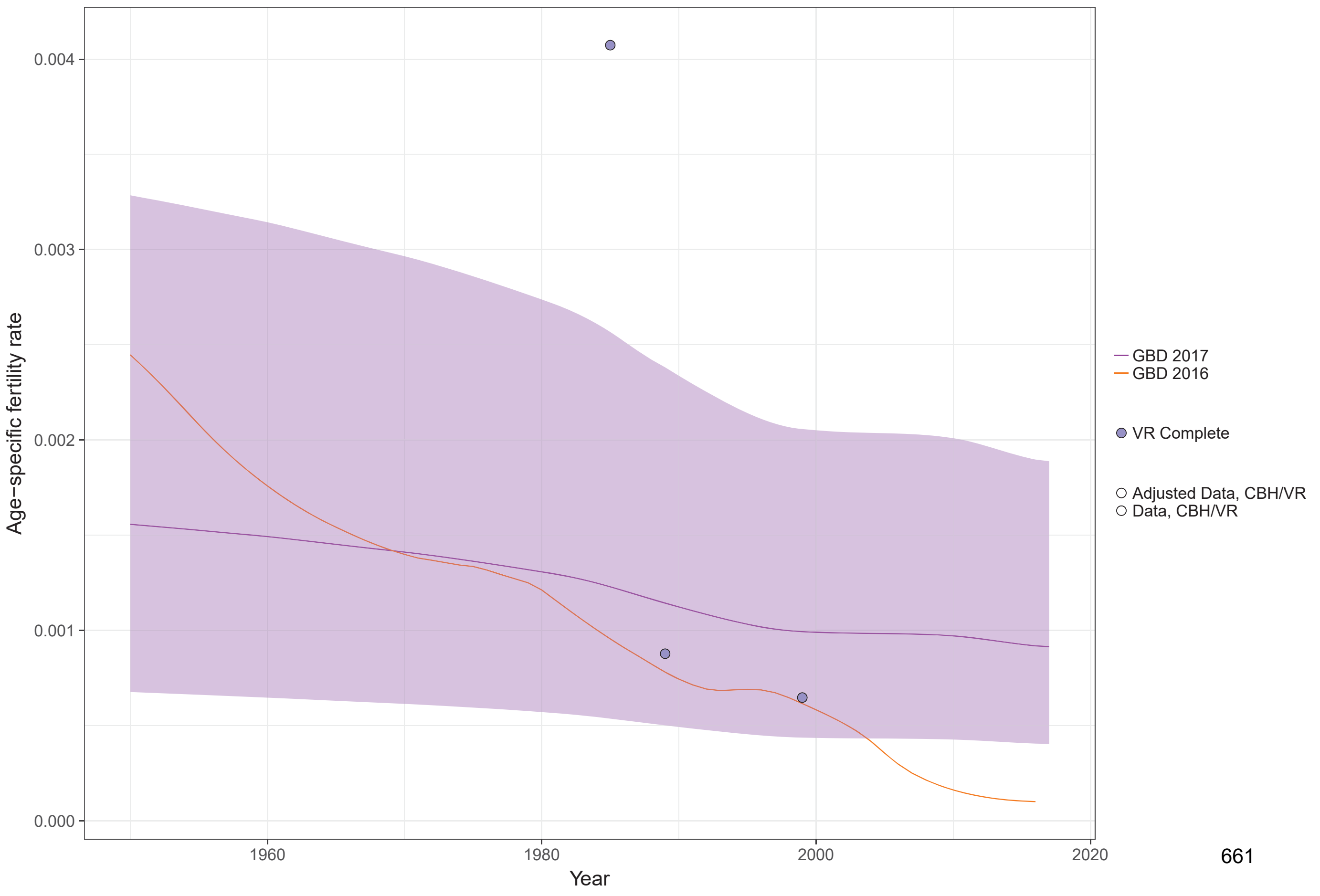

Age−specific fertility fitted trend lines, 1950−2017: Northern Mariana Islands, Ages: 15−19
Data points enclosed with a diamond are reference sources

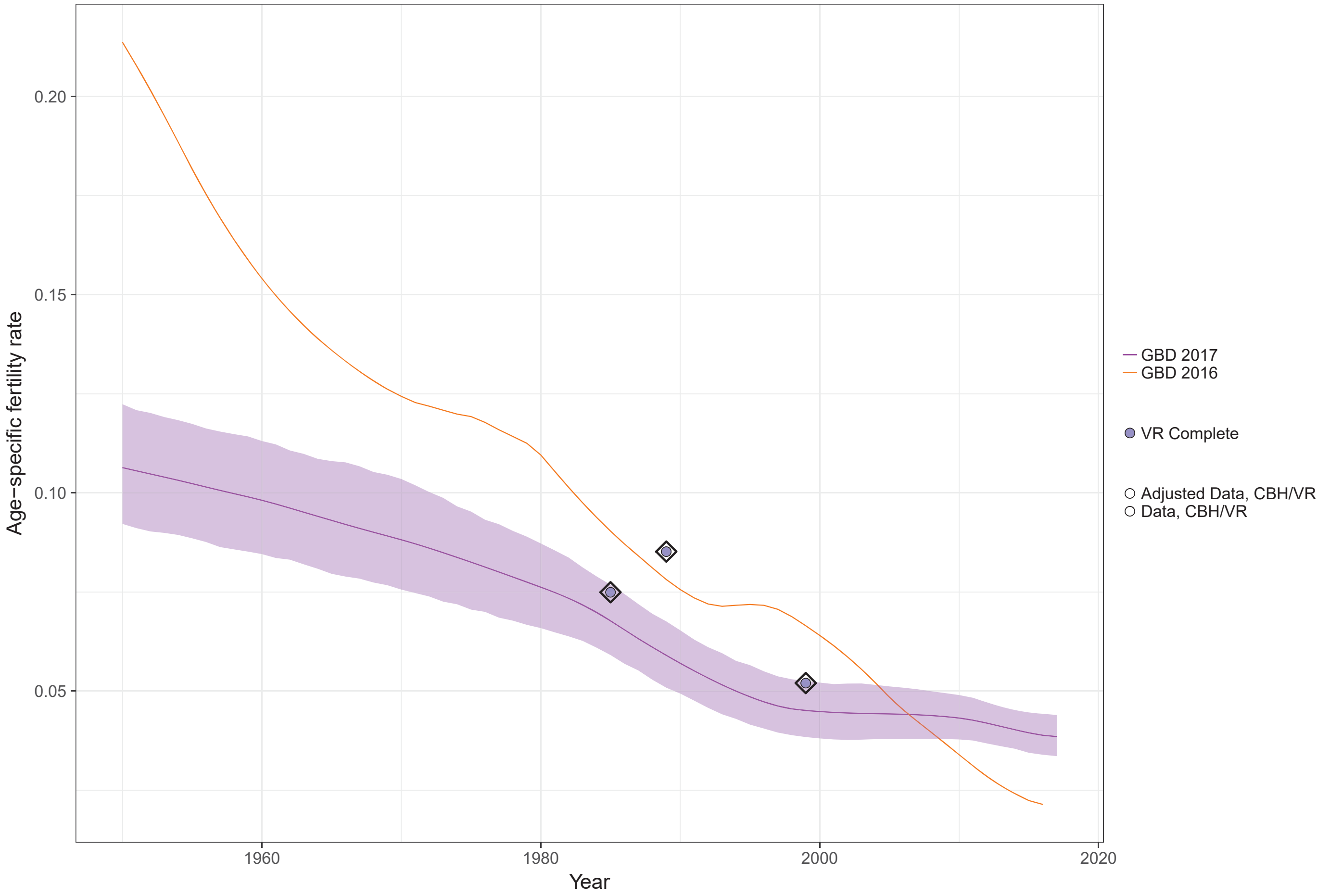

Age−specific fertility fitted trend lines, 1950−2017: Northern Mariana Islands, Ages: 20−24
Data points enclosed with a diamond are reference sources

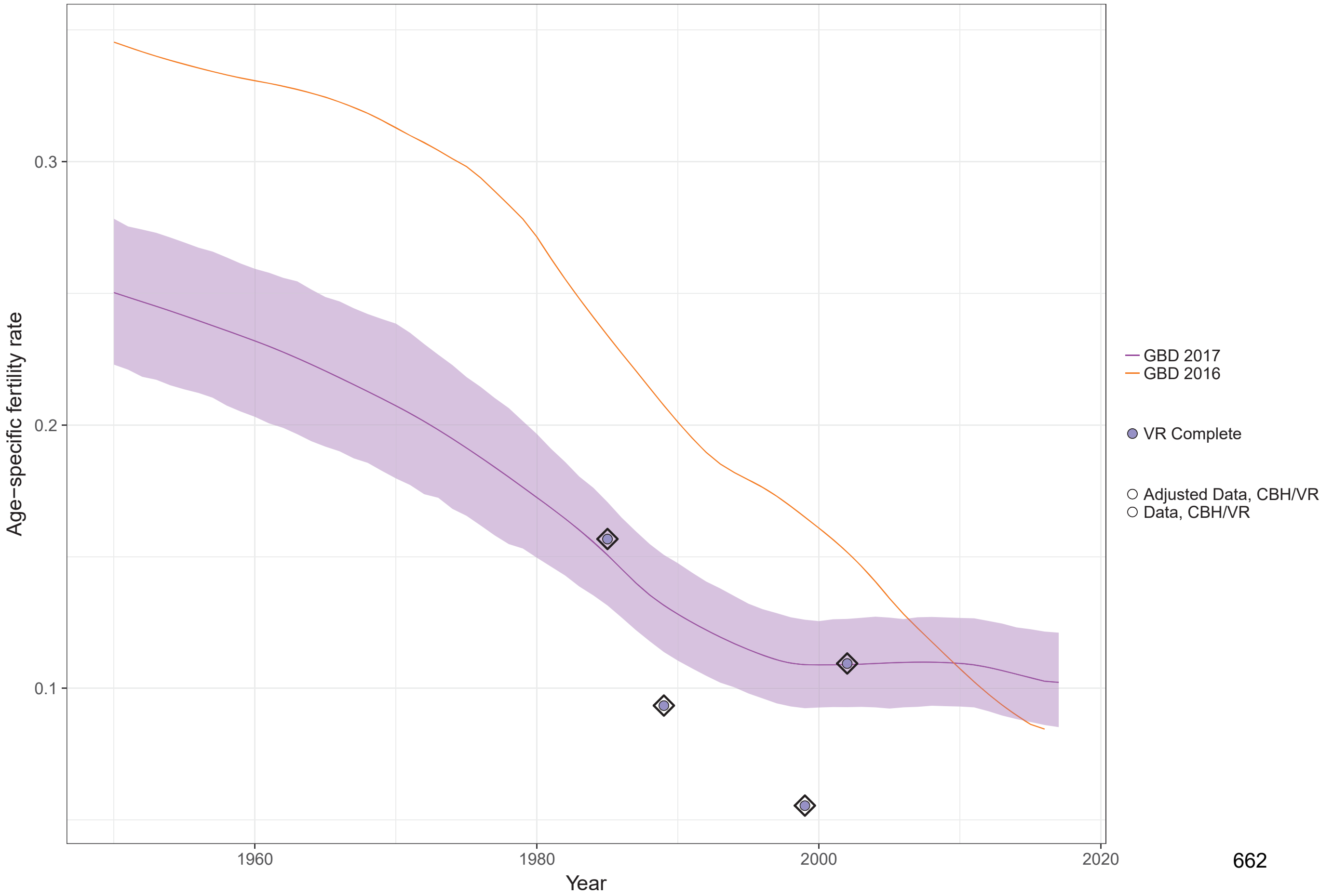

Age−specific fertility fitted trend lines, 1950−2017: Northern Mariana Islands, Ages: 25−29
Data points enclosed with a diamond are reference sources

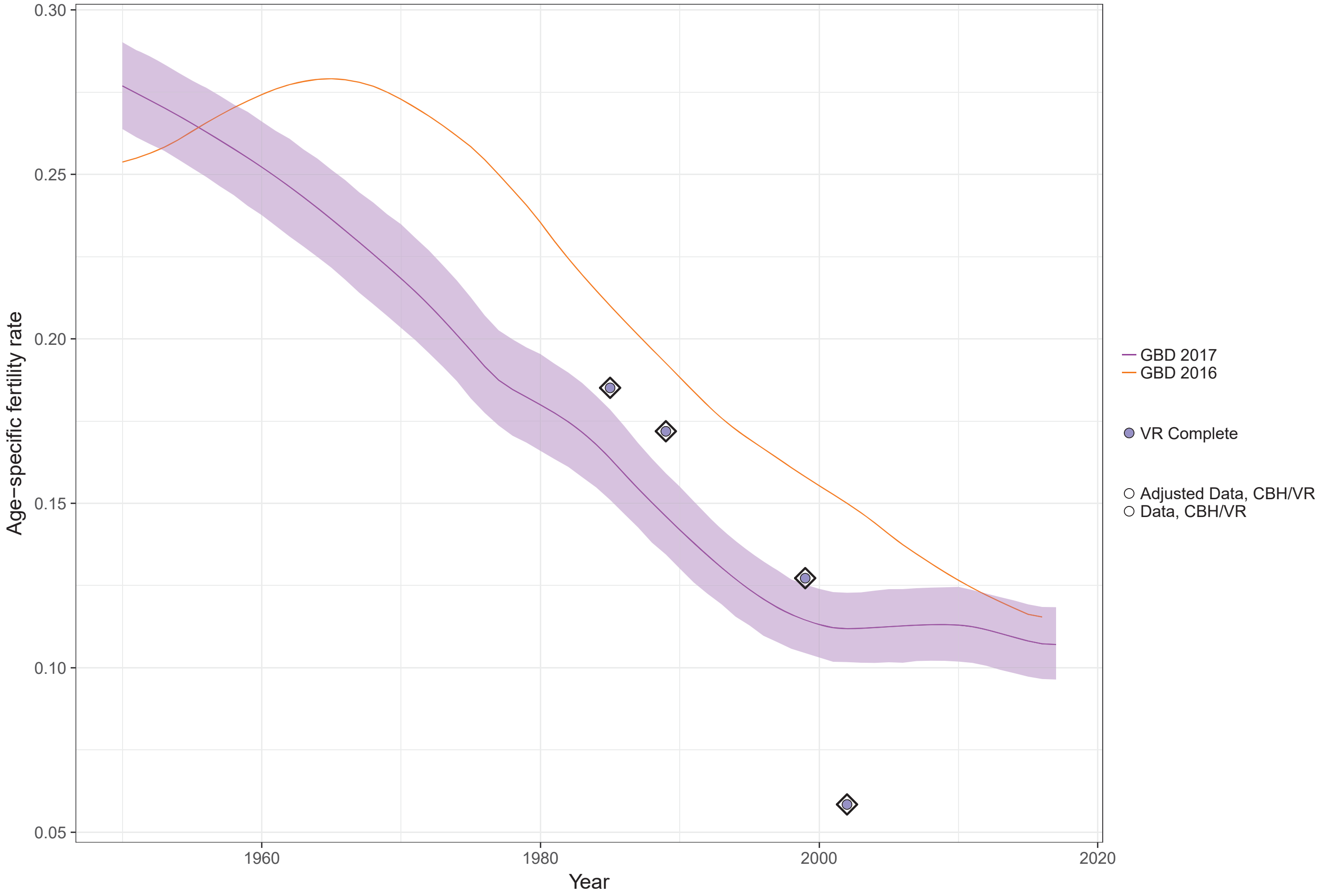

Age−specific fertility fitted trend lines, 1950−2017: Northern Mariana Islands, Ages: 30−34
Data points enclosed with a diamond are reference sources

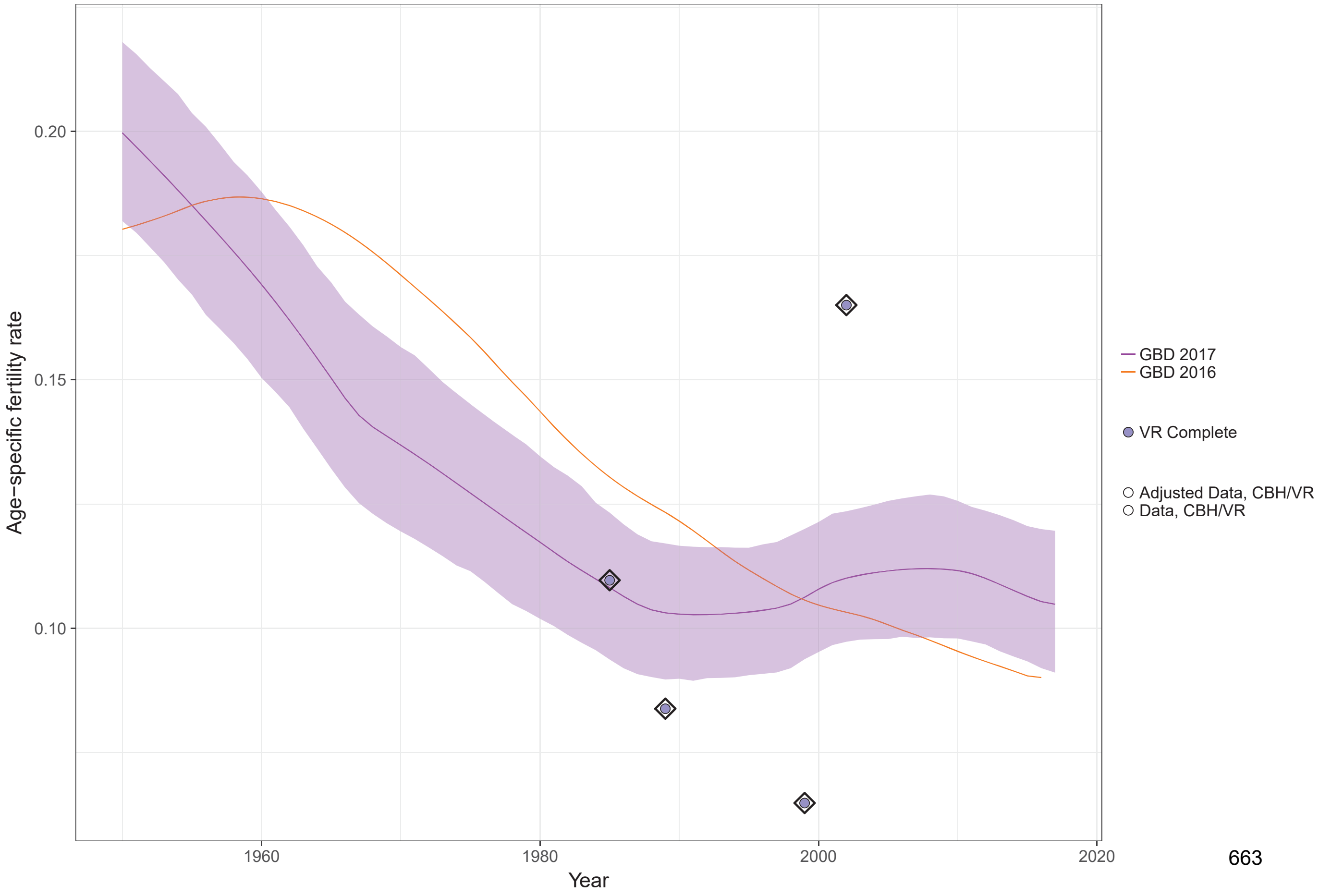

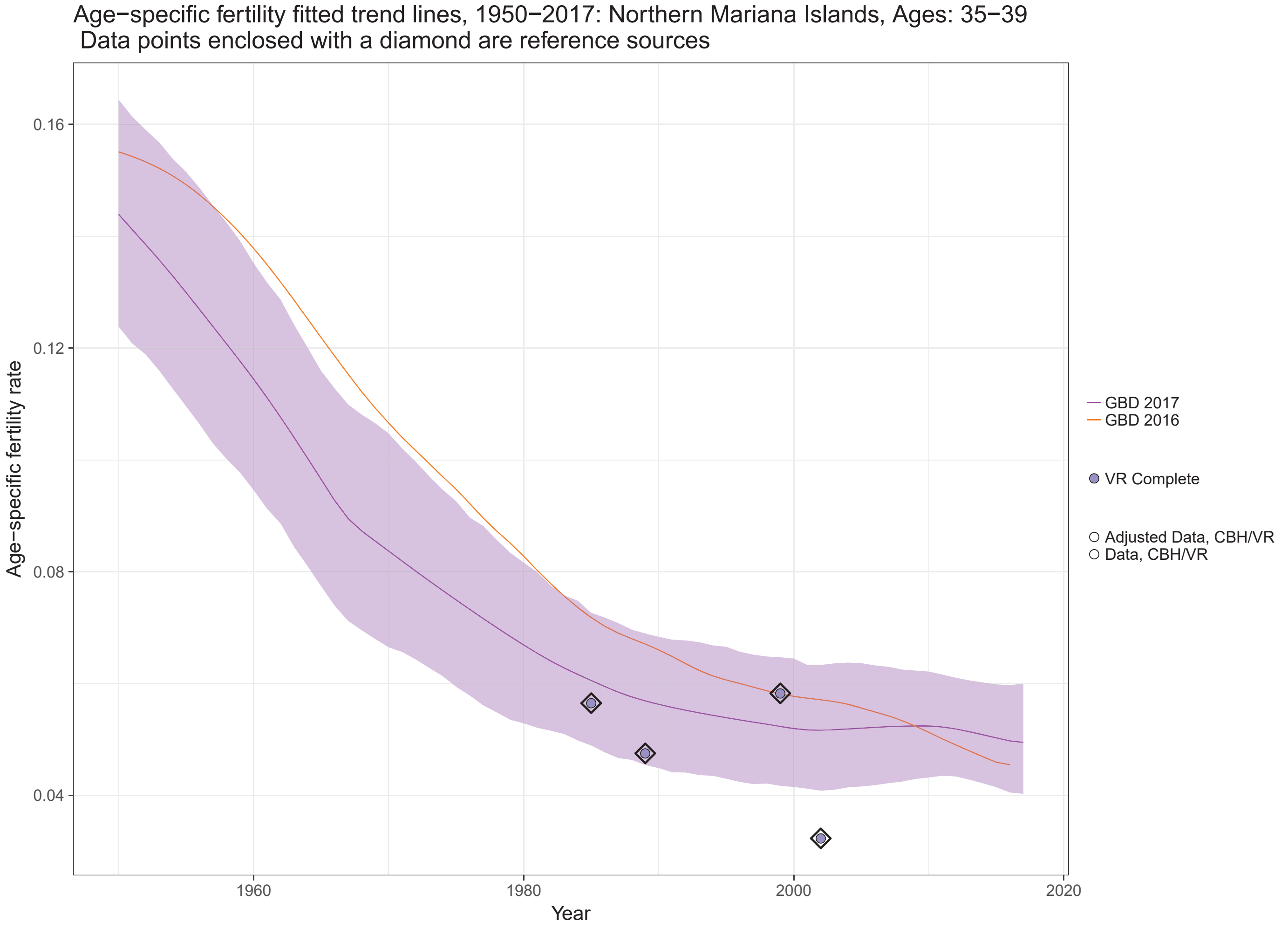

Age−specific fertility fitted trend lines, 1950−2017: Northern Mariana Islands, Ages: 35−39
Data points enclosed with a diamond are reference sources
Age−specific fertility rate
0.16
0.12
0.08
0.04
1960
1980
2000
2020
Year
GBD 2017
GBD 2016
VR Complete
Adjusted Data, CBH/VR
Data, CBH/VR

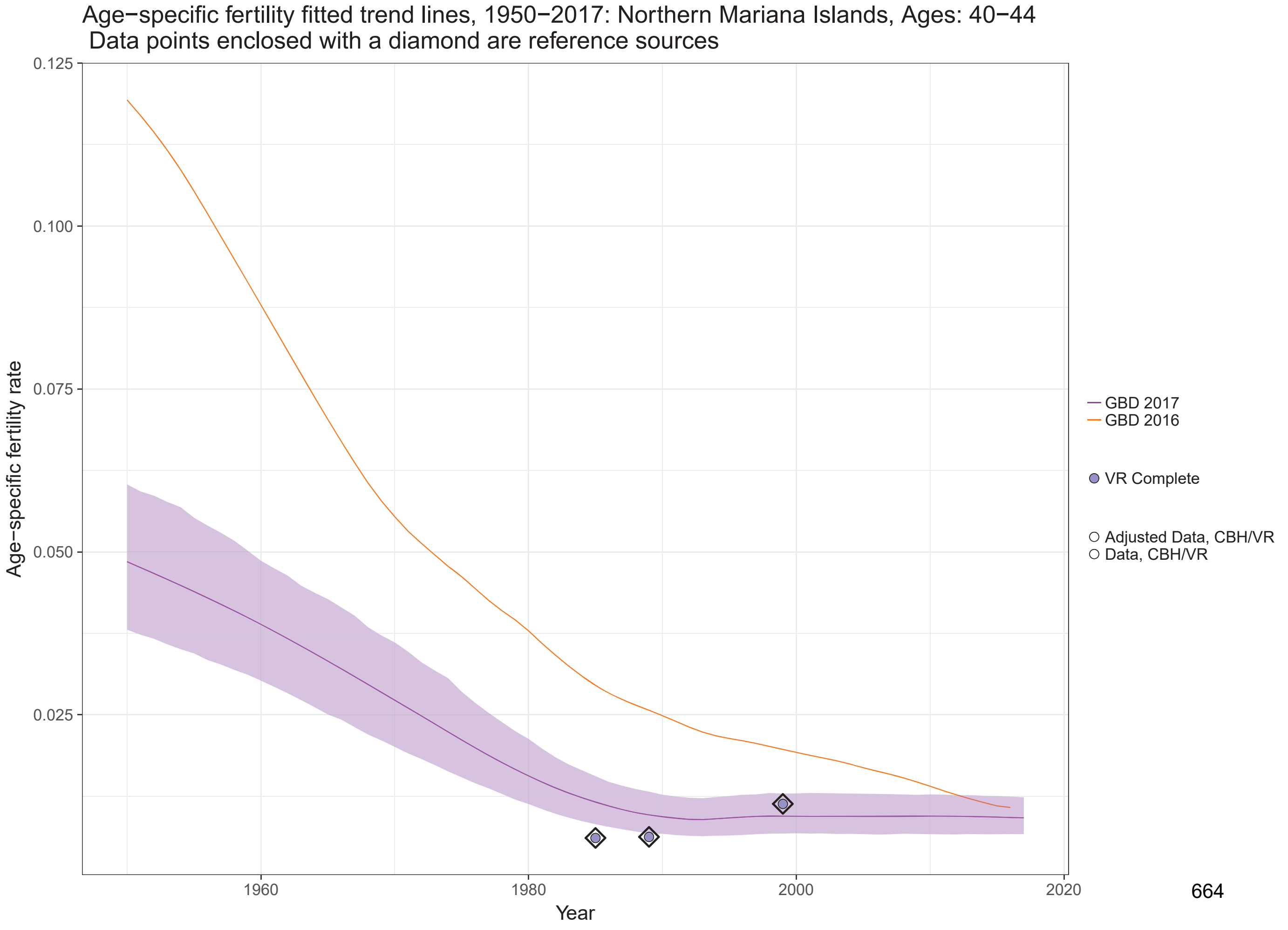

Age−specific fertility fitted trend lines, 1950−2017: Northern Mariana Islands, Ages: 40−44
Data points enclosed with a diamond are reference sources
Age−specific fertility rate
0.125
0.100
0.075
0.050
0.025
1960
1980
2000
2020
Year
GBD 2017
GBD 2016
VR Complete
Adjusted Data, CBH/VR
Data, CBH/VR

Age−specific fertility fitted trend lines, 1950−2017: Northern Mariana Islands, Ages: 45−49
Data points enclosed with a diamond are reference sources

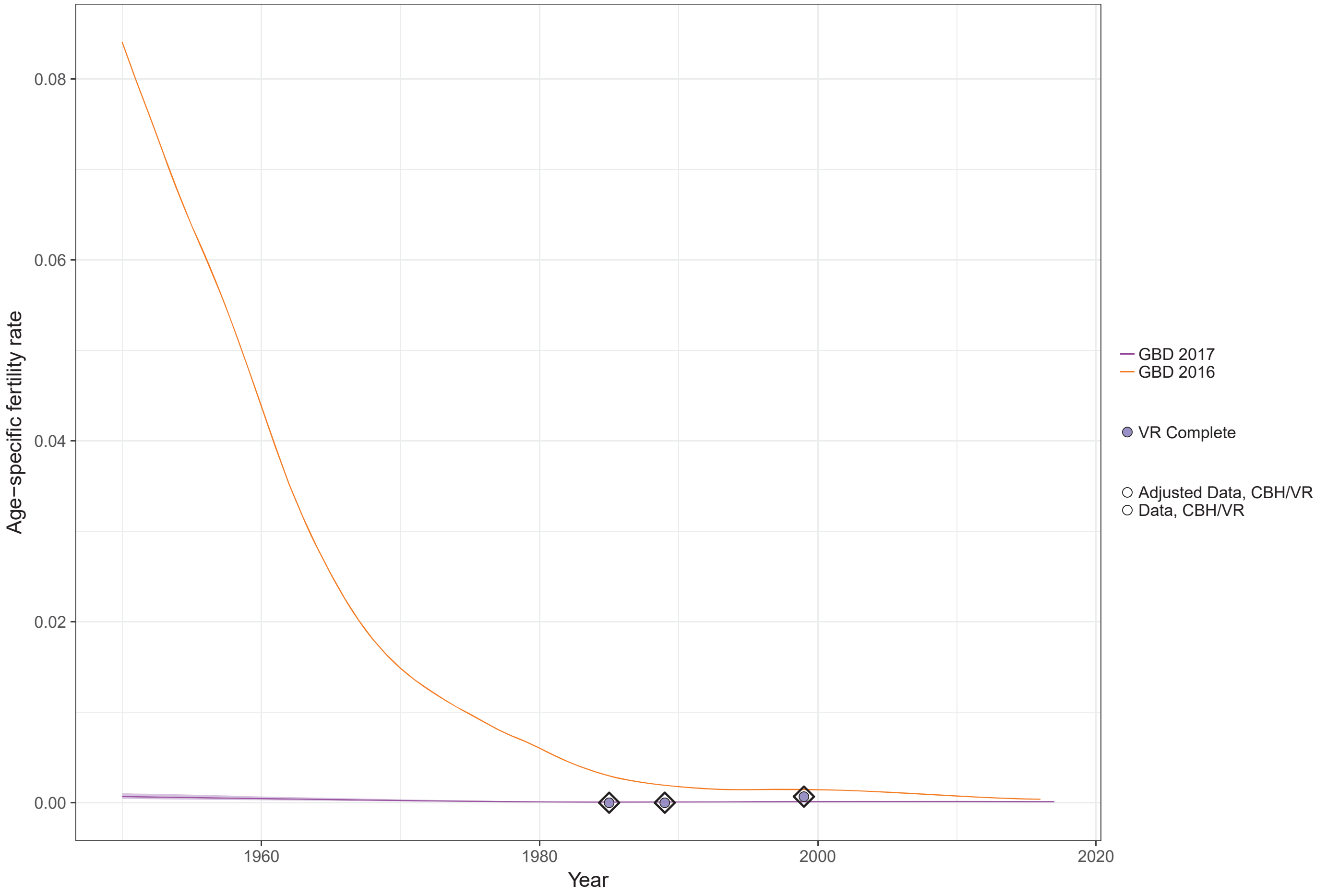

Age−specific fertility fitted trend lines, 1950−2017: Northern Mariana Islands, Ages: 50−54
Data points enclosed with a diamond are reference sources

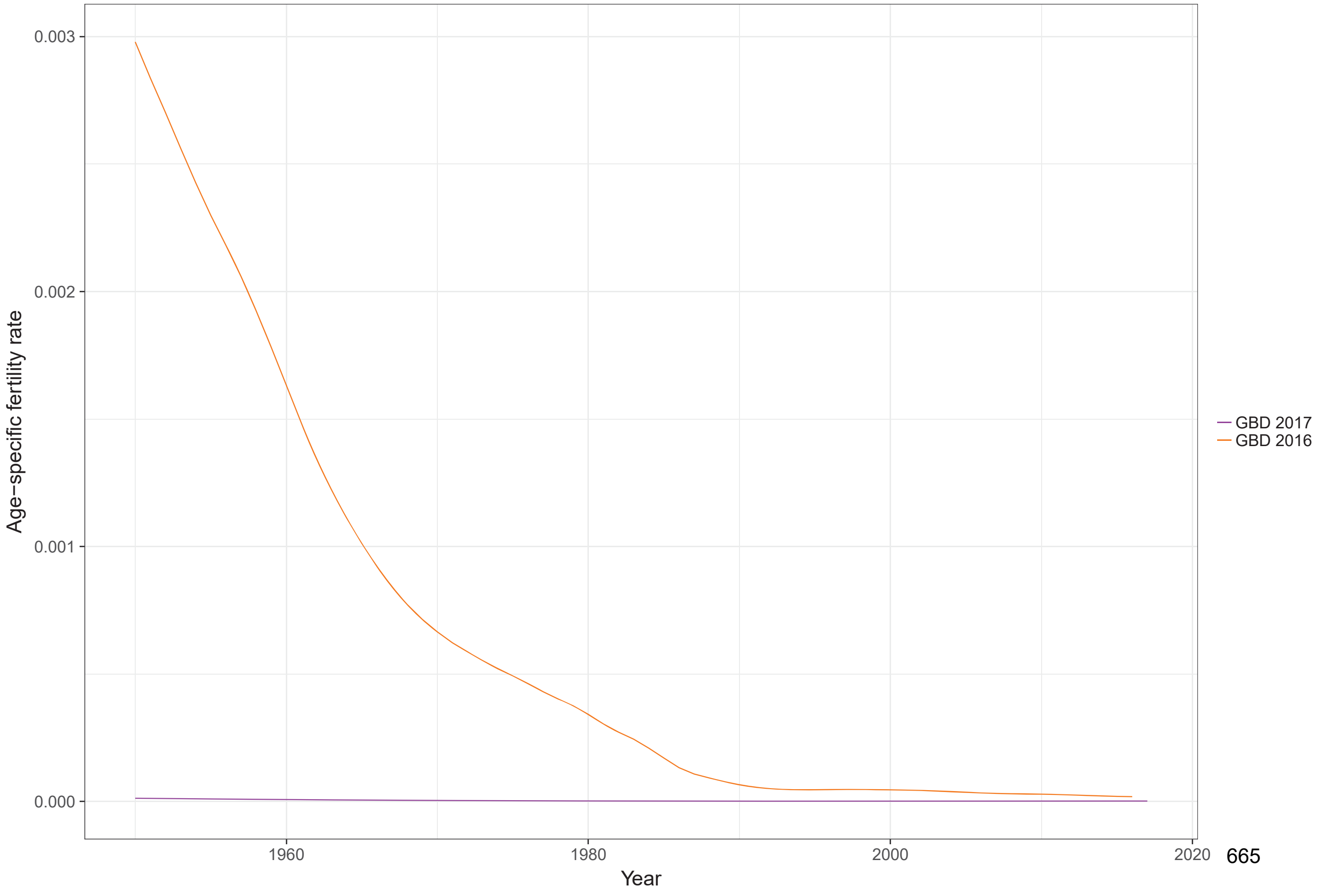

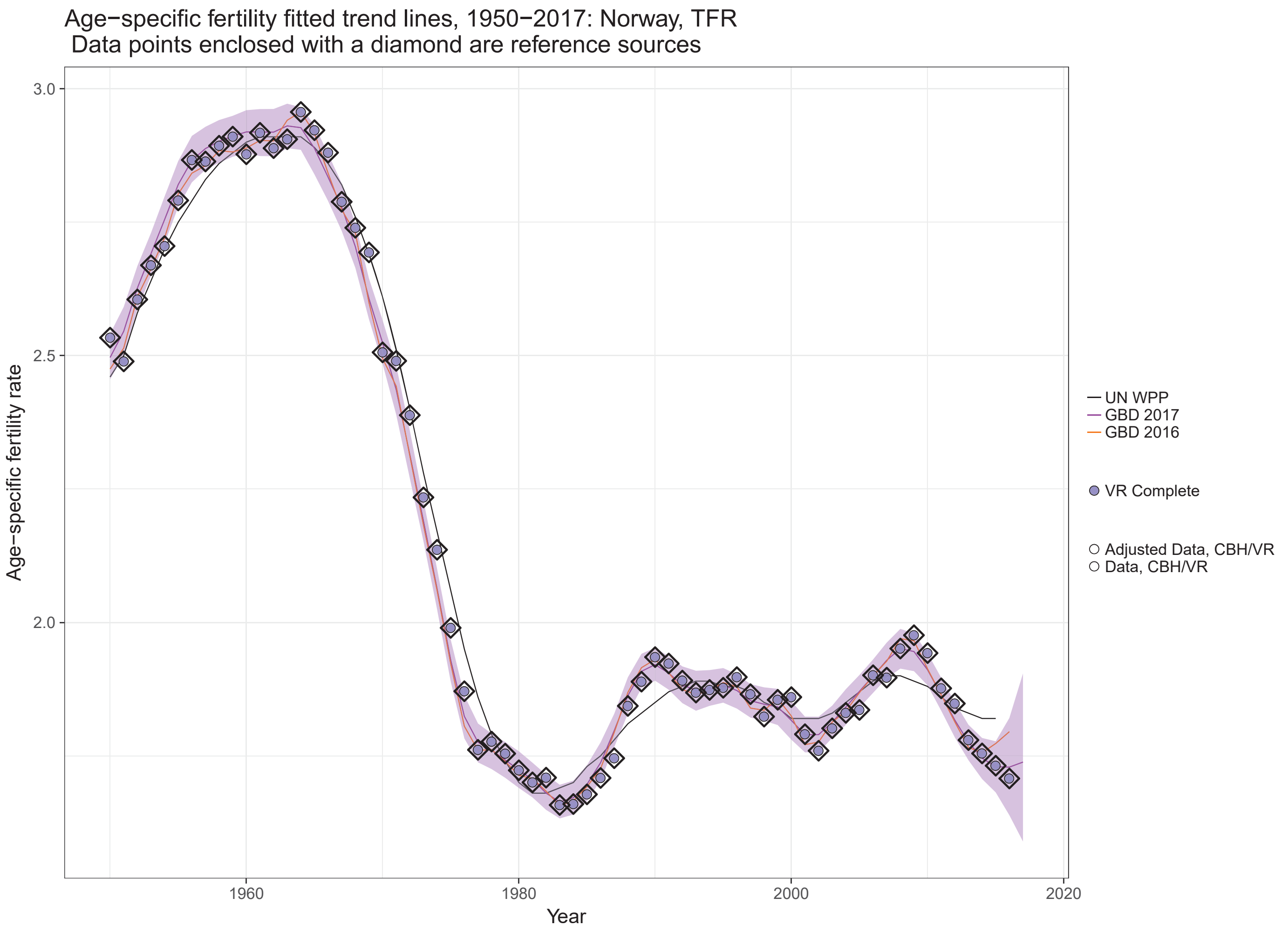

Age−specific fertility fitted trend lines, 1950−2017: Norway, TFR
Data points enclosed with a diamond are reference sources
Age−specific fertility rate
3.0
2.5
2.0
1960
1980
2000
2020
Year
UN WPP
GBD 2017
GBD 2016
VR Complete
Adjusted Data, CBH/VR
Data, CBH/VR

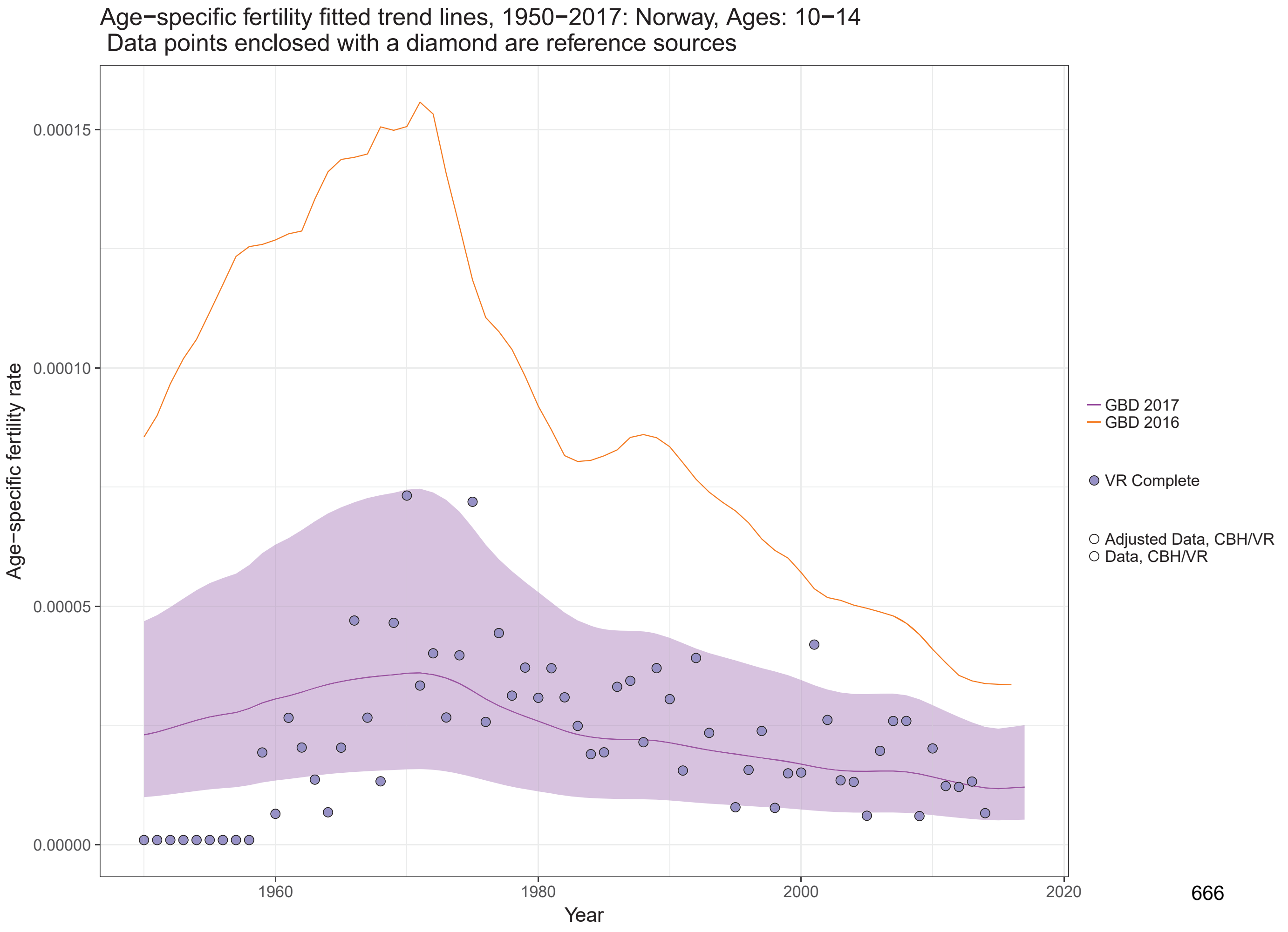

Age−specific fertility fitted trend lines, 1950−2017: Norway, Ages: 10−14
Data points enclosed with a diamond are reference sources
Age−specific fertility rate
0.00015
0.00010
0.00005
0.00000
1960
1980
2000
2020
Year
GBD 2017
GBD 2016
VR Complete
Adjusted Data, CBH/VR
Data, CBH/VR

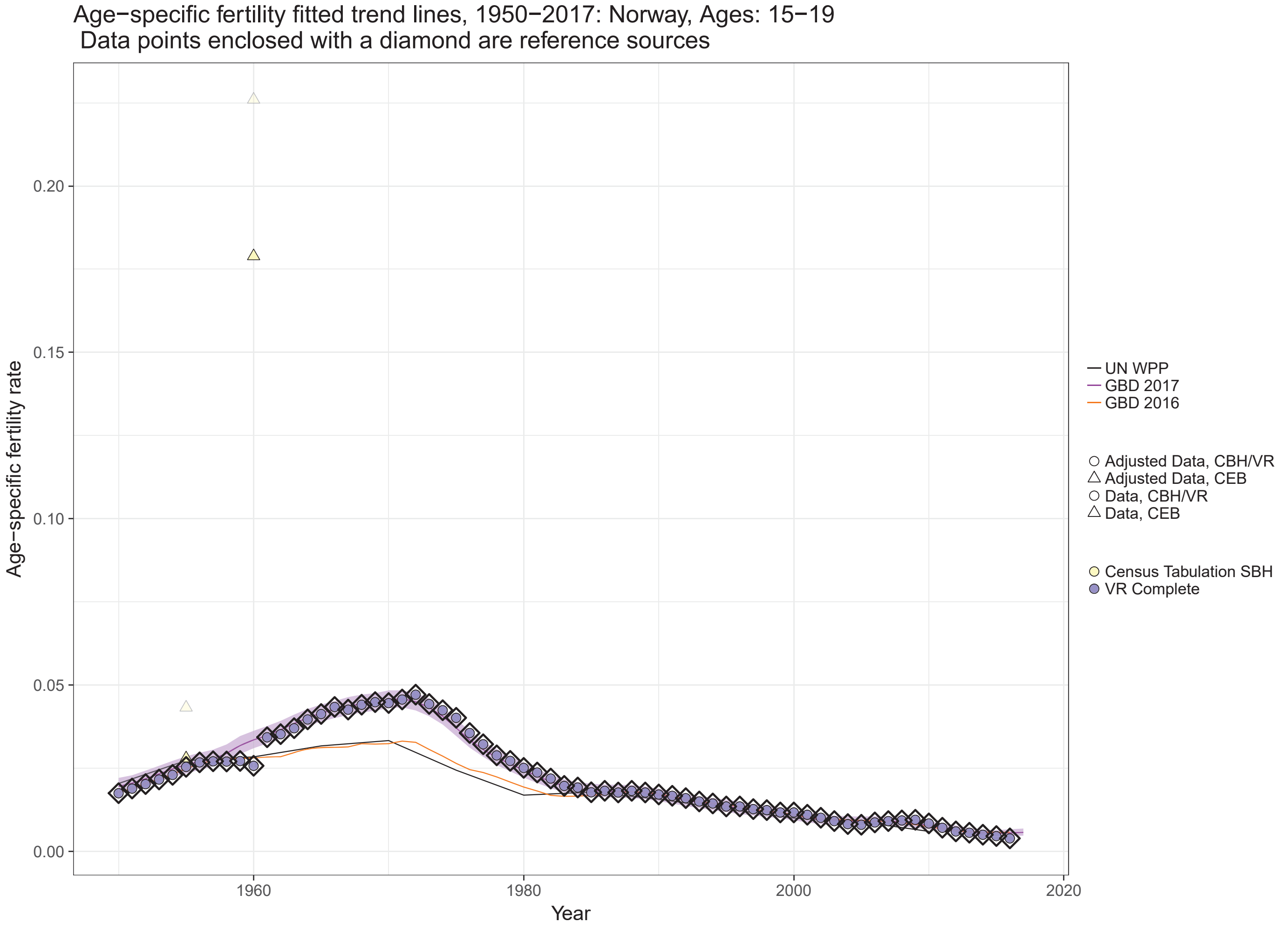

Age−specific fertility fitted trend lines, 1950−2017: Norway, Ages: 15−19
Data points enclosed with a diamond are reference sources
Age−specific fertility rate
0.00
0.05
0.10
0.15
0.20
1960
1980
2000
2020
Year
UN WPP
GBD 2017
GBD 2016
Adjusted Data, CBH/VR
Adjusted Data, CEB
Data, CBH/VR
Data, CEB
Census Tabulation SBH
VR Complete

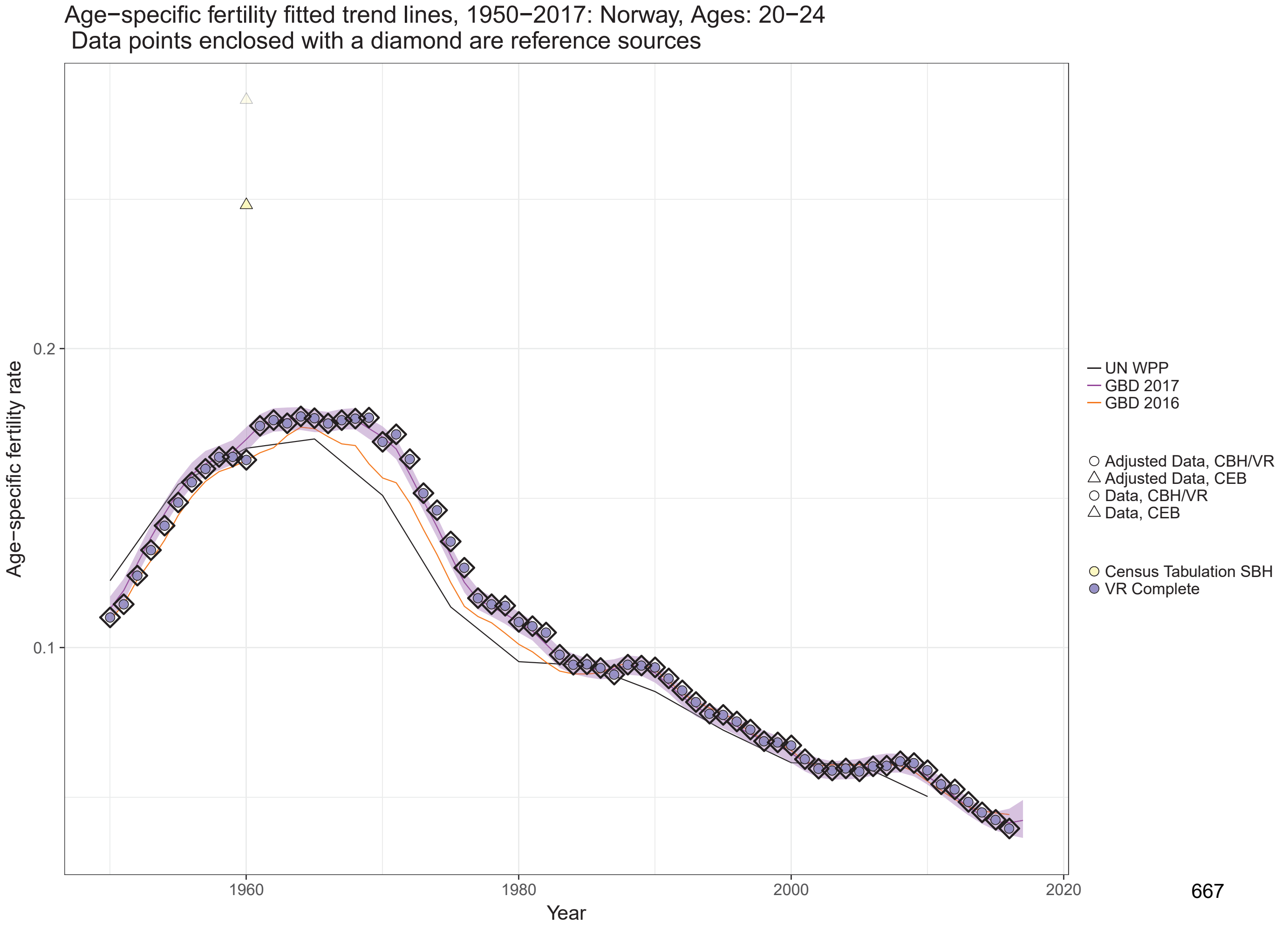

Age−specific fertility fitted trend lines, 1950−2017: Norway, Ages: 20−24
Data points enclosed with a diamond are reference sources
Age−specific fertility rate
0.1
0.2
1960
1980
2000
2020
Year
UN WPP
GBD 2017
GBD 2016
Adjusted Data, CBH/VR
Adjusted Data, CEB
Data, CBH/VR
Data, CEB
Census Tabulation SBH
VR Complete

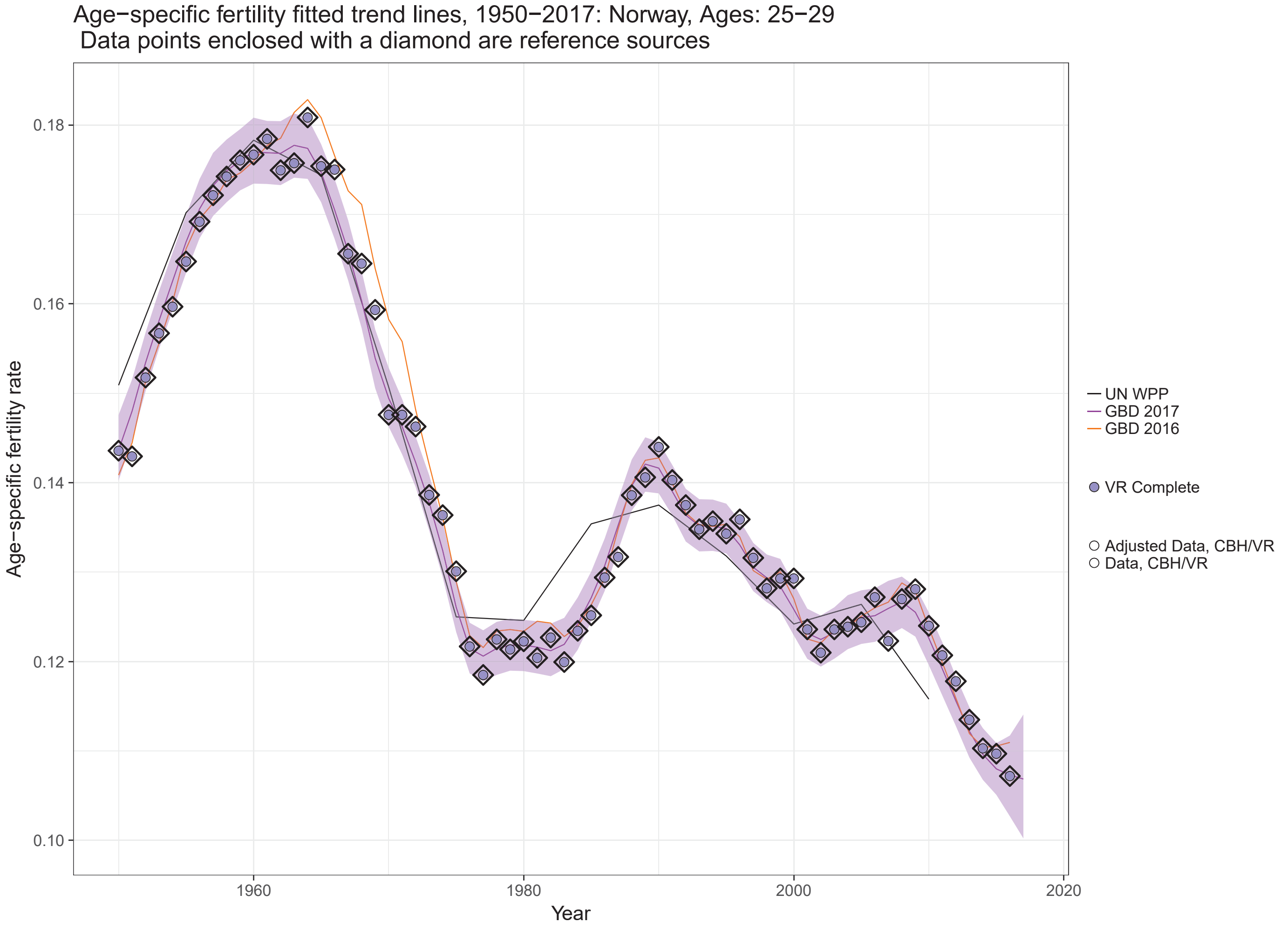

Age−specific fertility fitted trend lines, 1950−2017: Norway, Ages: 25−29
Data points enclosed with a diamond are reference sources
Age−specific fertility rate
0.18
0.16
0.14
0.12
0.10
1960
1980
2000
2020
Year
UN WPP
GBD 2017
GBD 2016
VR Complete
Adjusted Data, CBH/VR
Data, CBH/VR

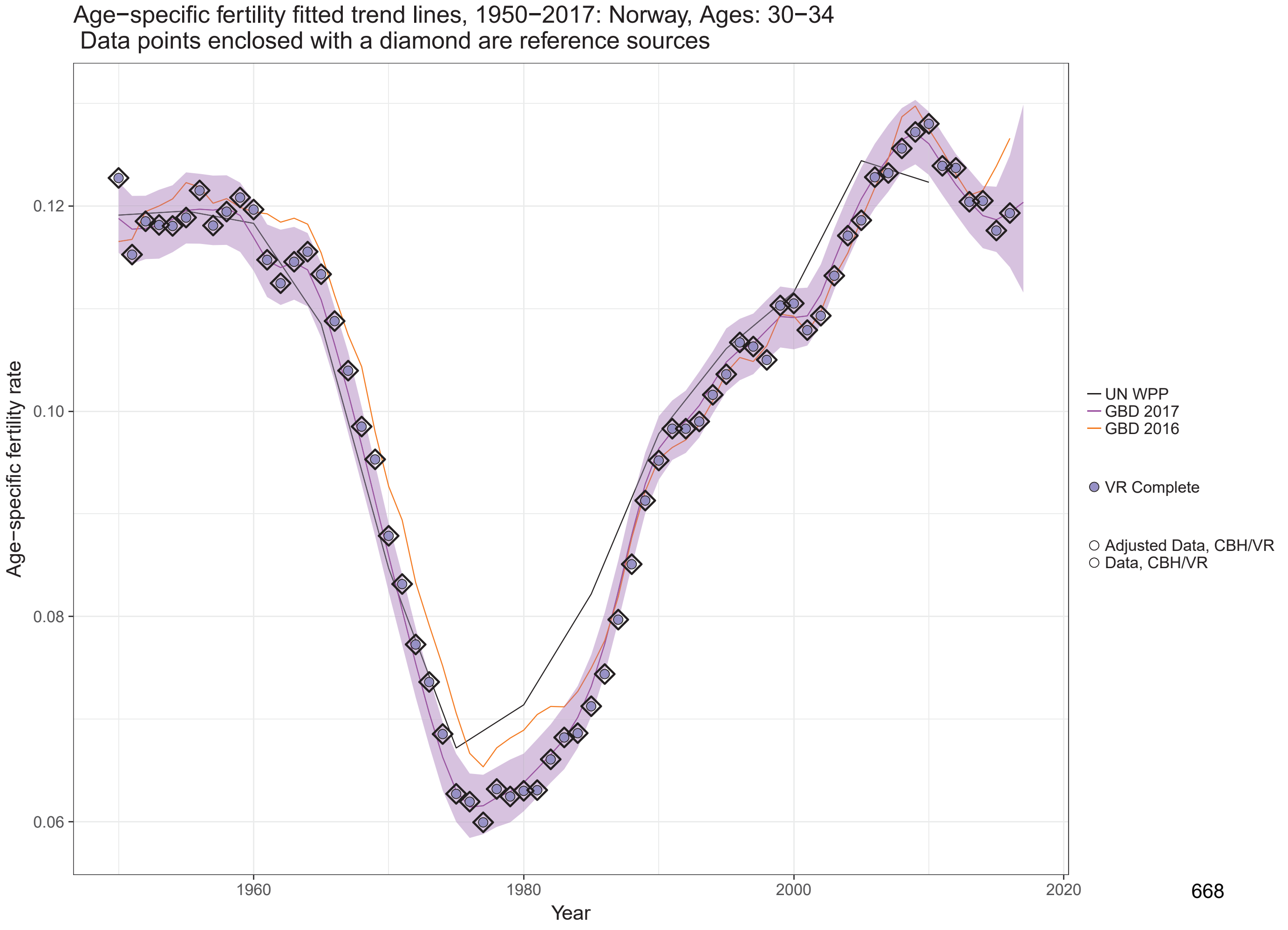

Age−specific fertility fitted trend lines, 1950−2017: Norway, Ages: 30−34
Data points enclosed with a diamond are reference sources
Age−specific fertility rate
0.12
0.10
0.08
0.06
1960
1980
2000
2020
Year
UN WPP
GBD 2017
GBD 2016
VR Complete
Adjusted Data, CBH/VR
Data, CBH/VR

Age−specific fertility fitted trend lines, 1950−2017: Norway, Ages: 35−39
Data points enclosed with a diamond are reference sources

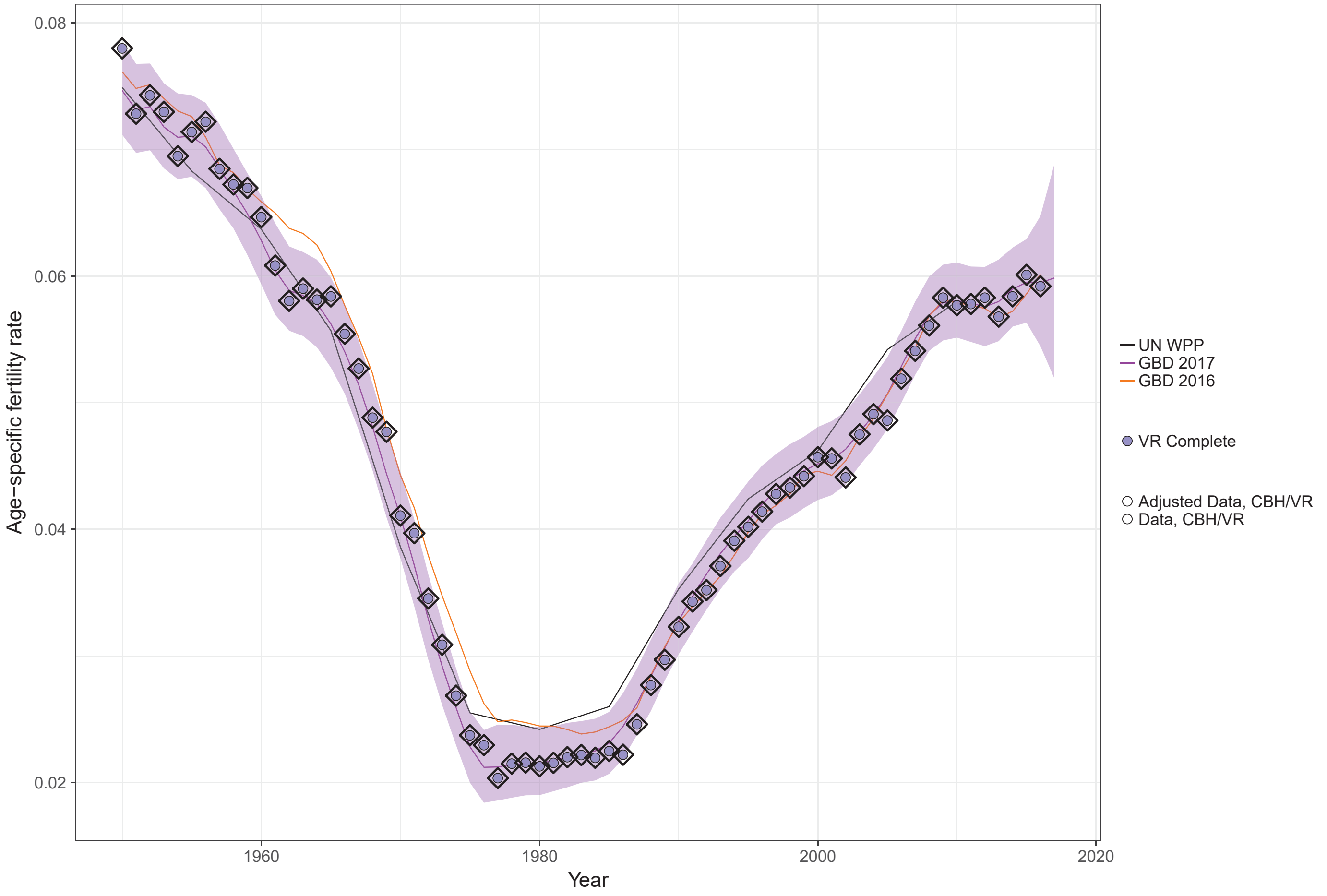

Age−specific fertility fitted trend lines, 1950−2017: Norway, Ages: 40−44
Data points enclosed with a diamond are reference sources

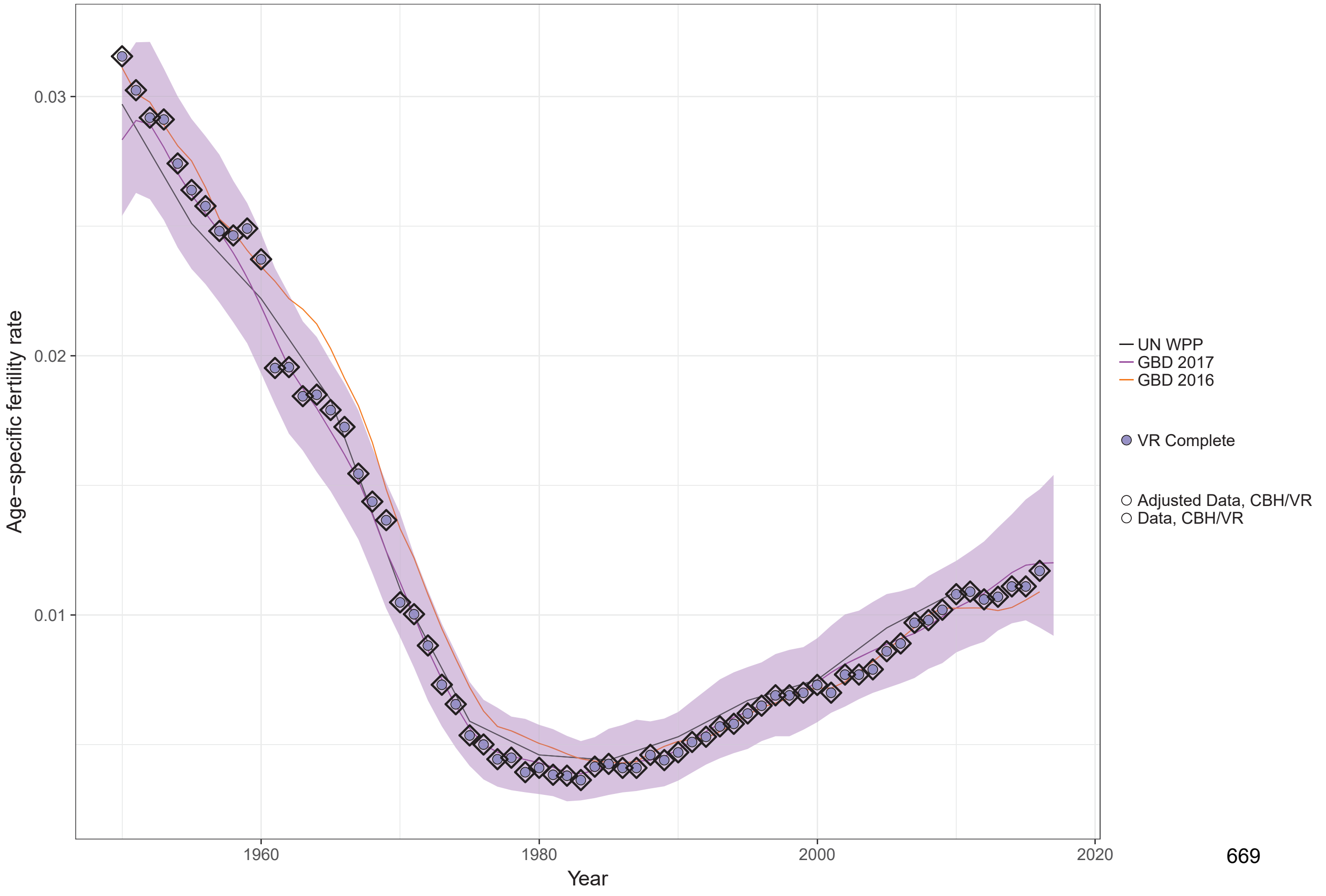

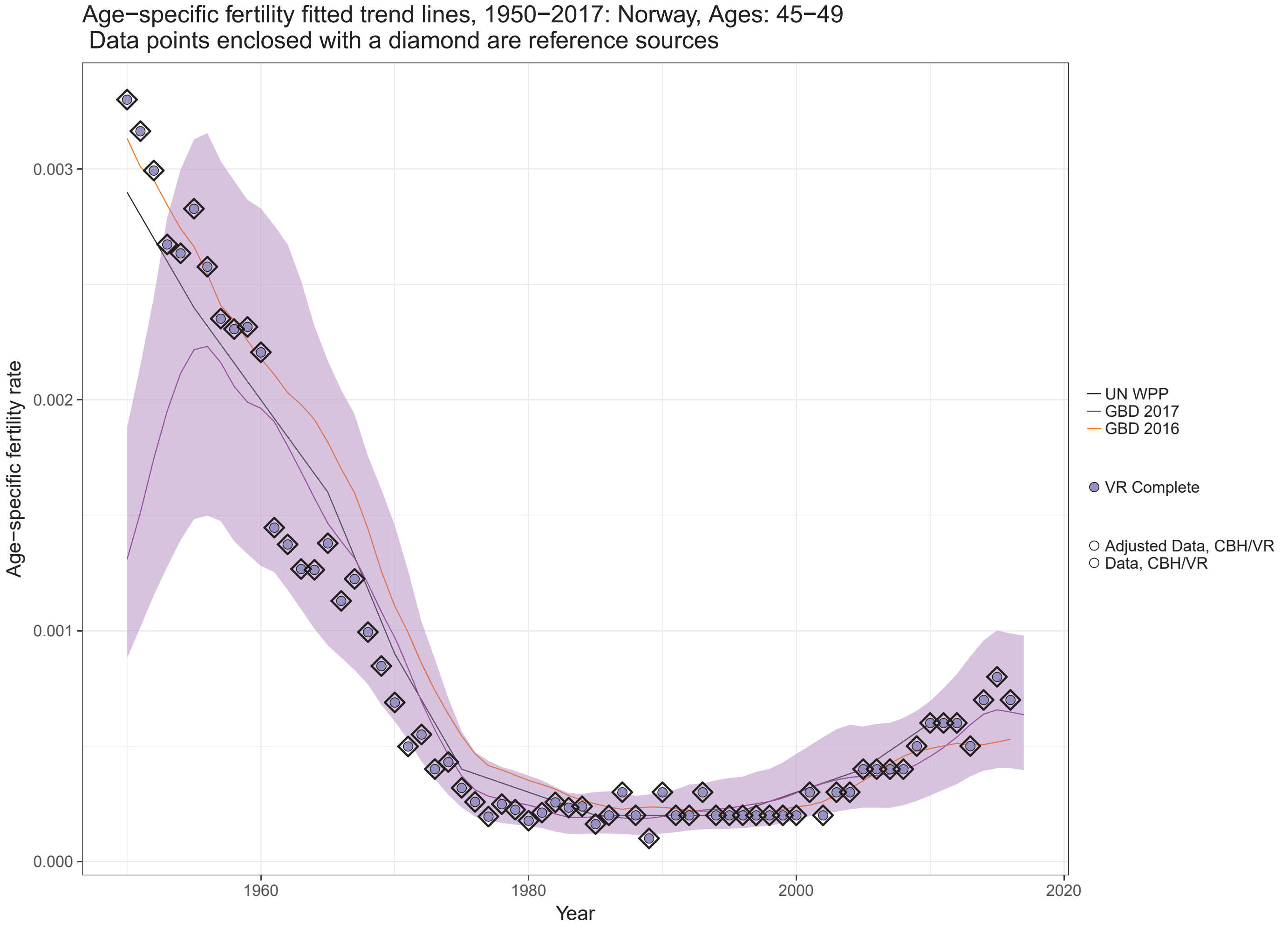

Age−specific fertility fitted trend lines, 1950−2017: Norway, Ages: 45−49
Data points enclosed with a diamond are reference sources
Age−specific fertility rate
0.000
0.001
0.002
0.003
1960
1980
2000
2020
Year
UN WPP
GBD 2017
GBD 2016
VR Complete
Adjusted Data, CBH/VR
Data, CBH/VR

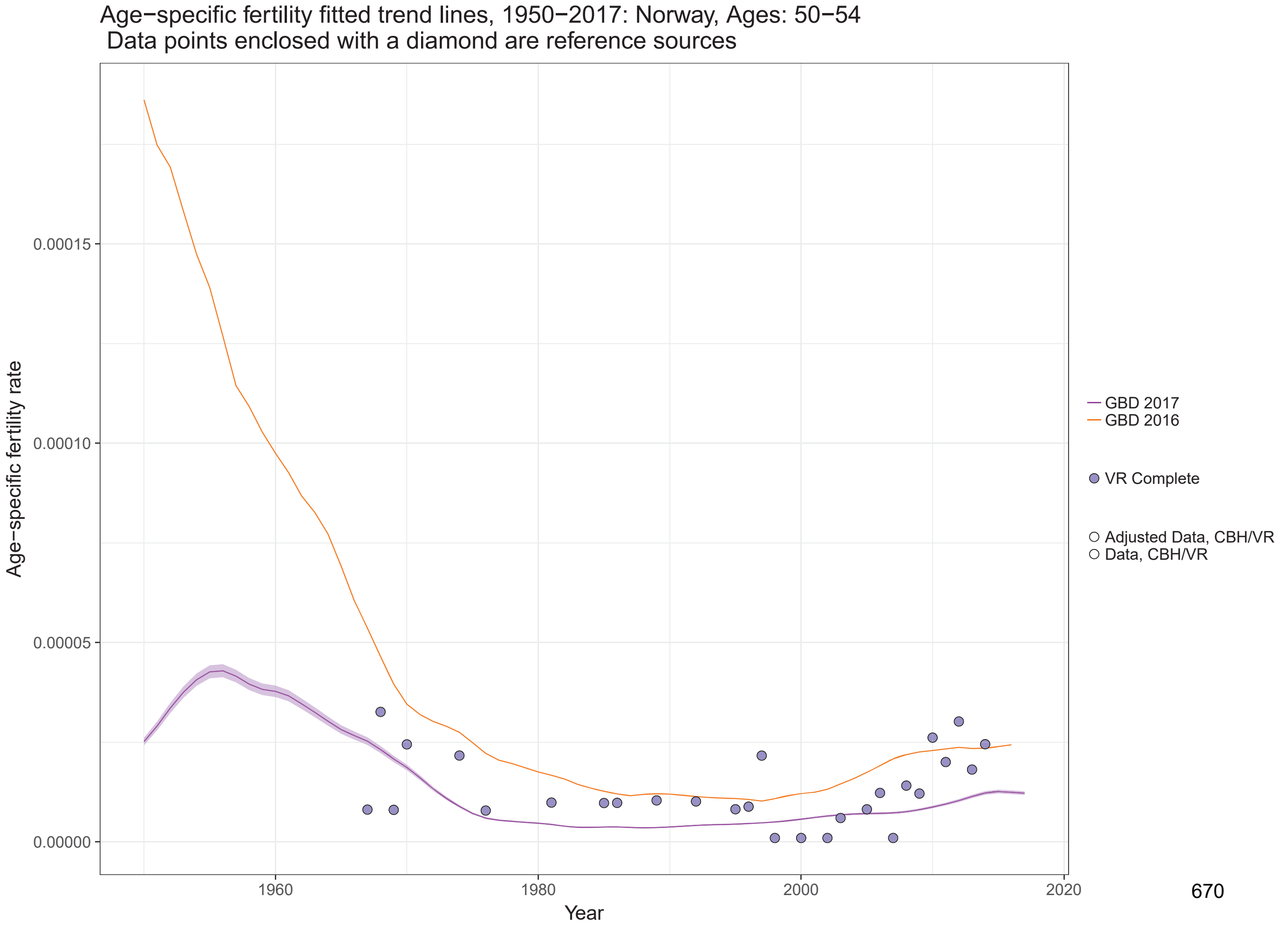

Age−specific fertility fitted trend lines, 1950−2017: Norway, Ages: 50−54
Data points enclosed with a diamond are reference sources
Age−specific fertility rate
0.00000
0.00005
0.00010
0.00015
1960
1980
2000
2020
Year
GBD 2017
GBD 2016
VR Complete
Adjusted Data, CBH/VR
Data, CBH/VR

Age−specific fertility fitted trend lines, 1950−2017: Oman, TFR
Data points enclosed with a diamond are reference sources

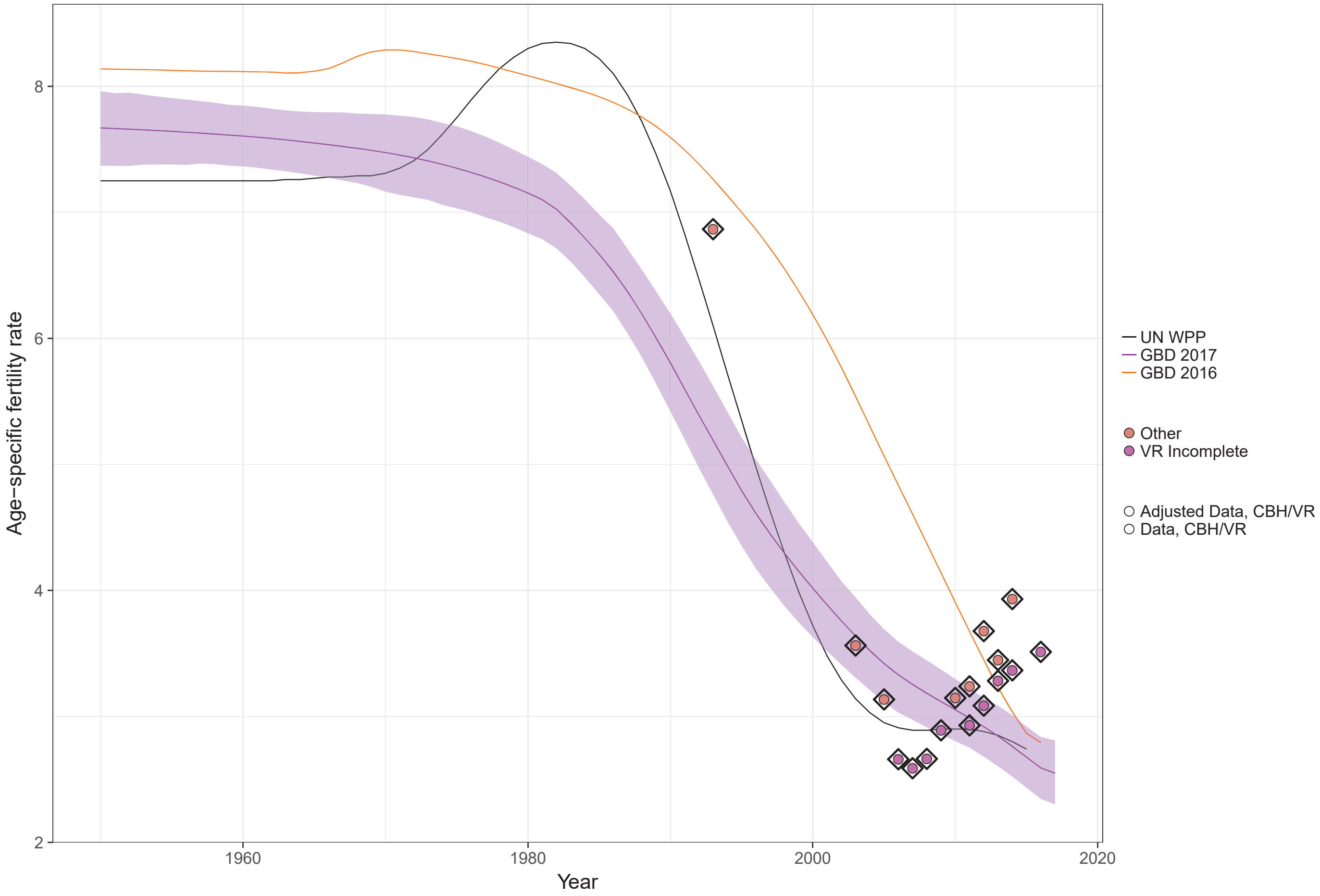

Age−specific fertility fitted trend lines, 1950−2017: Oman, Ages: 10−14
Data points enclosed with a diamond are reference sources

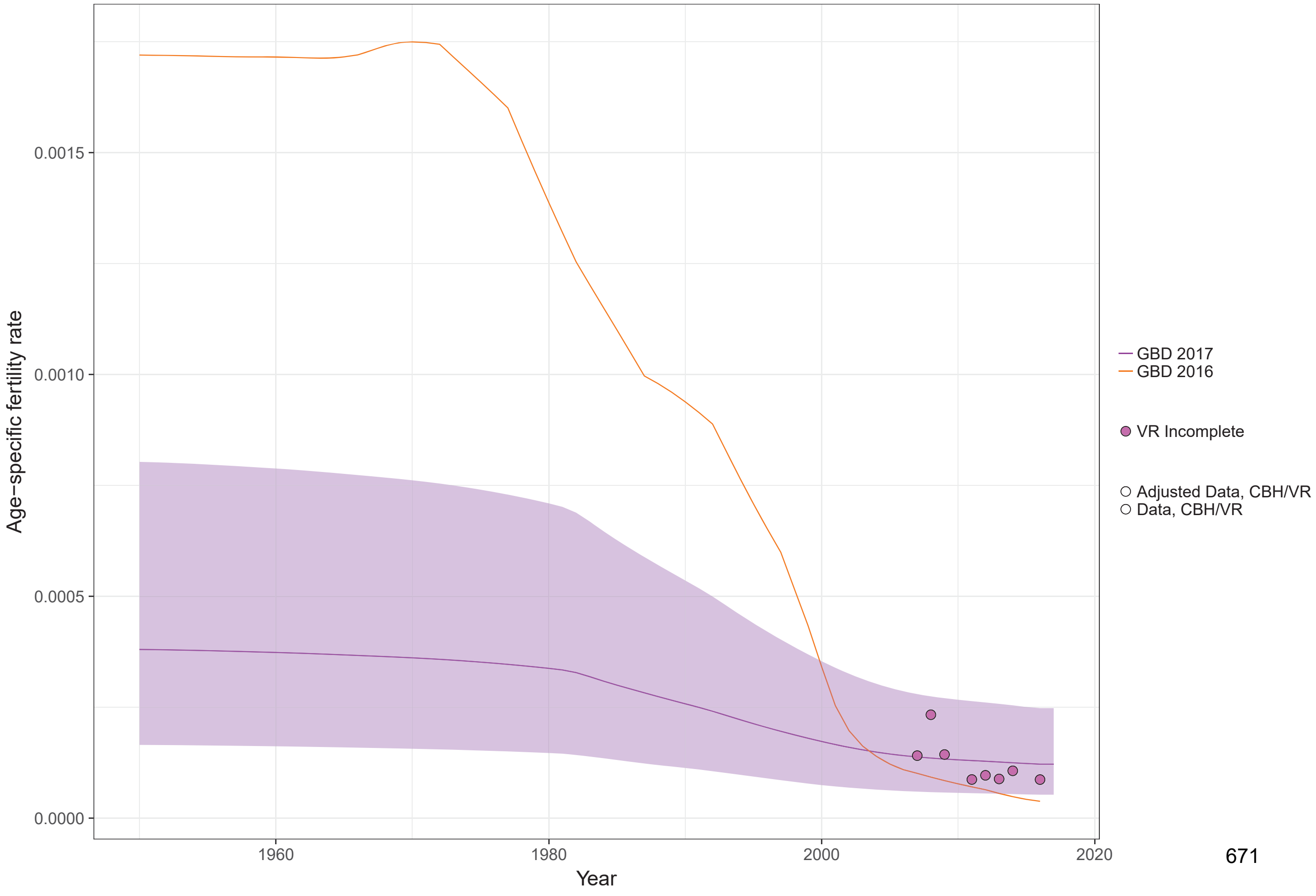

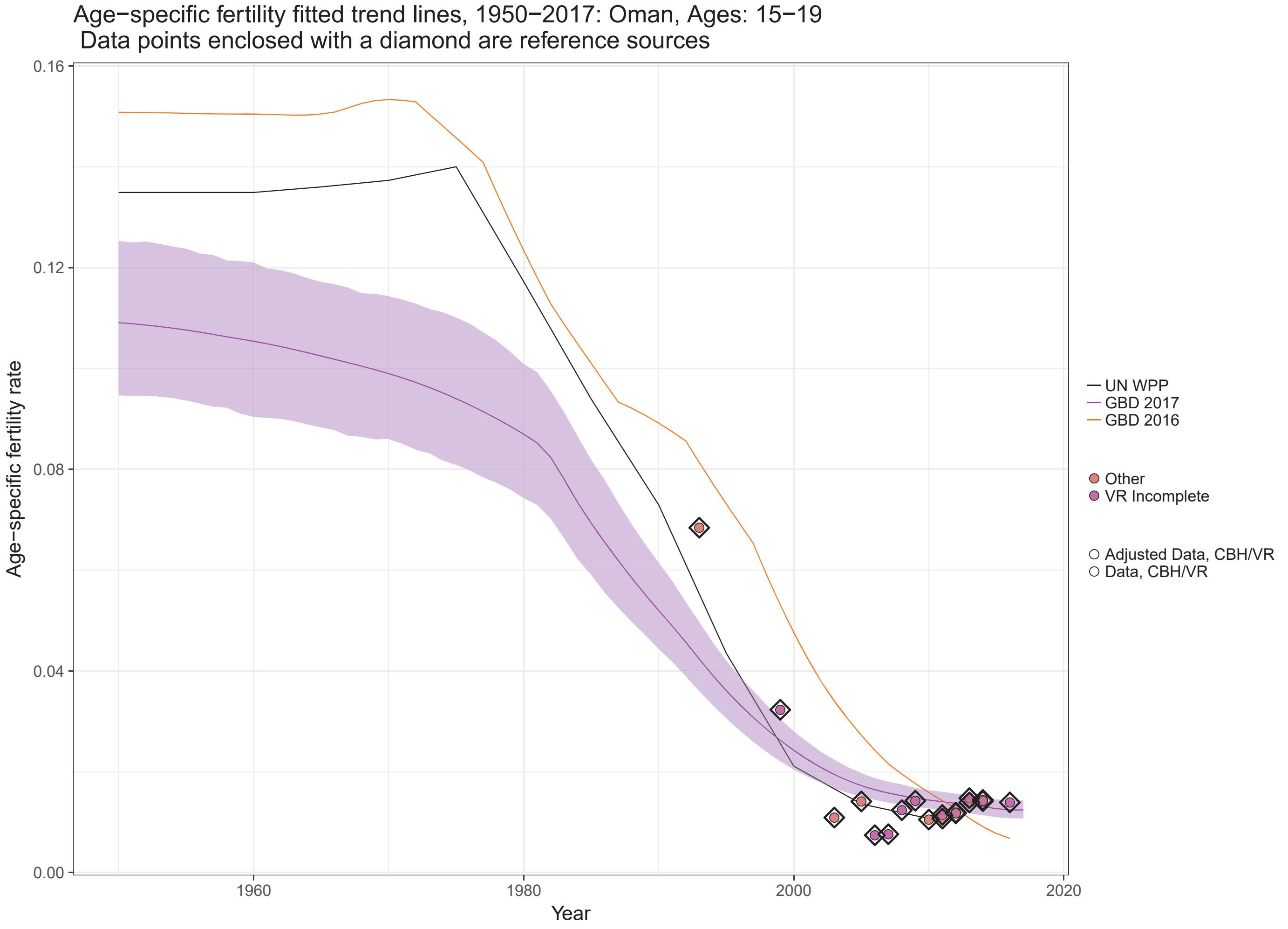
Age−specific fertility fitted trend lines, 1950−2017: Oman, Ages: 15−19
Data points enclosed with a diamond are reference sources
Age−specific fertility rate
Year
0.00
0.04
0.08
0.12
0.16
1960
1980
2000
2020
UN WPP
GBD 2017
GBD 2016
Other
VR Incomplete
Adjusted Data, CBH/VR
Data, CBH/VR

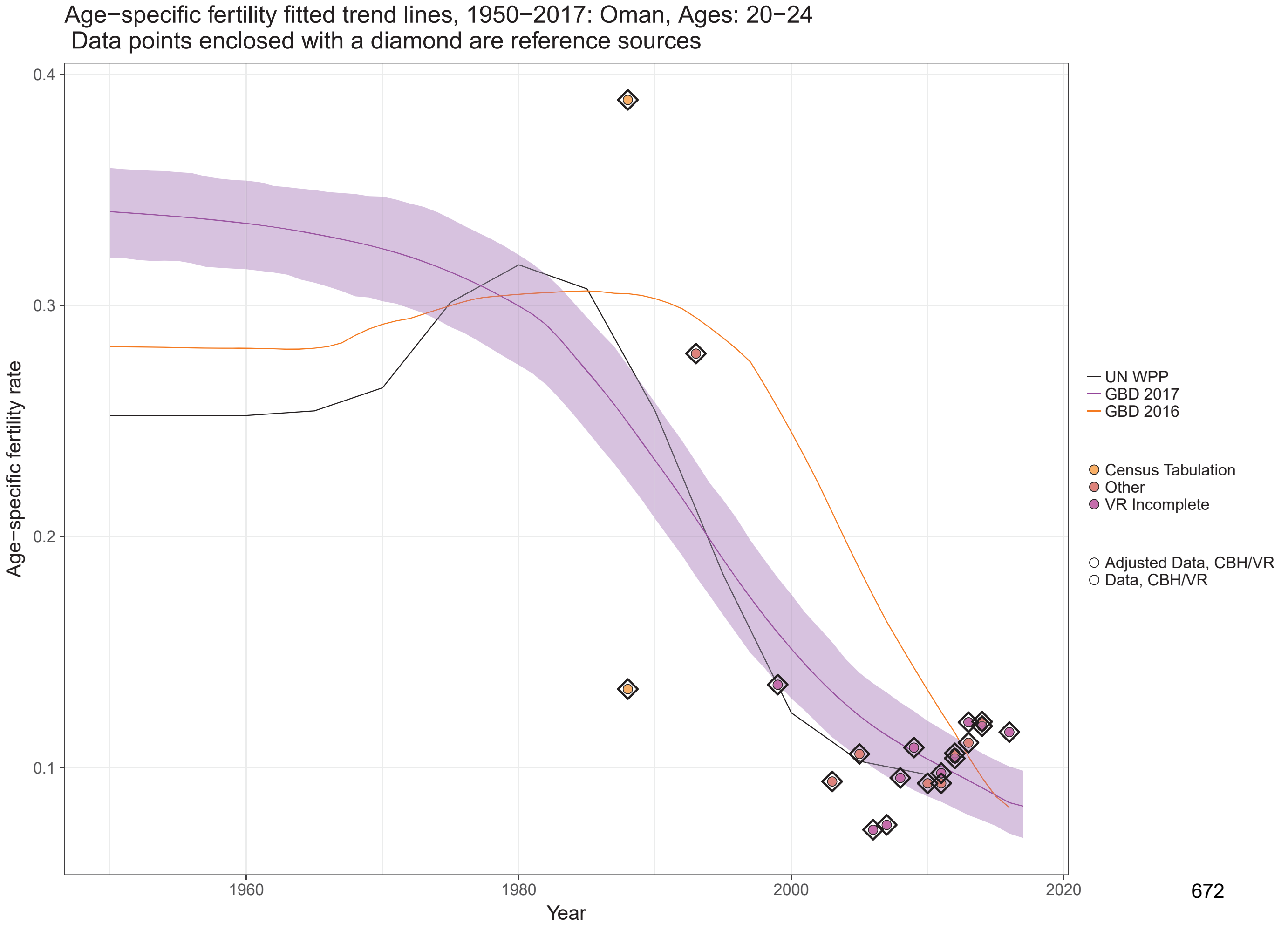
Age−specific fertility fitted trend lines, 1950−2017: Oman, Ages: 20−24
Data points enclosed with a diamond are reference sources
Age−specific fertility rate
Year
0.1
0.2
0.3
0.4
1960
1980
2000
2020
UN WPP
GBD 2017
GBD 2016
Census Tabulation
Other
VR Incomplete
Adjusted Data, CBH/VR
Data, CBH/VR

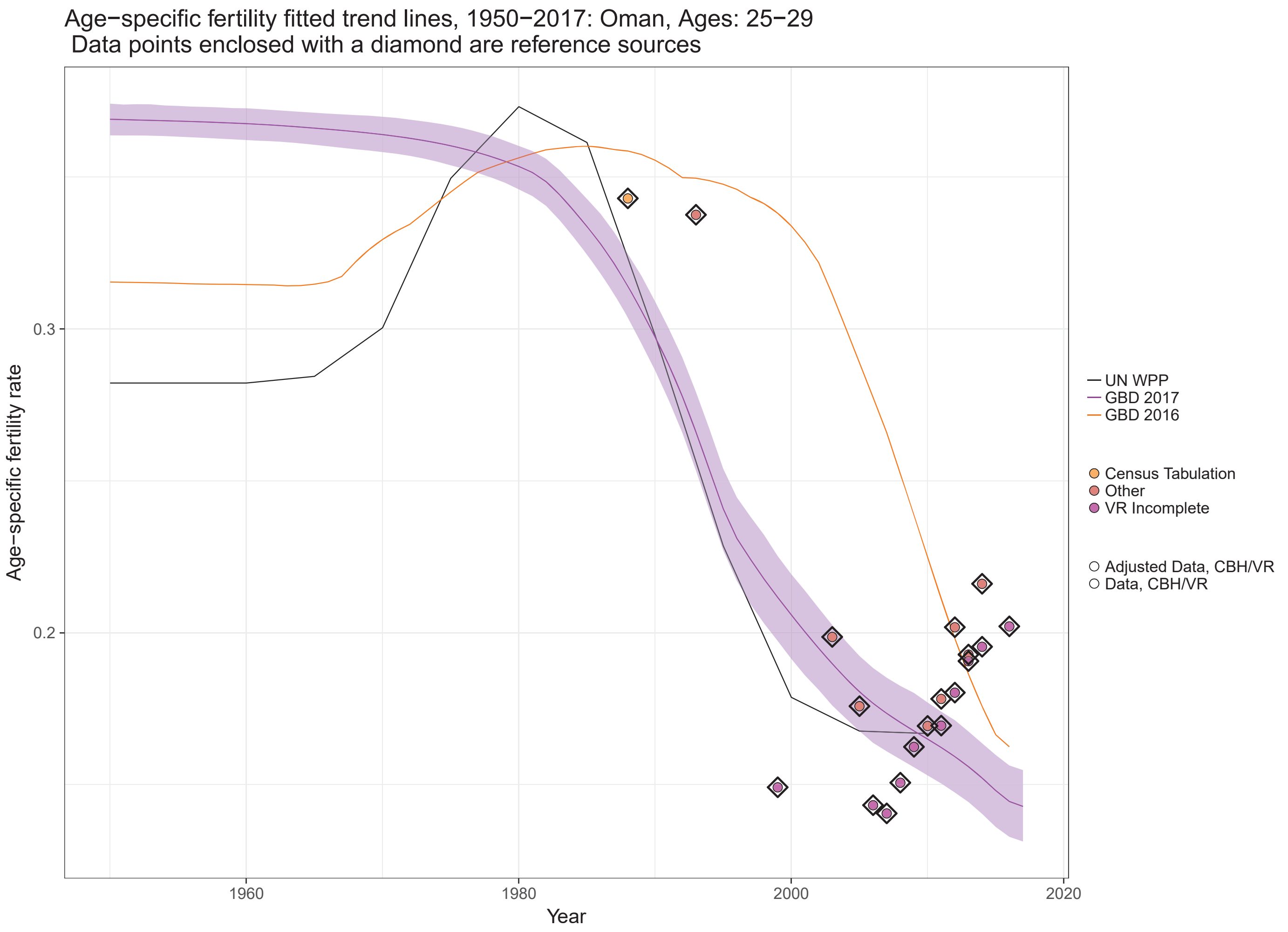
Age−specific fertility fitted trend lines, 1950−2017: Oman, Ages: 25−29
Data points enclosed with a diamond are reference sources
Age−specific fertility rate
0.3
0.2
1960
1980
2000
2020
Year
UN WPP
GBD 2017
GBD 2016
Census Tabulation
Other
VR Incomplete
Adjusted Data, CBH/VR
Data, CBH/VR

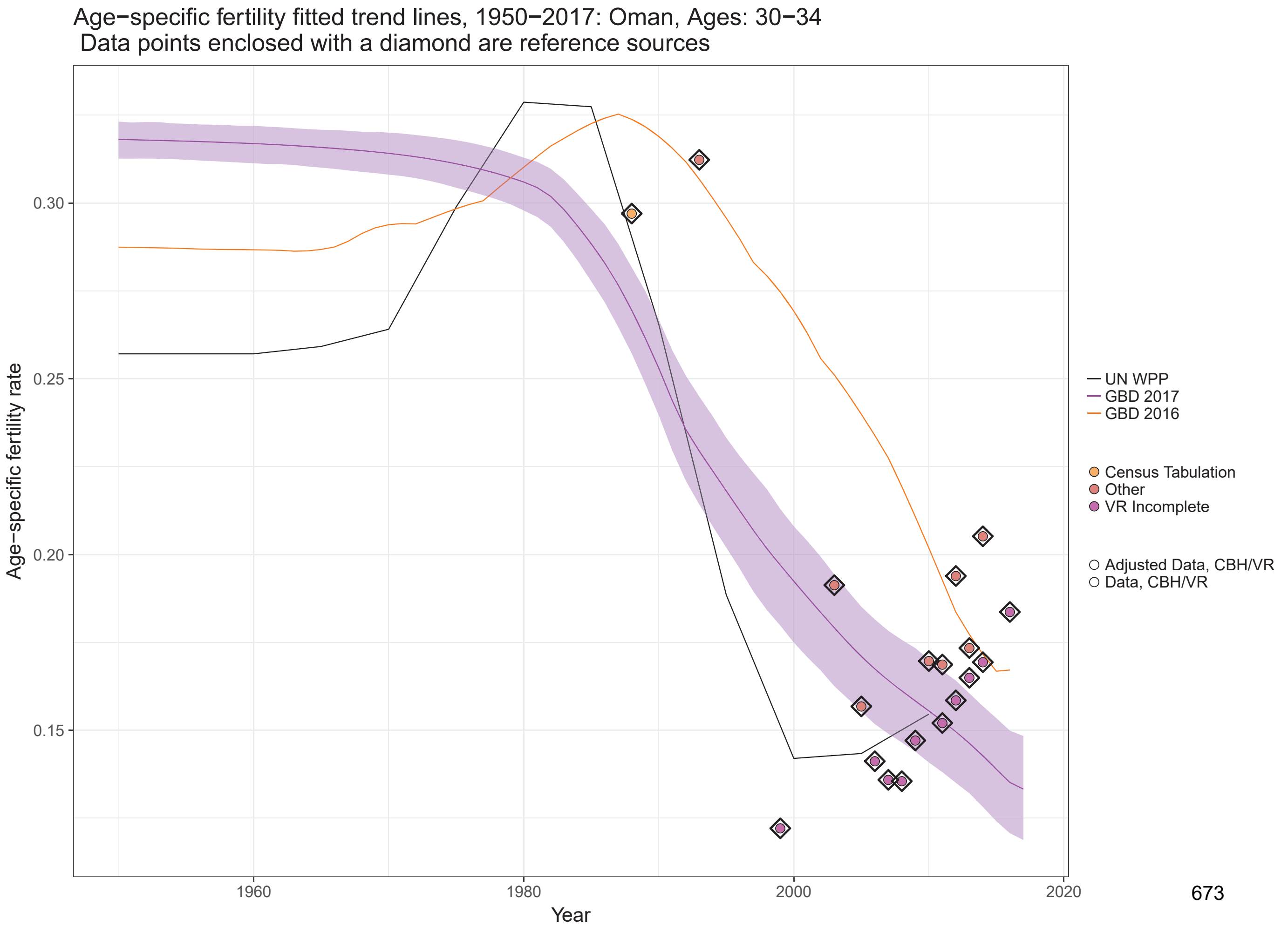
Age−specific fertility fitted trend lines, 1950−2017: Oman, Ages: 30−34
Data points enclosed with a diamond are reference sources
Age−specific fertility rate
0.30
0.25
0.20
0.15
1960
1980
2000
2020
Year
UN WPP
GBD 2017
GBD 2016
Census Tabulation
Other
VR Incomplete
Adjusted Data, CBH/VR
Data, CBH/VR

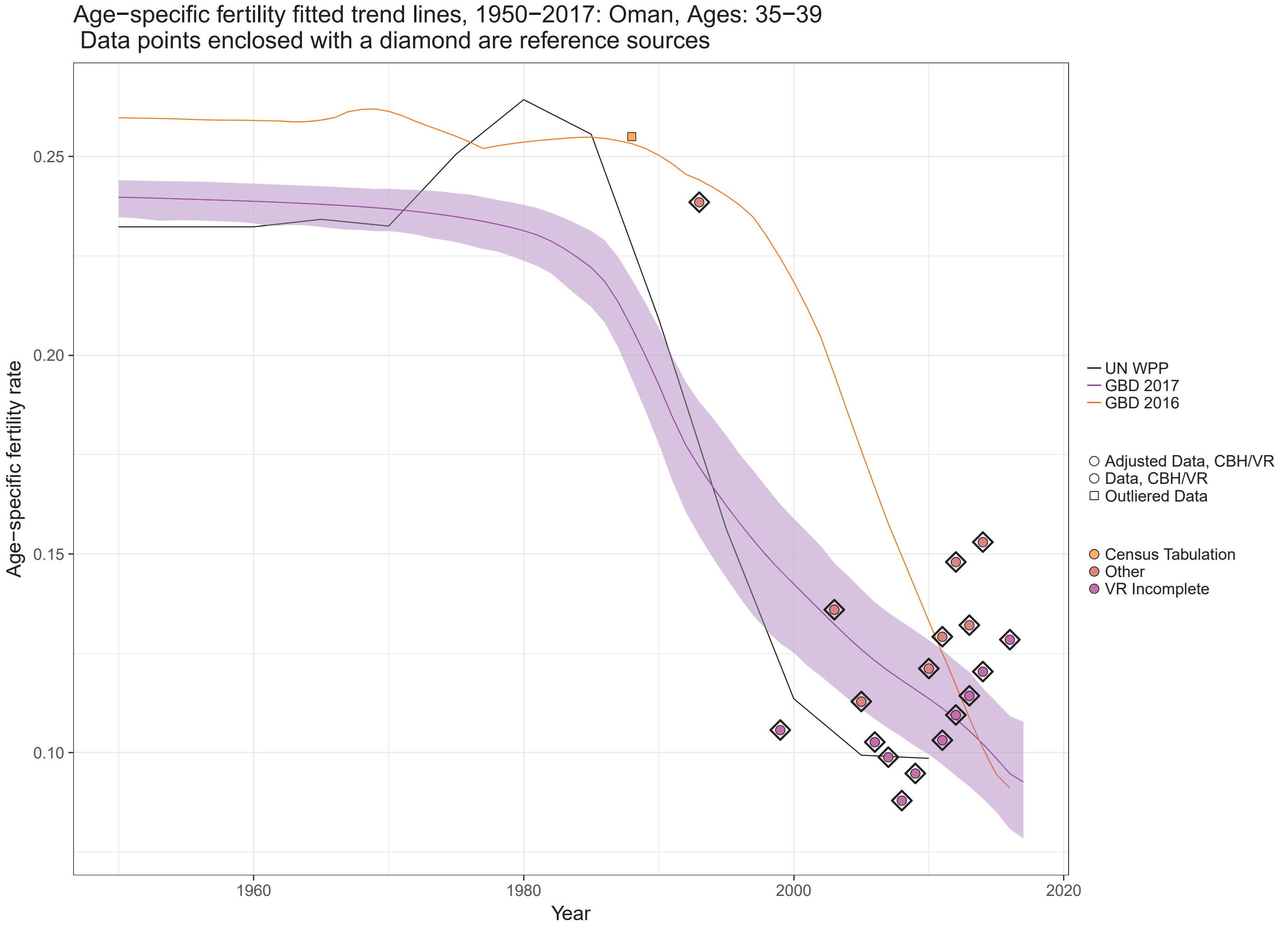
Age−specific fertility fitted trend lines, 1950−2017: Oman, Ages: 35−39
Data points enclosed with a diamond are reference sources
Age−specific fertility rate
Year
UN WPP
GBD 2017
GBD 2016
Adjusted Data, CBH/VR
Data, CBH/VR
Outliered Data
Census Tabulation
Other
VR Incomplete

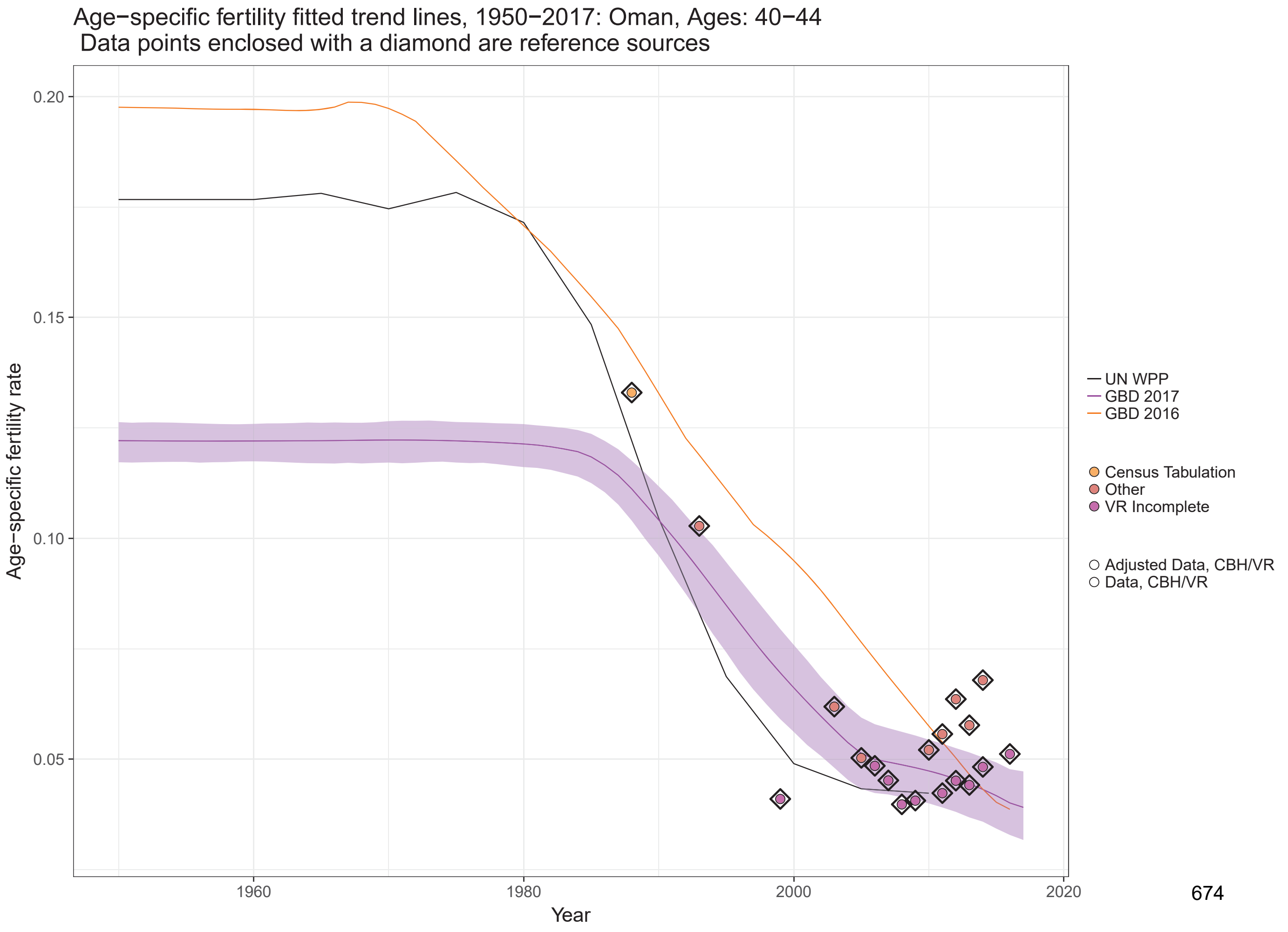
Age−specific fertility fitted trend lines, 1950−2017: Oman, Ages: 40−44
Data points enclosed with a diamond are reference sources
Age−specific fertility rate
Year
UN WPP
GBD 2017
GBD 2016
Census Tabulation
Other
VR Incomplete
Adjusted Data, CBH/VR
Data, CBH/VR

Age−specific fertility fitted trend lines, 1950−2017: Oman, Ages: 45−49
Data points enclosed with a diamond are reference sources

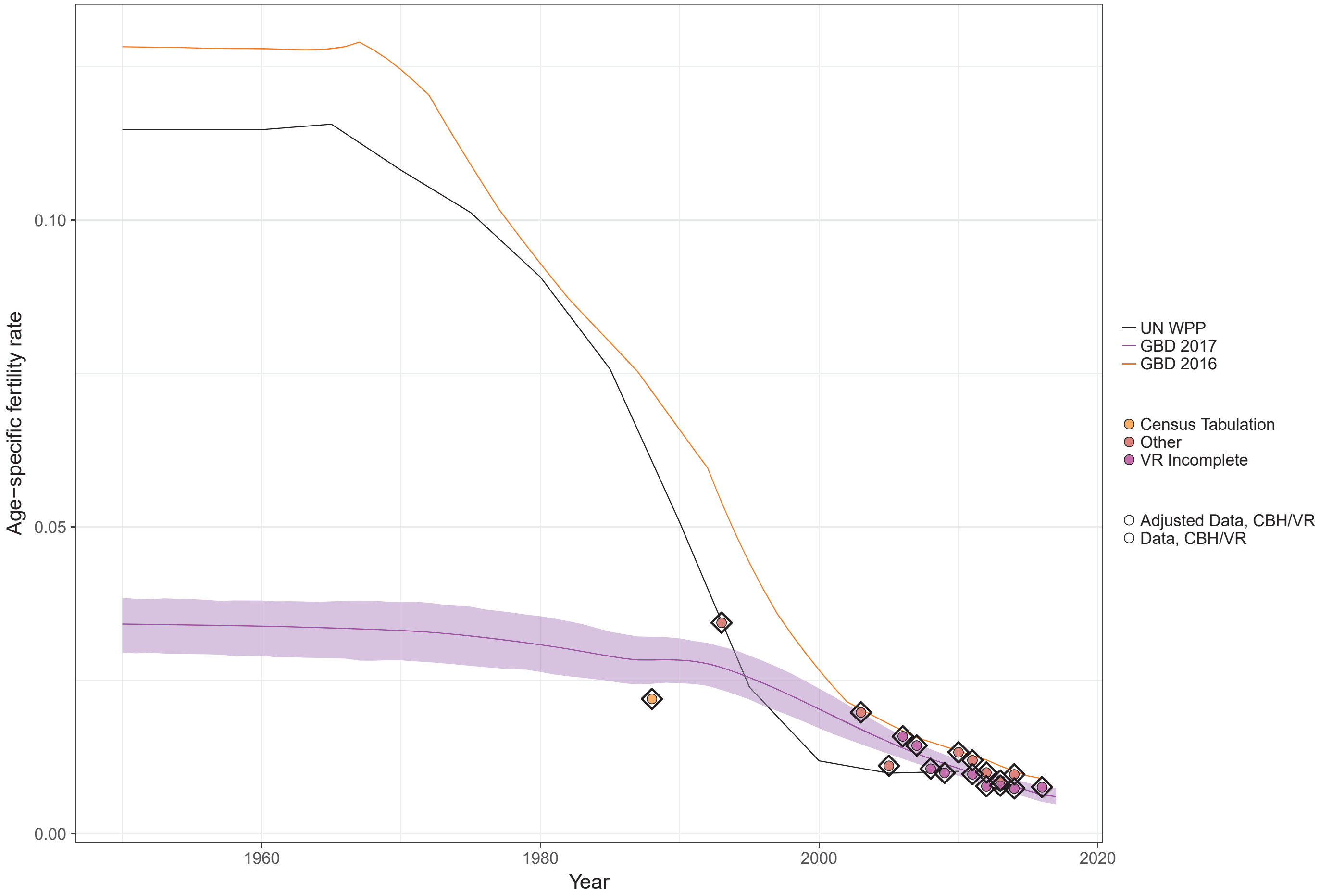

Age−specific fertility fitted trend lines, 1950−2017: Oman, Ages: 50−54
Data points enclosed with a diamond are reference sources

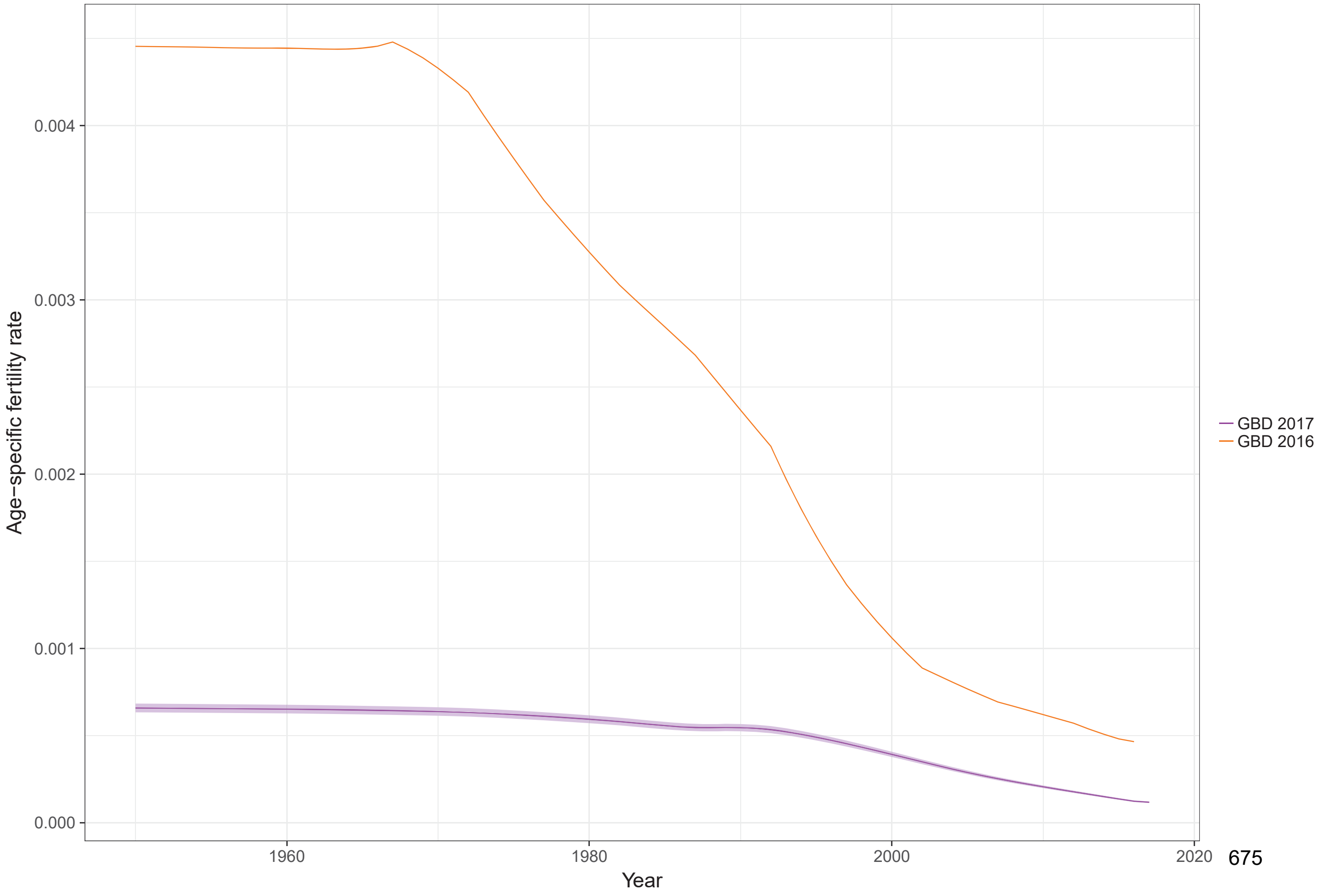

Age−specific fertility fitted trend lines, 1950−2017: Pakistan, TFR
Data points enclosed with a diamond are reference sources

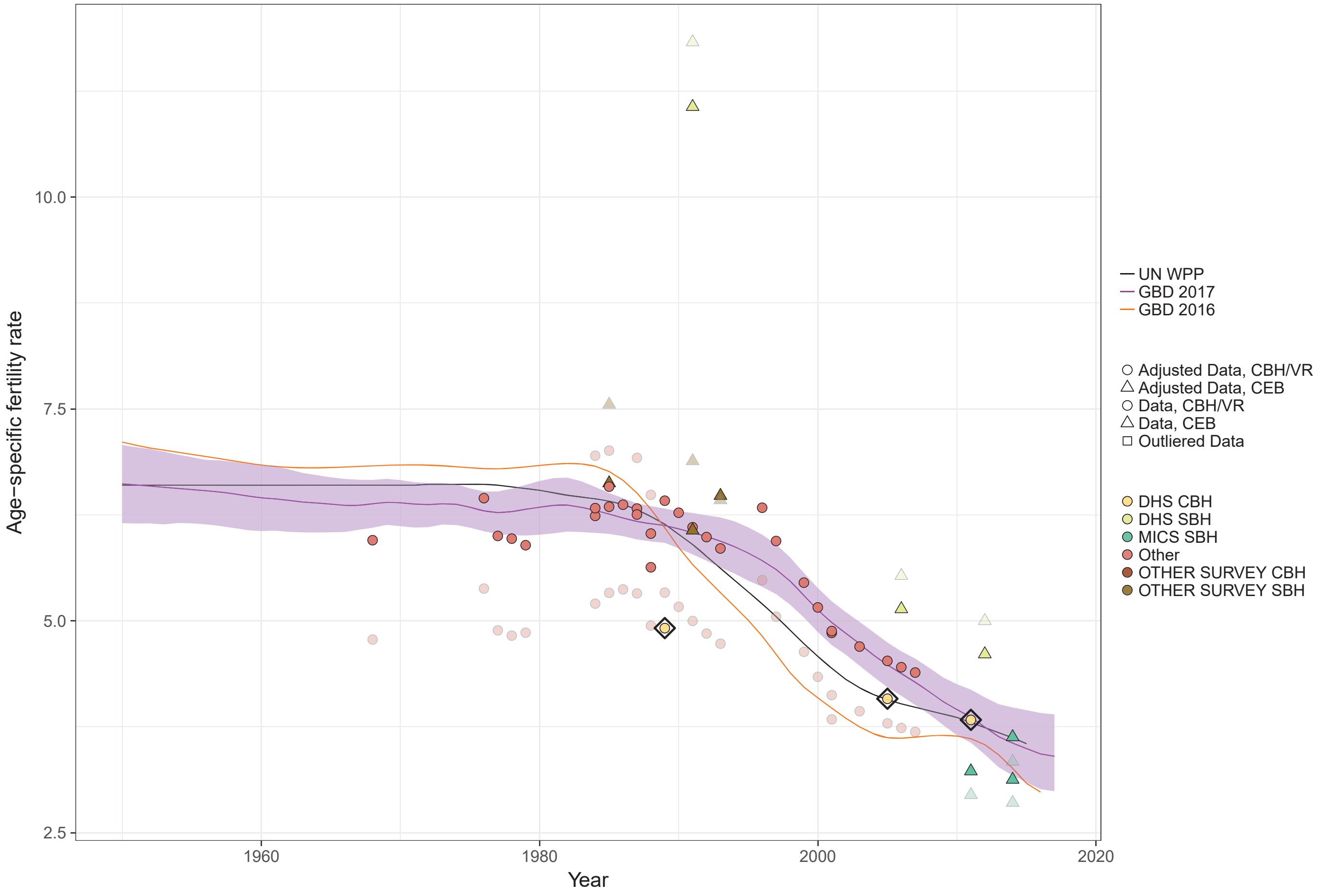

Age−specific fertility fitted trend lines, 1950−2017: Pakistan, Ages: 10−14
Data points enclosed with a diamond are reference sources

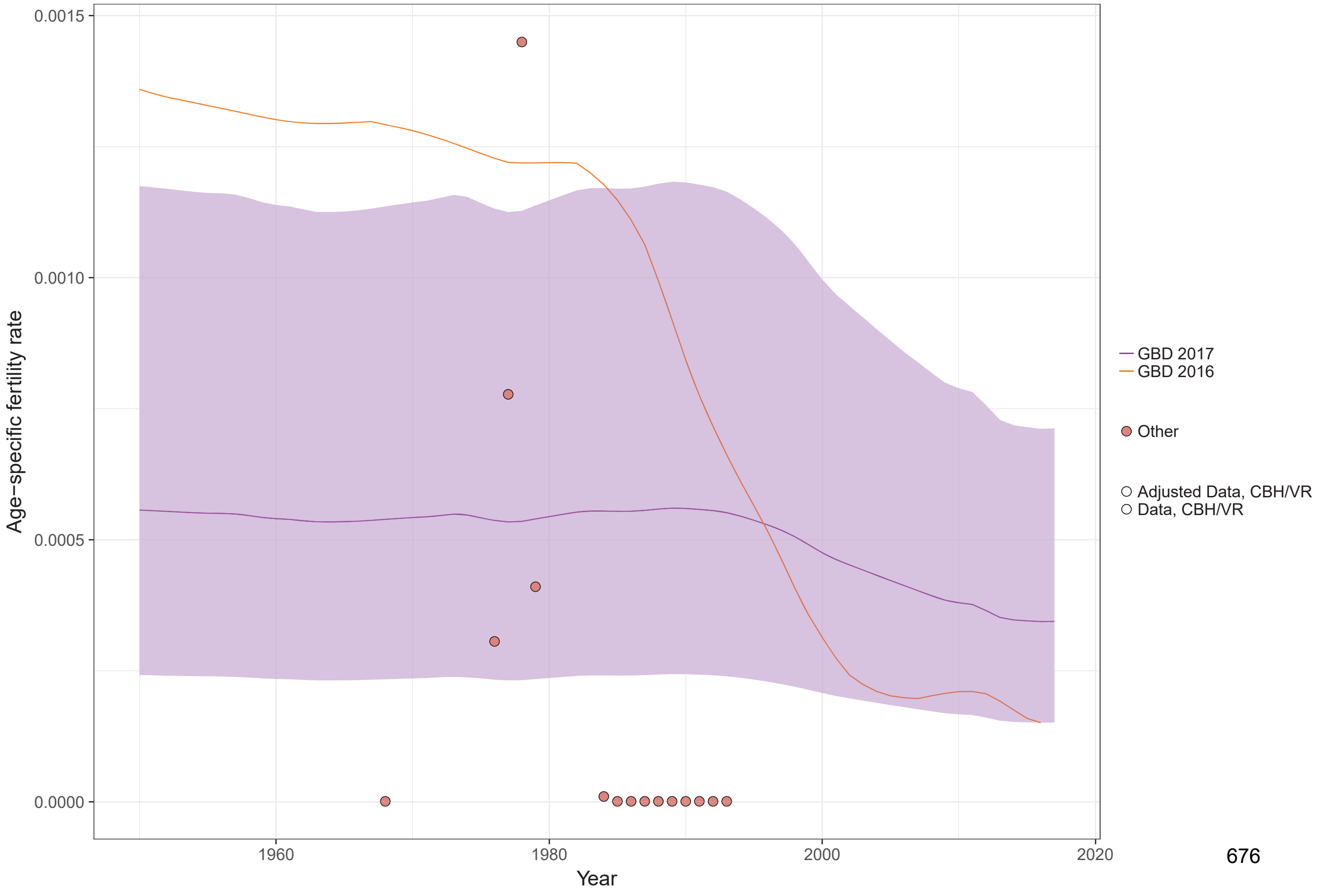

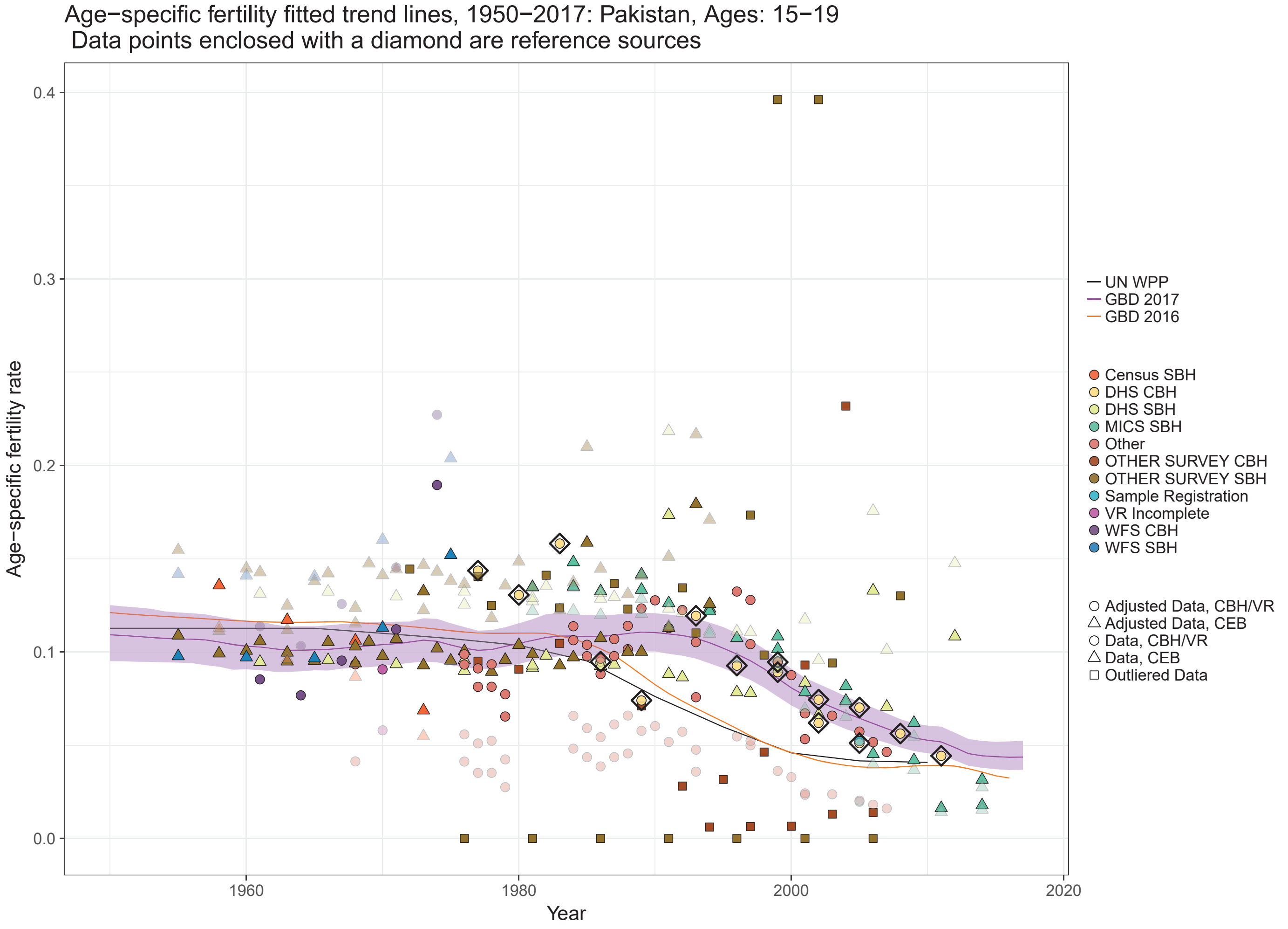

Age−specific fertility fitted trend lines, 1950−2017: Pakistan, Ages: 15−19
Data points enclosed with a diamond are reference sources
Age-specific fertility rate
Year
UN WPP
GBD 2017
GBD 2016
Census SBH
DHS CBH
DHS SBH
MICS SBH
Other
OTHER SURVEY CBH
OTHER SURVEY SBH
Sample Registration
VR Incomplete
WFS CBH
WFS SBH
Adjusted Data, CBH/VR
Adjusted Data, CEB
Data, CBH/VR
Data, CEB
Outliered Data

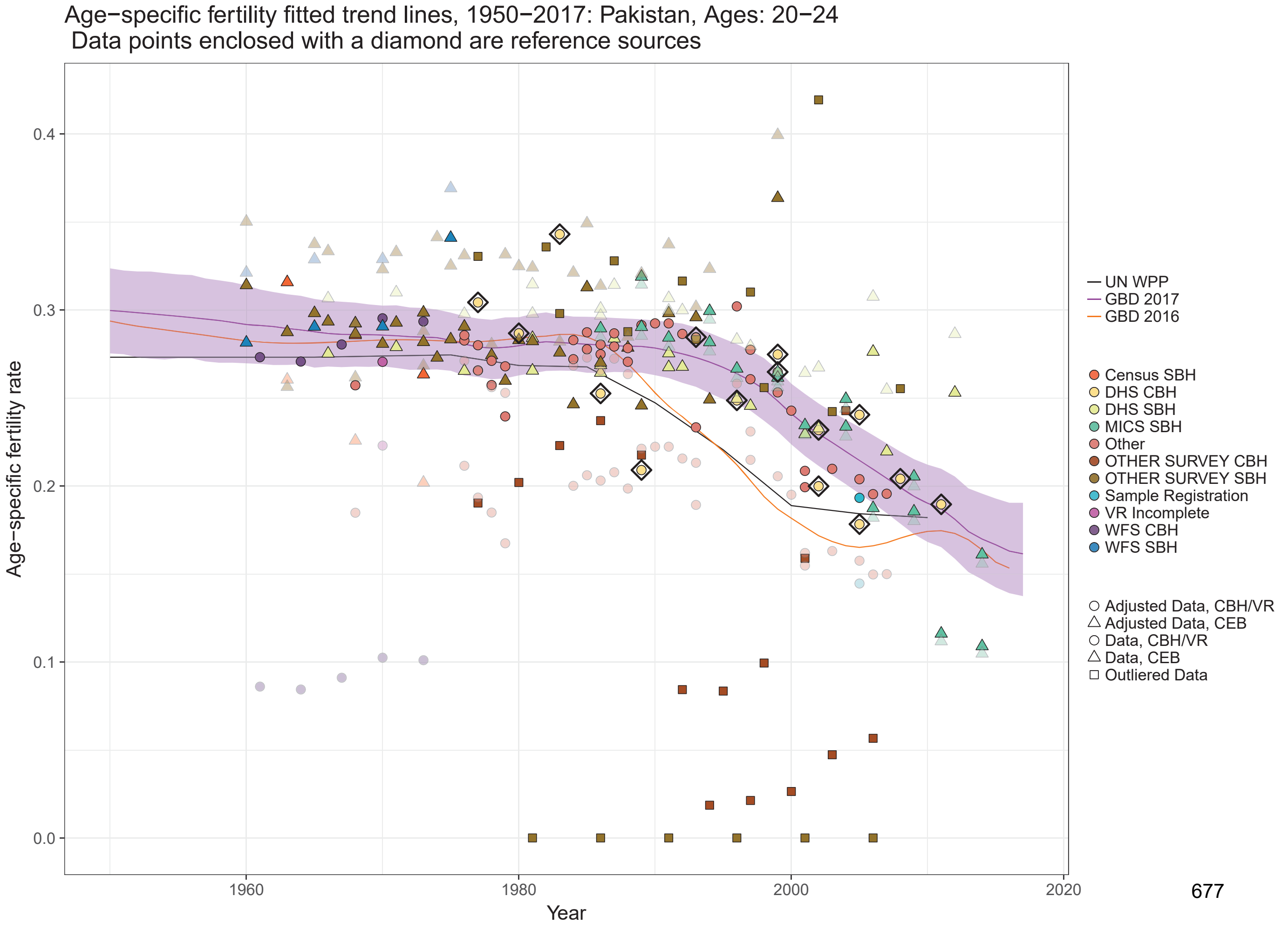

Age−specific fertility fitted trend lines, 1950−2017: Pakistan, Ages: 20−24
Data points enclosed with a diamond are reference sources
Age-specific fertility rate
Year
UN WPP
GBD 2017
GBD 2016
Census SBH
DHS CBH
DHS SBH
MICS SBH
Other
OTHER SURVEY CBH
OTHER SURVEY SBH
Sample Registration
VR Incomplete
WFS CBH
WFS SBH
Adjusted Data, CBH/VR
Adjusted Data, CEB
Data, CBH/VR
Data, CEB
Outliered Data

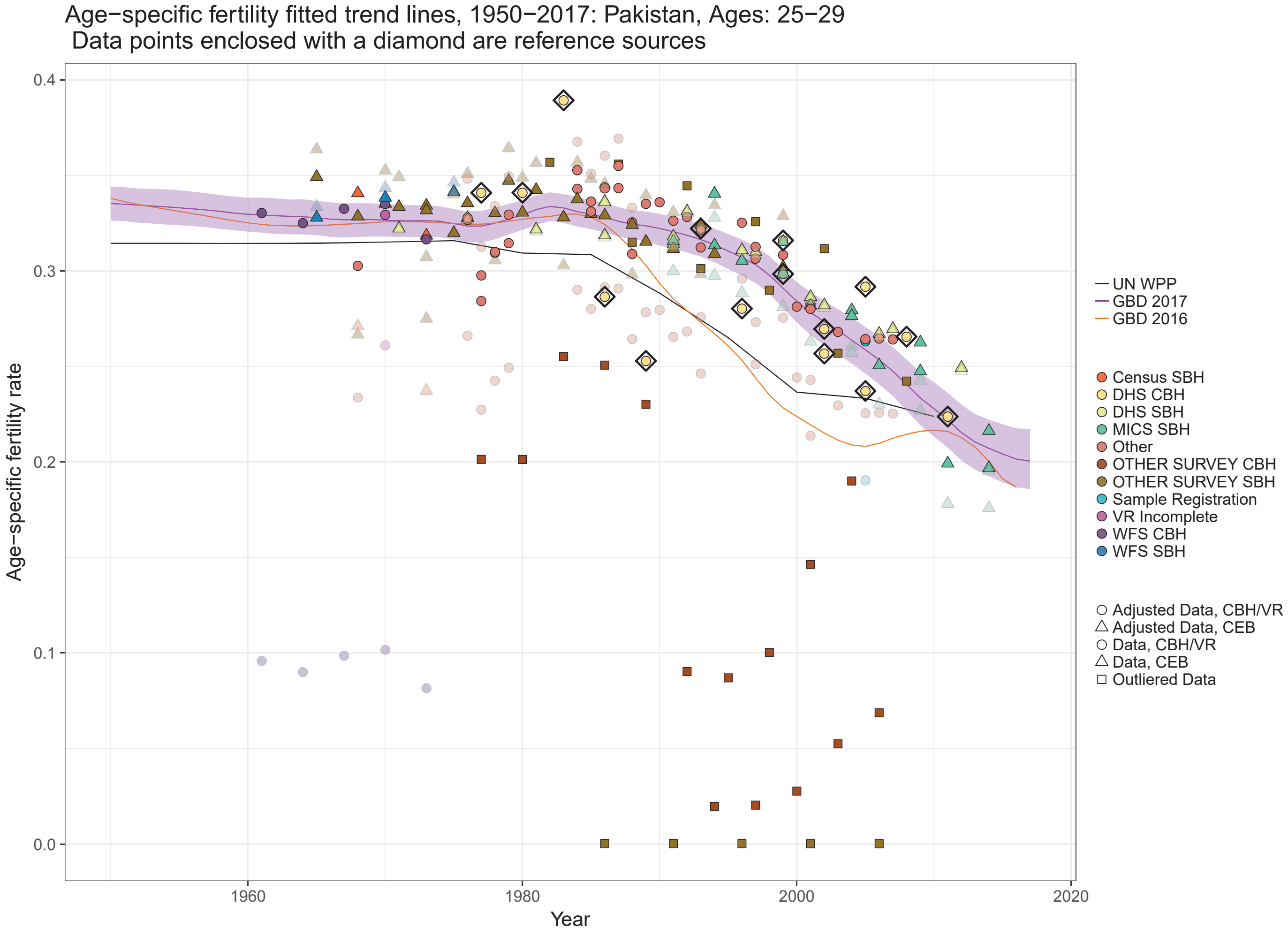
Age−specific fertility fitted trend lines, 1950−2017: Pakistan, Ages: 25−29
Data points enclosed with a diamond are reference sources
Age-specific fertility rate
Year
UN WPP
GBD 2017
GBD 2016
Census SBH
DHS CBH
DHS SBH
MICS SBH
Other
OTHER SURVEY CBH
OTHER SURVEY SBH
Sample Registration
VR Incomplete
WFS CBH
WFS SBH
Adjusted Data, CBH/VR
Adjusted Data, CEB
Data, CBH/VR
Data, CEB
Outliered Data

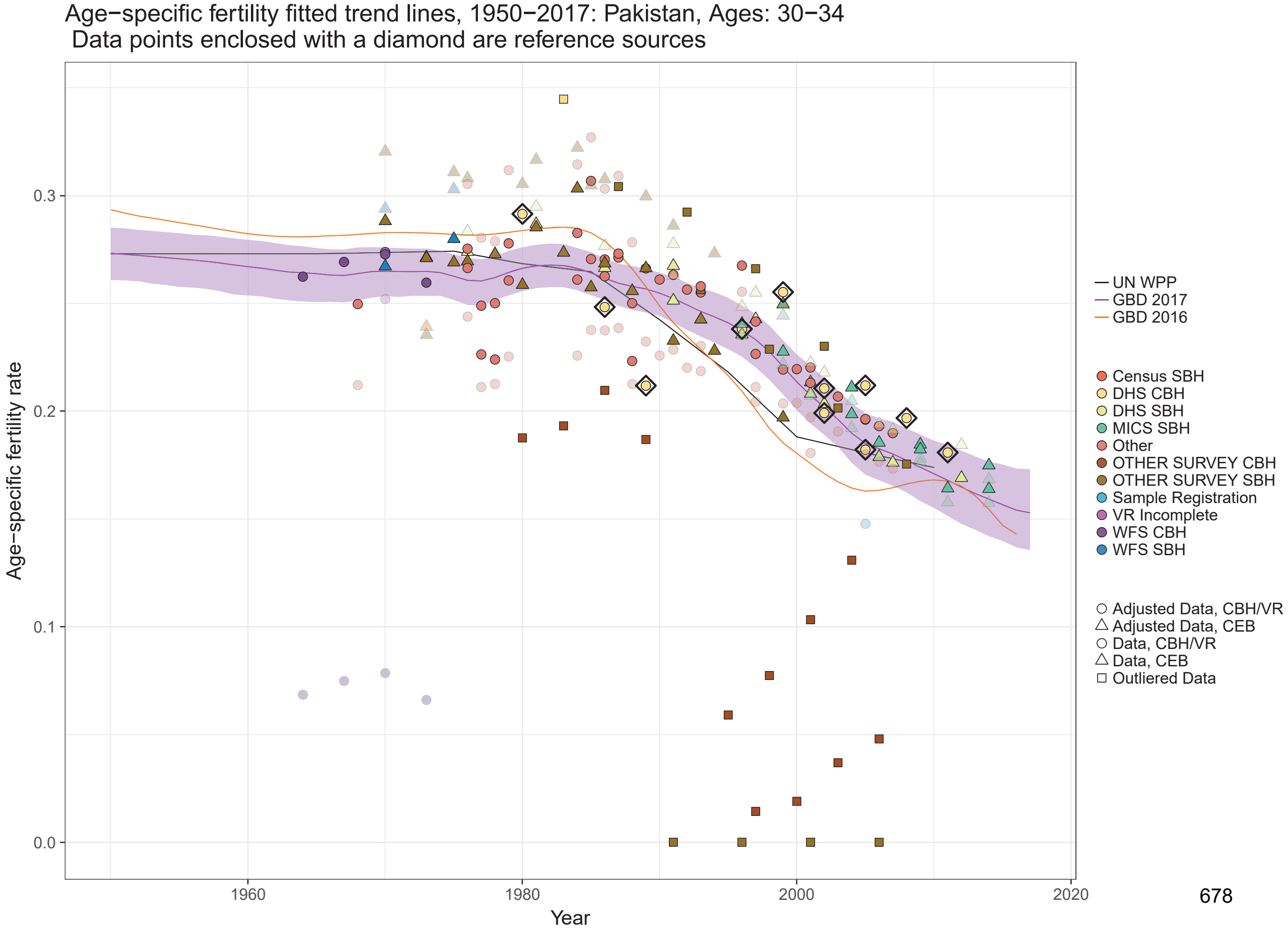
Age−specific fertility fitted trend lines, 1950−2017: Pakistan, Ages: 30−34
Data points enclosed with a diamond are reference sources
Age-specific fertility rate
Year
UN WPP
GBD 2017
GBD 2016
Census SBH
DHS CBH
DHS SBH
MICS SBH
Other
OTHER SURVEY CBH
OTHER SURVEY SBH
Sample Registration
VR Incomplete
WFS CBH
WFS SBH
Adjusted Data, CBH/VR
Adjusted Data, CEB
Data, CBH/VR
Data, CEB
Outliered Data

Age−specific fertility fitted trend lines, 1950−2017: Pakistan, Ages: 35−39
Data points enclosed with a diamond are reference sources

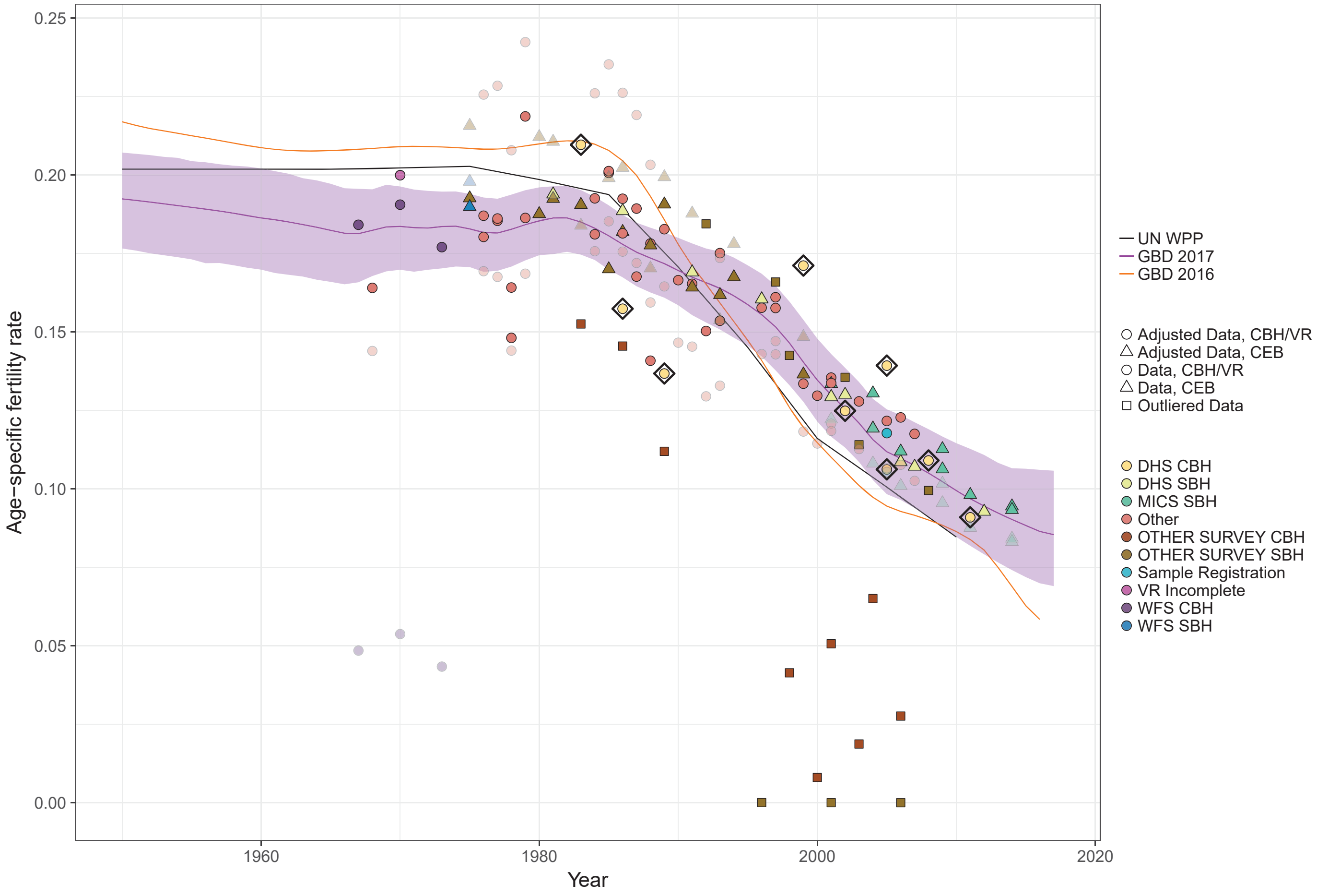

Age−specific fertility fitted trend lines, 1950−2017: Pakistan, Ages: 40−44
Data points enclosed with a diamond are reference sources

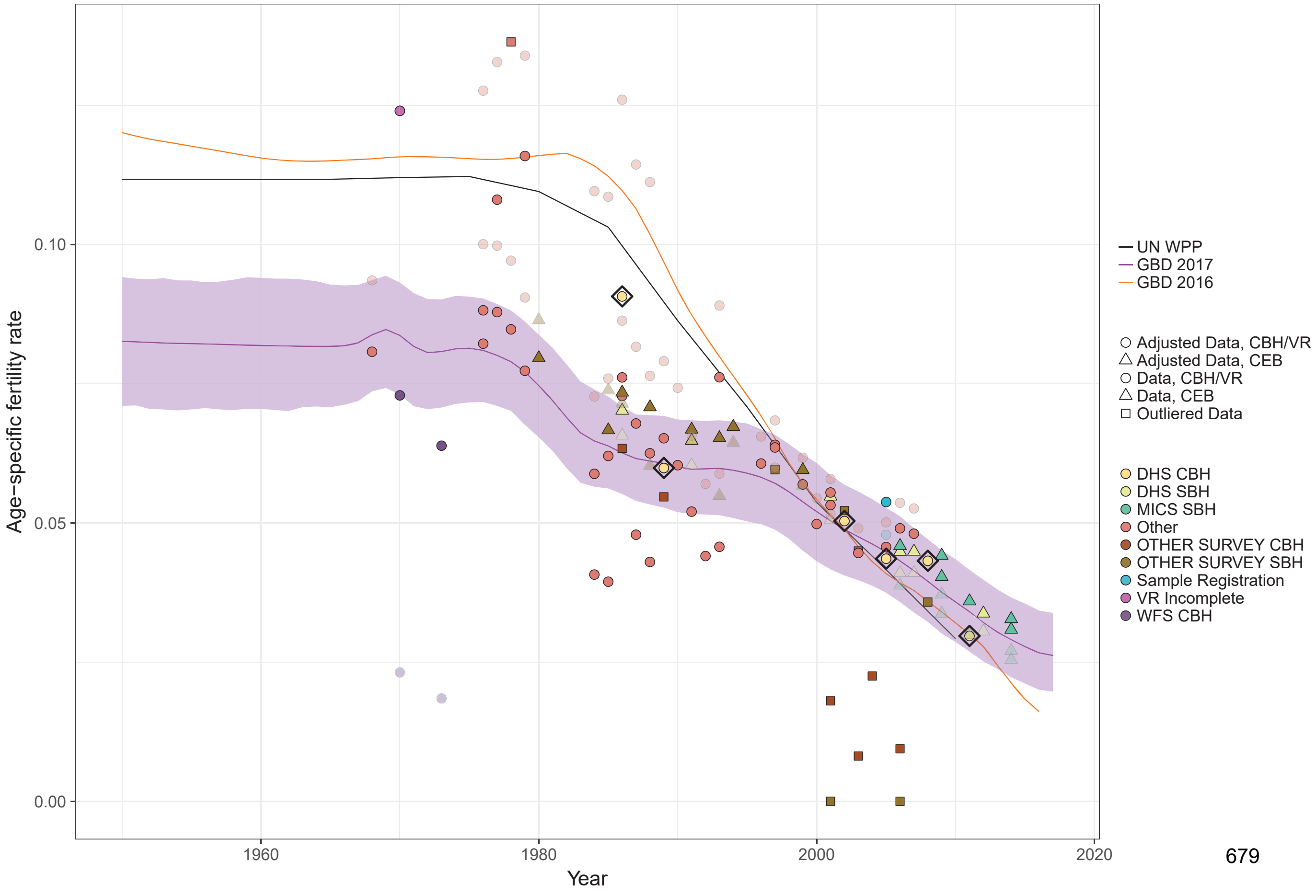

Age−specific fertility fitted trend lines, 1950−2017: Pakistan, Ages: 45−49
Data points enclosed with a diamond are reference sources

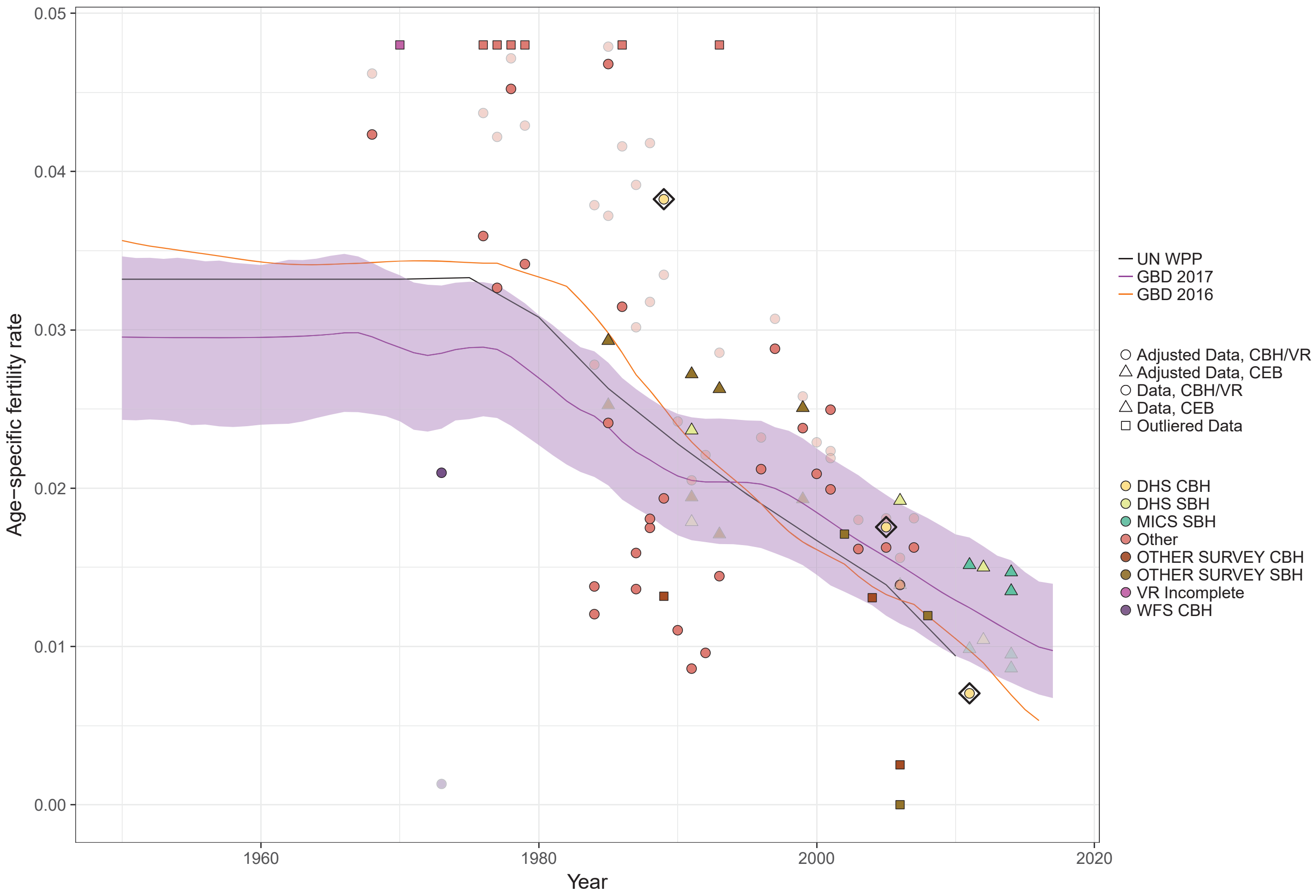

Age−specific fertility fitted trend lines, 1950−2017: Pakistan, Ages: 50−54
Data points enclosed with a diamond are reference sources

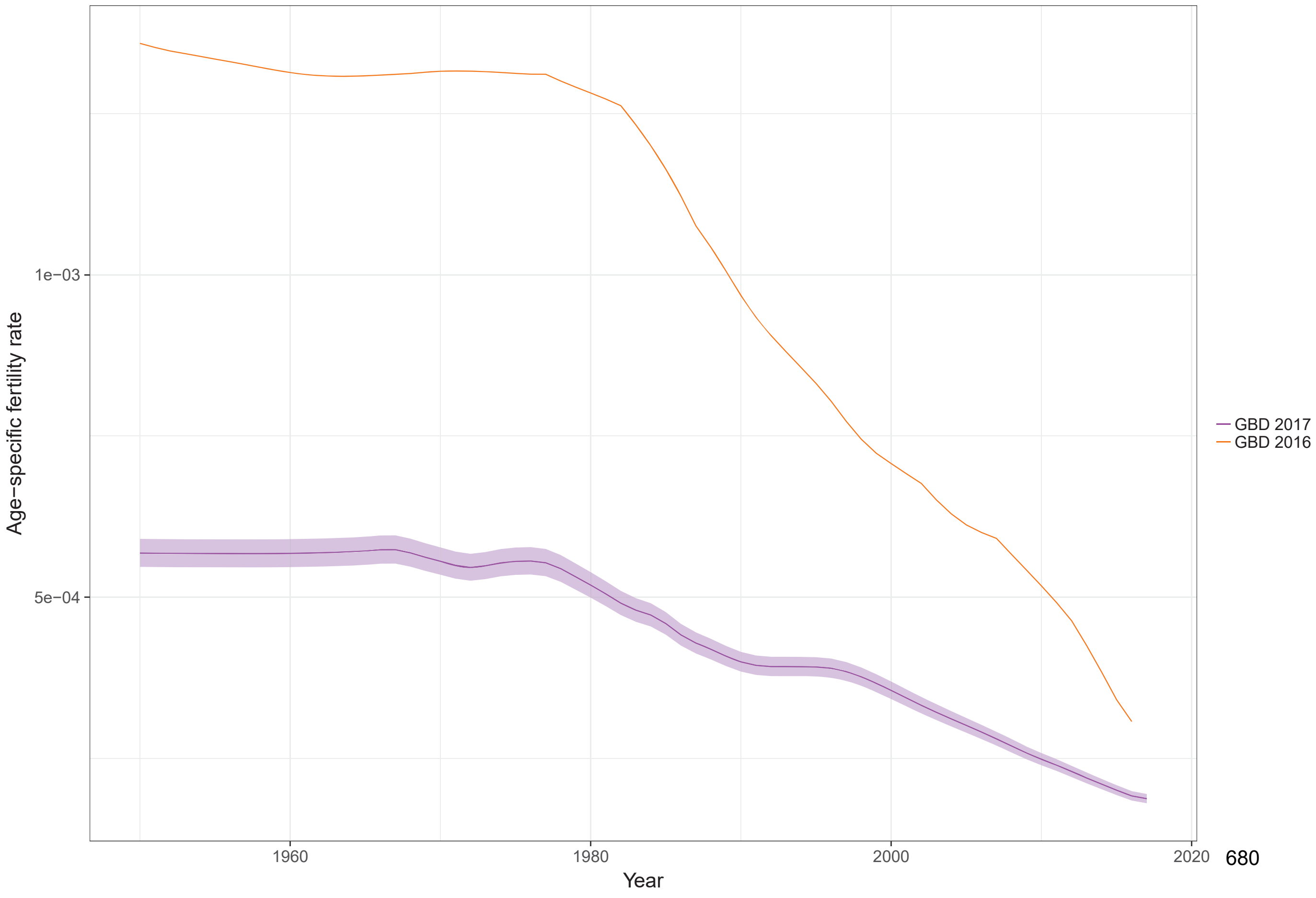

Age−specific fertility fitted trend lines, 1950−2017: Palestine, TFR
Data points enclosed with a diamond are reference sources

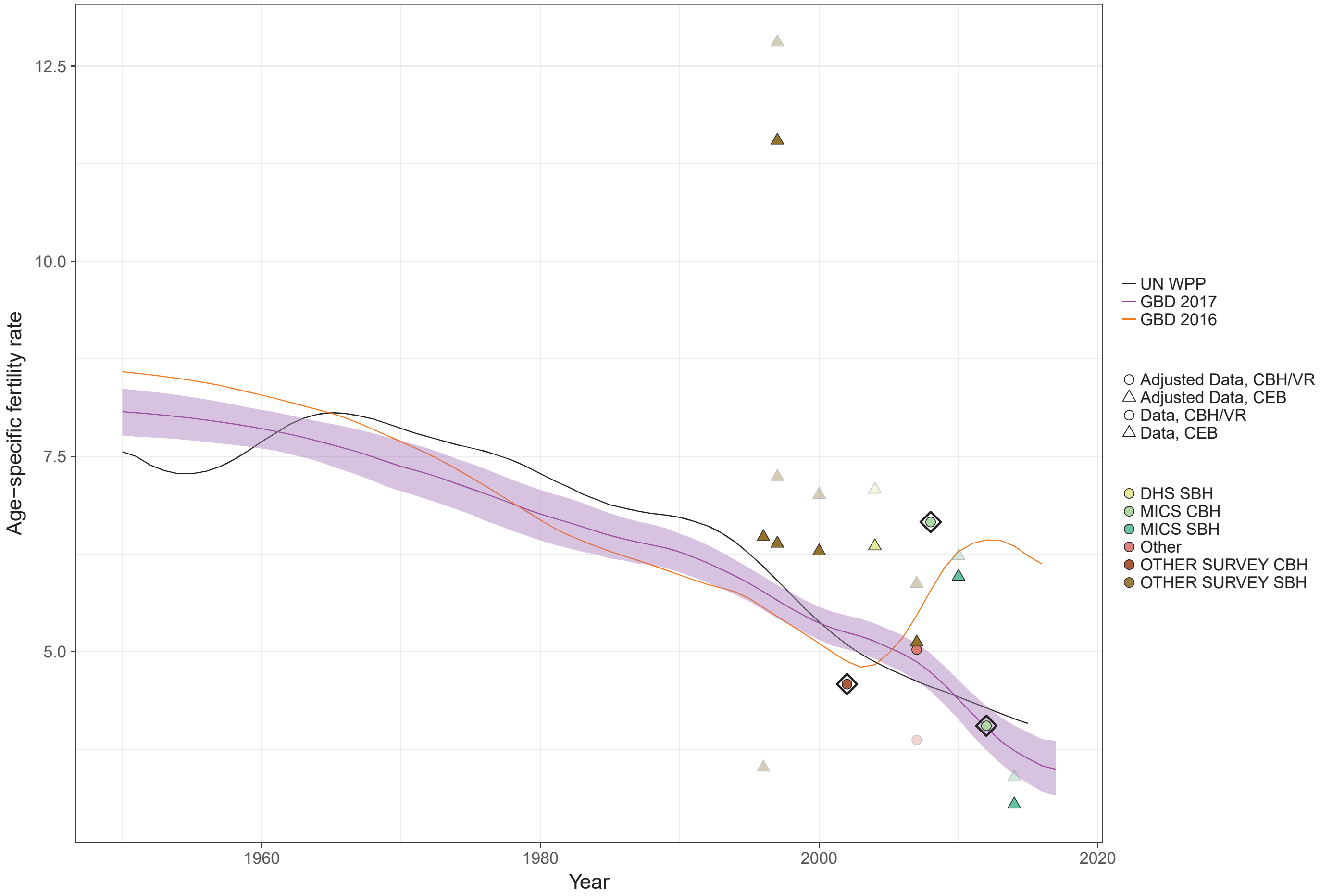

Age−specific fertility fitted trend lines, 1950−2017: Palestine, Ages: 10−14
Data points enclosed with a diamond are reference sources

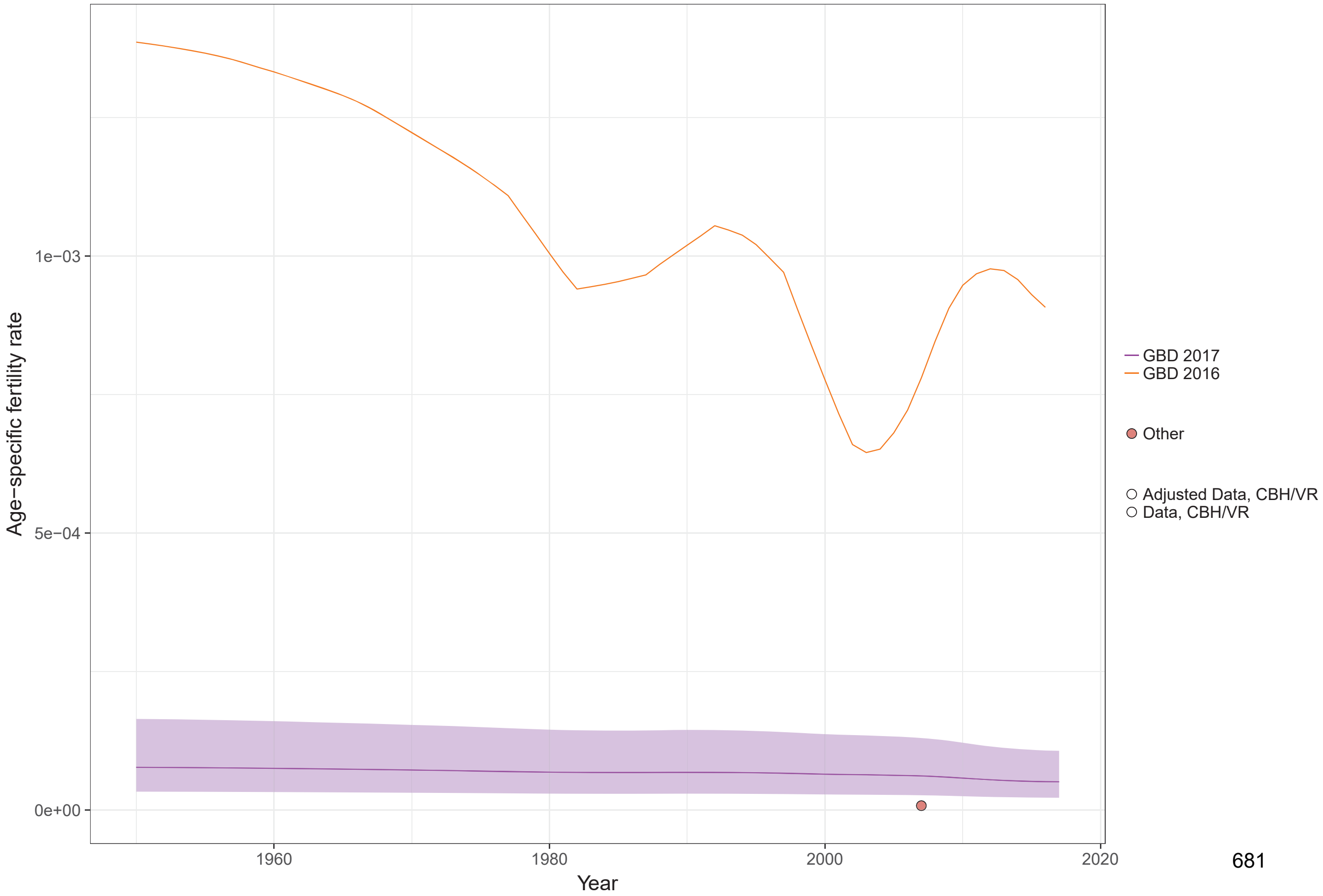

Age−specific fertility fitted trend lines, 1950−2017: Palestine, Ages: 15−19
Data points enclosed with a diamond are reference sources

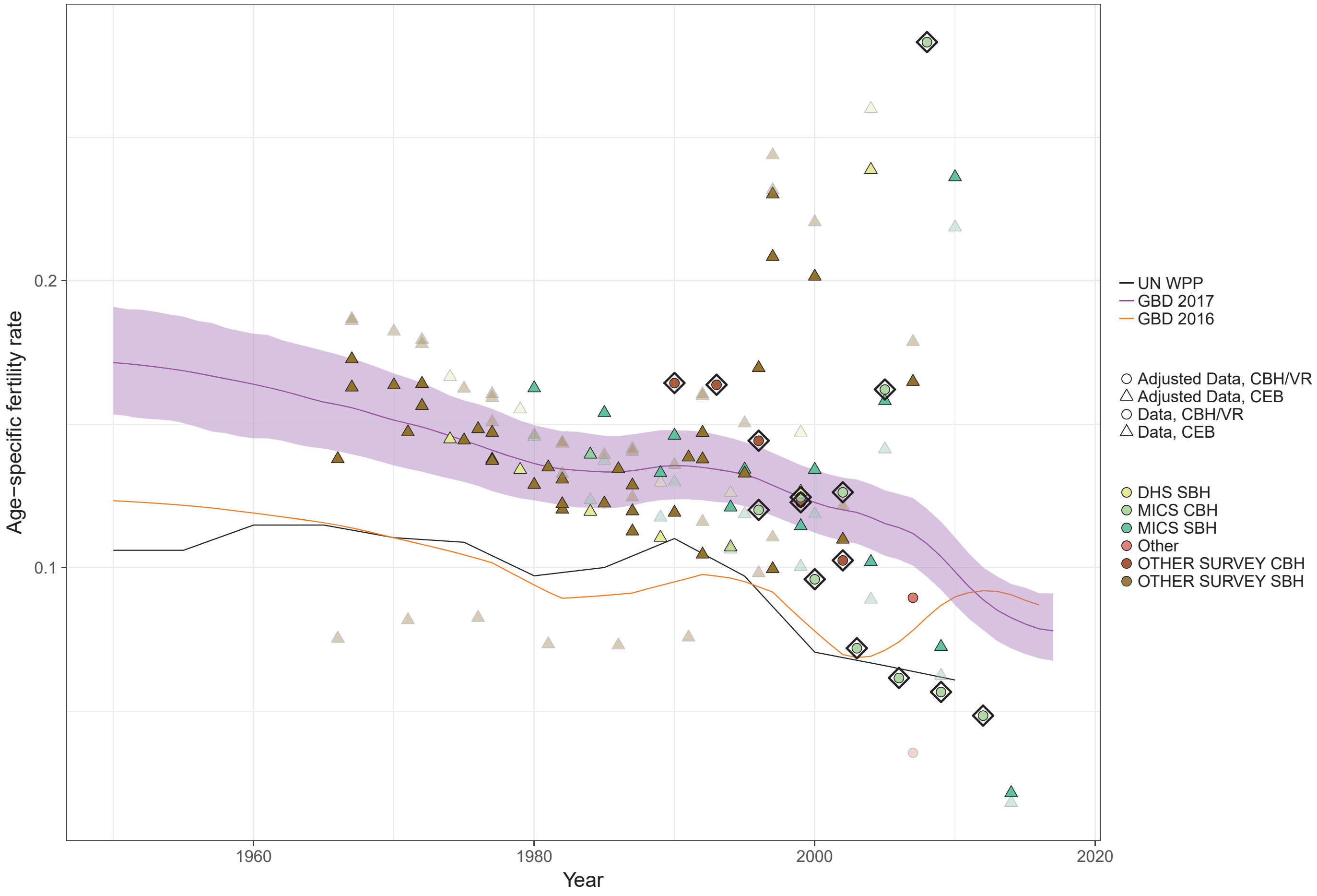

Age−specific fertility fitted trend lines, 1950−2017: Palestine, Ages: 20−24
Data points enclosed with a diamond are reference sources

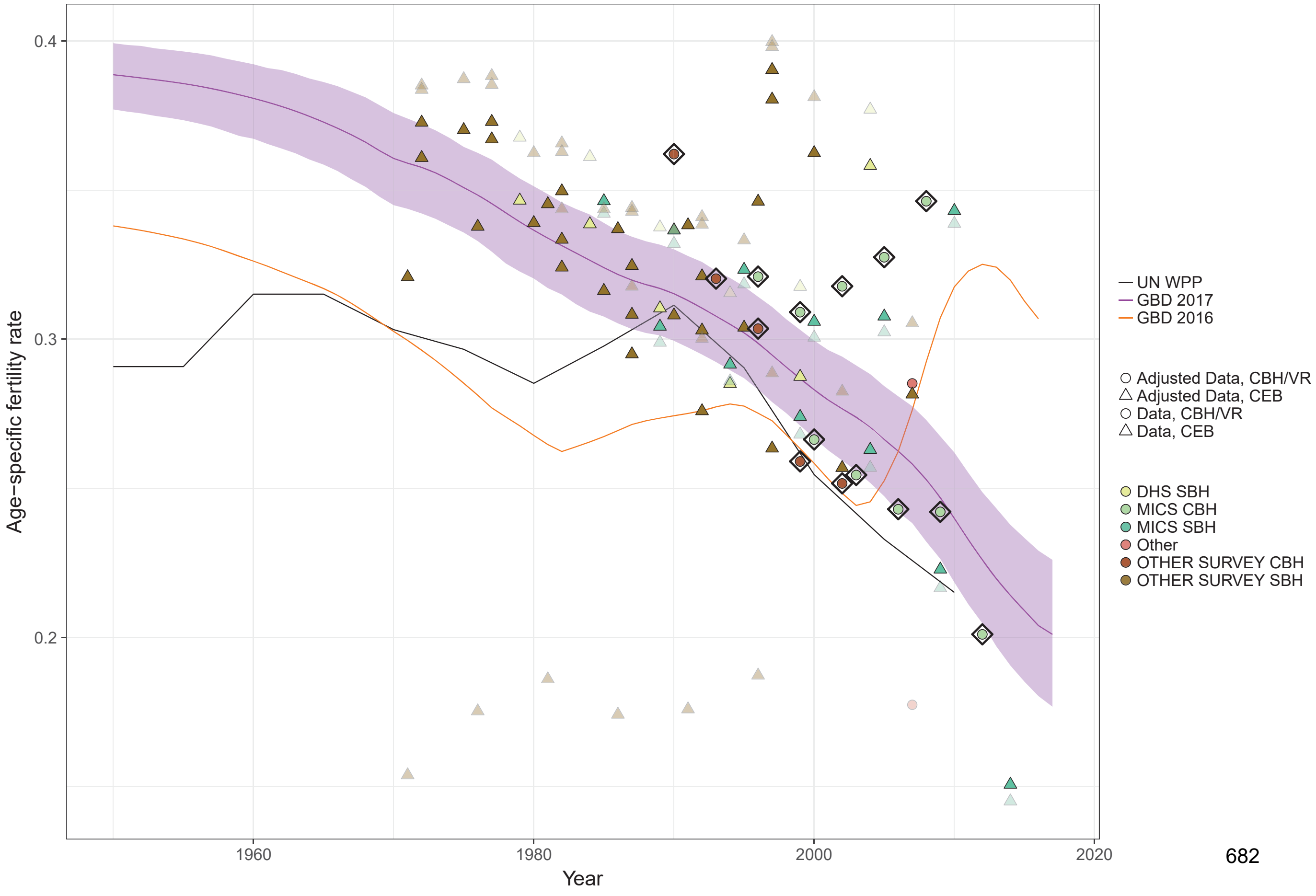

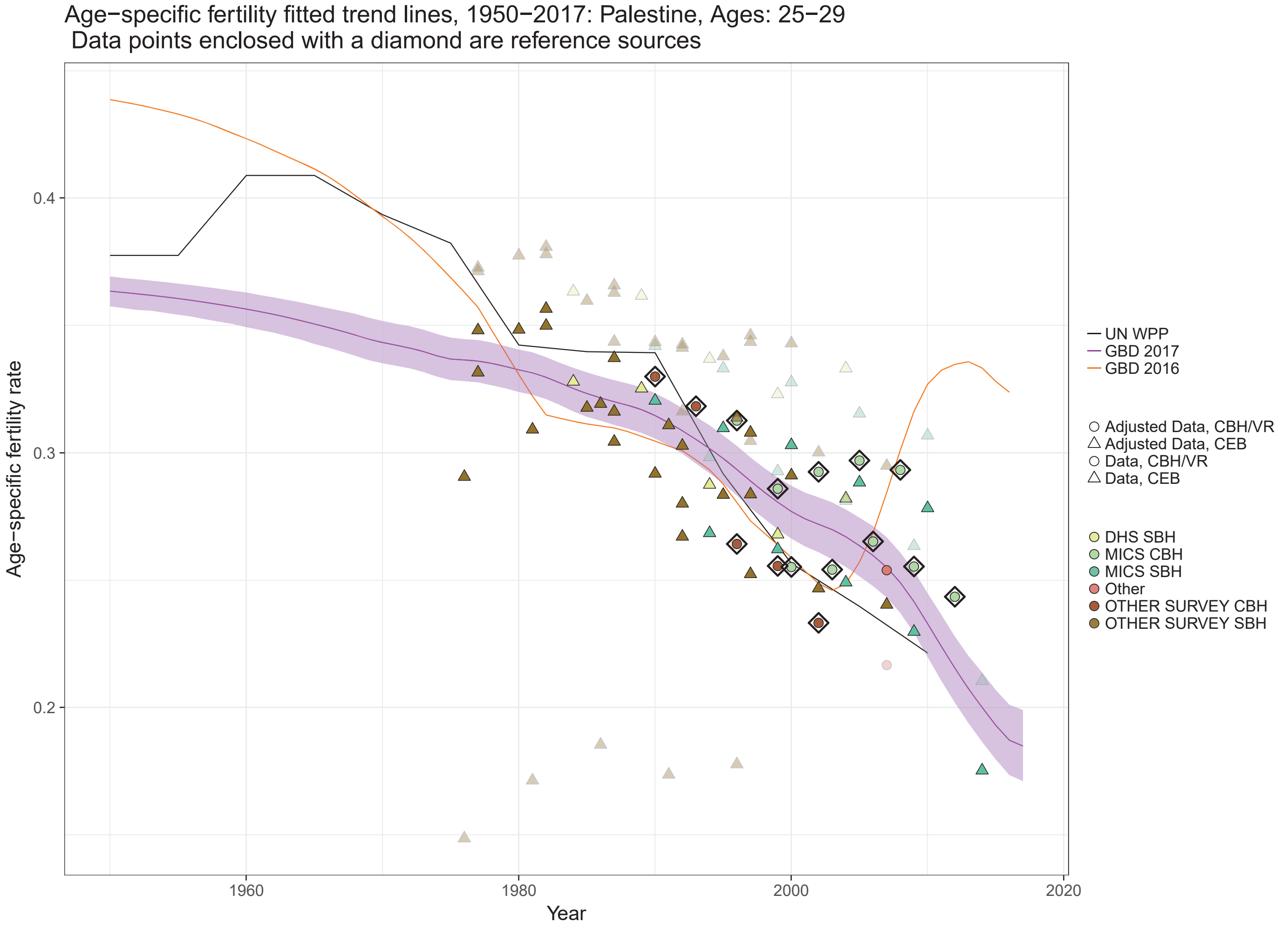

Age−specific fertility fitted trend lines, 1950−2017: Palestine, Ages: 25−29
Data points enclosed with a diamond are reference sources
Age−specific fertility rate
0.4
0.3
0.2
1960
1980
2000
2020
Year
UN WPP
GBD 2017
GBD 2016
Adjusted Data, CBH/VR
Adjusted Data, CEB
Data, CBH/VR
Data, CEB
DHS SBH
MICS CBH
MICS SBH
Other
OTHER SURVEY CBH
OTHER SURVEY SBH

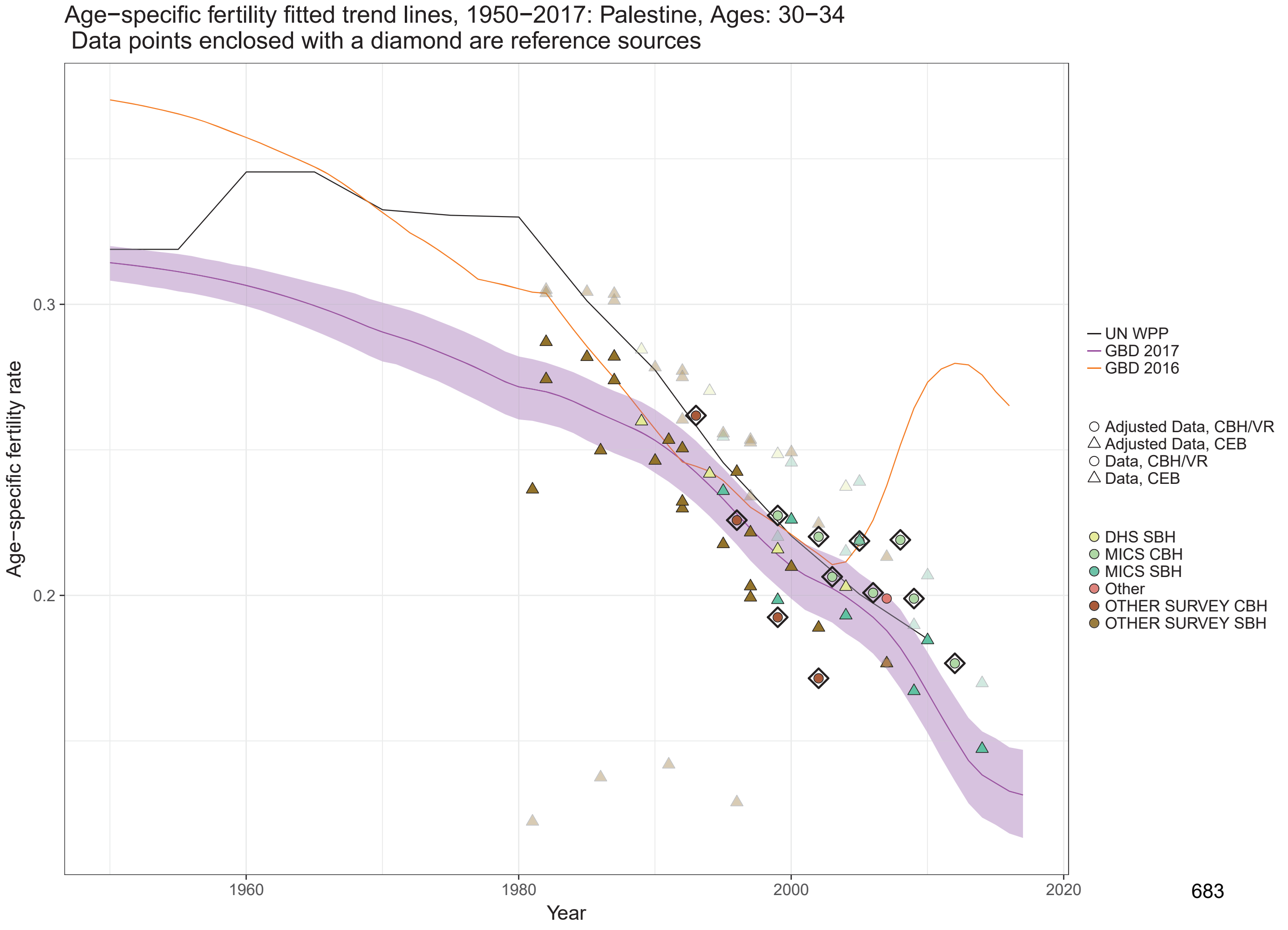

Age−specific fertility fitted trend lines, 1950−2017: Palestine, Ages: 30−34
Data points enclosed with a diamond are reference sources
Age−specific fertility rate
0.3
0.2
1960
1980
2000
2020
Year
UN WPP
GBD 2017
GBD 2016
Adjusted Data, CBH/VR
Adjusted Data, CEB
Data, CBH/VR
Data, CEB
DHS SBH
MICS CBH
MICS SBH
Other
OTHER SURVEY CBH
OTHER SURVEY SBH

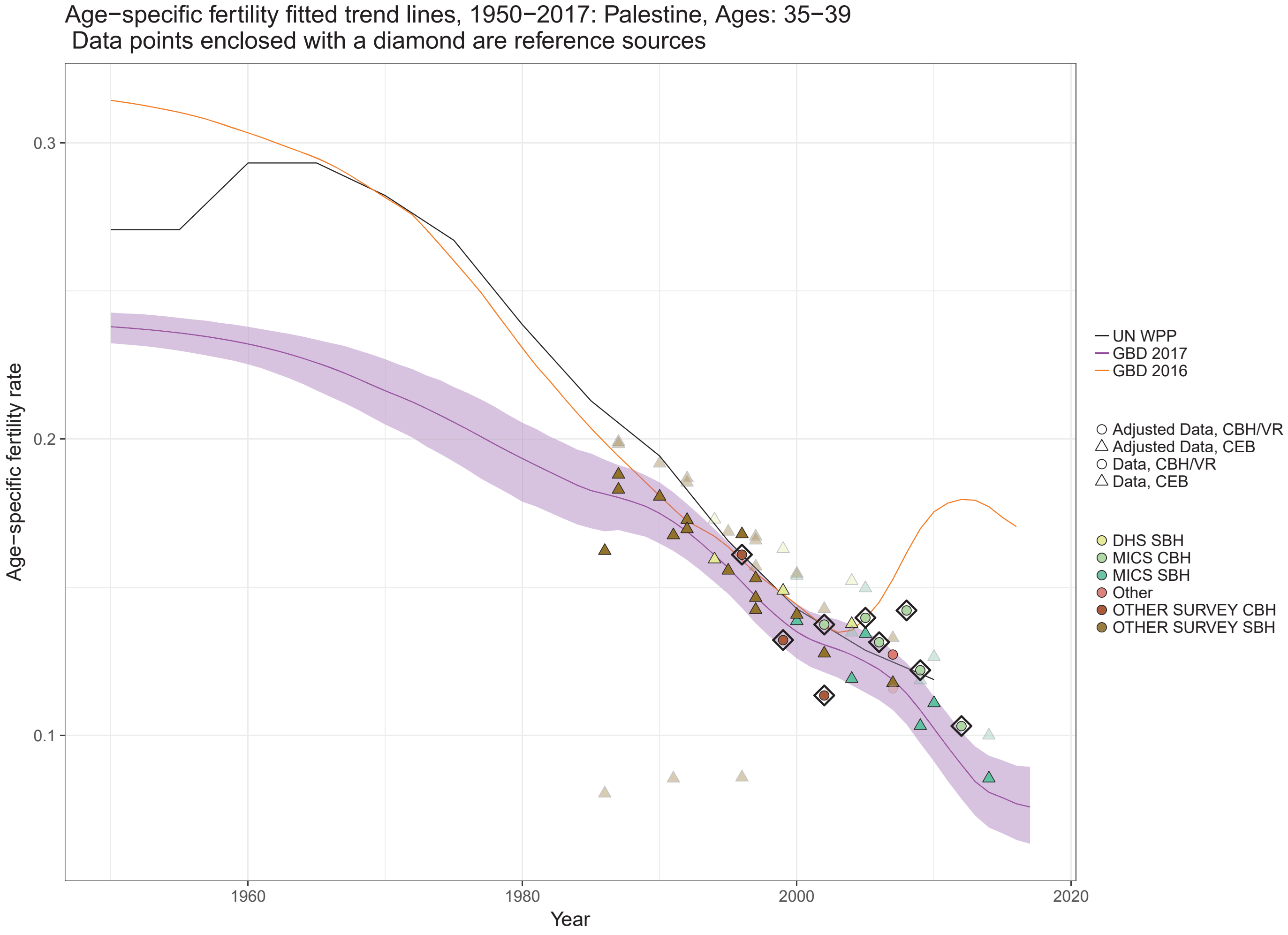

Age−specific fertility fitted trend lines, 1950−2017: Palestine, Ages: 35−39
Data points enclosed with a diamond are reference sources
Age−specific fertility rate
0.3
0.2
0.1
1960
1980
2000
2020
Year
UN WPP
GBD 2017
GBD 2016
Adjusted Data, CBH/VR
Adjusted Data, CEB
Data, CBH/VR
Data, CEB
DHS SBH
MICS CBH
MICS SBH
Other
OTHER SURVEY CBH
OTHER SURVEY SBH

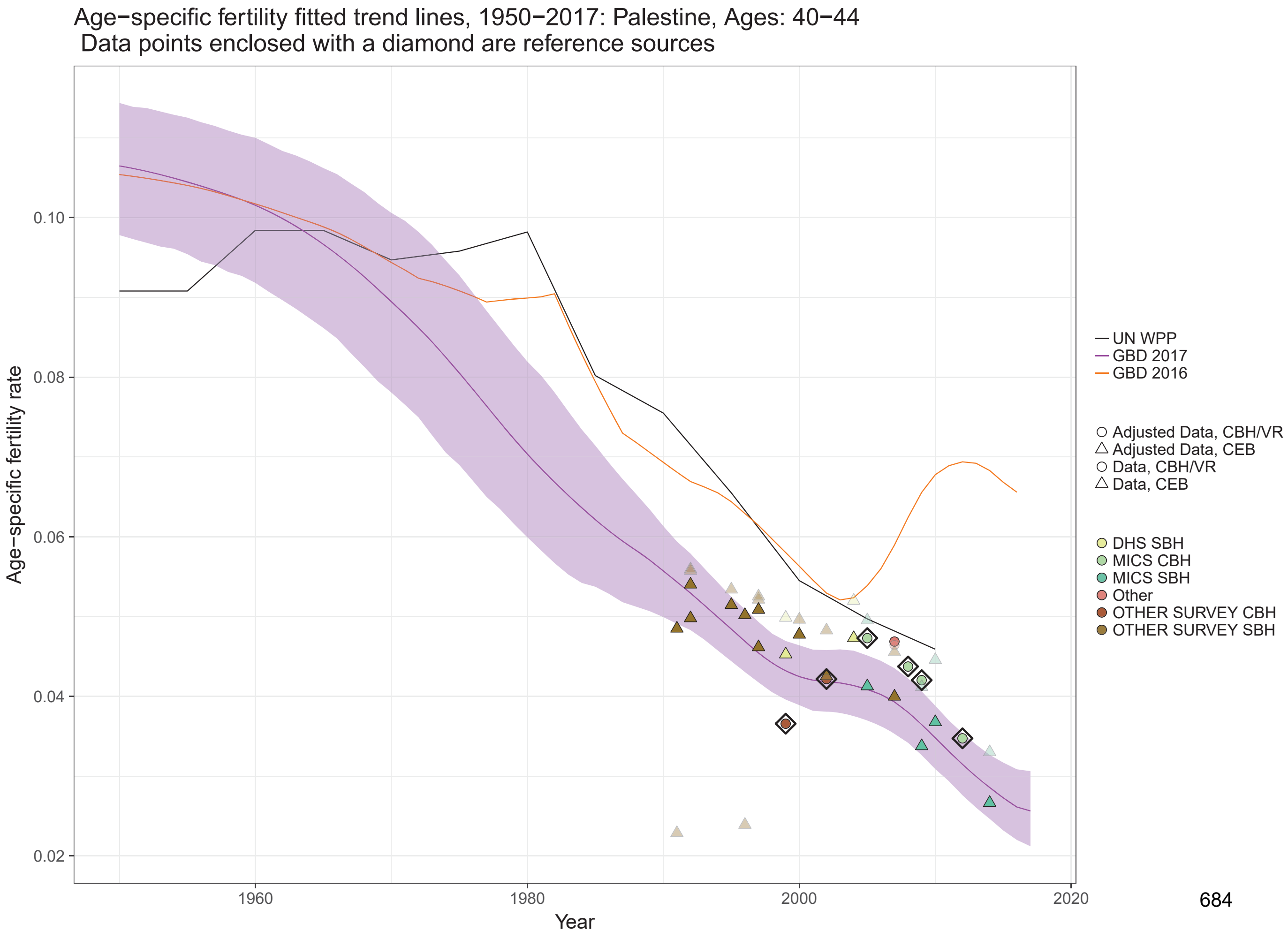

Age−specific fertility fitted trend lines, 1950−2017: Palestine, Ages: 40−44
Data points enclosed with a diamond are reference sources
Age−specific fertility rate
0.10
0.08
0.06
0.04
0.02
1960
1980
2000
2020
Year
UN WPP
GBD 2017
GBD 2016
Adjusted Data, CBH/VR
Adjusted Data, CEB
Data, CBH/VR
Data, CEB
DHS SBH
MICS CBH
MICS SBH
Other
OTHER SURVEY CBH
OTHER SURVEY SBH

Age−specific fertility fitted trend lines, 1950−2017: Palestine, Ages: 45−49
Data points enclosed with a diamond are reference sources

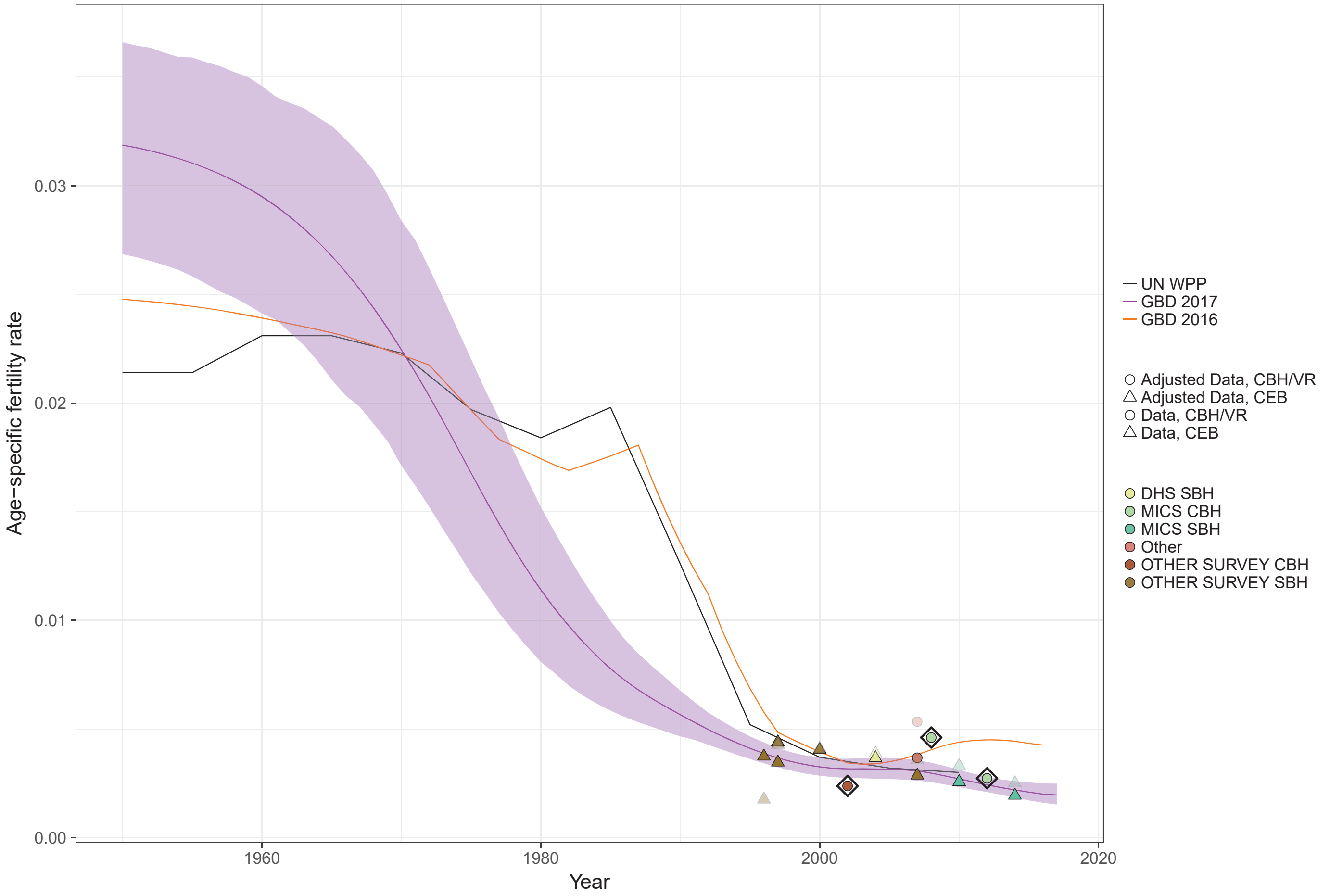

Age−specific fertility fitted trend lines, 1950−2017: Palestine, Ages: 50−54
Data points enclosed with a diamond are reference sources

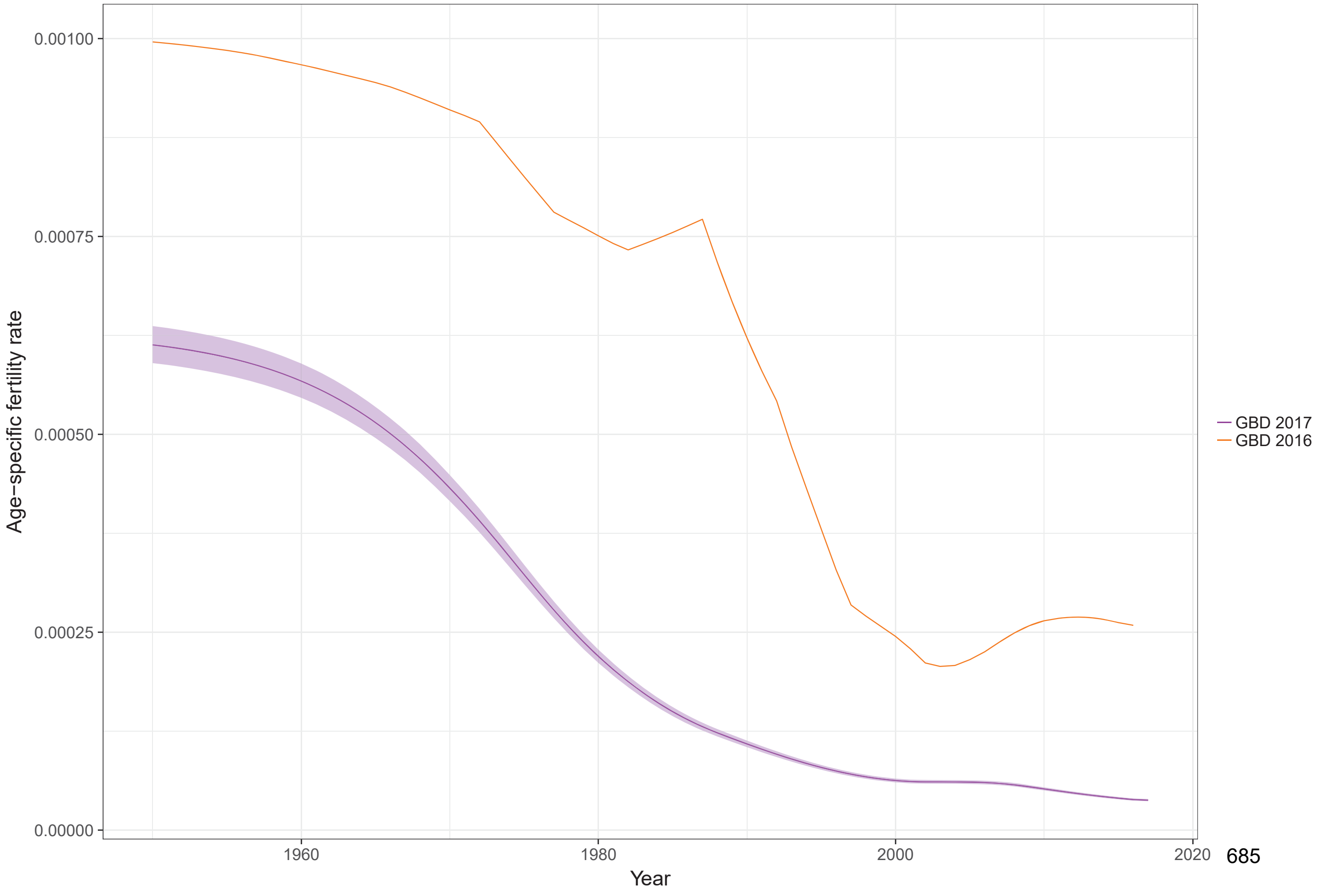

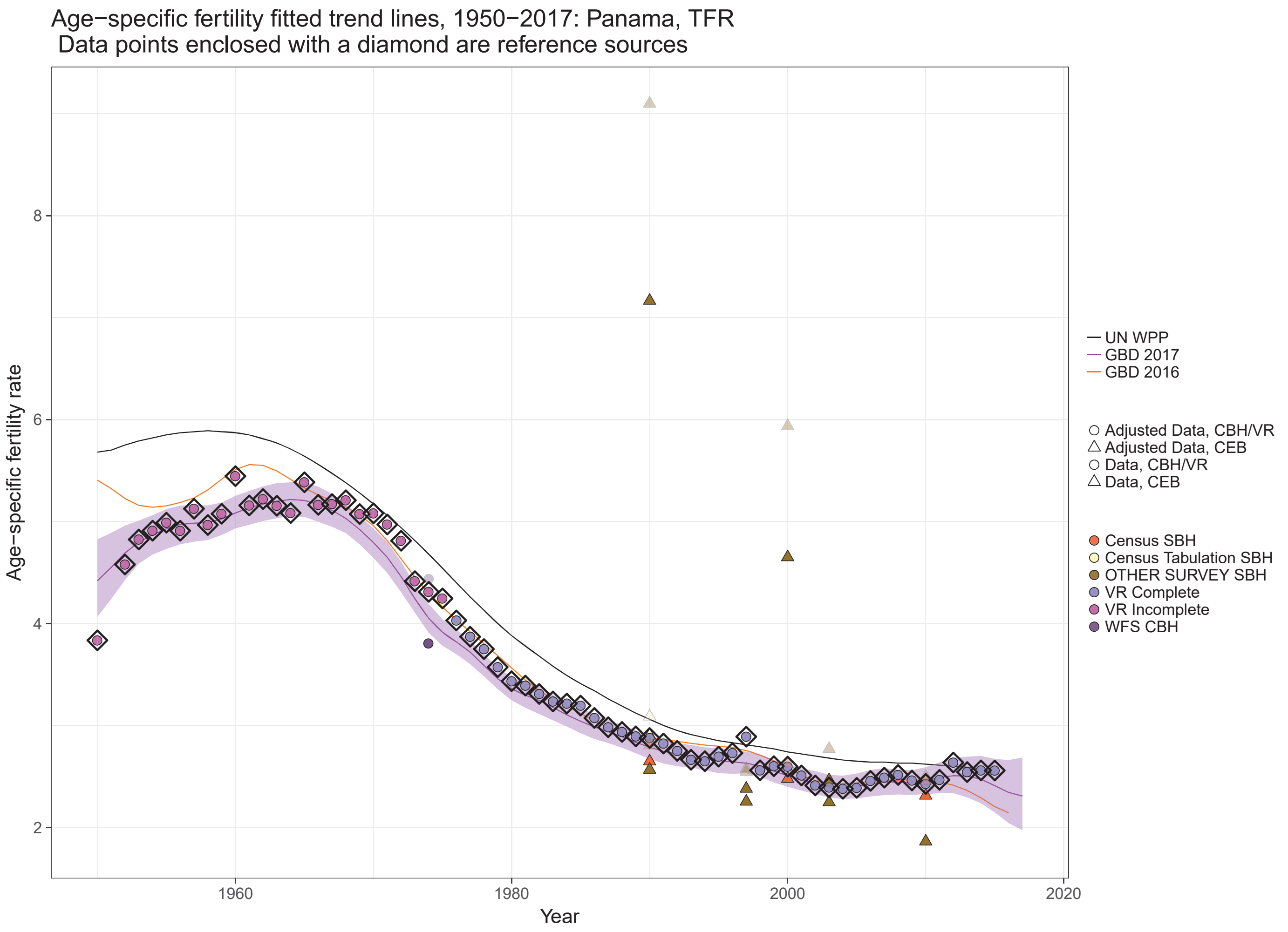

Age−specific fertility fitted trend lines, 1950−2017: Panama, TFR
Data points enclosed with a diamond are reference sources
Age−specific fertility rate
Year
2
4
6
8
1960
1980
2000
2020
UN WPP
GBD 2017
GBD 2016
Adjusted Data, CBH/VR
Adjusted Data, CEB
Data, CBH/VR
Data, CEB
Census SBH
Census Tabulation SBH
OTHER SURVEY SBH
VR Complete
VR Incomplete
WFS CBH

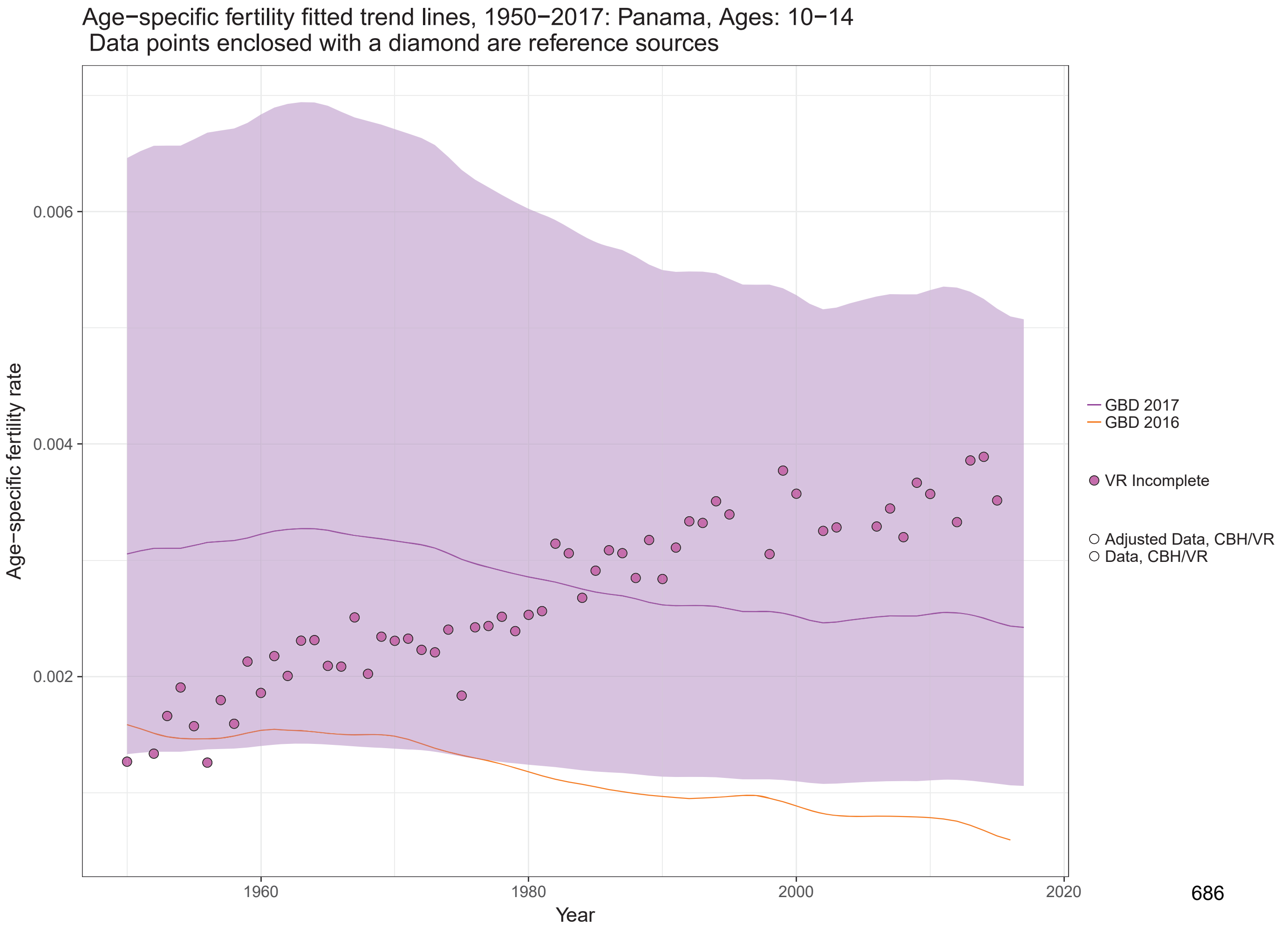

Age−specific fertility fitted trend lines, 1950−2017: Panama, Ages: 10−14
Data points enclosed with a diamond are reference sources
Age−specific fertility rate
Year
0.002
0.004
0.006
1960
1980
2000
2020
GBD 2017
GBD 2016
VR Incomplete
Adjusted Data, CBH/VR
Data, CBH/VR

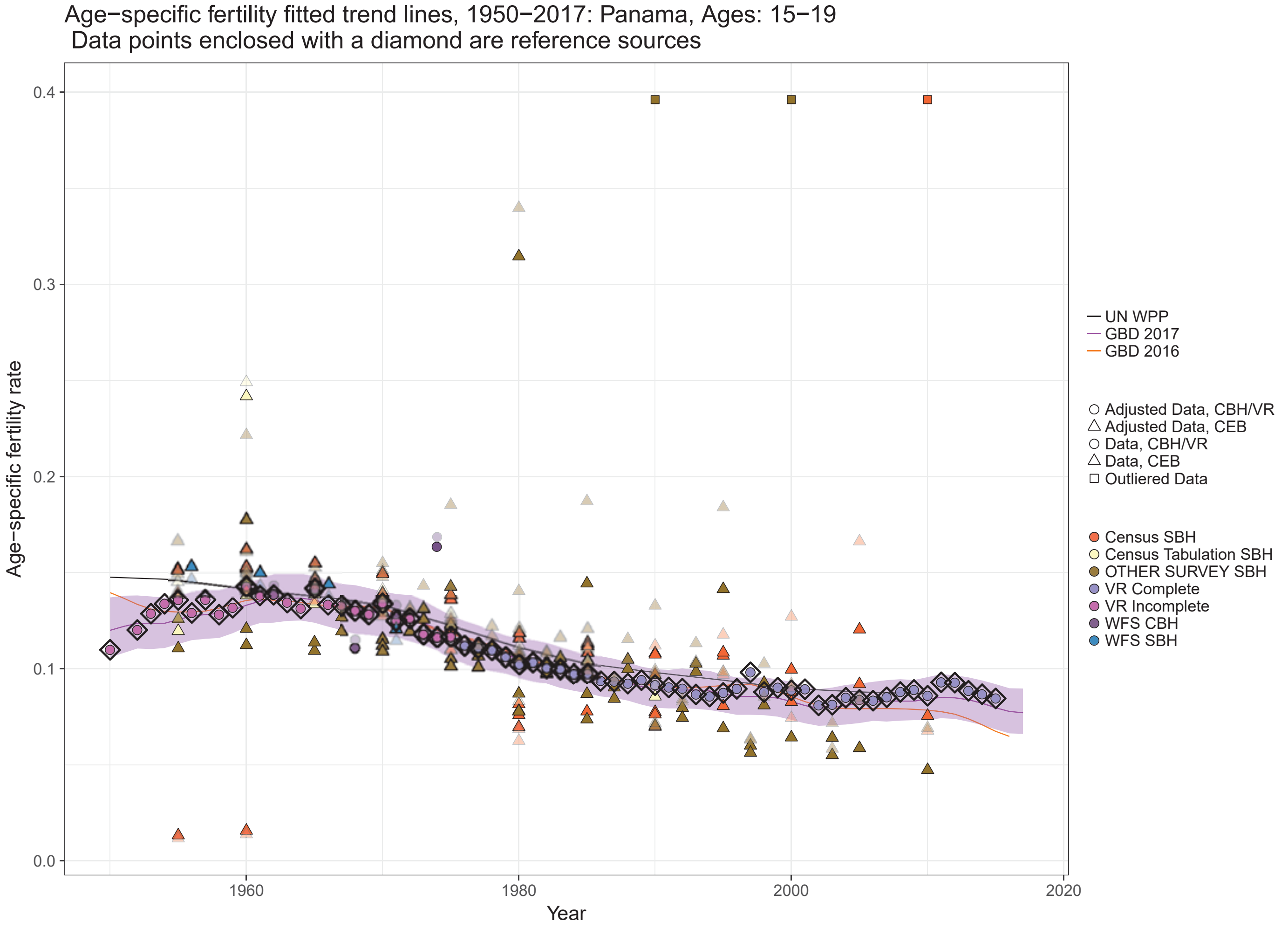
Age−specific fertility fitted trend lines, 1950−2017: Panama, Ages: 15−19
Data points enclosed with a diamond are reference sources
Age−specific fertility rate
Year
UN WPP
GBD 2017
GBD 2016
Adjusted Data, CBH/VR
Adjusted Data, CEB
Data, CBH/VR
Data, CEB
Outliered Data
Census SBH
Census Tabulation SBH
OTHER SURVEY SBH
VR Complete
VR Incomplete
WFS CBH
WFS SBH

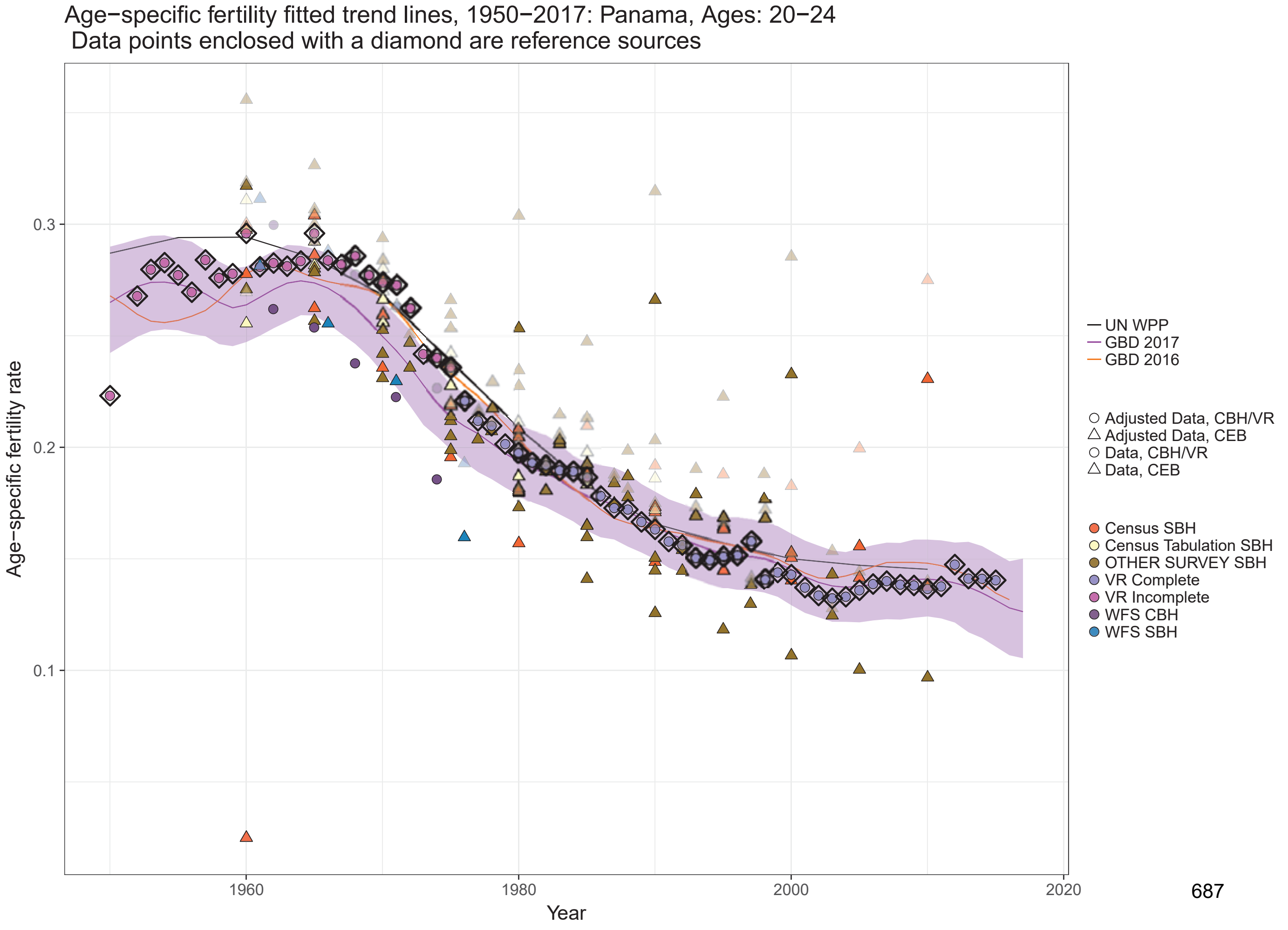
Age−specific fertility fitted trend lines, 1950−2017: Panama, Ages: 20−24
Data points enclosed with a diamond are reference sources
Age−specific fertility rate
Year
UN WPP
GBD 2017
GBD 2016
Adjusted Data, CBH/VR
Adjusted Data, CEB
Data, CBH/VR
Data, CEB
Census SBH
Census Tabulation SBH
OTHER SURVEY SBH
VR Complete
VR Incomplete
WFS CBH
WFS SBH

Age−specific fertility fitted trend lines, 1950−2017: Panama, Ages: 25−29
Data points enclosed with a diamond are reference sources

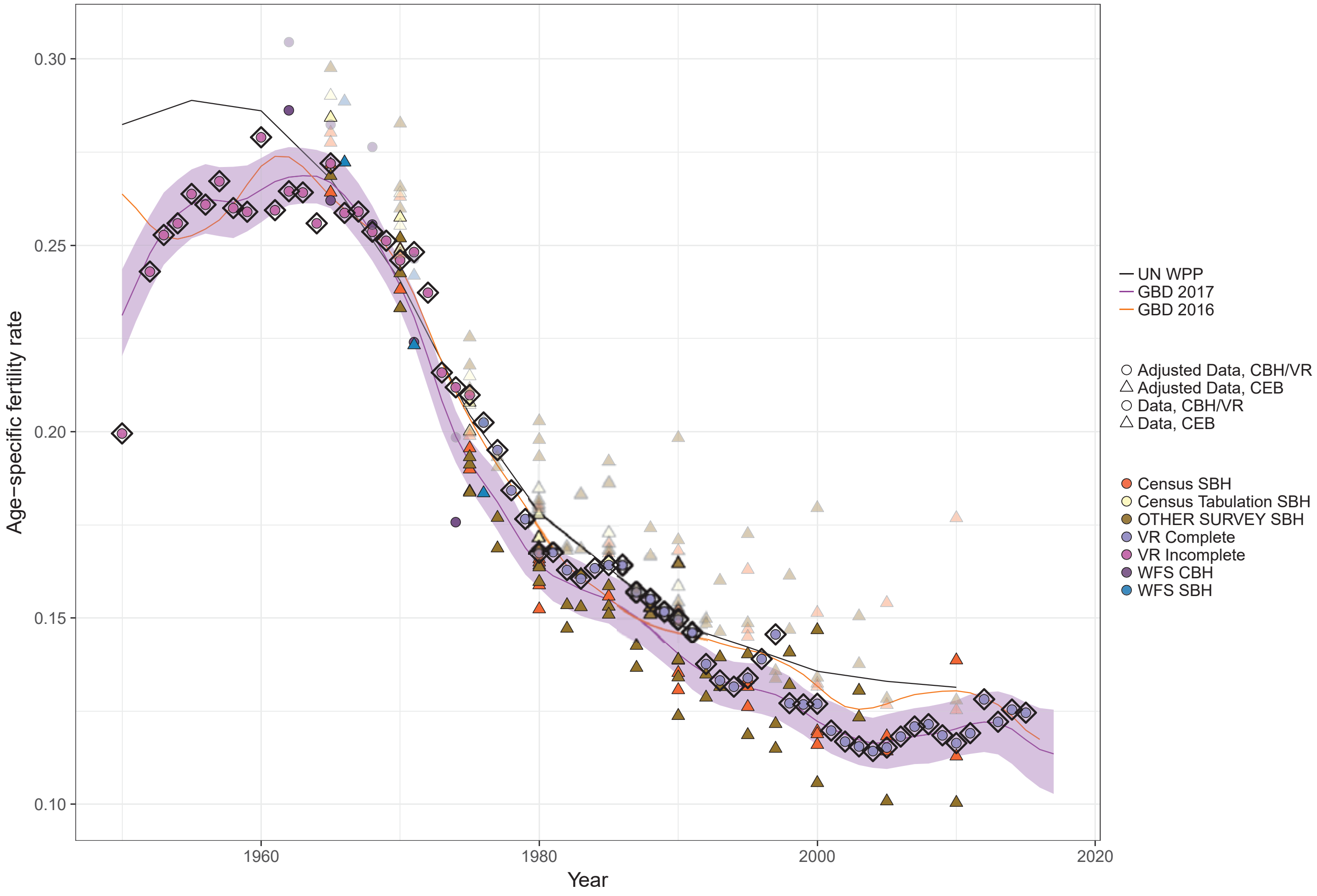

Age−specific fertility fitted trend lines, 1950−2017: Panama, Ages: 30−34
Data points enclosed with a diamond are reference sources

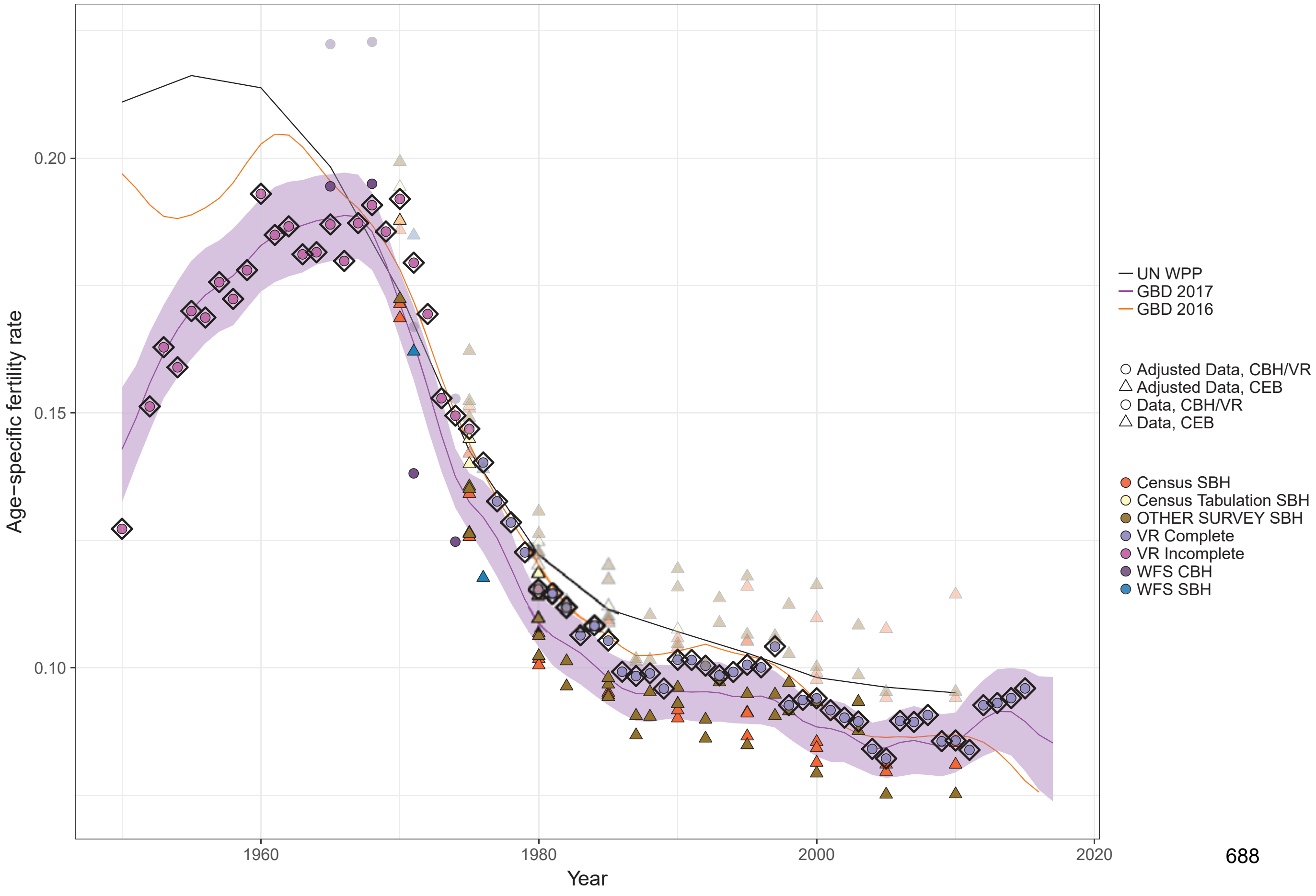

Age−specific fertility fitted trend lines, 1950−2017: Panama, Ages: 35−39
Data points enclosed with a diamond are reference sources

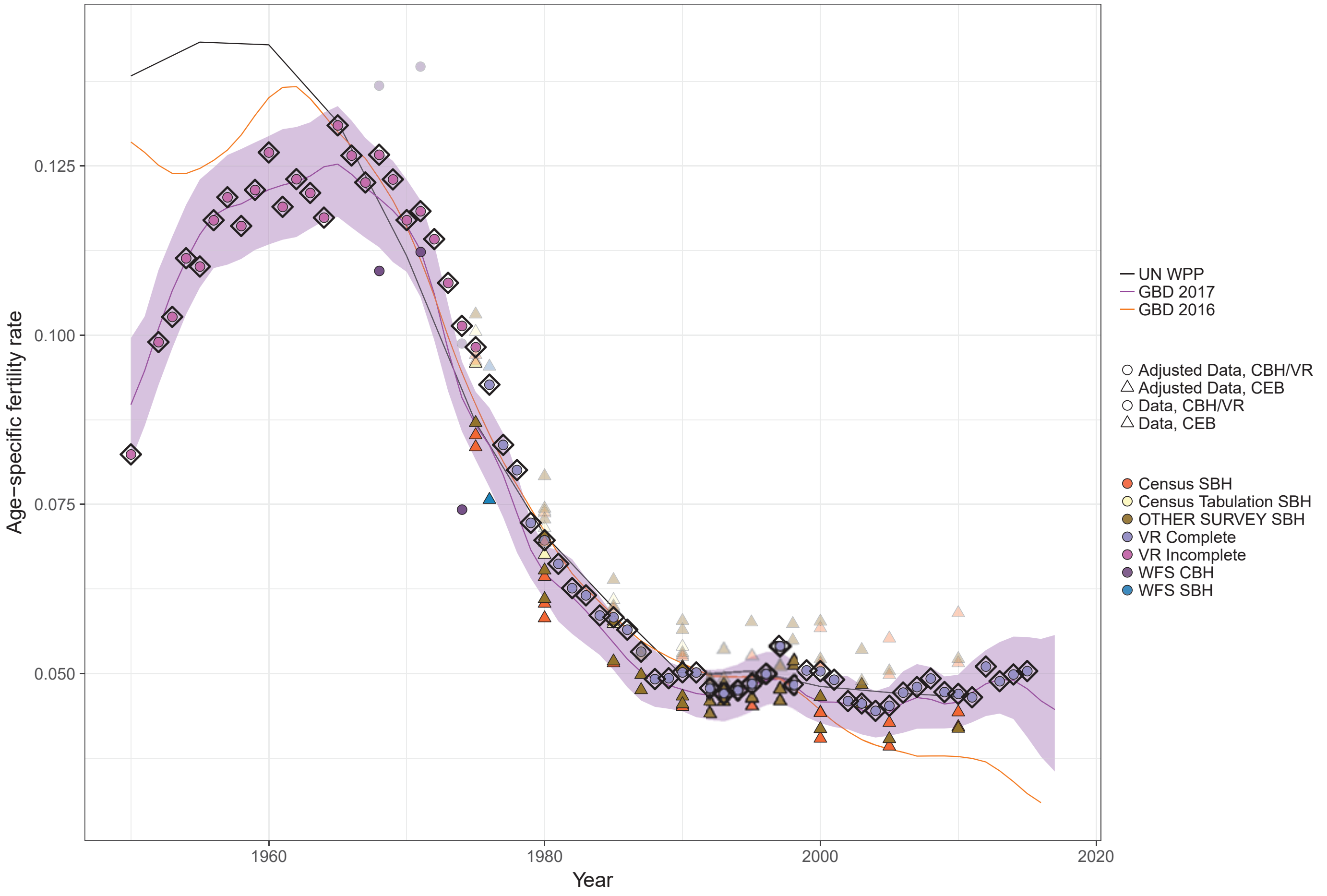

Age−specific fertility fitted trend lines, 1950−2017: Panama, Ages: 40−44
Data points enclosed with a diamond are reference sources

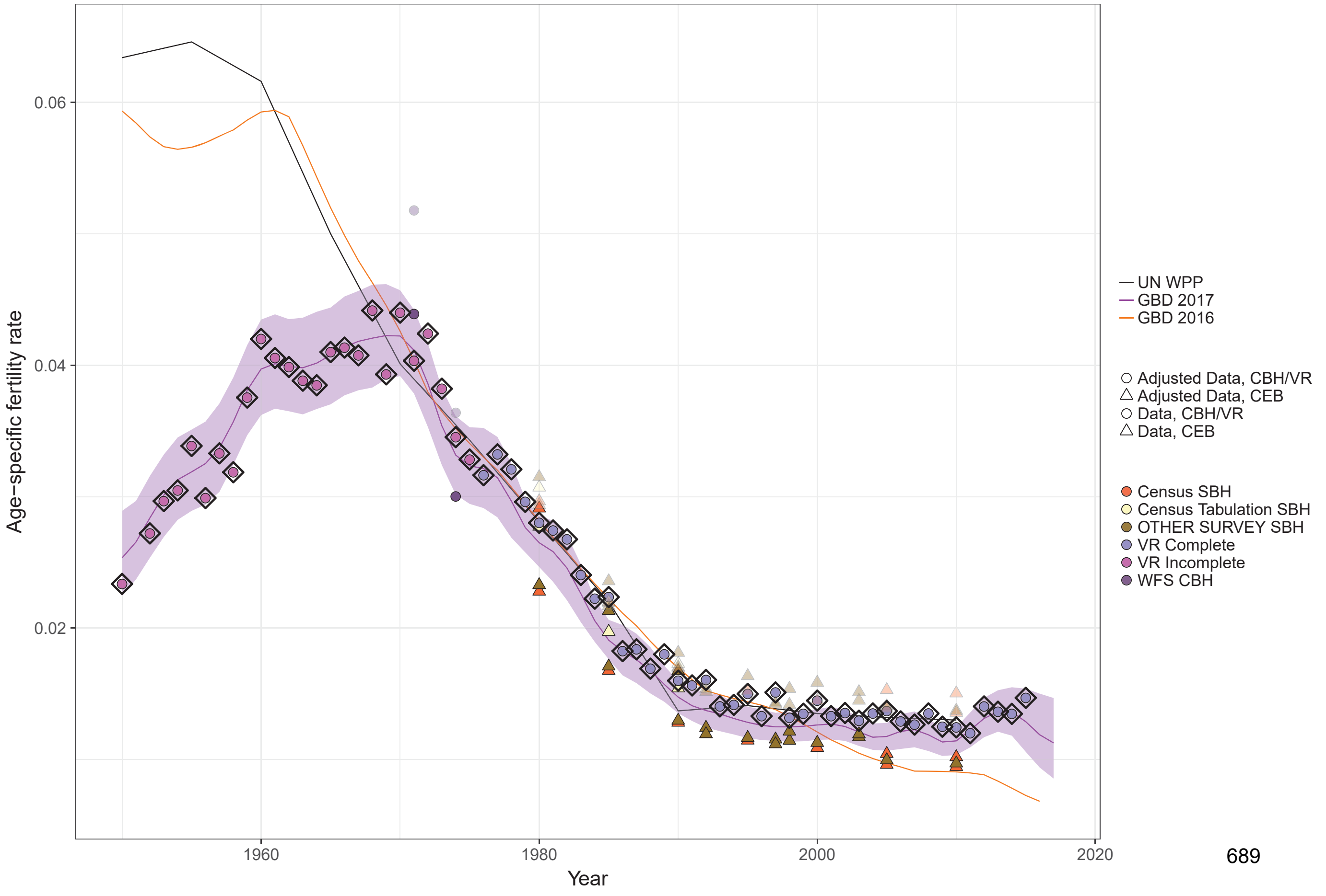

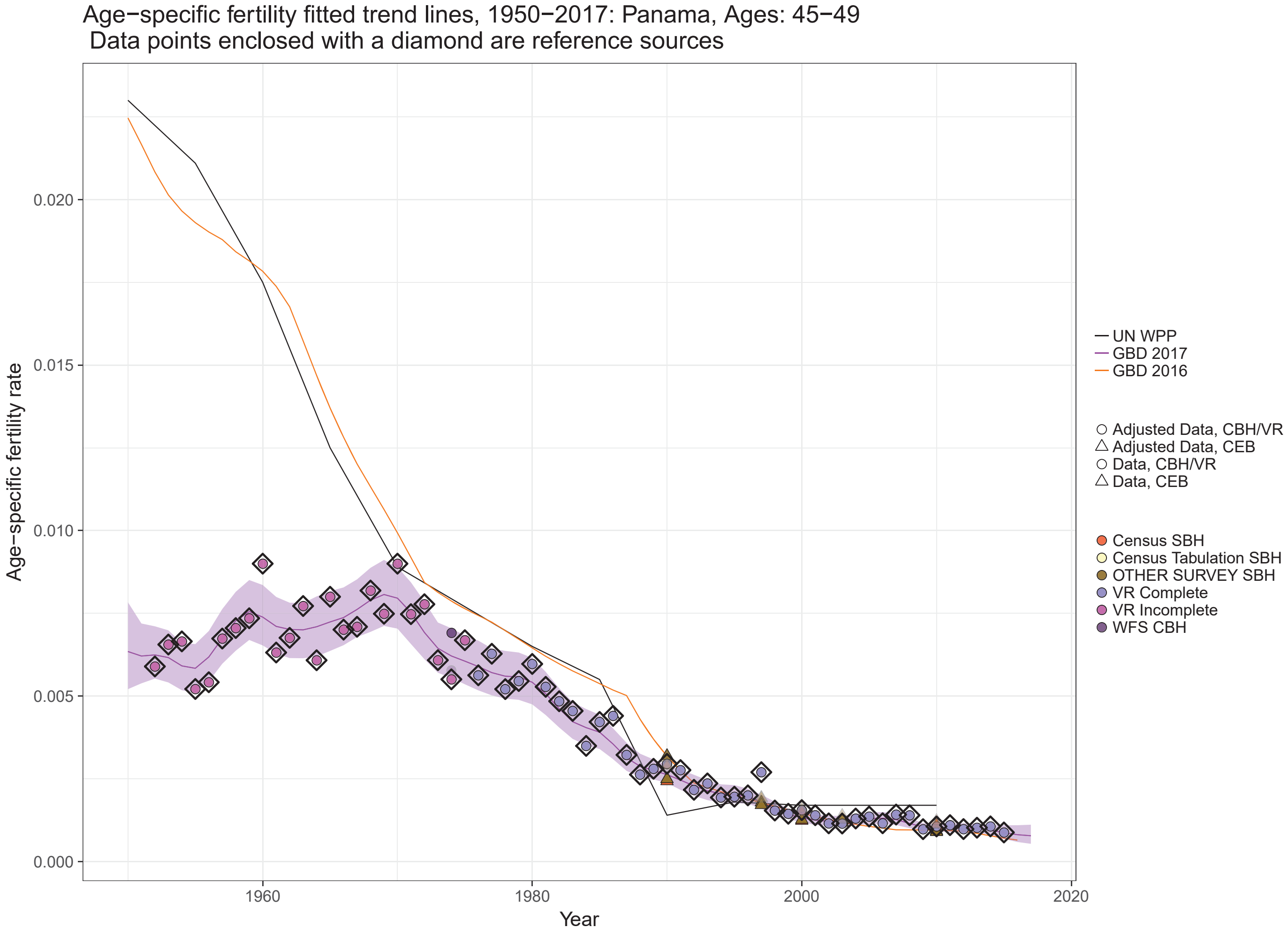

Age−specific fertility fitted trend lines, 1950−2017: Panama, Ages: 45−49
Data points enclosed with a diamond are reference sources
Age−specific fertility rate
0.000
0.005
0.010
0.015
0.020
1960
1980
2000
2020
Year
UN WPP
GBD 2017
GBD 2016
Adjusted Data, CBH/VR
Adjusted Data, CEB
Data, CBH/VR
Data, CEB
Census SBH
Census Tabulation SBH
OTHER SURVEY SBH
VR Complete
VR Incomplete
WFS CBH

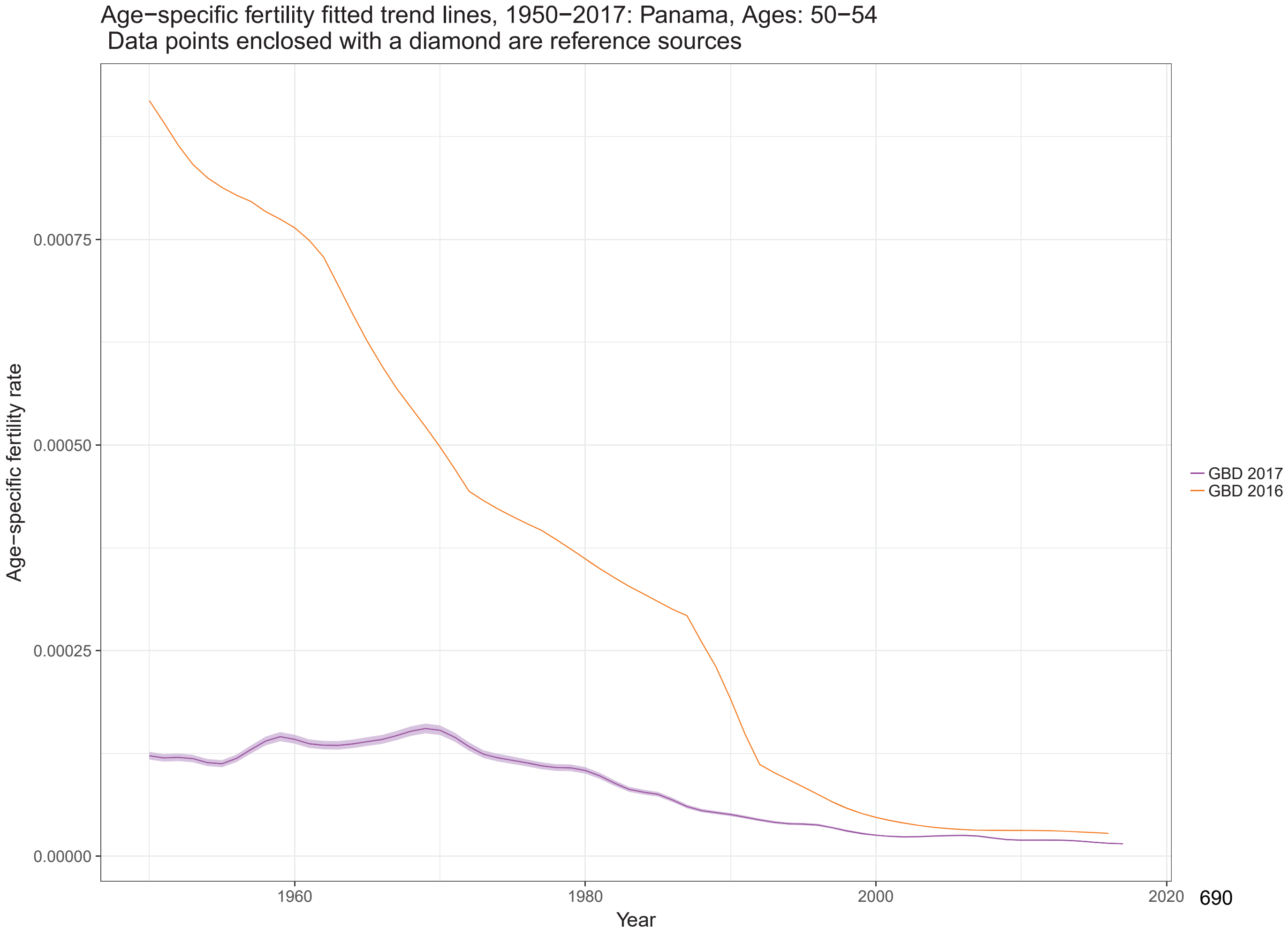

Age−specific fertility fitted trend lines, 1950−2017: Panama, Ages: 50−54
Data points enclosed with a diamond are reference sources
Age−specific fertility rate
0.00000
0.00025
0.00050
0.00075
1960
1980
2000
2020
Year
GBD 2017
GBD 2016

Age−specific fertility fitted trend lines, 1950−2017: Papua New Guinea, TFR
Data points enclosed with a diamond are reference sources

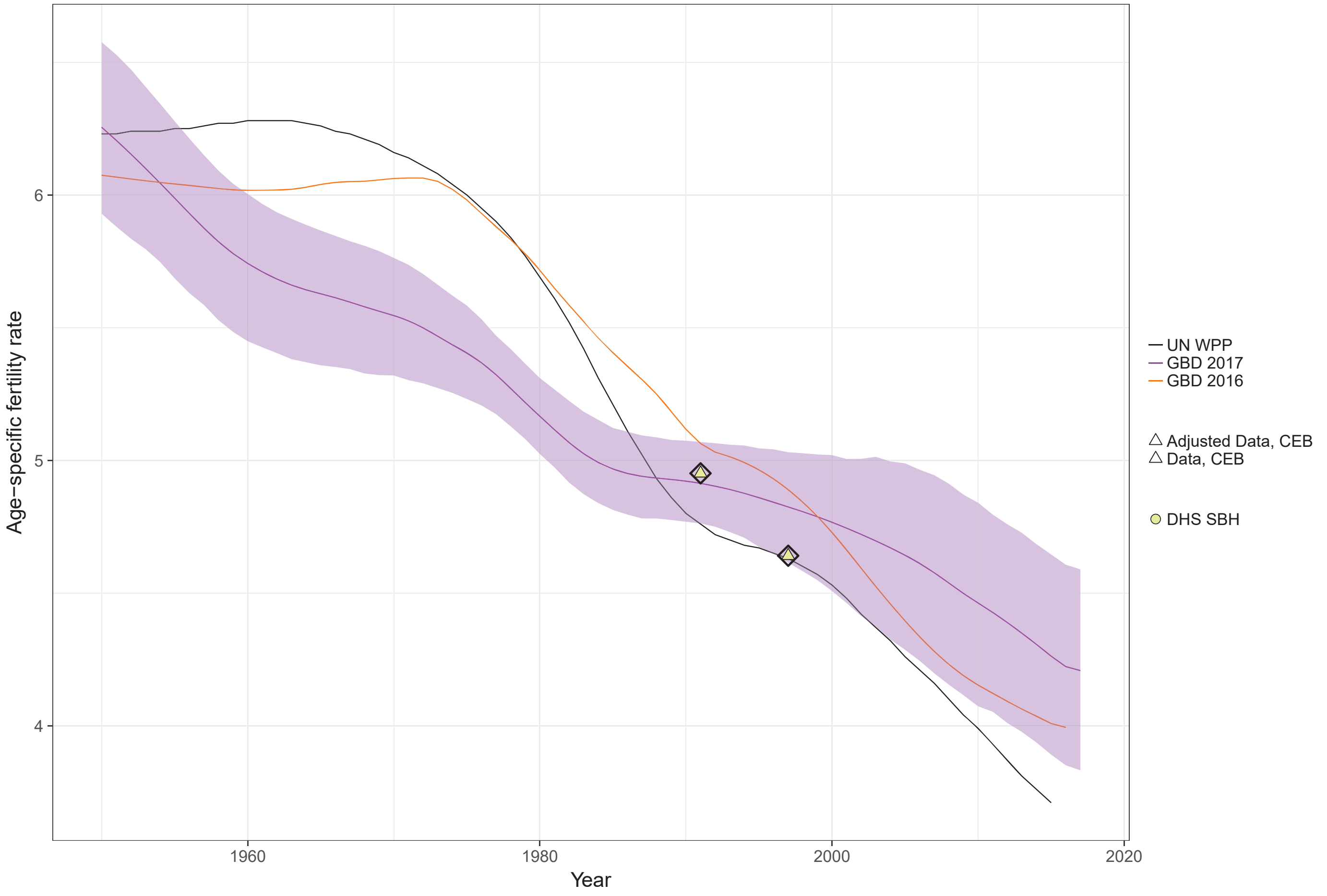

Age−specific fertility fitted trend lines, 1950−2017: Papua New Guinea, Ages: 10−14
Data points enclosed with a diamond are reference sources

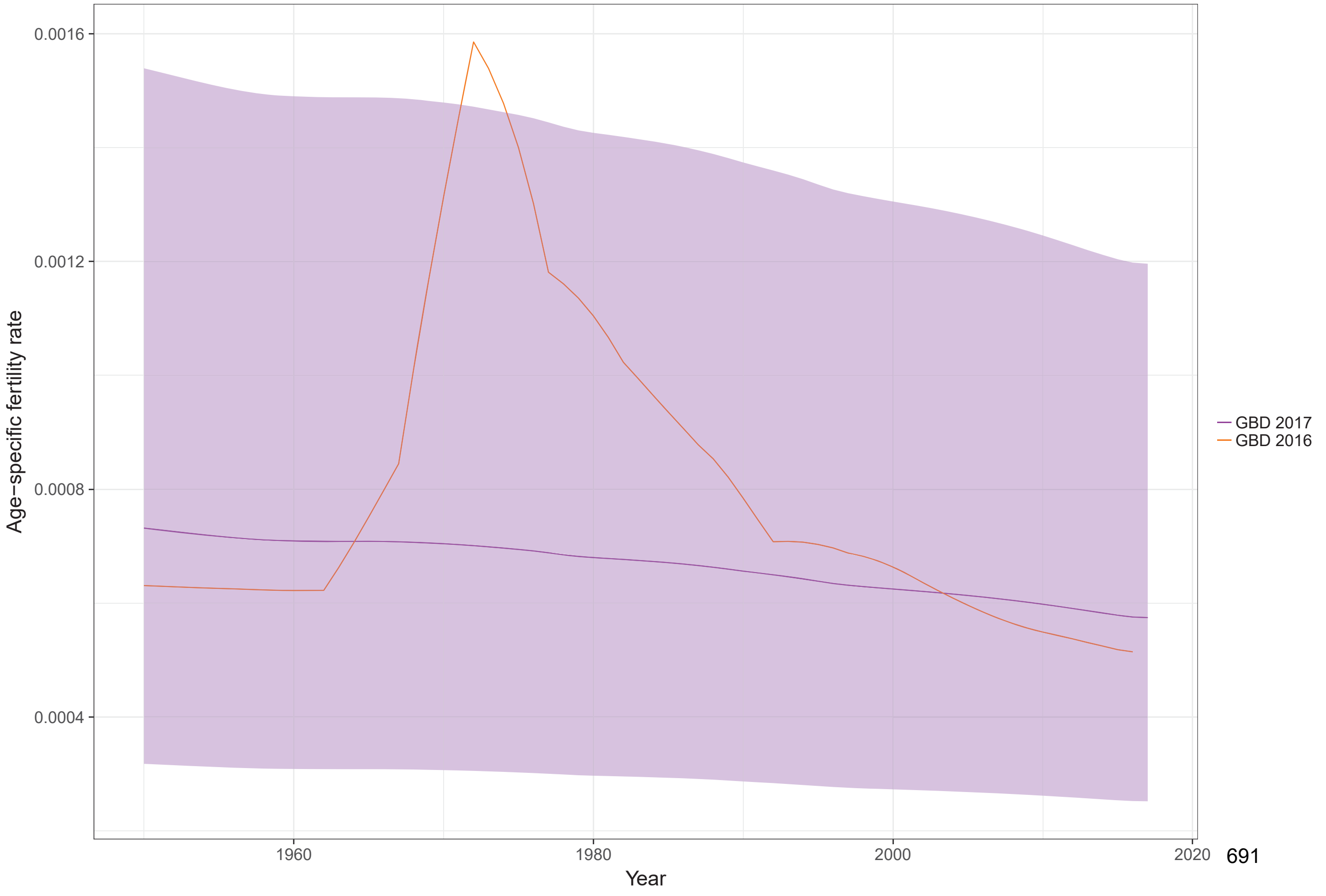

Age−specific fertility fitted trend lines, 1950−2017: Papua New Guinea, Ages: 15−19
Data points enclosed with a diamond are reference sources

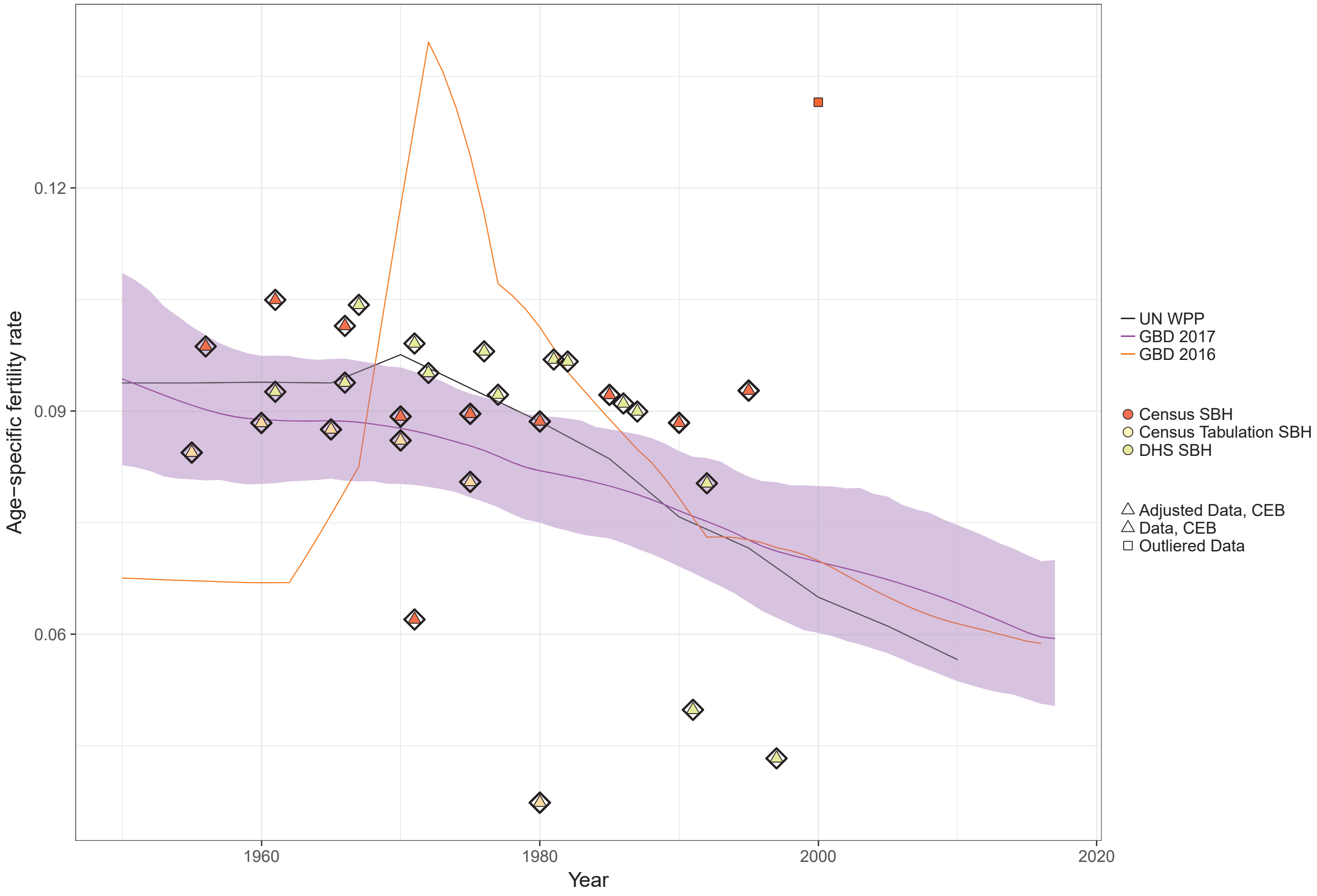

Age−specific fertility fitted trend lines, 1950−2017: Papua New Guinea, Ages: 20−24
Data points enclosed with a diamond are reference sources

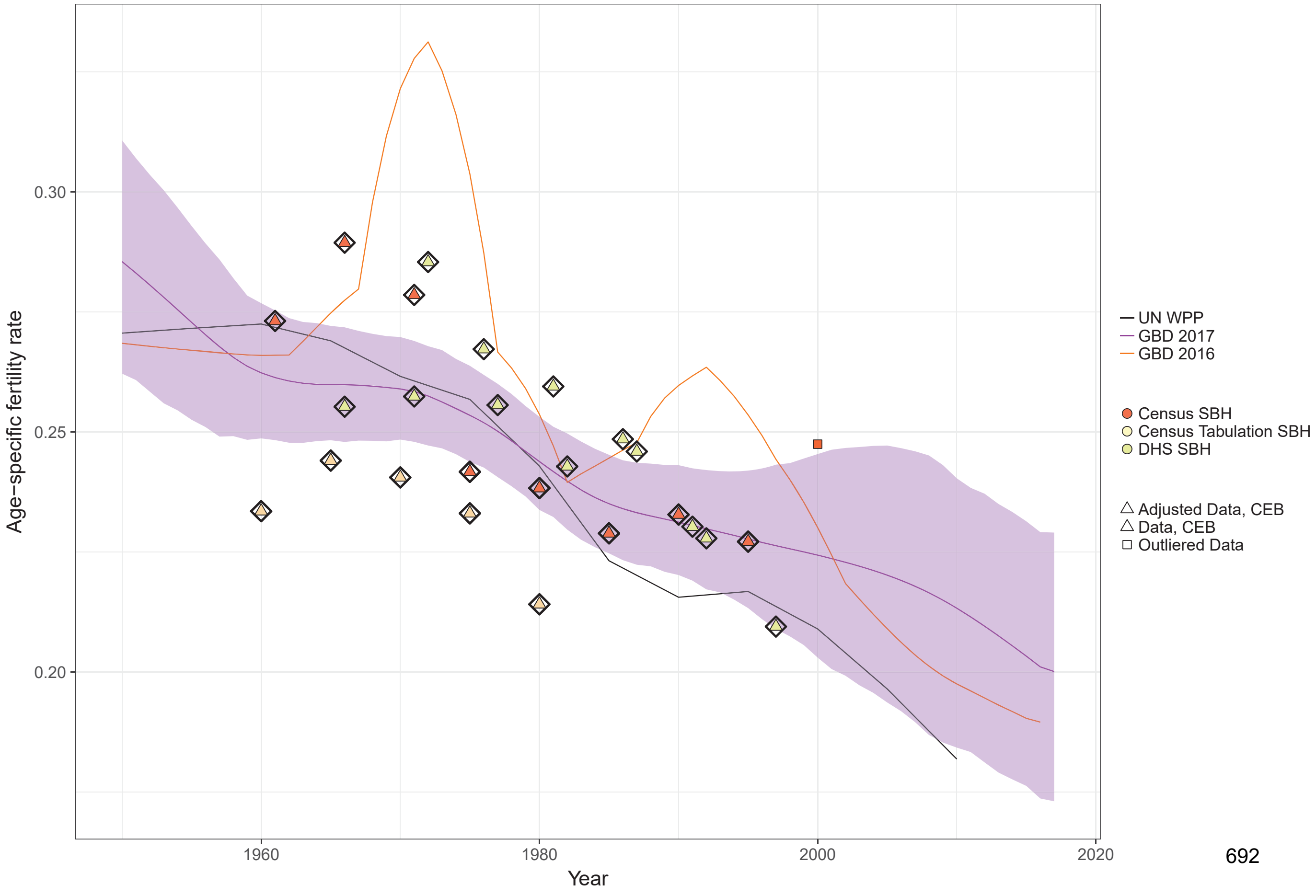

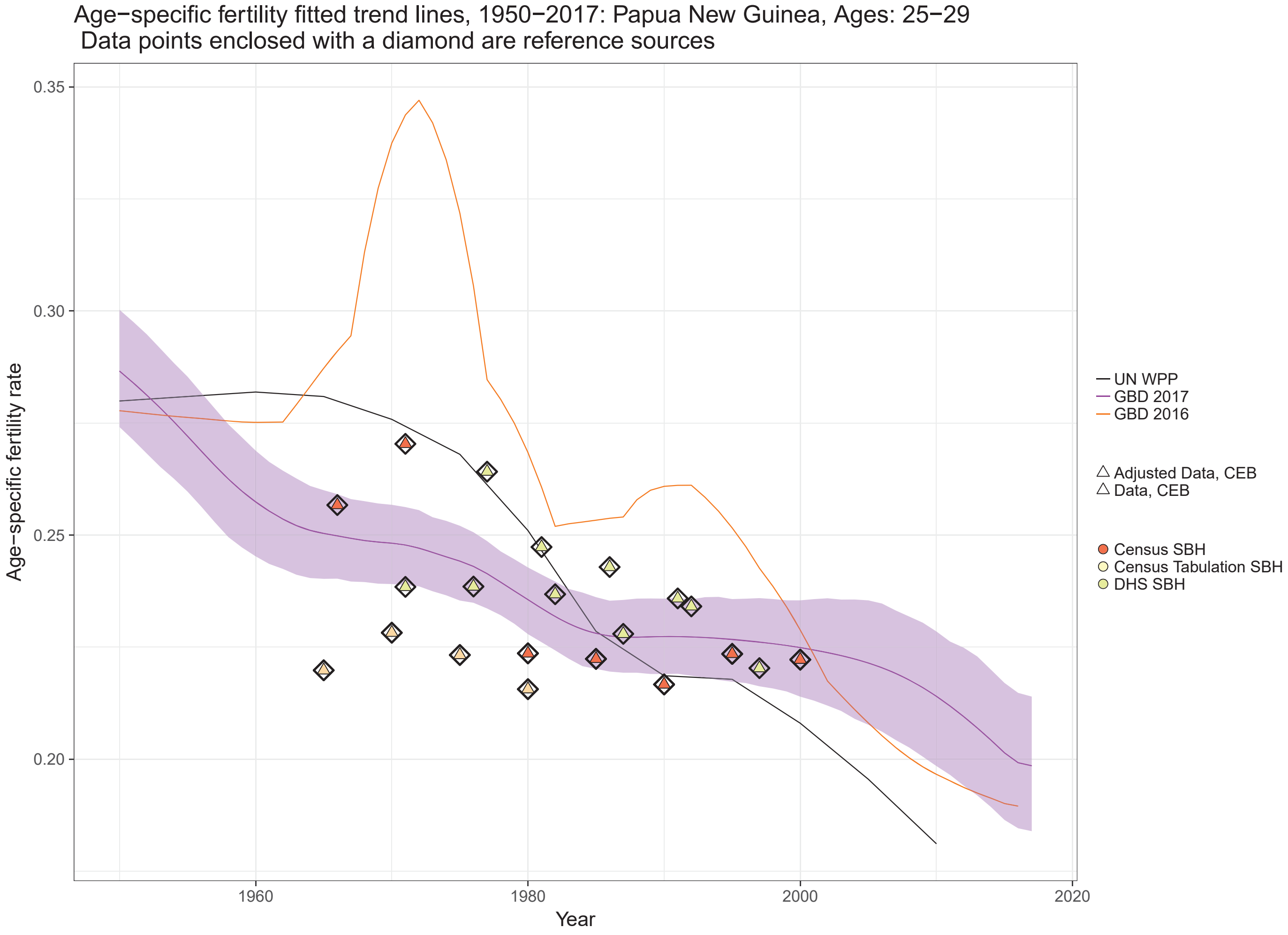

Age−specific fertility fitted trend lines, 1950−2017: Papua New Guinea, Ages: 25−29
Data points enclosed with a diamond are reference sources
Age−specific fertility rate
0.35
0.30
0.25
0.20
1960
1980
2000
2020
Year
UN WPP
GBD 2017
GBD 2016
Adjusted Data, CEB
Data, CEB
Census SBH
Census Tabulation SBH
DHS SBH

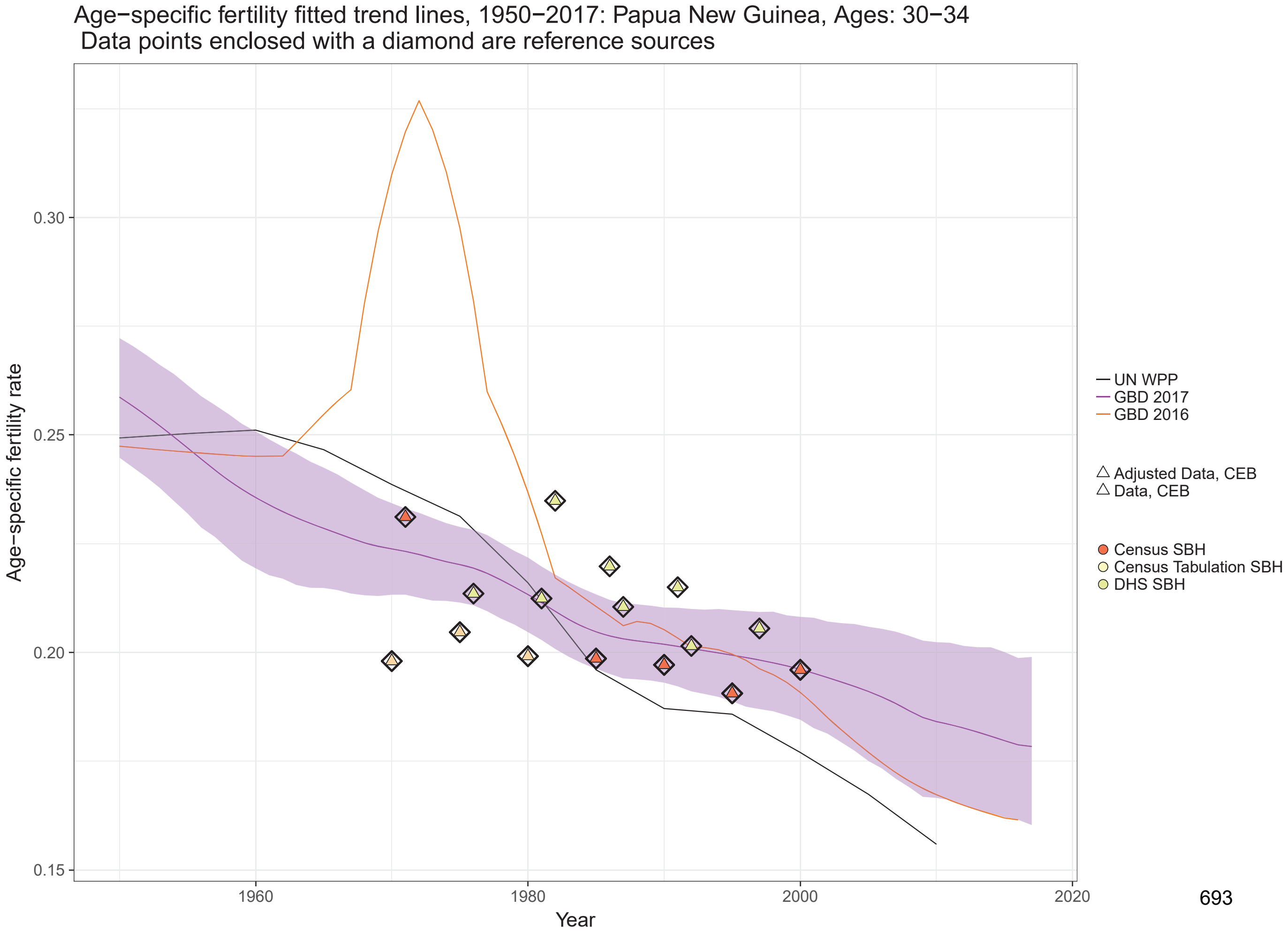

Age−specific fertility fitted trend lines, 1950−2017: Papua New Guinea, Ages: 30−34
Data points enclosed with a diamond are reference sources
Age−specific fertility rate
0.30
0.25
0.20
0.15
1960
1980
2000
2020
Year
UN WPP
GBD 2017
GBD 2016
Adjusted Data, CEB
Data, CEB
Census SBH
Census Tabulation SBH
DHS SBH

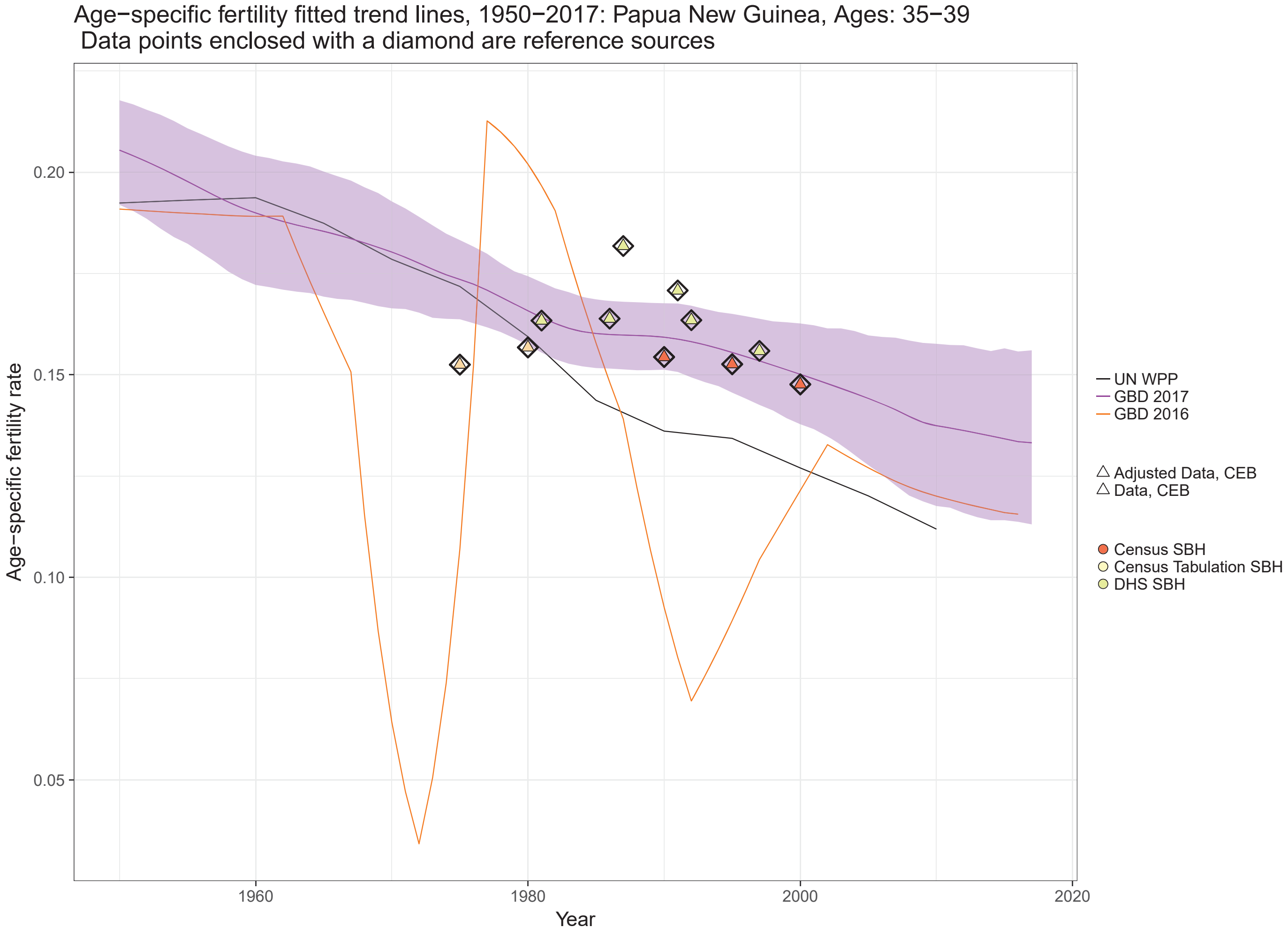
Age−specific fertility fitted trend lines, 1950−2017: Papua New Guinea, Ages: 35−39
Data points enclosed with a diamond are reference sources
Age−specific fertility rate
0.20
0.15
0.10
0.05
1960
1980
2000
2020
Year
UN WPP
GBD 2017
GBD 2016
Adjusted Data, CEB
Data, CEB
Census SBH
Census Tabulation SBH
DHS SBH

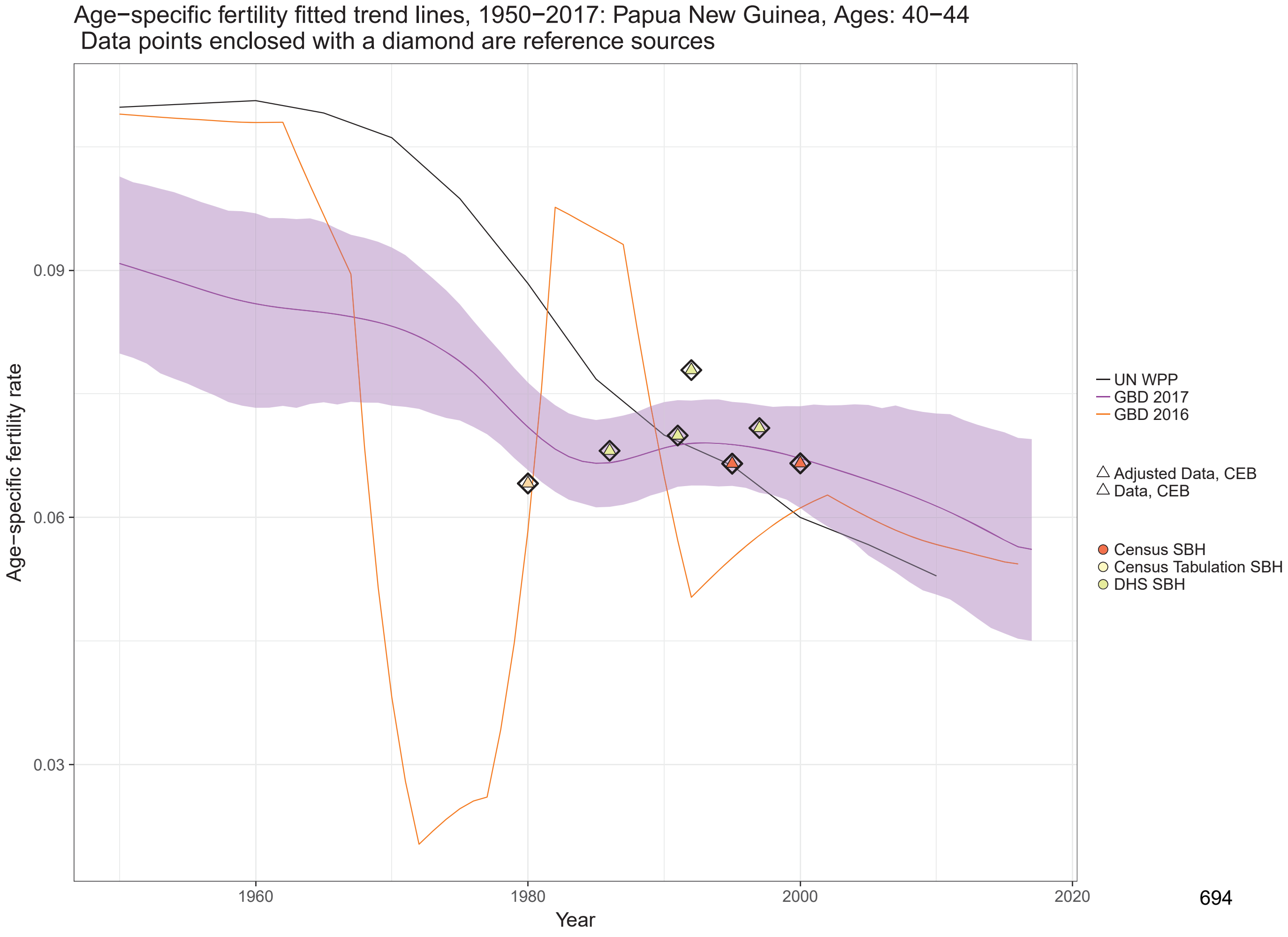
Age−specific fertility fitted trend lines, 1950−2017: Papua New Guinea, Ages: 40−44
Data points enclosed with a diamond are reference sources
Age−specific fertility rate
0.09
0.06
0.03
1960
1980
2000
2020
Year
UN WPP
GBD 2017
GBD 2016
Adjusted Data, CEB
Data, CEB
Census SBH
Census Tabulation SBH
DHS SBH

Age−specific fertility fitted trend lines, 1950−2017: Papua New Guinea, Ages: 45−49
Data points enclosed with a diamond are reference sources

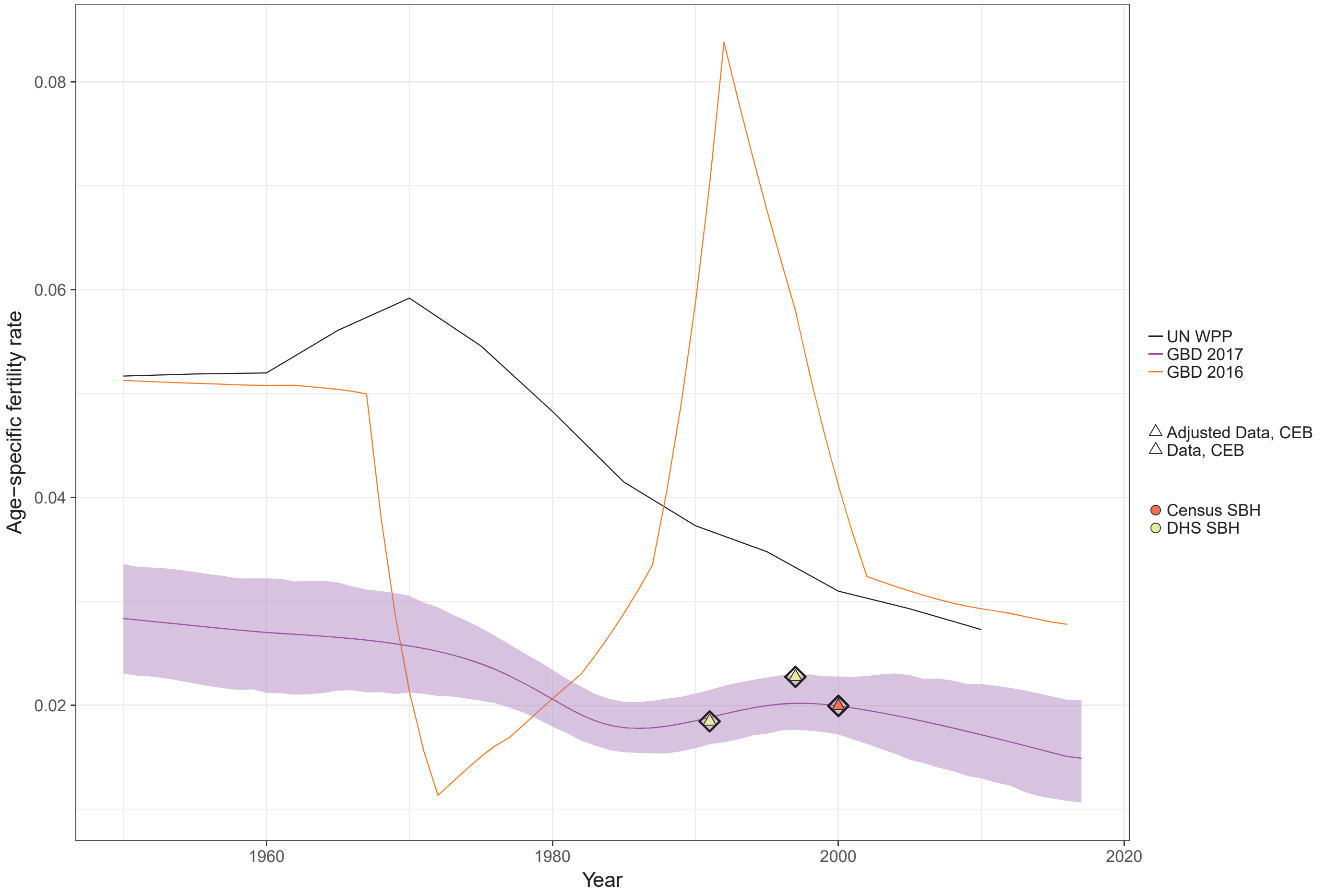

Age−specific fertility fitted trend lines, 1950−2017: Papua New Guinea, Ages: 50−54
Data points enclosed with a diamond are reference sources

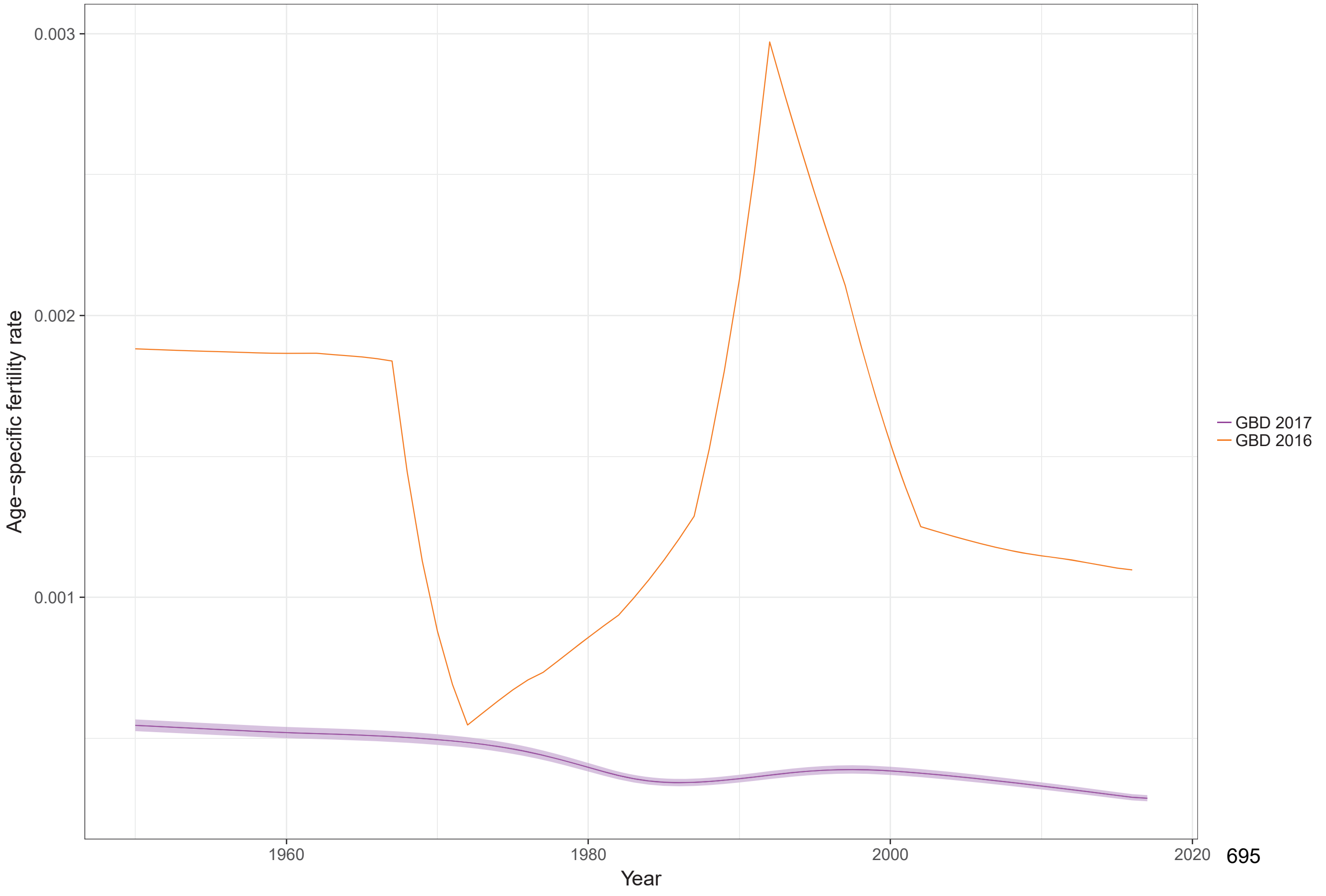

Age−specific fertility fitted trend lines, 1950−2017: Paraguay, TFR
Data points enclosed with a diamond are reference sources

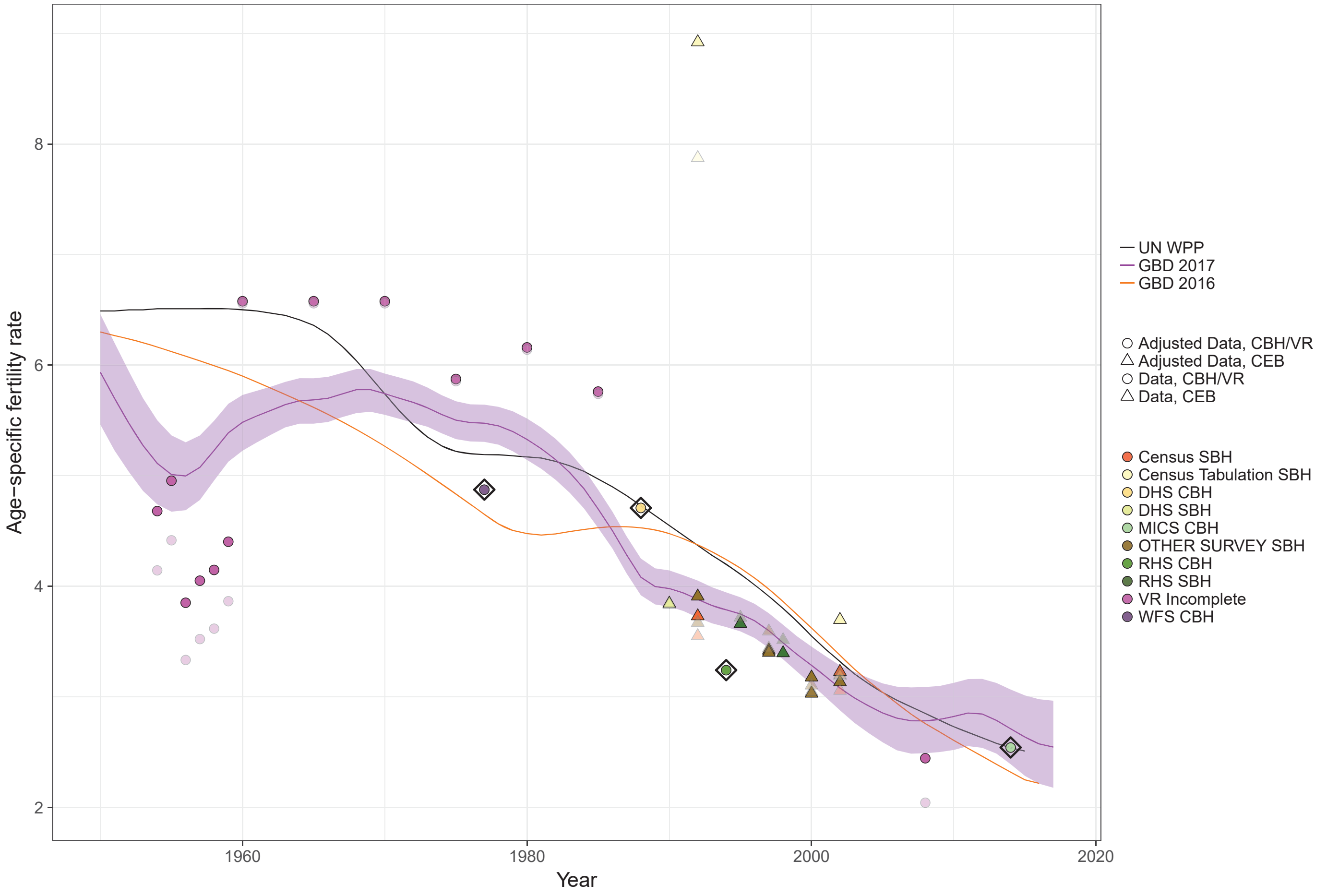

Age−specific fertility fitted trend lines, 1950−2017: Paraguay, Ages: 10−14
Data points enclosed with a diamond are reference sources

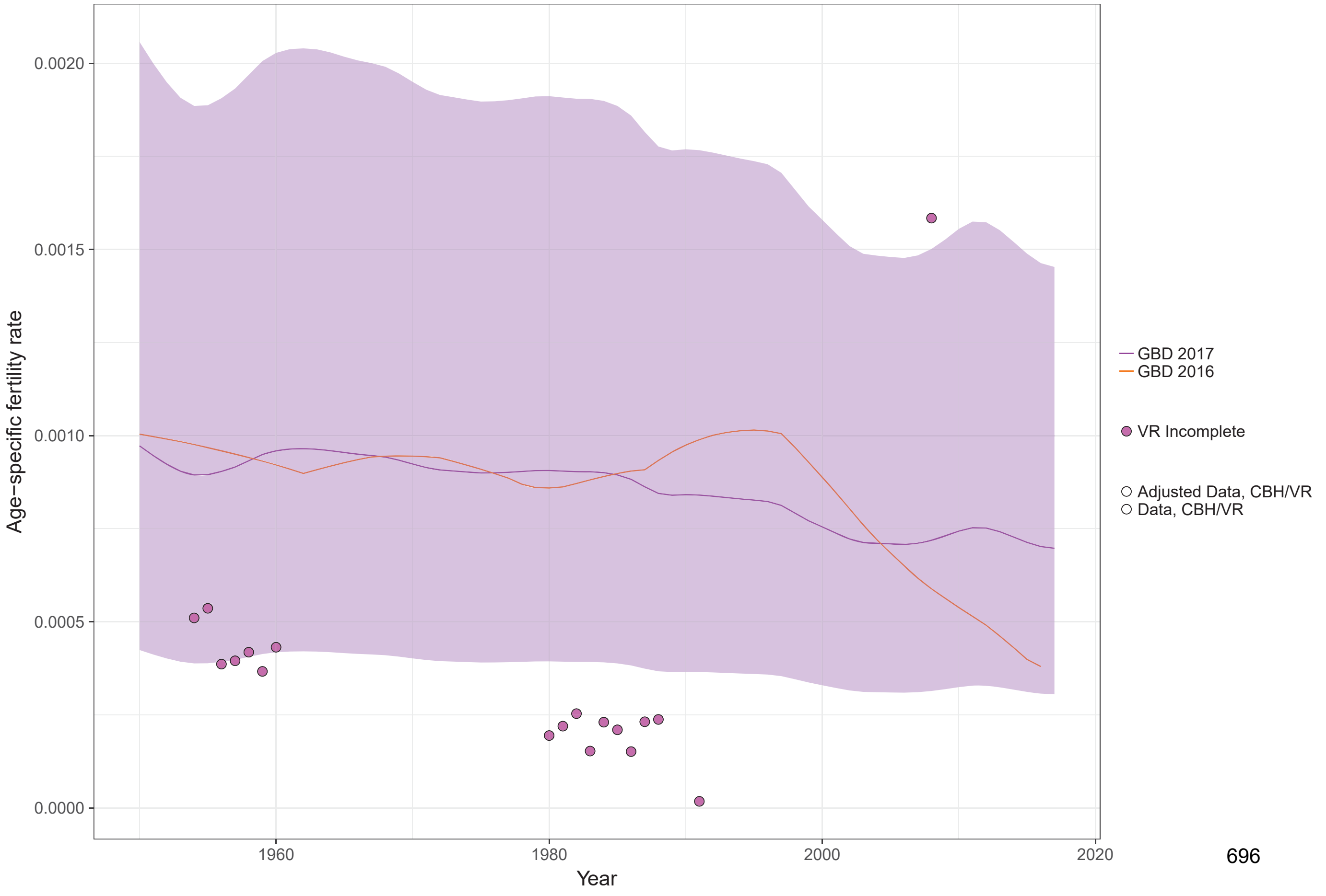

Age−specific fertility fitted trend lines, 1950−2017: Paraguay, Ages: 15−19
Data points enclosed with a diamond are reference sources

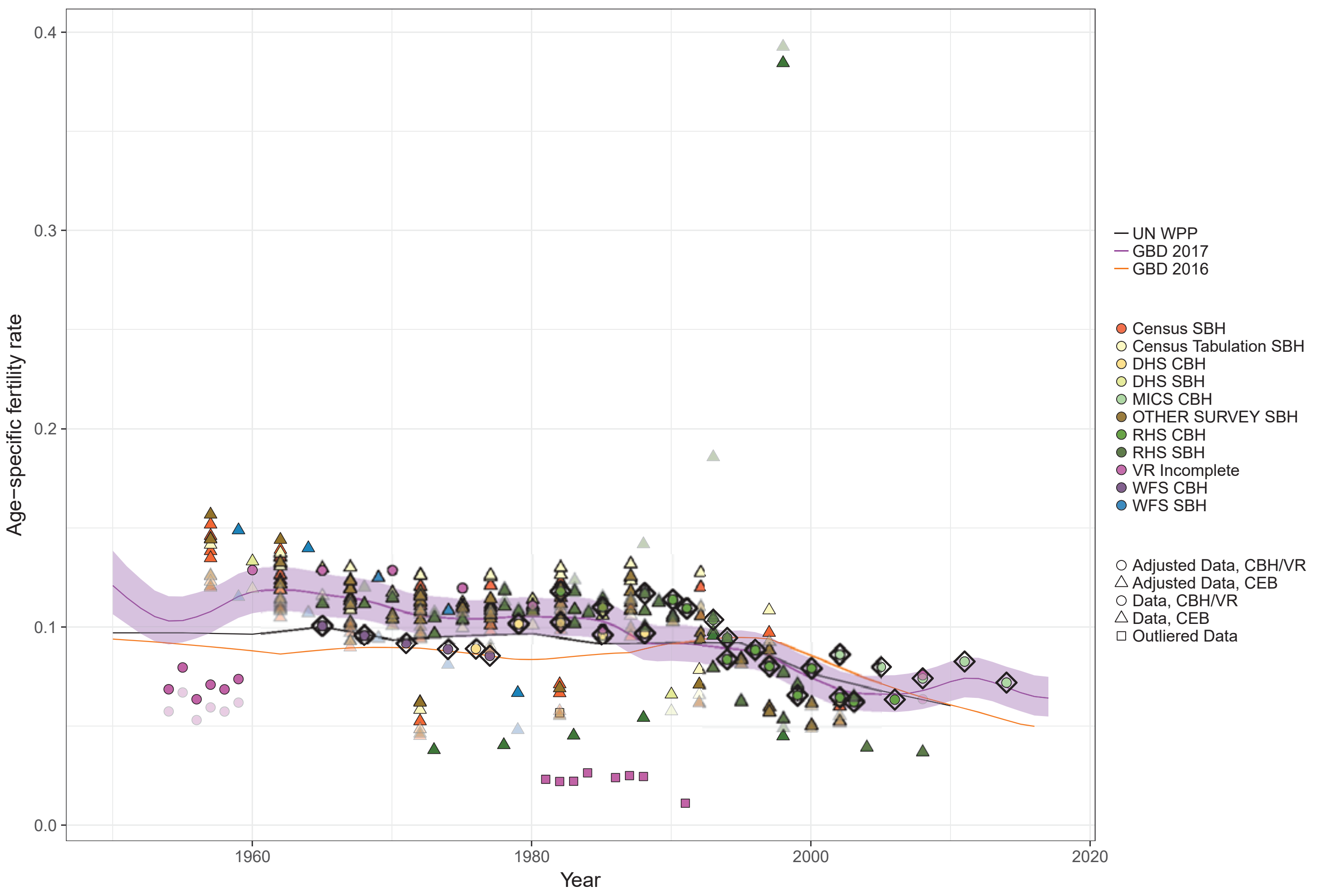

Age−specific fertility fitted trend lines, 1950−2017: Paraguay, Ages: 20−24
Data points enclosed with a diamond are reference sources

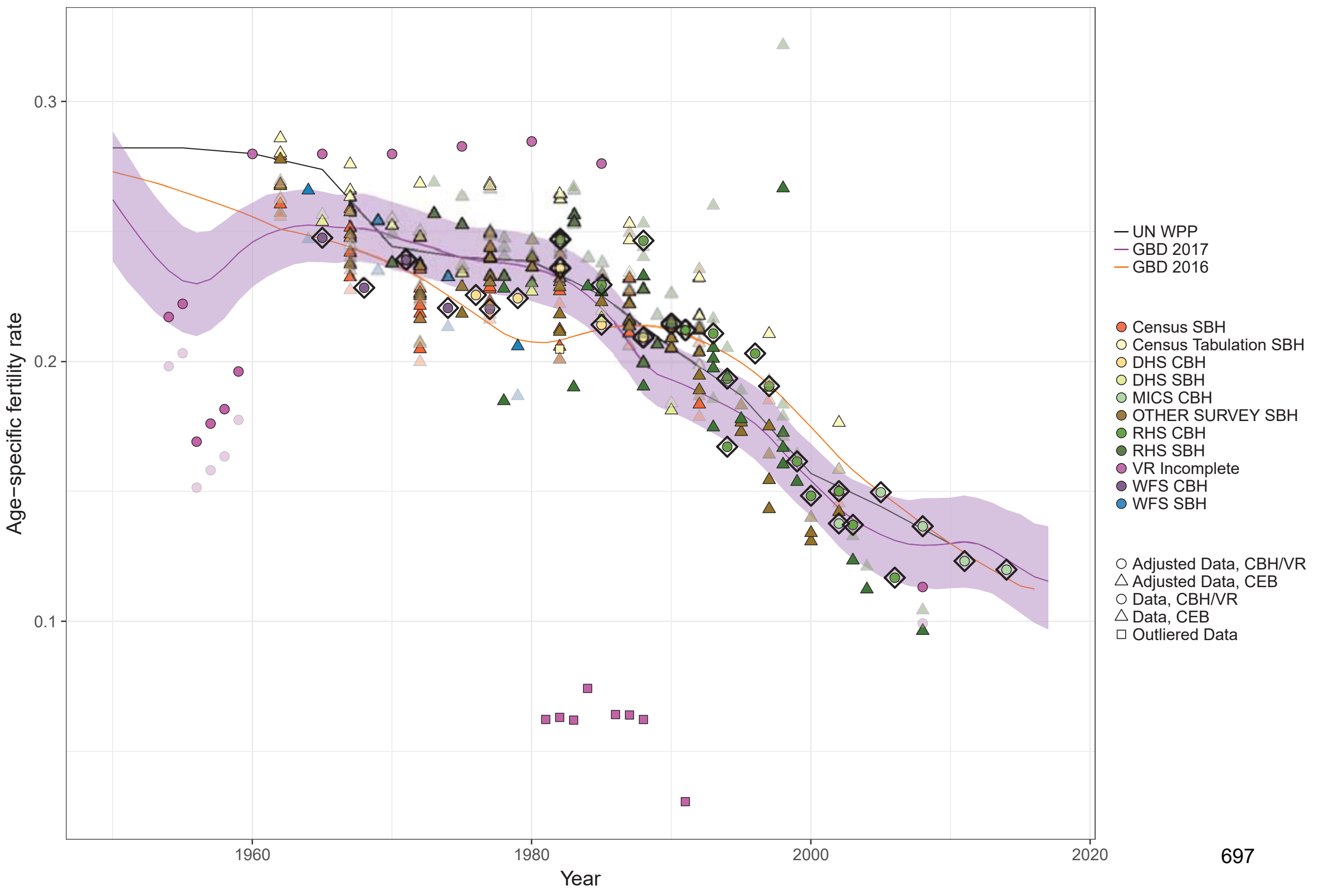

Age−specific fertility fitted trend lines, 1950−2017: Paraguay, Ages: 25−29
Data points enclosed with a diamond are reference sources

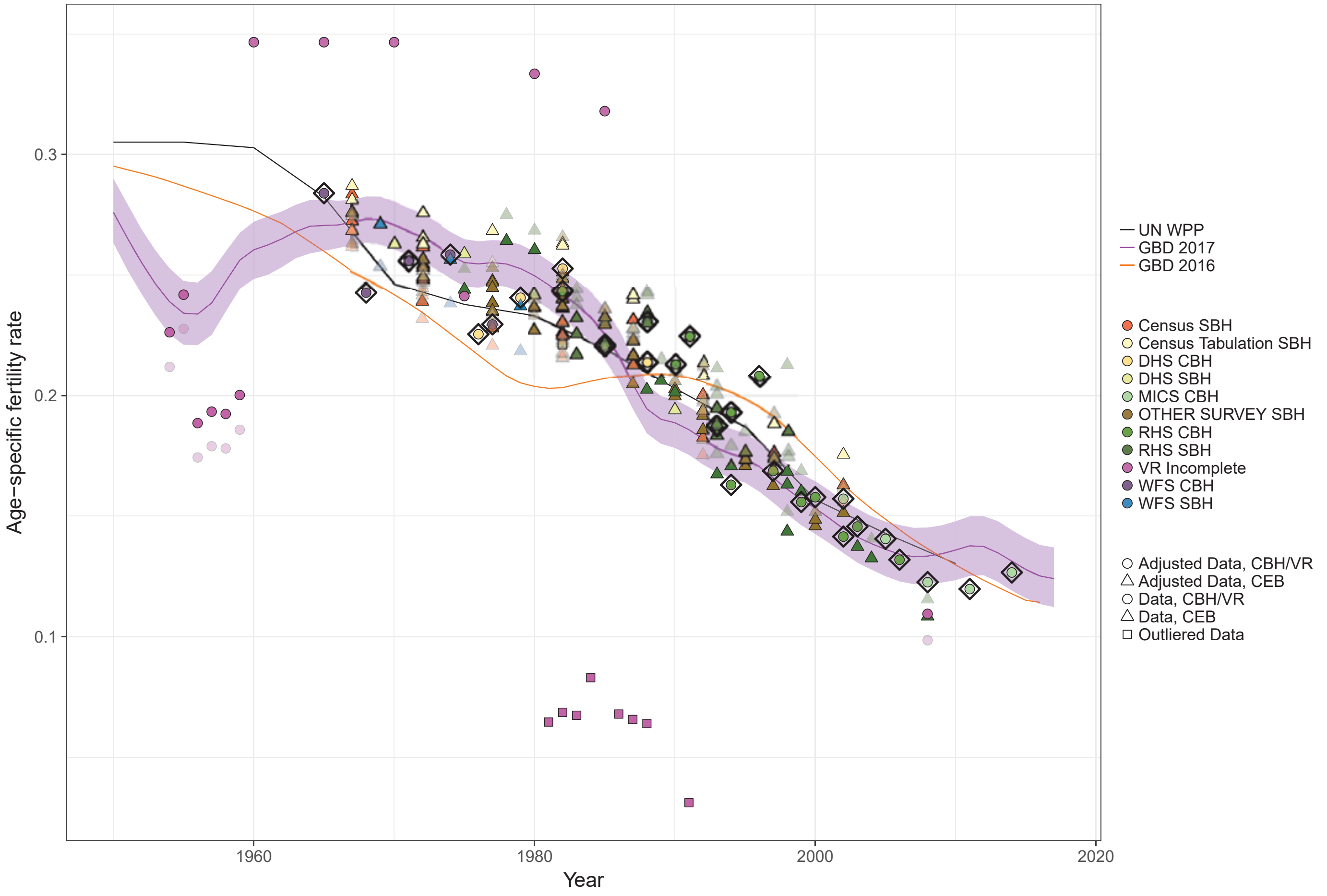

Age−specific fertility fitted trend lines, 1950−2017: Paraguay, Ages: 30−34
Data points enclosed with a diamond are reference sources

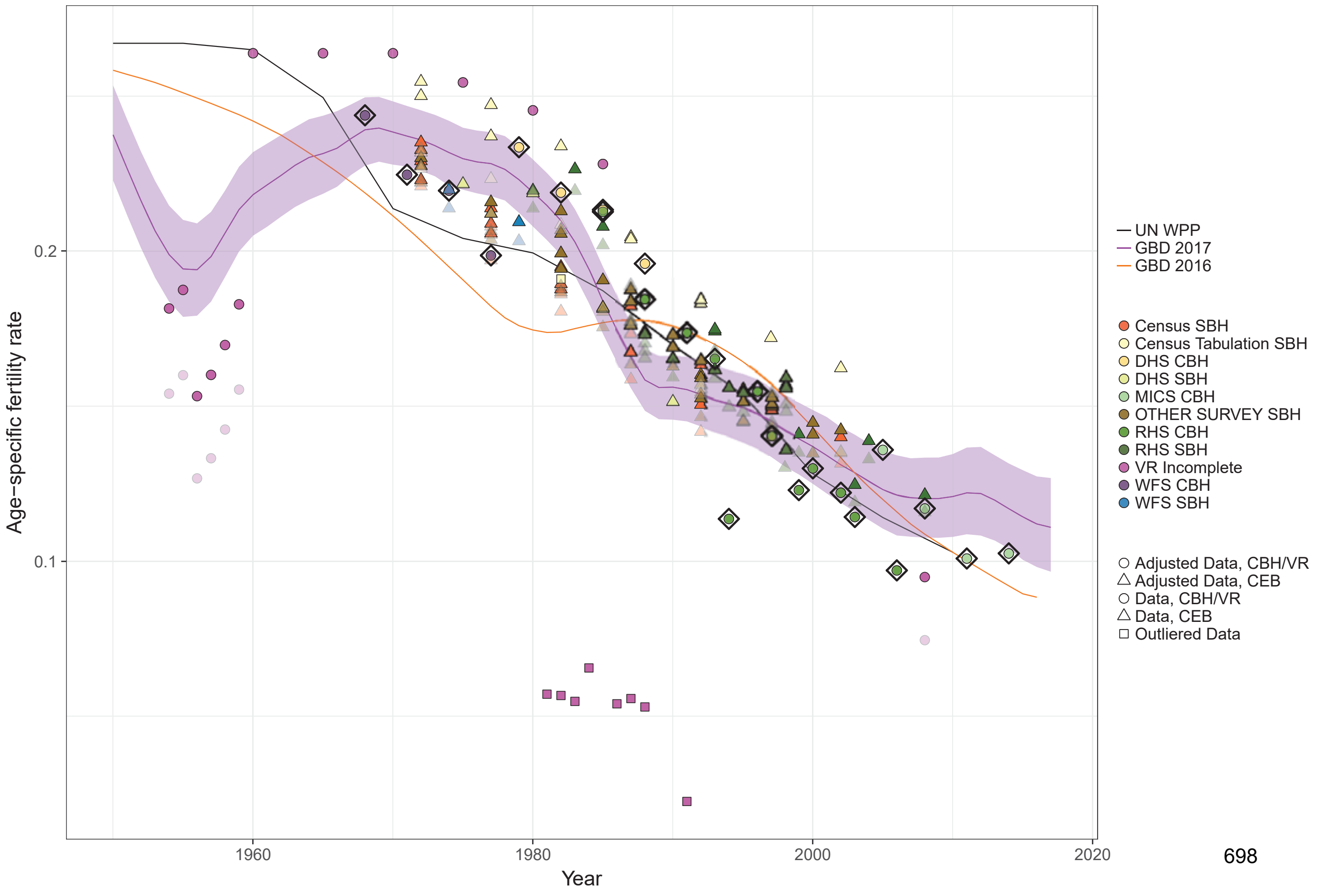

Age−specific fertility fitted trend lines, 1950−2017: Paraguay, Ages: 35−39
Data points enclosed with a diamond are reference sources

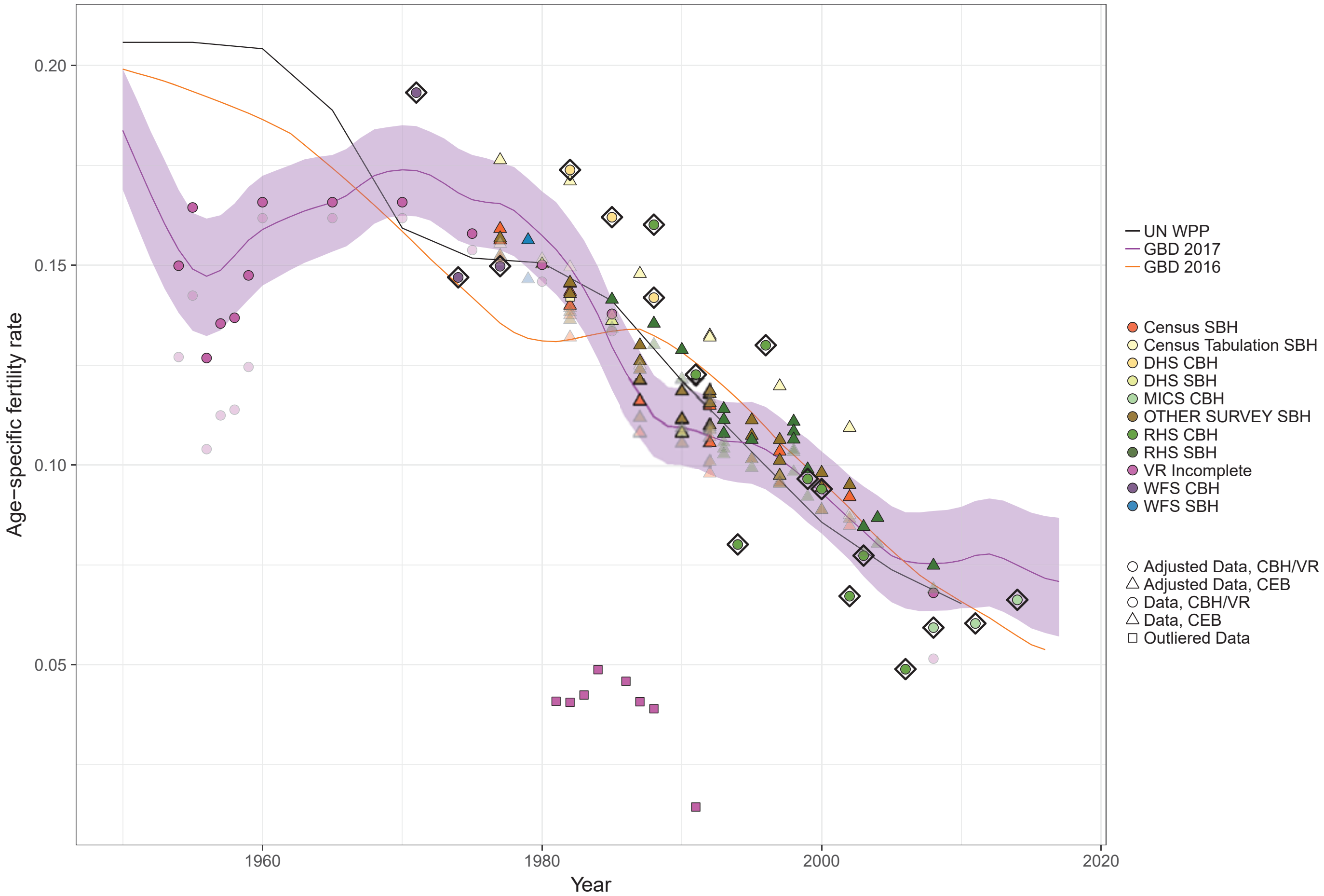

Age−specific fertility fitted trend lines, 1950−2017: Paraguay, Ages: 40−44
Data points enclosed with a diamond are reference sources

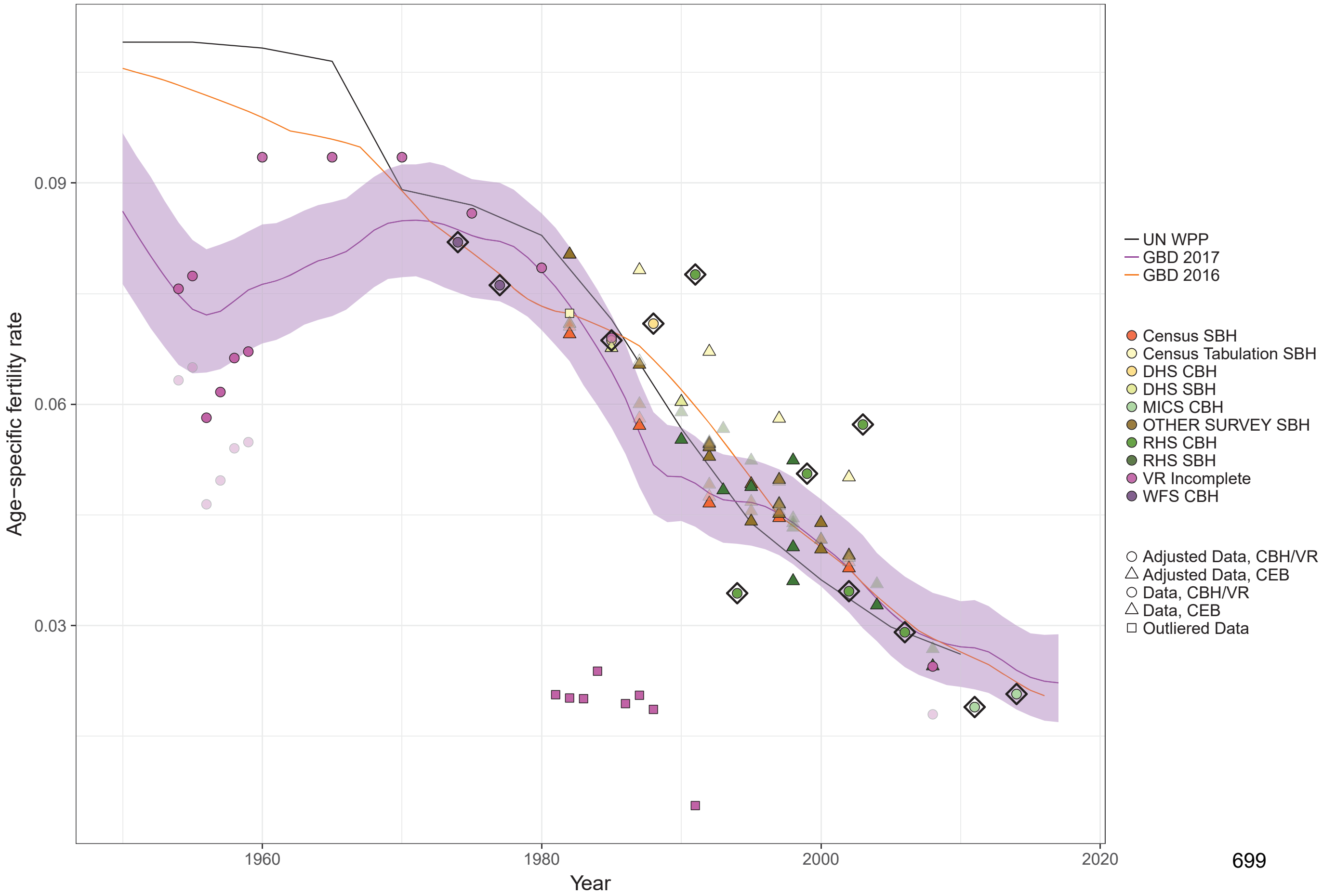

Age−specific fertility fitted trend lines, 1950−2017: Paraguay, Ages: 45−49
Data points enclosed with a diamond are reference sources

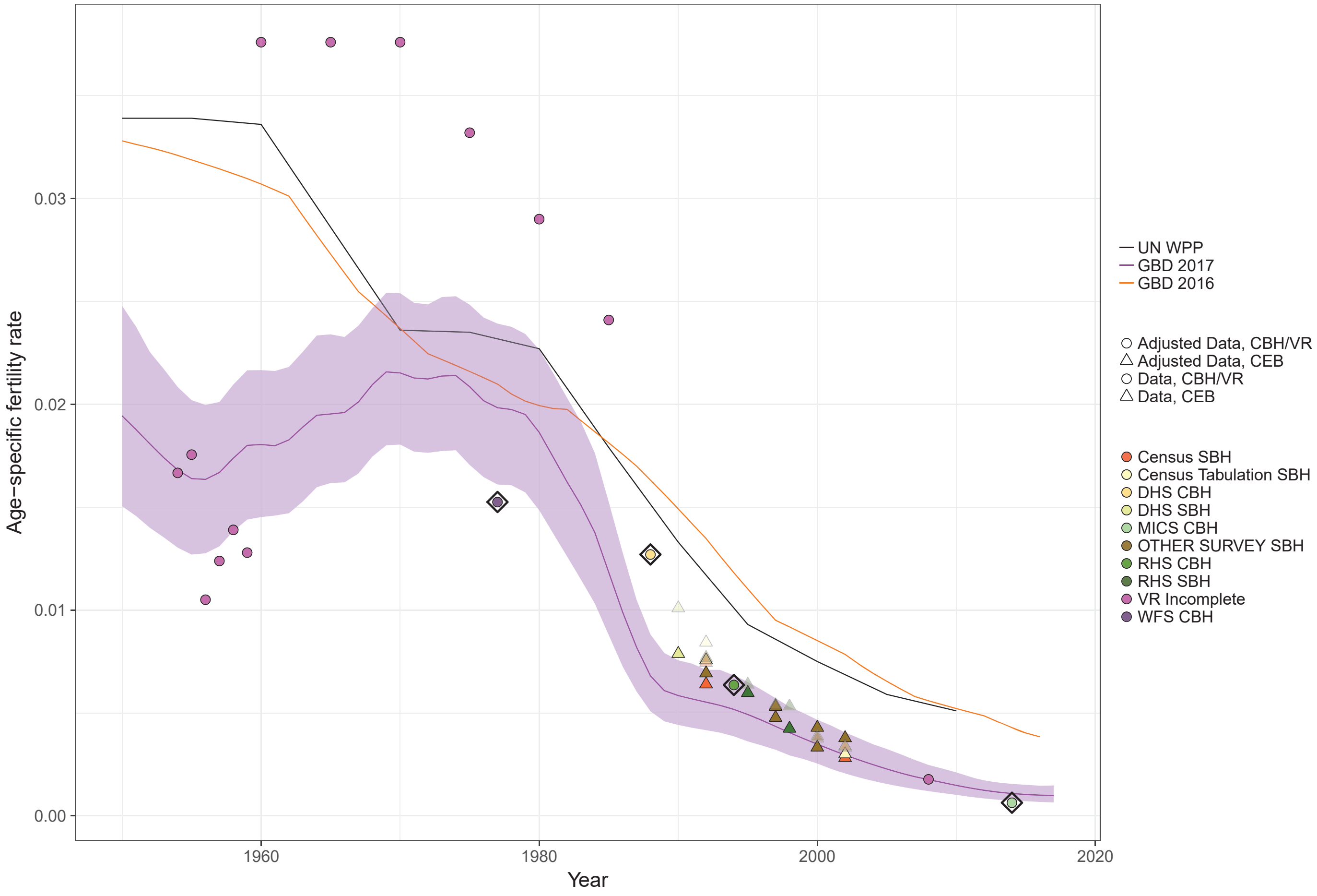

Age−specific fertility fitted trend lines, 1950−2017: Paraguay, Ages: 50−54
Data points enclosed with a diamond are reference sources

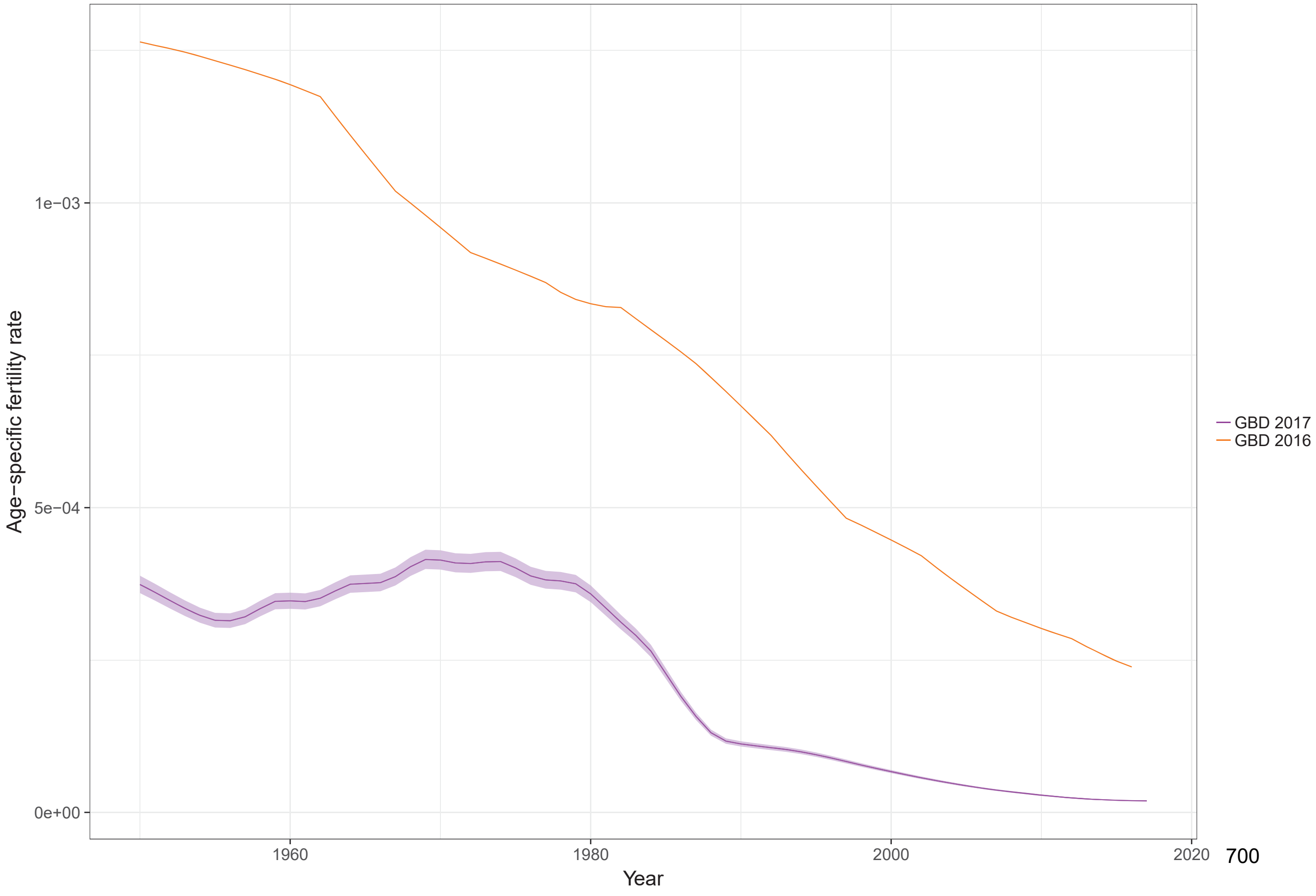

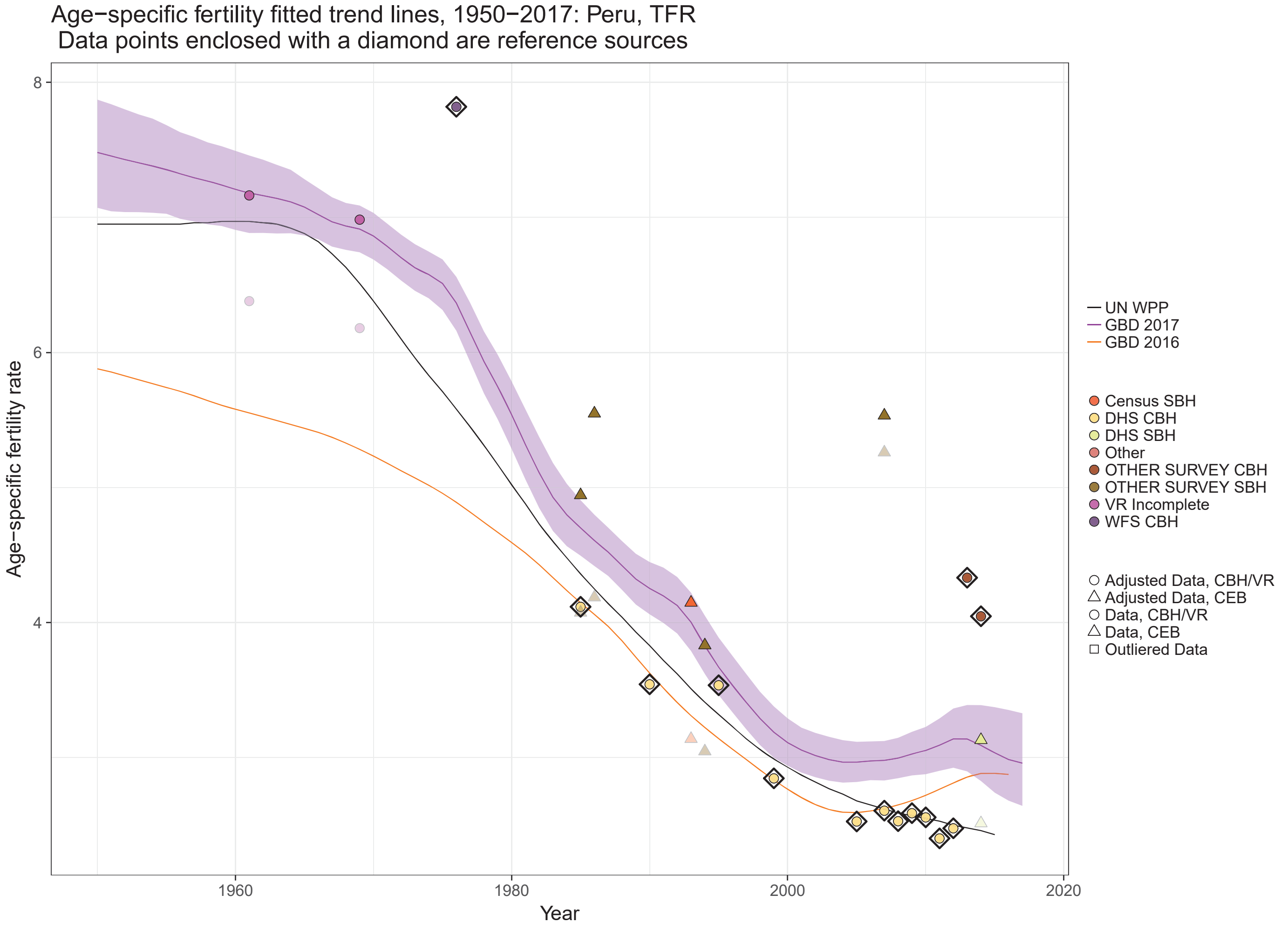

Age−specific fertility fitted trend lines, 1950−2017: Peru, TFR
Data points enclosed with a diamond are reference sources
Age−specific fertility rate
Year
UN WPP
GBD 2017
GBD 2016
Census SBH
DHS CBH
DHS SBH
Other
OTHER SURVEY CBH
OTHER SURVEY SBH
VR Incomplete
WFS CBH
Adjusted Data, CBH/VR
Adjusted Data, CEB
Data, CBH/VR
Data, CEB
Outliered Data

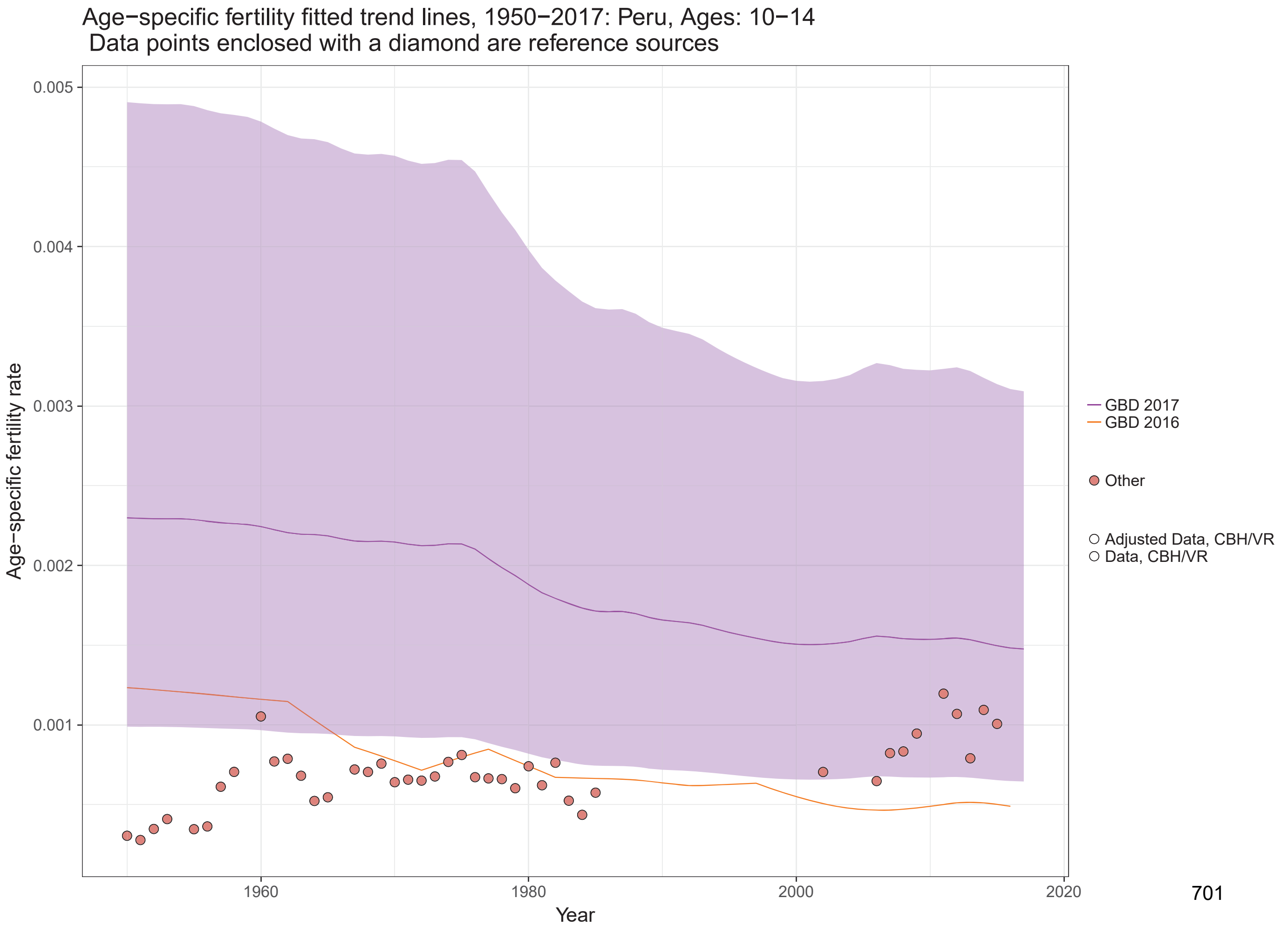

Age−specific fertility fitted trend lines, 1950−2017: Peru, Ages: 10−14
Data points enclosed with a diamond are reference sources
Age−specific fertility rate
Year
GBD 2017
GBD 2016
Other
Adjusted Data, CBH/VR
Data, CBH/VR

Age−specific fertility fitted trend lines, 1950−2017: Peru, Ages: 15−19
Data points enclosed with a diamond are reference sources

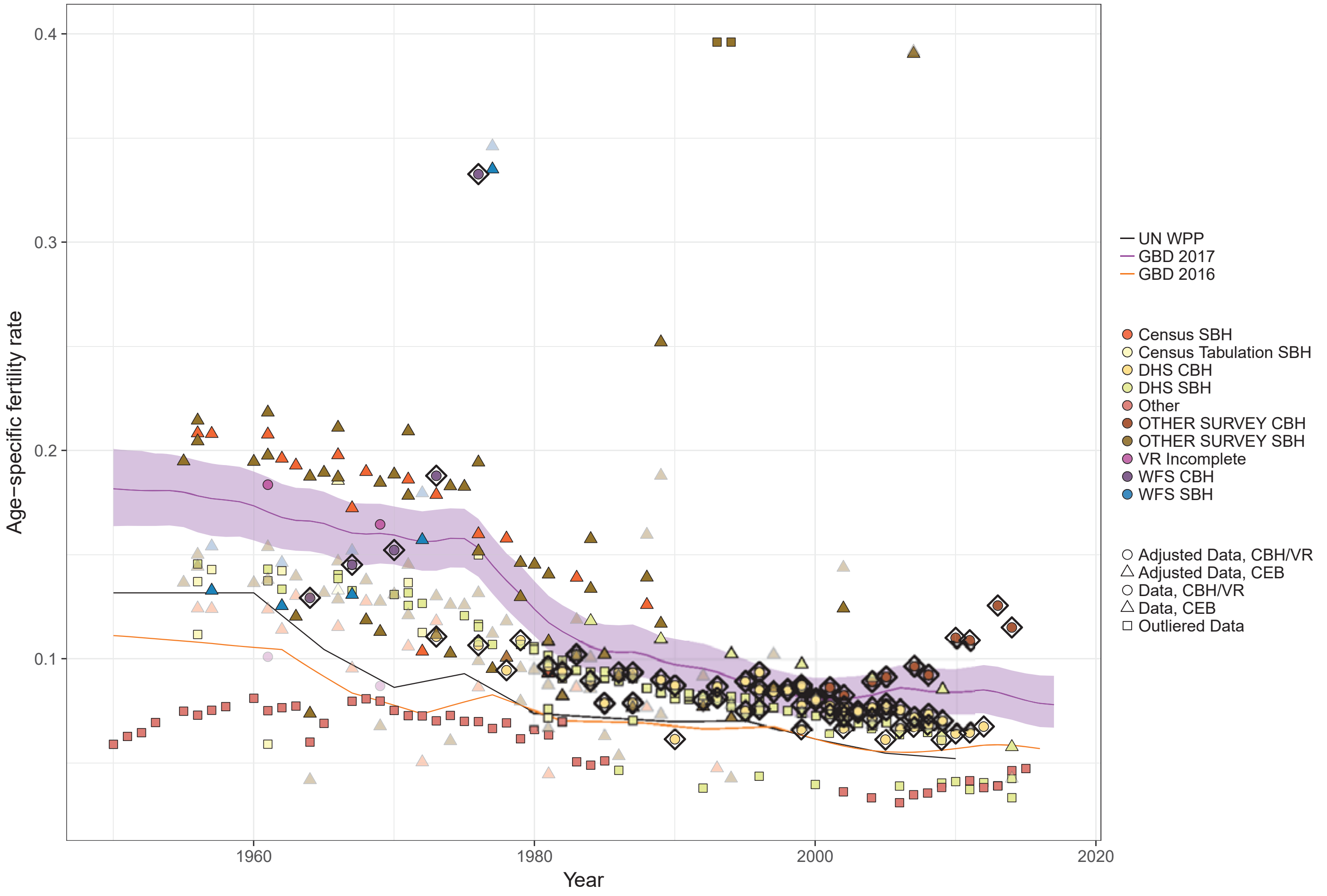

Age−specific fertility fitted trend lines, 1950−2017: Peru, Ages: 20−24
Data points enclosed with a diamond are reference sources

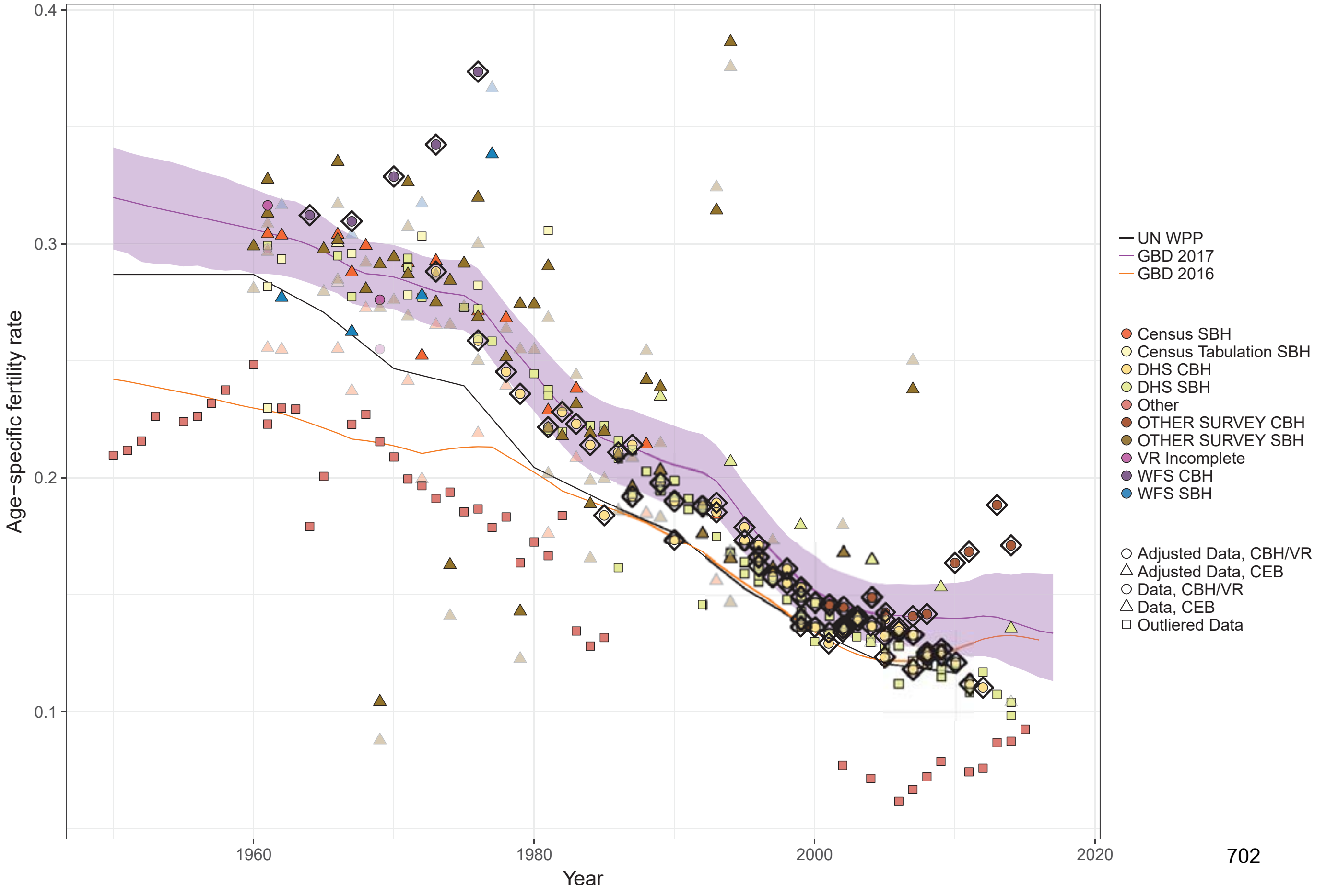

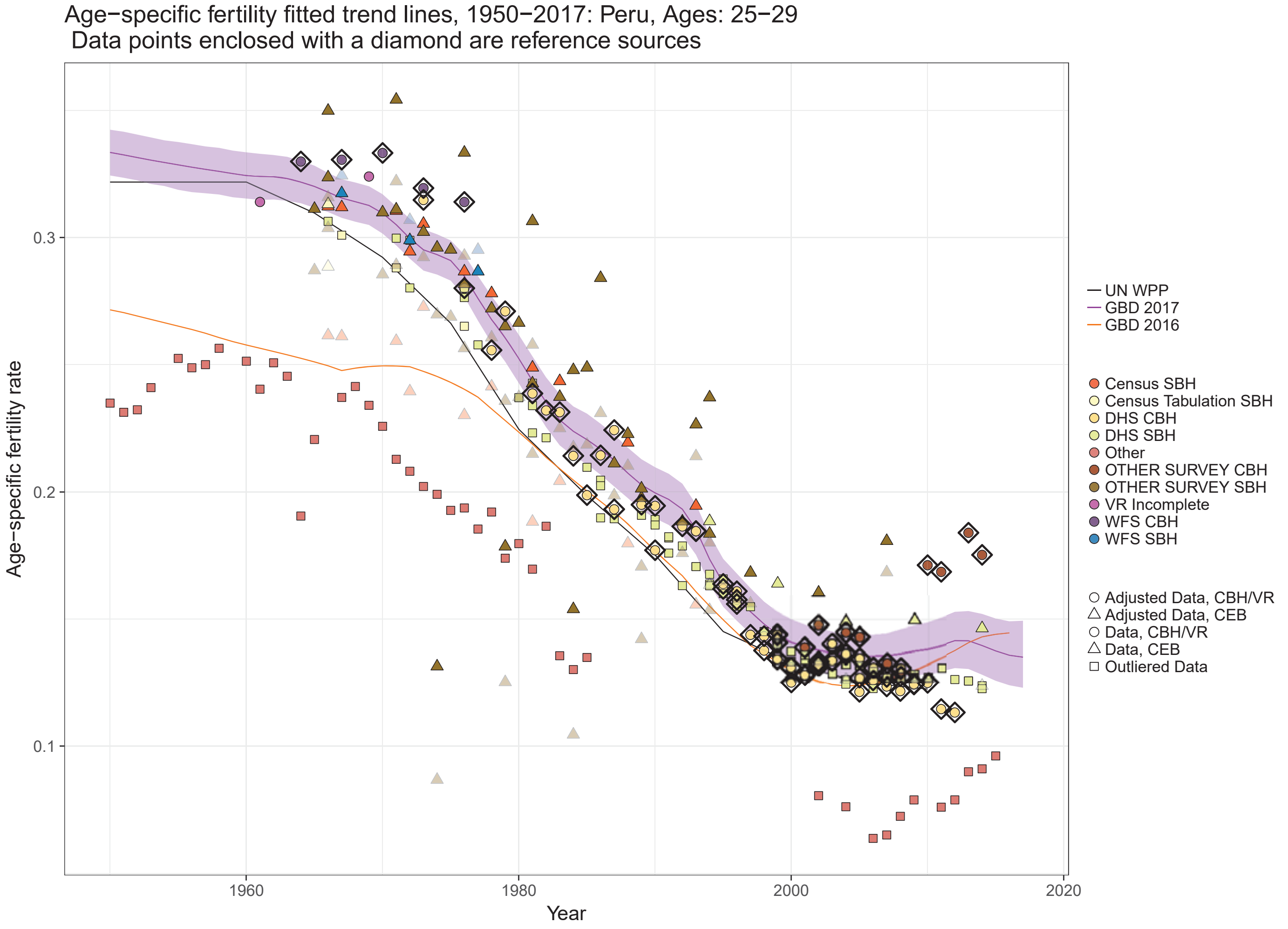
Age−specific fertility fitted trend lines, 1950−2017: Peru, Ages: 25−29
Data points enclosed with a diamond are reference sources
Age-specific fertility rate
Year
UN WPP
GBD 2017
GBD 2016
Census SBH
Census Tabulation SBH
DHS CBH
DHS SBH
Other
OTHER SURVEY CBH
OTHER SURVEY SBH
VR Incomplete
WFS CBH
WFS SBH
Adjusted Data, CBH/VR
Adjusted Data, CEB
Data, CBH/VR
Data, CEB
Outliered Data

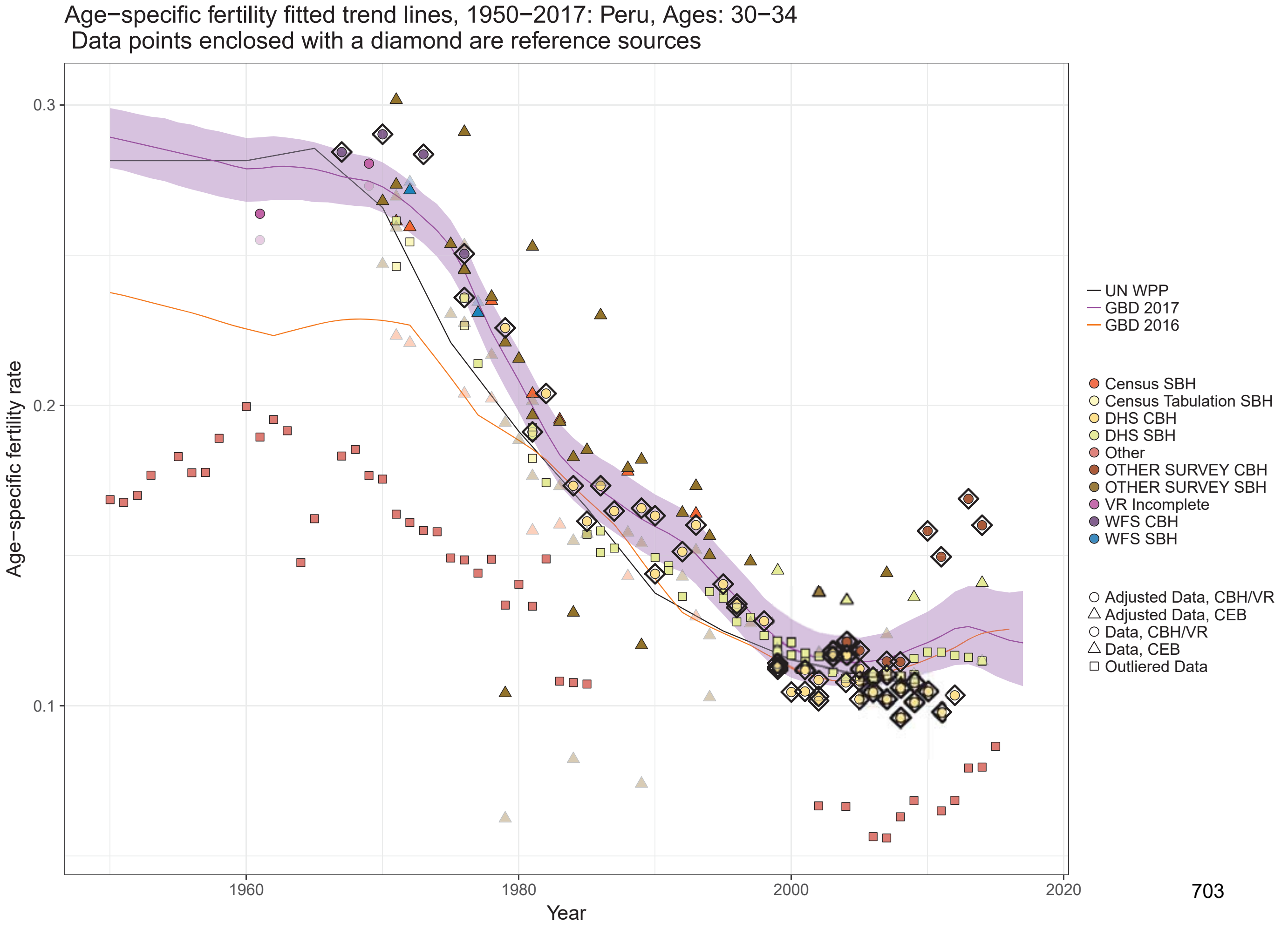
Age−specific fertility fitted trend lines, 1950−2017: Peru, Ages: 30−34
Data points enclosed with a diamond are reference sources
Age-specific fertility rate
Year
UN WPP
GBD 2017
GBD 2016
Census SBH
Census Tabulation SBH
DHS CBH
DHS SBH
Other
OTHER SURVEY CBH
OTHER SURVEY SBH
VR Incomplete
WFS CBH
WFS SBH
Adjusted Data, CBH/VR
Adjusted Data, CEB
Data, CBH/VR
Data, CEB
Outliered Data

Age−specific fertility fitted trend lines, 1950−2017: Peru, Ages: 35−39
Data points enclosed with a diamond are reference sources

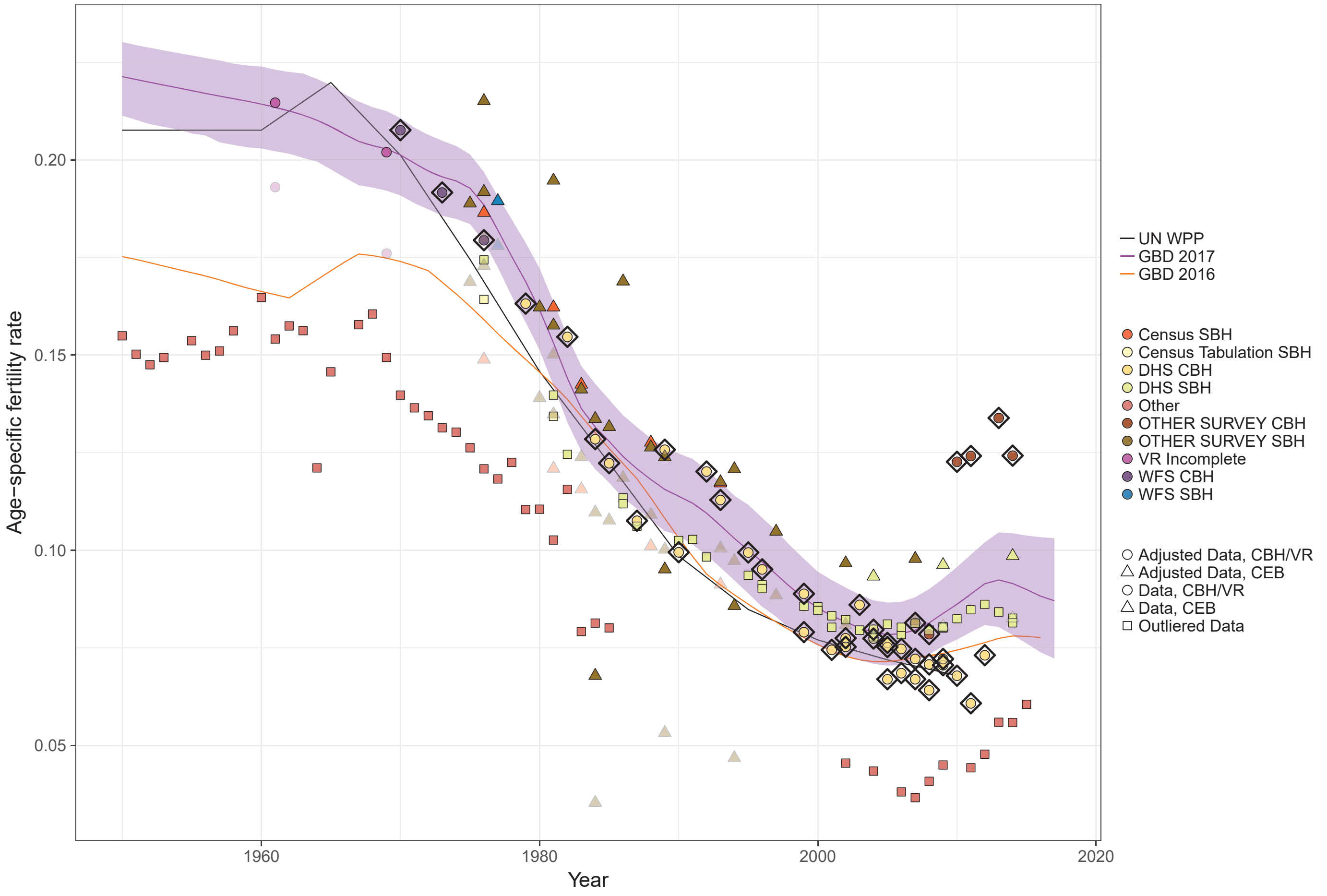

Age−specific fertility fitted trend lines, 1950−2017: Peru, Ages: 40−44
Data points enclosed with a diamond are reference sources

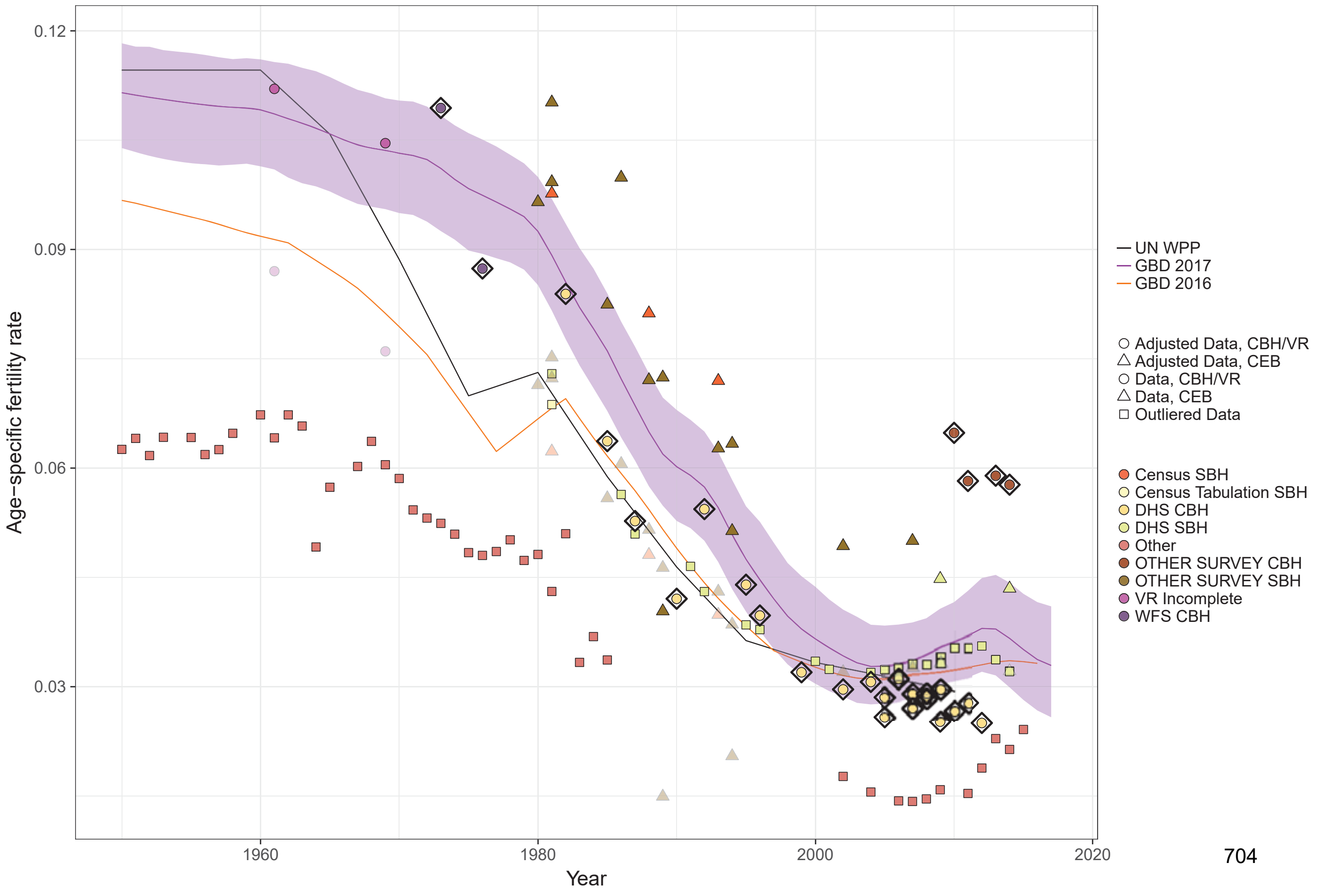

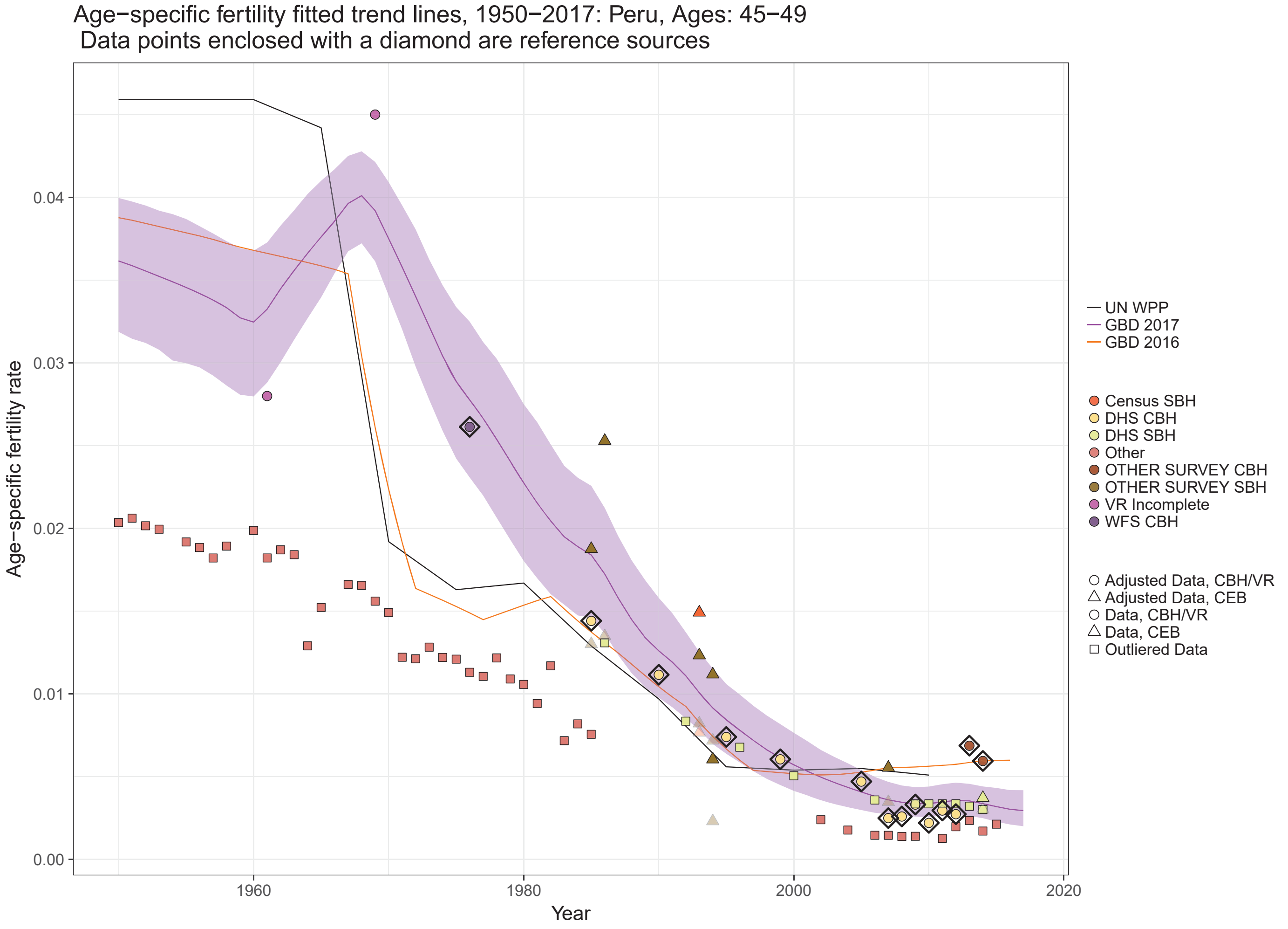
Age−specific fertility fitted trend lines, 1950−2017: Peru, Ages: 45−49
Data points enclosed with a diamond are reference sources
Age−specific fertility rate
Year
UN WPP
GBD 2017
GBD 2016
Census SBH
DHS CBH
DHS SBH
Other
OTHER SURVEY CBH
OTHER SURVEY SBH
VR Incomplete
WFS CBH
Adjusted Data, CBH/VR
Adjusted Data, CEB
Data, CBH/VR
Data, CEB
Outliered Data

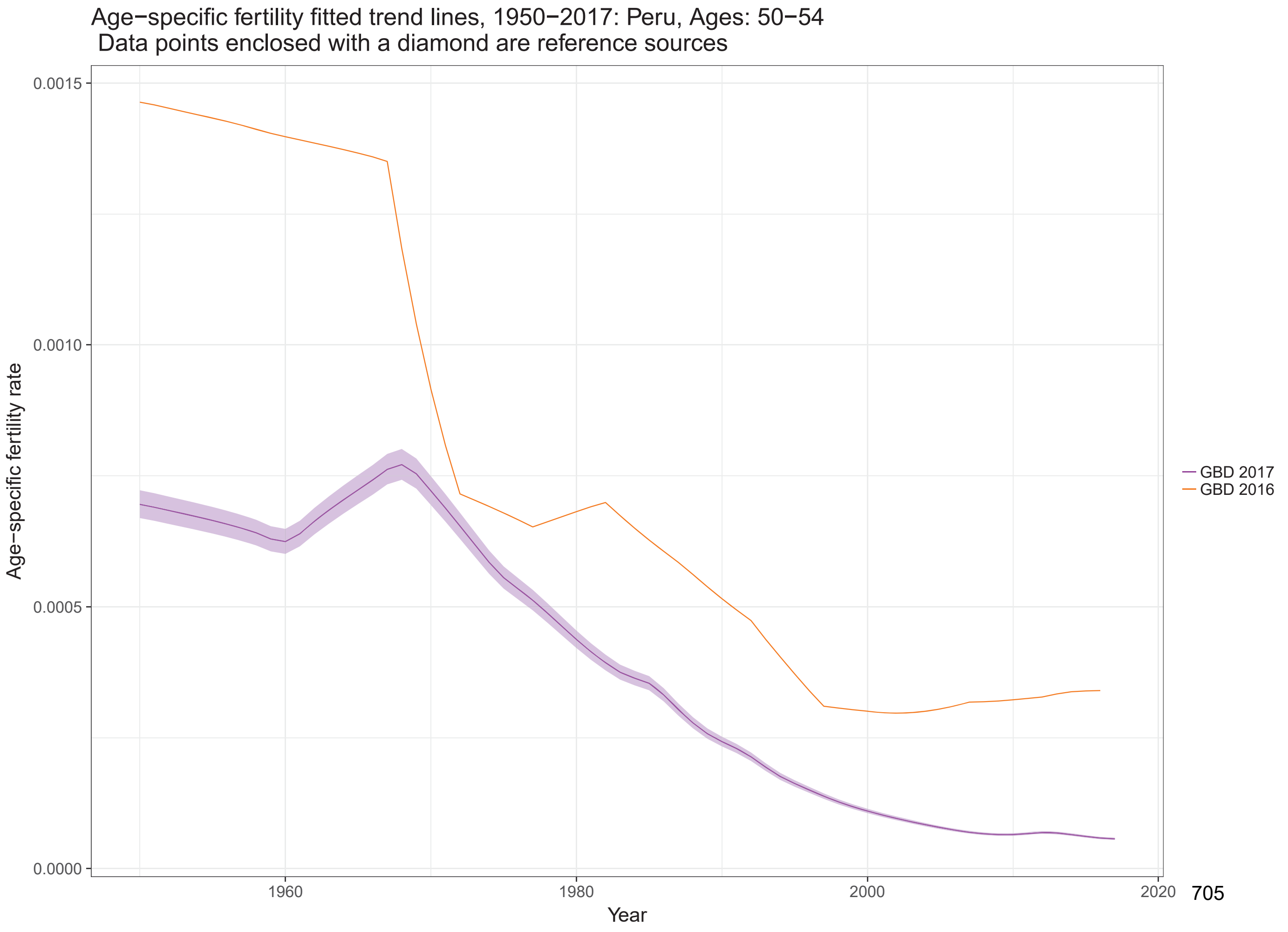
Age−specific fertility fitted trend lines, 1950−2017: Peru, Ages: 50−54
Data points enclosed with a diamond are reference sources
Age−specific fertility rate
Year
GBD 2017
GBD 2016

Age−specific fertility fitted trend lines, 1950−2017: Philippines, TFR
Data points enclosed with a diamond are reference sources

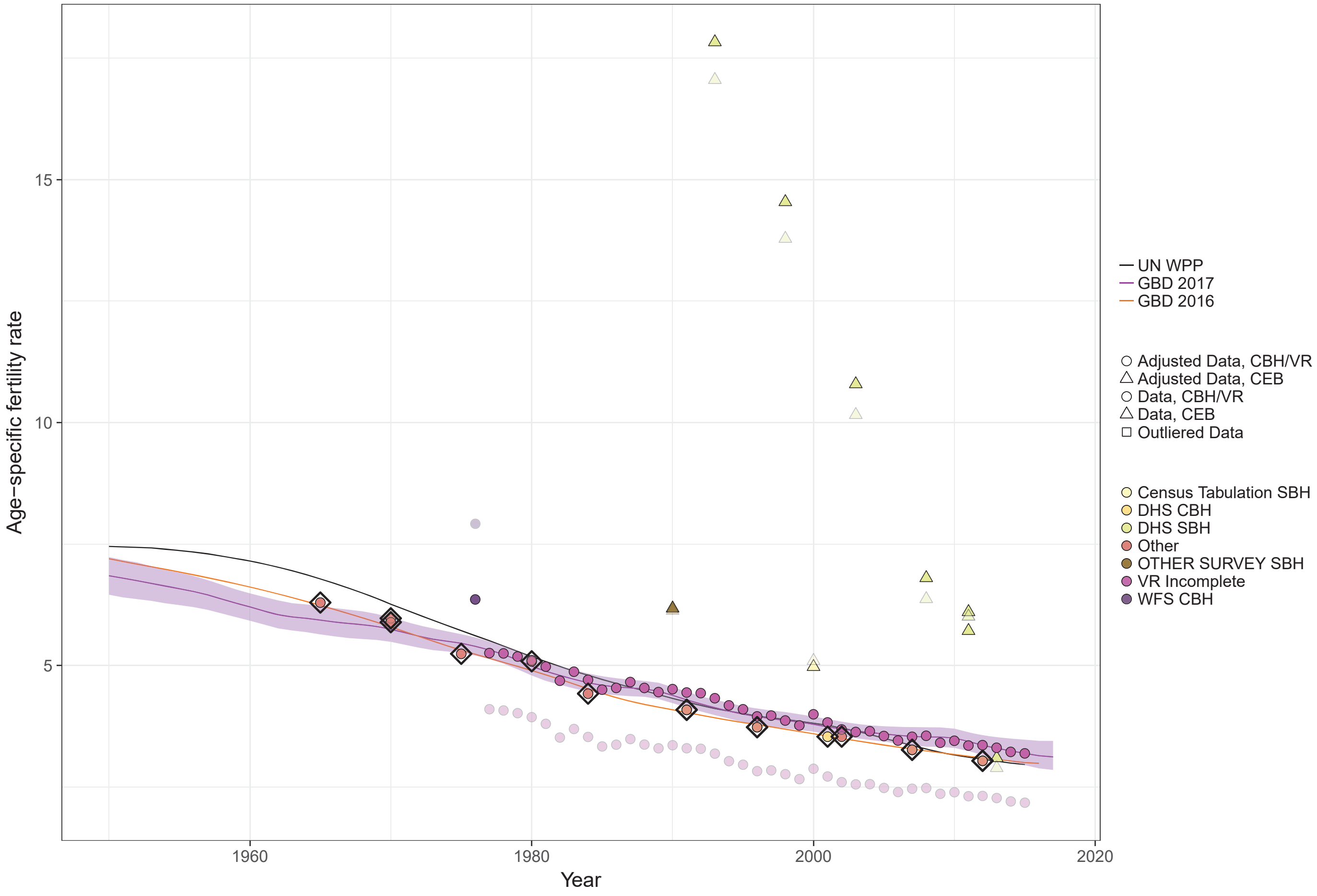

Age−specific fertility fitted trend lines, 1950−2017: Philippines, Ages: 10−14
Data points enclosed with a diamond are reference sources

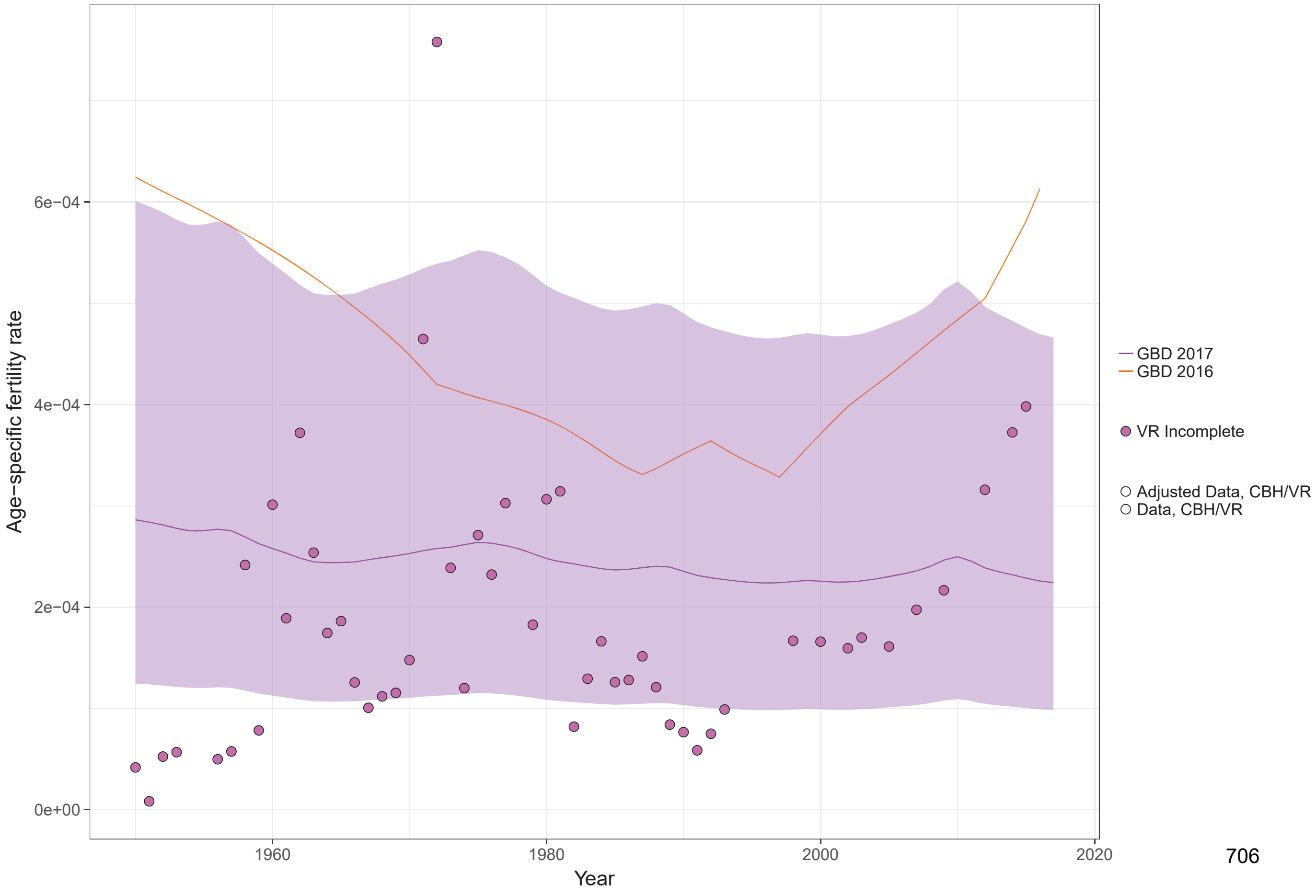

Age−specific fertility fitted trend lines, 1950−2017: Philippines, Ages: 15−19
Data points enclosed with a diamond are reference sources

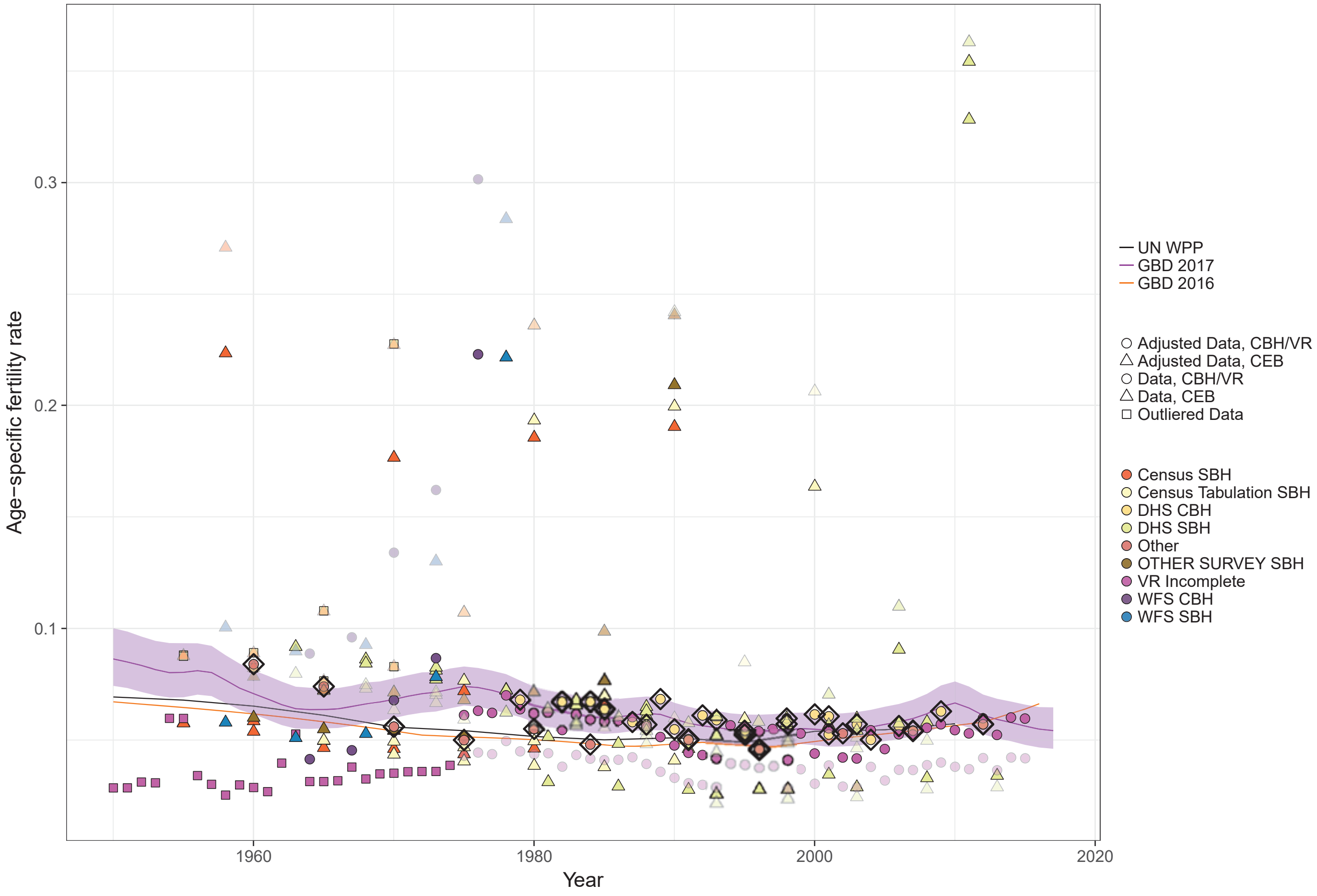

Age−specific fertility fitted trend lines, 1950−2017: Philippines, Ages: 20−24
Data points enclosed with a diamond are reference sources

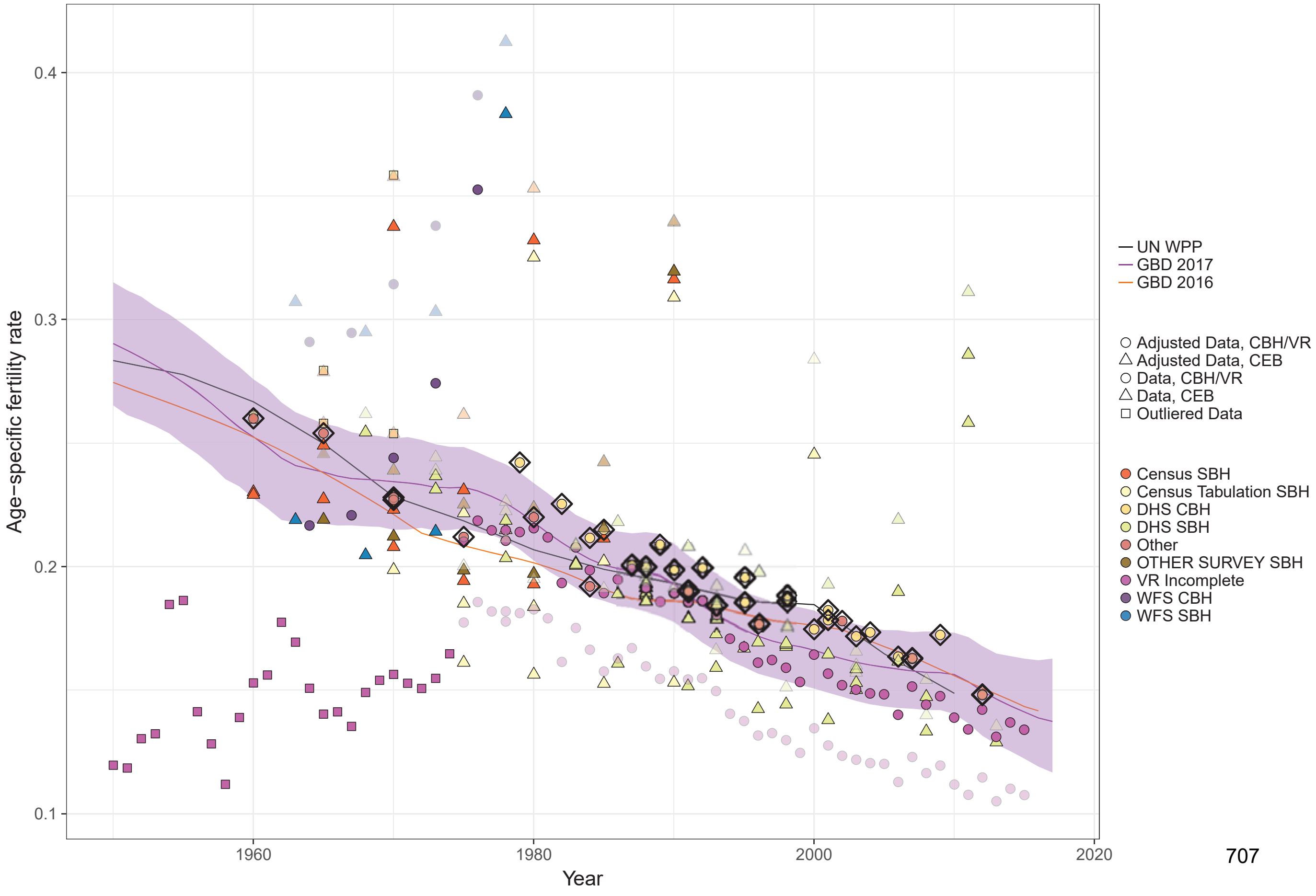

Age−specific fertility fitted trend lines, 1950−2017: Philippines, Ages: 25−29
Data points enclosed with a diamond are reference sources

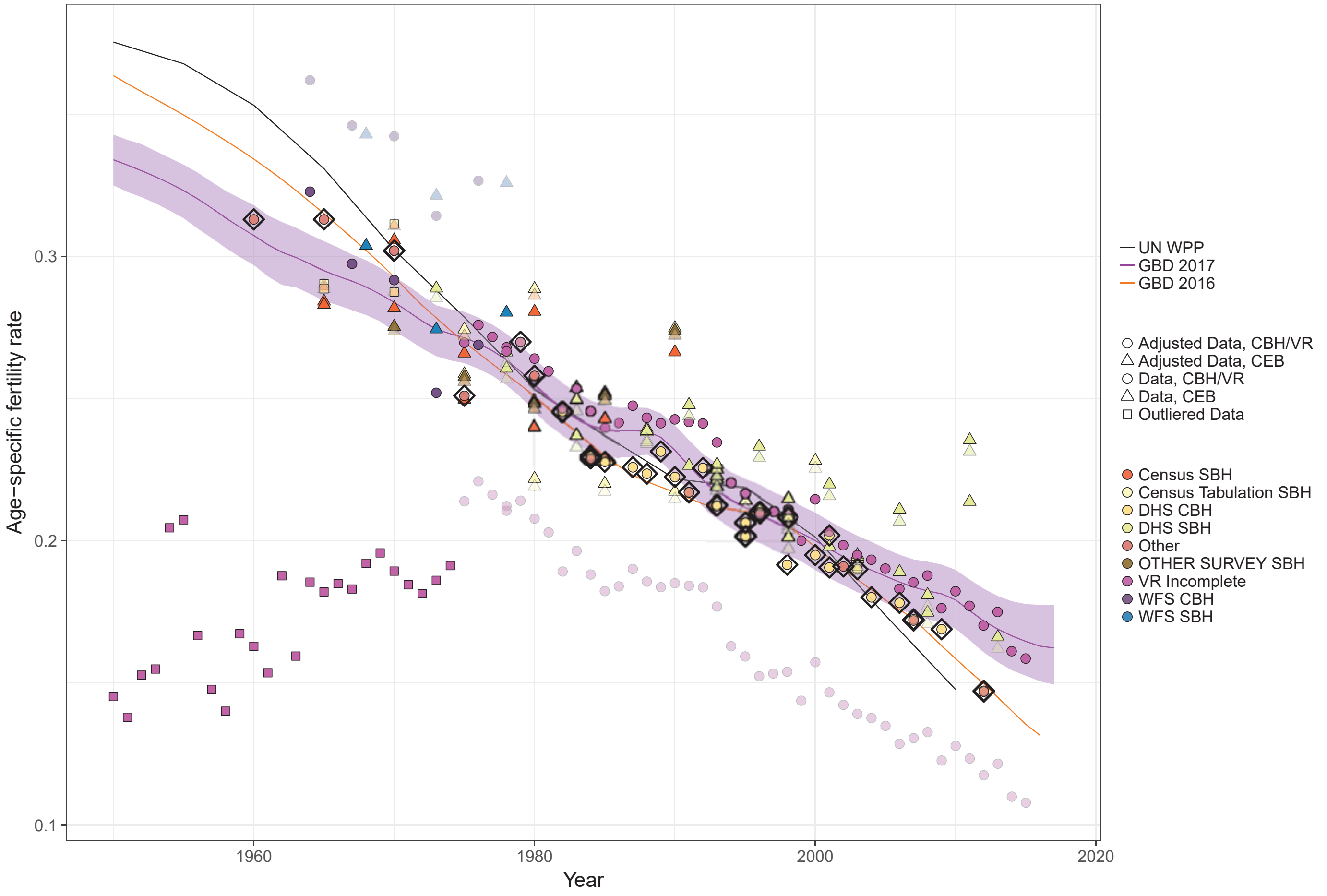

Age−specific fertility fitted trend lines, 1950−2017: Philippines, Ages: 30−34
Data points enclosed with a diamond are reference sources

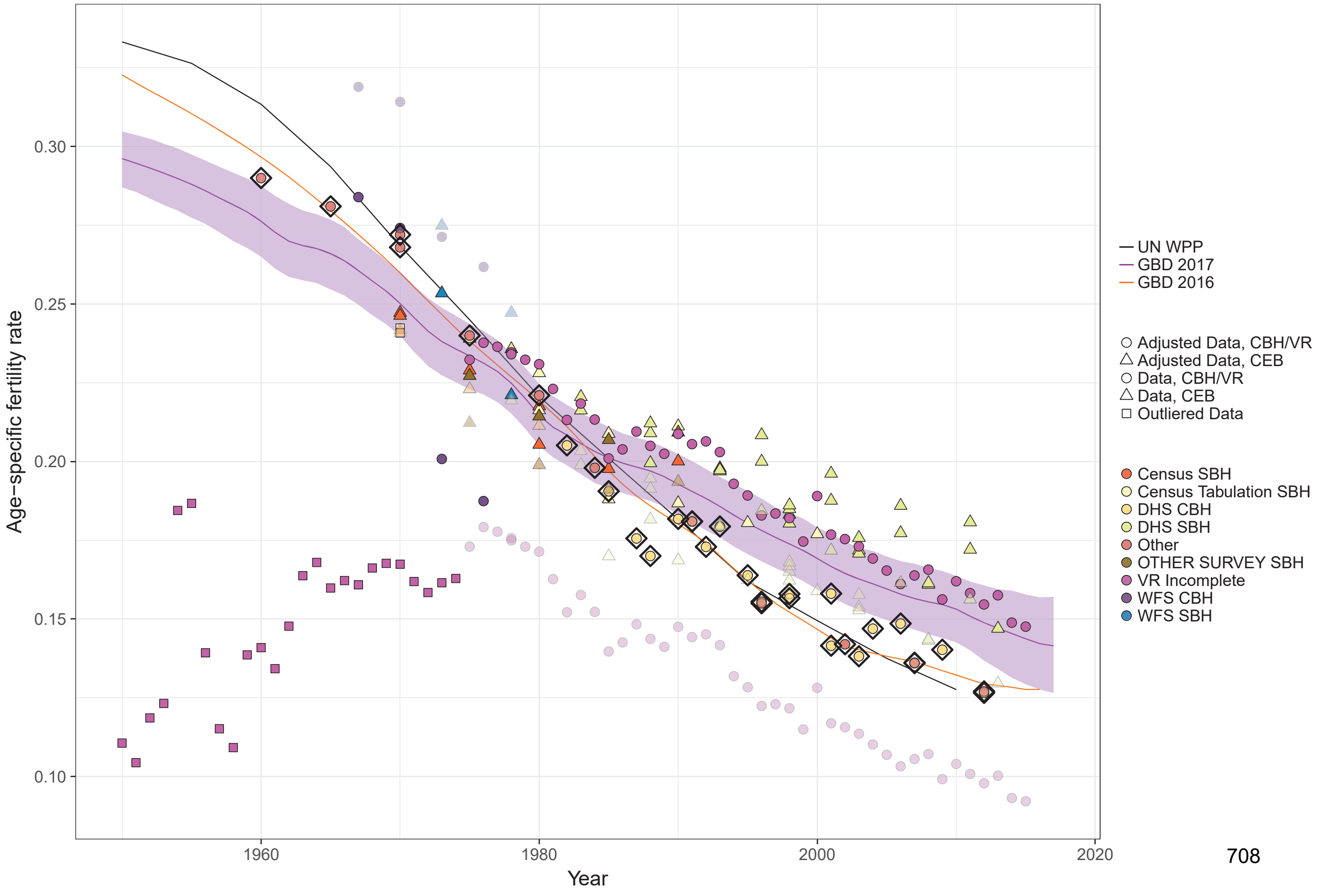

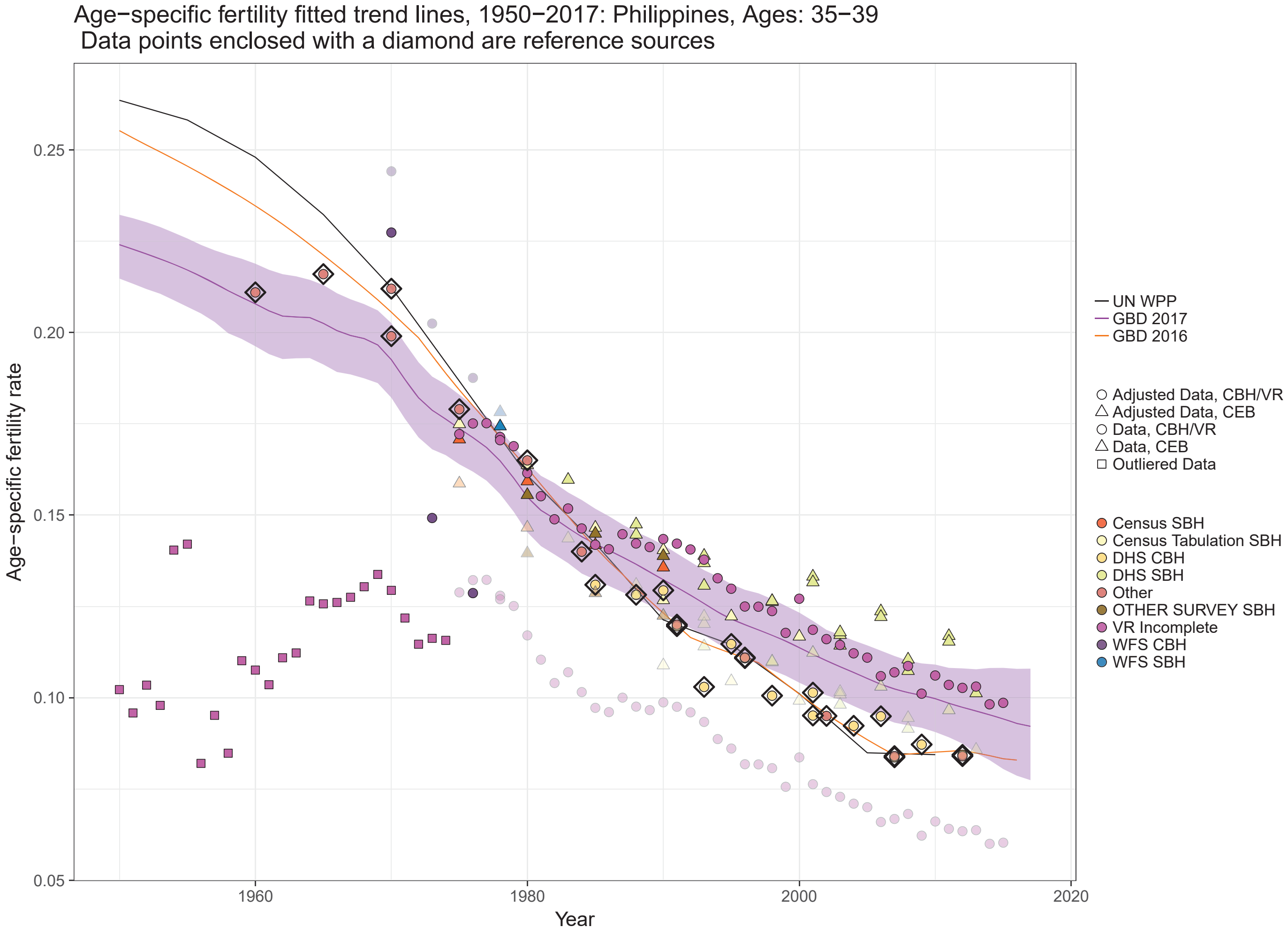

Age−specific fertility fitted trend lines, 1950−2017: Philippines, Ages: 35−39
Data points enclosed with a diamond are reference sources
Age−specific fertility rate
Year
0.05
0.10
0.15
0.20
0.25
1960
1980
2000
2020
UN WPP
GBD 2017
GBD 2016
Adjusted Data, CBH/VR
Adjusted Data, CEB
Data, CBH/VR
Data, CEB
Outliered Data
Census SBH
Census Tabulation SBH
DHS CBH
DHS SBH
Other
OTHER SURVEY SBH
VR Incomplete
WFS CBH
WFS SBH

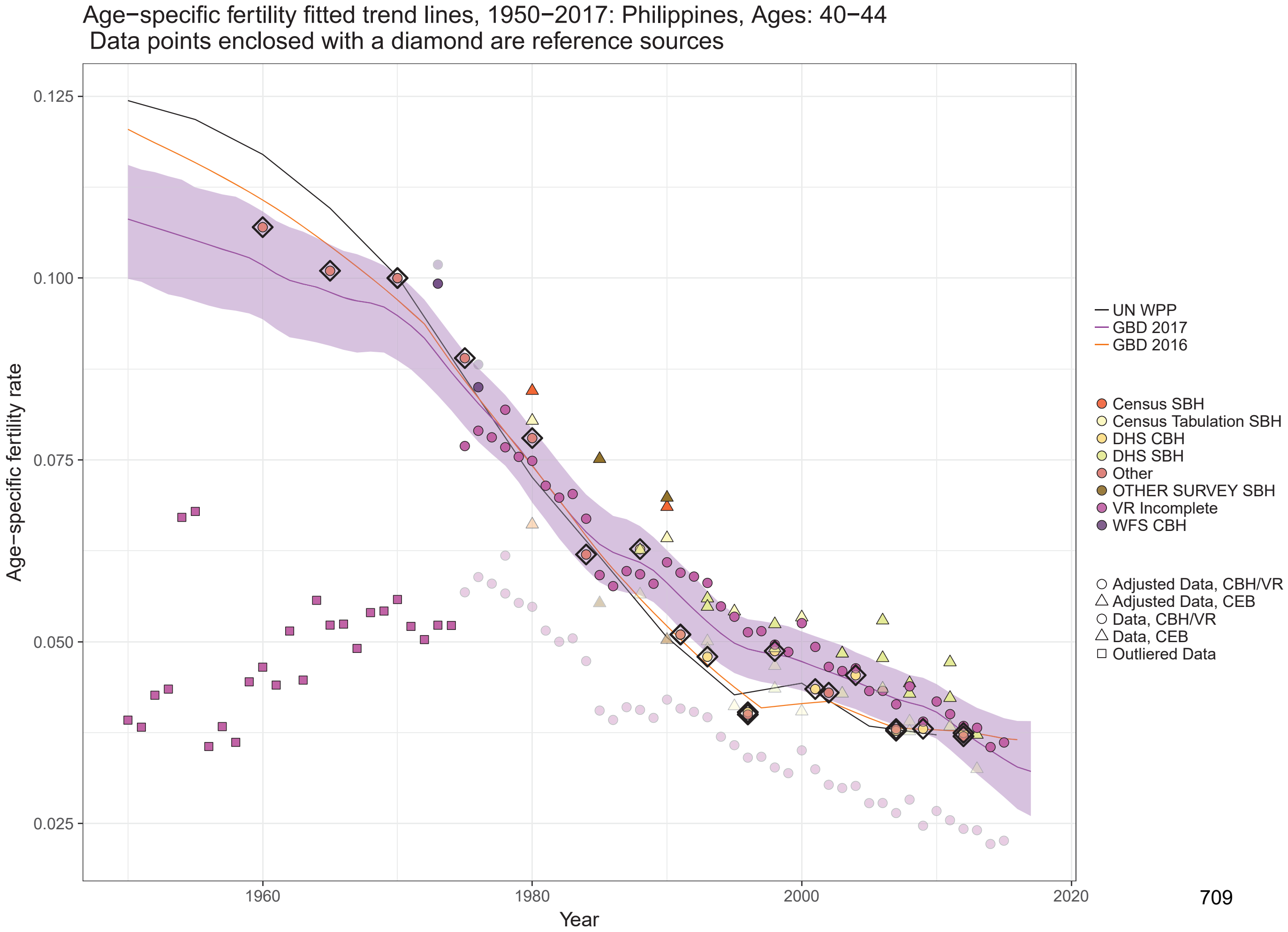

Age−specific fertility fitted trend lines, 1950−2017: Philippines, Ages: 40−44
Data points enclosed with a diamond are reference sources
Age−specific fertility rate
Year
0.025
0.050
0.075
0.100
0.125
1960
1980
2000
2020
UN WPP
GBD 2017
GBD 2016
Census SBH
Census Tabulation SBH
DHS CBH
DHS SBH
Other
OTHER SURVEY SBH
VR Incomplete
WFS CBH
Adjusted Data, CBH/VR
Adjusted Data, CEB
Data, CBH/VR
Data, CEB
Outliered Data

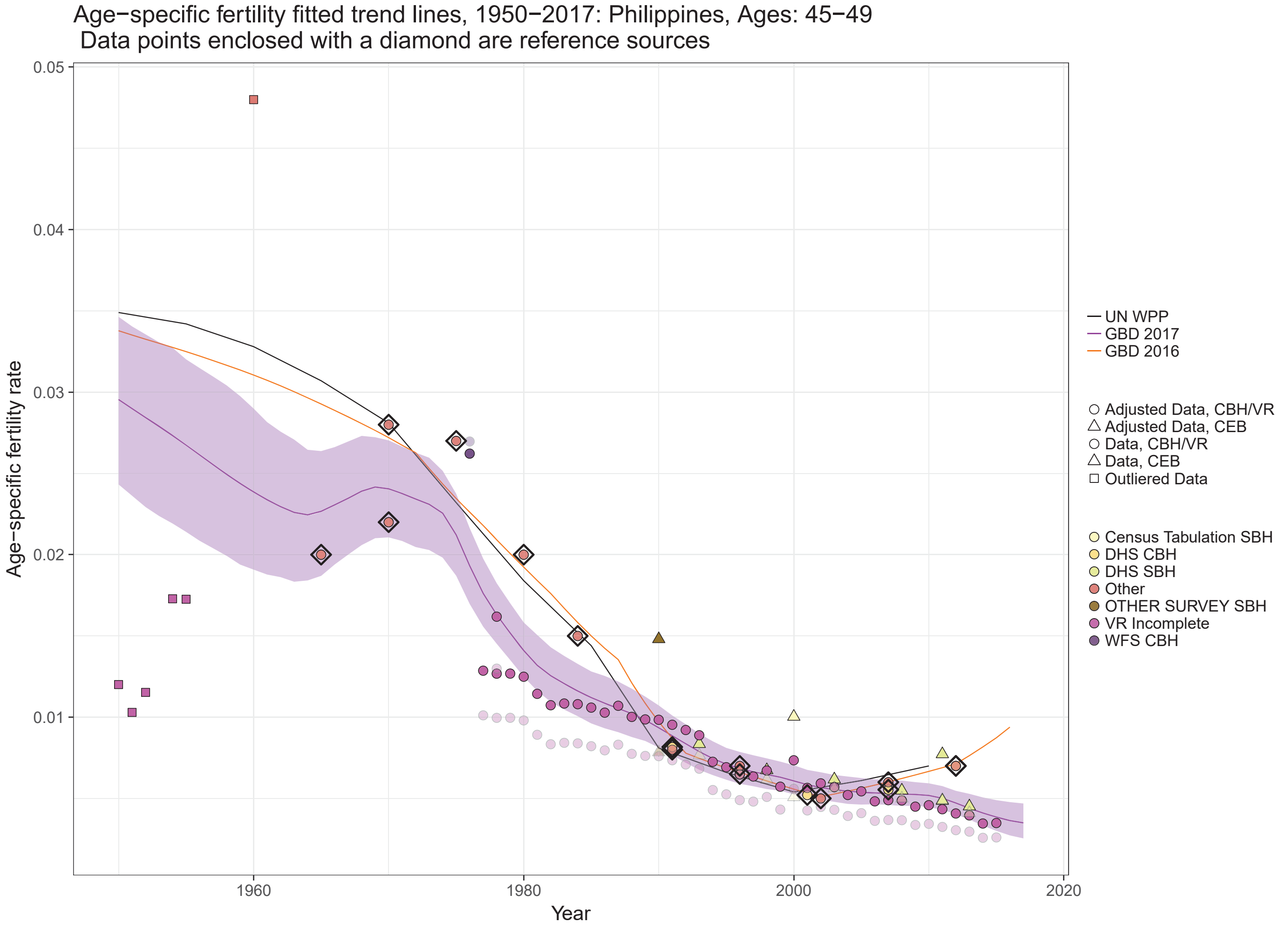

Age−specific fertility fitted trend lines, 1950−2017: Philippines, Ages: 45−49
Data points enclosed with a diamond are reference sources
Age−specific fertility rate
Year
0.05
0.04
0.03
0.02
0.01
1960
1980
2000
2020
UN WPP
GBD 2017
GBD 2016
Adjusted Data, CBH/VR
Adjusted Data, CEB
Data, CBH/VR
Data, CEB
Outliered Data
Census Tabulation SBH
DHS CBH
DHS SBH
Other
OTHER SURVEY SBH
VR Incomplete
WFS CBH

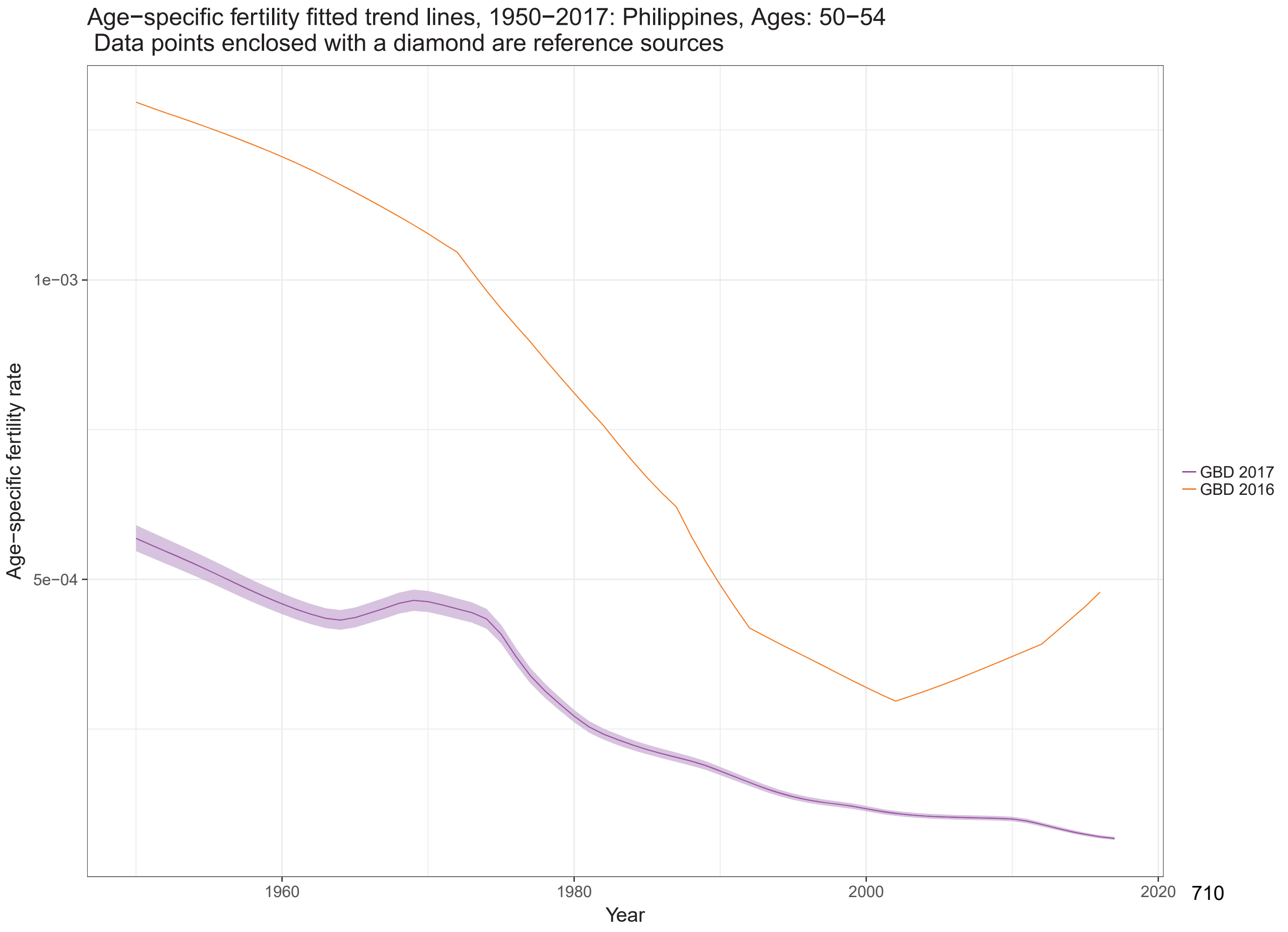

Age−specific fertility fitted trend lines, 1950−2017: Philippines, Ages: 50−54
Data points enclosed with a diamond are reference sources
Age−specific fertility rate
Year
1e−03
5e−04
1960
1980
2000
2020
GBD 2017
GBD 2016

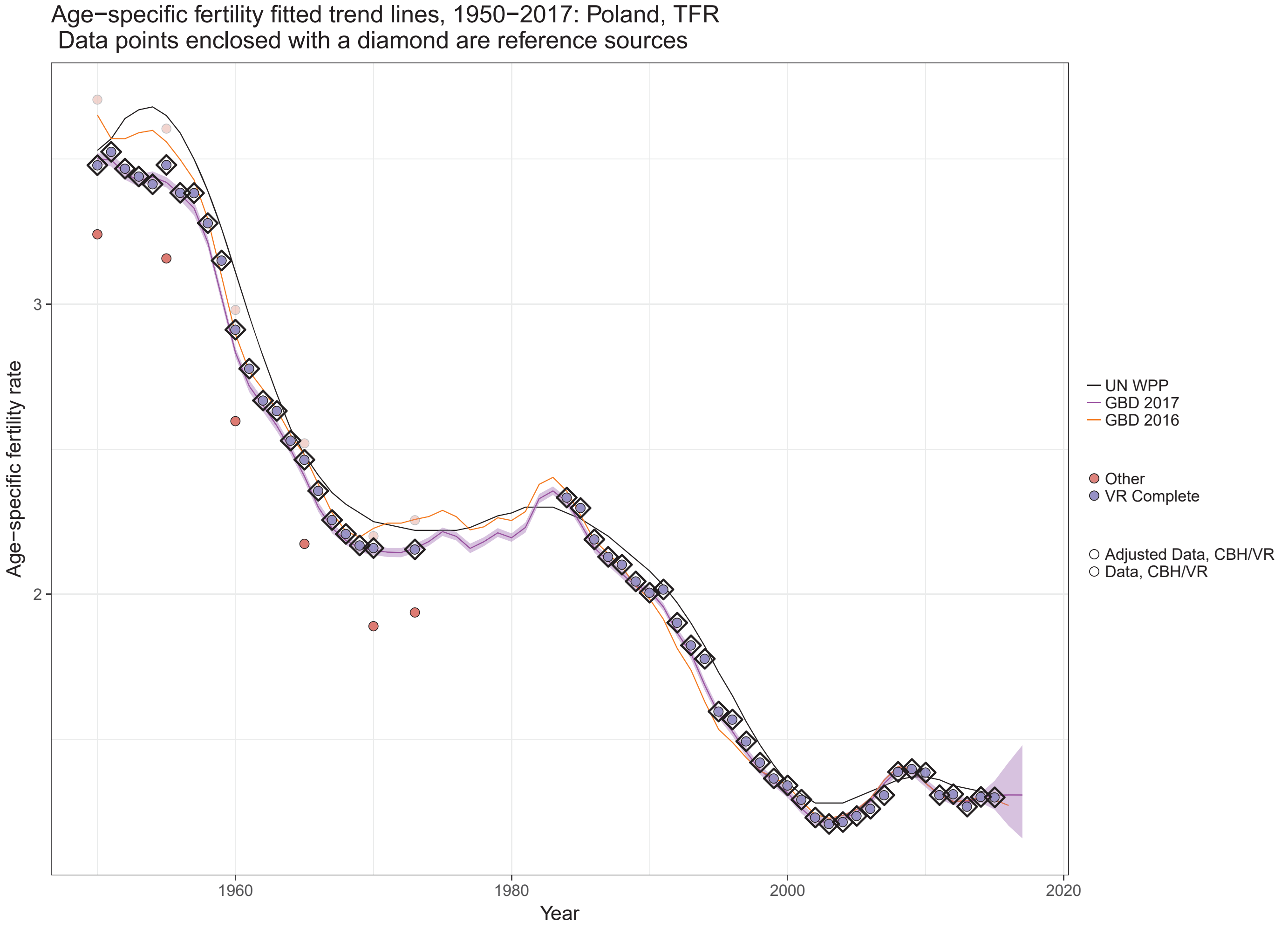

Age−specific fertility fitted trend lines, 1950−2017: Poland, TFR
Data points enclosed with a diamond are reference sources
Age−specific fertility rate
3
2
1960
1980
2000
2020
Year
UN WPP
GBD 2017
GBD 2016
Other
VR Complete
Adjusted Data, CBH/VR
Data, CBH/VR

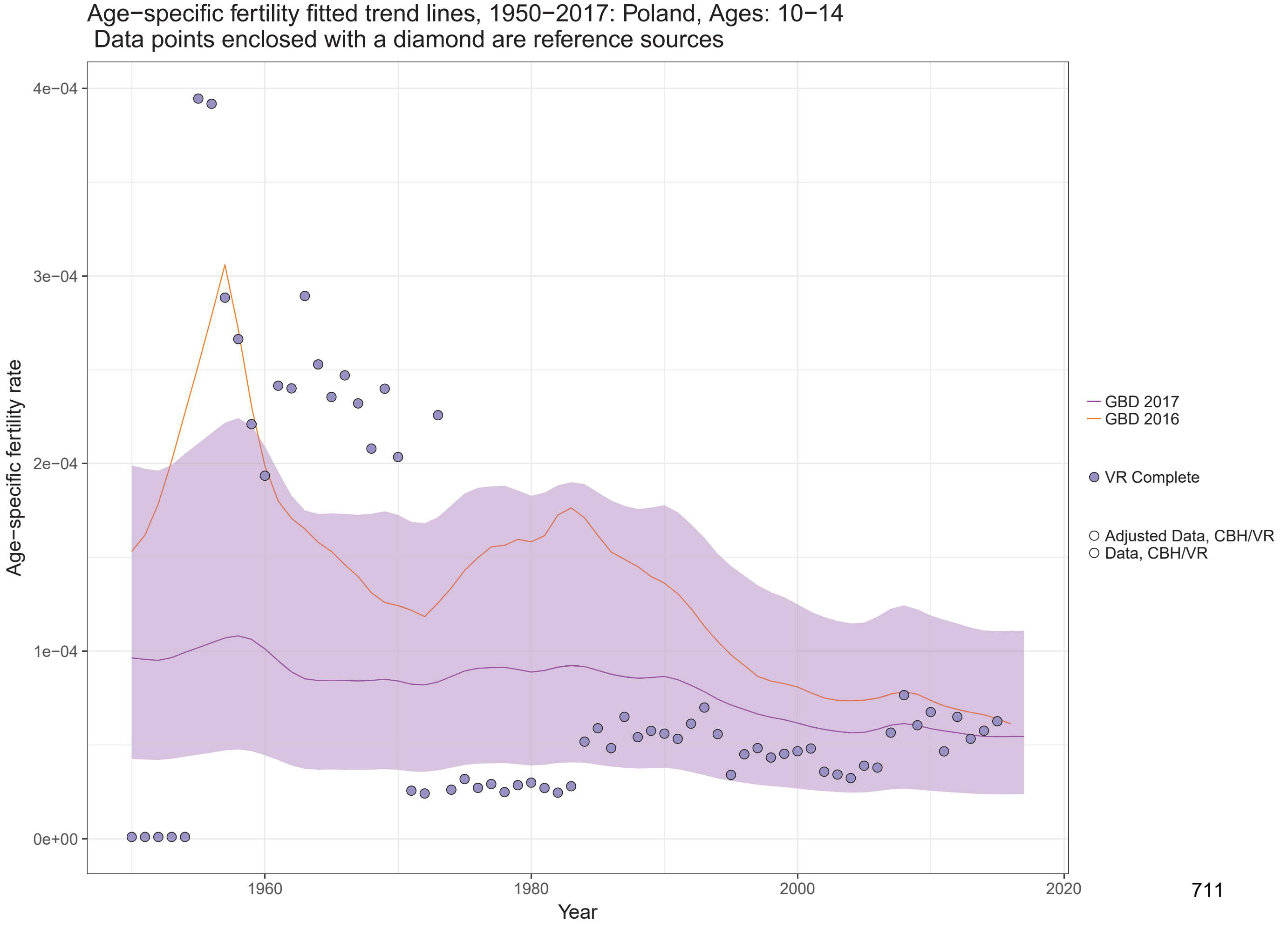

Age−specific fertility fitted trend lines, 1950−2017: Poland, Ages: 10−14
Data points enclosed with a diamond are reference sources
Age−specific fertility rate
4e−04
3e−04
2e−04
1e−04
0e+00
1960
1980
2000
2020
Year
GBD 2017
GBD 2016
VR Complete
Adjusted Data, CBH/VR
Data, CBH/VR

Age−specific fertility fitted trend lines, 1950−2017: Poland, Ages: 15−19
Data points enclosed with a diamond are reference sources

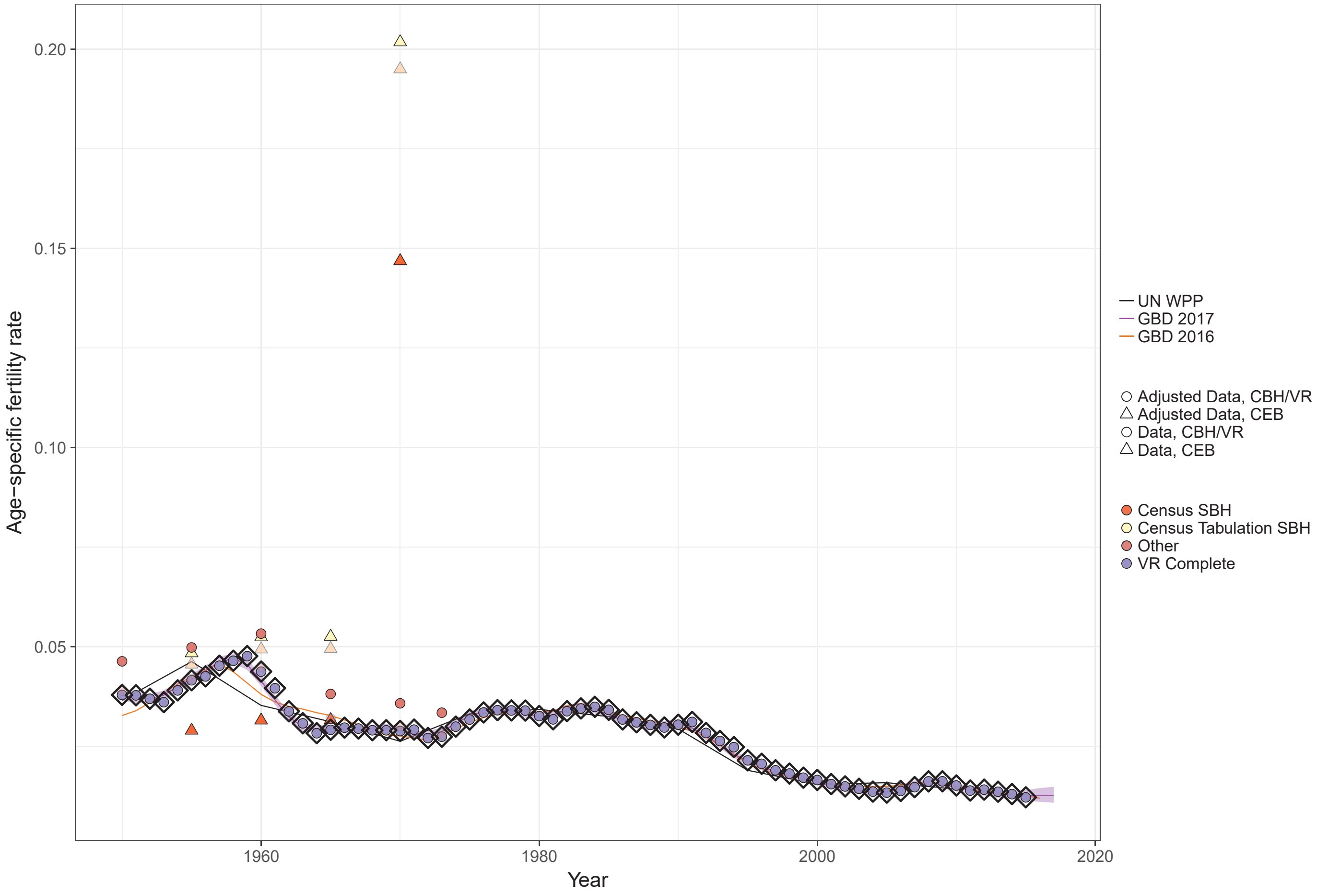

Age−specific fertility fitted trend lines, 1950−2017: Poland, Ages: 20−24
Data points enclosed with a diamond are reference sources

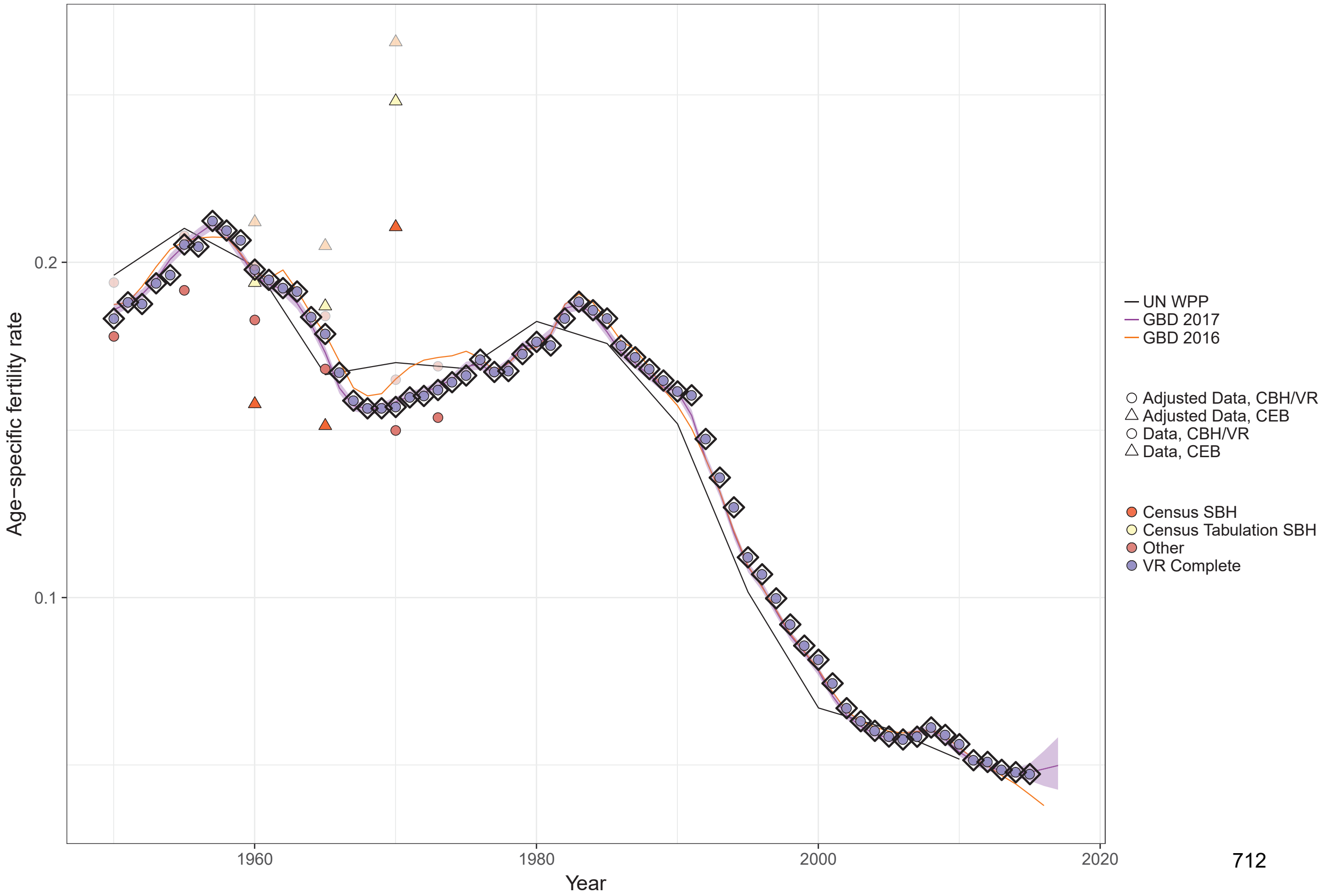

Age−specific fertility fitted trend lines, 1950−2017: Poland, Ages: 25−29
Data points enclosed with a diamond are reference sources

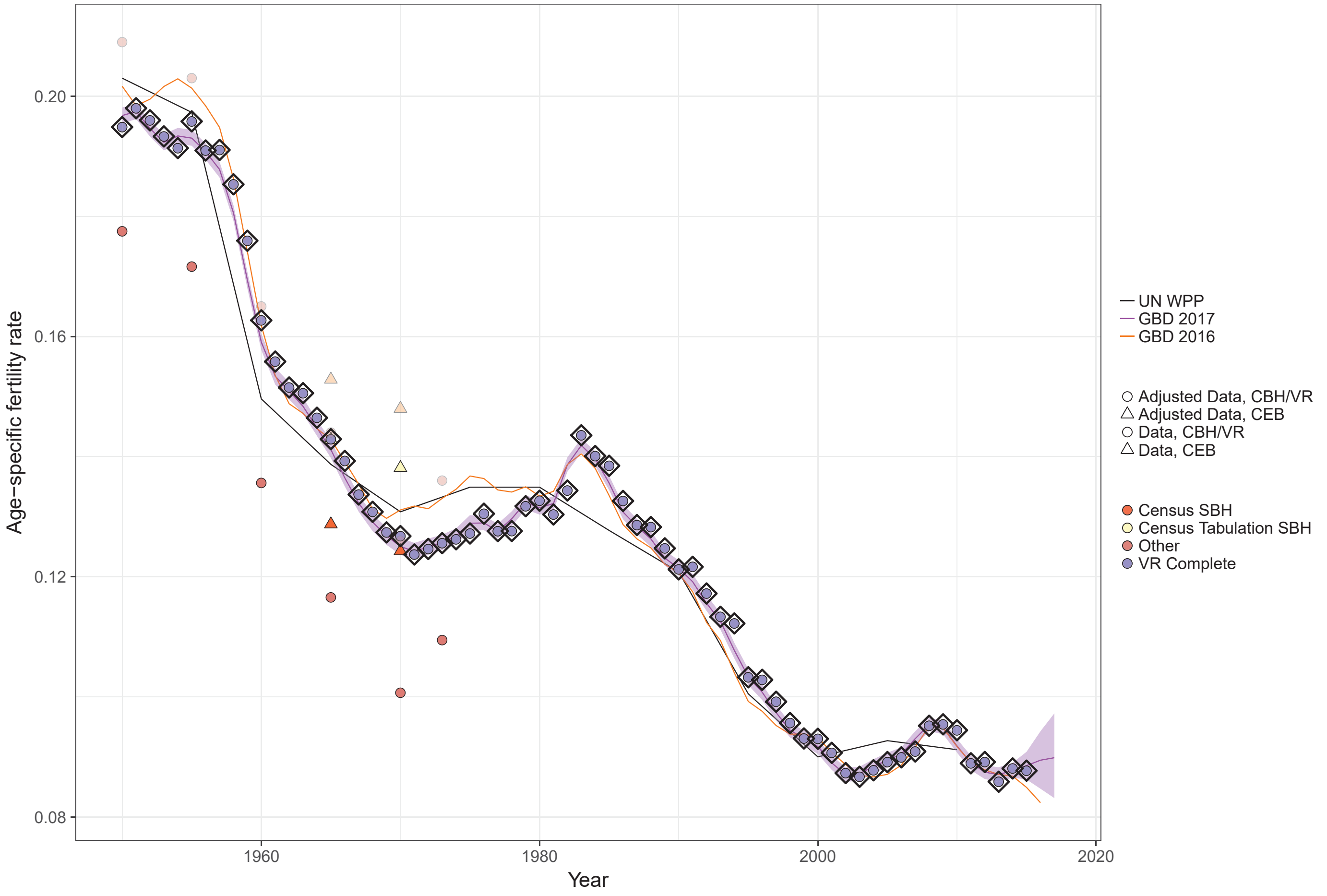

Age−specific fertility fitted trend lines, 1950−2017: Poland, Ages: 30−34
Data points enclosed with a diamond are reference sources

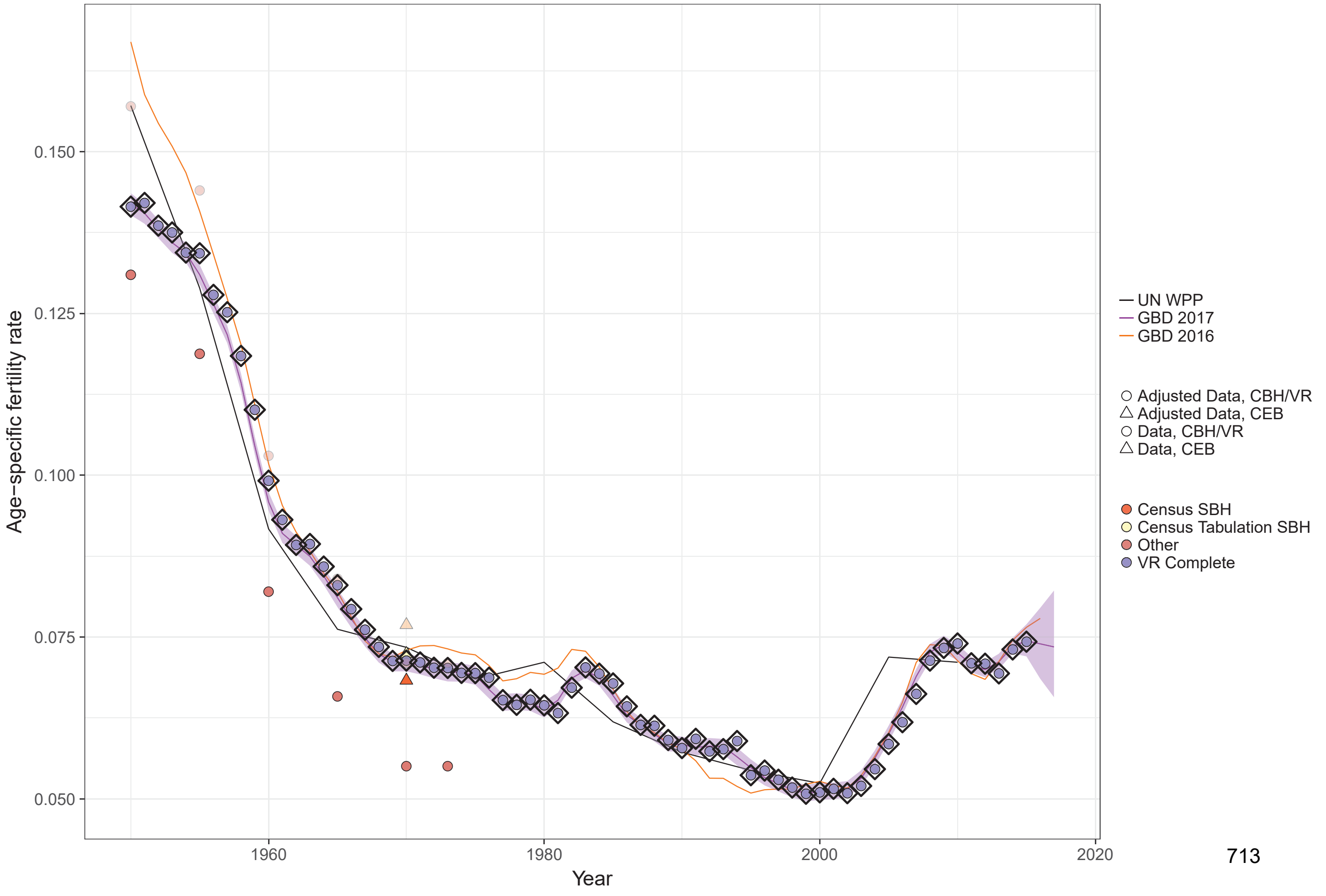

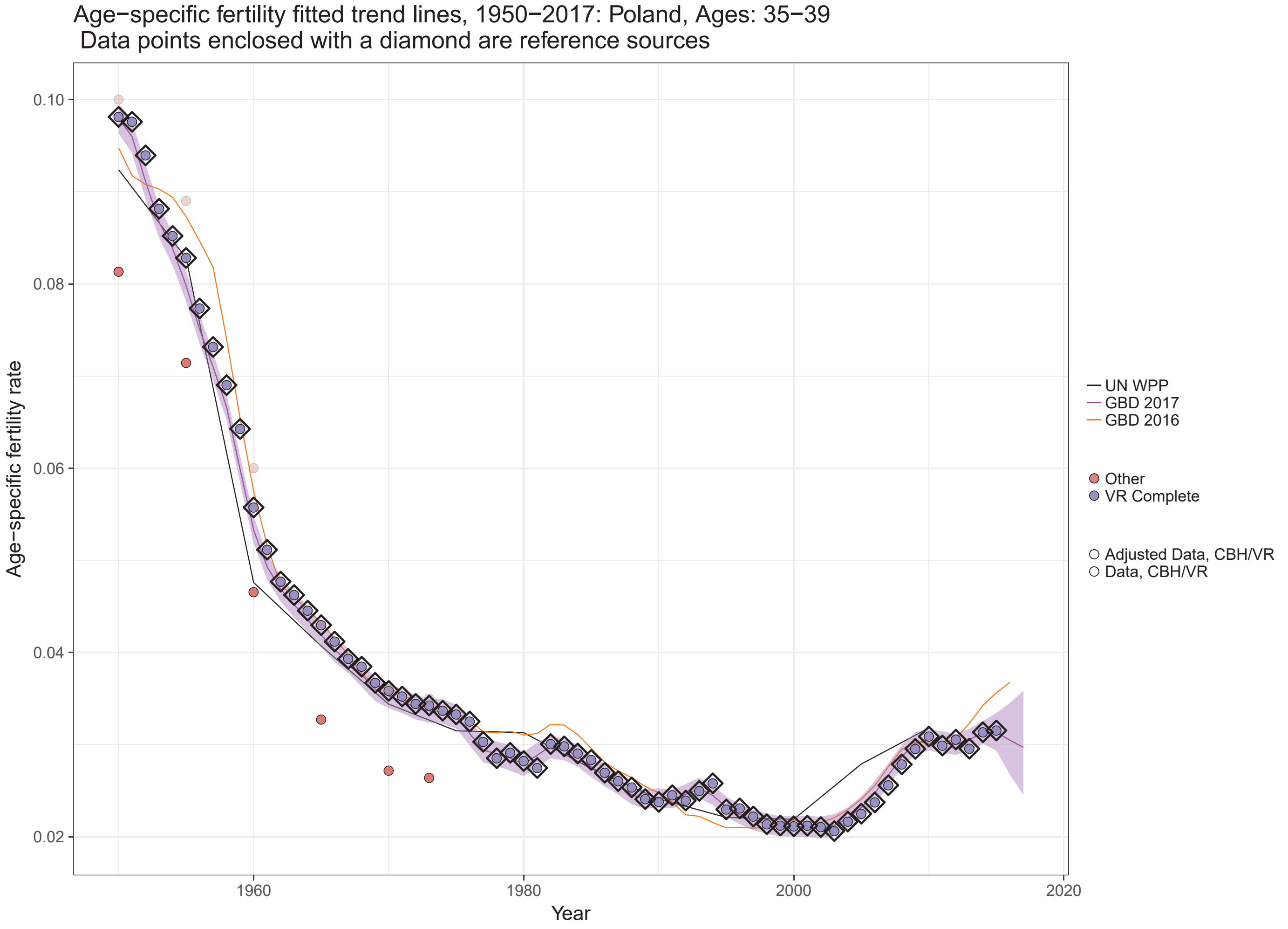
Age−specific fertility fitted trend lines, 1950−2017: Poland, Ages: 35−39
Data points enclosed with a diamond are reference sources
Age−specific fertility rate
0.10
0.08
0.06
0.04
0.02
1960
1980
2000
2020
Year
UN WPP
GBD 2017
GBD 2016
Other
VR Complete
Adjusted Data, CBH/VR
Data, CBH/VR

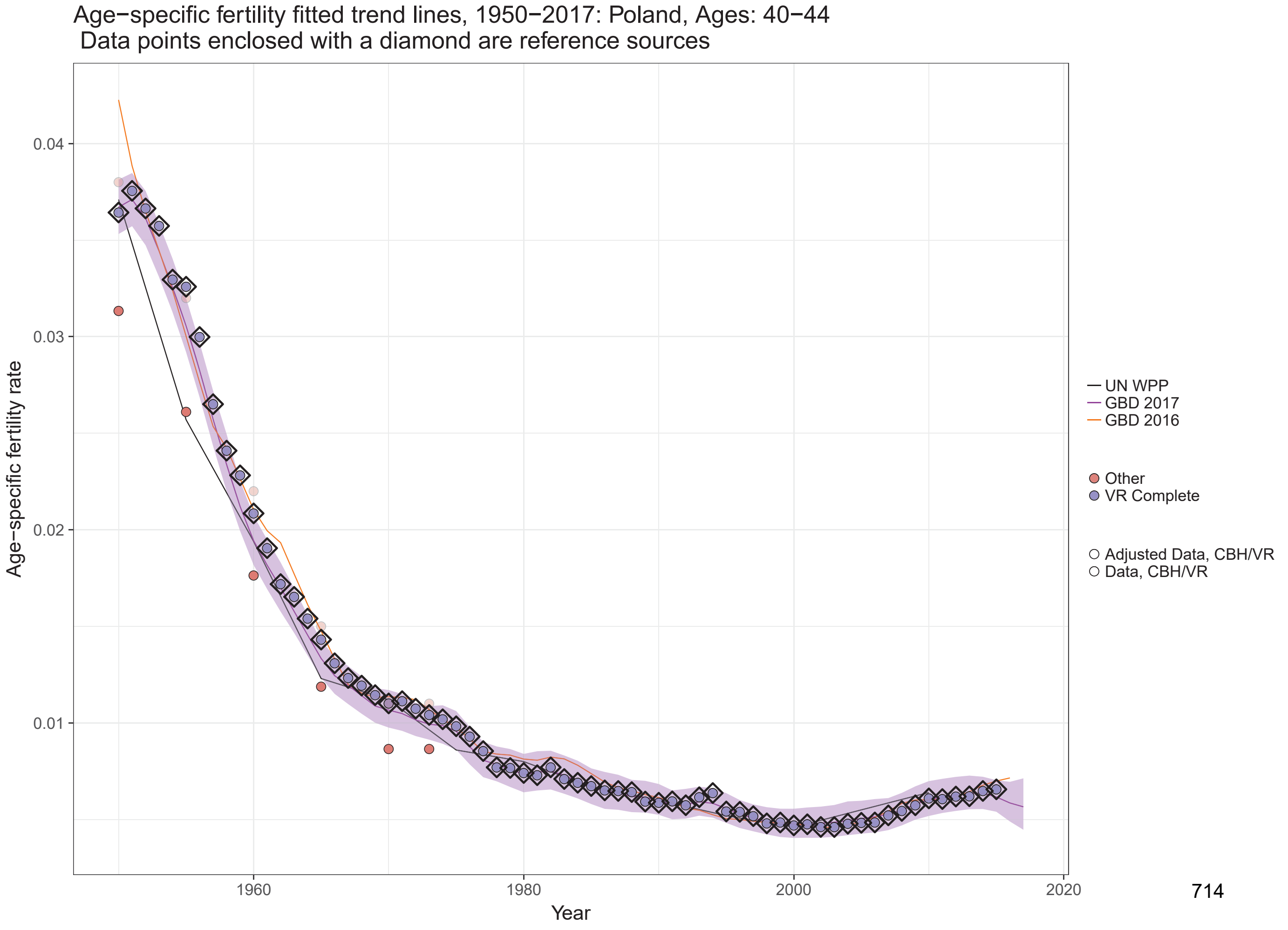
Age−specific fertility fitted trend lines, 1950−2017: Poland, Ages: 40−44
Data points enclosed with a diamond are reference sources
Age−specific fertility rate
0.04
0.03
0.02
0.01
1960
1980
2000
2020
Year
UN WPP
GBD 2017
GBD 2016
Other
VR Complete
Adjusted Data, CBH/VR
Data, CBH/VR

Age−specific fertility fitted trend lines, 1950−2017: Poland, Ages: 45−49
Data points enclosed with a diamond are reference sources

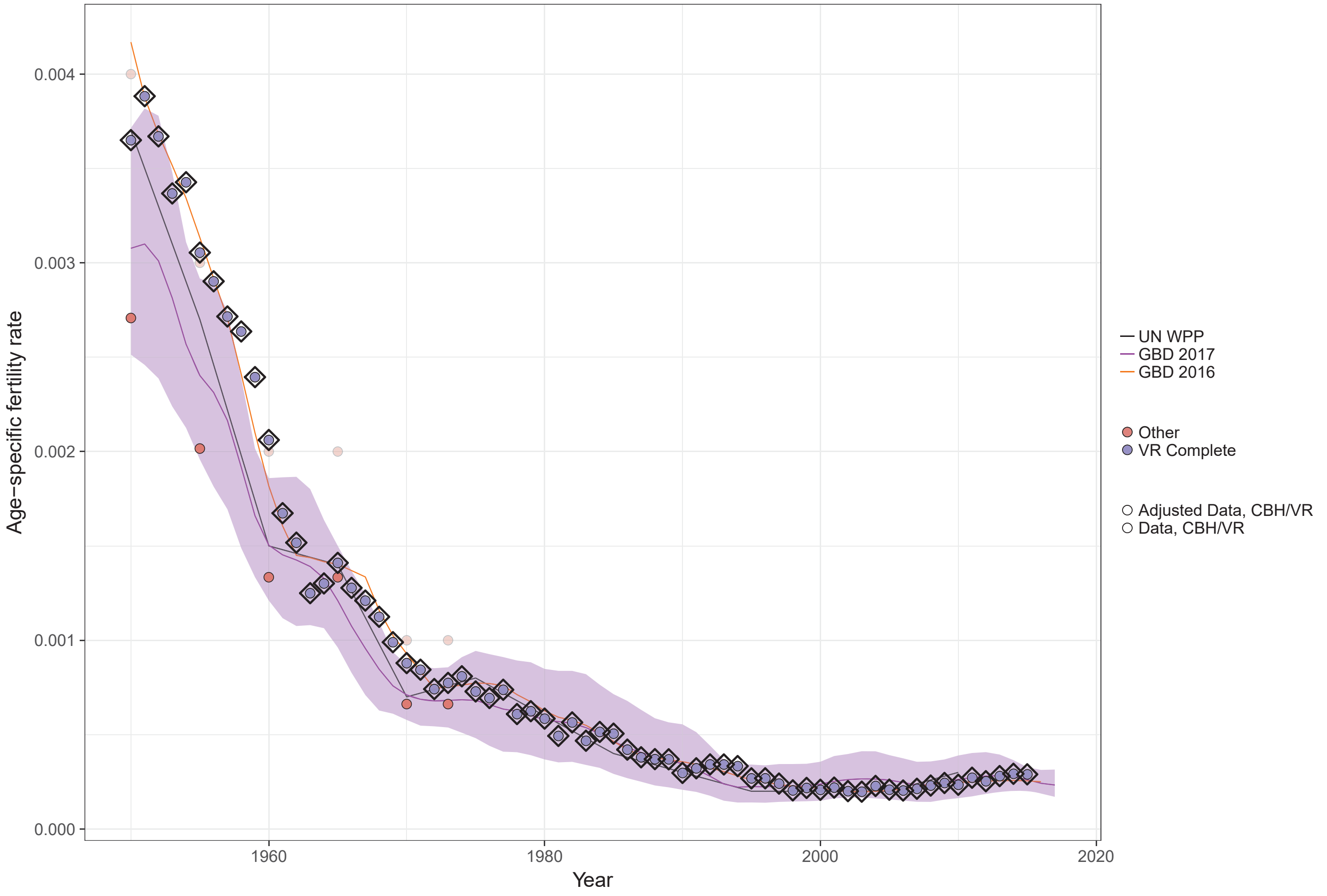

Age−specific fertility fitted trend lines, 1950−2017: Poland, Ages: 50−54
Data points enclosed with a diamond are reference sources

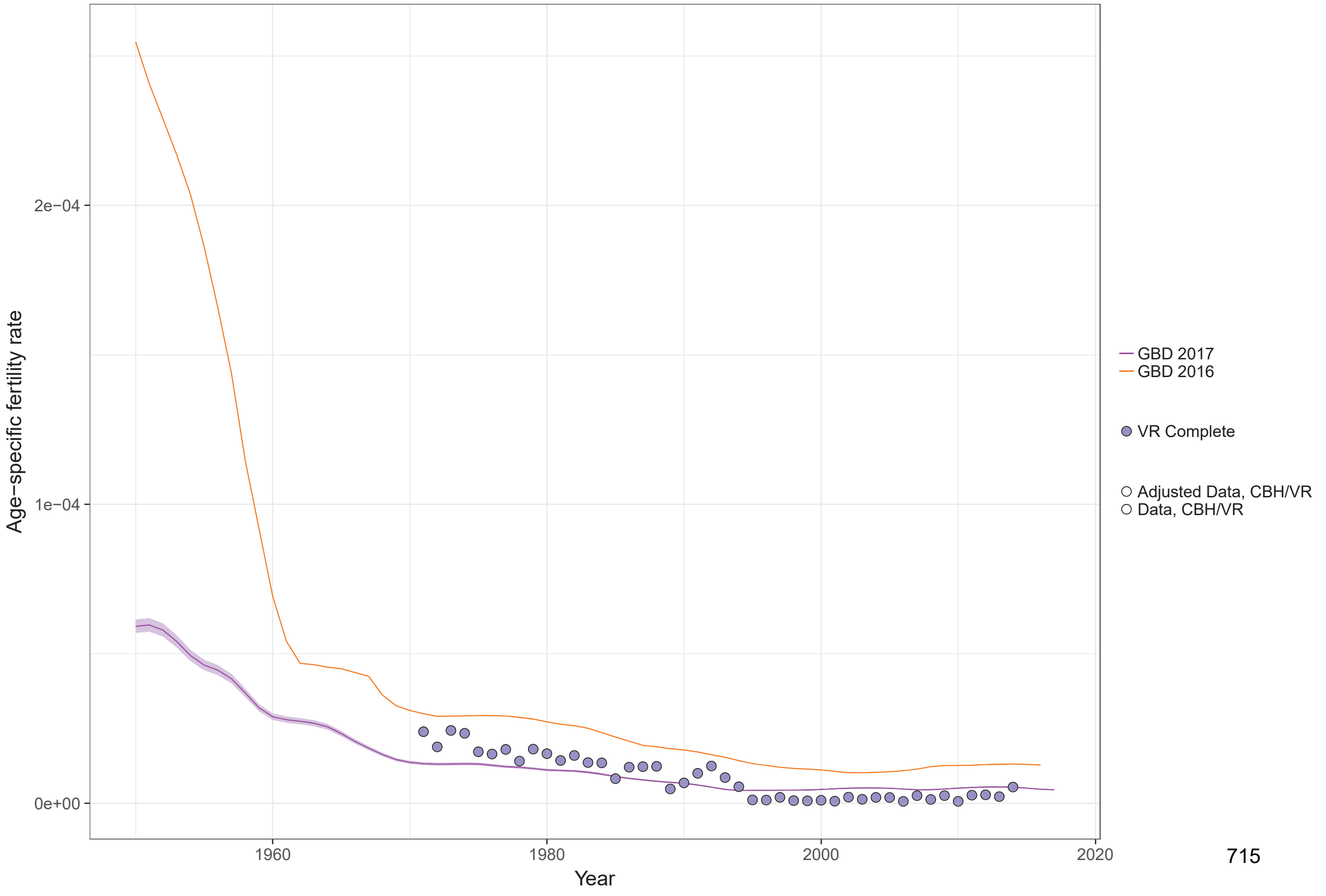

Age−specific fertility fitted trend lines, 1950−2017: Portugal, TFR
Data points enclosed with a diamond are reference sources

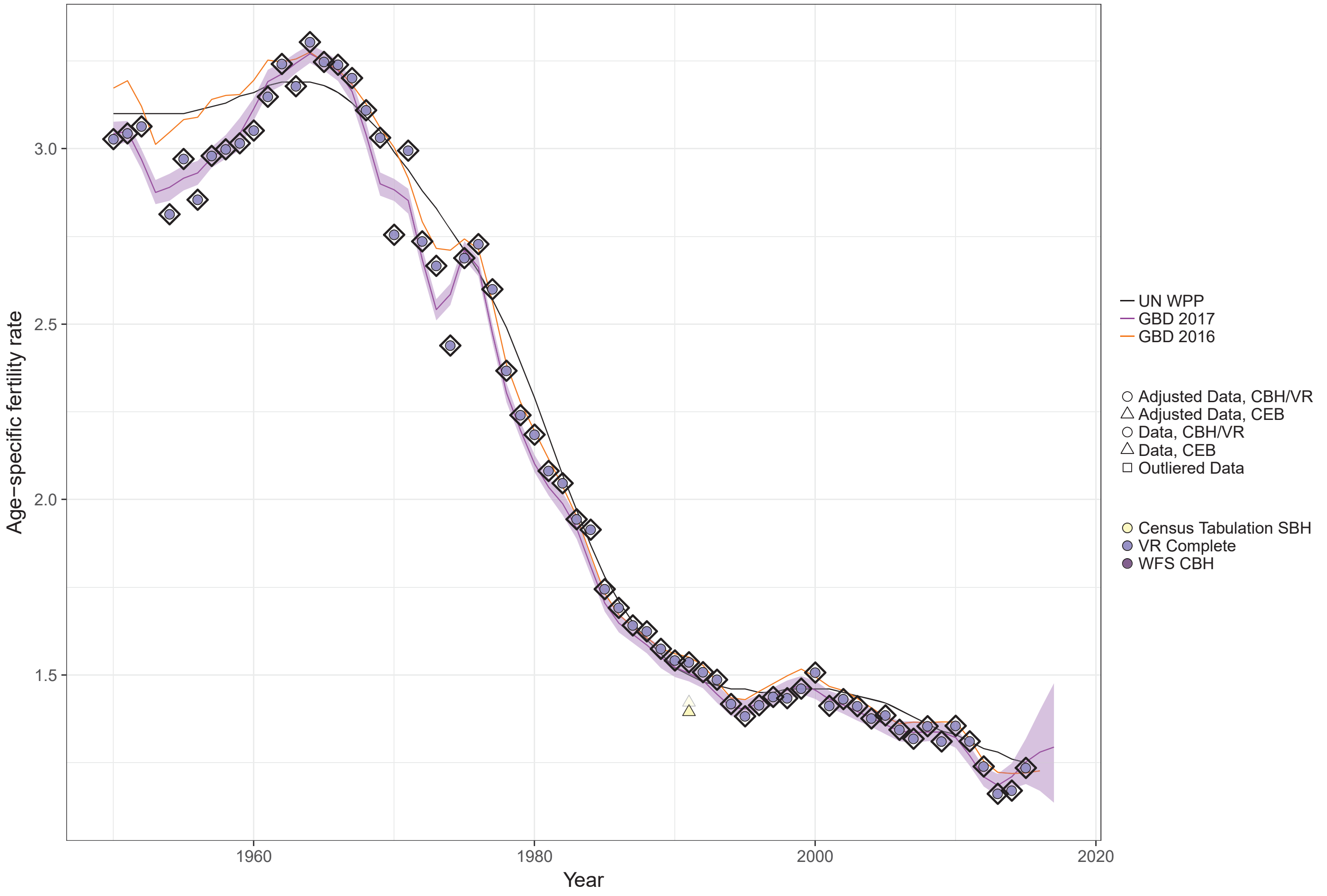

Age−specific fertility fitted trend lines, 1950−2017: Portugal, Ages: 10−14
Data points enclosed with a diamond are reference sources

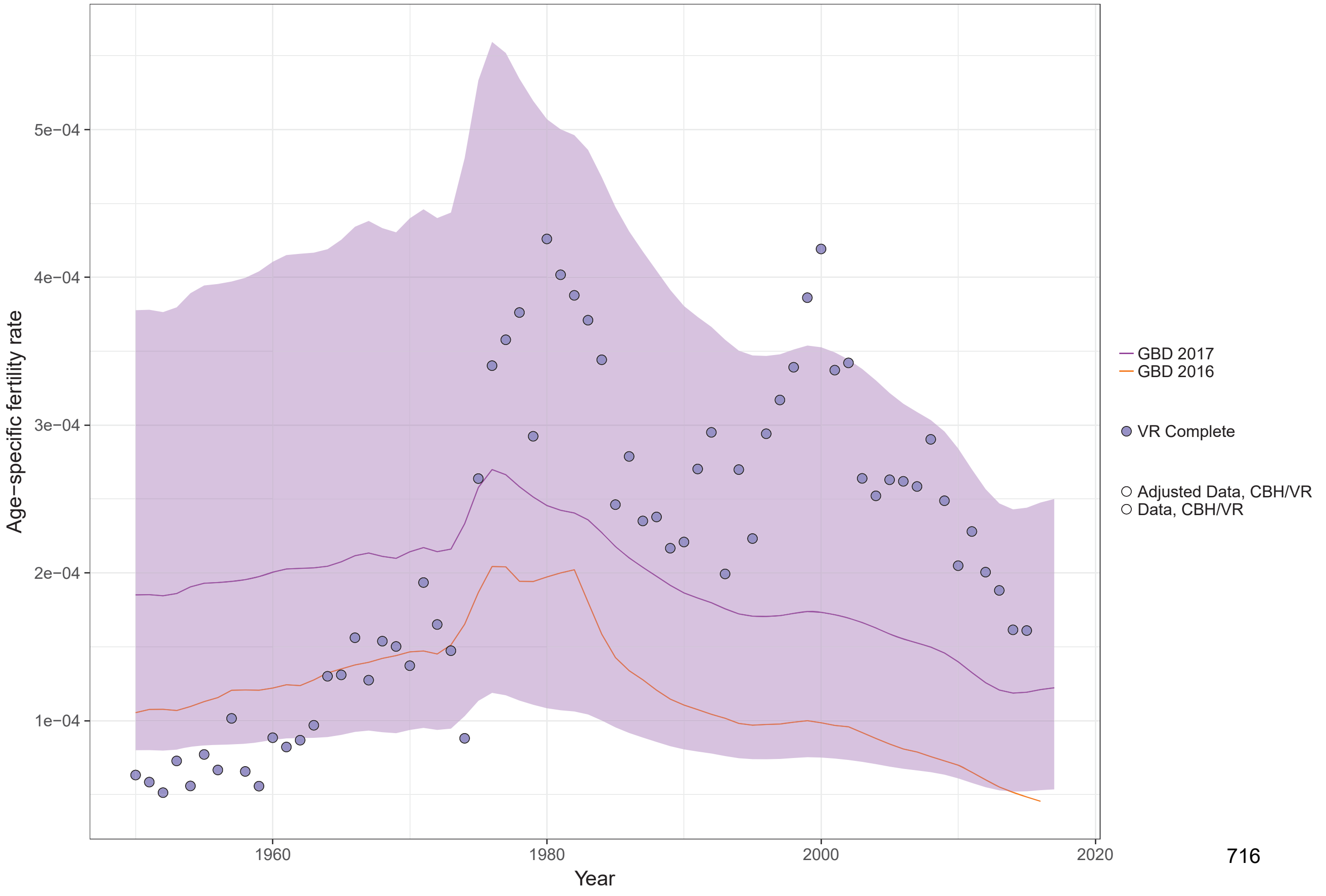

Age−specific fertility fitted trend lines, 1950−2017: Portugal, Ages: 15−19
Data points enclosed with a diamond are reference sources

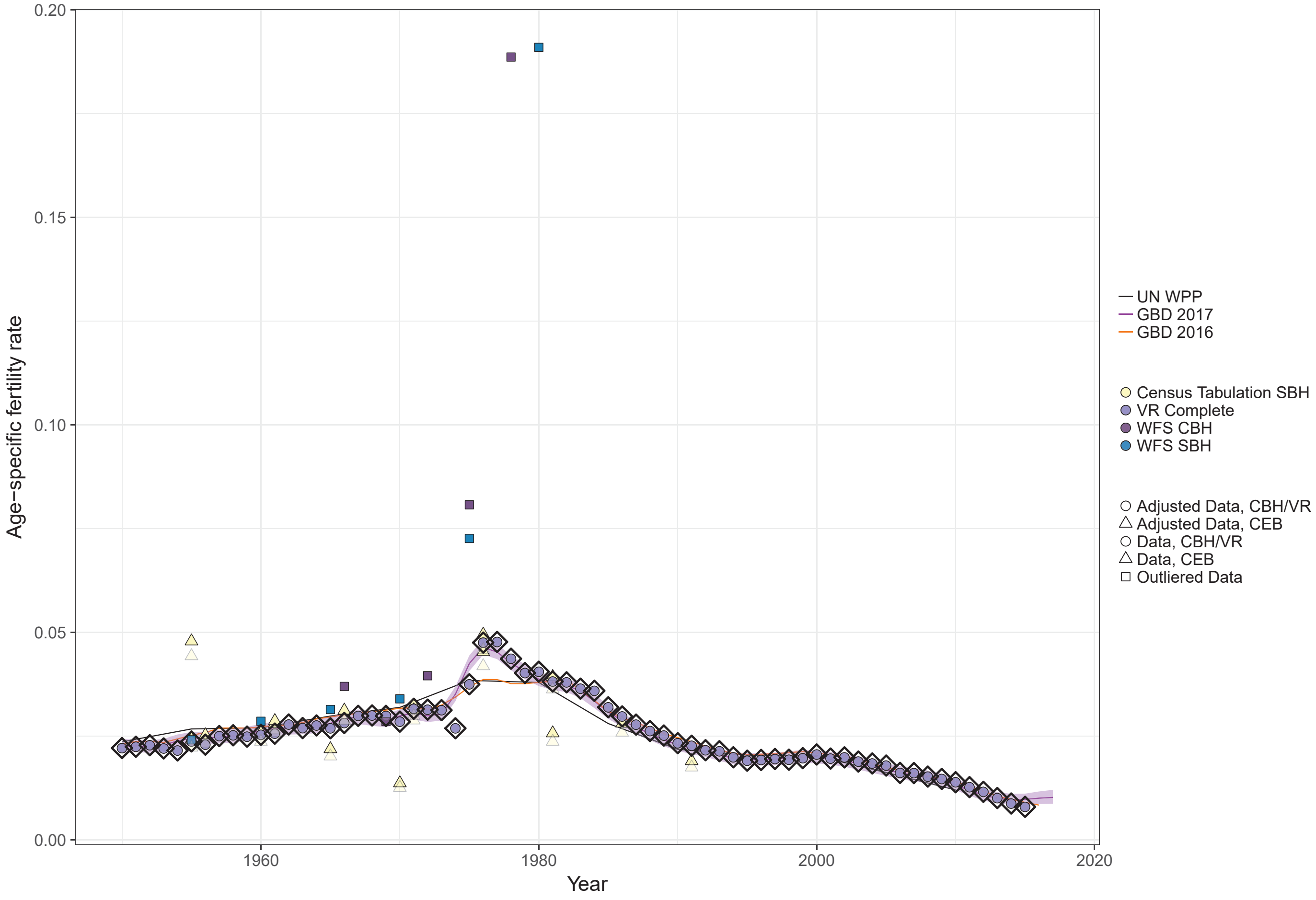

Age−specific fertility fitted trend lines, 1950−2017: Portugal, Ages: 20−24
Data points enclosed with a diamond are reference sources

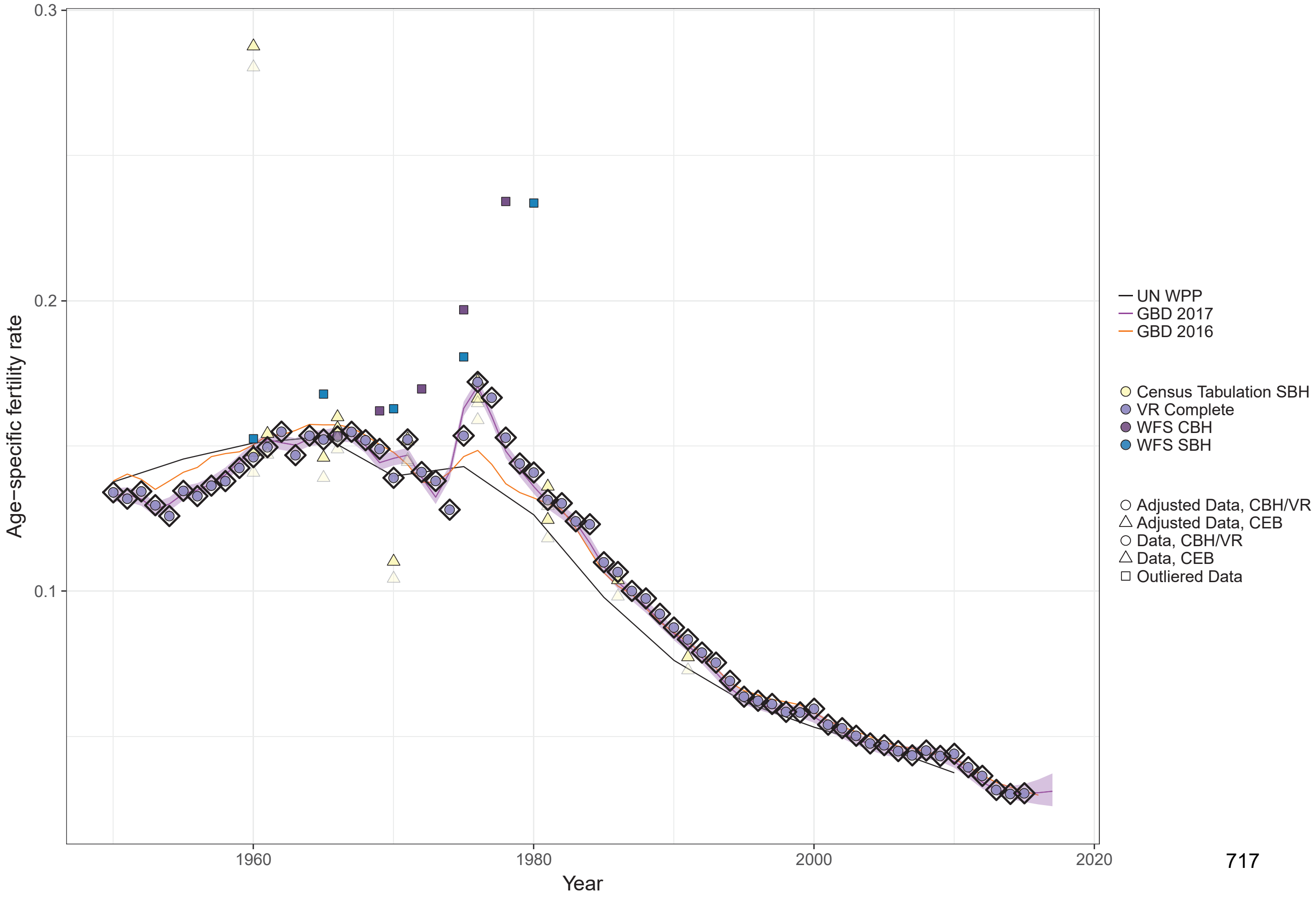

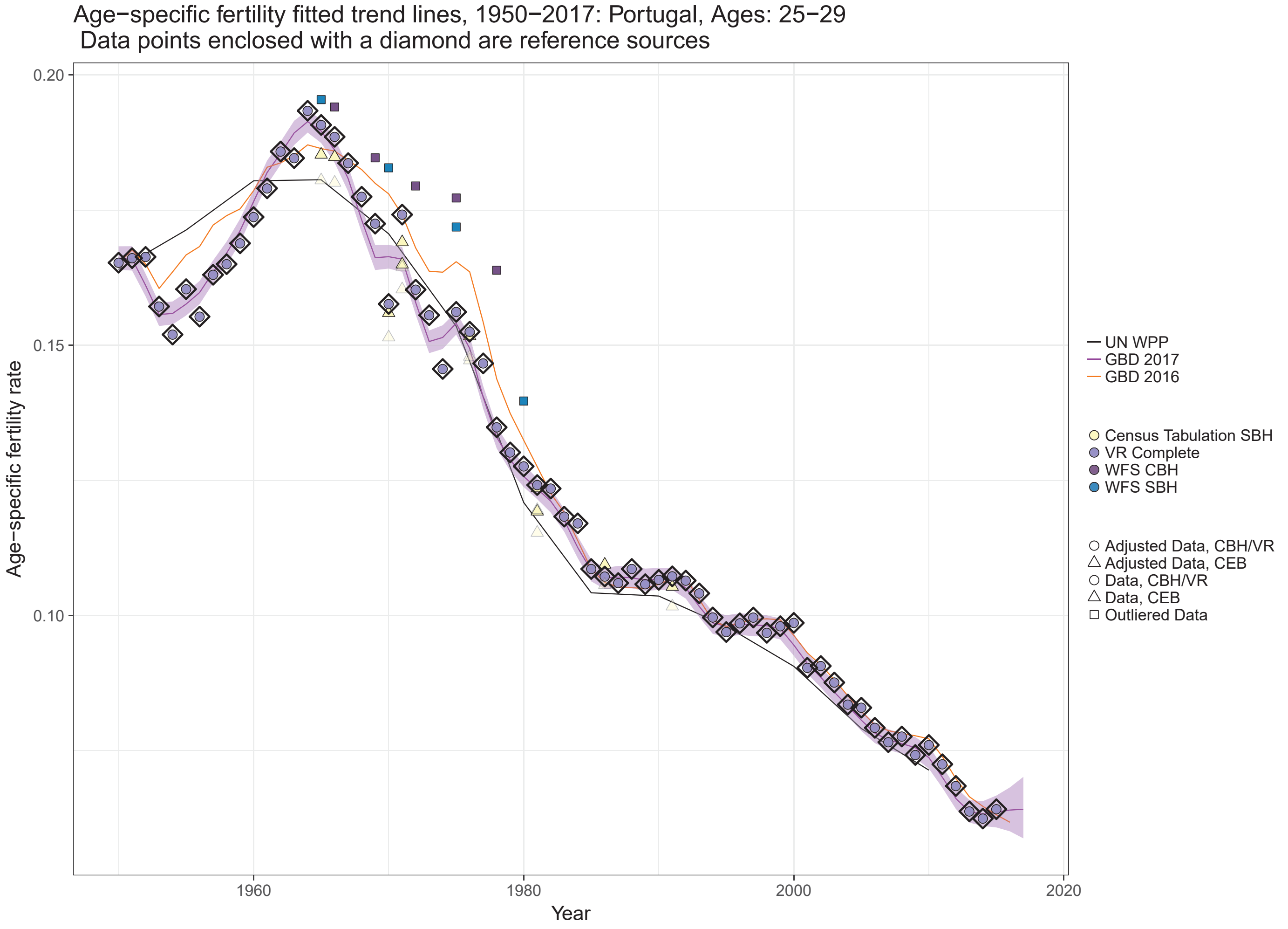

Age−specific fertility fitted trend lines, 1950−2017: Portugal, Ages: 25−29
Data points enclosed with a diamond are reference sources
Age−specific fertility rate
Year
UN WPP
GBD 2017
GBD 2016
Census Tabulation SBH
VR Complete
WFS CBH
WFS SBH
Adjusted Data, CBH/VR
Adjusted Data, CEB
Data, CBH/VR
Data, CEB
Outliered Data

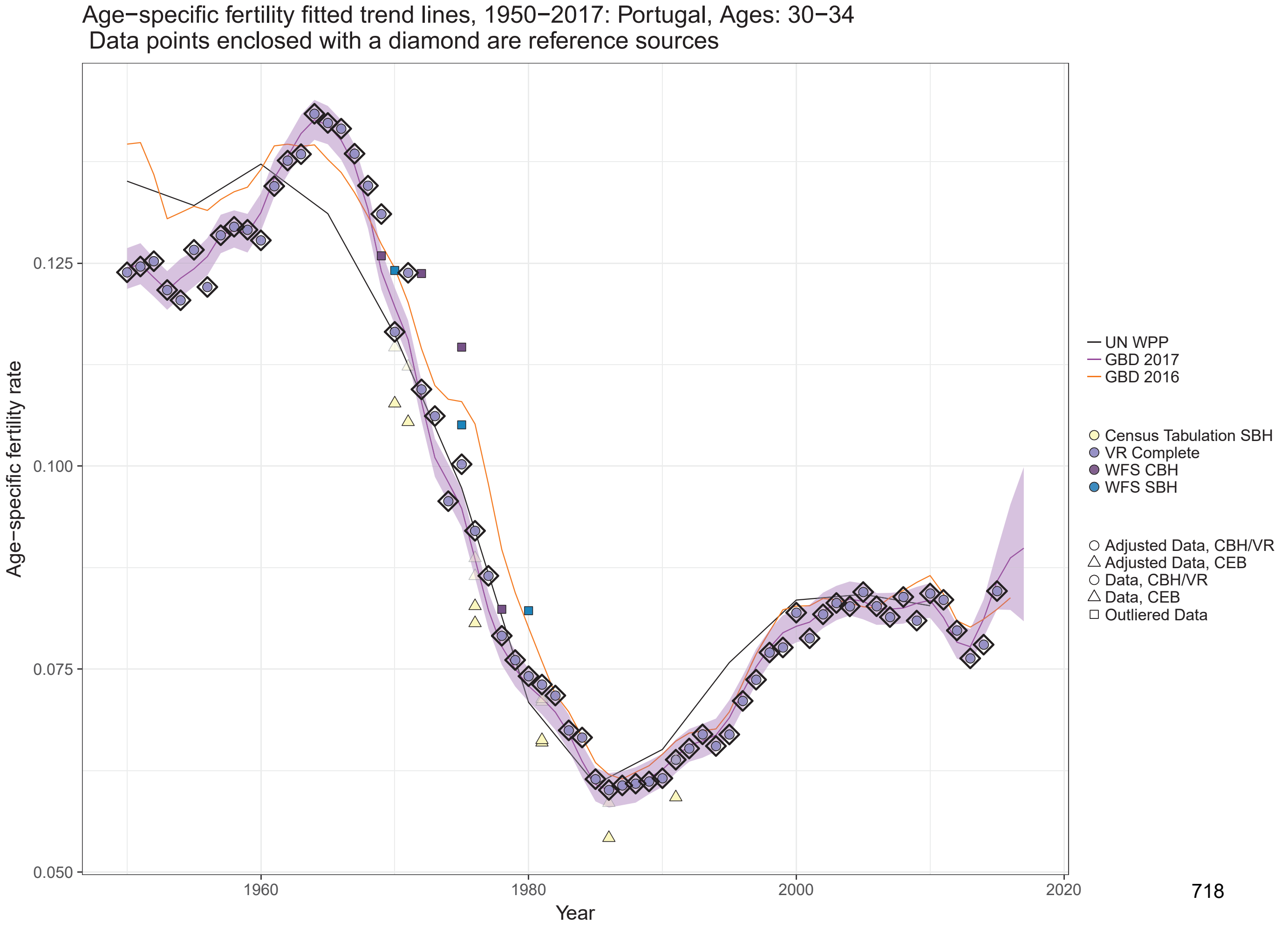

Age−specific fertility fitted trend lines, 1950−2017: Portugal, Ages: 30−34
Data points enclosed with a diamond are reference sources
Age−specific fertility rate
Year
UN WPP
GBD 2017
GBD 2016
Census Tabulation SBH
VR Complete
WFS CBH
WFS SBH
Adjusted Data, CBH/VR
Adjusted Data, CEB
Data, CBH/VR
Data, CEB
Outliered Data

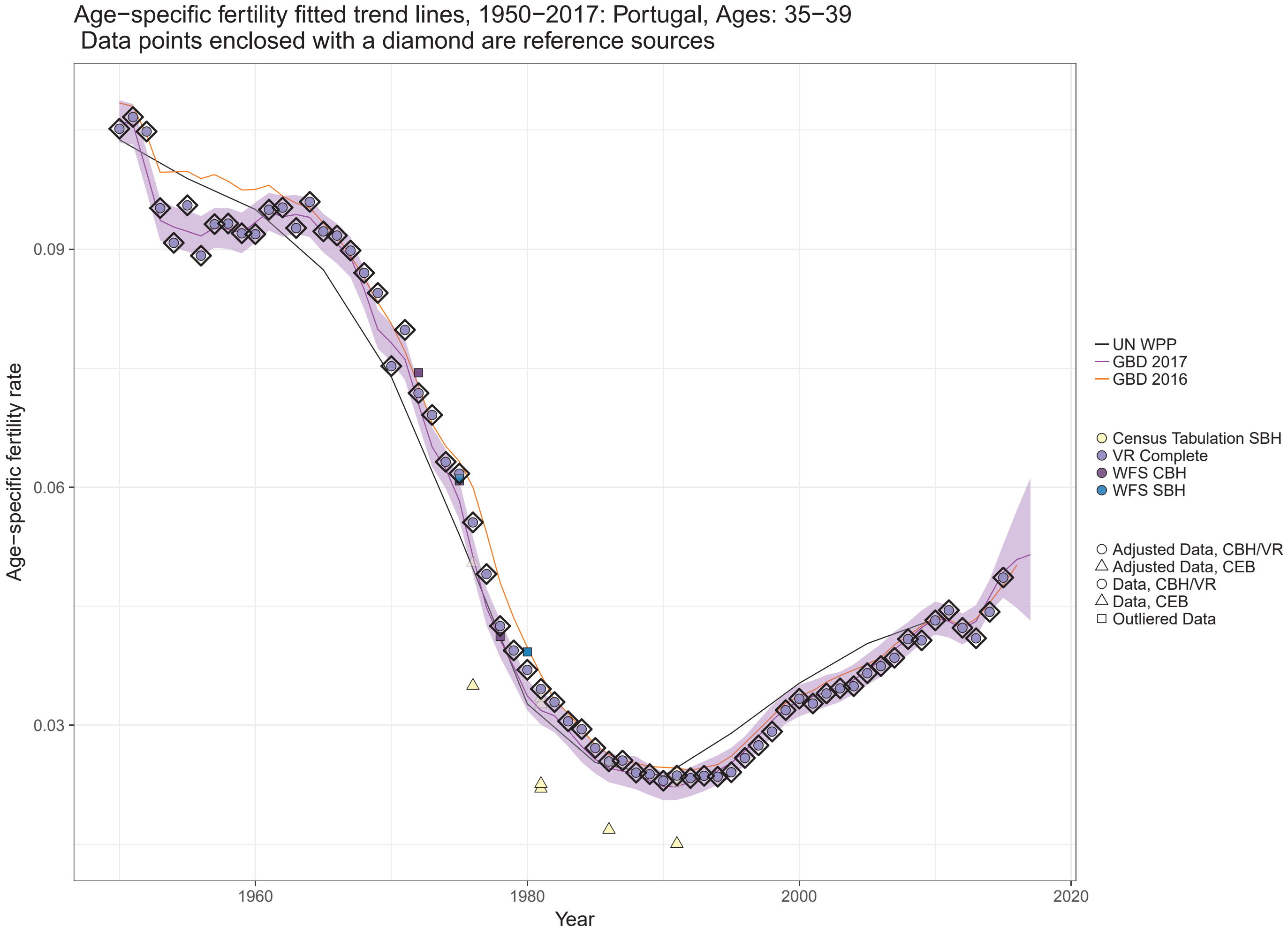

Age−specific fertility fitted trend lines, 1950−2017: Portugal, Ages: 35−39
Data points enclosed with a diamond are reference sources
0.09
0.06
0.03
Age−specific fertility rate
1960
1980
2000
2020
Year
UN WPP
GBD 2017
GBD 2016
Census Tabulation SBH
VR Complete
WFS CBH
WFS SBH
Adjusted Data, CBH/VR
Adjusted Data, CEB
Data, CBH/VR
Data, CEB
Outliered Data

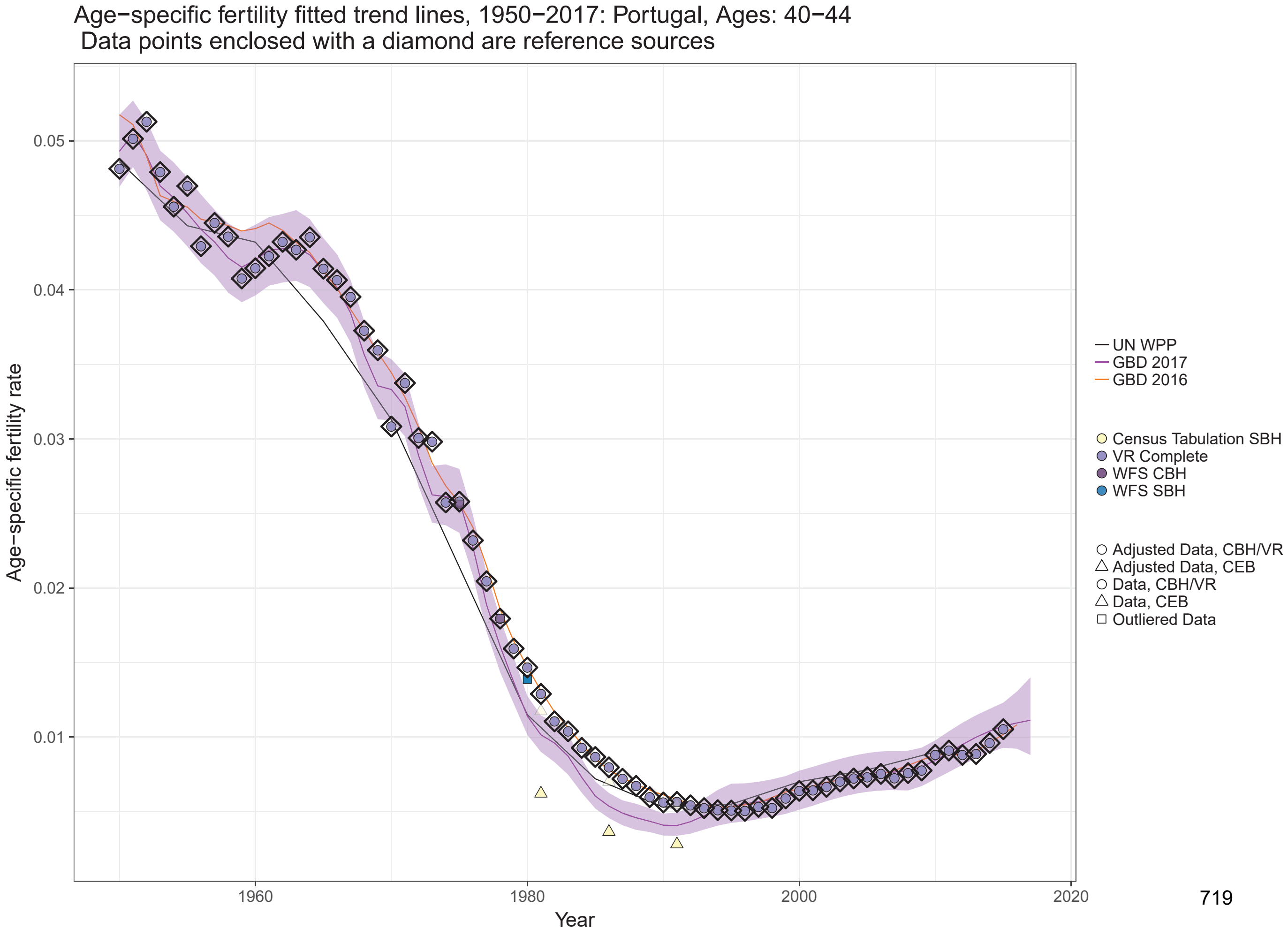

Age−specific fertility fitted trend lines, 1950−2017: Portugal, Ages: 40−44
Data points enclosed with a diamond are reference sources
0.05
0.04
0.03
0.02
0.01
Age−specific fertility rate
1960
1980
2000
2020
Year
UN WPP
GBD 2017
GBD 2016
Census Tabulation SBH
VR Complete
WFS CBH
WFS SBH
Adjusted Data, CBH/VR
Adjusted Data, CEB
Data, CBH/VR
Data, CEB
Outliered Data

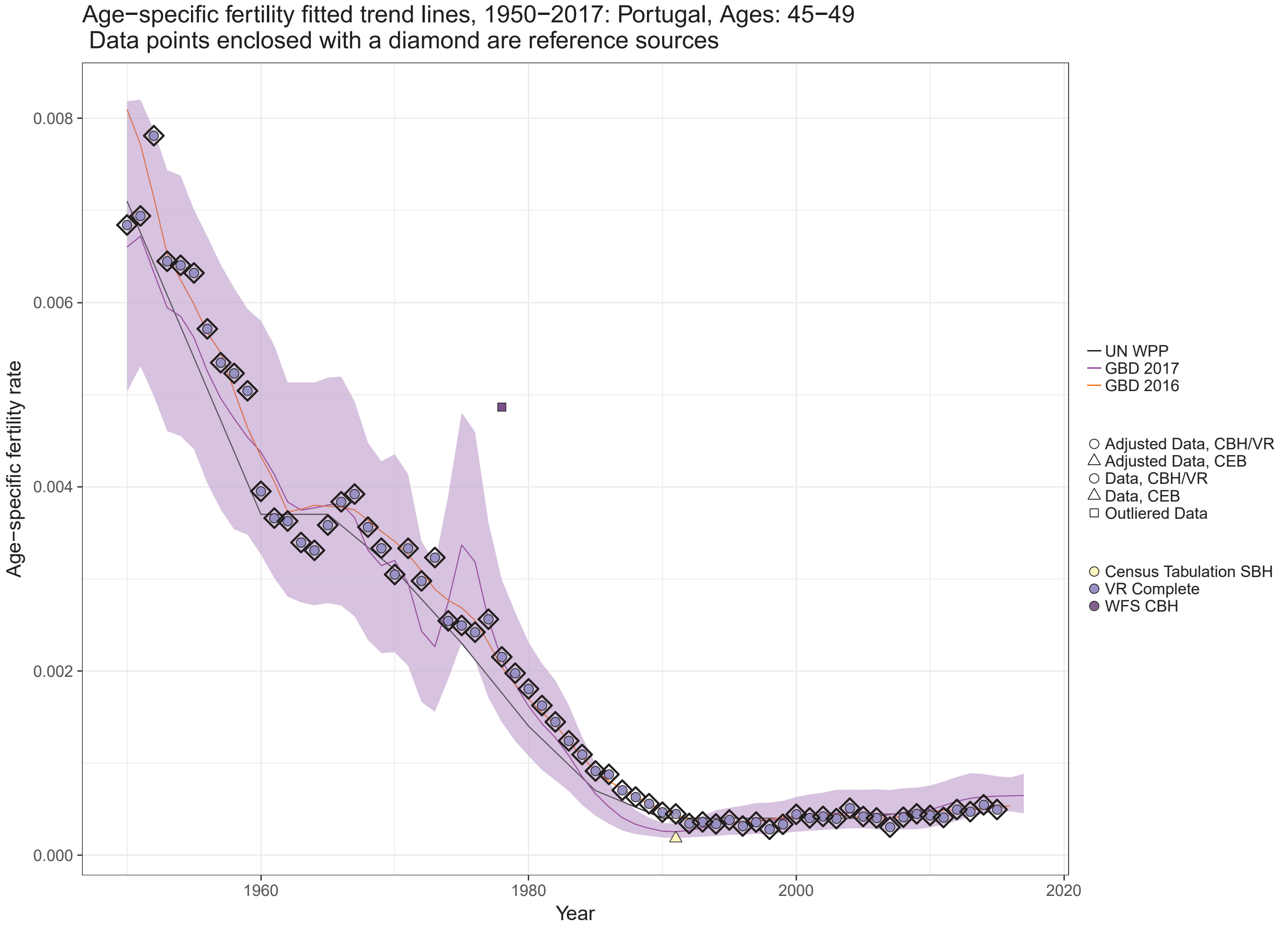
Age−specific fertility fitted trend lines, 1950−2017: Portugal, Ages: 45−49
Data points enclosed with a diamond are reference sources
Age−specific fertility rate
0.000
0.002
0.004
0.006
0.008
1960
1980
2000
2020
Year
UN WPP
GBD 2017
GBD 2016
Adjusted Data, CBH/VR
Adjusted Data, CEB
Data, CBH/VR
Data, CEB
Outliered Data
Census Tabulation SBH
VR Complete
WFS CBH

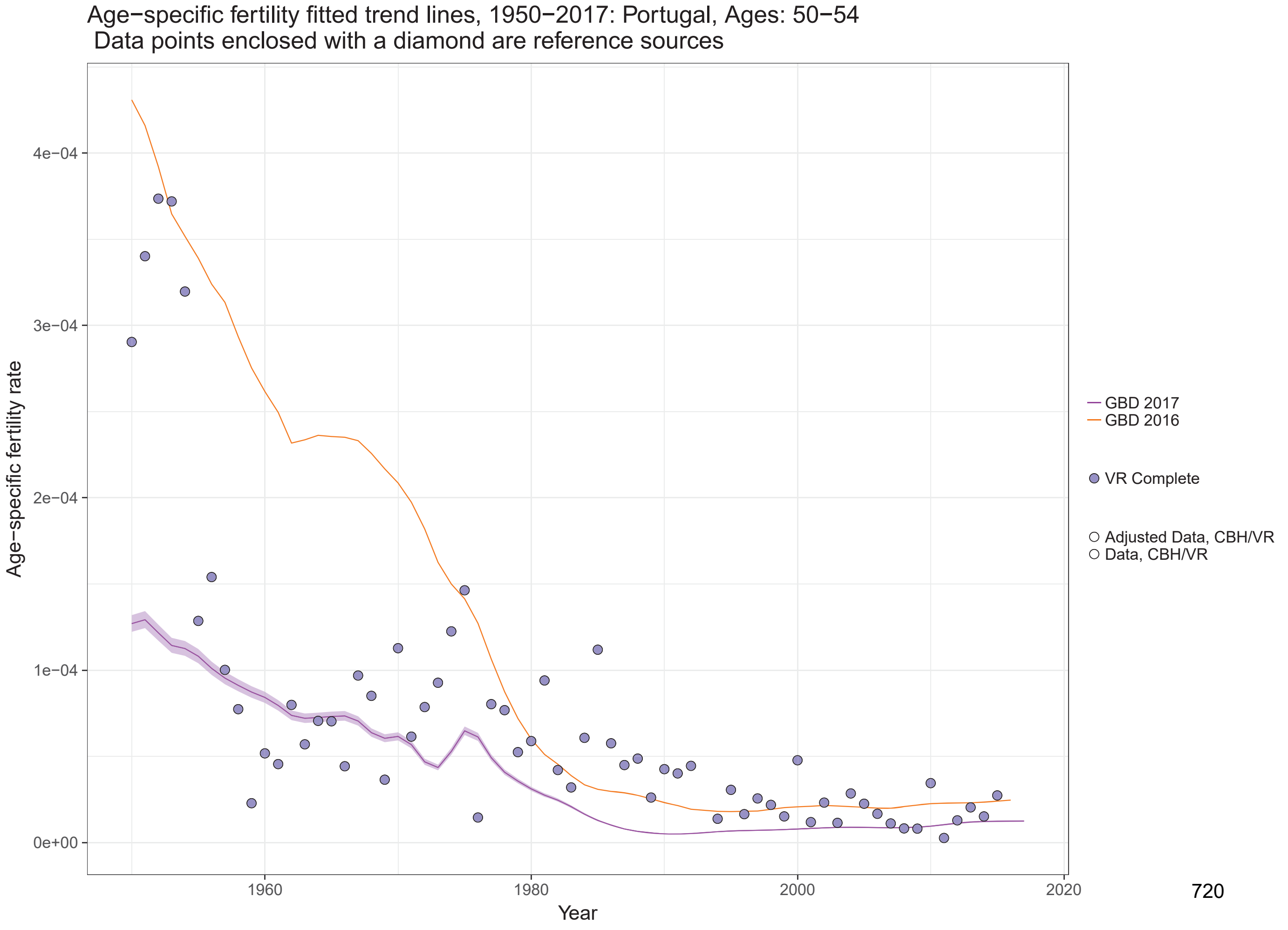
Age−specific fertility fitted trend lines, 1950−2017: Portugal, Ages: 50−54
Data points enclosed with a diamond are reference sources
Age−specific fertility rate
0e+00
1e−04
2e−04
3e−04
4e−04
1960
1980
2000
2020
Year
GBD 2017
GBD 2016
VR Complete
Adjusted Data, CBH/VR
Data, CBH/VR

Age−specific fertility fitted trend lines, 1950−2017: Puerto Rico, TFR
Data points enclosed with a diamond are reference sources

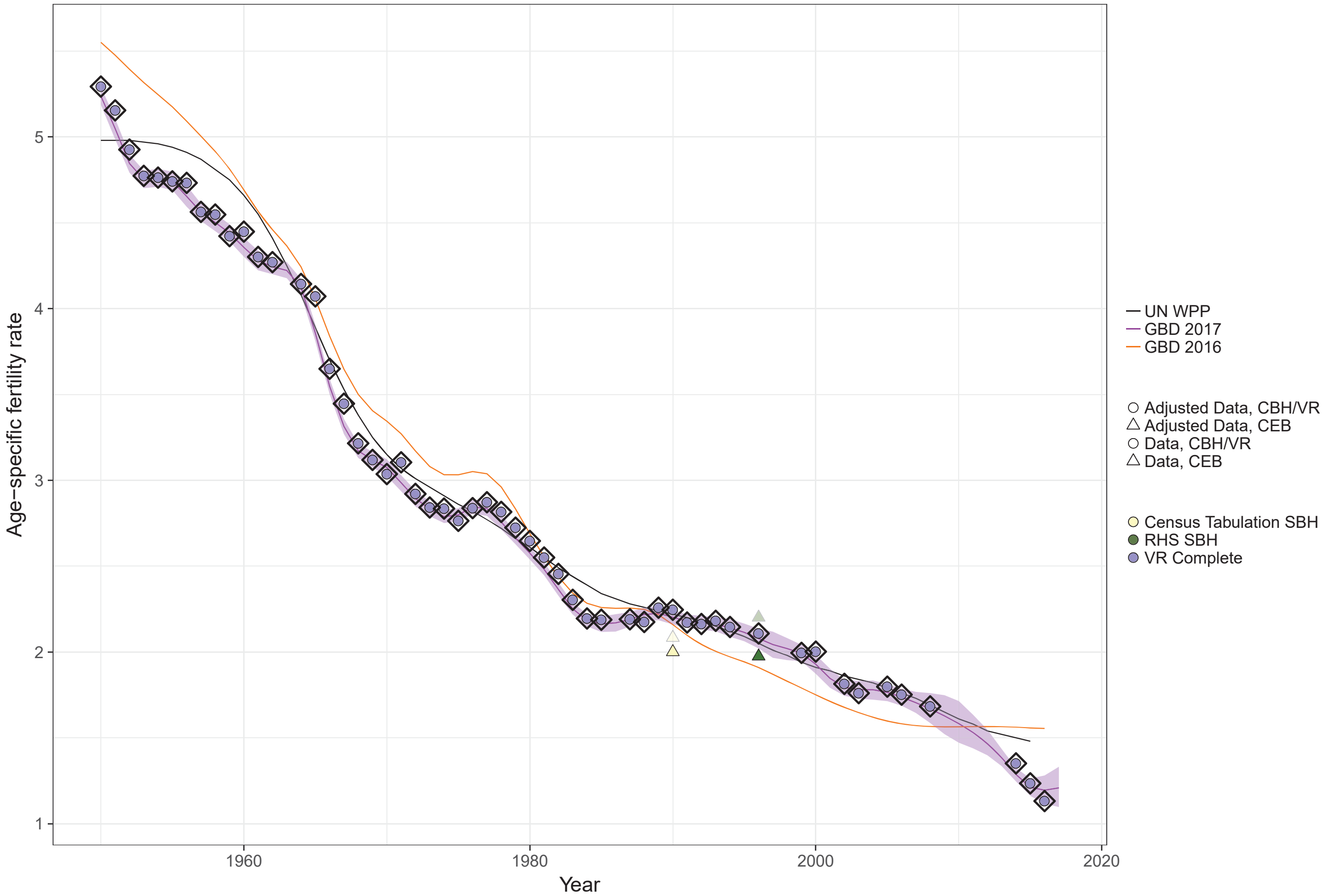

Age−specific fertility fitted trend lines, 1950−2017: Puerto Rico, Ages: 10−14
Data points enclosed with a diamond are reference sources

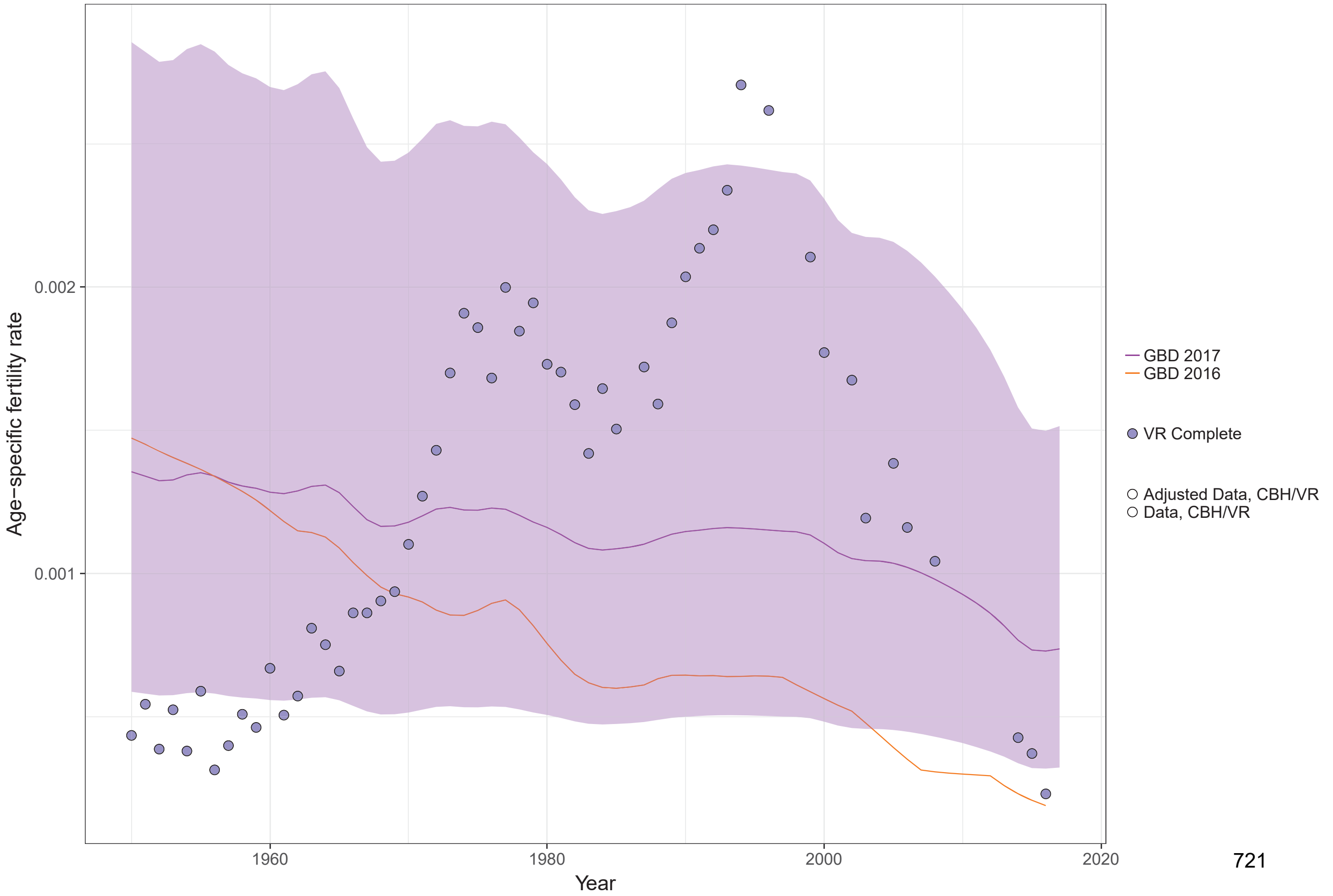

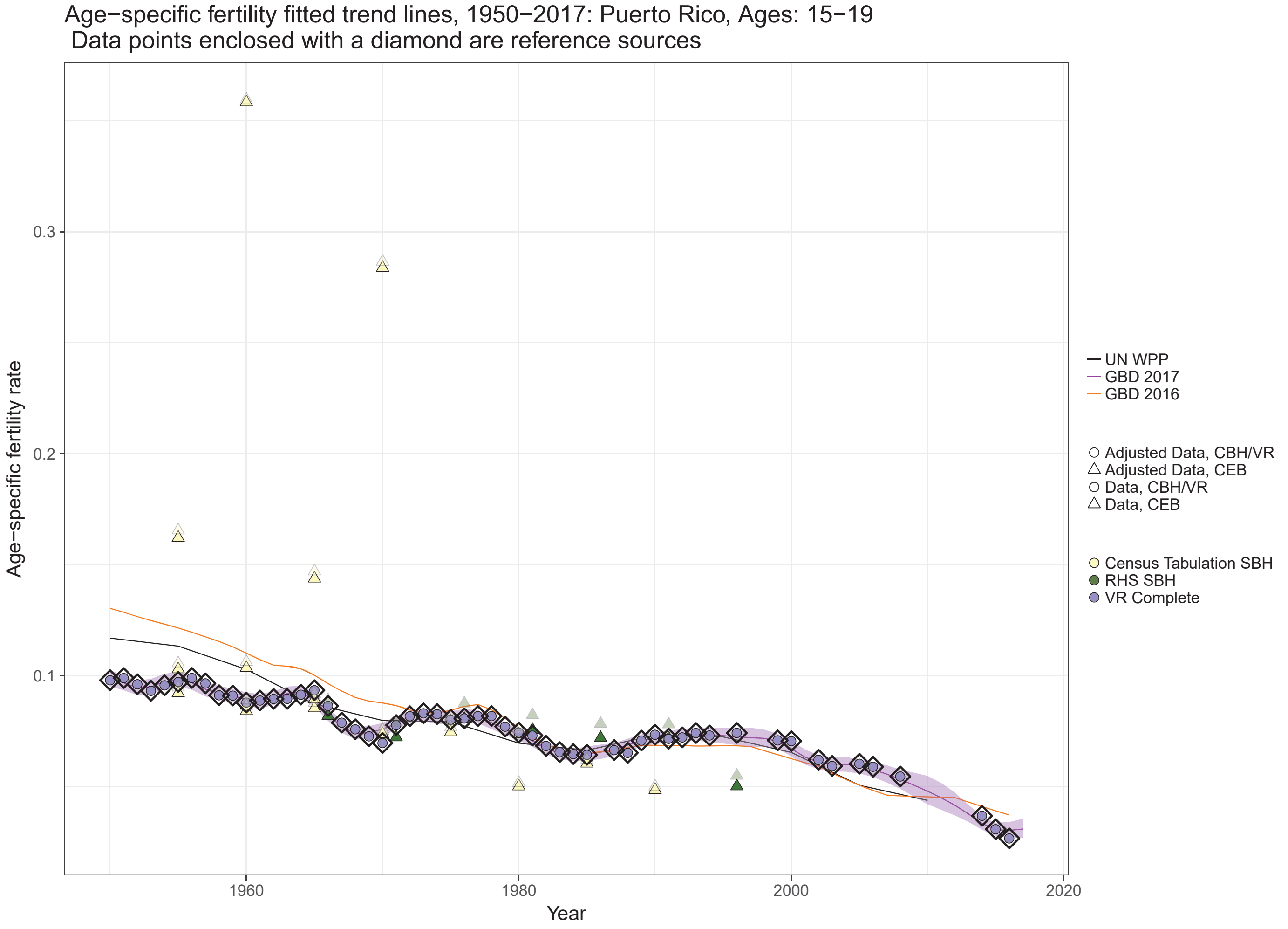

Age−specific fertility fitted trend lines, 1950−2017: Puerto Rico, Ages: 15−19
Data points enclosed with a diamond are reference sources
Age−specific fertility rate
0.1
0.2
0.3
1960
1980
2000
2020
Year
UN WPP
GBD 2017
GBD 2016
Adjusted Data, CBH/VR
Adjusted Data, CEB
Data, CBH/VR
Data, CEB
Census Tabulation SBH
RHS SBH
VR Complete

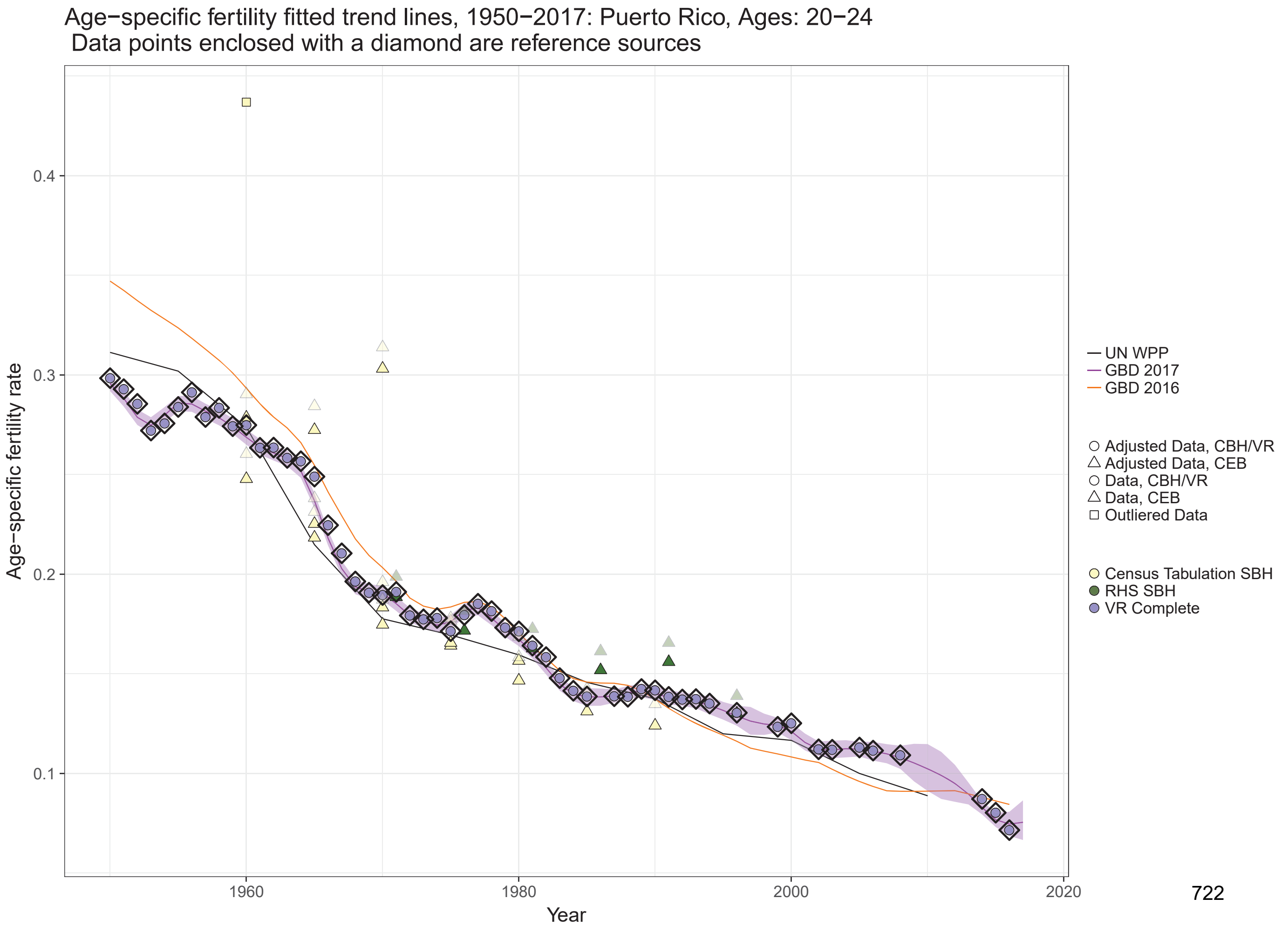

Age−specific fertility fitted trend lines, 1950−2017: Puerto Rico, Ages: 20−24
Data points enclosed with a diamond are reference sources
Age−specific fertility rate
0.1
0.2
0.3
0.4
1960
1980
2000
2020
Year
UN WPP
GBD 2017
GBD 2016
Adjusted Data, CBH/VR
Adjusted Data, CEB
Data, CBH/VR
Data, CEB
Outliered Data
Census Tabulation SBH
RHS SBH
VR Complete

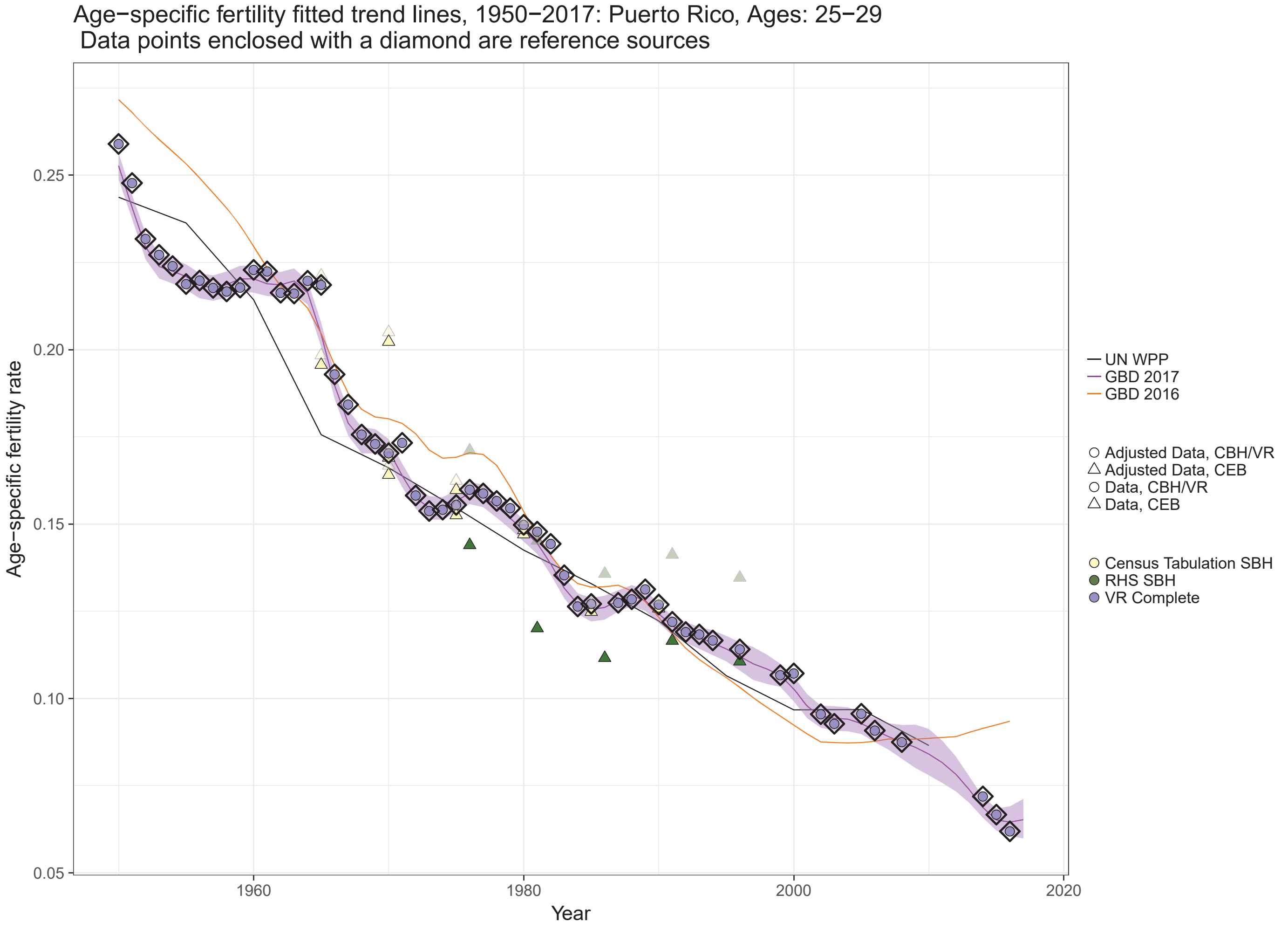

Age−specific fertility fitted trend lines, 1950−2017: Puerto Rico, Ages: 25−29
Data points enclosed with a diamond are reference sources
Age−specific fertility rate
0.25
0.20
0.15
0.10
0.05
1960
1980
2000
2020
Year
UN WPP
GBD 2017
GBD 2016
Adjusted Data, CBH/VR
Adjusted Data, CEB
Data, CBH/VR
Data, CEB
Census Tabulation SBH
RHS SBH
VR Complete

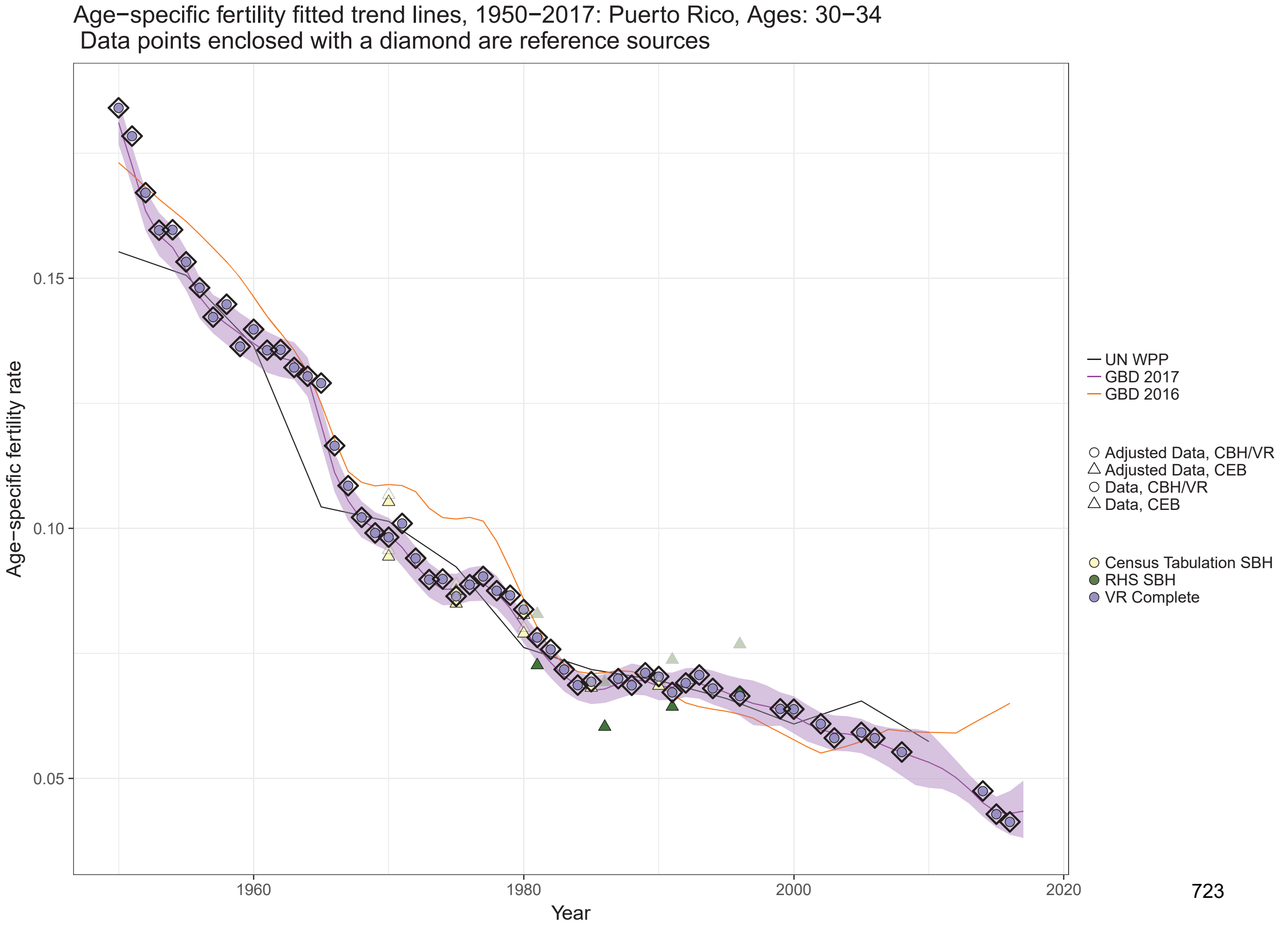

Age−specific fertility fitted trend lines, 1950−2017: Puerto Rico, Ages: 30−34
Data points enclosed with a diamond are reference sources
Age−specific fertility rate
0.15
0.10
0.05
1960
1980
2000
2020
Year
UN WPP
GBD 2017
GBD 2016
Adjusted Data, CBH/VR
Adjusted Data, CEB
Data, CBH/VR
Data, CEB
Census Tabulation SBH
RHS SBH
VR Complete

Age−specific fertility fitted trend lines, 1950−2017: Puerto Rico, Ages: 35−39
Data points enclosed with a diamond are reference sources

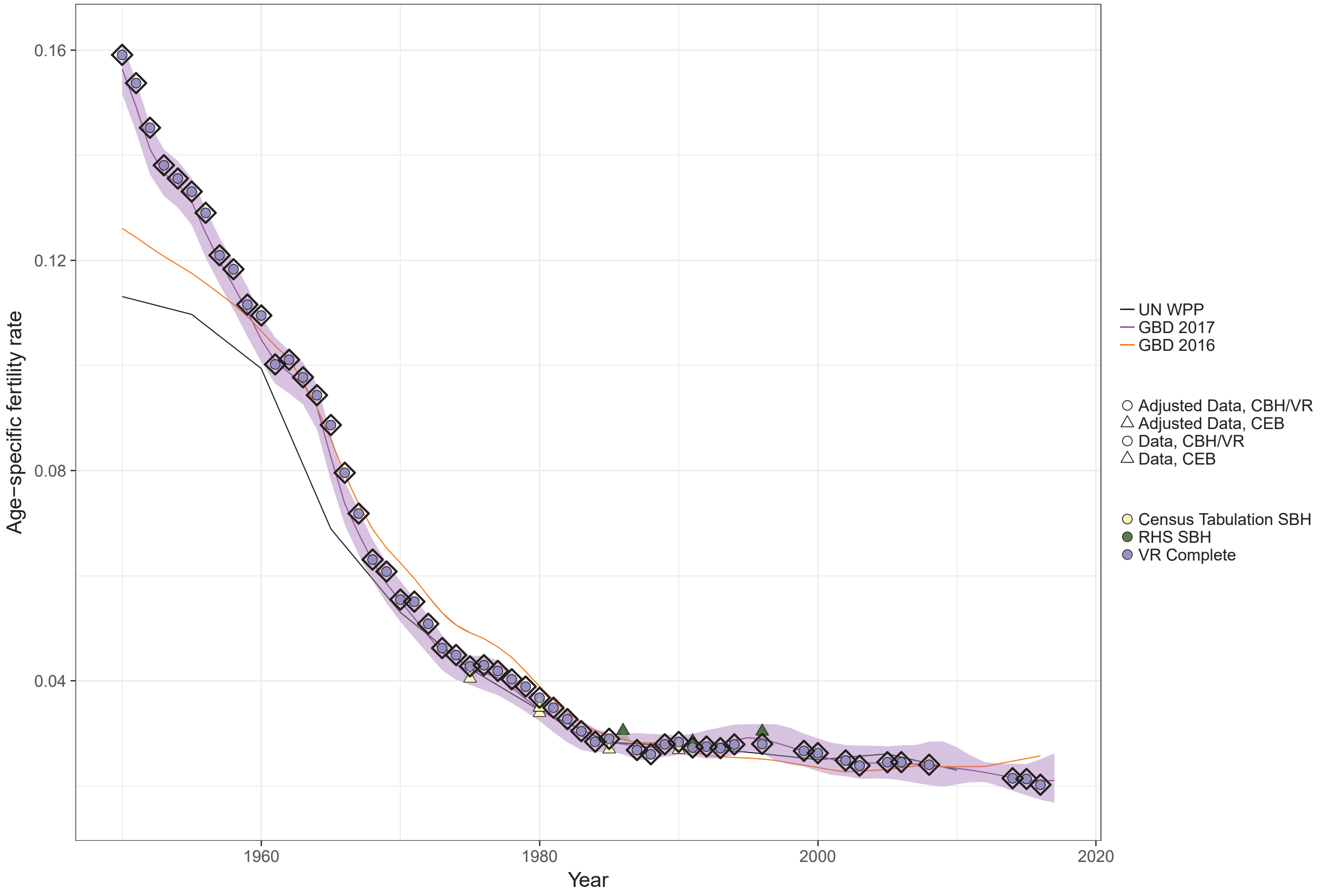

Age−specific fertility fitted trend lines, 1950−2017: Puerto Rico, Ages: 40−44
Data points enclosed with a diamond are reference sources

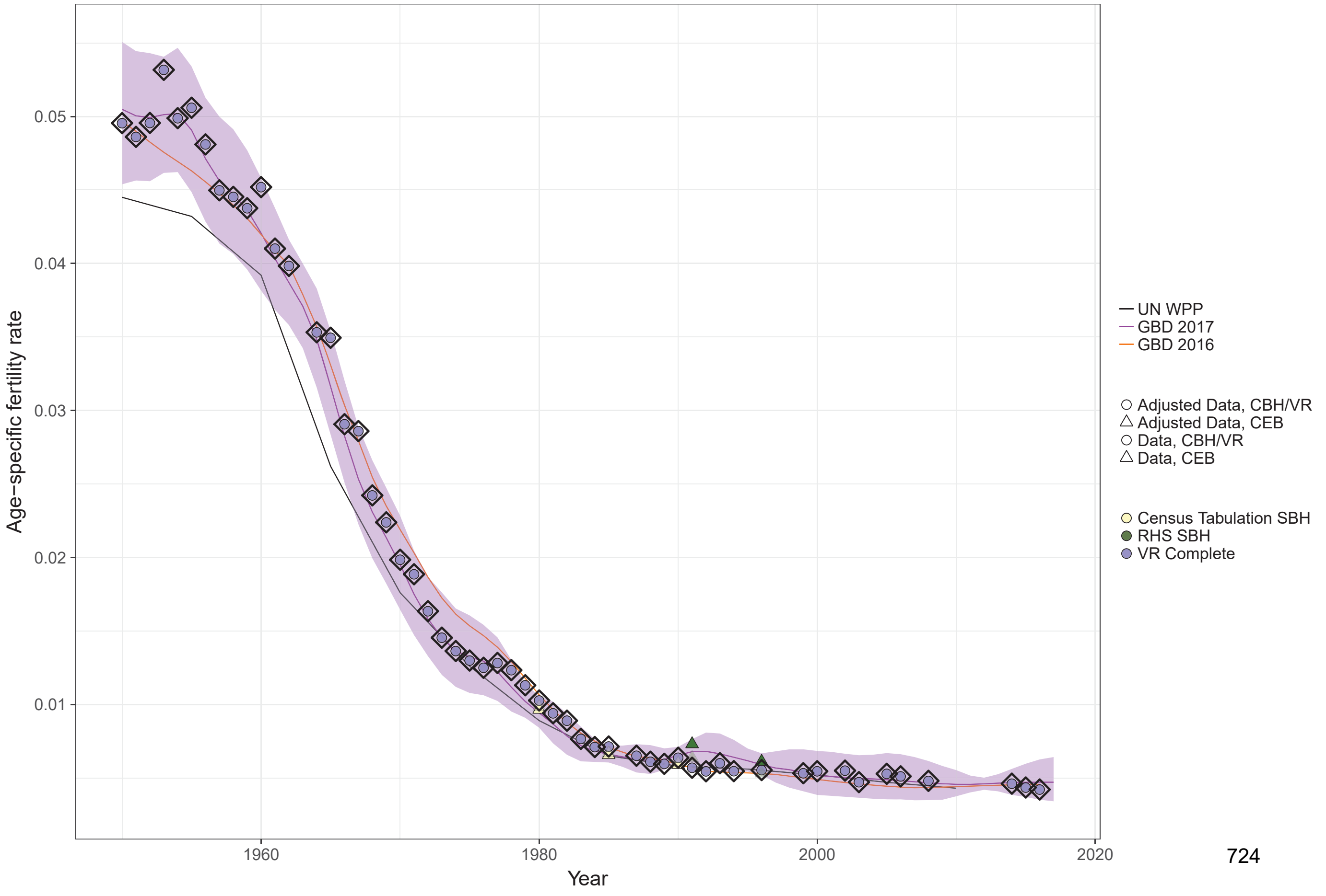

Age−specific fertility fitted trend lines, 1950−2017: Puerto Rico, Ages: 45−49
Data points enclosed with a diamond are reference sources

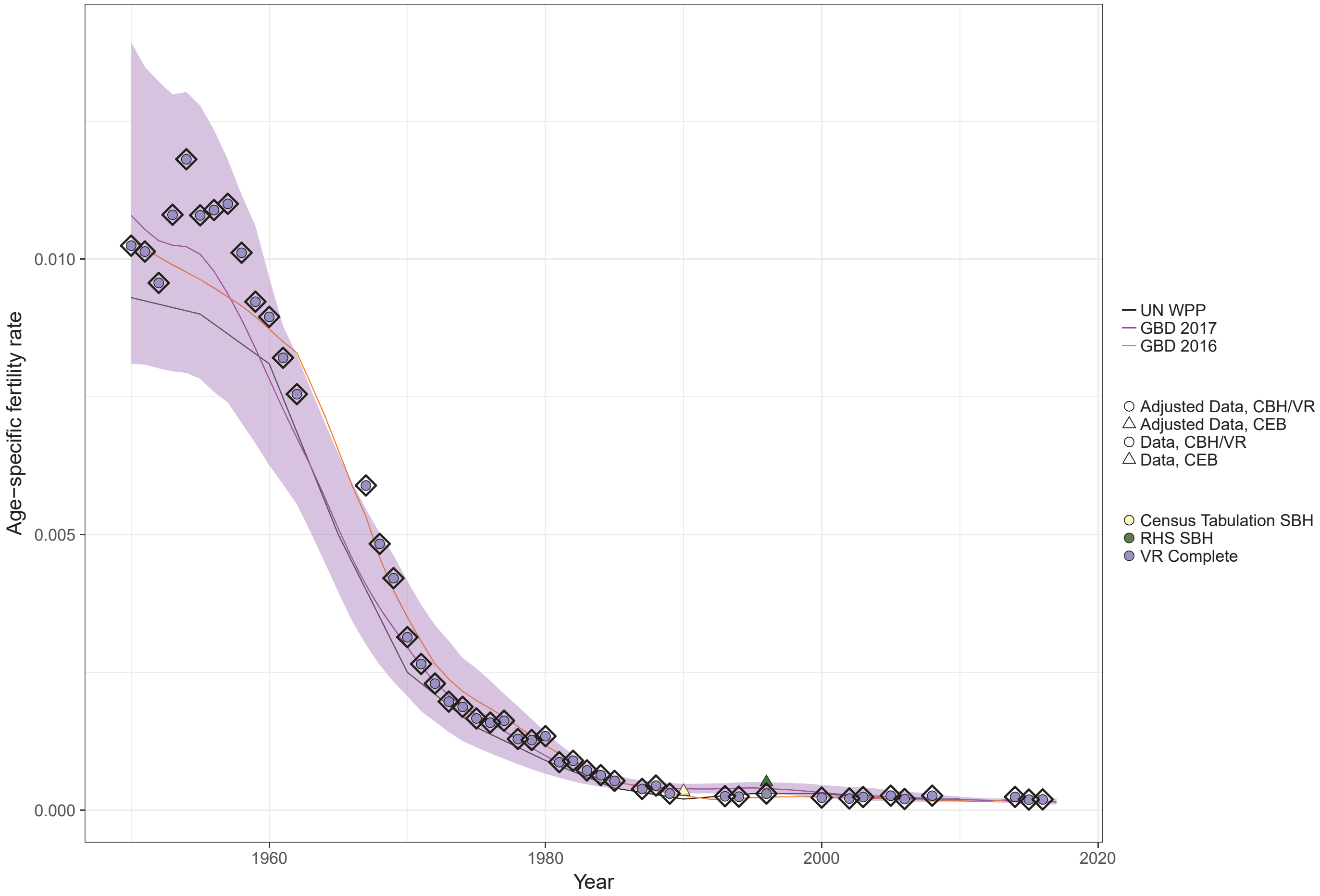

Age−specific fertility fitted trend lines, 1950−2017: Puerto Rico, Ages: 50−54
Data points enclosed with a diamond are reference sources

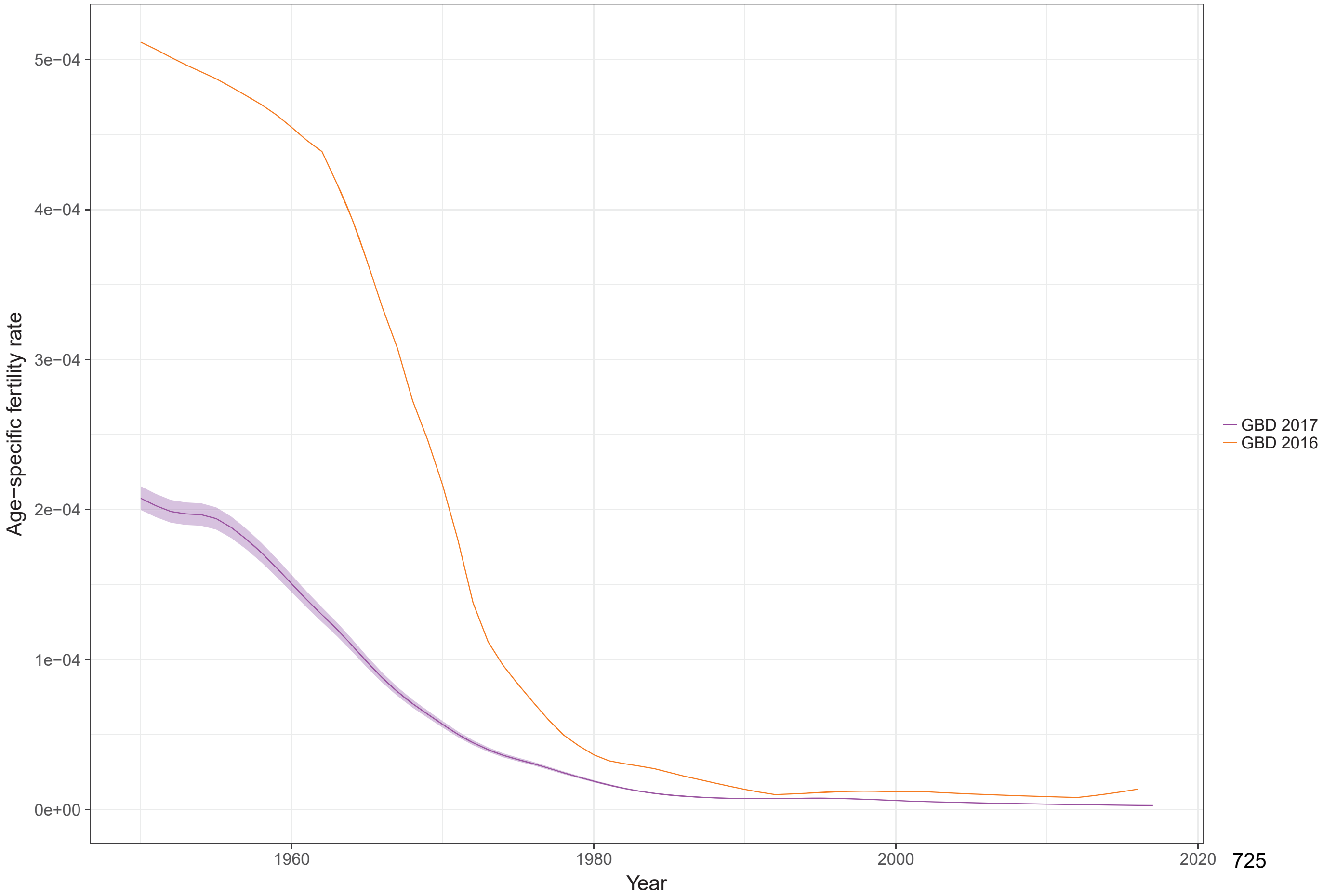

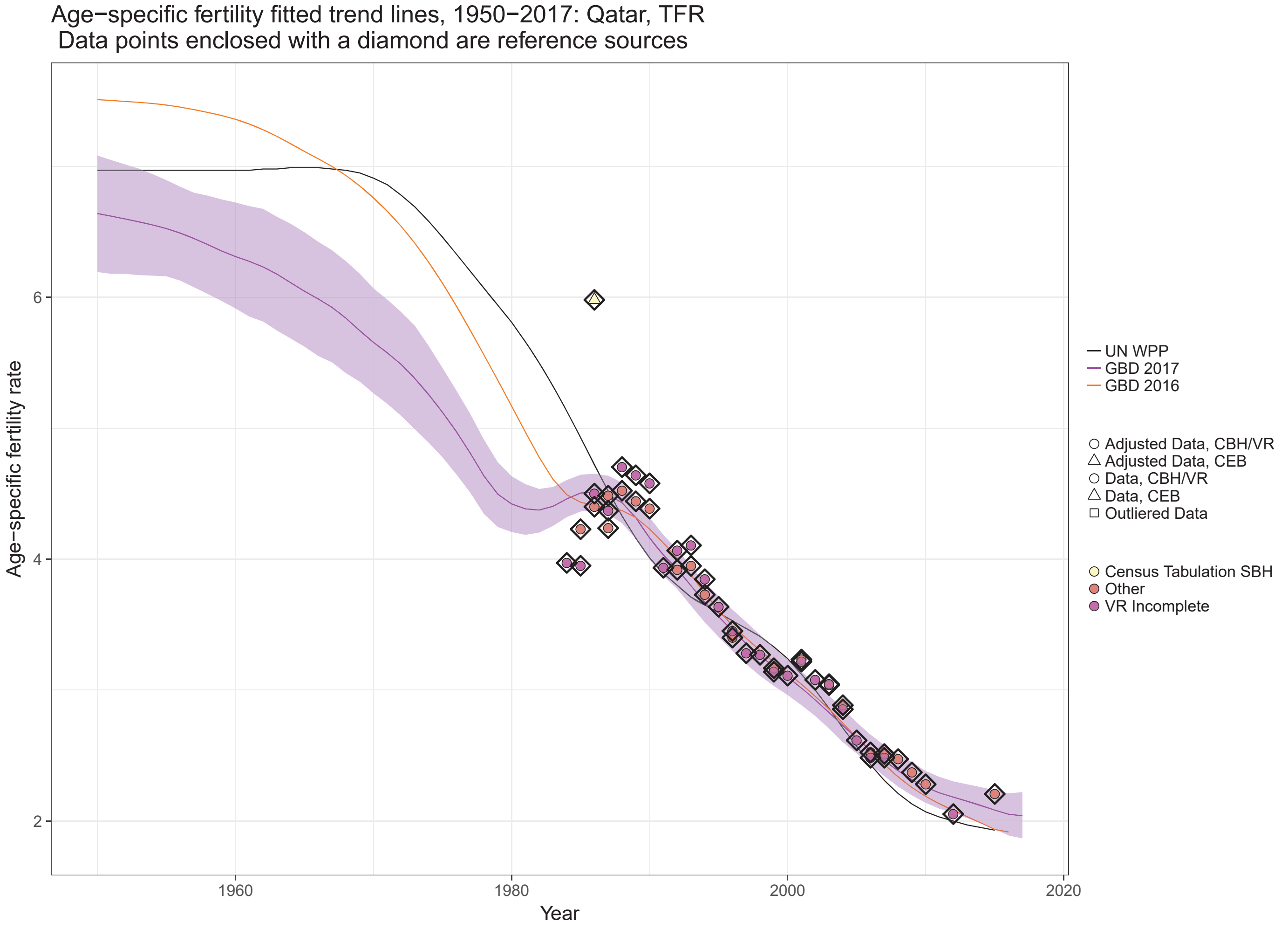

Age−specific fertility fitted trend lines, 1950−2017: Qatar, TFR
Data points enclosed with a diamond are reference sources
Age−specific fertility rate
Year
UN WPP
GBD 2017
GBD 2016
Adjusted Data, CBH/VR
Adjusted Data, CEB
Data, CBH/VR
Data, CEB
Outliered Data
Census Tabulation SBH
Other
VR Incomplete

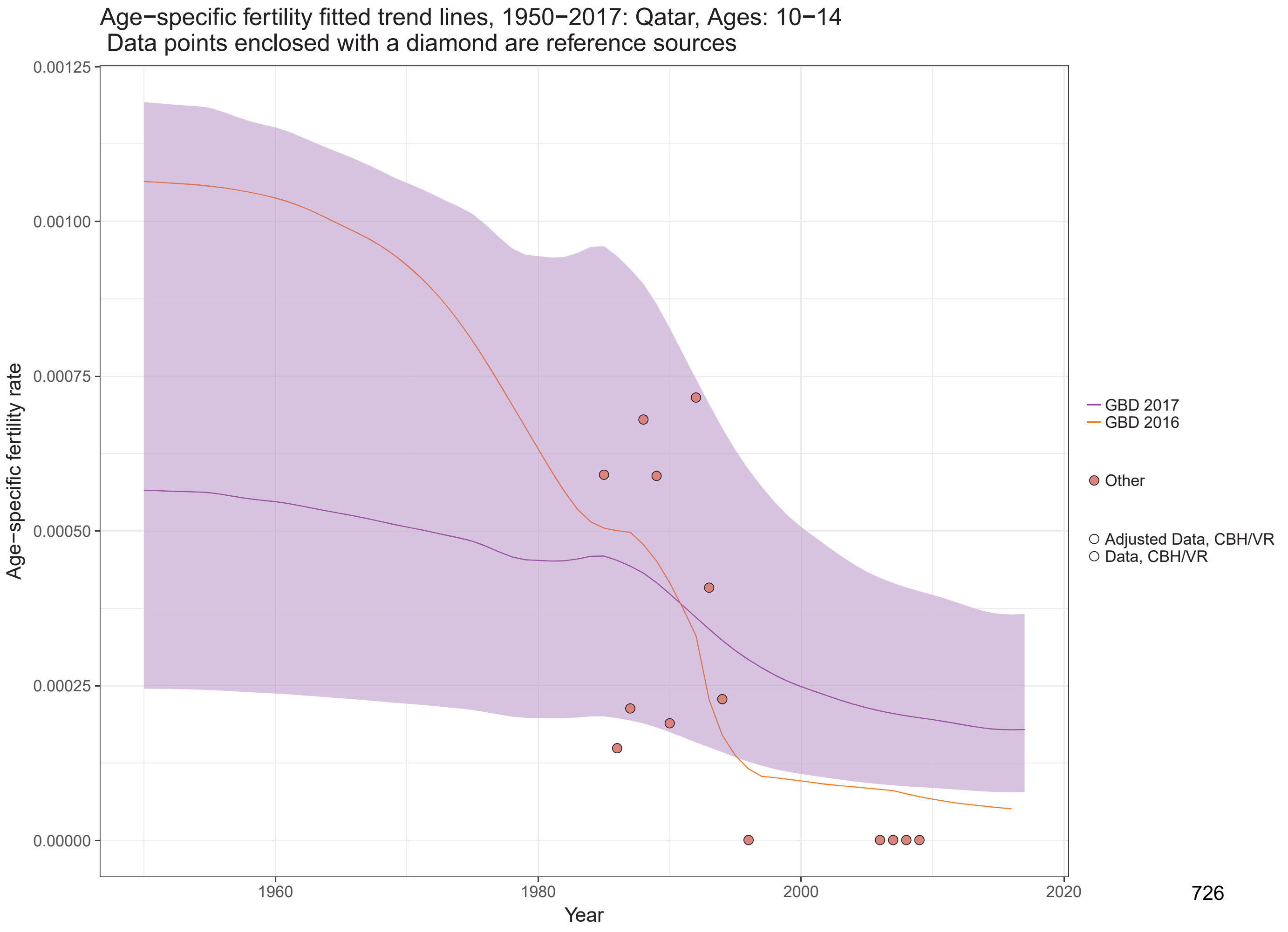

Age−specific fertility fitted trend lines, 1950−2017: Qatar, Ages: 10−14
Data points enclosed with a diamond are reference sources
Age−specific fertility rate
Year
GBD 2017
GBD 2016
Other
Adjusted Data, CBH/VR
Data, CBH/VR

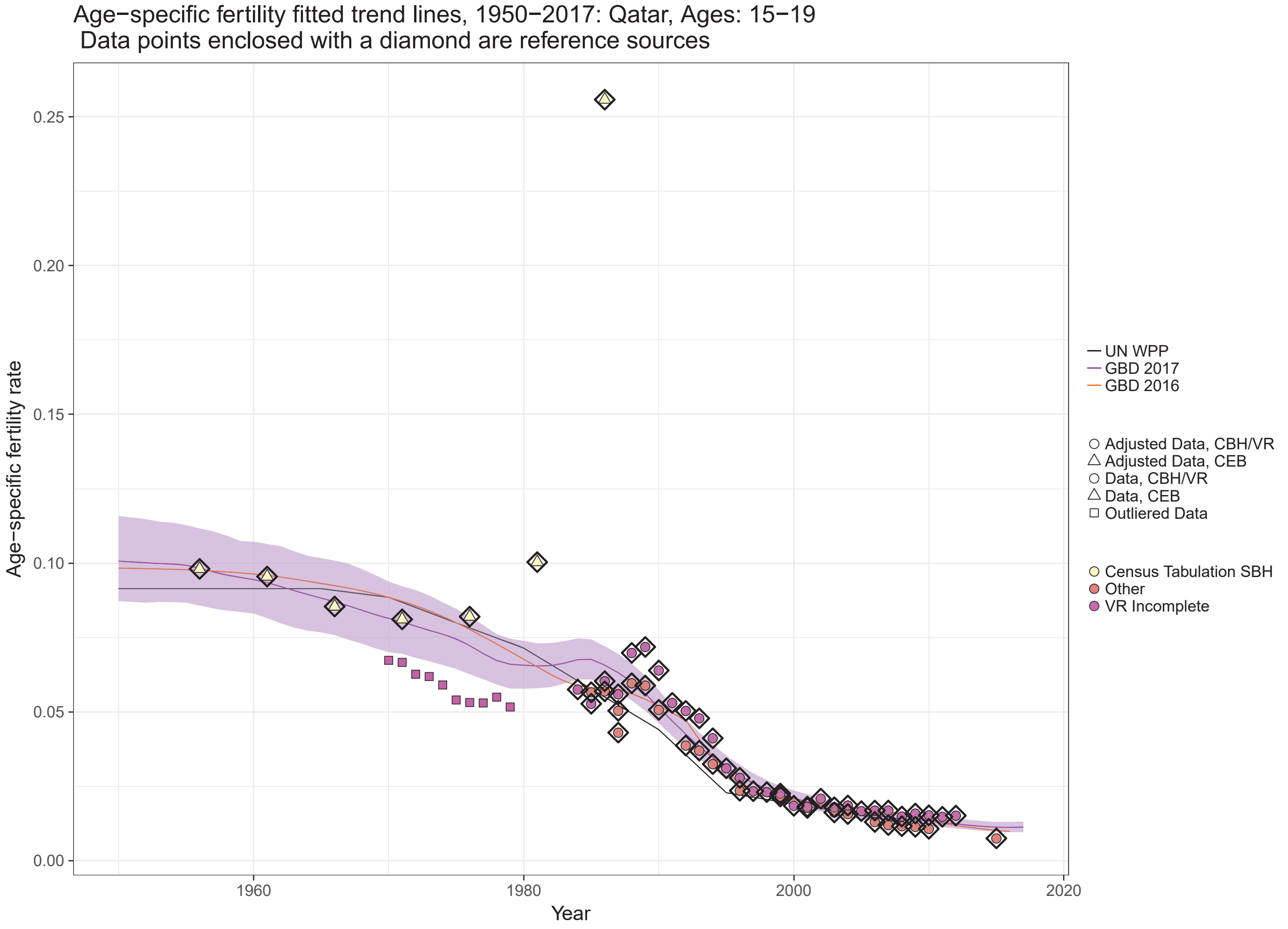

Age−specific fertility fitted trend lines, 1950−2017: Qatar, Ages: 15−19
Data points enclosed with a diamond are reference sources
Age−specific fertility rate
Year
UN WPP
GBD 2017
GBD 2016
Adjusted Data, CBH/VR
Adjusted Data, CEB
Data, CBH/VR
Data, CEB
Outliered Data
Census Tabulation SBH
Other
VR Incomplete

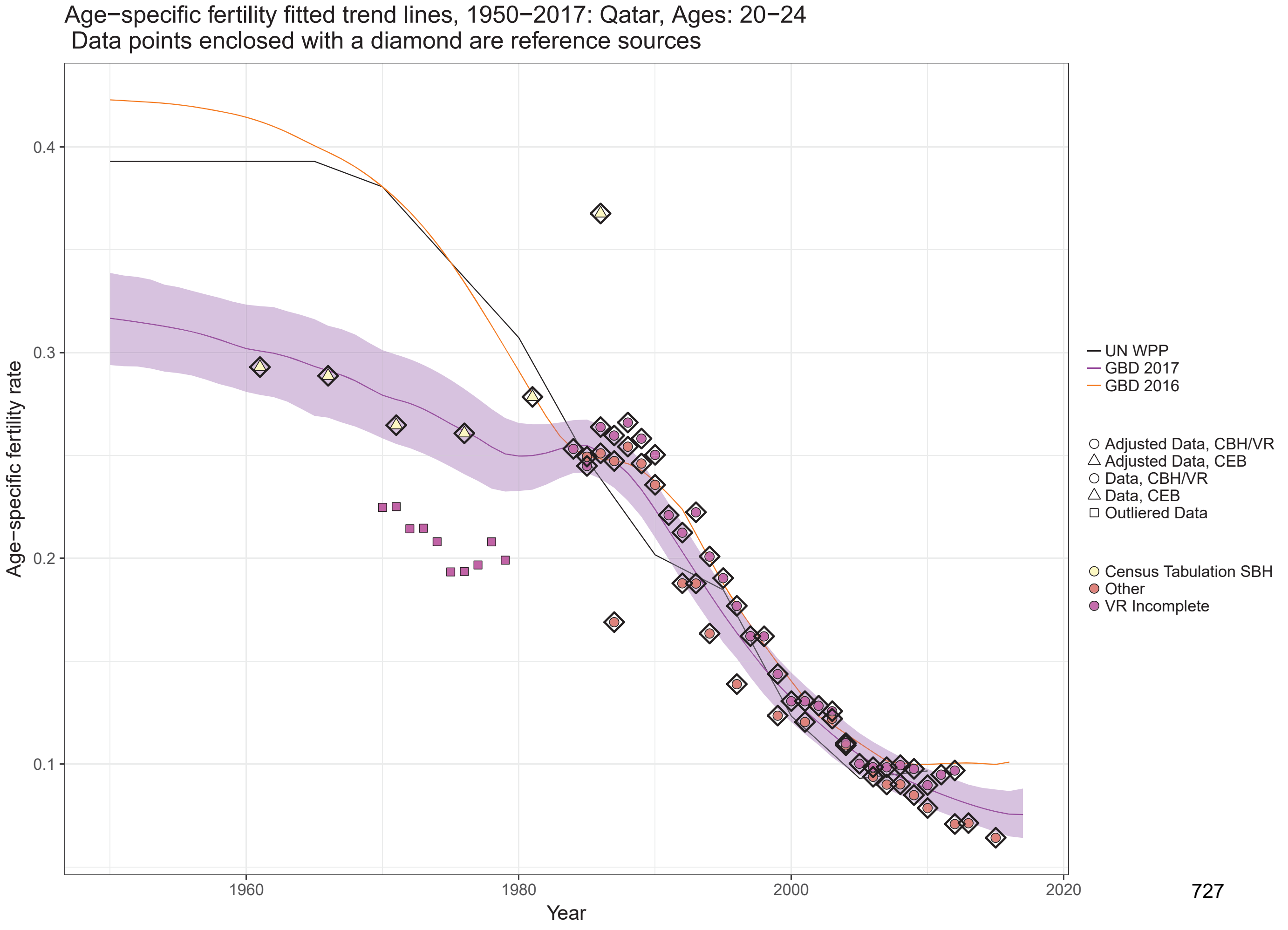

Age−specific fertility fitted trend lines, 1950−2017: Qatar, Ages: 20−24
Data points enclosed with a diamond are reference sources
Age−specific fertility rate
Year
UN WPP
GBD 2017
GBD 2016
Adjusted Data, CBH/VR
Adjusted Data, CEB
Data, CBH/VR
Data, CEB
Outliered Data
Census Tabulation SBH
Other
VR Incomplete

Age−specific fertility fitted trend lines, 1950−2017: Qatar, Ages: 25−29
Data points enclosed with a diamond are reference sources

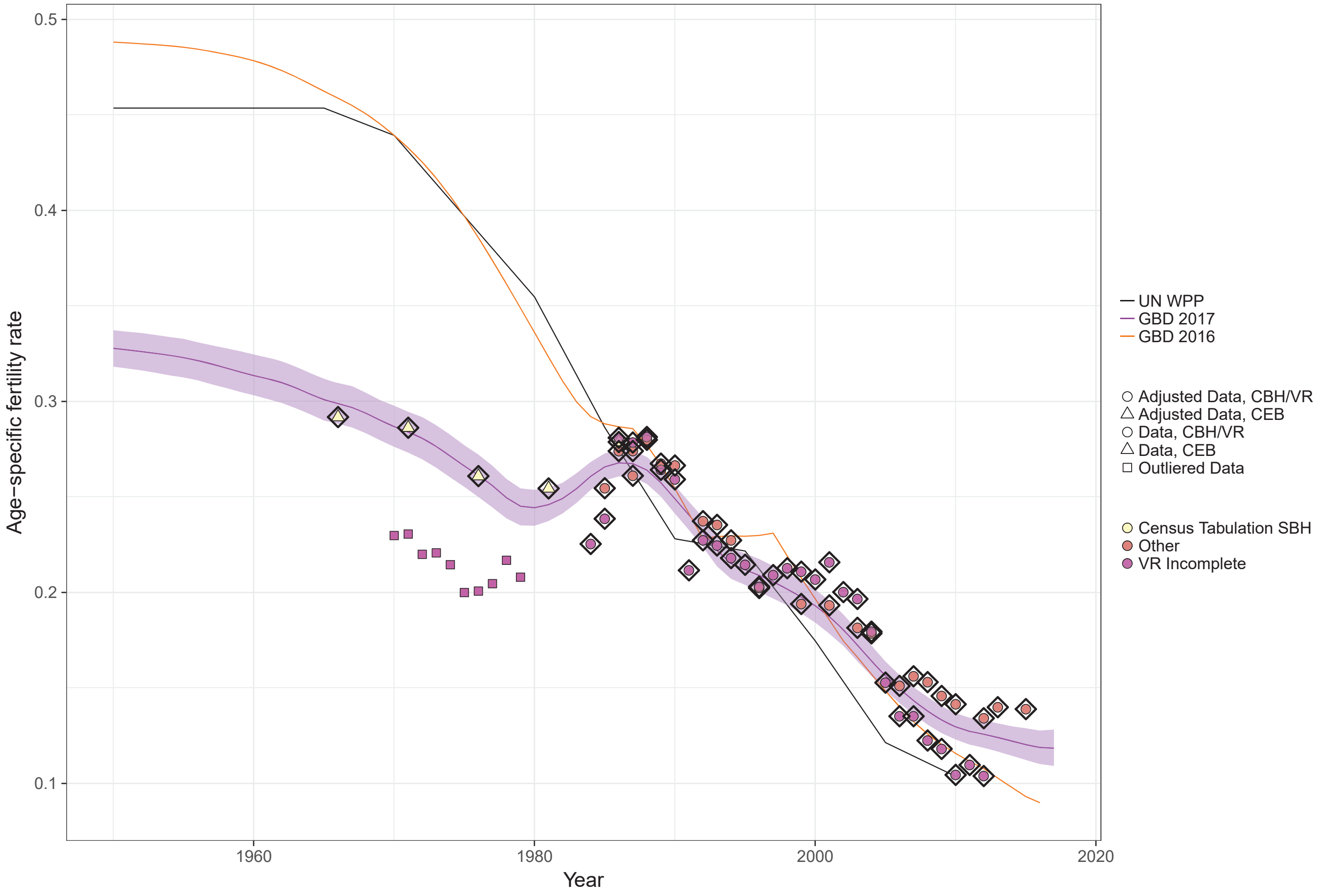

Age−specific fertility fitted trend lines, 1950−2017: Qatar, Ages: 30−34
Data points enclosed with a diamond are reference sources

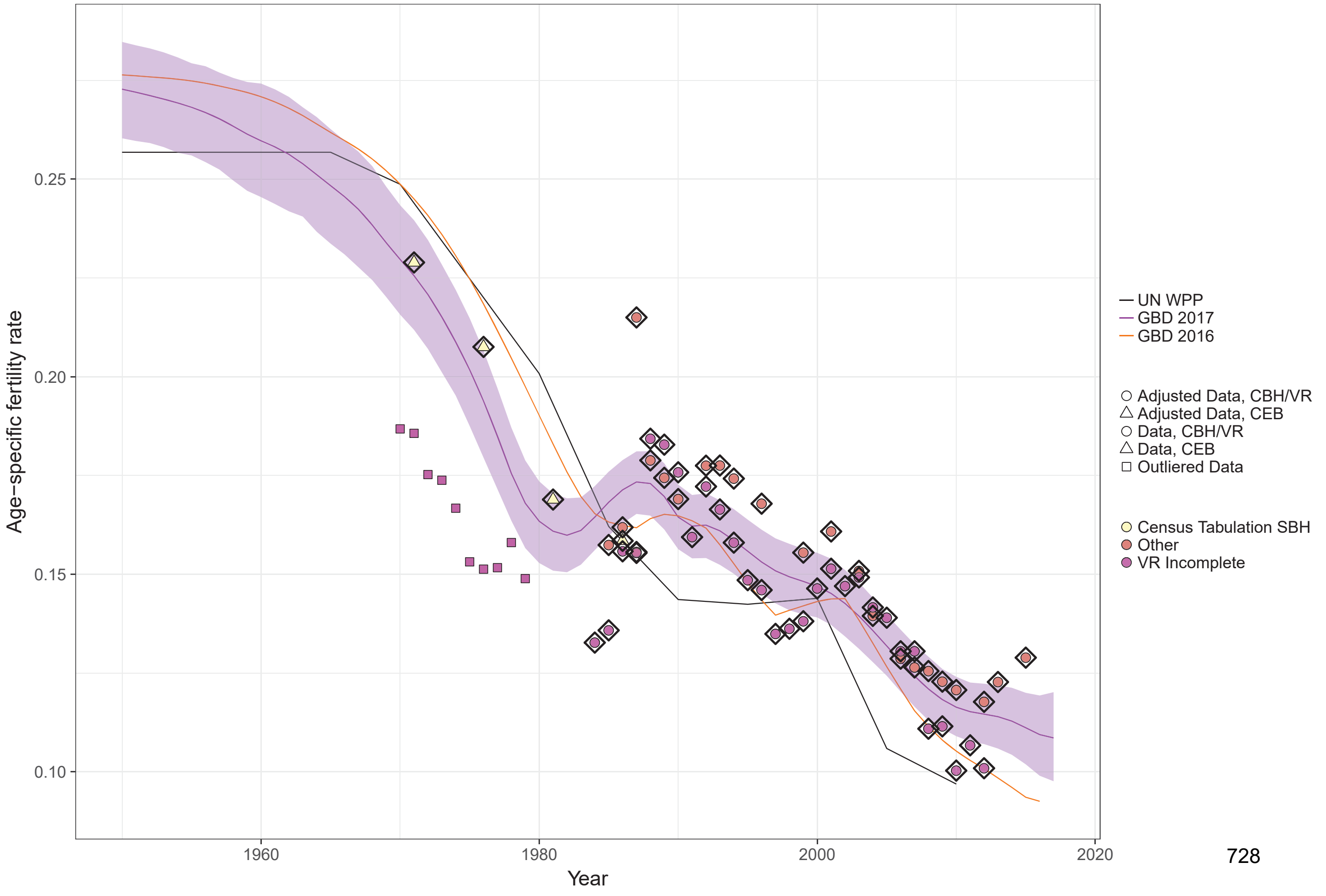

Age−specific fertility fitted trend lines, 1950−2017: Qatar, Ages: 35−39
Data points enclosed with a diamond are reference sources

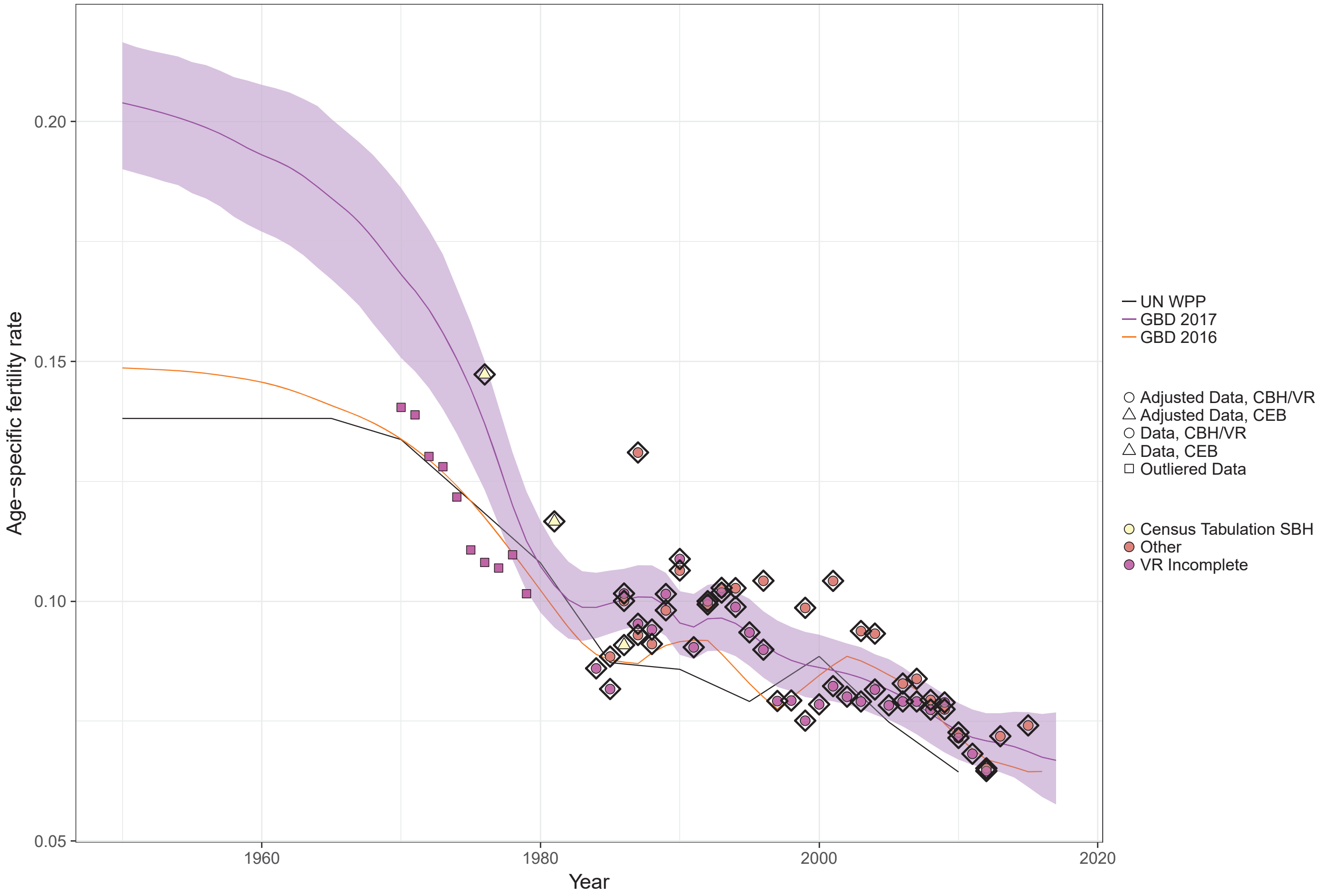

Age−specific fertility fitted trend lines, 1950−2017: Qatar, Ages: 40−44
Data points enclosed with a diamond are reference sources

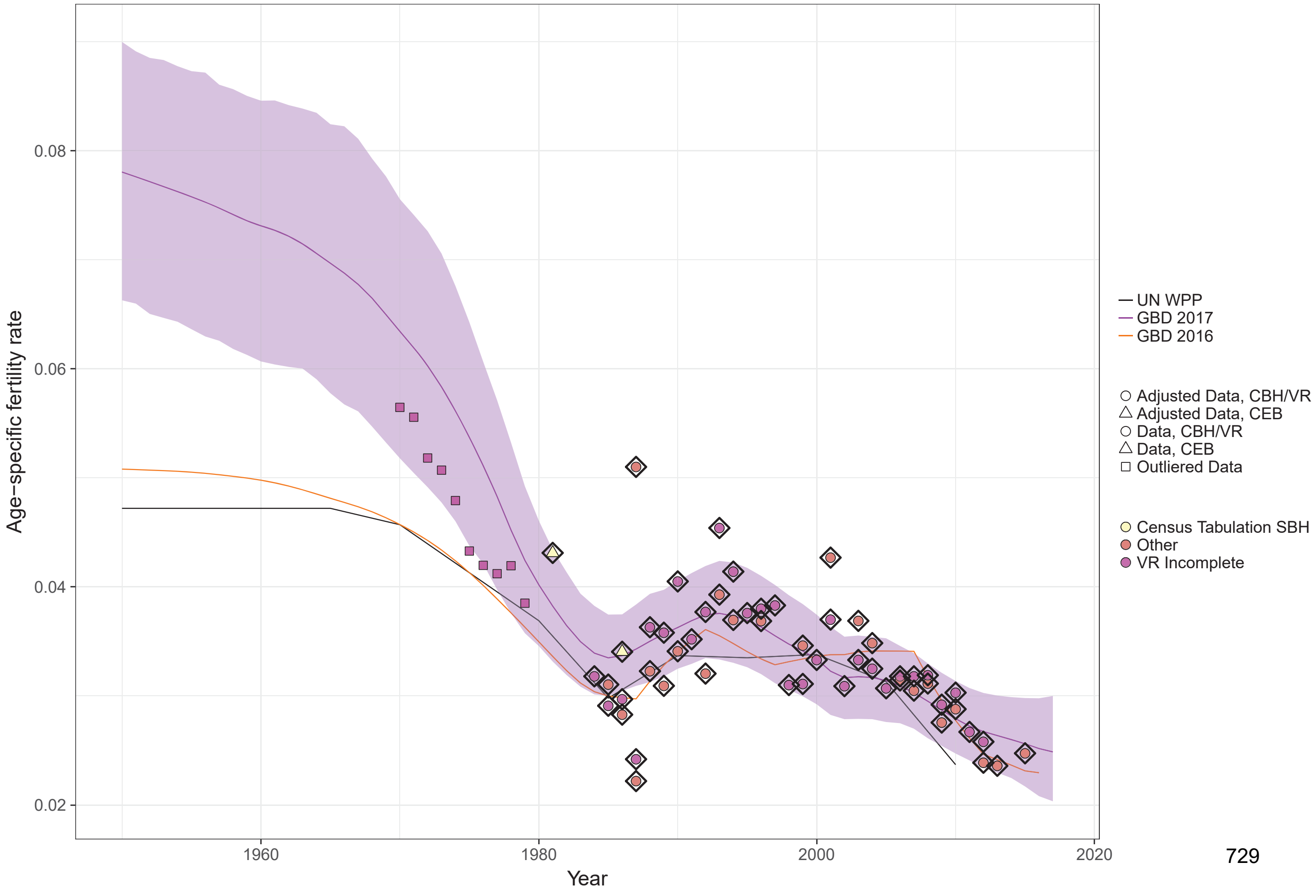

Age−specific fertility fitted trend lines, 1950−2017: Qatar, Ages: 45−49
Data points enclosed with a diamond are reference sources

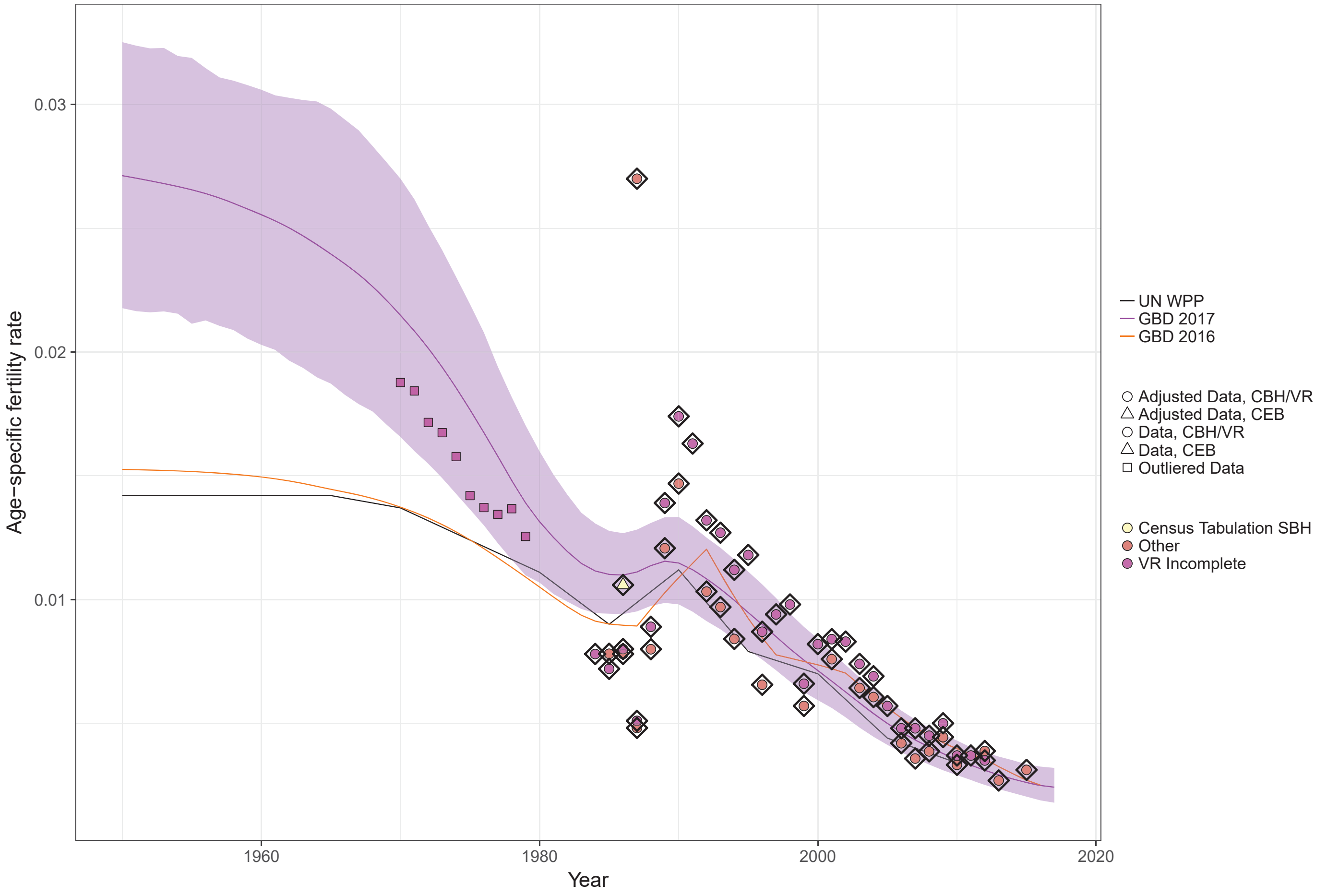

Age−specific fertility fitted trend lines, 1950−2017: Qatar, Ages: 50−54
Data points enclosed with a diamond are reference sources

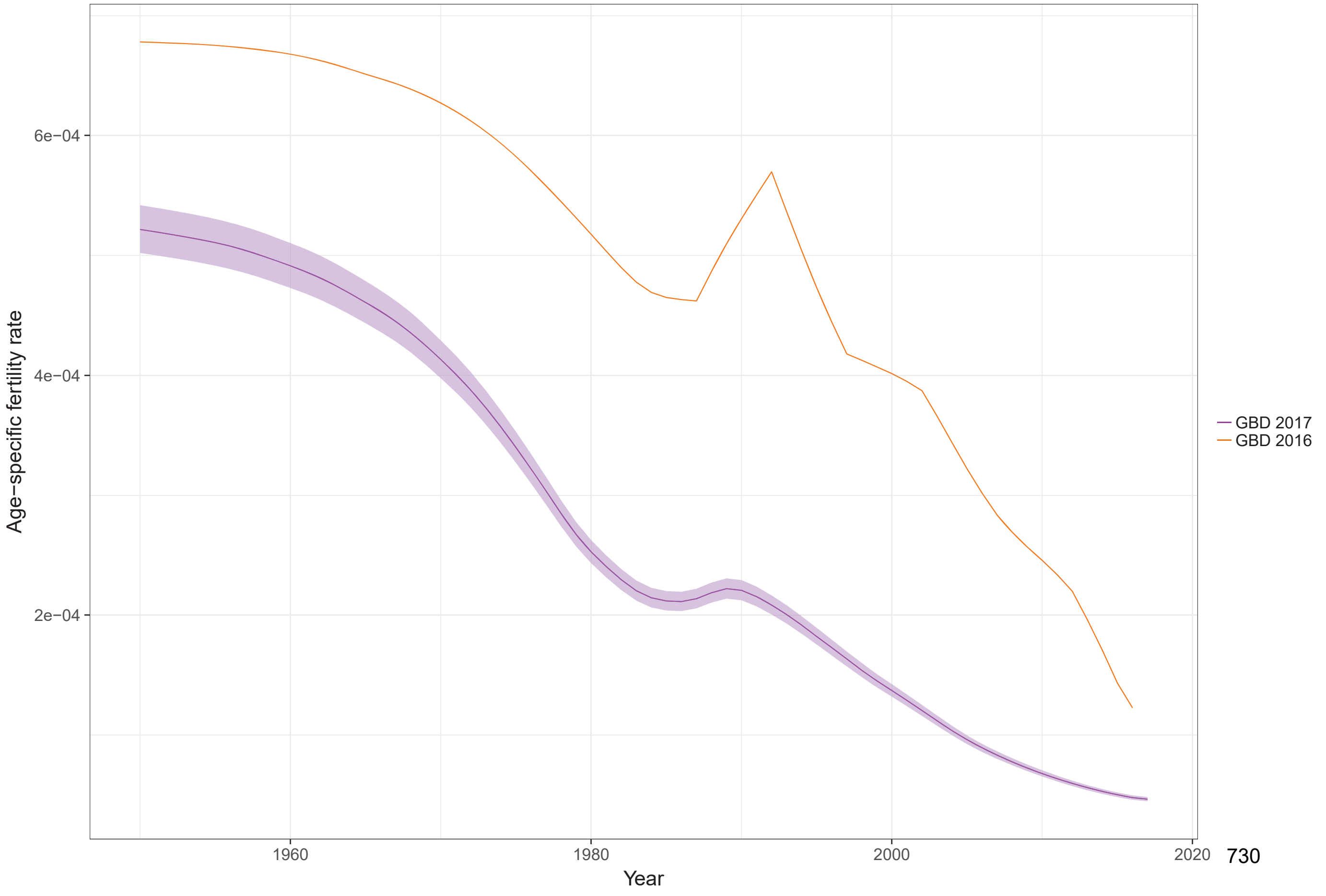

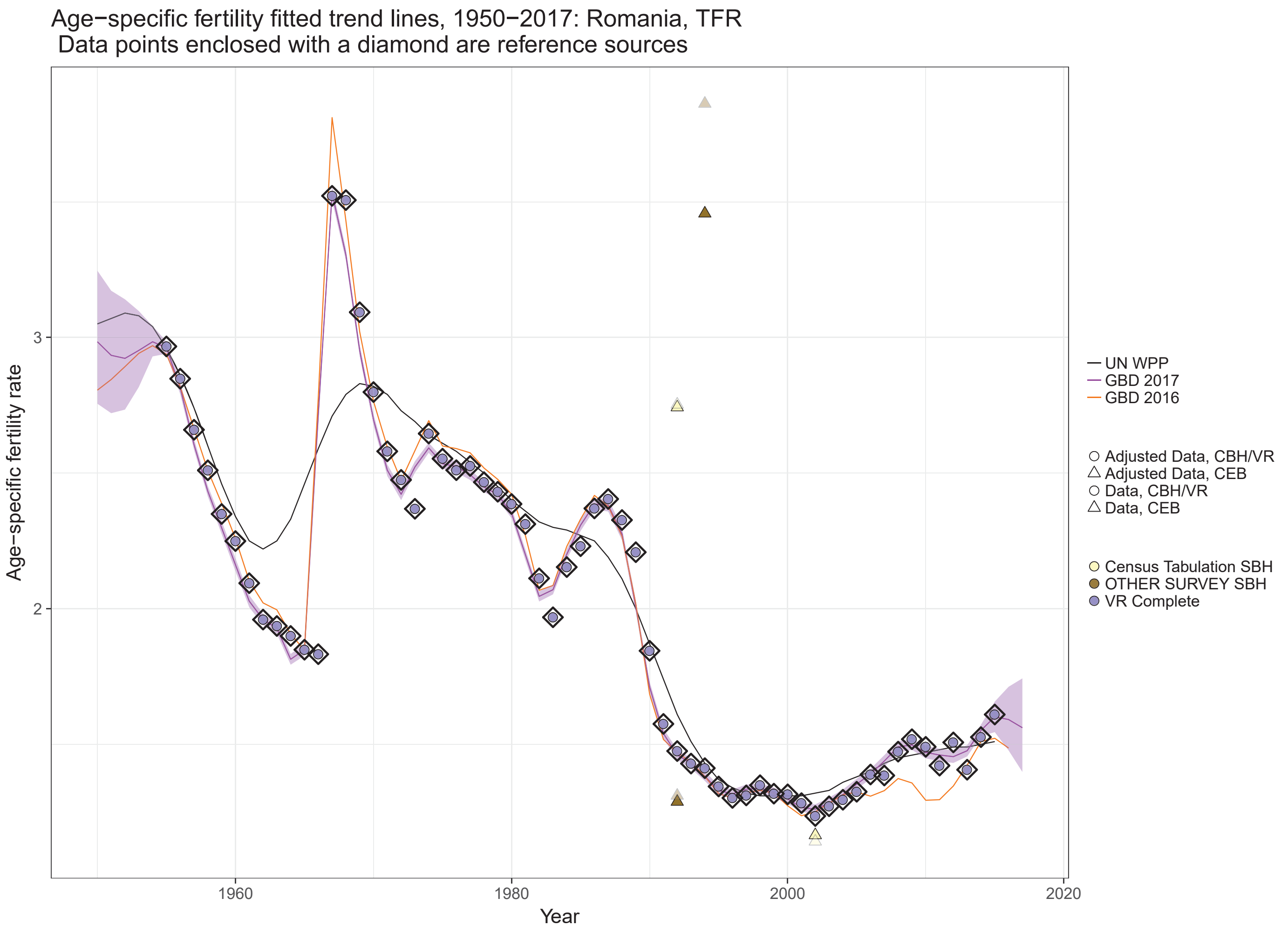

Age−specific fertility fitted trend lines, 1950−2017: Romania, TFR
Data points enclosed with a diamond are reference sources
Age−specific fertility rate
3
2
1960
1980
2000
2020
Year
UN WPP
GBD 2017
GBD 2016
Adjusted Data, CBH/VR
Adjusted Data, CEB
Data, CBH/VR
Data, CEB
Census Tabulation SBH
OTHER SURVEY SBH
VR Complete

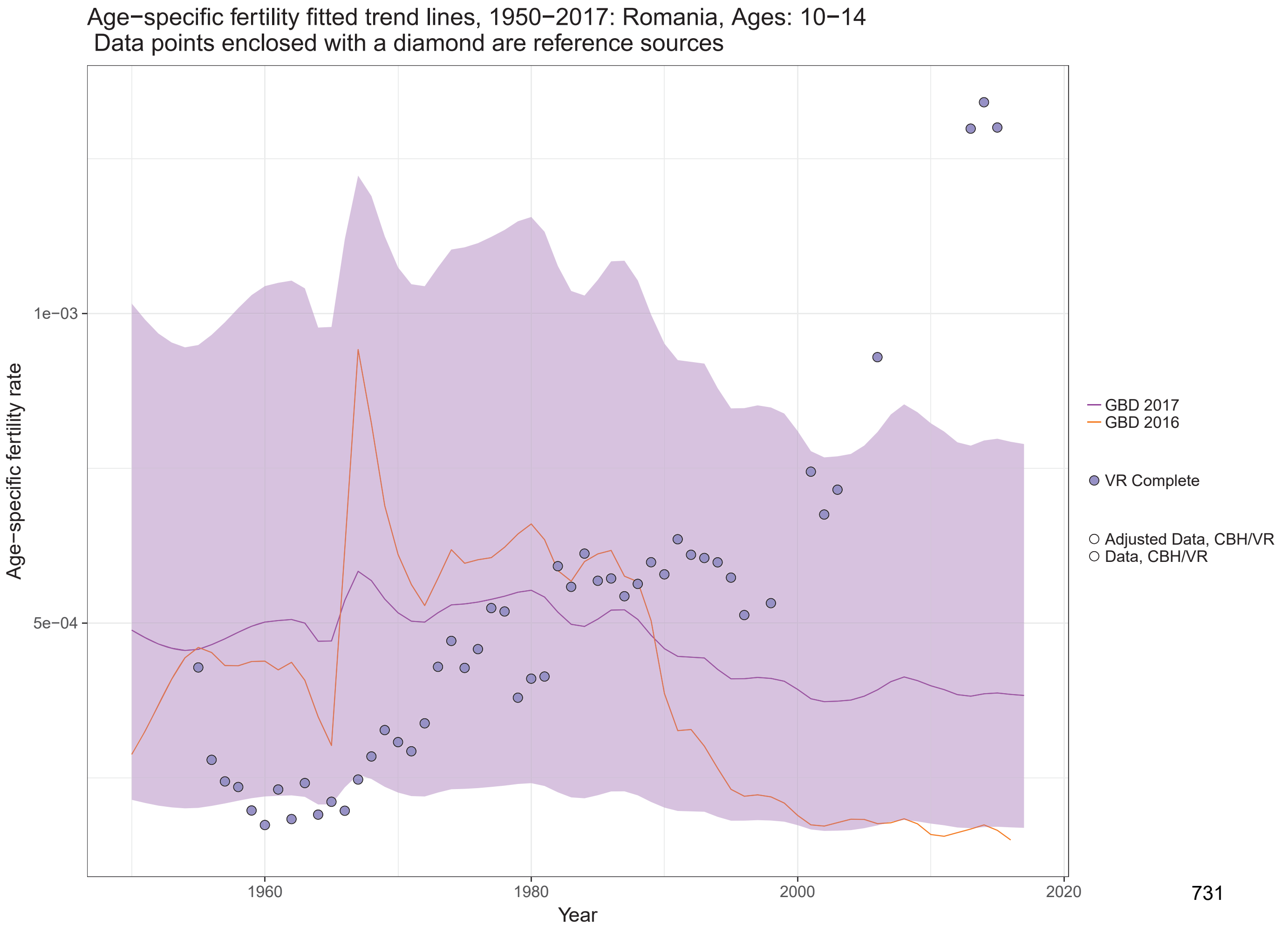

Age−specific fertility fitted trend lines, 1950−2017: Romania, Ages: 10−14
Data points enclosed with a diamond are reference sources
Age−specific fertility rate
1e−03
5e−04
1960
1980
2000
2020
Year
GBD 2017
GBD 2016
VR Complete
Adjusted Data, CBH/VR
Data, CBH/VR

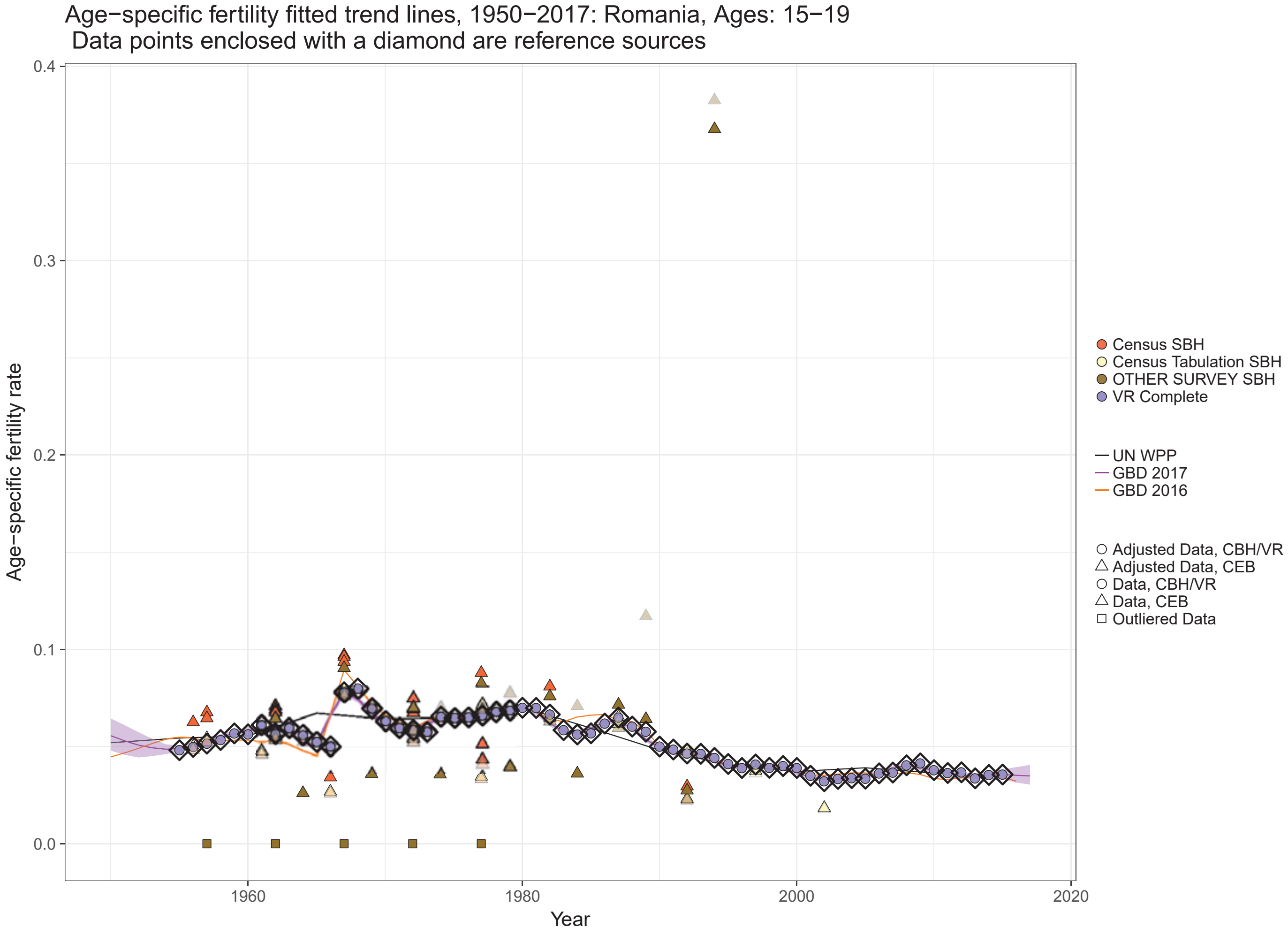
Age−specific fertility fitted trend lines, 1950−2017: Romania, Ages: 15−19
Data points enclosed with a diamond are reference sources
Age−specific fertility rate
0.0
0.1
0.2
0.3
0.4
1960
1980
2000
2020
Year
Census SBH
Census Tabulation SBH
OTHER SURVEY SBH
VR Complete
UN WPP
GBD 2017
GBD 2016
Adjusted Data, CBH/VR
Adjusted Data, CEB
Data, CBH/VR
Data, CEB
Outliered Data

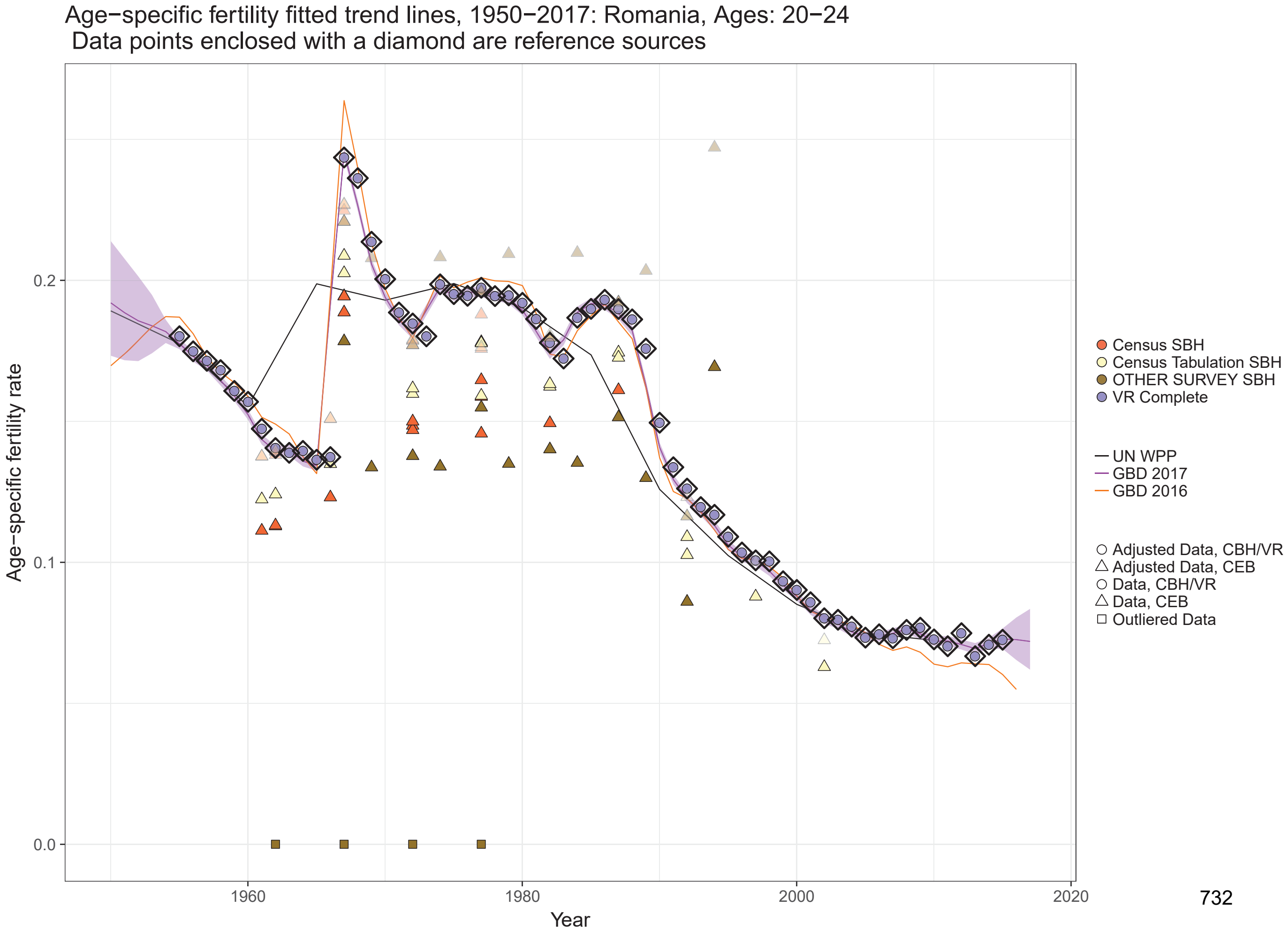
Age−specific fertility fitted trend lines, 1950−2017: Romania, Ages: 20−24
Data points enclosed with a diamond are reference sources
Age−specific fertility rate
0.0
0.1
0.2
1960
1980
2000
2020
Year
Census SBH
Census Tabulation SBH
OTHER SURVEY SBH
VR Complete
UN WPP
GBD 2017
GBD 2016
Adjusted Data, CBH/VR
Adjusted Data, CEB
Data, CBH/VR
Data, CEB
Outliered Data

Age−specific fertility fitted trend lines, 1950−2017: Romania, Ages: 25−29
Data points enclosed with a diamond are reference sources

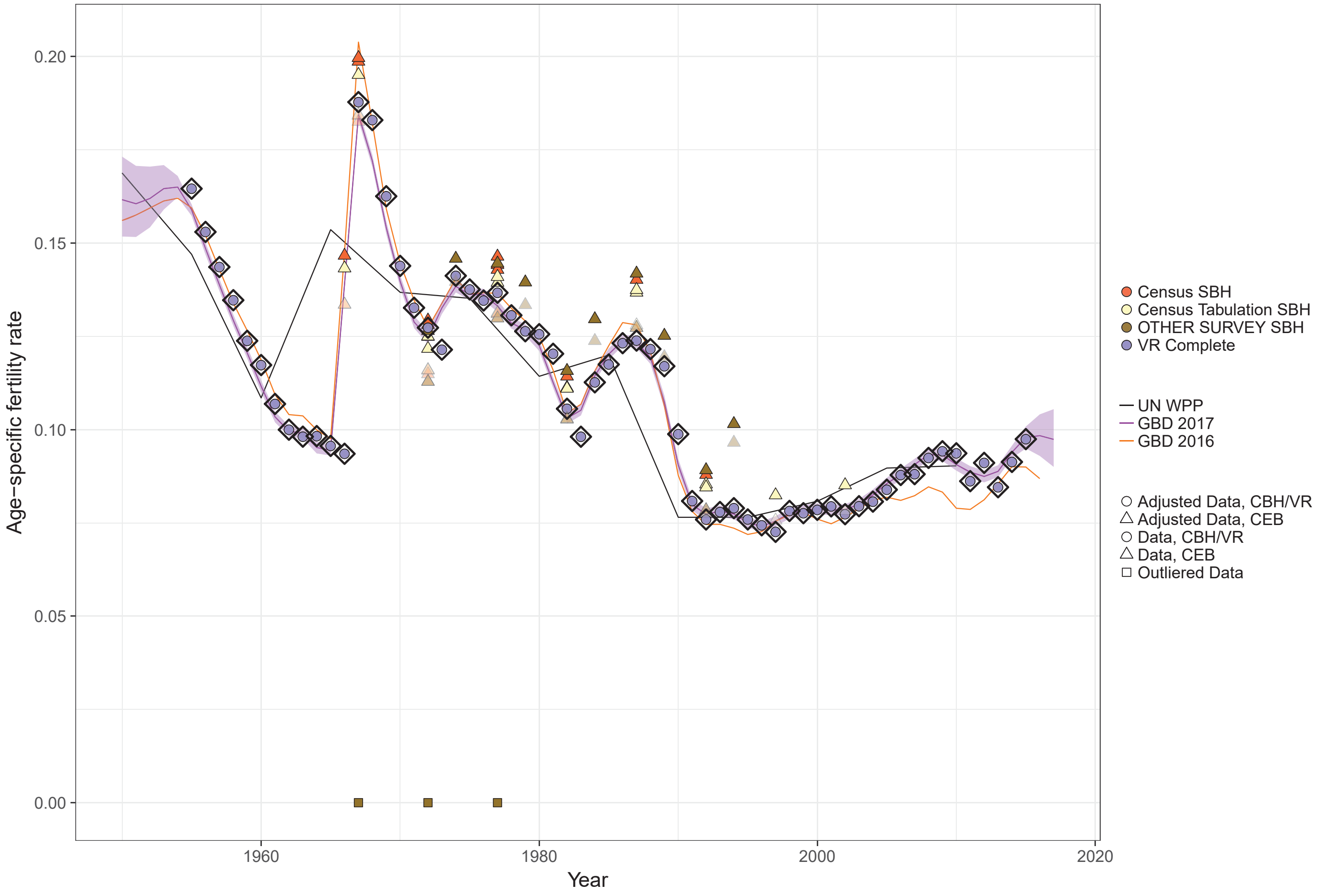

Age−specific fertility fitted trend lines, 1950−2017: Romania, Ages: 30−34
Data points enclosed with a diamond are reference sources

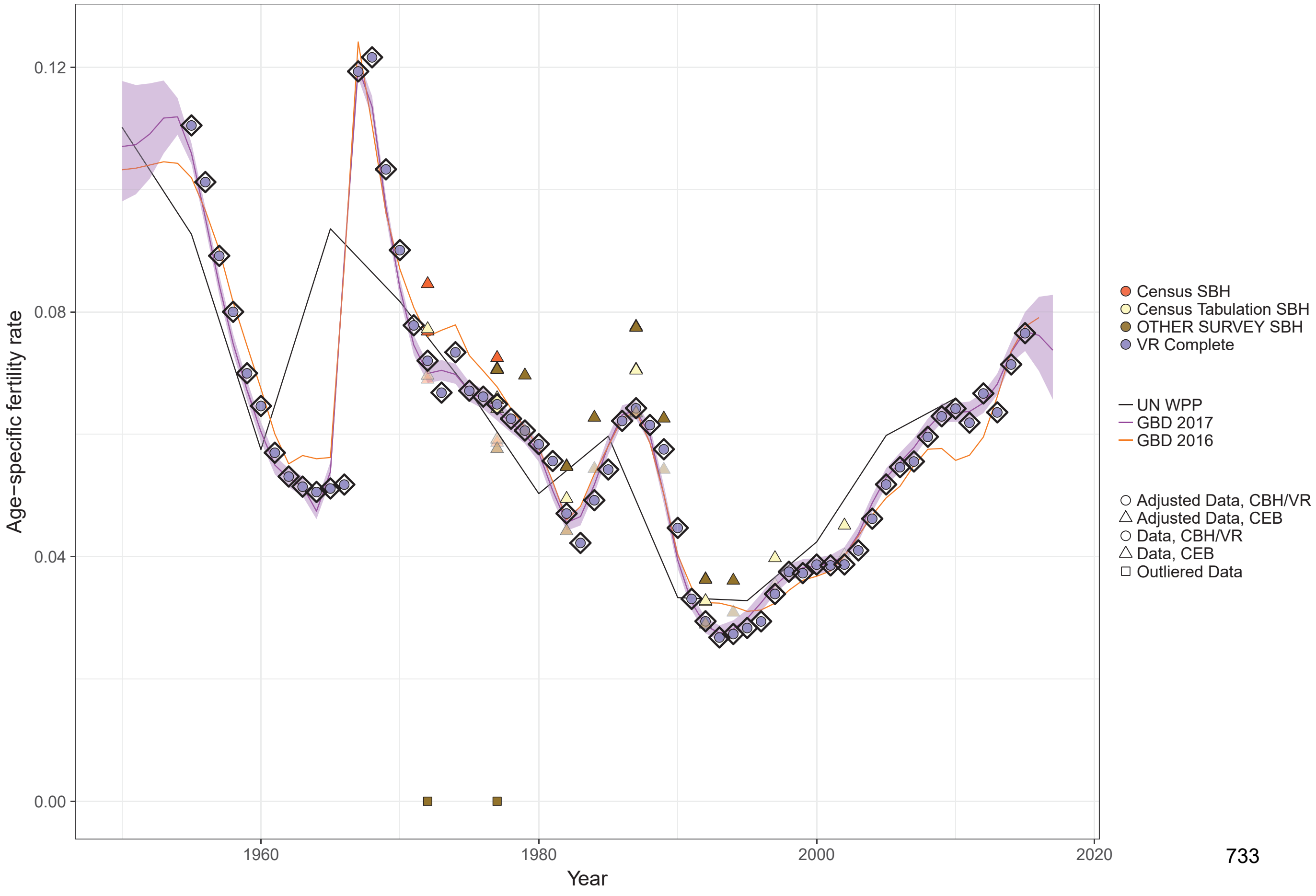

Age−specific fertility fitted trend lines, 1950−2017: Romania, Ages: 35−39
Data points enclosed with a diamond are reference sources

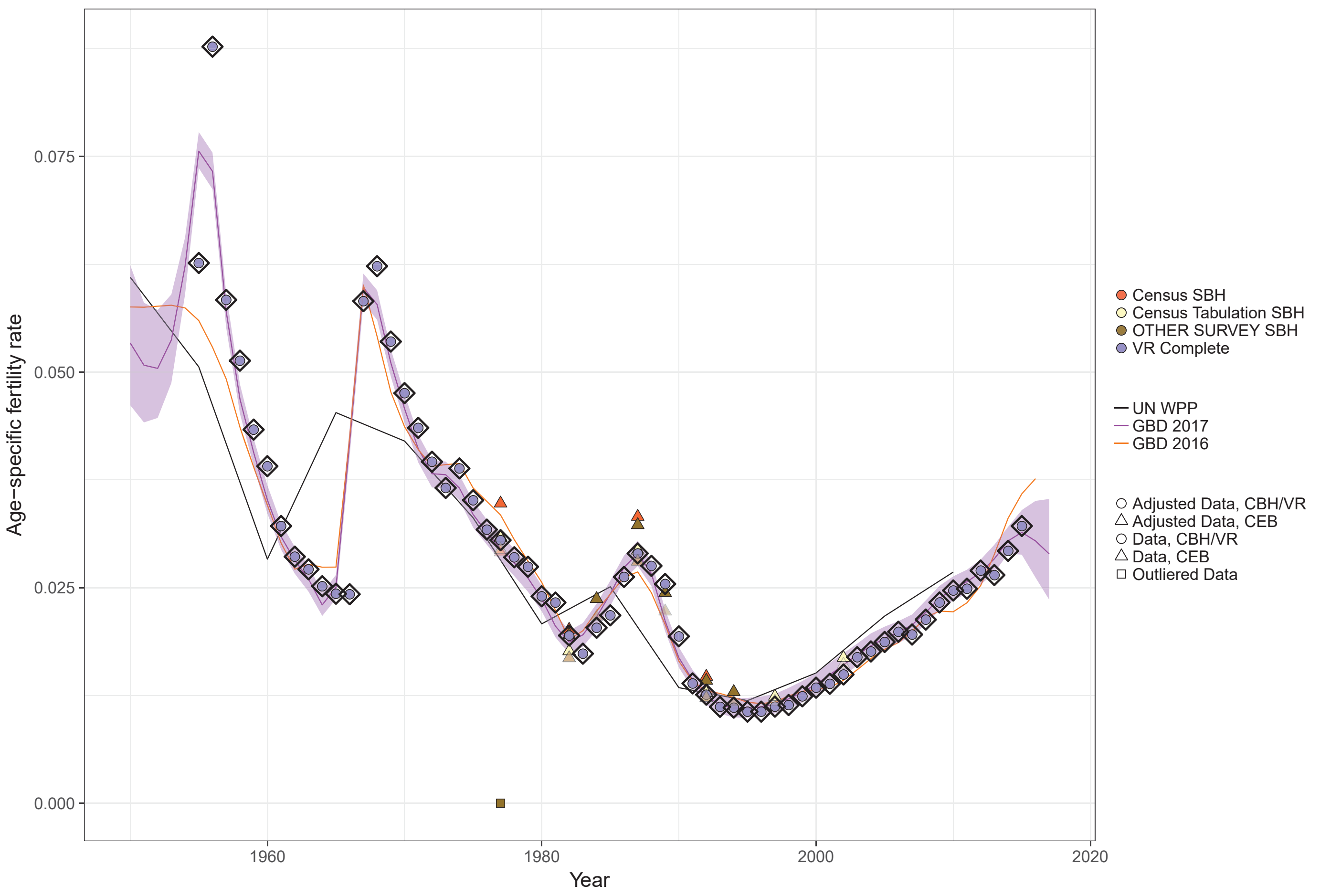

Age−specific fertility fitted trend lines, 1950−2017: Romania, Ages: 40−44
Data points enclosed with a diamond are reference sources

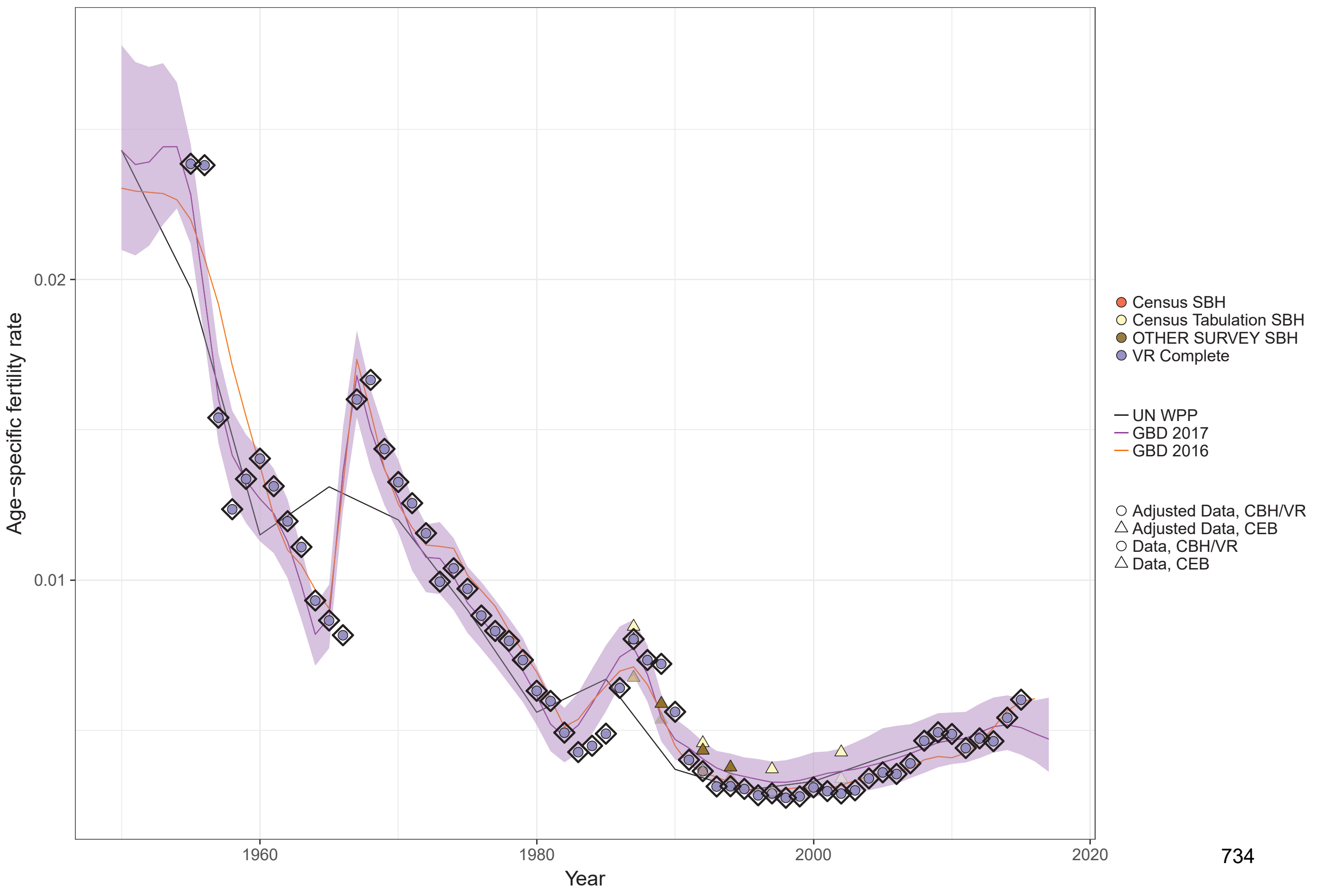

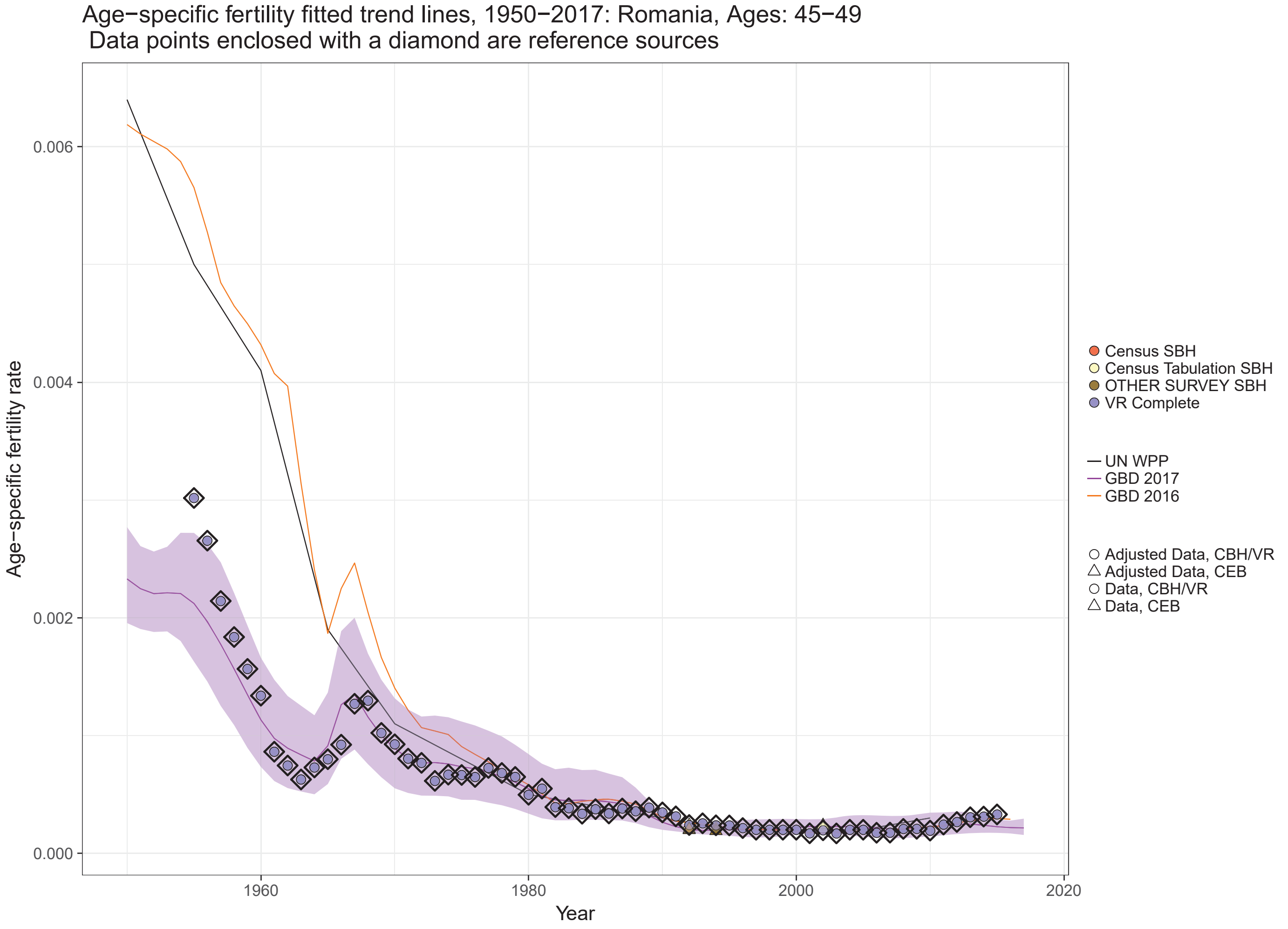
Age−specific fertility fitted trend lines, 1950−2017: Romania, Ages: 45−49
Data points enclosed with a diamond are reference sources
Age−specific fertility rate
0.000
0.002
0.004
0.006
1960
1980
2000
2020
Year
Census SBH
Census Tabulation SBH
OTHER SURVEY SBH
VR Complete
UN WPP
GBD 2017
GBD 2016
Adjusted Data, CBH/VR
Adjusted Data, CEB
Data, CBH/VR
Data, CEB

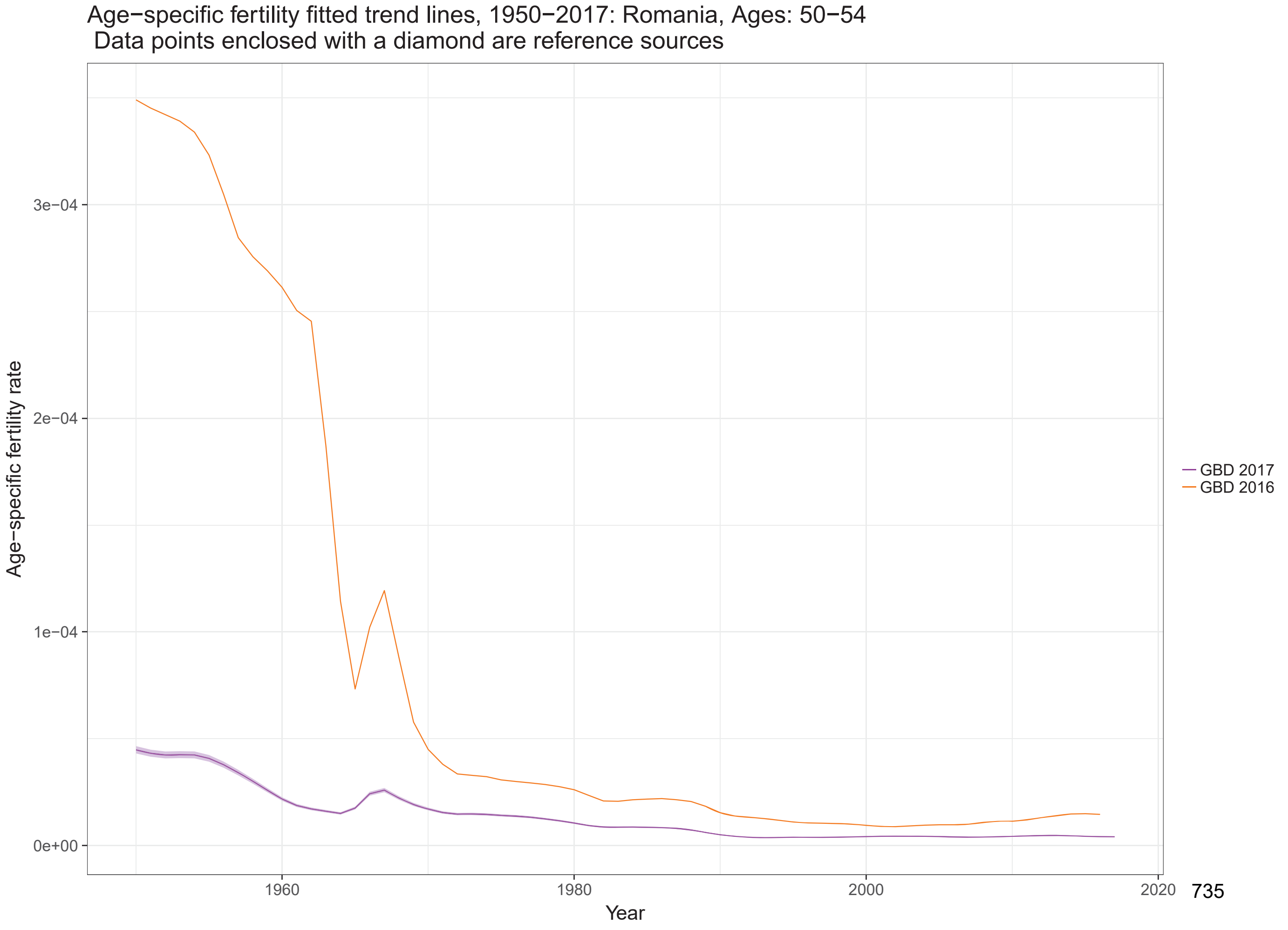
Age−specific fertility fitted trend lines, 1950−2017: Romania, Ages: 50−54
Data points enclosed with a diamond are reference sources
Age−specific fertility rate
0e+00
1e−04
2e−04
3e−04
1960
1980
2000
2020
Year
GBD 2017
GBD 2016

Age−specific fertility fitted trend lines, 1950−2017: Russian Federation, TFR
Data points enclosed with a diamond are reference sources

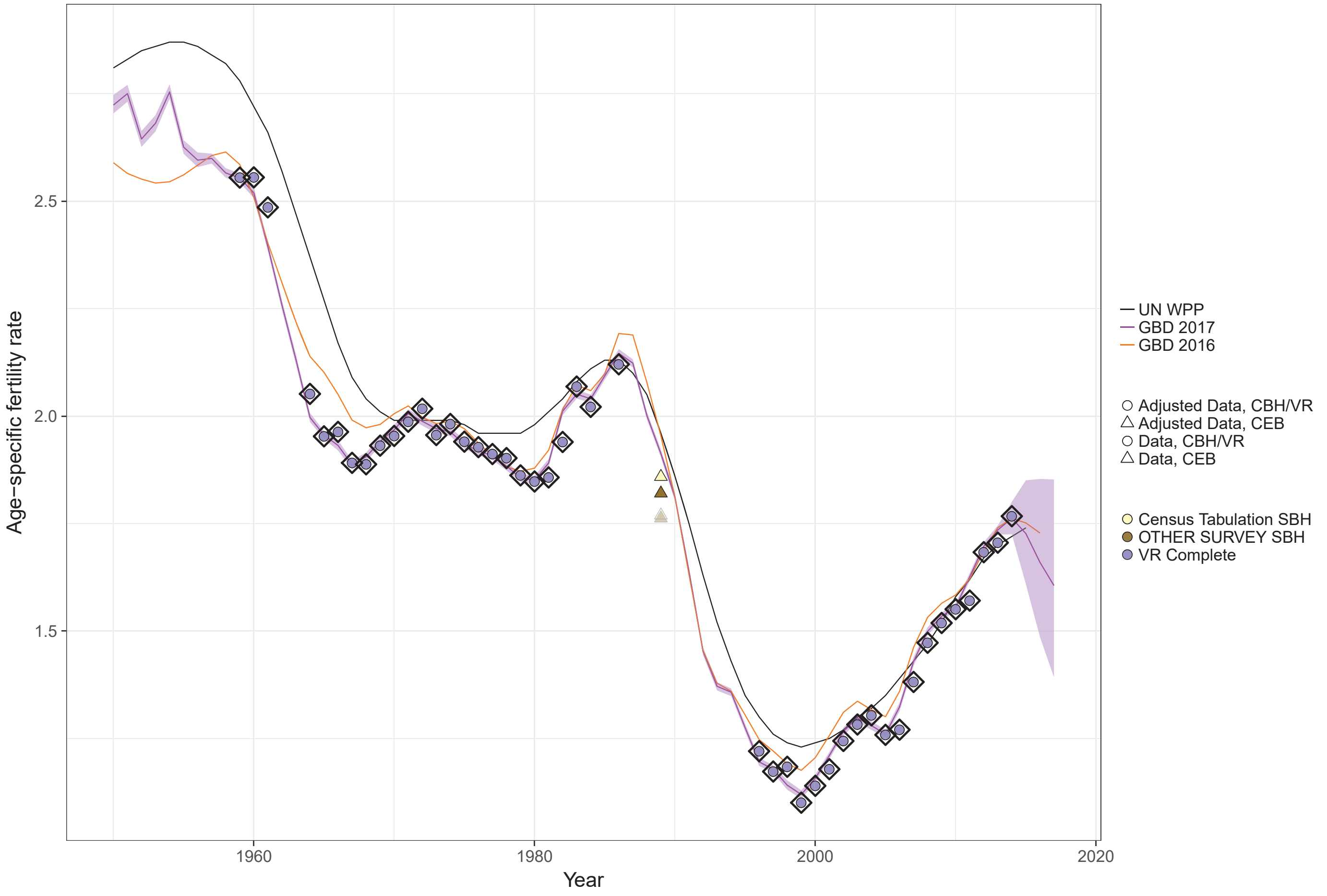

Age−specific fertility fitted trend lines, 1950−2017: Russian Federation, Ages: 10−14
Data points enclosed with a diamond are reference sources

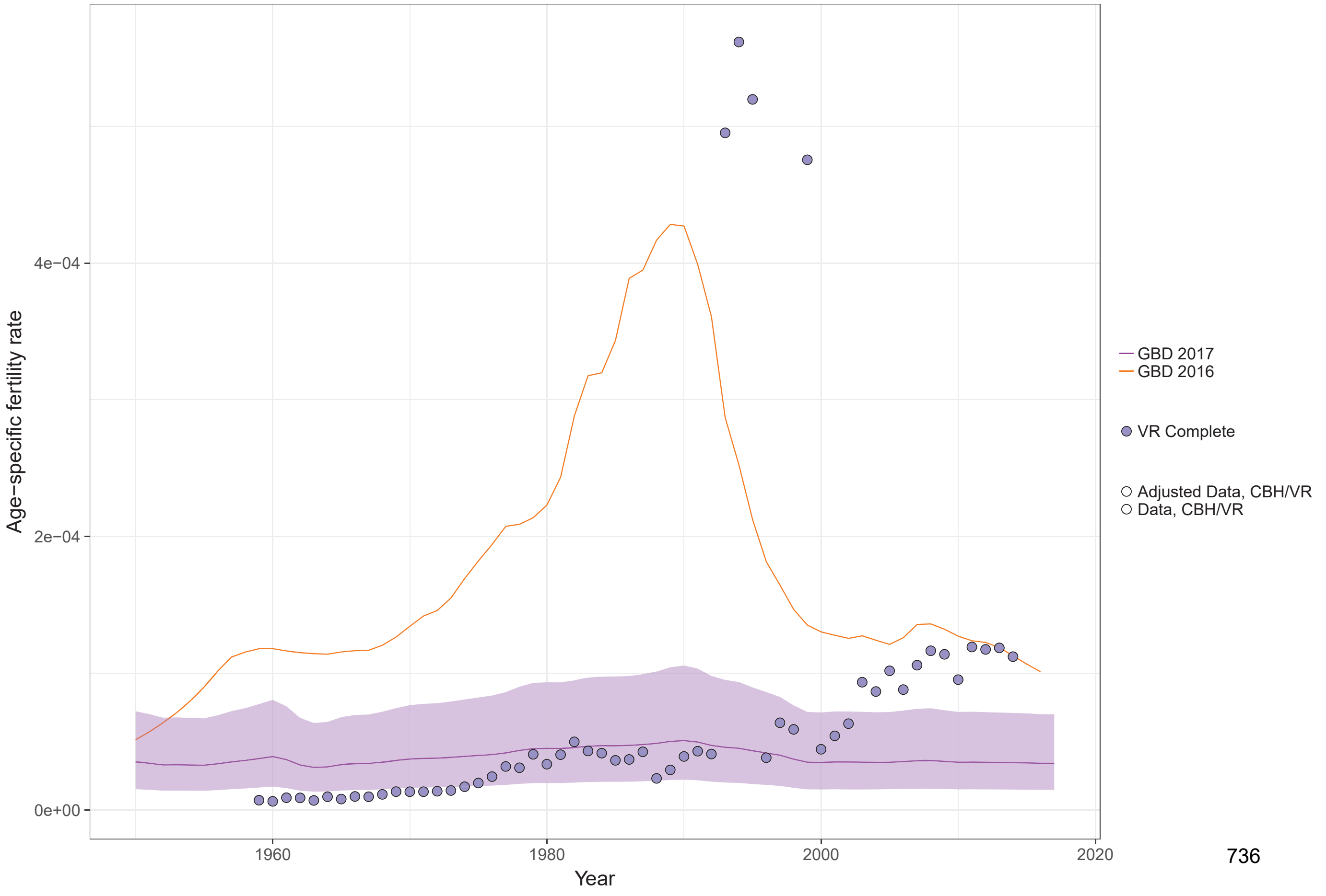

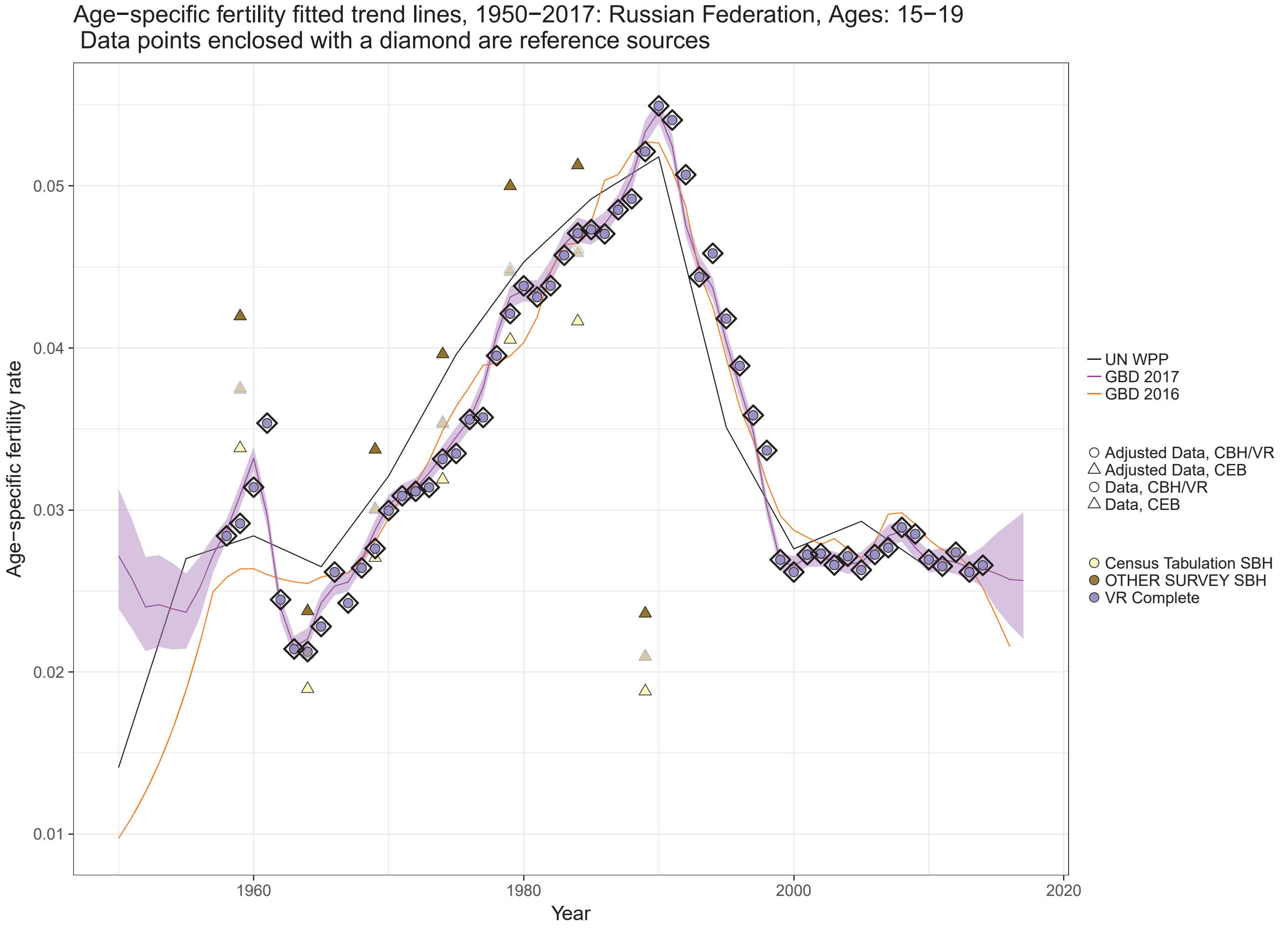
Age−specific fertility fitted trend lines, 1950−2017: Russian Federation, Ages: 15−19
Data points enclosed with a diamond are reference sources
Age-specific fertility rate
Year
UN WPP
GBD 2017
GBD 2016
Adjusted Data, CBH/VR
Adjusted Data, CEB
Data, CBH/VR
Data, CEB
Census Tabulation SBH
OTHER SURVEY SBH
VR Complete

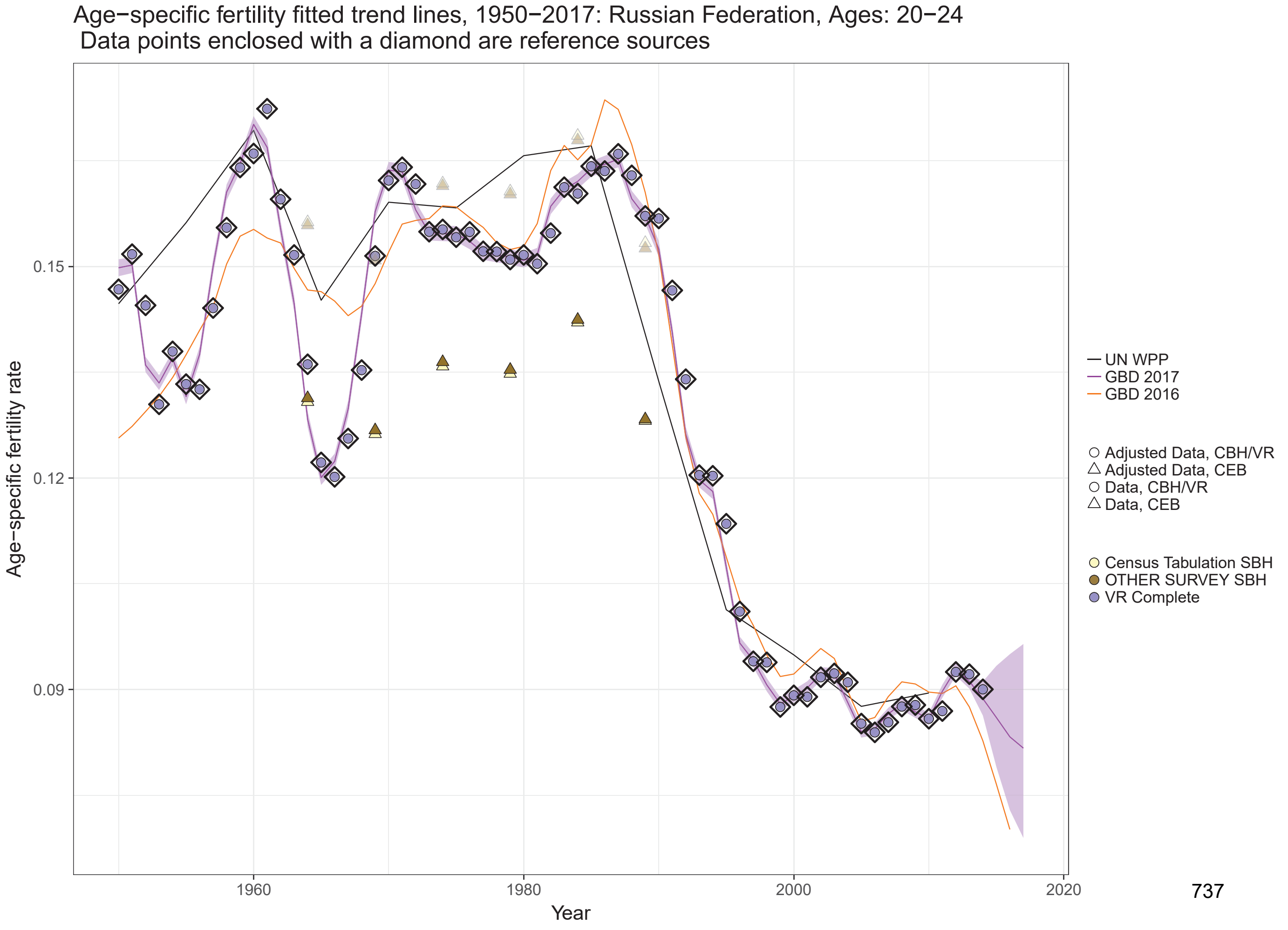
Age−specific fertility fitted trend lines, 1950−2017: Russian Federation, Ages: 20−24
Data points enclosed with a diamond are reference sources
Age-specific fertility rate
Year
UN WPP
GBD 2017
GBD 2016
Adjusted Data, CBH/VR
Adjusted Data, CEB
Data, CBH/VR
Data, CEB
Census Tabulation SBH
OTHER SURVEY SBH
VR Complete

Age−specific fertility fitted trend lines, 1950−2017: Russian Federation, Ages: 25−29
Data points enclosed with a diamond are reference sources

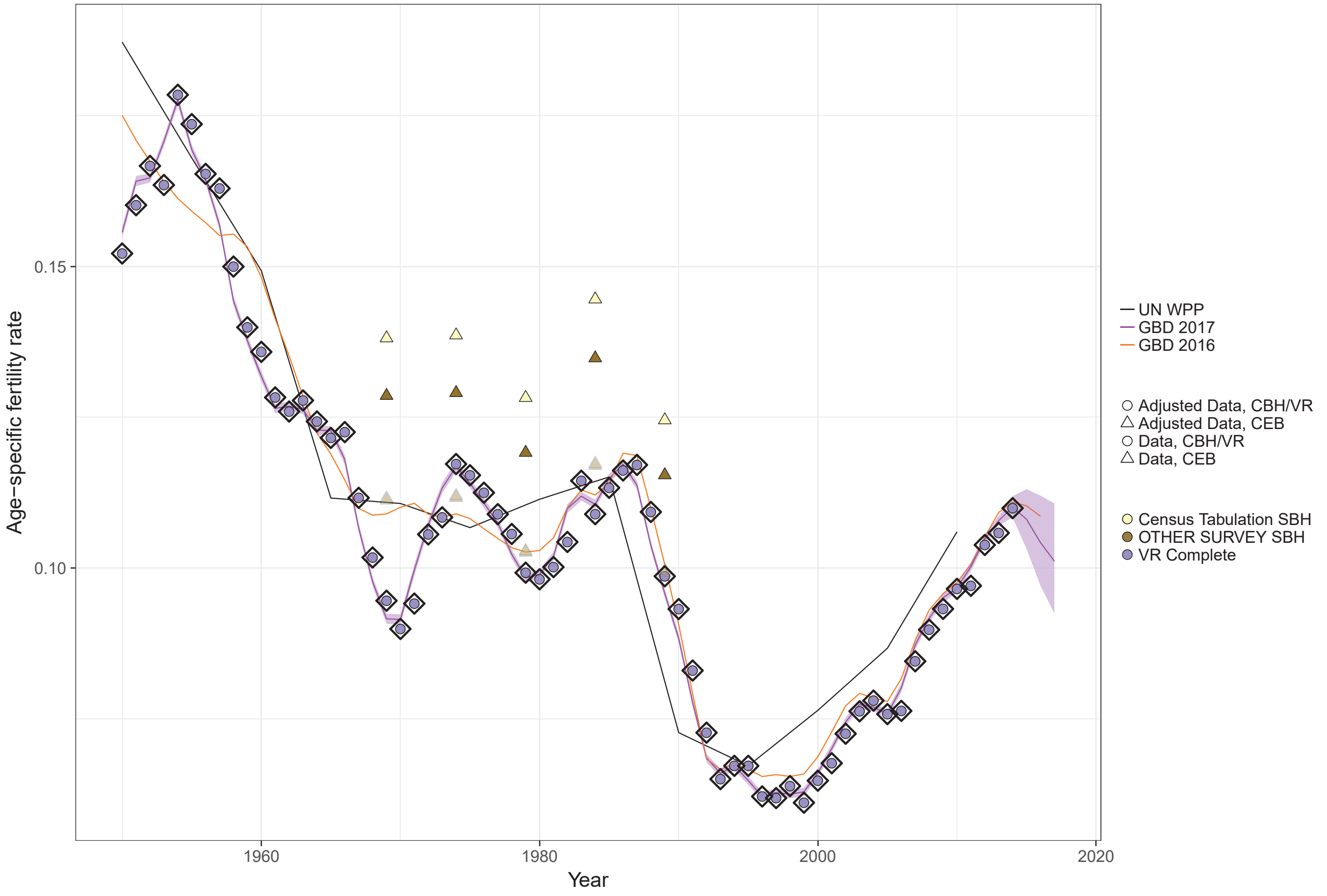

Age−specific fertility fitted trend lines, 1950−2017: Russian Federation, Ages: 30−34
Data points enclosed with a diamond are reference sources

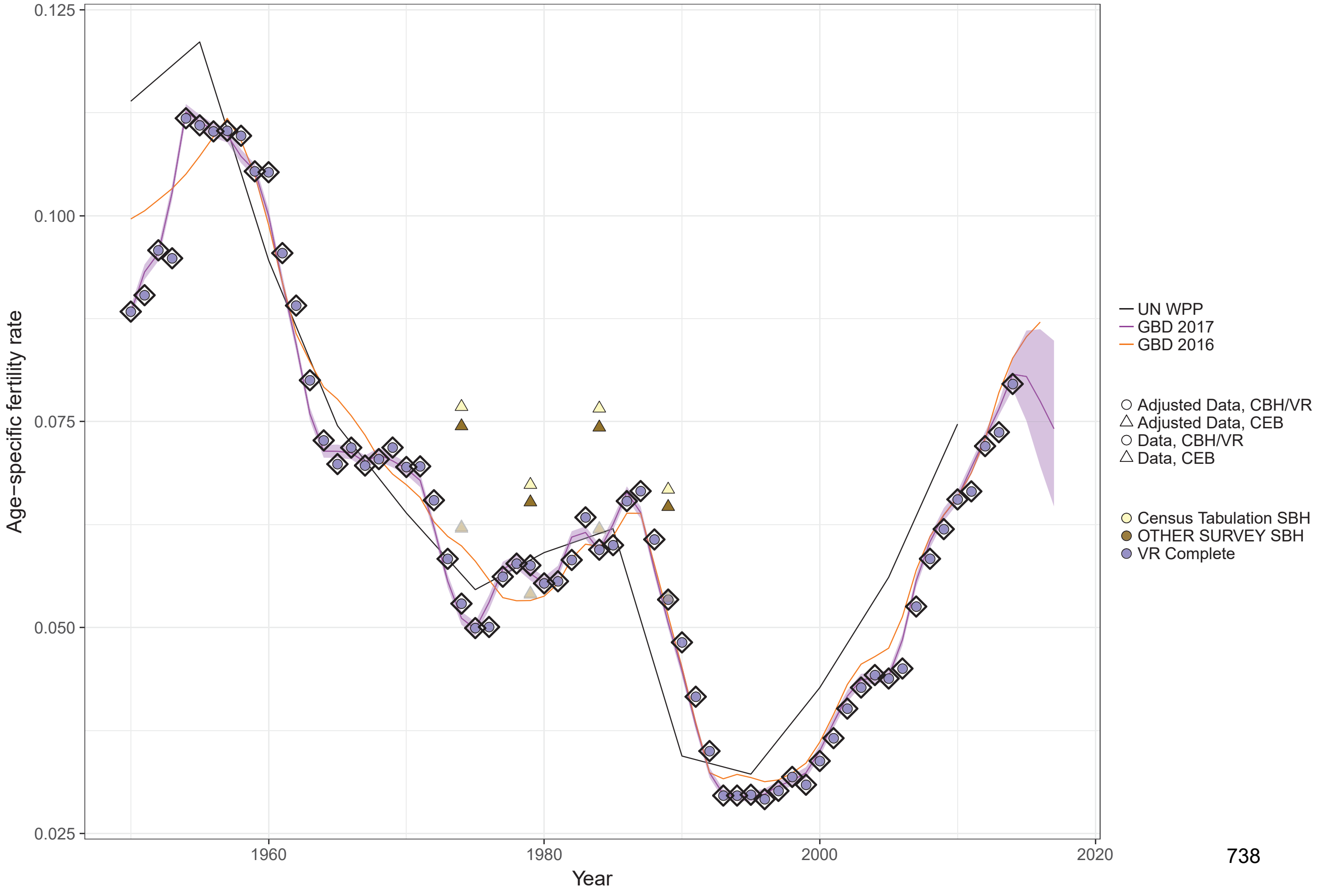

Age−specific fertility fitted trend lines, 1950−2017: Russian Federation, Ages: 35−39
Data points enclosed with a diamond are reference sources

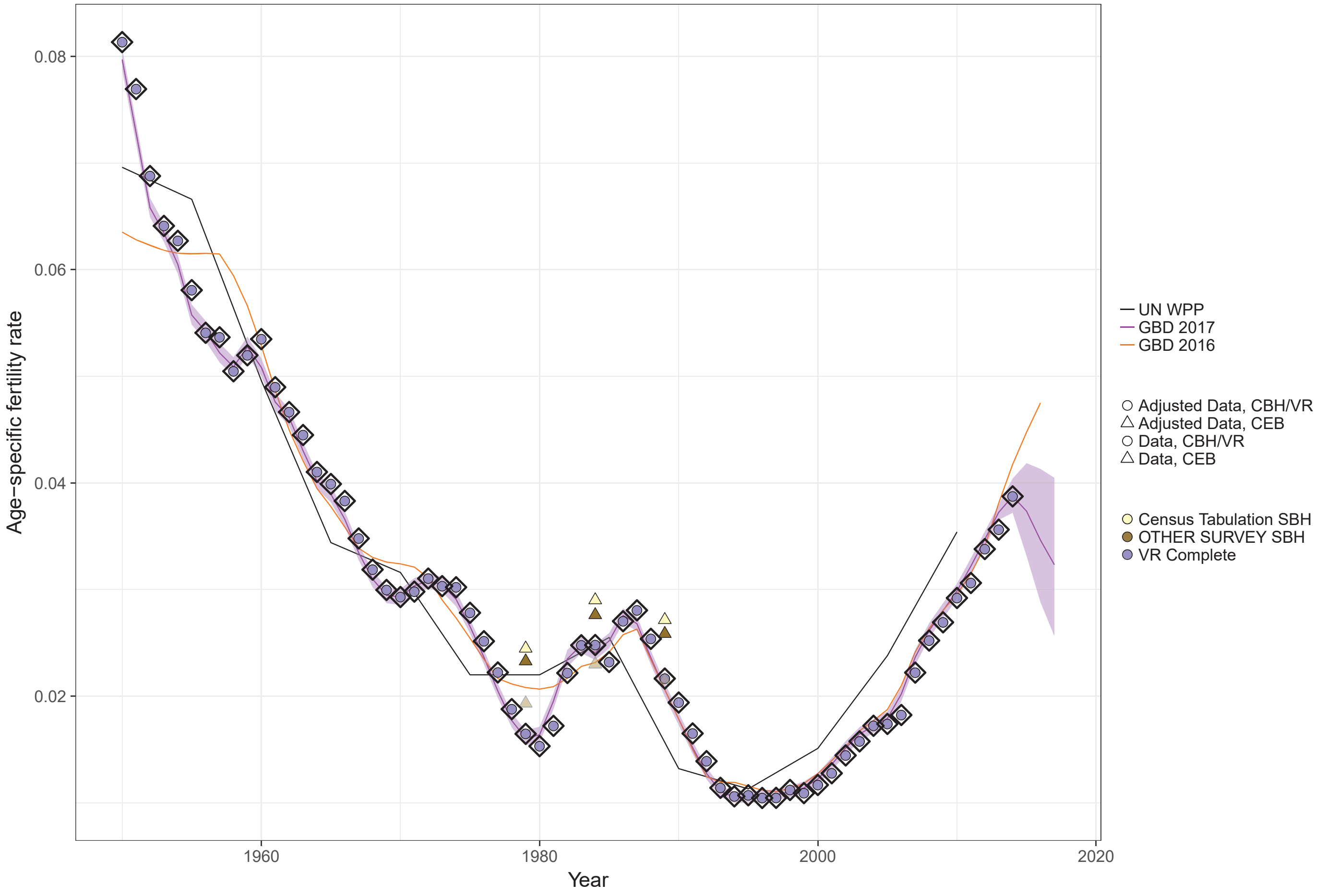

Age−specific fertility fitted trend lines, 1950−2017: Russian Federation, Ages: 40−44
Data points enclosed with a diamond are reference sources

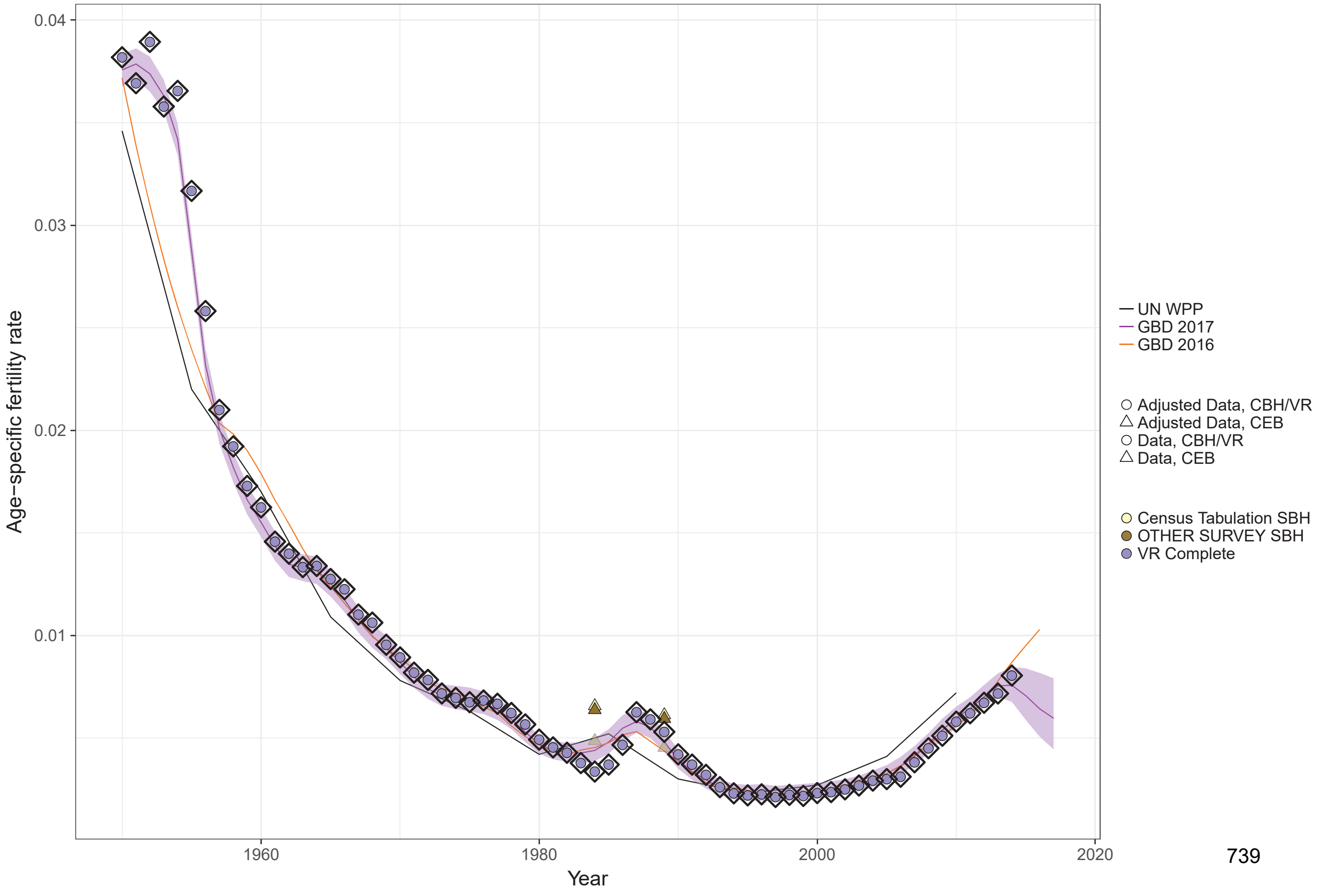

Age−specific fertility fitted trend lines, 1950−2017: Russian Federation, Ages: 45−49
Data points enclosed with a diamond are reference sources

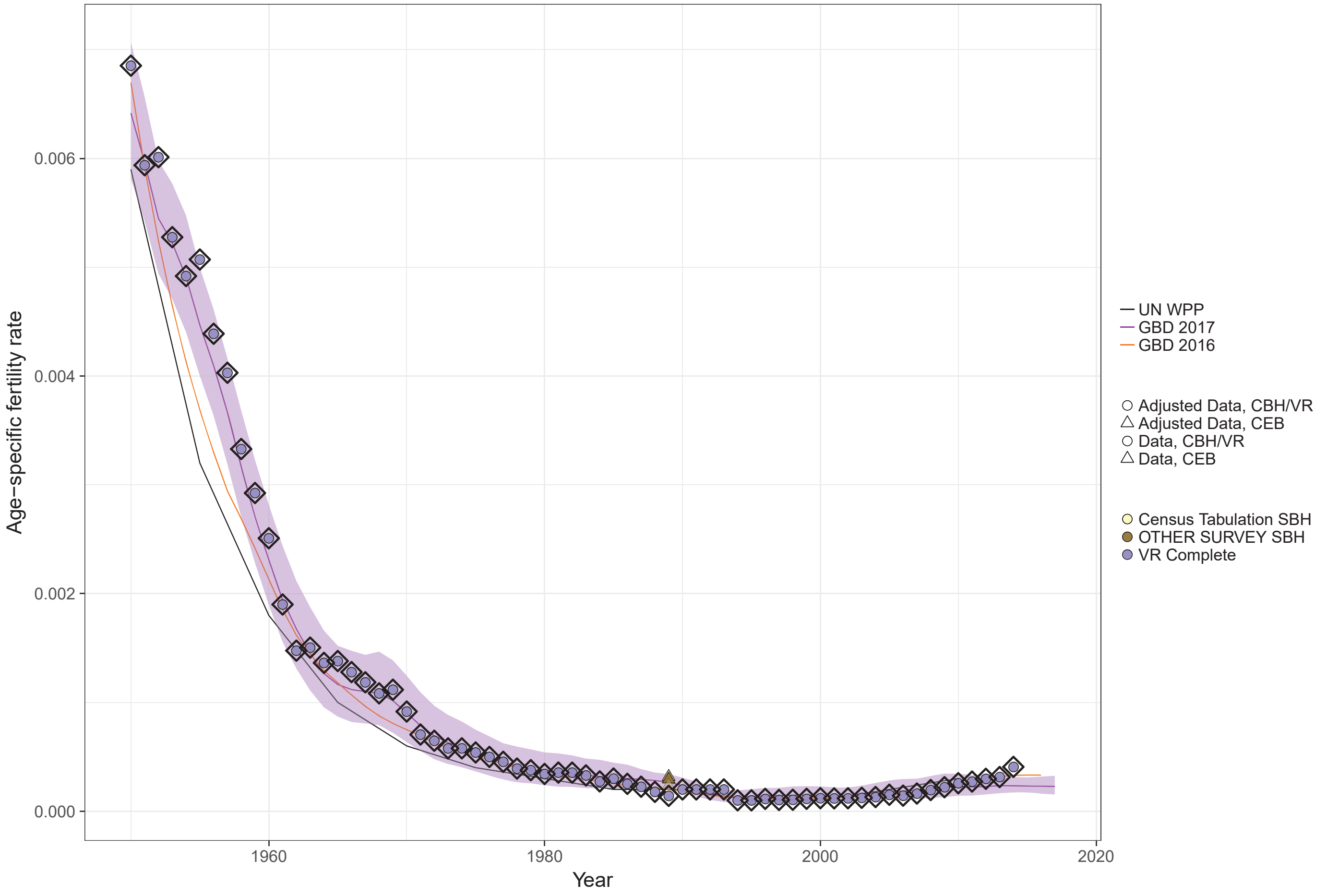

Age−specific fertility fitted trend lines, 1950−2017: Russian Federation, Ages: 50−54
Data points enclosed with a diamond are reference sources

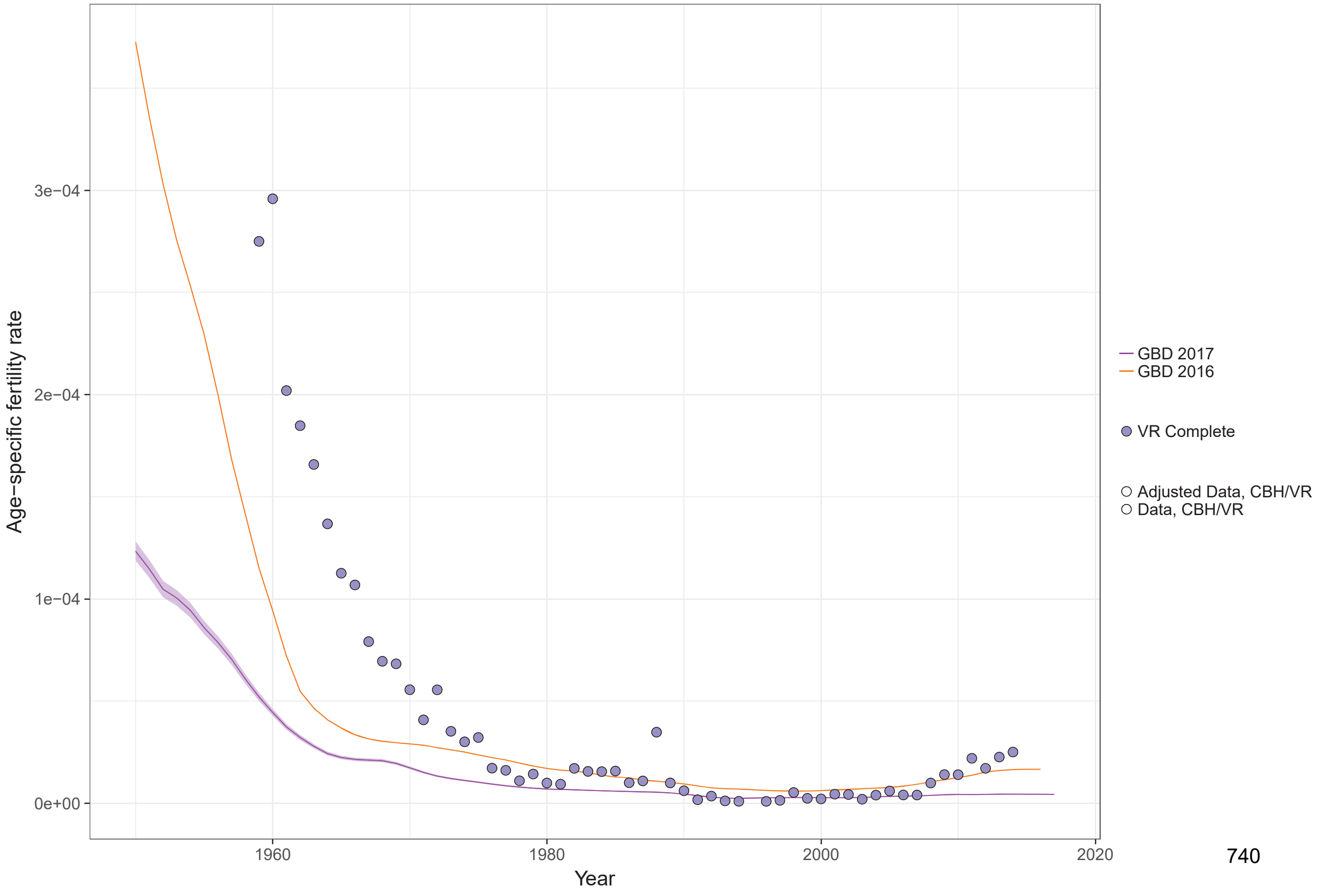

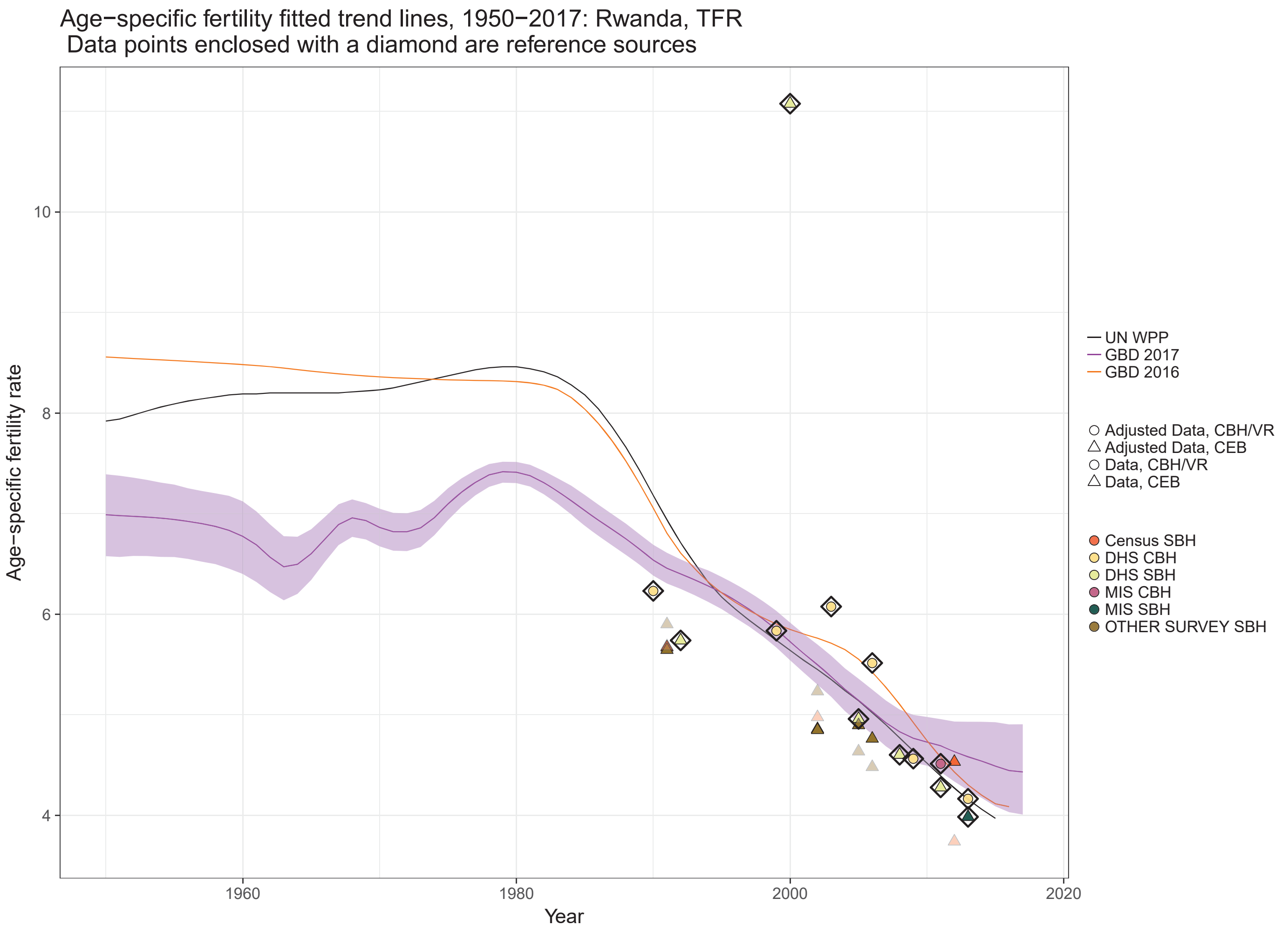

Age−specific fertility fitted trend lines, 1950−2017: Rwanda, TFR
Data points enclosed with a diamond are reference sources
Age−specific fertility rate
Year
4
6
8
10
1960
1980
2000
2020
UN WPP
GBD 2017
GBD 2016
Adjusted Data, CBH/VR
Adjusted Data, CEB
Data, CBH/VR
Data, CEB
Census SBH
DHS CBH
DHS SBH
MIS CBH
MIS SBH
OTHER SURVEY SBH

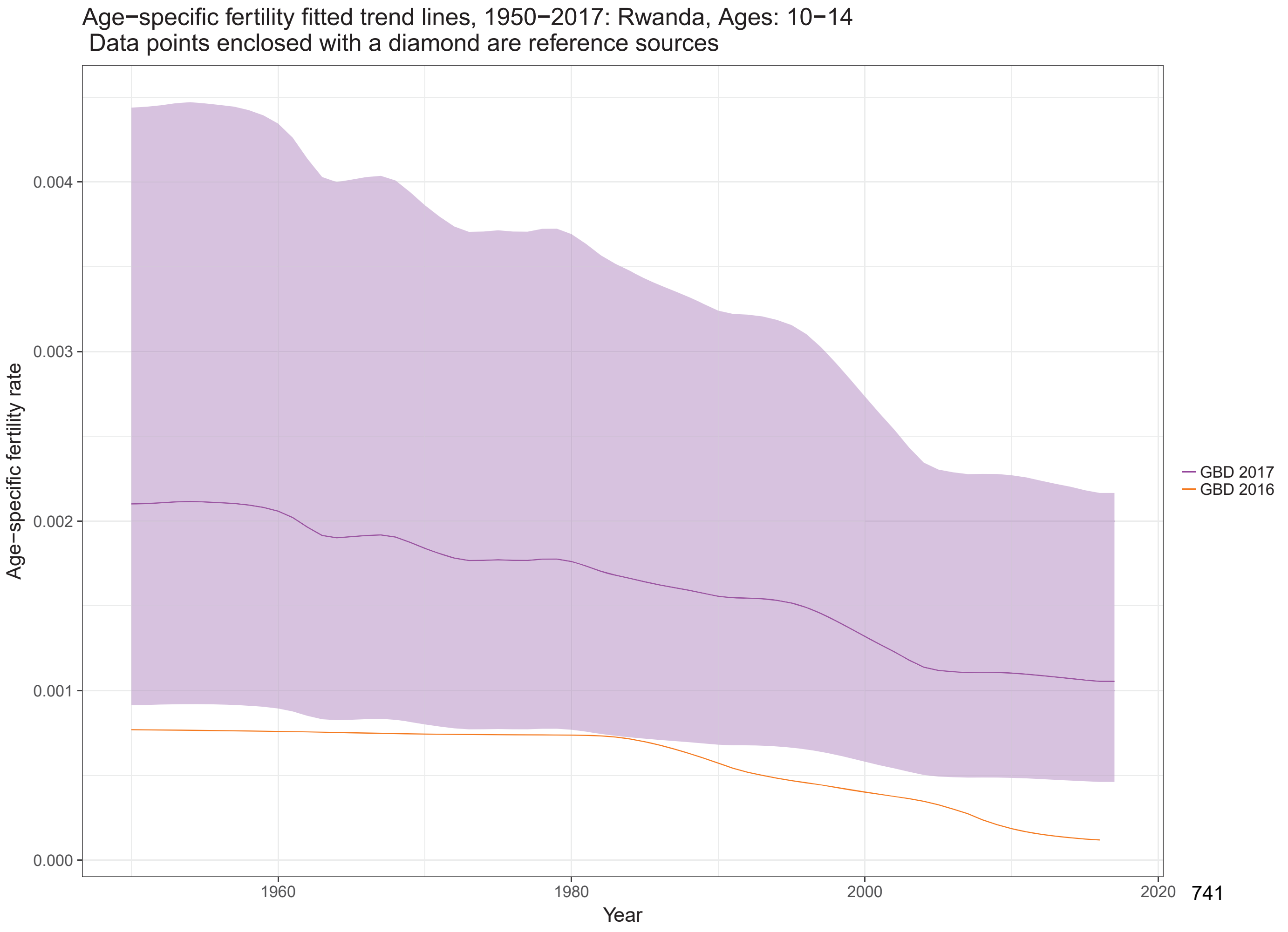

Age−specific fertility fitted trend lines, 1950−2017: Rwanda, Ages: 10−14
Data points enclosed with a diamond are reference sources
Age−specific fertility rate
Year
0.000
0.001
0.002
0.003
0.004
1960
1980
2000
2020
GBD 2017
GBD 2016

Age−specific fertility fitted trend lines, 1950−2017: Rwanda, Ages: 15−19
Data points enclosed with a diamond are reference sources

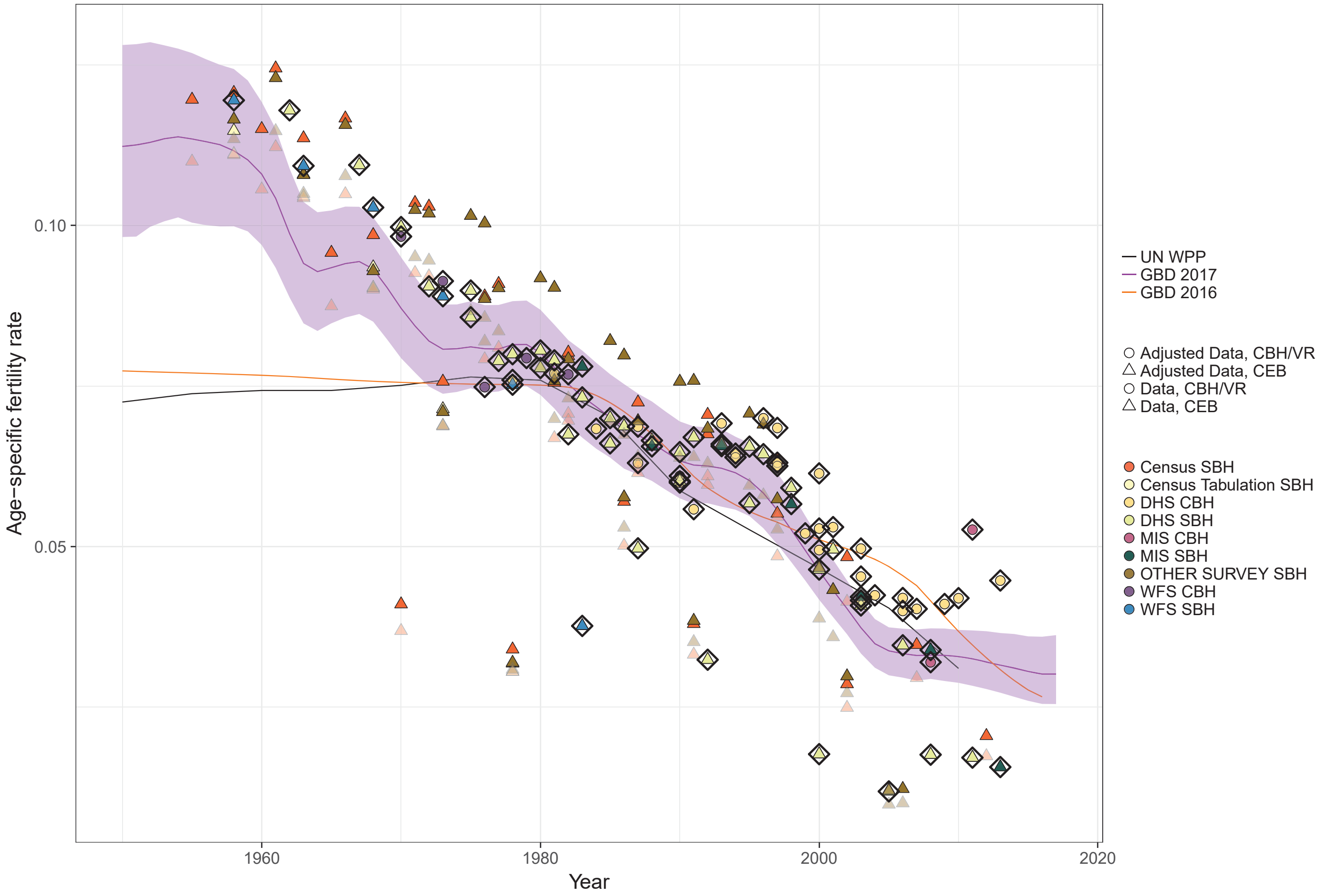

Age−specific fertility fitted trend lines, 1950−2017: Rwanda, Ages: 20−24
Data points enclosed with a diamond are reference sources

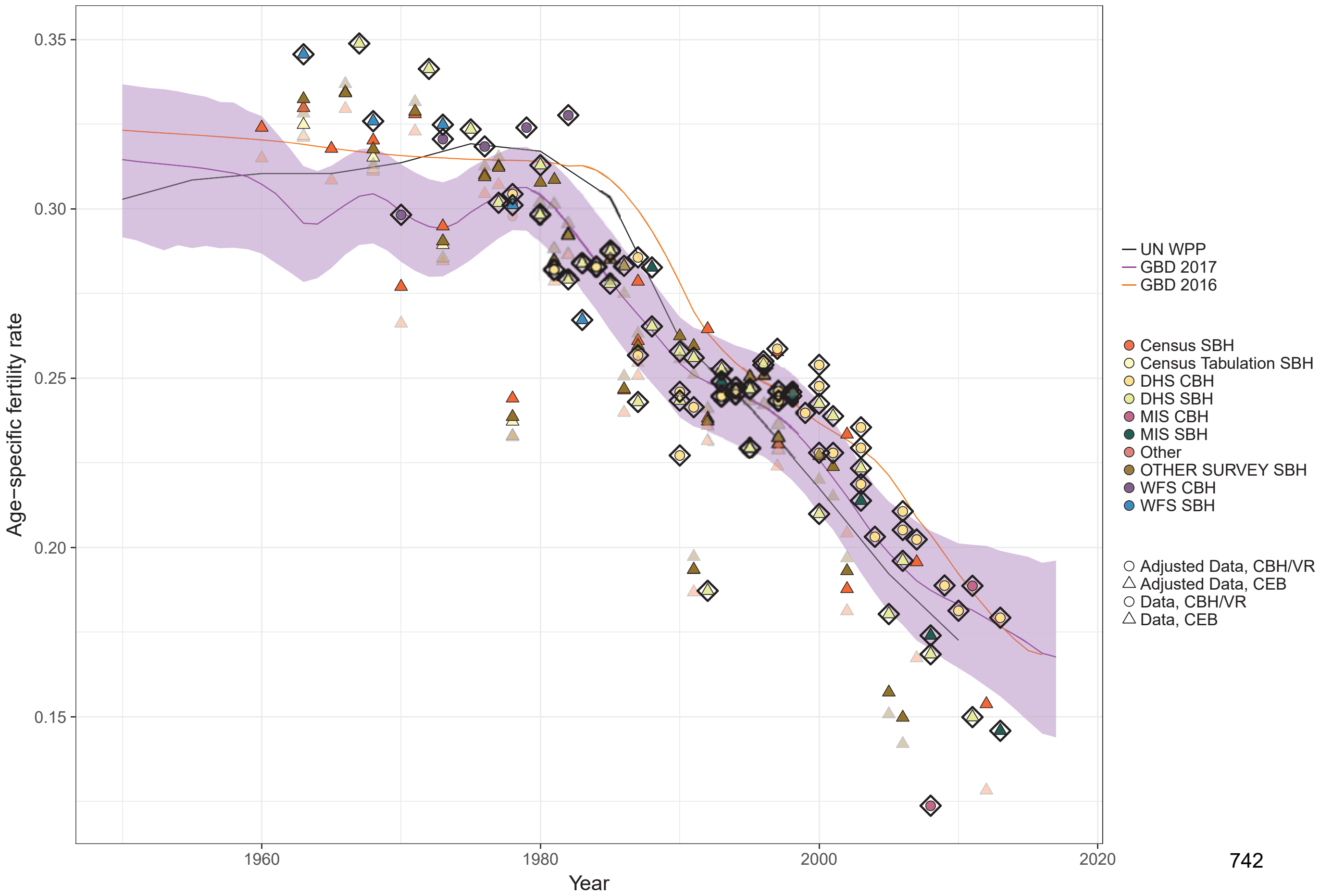

Age−specific fertility fitted trend lines, 1950−2017: Rwanda, Ages: 25−29
Data points enclosed with a diamond are reference sources

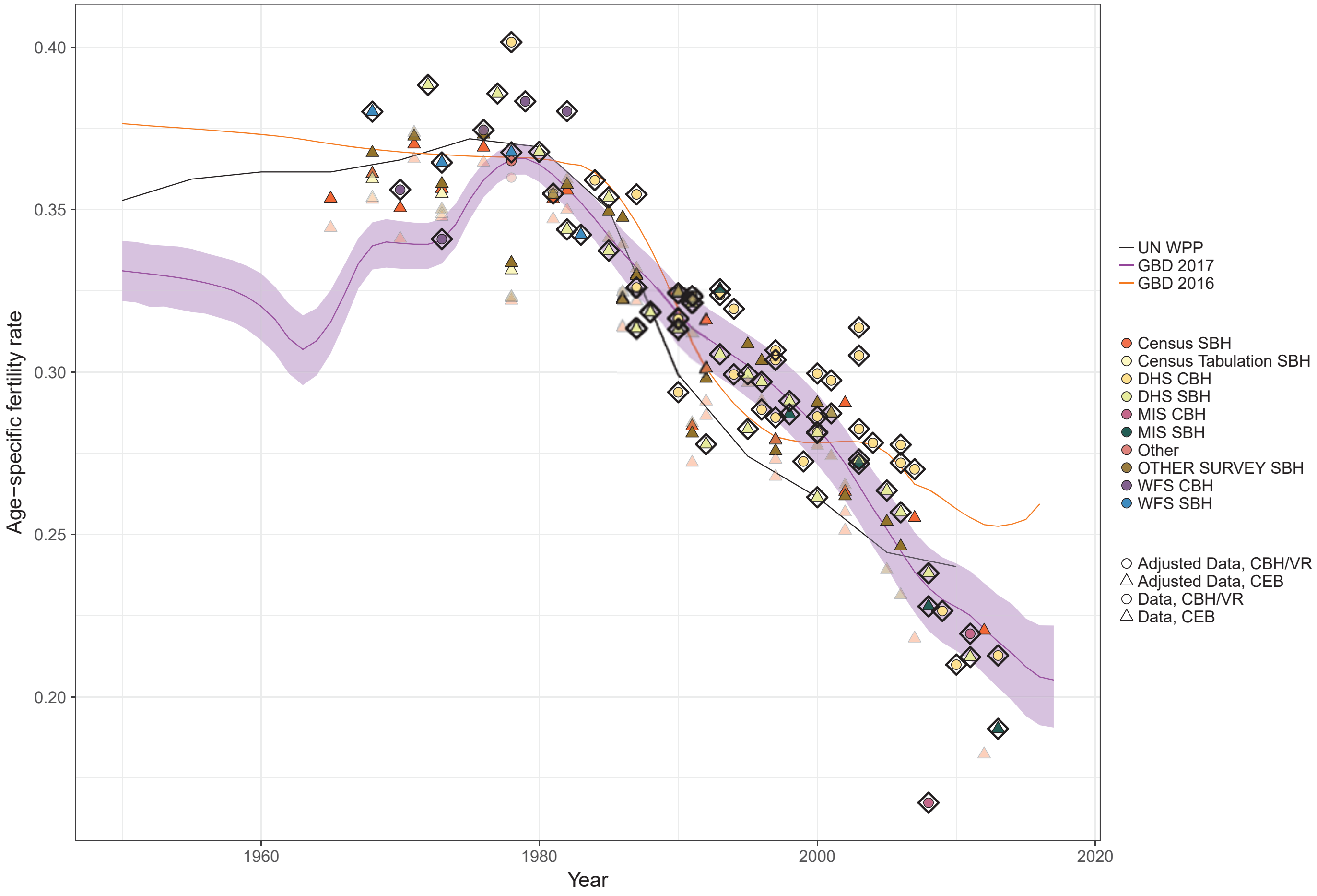

Age−specific fertility fitted trend lines, 1950−2017: Rwanda, Ages: 30−34
Data points enclosed with a diamond are reference sources

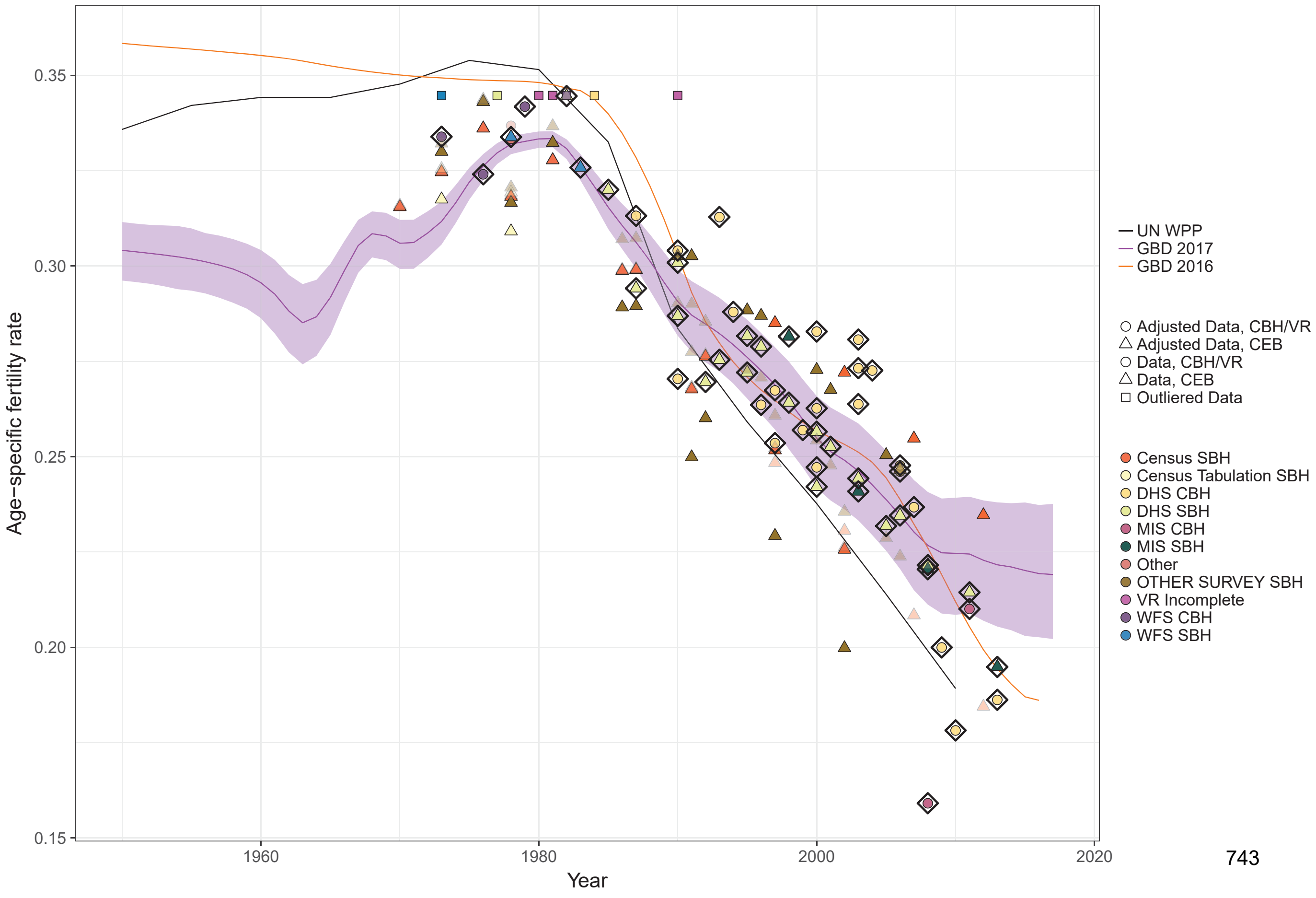

Age−specific fertility fitted trend lines, 1950−2017: Rwanda, Ages: 35−39
Data points enclosed with a diamond are reference sources

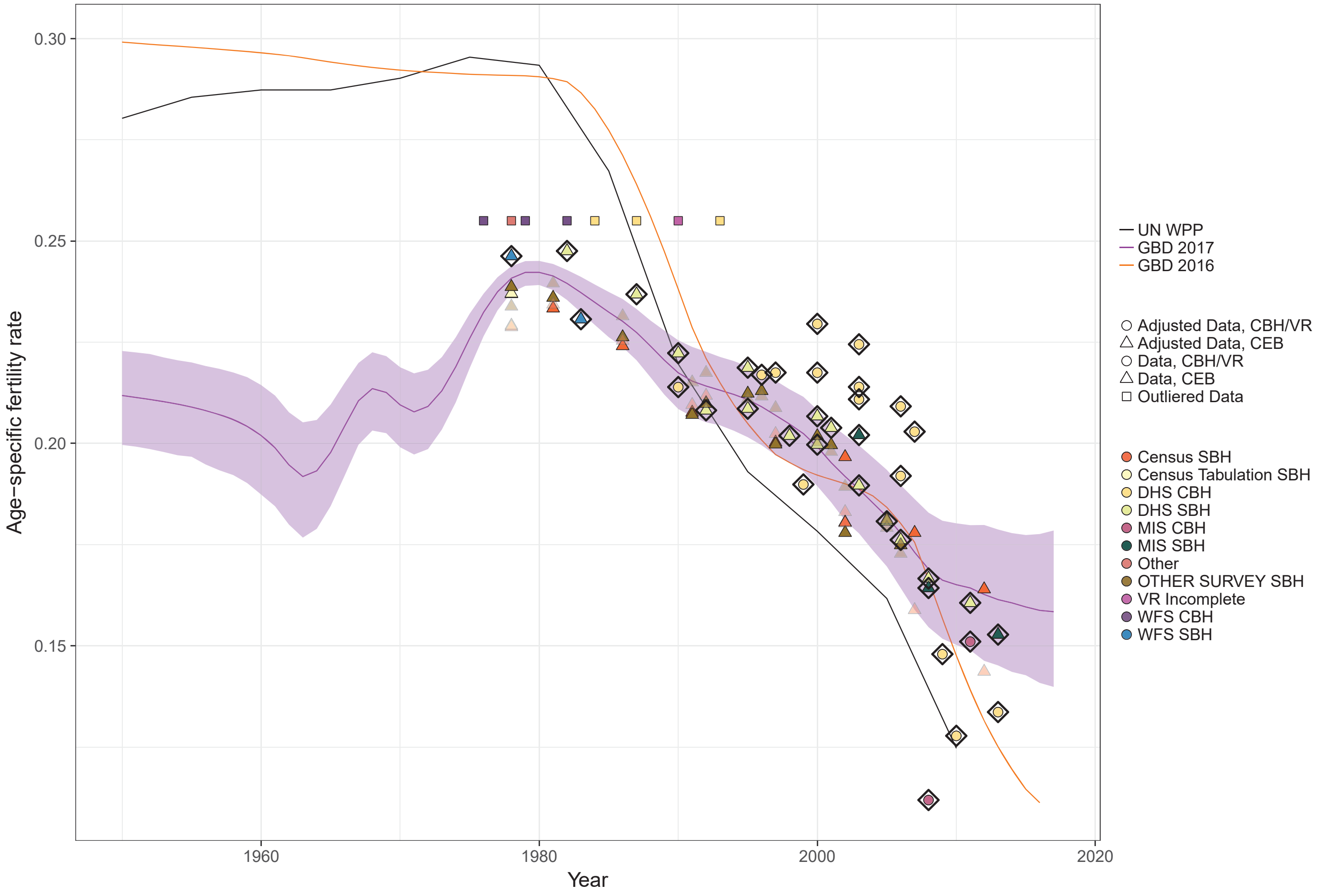

Age−specific fertility fitted trend lines, 1950−2017: Rwanda, Ages: 40−44
Data points enclosed with a diamond are reference sources

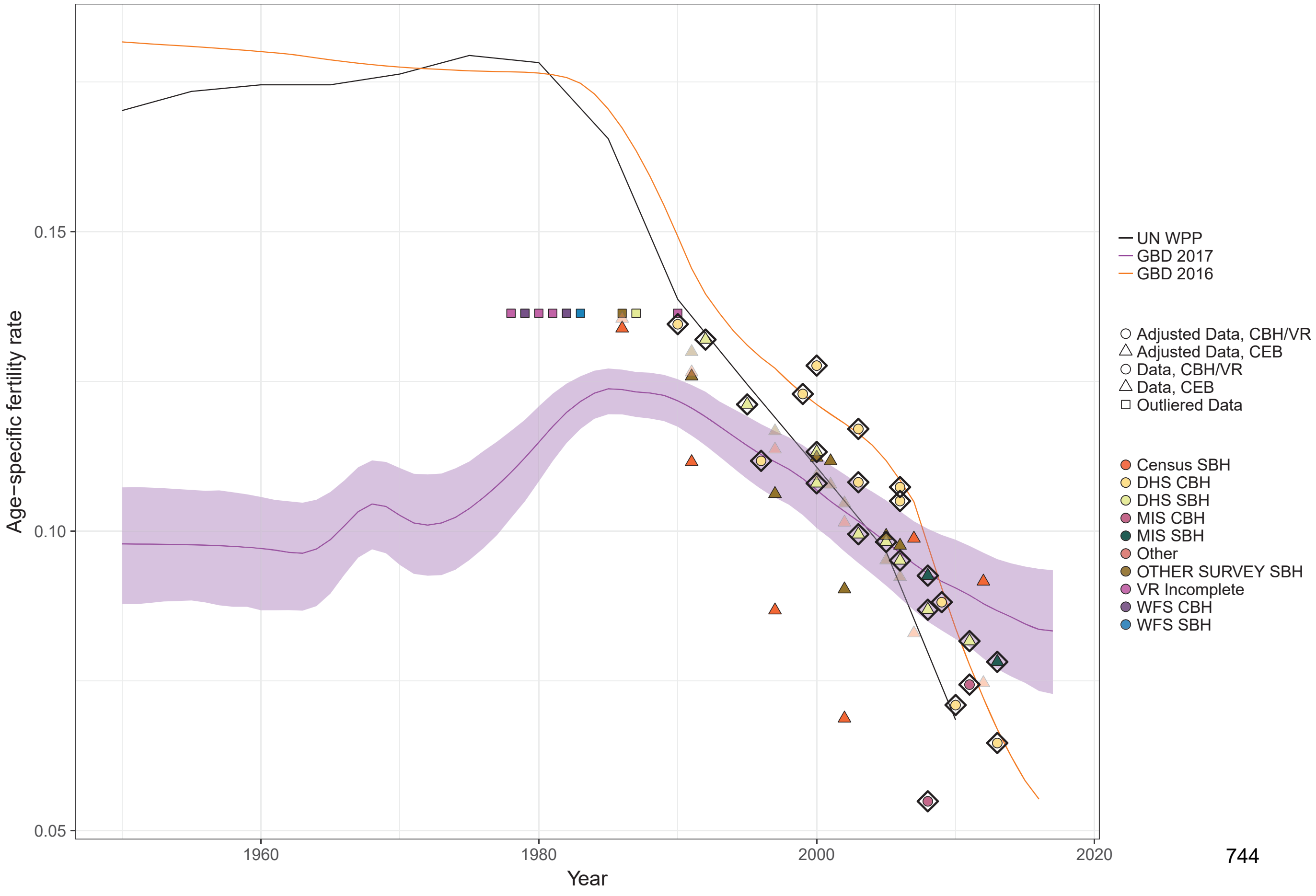

Age−specific fertility fitted trend lines, 1950−2017: Rwanda, Ages: 45−49
Data points enclosed with a diamond are reference sources

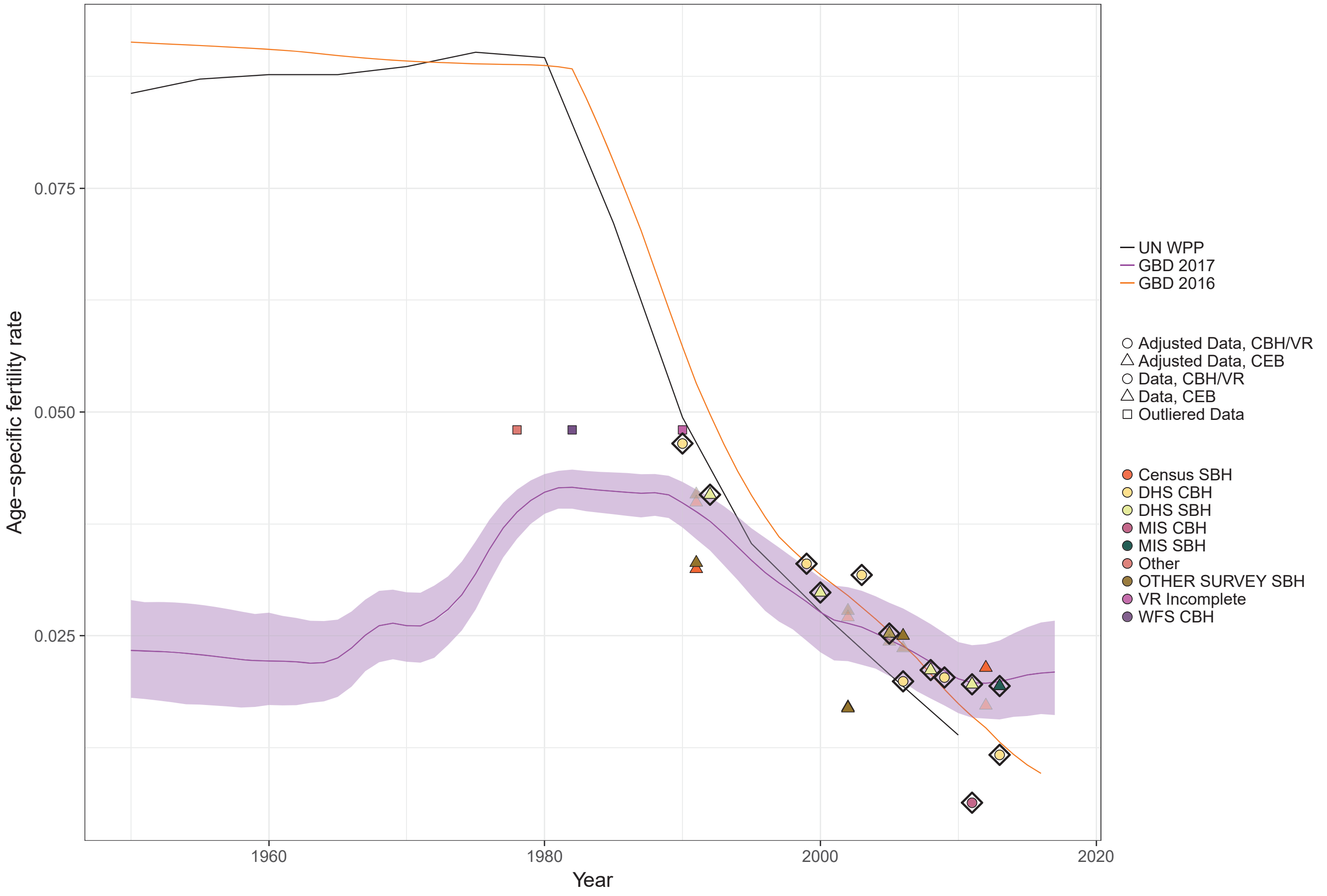

Age−specific fertility fitted trend lines, 1950−2017: Rwanda, Ages: 50−54
Data points enclosed with a diamond are reference sources

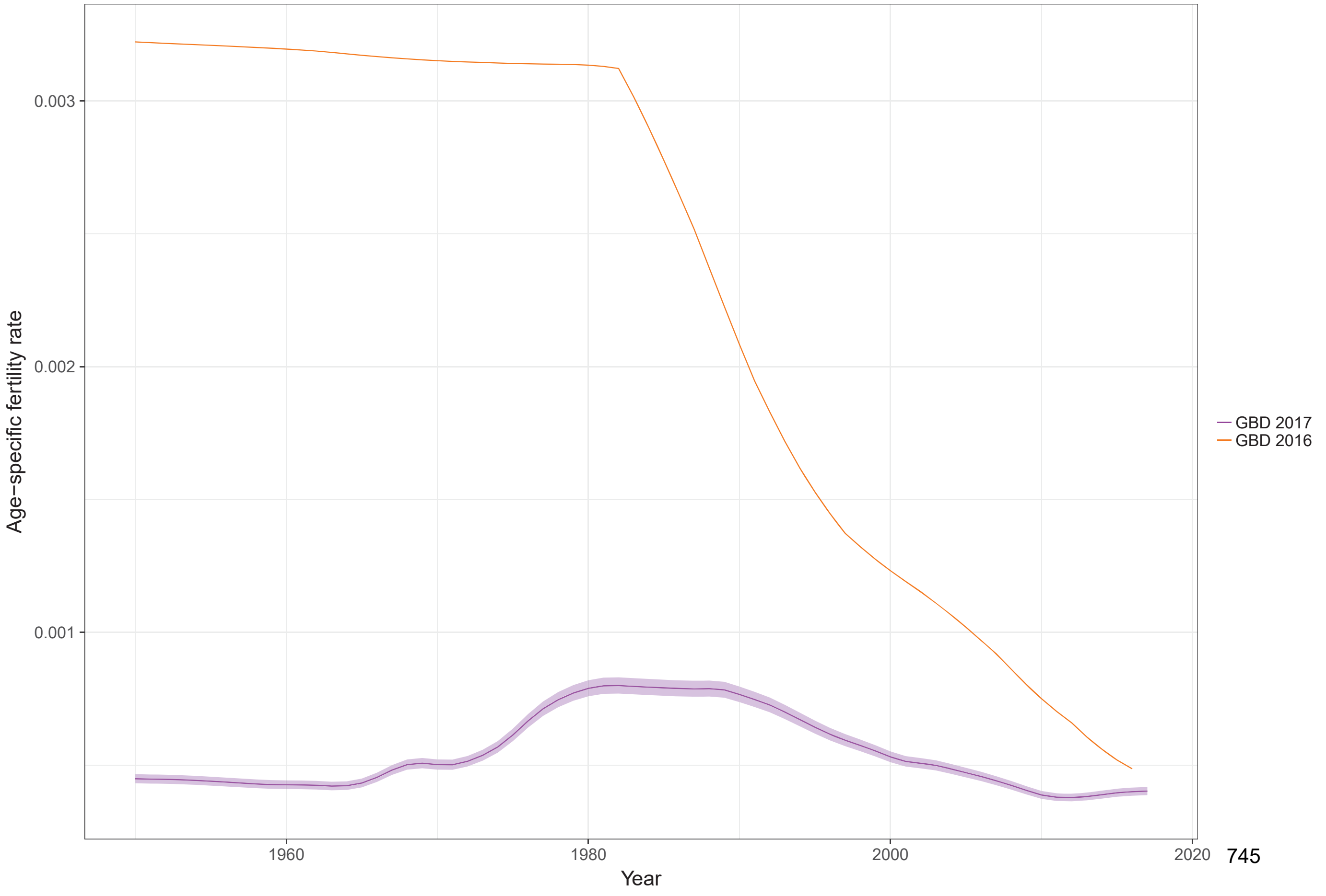

Age−specific fertility fitted trend lines, 1950−2017: Saint Lucia, TFR
Data points enclosed with a diamond are reference sources

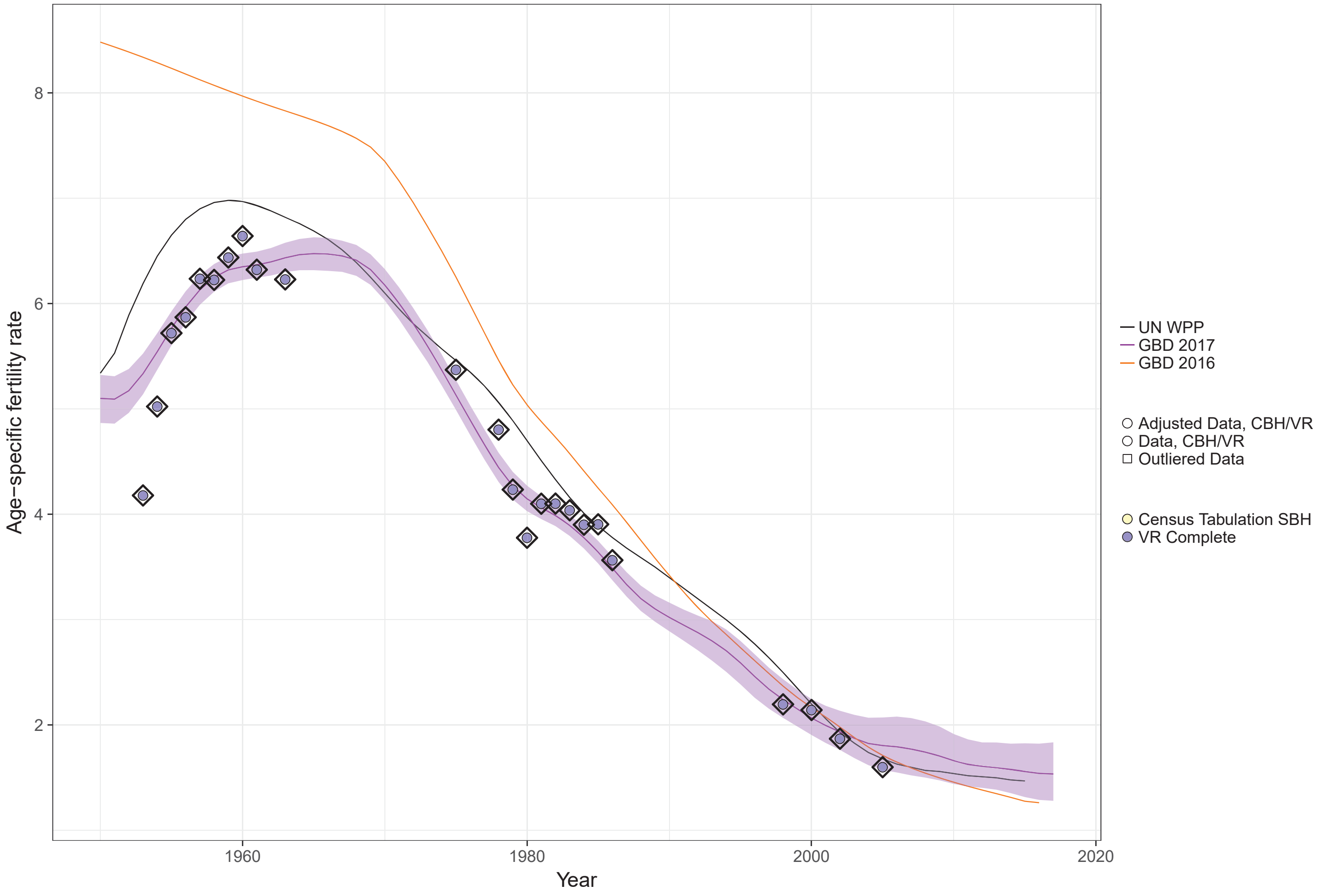

Age−specific fertility fitted trend lines, 1950−2017: Saint Lucia, Ages: 10−14
Data points enclosed with a diamond are reference sources

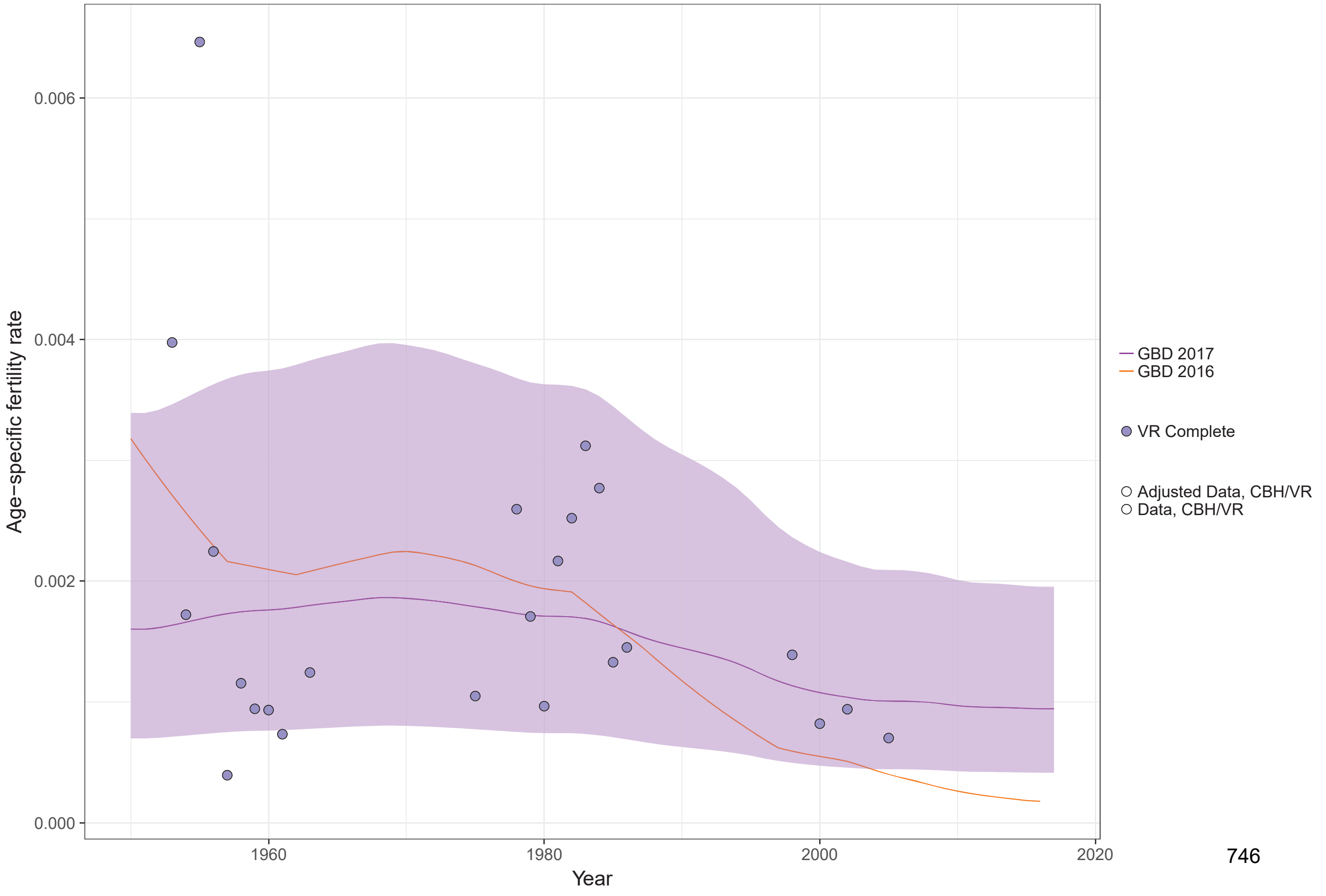

Age−specific fertility fitted trend lines, 1950−2017: Saint Lucia, Ages: 15−19
Data points enclosed with a diamond are reference sources

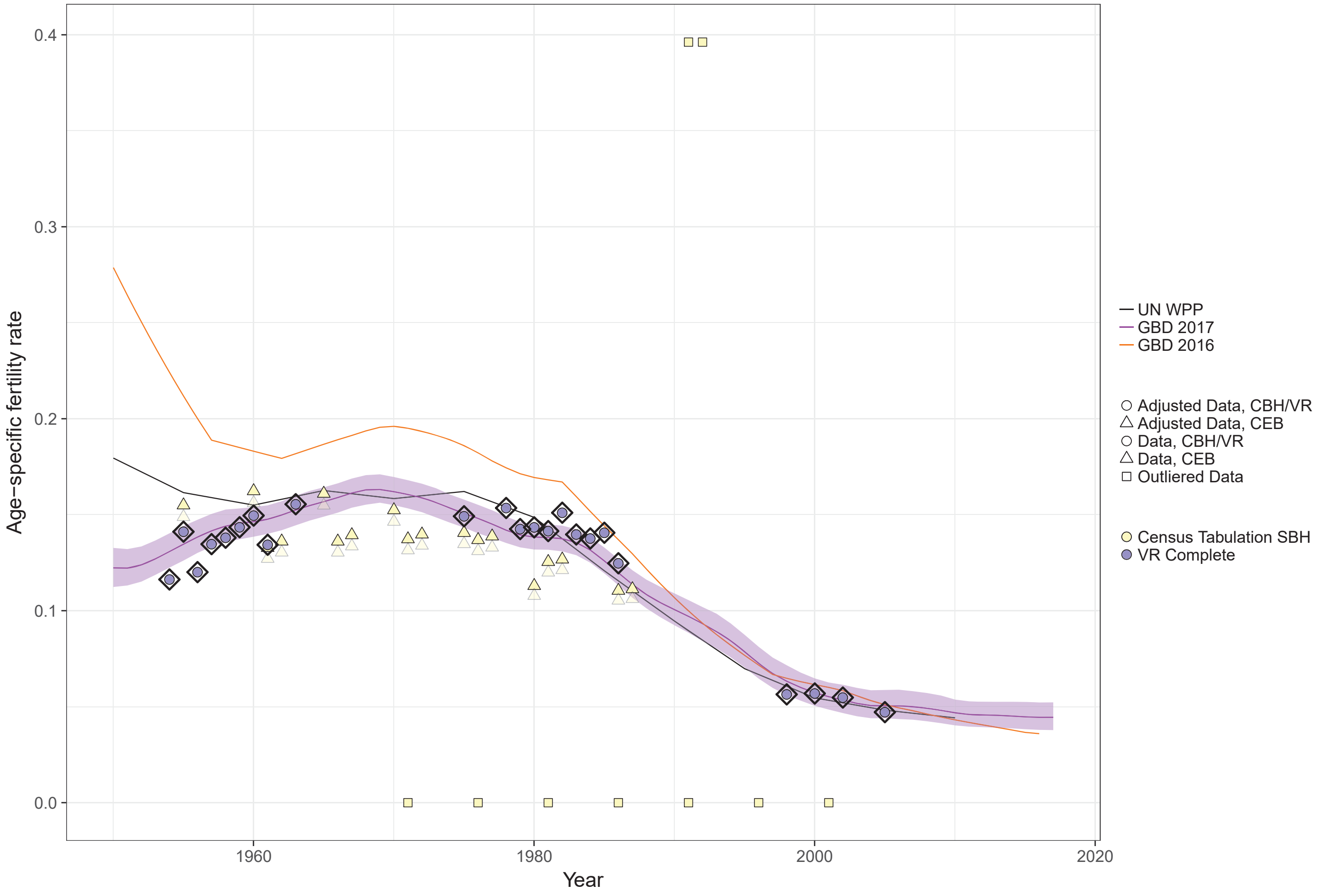

Age−specific fertility fitted trend lines, 1950−2017: Saint Lucia, Ages: 20−24
Data points enclosed with a diamond are reference sources

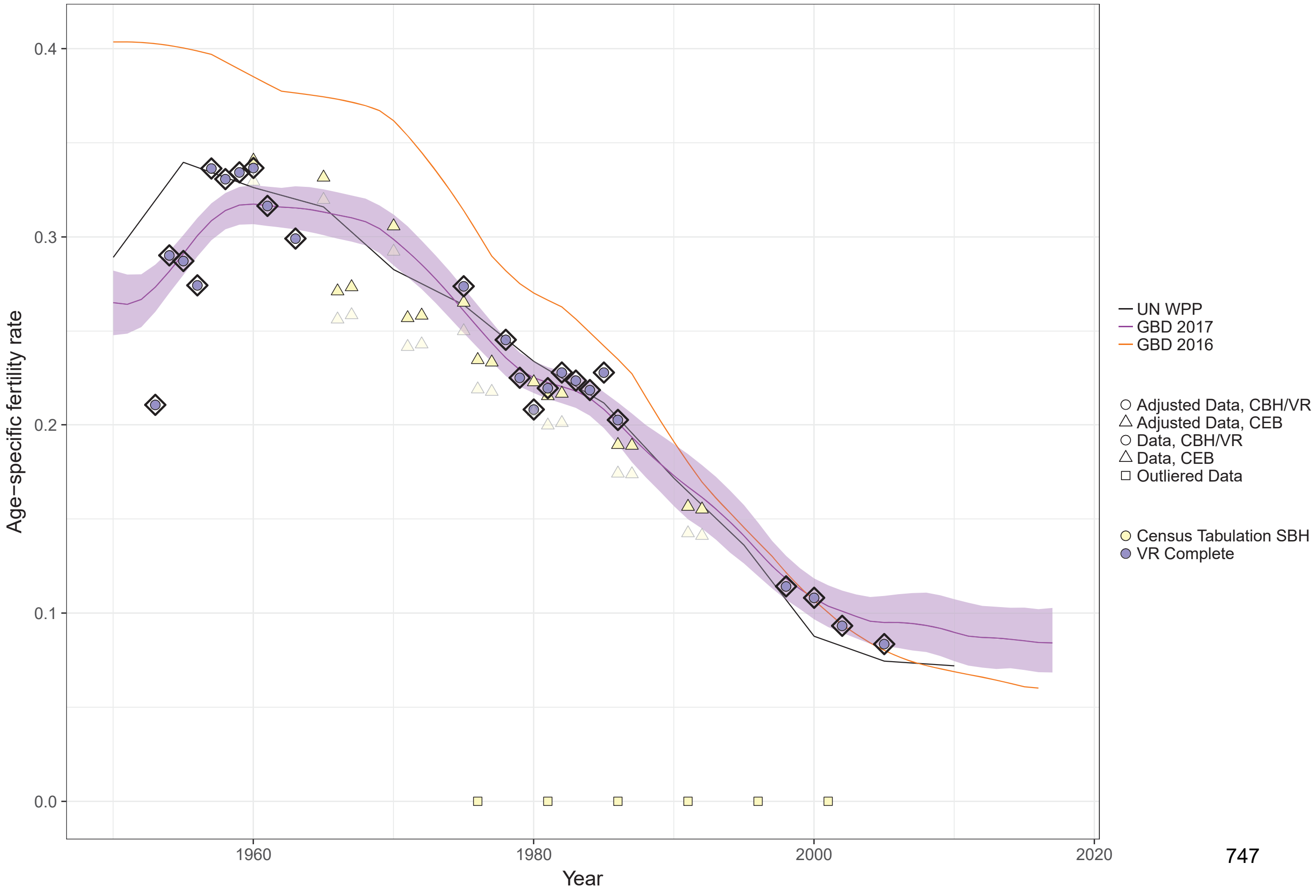

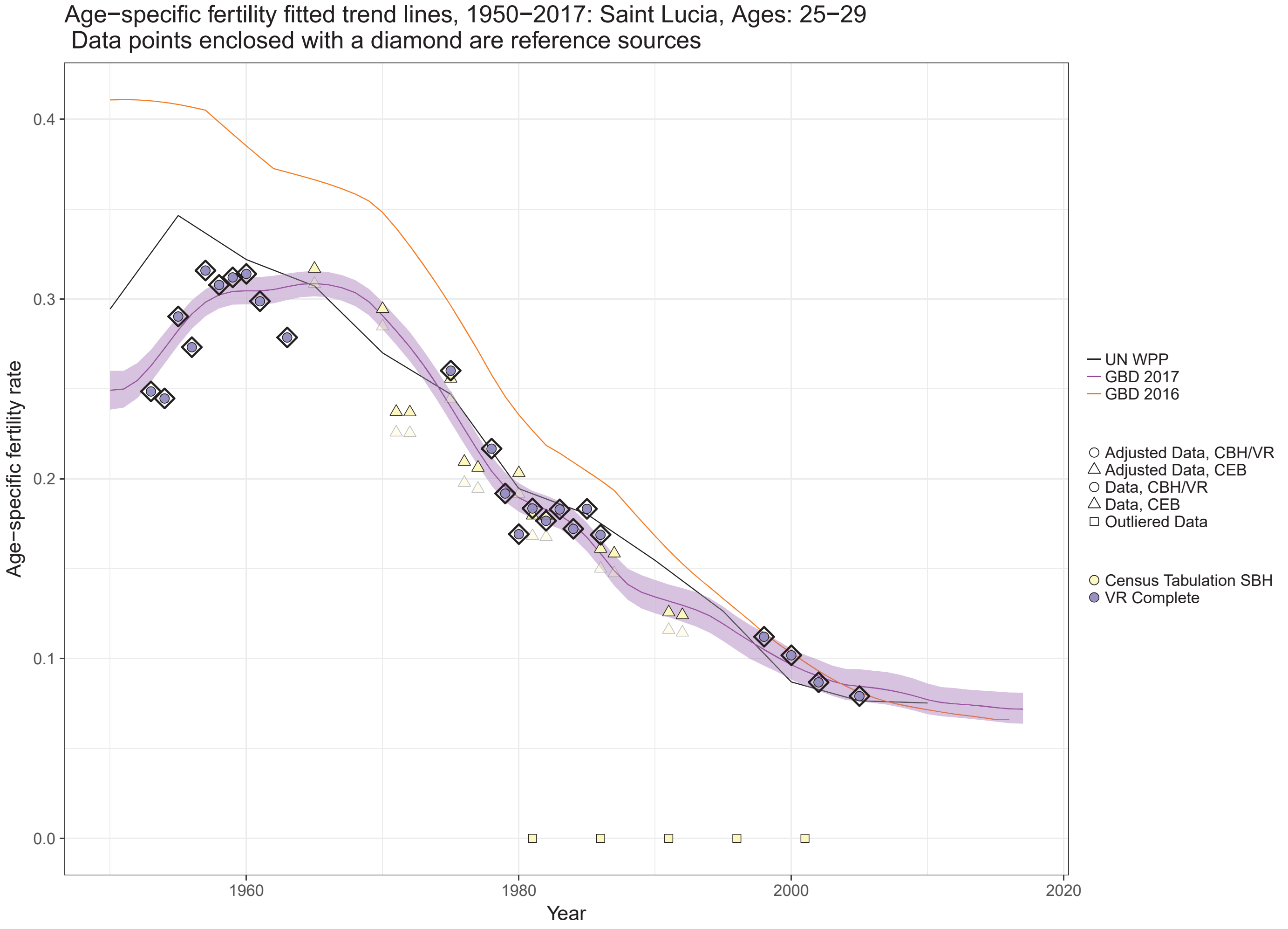

Age−specific fertility fitted trend lines, 1950−2017: Saint Lucia, Ages: 25−29
Data points enclosed with a diamond are reference sources
Age−specific fertility rate
Year
UN WPP
GBD 2017
GBD 2016
Adjusted Data, CBH/VR
Adjusted Data, CEB
Data, CBH/VR
Data, CEB
Outliered Data
Census Tabulation SBH
VR Complete

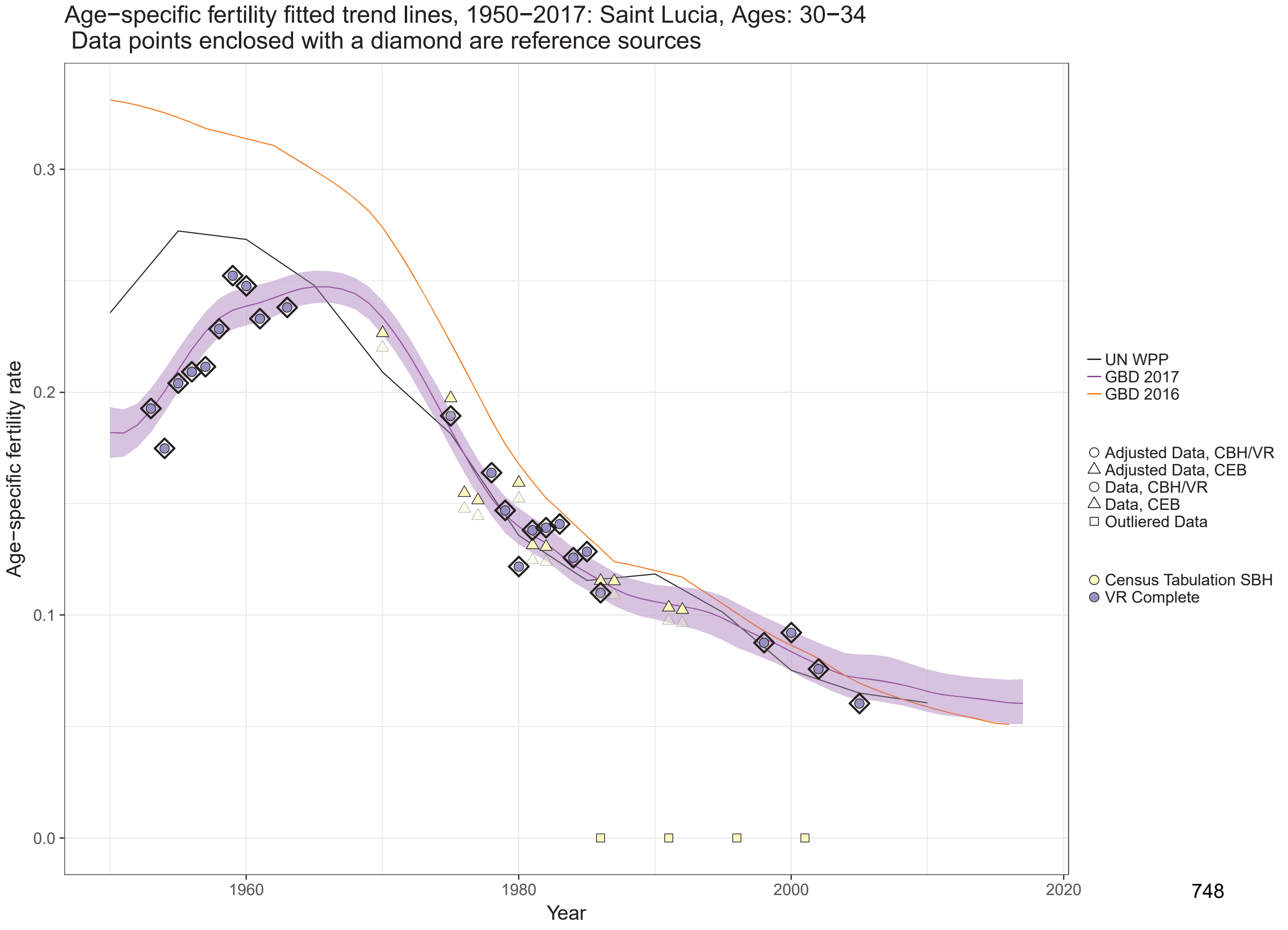

Age−specific fertility fitted trend lines, 1950−2017: Saint Lucia, Ages: 30−34
Data points enclosed with a diamond are reference sources
Age−specific fertility rate
Year
UN WPP
GBD 2017
GBD 2016
Adjusted Data, CBH/VR
Adjusted Data, CEB
Data, CBH/VR
Data, CEB
Outliered Data
Census Tabulation SBH
VR Complete

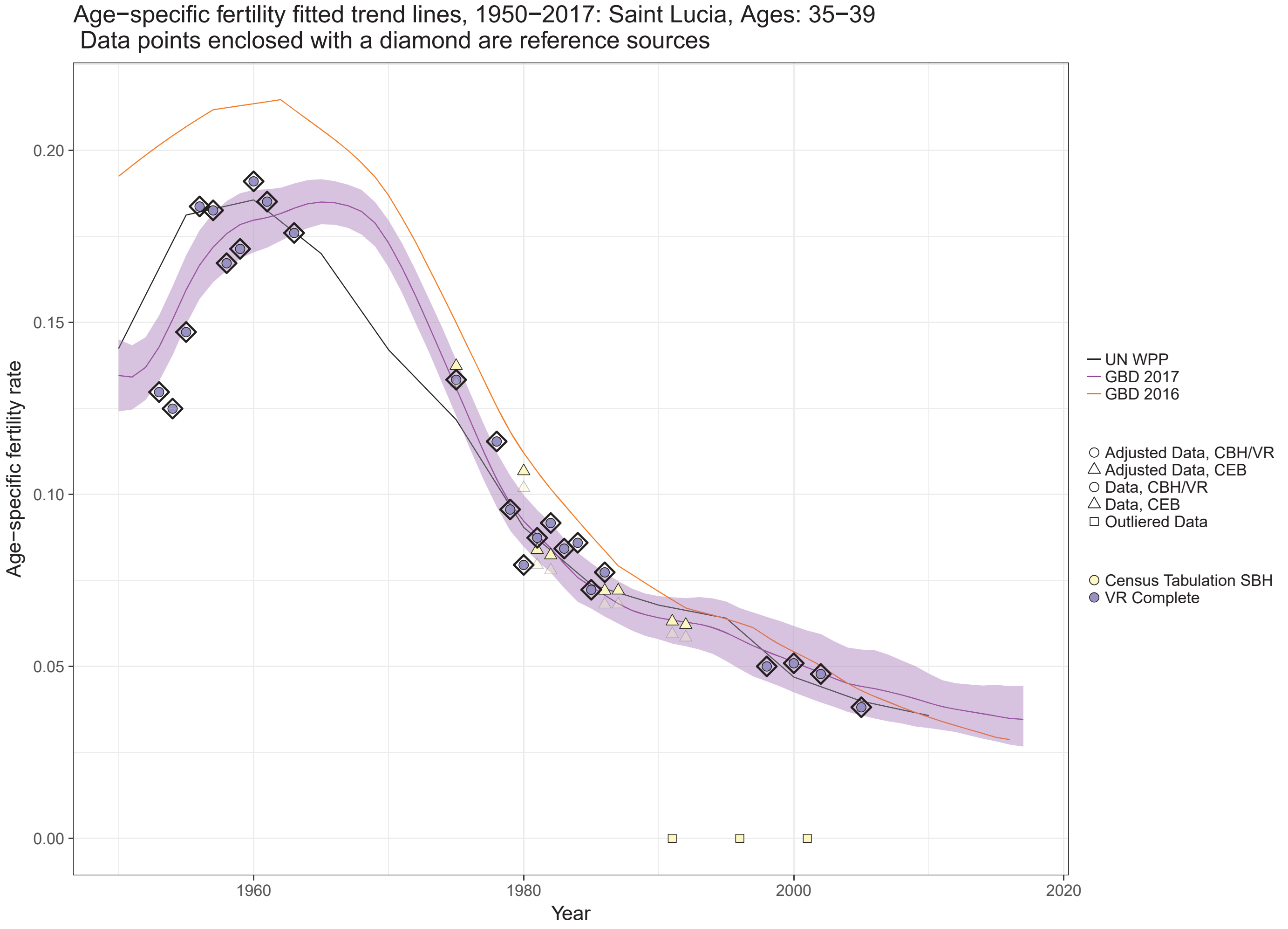

Age−specific fertility fitted trend lines, 1950−2017: Saint Lucia, Ages: 35−39
Data points enclosed with a diamond are reference sources
Age−specific fertility rate
0.00
0.05
0.10
0.15
0.20
1960
1980
2000
2020
Year
UN WPP
GBD 2017
GBD 2016
Adjusted Data, CBH/VR
Adjusted Data, CEB
Data, CBH/VR
Data, CEB
Outliered Data
Census Tabulation SBH
VR Complete

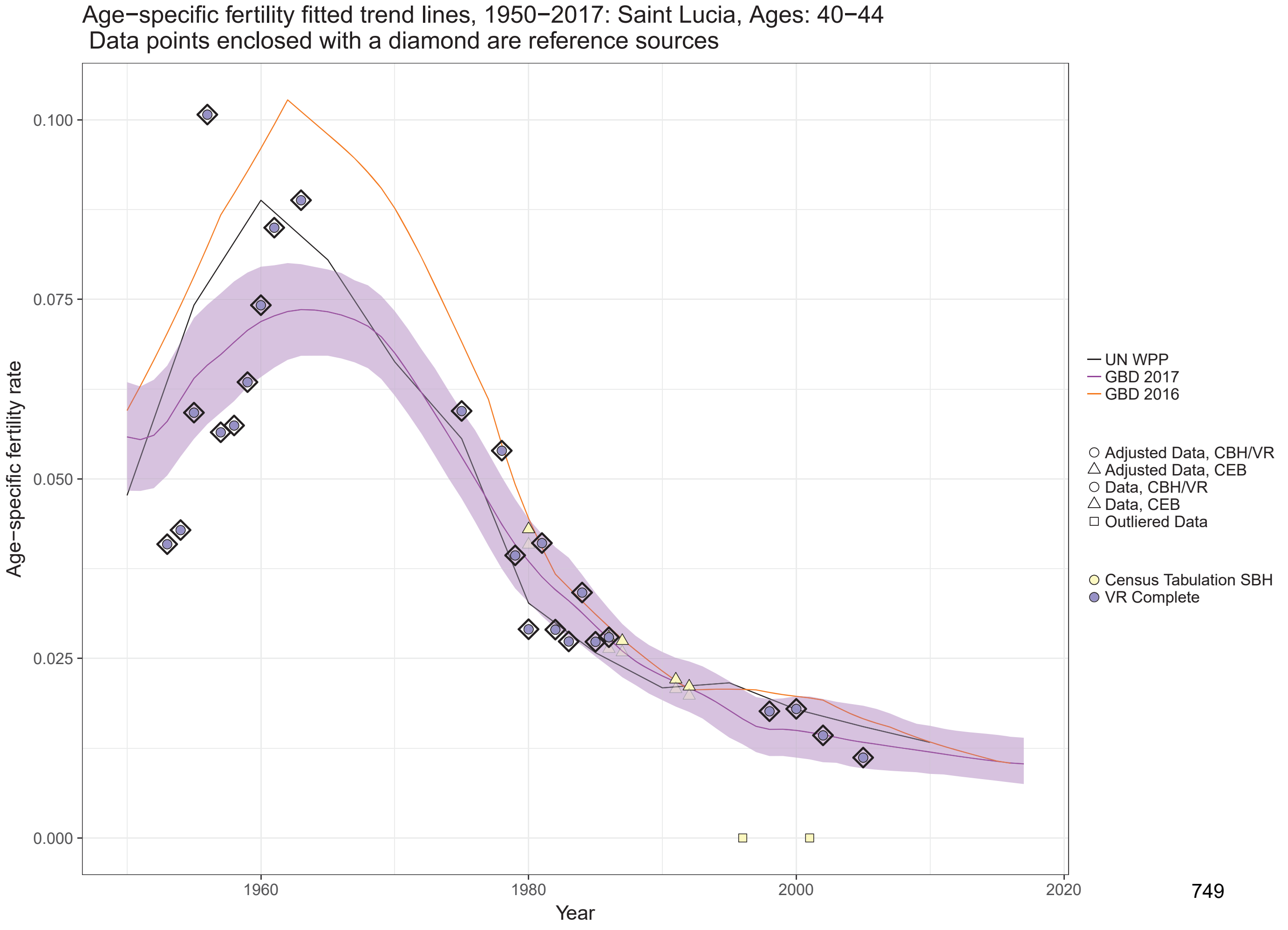

Age−specific fertility fitted trend lines, 1950−2017: Saint Lucia, Ages: 40−44
Data points enclosed with a diamond are reference sources
Age−specific fertility rate
0.000
0.025
0.050
0.075
0.100
1960
1980
2000
2020
Year
UN WPP
GBD 2017
GBD 2016
Adjusted Data, CBH/VR
Adjusted Data, CEB
Data, CBH/VR
Data, CEB
Outliered Data
Census Tabulation SBH
VR Complete

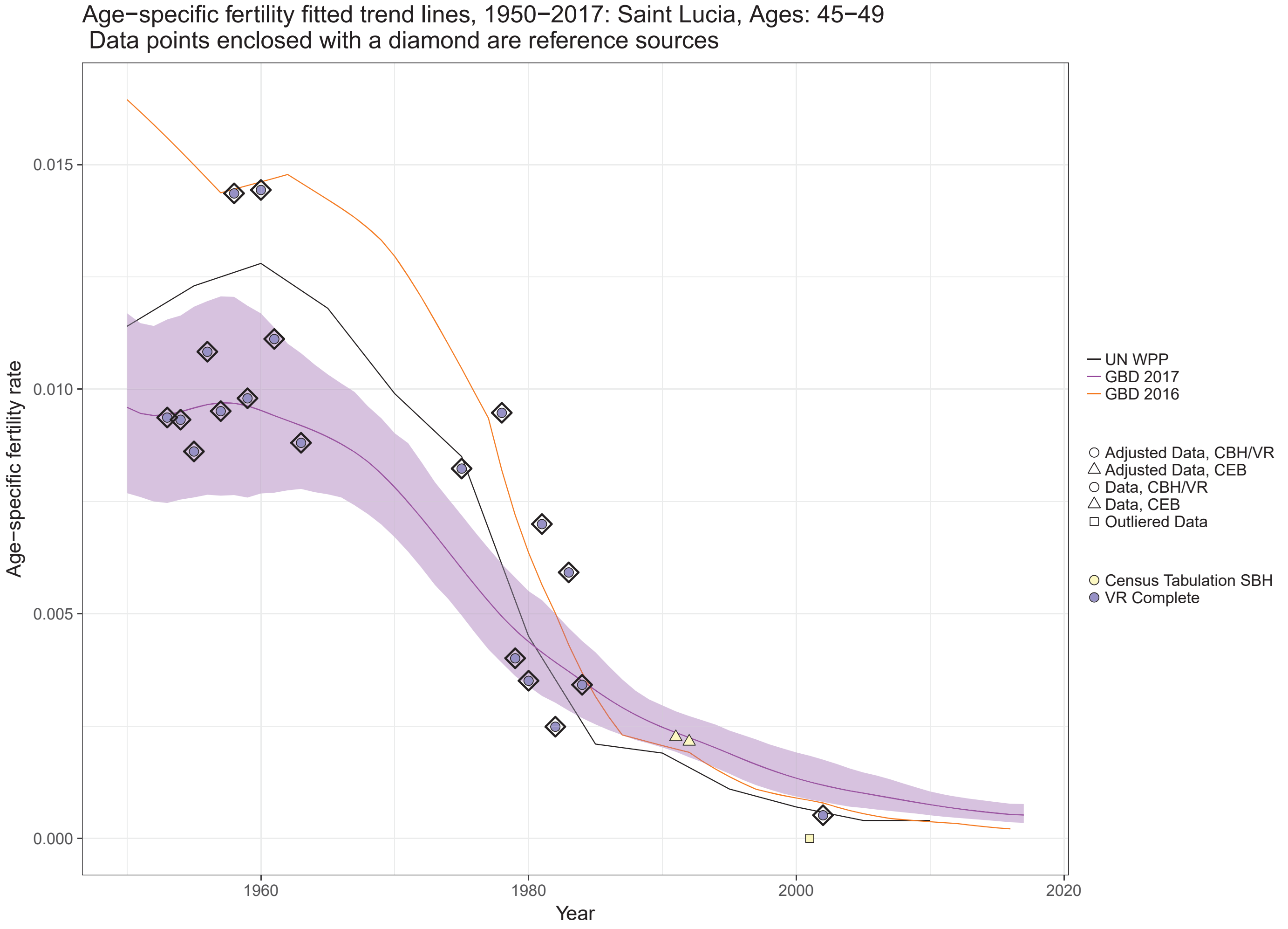
Age−specific fertility fitted trend lines, 1950−2017: Saint Lucia, Ages: 45−49
Data points enclosed with a diamond are reference sources
Age−specific fertility rate
0.015
0.010
0.005
0.000
1960
1980
2000
2020
Year
UN WPP
GBD 2017
GBD 2016
Adjusted Data, CBH/VR
Adjusted Data, CEB
Data, CBH/VR
Data, CEB
Outliered Data
Census Tabulation SBH
VR Complete

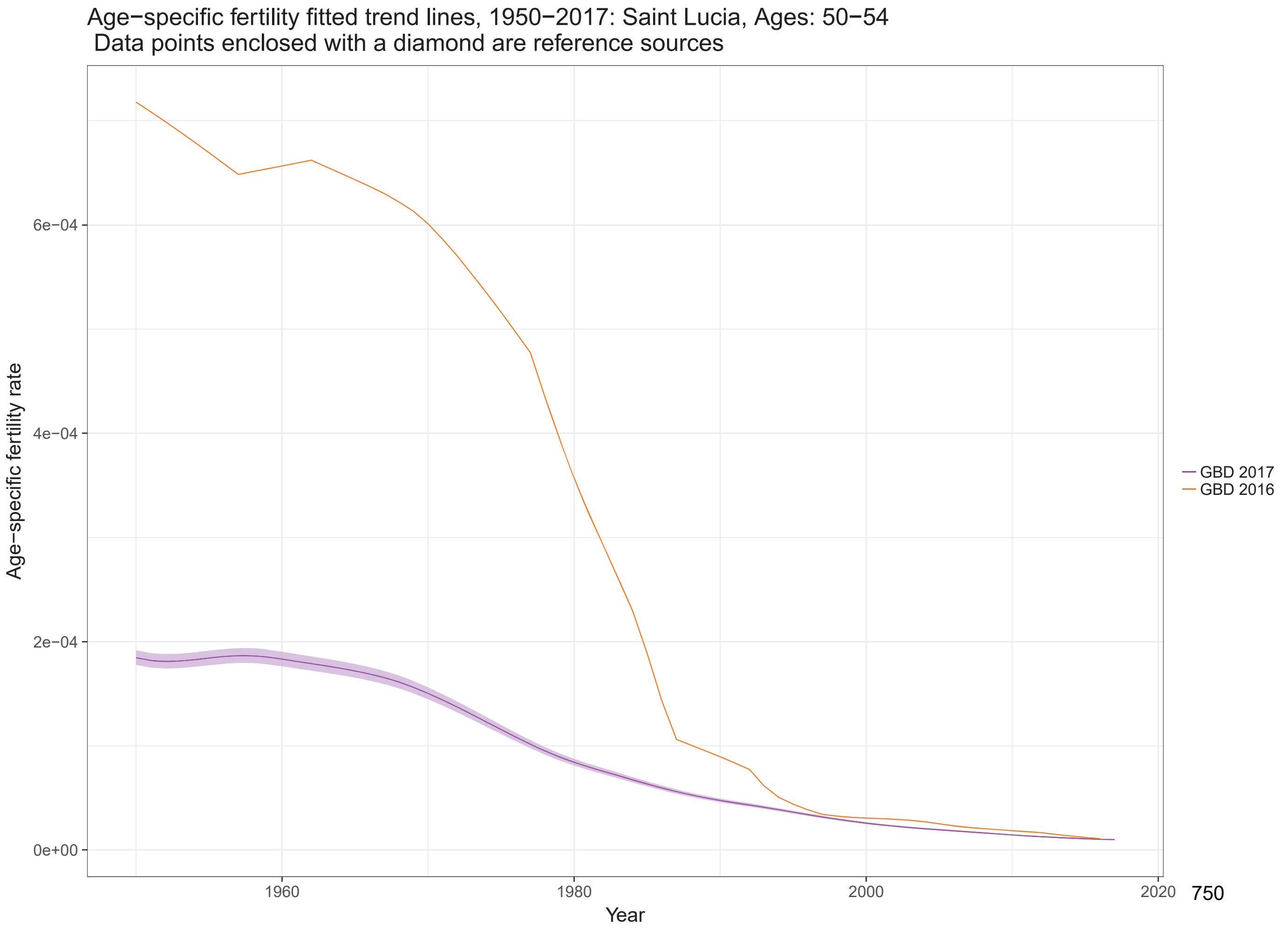
Age−specific fertility fitted trend lines, 1950−2017: Saint Lucia, Ages: 50−54
Data points enclosed with a diamond are reference sources
Age−specific fertility rate
6e−04
4e−04
2e−04
0e+00
1960
1980
2000
2020
Year
GBD 2017
GBD 2016

Age−specific fertility fitted trend lines, 1950−2017: Saint Vincent and the Grenadines, TFR
Data points enclosed with a diamond are reference sources

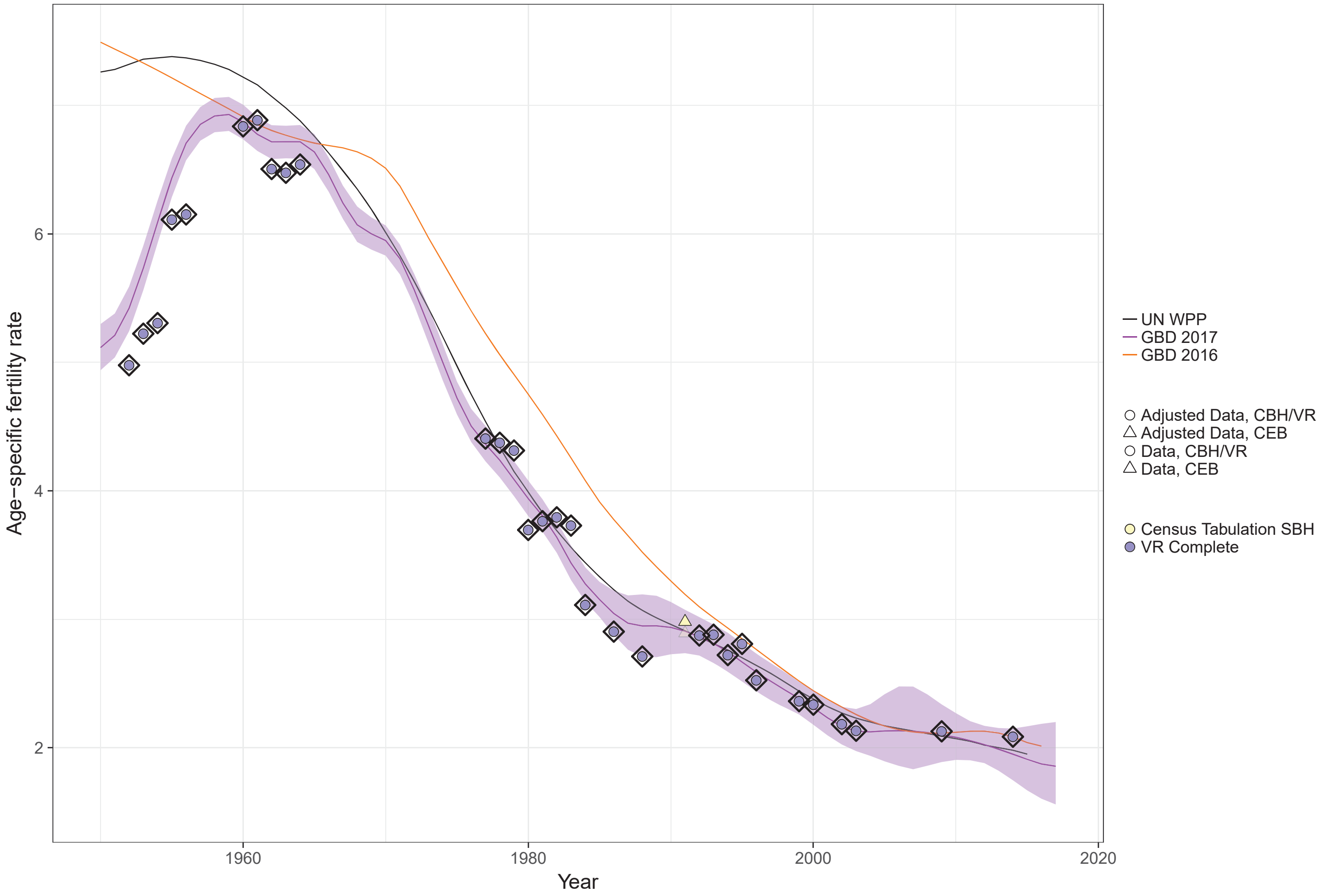

Age−specific fertility fitted trend lines, 1950−2017: Saint Vincent and the Grenadines, Ages: 10−14
Data points enclosed with a diamond are reference sources

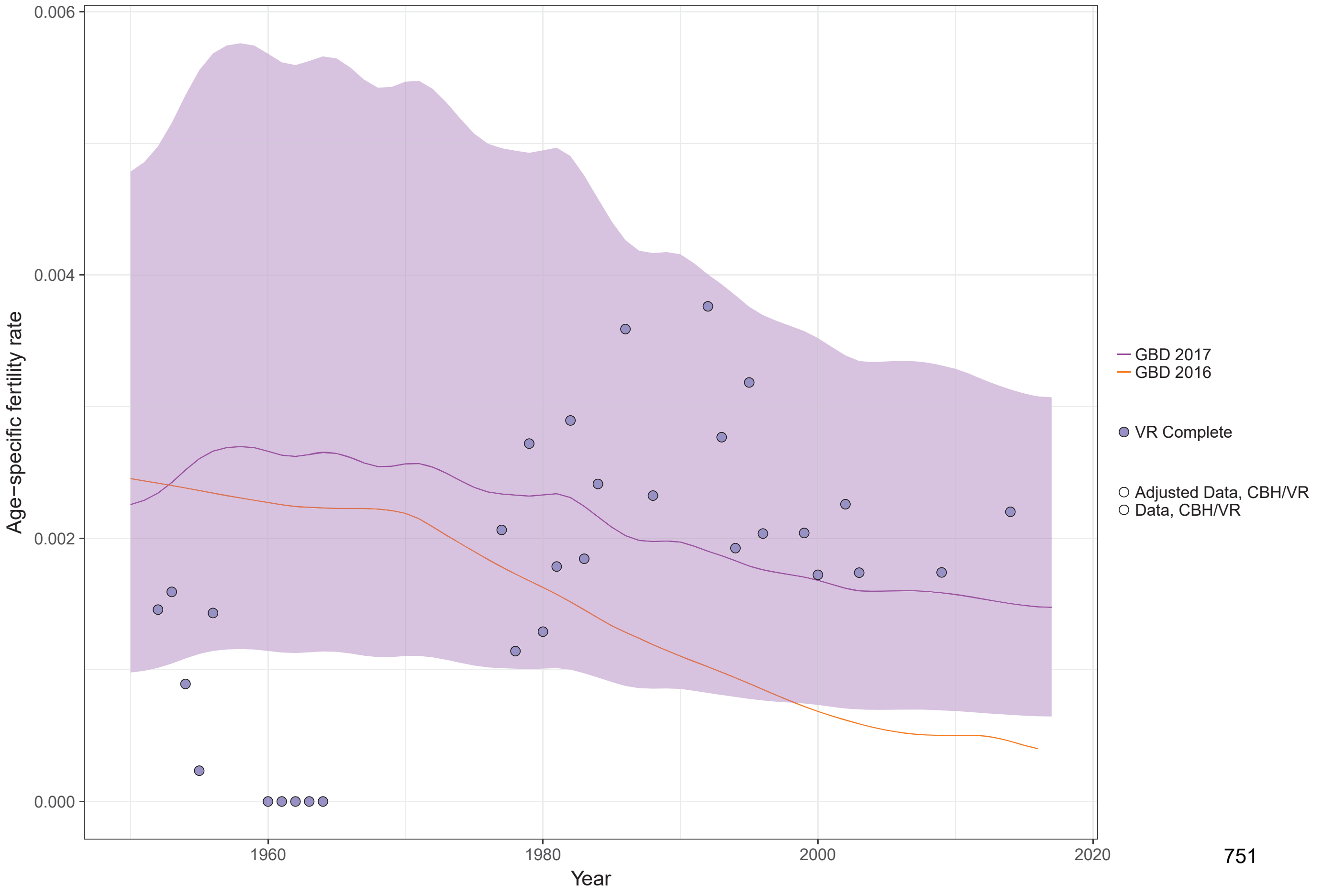

Age−specific fertility fitted trend lines, 1950−2017: Saint Vincent and the Grenadines, Ages: 15−19
Data points enclosed with a diamond are reference sources

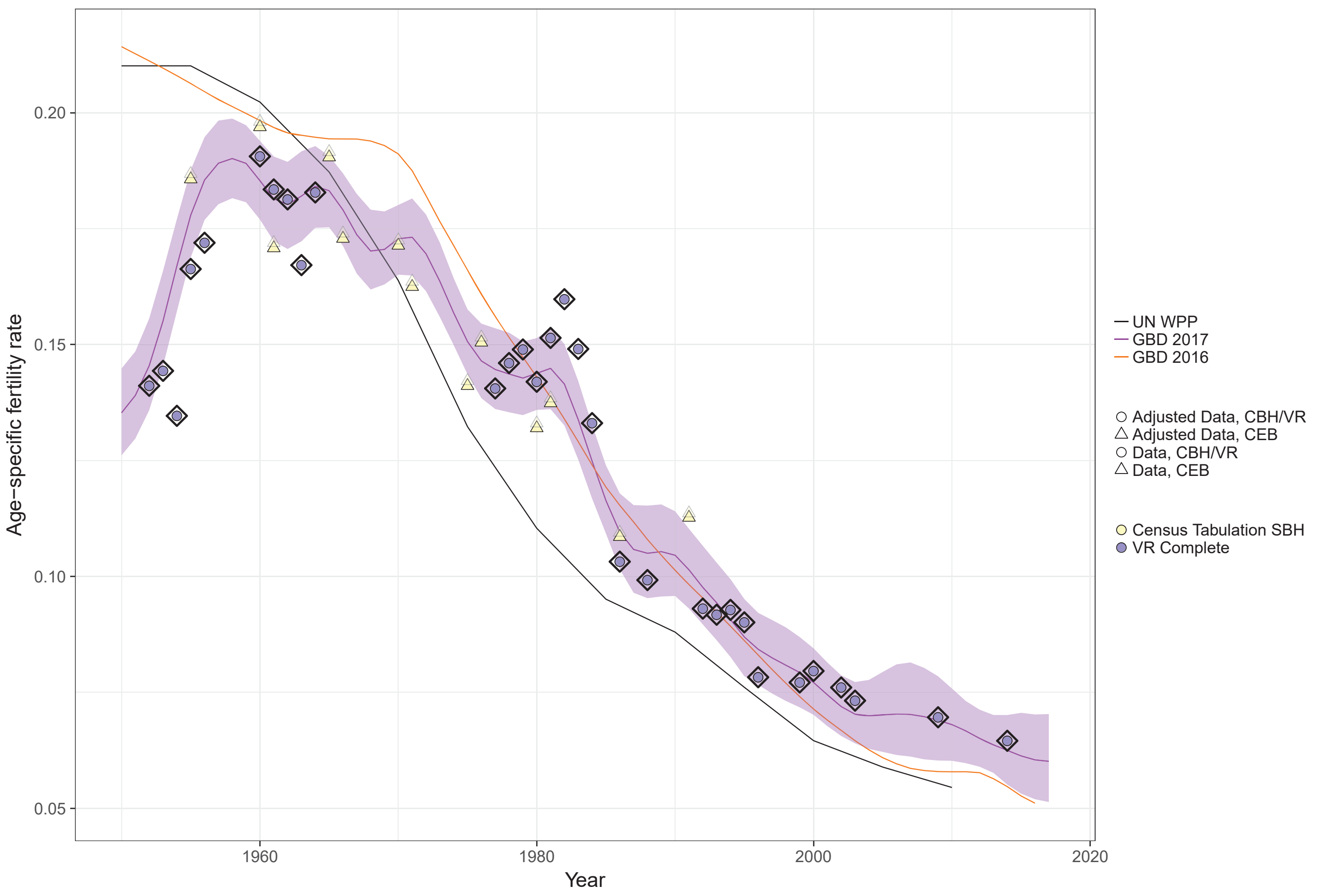

Age−specific fertility fitted trend lines, 1950−2017: Saint Vincent and the Grenadines, Ages: 20−24
Data points enclosed with a diamond are reference sources

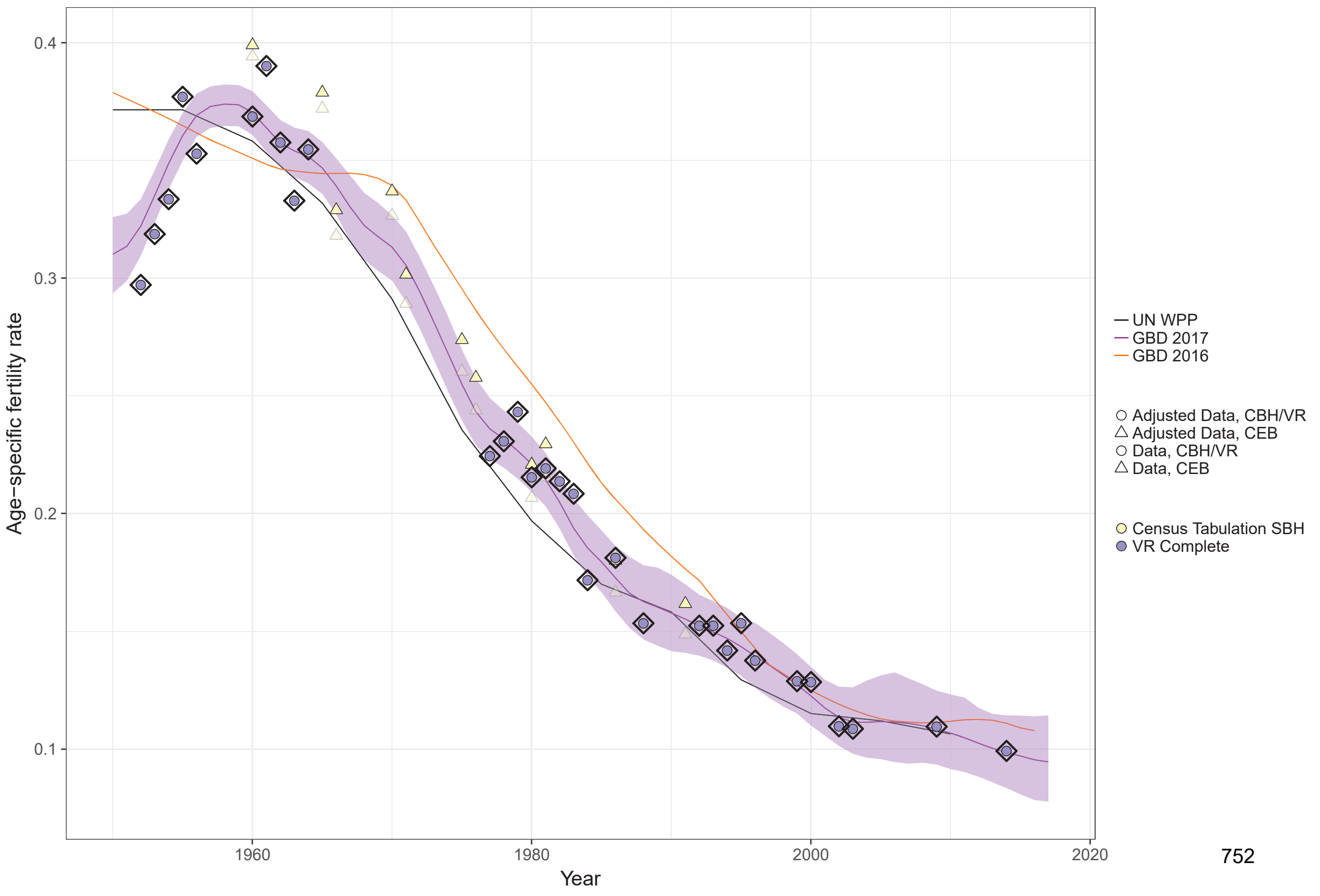

Age−specific fertility fitted trend lines, 1950−2017: Saint Vincent and the Grenadines, Ages: 25−29
Data points enclosed with a diamond are reference sources

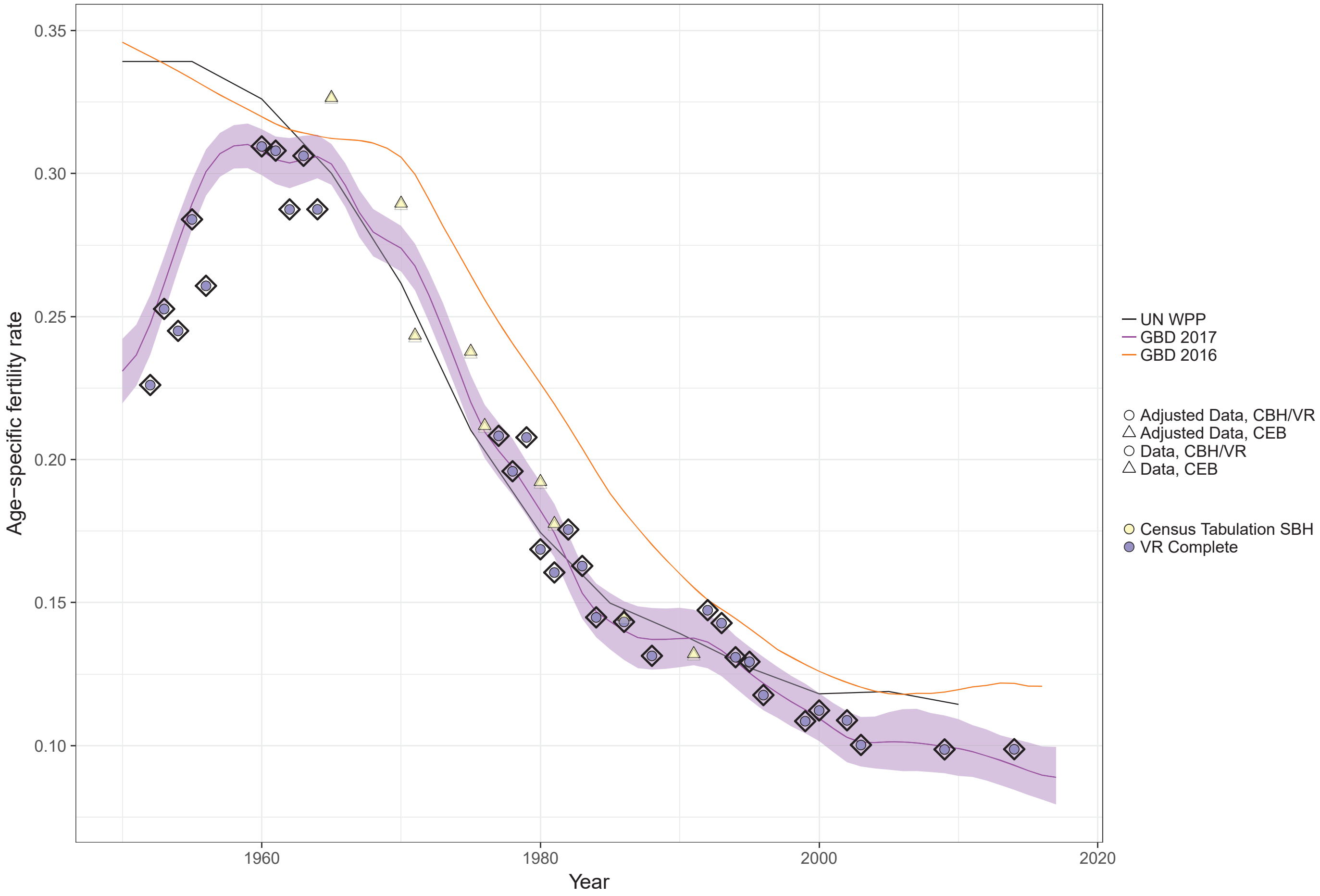

Age−specific fertility fitted trend lines, 1950−2017: Saint Vincent and the Grenadines, Ages: 30−34
Data points enclosed with a diamond are reference sources

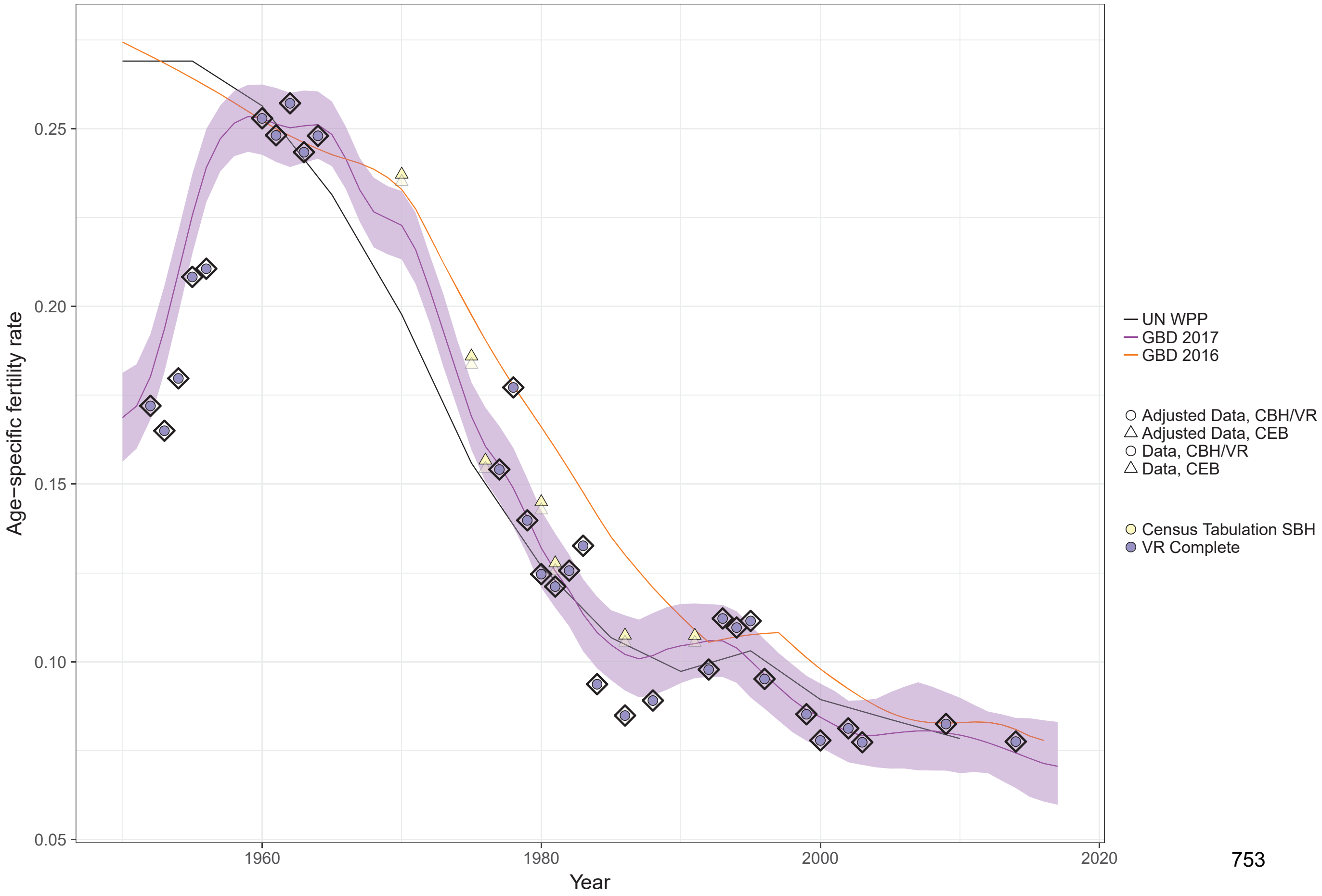

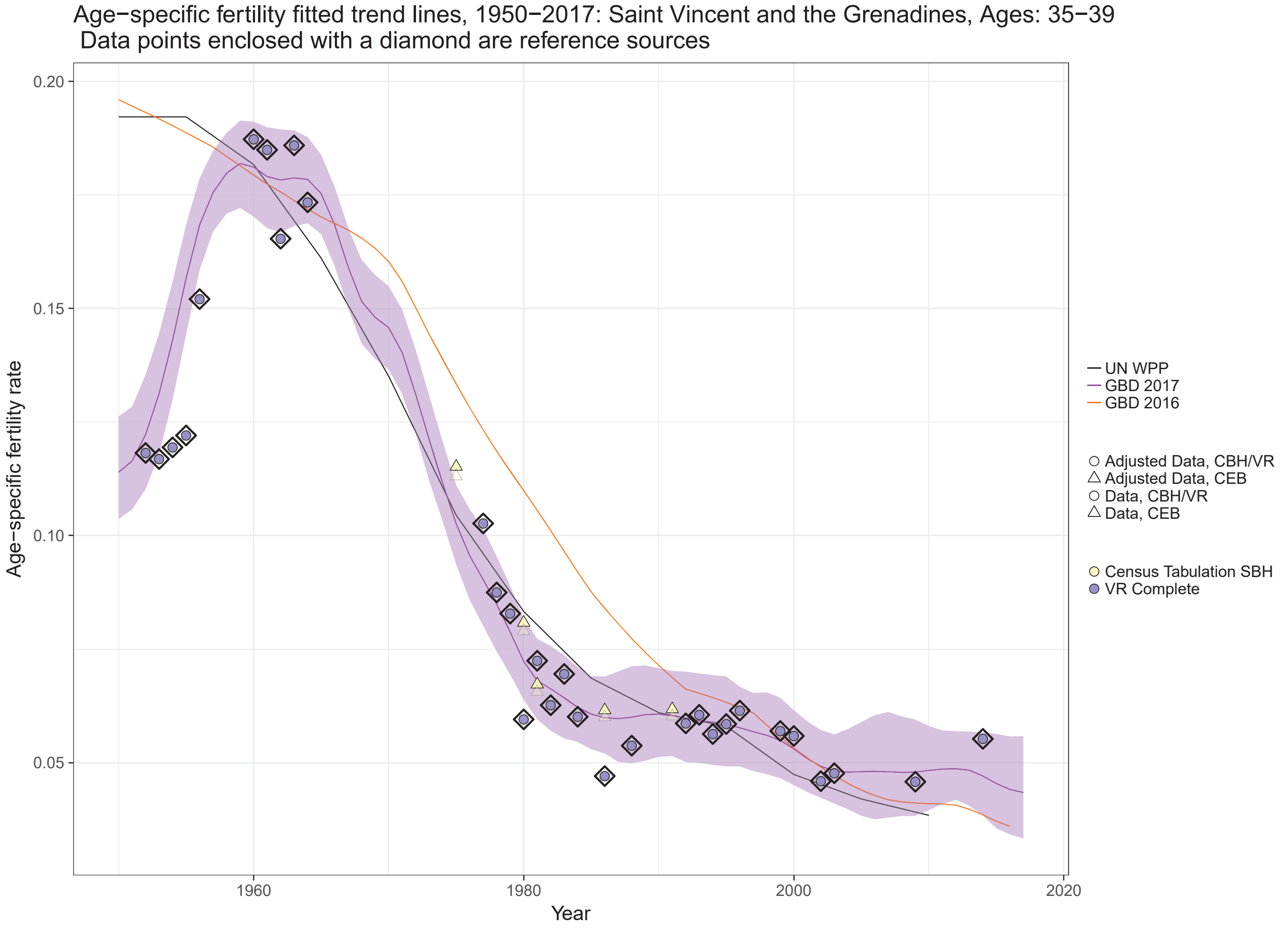

Age−specific fertility fitted trend lines, 1950−2017: Saint Vincent and the Grenadines, Ages: 35−39
Data points enclosed with a diamond are reference sources
Age−specific fertility rate
Year
0.20
0.15
0.10
0.05
1960
1980
2000
2020
UN WPP
GBD 2017
GBD 2016
Adjusted Data, CBH/VR
Adjusted Data, CEB
Data, CBH/VR
Data, CEB
Census Tabulation SBH
VR Complete

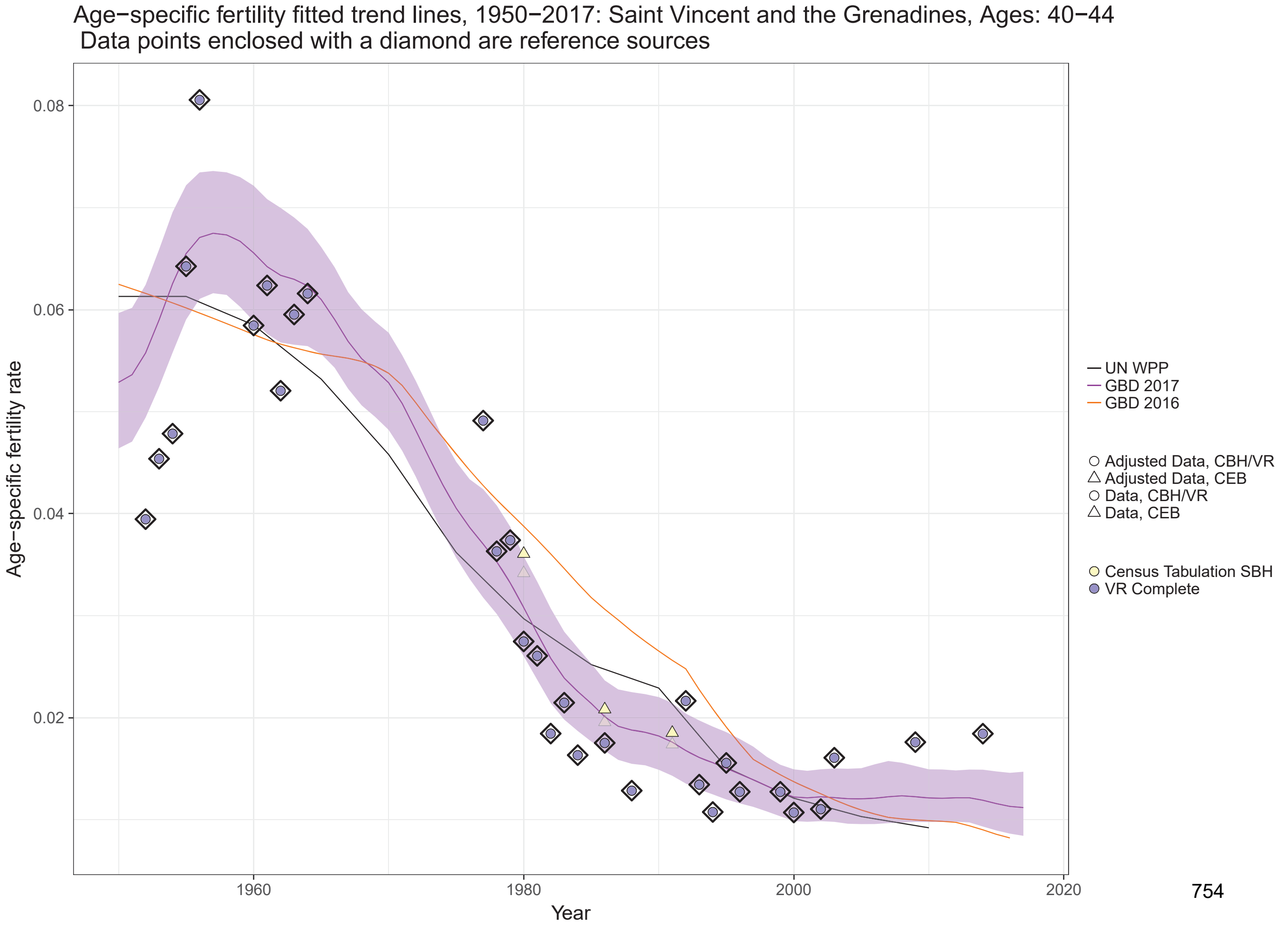

Age−specific fertility fitted trend lines, 1950−2017: Saint Vincent and the Grenadines, Ages: 40−44
Data points enclosed with a diamond are reference sources
Age−specific fertility rate
Year
0.08
0.06
0.04
0.02
1960
1980
2000
2020
UN WPP
GBD 2017
GBD 2016
Adjusted Data, CBH/VR
Adjusted Data, CEB
Data, CBH/VR
Data, CEB
Census Tabulation SBH
VR Complete

Age−specific fertility fitted trend lines, 1950−2017: Saint Vincent and the Grenadines, Ages: 45−49
Data points enclosed with a diamond are reference sources

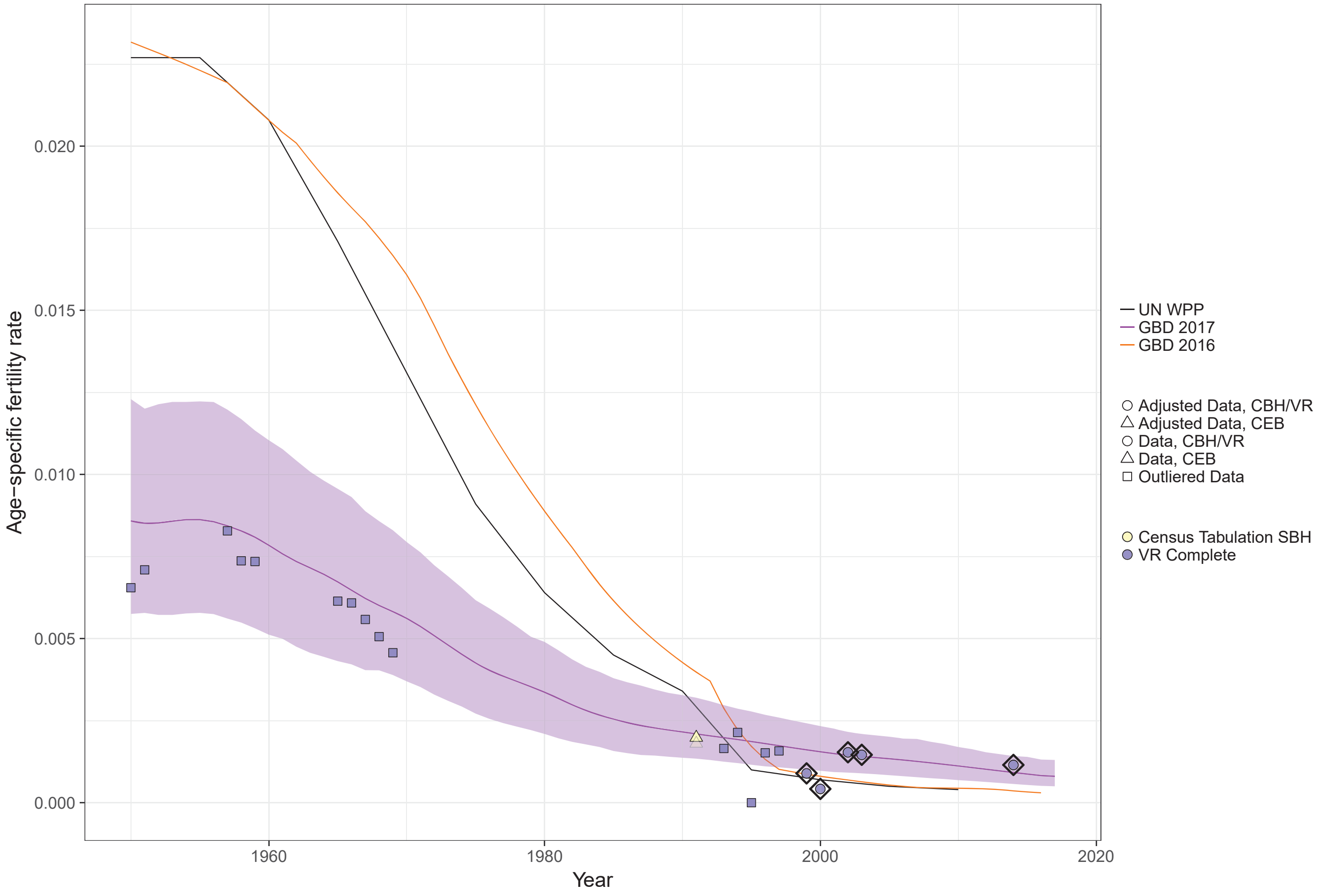

Age−specific fertility fitted trend lines, 1950−2017: Saint Vincent and the Grenadines, Ages: 50−54
Data points enclosed with a diamond are reference sources

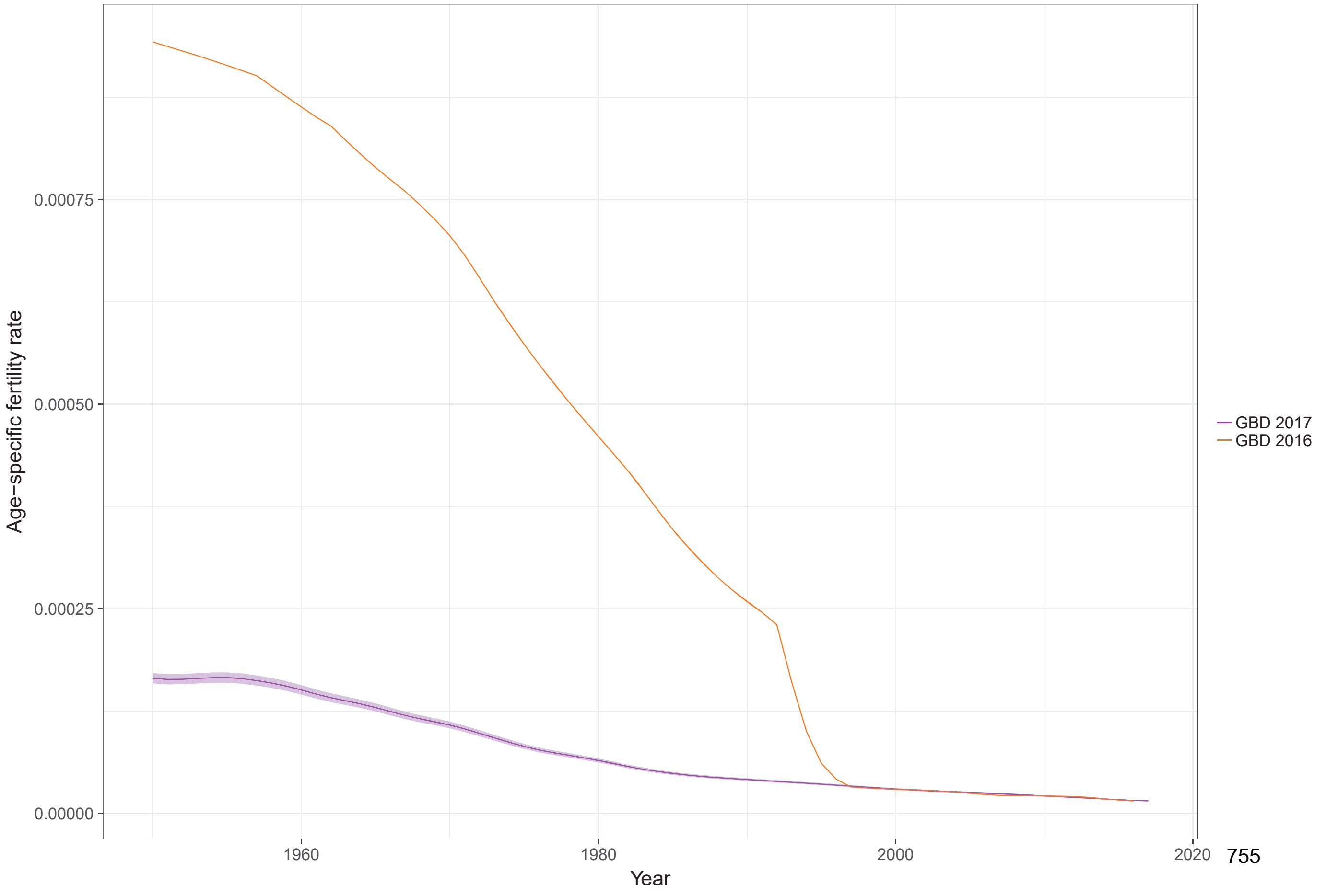

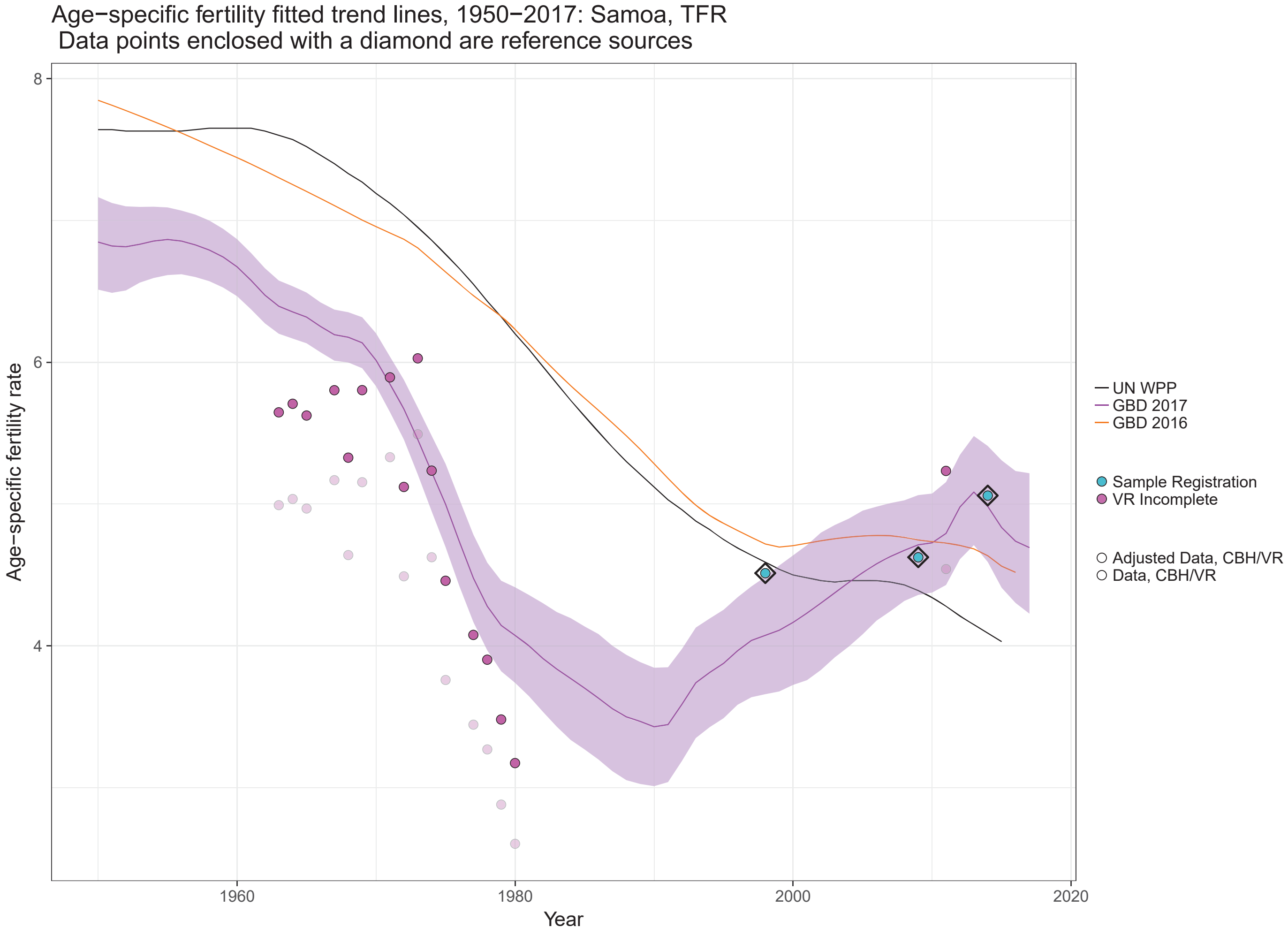

Age−specific fertility fitted trend lines, 1950−2017: Samoa, TFR
Data points enclosed with a diamond are reference sources
Age−specific fertility rate
Year
8
6
4
1960
1980
2000
2020
UN WPP
GBD 2017
GBD 2016
Sample Registration
VR Incomplete
Adjusted Data, CBH/VR
Data, CBH/VR

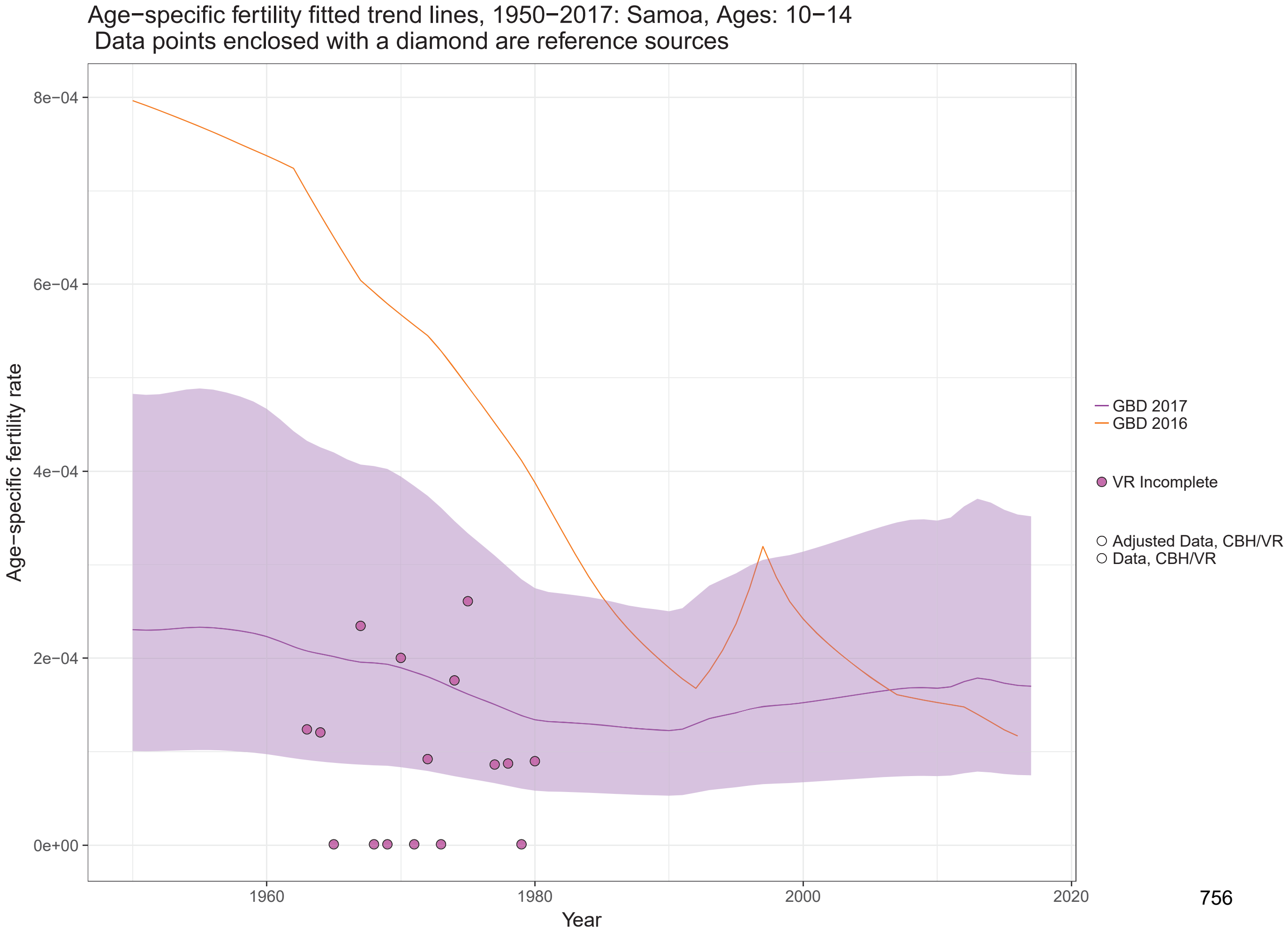

Age−specific fertility fitted trend lines, 1950−2017: Samoa, Ages: 10−14
Data points enclosed with a diamond are reference sources
Age−specific fertility rate
Year
8e−04
6e−04
4e−04
2e−04
0e+00
1960
1980
2000
2020
GBD 2017
GBD 2016
VR Incomplete
Adjusted Data, CBH/VR
Data, CBH/VR

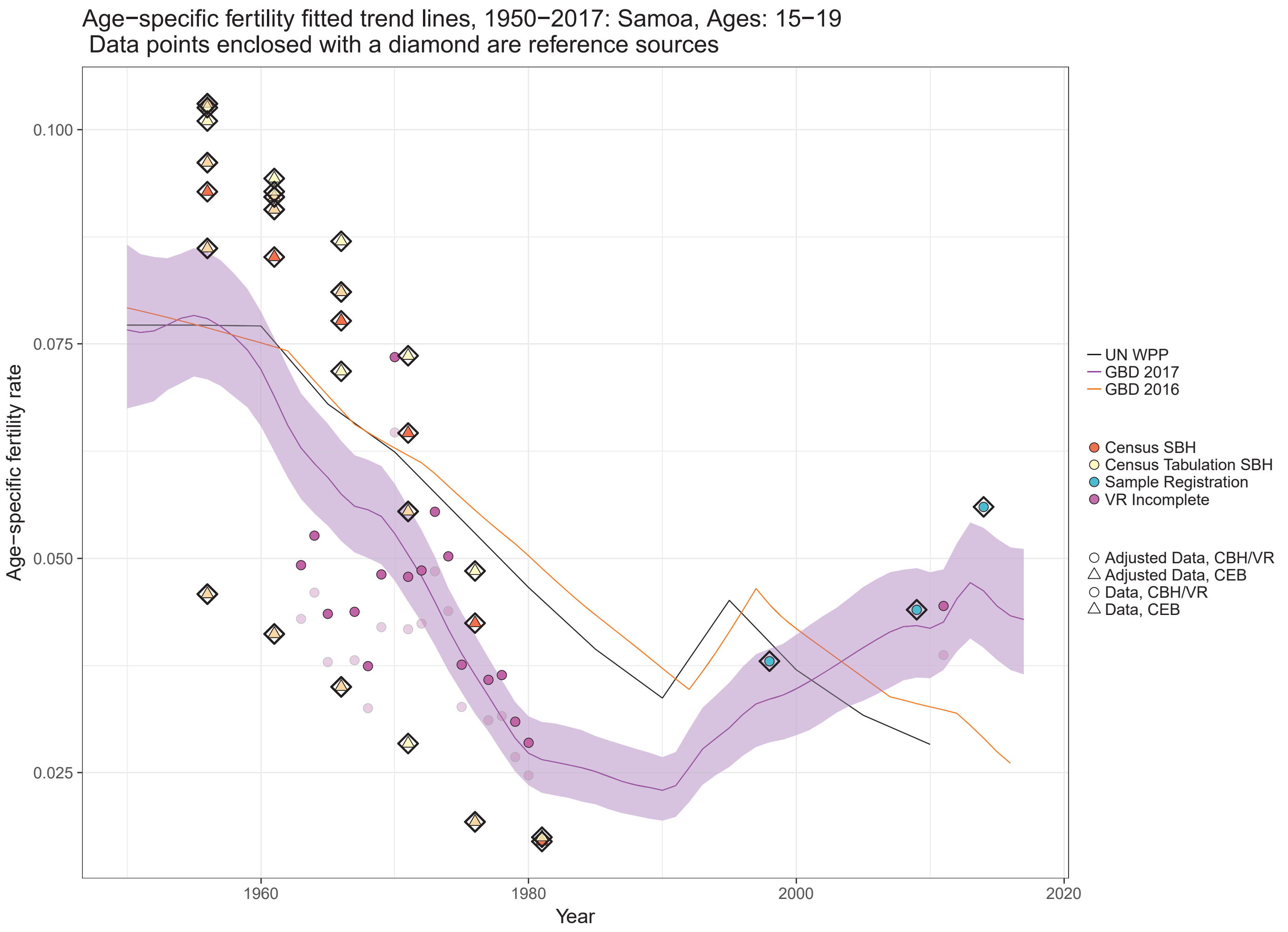

Age−specific fertility fitted trend lines, 1950−2017: Samoa, Ages: 15−19
Data points enclosed with a diamond are reference sources
Age-specific fertility rate
0.100
0.075
0.050
0.025
1960
1980
2000
2020
Year
UN WPP
GBD 2017
GBD 2016
Census SBH
Census Tabulation SBH
Sample Registration
VR Incomplete
Adjusted Data, CBH/VR
Adjusted Data, CEB
Data, CBH/VR
Data, CEB

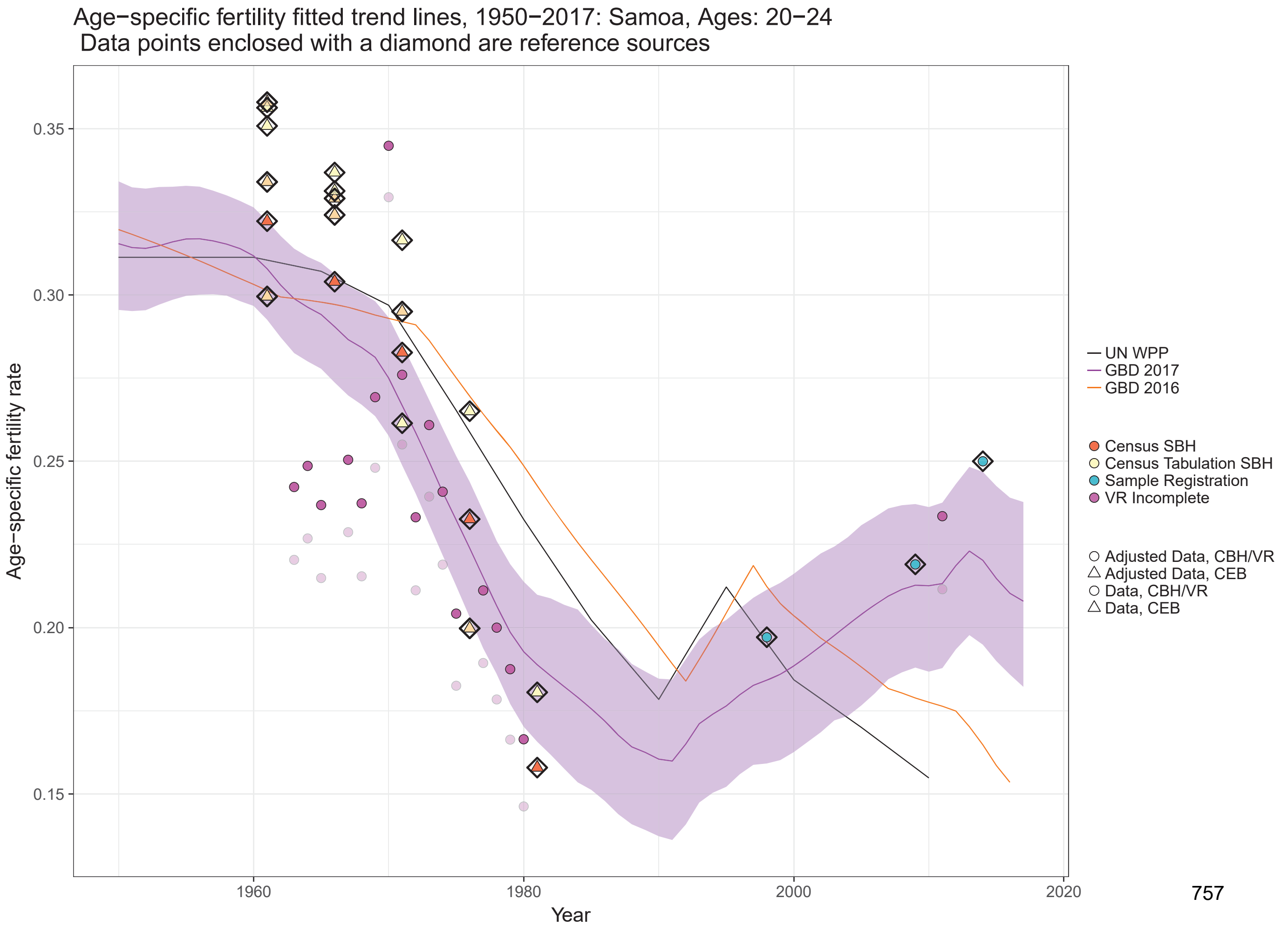

Age−specific fertility fitted trend lines, 1950−2017: Samoa, Ages: 20−24
Data points enclosed with a diamond are reference sources
Age-specific fertility rate
0.35
0.30
0.25
0.20
0.15
1960
1980
2000
2020
Year
UN WPP
GBD 2017
GBD 2016
Census SBH
Census Tabulation SBH
Sample Registration
VR Incomplete
Adjusted Data, CBH/VR
Adjusted Data, CEB
Data, CBH/VR
Data, CEB

Age−specific fertility fitted trend lines, 1950−2017: Samoa, Ages: 25−29
Data points enclosed with a diamond are reference sources

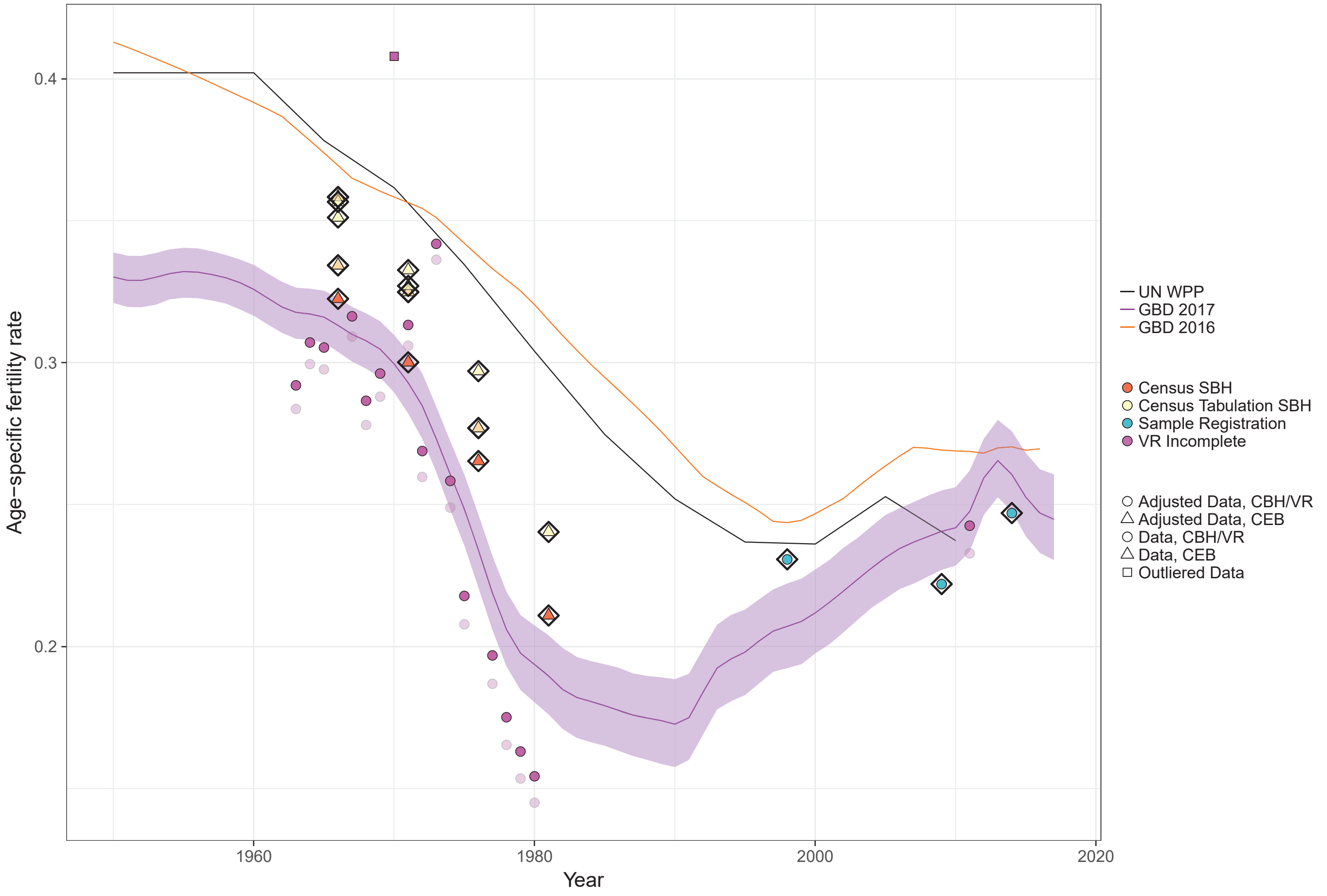

Age−specific fertility fitted trend lines, 1950−2017: Samoa, Ages: 30−34
Data points enclosed with a diamond are reference sources

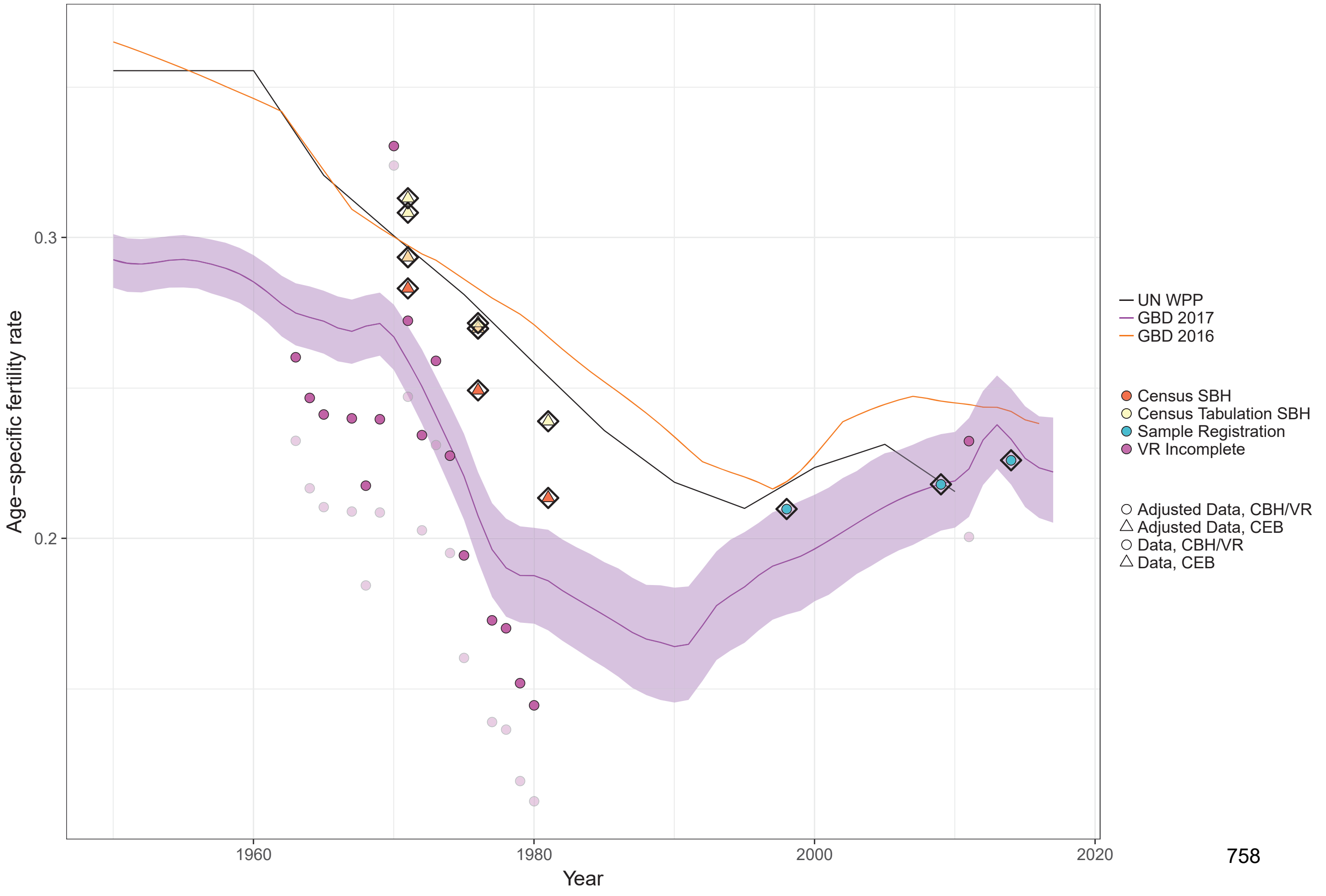

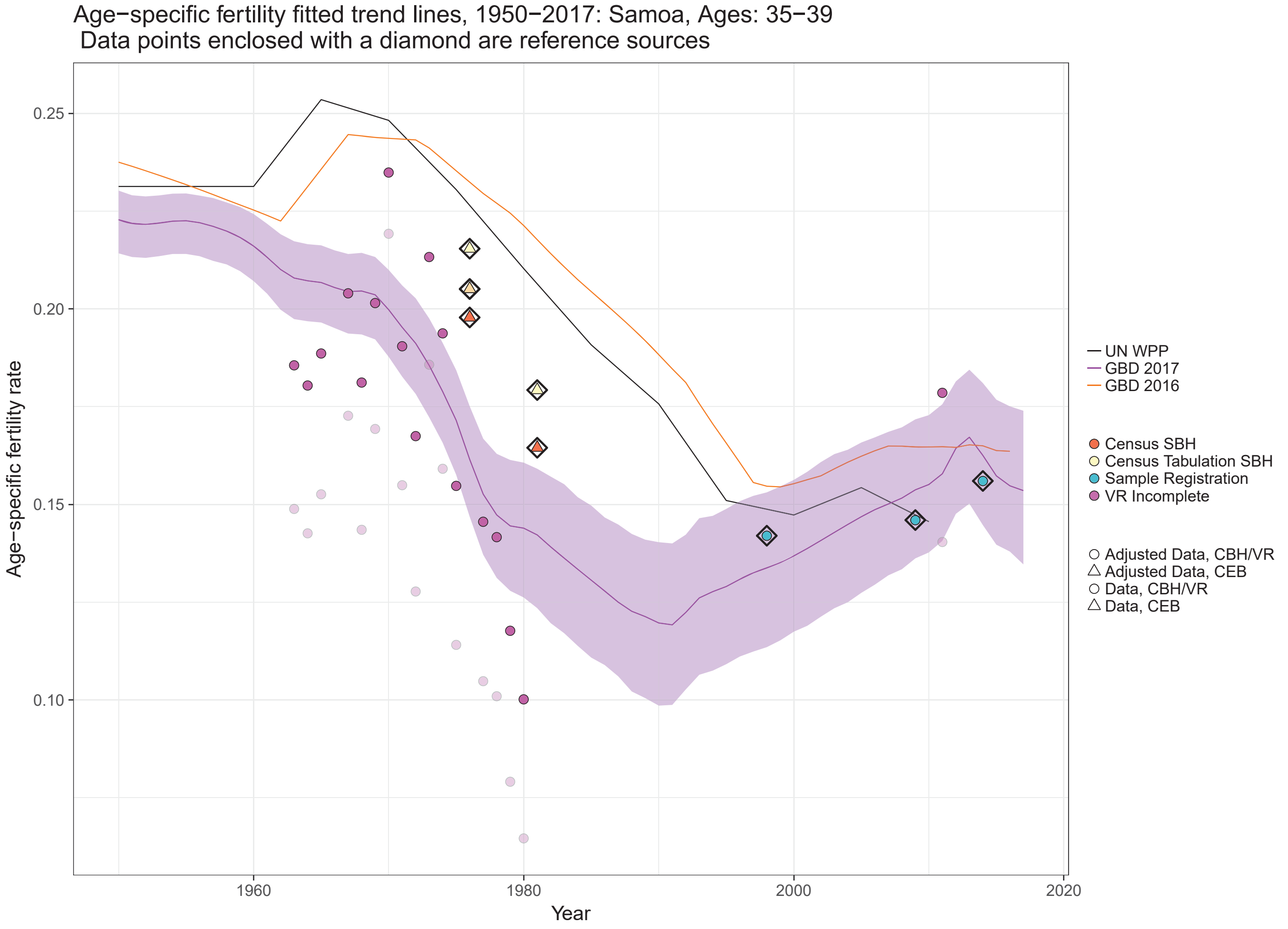
Age−specific fertility fitted trend lines, 1950−2017: Samoa, Ages: 35−39
Data points enclosed with a diamond are reference sources
Age−specific fertility rate
Year
0.25
0.20
0.15
0.10
1960
1980
2000
2020
UN WPP
GBD 2017
GBD 2016
Census SBH
Census Tabulation SBH
Sample Registration
VR Incomplete
Adjusted Data, CBH/VR
Adjusted Data, CEB
Data, CBH/VR
Data, CEB

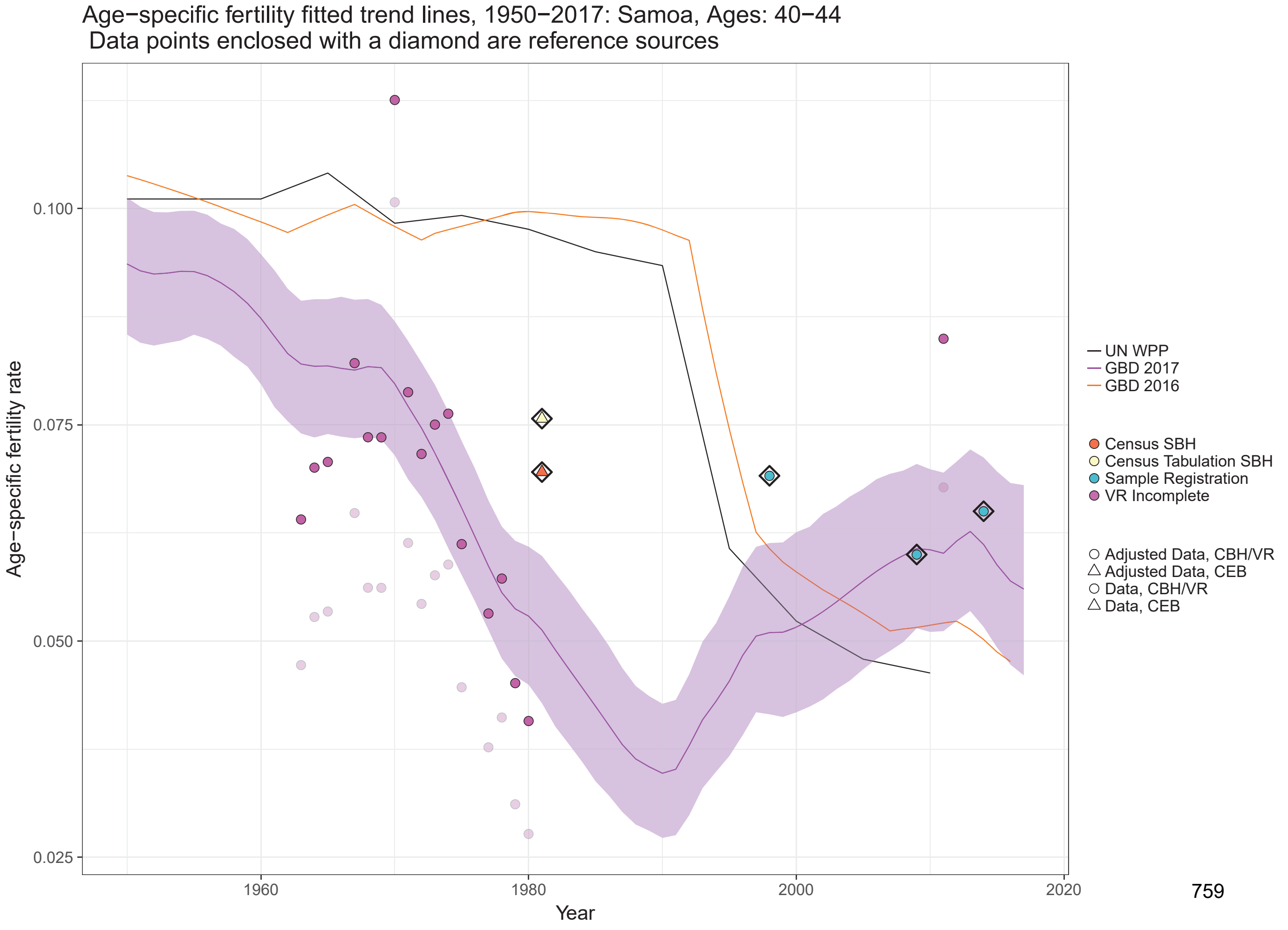
Age−specific fertility fitted trend lines, 1950−2017: Samoa, Ages: 40−44
Data points enclosed with a diamond are reference sources
Age−specific fertility rate
Year
0.100
0.075
0.050
0.025
1960
1980
2000
2020
UN WPP
GBD 2017
GBD 2016
Census SBH
Census Tabulation SBH
Sample Registration
VR Incomplete
Adjusted Data, CBH/VR
Adjusted Data, CEB
Data, CBH/VR
Data, CEB

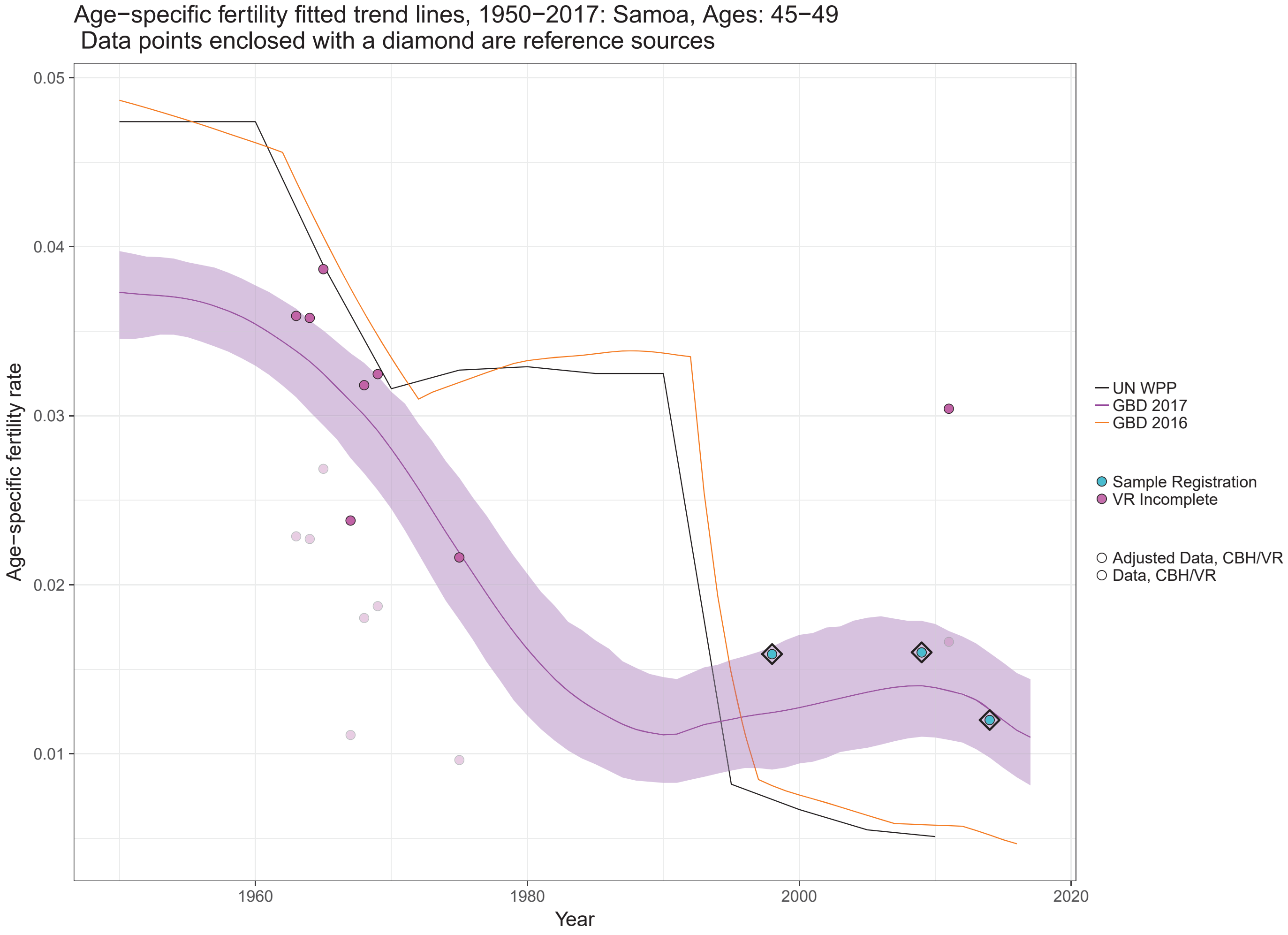
Age−specific fertility fitted trend lines, 1950−2017: Samoa, Ages: 45−49
Data points enclosed with a diamond are reference sources
Age−specific fertility rate
0.05
0.04
0.03
0.02
0.01
1960
1980
2000
2020
Year
UN WPP
GBD 2017
GBD 2016
Sample Registration
VR Incomplete
Adjusted Data, CBH/VR
Data, CBH/VR

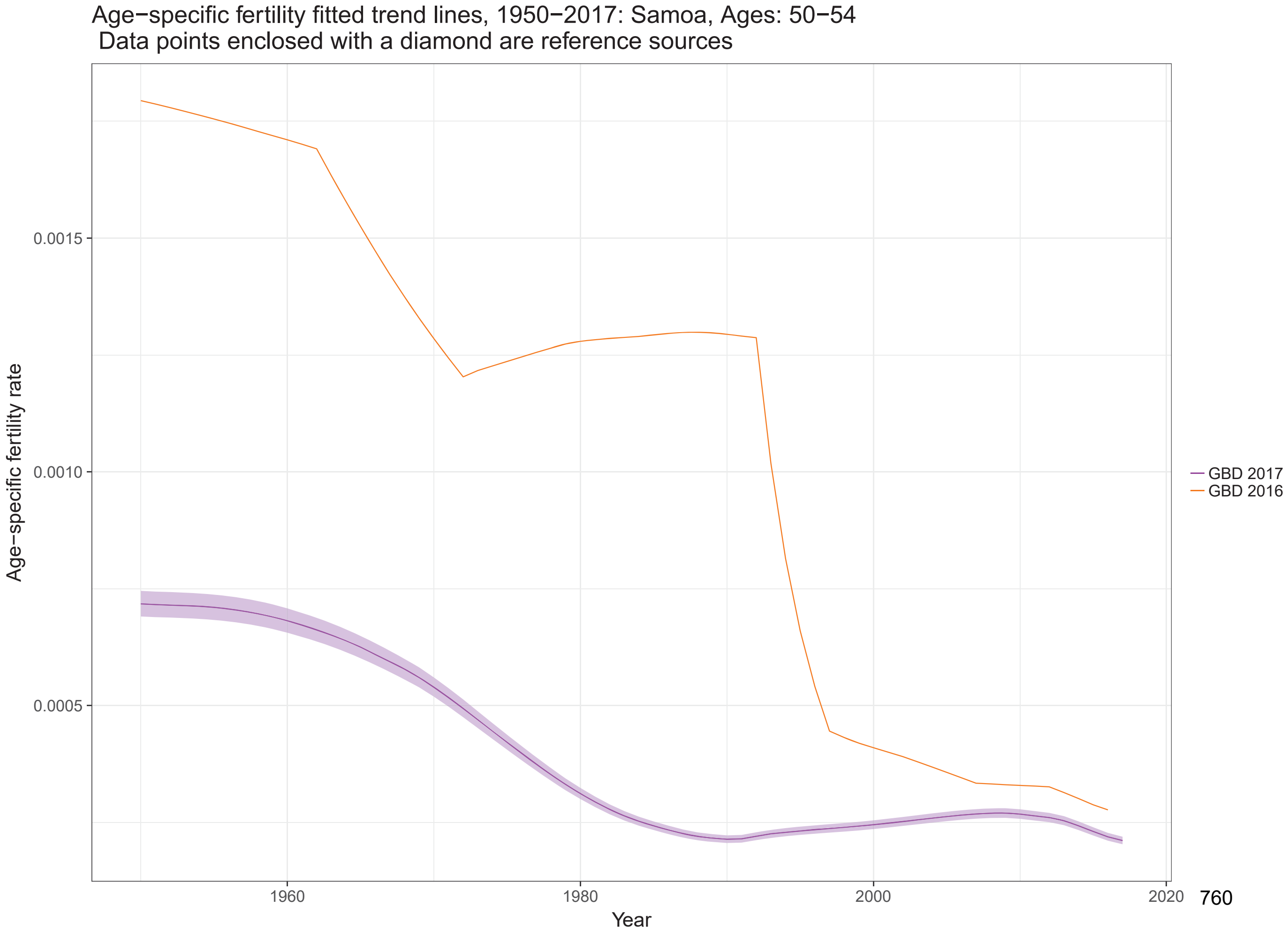
Age−specific fertility fitted trend lines, 1950−2017: Samoa, Ages: 50−54
Data points enclosed with a diamond are reference sources
Age−specific fertility rate
0.0015
0.0010
0.0005
1960
1980
2000
2020
Year
GBD 2017
GBD 2016

Age−specific fertility fitted trend lines, 1950−2017: Sao Tome and Principe, TFR
Data points enclosed with a diamond are reference sources

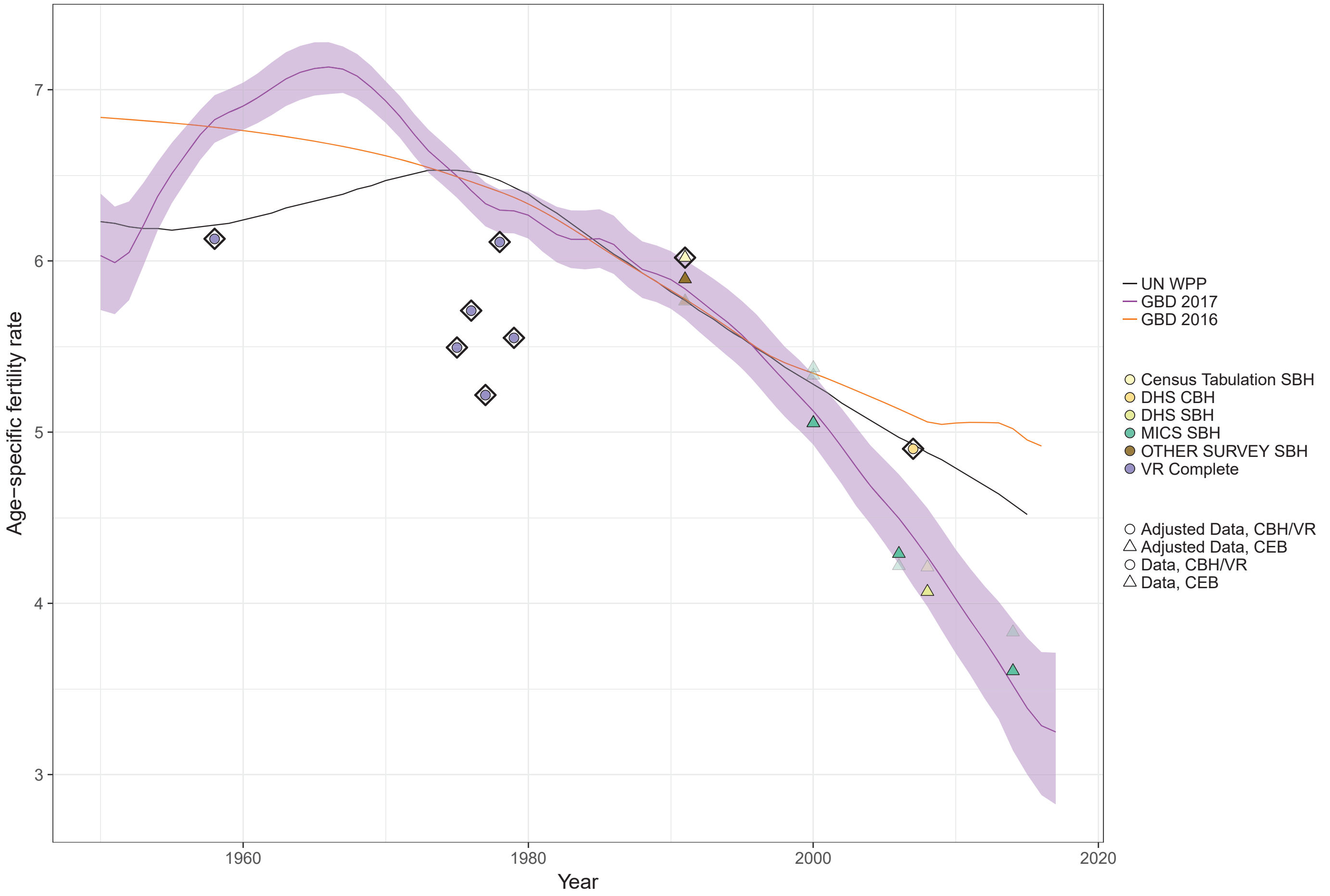

Age−specific fertility fitted trend lines, 1950−2017: Sao Tome and Principe, Ages: 10−14
Data points enclosed with a diamond are reference sources

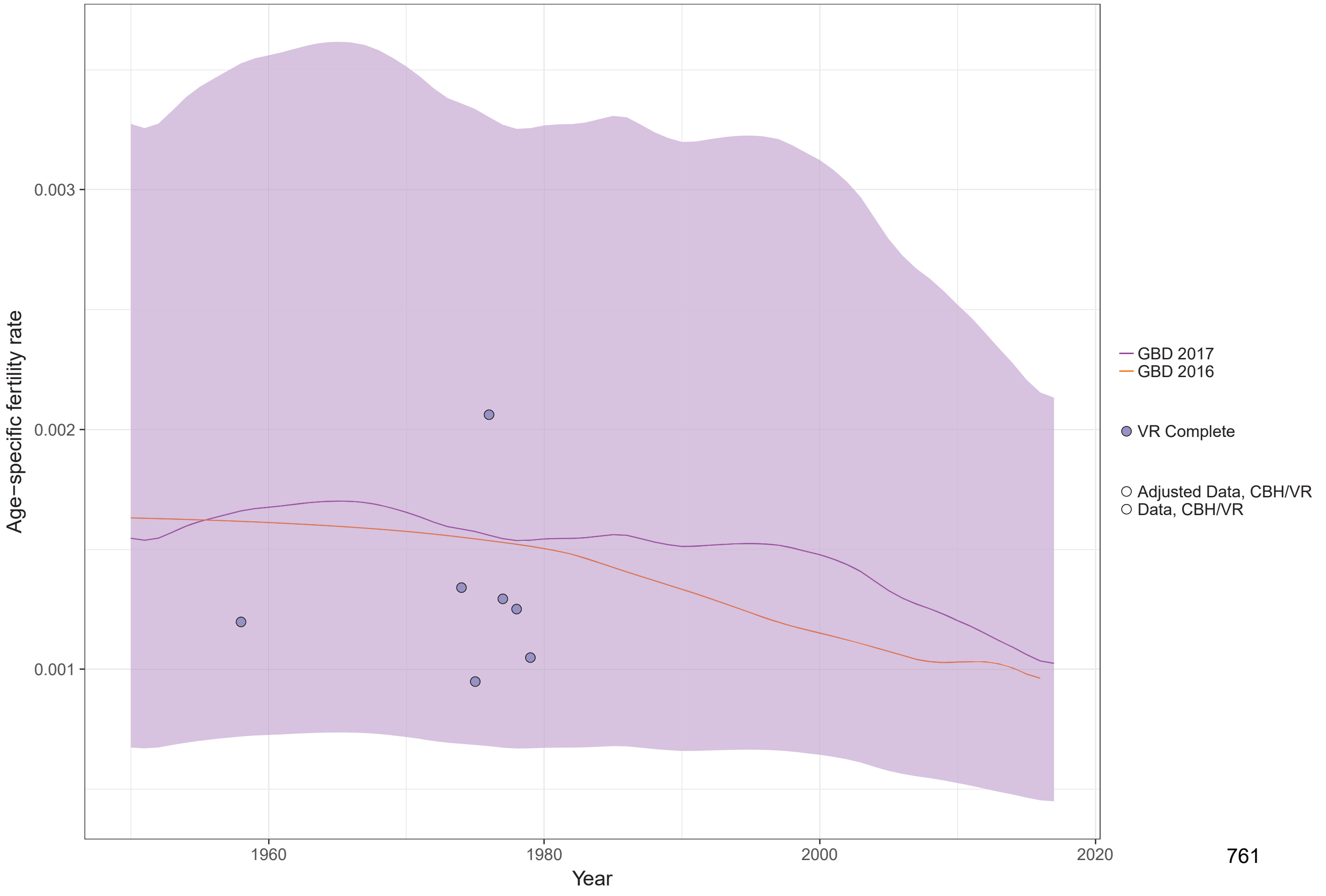

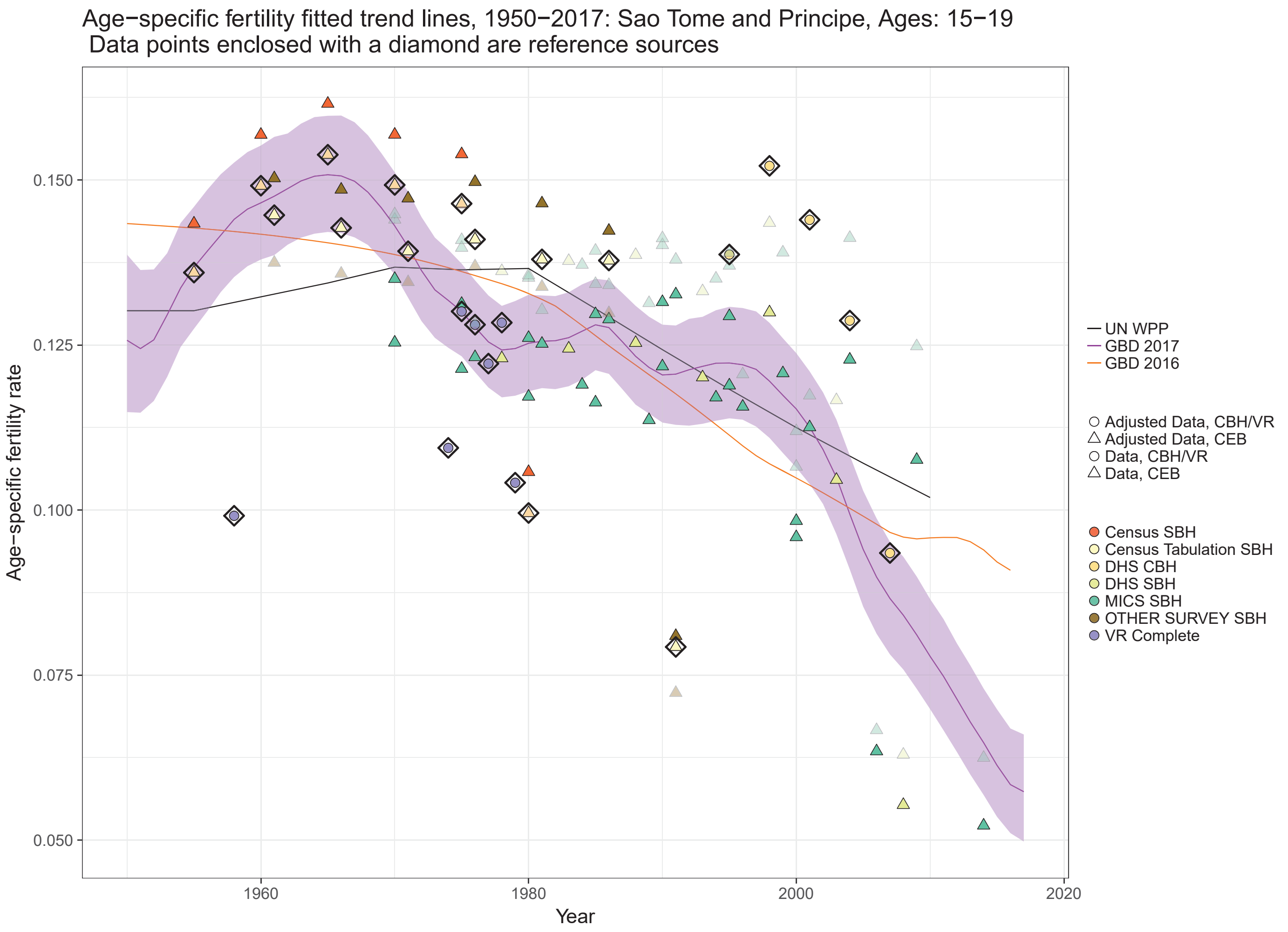

Age−specific fertility fitted trend lines, 1950−2017: Sao Tome and Principe, Ages: 15−19
Data points enclosed with a diamond are reference sources
Age−specific fertility rate
Year
UN WPP
GBD 2017
GBD 2016
Adjusted Data, CBH/VR
Adjusted Data, CEB
Data, CBH/VR
Data, CEB
Census SBH
Census Tabulation SBH
DHS CBH
DHS SBH
MICS SBH
OTHER SURVEY SBH
VR Complete

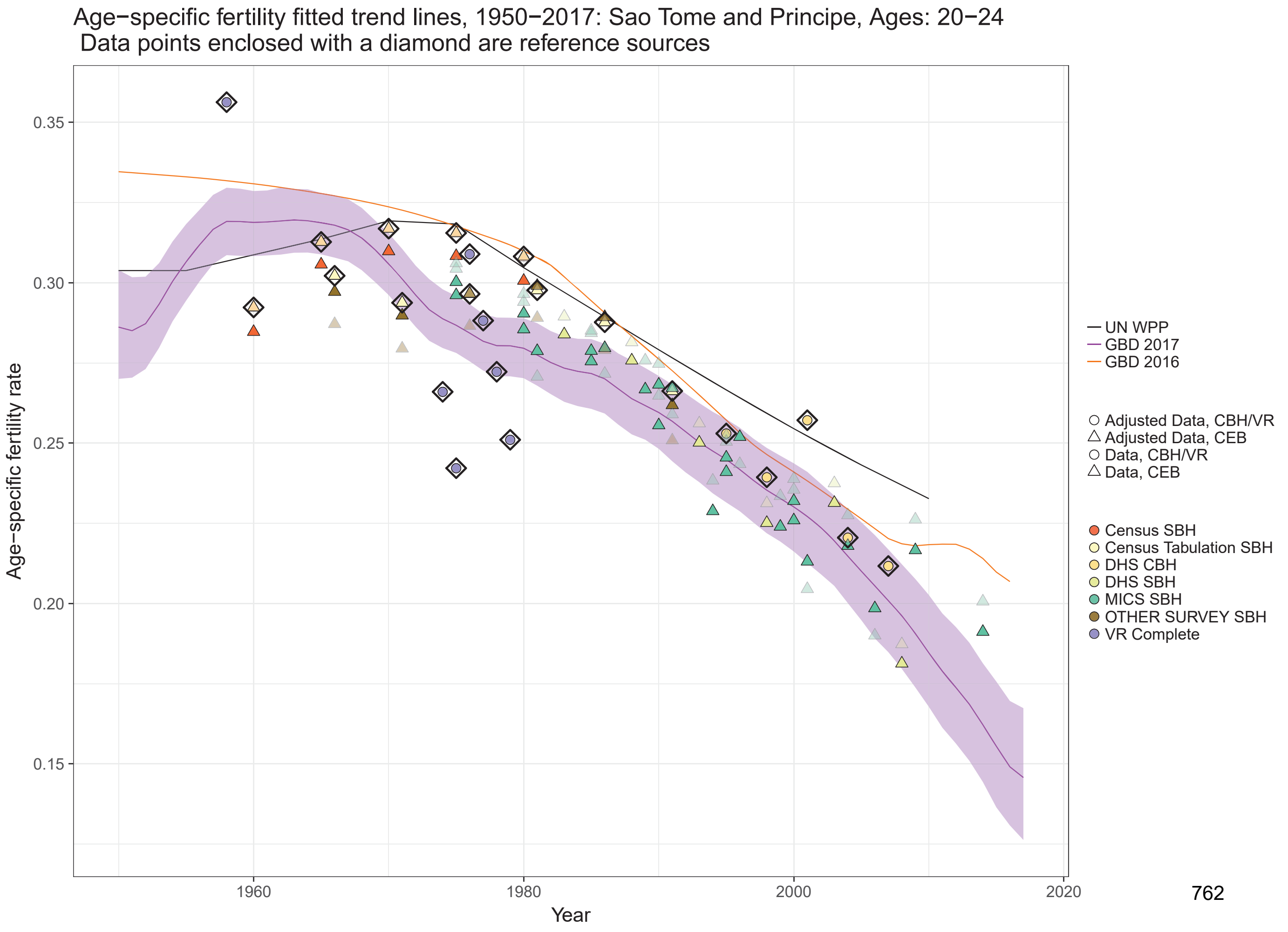

Age−specific fertility fitted trend lines, 1950−2017: Sao Tome and Principe, Ages: 20−24
Data points enclosed with a diamond are reference sources
Age−specific fertility rate
Year
UN WPP
GBD 2017
GBD 2016
Adjusted Data, CBH/VR
Adjusted Data, CEB
Data, CBH/VR
Data, CEB
Census SBH
Census Tabulation SBH
DHS CBH
DHS SBH
MICS SBH
OTHER SURVEY SBH
VR Complete

Age−specific fertility fitted trend lines, 1950−2017: Sao Tome and Principe, Ages: 25−29
Data points enclosed with a diamond are reference sources

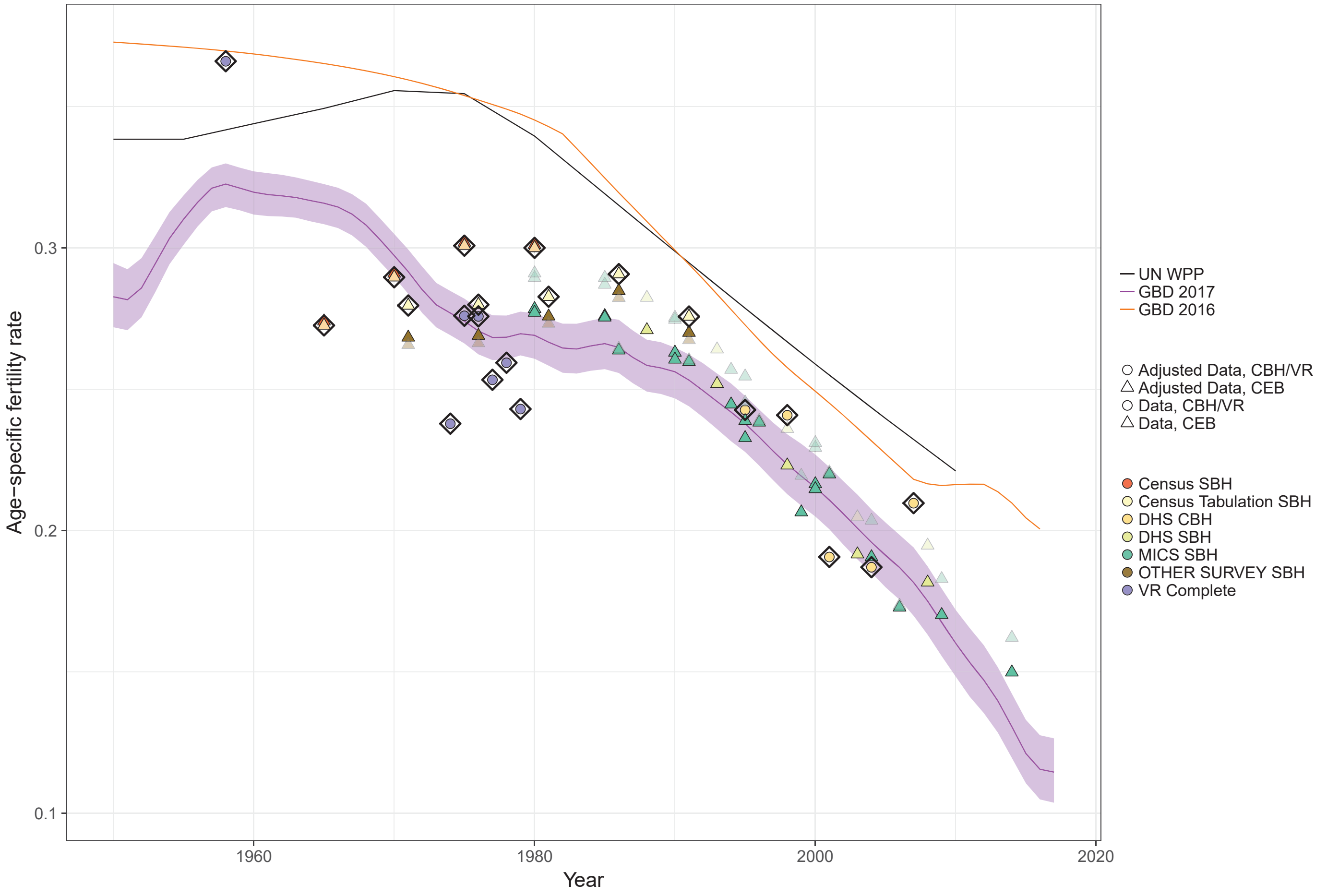

Age−specific fertility fitted trend lines, 1950−2017: Sao Tome and Principe, Ages: 30−34
Data points enclosed with a diamond are reference sources

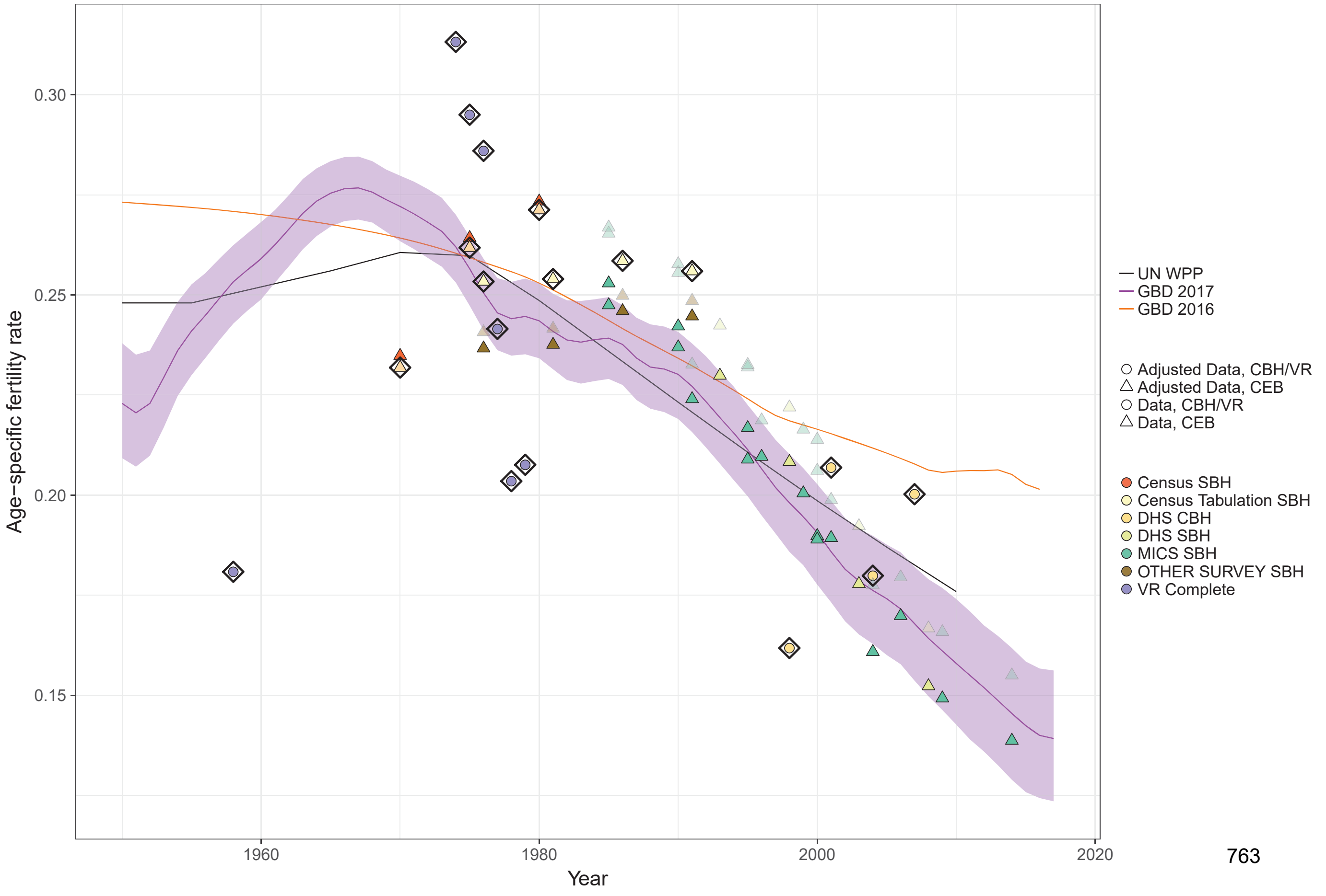

Age−specific fertility fitted trend lines, 1950−2017: Sao Tome and Principe, Ages: 35−39
Data points enclosed with a diamond are reference sources

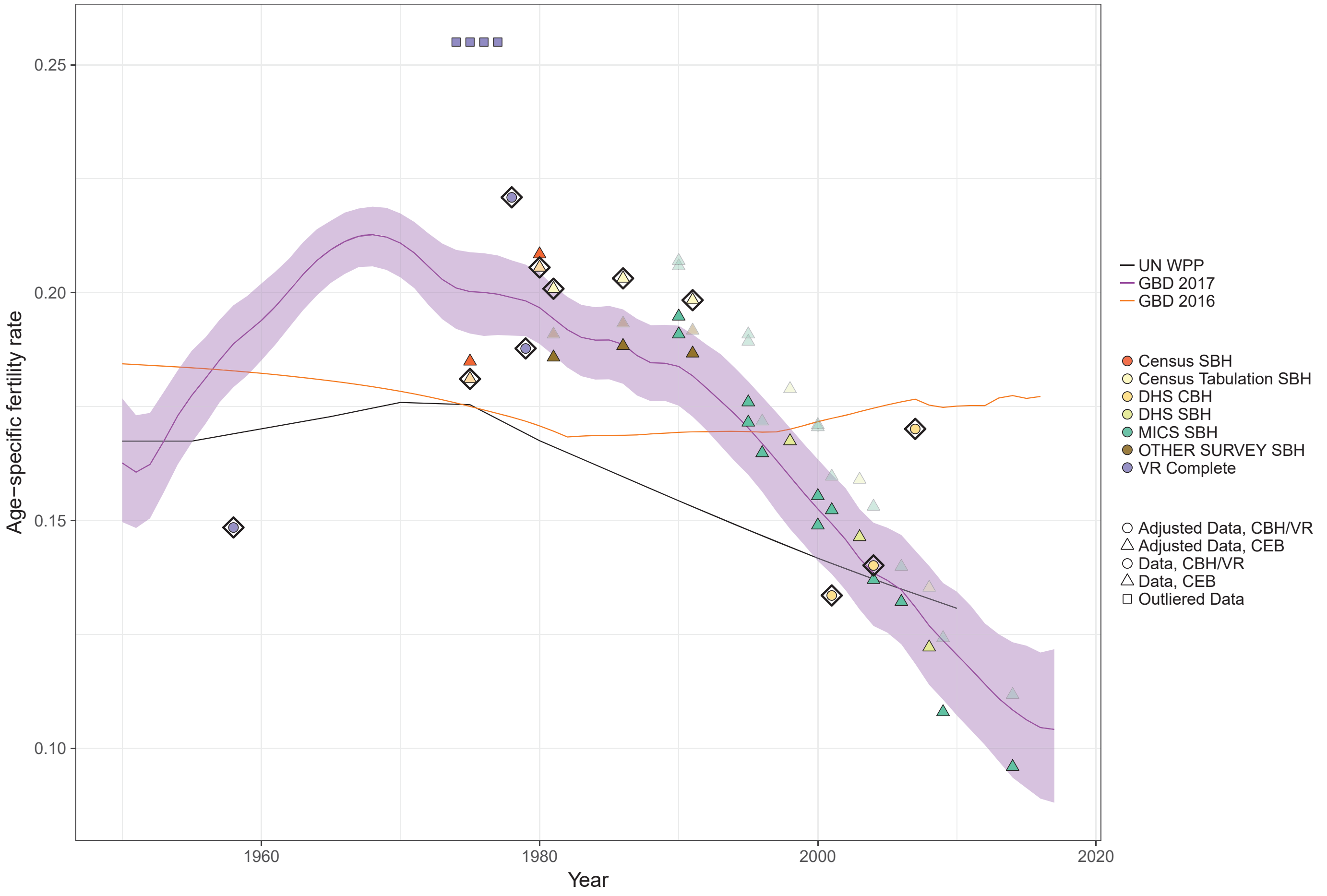

Age−specific fertility fitted trend lines, 1950−2017: Sao Tome and Principe, Ages: 40−44
Data points enclosed with a diamond are reference sources

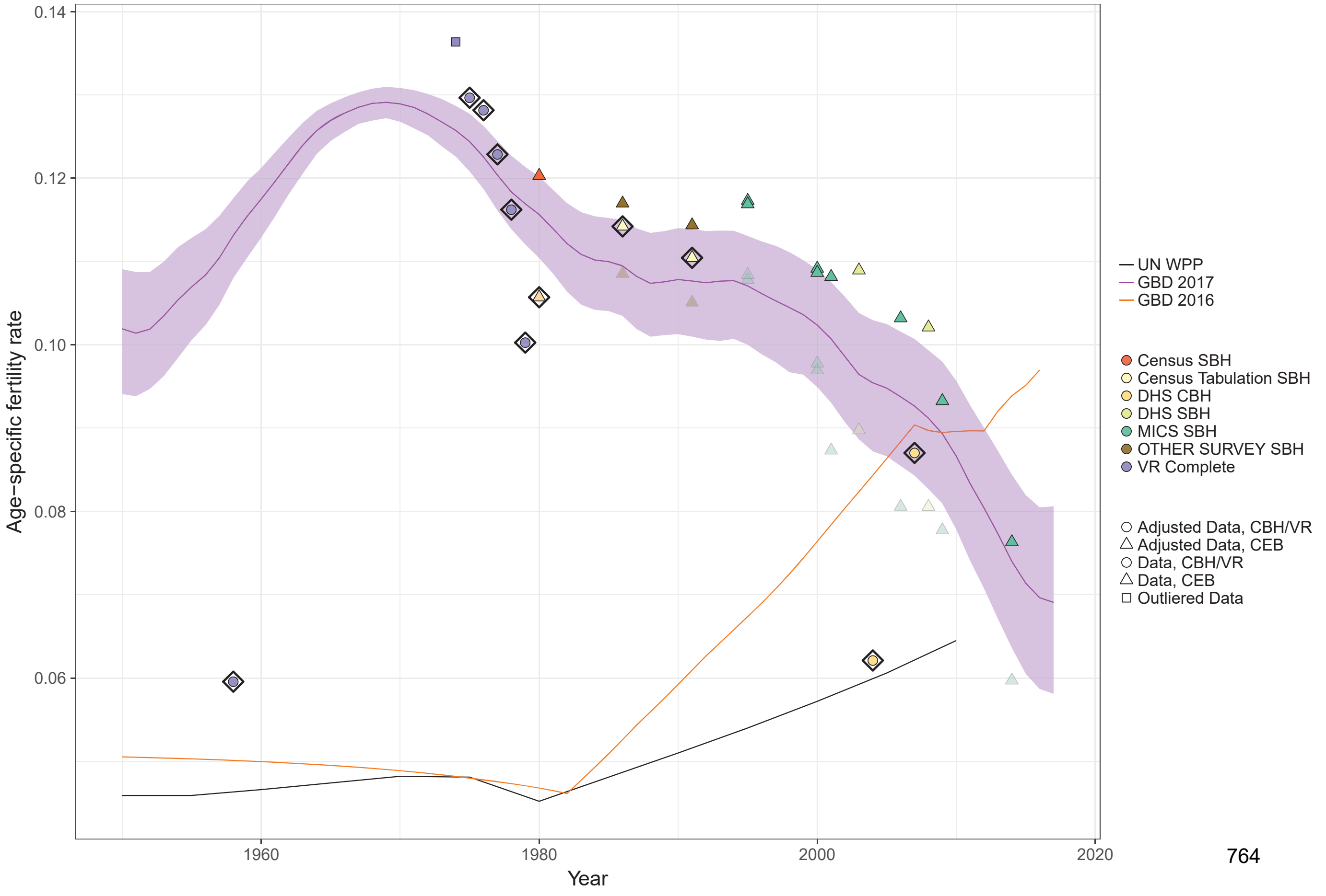

Age−specific fertility fitted trend lines, 1950−2017: Sao Tome and Principe, Ages: 45−49
Data points enclosed with a diamond are reference sources

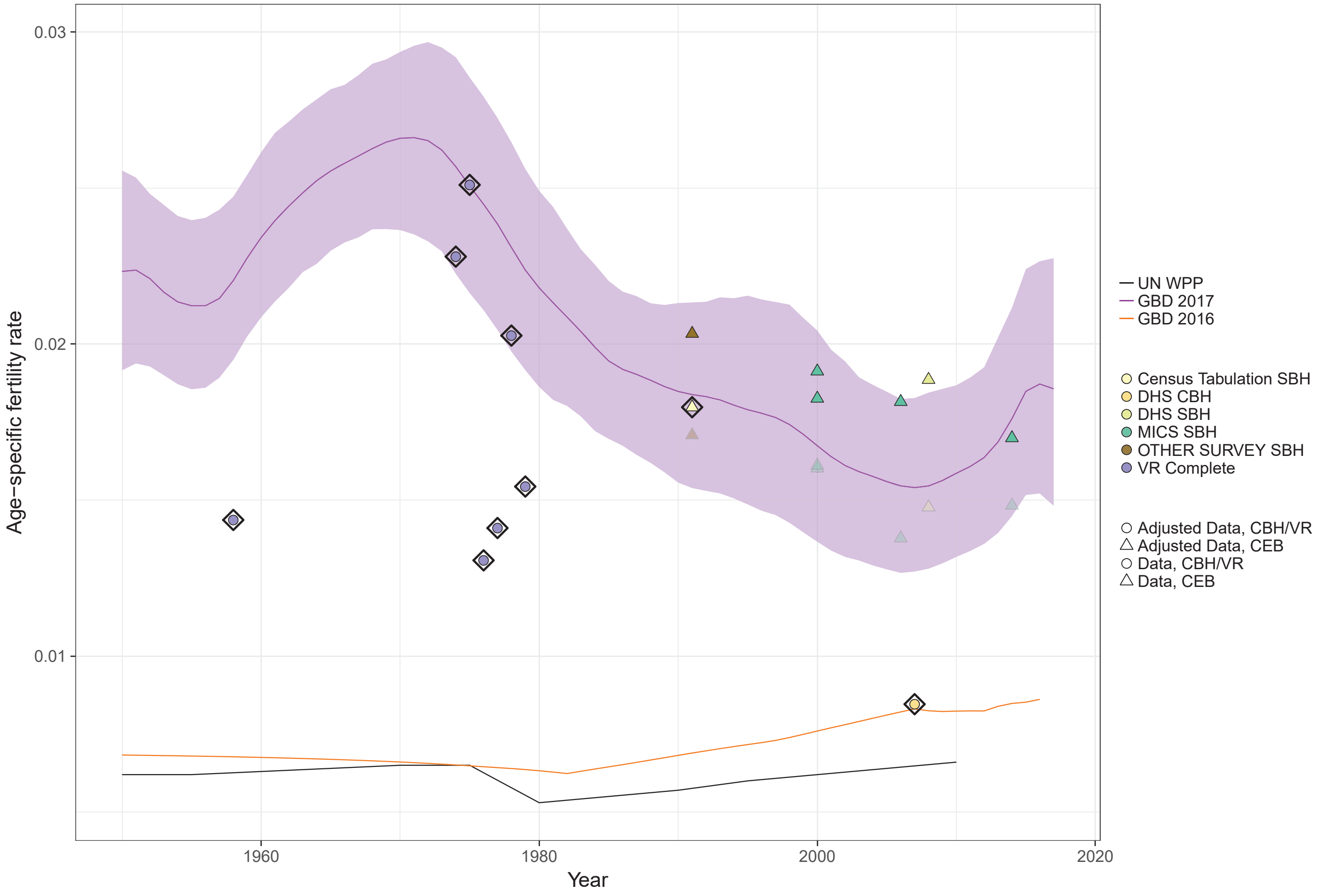

Age−specific fertility fitted trend lines, 1950−2017: Sao Tome and Principe, Ages: 50−54
Data points enclosed with a diamond are reference sources

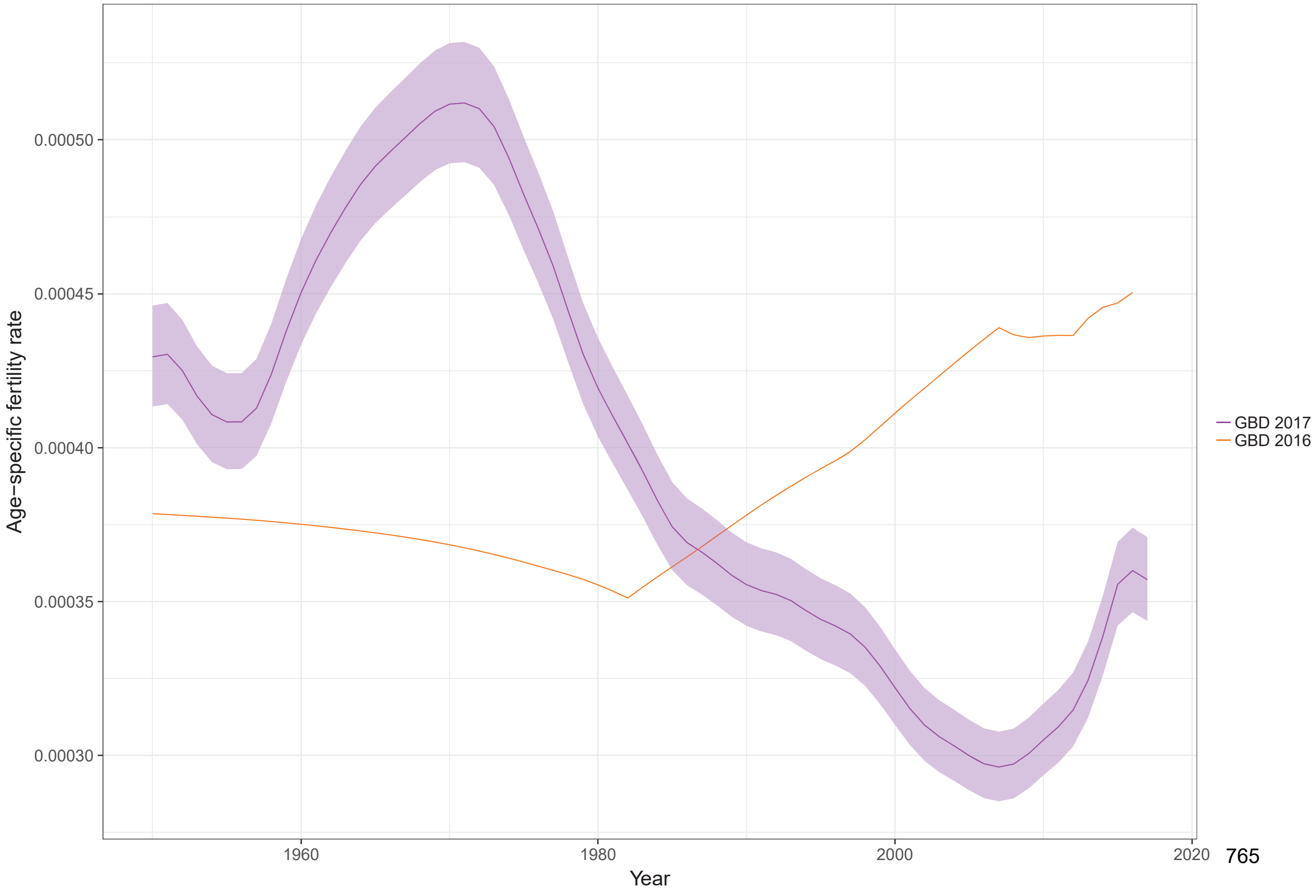

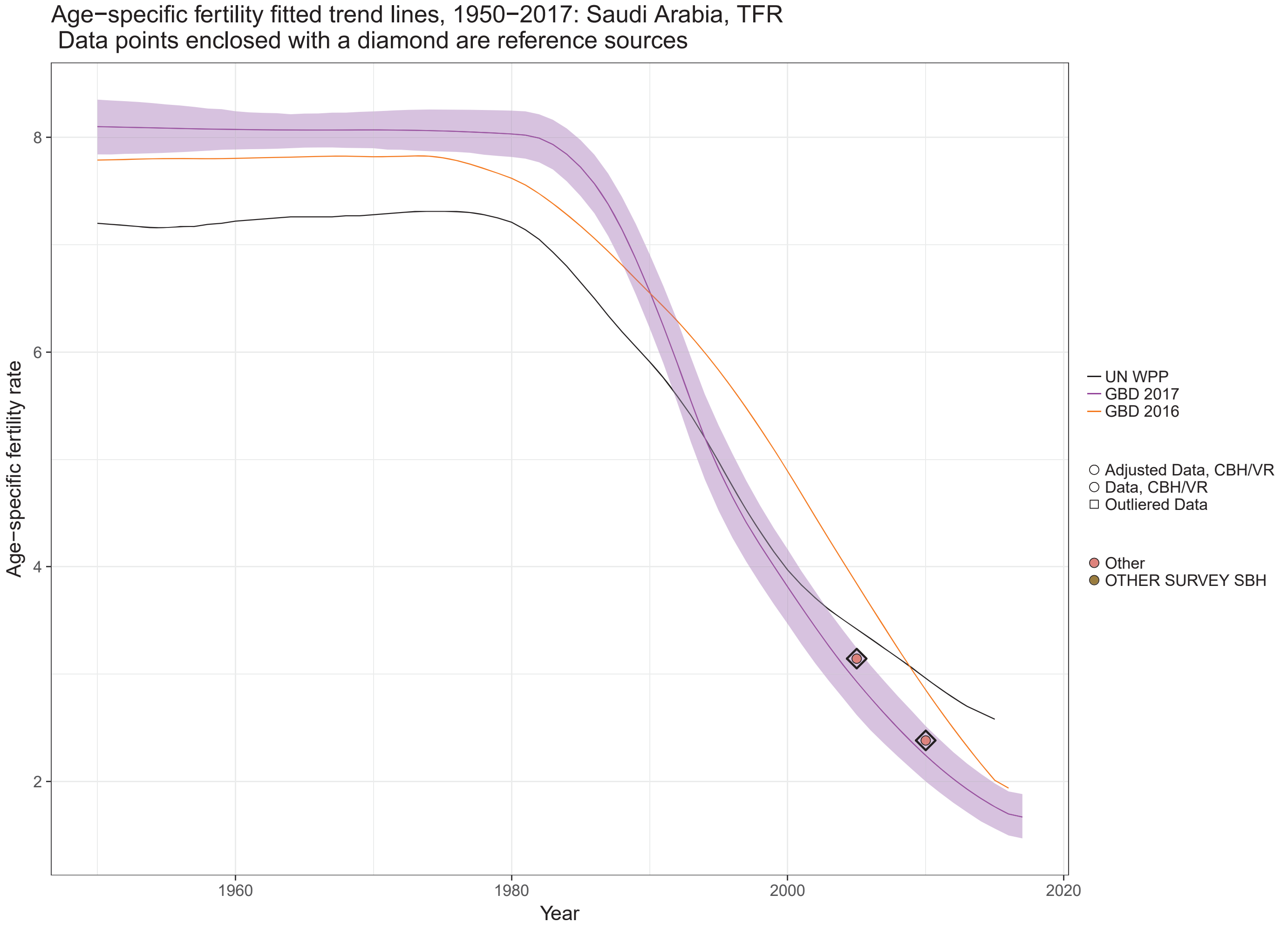

Age−specific fertility fitted trend lines, 1950−2017: Saudi Arabia, TFR
Data points enclosed with a diamond are reference sources
Age−specific fertility rate
Year
8
6
4
2
1960
1980
2000
2020
UN WPP
GBD 2017
GBD 2016
Adjusted Data, CBH/VR
Data, CBH/VR
Outliered Data
Other
OTHER SURVEY SBH

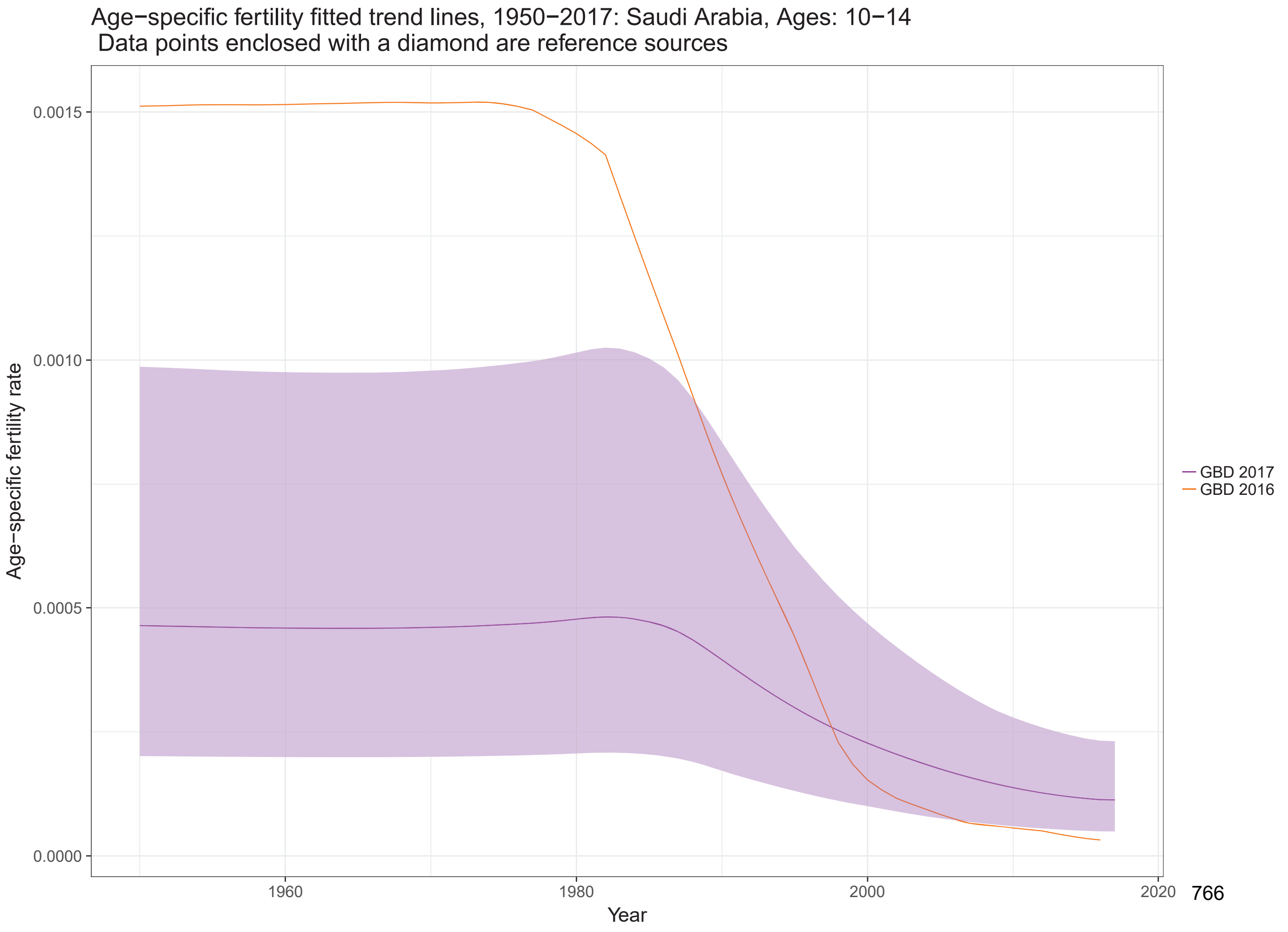

Age−specific fertility fitted trend lines, 1950−2017: Saudi Arabia, Ages: 10−14
Data points enclosed with a diamond are reference sources
Age−specific fertility rate
Year
0.0015
0.0010
0.0005
0.0000
1960
1980
2000
2020
GBD 2017
GBD 2016

Age−specific fertility fitted trend lines, 1950−2017: Saudi Arabia, Ages: 15−19
Data points enclosed with a diamond are reference sources

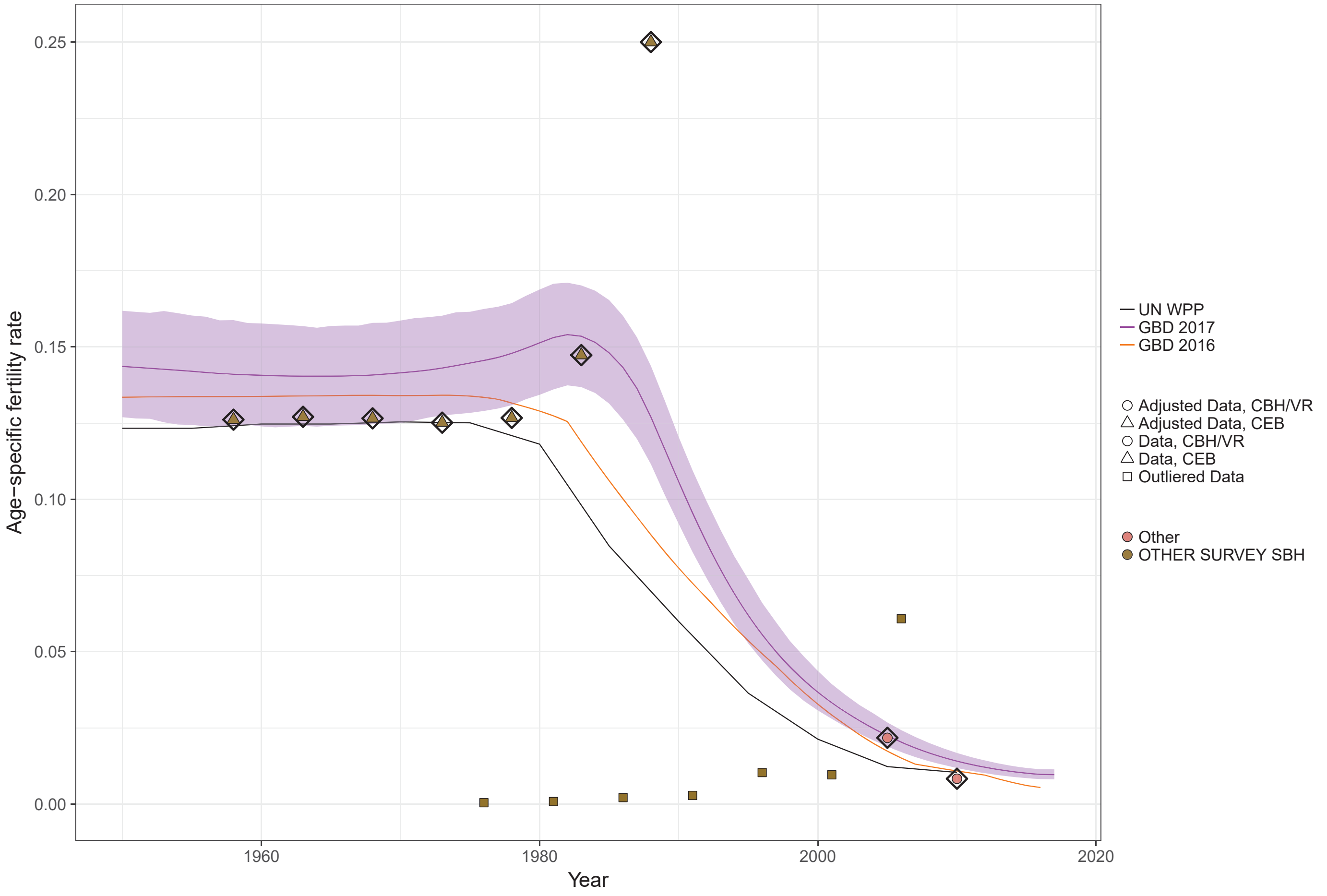

Age−specific fertility fitted trend lines, 1950−2017: Saudi Arabia, Ages: 20−24
Data points enclosed with a diamond are reference sources

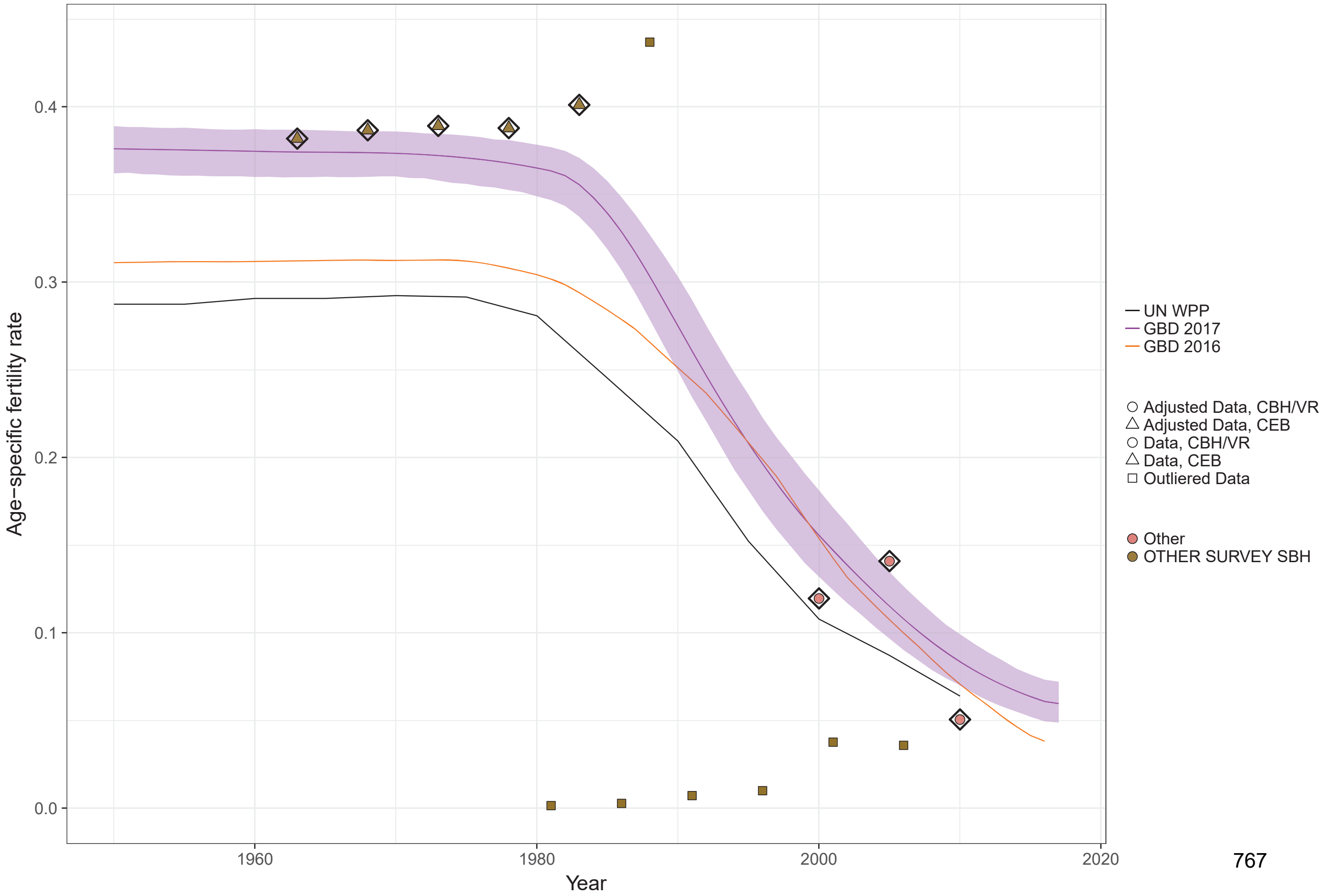

Age−specific fertility fitted trend lines, 1950−2017: Saudi Arabia, Ages: 25−29
Data points enclosed with a diamond are reference sources

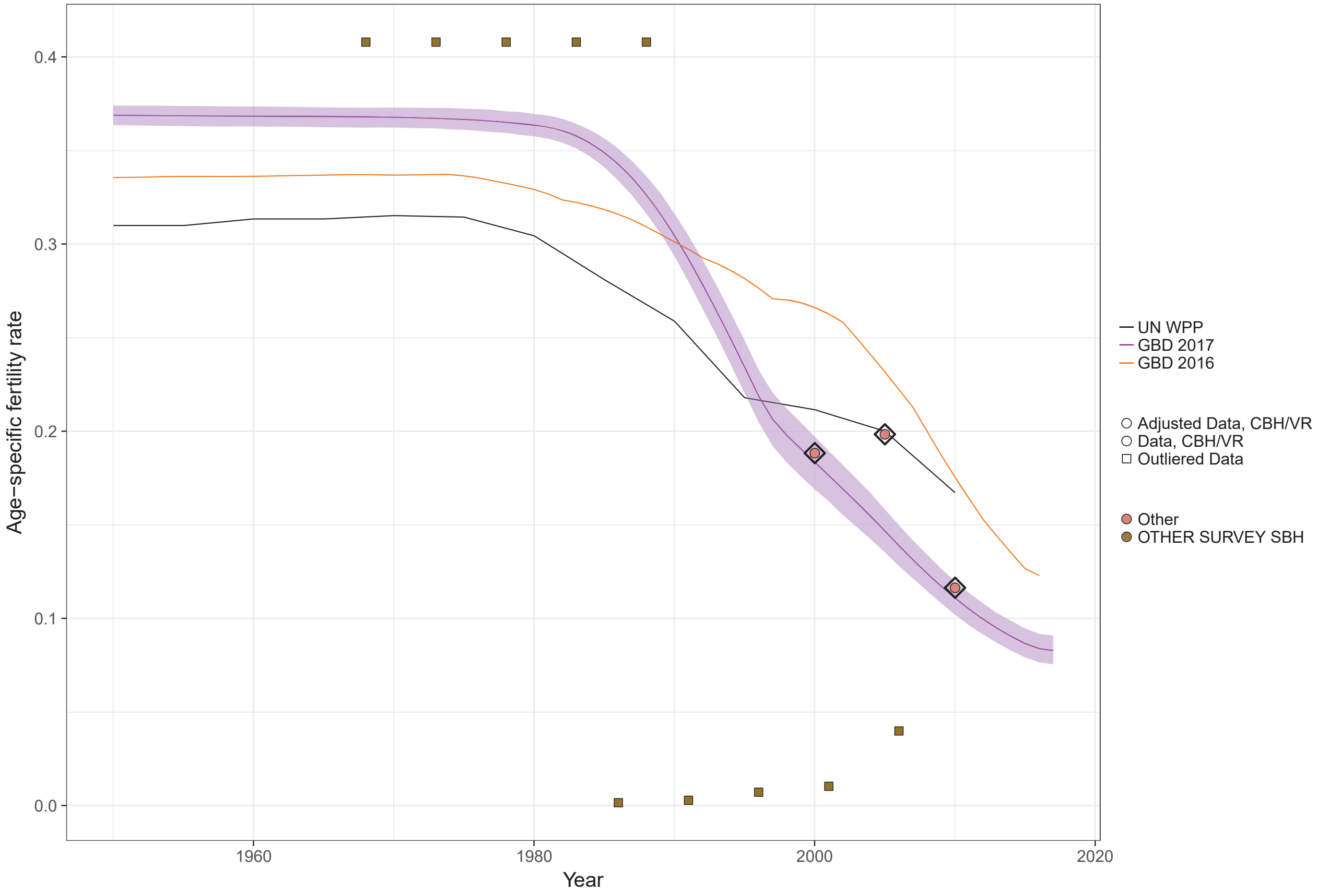

Age−specific fertility fitted trend lines, 1950−2017: Saudi Arabia, Ages: 30−34
Data points enclosed with a diamond are reference sources

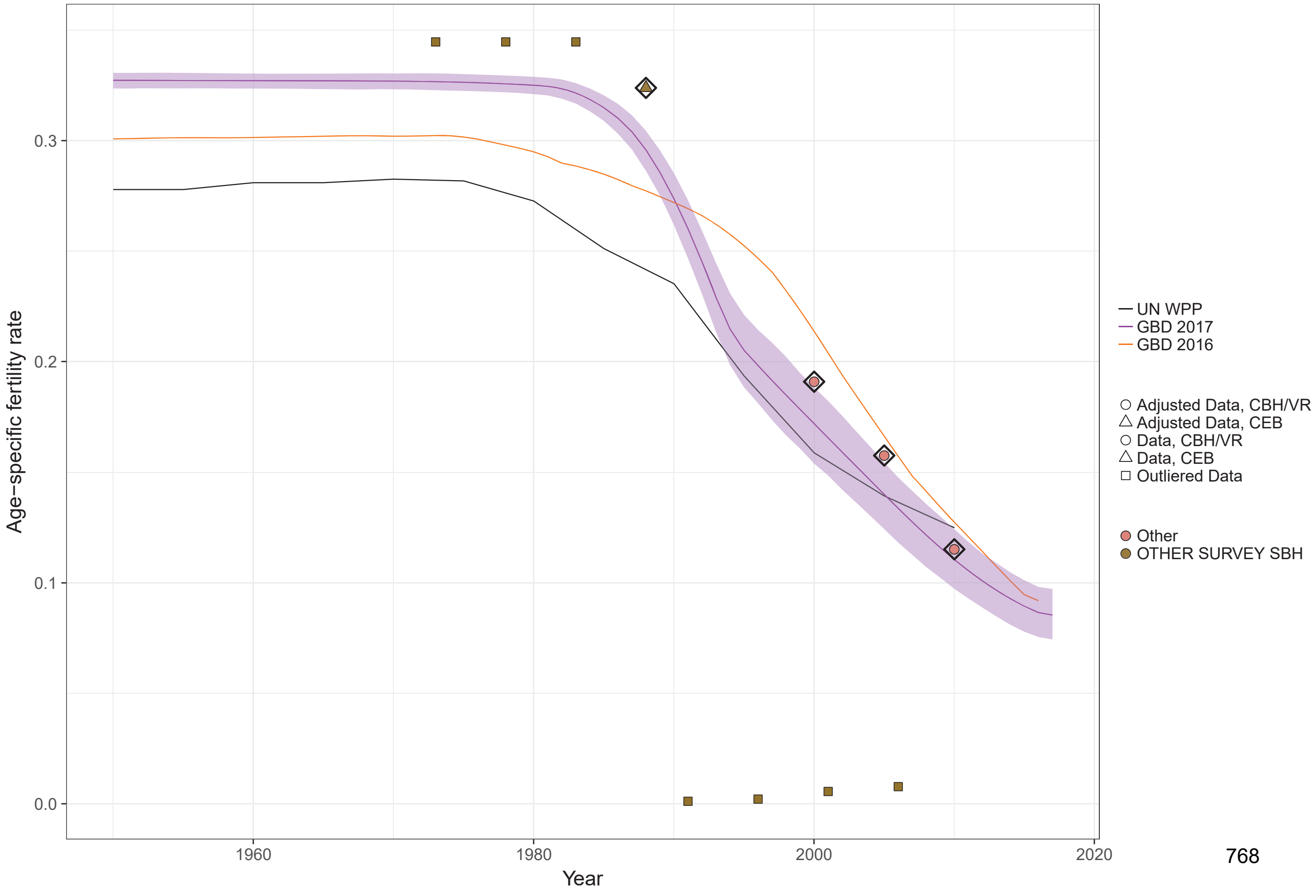

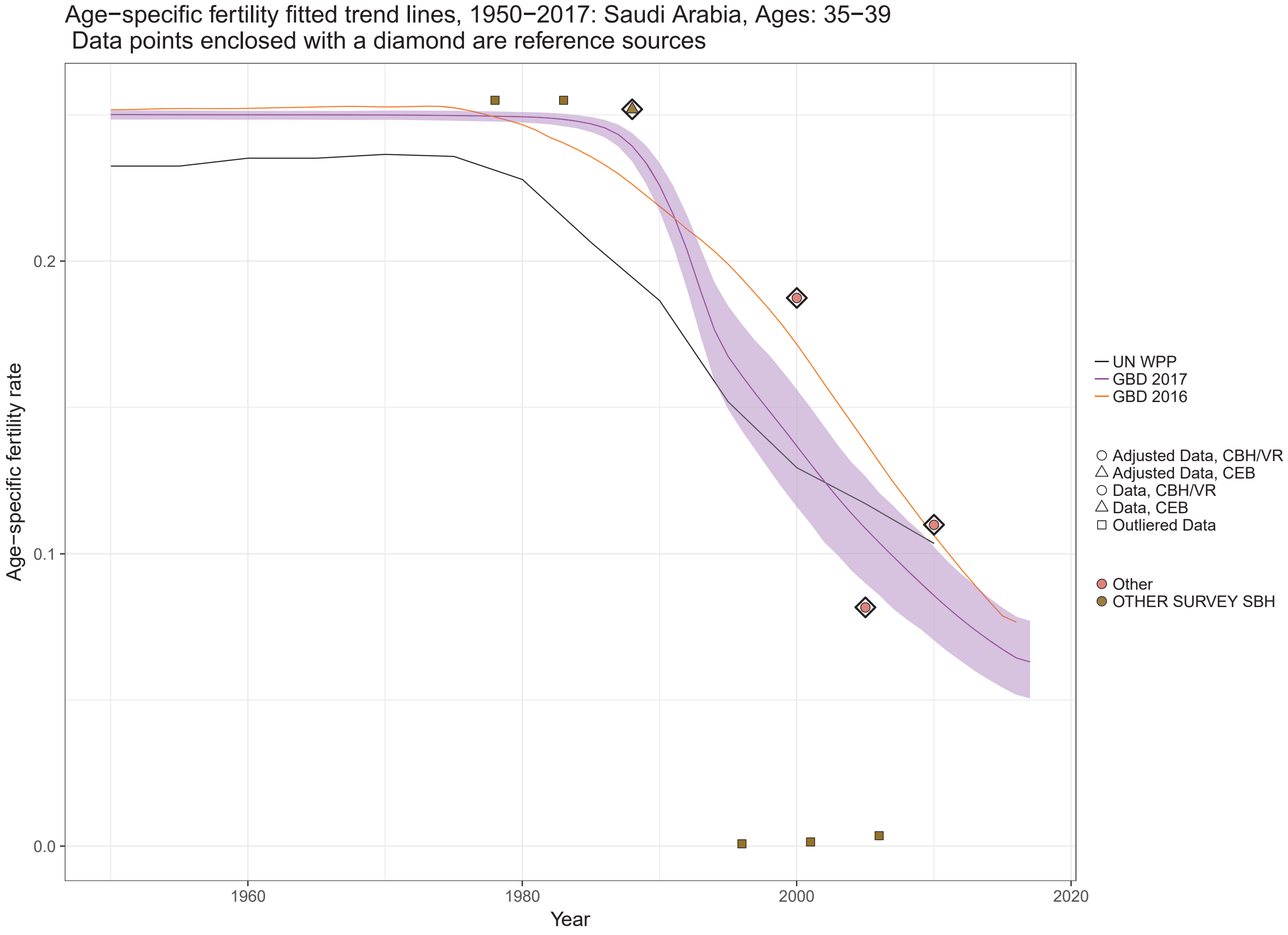
Age−specific fertility fitted trend lines, 1950−2017: Saudi Arabia, Ages: 35−39
Data points enclosed with a diamond are reference sources
Age−specific fertility rate
0.0
0.1
0.2
1960
1980
2000
2020
Year
UN WPP
GBD 2017
GBD 2016
Adjusted Data, CBH/VR
Adjusted Data, CEB
Data, CBH/VR
Data, CEB
Outliered Data
Other
OTHER SURVEY SBH

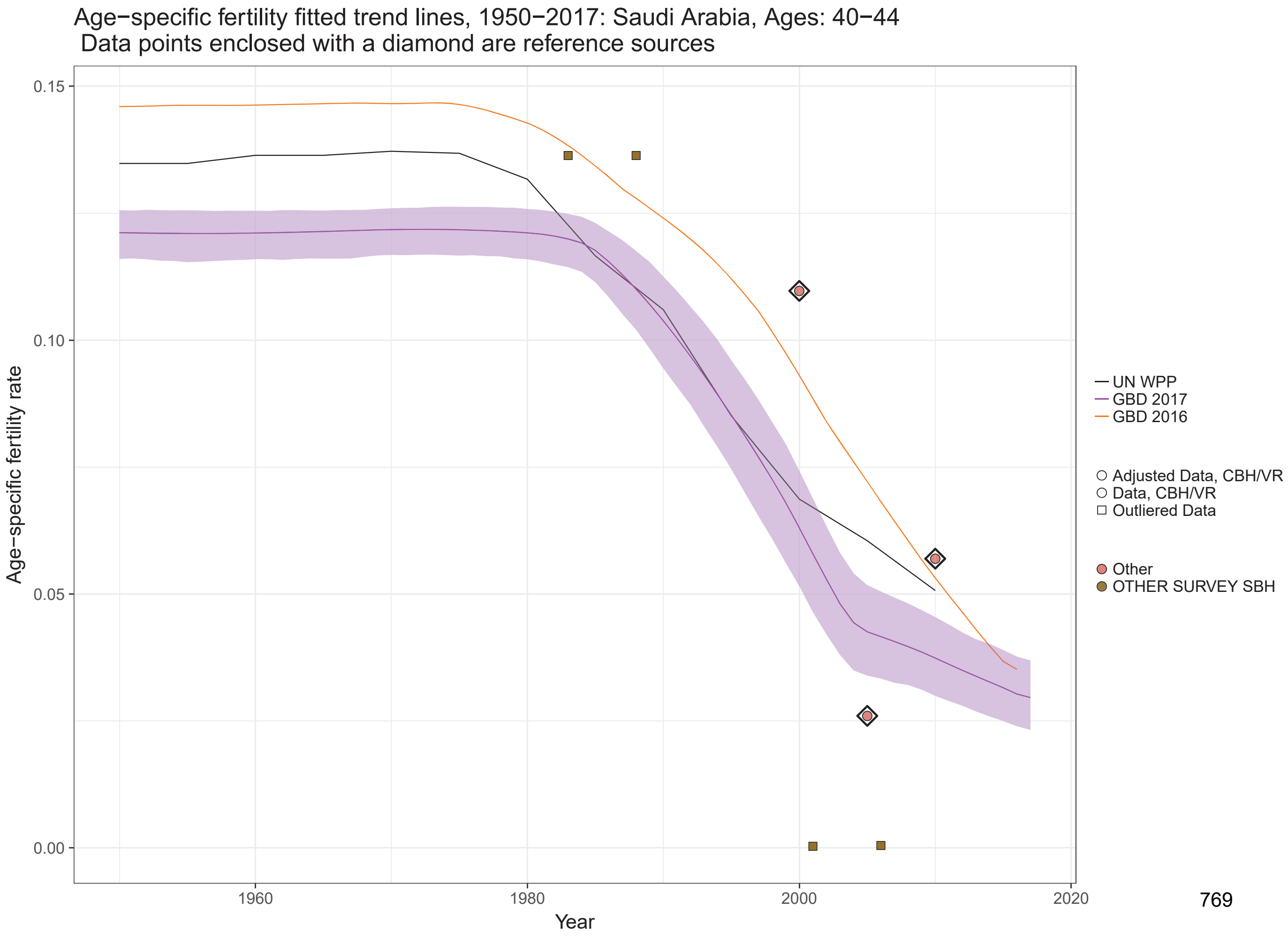
Age−specific fertility fitted trend lines, 1950−2017: Saudi Arabia, Ages: 40−44
Data points enclosed with a diamond are reference sources
Age−specific fertility rate
0.00
0.05
0.10
0.15
1960
1980
2000
2020
Year
UN WPP
GBD 2017
GBD 2016
Adjusted Data, CBH/VR
Data, CBH/VR
Outliered Data
Other
OTHER SURVEY SBH

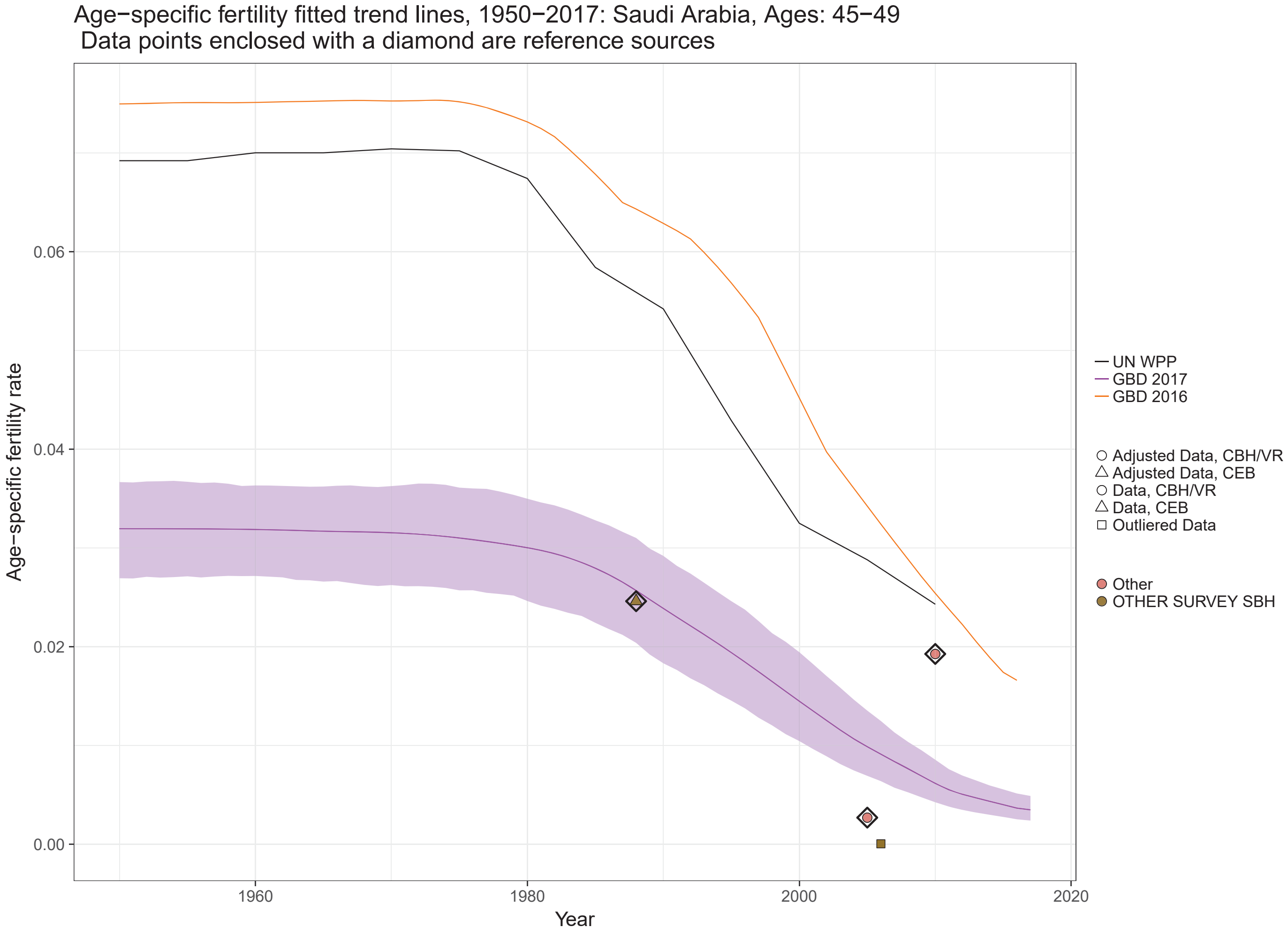

Age−specific fertility fitted trend lines, 1950−2017: Saudi Arabia, Ages: 45−49
Data points enclosed with a diamond are reference sources
Age−specific fertility rate
0.00
0.02
0.04
0.06
1960
1980
2000
2020
Year
UN WPP
GBD 2017
GBD 2016
Adjusted Data, CBH/VR
Adjusted Data, CEB
Data, CBH/VR
Data, CEB
Outliered Data
Other
OTHER SURVEY SBH

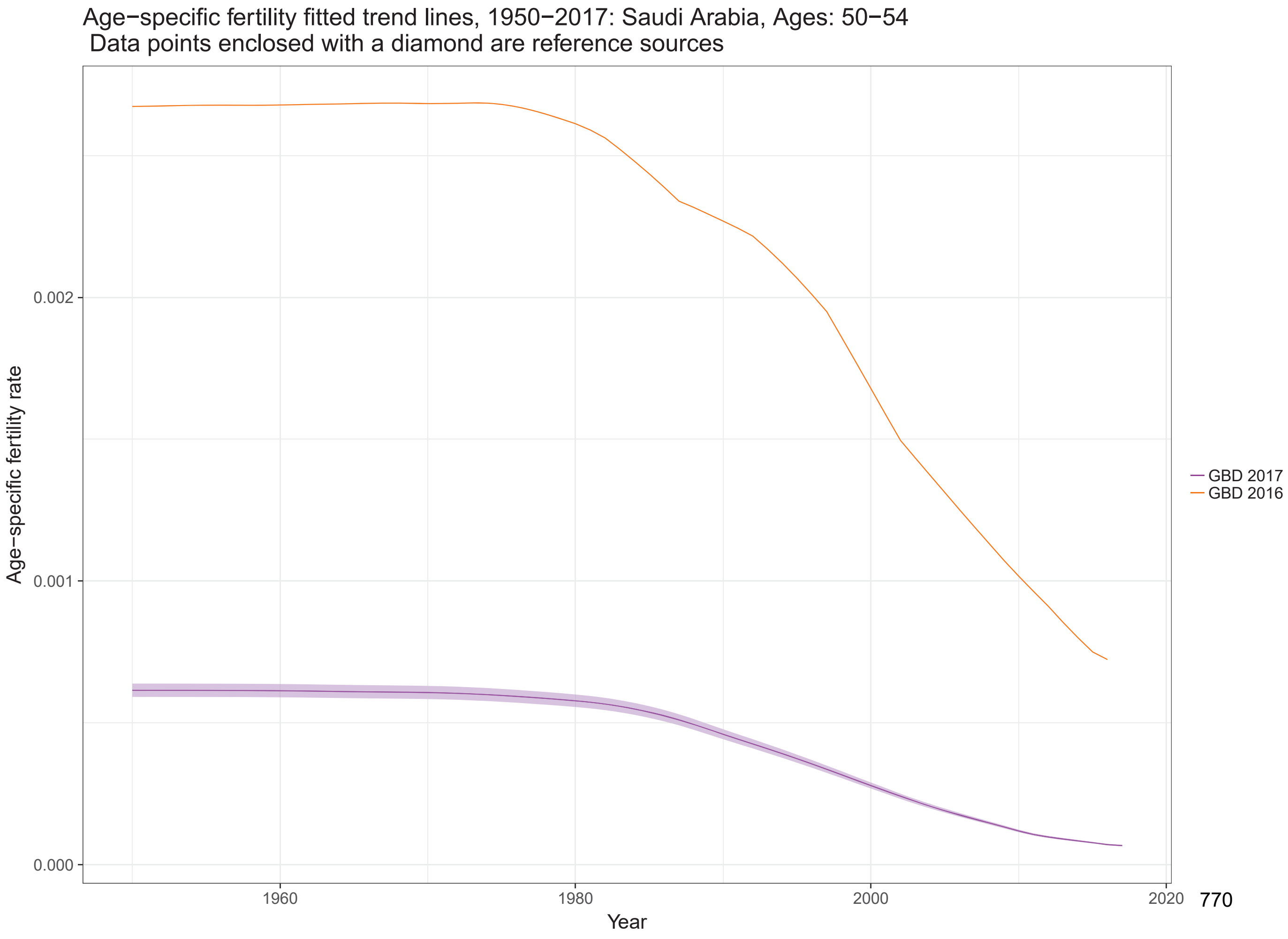

Age−specific fertility fitted trend lines, 1950−2017: Saudi Arabia, Ages: 50−54
Data points enclosed with a diamond are reference sources
Age−specific fertility rate
0.000
0.001
0.002
1960
1980
2000
2020
Year
GBD 2017
GBD 2016

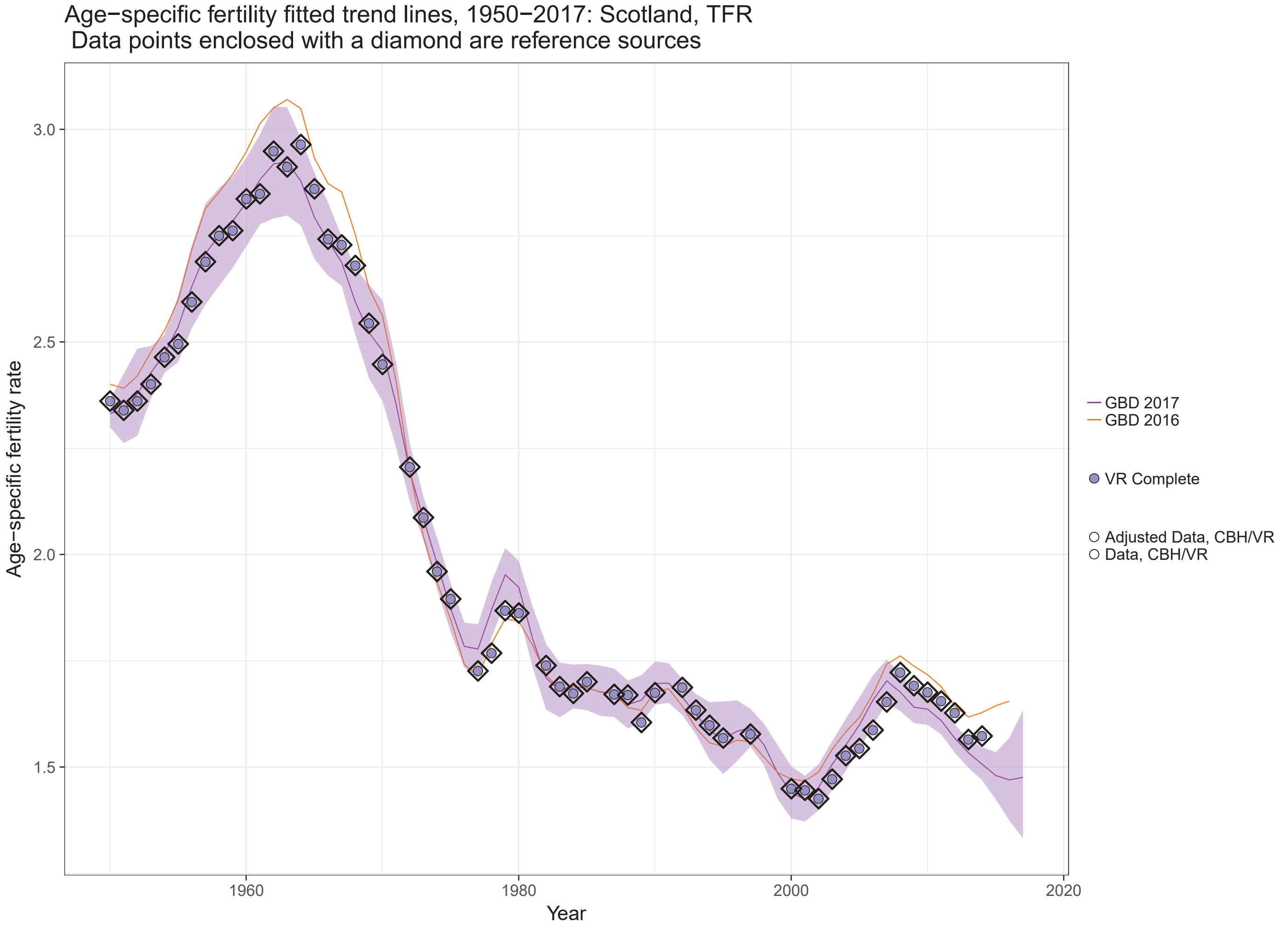

Age−specific fertility fitted trend lines, 1950−2017: Scotland, TFR
Data points enclosed with a diamond are reference sources
Age−specific fertility rate
3.0
2.5
2.0
1.5
1960
1980
2000
2020
Year
GBD 2017
GBD 2016
VR Complete
Adjusted Data, CBH/VR
Data, CBH/VR

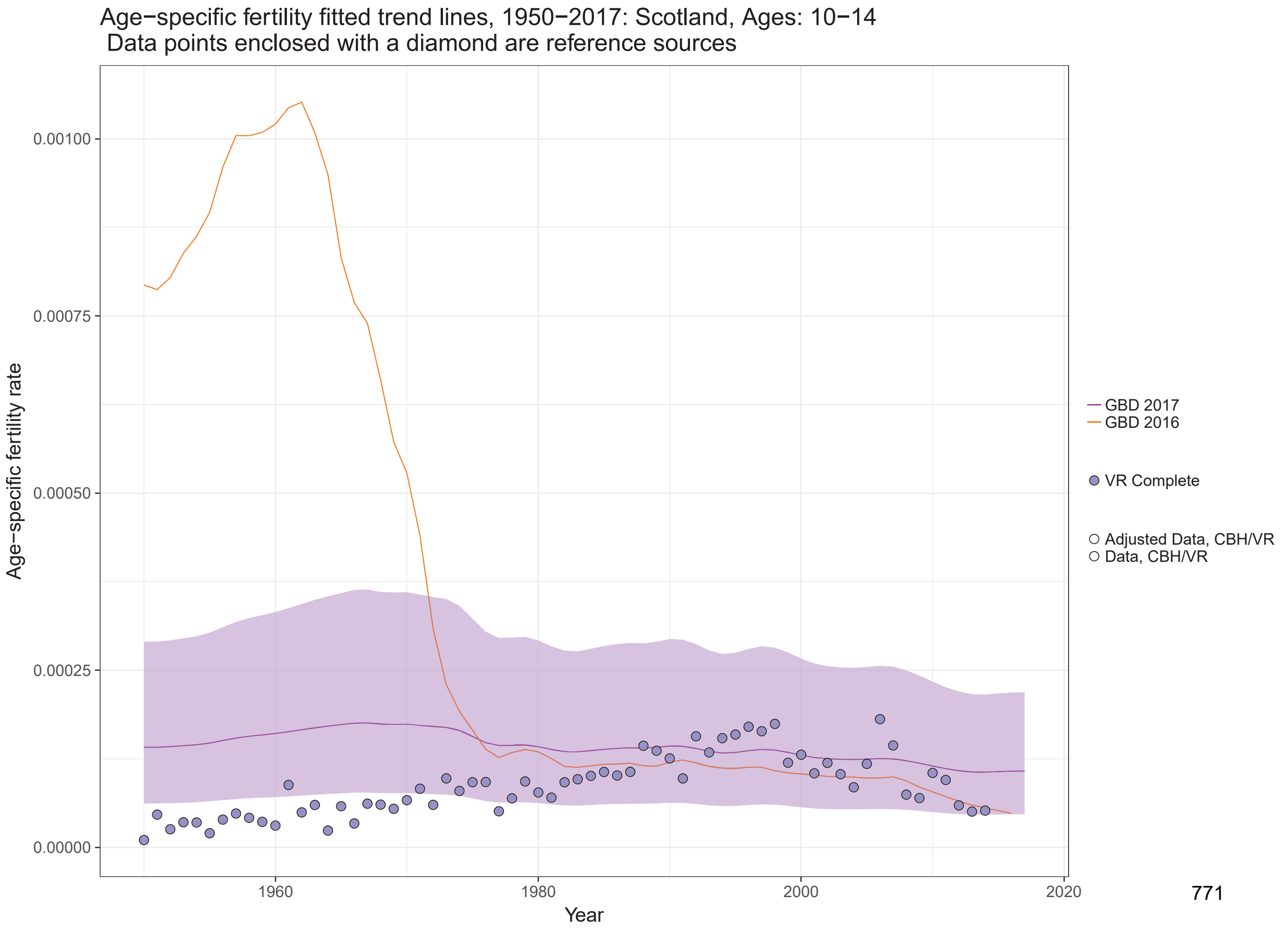

Age−specific fertility fitted trend lines, 1950−2017: Scotland, Ages: 10−14
Data points enclosed with a diamond are reference sources
Age−specific fertility rate
0.00100
0.00075
0.00050
0.00025
0.00000
1960
1980
2000
2020
Year
GBD 2017
GBD 2016
VR Complete
Adjusted Data, CBH/VR
Data, CBH/VR

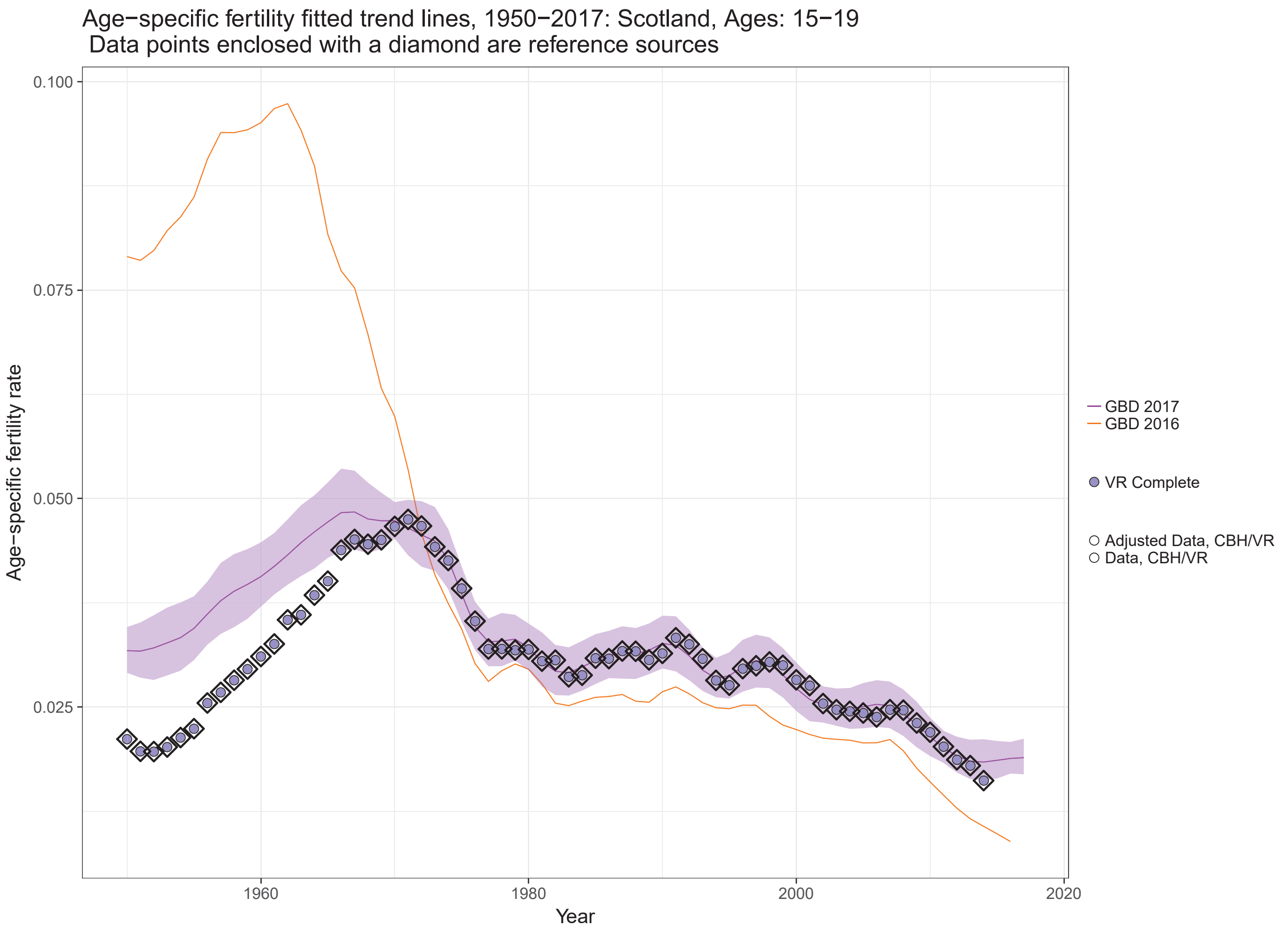

Age−specific fertility fitted trend lines, 1950−2017: Scotland, Ages: 15−19
Data points enclosed with a diamond are reference sources
0.100
0.075
0.050
0.025
Age−specific fertility rate
1960
1980
2000
2020
Year
GBD 2017
GBD 2016
VR Complete
Adjusted Data, CBH/VR
Data, CBH/VR

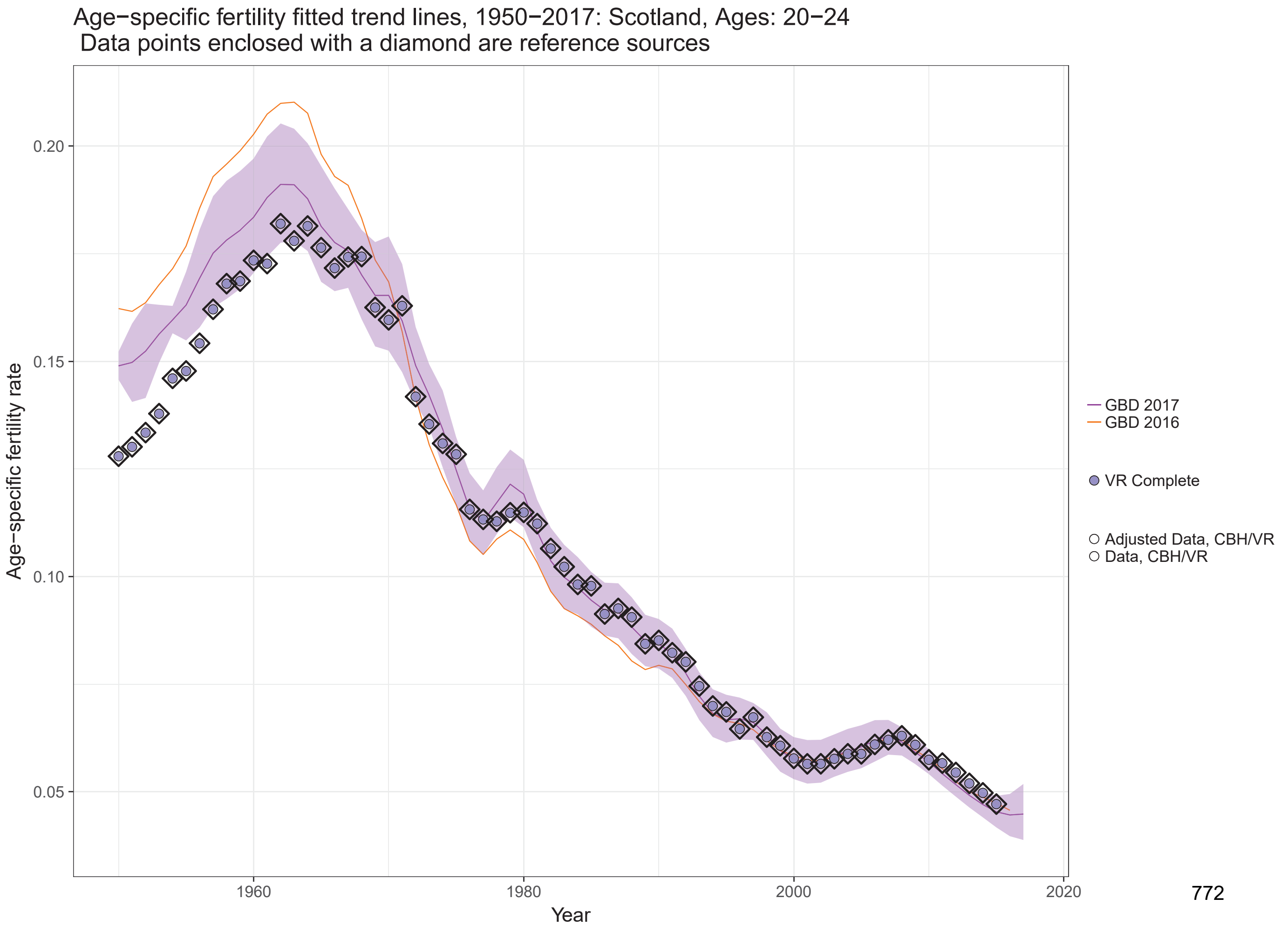

Age−specific fertility fitted trend lines, 1950−2017: Scotland, Ages: 20−24
Data points enclosed with a diamond are reference sources
0.20
0.15
0.10
0.05
Age−specific fertility rate
1960
1980
2000
2020
Year
GBD 2017
GBD 2016
VR Complete
Adjusted Data, CBH/VR
Data, CBH/VR

Age−specific fertility fitted trend lines, 1950−2017: Scotland, Ages: 25−29
Data points enclosed with a diamond are reference sources

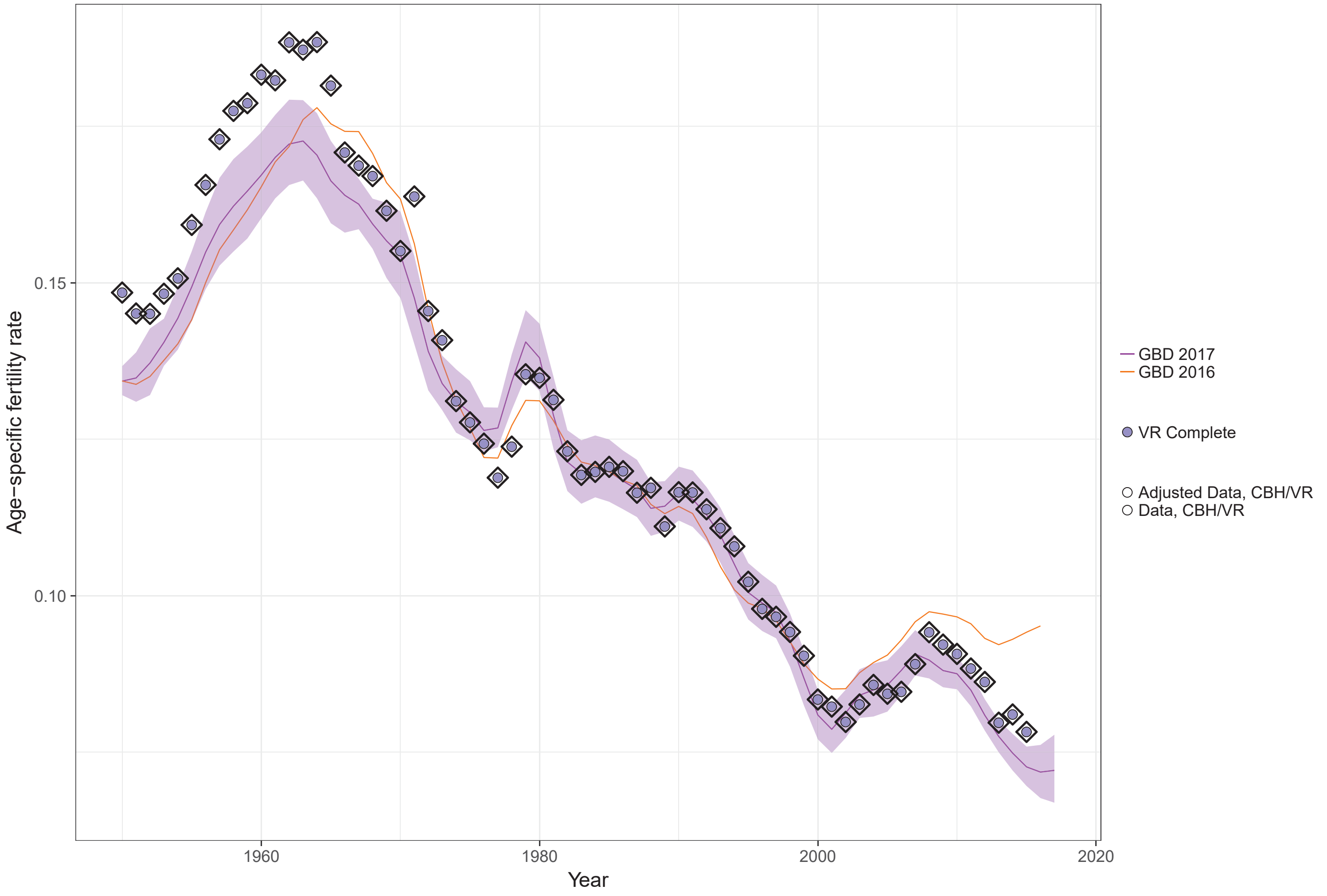

Age−specific fertility fitted trend lines, 1950−2017: Scotland, Ages: 30−34
Data points enclosed with a diamond are reference sources

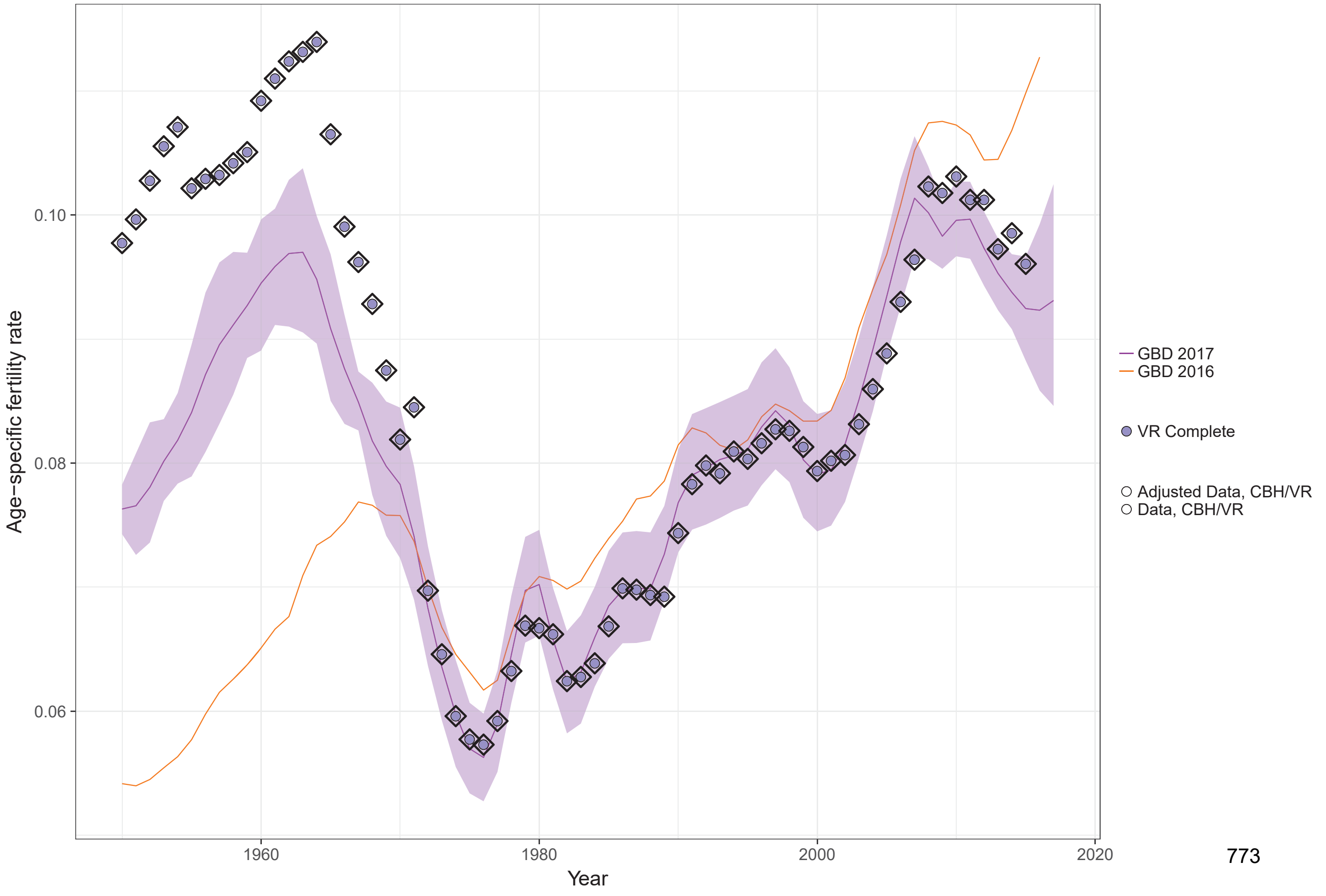

Age−specific fertility fitted trend lines, 1950−2017: Scotland, Ages: 35−39
Data points enclosed with a diamond are reference sources

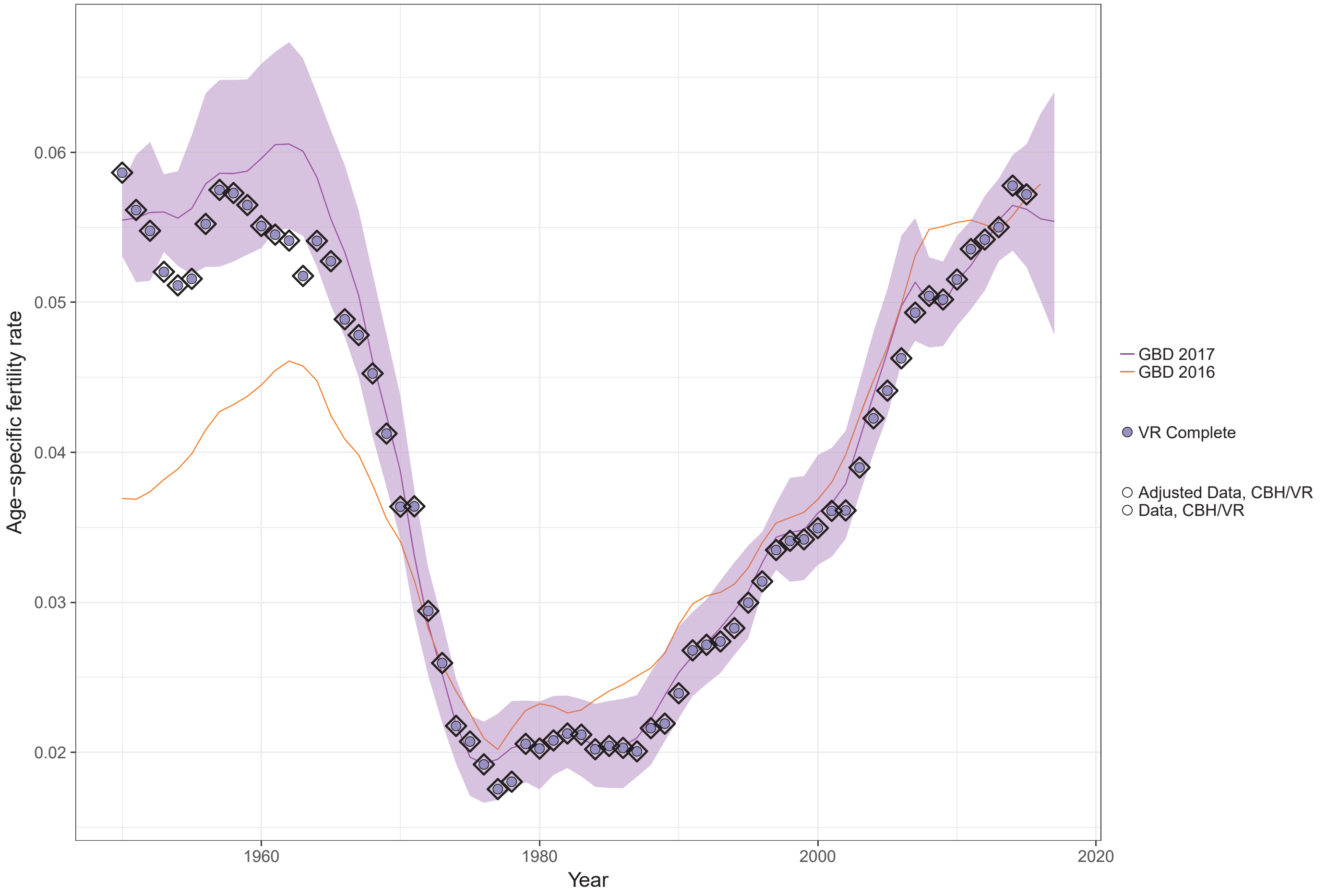

Age−specific fertility fitted trend lines, 1950−2017: Scotland, Ages: 40−44
Data points enclosed with a diamond are reference sources

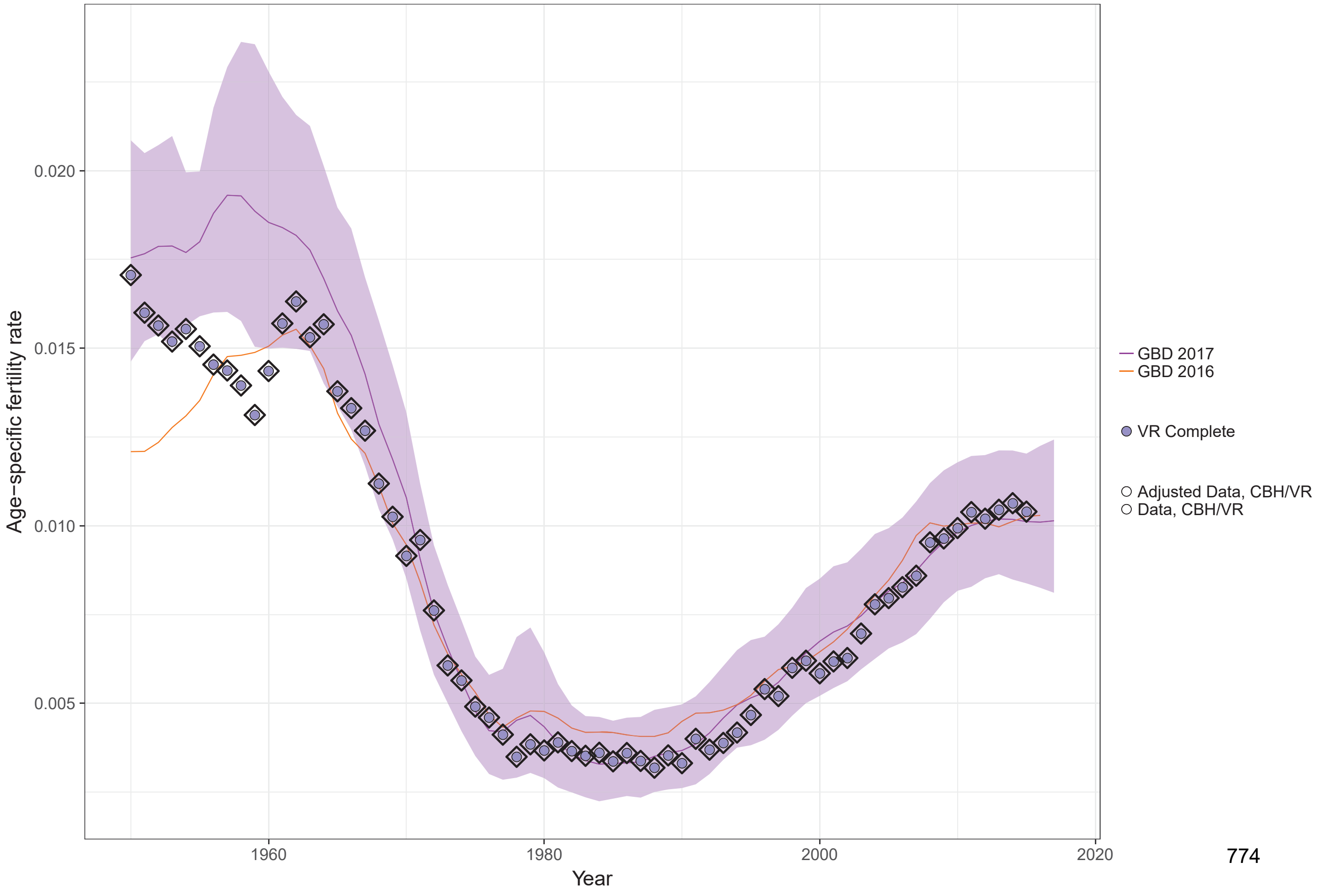

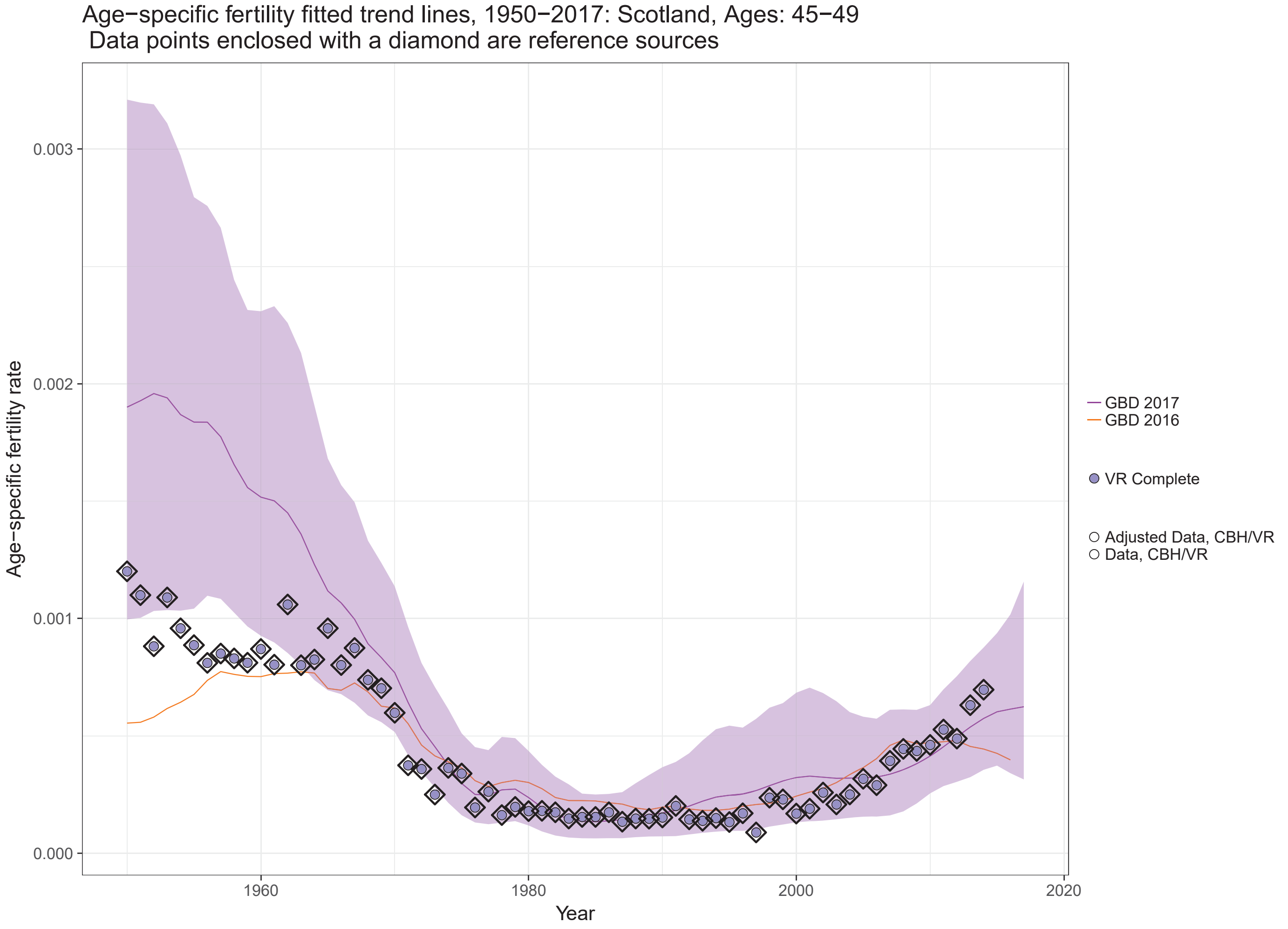

Age−specific fertility fitted trend lines, 1950−2017: Scotland, Ages: 45−49
Data points enclosed with a diamond are reference sources
Age−specific fertility rate
0.000
0.001
0.002
0.003
1960
1980
2000
2020
Year
GBD 2017
GBD 2016
VR Complete
Adjusted Data, CBH/VR
Data, CBH/VR

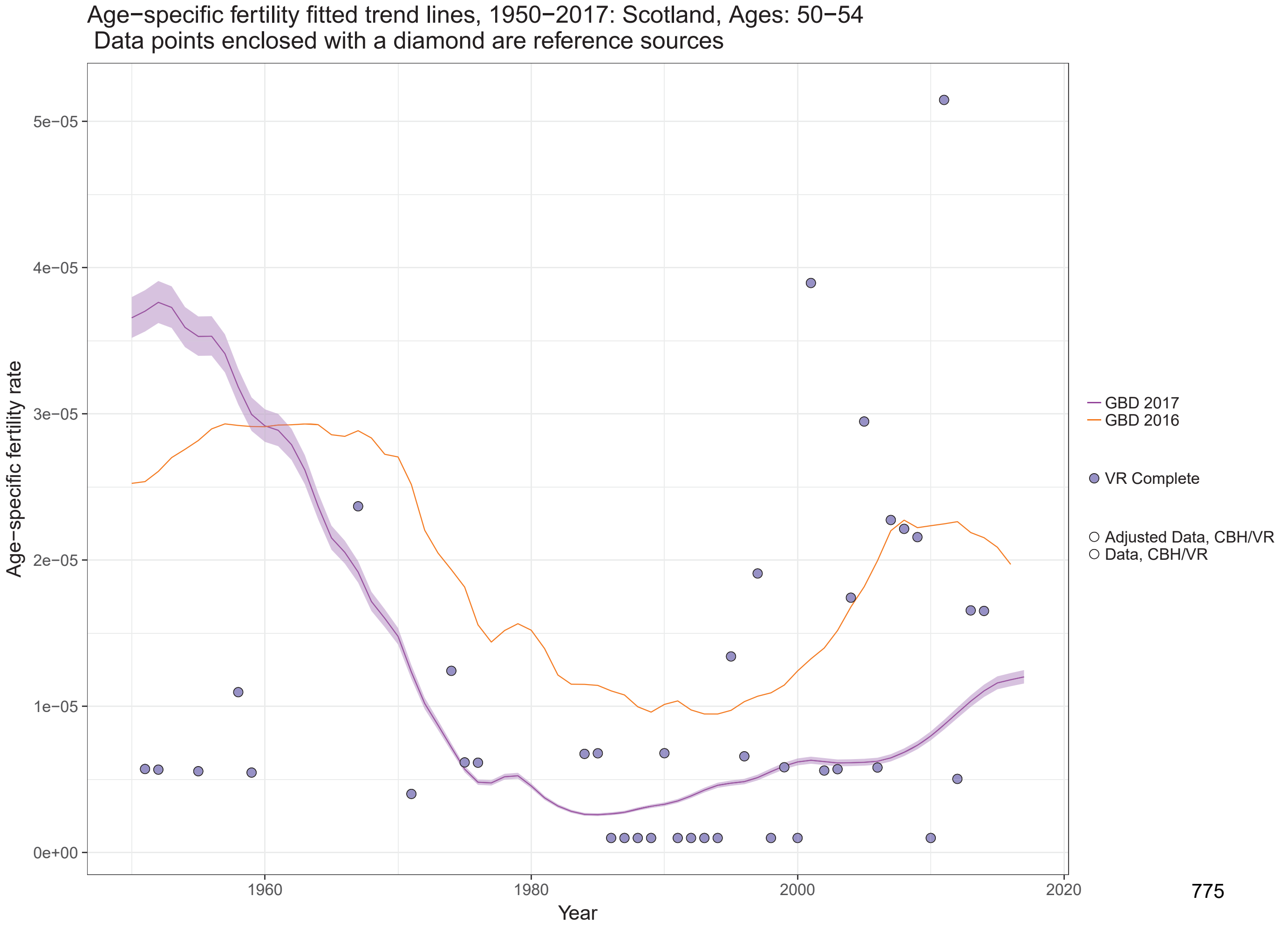

Age−specific fertility fitted trend lines, 1950−2017: Scotland, Ages: 50−54
Data points enclosed with a diamond are reference sources
Age−specific fertility rate
0e+00
1e−05
2e−05
3e−05
4e−05
5e−05
1960
1980
2000
2020
Year
GBD 2017
GBD 2016
VR Complete
Adjusted Data, CBH/VR
Data, CBH/VR

Age−specific fertility fitted trend lines, 1950−2017: Senegal, TFR
Data points enclosed with a diamond are reference sources

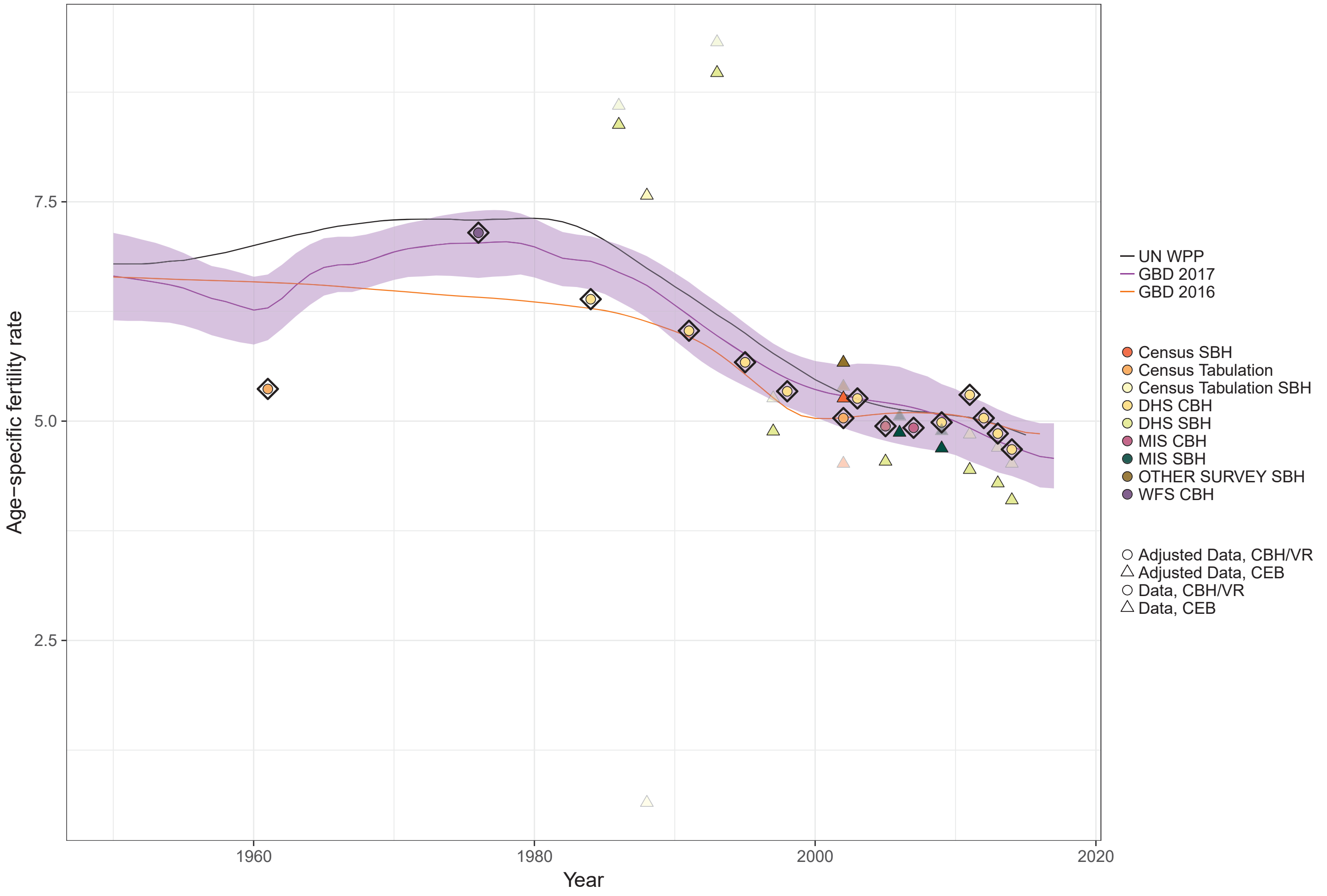

Age−specific fertility fitted trend lines, 1950−2017: Senegal, Ages: 10−14
Data points enclosed with a diamond are reference sources

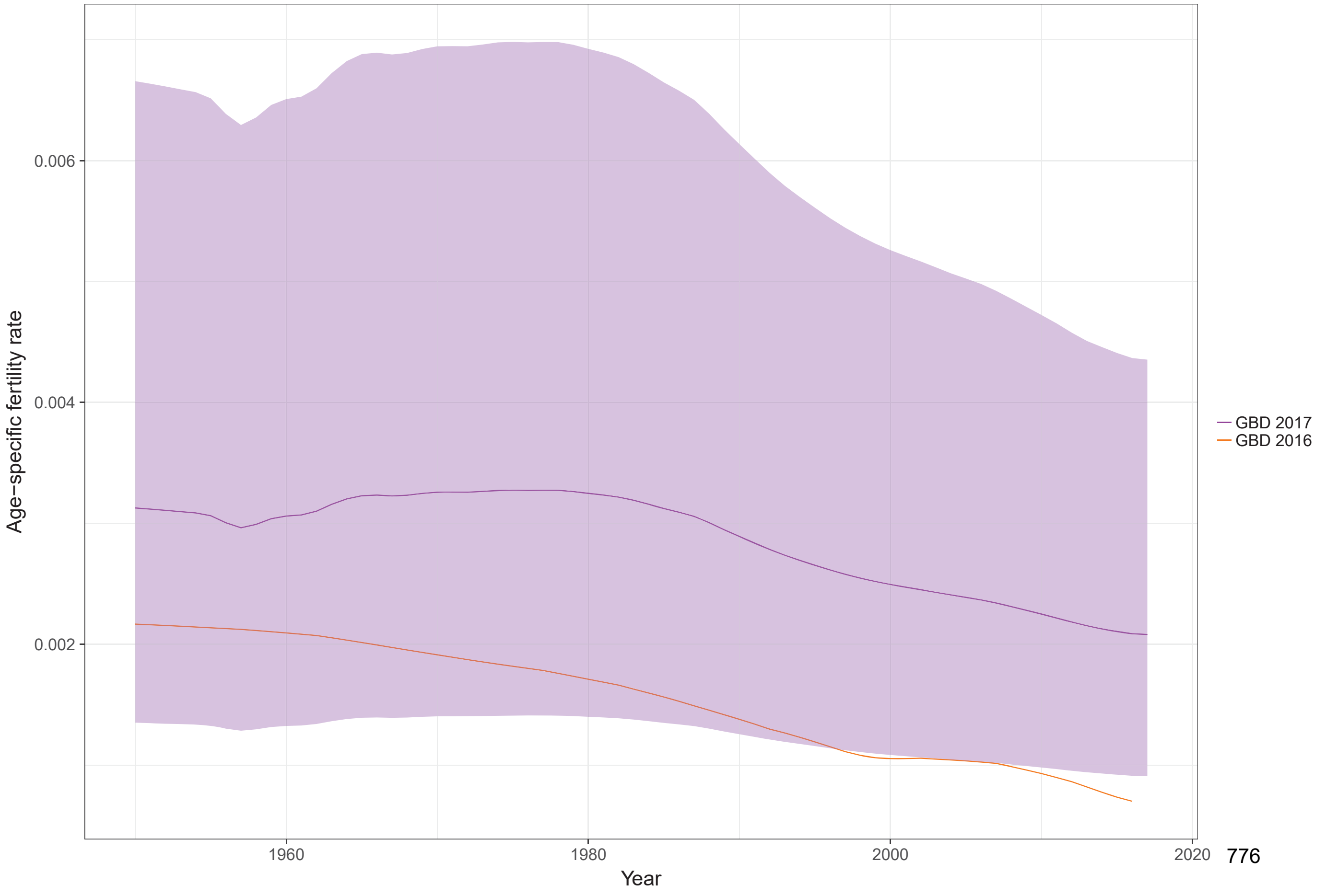

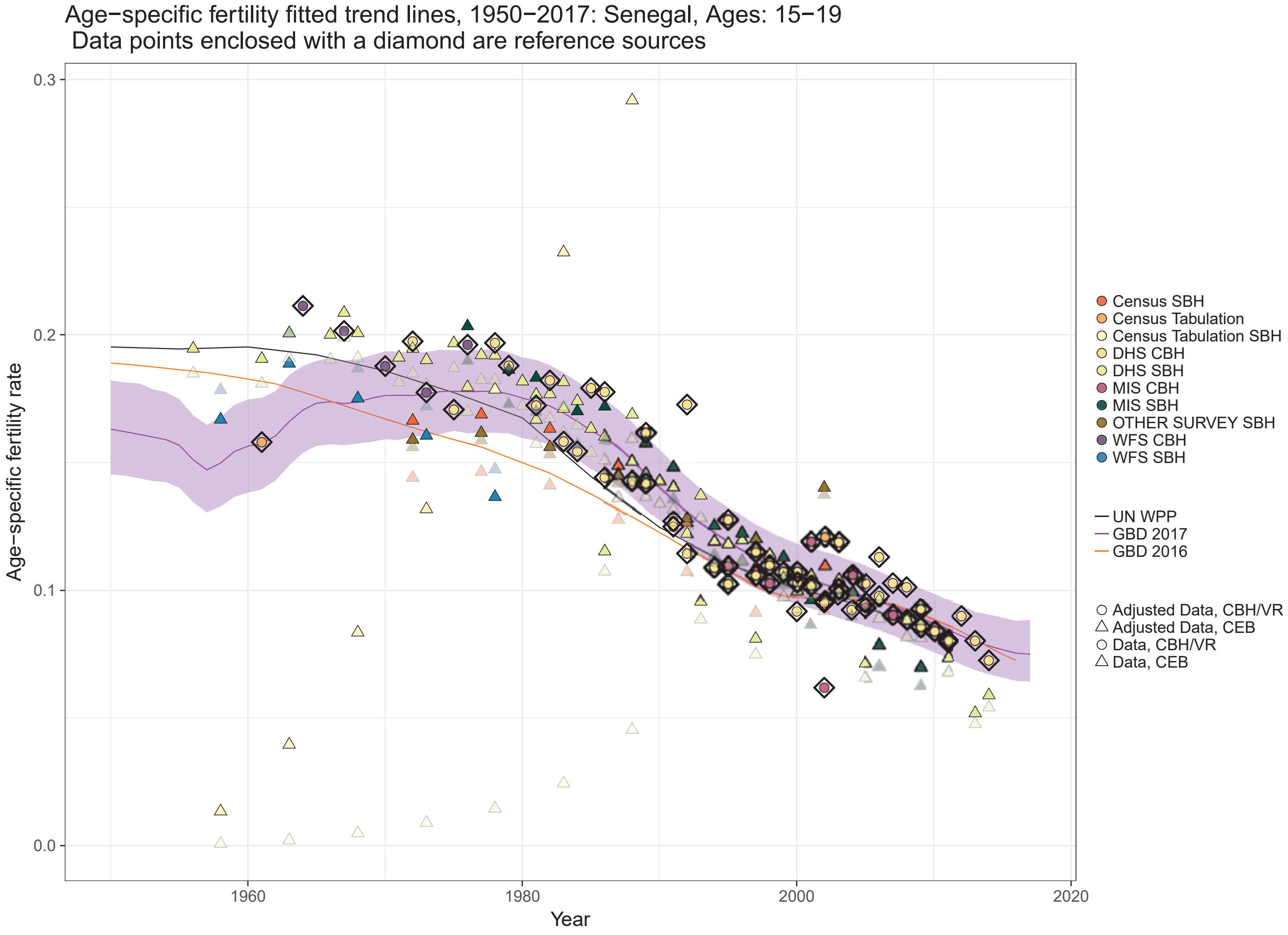
Age−specific fertility fitted trend lines, 1950−2017: Senegal, Ages: 15−19
Data points enclosed with a diamond are reference sources
Age−specific fertility rate
Year
0.0
0.1
0.2
0.3
1960
1980
2000
2020
Census SBH
Census Tabulation
Census Tabulation SBH
DHS CBH
DHS SBH
MIS CBH
MIS SBH
OTHER SURVEY SBH
WFS CBH
WFS SBH
UN WPP
GBD 2017
GBD 2016
Adjusted Data, CBH/VR
Adjusted Data, CEB
Data, CBH/VR
Data, CEB

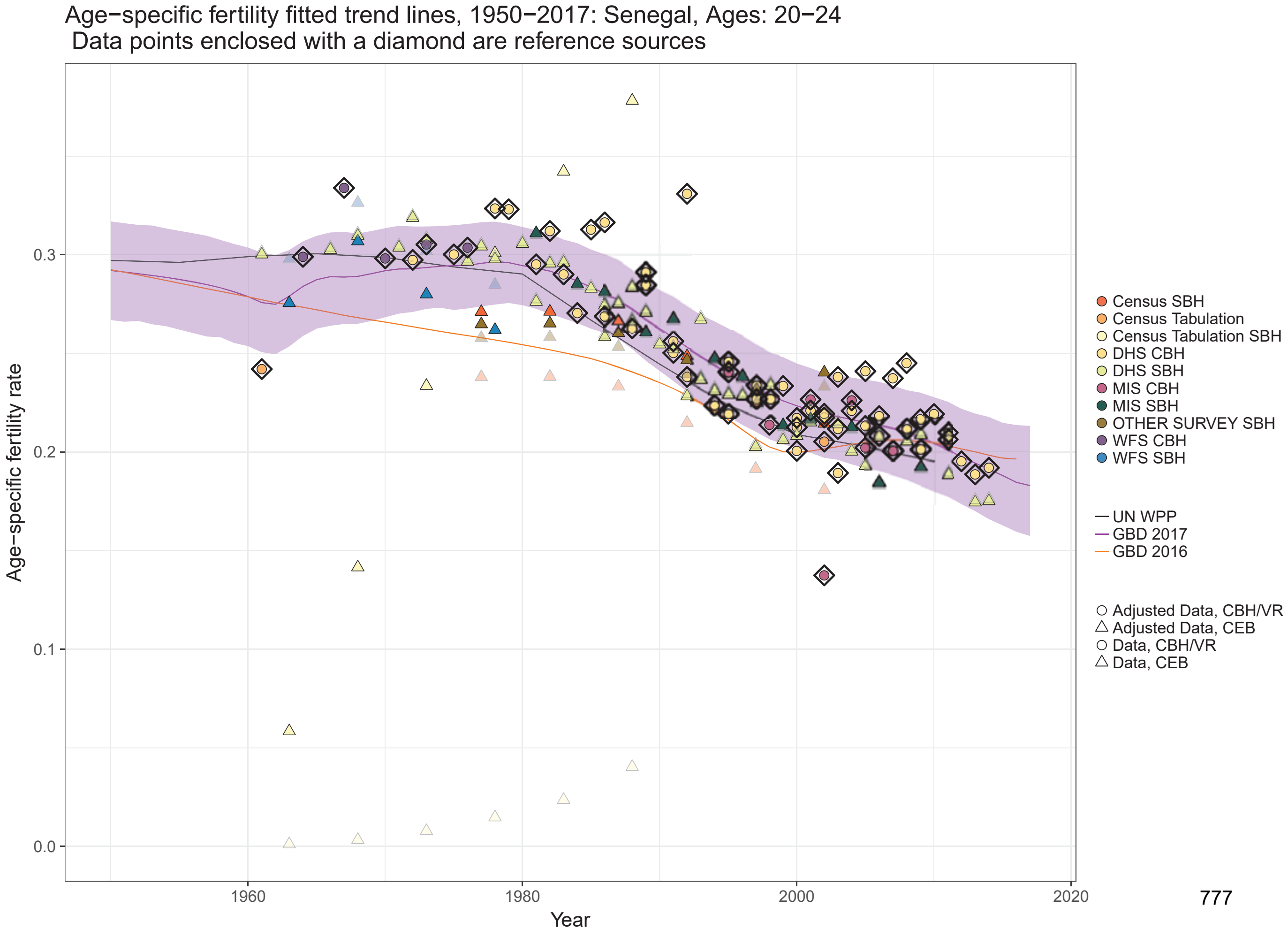
Age−specific fertility fitted trend lines, 1950−2017: Senegal, Ages: 20−24
Data points enclosed with a diamond are reference sources
Age−specific fertility rate
Year
0.0
0.1
0.2
0.3
1960
1980
2000
2020
Census SBH
Census Tabulation
Census Tabulation SBH
DHS CBH
DHS SBH
MIS CBH
MIS SBH
OTHER SURVEY SBH
WFS CBH
WFS SBH
UN WPP
GBD 2017
GBD 2016
Adjusted Data, CBH/VR
Adjusted Data, CEB
Data, CBH/VR
Data, CEB

Age−specific fertility fitted trend lines, 1950−2017: Senegal, Ages: 25−29
Data points enclosed with a diamond are reference sources

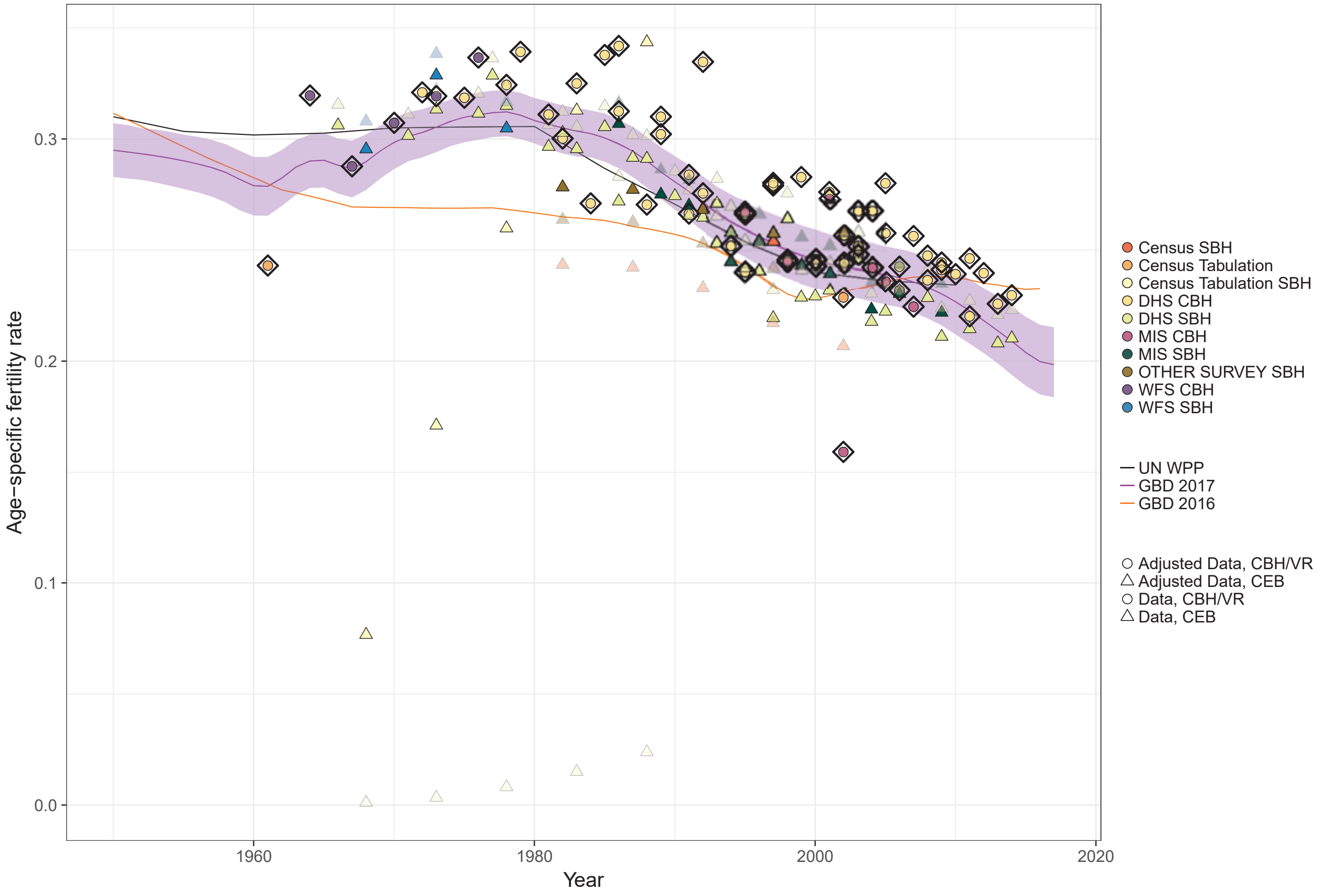

Age−specific fertility fitted trend lines, 1950−2017: Senegal, Ages: 30−34
Data points enclosed with a diamond are reference sources

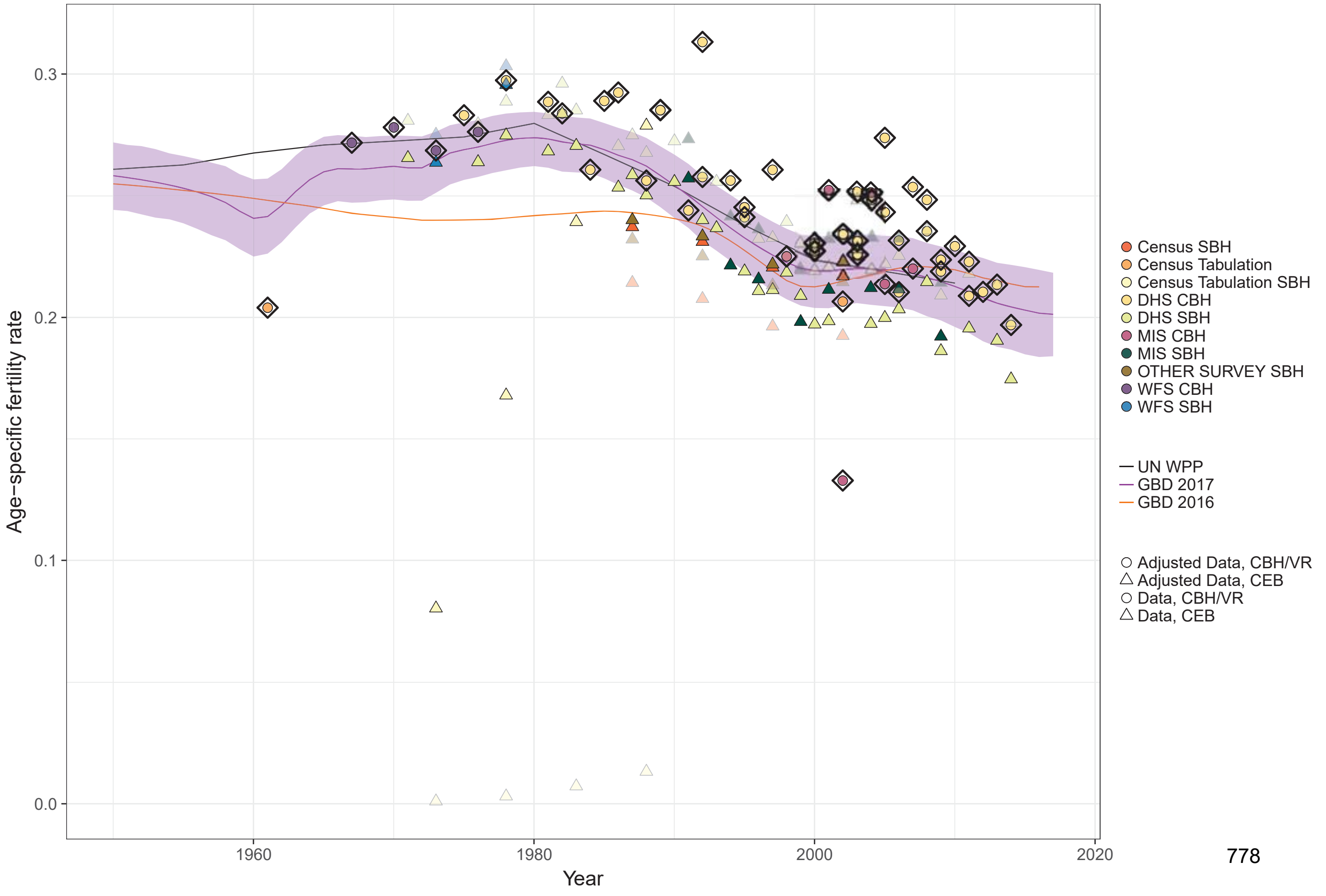

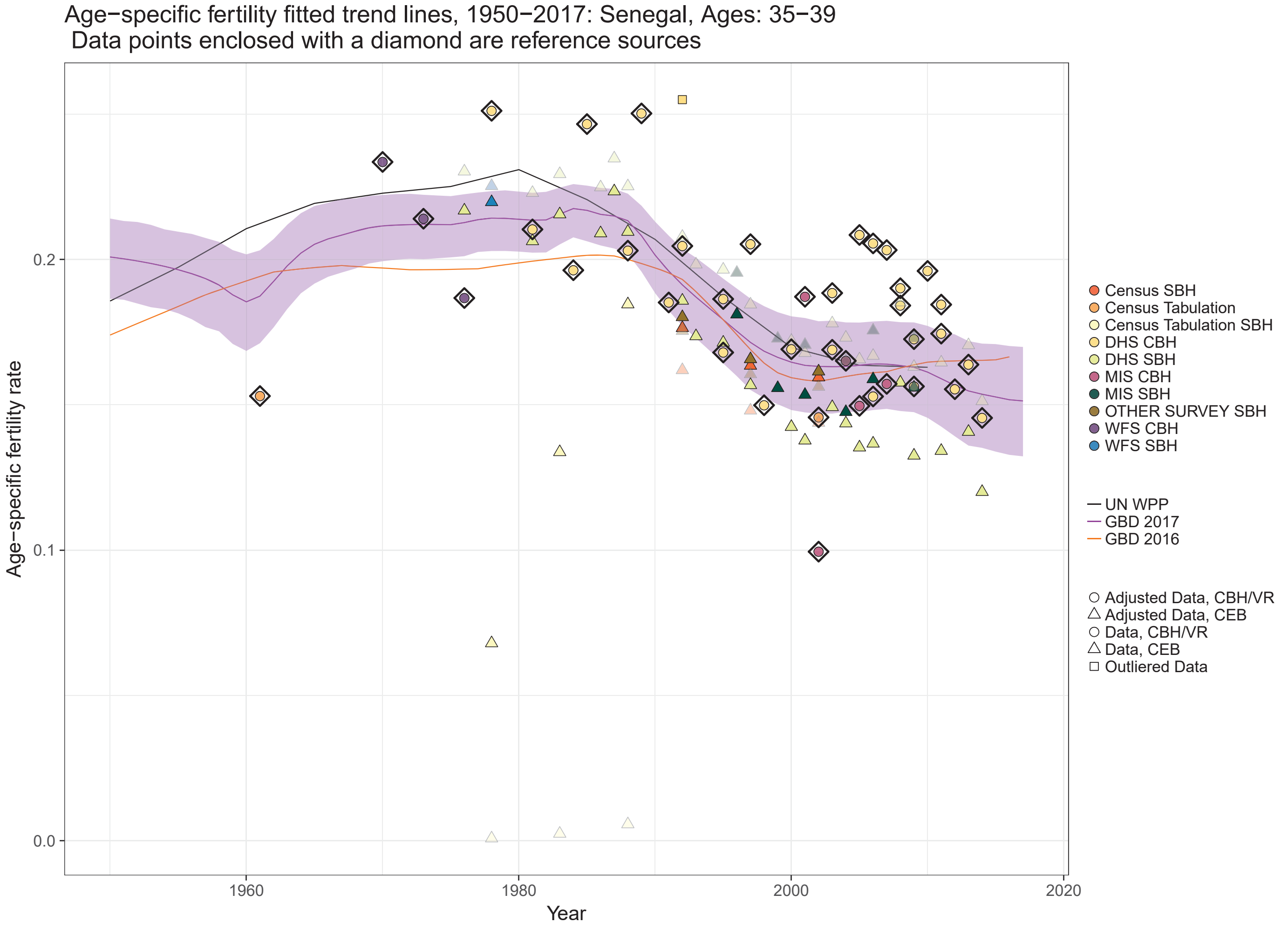
Age−specific fertility fitted trend lines, 1950−2017: Senegal, Ages: 35−39
Data points enclosed with a diamond are reference sources
Age−specific fertility rate
Year
0.0
0.1
0.2
1960
1980
2000
2020
Census SBH
Census Tabulation
Census Tabulation SBH
DHS CBH
DHS SBH
MIS CBH
MIS SBH
OTHER SURVEY SBH
WFS CBH
WFS SBH
UN WPP
GBD 2017
GBD 2016
Adjusted Data, CBH/VR
Adjusted Data, CEB
Data, CBH/VR
Data, CEB
Outliered Data

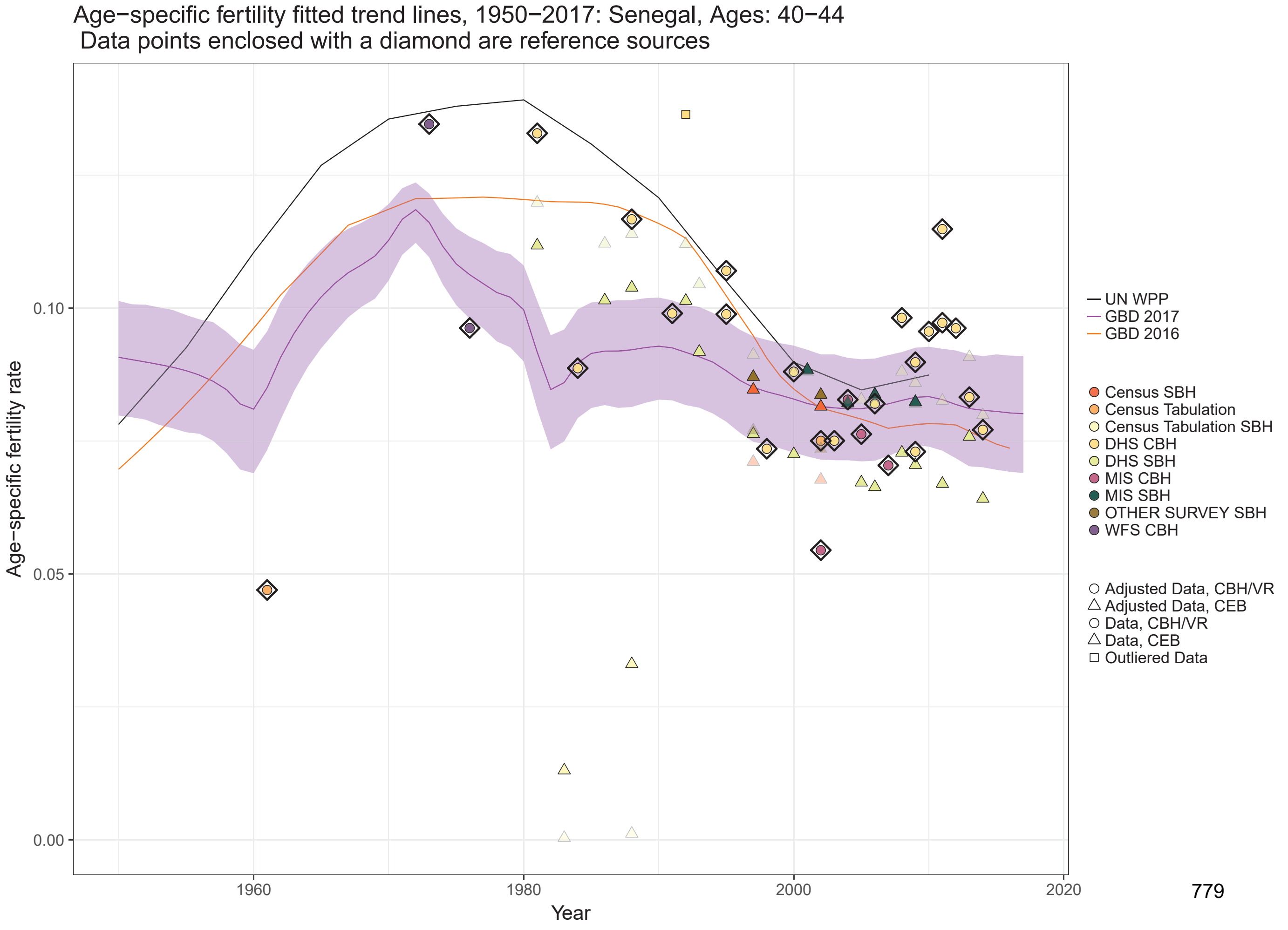
Age−specific fertility fitted trend lines, 1950−2017: Senegal, Ages: 40−44
Data points enclosed with a diamond are reference sources
Age−specific fertility rate
Year
0.00
0.05
0.10
1960
1980
2000
2020
UN WPP
GBD 2017
GBD 2016
Census SBH
Census Tabulation
Census Tabulation SBH
DHS CBH
DHS SBH
MIS CBH
MIS SBH
OTHER SURVEY SBH
WFS CBH
Adjusted Data, CBH/VR
Adjusted Data, CEB
Data, CBH/VR
Data, CEB
Outliered Data

Age−specific fertility fitted trend lines, 1950−2017: Senegal, Ages: 45−49
Data points enclosed with a diamond are reference sources

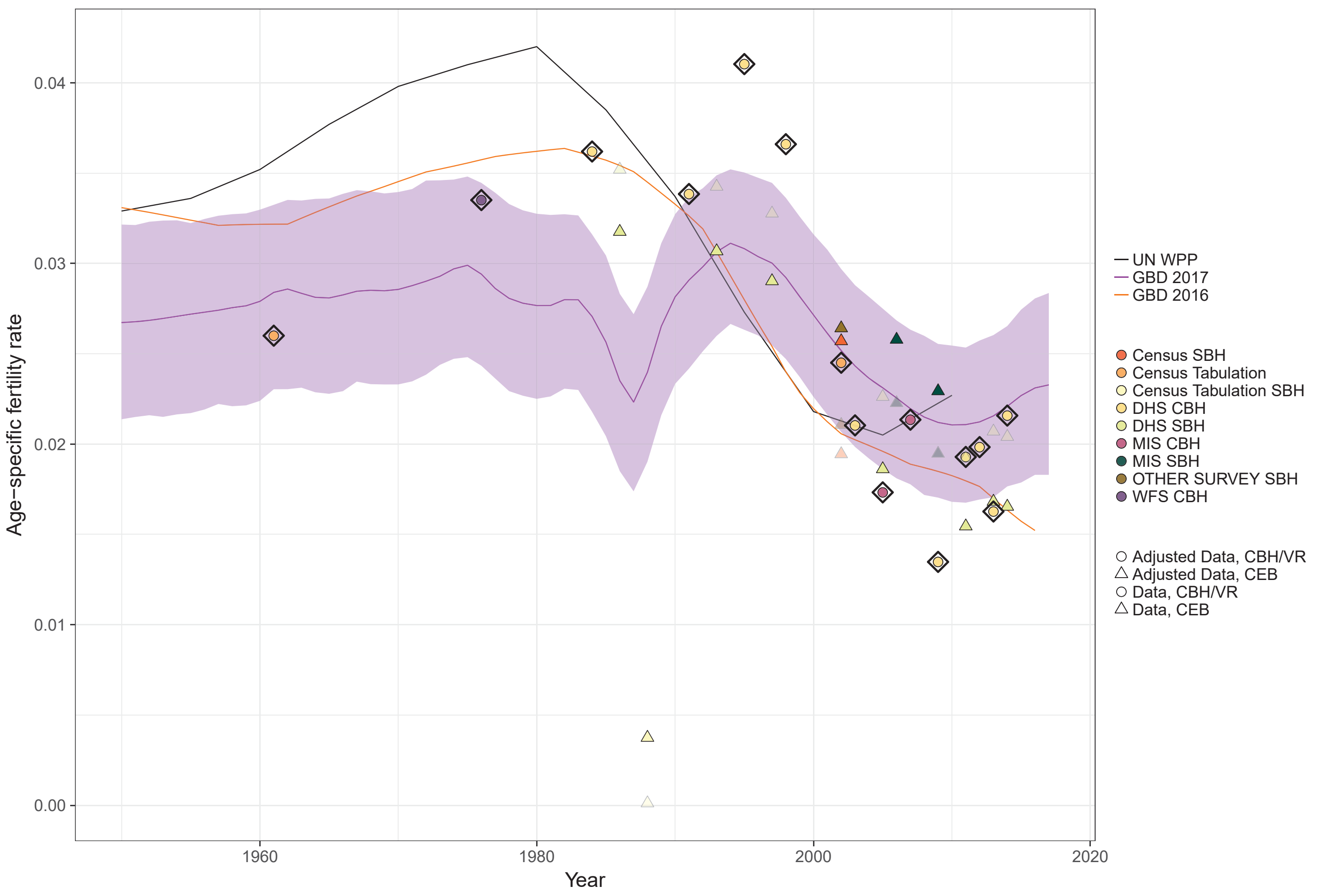

Age−specific fertility fitted trend lines, 1950−2017: Senegal, Ages: 50−54
Data points enclosed with a diamond are reference sources

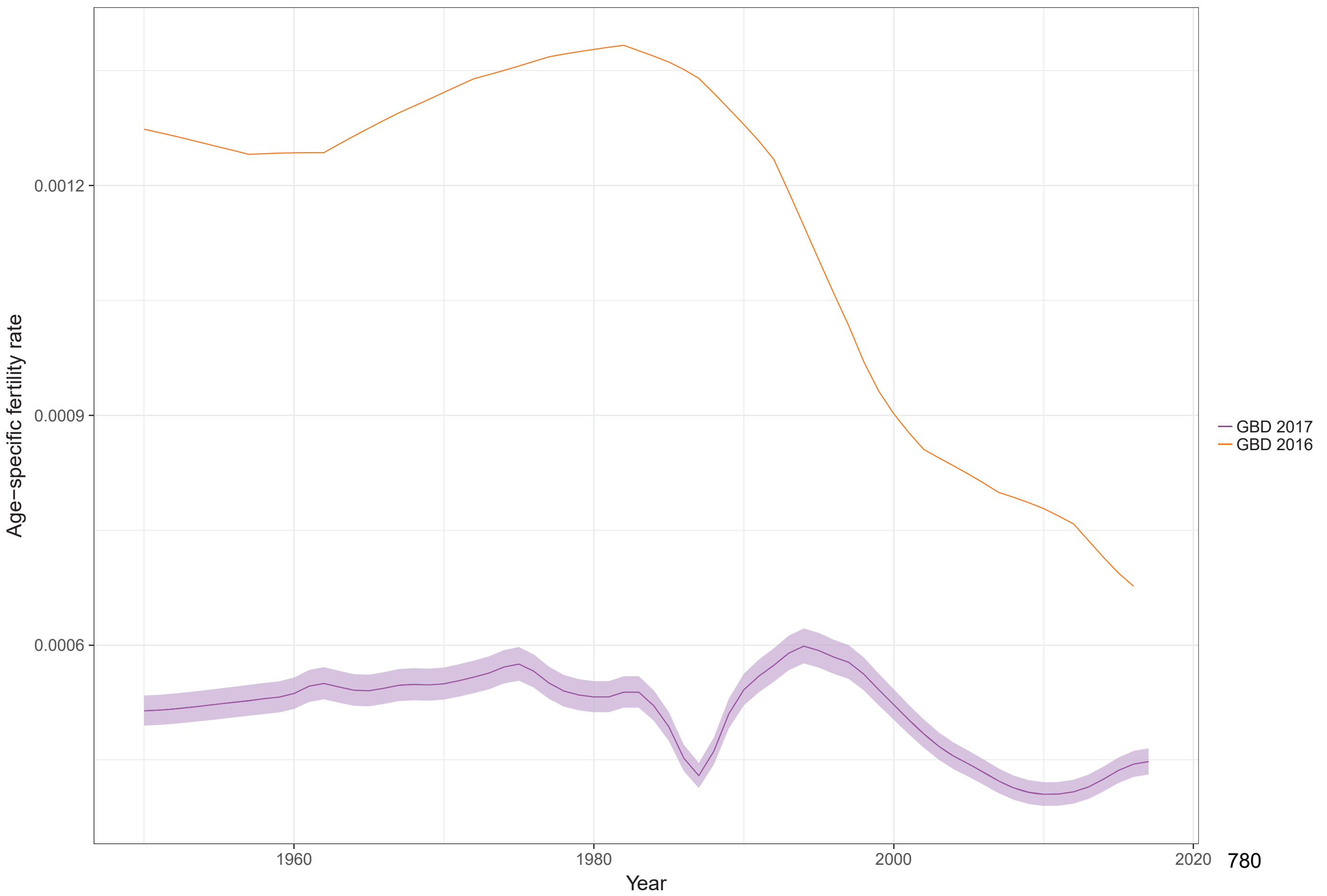

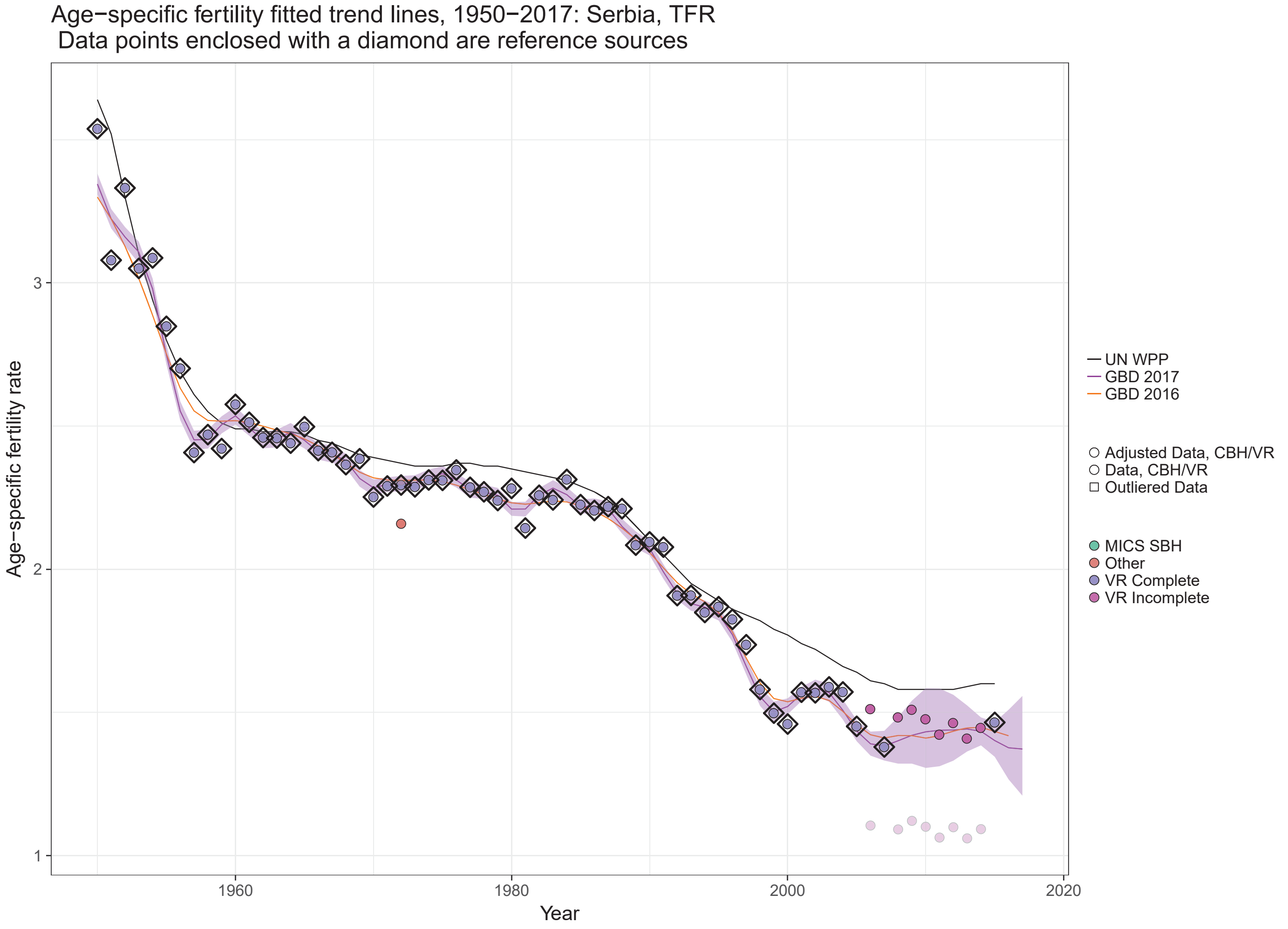
Age−specific fertility fitted trend lines, 1950−2017: Serbia, TFR
Data points enclosed with a diamond are reference sources
Age−specific fertility rate
3
2
1
1960
1980
2000
2020
Year
UN WPP
GBD 2017
GBD 2016
Adjusted Data, CBH/VR
Data, CBH/VR
Outliered Data
MICS SBH
Other
VR Complete
VR Incomplete

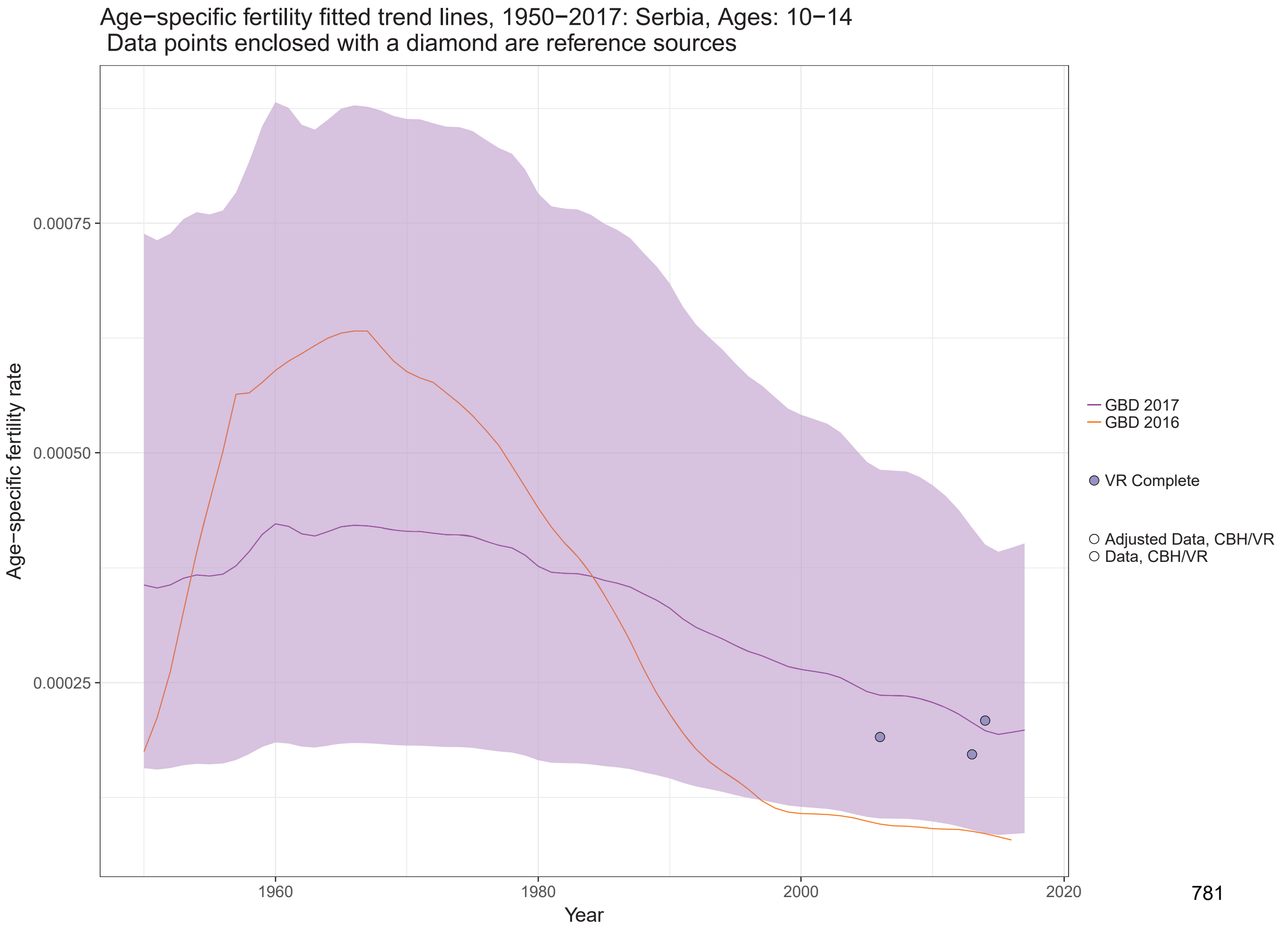
Age−specific fertility fitted trend lines, 1950−2017: Serbia, Ages: 10−14
Data points enclosed with a diamond are reference sources
Age−specific fertility rate
0.00075
0.00050
0.00025
1960
1980
2000
2020
Year
GBD 2017
GBD 2016
VR Complete
Adjusted Data, CBH/VR
Data, CBH/VR

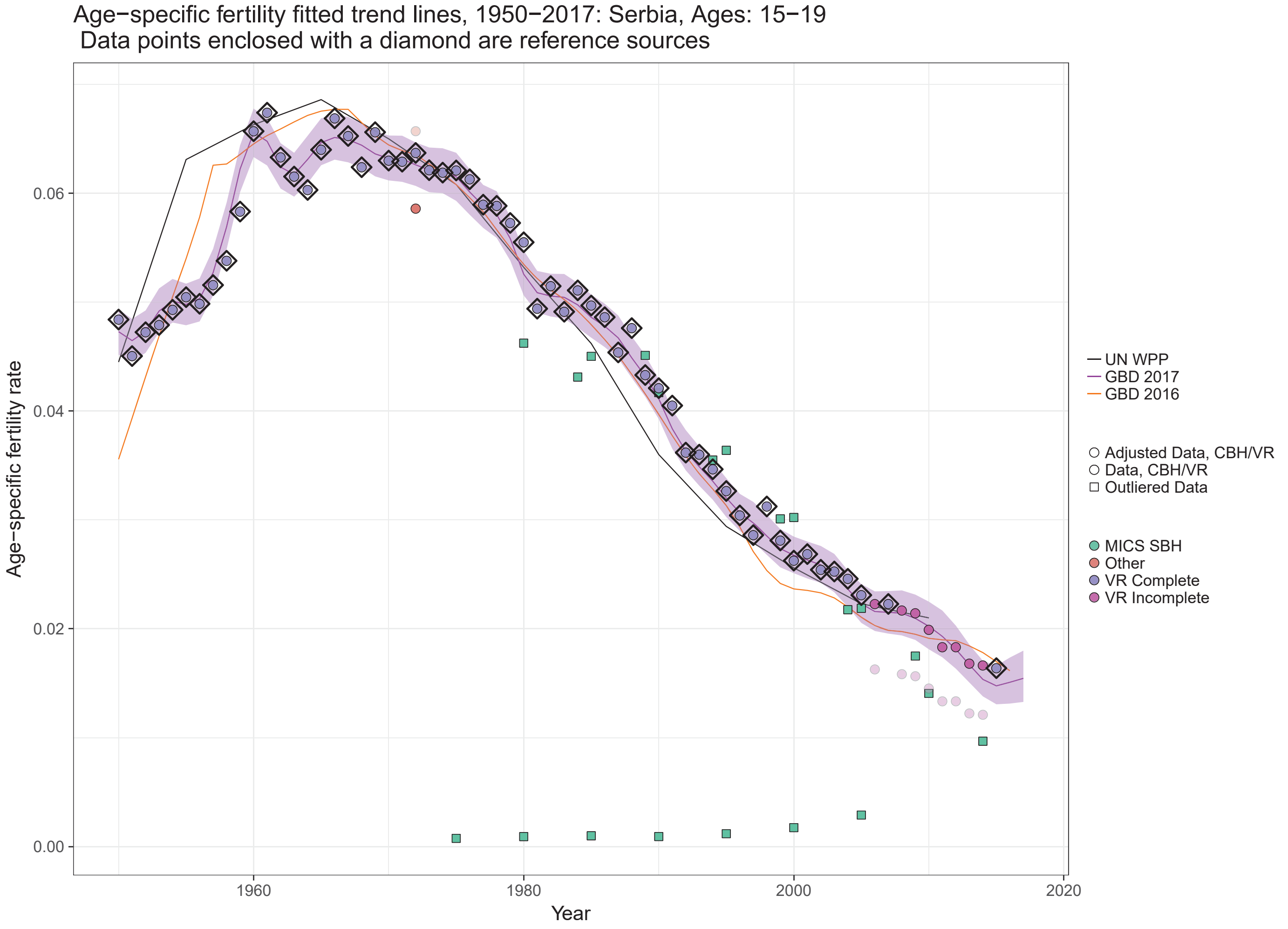

Age−specific fertility fitted trend lines, 1950−2017: Serbia, Ages: 15−19
Data points enclosed with a diamond are reference sources
Age-specific fertility rate
0.00
0.02
0.04
0.06
1960
1980
2000
2020
Year
UN WPP
GBD 2017
GBD 2016
Adjusted Data, CBH/VR
Data, CBH/VR
Outliered Data
MICS SBH
Other
VR Complete
VR Incomplete

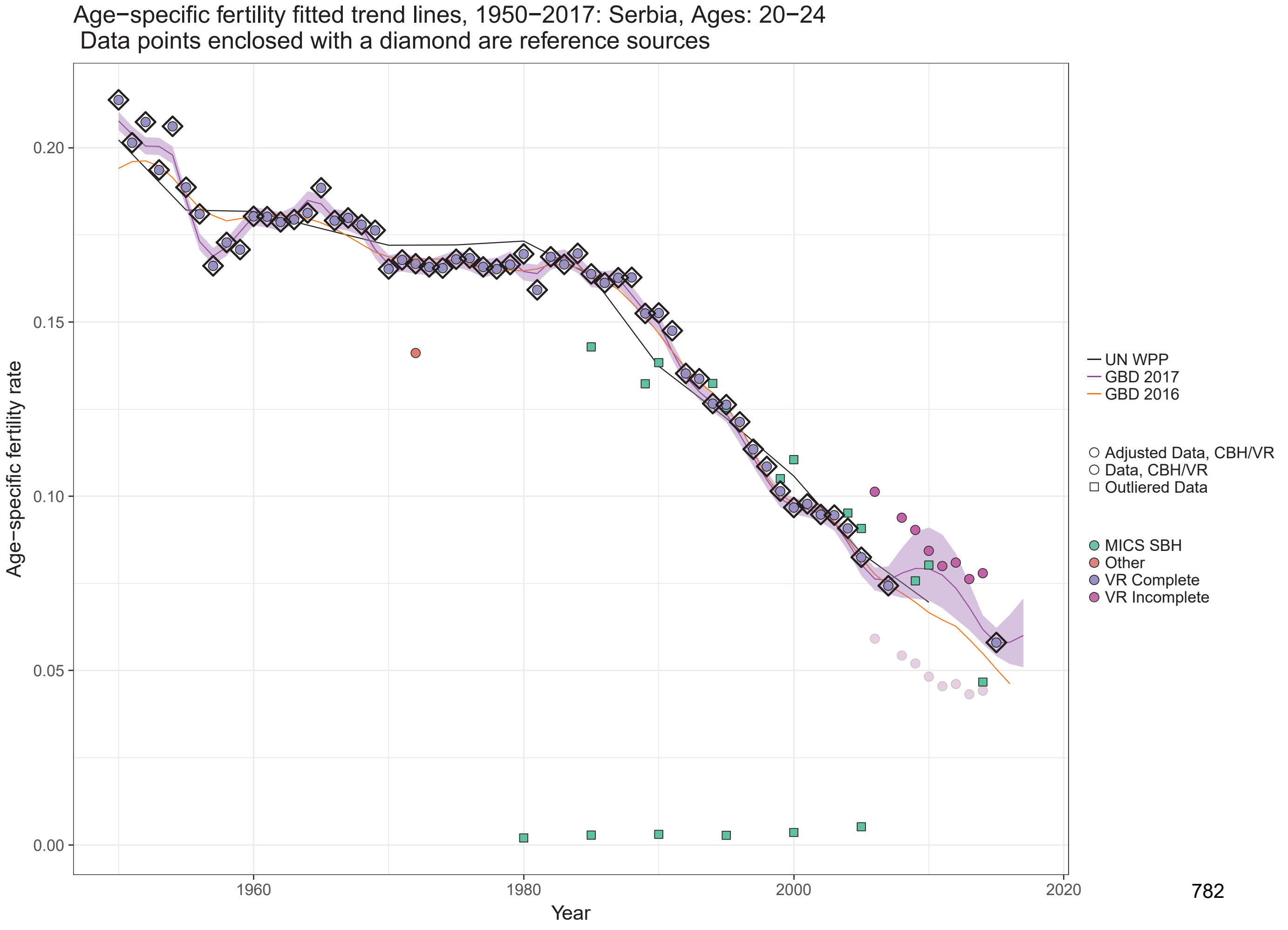

Age−specific fertility fitted trend lines, 1950−2017: Serbia, Ages: 20−24
Data points enclosed with a diamond are reference sources
Age-specific fertility rate
0.00
0.05
0.10
0.15
0.20
1960
1980
2000
2020
Year
UN WPP
GBD 2017
GBD 2016
Adjusted Data, CBH/VR
Data, CBH/VR
Outliered Data
MICS SBH
Other
VR Complete
VR Incomplete

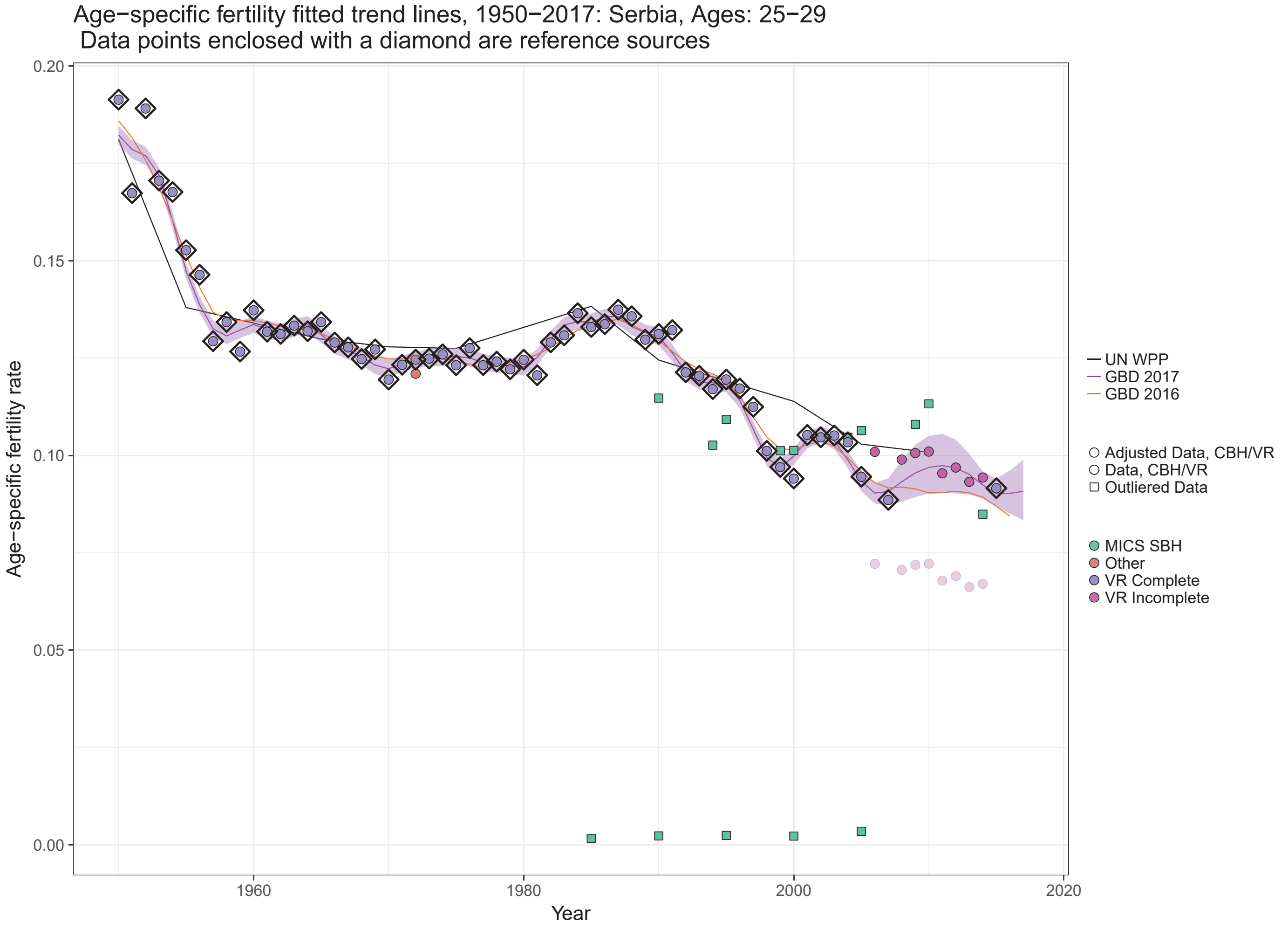
Age−specific fertility fitted trend lines, 1950−2017: Serbia, Ages: 25−29
Data points enclosed with a diamond are reference sources
Age-specific fertility rate
Year
0.20
0.15
0.10
0.05
0.00
1960
1980
2000
2020
UN WPP
GBD 2017
GBD 2016
Adjusted Data, CBH/VR
Data, CBH/VR
Outliered Data
MICS SBH
Other
VR Complete
VR Incomplete

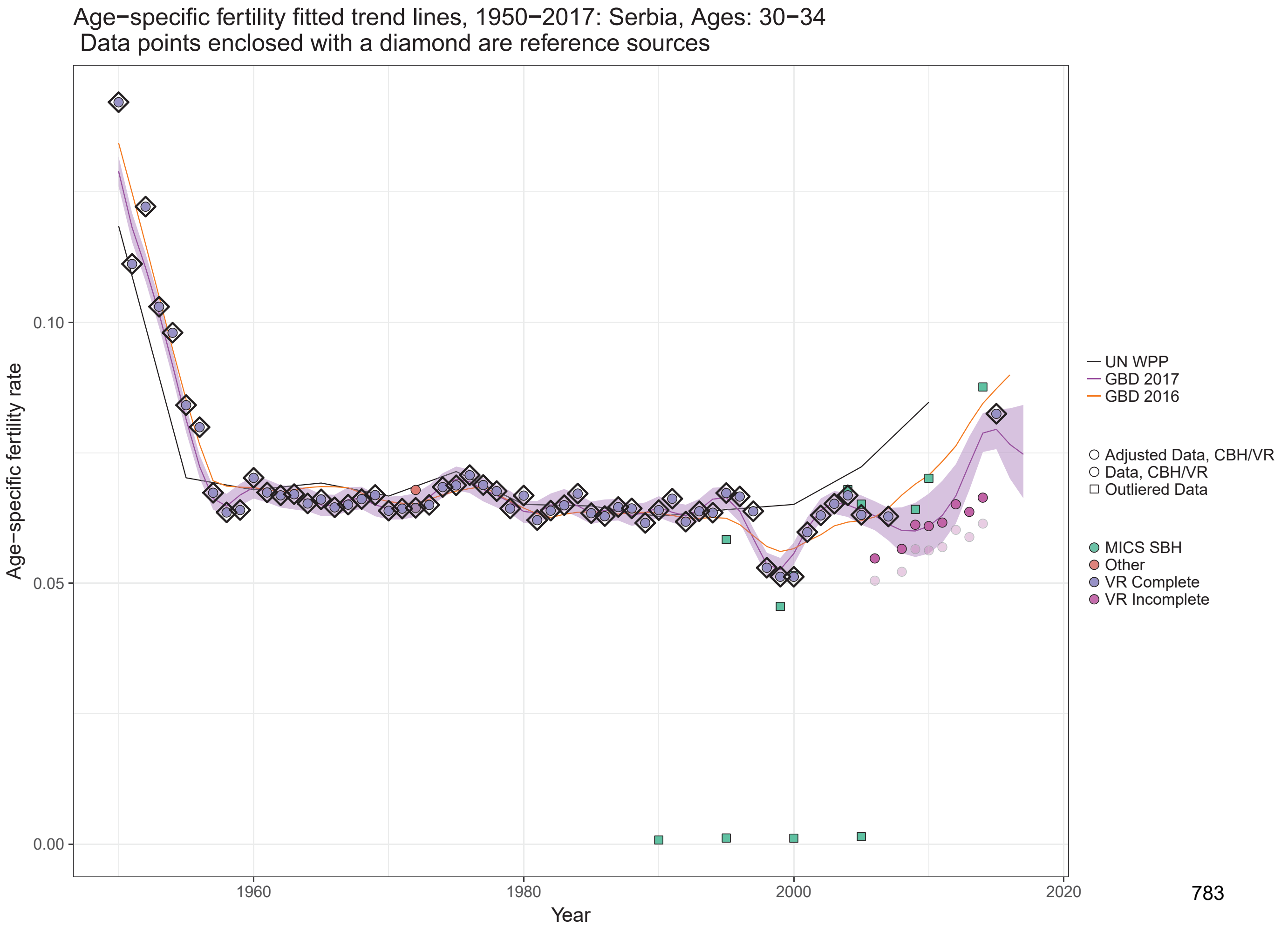
Age−specific fertility fitted trend lines, 1950−2017: Serbia, Ages: 30−34
Data points enclosed with a diamond are reference sources
Age-specific fertility rate
Year
0.10
0.05
0.00
1960
1980
2000
2020
UN WPP
GBD 2017
GBD 2016
Adjusted Data, CBH/VR
Data, CBH/VR
Outliered Data
MICS SBH
Other
VR Complete
VR Incomplete

Age−specific fertility fitted trend lines, 1950−2017: Serbia, Ages: 35−39
Data points enclosed with a diamond are reference sources

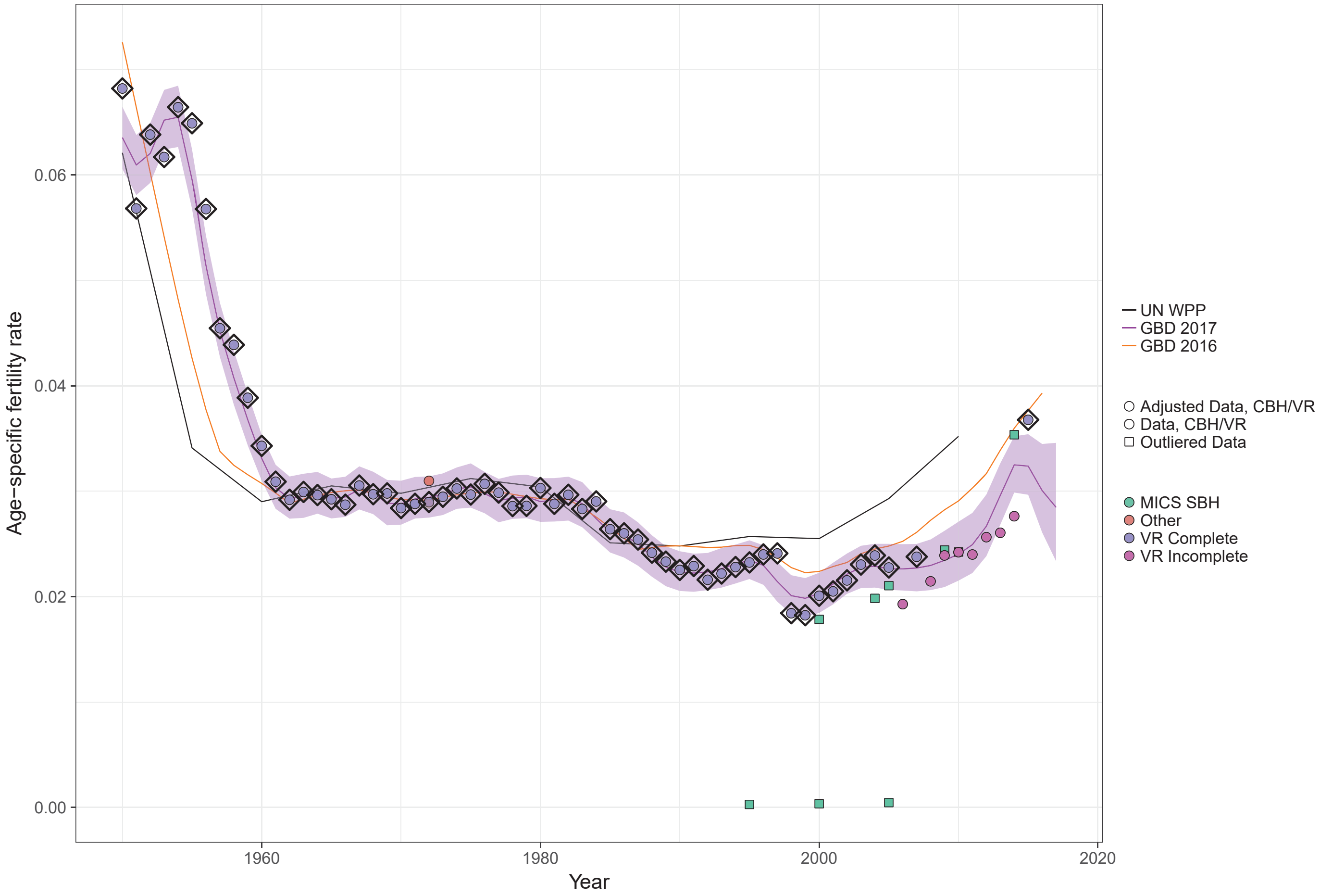

Age−specific fertility fitted trend lines, 1950−2017: Serbia, Ages: 40−44
Data points enclosed with a diamond are reference sources

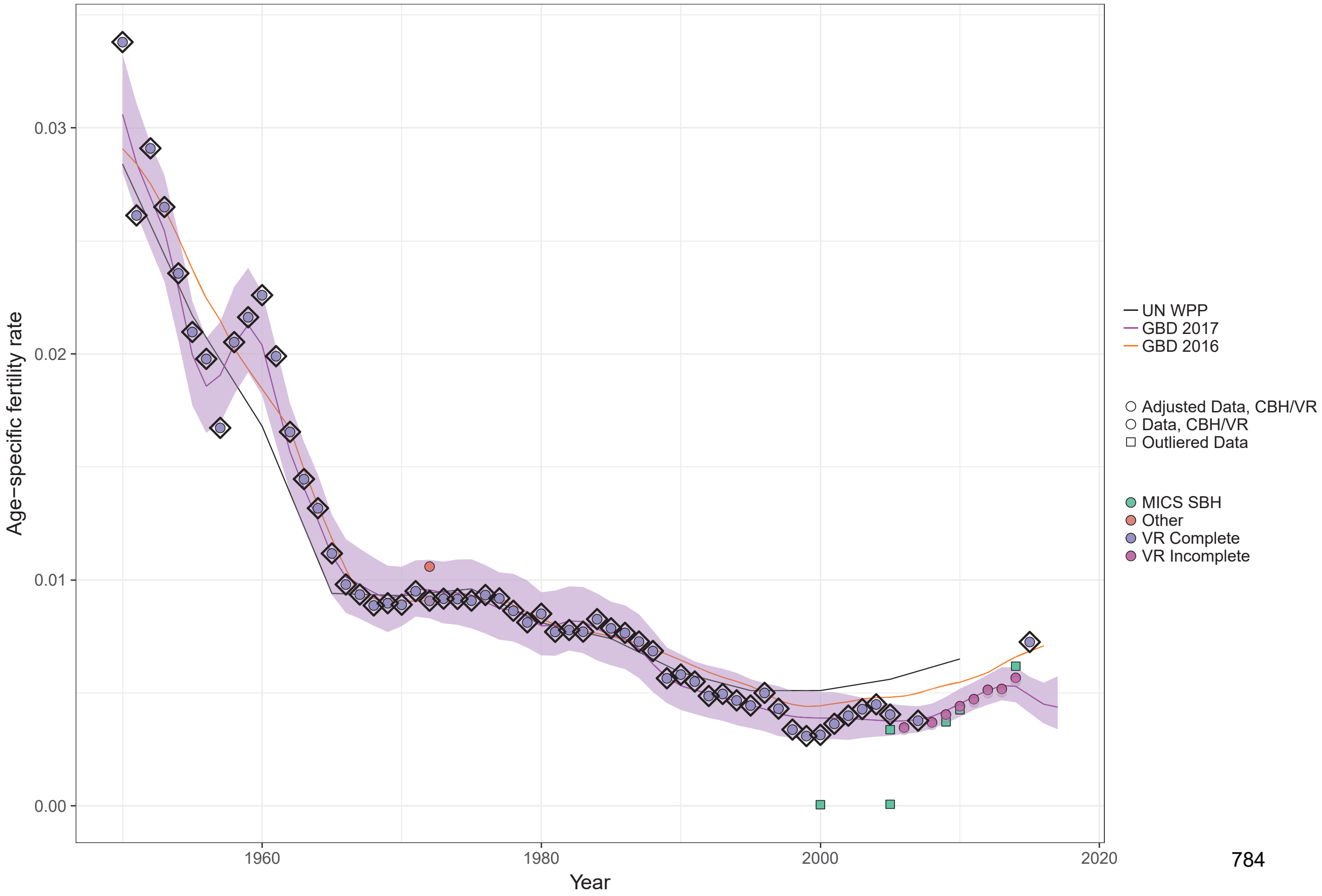

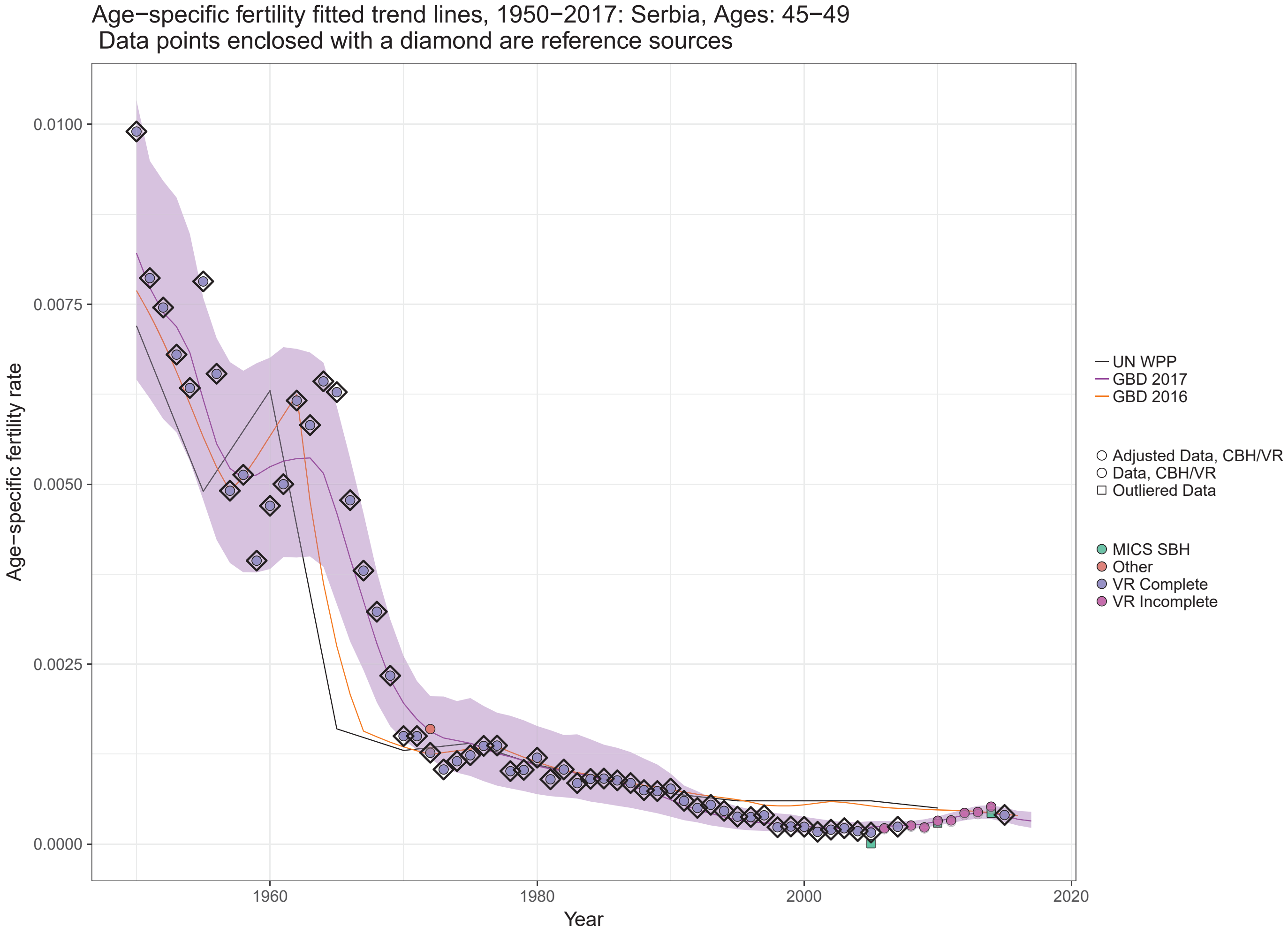
Age−specific fertility fitted trend lines, 1950−2017: Serbia, Ages: 45−49
Data points enclosed with a diamond are reference sources
Age−specific fertility rate
0.0100
0.0075
0.0050
0.0025
0.0000
1960
1980
2000
2020
Year
UN WPP
GBD 2017
GBD 2016
Adjusted Data, CBH/VR
Data, CBH/VR
Outliered Data
MICS SBH
Other
VR Complete
VR Incomplete

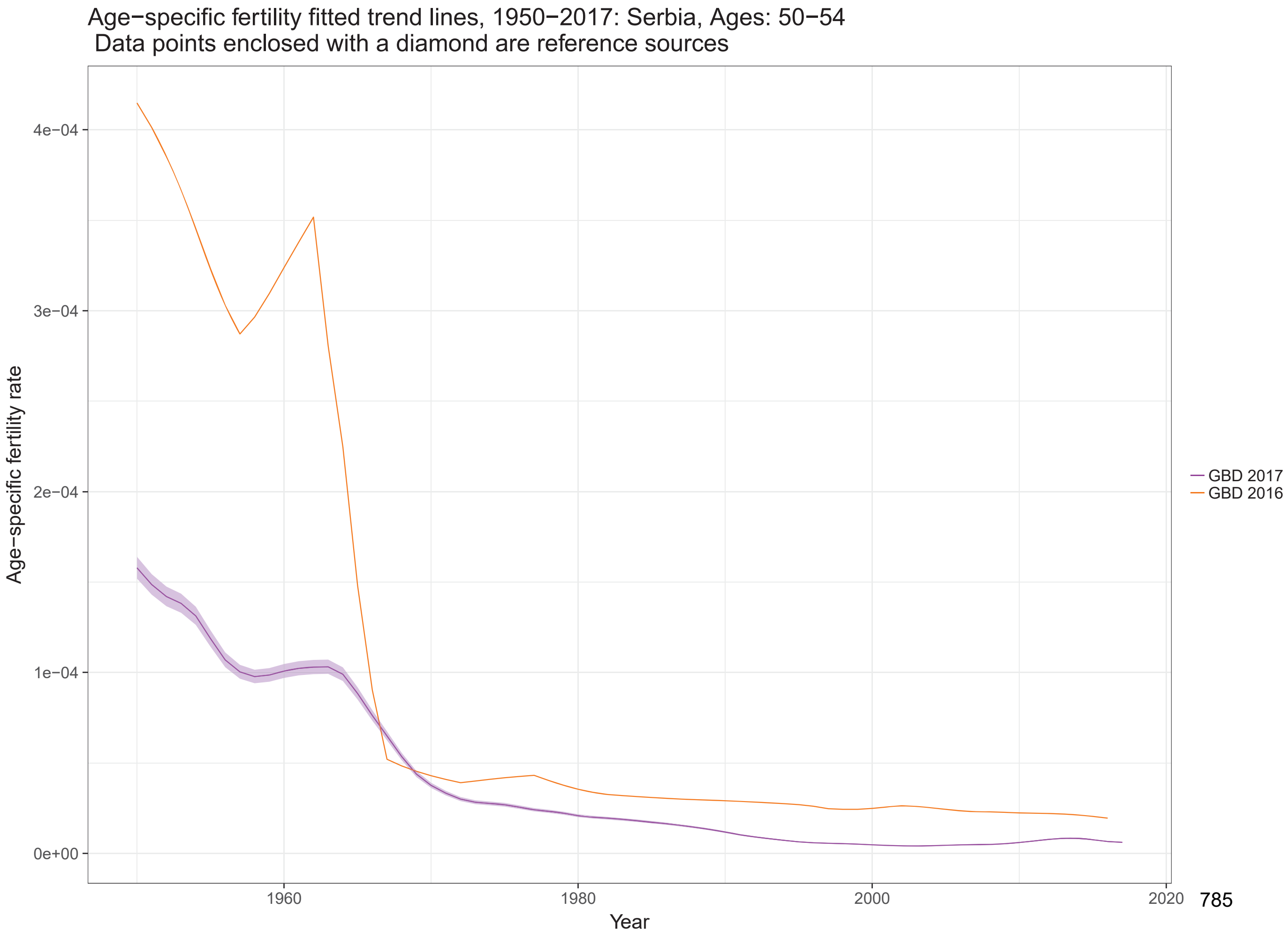
Age−specific fertility fitted trend lines, 1950−2017: Serbia, Ages: 50−54
Data points enclosed with a diamond are reference sources
Age−specific fertility rate
4e−04
3e−04
2e−04
1e−04
0e+00
1960
1980
2000
2020
Year
GBD 2017
GBD 2016

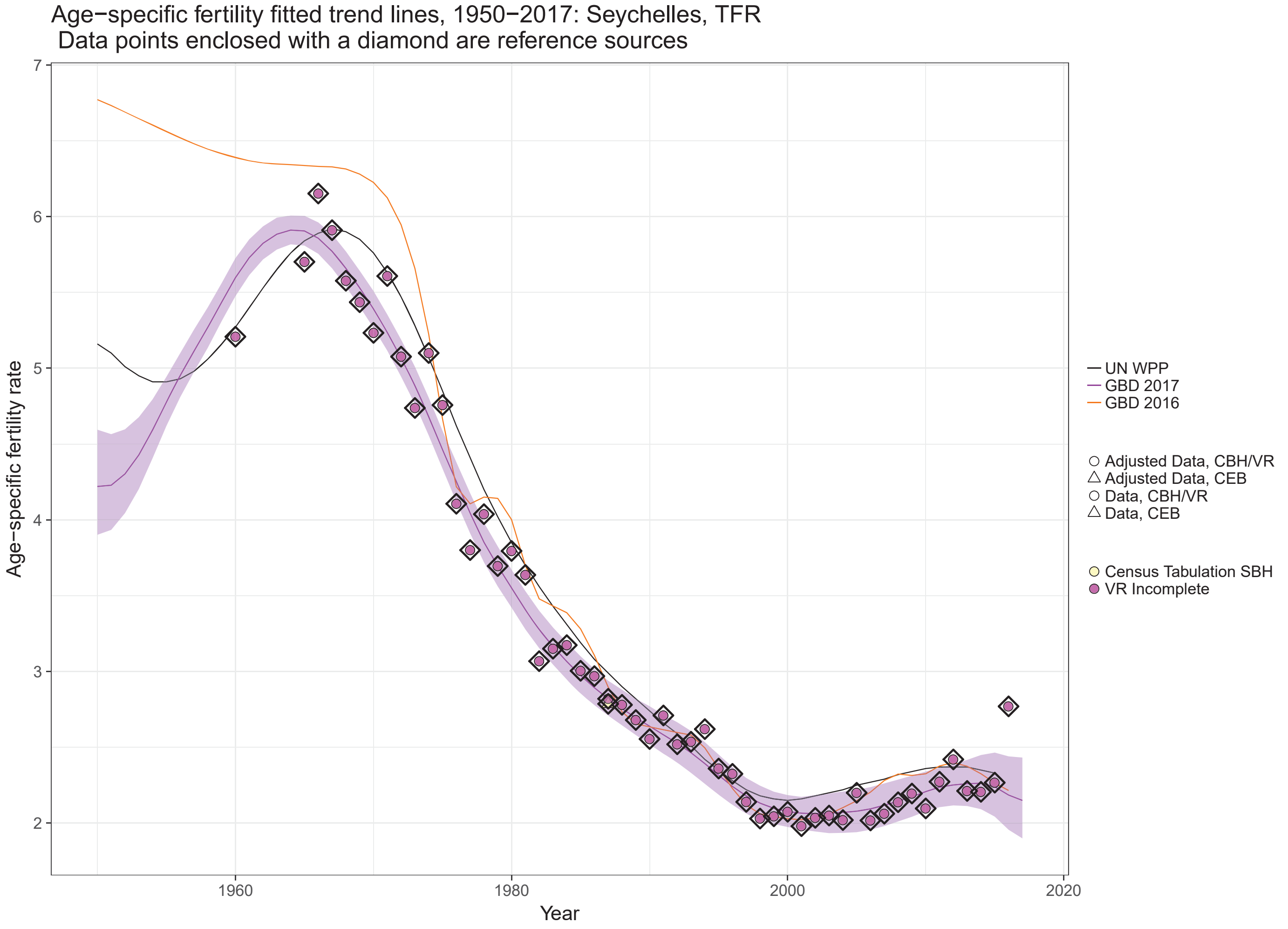

Age−specific fertility fitted trend lines, 1950−2017: Seychelles, TFR
Data points enclosed with a diamond are reference sources
Age−specific fertility rate
Year
UN WPP
GBD 2017
GBD 2016
Adjusted Data, CBH/VR
Adjusted Data, CEB
Data, CBH/VR
Data, CEB
Census Tabulation SBH
VR Incomplete

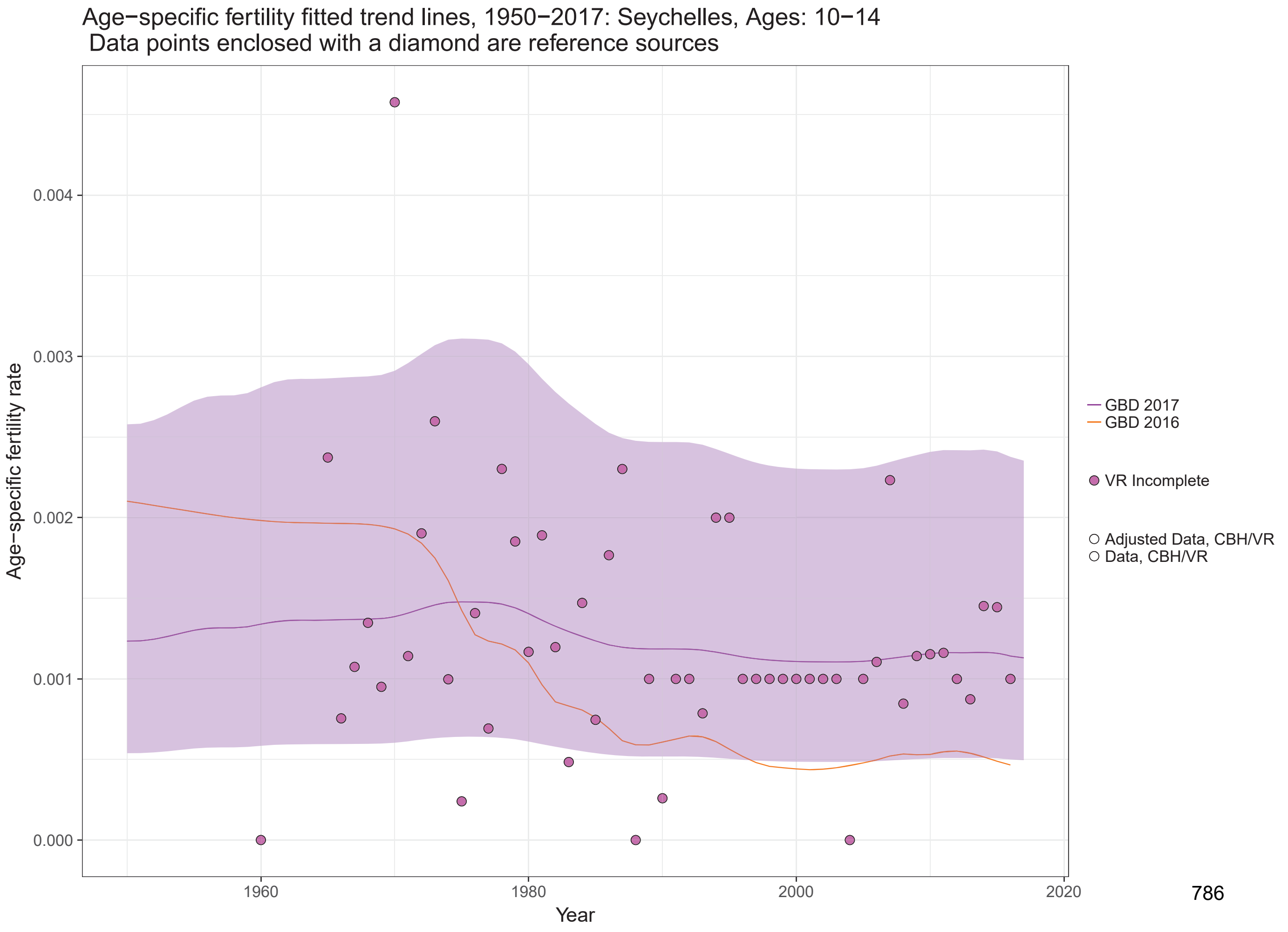

Age−specific fertility fitted trend lines, 1950−2017: Seychelles, Ages: 10−14
Data points enclosed with a diamond are reference sources
Age−specific fertility rate
Year
GBD 2017
GBD 2016
VR Incomplete
Adjusted Data, CBH/VR
Data, CBH/VR

Age−specific fertility fitted trend lines, 1950−2017: Seychelles, Ages: 15−19
Data points enclosed with a diamond are reference sources

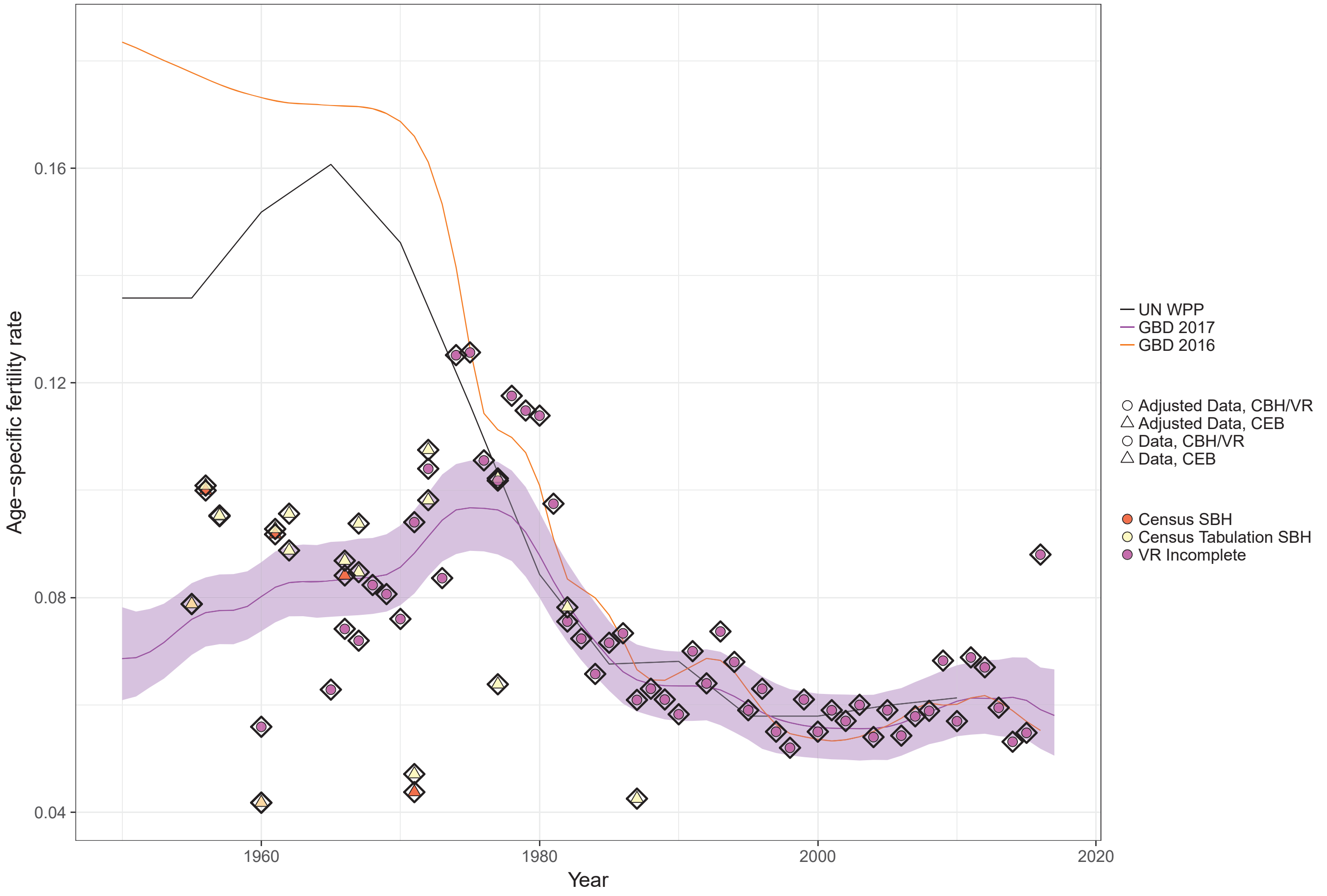

Age−specific fertility fitted trend lines, 1950−2017: Seychelles, Ages: 20−24
Data points enclosed with a diamond are reference sources

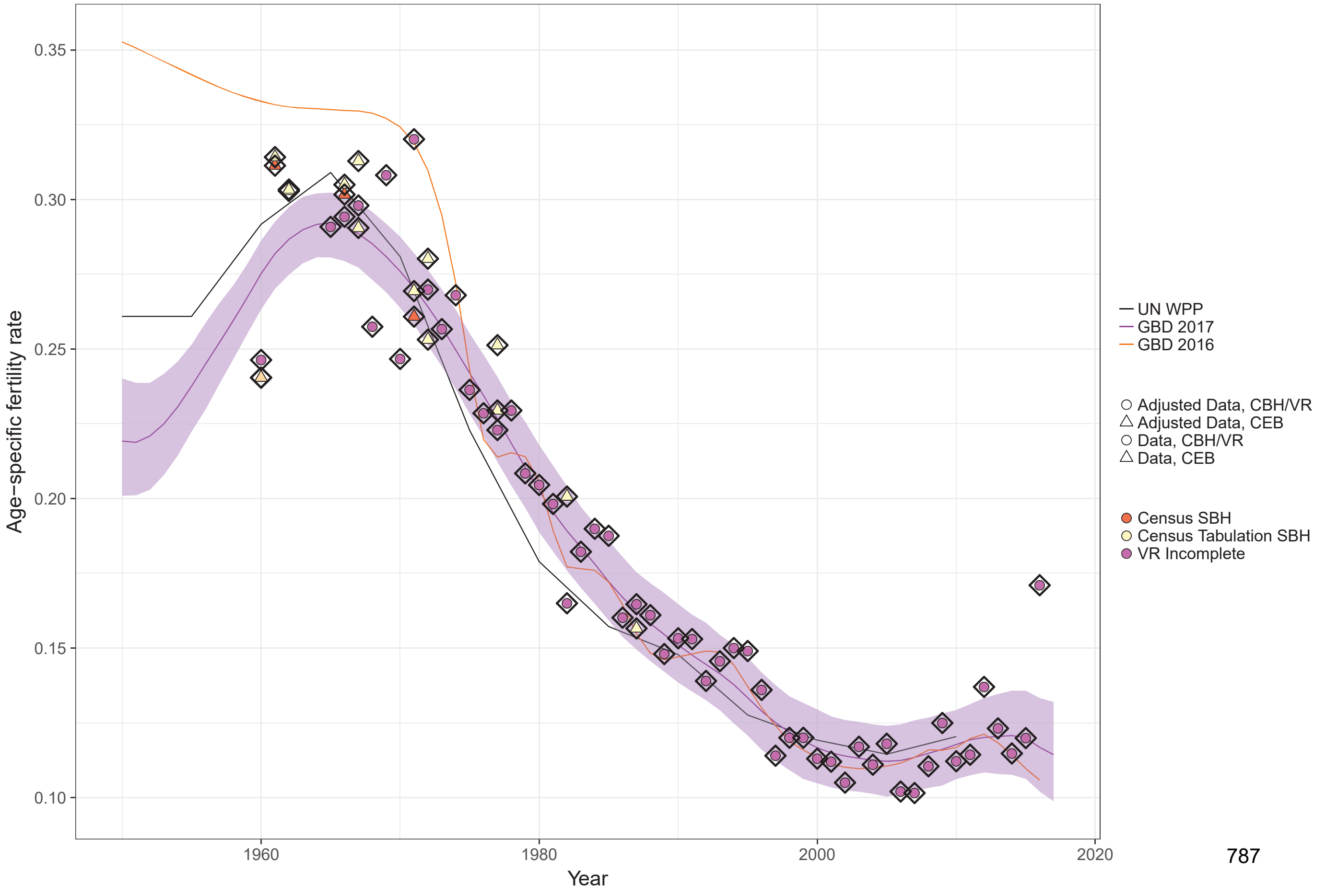

Age−specific fertility fitted trend lines, 1950−2017: Seychelles, Ages: 25−29
Data points enclosed with a diamond are reference sources

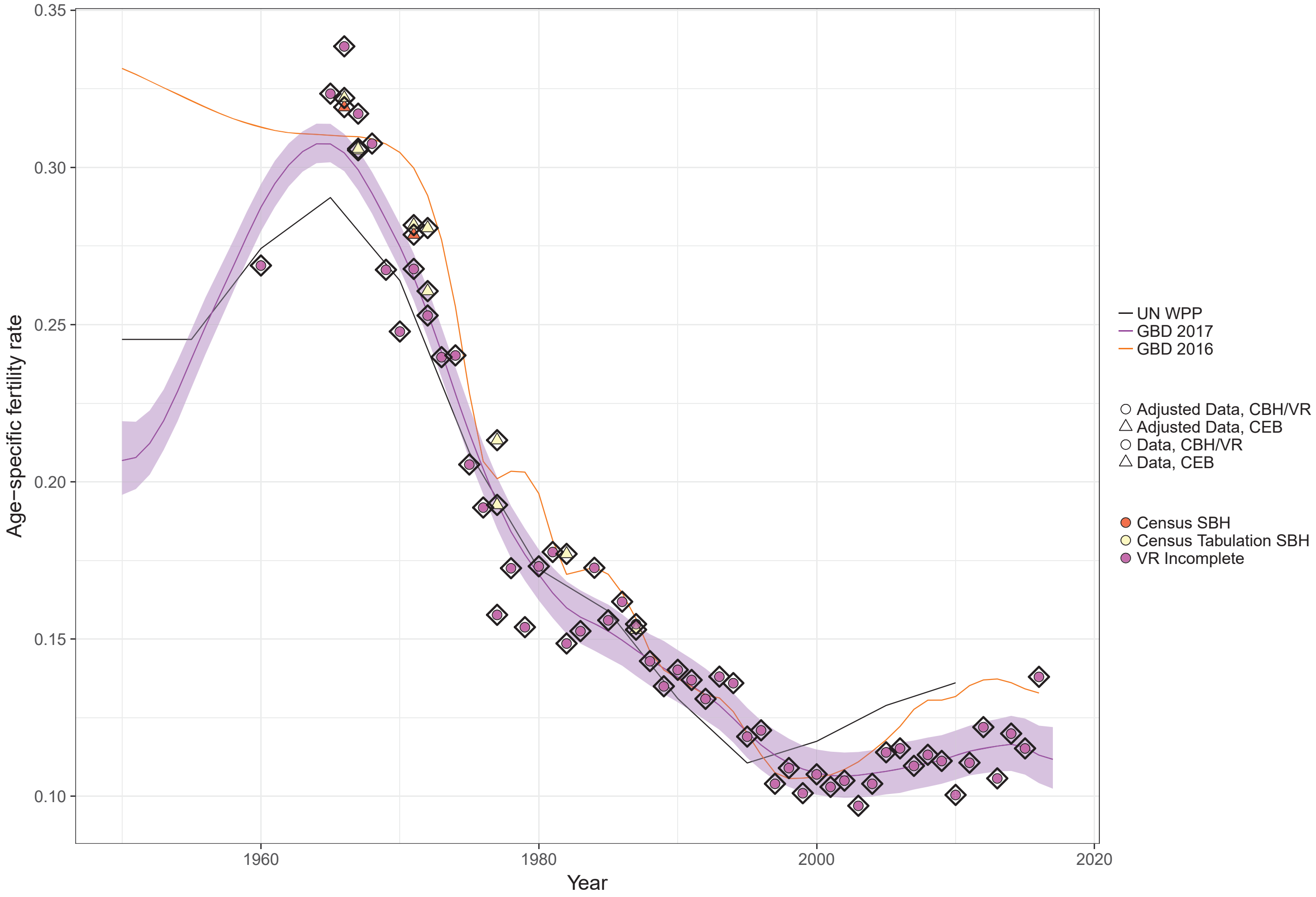

Age−specific fertility fitted trend lines, 1950−2017: Seychelles, Ages: 30−34
Data points enclosed with a diamond are reference sources

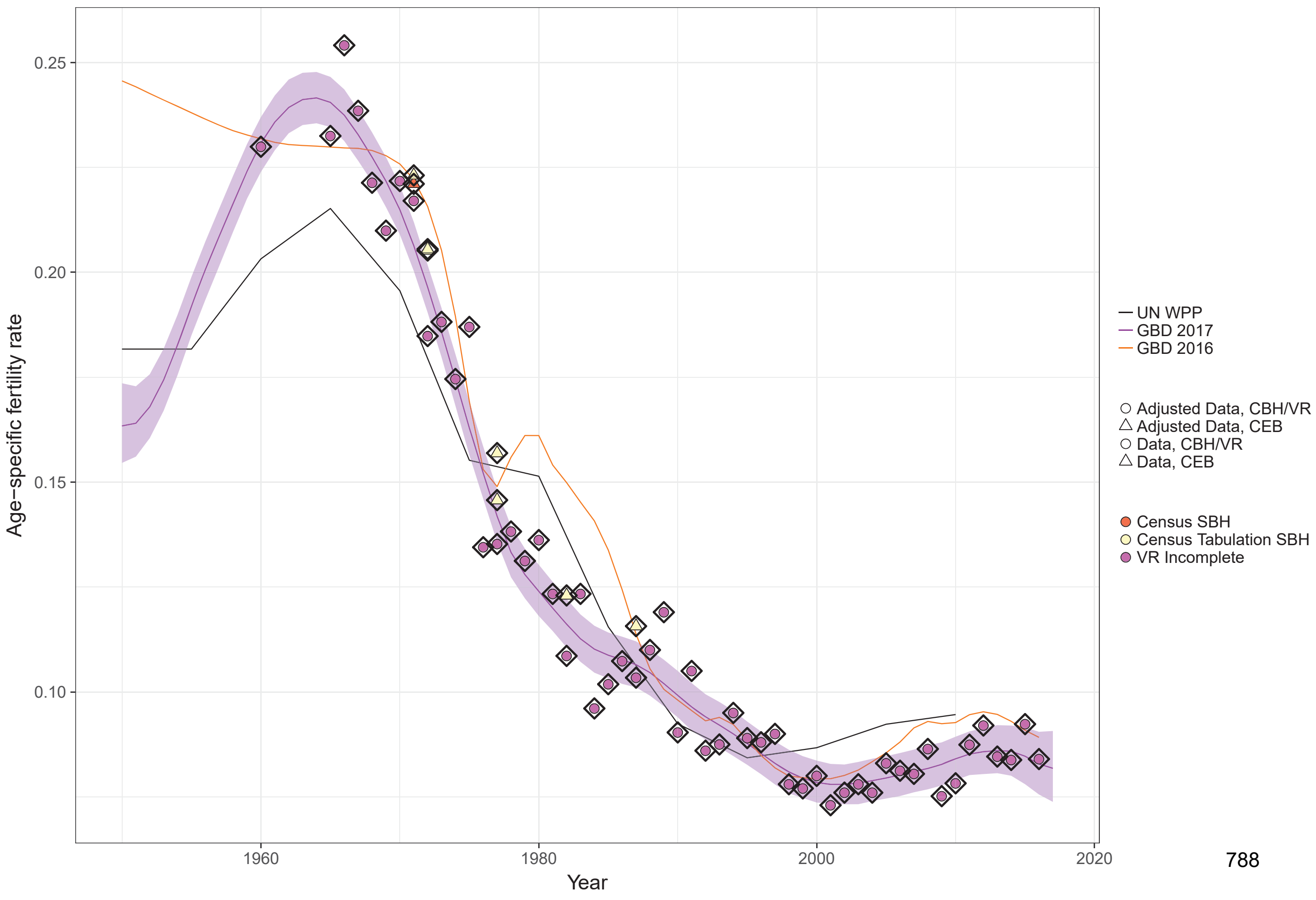

Age−specific fertility fitted trend lines, 1950−2017: Seychelles, Ages: 35−39
Data points enclosed with a diamond are reference sources

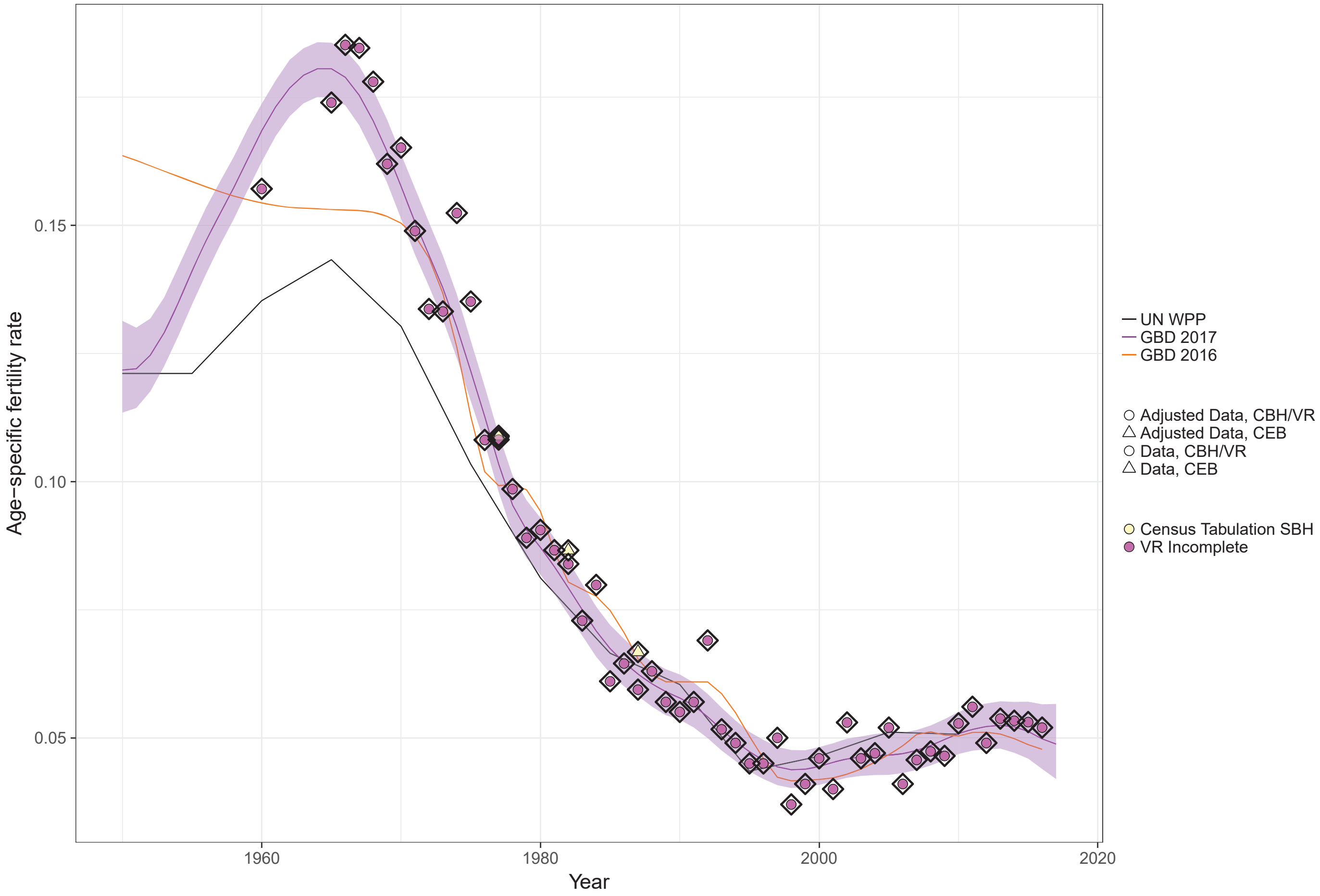

Age−specific fertility fitted trend lines, 1950−2017: Seychelles, Ages: 40−44
Data points enclosed with a diamond are reference sources

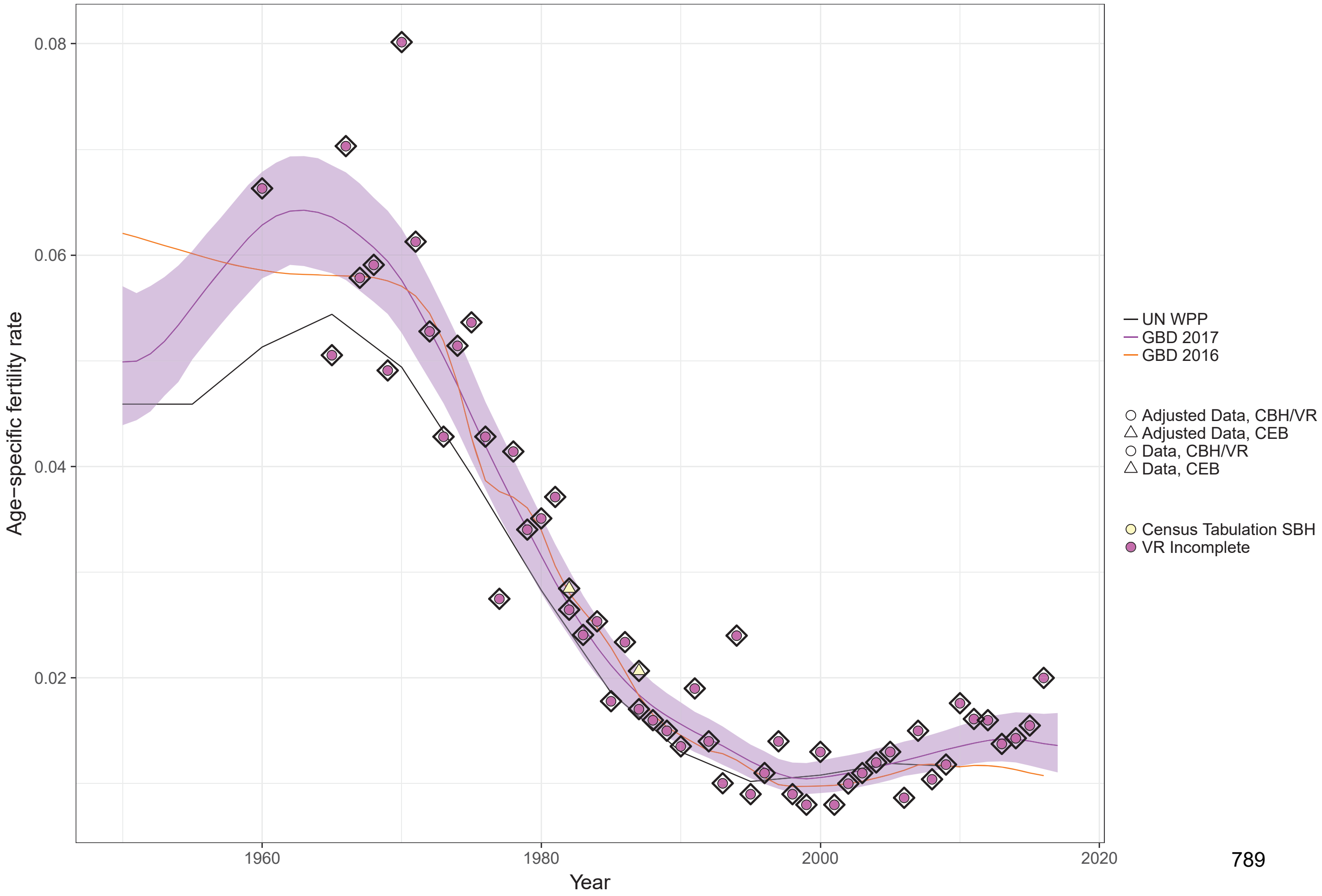

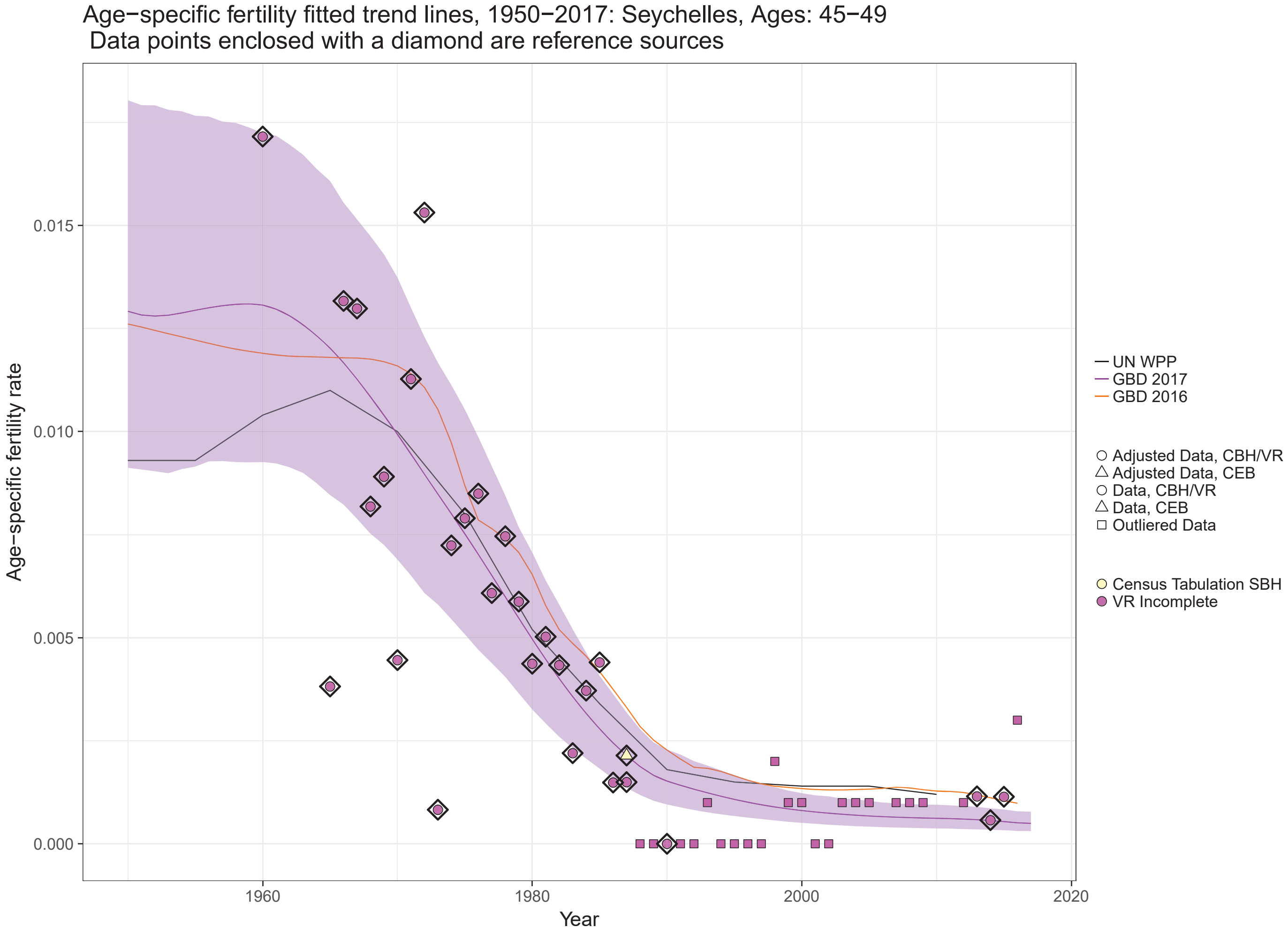
Age−specific fertility fitted trend lines, 1950−2017: Seychelles, Ages: 45−49
Data points enclosed with a diamond are reference sources
Age−specific fertility rate
0.015
0.010
0.005
0.000
1960
1980
2000
2020
Year
UN WPP
GBD 2017
GBD 2016
Adjusted Data, CBH/VR
Adjusted Data, CEB
Data, CBH/VR
Data, CEB
Outliered Data
Census Tabulation SBH
VR Incomplete

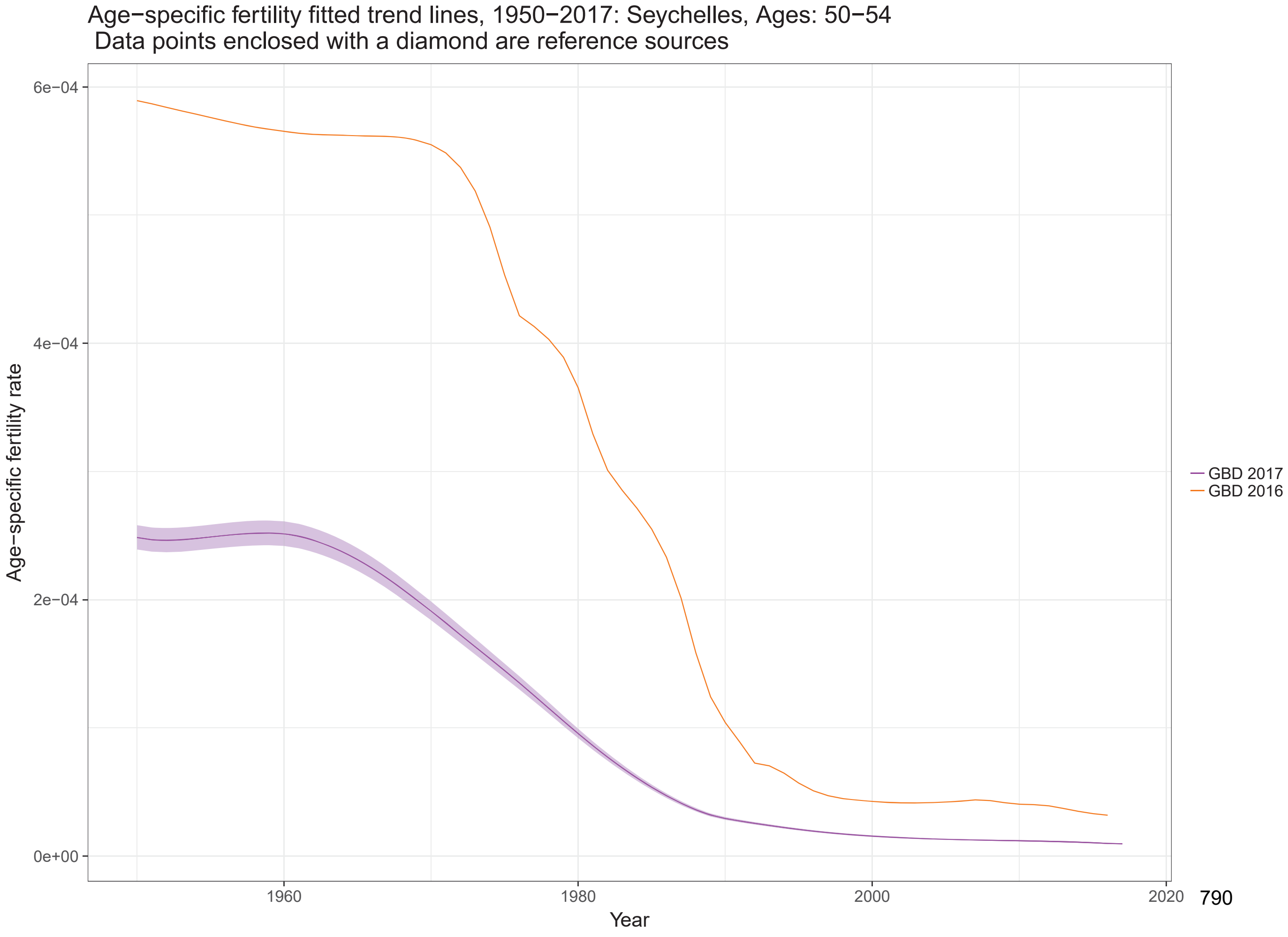
Age−specific fertility fitted trend lines, 1950−2017: Seychelles, Ages: 50−54
Data points enclosed with a diamond are reference sources
Age−specific fertility rate
6e−04
4e−04
2e−04
0e+00
1960
1980
2000
2020
Year
GBD 2017
GBD 2016

Age−specific fertility fitted trend lines, 1950−2017: Sierra Leone, TFR
Data points enclosed with a diamond are reference sources

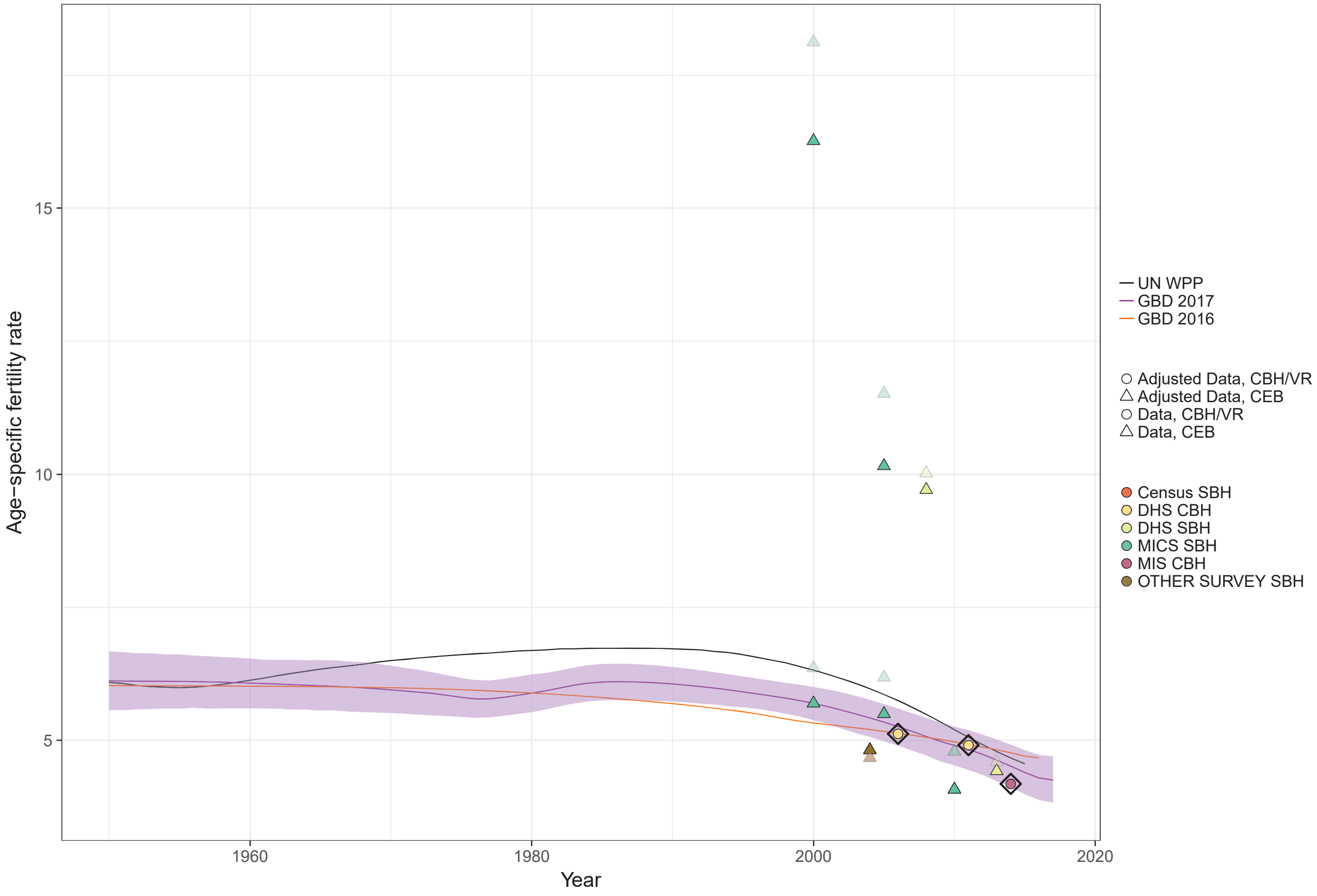

Age−specific fertility fitted trend lines, 1950−2017: Sierra Leone, Ages: 10−14
Data points enclosed with a diamond are reference sources

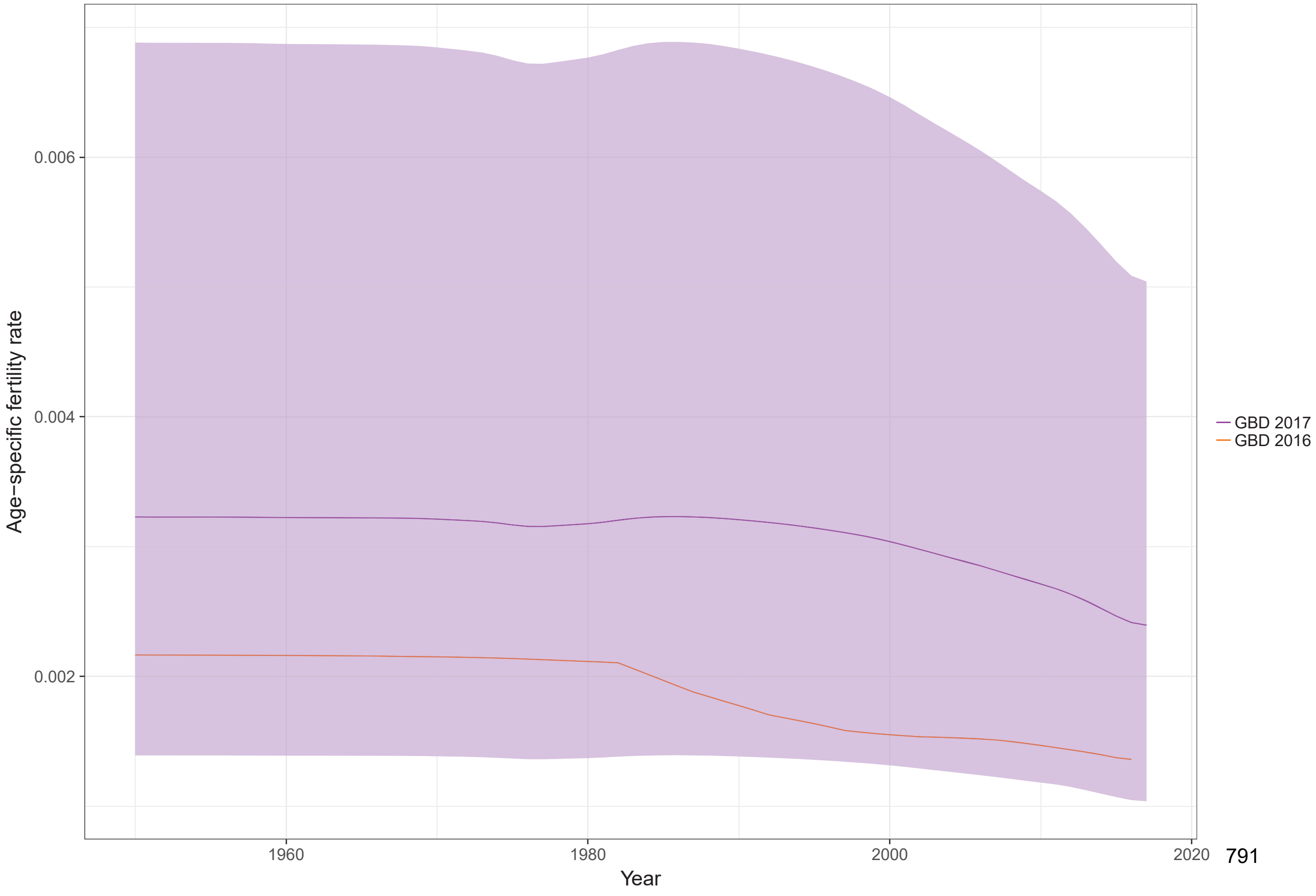

Age−specific fertility fitted trend lines, 1950−2017: Sierra Leone, Ages: 15−19
Data points enclosed with a diamond are reference sources

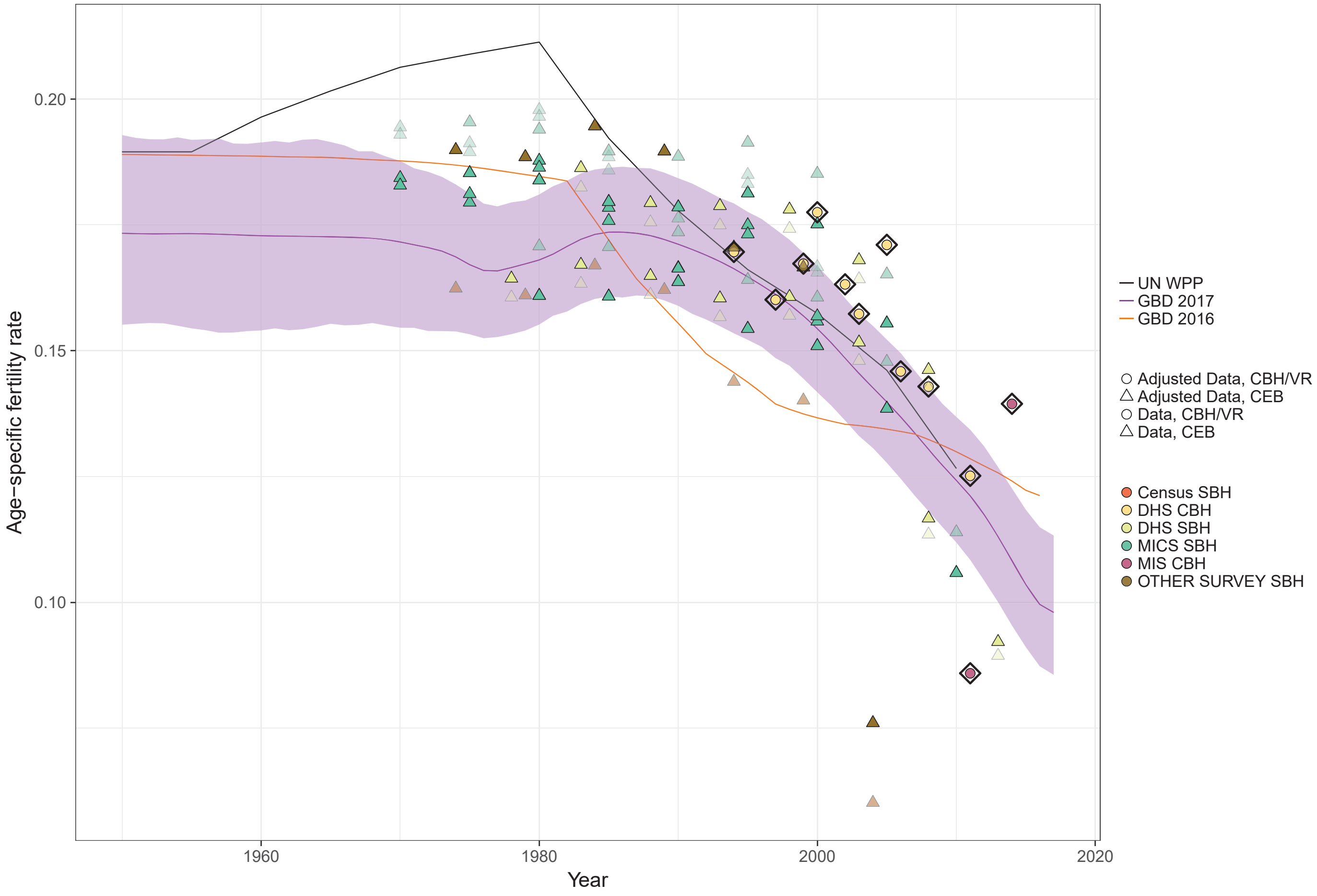

Age−specific fertility fitted trend lines, 1950−2017: Sierra Leone, Ages: 20−24
Data points enclosed with a diamond are reference sources

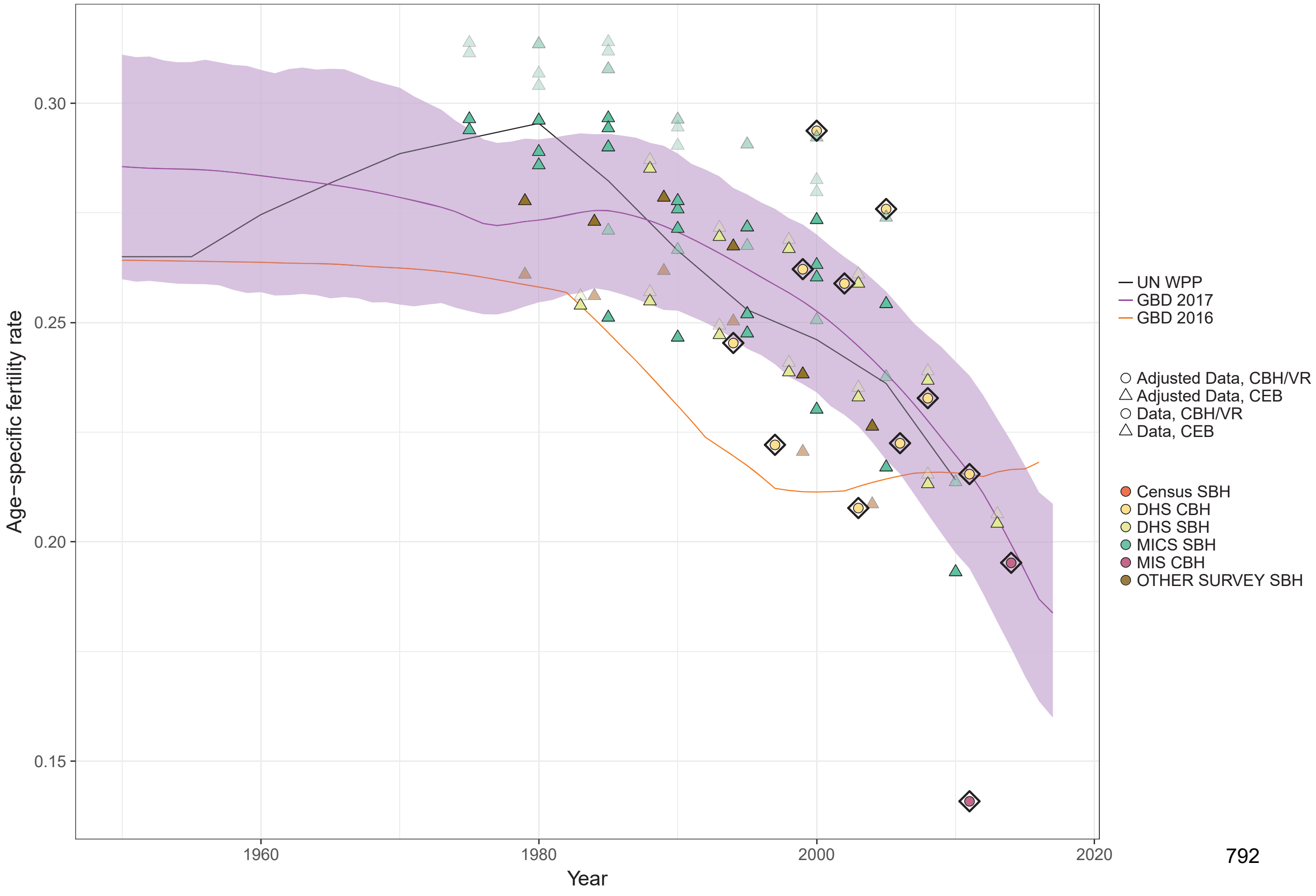

Age−specific fertility fitted trend lines, 1950−2017: Sierra Leone, Ages: 25−29
Data points enclosed with a diamond are reference sources

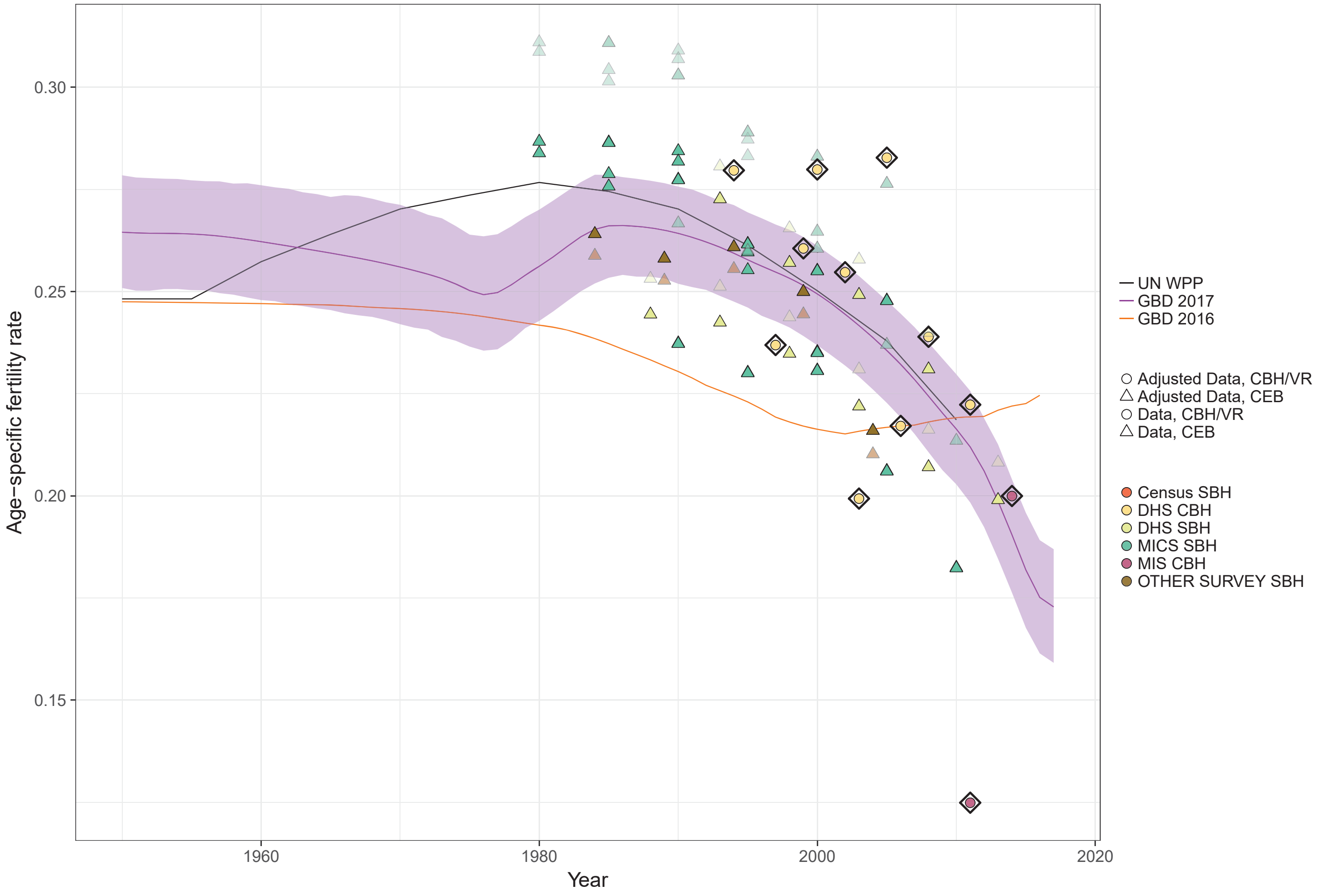

Age−specific fertility fitted trend lines, 1950−2017: Sierra Leone, Ages: 30−34
Data points enclosed with a diamond are reference sources

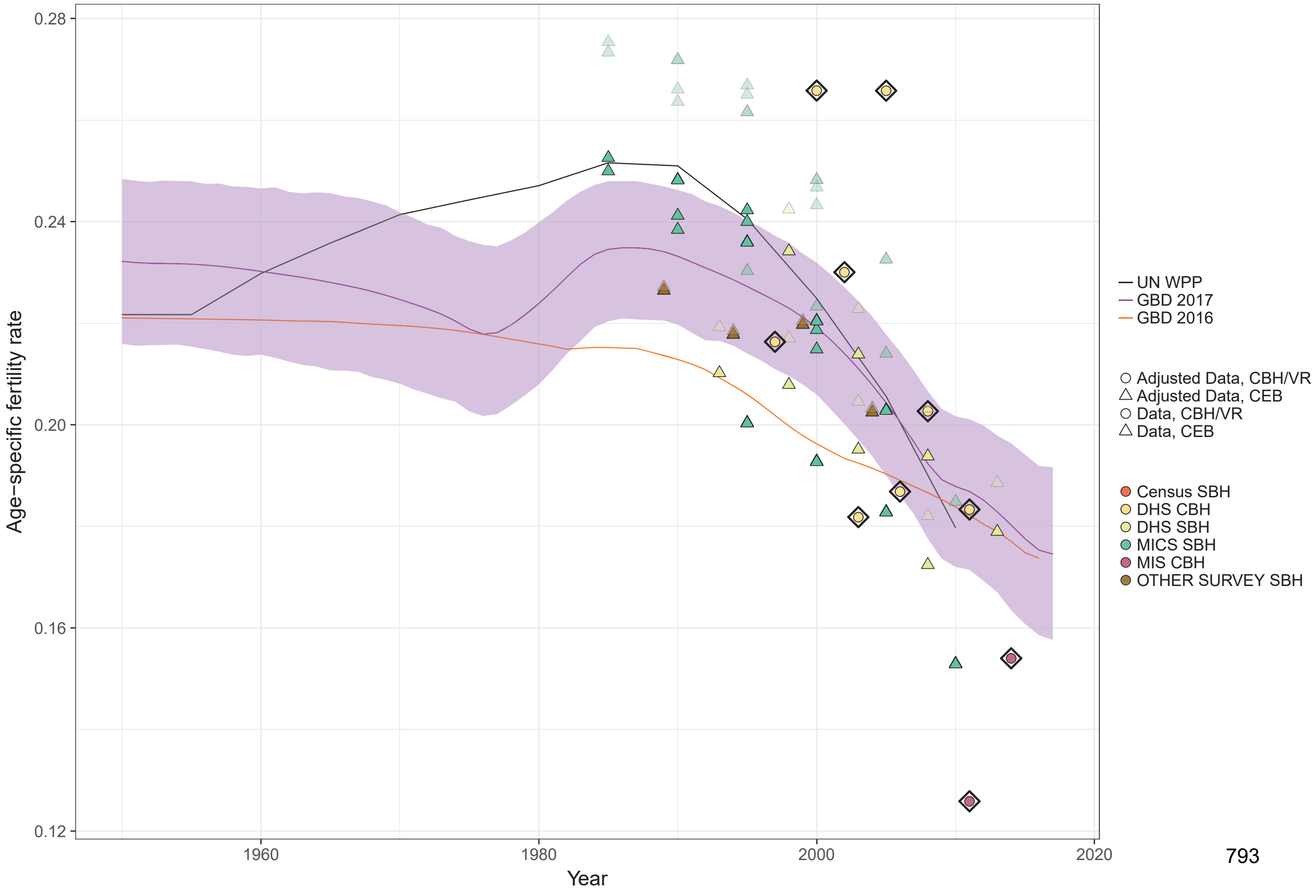

Age−specific fertility fitted trend lines, 1950−2017: Sierra Leone, Ages: 35−39
Data points enclosed with a diamond are reference sources

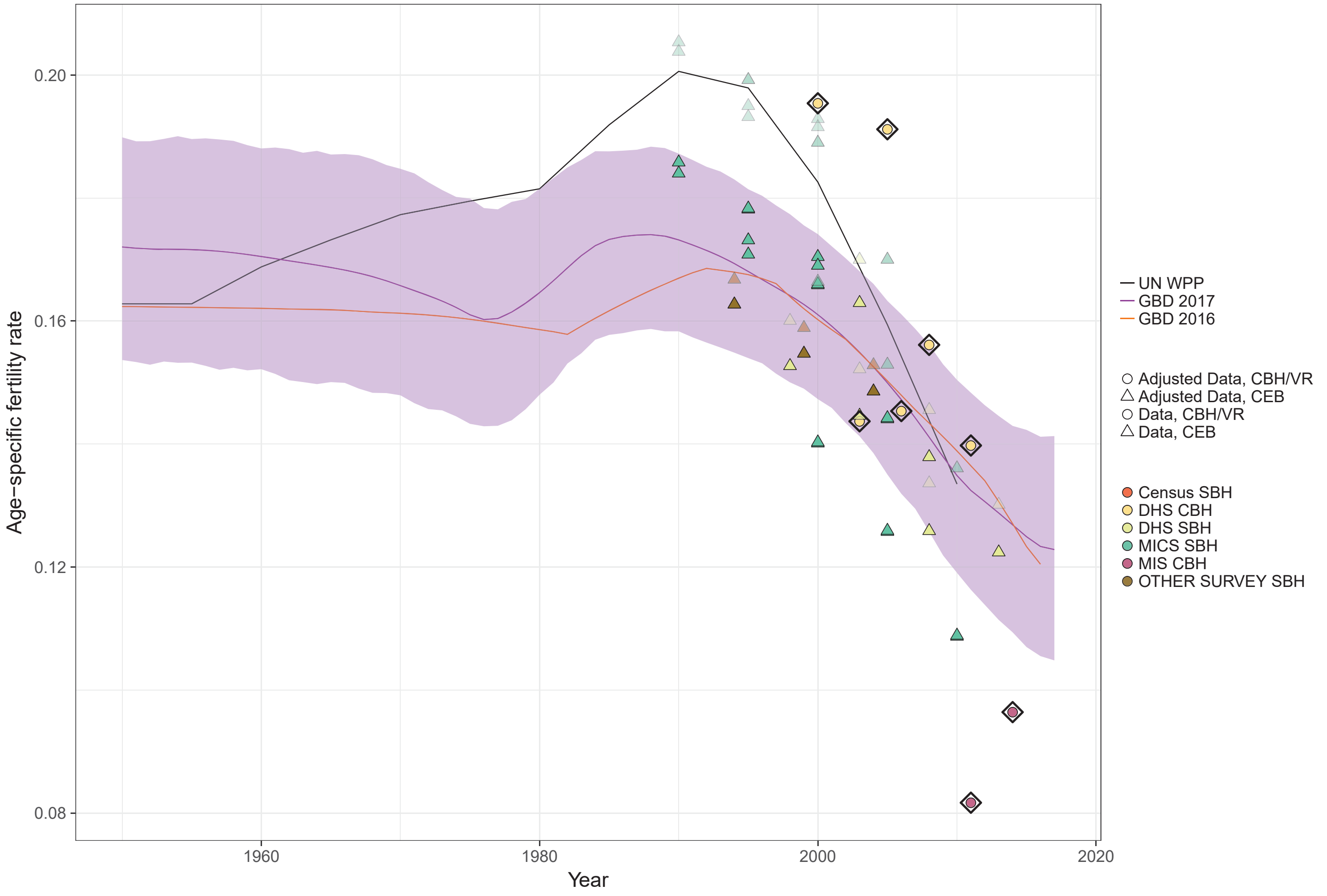

Age−specific fertility fitted trend lines, 1950−2017: Sierra Leone, Ages: 40−44
Data points enclosed with a diamond are reference sources

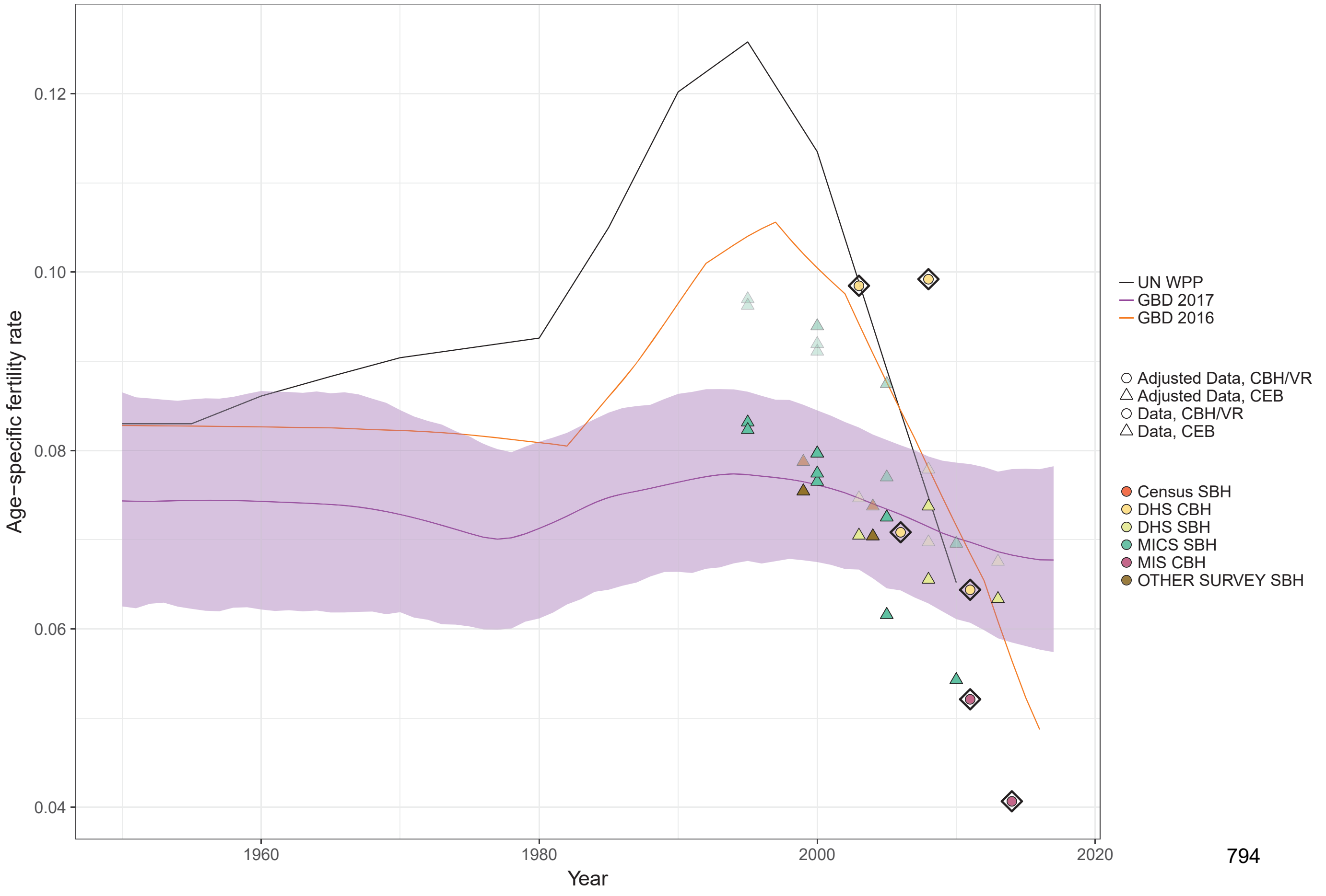

Age−specific fertility fitted trend lines, 1950−2017: Sierra Leone, Ages: 45−49
Data points enclosed with a diamond are reference sources

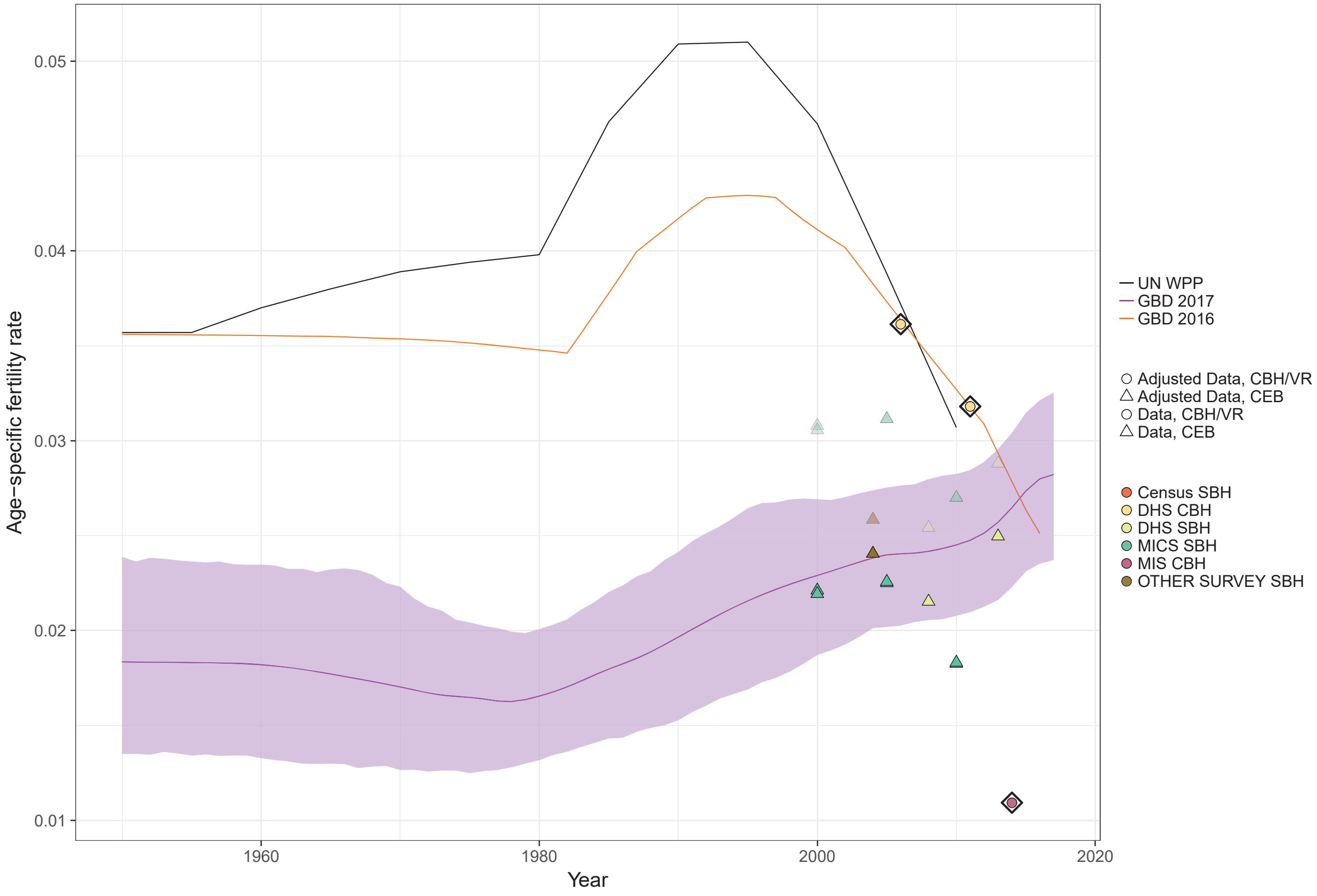

Age−specific fertility fitted trend lines, 1950−2017: Sierra Leone, Ages: 50−54
Data points enclosed with a diamond are reference sources

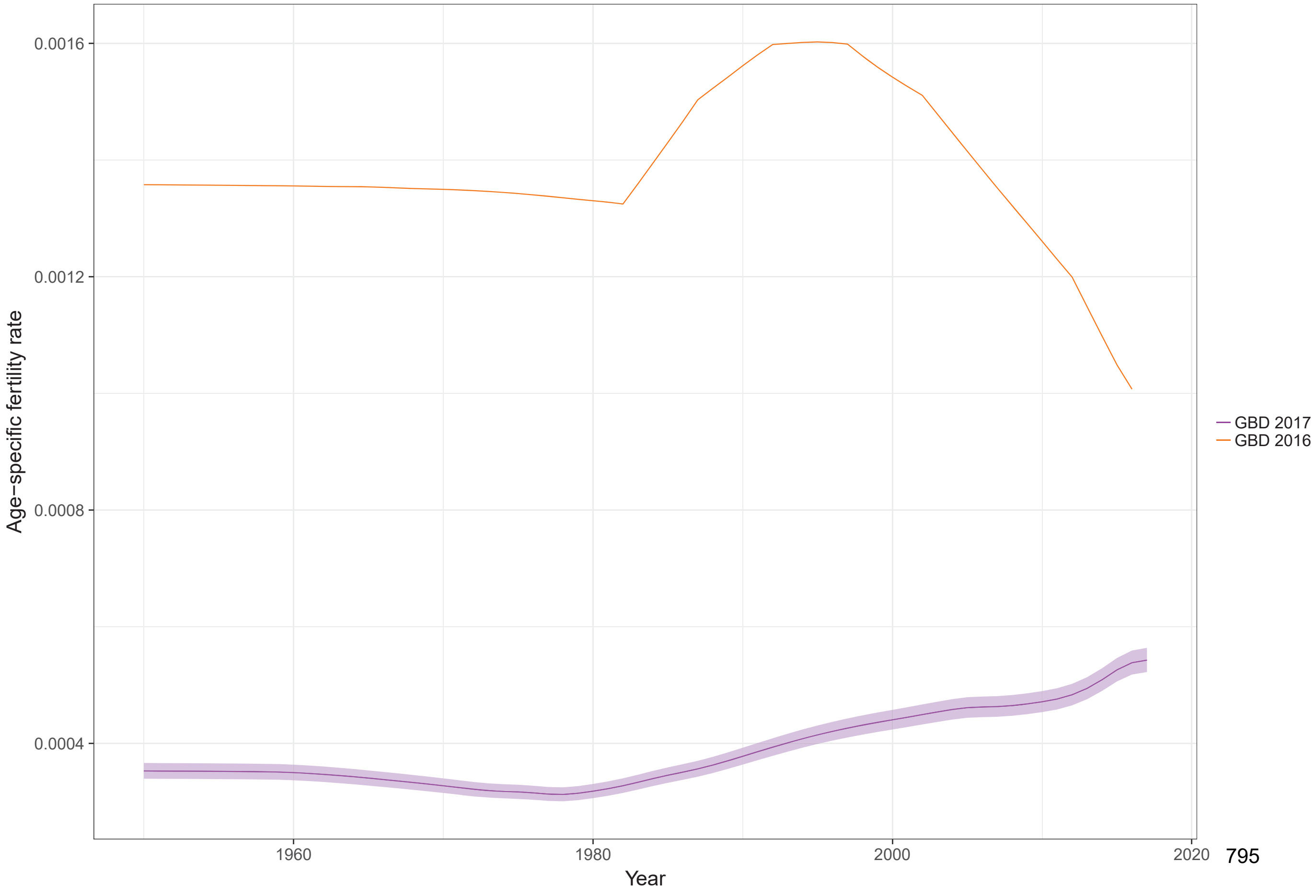

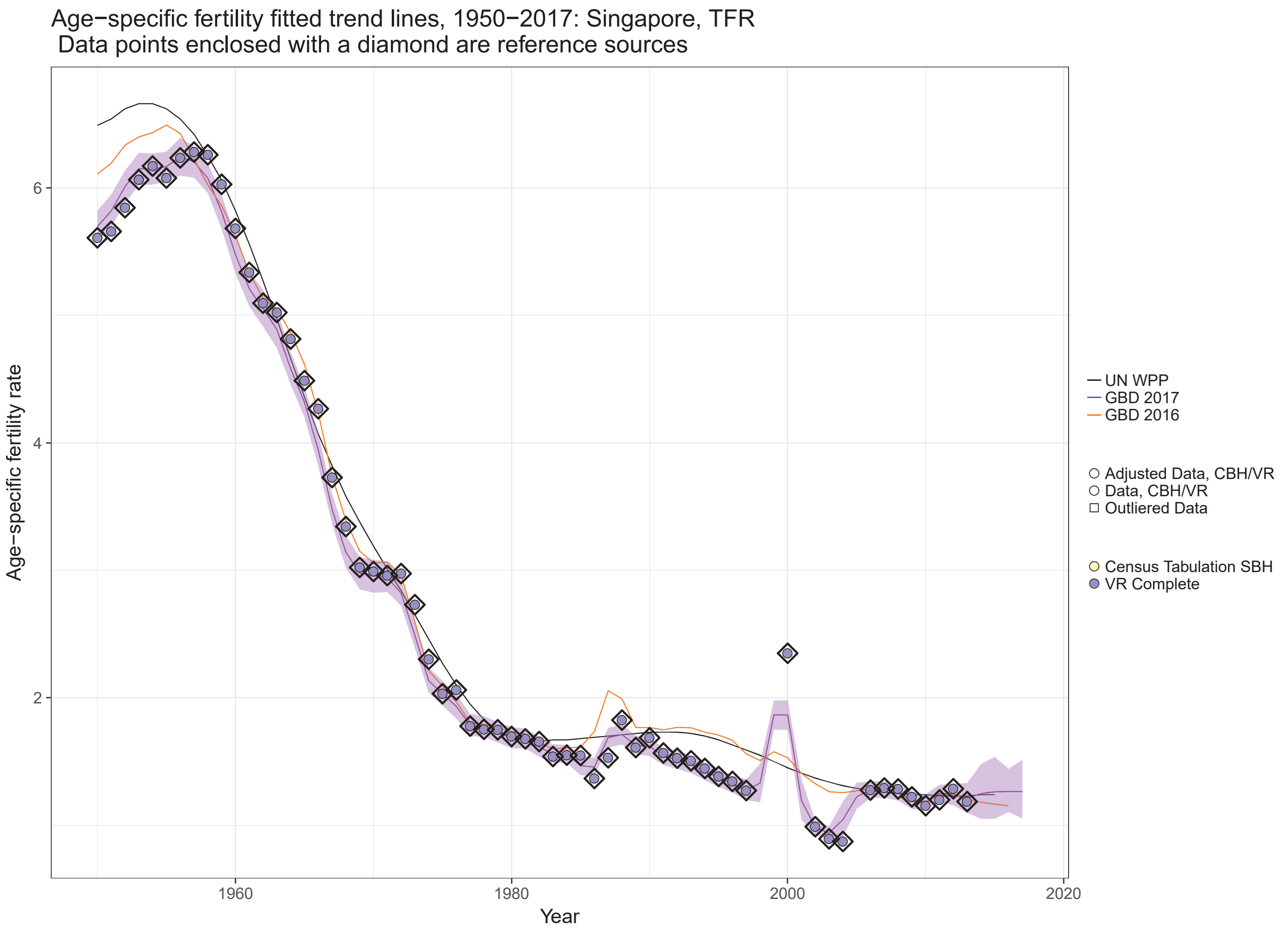
Age−specific fertility fitted trend lines, 1950−2017: Singapore, TFR
Data points enclosed with a diamond are reference sources
Age−specific fertility rate
Year
UN WPP
GBD 2017
GBD 2016
Adjusted Data, CBH/VR
Data, CBH/VR
Outliered Data
Census Tabulation SBH
VR Complete

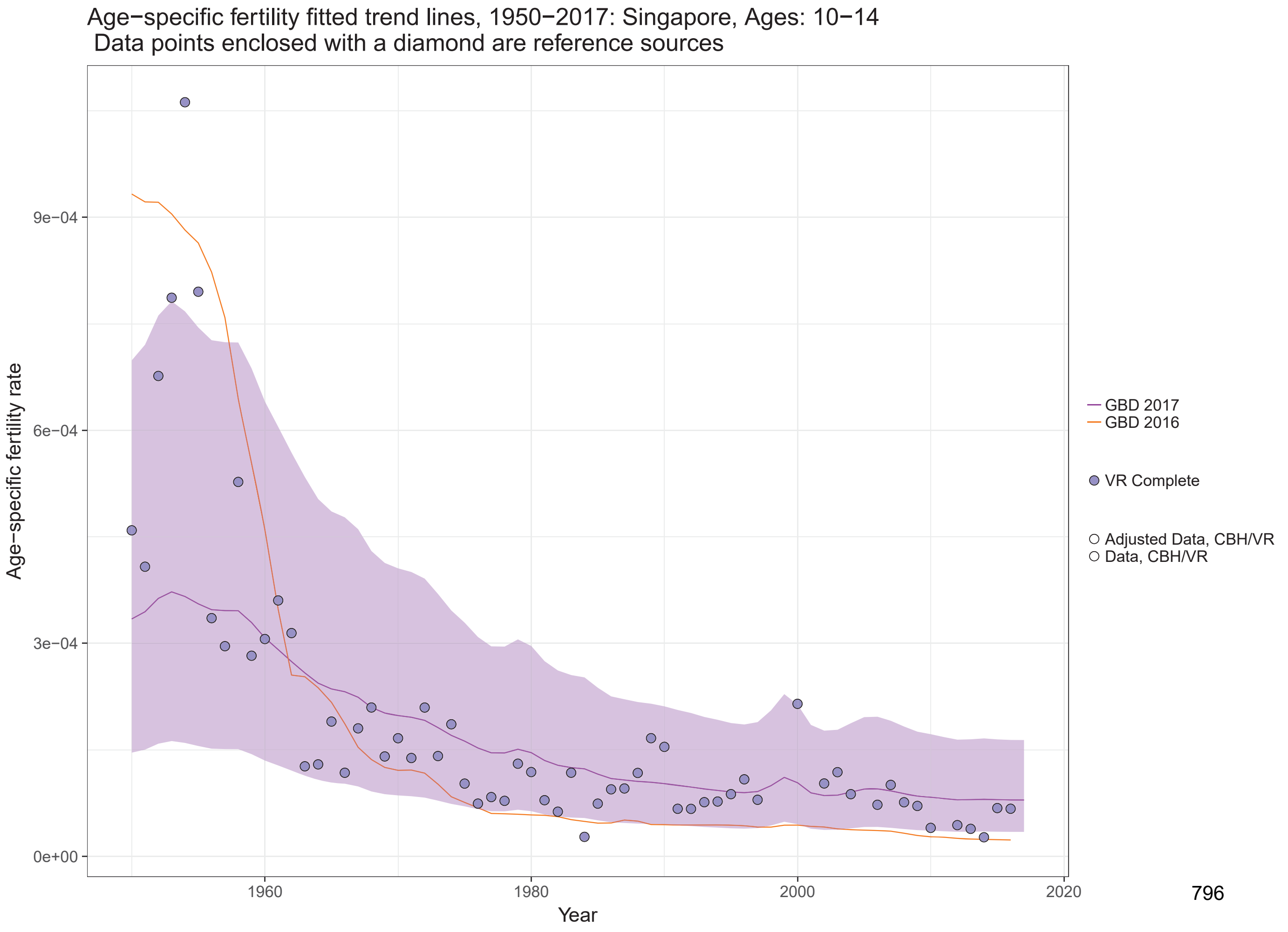
Age−specific fertility fitted trend lines, 1950−2017: Singapore, Ages: 10−14
Data points enclosed with a diamond are reference sources
Age−specific fertility rate
Year
GBD 2017
GBD 2016
VR Complete
Adjusted Data, CBH/VR
Data, CBH/VR

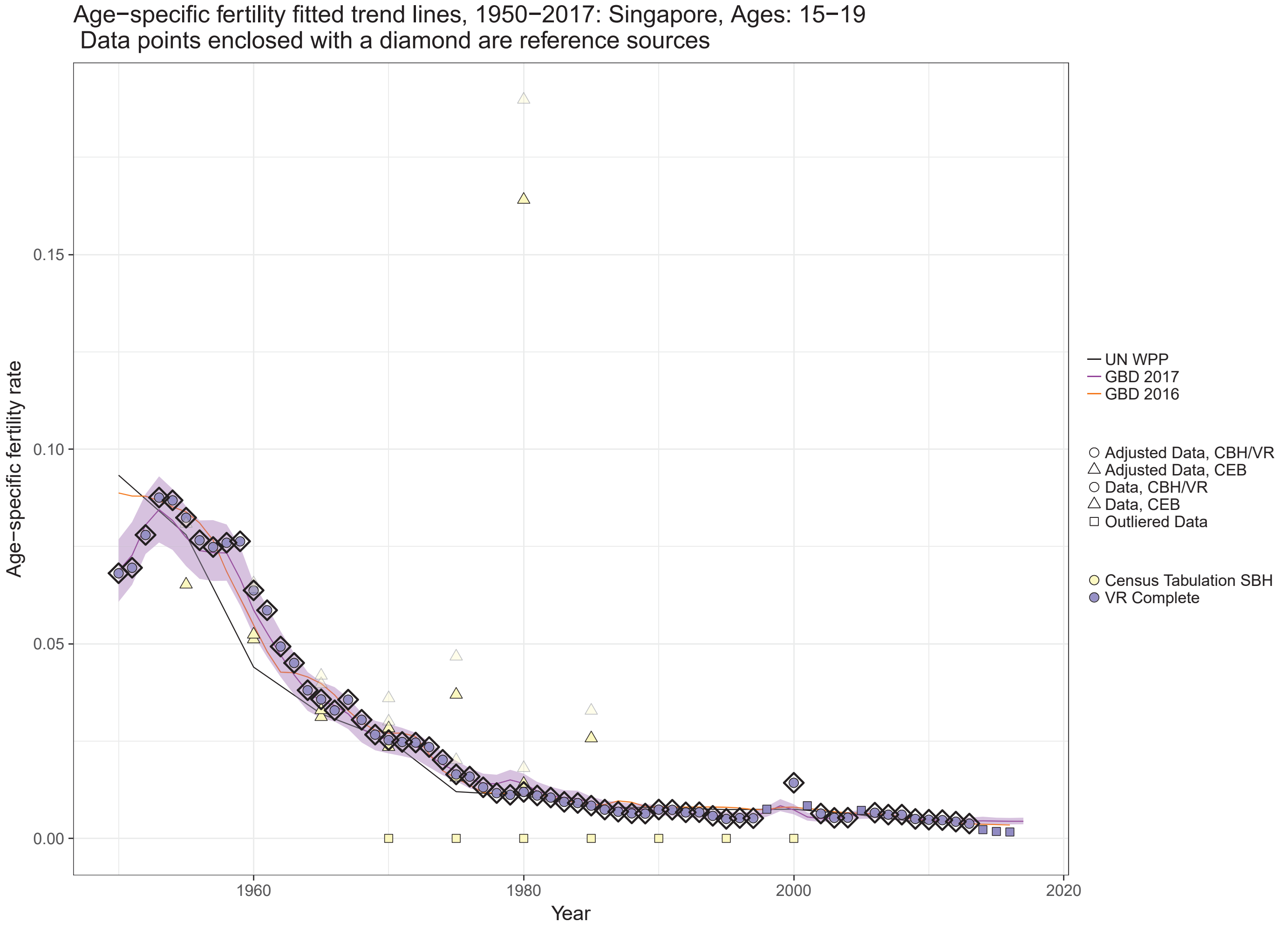
Age−specific fertility fitted trend lines, 1950−2017: Singapore, Ages: 15−19
Data points enclosed with a diamond are reference sources
Age−specific fertility rate
Year
UN WPP
GBD 2017
GBD 2016
Adjusted Data, CBH/VR
Adjusted Data, CEB
Data, CBH/VR
Data, CEB
Outliered Data
Census Tabulation SBH
VR Complete

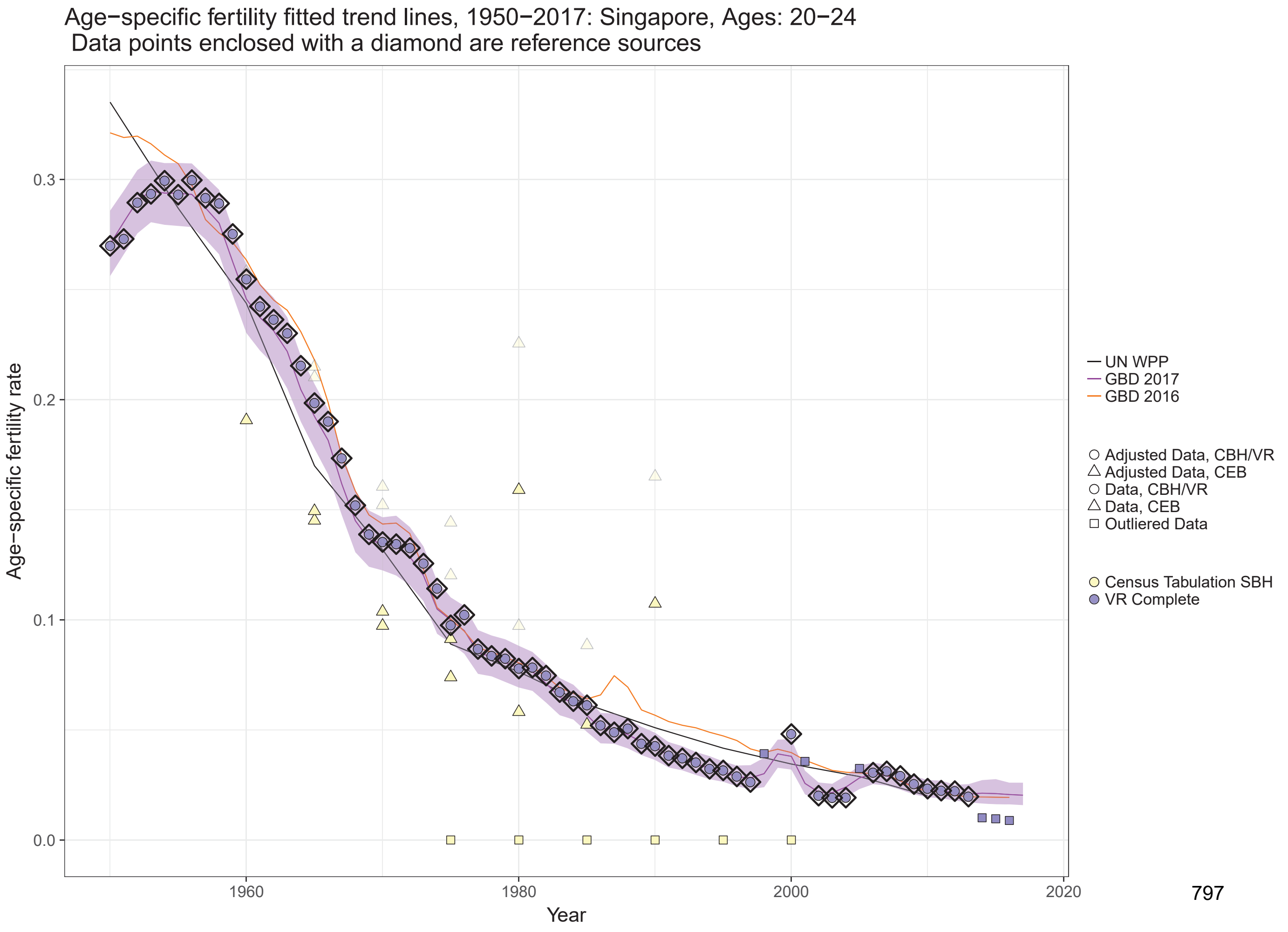
Age−specific fertility fitted trend lines, 1950−2017: Singapore, Ages: 20−24
Data points enclosed with a diamond are reference sources
Age−specific fertility rate
Year
UN WPP
GBD 2017
GBD 2016
Adjusted Data, CBH/VR
Adjusted Data, CEB
Data, CBH/VR
Data, CEB
Outliered Data
Census Tabulation SBH
VR Complete

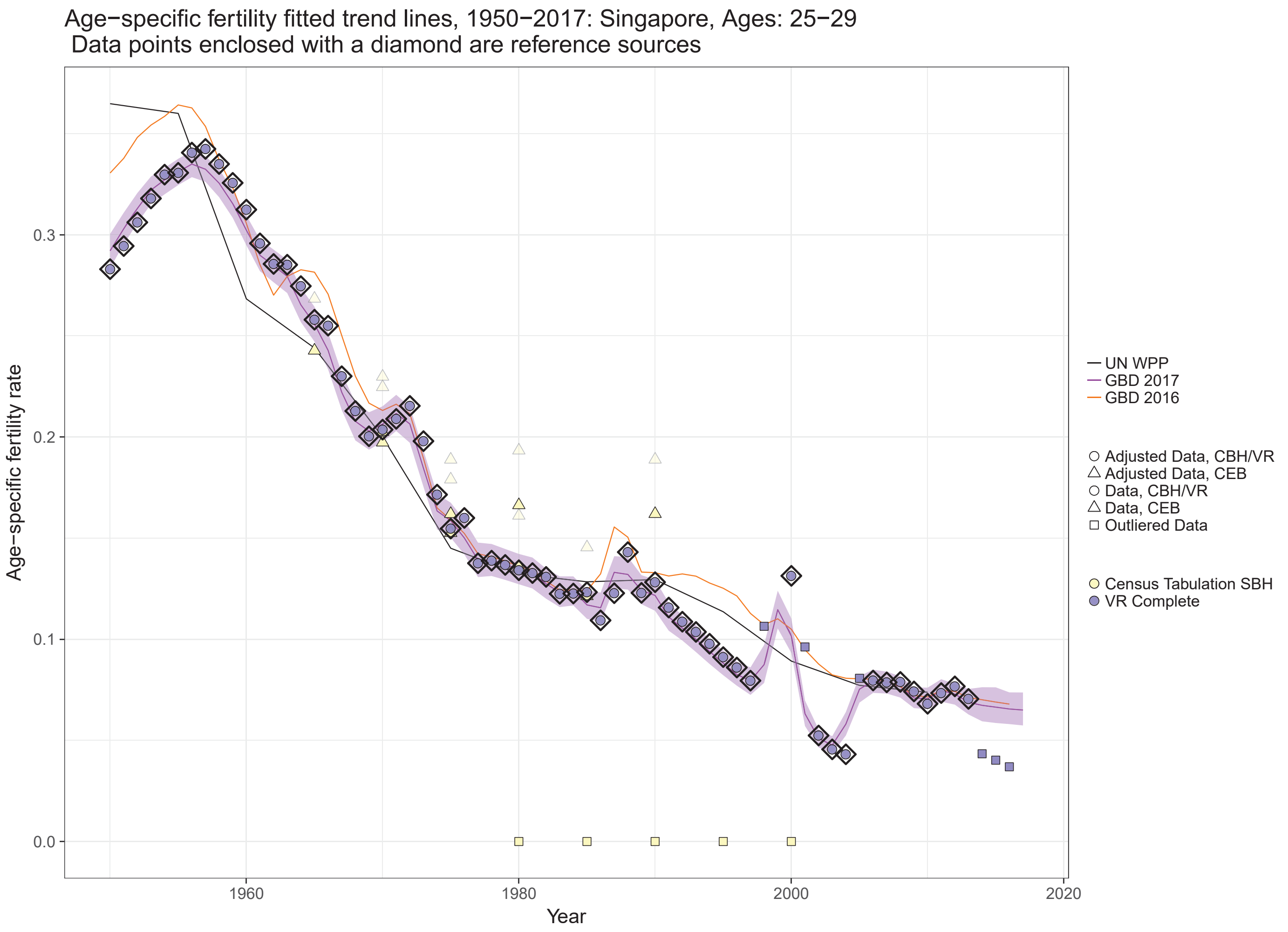
Age−specific fertility fitted trend lines, 1950−2017: Singapore, Ages: 25−29
Data points enclosed with a diamond are reference sources
Age−specific fertility rate
0.0
0.1
0.2
0.3
1960
1980
2000
2020
Year
UN WPP
GBD 2017
GBD 2016
Adjusted Data, CBH/VR
Adjusted Data, CEB
Data, CBH/VR
Data, CEB
Outliered Data
Census Tabulation SBH
VR Complete

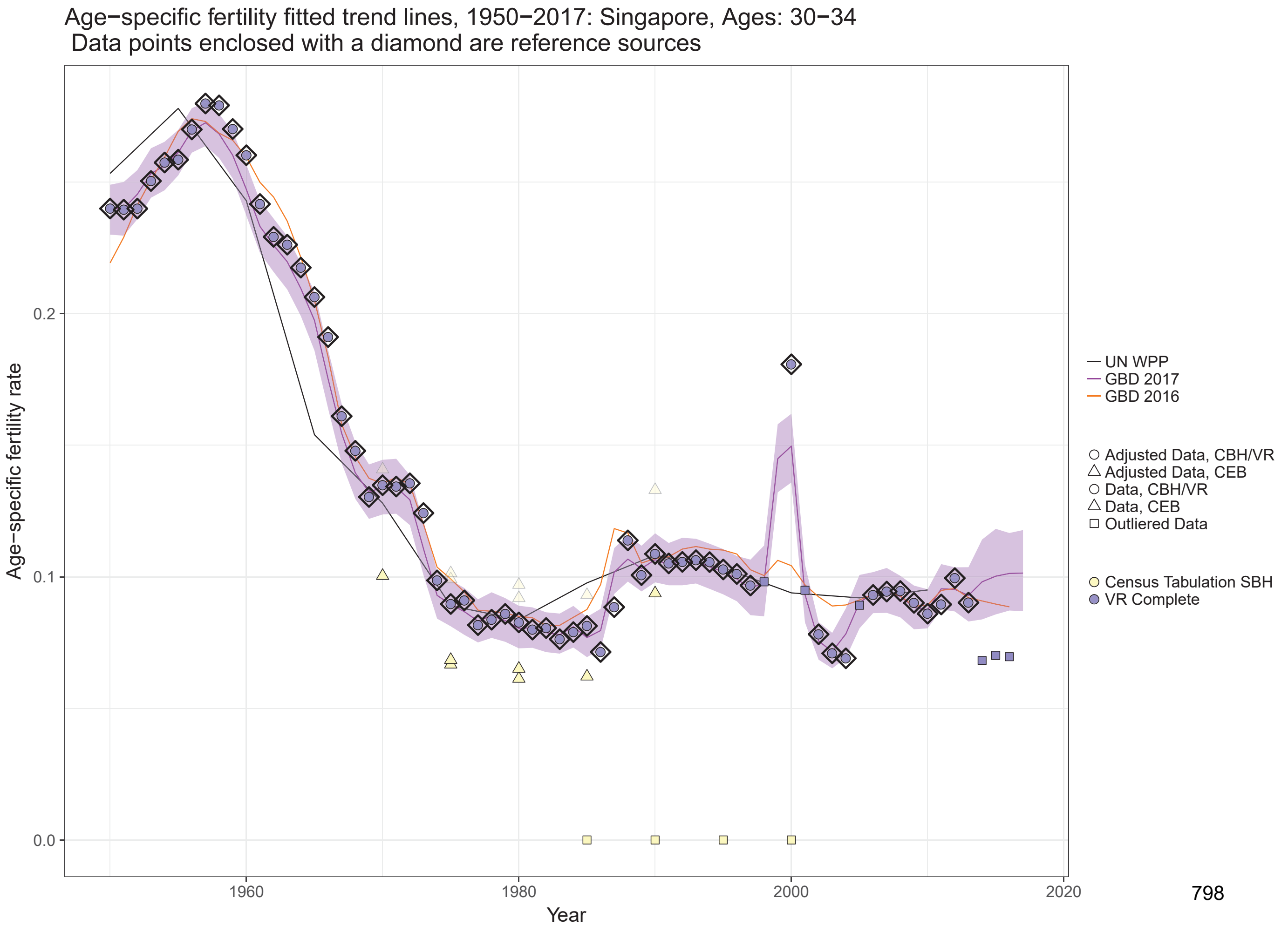
Age−specific fertility fitted trend lines, 1950−2017: Singapore, Ages: 30−34
Data points enclosed with a diamond are reference sources
Age−specific fertility rate
0.0
0.1
0.2
1960
1980
2000
2020
Year
UN WPP
GBD 2017
GBD 2016
Adjusted Data, CBH/VR
Adjusted Data, CEB
Data, CBH/VR
Data, CEB
Outliered Data
Census Tabulation SBH
VR Complete

Age−specific fertility fitted trend lines, 1950−2017: Singapore, Ages: 35−39
Data points enclosed with a diamond are reference sources

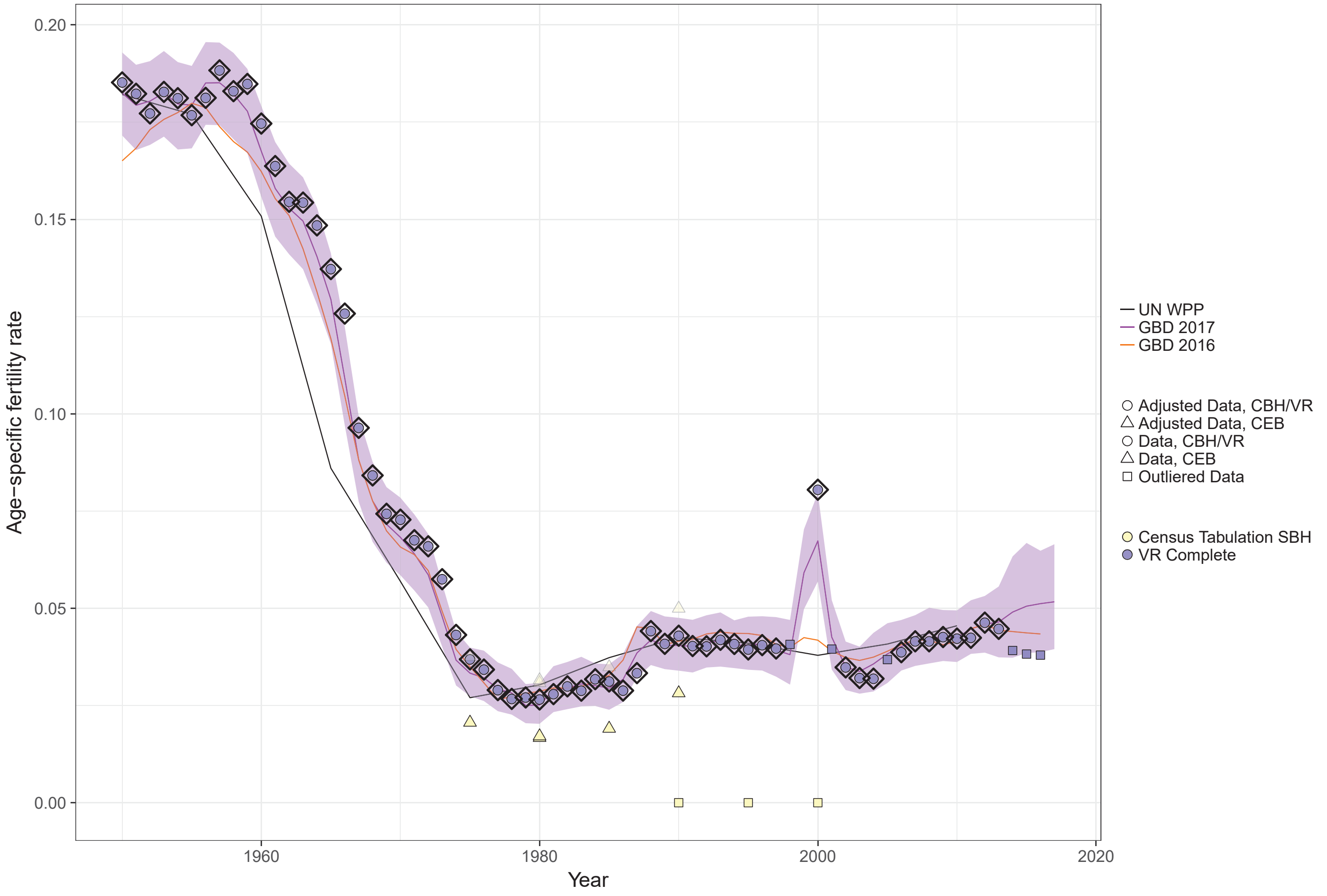

Age−specific fertility fitted trend lines, 1950−2017: Singapore, Ages: 40−44
Data points enclosed with a diamond are reference sources

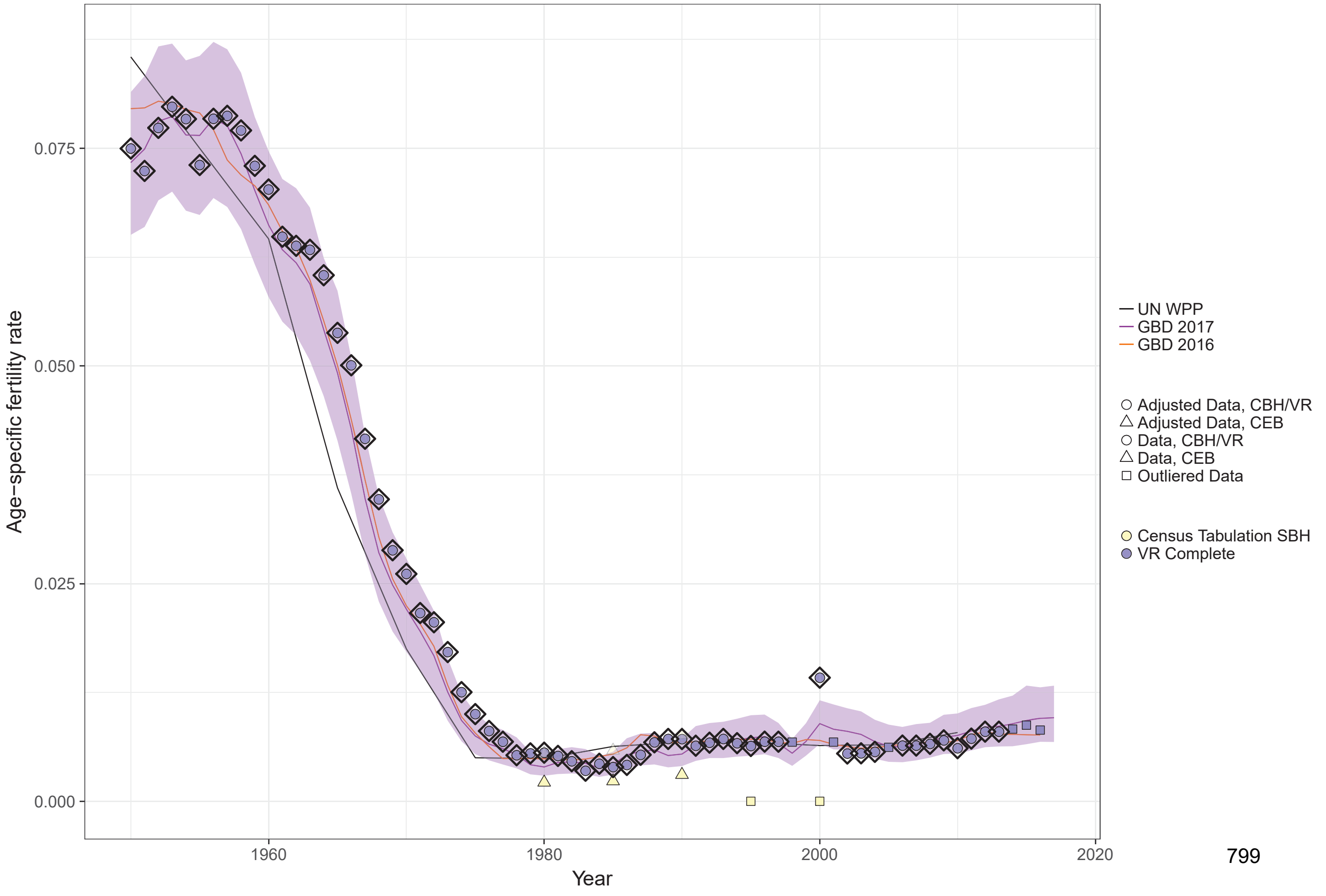

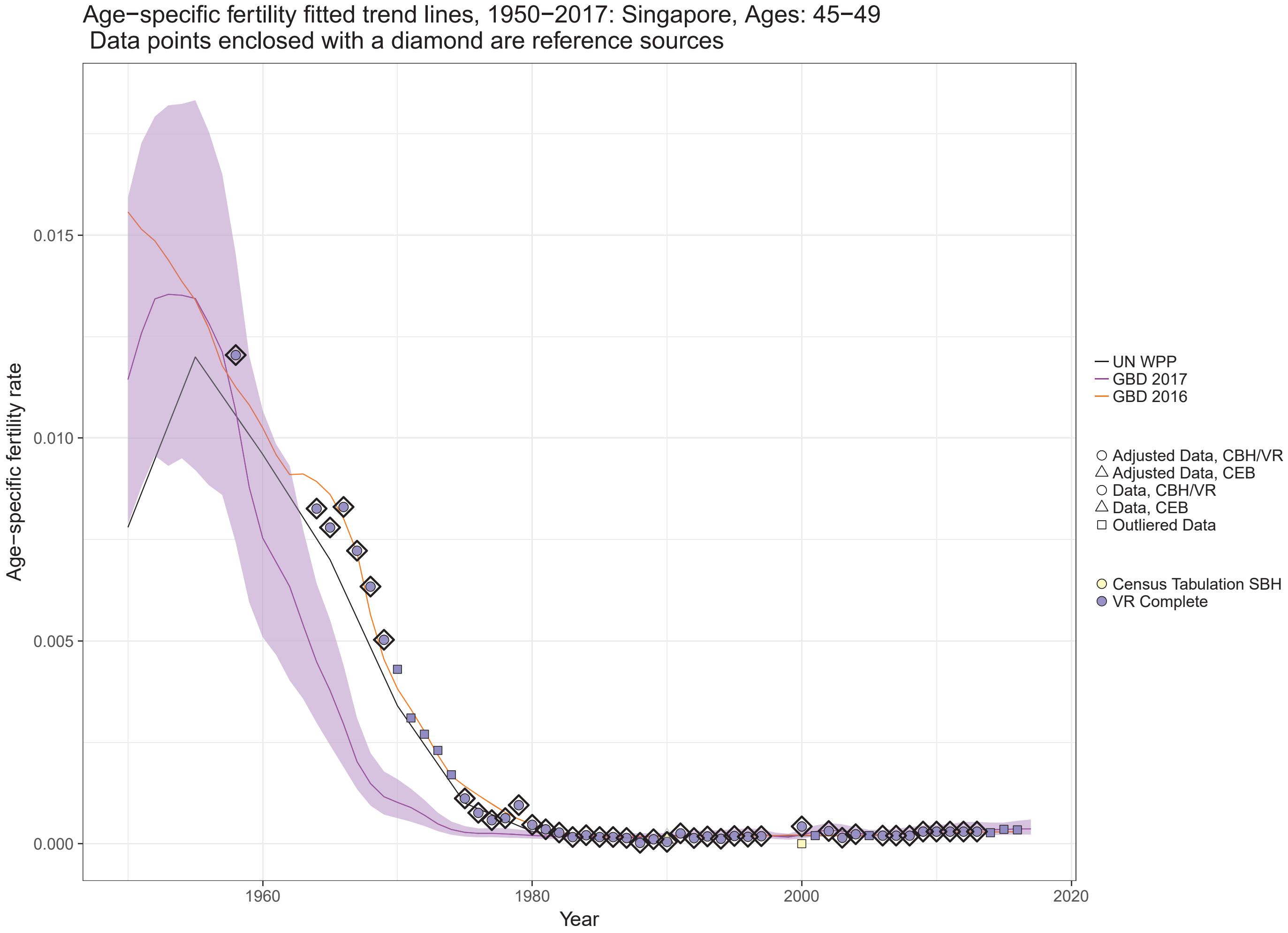

Age−specific fertility fitted trend lines, 1950−2017: Singapore, Ages: 45−49
Data points enclosed with a diamond are reference sources
Age−specific fertility rate
0.015
0.010
0.005
0.000
1960
1980
2000
2020
Year
UN WPP
GBD 2017
GBD 2016
Adjusted Data, CBH/VR
Adjusted Data, CEB
Data, CBH/VR
Data, CEB
Outliered Data
Census Tabulation SBH
VR Complete

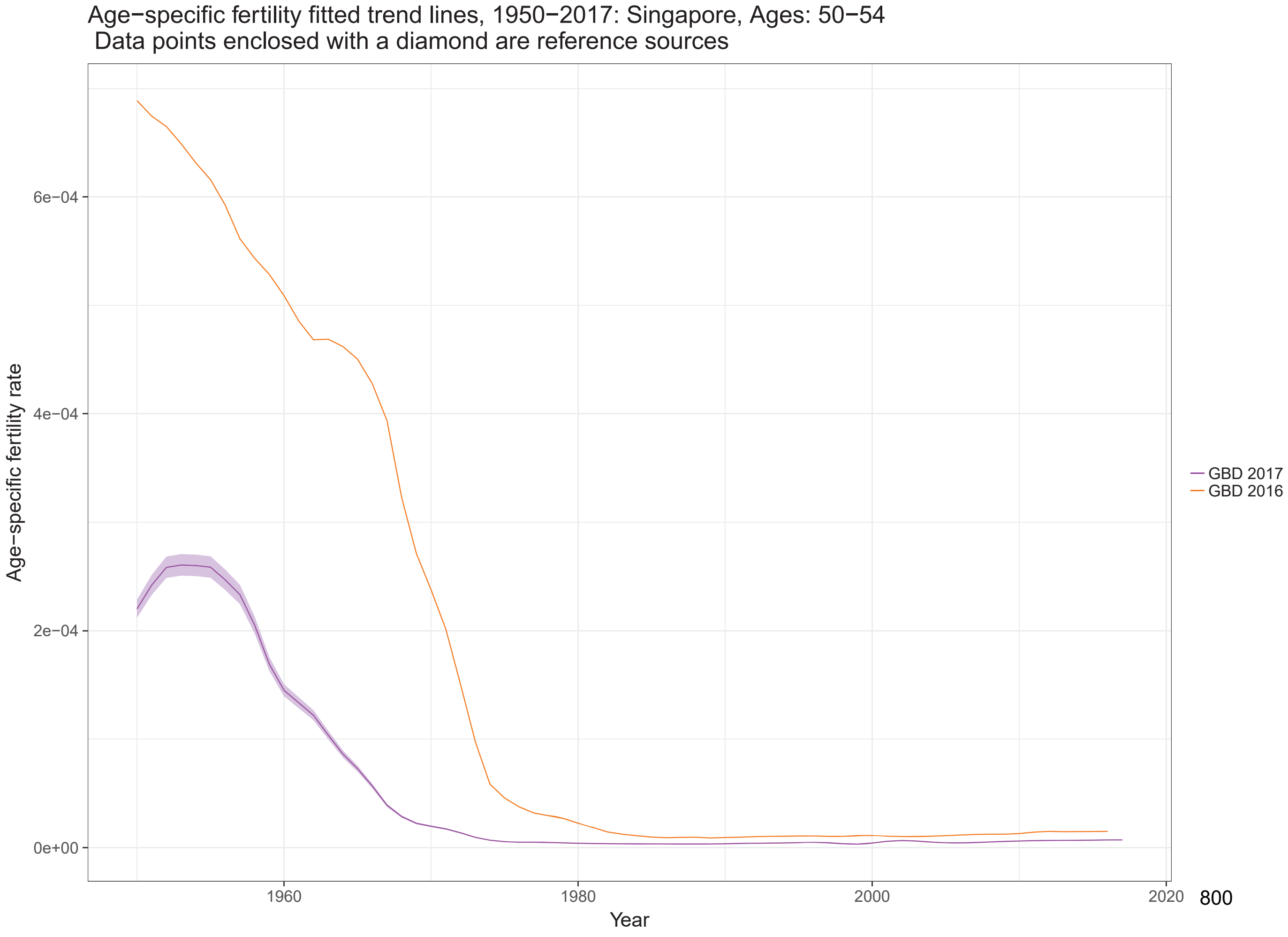

Age−specific fertility fitted trend lines, 1950−2017: Singapore, Ages: 50−54
Data points enclosed with a diamond are reference sources
Age−specific fertility rate
6e−04
4e−04
2e−04
0e+00
1960
1980
2000
2020
Year
GBD 2017
GBD 2016

Age−specific fertility fitted trend lines, 1950−2017: Slovakia, TFR
Data points enclosed with a diamond are reference sources

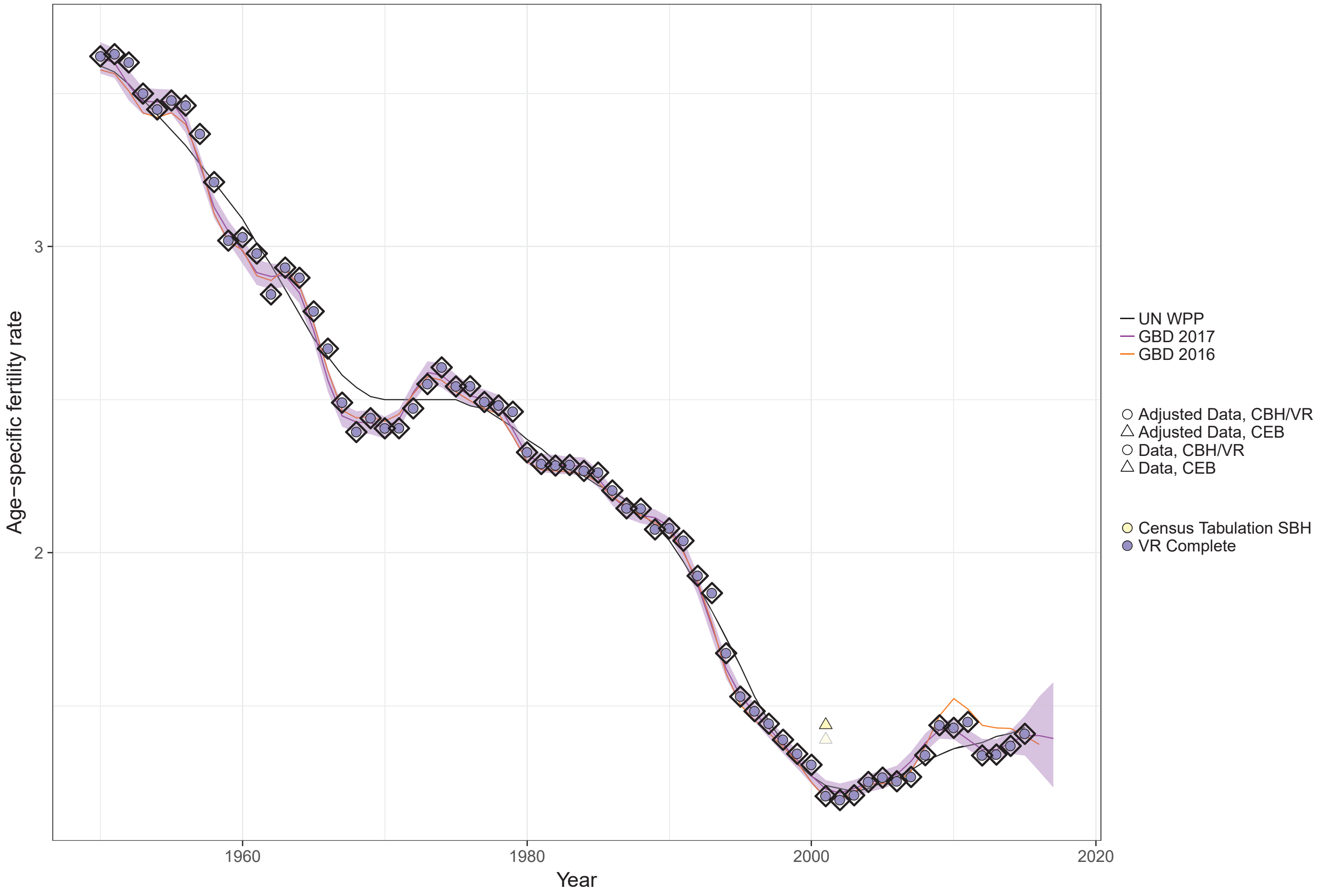

Age−specific fertility fitted trend lines, 1950−2017: Slovakia, Ages: 10−14
Data points enclosed with a diamond are reference sources

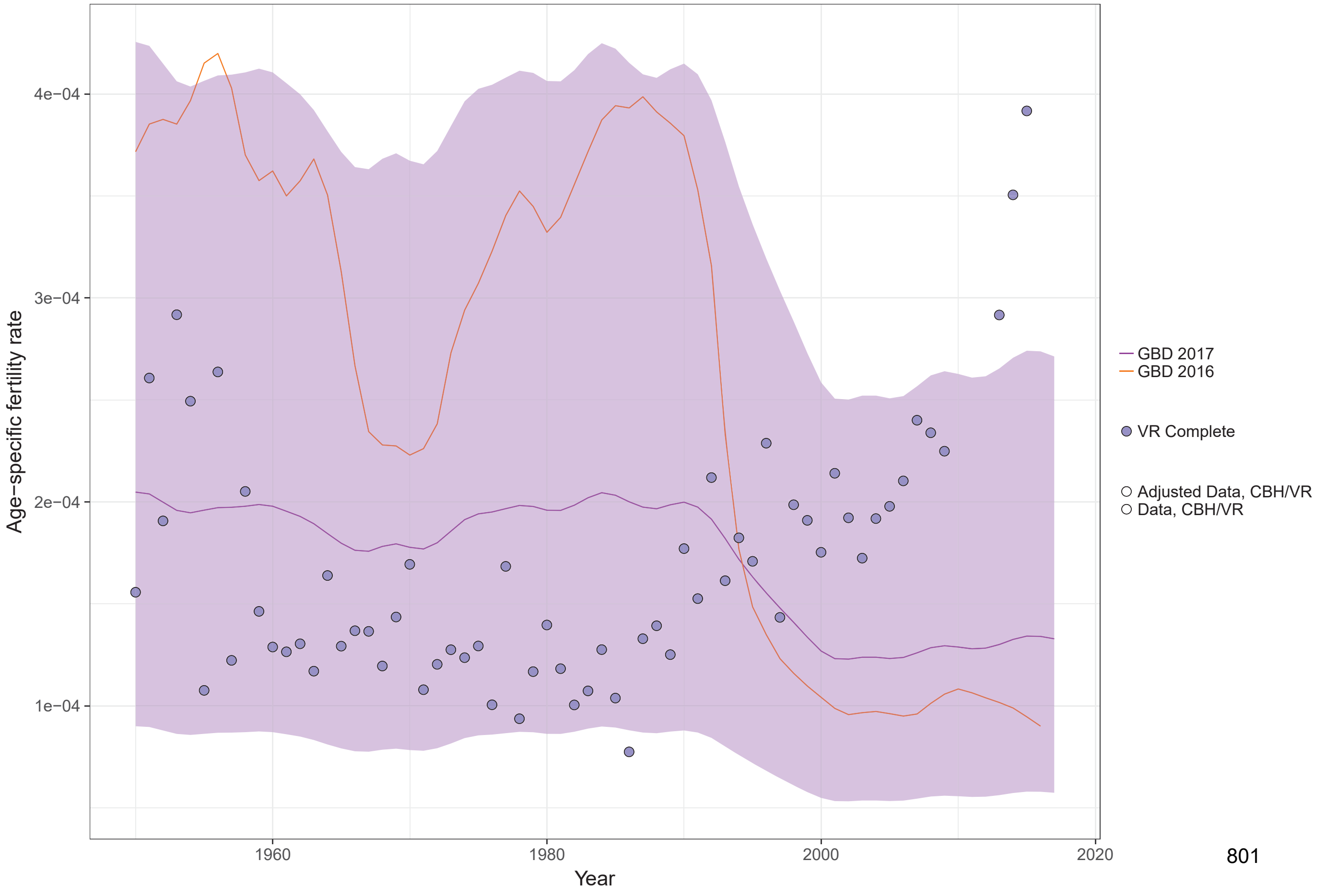

Age−specific fertility fitted trend lines, 1950−2017: Slovakia, Ages: 15−19
Data points enclosed with a diamond are reference sources

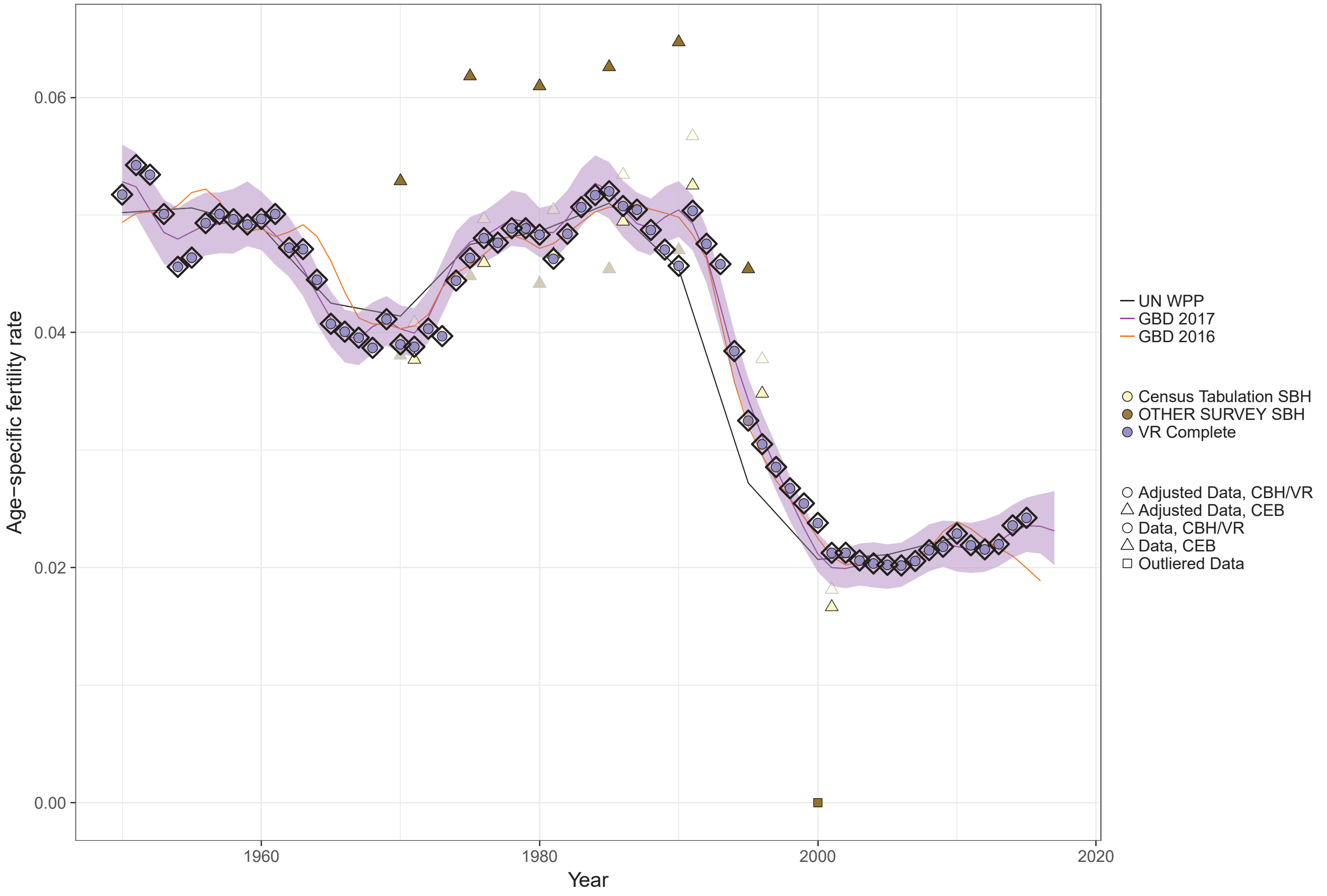

Age−specific fertility fitted trend lines, 1950−2017: Slovakia, Ages: 20−24
Data points enclosed with a diamond are reference sources

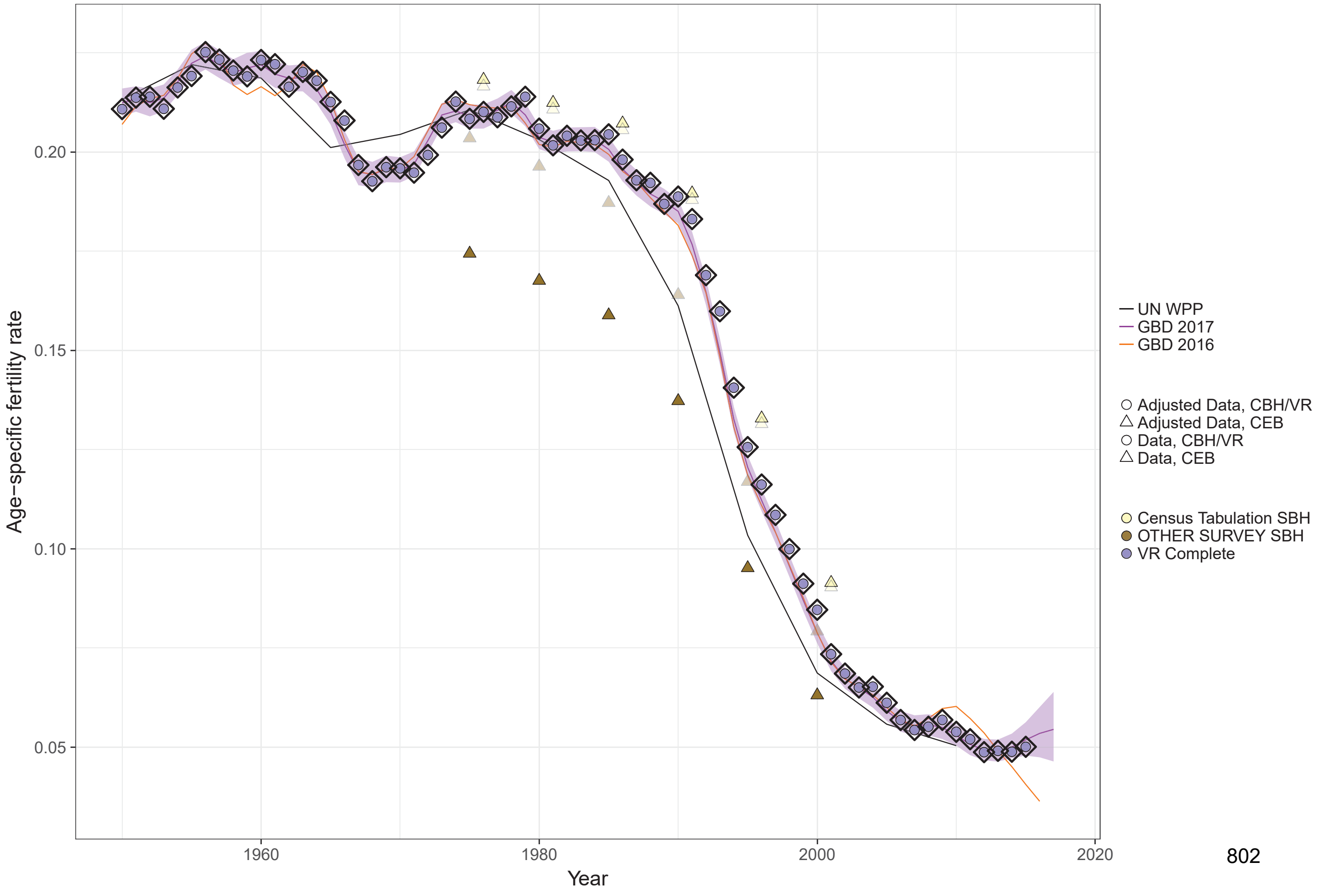

Age−specific fertility fitted trend lines, 1950−2017: Slovakia, Ages: 25−29
Data points enclosed with a diamond are reference sources

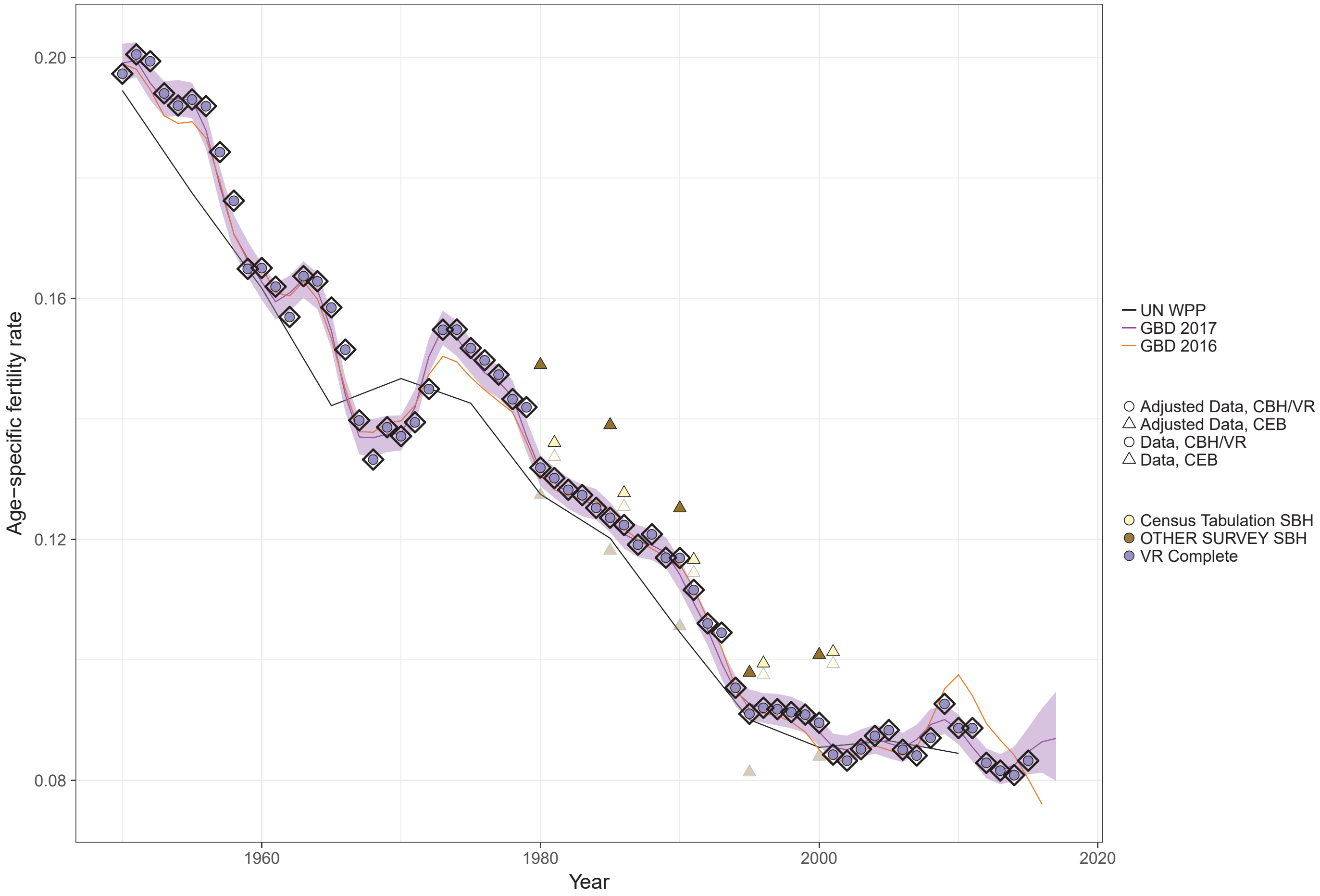

Age−specific fertility fitted trend lines, 1950−2017: Slovakia, Ages: 30−34
Data points enclosed with a diamond are reference sources

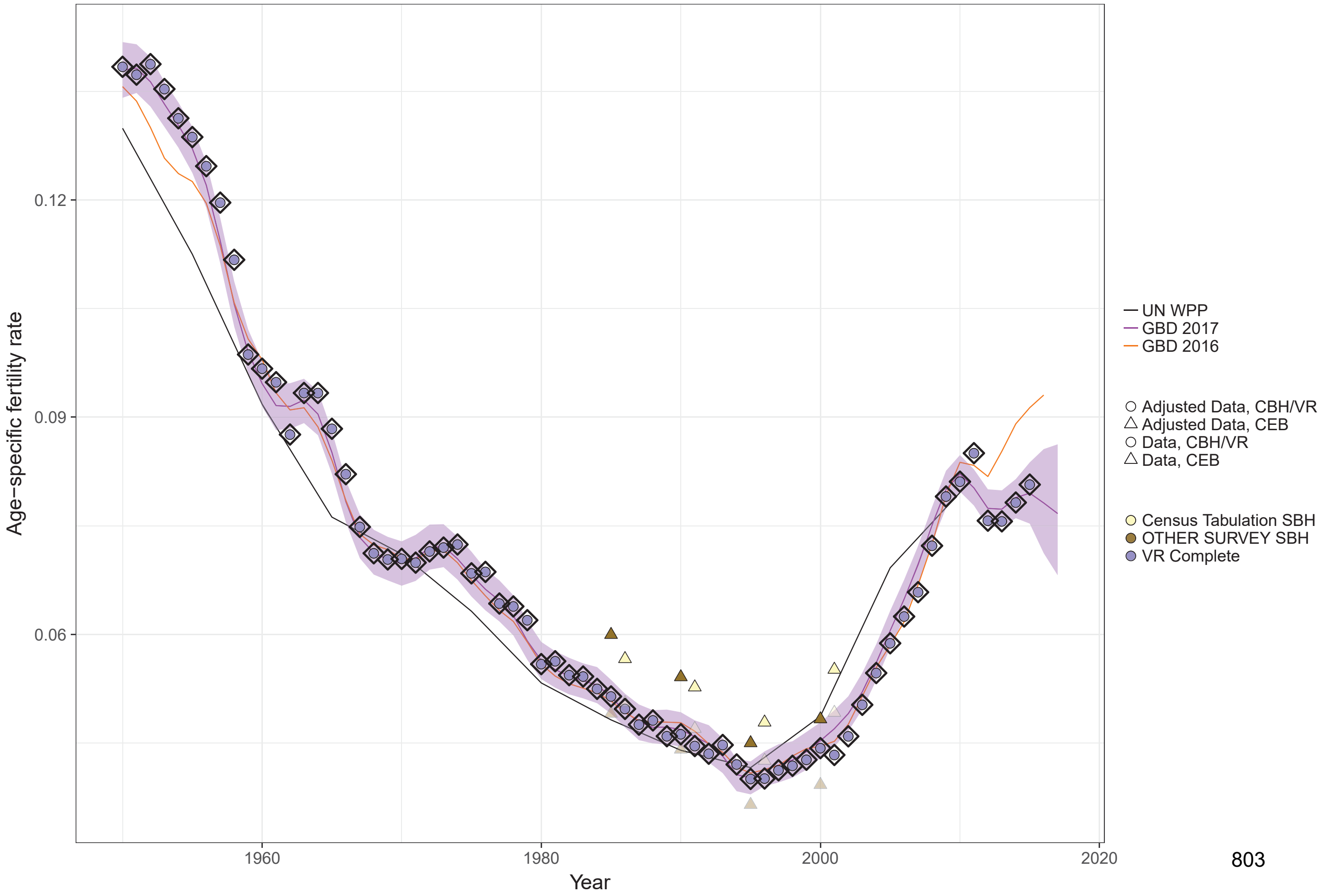

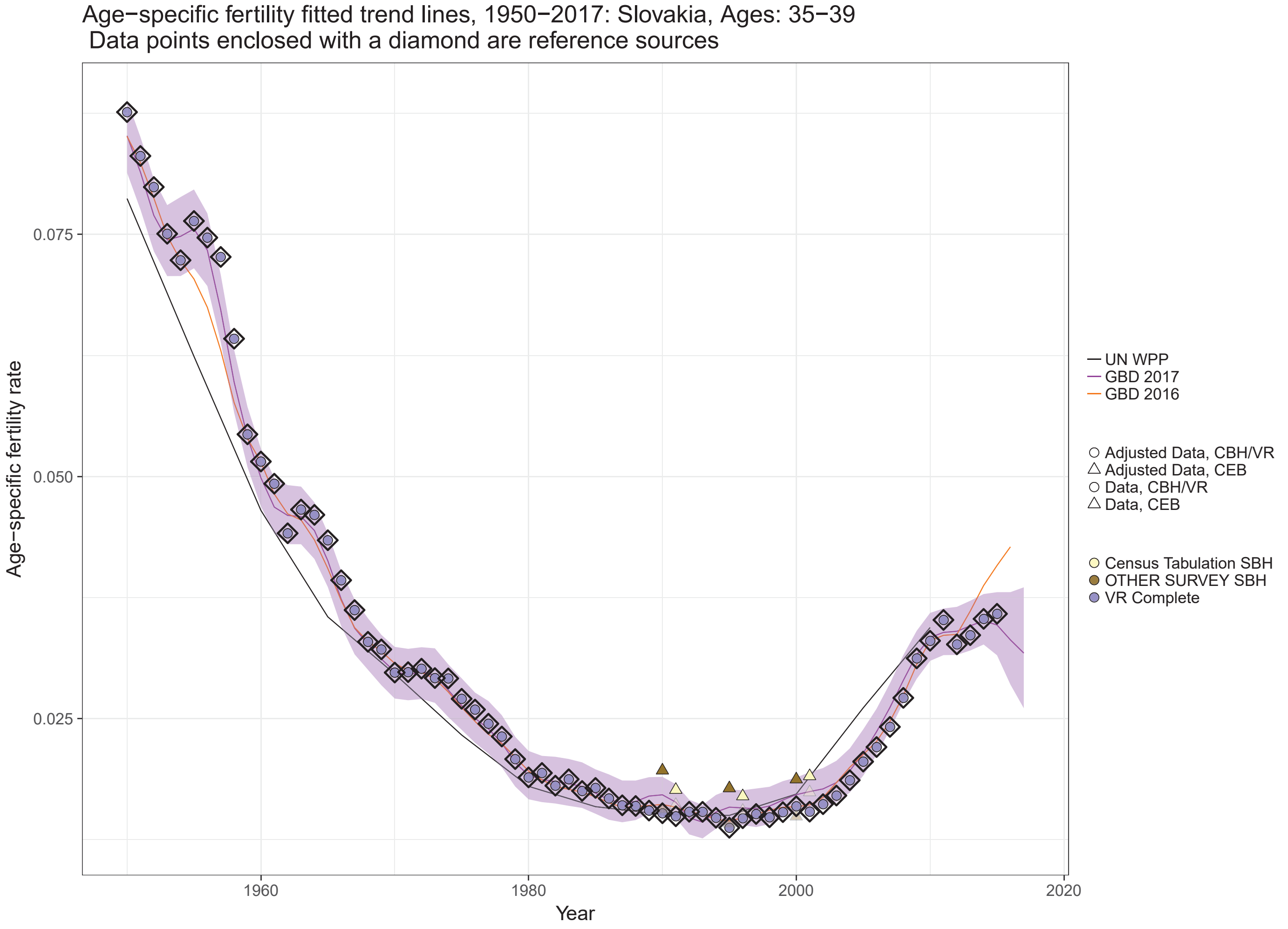

Age−specific fertility fitted trend lines, 1950−2017: Slovakia, Ages: 35−39
Data points enclosed with a diamond are reference sources
Age−specific fertility rate
0.075
0.050
0.025
1960
1980
2000
2020
Year
UN WPP
GBD 2017
GBD 2016
Adjusted Data, CBH/VR
Adjusted Data, CEB
Data, CBH/VR
Data, CEB
Census Tabulation SBH
OTHER SURVEY SBH
VR Complete

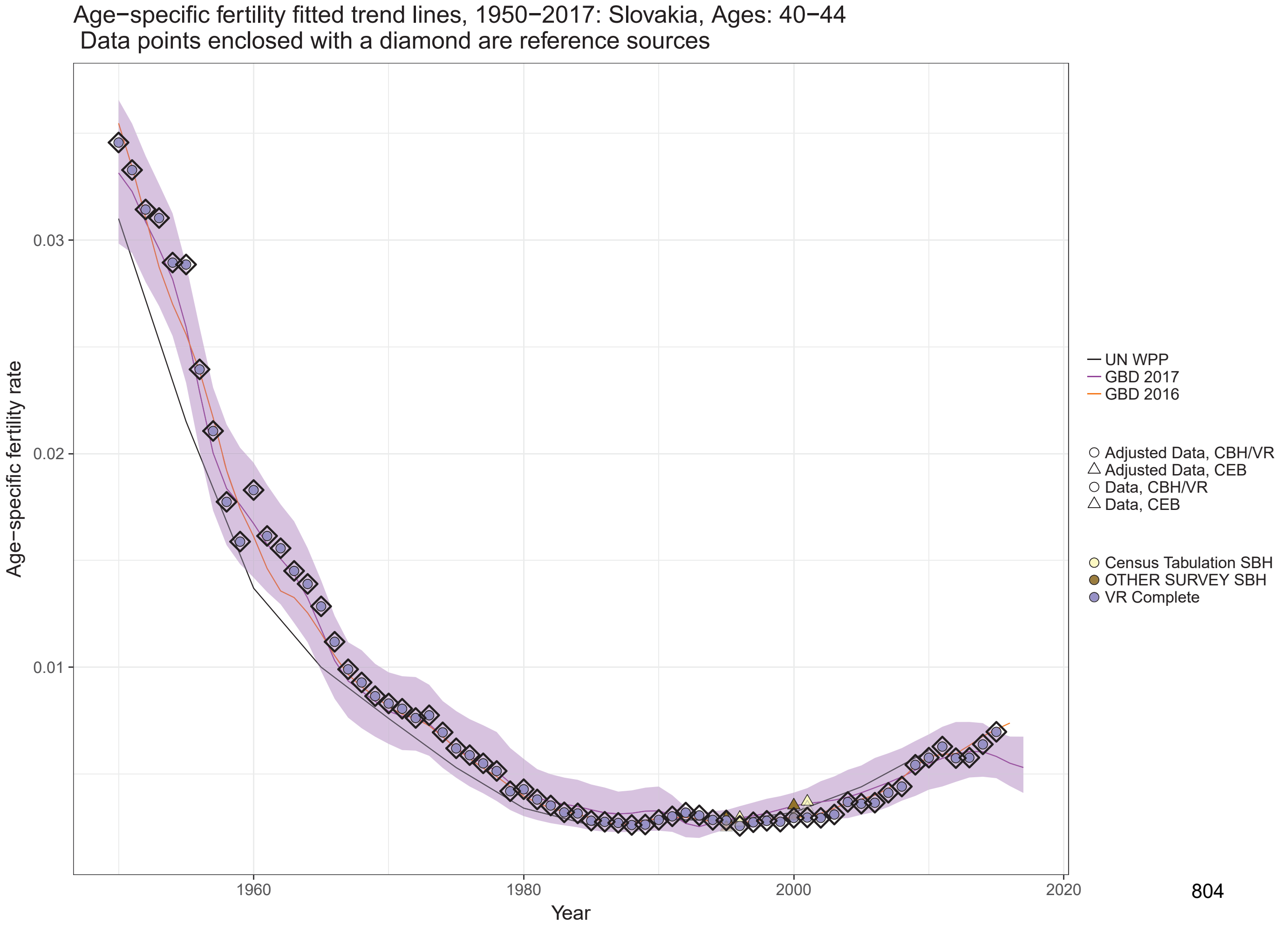

Age−specific fertility fitted trend lines, 1950−2017: Slovakia, Ages: 40−44
Data points enclosed with a diamond are reference sources
Age−specific fertility rate
0.03
0.02
0.01
1960
1980
2000
2020
Year
UN WPP
GBD 2017
GBD 2016
Adjusted Data, CBH/VR
Adjusted Data, CEB
Data, CBH/VR
Data, CEB
Census Tabulation SBH
OTHER SURVEY SBH
VR Complete

Age−specific fertility fitted trend lines, 1950−2017: Slovakia, Ages: 45−49
Data points enclosed with a diamond are reference sources

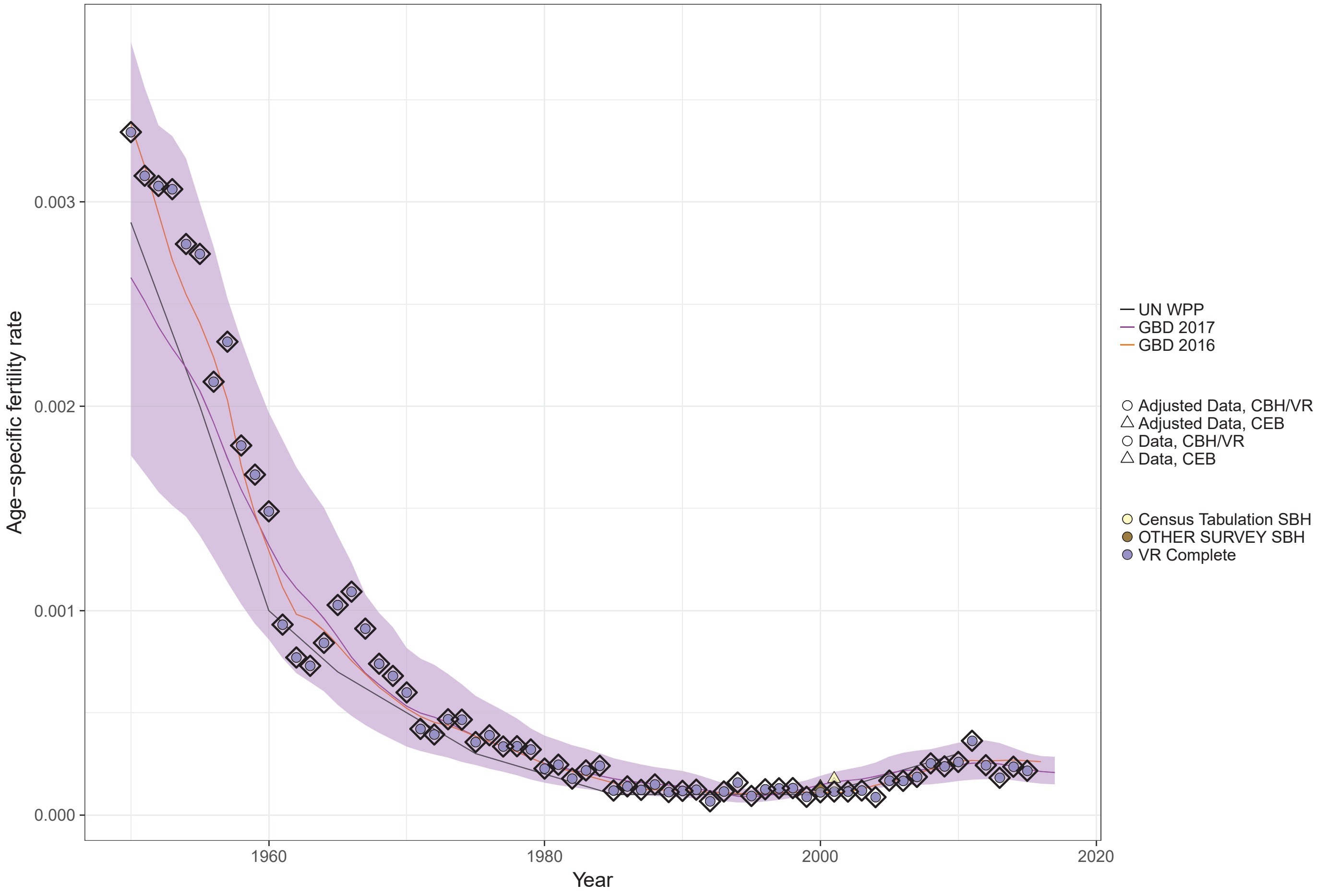

Age−specific fertility fitted trend lines, 1950−2017: Slovakia, Ages: 50−54
Data points enclosed with a diamond are reference sources

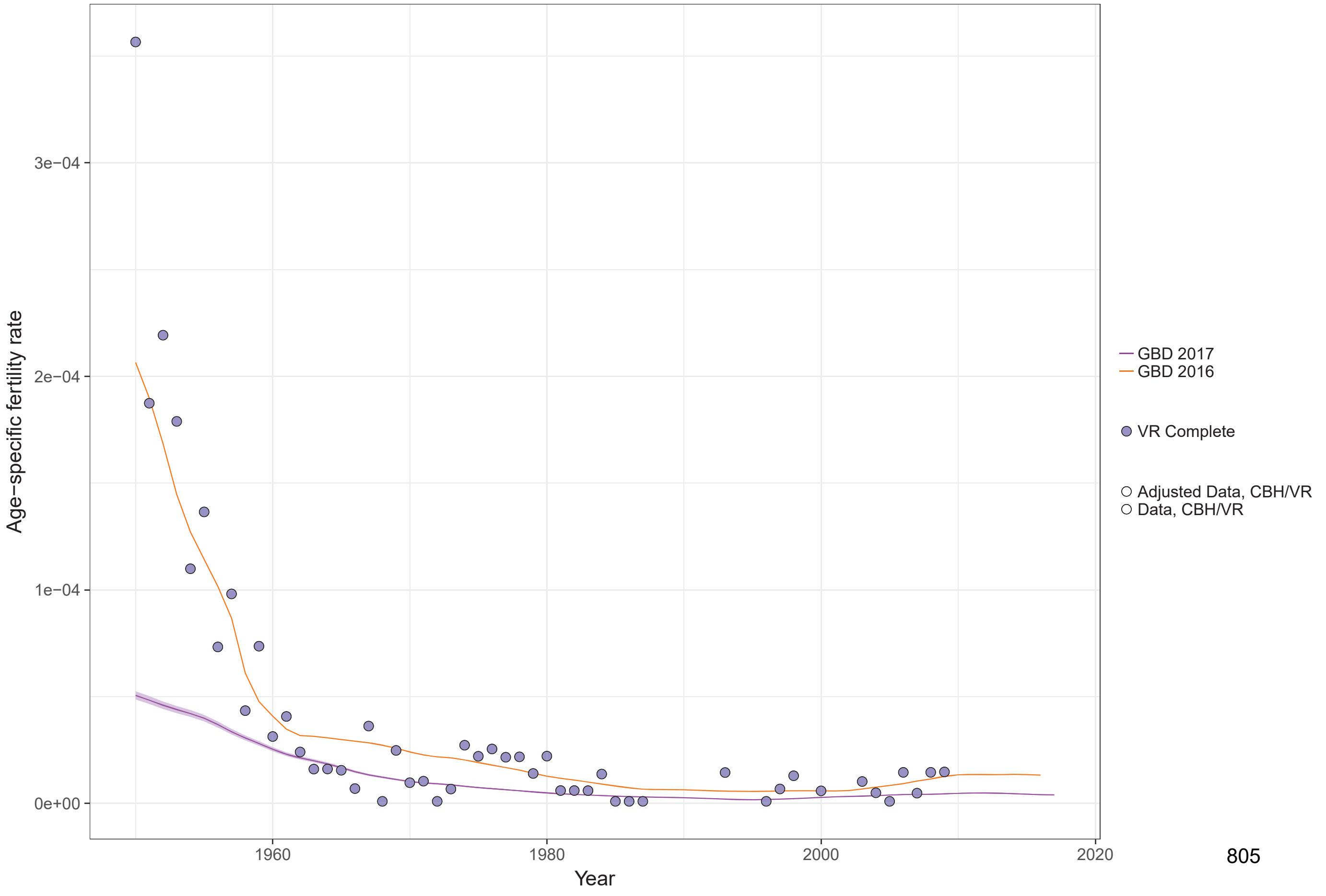

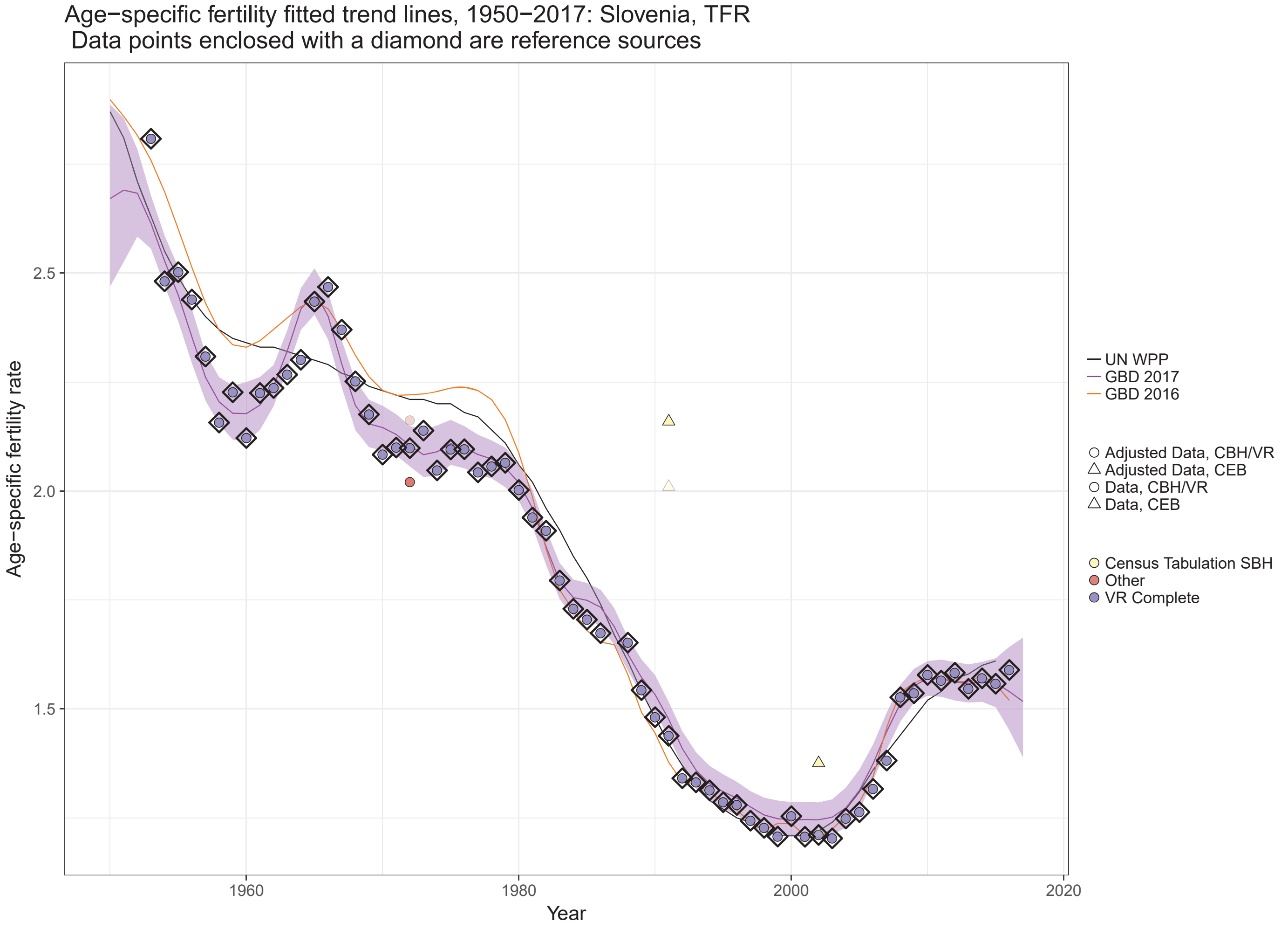

Age−specific fertility fitted trend lines, 1950−2017: Slovenia, TFR
Data points enclosed with a diamond are reference sources
Age−specific fertility rate
2.5
2.0
1.5
1960
1980
2000
2020
Year
UN WPP
GBD 2017
GBD 2016
Adjusted Data, CBH/VR
Adjusted Data, CEB
Data, CBH/VR
Data, CEB
Census Tabulation SBH
Other
VR Complete

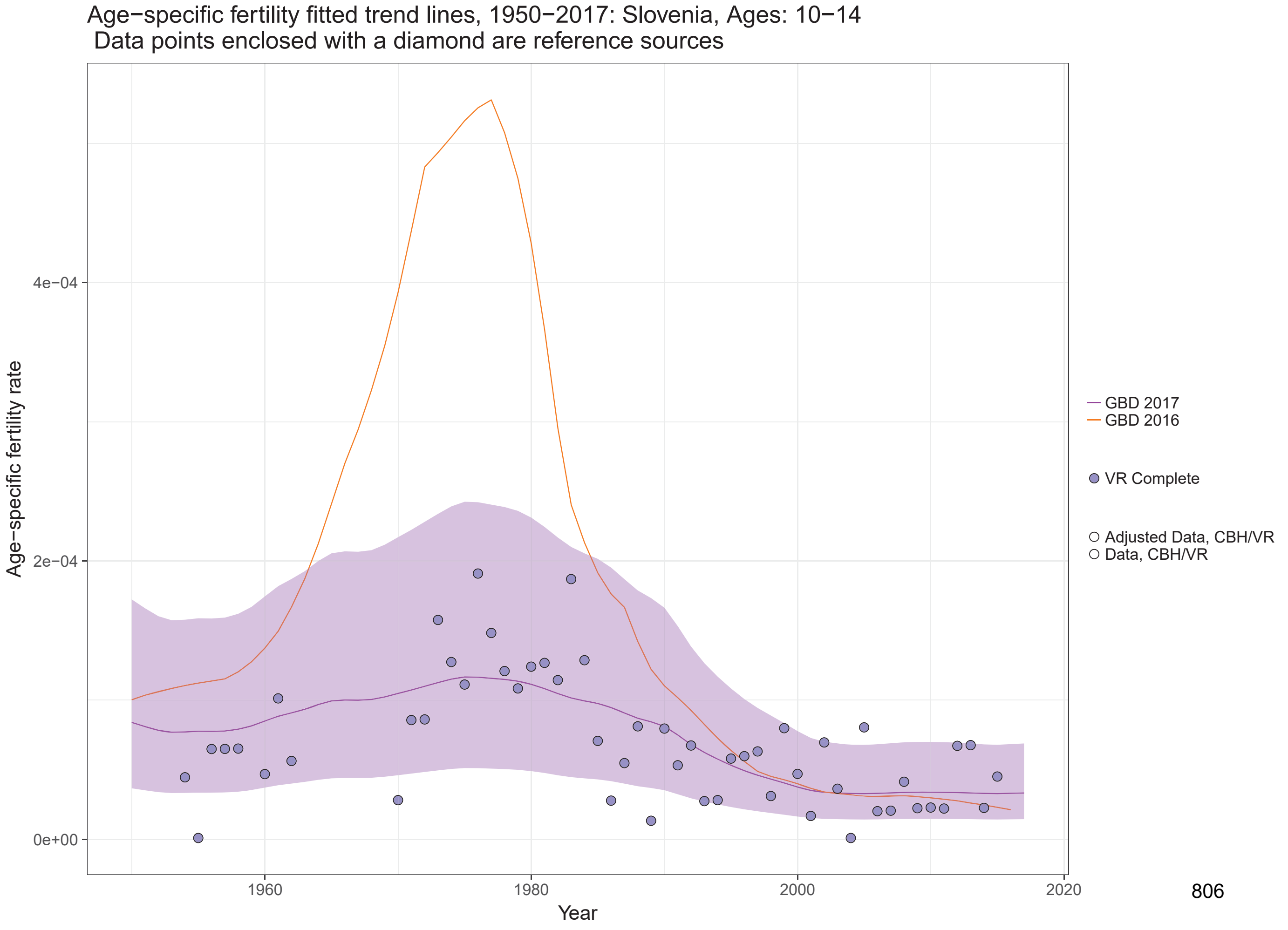

Age−specific fertility fitted trend lines, 1950−2017: Slovenia, Ages: 10−14
Data points enclosed with a diamond are reference sources
Age−specific fertility rate
4e−04
2e−04
0e+00
1960
1980
2000
2020
Year
GBD 2017
GBD 2016
VR Complete
Adjusted Data, CBH/VR
Data, CBH/VR

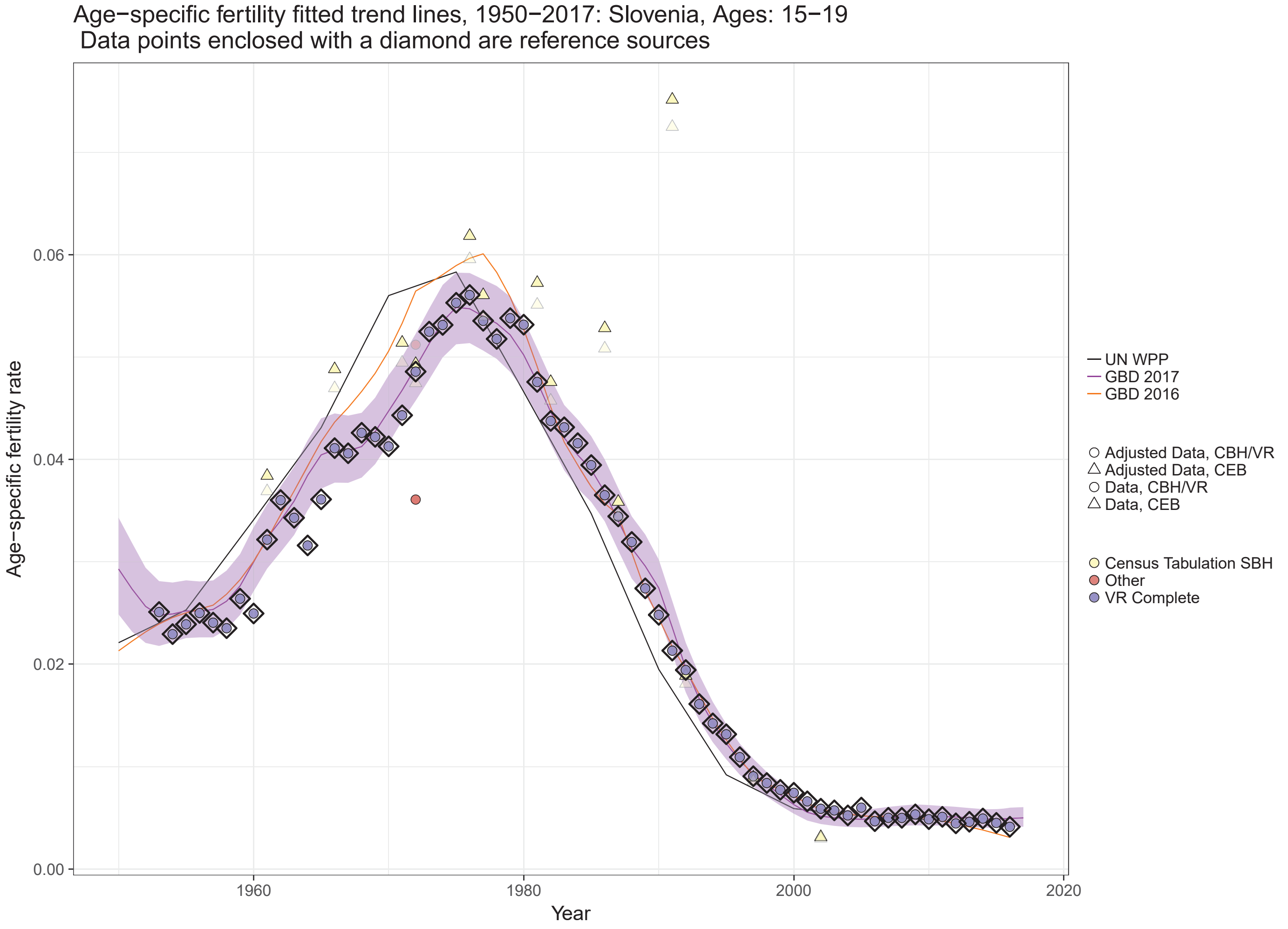

Age−specific fertility fitted trend lines, 1950−2017: Slovenia, Ages: 15−19
Data points enclosed with a diamond are reference sources
Age-specific fertility rate
0.00
0.02
0.04
0.06
1960
1980
2000
2020
Year
UN WPP
GBD 2017
GBD 2016
Adjusted Data, CBH/VR
Adjusted Data, CEB
Data, CBH/VR
Data, CEB
Census Tabulation SBH
Other
VR Complete

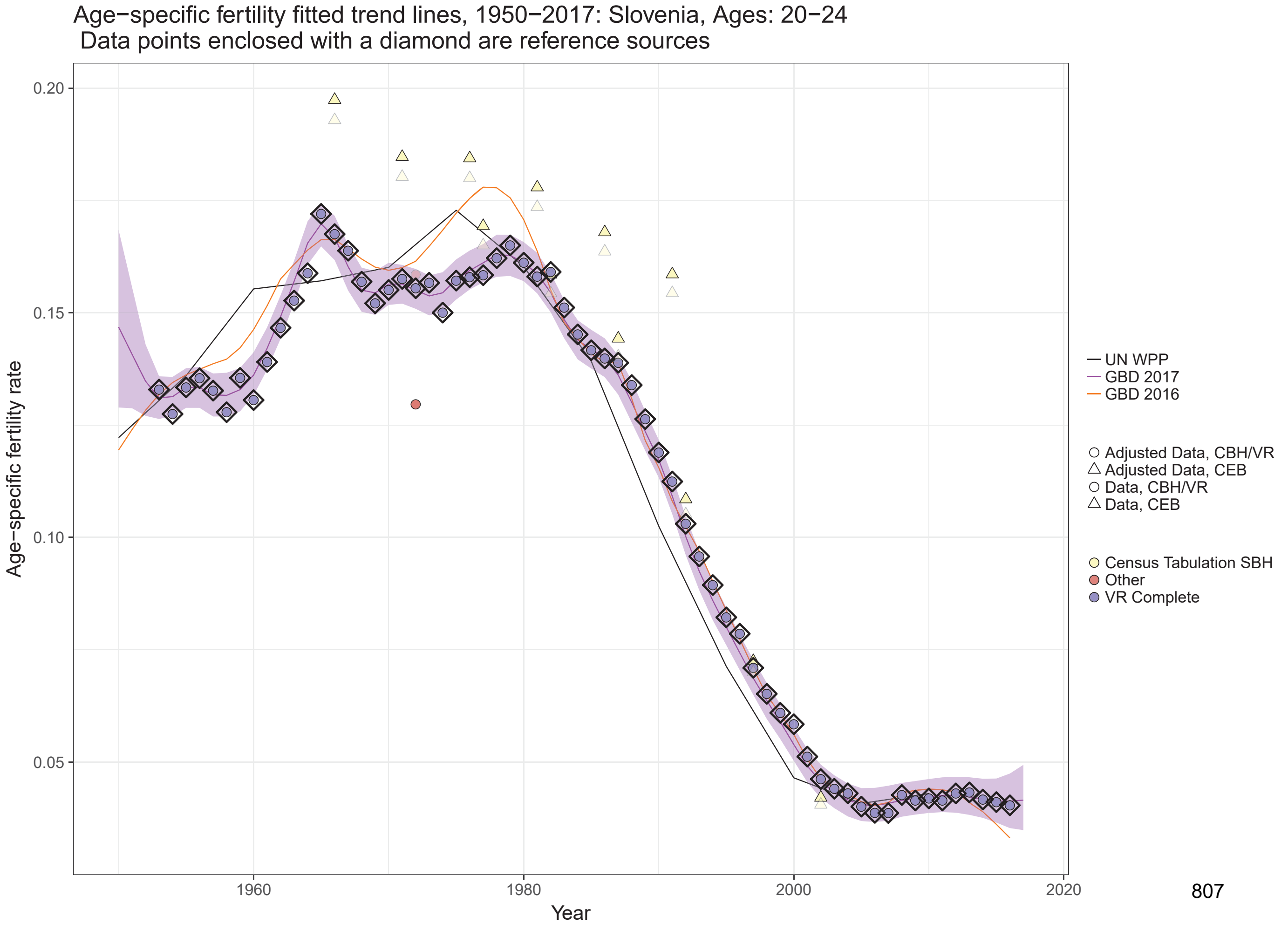

Age−specific fertility fitted trend lines, 1950−2017: Slovenia, Ages: 20−24
Data points enclosed with a diamond are reference sources
Age-specific fertility rate
0.05
0.10
0.15
0.20
1960
1980
2000
2020
Year
UN WPP
GBD 2017
GBD 2016
Adjusted Data, CBH/VR
Adjusted Data, CEB
Data, CBH/VR
Data, CEB
Census Tabulation SBH
Other
VR Complete

Age−specific fertility fitted trend lines, 1950−2017: Slovenia, Ages: 25−29
Data points enclosed with a diamond are reference sources

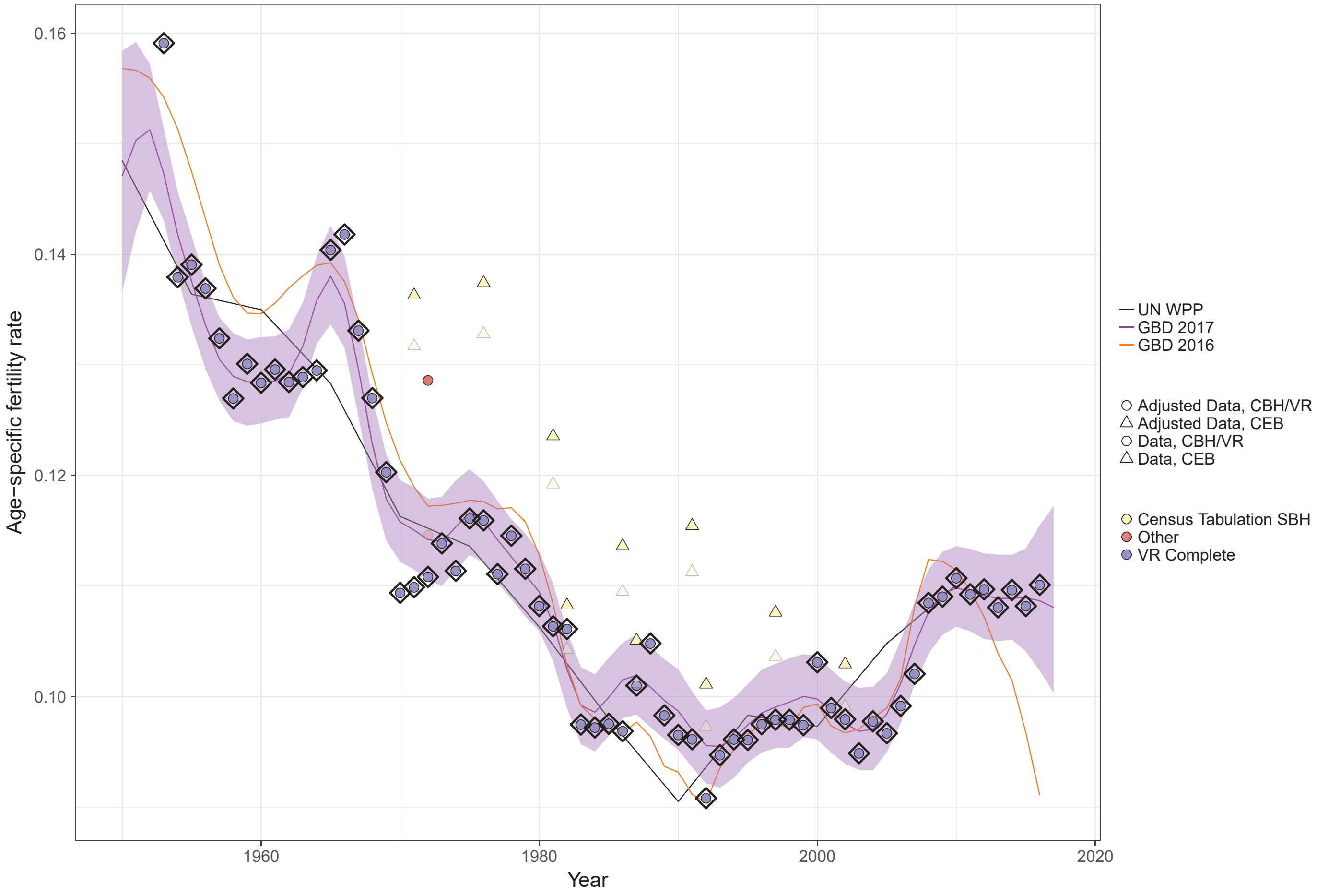

Age−specific fertility fitted trend lines, 1950−2017: Slovenia, Ages: 30−34
Data points enclosed with a diamond are reference sources

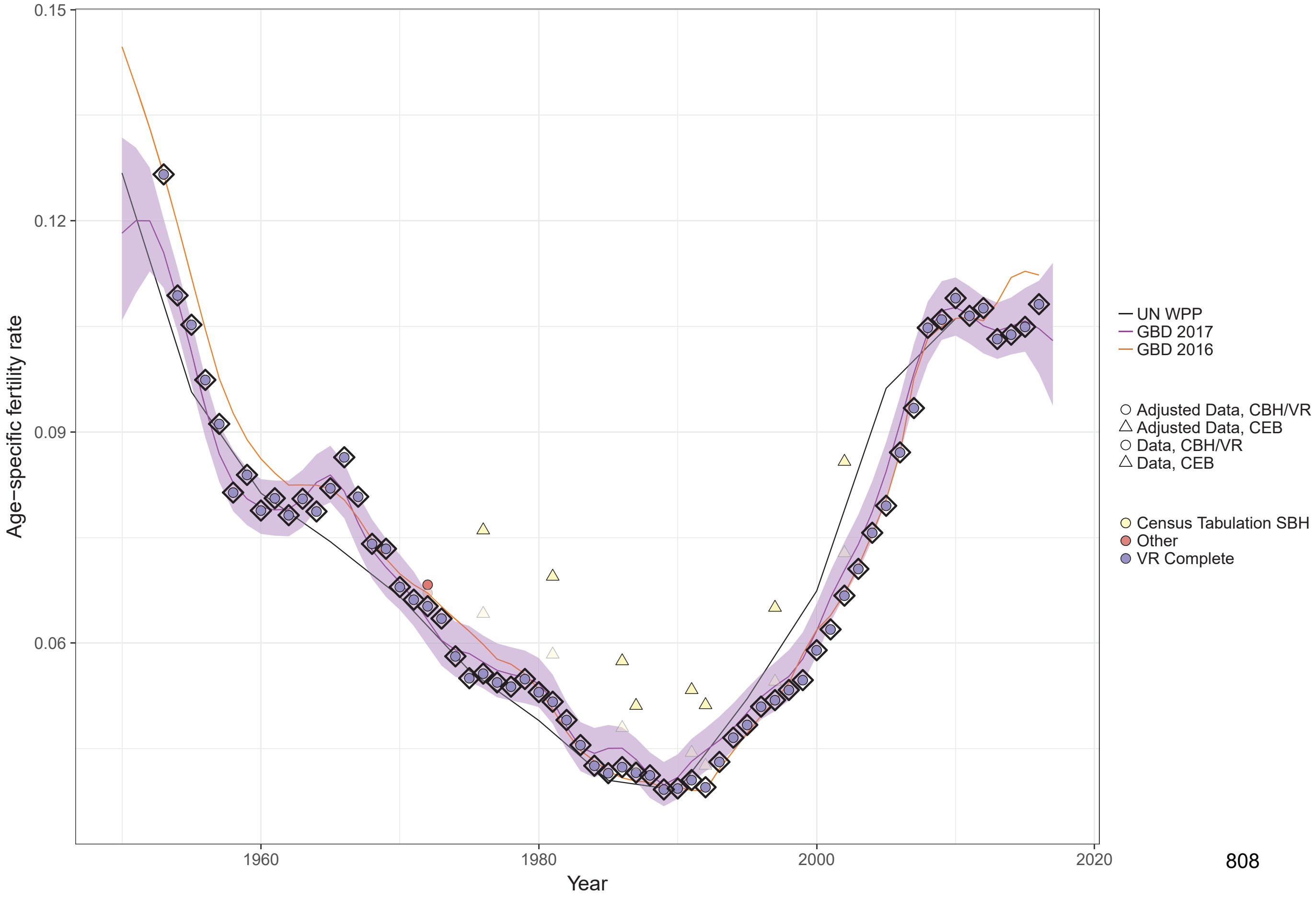

Age−specific fertility fitted trend lines, 1950−2017: Slovenia, Ages: 35−39
Data points enclosed with a diamond are reference sources

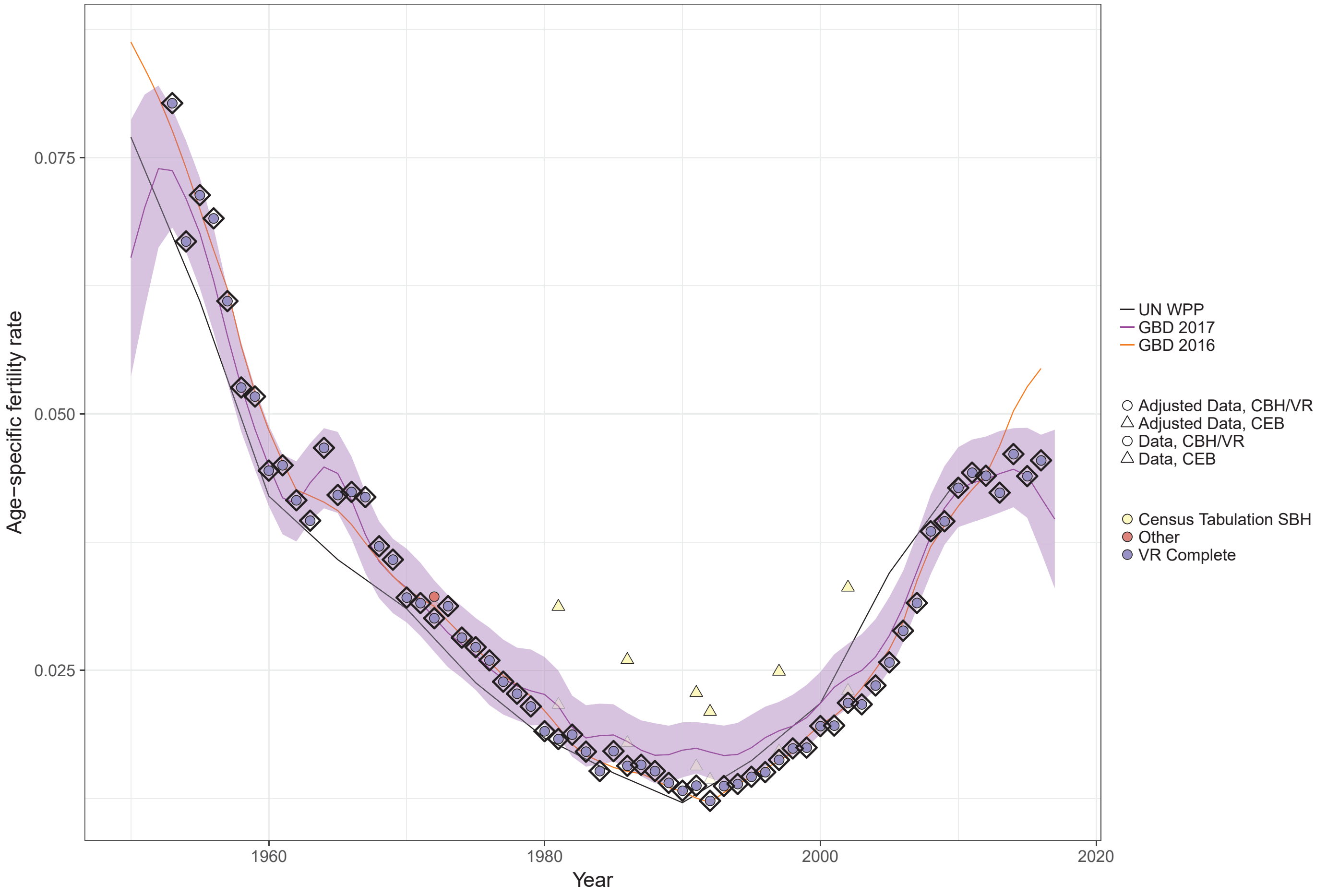

Age−specific fertility fitted trend lines, 1950−2017: Slovenia, Ages: 40−44
Data points enclosed with a diamond are reference sources

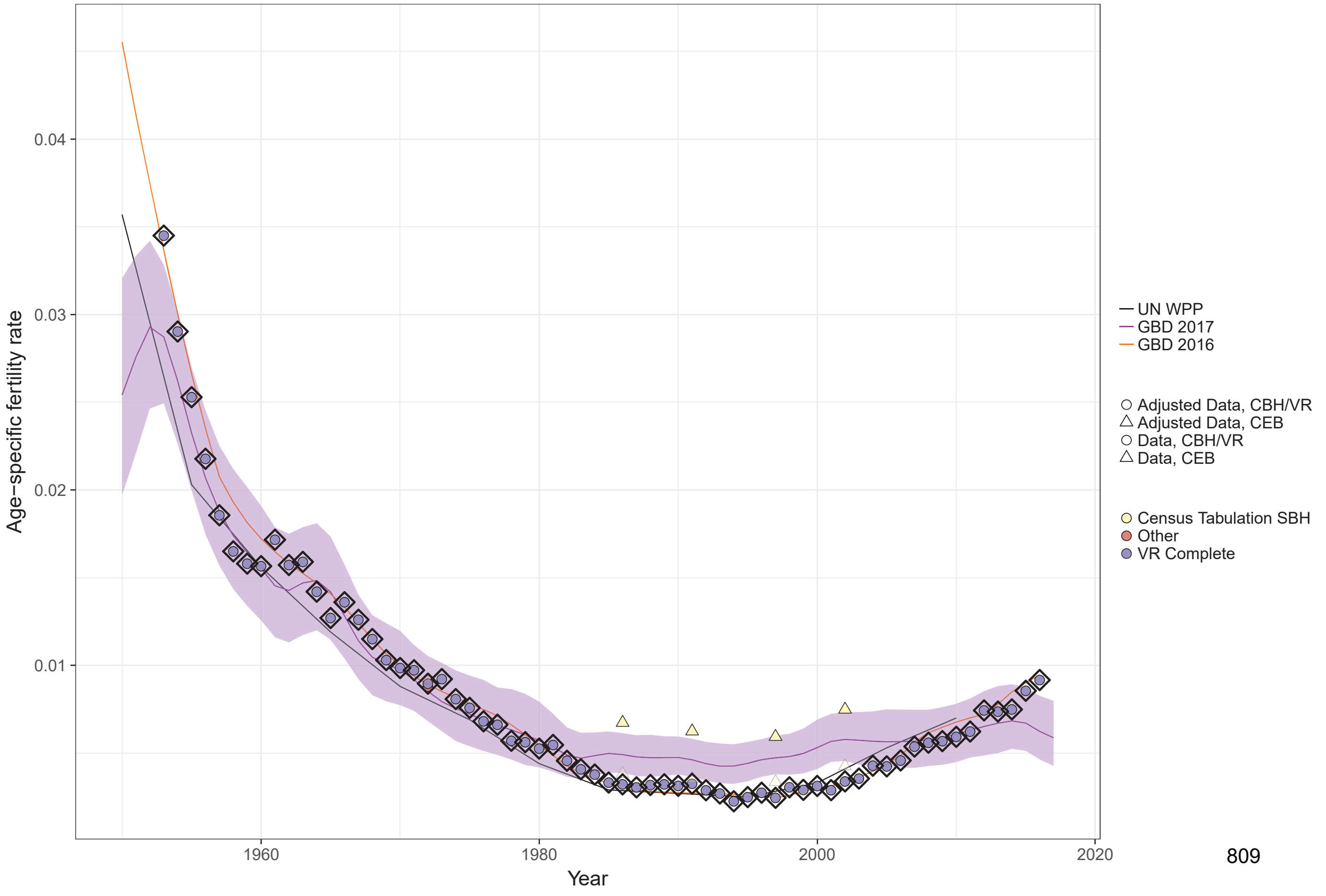

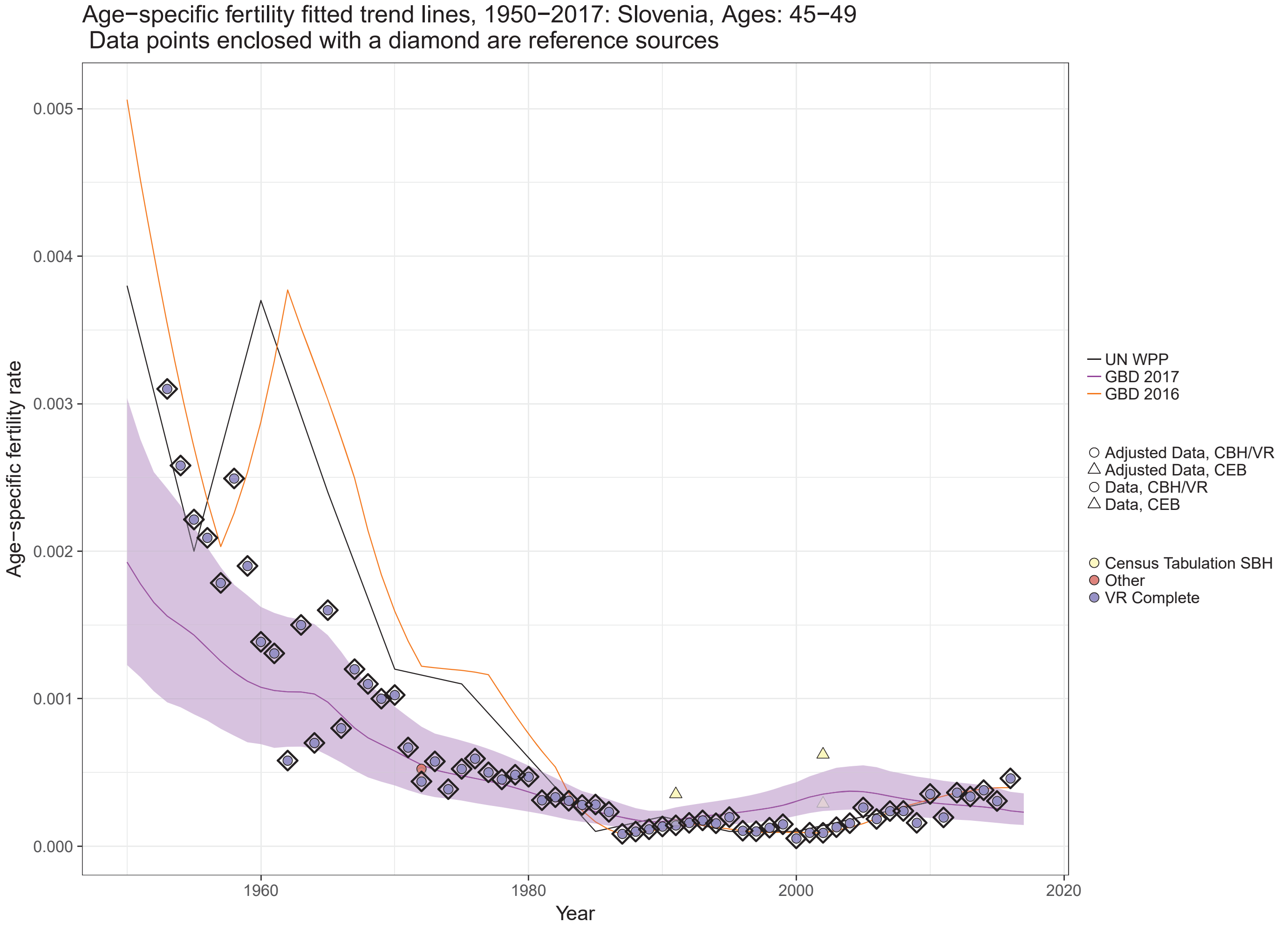

Age−specific fertility fitted trend lines, 1950−2017: Slovenia, Ages: 45−49
Data points enclosed with a diamond are reference sources
Age−specific fertility rate
0.005
0.004
0.003
0.002
0.001
0.000
1960
1980
2000
2020
Year
UN WPP
GBD 2017
GBD 2016
Adjusted Data, CBH/VR
Adjusted Data, CEB
Data, CBH/VR
Data, CEB
Census Tabulation SBH
Other
VR Complete

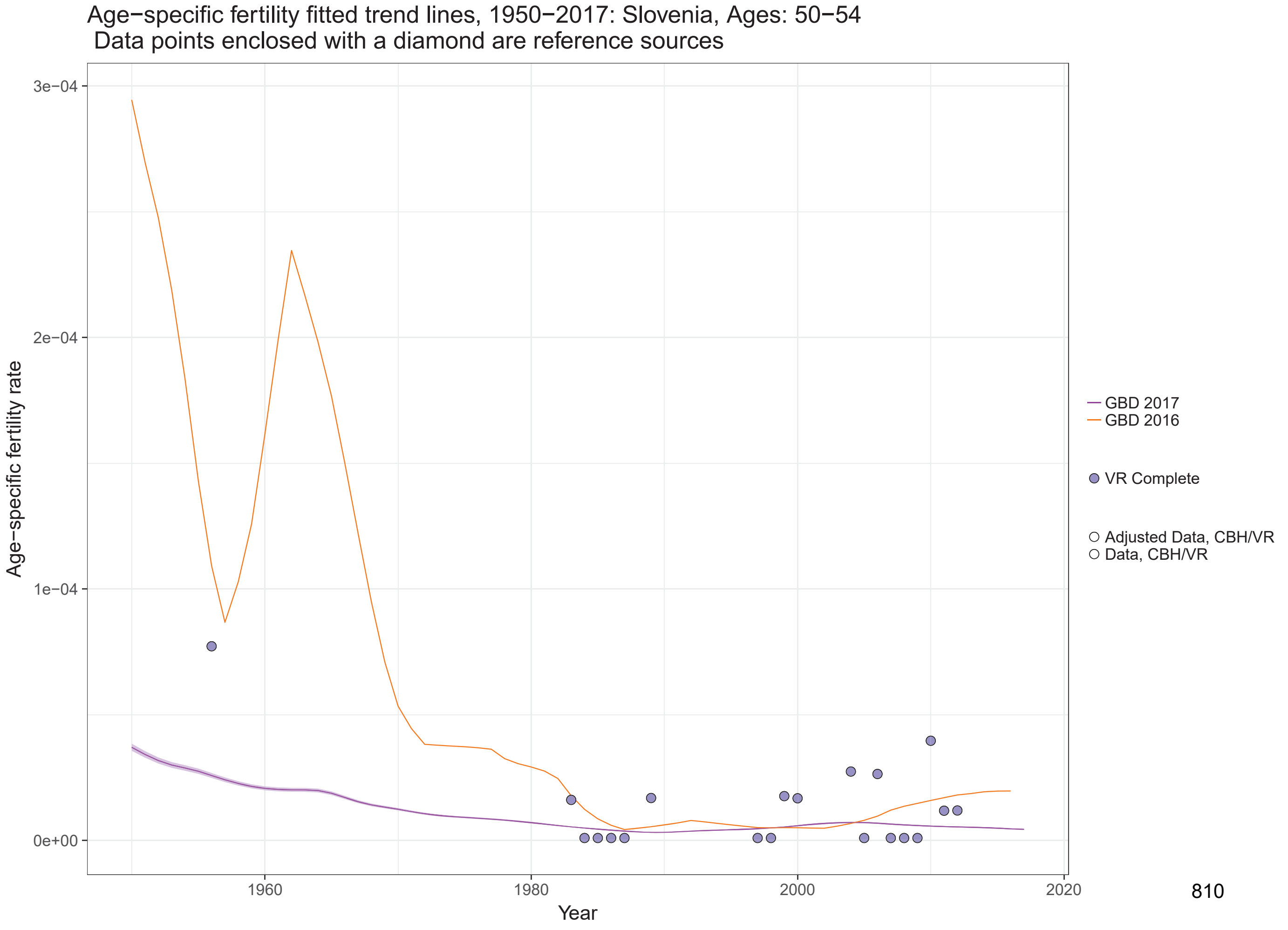

Age−specific fertility fitted trend lines, 1950−2017: Slovenia, Ages: 50−54
Data points enclosed with a diamond are reference sources
Age−specific fertility rate
3e−04
2e−04
1e−04
0e+00
1960
1980
2000
2020
Year
GBD 2017
GBD 2016
VR Complete
Adjusted Data, CBH/VR
Data, CBH/VR

Age−specific fertility fitted trend lines, 1950−2017: Solomon Islands, TFR
Data points enclosed with a diamond are reference sources

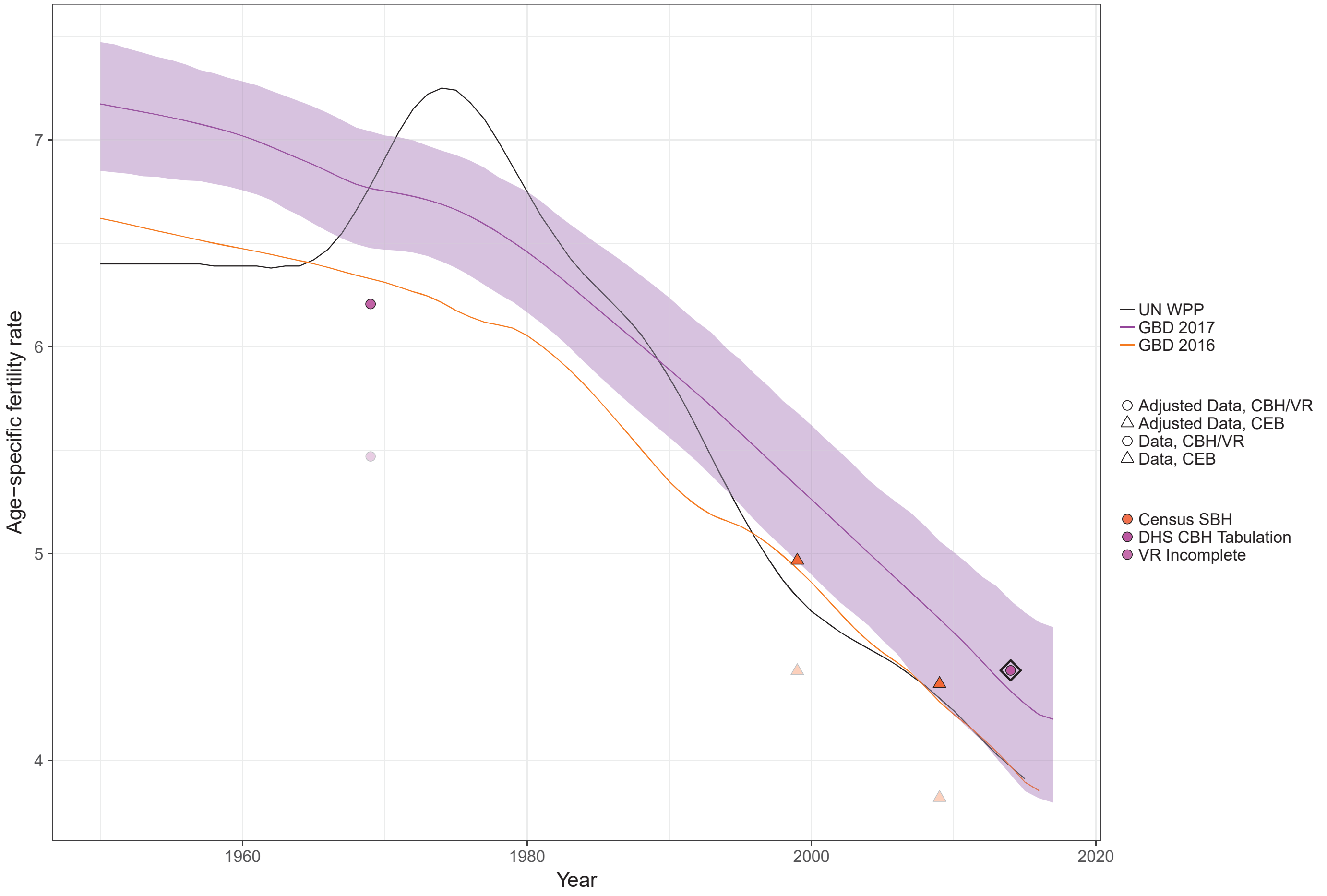

Age−specific fertility fitted trend lines, 1950−2017: Solomon Islands, Ages: 10−14
Data points enclosed with a diamond are reference sources

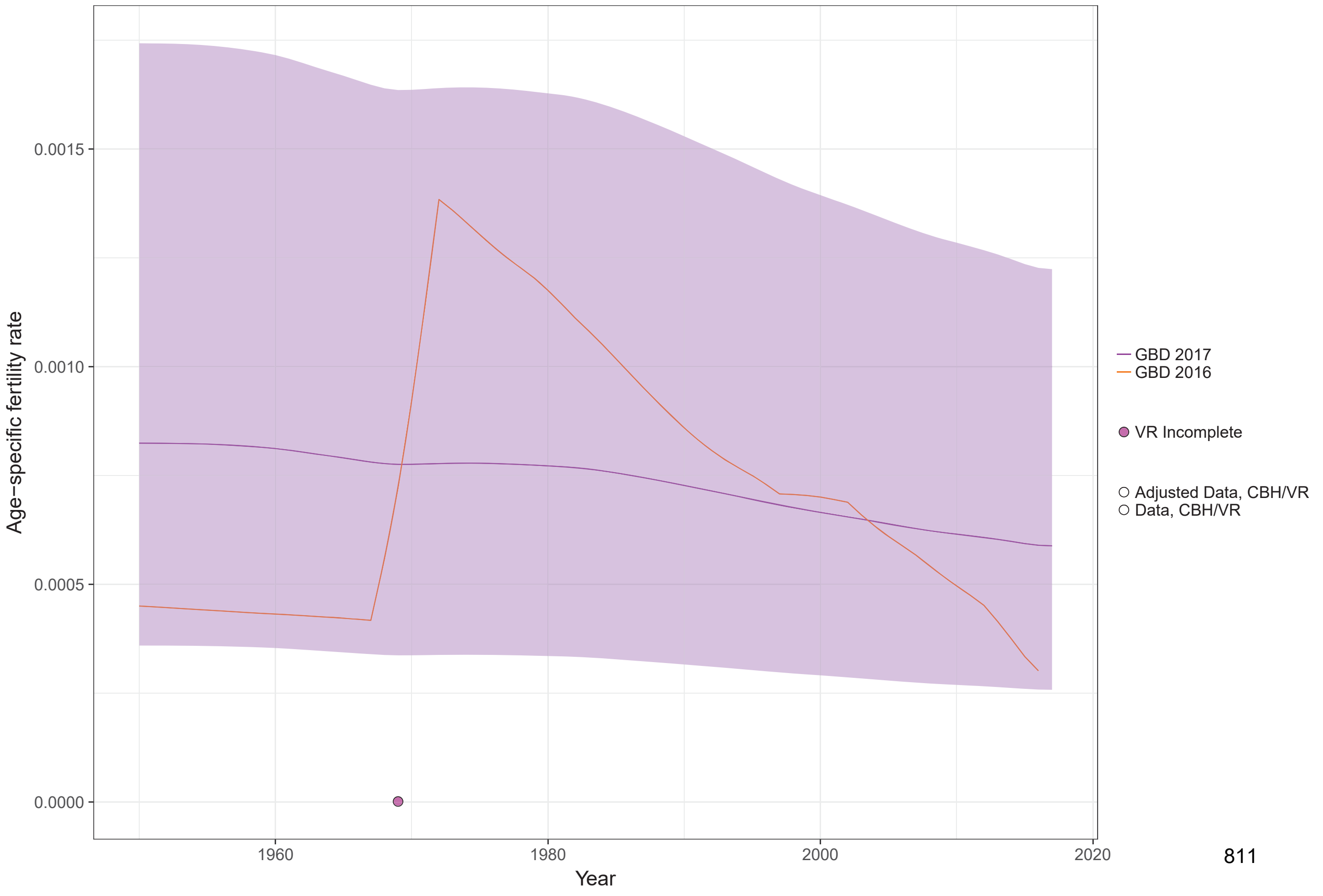

Age−specific fertility fitted trend lines, 1950−2017: Solomon Islands, Ages: 15−19
Data points enclosed with a diamond are reference sources

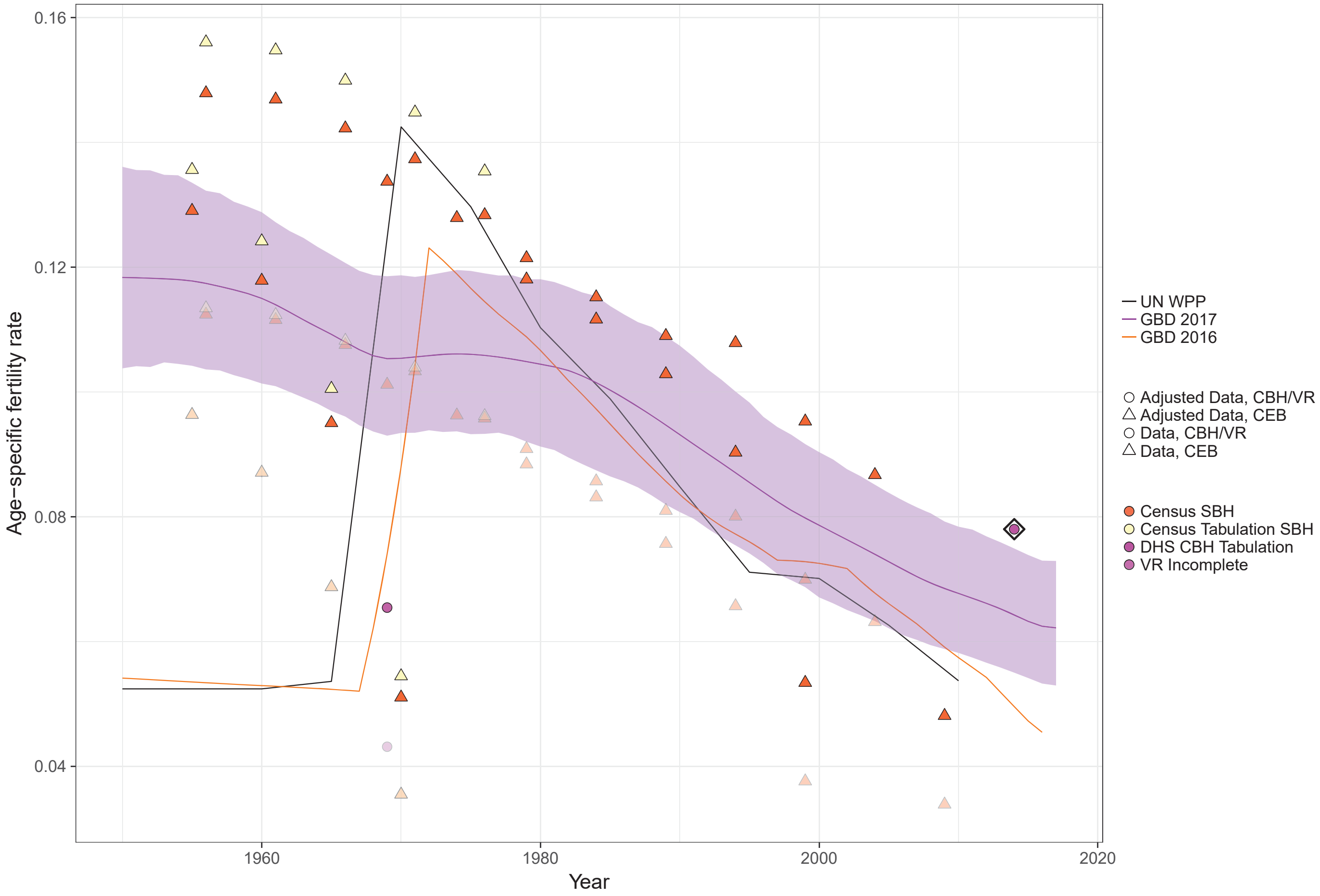

Age−specific fertility fitted trend lines, 1950−2017: Solomon Islands, Ages: 20−24
Data points enclosed with a diamond are reference sources

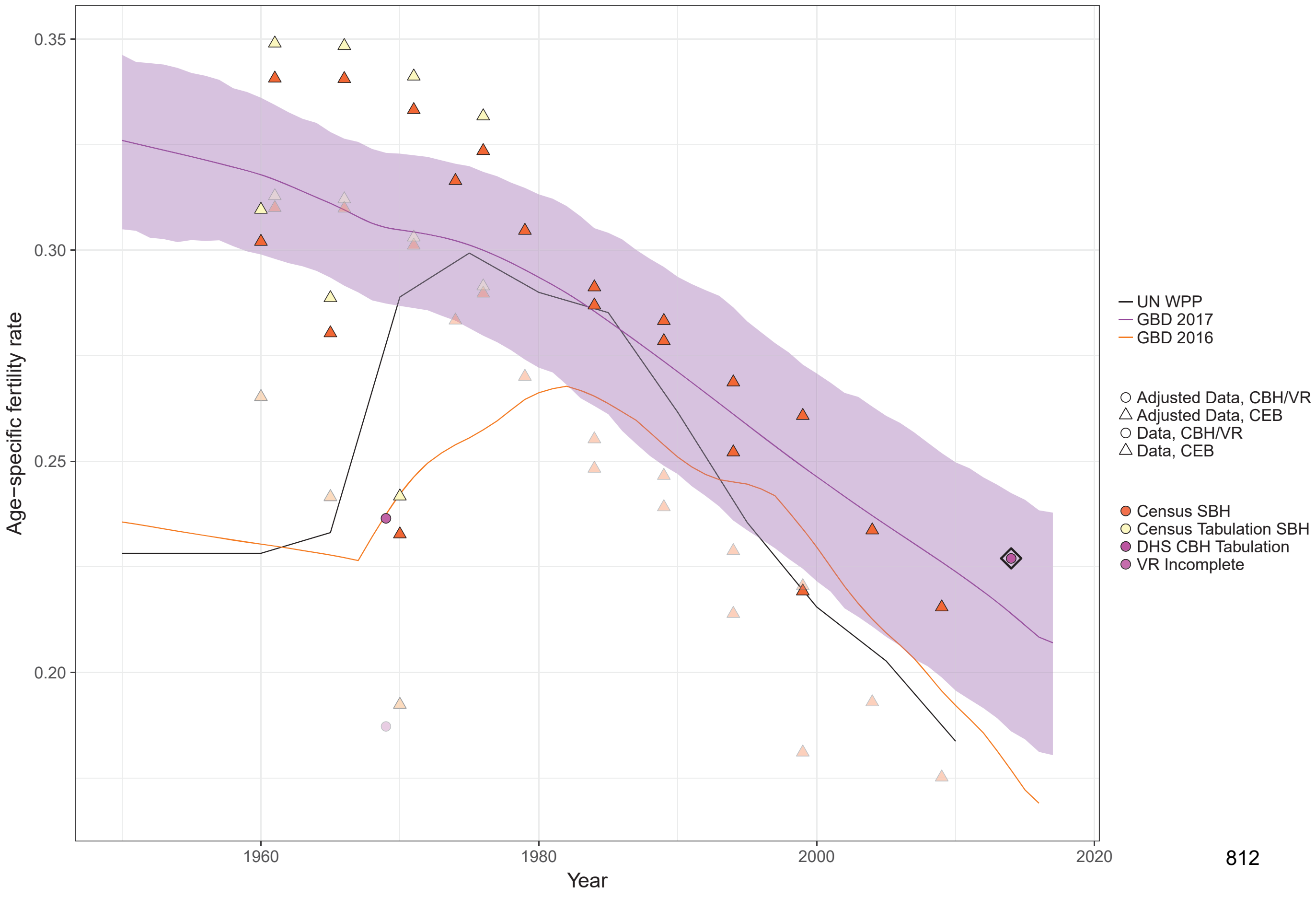

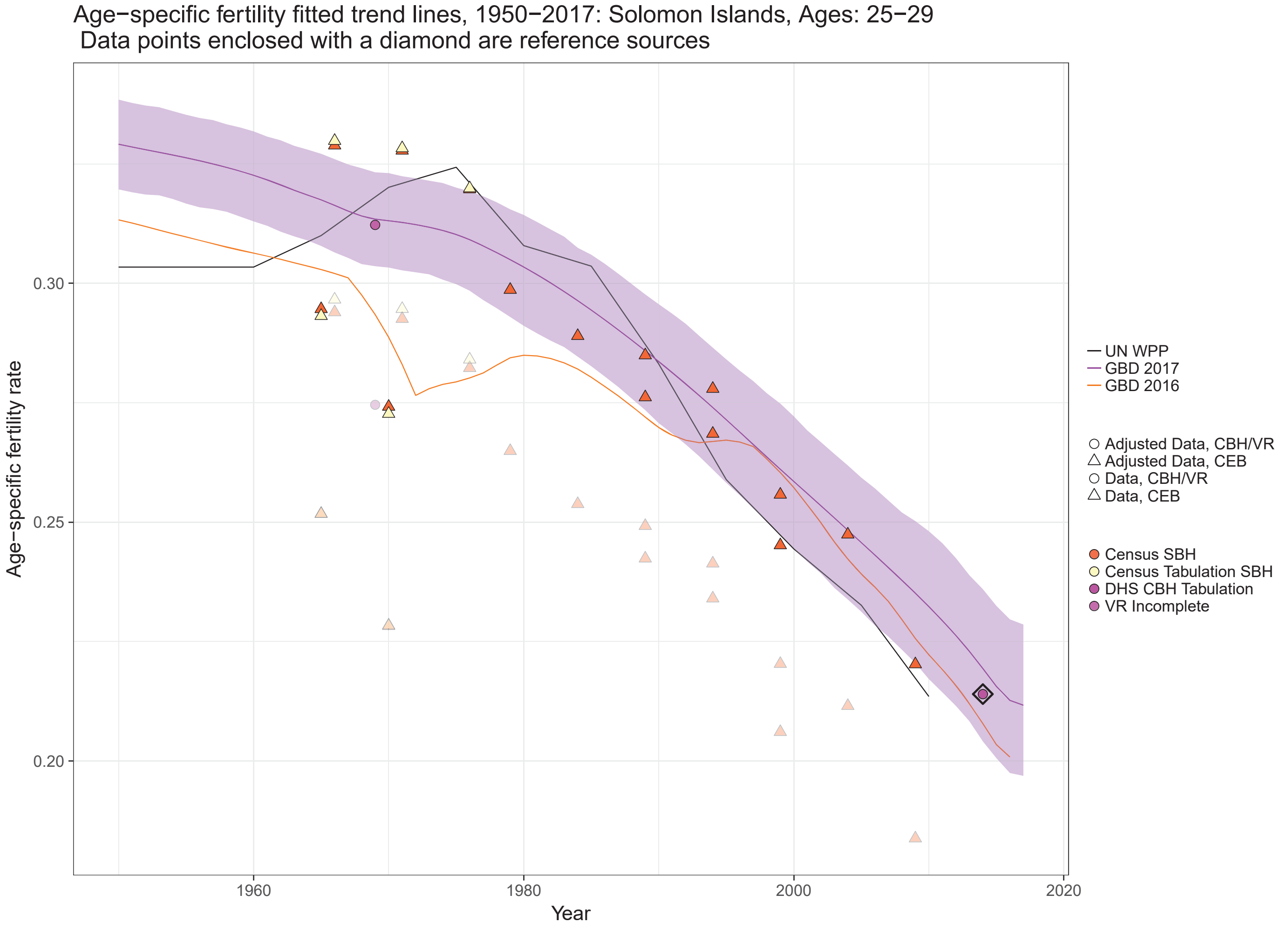
Age−specific fertility fitted trend lines, 1950−2017: Solomon Islands, Ages: 25−29
Data points enclosed with a diamond are reference sources
Age−specific fertility rate
0.30
0.25
0.20
1960
1980
2000
2020
Year
UN WPP
GBD 2017
GBD 2016
Adjusted Data, CBH/VR
Adjusted Data, CEB
Data, CBH/VR
Data, CEB
Census SBH
Census Tabulation SBH
DHS CBH Tabulation
VR Incomplete

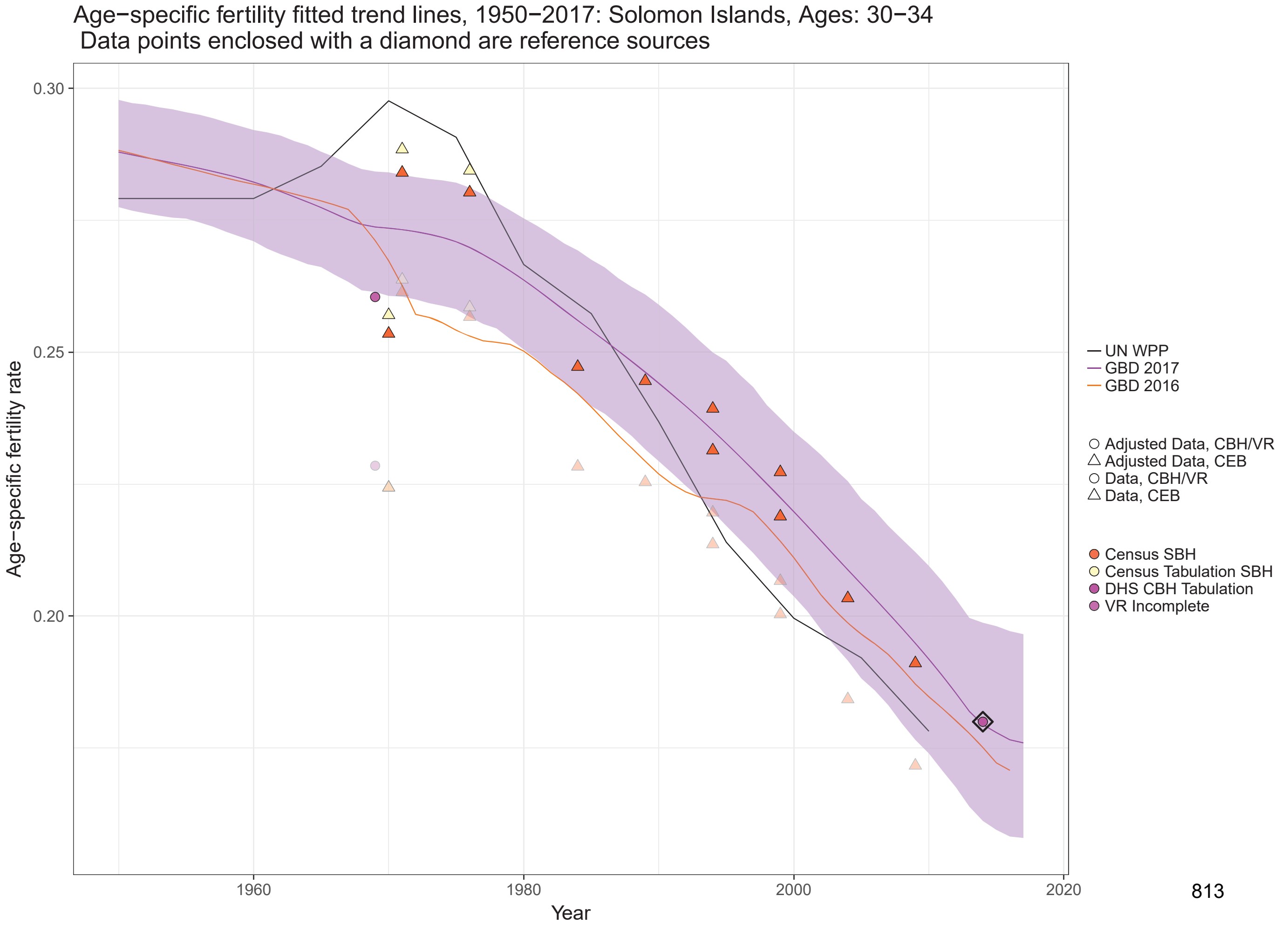
Age−specific fertility fitted trend lines, 1950−2017: Solomon Islands, Ages: 30−34
Data points enclosed with a diamond are reference sources
Age−specific fertility rate
0.30
0.25
0.20
1960
1980
2000
2020
Year
UN WPP
GBD 2017
GBD 2016
Adjusted Data, CBH/VR
Adjusted Data, CEB
Data, CBH/VR
Data, CEB
Census SBH
Census Tabulation SBH
DHS CBH Tabulation
VR Incomplete

Age−specific fertility fitted trend lines, 1950−2017: Solomon Islands, Ages: 35−39
Data points enclosed with a diamond are reference sources

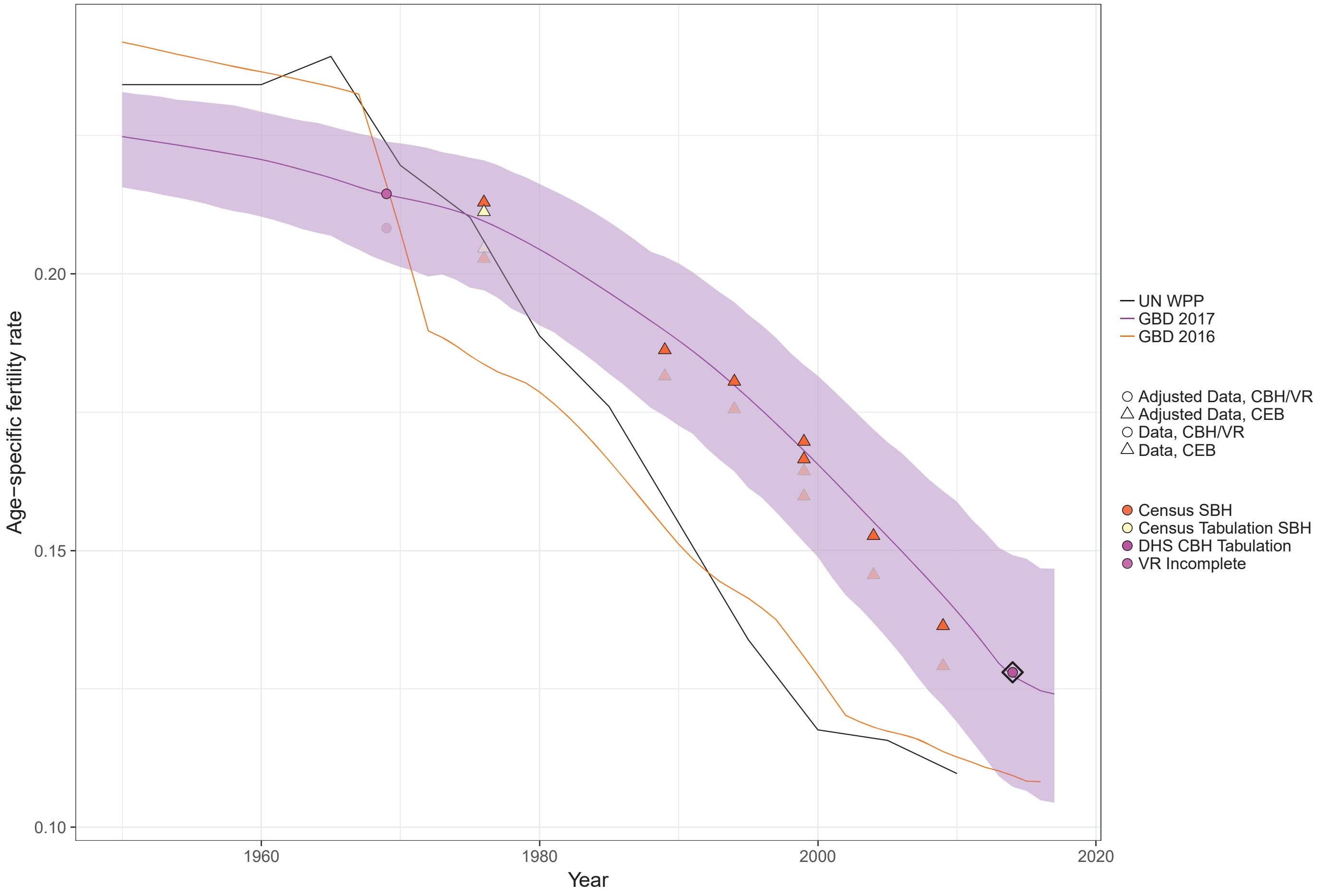

Age−specific fertility fitted trend lines, 1950−2017: Solomon Islands, Ages: 40−44
Data points enclosed with a diamond are reference sources

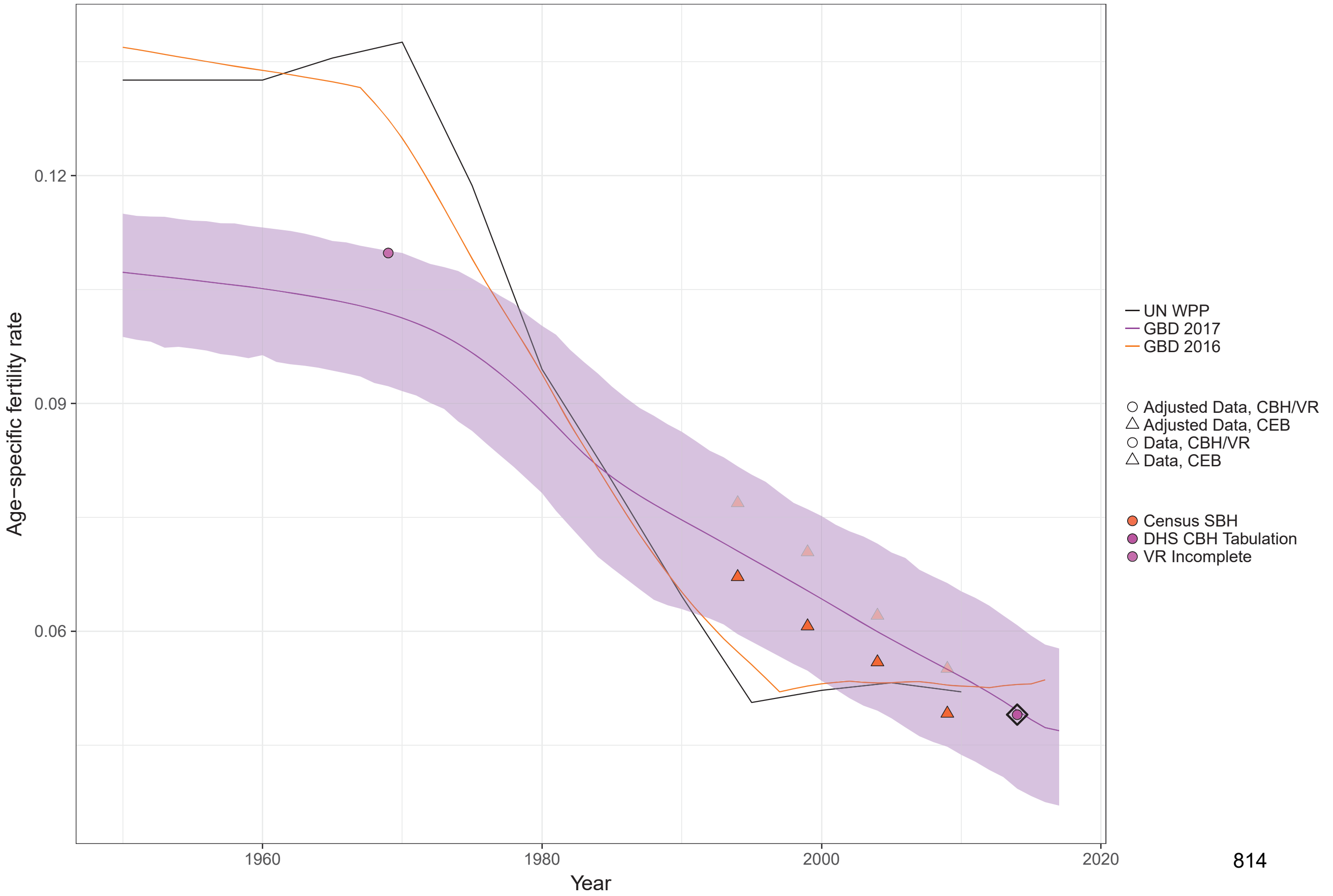

Age−specific fertility fitted trend lines, 1950−2017: Solomon Islands, Ages: 45−49
Data points enclosed with a diamond are reference sources

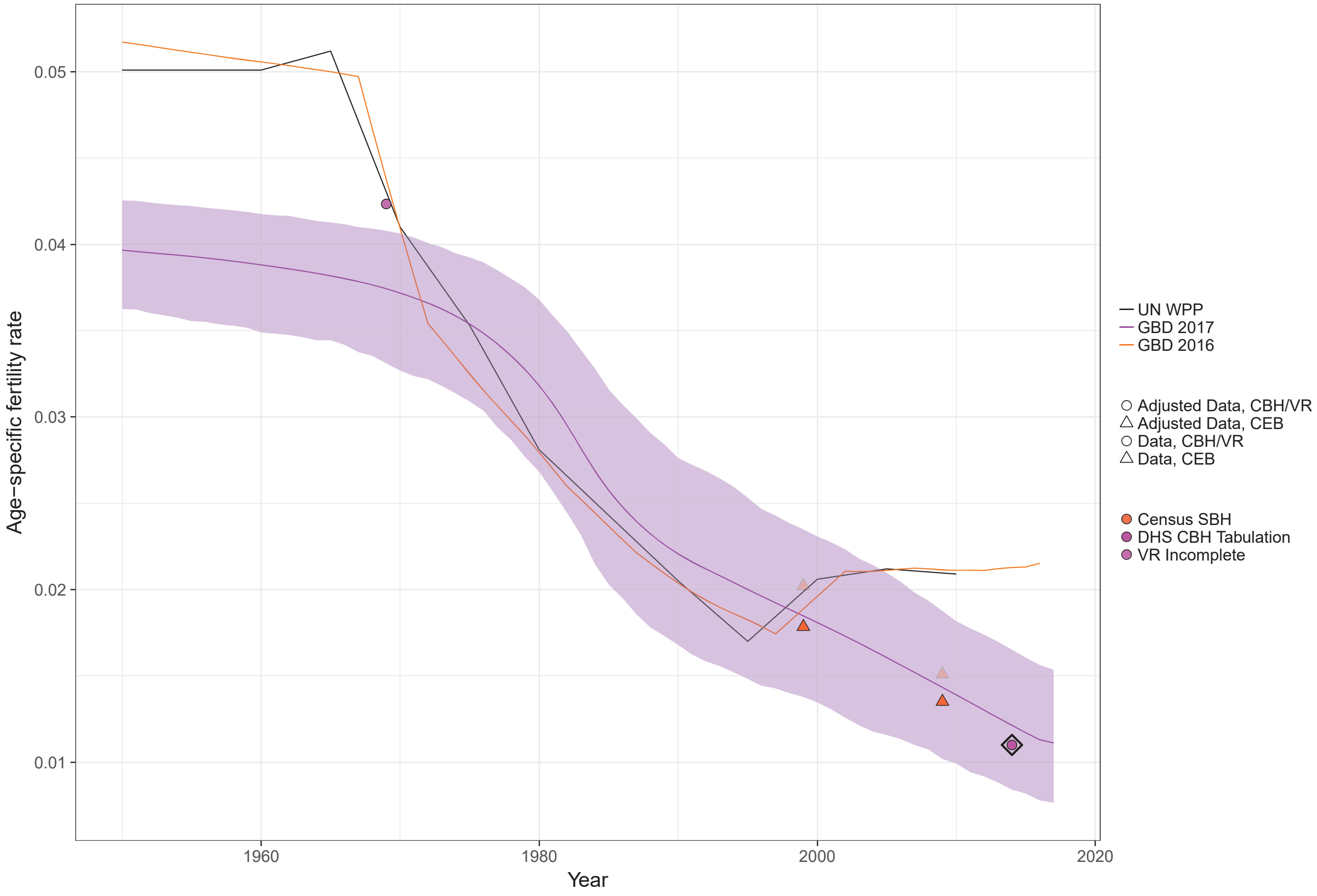

Age−specific fertility fitted trend lines, 1950−2017: Solomon Islands, Ages: 50−54
Data points enclosed with a diamond are reference sources

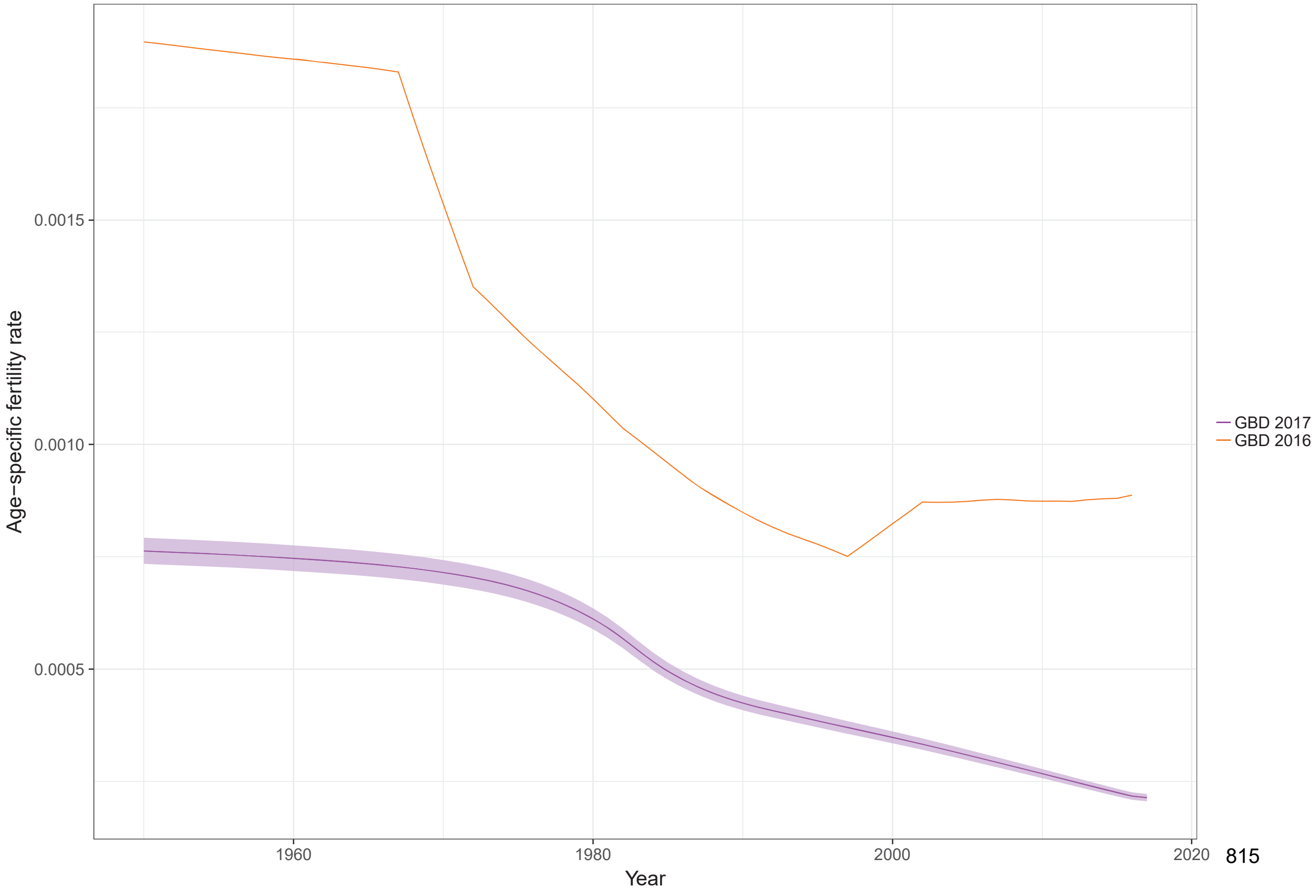

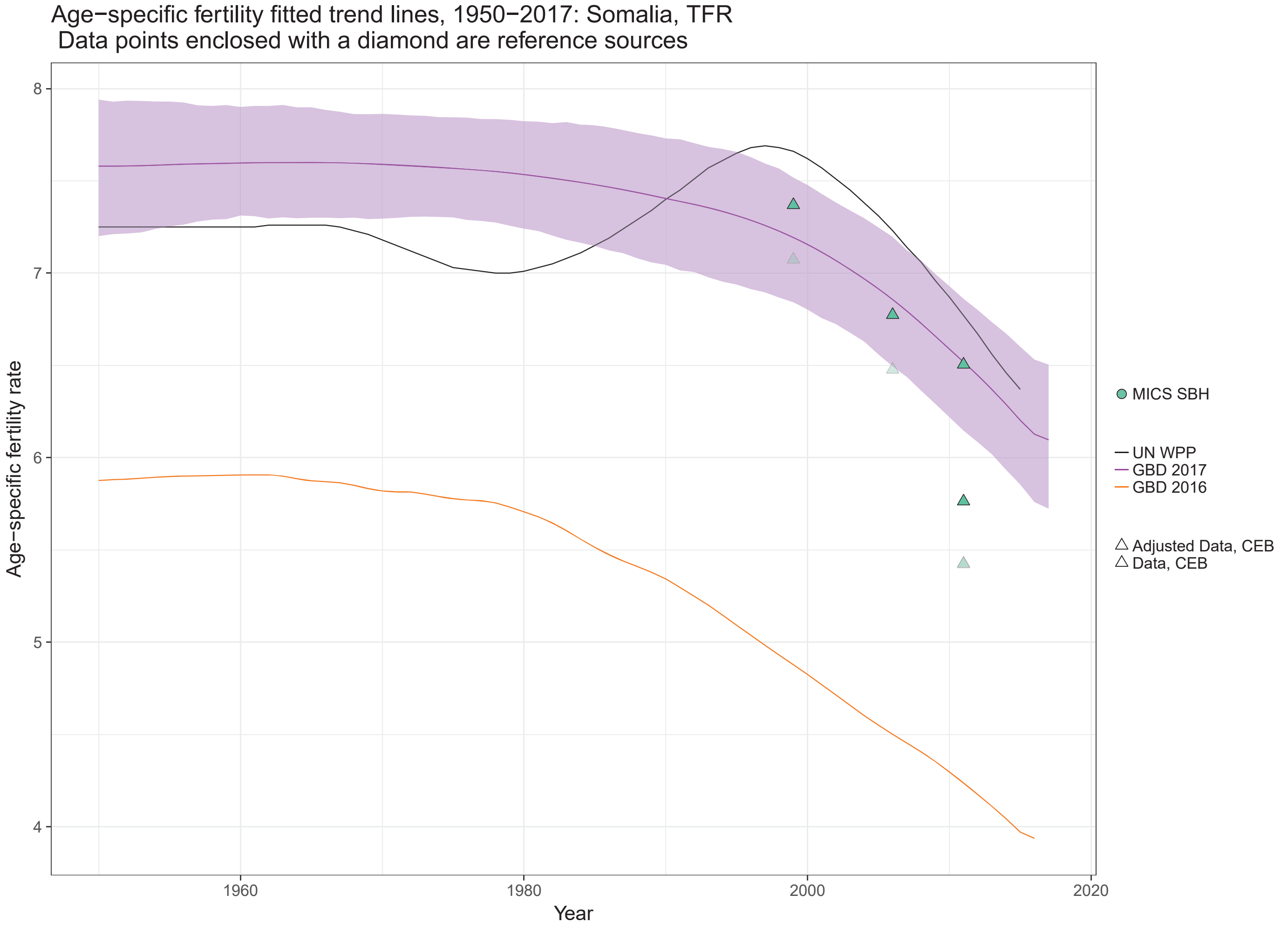

Age−specific fertility fitted trend lines, 1950−2017: Somalia, TFR
Data points enclosed with a diamond are reference sources
Age−specific fertility rate
Year
8
7
6
5
4
1960
1980
2000
2020
MICS SBH
UN WPP
GBD 2017
GBD 2016
Adjusted Data, CEB
Data, CEB

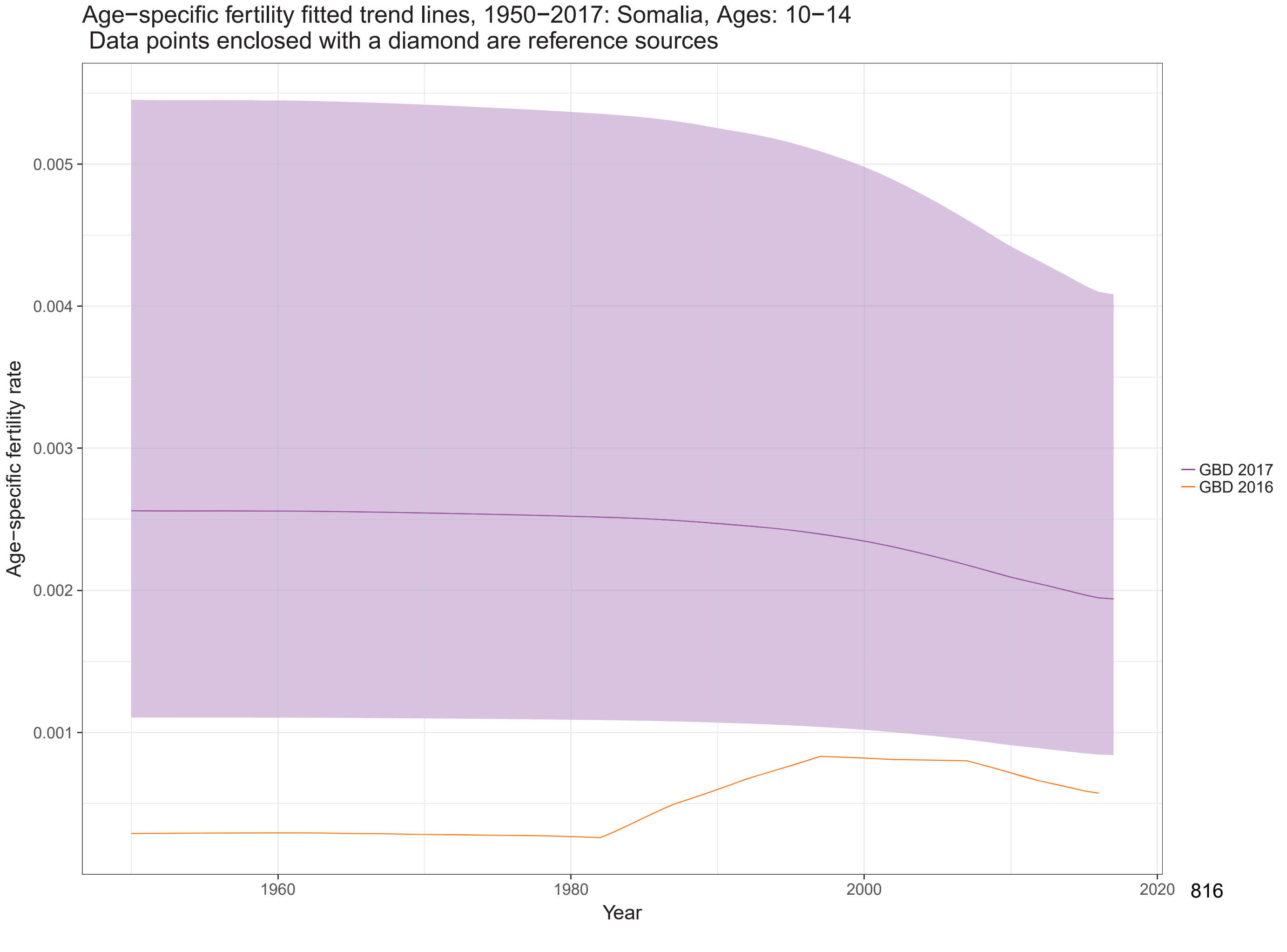

Age−specific fertility fitted trend lines, 1950−2017: Somalia, Ages: 10−14
Data points enclosed with a diamond are reference sources
Age−specific fertility rate
Year
0.005
0.004
0.003
0.002
0.001
1960
1980
2000
2020
GBD 2017
GBD 2016

Age−specific fertility fitted trend lines, 1950−2017: Somalia, Ages: 15−19
Data points enclosed with a diamond are reference sources

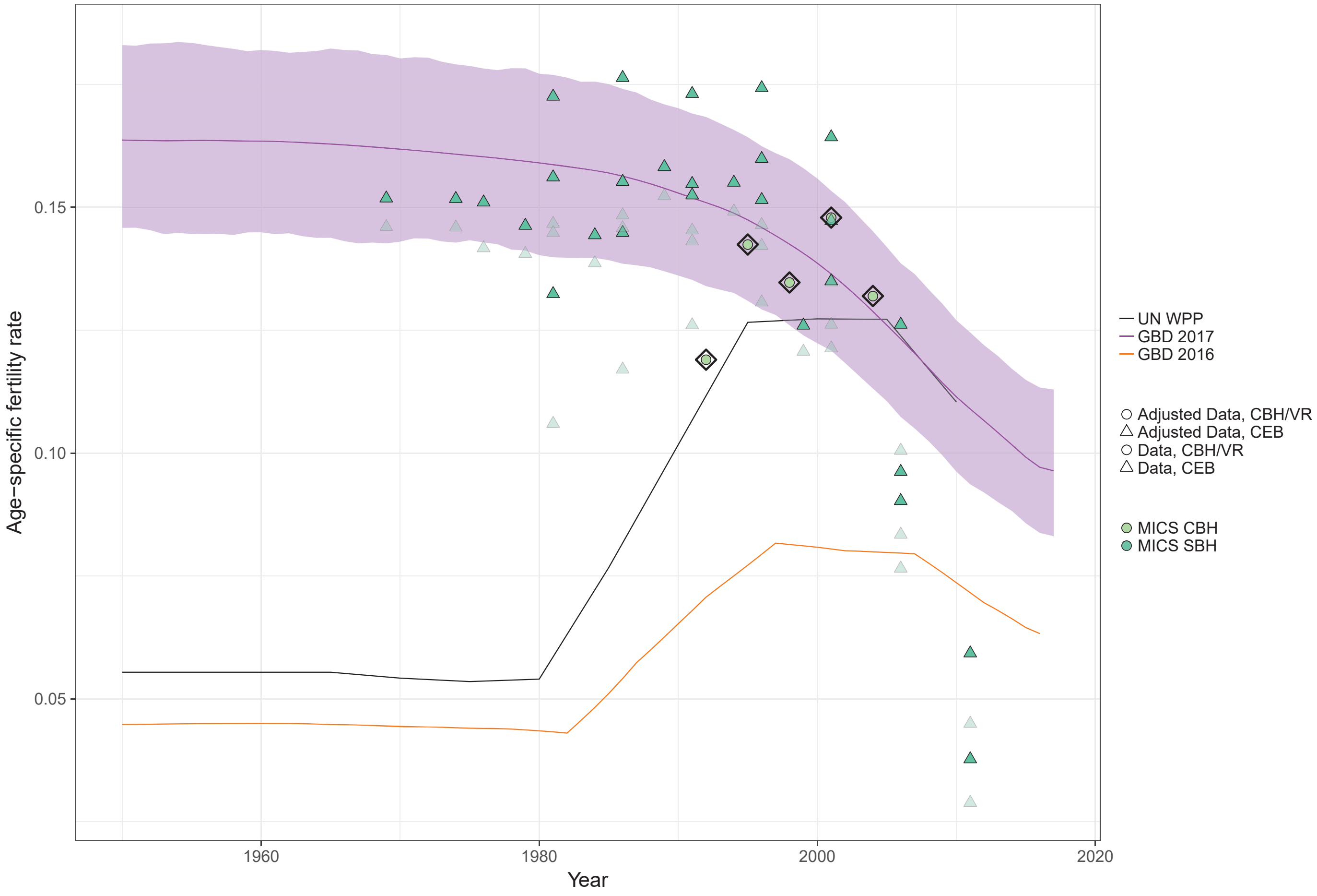

Age−specific fertility fitted trend lines, 1950−2017: Somalia, Ages: 20−24
Data points enclosed with a diamond are reference sources

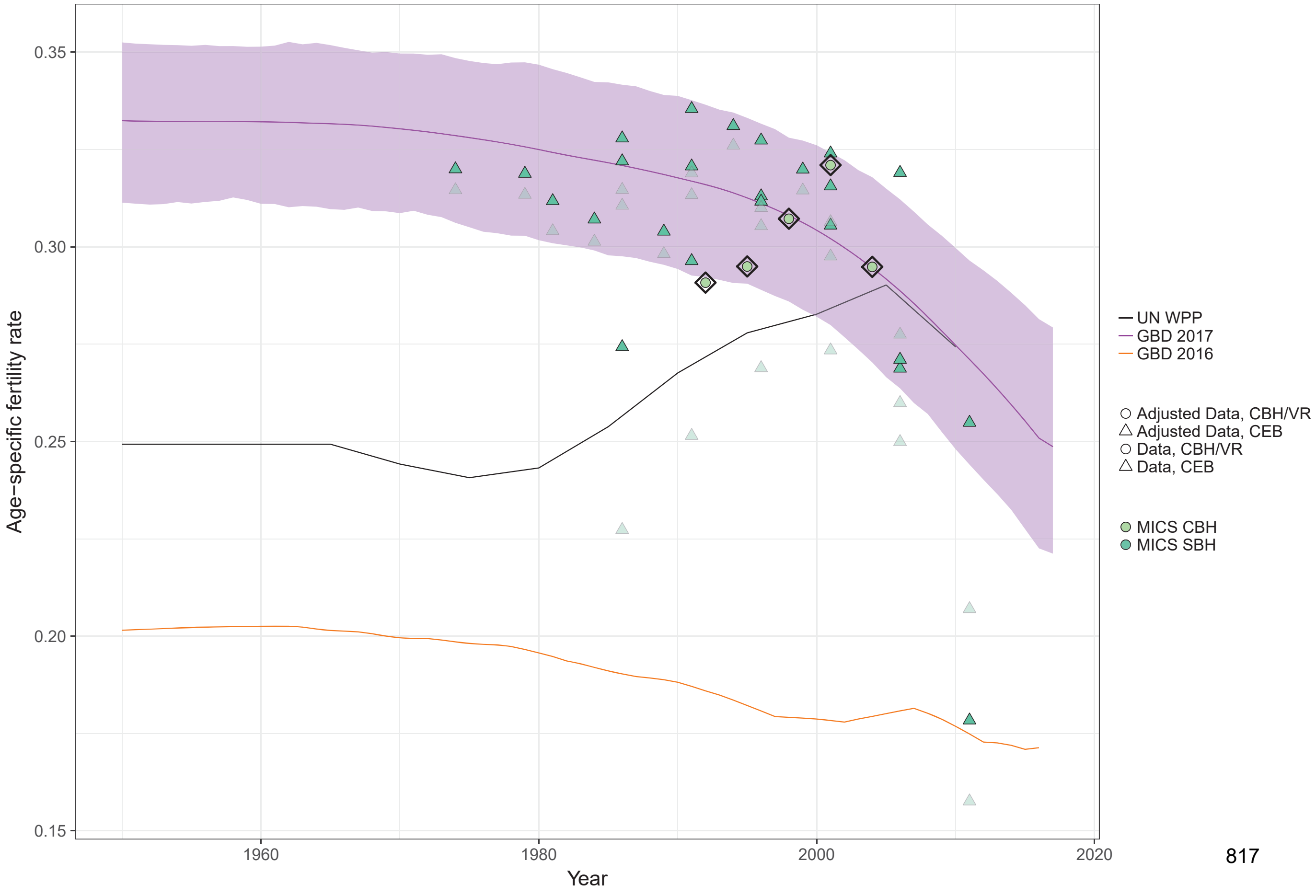

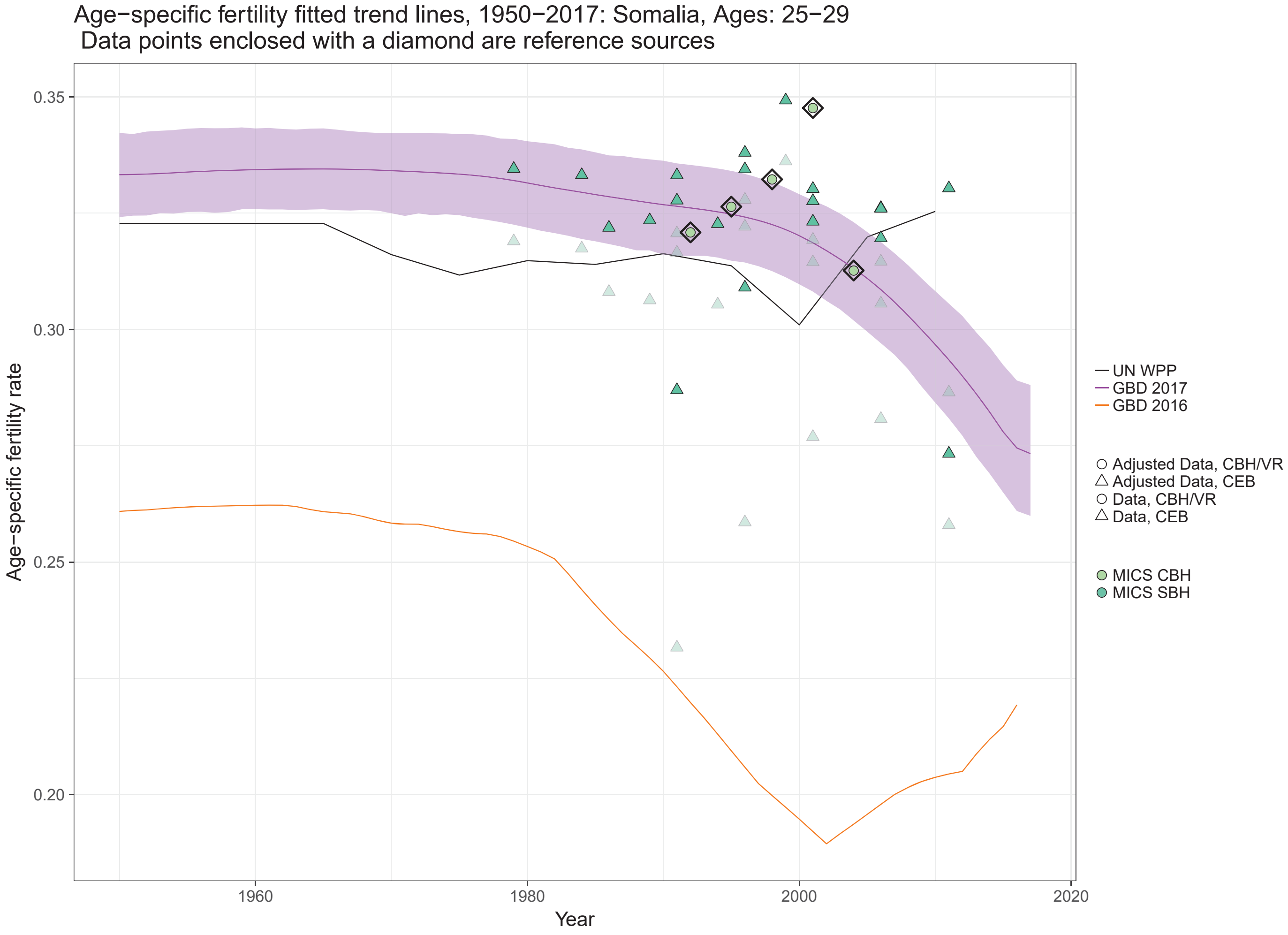

Age−specific fertility fitted trend lines, 1950−2017: Somalia, Ages: 25−29
Data points enclosed with a diamond are reference sources
Age−specific fertility rate
Year
UN WPP
GBD 2017
GBD 2016
Adjusted Data, CBH/VR
Adjusted Data, CEB
Data, CBH/VR
Data, CEB
MICS CBH
MICS SBH

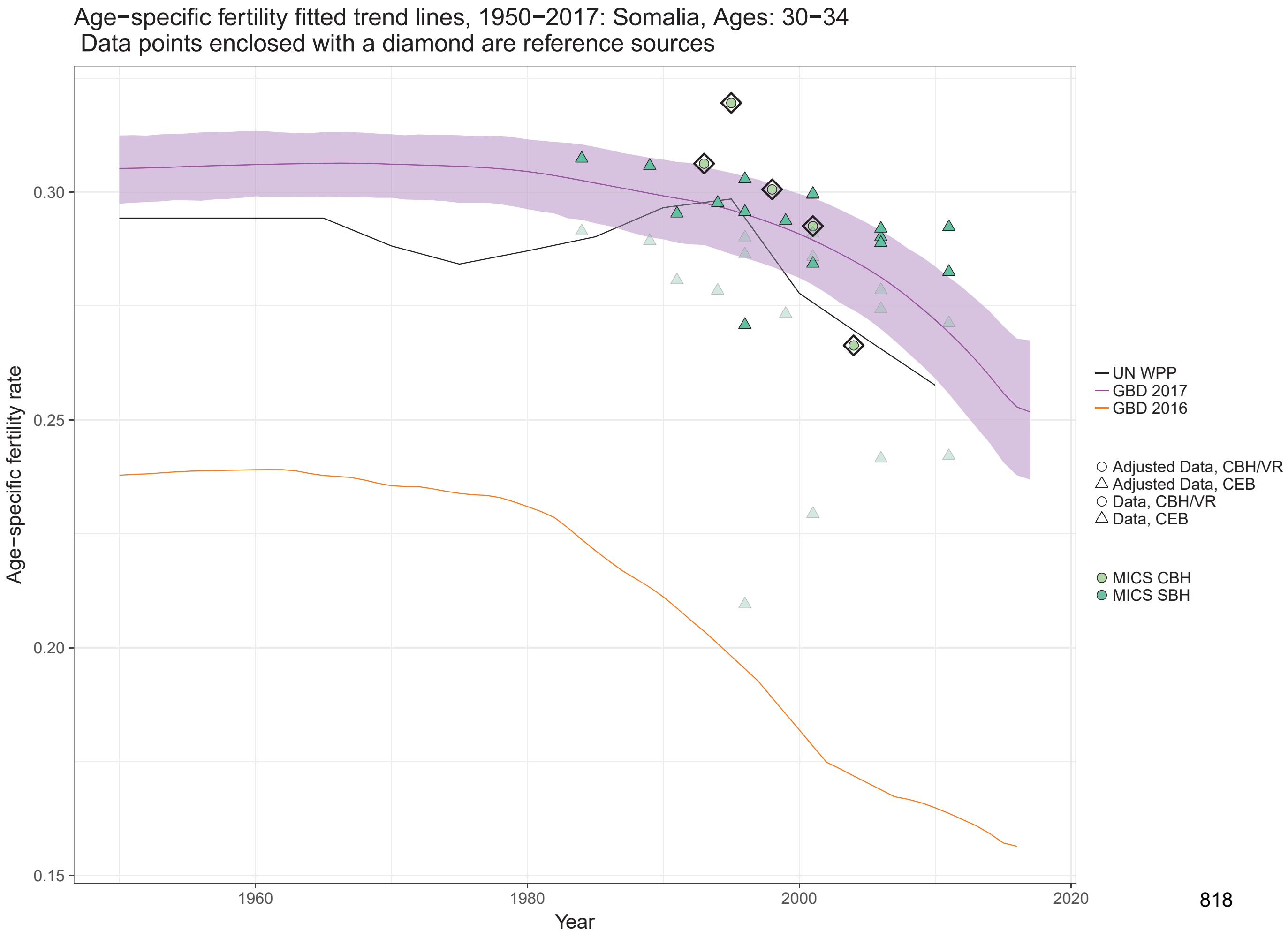

Age−specific fertility fitted trend lines, 1950−2017: Somalia, Ages: 30−34
Data points enclosed with a diamond are reference sources
Age−specific fertility rate
Year
UN WPP
GBD 2017
GBD 2016
Adjusted Data, CBH/VR
Adjusted Data, CEB
Data, CBH/VR
Data, CEB
MICS CBH
MICS SBH

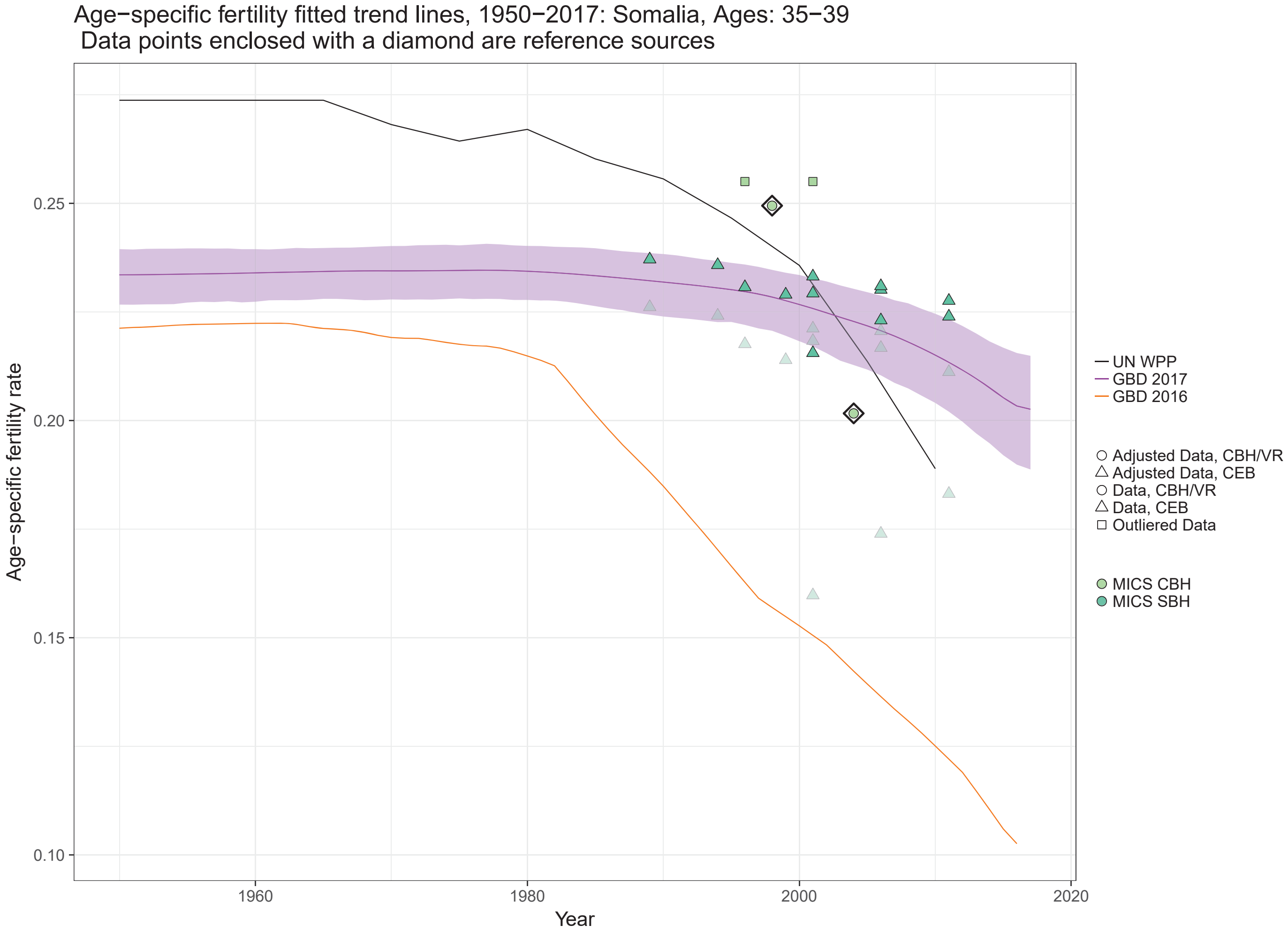

Age−specific fertility fitted trend lines, 1950−2017: Somalia, Ages: 35−39
Data points enclosed with a diamond are reference sources
Age−specific fertility rate
Year
0.10
0.15
0.20
0.25
1960
1980
2000
2020
UN WPP
GBD 2017
GBD 2016
Adjusted Data, CBH/VR
Adjusted Data, CEB
Data, CBH/VR
Data, CEB
Outliered Data
MICS CBH
MICS SBH

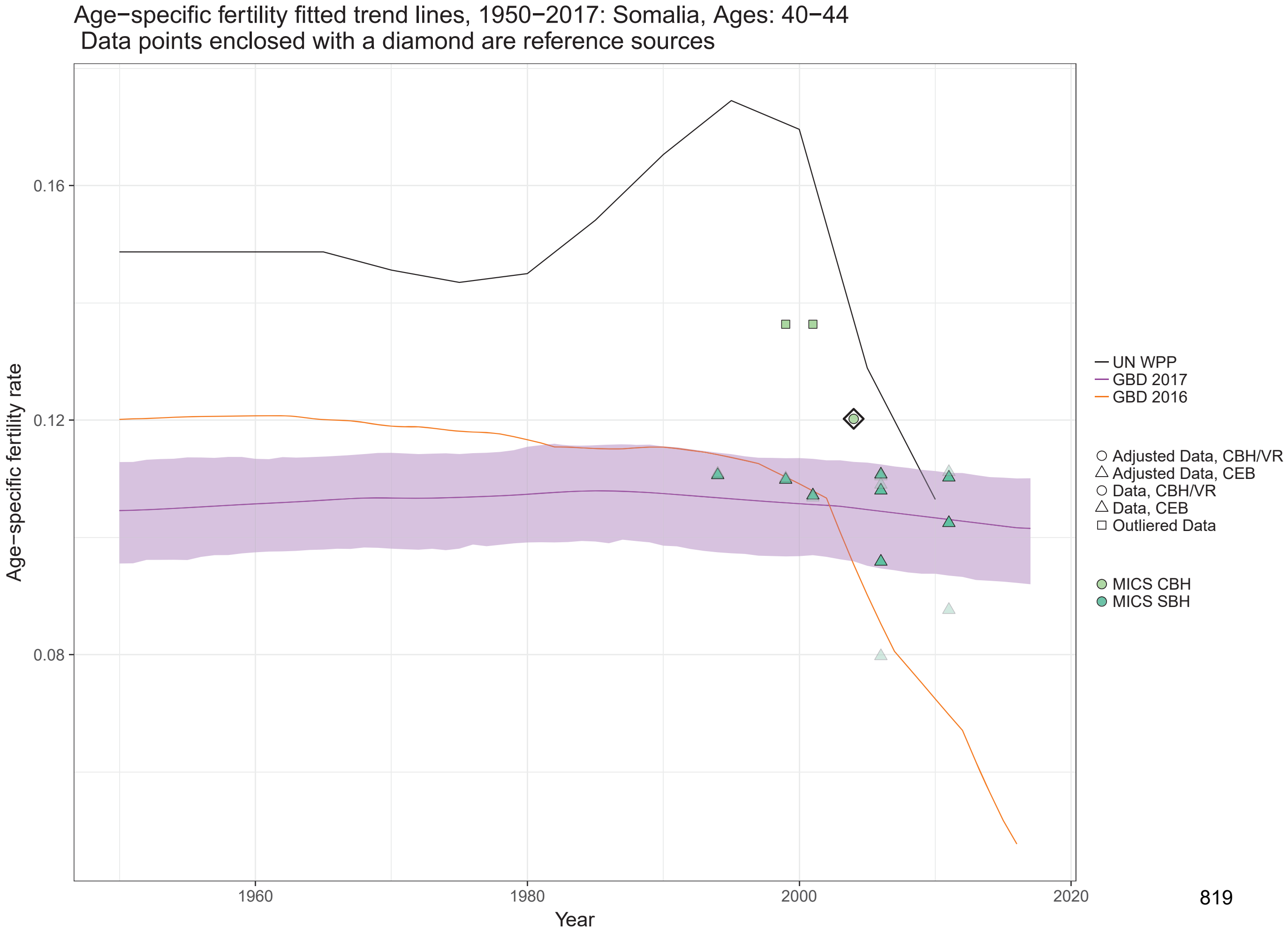

Age−specific fertility fitted trend lines, 1950−2017: Somalia, Ages: 40−44
Data points enclosed with a diamond are reference sources
Age−specific fertility rate
Year
0.08
0.12
0.16
1960
1980
2000
2020
UN WPP
GBD 2017
GBD 2016
Adjusted Data, CBH/VR
Adjusted Data, CEB
Data, CBH/VR
Data, CEB
Outliered Data
MICS CBH
MICS SBH

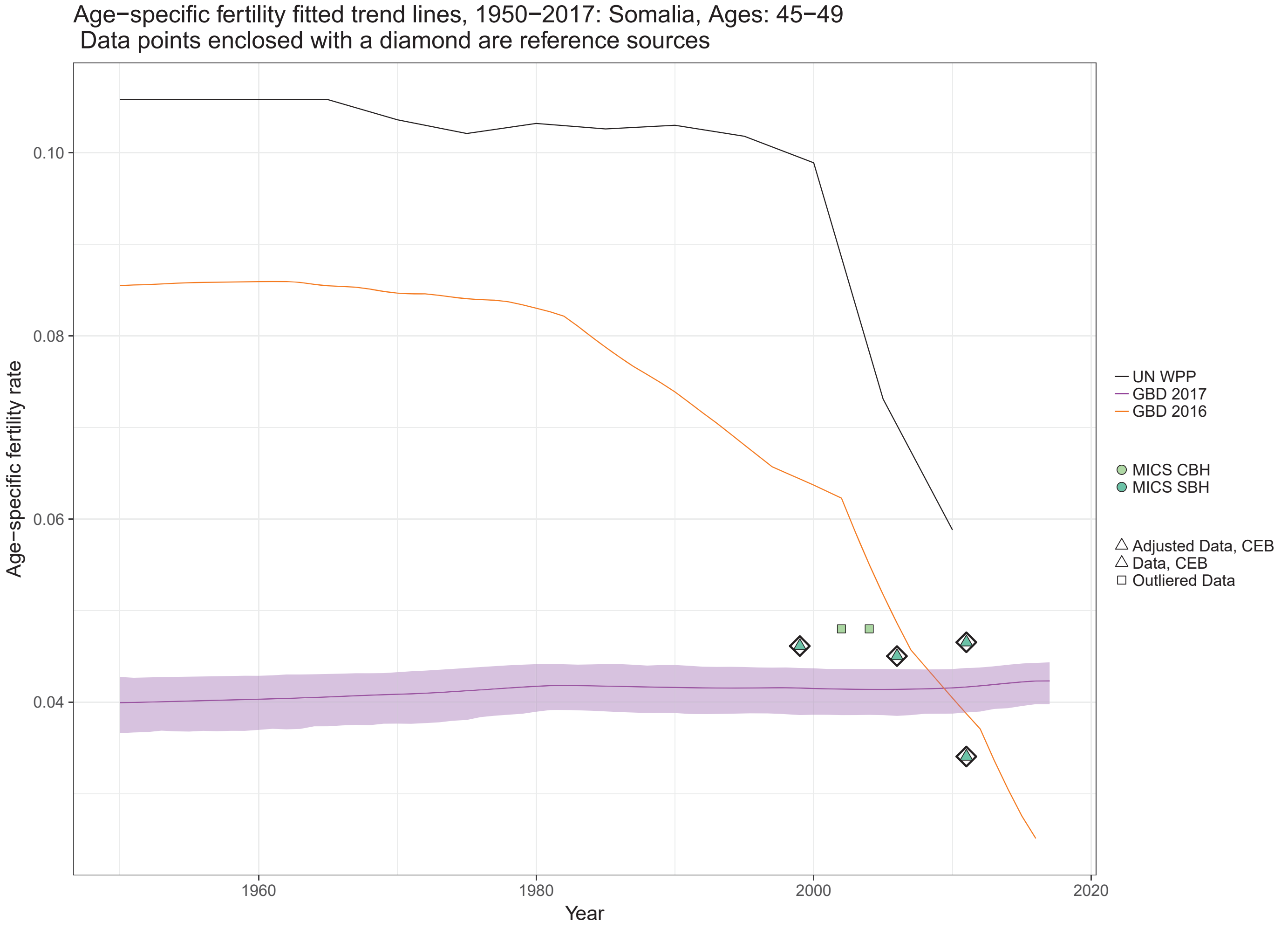

Age−specific fertility fitted trend lines, 1950−2017: Somalia, Ages: 45−49
Data points enclosed with a diamond are reference sources
Age−specific fertility rate
Year
0.10
0.08
0.06
0.04
1960
1980
2000
2020
UN WPP
GBD 2017
GBD 2016
MICS CBH
MICS SBH
Adjusted Data, CEB
Data, CEB
Outliered Data

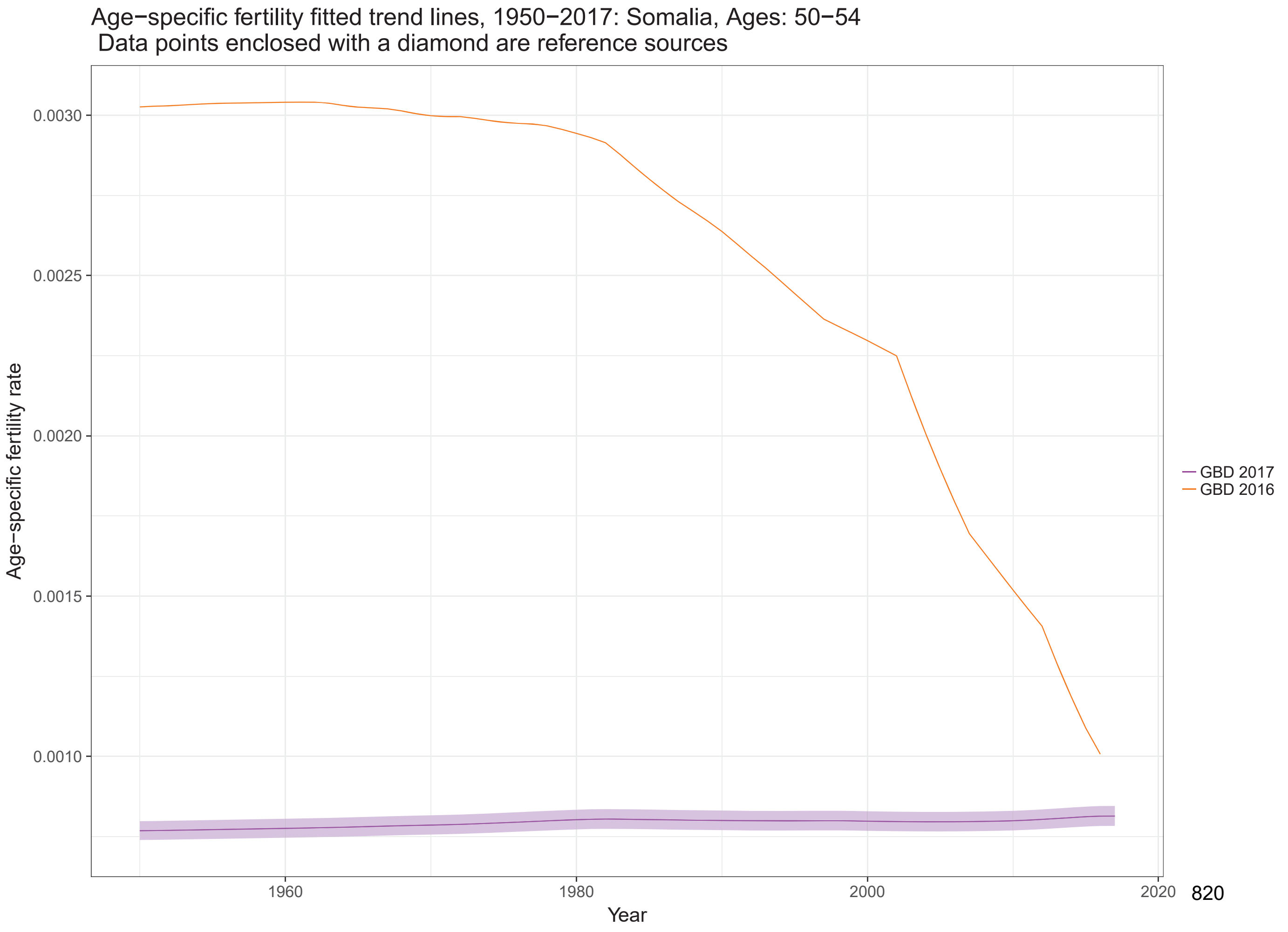

Age−specific fertility fitted trend lines, 1950−2017: Somalia, Ages: 50−54
Data points enclosed with a diamond are reference sources
Age−specific fertility rate
Year
0.0030
0.0025
0.0020
0.0015
0.0010
1960
1980
2000
2020
GBD 2017
GBD 2016

Age−specific fertility fitted trend lines, 1950−2017: South Africa, TFR
Data points enclosed with a diamond are reference sources

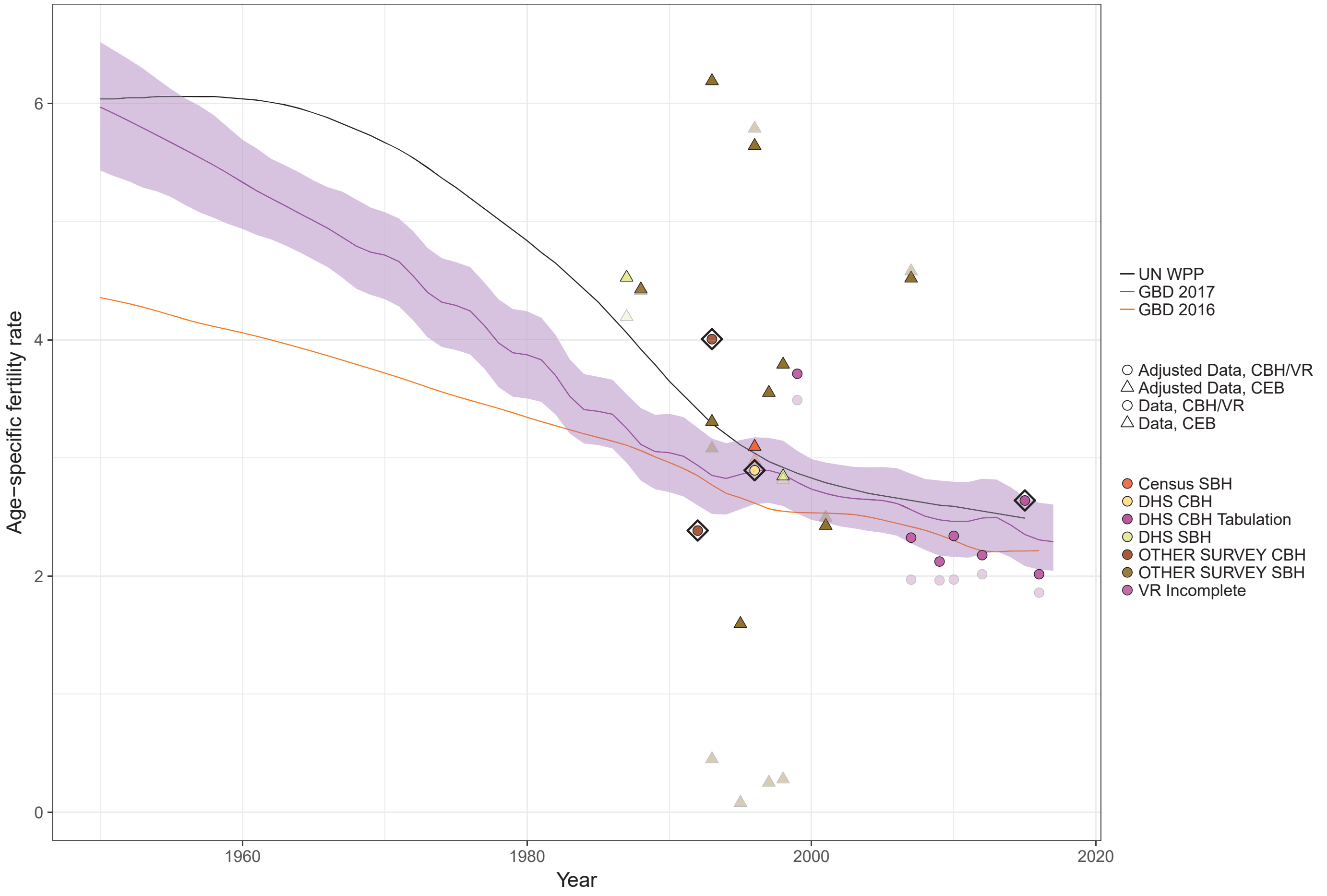

Age−specific fertility fitted trend lines, 1950−2017: South Africa, Ages: 10−14
Data points enclosed with a diamond are reference sources

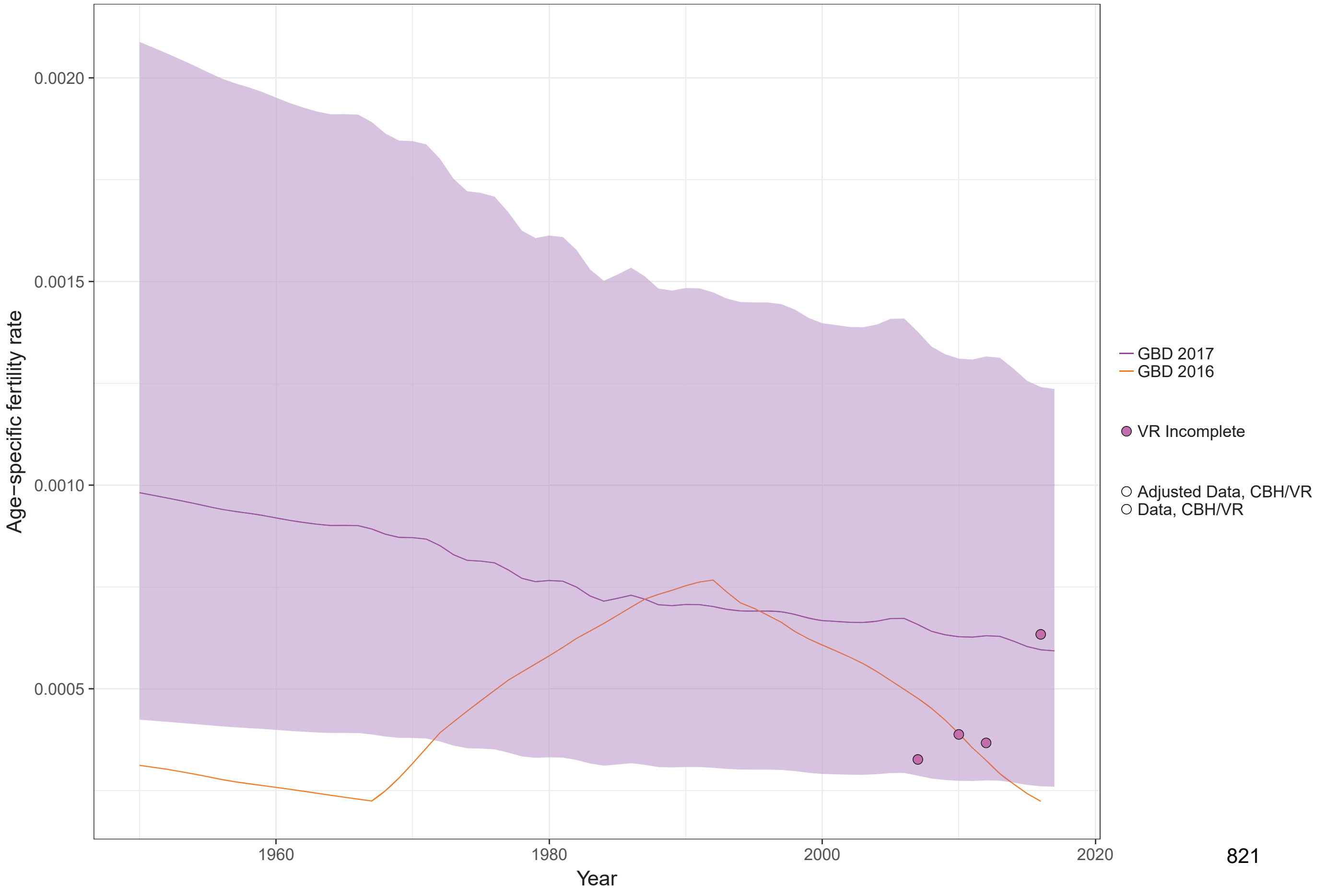

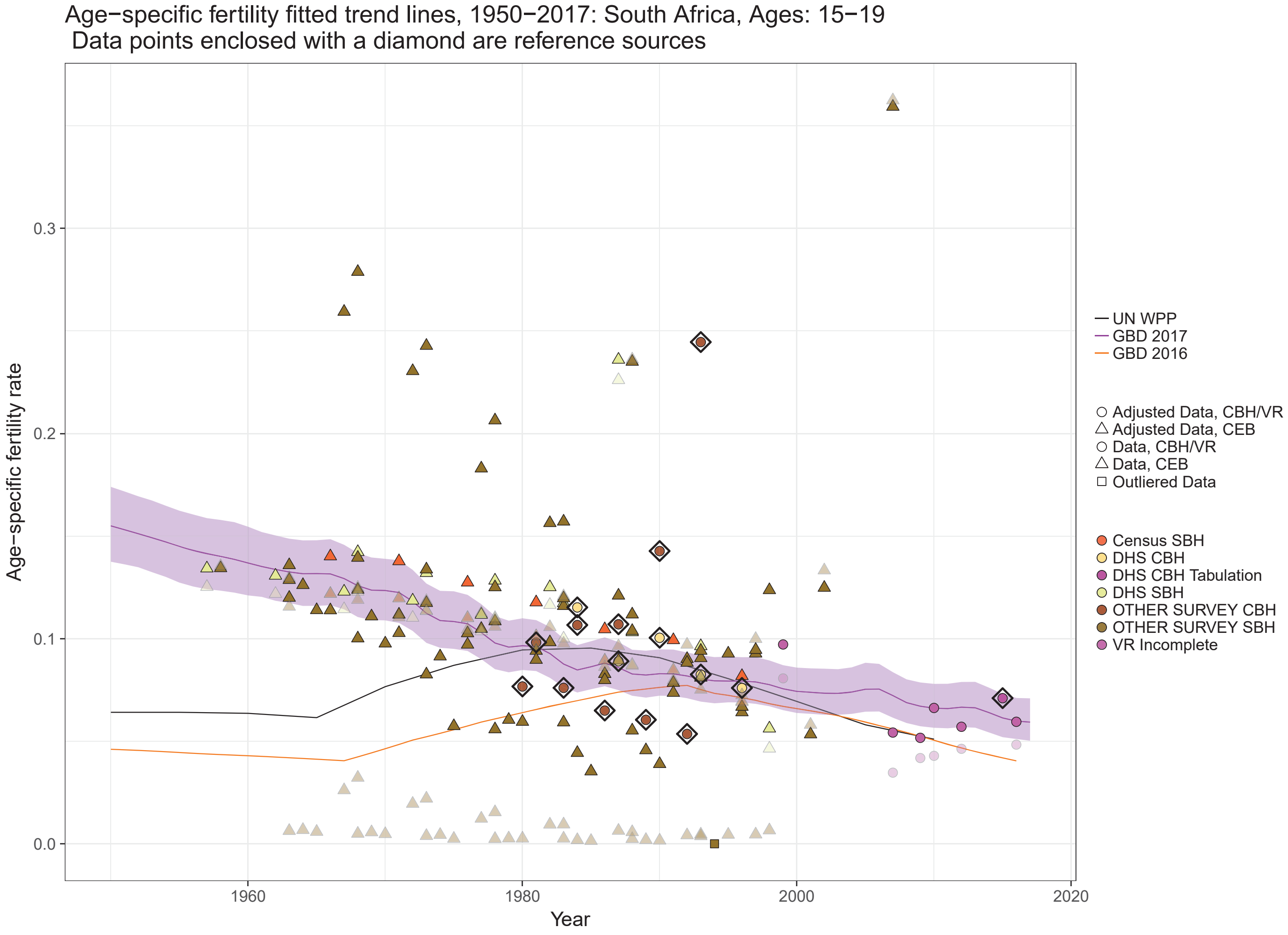

Age−specific fertility fitted trend lines, 1950−2017: South Africa, Ages: 15−19
Data points enclosed with a diamond are reference sources
Age-specific fertility rate
Year
UN WPP
GBD 2017
GBD 2016
Adjusted Data, CBH/VR
Adjusted Data, CEB
Data, CBH/VR
Data, CEB
Outliered Data
Census SBH
DHS CBH
DHS CBH Tabulation
DHS SBH
OTHER SURVEY CBH
OTHER SURVEY SBH
VR Incomplete

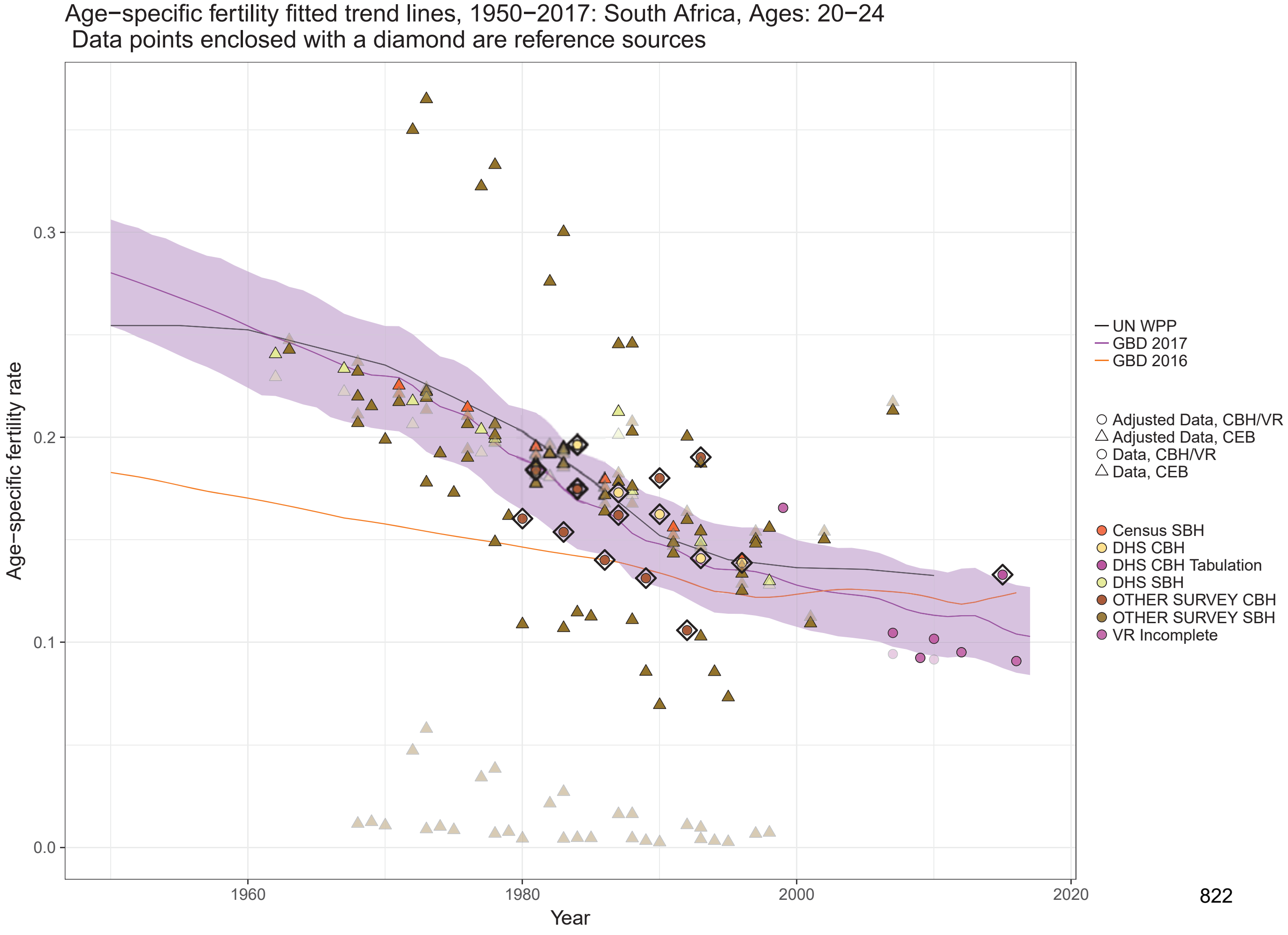

Age−specific fertility fitted trend lines, 1950−2017: South Africa, Ages: 20−24
Data points enclosed with a diamond are reference sources
Age-specific fertility rate
Year
UN WPP
GBD 2017
GBD 2016
Adjusted Data, CBH/VR
Adjusted Data, CEB
Data, CBH/VR
Data, CEB
Census SBH
DHS CBH
DHS CBH Tabulation
DHS SBH
OTHER SURVEY CBH
OTHER SURVEY SBH
VR Incomplete

Age−specific fertility fitted trend lines, 1950−2017: South Africa, Ages: 25−29
Data points enclosed with a diamond are reference sources

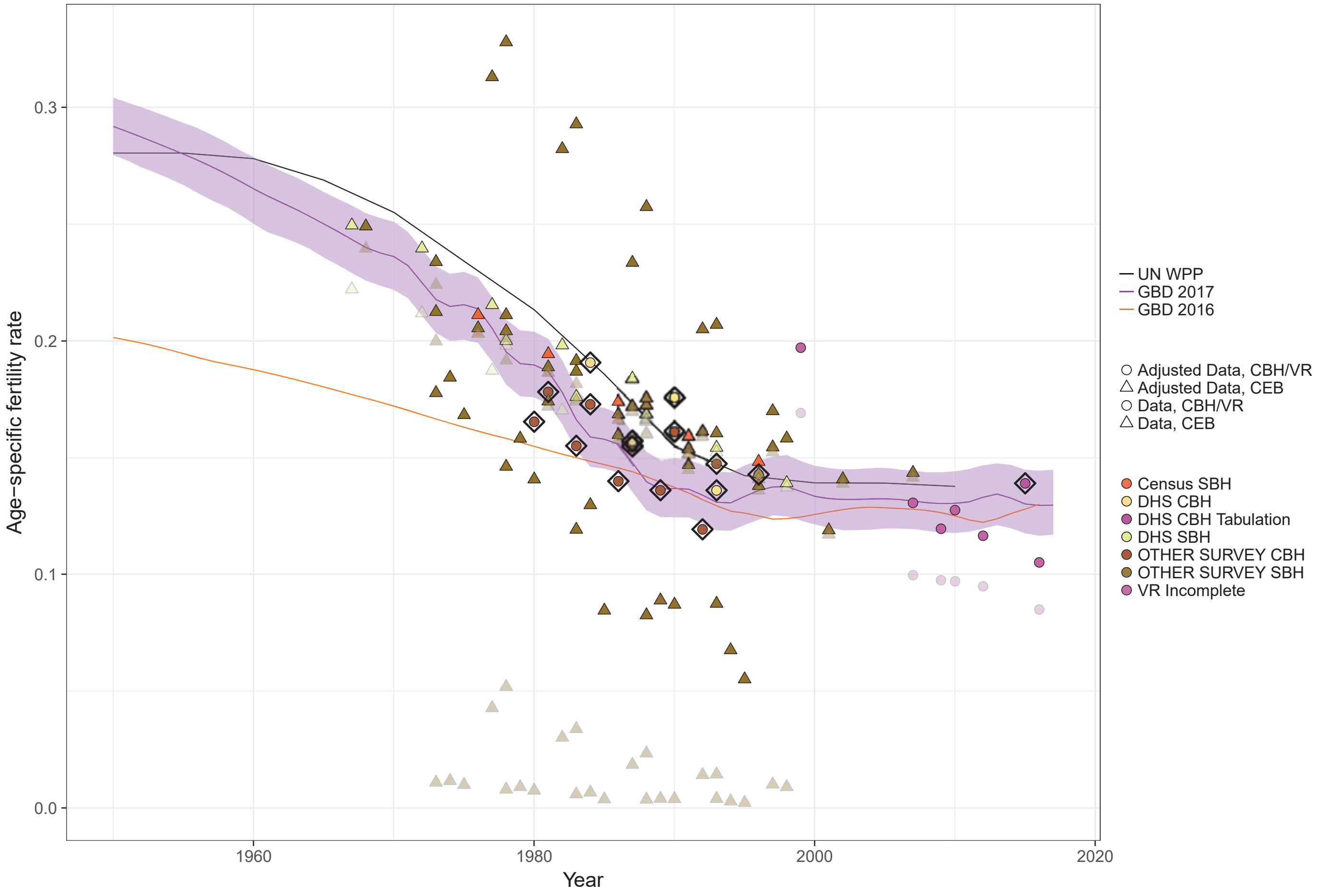

Age−specific fertility fitted trend lines, 1950−2017: South Africa, Ages: 30−34
Data points enclosed with a diamond are reference sources

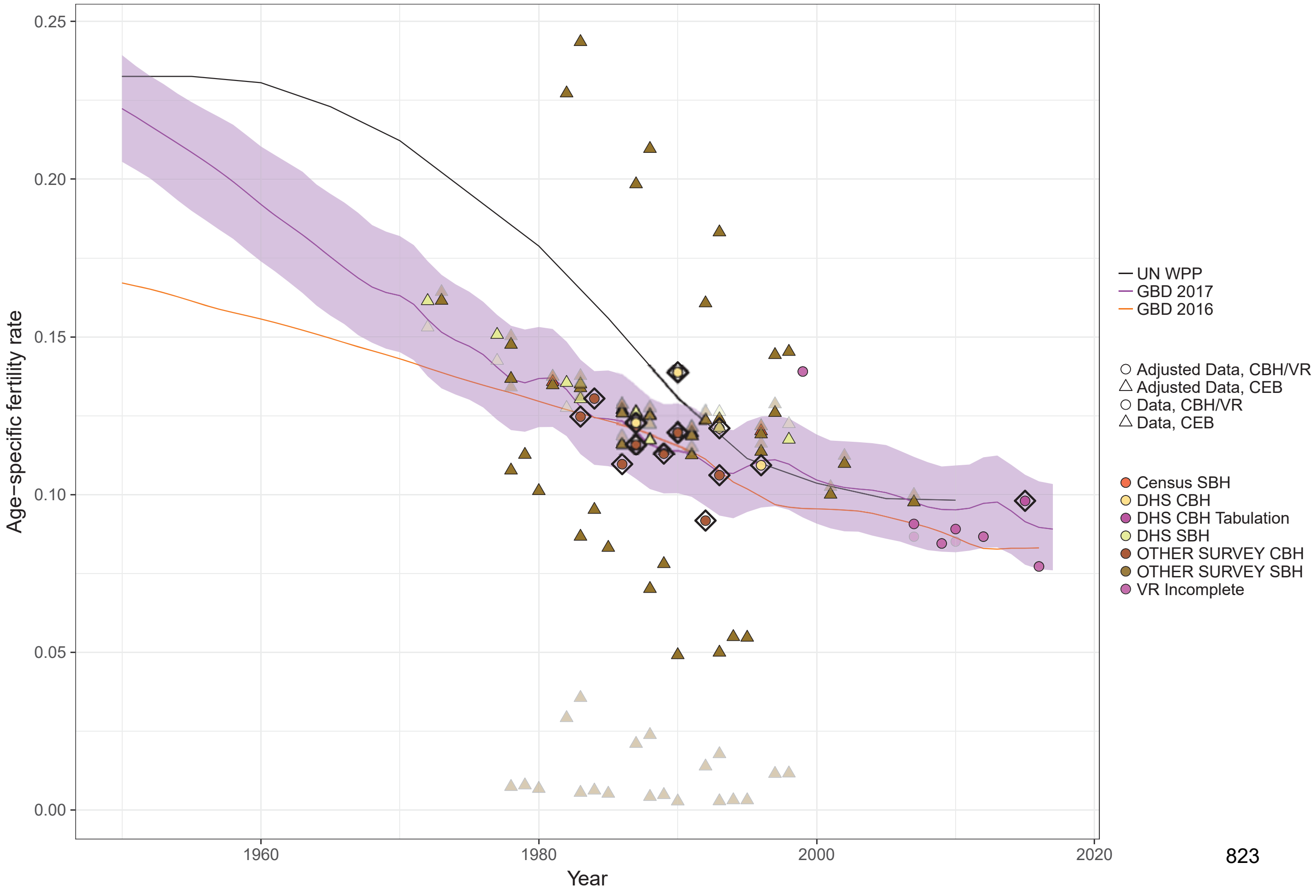

Age−specific fertility fitted trend lines, 1950−2017: South Africa, Ages: 35−39
Data points enclosed with a diamond are reference sources

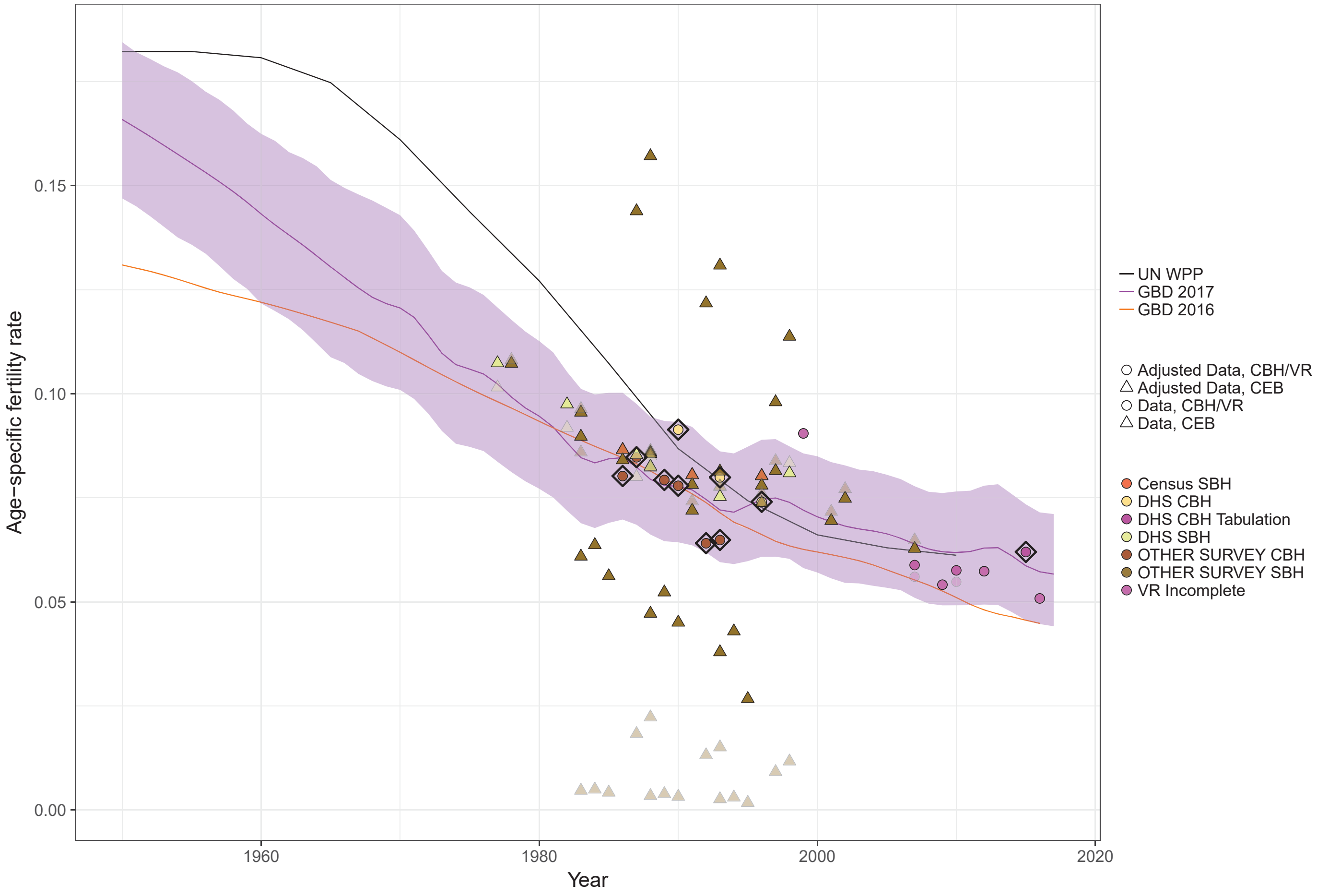

Age−specific fertility fitted trend lines, 1950−2017: South Africa, Ages: 40−44
Data points enclosed with a diamond are reference sources

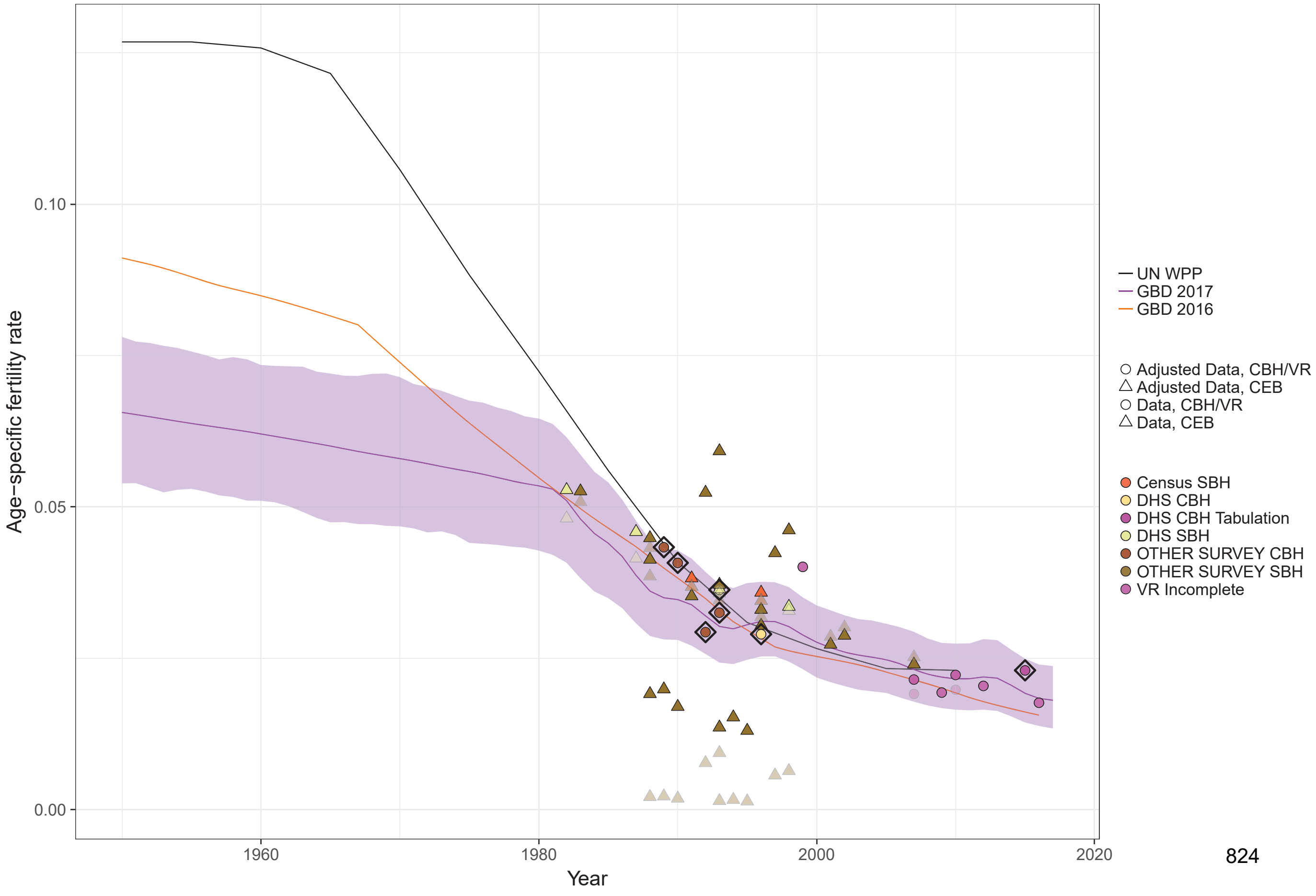

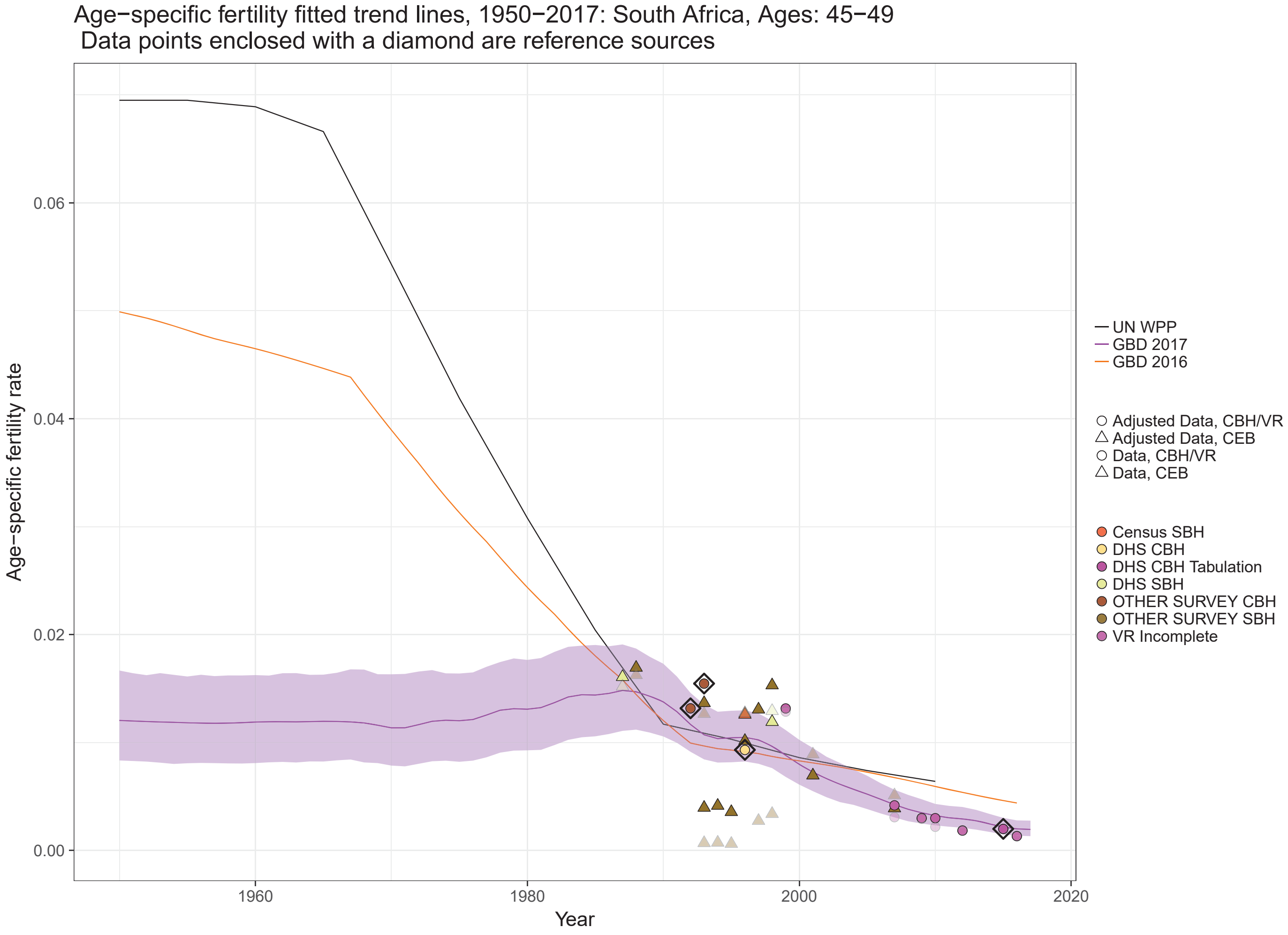

Age−specific fertility fitted trend lines, 1950−2017: South Africa, Ages: 45−49
Data points enclosed with a diamond are reference sources
Age−specific fertility rate
0.06
0.04
0.02
0.00
1960
1980
2000
2020
Year
UN WPP
GBD 2017
GBD 2016
Adjusted Data, CBH/VR
Adjusted Data, CEB
Data, CBH/VR
Data, CEB
Census SBH
DHS CBH
DHS CBH Tabulation
DHS SBH
OTHER SURVEY CBH
OTHER SURVEY SBH
VR Incomplete

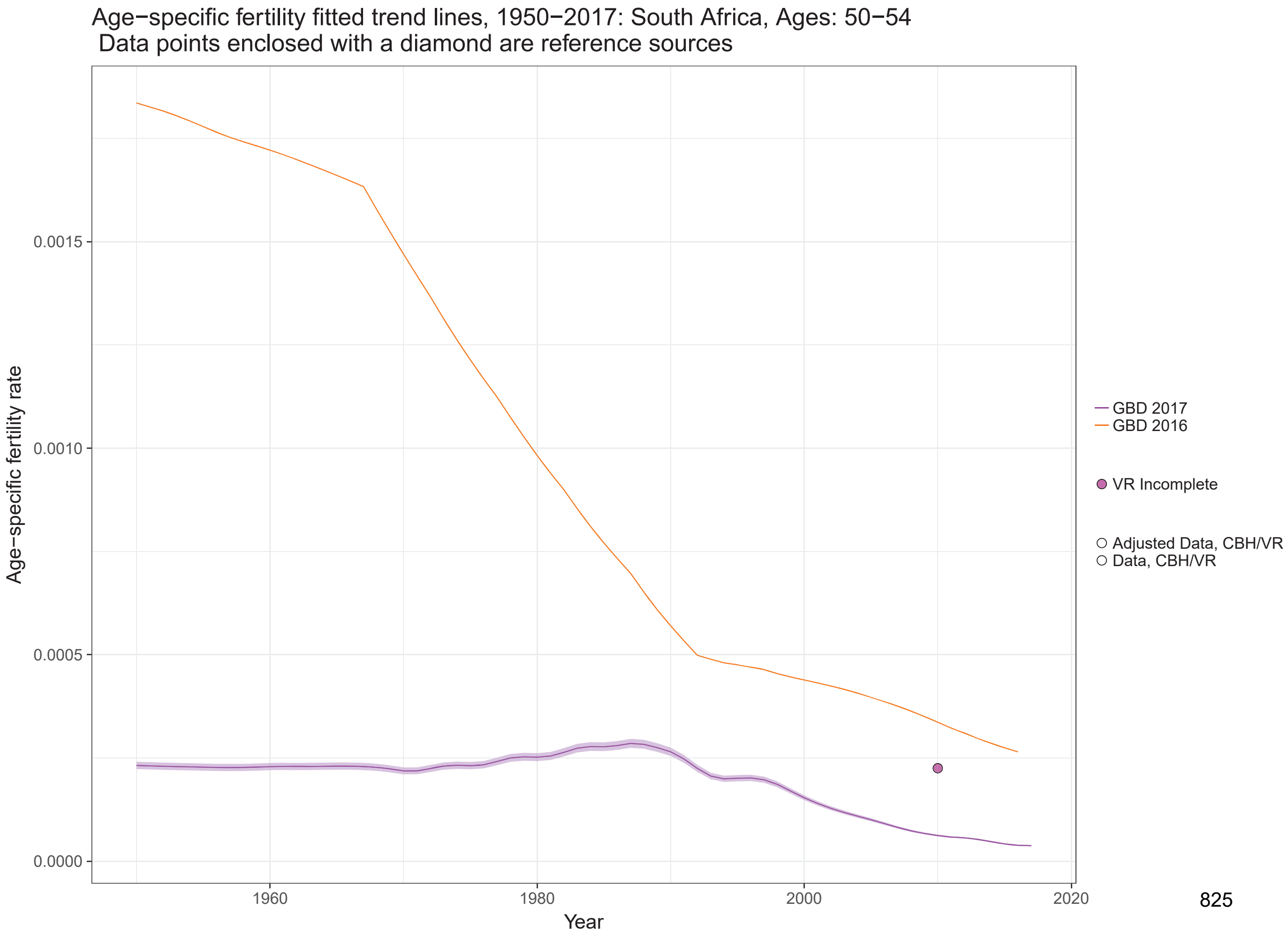

Age−specific fertility fitted trend lines, 1950−2017: South Africa, Ages: 50−54
Data points enclosed with a diamond are reference sources
Age−specific fertility rate
0.0015
0.0010
0.0005
0.0000
1960
1980
2000
2020
Year
GBD 2017
GBD 2016
VR Incomplete
Adjusted Data, CBH/VR
Data, CBH/VR

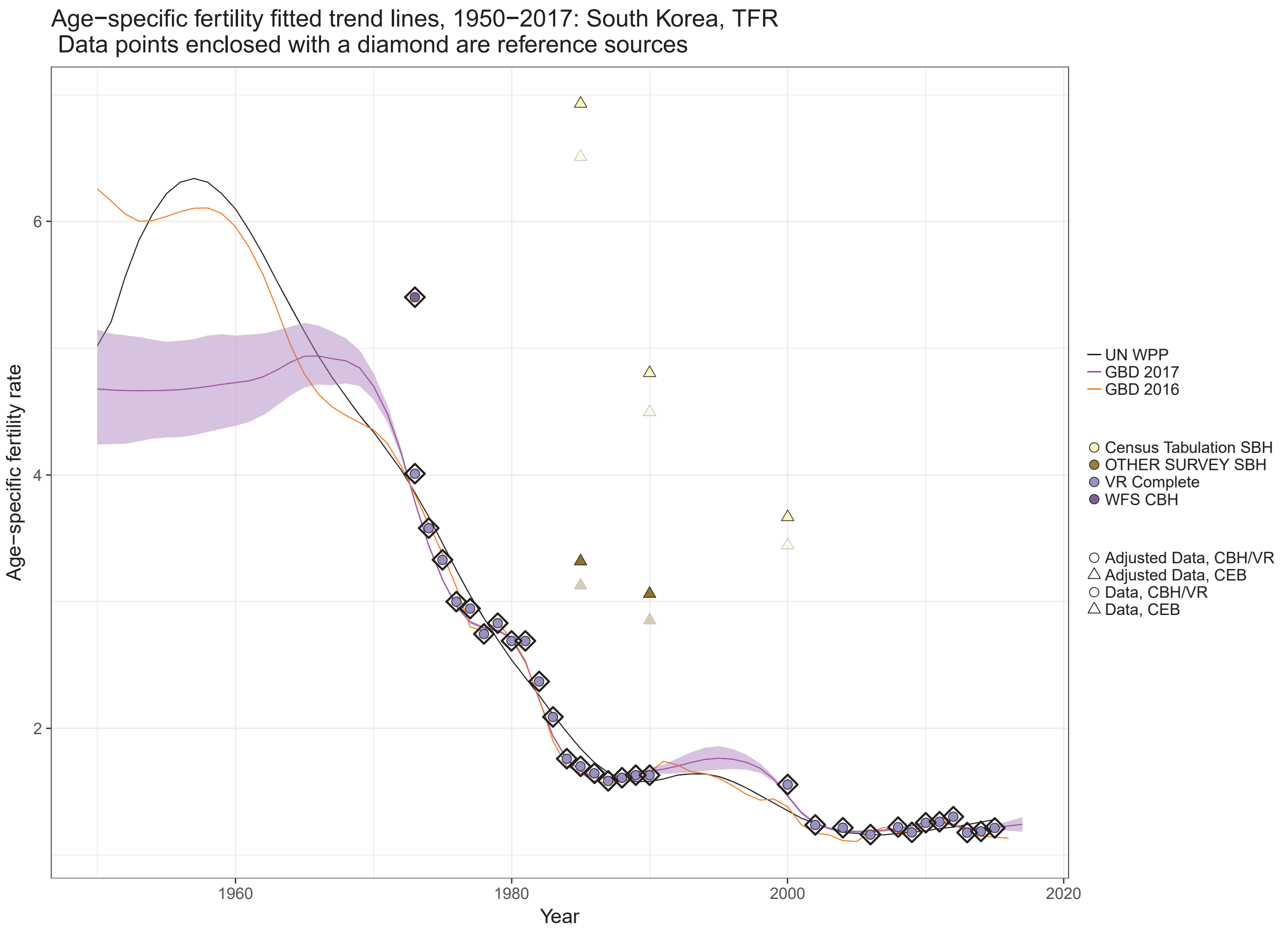
Age−specific fertility fitted trend lines, 1950−2017: South Korea, TFR
Data points enclosed with a diamond are reference sources
Age−specific fertility rate
Year
UN WPP
GBD 2017
GBD 2016
Census Tabulation SBH
OTHER SURVEY SBH
VR Complete
WFS CBH
Adjusted Data, CBH/VR
Adjusted Data, CEB
Data, CBH/VR
Data, CEB

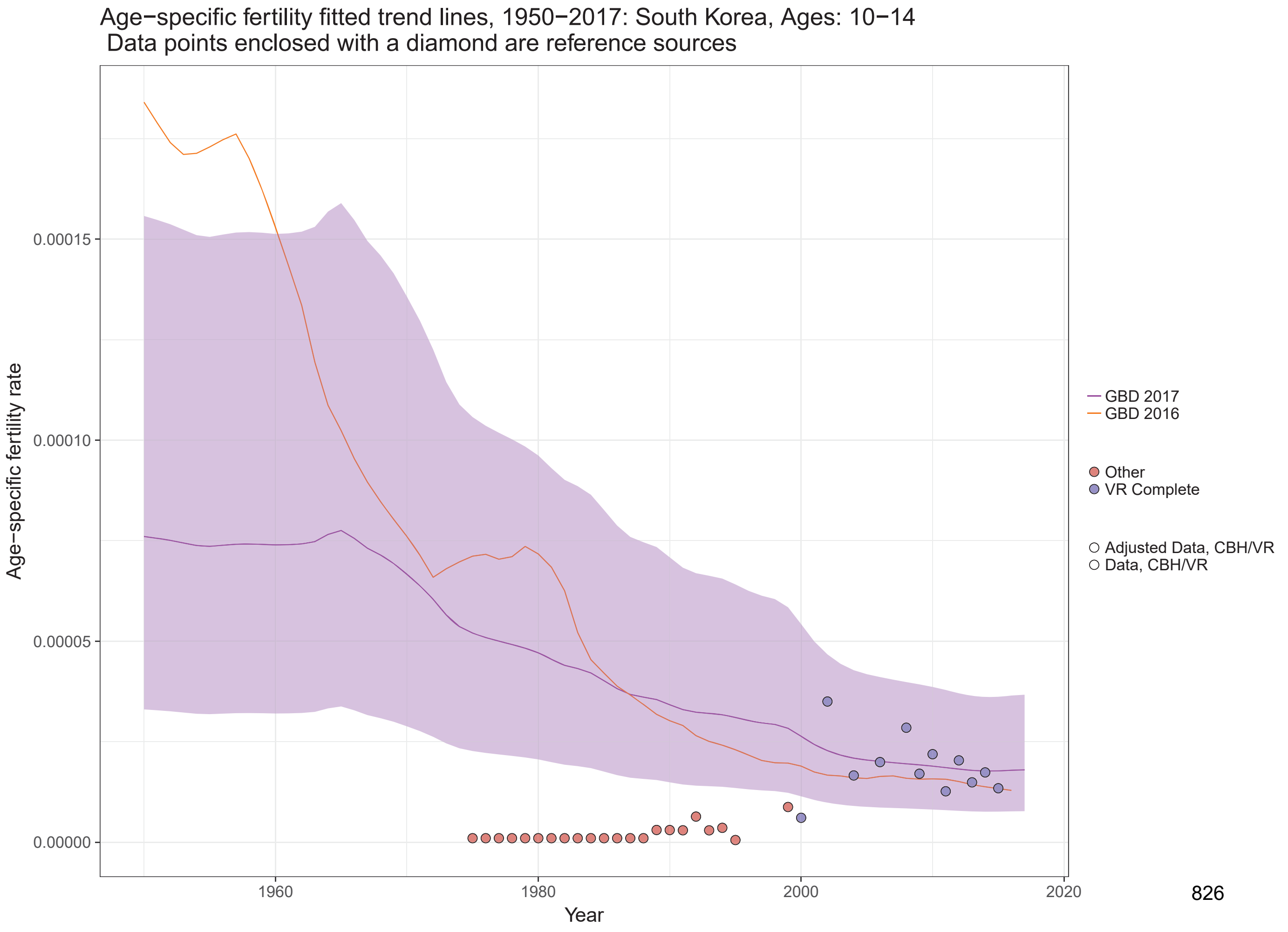
Age−specific fertility fitted trend lines, 1950−2017: South Korea, Ages: 10−14
Data points enclosed with a diamond are reference sources
Age−specific fertility rate
Year
GBD 2017
GBD 2016
Other
VR Complete
Adjusted Data, CBH/VR
Data, CBH/VR

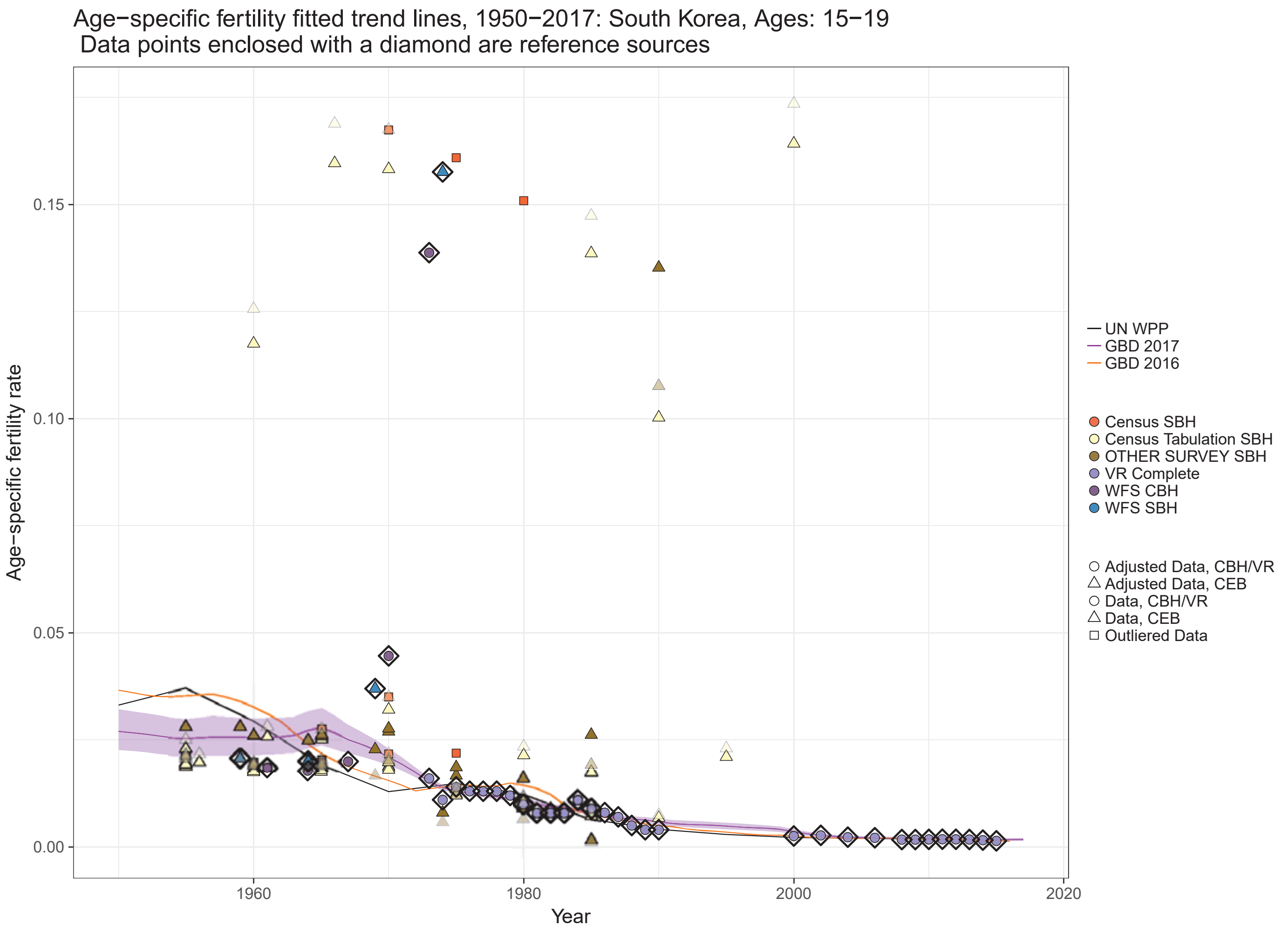
Age−specific fertility fitted trend lines, 1950−2017: South Korea, Ages: 15−19
Data points enclosed with a diamond are reference sources
Age−specific fertility rate
Year
0.00
0.05
0.10
0.15
1960
1980
2000
2020
UN WPP
GBD 2017
GBD 2016
Census SBH
Census Tabulation SBH
OTHER SURVEY SBH
VR Complete
WFS CBH
WFS SBH
Adjusted Data, CBH/VR
Adjusted Data, CEB
Data, CBH/VR
Data, CEB
Outliered Data

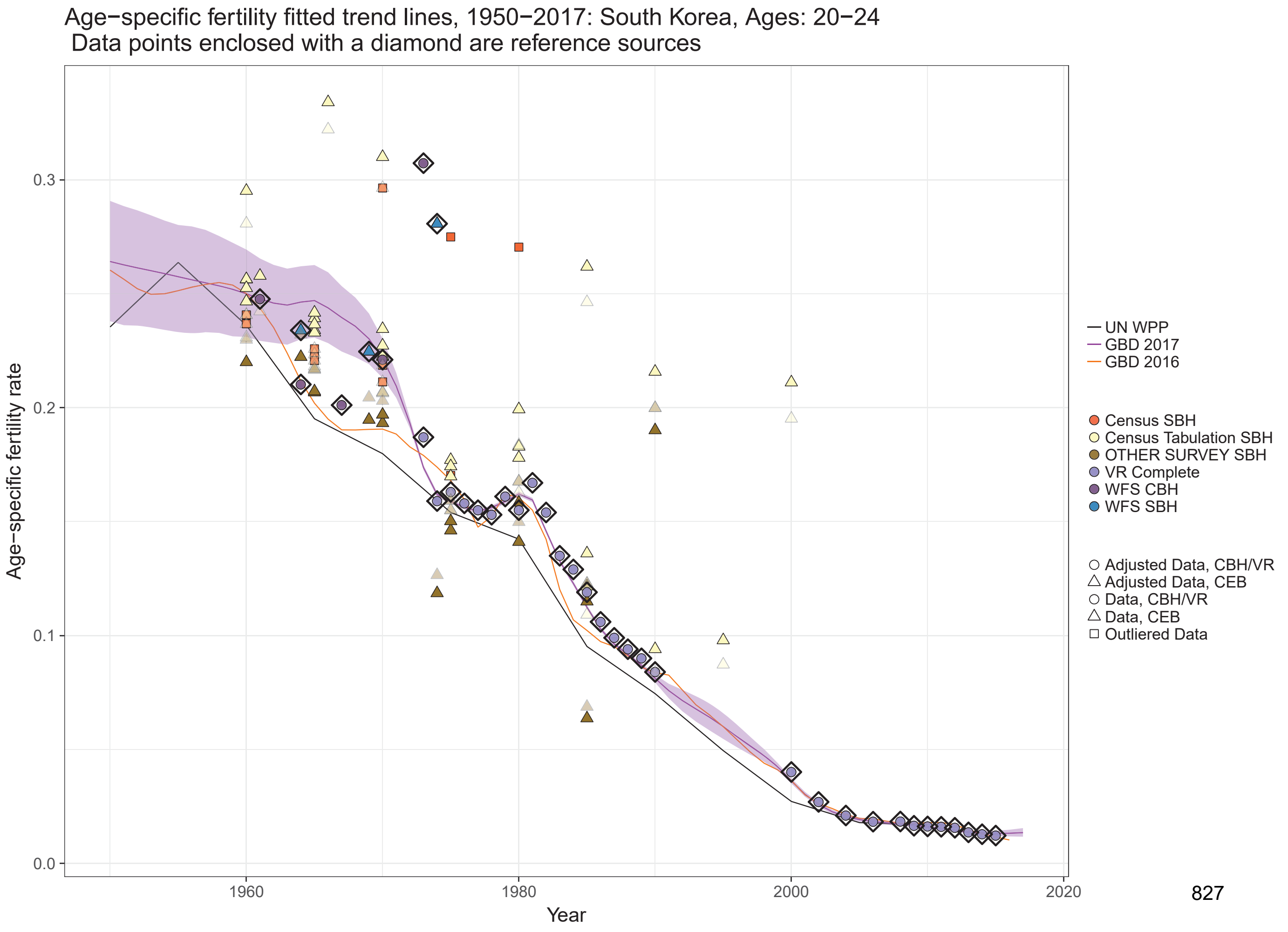
Age−specific fertility fitted trend lines, 1950−2017: South Korea, Ages: 20−24
Data points enclosed with a diamond are reference sources
Age−specific fertility rate
Year
0.0
0.1
0.2
0.3
1960
1980
2000
2020
UN WPP
GBD 2017
GBD 2016
Census SBH
Census Tabulation SBH
OTHER SURVEY SBH
VR Complete
WFS CBH
WFS SBH
Adjusted Data, CBH/VR
Adjusted Data, CEB
Data, CBH/VR
Data, CEB
Outliered Data

Age−specific fertility fitted trend lines, 1950−2017: South Korea, Ages: 25−29
Data points enclosed with a diamond are reference sources

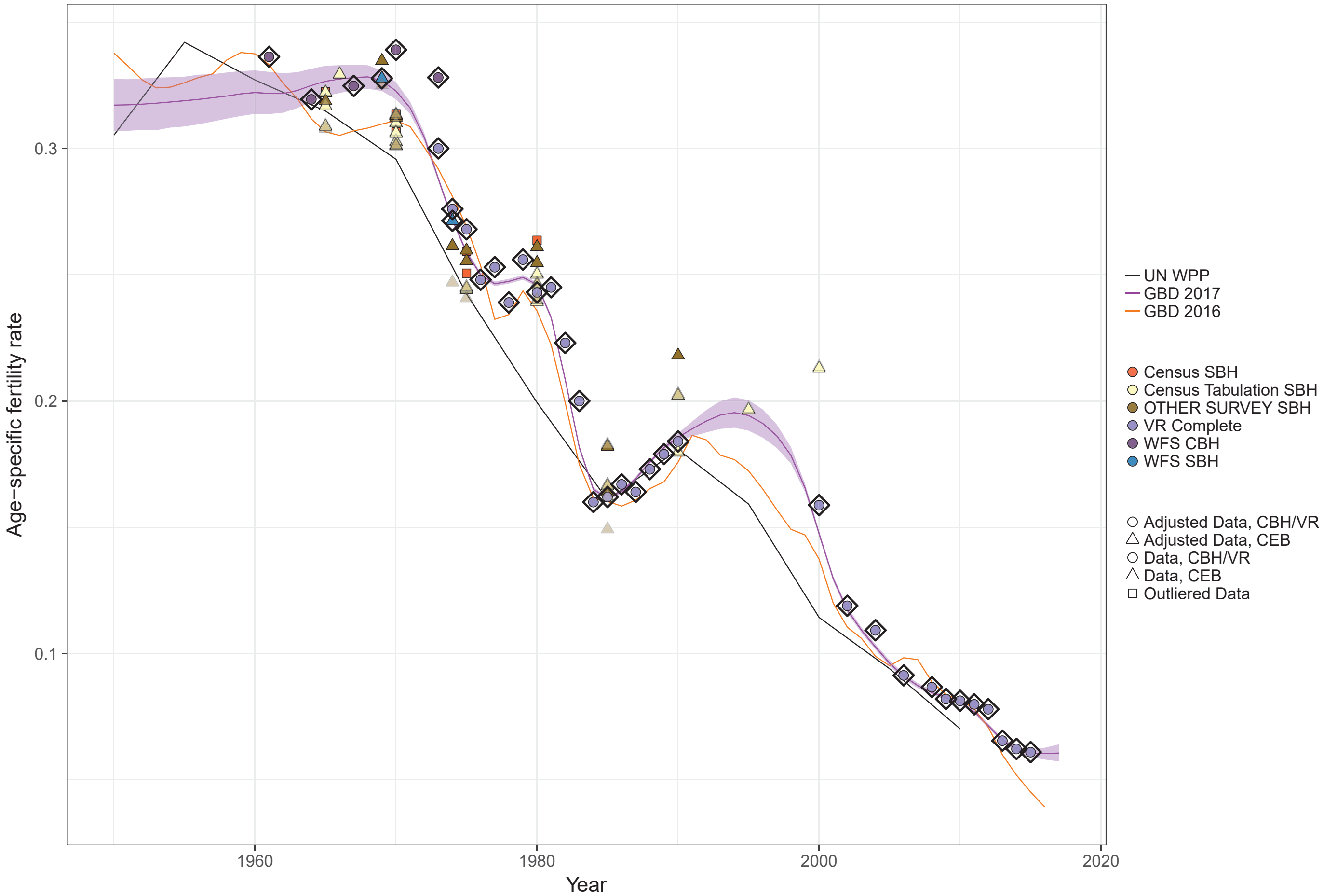

Age−specific fertility fitted trend lines, 1950−2017: South Korea, Ages: 30−34
Data points enclosed with a diamond are reference sources

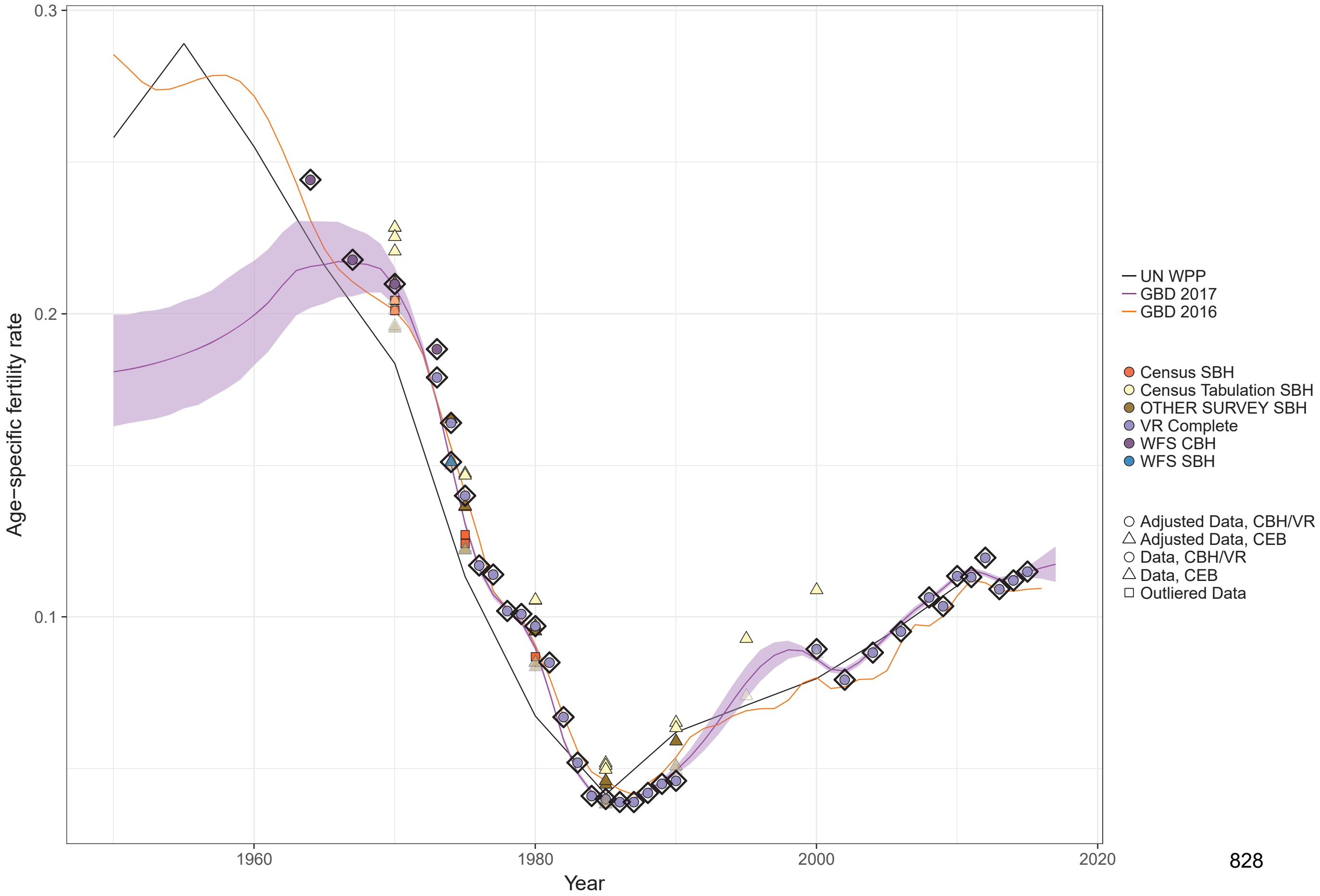

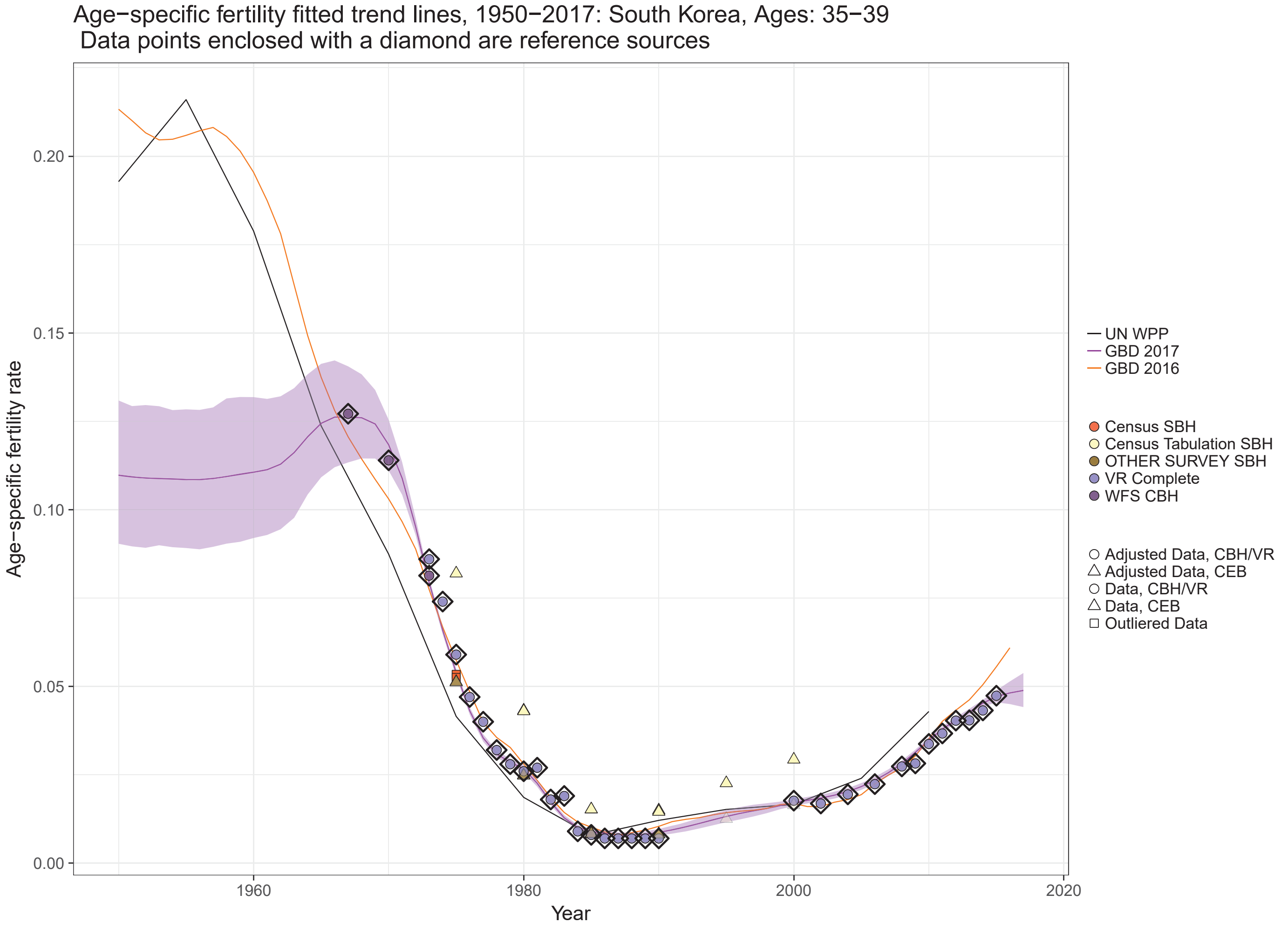

Age−specific fertility fitted trend lines, 1950−2017: South Korea, Ages: 35−39
Data points enclosed with a diamond are reference sources
Age−specific fertility rate
0.00
0.05
0.10
0.15
0.20
1960
1980
2000
2020
Year
UN WPP
GBD 2017
GBD 2016
Census SBH
Census Tabulation SBH
OTHER SURVEY SBH
VR Complete
WFS CBH
Adjusted Data, CBH/VR
Adjusted Data, CEB
Data, CBH/VR
Data, CEB
Outliered Data

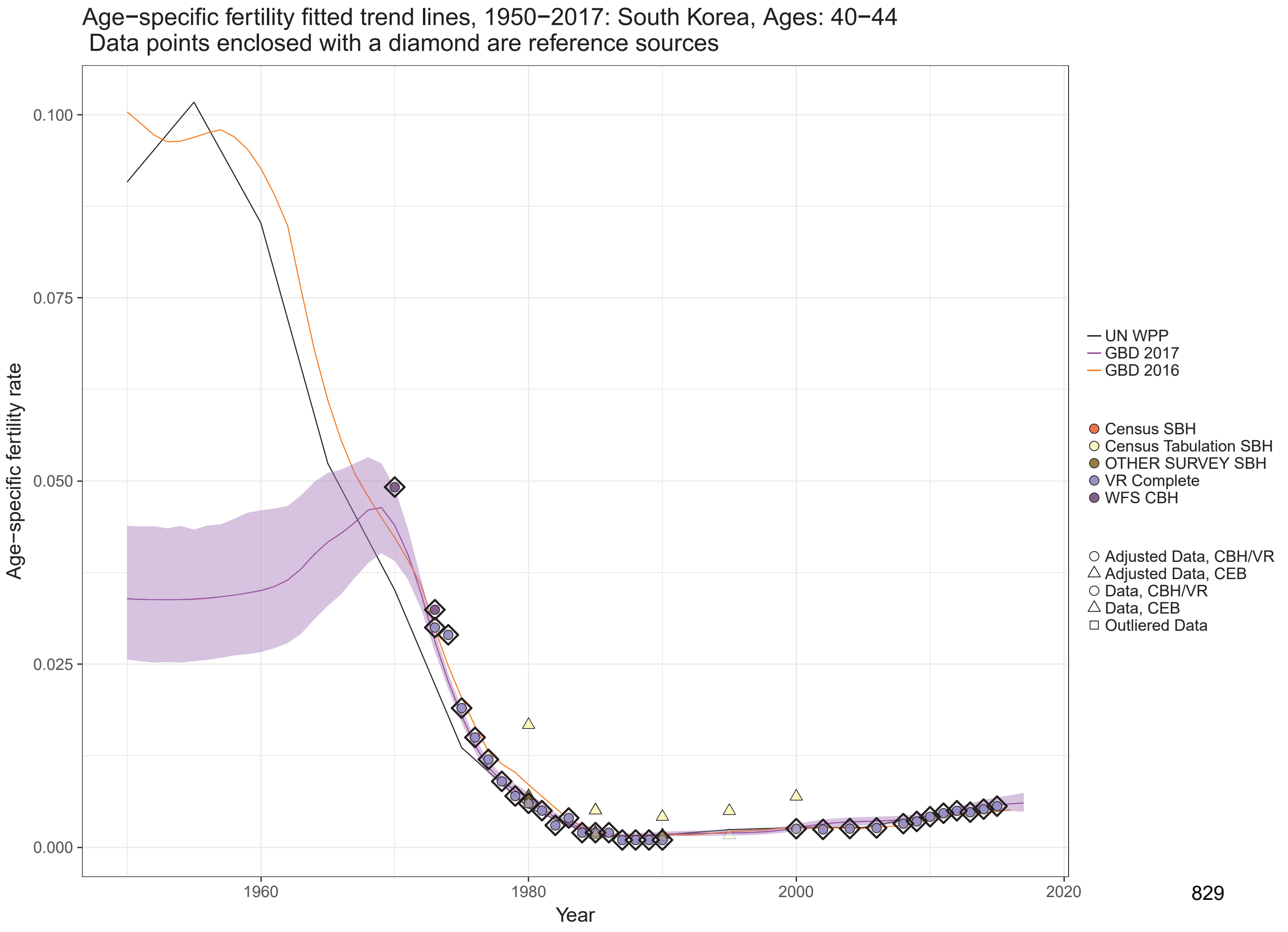

Age−specific fertility fitted trend lines, 1950−2017: South Korea, Ages: 40−44
Data points enclosed with a diamond are reference sources
Age−specific fertility rate
0.000
0.025
0.050
0.075
0.100
1960
1980
2000
2020
Year
UN WPP
GBD 2017
GBD 2016
Census SBH
Census Tabulation SBH
OTHER SURVEY SBH
VR Complete
WFS CBH
Adjusted Data, CBH/VR
Adjusted Data, CEB
Data, CBH/VR
Data, CEB
Outliered Data

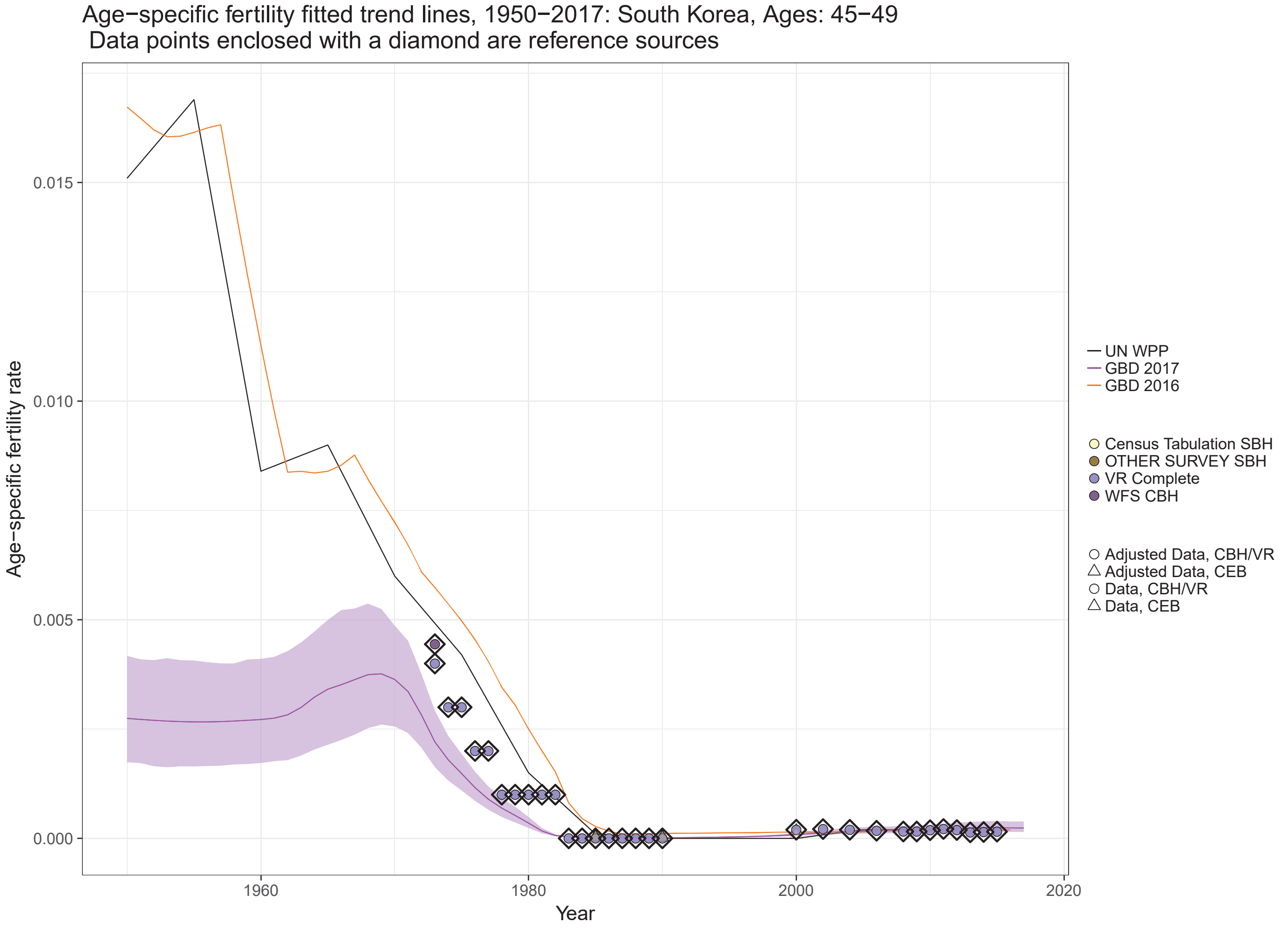
Age−specific fertility fitted trend lines, 1950−2017: South Korea, Ages: 45−49
Data points enclosed with a diamond are reference sources
Age−specific fertility rate
0.015
0.010
0.005
0.000
1960
1980
2000
2020
Year
UN WPP
GBD 2017
GBD 2016
Census Tabulation SBH
OTHER SURVEY SBH
VR Complete
WFS CBH
Adjusted Data, CBH/VR
Adjusted Data, CEB
Data, CBH/VR
Data, CEB

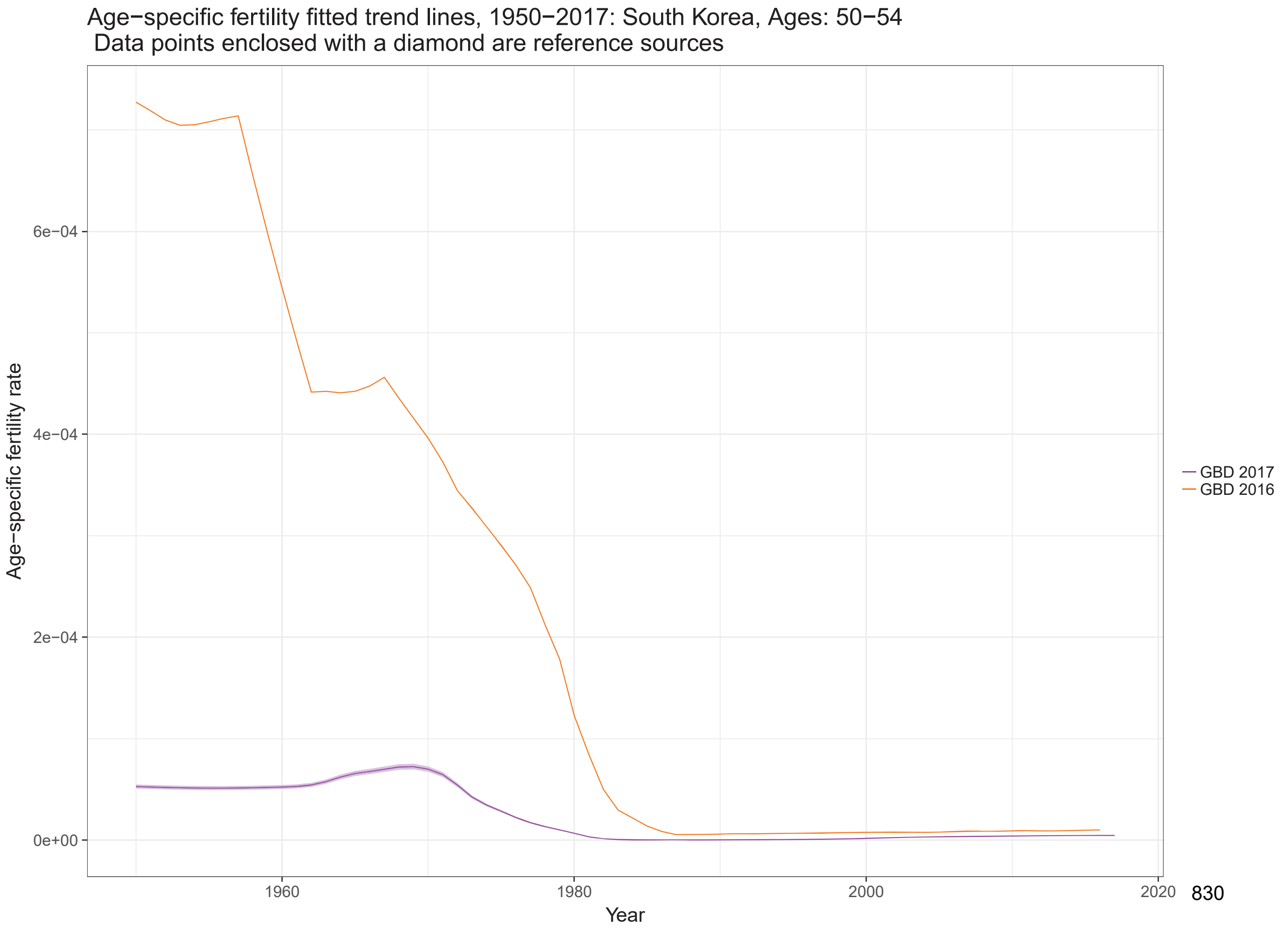
Age−specific fertility fitted trend lines, 1950−2017: South Korea, Ages: 50−54
Data points enclosed with a diamond are reference sources
Age−specific fertility rate
6e−04
4e−04
2e−04
0e+00
1960
1980
2000
2020
Year
GBD 2017
GBD 2016

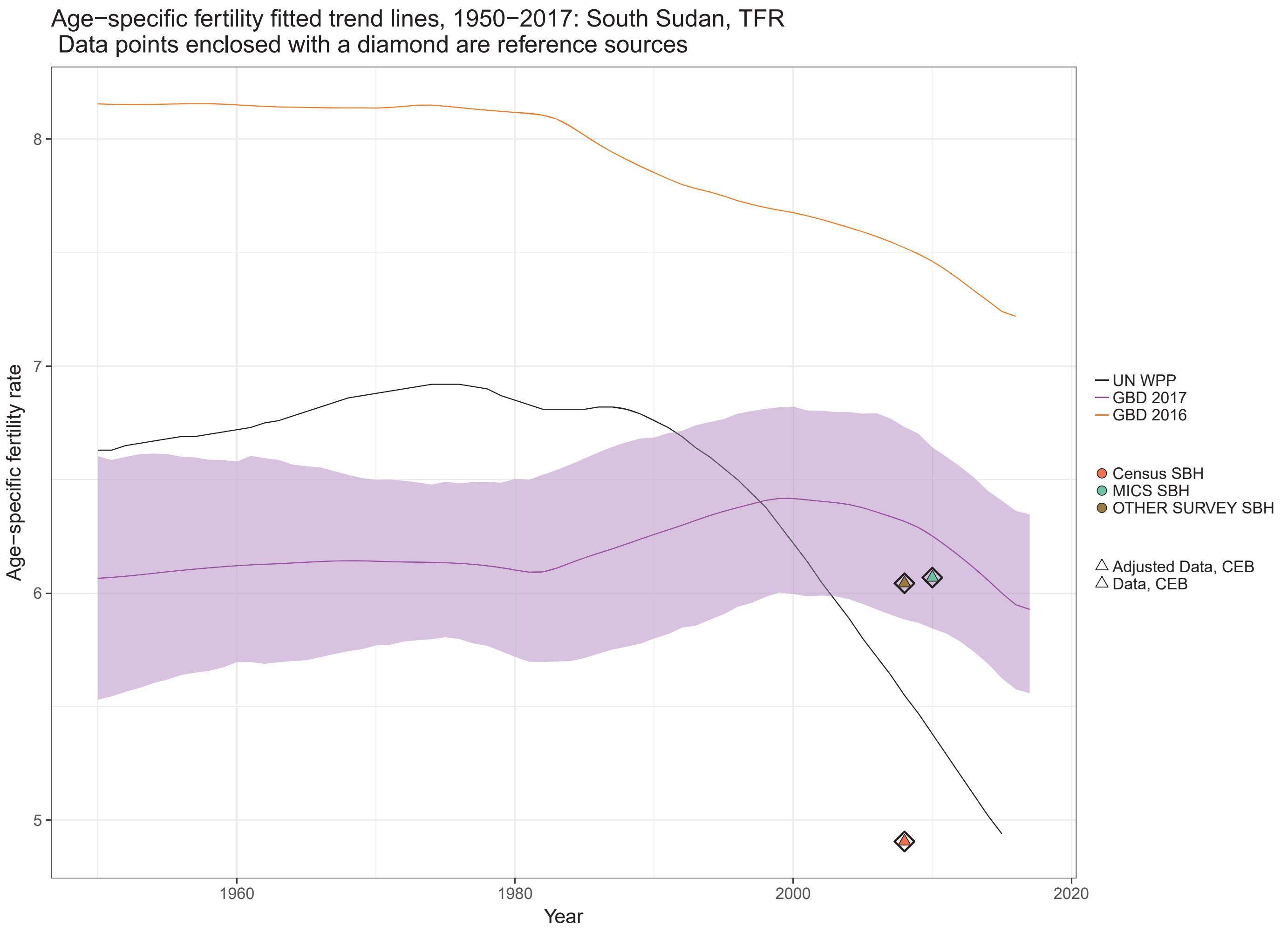
Age−specific fertility fitted trend lines, 1950−2017: South Sudan, TFR
Data points enclosed with a diamond are reference sources
Age-specific fertility rate
8
7
6
5
1960
1980
2000
2020
Year
UN WPP
GBD 2017
GBD 2016
Census SBH
MICS SBH
OTHER SURVEY SBH
Adjusted Data, CEB
Data, CEB

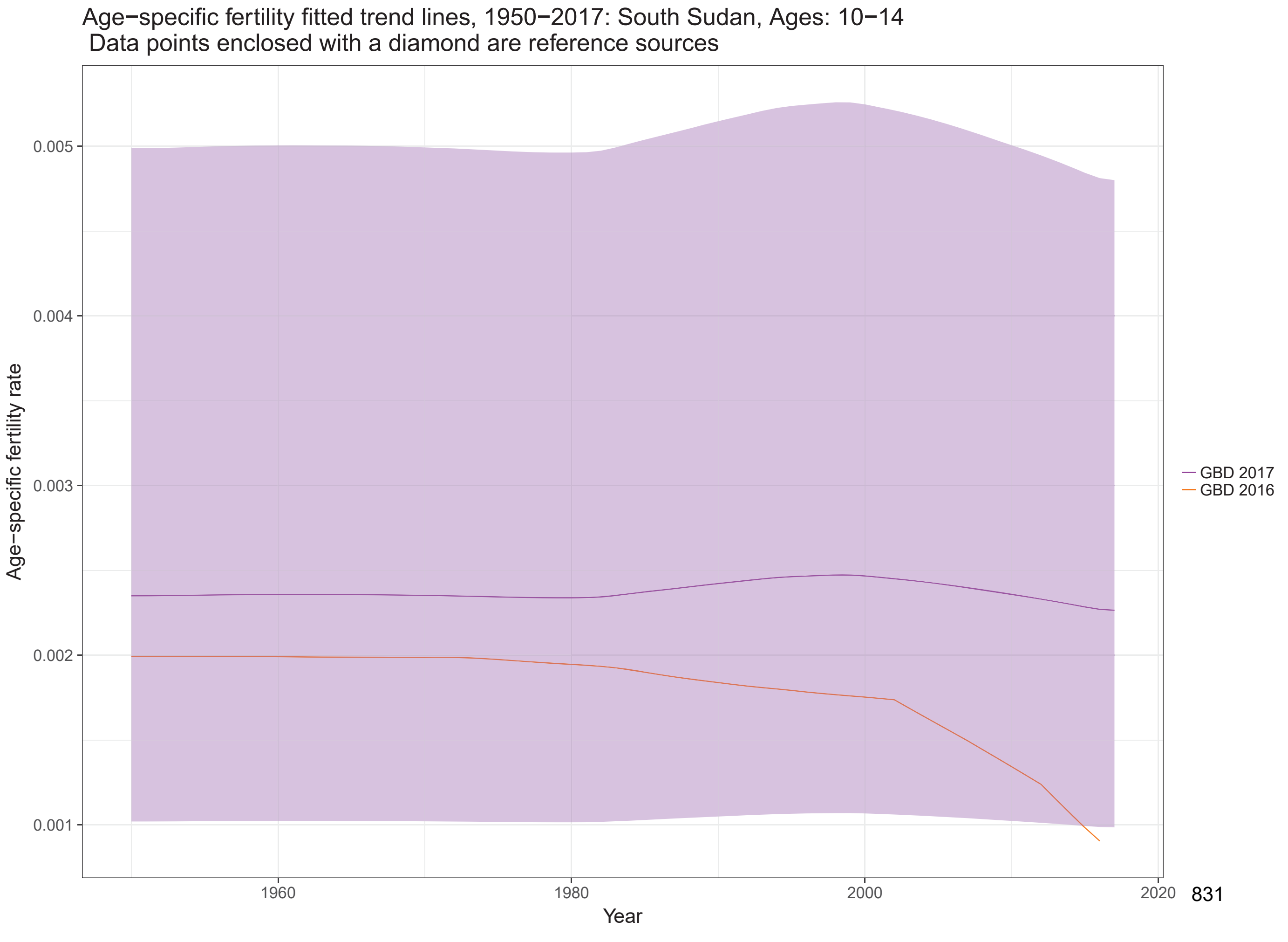
Age−specific fertility fitted trend lines, 1950−2017: South Sudan, Ages: 10−14
Data points enclosed with a diamond are reference sources
Age-specific fertility rate
0.005
0.004
0.003
0.002
0.001
1960
1980
2000
2020
Year
GBD 2017
GBD 2016

Age−specific fertility fitted trend lines, 1950−2017: South Sudan, Ages: 15−19
Data points enclosed with a diamond are reference sources

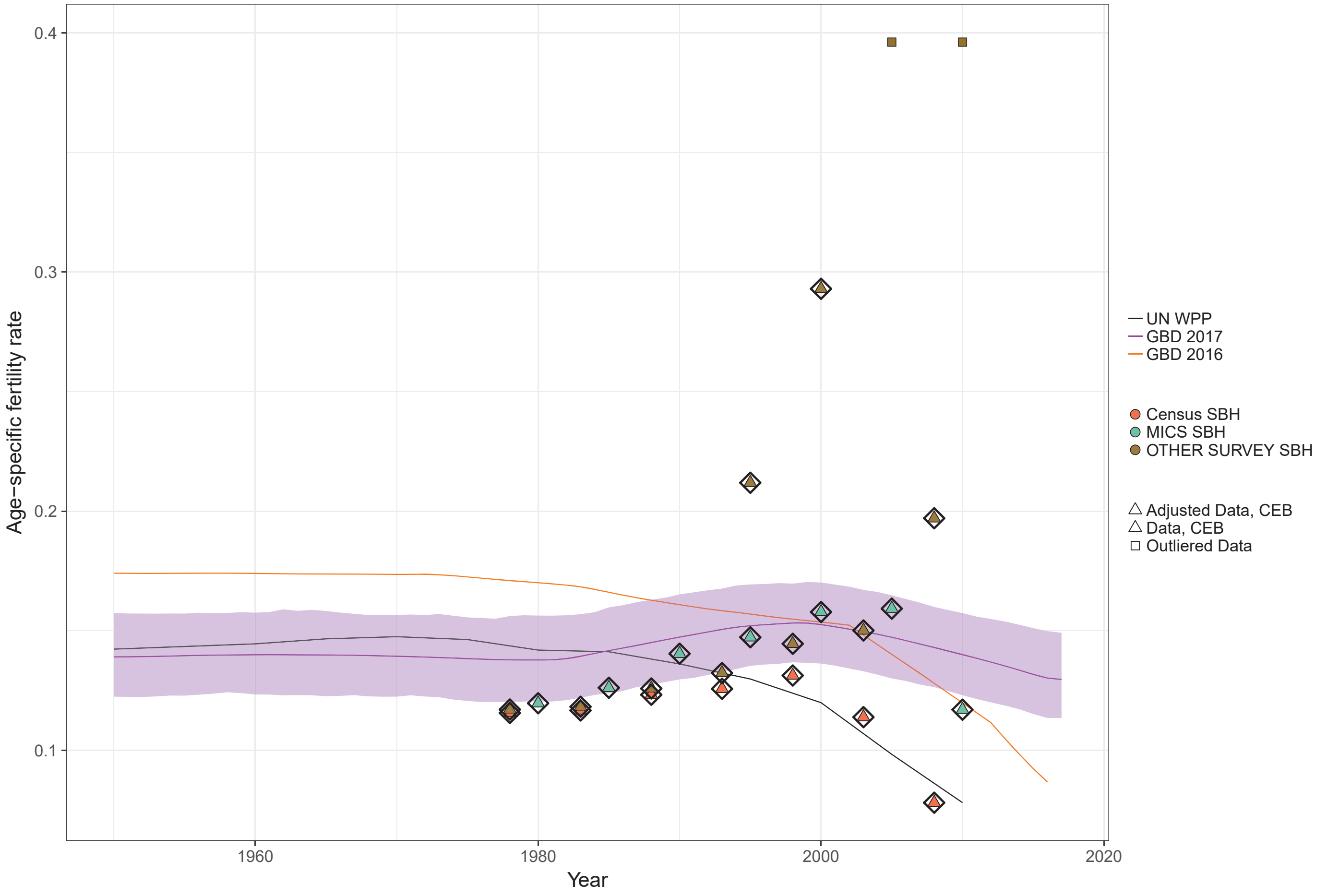

Age−specific fertility fitted trend lines, 1950−2017: South Sudan, Ages: 20−24
Data points enclosed with a diamond are reference sources

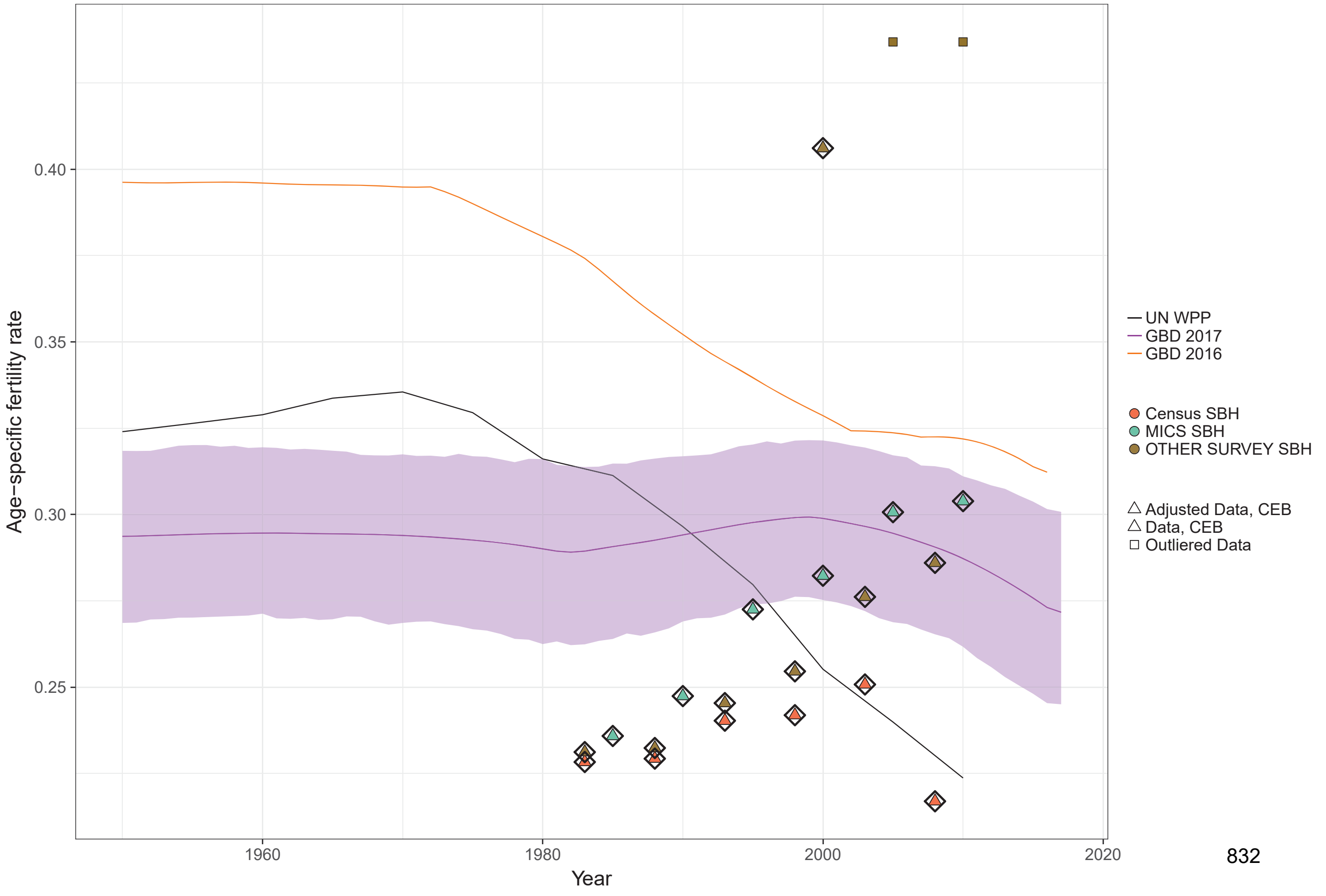

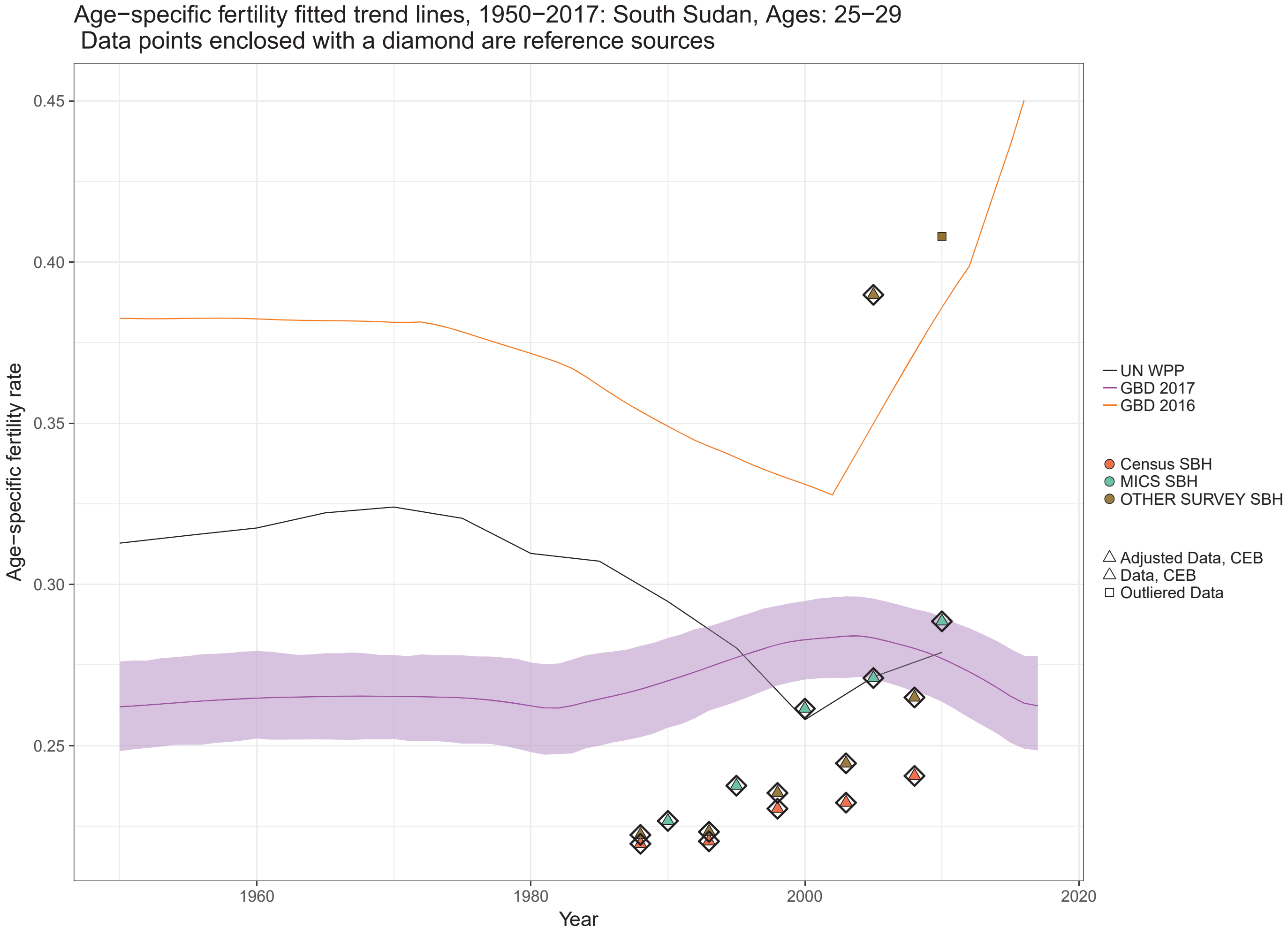
Age−specific fertility fitted trend lines, 1950−2017: South Sudan, Ages: 25−29
Data points enclosed with a diamond are reference sources
Age−specific fertility rate
Year
0.45
0.40
0.35
0.30
0.25
1960
1980
2000
2020
UN WPP
GBD 2017
GBD 2016
Census SBH
MICS SBH
OTHER SURVEY SBH
Adjusted Data, CEB
Data, CEB
Outliered Data

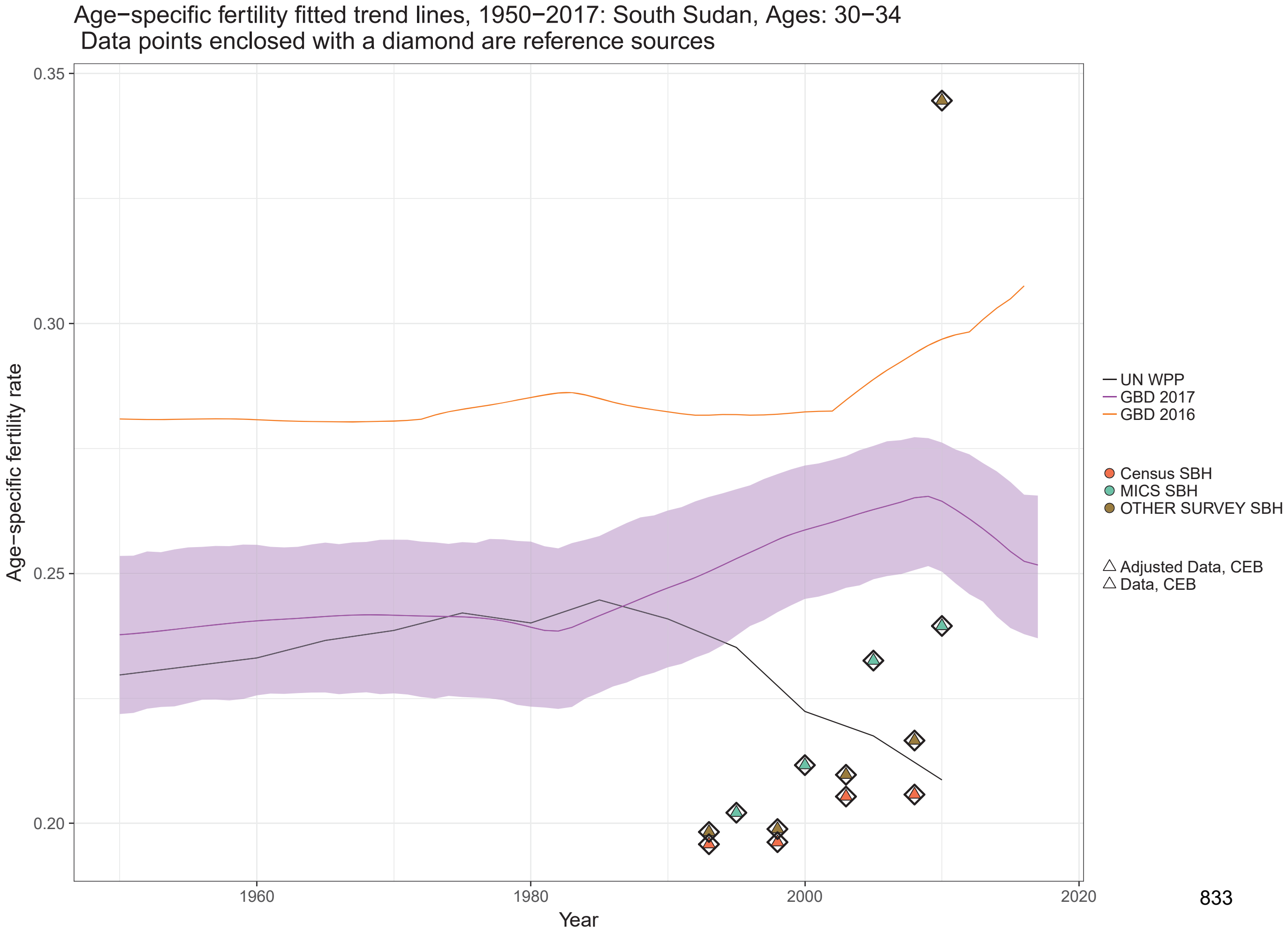
Age−specific fertility fitted trend lines, 1950−2017: South Sudan, Ages: 30−34
Data points enclosed with a diamond are reference sources
Age−specific fertility rate
Year
0.35
0.30
0.25
0.20
1960
1980
2000
2020
UN WPP
GBD 2017
GBD 2016
Census SBH
MICS SBH
OTHER SURVEY SBH
Adjusted Data, CEB
Data, CEB

Age−specific fertility fitted trend lines, 1950−2017: South Sudan, Ages: 35−39
Data points enclosed with a diamond are reference sources

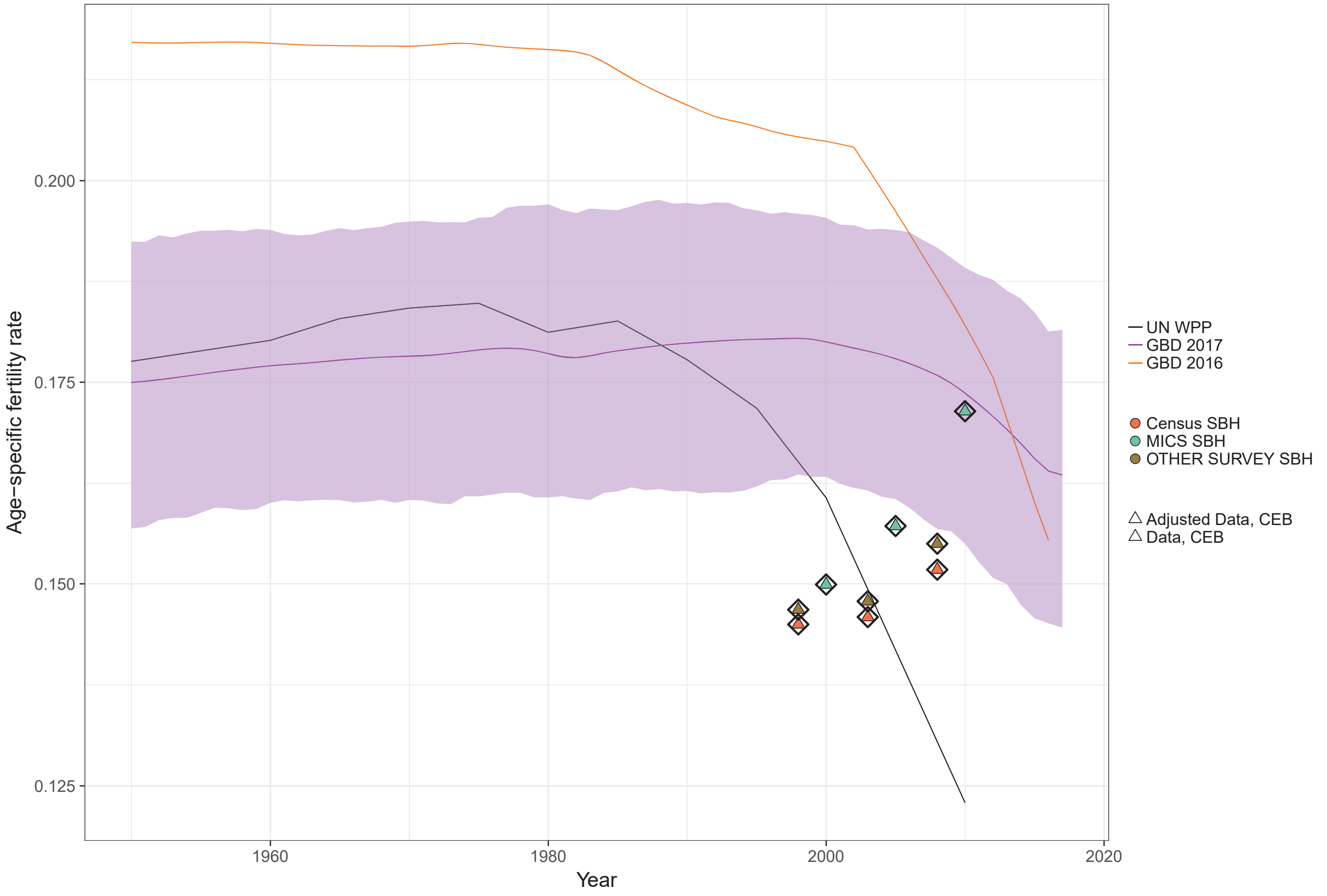

Age−specific fertility fitted trend lines, 1950−2017: South Sudan, Ages: 40−44
Data points enclosed with a diamond are reference sources

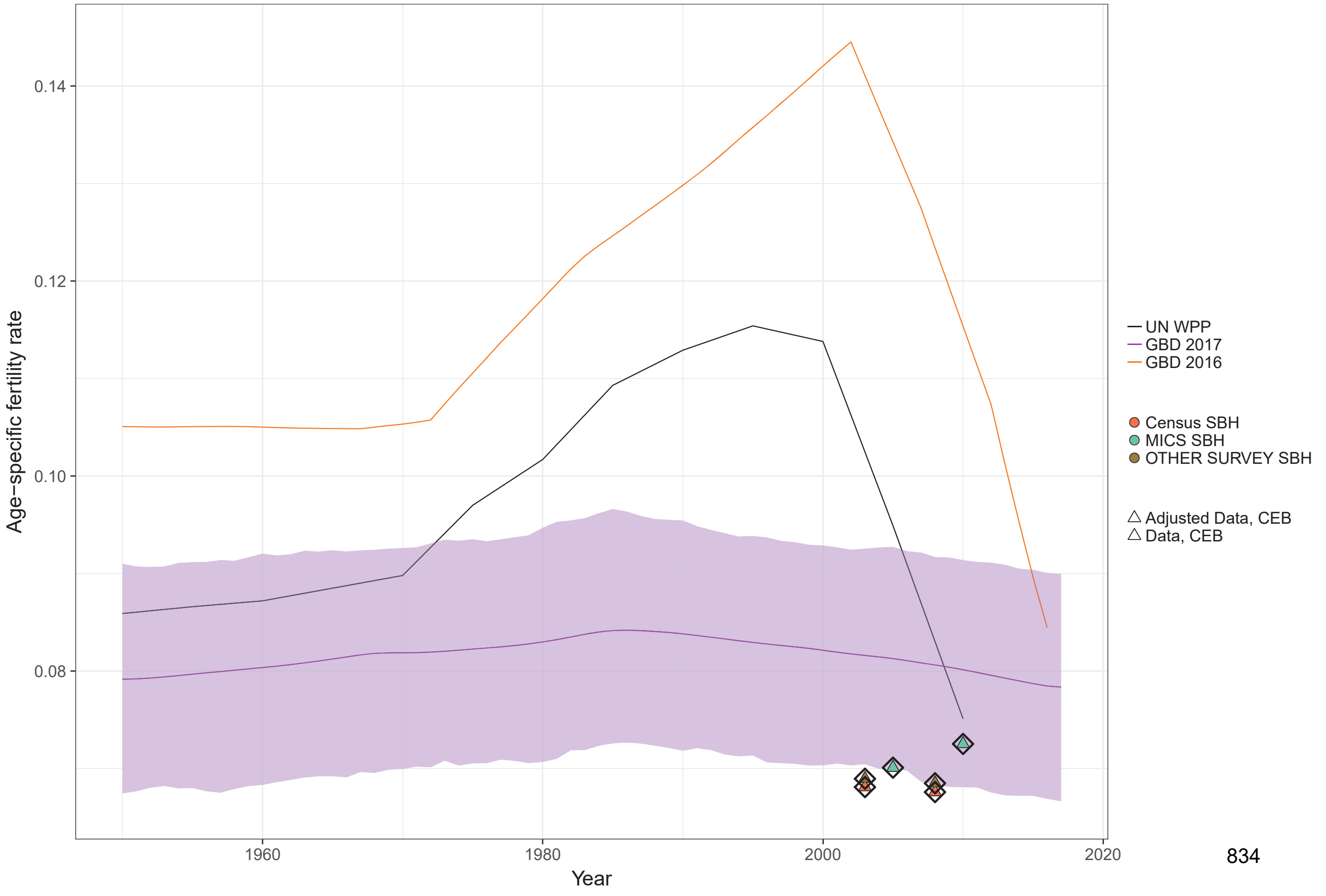

Age−specific fertility fitted trend lines, 1950−2017: South Sudan, Ages: 45−49
Data points enclosed with a diamond are reference sources

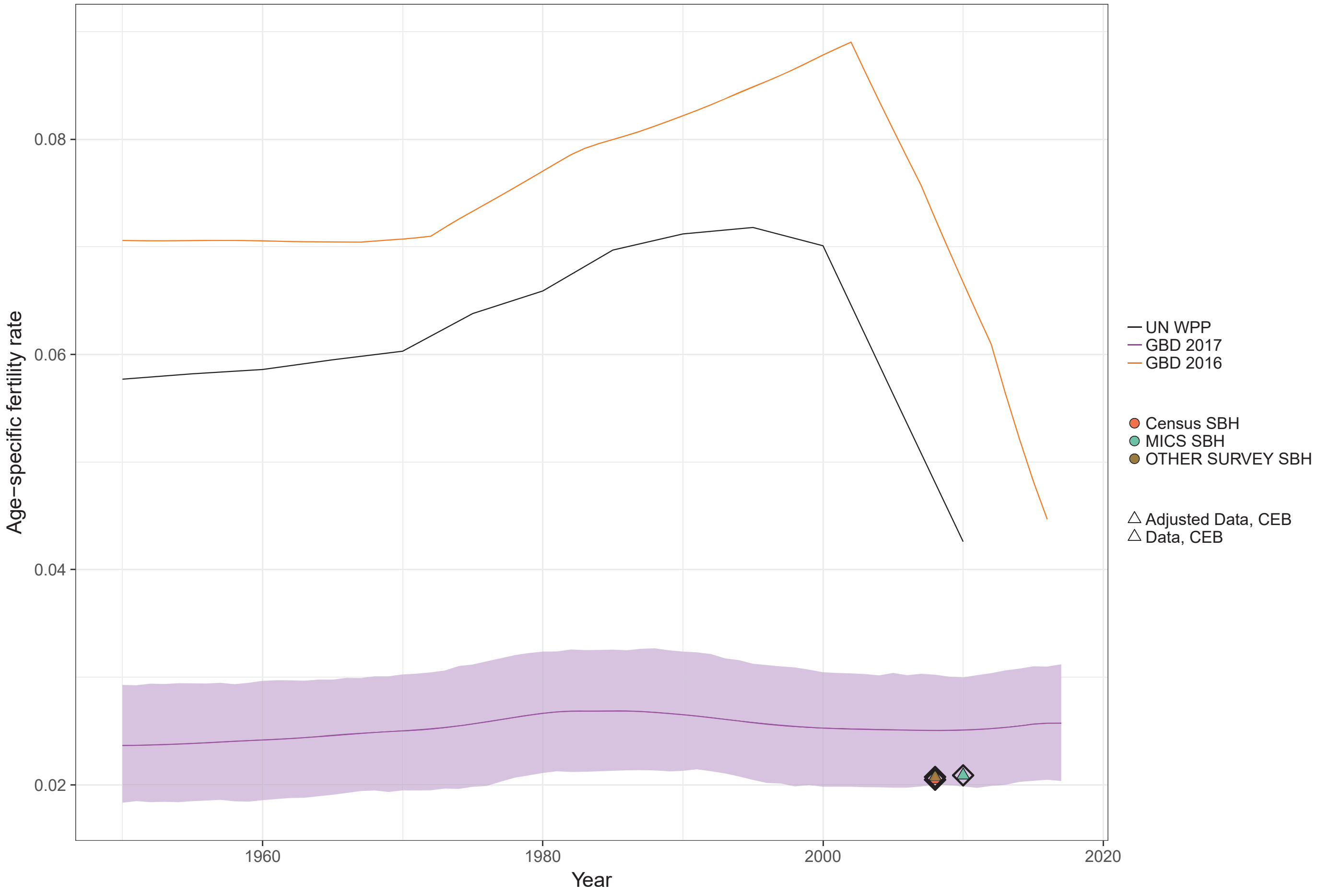

Age−specific fertility fitted trend lines, 1950−2017: South Sudan, Ages: 50−54
Data points enclosed with a diamond are reference sources

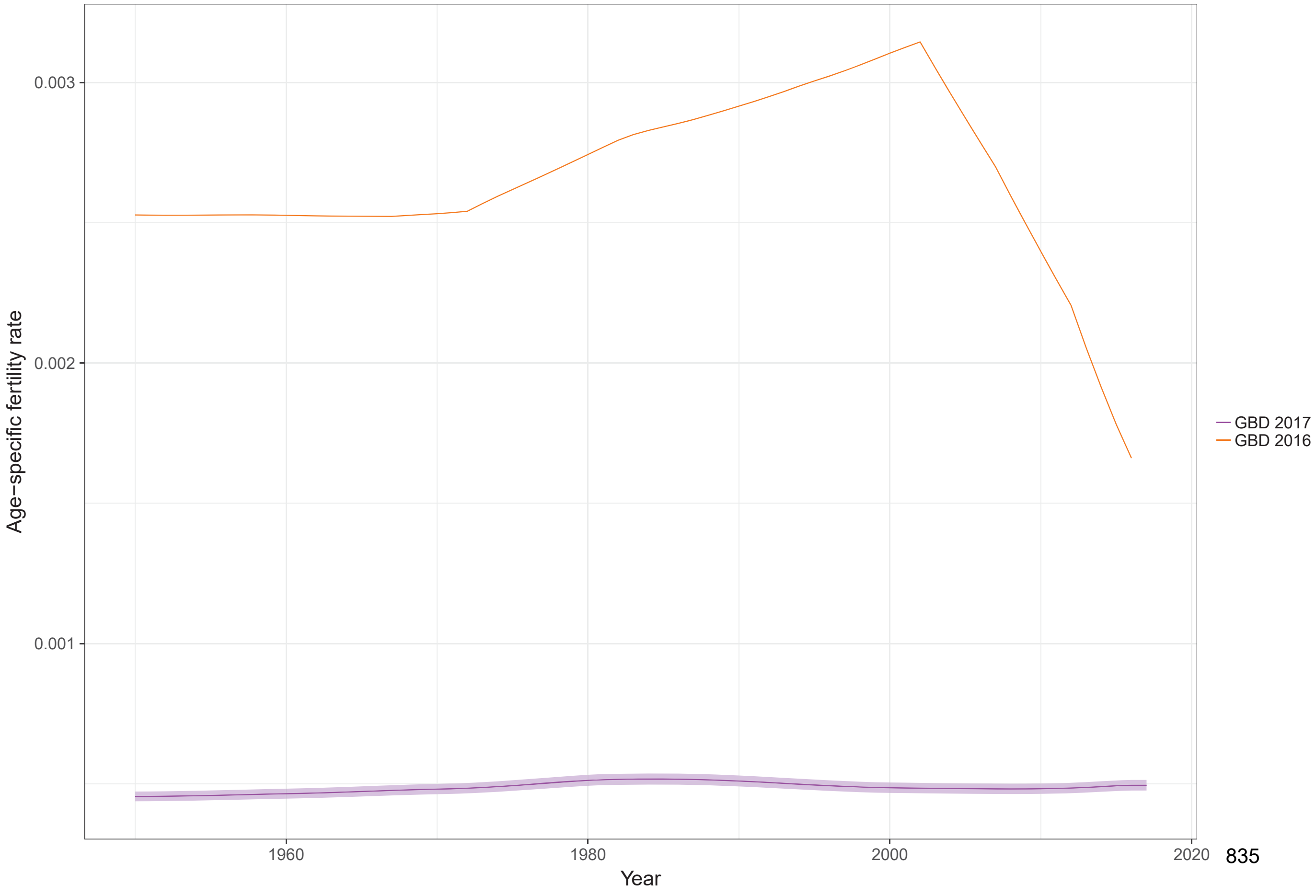

Age−specific fertility fitted trend lines, 1950−2017: Spain, TFR
Data points enclosed with a diamond are reference sources

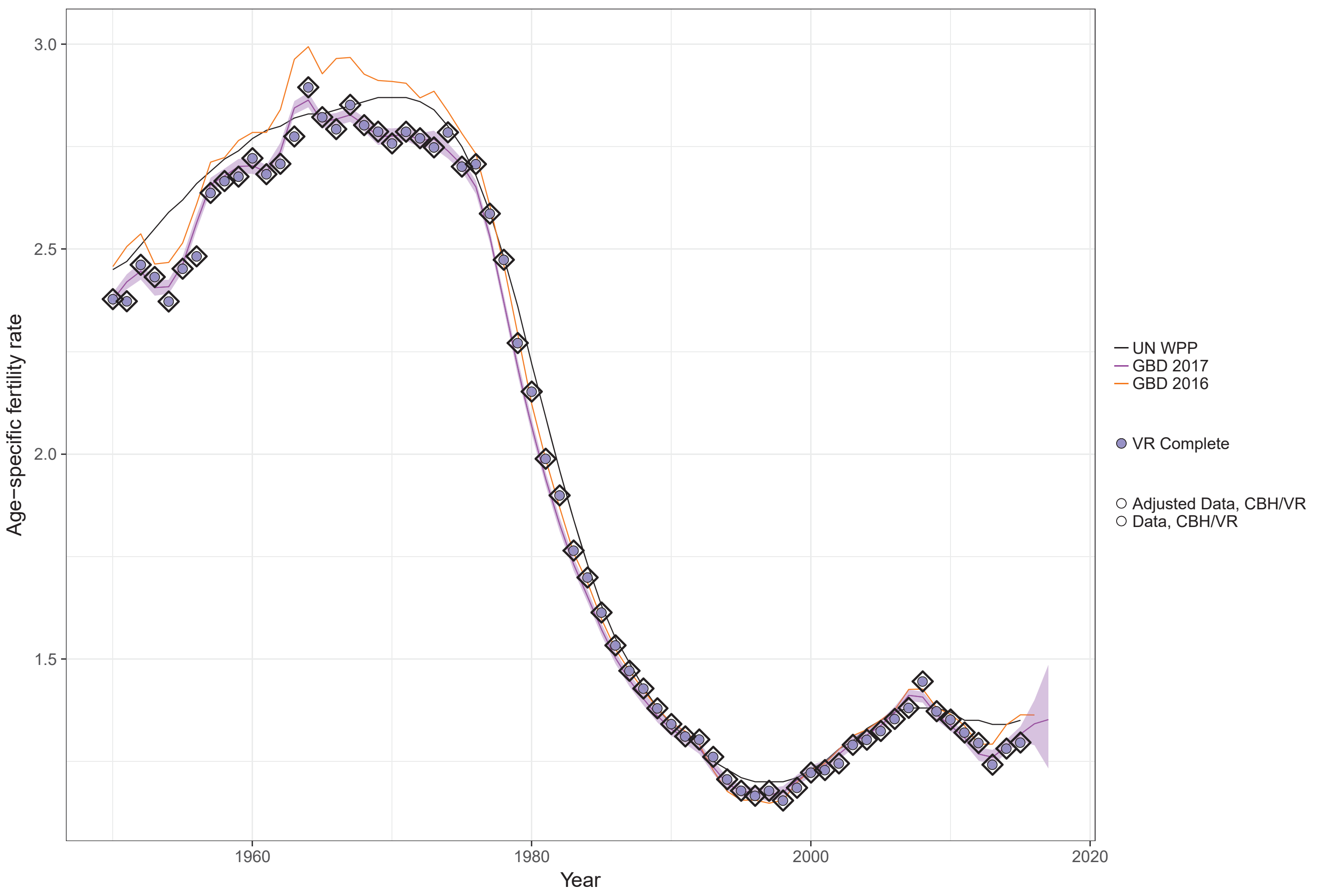

Age−specific fertility fitted trend lines, 1950−2017: Spain, Ages: 10−14
Data points enclosed with a diamond are reference sources

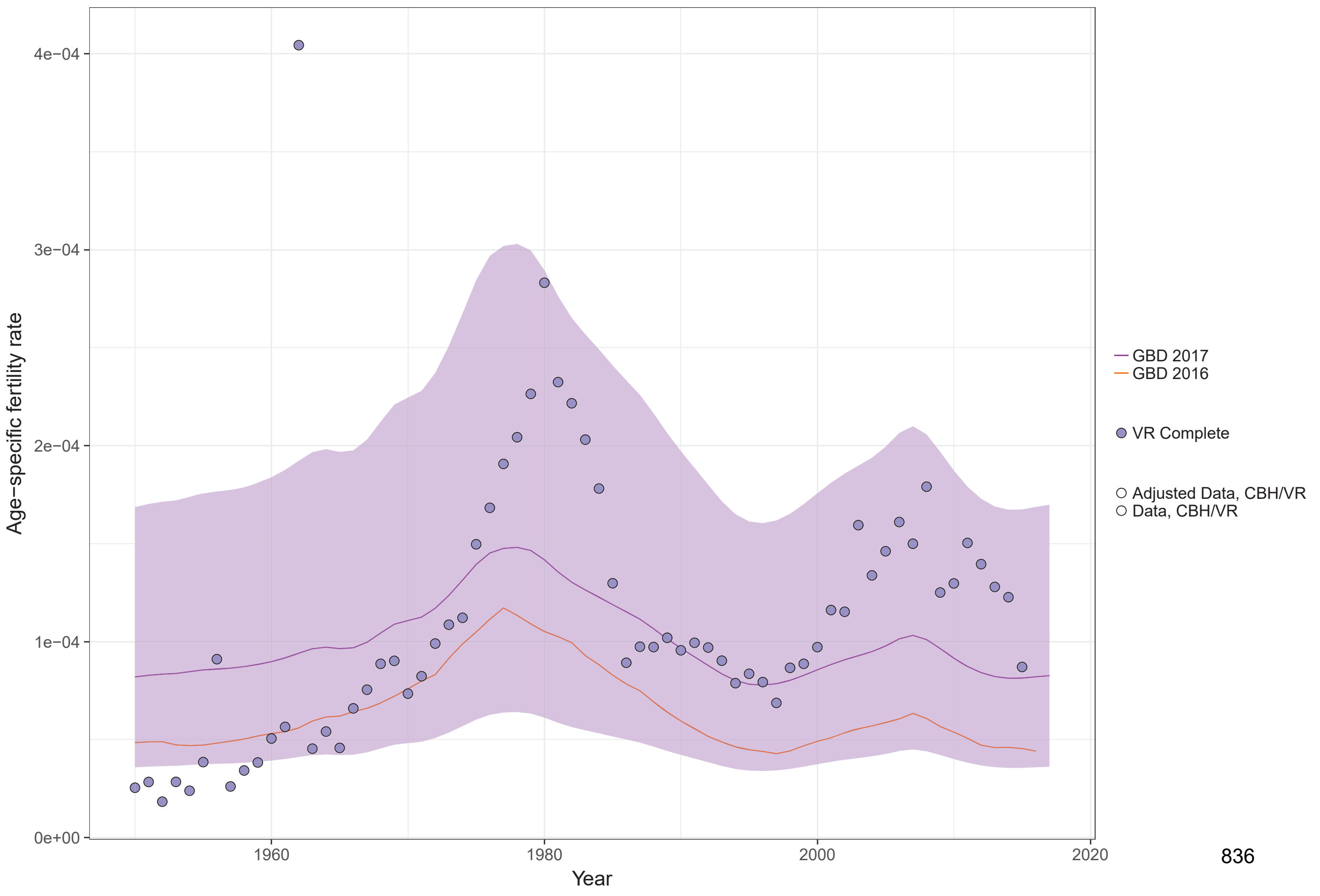

Age−specific fertility fitted trend lines, 1950−2017: Spain, Ages: 15−19
Data points enclosed with a diamond are reference sources

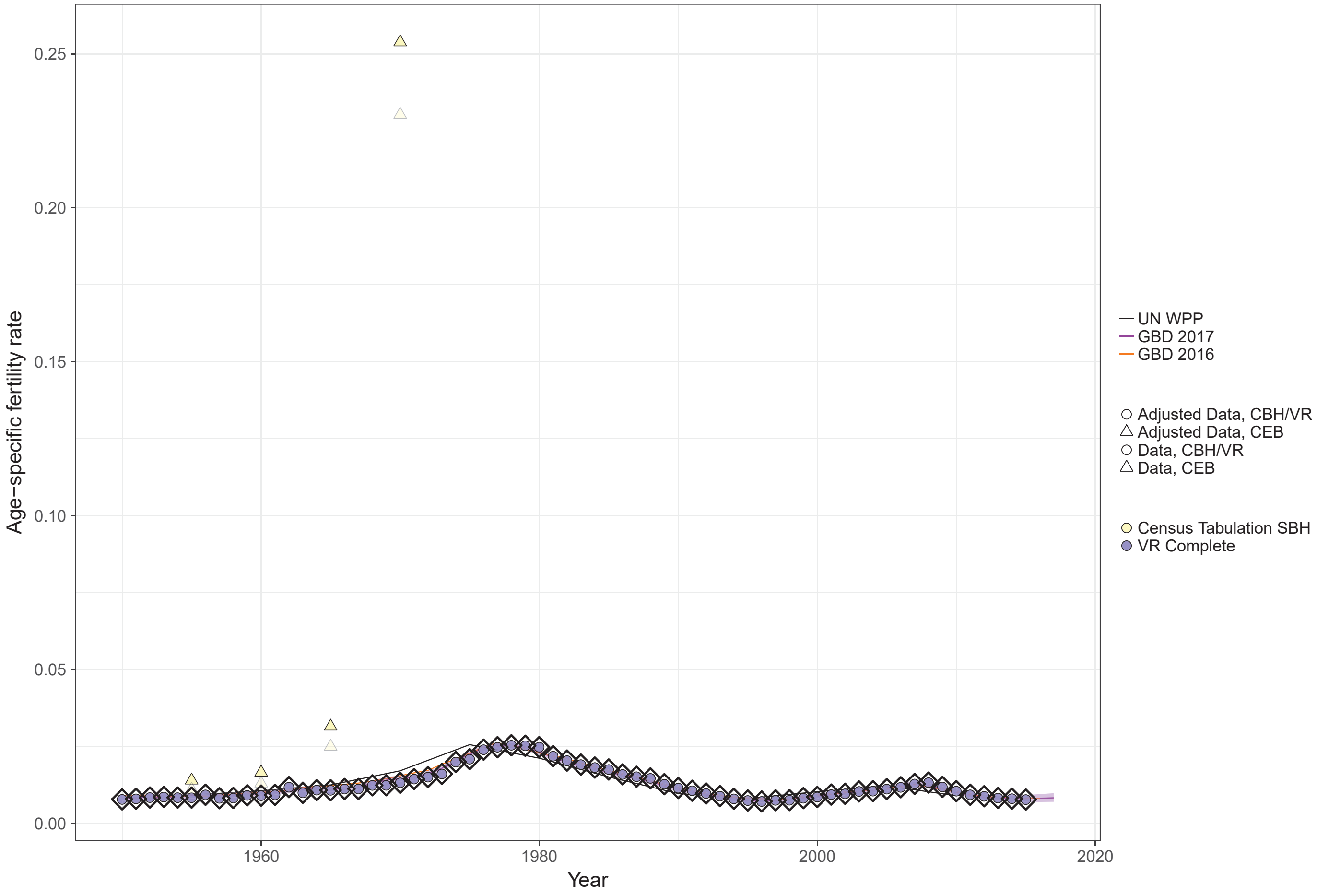

Age−specific fertility fitted trend lines, 1950−2017: Spain, Ages: 20−24
Data points enclosed with a diamond are reference sources

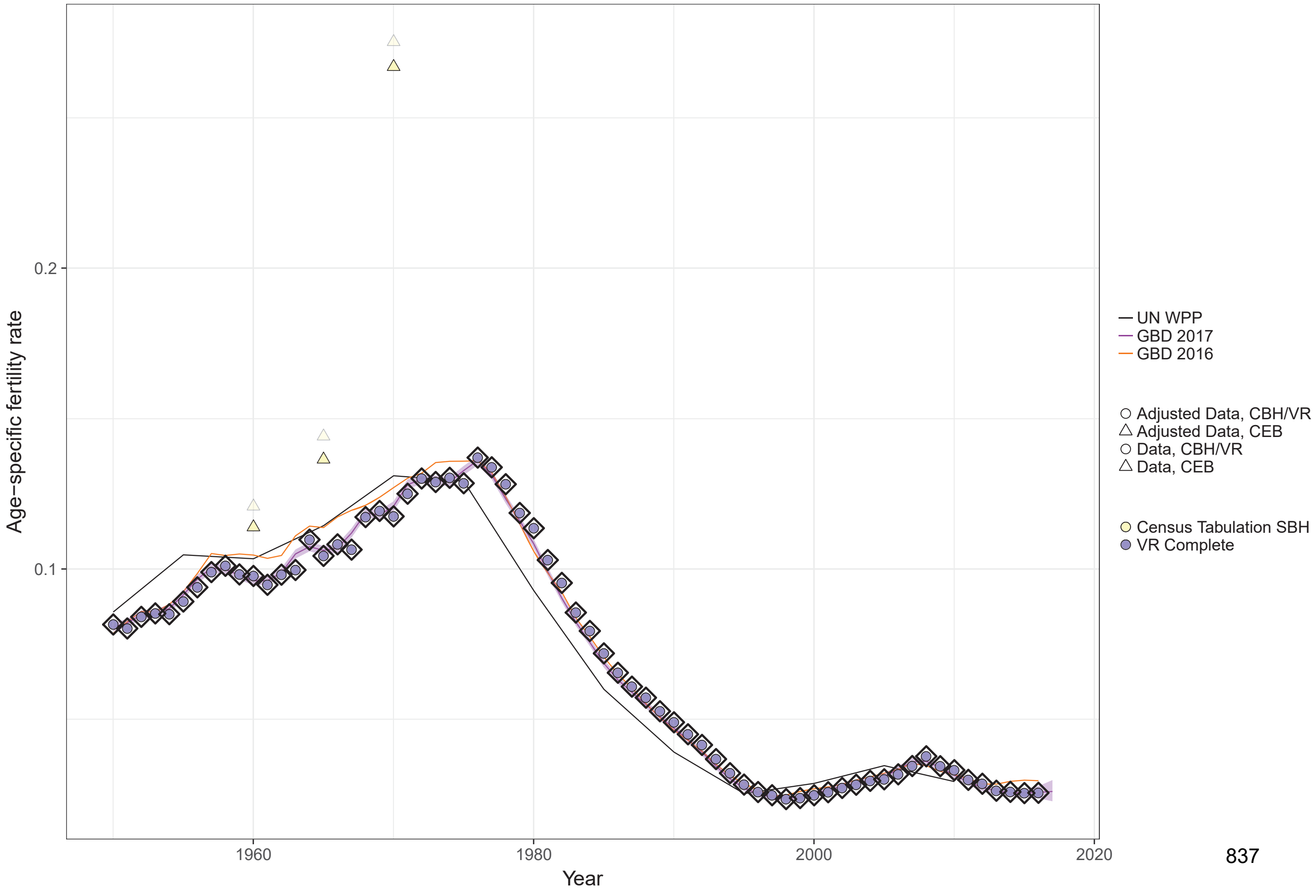

Age−specific fertility fitted trend lines, 1950−2017: Spain, Ages: 25−29
Data points enclosed with a diamond are reference sources

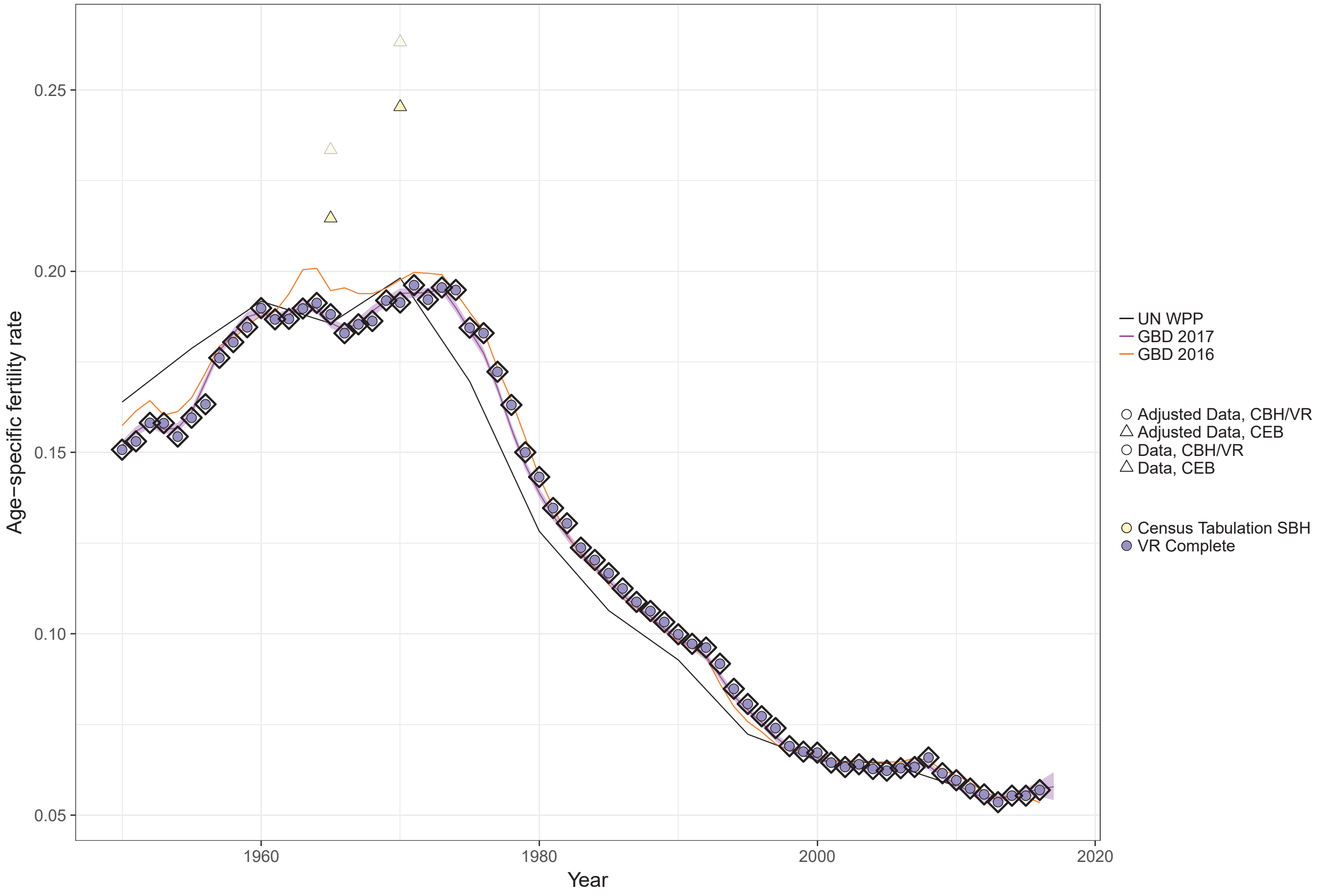

Age−specific fertility fitted trend lines, 1950−2017: Spain, Ages: 30−34
Data points enclosed with a diamond are reference sources

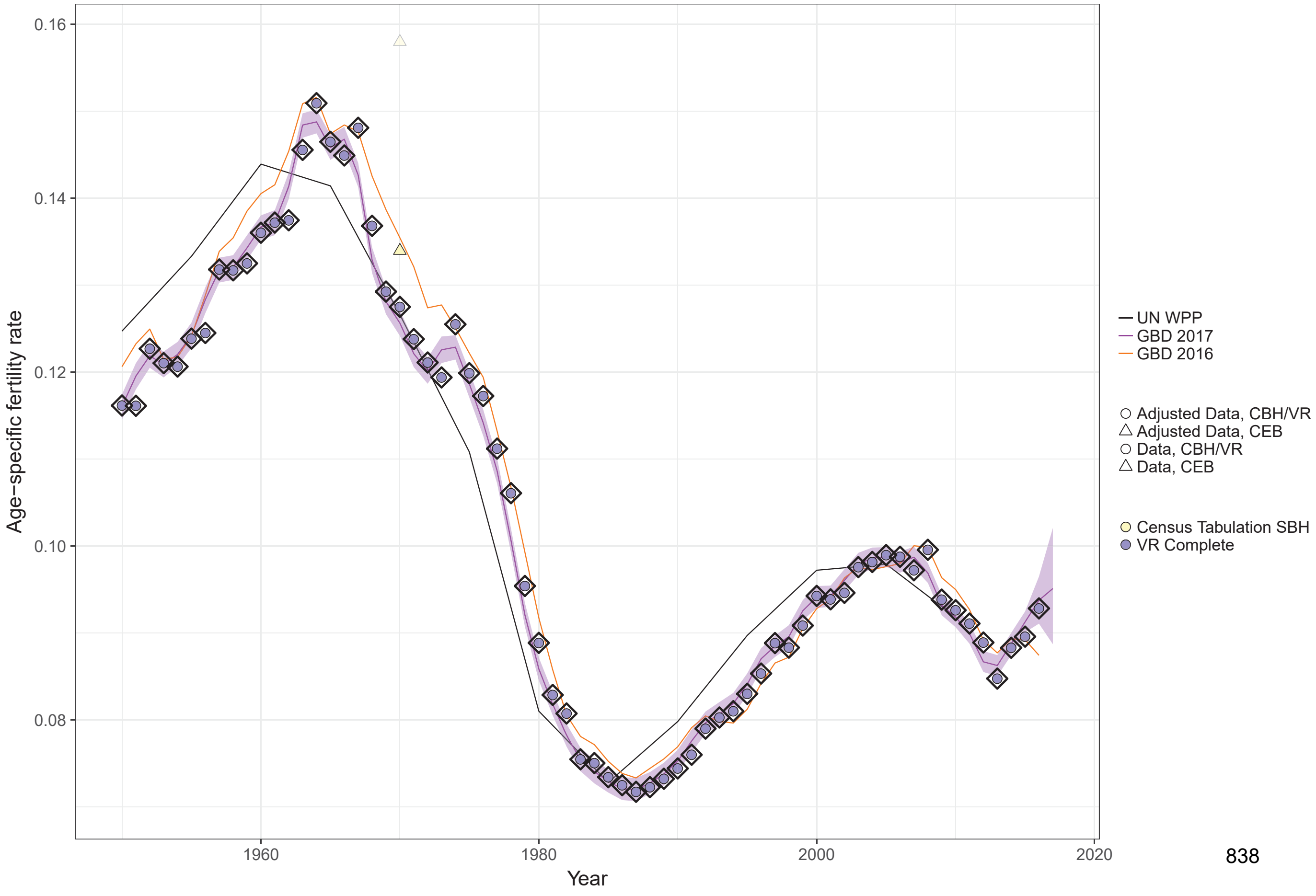

Age−specific fertility fitted trend lines, 1950−2017: Spain, Ages: 35−39
Data points enclosed with a diamond are reference sources

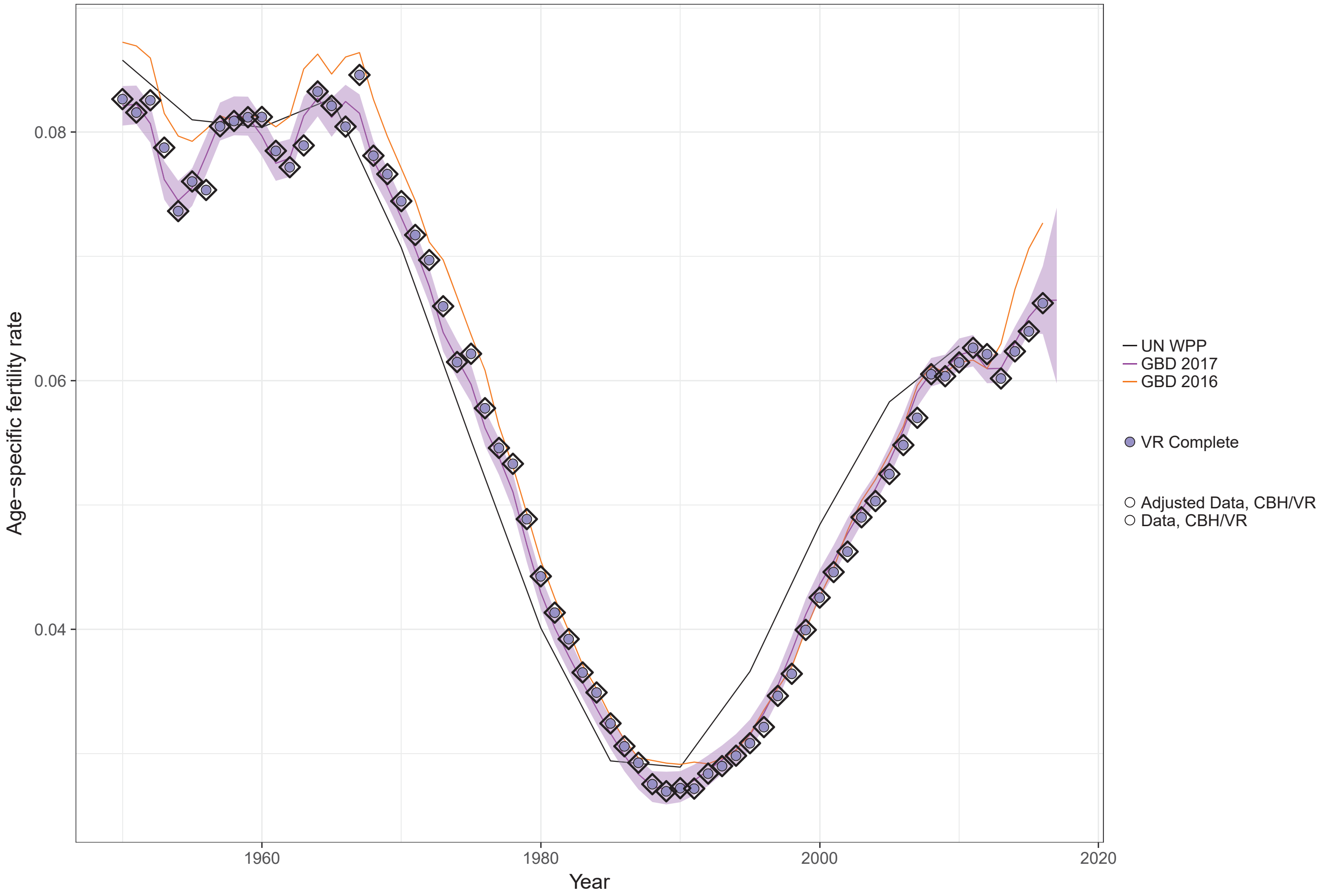

Age−specific fertility fitted trend lines, 1950−2017: Spain, Ages: 40−44
Data points enclosed with a diamond are reference sources

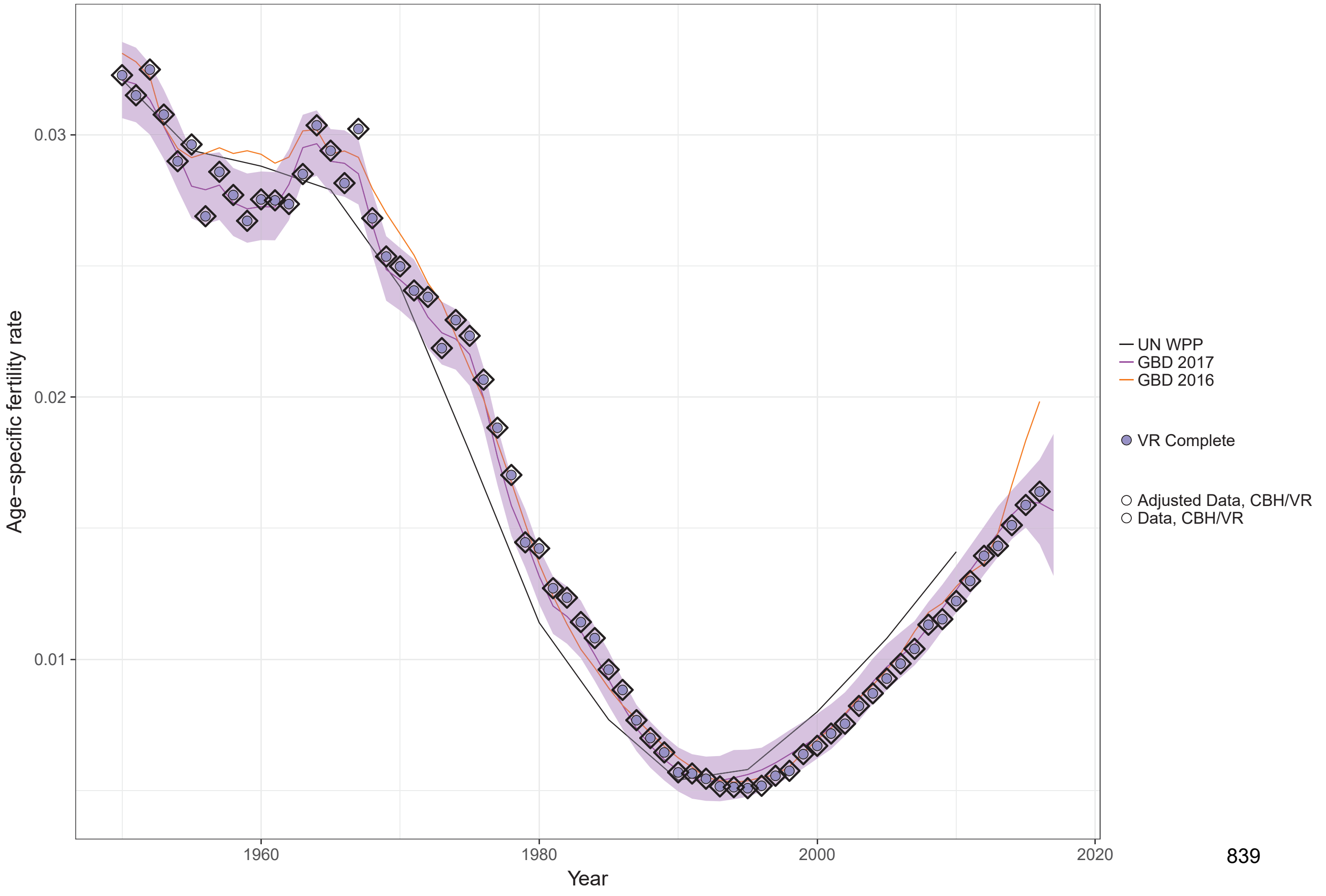

Age−specific fertility fitted trend lines, 1950−2017: Spain, Ages: 45−49
Data points enclosed with a diamond are reference sources

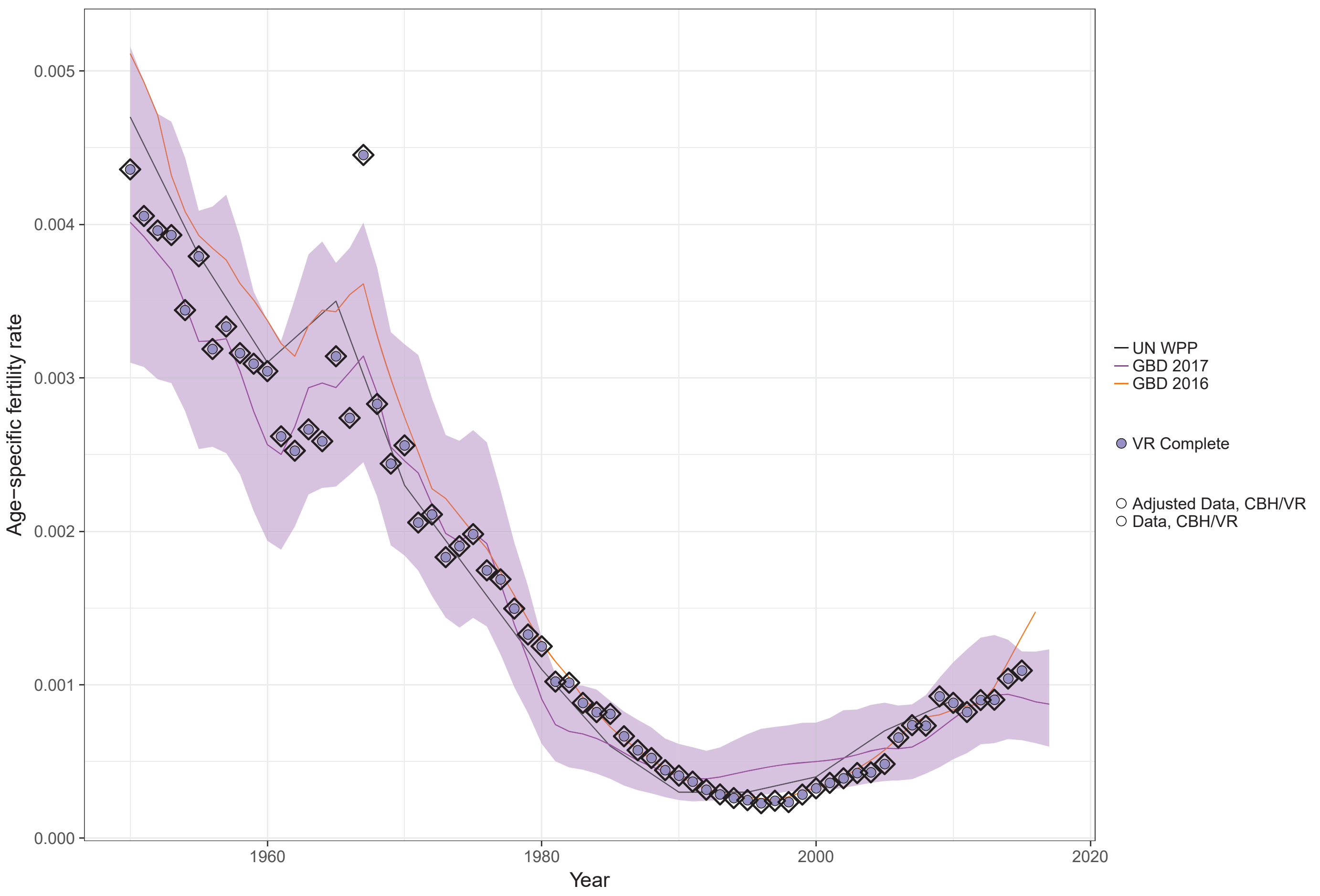

Age−specific fertility fitted trend lines, 1950−2017: Spain, Ages: 50−54
Data points enclosed with a diamond are reference sources

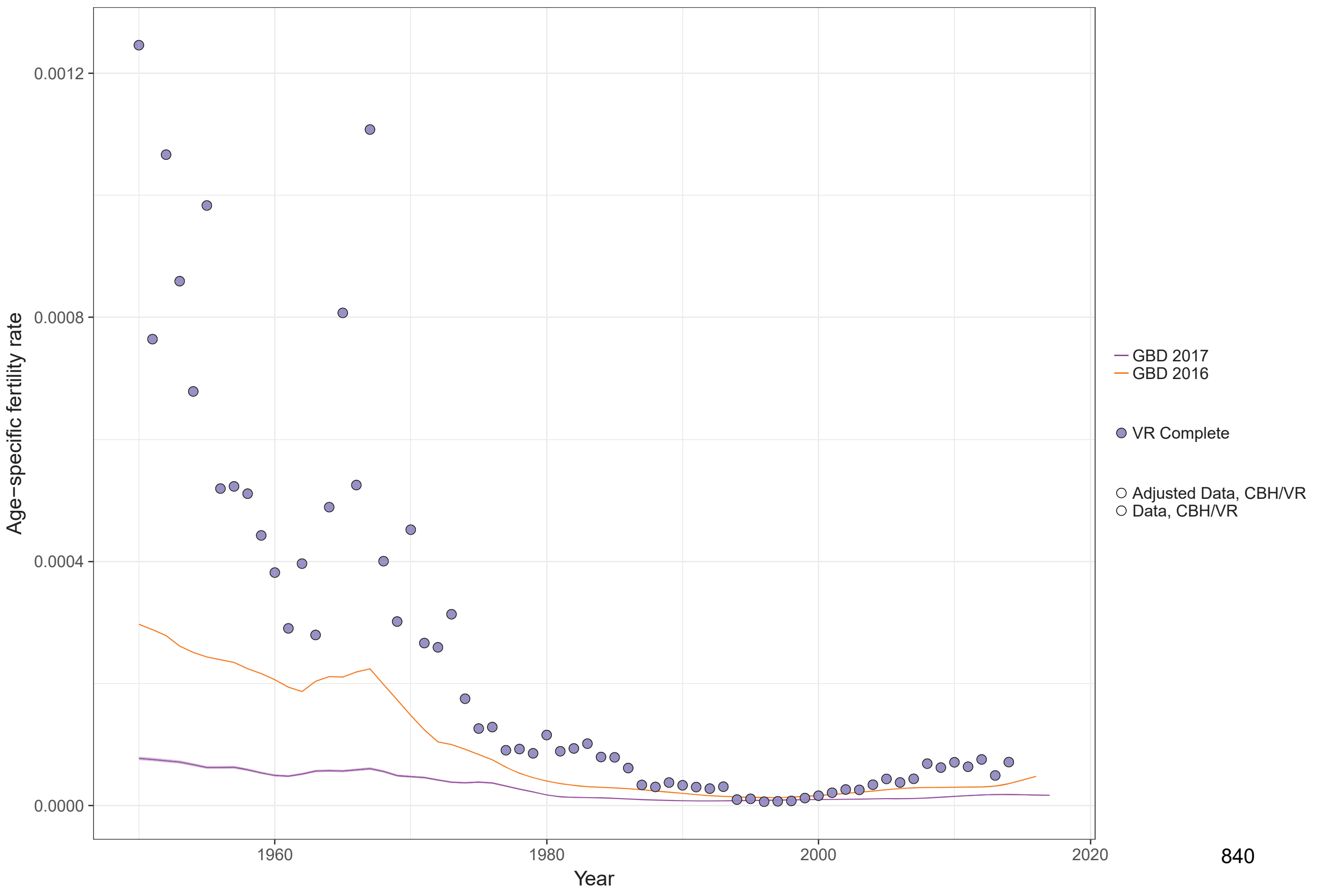

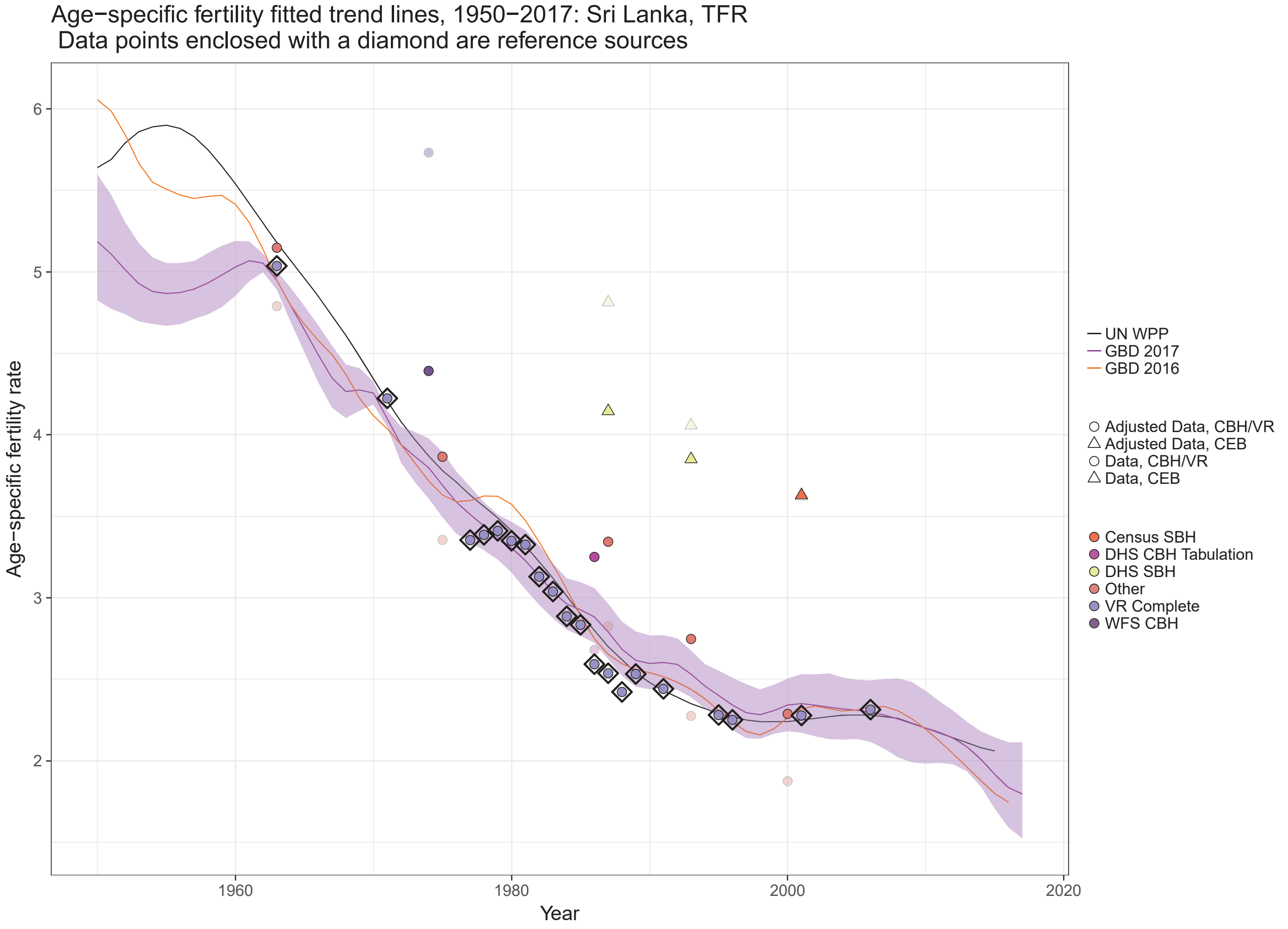
Age−specific fertility fitted trend lines, 1950−2017: Sri Lanka, TFR
Data points enclosed with a diamond are reference sources
Age−specific fertility rate
Year
UN WPP
GBD 2017
GBD 2016
Adjusted Data, CBH/VR
Adjusted Data, CEB
Data, CBH/VR
Data, CEB
Census SBH
DHS CBH Tabulation
DHS SBH
Other
VR Complete
WFS CBH

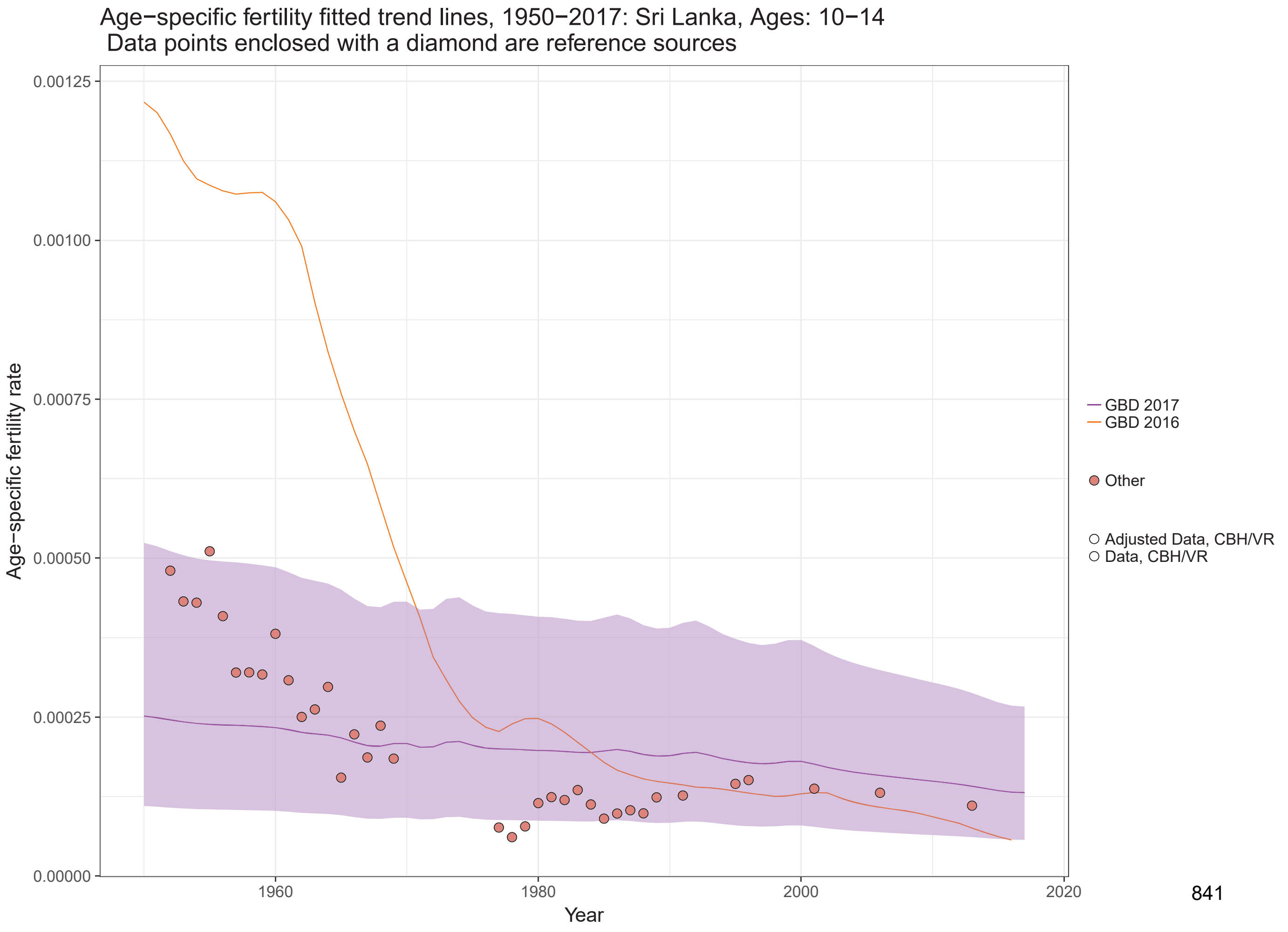
Age−specific fertility fitted trend lines, 1950−2017: Sri Lanka, Ages: 10−14
Data points enclosed with a diamond are reference sources
Age−specific fertility rate
Year
GBD 2017
GBD 2016
Other
Adjusted Data, CBH/VR
Data, CBH/VR

Age−specific fertility fitted trend lines, 1950−2017: Sri Lanka, Ages: 15−19
Data points enclosed with a diamond are reference sources

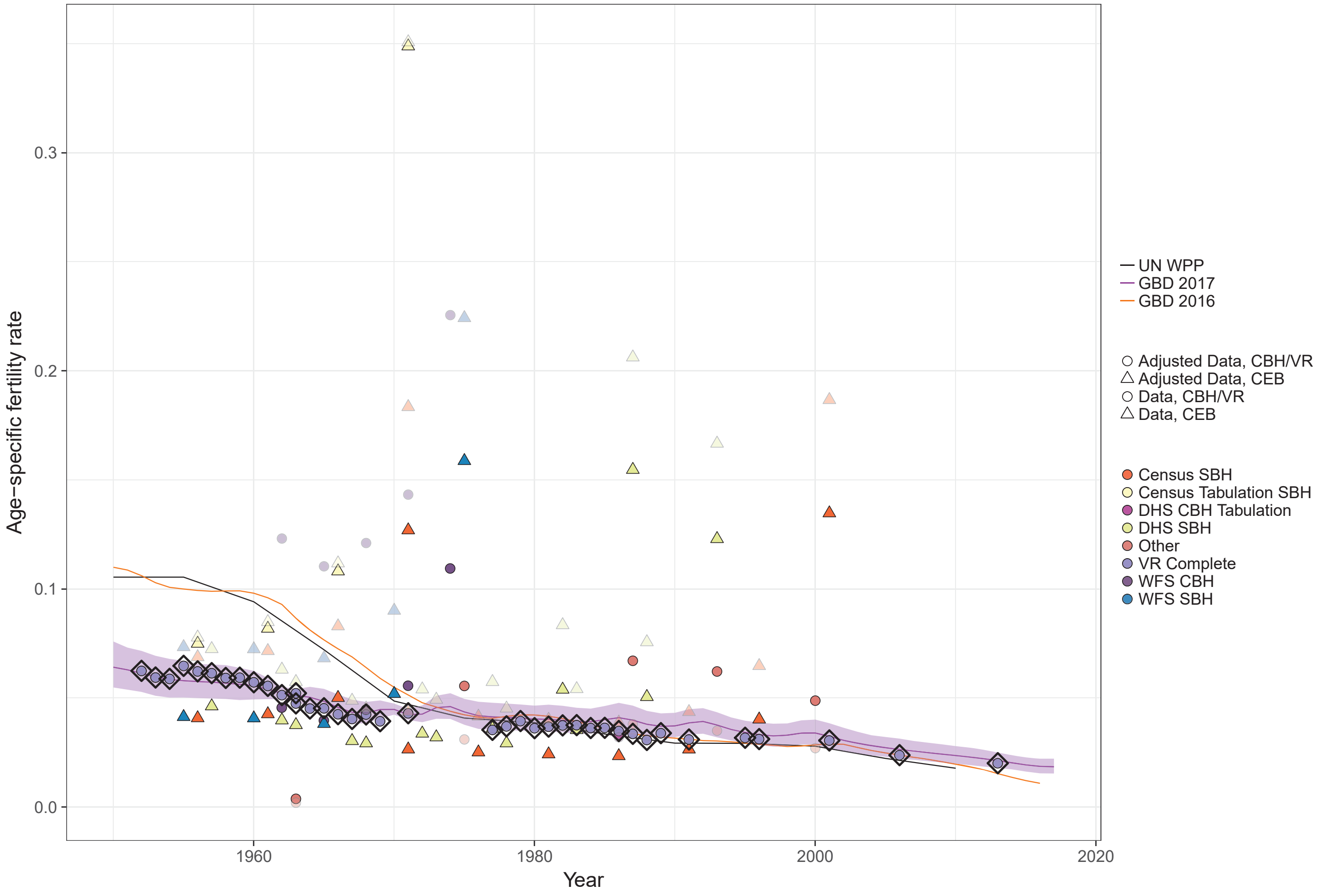

Age−specific fertility fitted trend lines, 1950−2017: Sri Lanka, Ages: 20−24
Data points enclosed with a diamond are reference sources

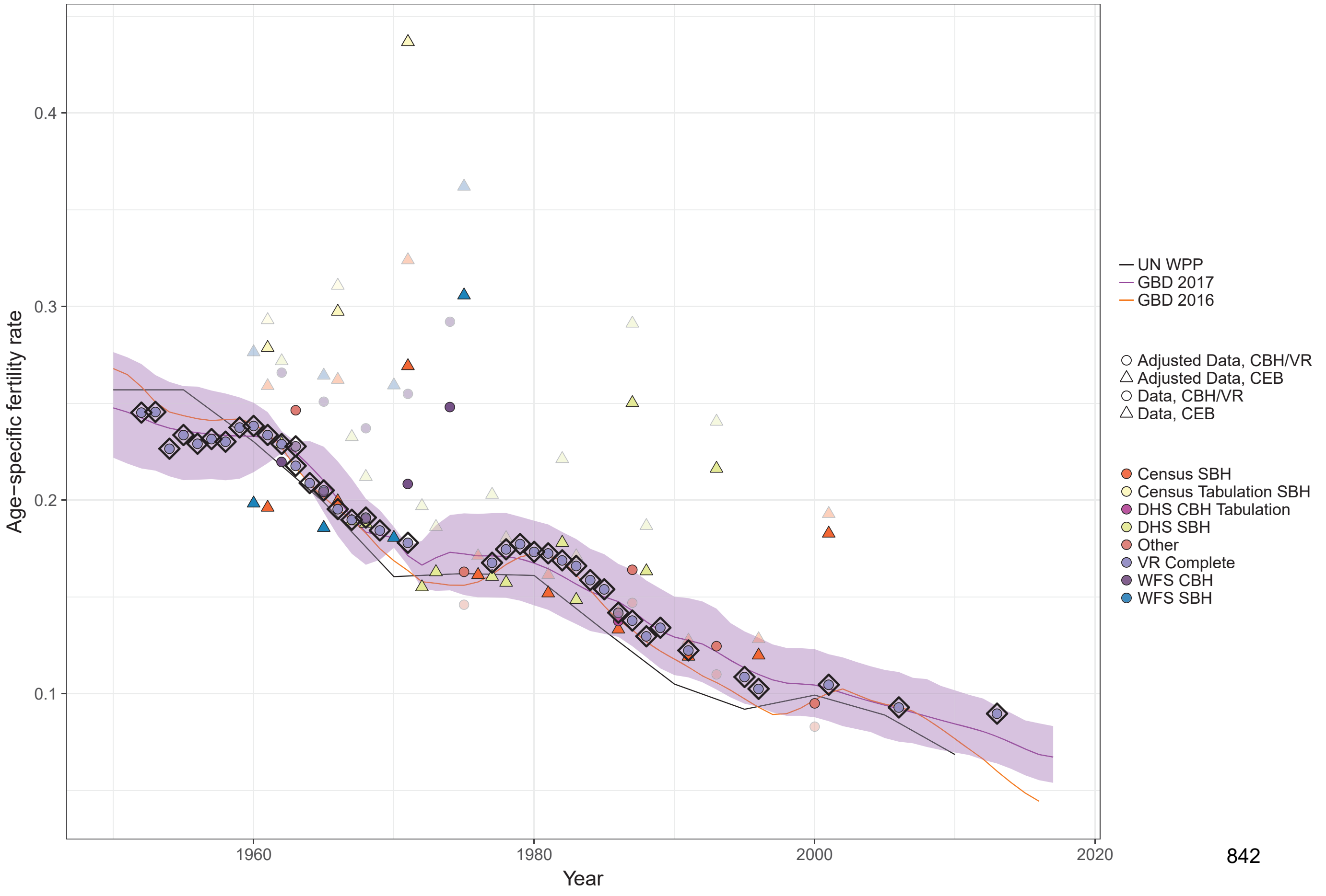

Age−specific fertility fitted trend lines, 1950−2017: Sri Lanka, Ages: 25−29
Data points enclosed with a diamond are reference sources

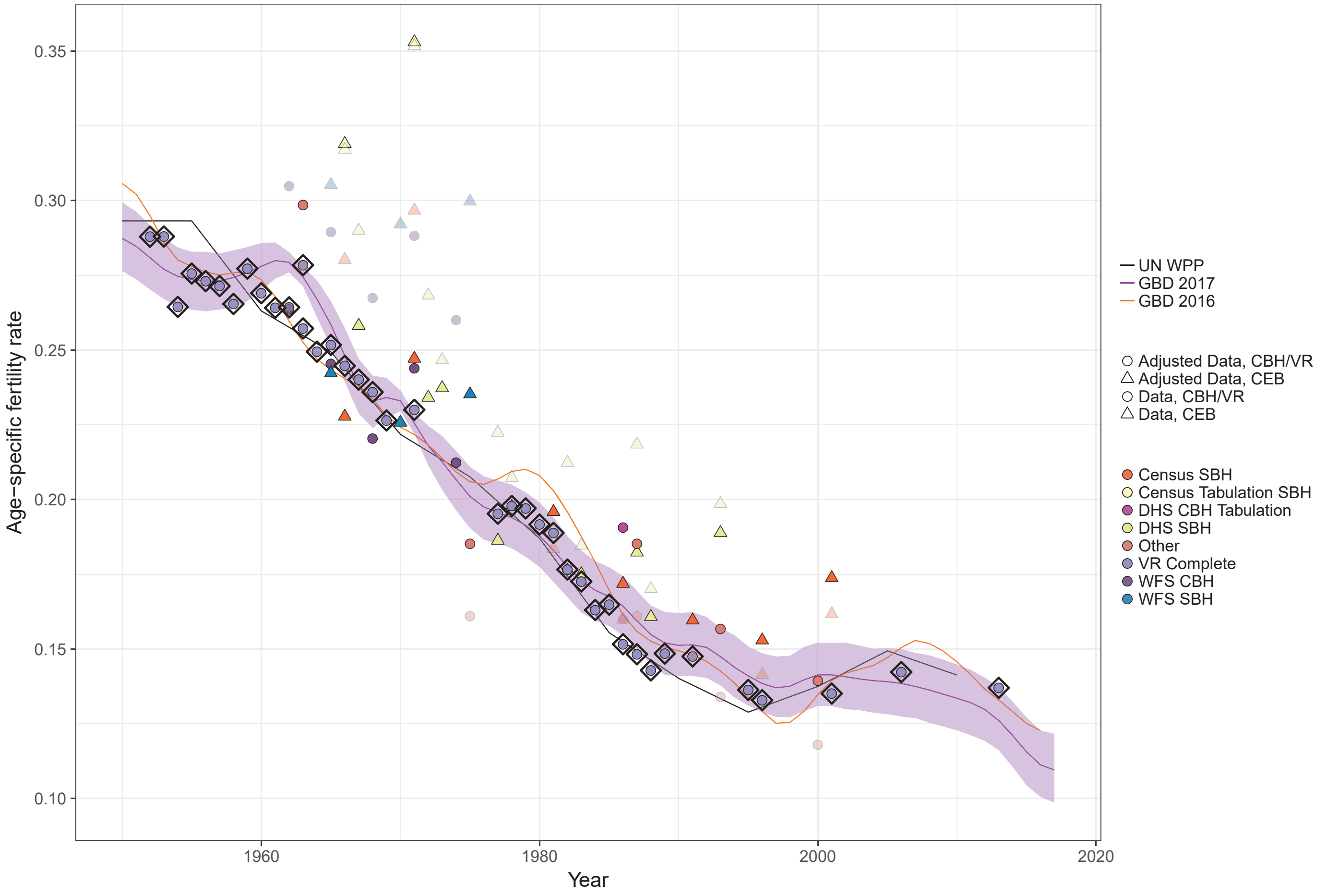

Age−specific fertility fitted trend lines, 1950−2017: Sri Lanka, Ages: 30−34
Data points enclosed with a diamond are reference sources

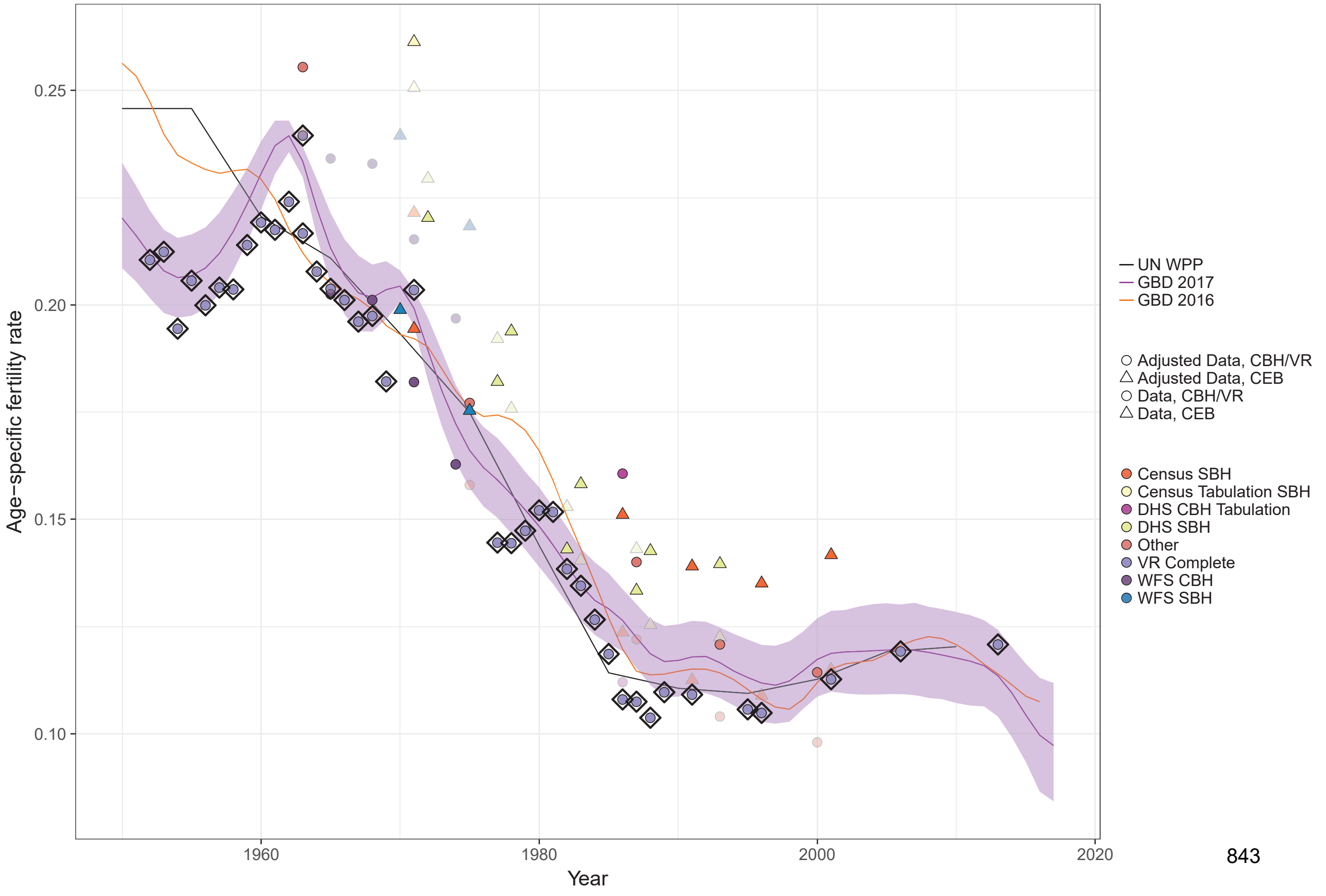

Age−specific fertility fitted trend lines, 1950−2017: Sri Lanka, Ages: 35−39
Data points enclosed with a diamond are reference sources

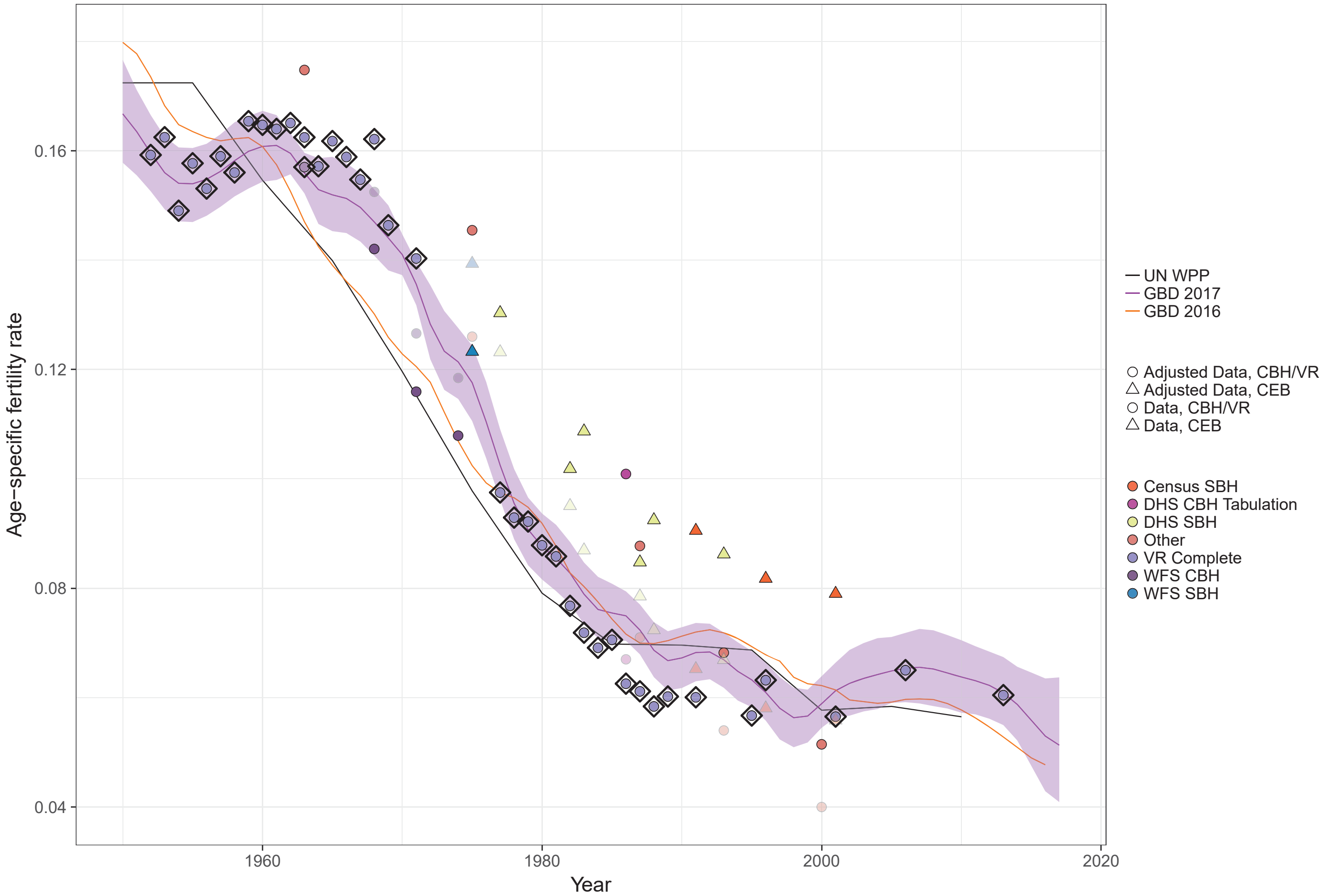

Age−specific fertility fitted trend lines, 1950−2017: Sri Lanka, Ages: 40−44
Data points enclosed with a diamond are reference sources

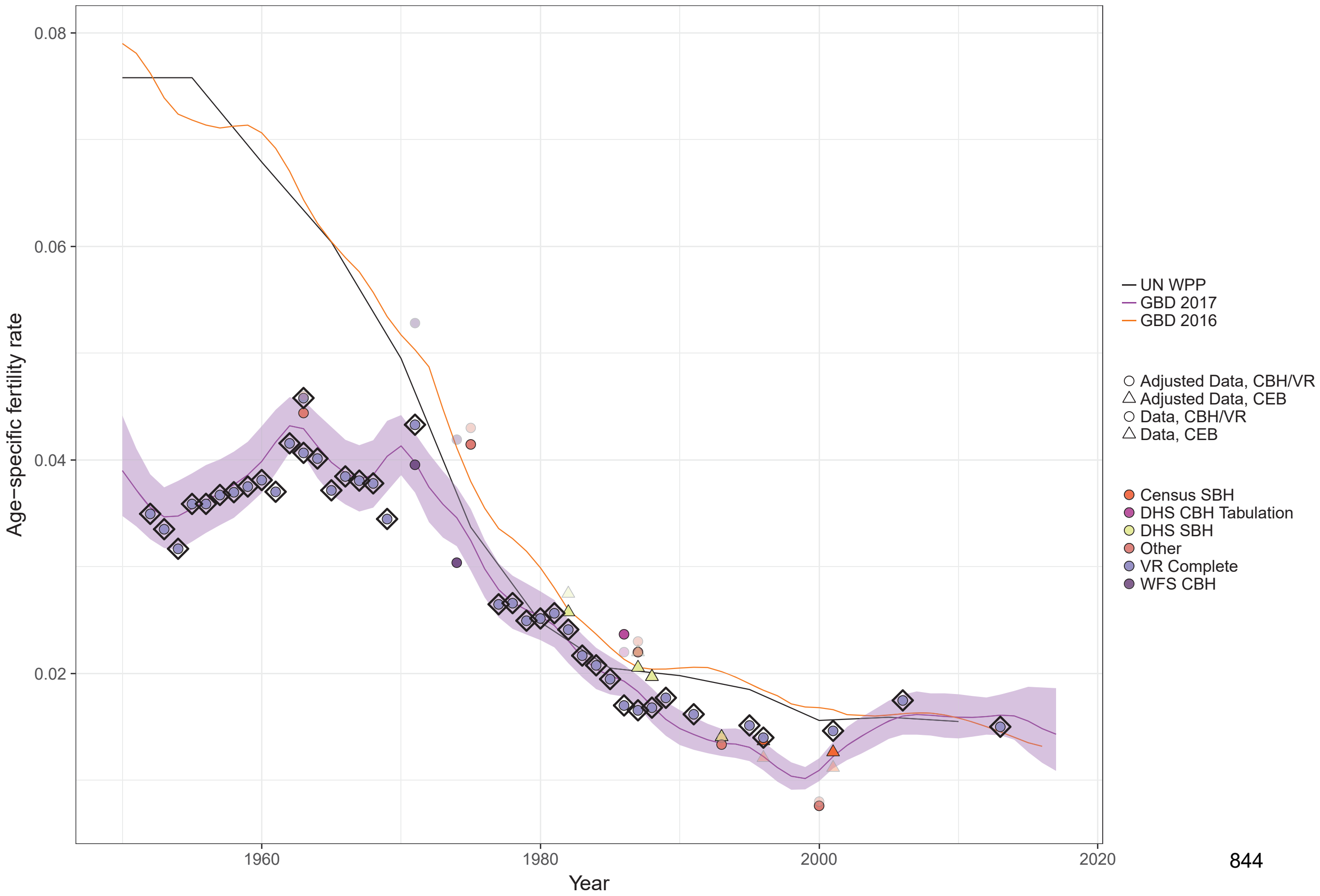

Age−specific fertility fitted trend lines, 1950−2017: Sri Lanka, Ages: 45−49
Data points enclosed with a diamond are reference sources

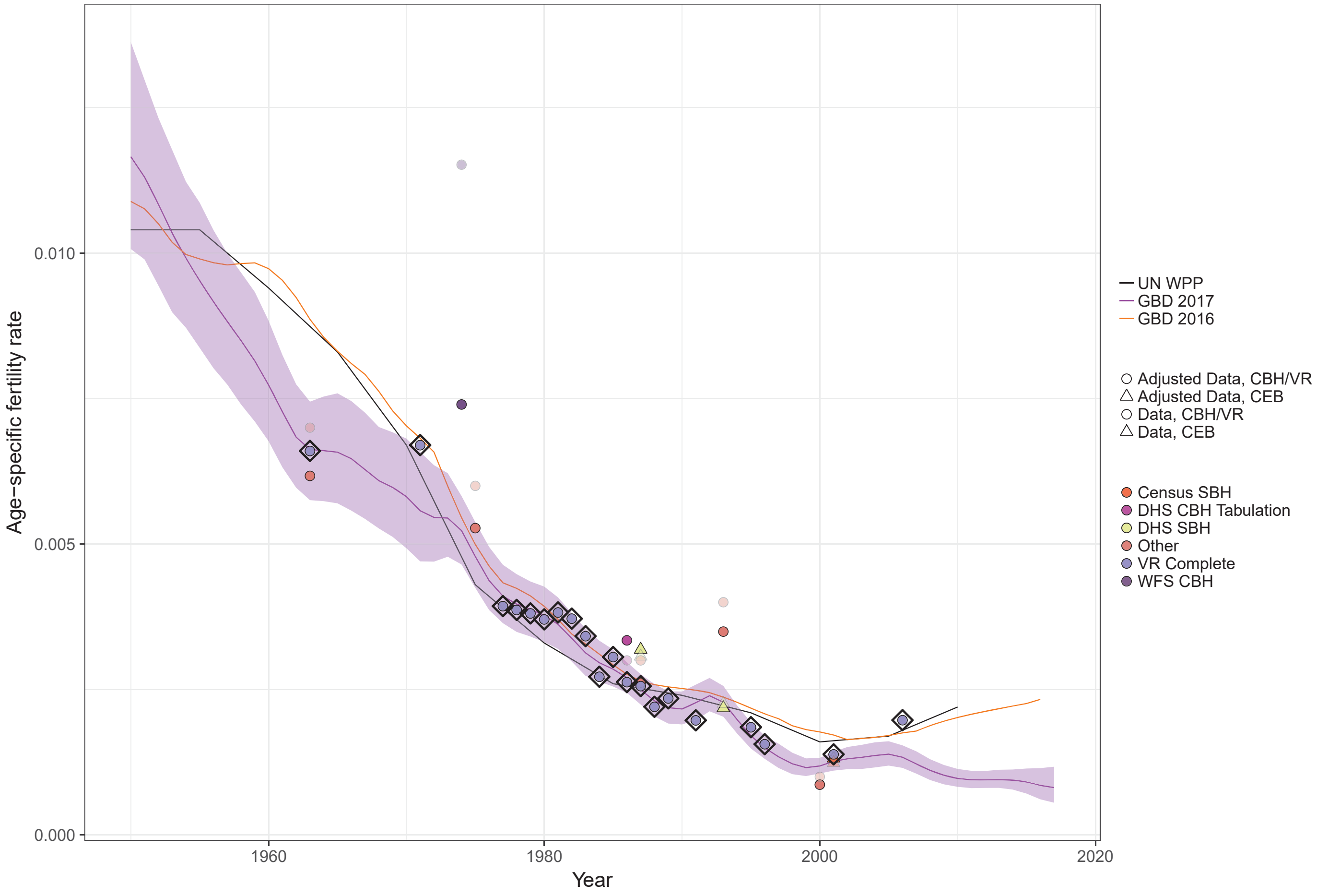

Age−specific fertility fitted trend lines, 1950−2017: Sri Lanka, Ages: 50−54
Data points enclosed with a diamond are reference sources

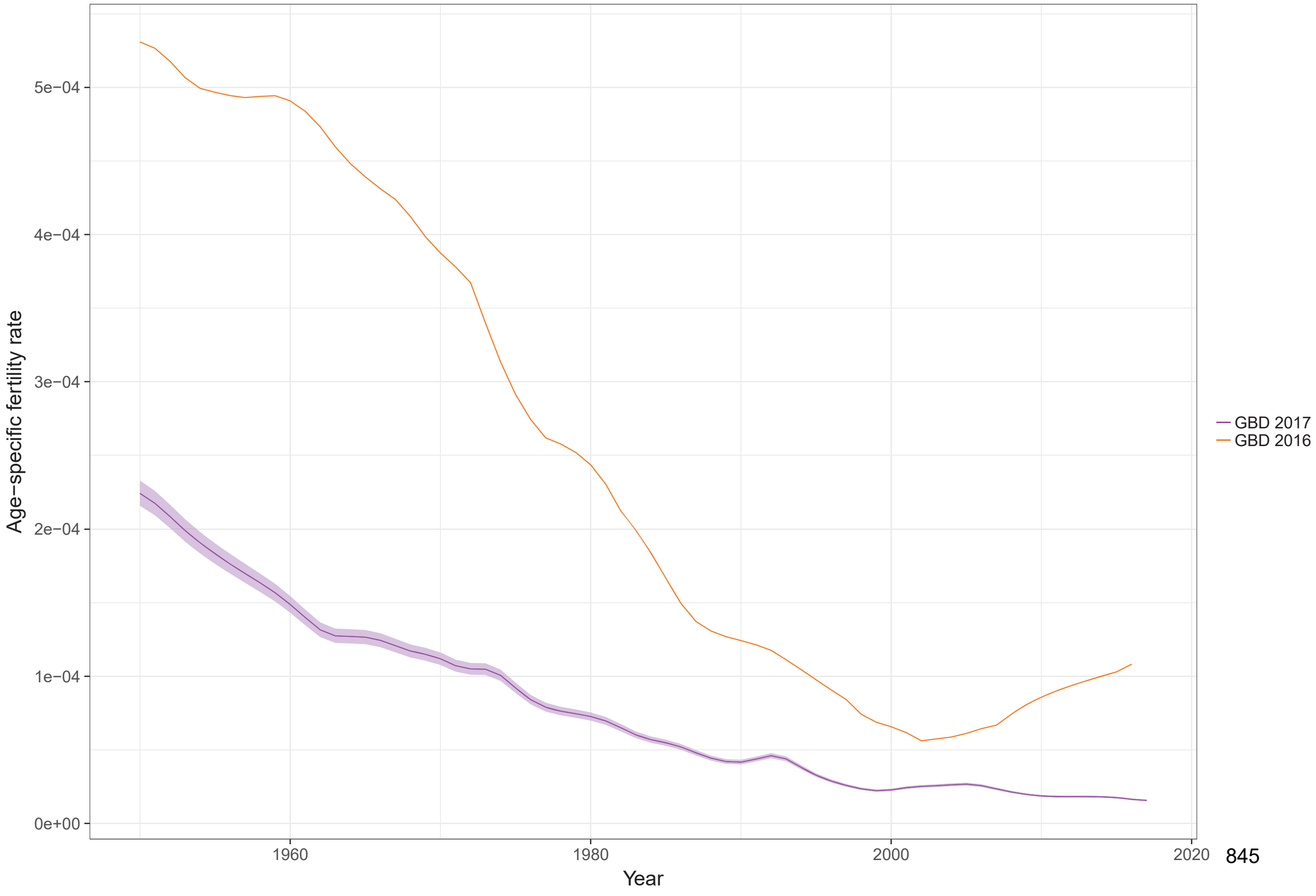

Age−specific fertility fitted trend lines, 1950−2017: Sudan, TFR
Data points enclosed with a diamond are reference sources

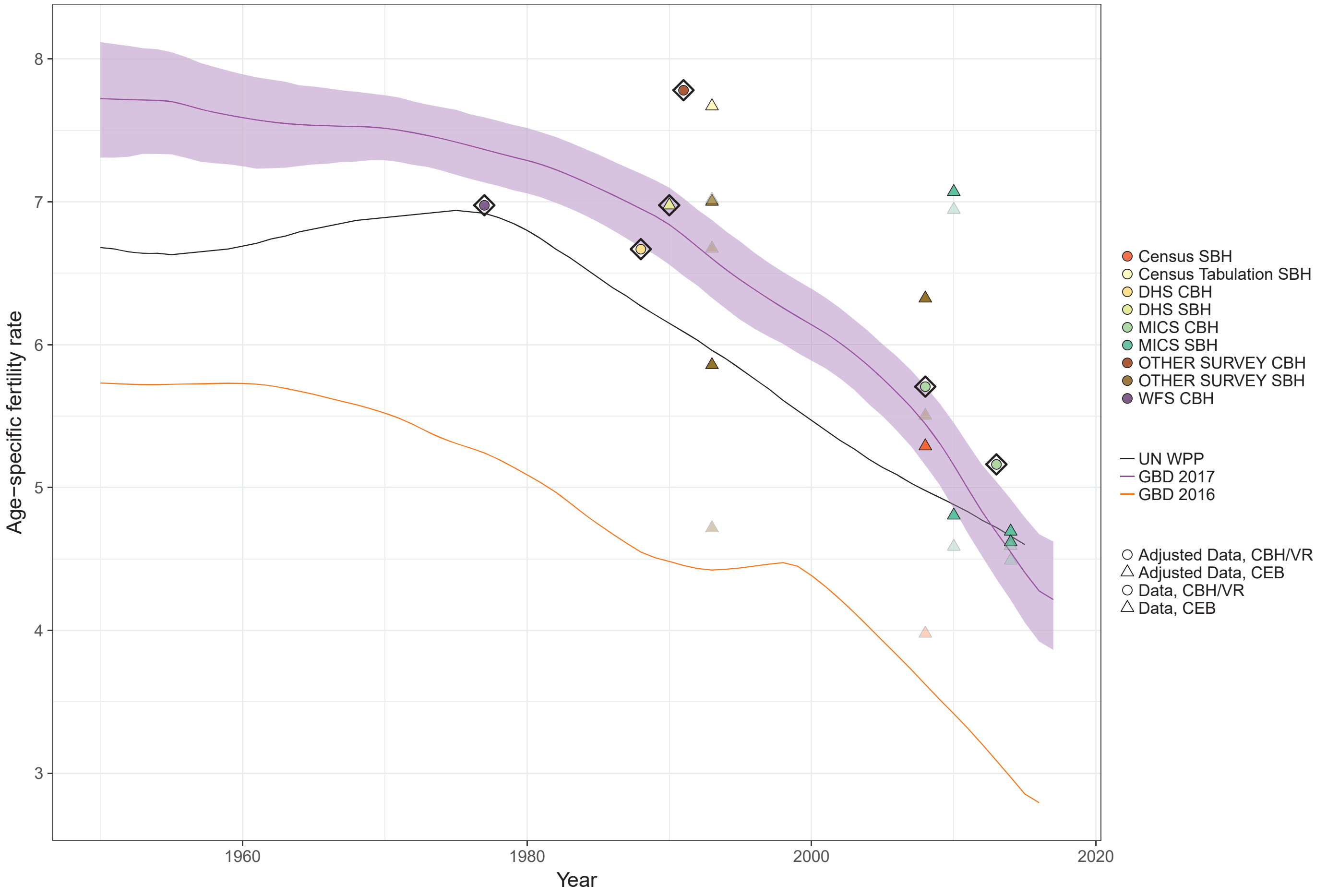

Age−specific fertility fitted trend lines, 1950−2017: Sudan, Ages: 10−14
Data points enclosed with a diamond are reference sources

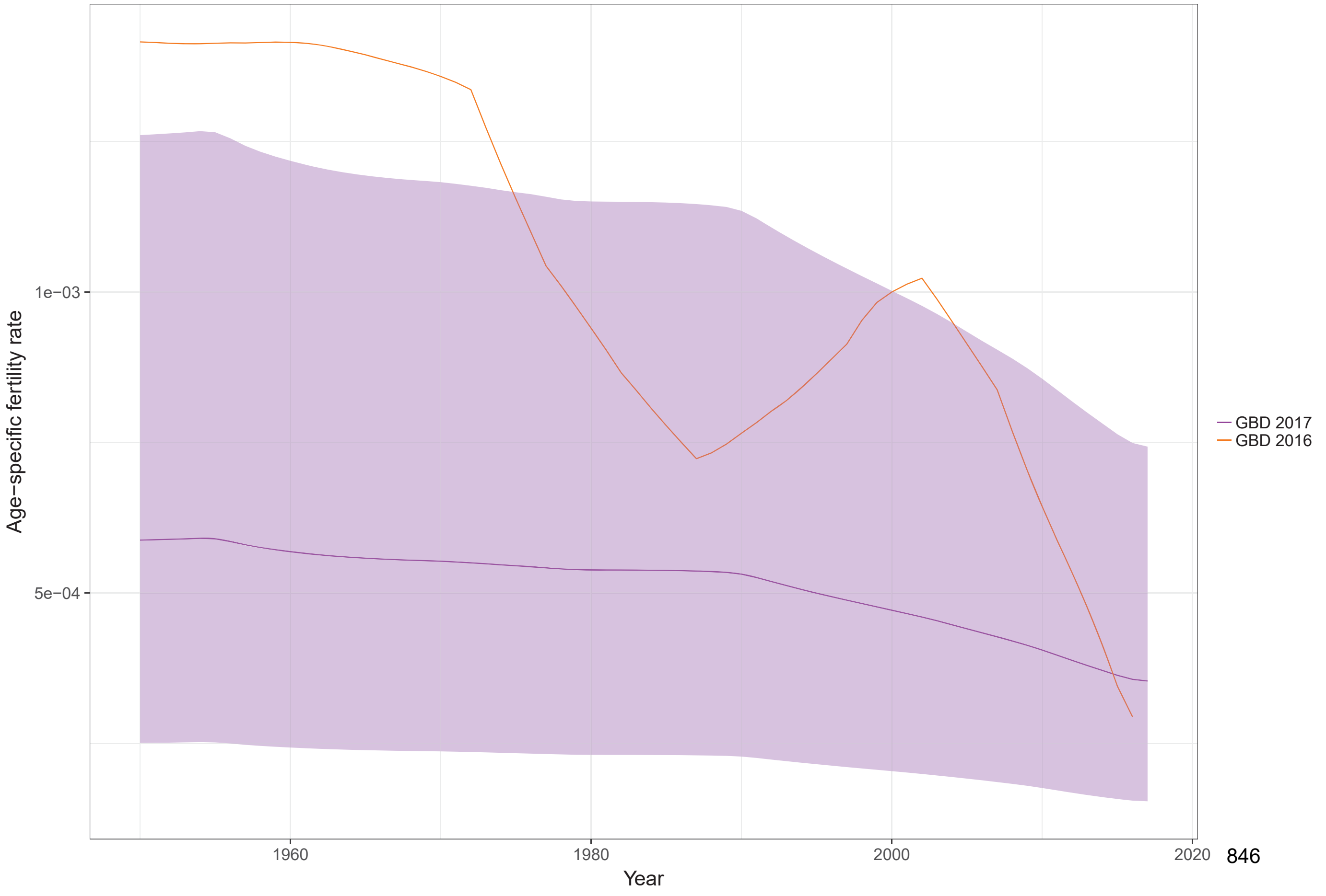

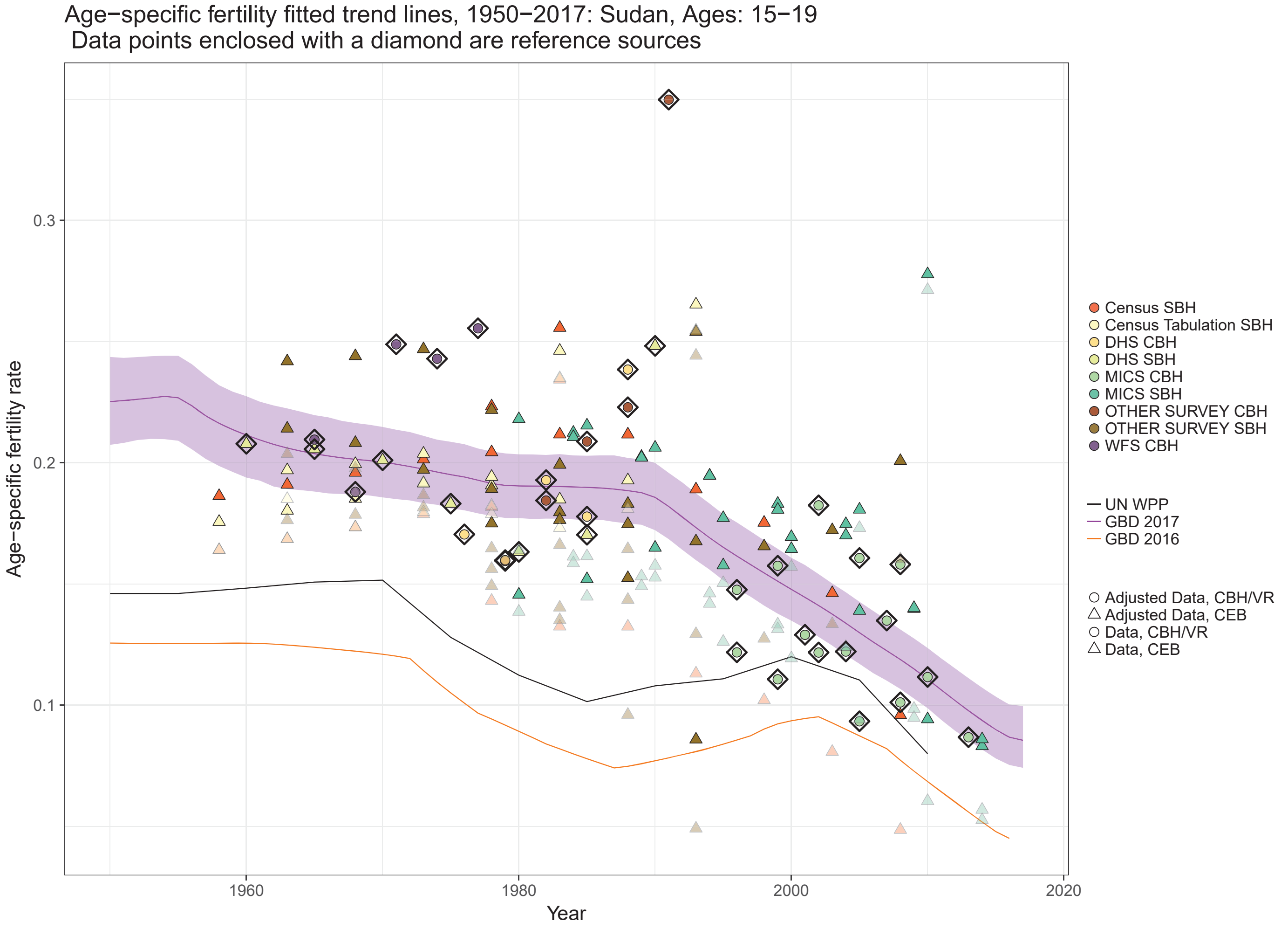

Age−specific fertility fitted trend lines, 1950−2017: Sudan, Ages: 15−19
Data points enclosed with a diamond are reference sources
Age−specific fertility rate
0.1
0.2
0.3
1960
1980
2000
2020
Year
Census SBH
Census Tabulation SBH
DHS CBH
DHS SBH
MICS CBH
MICS SBH
OTHER SURVEY CBH
OTHER SURVEY SBH
WFS CBH
UN WPP
GBD 2017
GBD 2016
Adjusted Data, CBH/VR
Adjusted Data, CEB
Data, CBH/VR
Data, CEB

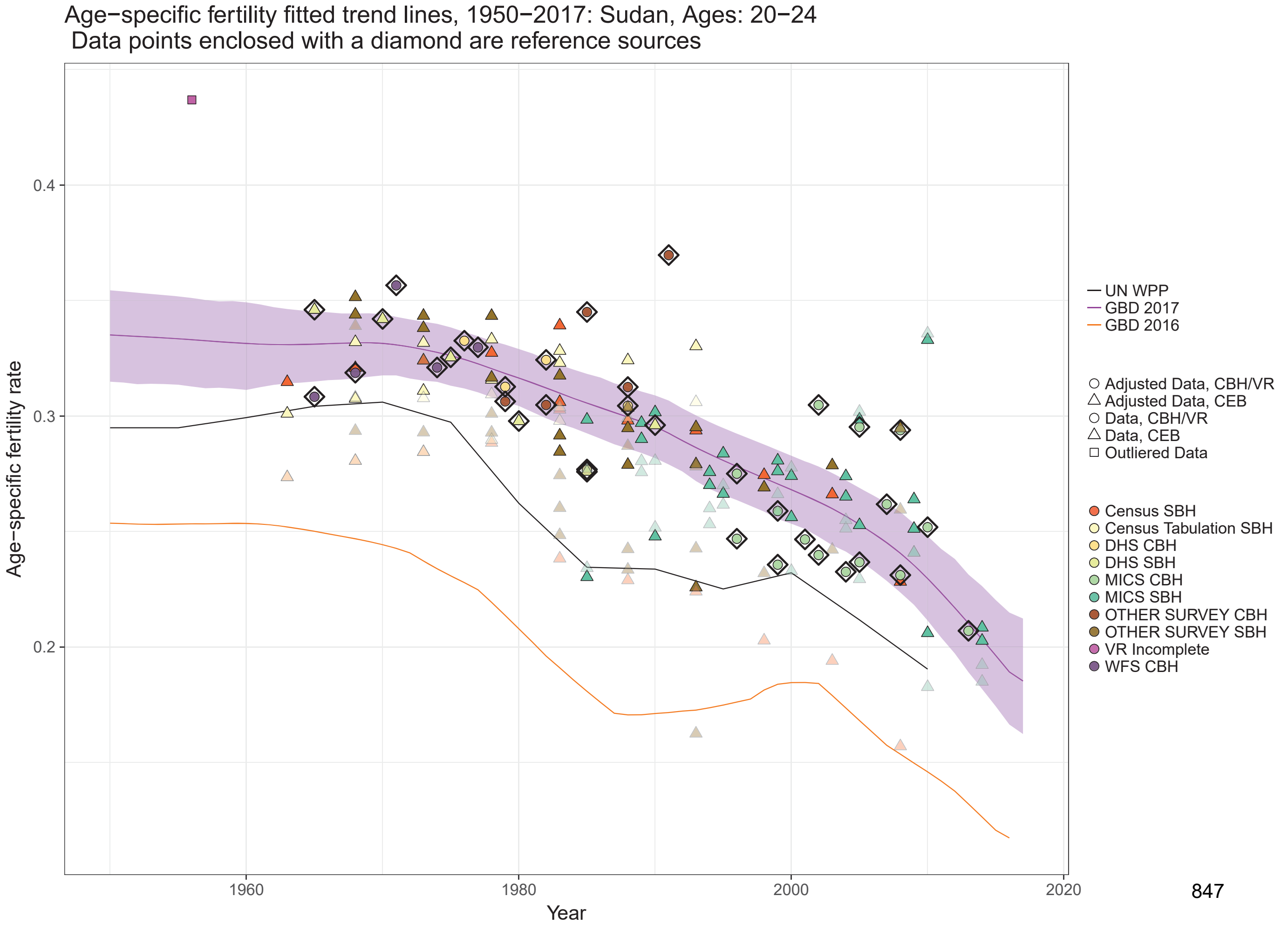

Age−specific fertility fitted trend lines, 1950−2017: Sudan, Ages: 20−24
Data points enclosed with a diamond are reference sources
Age−specific fertility rate
0.2
0.3
0.4
1960
1980
2000
2020
Year
UN WPP
GBD 2017
GBD 2016
Adjusted Data, CBH/VR
Adjusted Data, CEB
Data, CBH/VR
Data, CEB
Outliered Data
Census SBH
Census Tabulation SBH
DHS CBH
DHS SBH
MICS CBH
MICS SBH
OTHER SURVEY CBH
OTHER SURVEY SBH
VR Incomplete
WFS CBH

Age−specific fertility fitted trend lines, 1950−2017: Sudan, Ages: 25−29
Data points enclosed with a diamond are reference sources

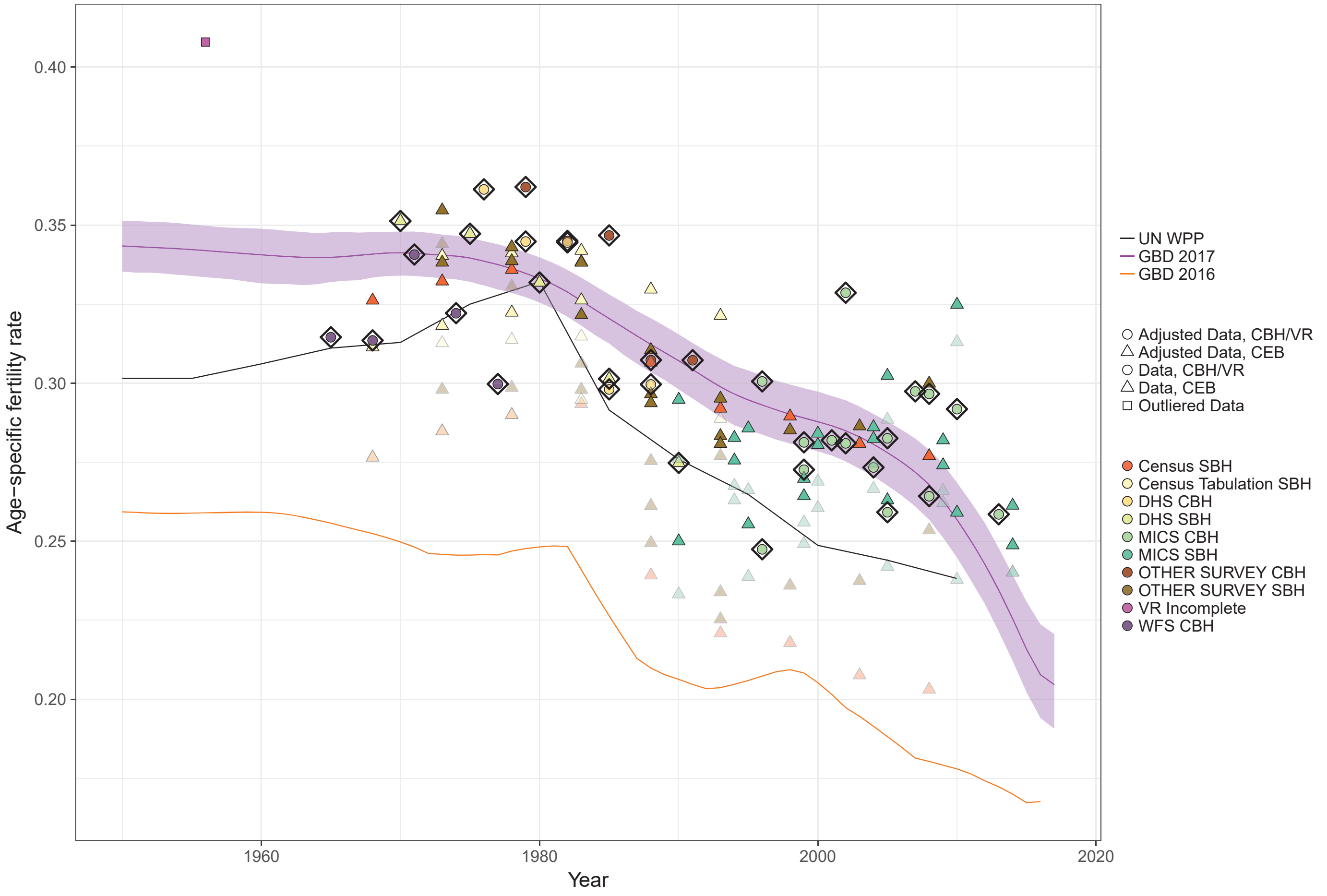

Age−specific fertility fitted trend lines, 1950−2017: Sudan, Ages: 30−34
Data points enclosed with a diamond are reference sources

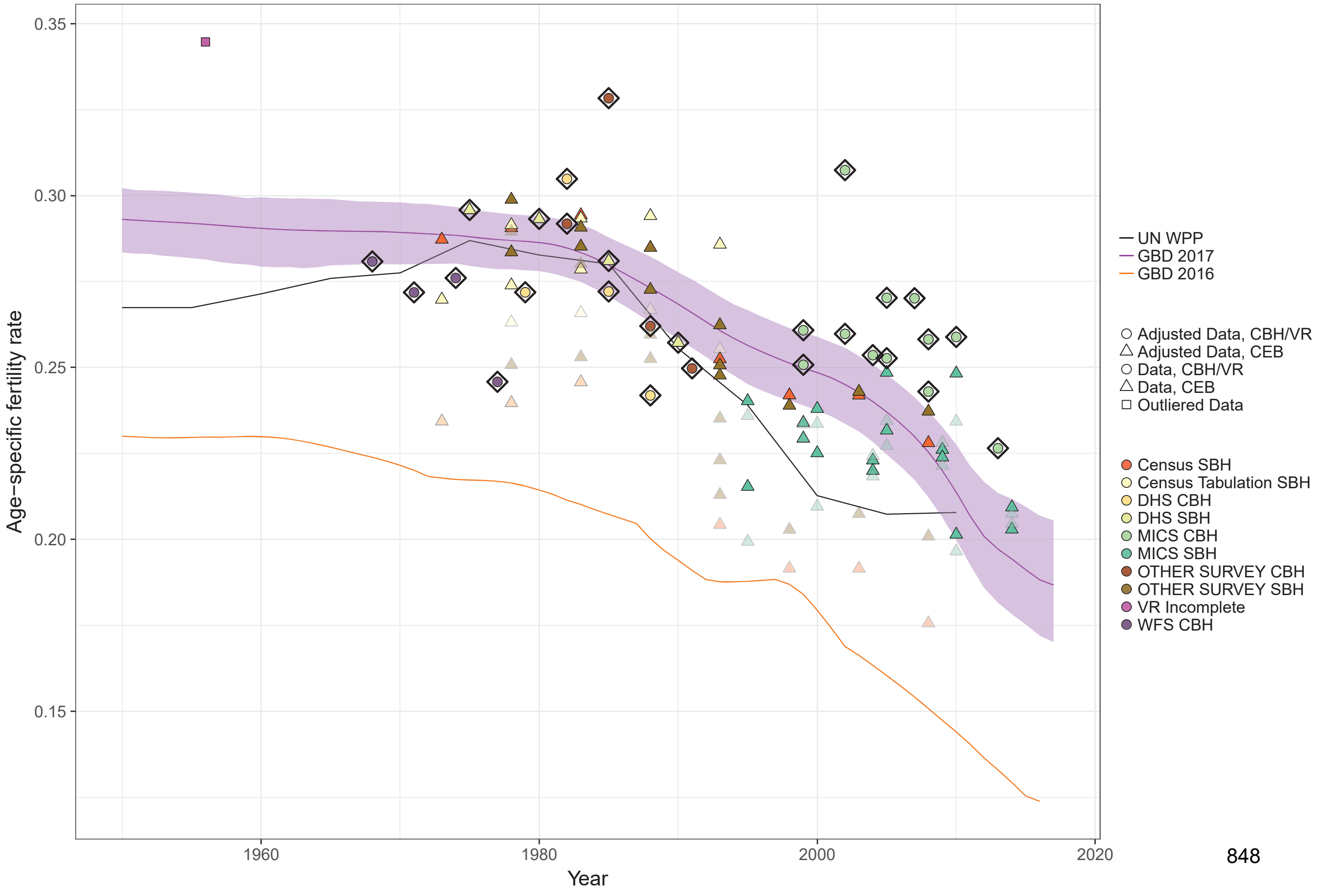

Age−specific fertility fitted trend lines, 1950−2017: Sudan, Ages: 35−39
Data points enclosed with a diamond are reference sources

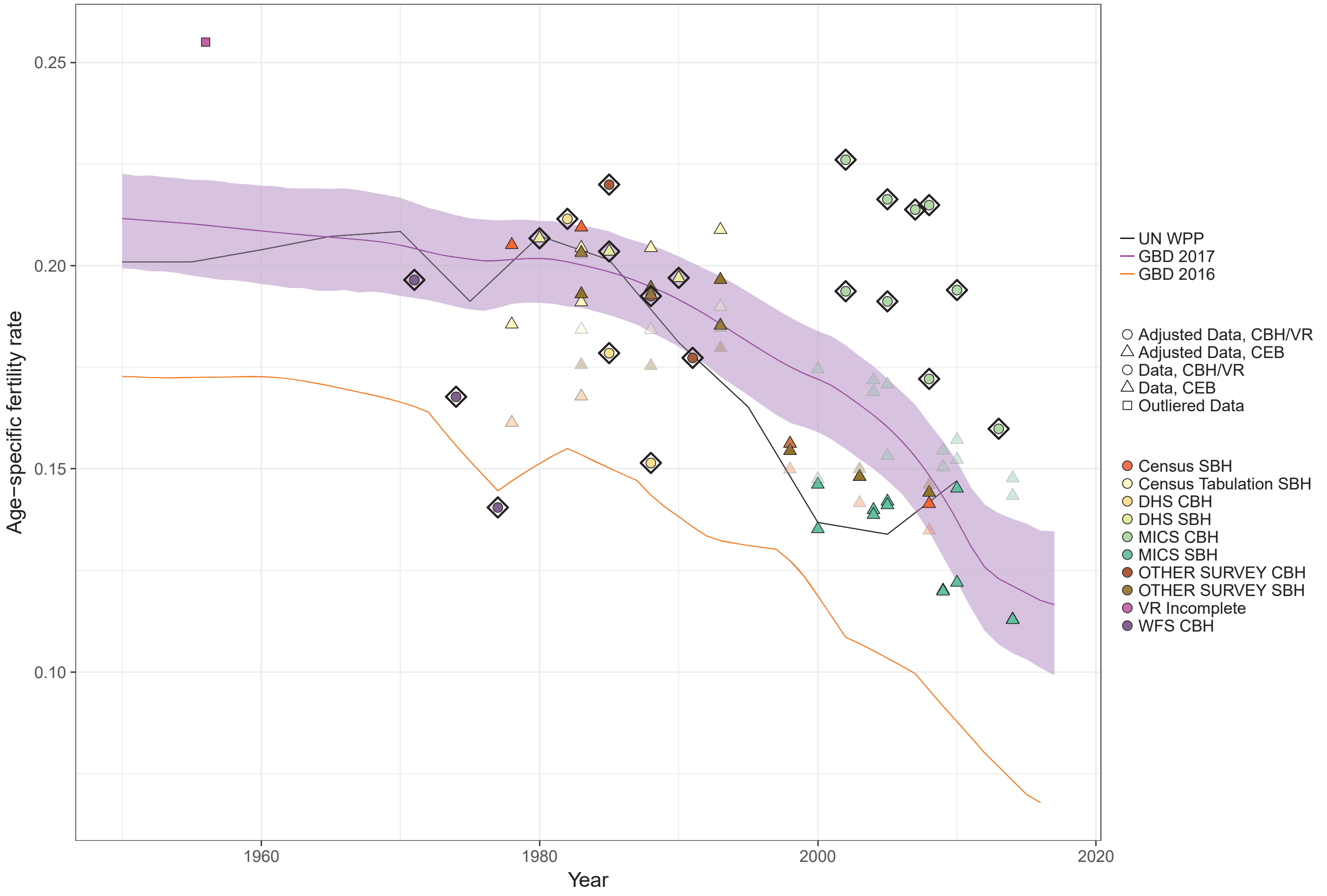

Age−specific fertility fitted trend lines, 1950−2017: Sudan, Ages: 40−44
Data points enclosed with a diamond are reference sources

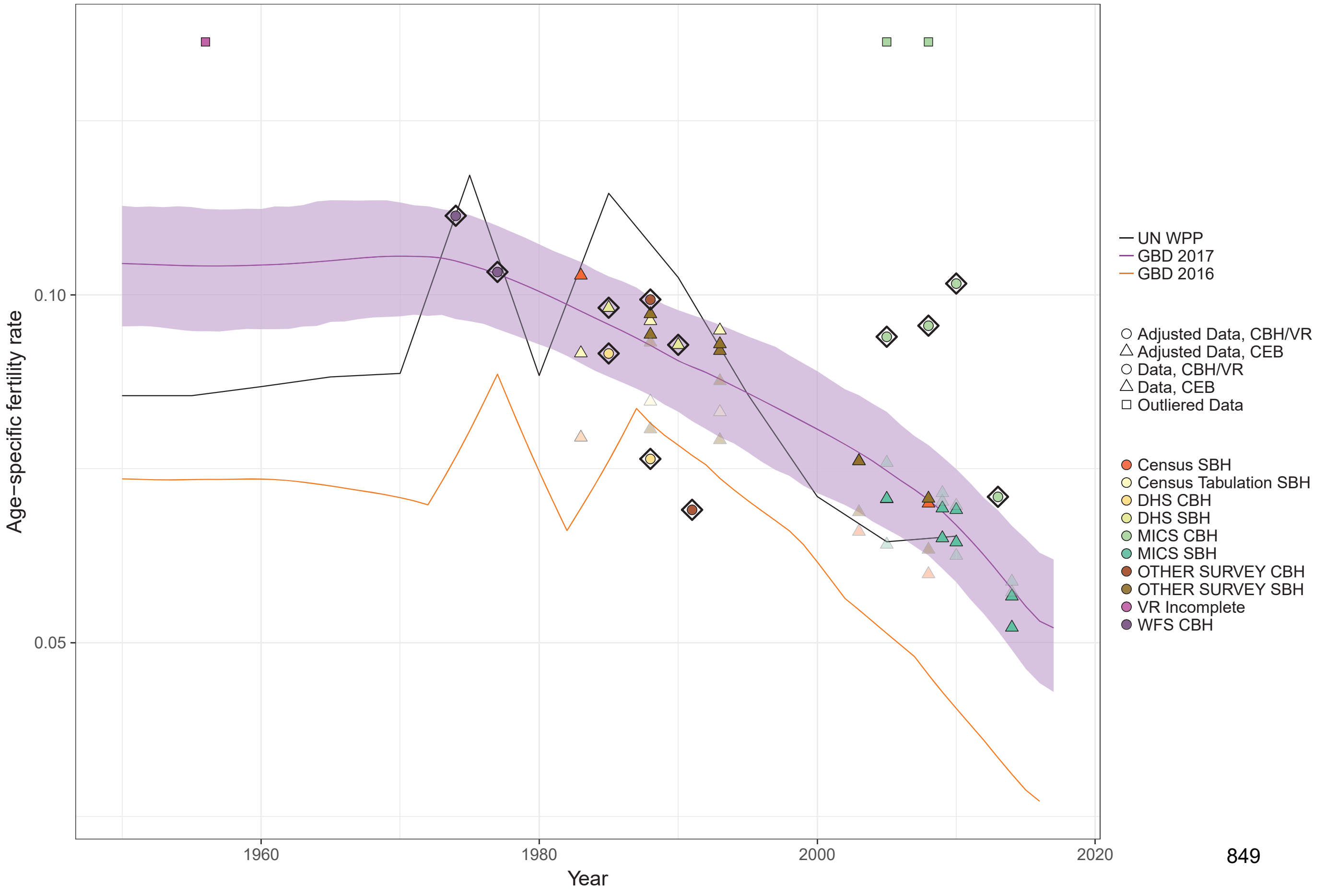

Age−specific fertility fitted trend lines, 1950−2017: Sudan, Ages: 45−49
Data points enclosed with a diamond are reference sources

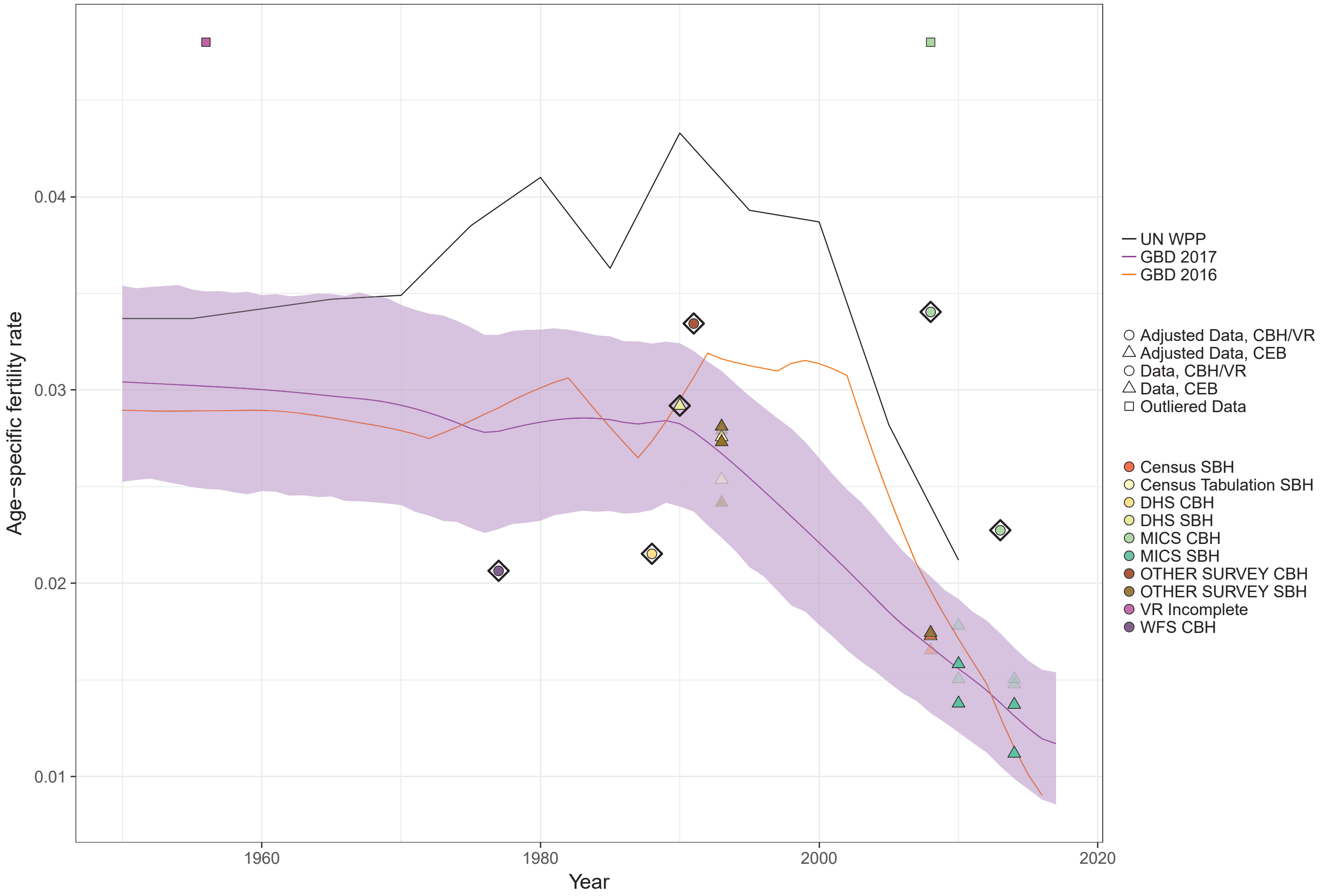

Age−specific fertility fitted trend lines, 1950−2017: Sudan, Ages: 50−54
Data points enclosed with a diamond are reference sources

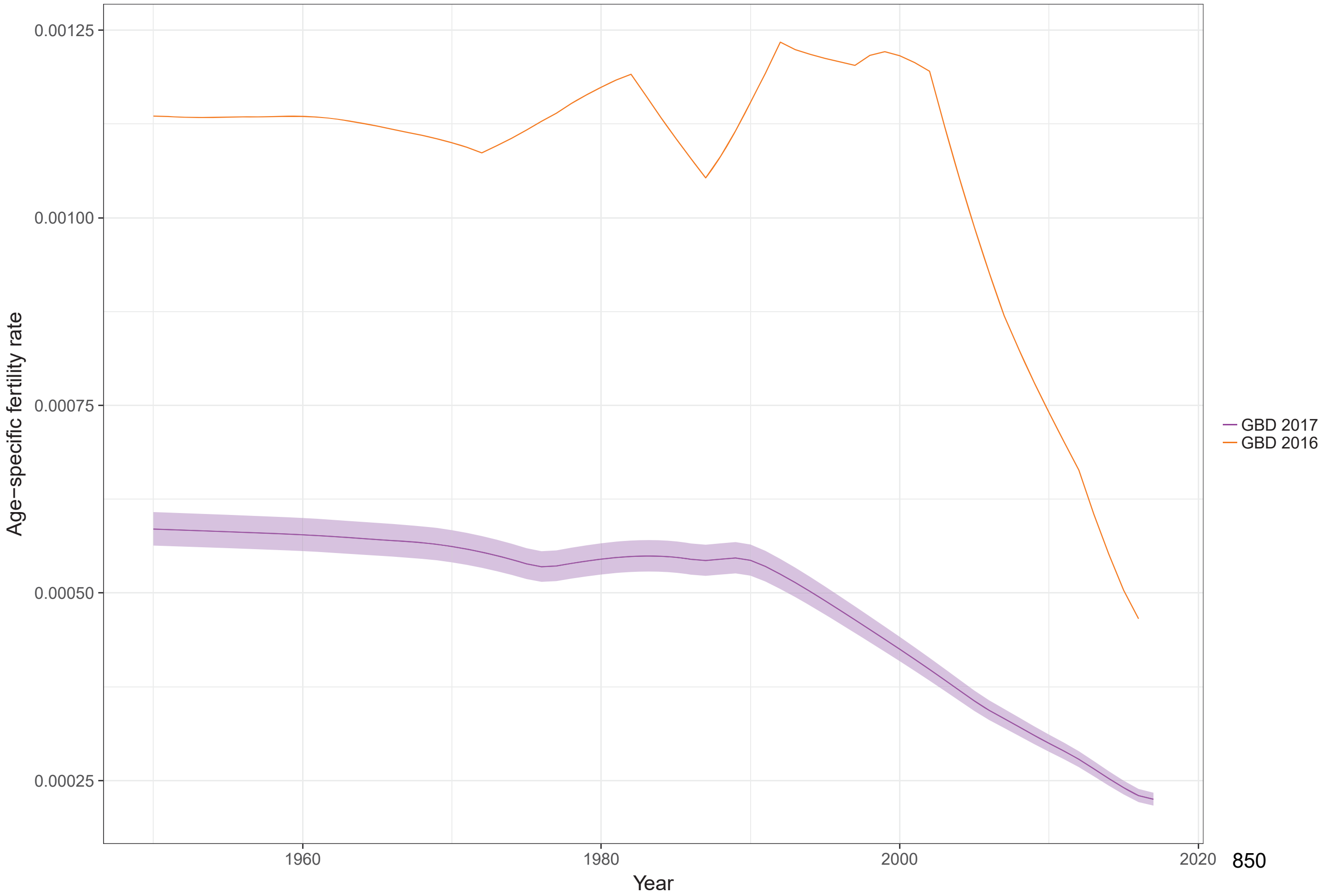

Age−specific fertility fitted trend lines, 1950−2017: Suriname, TFR
Data points enclosed with a diamond are reference sources

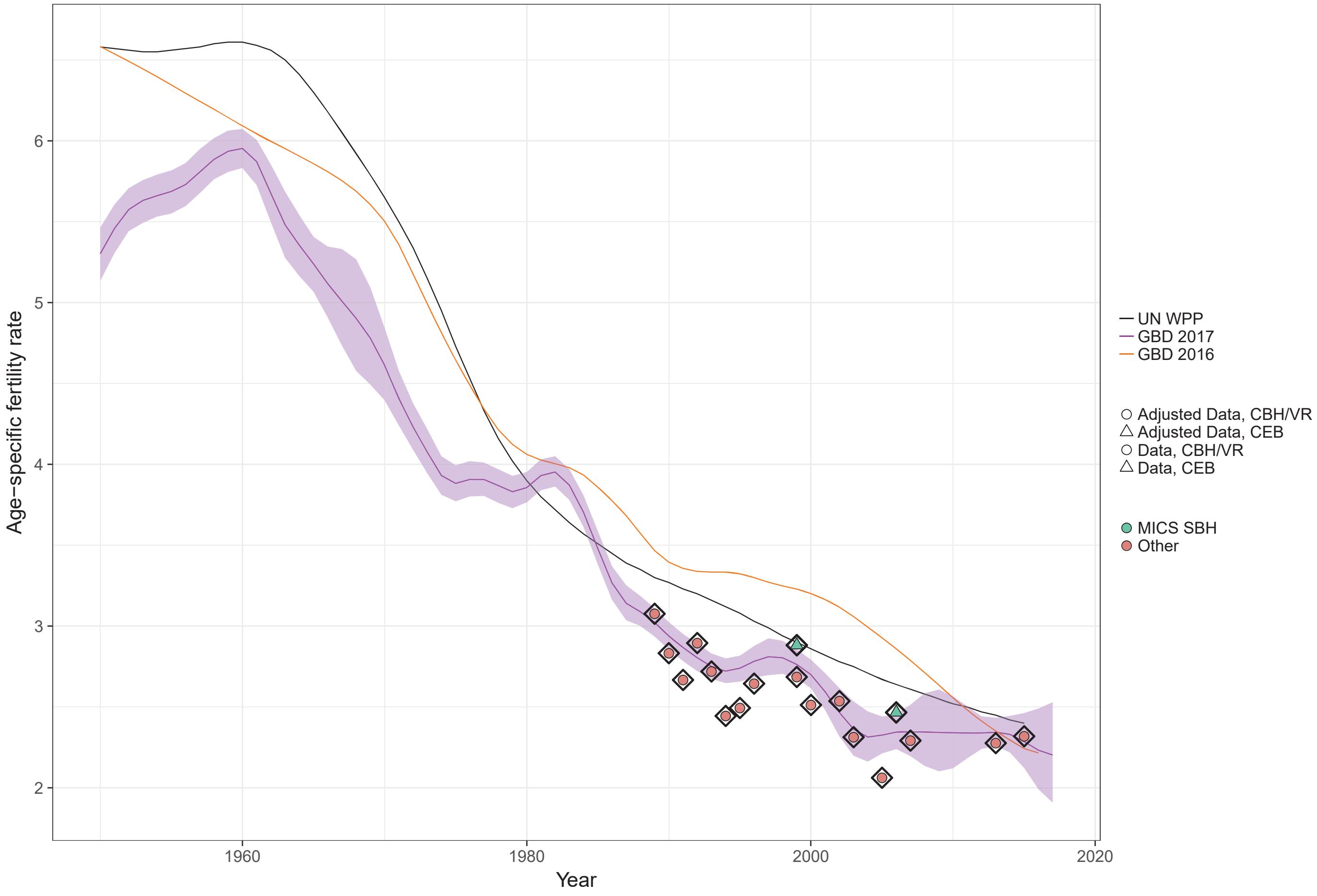

Age−specific fertility fitted trend lines, 1950−2017: Suriname, Ages: 10−14
Data points enclosed with a diamond are reference sources

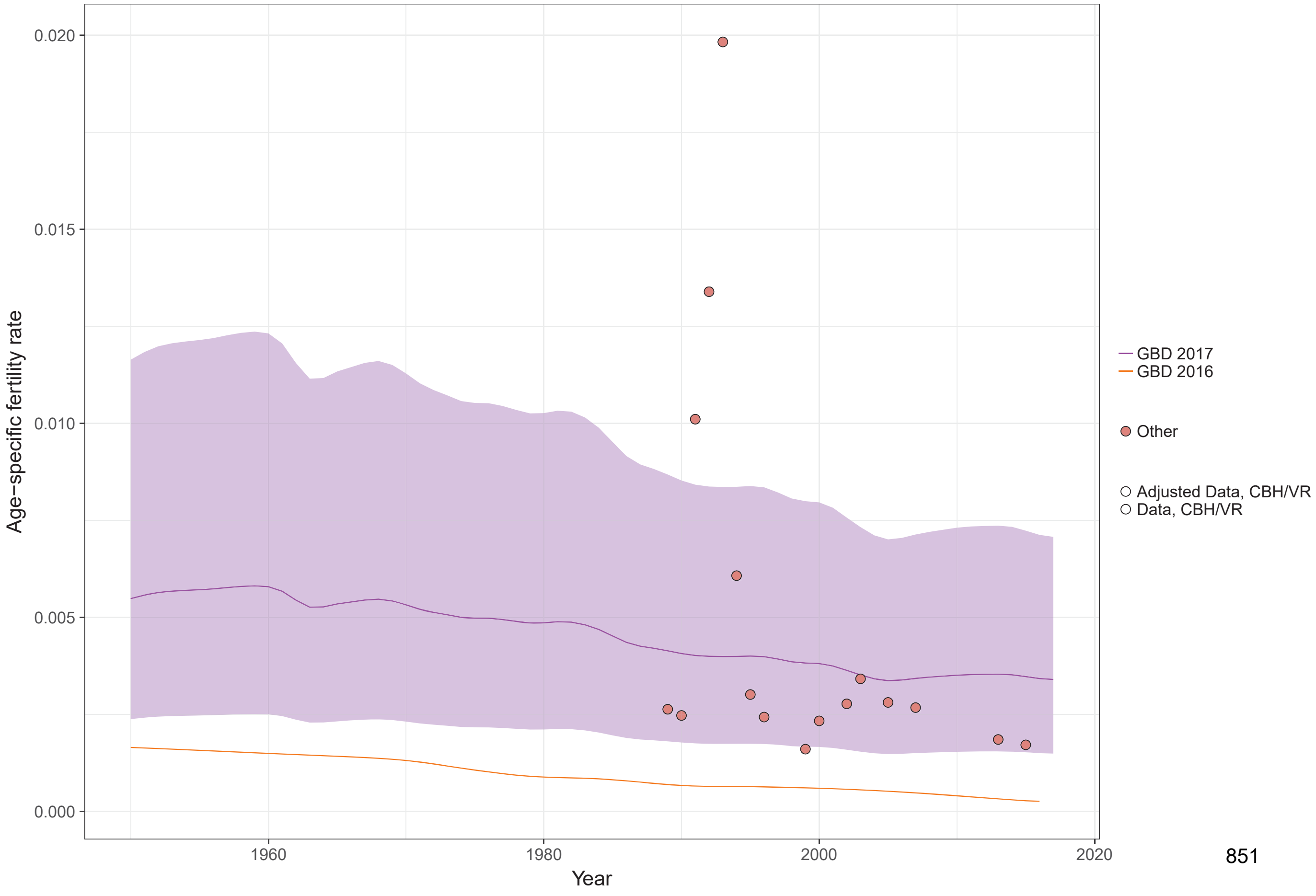

Age−specific fertility fitted trend lines, 1950−2017: Suriname, Ages: 15−19
Data points enclosed with a diamond are reference sources

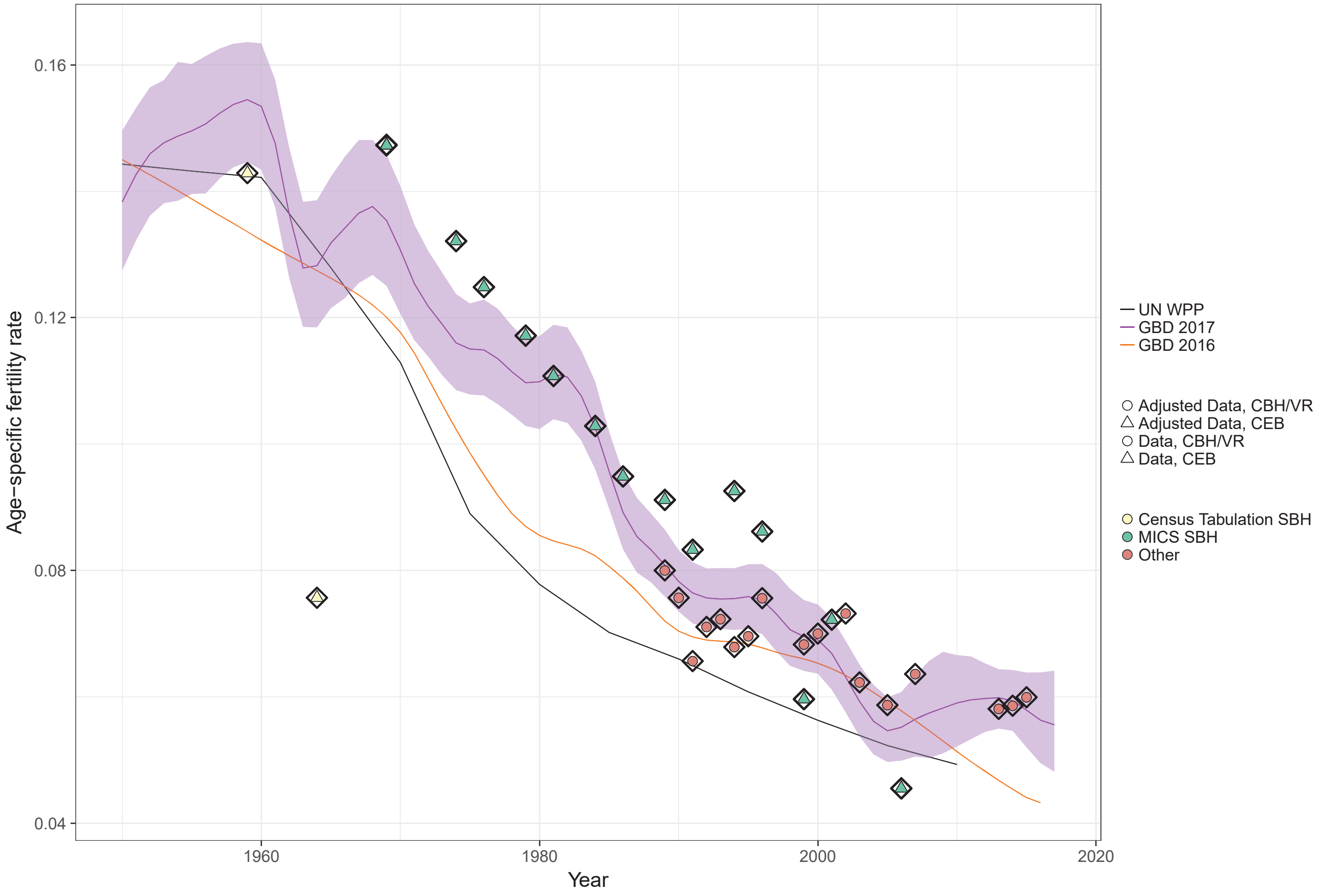

Age−specific fertility fitted trend lines, 1950−2017: Suriname, Ages: 20−24
Data points enclosed with a diamond are reference sources

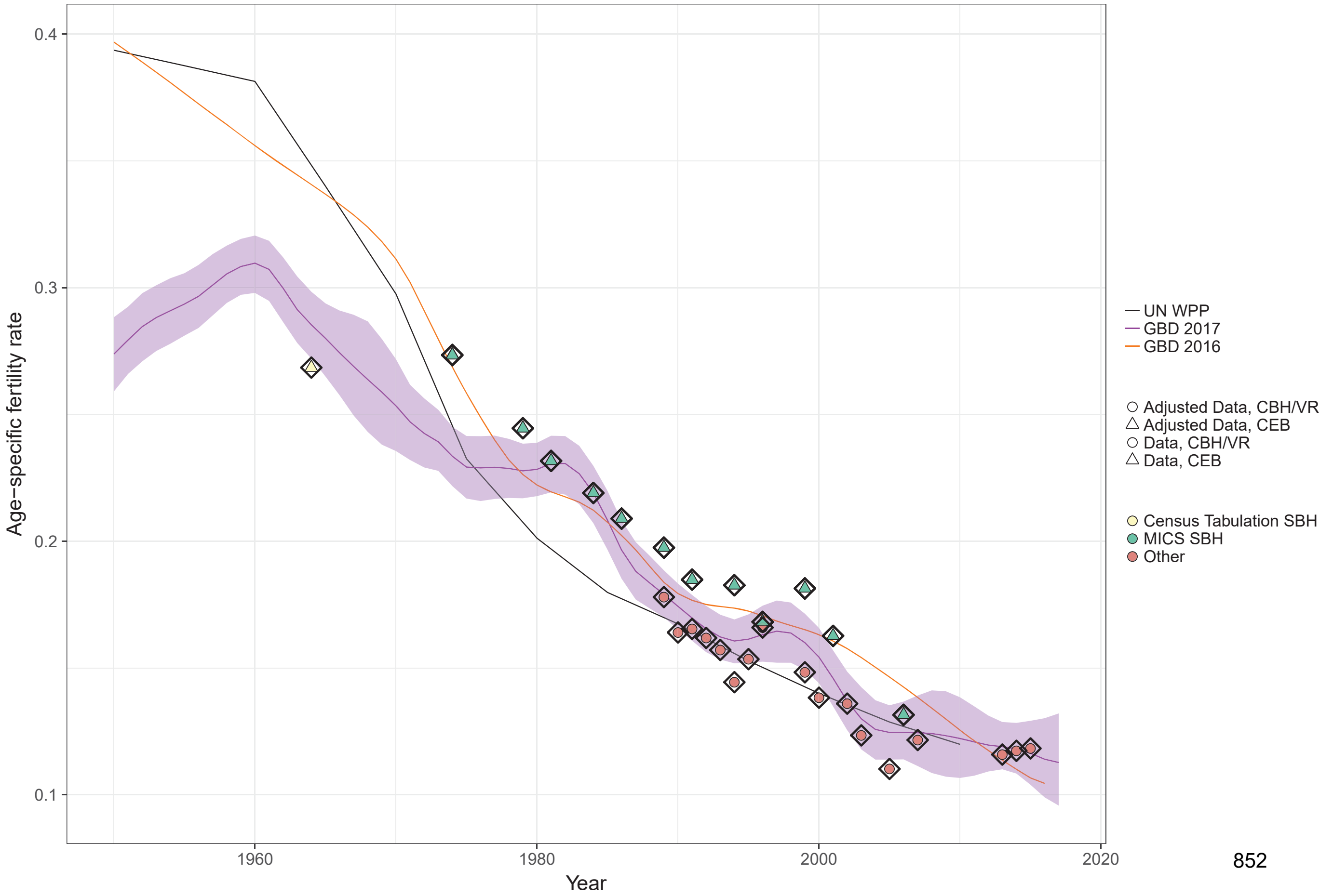

Age−specific fertility fitted trend lines, 1950−2017: Suriname, Ages: 25−29
Data points enclosed with a diamond are reference sources

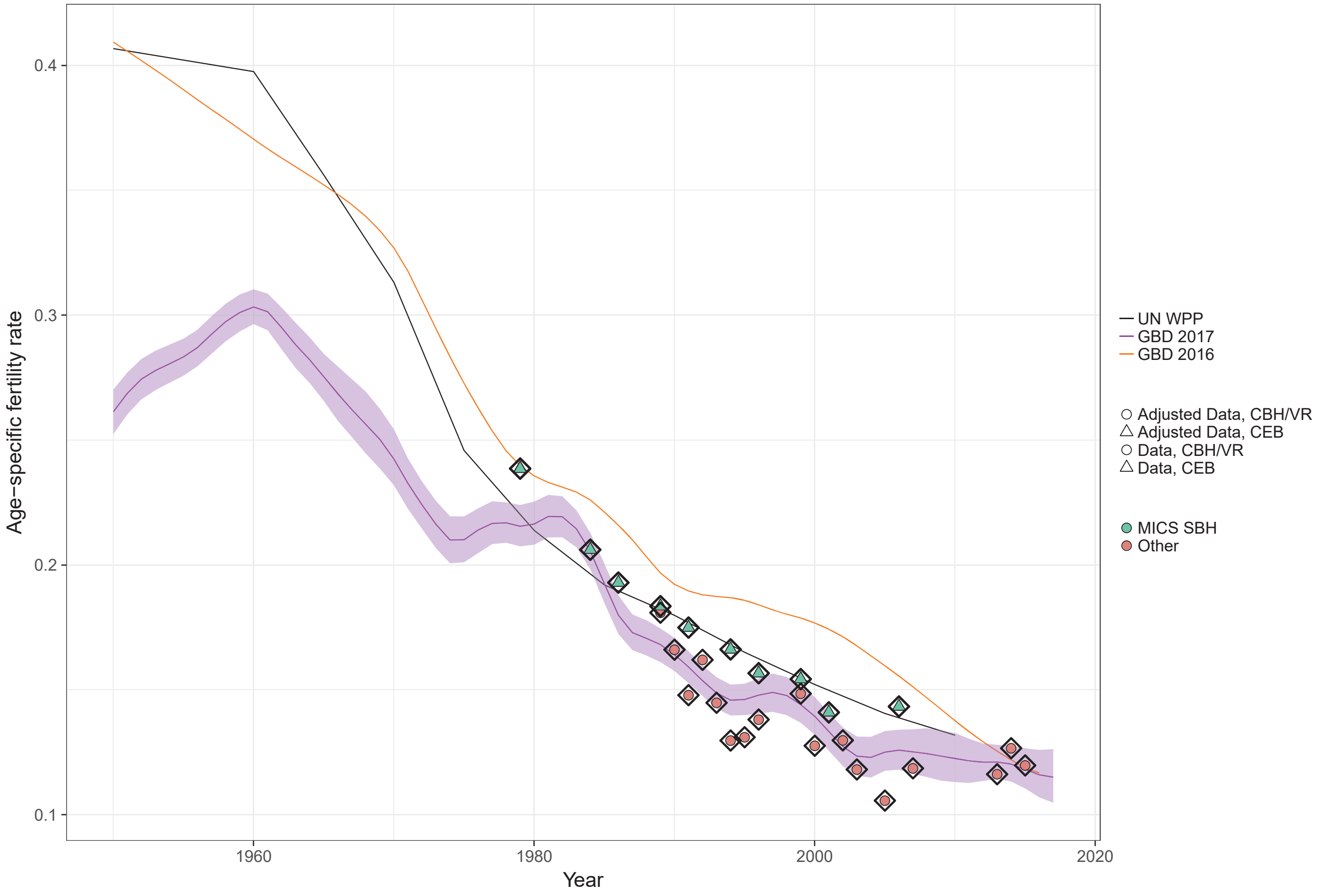

Age−specific fertility fitted trend lines, 1950−2017: Suriname, Ages: 30−34
Data points enclosed with a diamond are reference sources

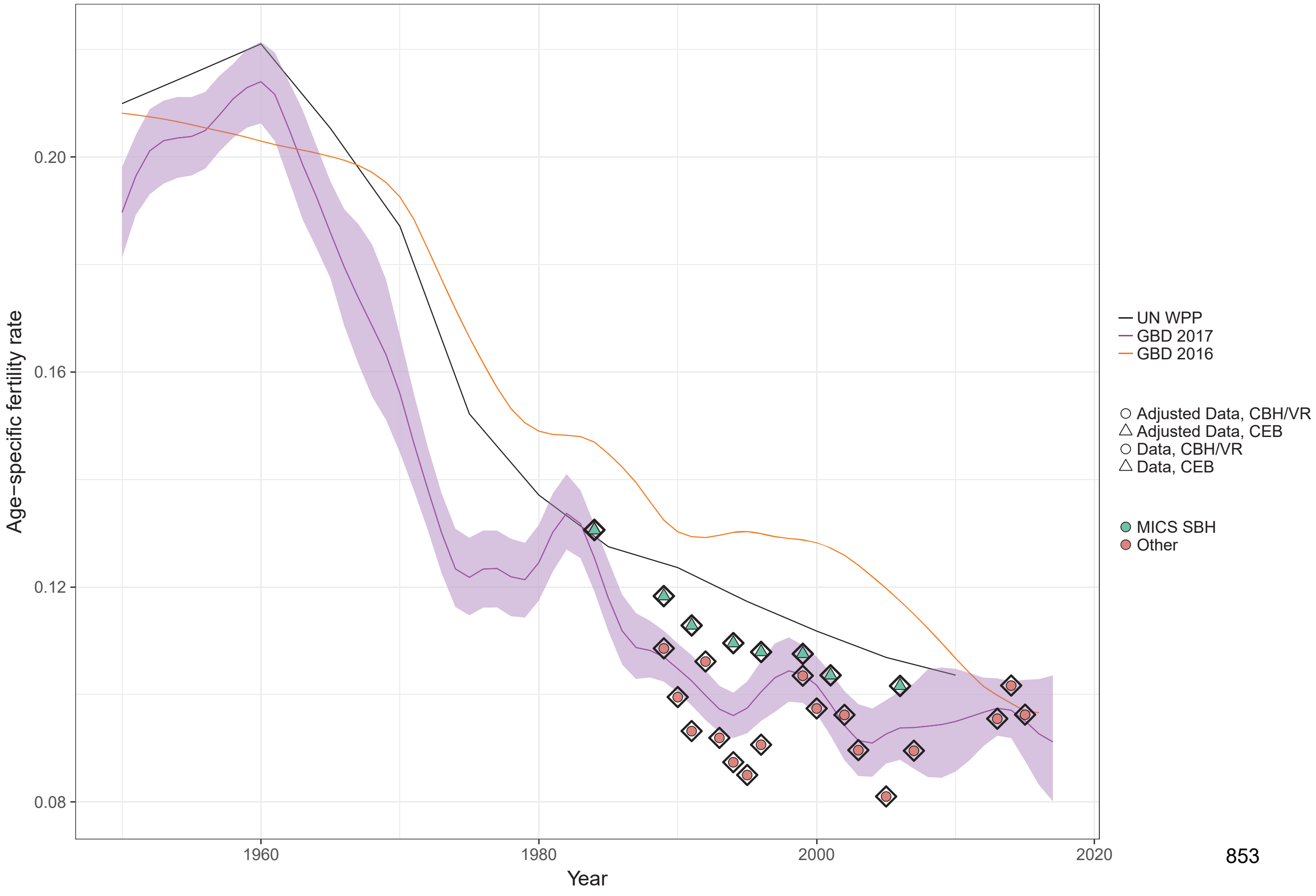

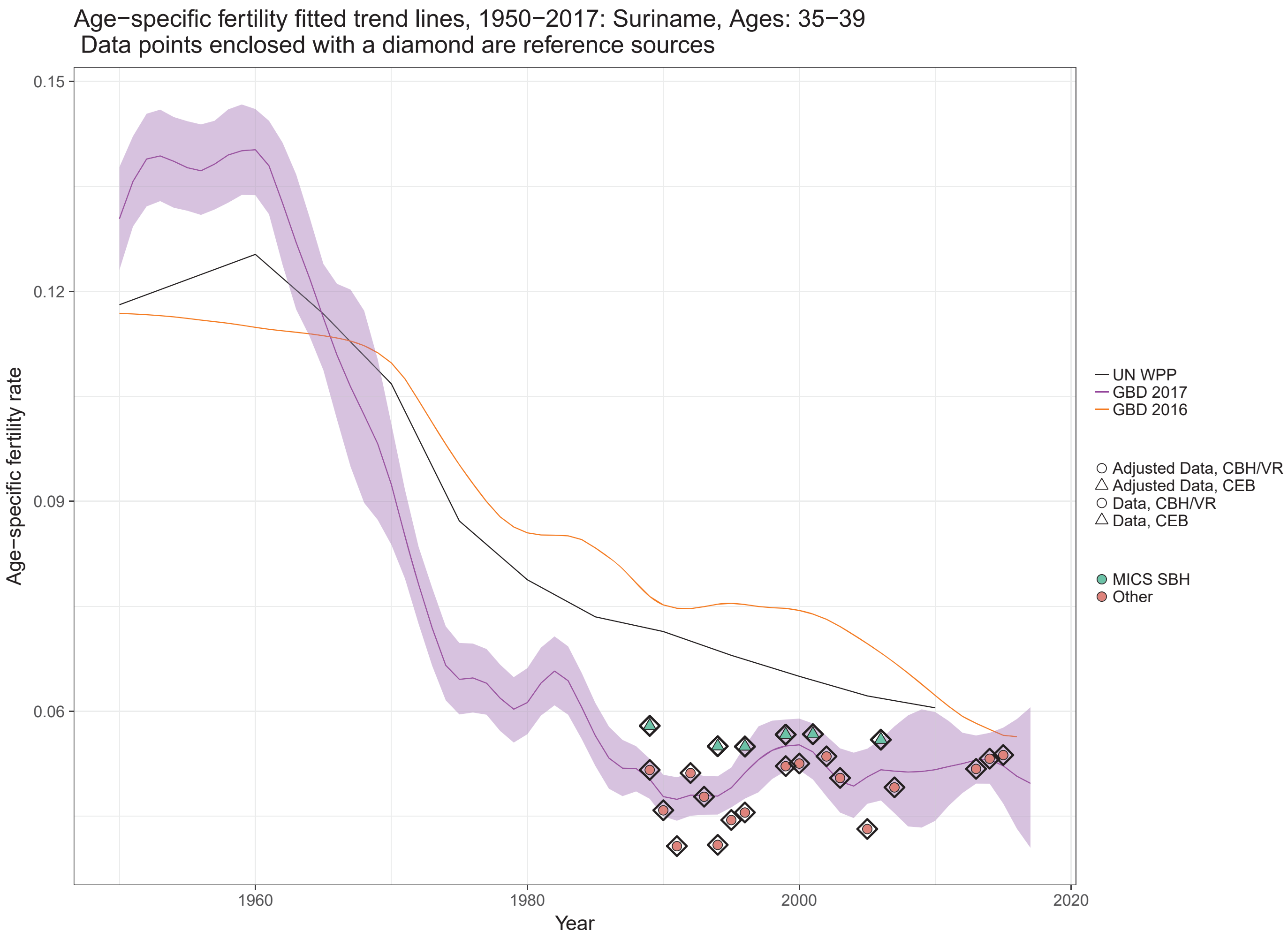
Age−specific fertility fitted trend lines, 1950−2017: Suriname, Ages: 35−39
Data points enclosed with a diamond are reference sources
Age−specific fertility rate
Year
0.15
0.12
0.09
0.06
1960
1980
2000
2020
UN WPP
GBD 2017
GBD 2016
Adjusted Data, CBH/VR
Adjusted Data, CEB
Data, CBH/VR
Data, CEB
MICS SBH
Other

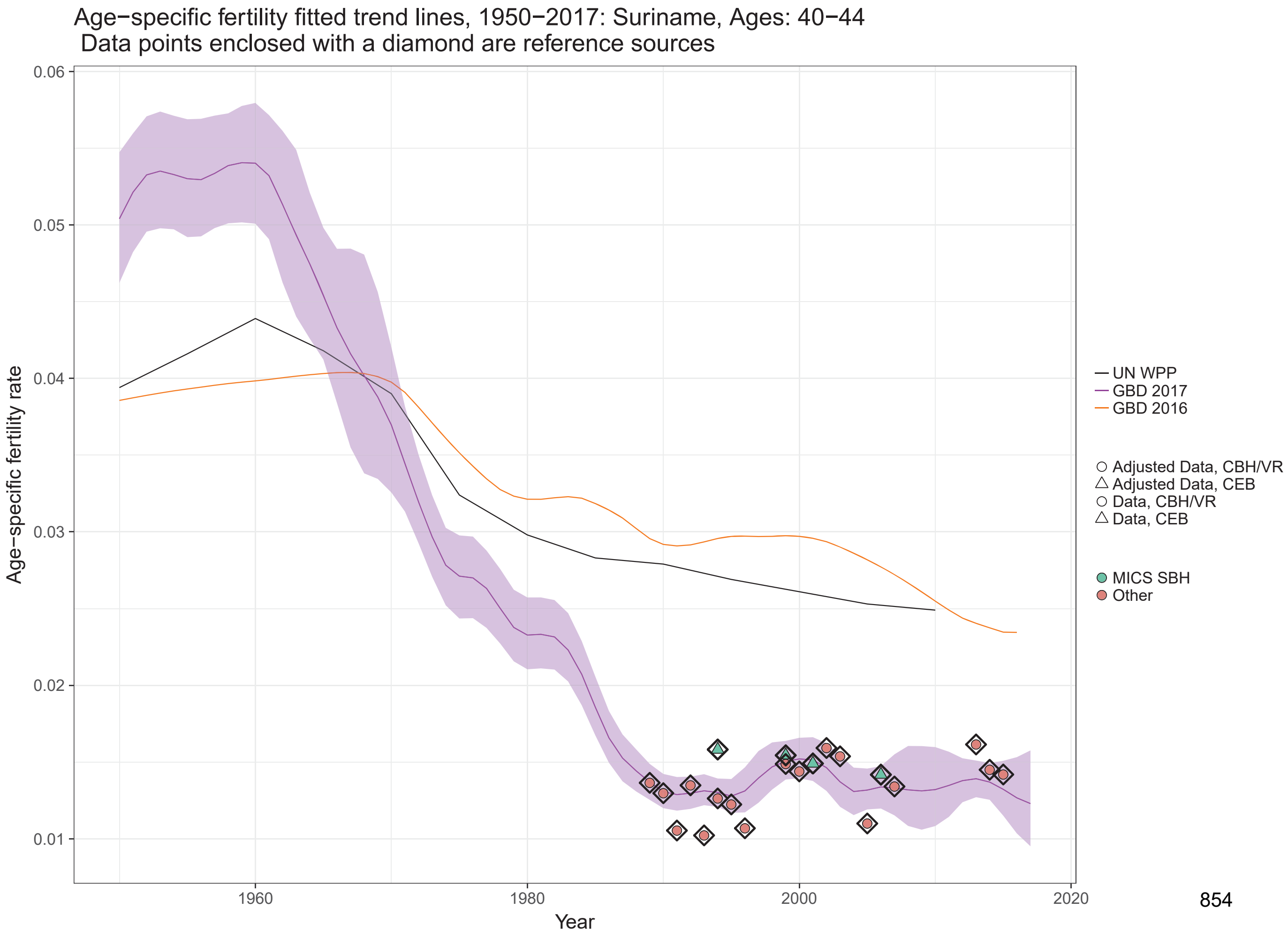
Age−specific fertility fitted trend lines, 1950−2017: Suriname, Ages: 40−44
Data points enclosed with a diamond are reference sources
Age−specific fertility rate
Year
0.06
0.05
0.04
0.03
0.02
0.01
1960
1980
2000
2020
UN WPP
GBD 2017
GBD 2016
Adjusted Data, CBH/VR
Adjusted Data, CEB
Data, CBH/VR
Data, CEB
MICS SBH
Other

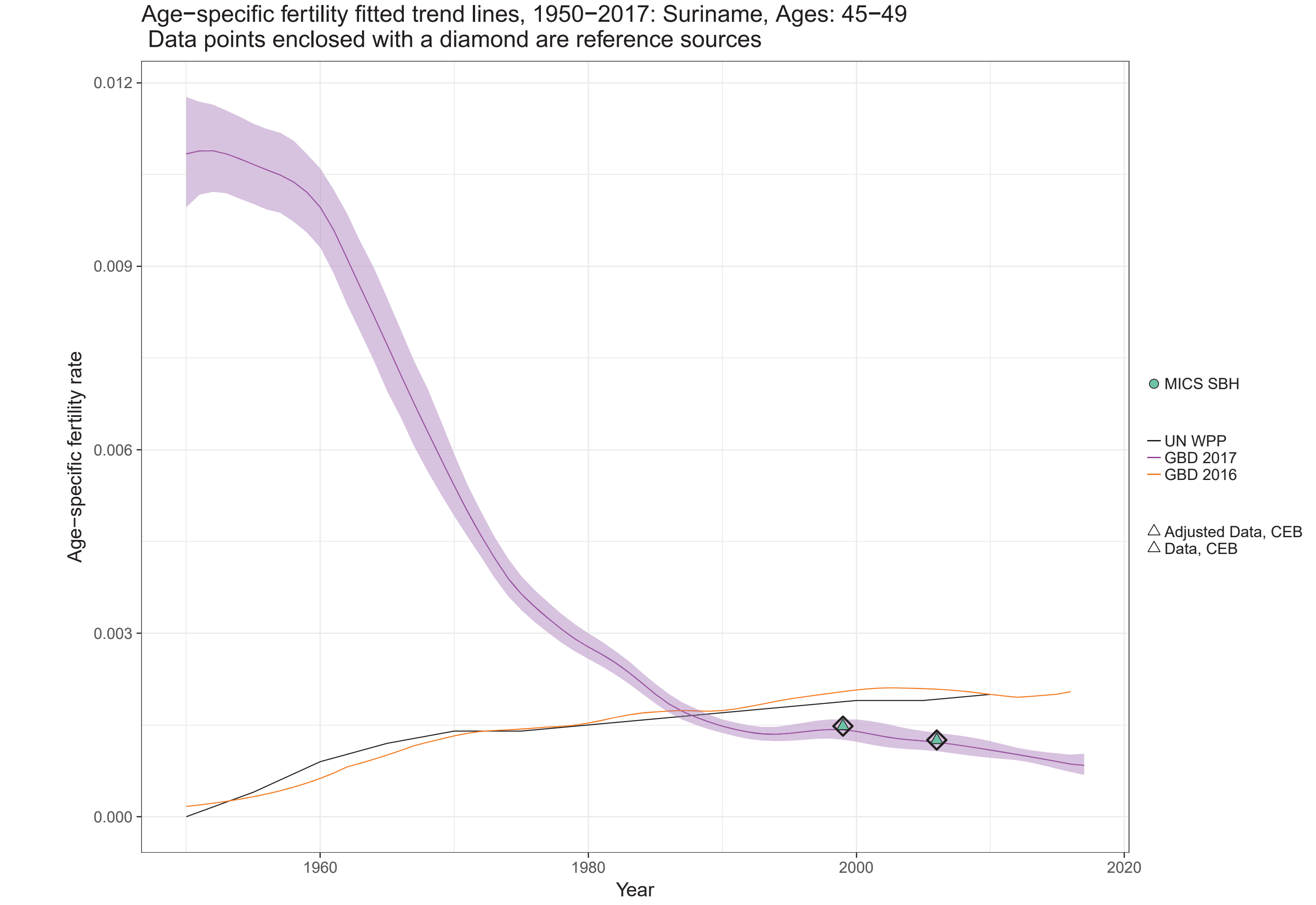

Age−specific fertility fitted trend lines, 1950−2017: Suriname, Ages: 45−49
Data points enclosed with a diamond are reference sources
Age−specific fertility rate
0.012
0.009
0.006
0.003
0.000
1960
1980
2000
2020
Year
MICS SBH
UN WPP
GBD 2017
GBD 2016
Adjusted Data, CEB
Data, CEB

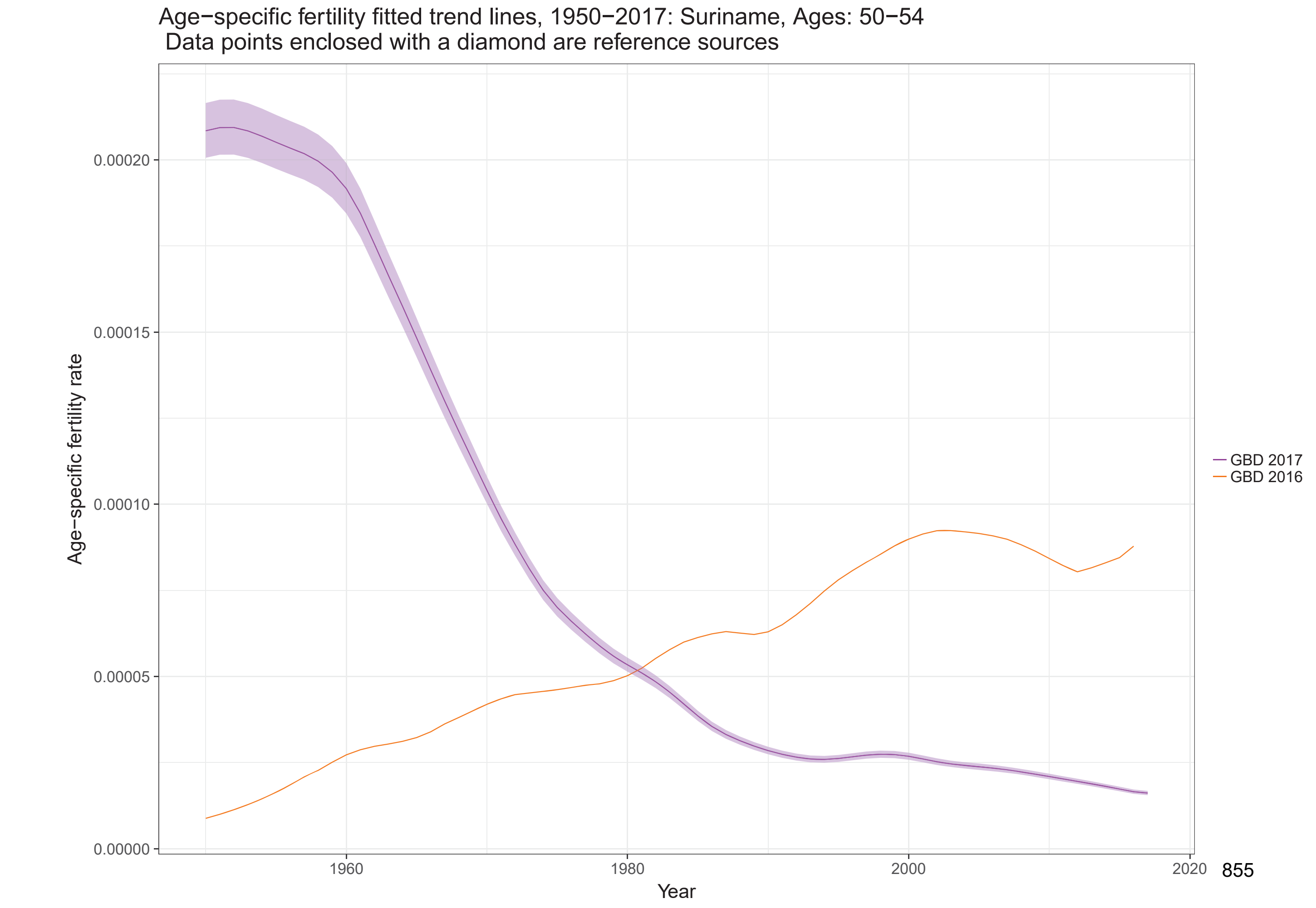

Age−specific fertility fitted trend lines, 1950−2017: Suriname, Ages: 50−54
Data points enclosed with a diamond are reference sources
Age−specific fertility rate
0.00020
0.00015
0.00010
0.00005
0.00000
1960
1980
2000
2020
Year
GBD 2017
GBD 2016

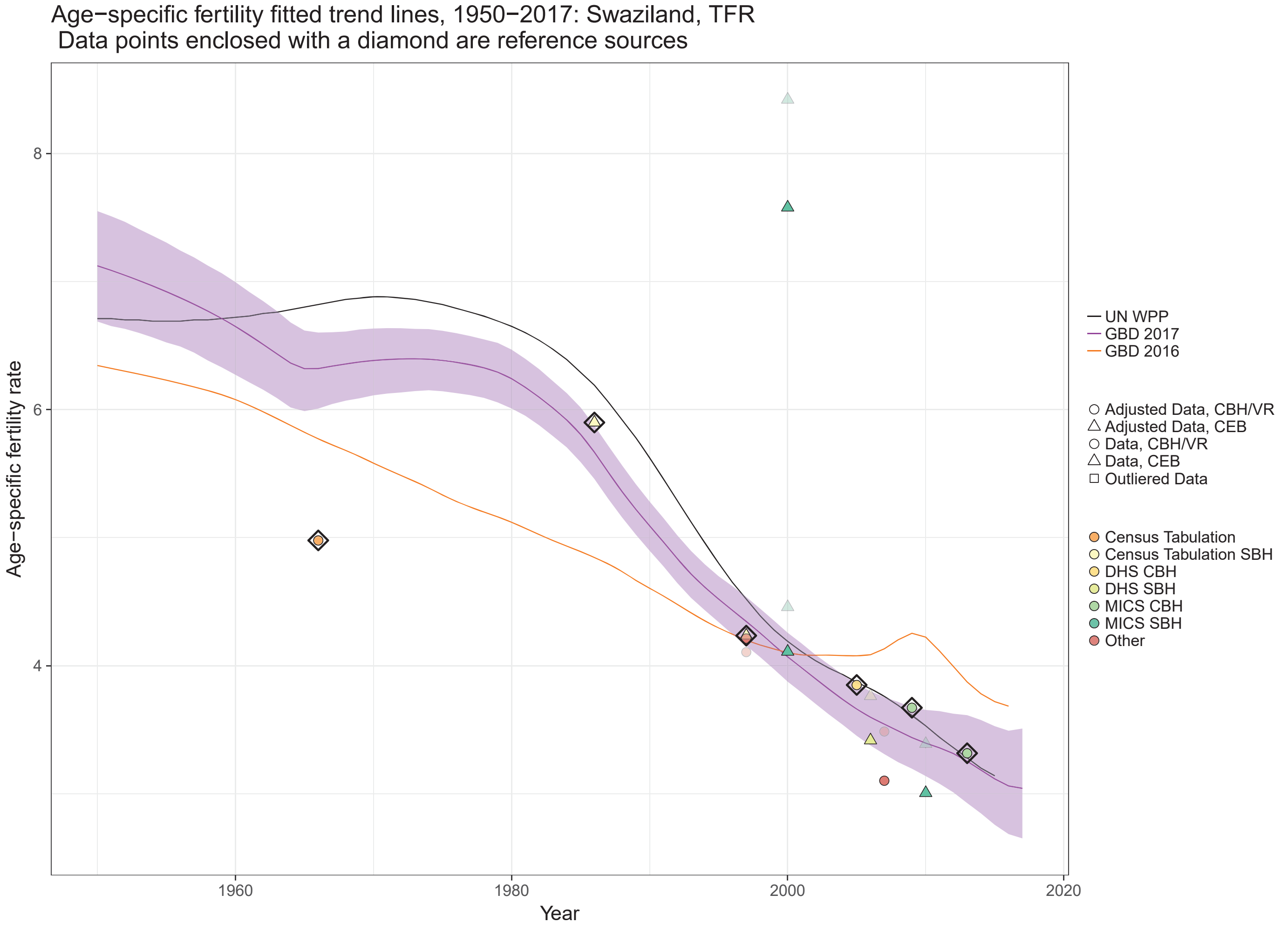

Age−specific fertility fitted trend lines, 1950−2017: Swaziland, TFR
Data points enclosed with a diamond are reference sources
Age−specific fertility rate
8
6
4
1960
1980
2000
2020
Year
UN WPP
GBD 2017
GBD 2016
Adjusted Data, CBH/VR
Adjusted Data, CEB
Data, CBH/VR
Data, CEB
Outliered Data
Census Tabulation
Census Tabulation SBH
DHS CBH
DHS SBH
MICS CBH
MICS SBH
Other

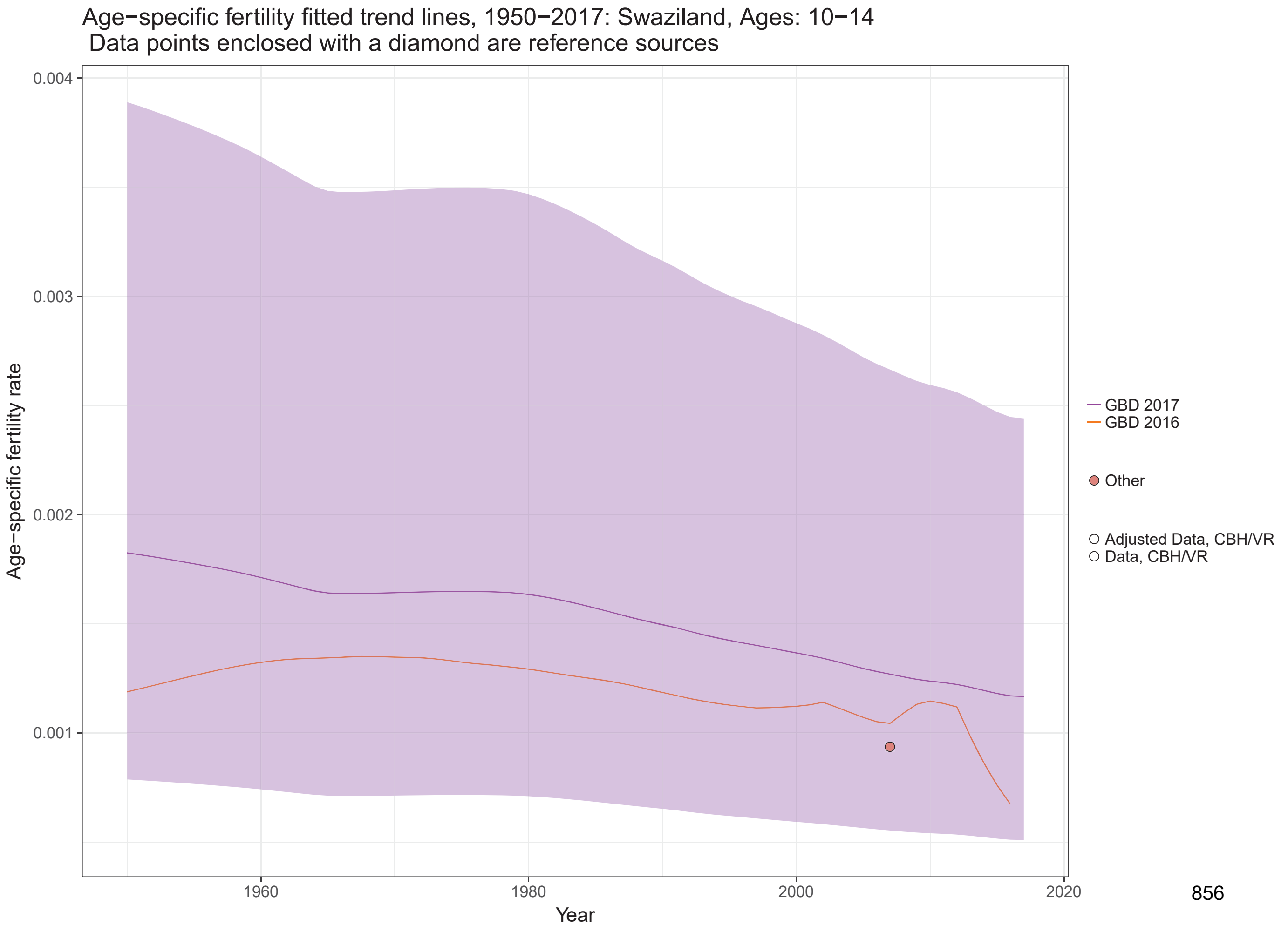

Age−specific fertility fitted trend lines, 1950−2017: Swaziland, Ages: 10−14
Data points enclosed with a diamond are reference sources
Age−specific fertility rate
0.004
0.003
0.002
0.001
1960
1980
2000
2020
Year
GBD 2017
GBD 2016
Other
Adjusted Data, CBH/VR
Data, CBH/VR

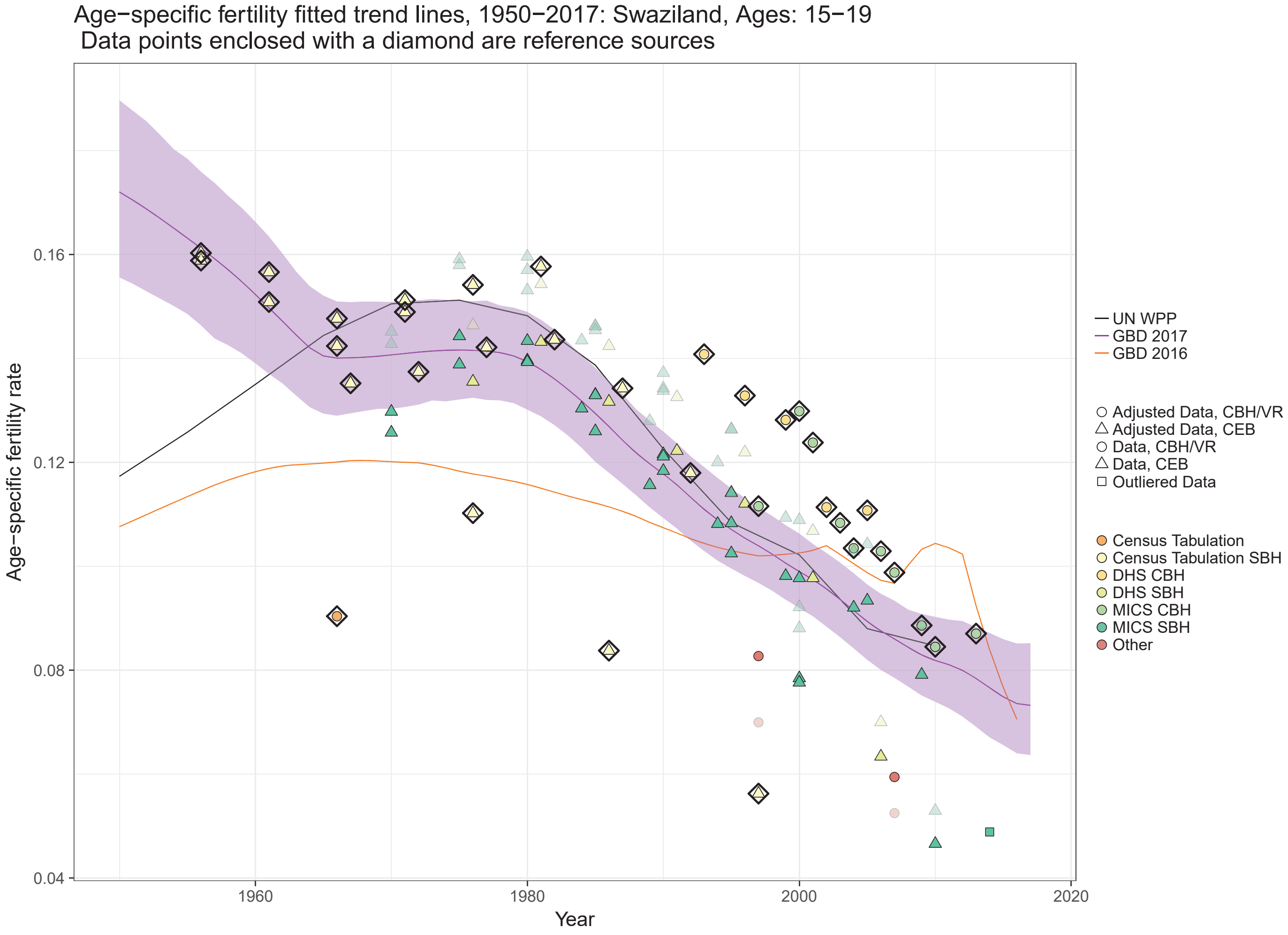
Age−specific fertility fitted trend lines, 1950−2017: Swaziland, Ages: 15−19
Data points enclosed with a diamond are reference sources
Age−specific fertility rate
0.04
0.08
0.12
0.16
1960
1980
2000
2020
Year
UN WPP
GBD 2017
GBD 2016
Adjusted Data, CBH/VR
Adjusted Data, CEB
Data, CBH/VR
Data, CEB
Outliered Data
Census Tabulation
Census Tabulation SBH
DHS CBH
DHS SBH
MICS CBH
MICS SBH
Other

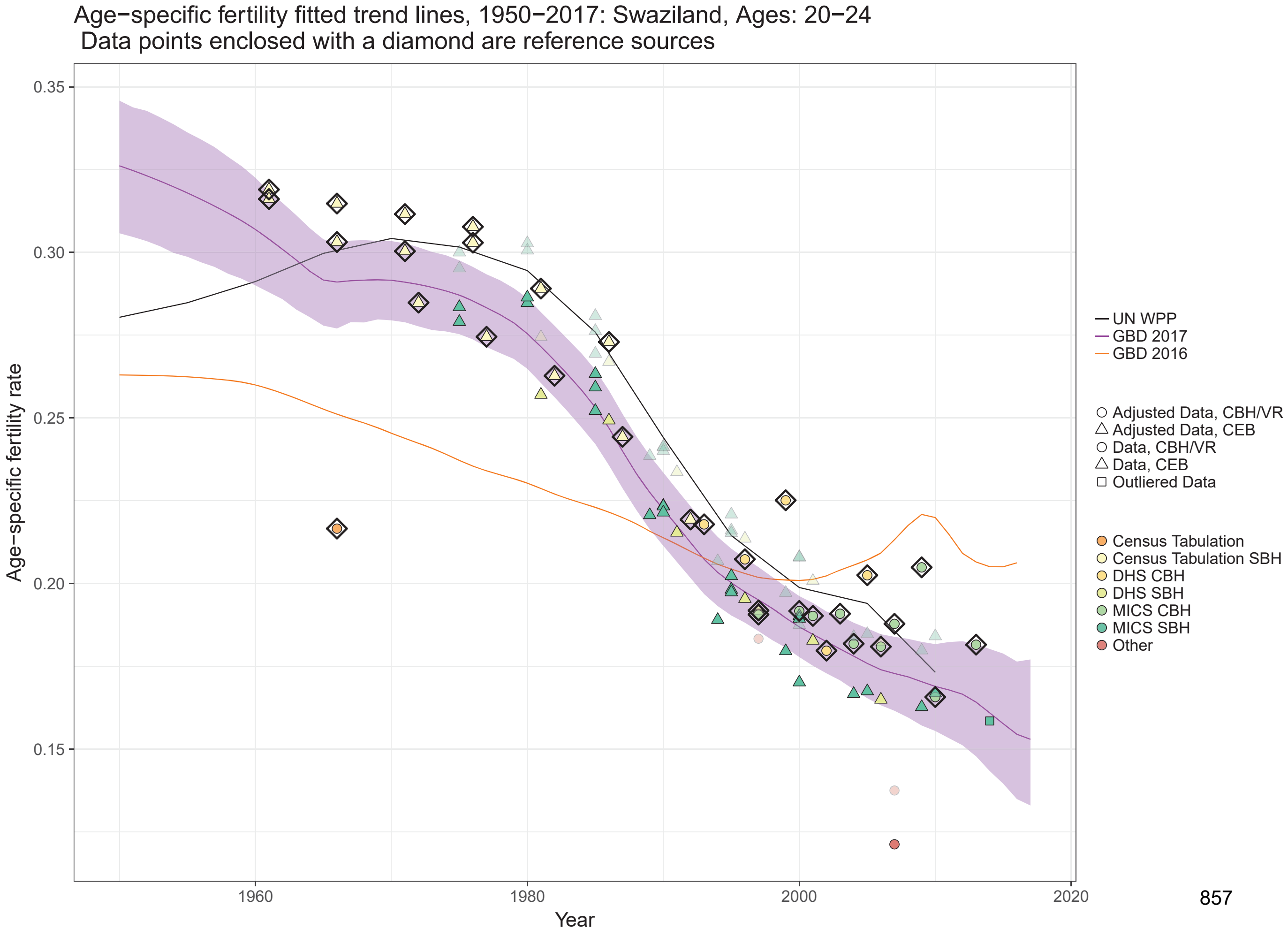
Age−specific fertility fitted trend lines, 1950−2017: Swaziland, Ages: 20−24
Data points enclosed with a diamond are reference sources
Age−specific fertility rate
0.15
0.20
0.25
0.30
0.35
1960
1980
2000
2020
Year
UN WPP
GBD 2017
GBD 2016
Adjusted Data, CBH/VR
Adjusted Data, CEB
Data, CBH/VR
Data, CEB
Outliered Data
Census Tabulation
Census Tabulation SBH
DHS CBH
DHS SBH
MICS CBH
MICS SBH
Other

Age−specific fertility fitted trend lines, 1950−2017: Swaziland, Ages: 25−29
Data points enclosed with a diamond are reference sources

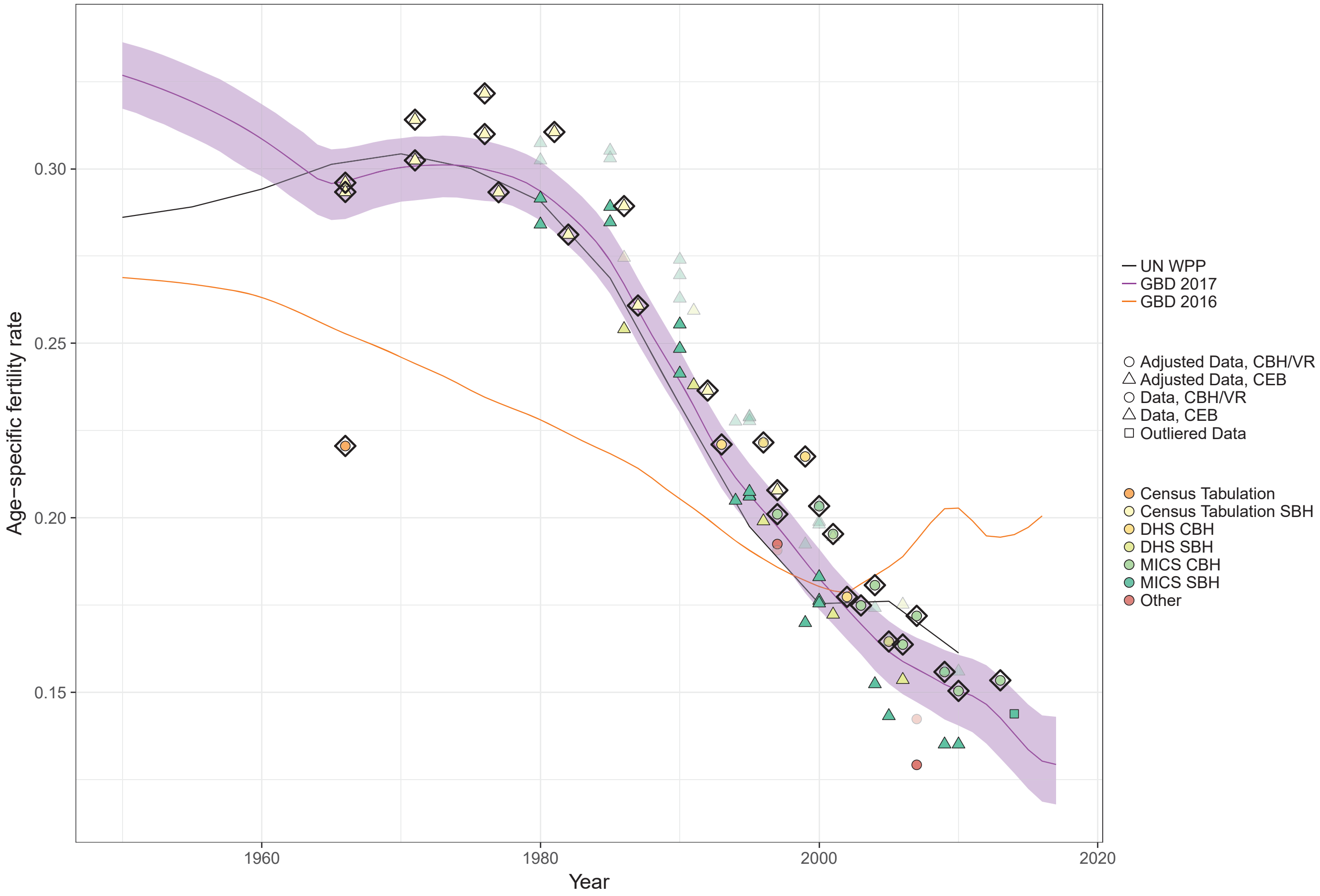

Age−specific fertility fitted trend lines, 1950−2017: Swaziland, Ages: 30−34
Data points enclosed with a diamond are reference sources

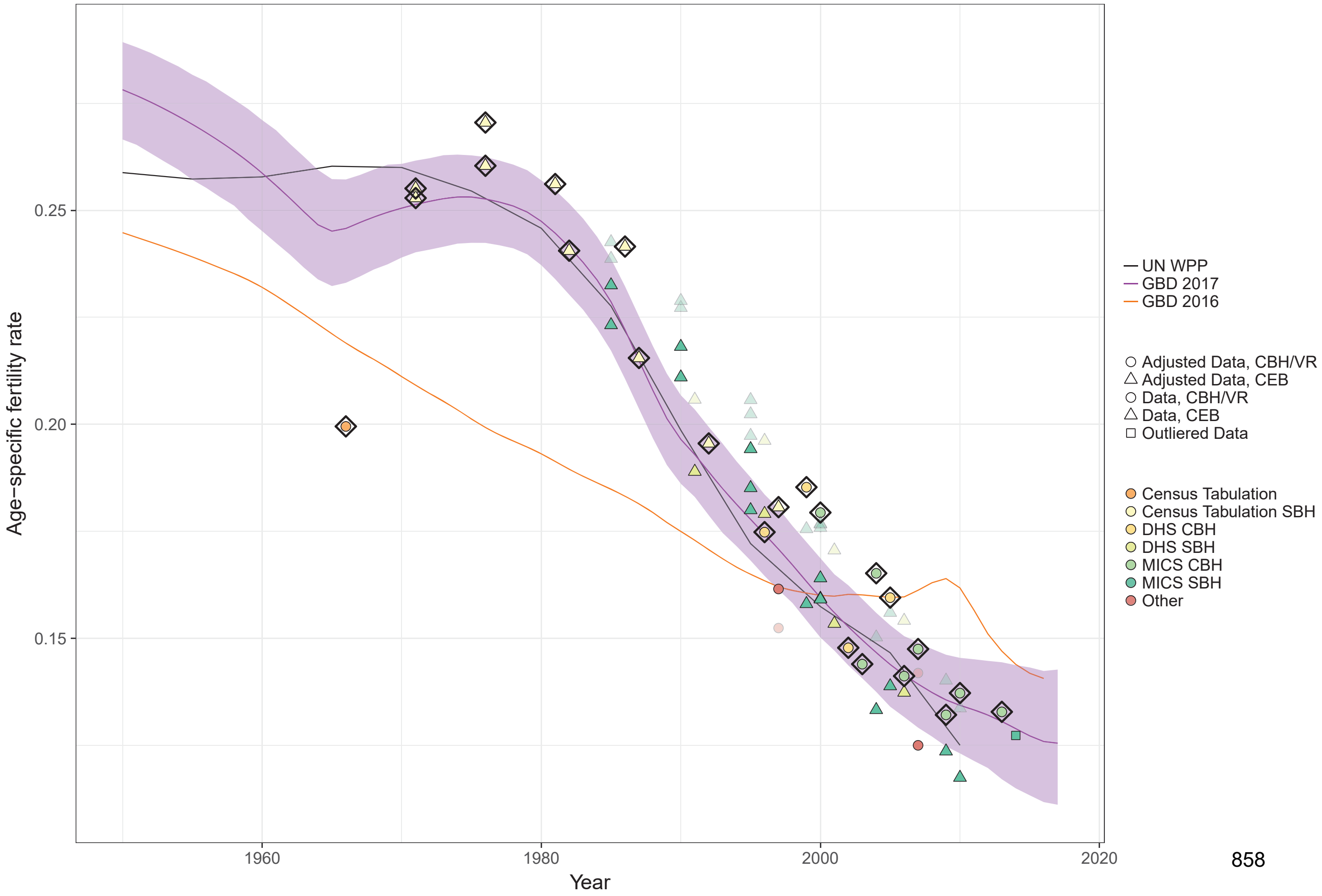

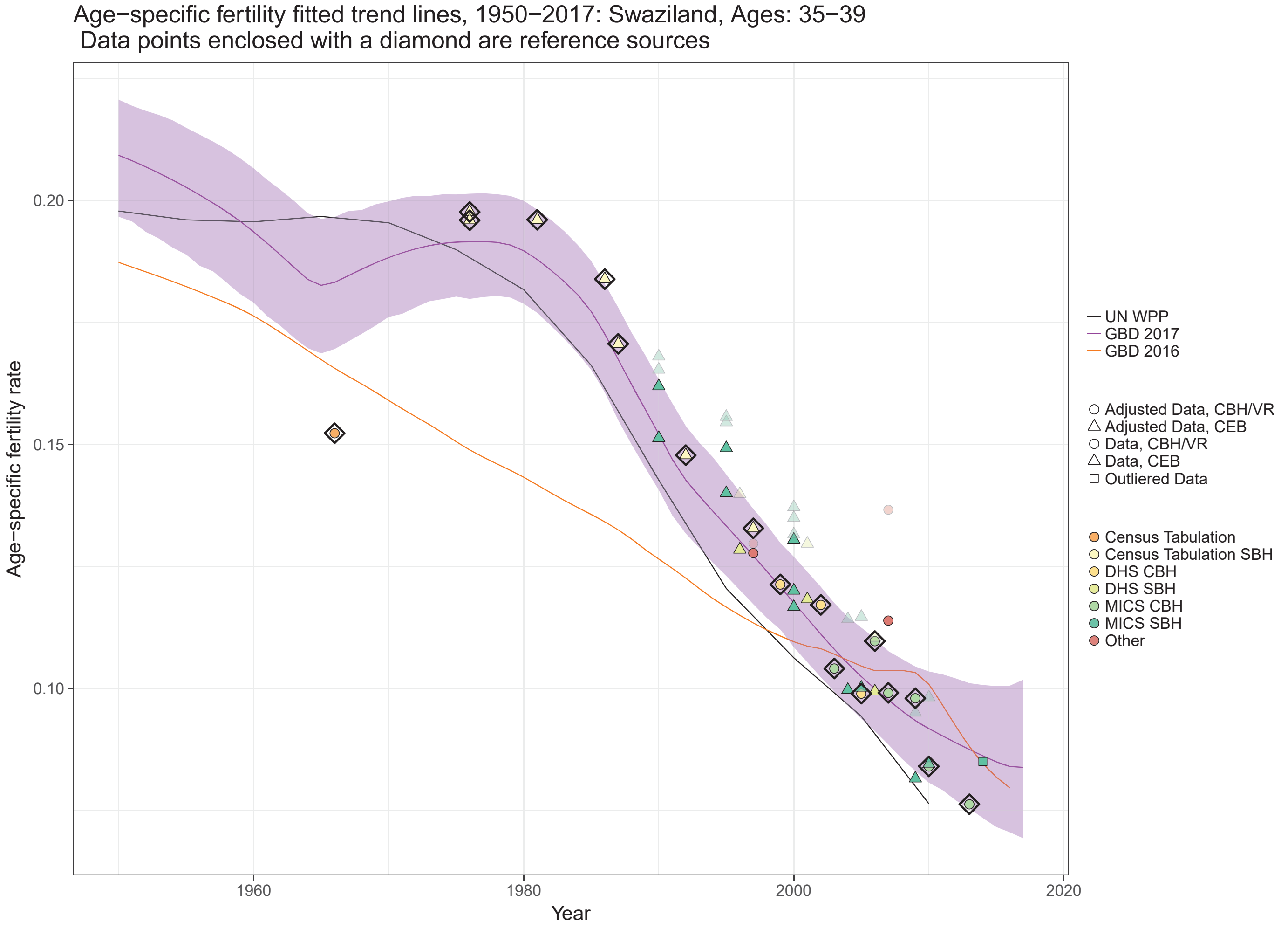

Age−specific fertility fitted trend lines, 1950−2017: Swaziland, Ages: 35−39
Data points enclosed with a diamond are reference sources
Age−specific fertility rate
Year
0.10
0.15
0.20
1960
1980
2000
2020
UN WPP
GBD 2017
GBD 2016
Adjusted Data, CBH/VR
Adjusted Data, CEB
Data, CBH/VR
Data, CEB
Outliered Data
Census Tabulation
Census Tabulation SBH
DHS CBH
DHS SBH
MICS CBH
MICS SBH
Other

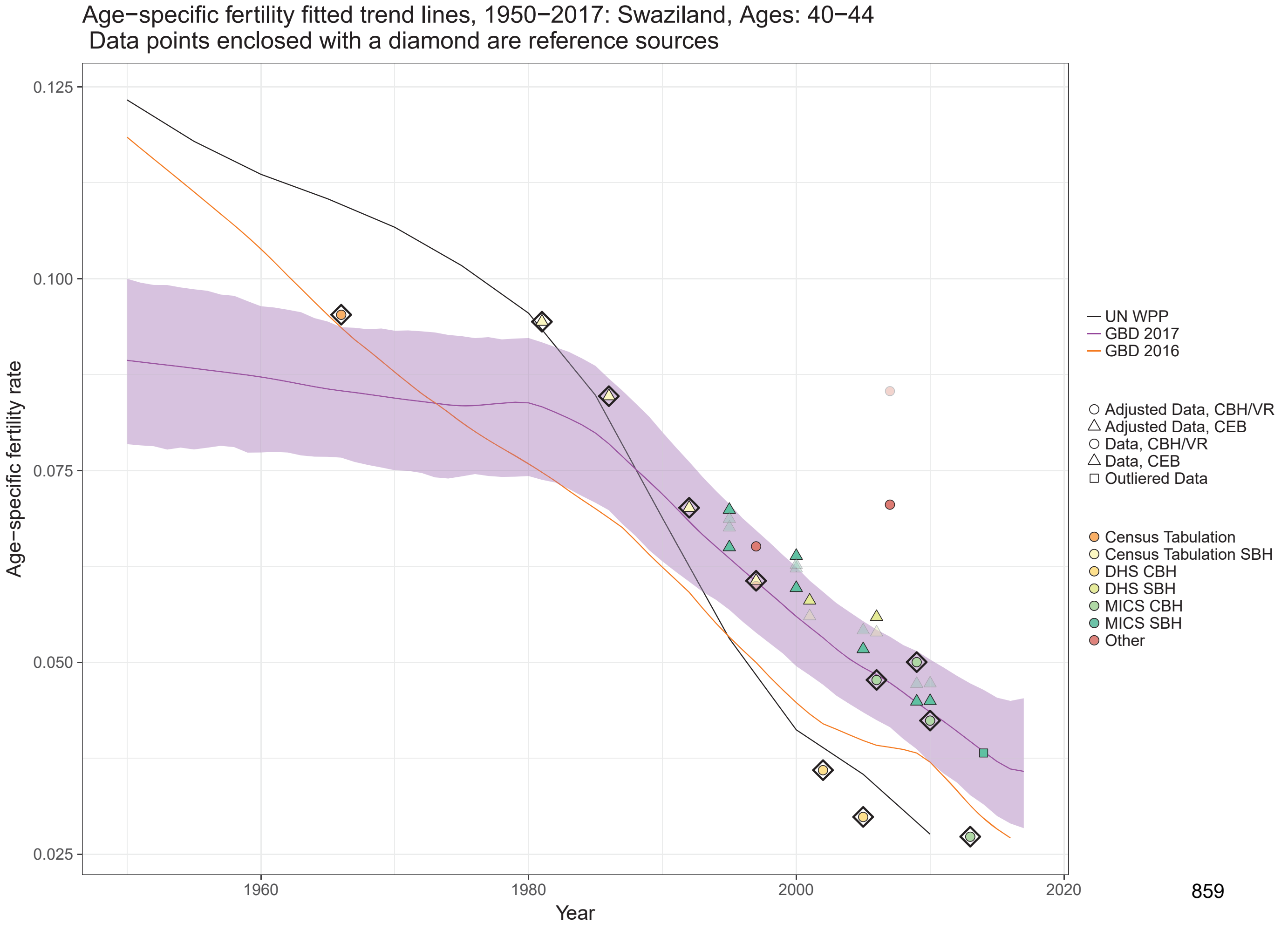

Age−specific fertility fitted trend lines, 1950−2017: Swaziland, Ages: 40−44
Data points enclosed with a diamond are reference sources
Age−specific fertility rate
Year
0.025
0.050
0.075
0.100
0.125
1960
1980
2000
2020
UN WPP
GBD 2017
GBD 2016
Adjusted Data, CBH/VR
Adjusted Data, CEB
Data, CBH/VR
Data, CEB
Outliered Data
Census Tabulation
Census Tabulation SBH
DHS CBH
DHS SBH
MICS CBH
MICS SBH
Other

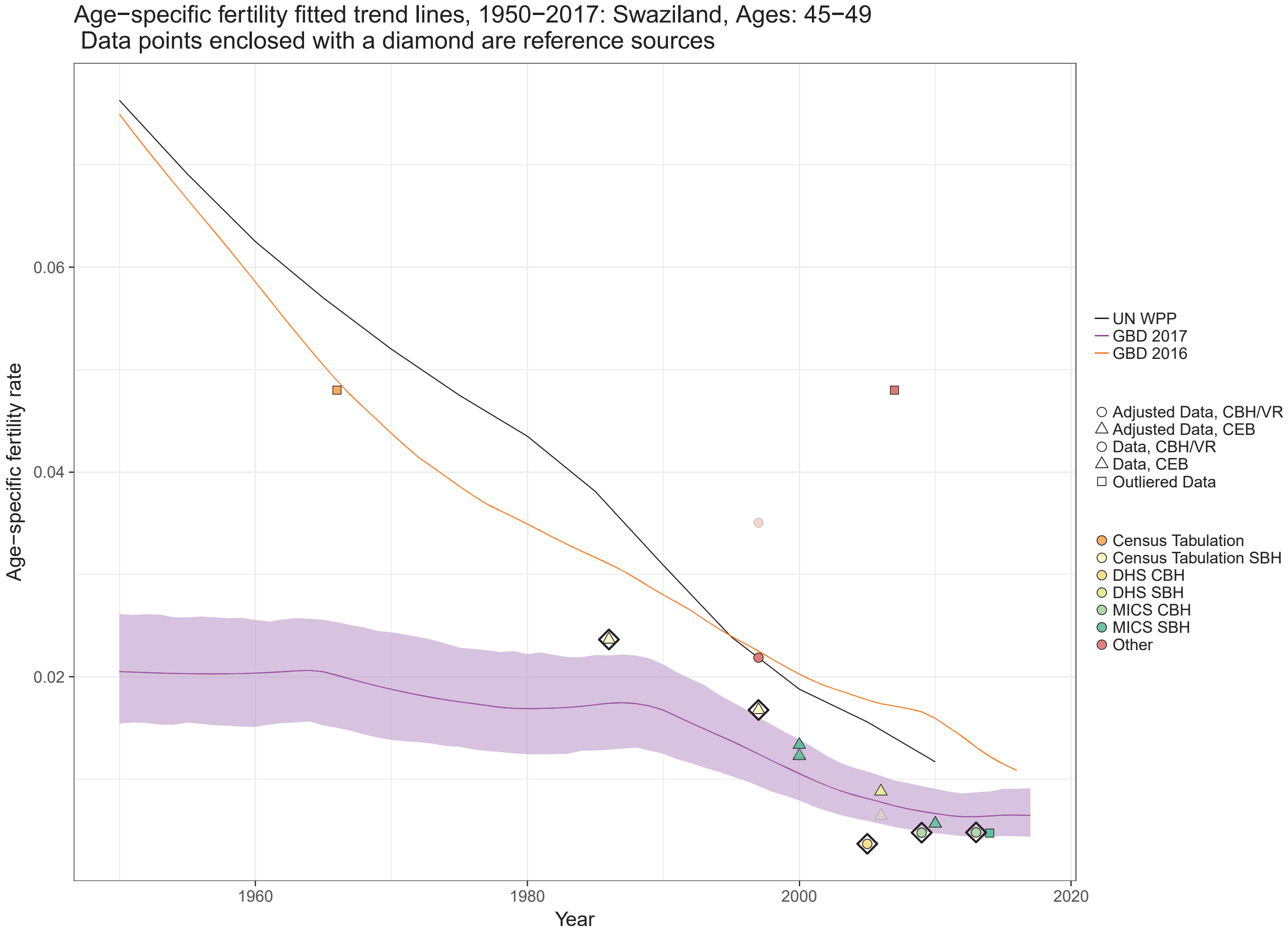

Age−specific fertility fitted trend lines, 1950−2017: Swaziland, Ages: 45−49
Data points enclosed with a diamond are reference sources
Age−specific fertility rate
0.06
0.04
0.02
1960
1980
2000
2020
Year
UN WPP
GBD 2017
GBD 2016
Adjusted Data, CBH/VR
Adjusted Data, CEB
Data, CBH/VR
Data, CEB
Outliered Data
Census Tabulation
Census Tabulation SBH
DHS CBH
DHS SBH
MICS CBH
MICS SBH
Other

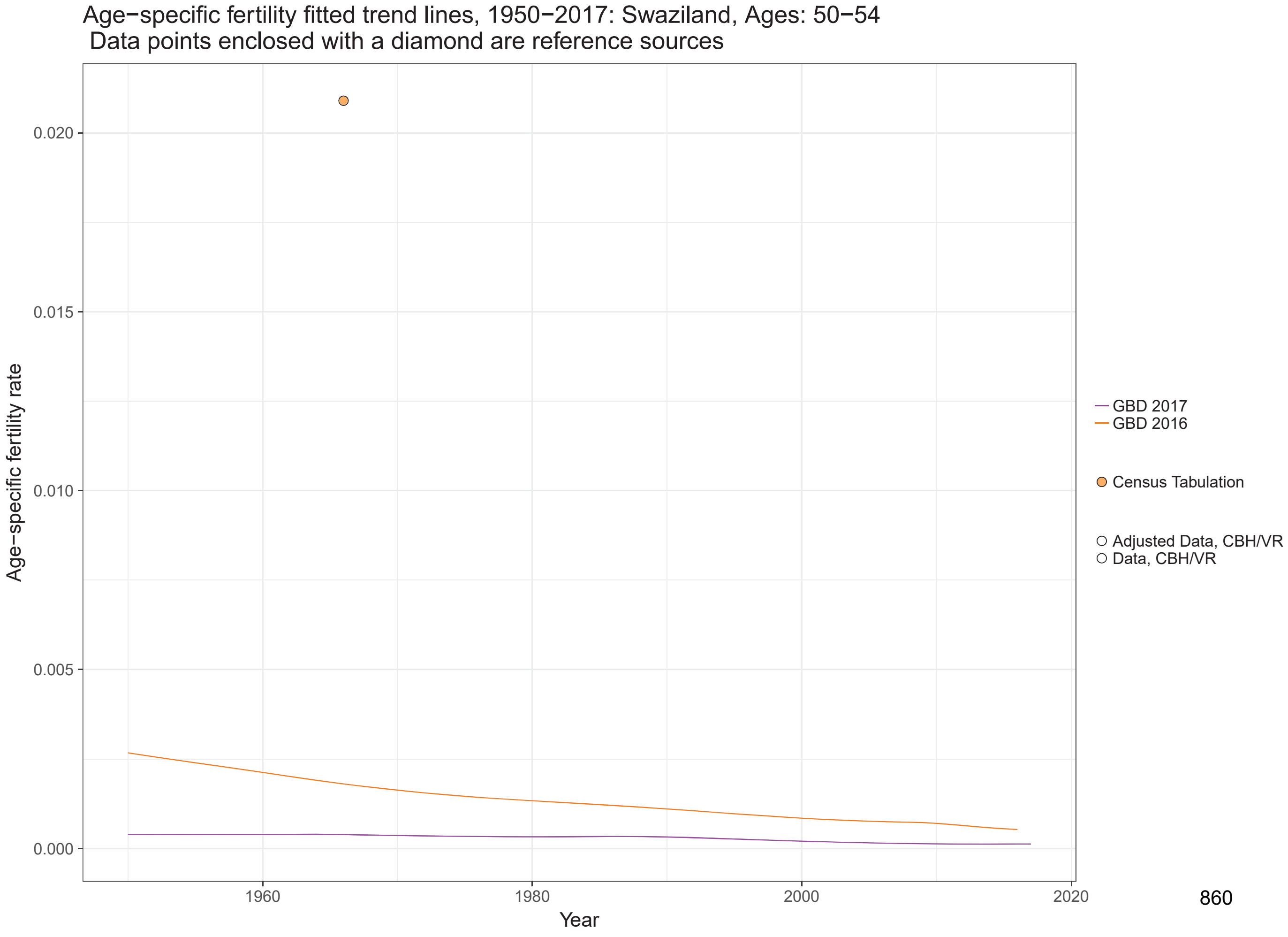

Age−specific fertility fitted trend lines, 1950−2017: Swaziland, Ages: 50−54
Data points enclosed with a diamond are reference sources
Age−specific fertility rate
0.020
0.015
0.010
0.005
0.000
1960
1980
2000
2020
Year
GBD 2017
GBD 2016
Census Tabulation
Adjusted Data, CBH/VR
Data, CBH/VR

Age−specific fertility fitted trend lines, 1950−2017: Sweden, TFR
Data points enclosed with a diamond are reference sources

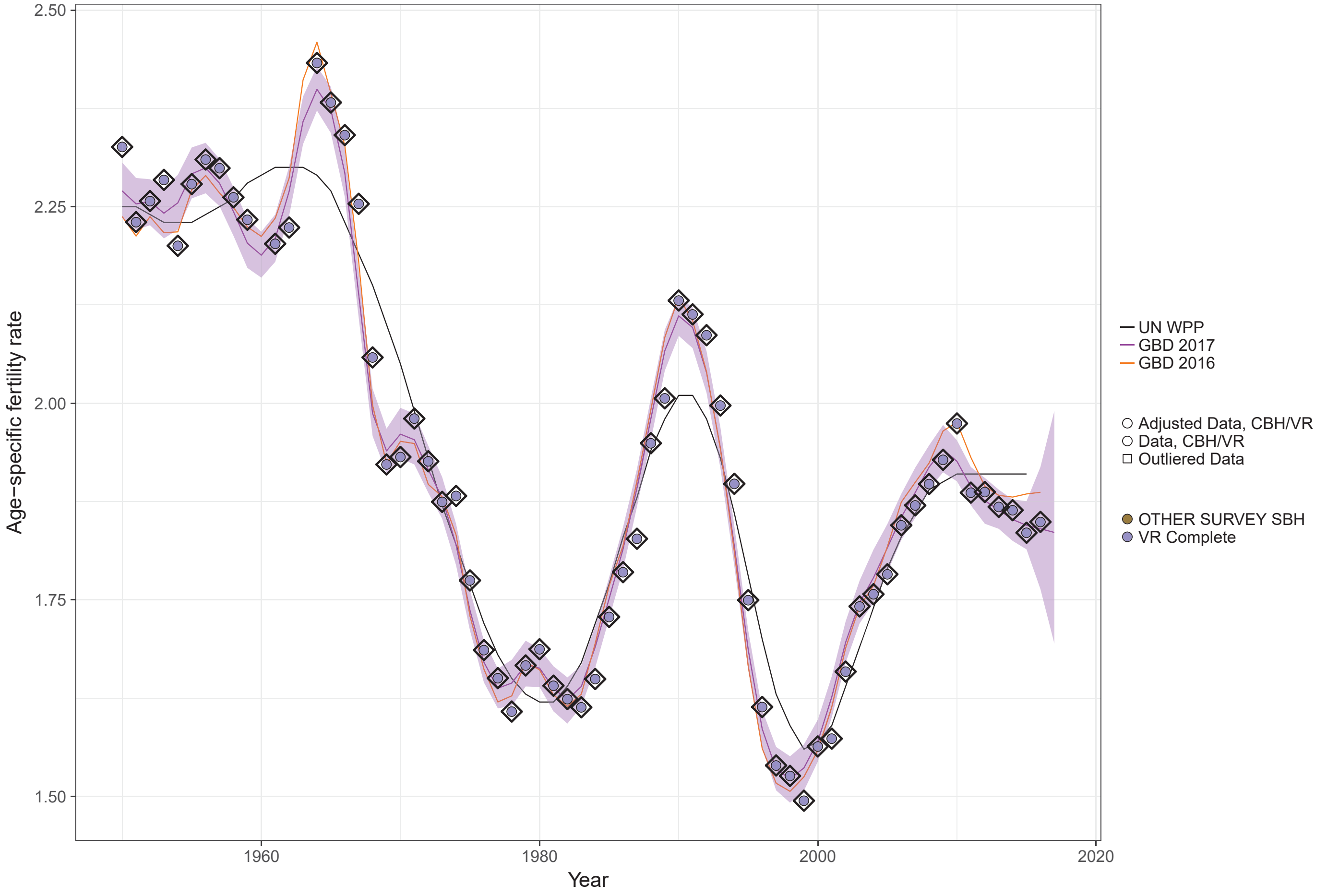

Age−specific fertility fitted trend lines, 1950−2017: Sweden, Ages: 10−14
Data points enclosed with a diamond are reference sources

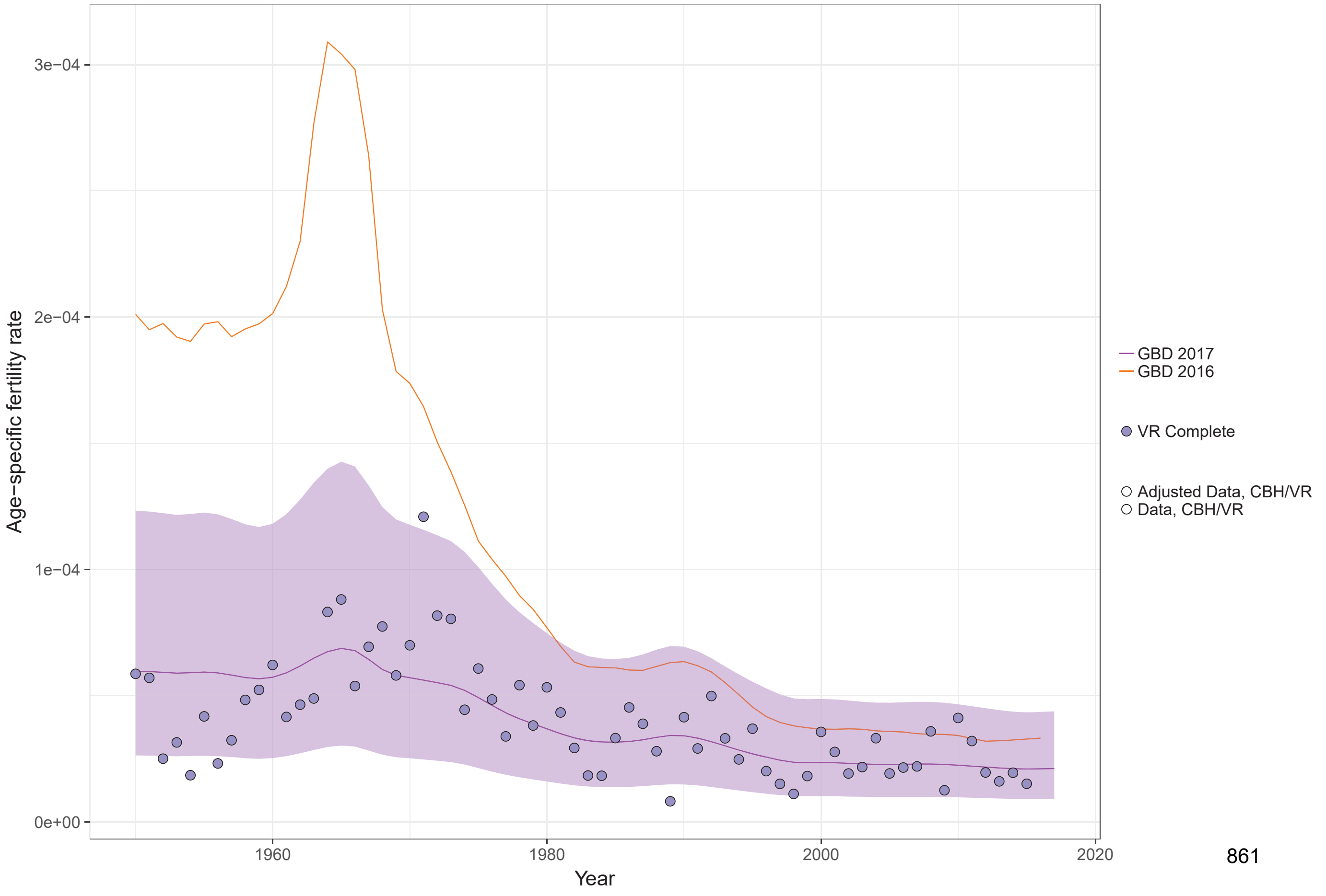

Age−specific fertility fitted trend lines, 1950−2017: Sweden, Ages: 15−19
Data points enclosed with a diamond are reference sources

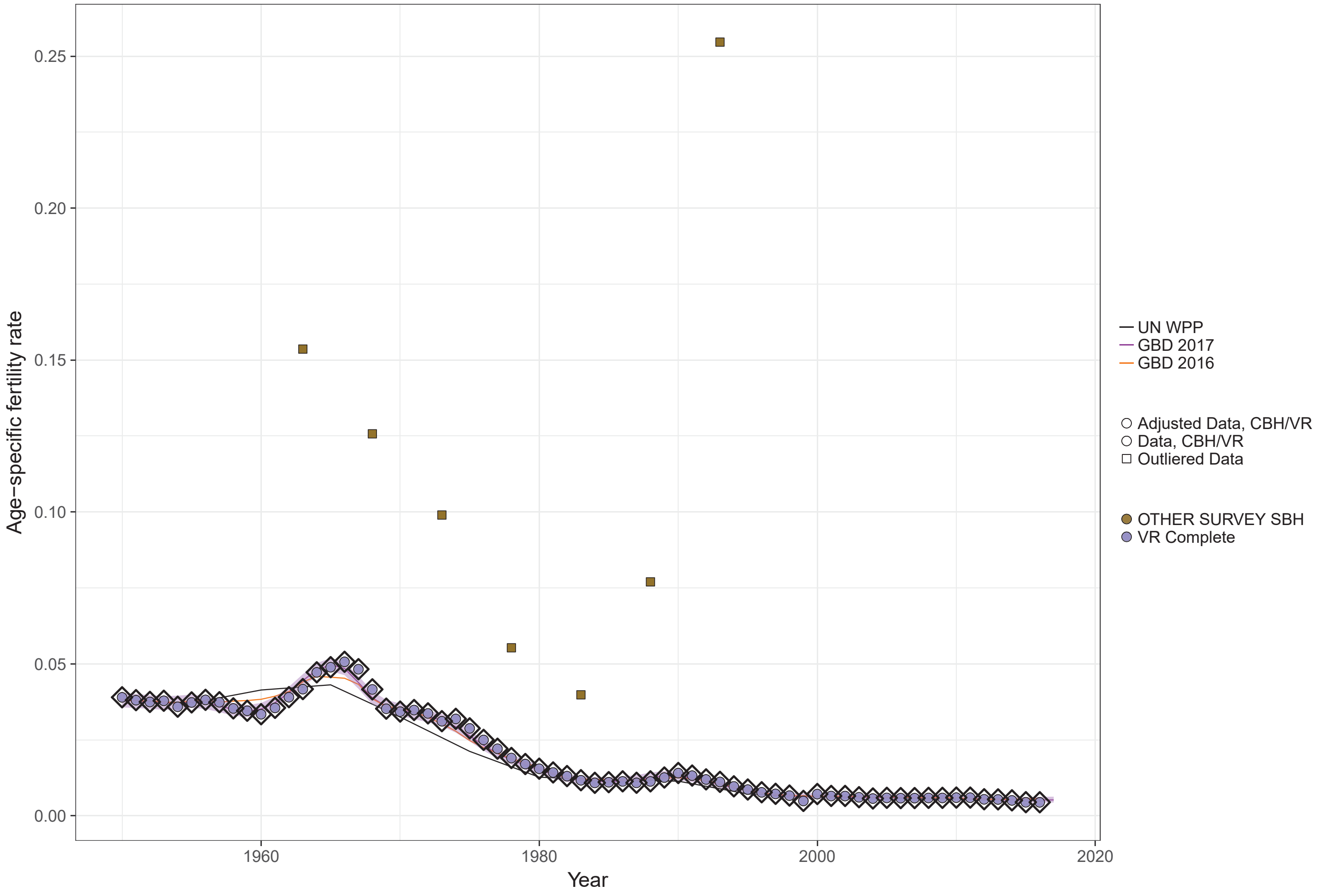

Age−specific fertility fitted trend lines, 1950−2017: Sweden, Ages: 20−24
Data points enclosed with a diamond are reference sources

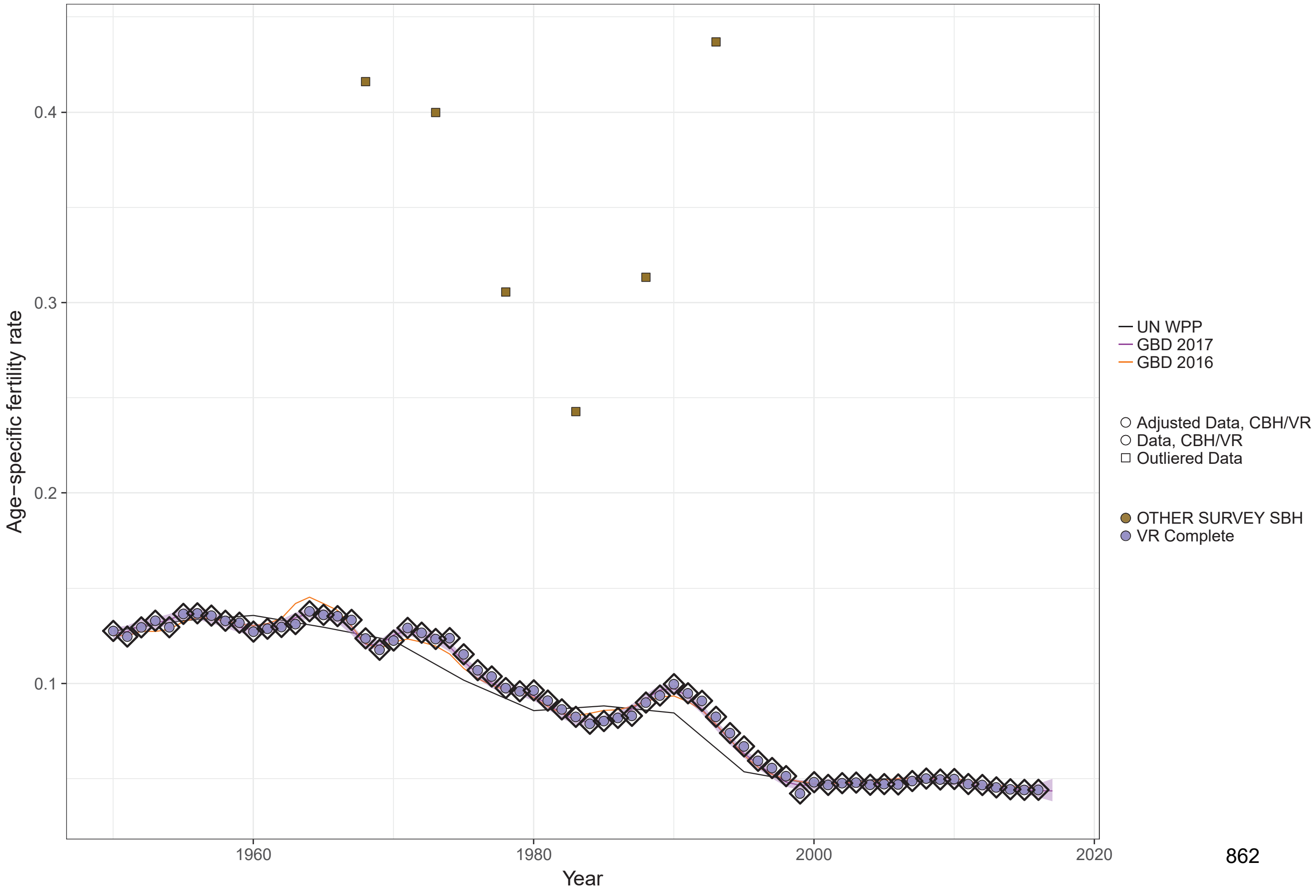

Age−specific fertility fitted trend lines, 1950−2017: Sweden, Ages: 25−29
Data points enclosed with a diamond are reference sources

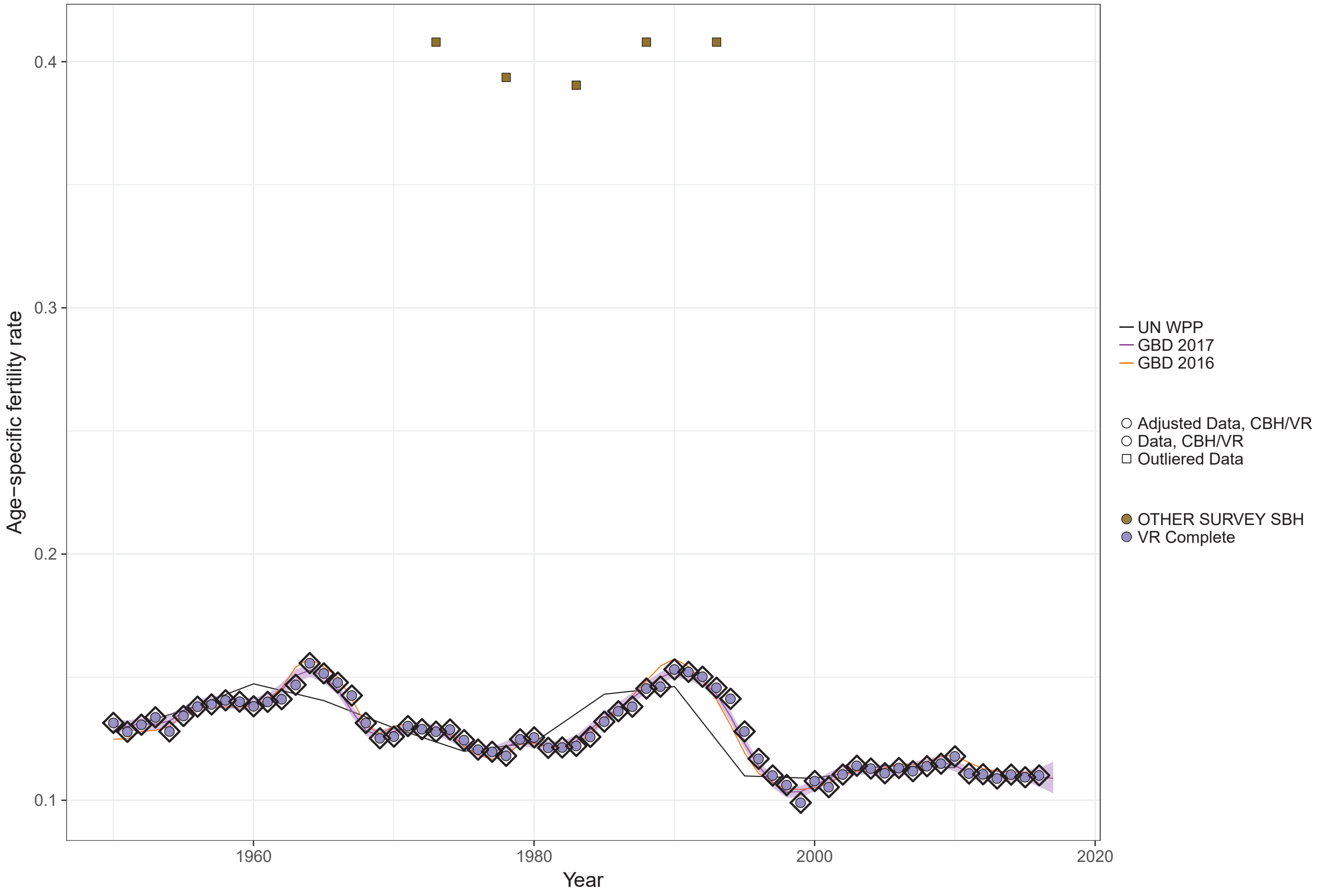

Age−specific fertility fitted trend lines, 1950−2017: Sweden, Ages: 30−34
Data points enclosed with a diamond are reference sources

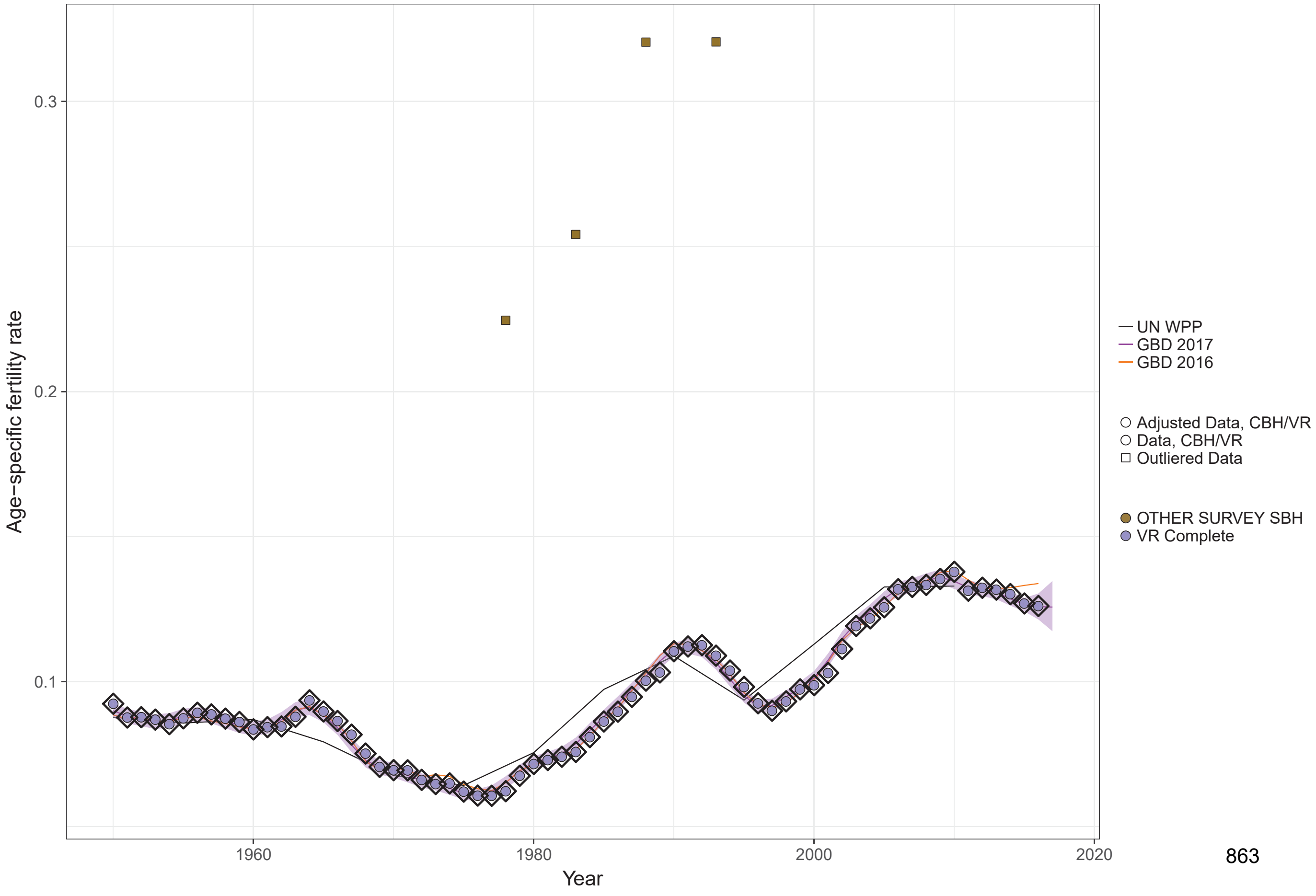

Age−specific fertility fitted trend lines, 1950−2017: Sweden, Ages: 35−39
Data points enclosed with a diamond are reference sources

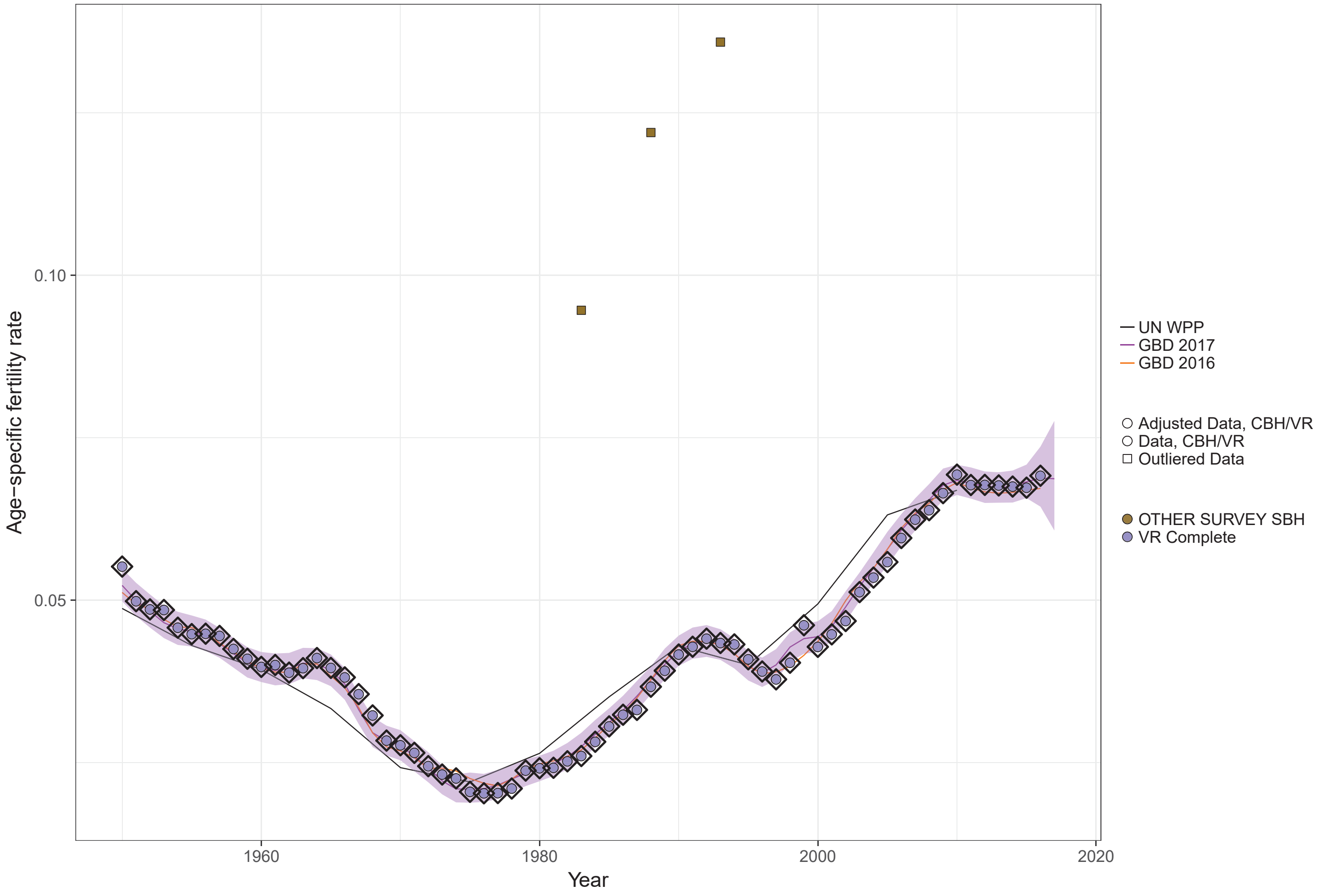

Age−specific fertility fitted trend lines, 1950−2017: Sweden, Ages: 40−44
Data points enclosed with a diamond are reference sources

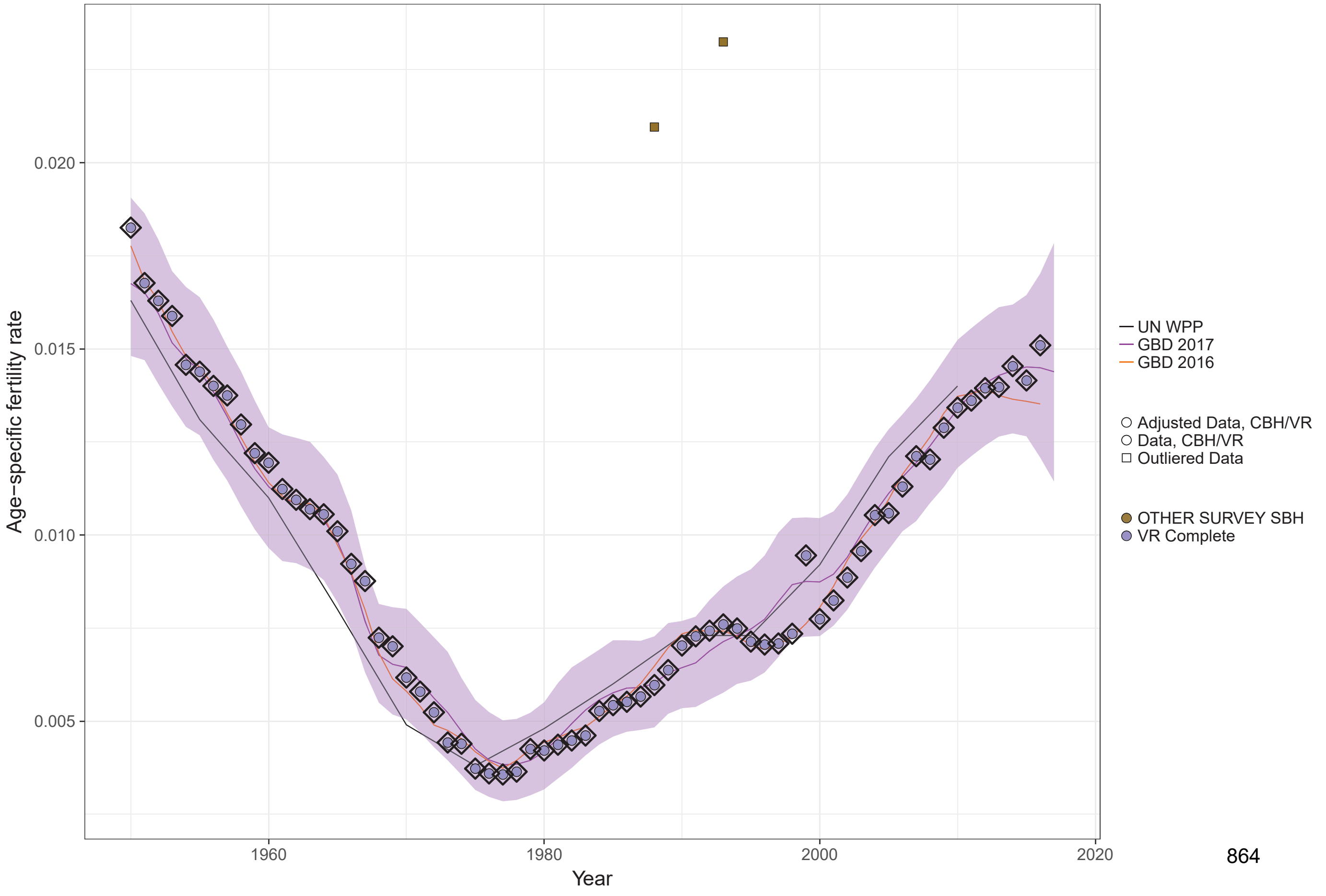

Age−specific fertility fitted trend lines, 1950−2017: Sweden, Ages: 45−49
Data points enclosed with a diamond are reference sources

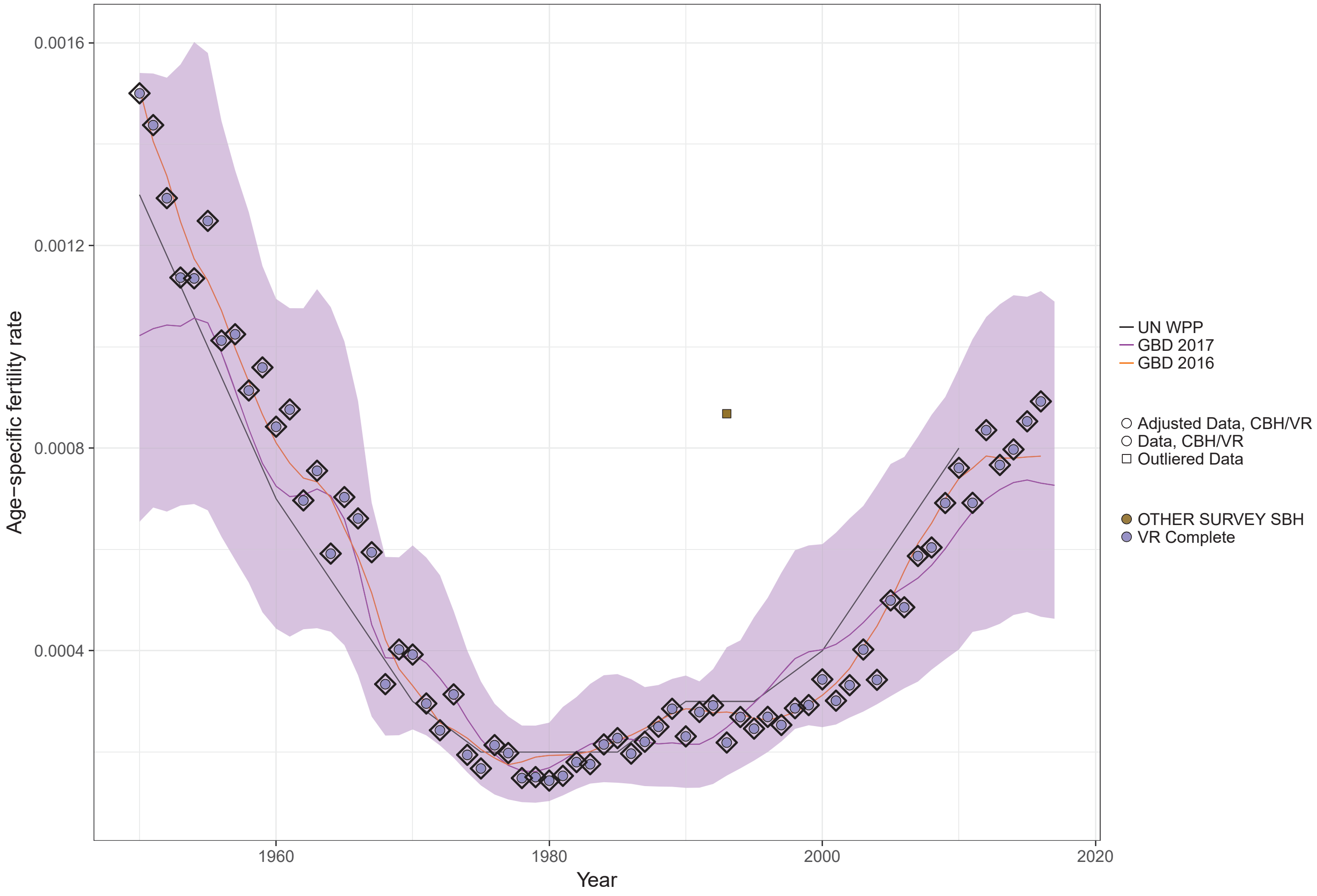

Age−specific fertility fitted trend lines, 1950−2017: Sweden, Ages: 50−54
Data points enclosed with a diamond are reference sources

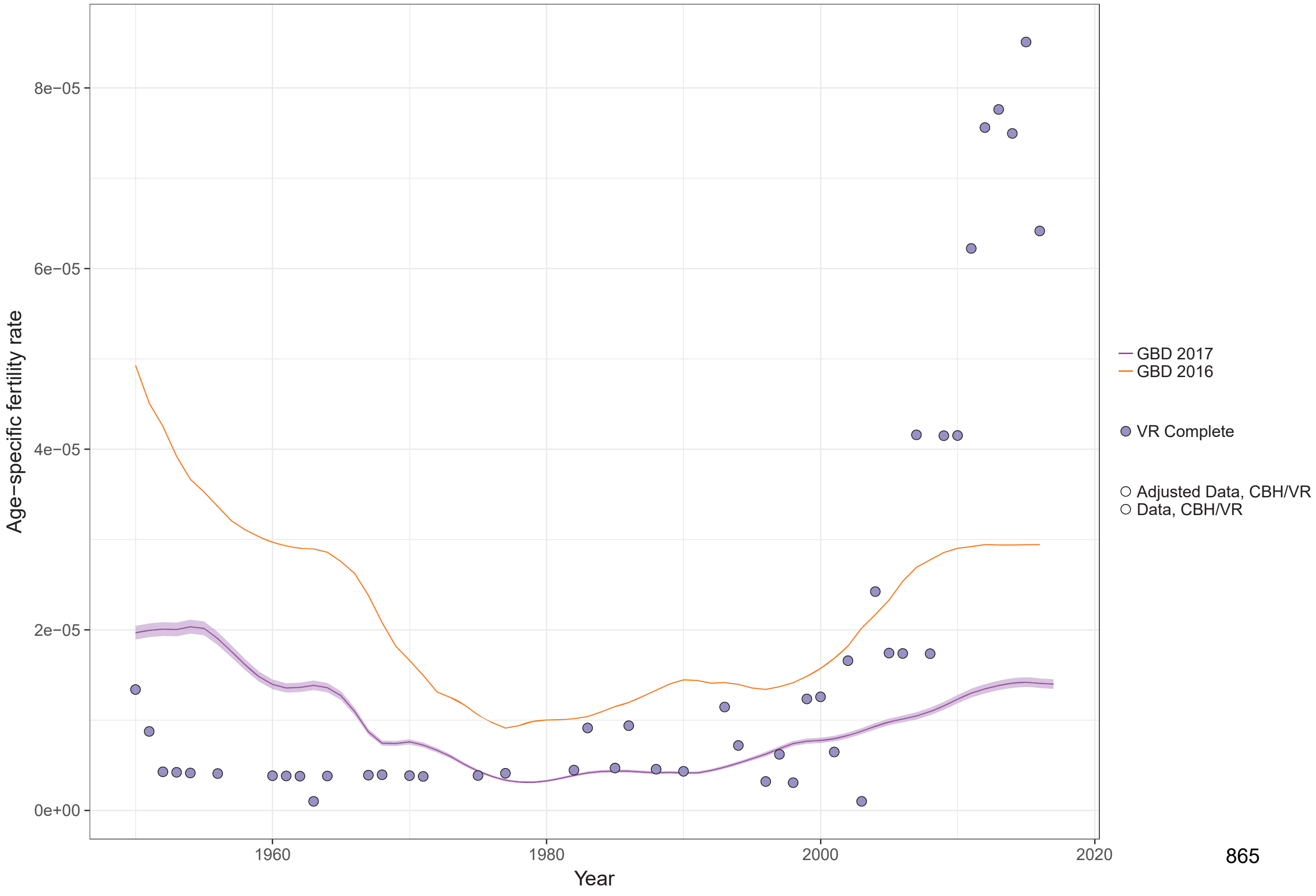

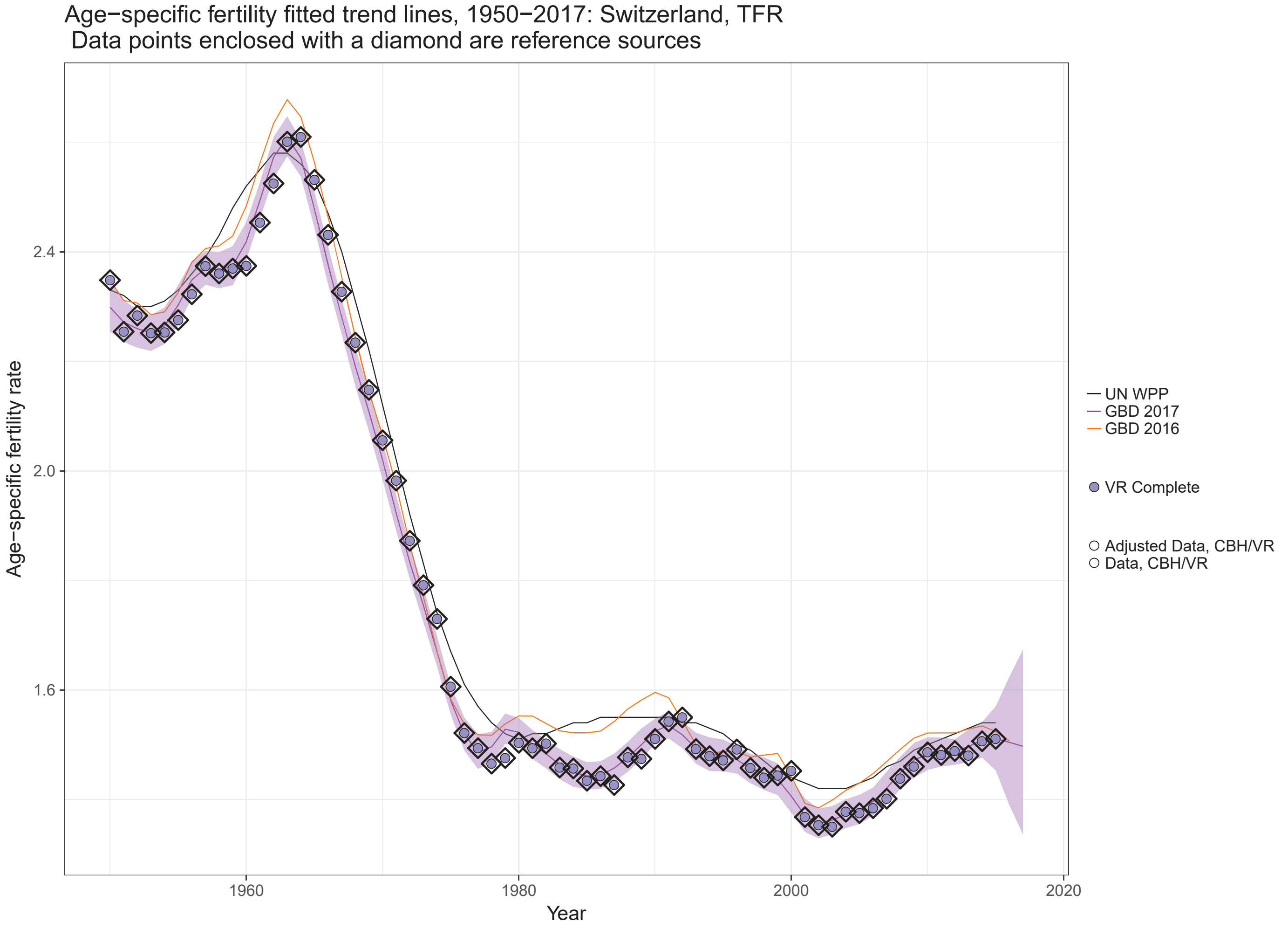
Age−specific fertility fitted trend lines, 1950−2017: Switzerland, TFR
Data points enclosed with a diamond are reference sources
Age−specific fertility rate
2.4
2.0
1.6
1960
1980
2000
2020
Year
UN WPP
GBD 2017
GBD 2016
VR Complete
Adjusted Data, CBH/VR
Data, CBH/VR

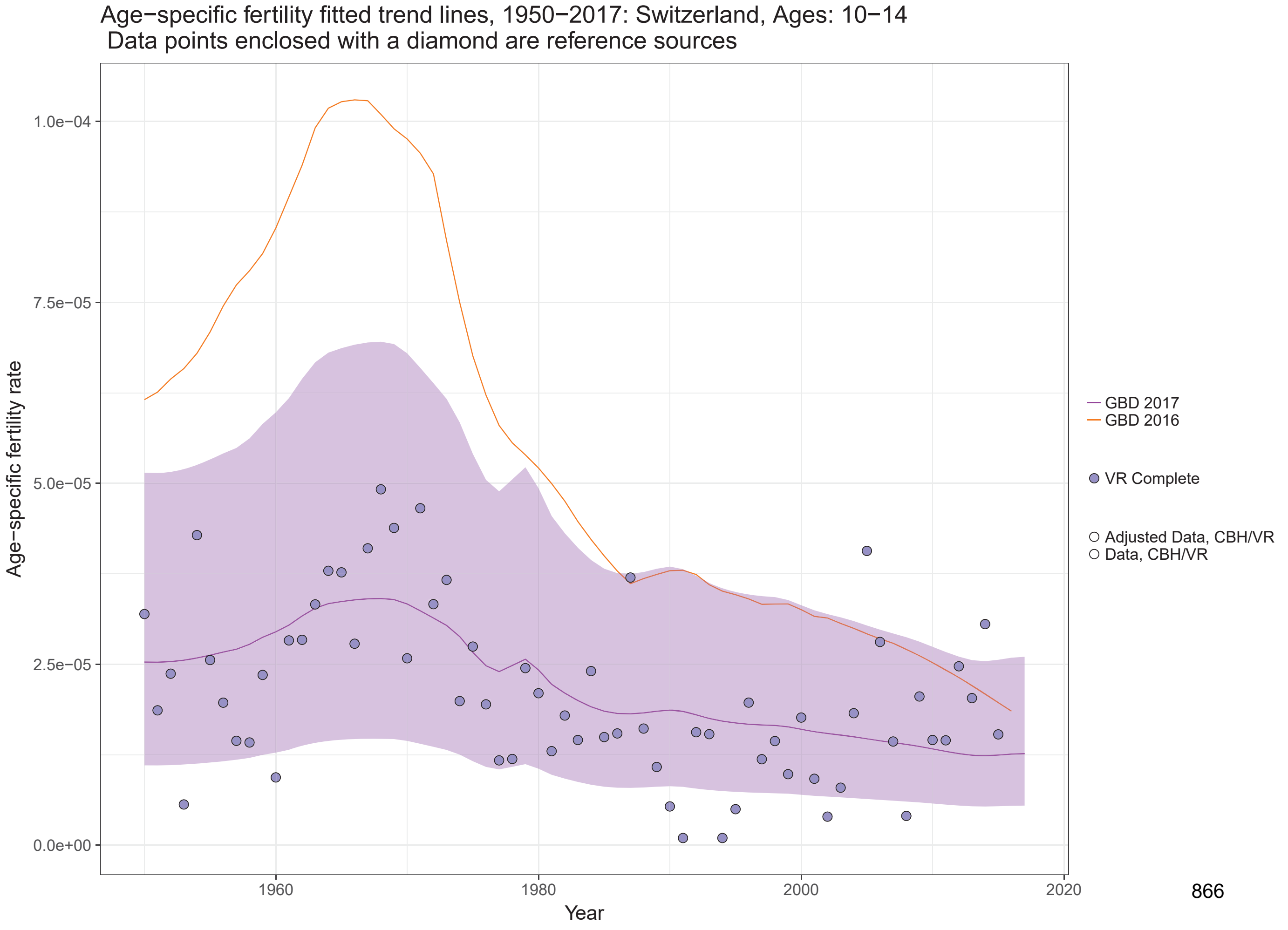
Age−specific fertility fitted trend lines, 1950−2017: Switzerland, Ages: 10−14
Data points enclosed with a diamond are reference sources
Age−specific fertility rate
1.0e−04
7.5e−05
5.0e−05
2.5e−05
0.0e+00
1960
1980
2000
2020
Year
GBD 2017
GBD 2016
VR Complete
Adjusted Data, CBH/VR
Data, CBH/VR

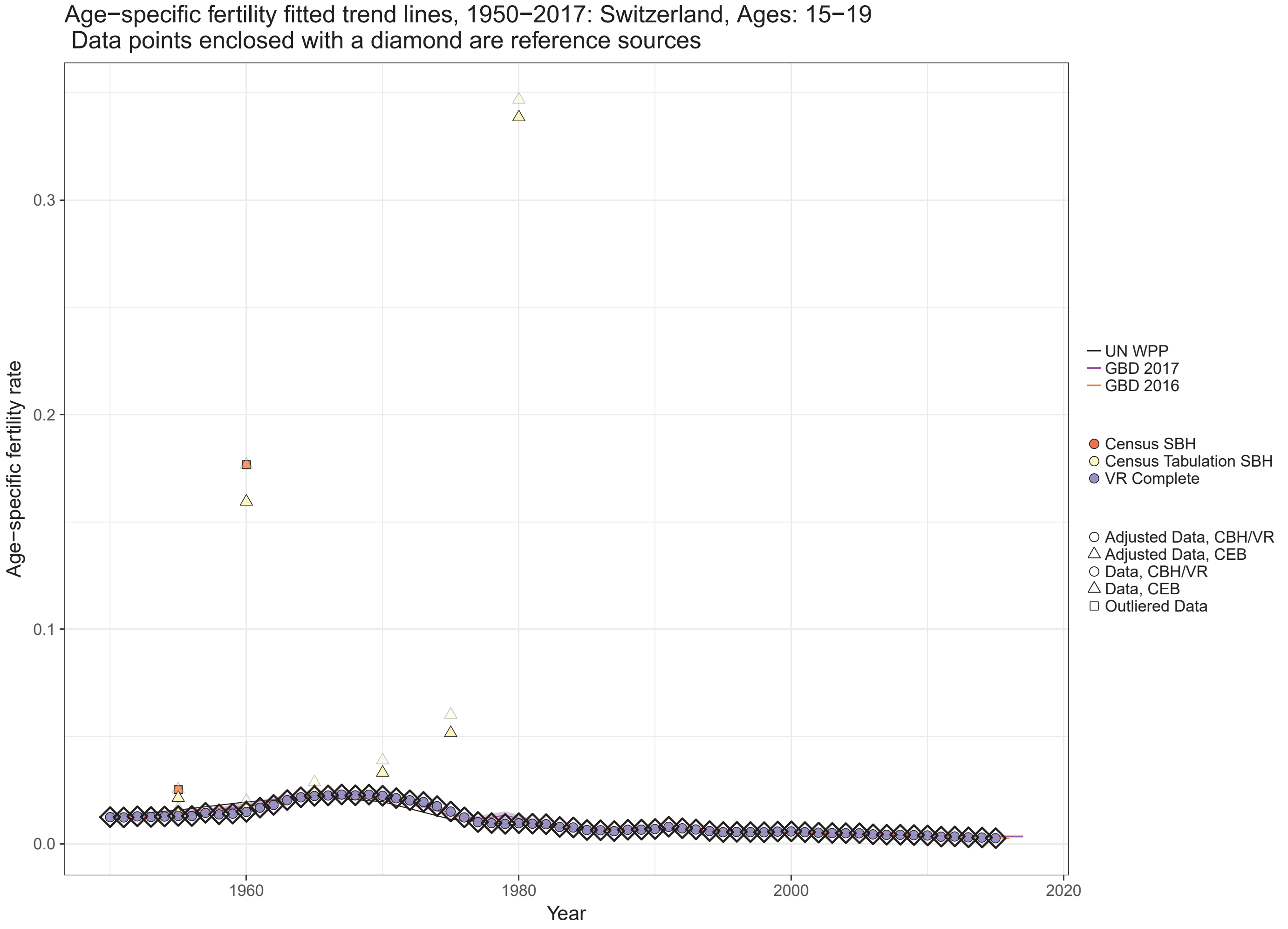
Age−specific fertility fitted trend lines, 1950−2017: Switzerland, Ages: 15−19
Data points enclosed with a diamond are reference sources
Age−specific fertility rate
Year
0.0
0.1
0.2
0.3
1960
1980
2000
2020
UN WPP
GBD 2017
GBD 2016
Census SBH
Census Tabulation SBH
VR Complete
Adjusted Data, CBH/VR
Adjusted Data, CEB
Data, CBH/VR
Data, CEB
Outliered Data

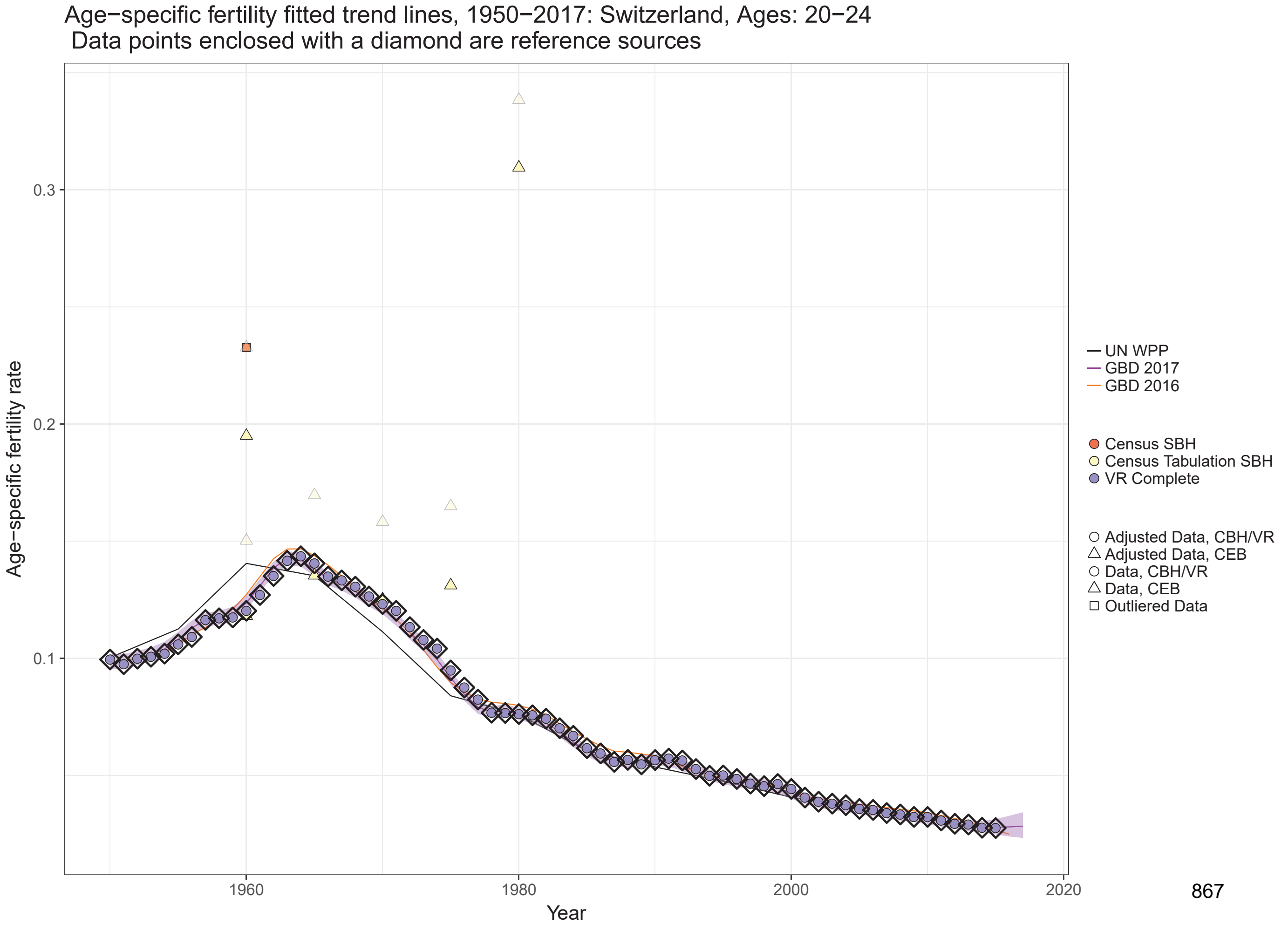
Age−specific fertility fitted trend lines, 1950−2017: Switzerland, Ages: 20−24
Data points enclosed with a diamond are reference sources
Age−specific fertility rate
Year
0.1
0.2
0.3
1960
1980
2000
2020
UN WPP
GBD 2017
GBD 2016
Census SBH
Census Tabulation SBH
VR Complete
Adjusted Data, CBH/VR
Adjusted Data, CEB
Data, CBH/VR
Data, CEB
Outliered Data

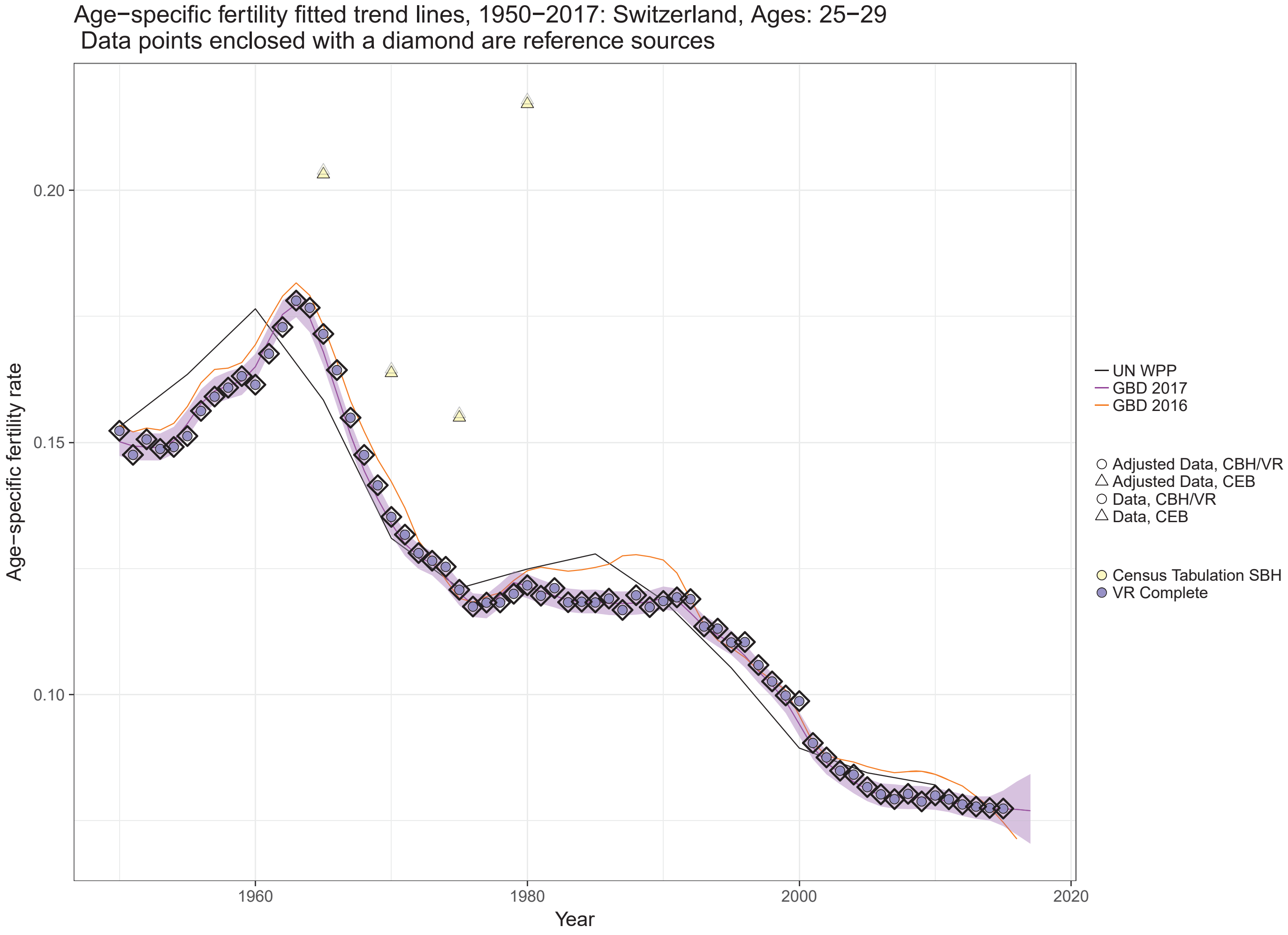

Age−specific fertility fitted trend lines, 1950−2017: Switzerland, Ages: 25−29
Data points enclosed with a diamond are reference sources
Age−specific fertility rate
0.20
0.15
0.10
1960
1980
2000
2020
Year
UN WPP
GBD 2017
GBD 2016
Adjusted Data, CBH/VR
Adjusted Data, CEB
Data, CBH/VR
Data, CEB
Census Tabulation SBH
VR Complete

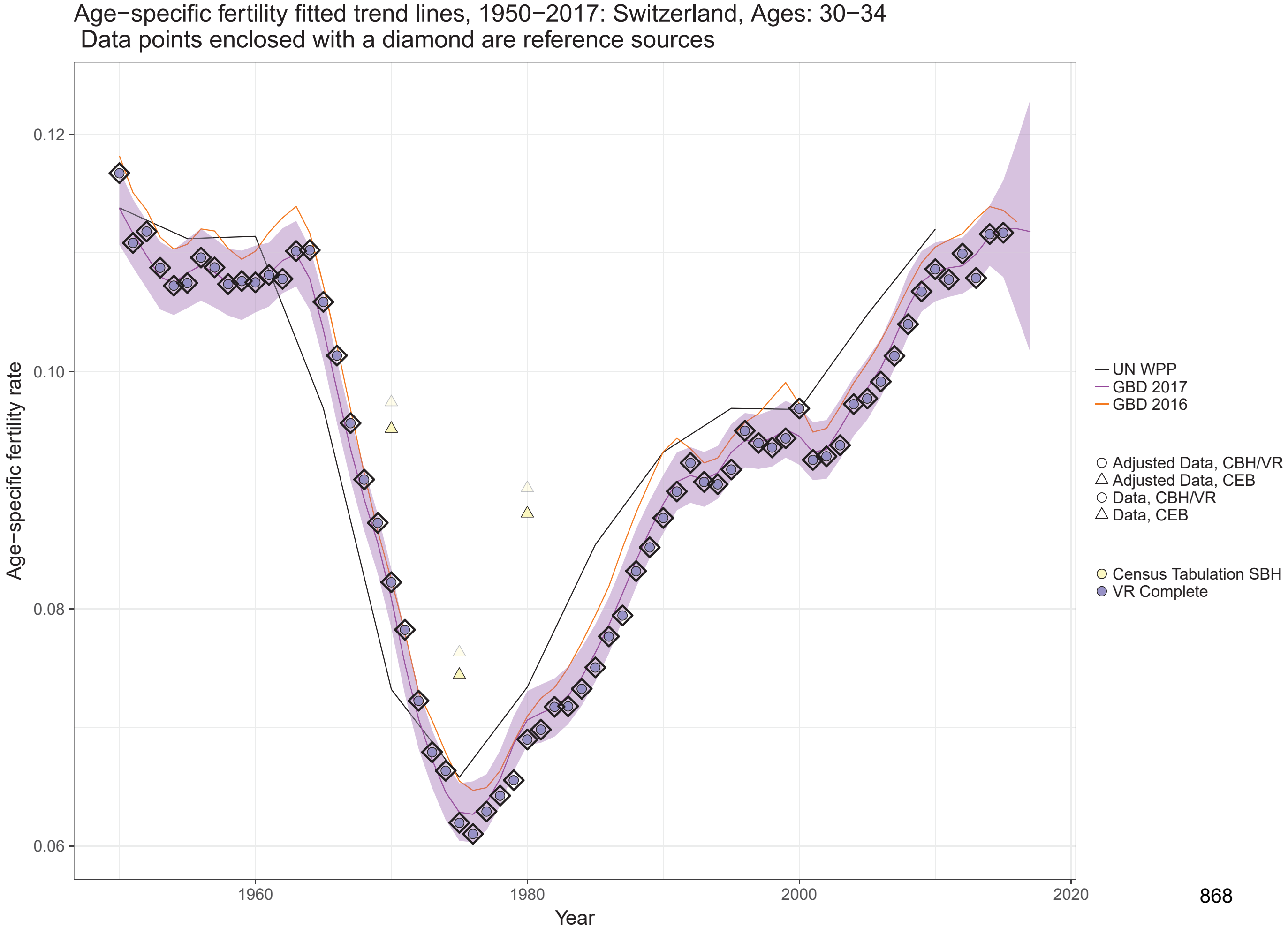

Age−specific fertility fitted trend lines, 1950−2017: Switzerland, Ages: 30−34
Data points enclosed with a diamond are reference sources
Age−specific fertility rate
0.12
0.10
0.08
0.06
1960
1980
2000
2020
Year
UN WPP
GBD 2017
GBD 2016
Adjusted Data, CBH/VR
Adjusted Data, CEB
Data, CBH/VR
Data, CEB
Census Tabulation SBH
VR Complete

Age−specific fertility fitted trend lines, 1950−2017: Switzerland, Ages: 35−39
Data points enclosed with a diamond are reference sources

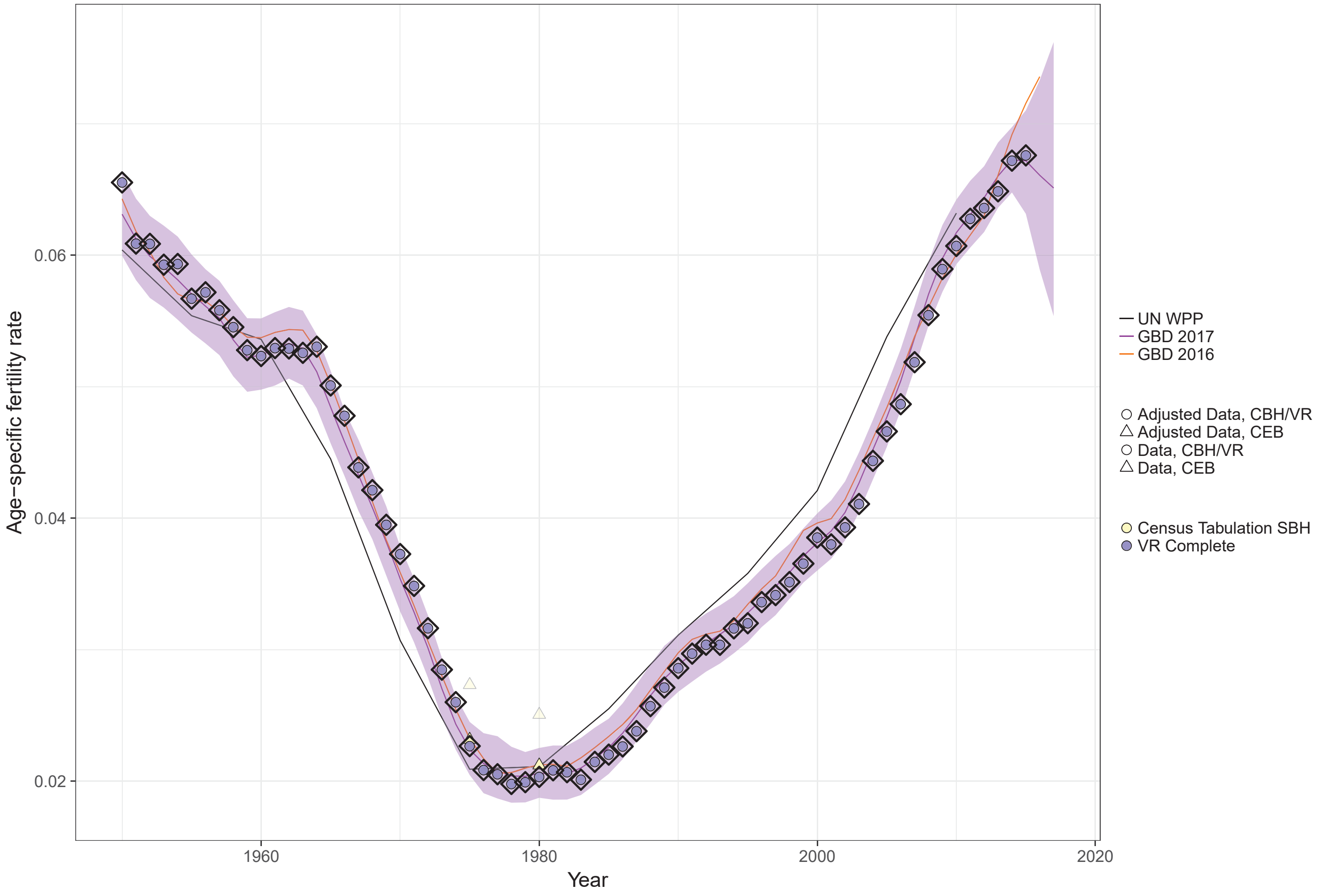

Age−specific fertility fitted trend lines, 1950−2017: Switzerland, Ages: 40−44
Data points enclosed with a diamond are reference sources

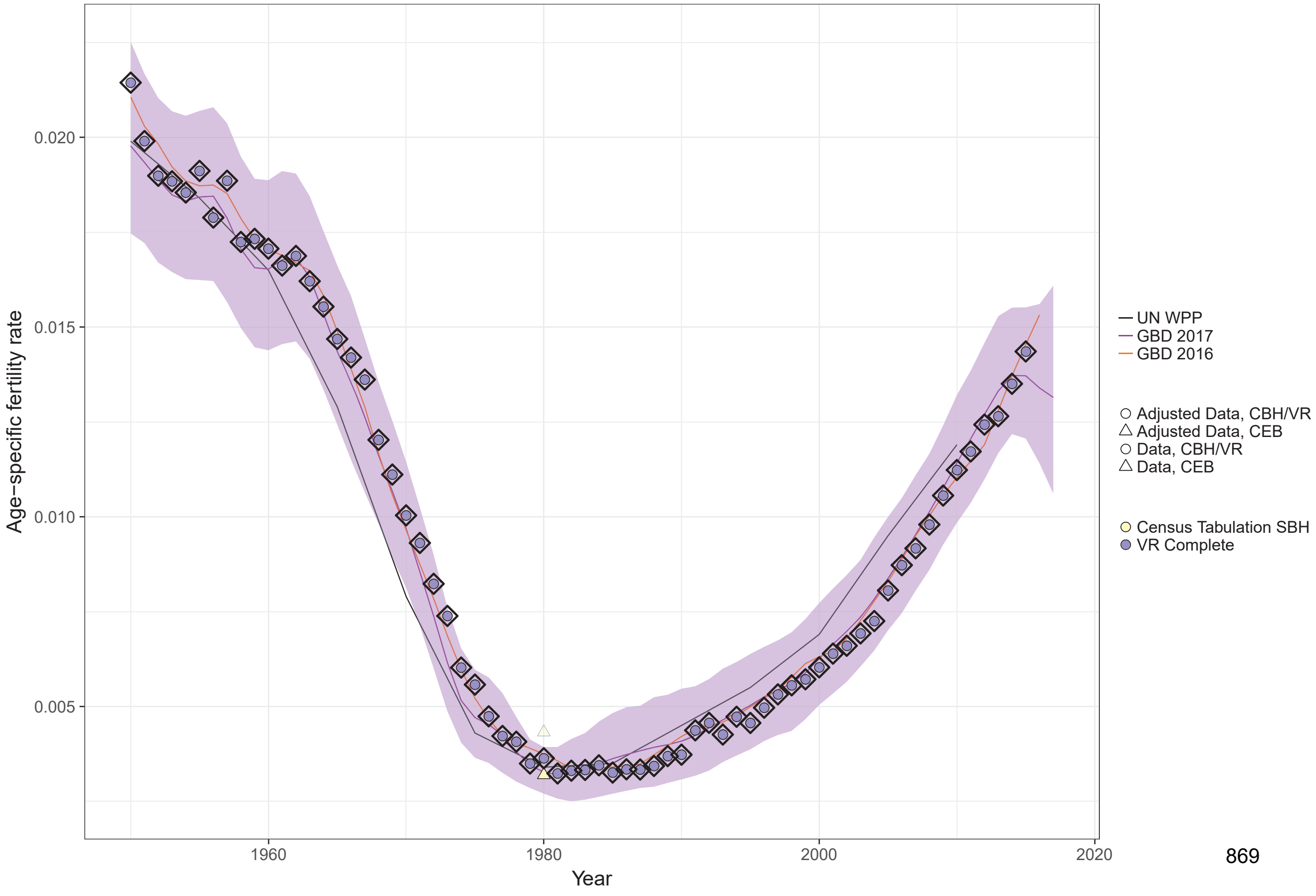

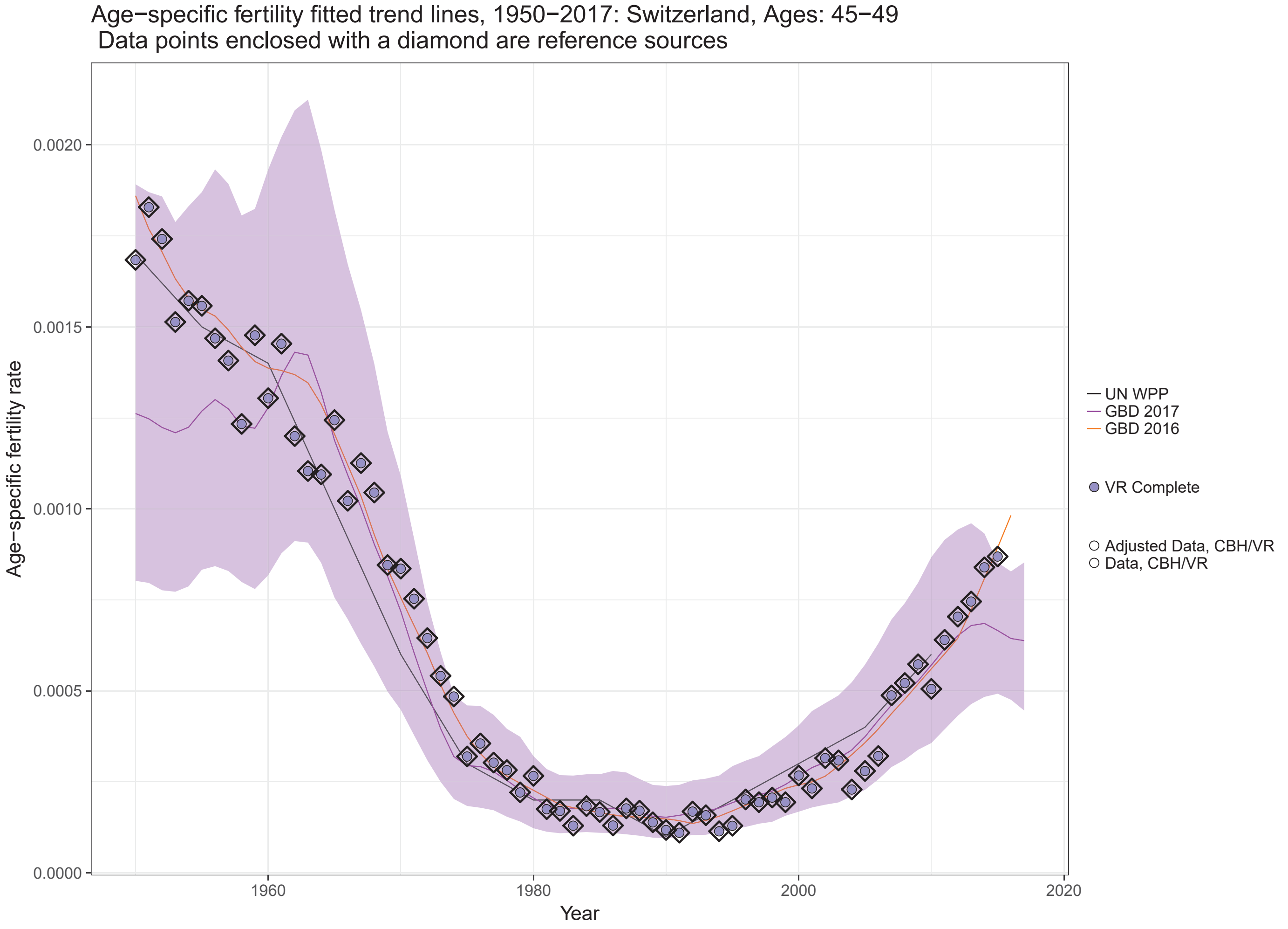

Age−specific fertility fitted trend lines, 1950−2017: Switzerland, Ages: 45−49
Data points enclosed with a diamond are reference sources
Age−specific fertility rate
0.0020
0.0015
0.0010
0.0005
0.0000
1960
1980
2000
2020
Year
UN WPP
GBD 2017
GBD 2016
VR Complete
Adjusted Data, CBH/VR
Data, CBH/VR

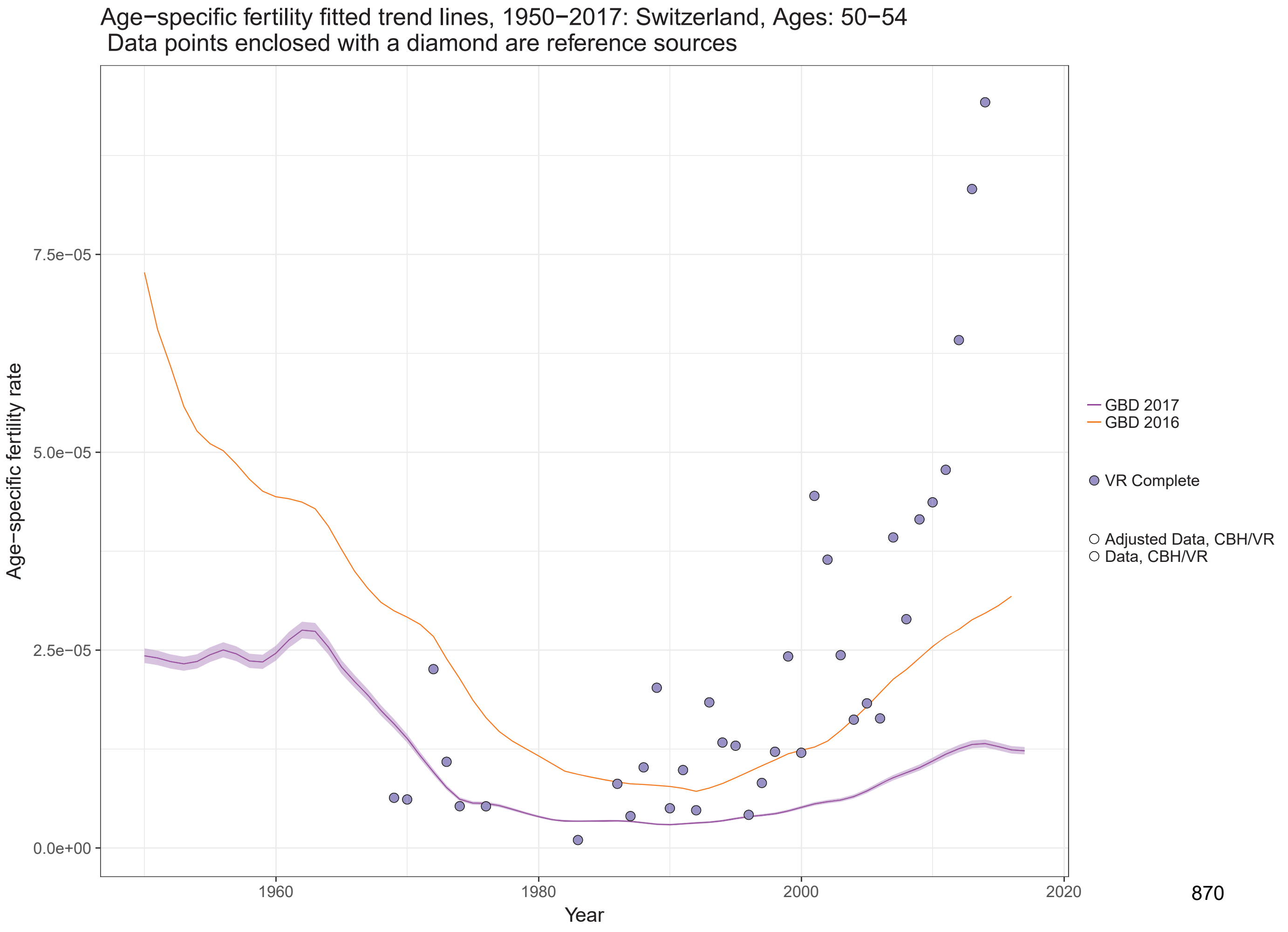

Age−specific fertility fitted trend lines, 1950−2017: Switzerland, Ages: 50−54
Data points enclosed with a diamond are reference sources
Age−specific fertility rate
7.5e−05
5.0e−05
2.5e−05
0.0e+00
1960
1980
2000
2020
Year
GBD 2017
GBD 2016
VR Complete
Adjusted Data, CBH/VR
Data, CBH/VR

Age−specific fertility fitted trend lines, 1950−2017: Syria, TFR
Data points enclosed with a diamond are reference sources

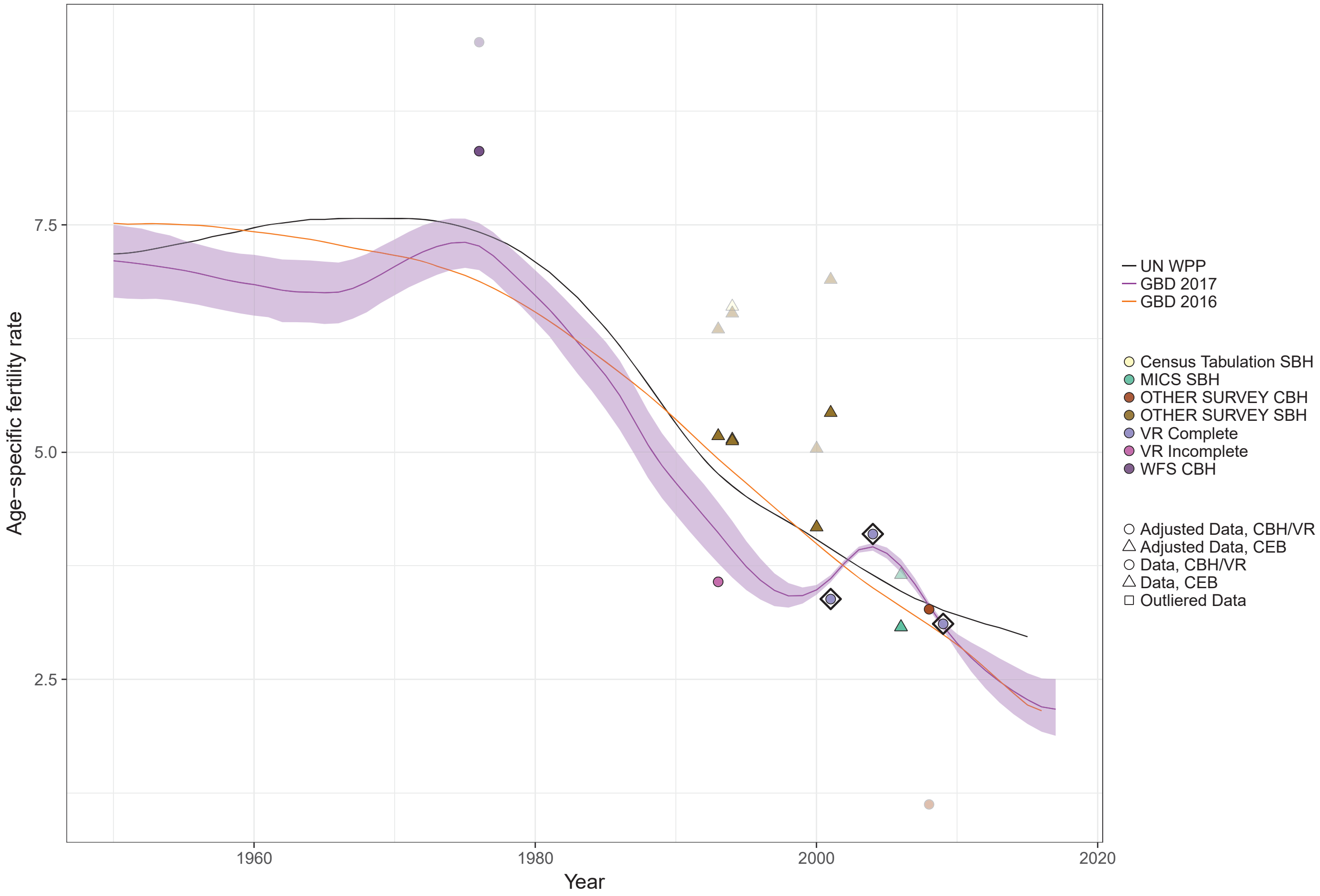

Age−specific fertility fitted trend lines, 1950−2017: Syria, Ages: 10−14
Data points enclosed with a diamond are reference sources

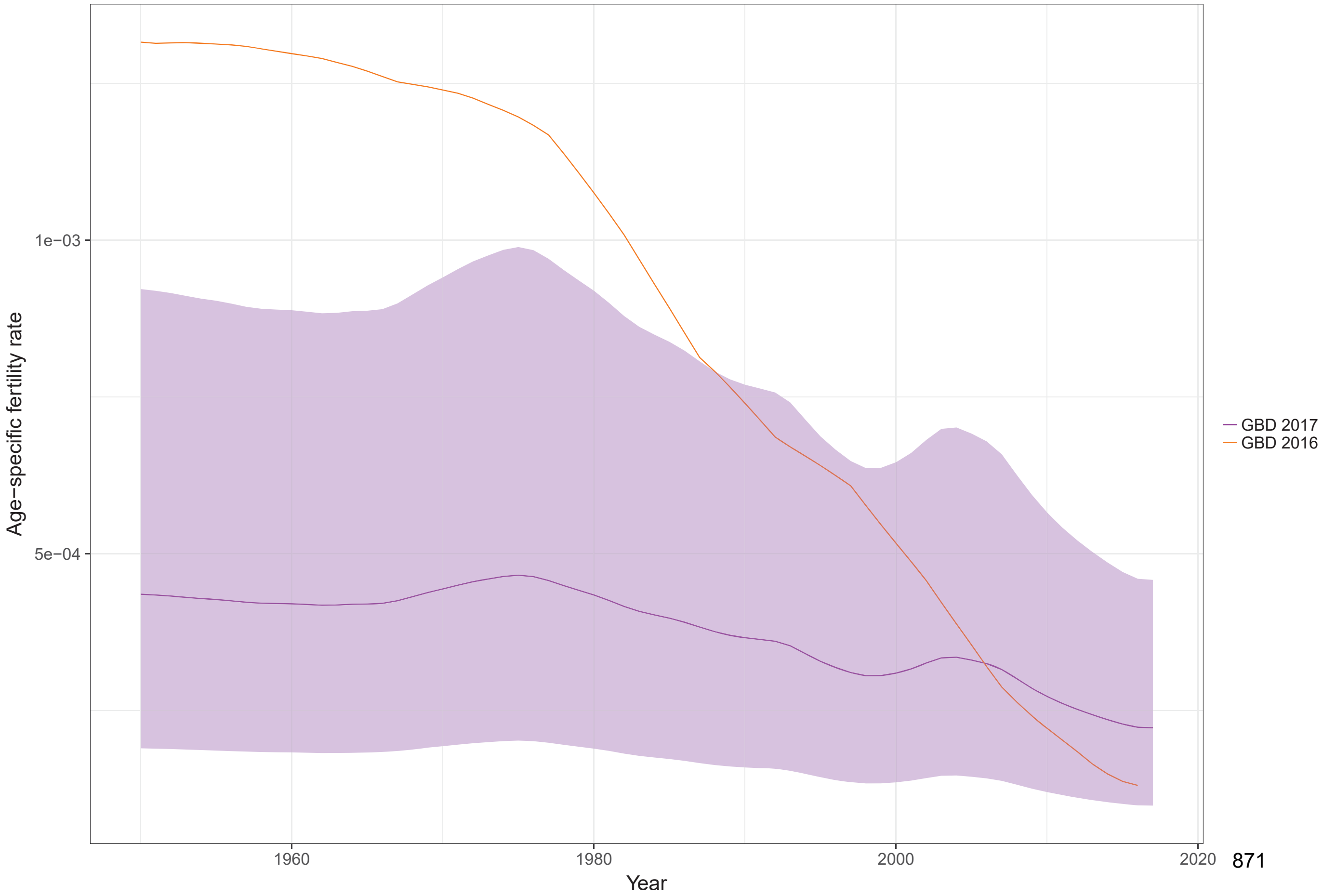

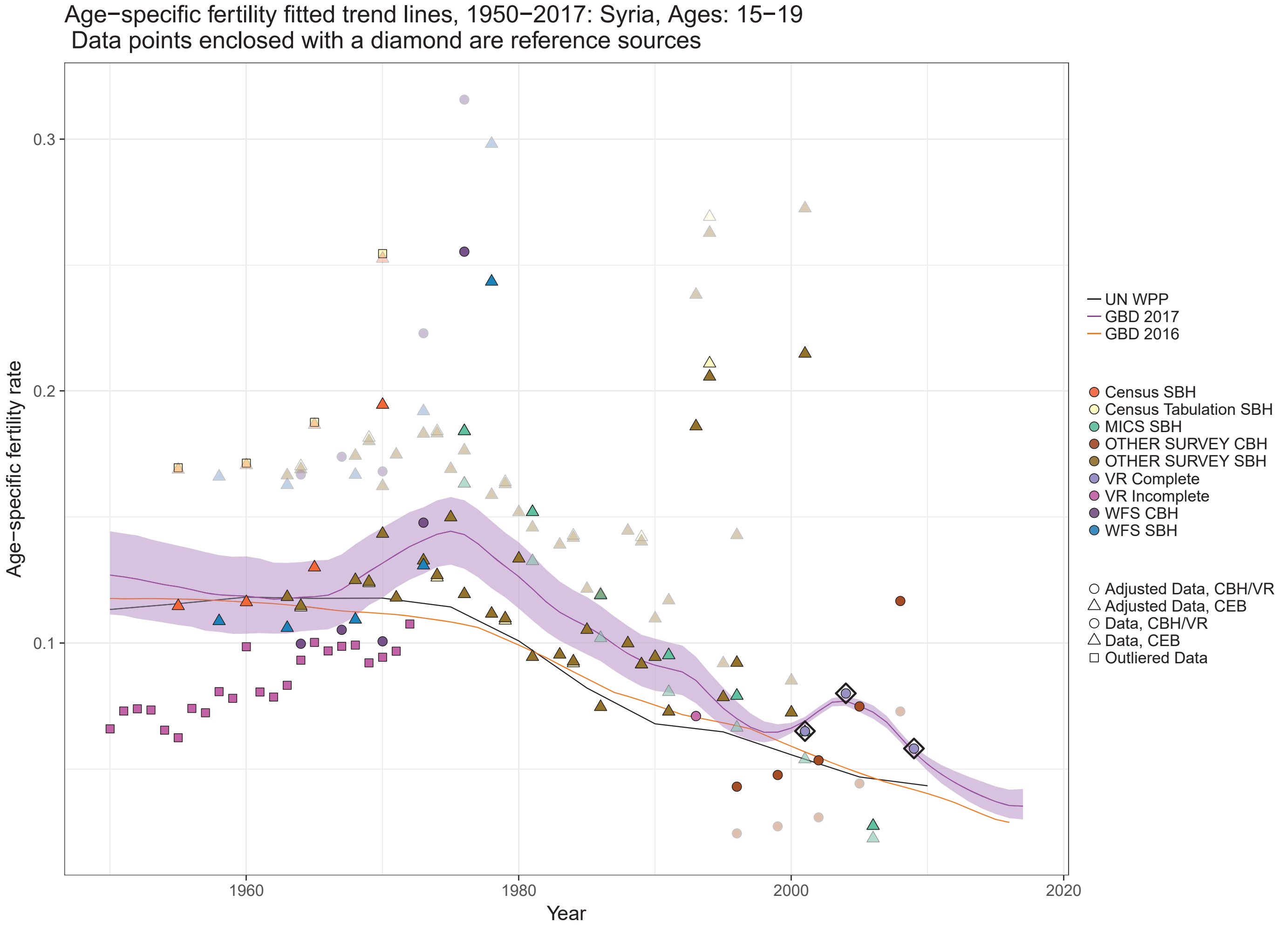
Age−specific fertility fitted trend lines, 1950−2017: Syria, Ages: 15−19
Data points enclosed with a diamond are reference sources
Age−specific fertility rate
0.3
0.2
0.1
1960
1980
2000
2020
Year
UN WPP
GBD 2017
GBD 2016
Census SBH
Census Tabulation SBH
MICS SBH
OTHER SURVEY CBH
OTHER SURVEY SBH
VR Complete
VR Incomplete
WFS CBH
WFS SBH
Adjusted Data, CBH/VR
Adjusted Data, CEB
Data, CBH/VR
Data, CEB
Outliered Data

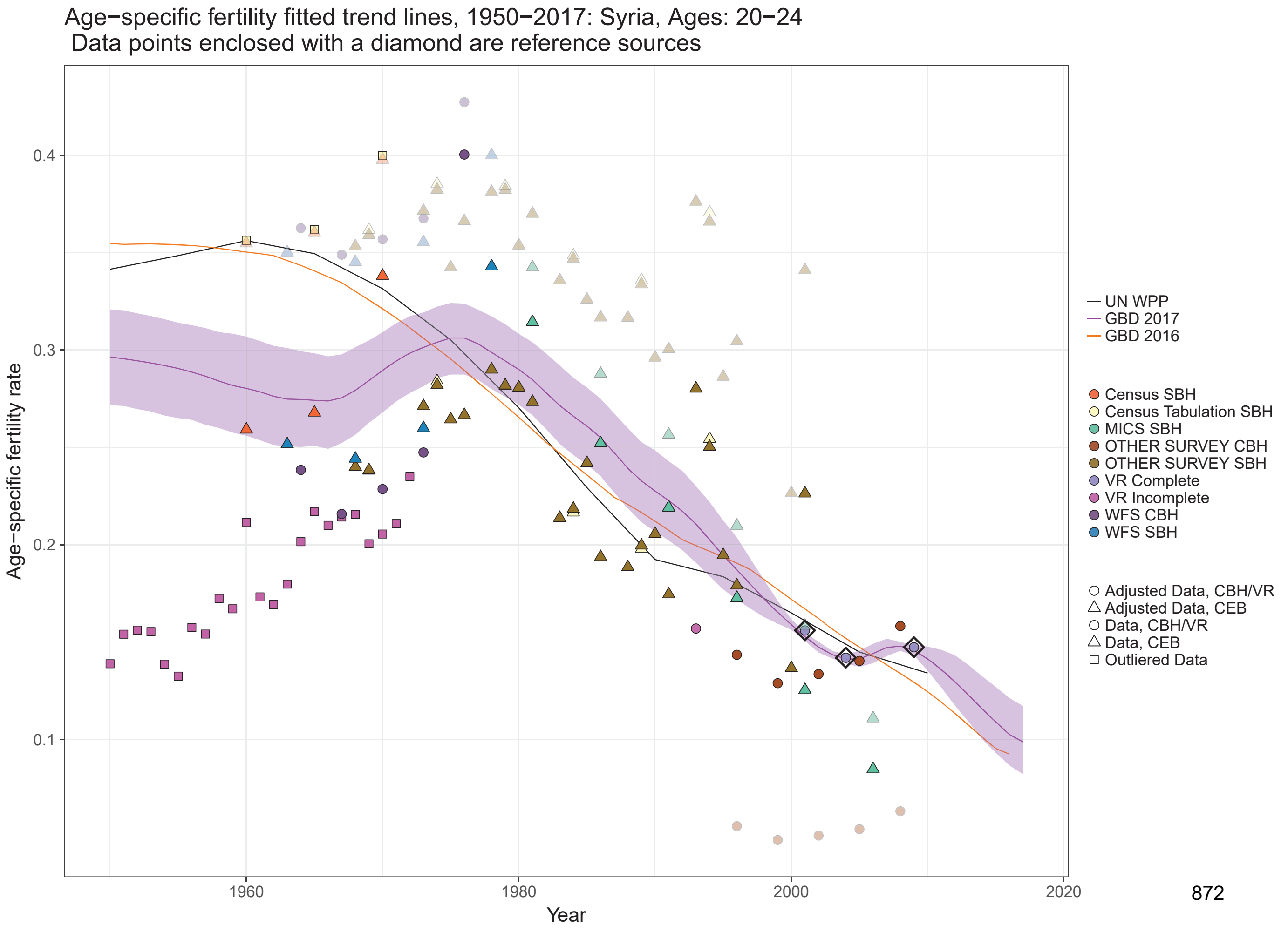
Age−specific fertility fitted trend lines, 1950−2017: Syria, Ages: 20−24
Data points enclosed with a diamond are reference sources
Age−specific fertility rate
0.4
0.3
0.2
0.1
1960
1980
2000
2020
Year
UN WPP
GBD 2017
GBD 2016
Census SBH
Census Tabulation SBH
MICS SBH
OTHER SURVEY CBH
OTHER SURVEY SBH
VR Complete
VR Incomplete
WFS CBH
WFS SBH
Adjusted Data, CBH/VR
Adjusted Data, CEB
Data, CBH/VR
Data, CEB
Outliered Data

Age−specific fertility fitted trend lines, 1950−2017: Syria, Ages: 25−29
Data points enclosed with a diamond are reference sources

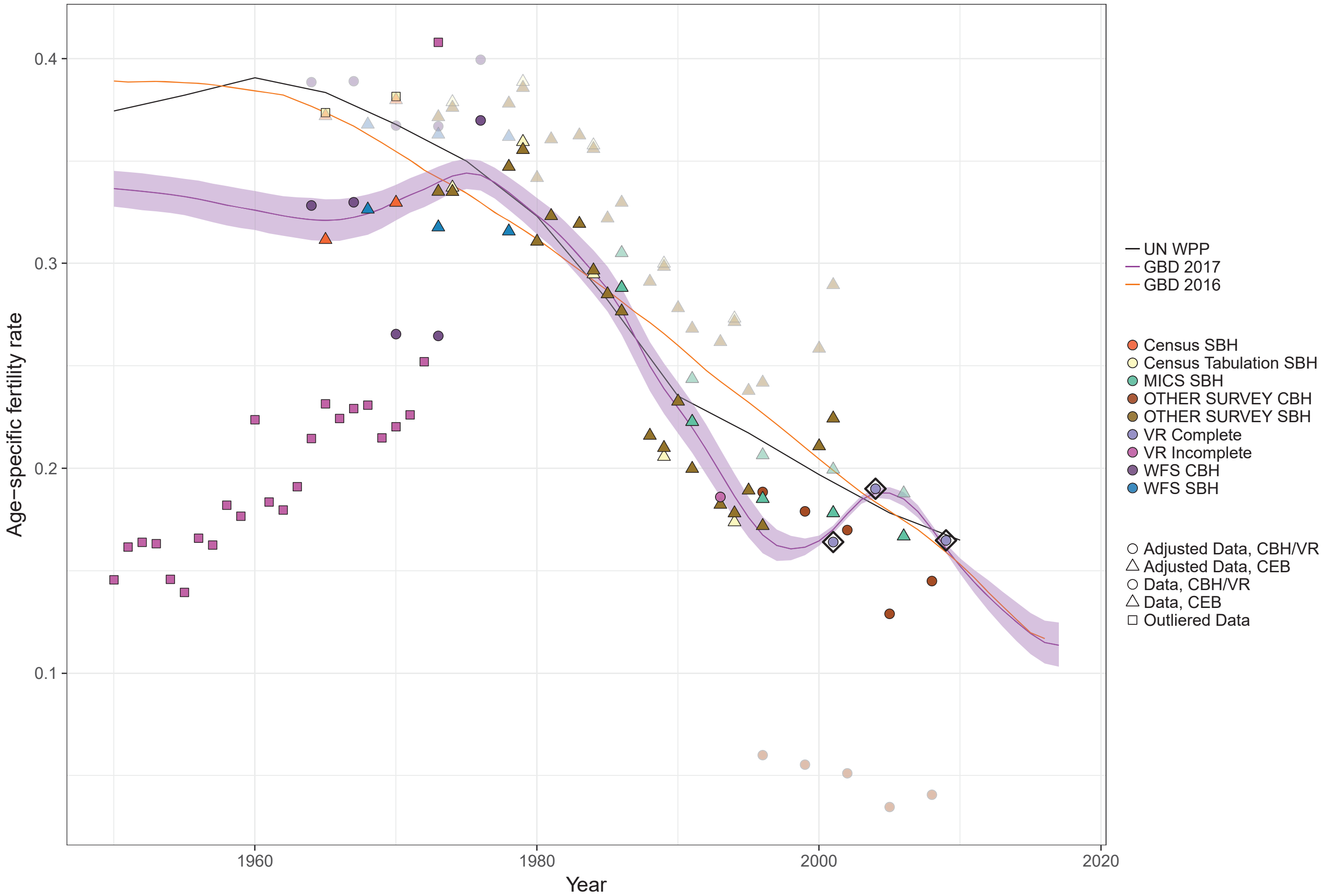

Age−specific fertility fitted trend lines, 1950−2017: Syria, Ages: 30−34
Data points enclosed with a diamond are reference sources

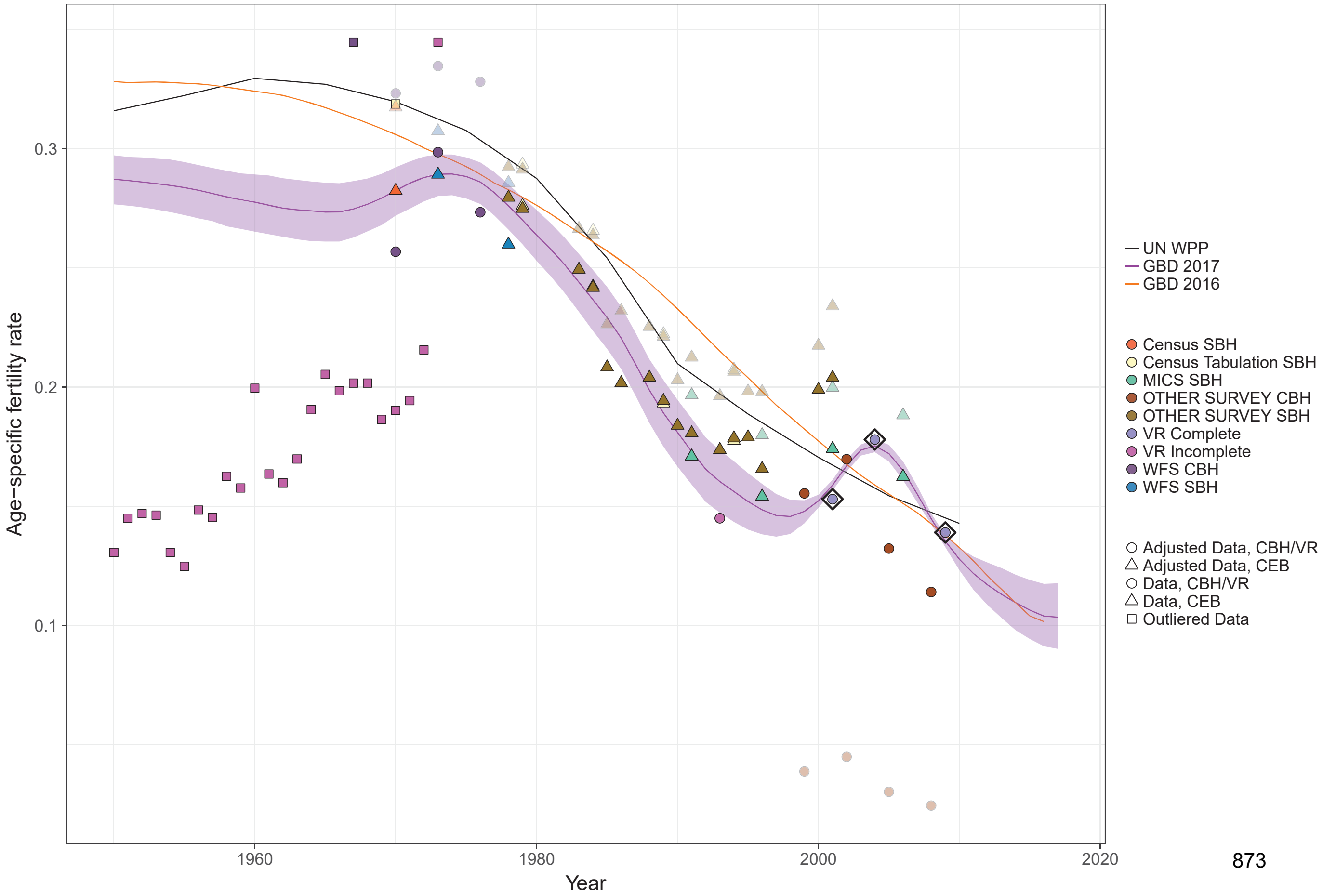

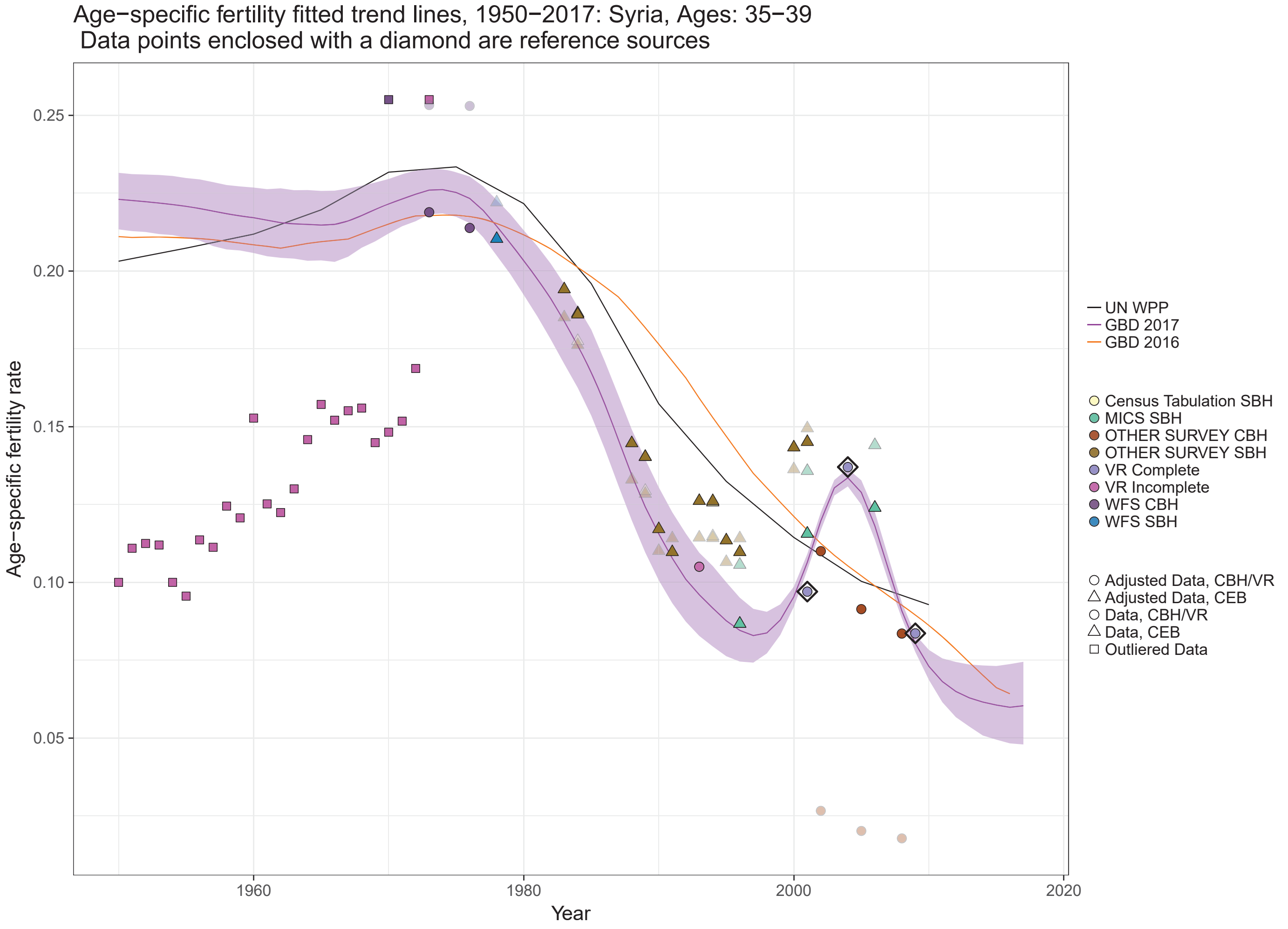

Age−specific fertility fitted trend lines, 1950−2017: Syria, Ages: 35−39
Data points enclosed with a diamond are reference sources
Age-specific fertility rate
0.25
0.20
0.15
0.10
0.05
Year
1960
1980
2000
2020
UN WPP
GBD 2017
GBD 2016
Census Tabulation SBH
MICS SBH
OTHER SURVEY CBH
OTHER SURVEY SBH
VR Complete
VR Incomplete
WFS CBH
WFS SBH
Adjusted Data, CBH/VR
Adjusted Data, CEB
Data, CBH/VR
Data, CEB
Outliered Data

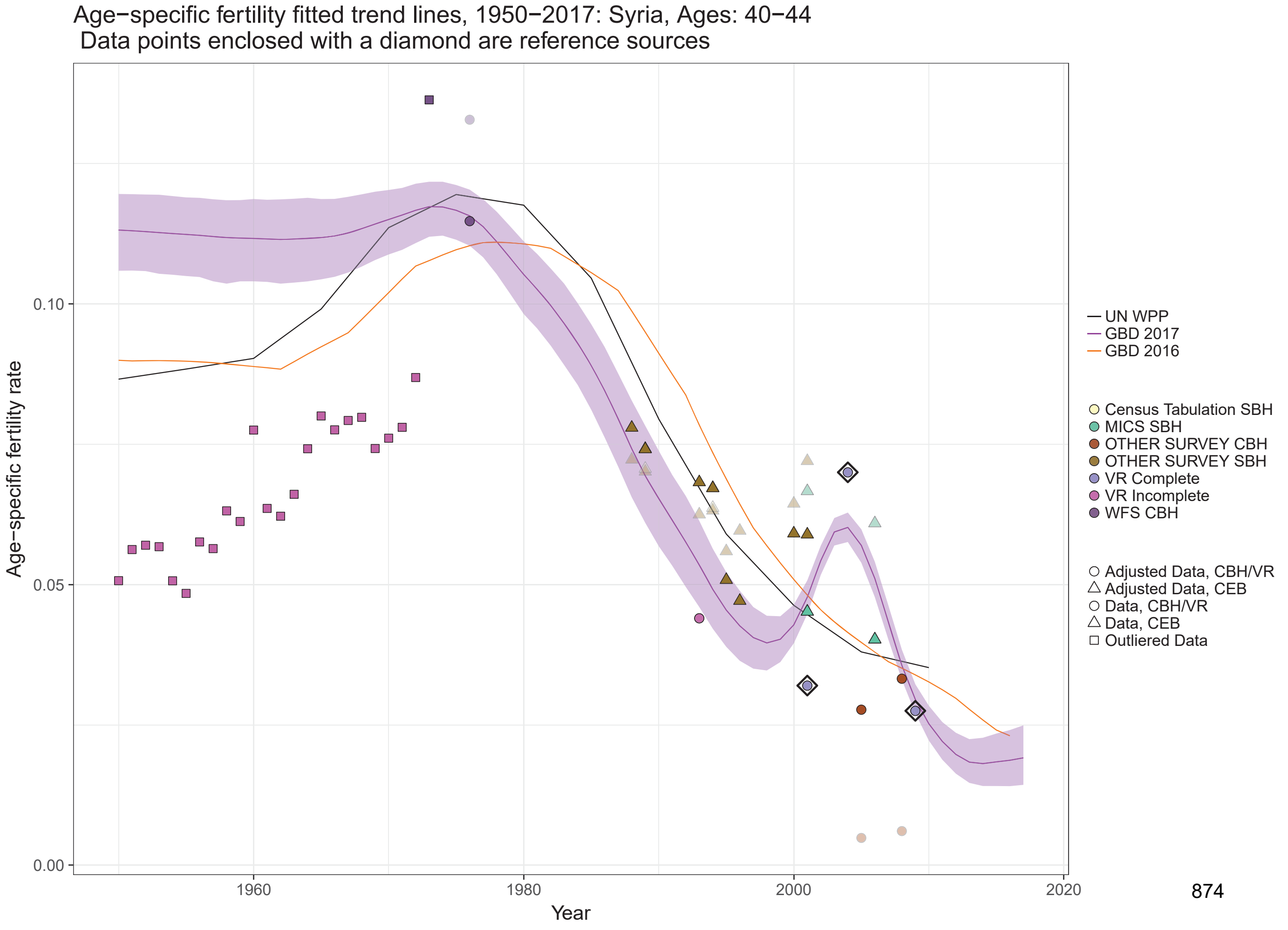

Age−specific fertility fitted trend lines, 1950−2017: Syria, Ages: 40−44
Data points enclosed with a diamond are reference sources
Age-specific fertility rate
0.10
0.05
0.00
Year
1960
1980
2000
2020
UN WPP
GBD 2017
GBD 2016
Census Tabulation SBH
MICS SBH
OTHER SURVEY CBH
OTHER SURVEY SBH
VR Complete
VR Incomplete
WFS CBH
Adjusted Data, CBH/VR
Adjusted Data, CEB
Data, CBH/VR
Data, CEB
Outliered Data

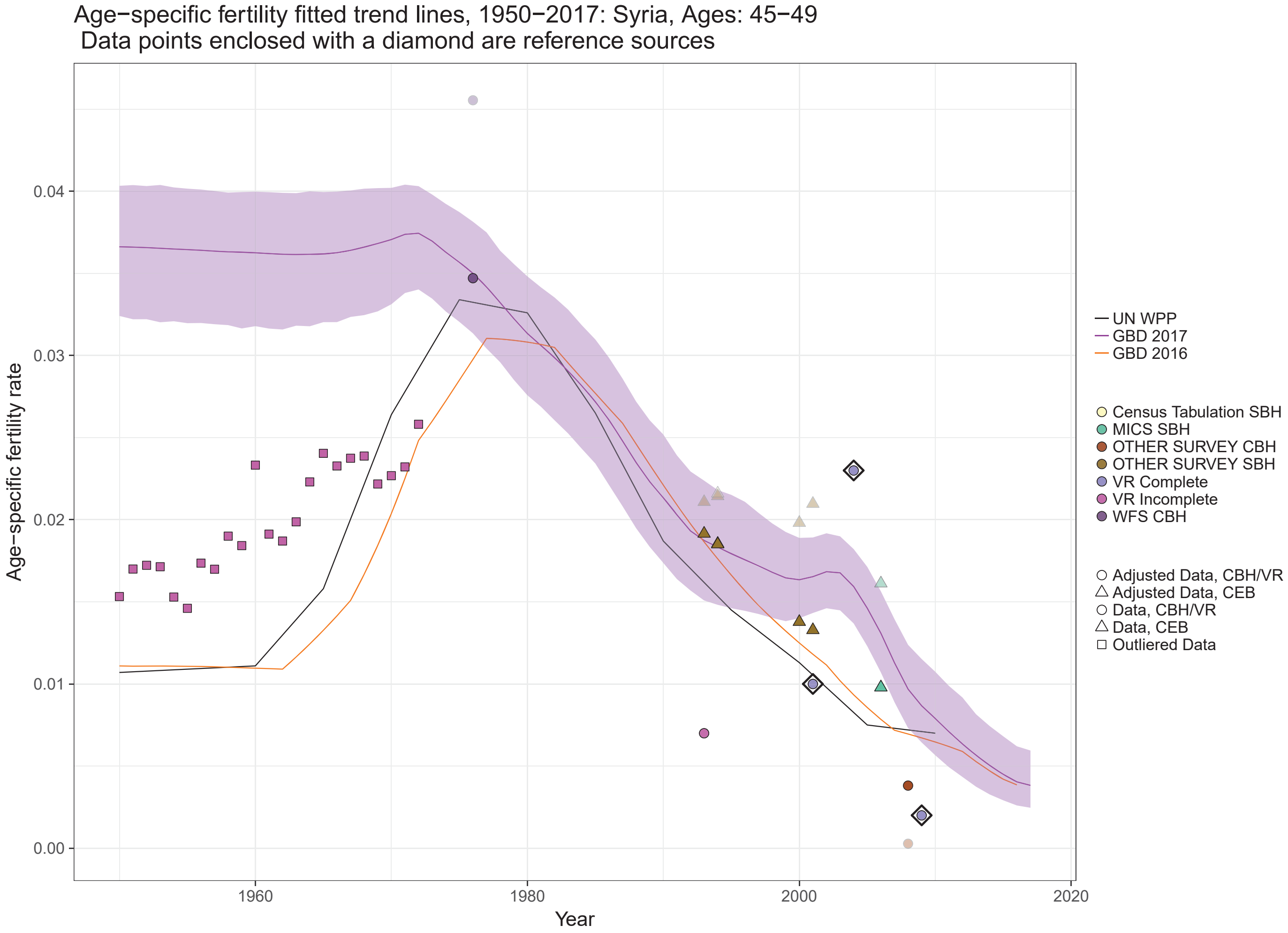

Age−specific fertility fitted trend lines, 1950−2017: Syria, Ages: 45−49
Data points enclosed with a diamond are reference sources
Age−specific fertility rate
Year
0.00
0.01
0.02
0.03
0.04
1960
1980
2000
2020
UN WPP
GBD 2017
GBD 2016
Census Tabulation SBH
MICS SBH
OTHER SURVEY CBH
OTHER SURVEY SBH
VR Complete
VR Incomplete
WFS CBH
Adjusted Data, CBH/VR
Adjusted Data, CEB
Data, CBH/VR
Data, CEB
Outliered Data

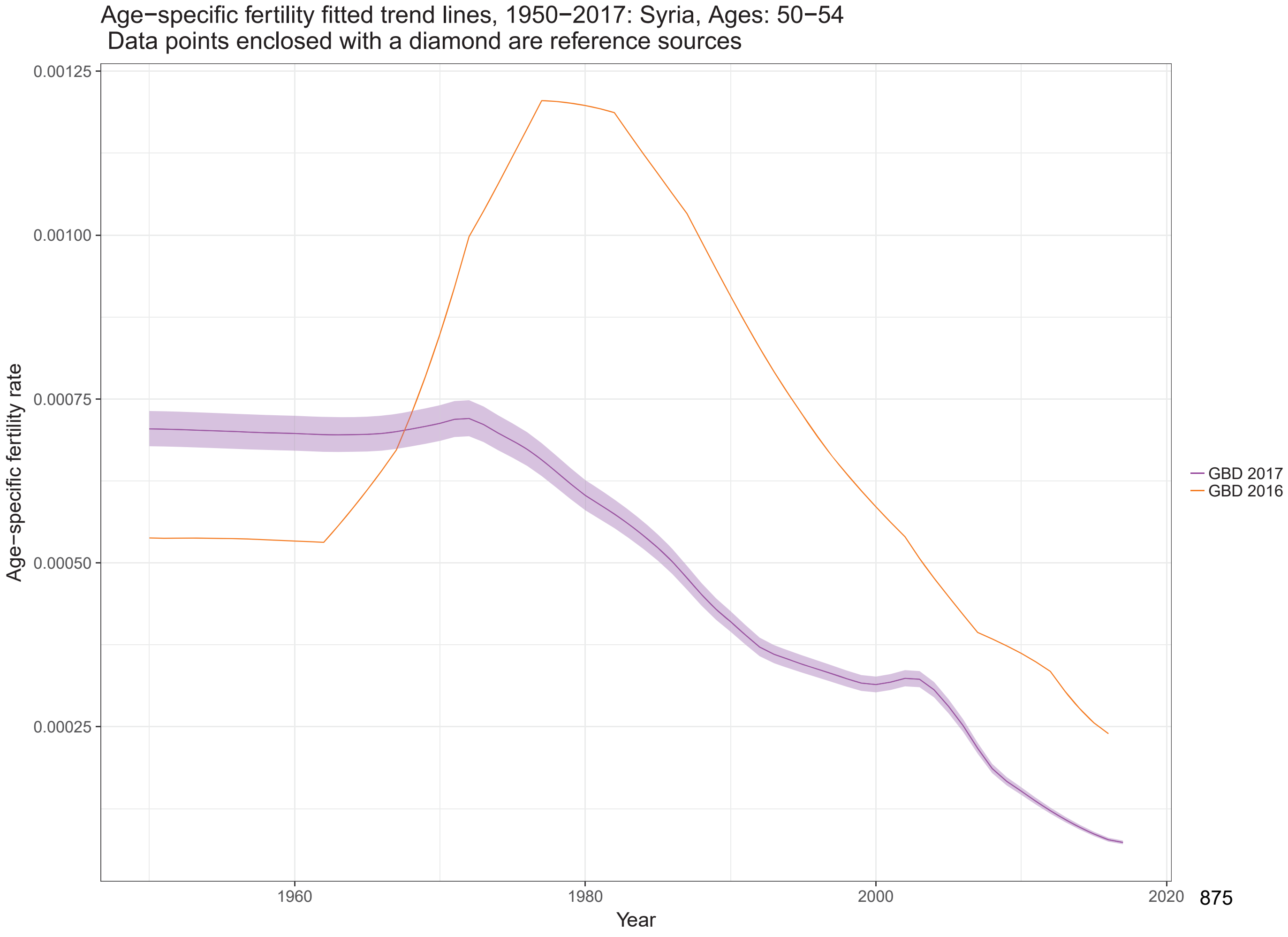

Age−specific fertility fitted trend lines, 1950−2017: Syria, Ages: 50−54
Data points enclosed with a diamond are reference sources
Age−specific fertility rate
Year
0.00025
0.00050
0.00075
0.00100
0.00125
1960
1980
2000
2020
GBD 2017
GBD 2016

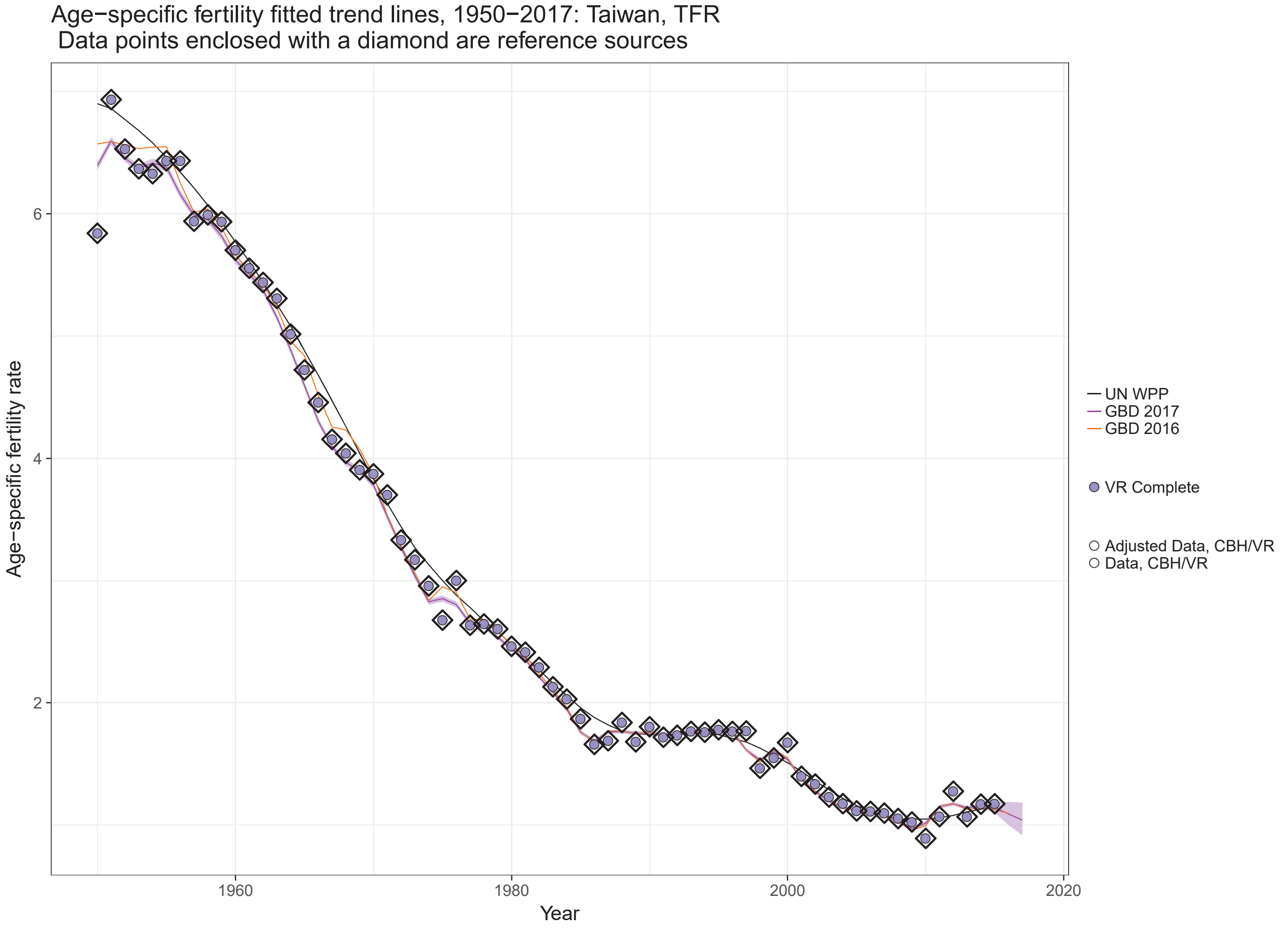
Age−specific fertility fitted trend lines, 1950−2017: Taiwan, TFR
Data points enclosed with a diamond are reference sources
Age−specific fertility rate
Year
6
4
2
1960
1980
2000
2020
UN WPP
GBD 2017
GBD 2016
VR Complete
Adjusted Data, CBH/VR
Data, CBH/VR

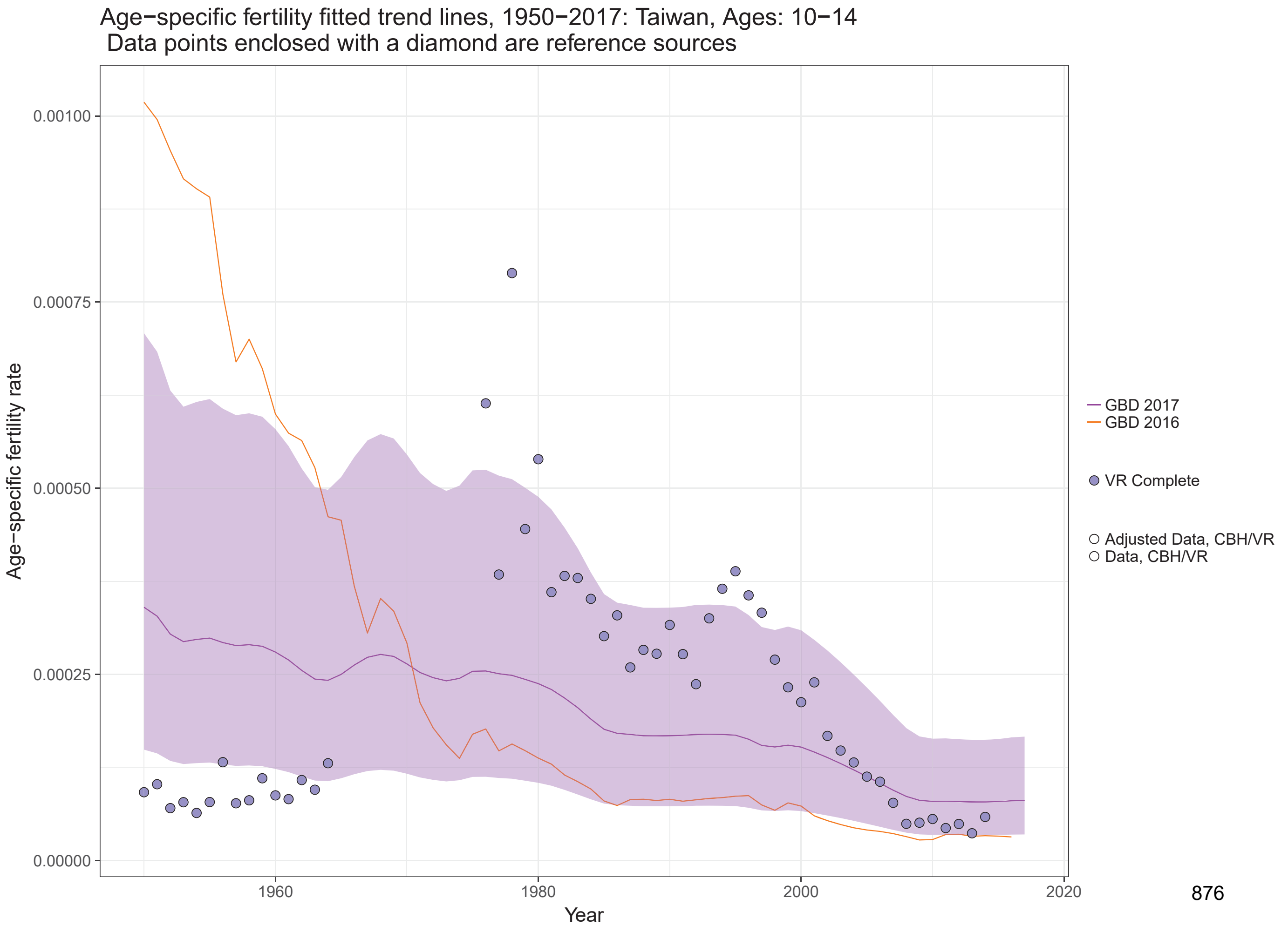
Age−specific fertility fitted trend lines, 1950−2017: Taiwan, Ages: 10−14
Data points enclosed with a diamond are reference sources
Age−specific fertility rate
Year
0.00100
0.00075
0.00050
0.00025
0.00000
1960
1980
2000
2020
GBD 2017
GBD 2016
VR Complete
Adjusted Data, CBH/VR
Data, CBH/VR

Age−specific fertility fitted trend lines, 1950−2017: Taiwan, Ages: 15−19
Data points enclosed with a diamond are reference sources

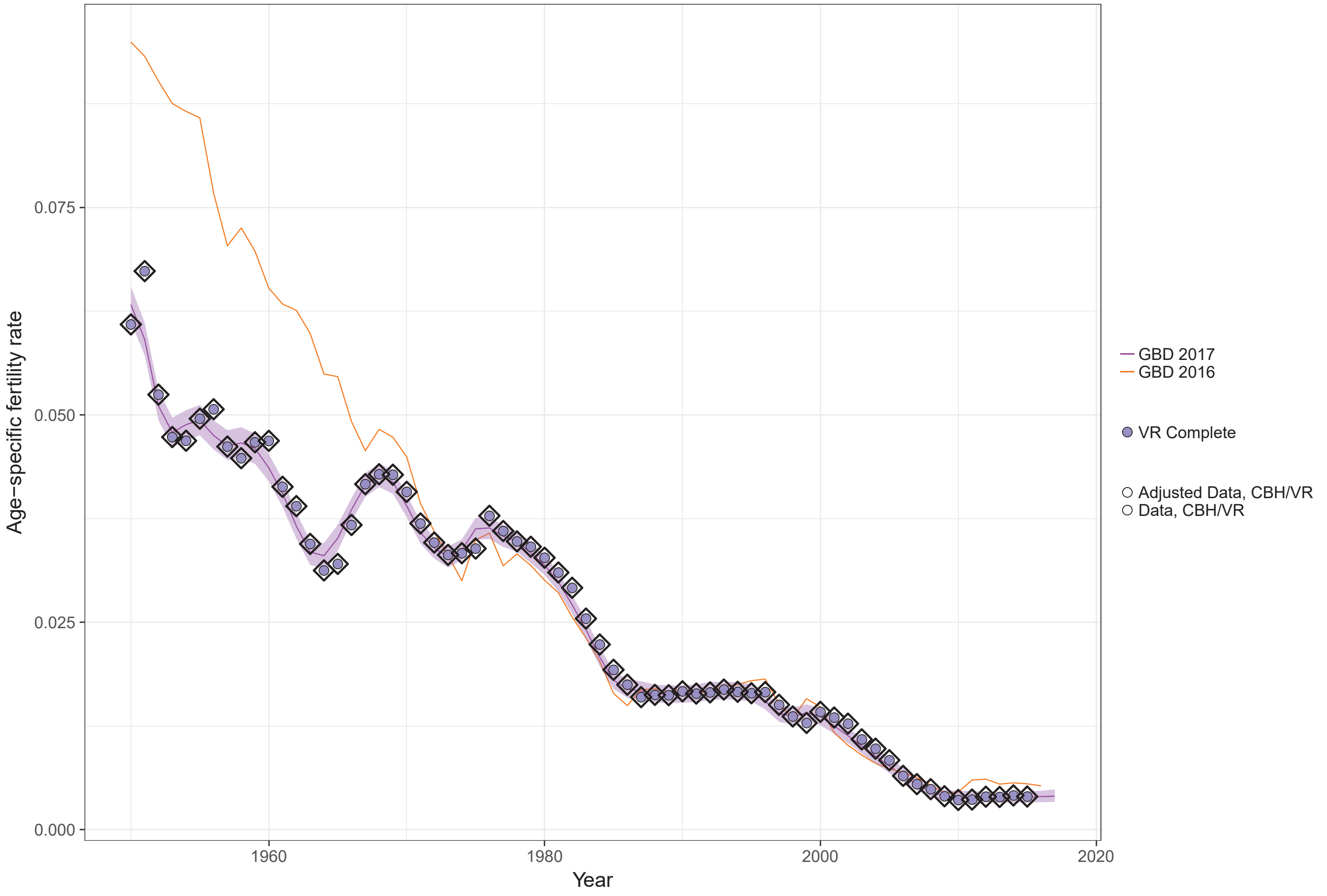

Age−specific fertility fitted trend lines, 1950−2017: Taiwan, Ages: 20−24
Data points enclosed with a diamond are reference sources

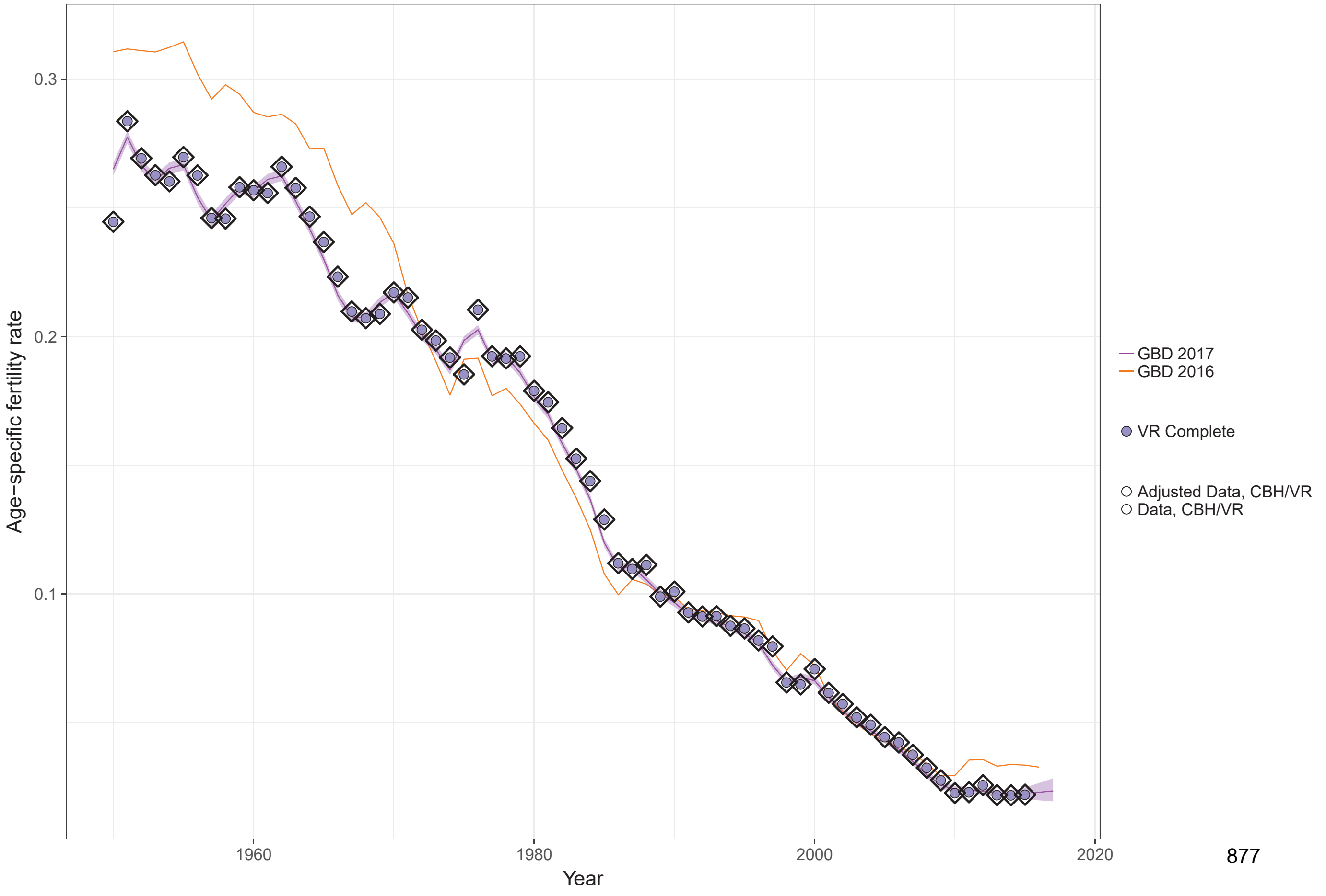

Age−specific fertility fitted trend lines, 1950−2017: Taiwan, Ages: 25−29
Data points enclosed with a diamond are reference sources

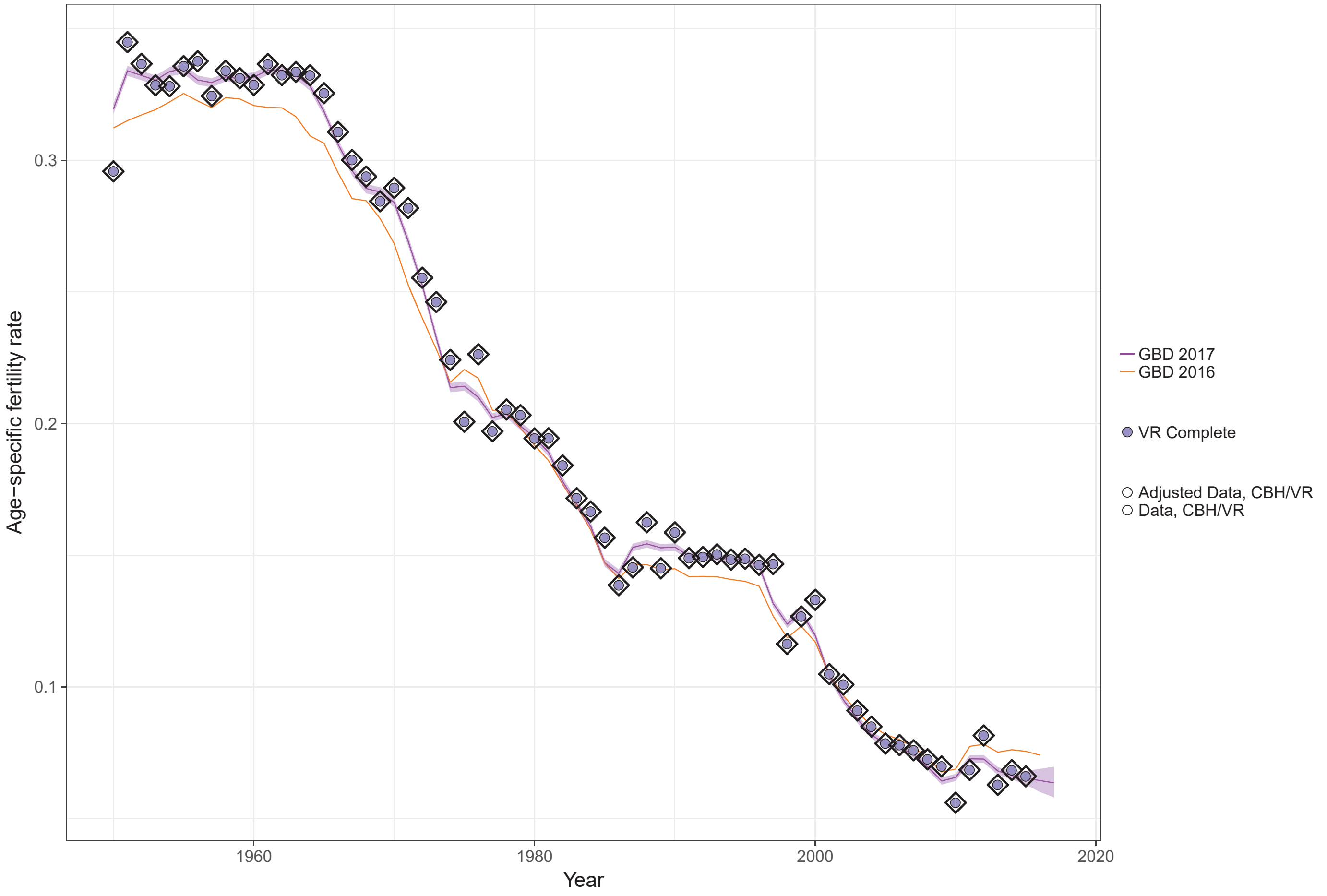

Age−specific fertility fitted trend lines, 1950−2017: Taiwan, Ages: 30−34
Data points enclosed with a diamond are reference sources

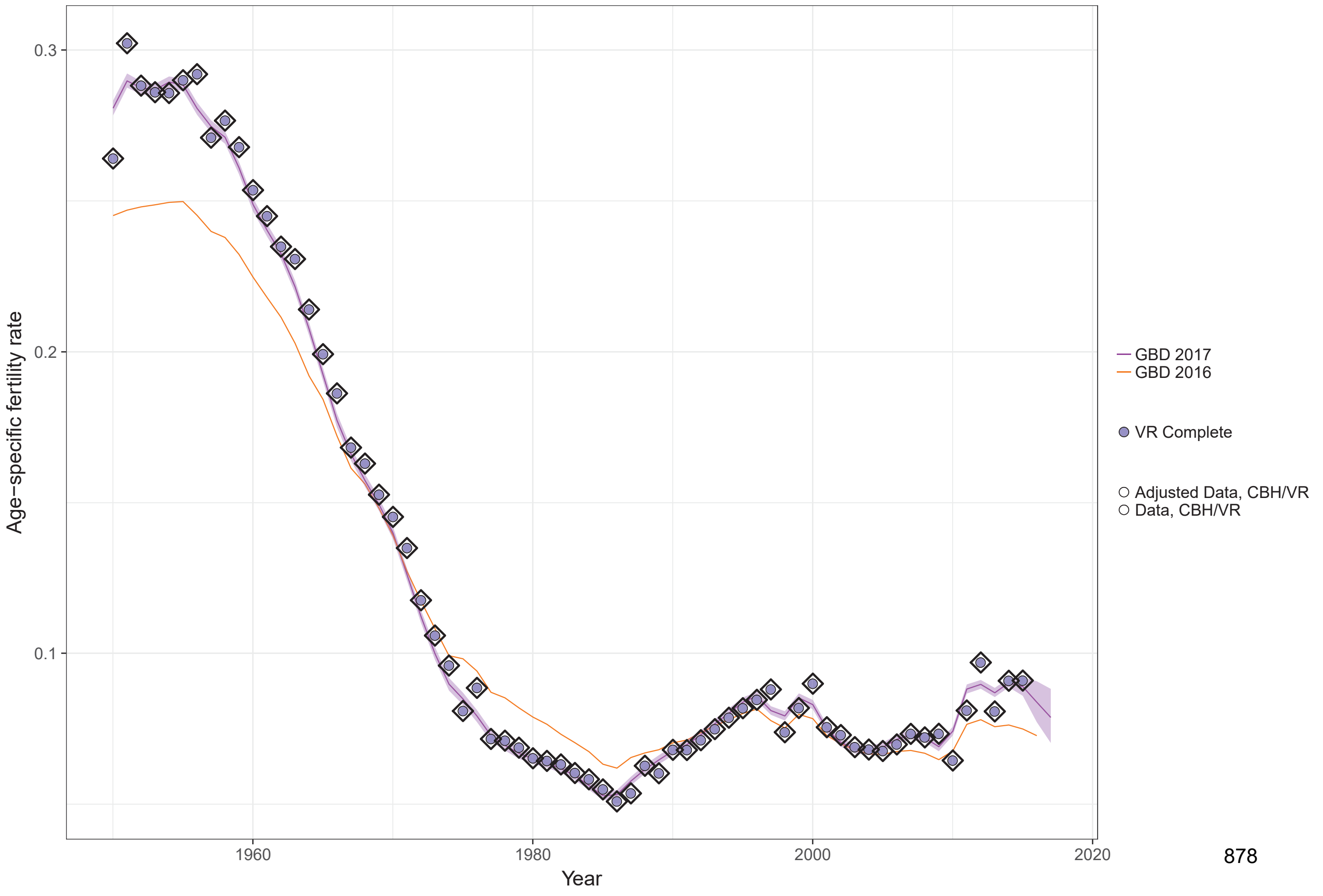

Age−specific fertility fitted trend lines, 1950−2017: Taiwan, Ages: 35−39
Data points enclosed with a diamond are reference sources

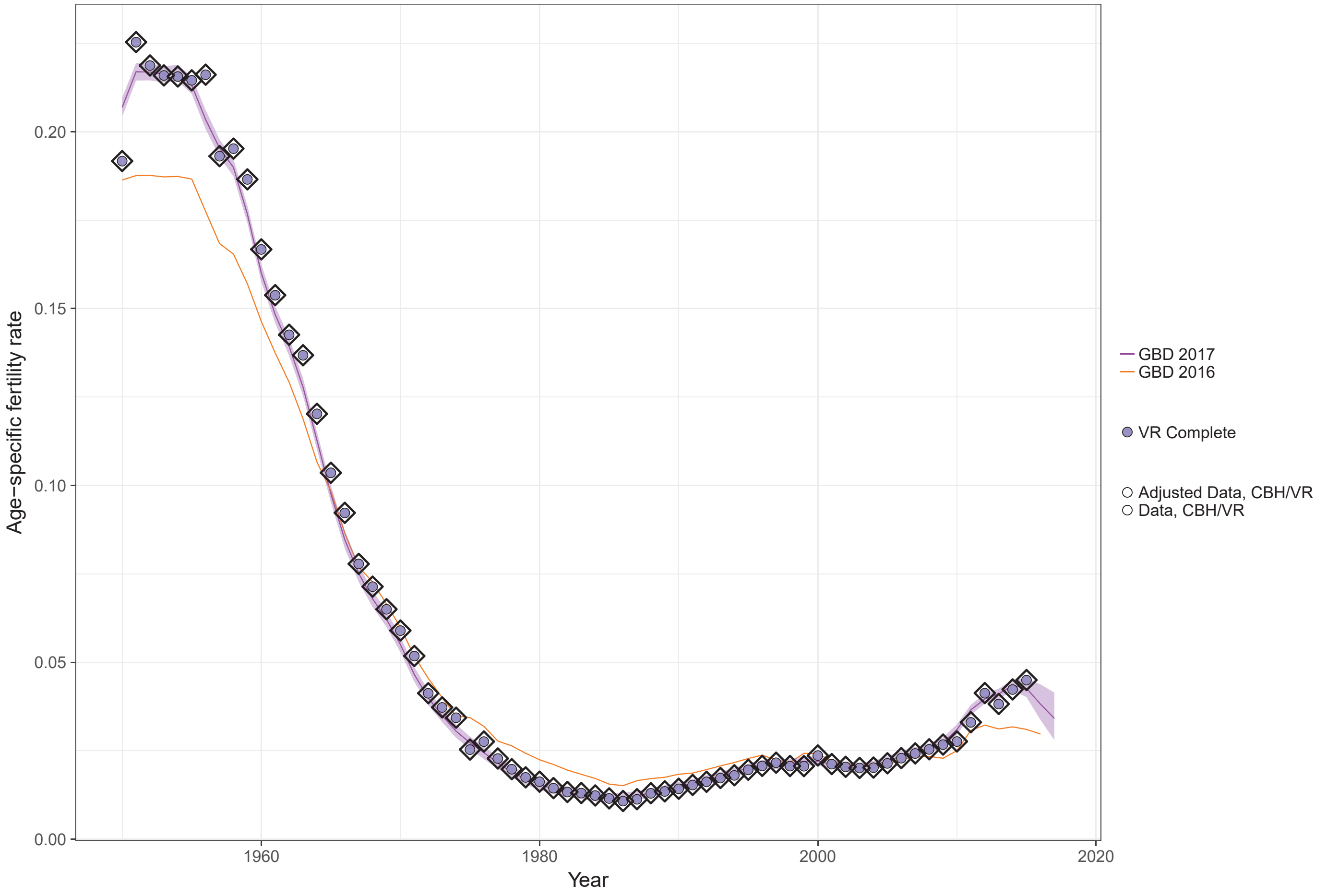

Age−specific fertility fitted trend lines, 1950−2017: Taiwan, Ages: 40−44
Data points enclosed with a diamond are reference sources

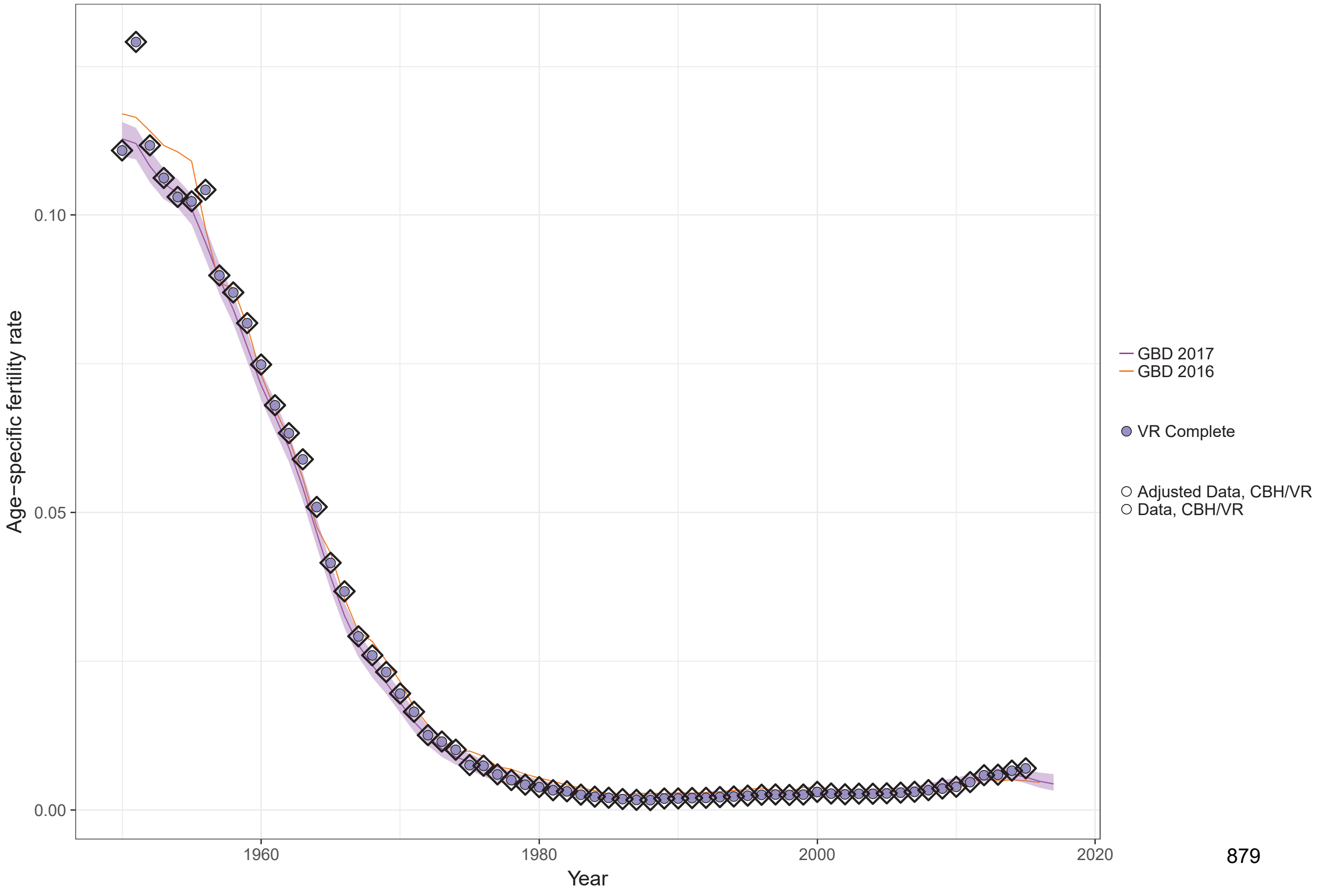

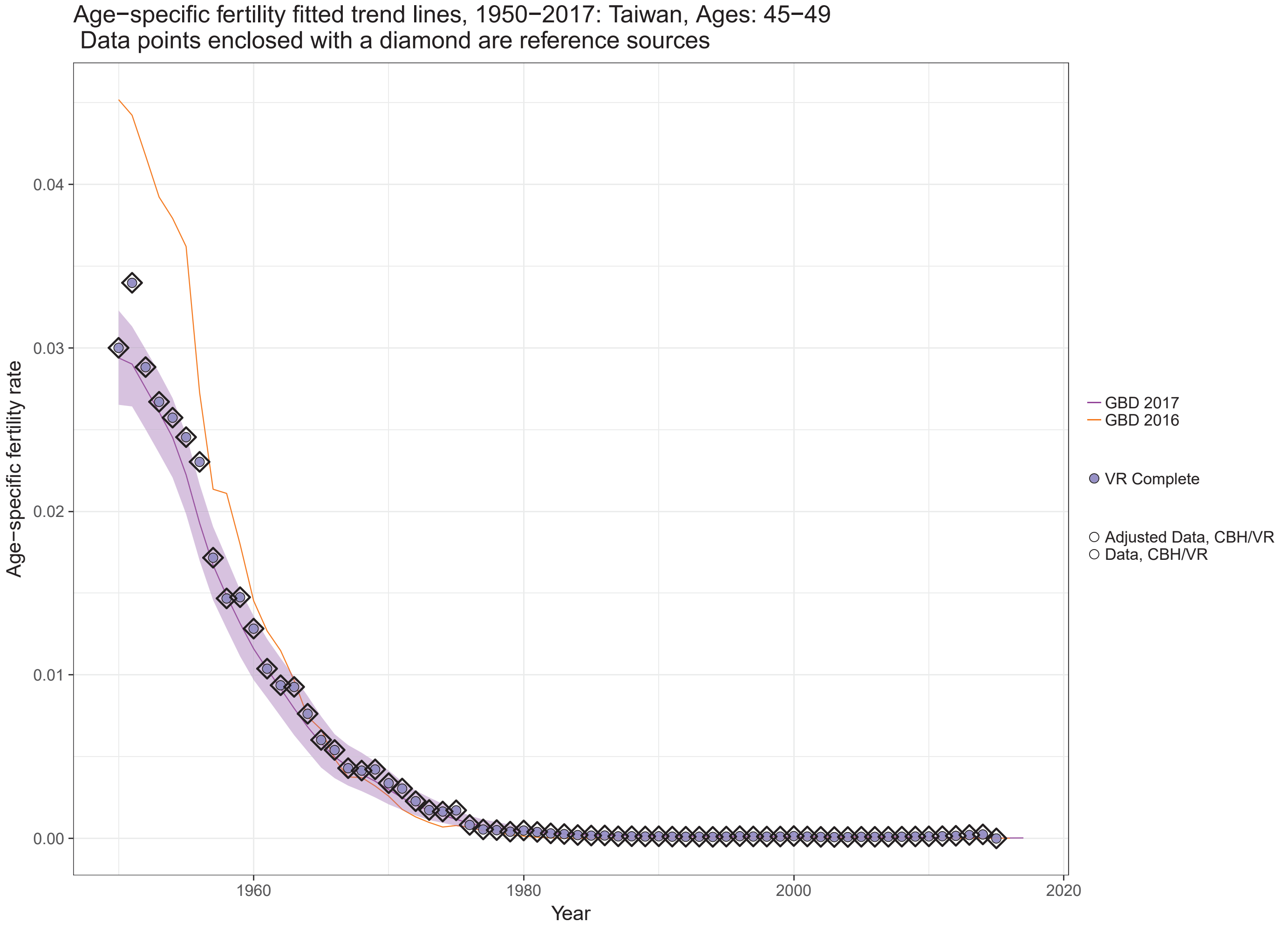

Age−specific fertility fitted trend lines, 1950−2017: Taiwan, Ages: 45−49
Data points enclosed with a diamond are reference sources
Age−specific fertility rate
0.04
0.03
0.02
0.01
0.00
1960
1980
2000
2020
Year
GBD 2017
GBD 2016
VR Complete
Adjusted Data, CBH/VR
Data, CBH/VR

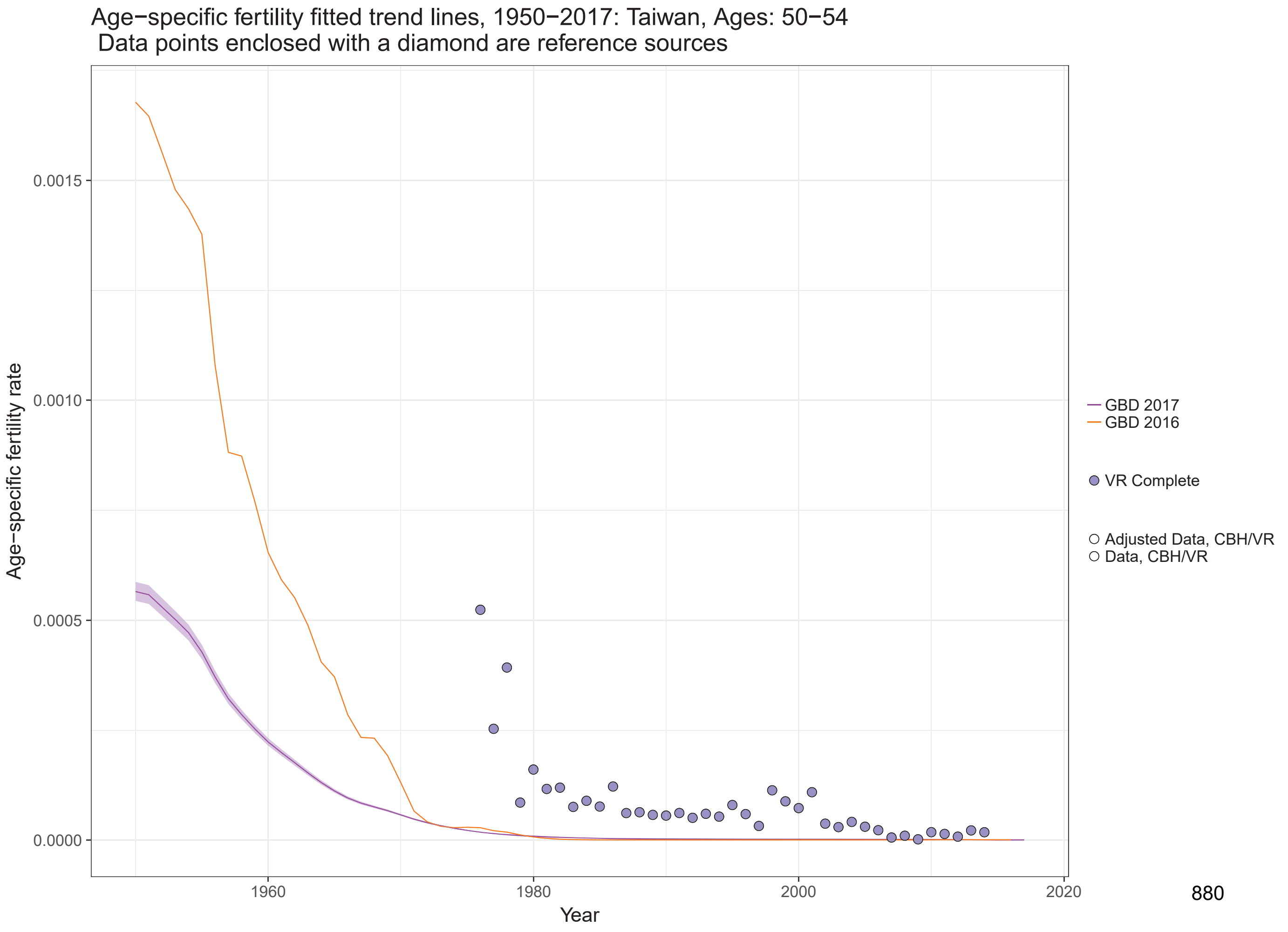

Age−specific fertility fitted trend lines, 1950−2017: Taiwan, Ages: 50−54
Data points enclosed with a diamond are reference sources
Age−specific fertility rate
0.0015
0.0010
0.0005
0.0000
1960
1980
2000
2020
Year
GBD 2017
GBD 2016
VR Complete
Adjusted Data, CBH/VR
Data, CBH/VR

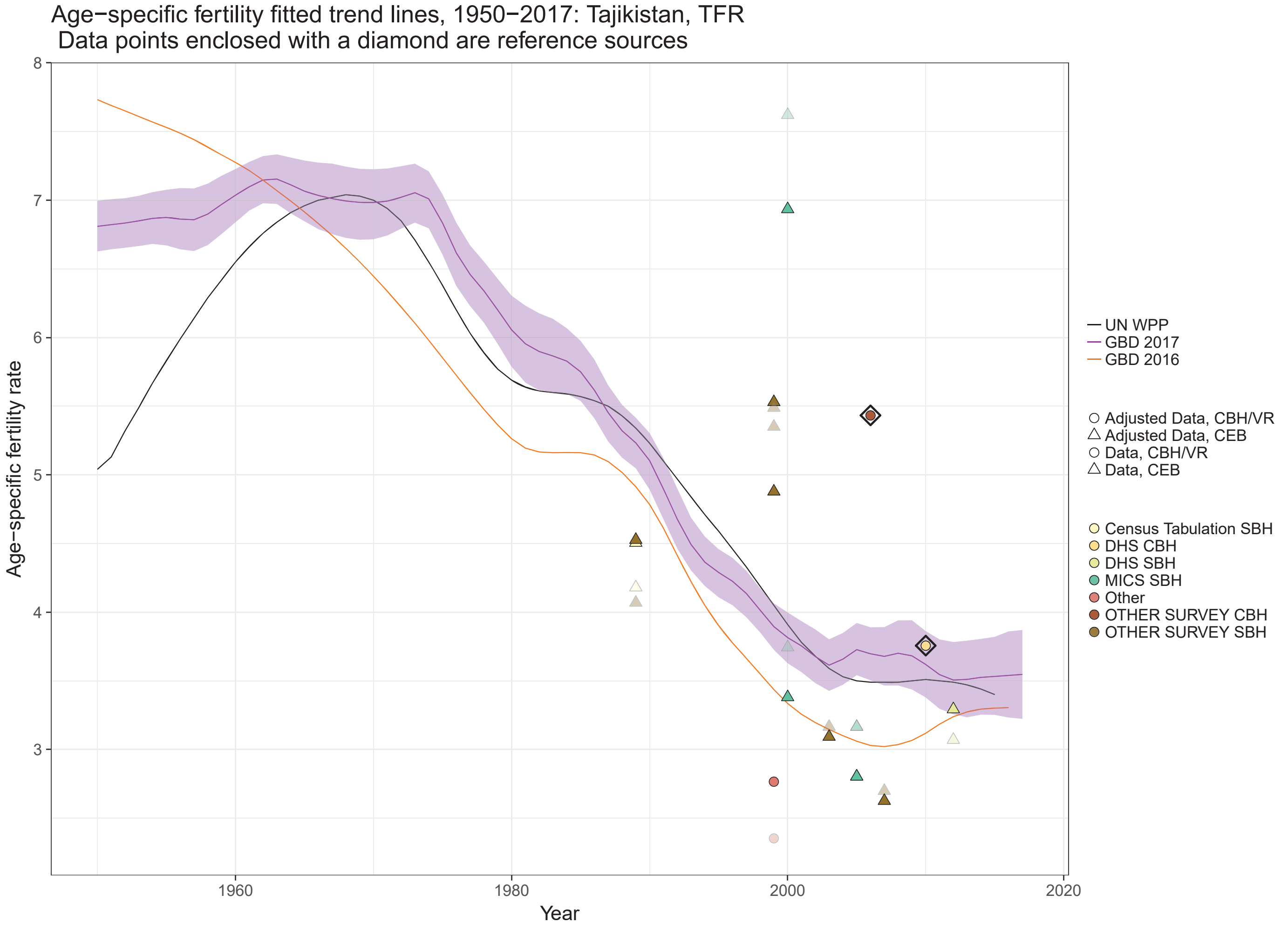
Age−specific fertility fitted trend lines, 1950−2017: Tajikistan, TFR
Data points enclosed with a diamond are reference sources
Age−specific fertility rate
Year
8
7
6
5
4
3
1960
1980
2000
2020
UN WPP
GBD 2017
GBD 2016
Adjusted Data, CBH/VR
Adjusted Data, CEB
Data, CBH/VR
Data, CEB
Census Tabulation SBH
DHS CBH
DHS SBH
MICS SBH
Other
OTHER SURVEY CBH
OTHER SURVEY SBH

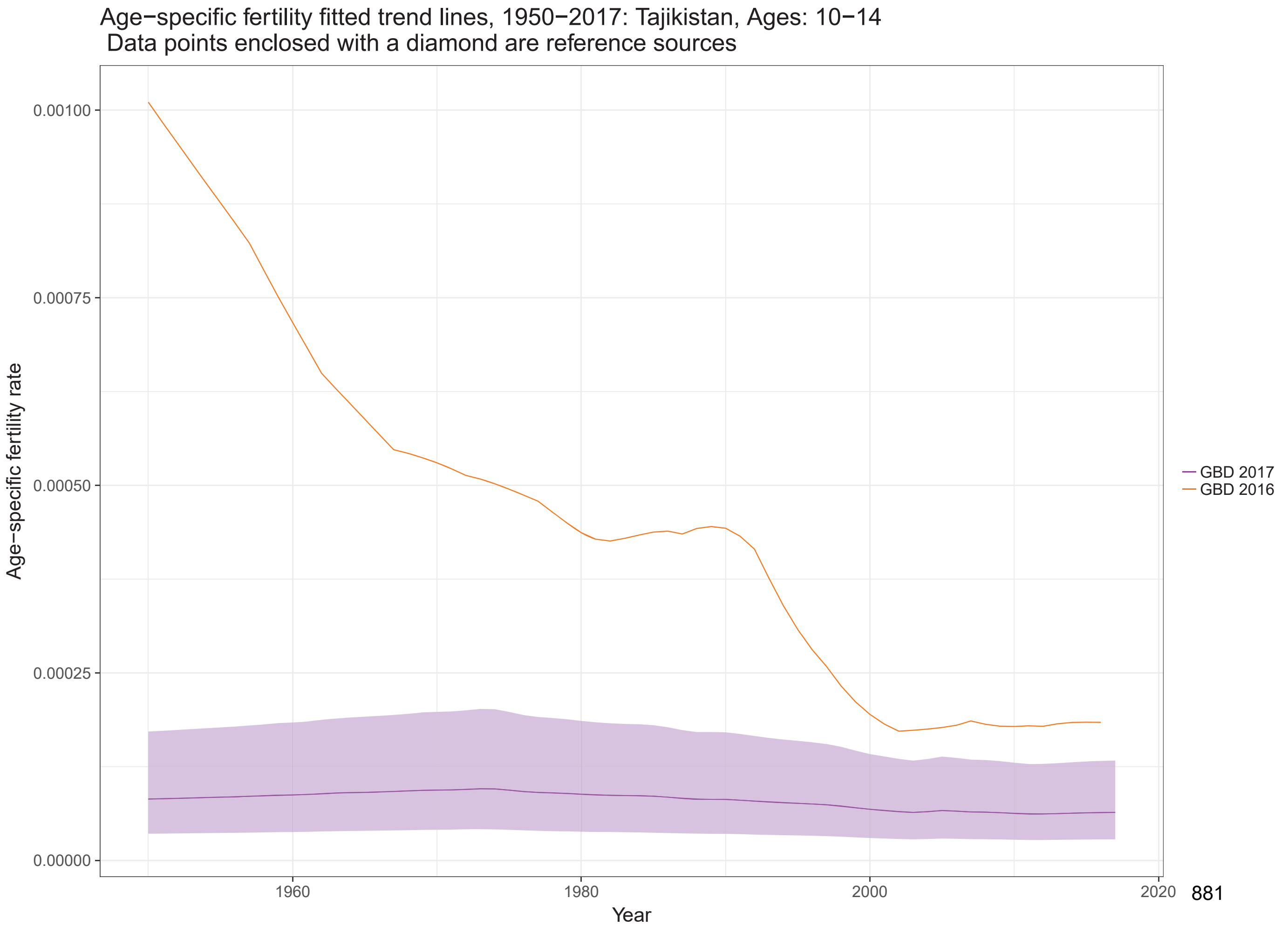
Age−specific fertility fitted trend lines, 1950−2017: Tajikistan, Ages: 10−14
Data points enclosed with a diamond are reference sources
Age−specific fertility rate
Year
0.00100
0.00075
0.00050
0.00025
0.00000
1960
1980
2000
2020
GBD 2017
GBD 2016

Age−specific fertility fitted trend lines, 1950−2017: Tajikistan, Ages: 15−19
Data points enclosed with a diamond are reference sources

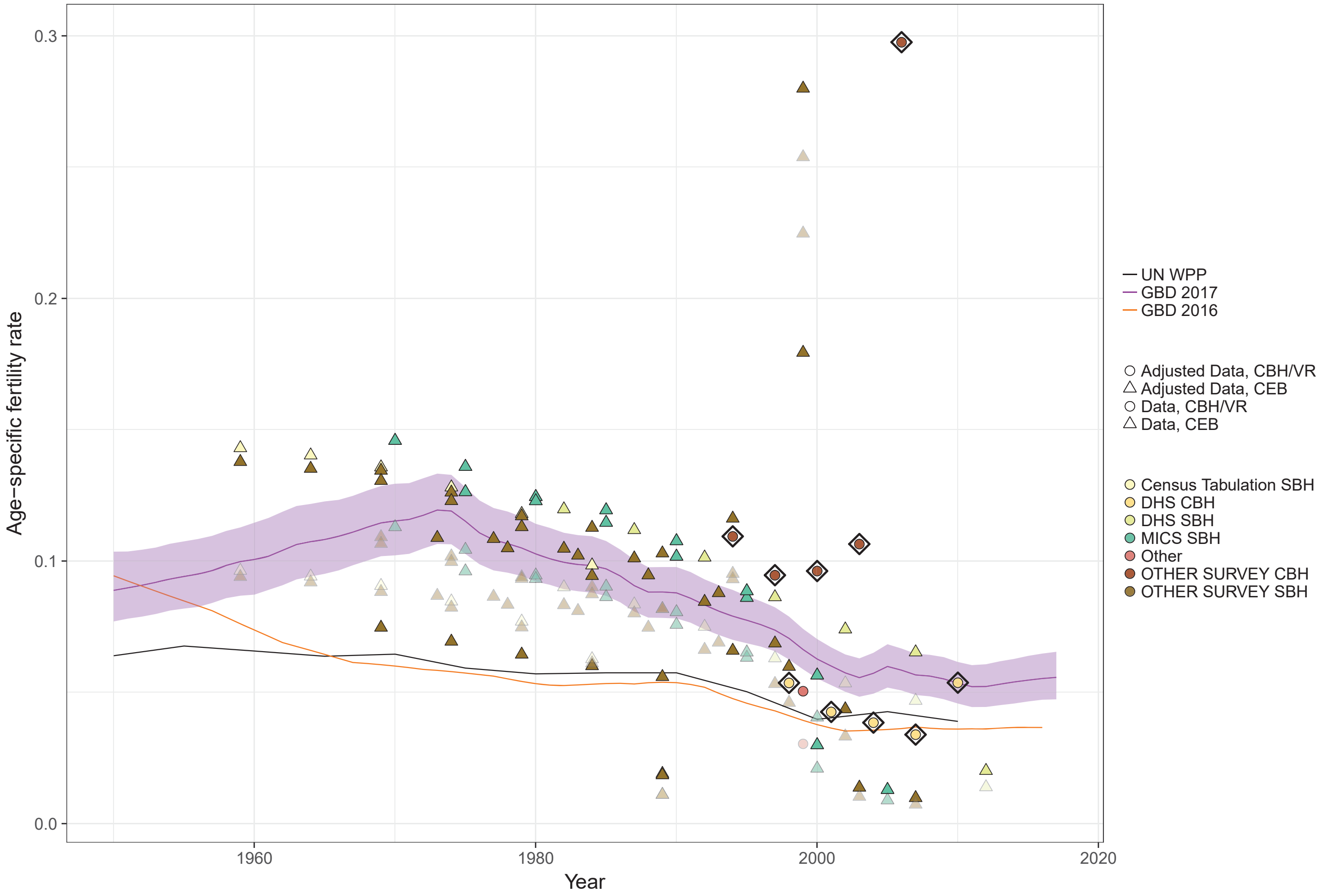

Age−specific fertility fitted trend lines, 1950−2017: Tajikistan, Ages: 20−24
Data points enclosed with a diamond are reference sources

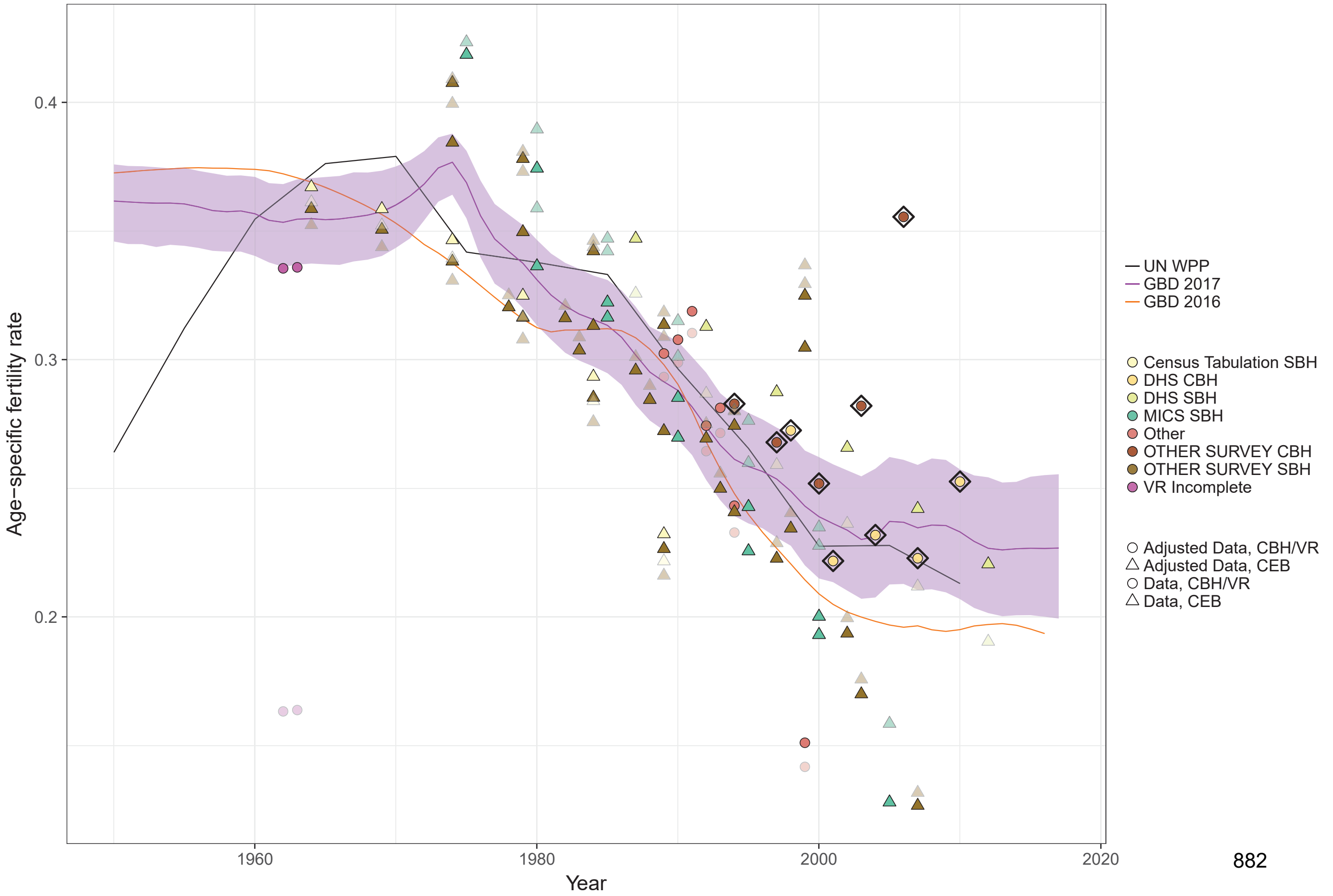

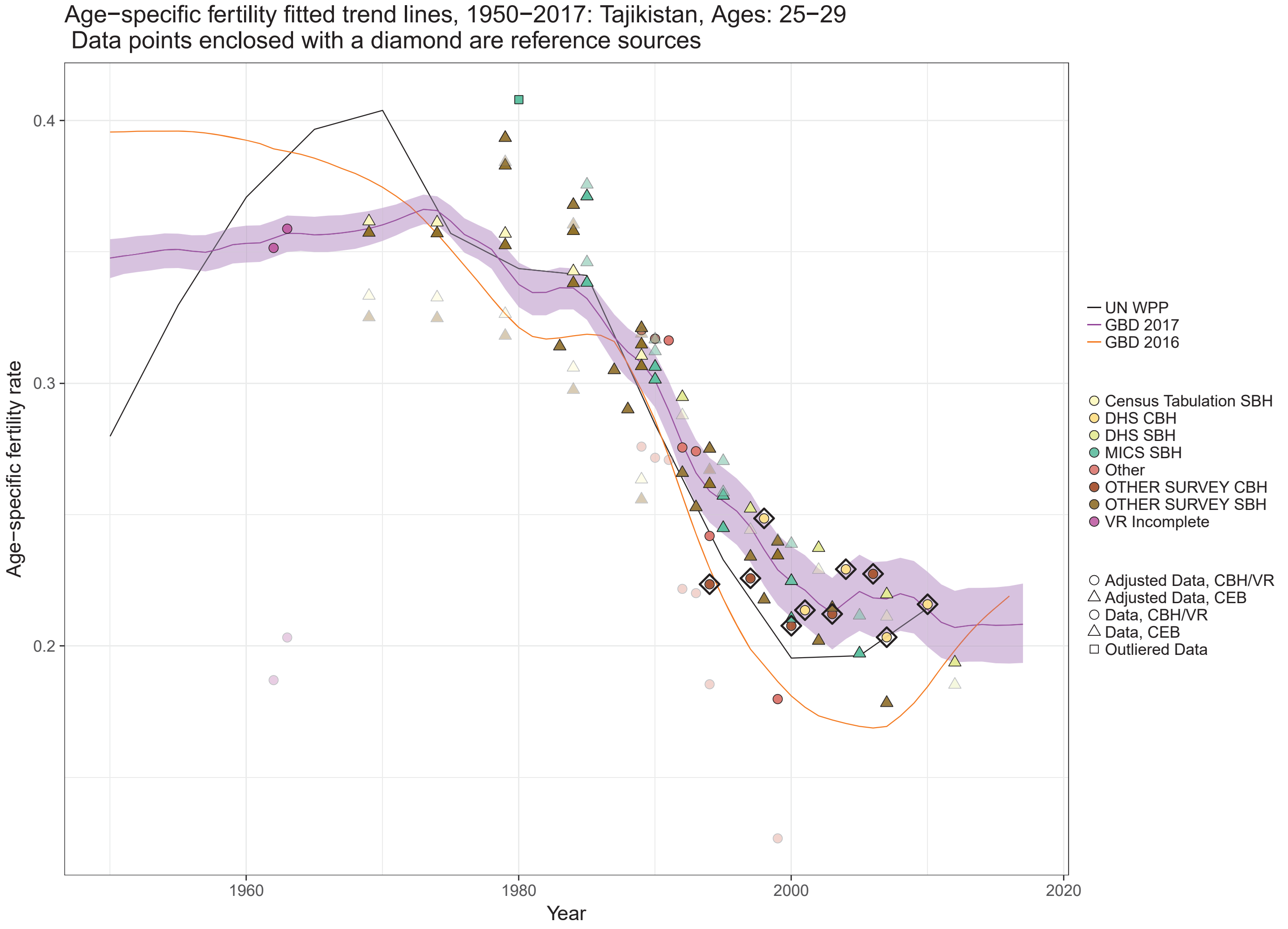
Age−specific fertility fitted trend lines, 1950−2017: Tajikistan, Ages: 25−29
Data points enclosed with a diamond are reference sources
Age-specific fertility rate
0.4
0.3
0.2
1960
1980
2000
2020
Year
UN WPP
GBD 2017
GBD 2016
Census Tabulation SBH
DHS CBH
DHS SBH
MICS SBH
Other
OTHER SURVEY CBH
OTHER SURVEY SBH
VR Incomplete
Adjusted Data, CBH/VR
Adjusted Data, CEB
Data, CBH/VR
Data, CEB
Outliered Data

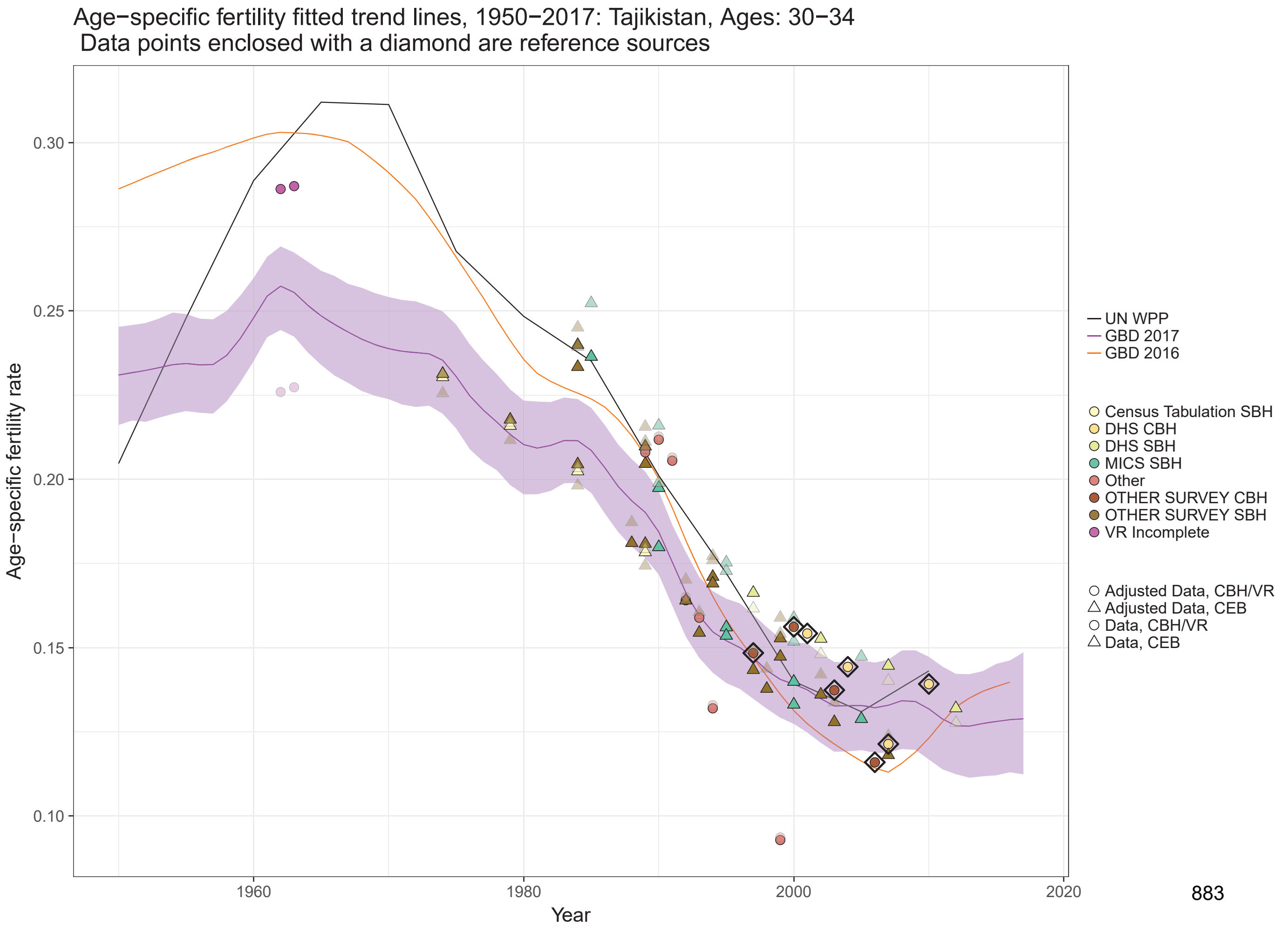
Age−specific fertility fitted trend lines, 1950−2017: Tajikistan, Ages: 30−34
Data points enclosed with a diamond are reference sources
Age-specific fertility rate
0.30
0.25
0.20
0.15
0.10
1960
1980
2000
2020
Year
UN WPP
GBD 2017
GBD 2016
Census Tabulation SBH
DHS CBH
DHS SBH
MICS SBH
Other
OTHER SURVEY CBH
OTHER SURVEY SBH
VR Incomplete
Adjusted Data, CBH/VR
Adjusted Data, CEB
Data, CBH/VR
Data, CEB

Age−specific fertility fitted trend lines, 1950−2017: Tajikistan, Ages: 35−39
Data points enclosed with a diamond are reference sources

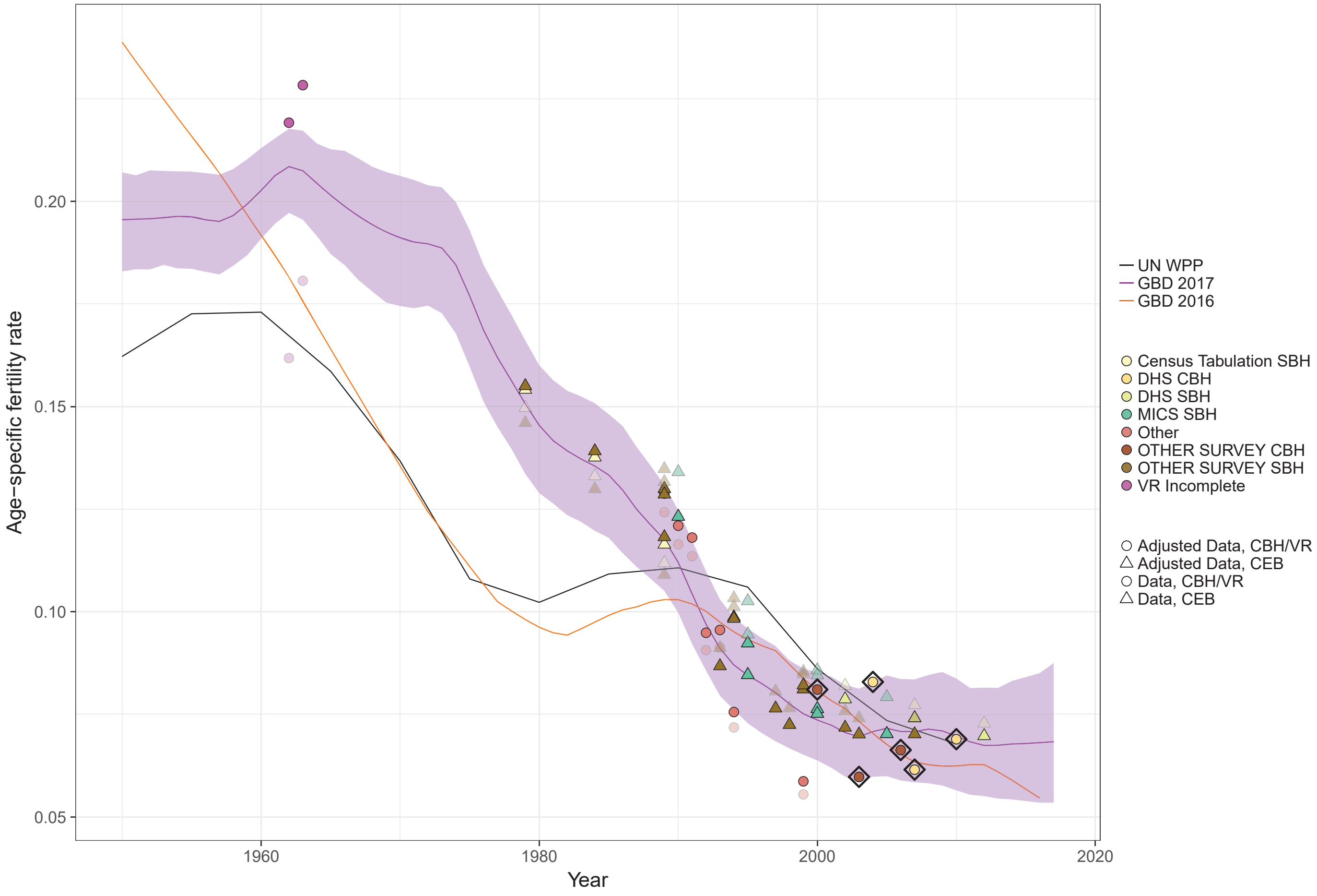

Age−specific fertility fitted trend lines, 1950−2017: Tajikistan, Ages: 40−44
Data points enclosed with a diamond are reference sources

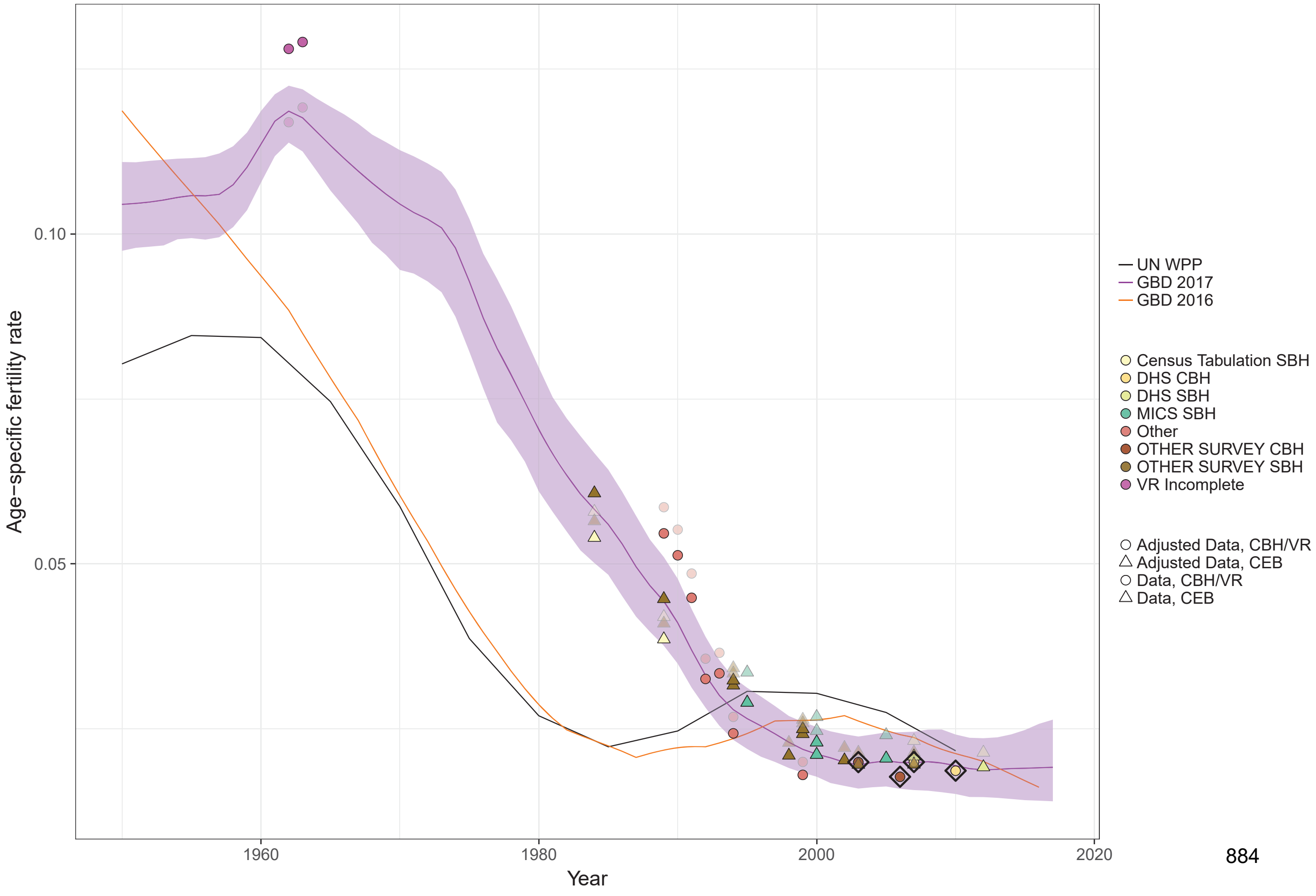

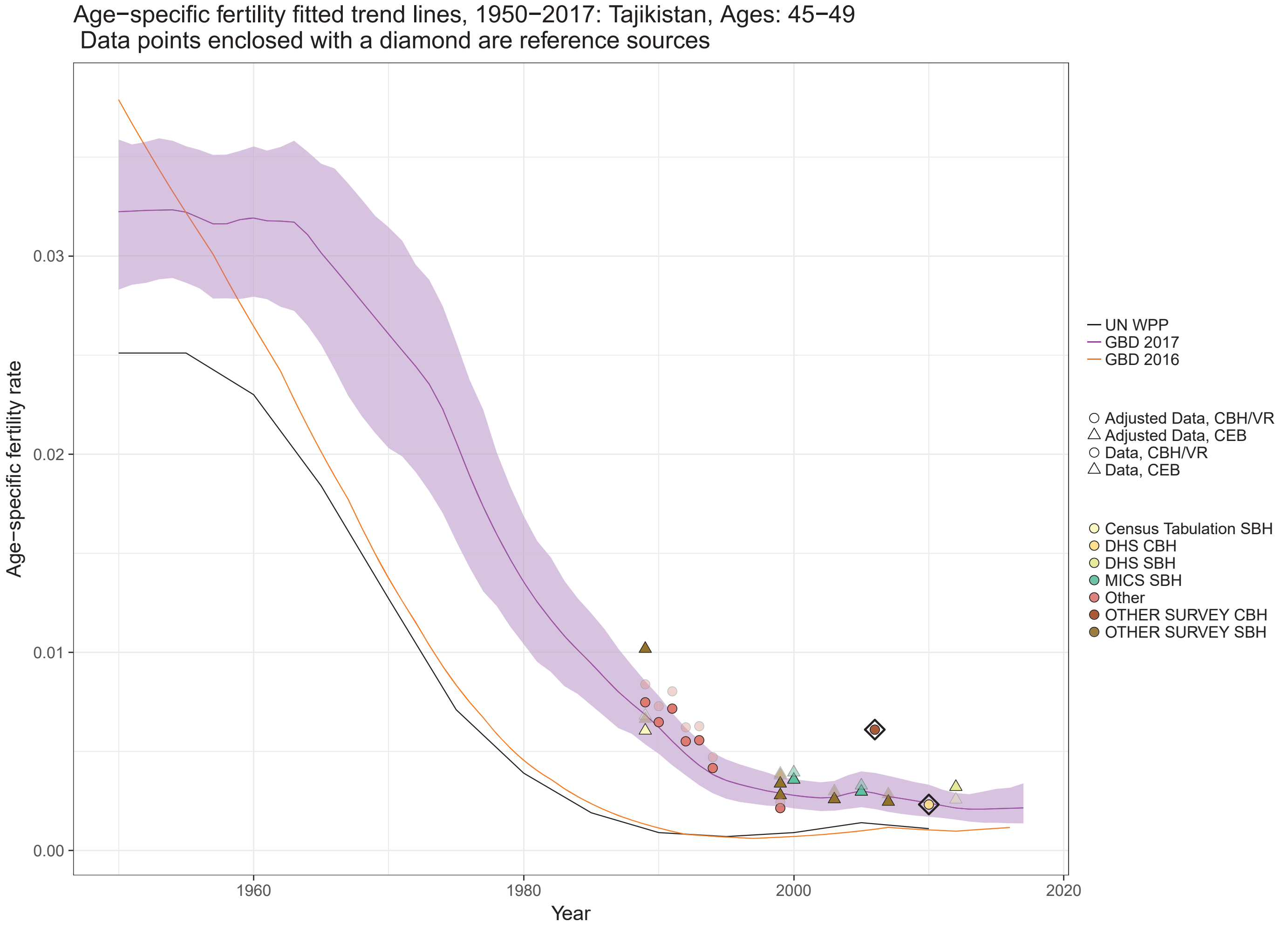

Age−specific fertility fitted trend lines, 1950−2017: Tajikistan, Ages: 45−49
Data points enclosed with a diamond are reference sources
Age−specific fertility rate
Year
0.00
0.01
0.02
0.03
1960
1980
2000
2020
UN WPP
GBD 2017
GBD 2016
Adjusted Data, CBH/VR
Adjusted Data, CEB
Data, CBH/VR
Data, CEB
Census Tabulation SBH
DHS CBH
DHS SBH
MICS SBH
Other
OTHER SURVEY CBH
OTHER SURVEY SBH

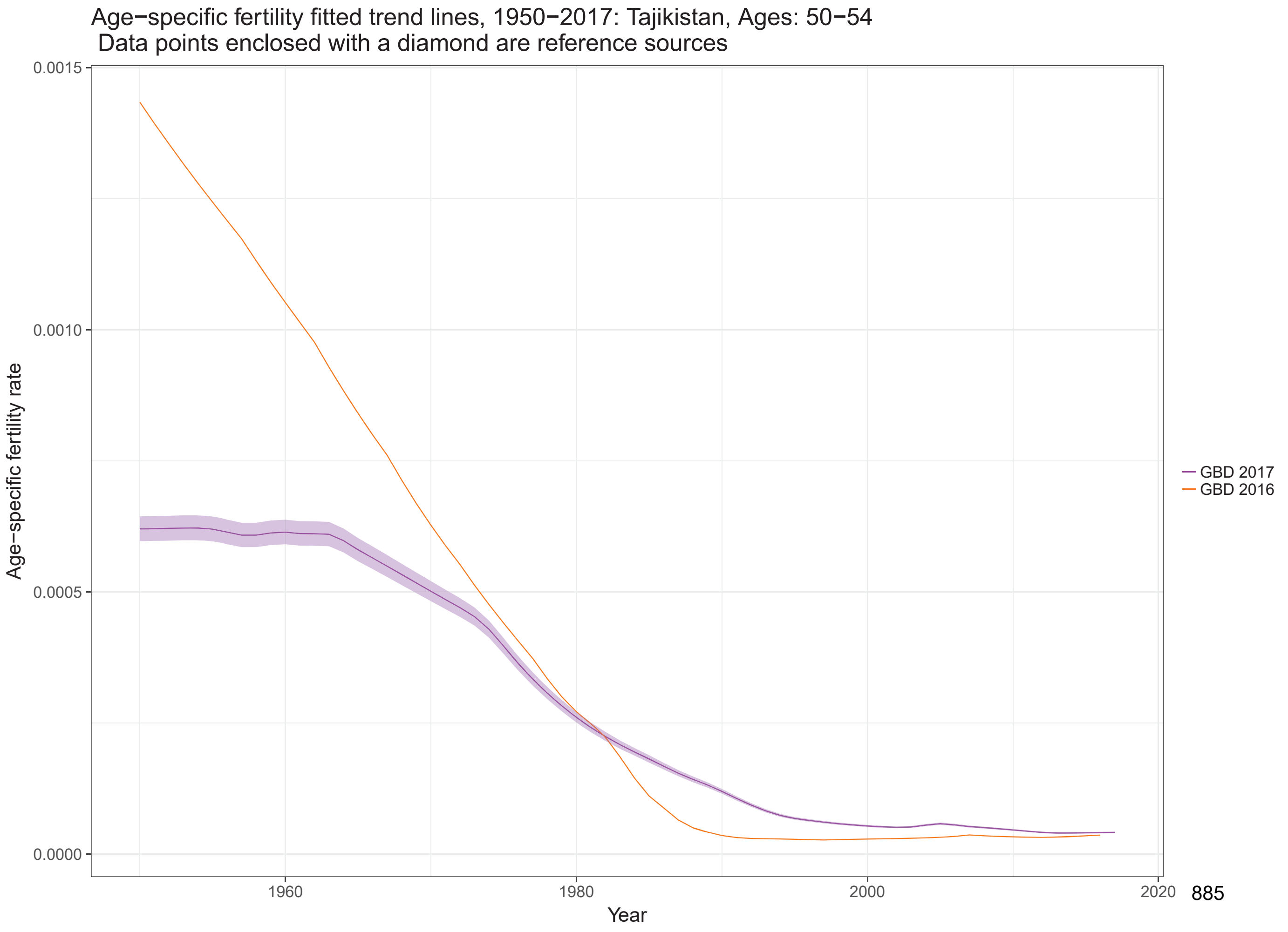

Age−specific fertility fitted trend lines, 1950−2017: Tajikistan, Ages: 50−54
Data points enclosed with a diamond are reference sources
Age−specific fertility rate
Year
0.0000
0.0005
0.0010
0.0015
1960
1980
2000
2020
GBD 2017
GBD 2016

Age−specific fertility fitted trend lines, 1950−2017: Tanzania, TFR
Data points enclosed with a diamond are reference sources

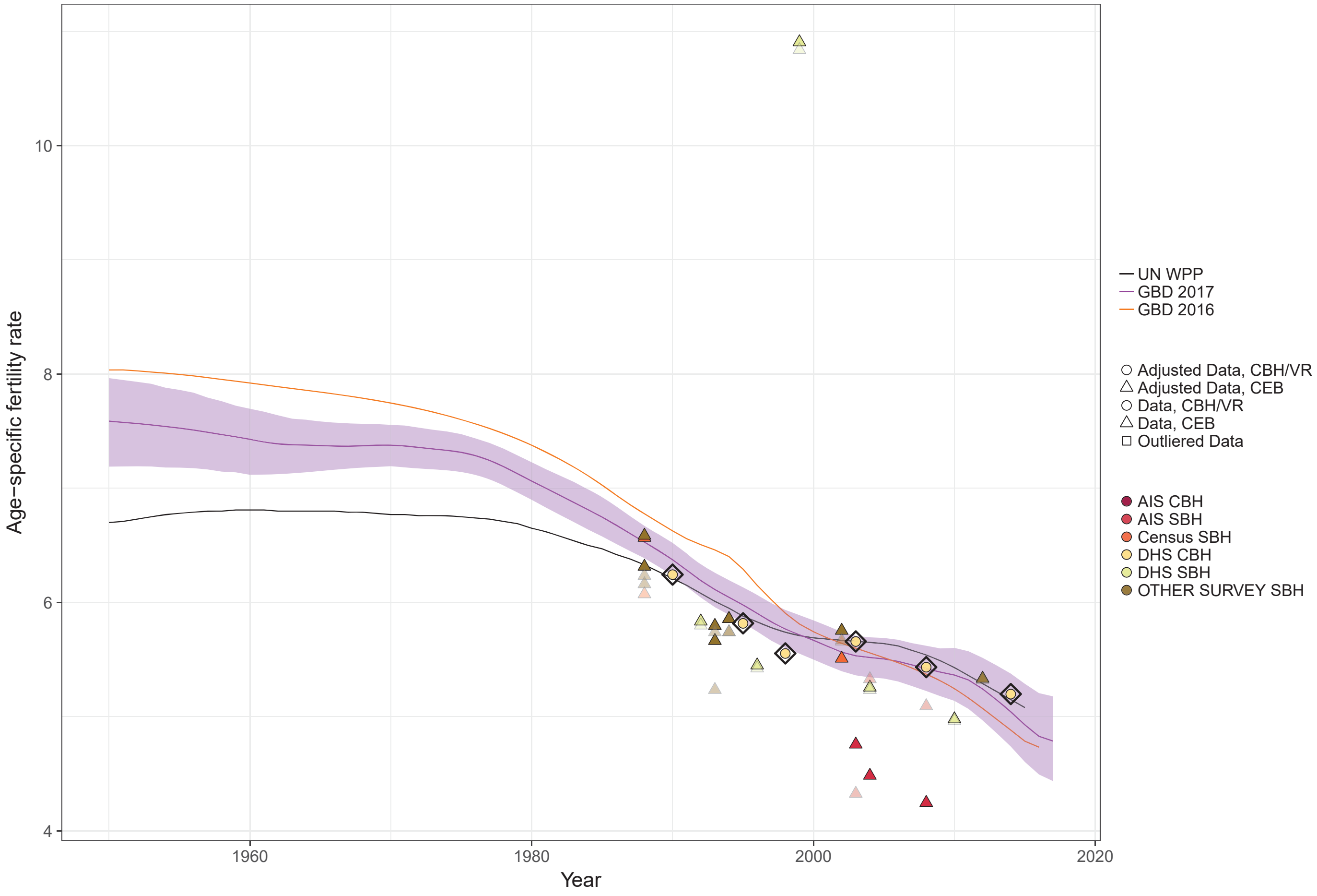

Age−specific fertility fitted trend lines, 1950−2017: Tanzania, Ages: 10−14
Data points enclosed with a diamond are reference sources

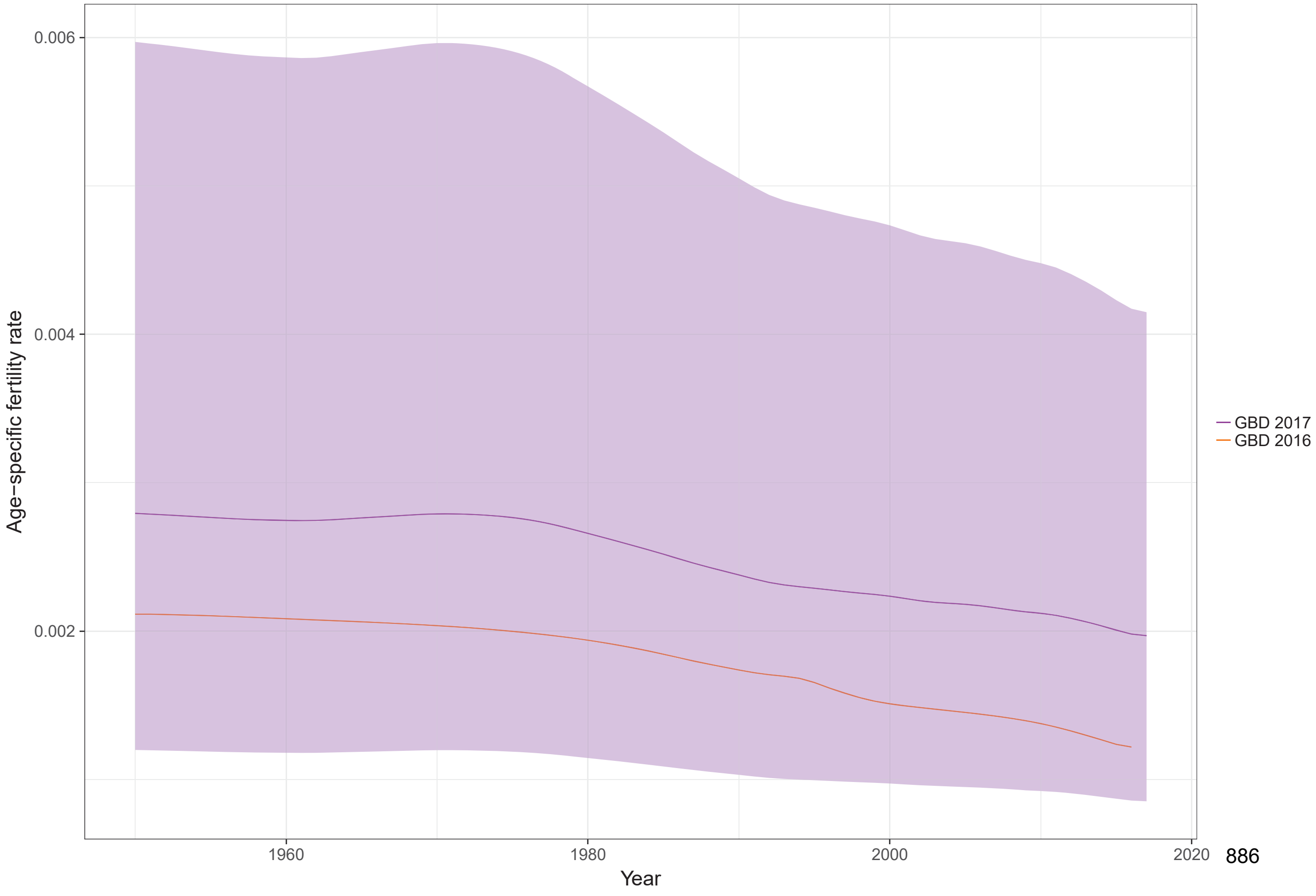

Age−specific fertility fitted trend lines, 1950−2017: Tanzania, Ages: 15−19
Data points enclosed with a diamond are reference sources

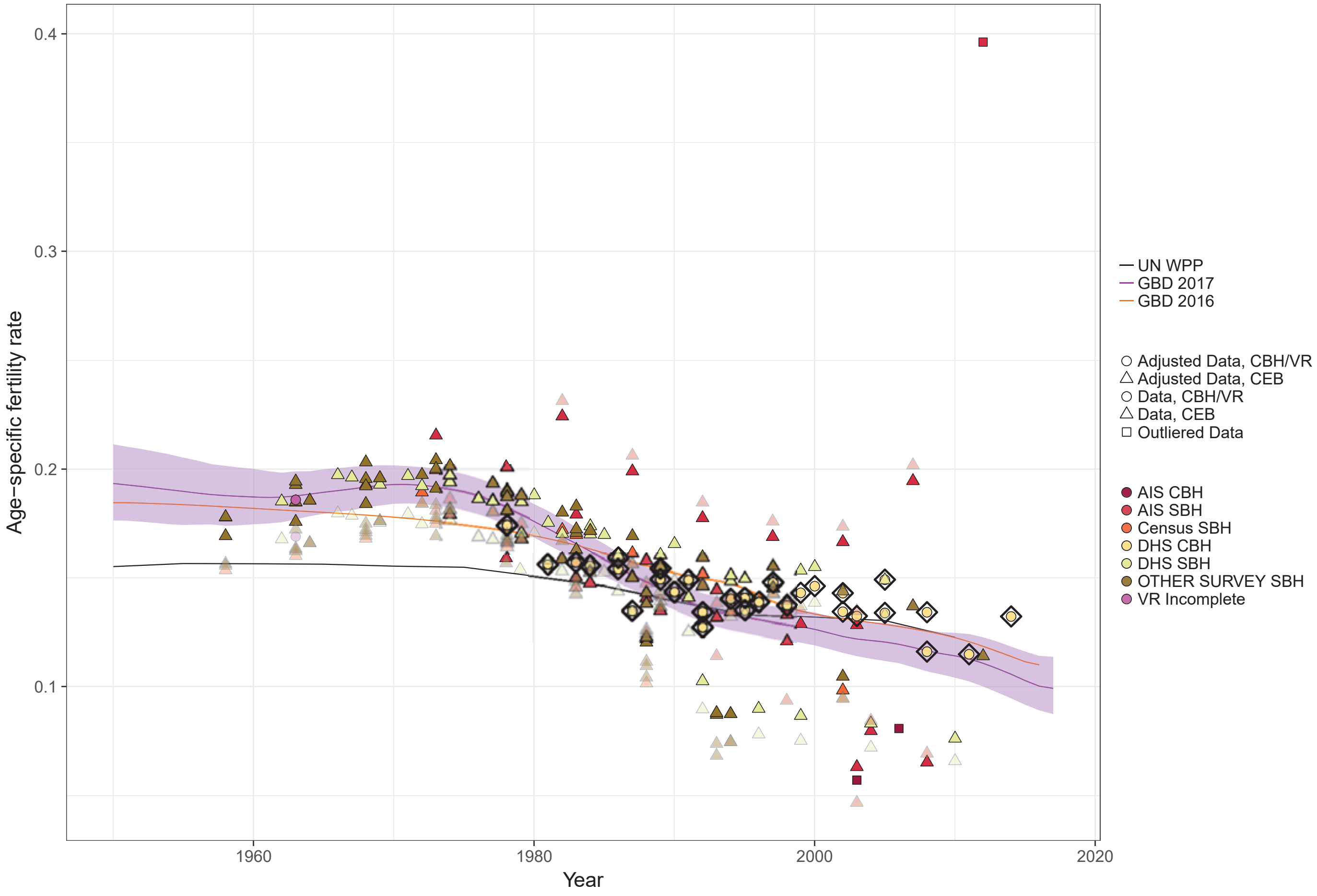

Age−specific fertility fitted trend lines, 1950−2017: Tanzania, Ages: 20−24
Data points enclosed with a diamond are reference sources

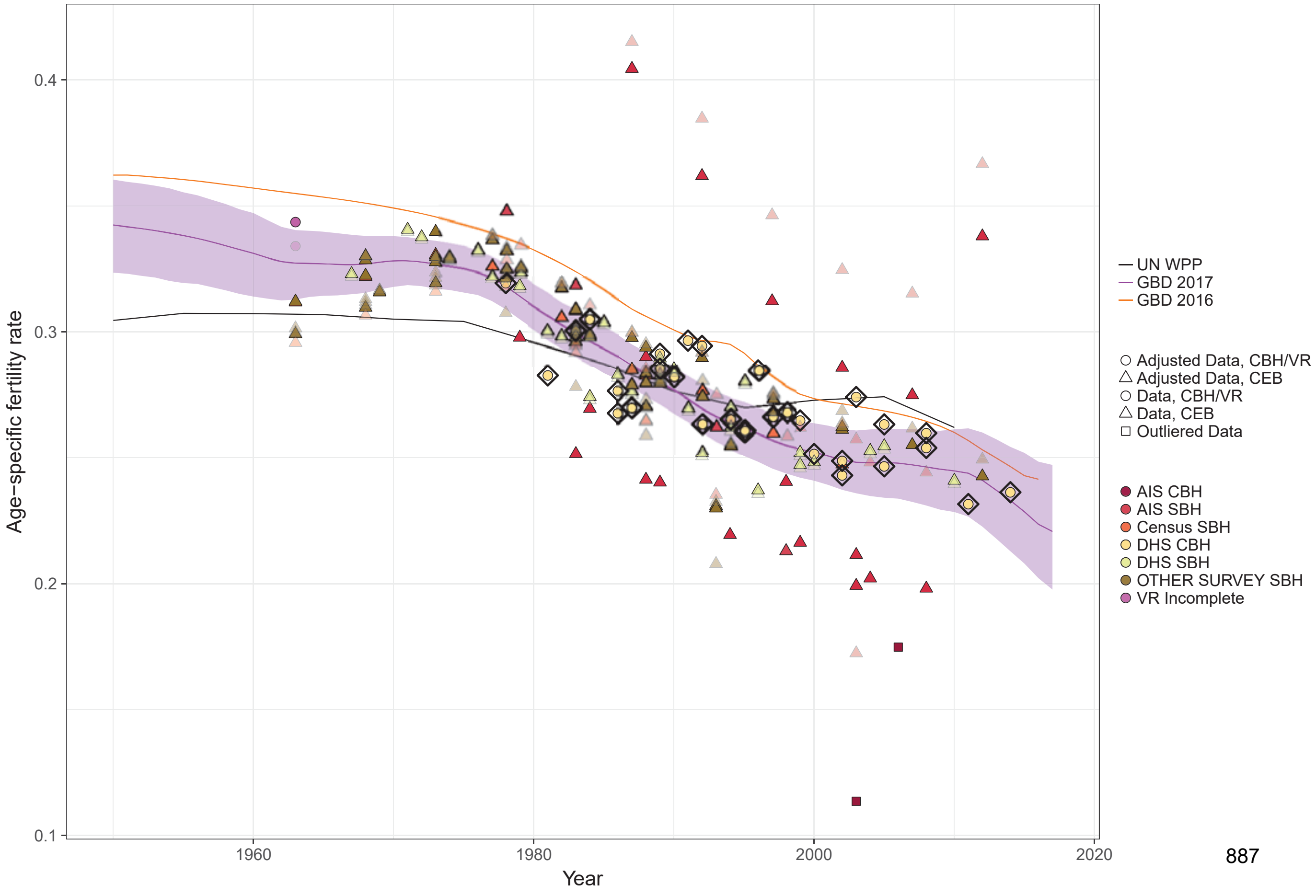

Age−specific fertility fitted trend lines, 1950−2017: Tanzania, Ages: 25−29
Data points enclosed with a diamond are reference sources

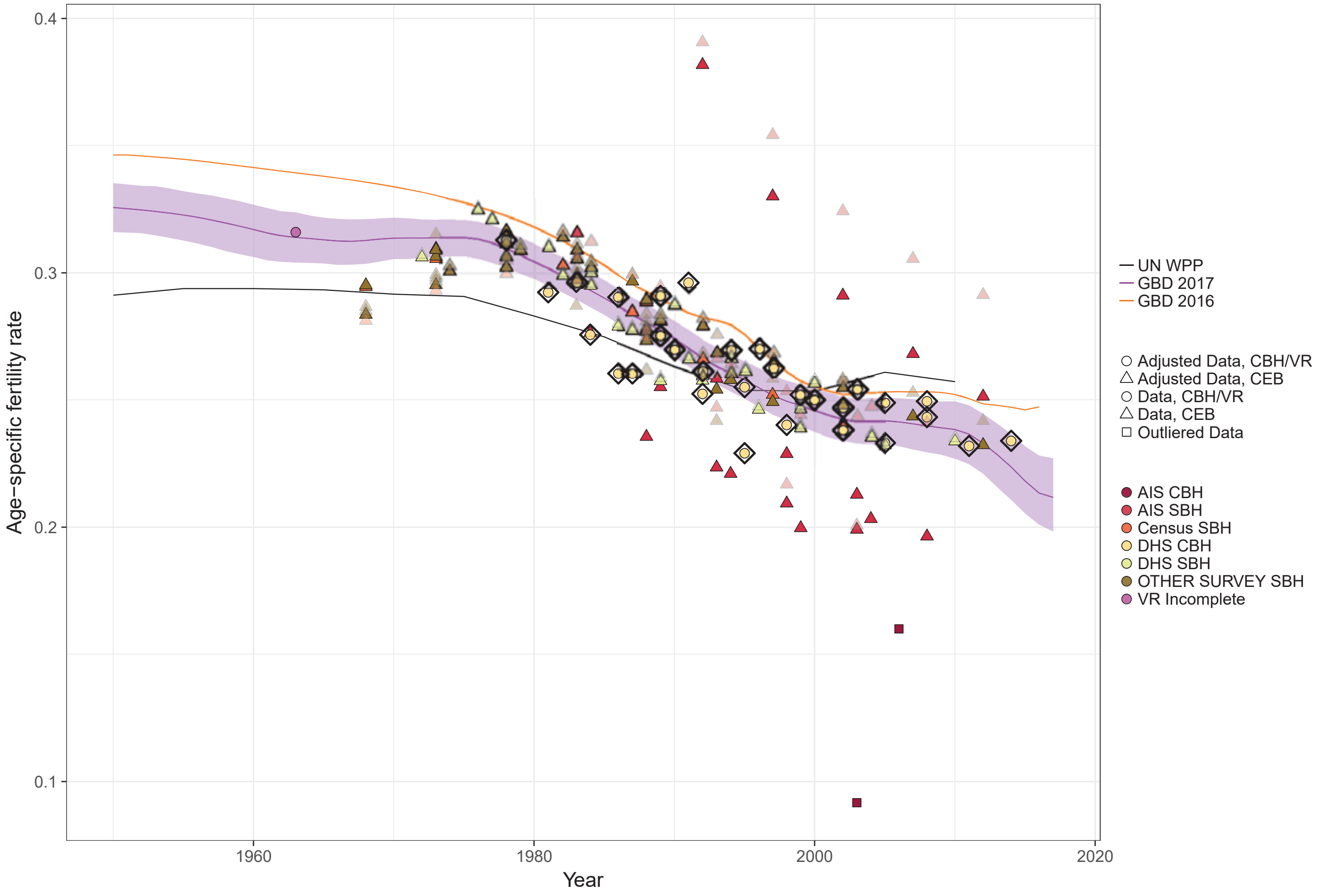

Age−specific fertility fitted trend lines, 1950−2017: Tanzania, Ages: 30−34
Data points enclosed with a diamond are reference sources

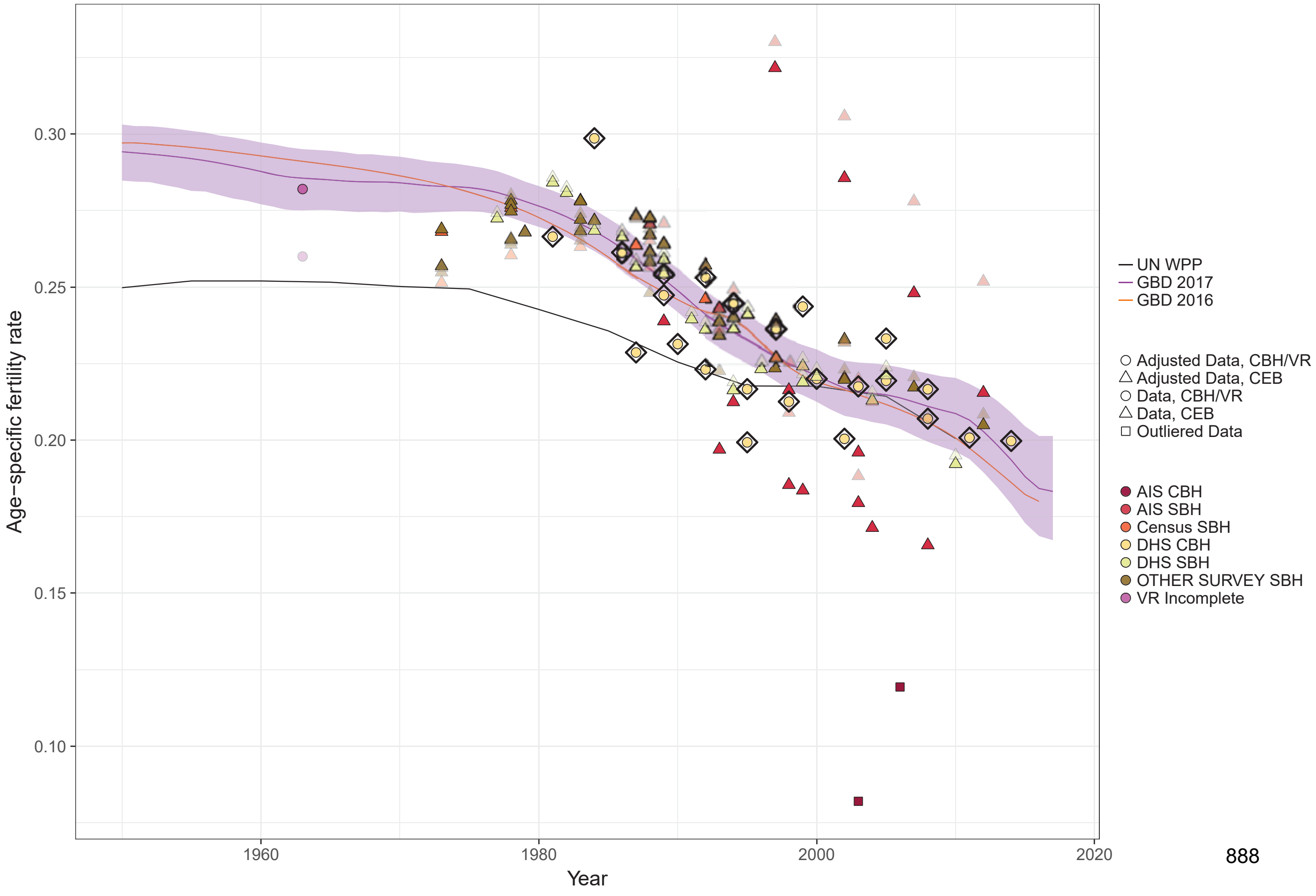

Age−specific fertility fitted trend lines, 1950−2017: Tanzania, Ages: 35−39
Data points enclosed with a diamond are reference sources

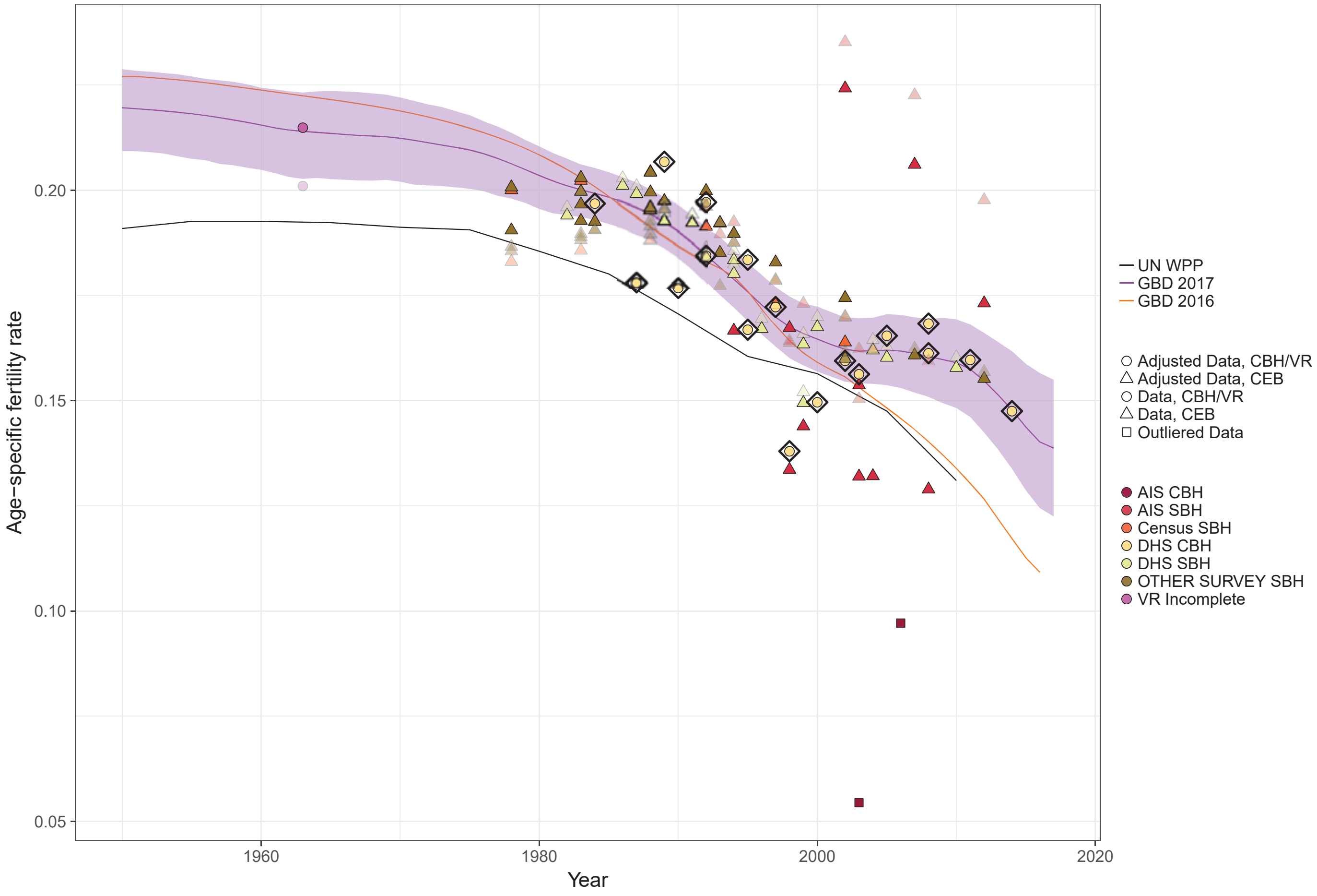

Age−specific fertility fitted trend lines, 1950−2017: Tanzania, Ages: 40−44
Data points enclosed with a diamond are reference sources

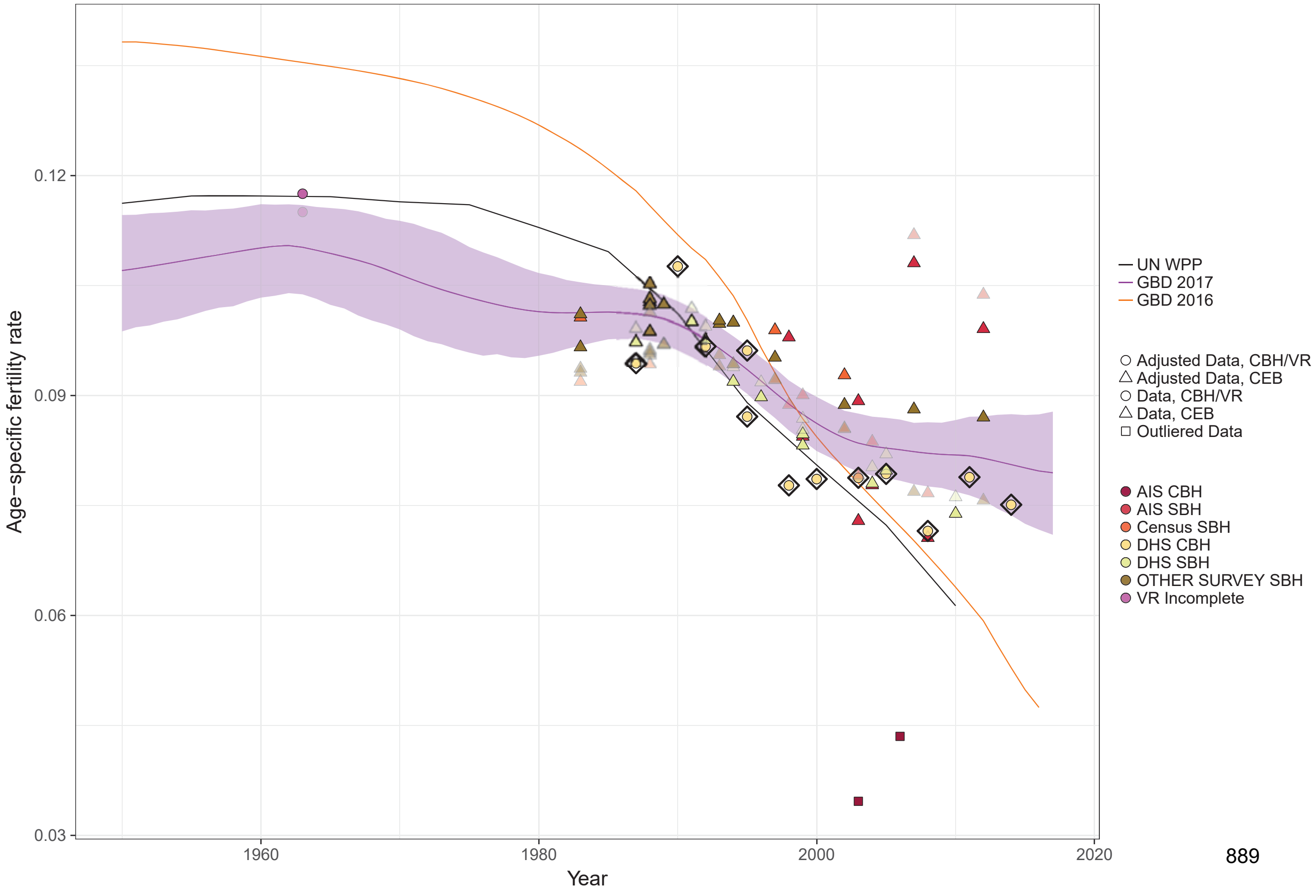

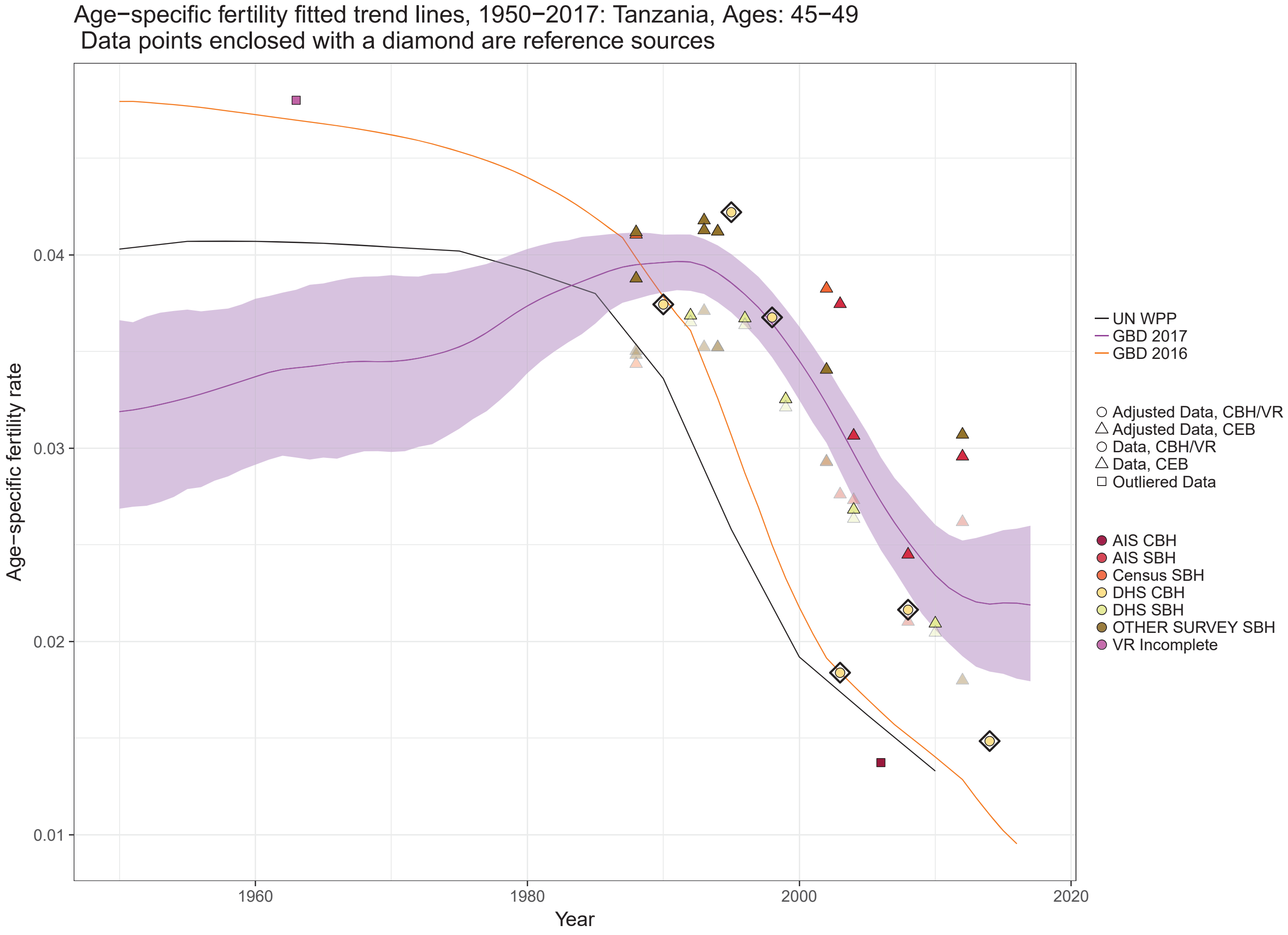
Age−specific fertility fitted trend lines, 1950−2017: Tanzania, Ages: 45−49
Data points enclosed with a diamond are reference sources
Age−specific fertility rate
Year
0.01
0.02
0.03
0.04
1960
1980
2000
2020
UN WPP
GBD 2017
GBD 2016
Adjusted Data, CBH/VR
Adjusted Data, CEB
Data, CBH/VR
Data, CEB
Outliered Data
AIS CBH
AIS SBH
Census SBH
DHS CBH
DHS SBH
OTHER SURVEY SBH
VR Incomplete

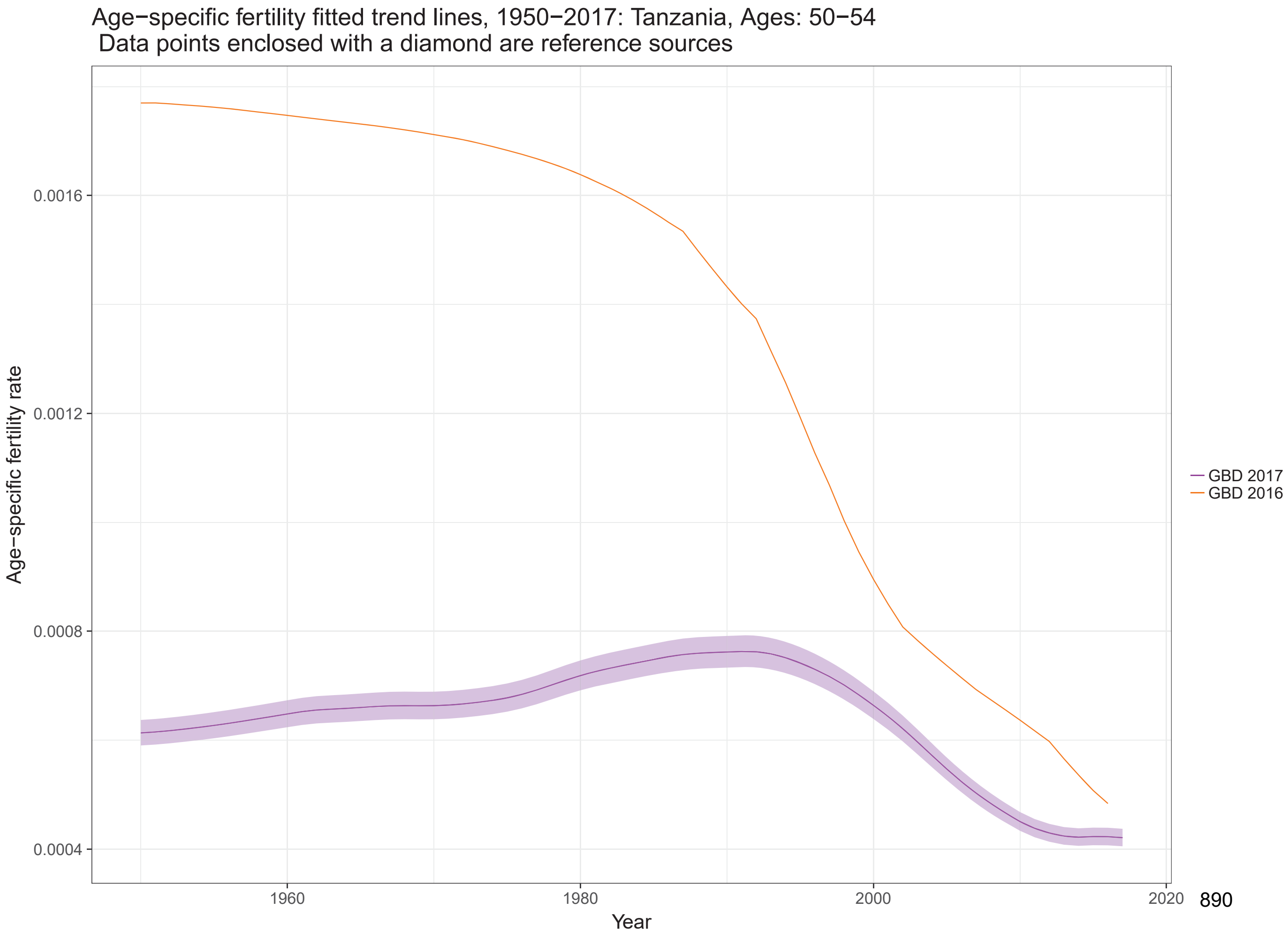
Age−specific fertility fitted trend lines, 1950−2017: Tanzania, Ages: 50−54
Data points enclosed with a diamond are reference sources
Age−specific fertility rate
Year
0.0004
0.0008
0.0012
0.0016
1960
1980
2000
2020
GBD 2017
GBD 2016

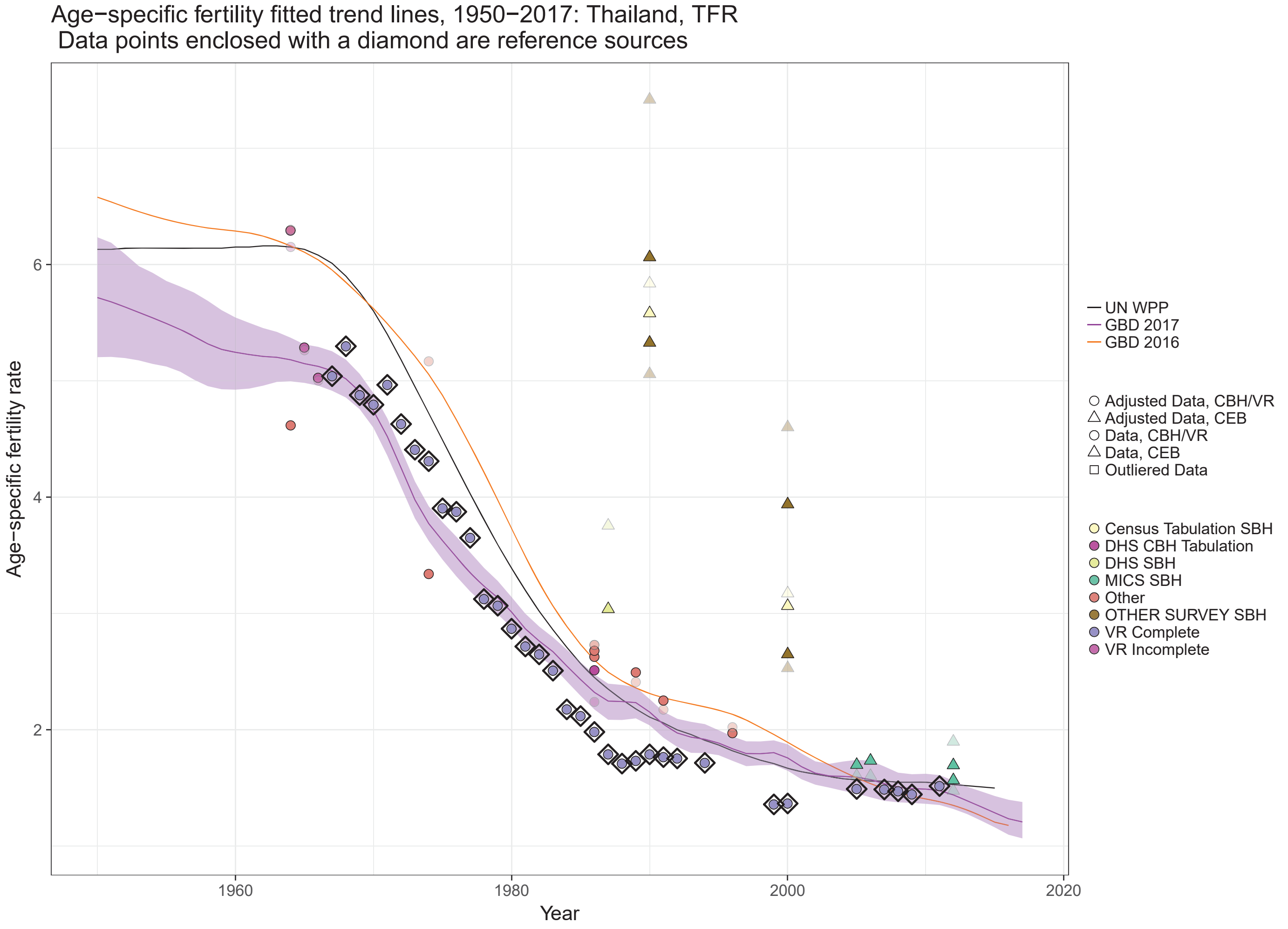

Age−specific fertility fitted trend lines, 1950−2017: Thailand, TFR
Data points enclosed with a diamond are reference sources
Age-specific fertility rate
Year
UN WPP
GBD 2017
GBD 2016
Adjusted Data, CBH/VR
Adjusted Data, CEB
Data, CBH/VR
Data, CEB
Outliered Data
Census Tabulation SBH
DHS CBH Tabulation
DHS SBH
MICS SBH
Other
OTHER SURVEY SBH
VR Complete
VR Incomplete

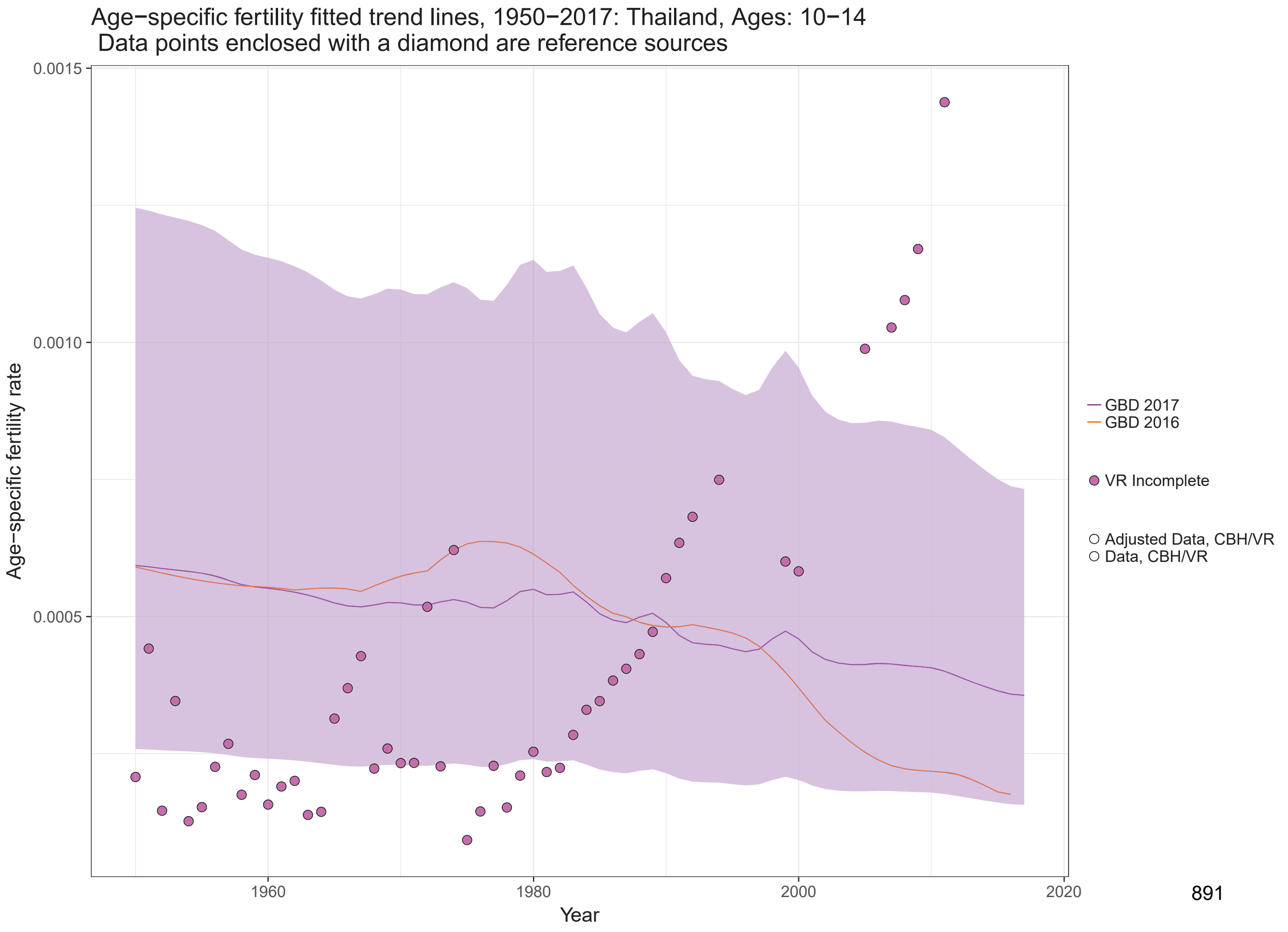

Age−specific fertility fitted trend lines, 1950−2017: Thailand, Ages: 10−14
Data points enclosed with a diamond are reference sources
Age-specific fertility rate
Year
GBD 2017
GBD 2016
VR Incomplete
Adjusted Data, CBH/VR
Data, CBH/VR

Age−specific fertility fitted trend lines, 1950−2017: Thailand, Ages: 15−19
Data points enclosed with a diamond are reference sources

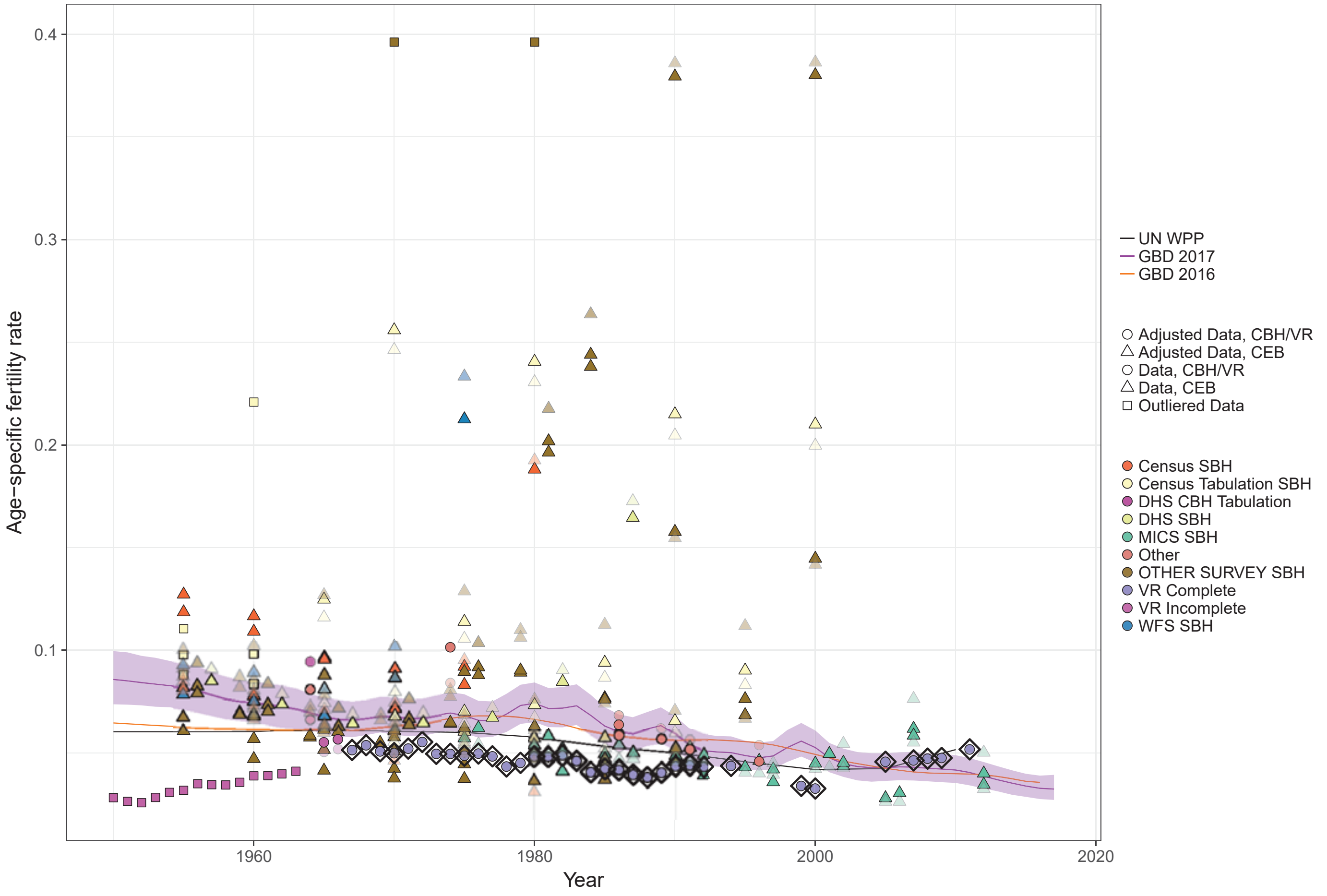

Age−specific fertility fitted trend lines, 1950−2017: Thailand, Ages: 20−24
Data points enclosed with a diamond are reference sources

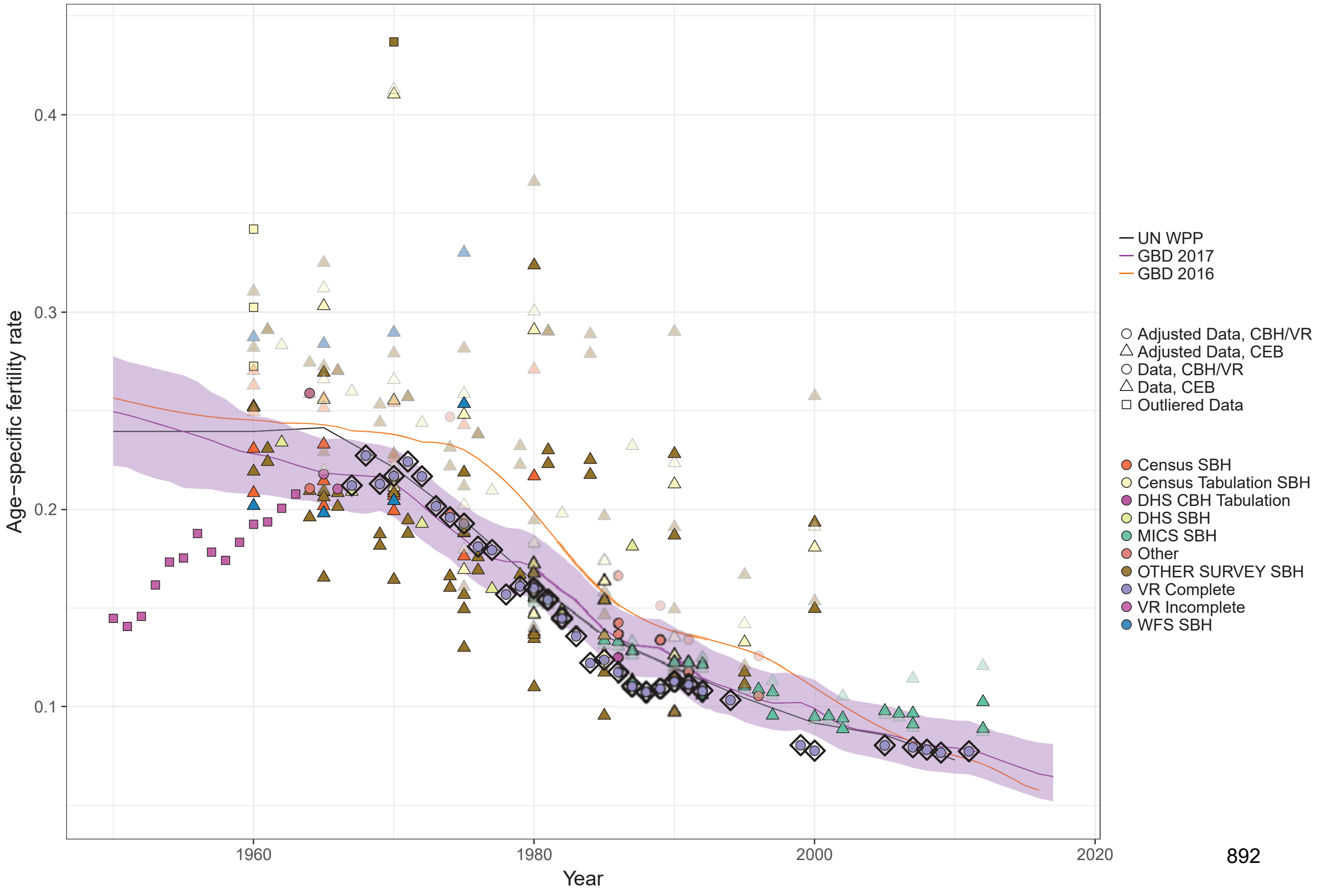

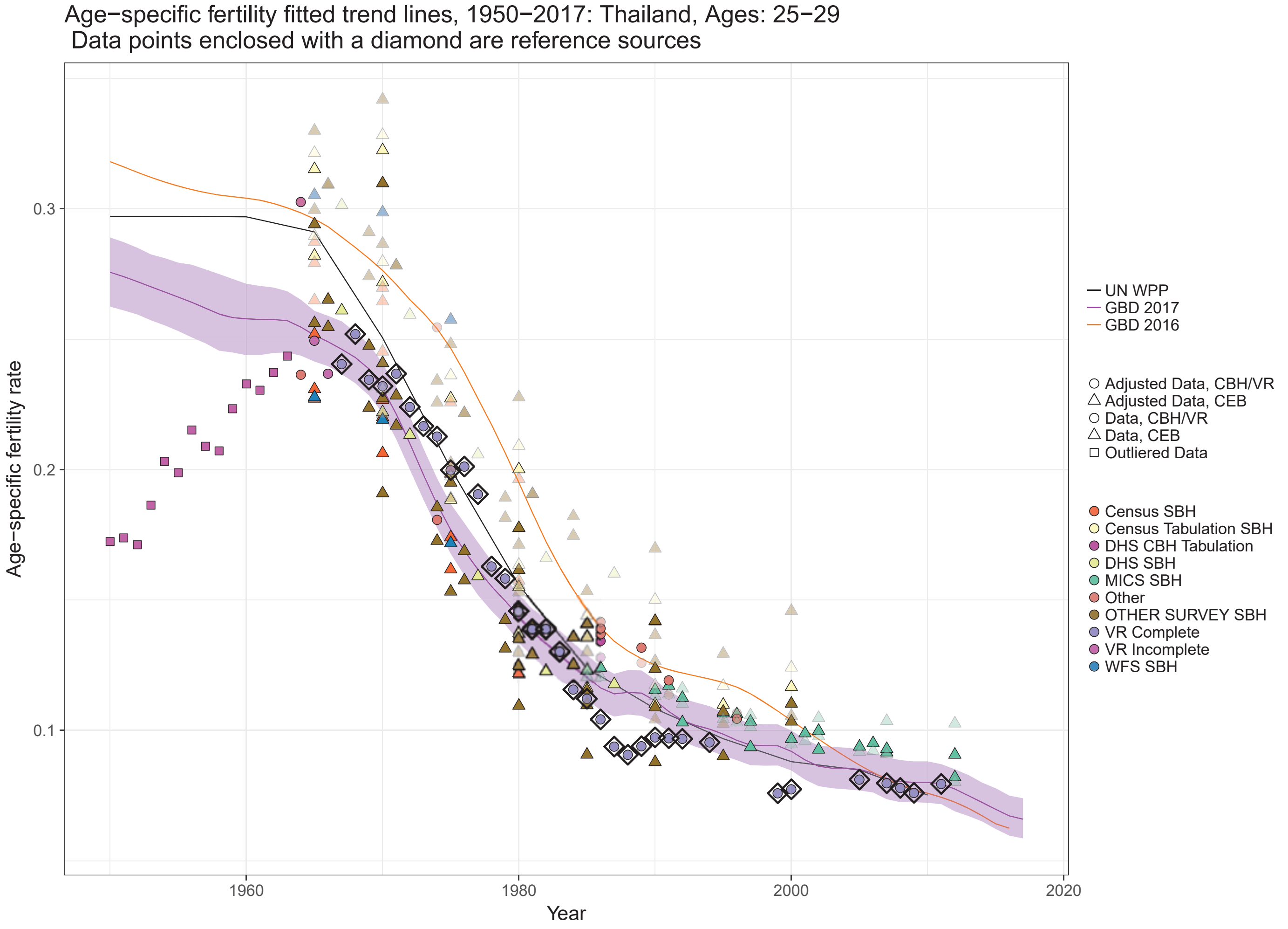
Age−specific fertility fitted trend lines, 1950−2017: Thailand, Ages: 25−29
Data points enclosed with a diamond are reference sources
Age−specific fertility rate
0.1
0.2
0.3
1960
1980
2000
2020
Year
UN WPP
GBD 2017
GBD 2016
Adjusted Data, CBH/VR
Adjusted Data, CEB
Data, CBH/VR
Data, CEB
Outliered Data
Census SBH
Census Tabulation SBH
DHS CBH Tabulation
DHS SBH
MICS SBH
Other
OTHER SURVEY SBH
VR Complete
VR Incomplete
WFS SBH

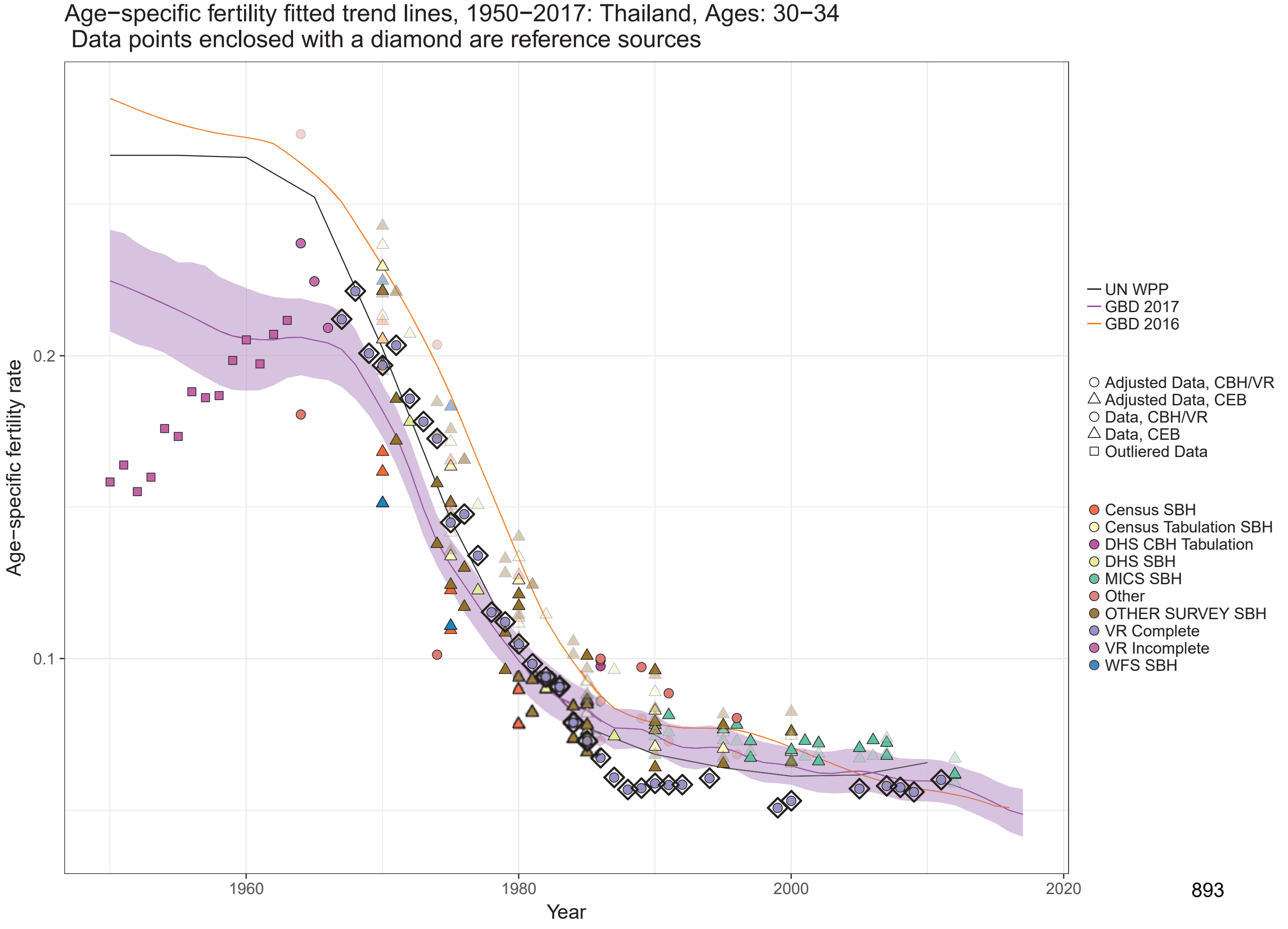
Age−specific fertility fitted trend lines, 1950−2017: Thailand, Ages: 30−34
Data points enclosed with a diamond are reference sources
Age−specific fertility rate
0.1
0.2
1960
1980
2000
2020
Year
UN WPP
GBD 2017
GBD 2016
Adjusted Data, CBH/VR
Adjusted Data, CEB
Data, CBH/VR
Data, CEB
Outliered Data
Census SBH
Census Tabulation SBH
DHS CBH Tabulation
DHS SBH
MICS SBH
Other
OTHER SURVEY SBH
VR Complete
VR Incomplete
WFS SBH

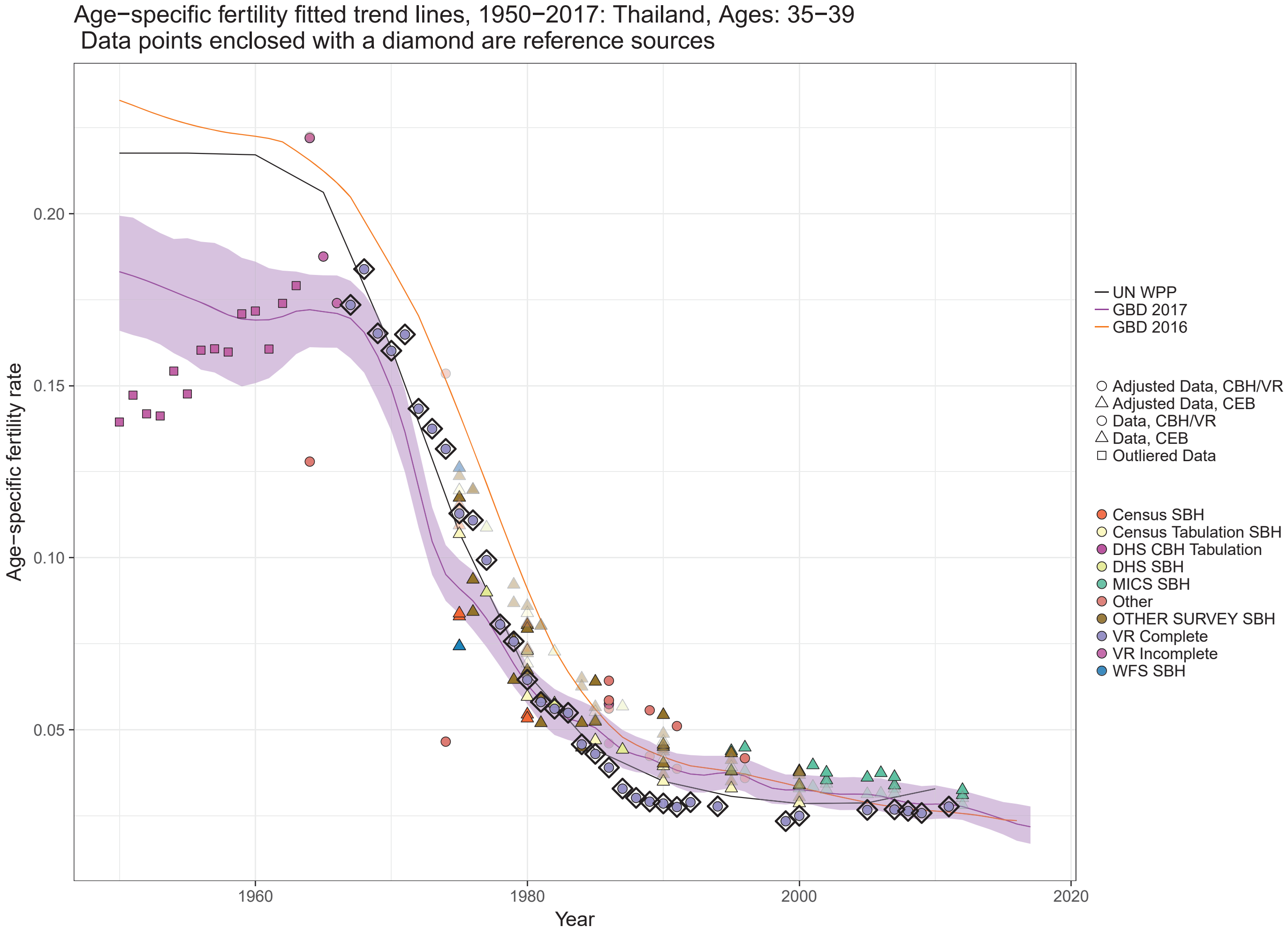
Age−specific fertility fitted trend lines, 1950−2017: Thailand, Ages: 35−39
Data points enclosed with a diamond are reference sources
Age−specific fertility rate
0.20
0.15
0.10
0.05
1960
1980
2000
2020
Year
UN WPP
GBD 2017
GBD 2016
Adjusted Data, CBH/VR
Adjusted Data, CEB
Data, CBH/VR
Data, CEB
Outliered Data
Census SBH
Census Tabulation SBH
DHS CBH Tabulation
DHS SBH
MICS SBH
Other
OTHER SURVEY SBH
VR Complete
VR Incomplete
WFS SBH

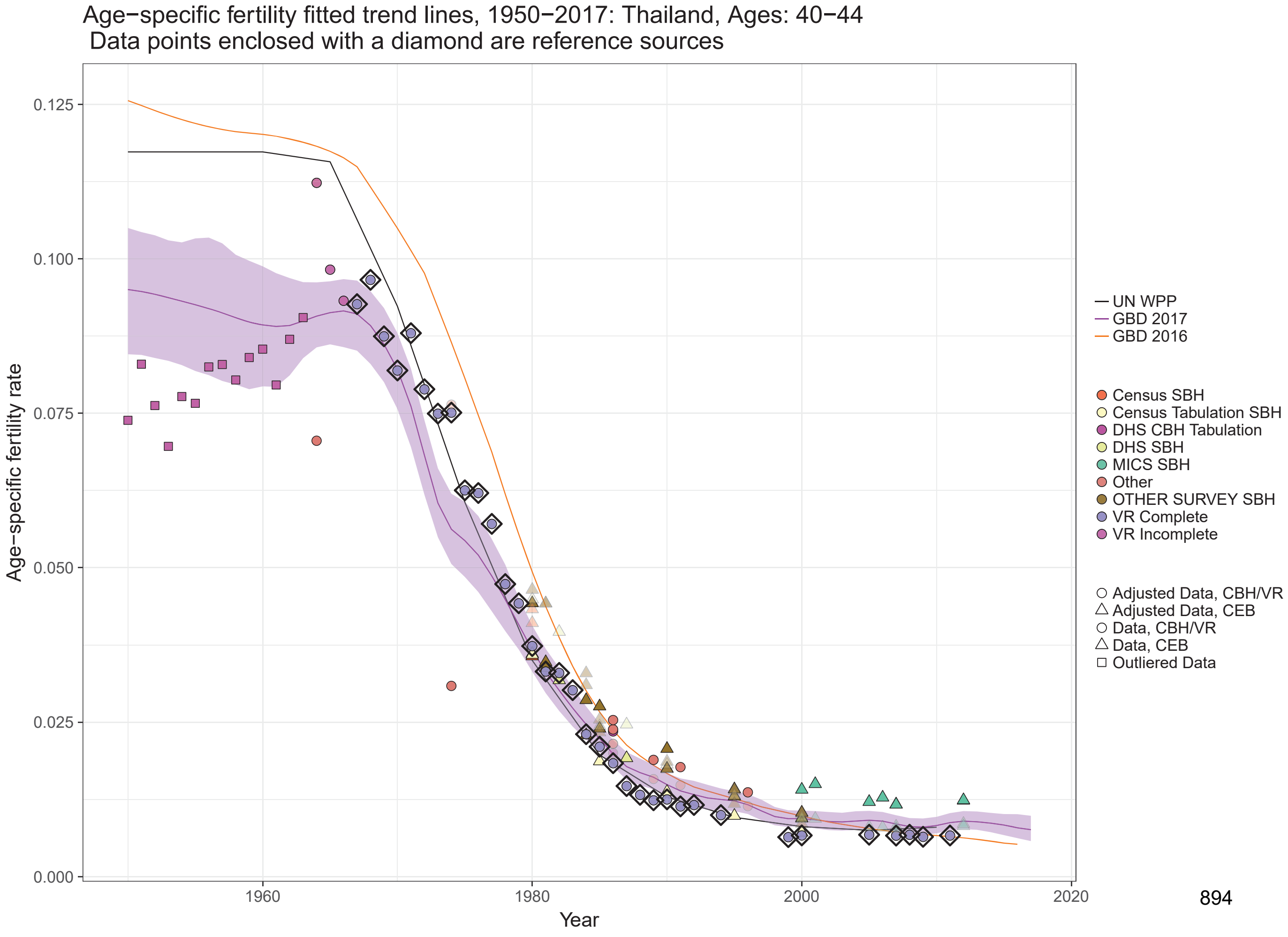
Age−specific fertility fitted trend lines, 1950−2017: Thailand, Ages: 40−44
Data points enclosed with a diamond are reference sources
Age−specific fertility rate
0.125
0.100
0.075
0.050
0.025
0.000
1960
1980
2000
2020
Year
UN WPP
GBD 2017
GBD 2016
Census SBH
Census Tabulation SBH
DHS CBH Tabulation
DHS SBH
MICS SBH
Other
OTHER SURVEY SBH
VR Complete
VR Incomplete
Adjusted Data, CBH/VR
Adjusted Data, CEB
Data, CBH/VR
Data, CEB
Outliered Data

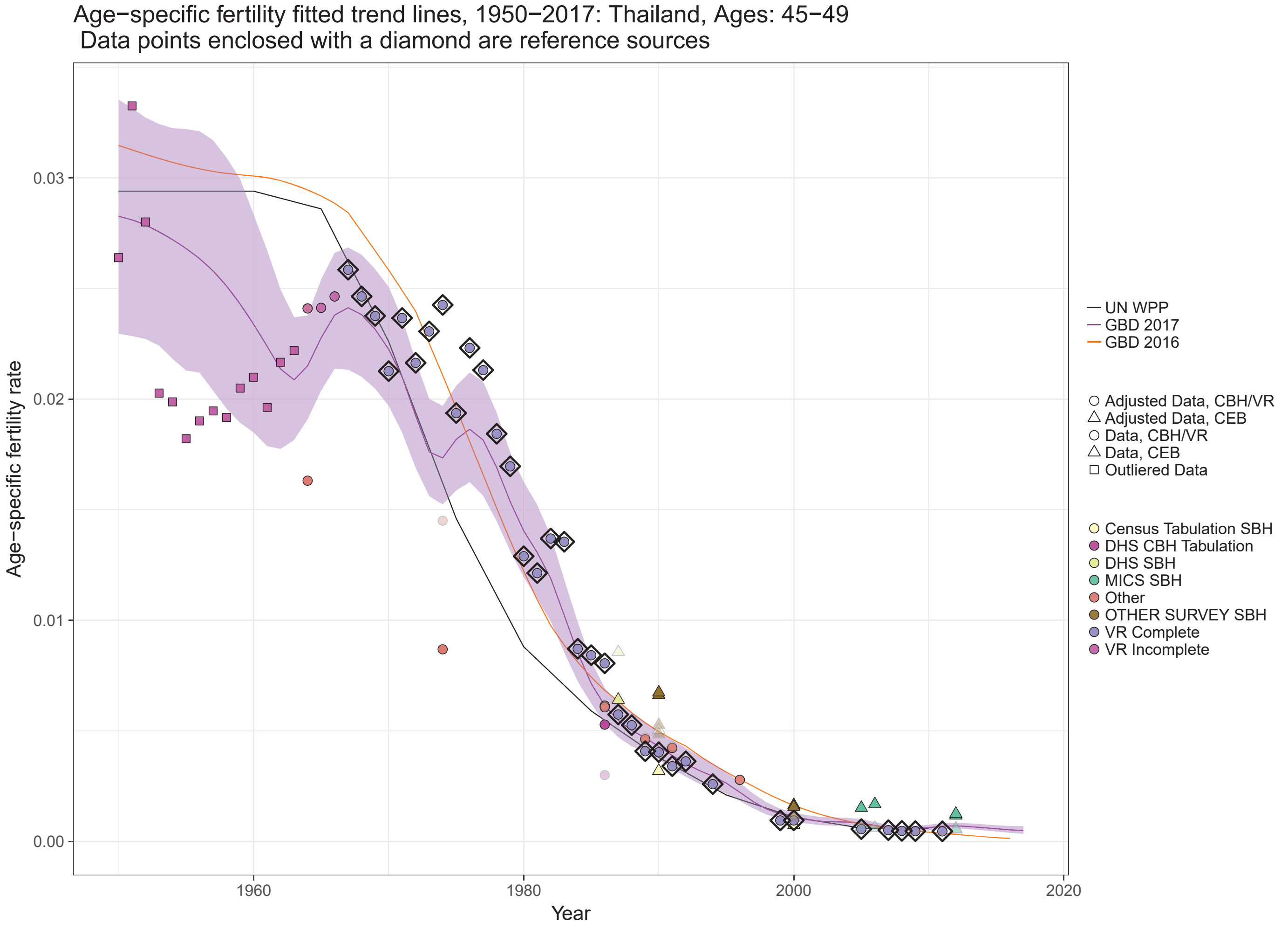

Age−specific fertility fitted trend lines, 1950−2017: Thailand, Ages: 45−49
Data points enclosed with a diamond are reference sources
Age−specific fertility rate
Year
0.00
0.01
0.02
0.03
1960
1980
2000
2020
UN WPP
GBD 2017
GBD 2016
Adjusted Data, CBH/VR
Adjusted Data, CEB
Data, CBH/VR
Data, CEB
Outliered Data
Census Tabulation SBH
DHS CBH Tabulation
DHS SBH
MICS SBH
Other
OTHER SURVEY SBH
VR Complete
VR Incomplete

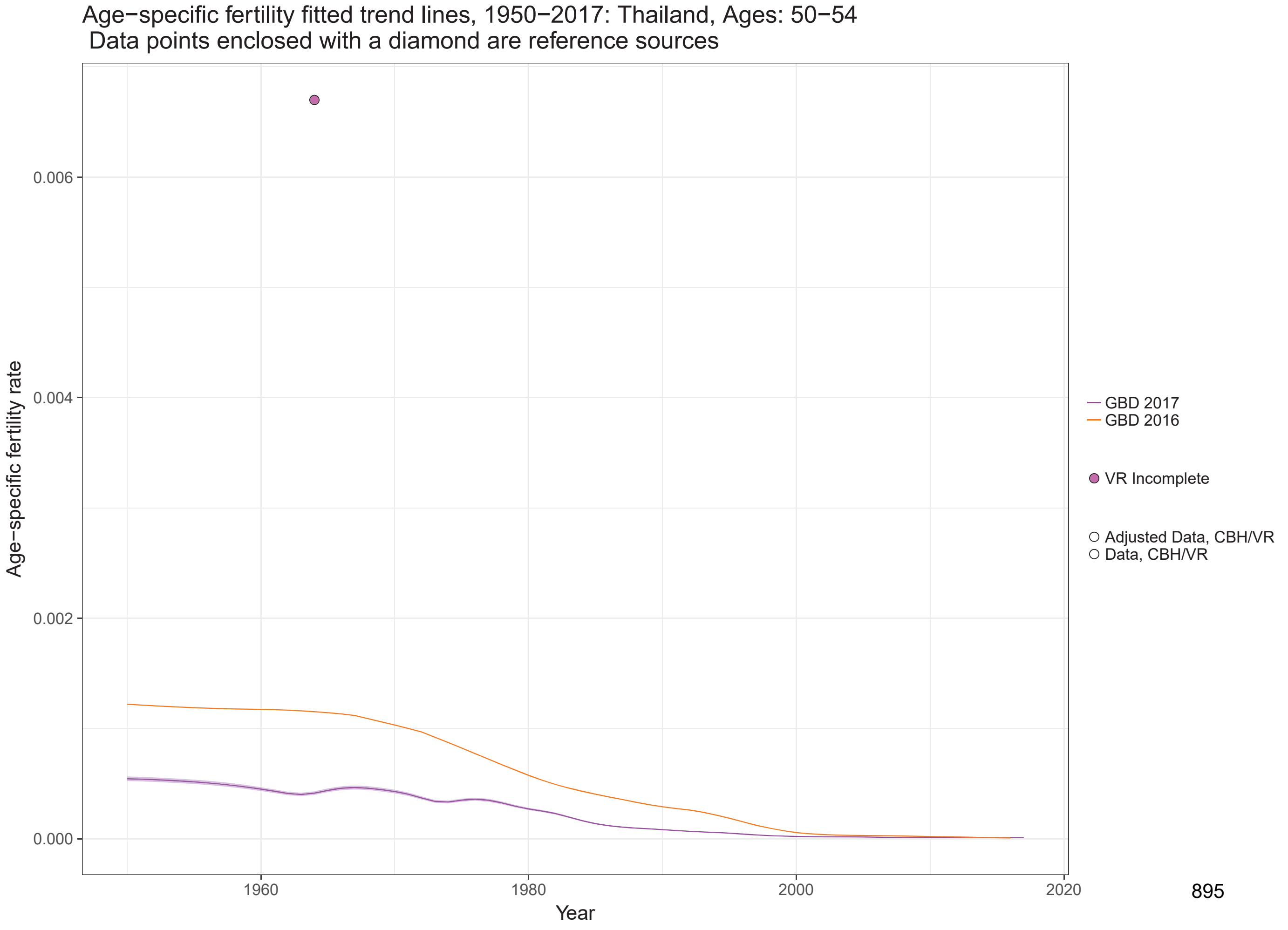

Age−specific fertility fitted trend lines, 1950−2017: Thailand, Ages: 50−54
Data points enclosed with a diamond are reference sources
Age−specific fertility rate
Year
0.000
0.002
0.004
0.006
1960
1980
2000
2020
GBD 2017
GBD 2016
VR Incomplete
Adjusted Data, CBH/VR
Data, CBH/VR

Age−specific fertility fitted trend lines, 1950−2017: The Bahamas, TFR
Data points enclosed with a diamond are reference sources

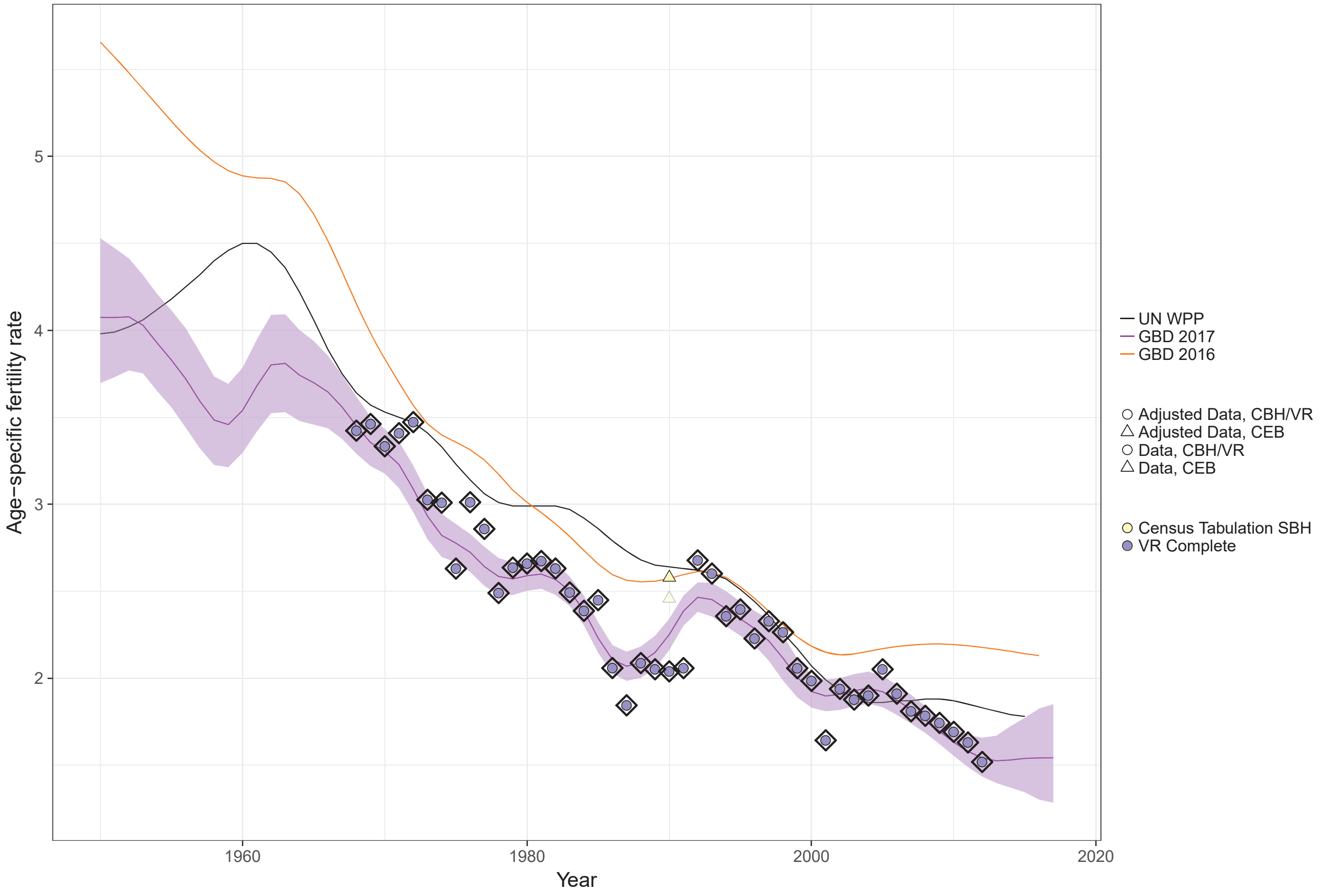

Age−specific fertility fitted trend lines, 1950−2017: The Bahamas, Ages: 10−14
Data points enclosed with a diamond are reference sources

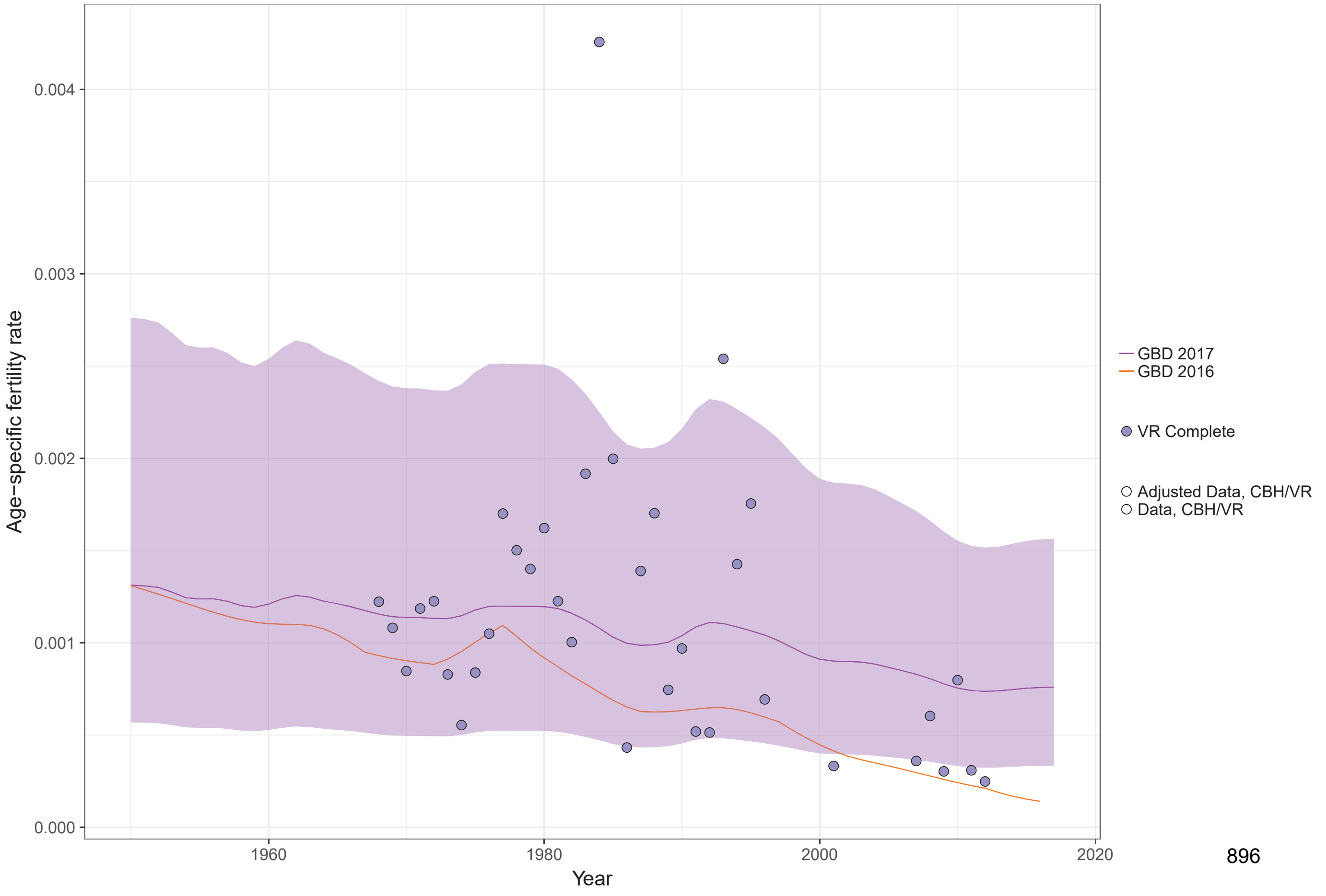

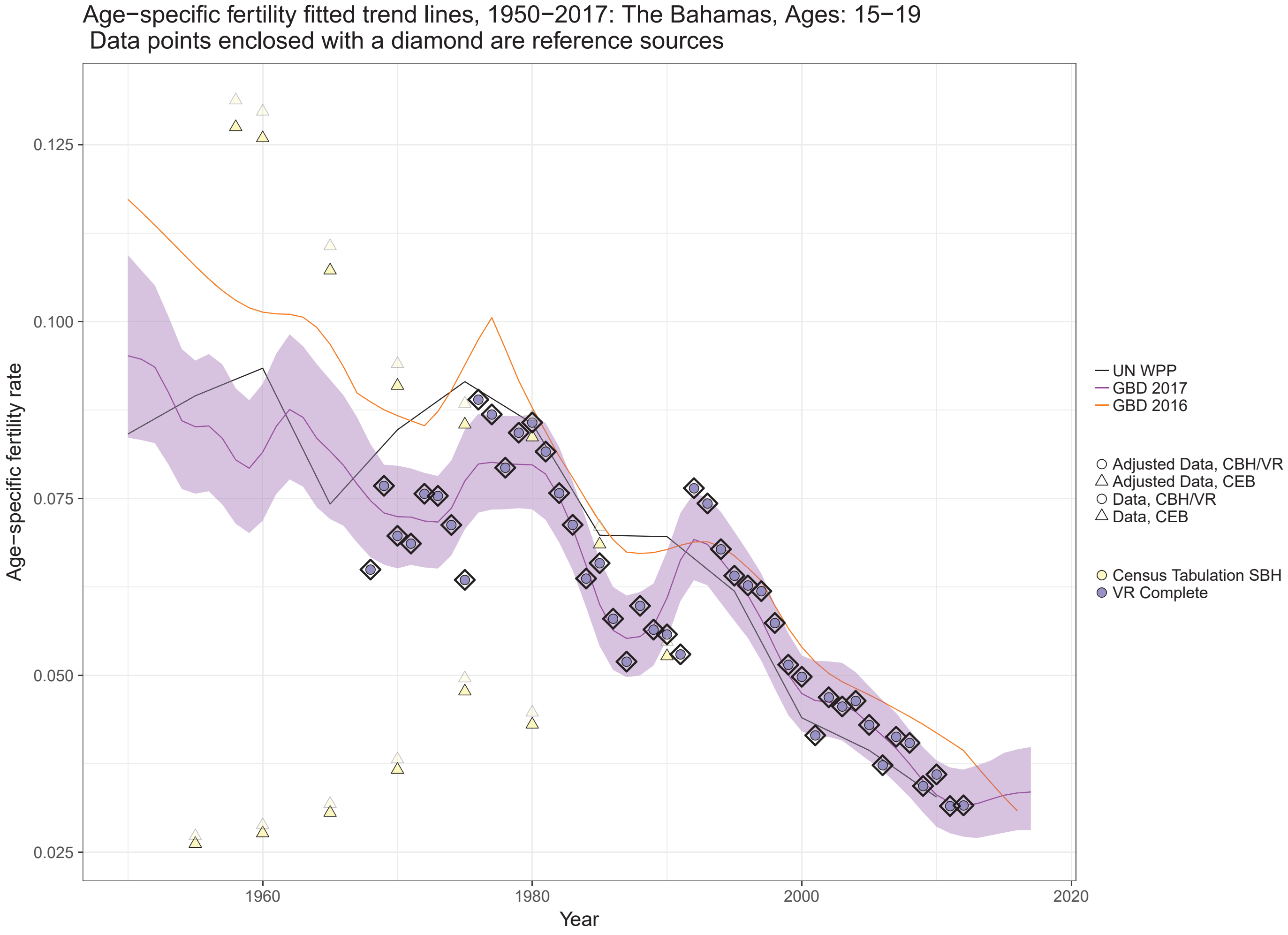
Age−specific fertility fitted trend lines, 1950−2017: The Bahamas, Ages: 15−19
Data points enclosed with a diamond are reference sources
Age−specific fertility rate
Year
UN WPP
GBD 2017
GBD 2016
Adjusted Data, CBH/VR
Adjusted Data, CEB
Data, CBH/VR
Data, CEB
Census Tabulation SBH
VR Complete

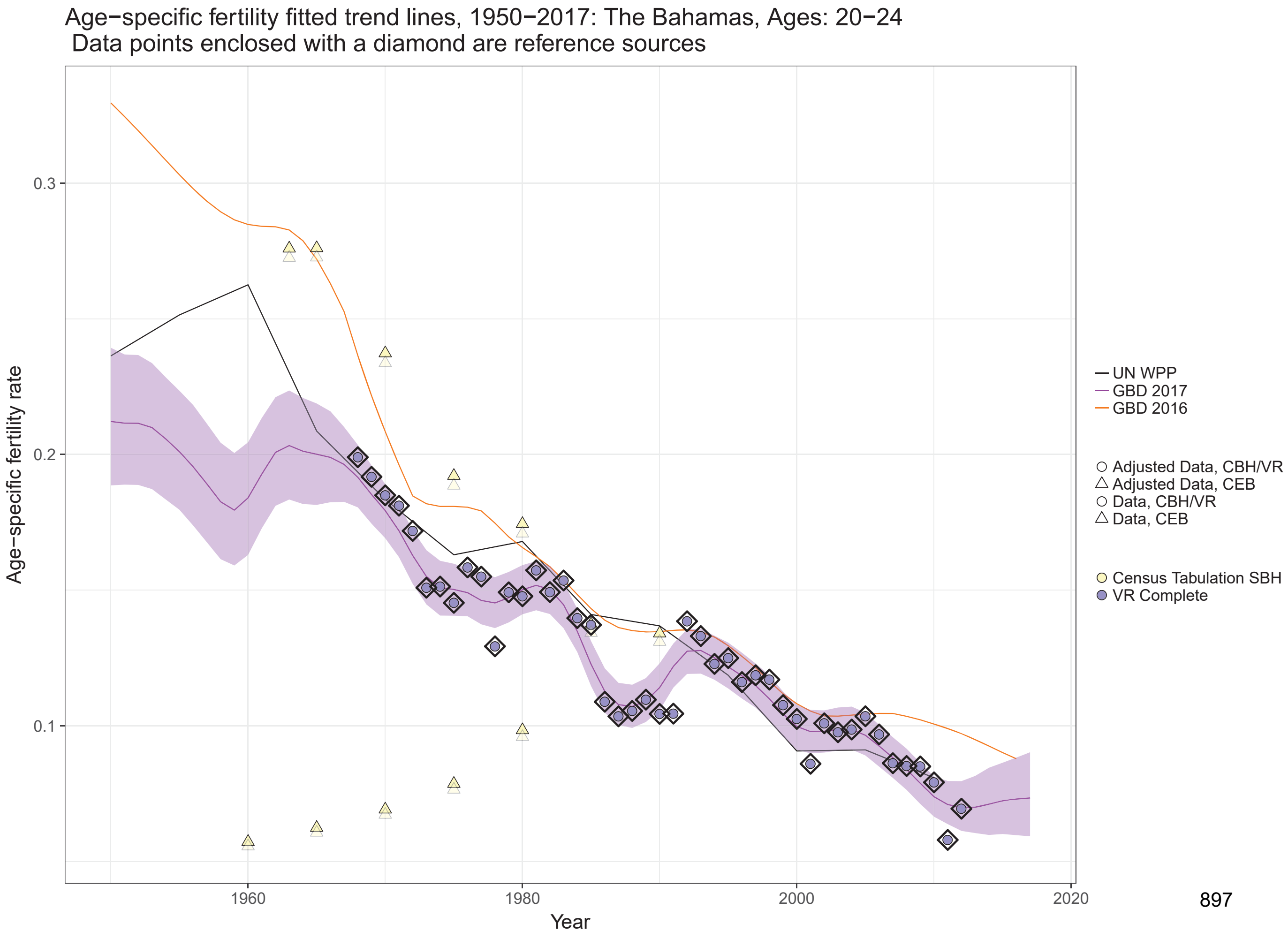
Age−specific fertility fitted trend lines, 1950−2017: The Bahamas, Ages: 20−24
Data points enclosed with a diamond are reference sources
Age−specific fertility rate
Year
UN WPP
GBD 2017
GBD 2016
Adjusted Data, CBH/VR
Adjusted Data, CEB
Data, CBH/VR
Data, CEB
Census Tabulation SBH
VR Complete

Age−specific fertility fitted trend lines, 1950−2017: The Bahamas, Ages: 25−29
Data points enclosed with a diamond are reference sources

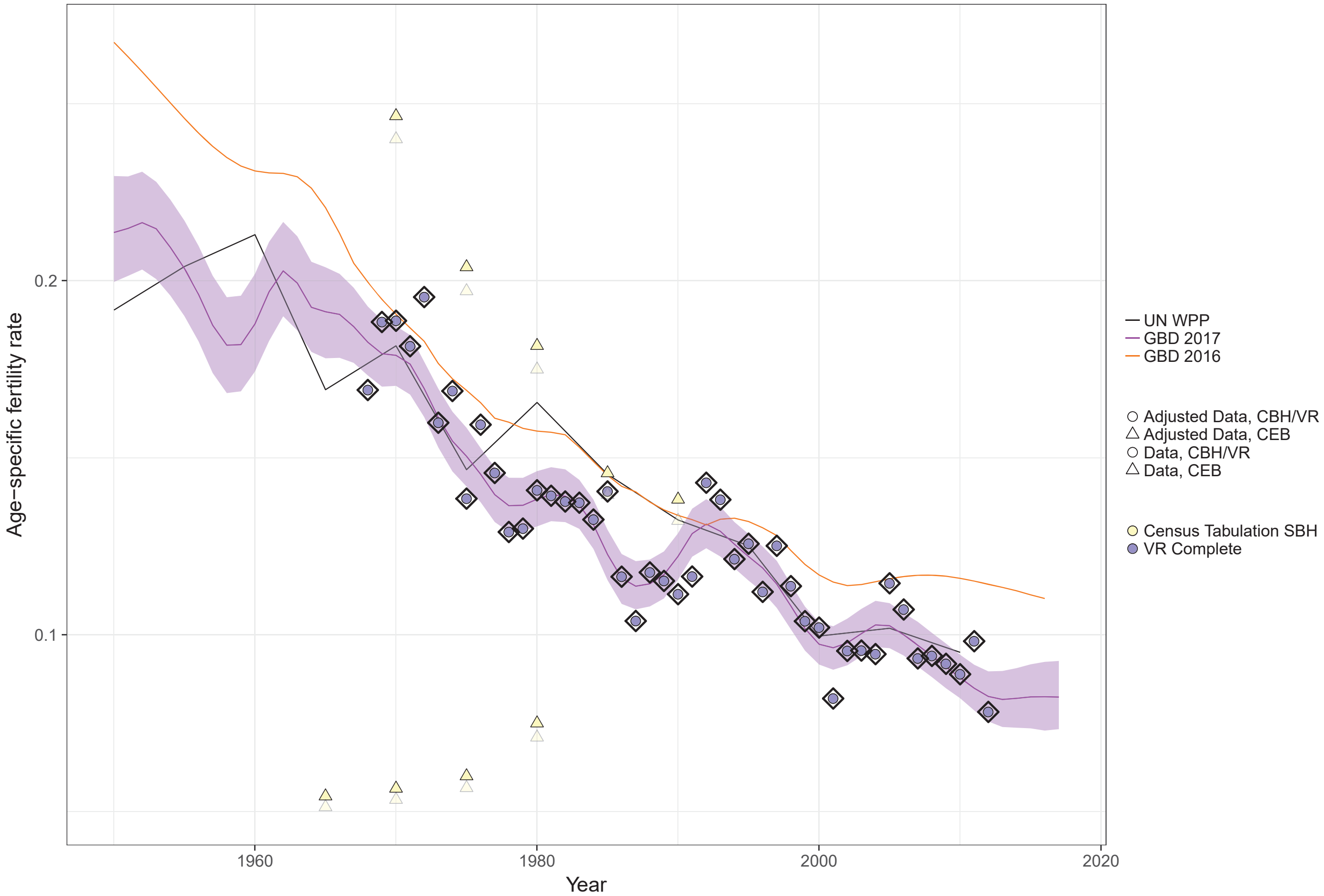

Age−specific fertility fitted trend lines, 1950−2017: The Bahamas, Ages: 30−34
Data points enclosed with a diamond are reference sources

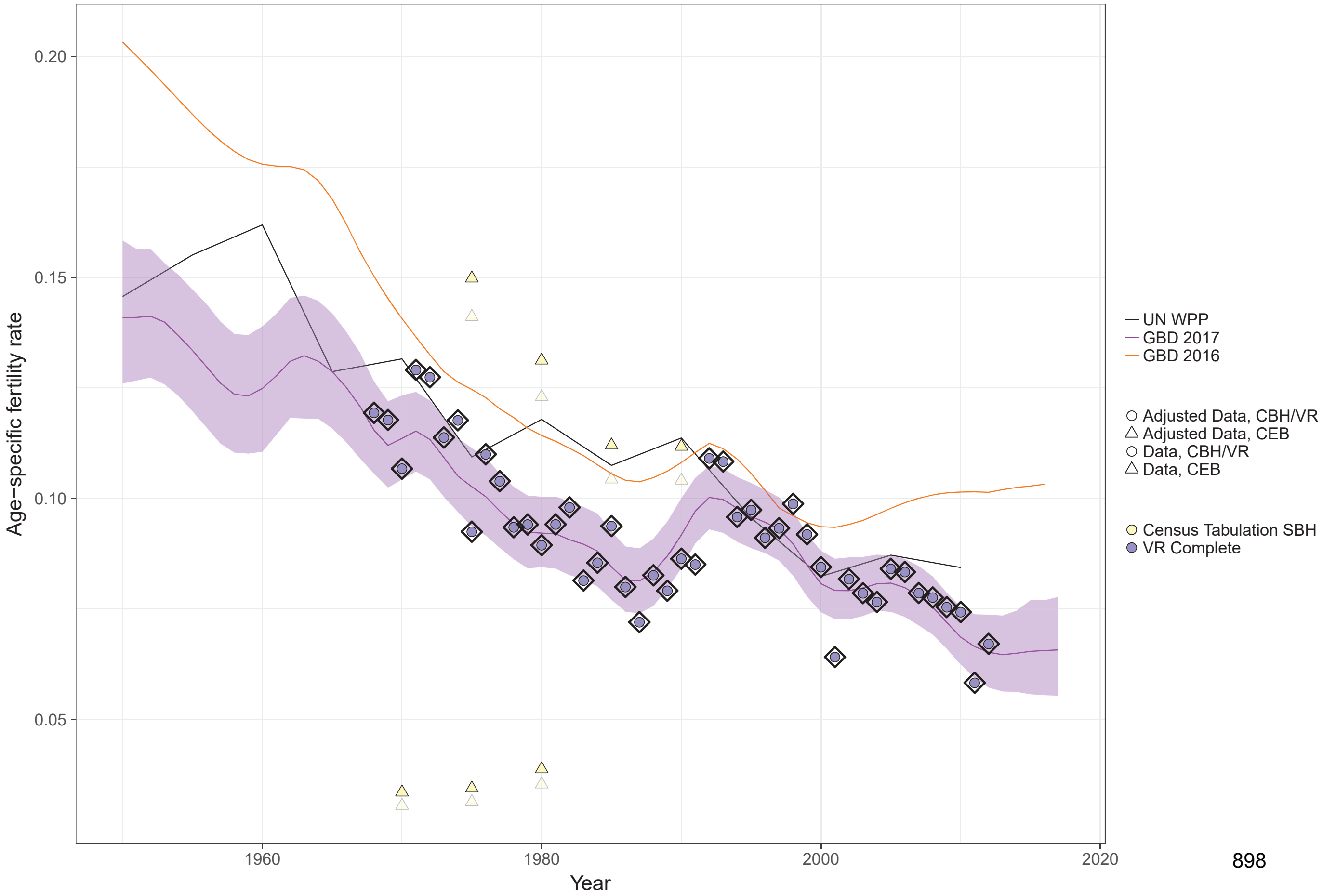

Age−specific fertility fitted trend lines, 1950−2017: The Bahamas, Ages: 35−39
Data points enclosed with a diamond are reference sources

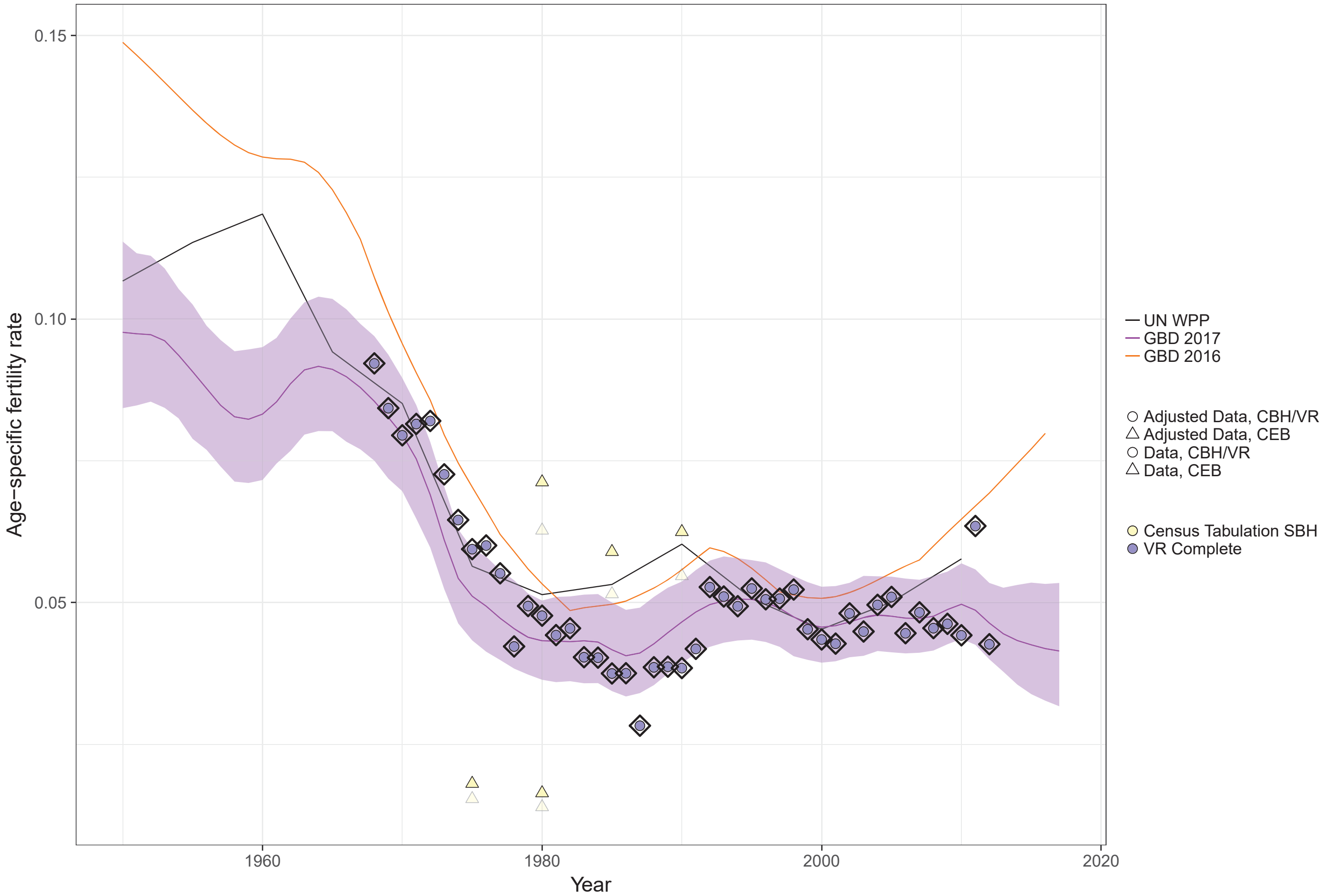

Age−specific fertility fitted trend lines, 1950−2017: The Bahamas, Ages: 40−44
Data points enclosed with a diamond are reference sources

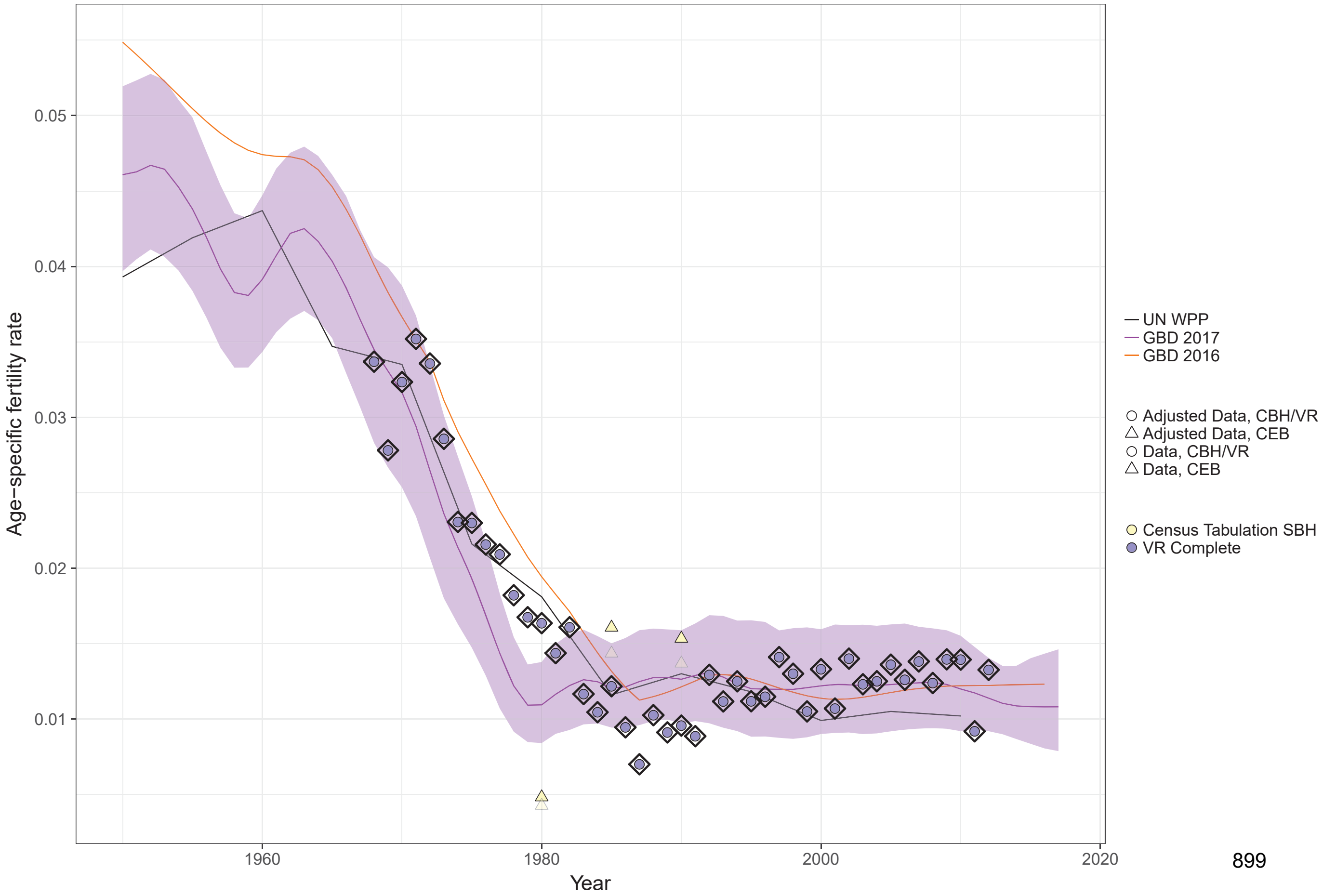

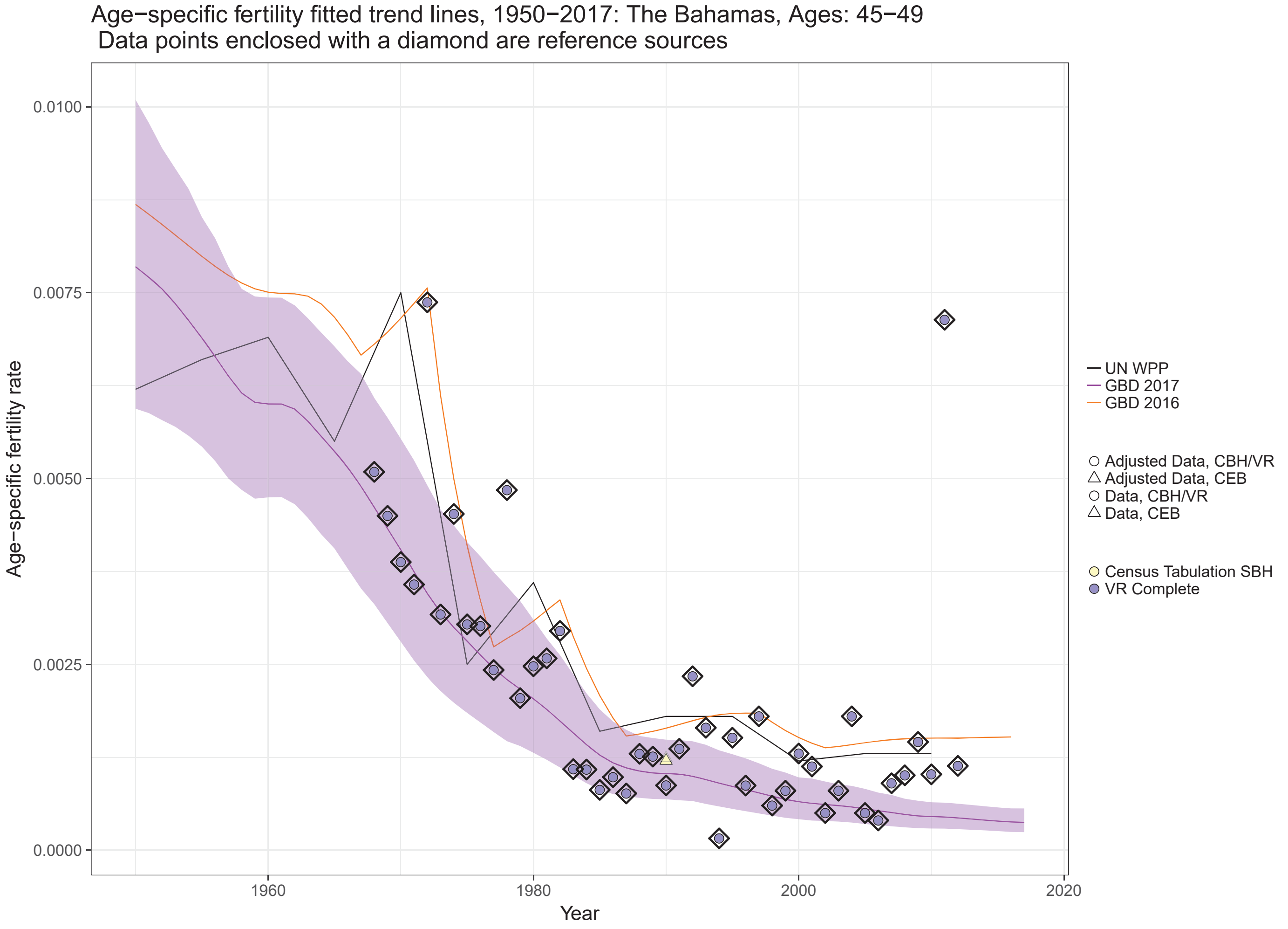

Age−specific fertility fitted trend lines, 1950−2017: The Bahamas, Ages: 45−49
Data points enclosed with a diamond are reference sources
Age−specific fertility rate
0.0100
0.0075
0.0050
0.0025
0.0000
1960
1980
2000
2020
Year
UN WPP
GBD 2017
GBD 2016
Adjusted Data, CBH/VR
Adjusted Data, CEB
Data, CBH/VR
Data, CEB
Census Tabulation SBH
VR Complete

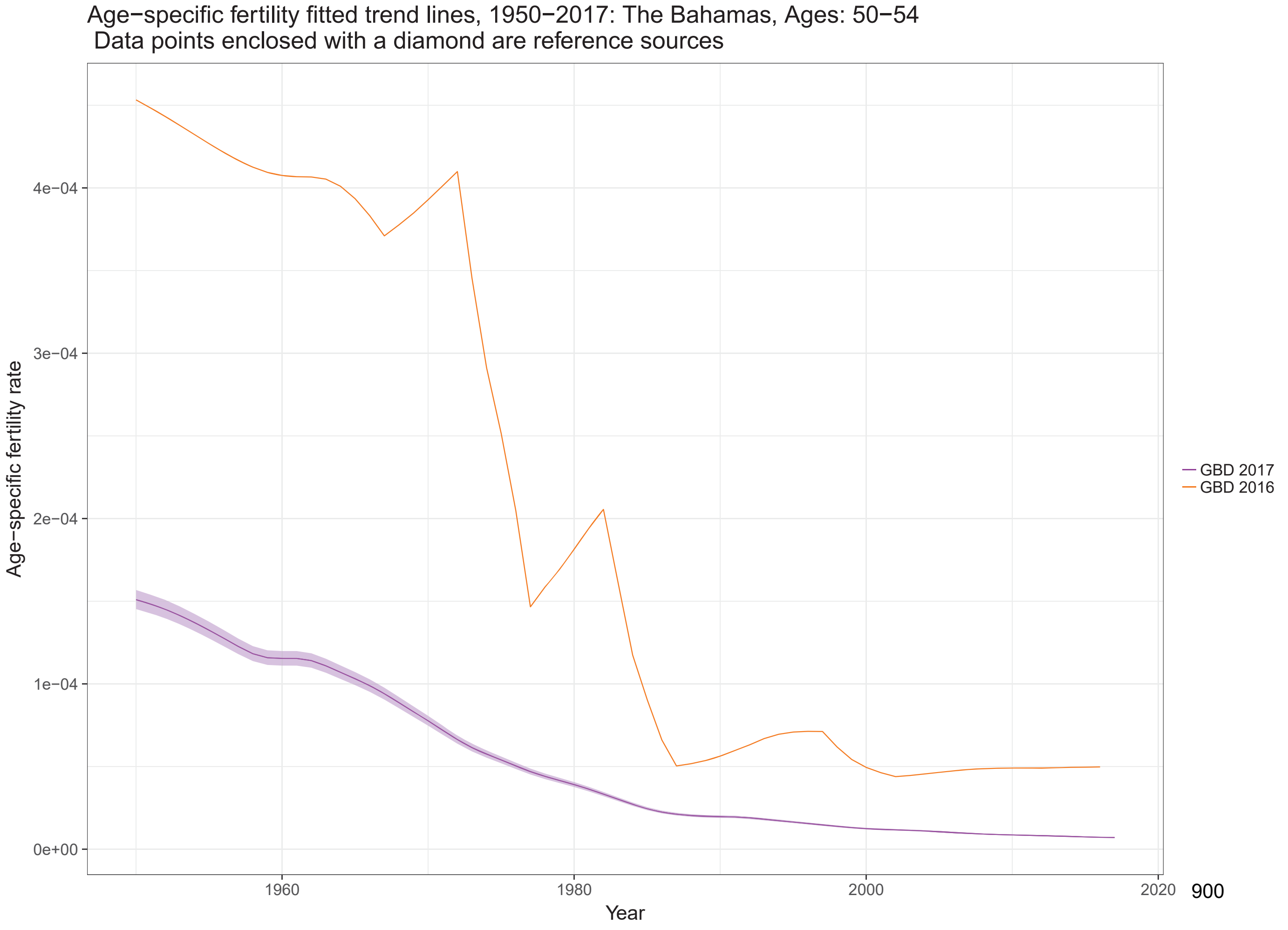

Age−specific fertility fitted trend lines, 1950−2017: The Bahamas, Ages: 50−54
Data points enclosed with a diamond are reference sources
Age−specific fertility rate
4e−04
3e−04
2e−04
1e−04
0e+00
1960
1980
2000
2020
Year
GBD 2017
GBD 2016

Age−specific fertility fitted trend lines, 1950−2017: The Gambia, TFR
Data points enclosed with a diamond are reference sources

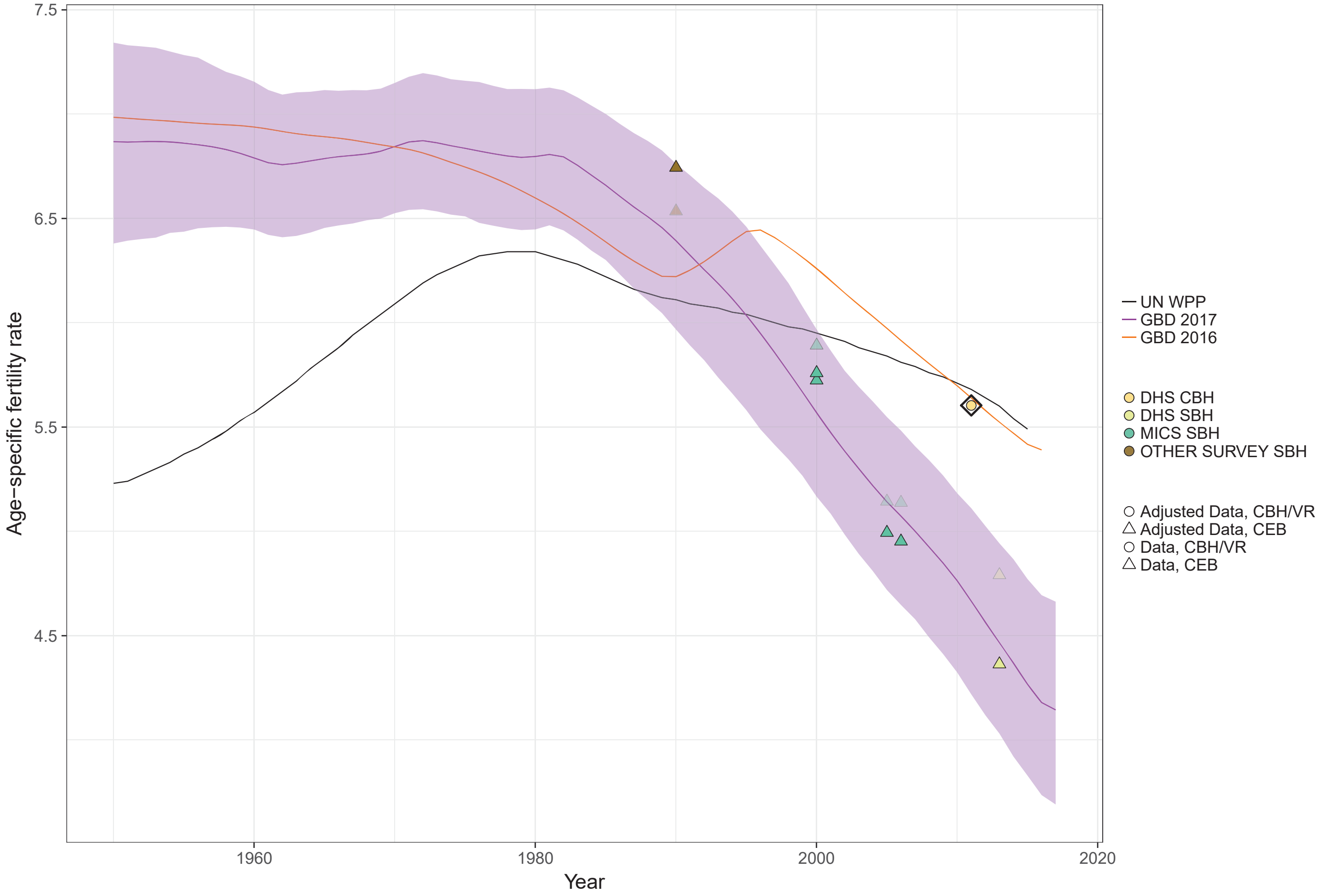

Age−specific fertility fitted trend lines, 1950−2017: The Gambia, Ages: 10−14
Data points enclosed with a diamond are reference sources

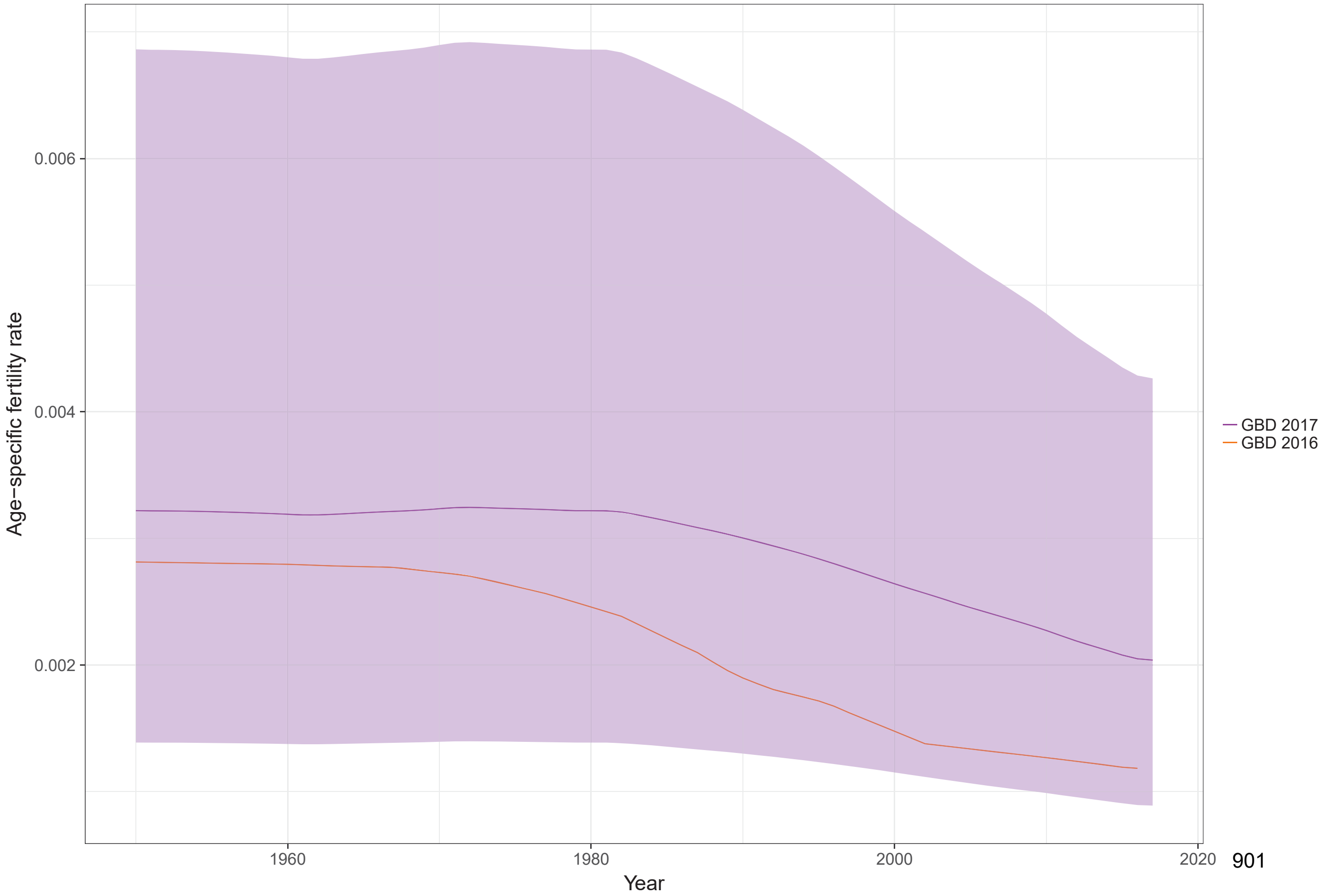

Age−specific fertility fitted trend lines, 1950−2017: The Gambia, Ages: 15−19
Data points enclosed with a diamond are reference sources

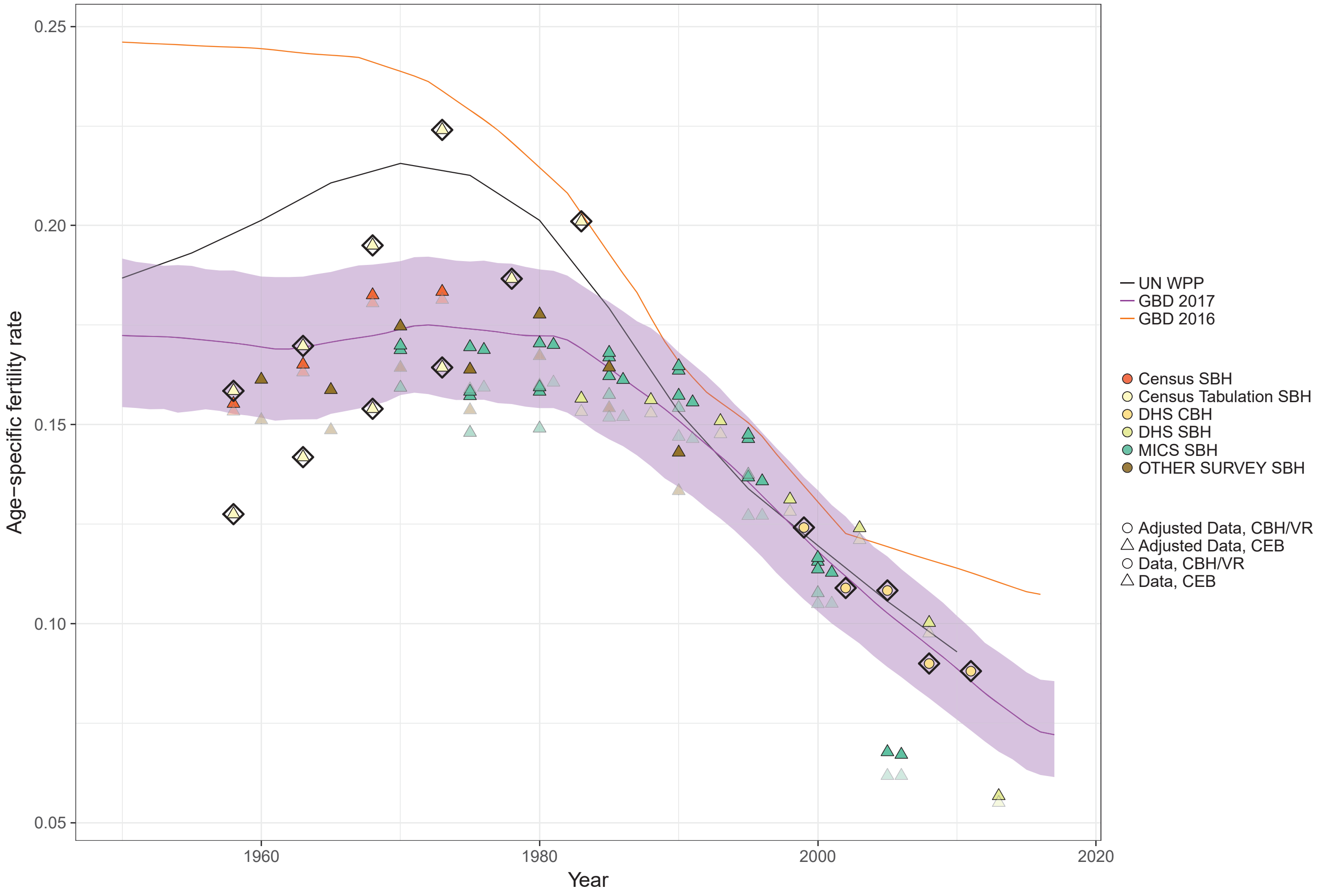

Age−specific fertility fitted trend lines, 1950−2017: The Gambia, Ages: 20−24
Data points enclosed with a diamond are reference sources

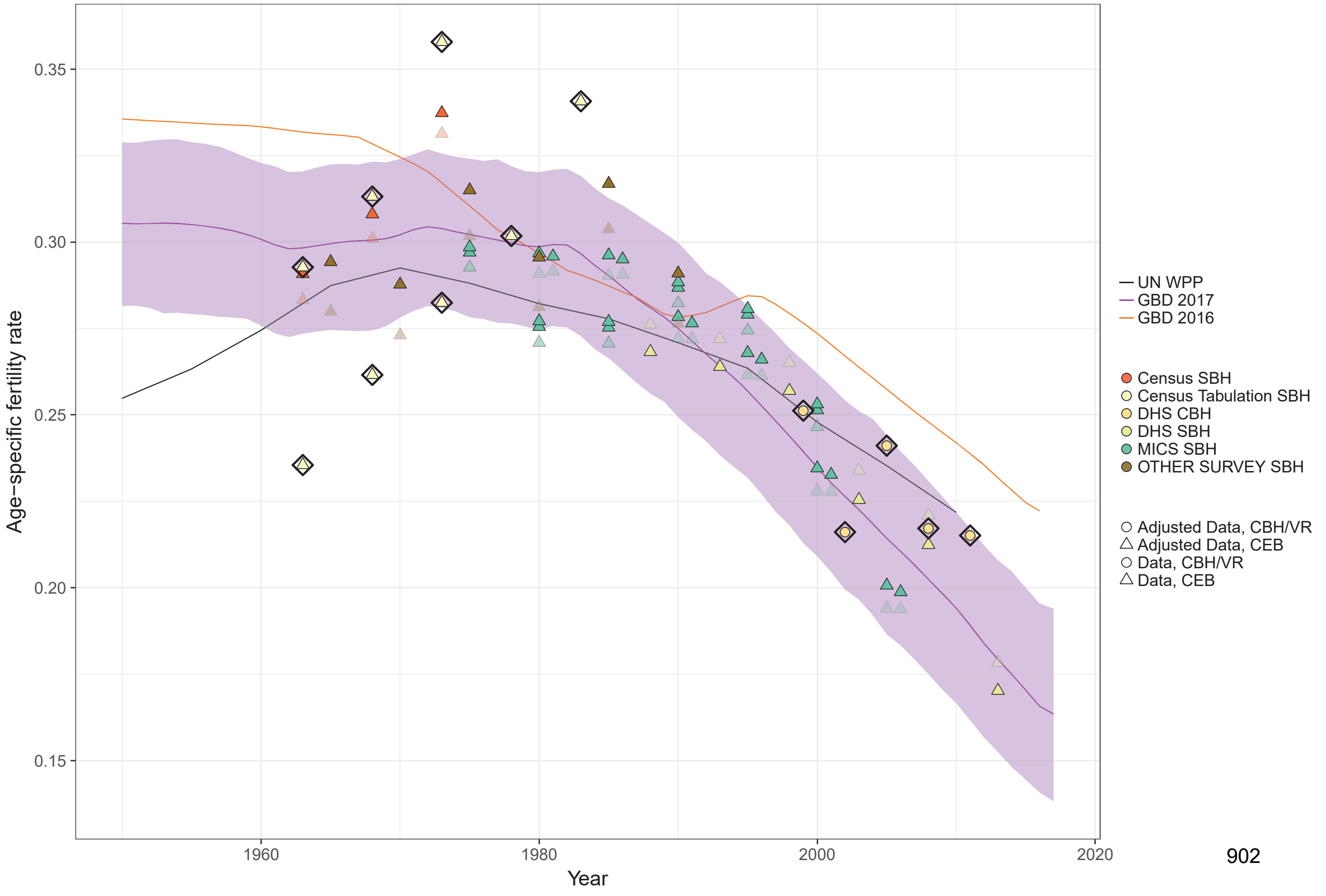

Age−specific fertility fitted trend lines, 1950−2017: The Gambia, Ages: 25−29
Data points enclosed with a diamond are reference sources

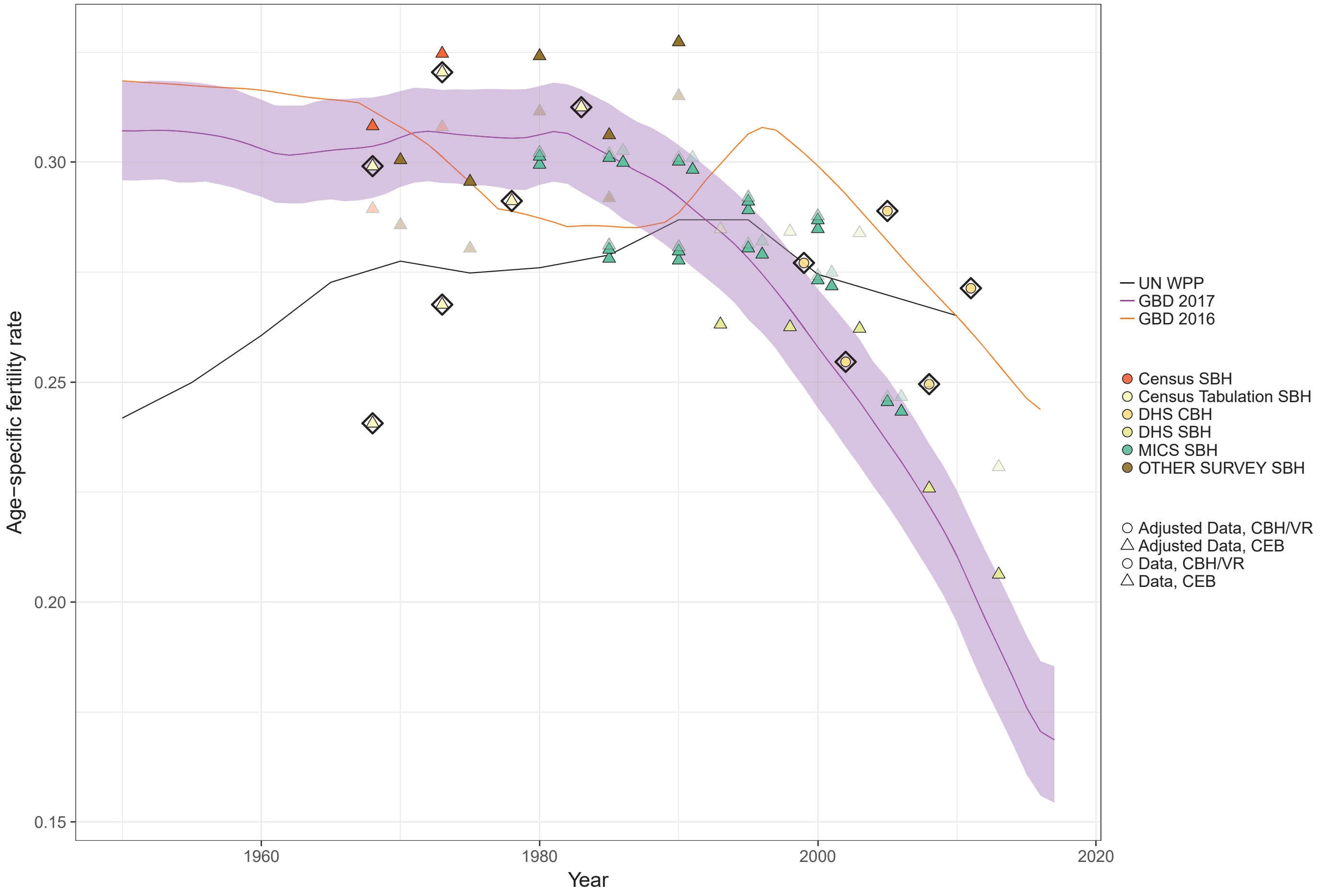

Age−specific fertility fitted trend lines, 1950−2017: The Gambia, Ages: 30−34
Data points enclosed with a diamond are reference sources

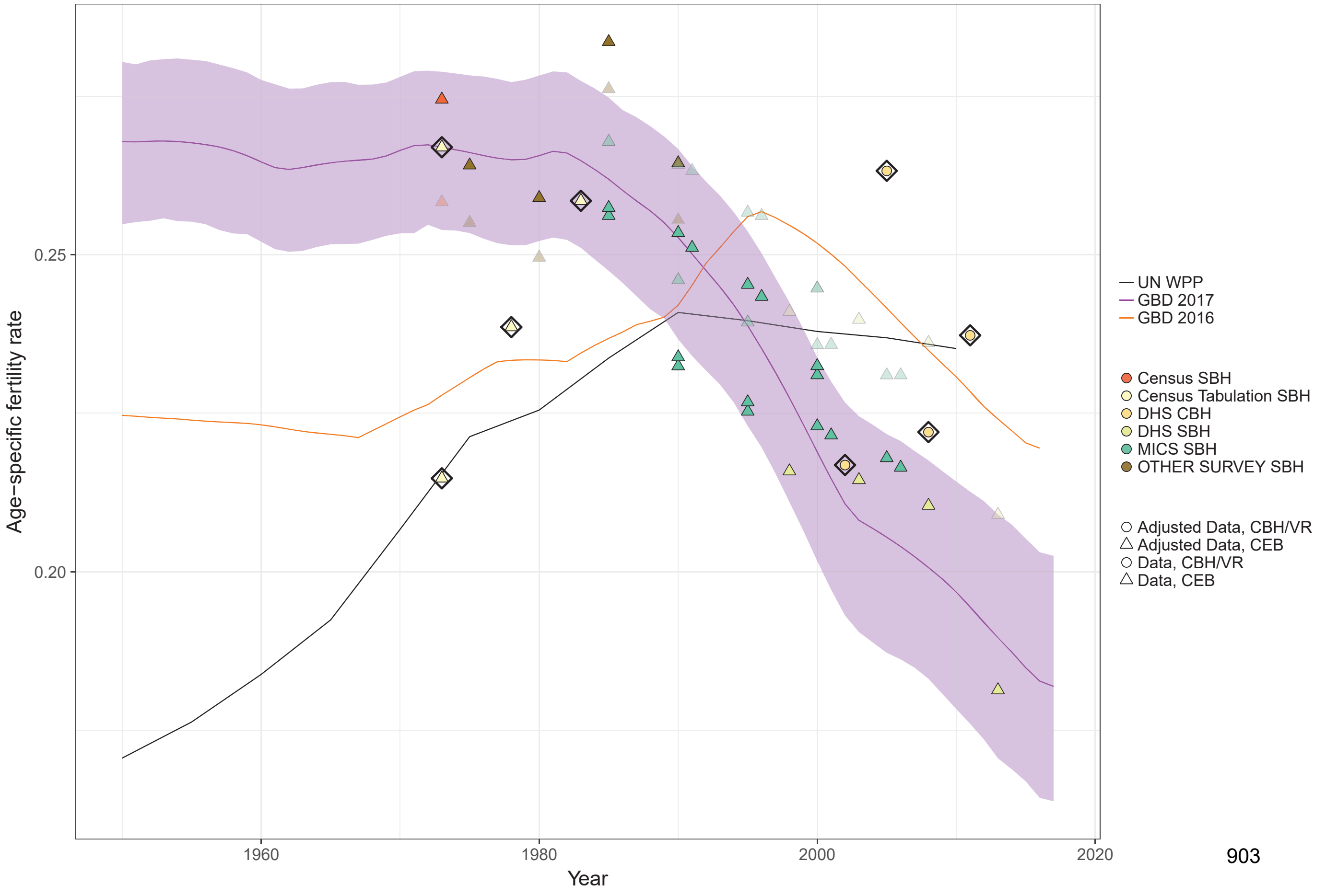

Age−specific fertility fitted trend lines, 1950−2017: The Gambia, Ages: 35−39
Data points enclosed with a diamond are reference sources

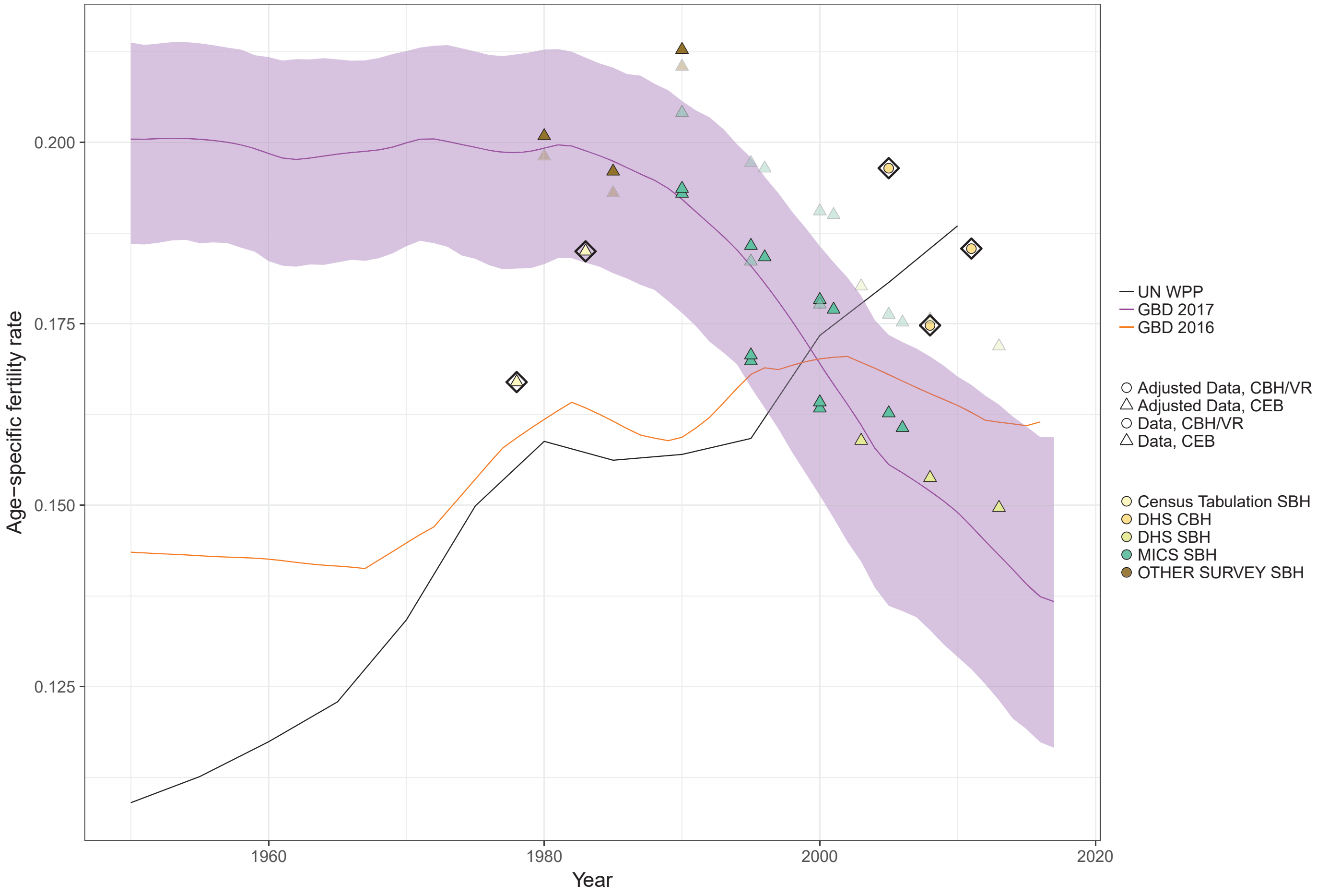

Age−specific fertility fitted trend lines, 1950−2017: The Gambia, Ages: 40−44
Data points enclosed with a diamond are reference sources

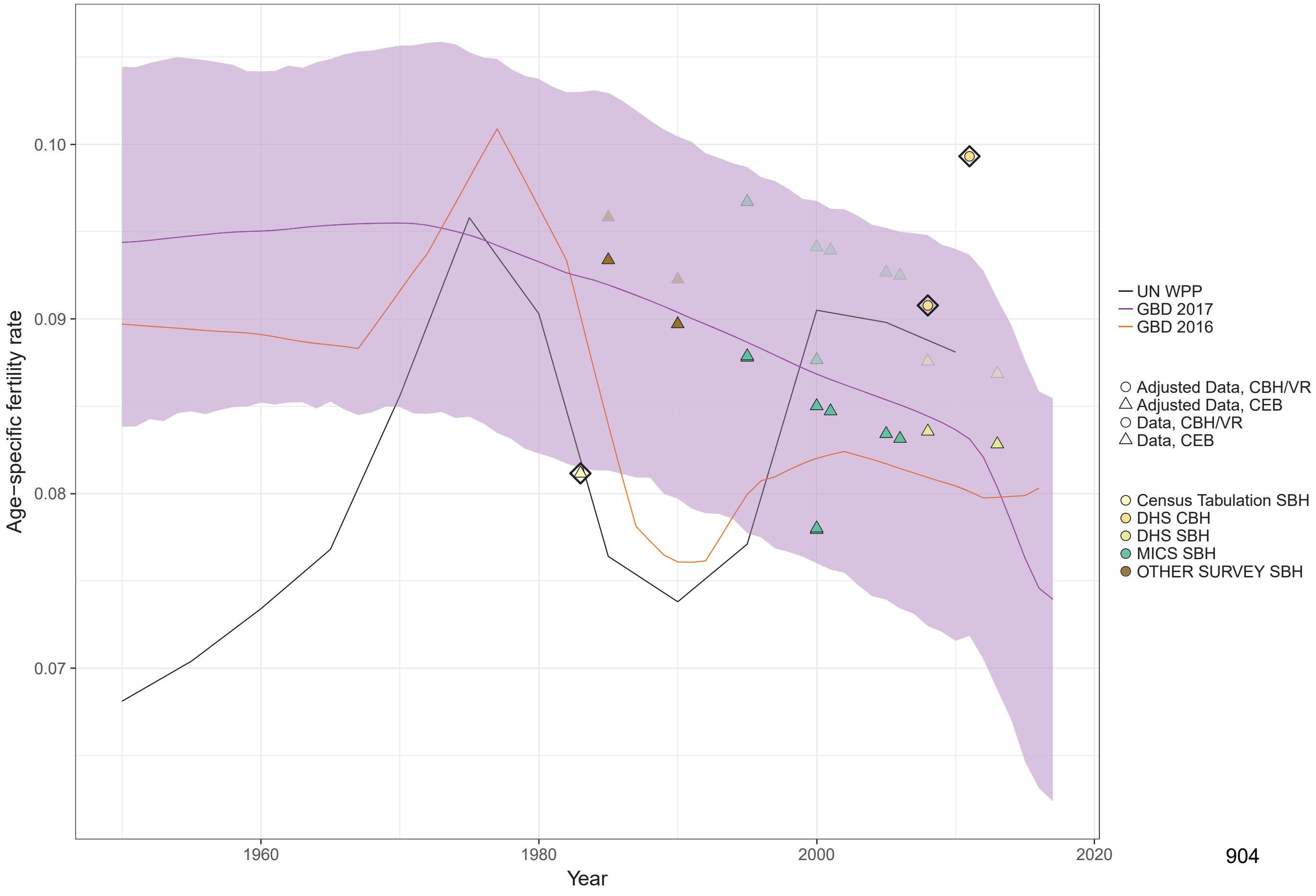

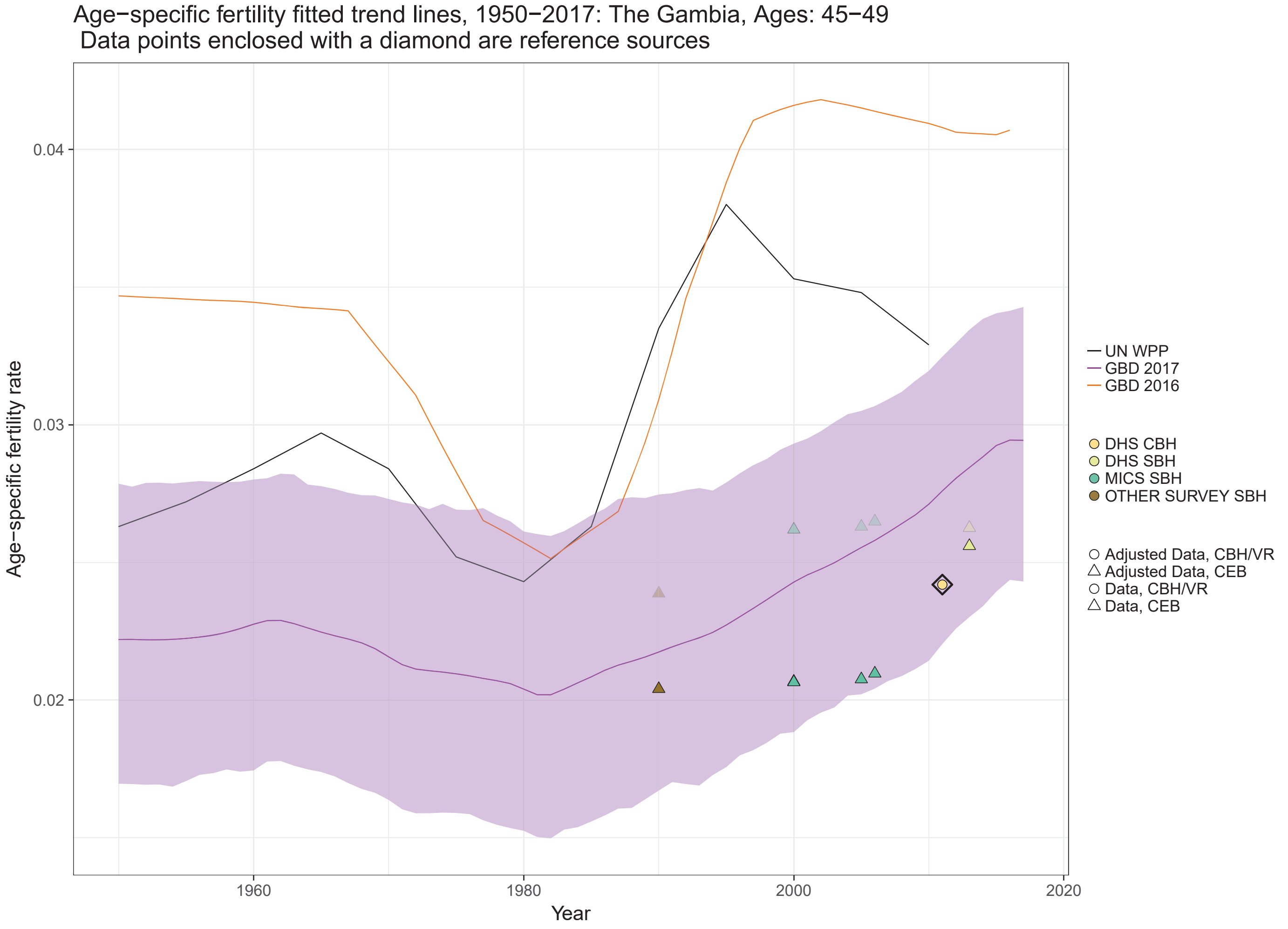

Age−specific fertility fitted trend lines, 1950−2017: The Gambia, Ages: 45−49
Data points enclosed with a diamond are reference sources
Age−specific fertility rate
0.04
0.03
0.02
1960
1980
2000
2020
Year
UN WPP
GBD 2017
GBD 2016
DHS CBH
DHS SBH
MICS SBH
OTHER SURVEY SBH
Adjusted Data, CBH/VR
Adjusted Data, CEB
Data, CBH/VR
Data, CEB

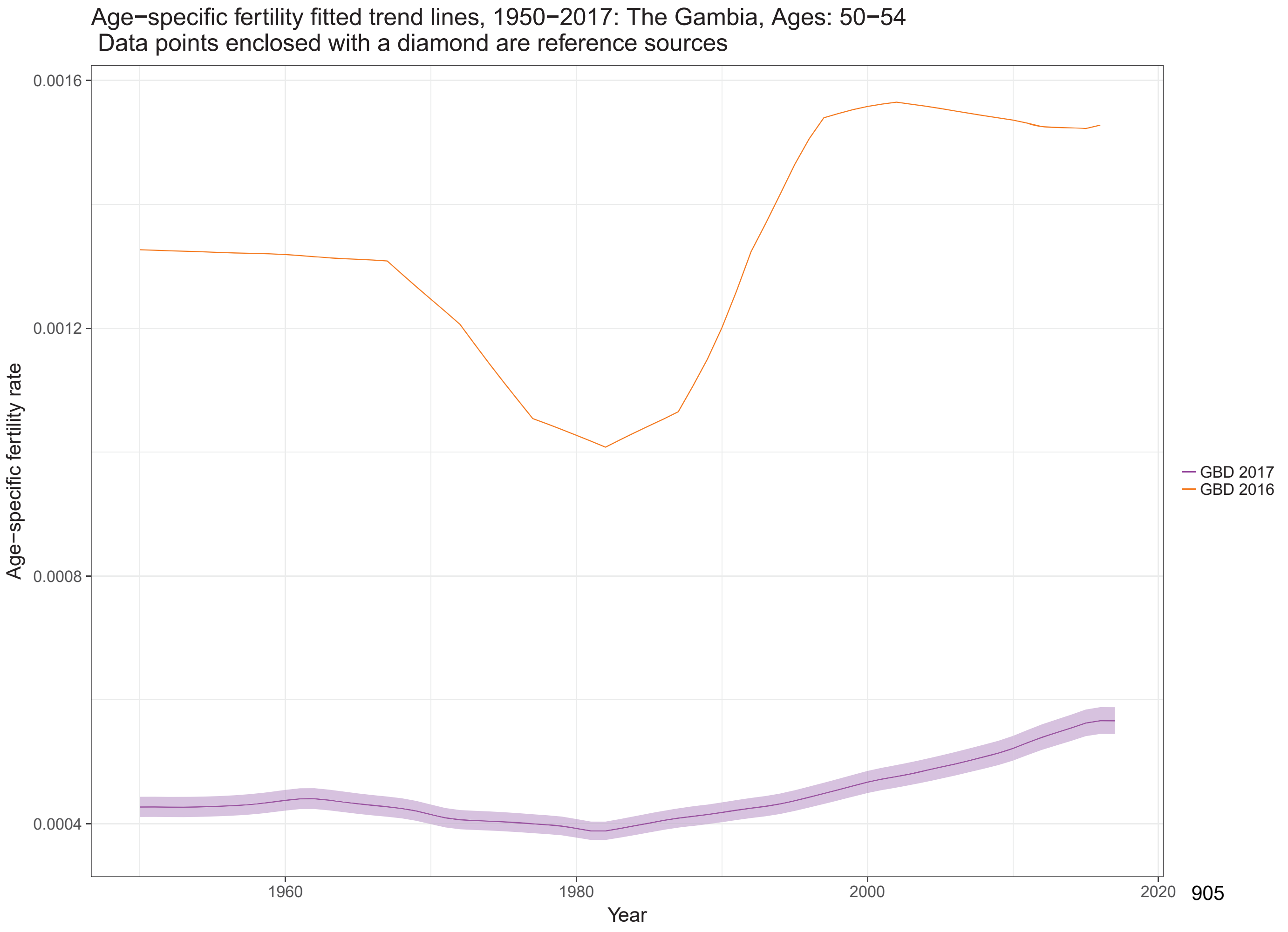

Age−specific fertility fitted trend lines, 1950−2017: The Gambia, Ages: 50−54
Data points enclosed with a diamond are reference sources
Age−specific fertility rate
0.0016
0.0012
0.0008
0.0004
1960
1980
2000
2020
Year
GBD 2017
GBD 2016

Age−specific fertility fitted trend lines, 1950−2017: Timor−Leste, TFR
Data points enclosed with a diamond are reference sources

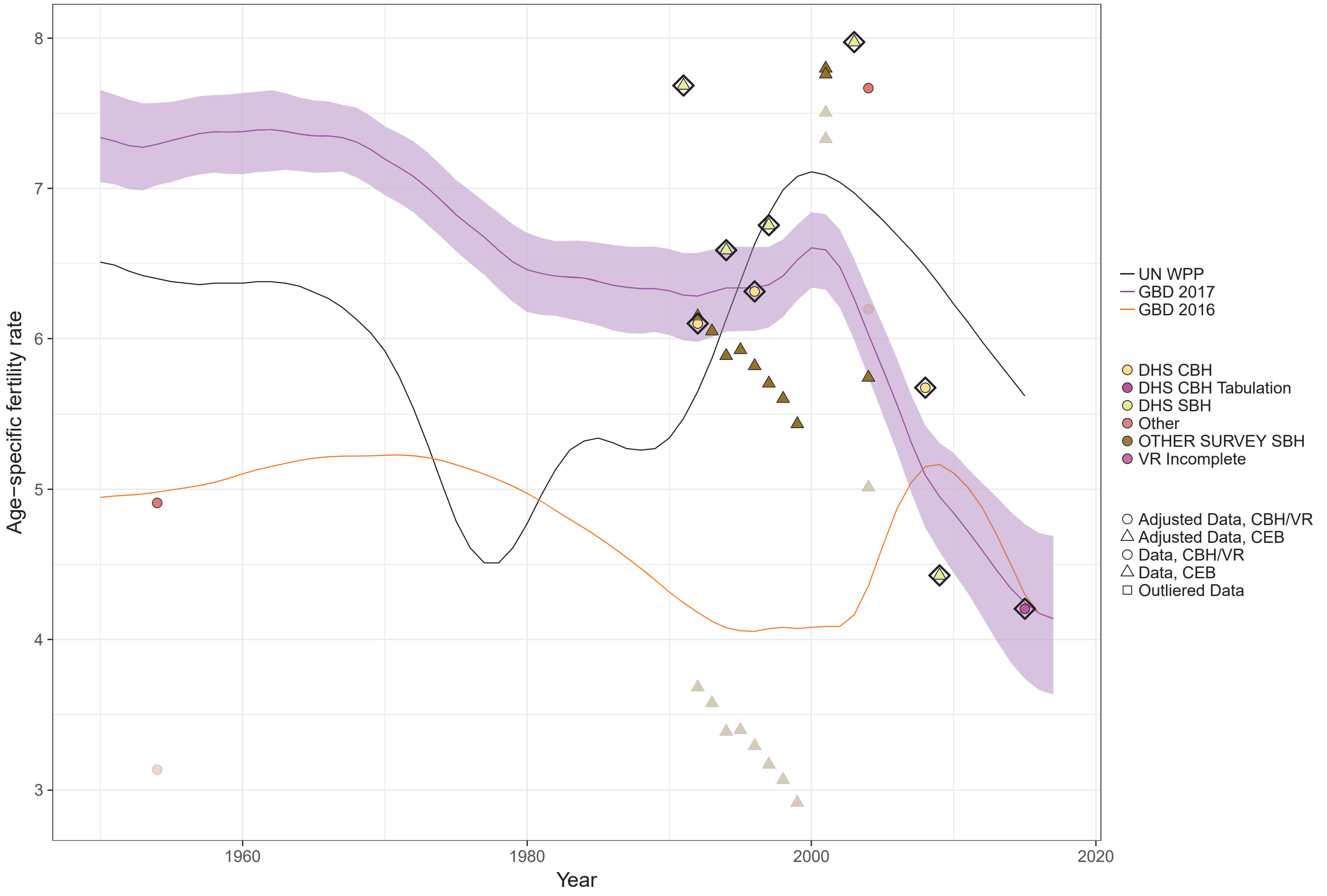

Age−specific fertility fitted trend lines, 1950−2017: Timor−Leste, Ages: 10−14
Data points enclosed with a diamond are reference sources

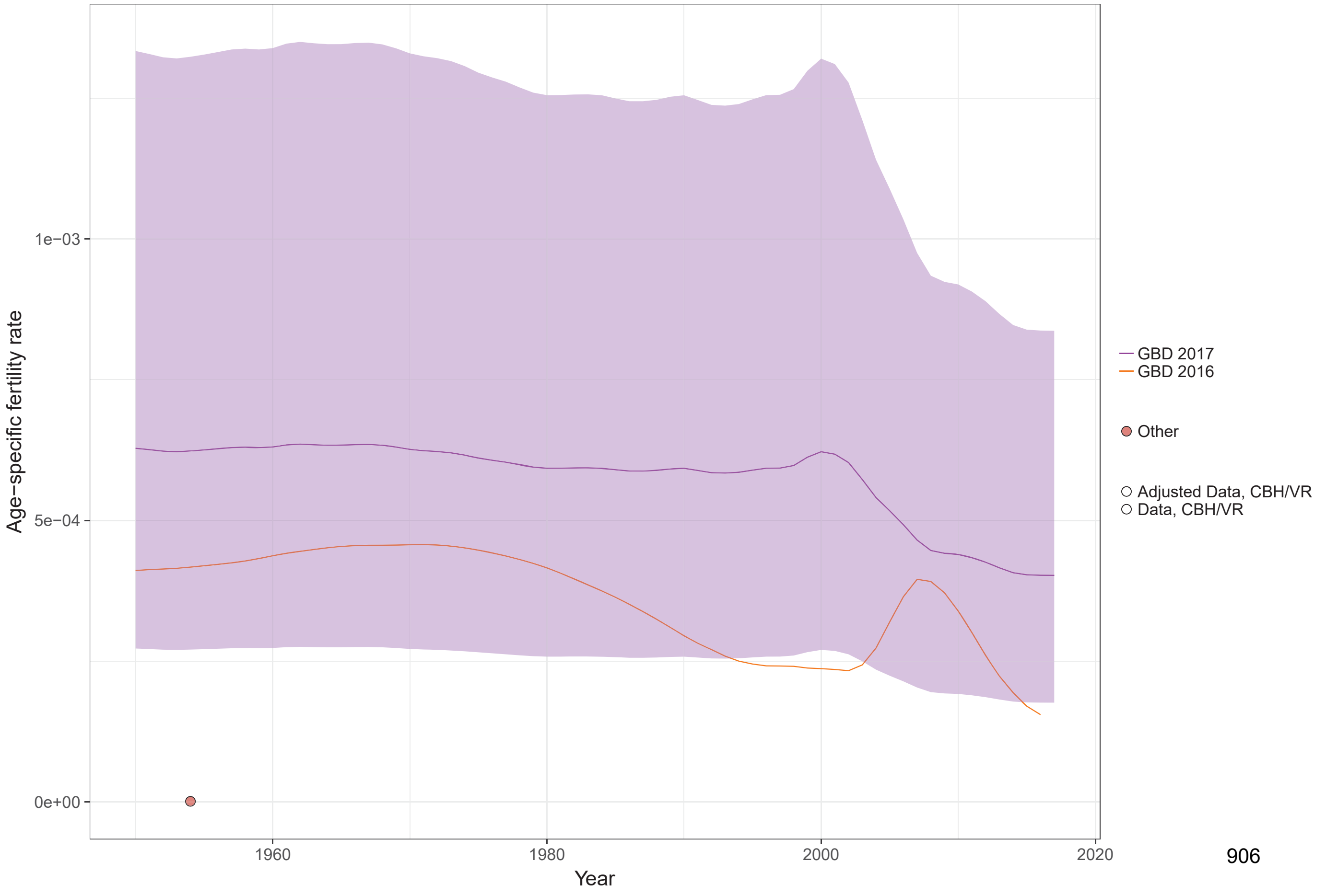

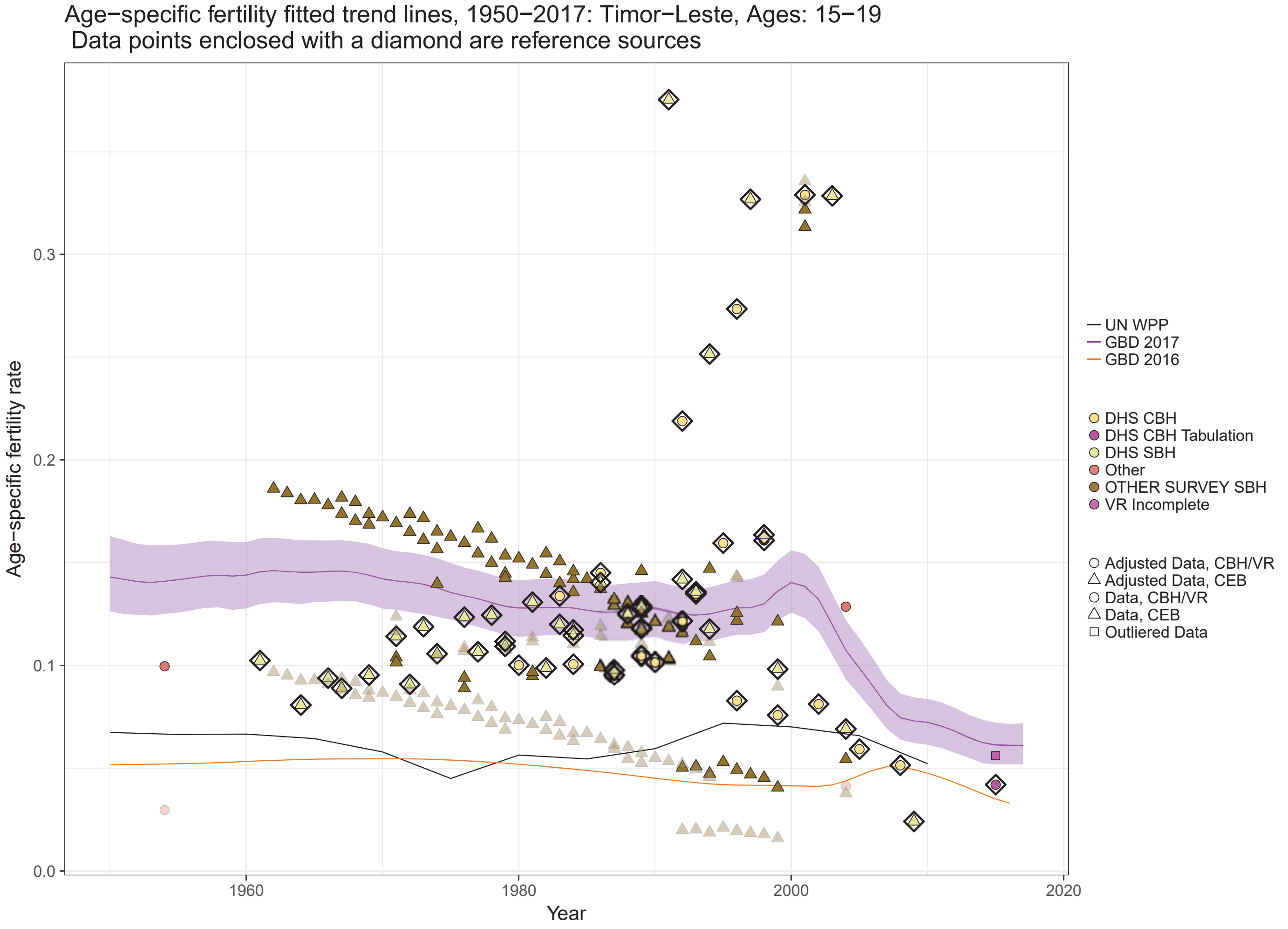

Age−specific fertility fitted trend lines, 1950−2017: Timor−Leste, Ages: 15−19
Data points enclosed with a diamond are reference sources
Age-specific fertility rate
Year
0.0
0.1
0.2
0.3
1960
1980
2000
2020
UN WPP
GBD 2017
GBD 2016
DHS CBH
DHS CBH Tabulation
DHS SBH
Other
OTHER SURVEY SBH
VR Incomplete
Adjusted Data, CBH/VR
Adjusted Data, CEB
Data, CBH/VR
Data, CEB
Outliered Data

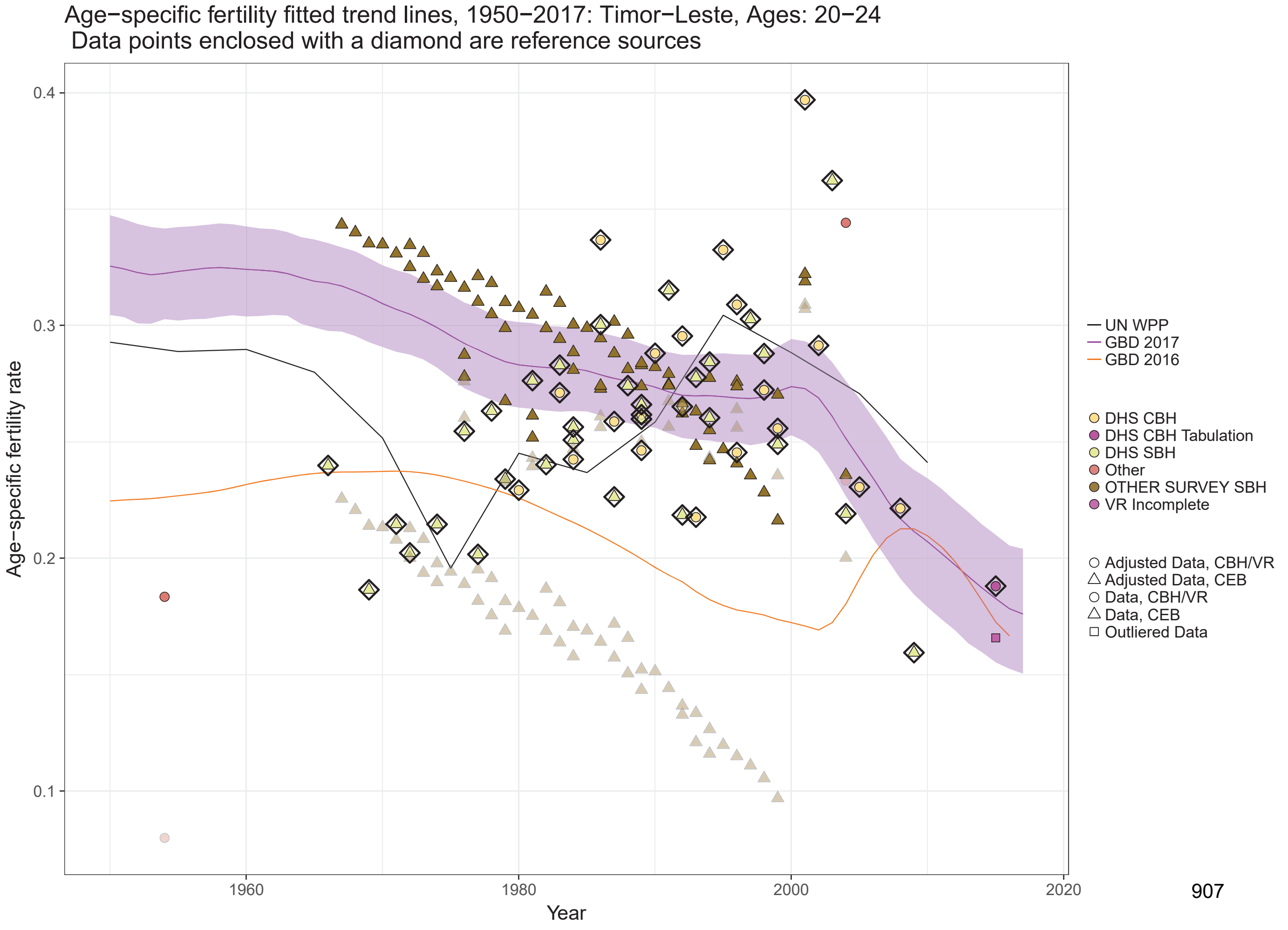

Age−specific fertility fitted trend lines, 1950−2017: Timor−Leste, Ages: 20−24
Data points enclosed with a diamond are reference sources
Age-specific fertility rate
Year
0.1
0.2
0.3
0.4
1960
1980
2000
2020
UN WPP
GBD 2017
GBD 2016
DHS CBH
DHS CBH Tabulation
DHS SBH
Other
OTHER SURVEY SBH
VR Incomplete
Adjusted Data, CBH/VR
Adjusted Data, CEB
Data, CBH/VR
Data, CEB
Outliered Data

Age−specific fertility fitted trend lines, 1950−2017: Timor−Leste, Ages: 25−29
Data points enclosed with a diamond are reference sources

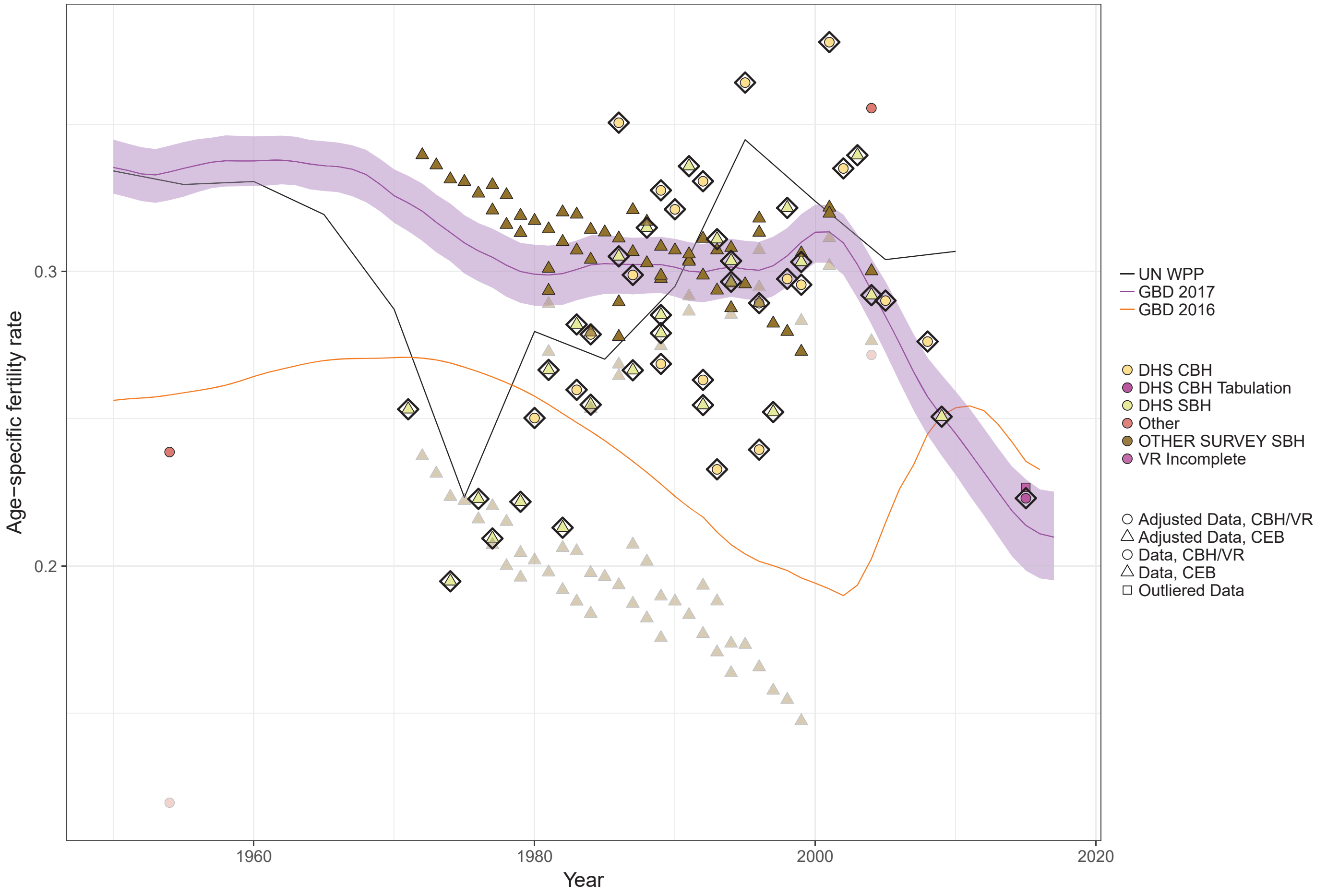

Age−specific fertility fitted trend lines, 1950−2017: Timor−Leste, Ages: 30−34
Data points enclosed with a diamond are reference sources

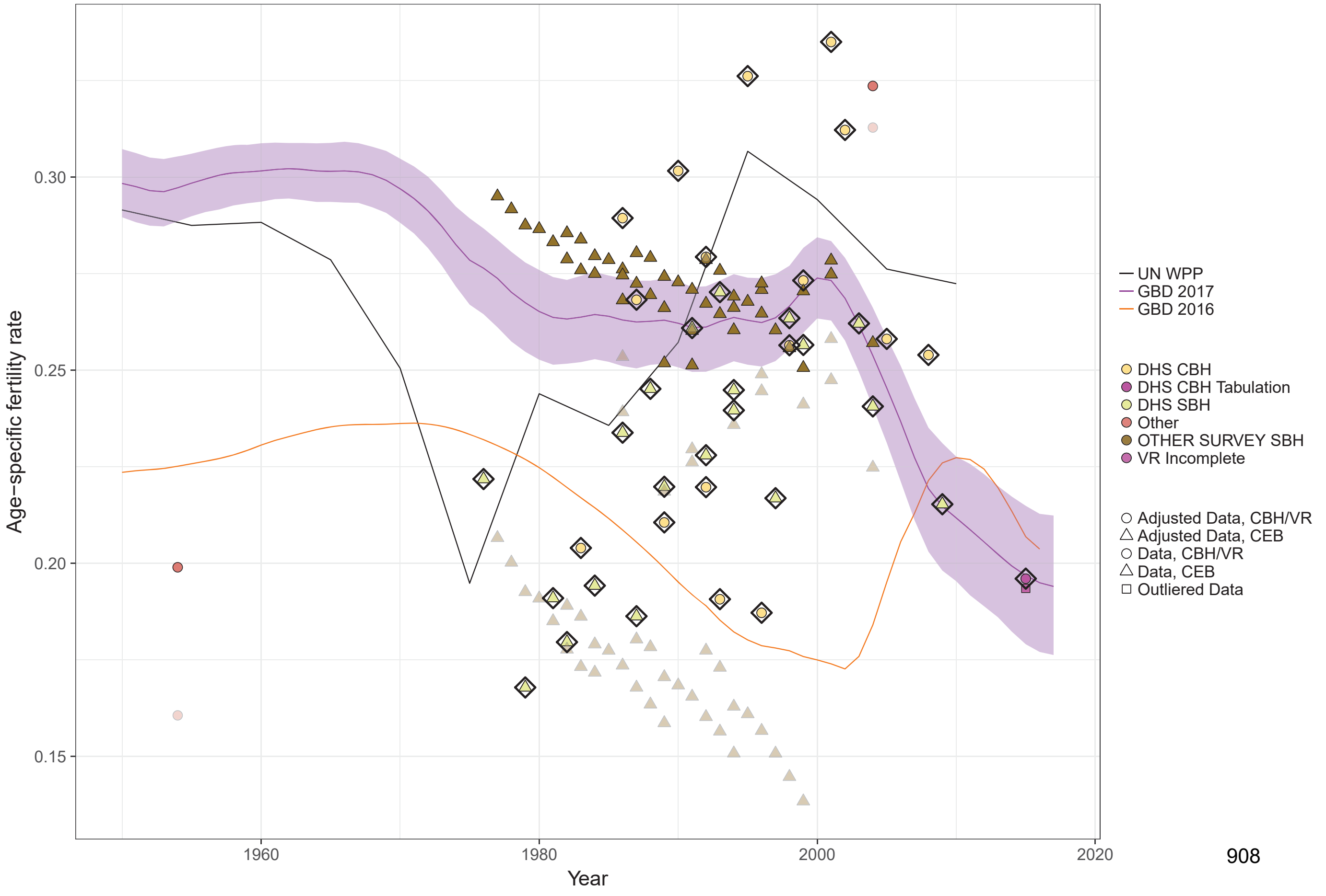

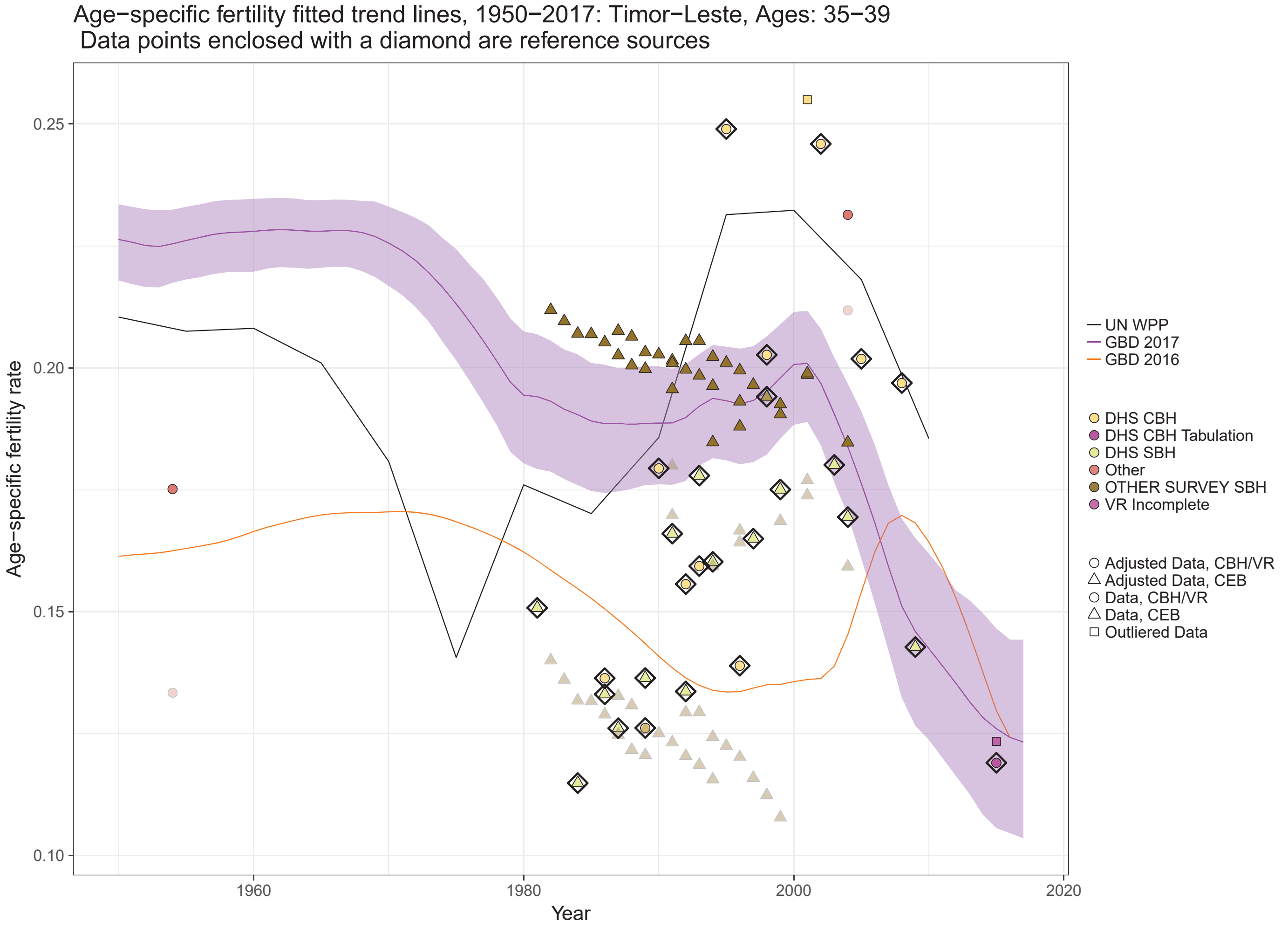
Age−specific fertility fitted trend lines, 1950−2017: Timor−Leste, Ages: 35−39
Data points enclosed with a diamond are reference sources
Age−specific fertility rate
Year
0.10
0.15
0.20
0.25
1960
1980
2000
2020
UN WPP
GBD 2017
GBD 2016
DHS CBH
DHS CBH Tabulation
DHS SBH
Other
OTHER SURVEY SBH
VR Incomplete
Adjusted Data, CBH/VR
Adjusted Data, CEB
Data, CBH/VR
Data, CEB
Outliered Data

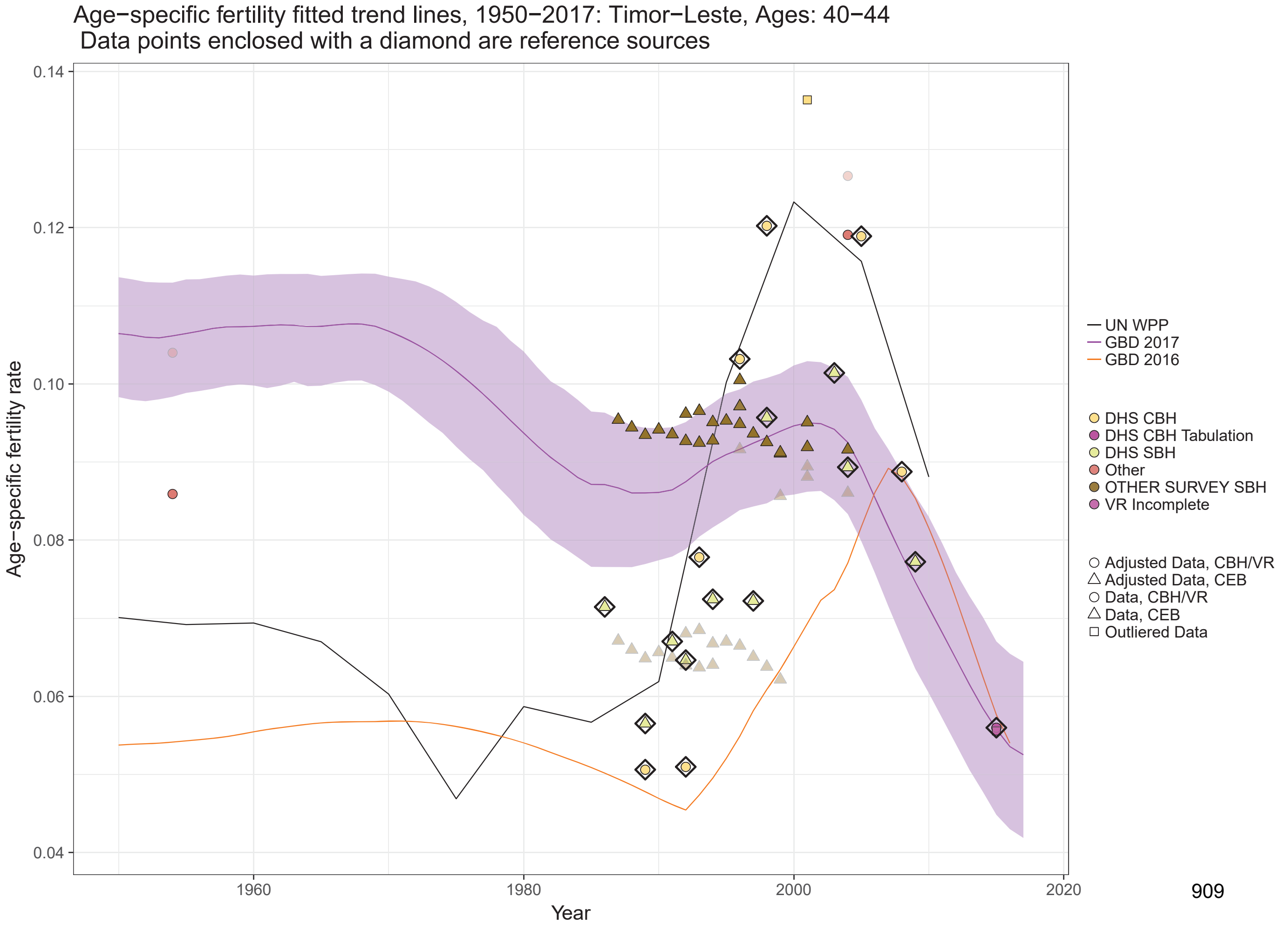
Age−specific fertility fitted trend lines, 1950−2017: Timor−Leste, Ages: 40−44
Data points enclosed with a diamond are reference sources
Age−specific fertility rate
Year
0.04
0.06
0.08
0.10
0.12
0.14
1960
1980
2000
2020
UN WPP
GBD 2017
GBD 2016
DHS CBH
DHS CBH Tabulation
DHS SBH
Other
OTHER SURVEY SBH
VR Incomplete
Adjusted Data, CBH/VR
Adjusted Data, CEB
Data, CBH/VR
Data, CEB
Outliered Data

Age−specific fertility fitted trend lines, 1950−2017: Timor−Leste, Ages: 45−49
Data points enclosed with a diamond are reference sources

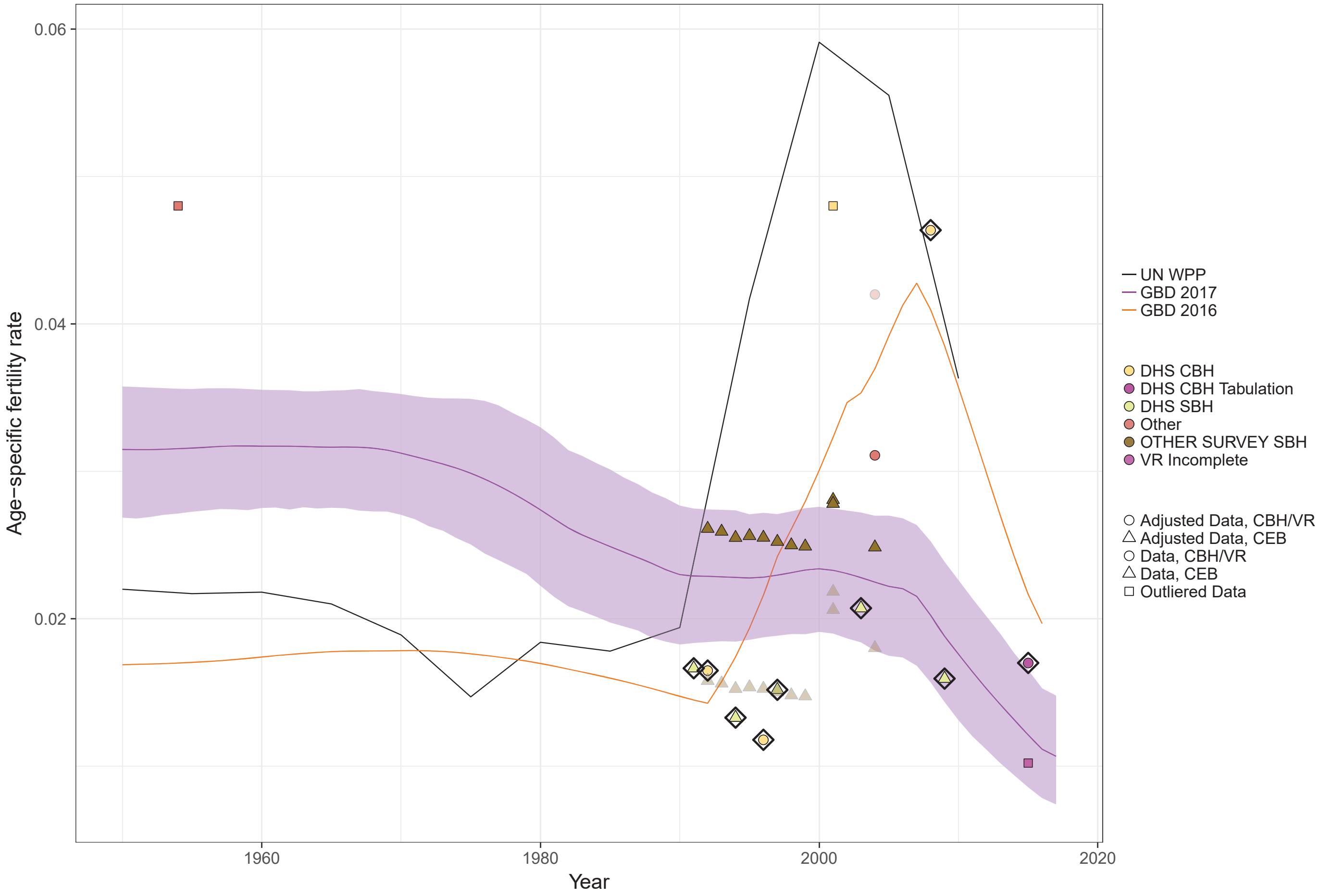

Age−specific fertility fitted trend lines, 1950−2017: Timor−Leste, Ages: 50−54
Data points enclosed with a diamond are reference sources

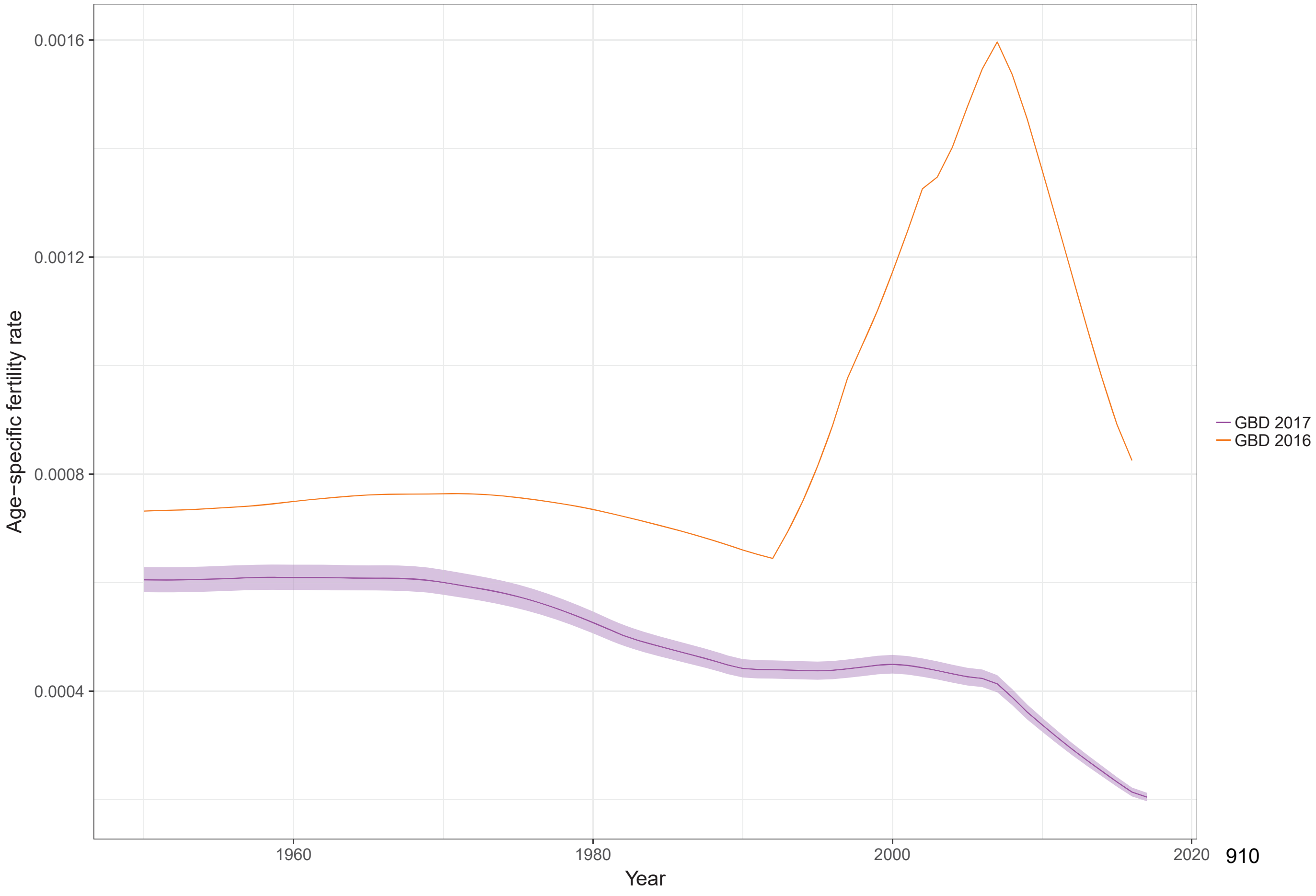

Age−specific fertility fitted trend lines, 1950−2017: Togo, TFR
Data points enclosed with a diamond are reference sources

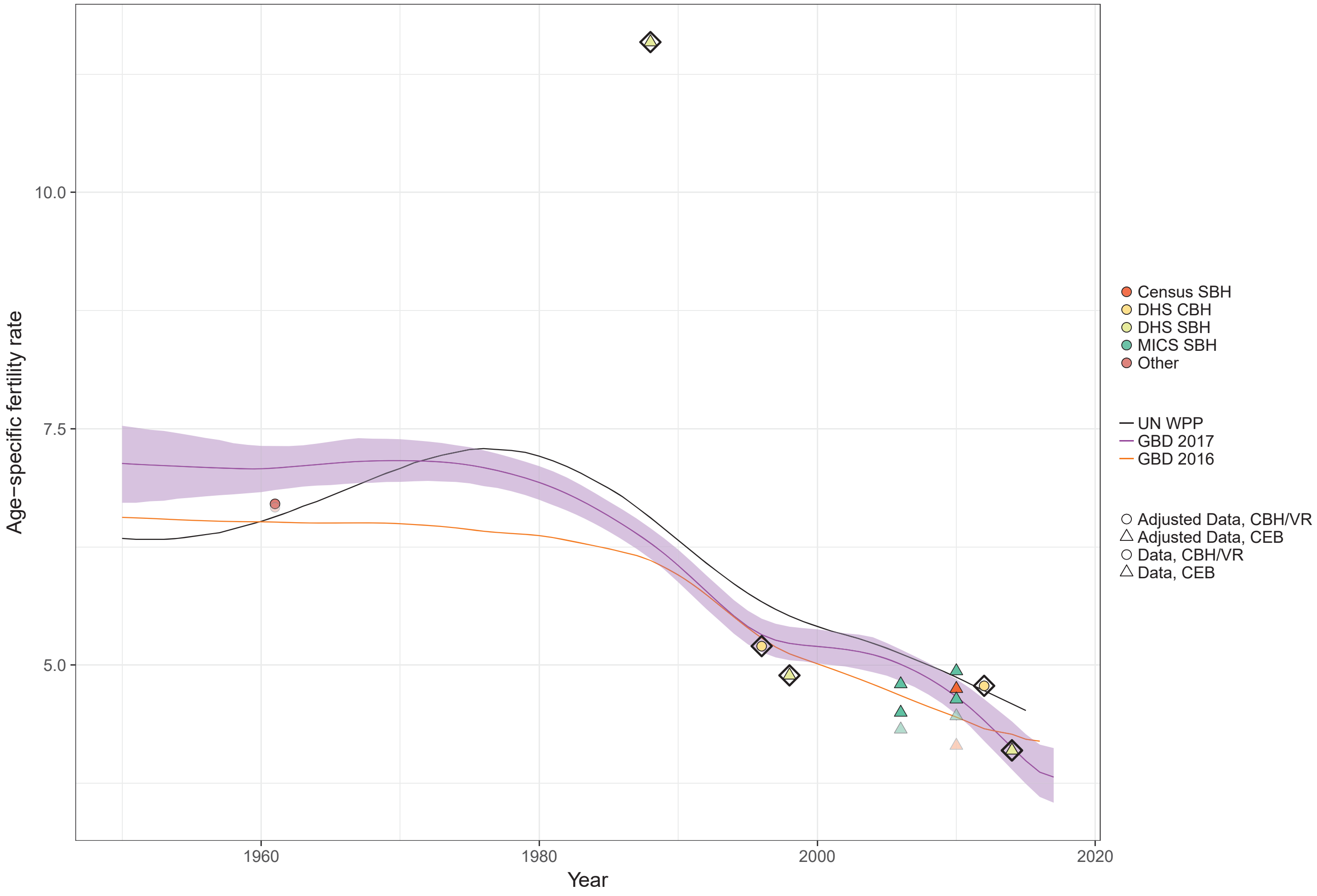

Age−specific fertility fitted trend lines, 1950−2017: Togo, Ages: 10−14
Data points enclosed with a diamond are reference sources

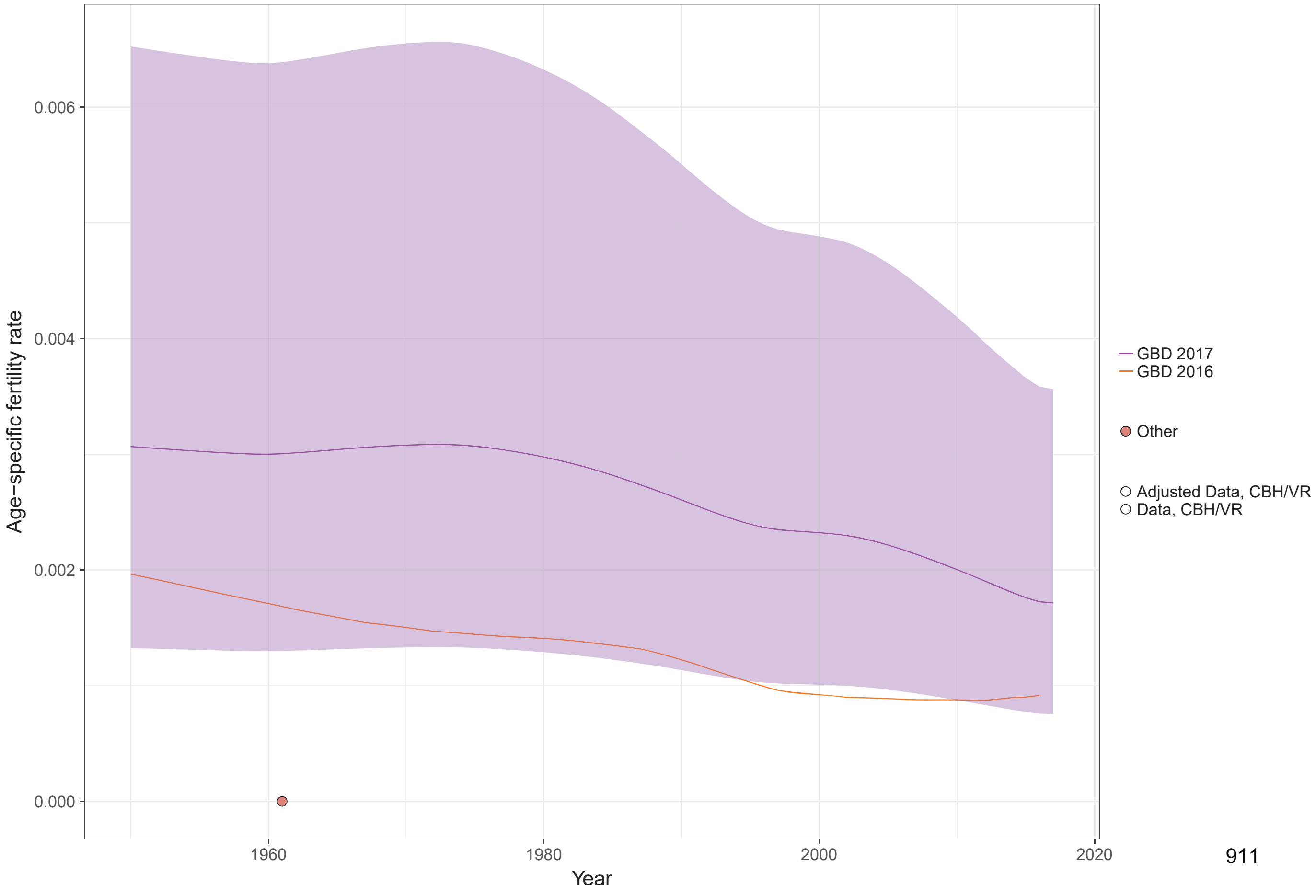

Age−specific fertility fitted trend lines, 1950−2017: Togo, Ages: 15−19
Data points enclosed with a diamond are reference sources

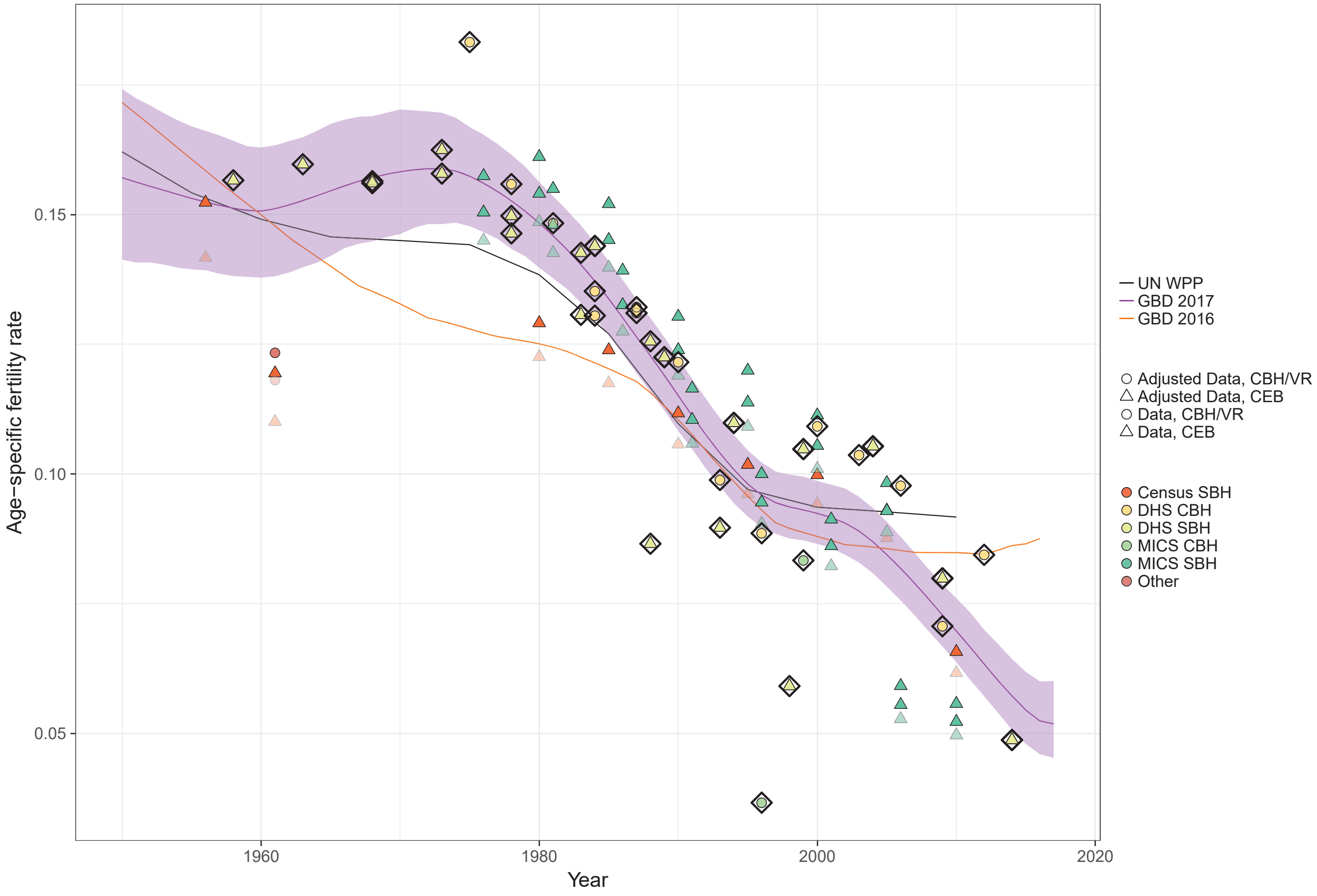

Age−specific fertility fitted trend lines, 1950−2017: Togo, Ages: 20−24
Data points enclosed with a diamond are reference sources

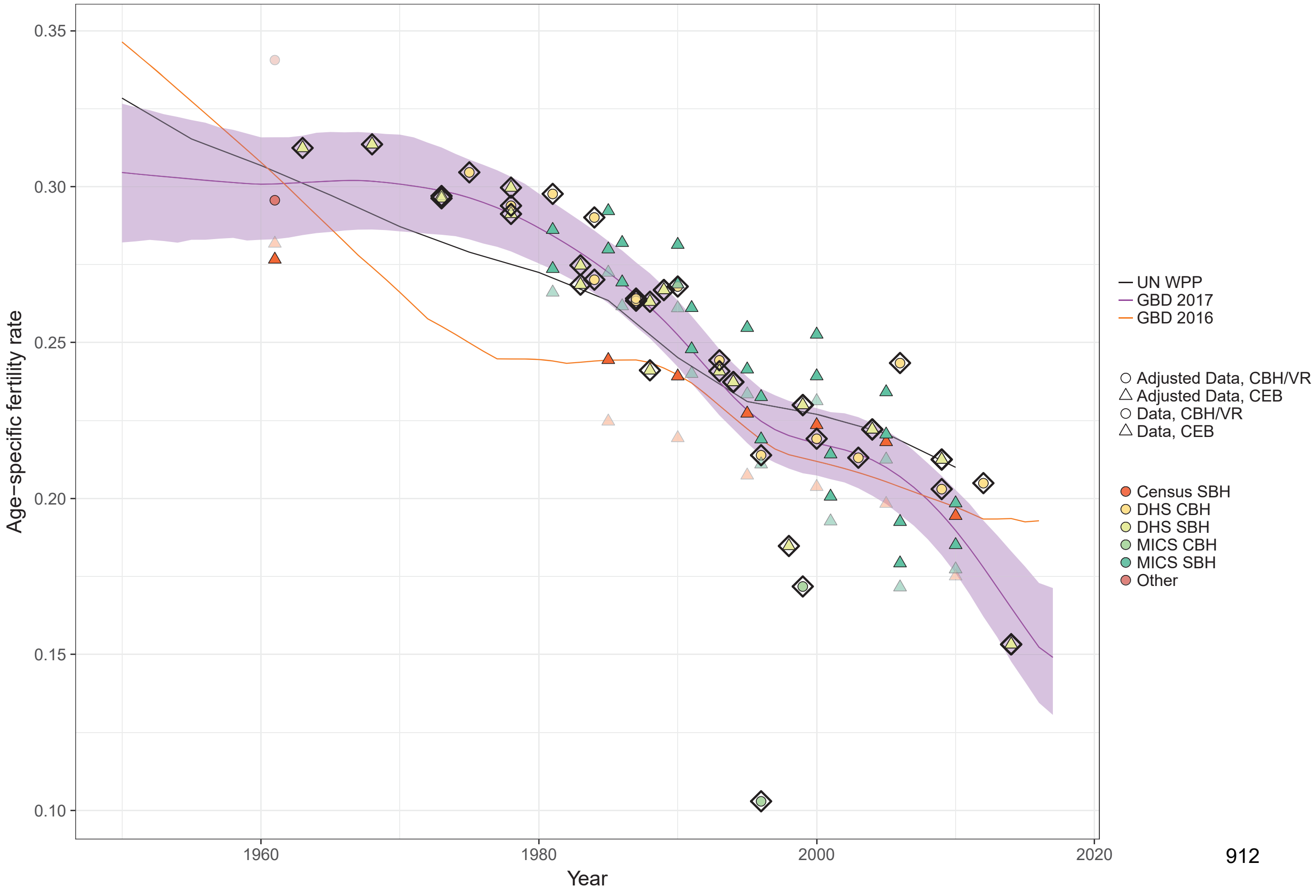

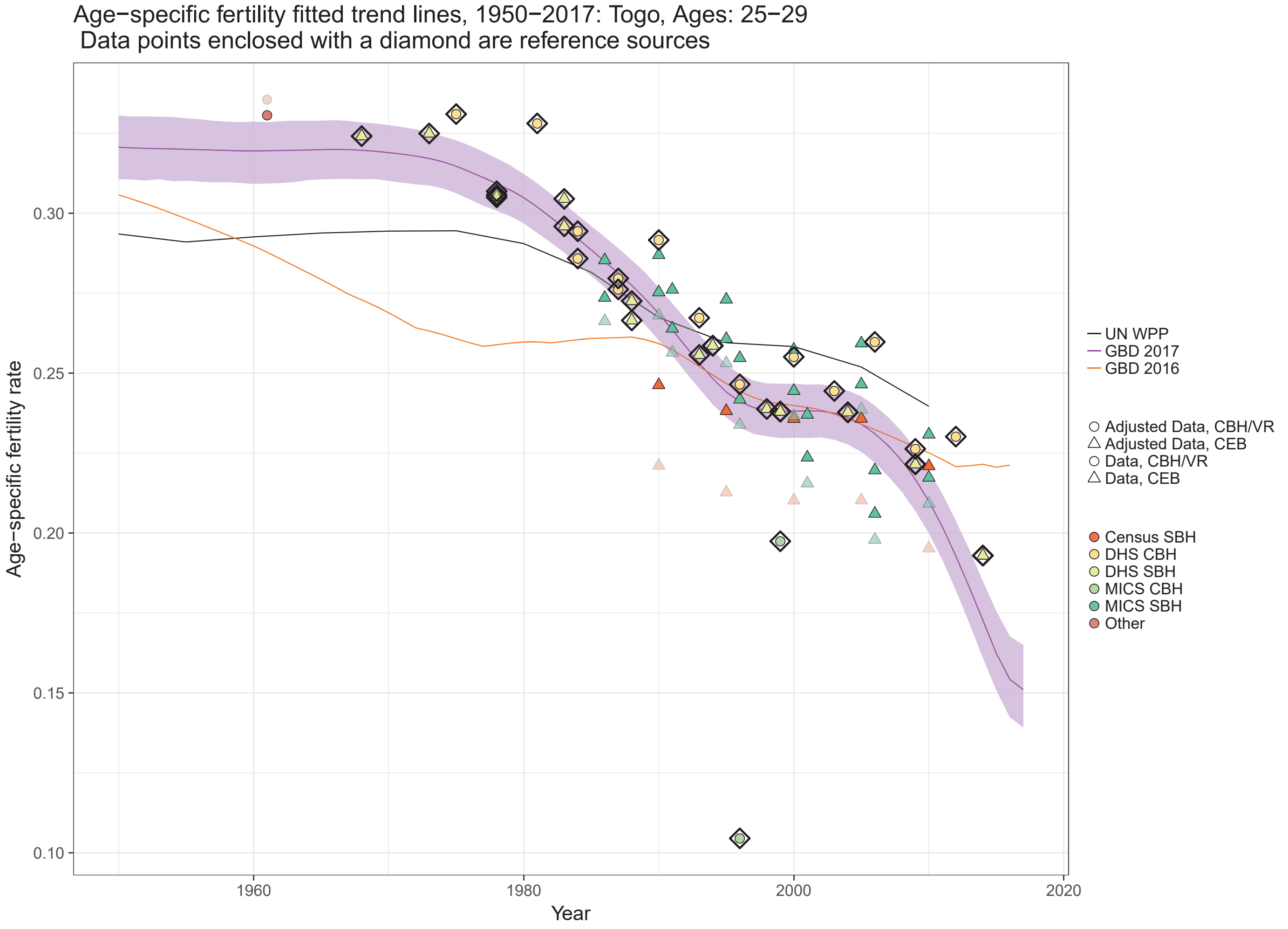
Age−specific fertility fitted trend lines, 1950−2017: Togo, Ages: 25−29
Data points enclosed with a diamond are reference sources
Age−specific fertility rate
Year
0.10
0.15
0.20
0.25
0.30
1960
1980
2000
2020
UN WPP
GBD 2017
GBD 2016
Adjusted Data, CBH/VR
Adjusted Data, CEB
Data, CBH/VR
Data, CEB
Census SBH
DHS CBH
DHS SBH
MICS CBH
MICS SBH
Other

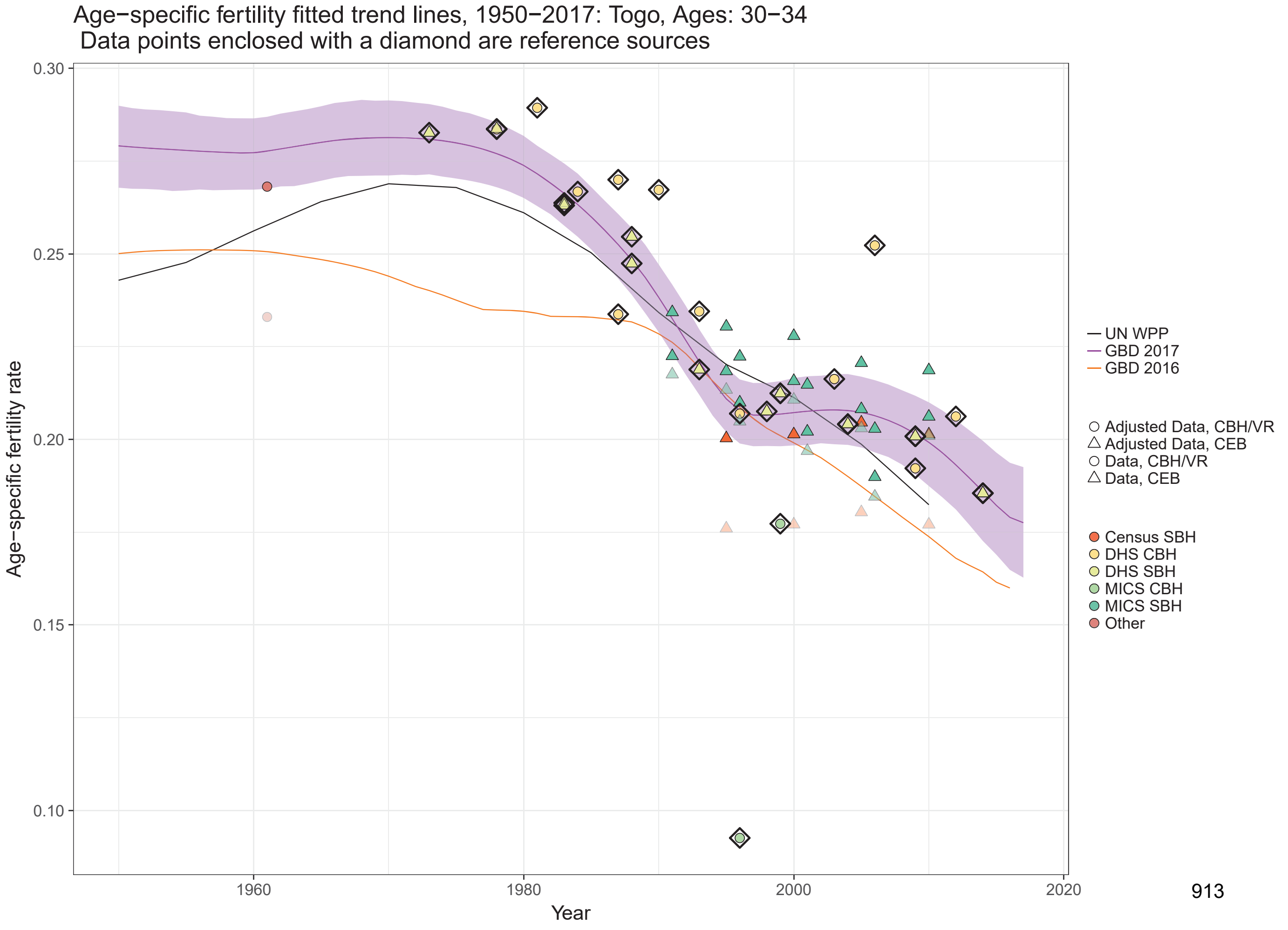
Age−specific fertility fitted trend lines, 1950−2017: Togo, Ages: 30−34
Data points enclosed with a diamond are reference sources
Age−specific fertility rate
Year
0.10
0.15
0.20
0.25
0.30
1960
1980
2000
2020
UN WPP
GBD 2017
GBD 2016
Adjusted Data, CBH/VR
Adjusted Data, CEB
Data, CBH/VR
Data, CEB
Census SBH
DHS CBH
DHS SBH
MICS CBH
MICS SBH
Other

Age−specific fertility fitted trend lines, 1950−2017: Togo, Ages: 35−39
Data points enclosed with a diamond are reference sources

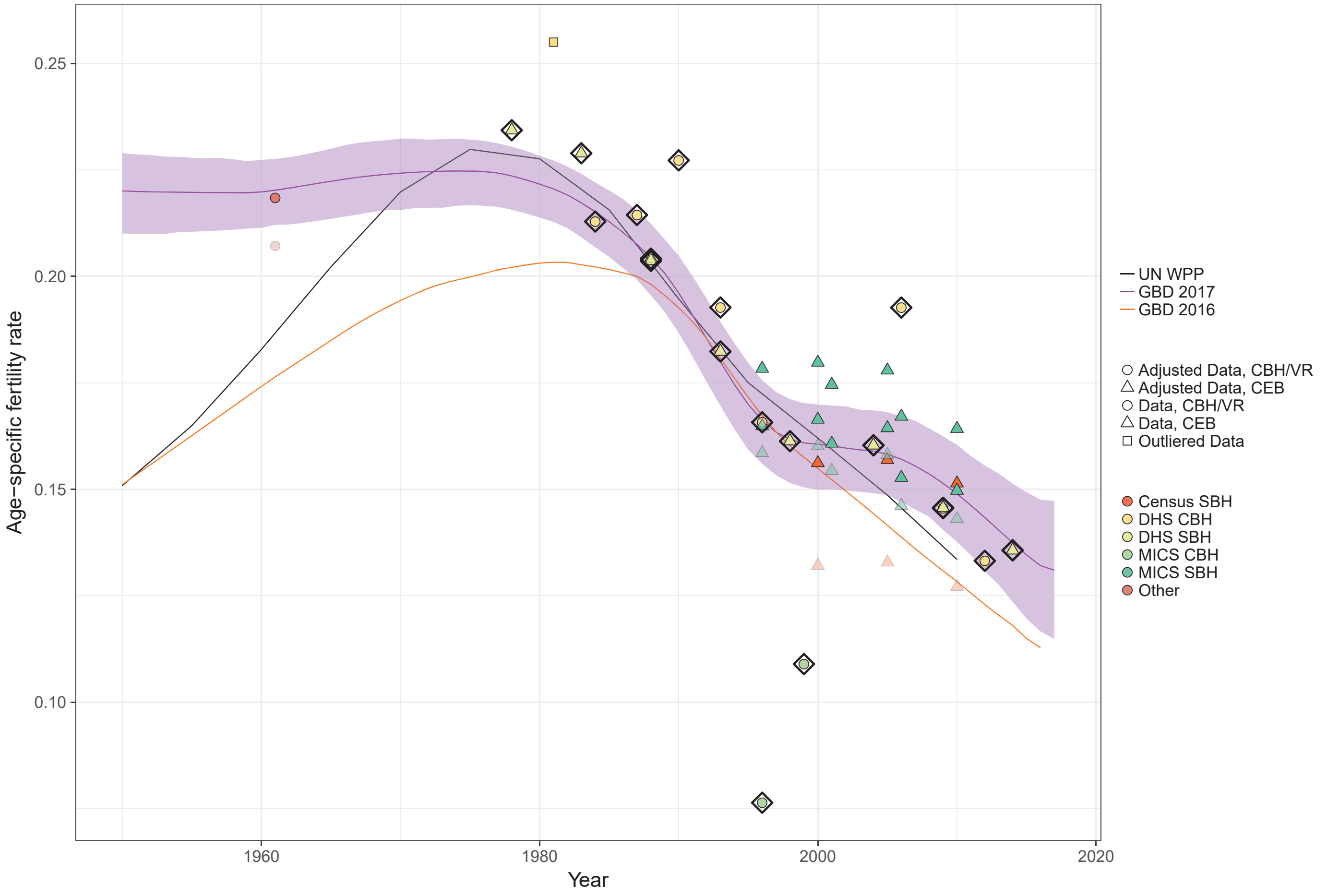

Age−specific fertility fitted trend lines, 1950−2017: Togo, Ages: 40−44
Data points enclosed with a diamond are reference sources

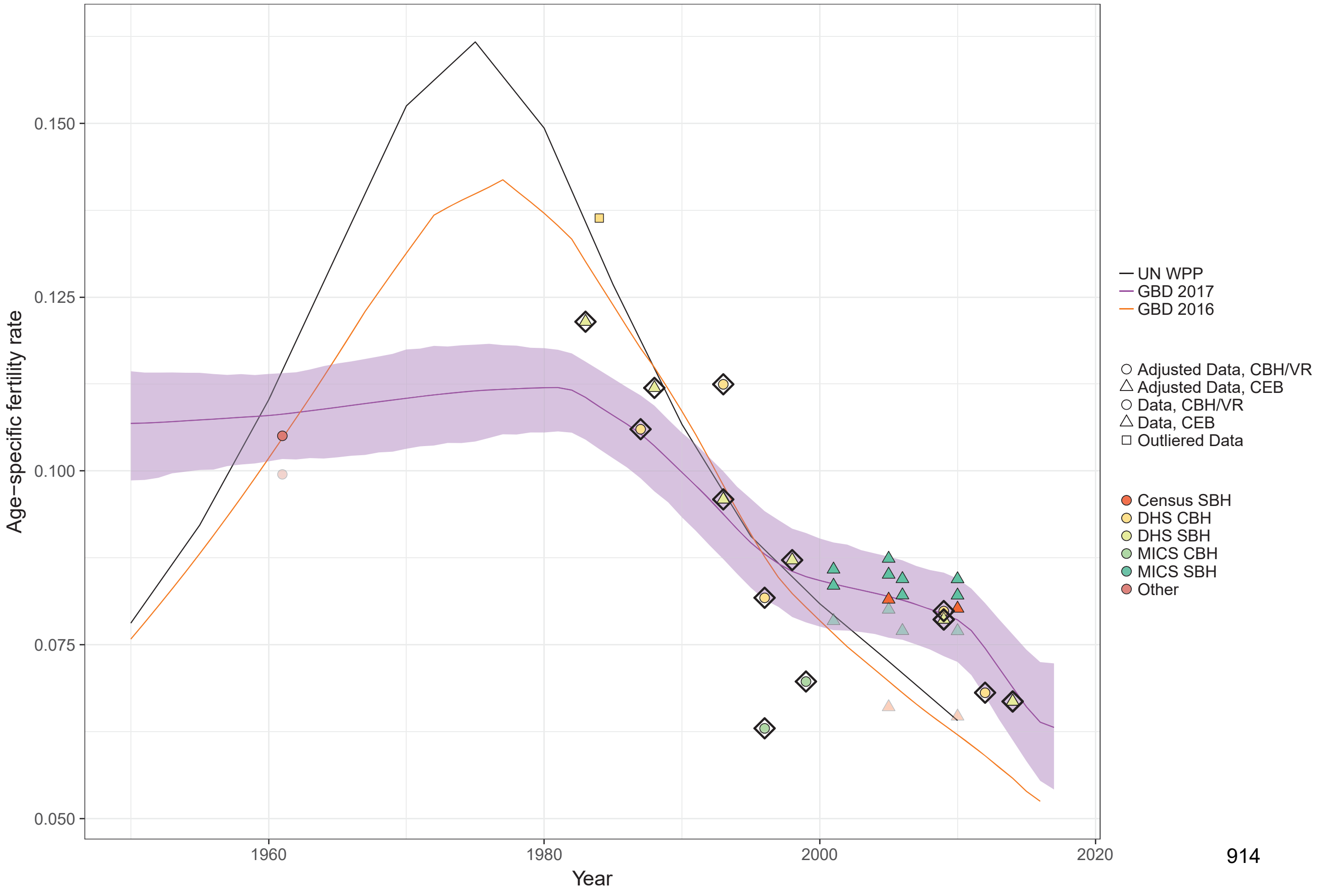

Age−specific fertility fitted trend lines, 1950−2017: Togo, Ages: 45−49
Data points enclosed with a diamond are reference sources

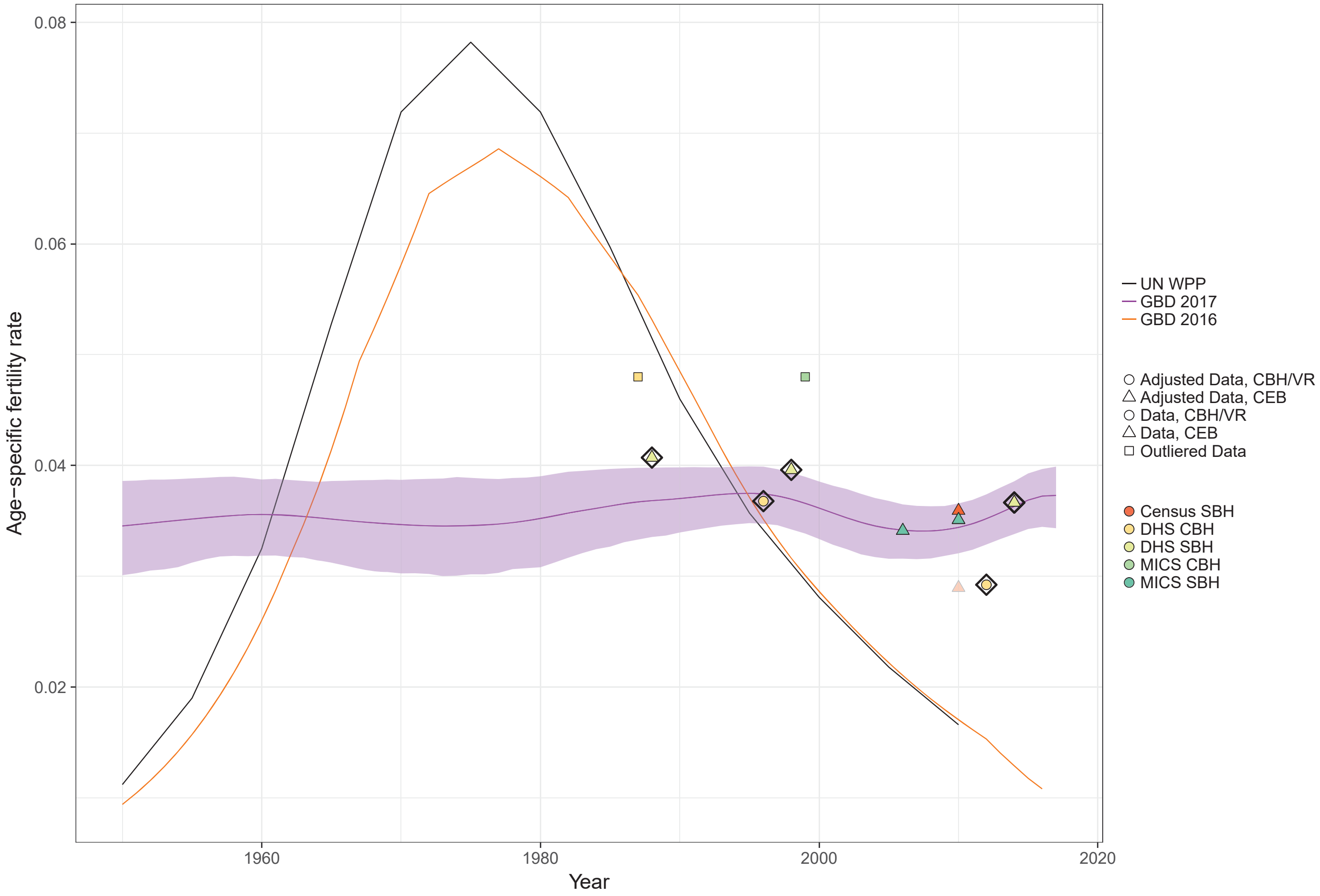

Age−specific fertility fitted trend lines, 1950−2017: Togo, Ages: 50−54
Data points enclosed with a diamond are reference sources

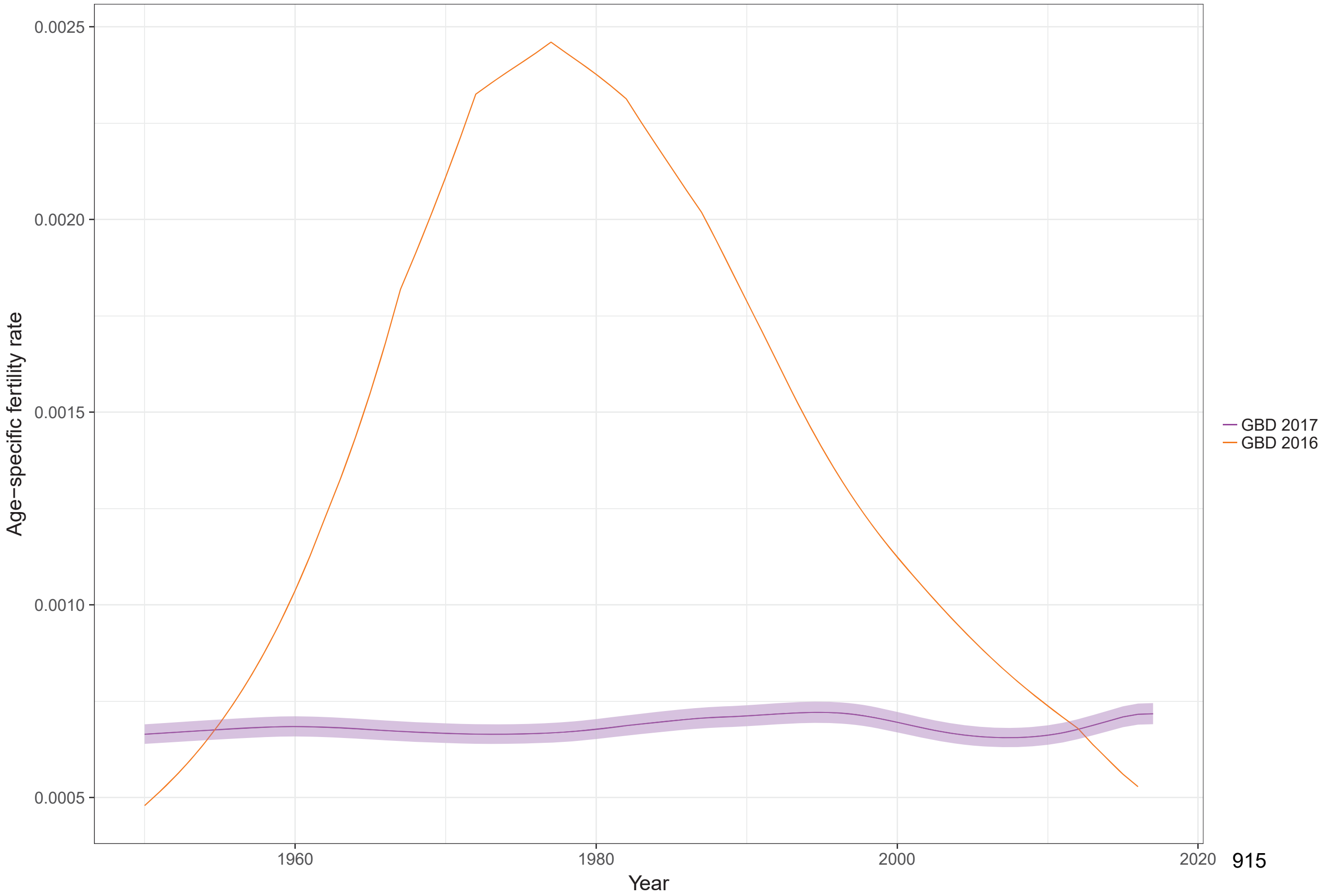

Age−specific fertility fitted trend lines, 1950−2017: Tonga, TFR
Data points enclosed with a diamond are reference sources

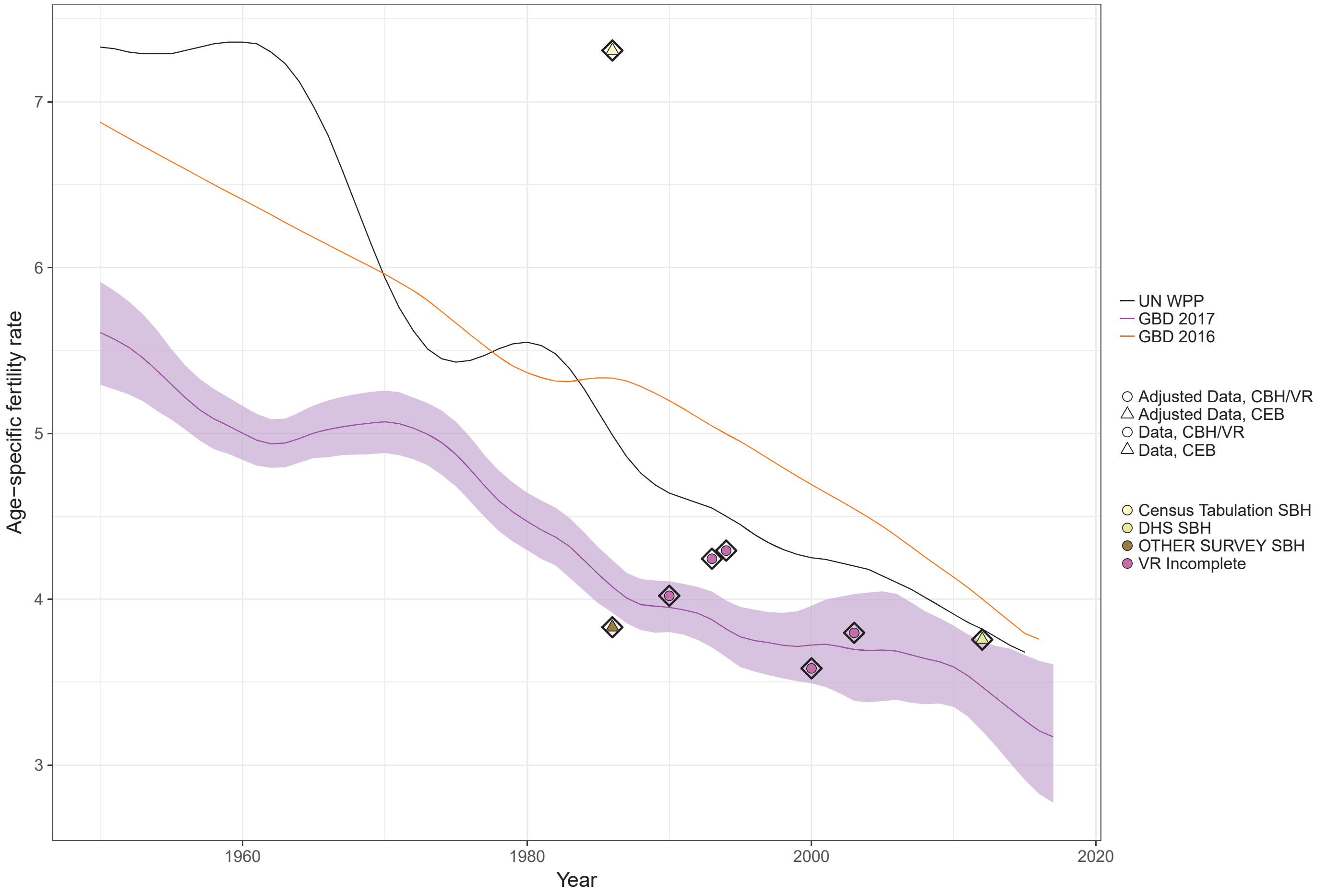

Age−specific fertility fitted trend lines, 1950−2017: Tonga, Ages: 10−14
Data points enclosed with a diamond are reference sources

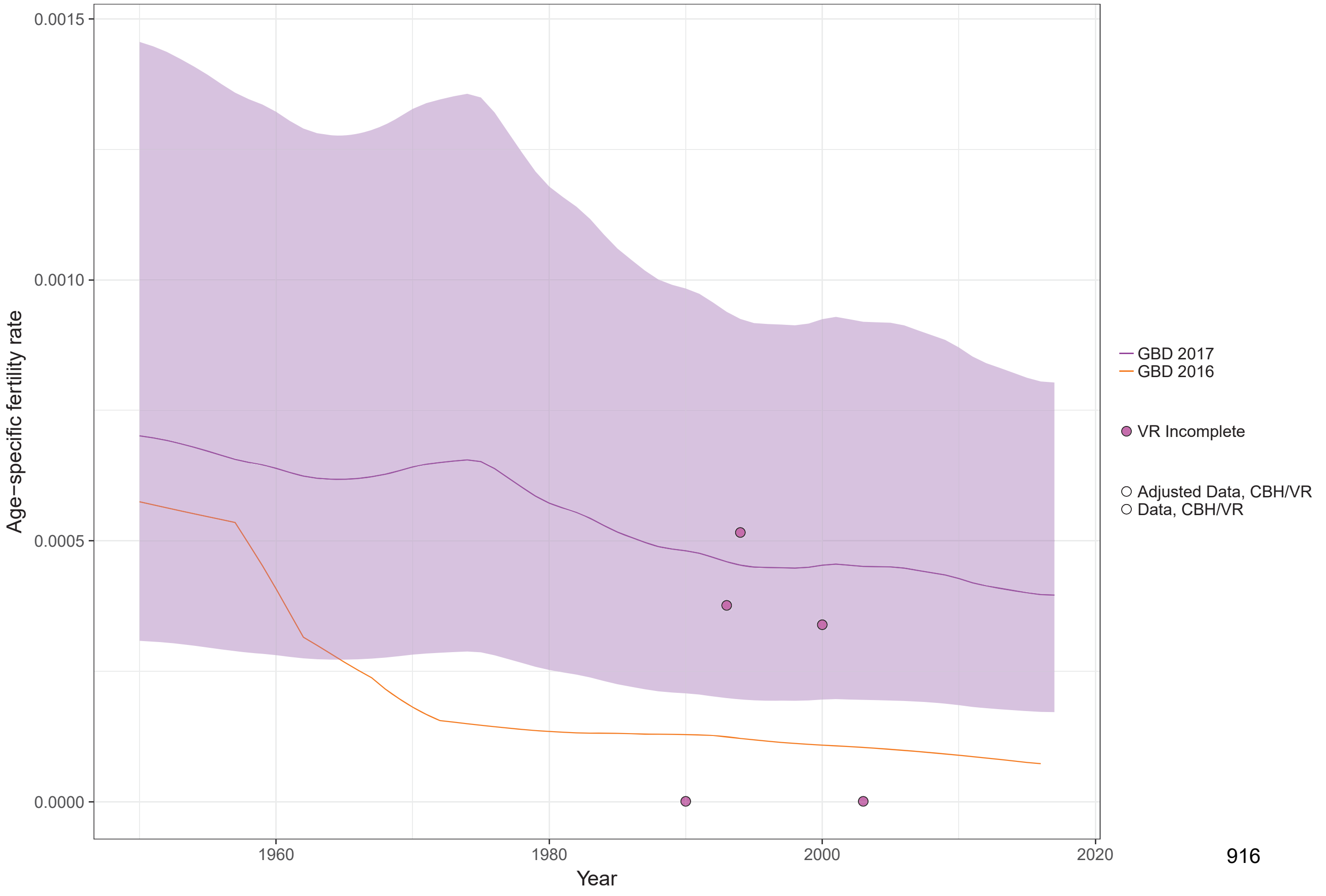

Age−specific fertility fitted trend lines, 1950−2017: Tonga, Ages: 15−19
Data points enclosed with a diamond are reference sources

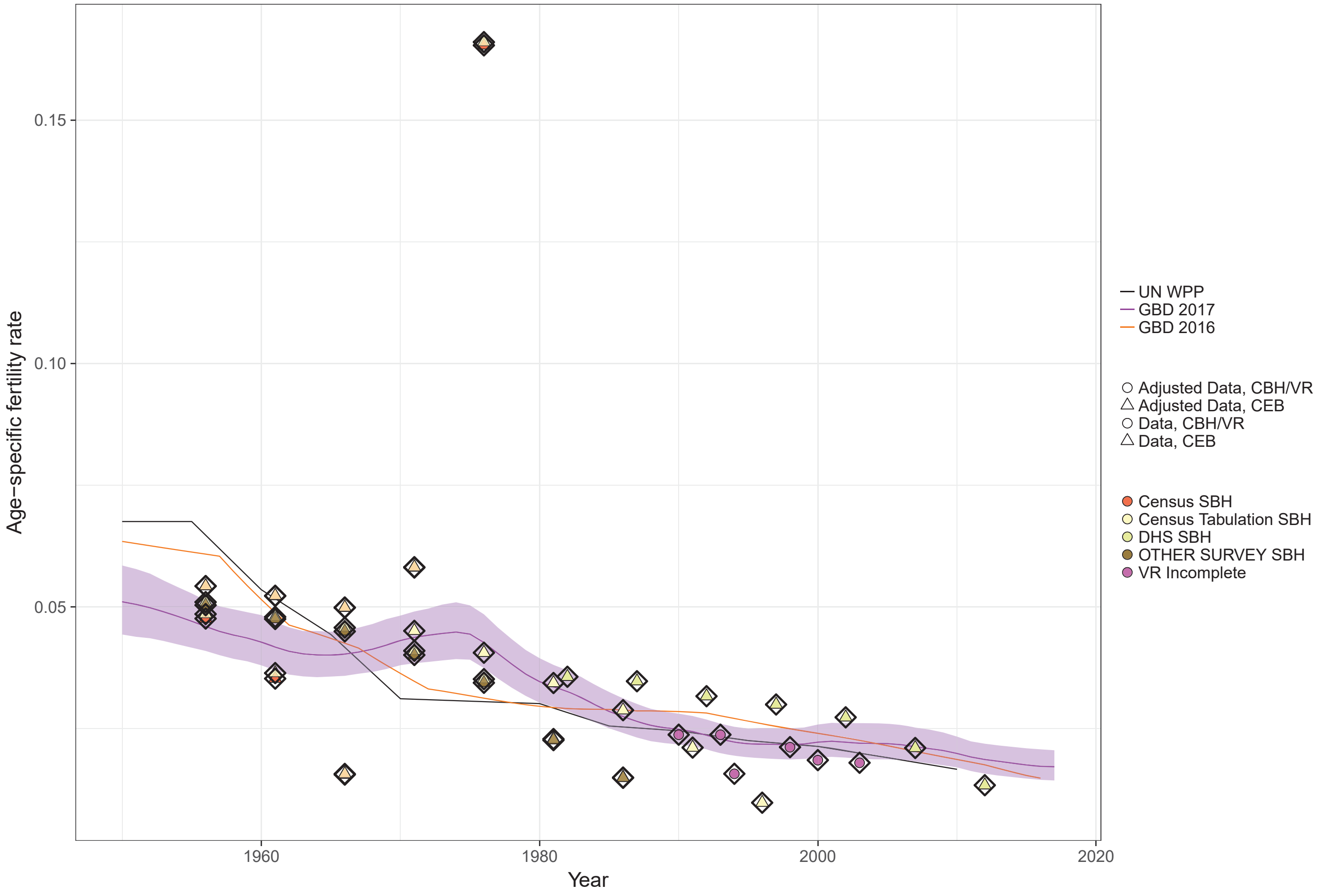

Age−specific fertility fitted trend lines, 1950−2017: Tonga, Ages: 20−24
Data points enclosed with a diamond are reference sources

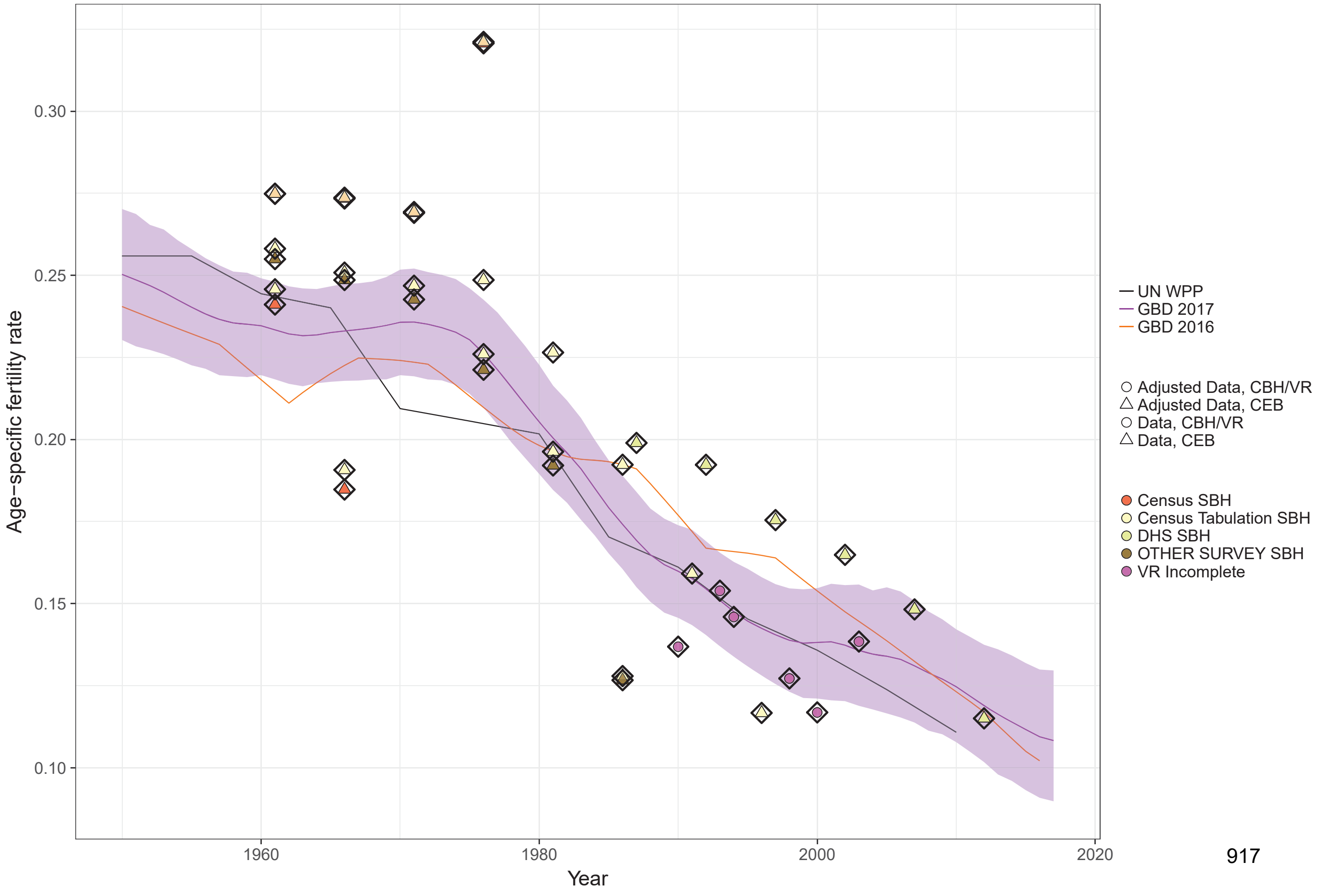

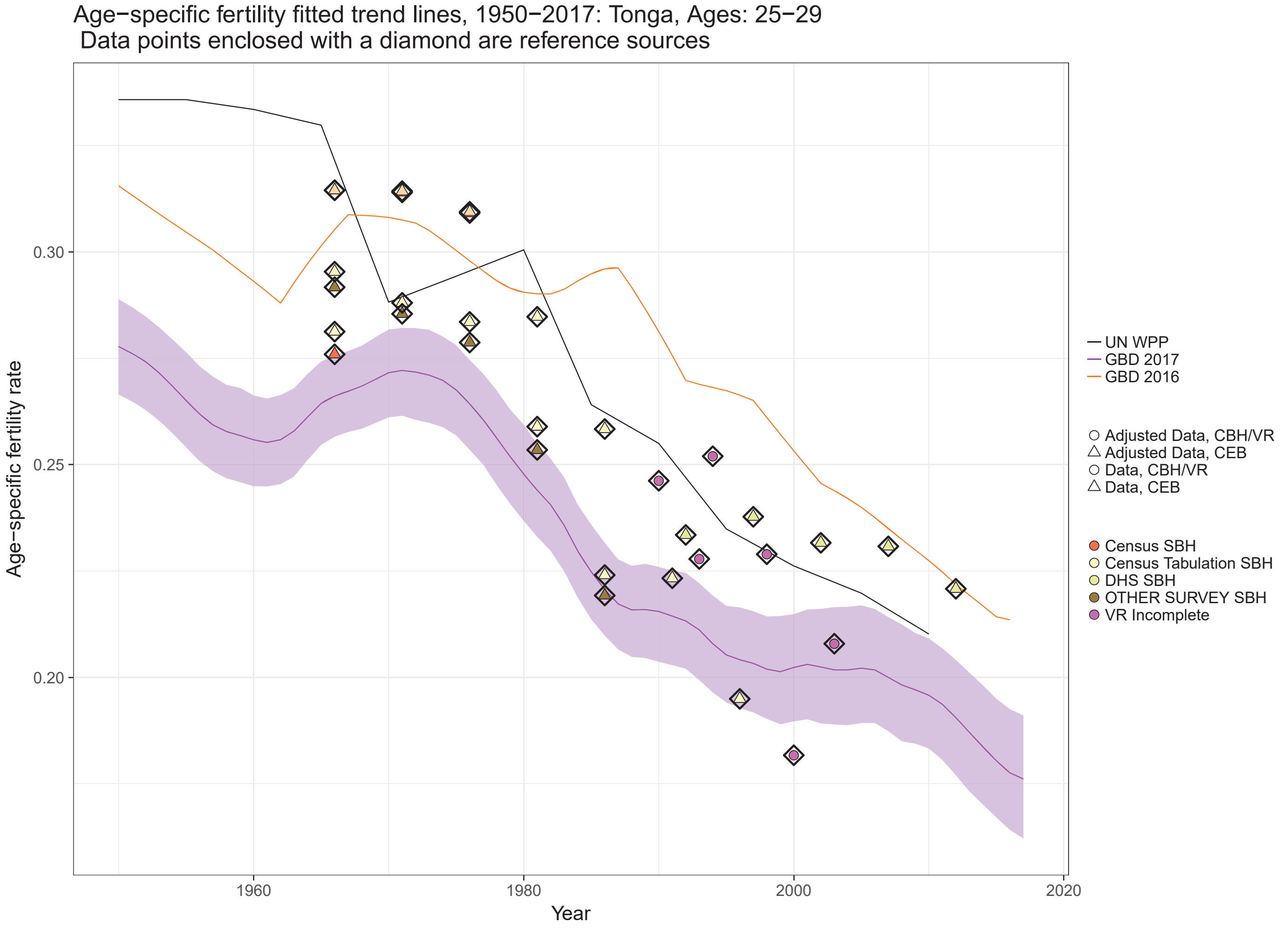
Age−specific fertility fitted trend lines, 1950−2017: Tonga, Ages: 25−29
Data points enclosed with a diamond are reference sources
Age−specific fertility rate
0.20
0.25
0.30
Year
1960
1980
2000
2020
UN WPP
GBD 2017
GBD 2016
Adjusted Data, CBH/VR
Adjusted Data, CEB
Data, CBH/VR
Data, CEB
Census SBH
Census Tabulation SBH
DHS SBH
OTHER SURVEY SBH
VR Incomplete

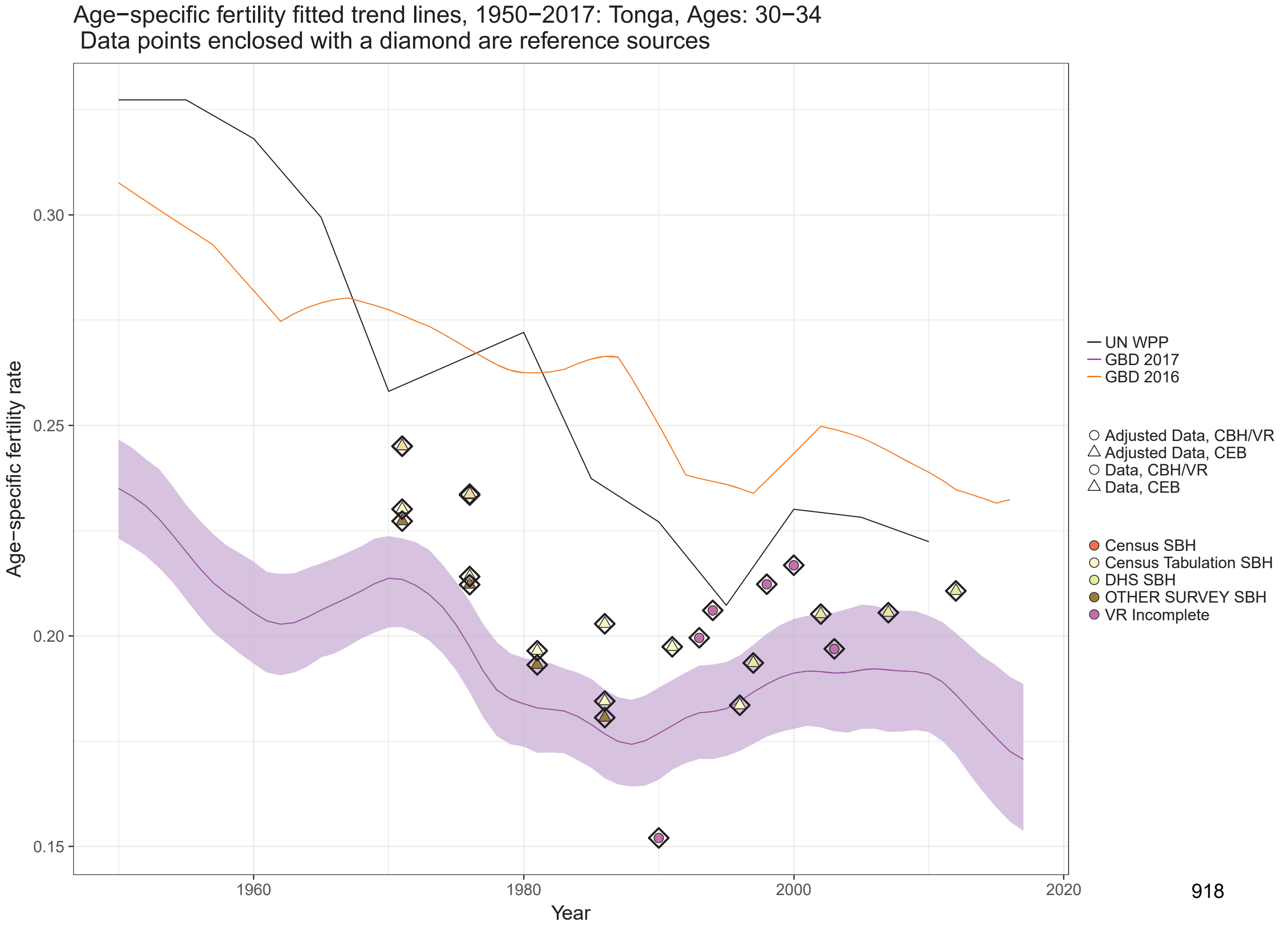
Age−specific fertility fitted trend lines, 1950−2017: Tonga, Ages: 30−34
Data points enclosed with a diamond are reference sources
Age−specific fertility rate
0.15
0.20
0.25
0.30
Year
1960
1980
2000
2020
UN WPP
GBD 2017
GBD 2016
Adjusted Data, CBH/VR
Adjusted Data, CEB
Data, CBH/VR
Data, CEB
Census SBH
Census Tabulation SBH
DHS SBH
OTHER SURVEY SBH
VR Incomplete

Age−specific fertility fitted trend lines, 1950−2017: Tonga, Ages: 35−39
Data points enclosed with a diamond are reference sources

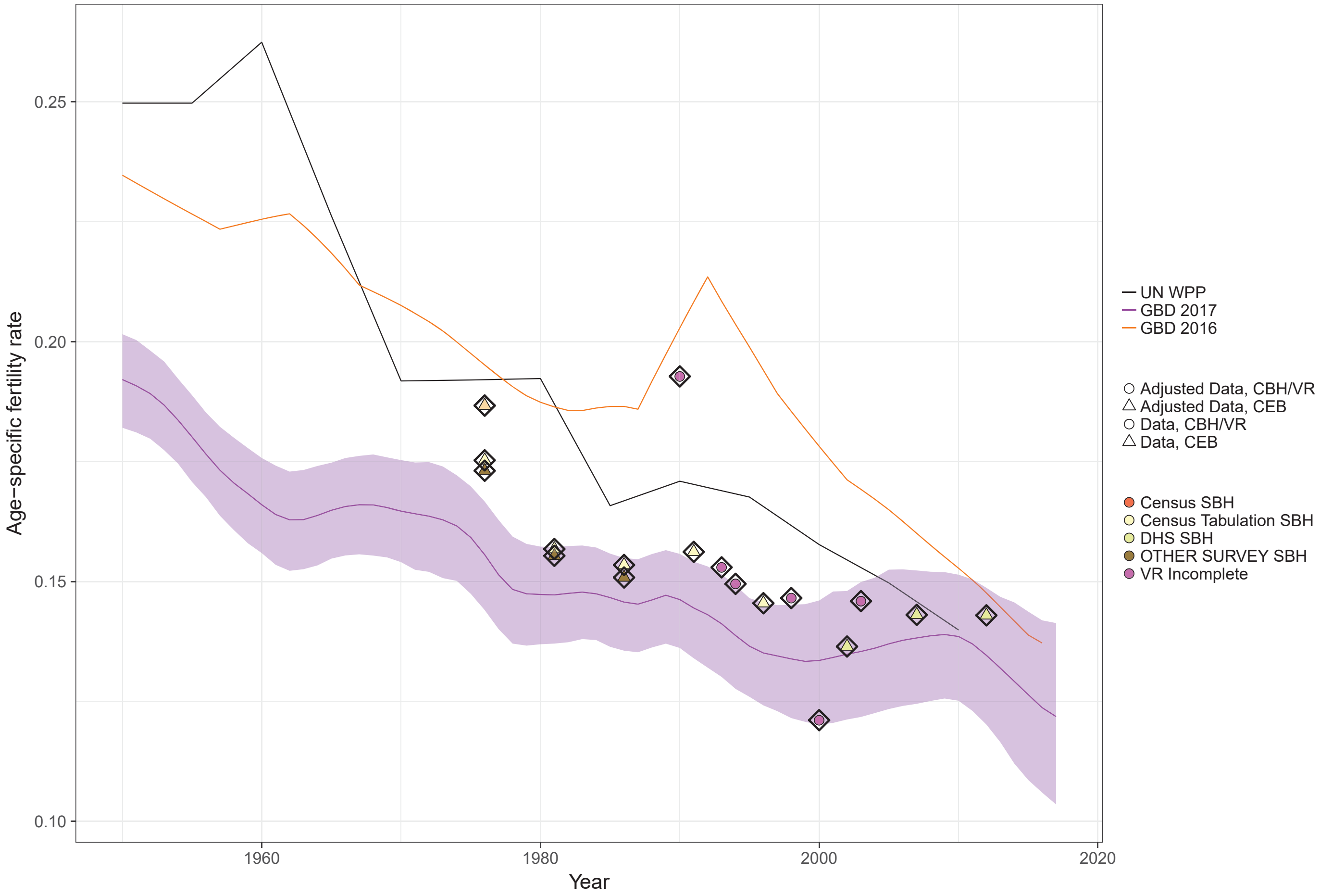

Age−specific fertility fitted trend lines, 1950−2017: Tonga, Ages: 40−44
Data points enclosed with a diamond are reference sources

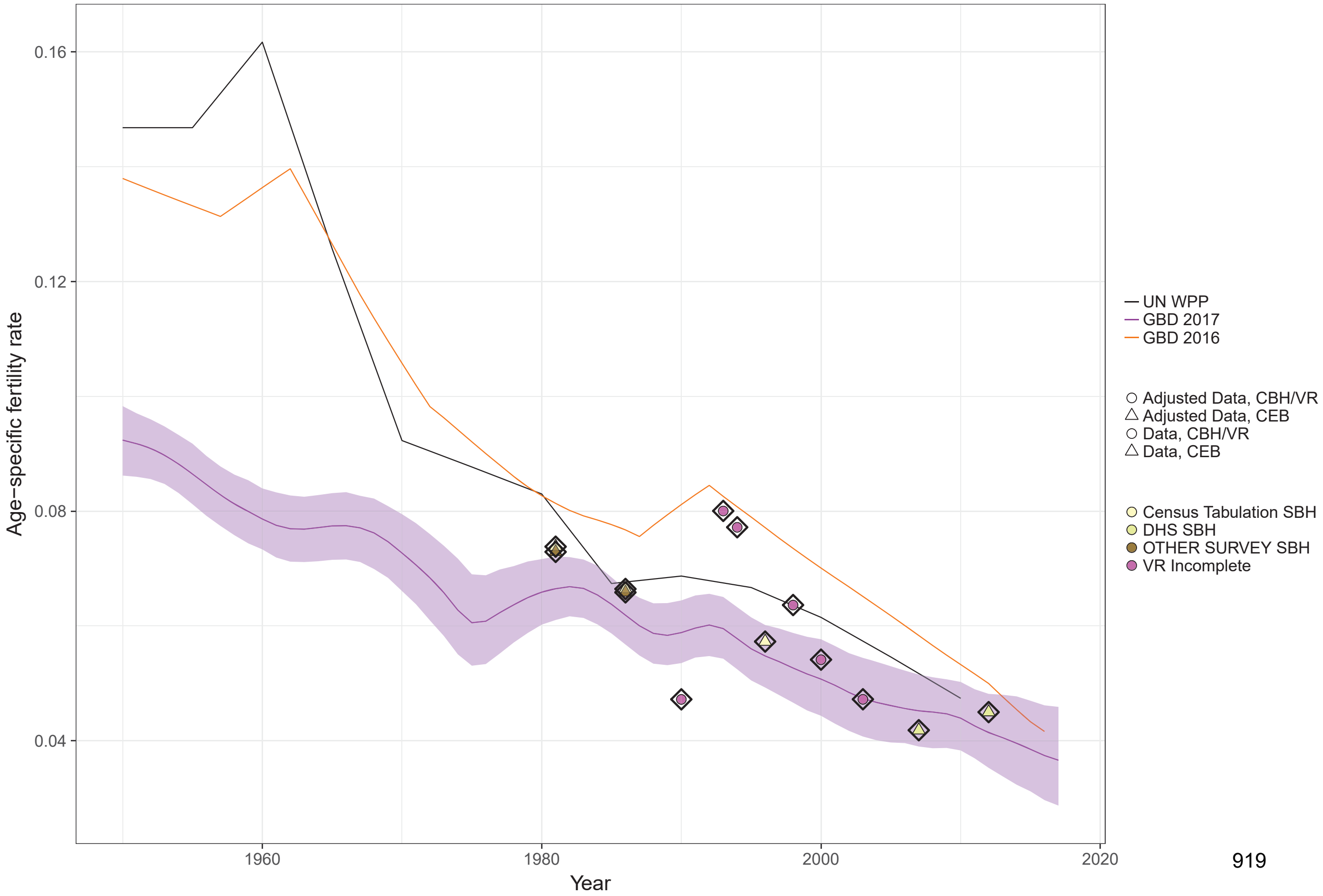

Age−specific fertility fitted trend lines, 1950−2017: Tonga, Ages: 45−49
Data points enclosed with a diamond are reference sources

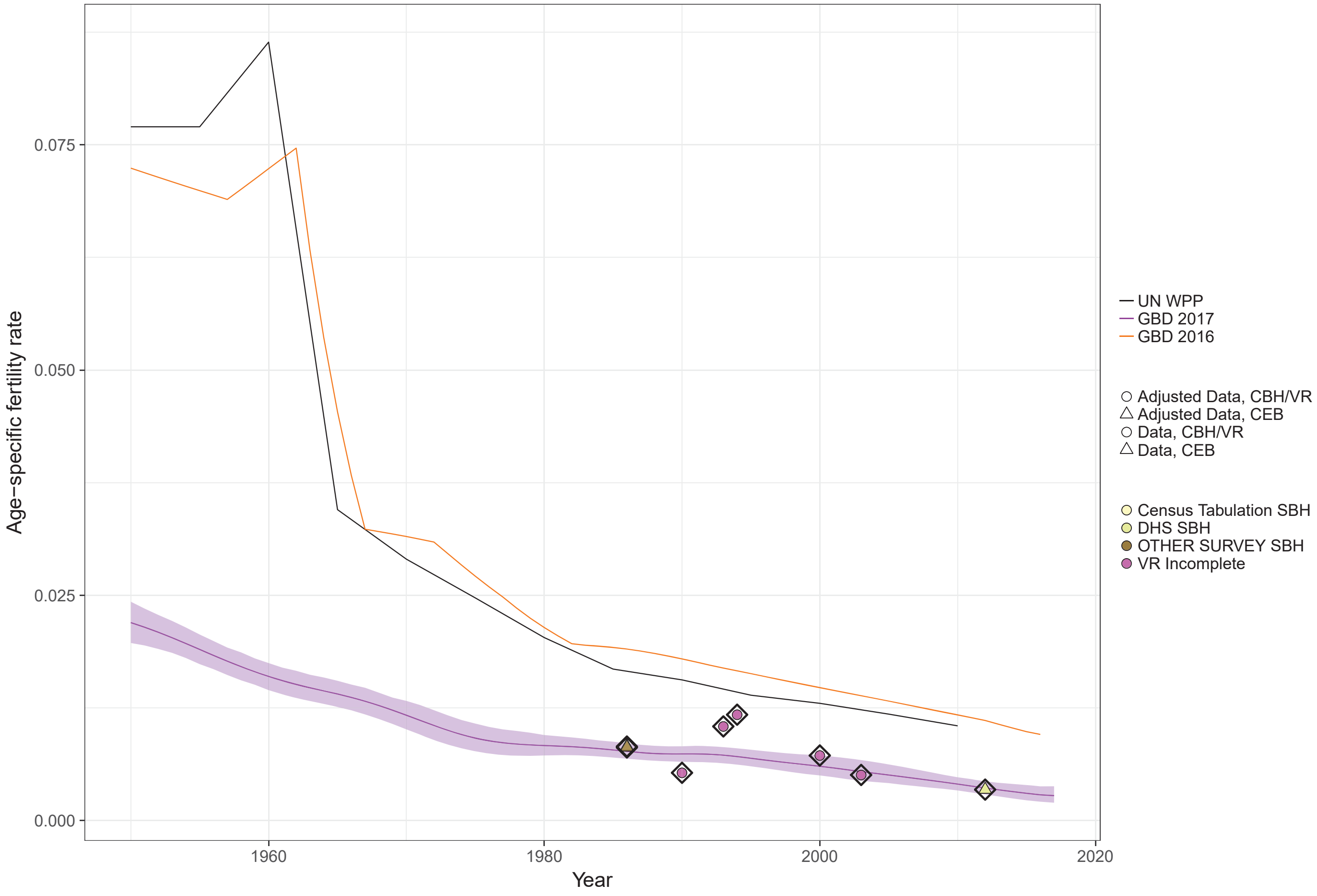

Age−specific fertility fitted trend lines, 1950−2017: Tonga, Ages: 50−54
Data points enclosed with a diamond are reference sources

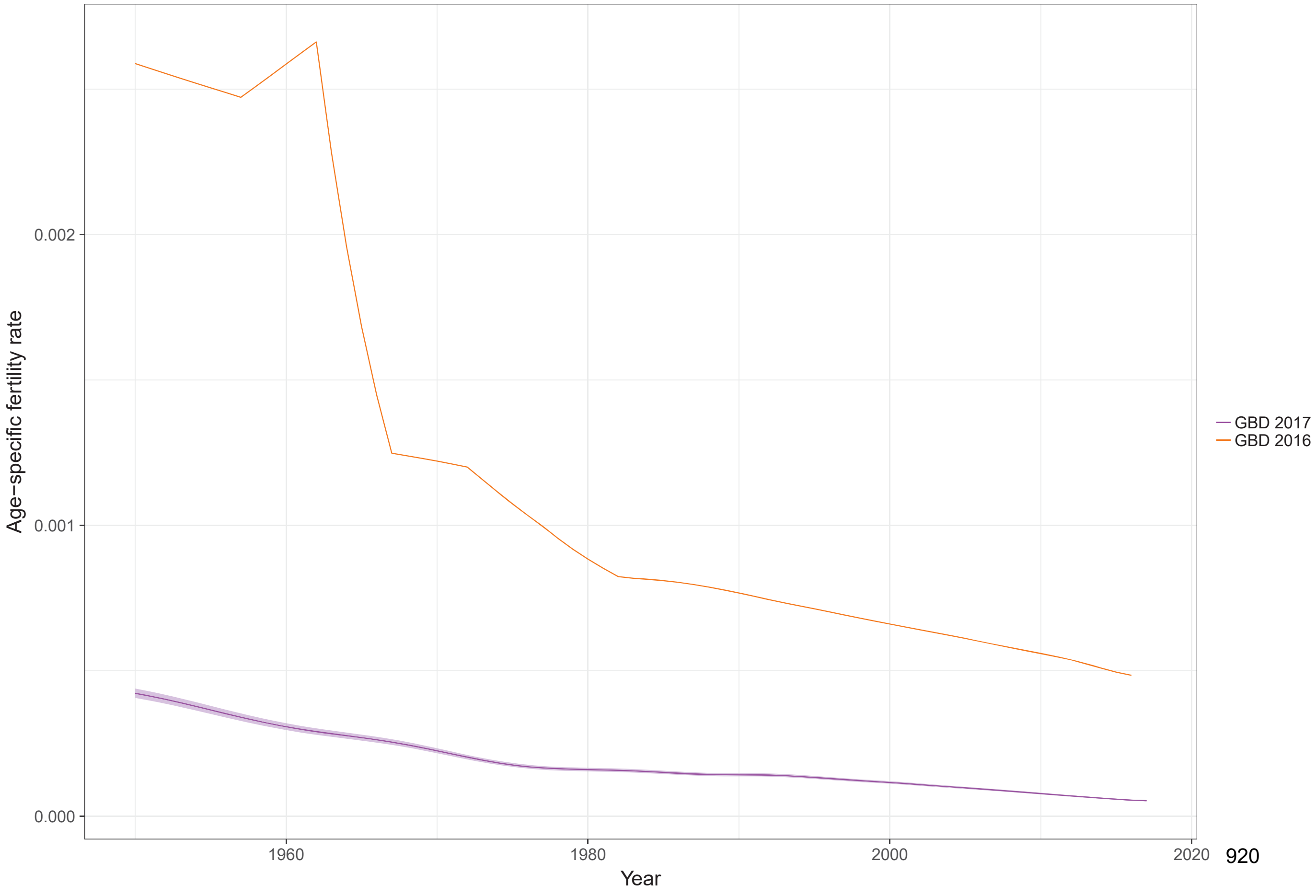

Age−specific fertility fitted trend lines, 1950−2017: Trinidad and Tobago, TFR
Data points enclosed with a diamond are reference sources

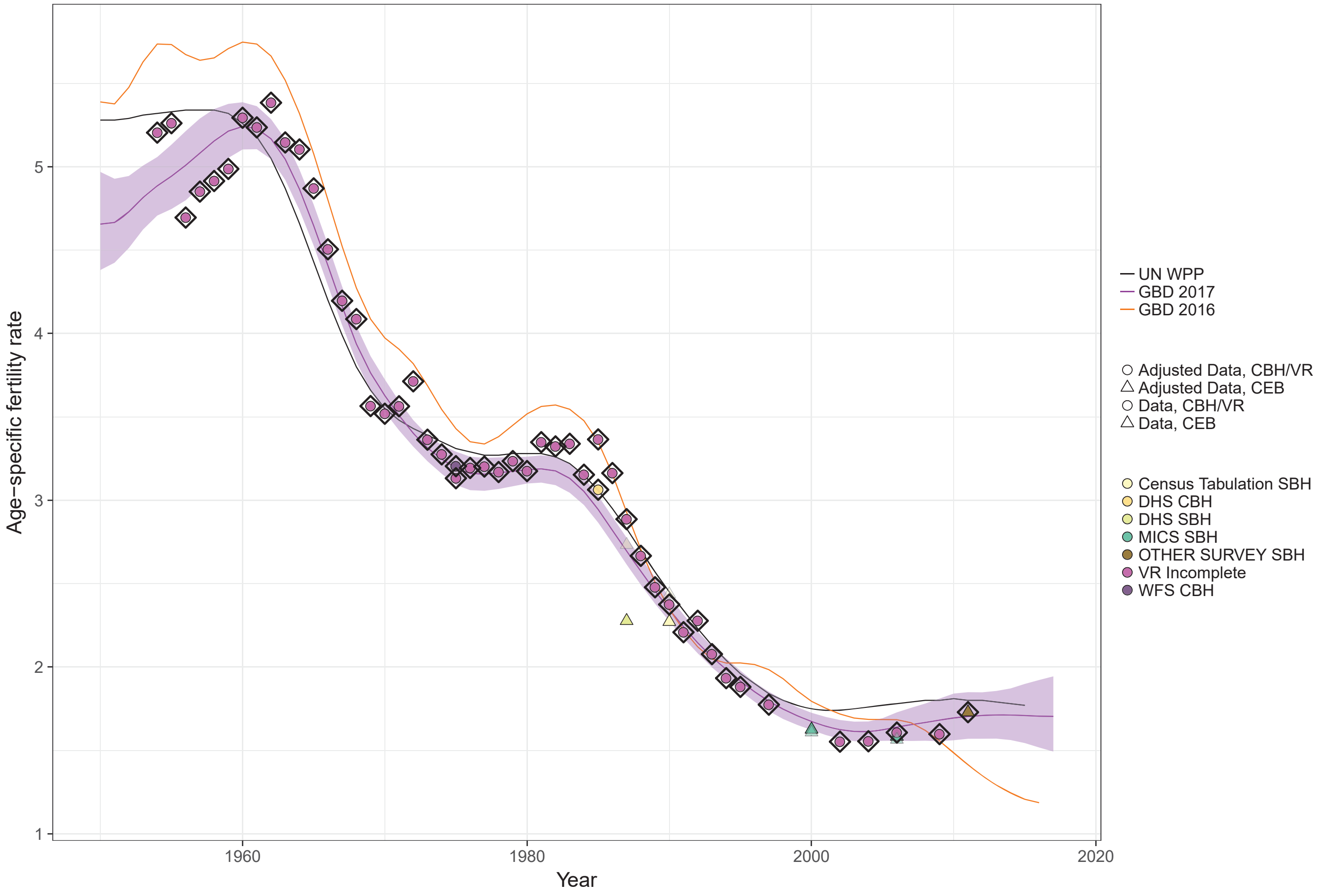

Age−specific fertility fitted trend lines, 1950−2017: Trinidad and Tobago, Ages: 10−14
Data points enclosed with a diamond are reference sources

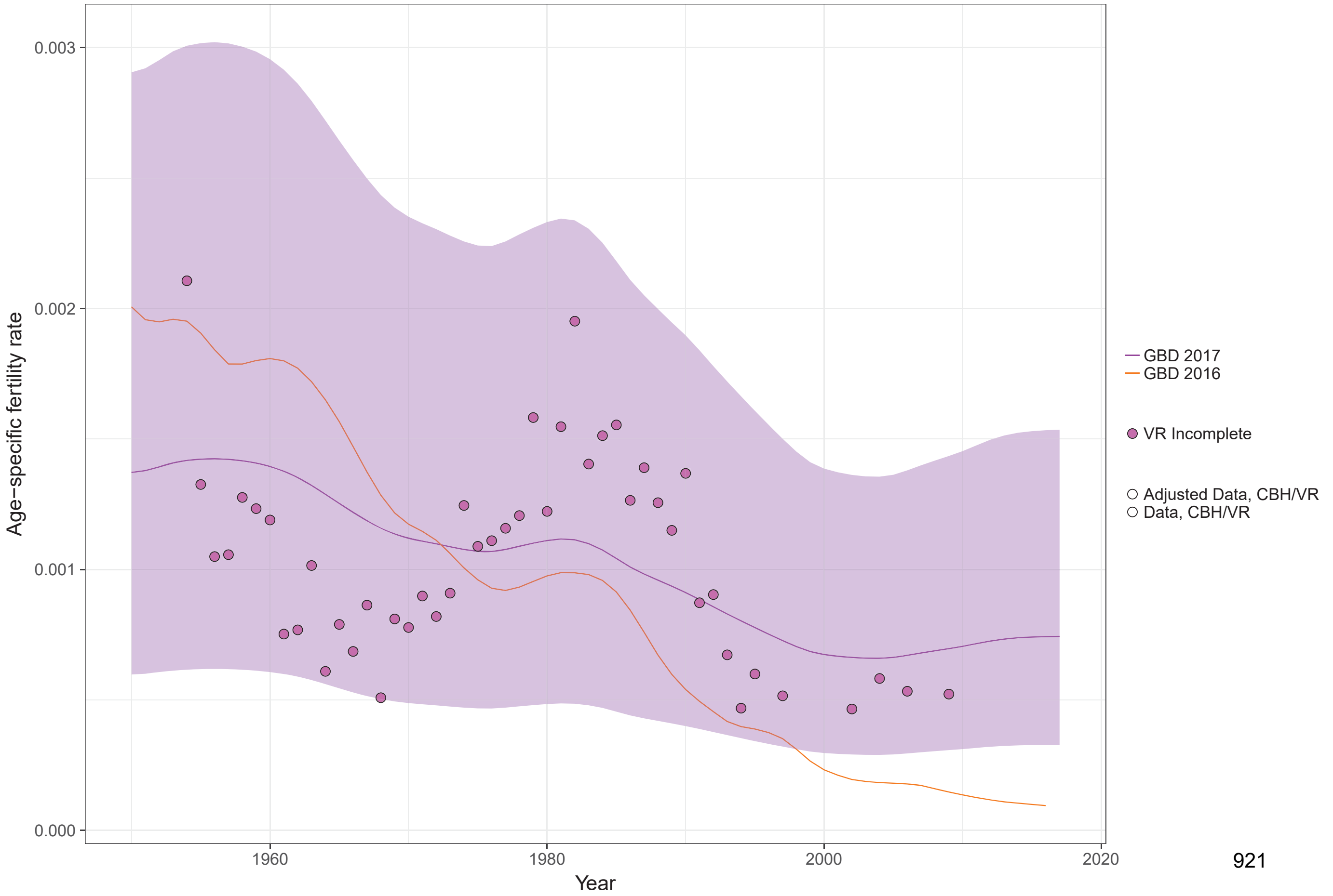

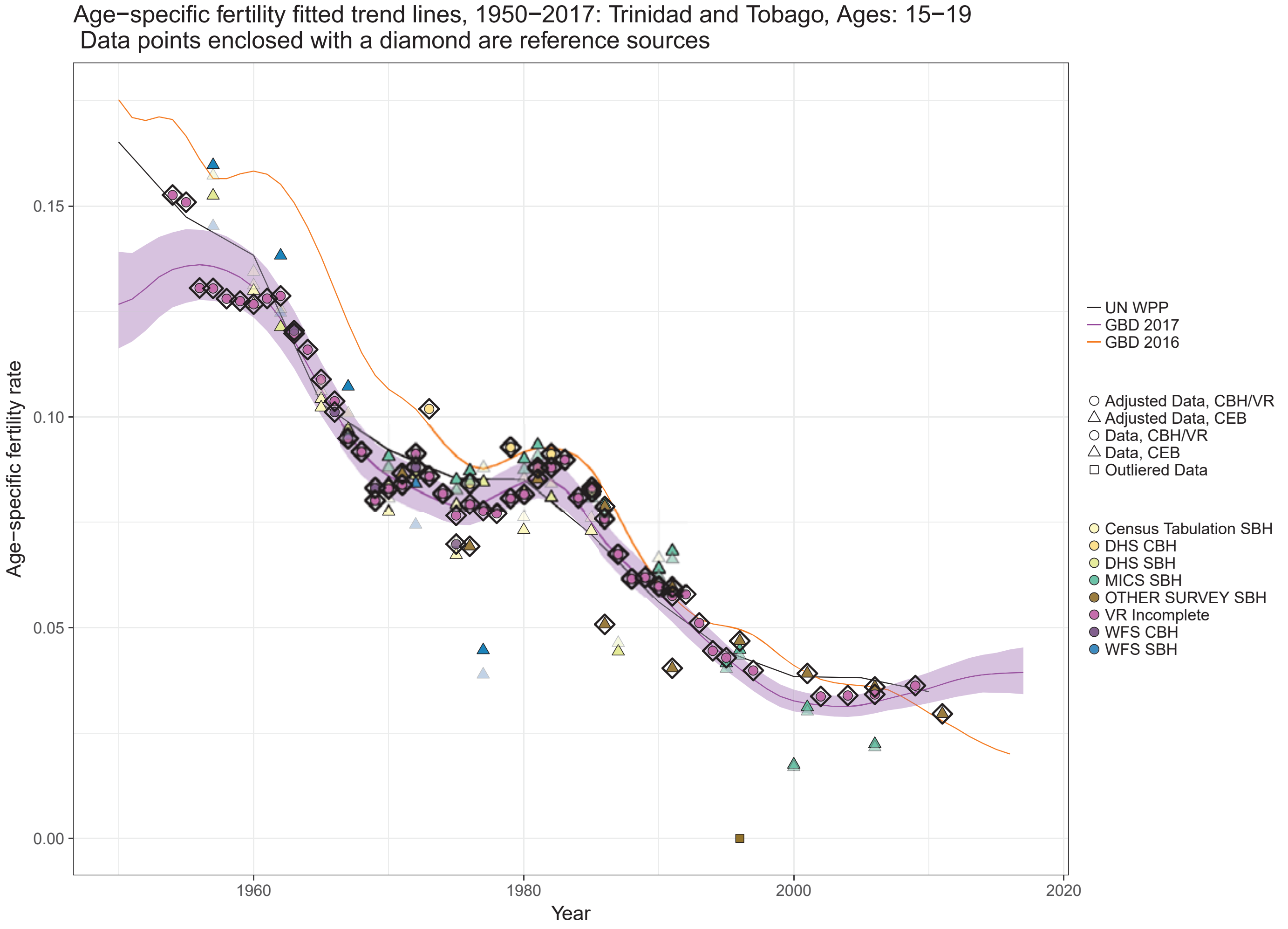

Age−specific fertility fitted trend lines, 1950−2017: Trinidad and Tobago, Ages: 15−19
Data points enclosed with a diamond are reference sources
Age-specific fertility rate
Year
0.00
0.05
0.10
0.15
1960
1980
2000
2020
UN WPP
GBD 2017
GBD 2016
Adjusted Data, CBH/VR
Adjusted Data, CEB
Data, CBH/VR
Data, CEB
Outliered Data
Census Tabulation SBH
DHS CBH
DHS SBH
MICS SBH
OTHER SURVEY SBH
VR Incomplete
WFS CBH
WFS SBH

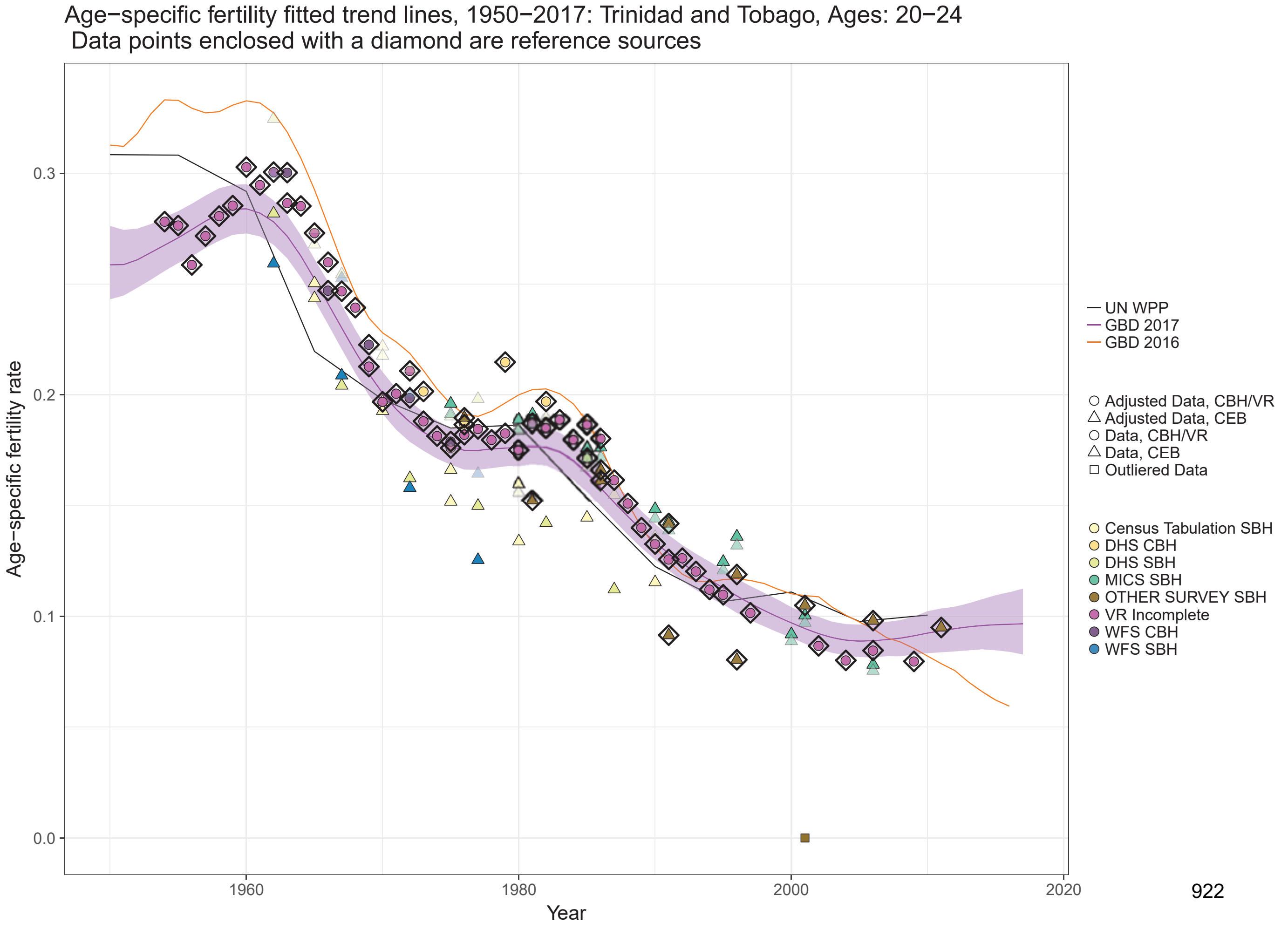

Age−specific fertility fitted trend lines, 1950−2017: Trinidad and Tobago, Ages: 20−24
Data points enclosed with a diamond are reference sources
Age-specific fertility rate
Year
0.0
0.1
0.2
0.3
1960
1980
2000
2020
UN WPP
GBD 2017
GBD 2016
Adjusted Data, CBH/VR
Adjusted Data, CEB
Data, CBH/VR
Data, CEB
Outliered Data
Census Tabulation SBH
DHS CBH
DHS SBH
MICS SBH
OTHER SURVEY SBH
VR Incomplete
WFS CBH
WFS SBH

Age−specific fertility fitted trend lines, 1950−2017: Trinidad and Tobago, Ages: 25−29
Data points enclosed with a diamond are reference sources

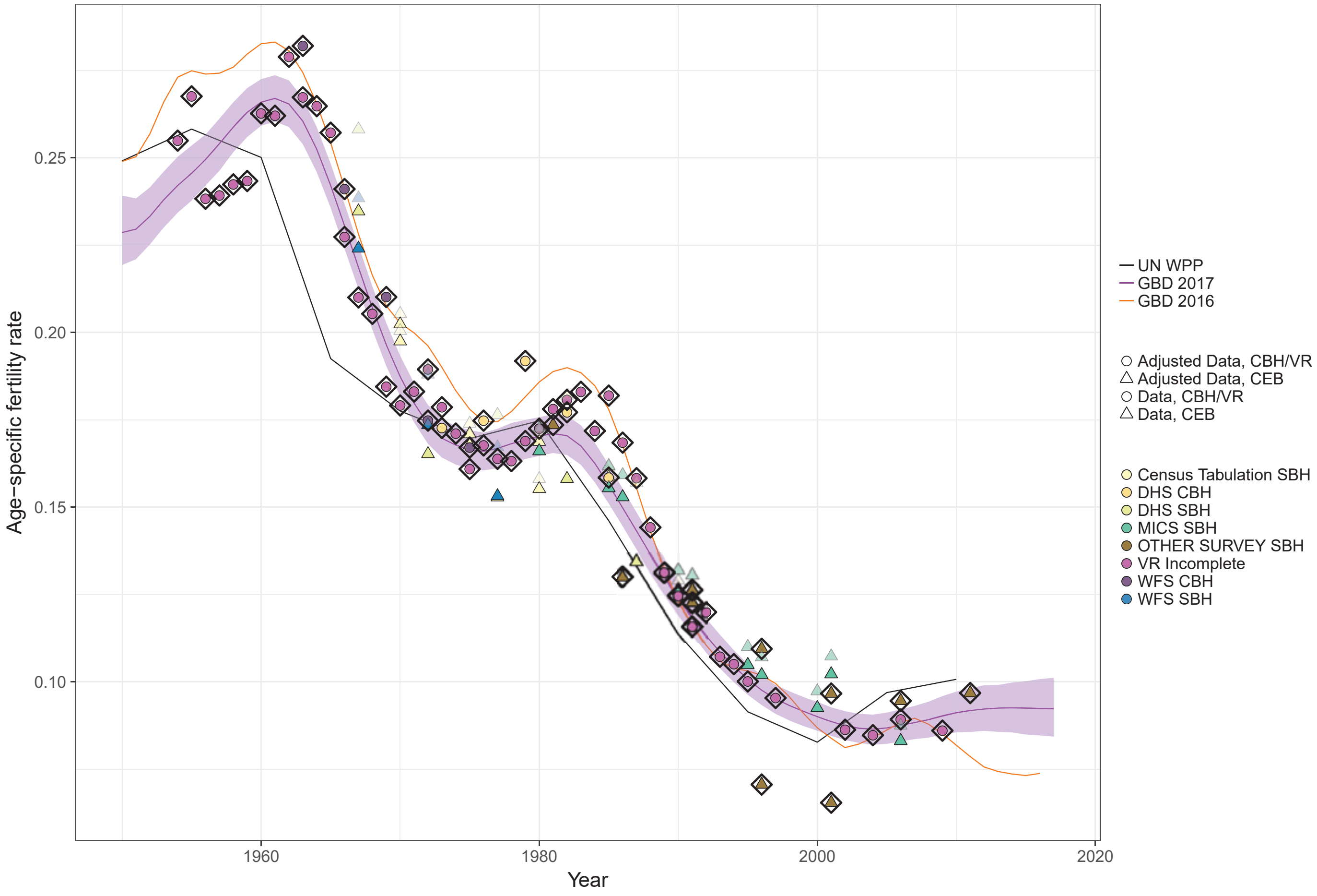

Age−specific fertility fitted trend lines, 1950−2017: Trinidad and Tobago, Ages: 30−34
Data points enclosed with a diamond are reference sources

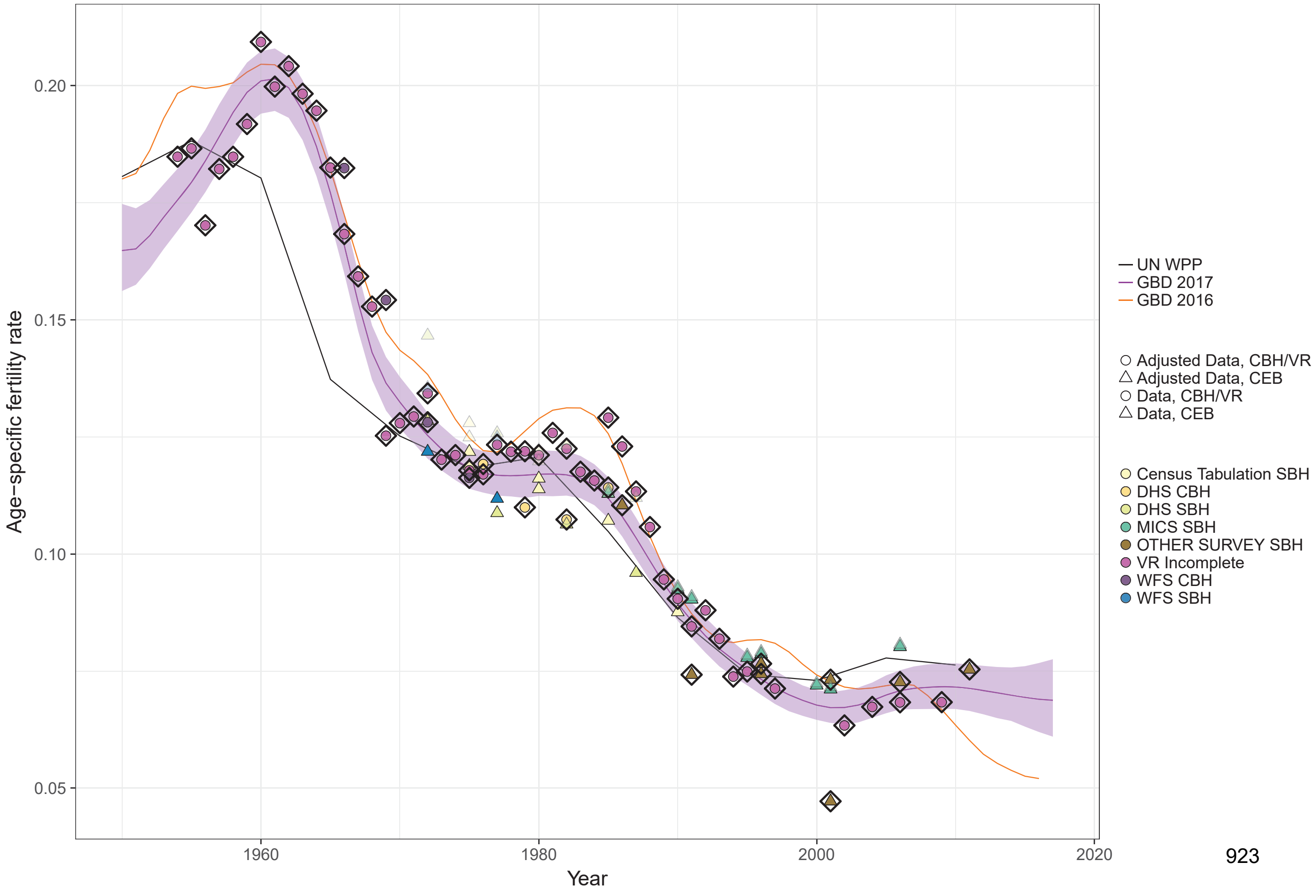

Age−specific fertility fitted trend lines, 1950−2017: Trinidad and Tobago, Ages: 35−39
Data points enclosed with a diamond are reference sources

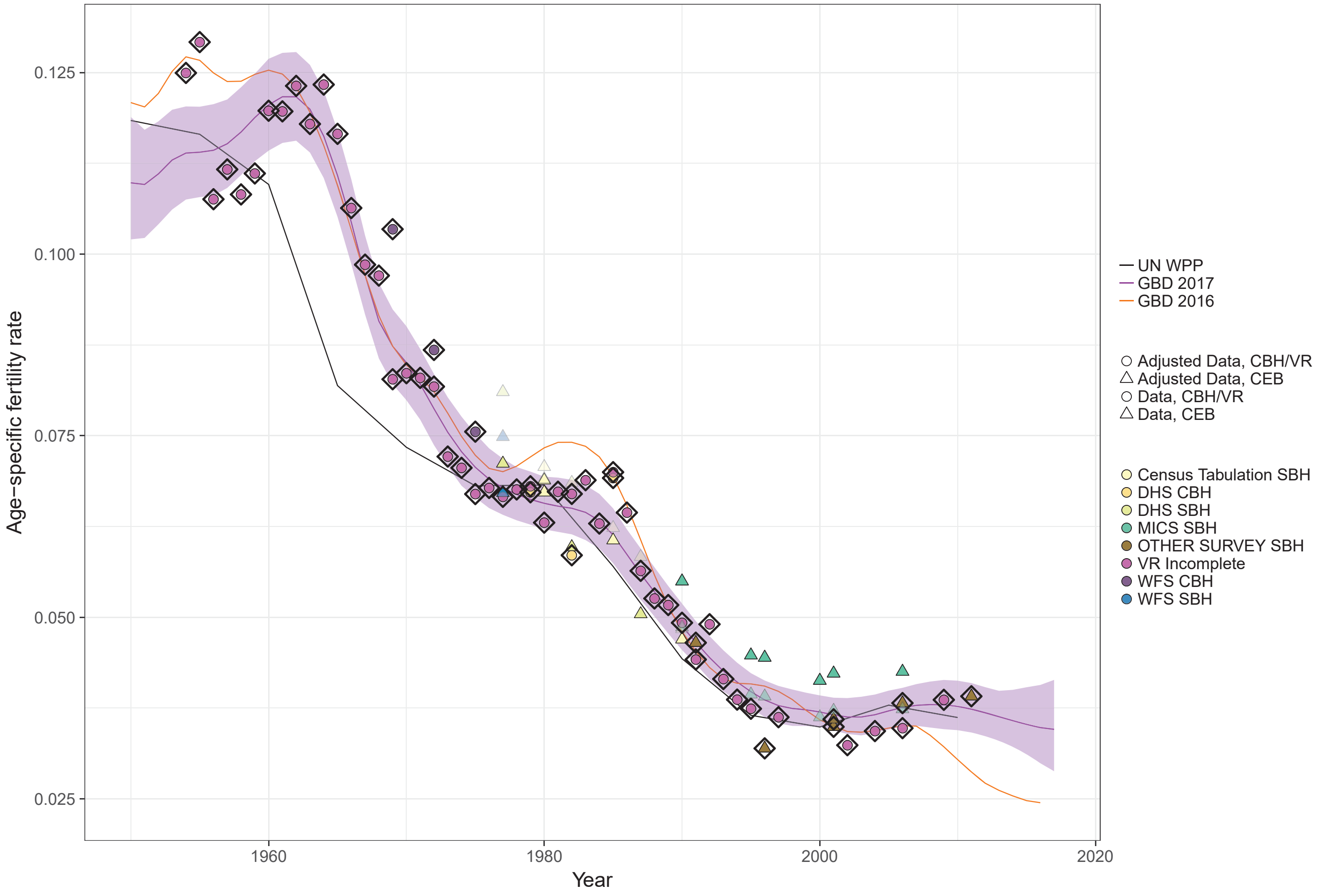

Age−specific fertility fitted trend lines, 1950−2017: Trinidad and Tobago, Ages: 40−44
Data points enclosed with a diamond are reference sources

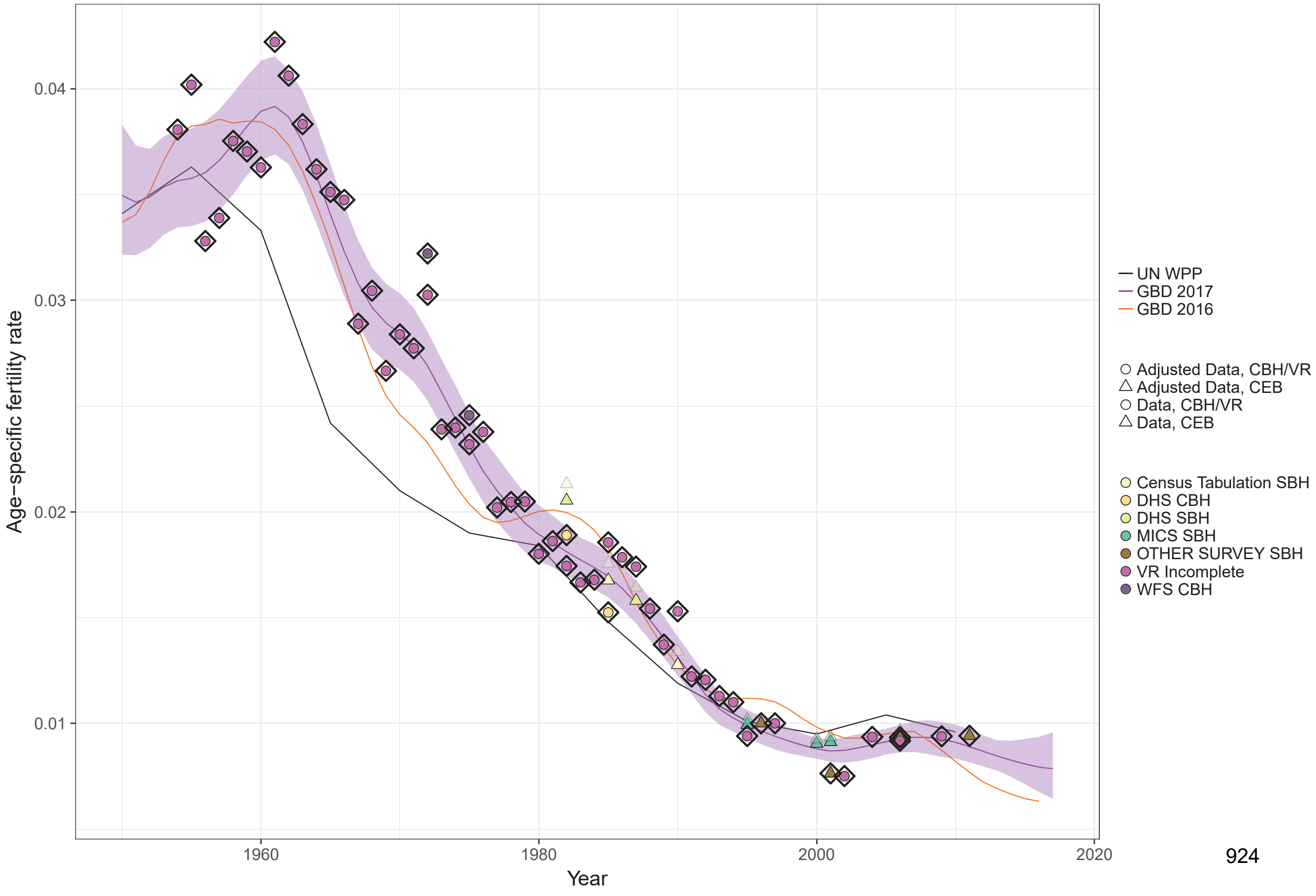

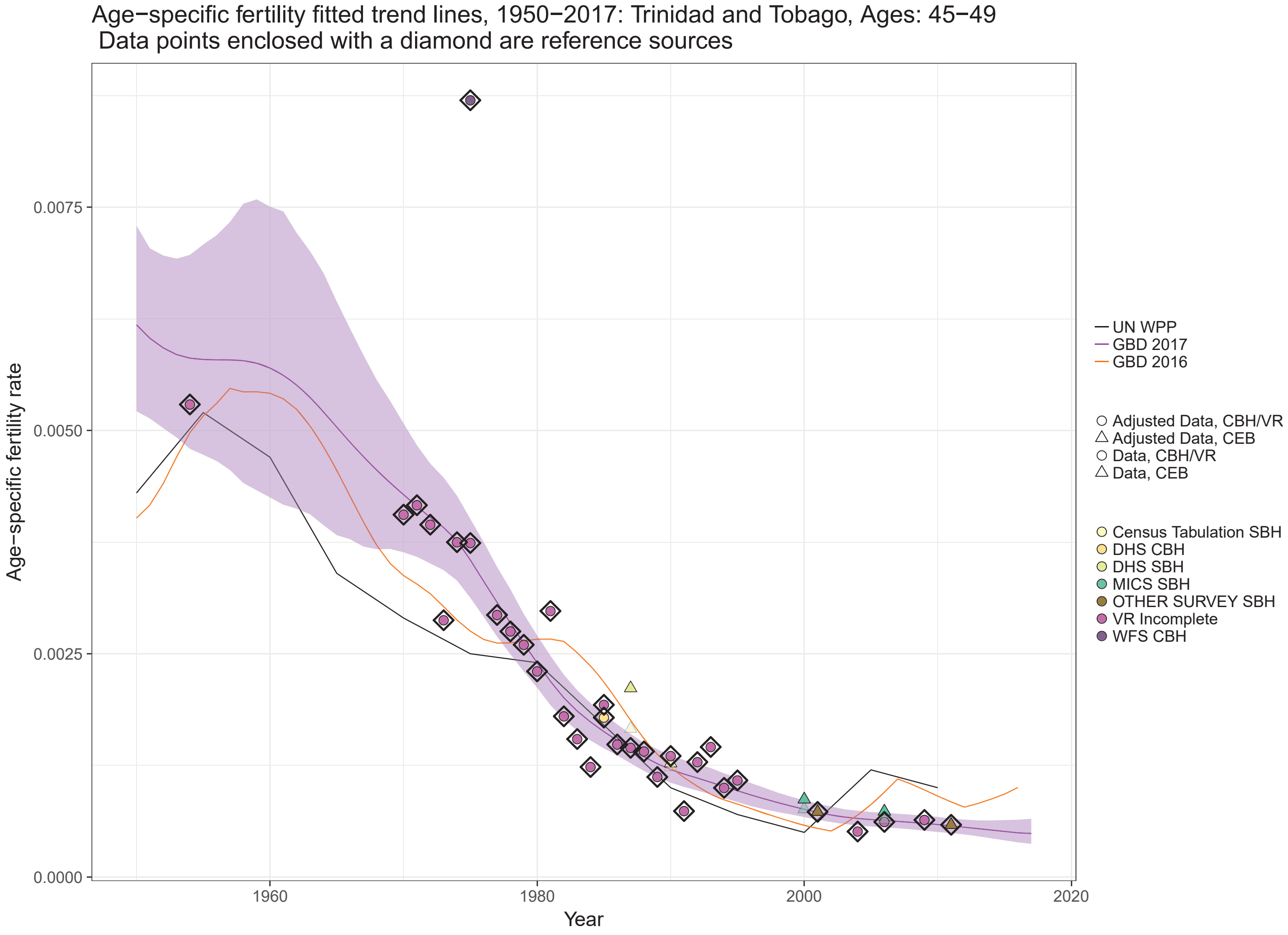
Age−specific fertility fitted trend lines, 1950−2017: Trinidad and Tobago, Ages: 45−49
Data points enclosed with a diamond are reference sources
Age−specific fertility rate
0.0075
0.0050
0.0025
0.0000
1960
1980
2000
2020
Year
UN WPP
GBD 2017
GBD 2016
Adjusted Data, CBH/VR
Adjusted Data, CEB
Data, CBH/VR
Data, CEB
Census Tabulation SBH
DHS CBH
DHS SBH
MICS SBH
OTHER SURVEY SBH
VR Incomplete
WFS CBH

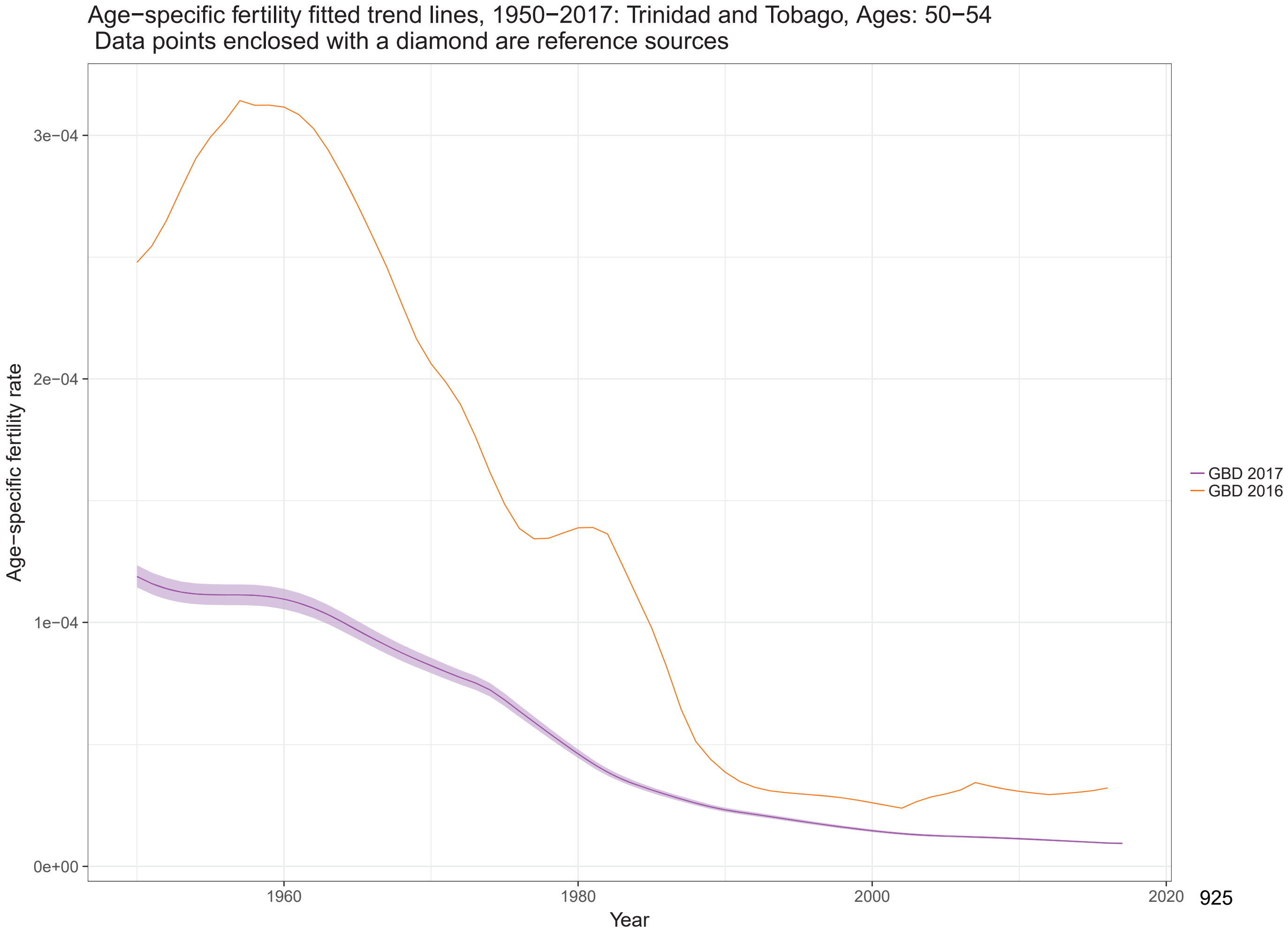
Age−specific fertility fitted trend lines, 1950−2017: Trinidad and Tobago, Ages: 50−54
Data points enclosed with a diamond are reference sources
Age−specific fertility rate
3e−04
2e−04
1e−04
0e+00
1960
1980
2000
2020
Year
GBD 2017
GBD 2016

Age−specific fertility fitted trend lines, 1950−2017: Tunisia, TFR
Data points enclosed with a diamond are reference sources

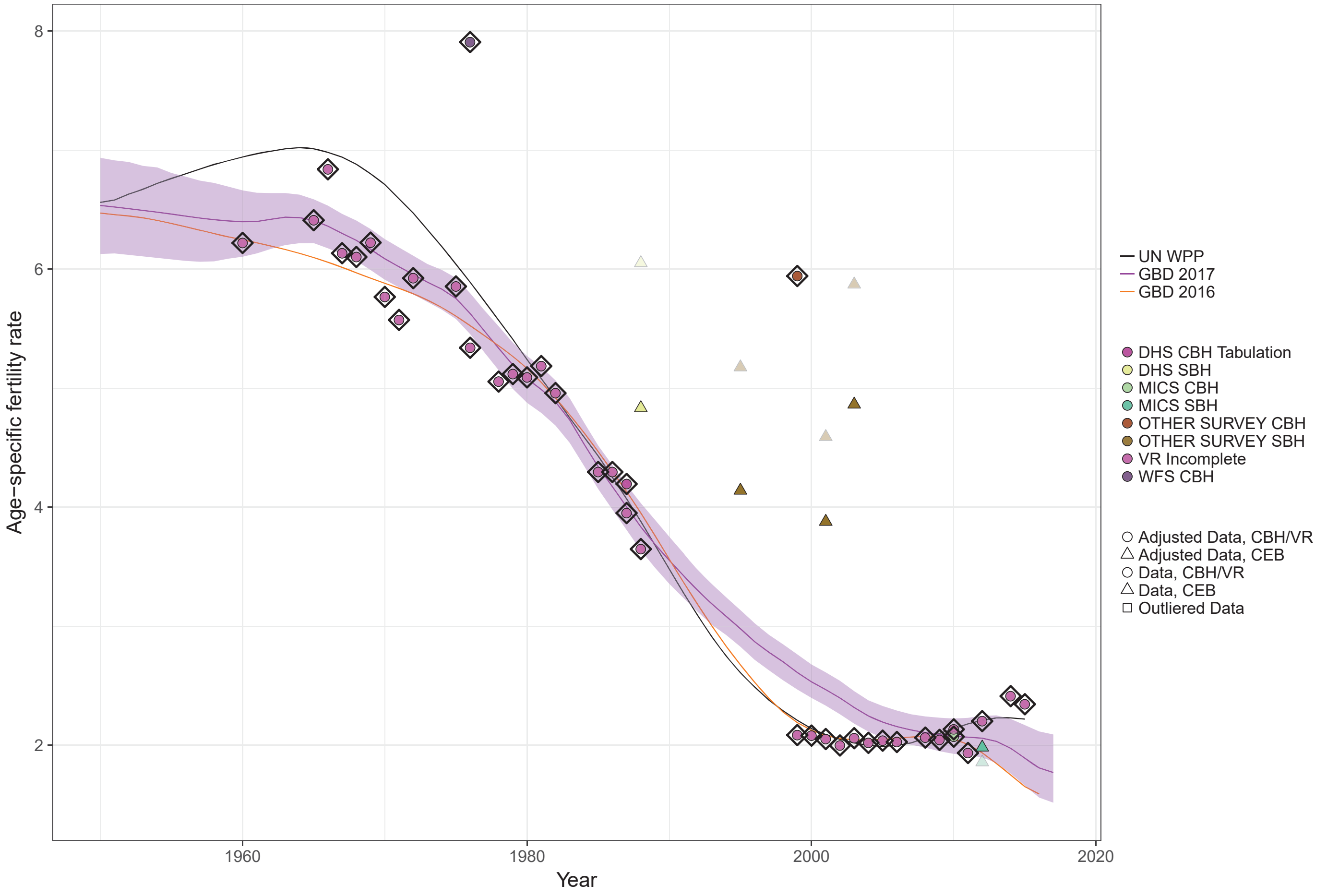

Age−specific fertility fitted trend lines, 1950−2017: Tunisia, Ages: 10−14
Data points enclosed with a diamond are reference sources

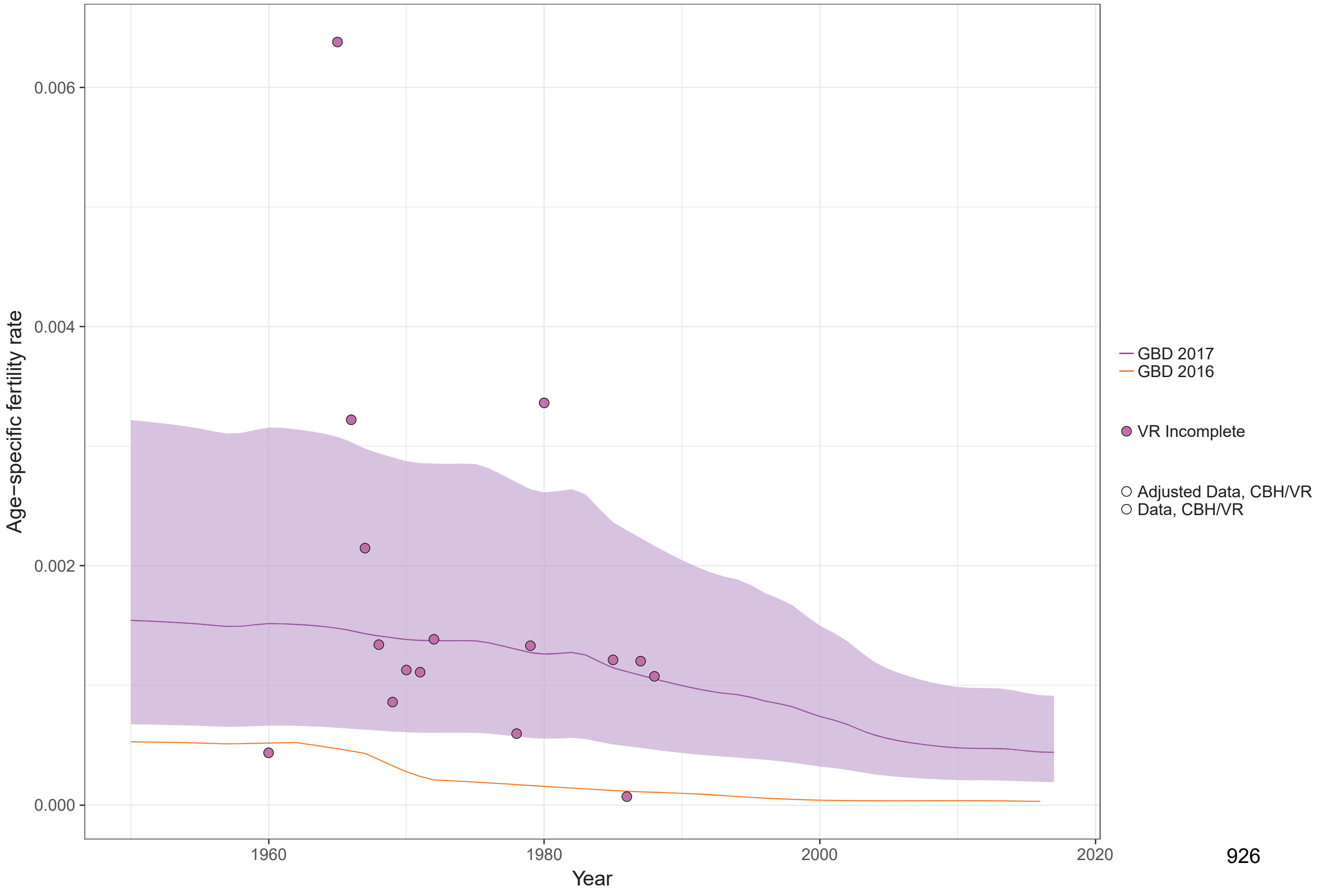

Age−specific fertility fitted trend lines, 1950−2017: Tunisia, Ages: 15−19
Data points enclosed with a diamond are reference sources

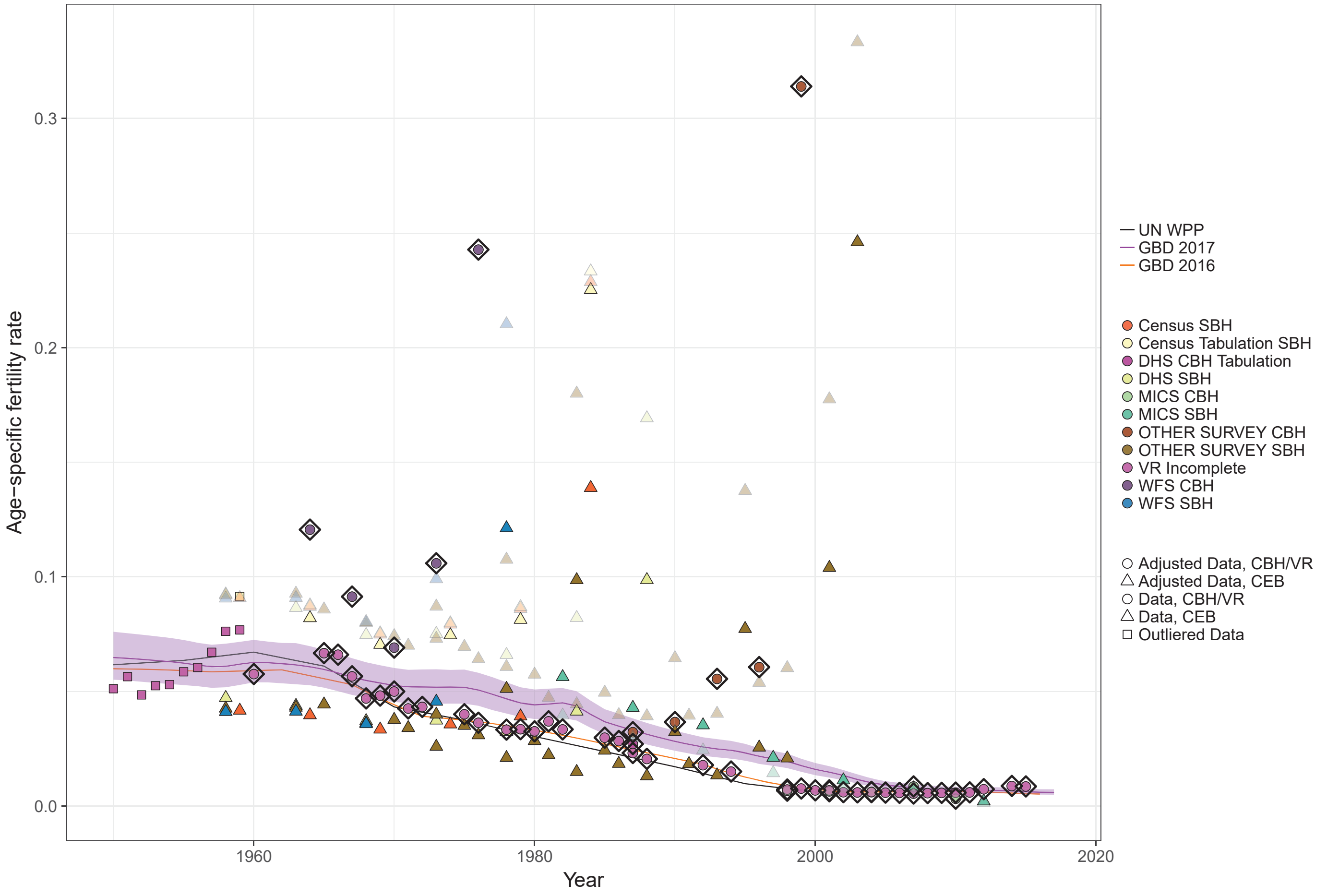

Age−specific fertility fitted trend lines, 1950−2017: Tunisia, Ages: 20−24
Data points enclosed with a diamond are reference sources

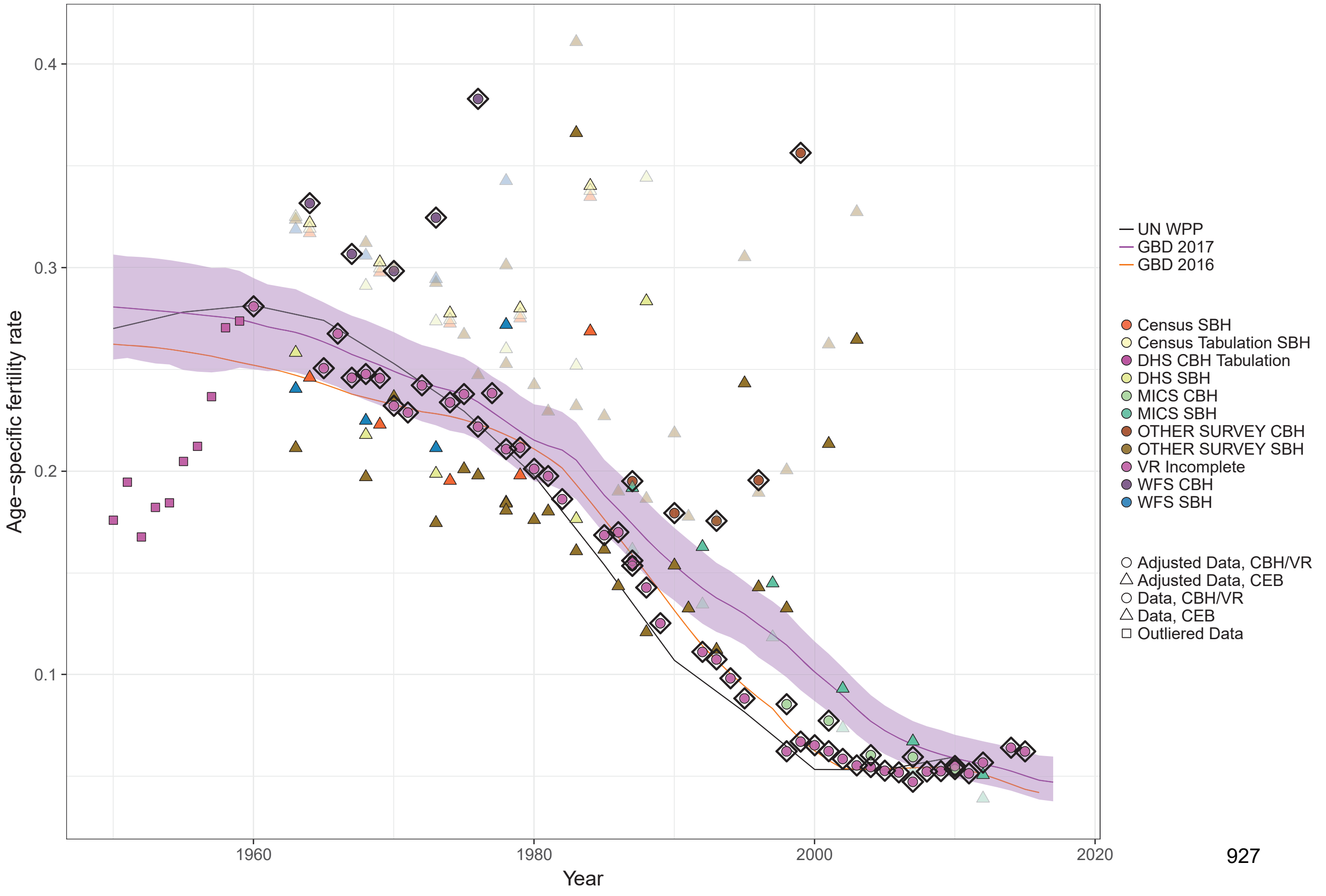

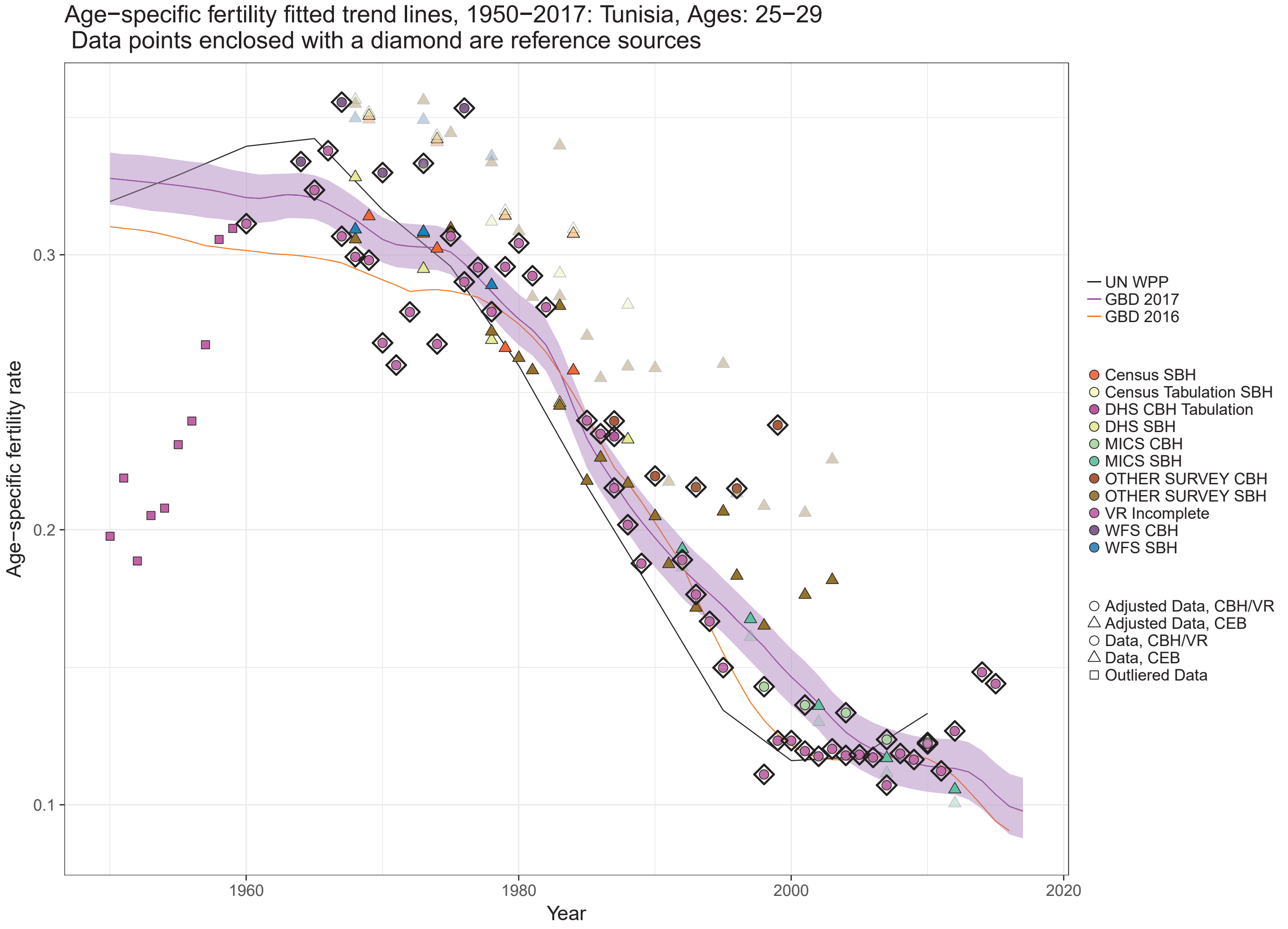

Age−specific fertility fitted trend lines, 1950−2017: Tunisia, Ages: 25−29
Data points enclosed with a diamond are reference sources
Age−specific fertility rate
Year
0.1
0.2
0.3
1960
1980
2000
2020
UN WPP
GBD 2017
GBD 2016
Census SBH
Census Tabulation SBH
DHS CBH Tabulation
DHS SBH
MICS CBH
MICS SBH
OTHER SURVEY CBH
OTHER SURVEY SBH
VR Incomplete
WFS CBH
WFS SBH
Adjusted Data, CBH/VR
Adjusted Data, CEB
Data, CBH/VR
Data, CEB
Outliered Data

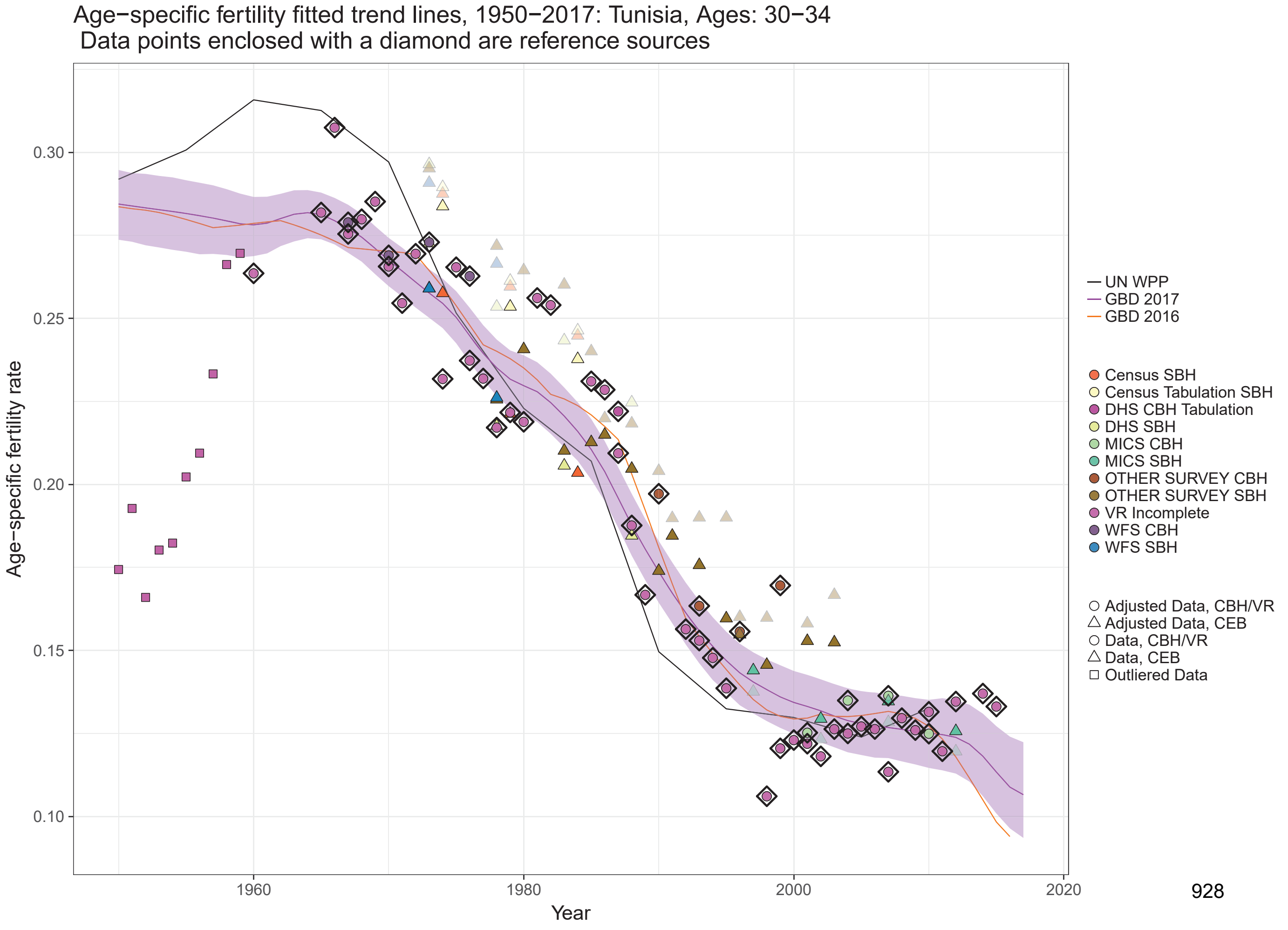

Age−specific fertility fitted trend lines, 1950−2017: Tunisia, Ages: 30−34
Data points enclosed with a diamond are reference sources
Age−specific fertility rate
Year
0.10
0.15
0.20
0.25
0.30
1960
1980
2000
2020
UN WPP
GBD 2017
GBD 2016
Census SBH
Census Tabulation SBH
DHS CBH Tabulation
DHS SBH
MICS CBH
MICS SBH
OTHER SURVEY CBH
OTHER SURVEY SBH
VR Incomplete
WFS CBH
WFS SBH
Adjusted Data, CBH/VR
Adjusted Data, CEB
Data, CBH/VR
Data, CEB
Outliered Data

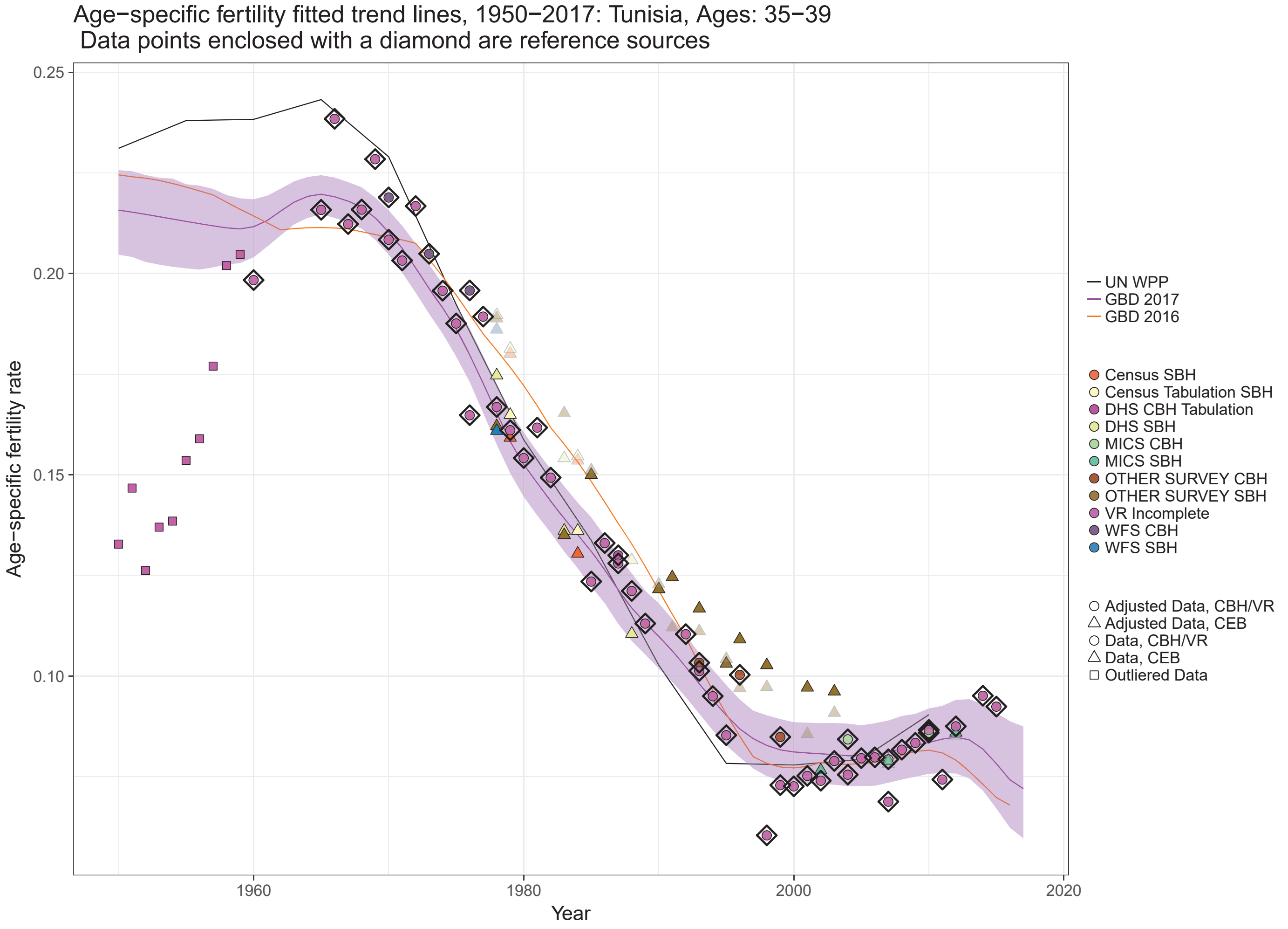
Age−specific fertility fitted trend lines, 1950−2017: Tunisia, Ages: 35−39
Data points enclosed with a diamond are reference sources
Age−specific fertility rate
Year
UN WPP
GBD 2017
GBD 2016
Census SBH
Census Tabulation SBH
DHS CBH Tabulation
DHS SBH
MICS CBH
MICS SBH
OTHER SURVEY CBH
OTHER SURVEY SBH
VR Incomplete
WFS CBH
WFS SBH
Adjusted Data, CBH/VR
Adjusted Data, CEB
Data, CBH/VR
Data, CEB
Outliered Data

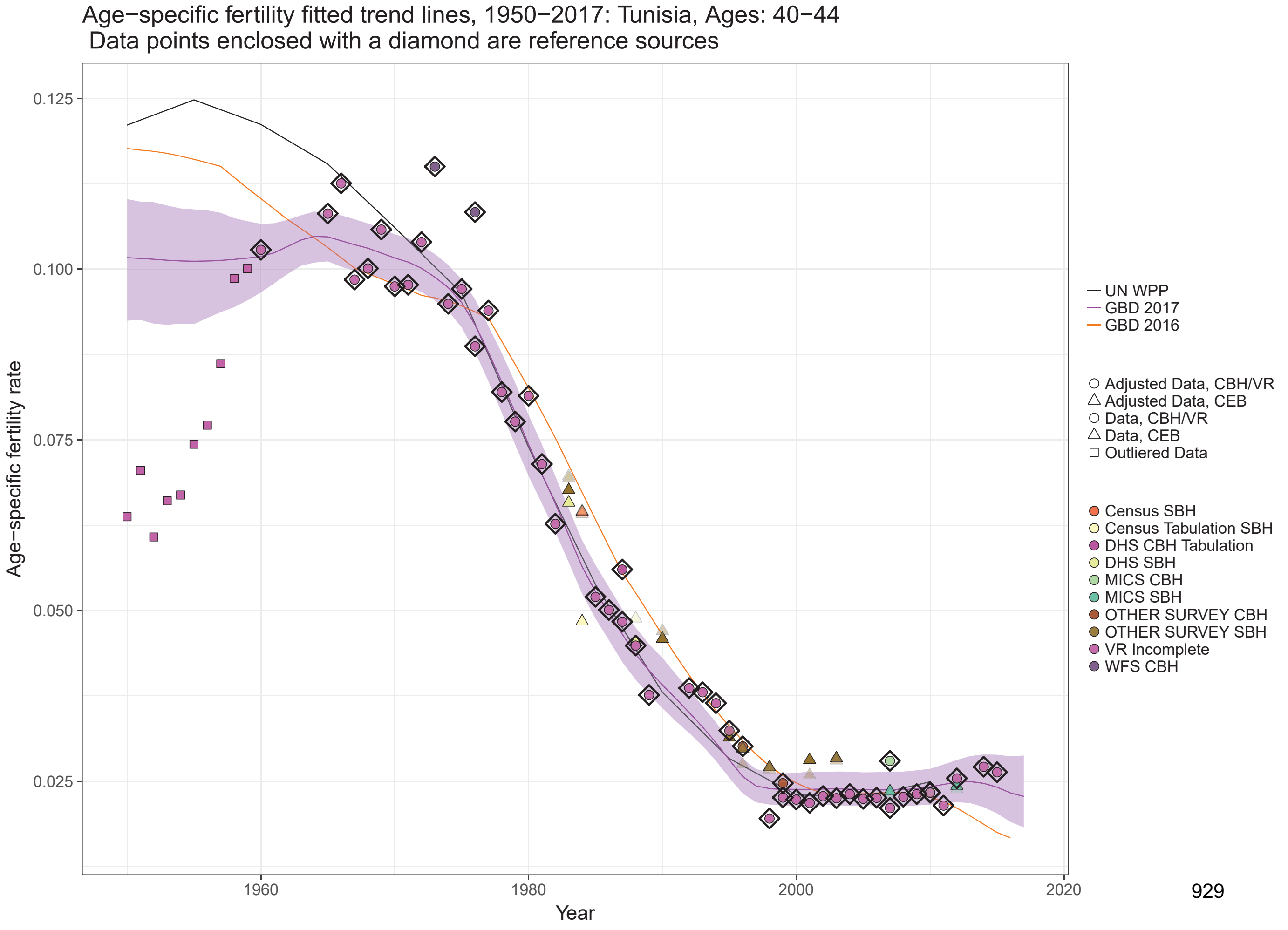
Age−specific fertility fitted trend lines, 1950−2017: Tunisia, Ages: 40−44
Data points enclosed with a diamond are reference sources
Age−specific fertility rate
Year
UN WPP
GBD 2017
GBD 2016
Adjusted Data, CBH/VR
Adjusted Data, CEB
Data, CBH/VR
Data, CEB
Outliered Data
Census SBH
Census Tabulation SBH
DHS CBH Tabulation
DHS SBH
MICS CBH
MICS SBH
OTHER SURVEY CBH
OTHER SURVEY SBH
VR Incomplete
WFS CBH

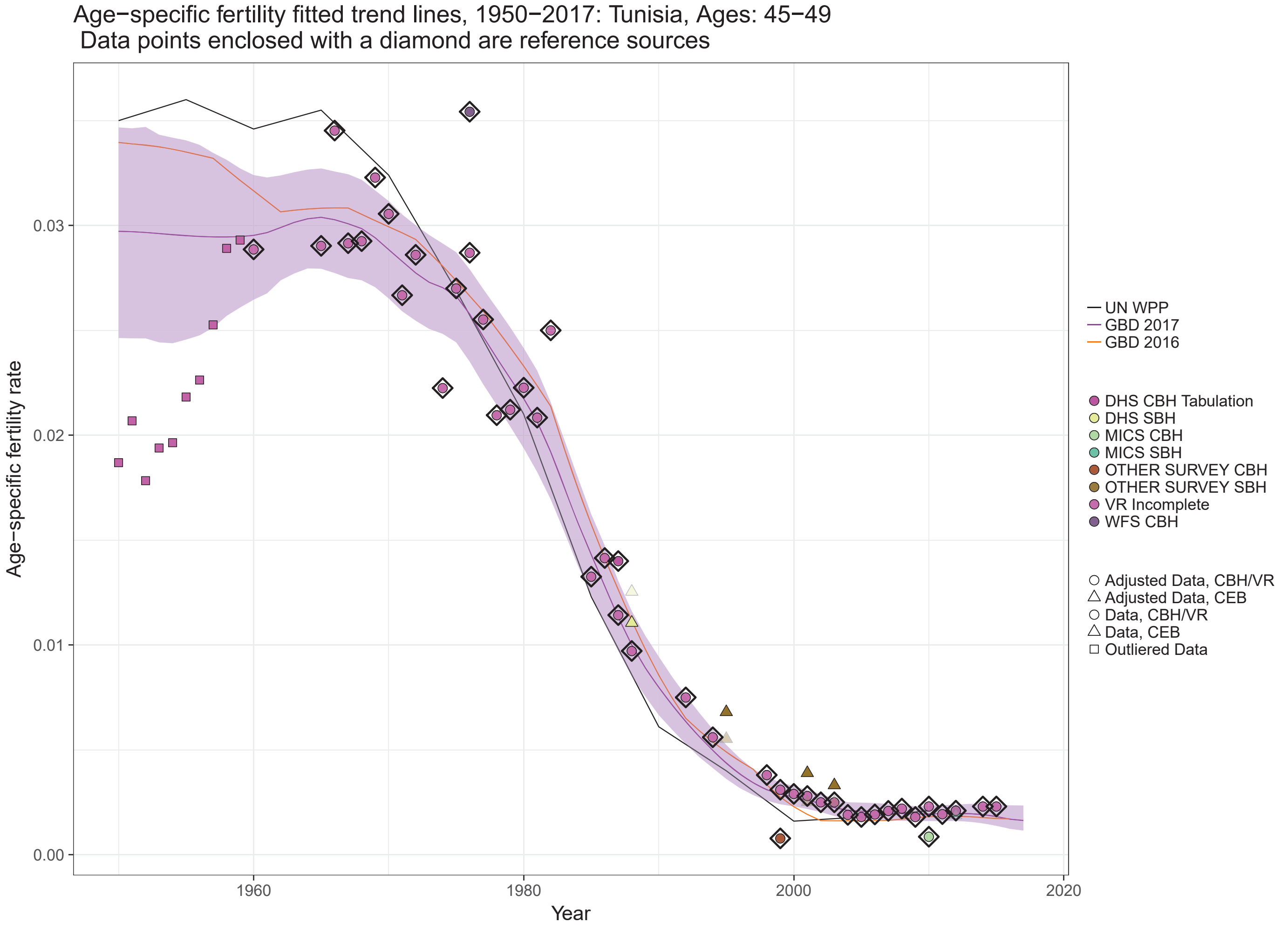

Age−specific fertility fitted trend lines, 1950−2017: Tunisia, Ages: 45−49
Data points enclosed with a diamond are reference sources
Age−specific fertility rate
0.00
0.01
0.02
0.03
1960
1980
2000
2020
Year
UN WPP
GBD 2017
GBD 2016
DHS CBH Tabulation
DHS SBH
MICS CBH
MICS SBH
OTHER SURVEY CBH
OTHER SURVEY SBH
VR Incomplete
WFS CBH
Adjusted Data, CBH/VR
Adjusted Data, CEB
Data, CBH/VR
Data, CEB
Outliered Data

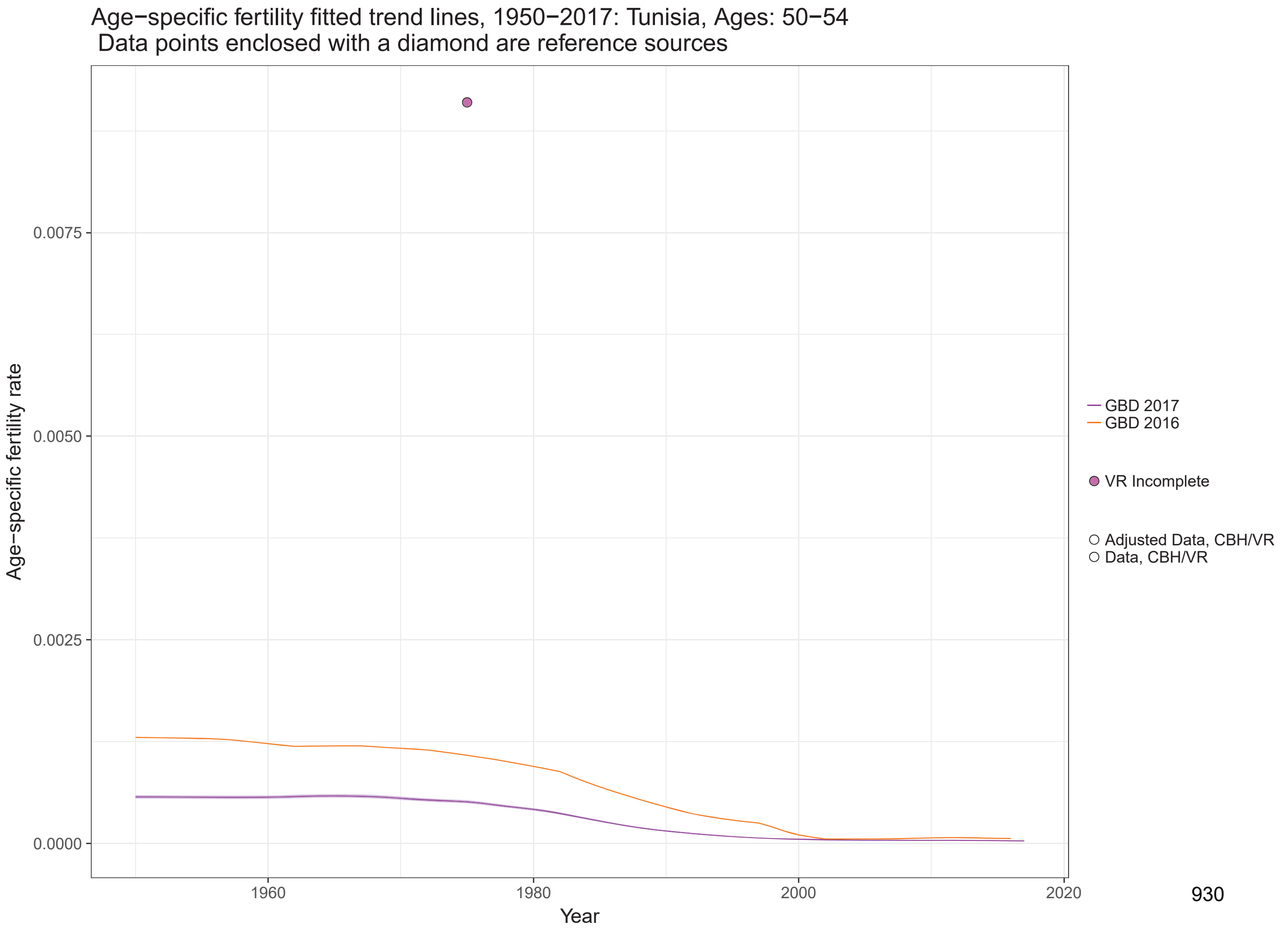

Age−specific fertility fitted trend lines, 1950−2017: Tunisia, Ages: 50−54
Data points enclosed with a diamond are reference sources
Age−specific fertility rate
0.0000
0.0025
0.0050
0.0075
1960
1980
2000
2020
Year
GBD 2017
GBD 2016
VR Incomplete
Adjusted Data, CBH/VR
Data, CBH/VR

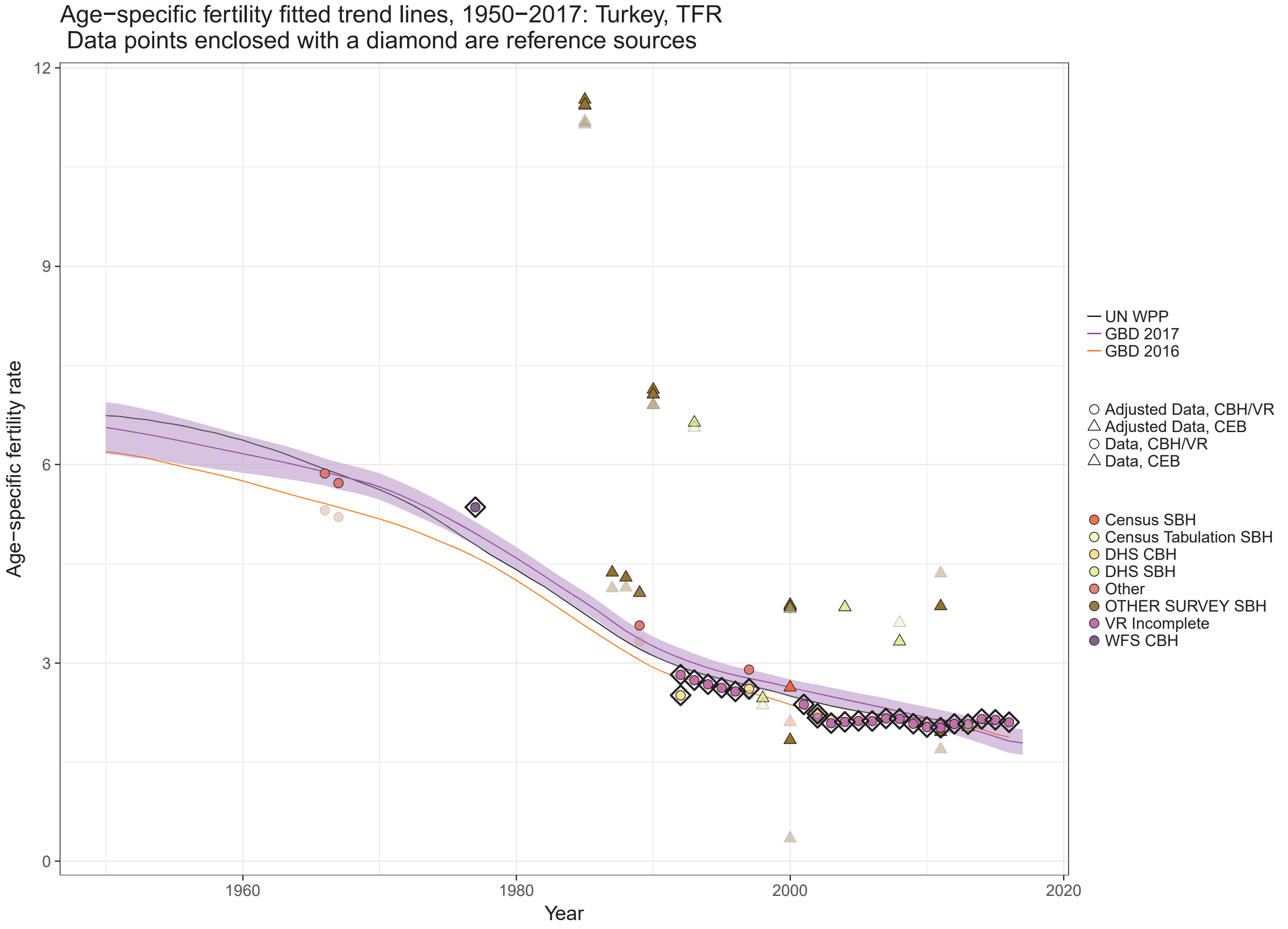

Age−specific fertility fitted trend lines, 1950−2017: Turkey, TFR
Data points enclosed with a diamond are reference sources
Age−specific fertility rate
Year
0
3
6
9
12
1960
1980
2000
2020
UN WPP
GBD 2017
GBD 2016
Adjusted Data, CBH/VR
Adjusted Data, CEB
Data, CBH/VR
Data, CEB
Census SBH
Census Tabulation SBH
DHS CBH
DHS SBH
Other
OTHER SURVEY SBH
VR Incomplete
WFS CBH

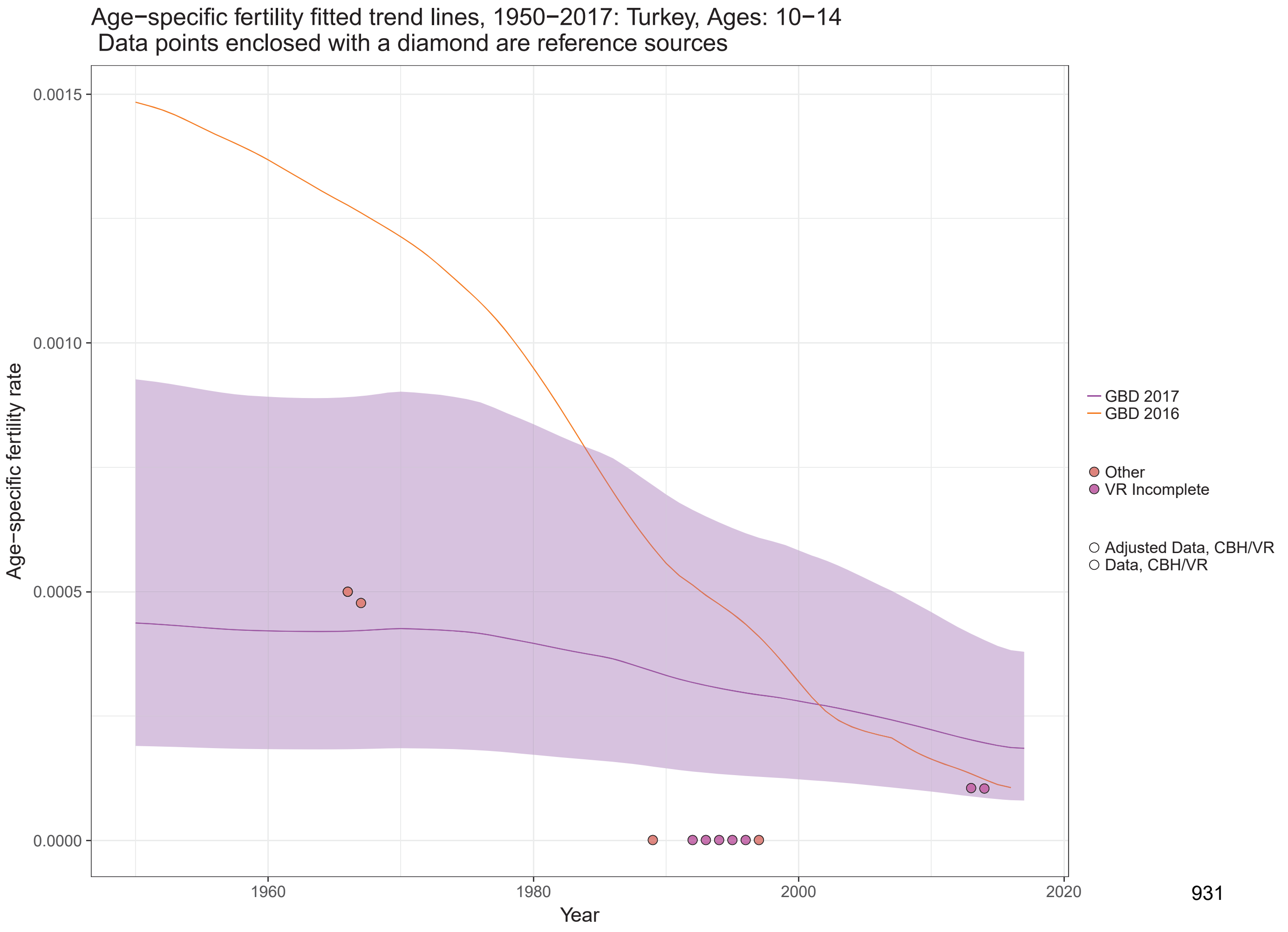

Age−specific fertility fitted trend lines, 1950−2017: Turkey, Ages: 10−14
Data points enclosed with a diamond are reference sources
Age−specific fertility rate
Year
0.0000
0.0005
0.0010
0.0015
1960
1980
2000
2020
GBD 2017
GBD 2016
Other
VR Incomplete
Adjusted Data, CBH/VR
Data, CBH/VR

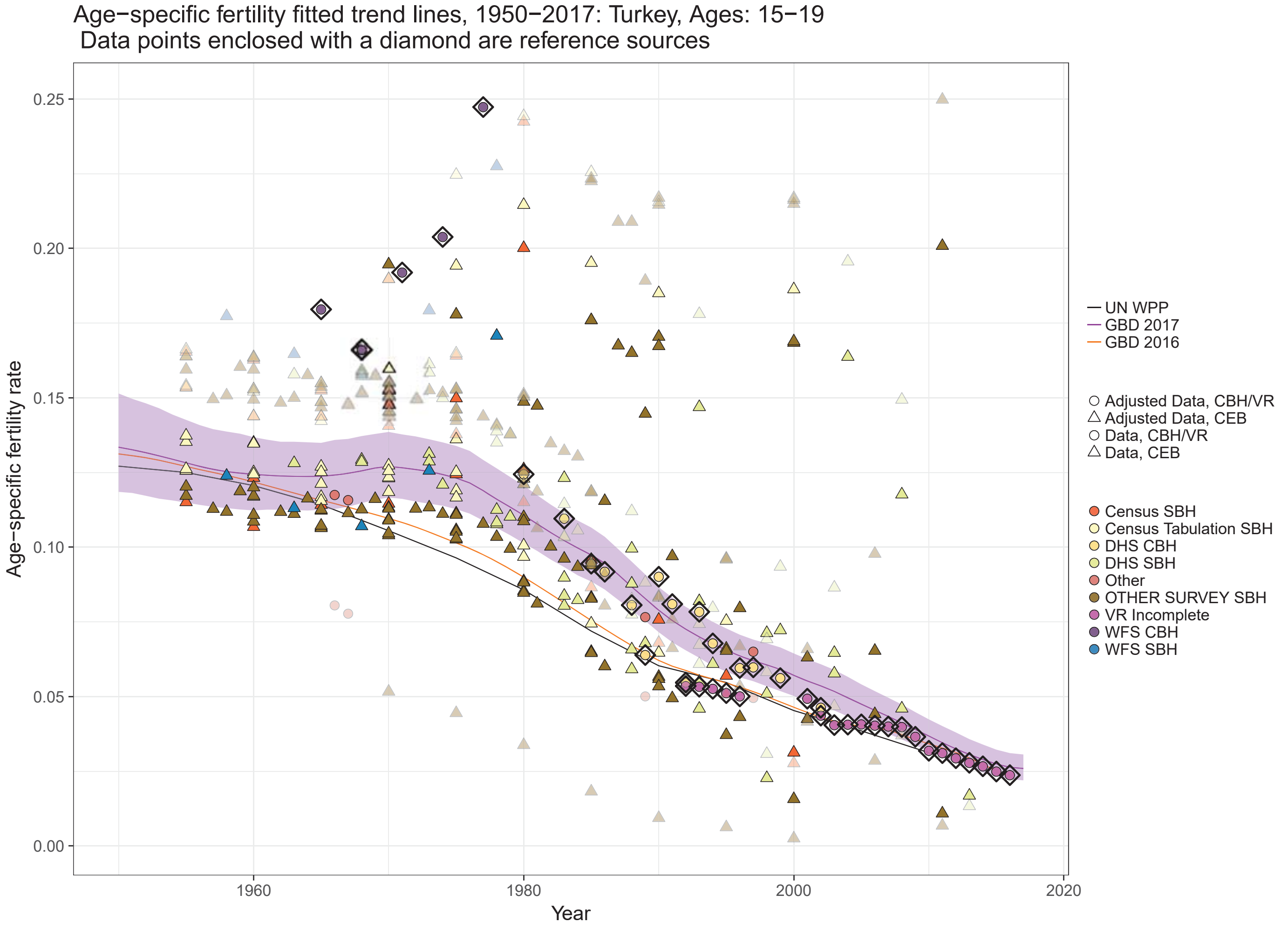

Age−specific fertility fitted trend lines, 1950−2017: Turkey, Ages: 15−19
Data points enclosed with a diamond are reference sources
Age−specific fertility rate
0.00
0.05
0.10
0.15
0.20
0.25
1960
1980
2000
2020
Year
UN WPP
GBD 2017
GBD 2016
Adjusted Data, CBH/VR
Adjusted Data, CEB
Data, CBH/VR
Data, CEB
Census SBH
Census Tabulation SBH
DHS CBH
DHS SBH
Other
OTHER SURVEY SBH
VR Incomplete
WFS CBH
WFS SBH

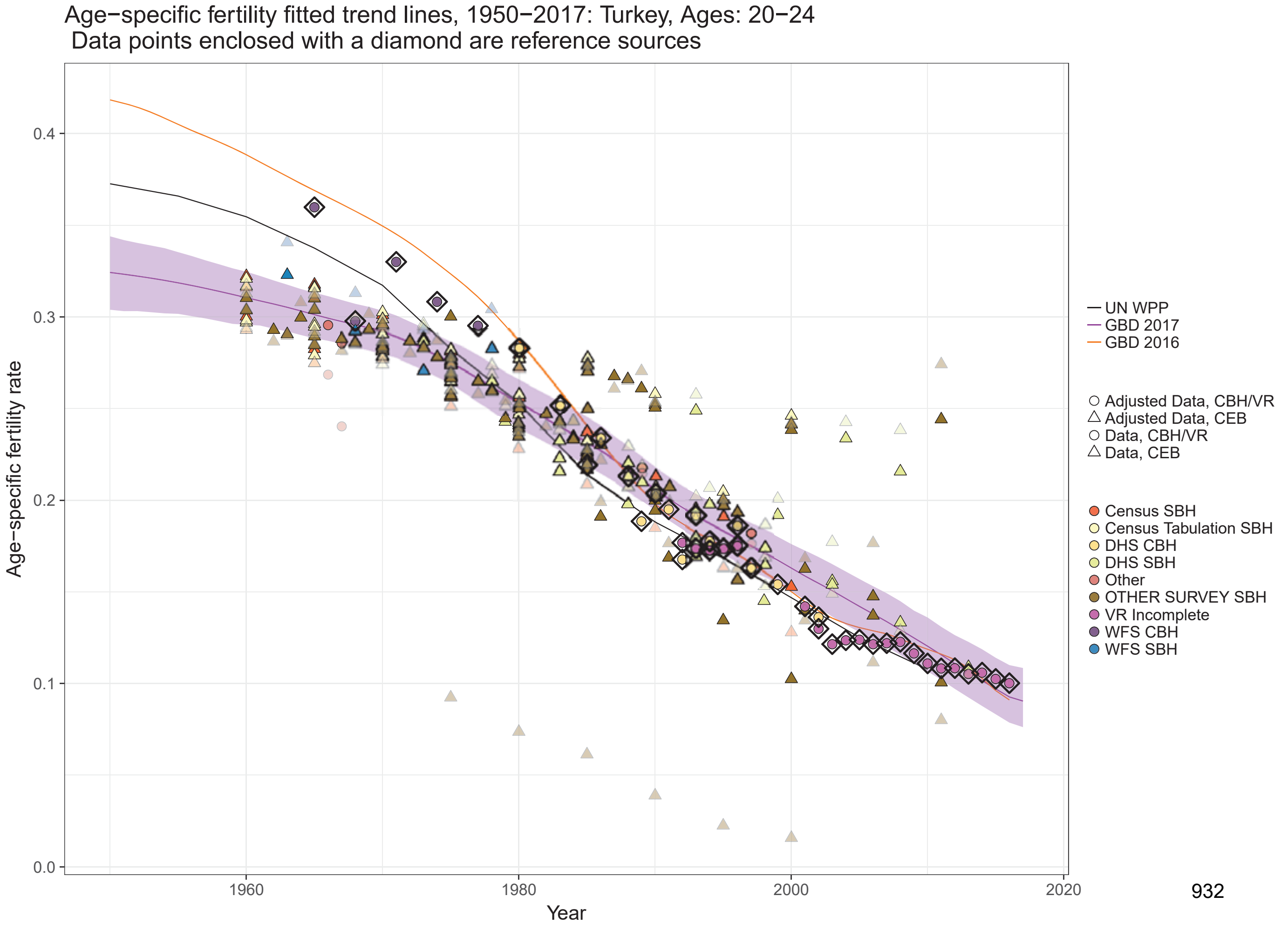

Age−specific fertility fitted trend lines, 1950−2017: Turkey, Ages: 20−24
Data points enclosed with a diamond are reference sources
Age−specific fertility rate
0.0
0.1
0.2
0.3
0.4
1960
1980
2000
2020
Year
UN WPP
GBD 2017
GBD 2016
Adjusted Data, CBH/VR
Adjusted Data, CEB
Data, CBH/VR
Data, CEB
Census SBH
Census Tabulation SBH
DHS CBH
DHS SBH
Other
OTHER SURVEY SBH
VR Incomplete
WFS CBH
WFS SBH

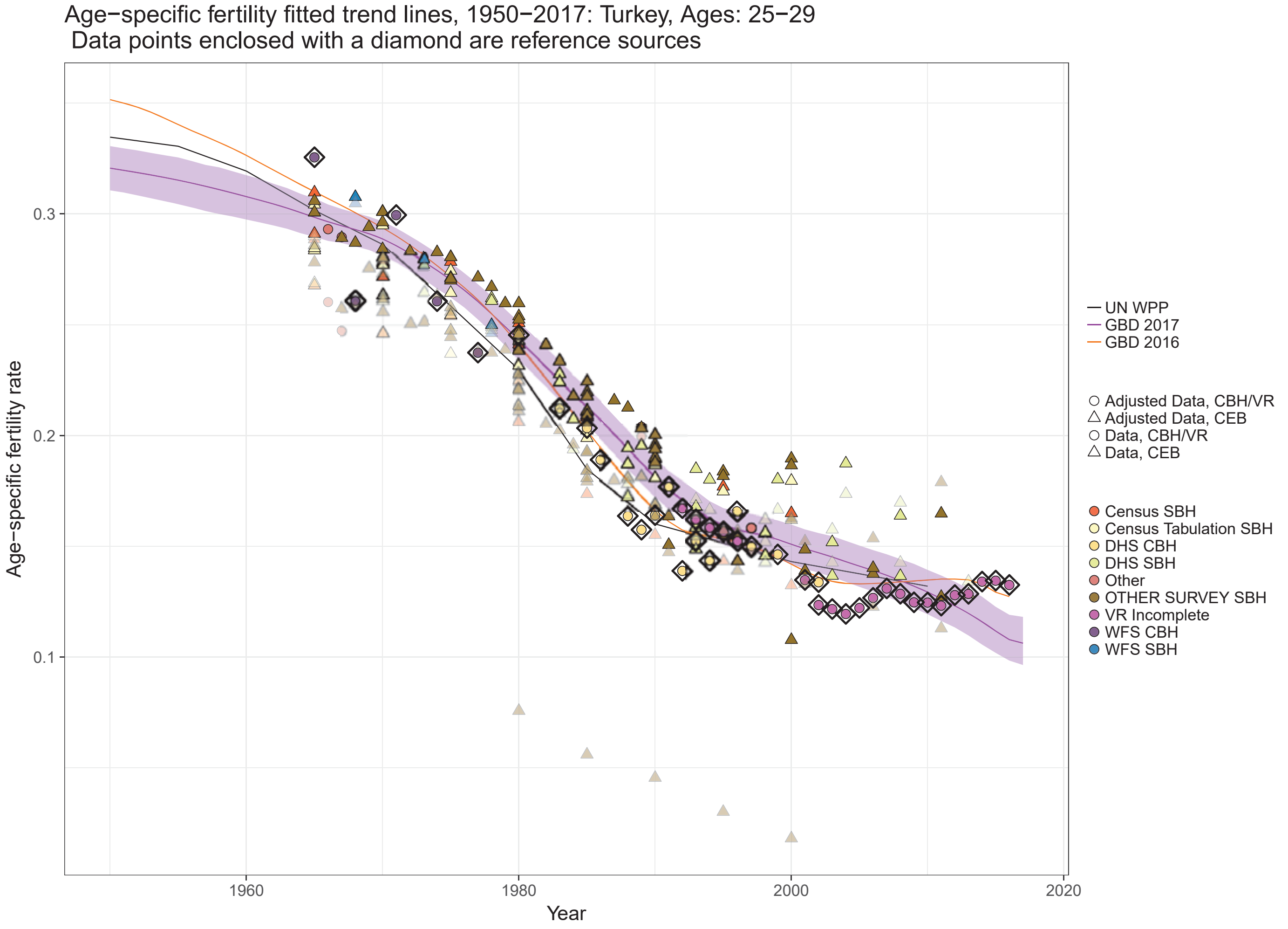

Age−specific fertility fitted trend lines, 1950−2017: Turkey, Ages: 25−29
Data points enclosed with a diamond are reference sources
Age−specific fertility rate
0.3
0.2
0.1
1960
1980
2000
2020
Year
UN WPP
GBD 2017
GBD 2016
Adjusted Data, CBH/VR
Adjusted Data, CEB
Data, CBH/VR
Data, CEB
Census SBH
Census Tabulation SBH
DHS CBH
DHS SBH
Other
OTHER SURVEY SBH
VR Incomplete
WFS CBH
WFS SBH

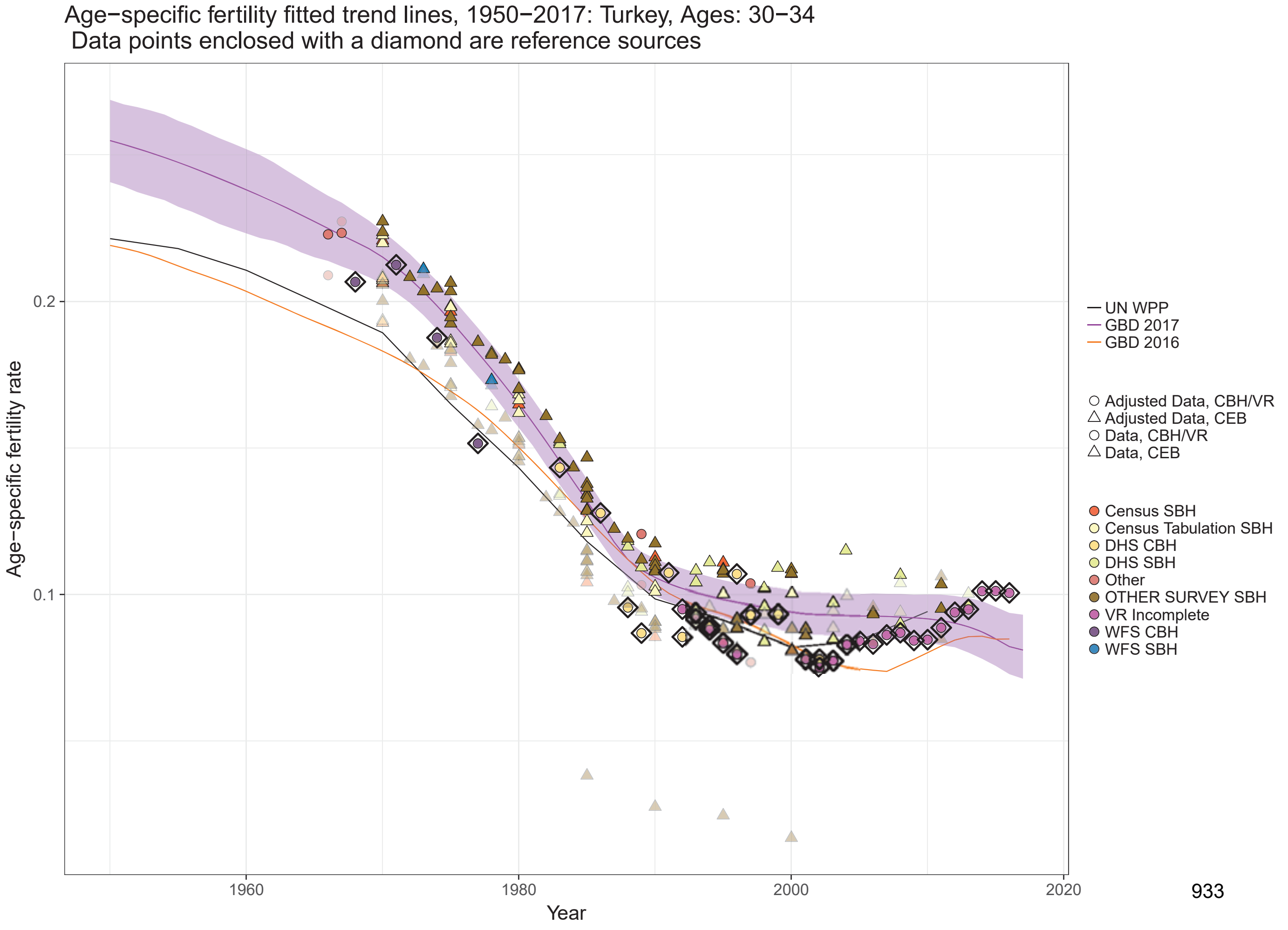

Age−specific fertility fitted trend lines, 1950−2017: Turkey, Ages: 30−34
Data points enclosed with a diamond are reference sources
Age−specific fertility rate
0.2
0.1
1960
1980
2000
2020
Year
UN WPP
GBD 2017
GBD 2016
Adjusted Data, CBH/VR
Adjusted Data, CEB
Data, CBH/VR
Data, CEB
Census SBH
Census Tabulation SBH
DHS CBH
DHS SBH
Other
OTHER SURVEY SBH
VR Incomplete
WFS CBH
WFS SBH

Age−specific fertility fitted trend lines, 1950−2017: Turkey, Ages: 35−39
Data points enclosed with a diamond are reference sources

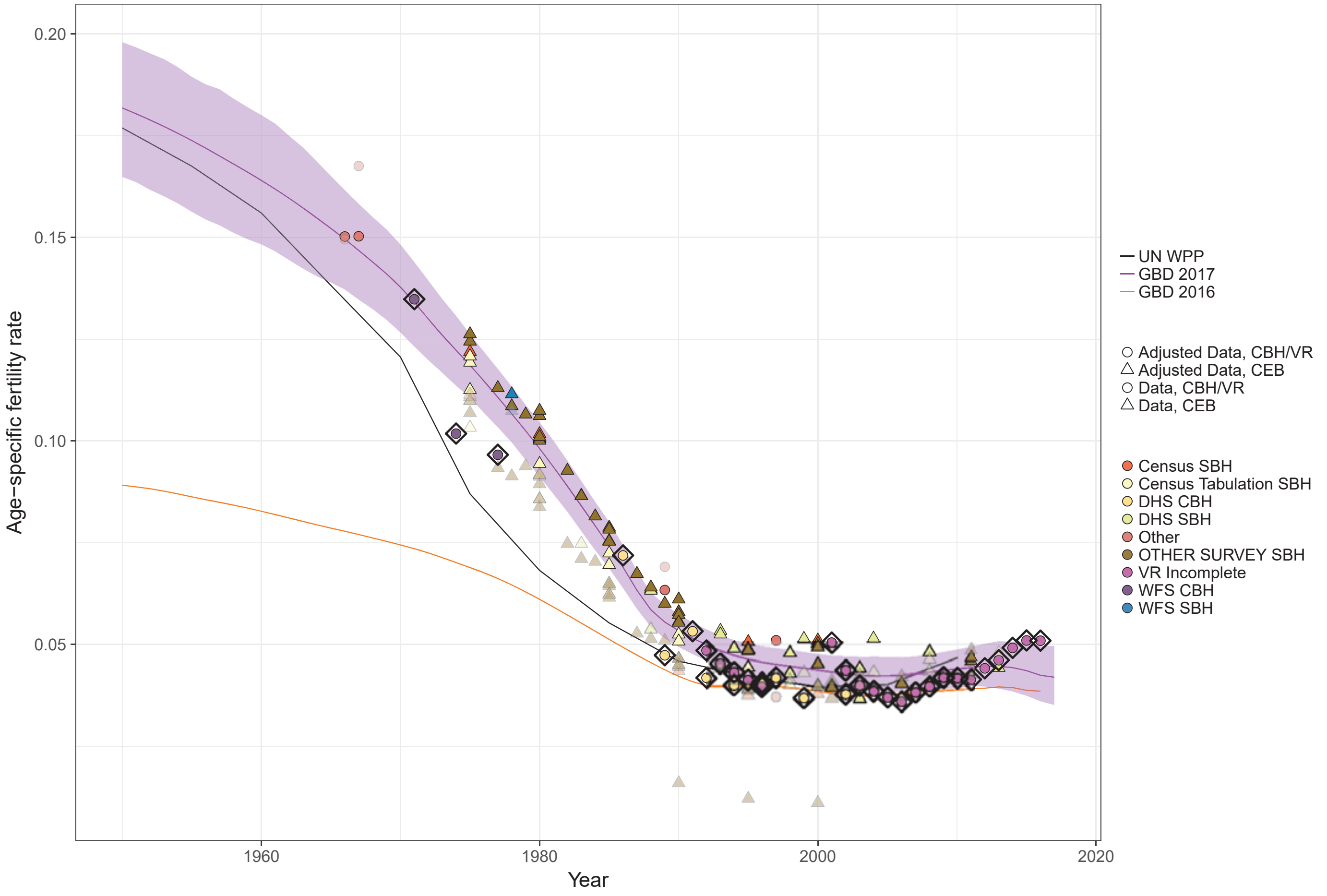

Age−specific fertility fitted trend lines, 1950−2017: Turkey, Ages: 40−44
Data points enclosed with a diamond are reference sources

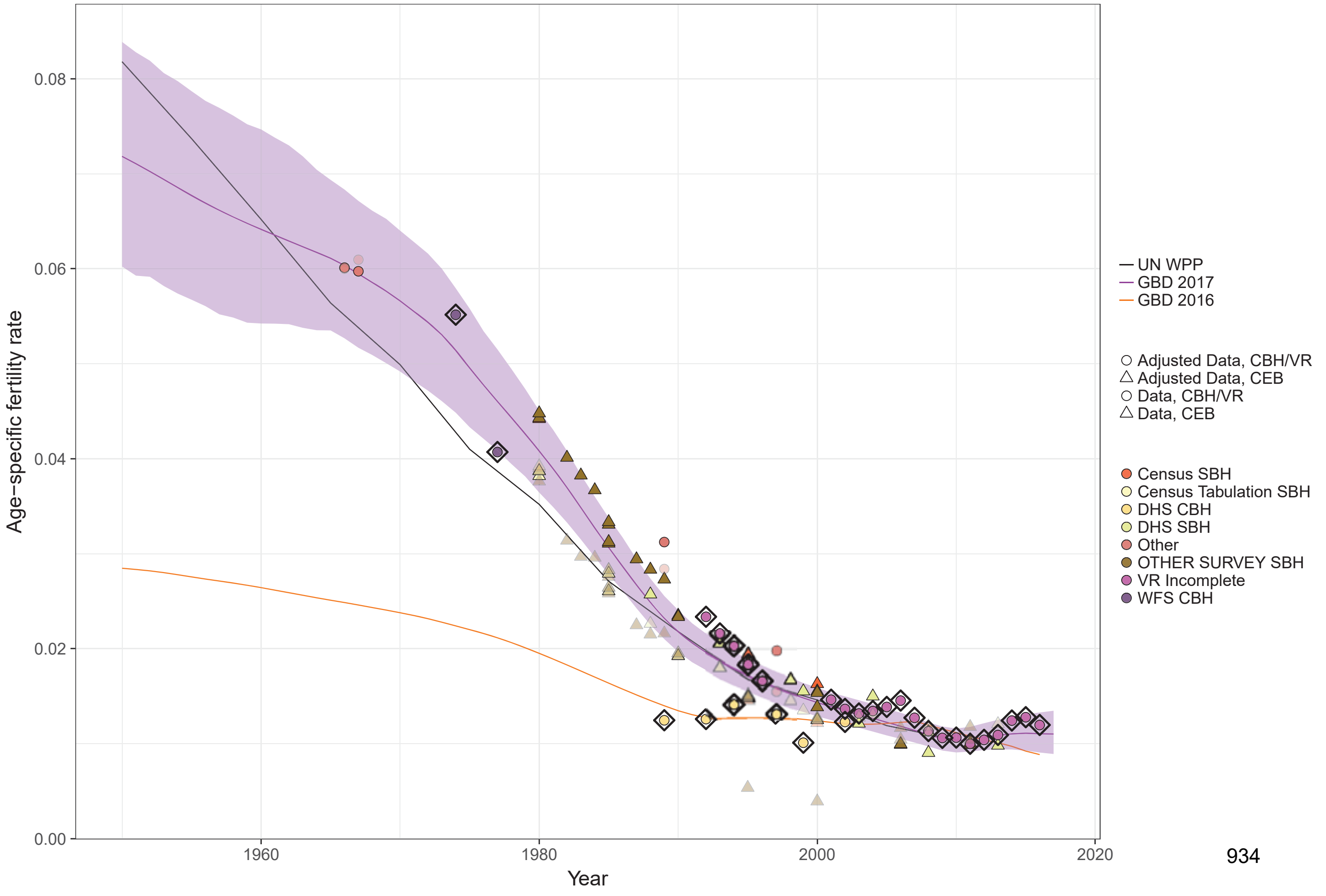

Age−specific fertility fitted trend lines, 1950−2017: Turkey, Ages: 45−49
Data points enclosed with a diamond are reference sources

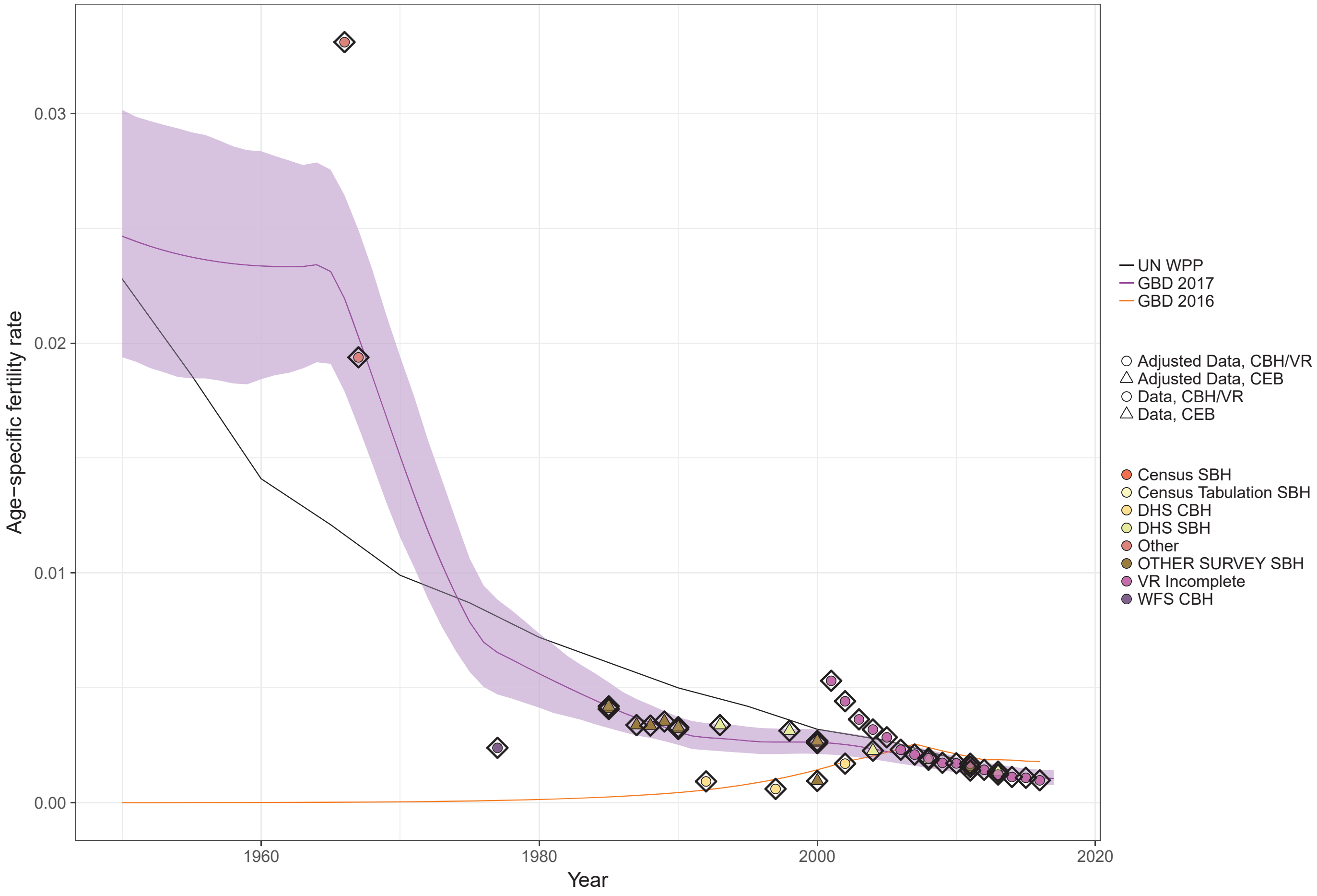

Age−specific fertility fitted trend lines, 1950−2017: Turkey, Ages: 50−54
Data points enclosed with a diamond are reference sources

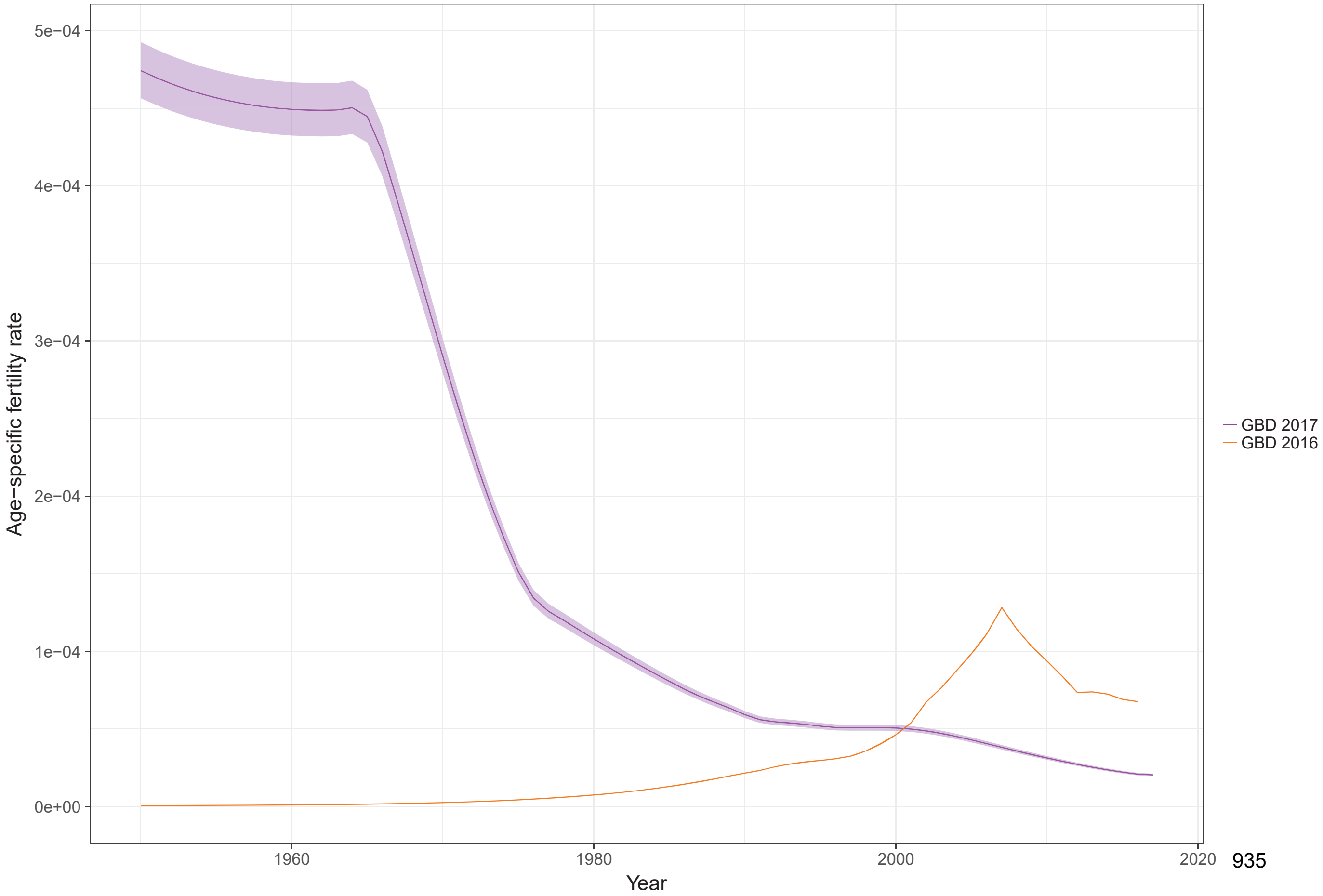

Age−specific fertility fitted trend lines, 1950−2017: Turkmenistan, TFR
Data points enclosed with a diamond are reference sources

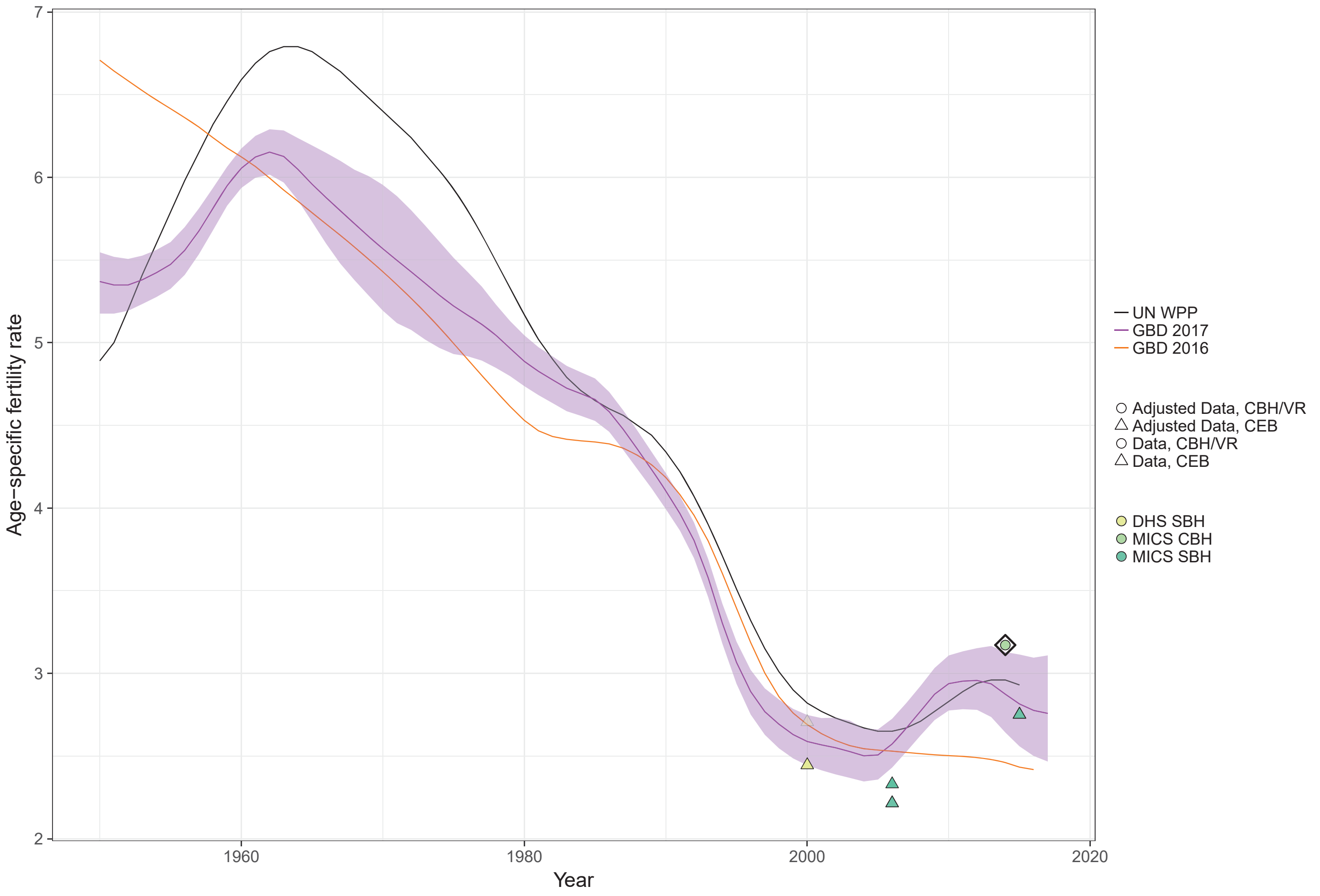

Age−specific fertility fitted trend lines, 1950−2017: Turkmenistan, Ages: 10−14
Data points enclosed with a diamond are reference sources

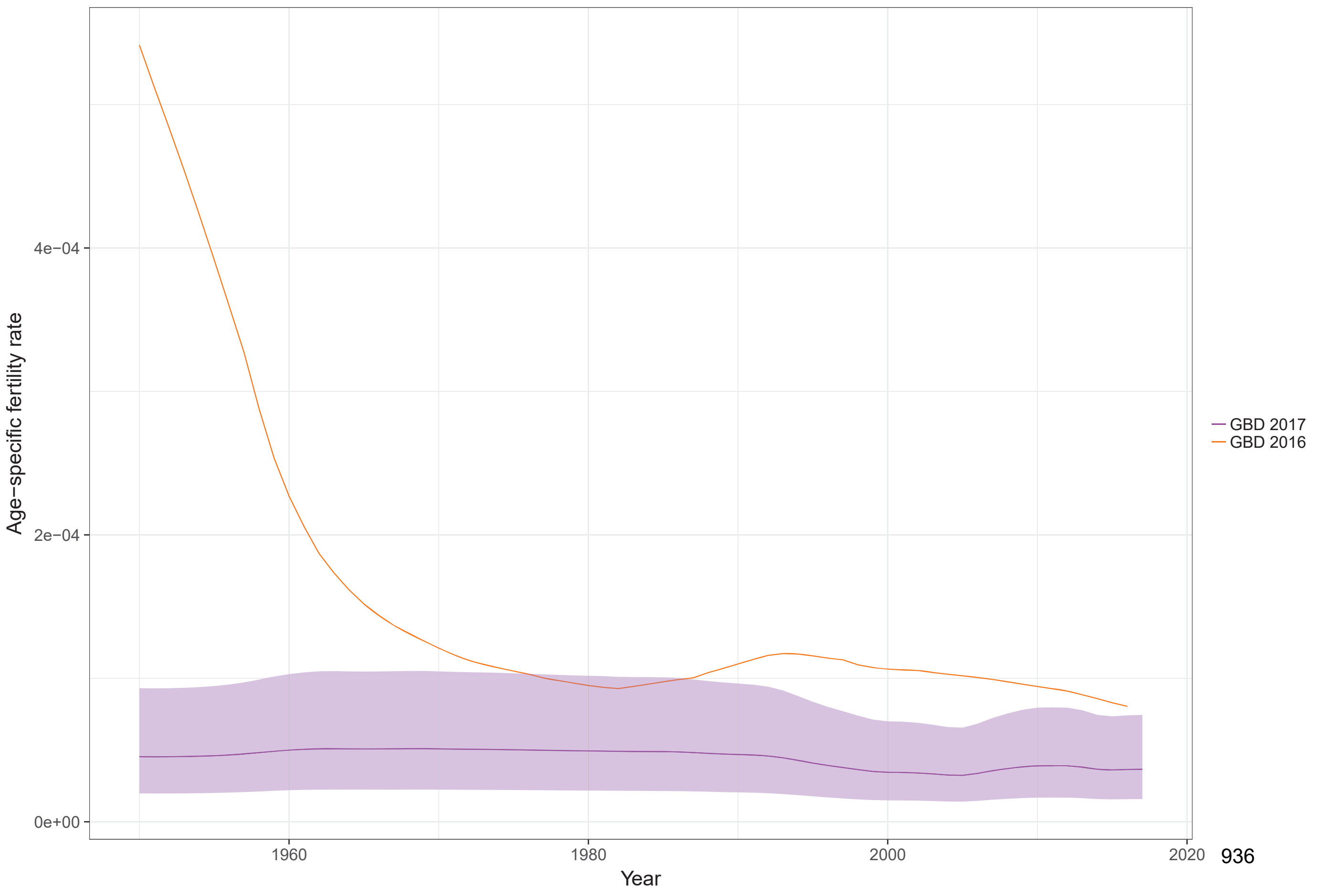

Age−specific fertility fitted trend lines, 1950−2017: Turkmenistan, Ages: 15−19
Data points enclosed with a diamond are reference sources

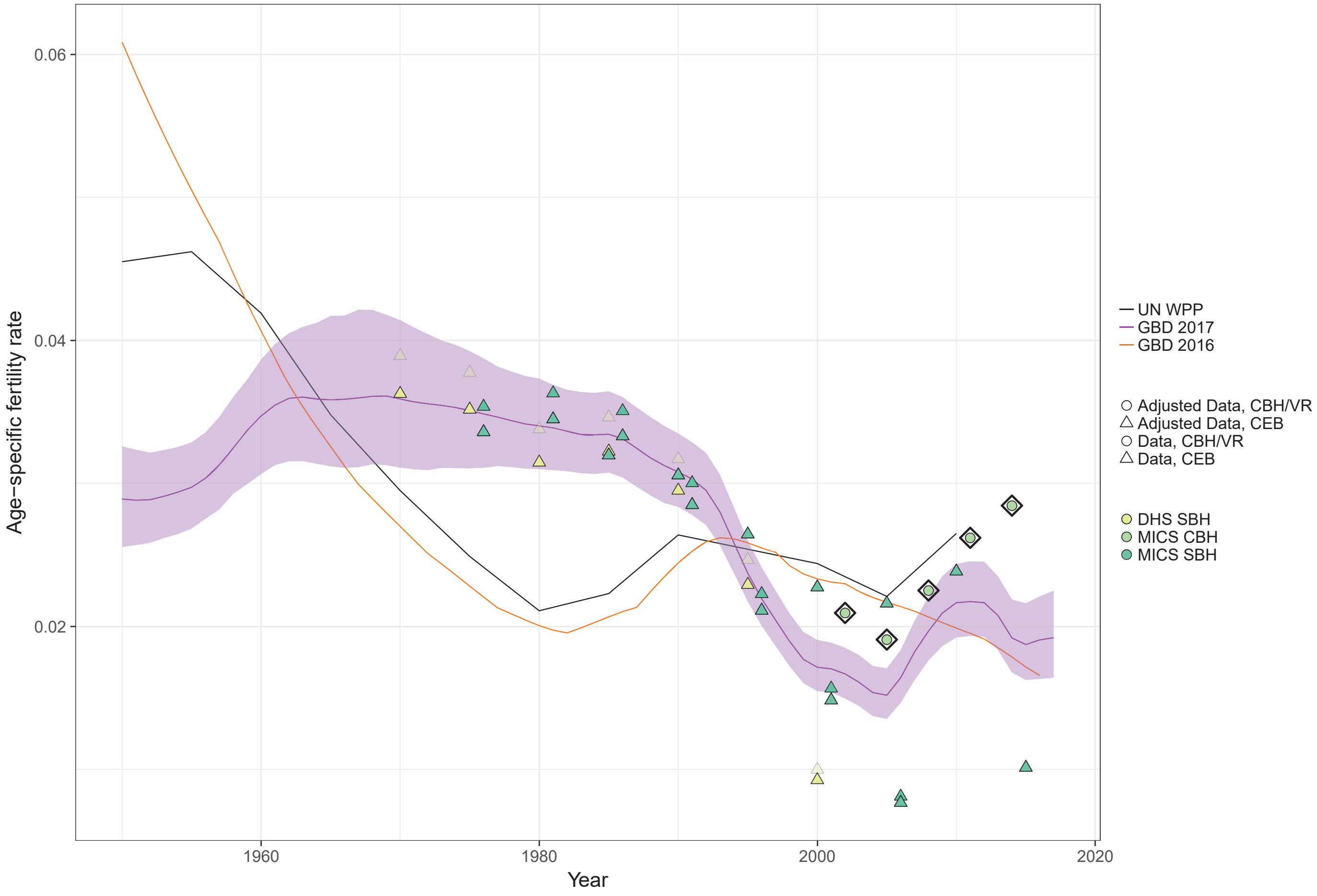

Age−specific fertility fitted trend lines, 1950−2017: Turkmenistan, Ages: 20−24
Data points enclosed with a diamond are reference sources

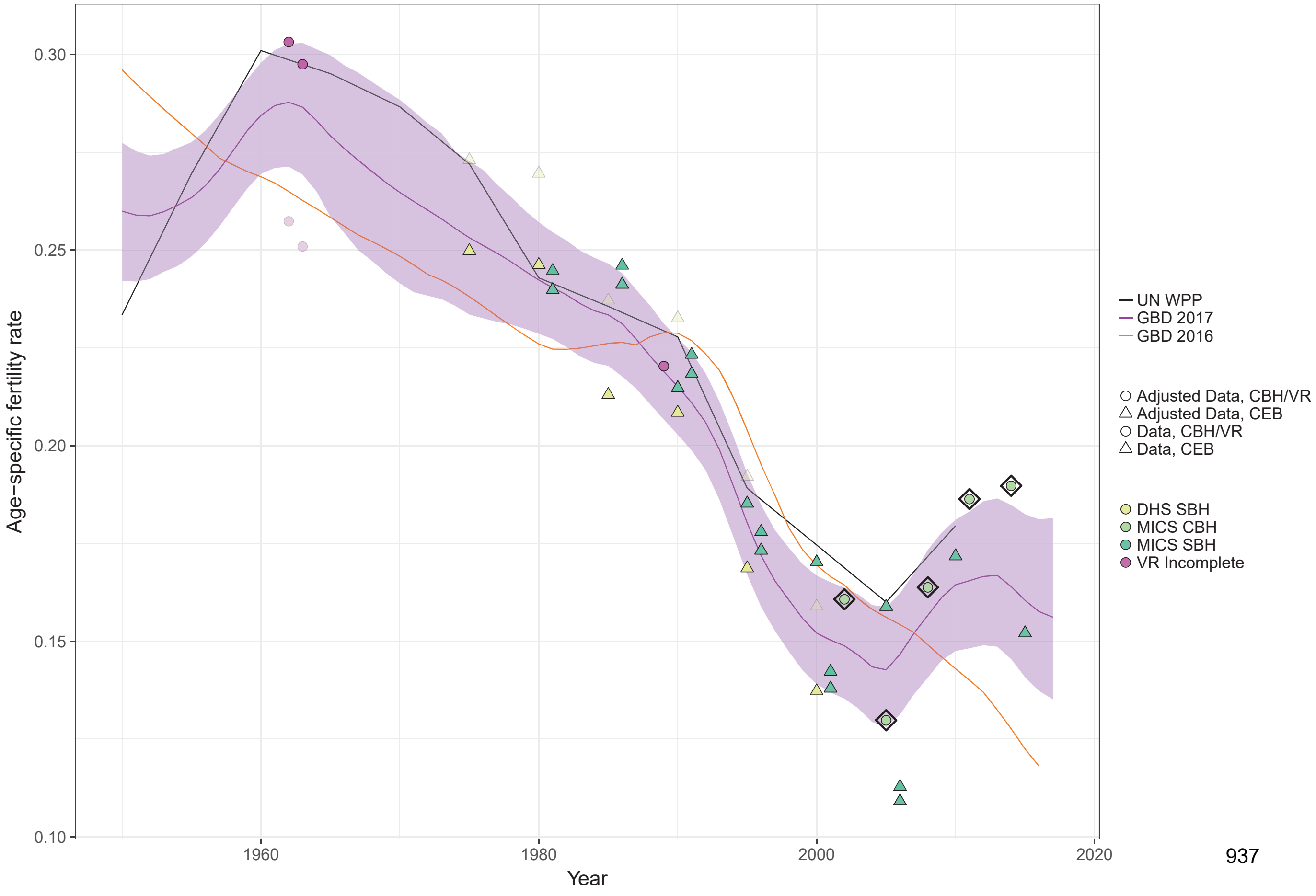

Age−specific fertility fitted trend lines, 1950−2017: Turkmenistan, Ages: 25−29
Data points enclosed with a diamond are reference sources

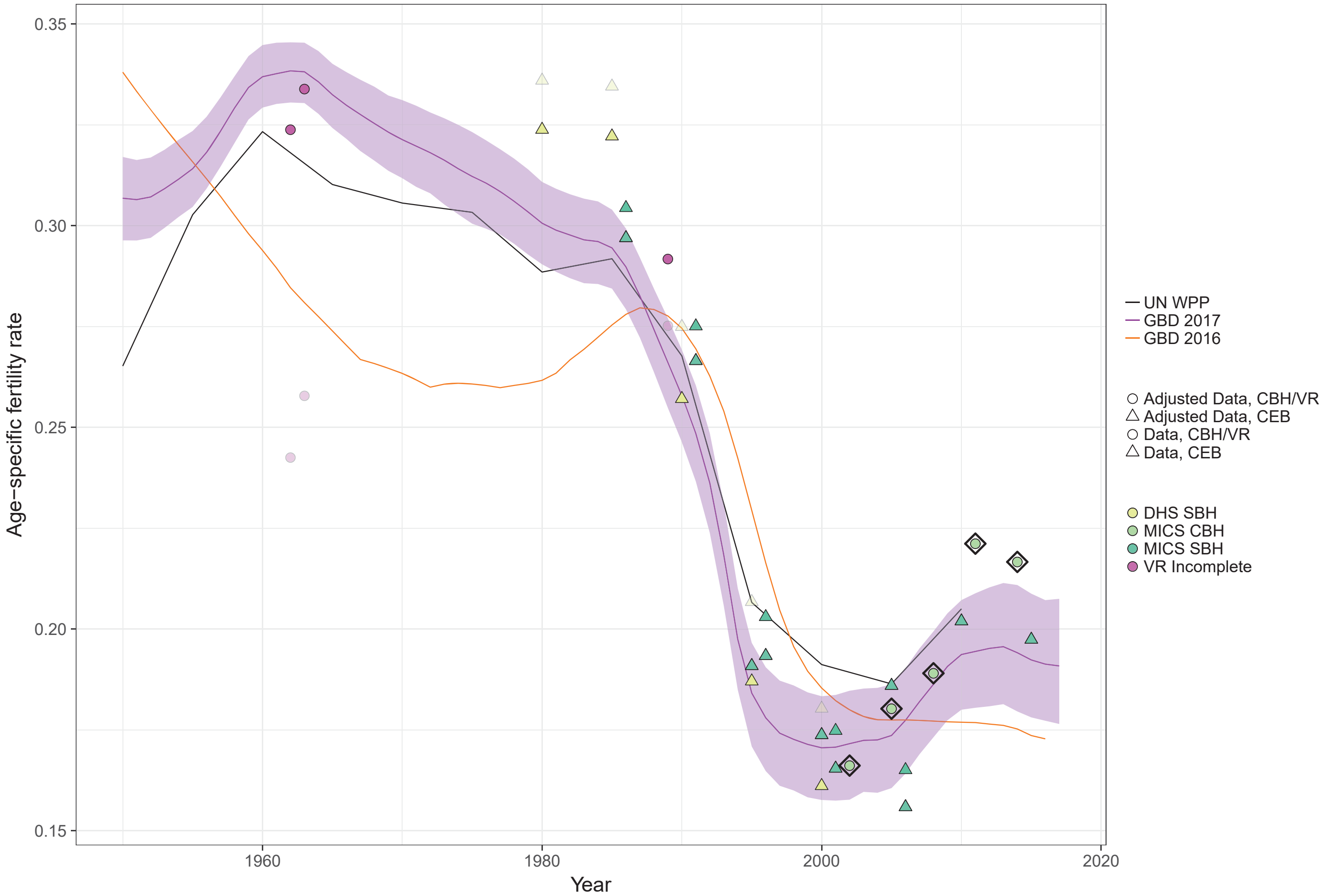

Age−specific fertility fitted trend lines, 1950−2017: Turkmenistan, Ages: 30−34
Data points enclosed with a diamond are reference sources

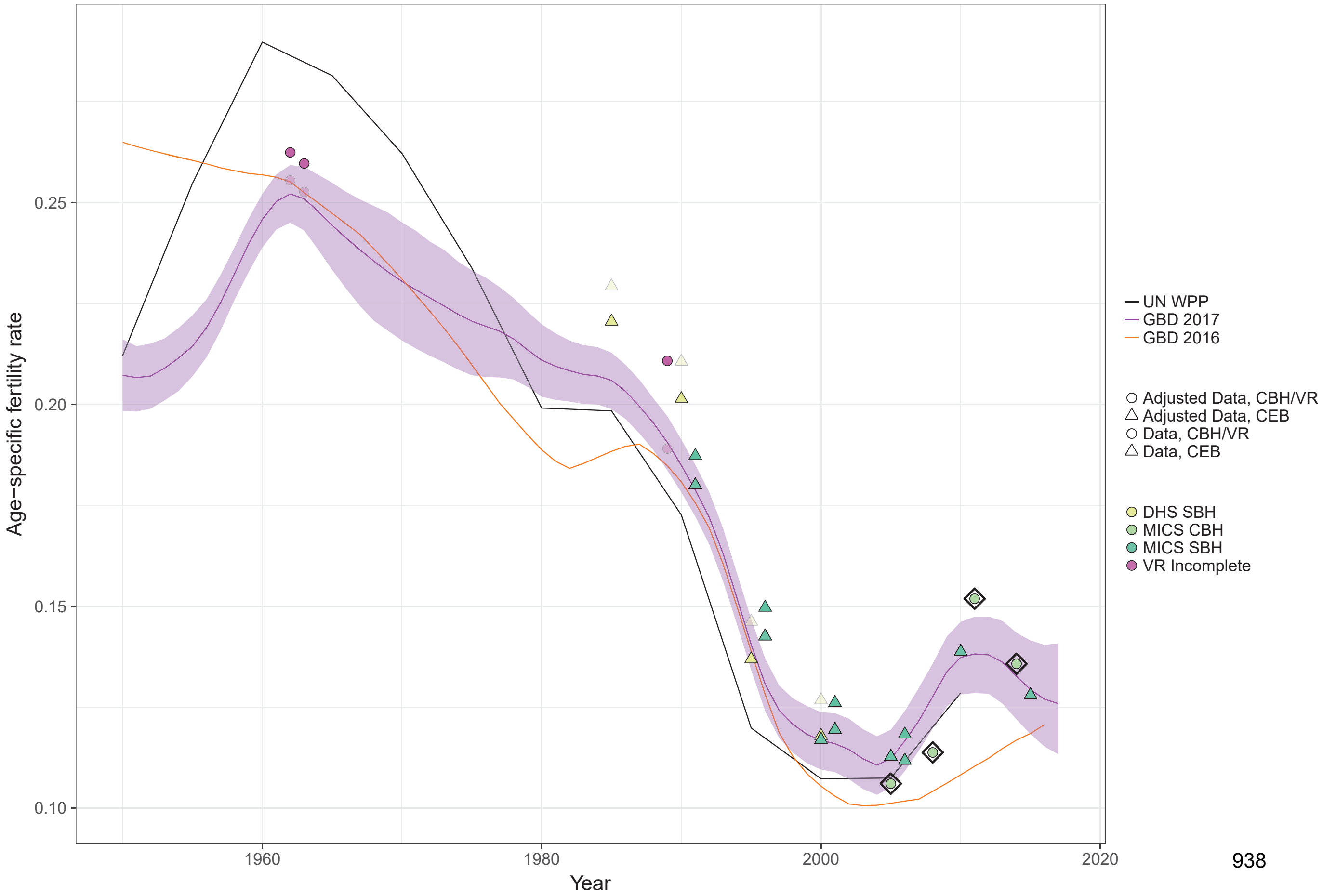

Age−specific fertility fitted trend lines, 1950−2017: Turkmenistan, Ages: 35−39
Data points enclosed with a diamond are reference sources

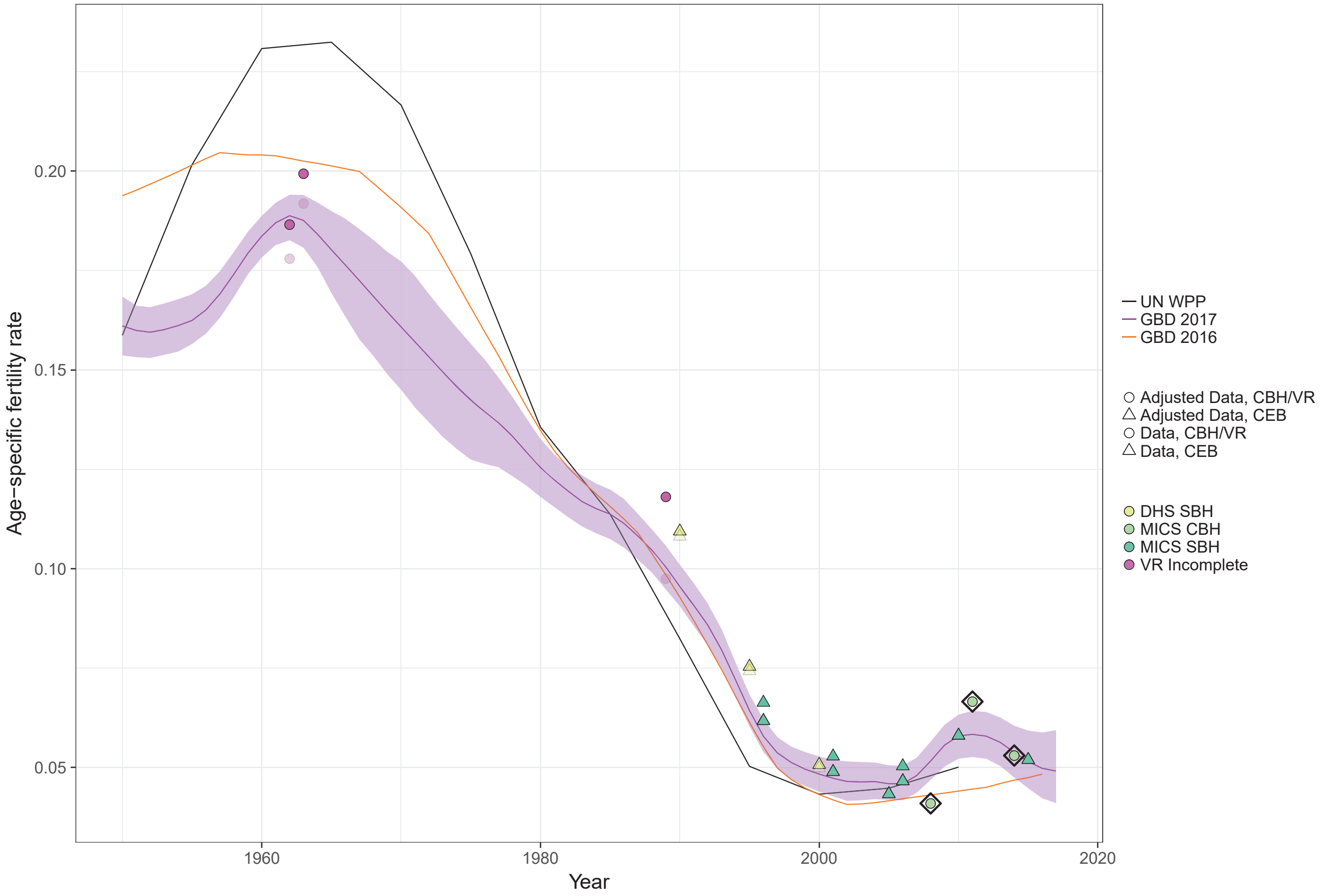

Age−specific fertility fitted trend lines, 1950−2017: Turkmenistan, Ages: 40−44
Data points enclosed with a diamond are reference sources

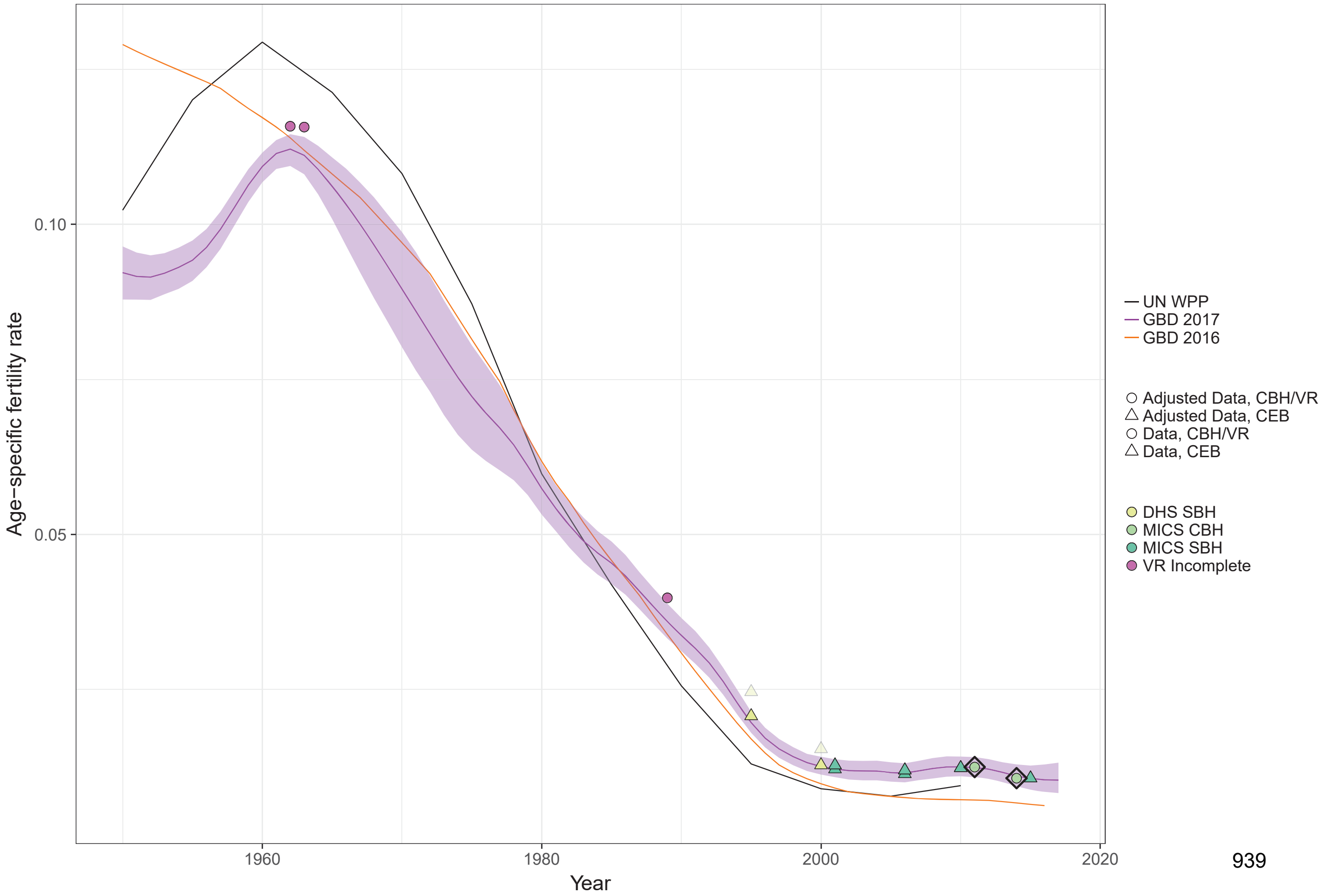

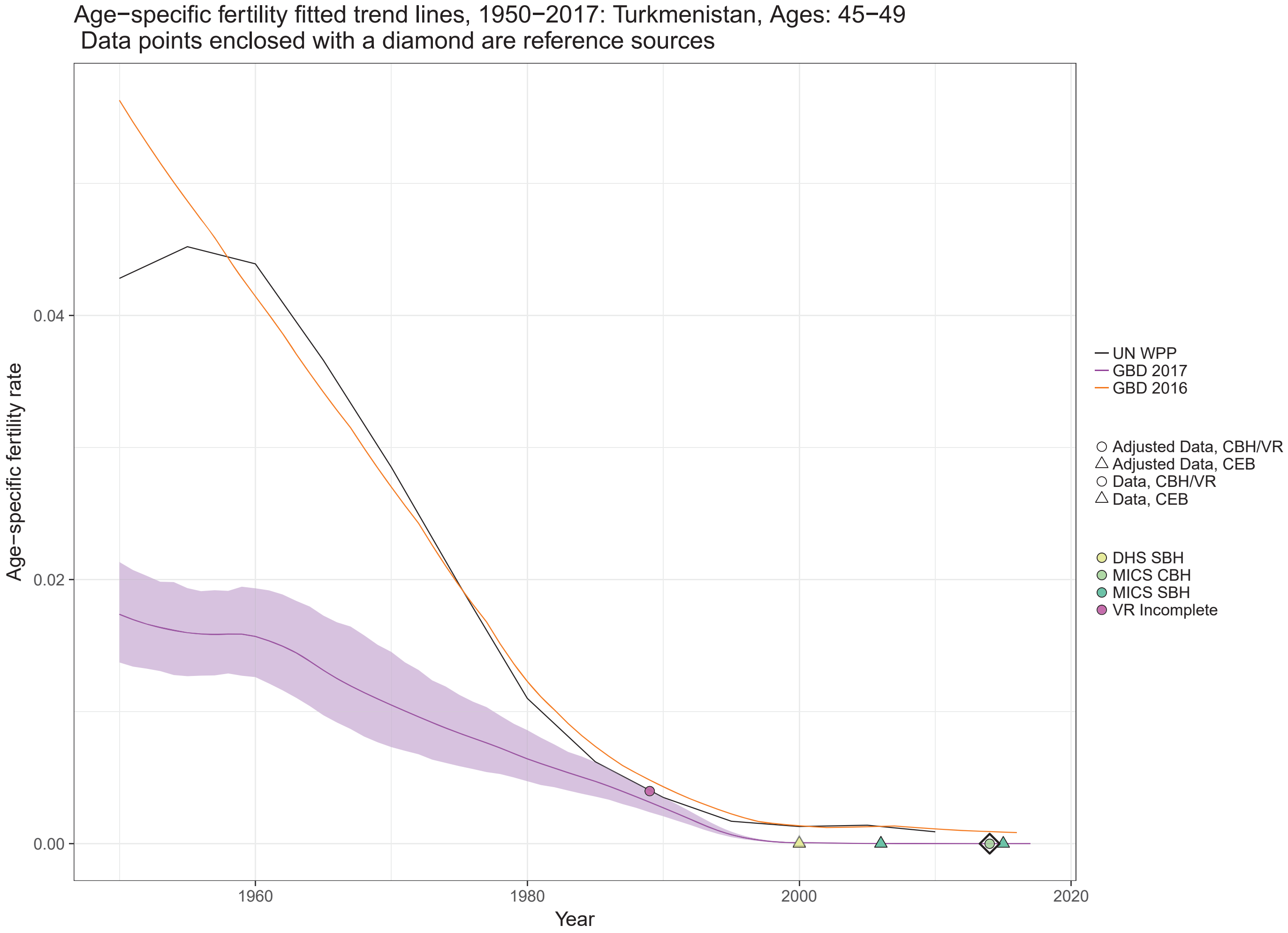
Age−specific fertility fitted trend lines, 1950−2017: Turkmenistan, Ages: 45−49
Data points enclosed with a diamond are reference sources
Age−specific fertility rate
Year
0.00
0.02
0.04
1960
1980
2000
2020
UN WPP
GBD 2017
GBD 2016
Adjusted Data, CBH/VR
Adjusted Data, CEB
Data, CBH/VR
Data, CEB
DHS SBH
MICS CBH
MICS SBH
VR Incomplete

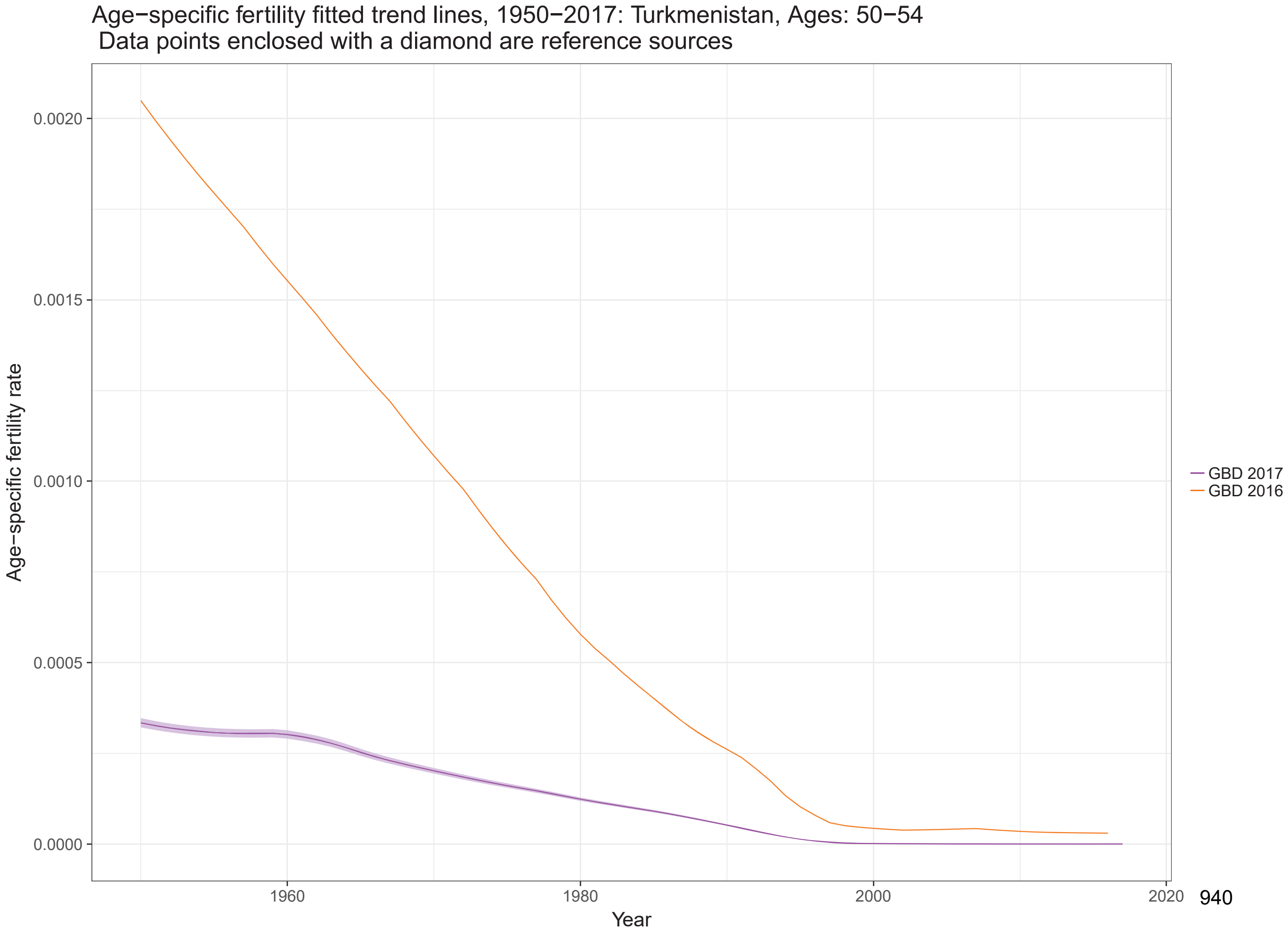
Age−specific fertility fitted trend lines, 1950−2017: Turkmenistan, Ages: 50−54
Data points enclosed with a diamond are reference sources
Age−specific fertility rate
Year
0.0000
0.0005
0.0010
0.0015
0.0020
1960
1980
2000
2020
GBD 2017
GBD 2016

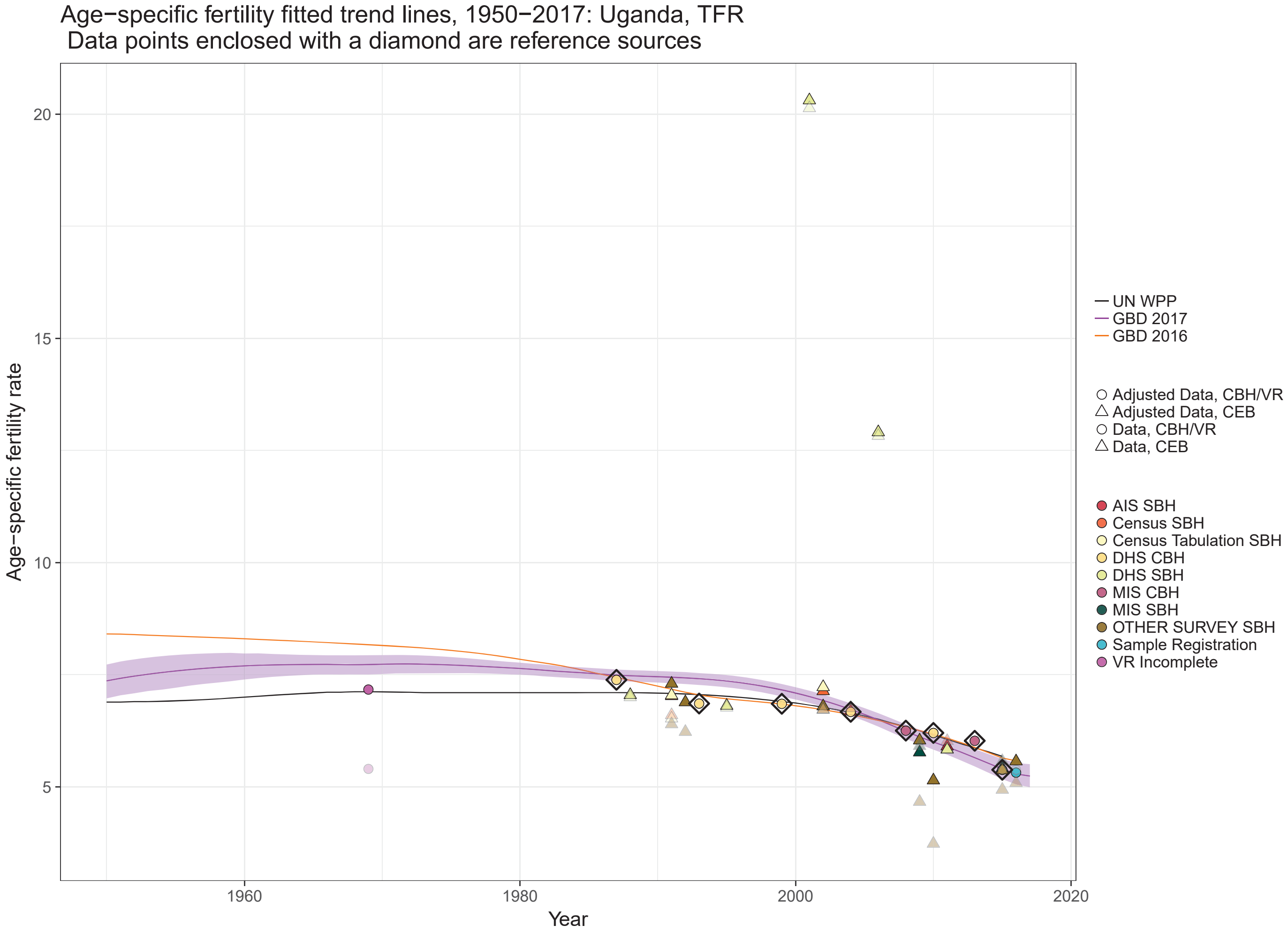

Age−specific fertility fitted trend lines, 1950−2017: Uganda, TFR
Data points enclosed with a diamond are reference sources
Age−specific fertility rate
Year
20
15
10
5
1960
1980
2000
2020
UN WPP
GBD 2017
GBD 2016
Adjusted Data, CBH/VR
Adjusted Data, CEB
Data, CBH/VR
Data, CEB
AIS SBH
Census SBH
Census Tabulation SBH
DHS CBH
DHS SBH
MIS CBH
MIS SBH
OTHER SURVEY SBH
Sample Registration
VR Incomplete

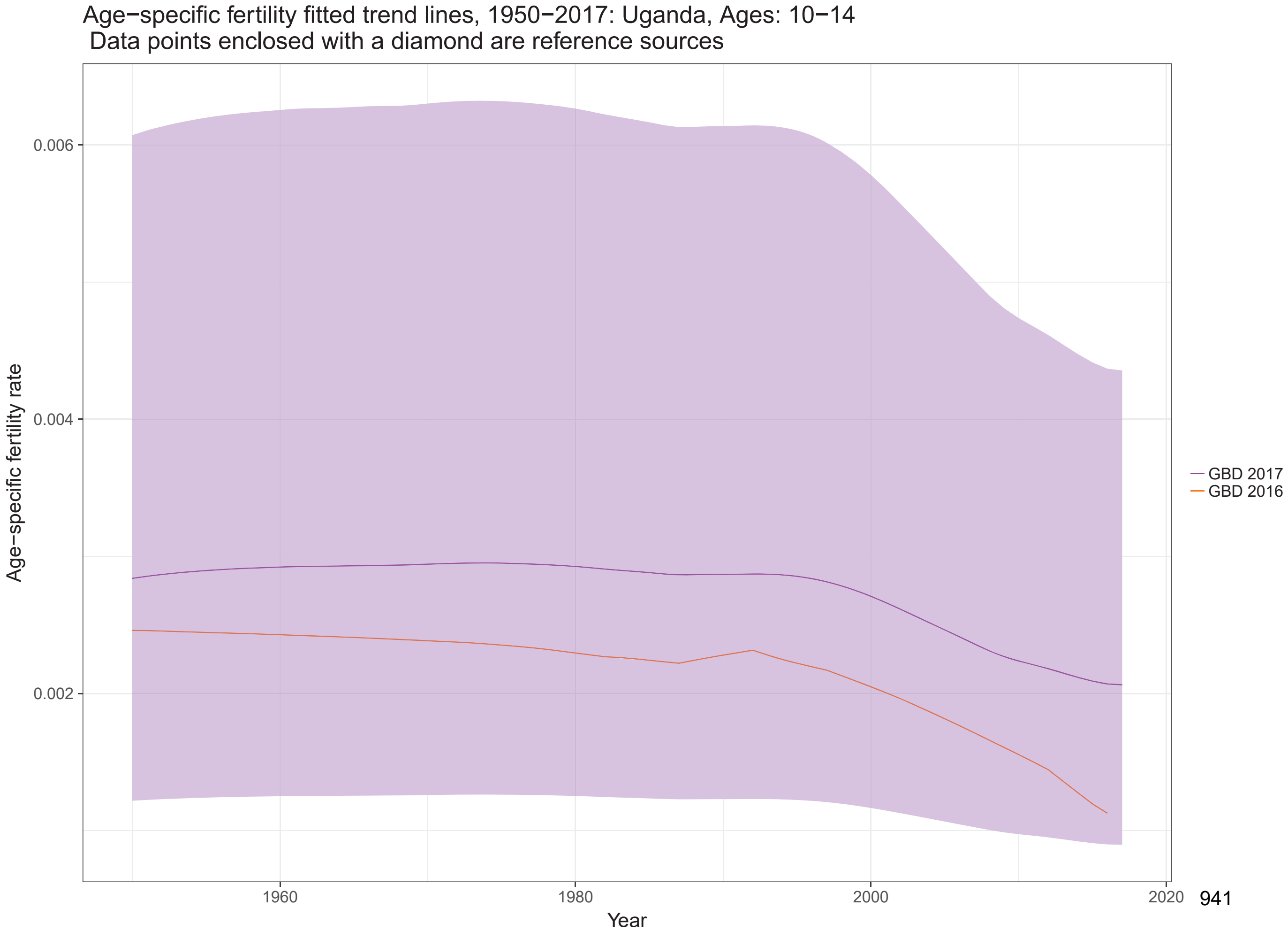

Age−specific fertility fitted trend lines, 1950−2017: Uganda, Ages: 10−14
Data points enclosed with a diamond are reference sources
Age−specific fertility rate
Year
0.006
0.004
0.002
1960
1980
2000
2020
GBD 2017
GBD 2016

Age−specific fertility fitted trend lines, 1950−2017: Uganda, Ages: 15−19
Data points enclosed with a diamond are reference sources

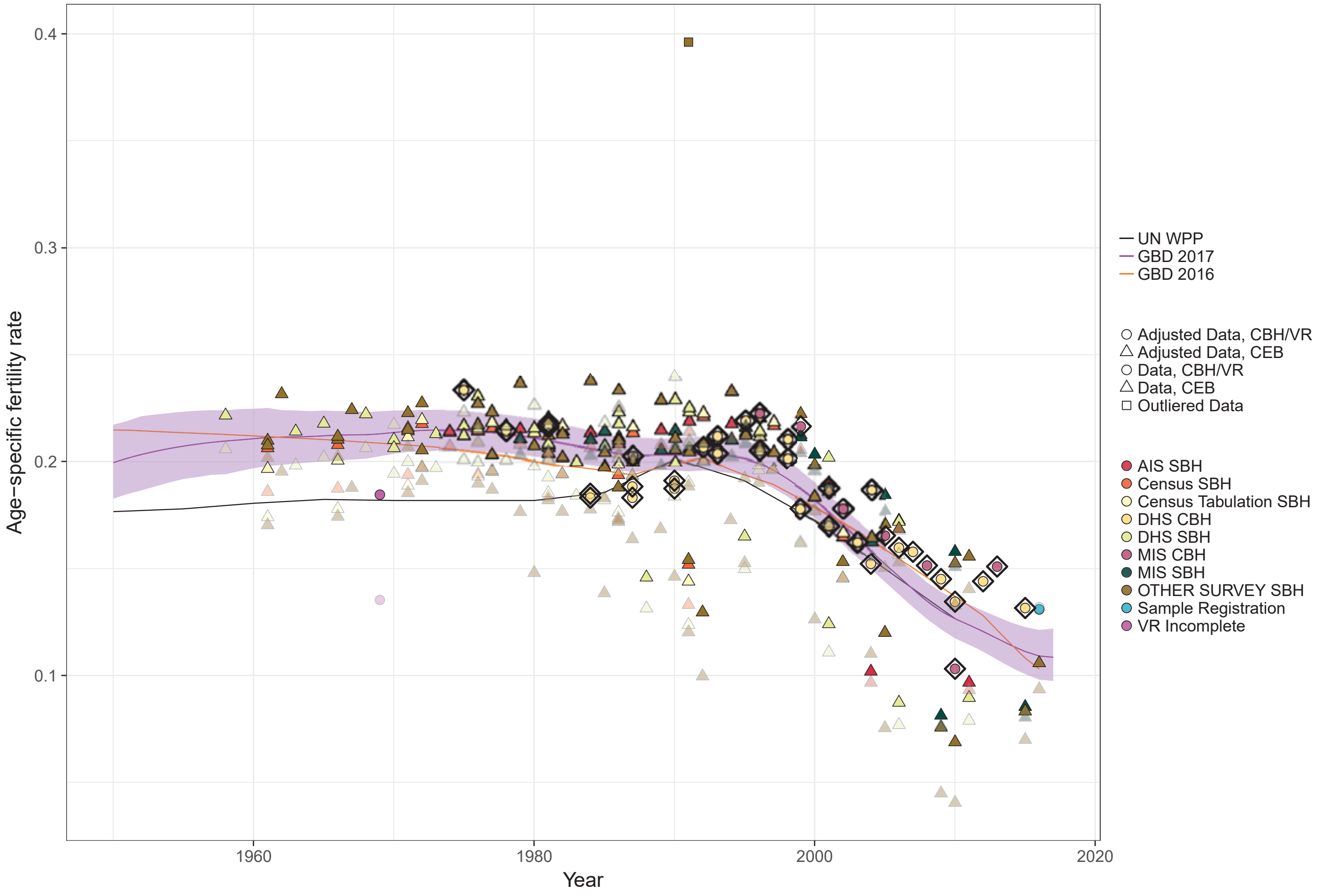

Age−specific fertility fitted trend lines, 1950−2017: Uganda, Ages: 20−24
Data points enclosed with a diamond are reference sources

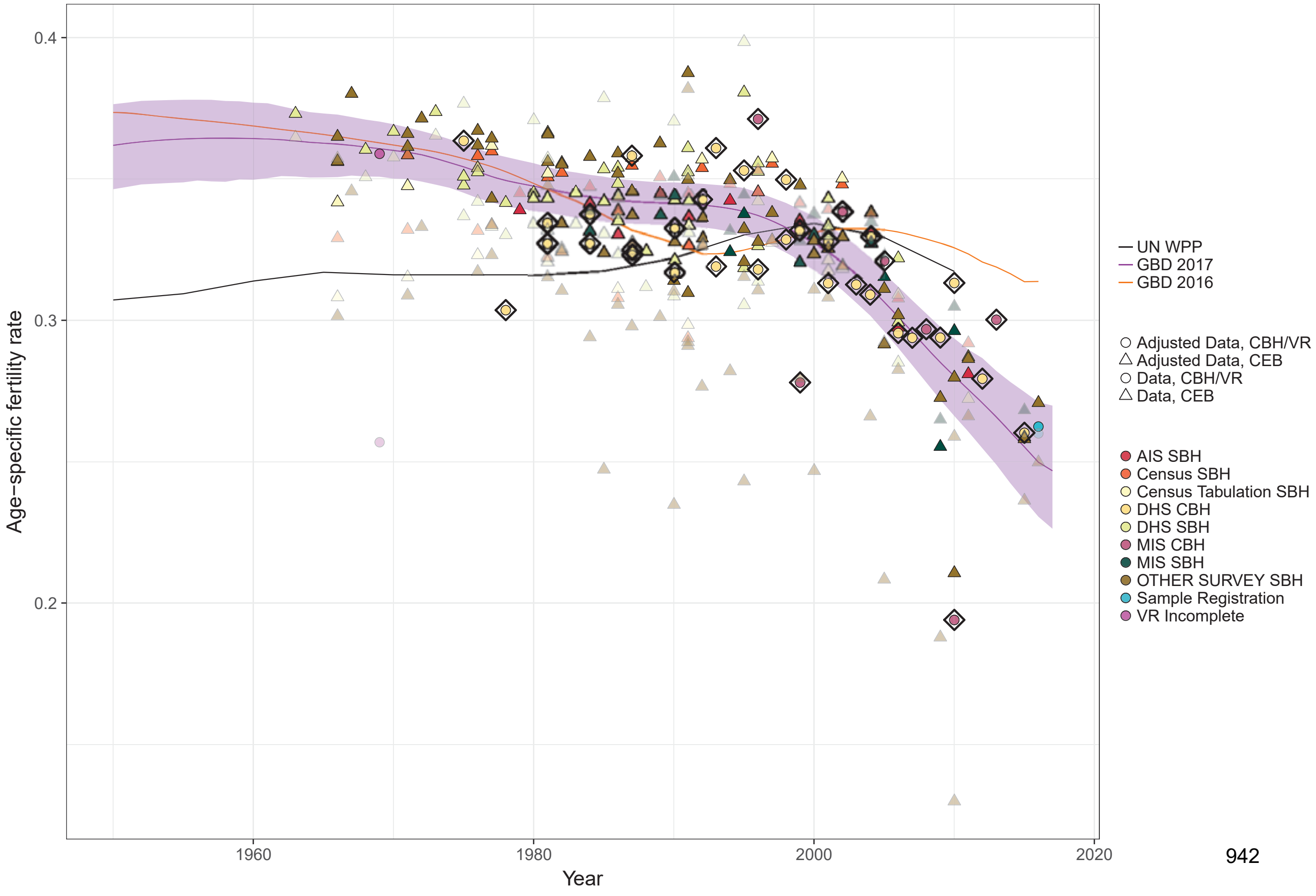

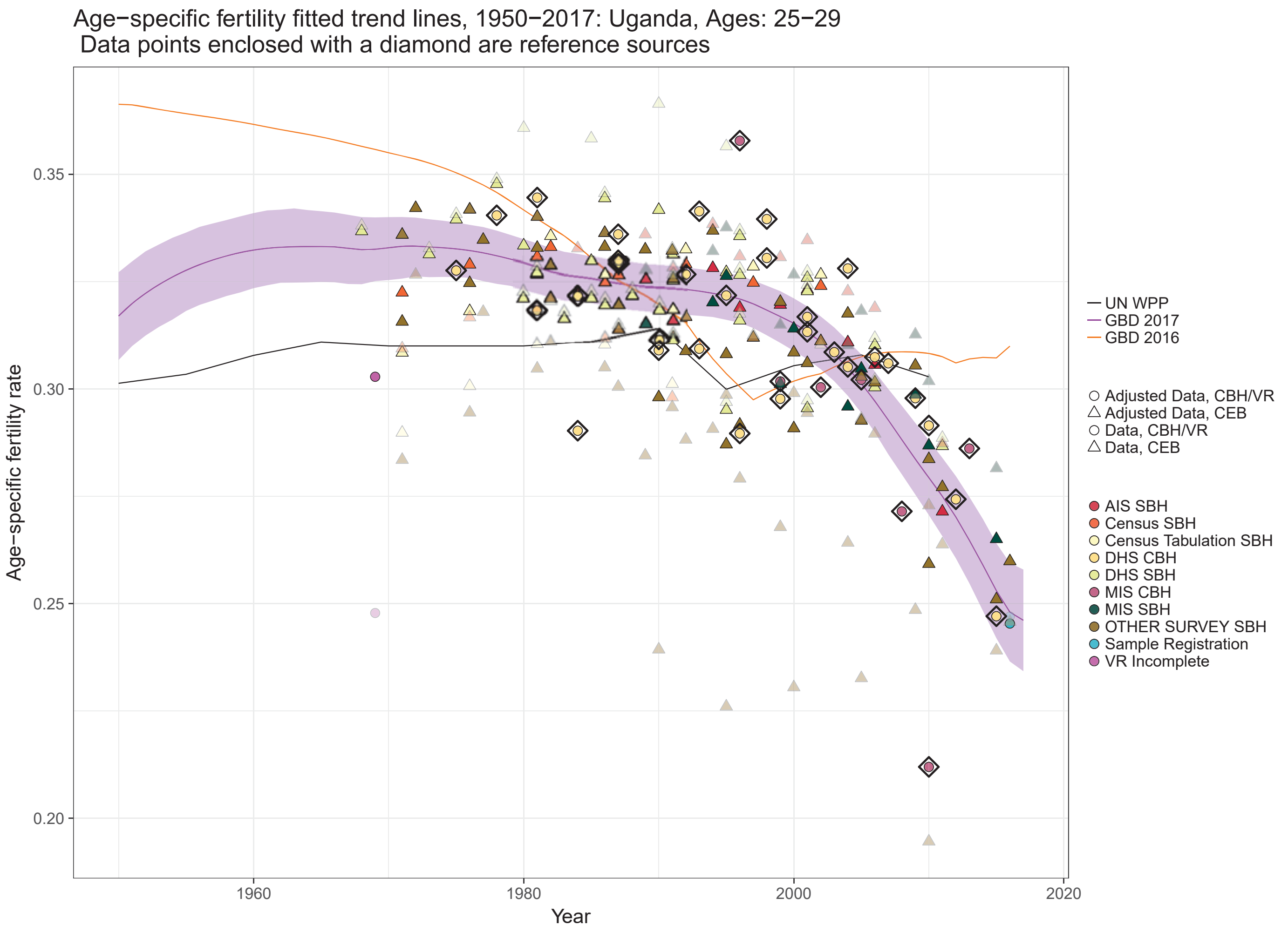
Age−specific fertility fitted trend lines, 1950−2017: Uganda, Ages: 25−29
Data points enclosed with a diamond are reference sources
Age−specific fertility rate
Year
0.35
0.30
0.25
0.20
1960
1980
2000
2020
UN WPP
GBD 2017
GBD 2016
Adjusted Data, CBH/VR
Adjusted Data, CEB
Data, CBH/VR
Data, CEB
AIS SBH
Census SBH
Census Tabulation SBH
DHS CBH
DHS SBH
MIS CBH
MIS SBH
OTHER SURVEY SBH
Sample Registration
VR Incomplete

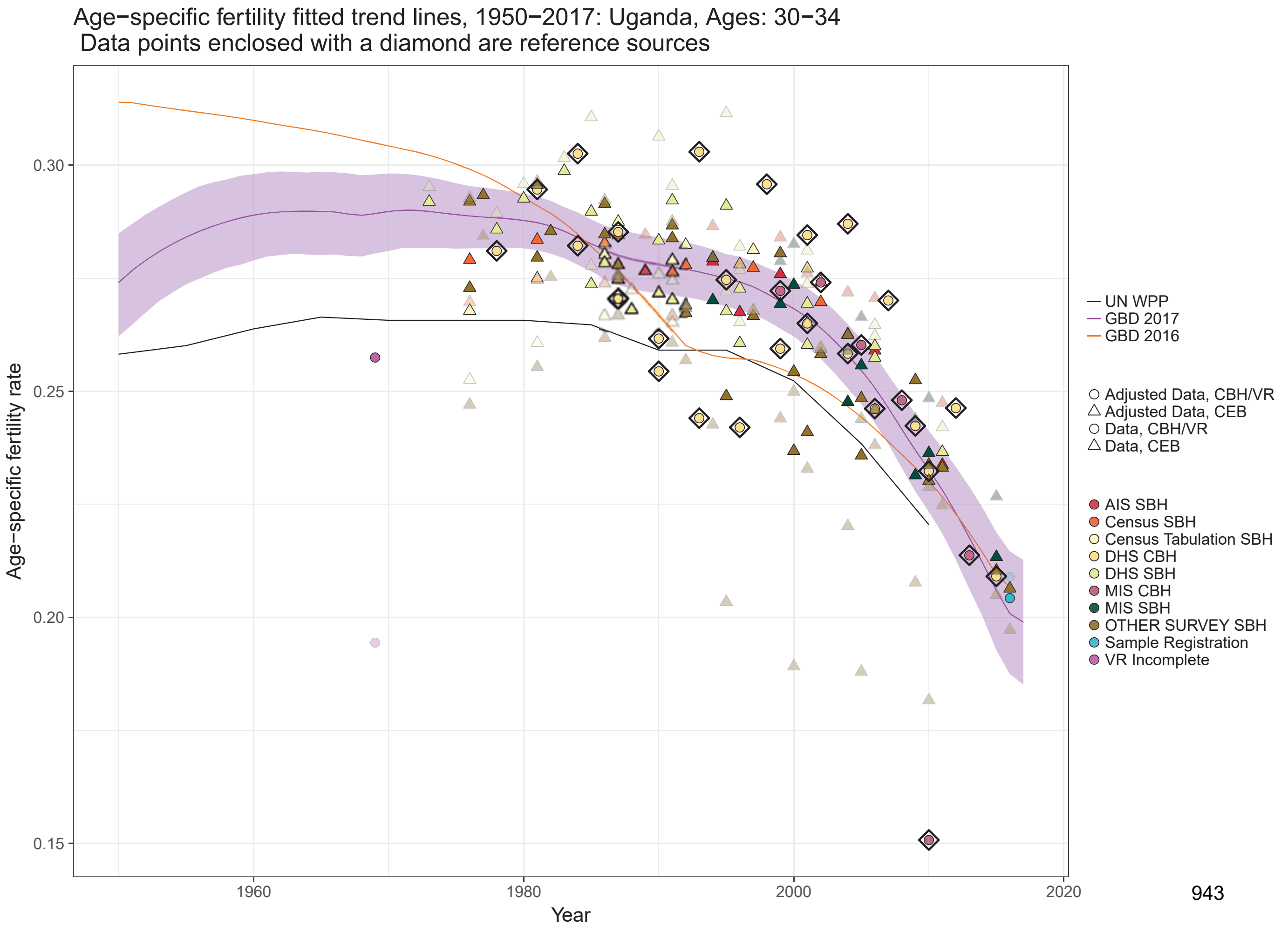
Age−specific fertility fitted trend lines, 1950−2017: Uganda, Ages: 30−34
Data points enclosed with a diamond are reference sources
Age−specific fertility rate
Year
0.30
0.25
0.20
0.15
1960
1980
2000
2020
UN WPP
GBD 2017
GBD 2016
Adjusted Data, CBH/VR
Adjusted Data, CEB
Data, CBH/VR
Data, CEB
AIS SBH
Census SBH
Census Tabulation SBH
DHS CBH
DHS SBH
MIS CBH
MIS SBH
OTHER SURVEY SBH
Sample Registration
VR Incomplete

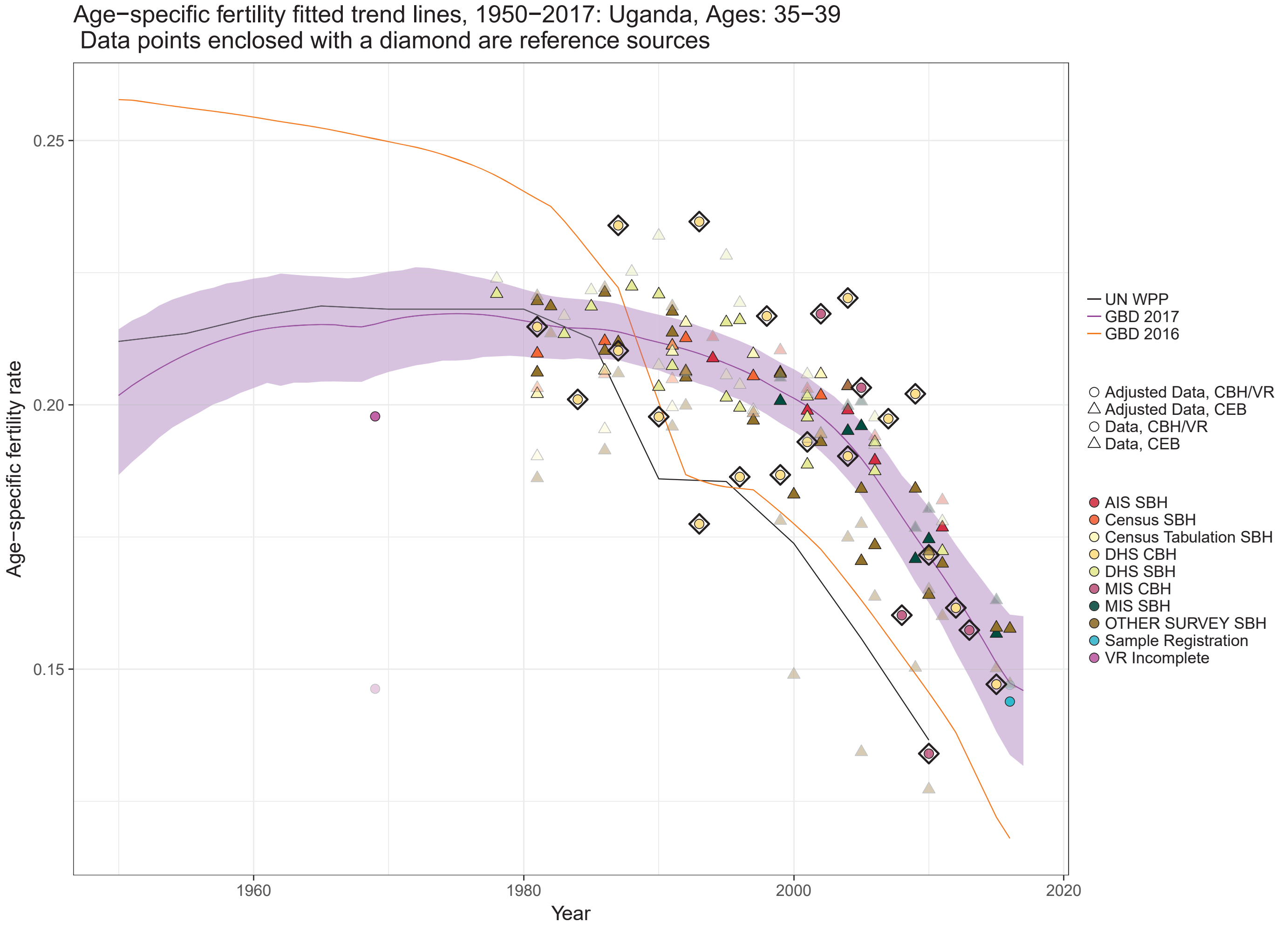

Age−specific fertility fitted trend lines, 1950−2017: Uganda, Ages: 35−39
Data points enclosed with a diamond are reference sources
Age−specific fertility rate
0.25
0.20
0.15
1960
1980
2000
2020
Year
UN WPP
GBD 2017
GBD 2016
Adjusted Data, CBH/VR
Adjusted Data, CEB
Data, CBH/VR
Data, CEB
AIS SBH
Census SBH
Census Tabulation SBH
DHS CBH
DHS SBH
MIS CBH
MIS SBH
OTHER SURVEY SBH
Sample Registration
VR Incomplete

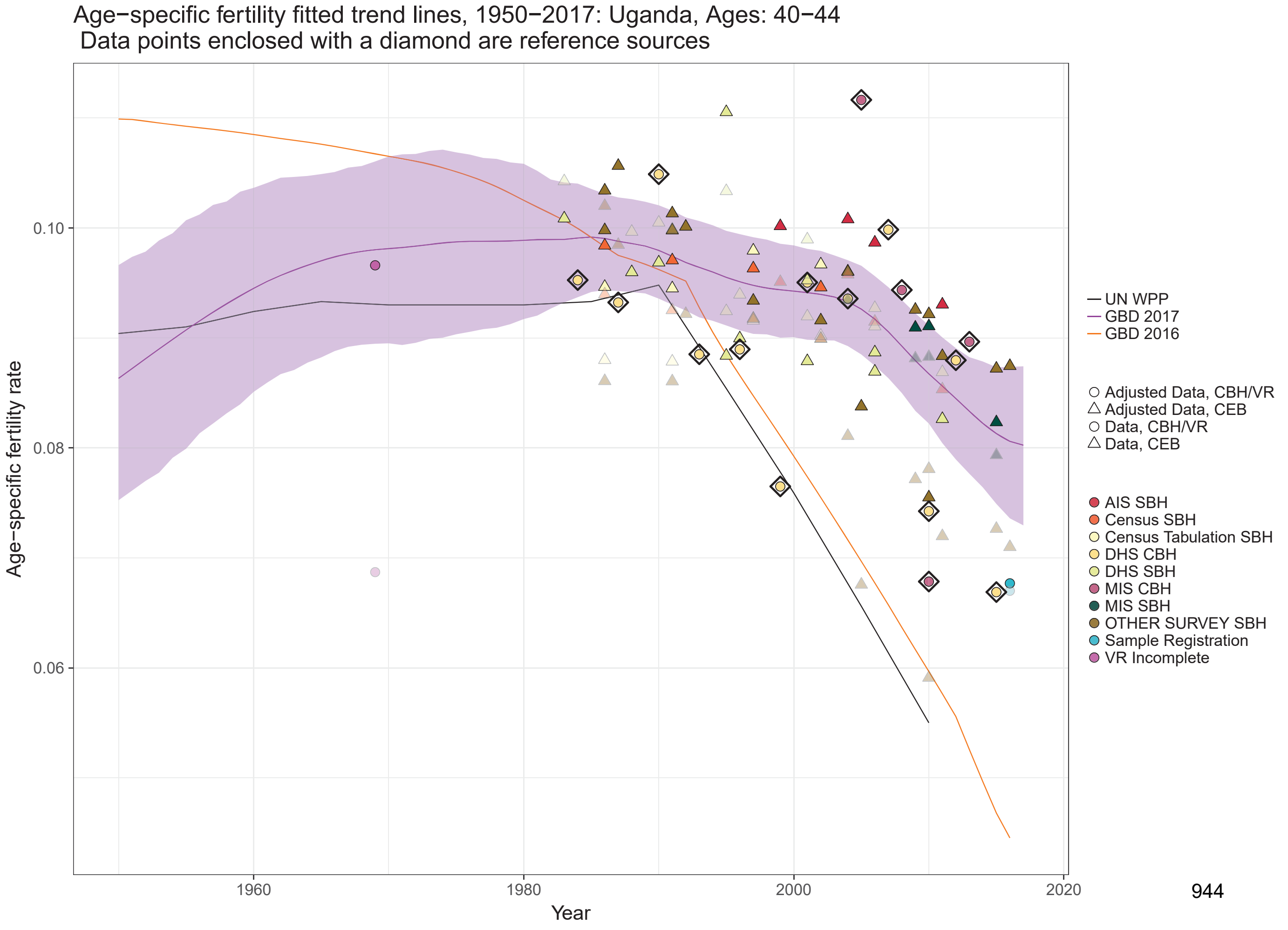

Age−specific fertility fitted trend lines, 1950−2017: Uganda, Ages: 40−44
Data points enclosed with a diamond are reference sources
Age−specific fertility rate
0.10
0.08
0.06
1960
1980
2000
2020
Year
UN WPP
GBD 2017
GBD 2016
Adjusted Data, CBH/VR
Adjusted Data, CEB
Data, CBH/VR
Data, CEB
AIS SBH
Census SBH
Census Tabulation SBH
DHS CBH
DHS SBH
MIS CBH
MIS SBH
OTHER SURVEY SBH
Sample Registration
VR Incomplete

Age−specific fertility fitted trend lines, 1950−2017: Uganda, Ages: 45−49
Data points enclosed with a diamond are reference sources

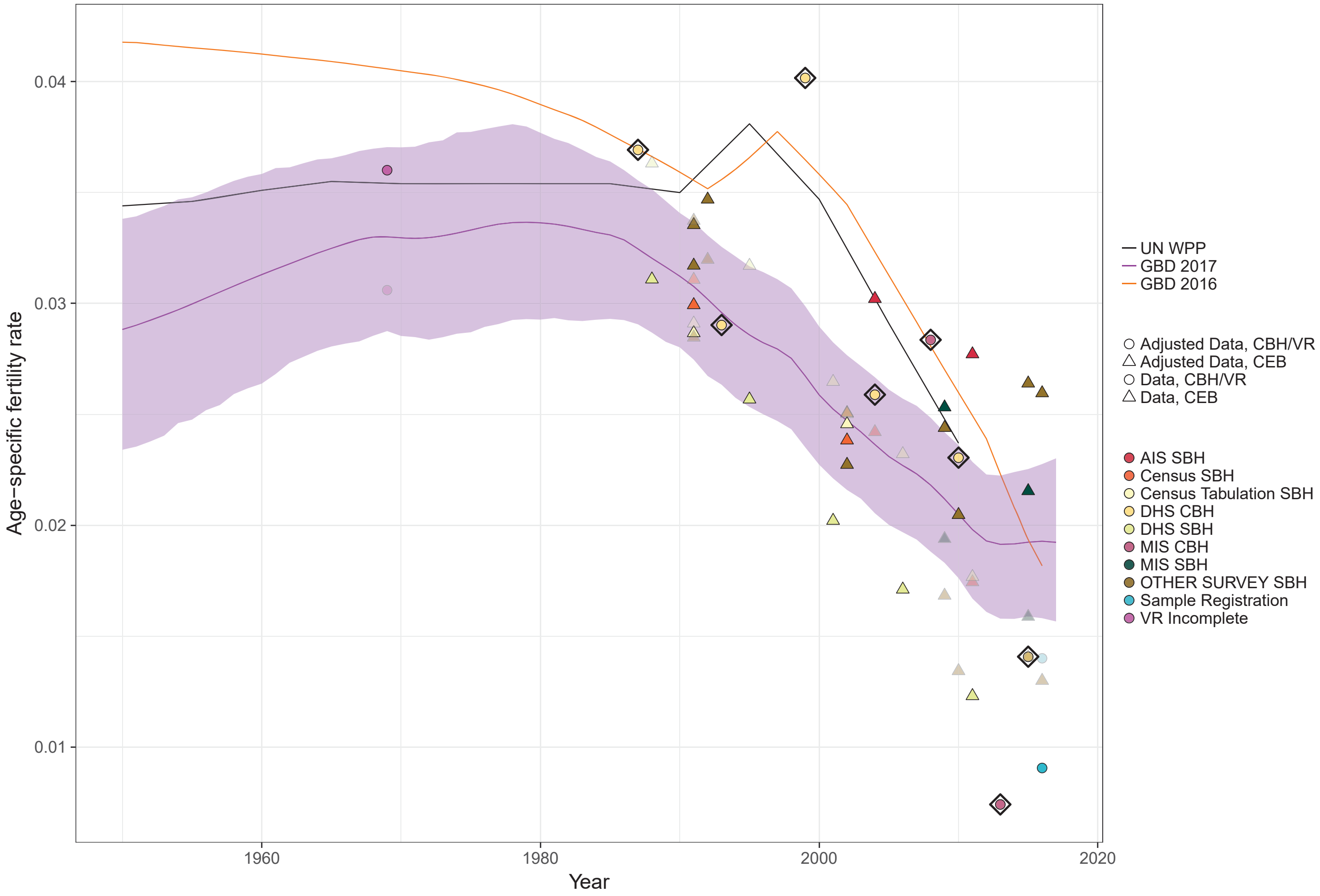

Age−specific fertility fitted trend lines, 1950−2017: Uganda, Ages: 50−54
Data points enclosed with a diamond are reference sources

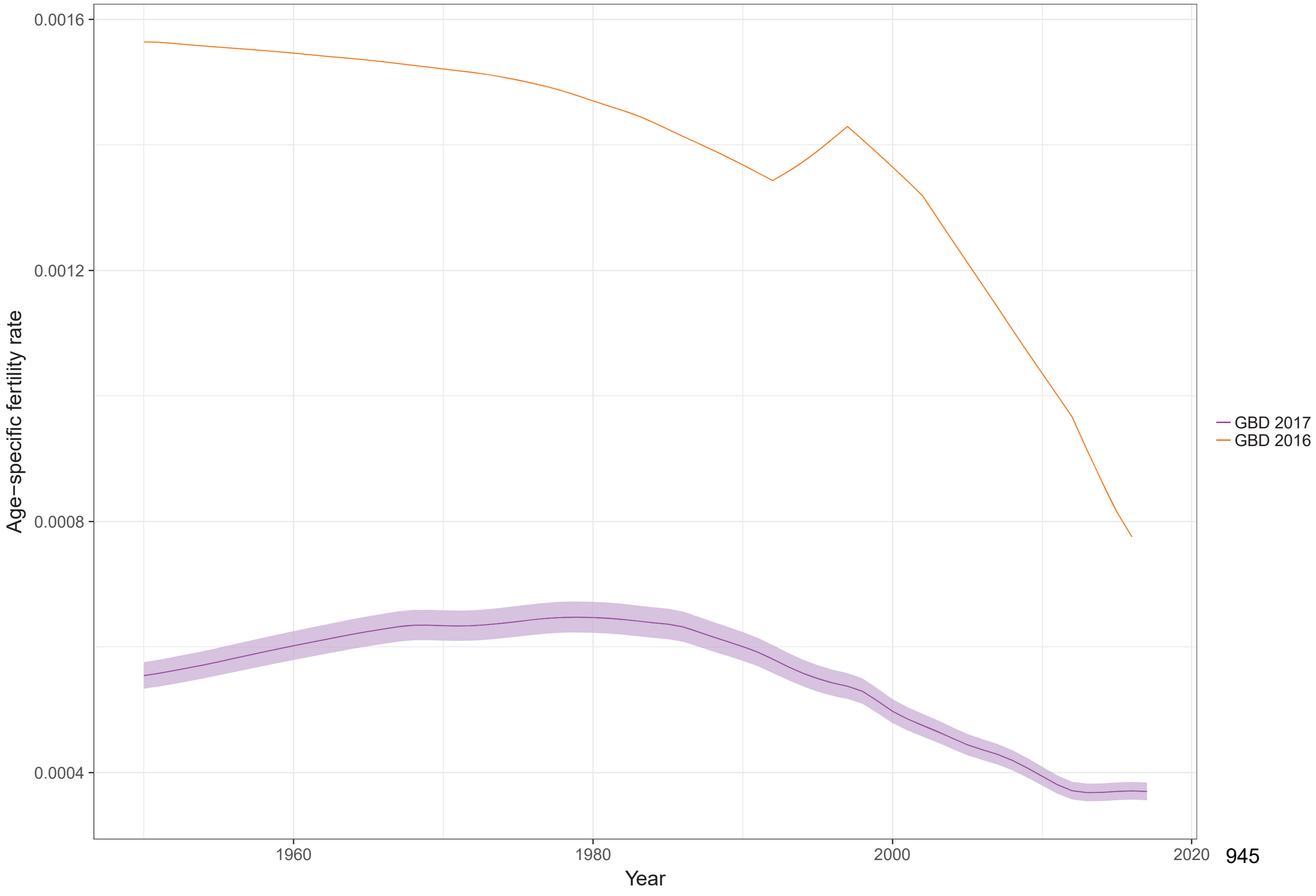

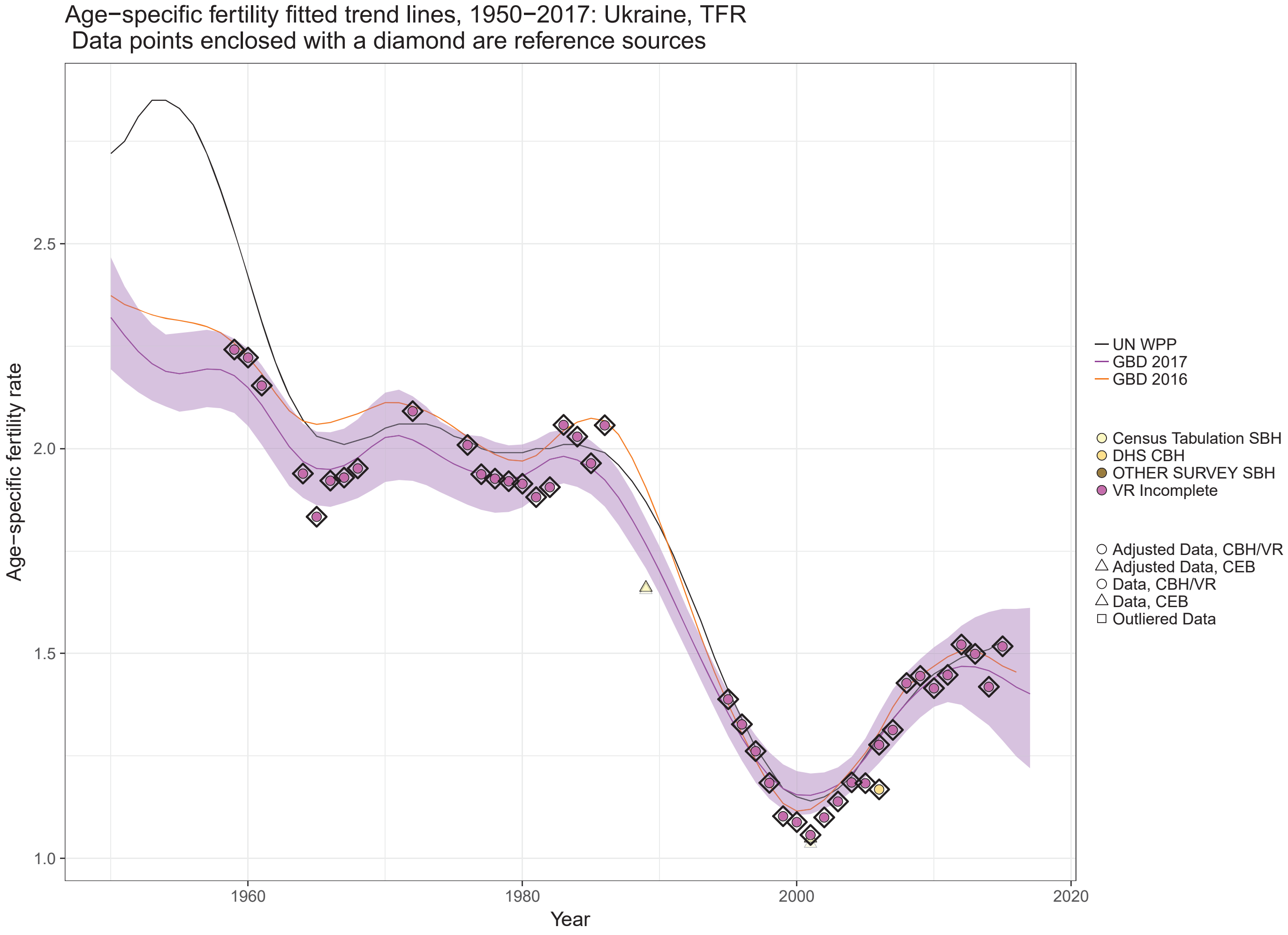

Age−specific fertility fitted trend lines, 1950−2017: Ukraine, TFR
Data points enclosed with a diamond are reference sources
Age−specific fertility rate
Year
1.0
1.5
2.0
2.5
1960
1980
2000
2020
UN WPP
GBD 2017
GBD 2016
Census Tabulation SBH
DHS CBH
OTHER SURVEY SBH
VR Incomplete
Adjusted Data, CBH/VR
Adjusted Data, CEB
Data, CBH/VR
Data, CEB
Outliered Data

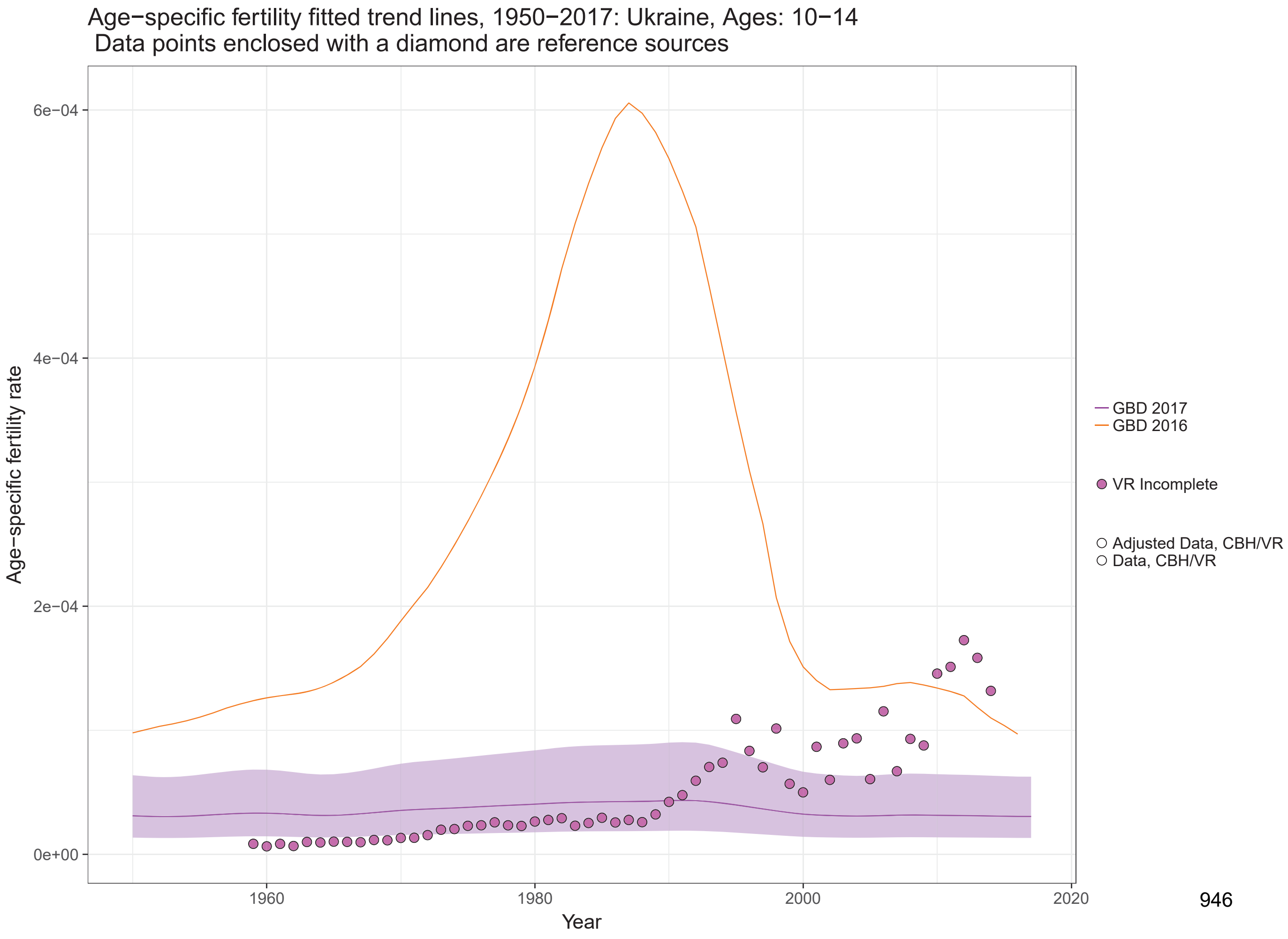

Age−specific fertility fitted trend lines, 1950−2017: Ukraine, Ages: 10−14
Data points enclosed with a diamond are reference sources
Age−specific fertility rate
Year
0e+00
2e−04
4e−04
6e−04
1960
1980
2000
2020
GBD 2017
GBD 2016
VR Incomplete
Adjusted Data, CBH/VR
Data, CBH/VR

Age−specific fertility fitted trend lines, 1950−2017: Ukraine, Ages: 15−19
Data points enclosed with a diamond are reference sources

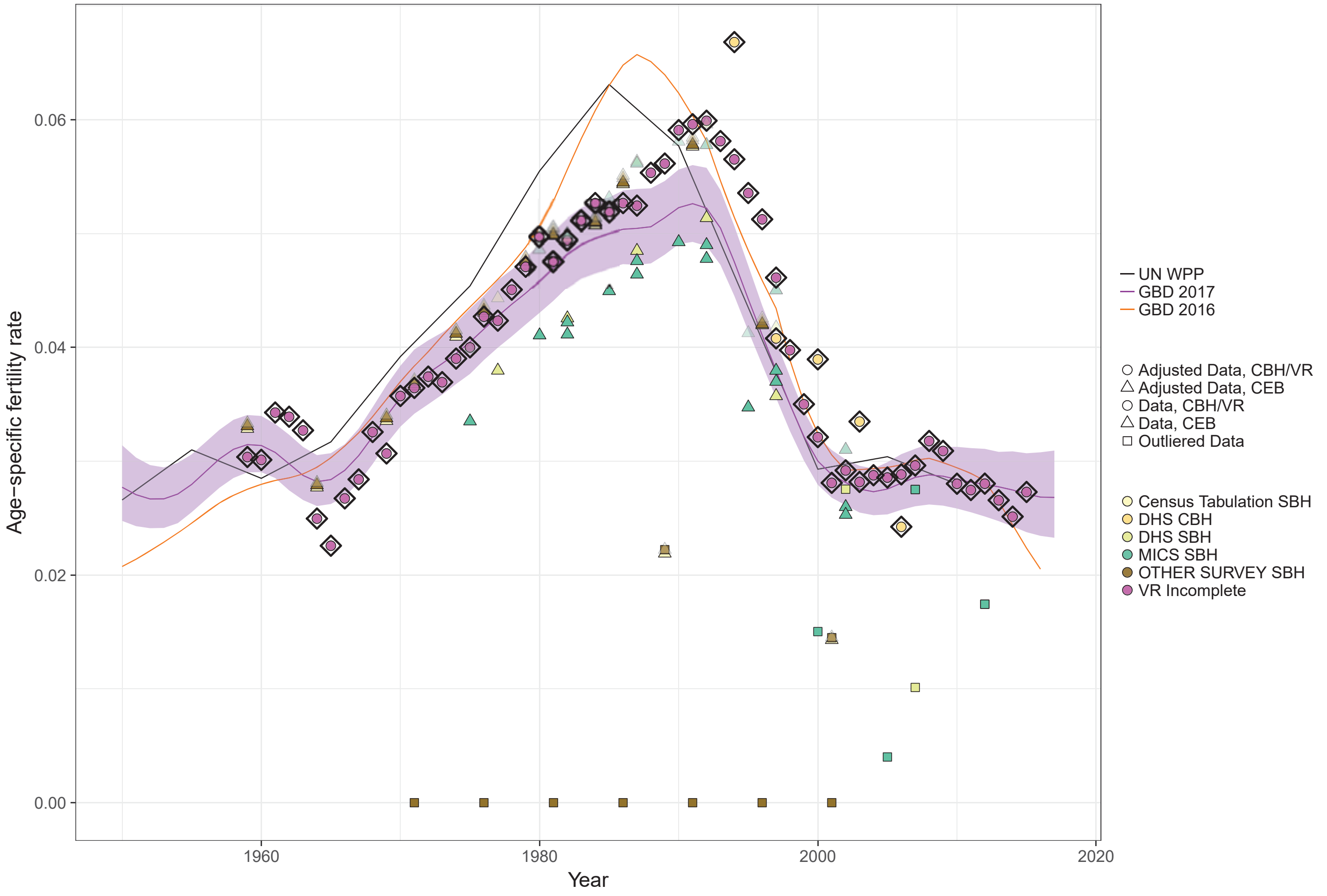

Age−specific fertility fitted trend lines, 1950−2017: Ukraine, Ages: 20−24
Data points enclosed with a diamond are reference sources

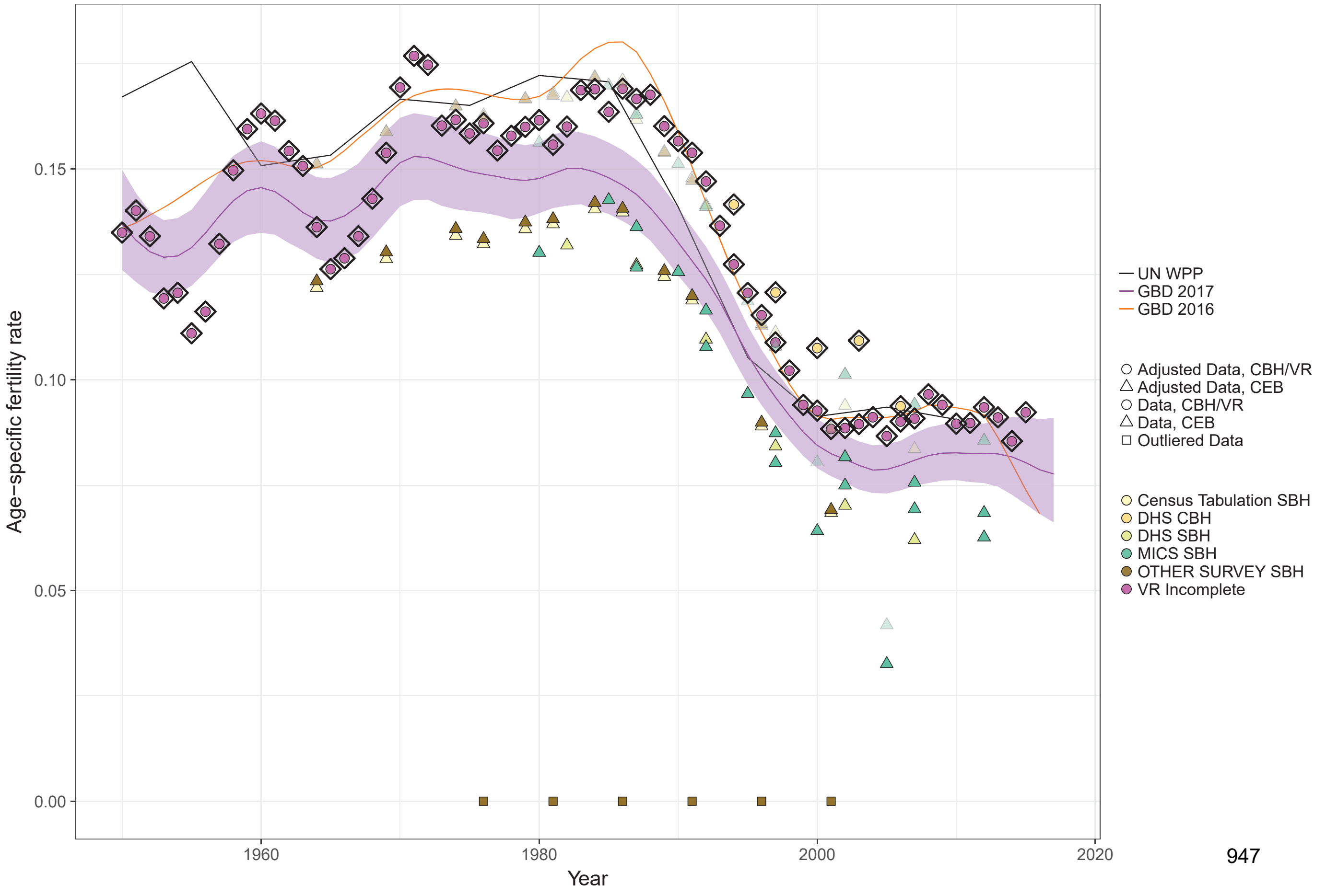

Age−specific fertility fitted trend lines, 1950−2017: Ukraine, Ages: 25−29
Data points enclosed with a diamond are reference sources

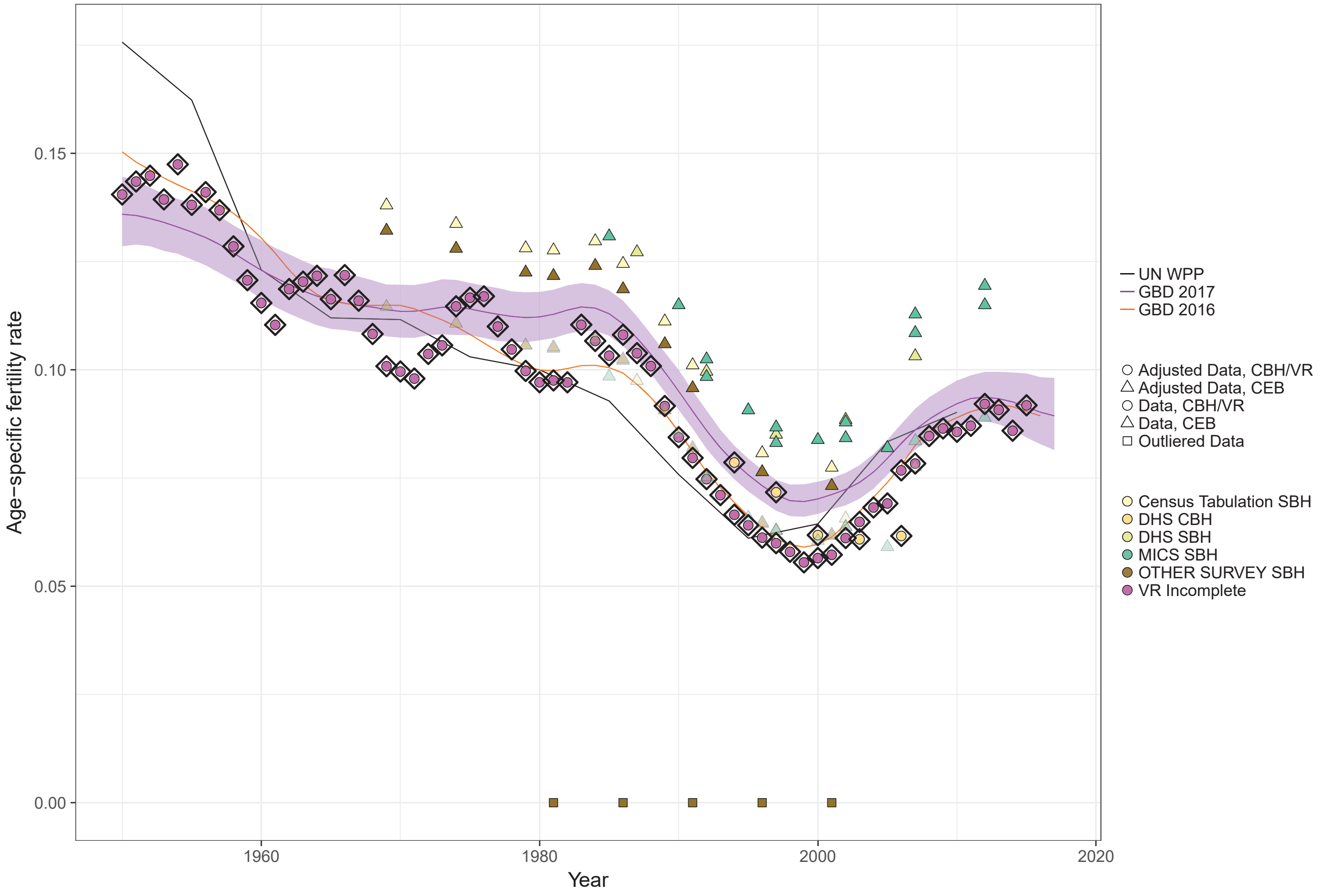

Age−specific fertility fitted trend lines, 1950−2017: Ukraine, Ages: 30−34
Data points enclosed with a diamond are reference sources

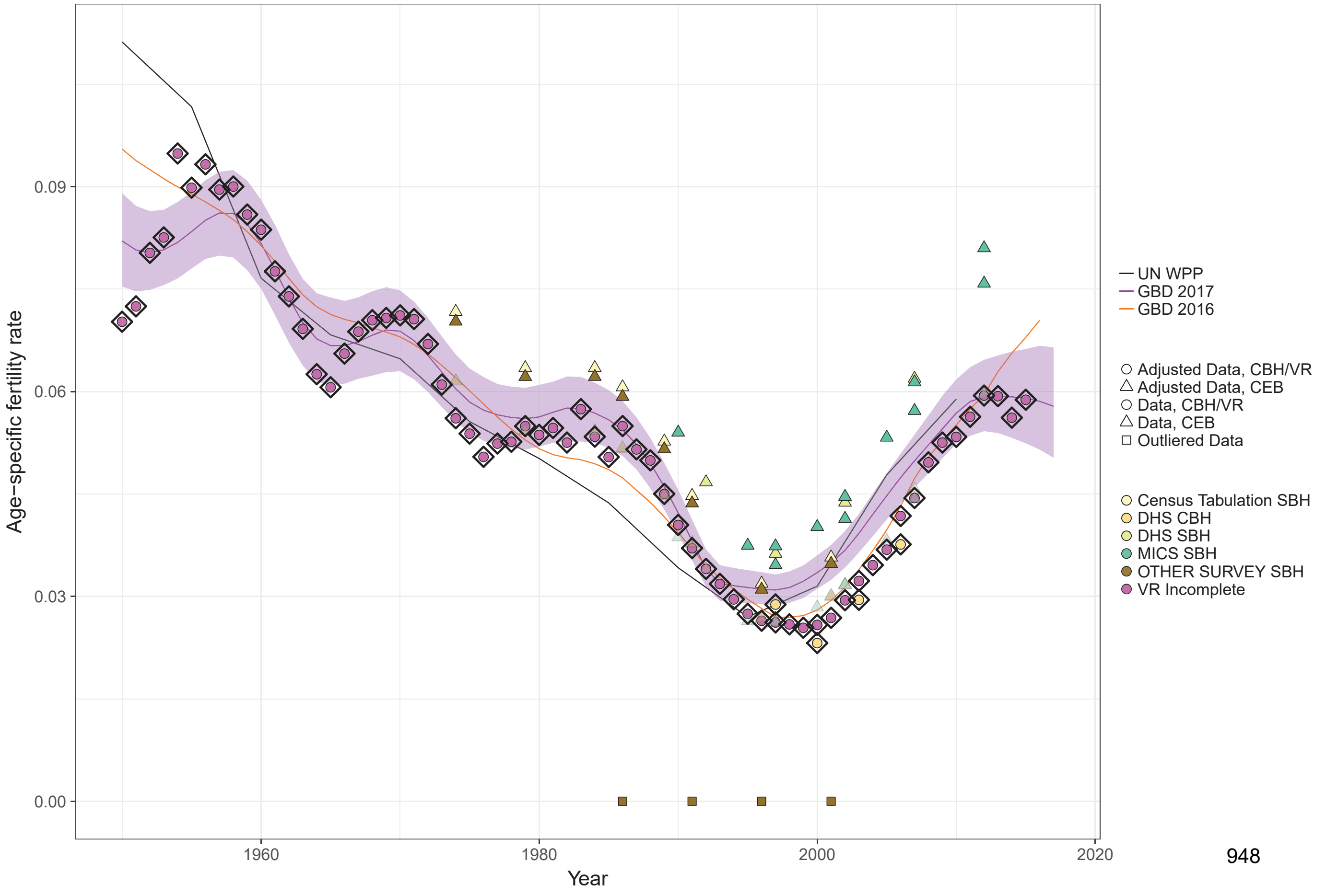

Age−specific fertility fitted trend lines, 1950−2017: Ukraine, Ages: 35−39
Data points enclosed with a diamond are reference sources

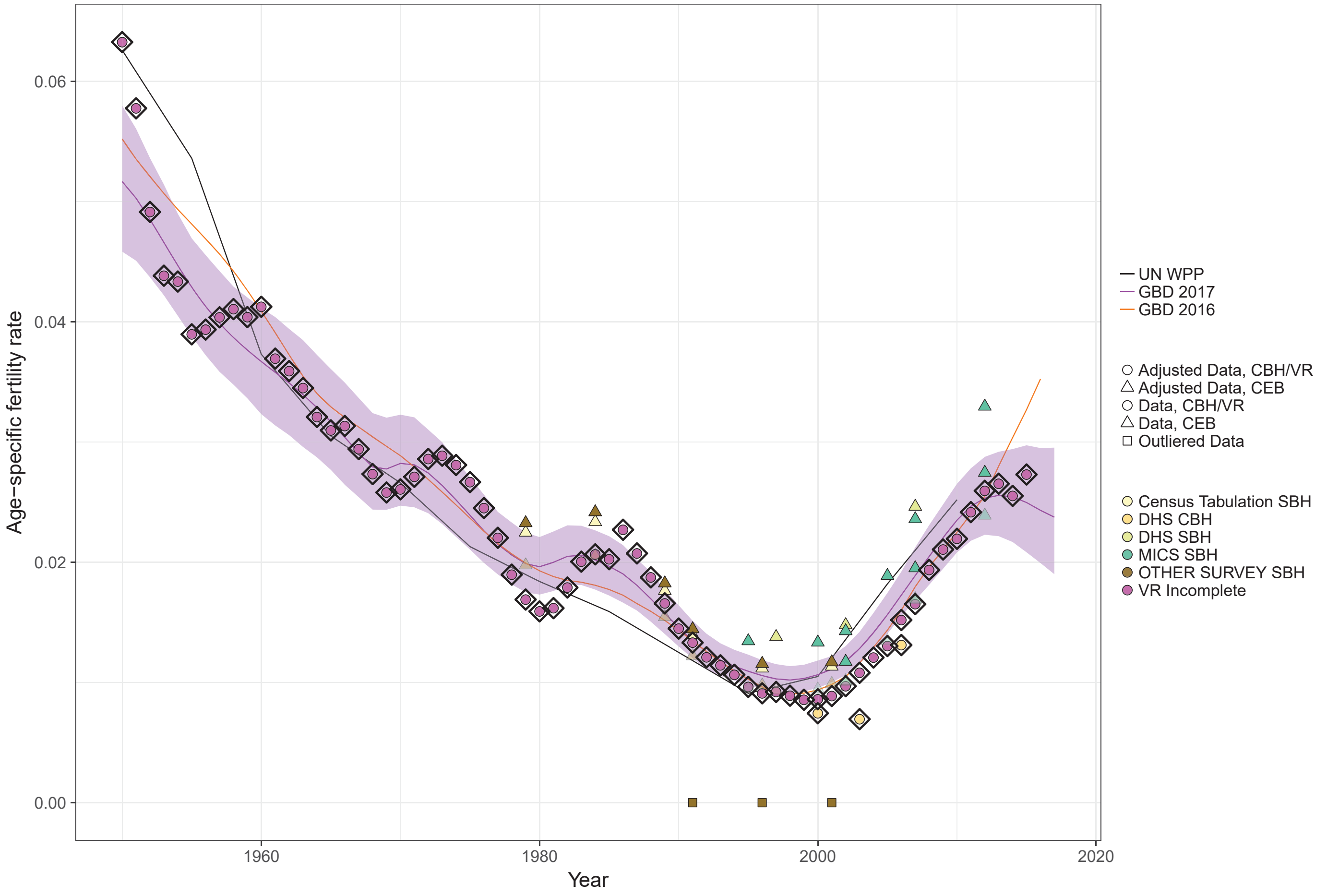

Age−specific fertility fitted trend lines, 1950−2017: Ukraine, Ages: 40−44
Data points enclosed with a diamond are reference sources

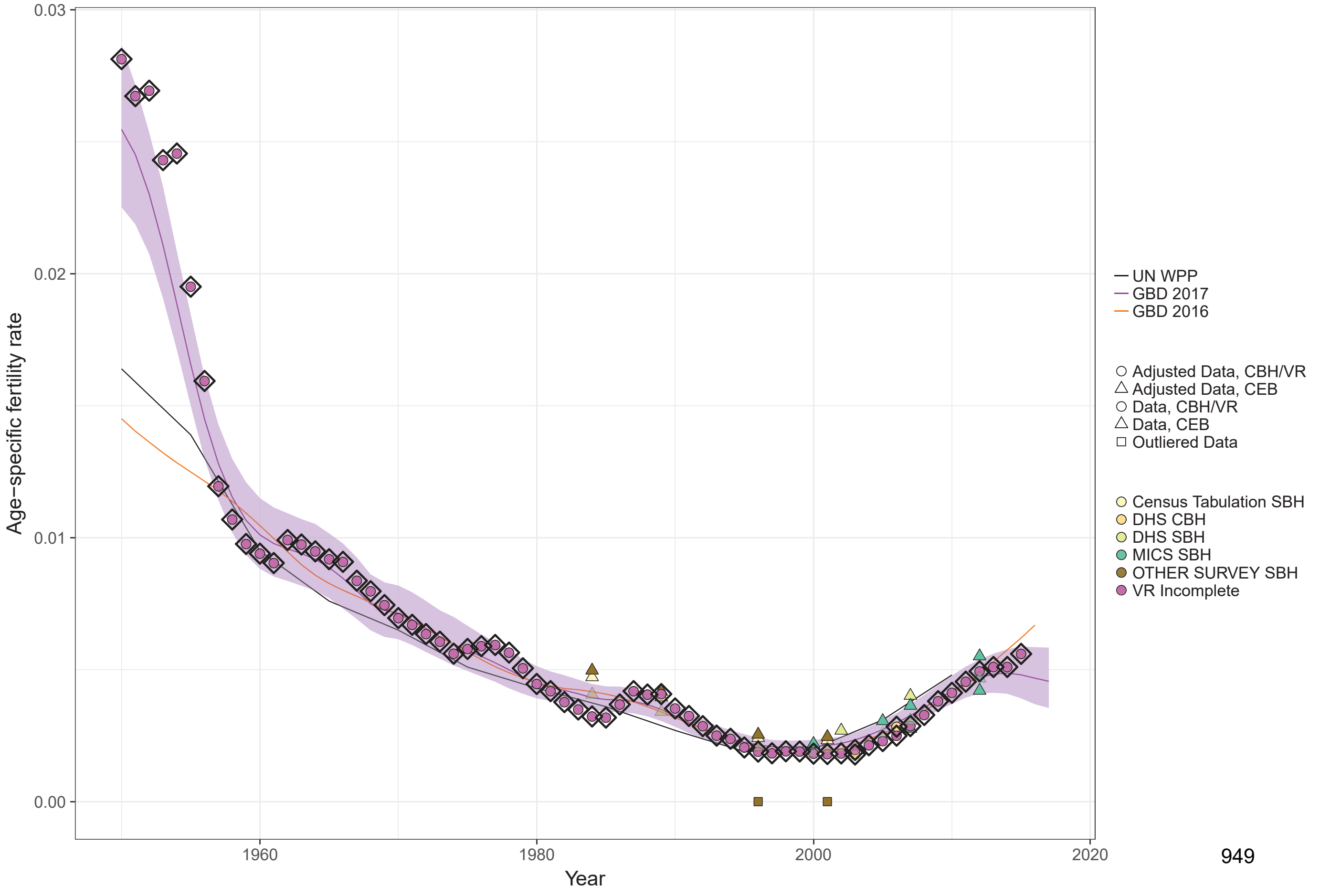

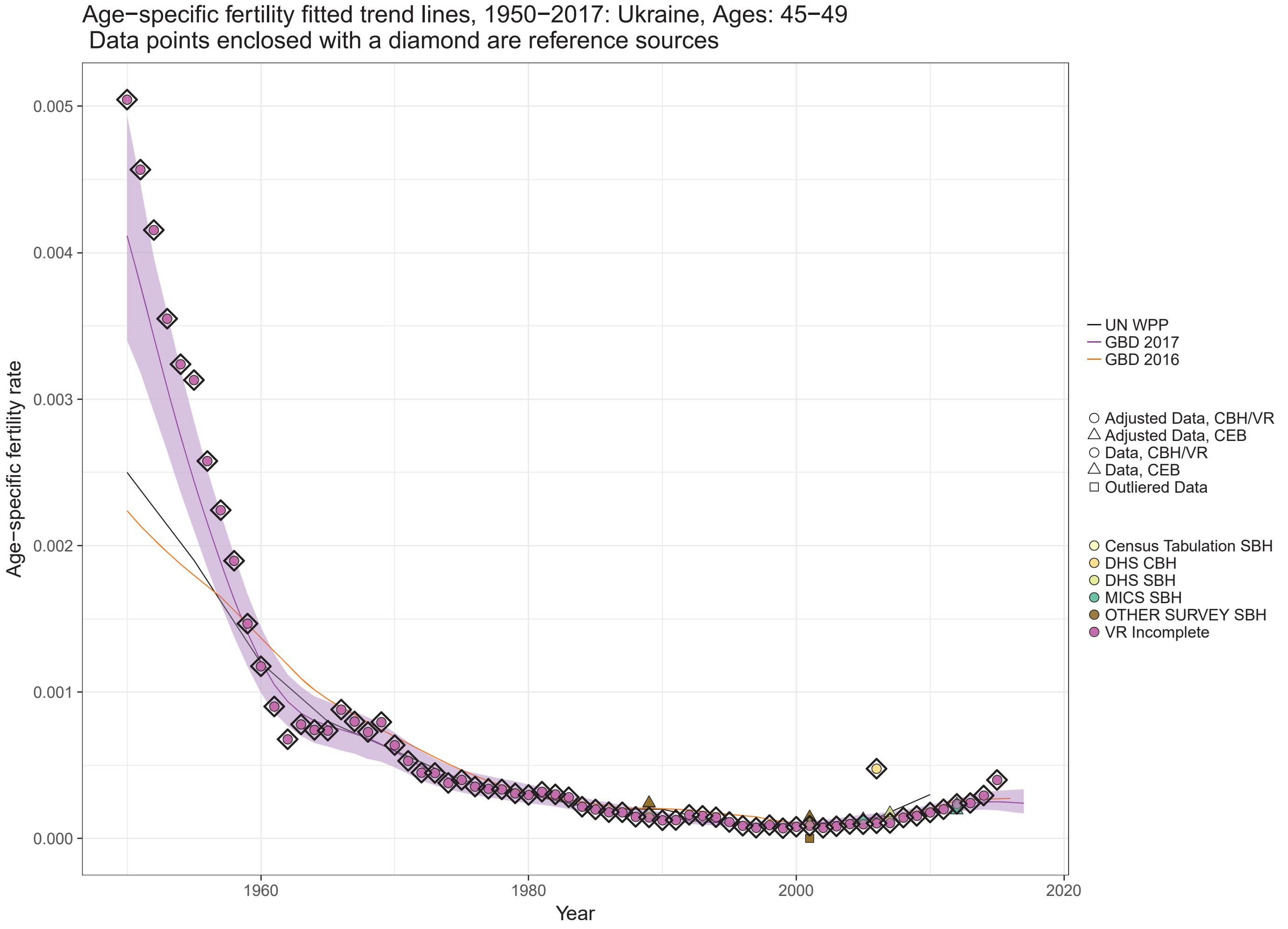
Age−specific fertility fitted trend lines, 1950−2017: Ukraine, Ages: 45−49
Data points enclosed with a diamond are reference sources
Age−specific fertility rate
0.000
0.001
0.002
0.003
0.004
0.005
1960
1980
2000
2020
Year
UN WPP
GBD 2017
GBD 2016
Adjusted Data, CBH/VR
Adjusted Data, CEB
Data, CBH/VR
Data, CEB
Outliered Data
Census Tabulation SBH
DHS CBH
DHS SBH
MICS SBH
OTHER SURVEY SBH
VR Incomplete

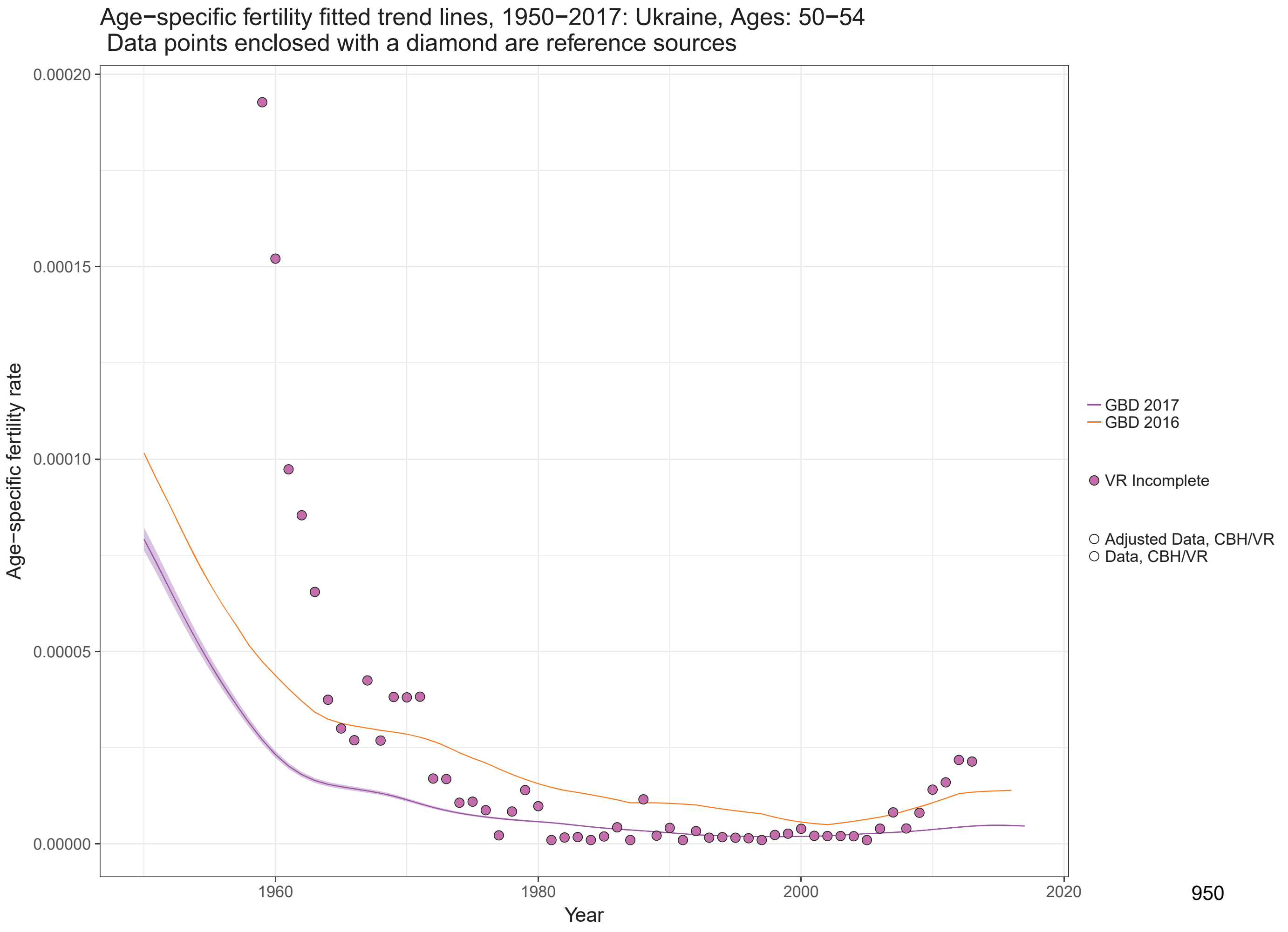
Age−specific fertility fitted trend lines, 1950−2017: Ukraine, Ages: 50−54
Data points enclosed with a diamond are reference sources
Age−specific fertility rate
0.00000
0.00005
0.00010
0.00015
0.00020
1960
1980
2000
2020
Year
GBD 2017
GBD 2016
VR Incomplete
Adjusted Data, CBH/VR
Data, CBH/VR

Age−specific fertility fitted trend lines, 1950−2017: United Arab Emirates, TFR
Data points enclosed with a diamond are reference sources

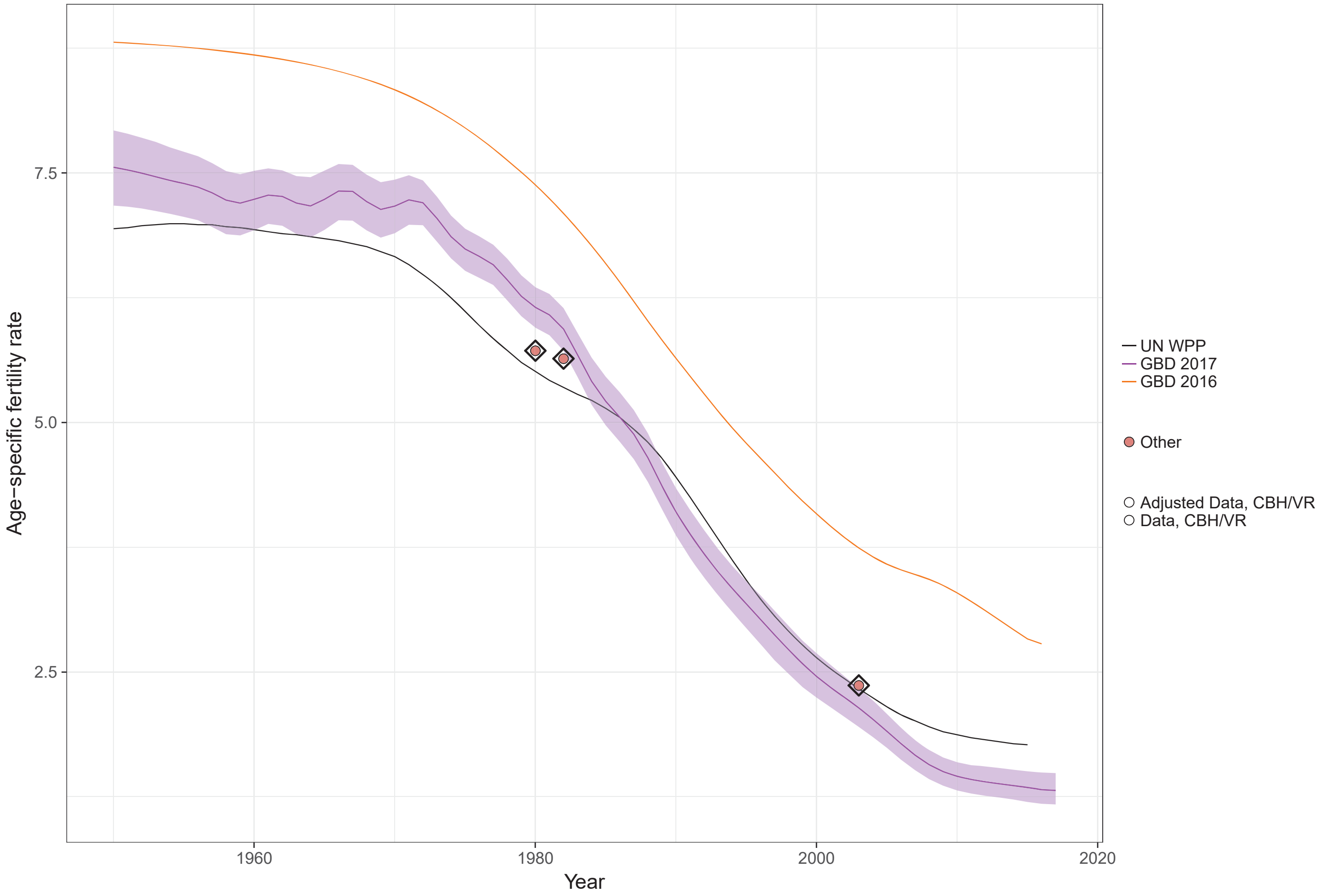

Age−specific fertility fitted trend lines, 1950−2017: United Arab Emirates, Ages: 10−14
Data points enclosed with a diamond are reference sources

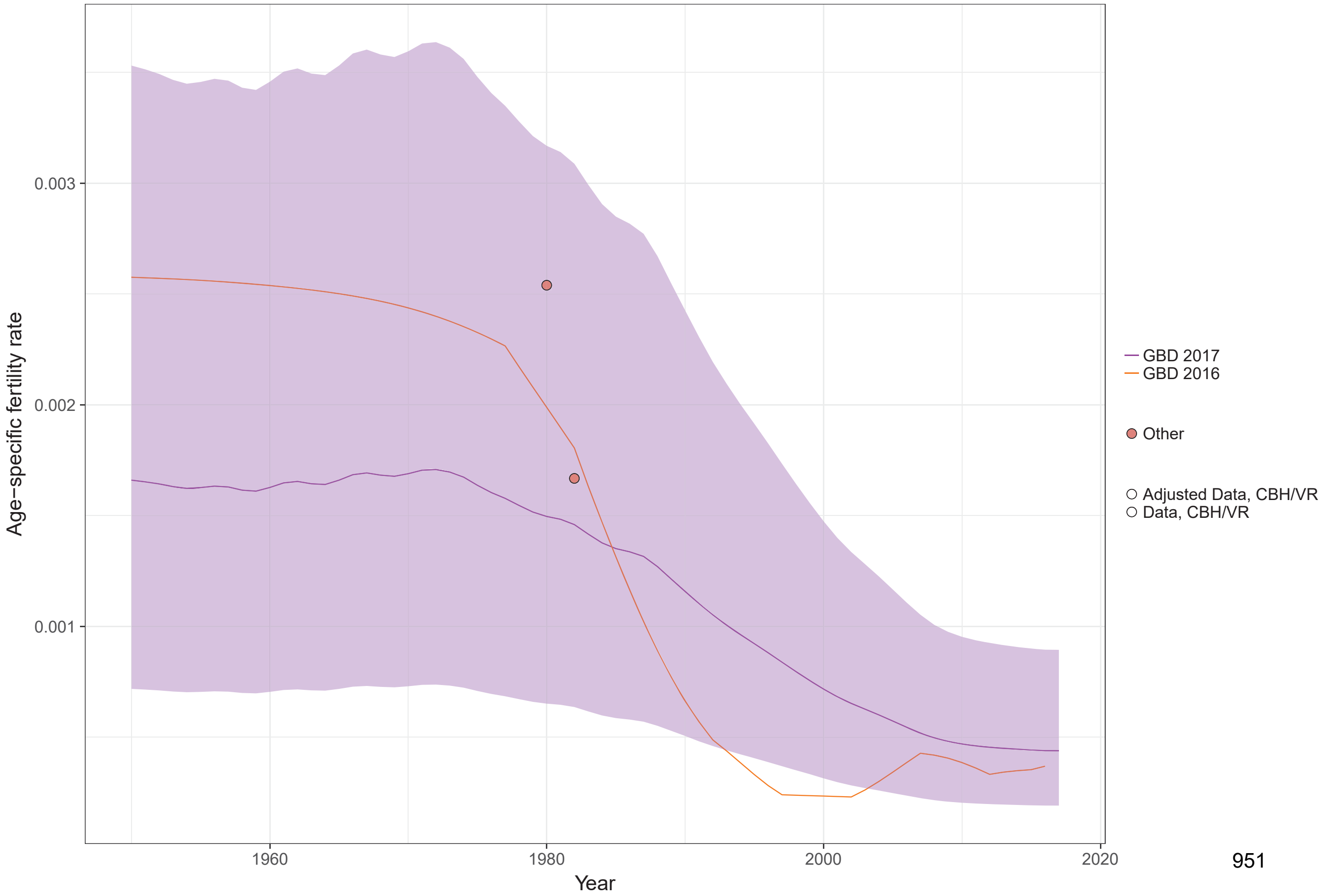

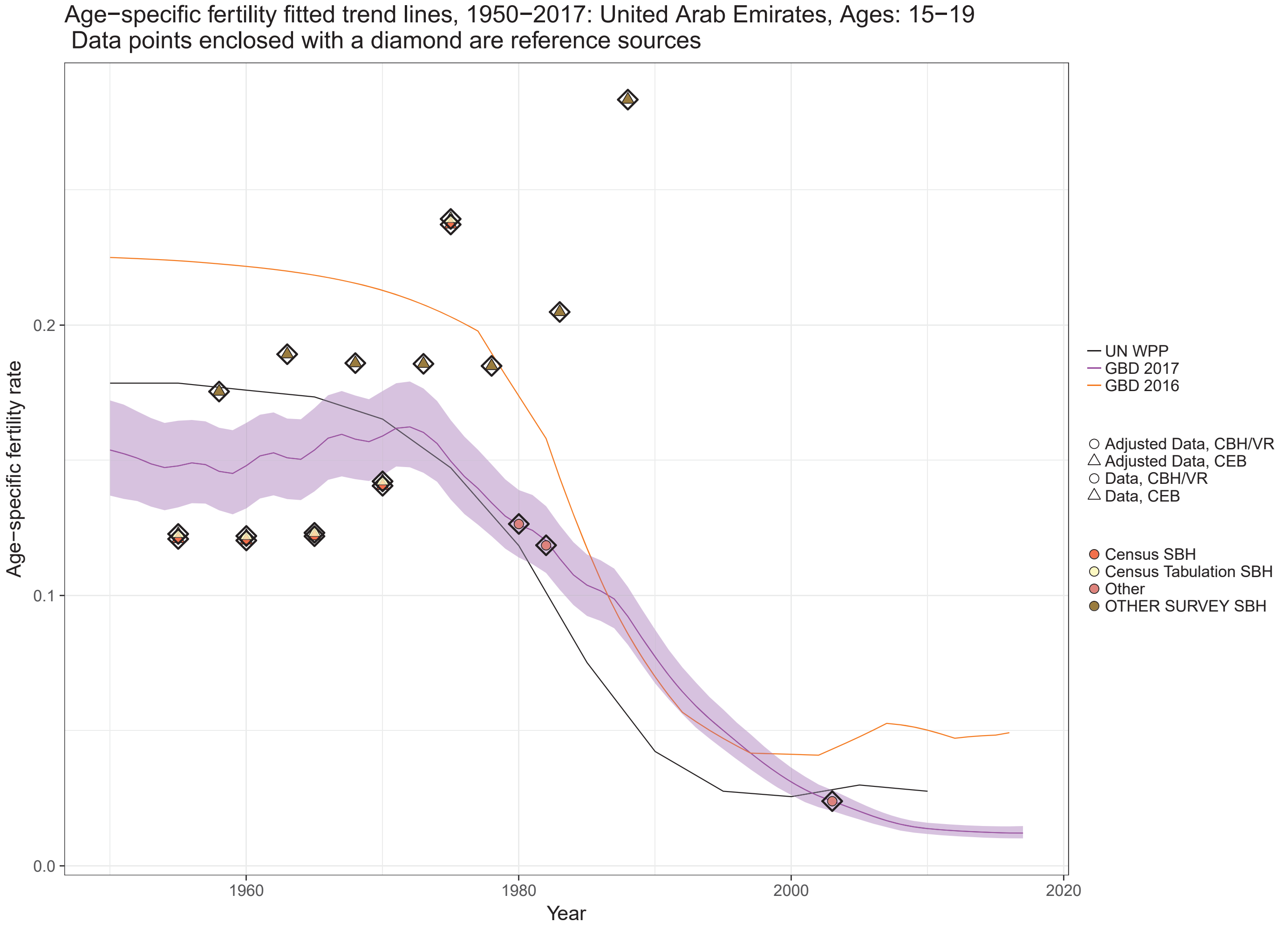

Age−specific fertility fitted trend lines, 1950−2017: United Arab Emirates, Ages: 15−19
Data points enclosed with a diamond are reference sources
Age−specific fertility rate
Year
0.0
0.1
0.2
1960
1980
2000
2020
UN WPP
GBD 2017
GBD 2016
Adjusted Data, CBH/VR
Adjusted Data, CEB
Data, CBH/VR
Data, CEB
Census SBH
Census Tabulation SBH
Other
OTHER SURVEY SBH

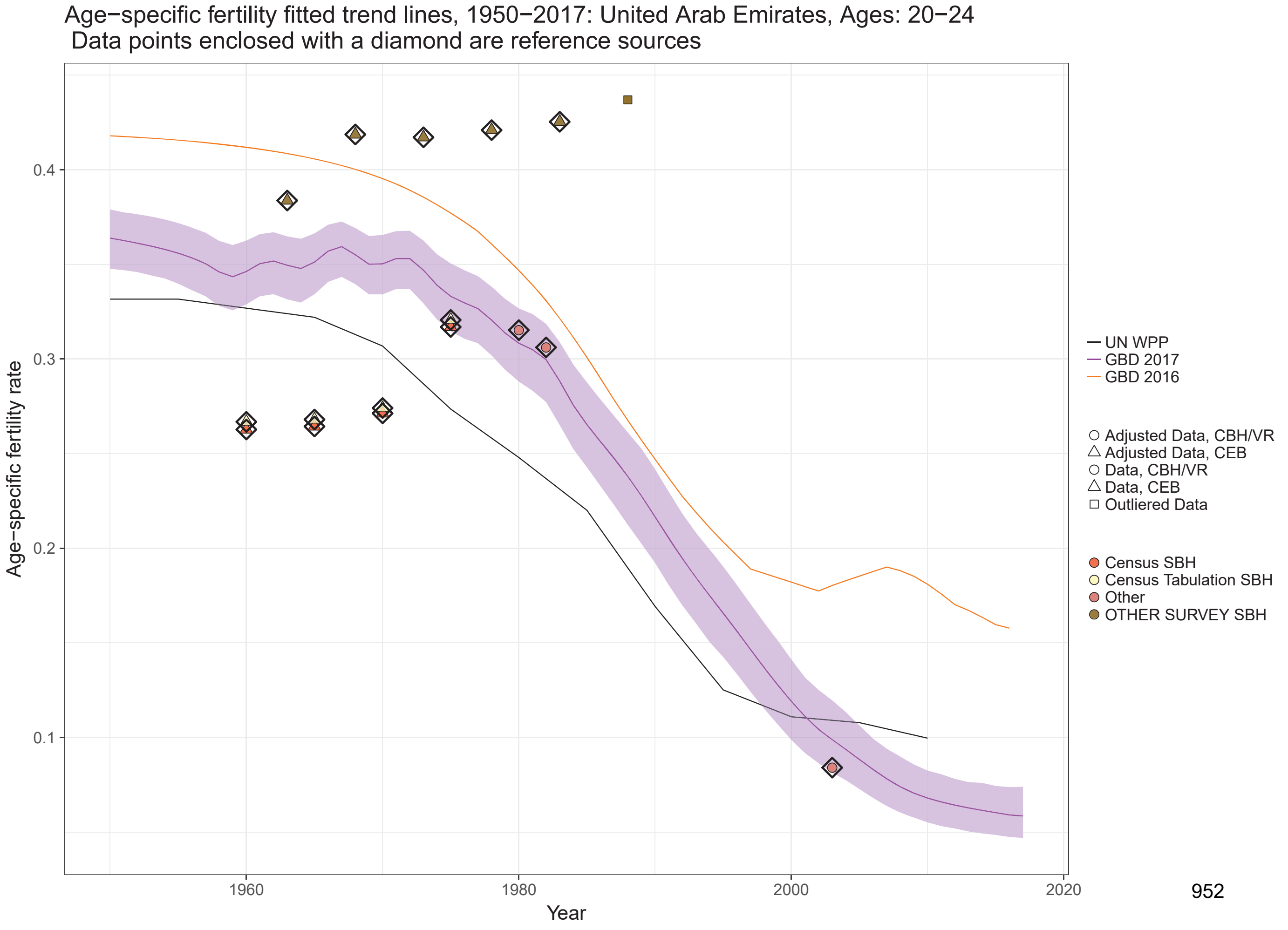

Age−specific fertility fitted trend lines, 1950−2017: United Arab Emirates, Ages: 20−24
Data points enclosed with a diamond are reference sources
Age−specific fertility rate
Year
0.1
0.2
0.3
0.4
1960
1980
2000
2020
UN WPP
GBD 2017
GBD 2016
Adjusted Data, CBH/VR
Adjusted Data, CEB
Data, CBH/VR
Data, CEB
Outliered Data
Census SBH
Census Tabulation SBH
Other
OTHER SURVEY SBH

Age−specific fertility fitted trend lines, 1950−2017: United Arab Emirates, Ages: 25−29
Data points enclosed with a diamond are reference sources

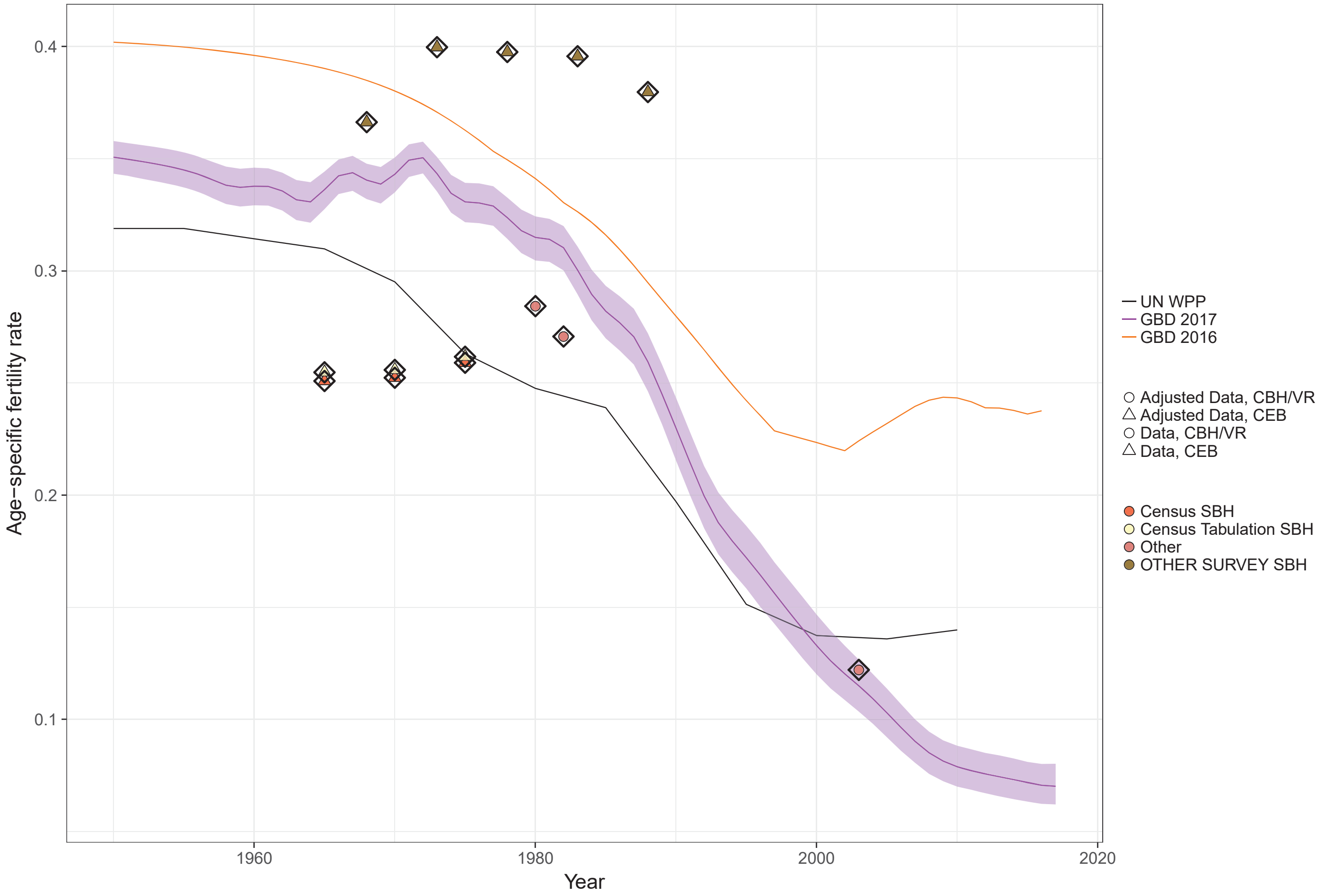

Age−specific fertility fitted trend lines, 1950−2017: United Arab Emirates, Ages: 30−34
Data points enclosed with a diamond are reference sources

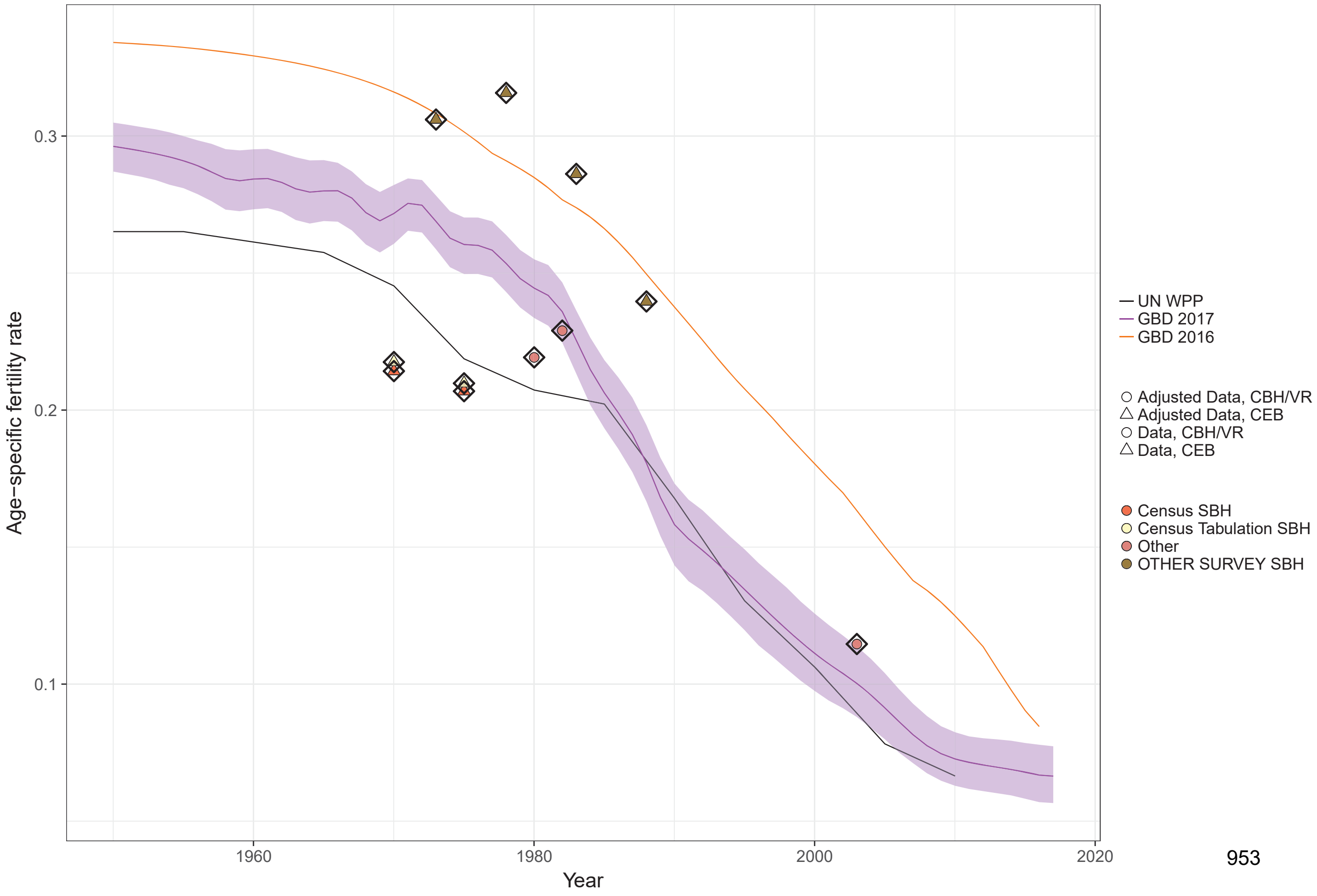

Age−specific fertility fitted trend lines, 1950−2017: United Arab Emirates, Ages: 35−39
Data points enclosed with a diamond are reference sources

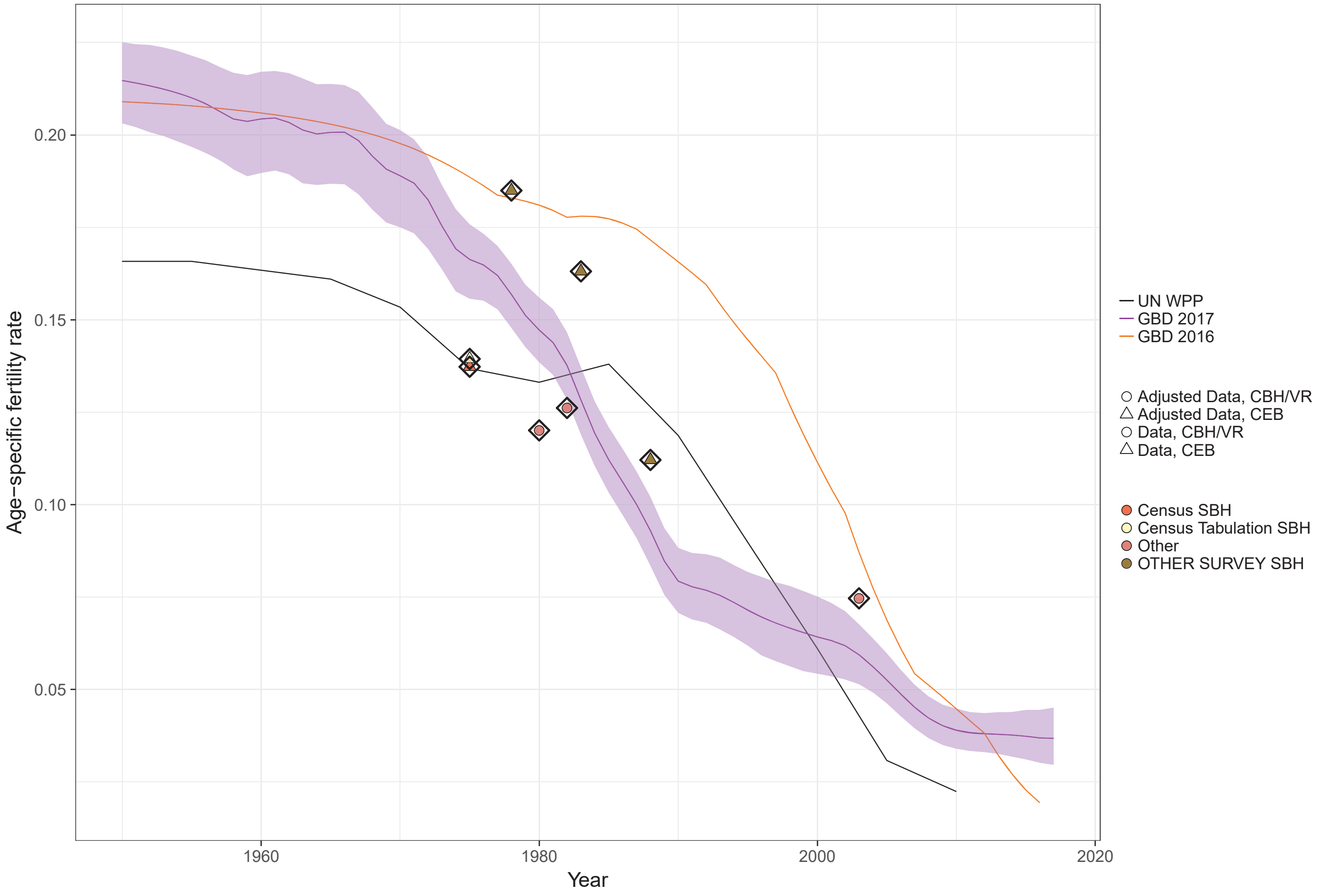

Age−specific fertility fitted trend lines, 1950−2017: United Arab Emirates, Ages: 40−44
Data points enclosed with a diamond are reference sources

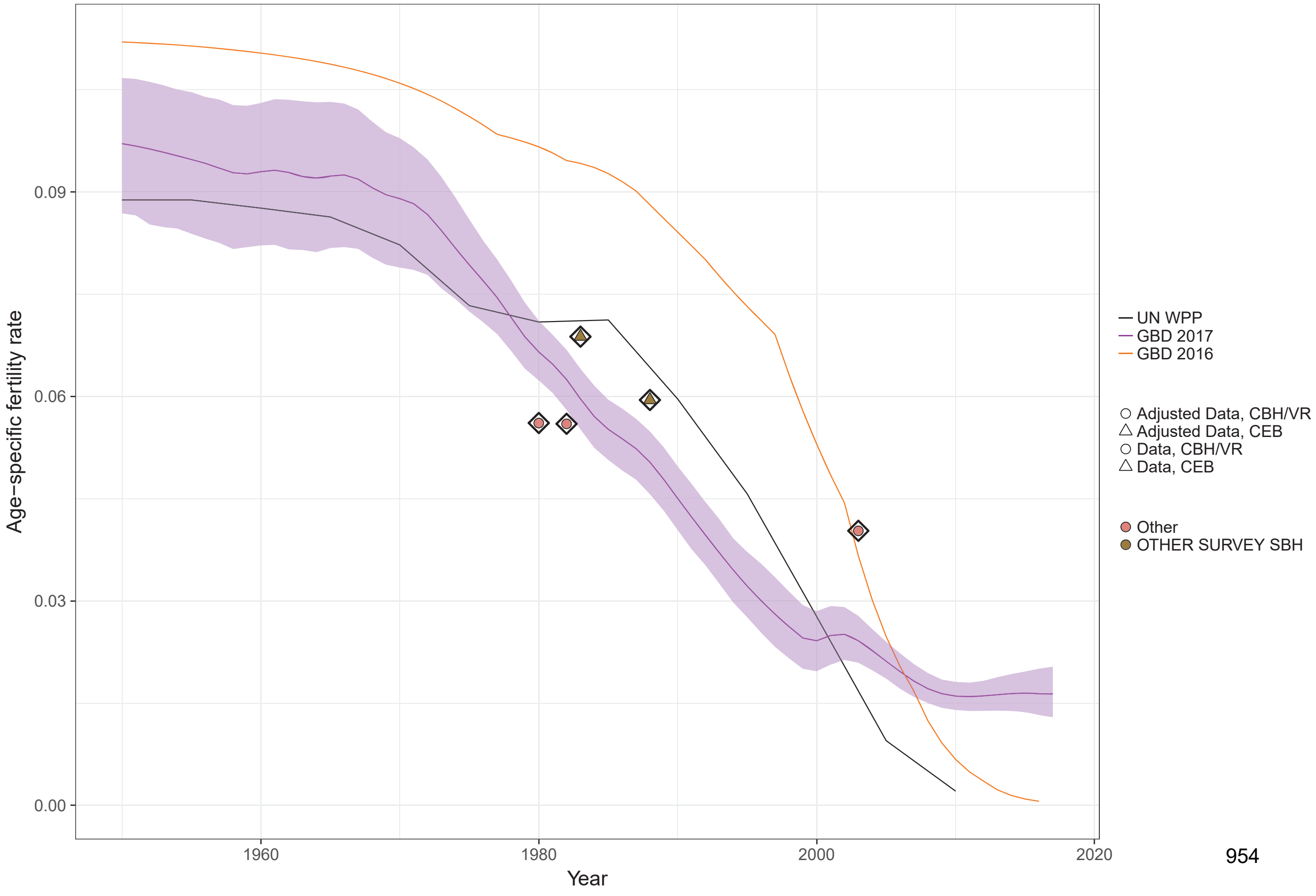

Age−specific fertility fitted trend lines, 1950−2017: United Arab Emirates, Ages: 45−49
Data points enclosed with a diamond are reference sources

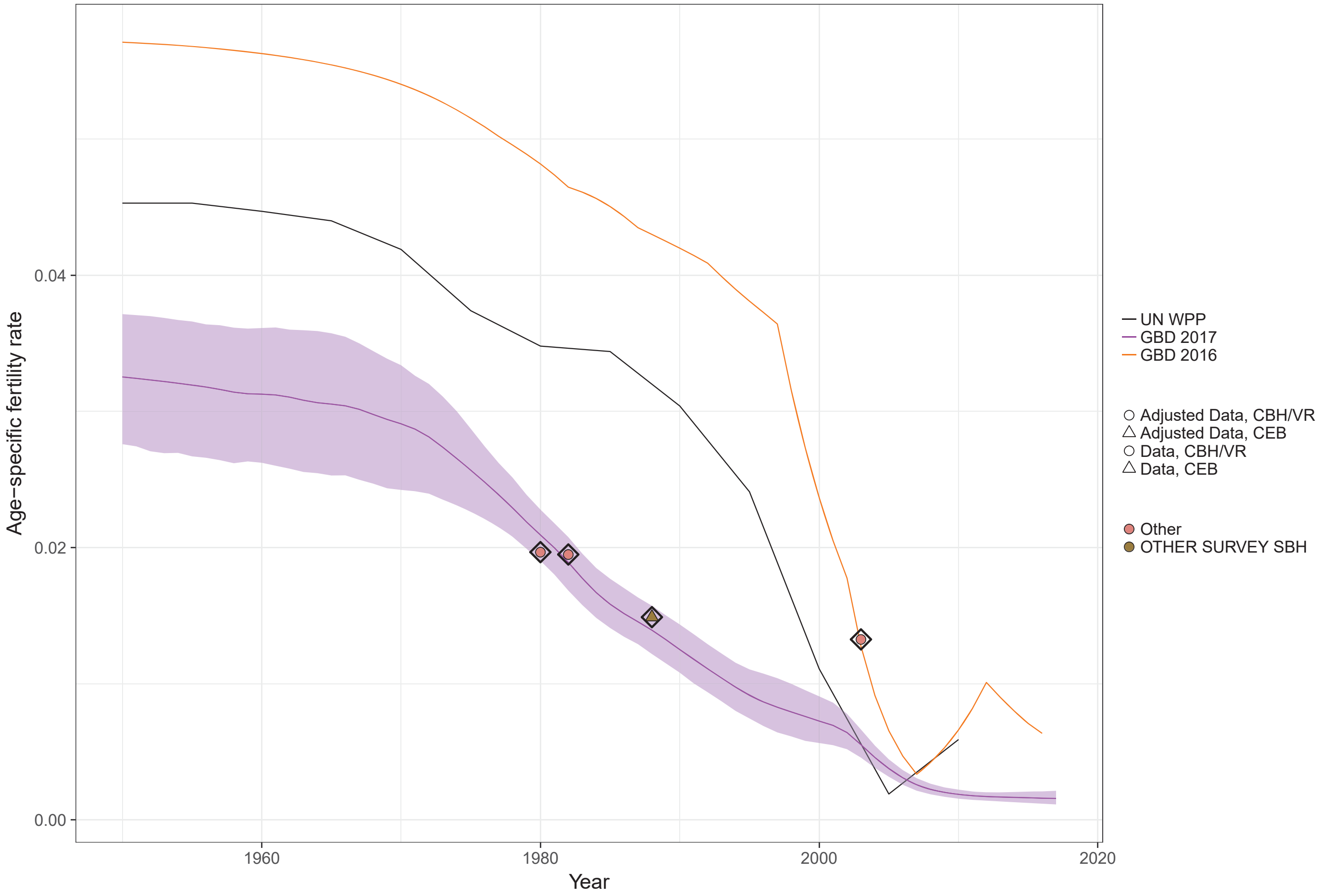

Age−specific fertility fitted trend lines, 1950−2017: United Arab Emirates, Ages: 50−54
Data points enclosed with a diamond are reference sources

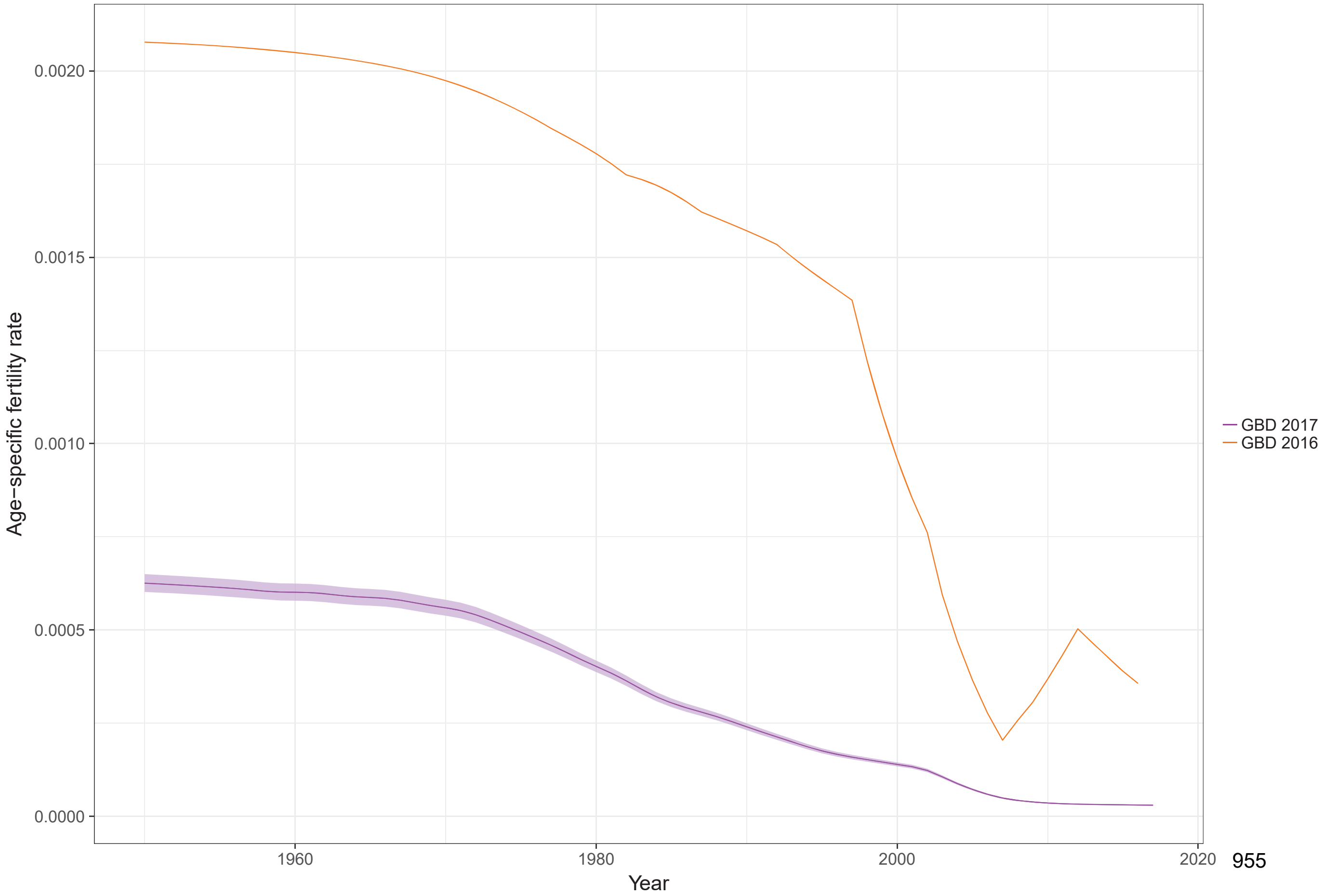

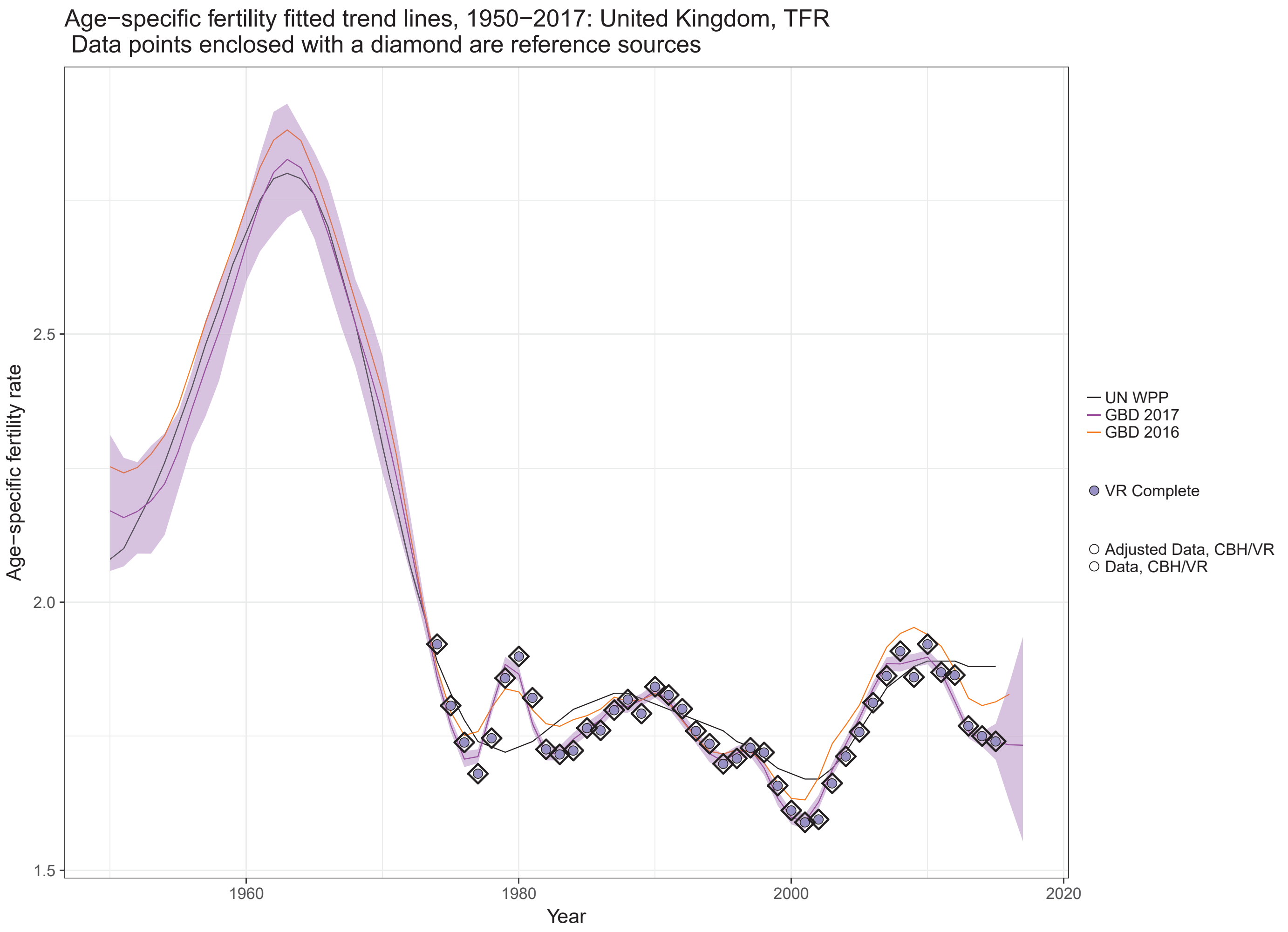

Age−specific fertility fitted trend lines, 1950−2017: United Kingdom, TFR
Data points enclosed with a diamond are reference sources
Age−specific fertility rate
2.5
2.0
1.5
1960
1980
2000
2020
Year
UN WPP
GBD 2017
GBD 2016
VR Complete
Adjusted Data, CBH/VR
Data, CBH/VR

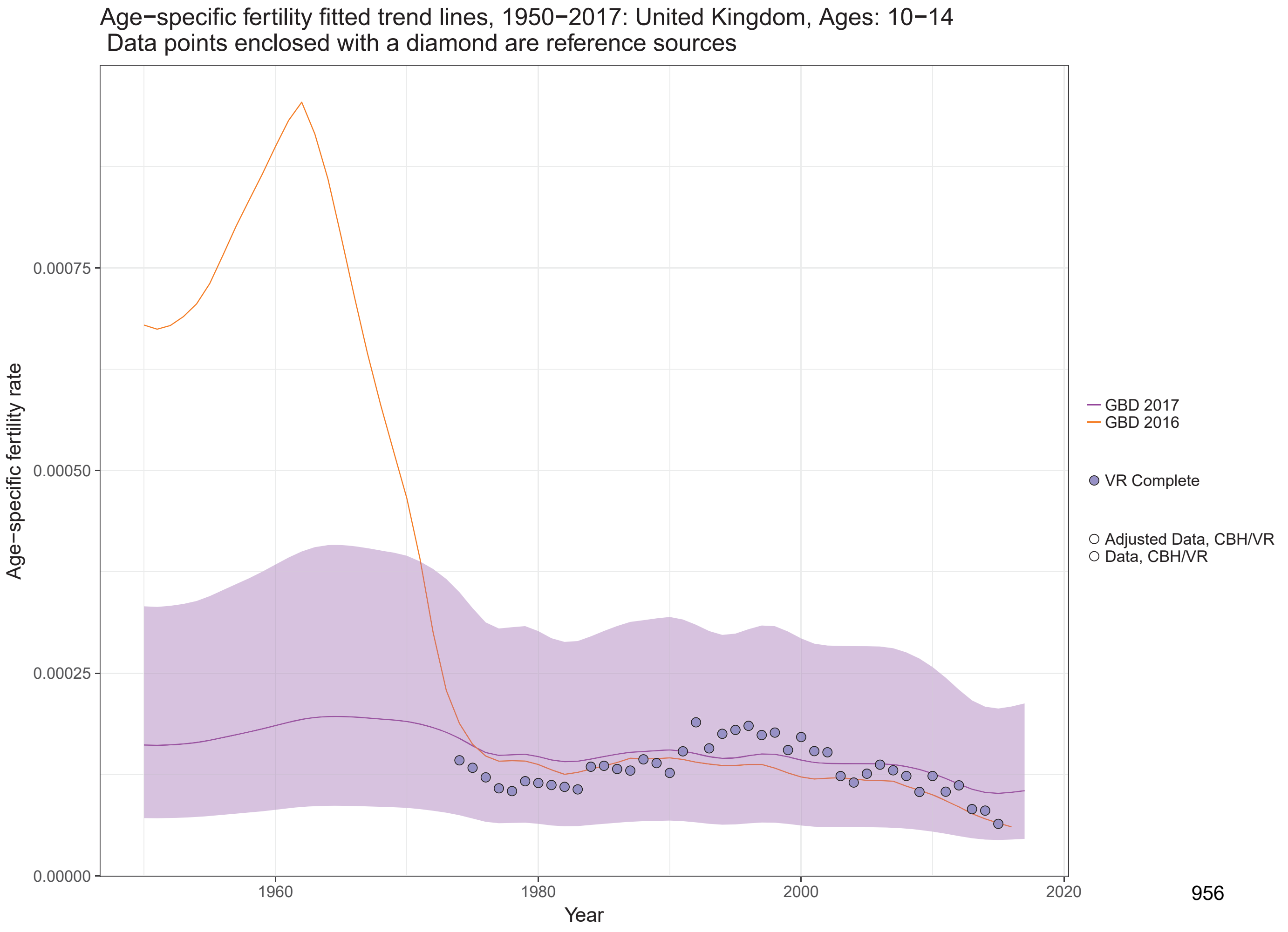

Age−specific fertility fitted trend lines, 1950−2017: United Kingdom, Ages: 10−14
Data points enclosed with a diamond are reference sources
Age−specific fertility rate
0.00075
0.00050
0.00025
0.00000
1960
1980
2000
2020
Year
GBD 2017
GBD 2016
VR Complete
Adjusted Data, CBH/VR
Data, CBH/VR

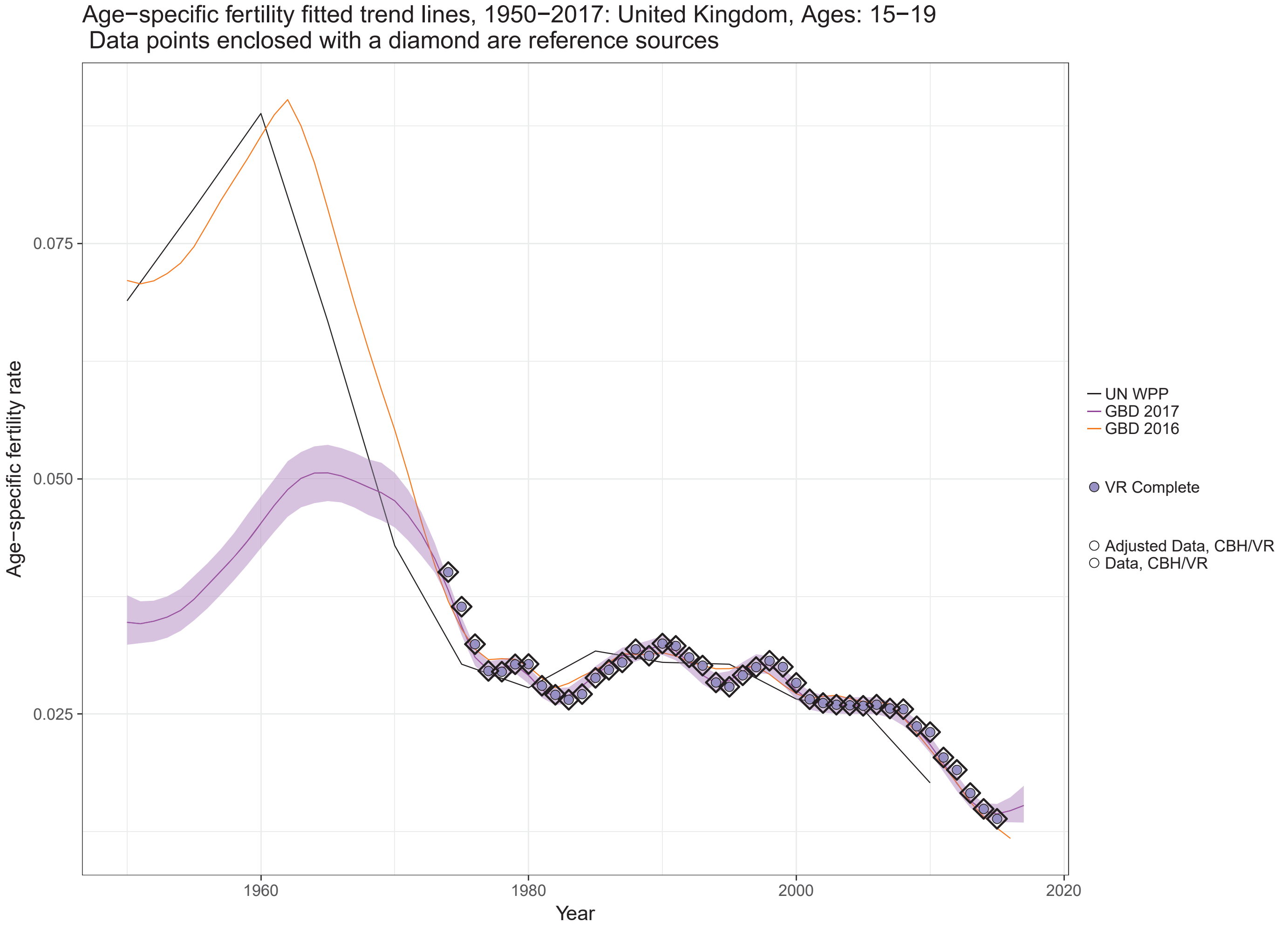

Age−specific fertility fitted trend lines, 1950−2017: United Kingdom, Ages: 15−19
Data points enclosed with a diamond are reference sources
Age−specific fertility rate
0.075
0.050
0.025
1960
1980
2000
2020
Year
UN WPP
GBD 2017
GBD 2016
VR Complete
Adjusted Data, CBH/VR
Data, CBH/VR

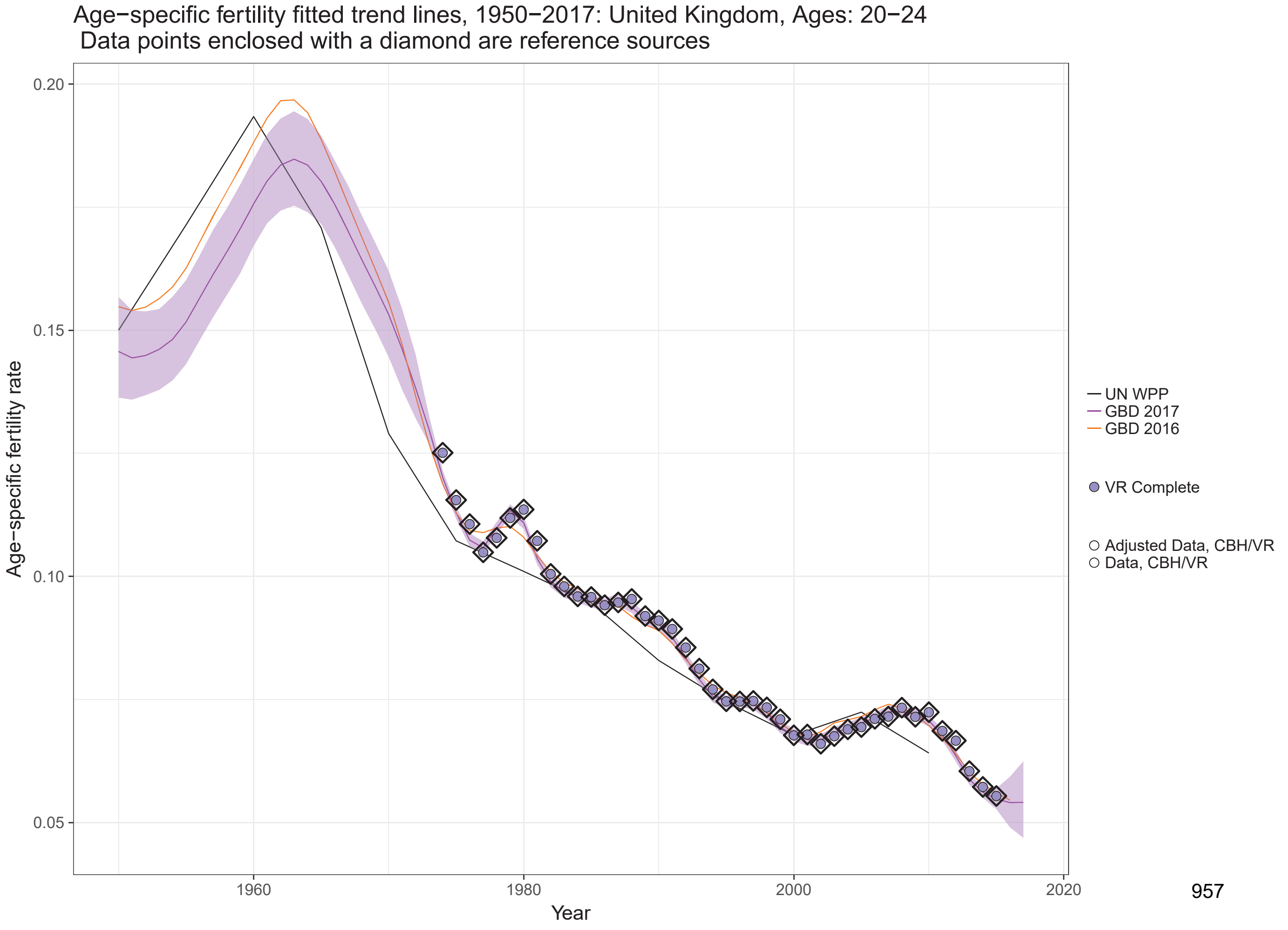

Age−specific fertility fitted trend lines, 1950−2017: United Kingdom, Ages: 20−24
Data points enclosed with a diamond are reference sources
Age−specific fertility rate
0.20
0.15
0.10
0.05
1960
1980
2000
2020
Year
UN WPP
GBD 2017
GBD 2016
VR Complete
Adjusted Data, CBH/VR
Data, CBH/VR

Age−specific fertility fitted trend lines, 1950−2017: United Kingdom, Ages: 25−29
Data points enclosed with a diamond are reference sources

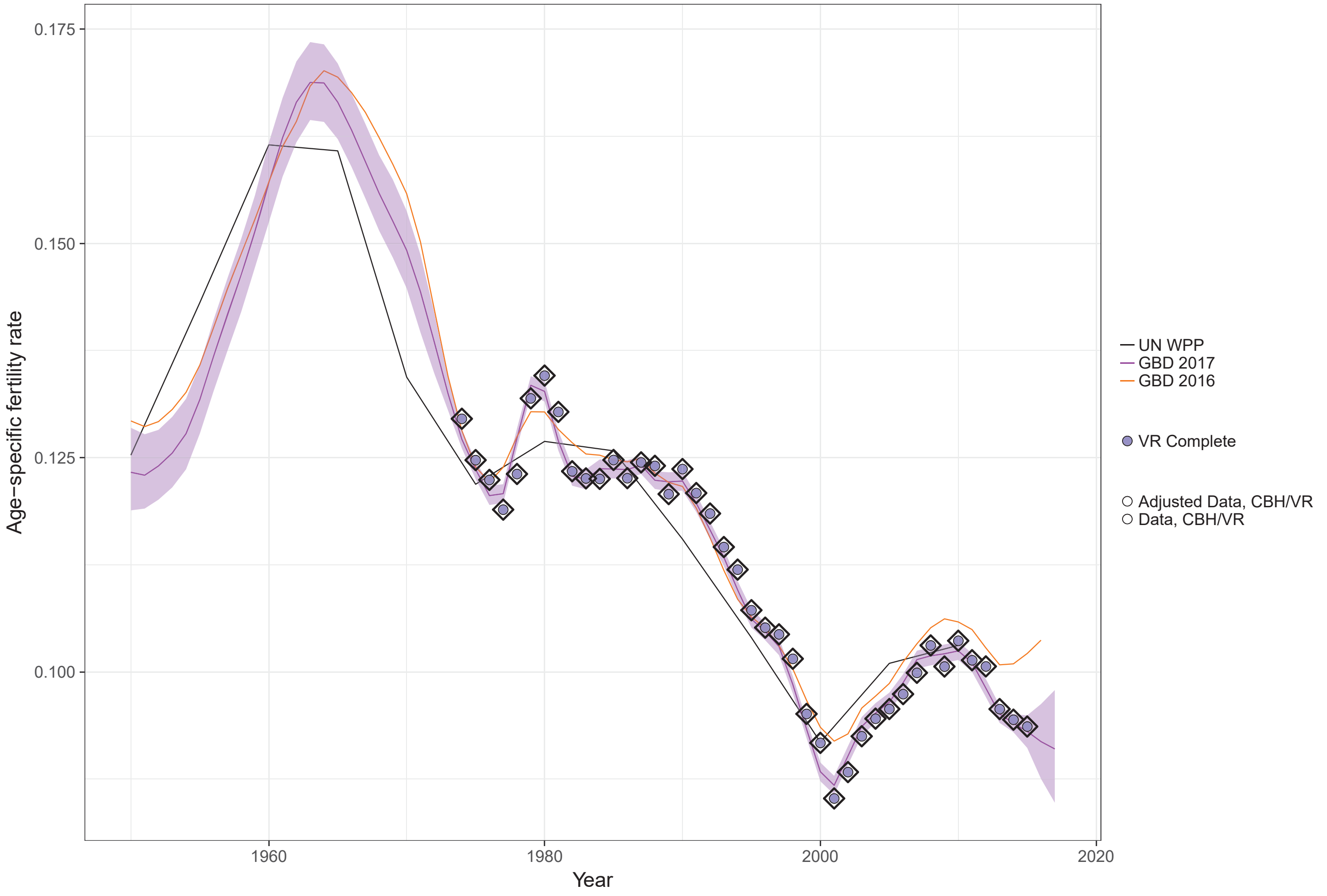

Age−specific fertility fitted trend lines, 1950−2017: United Kingdom, Ages: 30−34
Data points enclosed with a diamond are reference sources

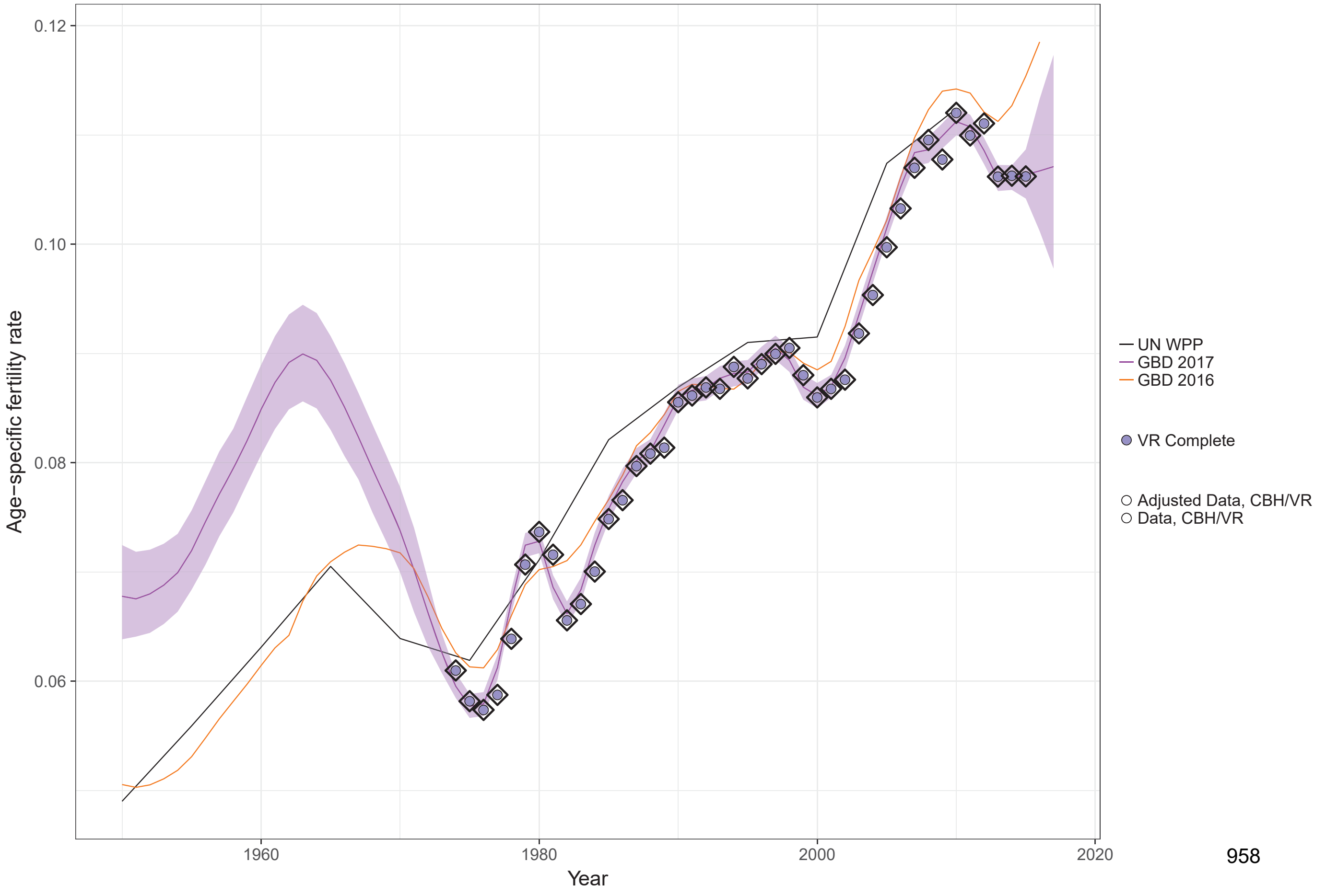

Age−specific fertility fitted trend lines, 1950−2017: United Kingdom, Ages: 35−39
Data points enclosed with a diamond are reference sources

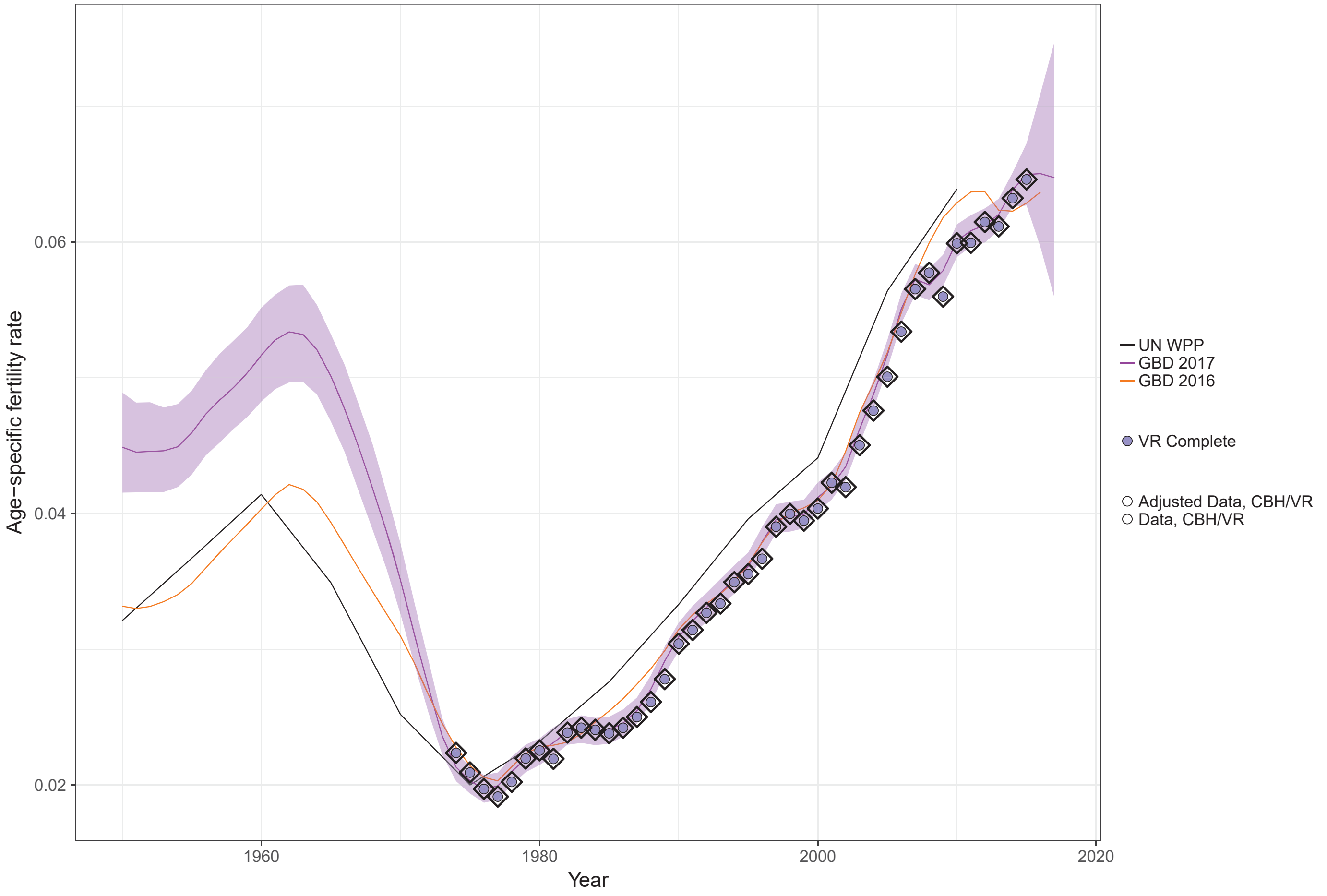

Age−specific fertility fitted trend lines, 1950−2017: United Kingdom, Ages: 40−44
Data points enclosed with a diamond are reference sources

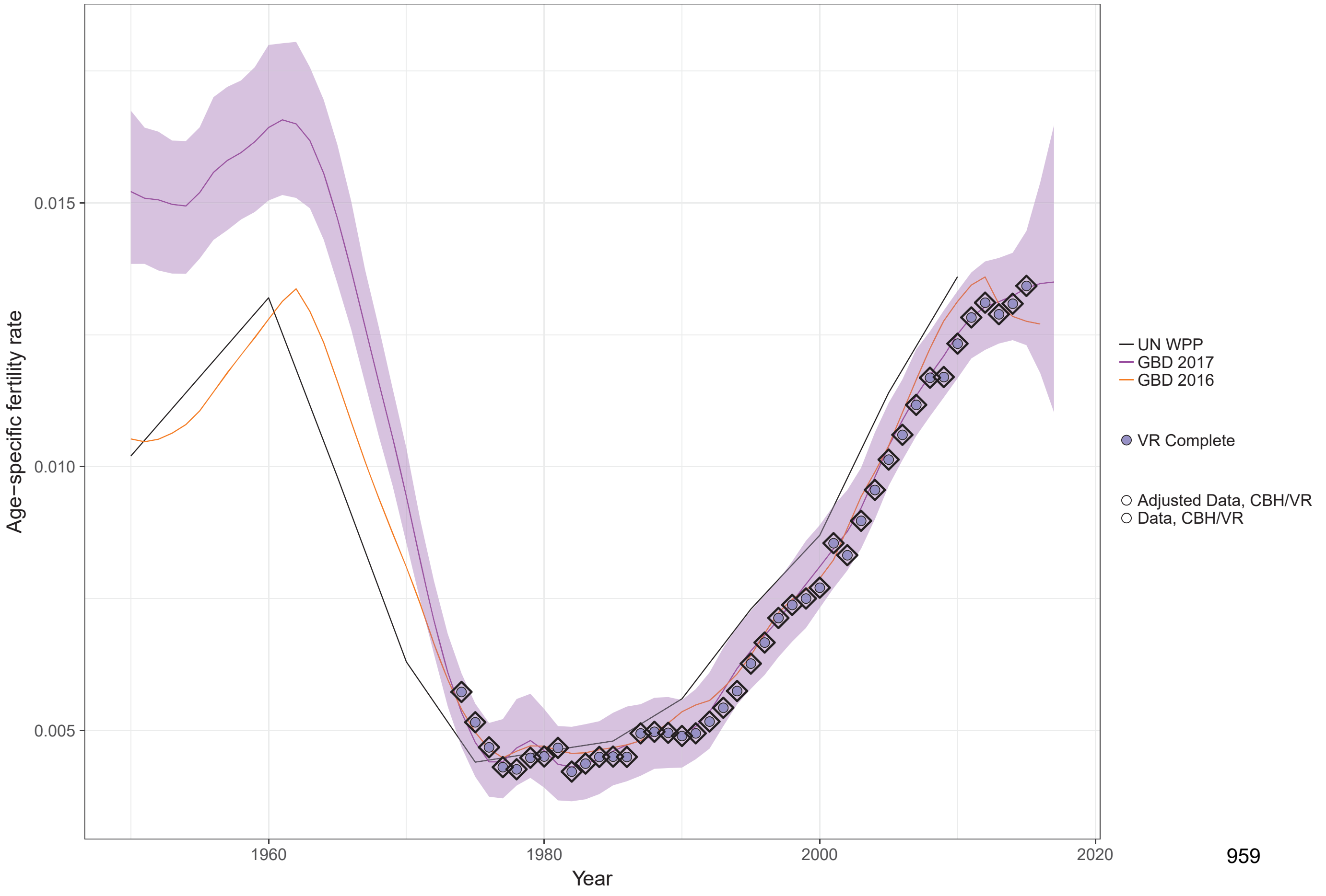

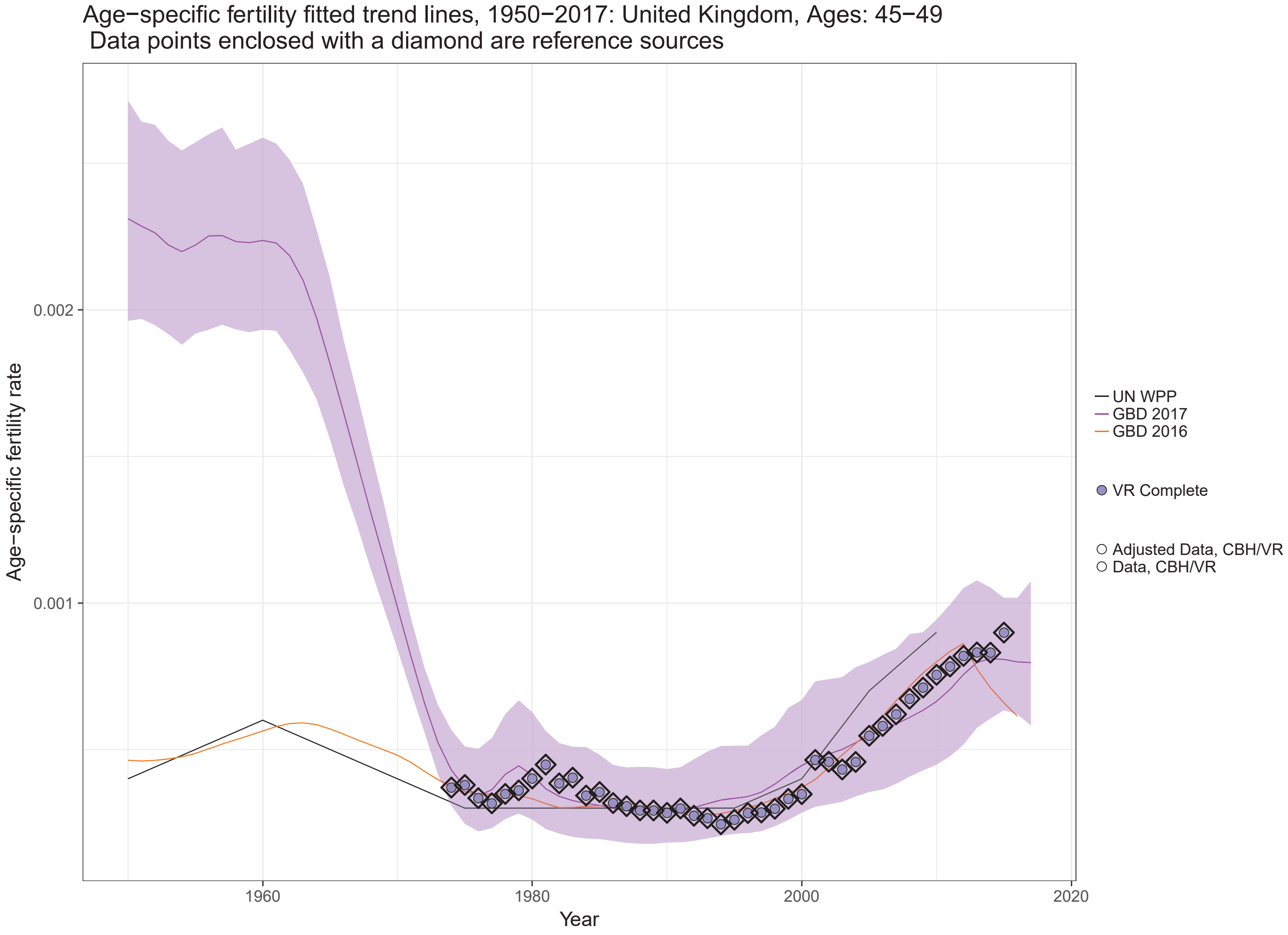

Age−specific fertility fitted trend lines, 1950−2017: United Kingdom, Ages: 45−49
Data points enclosed with a diamond are reference sources
0.002
0.001
Age−specific fertility rate
1960
1980
2000
2020
Year
UN WPP
GBD 2017
GBD 2016
VR Complete
Adjusted Data, CBH/VR
Data, CBH/VR

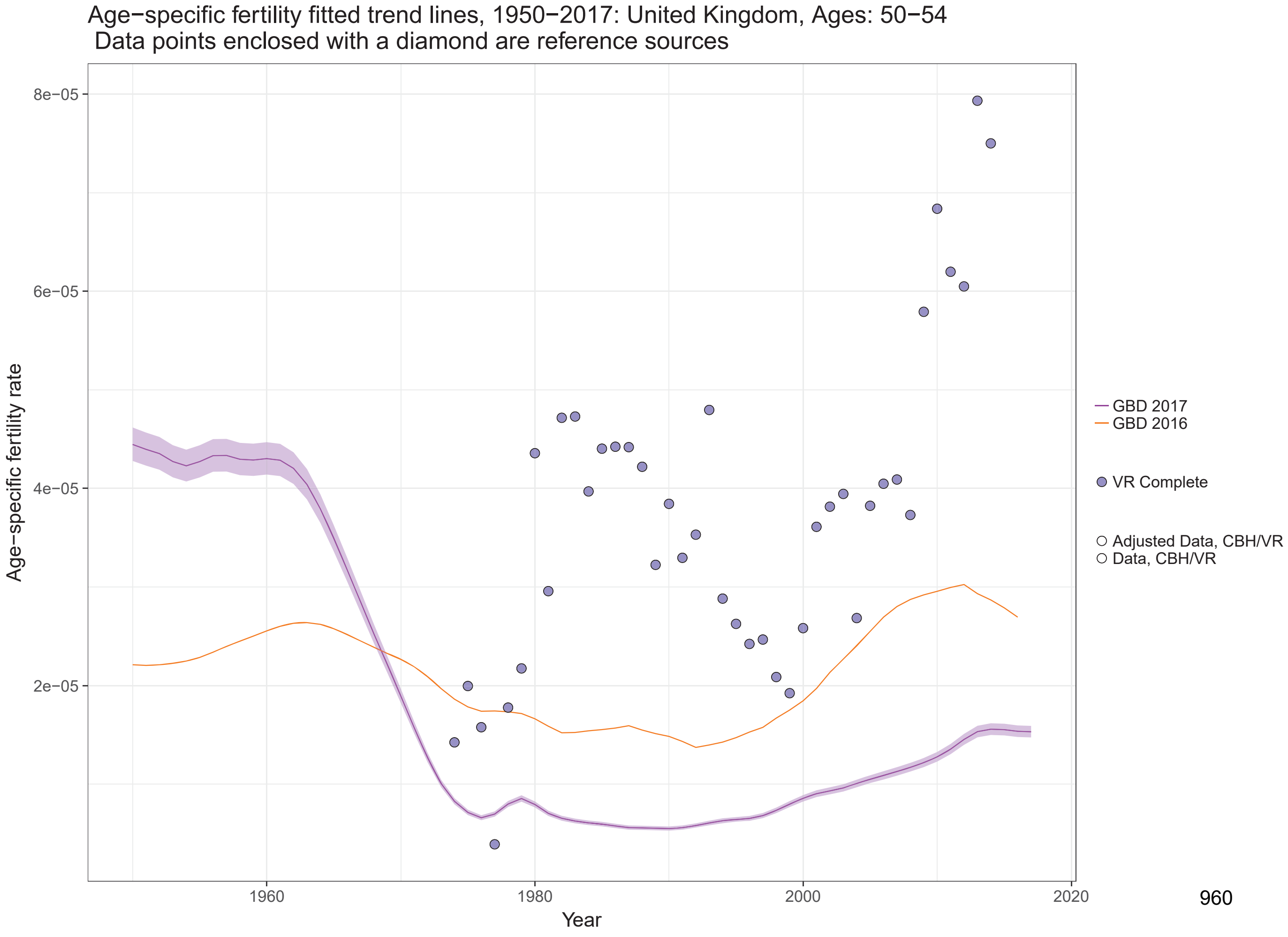

Age−specific fertility fitted trend lines, 1950−2017: United Kingdom, Ages: 50−54
Data points enclosed with a diamond are reference sources
8e−05
6e−05
4e−05
2e−05
Age−specific fertility rate
1960
1980
2000
2020
Year
GBD 2017
GBD 2016
VR Complete
Adjusted Data, CBH/VR
Data, CBH/VR

Age−specific fertility fitted trend lines, 1950−2017: United States, TFR
Data points enclosed with a diamond are reference sources

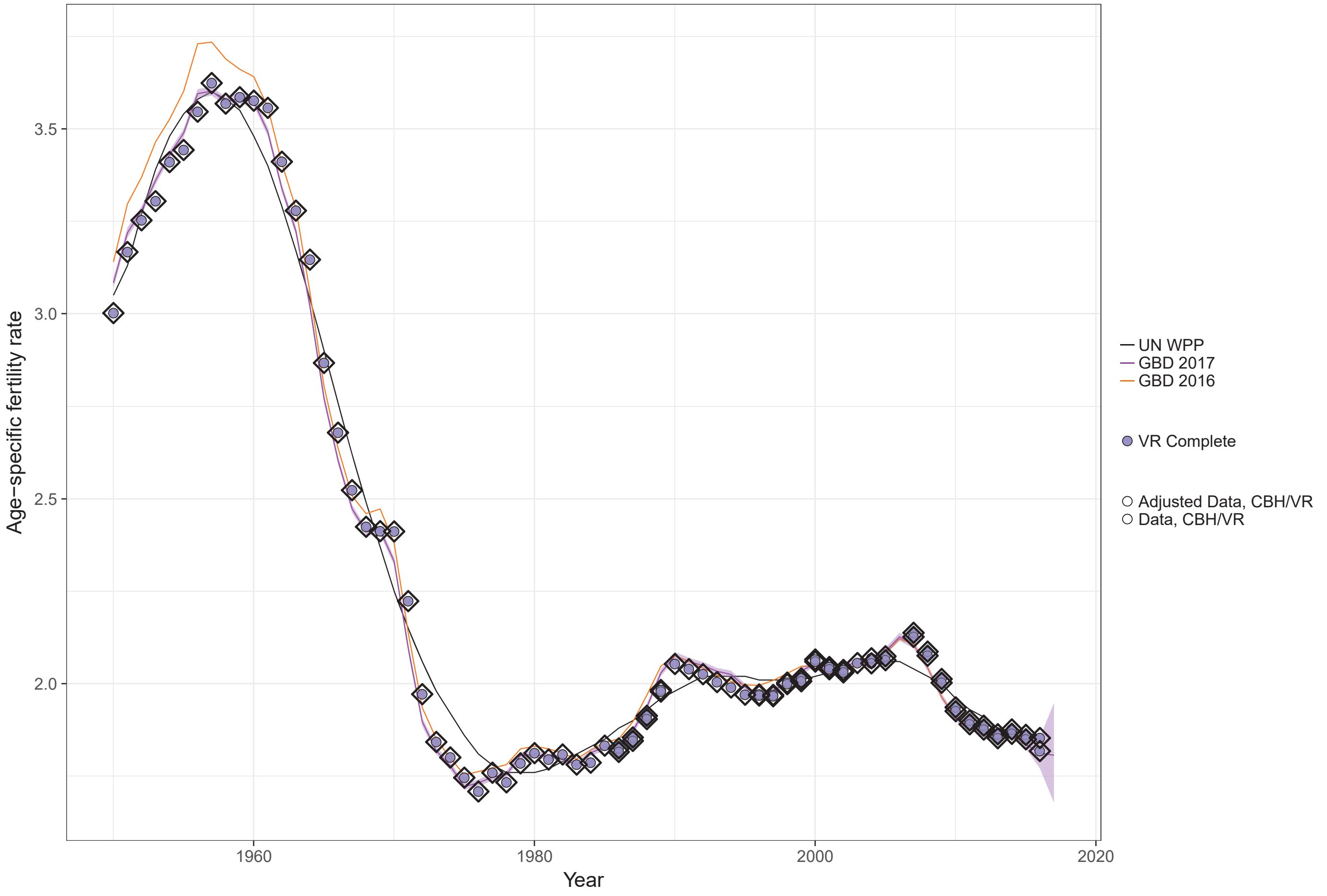

Age−specific fertility fitted trend lines, 1950−2017: United States, Ages: 10−14
Data points enclosed with a diamond are reference sources

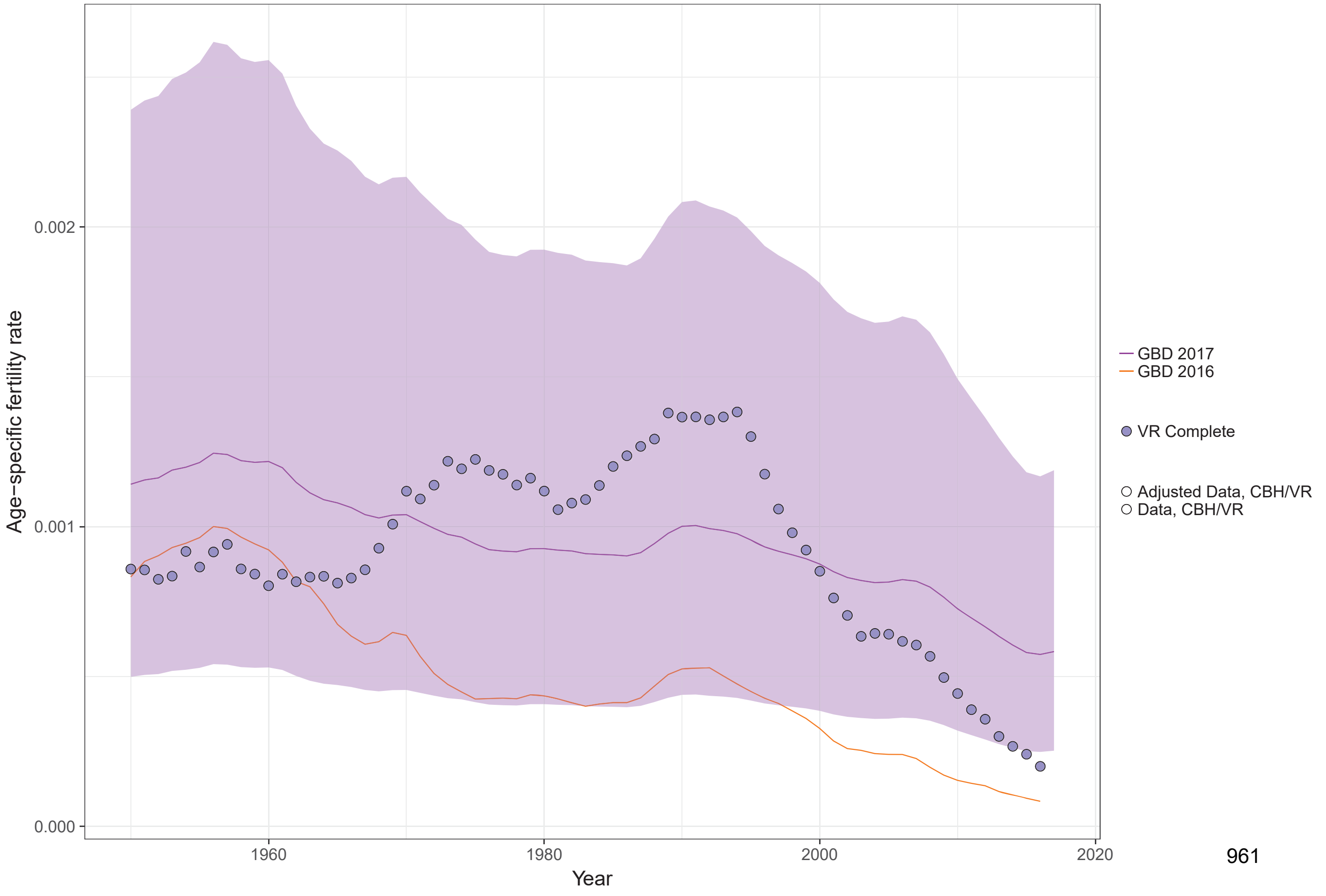

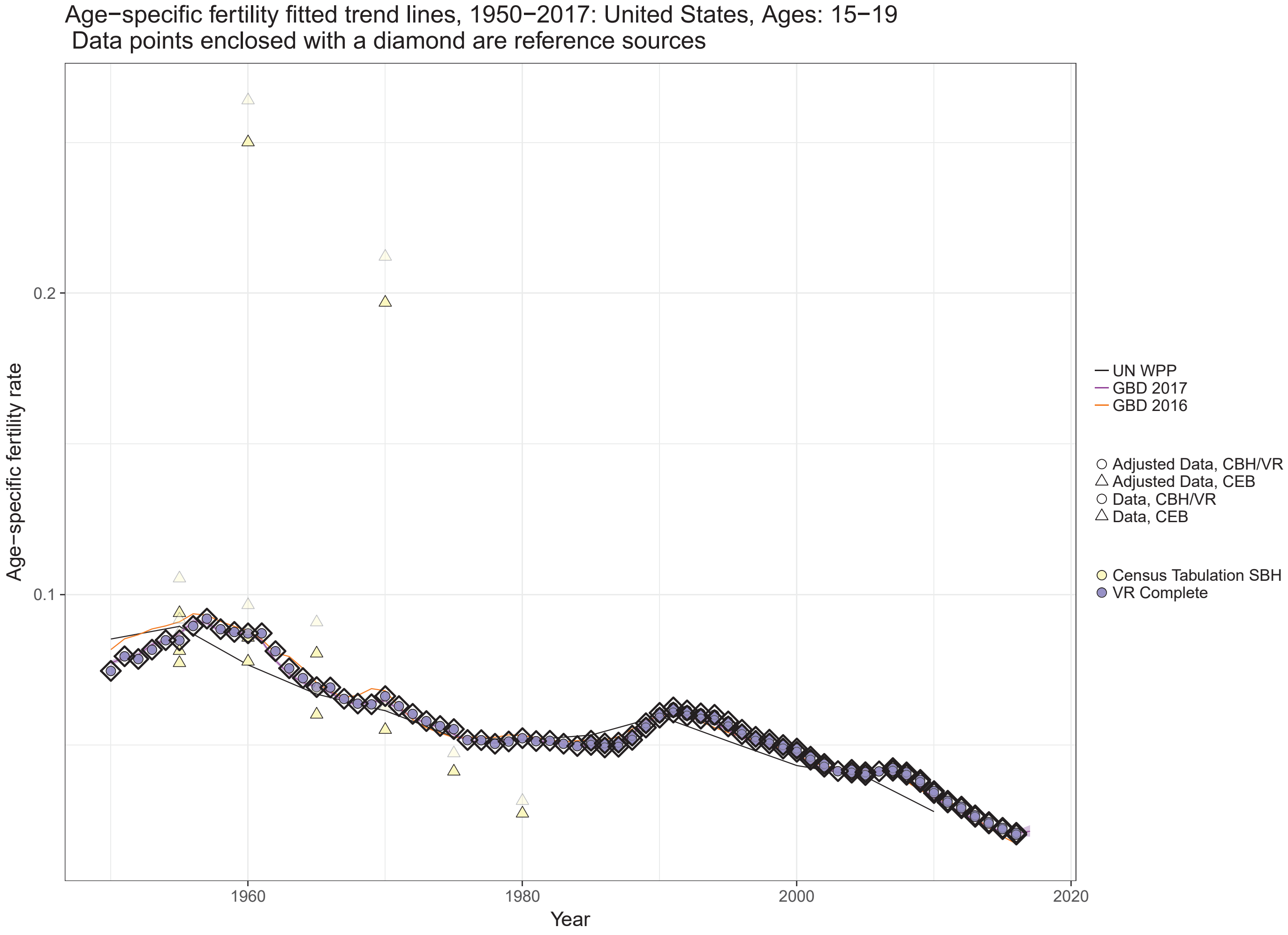

Age−specific fertility fitted trend lines, 1950−2017: United States, Ages: 15−19
Data points enclosed with a diamond are reference sources
Age−specific fertility rate
0.2
0.1
1960
1980
2000
2020
Year
UN WPP
GBD 2017
GBD 2016
Adjusted Data, CBH/VR
Adjusted Data, CEB
Data, CBH/VR
Data, CEB
Census Tabulation SBH
VR Complete

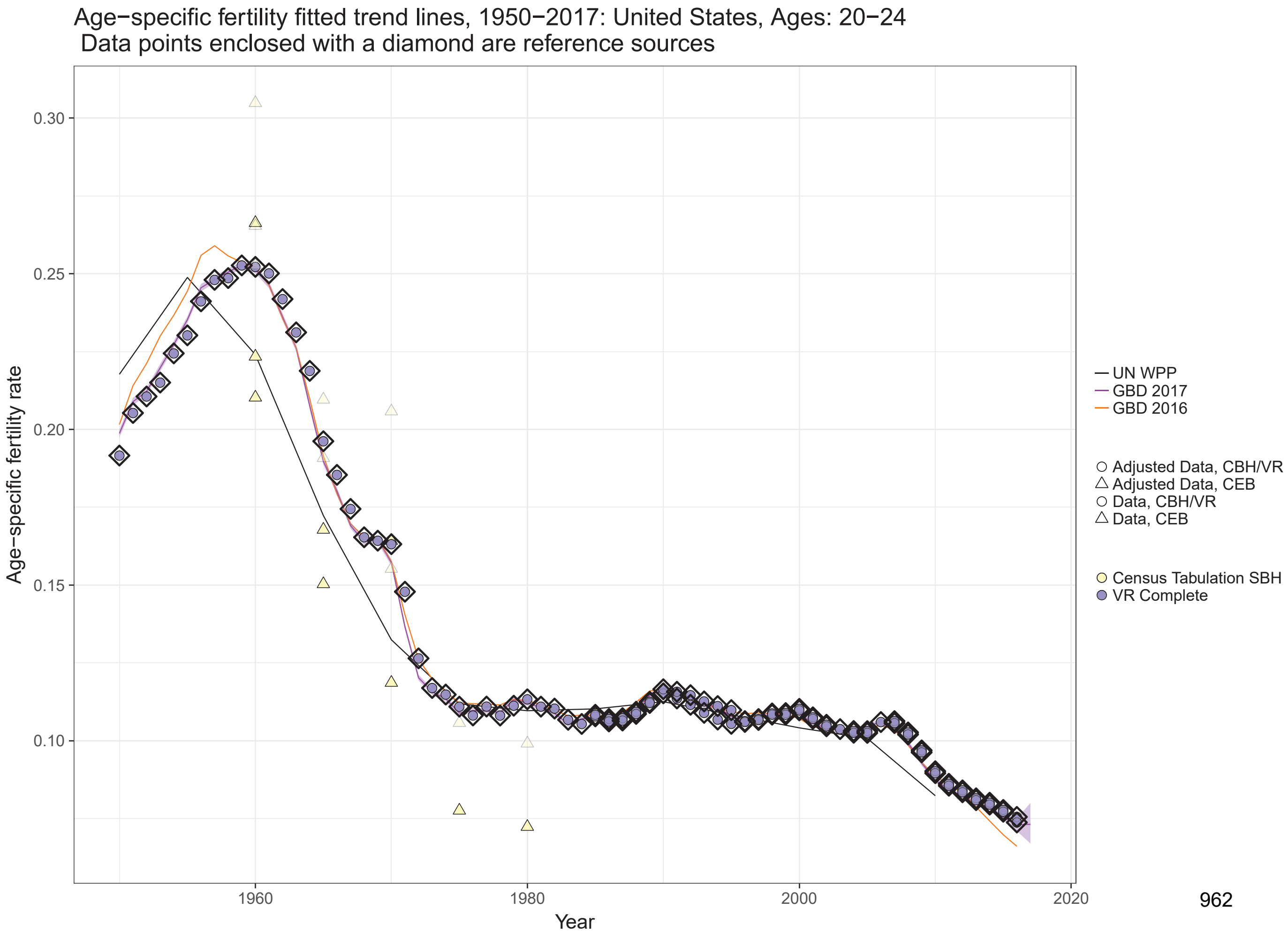

Age−specific fertility fitted trend lines, 1950−2017: United States, Ages: 20−24
Data points enclosed with a diamond are reference sources
Age−specific fertility rate
0.30
0.25
0.20
0.15
0.10
1960
1980
2000
2020
Year
UN WPP
GBD 2017
GBD 2016
Adjusted Data, CBH/VR
Adjusted Data, CEB
Data, CBH/VR
Data, CEB
Census Tabulation SBH
VR Complete

Age−specific fertility fitted trend lines, 1950−2017: United States, Ages: 25−29
Data points enclosed with a diamond are reference sources

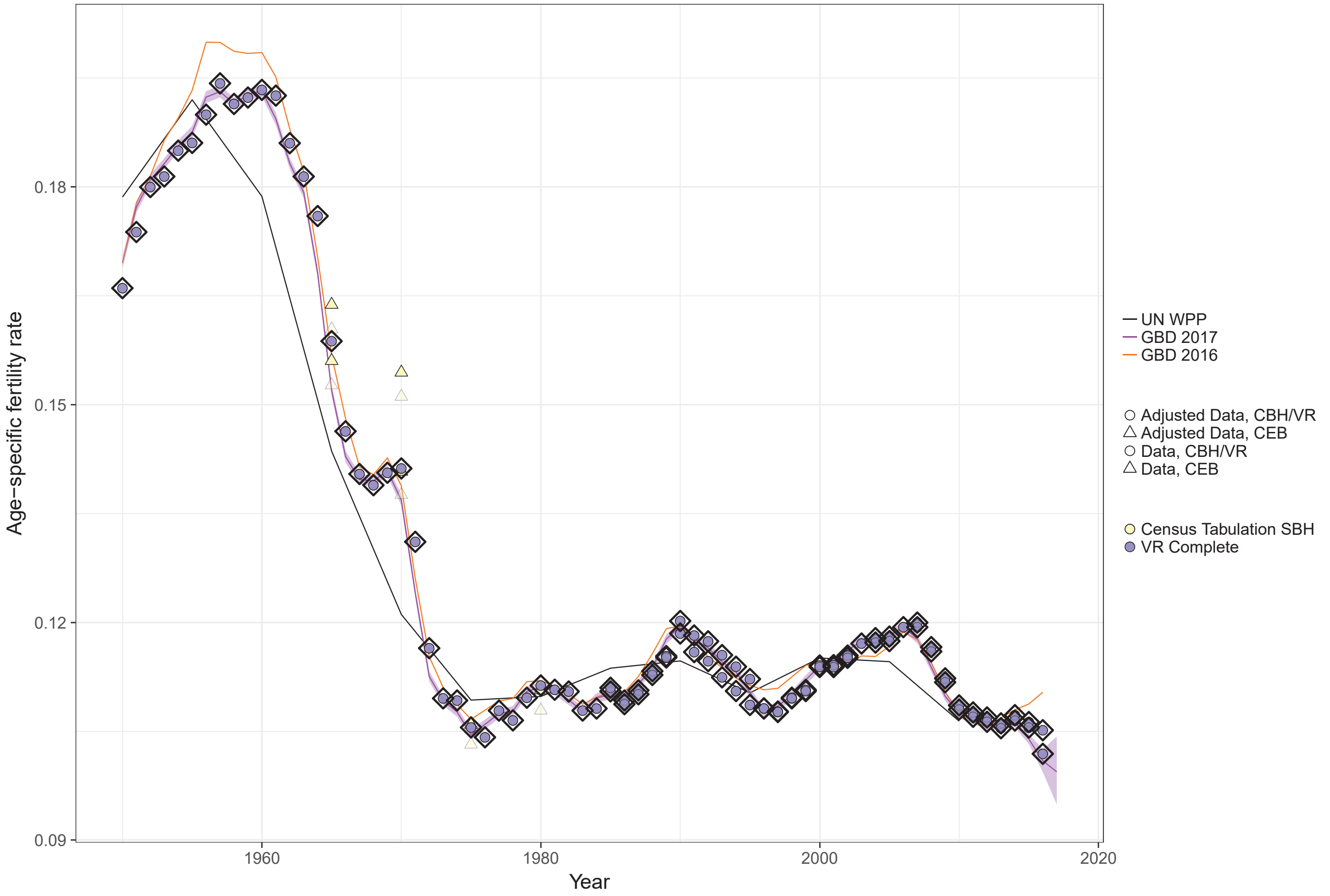

Age−specific fertility fitted trend lines, 1950−2017: United States, Ages: 30−34
Data points enclosed with a diamond are reference sources

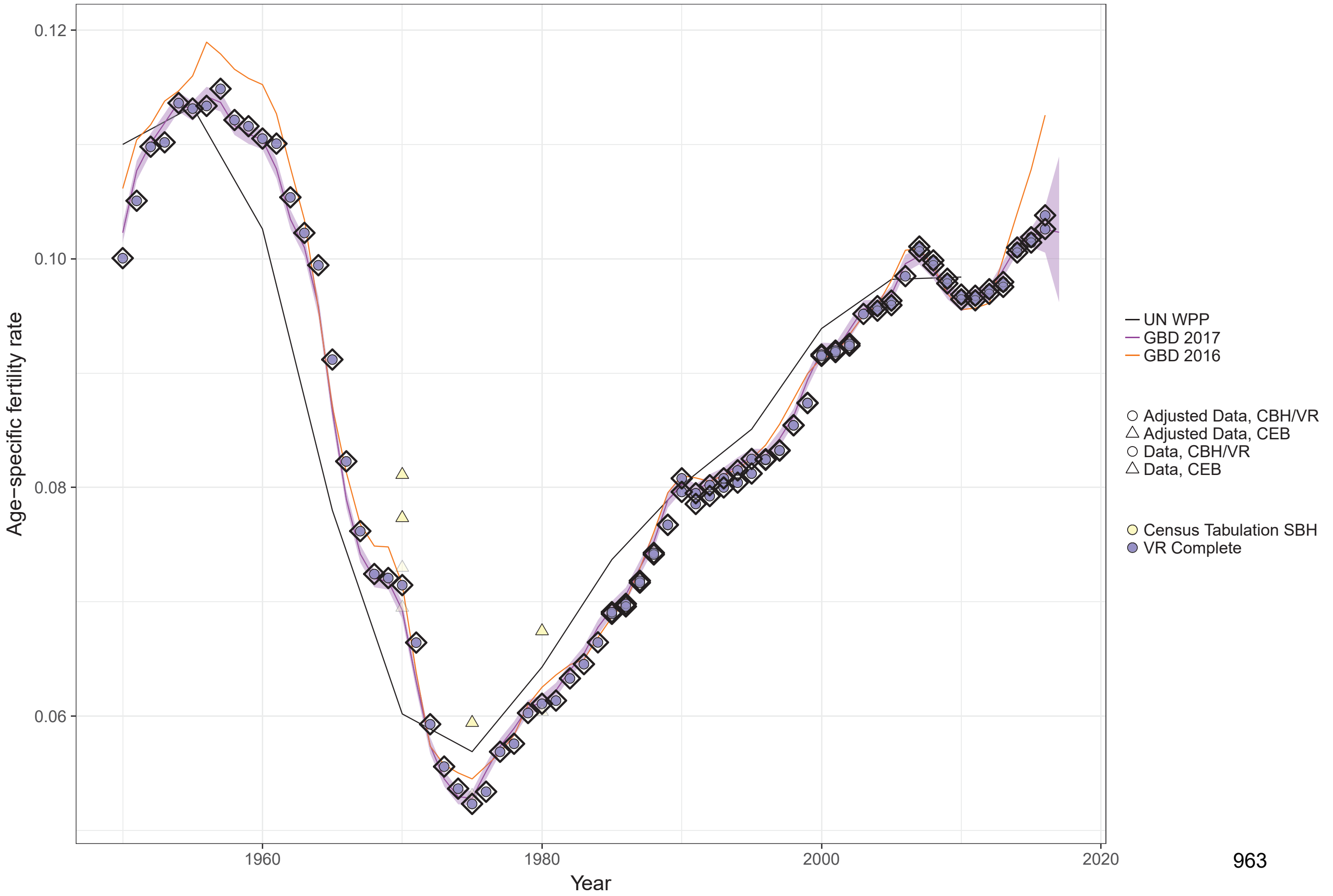

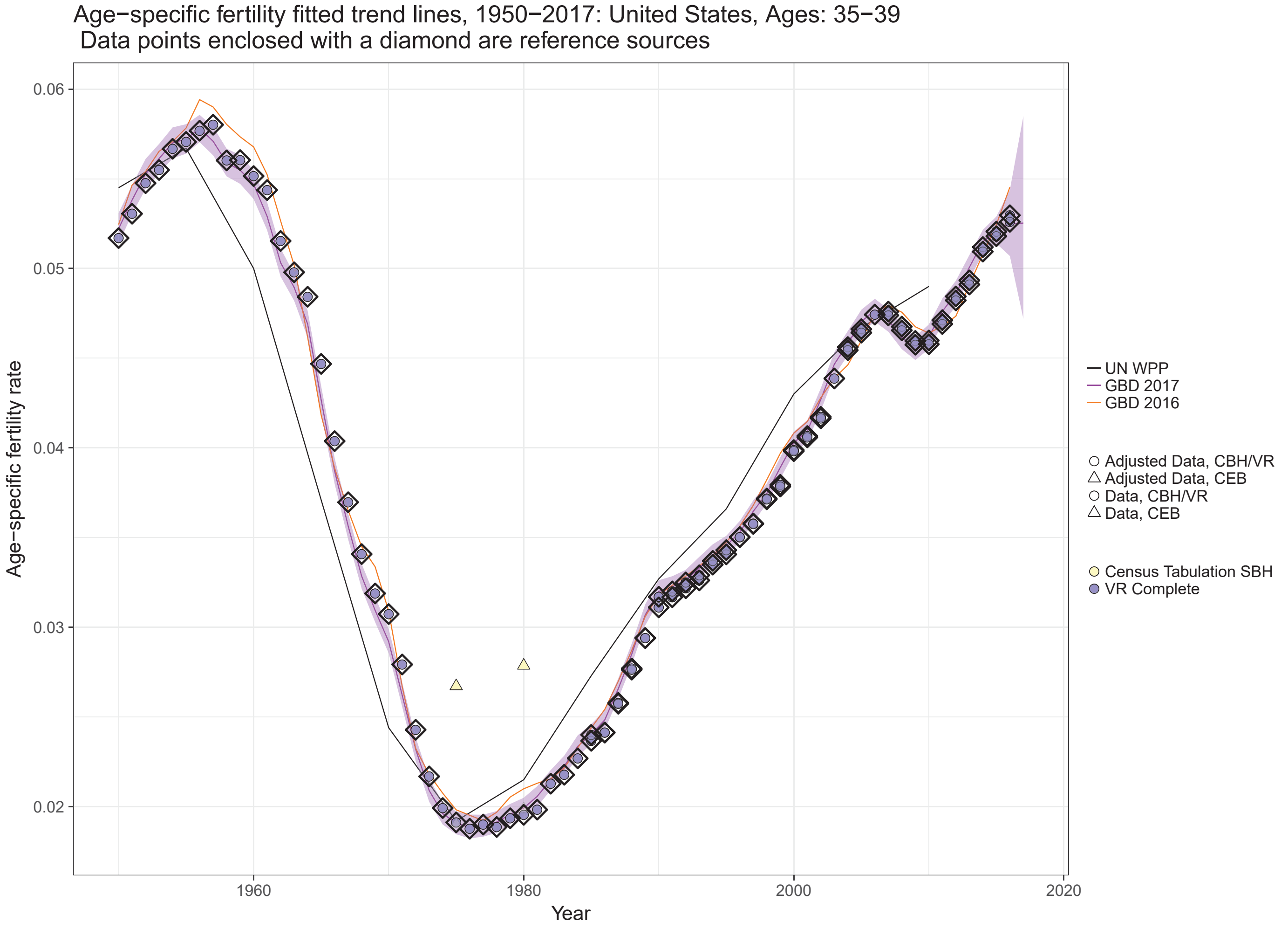
Age−specific fertility fitted trend lines, 1950−2017: United States, Ages: 35−39
Data points enclosed with a diamond are reference sources
Age−specific fertility rate
0.06
0.05
0.04
0.03
0.02
1960
1980
2000
2020
Year
UN WPP
GBD 2017
GBD 2016
Adjusted Data, CBH/VR
Adjusted Data, CEB
Data, CBH/VR
Data, CEB
Census Tabulation SBH
VR Complete

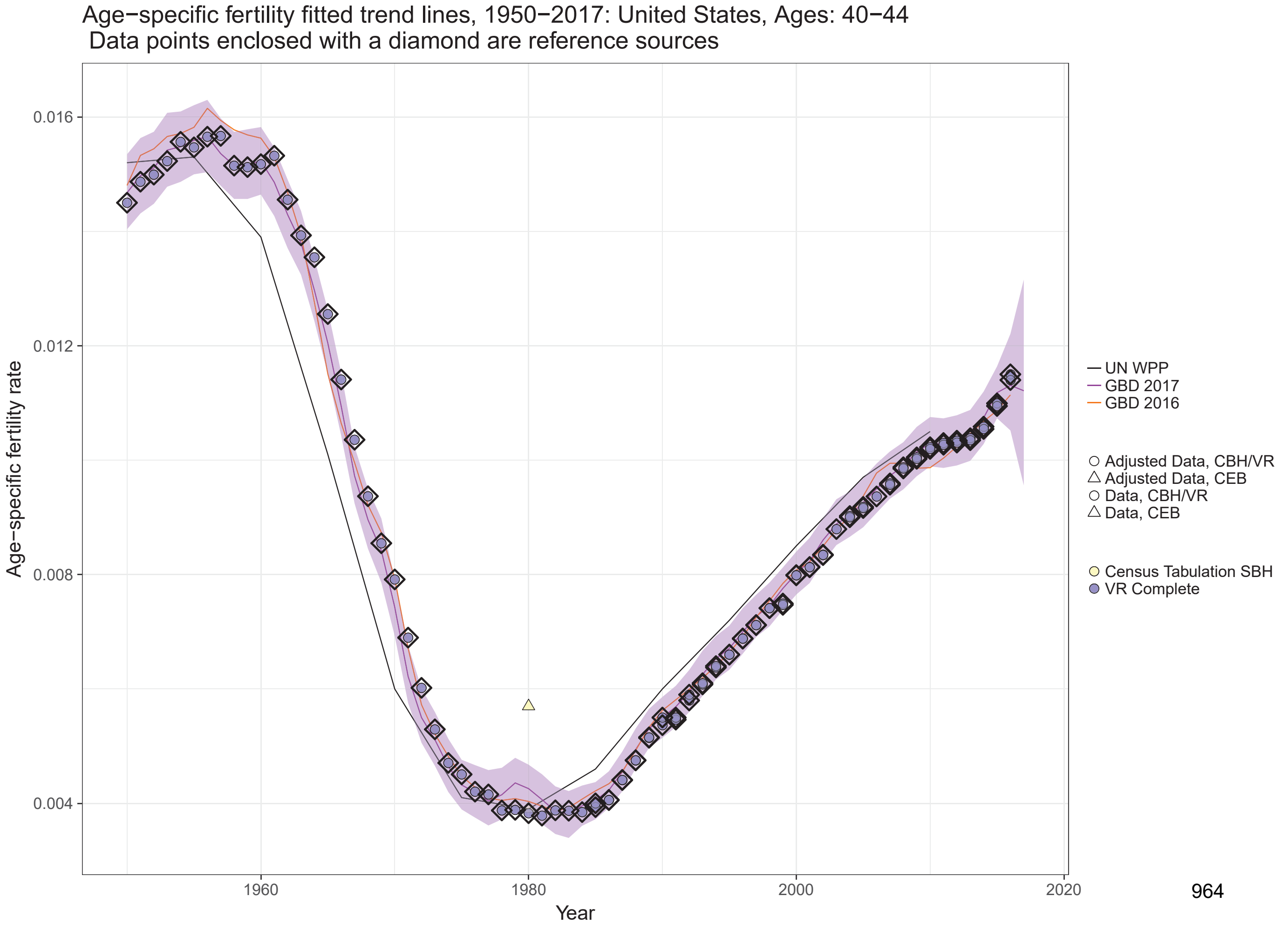
Age−specific fertility fitted trend lines, 1950−2017: United States, Ages: 40−44
Data points enclosed with a diamond are reference sources
Age−specific fertility rate
0.016
0.012
0.008
0.004
1960
1980
2000
2020
Year
UN WPP
GBD 2017
GBD 2016
Adjusted Data, CBH/VR
Adjusted Data, CEB
Data, CBH/VR
Data, CEB
Census Tabulation SBH
VR Complete

Age−specific fertility fitted trend lines, 1950−2017: United States, Ages: 45−49
Data points enclosed with a diamond are reference sources

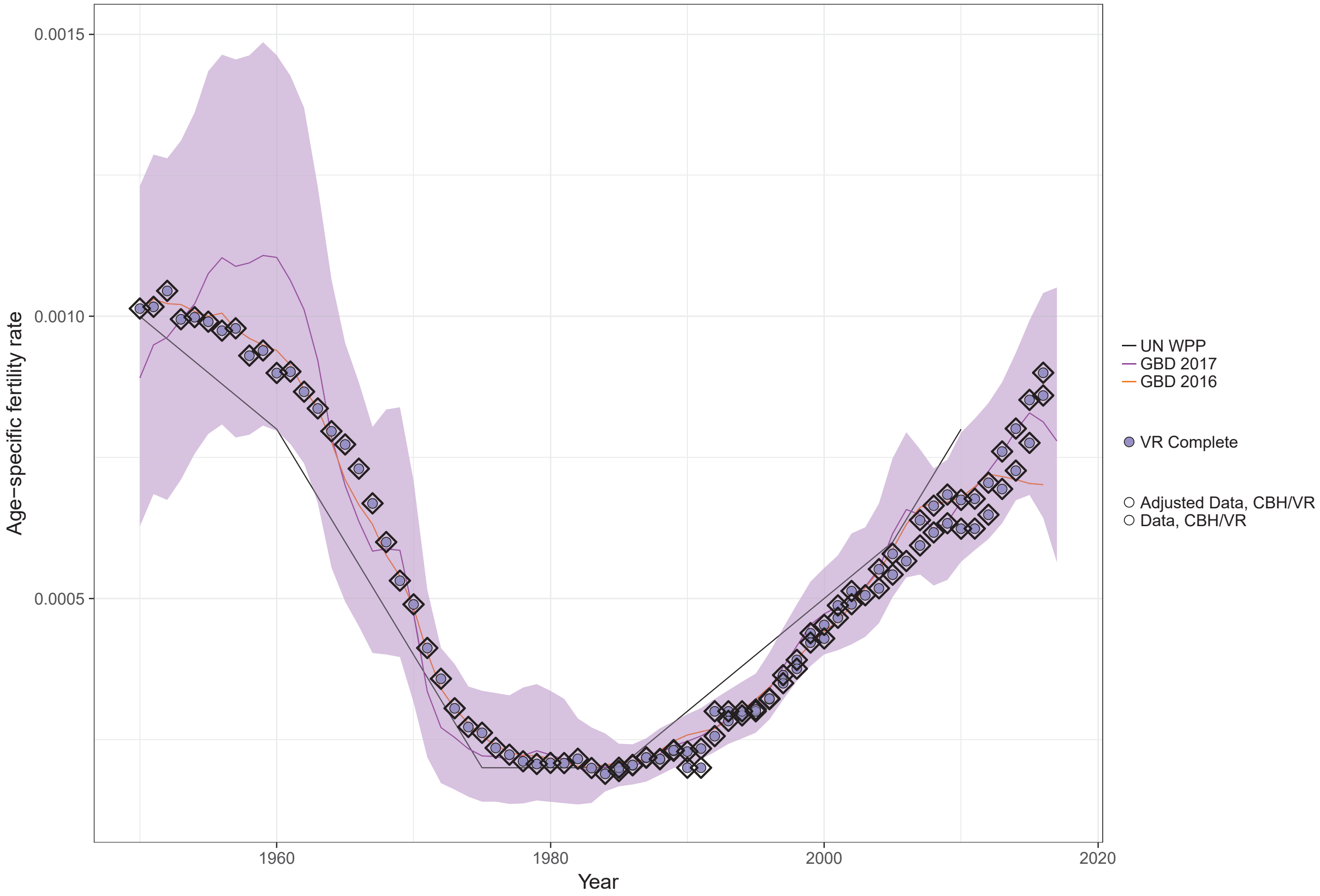

Age−specific fertility fitted trend lines, 1950−2017: United States, Ages: 50−54
Data points enclosed with a diamond are reference sources

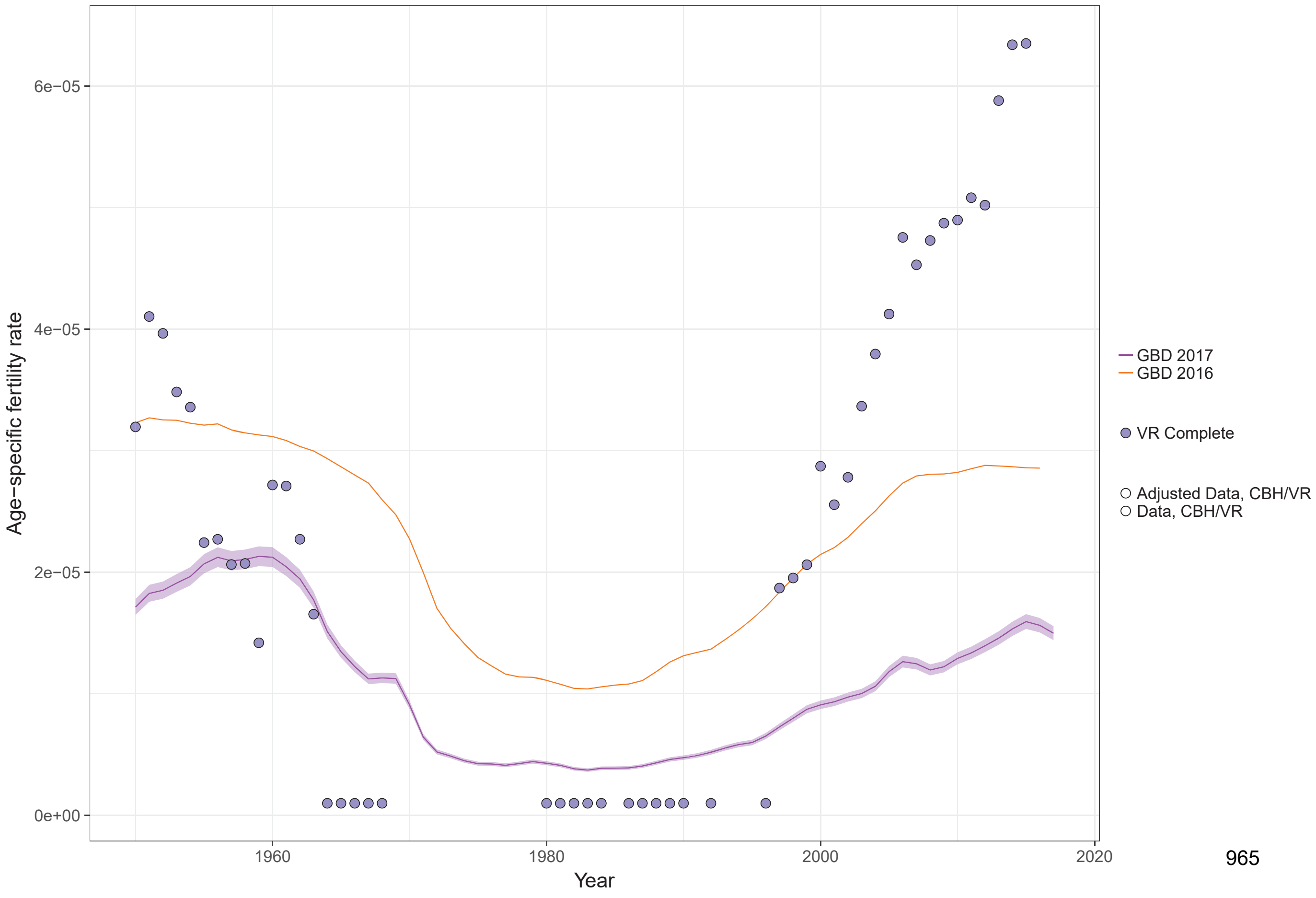

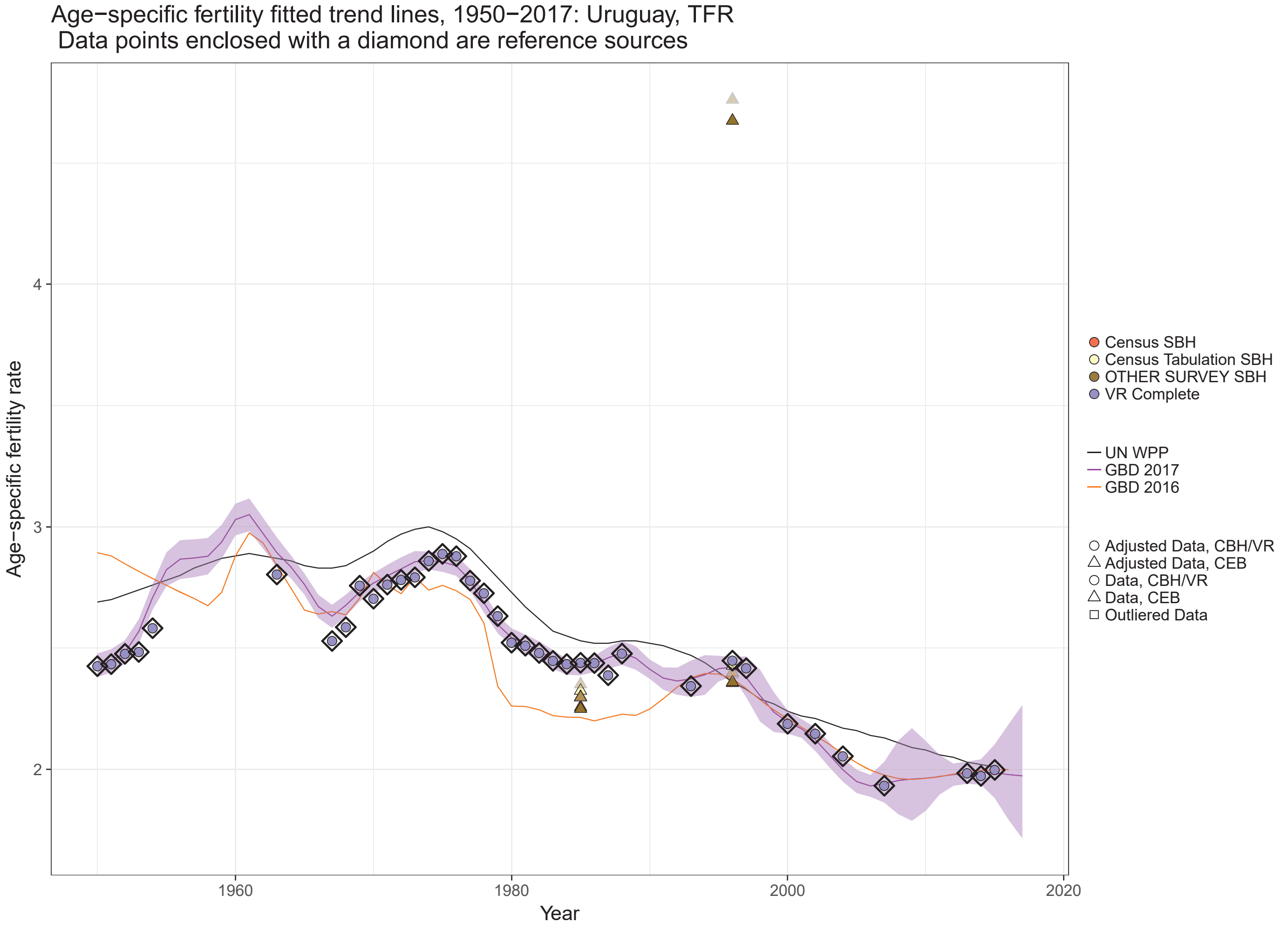

Age−specific fertility fitted trend lines, 1950−2017: Uruguay, TFR
Data points enclosed with a diamond are reference sources
Age−specific fertility rate
Year
1960
1980
2000
2020
2
3
4
Census SBH
Census Tabulation SBH
OTHER SURVEY SBH
VR Complete
UN WPP
GBD 2017
GBD 2016
Adjusted Data, CBH/VR
Adjusted Data, CEB
Data, CBH/VR
Data, CEB
Outliered Data

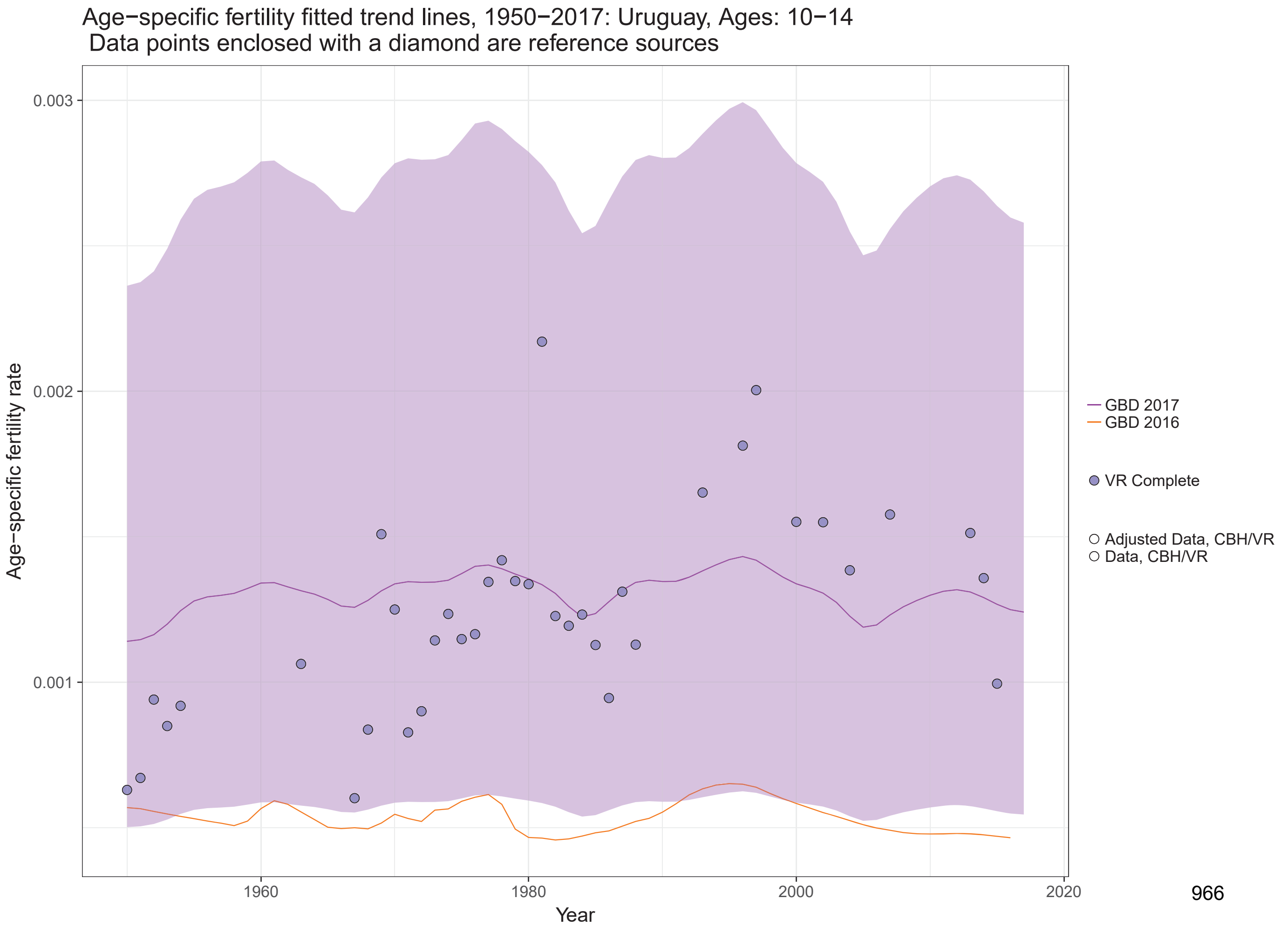

Age−specific fertility fitted trend lines, 1950−2017: Uruguay, Ages: 10−14
Data points enclosed with a diamond are reference sources
Age−specific fertility rate
Year
1960
1980
2000
2020
0.001
0.002
0.003
GBD 2017
GBD 2016
VR Complete
Adjusted Data, CBH/VR
Data, CBH/VR

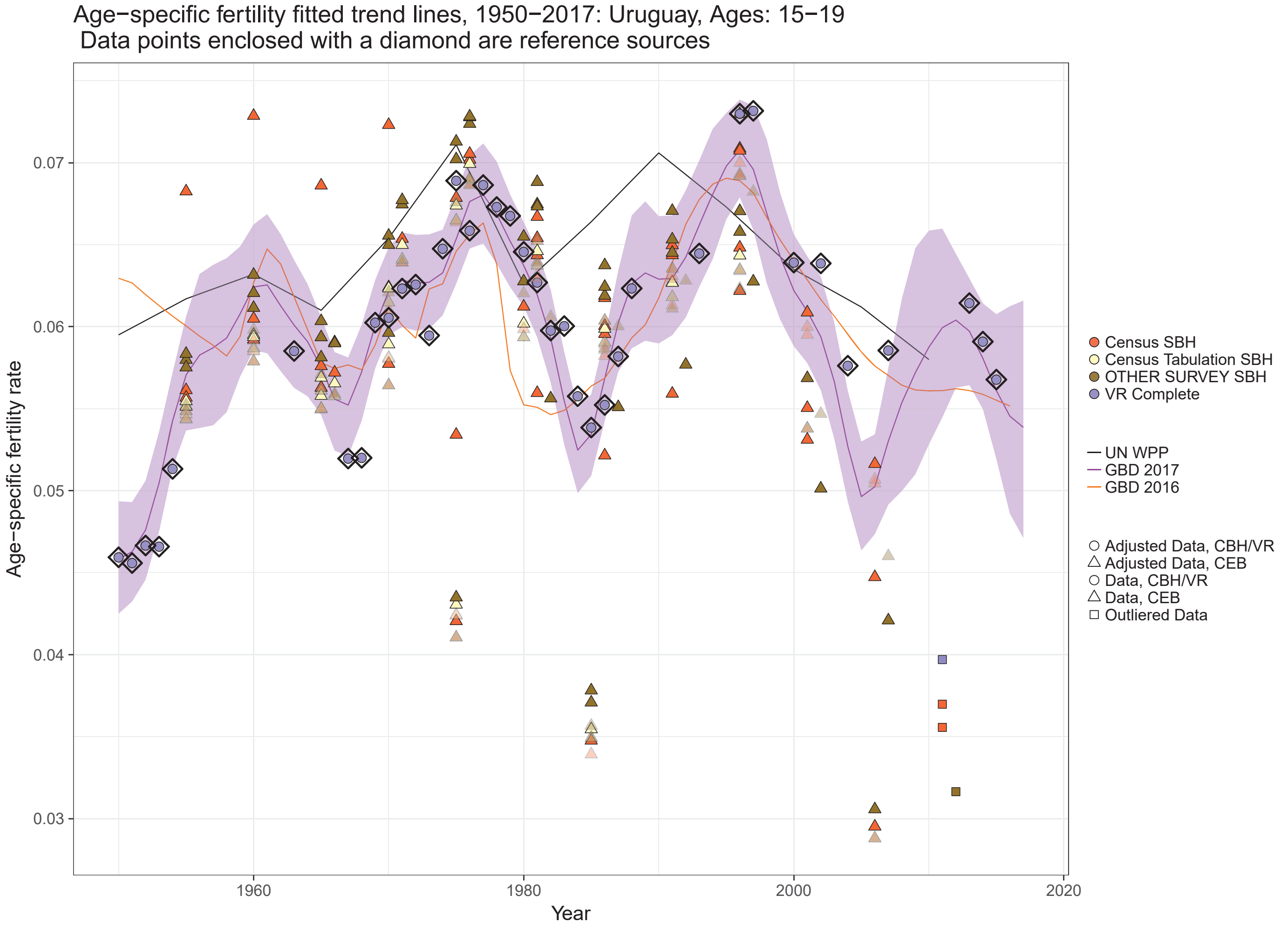

Age−specific fertility fitted trend lines, 1950−2017: Uruguay, Ages: 15−19
Data points enclosed with a diamond are reference sources
Age−specific fertility rate
0.07
0.06
0.05
0.04
0.03
1960
1980
2000
2020
Year
Census SBH
Census Tabulation SBH
OTHER SURVEY SBH
VR Complete
UN WPP
GBD 2017
GBD 2016
Adjusted Data, CBH/VR
Adjusted Data, CEB
Data, CBH/VR
Data, CEB
Outliered Data

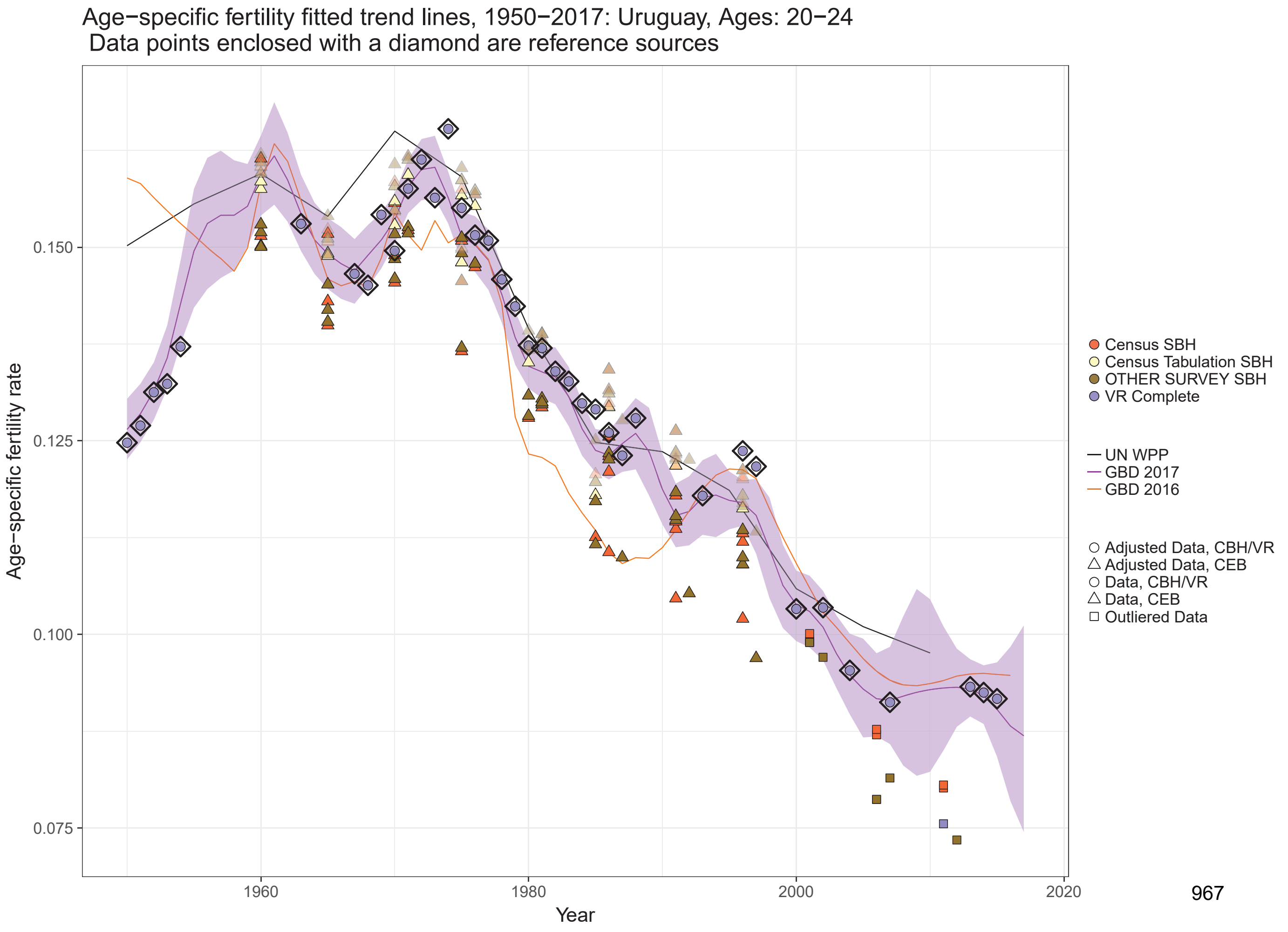

Age−specific fertility fitted trend lines, 1950−2017: Uruguay, Ages: 20−24
Data points enclosed with a diamond are reference sources
Age−specific fertility rate
0.150
0.125
0.100
0.075
1960
1980
2000
2020
Year
Census SBH
Census Tabulation SBH
OTHER SURVEY SBH
VR Complete
UN WPP
GBD 2017
GBD 2016
Adjusted Data, CBH/VR
Adjusted Data, CEB
Data, CBH/VR
Data, CEB
Outliered Data

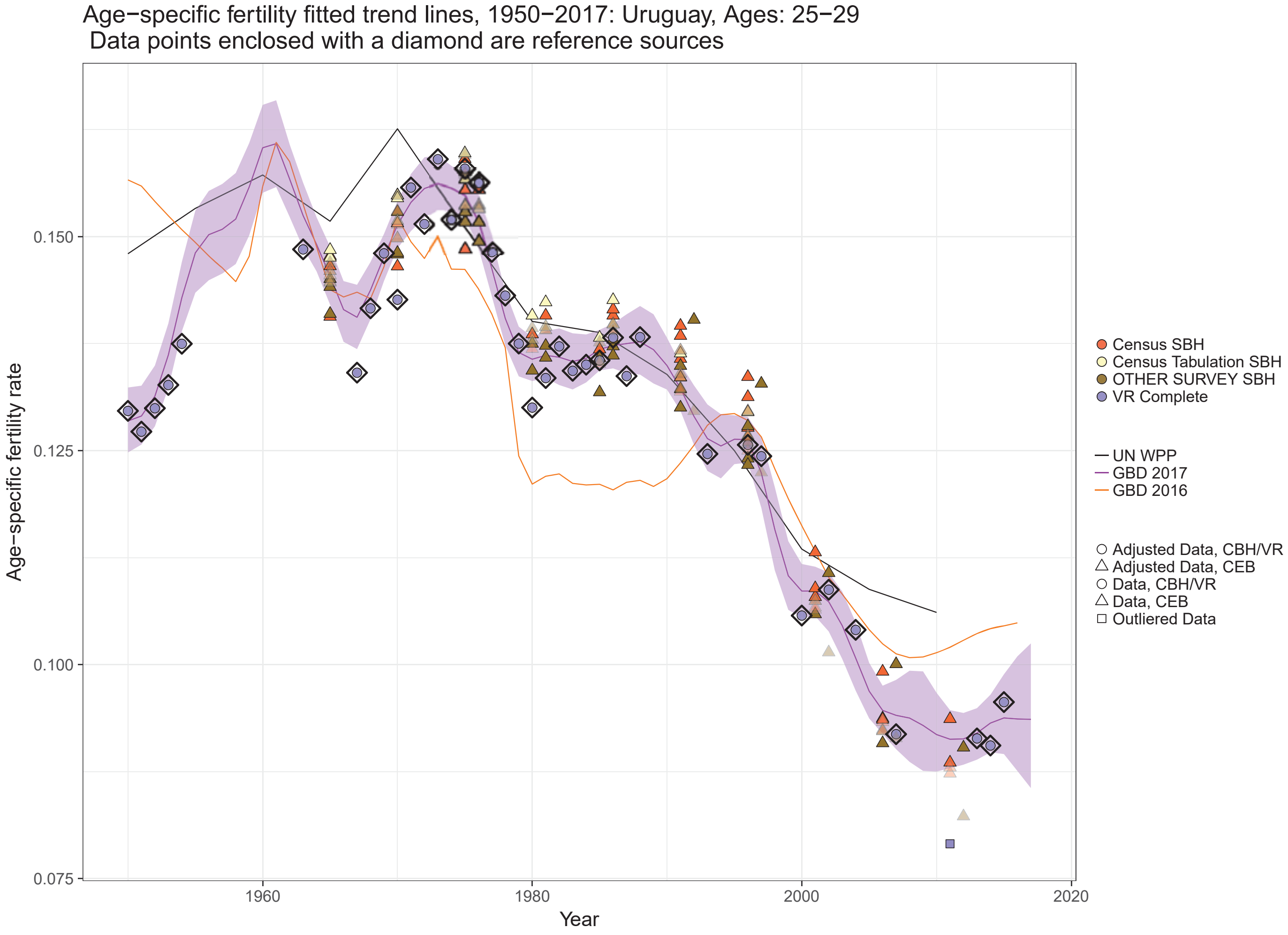

Age−specific fertility fitted trend lines, 1950−2017: Uruguay, Ages: 25−29
Data points enclosed with a diamond are reference sources
Age−specific fertility rate
Year
Census SBH
Census Tabulation SBH
OTHER SURVEY SBH
VR Complete
UN WPP
GBD 2017
GBD 2016
Adjusted Data, CBH/VR
Adjusted Data, CEB
Data, CBH/VR
Data, CEB
Outliered Data

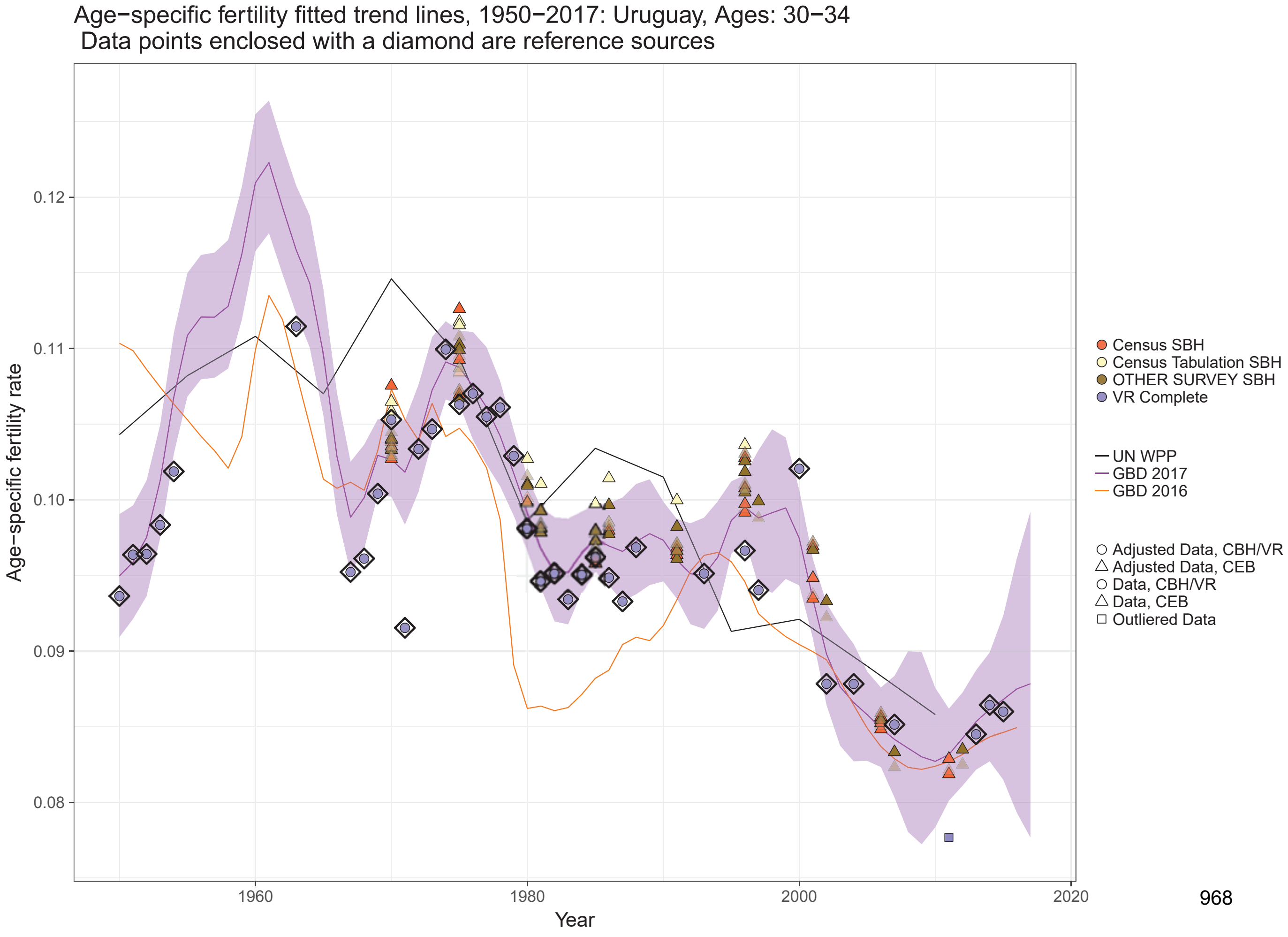

Age−specific fertility fitted trend lines, 1950−2017: Uruguay, Ages: 30−34
Data points enclosed with a diamond are reference sources
Age−specific fertility rate
Year
Census SBH
Census Tabulation SBH
OTHER SURVEY SBH
VR Complete
UN WPP
GBD 2017
GBD 2016
Adjusted Data, CBH/VR
Adjusted Data, CEB
Data, CBH/VR
Data, CEB
Outliered Data

Age−specific fertility fitted trend lines, 1950−2017: Uruguay, Ages: 35−39
Data points enclosed with a diamond are reference sources

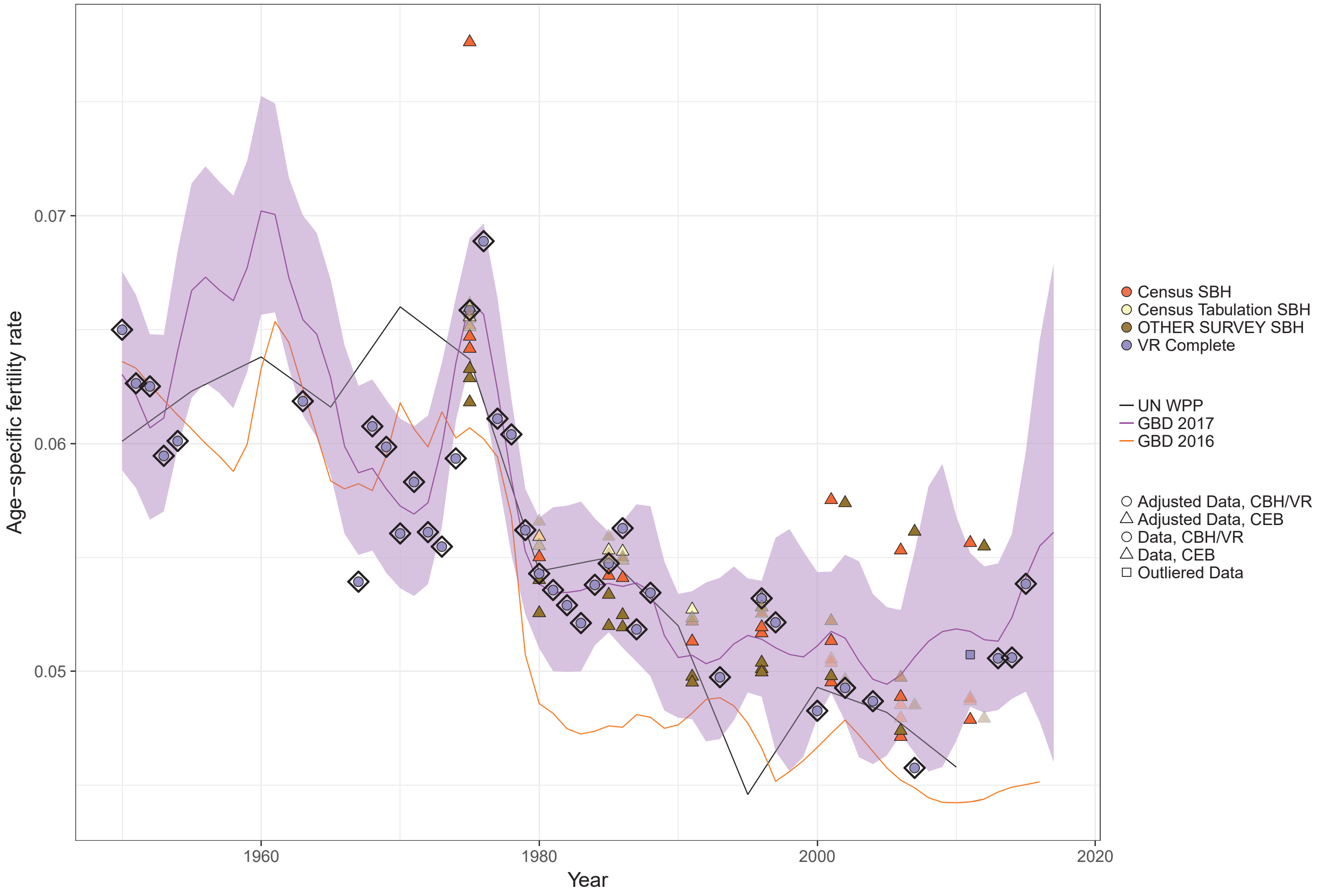

Age−specific fertility fitted trend lines, 1950−2017: Uruguay, Ages: 40−44
Data points enclosed with a diamond are reference sources

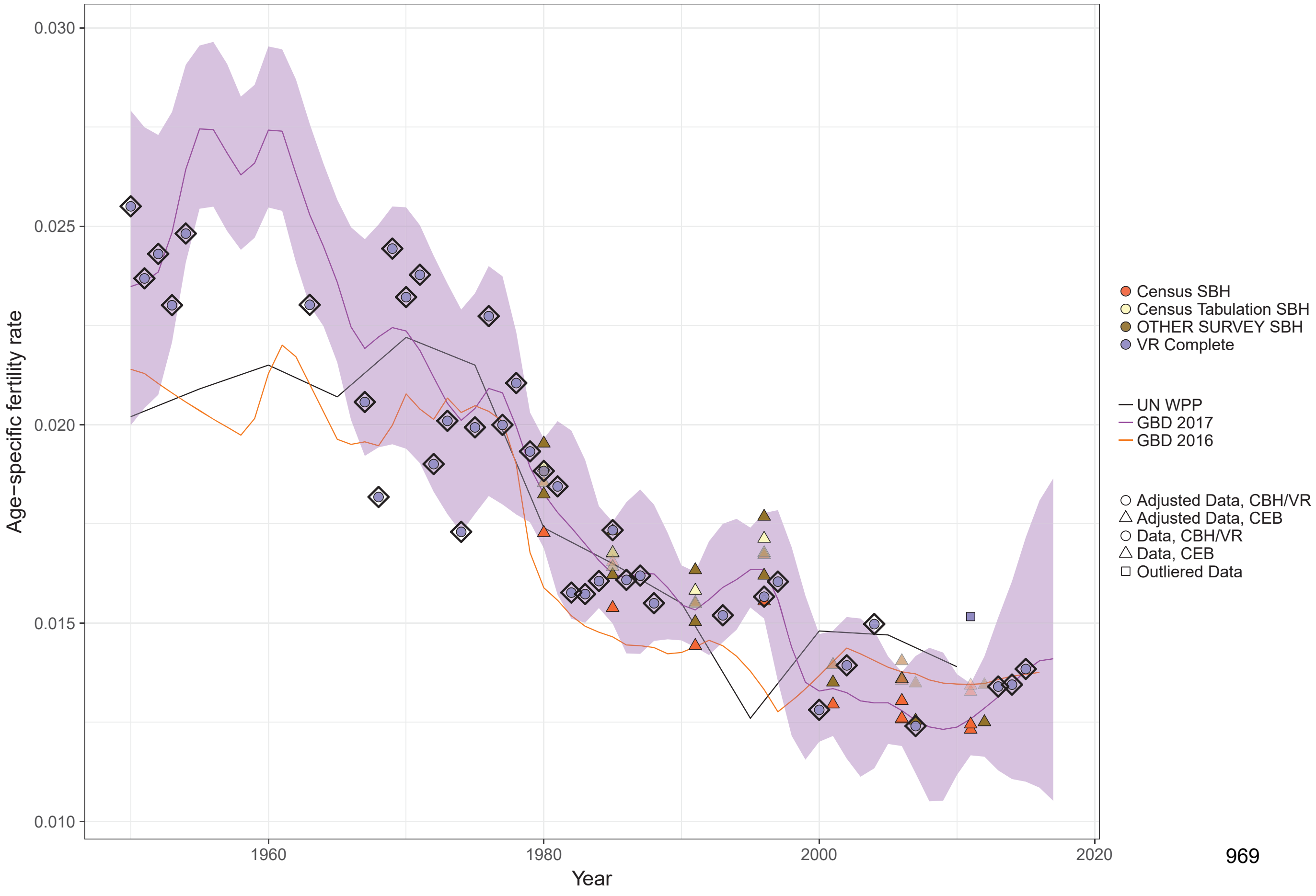

Age−specific fertility fitted trend lines, 1950−2017: Uruguay, Ages: 45−49
Data points enclosed with a diamond are reference sources

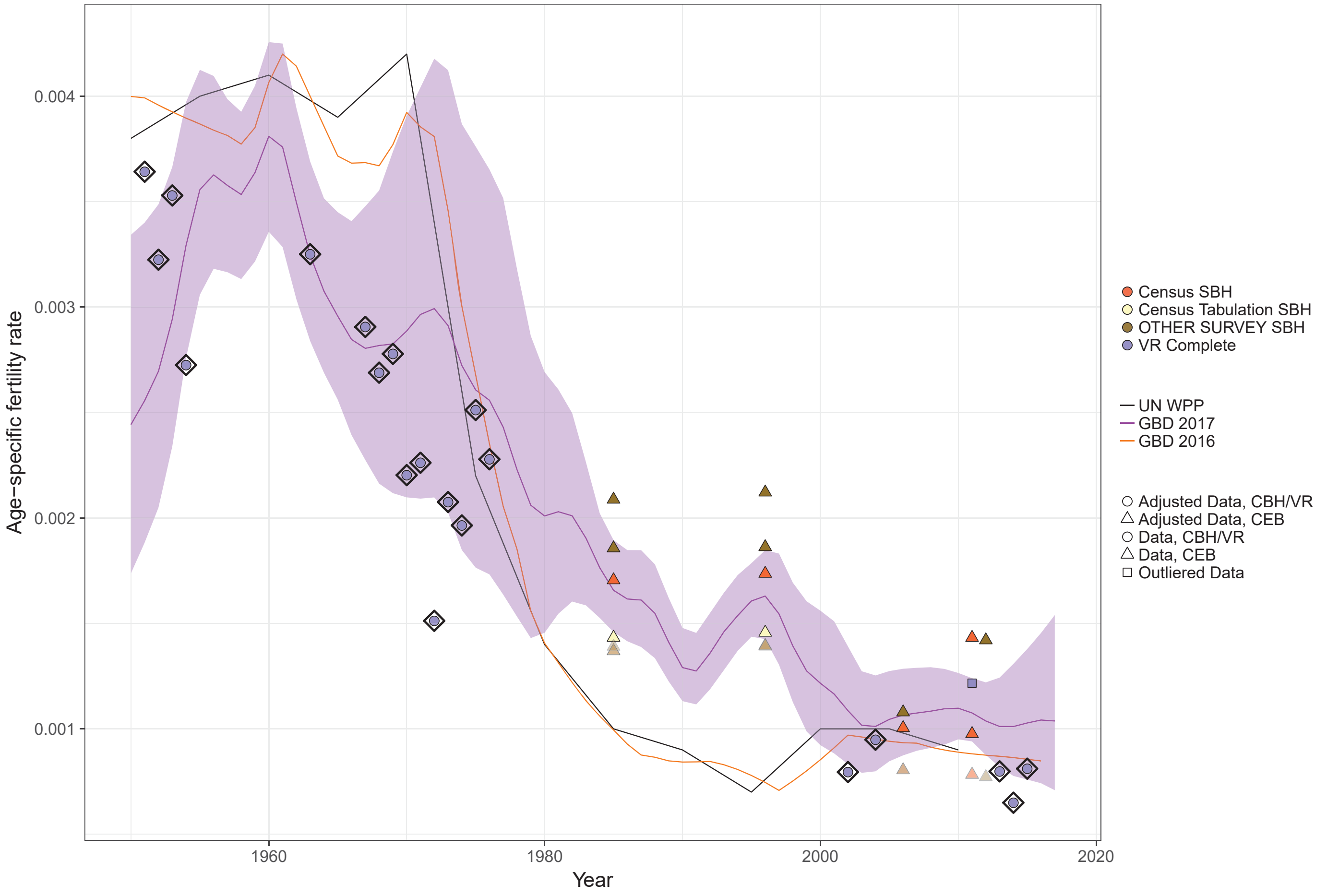

Age−specific fertility fitted trend lines, 1950−2017: Uruguay, Ages: 50−54
Data points enclosed with a diamond are reference sources

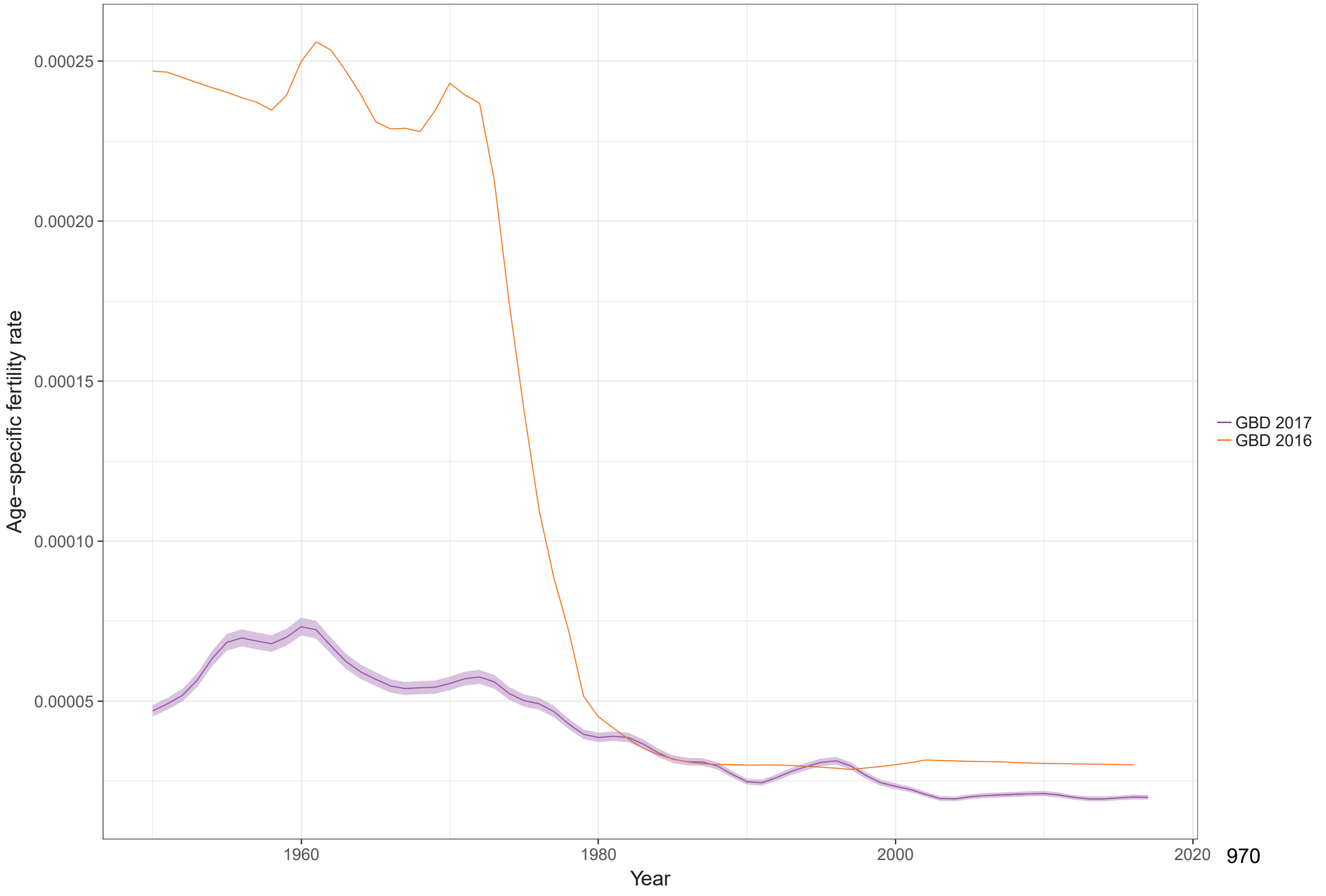

Age−specific fertility fitted trend lines, 1950−2017: Uzbekistan, TFR
Data points enclosed with a diamond are reference sources

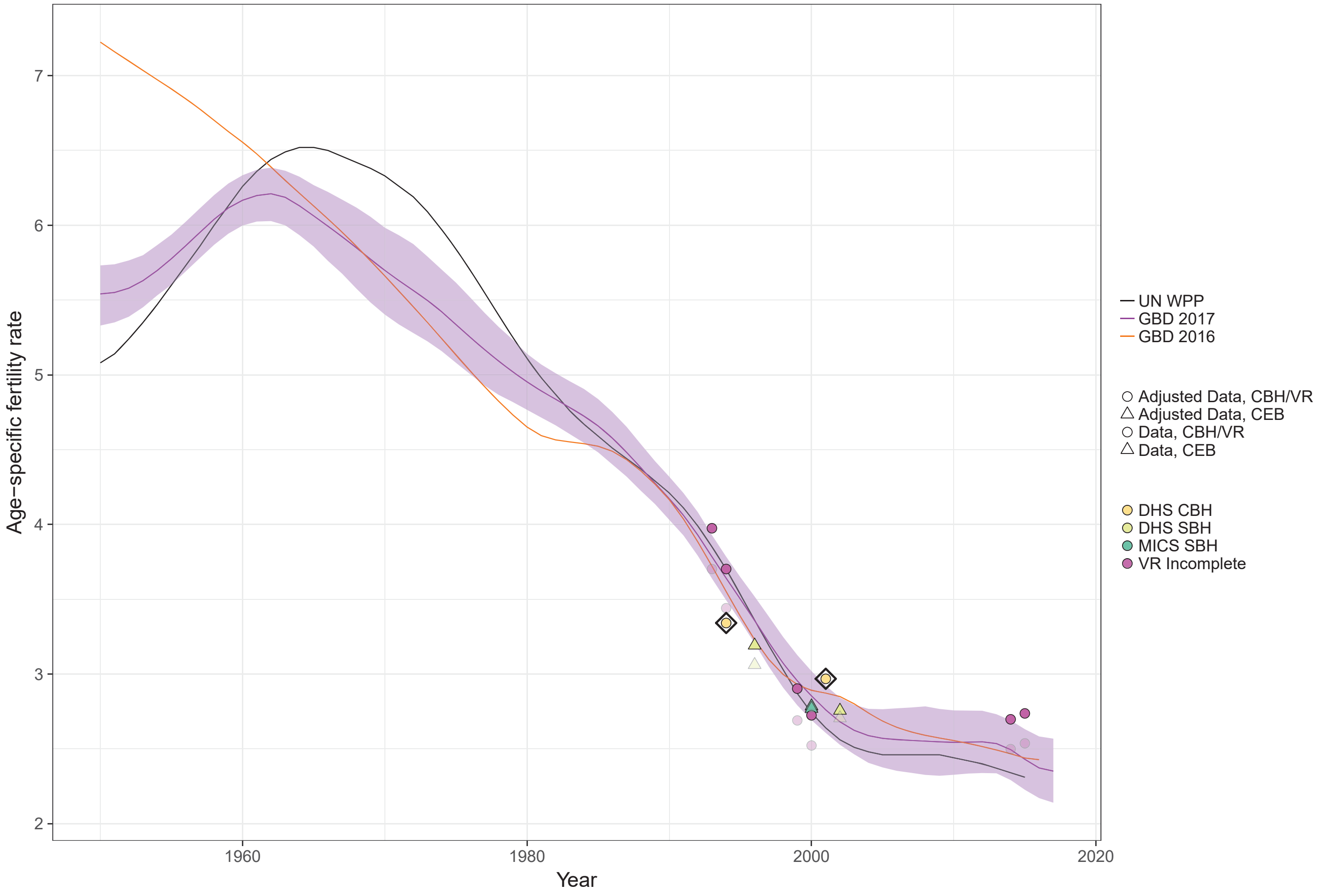

Age−specific fertility fitted trend lines, 1950−2017: Uzbekistan, Ages: 10−14
Data points enclosed with a diamond are reference sources

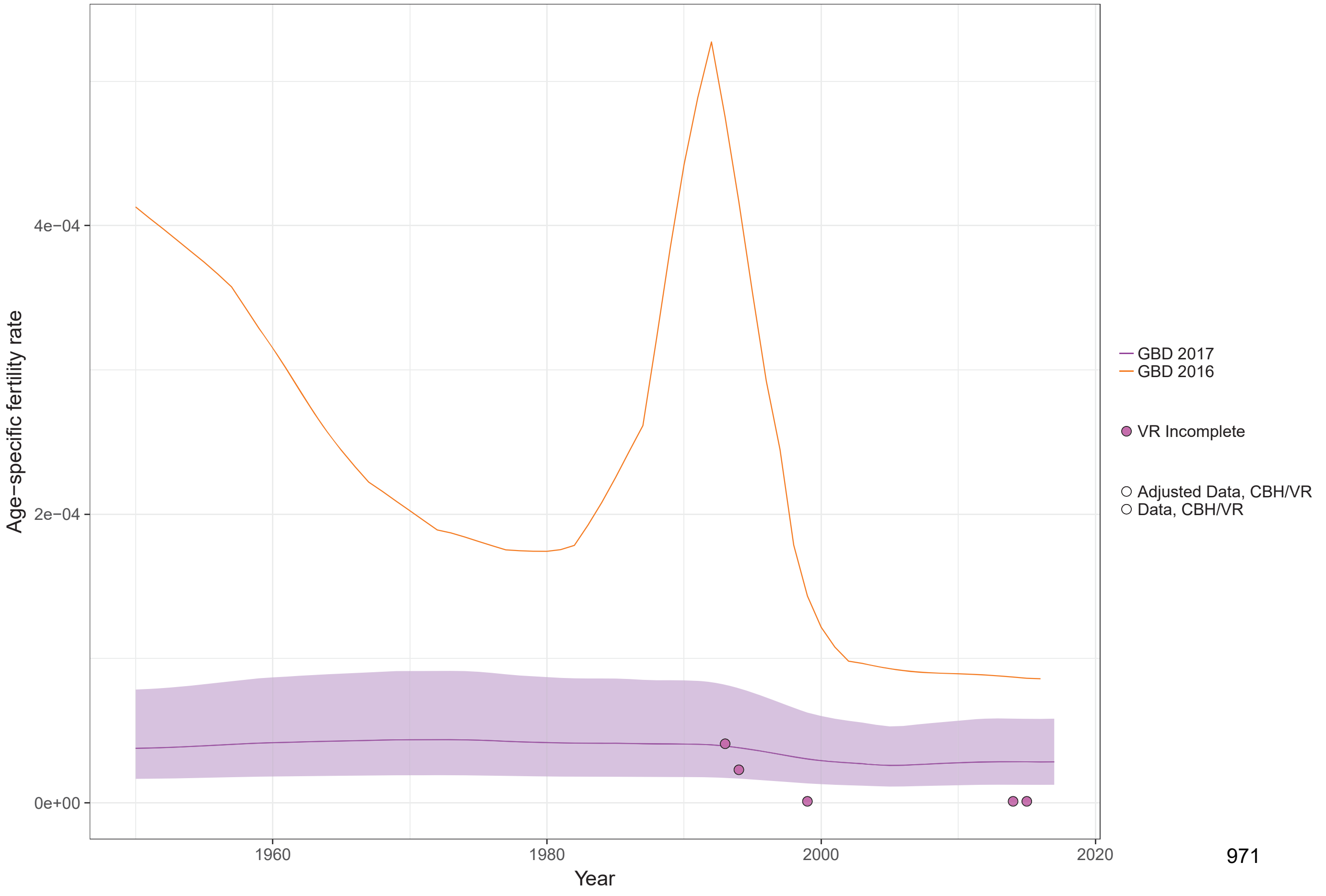

Age−specific fertility fitted trend lines, 1950−2017: Uzbekistan, Ages: 15−19
Data points enclosed with a diamond are reference sources

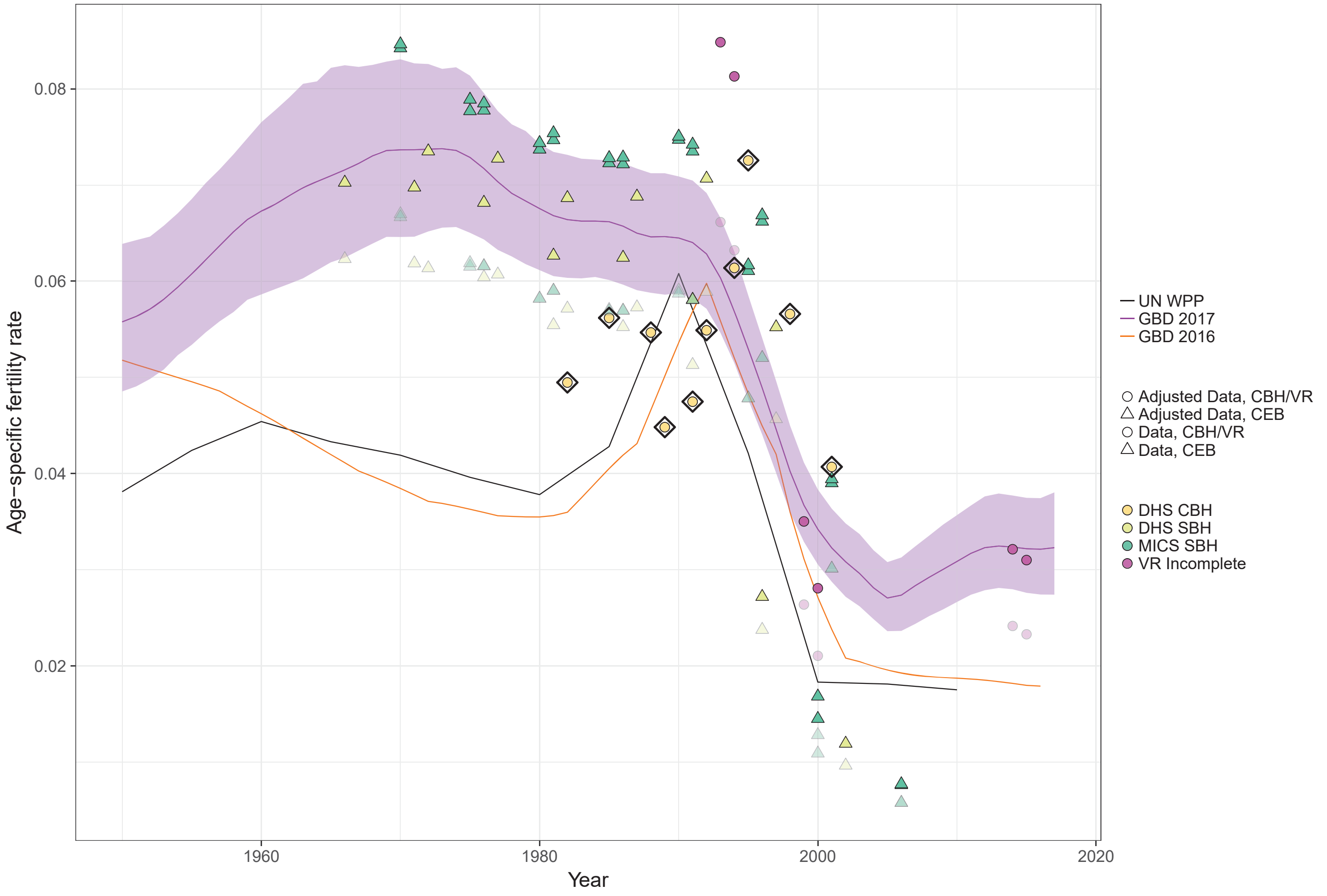

Age−specific fertility fitted trend lines, 1950−2017: Uzbekistan, Ages: 20−24
Data points enclosed with a diamond are reference sources

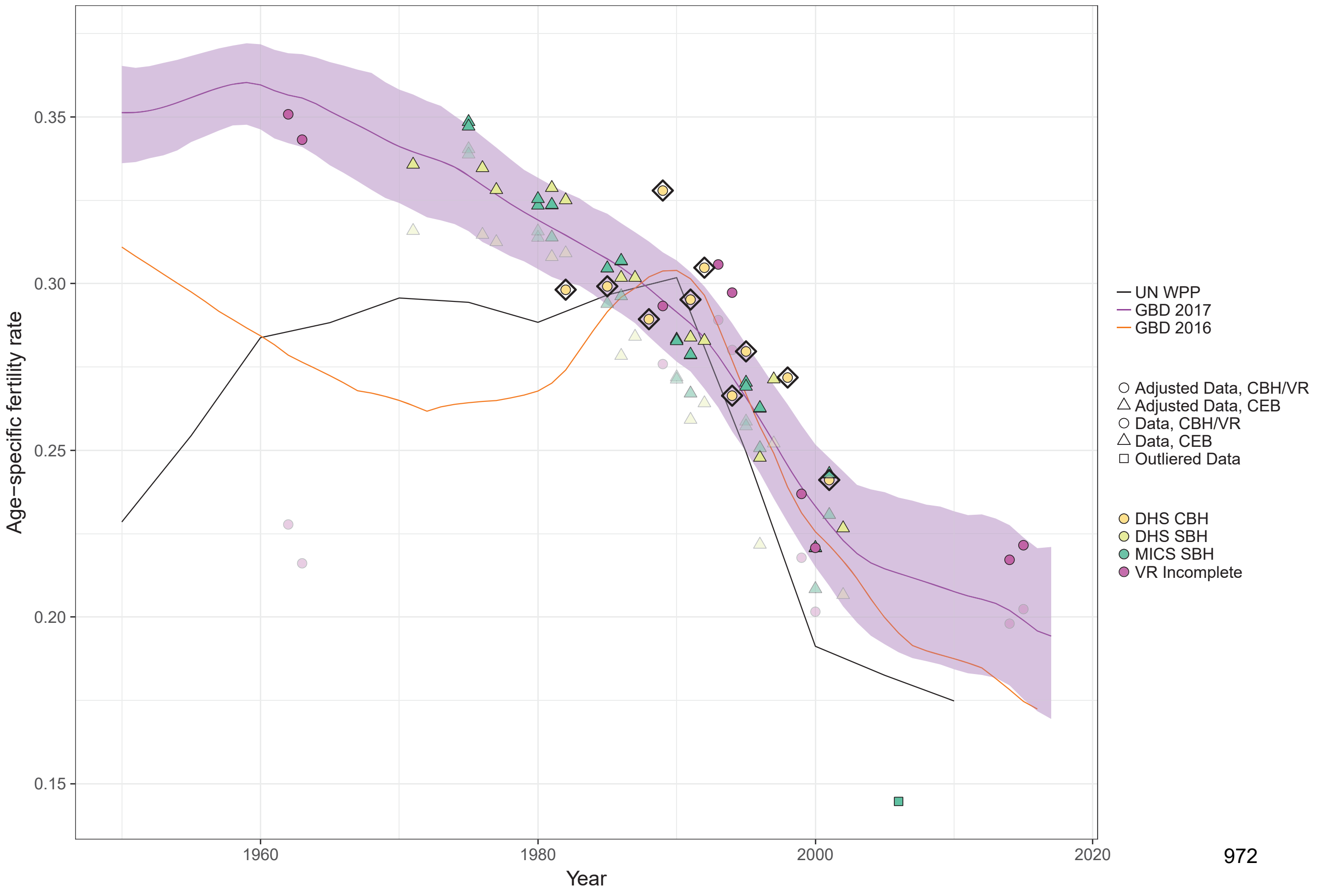

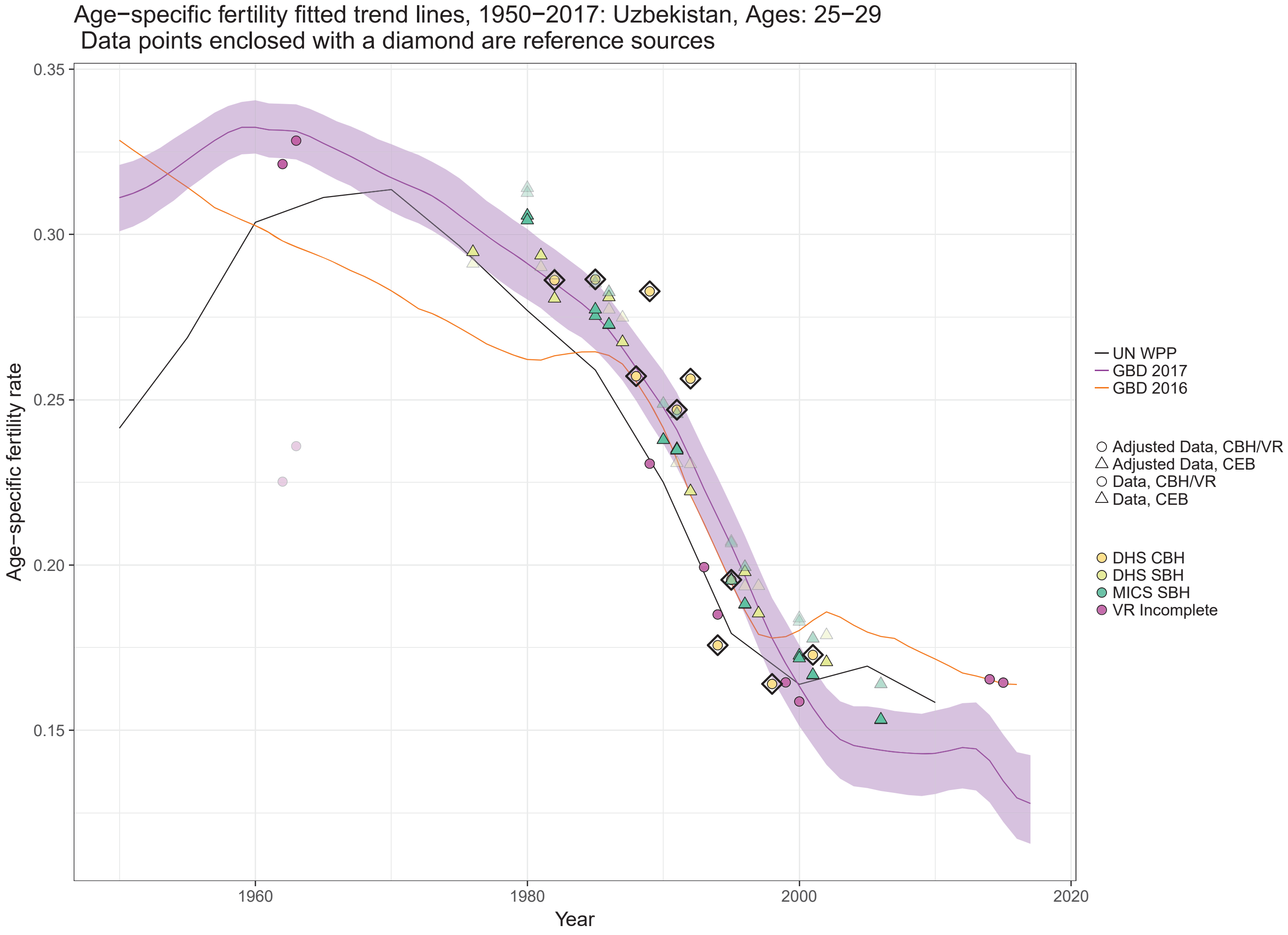
Age−specific fertility fitted trend lines, 1950−2017: Uzbekistan, Ages: 25−29
Data points enclosed with a diamond are reference sources
Age−specific fertility rate
Year
UN WPP
GBD 2017
GBD 2016
Adjusted Data, CBH/VR
Adjusted Data, CEB
Data, CBH/VR
Data, CEB
DHS CBH
DHS SBH
MICS SBH
VR Incomplete

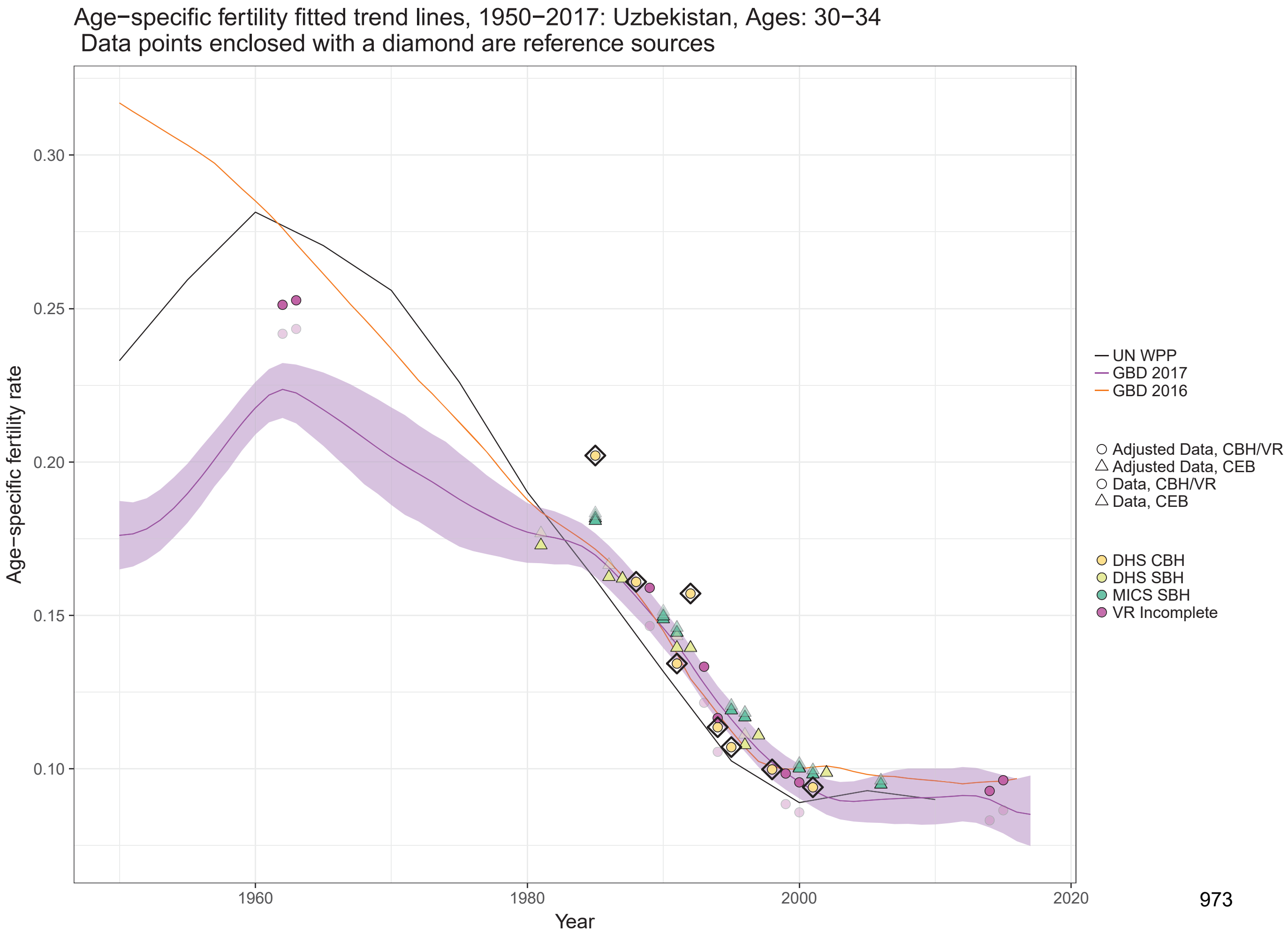
Age−specific fertility fitted trend lines, 1950−2017: Uzbekistan, Ages: 30−34
Data points enclosed with a diamond are reference sources
Age−specific fertility rate
Year
UN WPP
GBD 2017
GBD 2016
Adjusted Data, CBH/VR
Adjusted Data, CEB
Data, CBH/VR
Data, CEB
DHS CBH
DHS SBH
MICS SBH
VR Incomplete

Age−specific fertility fitted trend lines, 1950−2017: Uzbekistan, Ages: 35−39
Data points enclosed with a diamond are reference sources

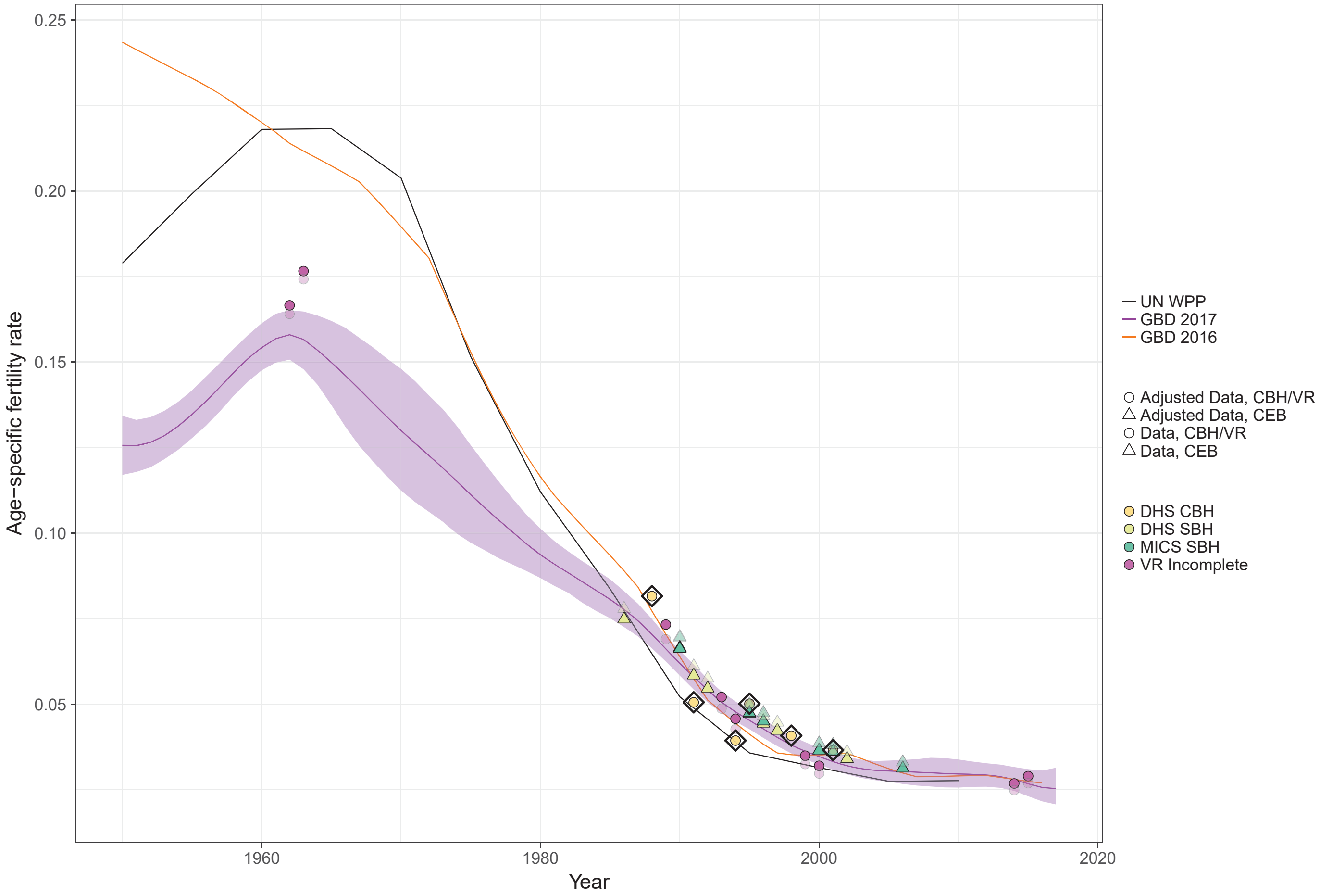

Age−specific fertility fitted trend lines, 1950−2017: Uzbekistan, Ages: 40−44
Data points enclosed with a diamond are reference sources

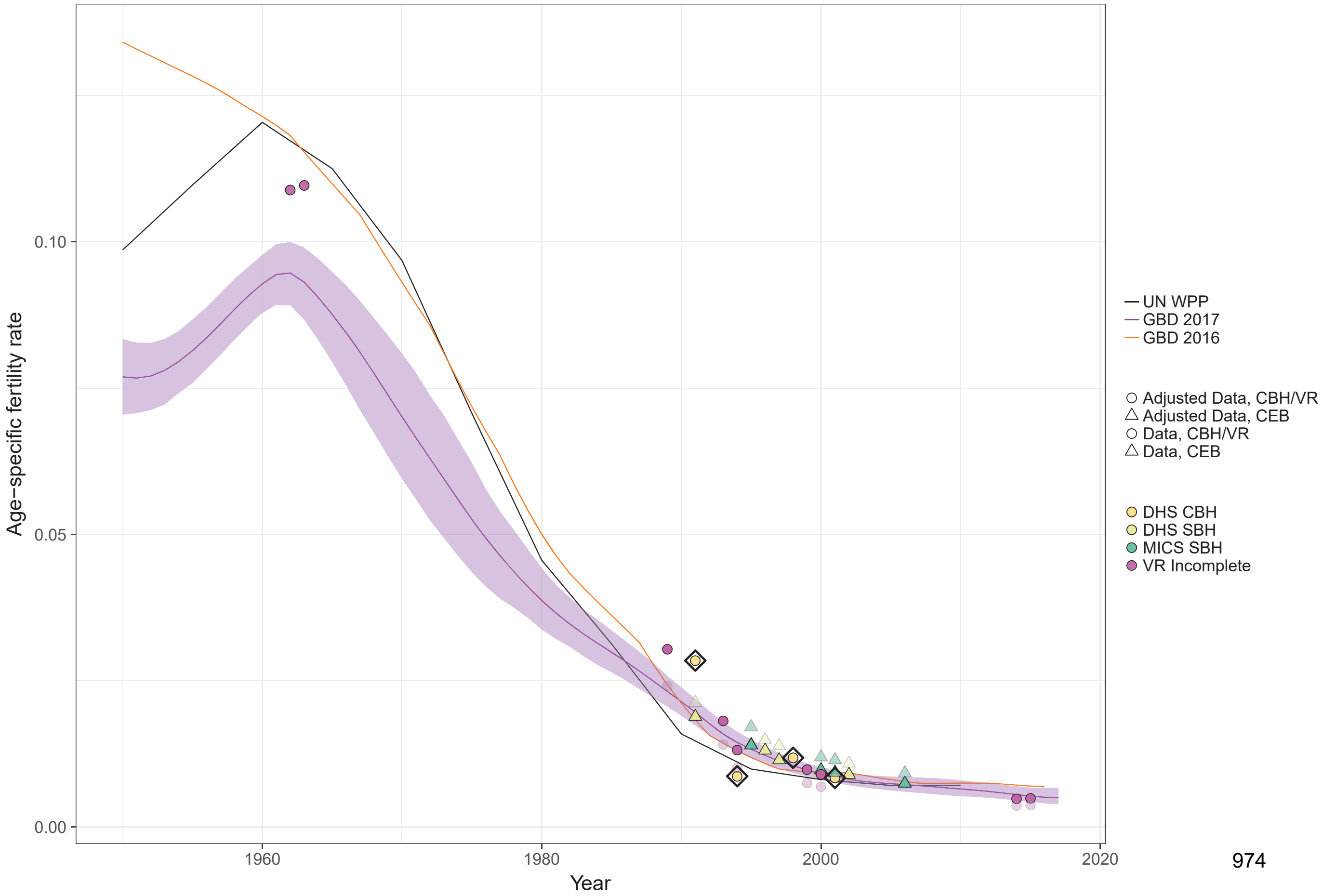

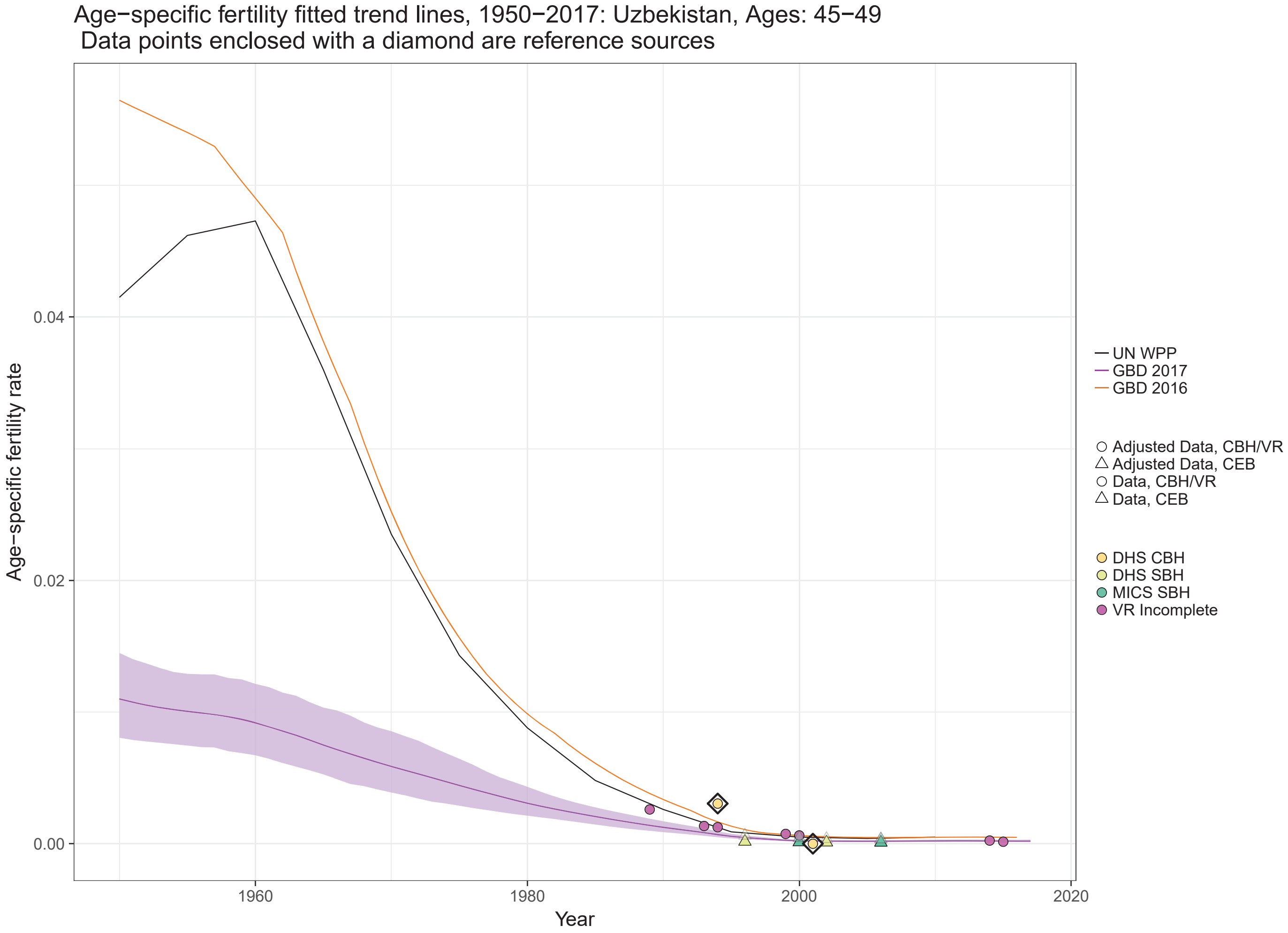
Age−specific fertility fitted trend lines, 1950−2017: Uzbekistan, Ages: 45−49
Data points enclosed with a diamond are reference sources
Age−specific fertility rate
0.04
0.02
0.00
1960
1980
2000
2020
Year
UN WPP
GBD 2017
GBD 2016
Adjusted Data, CBH/VR
Adjusted Data, CEB
Data, CBH/VR
Data, CEB
DHS CBH
DHS SBH
MICS SBH
VR Incomplete

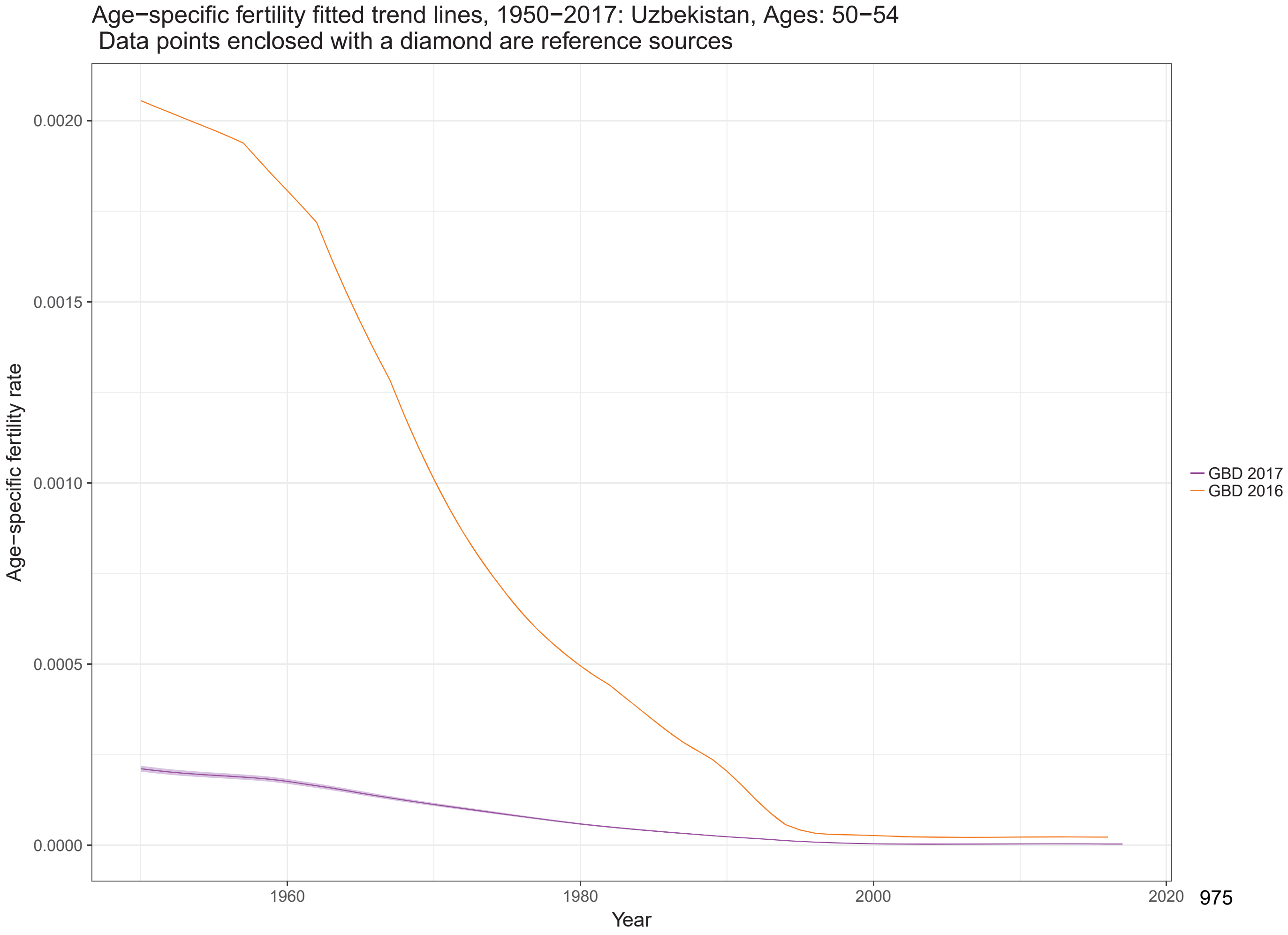
Age−specific fertility fitted trend lines, 1950−2017: Uzbekistan, Ages: 50−54
Data points enclosed with a diamond are reference sources
Age−specific fertility rate
0.0020
0.0015
0.0010
0.0005
0.0000
1960
1980
2000
2020
Year
GBD 2017
GBD 2016

Age−specific fertility fitted trend lines, 1950−2017: Vanuatu, TFR
Data points enclosed with a diamond are reference sources

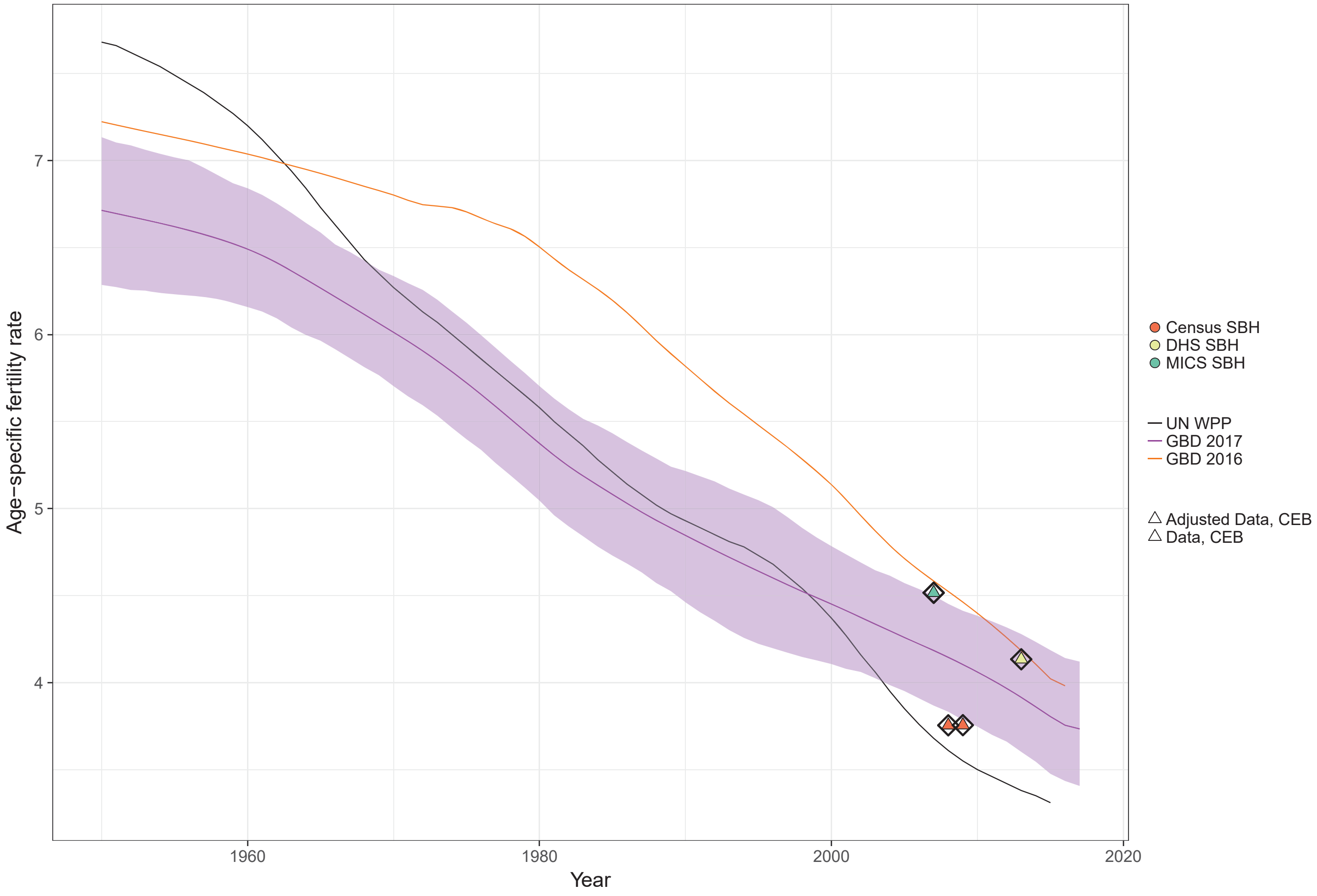

Age−specific fertility fitted trend lines, 1950−2017: Vanuatu, Ages: 10−14
Data points enclosed with a diamond are reference sources

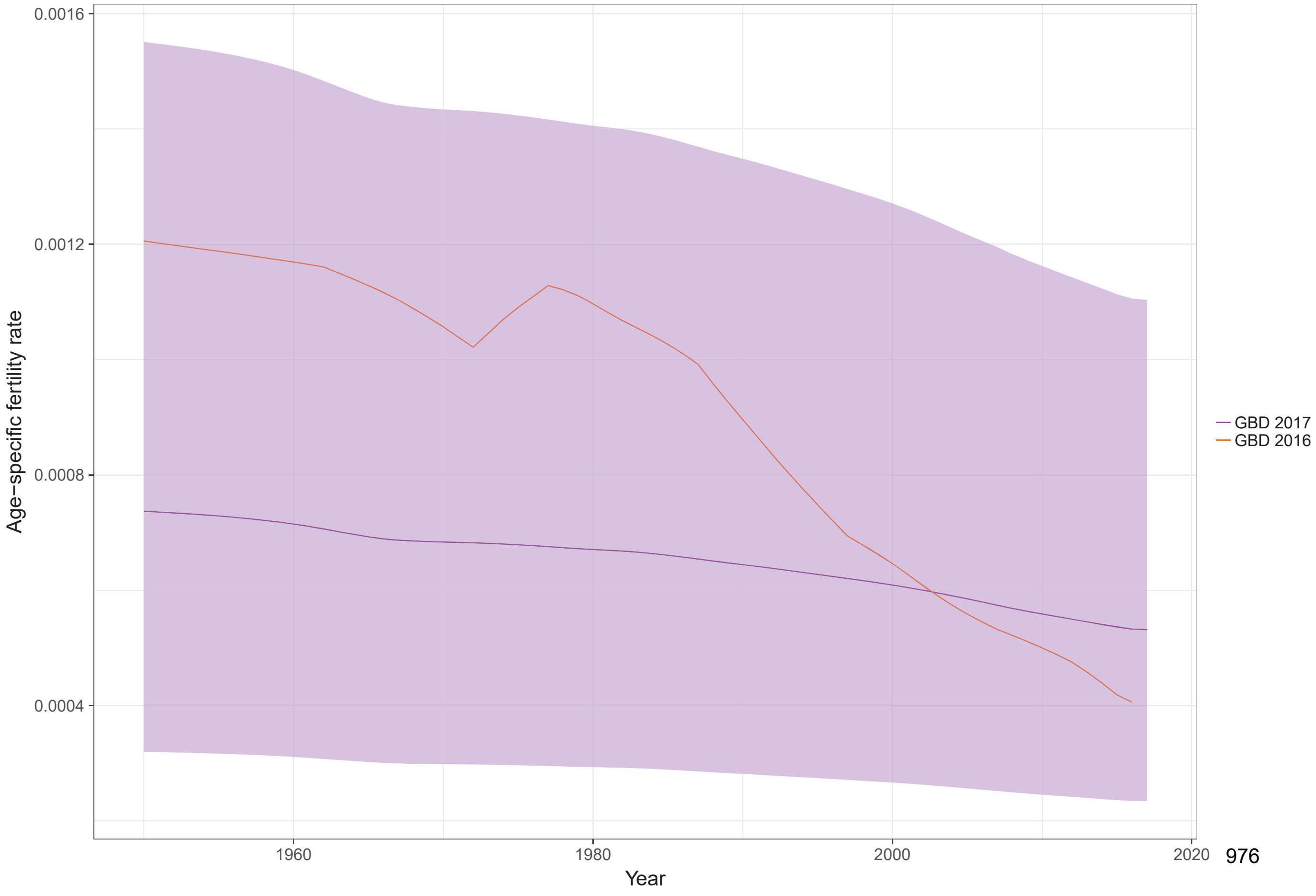

Age−specific fertility fitted trend lines, 1950−2017: Vanuatu, Ages: 15−19
Data points enclosed with a diamond are reference sources

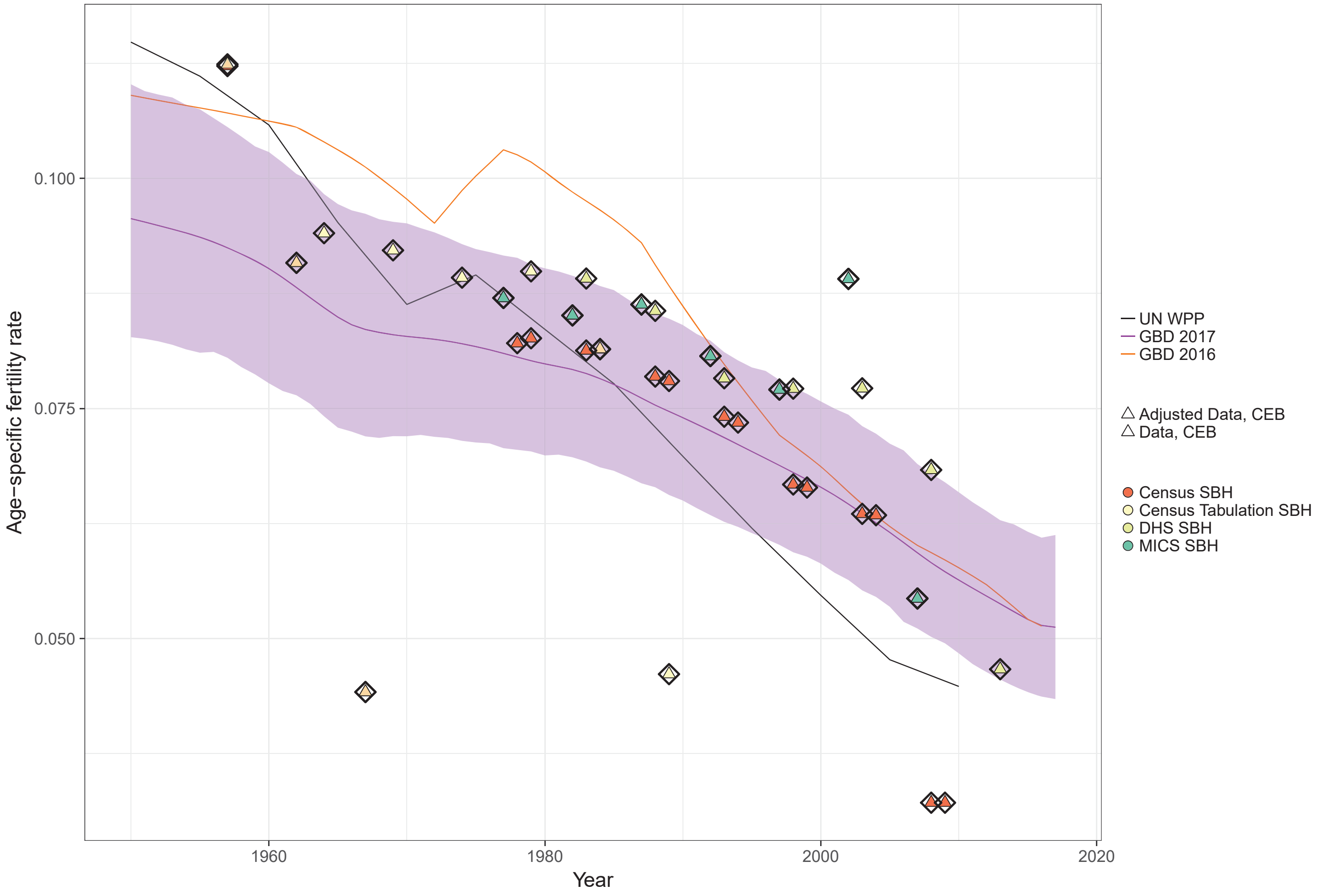

Age−specific fertility fitted trend lines, 1950−2017: Vanuatu, Ages: 20−24
Data points enclosed with a diamond are reference sources

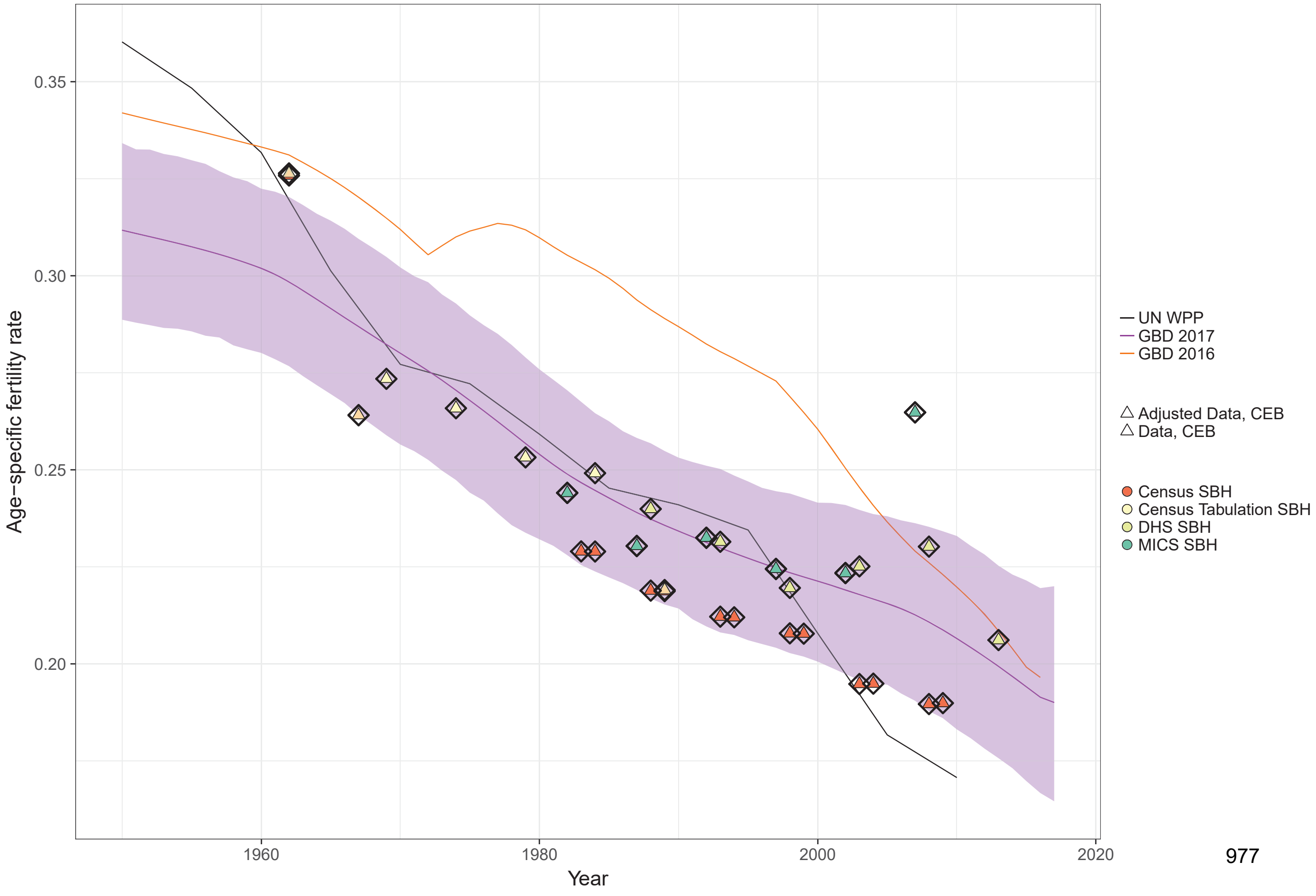

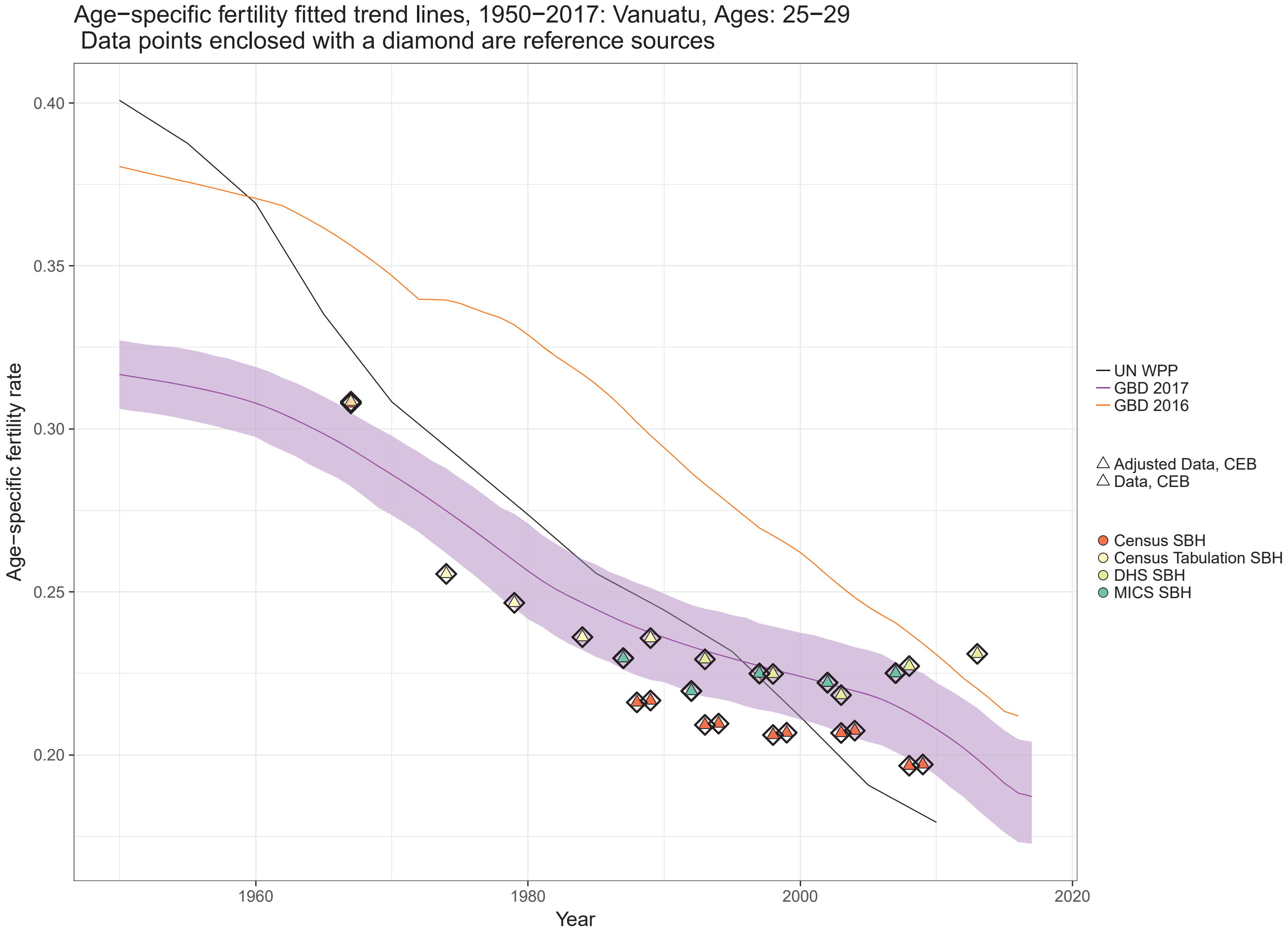

Age−specific fertility fitted trend lines, 1950−2017: Vanuatu, Ages: 25−29
Data points enclosed with a diamond are reference sources
Age−specific fertility rate
Year
0.40
0.35
0.30
0.25
0.20
1960
1980
2000
2020
UN WPP
GBD 2017
GBD 2016
Adjusted Data, CEB
Data, CEB
Census SBH
Census Tabulation SBH
DHS SBH
MICS SBH

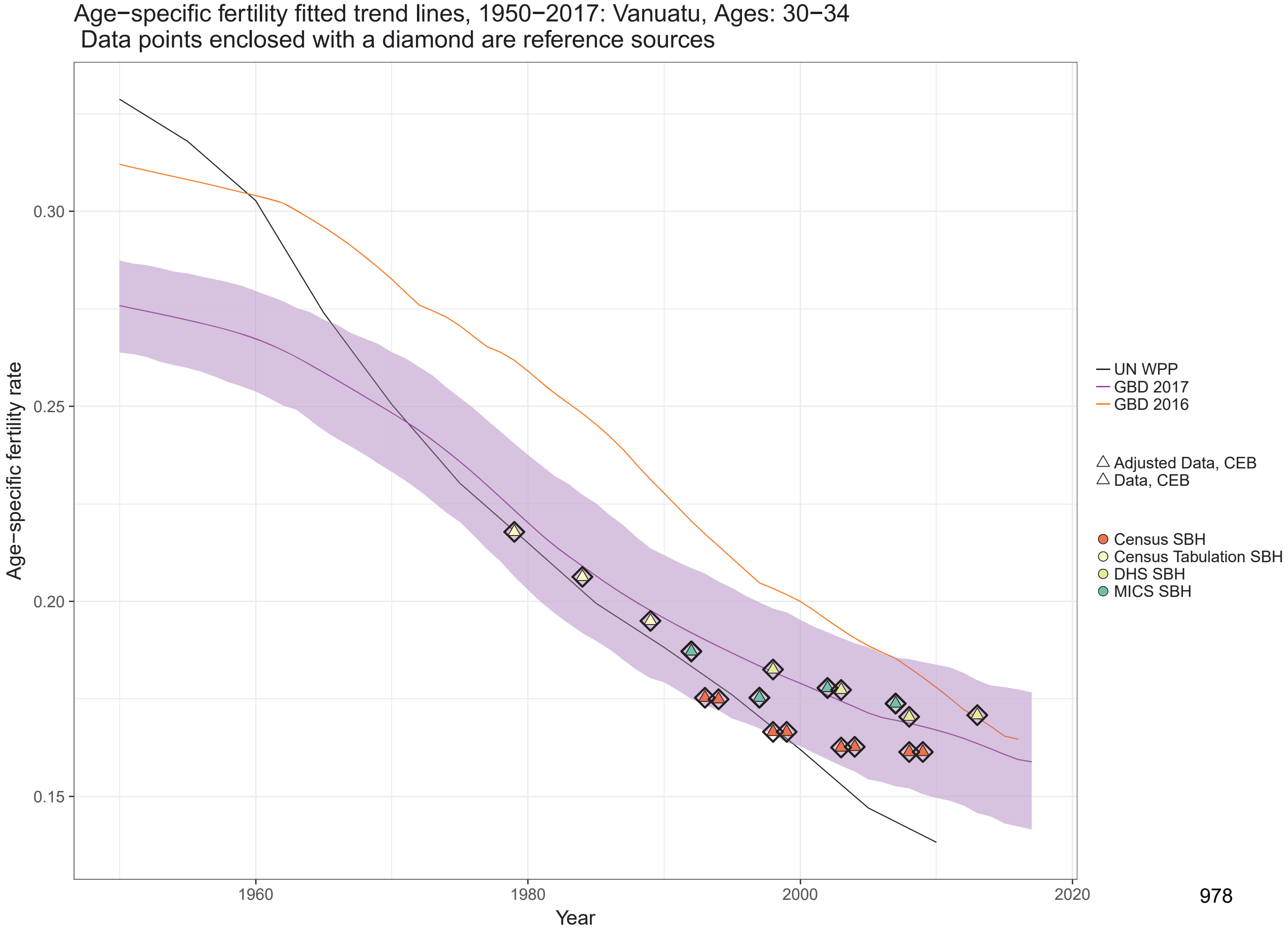

Age−specific fertility fitted trend lines, 1950−2017: Vanuatu, Ages: 30−34
Data points enclosed with a diamond are reference sources
Age−specific fertility rate
Year
0.30
0.25
0.20
0.15
1960
1980
2000
2020
UN WPP
GBD 2017
GBD 2016
Adjusted Data, CEB
Data, CEB
Census SBH
Census Tabulation SBH
DHS SBH
MICS SBH

Age−specific fertility fitted trend lines, 1950−2017: Vanuatu, Ages: 35−39
Data points enclosed with a diamond are reference sources

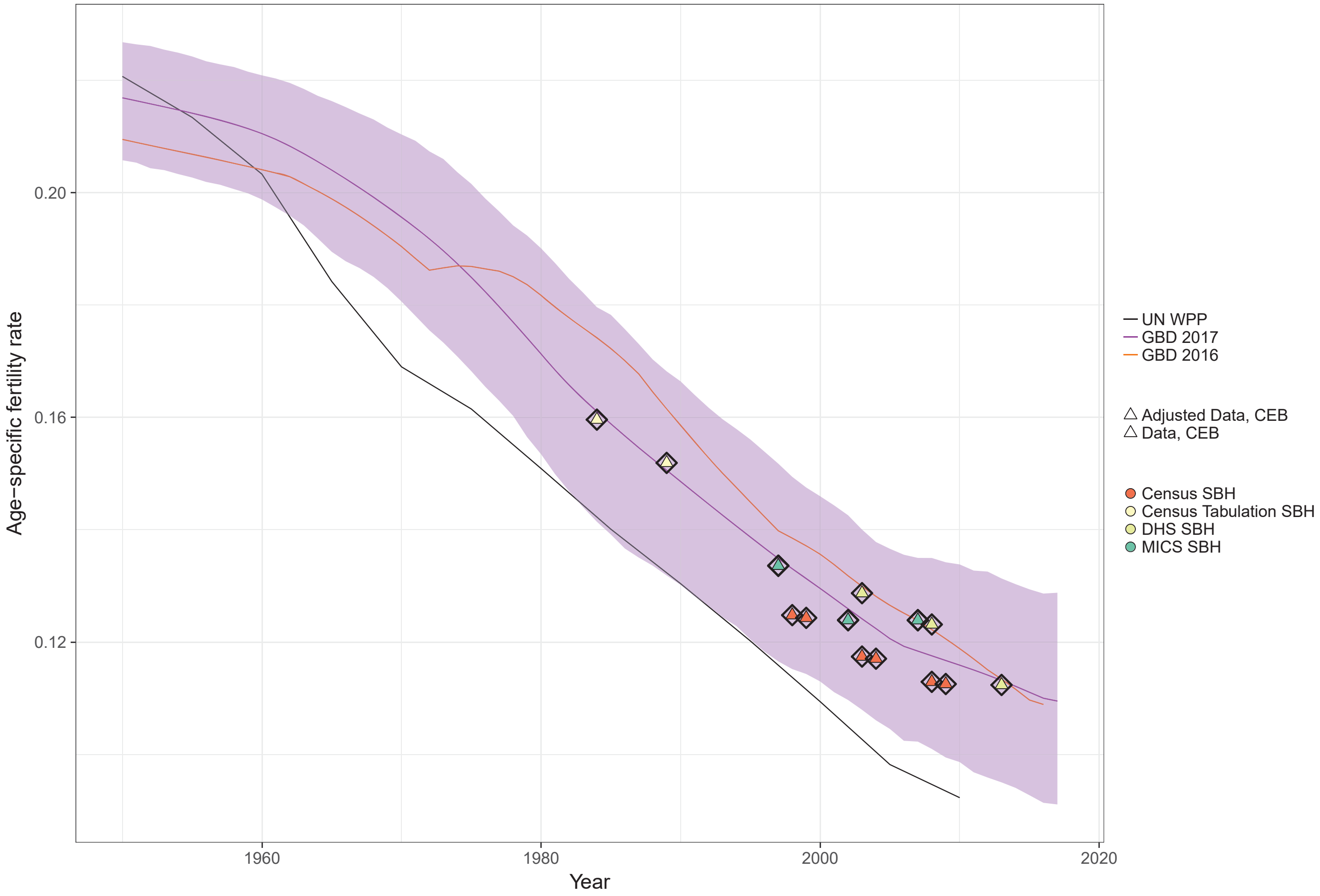

Age−specific fertility fitted trend lines, 1950−2017: Vanuatu, Ages: 40−44
Data points enclosed with a diamond are reference sources

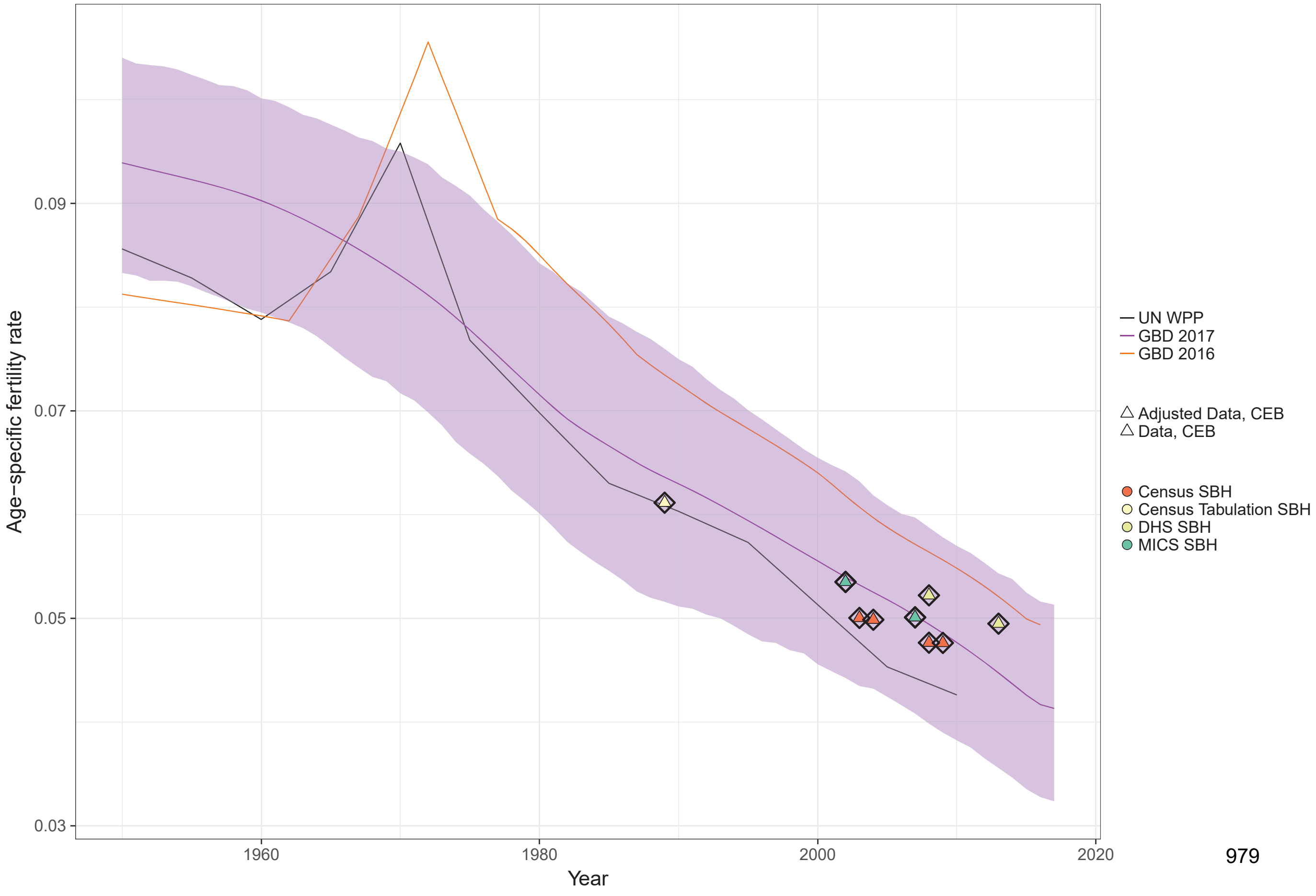

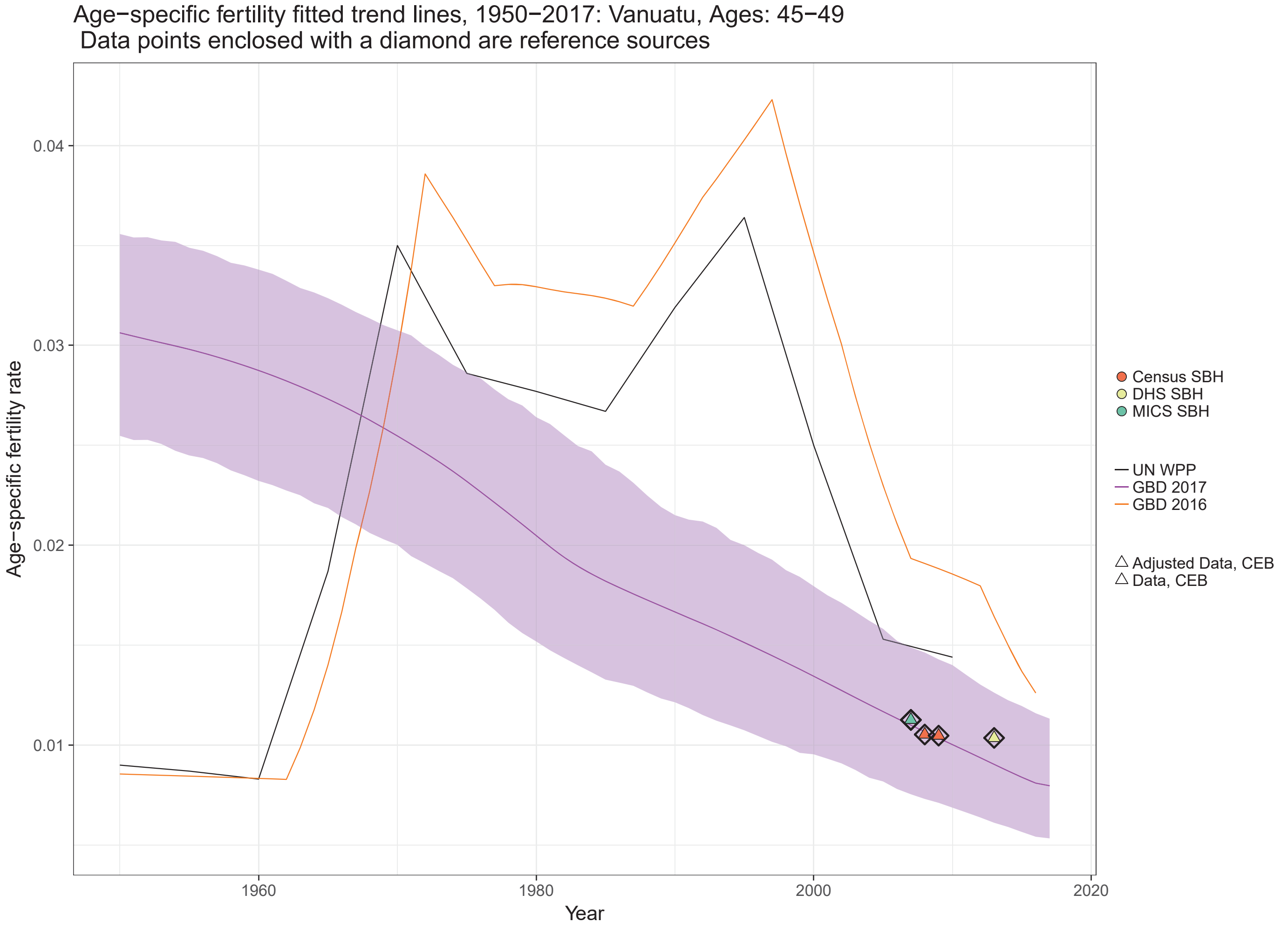
Age−specific fertility fitted trend lines, 1950−2017: Vanuatu, Ages: 45−49
Data points enclosed with a diamond are reference sources
Age−specific fertility rate
0.04
0.03
0.02
0.01
1960
1980
2000
2020
Year
Census SBH
DHS SBH
MICS SBH
UN WPP
GBD 2017
GBD 2016
Adjusted Data, CEB
Data, CEB

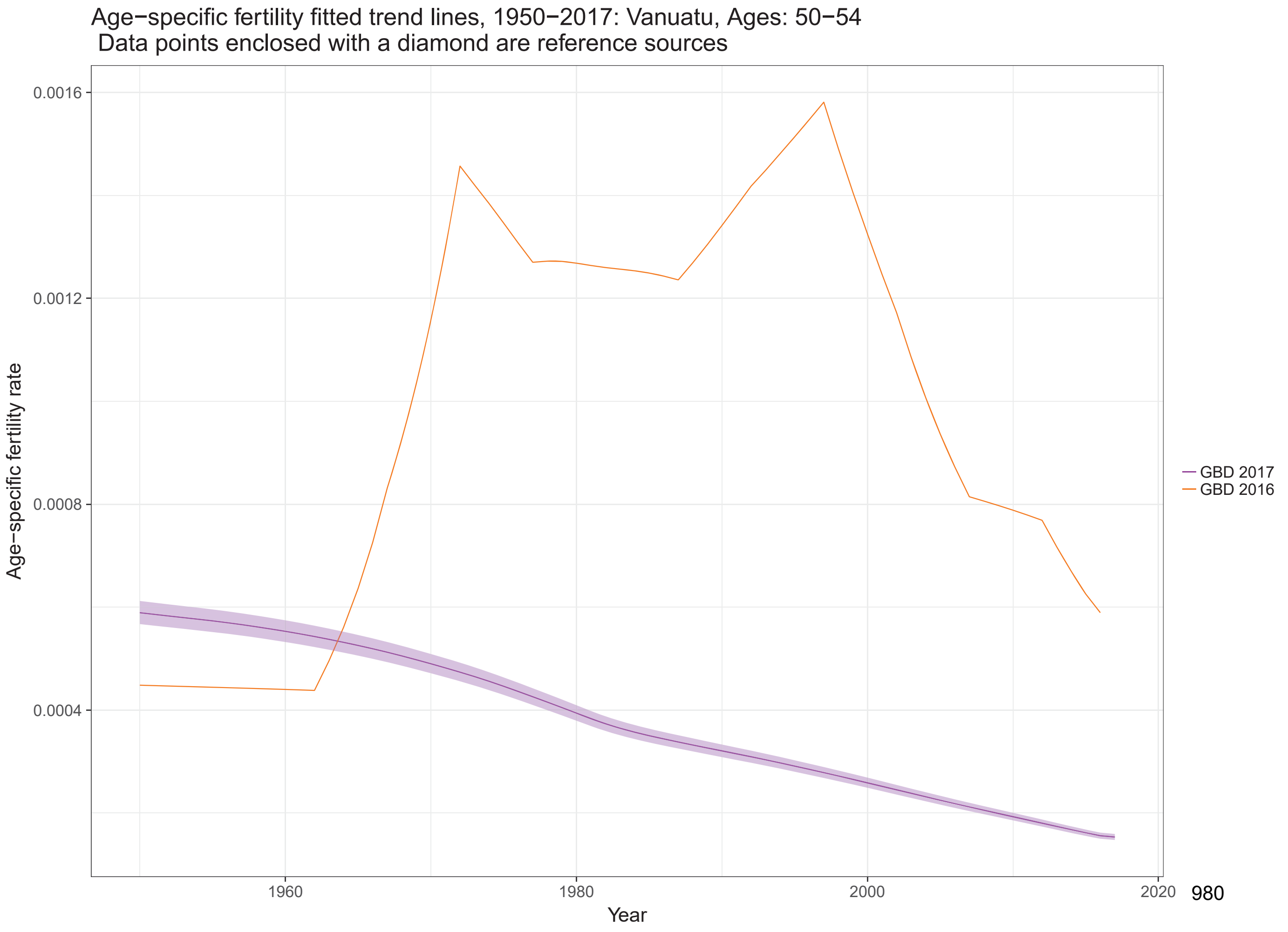
Age−specific fertility fitted trend lines, 1950−2017: Vanuatu, Ages: 50−54
Data points enclosed with a diamond are reference sources
Age−specific fertility rate
0.0016
0.0012
0.0008
0.0004
1960
1980
2000
2020
Year
GBD 2017
GBD 2016

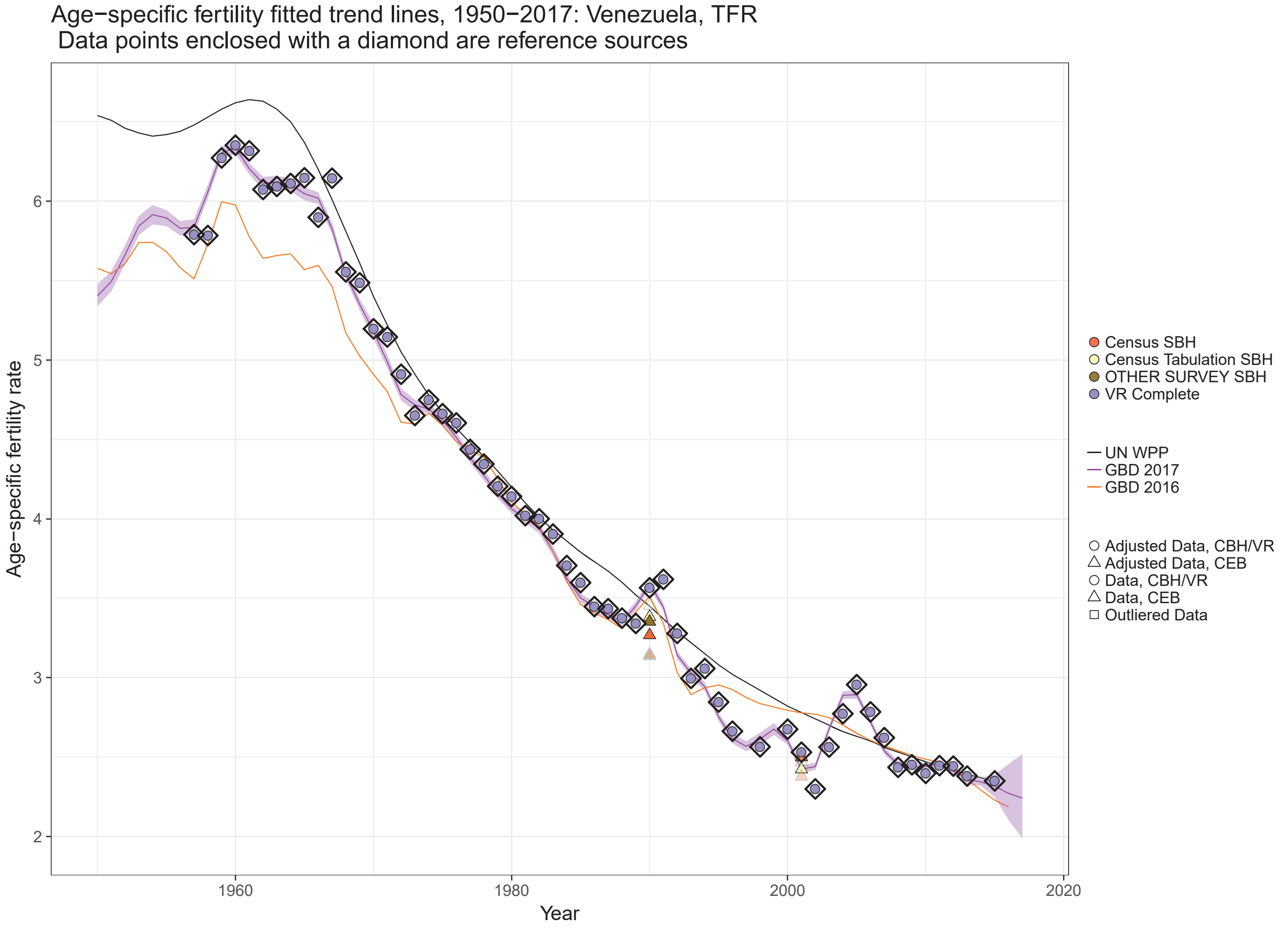

Age−specific fertility fitted trend lines, 1950−2017: Venezuela, TFR
Data points enclosed with a diamond are reference sources
Age−specific fertility rate
Year
Census SBH
Census Tabulation SBH
OTHER SURVEY SBH
VR Complete
UN WPP
GBD 2017
GBD 2016
Adjusted Data, CBH/VR
Adjusted Data, CEB
Data, CBH/VR
Data, CEB
Outliered Data

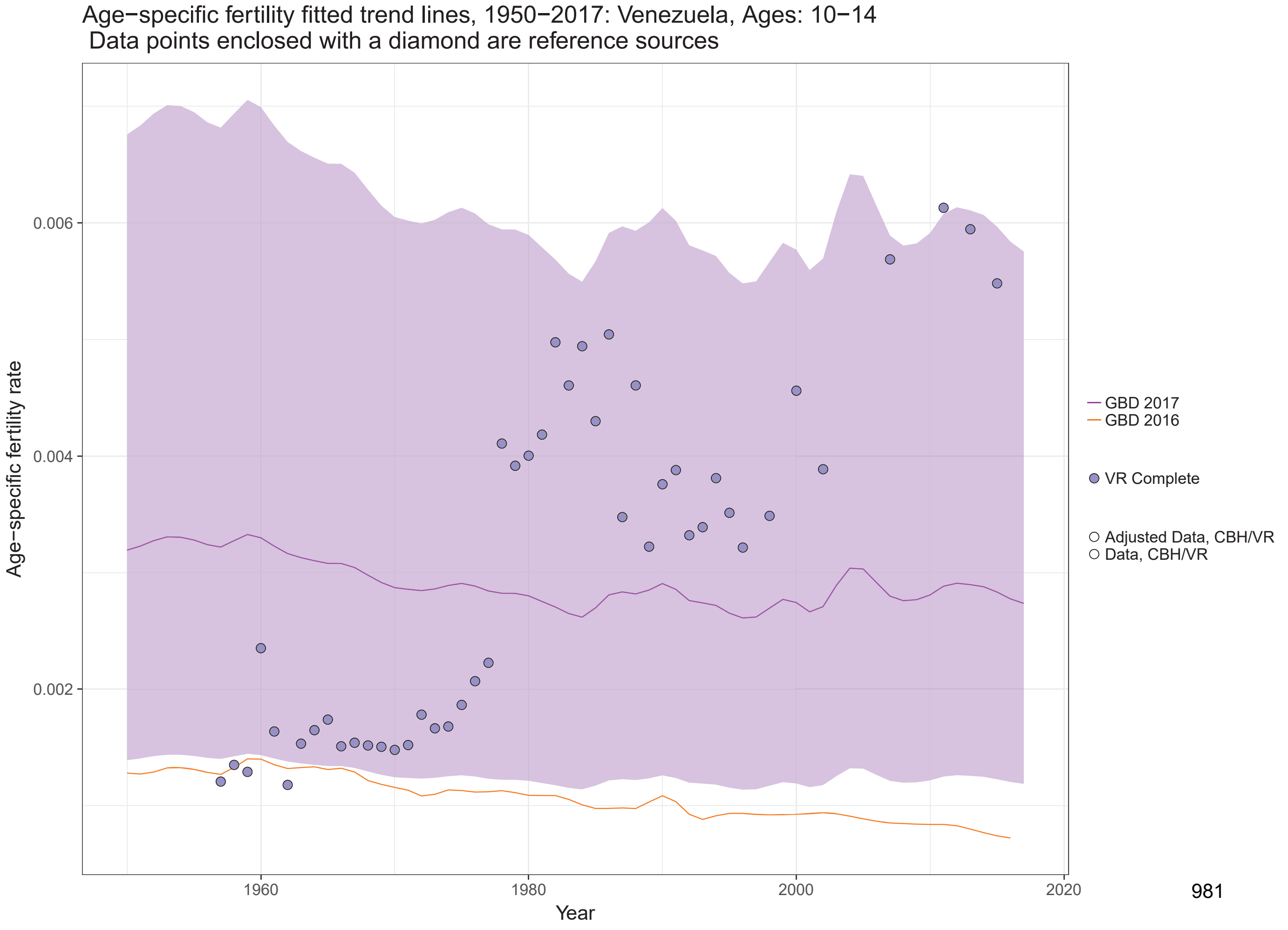

Age−specific fertility fitted trend lines, 1950−2017: Venezuela, Ages: 10−14
Data points enclosed with a diamond are reference sources
Age−specific fertility rate
Year
GBD 2017
GBD 2016
VR Complete
Adjusted Data, CBH/VR
Data, CBH/VR

Age−specific fertility fitted trend lines, 1950−2017: Venezuela, Ages: 15−19
Data points enclosed with a diamond are reference sources

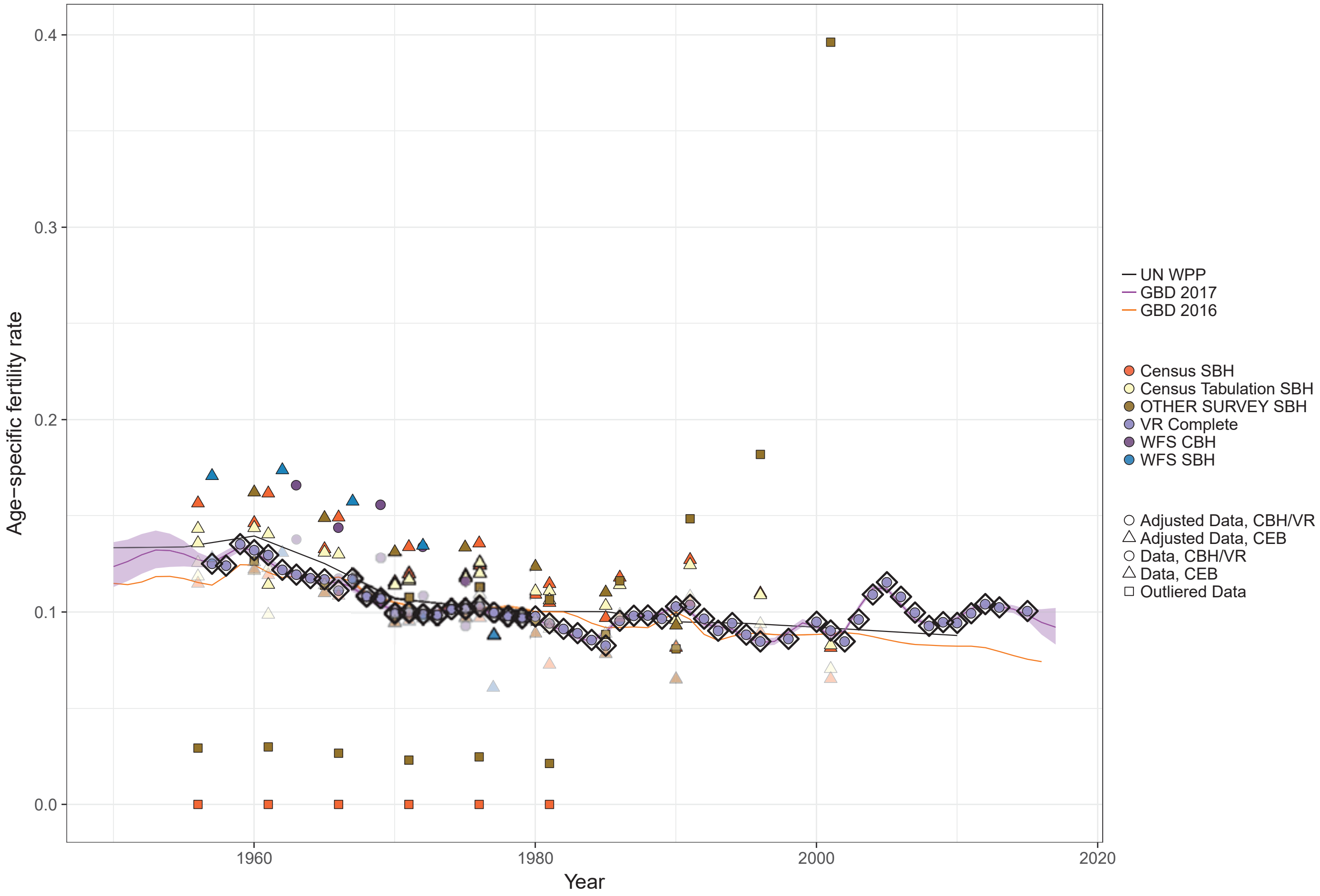

Age−specific fertility fitted trend lines, 1950−2017: Venezuela, Ages: 20−24
Data points enclosed with a diamond are reference sources

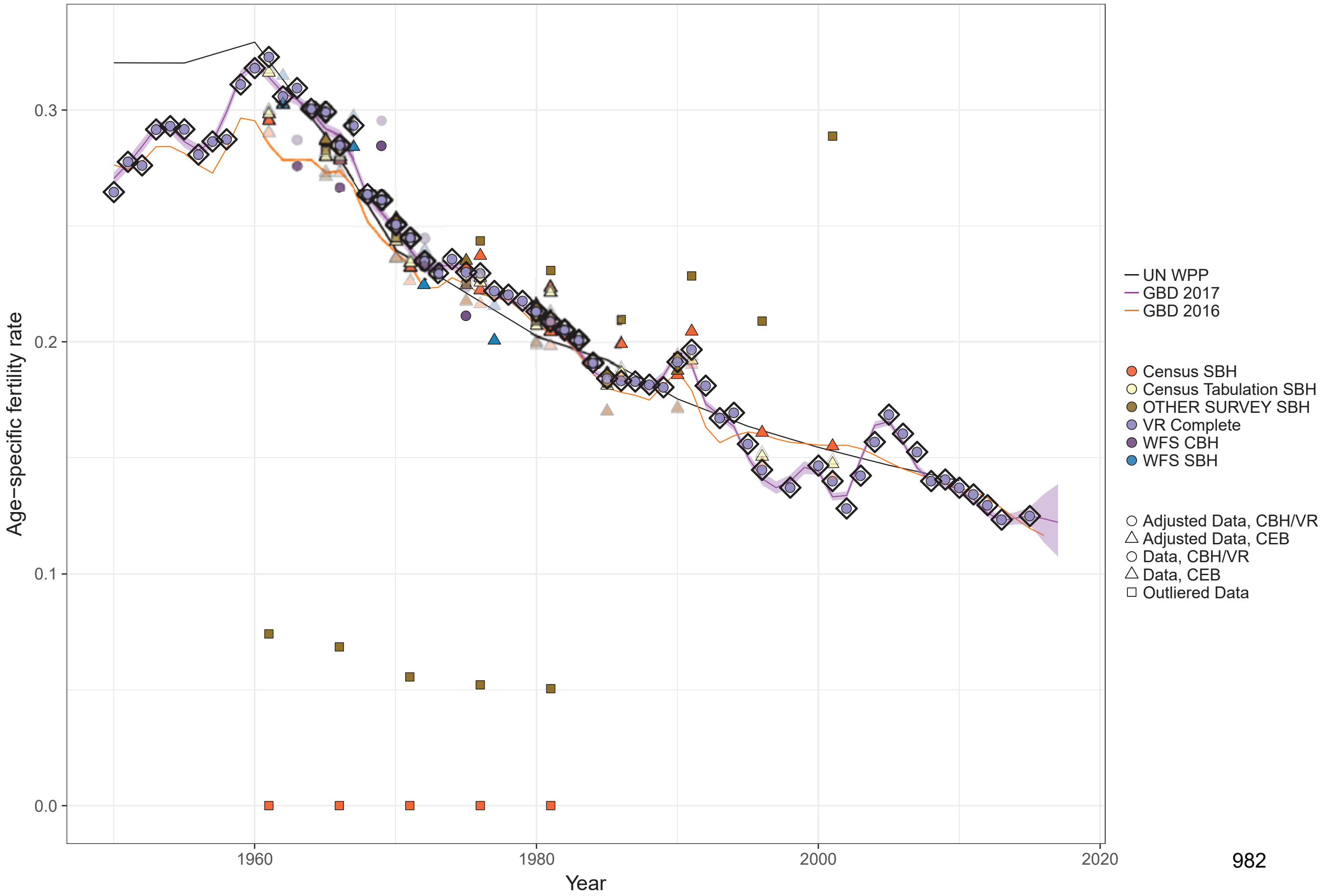

Age−specific fertility fitted trend lines, 1950−2017: Venezuela, Ages: 25−29
Data points enclosed with a diamond are reference sources

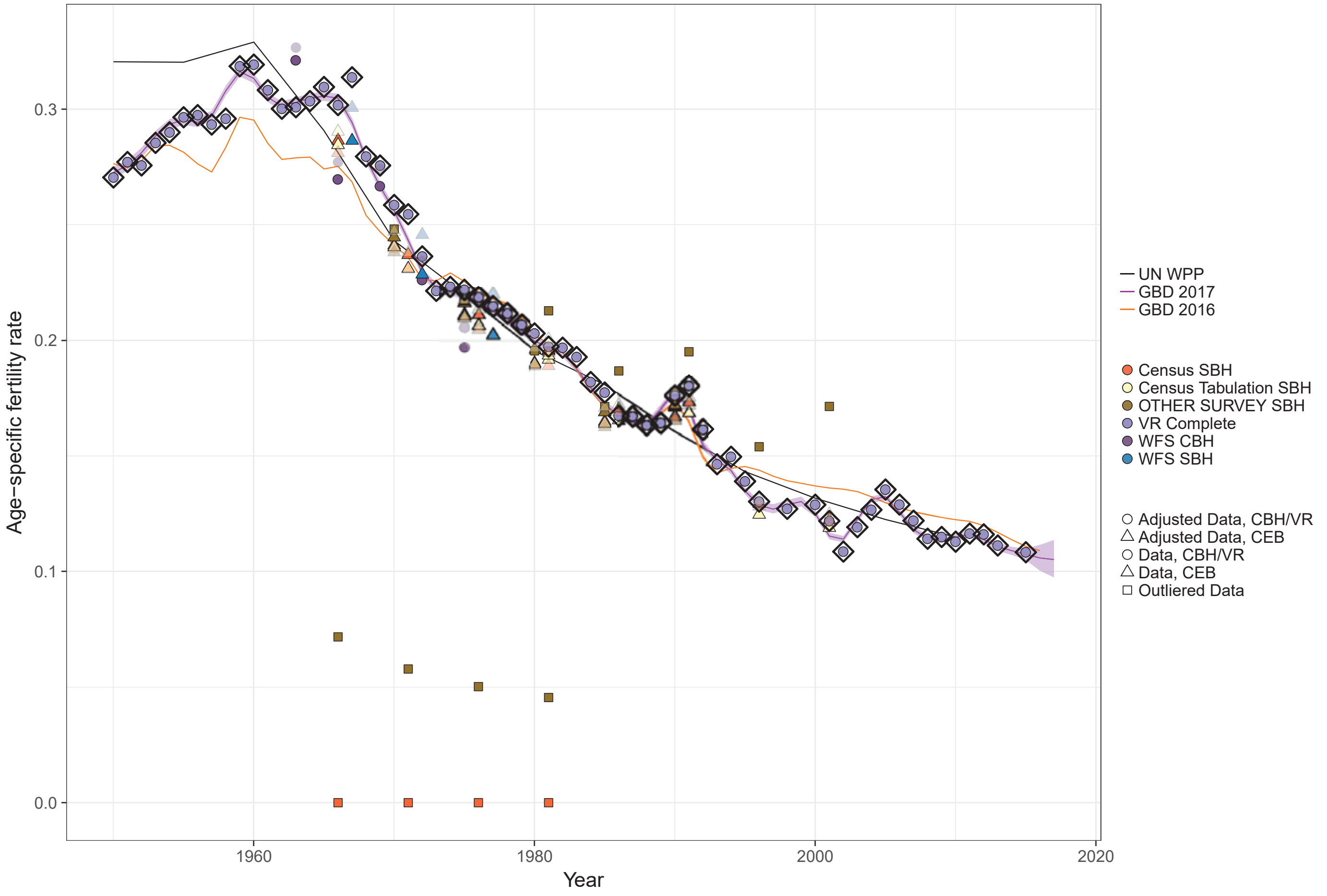

Age−specific fertility fitted trend lines, 1950−2017: Venezuela, Ages: 30−34
Data points enclosed with a diamond are reference sources

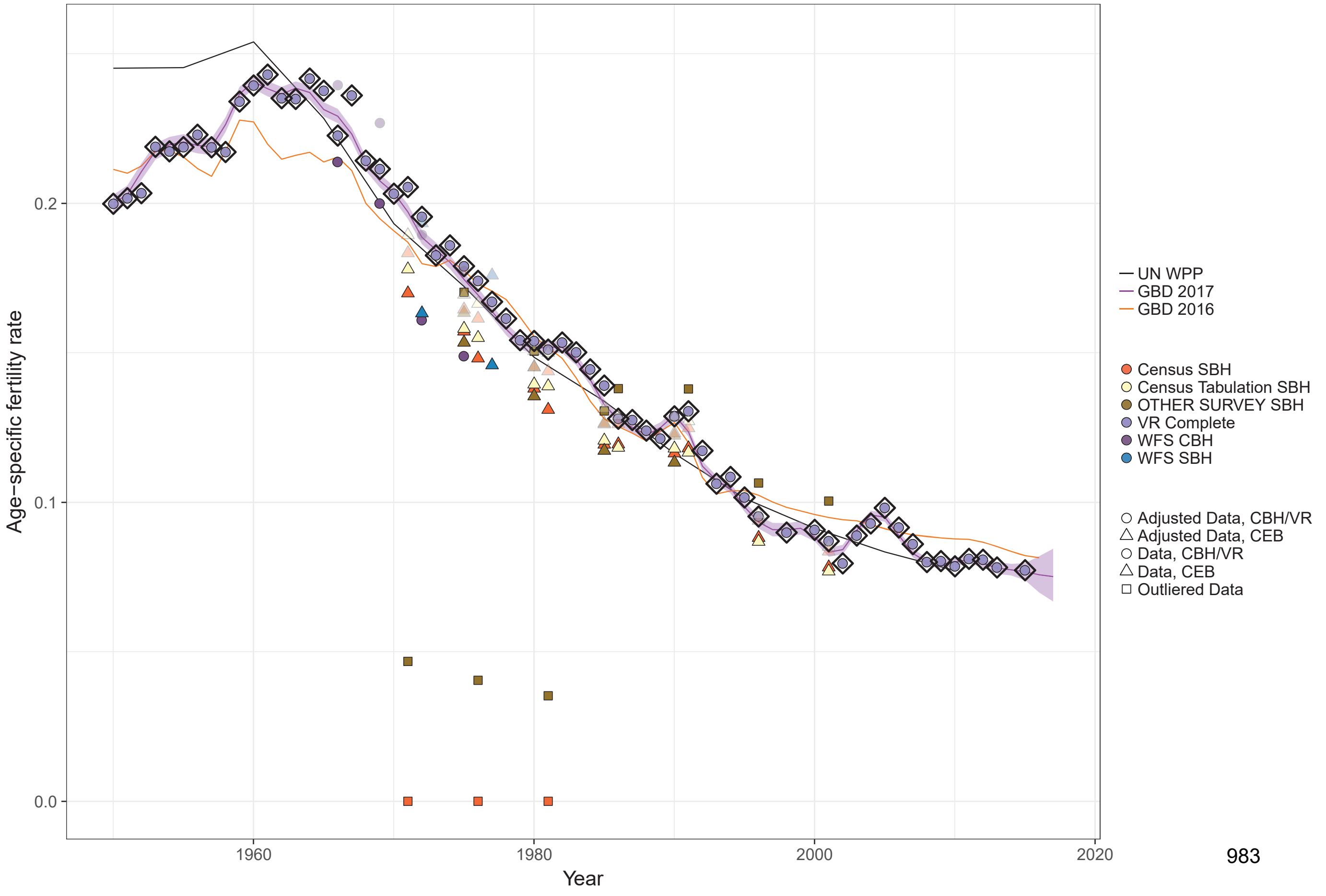

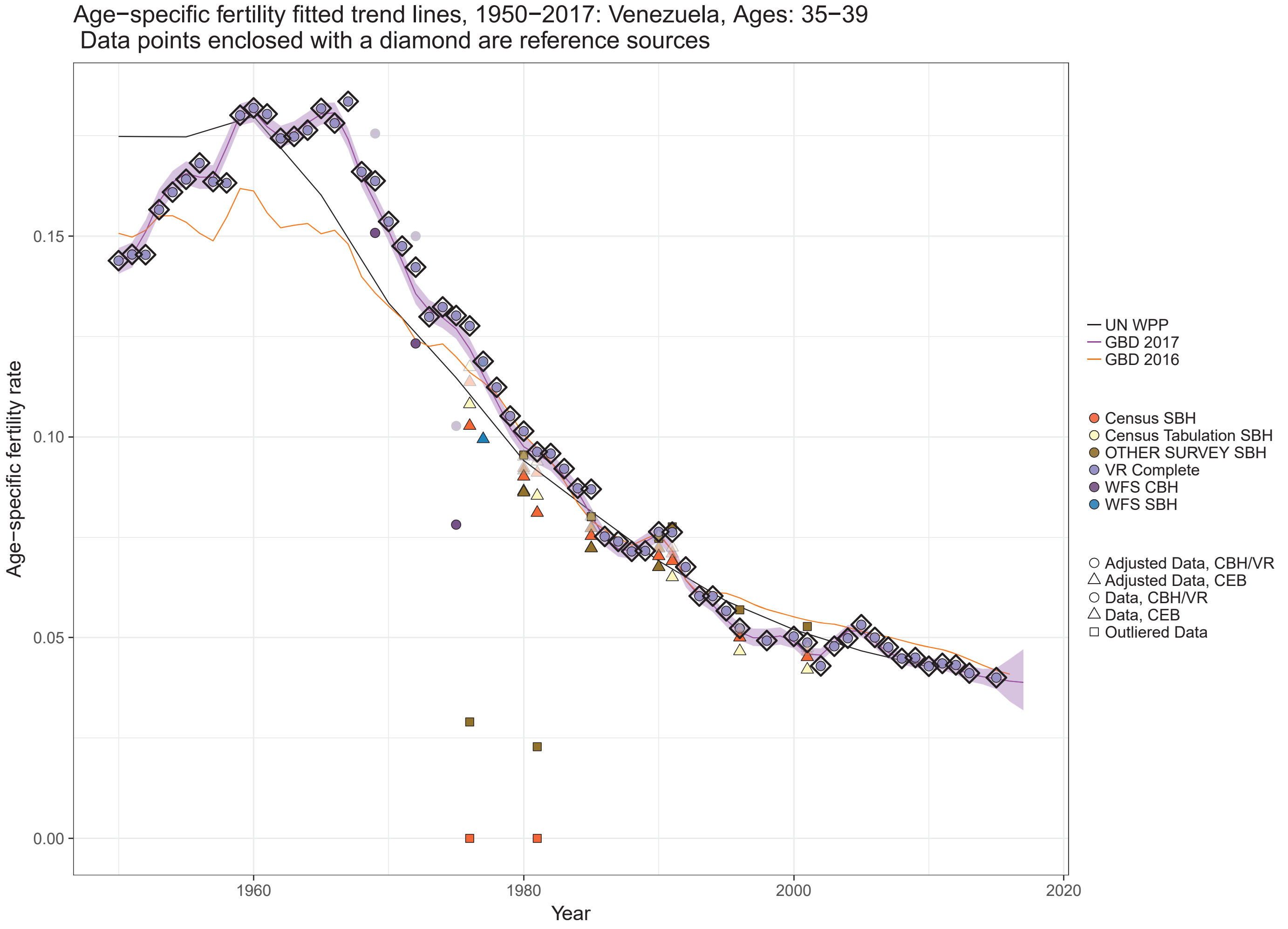
Age−specific fertility fitted trend lines, 1950−2017: Venezuela, Ages: 35−39
Data points enclosed with a diamond are reference sources
Age−specific fertility rate
0.00
0.05
0.10
0.15
1960
1980
2000
2020
Year
UN WPP
GBD 2017
GBD 2016
Census SBH
Census Tabulation SBH
OTHER SURVEY SBH
VR Complete
WFS CBH
WFS SBH
Adjusted Data, CBH/VR
Adjusted Data, CEB
Data, CBH/VR
Data, CEB
Outliered Data

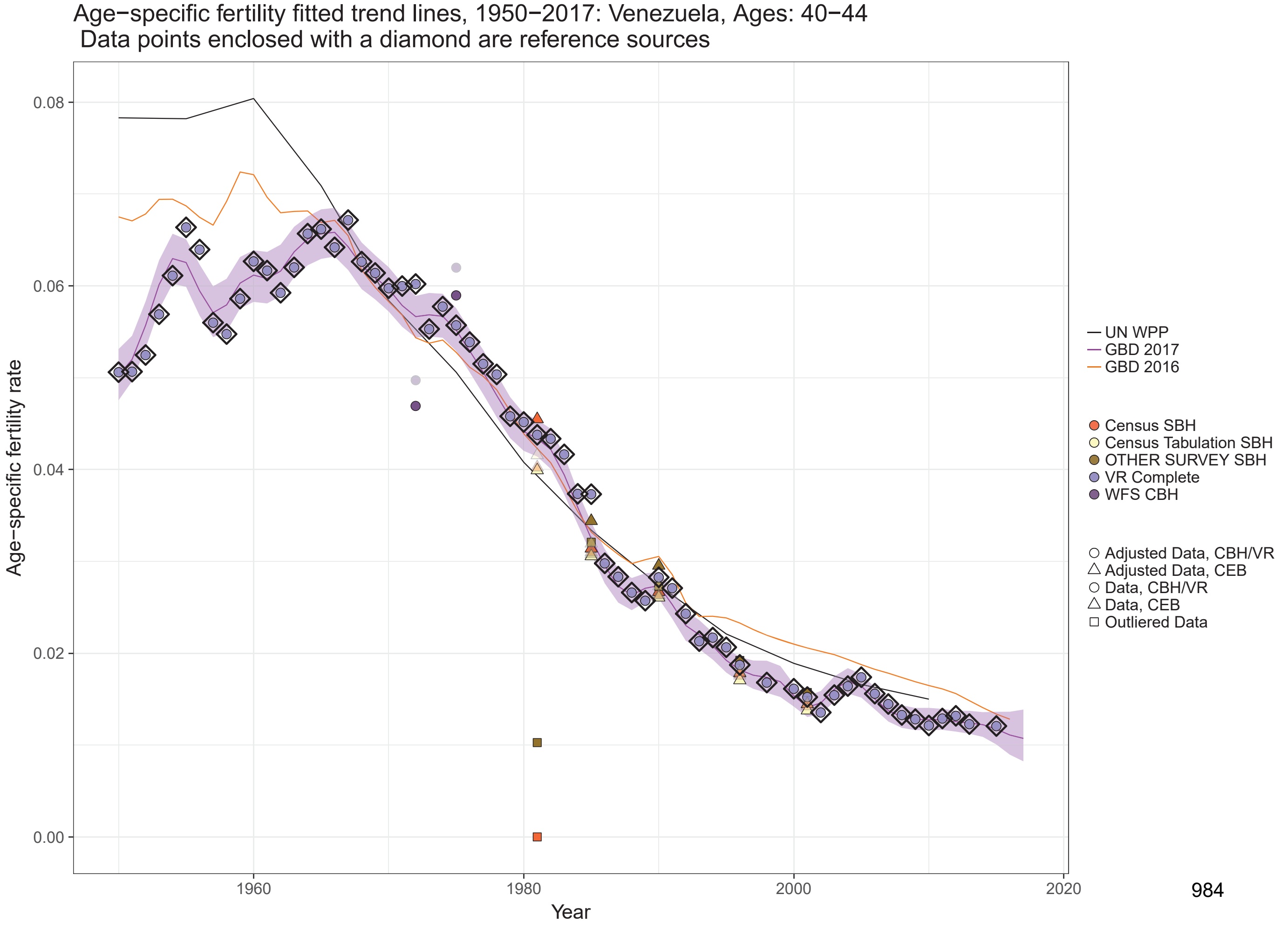
Age−specific fertility fitted trend lines, 1950−2017: Venezuela, Ages: 40−44
Data points enclosed with a diamond are reference sources
Age−specific fertility rate
0.00
0.02
0.04
0.06
0.08
1960
1980
2000
2020
Year
UN WPP
GBD 2017
GBD 2016
Census SBH
Census Tabulation SBH
OTHER SURVEY SBH
VR Complete
WFS CBH
Adjusted Data, CBH/VR
Adjusted Data, CEB
Data, CBH/VR
Data, CEB
Outliered Data

Age−specific fertility fitted trend lines, 1950−2017: Venezuela, Ages: 45−49
Data points enclosed with a diamond are reference sources

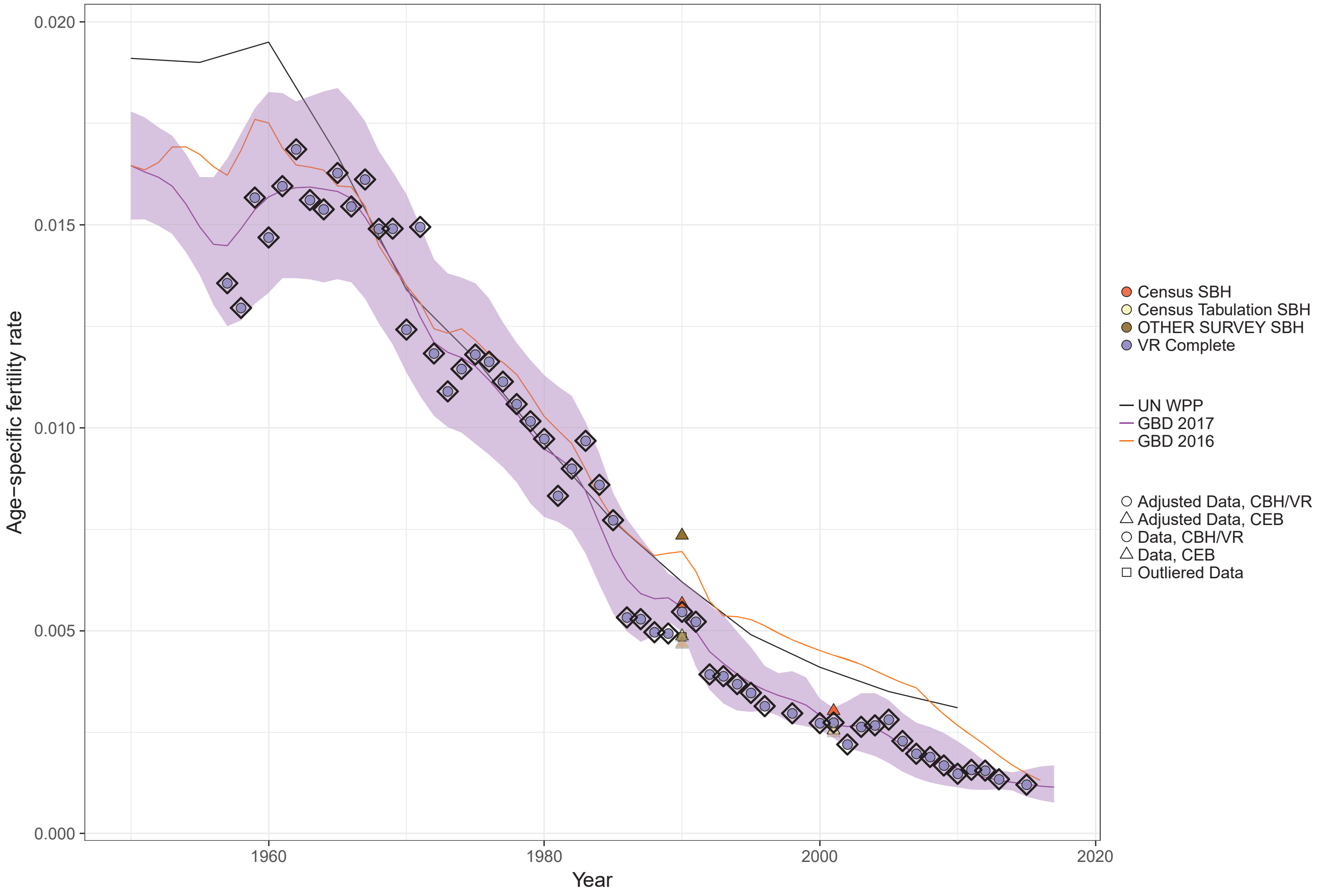

Age−specific fertility fitted trend lines, 1950−2017: Venezuela, Ages: 50−54
Data points enclosed with a diamond are reference sources

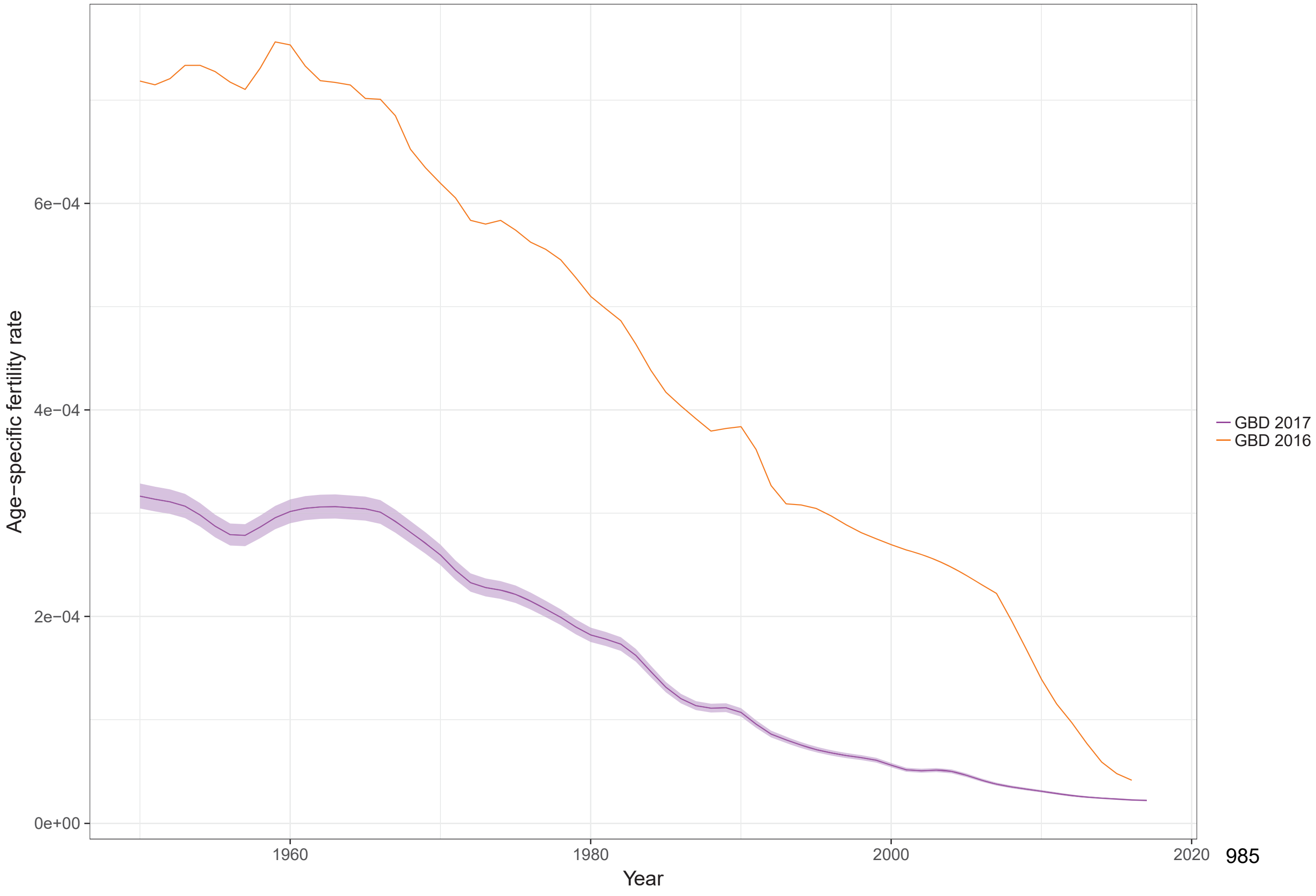

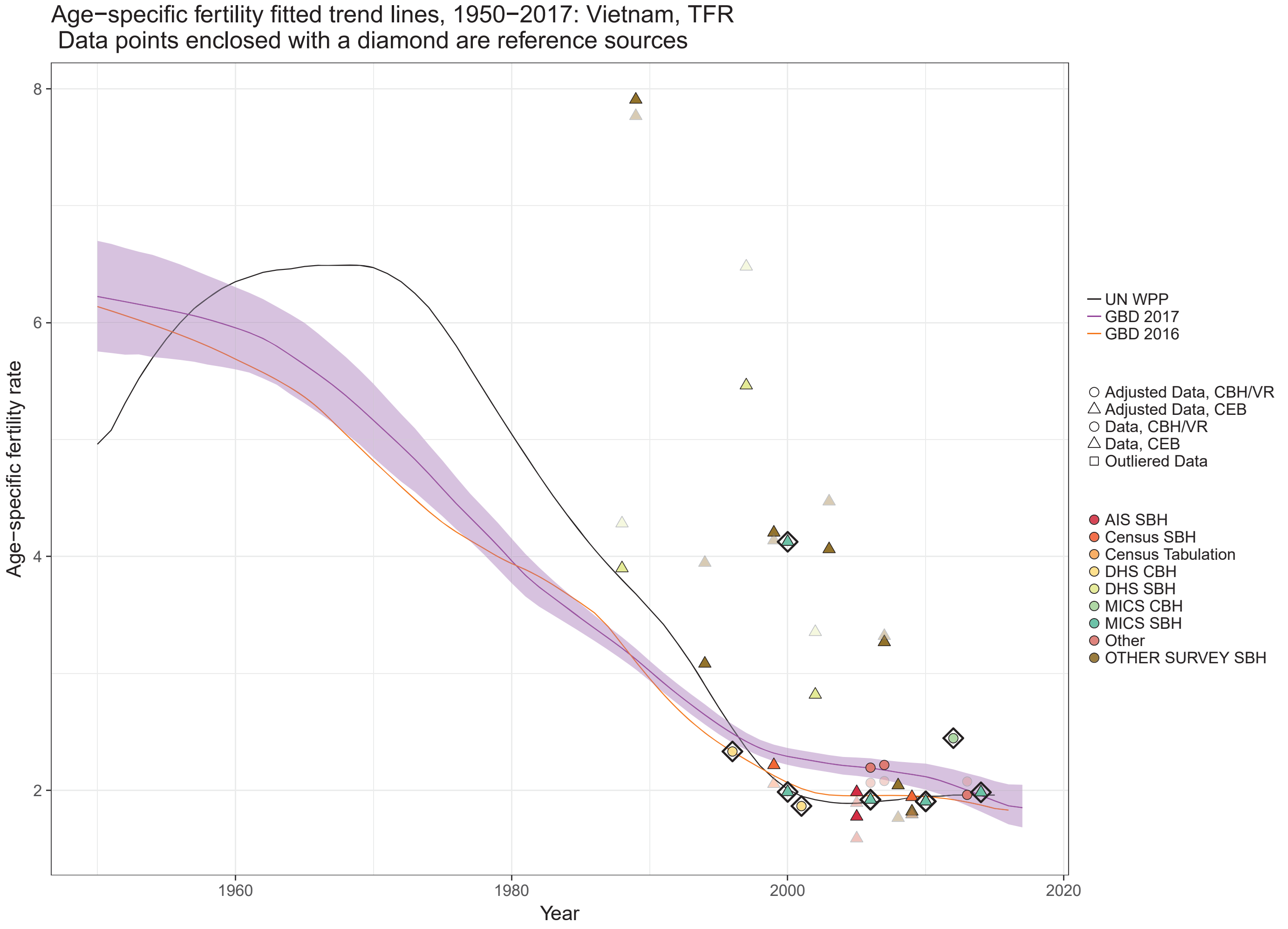

Age−specific fertility fitted trend lines, 1950−2017: Vietnam, TFR
Data points enclosed with a diamond are reference sources
Age−specific fertility rate
Year
8
6
4
2
1960
1980
2000
2020
UN WPP
GBD 2017
GBD 2016
Adjusted Data, CBH/VR
Adjusted Data, CEB
Data, CBH/VR
Data, CEB
Outliered Data
AIS SBH
Census SBH
Census Tabulation
DHS CBH
DHS SBH
MICS CBH
MICS SBH
Other
OTHER SURVEY SBH

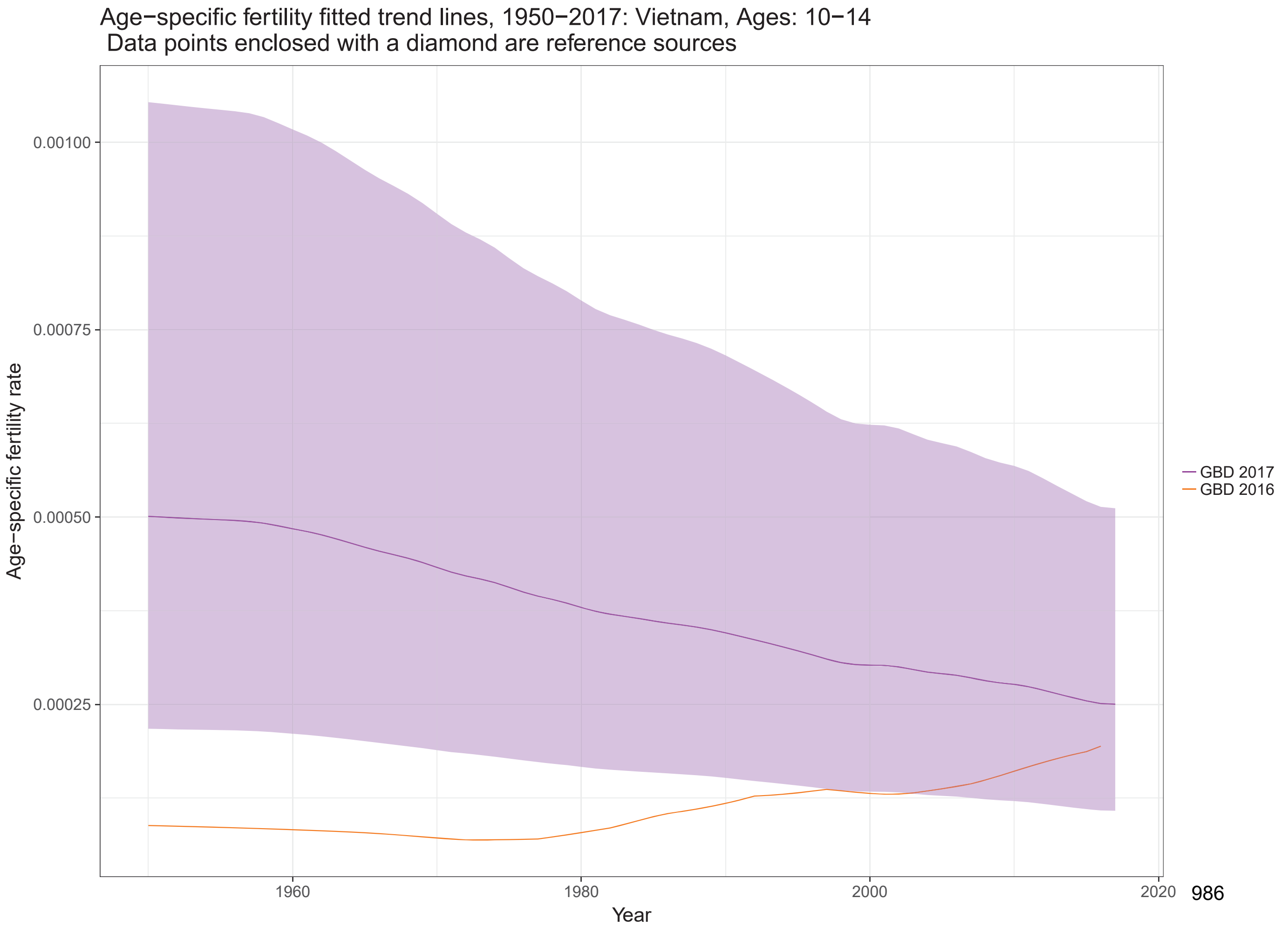

Age−specific fertility fitted trend lines, 1950−2017: Vietnam, Ages: 10−14
Data points enclosed with a diamond are reference sources
Age−specific fertility rate
Year
0.00100
0.00075
0.00050
0.00025
1960
1980
2000
2020
GBD 2017
GBD 2016

Age−specific fertility fitted trend lines, 1950−2017: Vietnam, Ages: 15−19
Data points enclosed with a diamond are reference sources

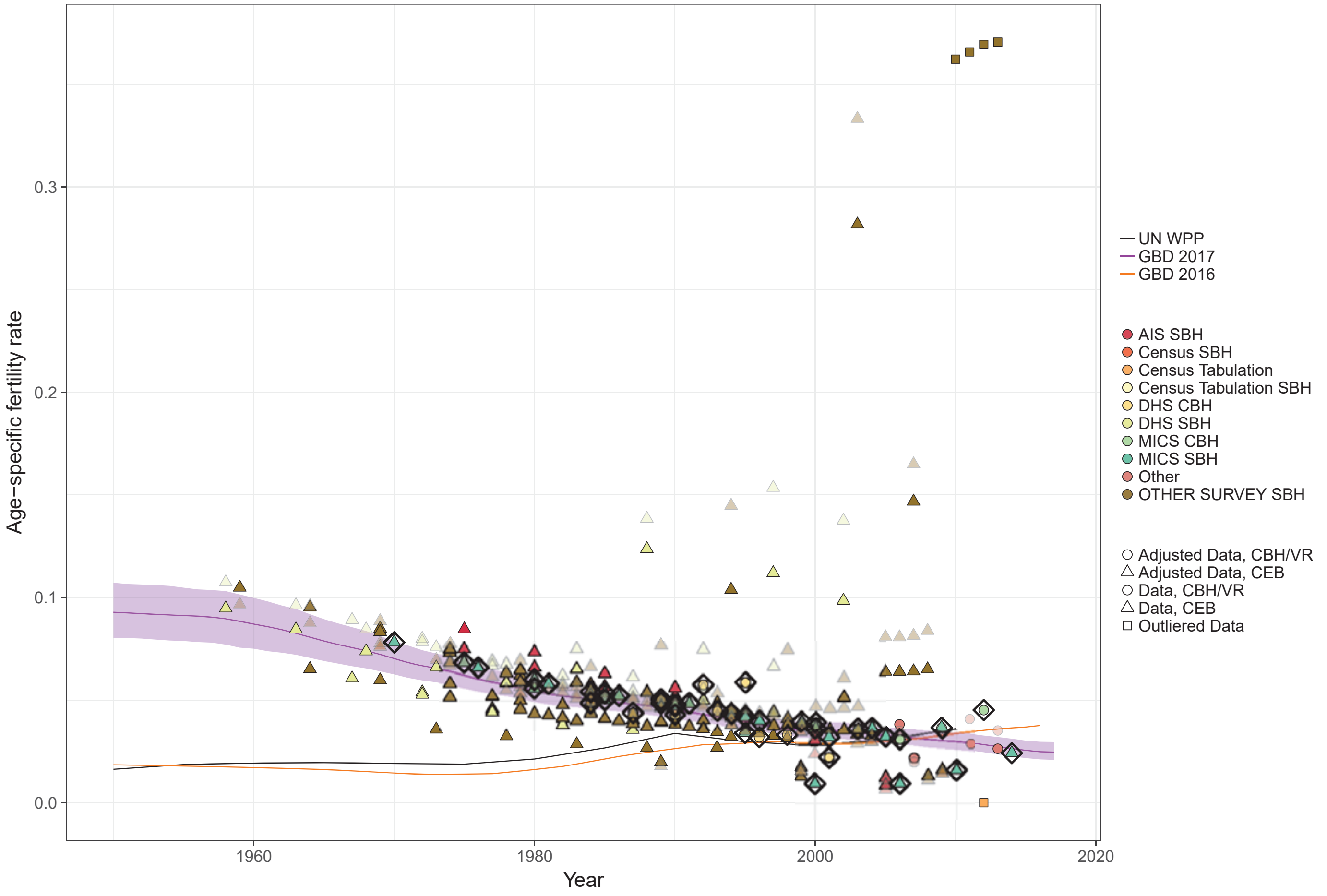

Age−specific fertility fitted trend lines, 1950−2017: Vietnam, Ages: 20−24
Data points enclosed with a diamond are reference sources

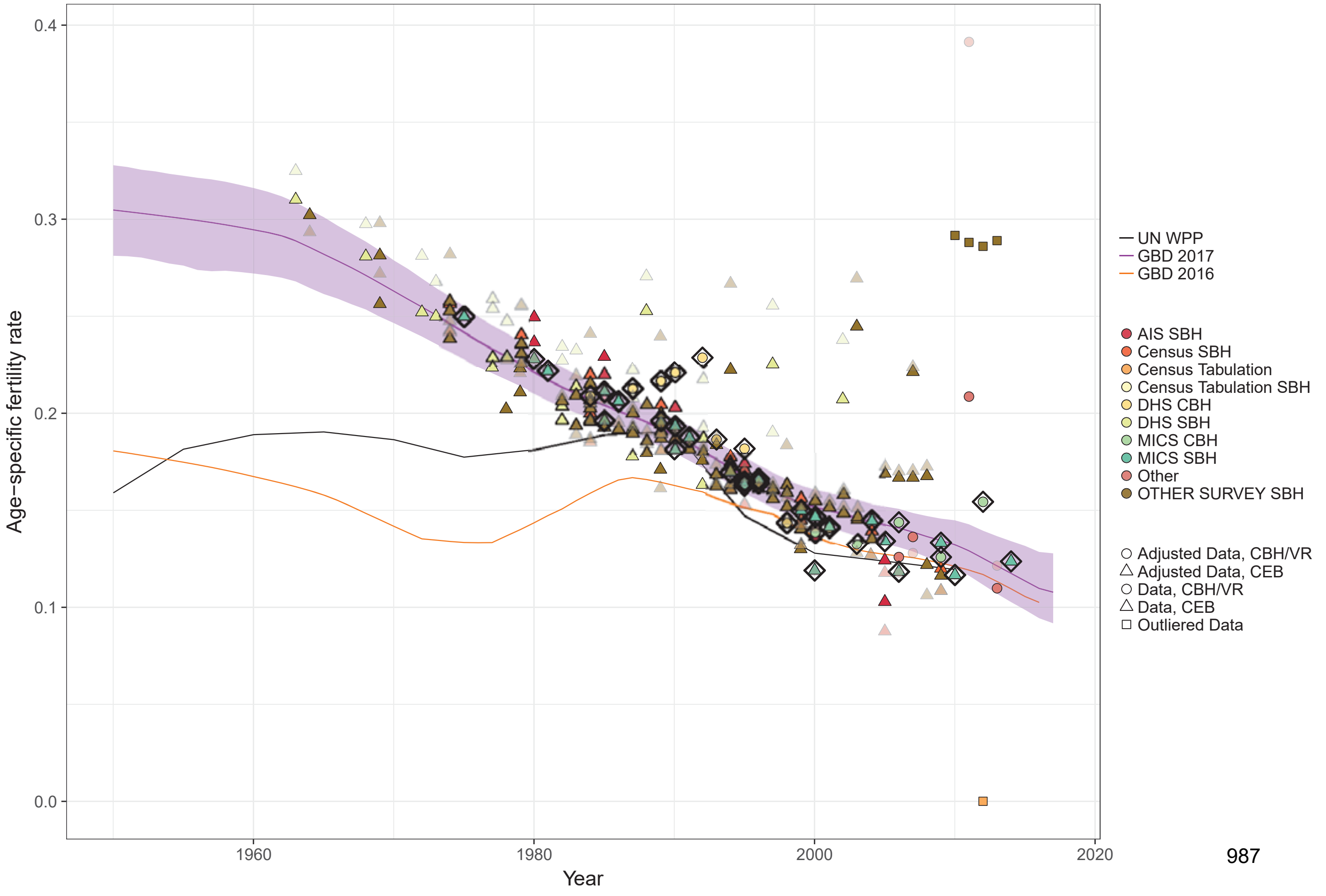

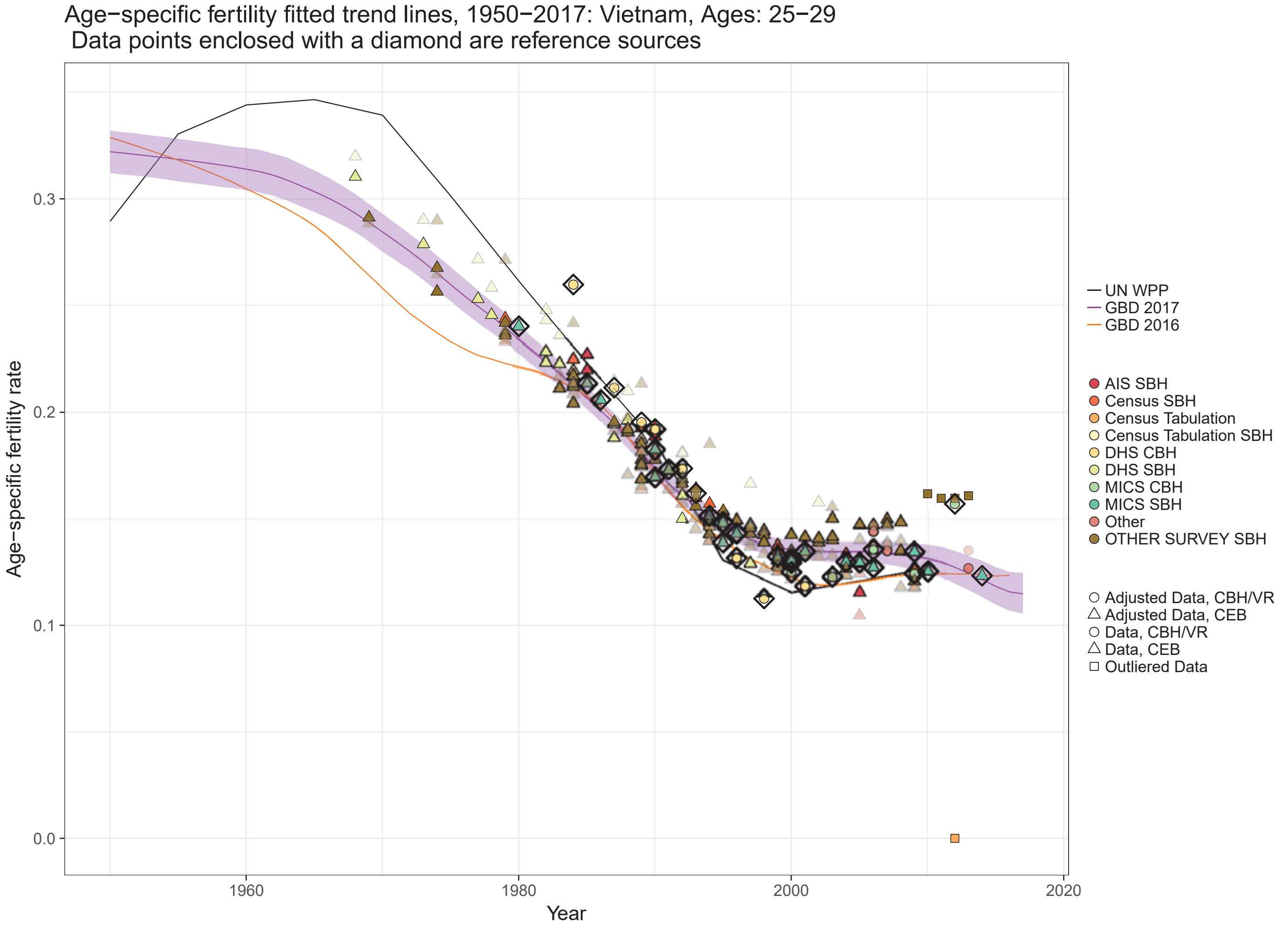

Age−specific fertility fitted trend lines, 1950−2017: Vietnam, Ages: 25−29
Data points enclosed with a diamond are reference sources
Age−specific fertility rate
0.0
0.1
0.2
0.3
1960
1980
2000
2020
Year
UN WPP
GBD 2017
GBD 2016
AIS SBH
Census SBH
Census Tabulation
Census Tabulation SBH
DHS CBH
DHS SBH
MICS CBH
MICS SBH
Other
OTHER SURVEY SBH
Adjusted Data, CBH/VR
Adjusted Data, CEB
Data, CBH/VR
Data, CEB
Outliered Data

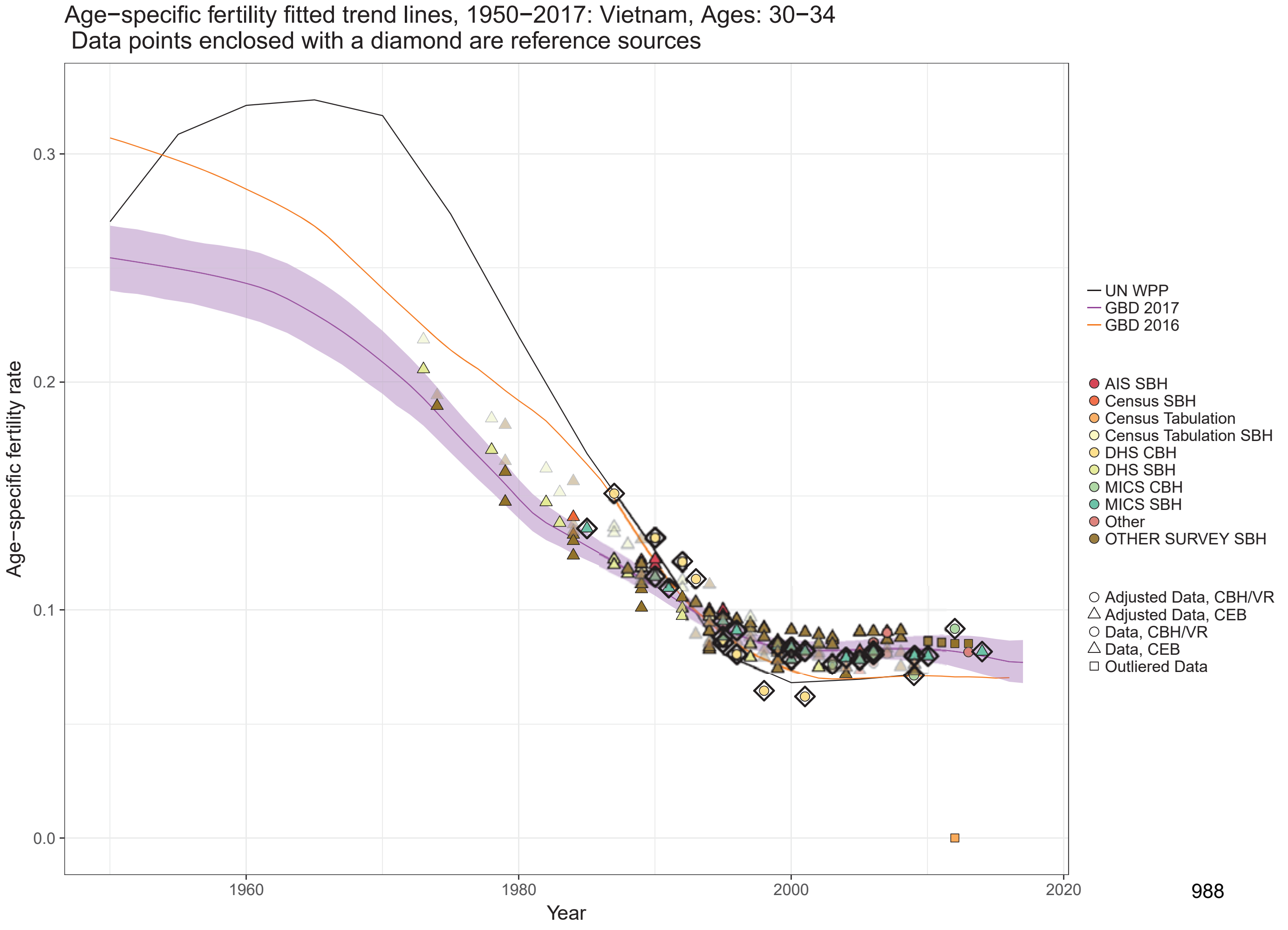

Age−specific fertility fitted trend lines, 1950−2017: Vietnam, Ages: 30−34
Data points enclosed with a diamond are reference sources
Age−specific fertility rate
0.0
0.1
0.2
0.3
1960
1980
2000
2020
Year
UN WPP
GBD 2017
GBD 2016
AIS SBH
Census SBH
Census Tabulation
Census Tabulation SBH
DHS CBH
DHS SBH
MICS CBH
MICS SBH
Other
OTHER SURVEY SBH
Adjusted Data, CBH/VR
Adjusted Data, CEB
Data, CBH/VR
Data, CEB
Outliered Data

Age−specific fertility fitted trend lines, 1950−2017: Vietnam, Ages: 35−39
Data points enclosed with a diamond are reference sources

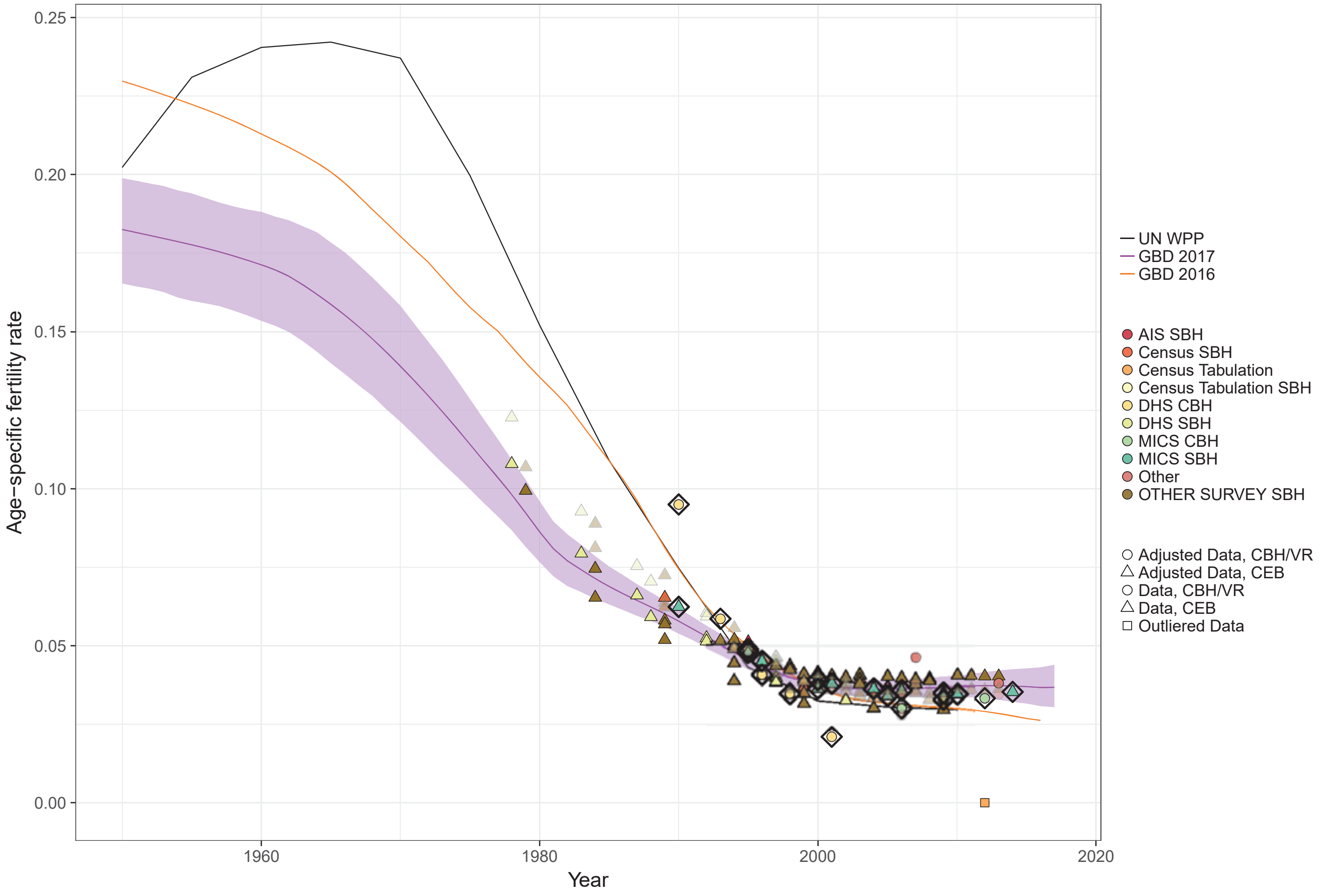

Age−specific fertility fitted trend lines, 1950−2017: Vietnam, Ages: 40−44
Data points enclosed with a diamond are reference sources

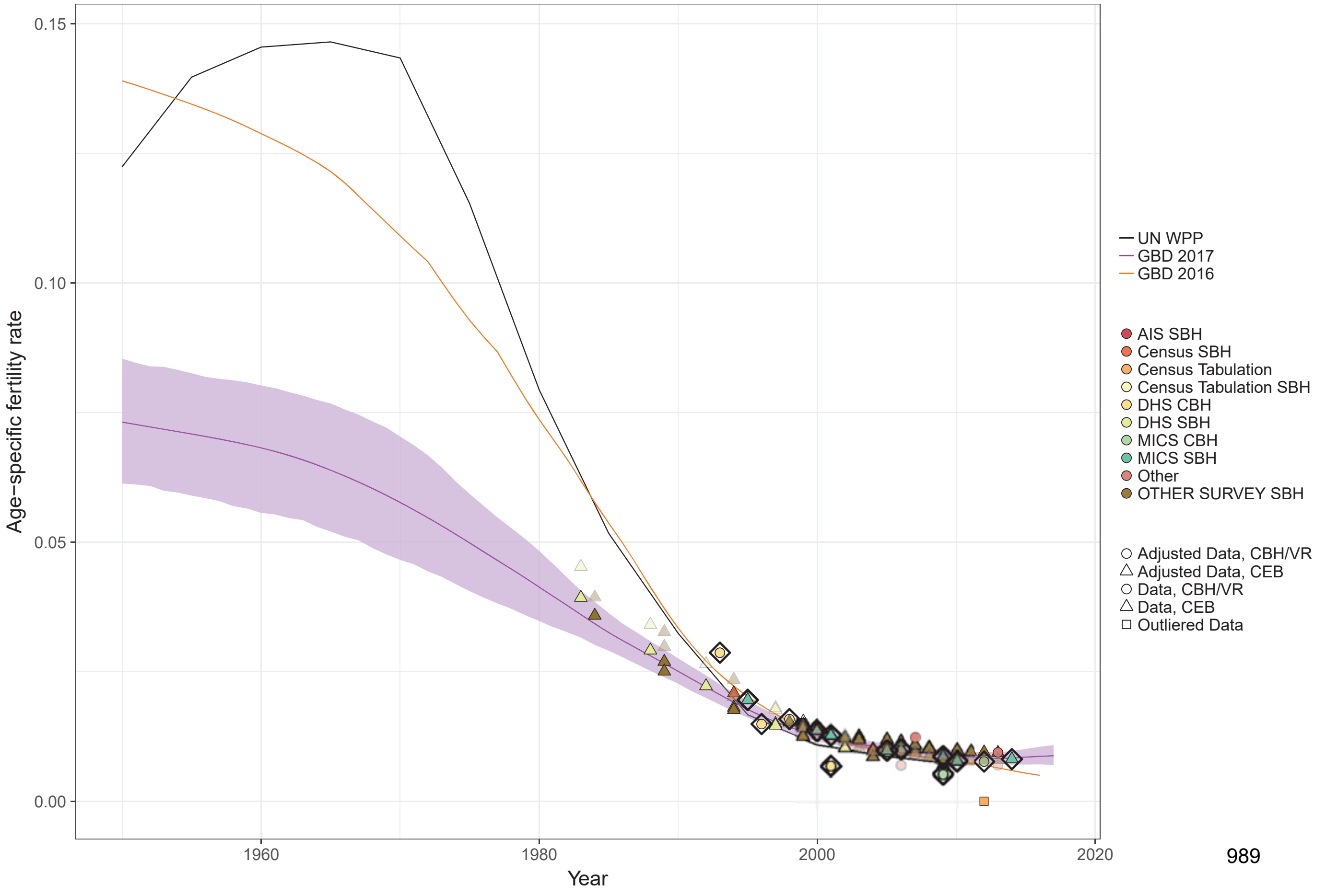

Age−specific fertility fitted trend lines, 1950−2017: Vietnam, Ages: 45−49
Data points enclosed with a diamond are reference sources

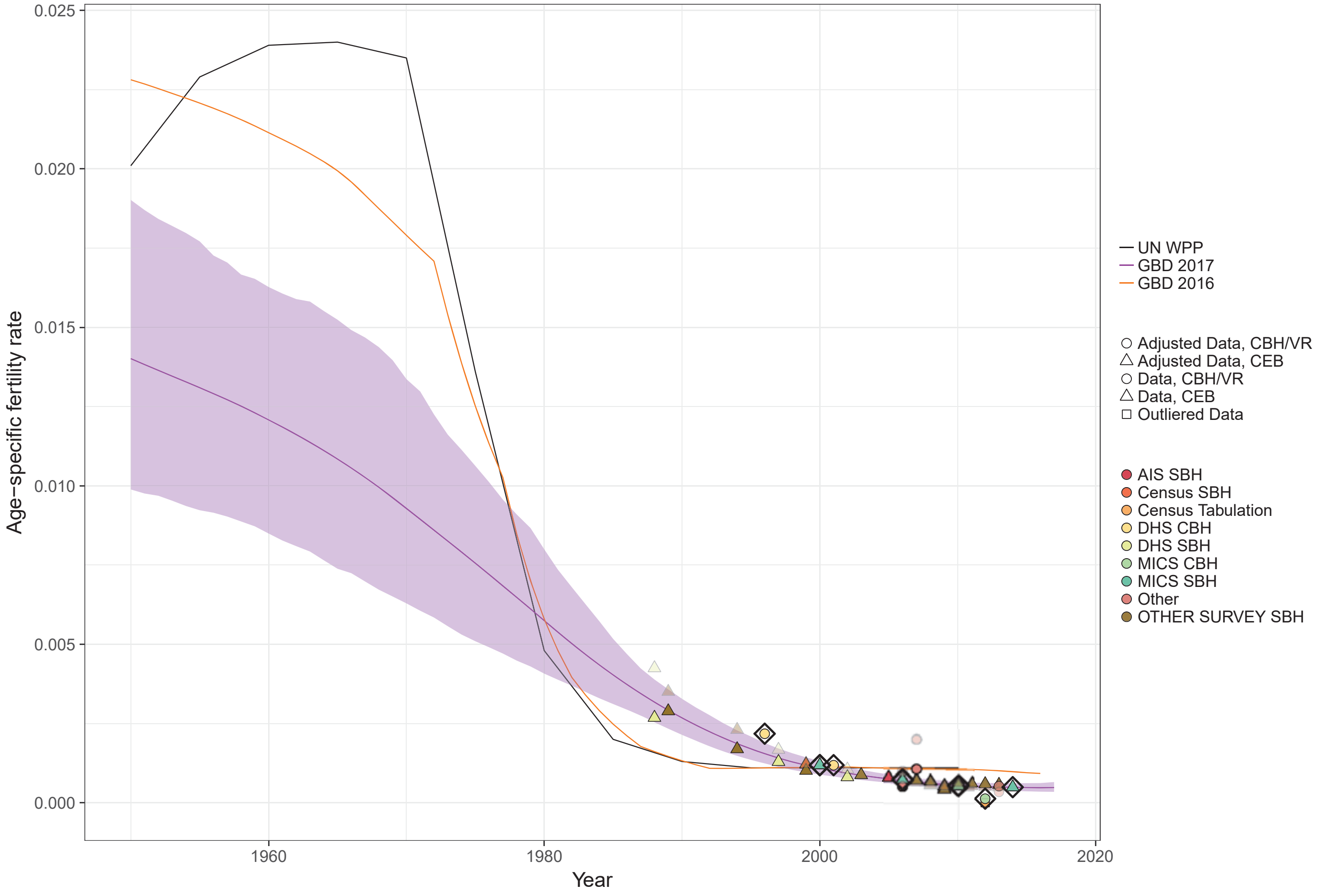

Age−specific fertility fitted trend lines, 1950−2017: Vietnam, Ages: 50−54
Data points enclosed with a diamond are reference sources

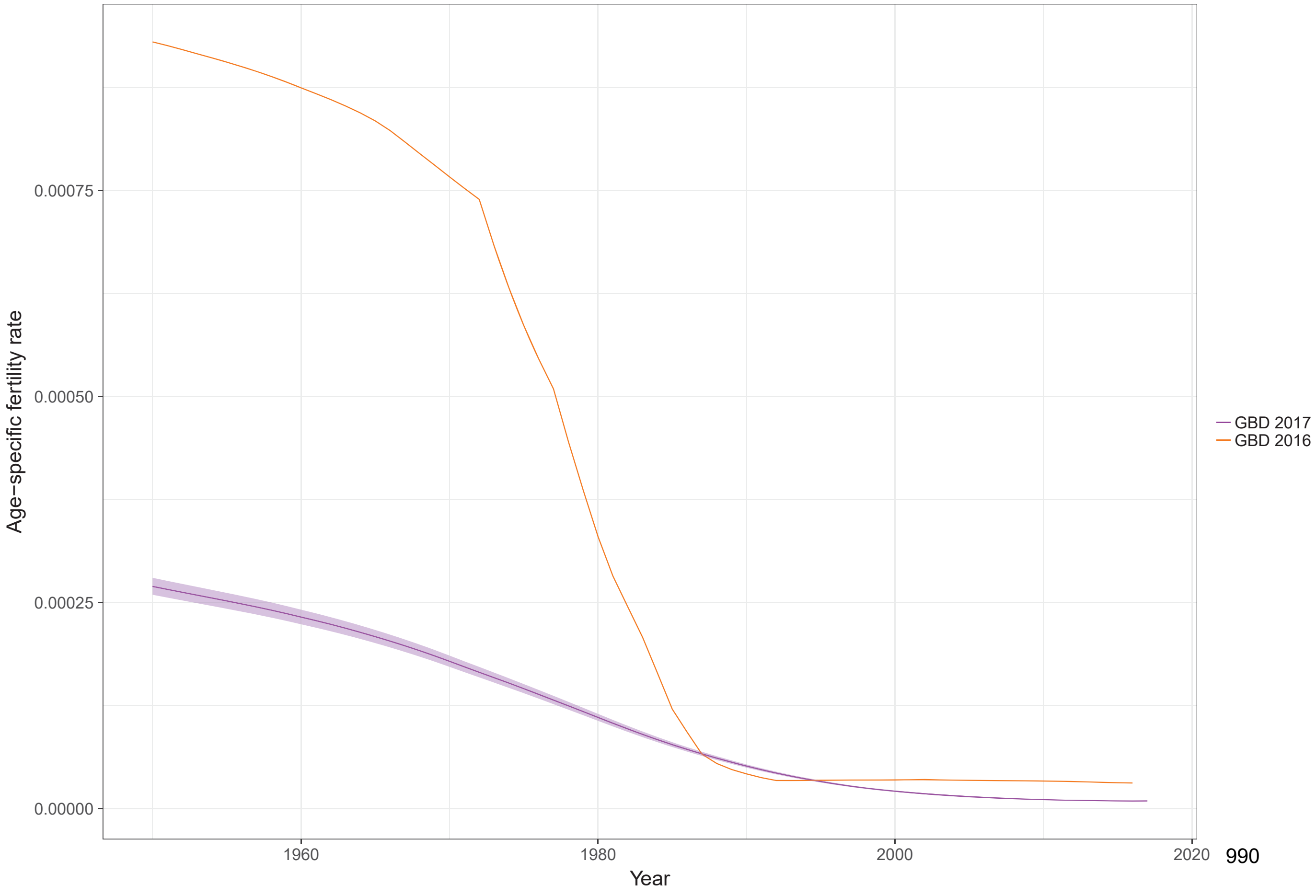

Age−specific fertility fitted trend lines, 1950−2017: Virgin Islands, U.S., TFR
Data points enclosed with a diamond are reference sources

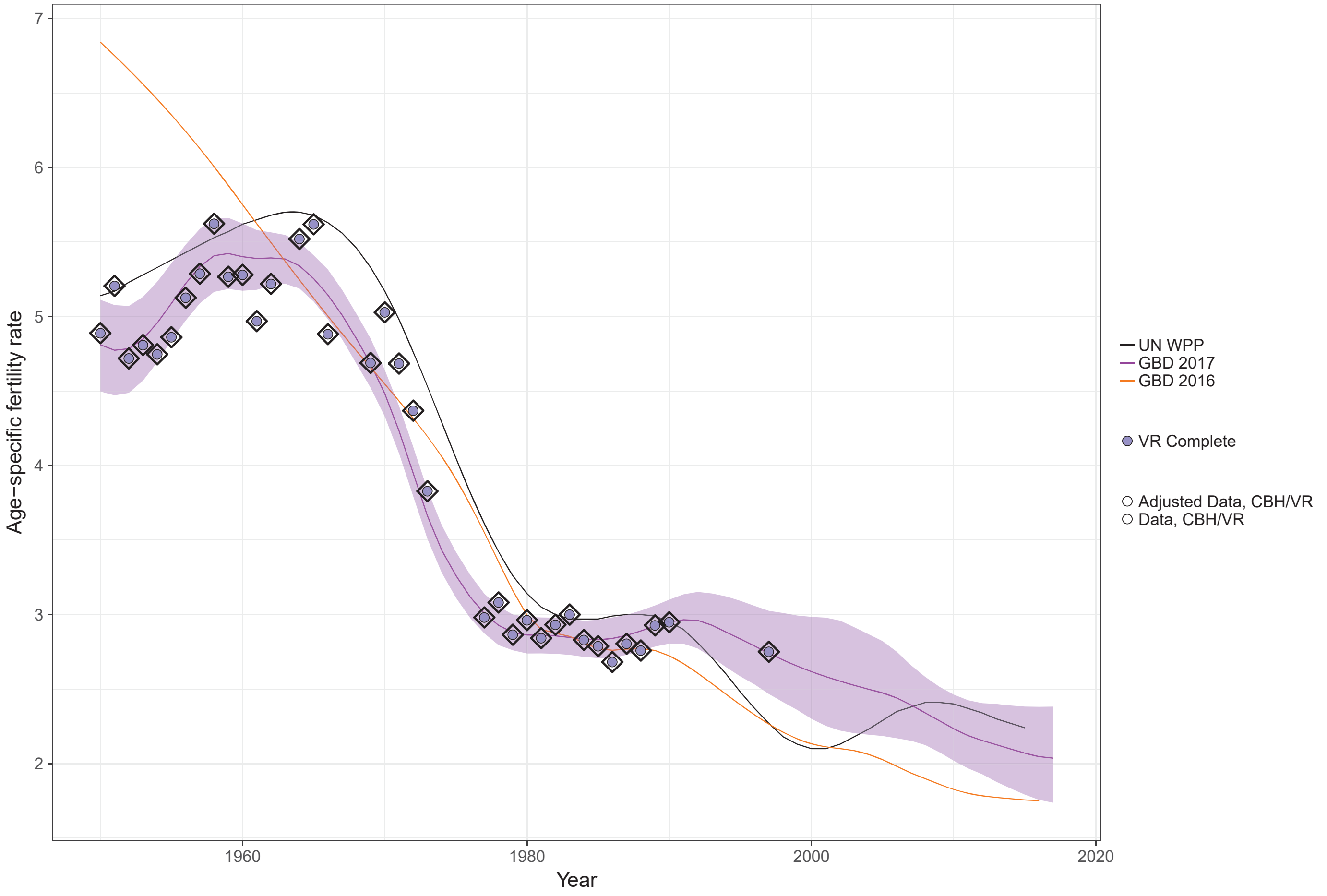

Age−specific fertility fitted trend lines, 1950−2017: Virgin Islands, U.S., Ages: 10−14
Data points enclosed with a diamond are reference sources

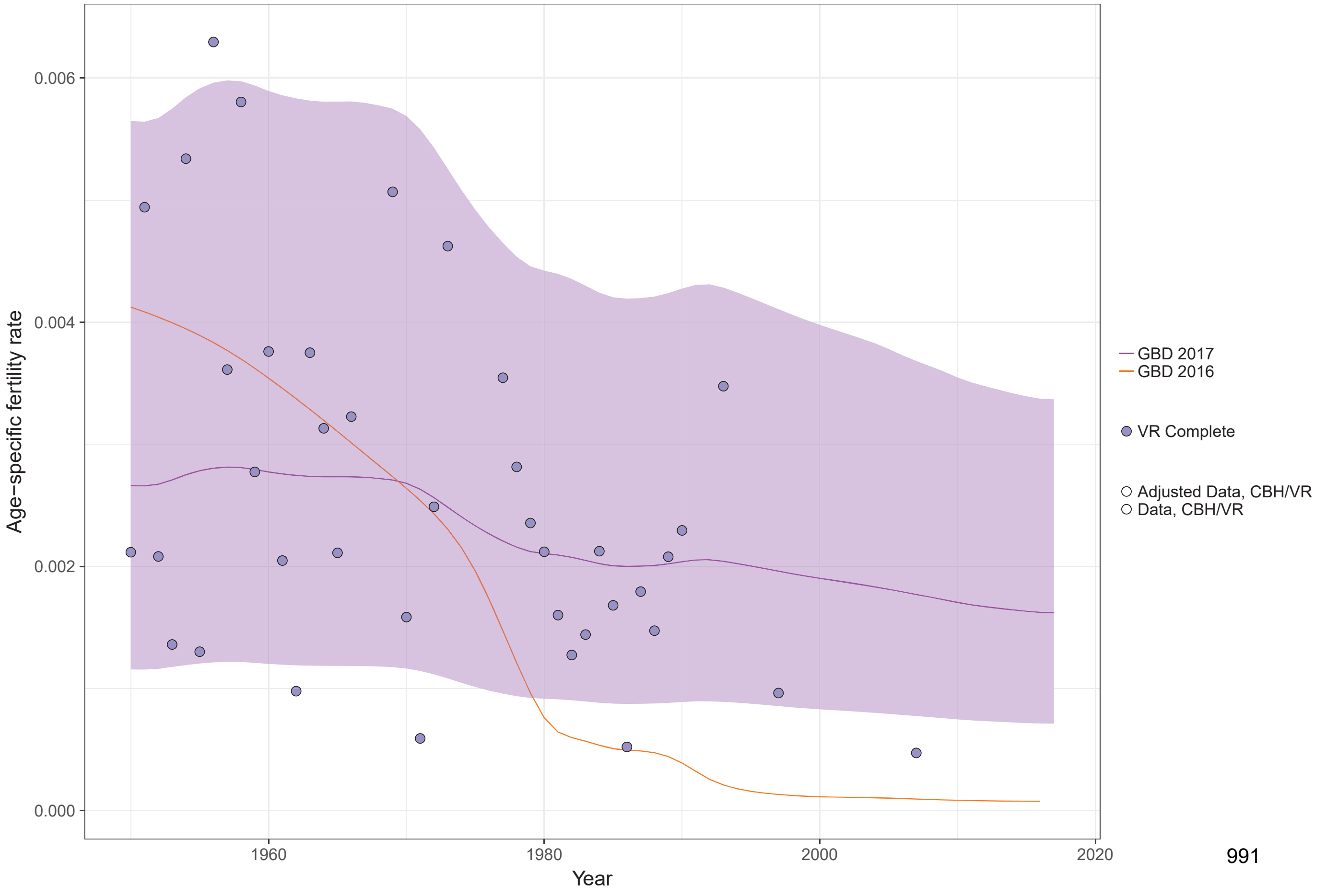

Age−specific fertility fitted trend lines, 1950−2017: Virgin Islands, U.S., Ages: 15−19
Data points enclosed with a diamond are reference sources

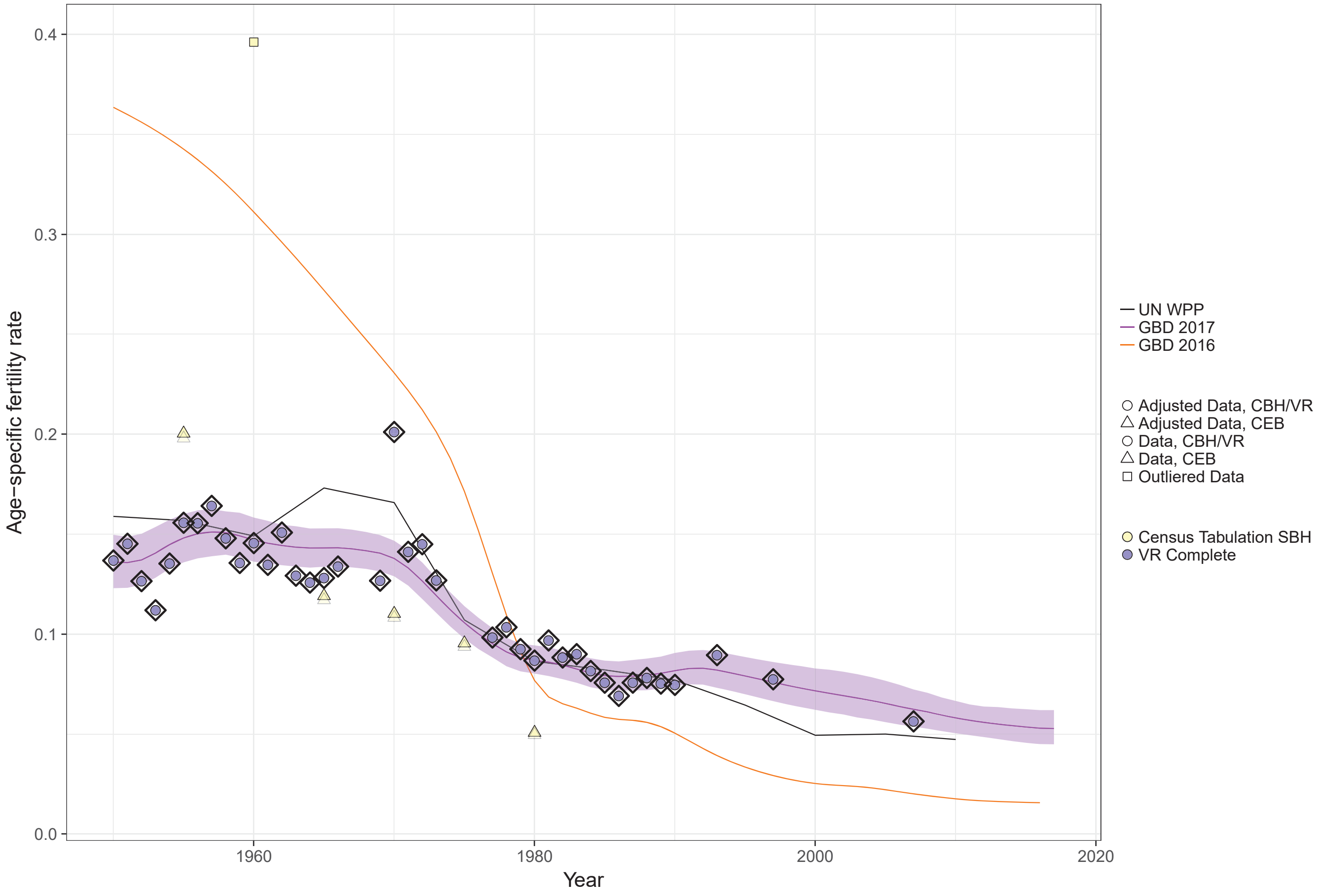

Age−specific fertility fitted trend lines, 1950−2017: Virgin Islands, U.S., Ages: 20−24
Data points enclosed with a diamond are reference sources

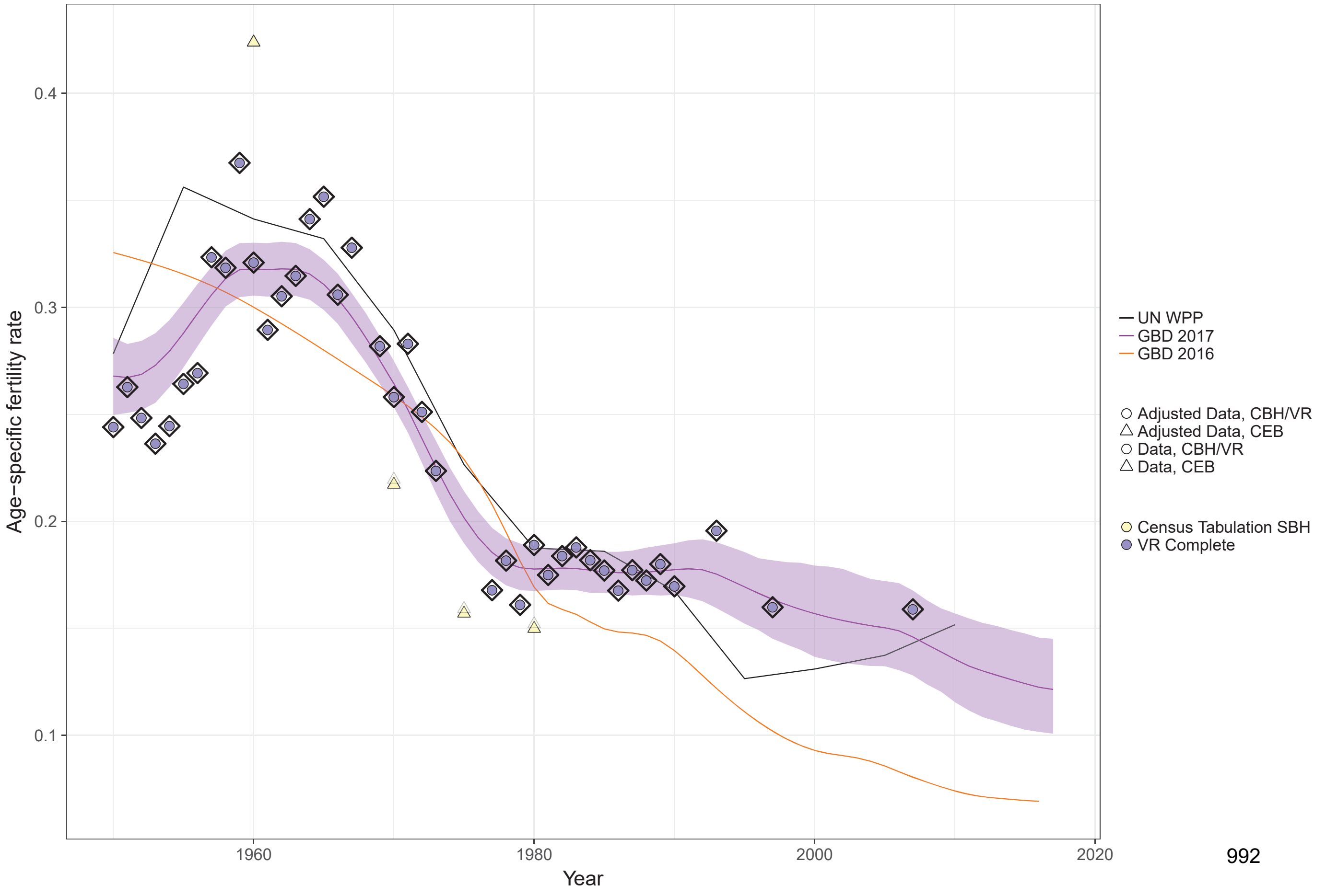

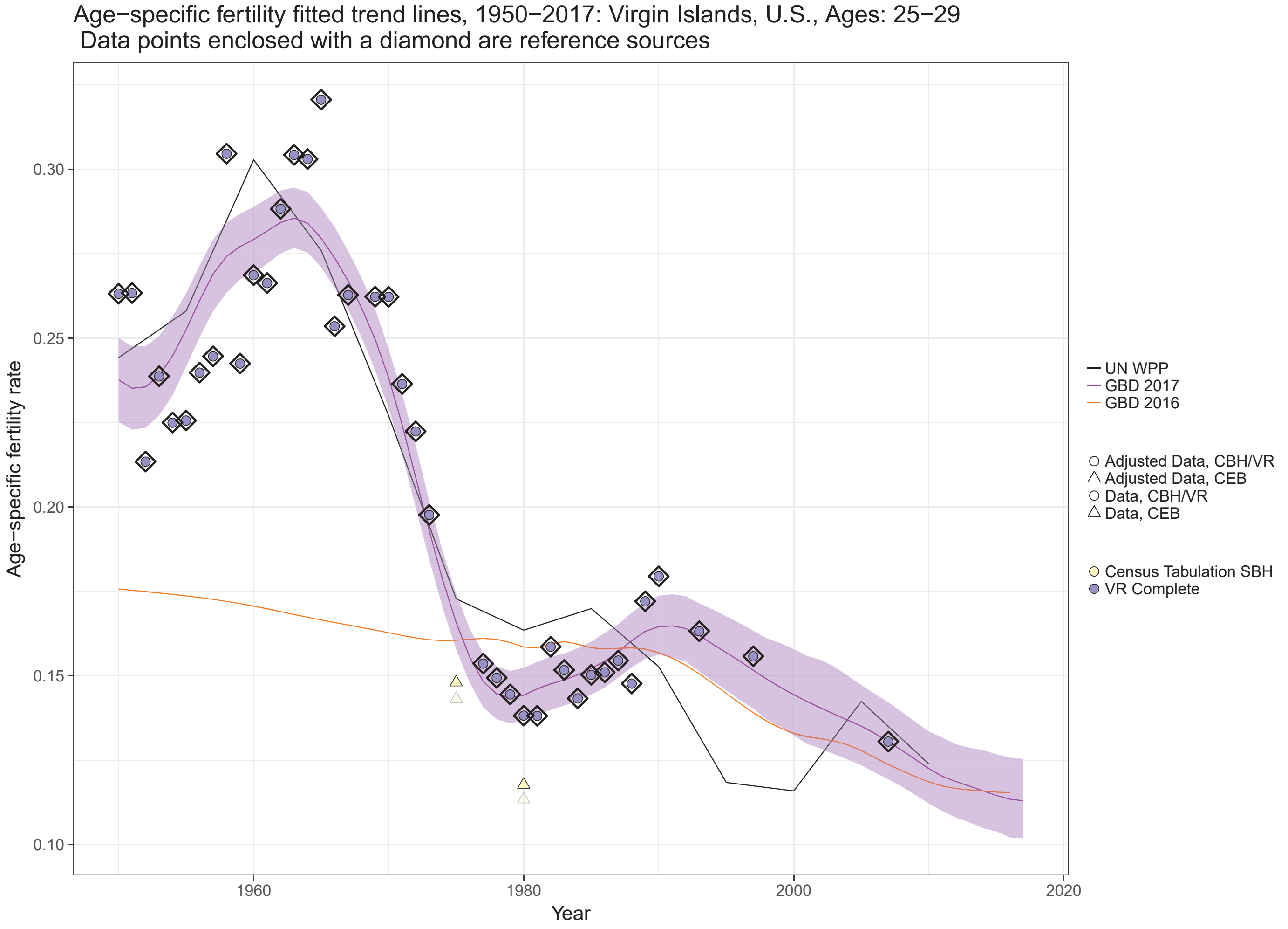

Age−specific fertility fitted trend lines, 1950−2017: Virgin Islands, U.S., Ages: 25−29
Data points enclosed with a diamond are reference sources
Age−specific fertility rate
0.10
0.15
0.20
0.25
0.30
1960
1980
2000
2020
Year
UN WPP
GBD 2017
GBD 2016
Adjusted Data, CBH/VR
Adjusted Data, CEB
Data, CBH/VR
Data, CEB
Census Tabulation SBH
VR Complete

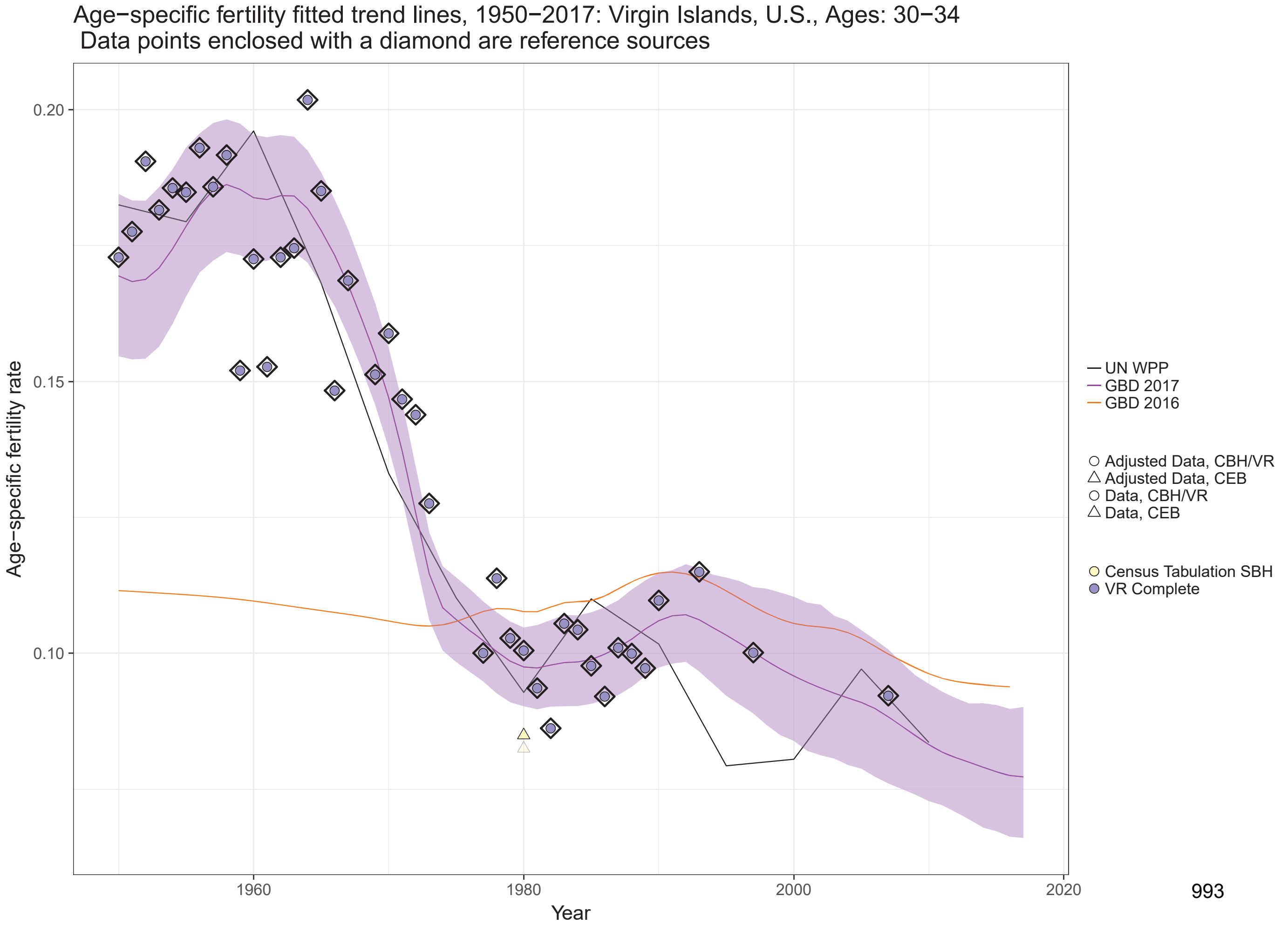

Age−specific fertility fitted trend lines, 1950−2017: Virgin Islands, U.S., Ages: 30−34
Data points enclosed with a diamond are reference sources
Age−specific fertility rate
0.10
0.15
0.20
1960
1980
2000
2020
Year
UN WPP
GBD 2017
GBD 2016
Adjusted Data, CBH/VR
Adjusted Data, CEB
Data, CBH/VR
Data, CEB
Census Tabulation SBH
VR Complete

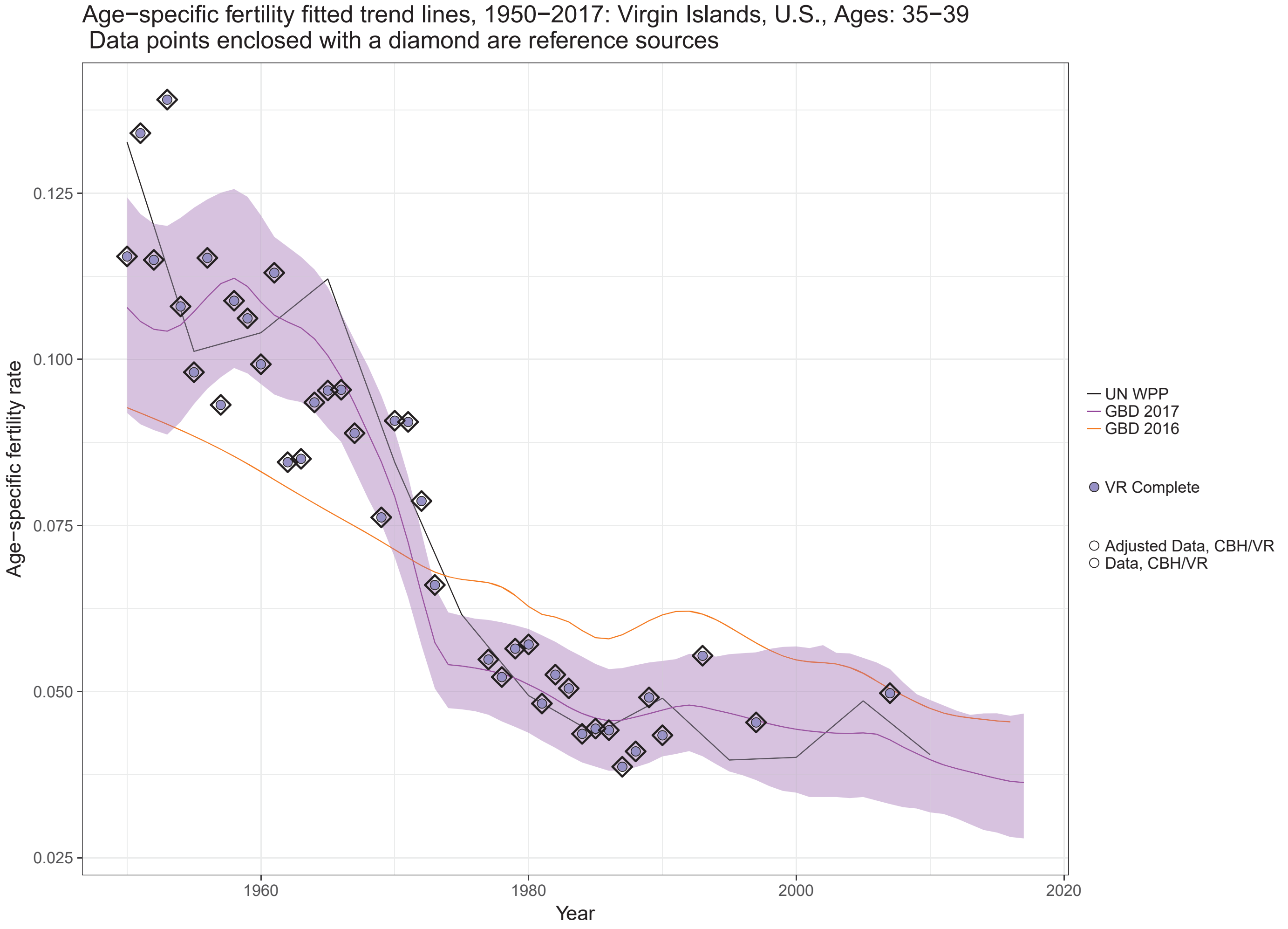

Age−specific fertility fitted trend lines, 1950−2017: Virgin Islands, U.S., Ages: 35−39
Data points enclosed with a diamond are reference sources
Age−specific fertility rate
0.125
0.100
0.075
0.050
0.025
1960
1980
2000
2020
Year
UN WPP
GBD 2017
GBD 2016
VR Complete
Adjusted Data, CBH/VR
Data, CBH/VR

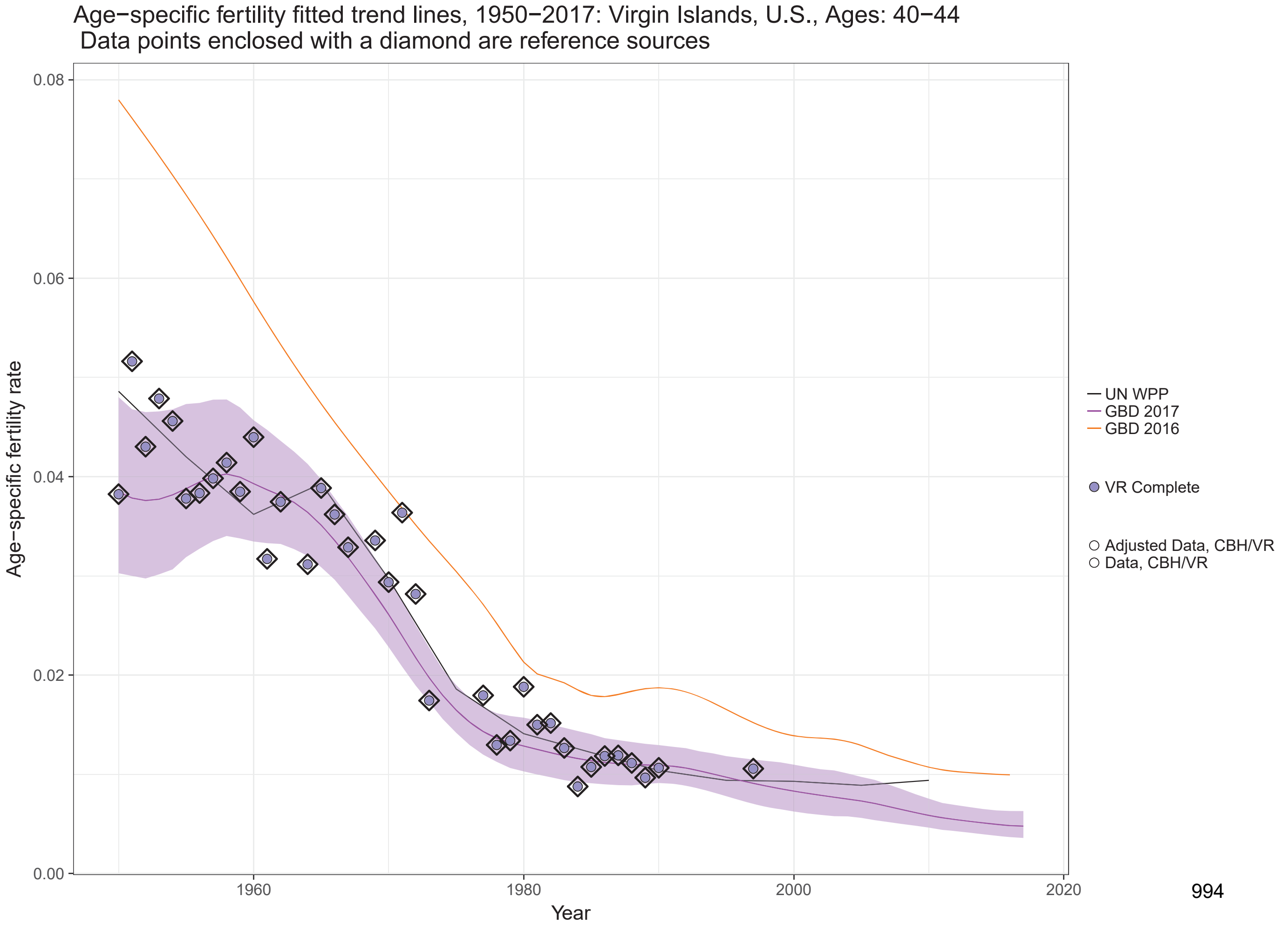

Age−specific fertility fitted trend lines, 1950−2017: Virgin Islands, U.S., Ages: 40−44
Data points enclosed with a diamond are reference sources
Age−specific fertility rate
0.08
0.06
0.04
0.02
0.00
1960
1980
2000
2020
Year
UN WPP
GBD 2017
GBD 2016
VR Complete
Adjusted Data, CBH/VR
Data, CBH/VR

Age−specific fertility fitted trend lines, 1950−2017: Virgin Islands, U.S., Ages: 45−49
Data points enclosed with a diamond are reference sources

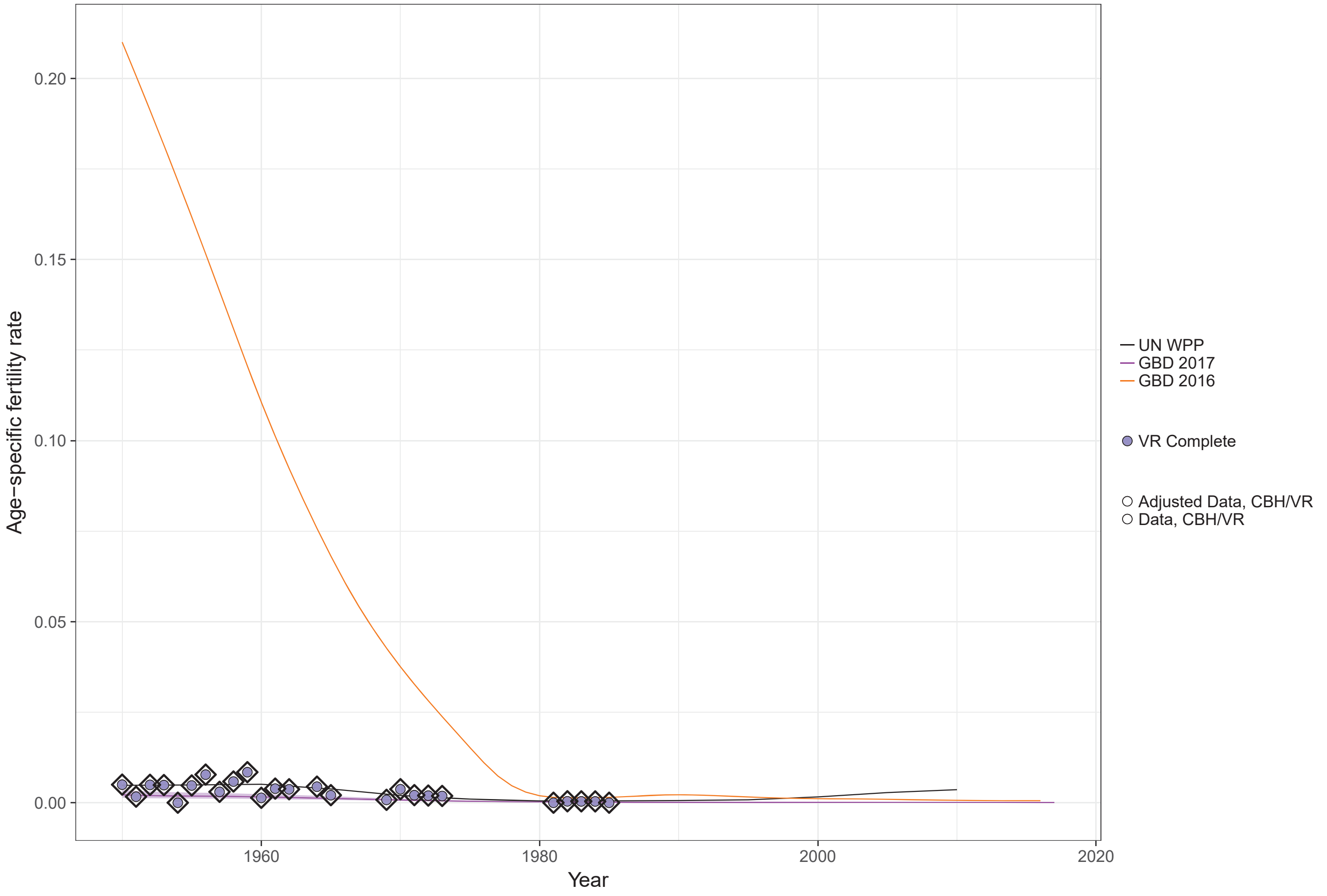

Age−specific fertility fitted trend lines, 1950−2017: Virgin Islands, U.S., Ages: 50−54
Data points enclosed with a diamond are reference sources

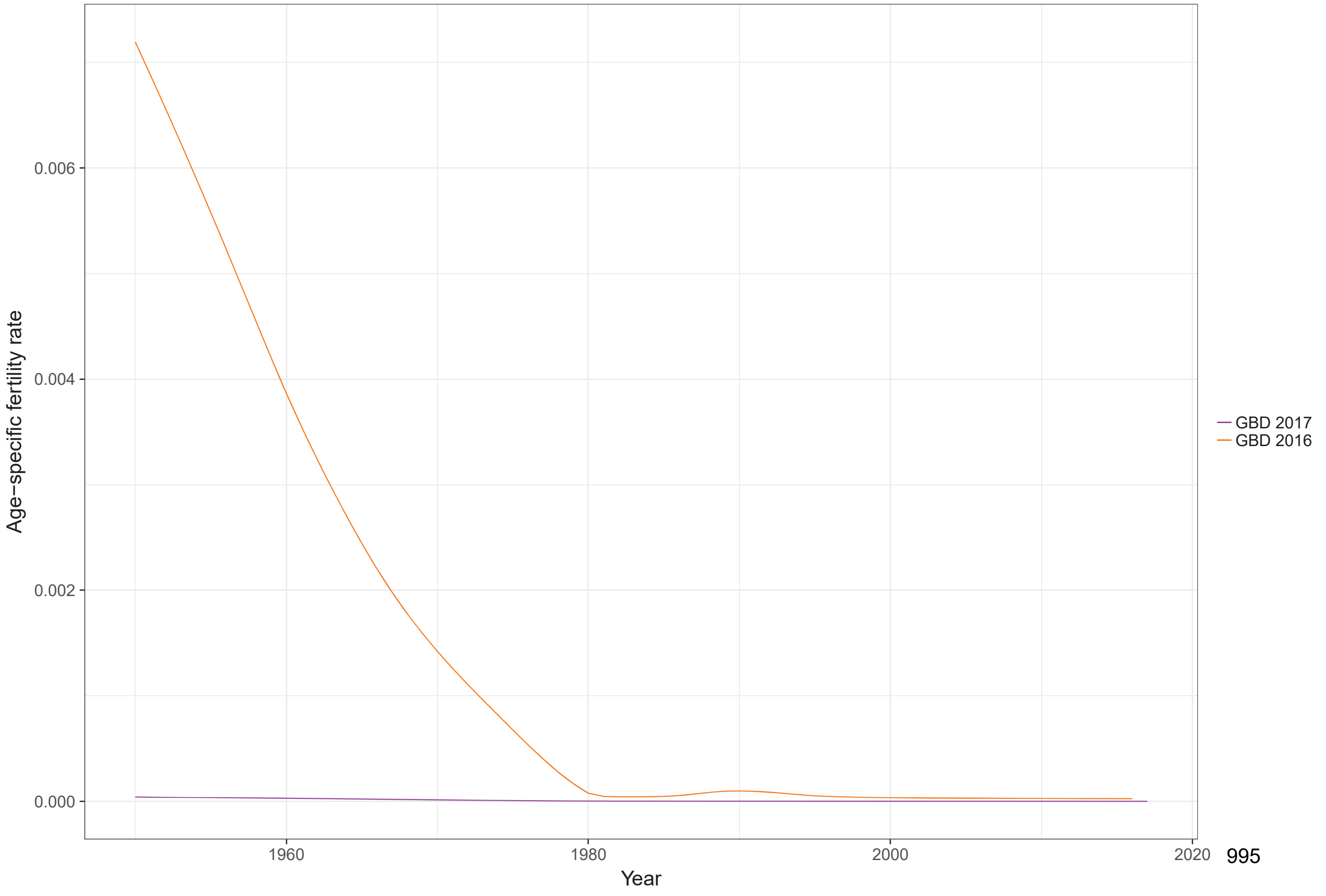

Age−specific fertility fitted trend lines, 1950−2017: Wales, TFR
Data points enclosed with a diamond are reference sources

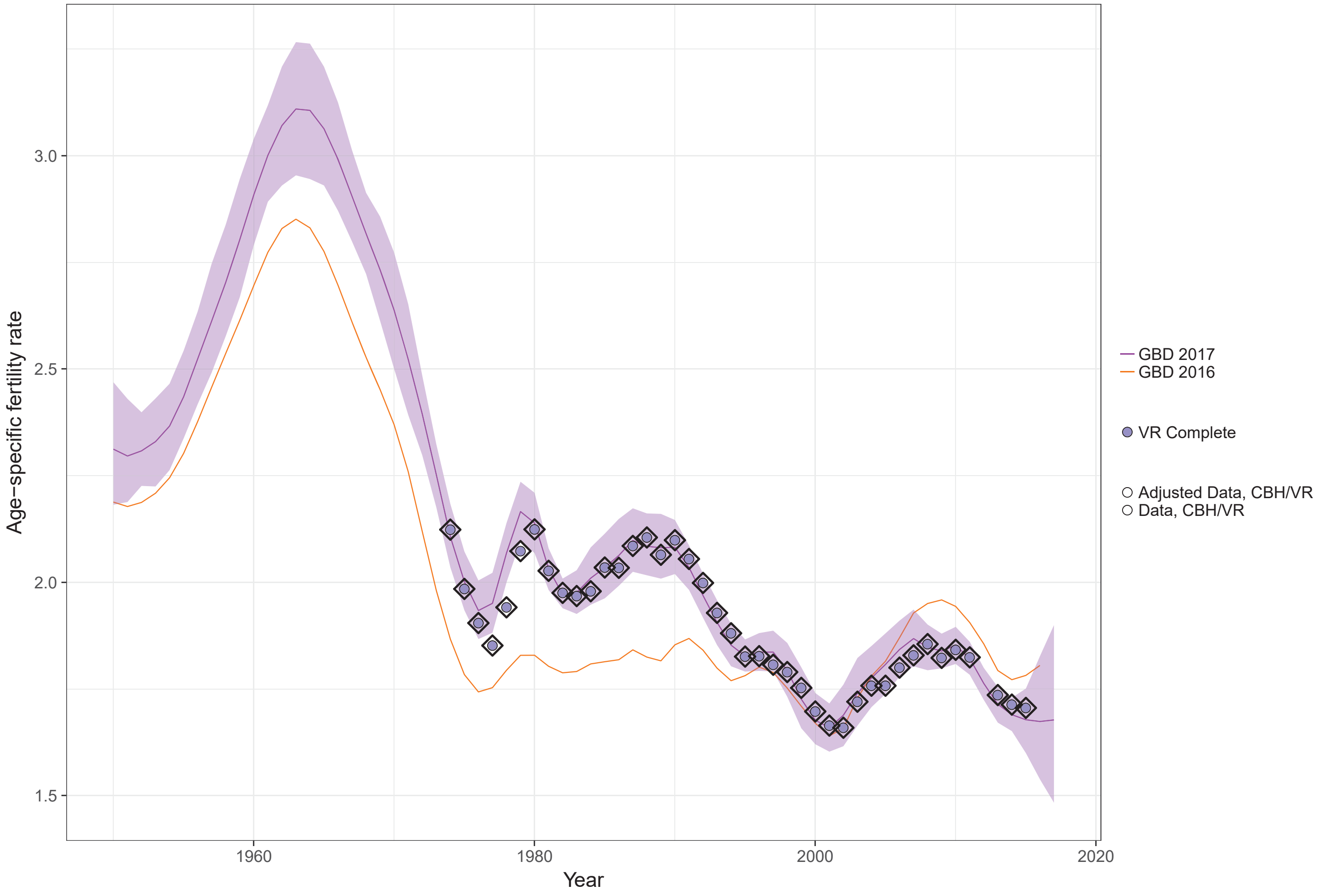

Age−specific fertility fitted trend lines, 1950−2017: Wales, Ages: 10−14
Data points enclosed with a diamond are reference sources

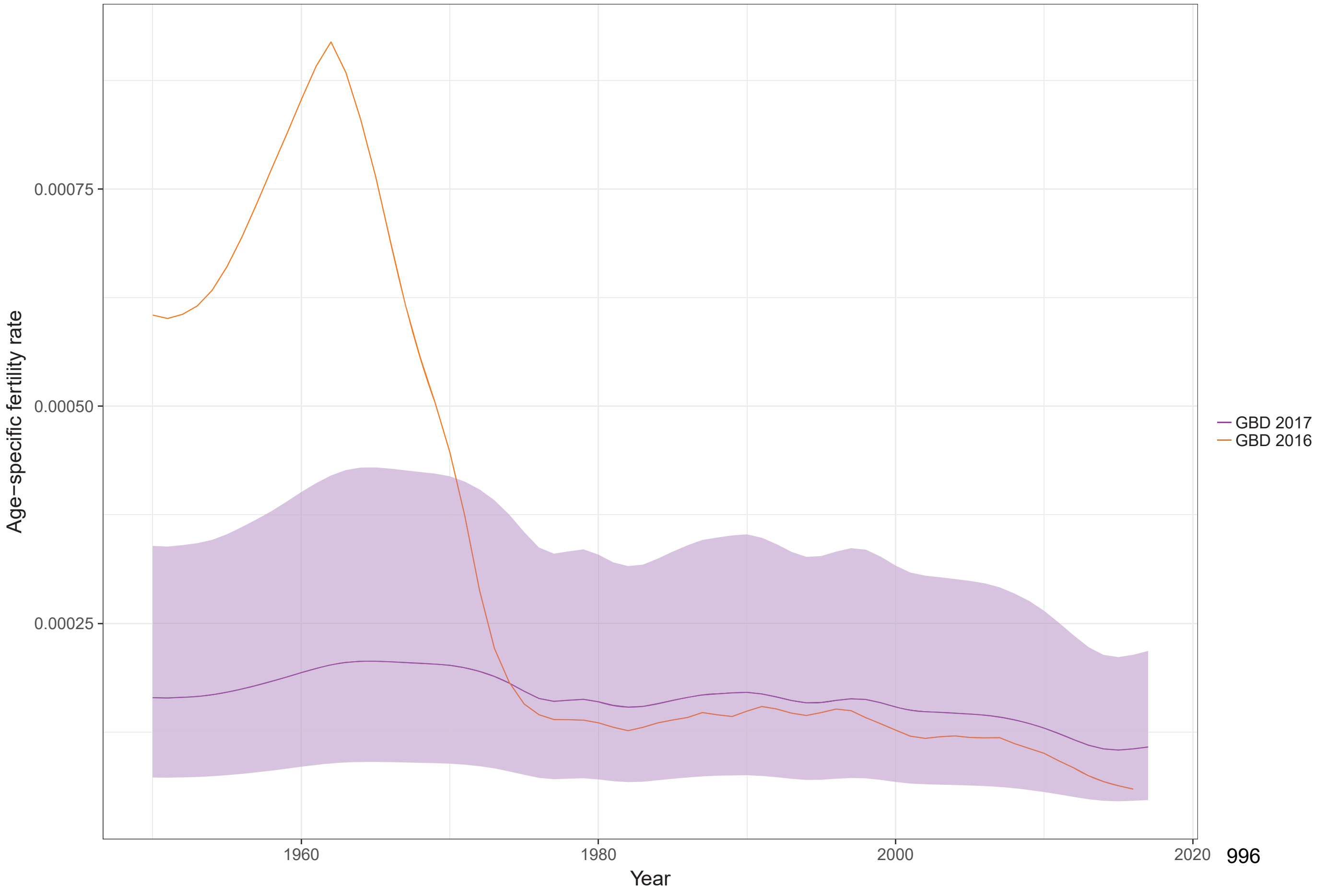

Age−specific fertility fitted trend lines, 1950−2017: Wales, Ages: 15−19
Data points enclosed with a diamond are reference sources

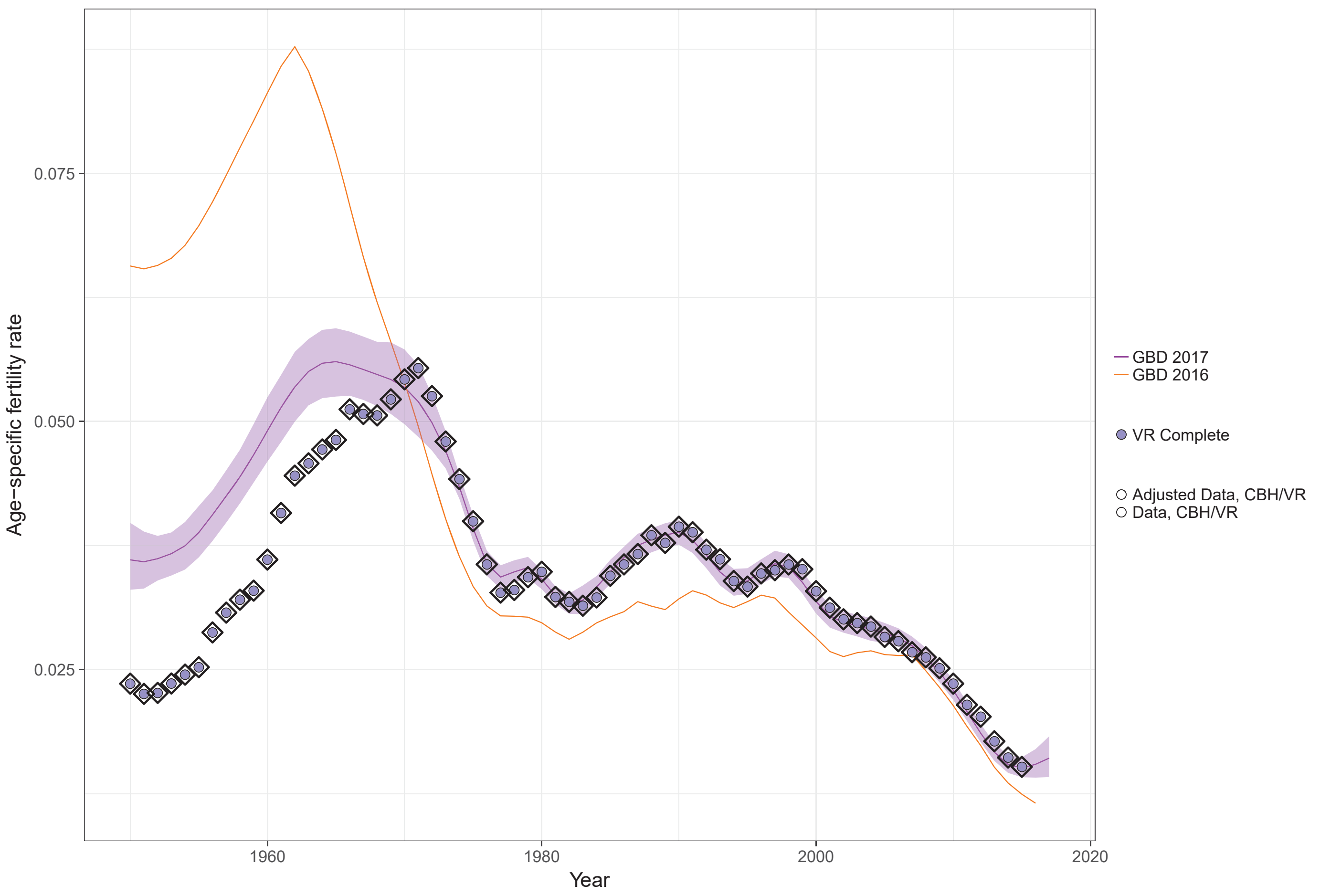

Age−specific fertility fitted trend lines, 1950−2017: Wales, Ages: 20−24
Data points enclosed with a diamond are reference sources

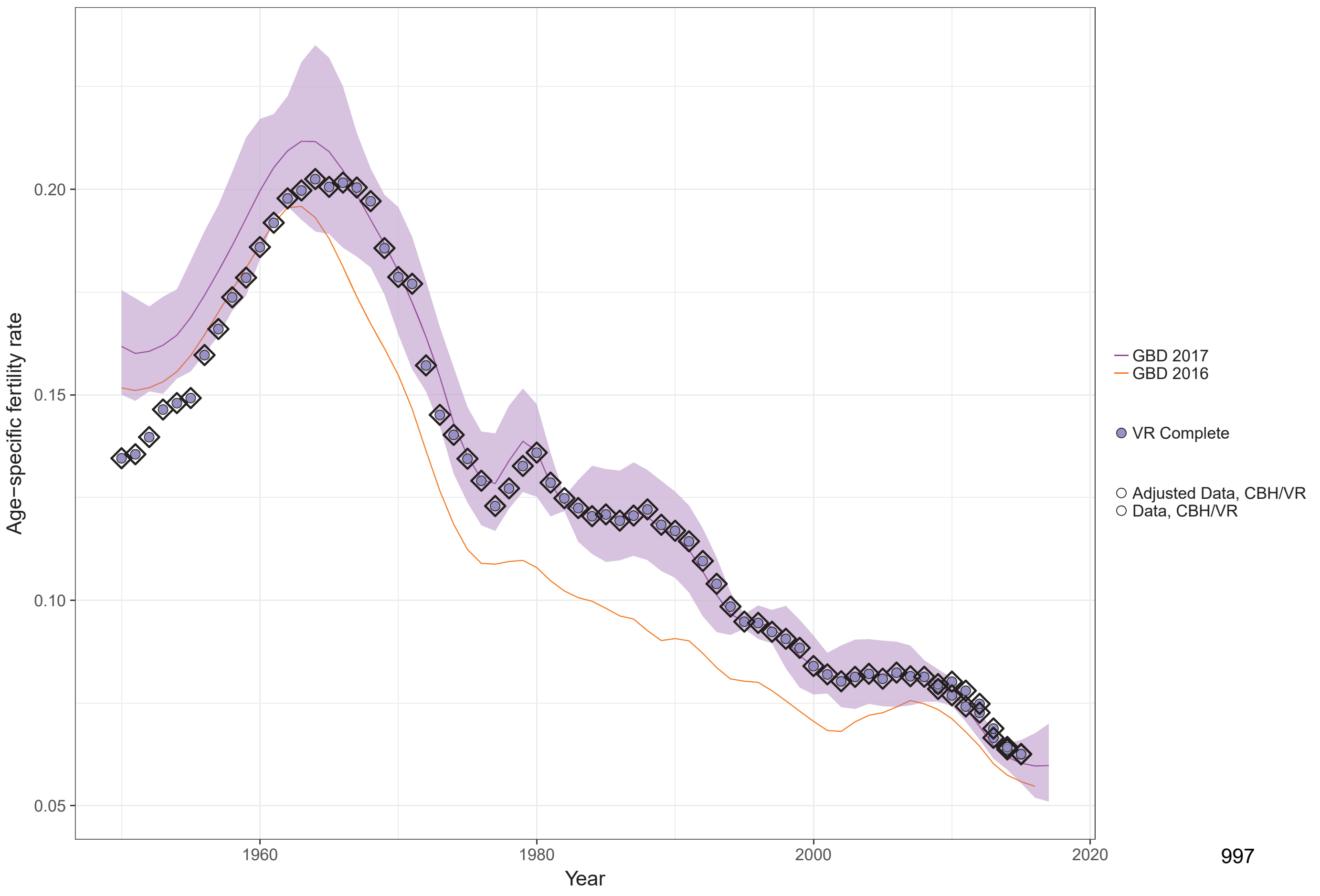

Age−specific fertility fitted trend lines, 1950−2017: Wales, Ages: 25−29
Data points enclosed with a diamond are reference sources

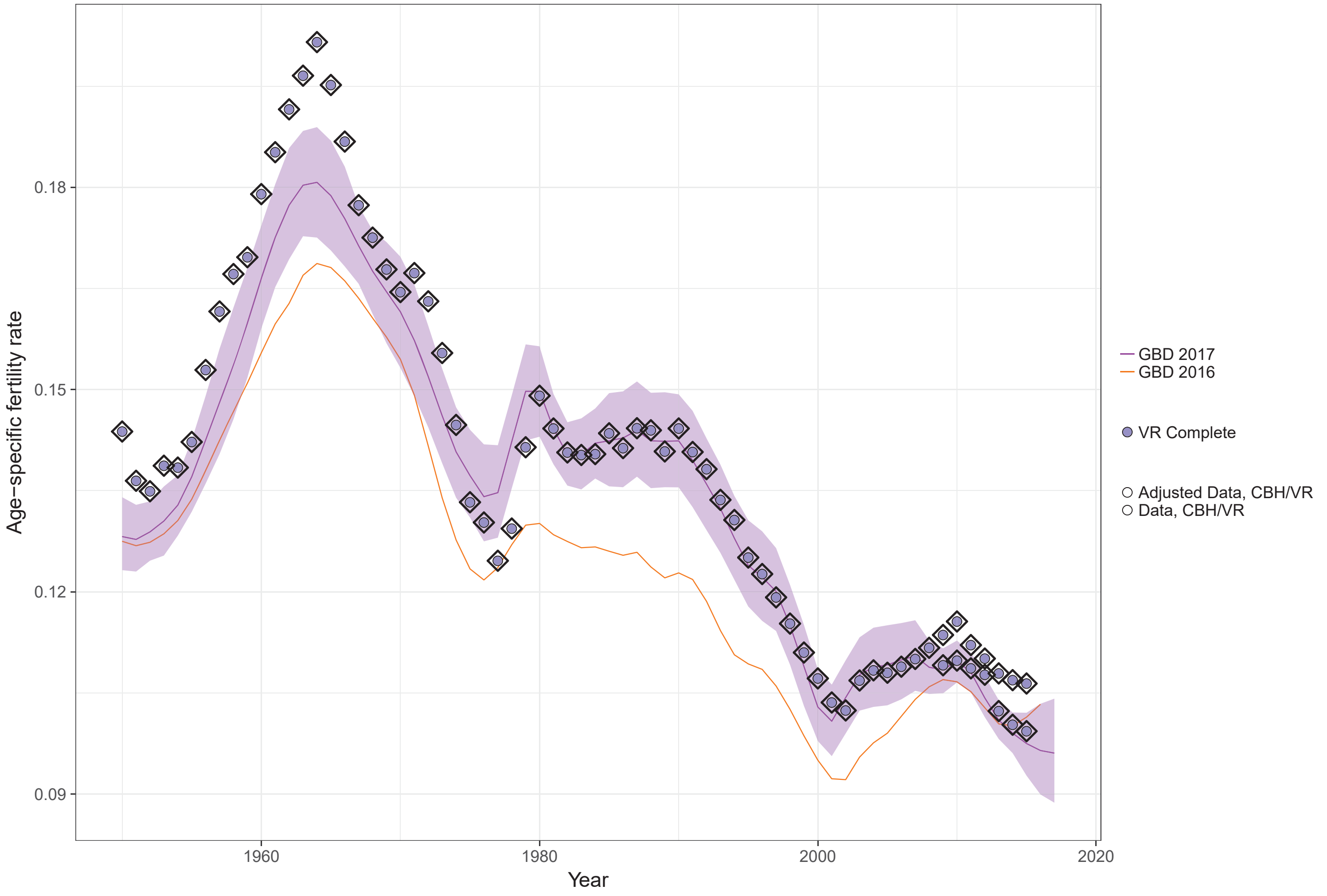

Age−specific fertility fitted trend lines, 1950−2017: Wales, Ages: 30−34
Data points enclosed with a diamond are reference sources

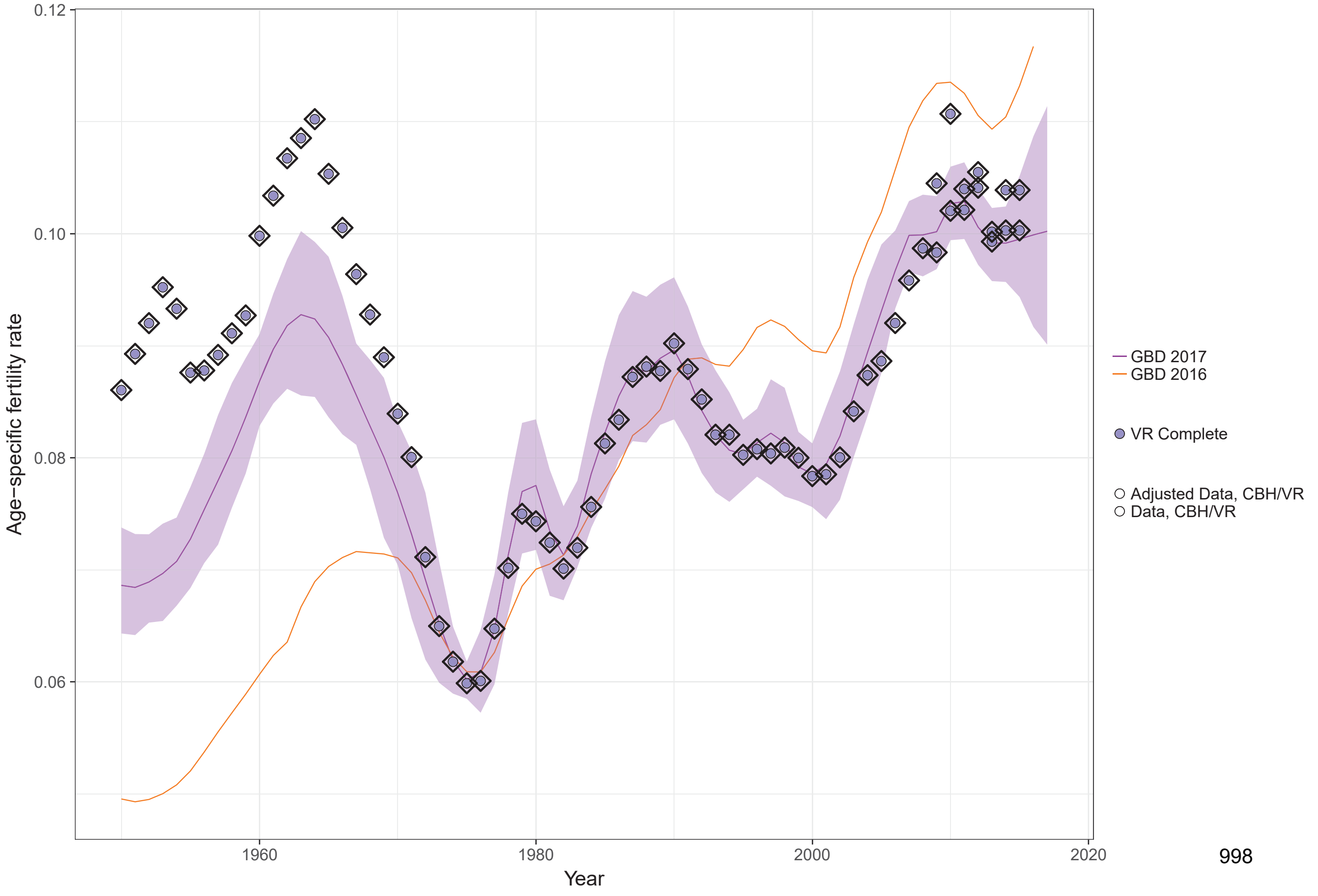

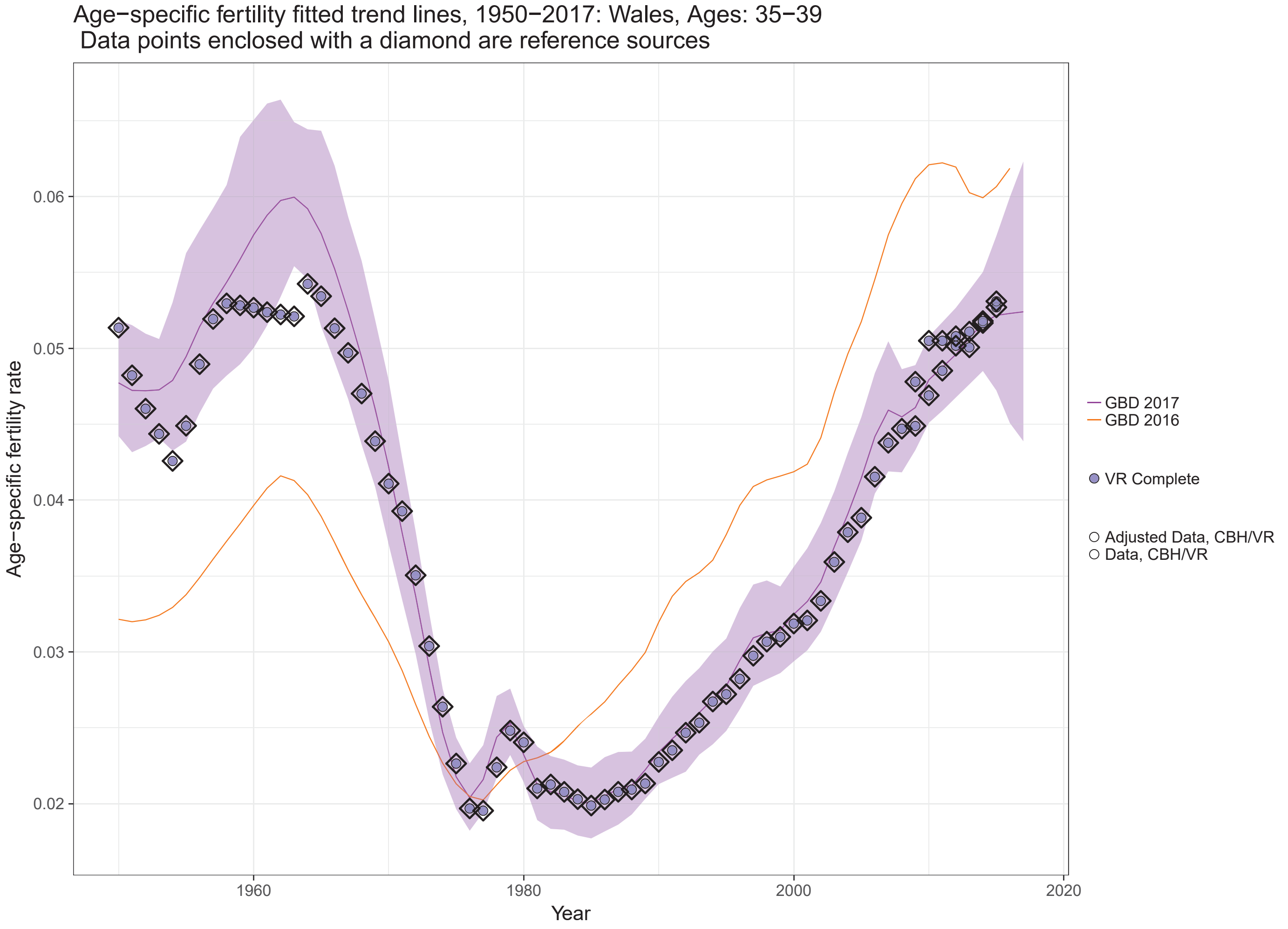

Age−specific fertility fitted trend lines, 1950−2017: Wales, Ages: 35−39
Data points enclosed with a diamond are reference sources
Age-specific fertility rate
0.06
0.05
0.04
0.03
0.02
1960
1980
2000
2020
Year
GBD 2017
GBD 2016
VR Complete
Adjusted Data, CBH/VR
Data, CBH/VR

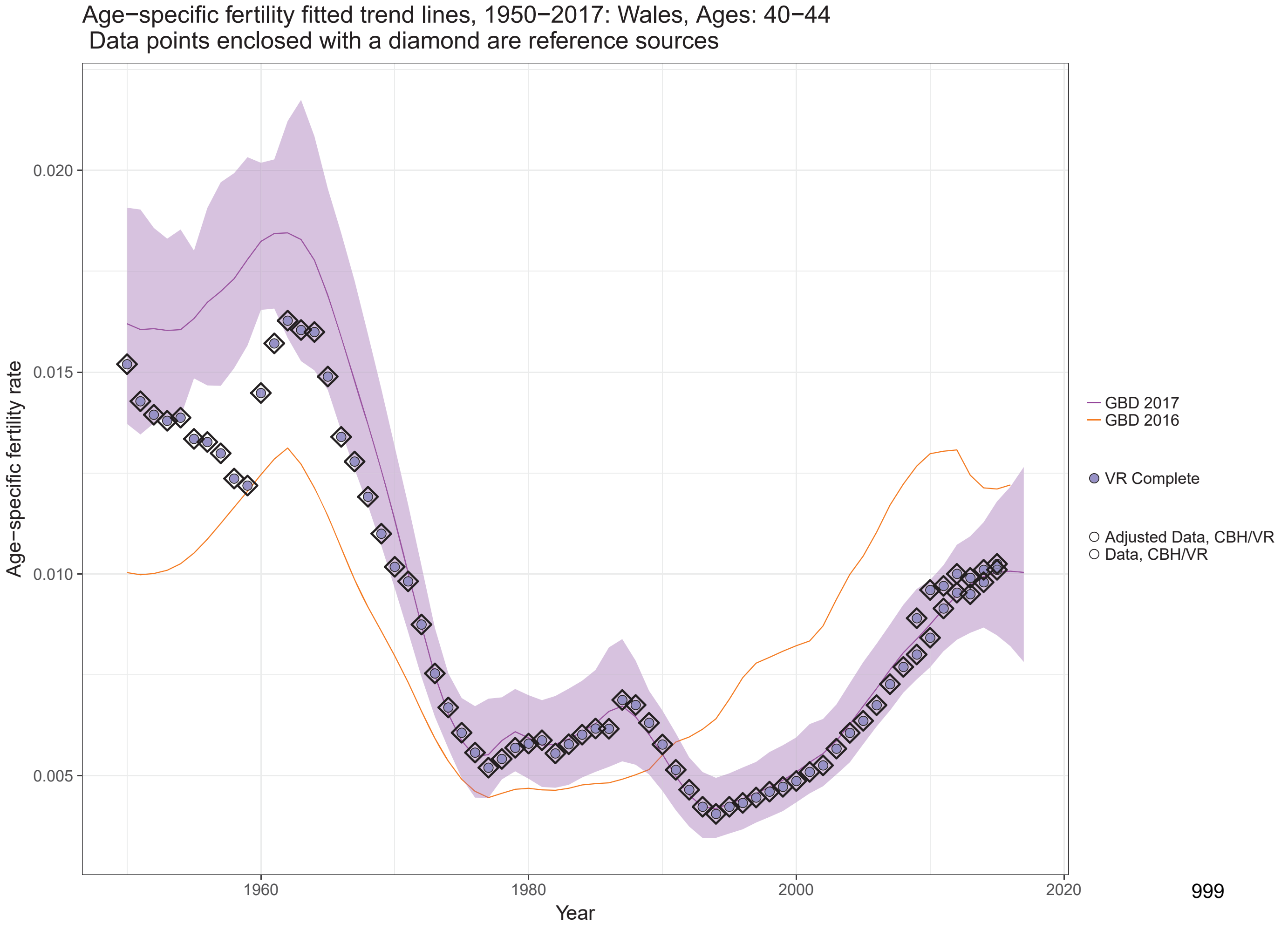

Age−specific fertility fitted trend lines, 1950−2017: Wales, Ages: 40−44
Data points enclosed with a diamond are reference sources
Age-specific fertility rate
0.020
0.015
0.010
0.005
1960
1980
2000
2020
Year
GBD 2017
GBD 2016
VR Complete
Adjusted Data, CBH/VR
Data, CBH/VR

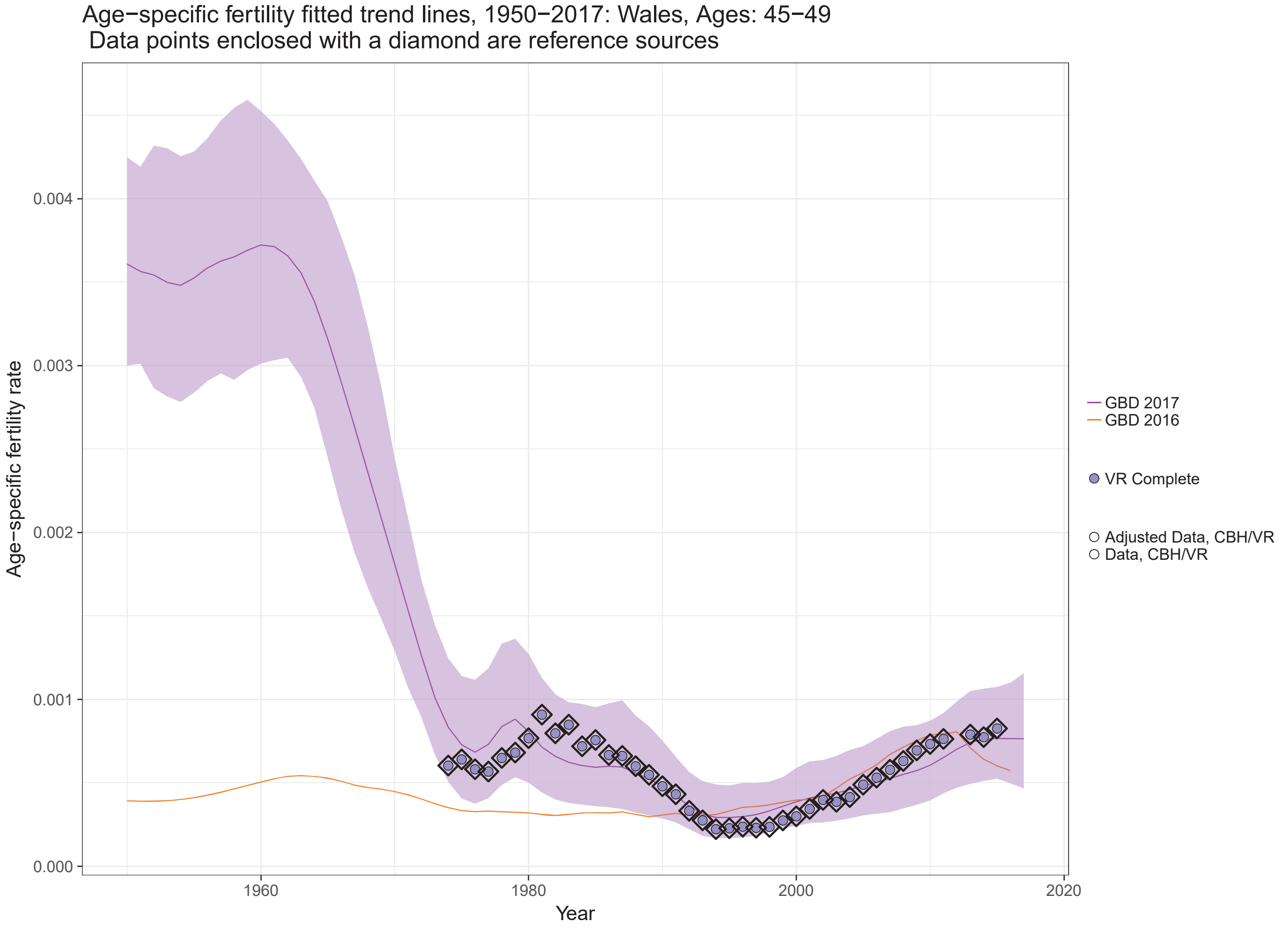
Age−specific fertility fitted trend lines, 1950−2017: Wales, Ages: 45−49
Data points enclosed with a diamond are reference sources
Age−specific fertility rate
0.000
0.001
0.002
0.003
0.004
1960
1980
2000
2020
Year
GBD 2017
GBD 2016
VR Complete
Adjusted Data, CBH/VR
Data, CBH/VR

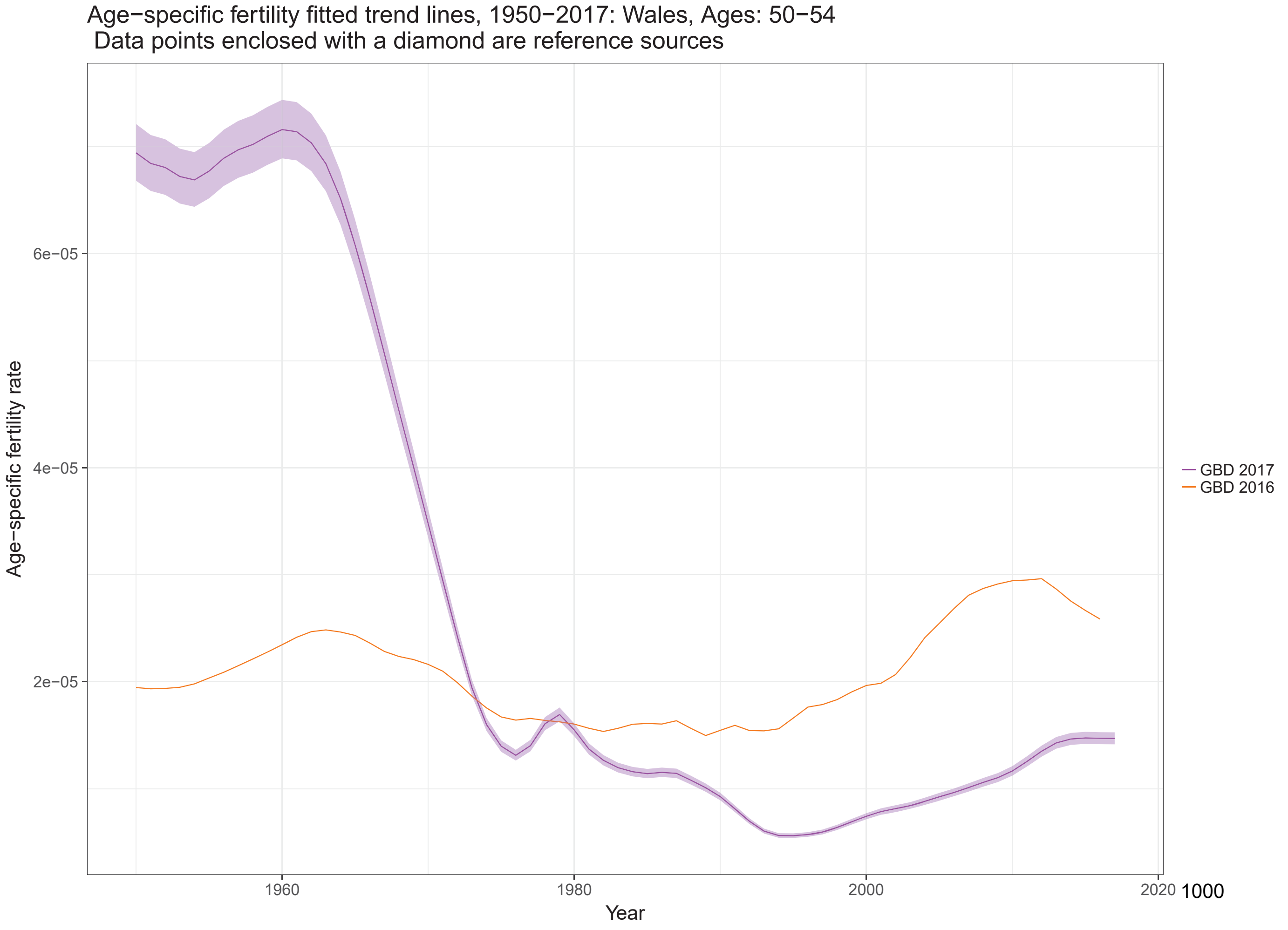
Age−specific fertility fitted trend lines, 1950−2017: Wales, Ages: 50−54
Data points enclosed with a diamond are reference sources
Age−specific fertility rate
2e−05
4e−05
6e−05
1960
1980
2000
2020
Year
GBD 2017
GBD 2016

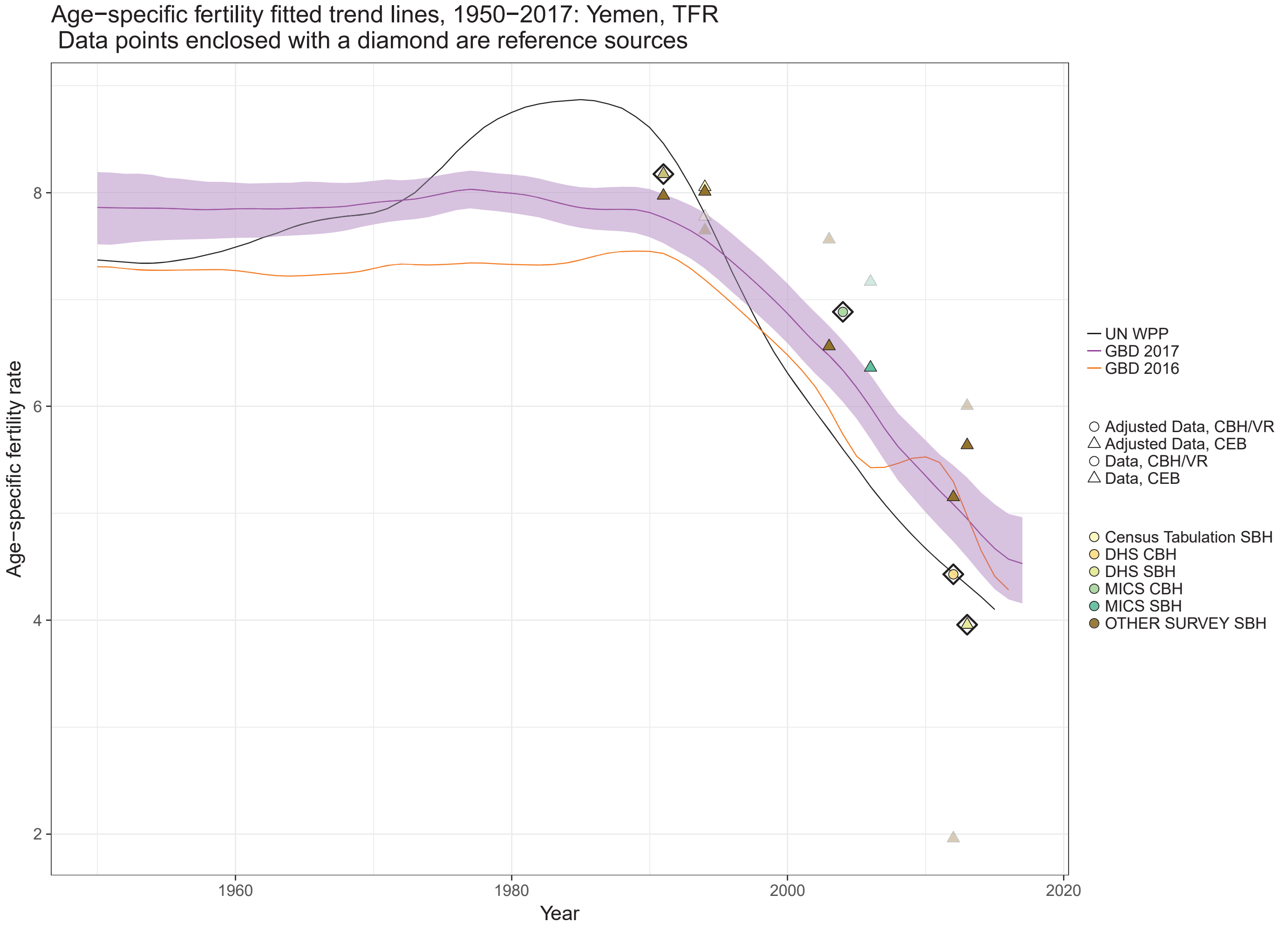
Age−specific fertility fitted trend lines, 1950−2017: Yemen, TFR
Data points enclosed with a diamond are reference sources
Age−specific fertility rate
Year
UN WPP
GBD 2017
GBD 2016
Adjusted Data, CBH/VR
Adjusted Data, CEB
Data, CBH/VR
Data, CEB
Census Tabulation SBH
DHS CBH
DHS SBH
MICS CBH
MICS SBH
OTHER SURVEY SBH

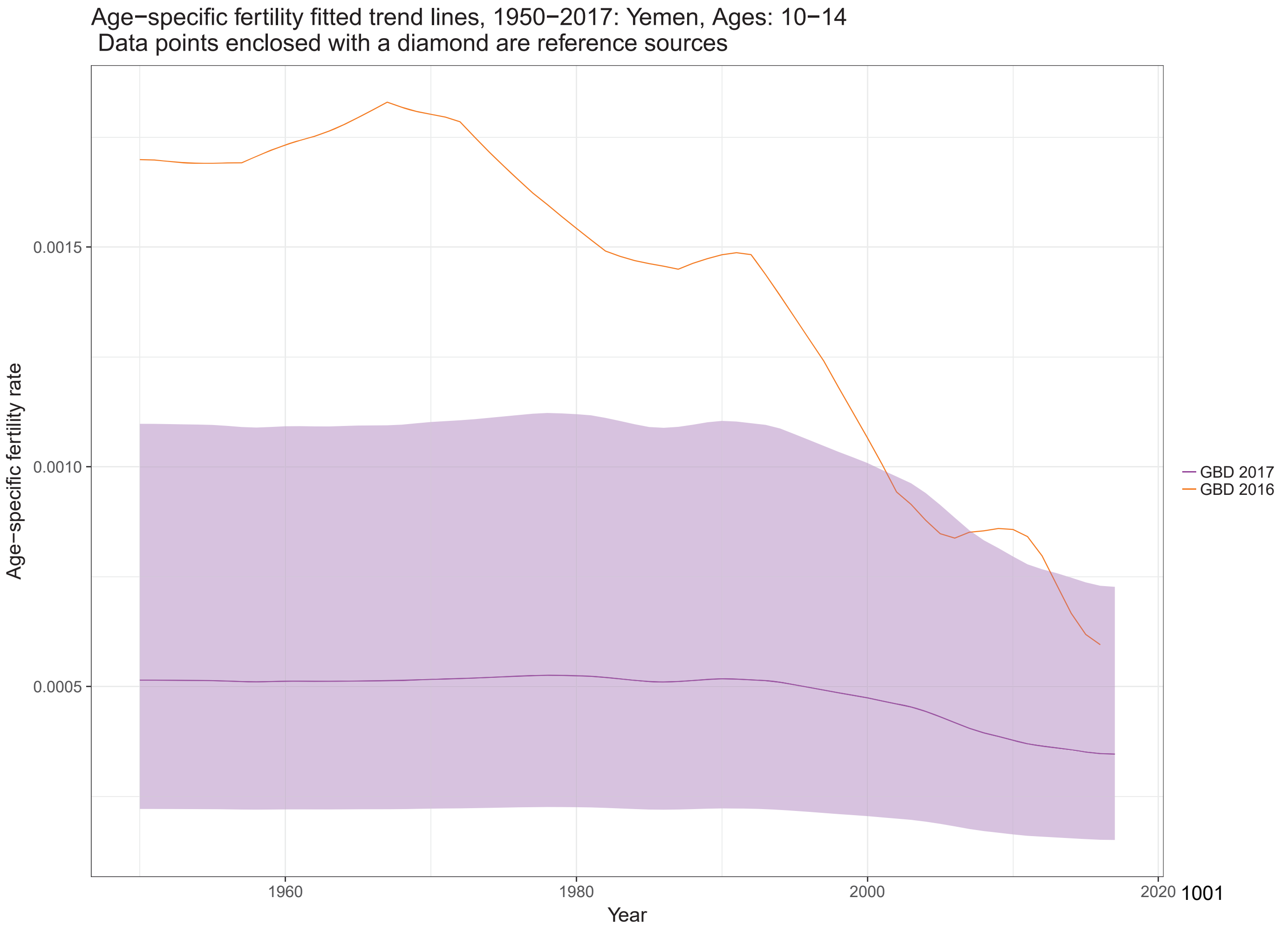
Age−specific fertility fitted trend lines, 1950−2017: Yemen, Ages: 10−14
Data points enclosed with a diamond are reference sources
Age−specific fertility rate
Year
GBD 2017
GBD 2016

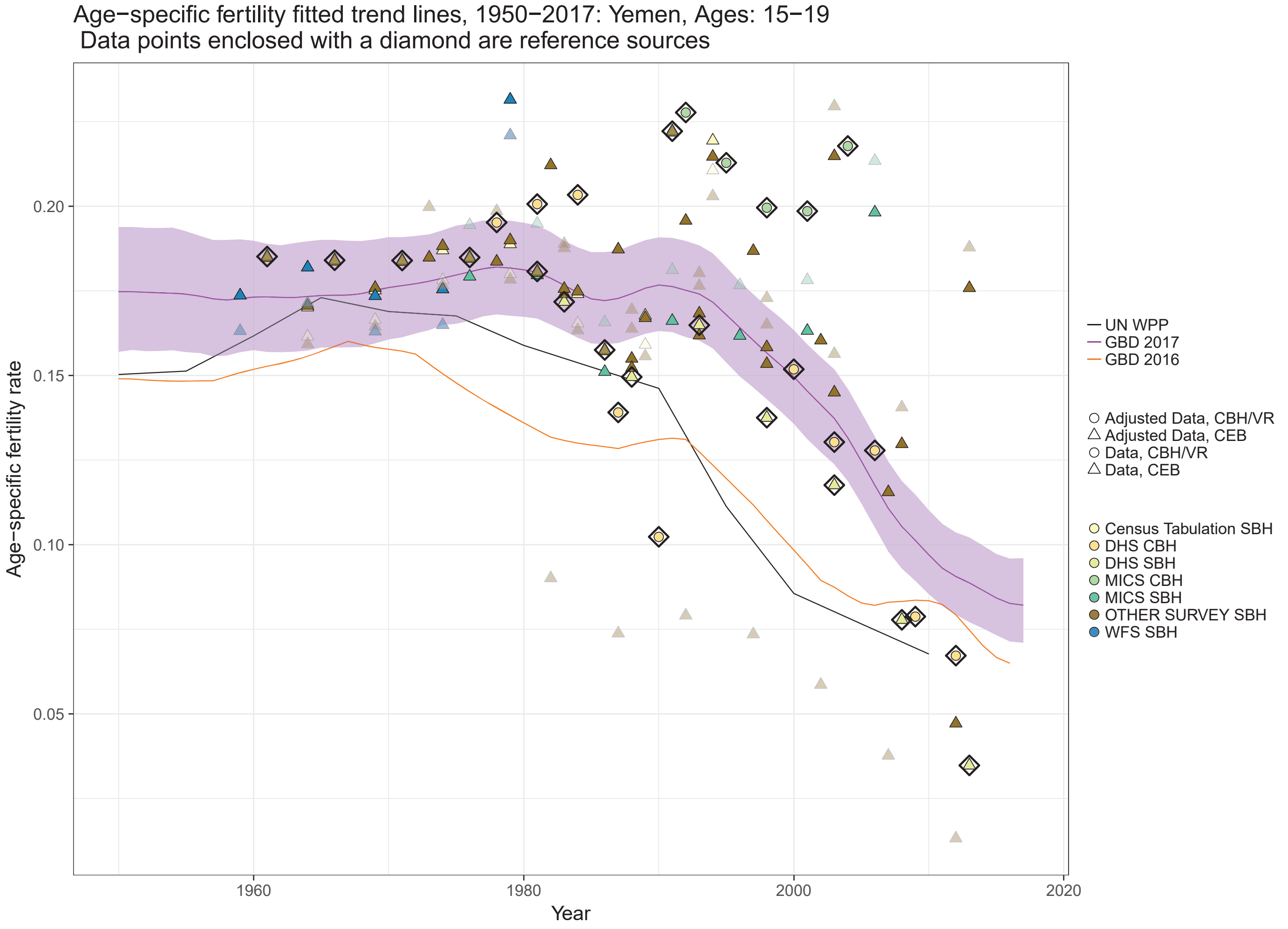
Age−specific fertility fitted trend lines, 1950−2017: Yemen, Ages: 15−19
Data points enclosed with a diamond are reference sources
Age−specific fertility rate
Year
UN WPP
GBD 2017
GBD 2016
Adjusted Data, CBH/VR
Adjusted Data, CEB
Data, CBH/VR
Data, CEB
Census Tabulation SBH
DHS CBH
DHS SBH
MICS CBH
MICS SBH
OTHER SURVEY SBH
WFS SBH

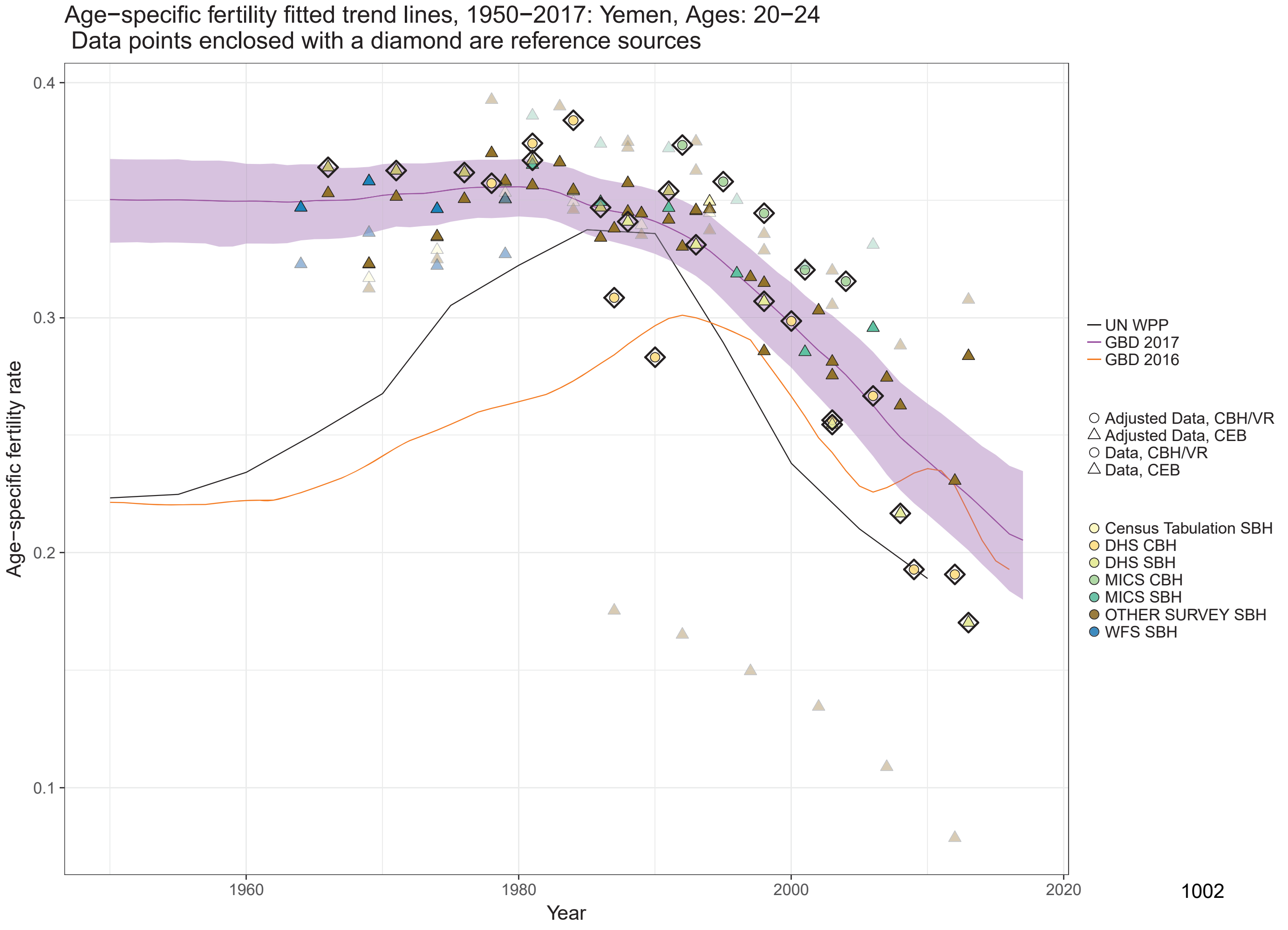
Age−specific fertility fitted trend lines, 1950−2017: Yemen, Ages: 20−24
Data points enclosed with a diamond are reference sources
Age−specific fertility rate
Year
UN WPP
GBD 2017
GBD 2016
Adjusted Data, CBH/VR
Adjusted Data, CEB
Data, CBH/VR
Data, CEB
Census Tabulation SBH
DHS CBH
DHS SBH
MICS CBH
MICS SBH
OTHER SURVEY SBH
WFS SBH

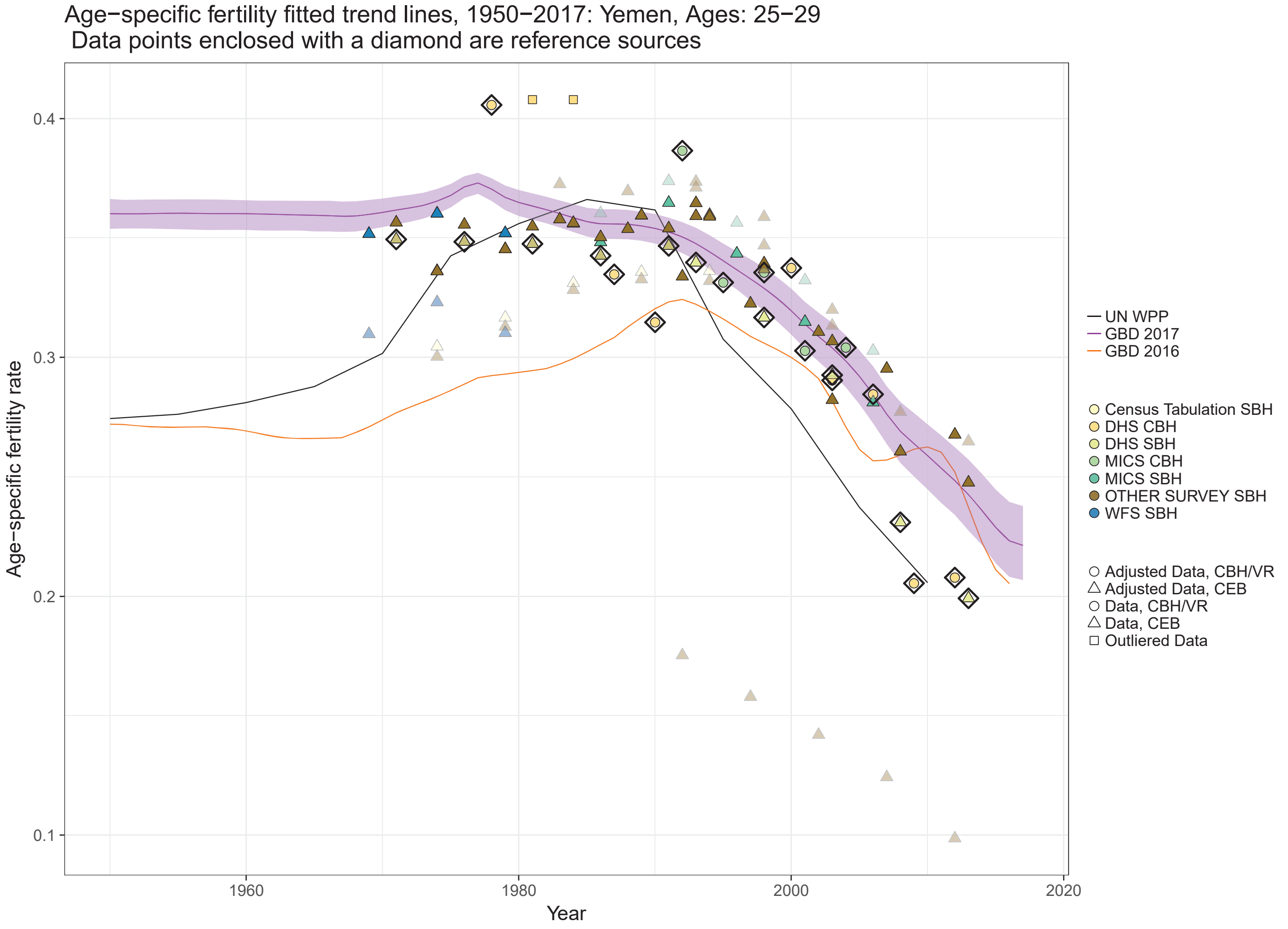

Age−specific fertility fitted trend lines, 1950−2017: Yemen, Ages: 25−29
Data points enclosed with a diamond are reference sources
Age−specific fertility rate
0.1
0.2
0.3
0.4
1960
1980
2000
2020
Year
UN WPP
GBD 2017
GBD 2016
Census Tabulation SBH
DHS CBH
DHS SBH
MICS CBH
MICS SBH
OTHER SURVEY SBH
WFS SBH
Adjusted Data, CBH/VR
Adjusted Data, CEB
Data, CBH/VR
Data, CEB
Outliered Data

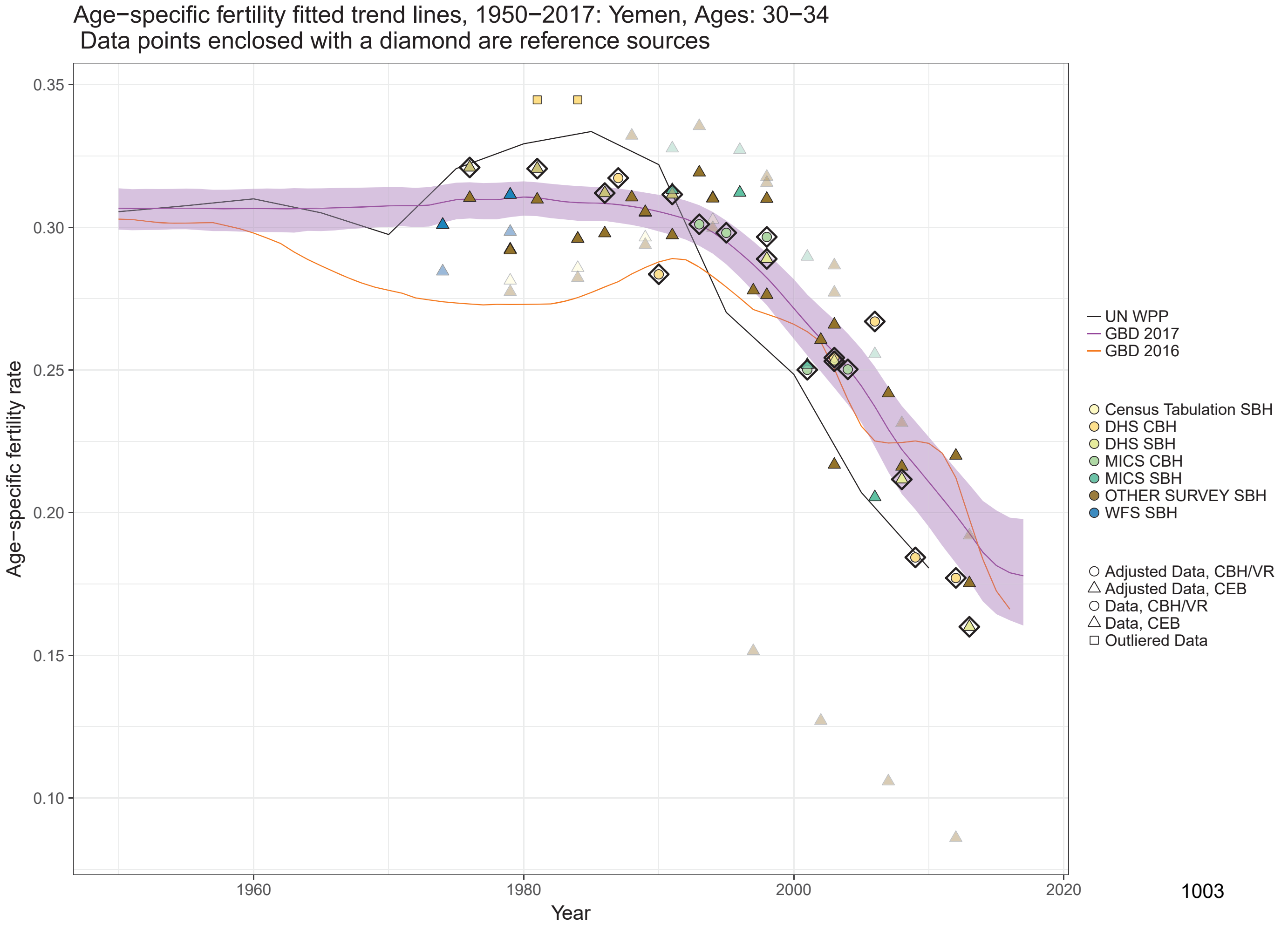

Age−specific fertility fitted trend lines, 1950−2017: Yemen, Ages: 30−34
Data points enclosed with a diamond are reference sources
Age−specific fertility rate
0.10
0.15
0.20
0.25
0.30
0.35
1960
1980
2000
2020
Year
UN WPP
GBD 2017
GBD 2016
Census Tabulation SBH
DHS CBH
DHS SBH
MICS CBH
MICS SBH
OTHER SURVEY SBH
WFS SBH
Adjusted Data, CBH/VR
Adjusted Data, CEB
Data, CBH/VR
Data, CEB
Outliered Data

Age−specific fertility fitted trend lines, 1950−2017: Yemen, Ages: 35−39
Data points enclosed with a diamond are reference sources

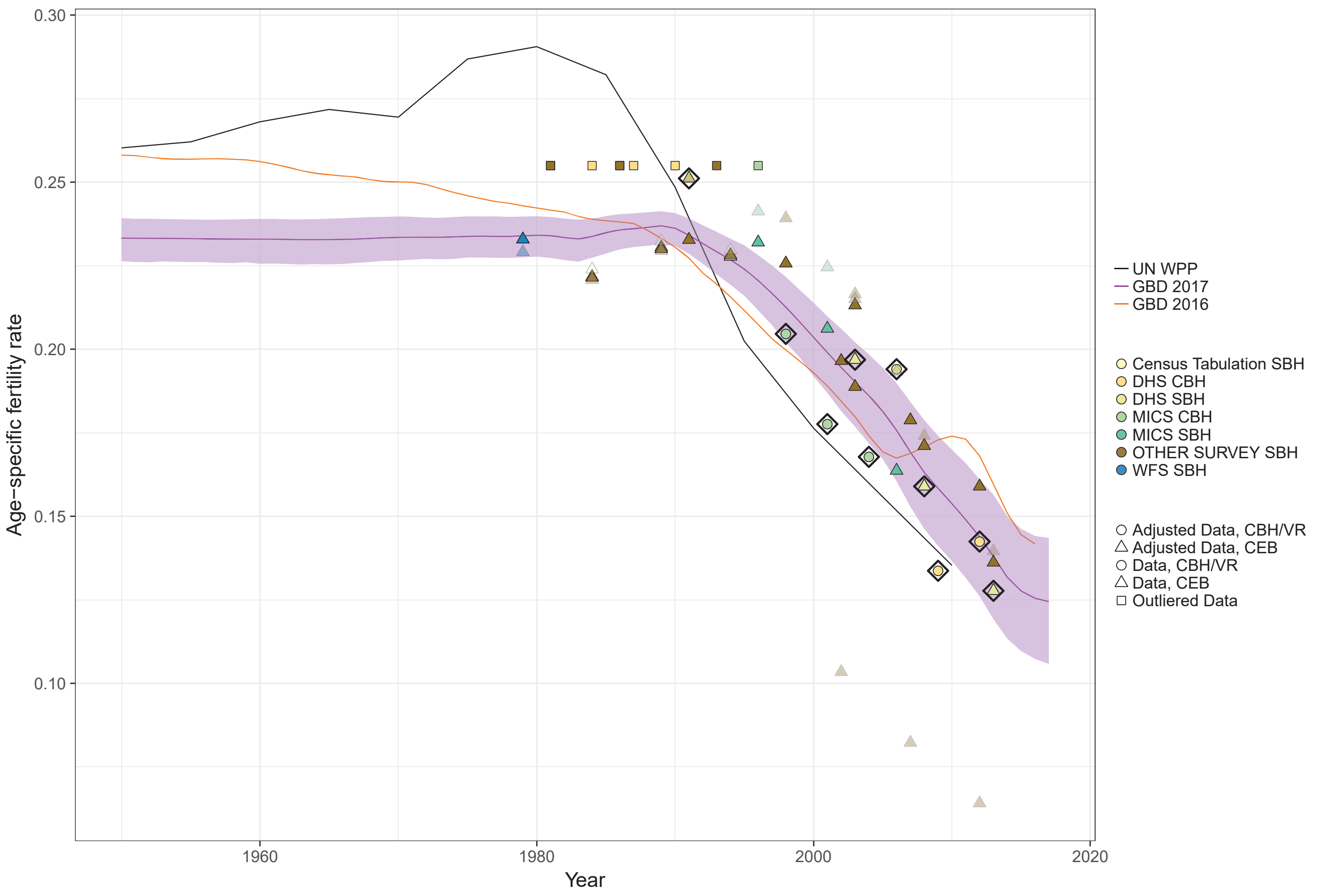

Age−specific fertility fitted trend lines, 1950−2017: Yemen, Ages: 40−44
Data points enclosed with a diamond are reference sources

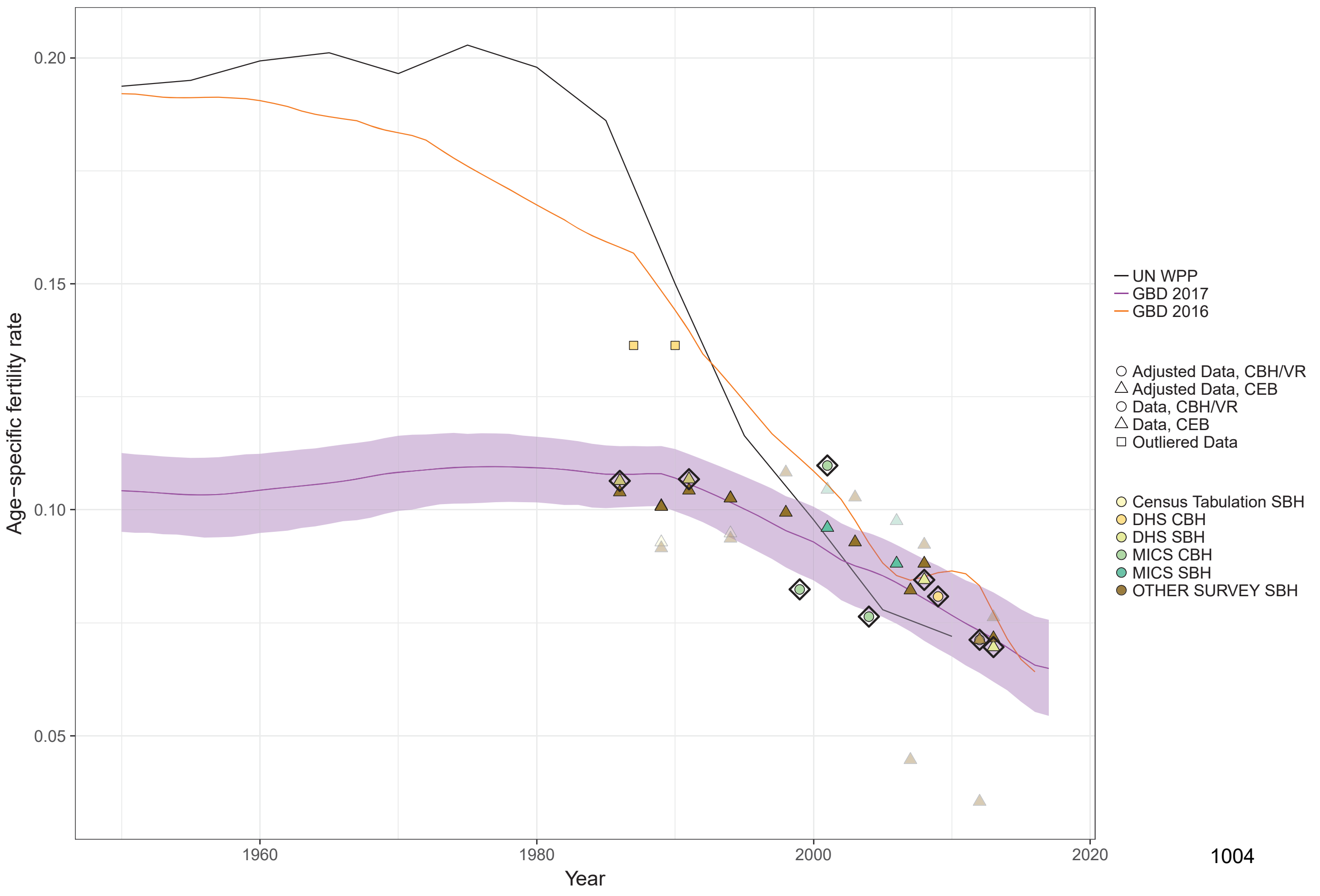

Age−specific fertility fitted trend lines, 1950−2017: Yemen, Ages: 45−49
Data points enclosed with a diamond are reference sources

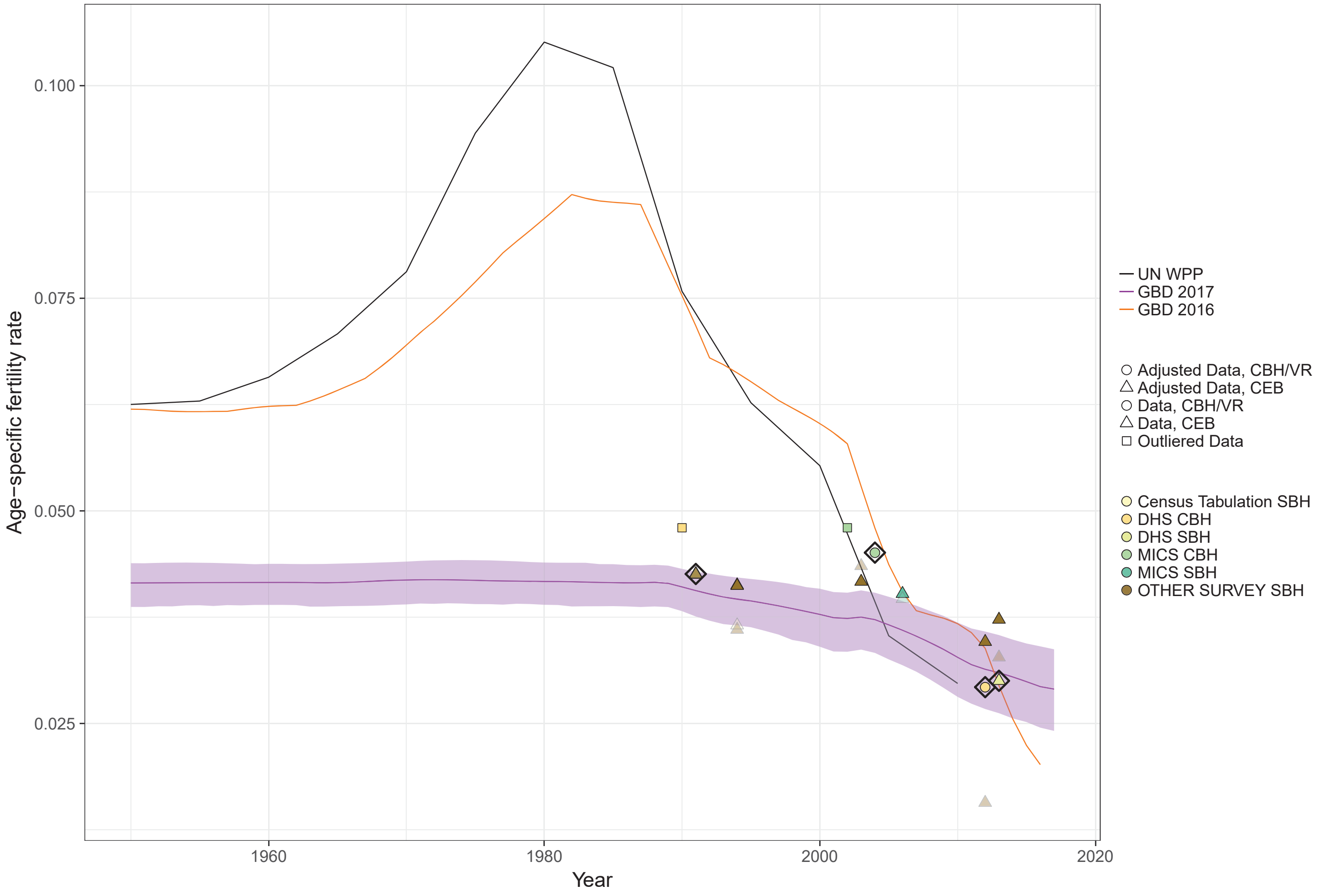

Age−specific fertility fitted trend lines, 1950−2017: Yemen, Ages: 50−54
Data points enclosed with a diamond are reference sources

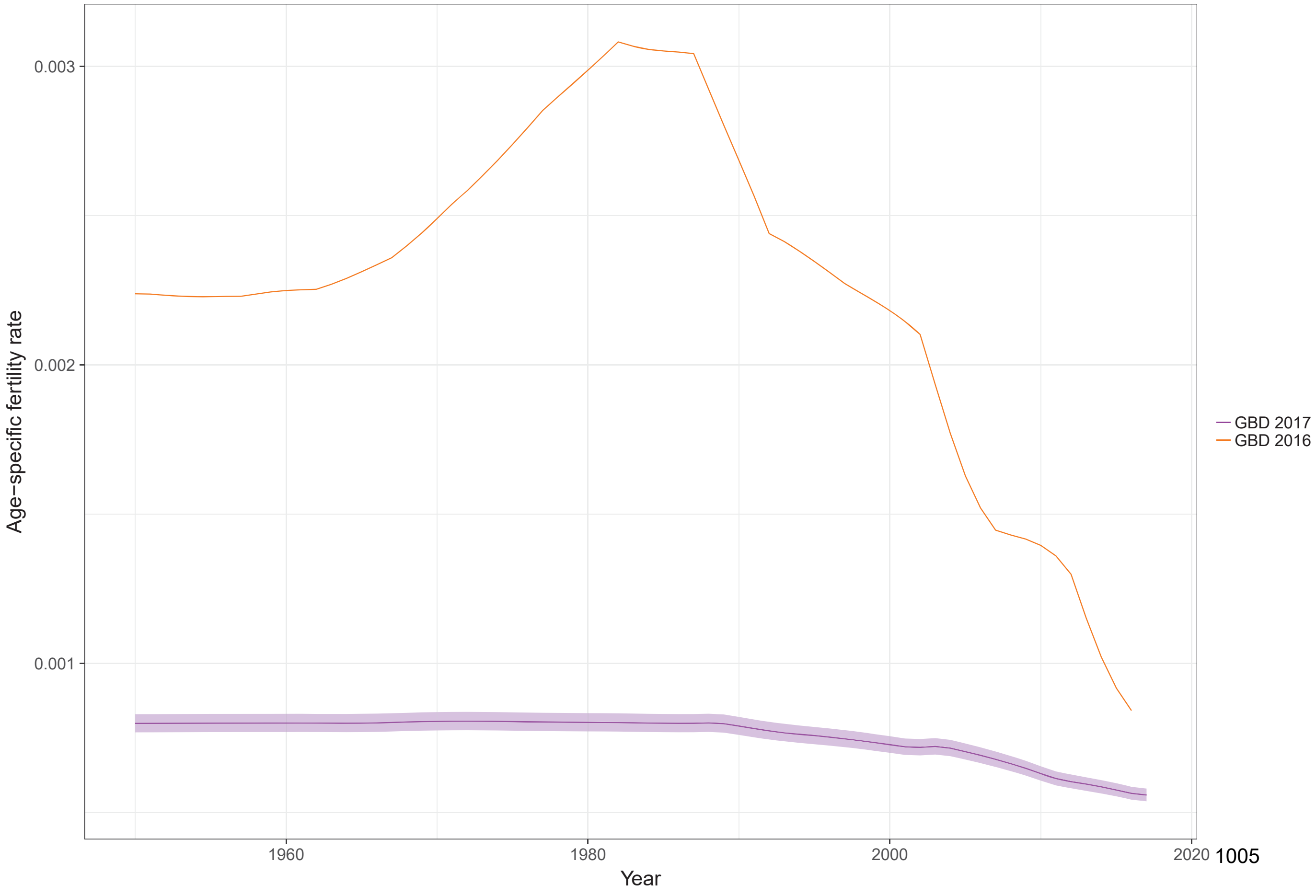

Age−specific fertility fitted trend lines, 1950−2017: Zambia, TFR
Data points enclosed with a diamond are reference sources

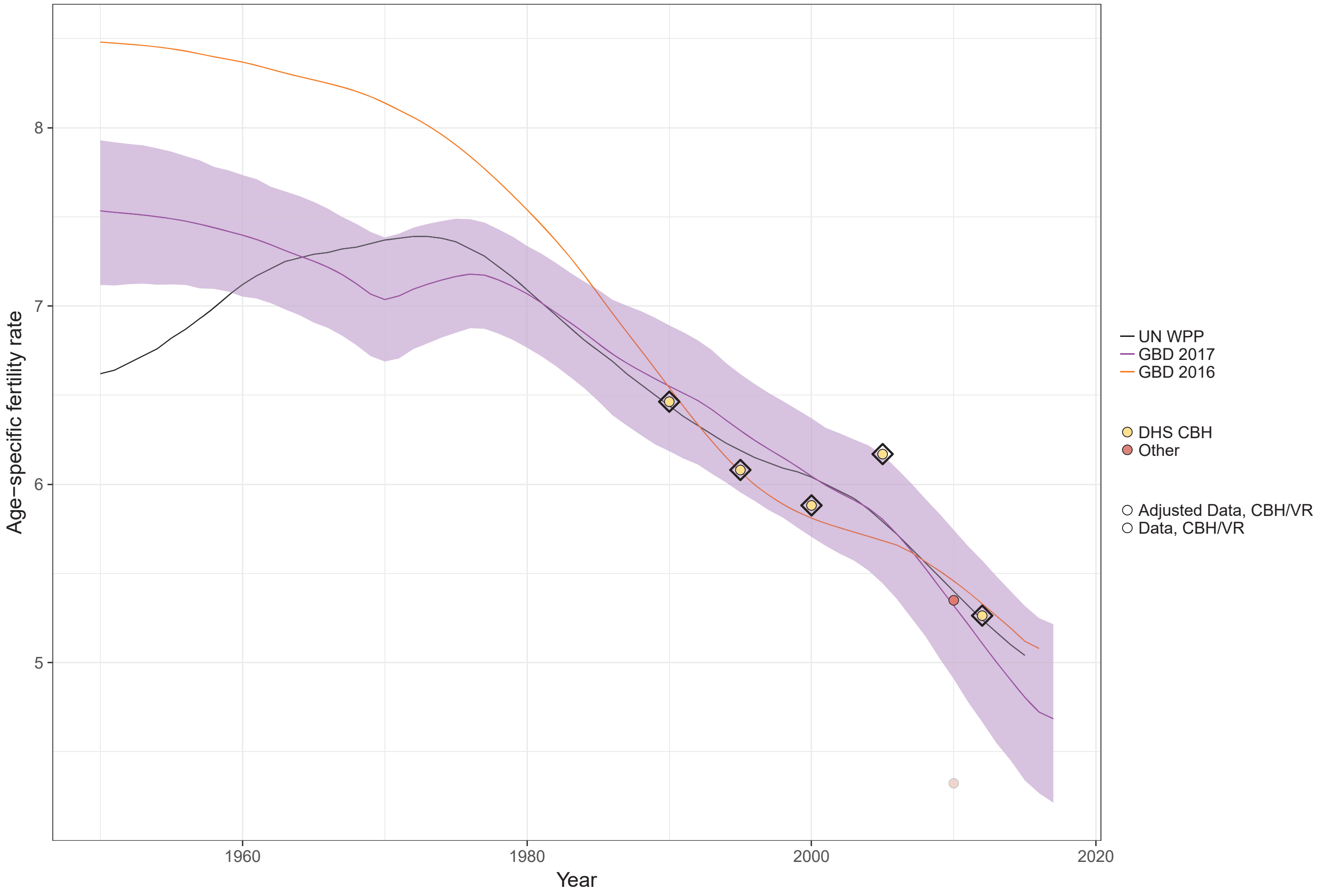

Age−specific fertility fitted trend lines, 1950−2017: Zambia, Ages: 10−14
Data points enclosed with a diamond are reference sources

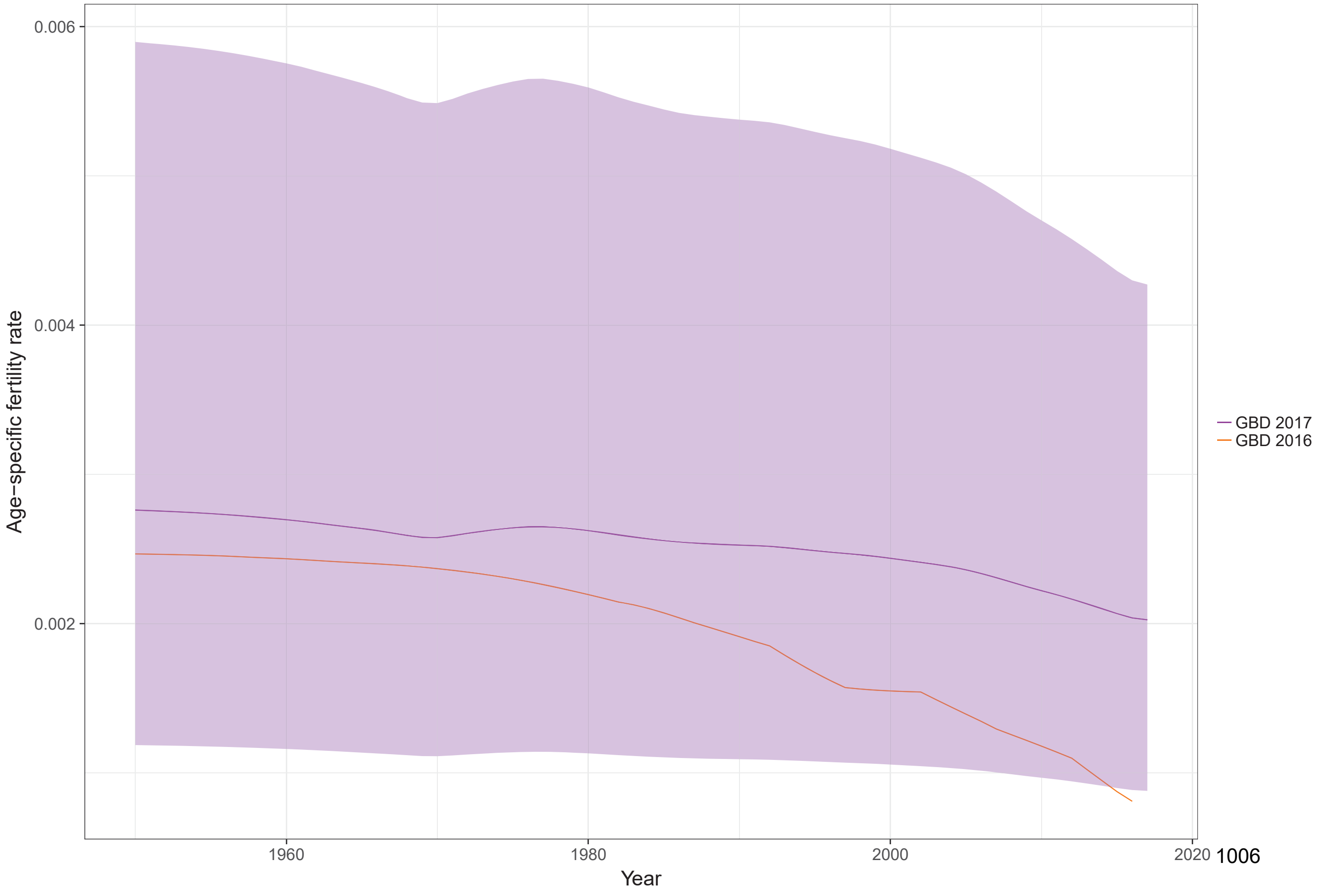

Age−specific fertility fitted trend lines, 1950−2017: Zambia, Ages: 15−19
Data points enclosed with a diamond are reference sources

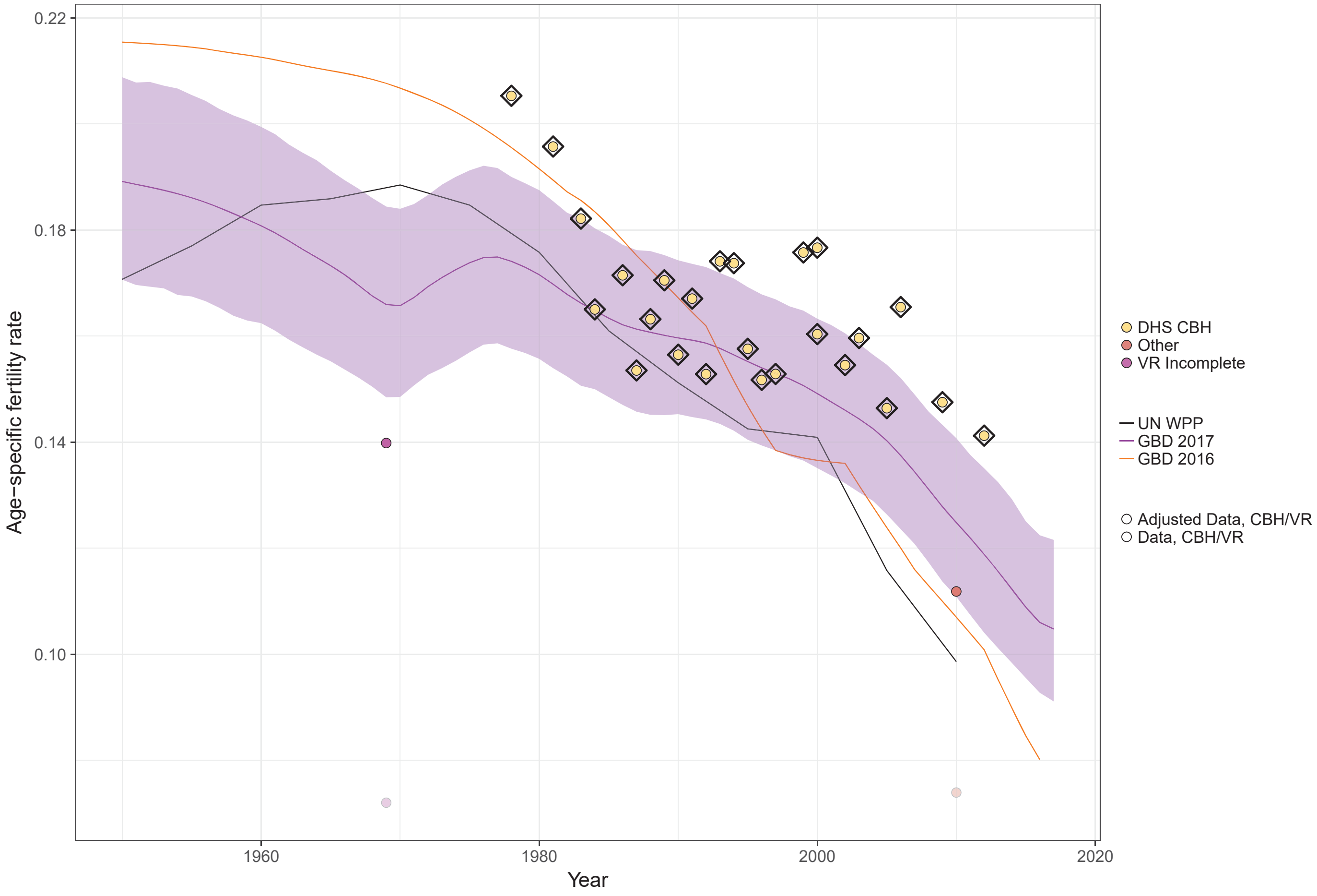

Age−specific fertility fitted trend lines, 1950−2017: Zambia, Ages: 20−24
Data points enclosed with a diamond are reference sources

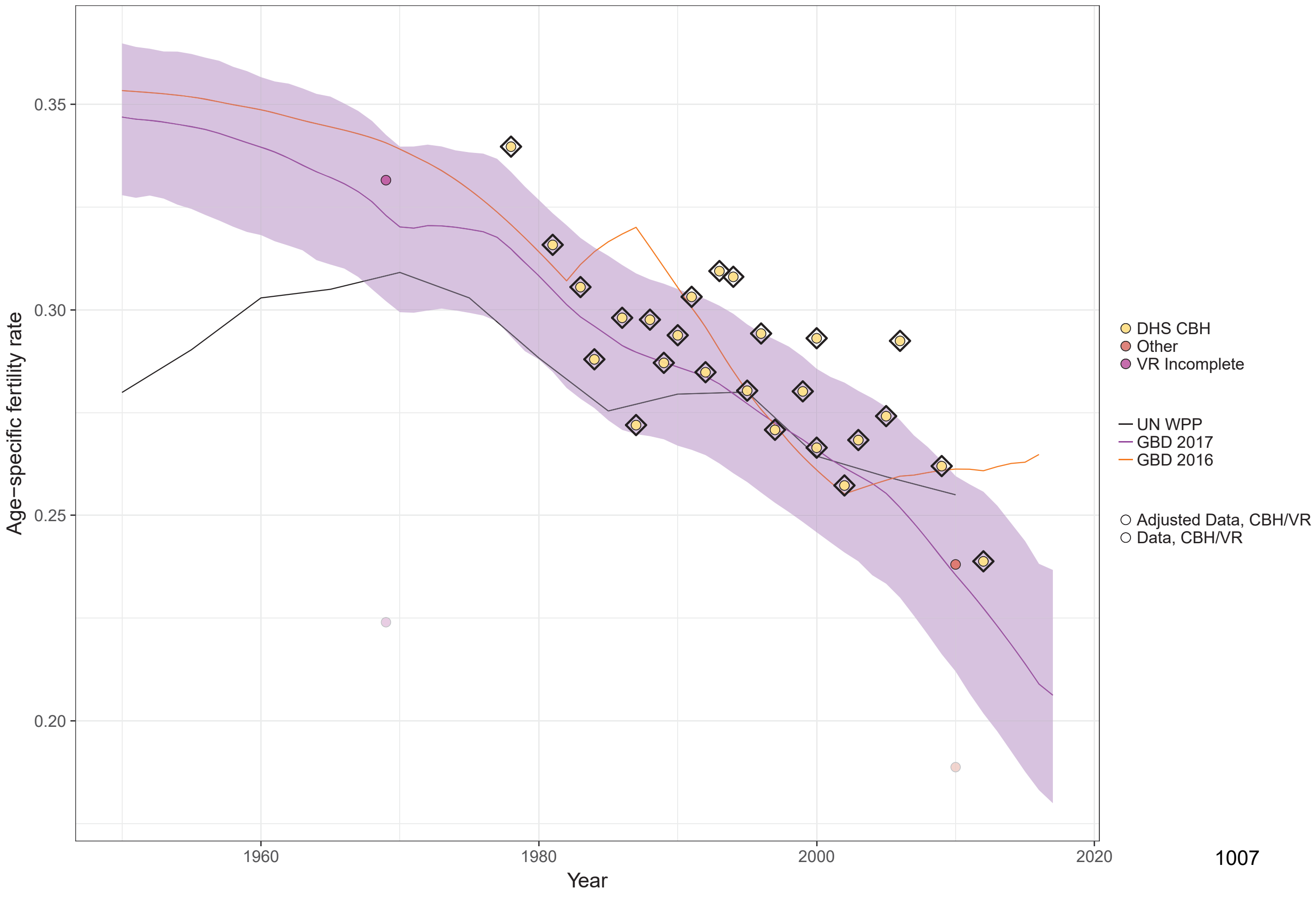

Age−specific fertility fitted trend lines, 1950−2017: Zambia, Ages: 25−29
Data points enclosed with a diamond are reference sources

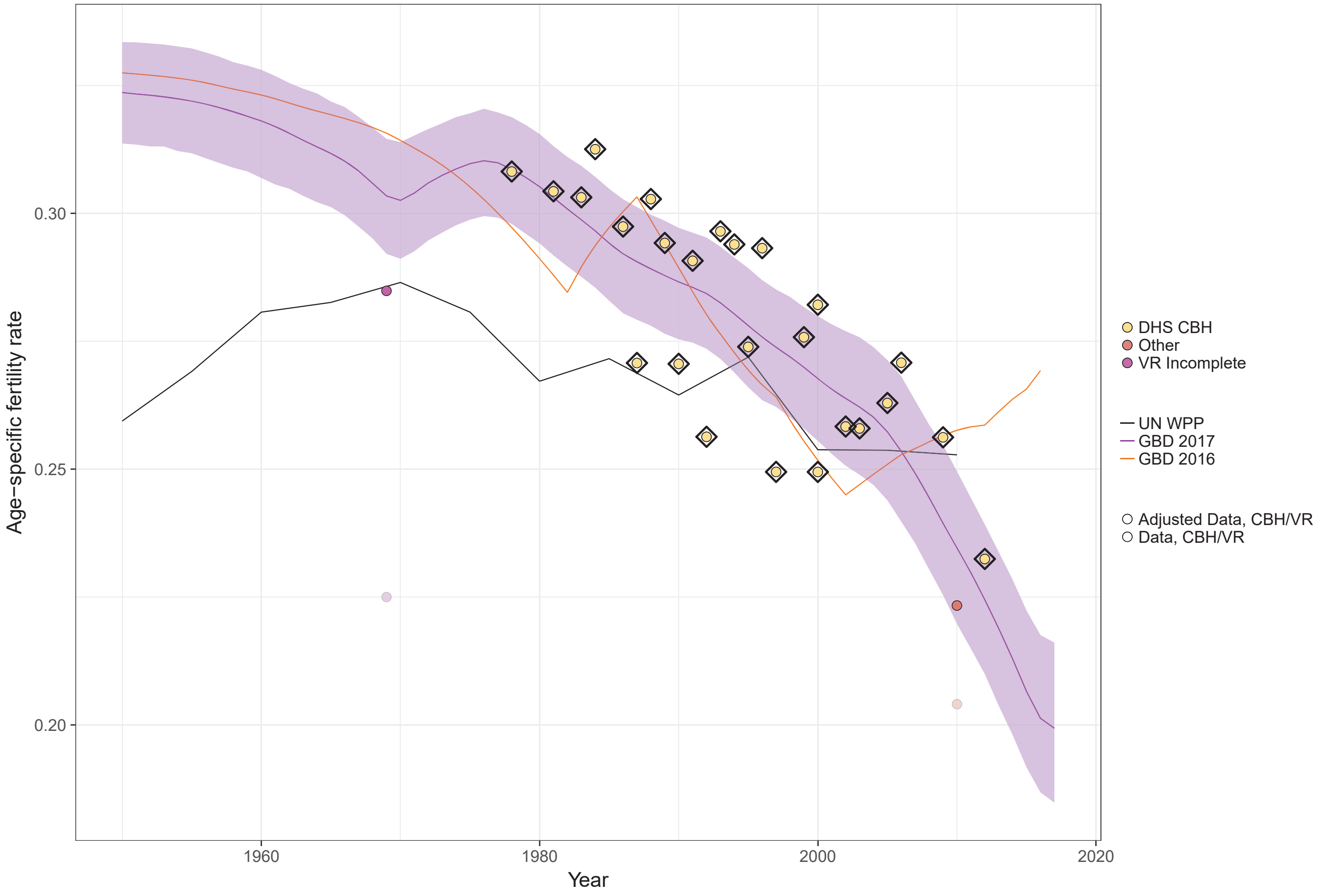

Age−specific fertility fitted trend lines, 1950−2017: Zambia, Ages: 30−34
Data points enclosed with a diamond are reference sources

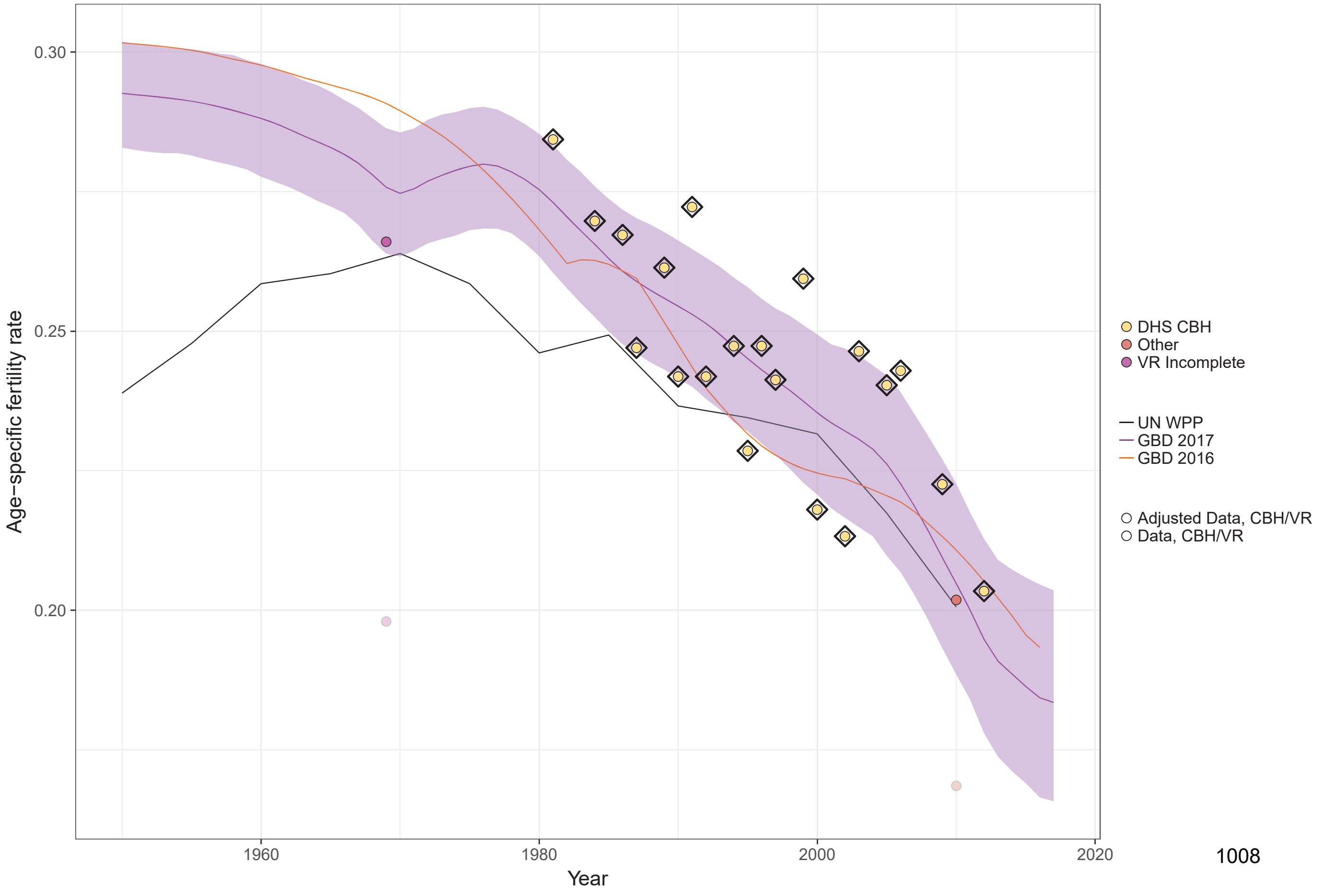

Age−specific fertility fitted trend lines, 1950−2017: Zambia, Ages: 35−39
Data points enclosed with a diamond are reference sources

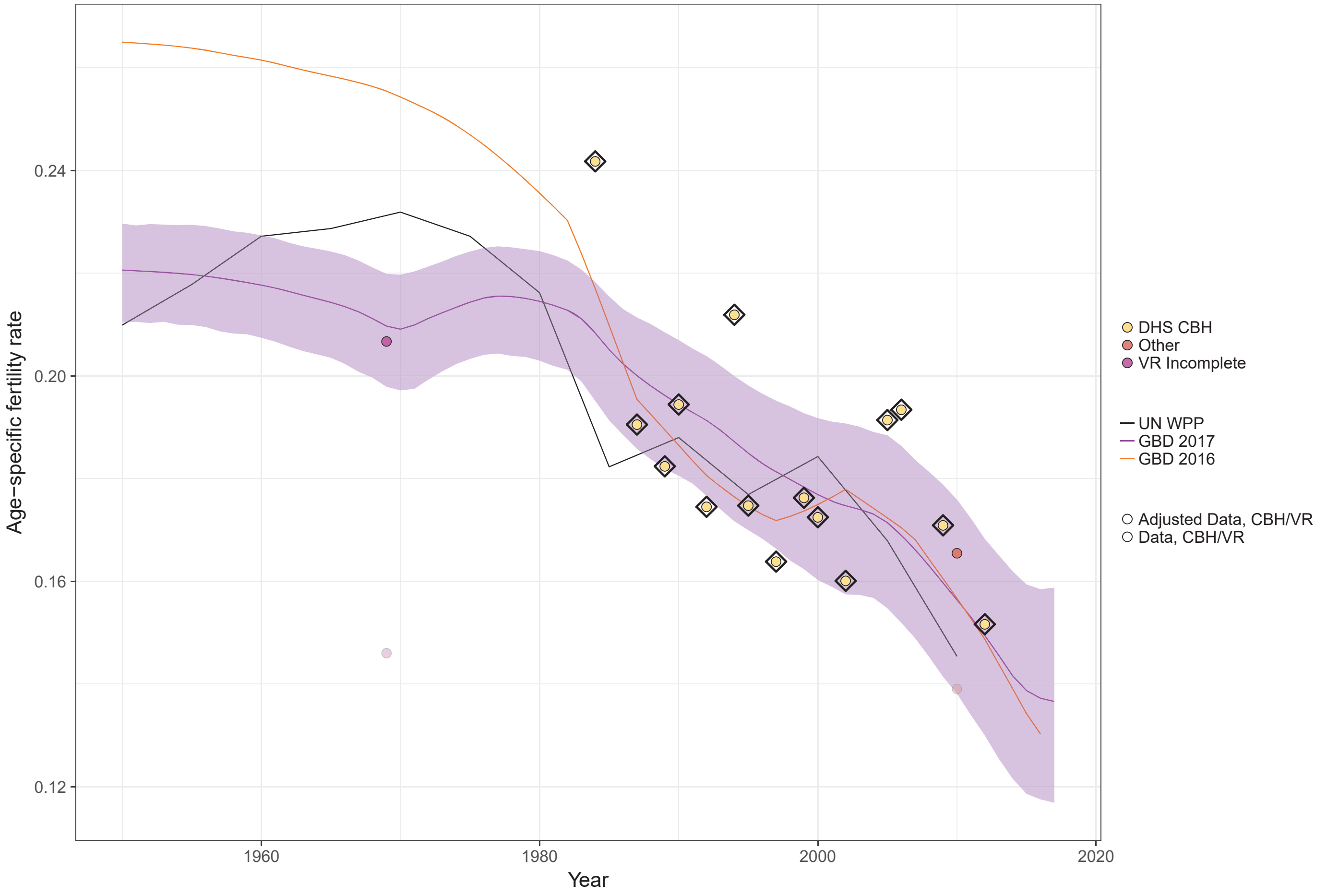

Age−specific fertility fitted trend lines, 1950−2017: Zambia, Ages: 40−44
Data points enclosed with a diamond are reference sources

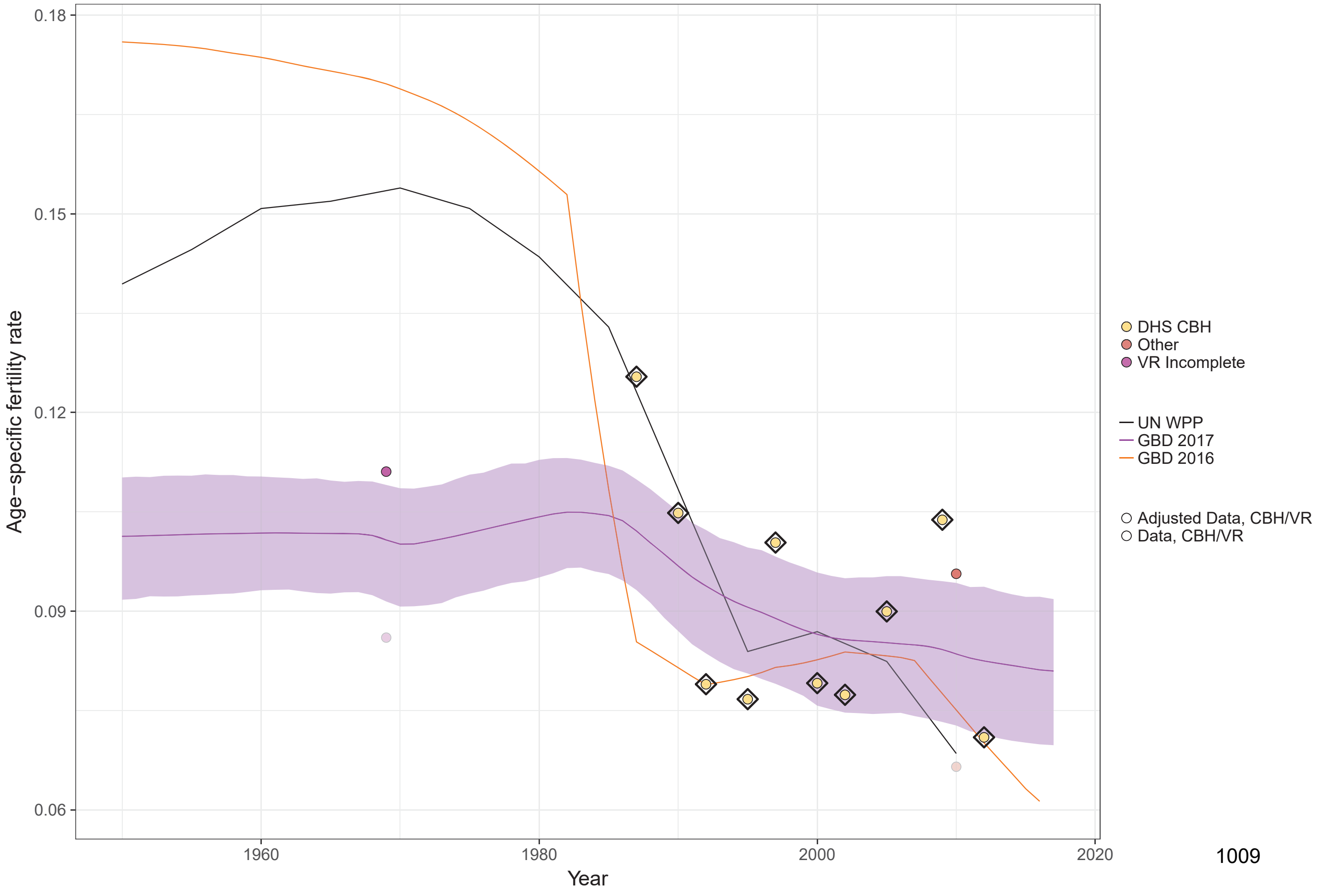

Age−specific fertility fitted trend lines, 1950−2017: Zambia, Ages: 45−49
Data points enclosed with a diamond are reference sources

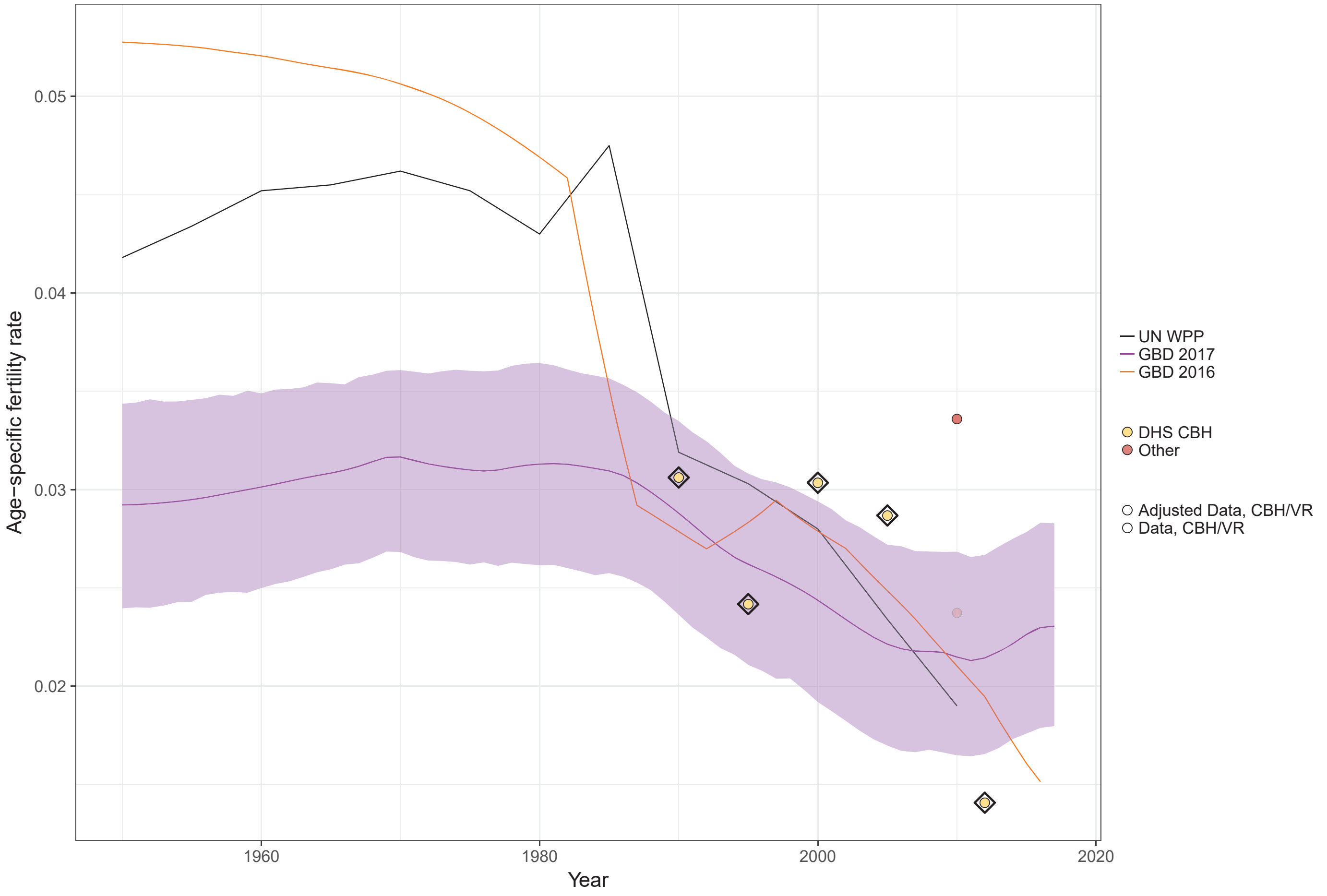

Age−specific fertility fitted trend lines, 1950−2017: Zambia, Ages: 50−54
Data points enclosed with a diamond are reference sources

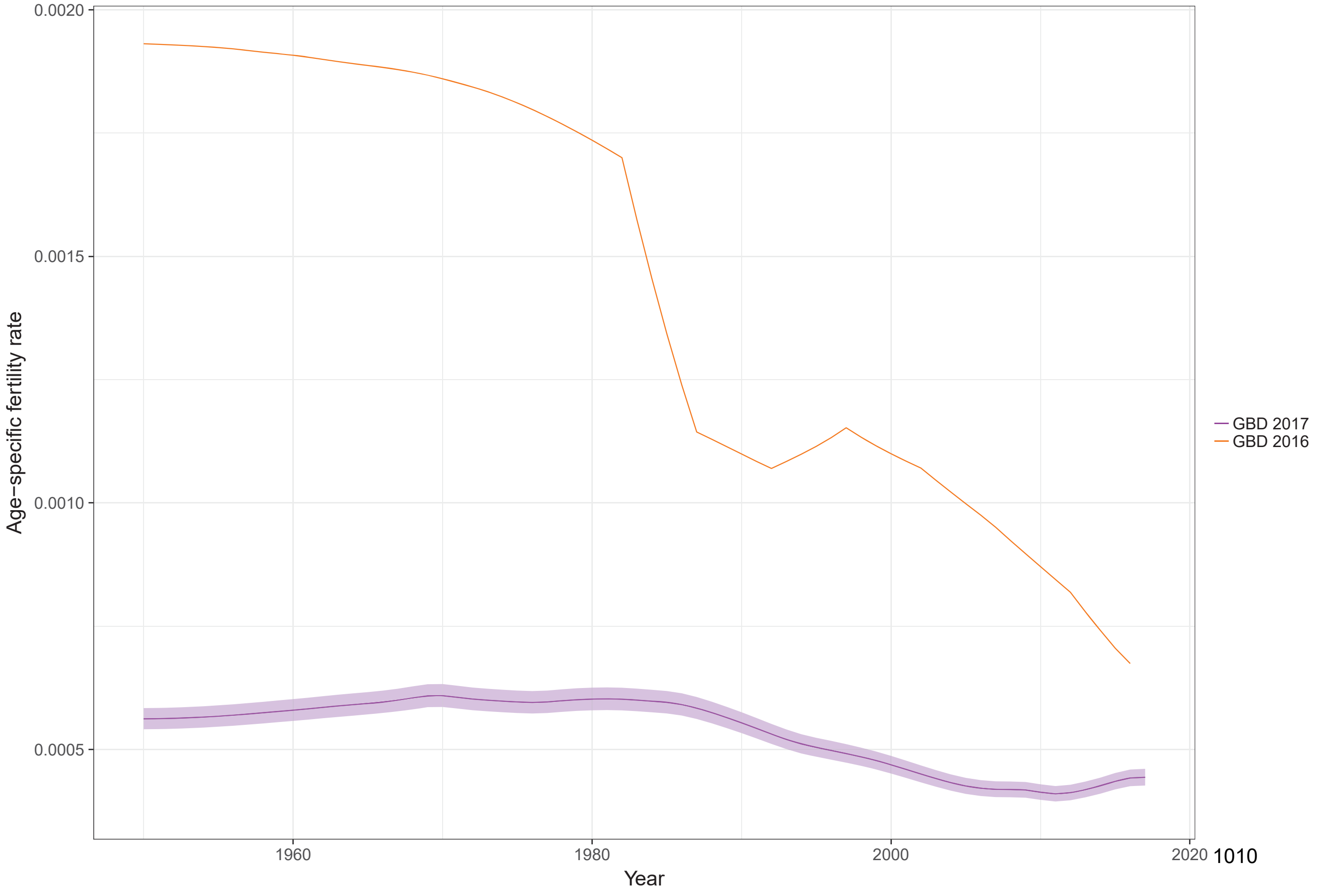

Age−specific fertility fitted trend lines, 1950−2017: Zimbabwe, TFR
Data points enclosed with a diamond are reference sources

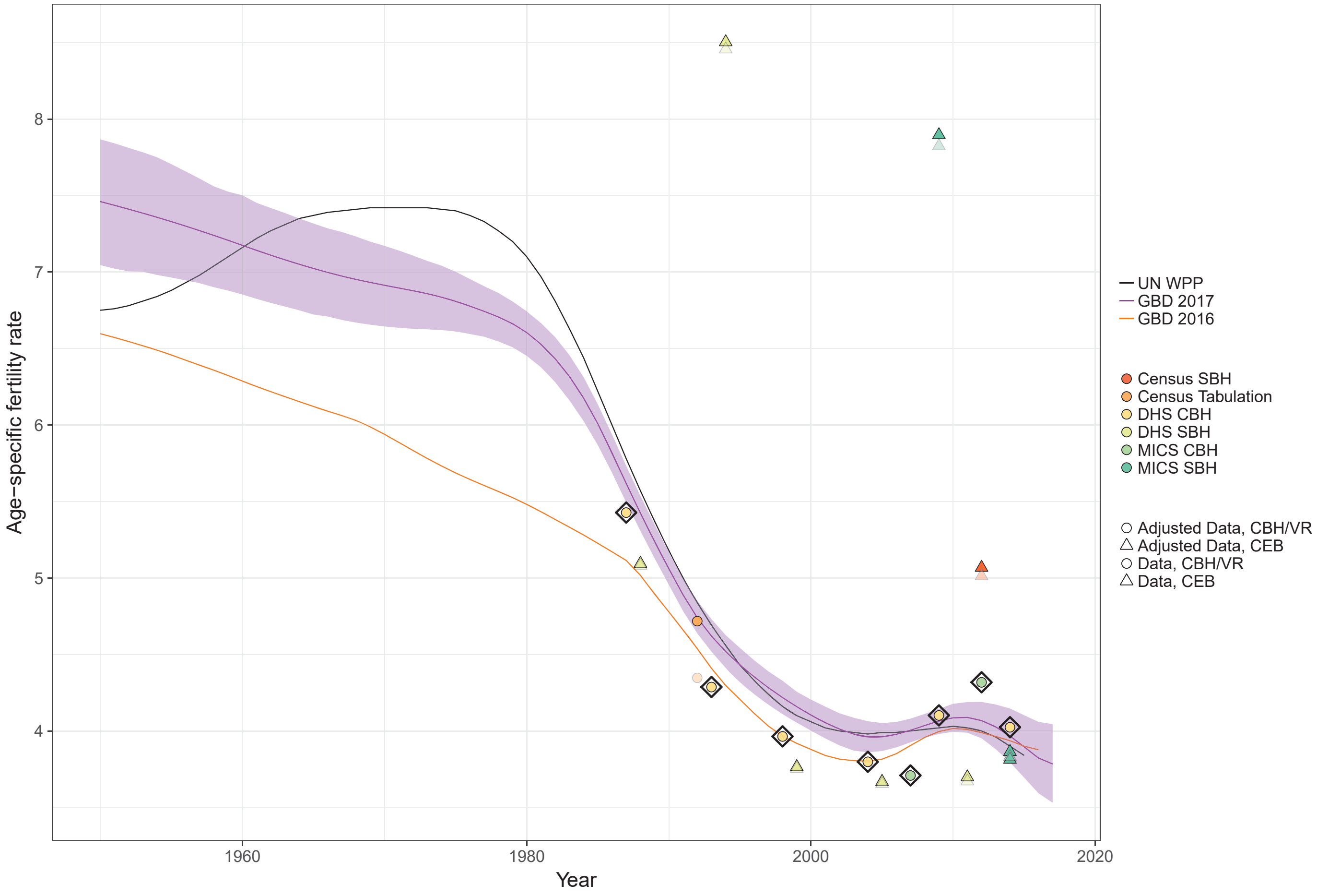

Age−specific fertility fitted trend lines, 1950−2017: Zimbabwe, Ages: 10−14
Data points enclosed with a diamond are reference sources

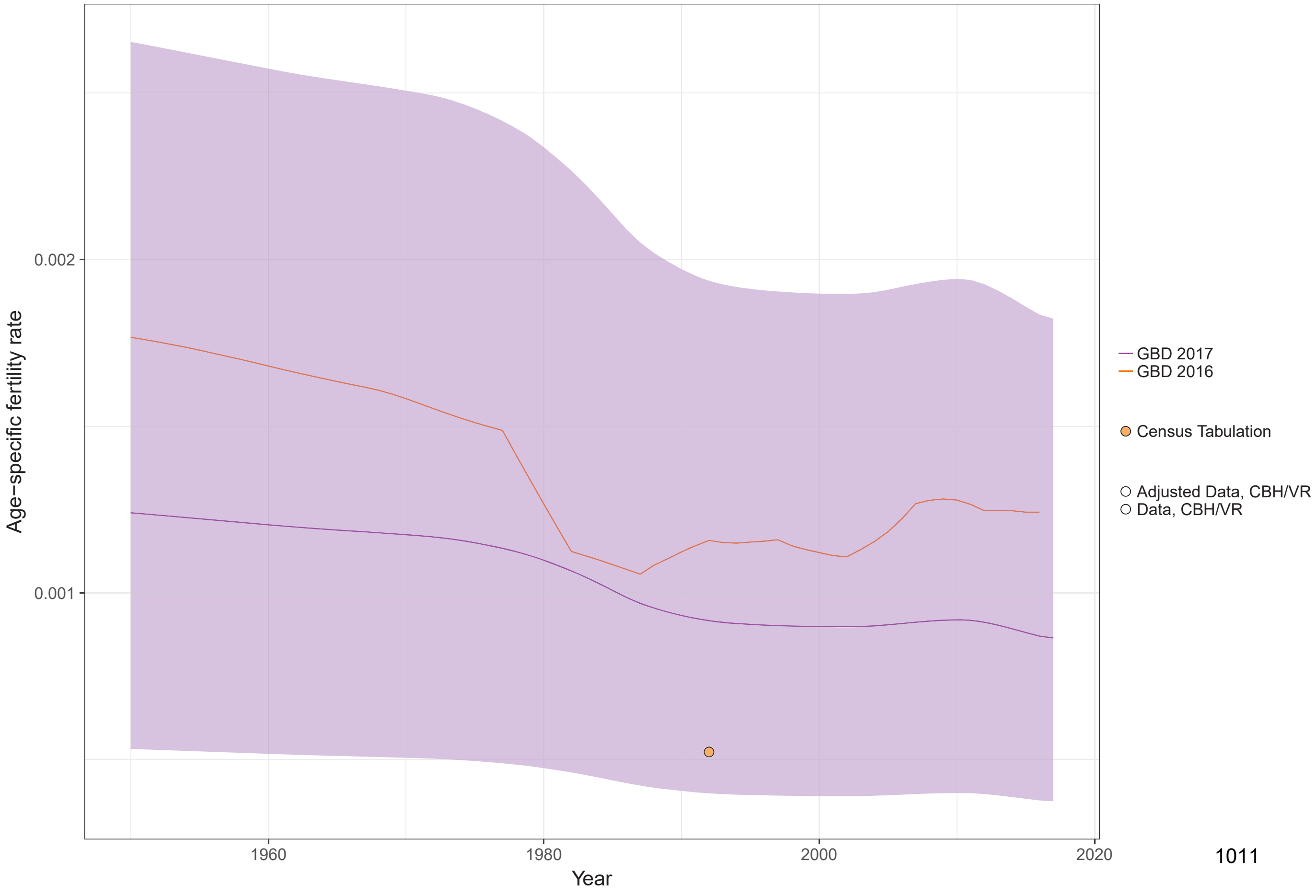

Age−specific fertility fitted trend lines, 1950−2017: Zimbabwe, Ages: 15−19
Data points enclosed with a diamond are reference sources

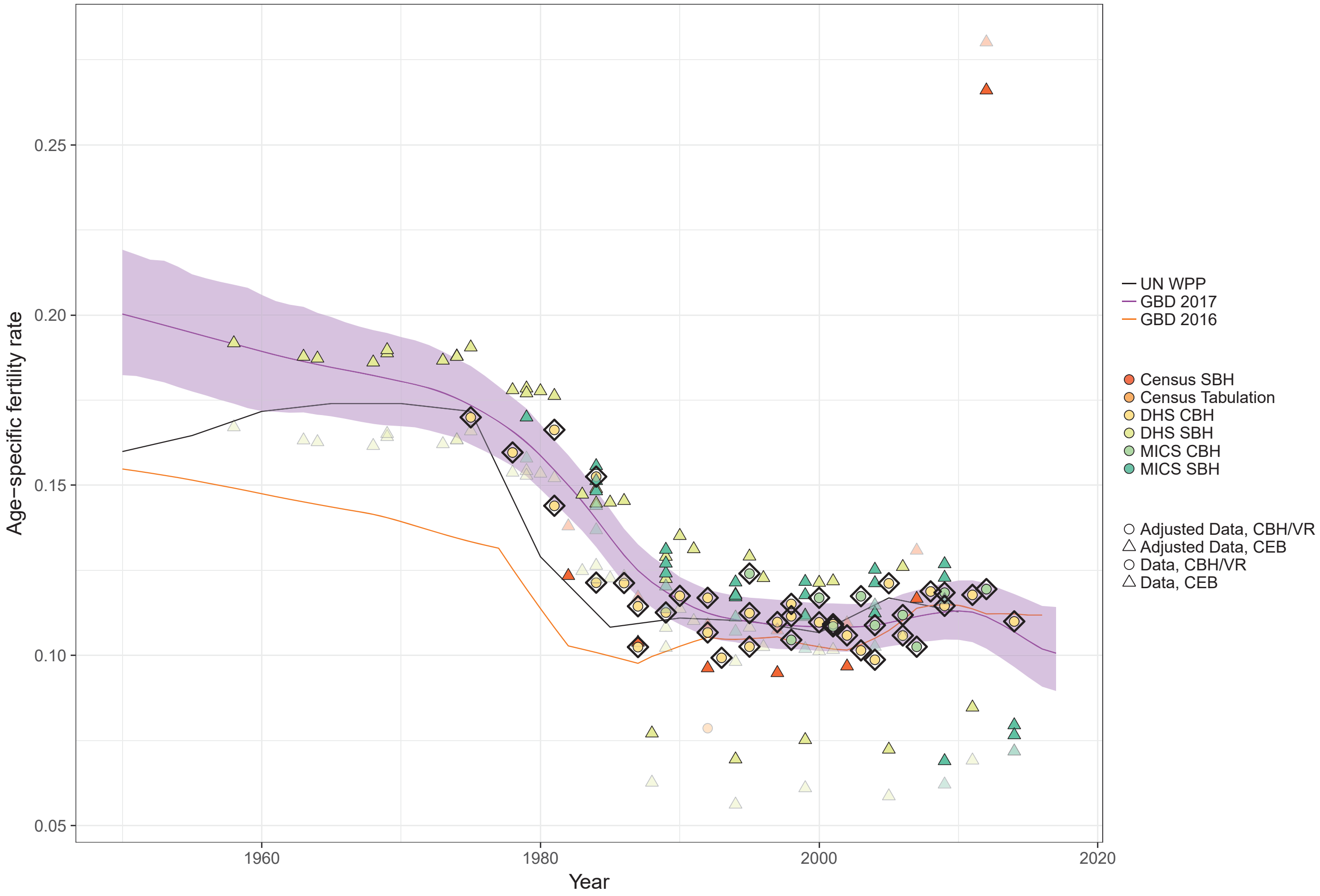

Age−specific fertility fitted trend lines, 1950−2017: Zimbabwe, Ages: 20−24
Data points enclosed with a diamond are reference sources

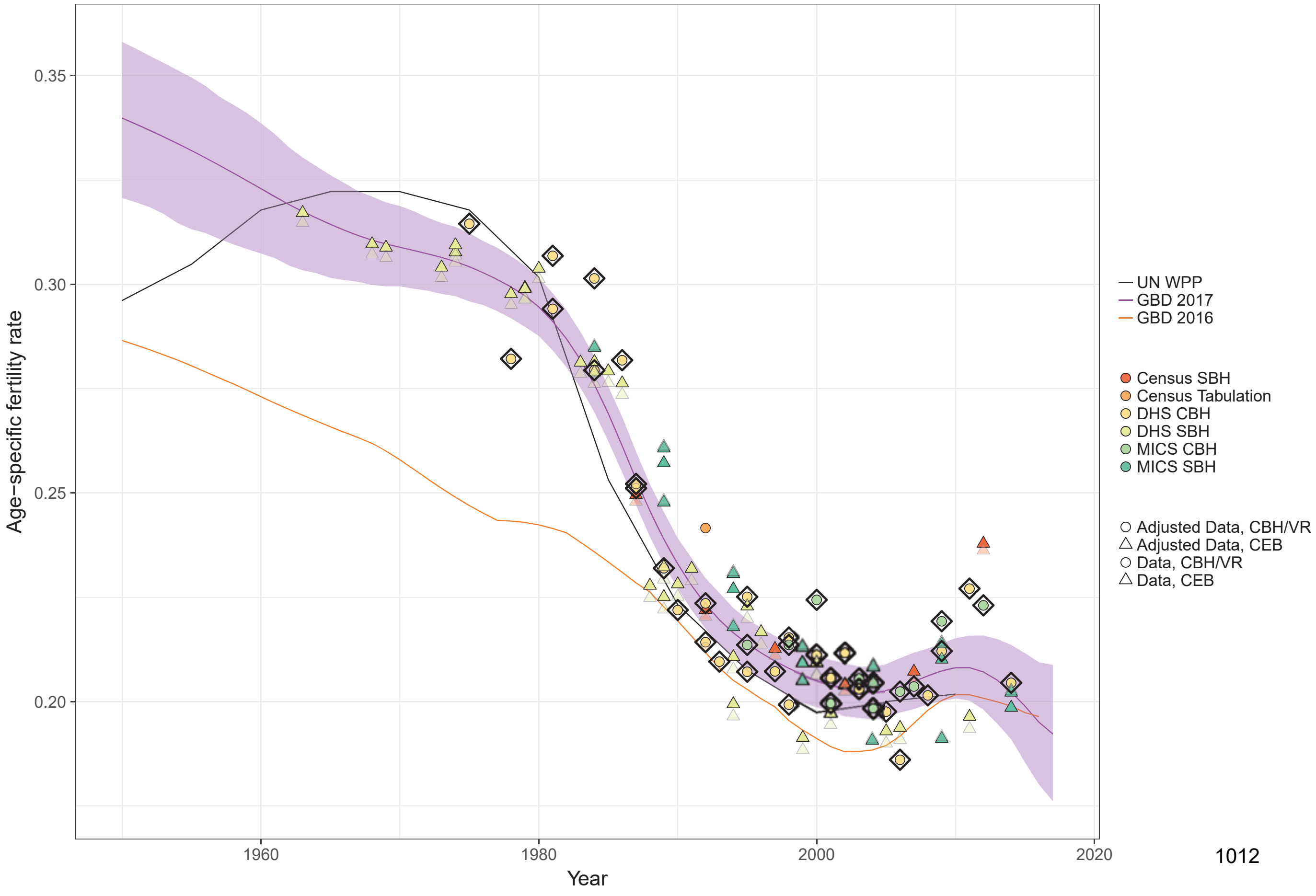

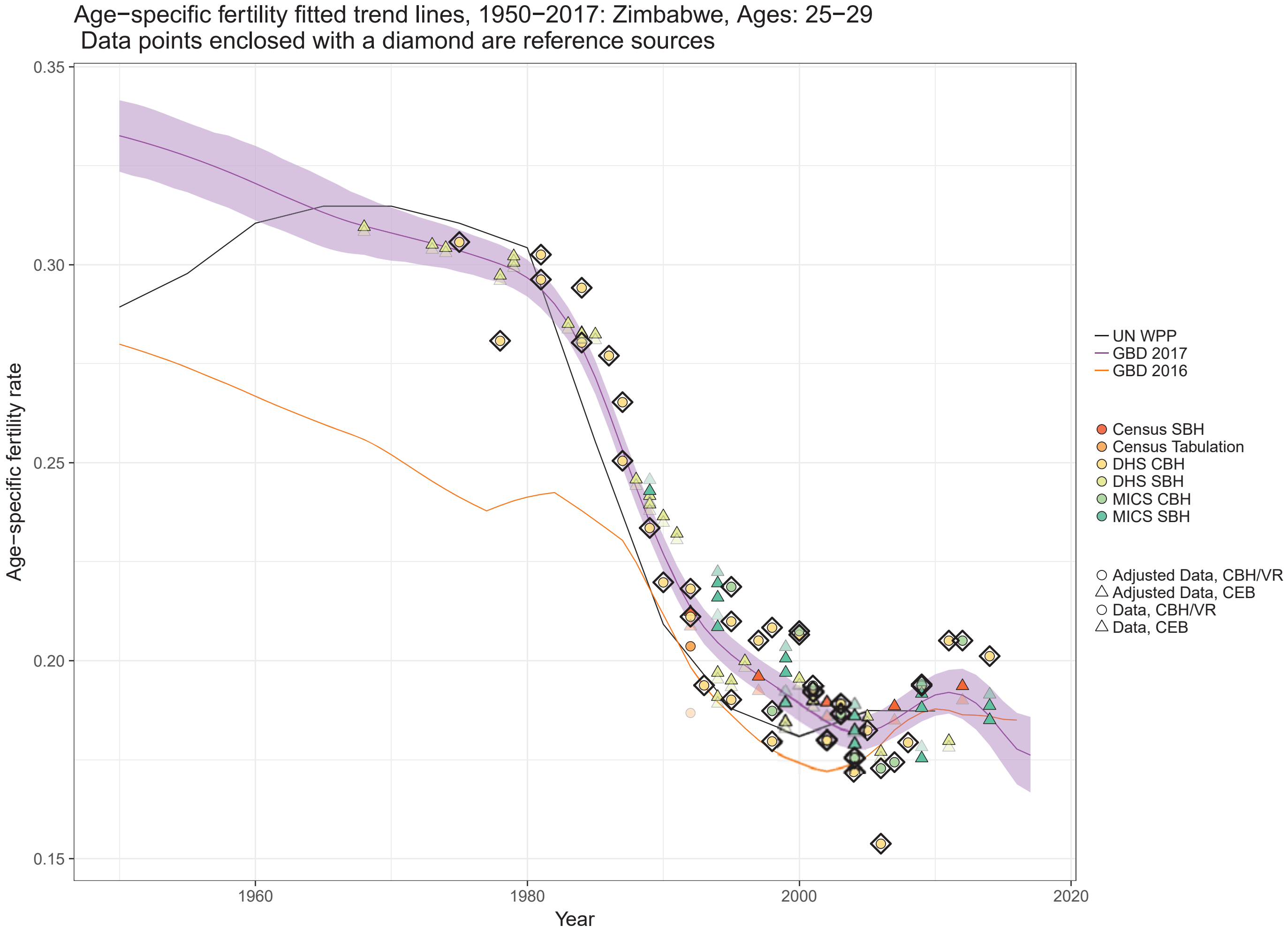

Age−specific fertility fitted trend lines, 1950−2017: Zimbabwe, Ages: 25−29
Data points enclosed with a diamond are reference sources
Age−specific fertility rate
0.35
0.30
0.25
0.20
0.15
1960
1980
2000
2020
Year
UN WPP
GBD 2017
GBD 2016
Census SBH
Census Tabulation
DHS CBH
DHS SBH
MICS CBH
MICS SBH
Adjusted Data, CBH/VR
Adjusted Data, CEB
Data, CBH/VR
Data, CEB

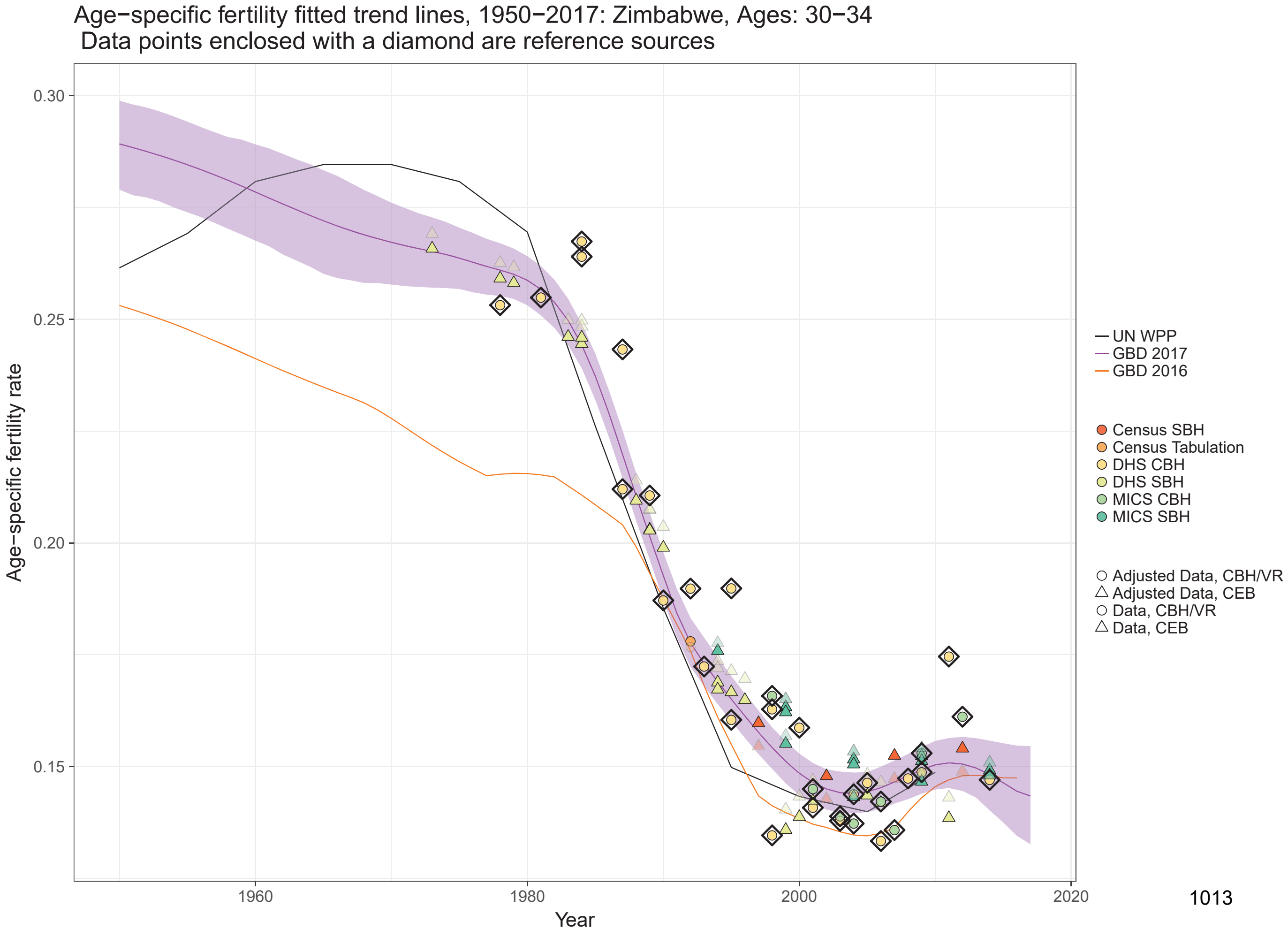

Age−specific fertility fitted trend lines, 1950−2017: Zimbabwe, Ages: 30−34
Data points enclosed with a diamond are reference sources
Age−specific fertility rate
0.30
0.25
0.20
0.15
1960
1980
2000
2020
Year
UN WPP
GBD 2017
GBD 2016
Census SBH
Census Tabulation
DHS CBH
DHS SBH
MICS CBH
MICS SBH
Adjusted Data, CBH/VR
Adjusted Data, CEB
Data, CBH/VR
Data, CEB

Age−specific fertility fitted trend lines, 1950−2017: Zimbabwe, Ages: 35−39
Data points enclosed with a diamond are reference sources

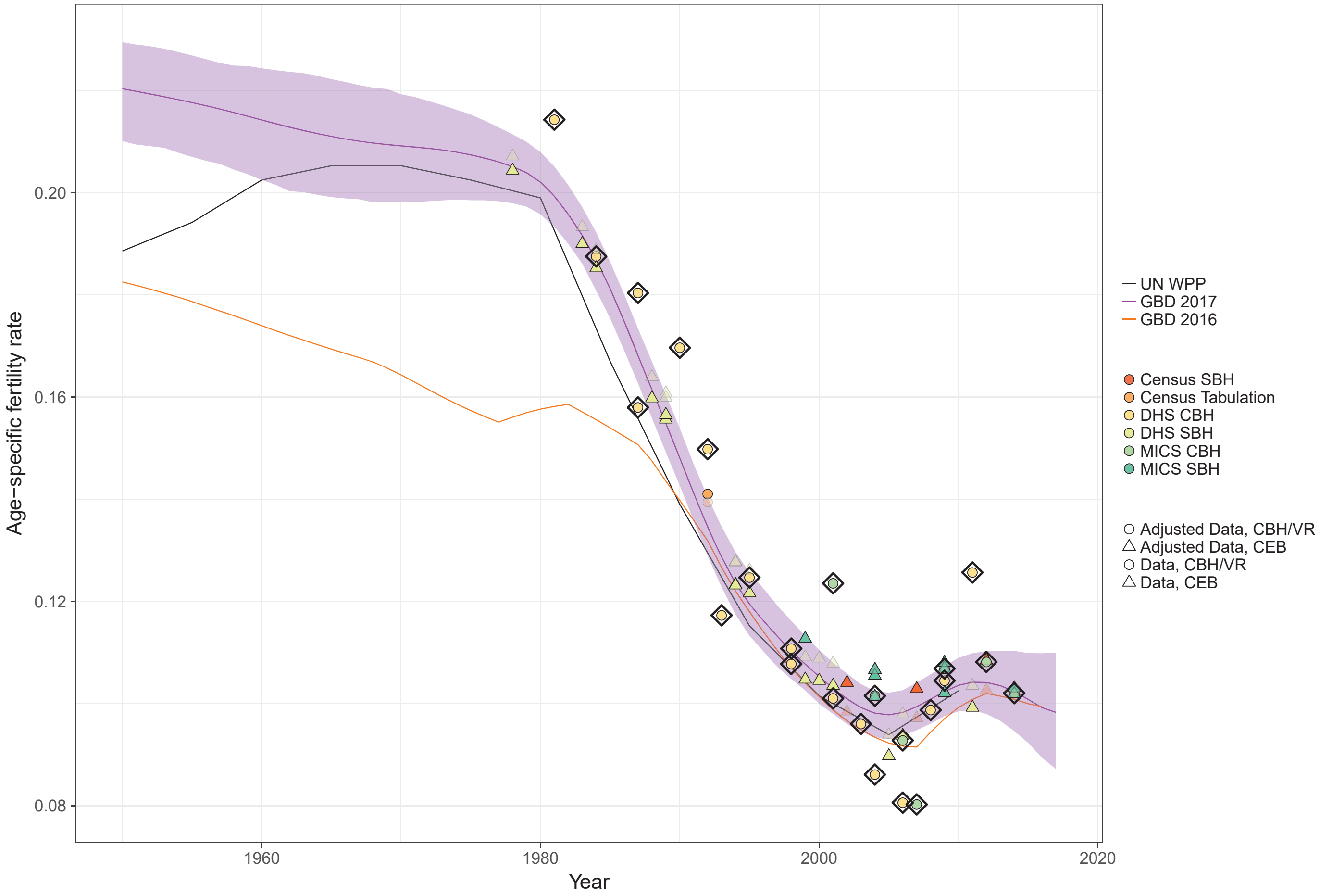

Age−specific fertility fitted trend lines, 1950−2017: Zimbabwe, Ages: 40−44
Data points enclosed with a diamond are reference sources

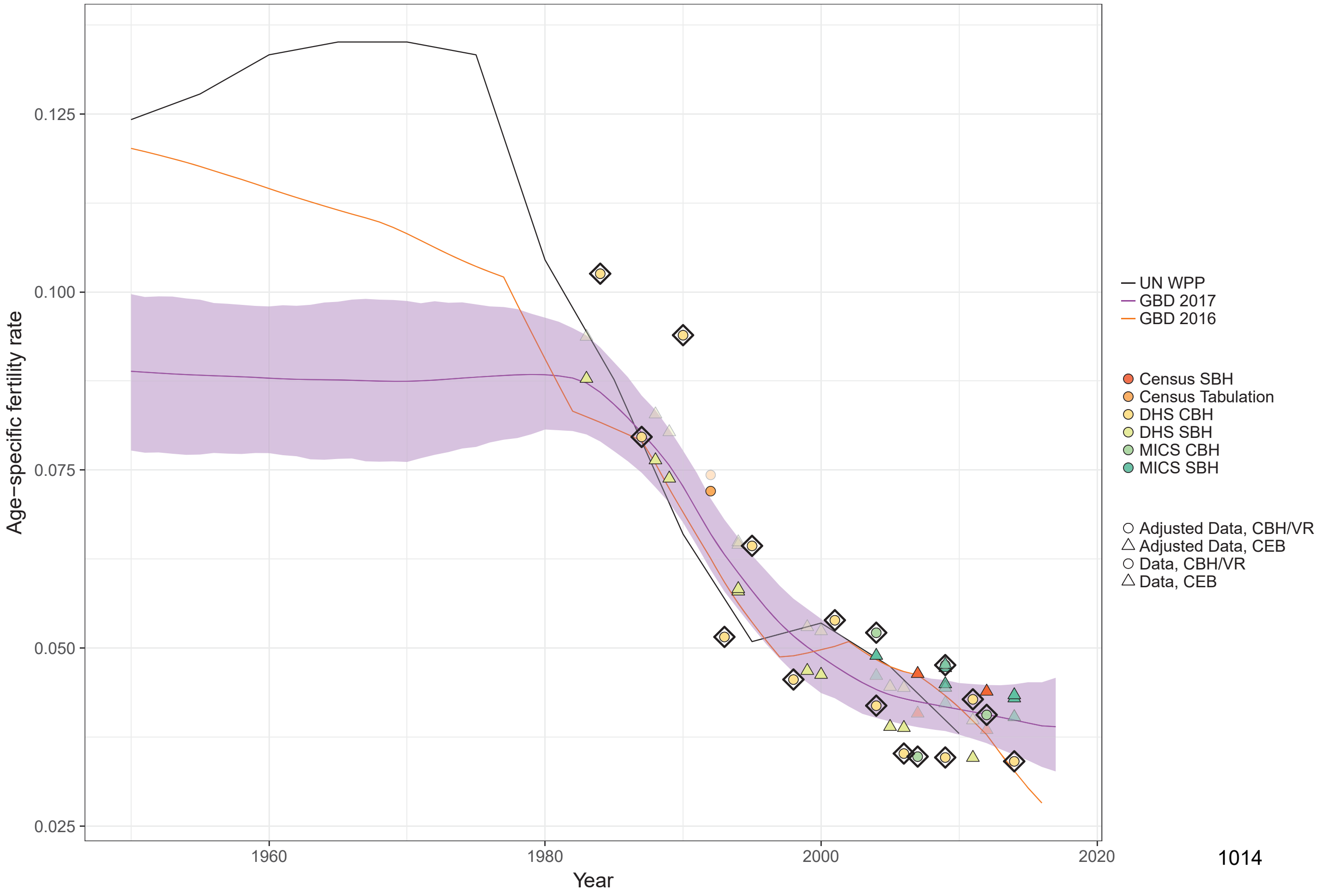

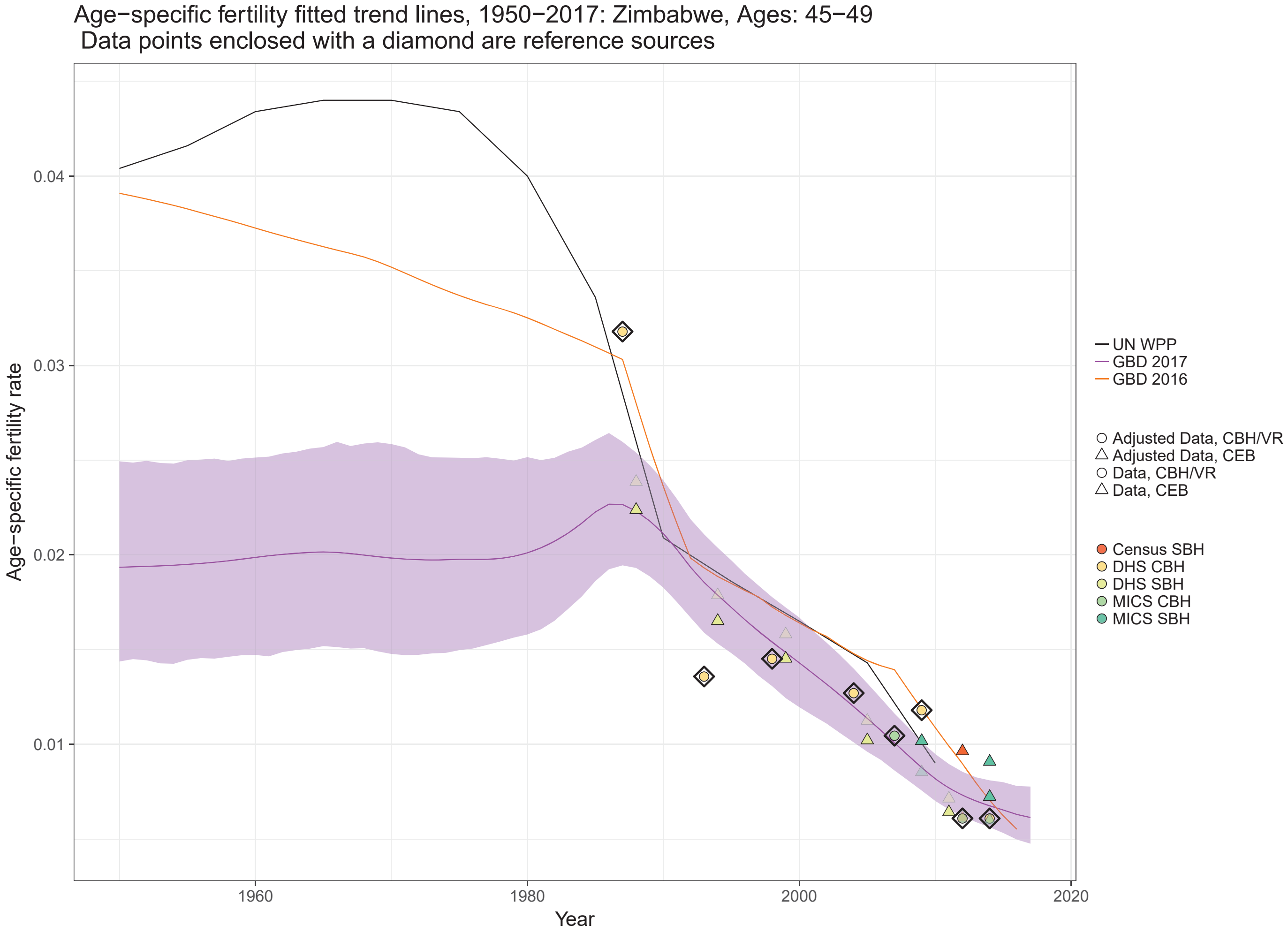
Age−specific fertility fitted trend lines, 1950−2017: Zimbabwe, Ages: 45−49
Data points enclosed with a diamond are reference sources
Age−specific fertility rate
0.04
0.03
0.02
0.01
1960
1980
2000
2020
Year
UN WPP
GBD 2017
GBD 2016
Adjusted Data, CBH/VR
Adjusted Data, CEB
Data, CBH/VR
Data, CEB
Census SBH
DHS CBH
DHS SBH
MICS CBH
MICS SBH

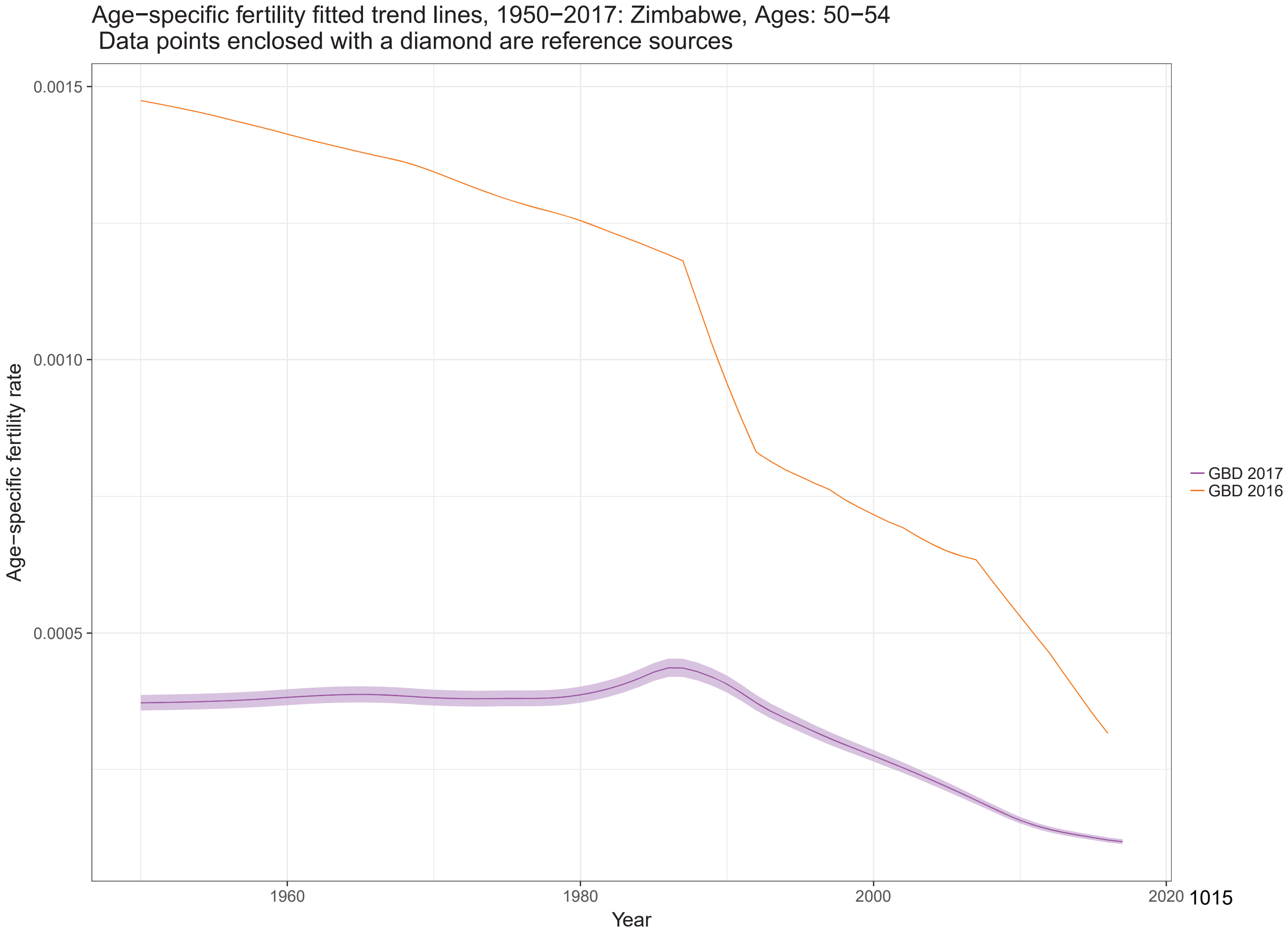
Age−specific fertility fitted trend lines, 1950−2017: Zimbabwe, Ages: 50−54
Data points enclosed with a diamond are reference sources
Age−specific fertility rate
0.0015
0.0010
0.0005
1960
1980
2000
2020
Year
GBD 2017
GBD 2016

Supplemental Results Figure 4: GBD Bayesian Demographic Balancing Model fit in Afghanistan for (a) total population of males, and (b) total population of females, 1950−2017.

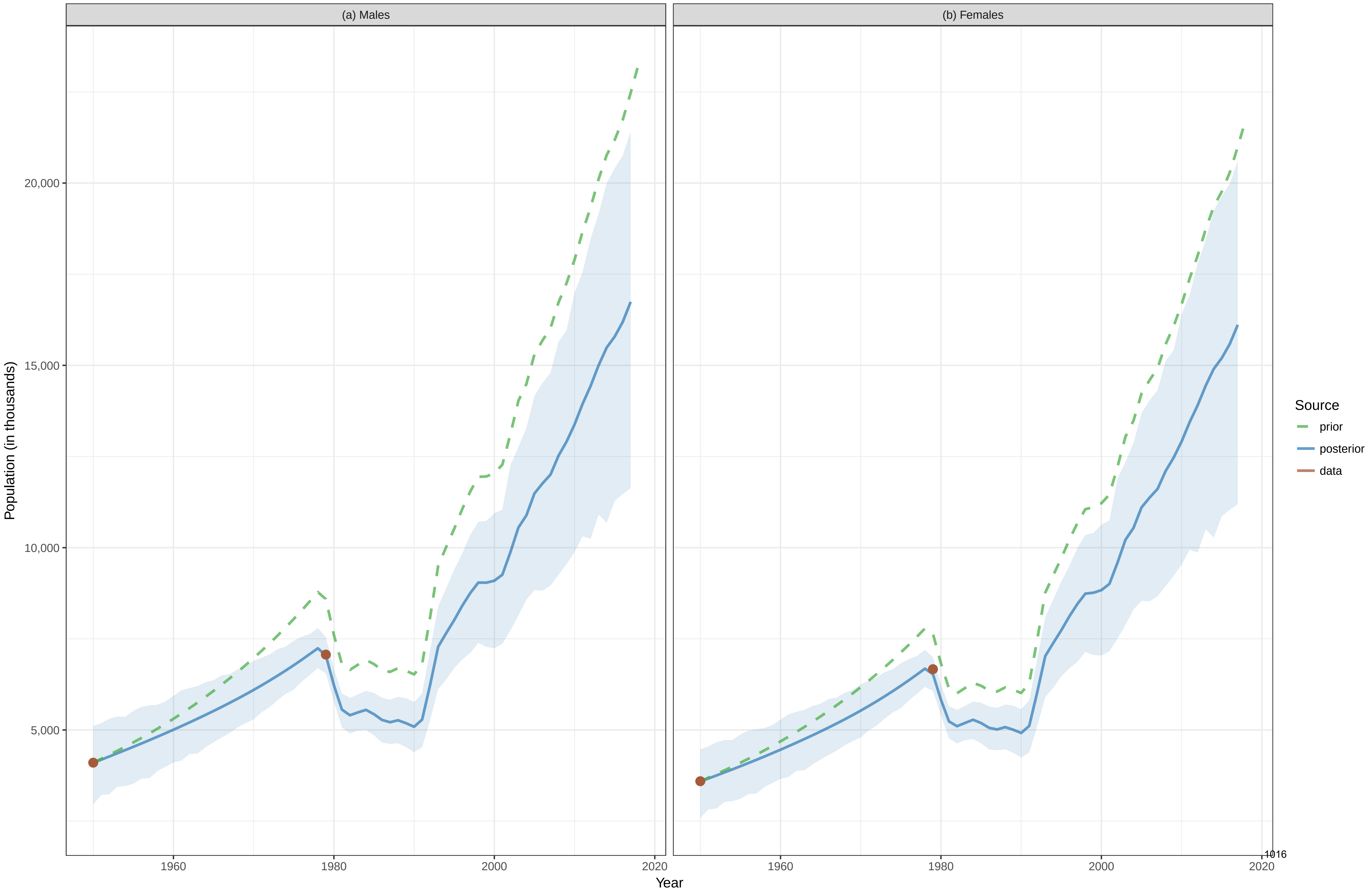

Supplemental Results Figure 4: GBD Bayesian Demographic Balancing Model fit in Albania for (a) total population of males, and (b) total population of females, 1950−2017.

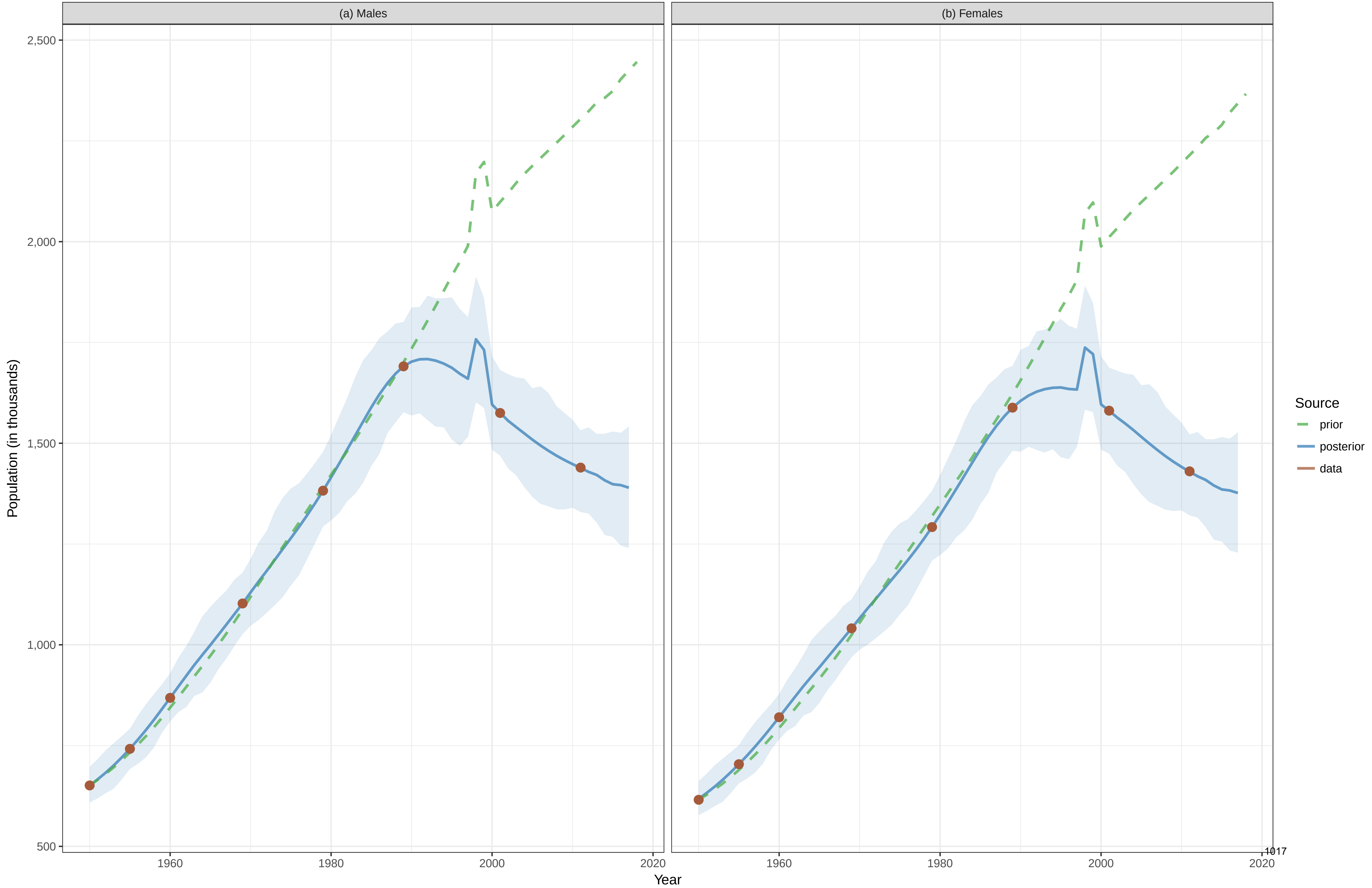

Supplemental Results Figure 4: GBD Bayesian Demographic Balancing Model fit in Algeria for (a) total population of males, and (b) total population of females, 1950−2017.

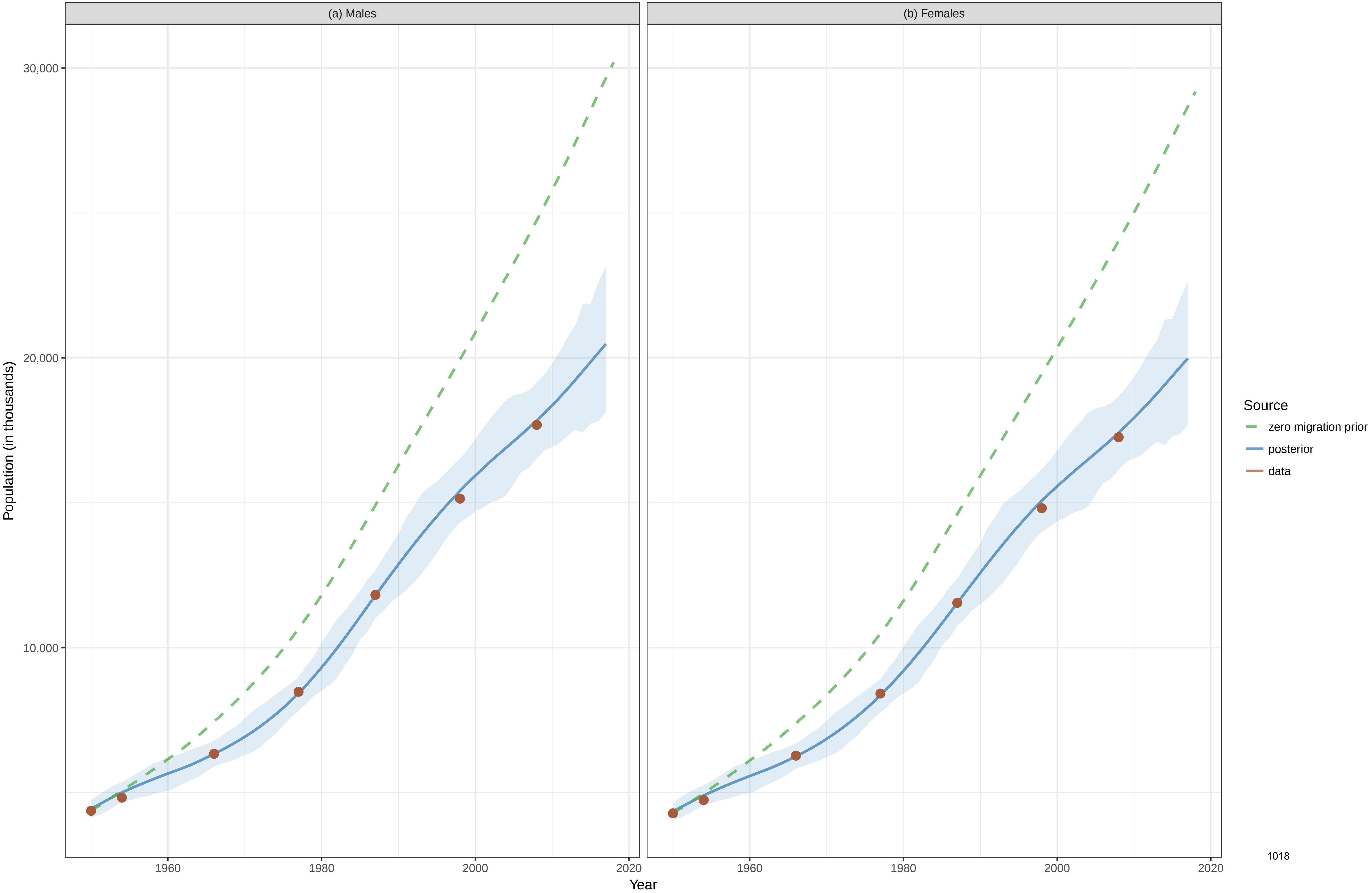

Supplemental Results Figure 4: GBD Bayesian Demographic Balancing Model fit in American Samoa for (a) total population of males, and (b) total population of females, 1950−2017.

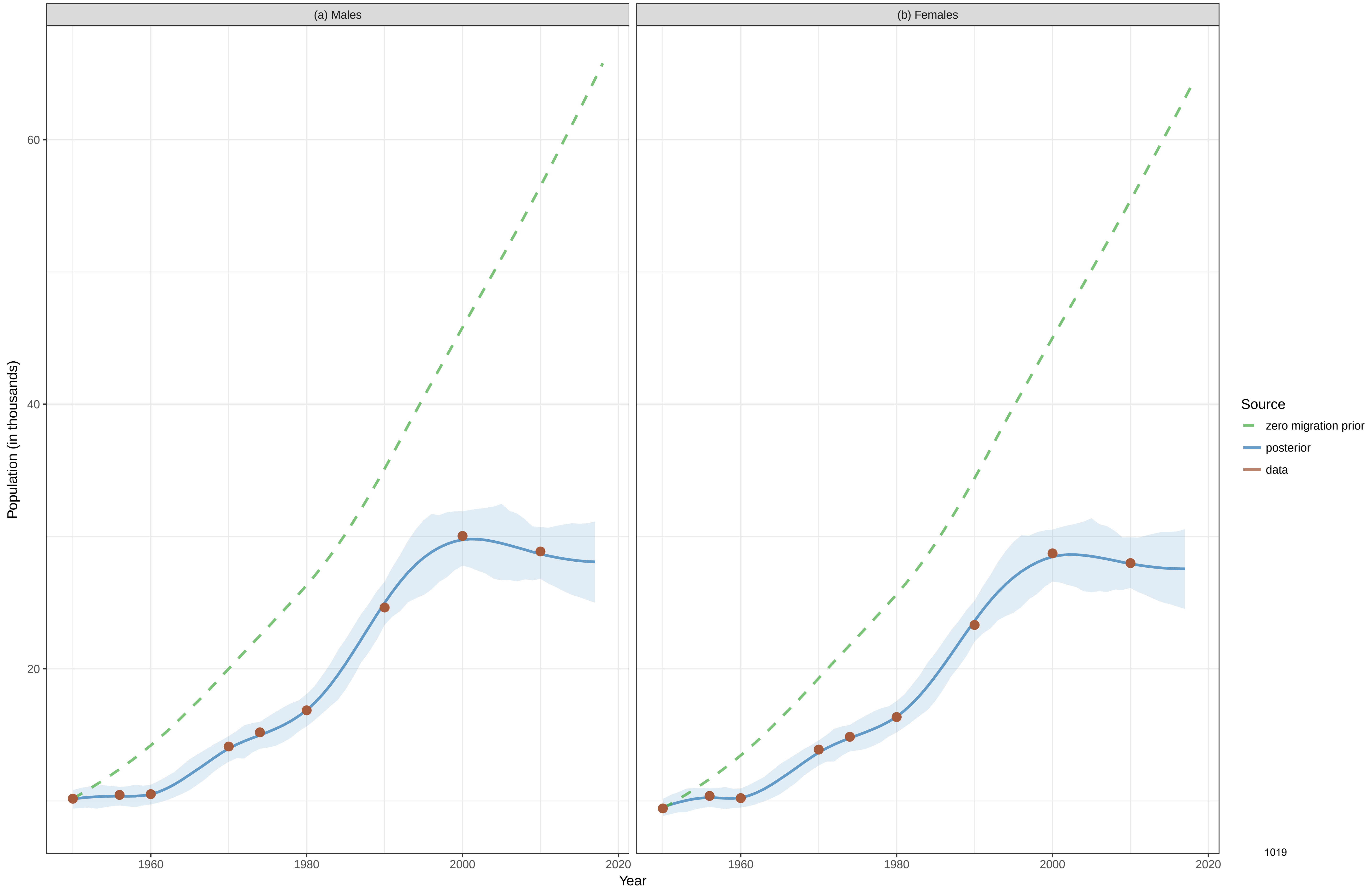

Supplemental Results Figure 4: GBD Bayesian Demographic Balancing Model fit in Andorra for (a) total population of males, and (b) total population of females, 1950−2017.

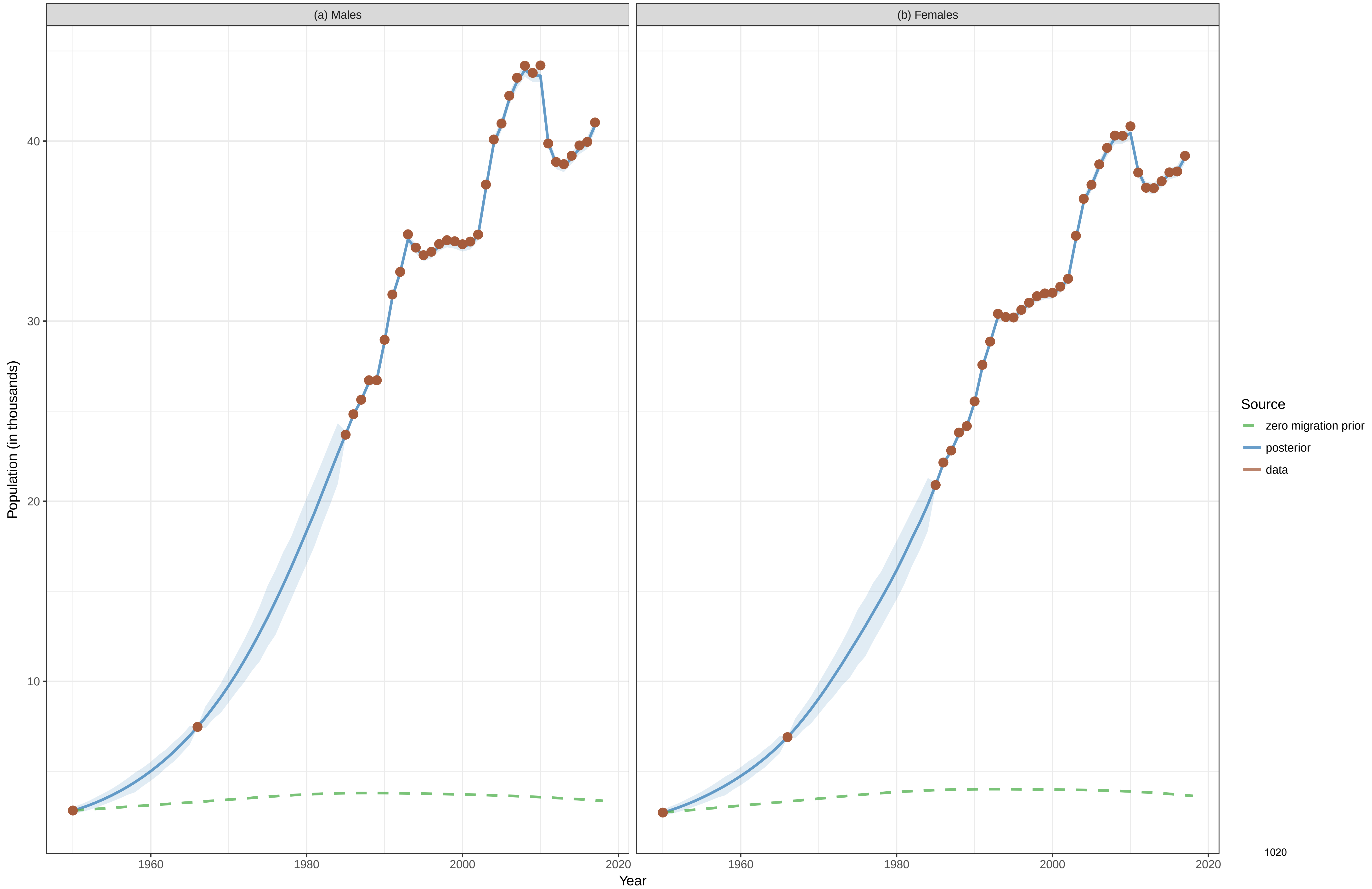

Supplemental Results Figure 4: GBD Bayesian Demographic Balancing Model fit in Angola for (a) total population of males, and (b) total population of females, 1950−2017.

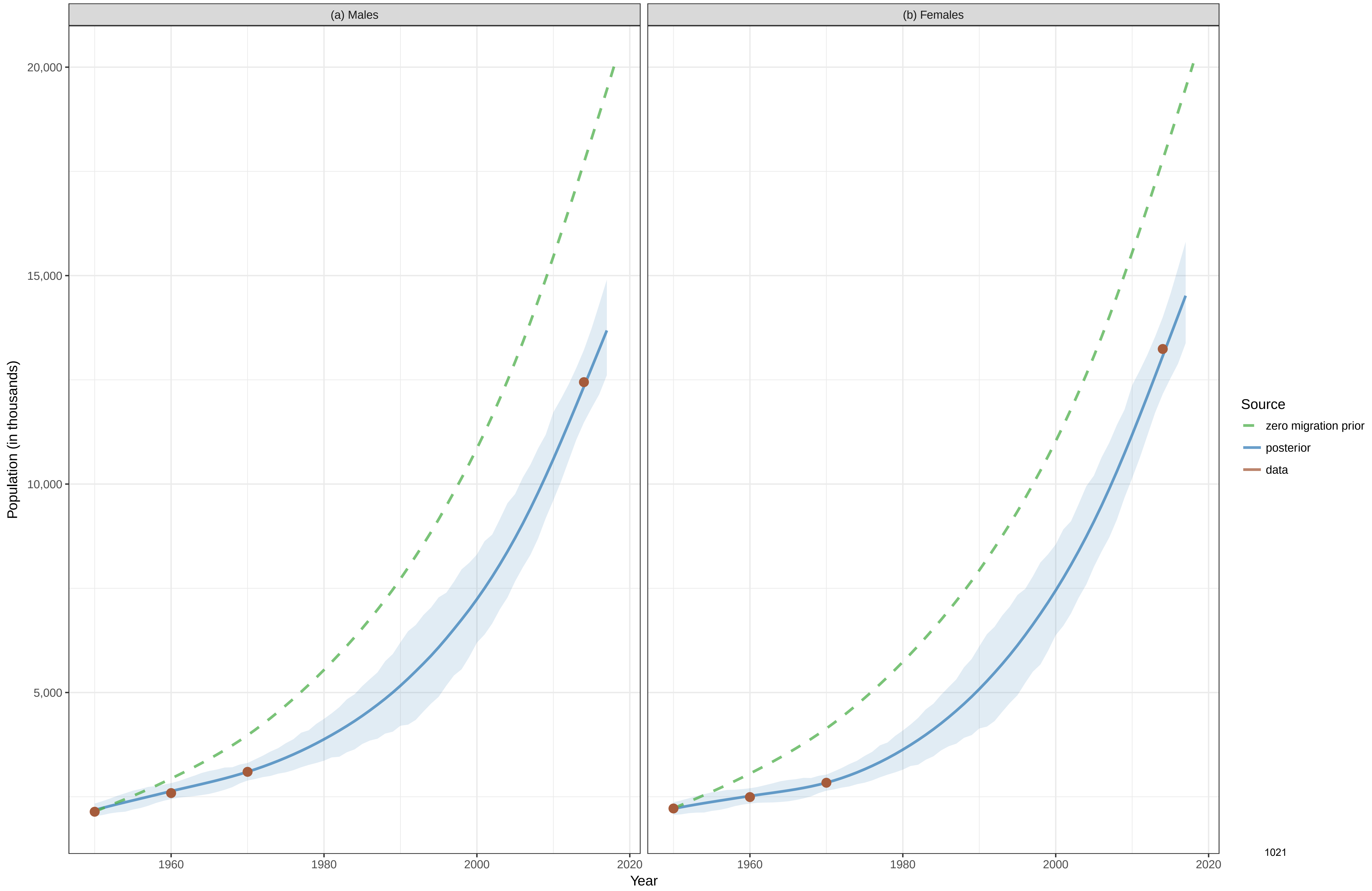

Supplemental Results Figure 4: GBD Bayesian Demographic Balancing Model fit in Antigua and Barbuda for (a) total population of males, and (b) total population of females, 1950−2017.

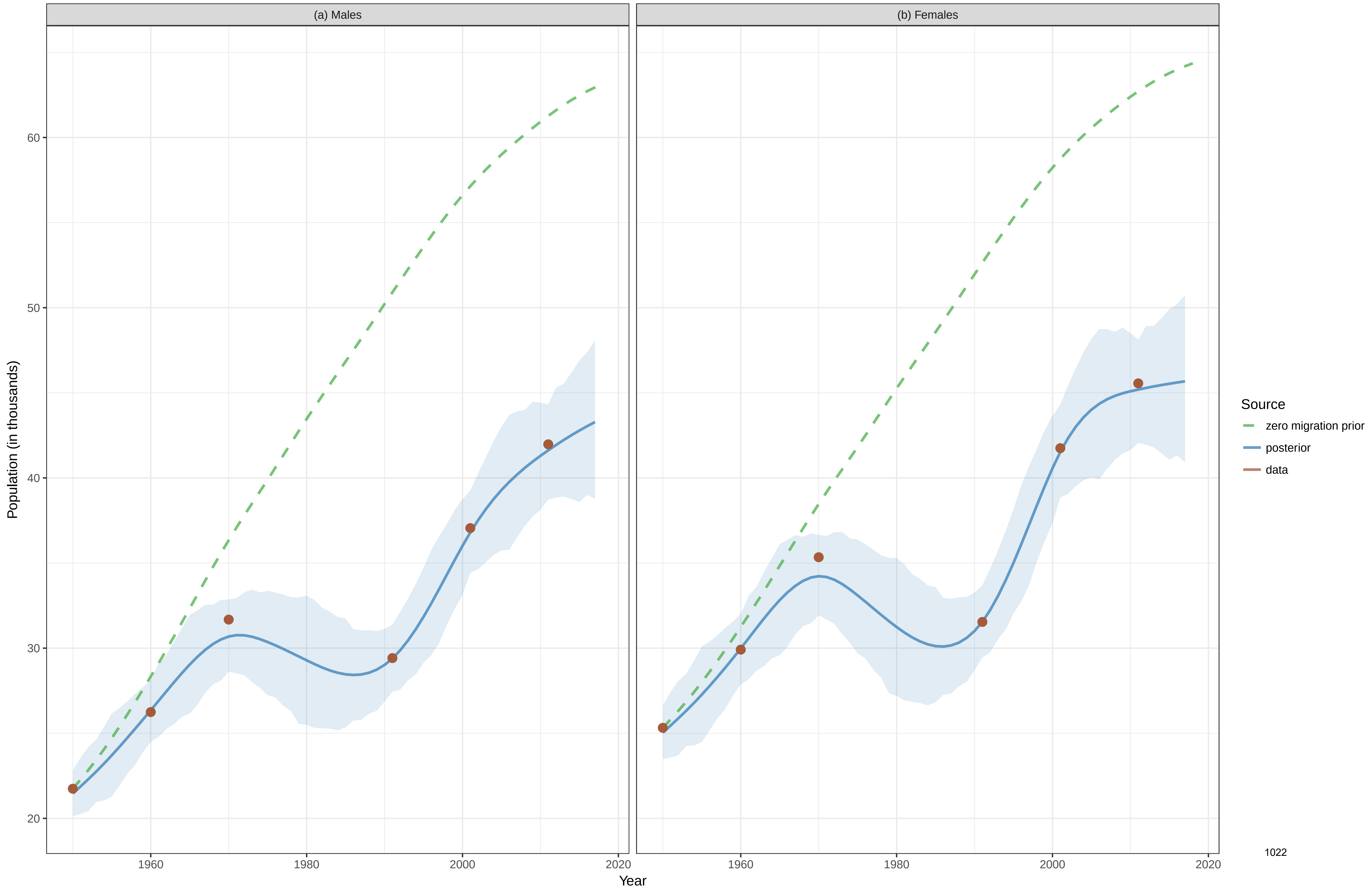

Supplemental Results Figure 4: GBD Bayesian Demographic Balancing Model fit in Argentina for (a) total population of males, and (b) total population of females, 1950−2017.

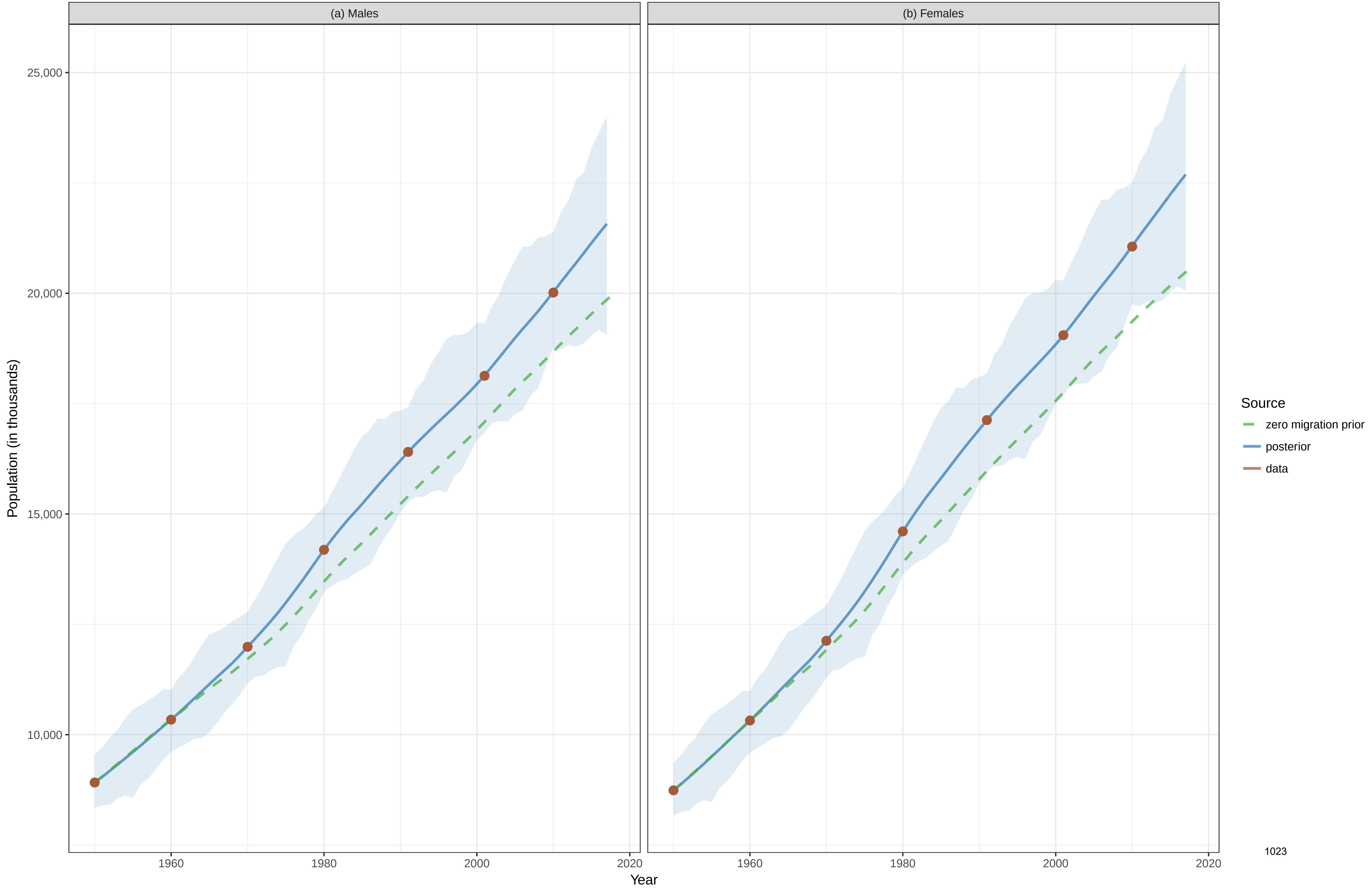

Supplemental Results Figure 4: GBD Bayesian Demographic Balancing Model fit in Armenia for (a) total population of males, and (b) total population of females, 1950−2017.

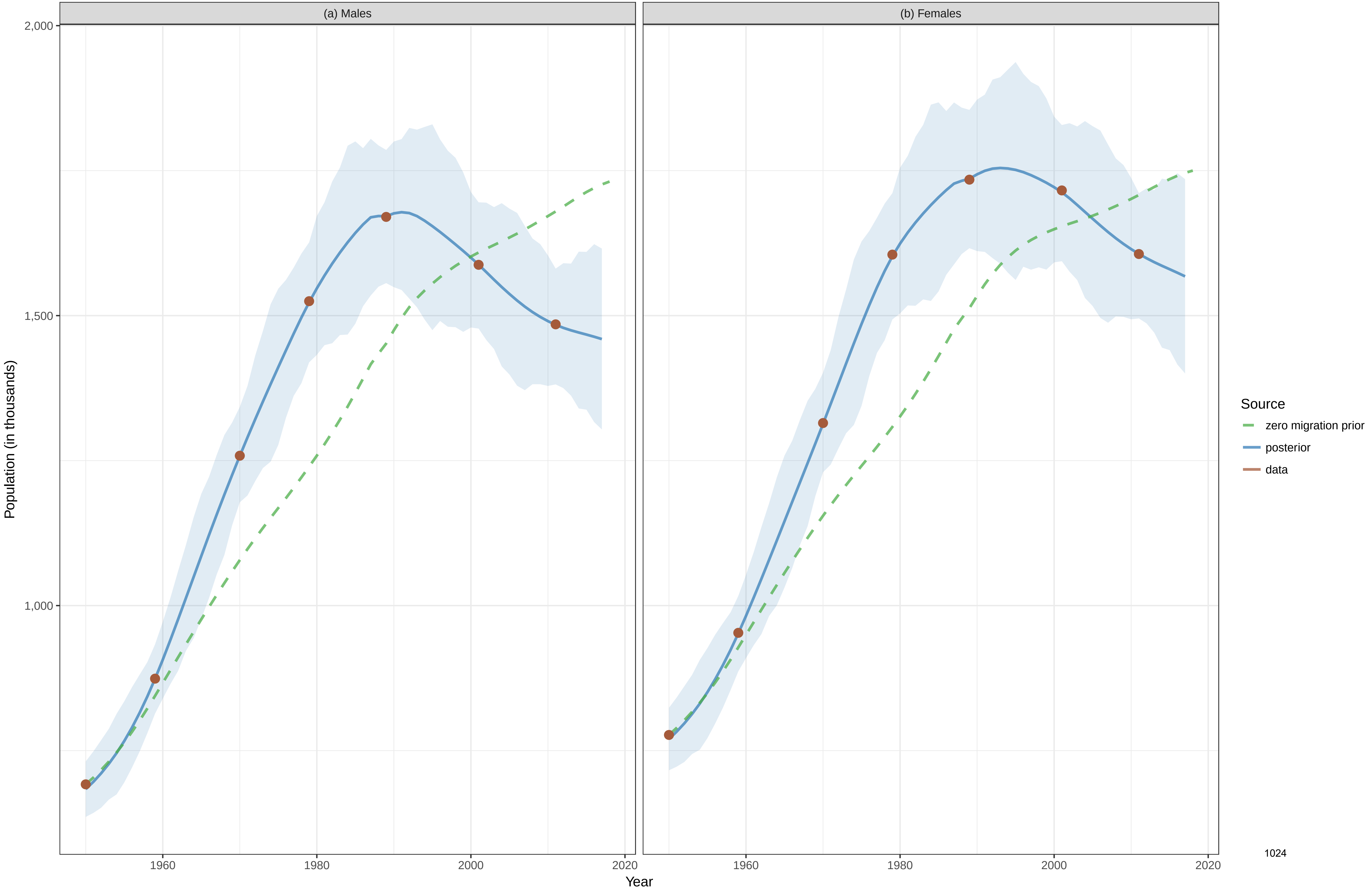

Supplemental Results Figure 4: GBD Bayesian Demographic Balancing Model fit in Australia for (a) total population of males, and (b) total population of females, 1950−2017.

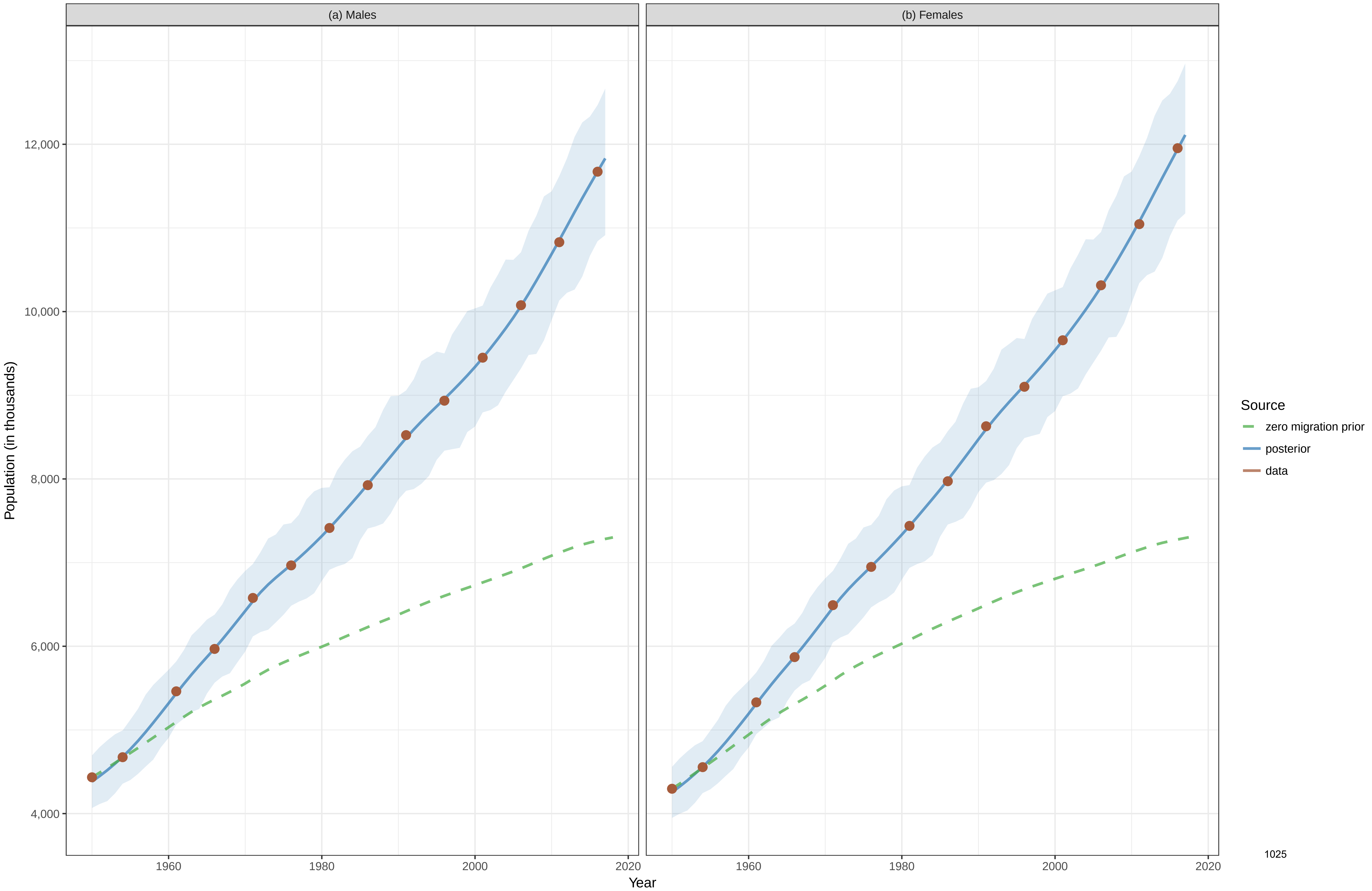

Supplemental Results Figure 4: GBD Bayesian Demographic Balancing Model fit in Austria for (a) total population of males, and (b) total population of females, 1950−2017.

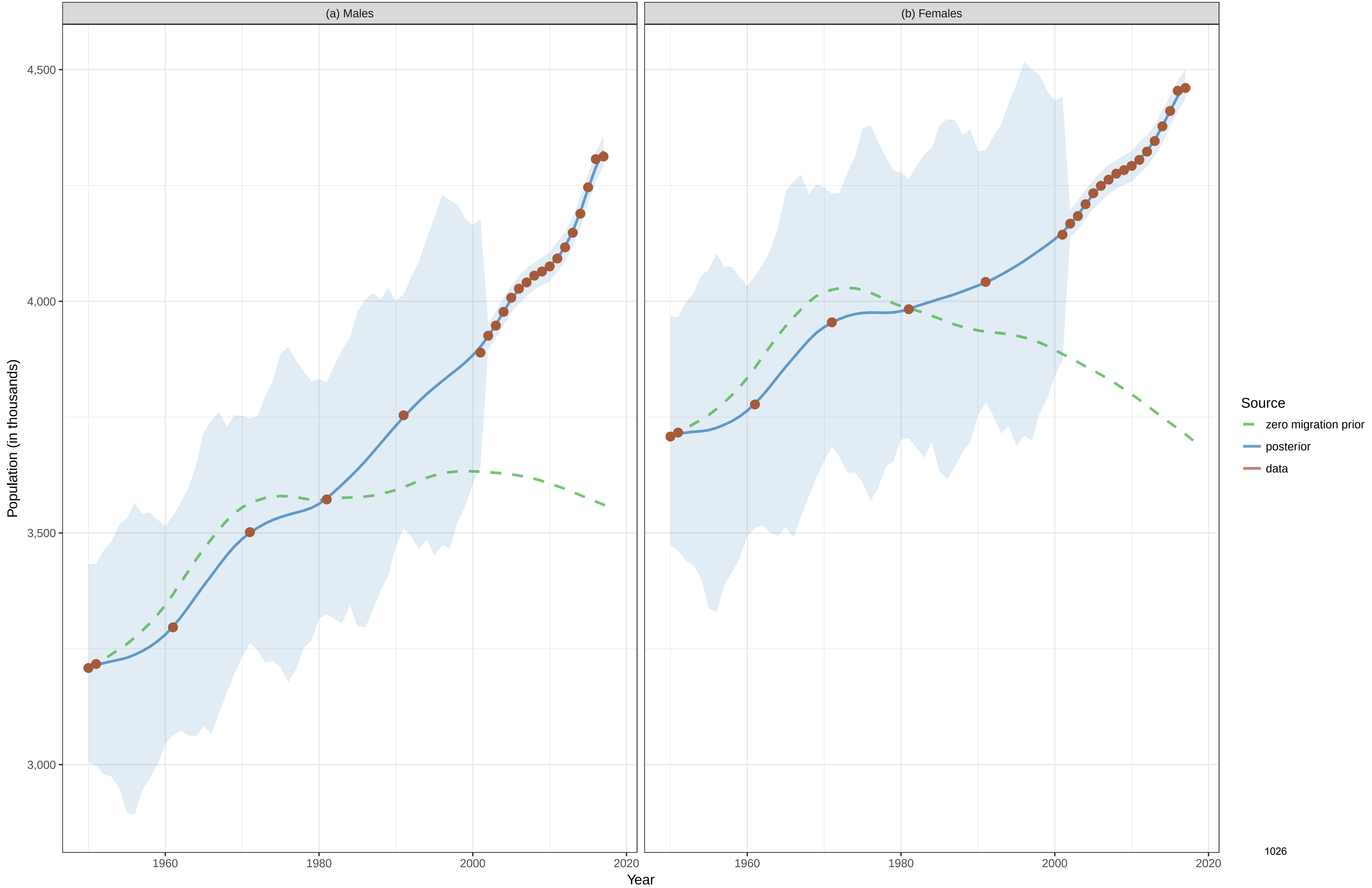

Supplemental Results Figure 4: GBD Bayesian Demographic Balancing Model fit in Azerbaijan for (a) total population of males, and (b) total population of females, 1950−2017.

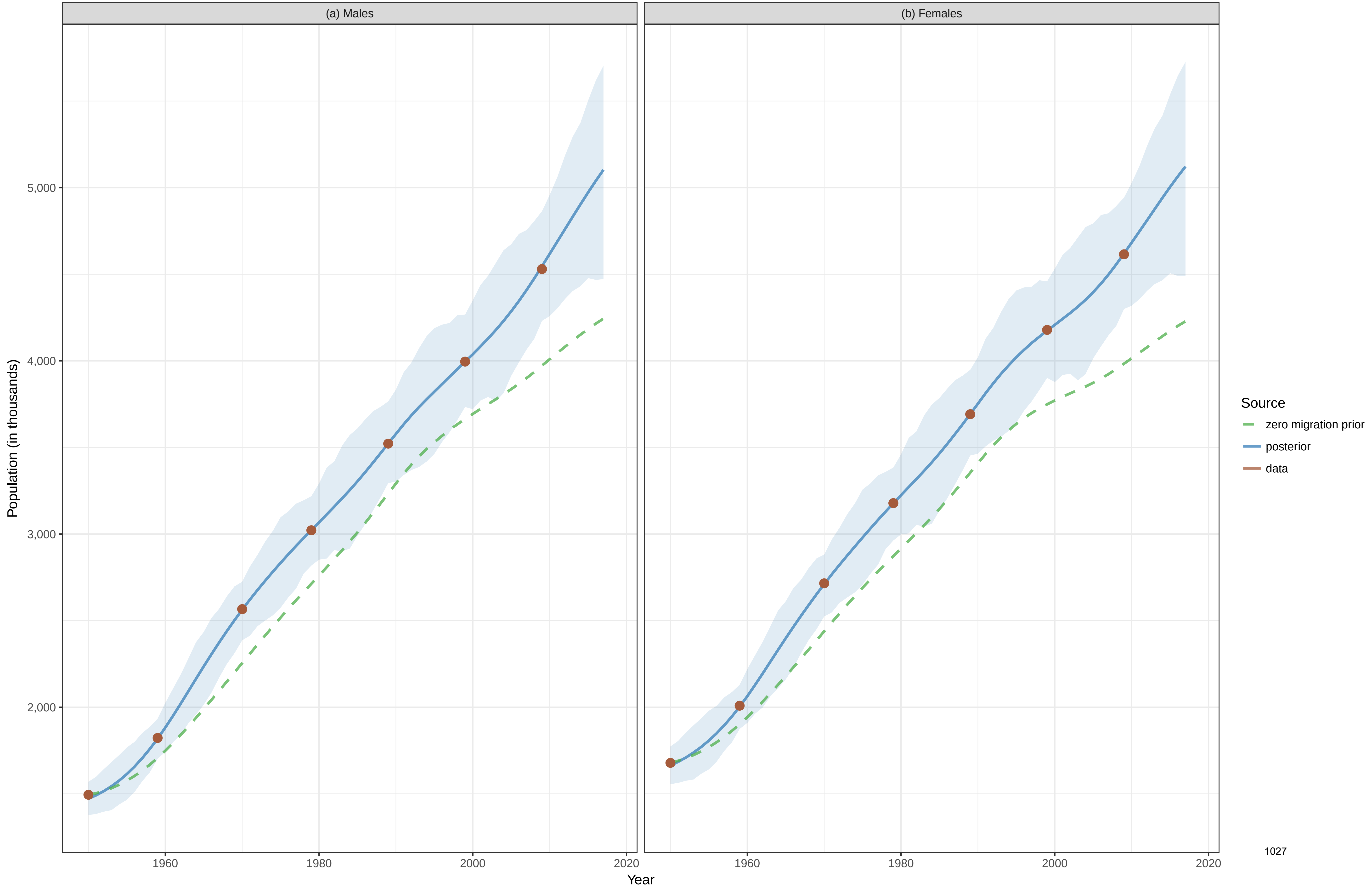

Supplemental Results Figure 4: GBD Bayesian Demographic Balancing Model fit in Bahrain for (a) total population of males, and (b) total population of females, 1950−2017.

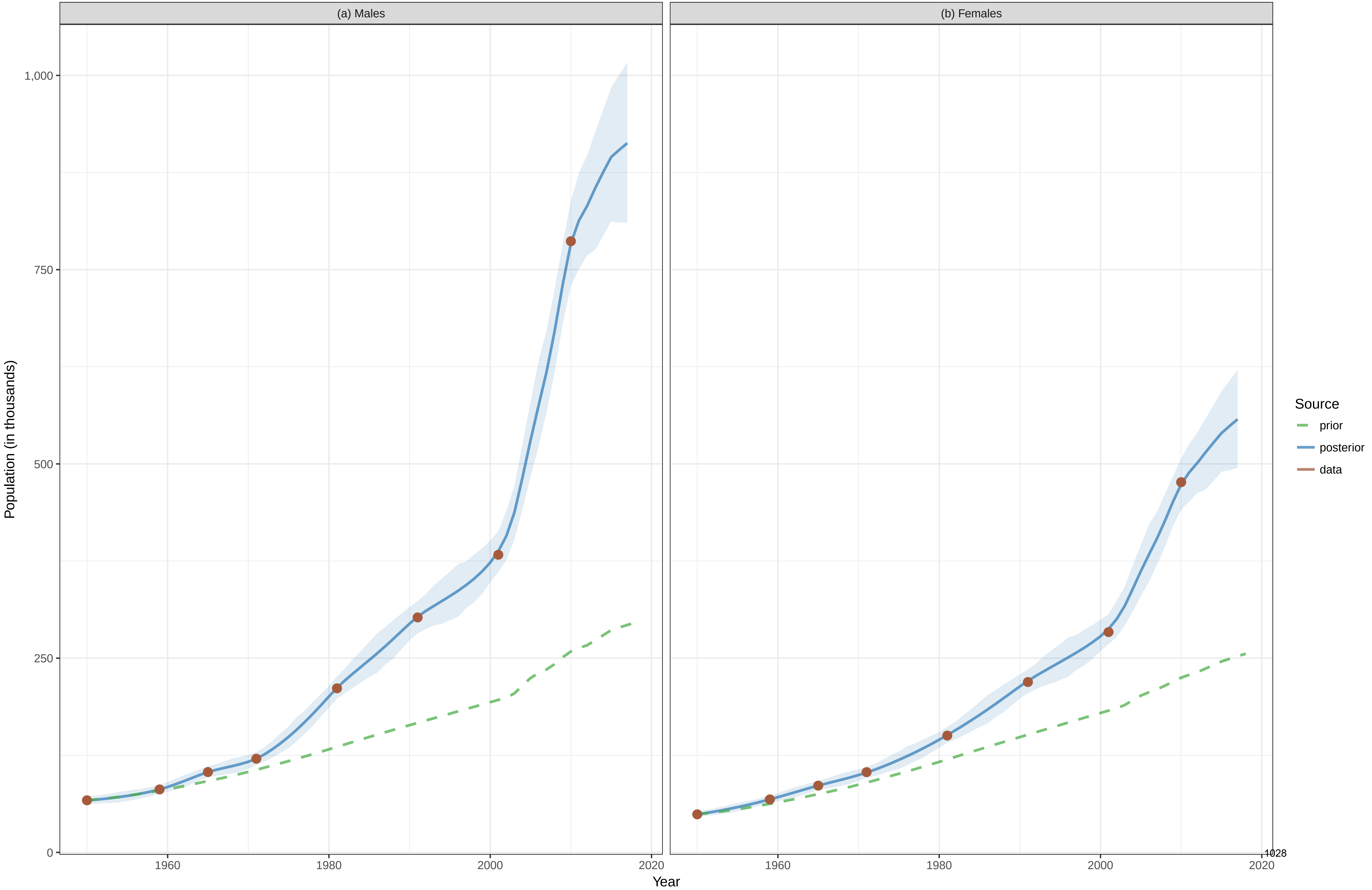

Supplemental Results Figure 4: GBD Bayesian Demographic Balancing Model fit in Bangladesh for (a) total population of males, and (b) total population of females, 1950−2017.

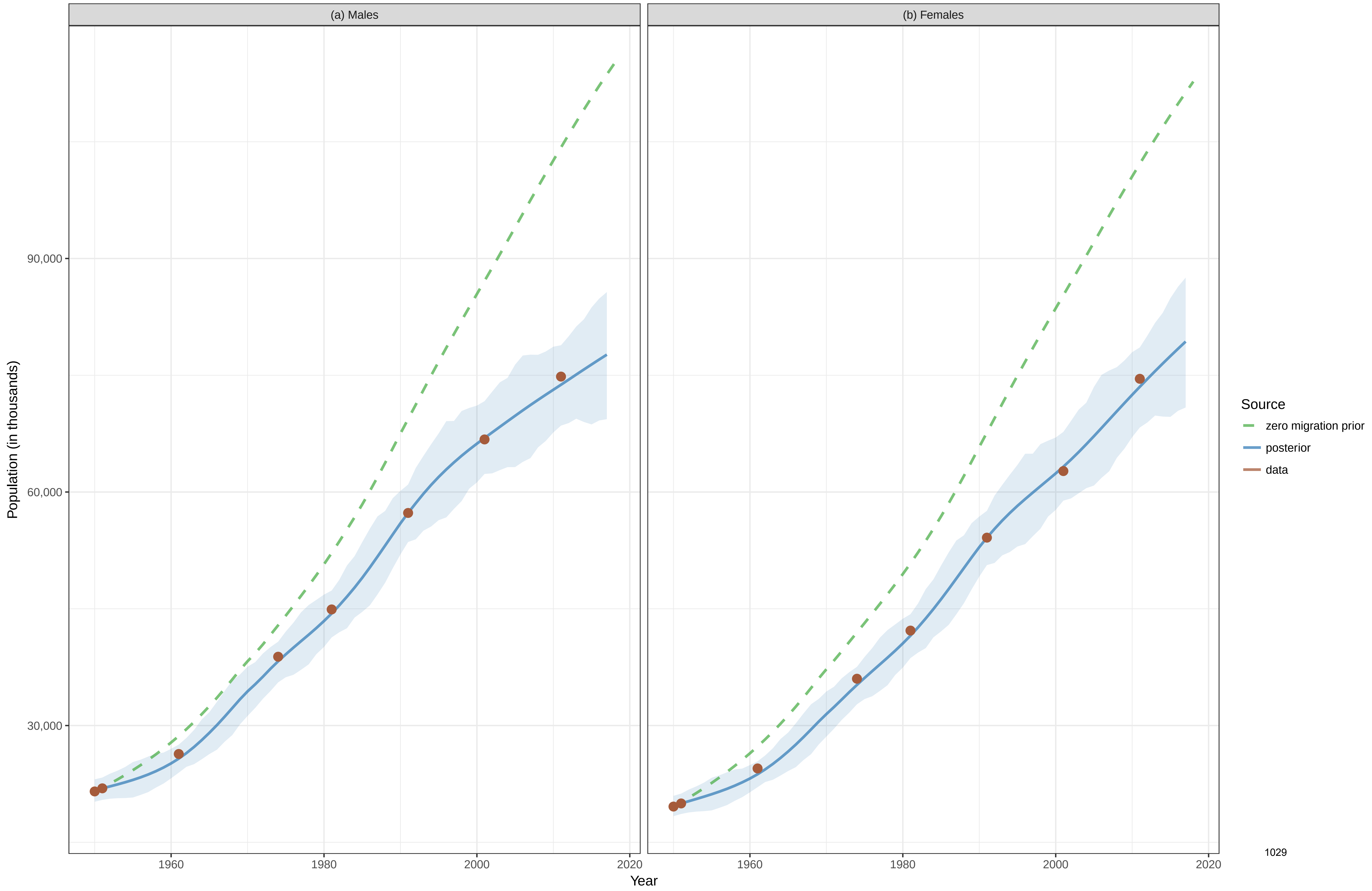

Supplemental Results Figure 4: GBD Bayesian Demographic Balancing Model fit in Barbados for (a) total population of males, and (b) total population of females, 1950−2017.

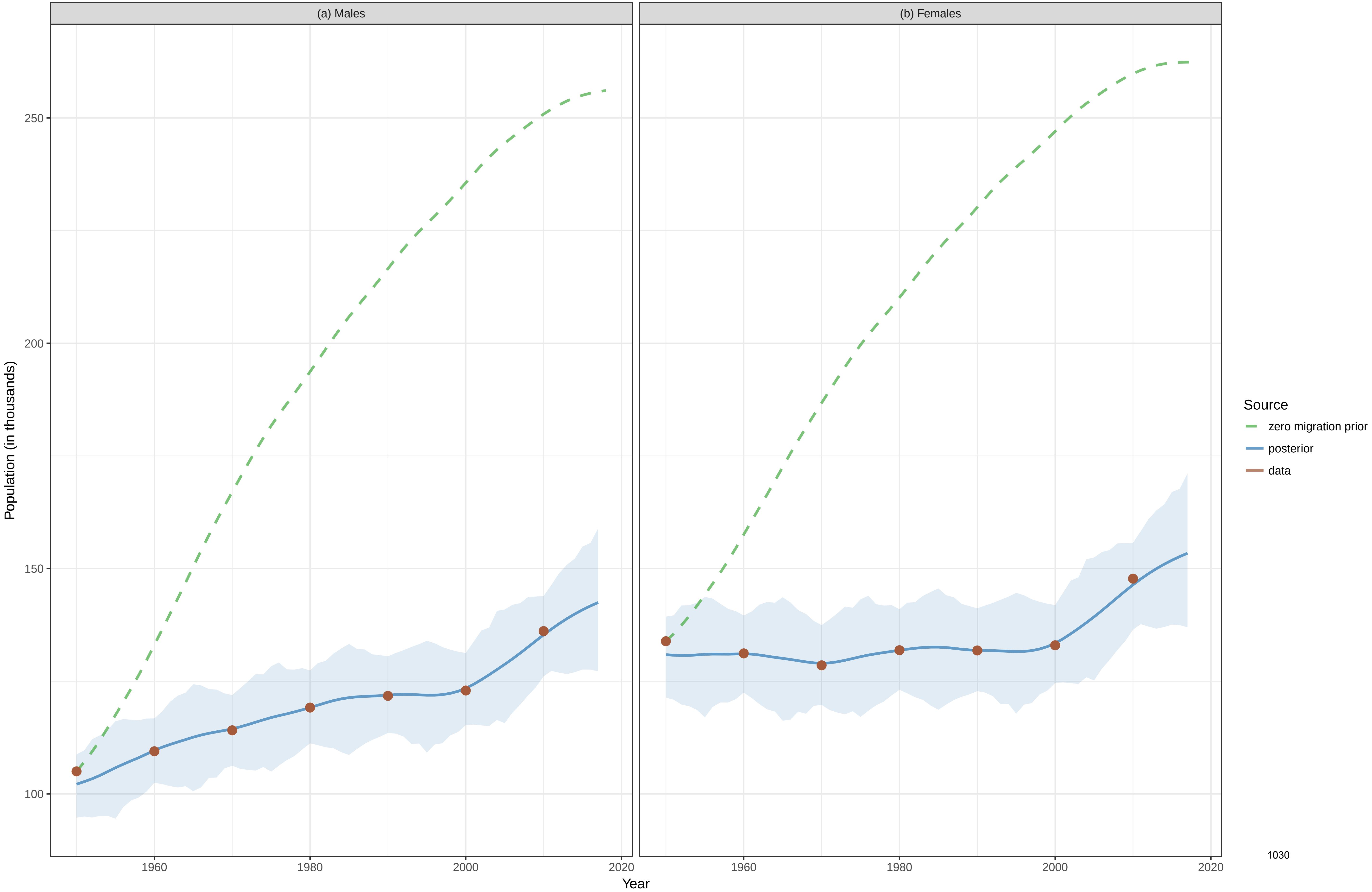

Supplemental Results Figure 4: GBD Bayesian Demographic Balancing Model fit in Belarus for (a) total population of males, and (b) total population of females, 1950−2017.

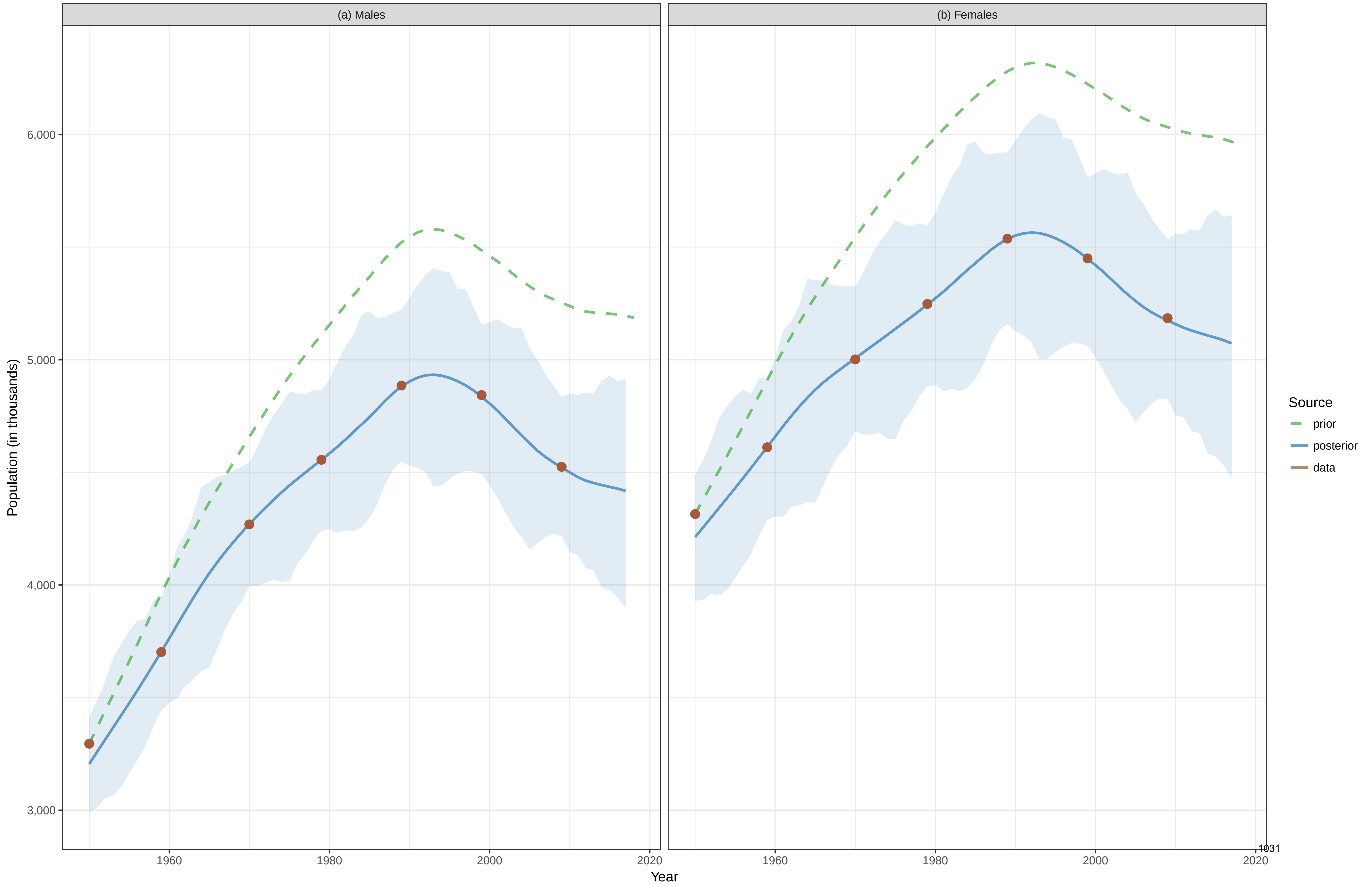

Supplemental Results Figure 4: GBD Bayesian Demographic Balancing Model fit in Belgium for (a) total population of males, and (b) total population of females, 1950−2017.

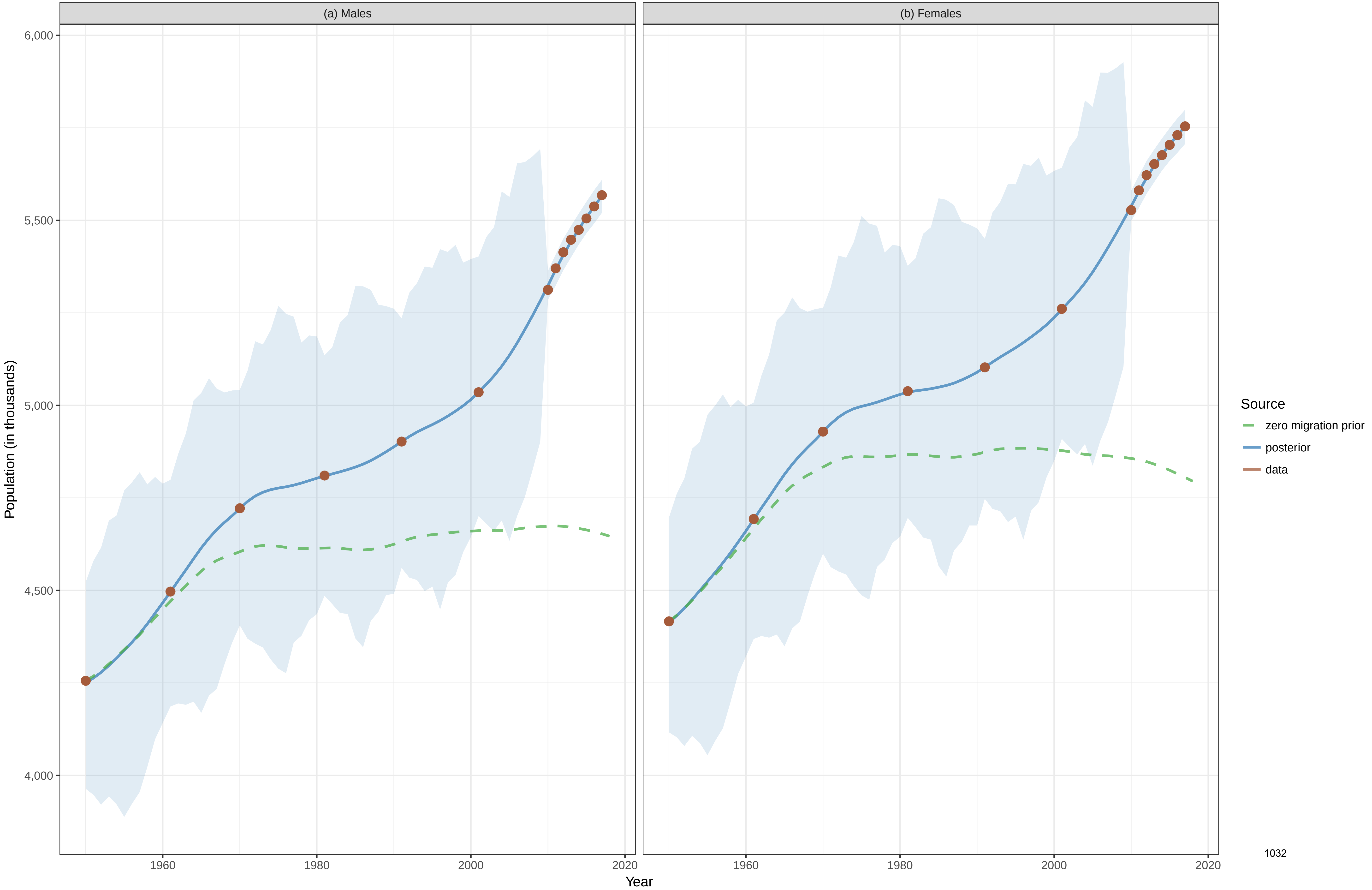

Supplemental Results Figure 4: GBD Bayesian Demographic Balancing Model fit in Belize for (a) total population of males, and (b) total population of females, 1950−2017.

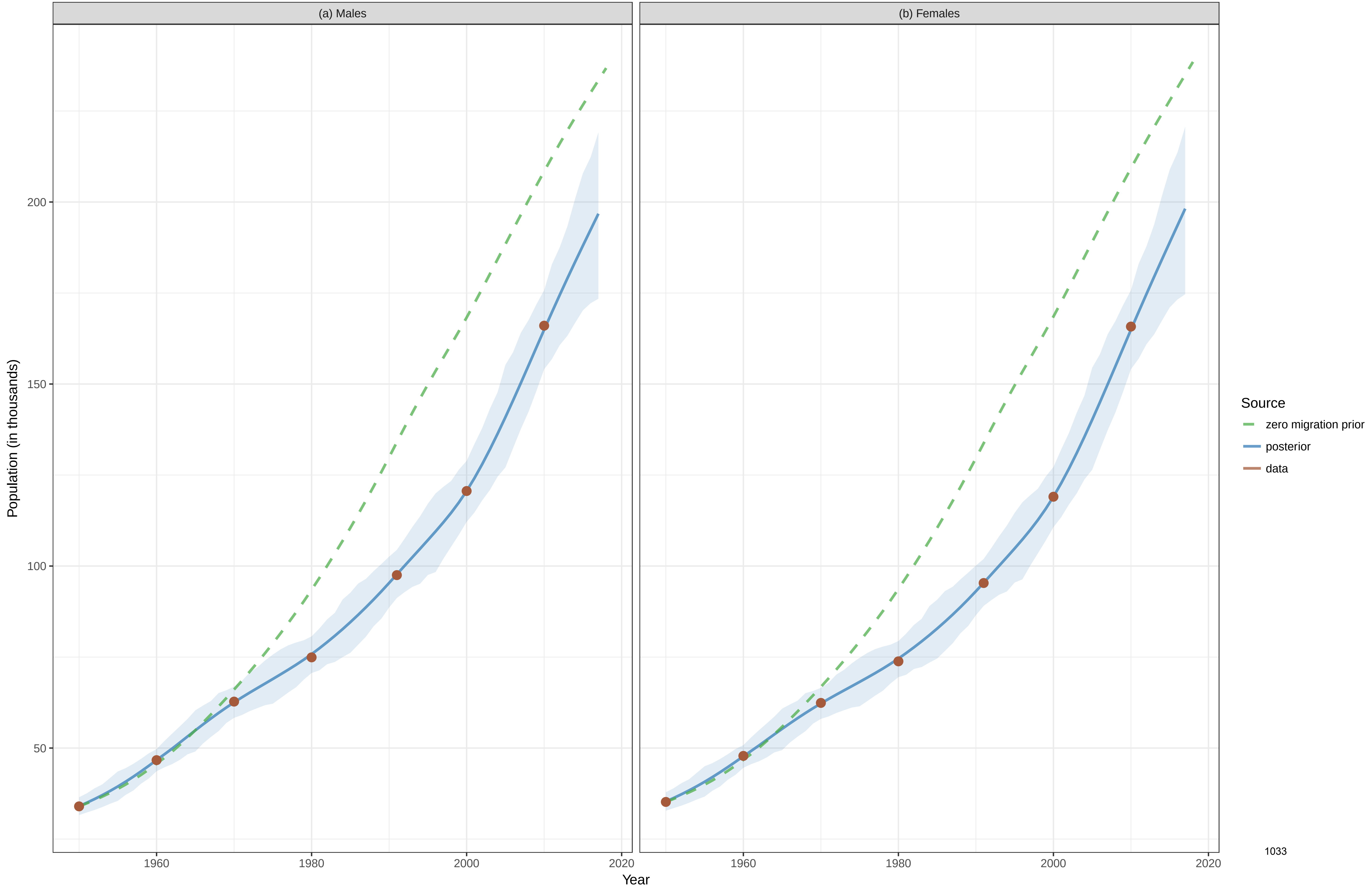

Supplemental Results Figure 4: GBD Bayesian Demographic Balancing Model fit in Benin for (a) total population of males, and (b) total population of females, 1950−2017.

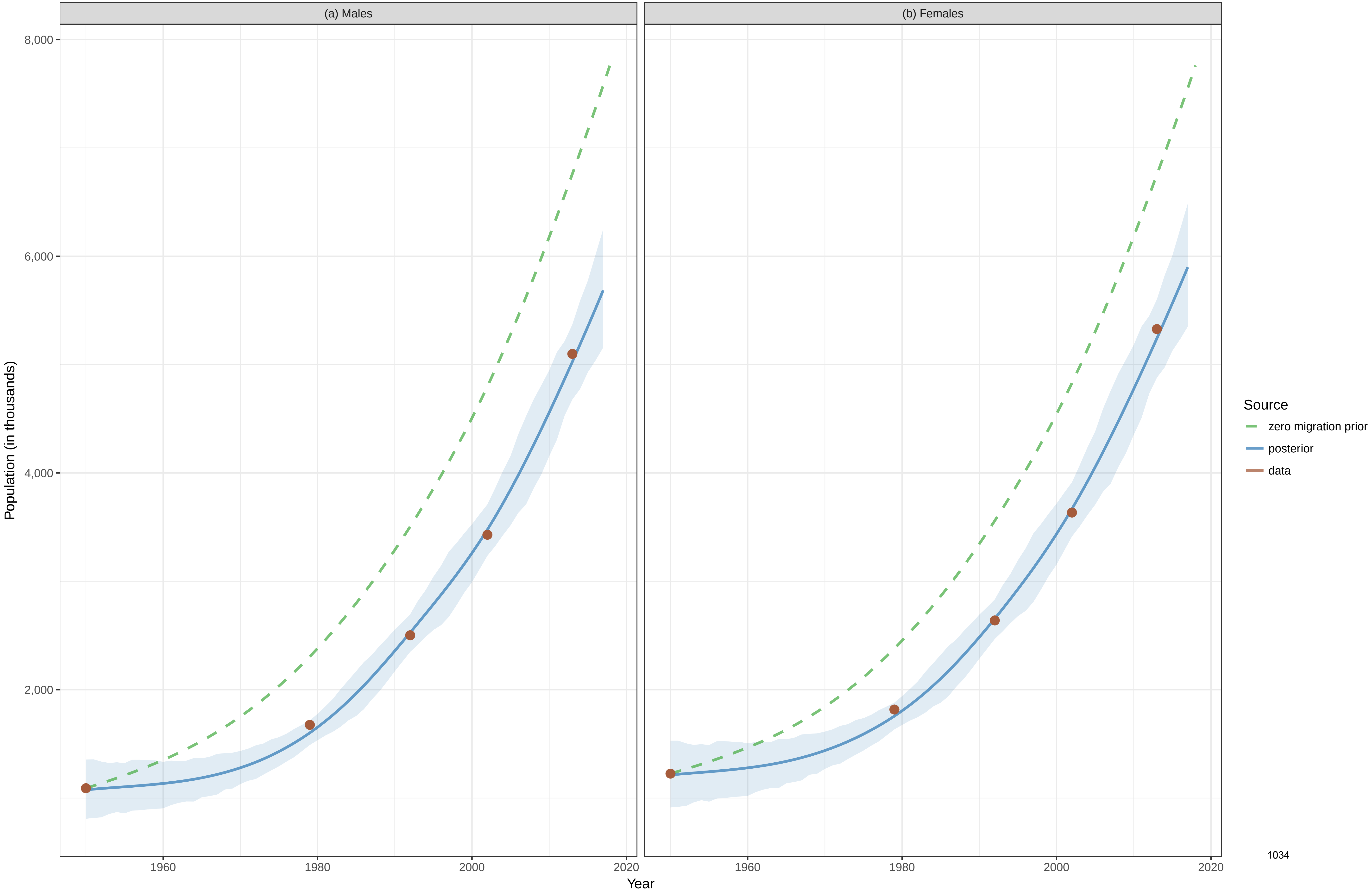

Supplemental Results Figure 4: GBD Bayesian Demographic Balancing Model fit in Bermuda for (a) total population of males, and (b) total population of females, 1950−2017.

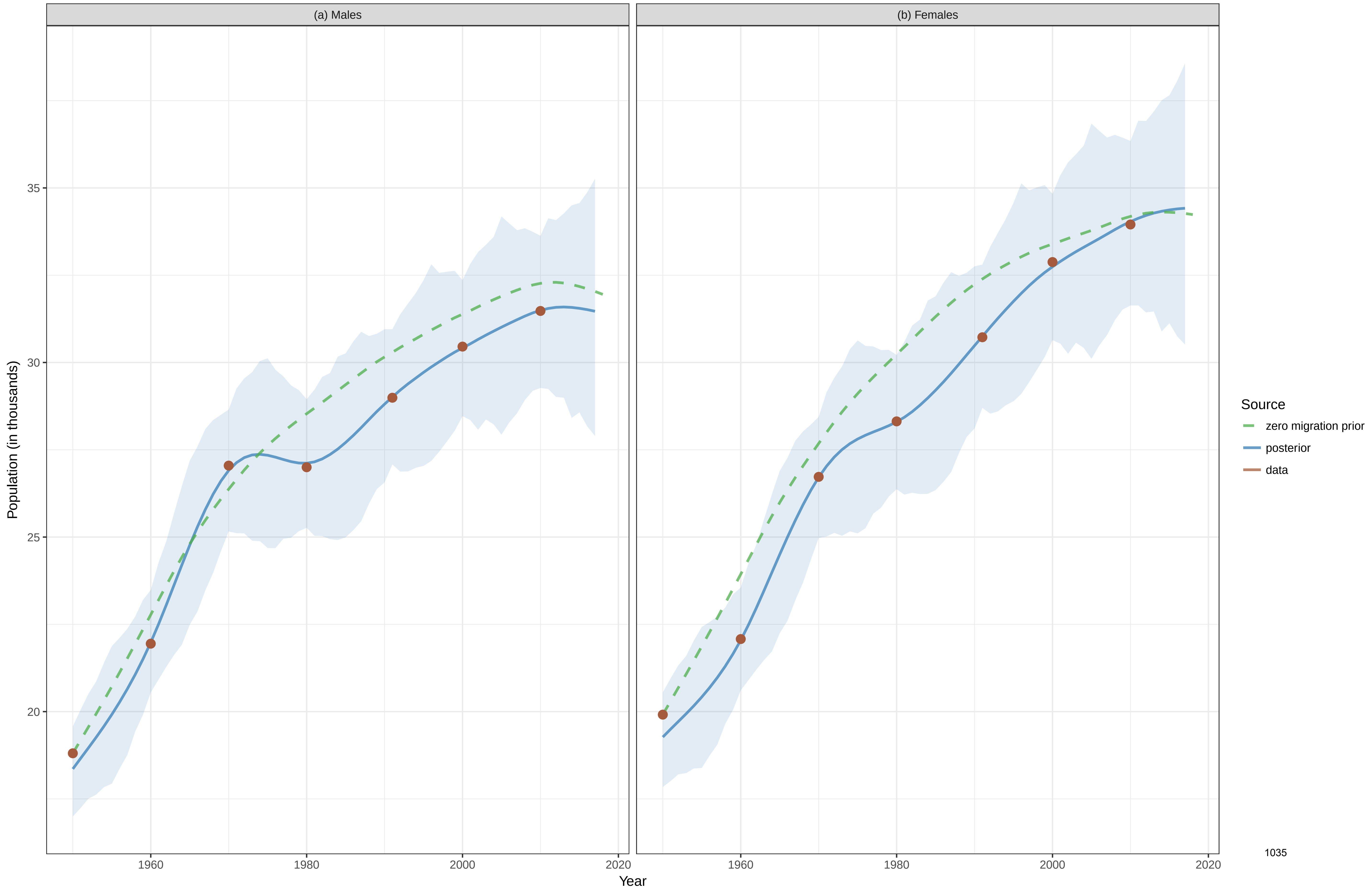

Supplemental Results Figure 4: GBD Bayesian Demographic Balancing Model fit in Bhutan for (a) total population of males, and (b) total population of females, 1950−2017.

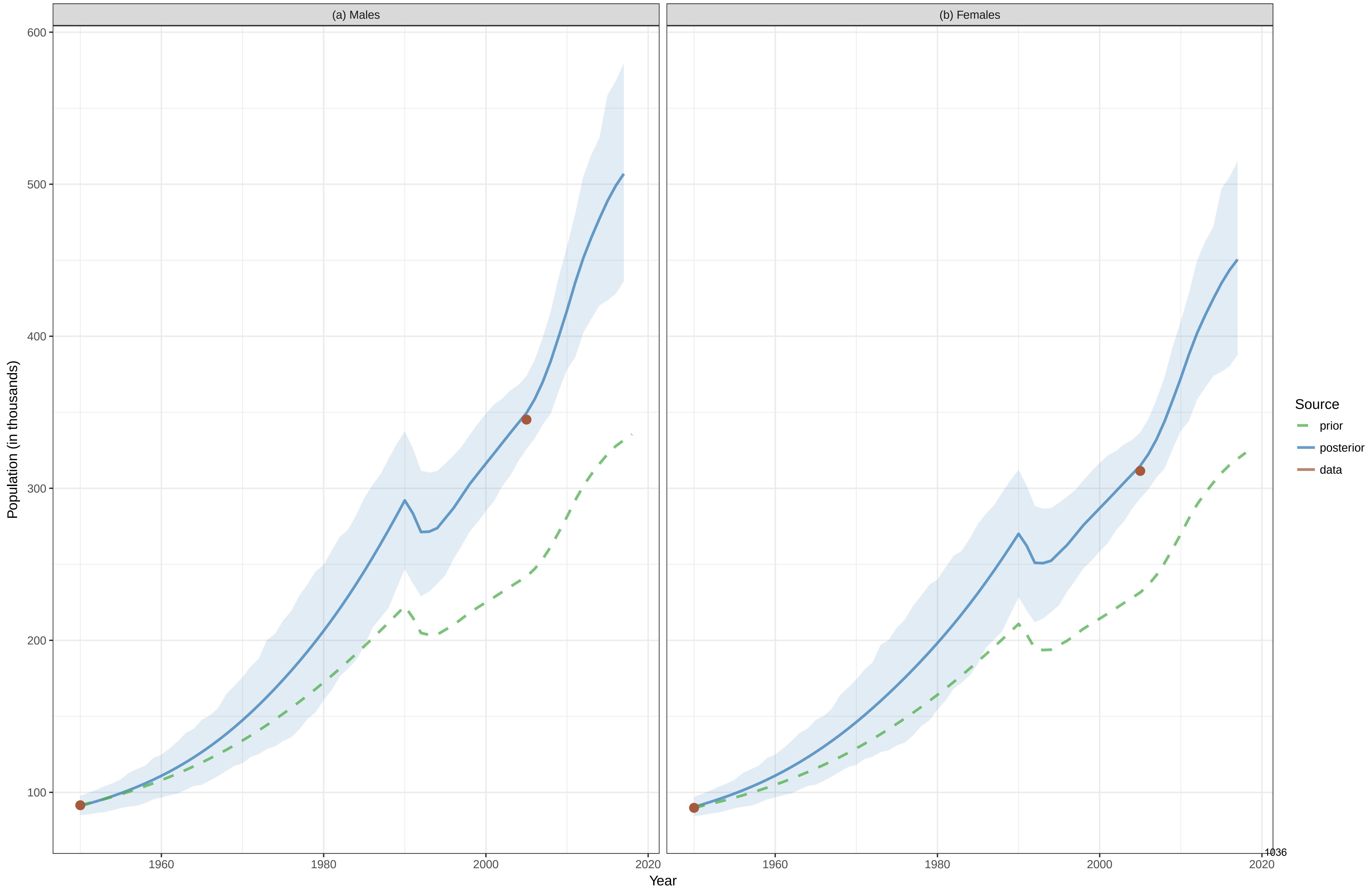

Supplemental Results Figure 4: GBD Bayesian Demographic Balancing Model fit in Bolivia for (a) total population of males, and (b) total population of females, 1950−2017.

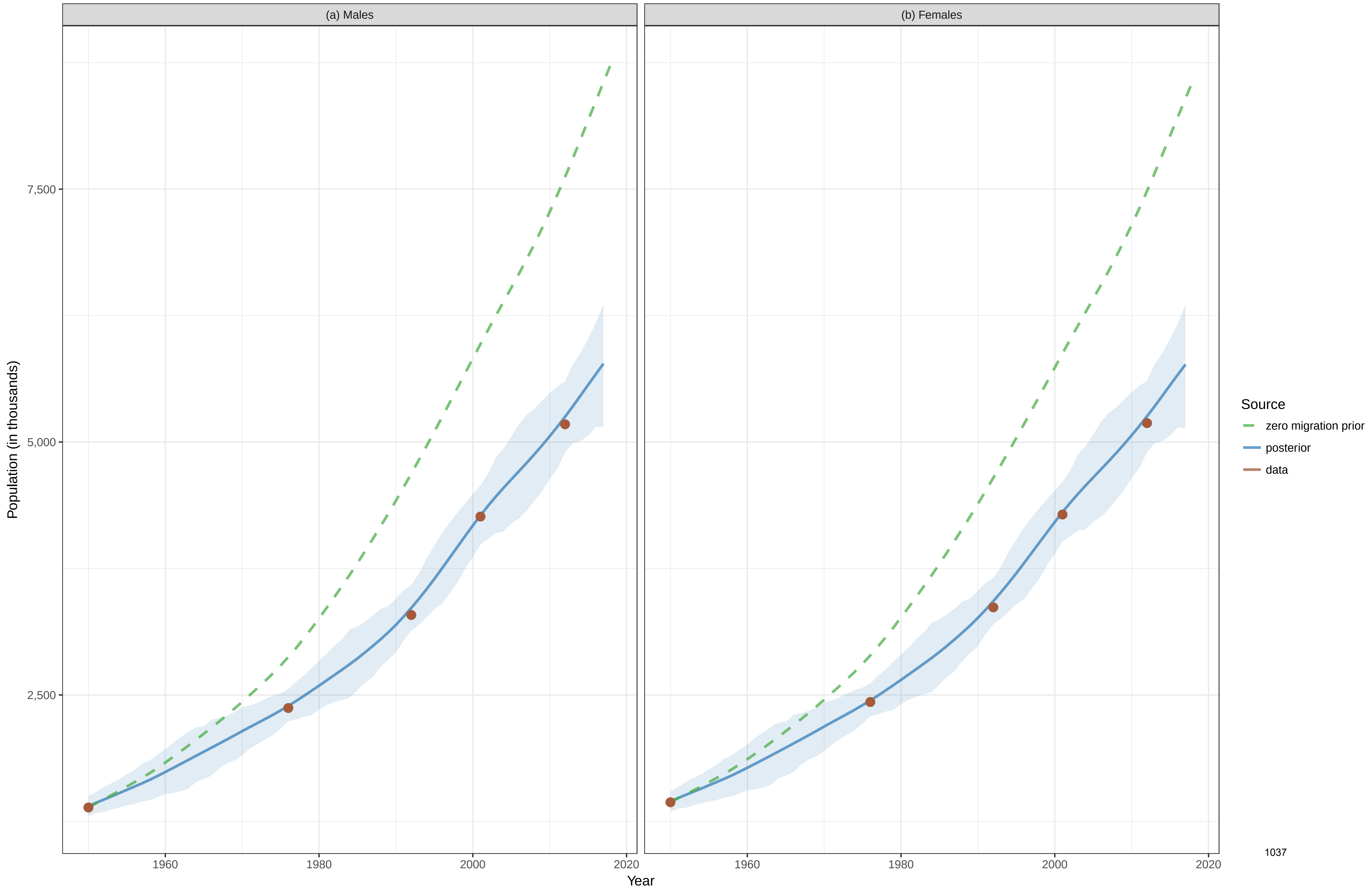

Supplemental Results Figure 4: GBD Bayesian Demographic Balancing Model fit in Bosnia and Herzegovina for (a) total population of males, and (b) total population of females, 1950−2017.

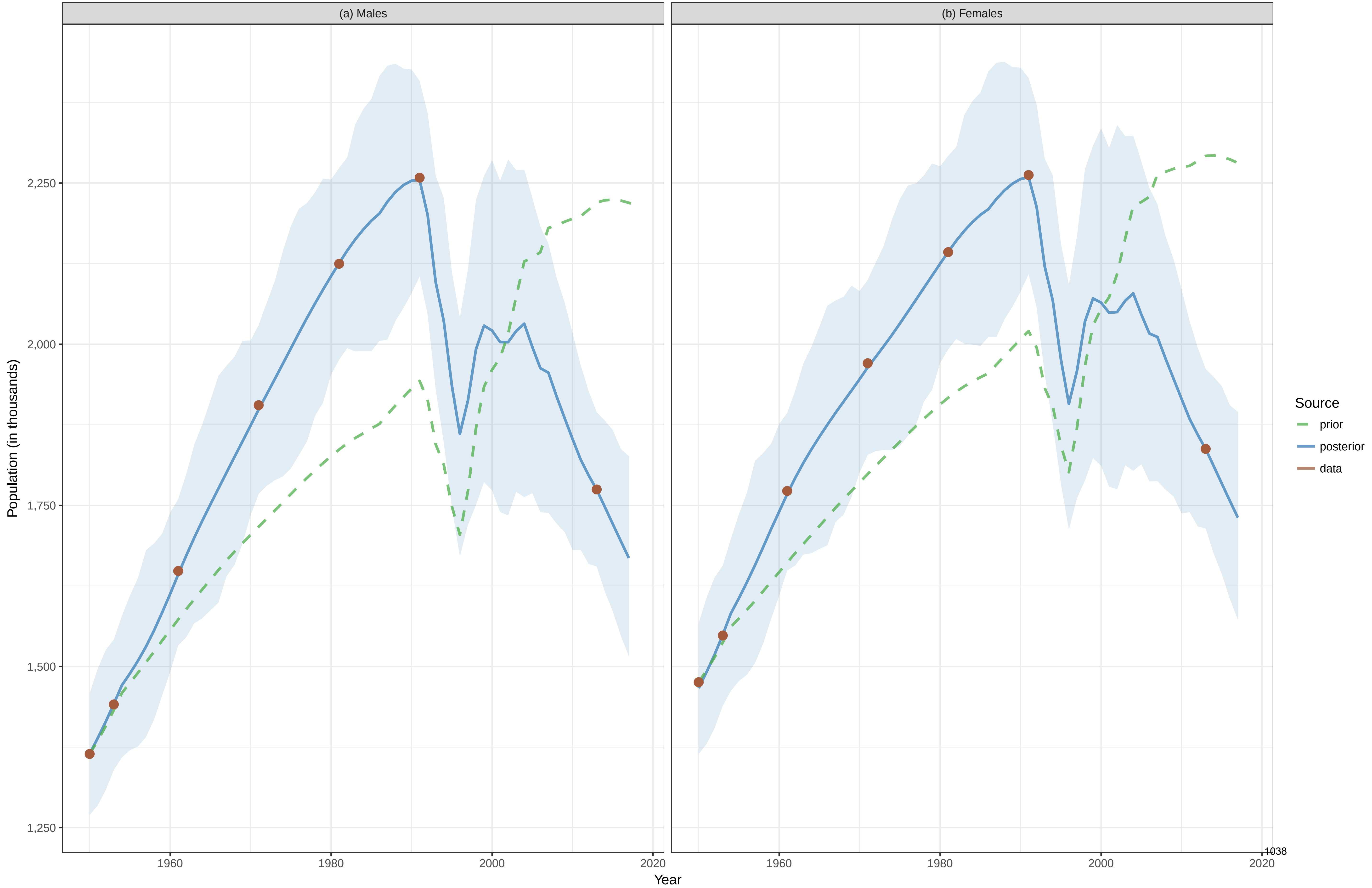

Supplemental Results Figure 4: GBD Bayesian Demographic Balancing Model fit in Botswana for (a) total population of males, and (b) total population of females, 1950−2017.

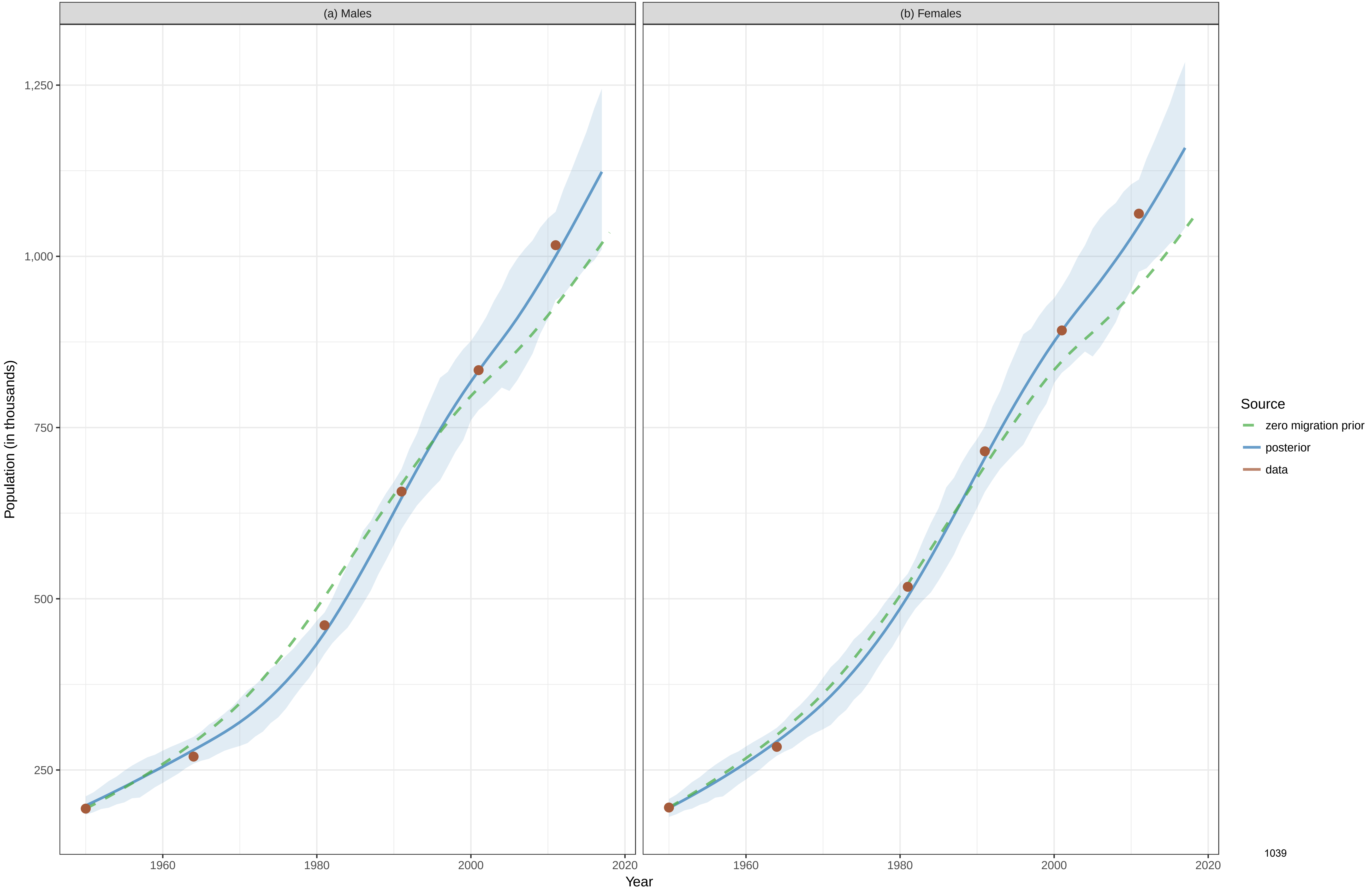

Supplemental Results Figure 4: GBD Bayesian Demographic Balancing Model fit in Brazil for (a) total population of males, and (b) total population of females, 1950−2017.

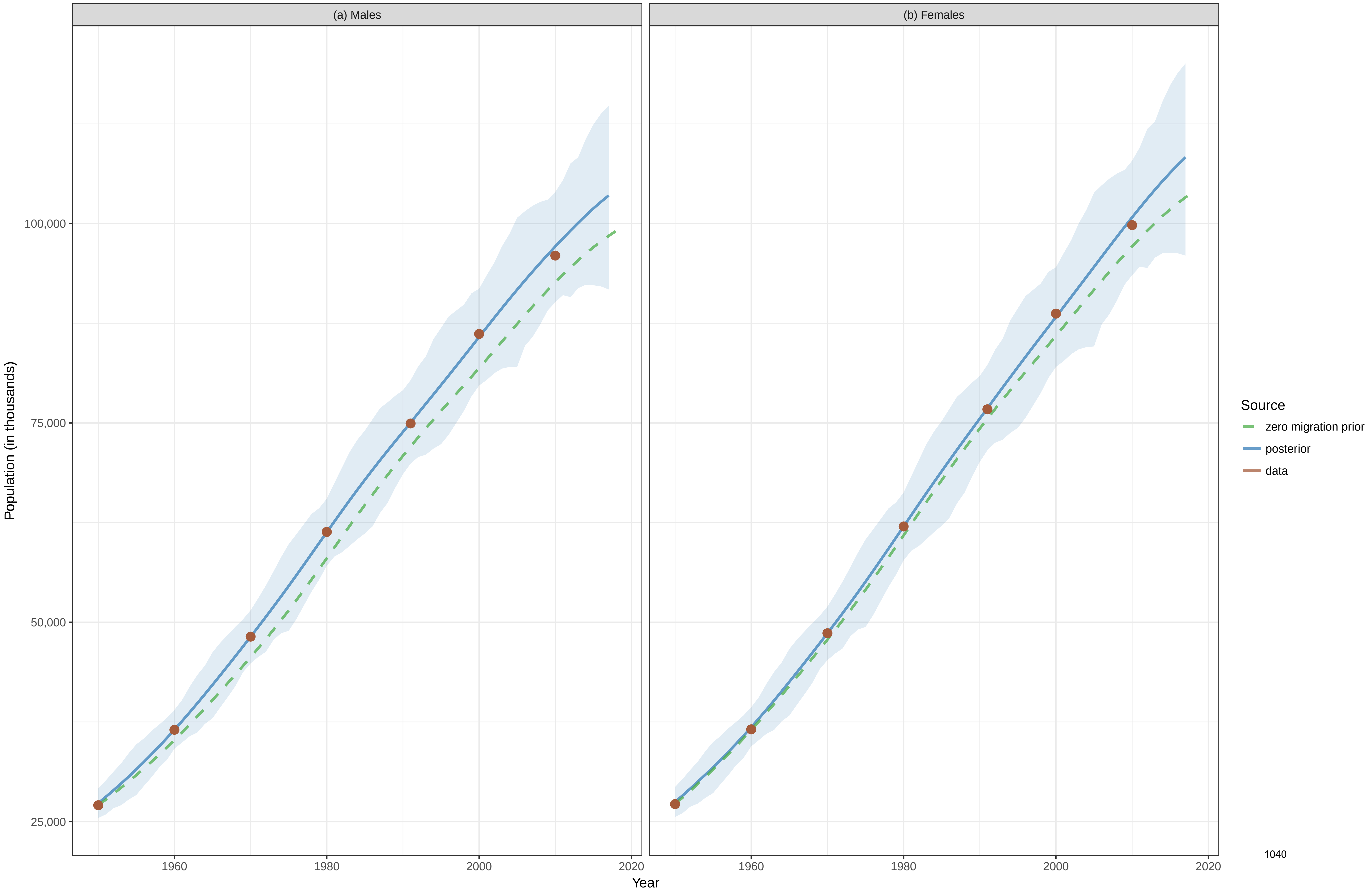

Supplemental Results Figure 4: GBD Bayesian Demographic Balancing Model fit in Brunei for (a) total population of males, and (b) total population of females, 1950−2017.

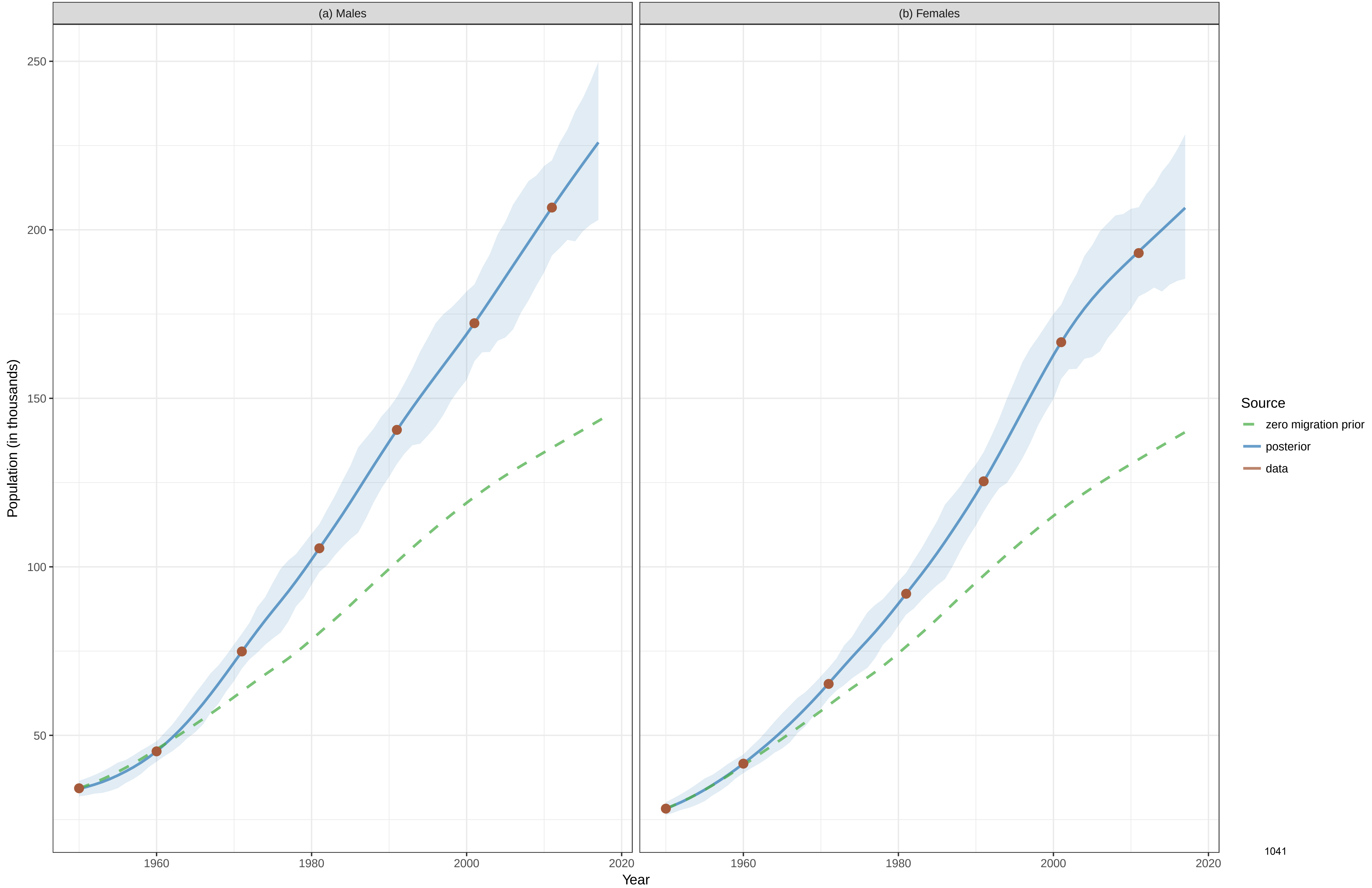

Supplemental Results Figure 4: GBD Bayesian Demographic Balancing Model fit in Bulgaria for (a) total population of males, and (b) total population of females, 1950−2017.

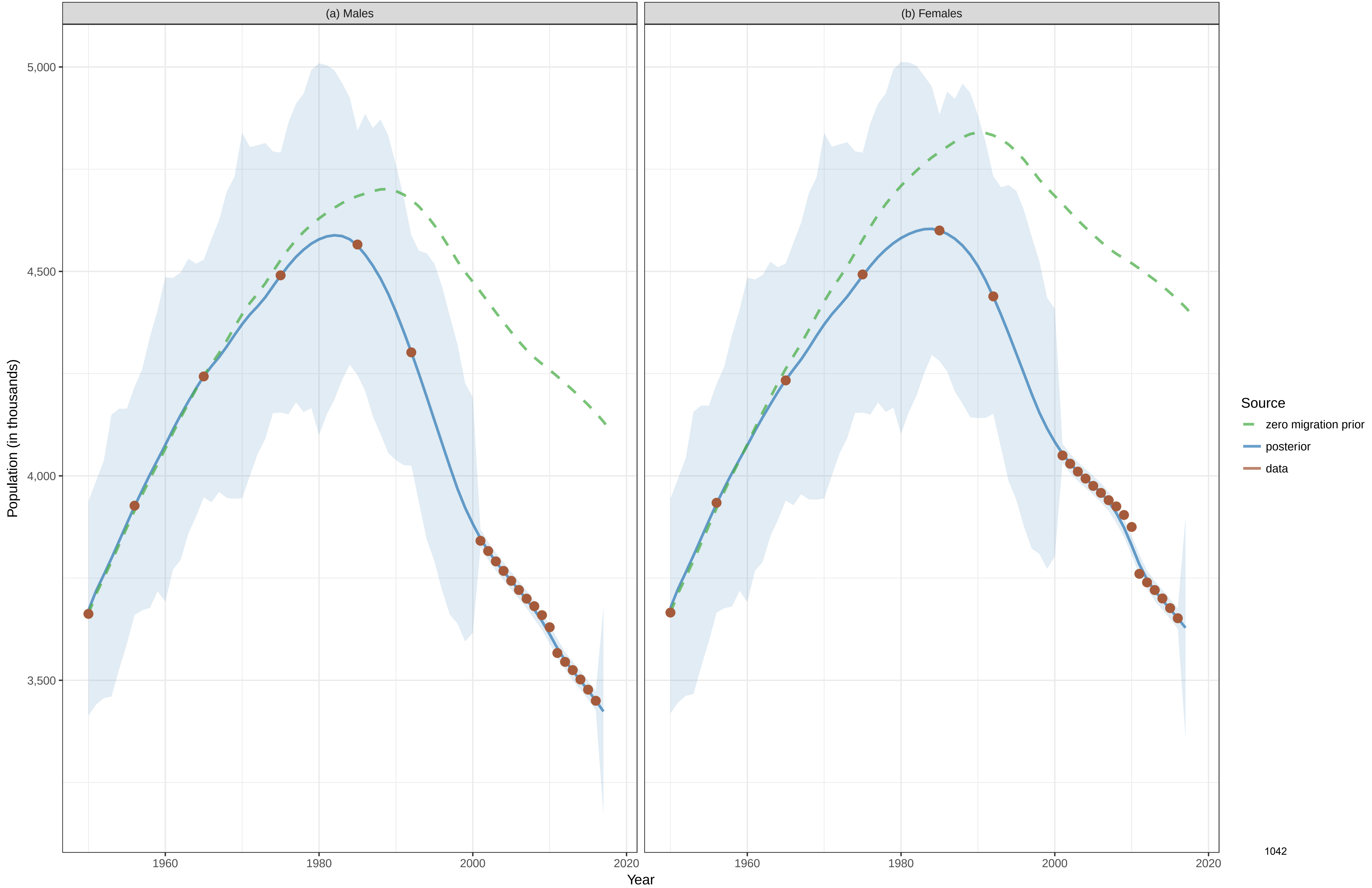

Supplemental Results Figure 4: GBD Bayesian Demographic Balancing Model fit in Burkina Faso for (a) total population of males, and (b) total population of females, 1950−2017.

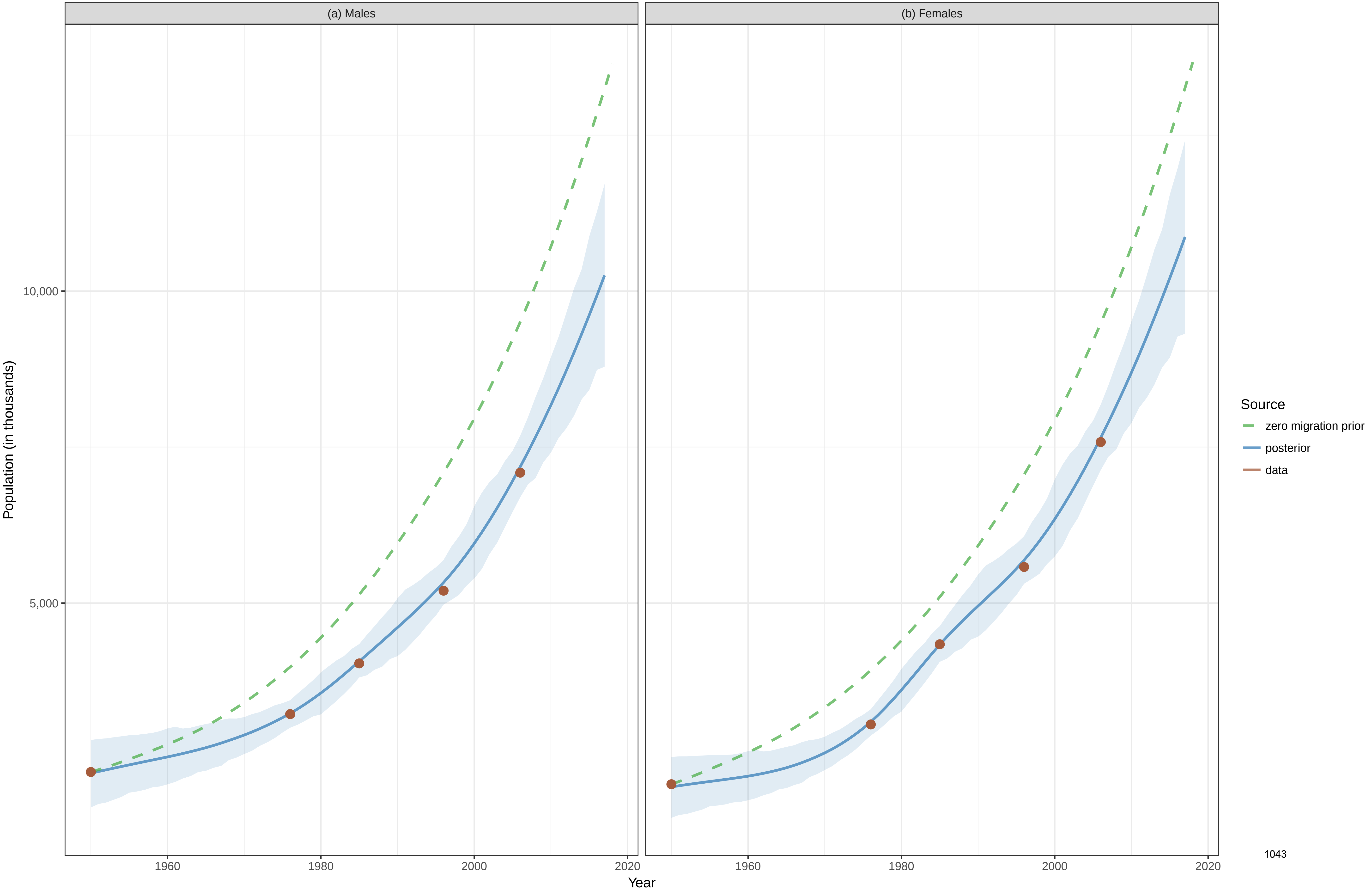

Supplemental Results Figure 4: GBD Bayesian Demographic Balancing Model fit in Burundi for (a) total population of males, and (b) total population of females, 1950−2017.

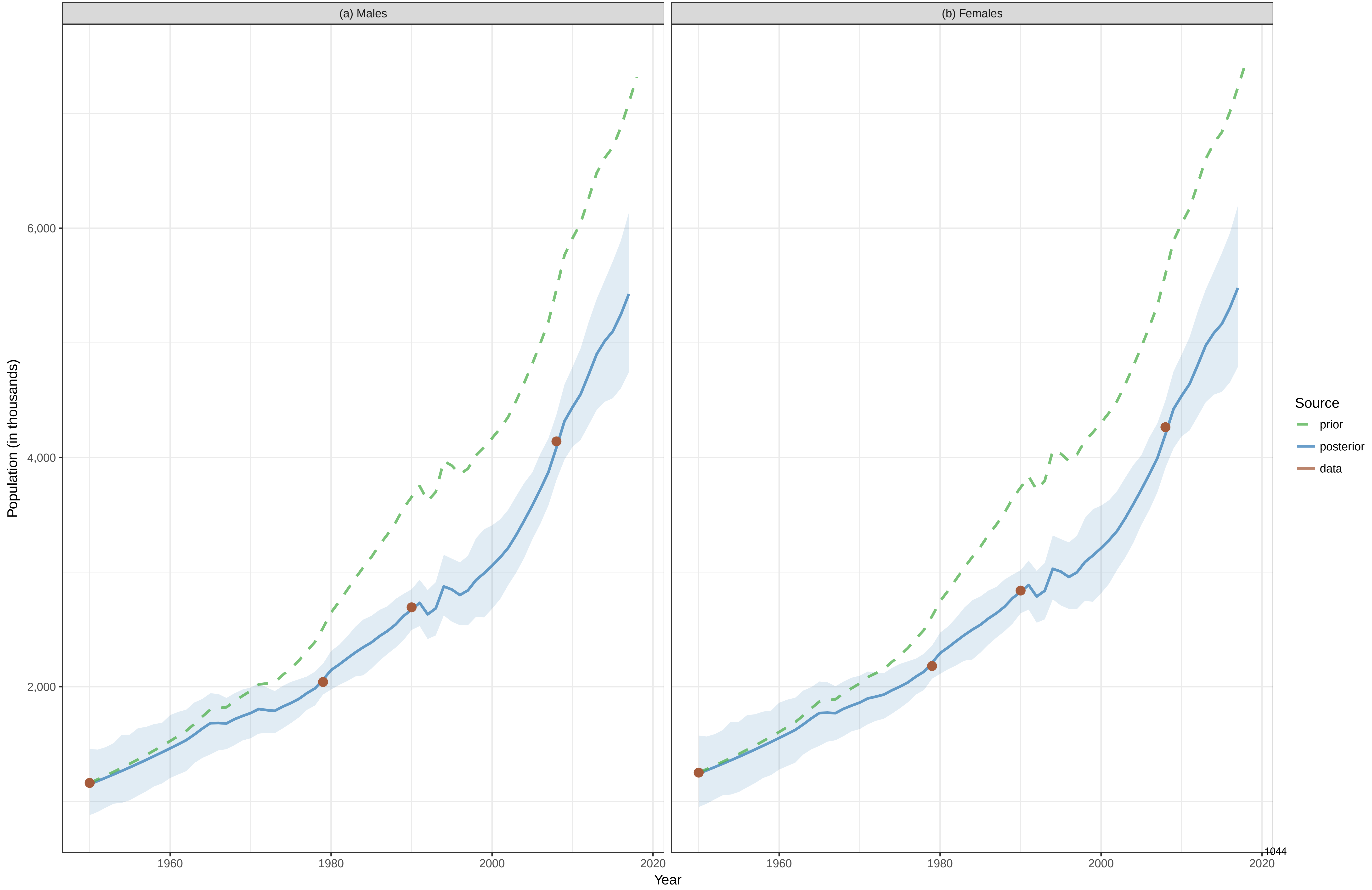

Supplemental Results Figure 4: GBD Bayesian Demographic Balancing Model fit in Cambodia for (a) total population of males, and (b) total population of females, 1950−2017.

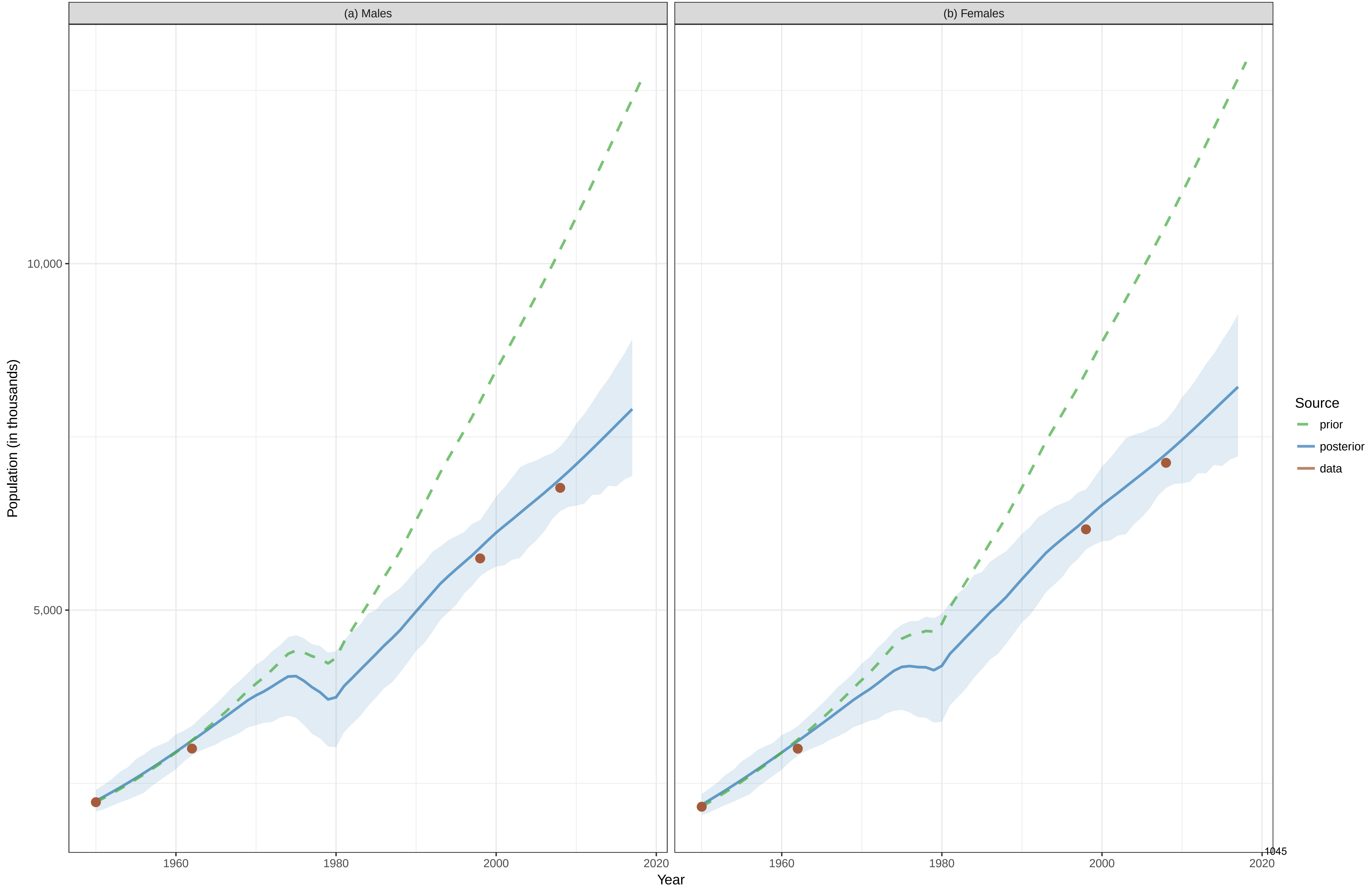

Supplemental Results Figure 4: GBD Bayesian Demographic Balancing Model fit in Cameroon for (a) total population of males, and (b) total population of females, 1950−2017.

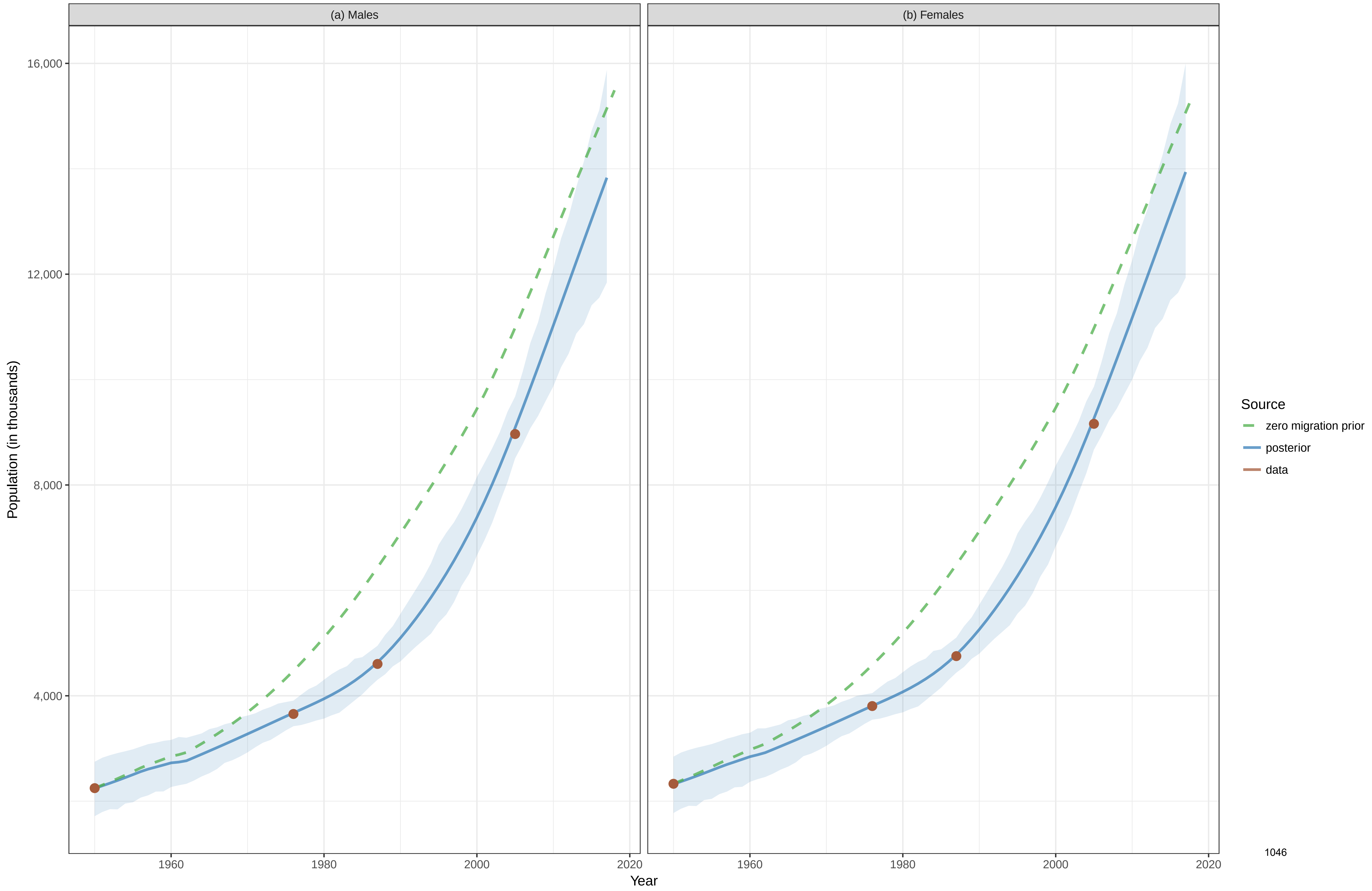

Supplemental Results Figure 4: GBD Bayesian Demographic Balancing Model fit in Canada for (a) total population of males, and (b) total population of females, 1950−2017.

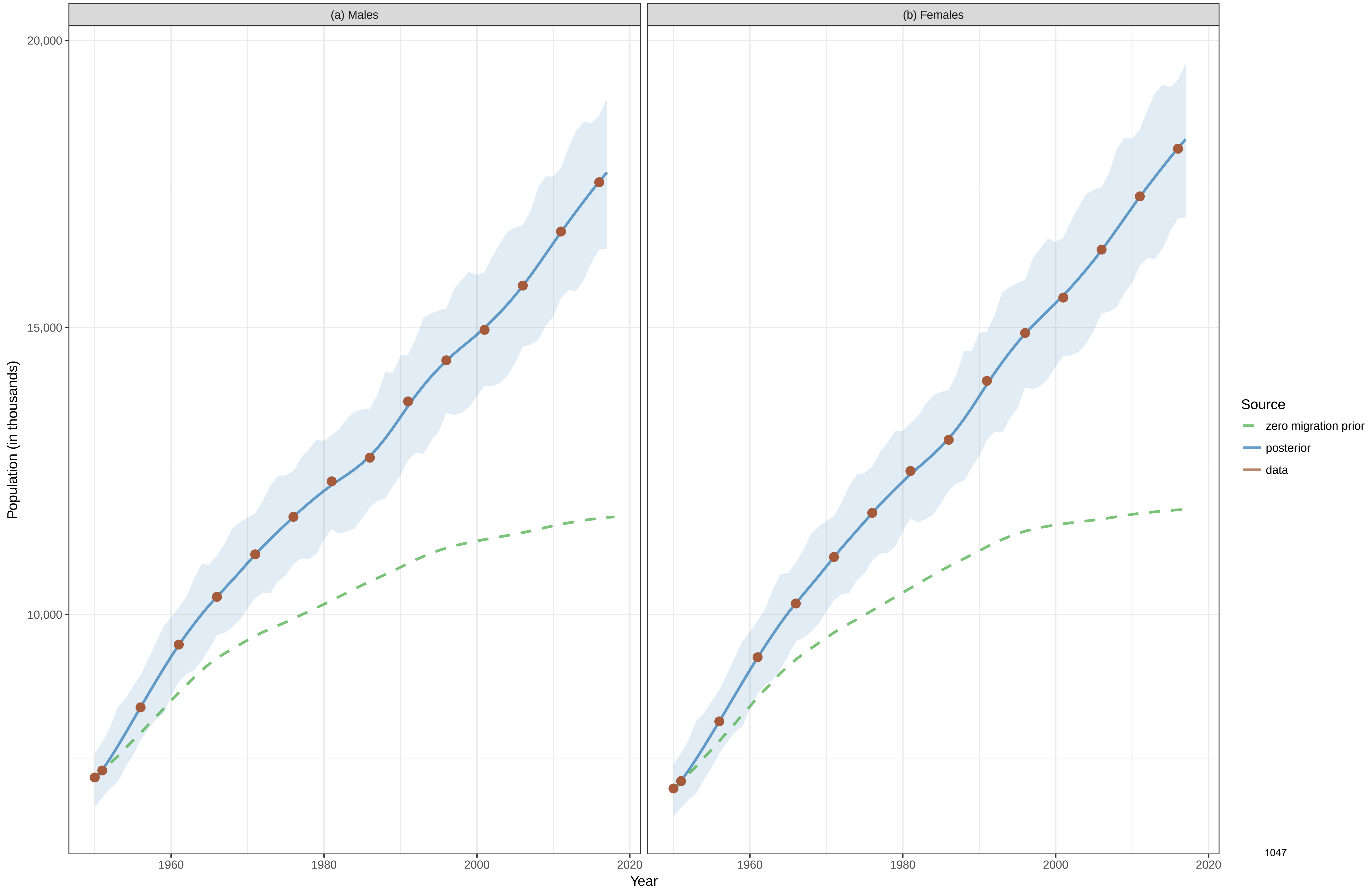

Supplemental Results Figure 4: GBD Bayesian Demographic Balancing Model fit in Cape Verde for (a) total population of males, and (b) total population of females, 1950−2017.

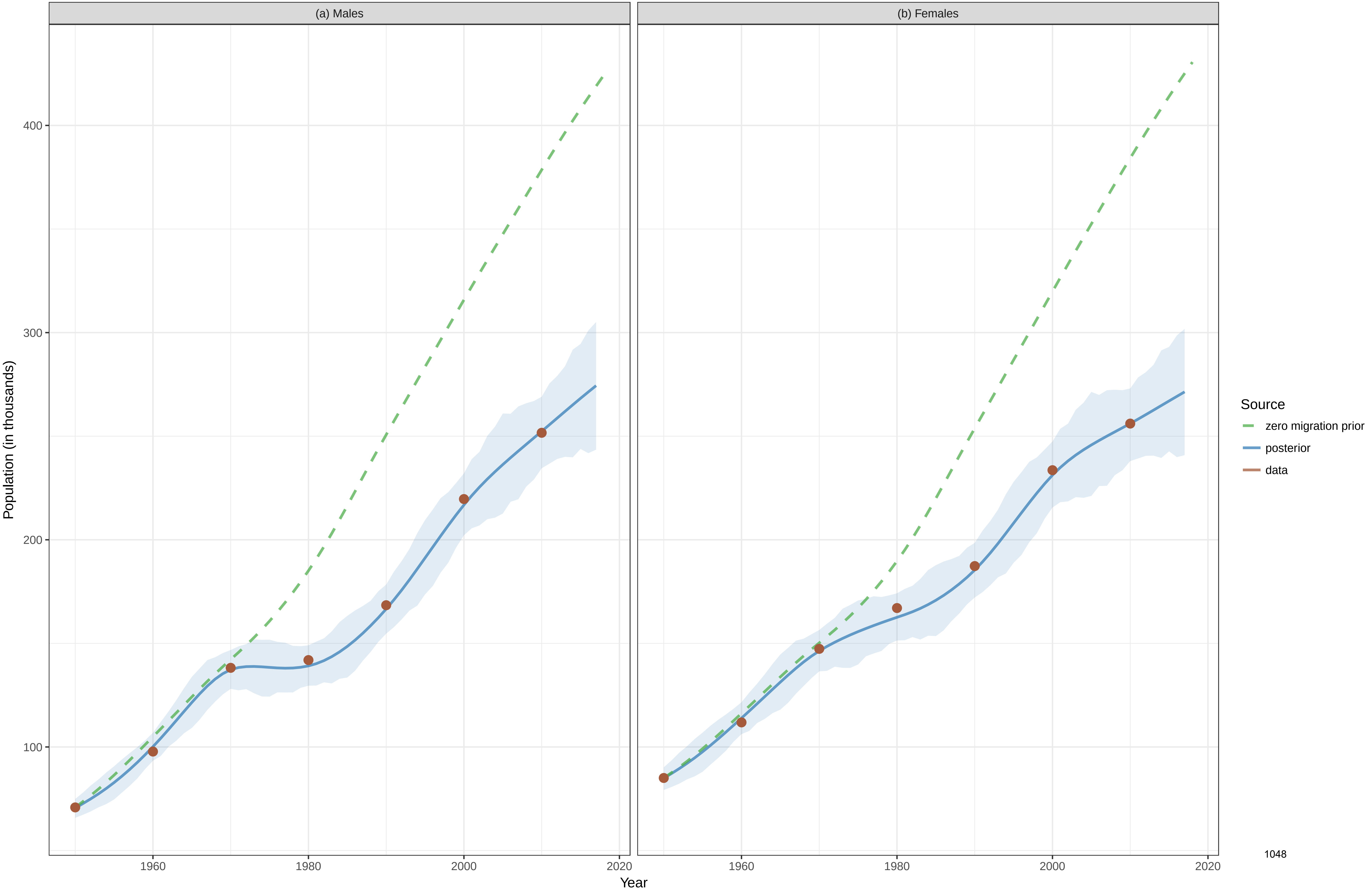

Supplemental Results Figure 4: GBD Bayesian Demographic Balancing Model fit in Central African Republic for (a) total population of males, and (b) total population of females, 1950−2017.

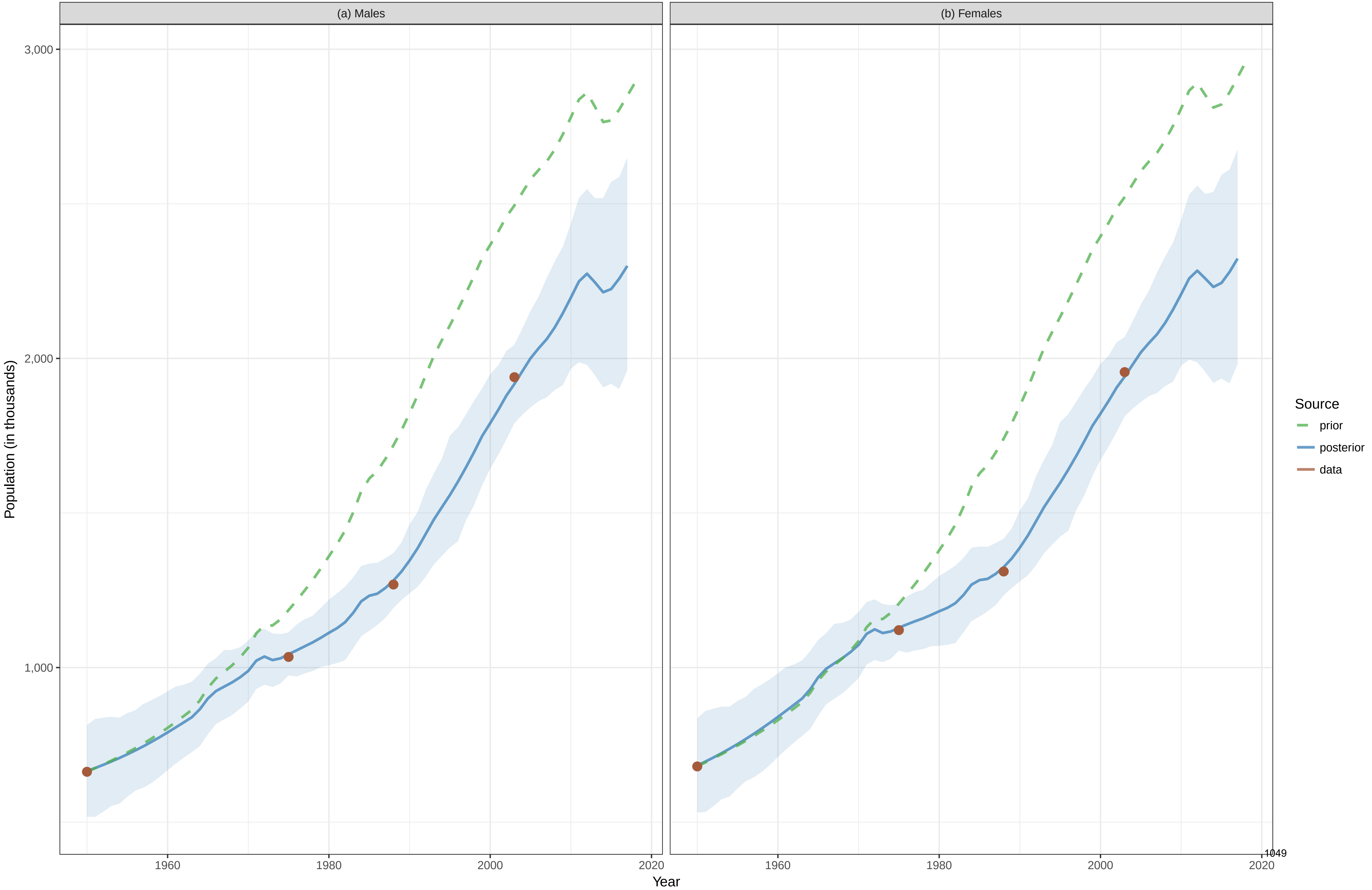

Supplemental Results Figure 4: GBD Bayesian Demographic Balancing Model fit in Chad for (a) total population of males, and (b) total population of females, 1950−2017.

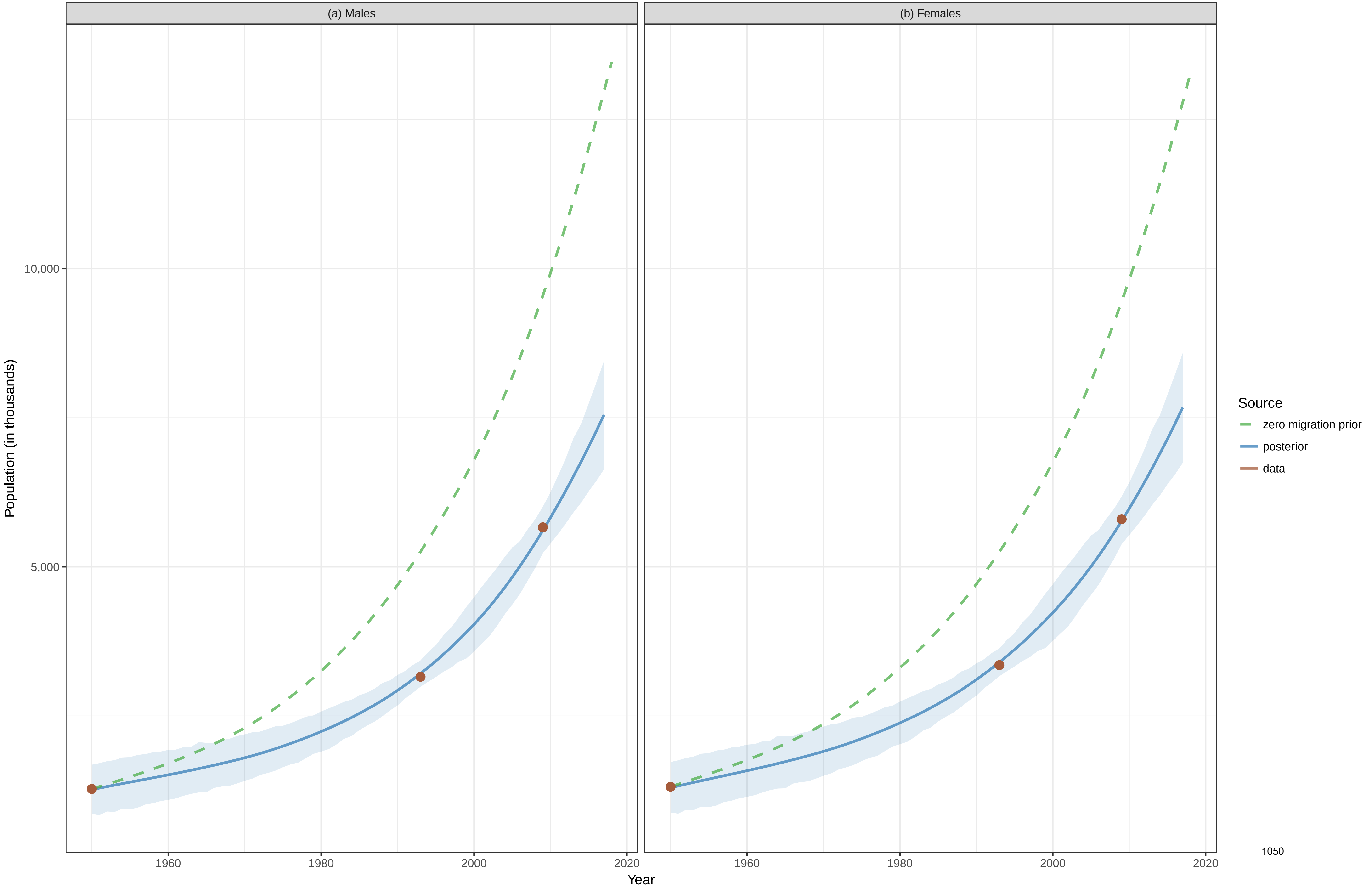

Supplemental Results Figure 4: GBD Bayesian Demographic Balancing Model fit in Chile for (a) total population of males, and (b) total population of females, 1950−2017.

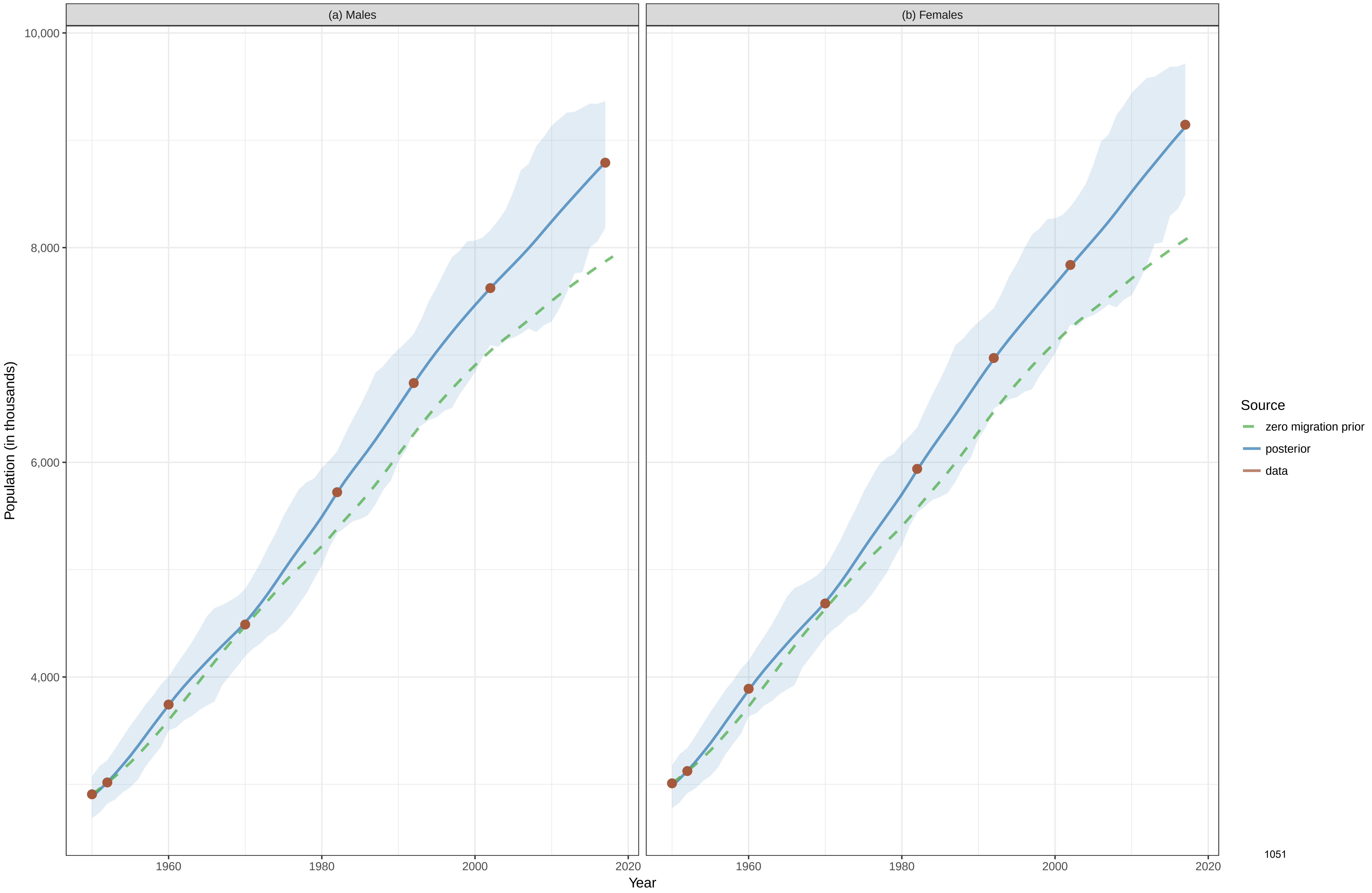

Supplemental Results Figure 4: GBD Bayesian Demographic Balancing Model fit in China (without Hong Kong and Macao) for (a) total population of males, and (b) total population of females, 1950−2017.

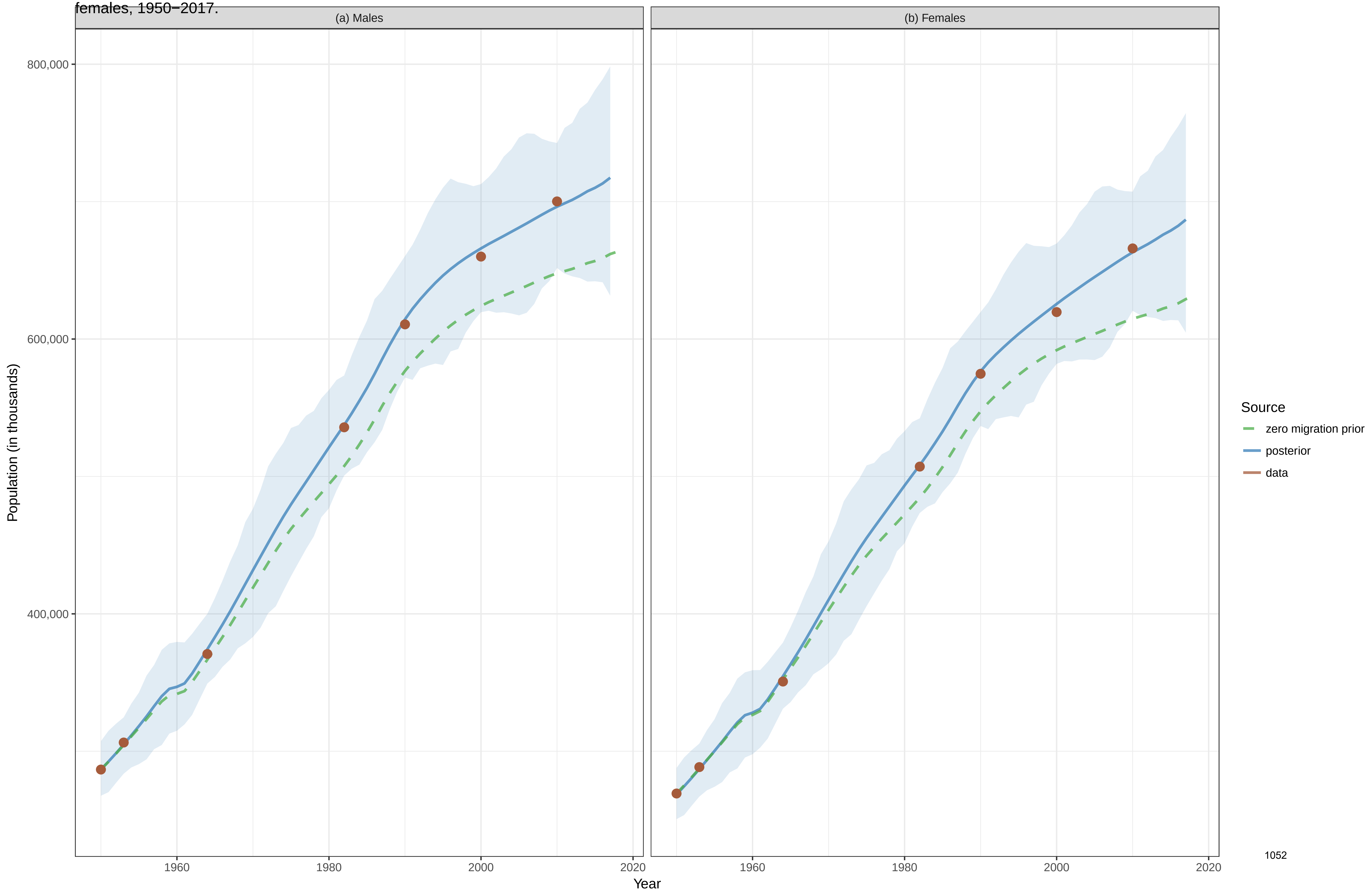

Supplemental Results Figure 4: GBD Bayesian Demographic Balancing Model fit in Colombia for (a) total population of males, and (b) total population of females, 1950−2017.

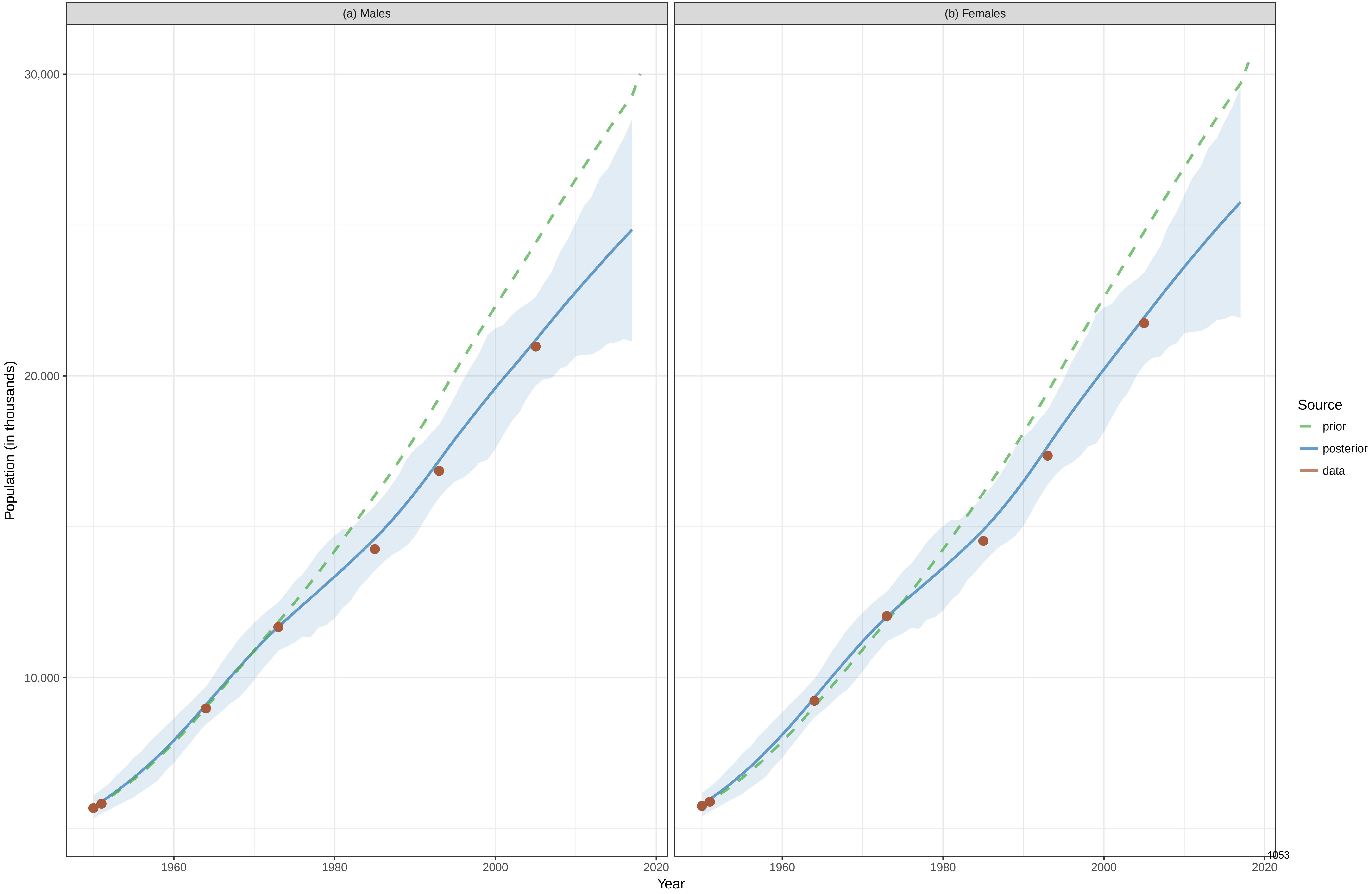

Supplemental Results Figure 4: GBD Bayesian Demographic Balancing Model fit in Comoros for (a) total population of males, and (b) total population of females, 1950−2017.

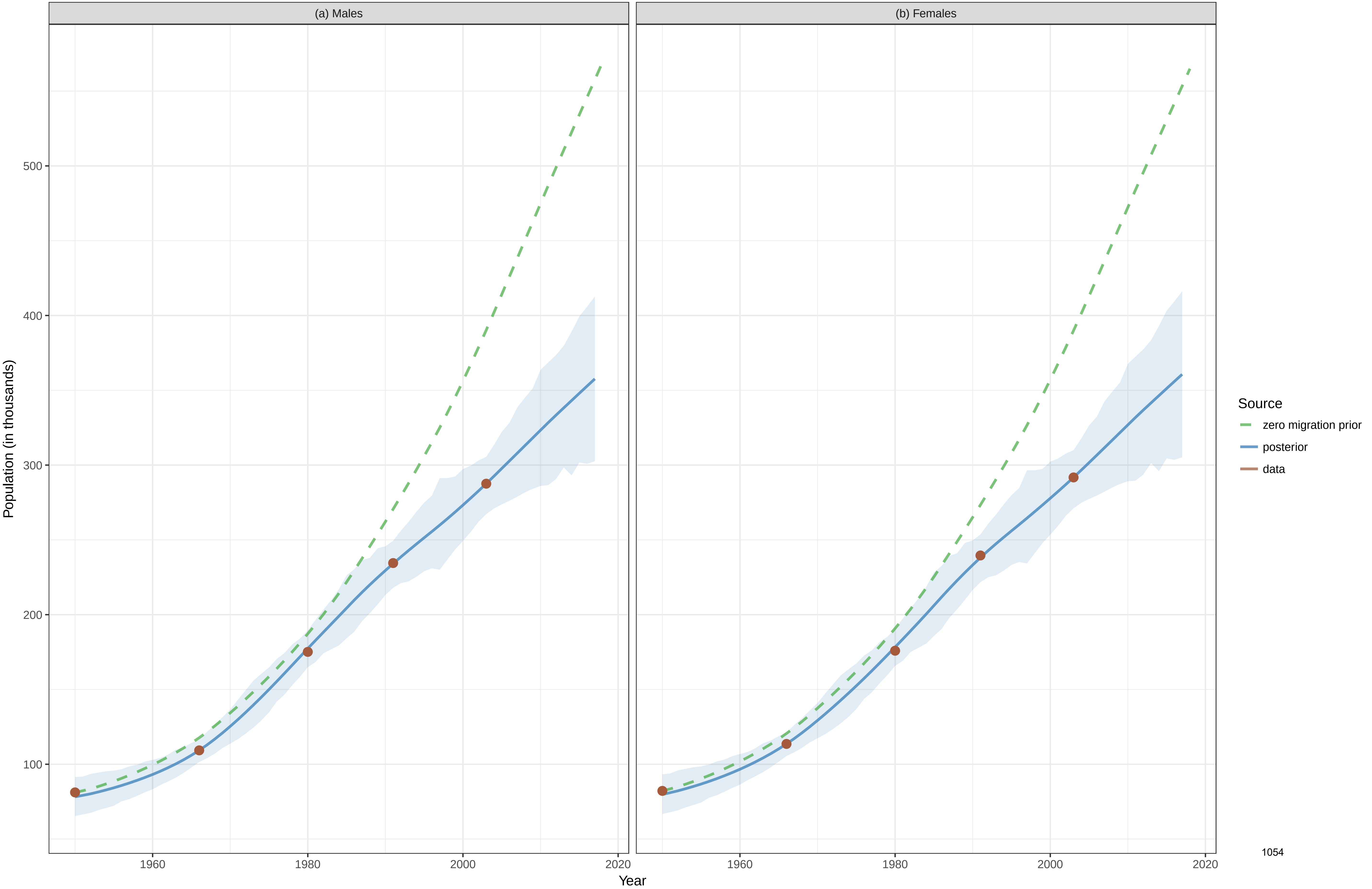

Supplemental Results Figure 4: GBD Bayesian Demographic Balancing Model fit in Congo for (a) total population of males, and (b) total population of females, 1950−2017.

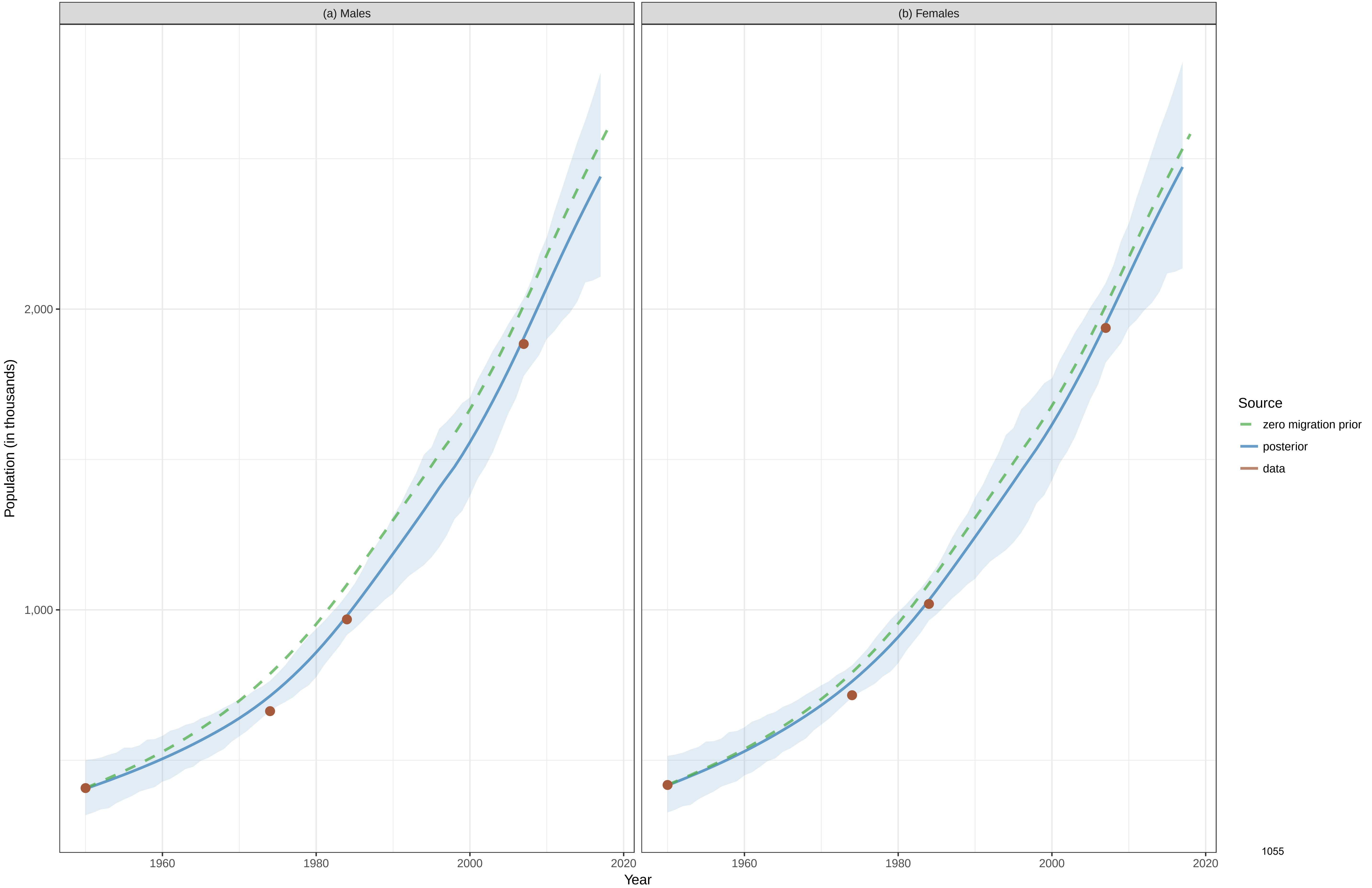

Supplemental Results Figure 4: GBD Bayesian Demographic Balancing Model fit in Costa Rica for (a) total population of males, and (b) total population of females, 1950−2017.

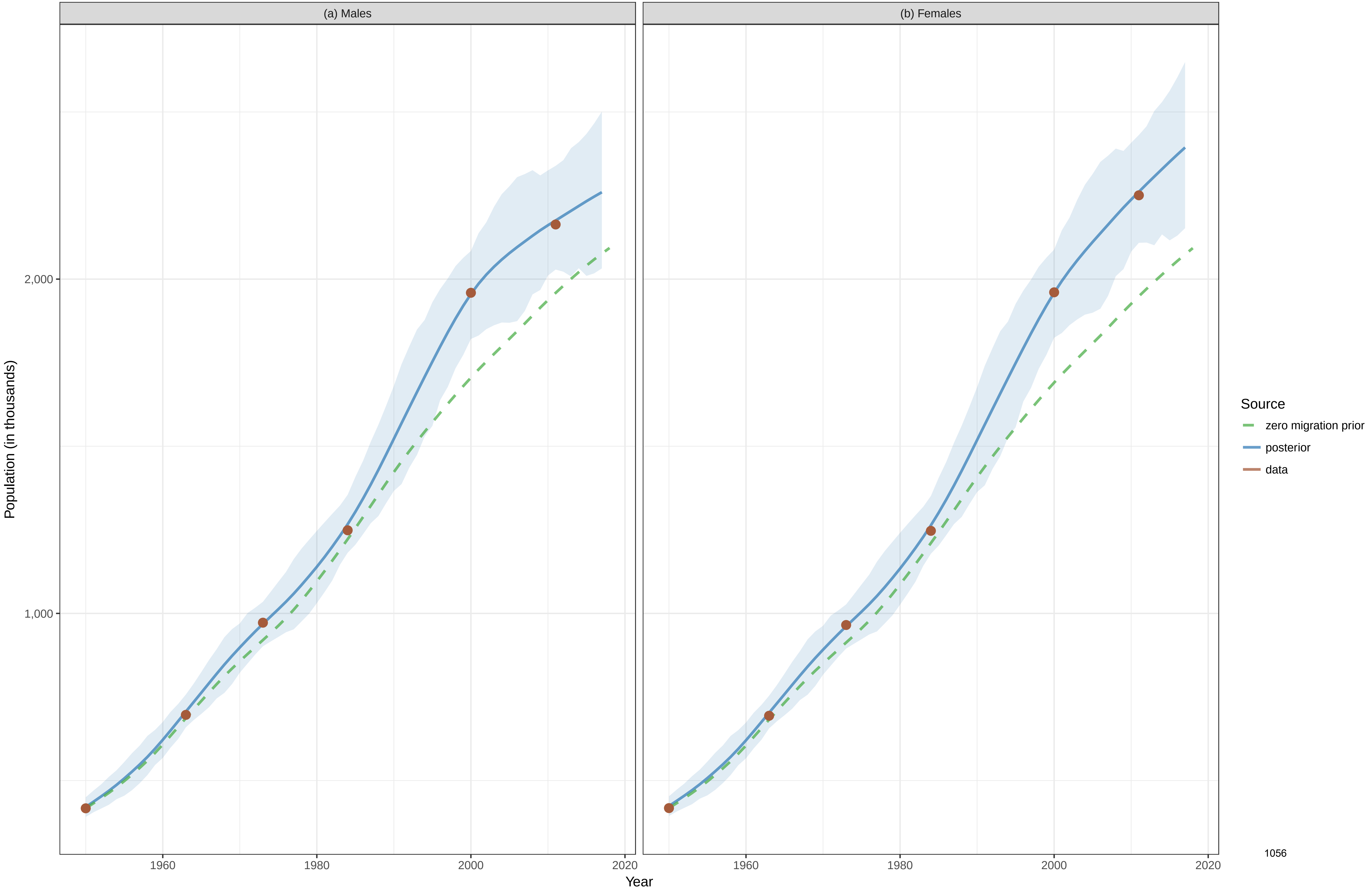

Supplemental Results Figure 4: GBD Bayesian Demographic Balancing Model fit in Cote d'Ivoire for (a) total population of males, and (b) total population of females, 1950−2017.

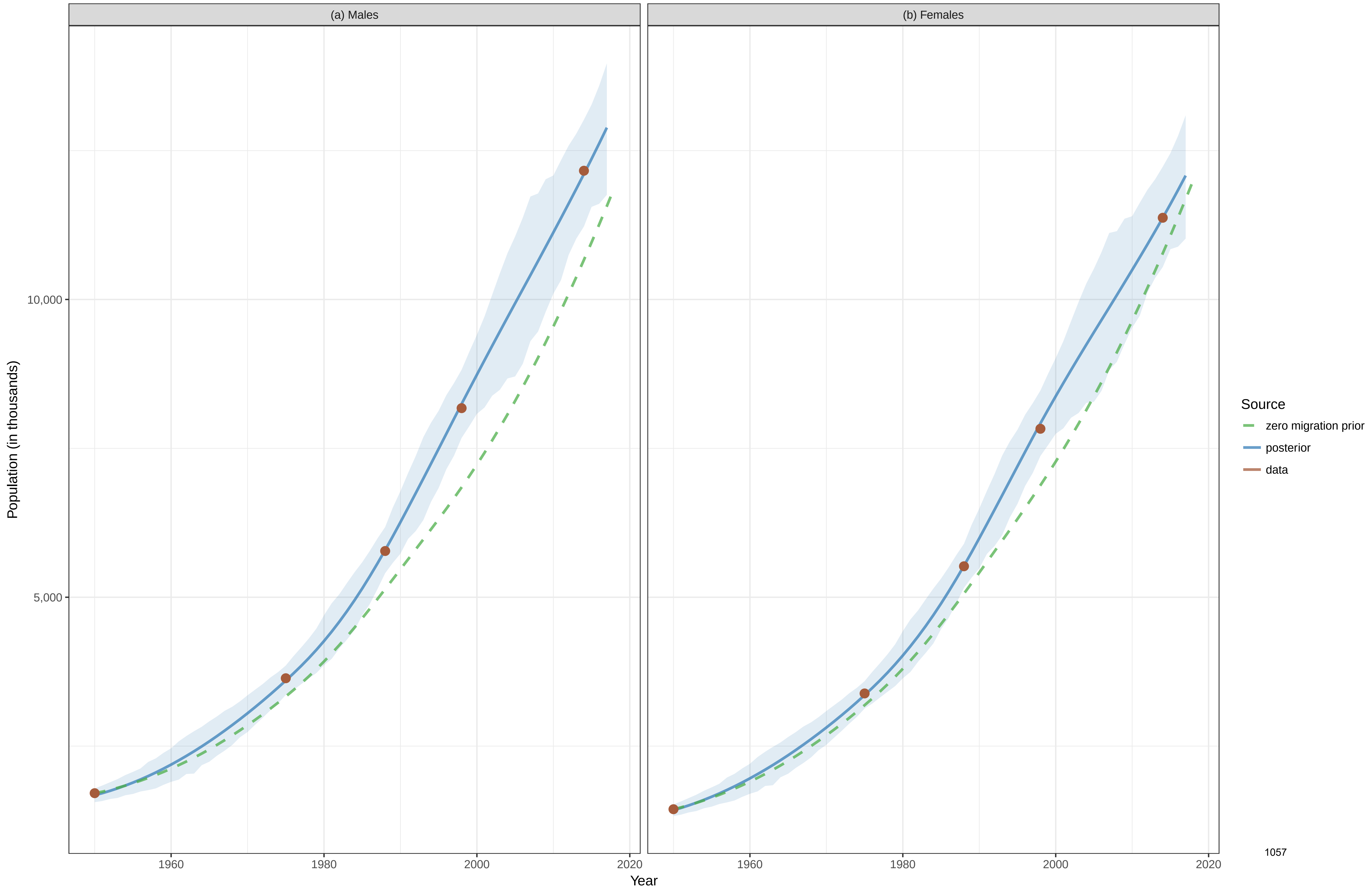

Supplemental Results Figure 4: GBD Bayesian Demographic Balancing Model fit in Croatia for (a) total population of males, and (b) total population of females, 1950−2017.

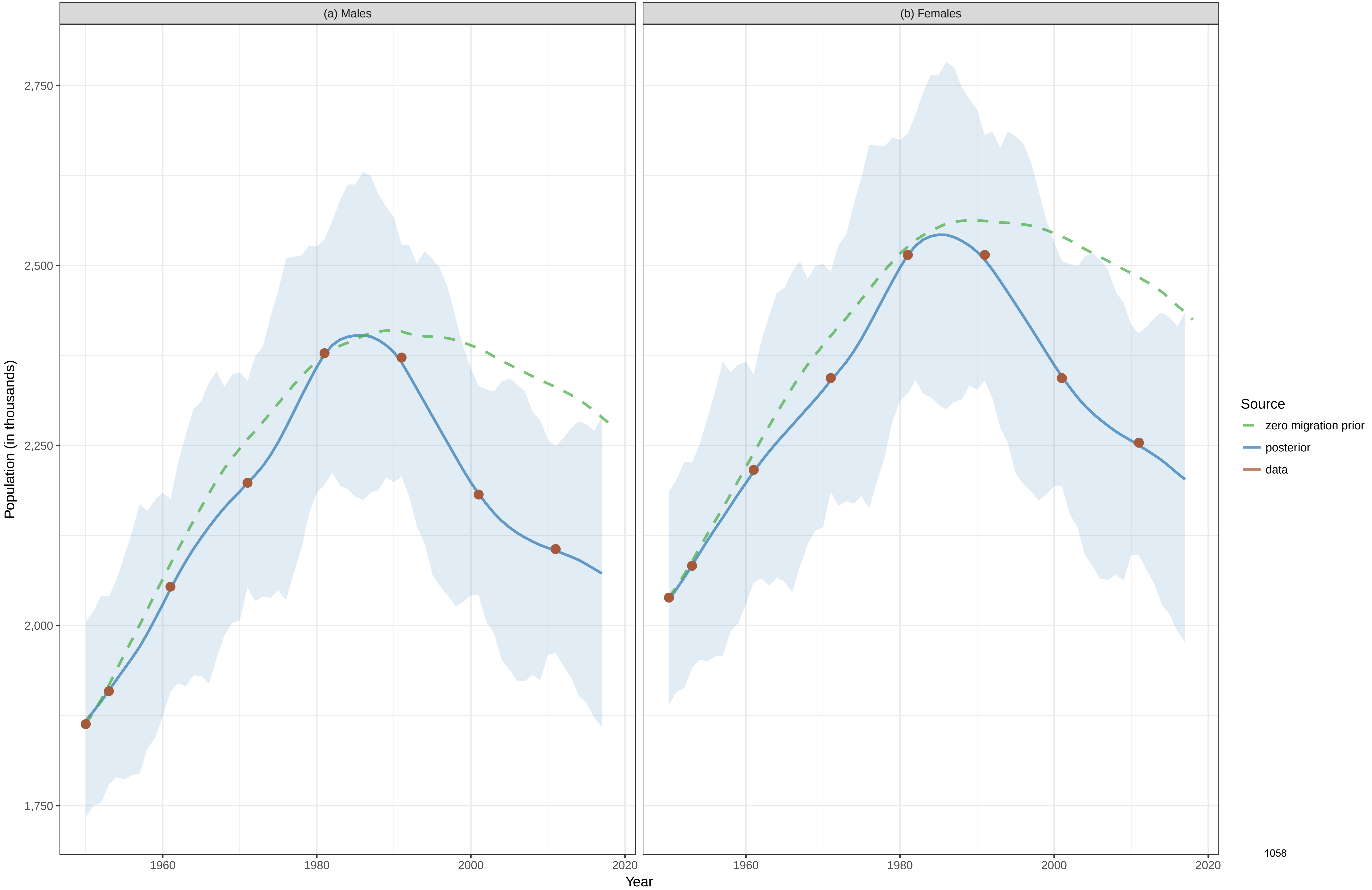

Supplemental Results Figure 4: GBD Bayesian Demographic Balancing Model fit in Cuba for (a) total population of males, and (b) total population of females, 1950−2017.

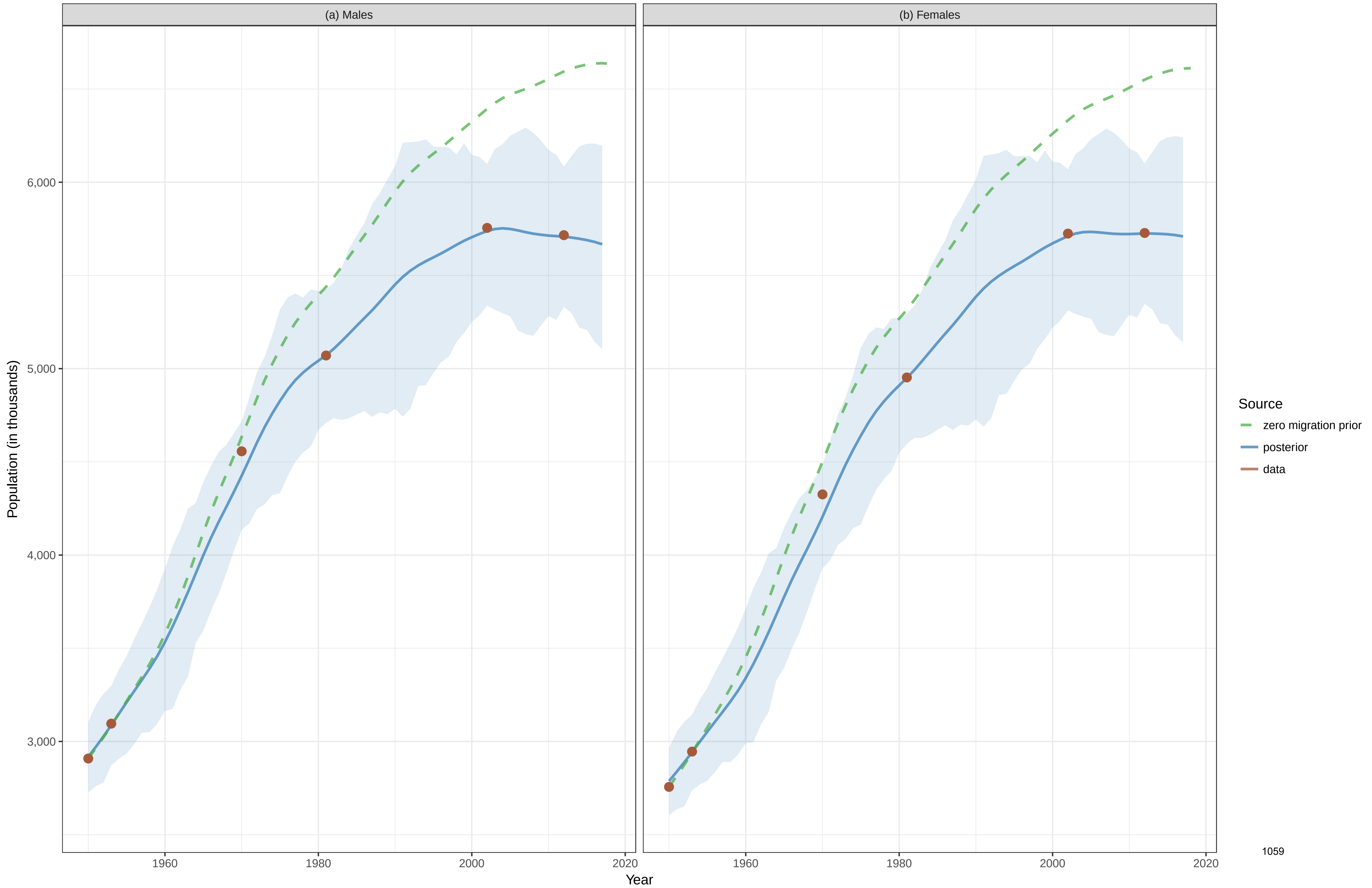

Supplemental Results Figure 4: GBD Bayesian Demographic Balancing Model fit in Cyprus for (a) total population of males, and (b) total population of females, 1950−2017.

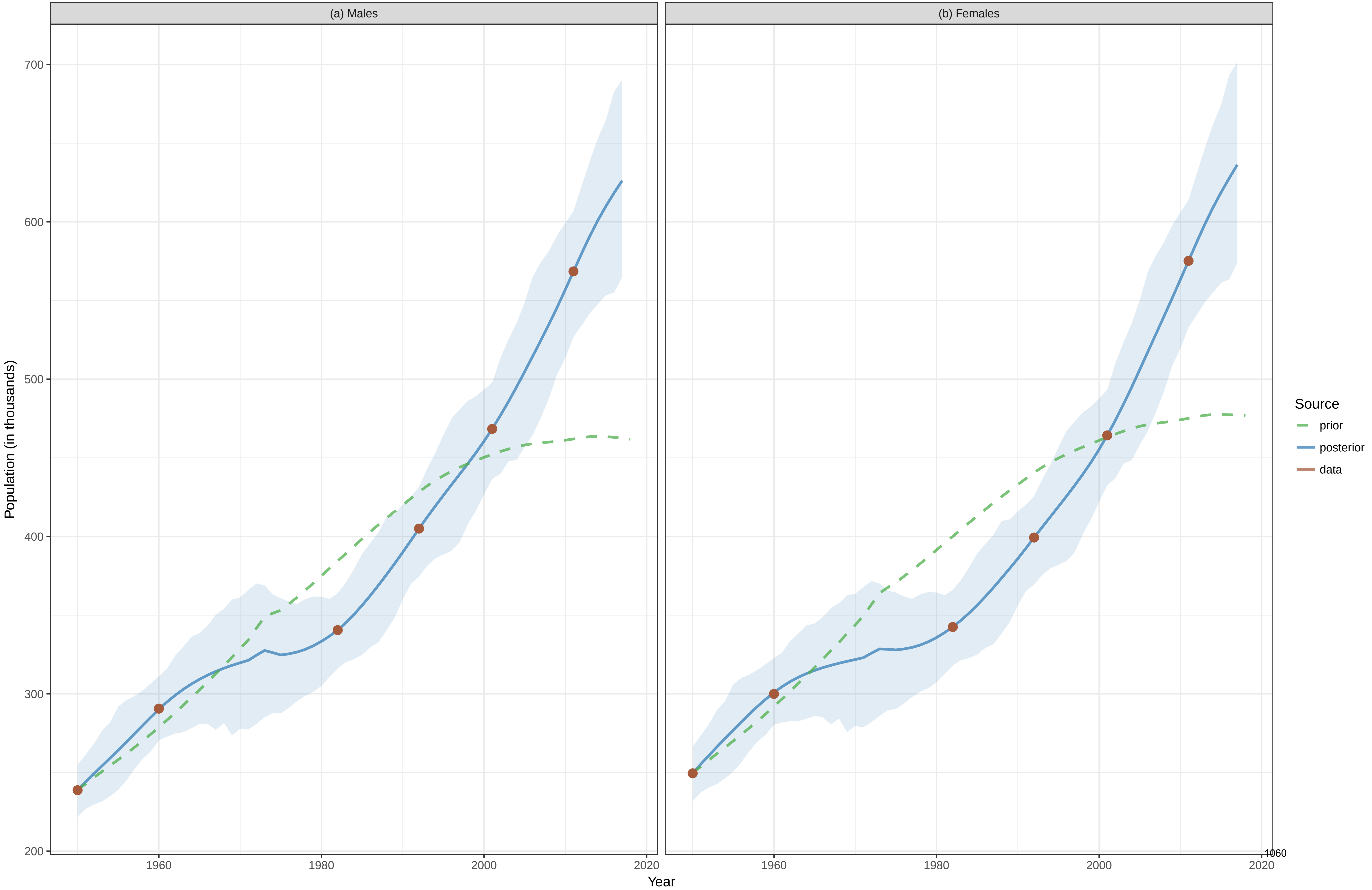

Supplemental Results Figure 4: GBD Bayesian Demographic Balancing Model fit in Czech Republic for (a) total population of males, and (b) total population of females, 1950−2017.

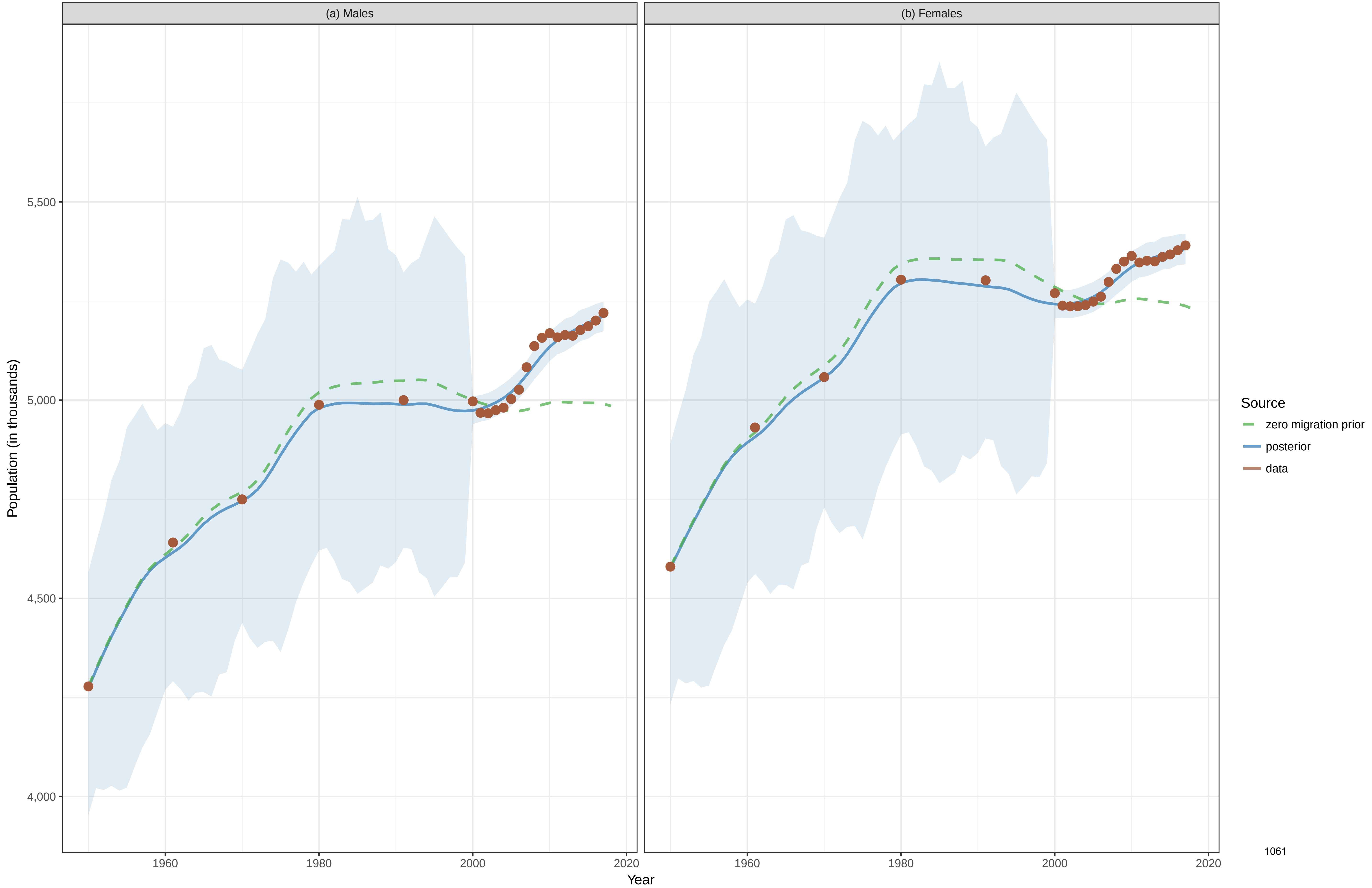

Supplemental Results Figure 4: GBD Bayesian Demographic Balancing Model fit in Democratic Republic of the Congo for (a) total population of males, and (b) total population of females, 1950−2017.

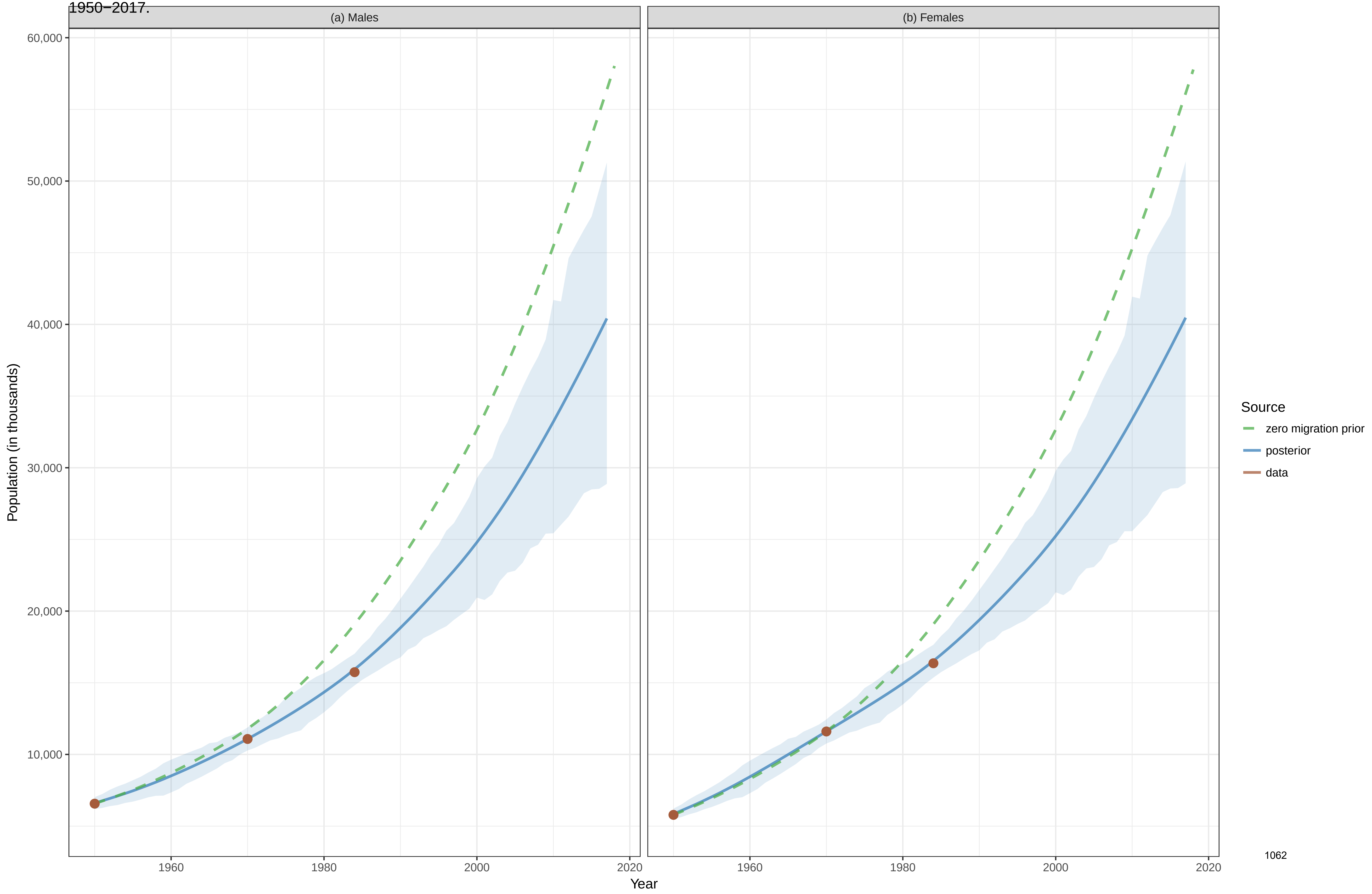

Supplemental Results Figure 4: GBD Bayesian Demographic Balancing Model fit in Denmark for (a) total population of males, and (b) total population of females, 1950−2017.

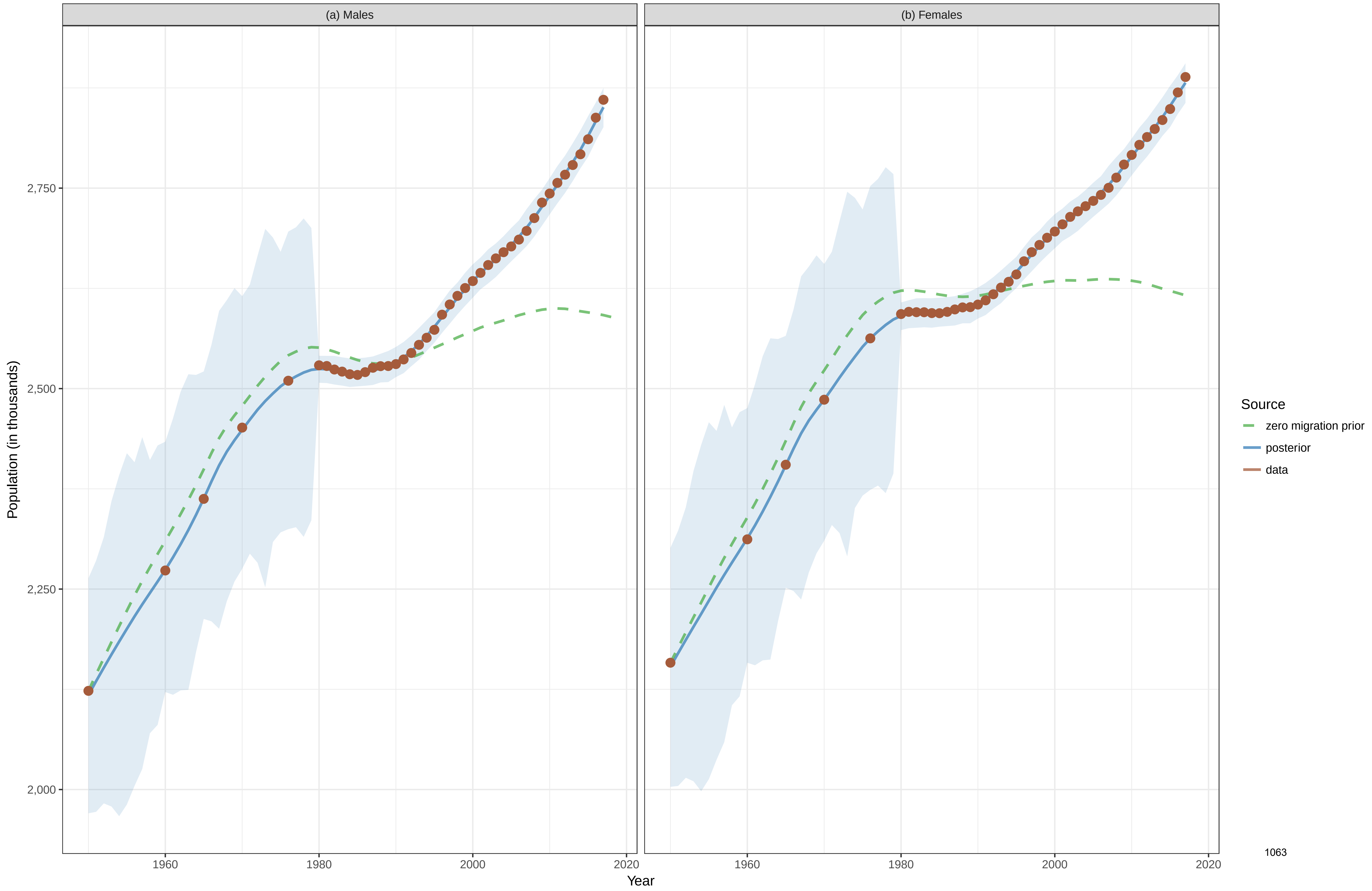

Supplemental Results Figure 4: GBD Bayesian Demographic Balancing Model fit in Djibouti for (a) total population of males, and (b) total population of females, 1950−2017.

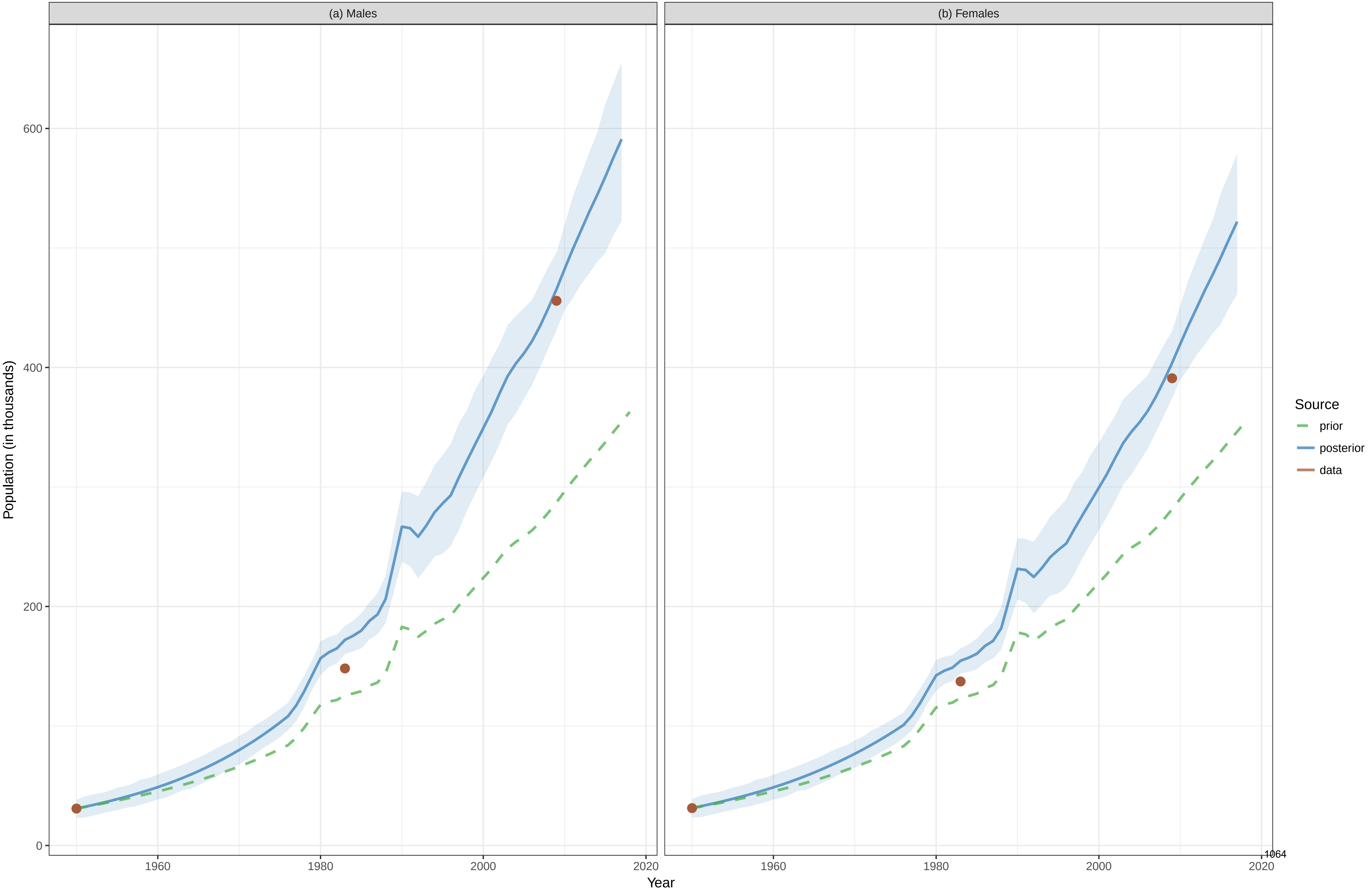

Supplemental Results Figure 4: GBD Bayesian Demographic Balancing Model fit in Dominica for (a) total population of males, and (b) total population of females, 1950−2017.

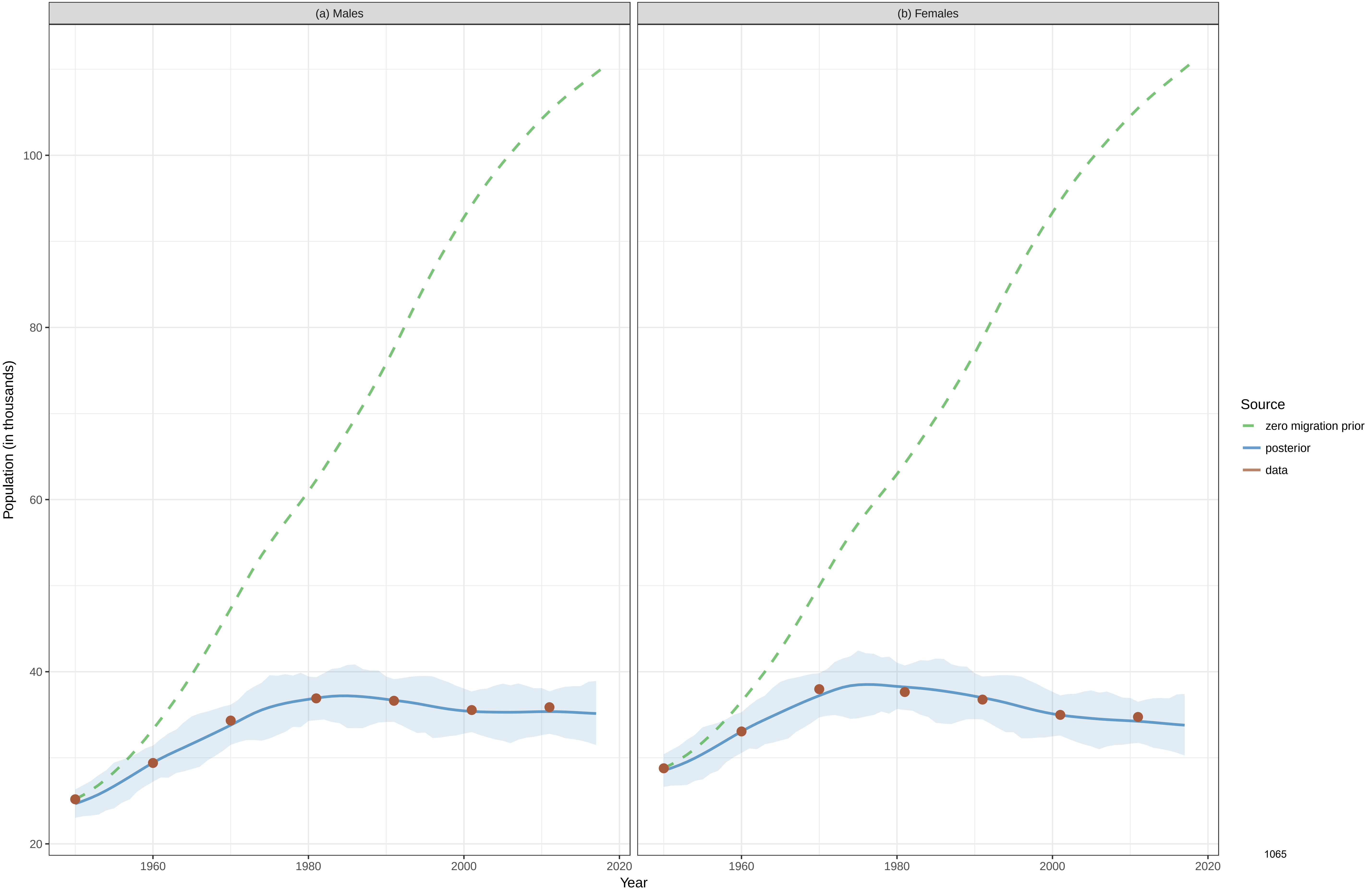

Supplemental Results Figure 4: GBD Bayesian Demographic Balancing Model fit in Dominican Republic for (a) total population of males, and (b) total population of females, 1950−2017.

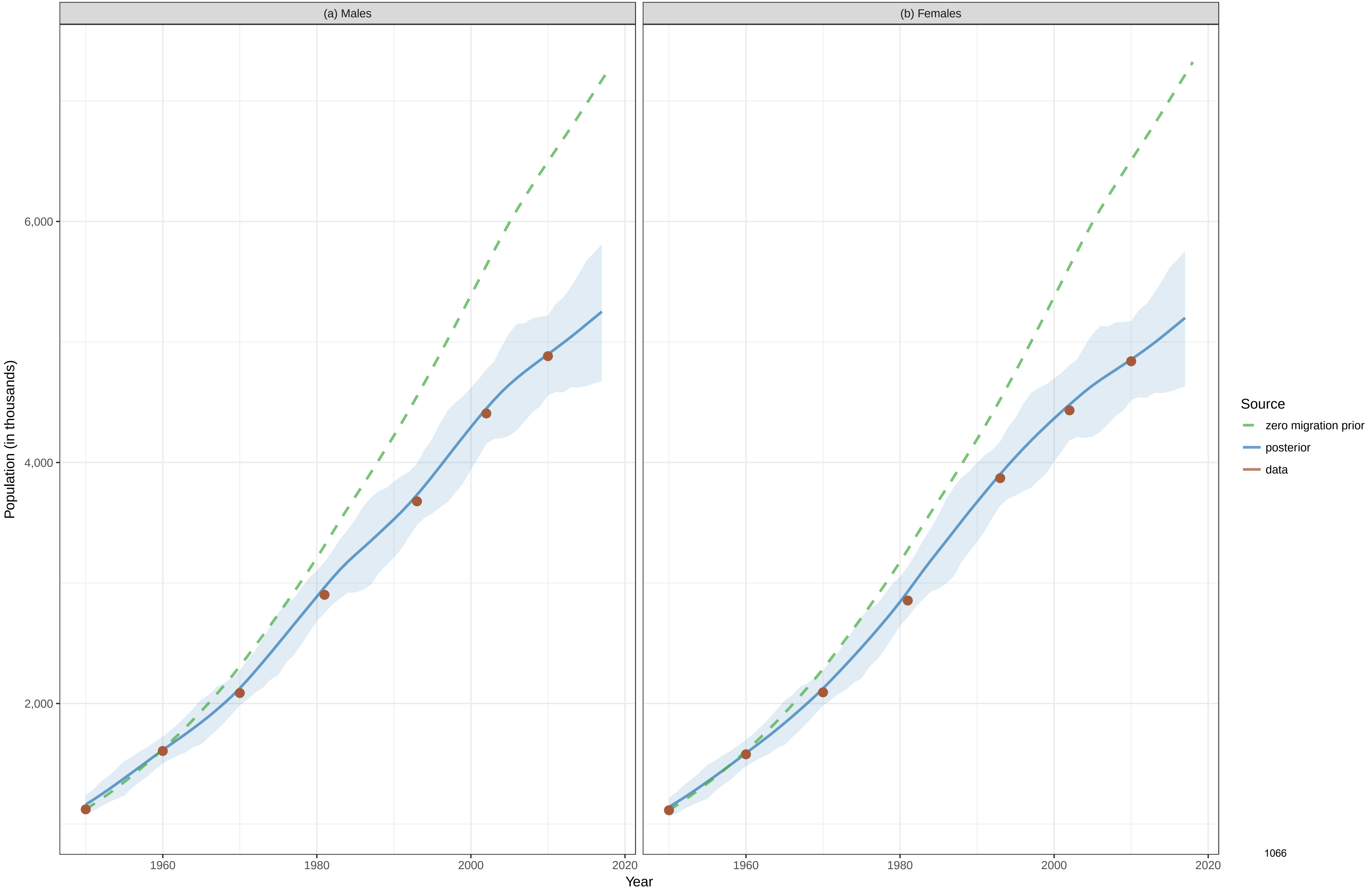

Supplemental Results Figure 4: GBD Bayesian Demographic Balancing Model fit in Ecuador for (a) total population of males, and (b) total population of females, 1950−2017.

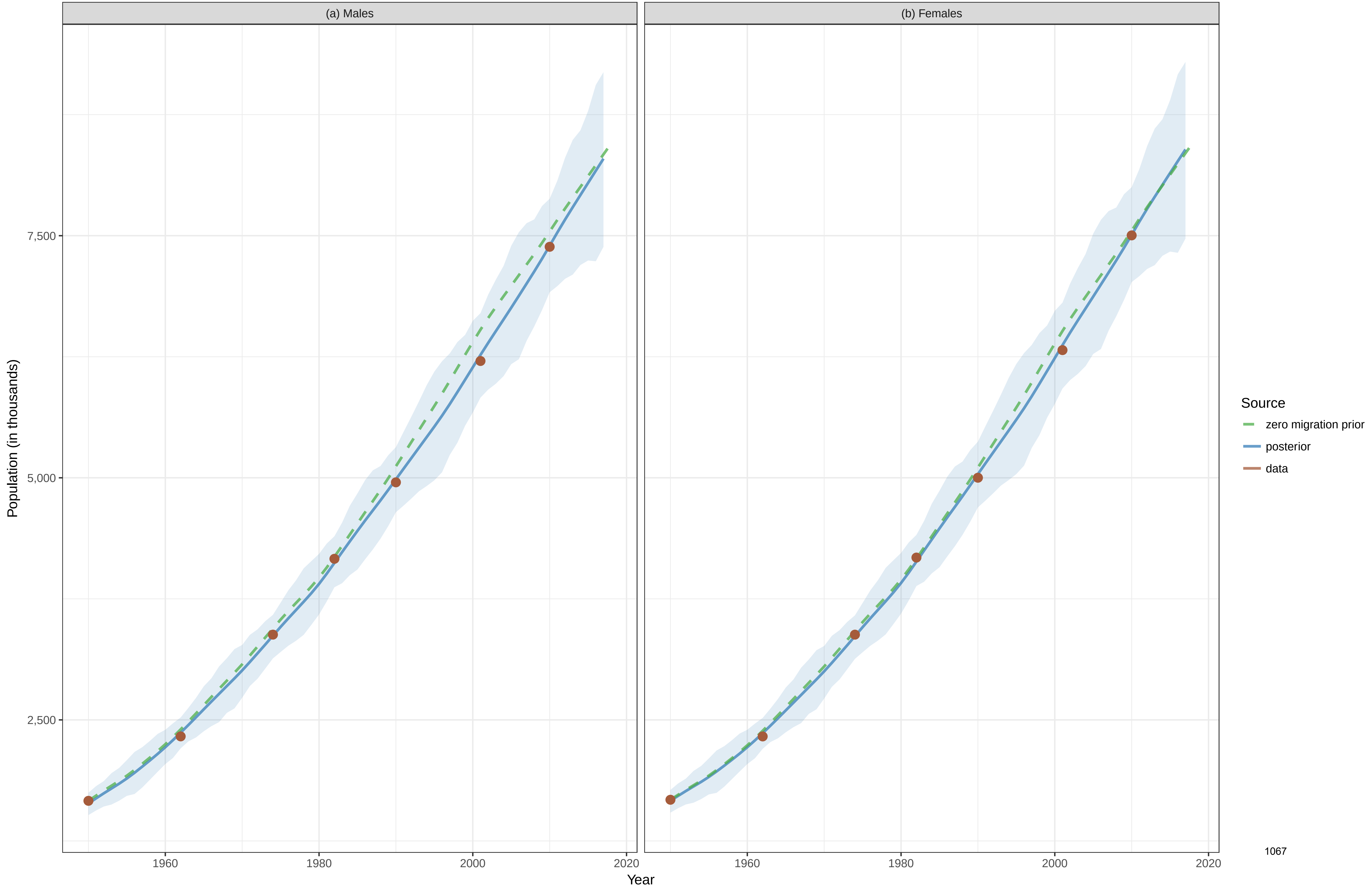

Supplemental Results Figure 4: GBD Bayesian Demographic Balancing Model fit in Egypt for (a) total population of males, and (b) total population of females, 1950−2017.

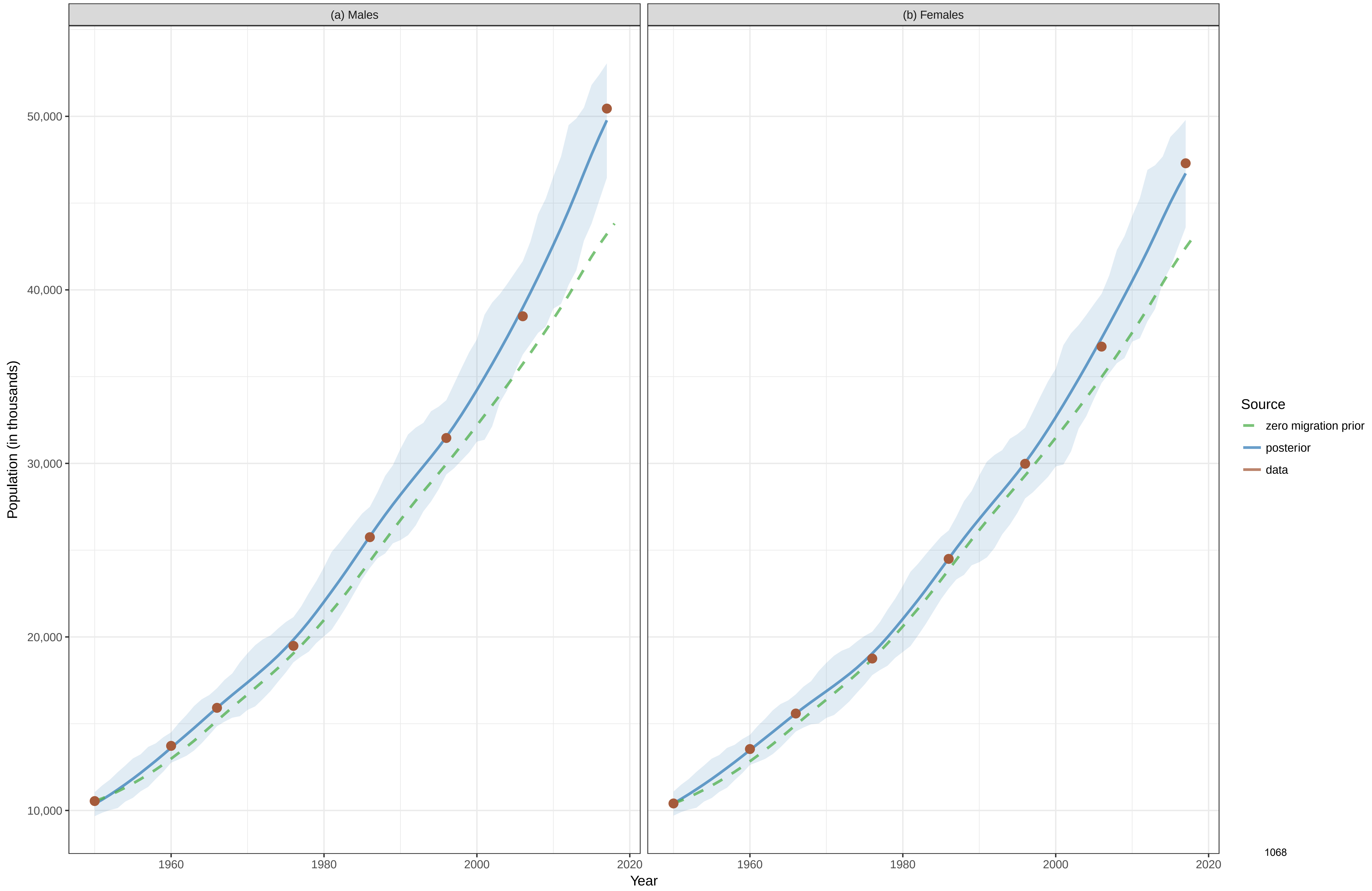

Supplemental Results Figure 4: GBD Bayesian Demographic Balancing Model fit in El Salvador for (a) total population of males, and (b) total population of females, 1950−2017.

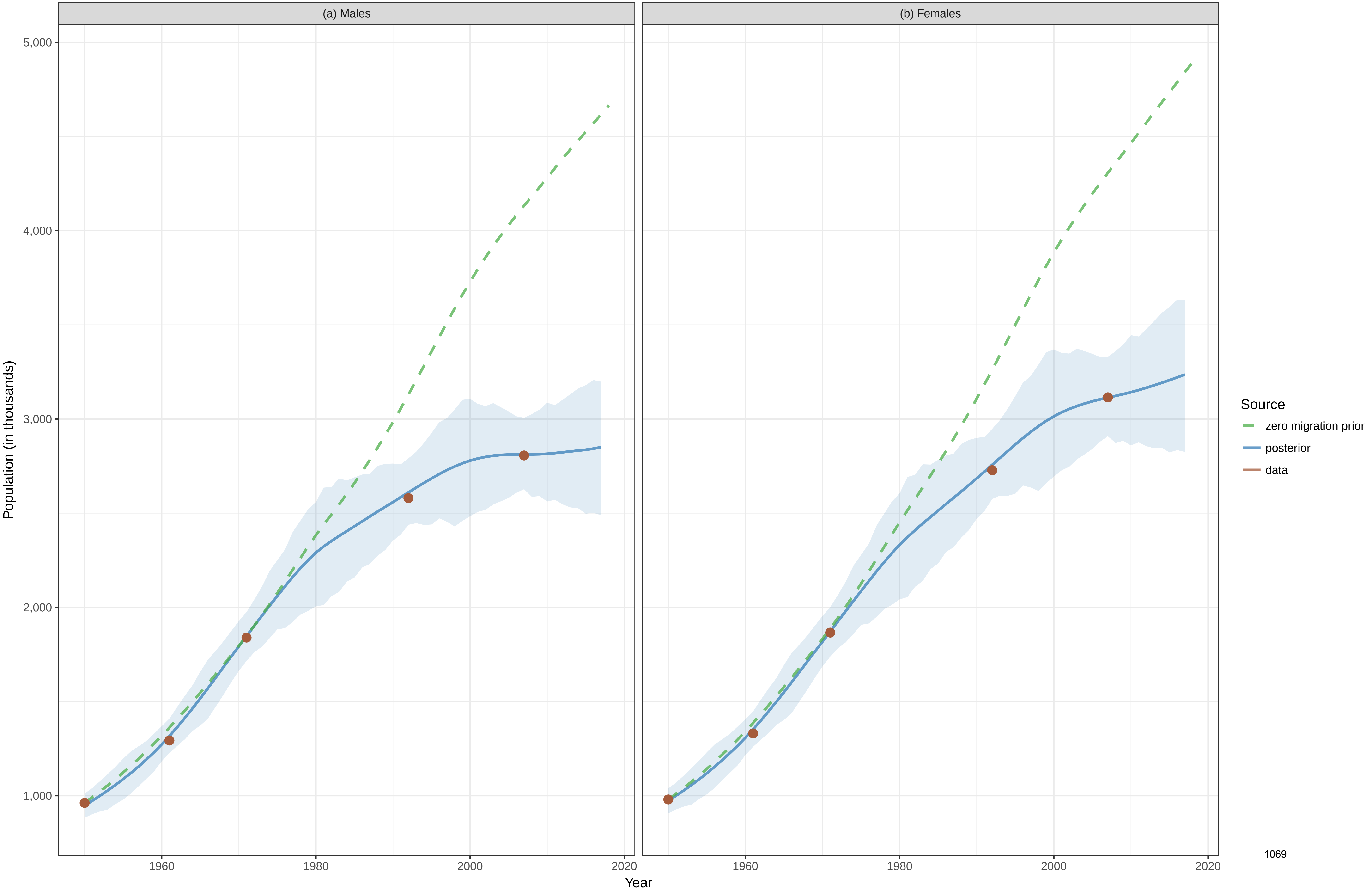

Supplemental Results Figure 4: GBD Bayesian Demographic Balancing Model fit in England for (a) total population of males, and (b) total population of females, 1950−2017.

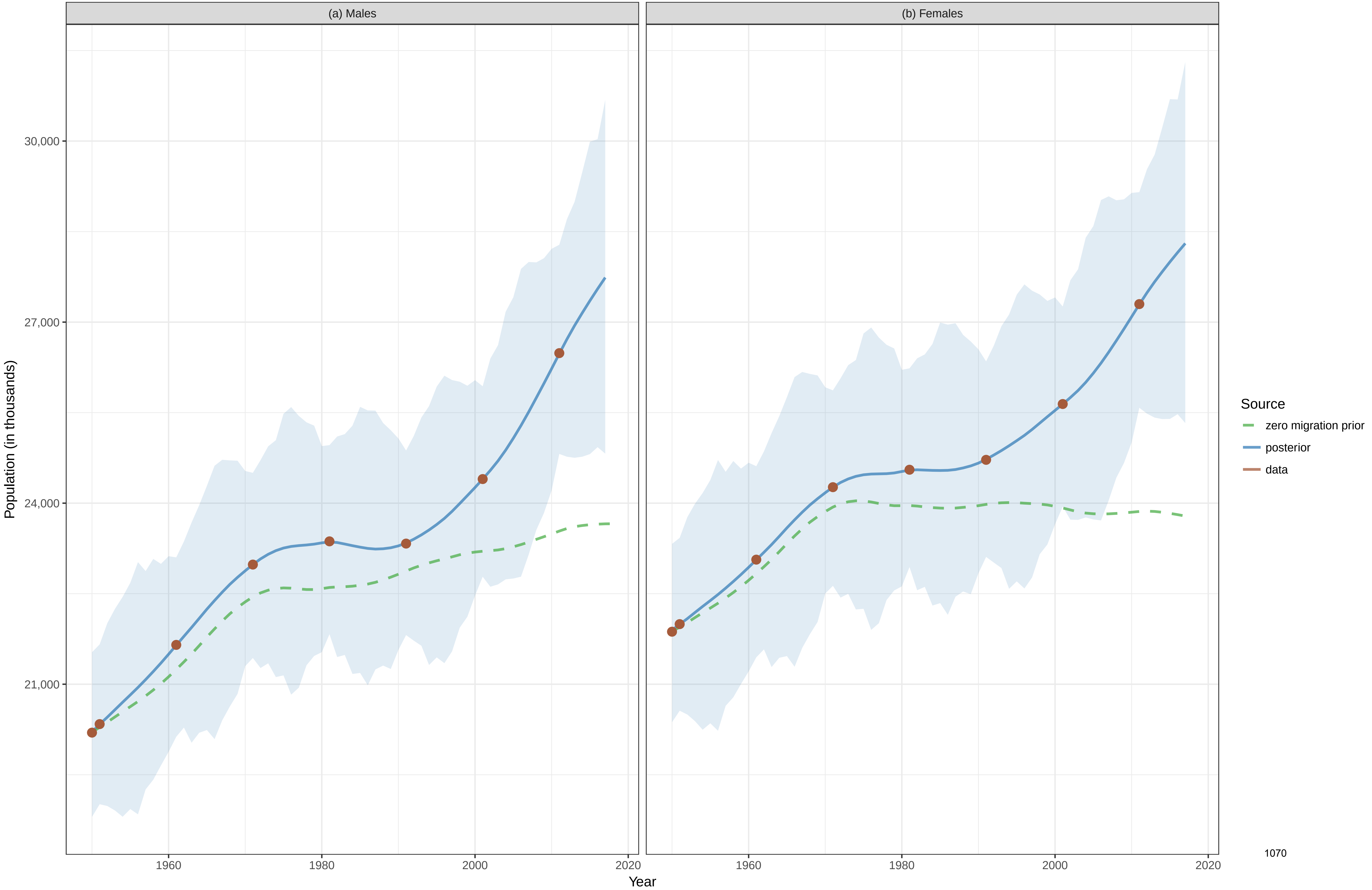

Supplemental Results Figure 4: GBD Bayesian Demographic Balancing Model fit in Equatorial Guinea for (a) total population of males, and (b) total population of females, 1950−2017.

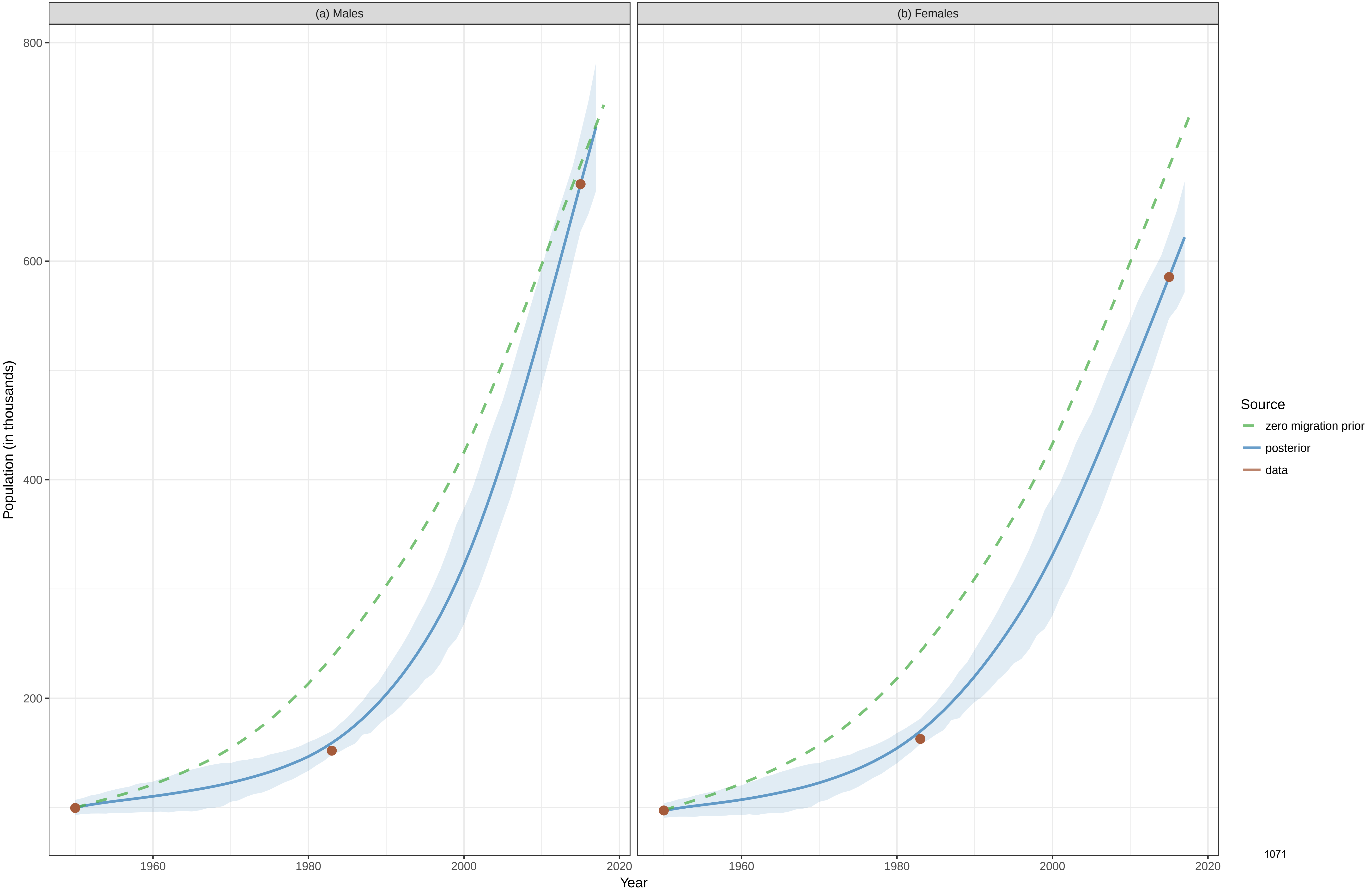

Supplemental Results Figure 4: GBD Bayesian Demographic Balancing Model fit in Eritrea for (a) total population of males, and (b) total population of females, 1950−2017.

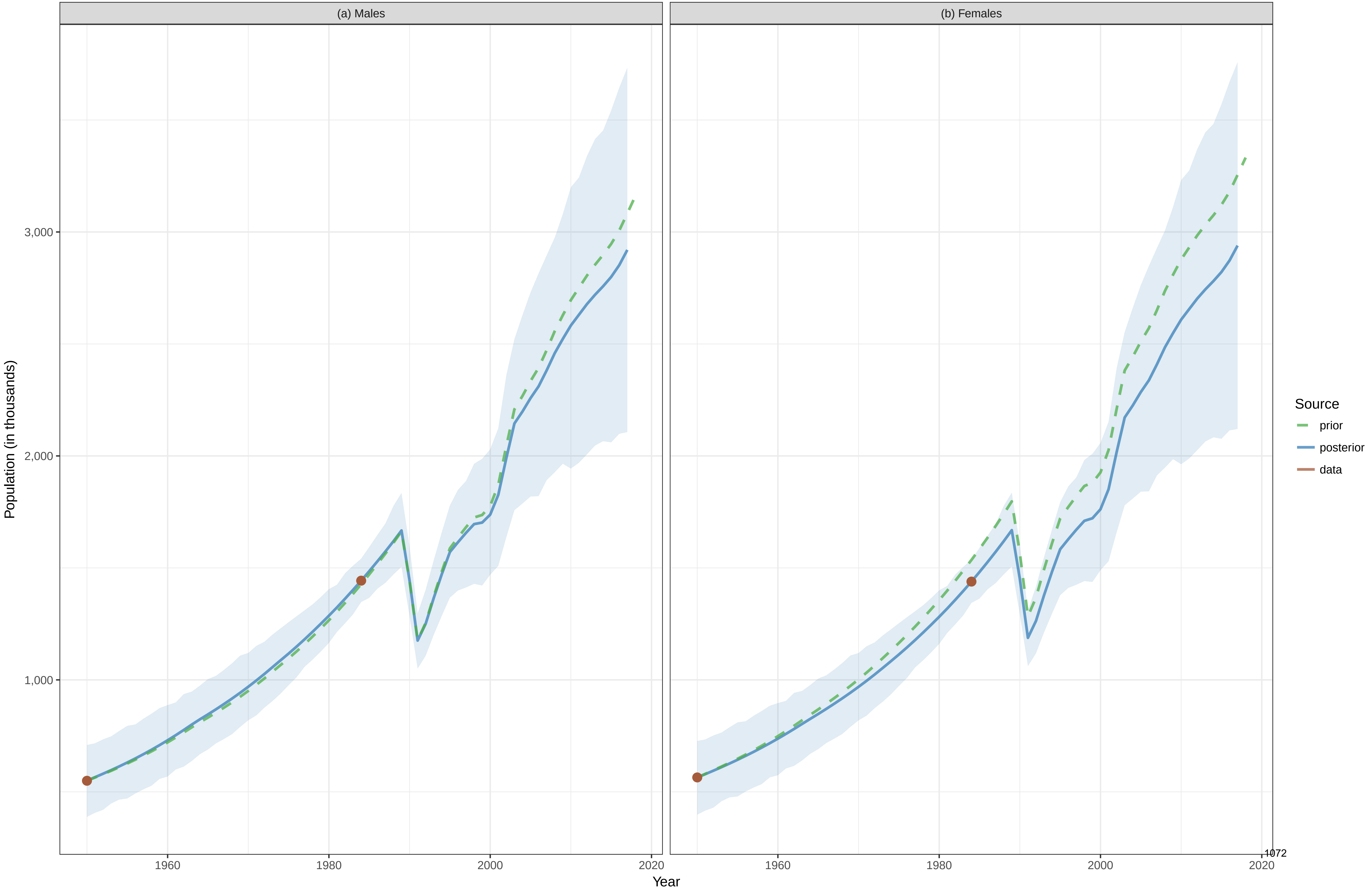

Supplemental Results Figure 4: GBD Bayesian Demographic Balancing Model fit in Estonia for (a) total population of males, and (b) total population of females, 1950−2017.

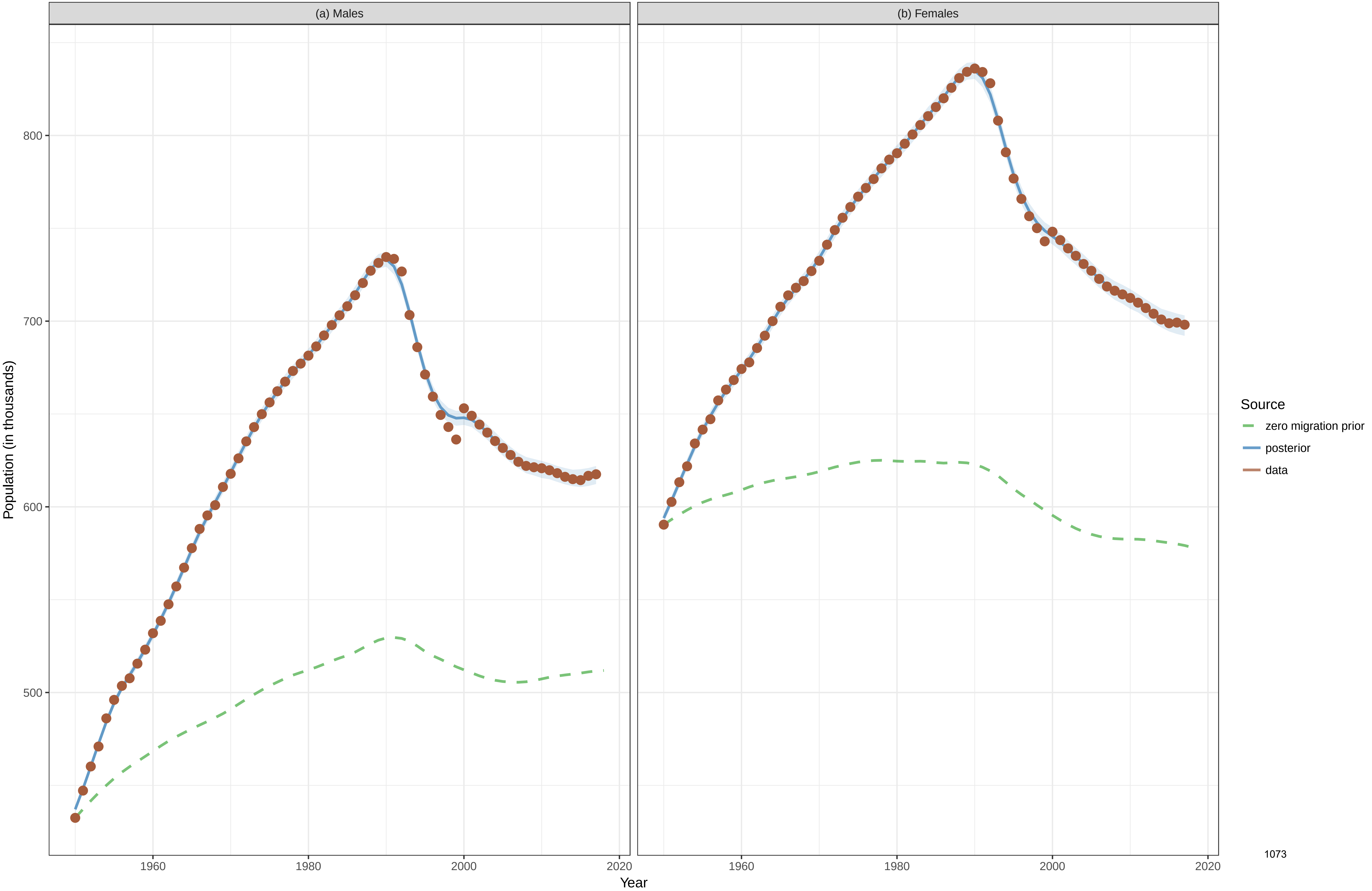

Supplemental Results Figure 4: GBD Bayesian Demographic Balancing Model fit in Ethiopia for (a) total population of males, and (b) total population of females, 1950−2017.

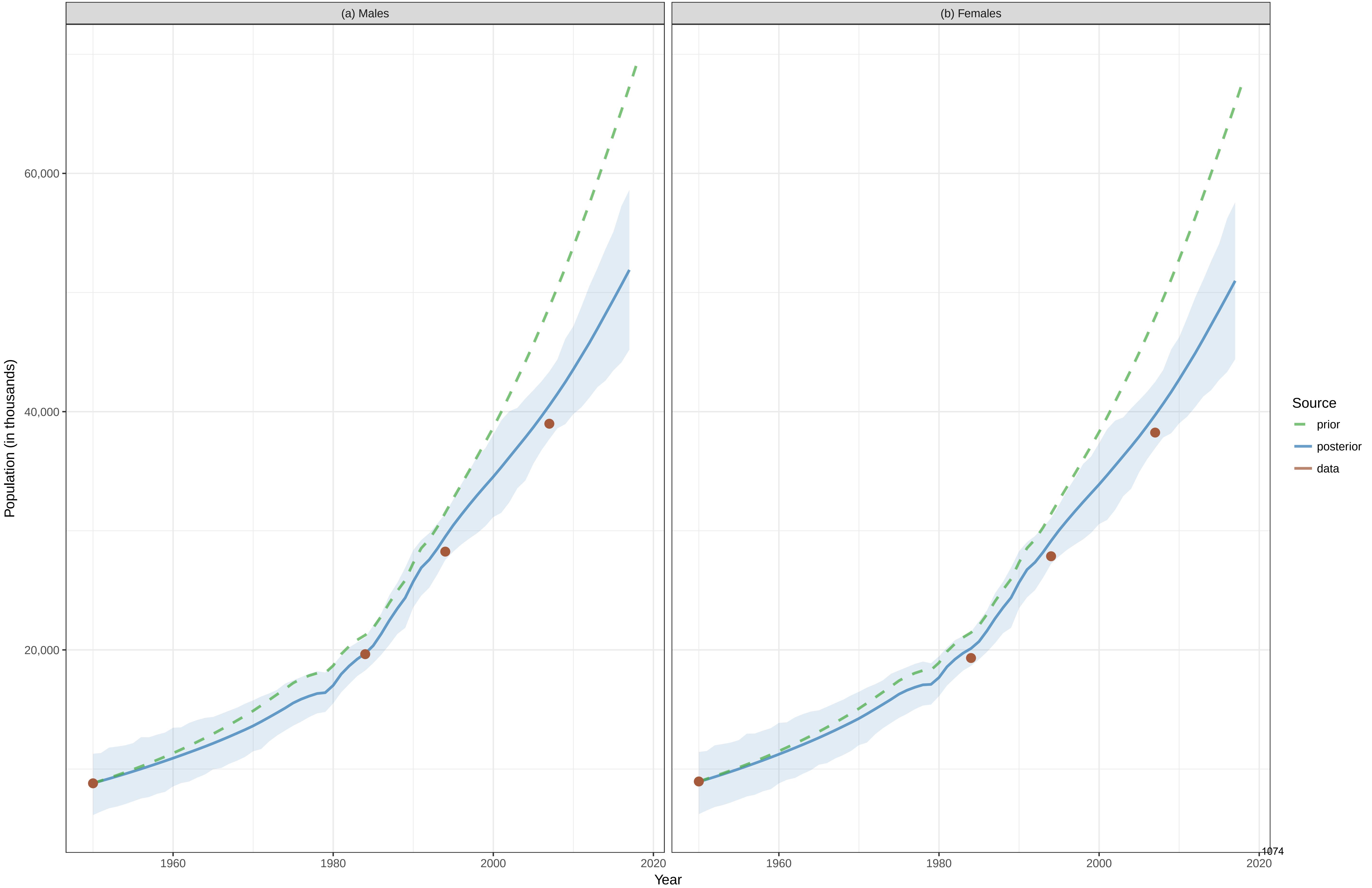

Supplemental Results Figure 4: GBD Bayesian Demographic Balancing Model fit in Federated States of Micronesia for (a) total population of males, and (b) total population of females, 1950–2017.

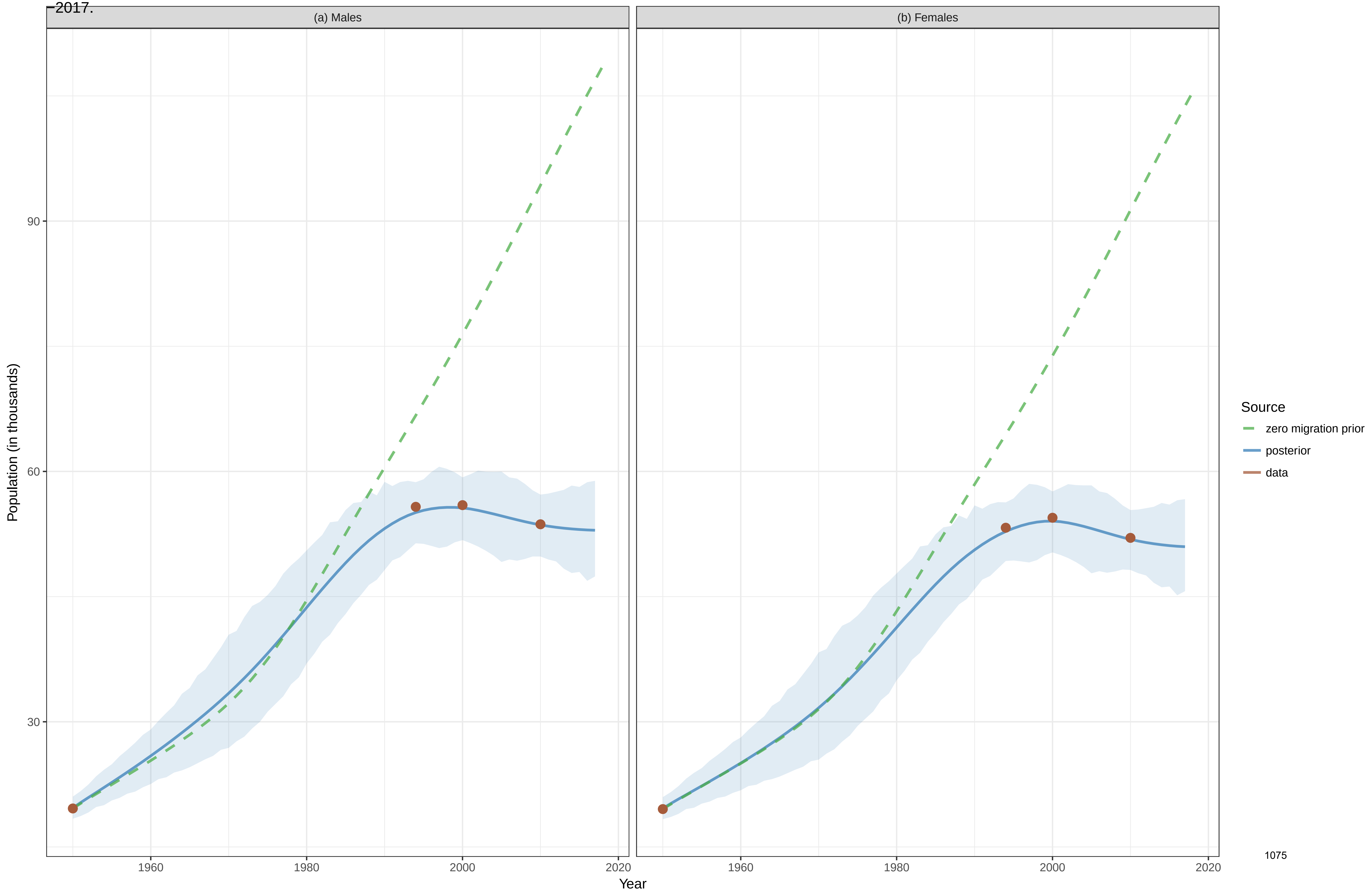

Supplemental Results Figure 4: GBD Bayesian Demographic Balancing Model fit in Fiji for (a) total population of males, and (b) total population of females, 1950−2017.

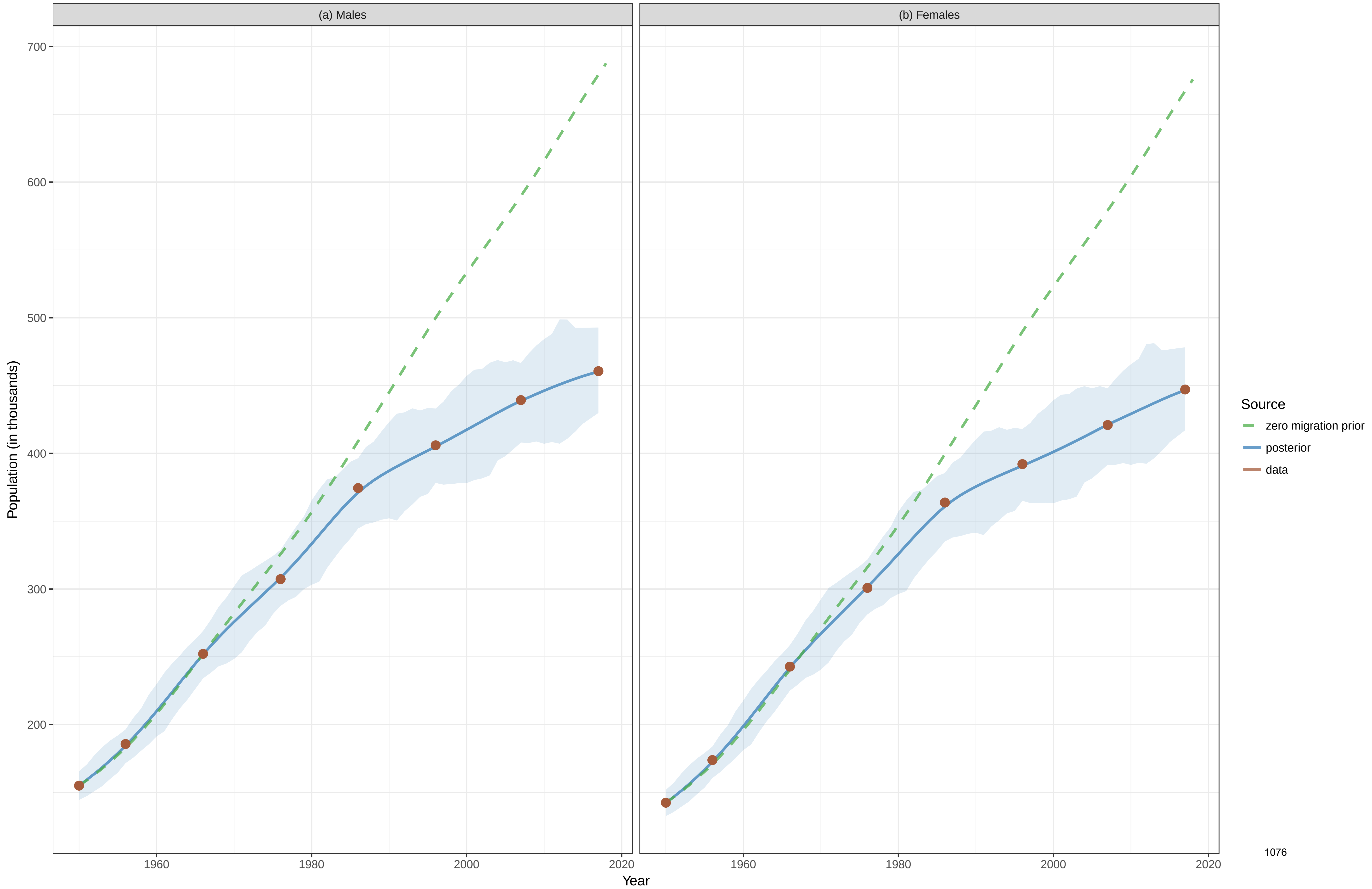

Supplemental Results Figure 4: GBD Bayesian Demographic Balancing Model fit in Finland for (a) total population of males, and (b) total population of females, 1950−2017.

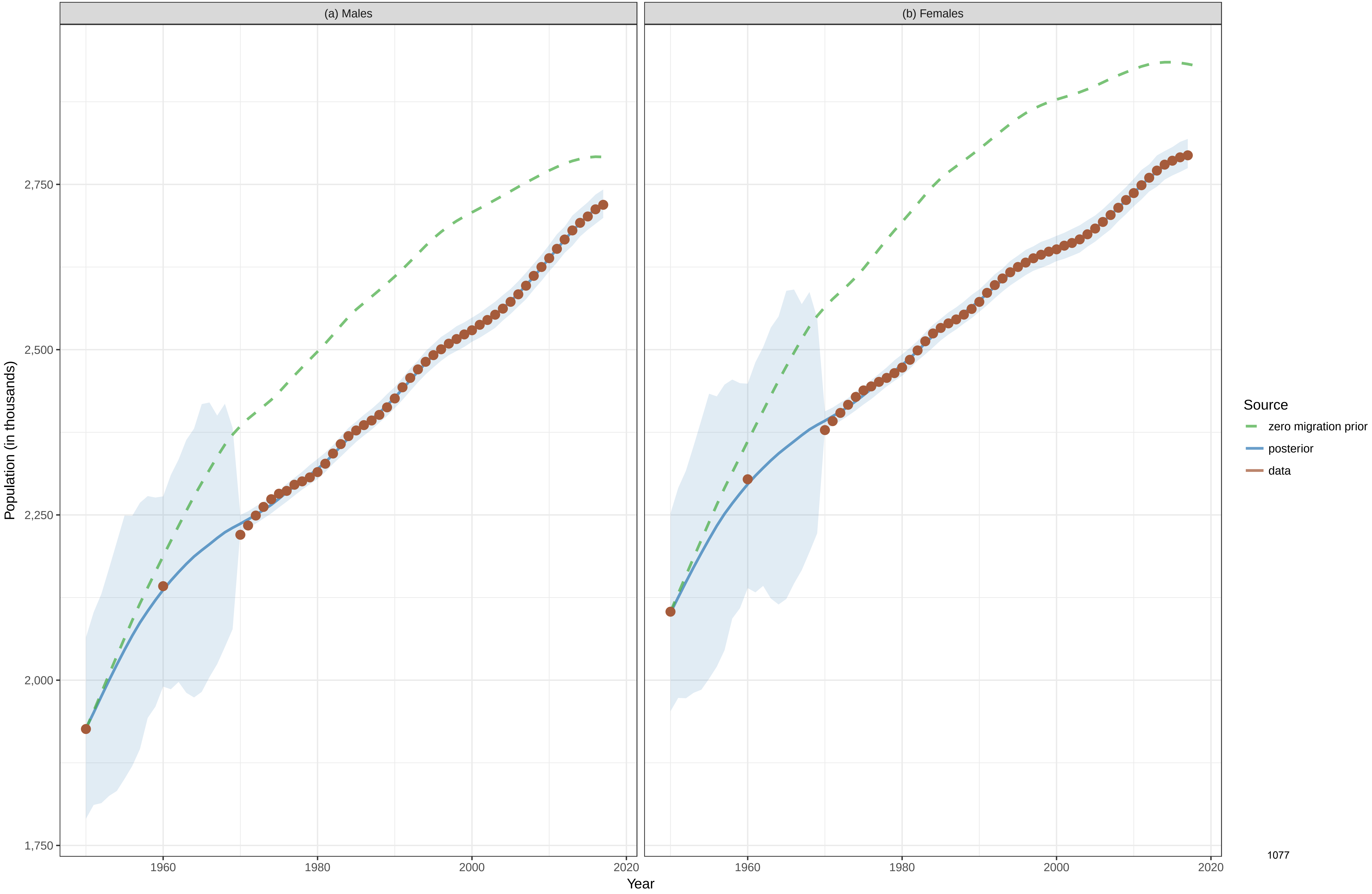

Supplemental Results Figure 4: GBD Bayesian Demographic Balancing Model fit in France for (a) total population of males, and (b) total population of females, 1950−2017.

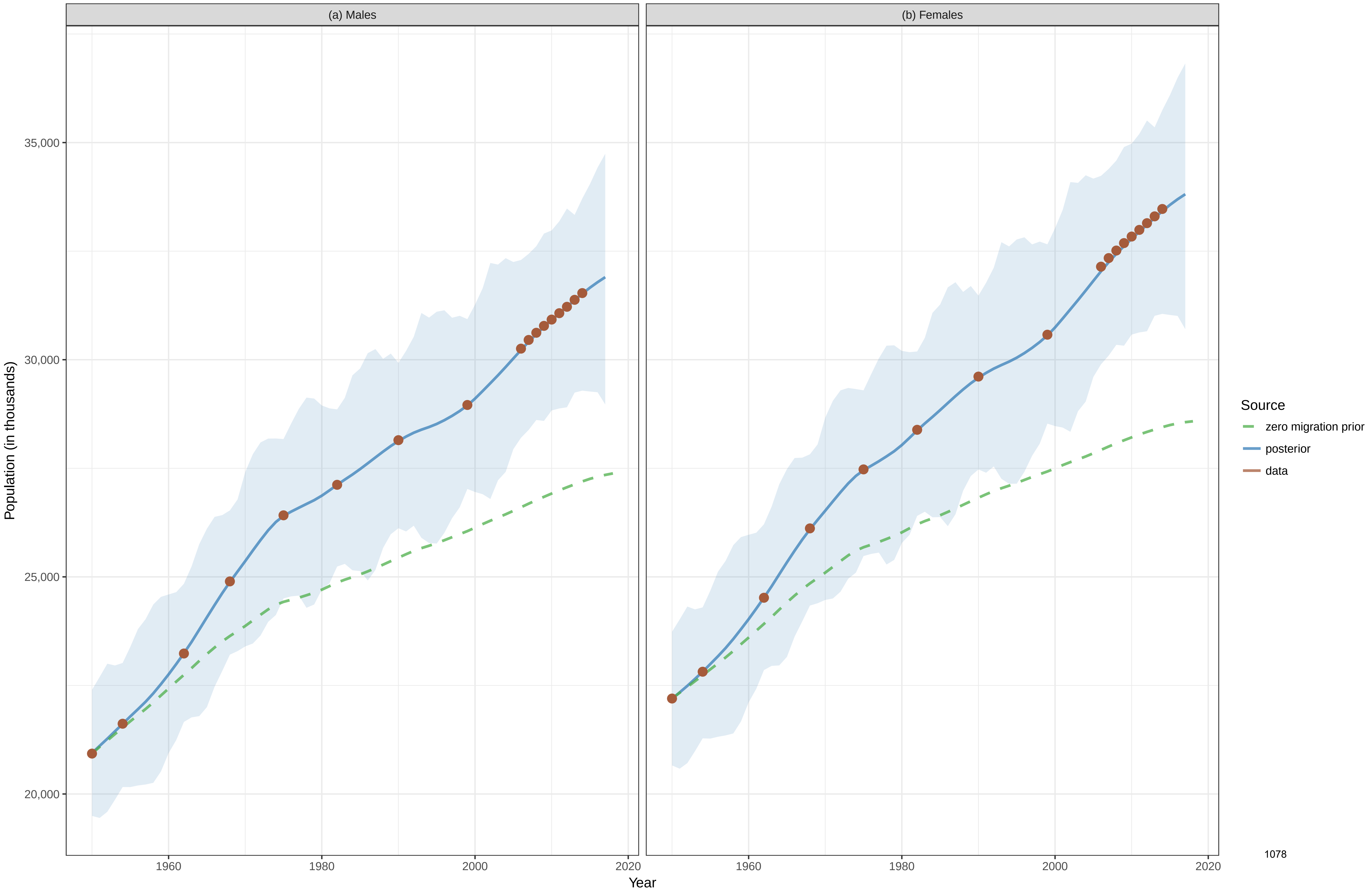

Supplemental Results Figure 4: GBD Bayesian Demographic Balancing Model fit in Gabon for (a) total population of males, and (b) total population of females, 1950−2017.

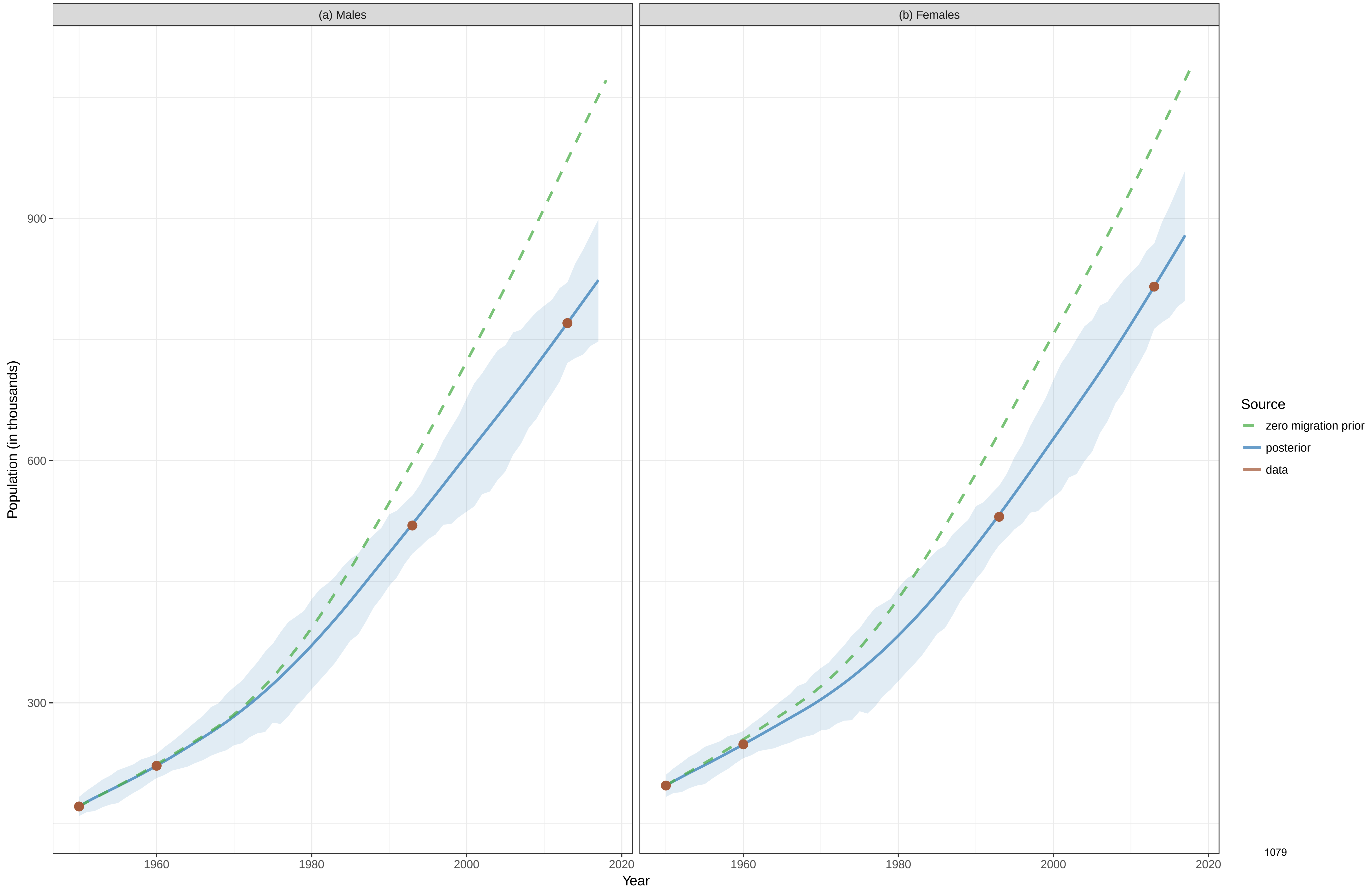

Supplemental Results Figure 4: GBD Bayesian Demographic Balancing Model fit in Georgia for (a) total population of males, and (b) total population of females, 1950−2017.

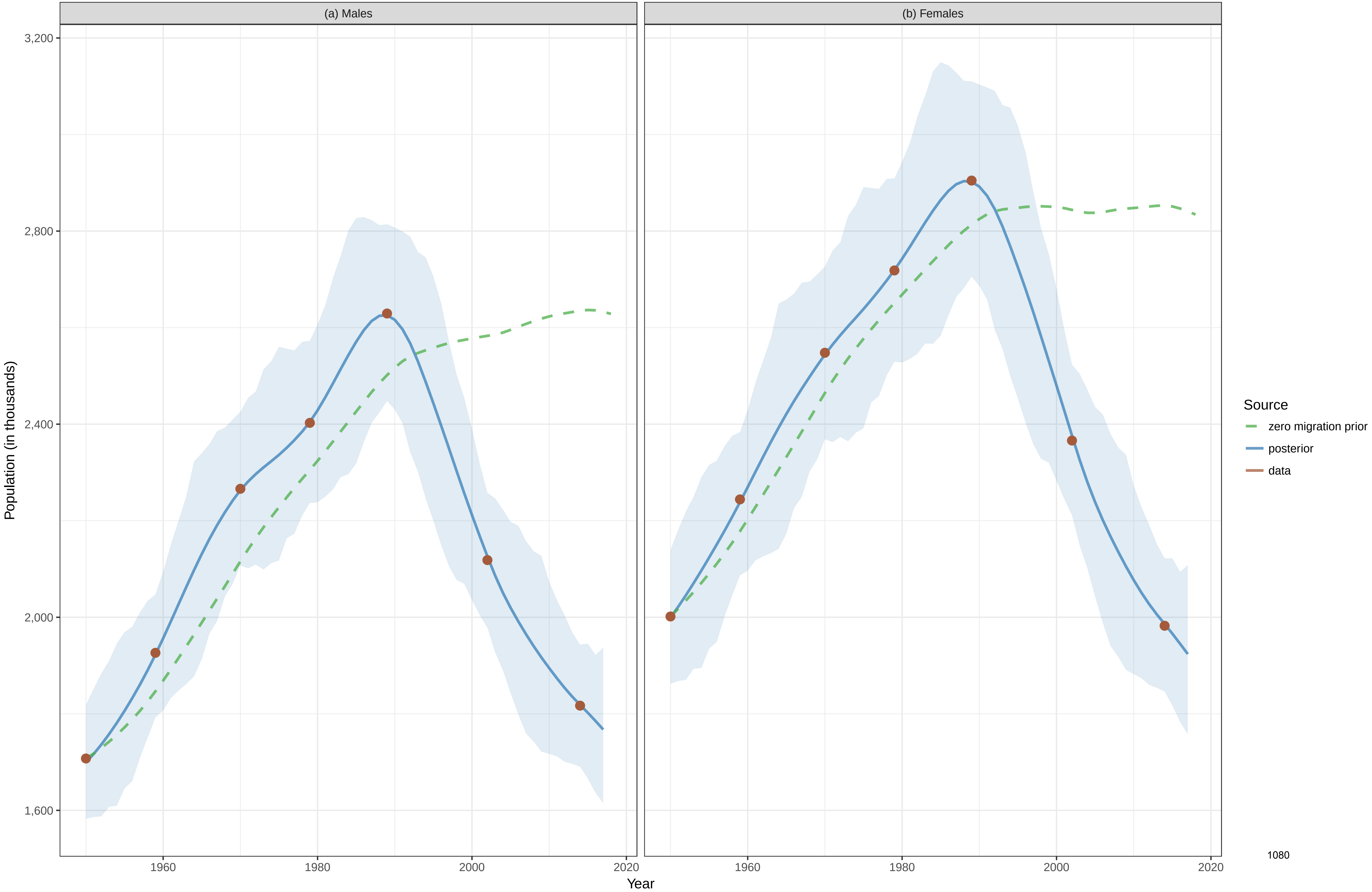

1080

Supplemental Results Figure 4: GBD Bayesian Demographic Balancing Model fit in Germany for (a) total population of males, and (b) total population of females, 1950−2017.

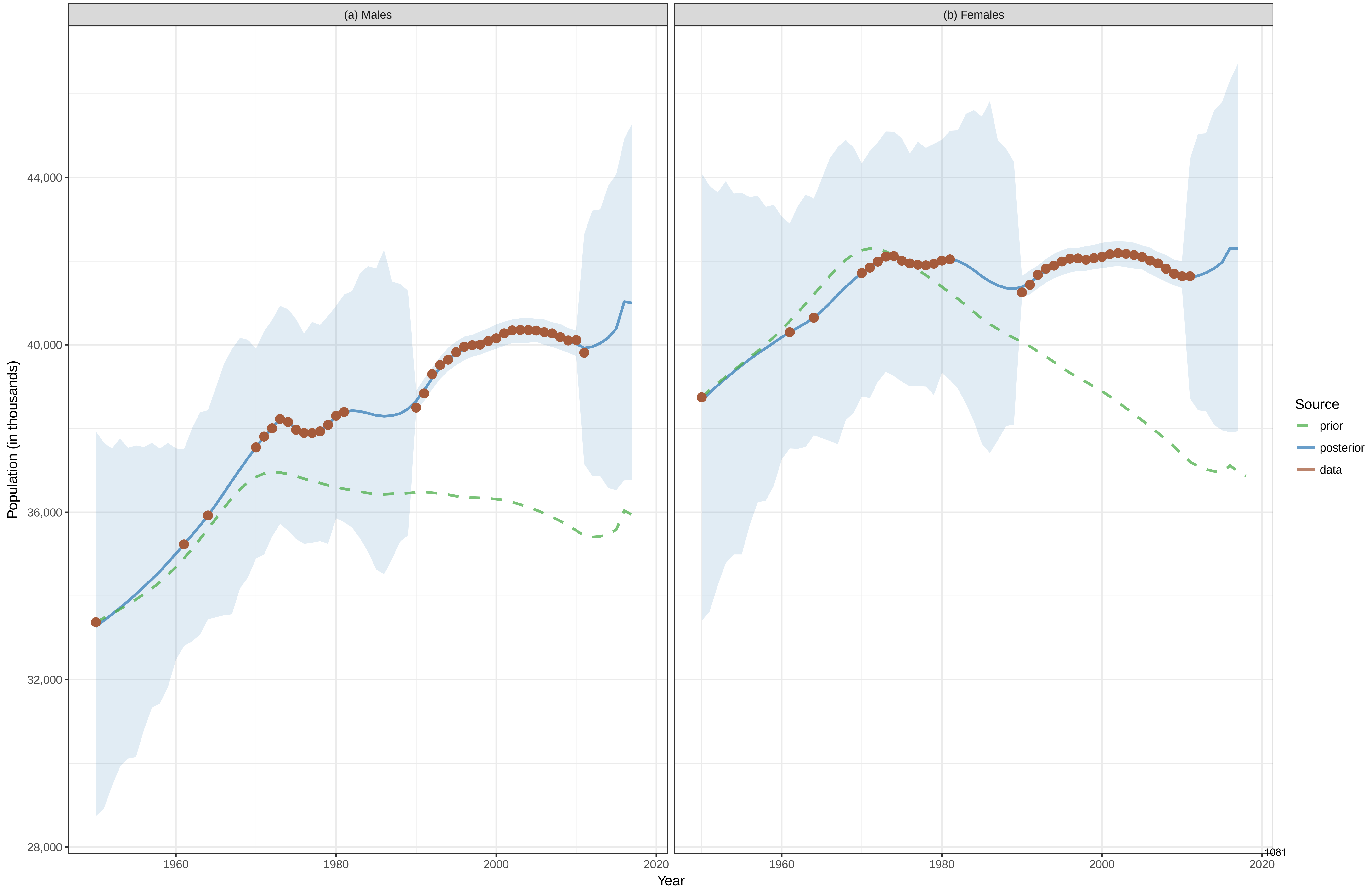

Supplemental Results Figure 4: GBD Bayesian Demographic Balancing Model fit in Ghana for (a) total population of males, and (b) total population of females, 1950−2017.

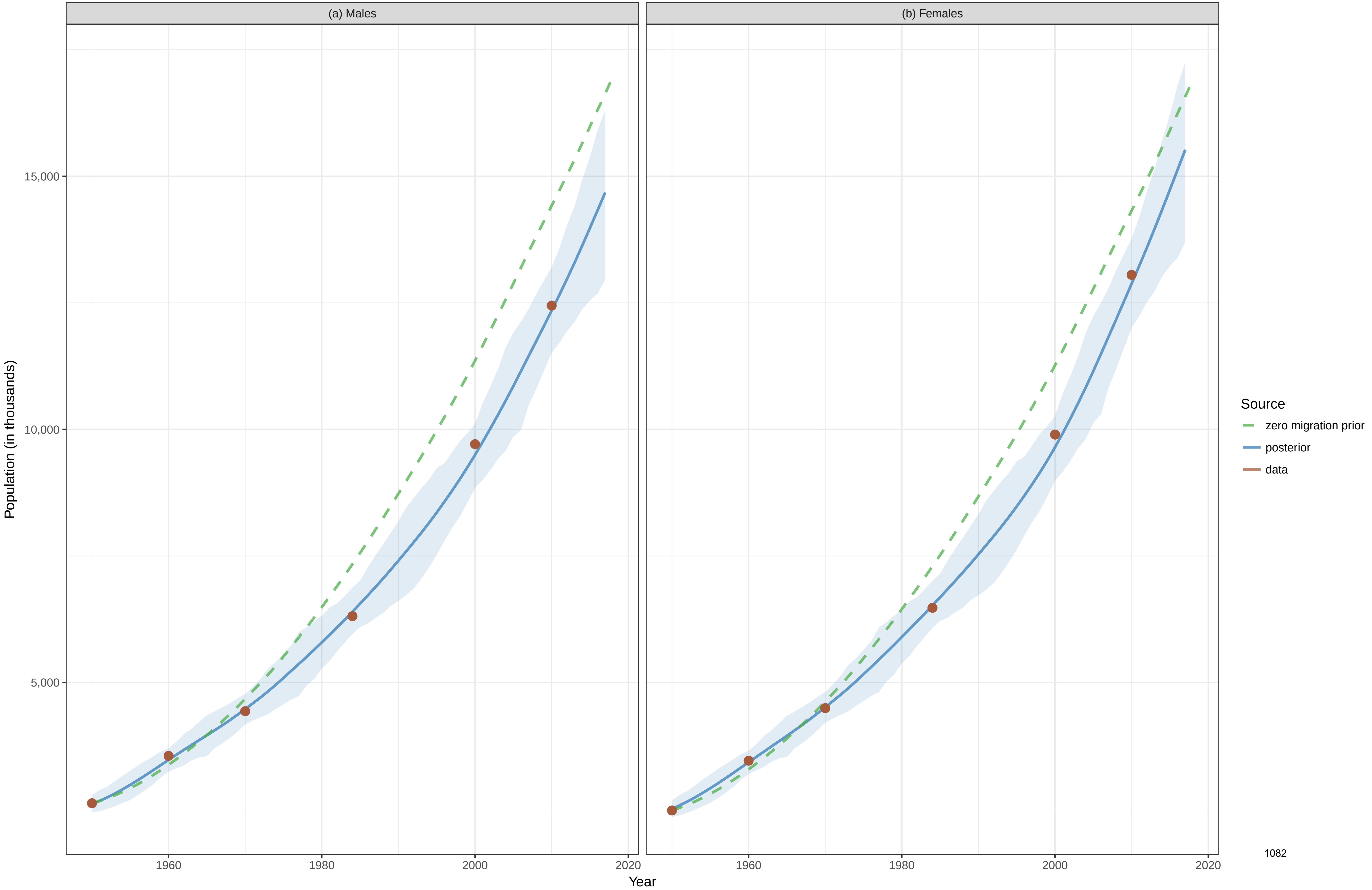

Supplemental Results Figure 4: GBD Bayesian Demographic Balancing Model fit in Greece for (a) total population of males, and (b) total population of females, 1950−2017.

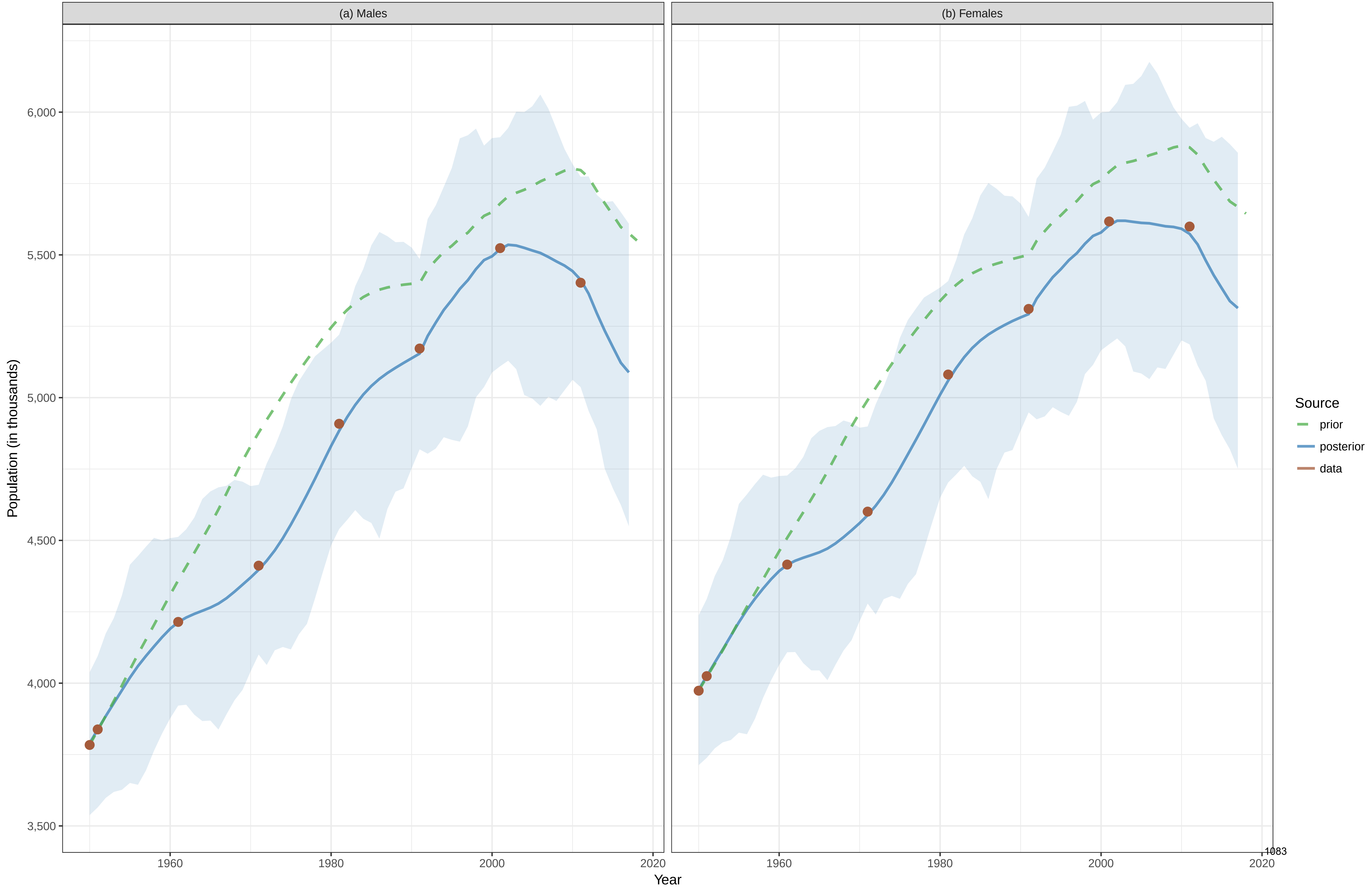

Supplemental Results Figure 4: GBD Bayesian Demographic Balancing Model fit in Greenland for (a) total population of males, and (b) total population of females, 1950−2017.

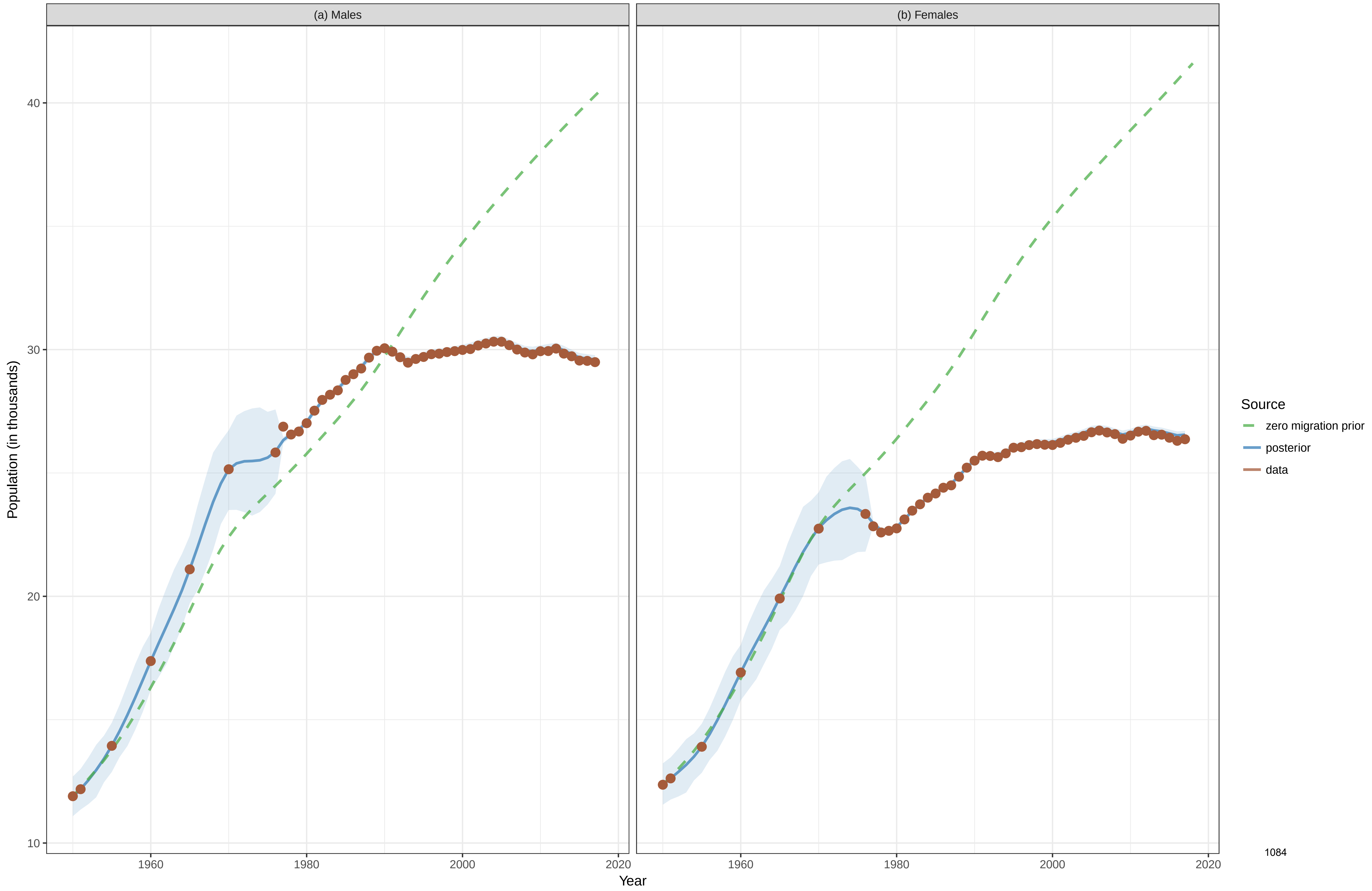

Supplemental Results Figure 4: GBD Bayesian Demographic Balancing Model fit in Grenada for (a) total population of males, and (b) total population of females, 1950−2017.

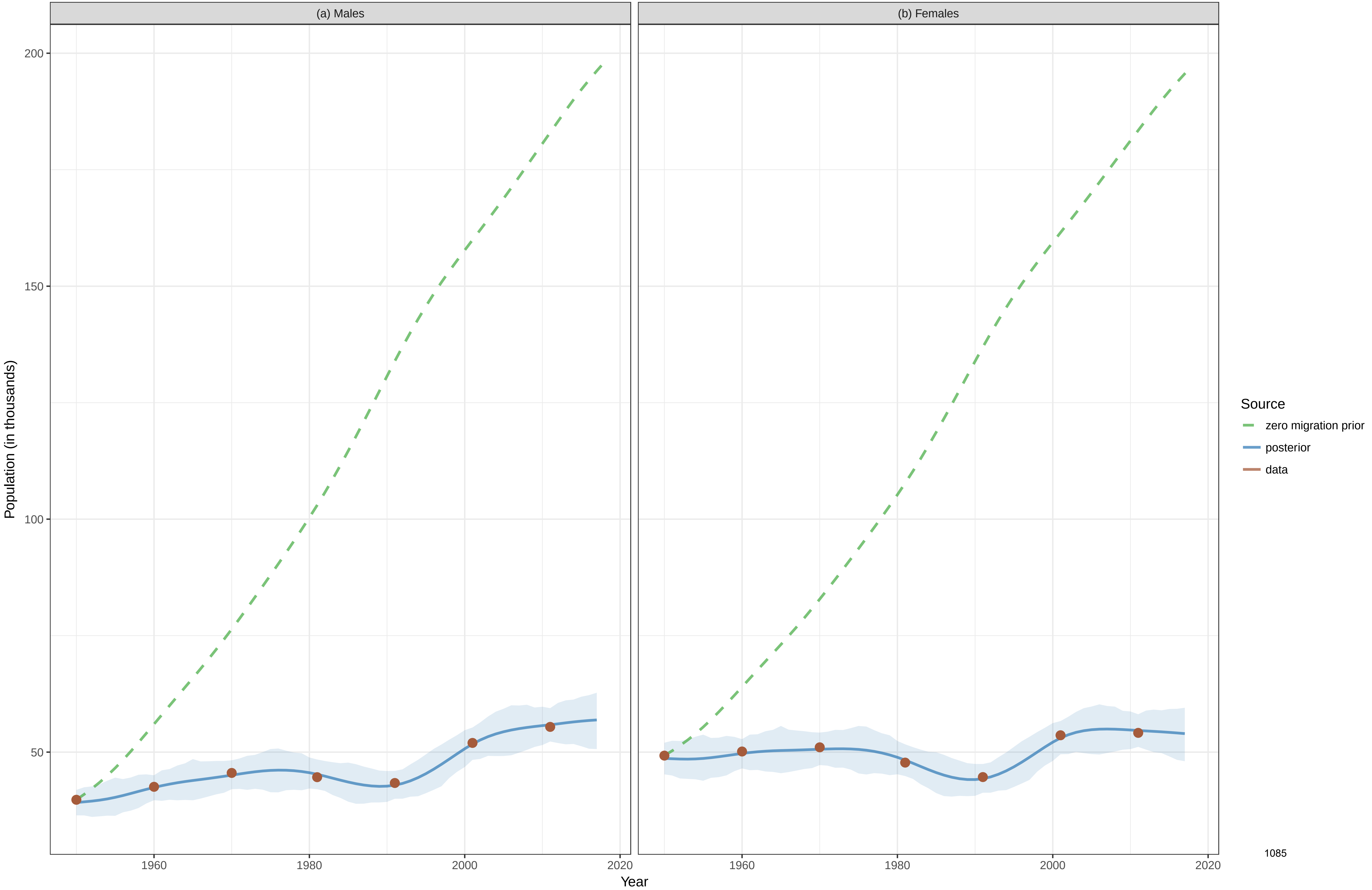

Supplemental Results Figure 4: GBD Bayesian Demographic Balancing Model fit in Guam for (a) total population of males, and (b) total population of females, 1950−2017.

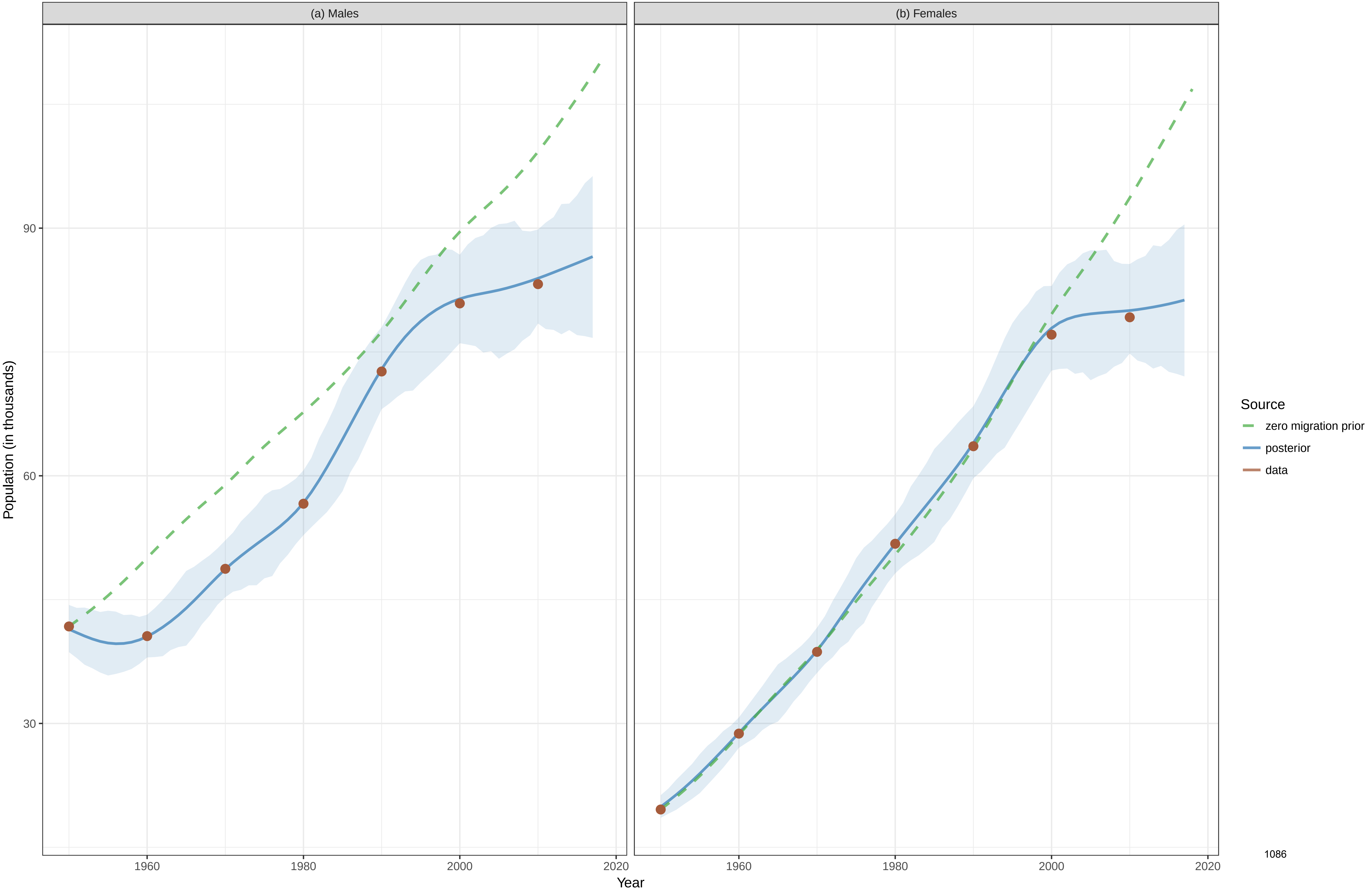

Supplemental Results Figure 4: GBD Bayesian Demographic Balancing Model fit in Guatemala for (a) total population of males, and (b) total population of females, 1950−2017.

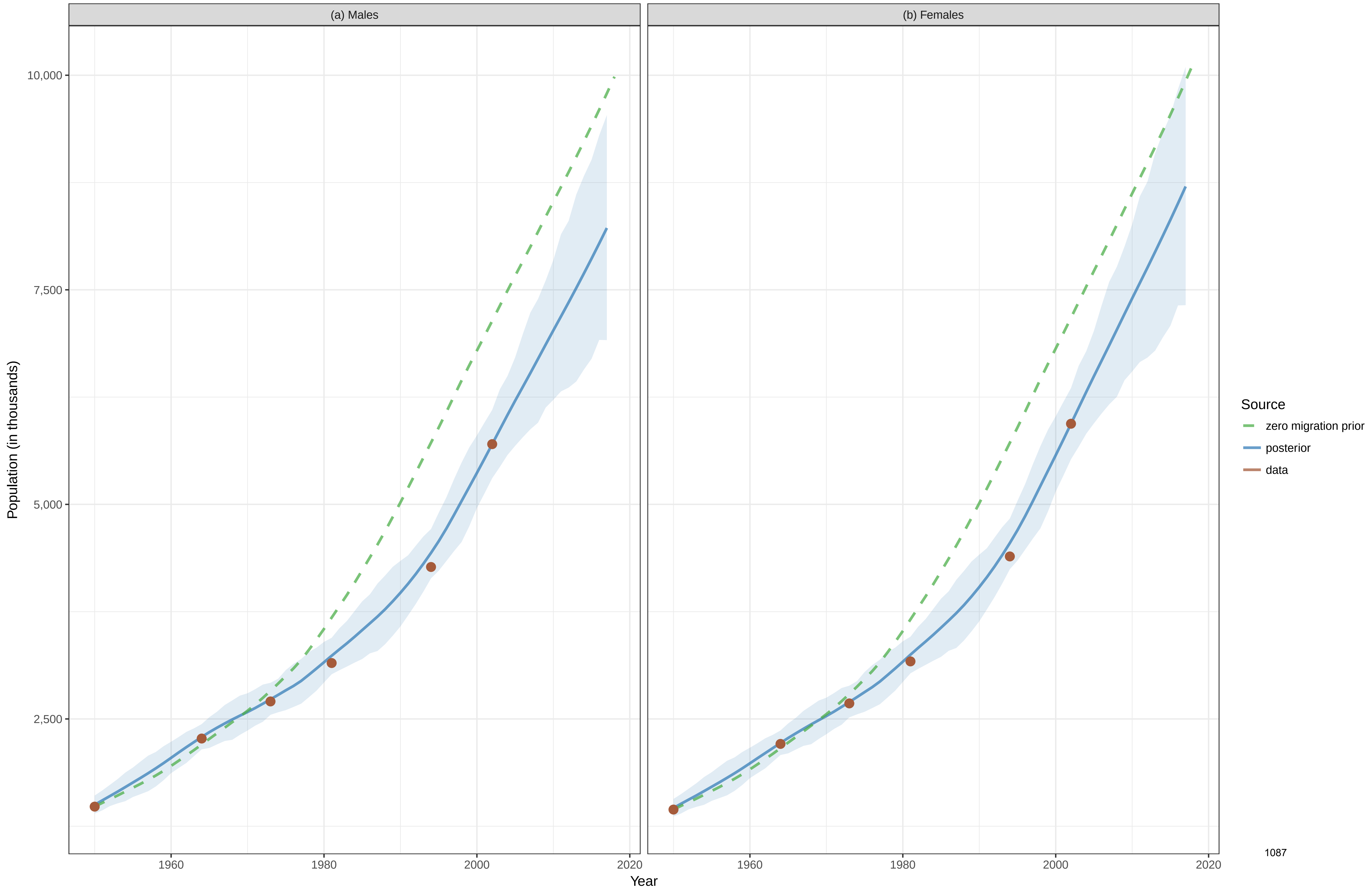

Supplemental Results Figure 4: GBD Bayesian Demographic Balancing Model fit in Guinea for (a) total population of males, and (b) total population of females, 1950−2017.

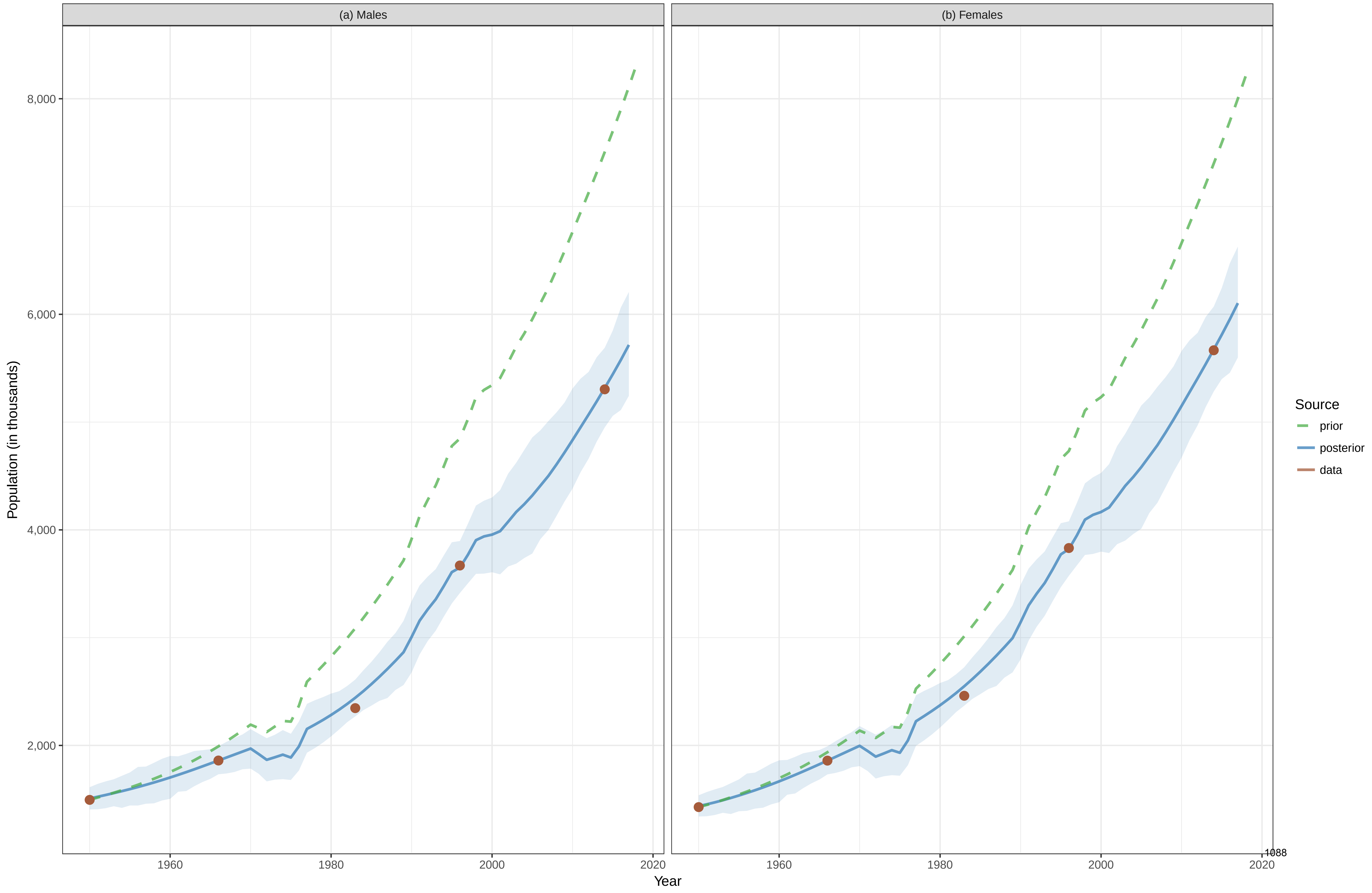

Supplemental Results Figure 4: GBD Bayesian Demographic Balancing Model fit in Guinea−Bissau for (a) total population of males, and (b) total population of females, 1950−2017.

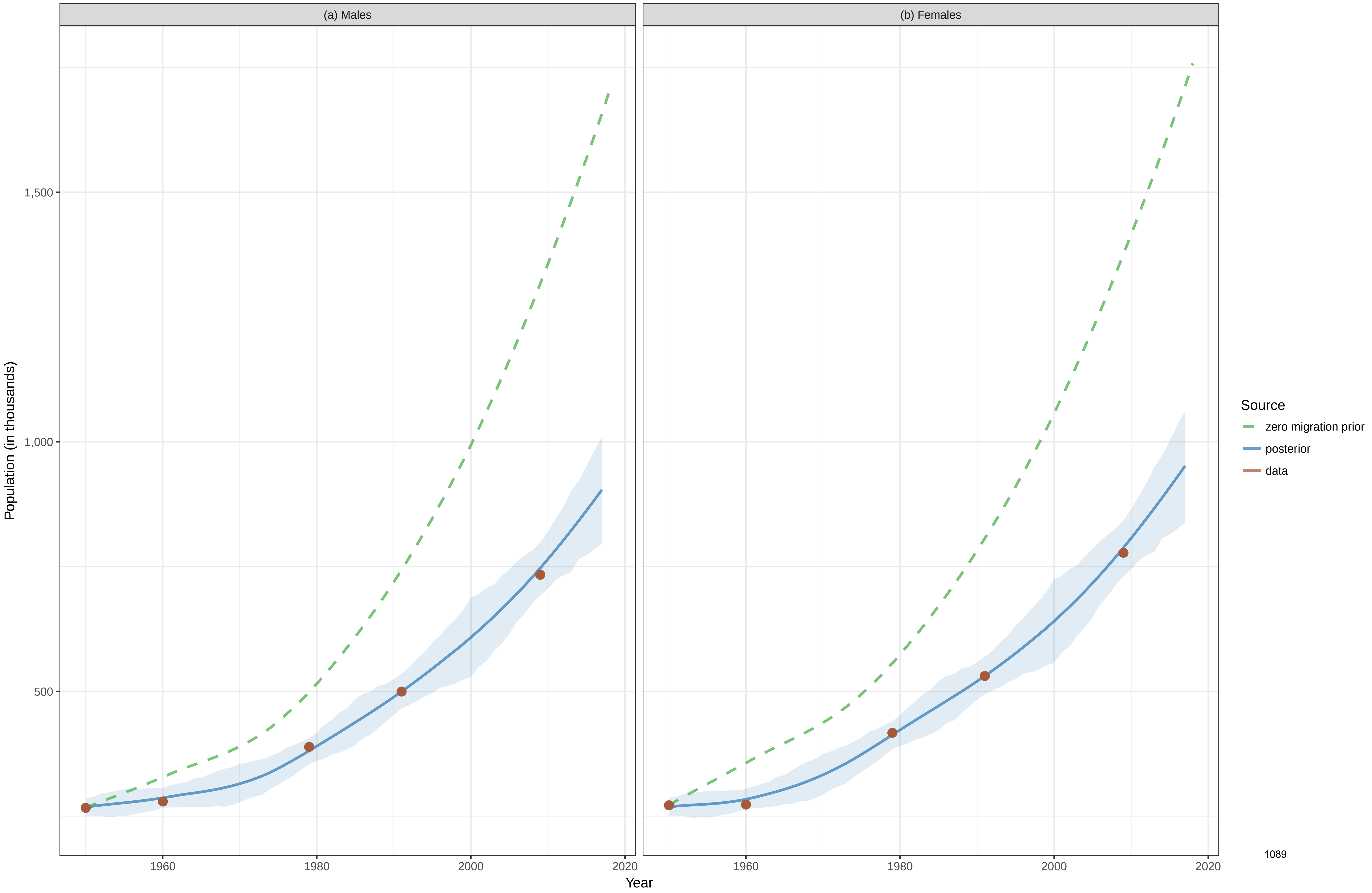

Supplemental Results Figure 4: GBD Bayesian Demographic Balancing Model fit in Guyana for (a) total population of males, and (b) total population of females, 1950−2017.

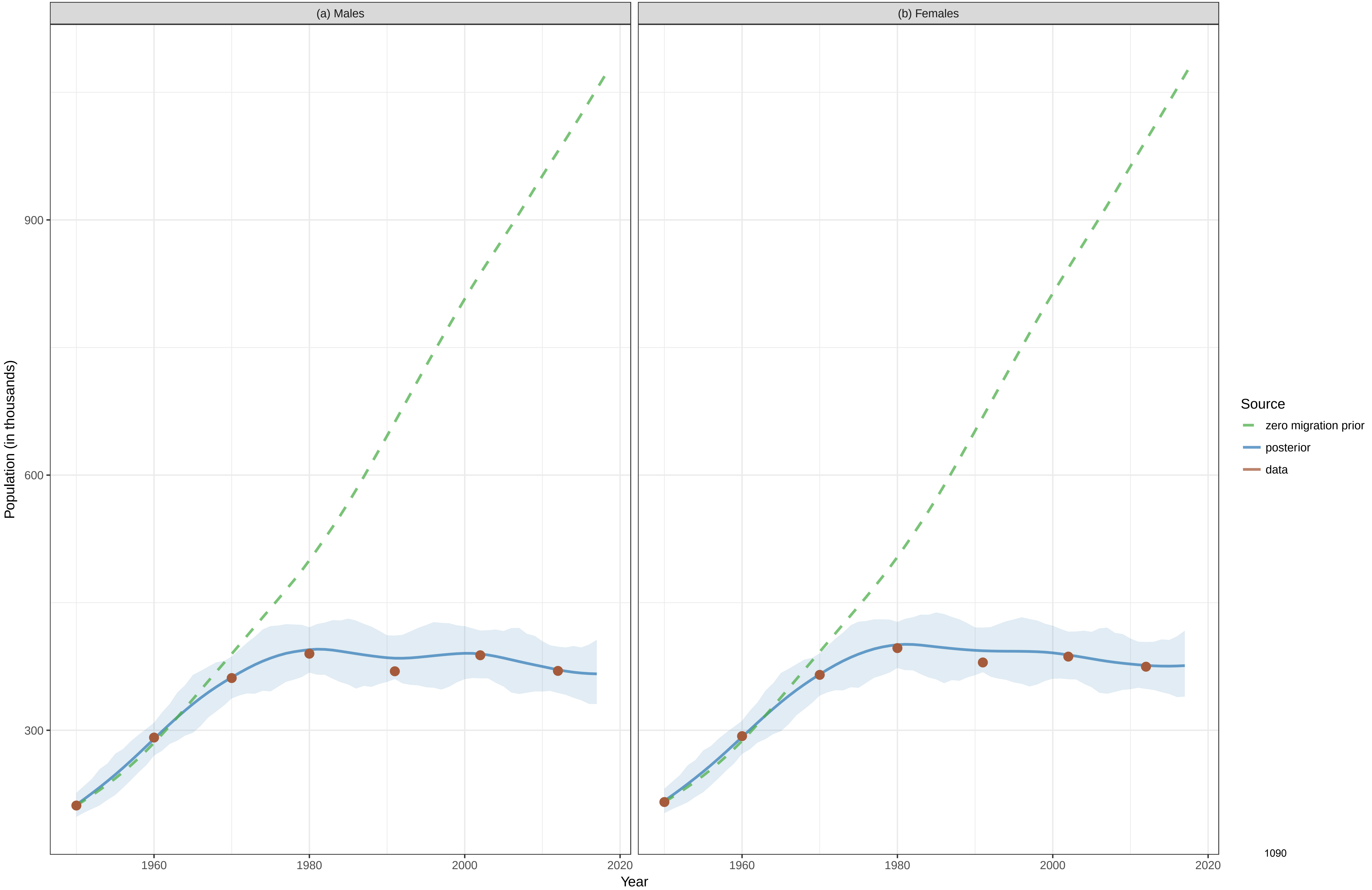

Supplemental Results Figure 4: GBD Bayesian Demographic Balancing Model fit in Haiti for (a) total population of males, and (b) total population of females, 1950−2017.

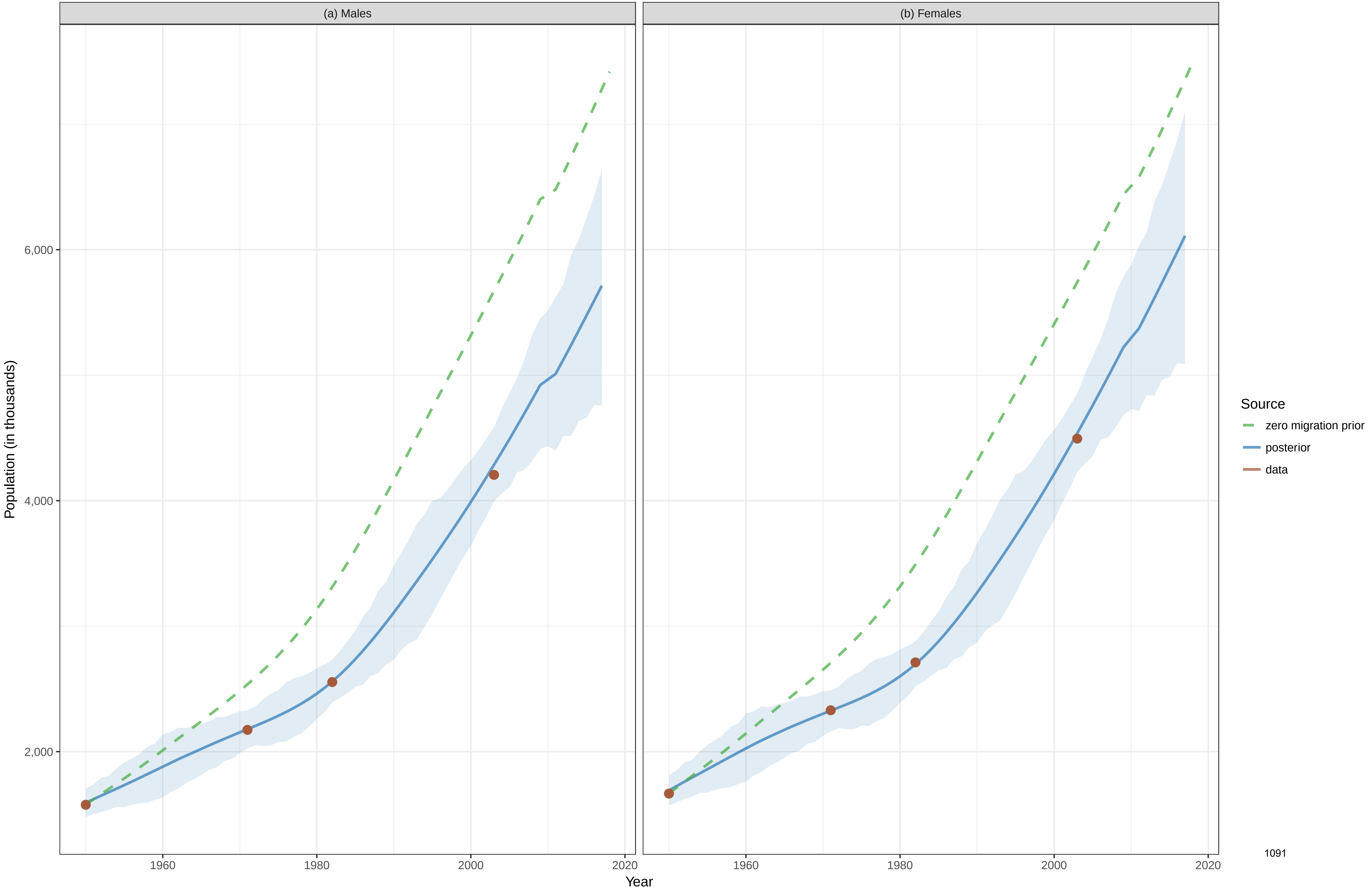

Supplemental Results Figure 4: GBD Bayesian Demographic Balancing Model fit in Honduras for (a) total population of males, and (b) total population of females, 1950−2017.

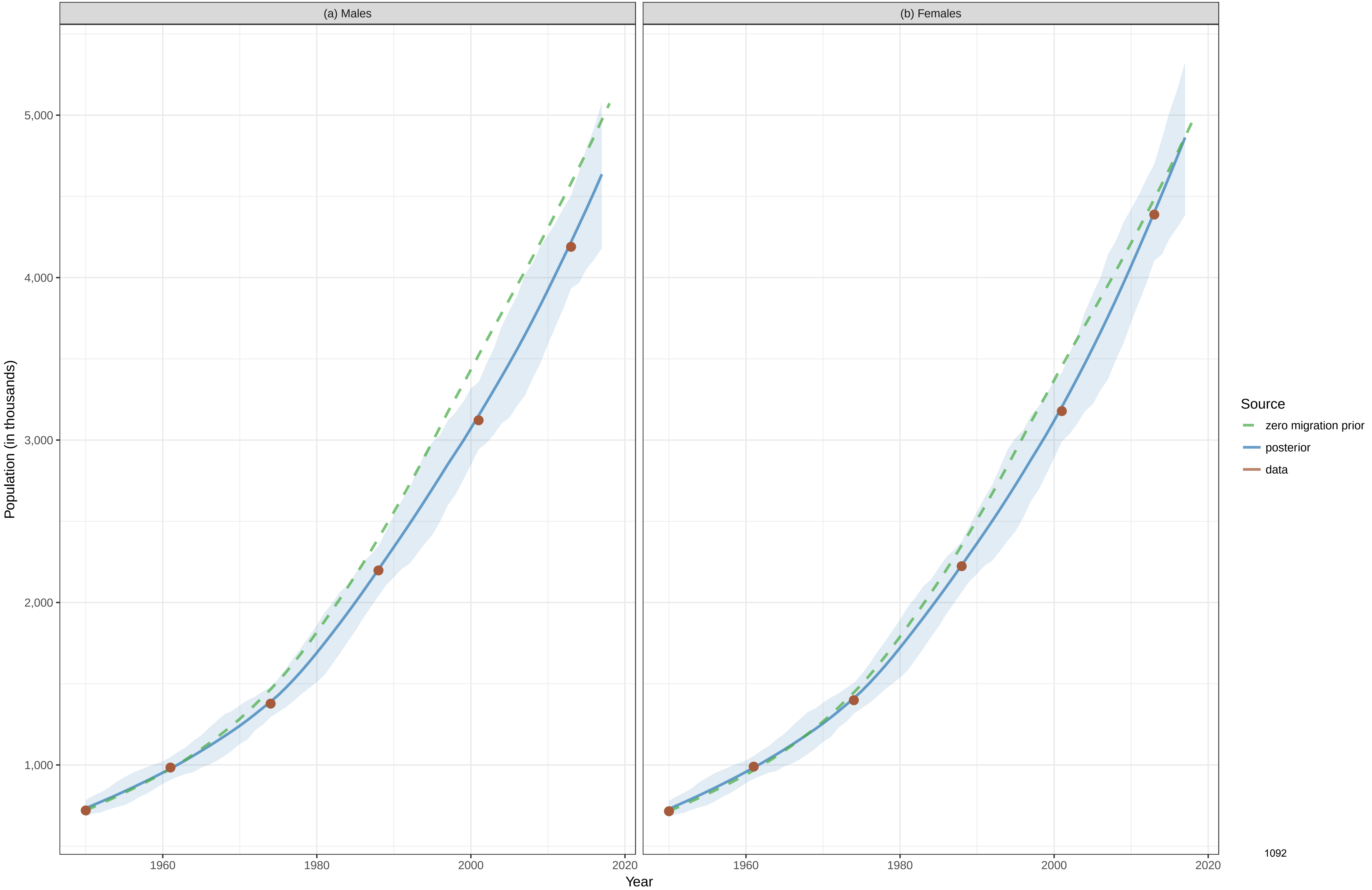

Supplemental Results Figure 4: GBD Bayesian Demographic Balancing Model fit in Hong Kong Special Administrative Region of China for (a) total population of males, and (b) total population of females, 1950−2017.

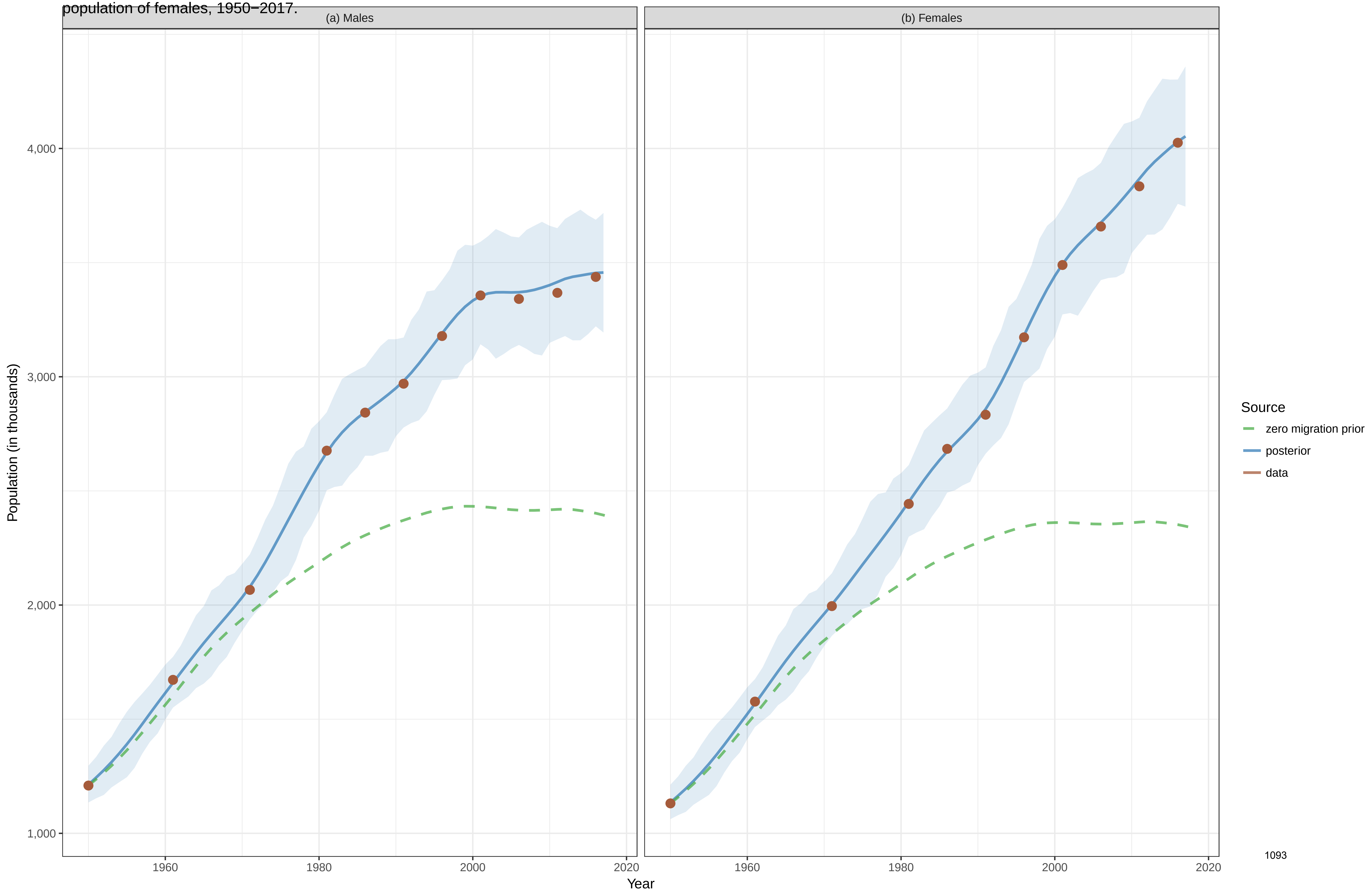

Supplemental Results Figure 4: GBD Bayesian Demographic Balancing Model fit in Hungary for (a) total population of males, and (b) total population of females, 1950−2017.

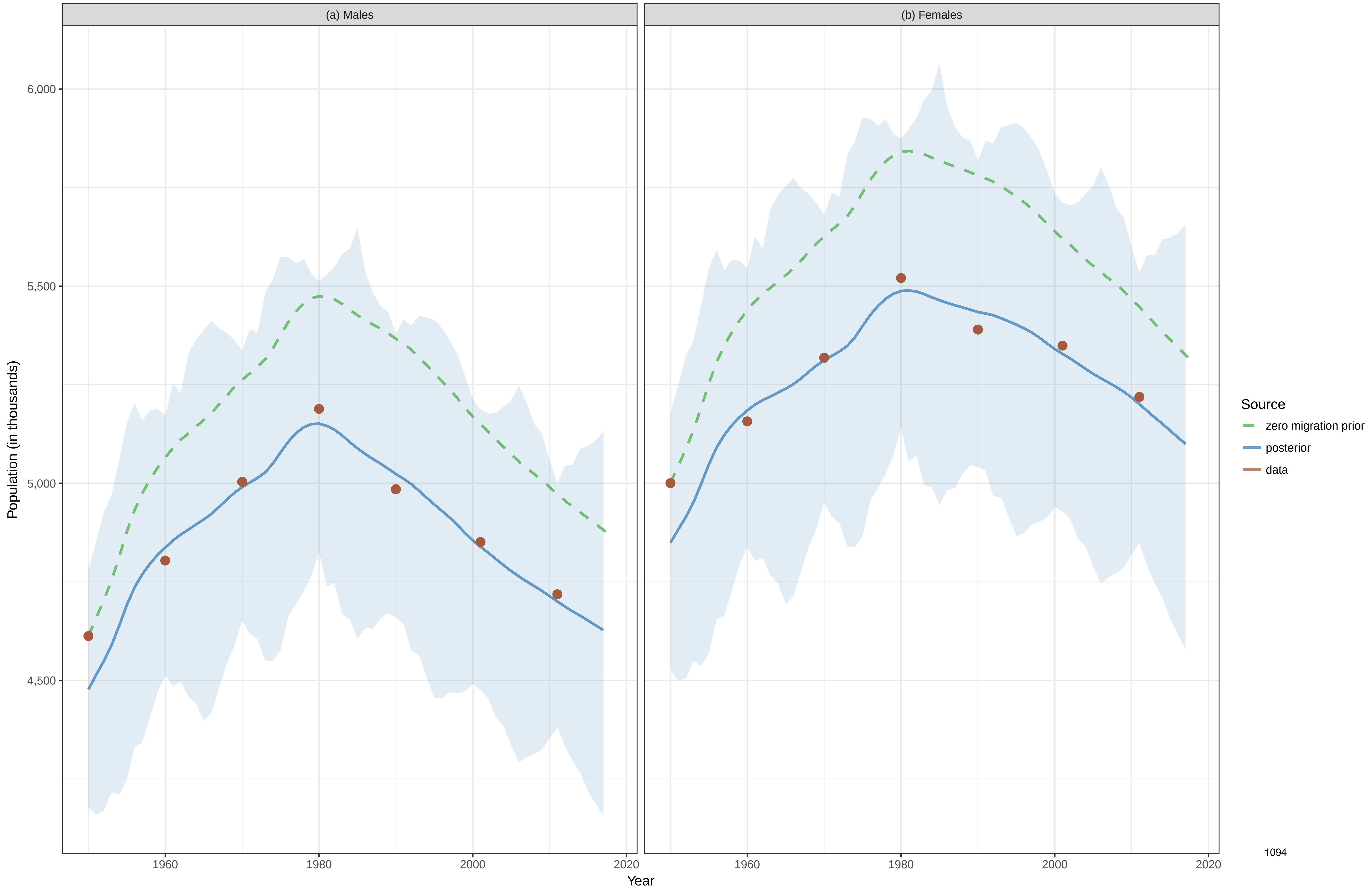

Supplemental Results Figure 4: GBD Bayesian Demographic Balancing Model fit in Iceland for (a) total population of males, and (b) total population of females, 1950−2017.

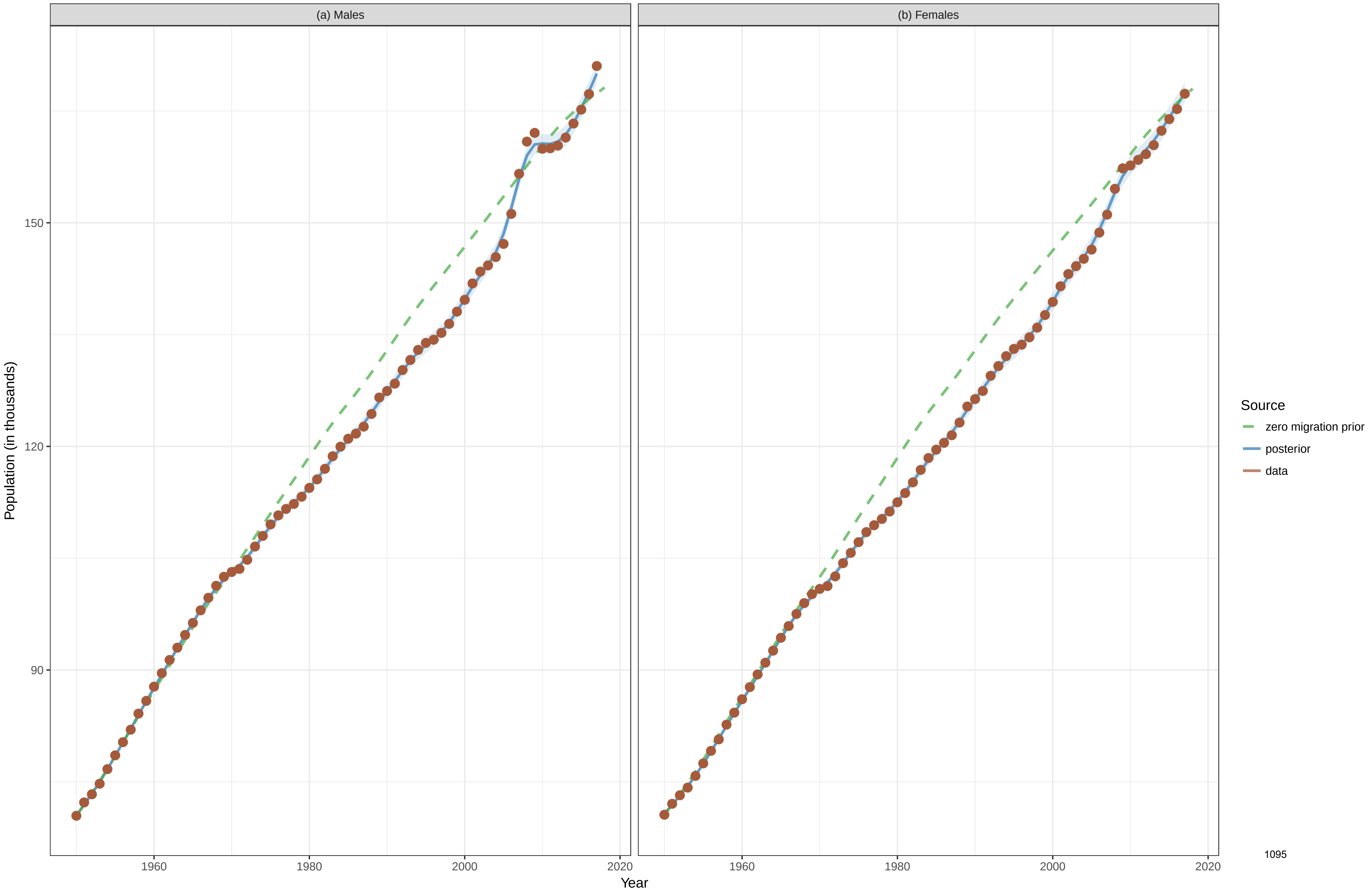

Supplemental Results Figure 4: GBD Bayesian Demographic Balancing Model fit in India for (a) total population of males, and (b) total population of females, 1950−2017.

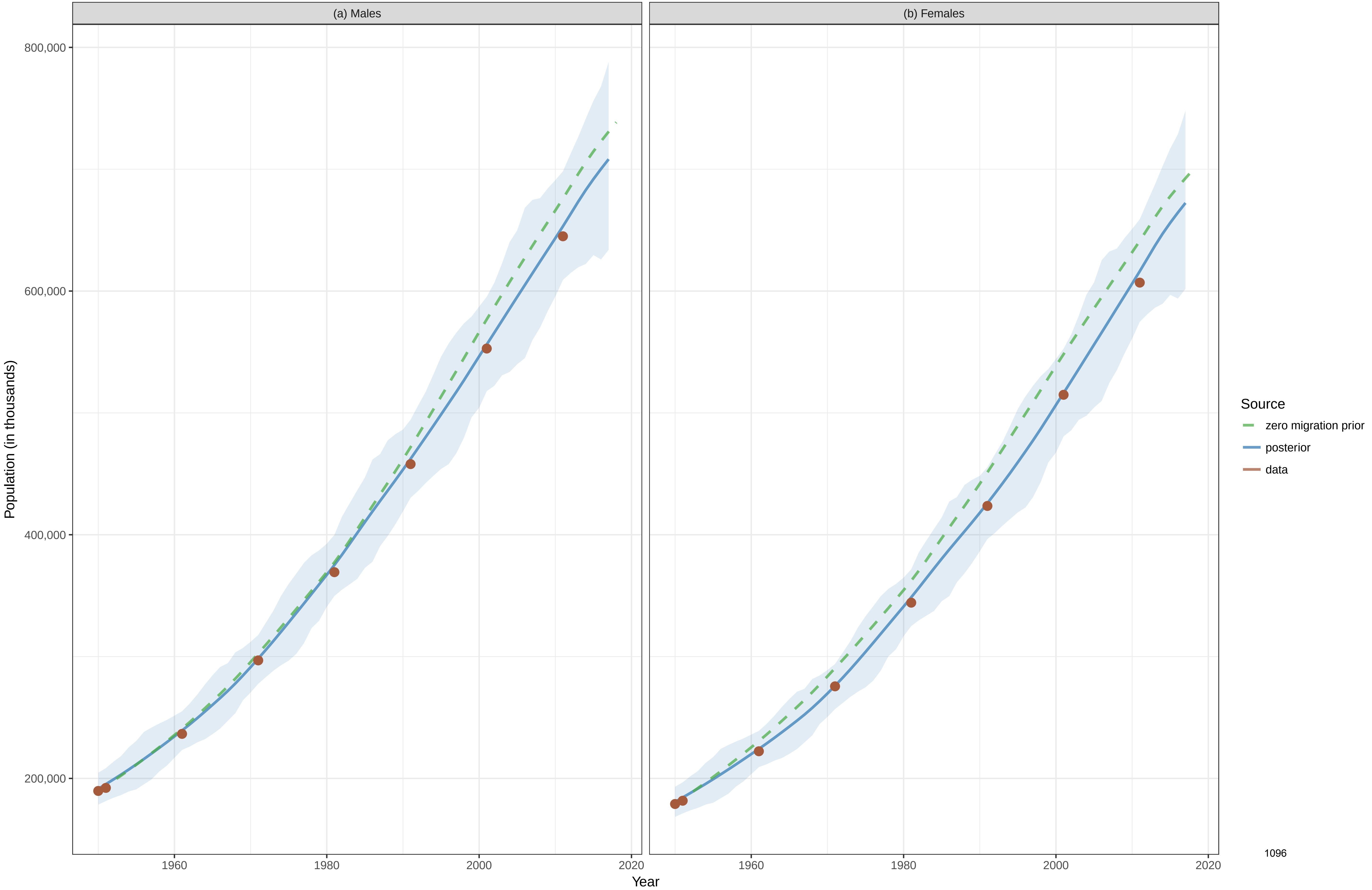

Supplemental Results Figure 4: GBD Bayesian Demographic Balancing Model fit in Indonesia for (a) total population of males, and (b) total population of females, 1950−2017.

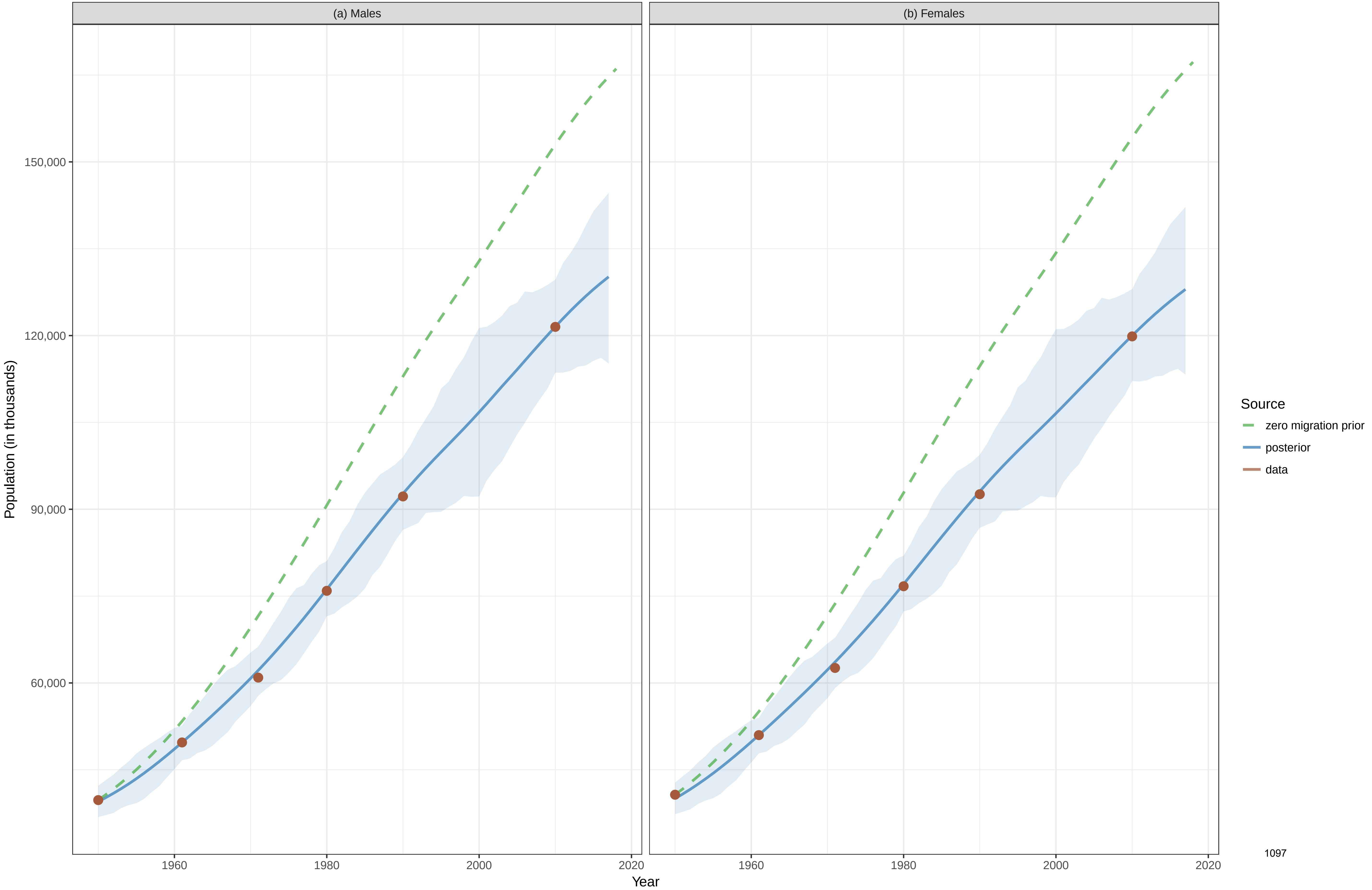

Supplemental Results Figure 4: GBD Bayesian Demographic Balancing Model fit in Iran for (a) total population of males, and (b) total population of females, 1950−2017.

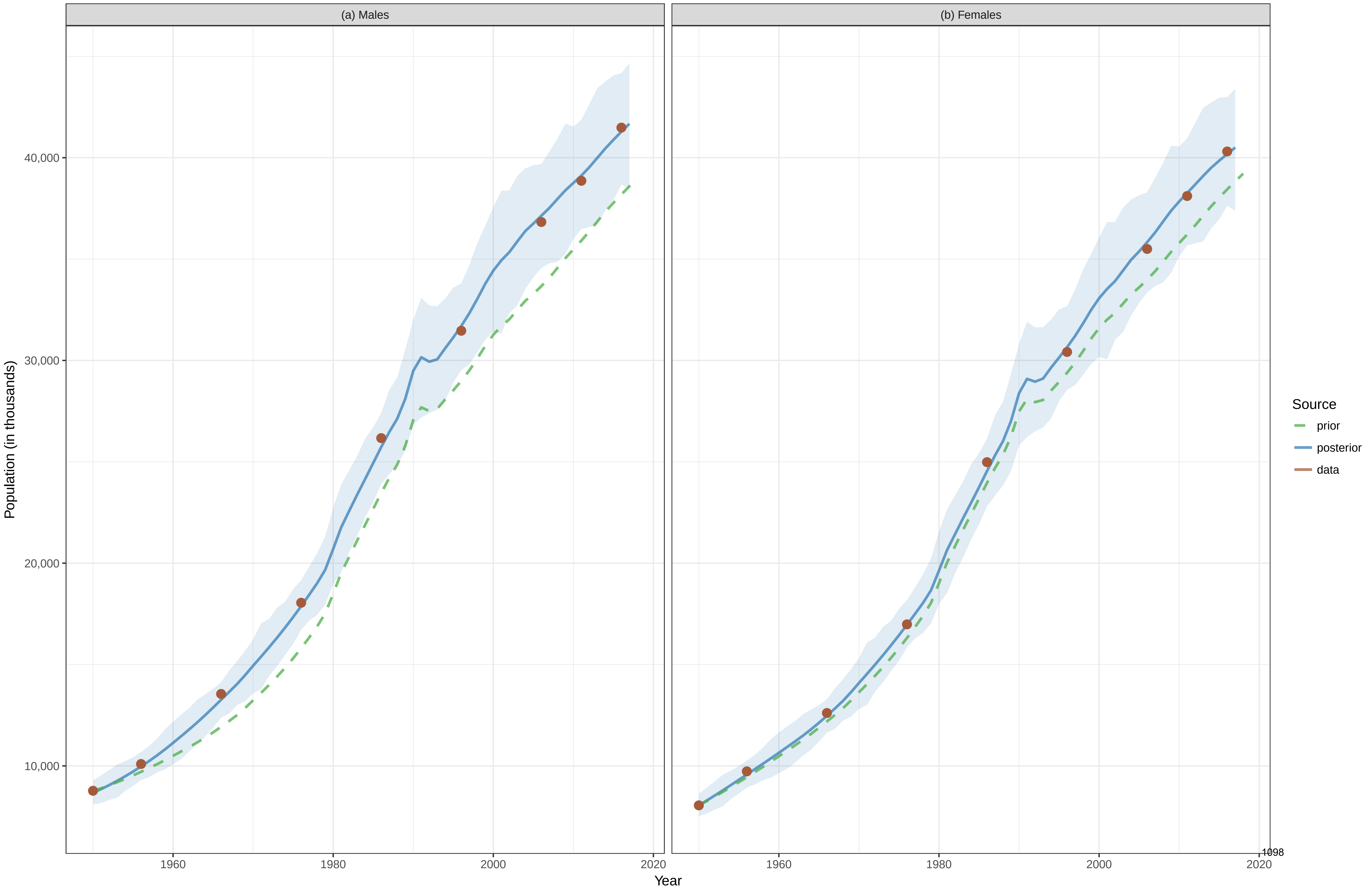

Supplemental Results Figure 4: GBD Bayesian Demographic Balancing Model fit in Iraq for (a) total population of males, and (b) total population of females, 1950−2017.

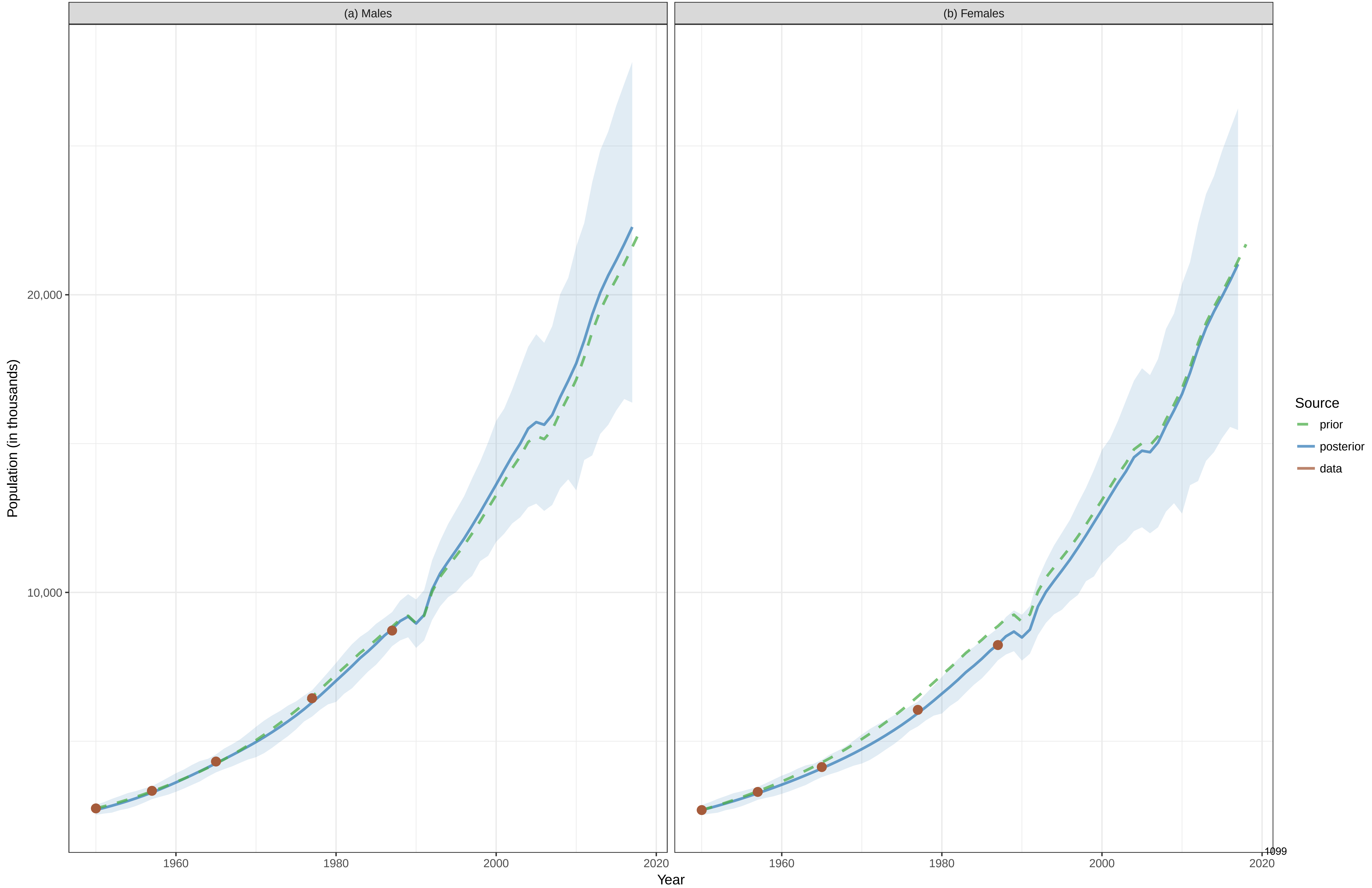

Supplemental Results Figure 4: GBD Bayesian Demographic Balancing Model fit in Ireland for (a) total population of males, and (b) total population of females, 1950−2017.

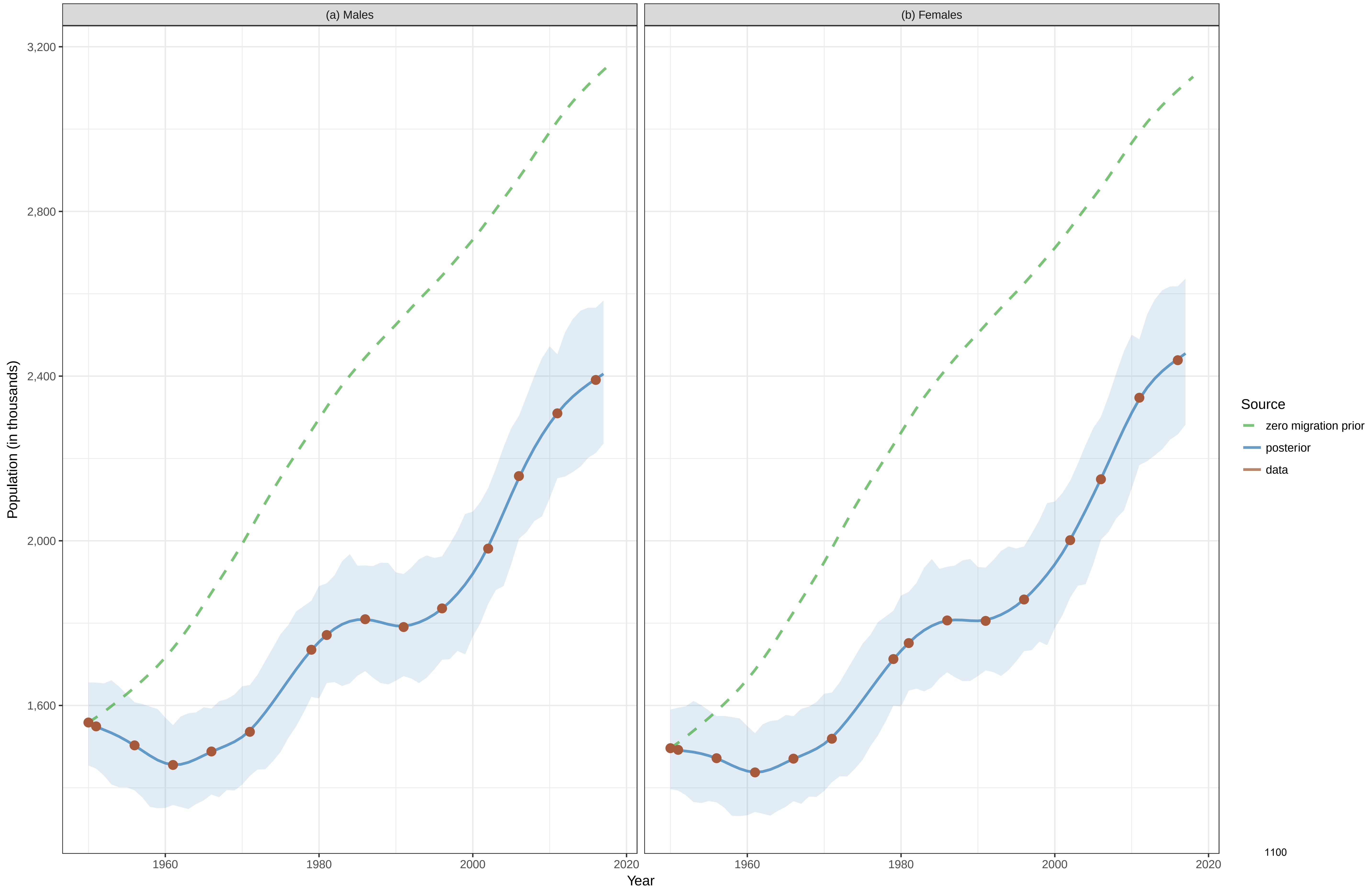

Supplemental Results Figure 4: GBD Bayesian Demographic Balancing Model fit in Israel for (a) total population of males, and (b) total population of females, 1950−2017.

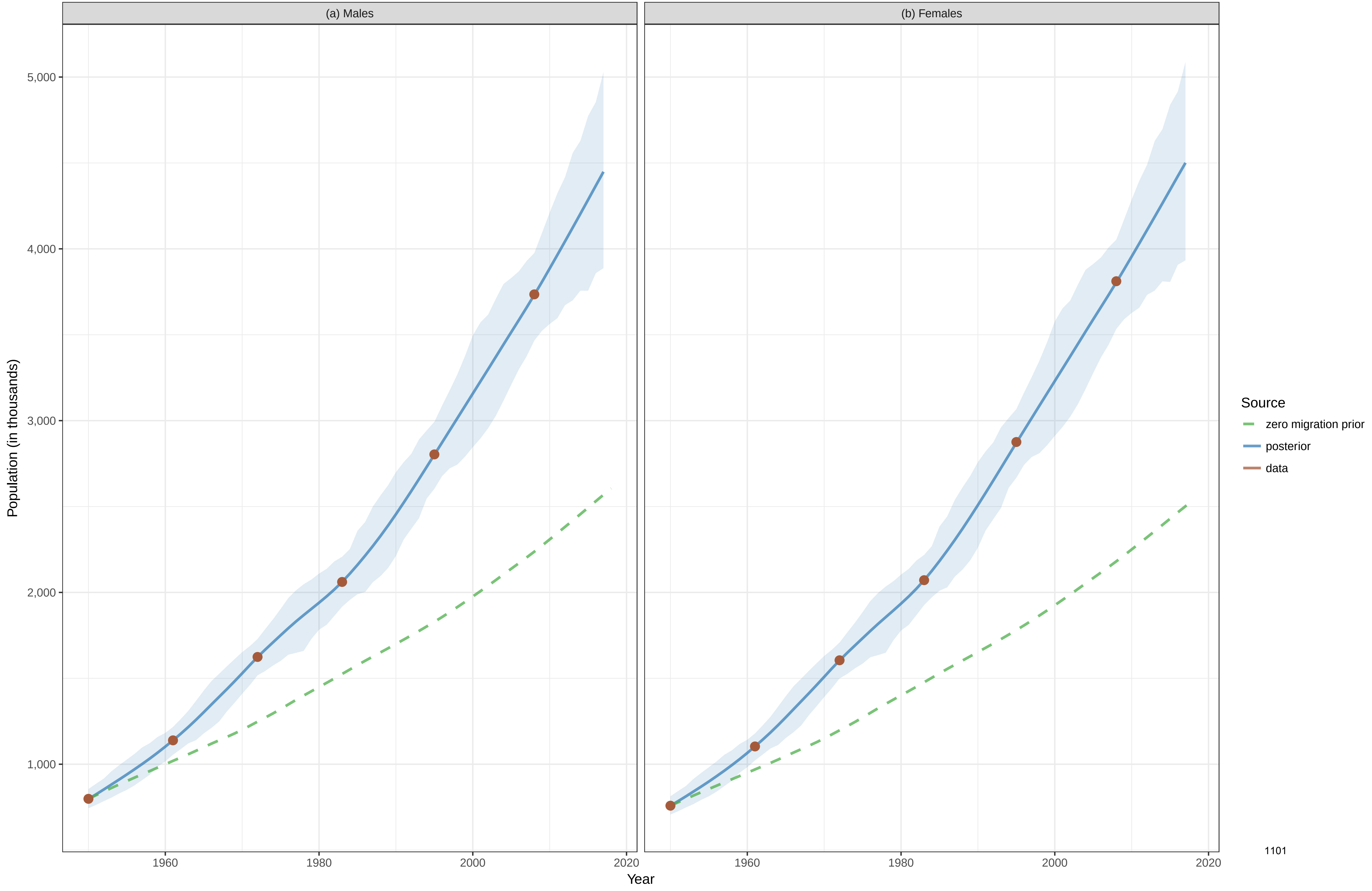

Supplemental Results Figure 4: GBD Bayesian Demographic Balancing Model fit in Italy for (a) total population of males, and (b) total population of females, 1950−2017.

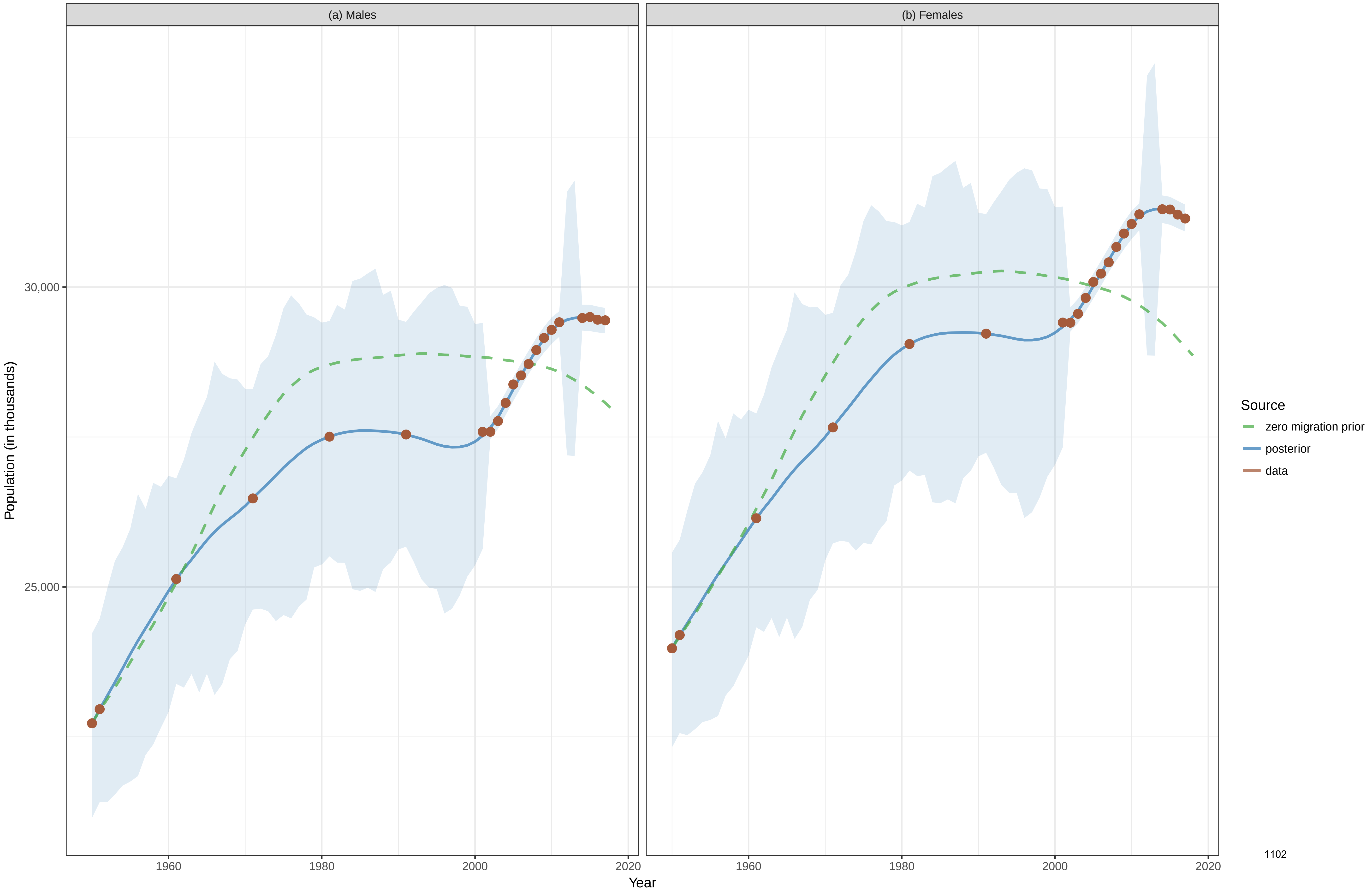

Supplemental Results Figure 4: GBD Bayesian Demographic Balancing Model fit in Jamaica for (a) total population of males, and (b) total population of females, 1950−2017.

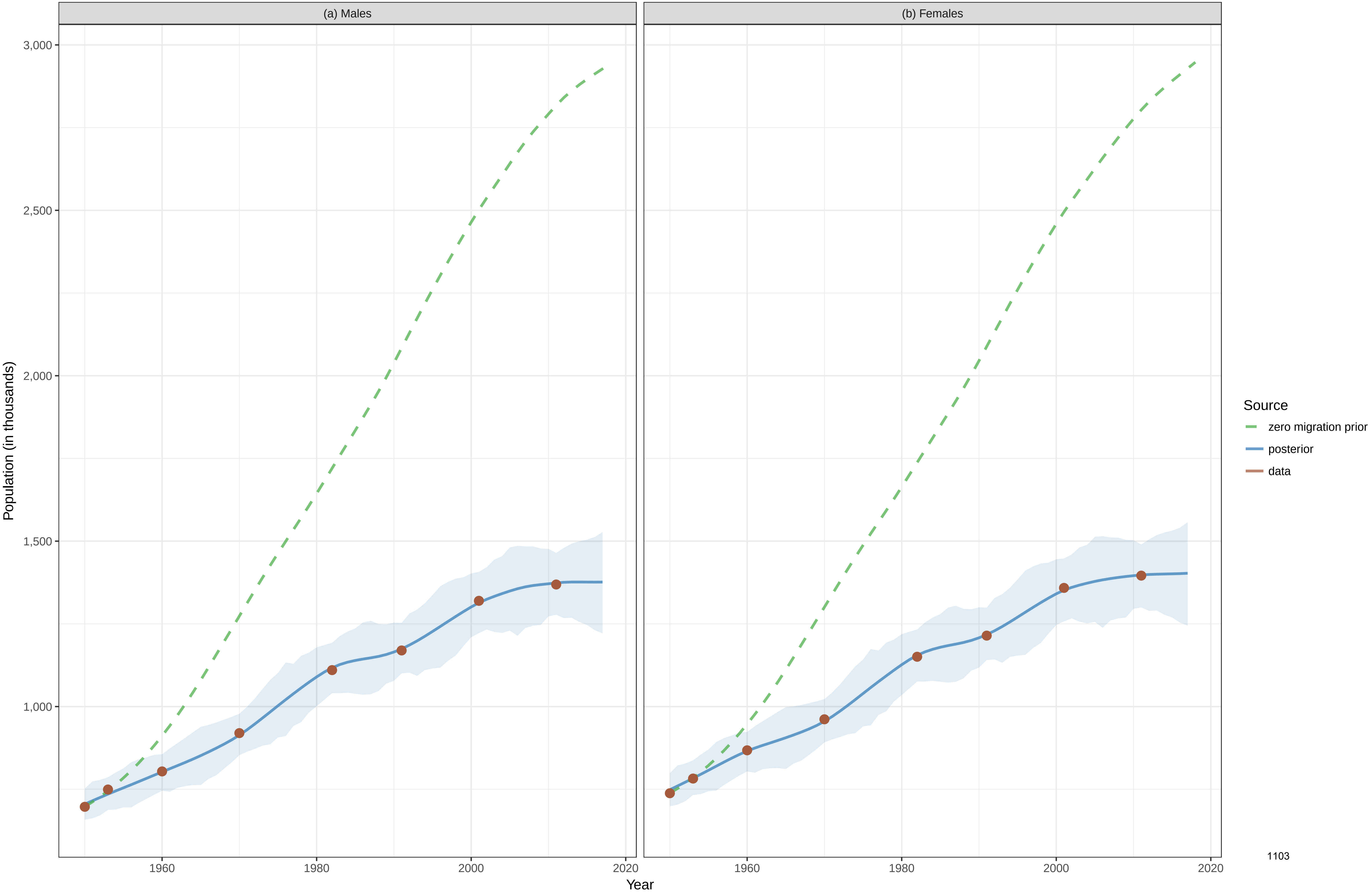

Supplemental Results Figure 4: GBD Bayesian Demographic Balancing Model fit in Japan for (a) total population of males, and (b) total population of females, 1950−2017.

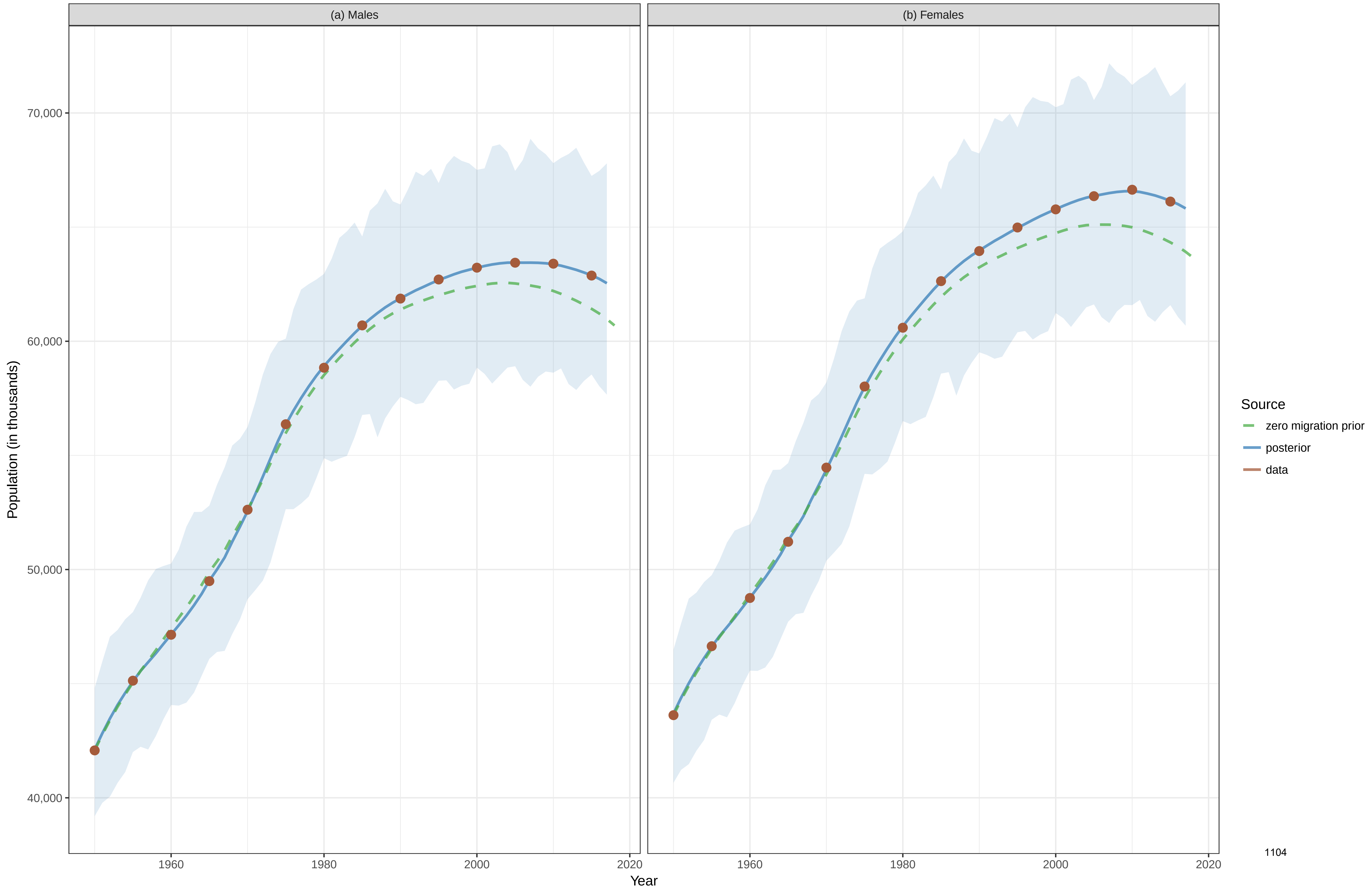

Supplemental Results Figure 4: GBD Bayesian Demographic Balancing Model fit in Jordan for (a) total population of males, and (b) total population of females, 1950−2017.

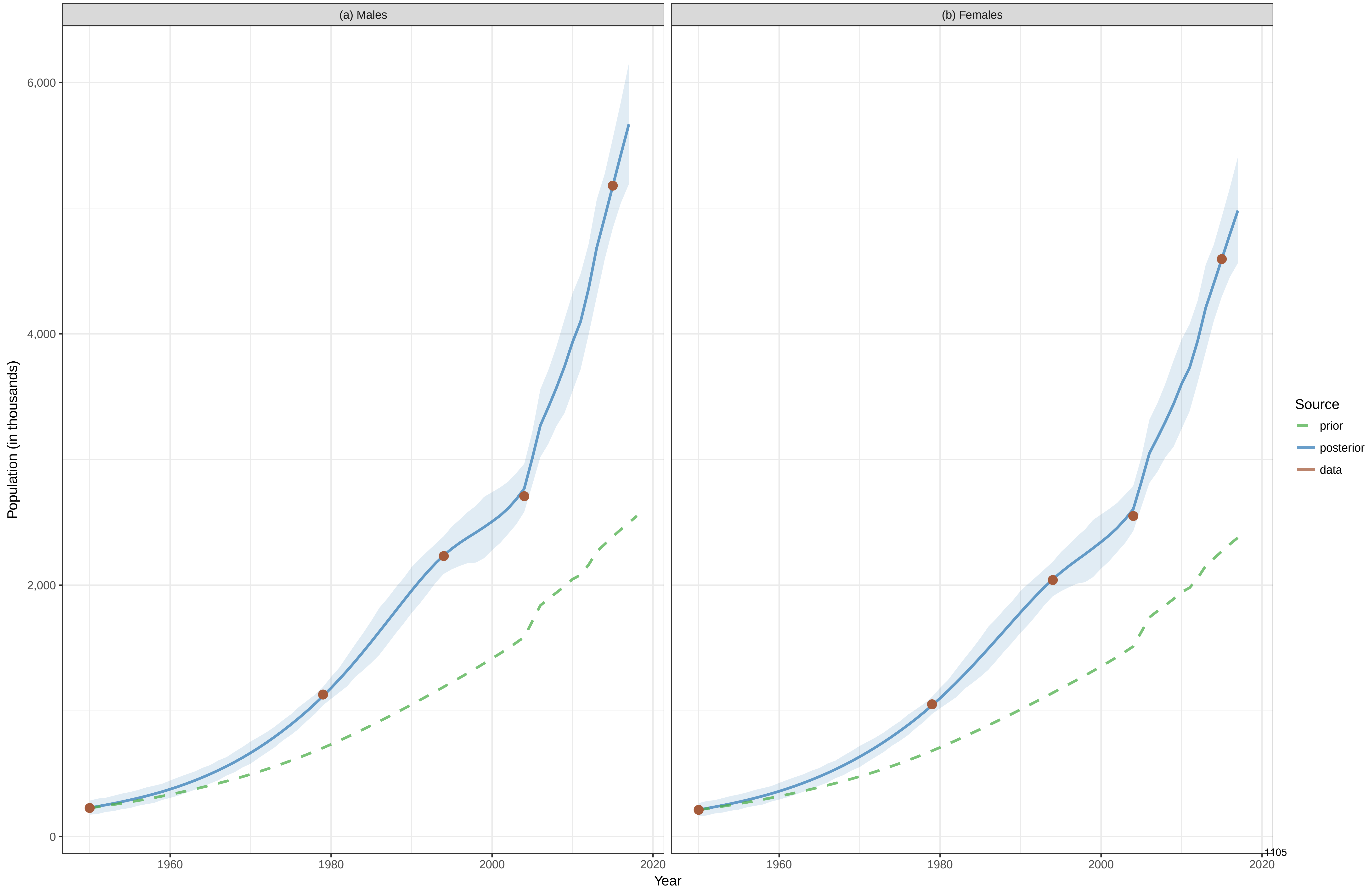

Supplemental Results Figure 4: GBD Bayesian Demographic Balancing Model fit in Kazakhstan for (a) total population of males, and (b) total population of females, 1950−2017.

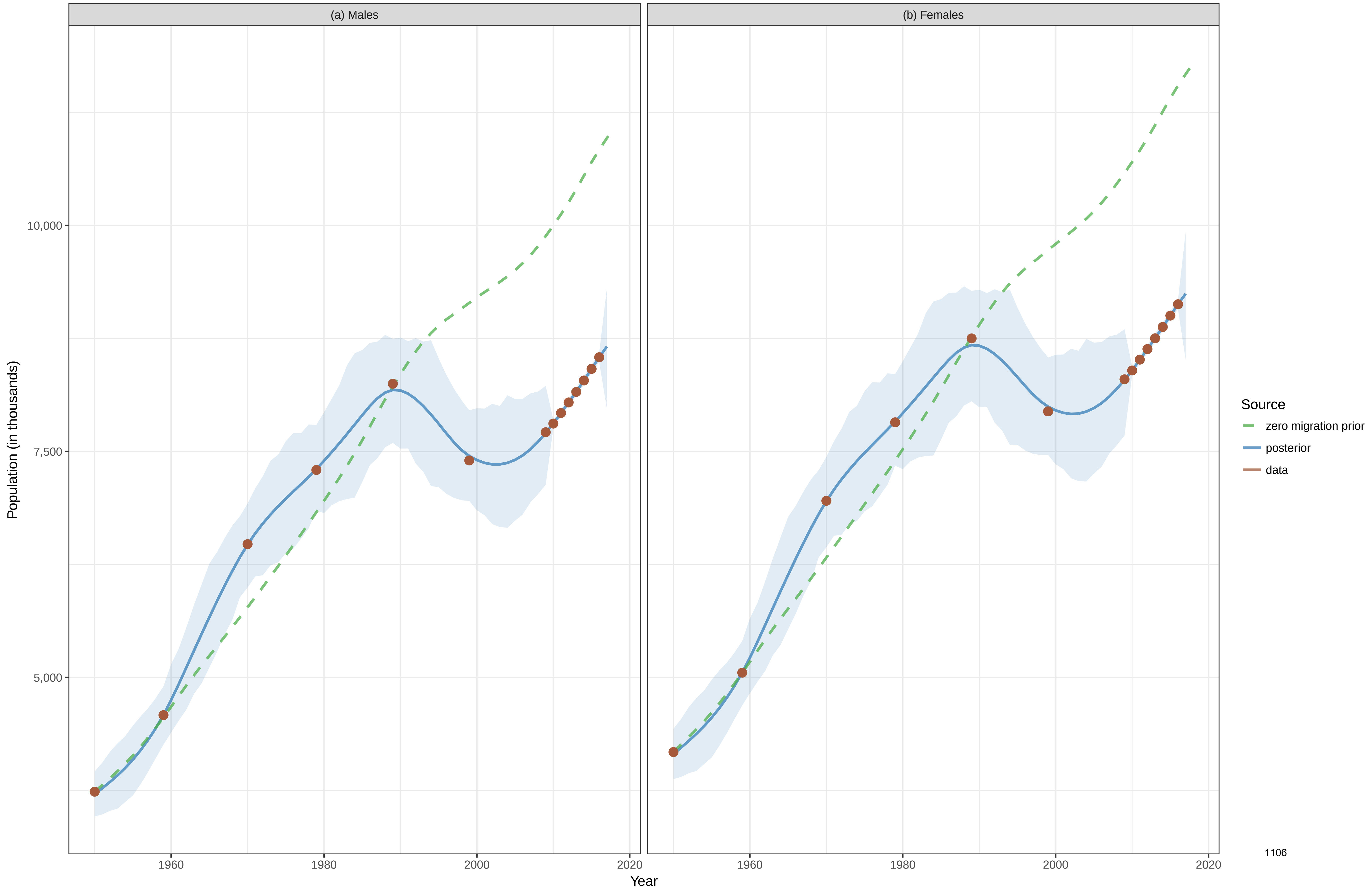

Supplemental Results Figure 4: GBD Bayesian Demographic Balancing Model fit in Kenya for (a) total population of males, and (b) total population of females, 1950−2017.

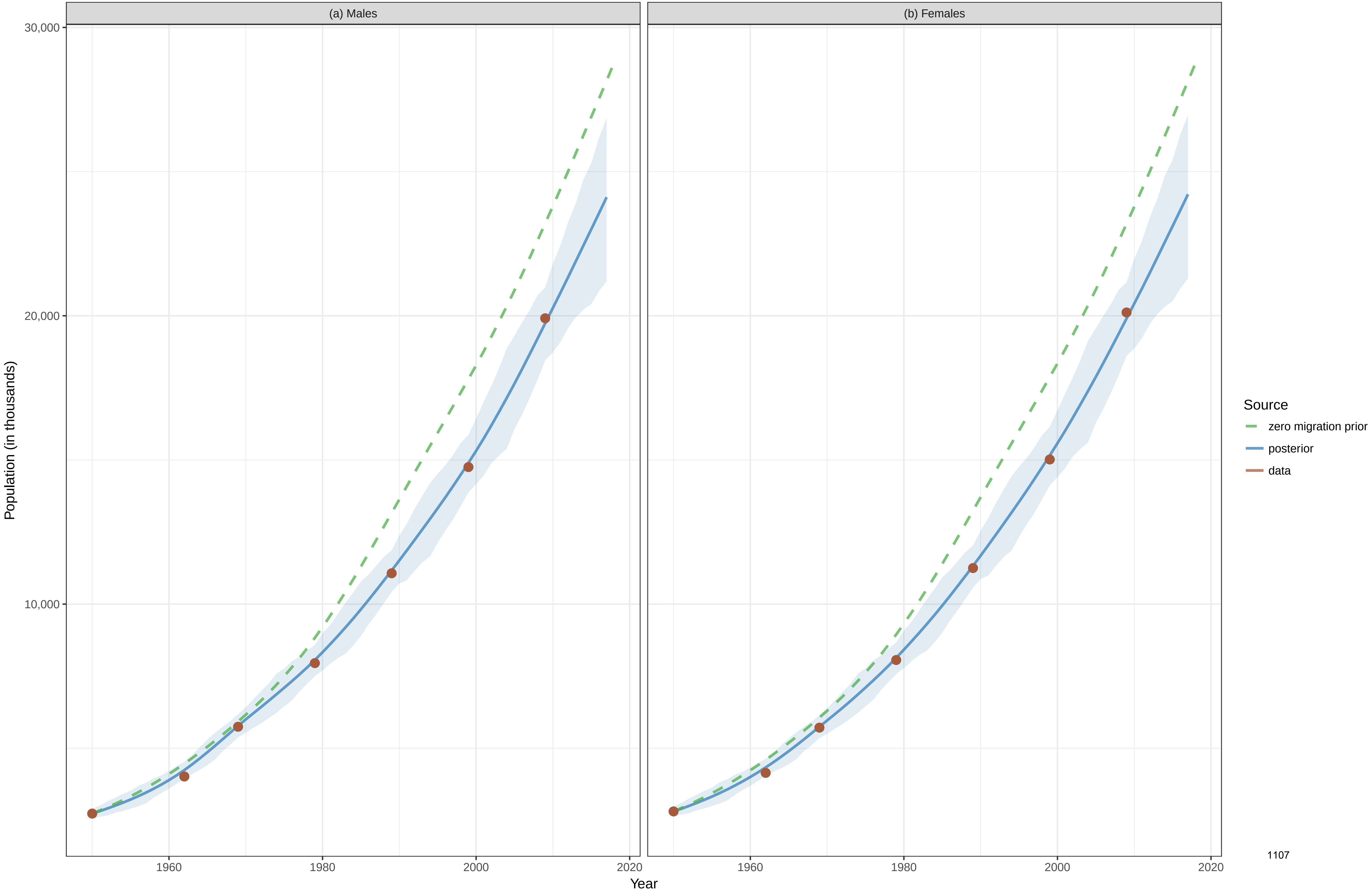

Supplemental Results Figure 4: GBD Bayesian Demographic Balancing Model fit in Kiribati for (a) total population of males, and (b) total population of females, 1950−2017.

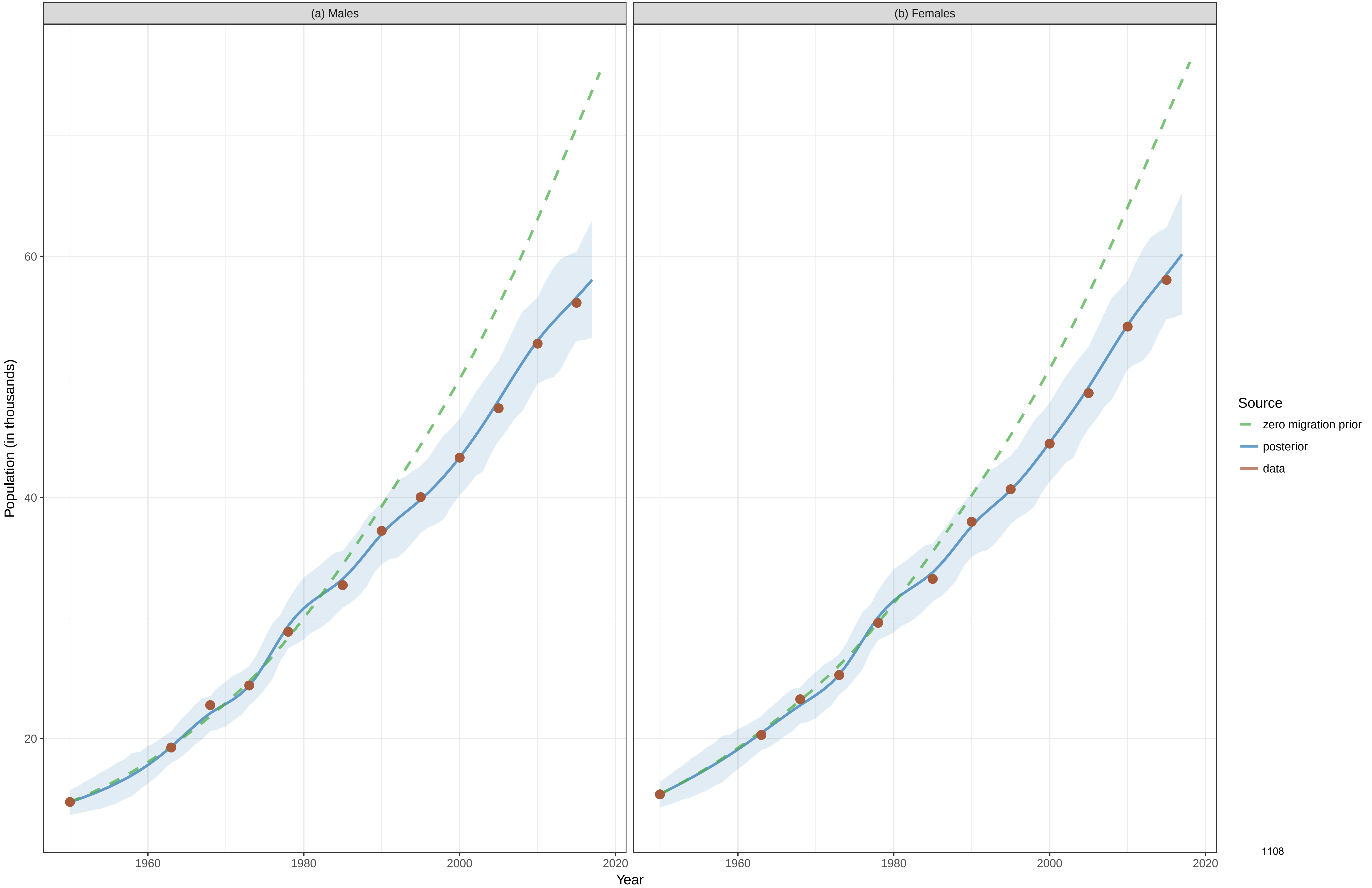

Supplemental Results Figure 4: GBD Bayesian Demographic Balancing Model fit in Kuwait for (a) total population of males, and (b) total population of females, 1950−2017.

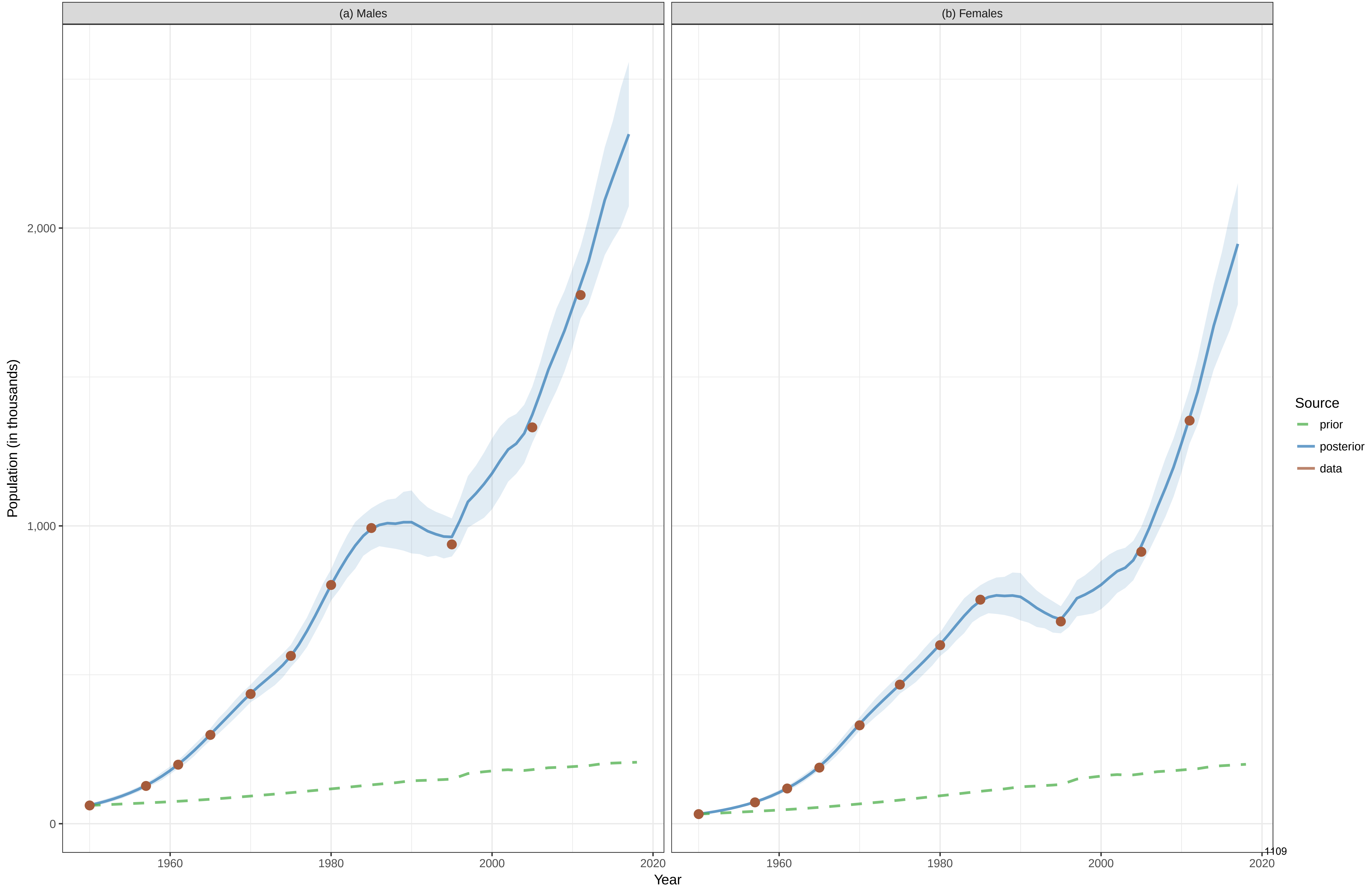

Supplemental Results Figure 4: GBD Bayesian Demographic Balancing Model fit in Kyrgyzstan for (a) total population of males, and (b) total population of females, 1950−2017.

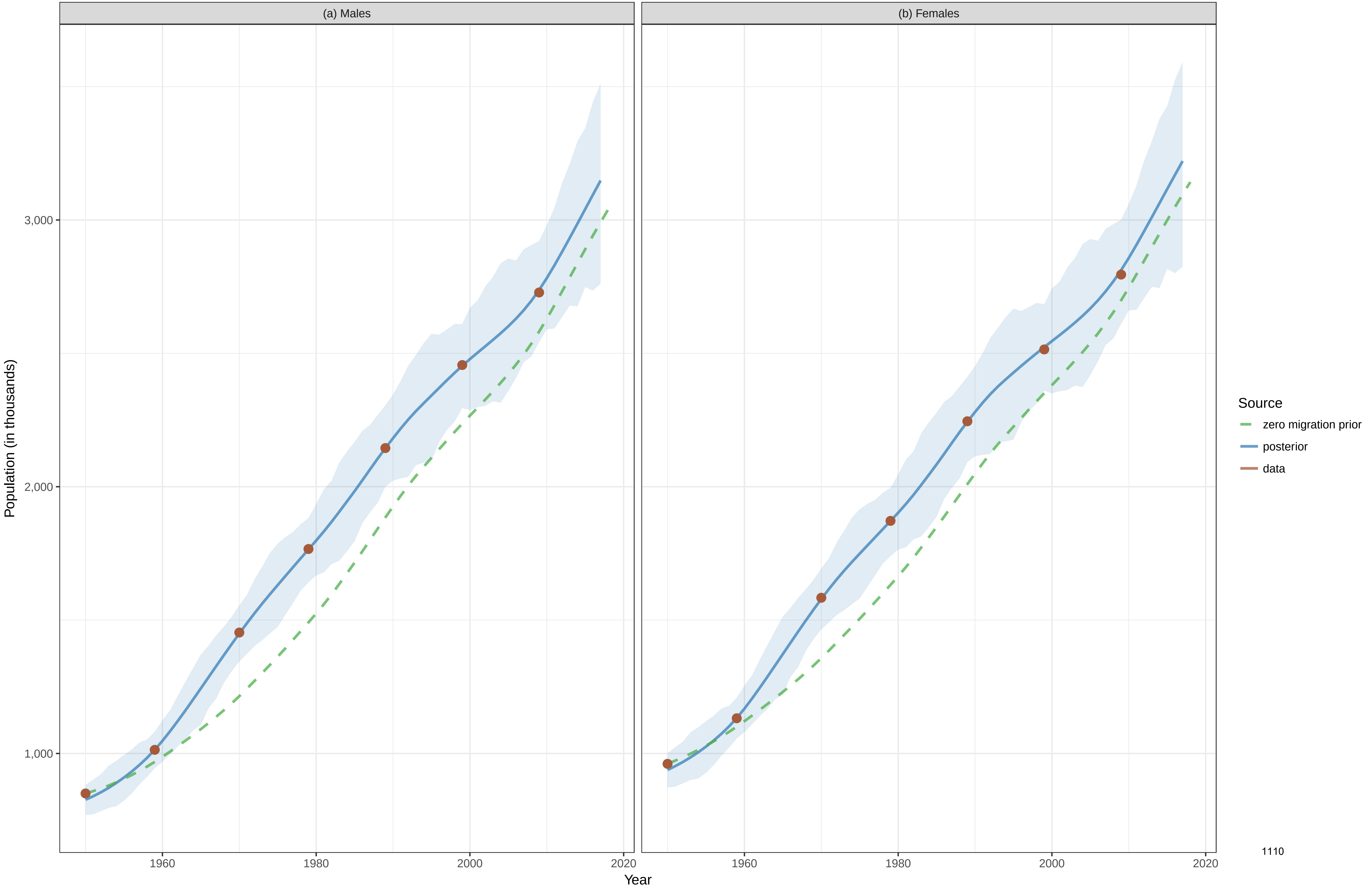

Supplemental Results Figure 4: GBD Bayesian Demographic Balancing Model fit in Laos for (a) total population of males, and (b) total population of females, 1950−2017.

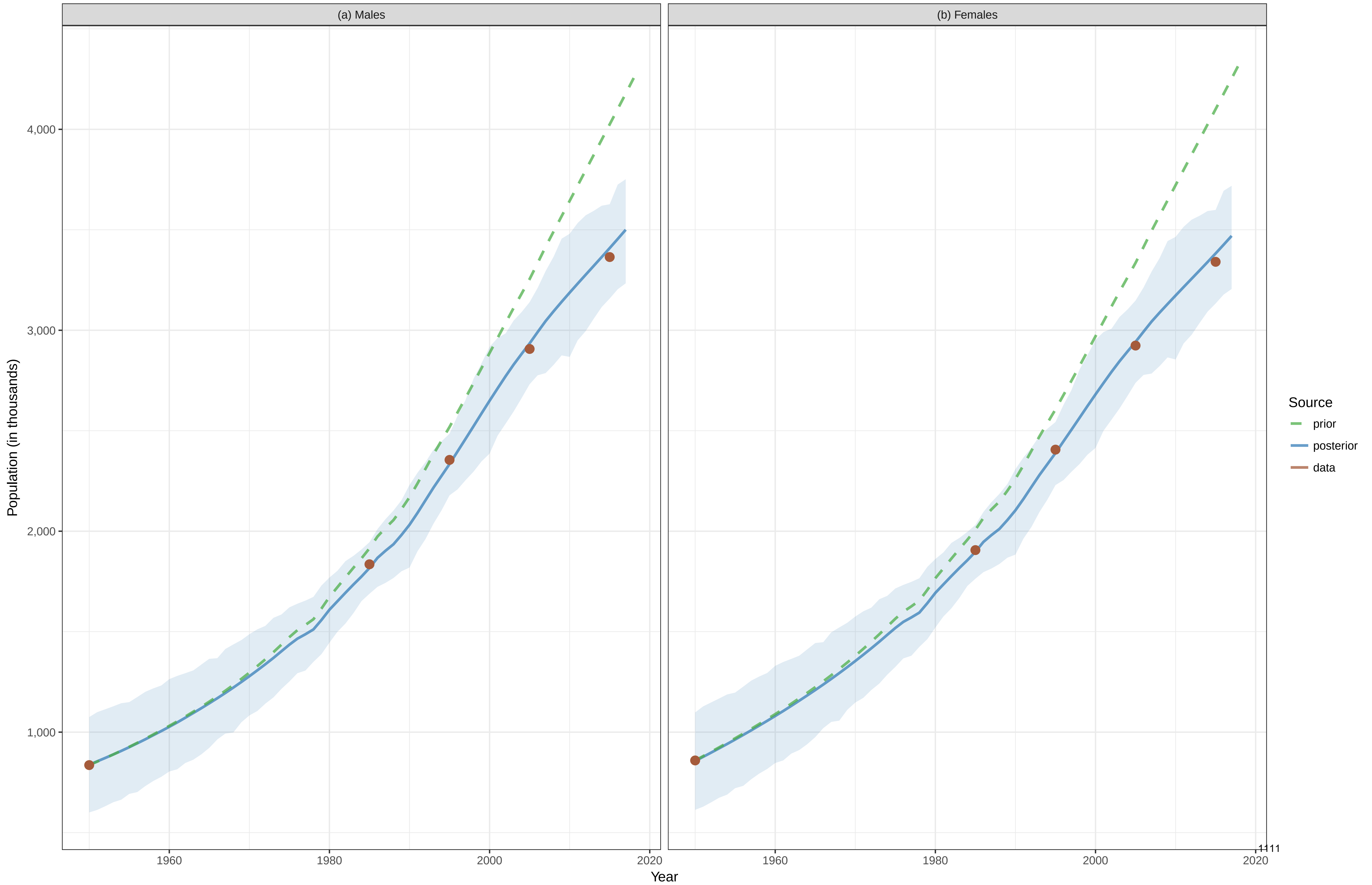

Supplemental Results Figure 4: GBD Bayesian Demographic Balancing Model fit in Latvia for (a) total population of males, and (b) total population of females, 1950−2017.

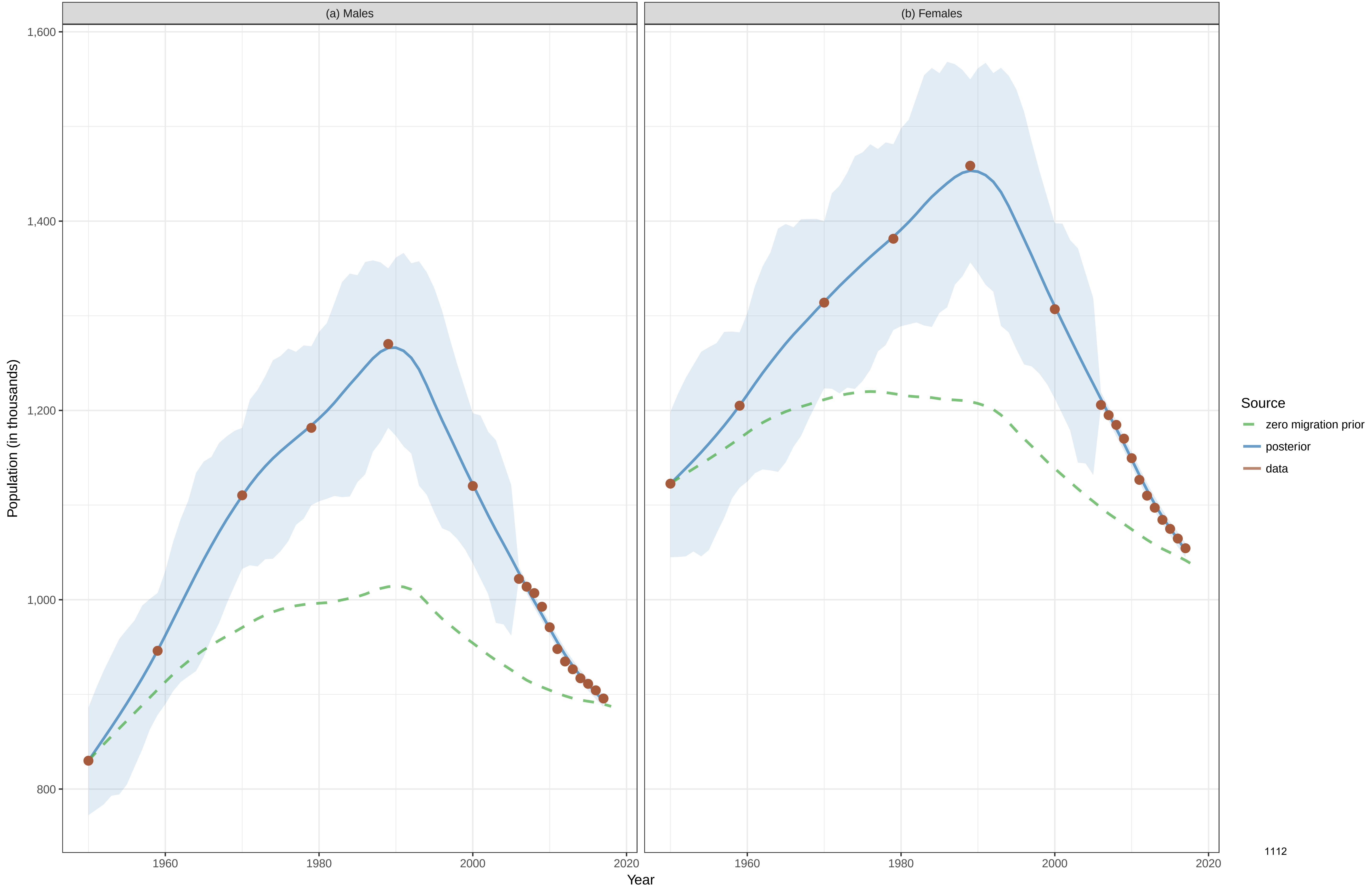

Supplemental Results Figure 4: GBD Bayesian Demographic Balancing Model fit in Lebanon for (a) total population of males, and (b) total population of females, 1950−2017.

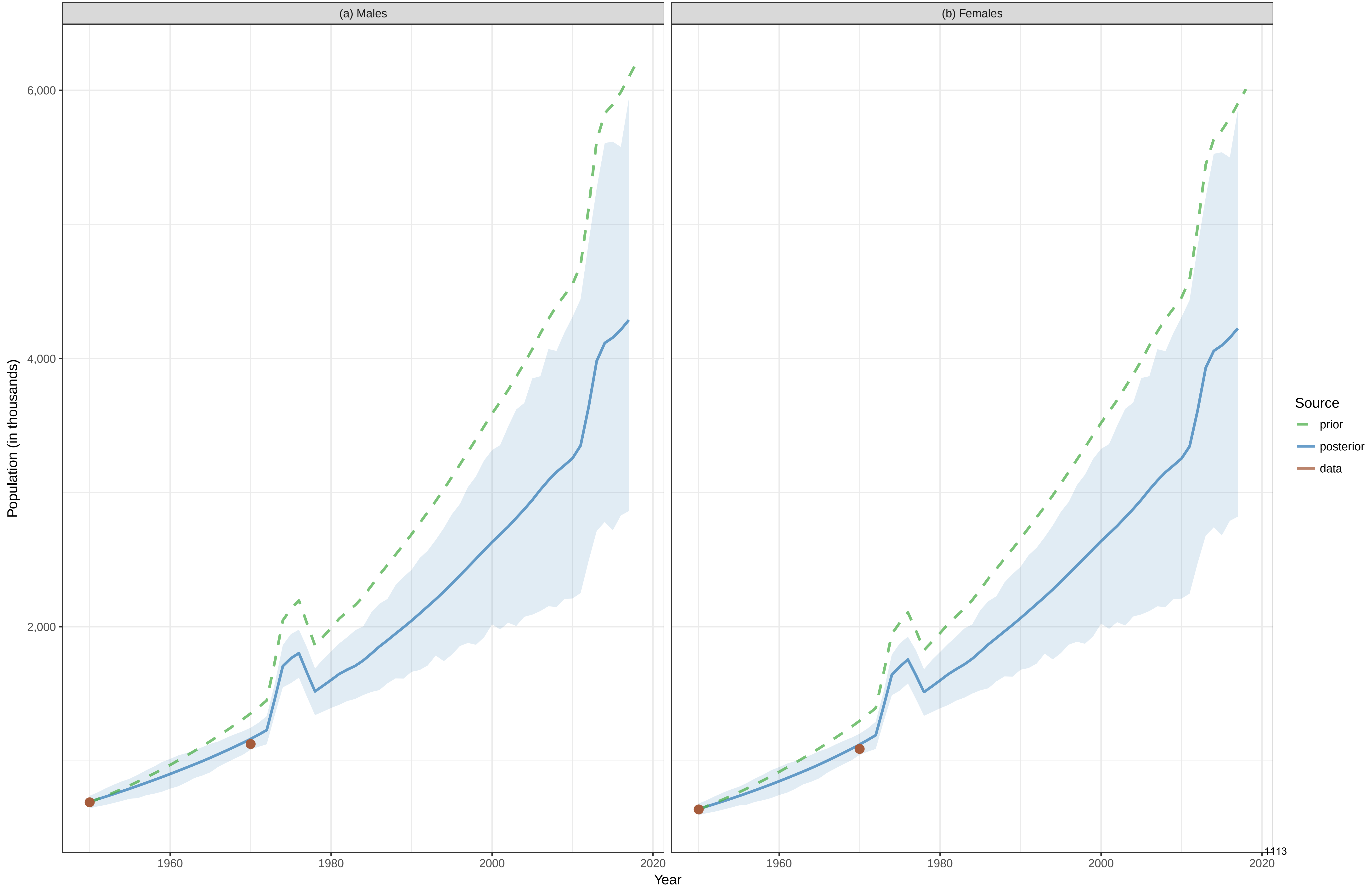

Supplemental Results Figure 4: GBD Bayesian Demographic Balancing Model fit in Lesotho for (a) total population of males, and (b) total population of females, 1950−2017.

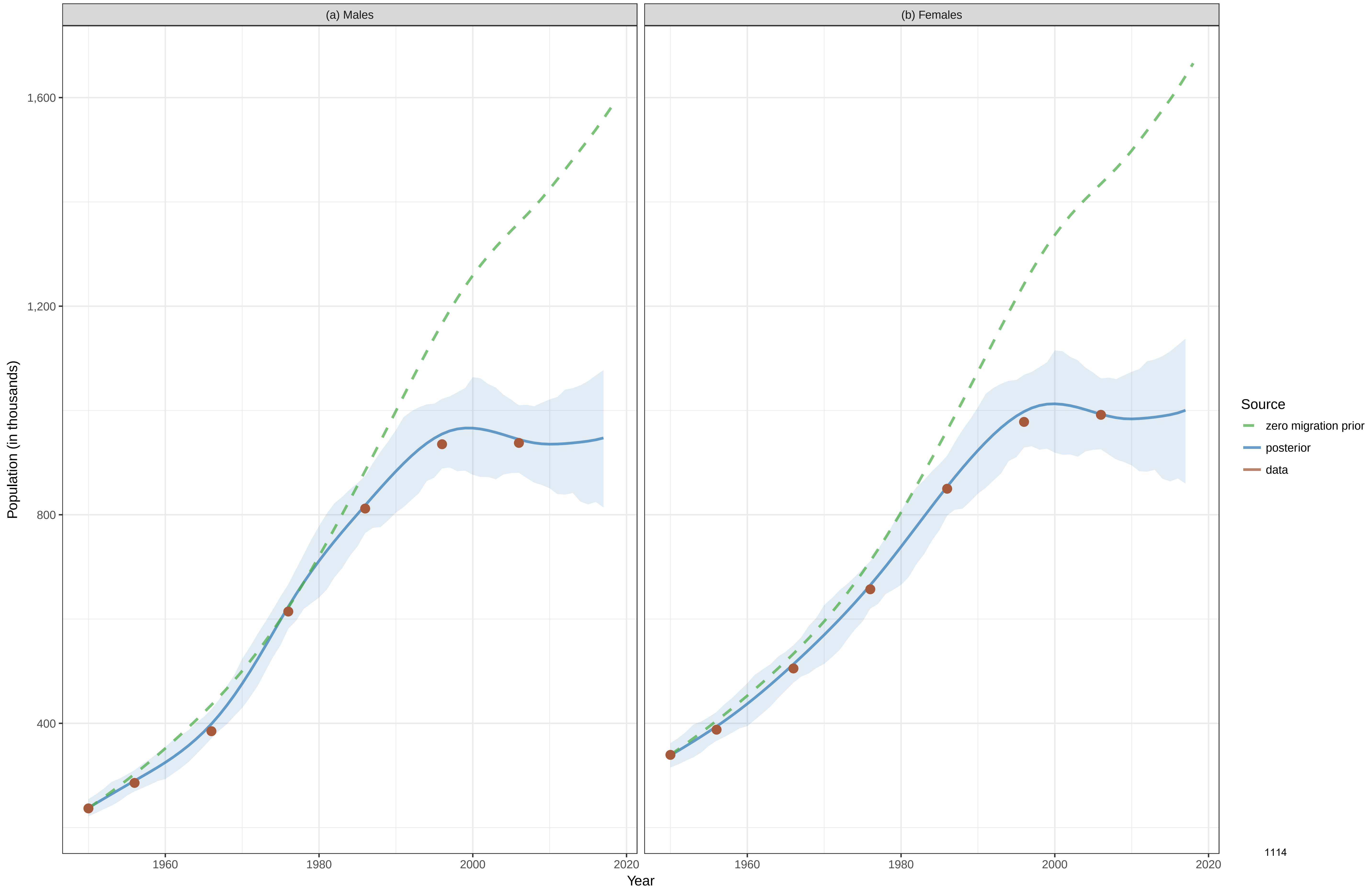

Supplemental Results Figure 4: GBD Bayesian Demographic Balancing Model fit in Liberia for (a) total population of males, and (b) total population of females, 1950−2017.

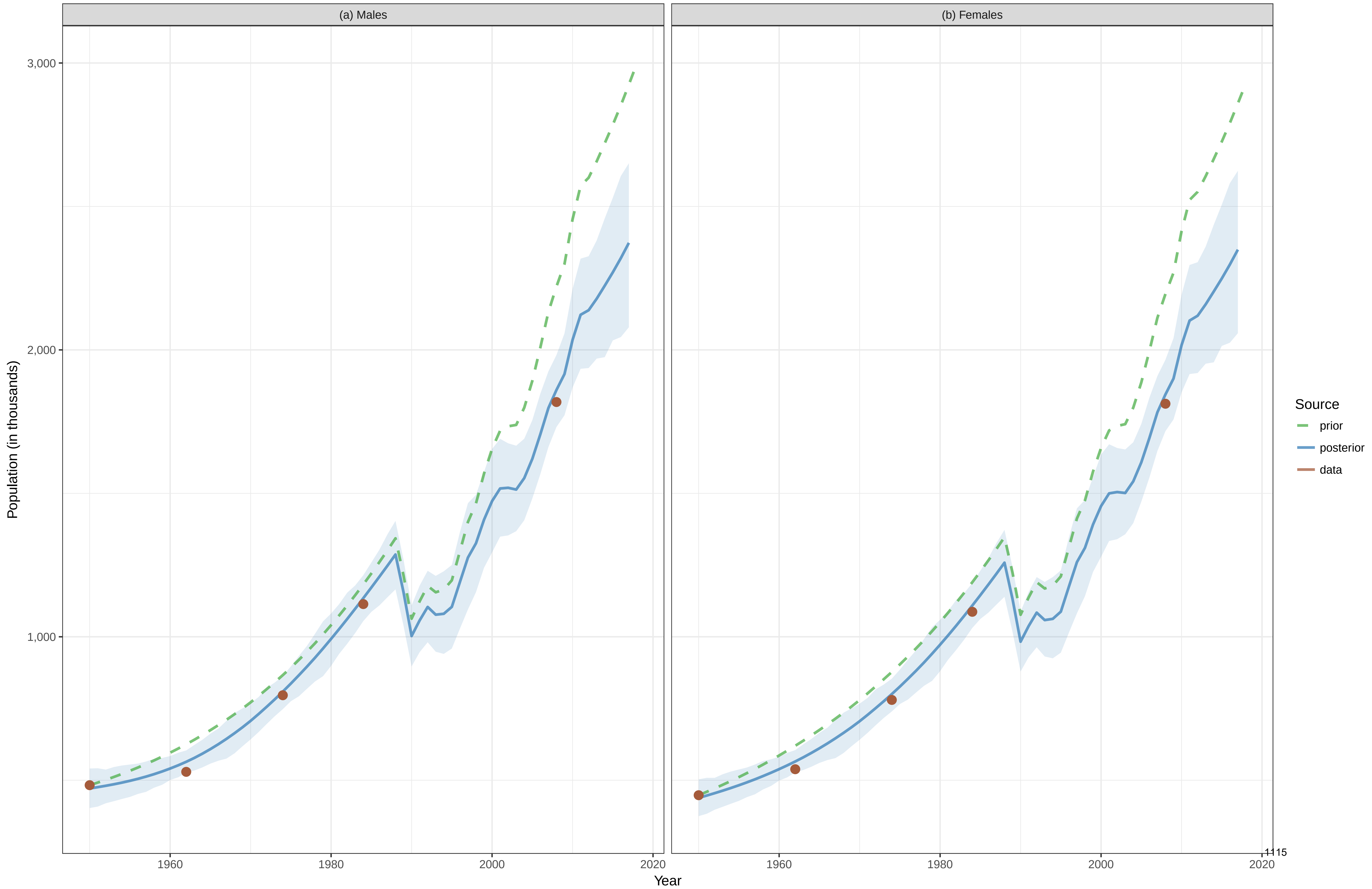

Supplemental Results Figure 4: GBD Bayesian Demographic Balancing Model fit in Libya for (a) total population of males, and (b) total population of females, 1950−2017.

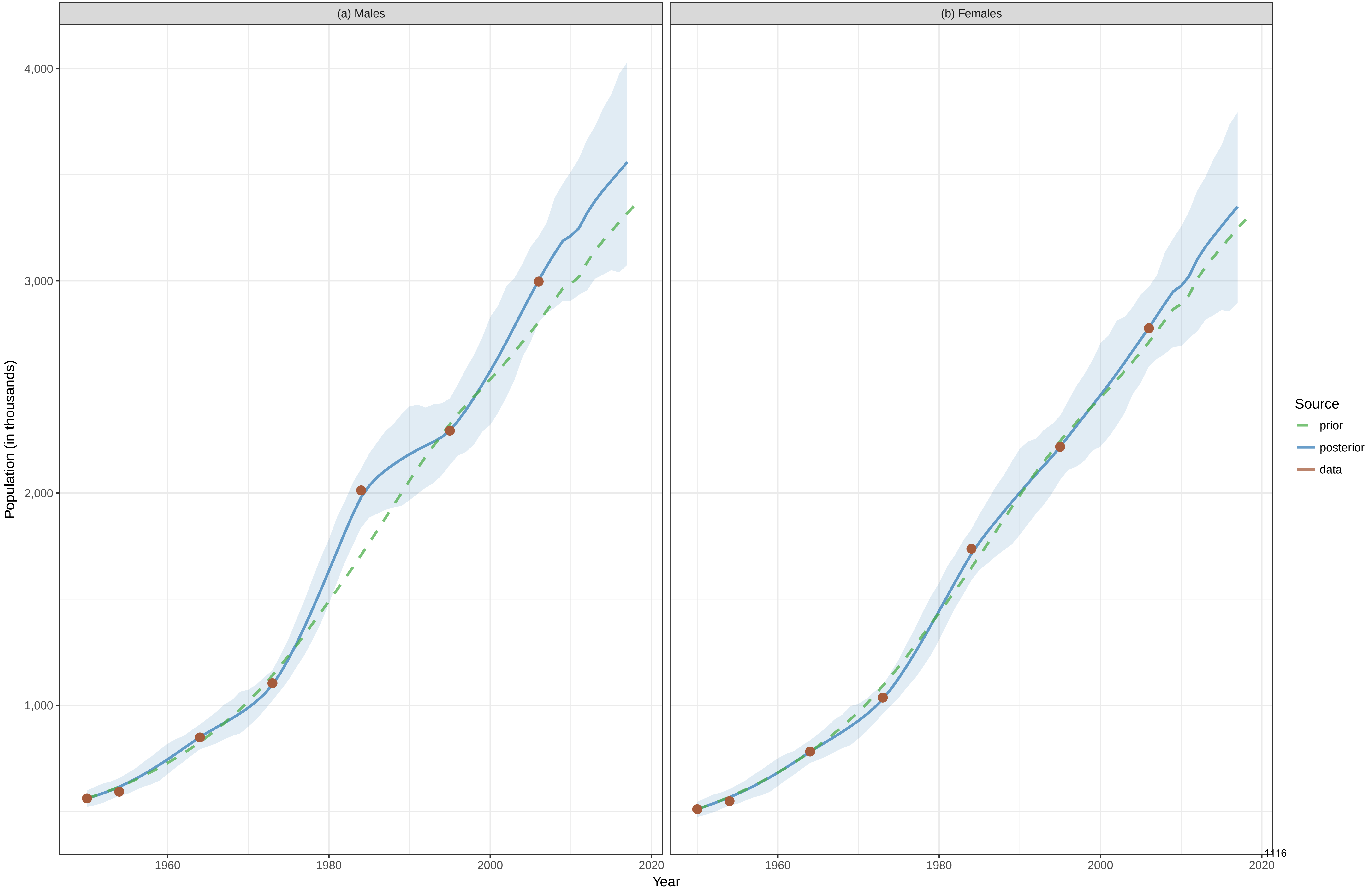

Supplemental Results Figure 4: GBD Bayesian Demographic Balancing Model fit in Lithuania for (a) total population of males, and (b) total population of females, 1950−2017.

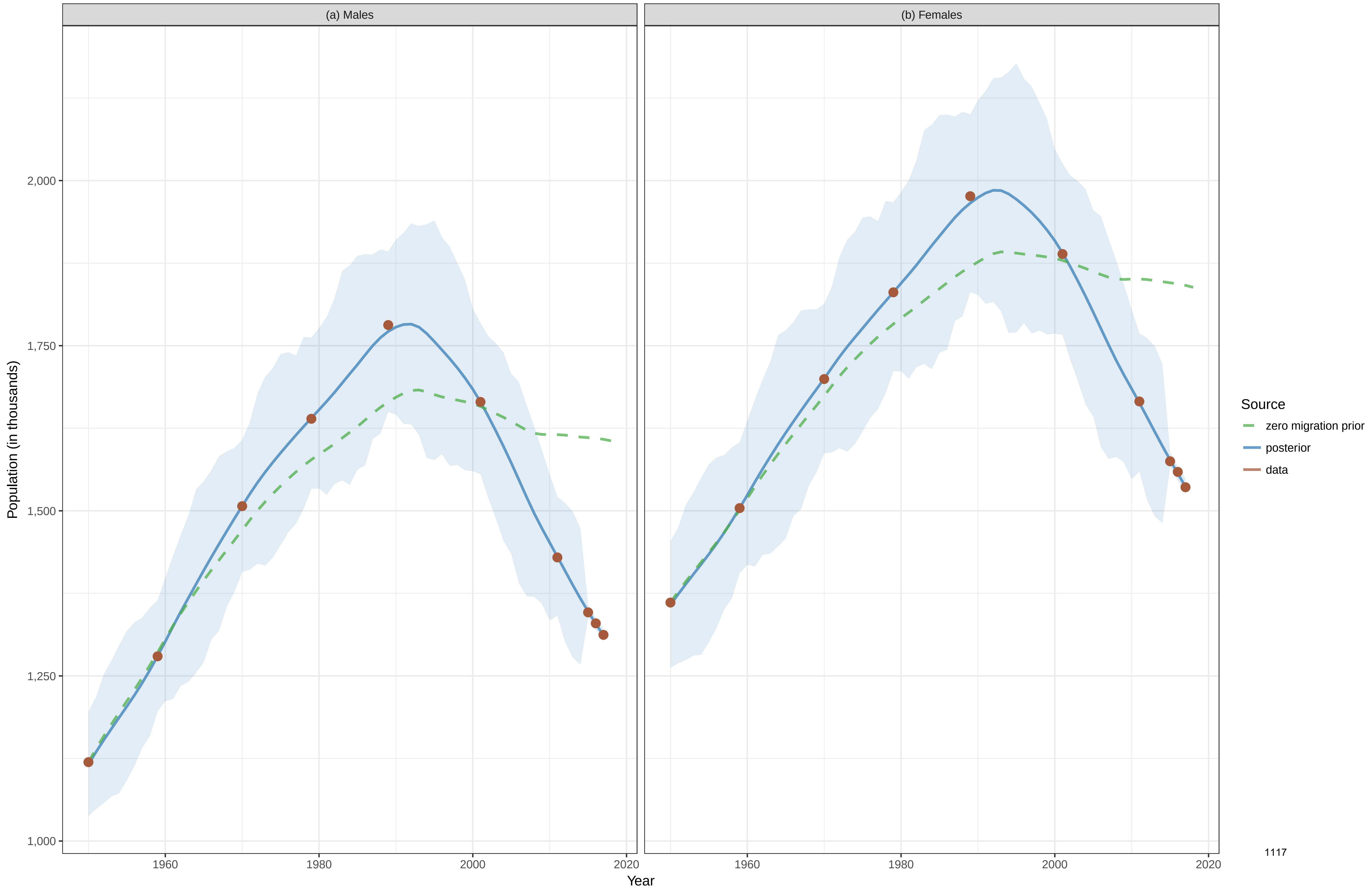

Supplemental Results Figure 4: GBD Bayesian Demographic Balancing Model fit in Luxembourg for (a) total population of males, and (b) total population of females, 1950−2017.

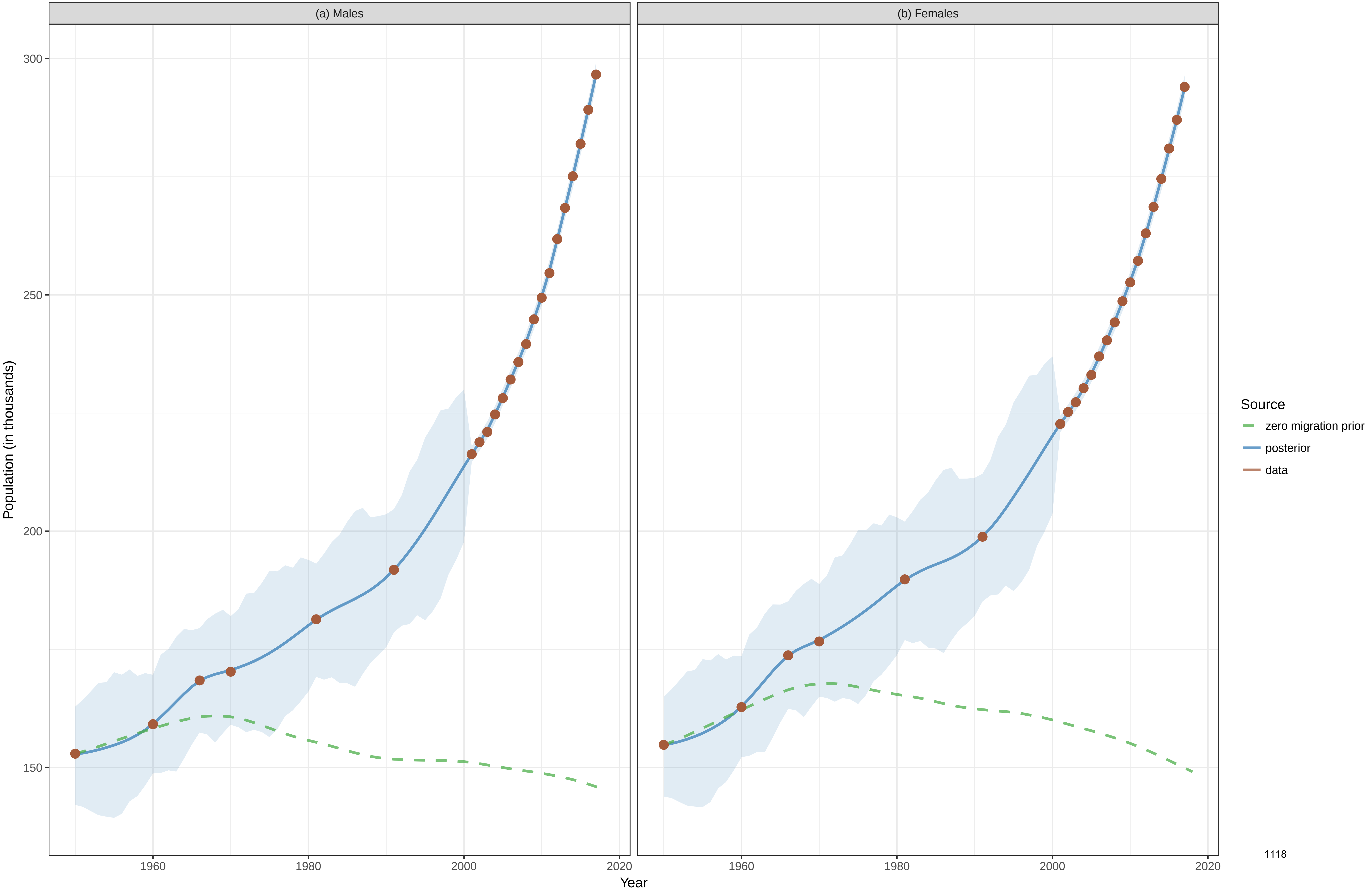

Supplemental Results Figure 4: GBD Bayesian Demographic Balancing Model fit in Macao Special Administrative Region of China for (a) total population of males, and (b) total population of females, 1950−2017.

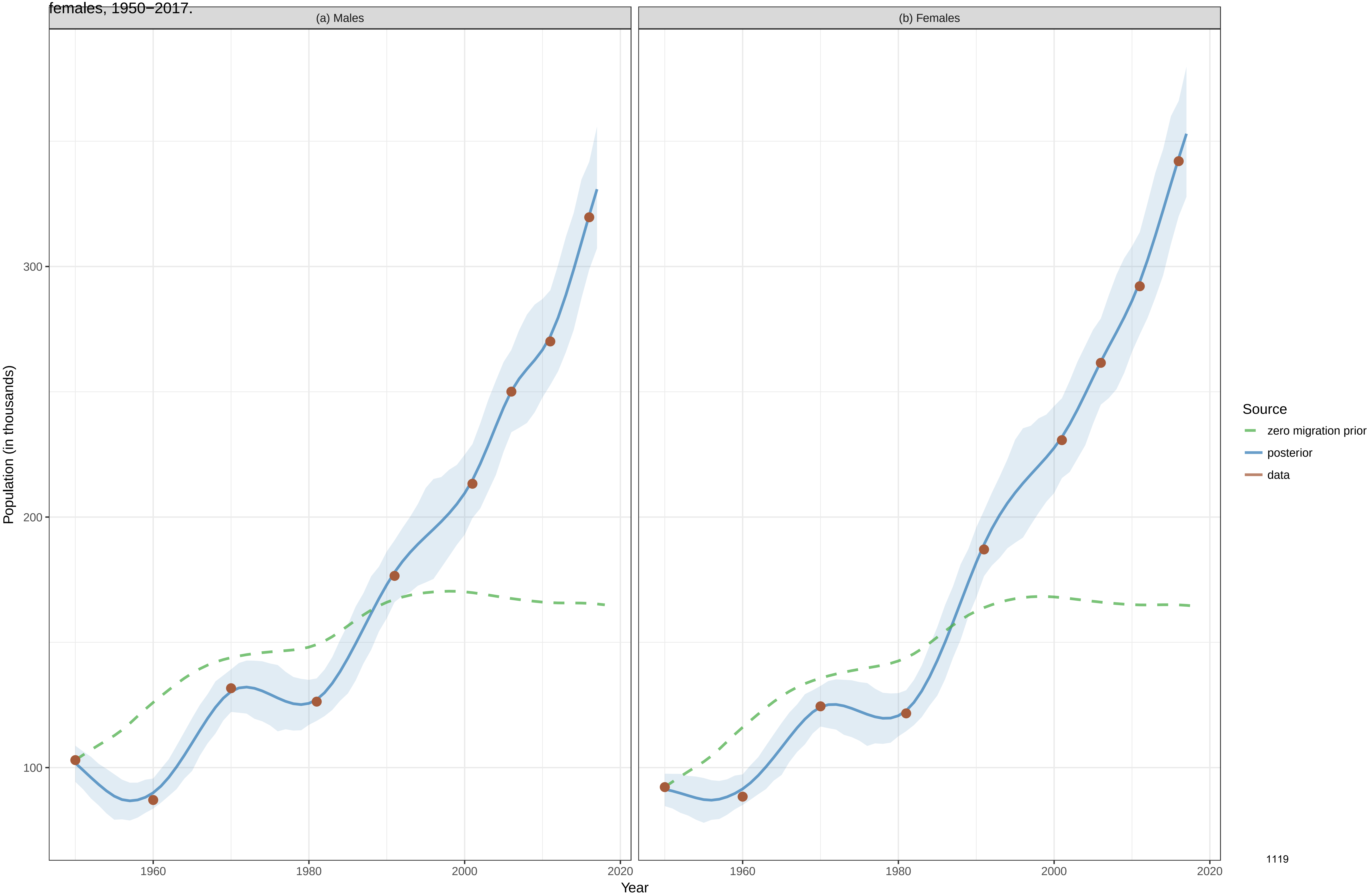

Supplemental Results Figure 4: GBD Bayesian Demographic Balancing Model fit in Macedonia for (a) total population of males, and (b) total population of females, 1950−2017.

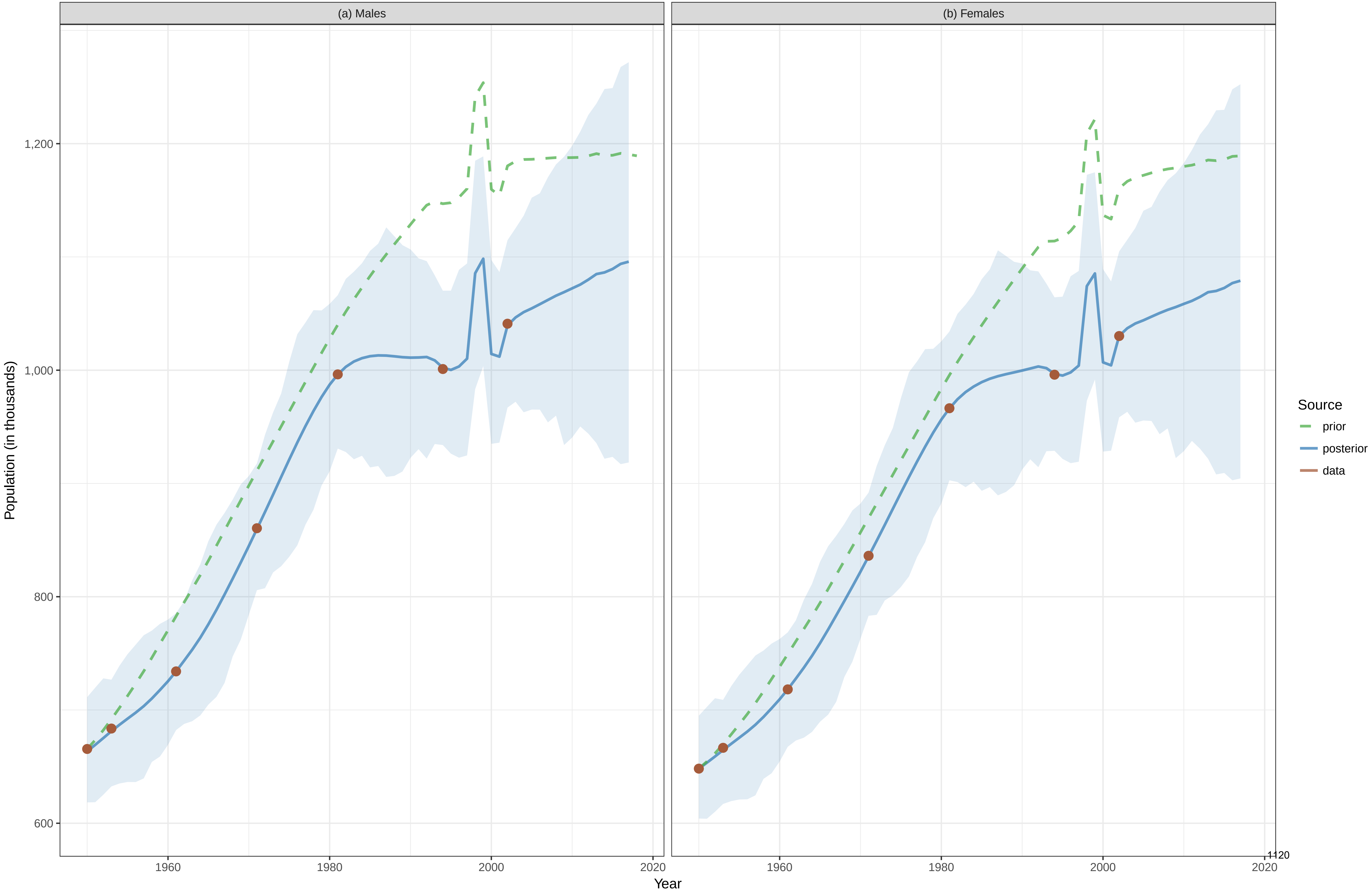

Supplemental Results Figure 4: GBD Bayesian Demographic Balancing Model fit in Madagascar for (a) total population of males, and (b) total population of females, 1950−2017.

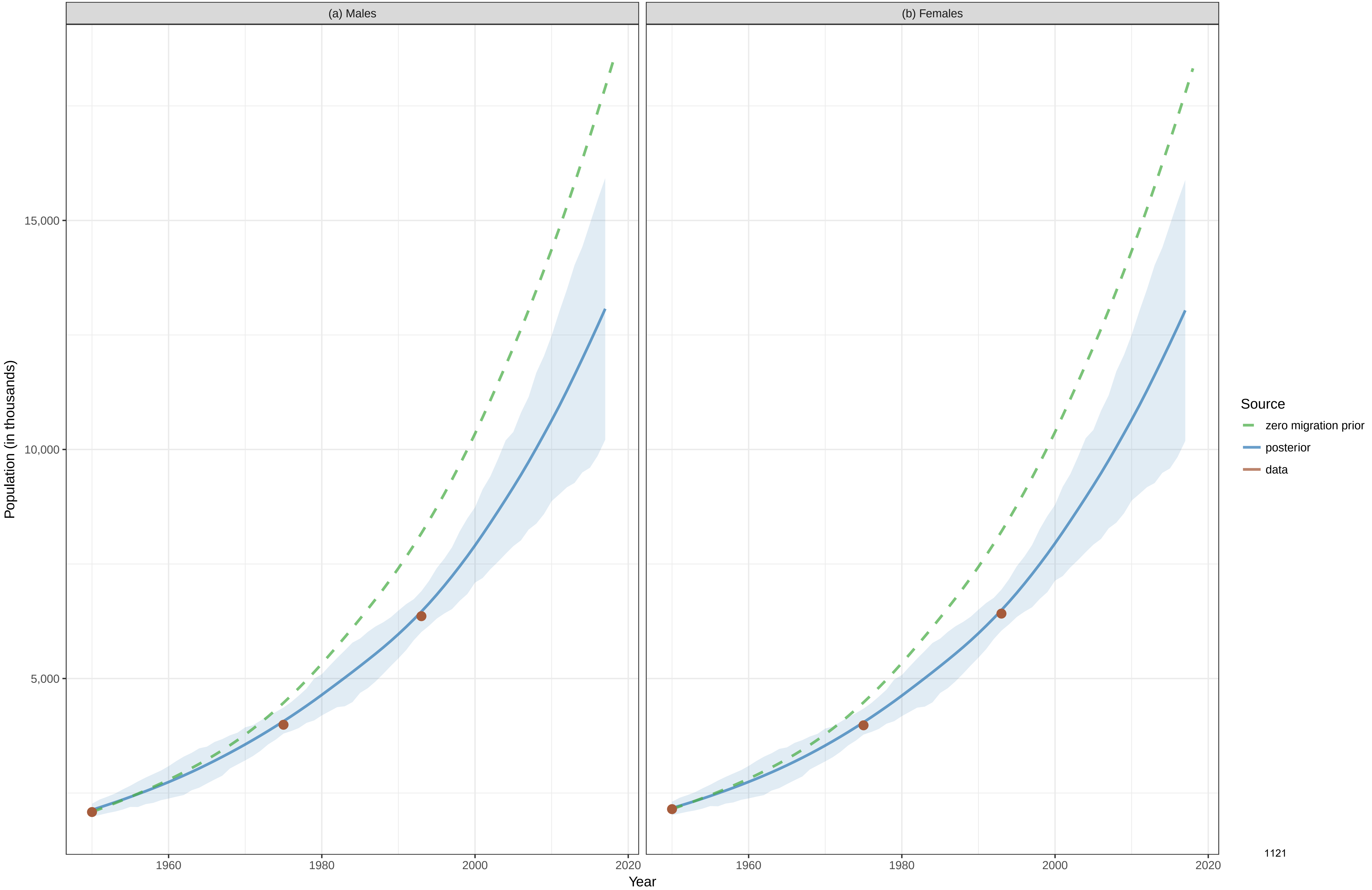

Supplemental Results Figure 4: GBD Bayesian Demographic Balancing Model fit in Malawi for (a) total population of males, and (b) total population of females, 1950−2017.

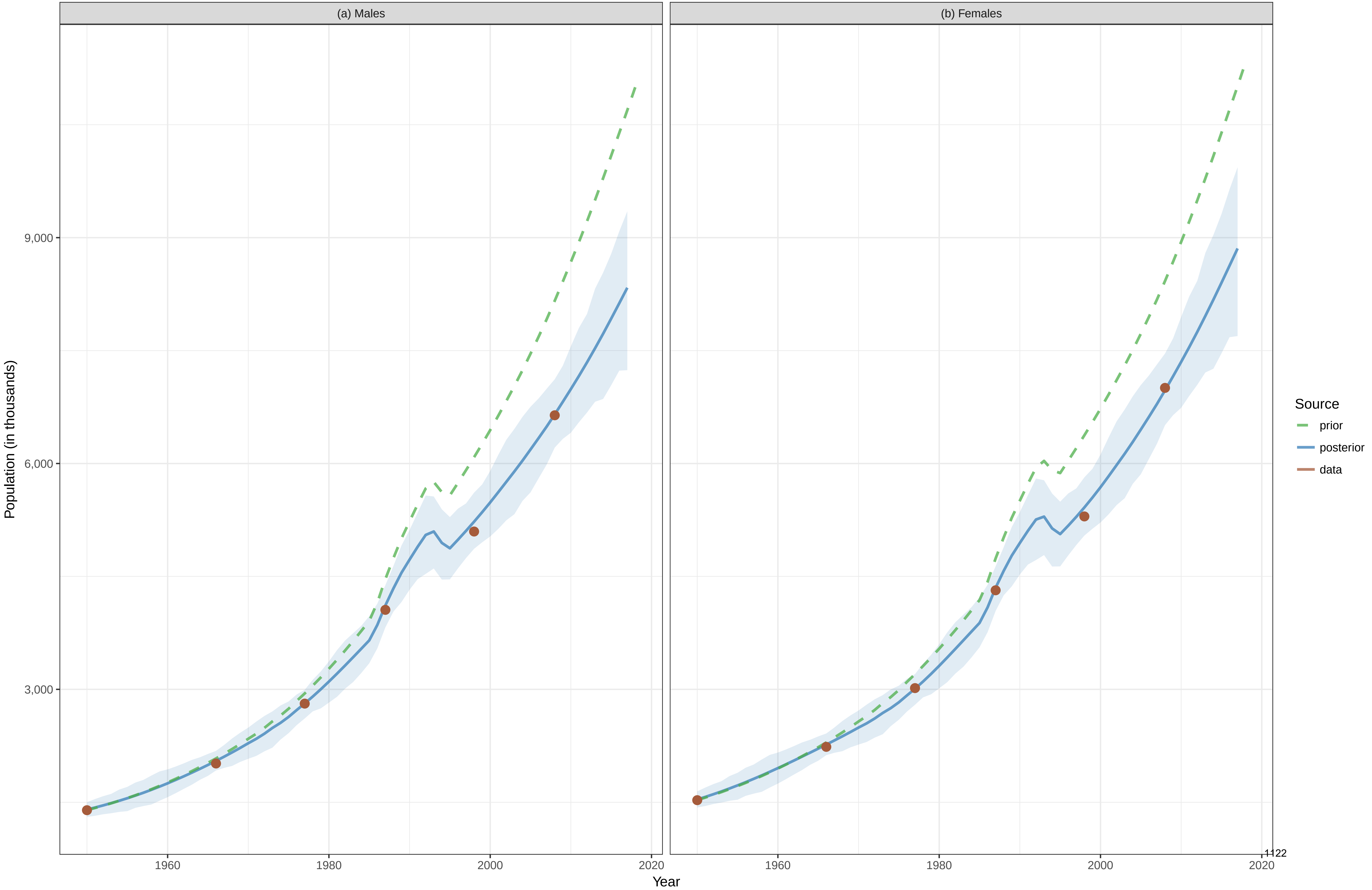

Supplemental Results Figure 4: GBD Bayesian Demographic Balancing Model fit in Malaysia for (a) total population of males, and (b) total population of females, 1950−2017.

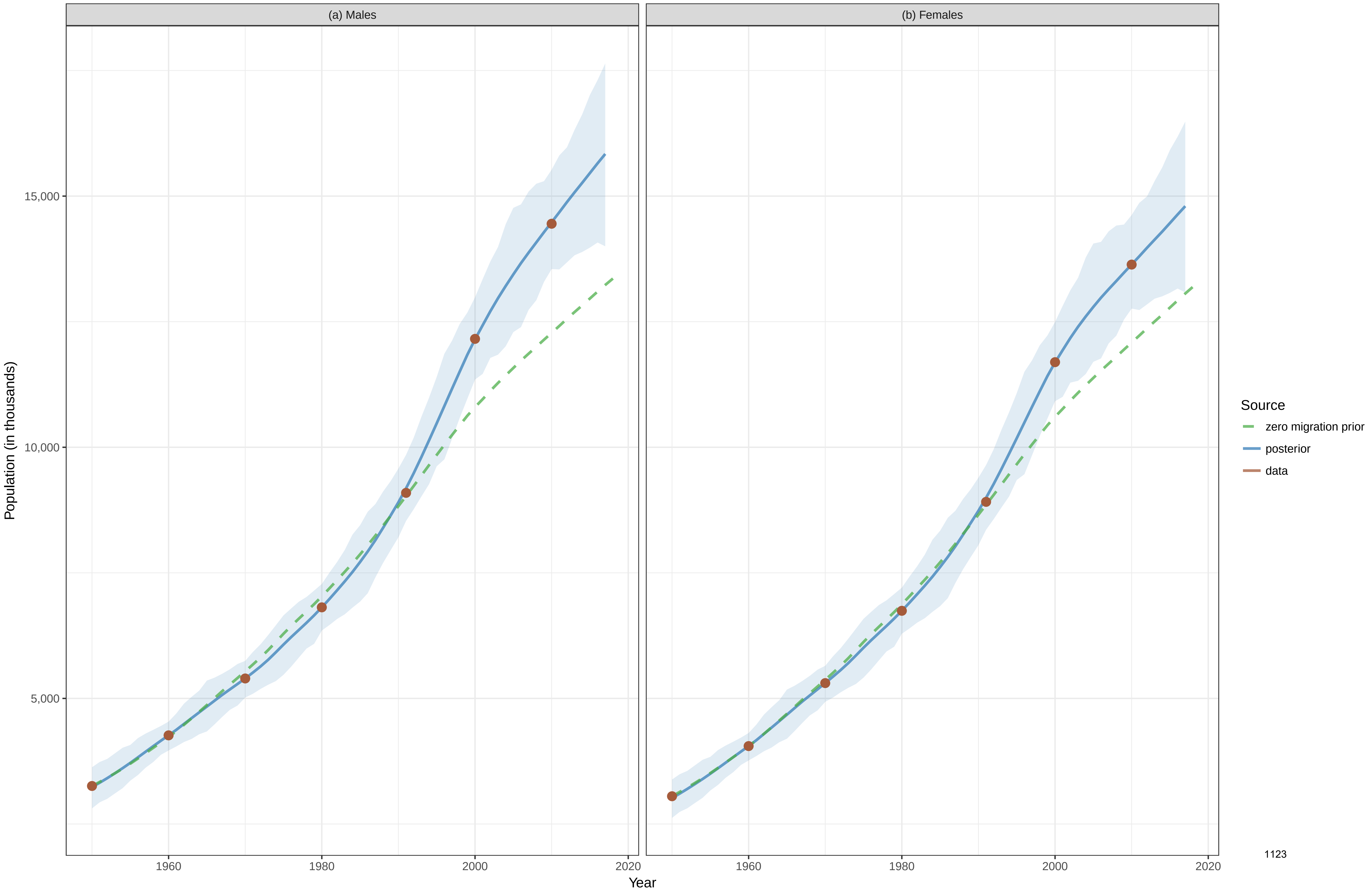

Supplemental Results Figure 4: GBD Bayesian Demographic Balancing Model fit in Maldives for (a) total population of males, and (b) total population of females, 1950−2017.

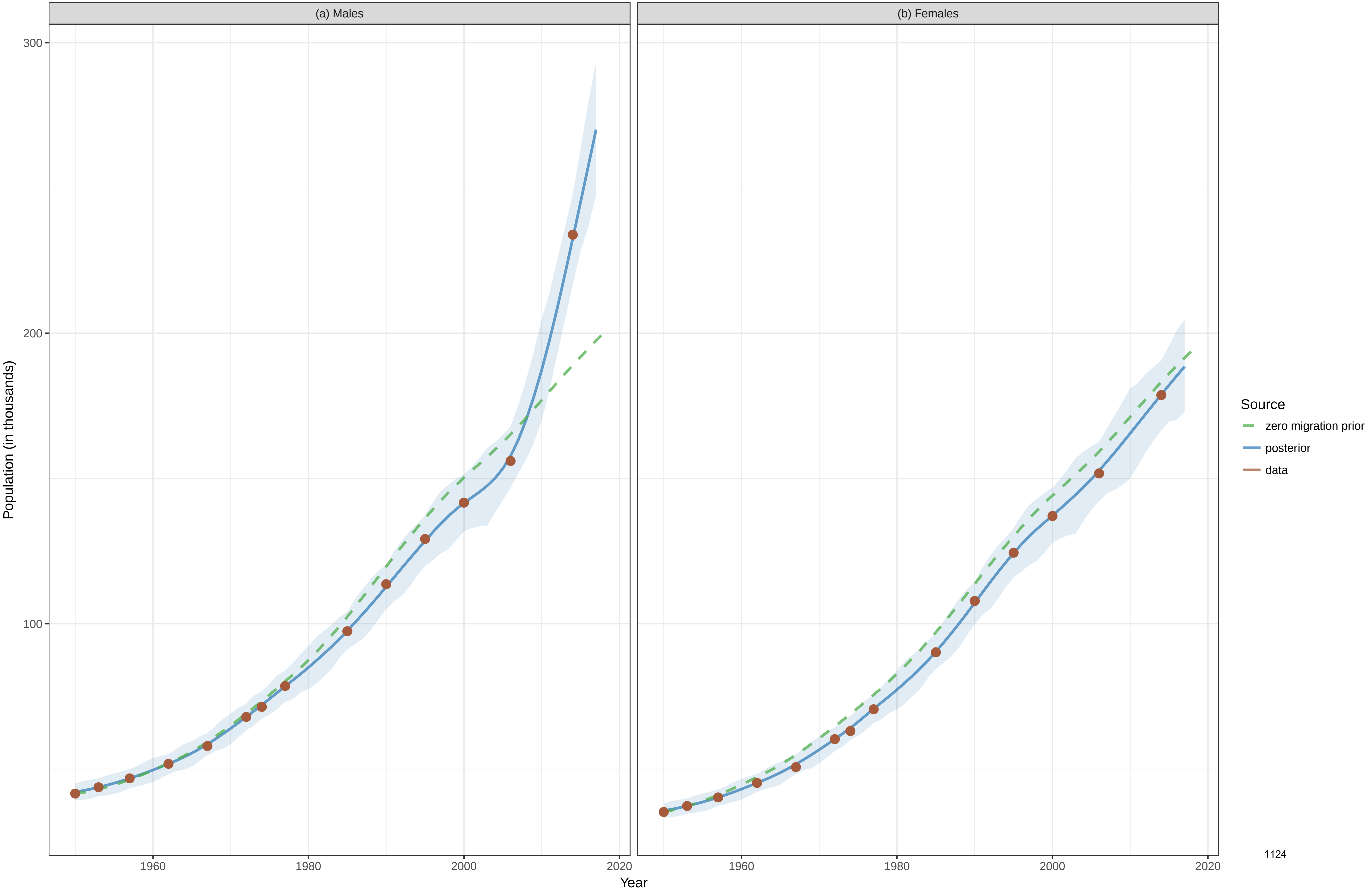

Supplemental Results Figure 4: GBD Bayesian Demographic Balancing Model fit in Mali for (a) total population of males, and (b) total population of females, 1950−2017.

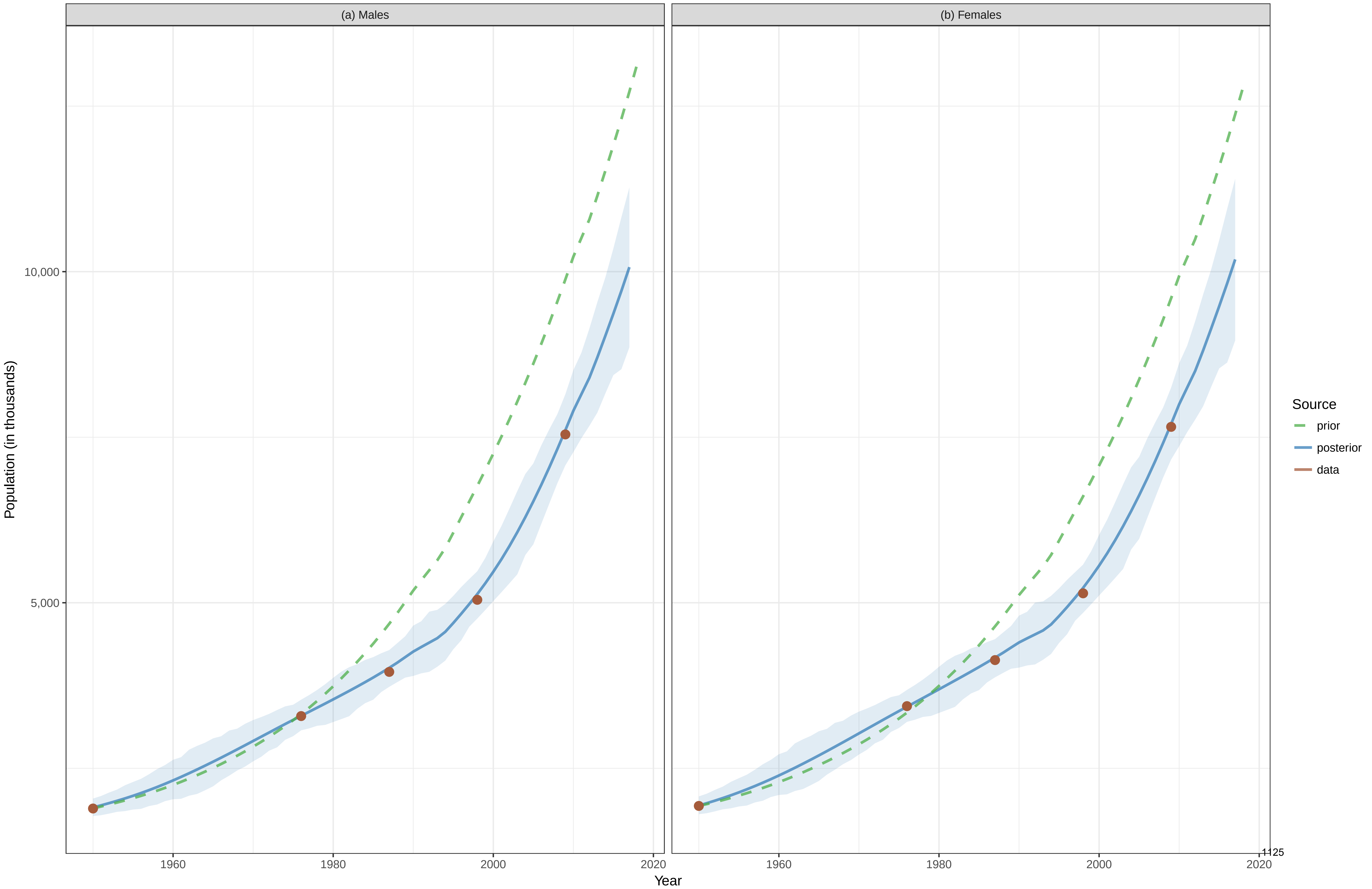

Supplemental Results Figure 4: GBD Bayesian Demographic Balancing Model fit in Malta for (a) total population of males, and (b) total population of females, 1950−2017.

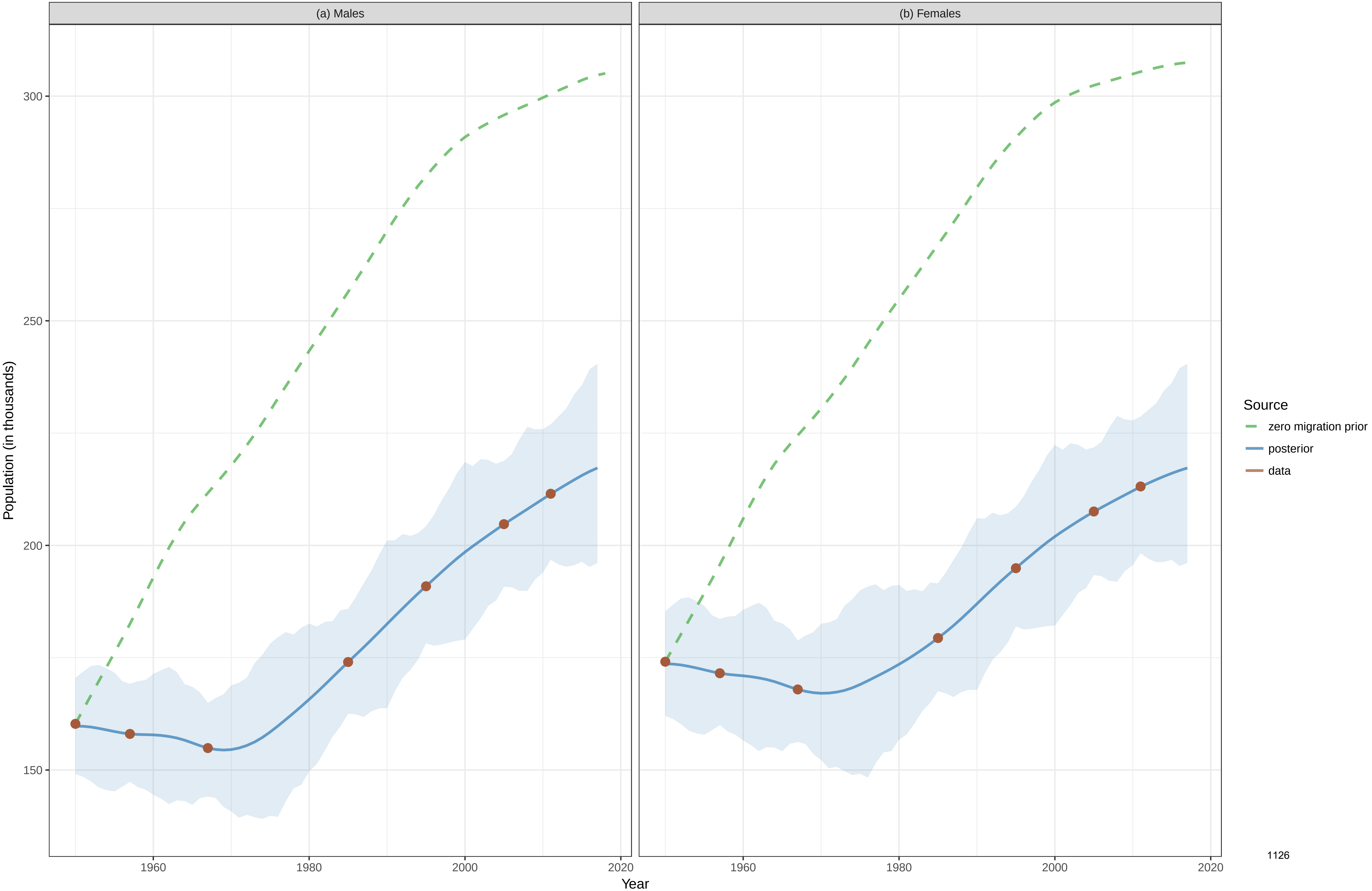

Supplemental Results Figure 4: GBD Bayesian Demographic Balancing Model fit in Marshall Islands for (a) total population of males, and (b) total population of females, 1950−2017.

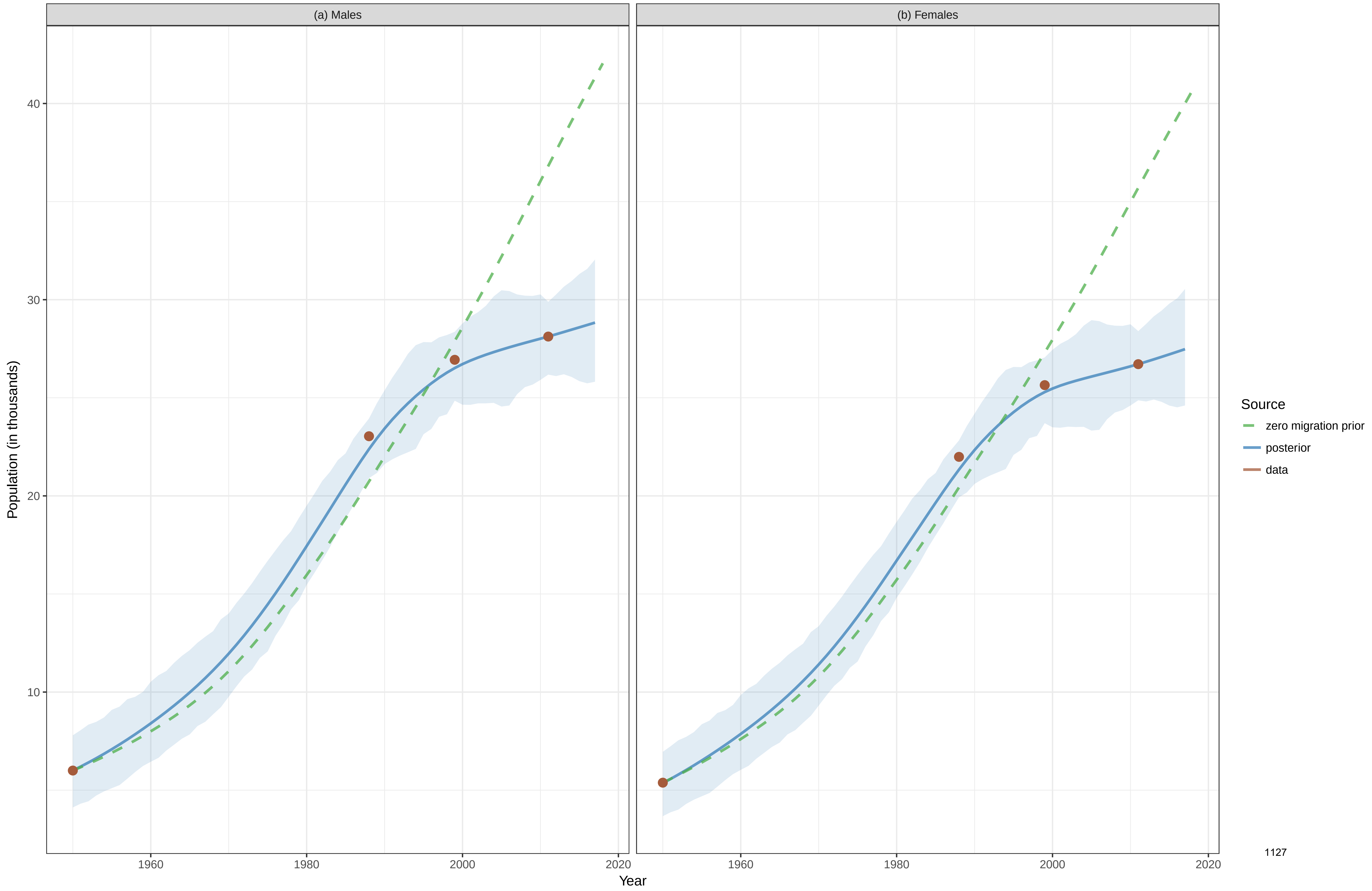

Supplemental Results Figure 4: GBD Bayesian Demographic Balancing Model fit in Mauritania for (a) total population of males, and (b) total population of females, 1950−2017.

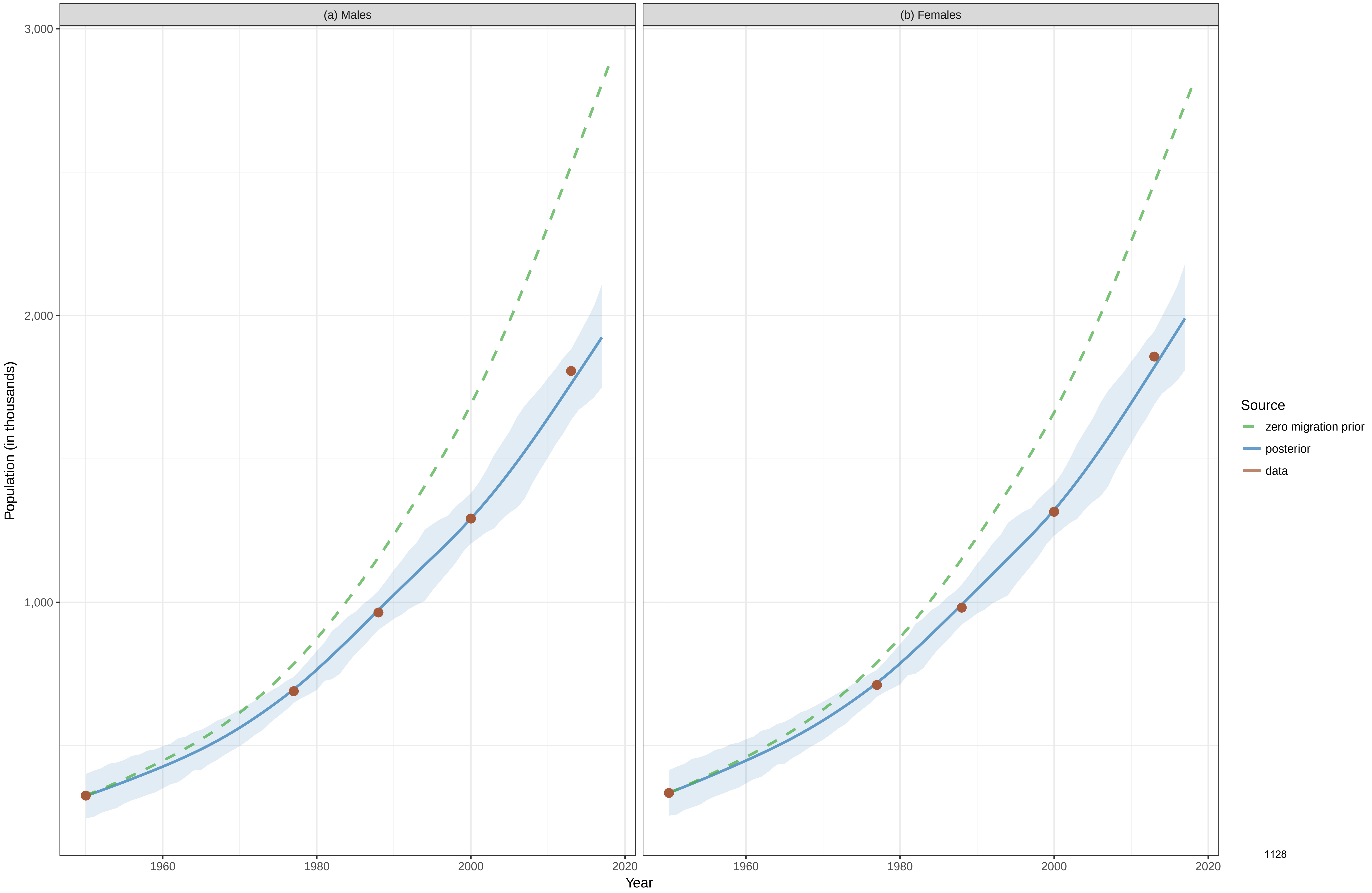

Supplemental Results Figure 4: GBD Bayesian Demographic Balancing Model fit in Mauritius for (a) total population of males, and (b) total population of females, 1950−2017.

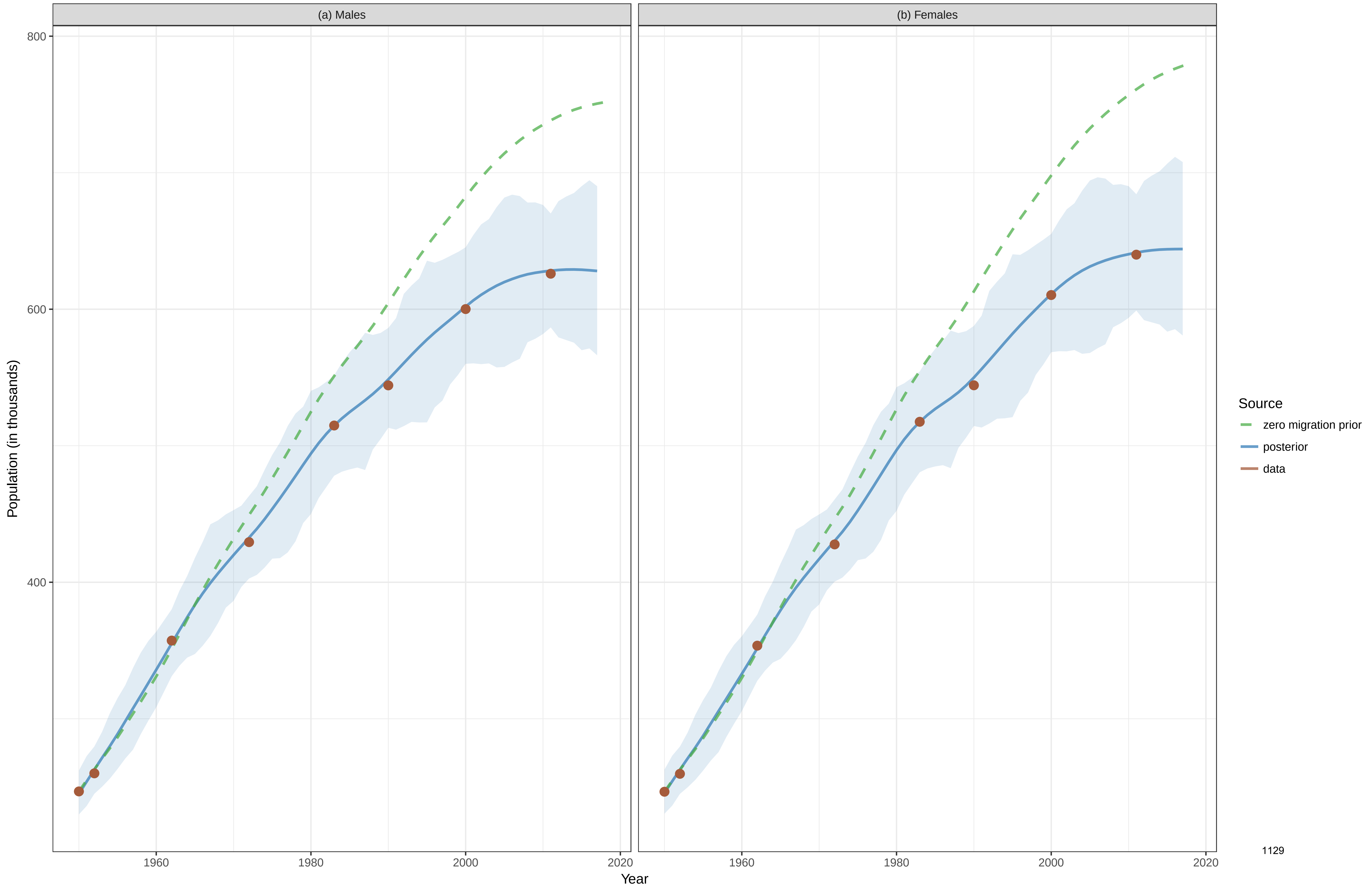

Supplemental Results Figure 4: GBD Bayesian Demographic Balancing Model fit in Mexico for (a) total population of males, and (b) total population of females, 1950−2017.

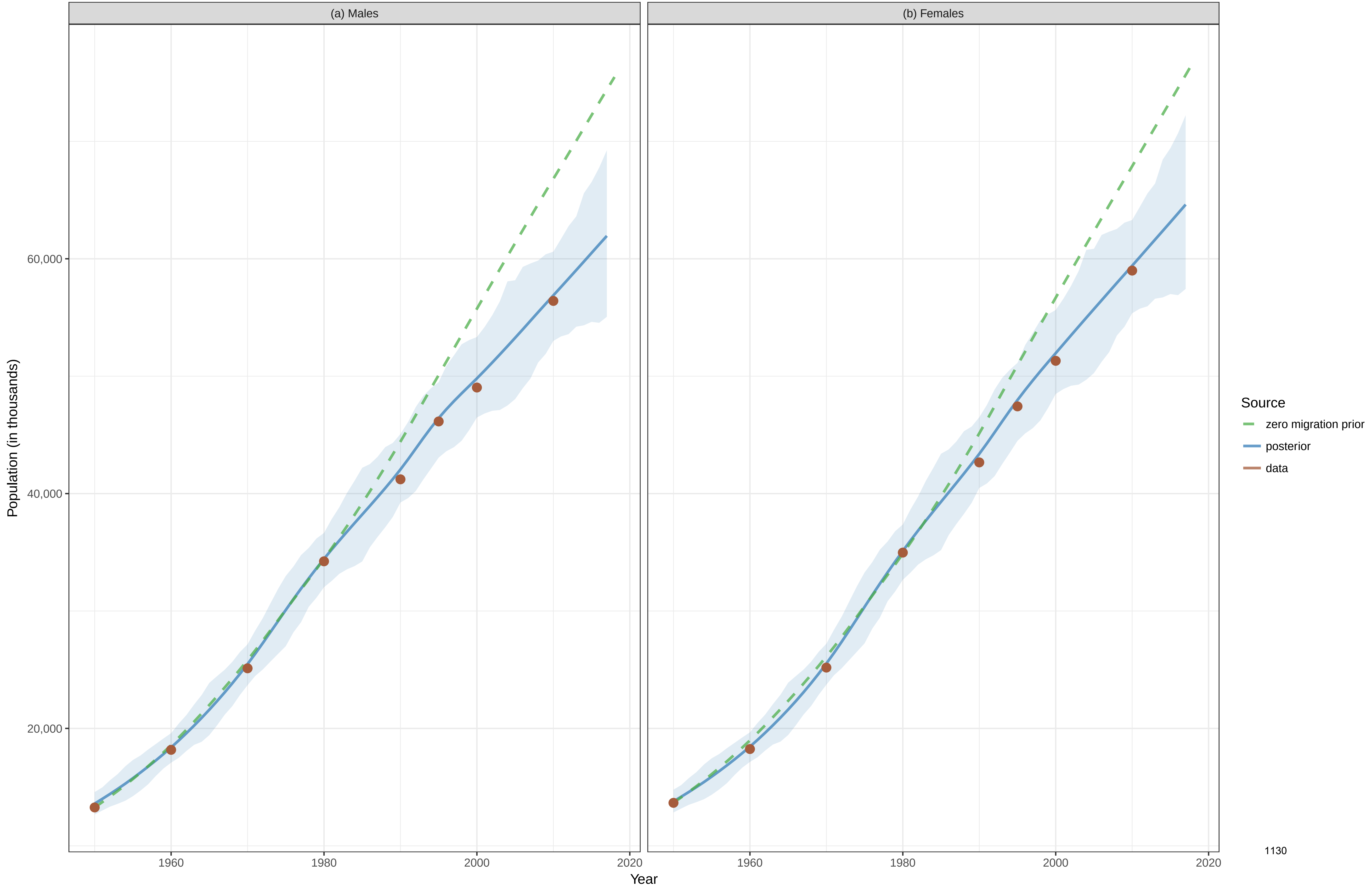

Supplemental Results Figure 4: GBD Bayesian Demographic Balancing Model fit in Moldova for (a) total population of males, and (b) total population of females, 1950−2017.

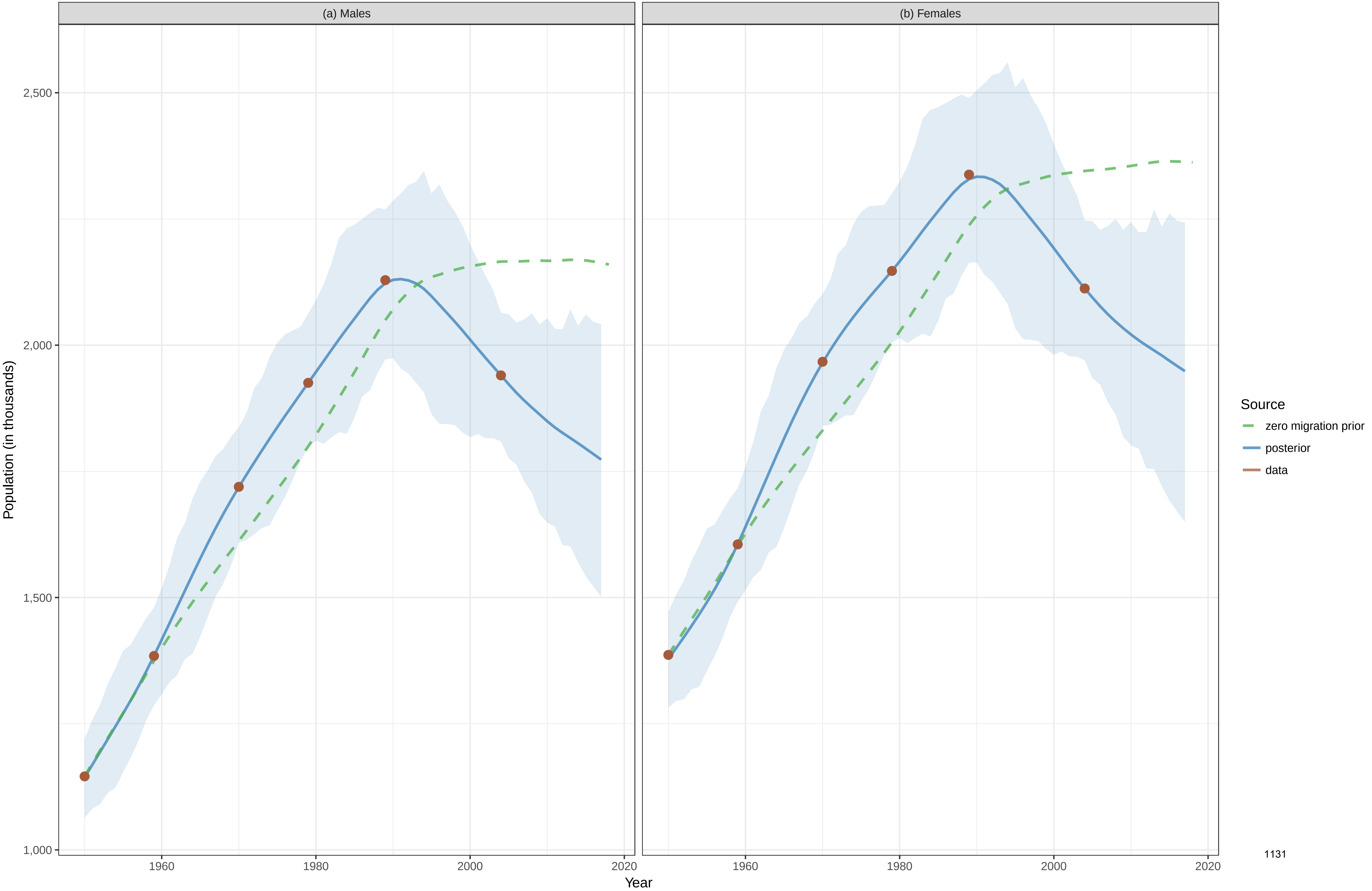

Supplemental Results Figure 4: GBD Bayesian Demographic Balancing Model fit in Mongolia for (a) total population of males, and (b) total population of females, 1950−2017.

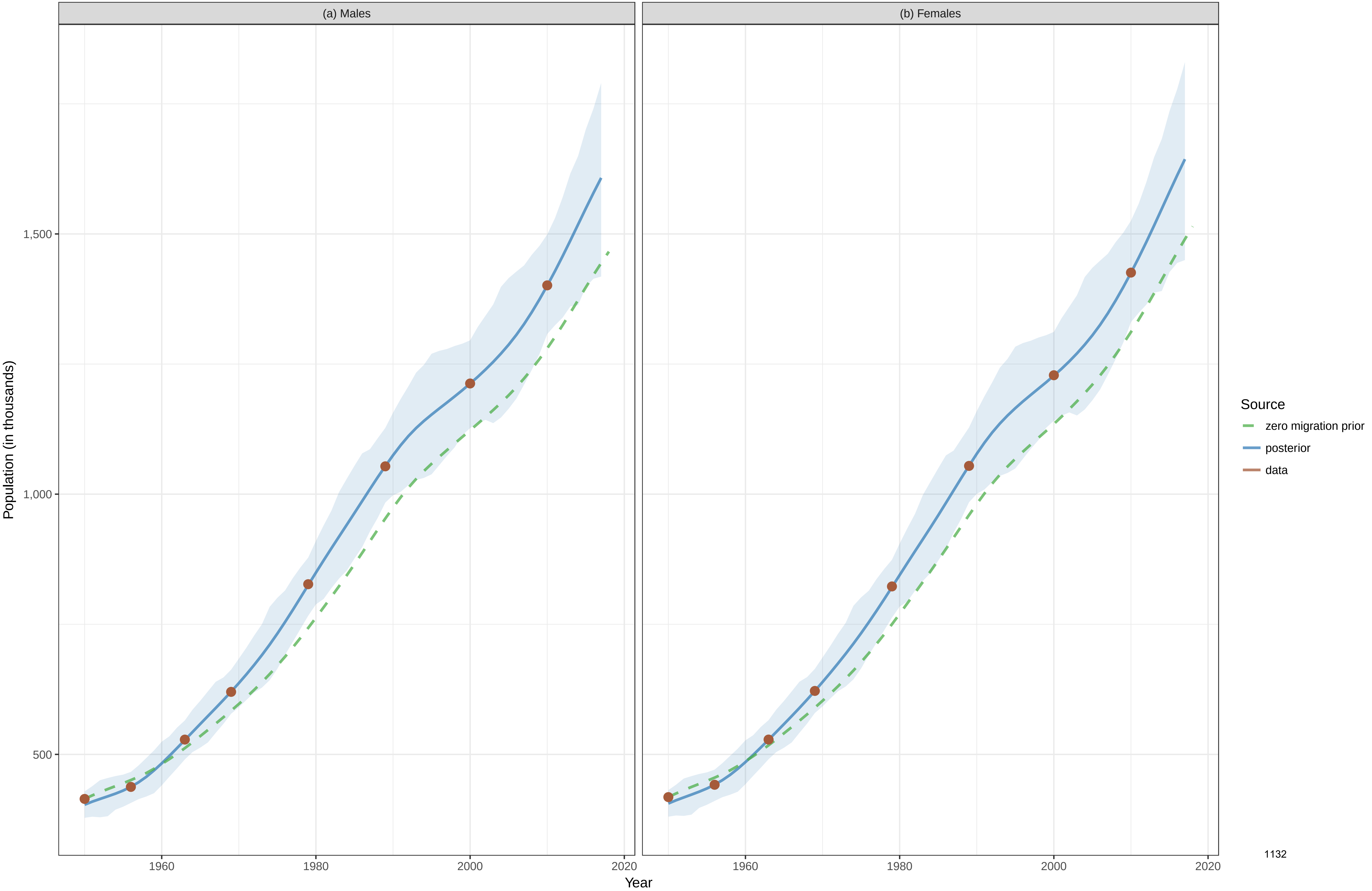

Supplemental Results Figure 4: GBD Bayesian Demographic Balancing Model fit in Montenegro for (a) total population of males, and (b) total population of females, 1950−2017.

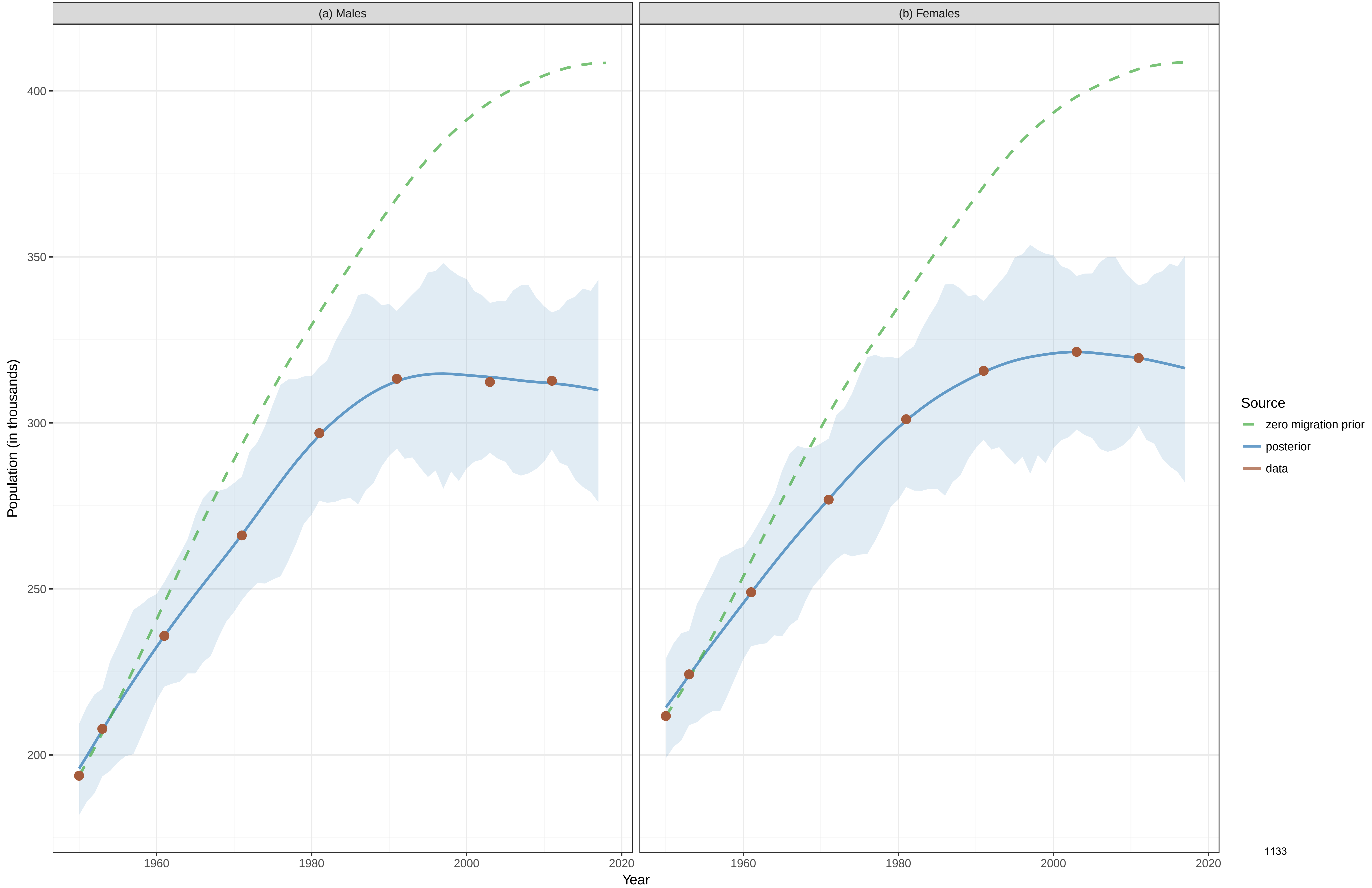

Supplemental Results Figure 4: GBD Bayesian Demographic Balancing Model fit in Morocco for (a) total population of males, and (b) total population of females, 1950−2017.

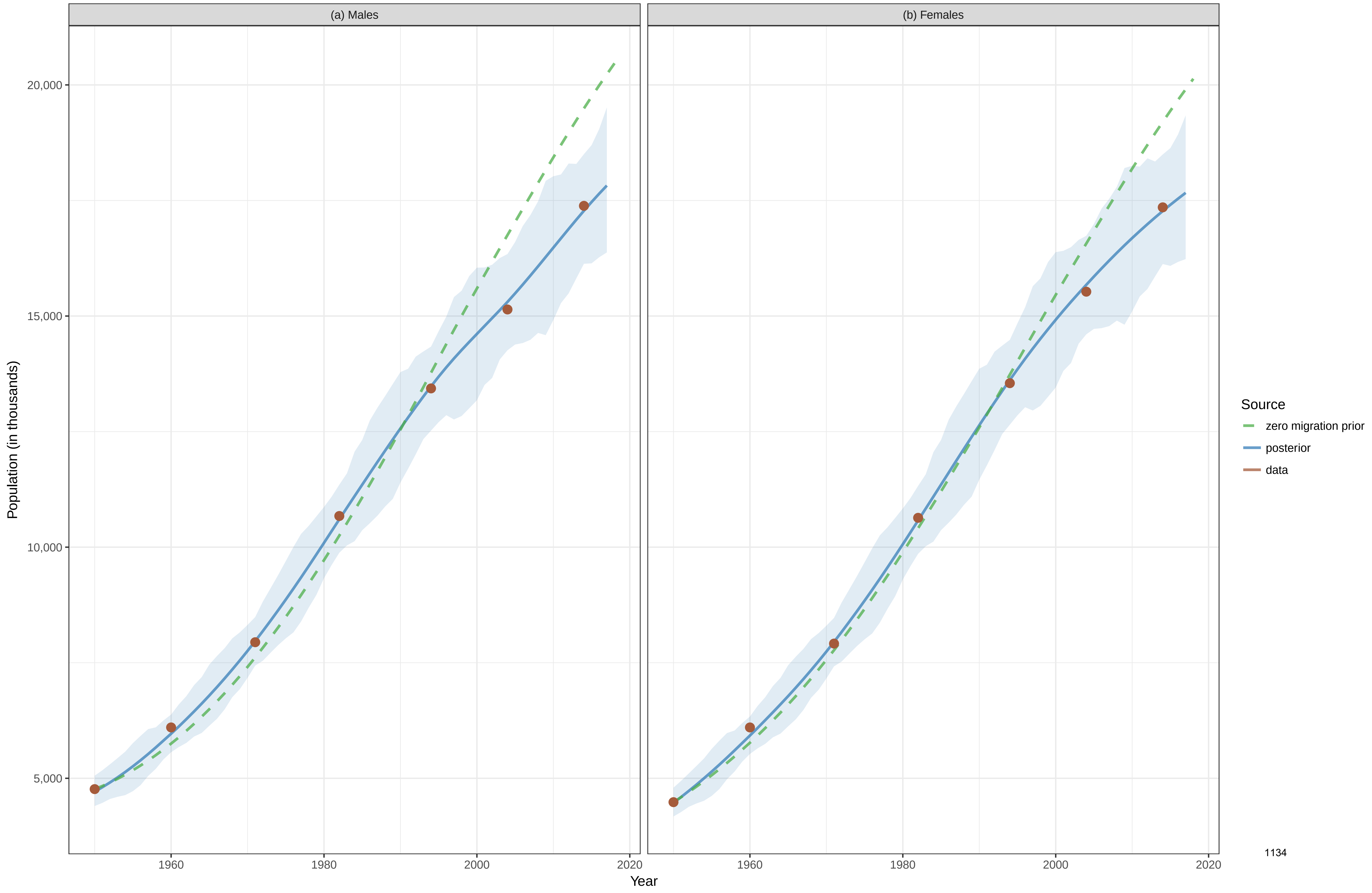

Supplemental Results Figure 4: GBD Bayesian Demographic Balancing Model fit in Mozambique for (a) total population of males, and (b) total population of females, 1950−2017.

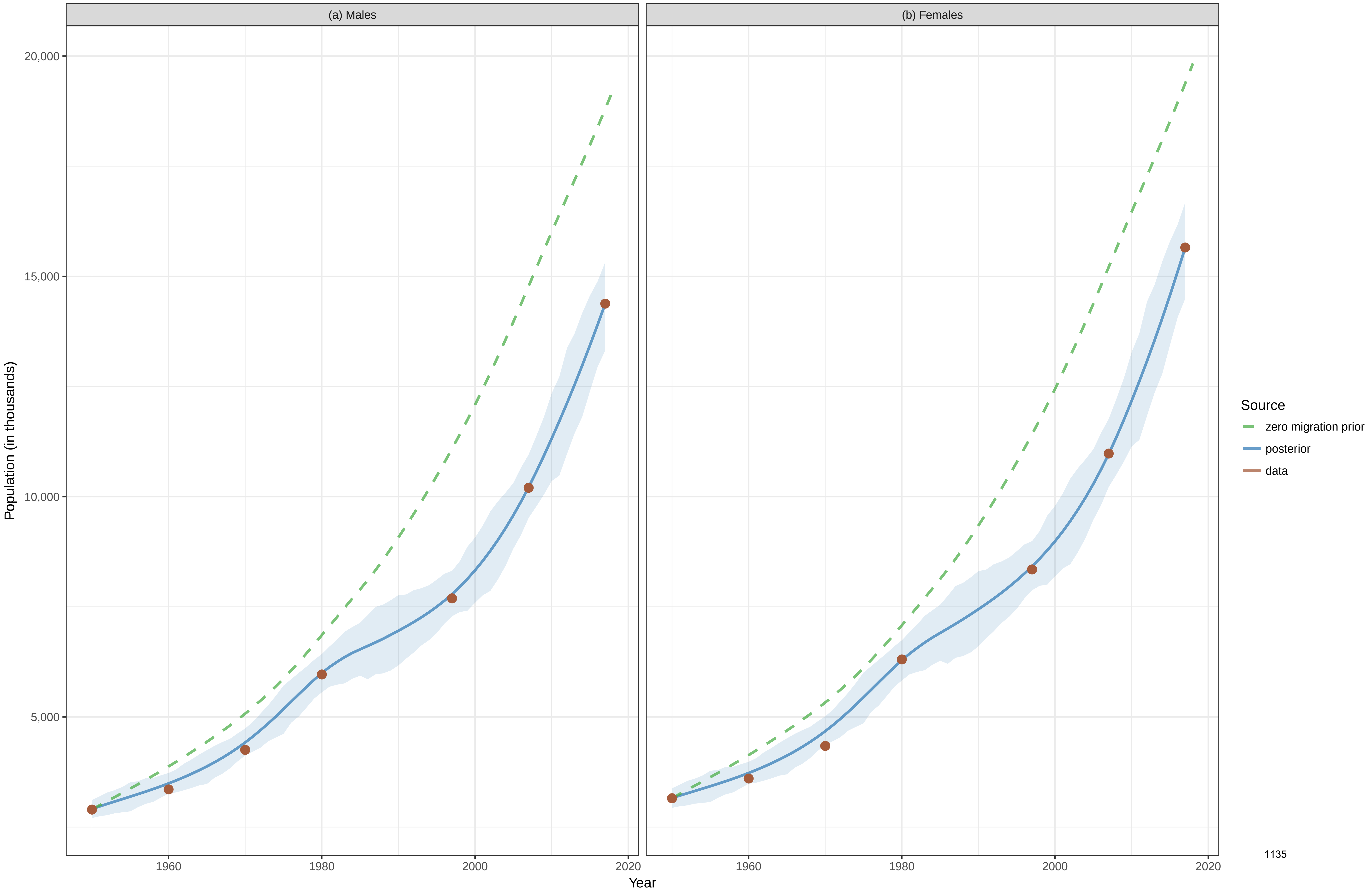

Supplemental Results Figure 4: GBD Bayesian Demographic Balancing Model fit in Myanmar for (a) total population of males, and (b) total population of females, 1950−2017.

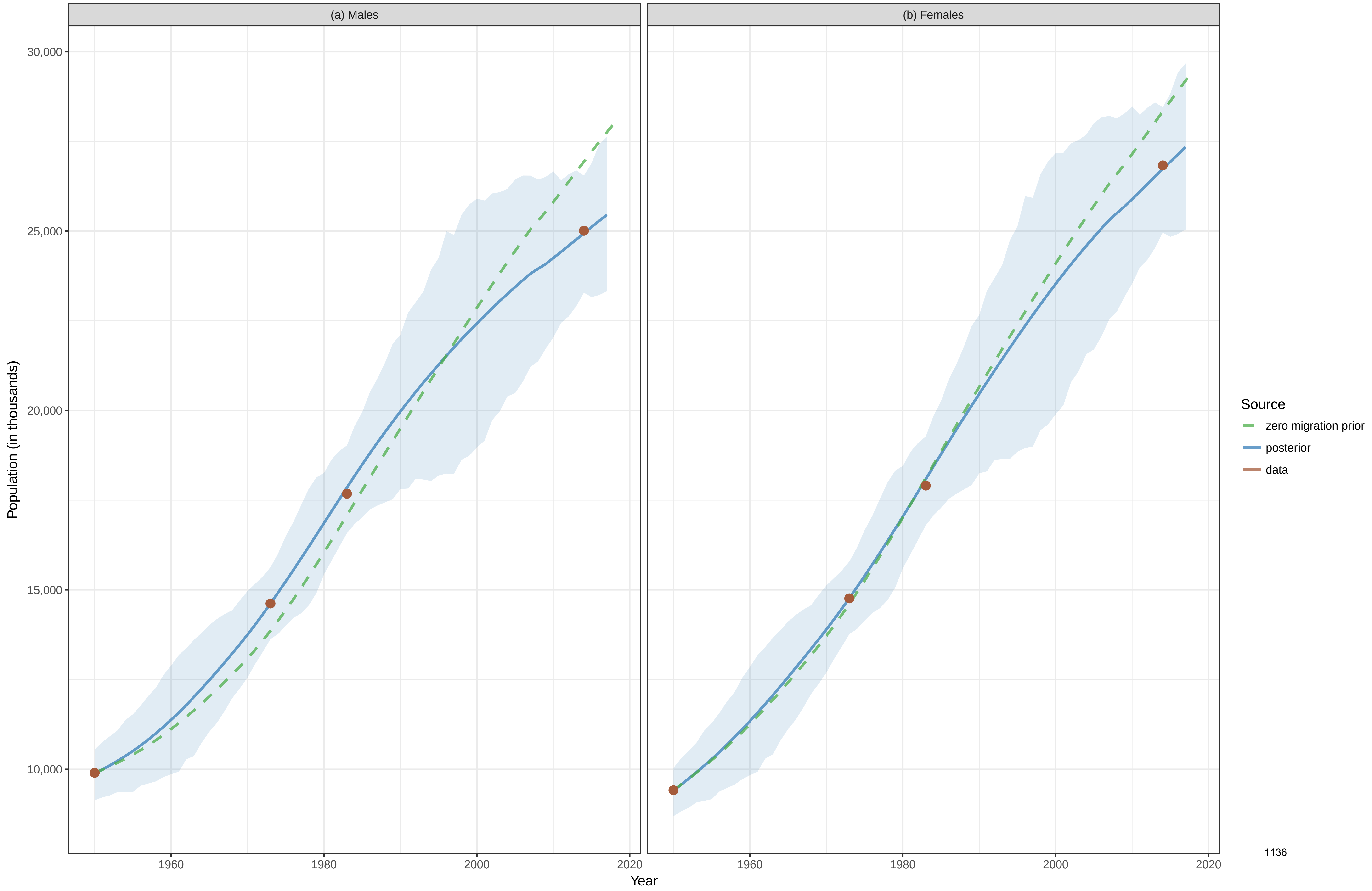

Supplemental Results Figure 4: GBD Bayesian Demographic Balancing Model fit in Namibia for (a) total population of males, and (b) total population of females, 1950−2017.

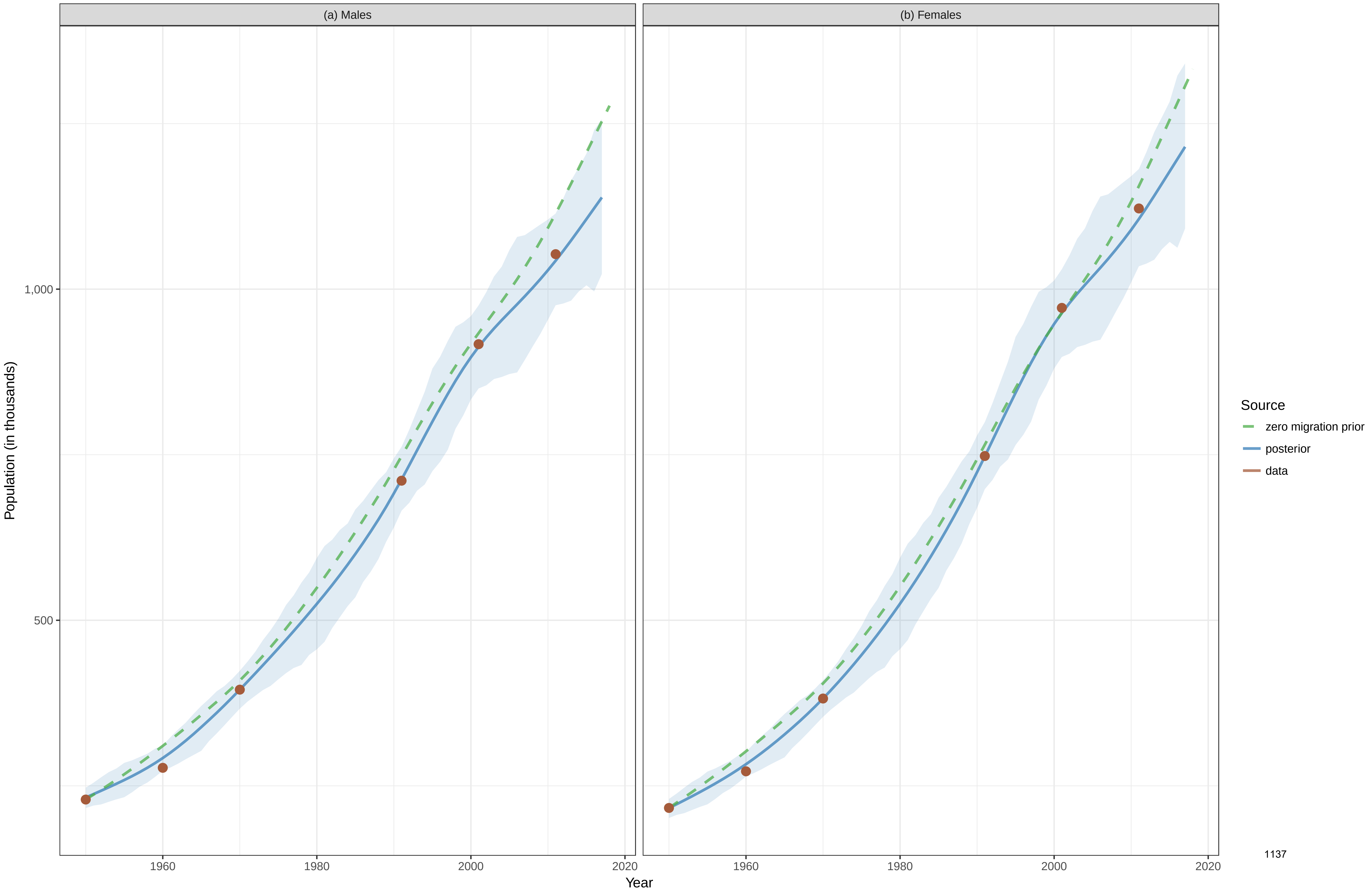

Supplemental Results Figure 4: GBD Bayesian Demographic Balancing Model fit in Nepal for (a) total population of males, and (b) total population of females, 1950−2017.

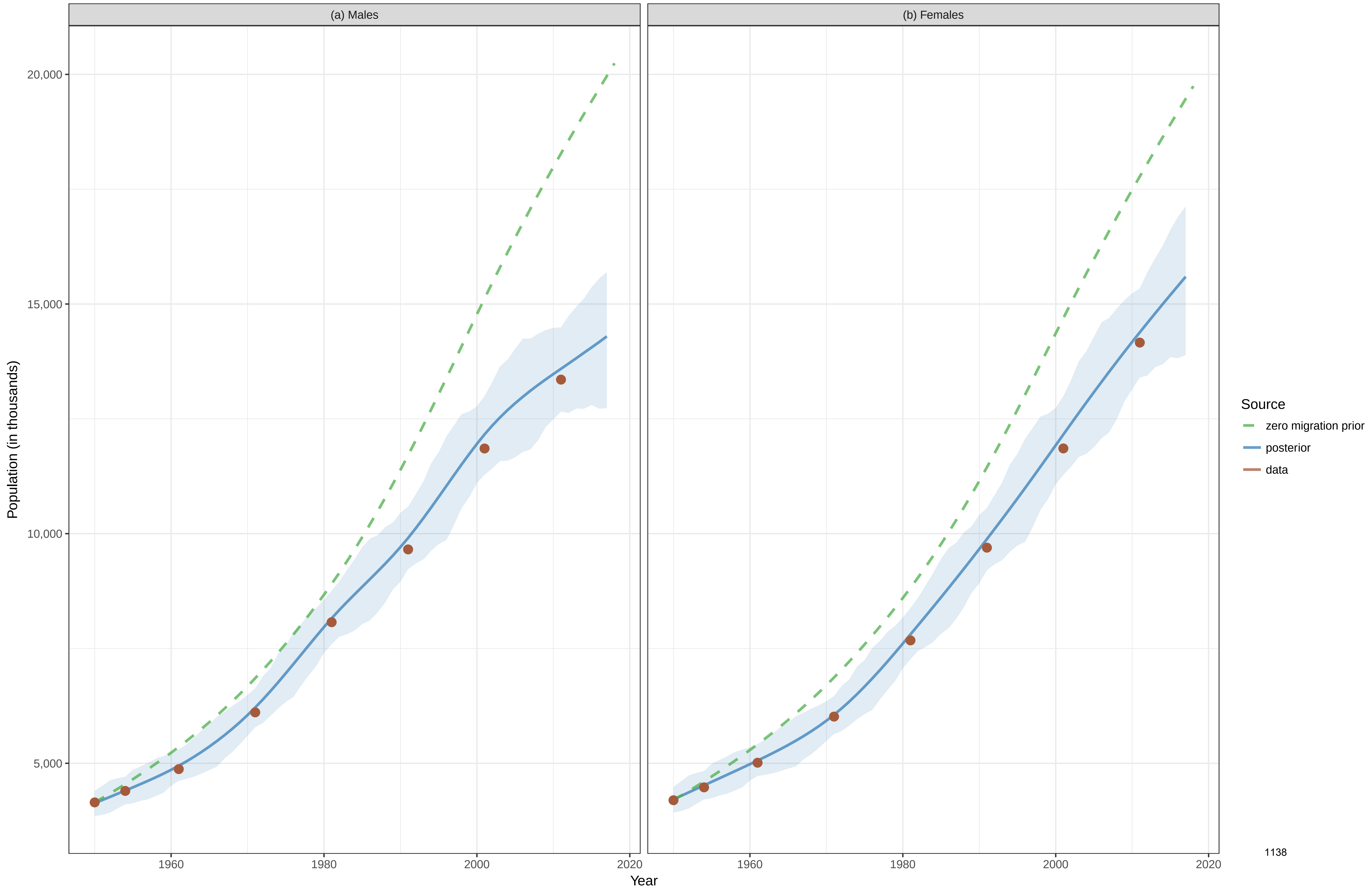

Supplemental Results Figure 4: GBD Bayesian Demographic Balancing Model fit in Netherlands for (a) total population of males, and (b) total population of females, 1950−2017.

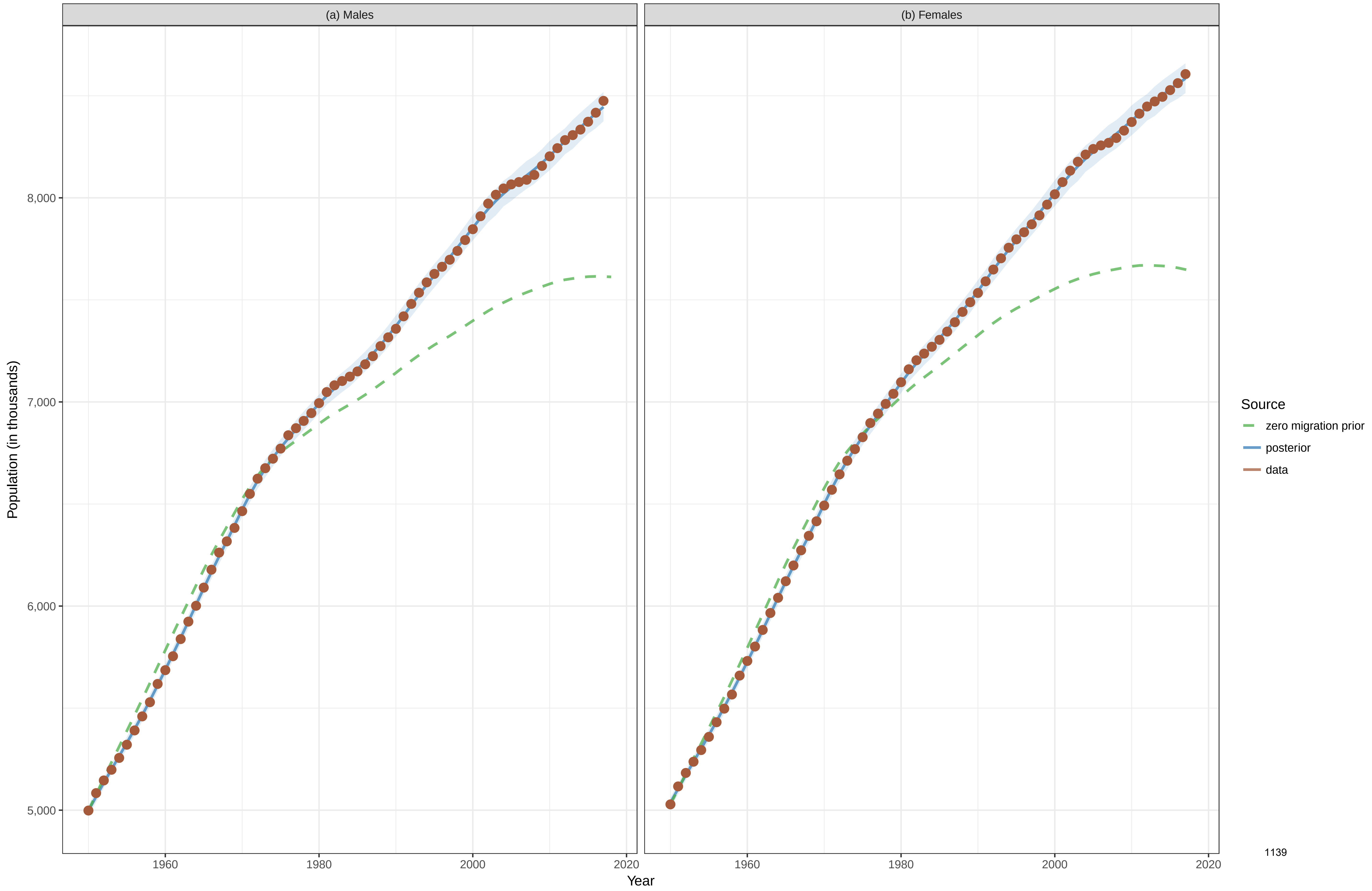

Supplemental Results Figure 4: GBD Bayesian Demographic Balancing Model fit in New Zealand for (a) total population of males, and (b) total population of females, 1950−2017.

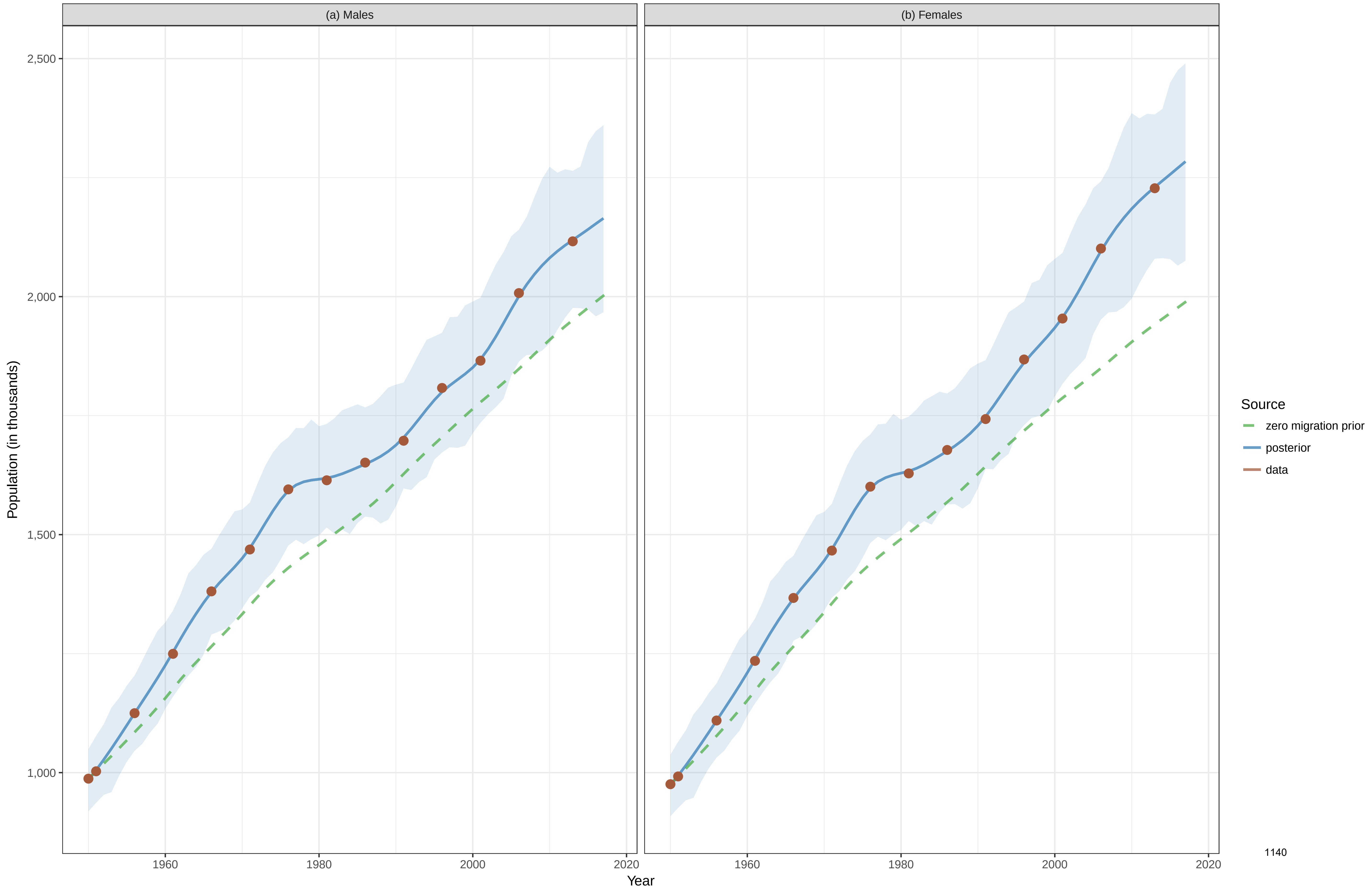

Supplemental Results Figure 4: GBD Bayesian Demographic Balancing Model fit in Nicaragua for (a) total population of males, and (b) total population of females, 1950−2017.

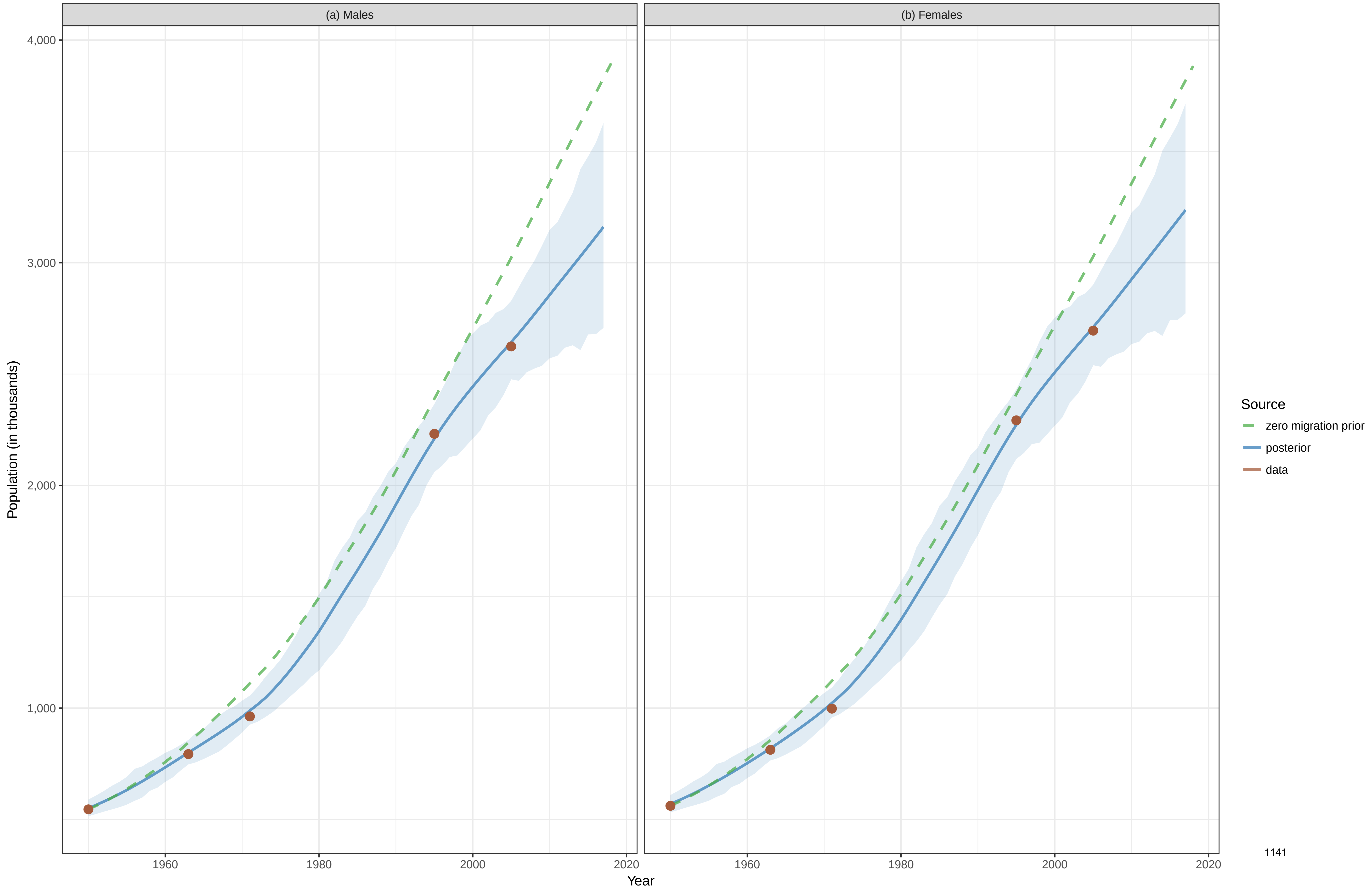

Supplemental Results Figure 4: GBD Bayesian Demographic Balancing Model fit in Niger for (a) total population of males, and (b) total population of females, 1950−2017.

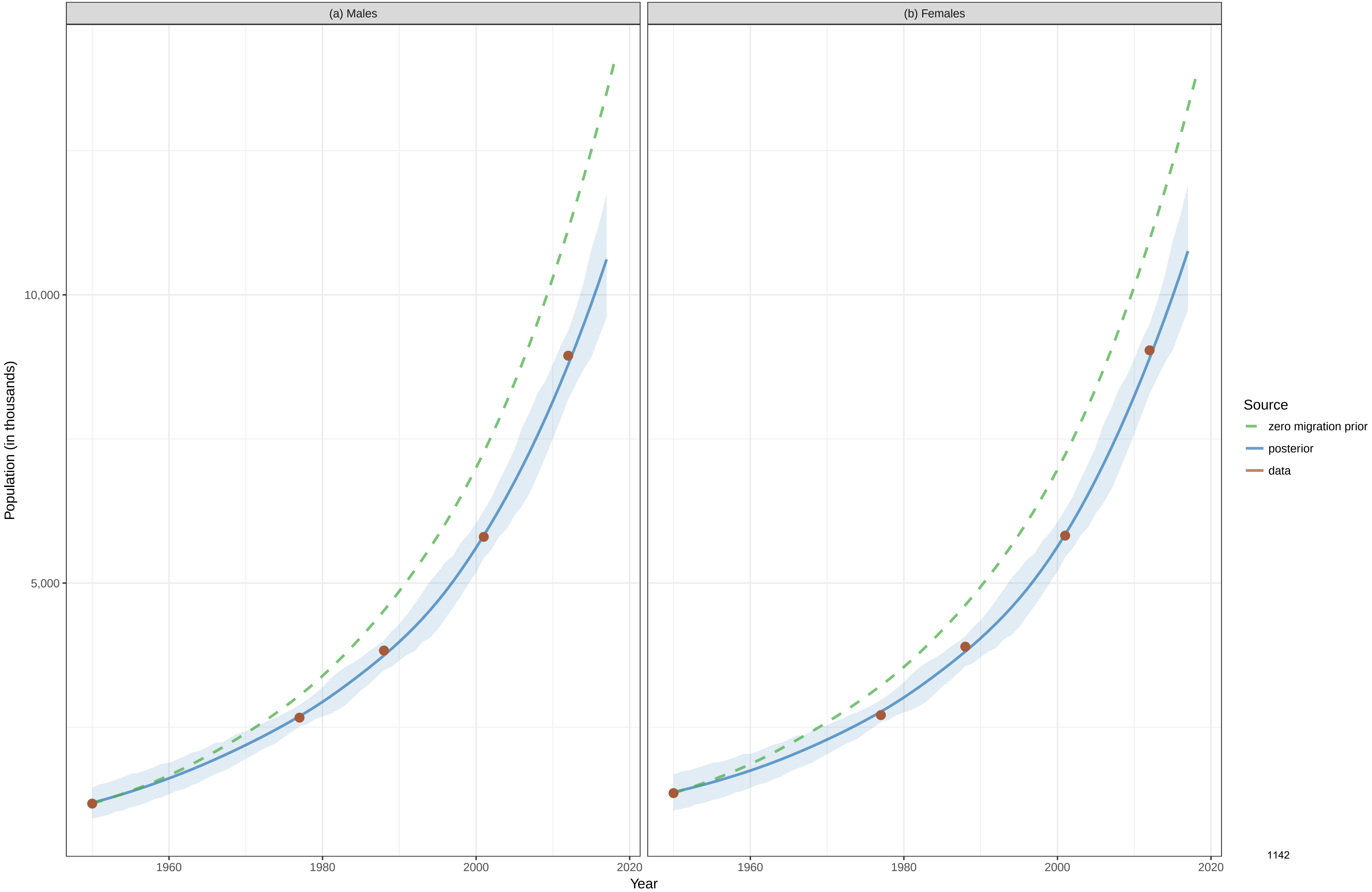

Supplemental Results Figure 4: GBD Bayesian Demographic Balancing Model fit in Nigeria for (a) total population of males, and (b) total population of females, 1950−2017.

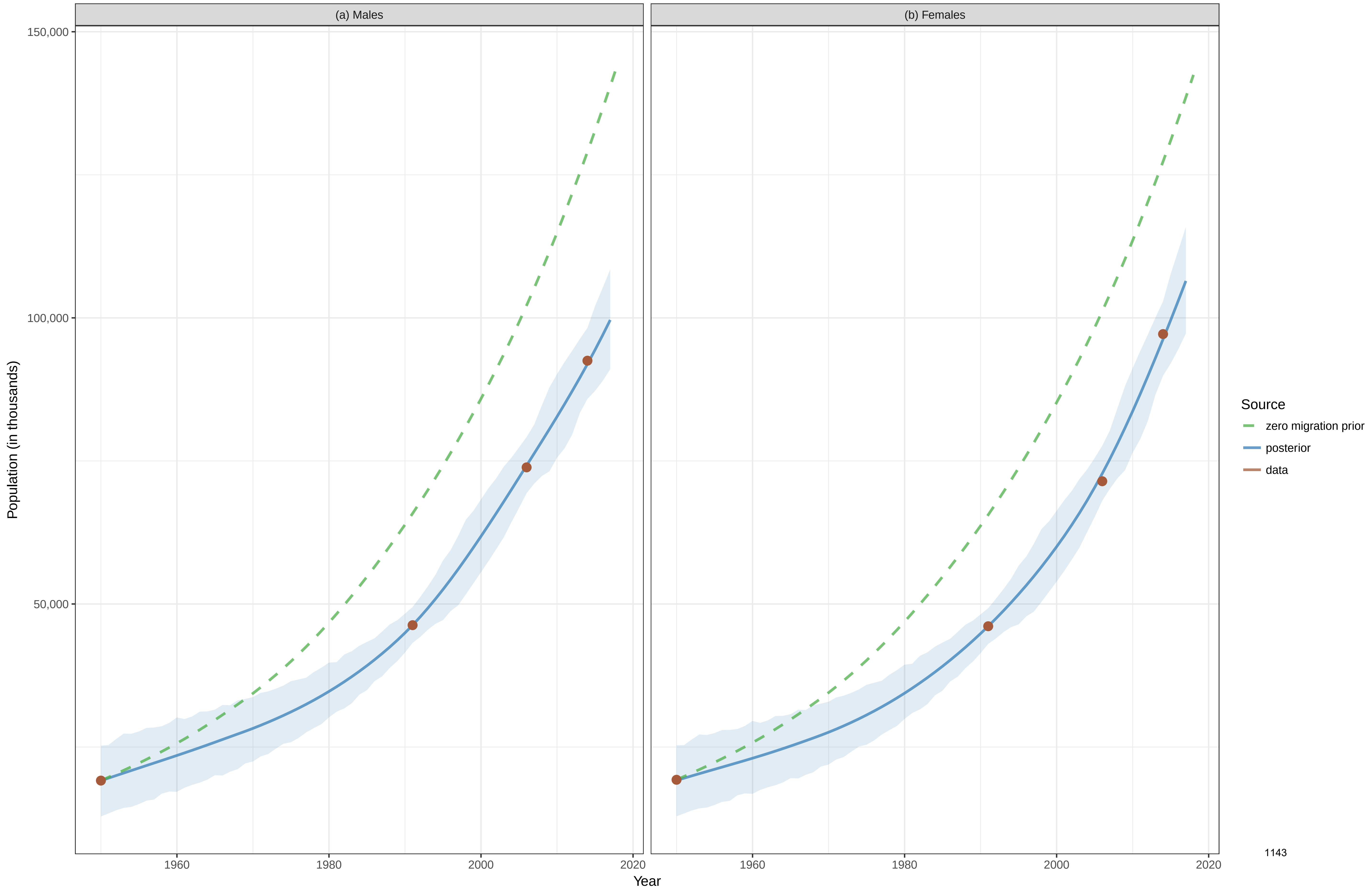

Supplemental Results Figure 4: GBD Bayesian Demographic Balancing Model fit in North Korea for (a) total population of males, and (b) total population of females, 1950−2017.

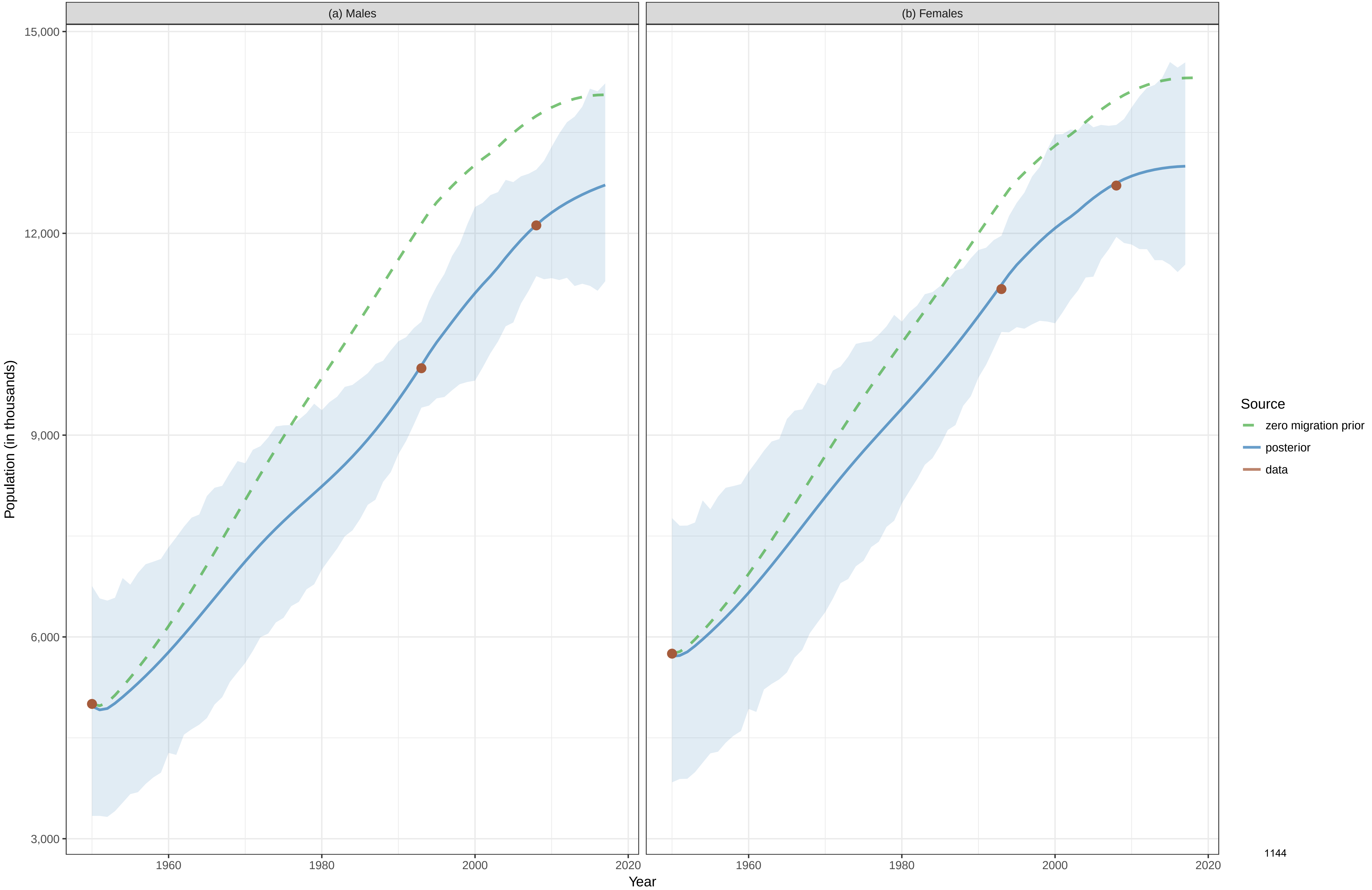

Supplemental Results Figure 4: GBD Bayesian Demographic Balancing Model fit in Northern Ireland for (a) total population of males, and (b) total population of females, 1950−2017.

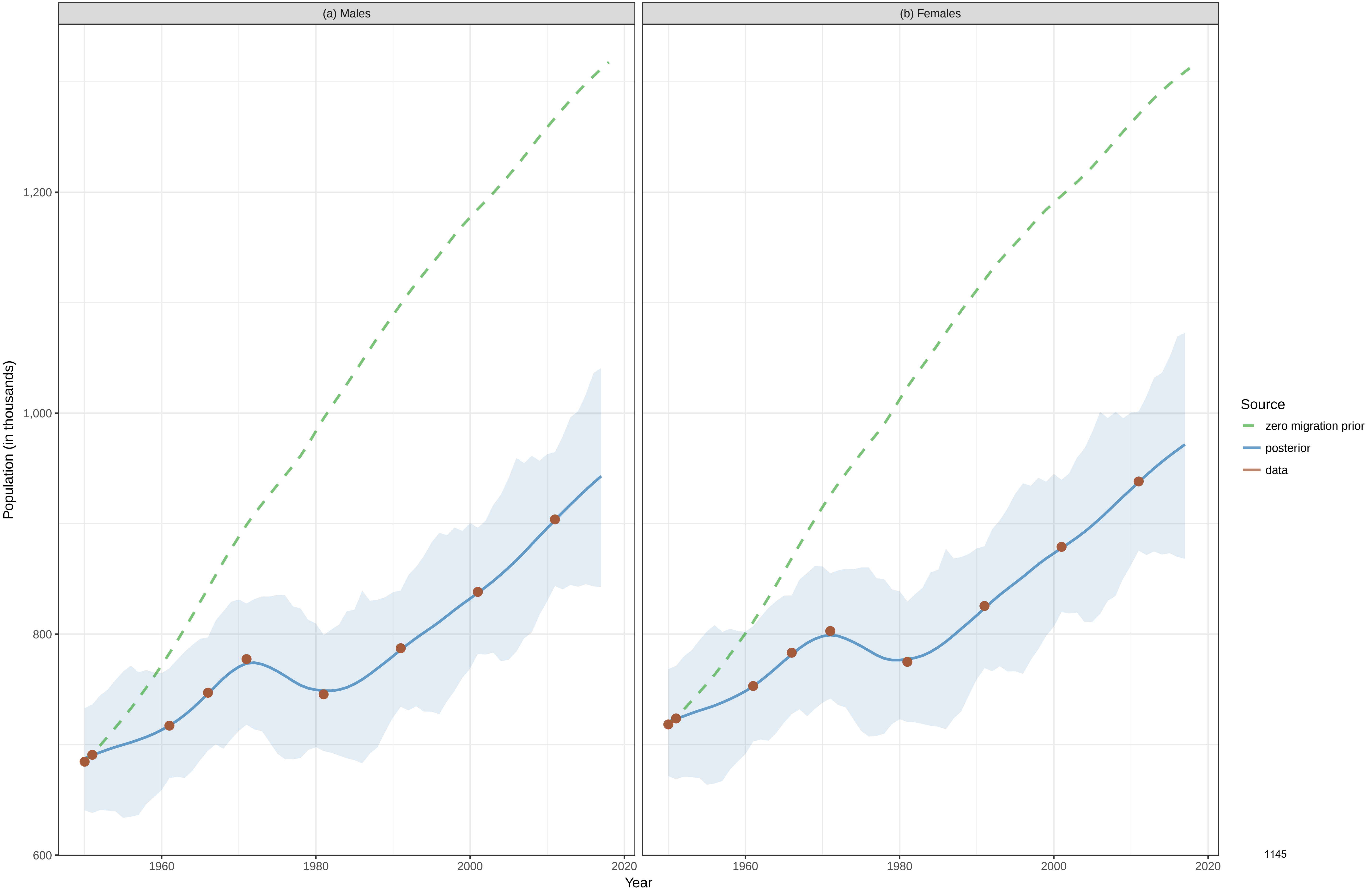

Supplemental Results Figure 4: GBD Bayesian Demographic Balancing Model fit in Northern Mariana Islands for (a) total population of males, and (b) total population of females, 1950−2017.

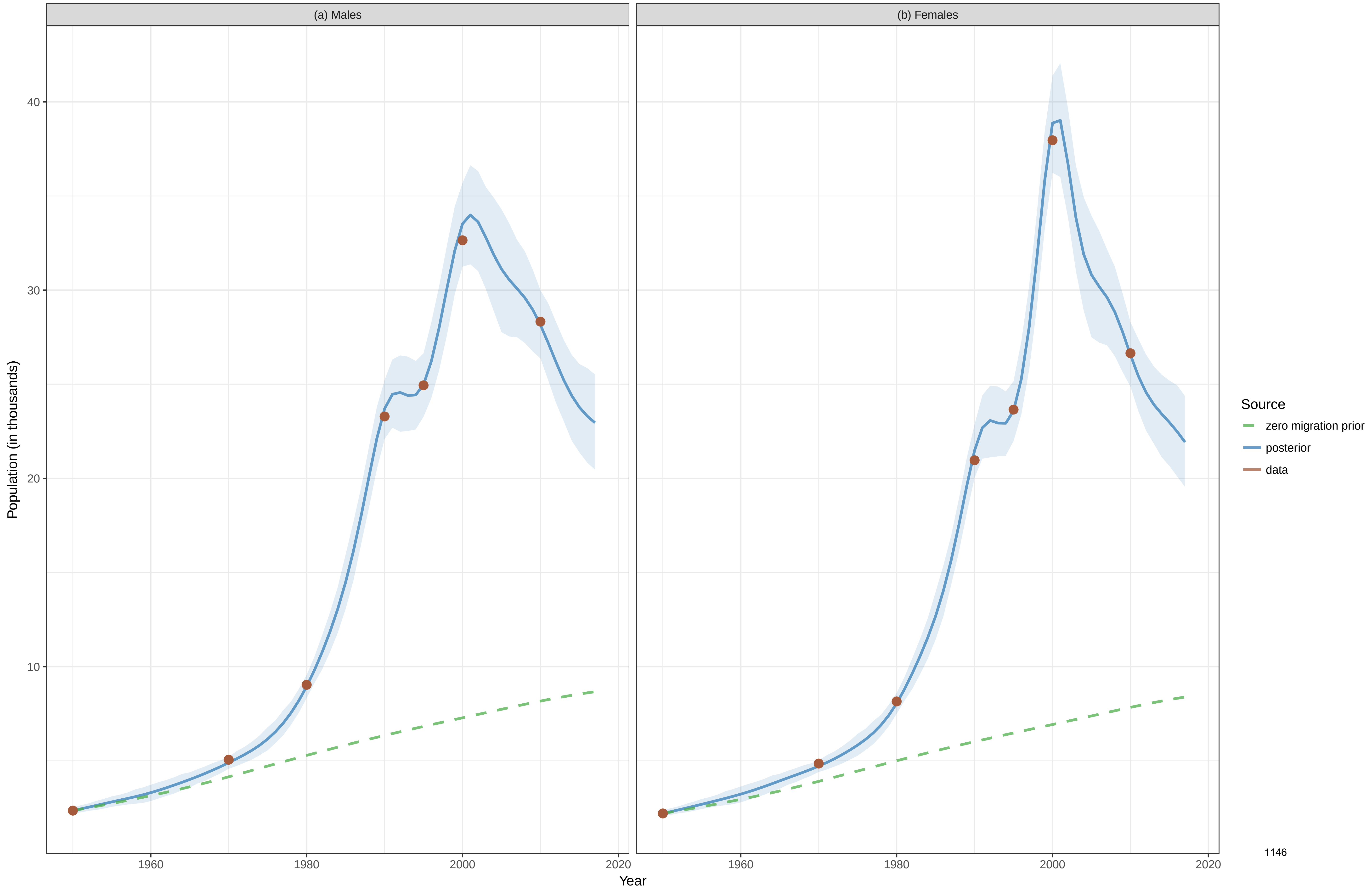

Supplemental Results Figure 4: GBD Bayesian Demographic Balancing Model fit in Norway for (a) total population of males, and (b) total population of females, 1950−2017.

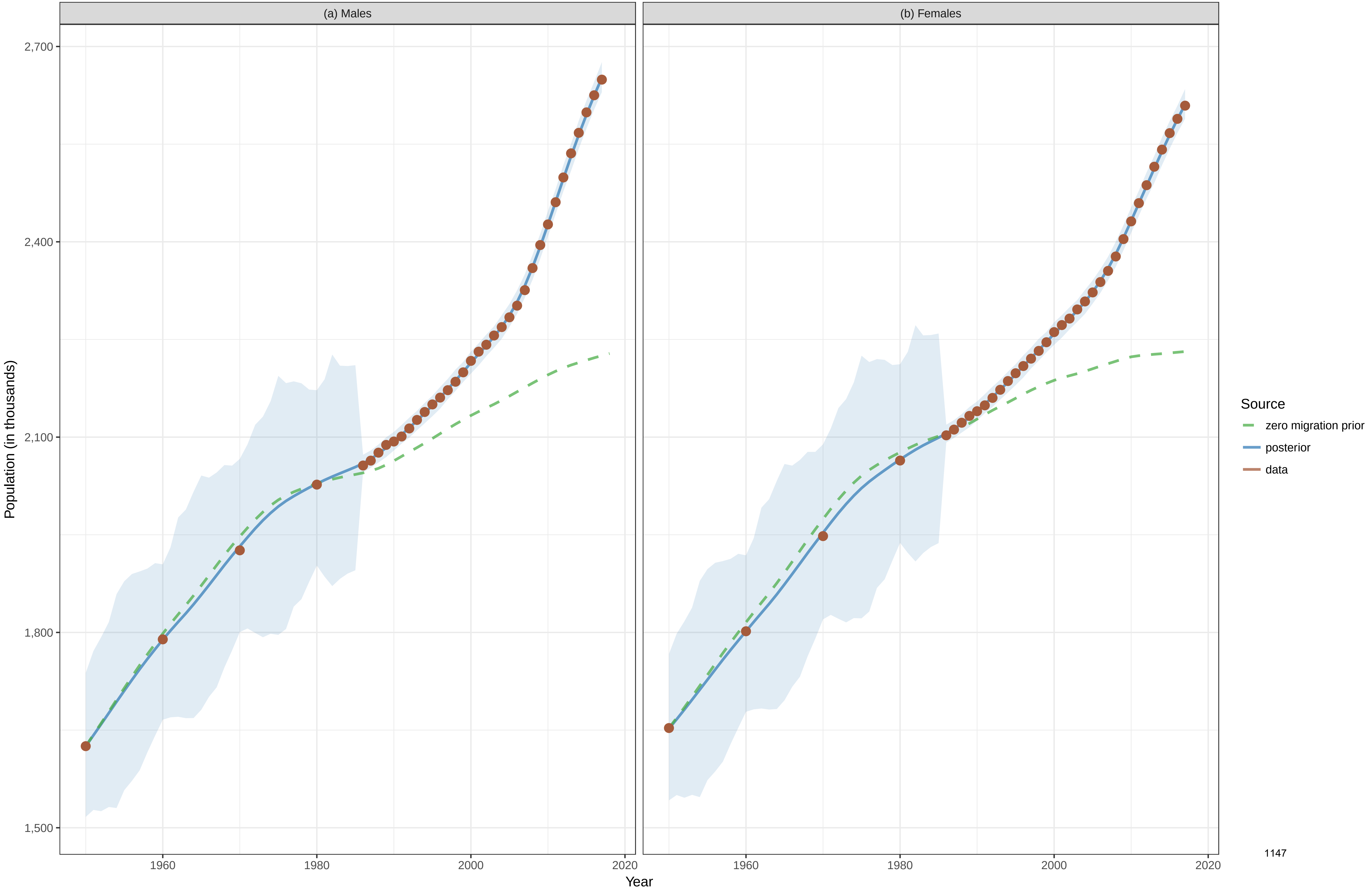

Supplemental Results Figure 4: GBD Bayesian Demographic Balancing Model fit in Oman for (a) total population of males, and (b) total population of females, 1950−2017.

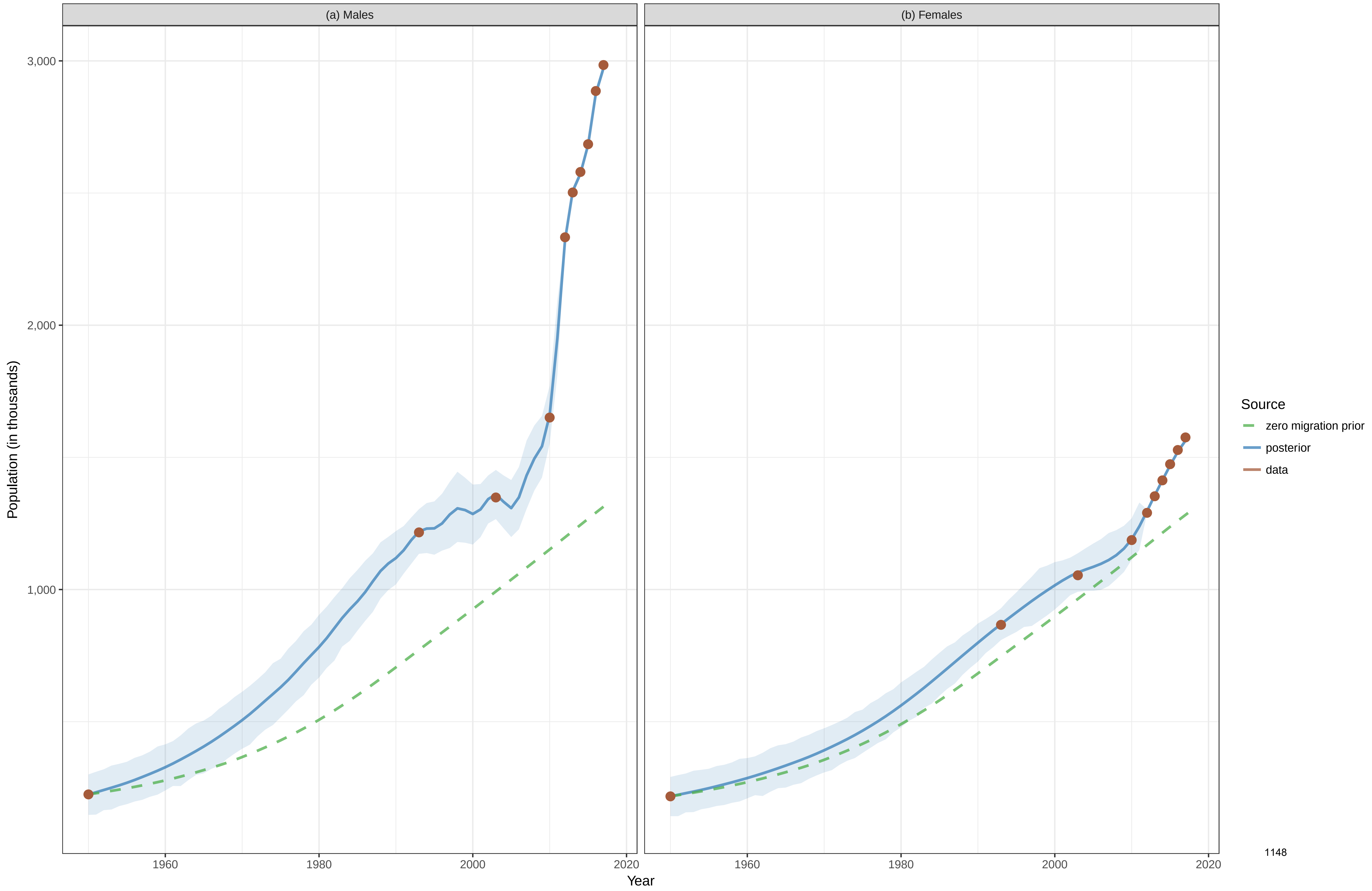

Supplemental Results Figure 4: GBD Bayesian Demographic Balancing Model fit in Pakistan for (a) total population of males, and (b) total population of females, 1950−2017.

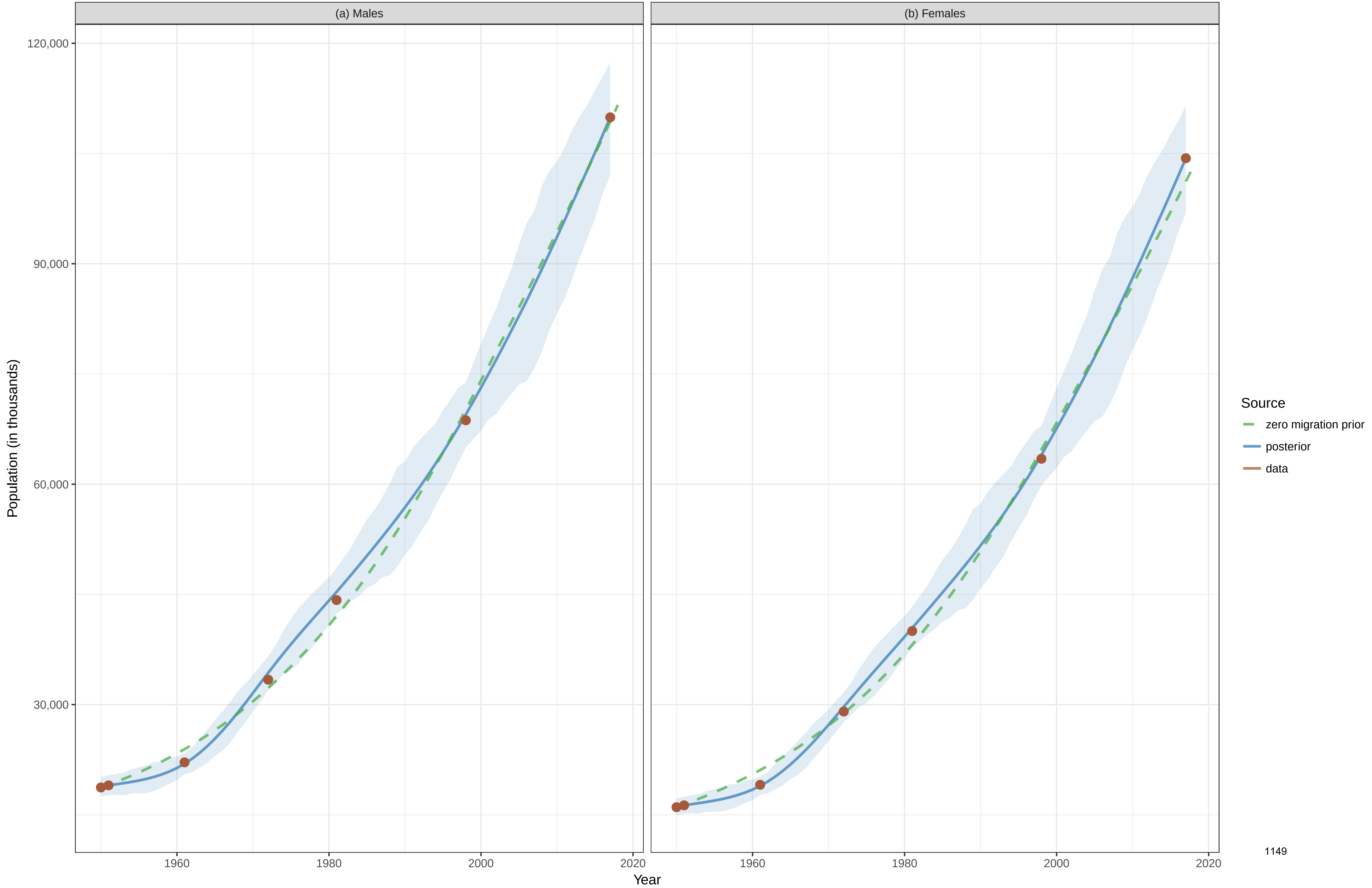

Supplemental Results Figure 4: GBD Bayesian Demographic Balancing Model fit in Palestine for (a) total population of males, and (b) total population of females, 1950−2017.

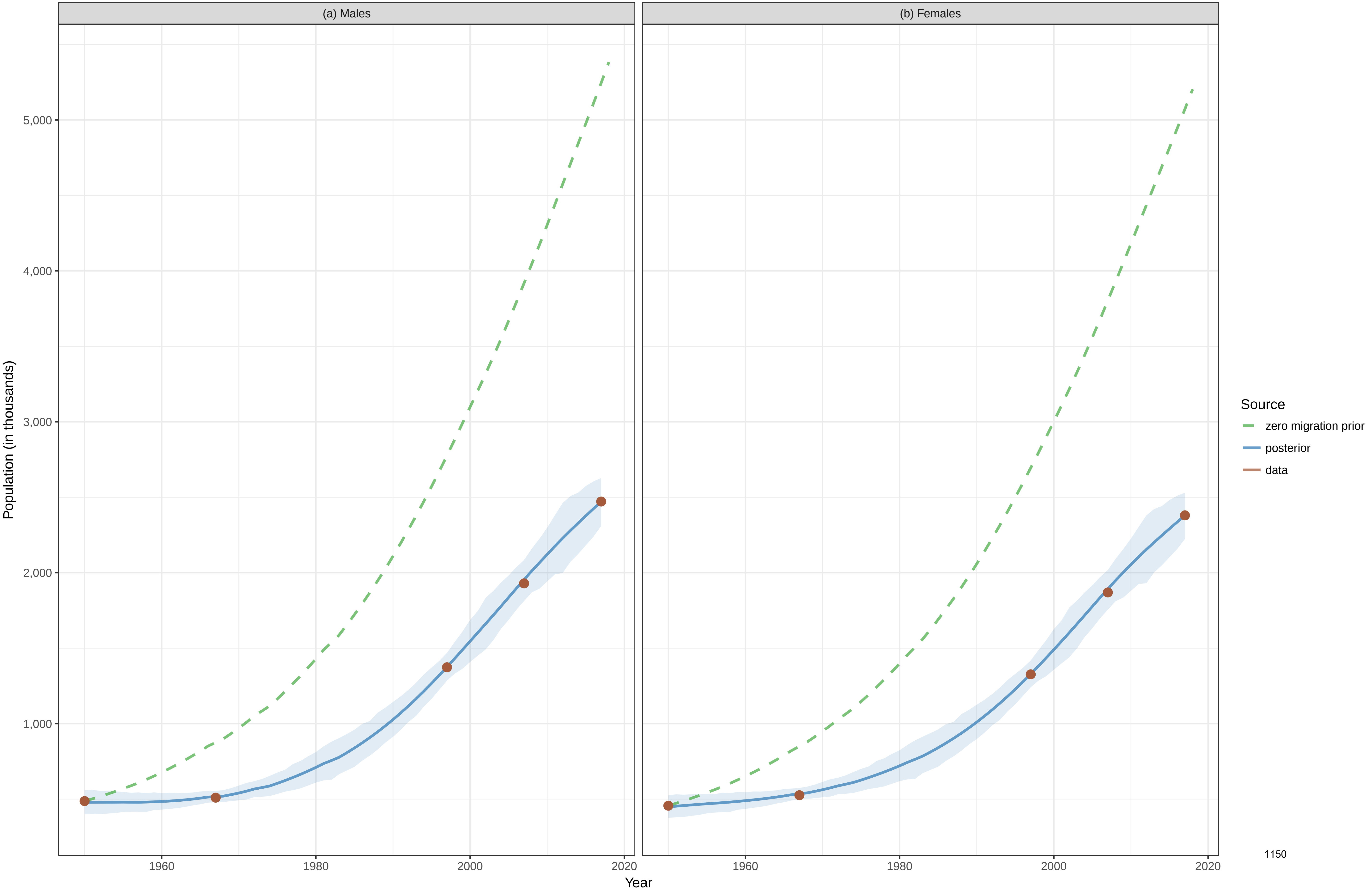

Supplemental Results Figure 4: GBD Bayesian Demographic Balancing Model fit in Panama for (a) total population of males, and (b) total population of females, 1950−2017.

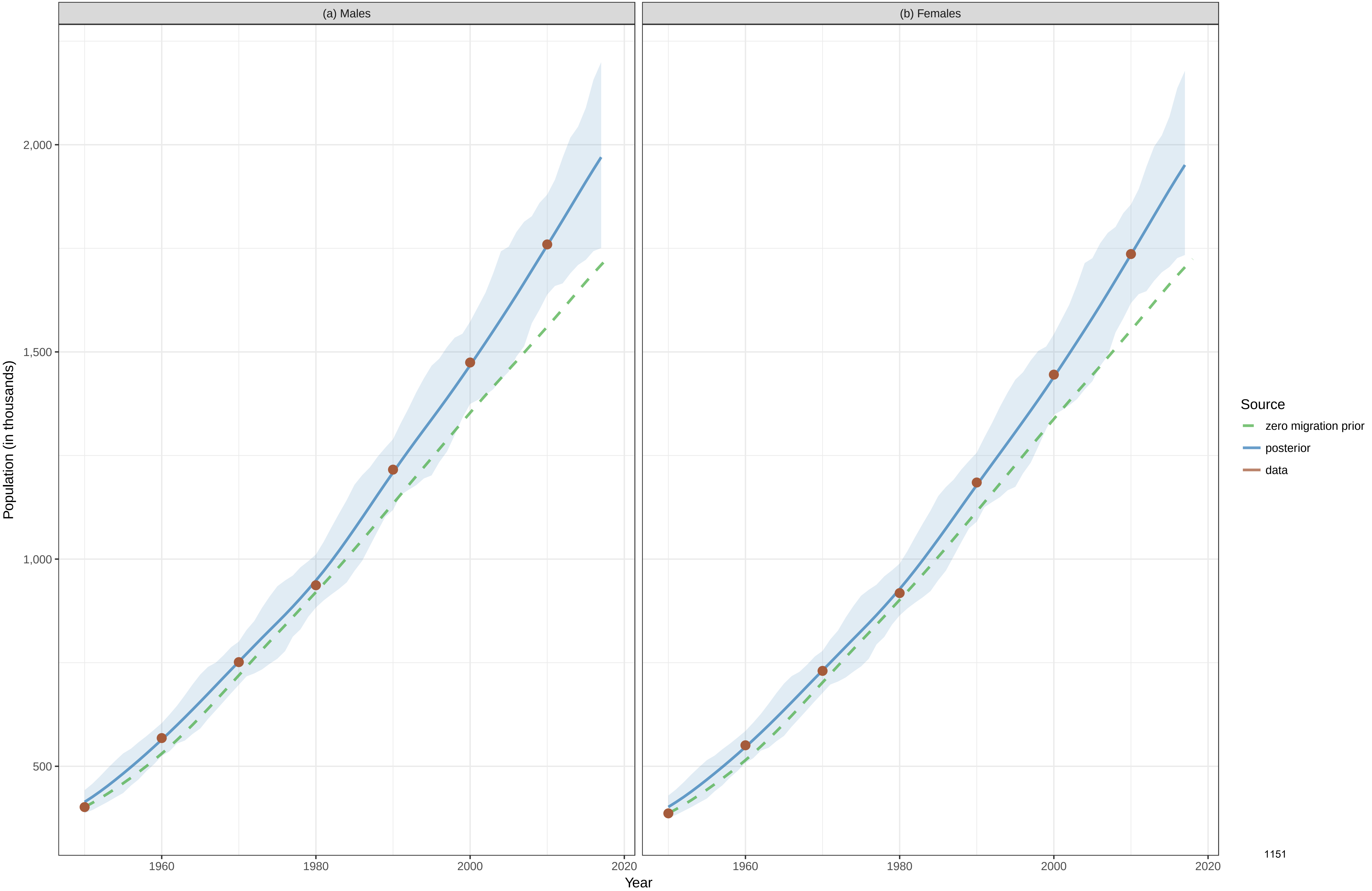

Supplemental Results Figure 4: GBD Bayesian Demographic Balancing Model fit in Papua New Guinea for (a) total population of males, and (b) total population of females, 1950−2017.

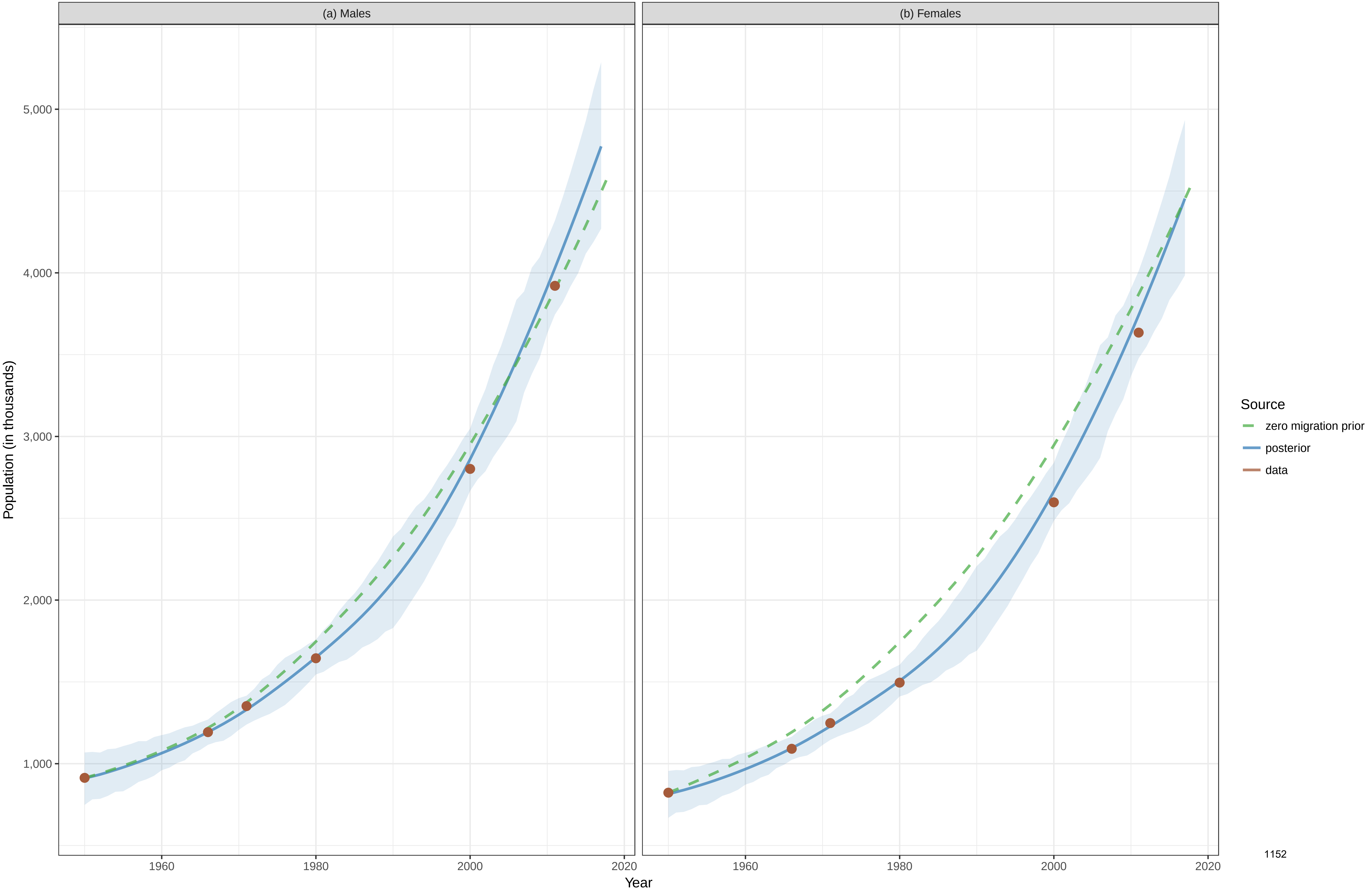

Supplemental Results Figure 4: GBD Bayesian Demographic Balancing Model fit in Paraguay for (a) total population of males, and (b) total population of females, 1950−2017.

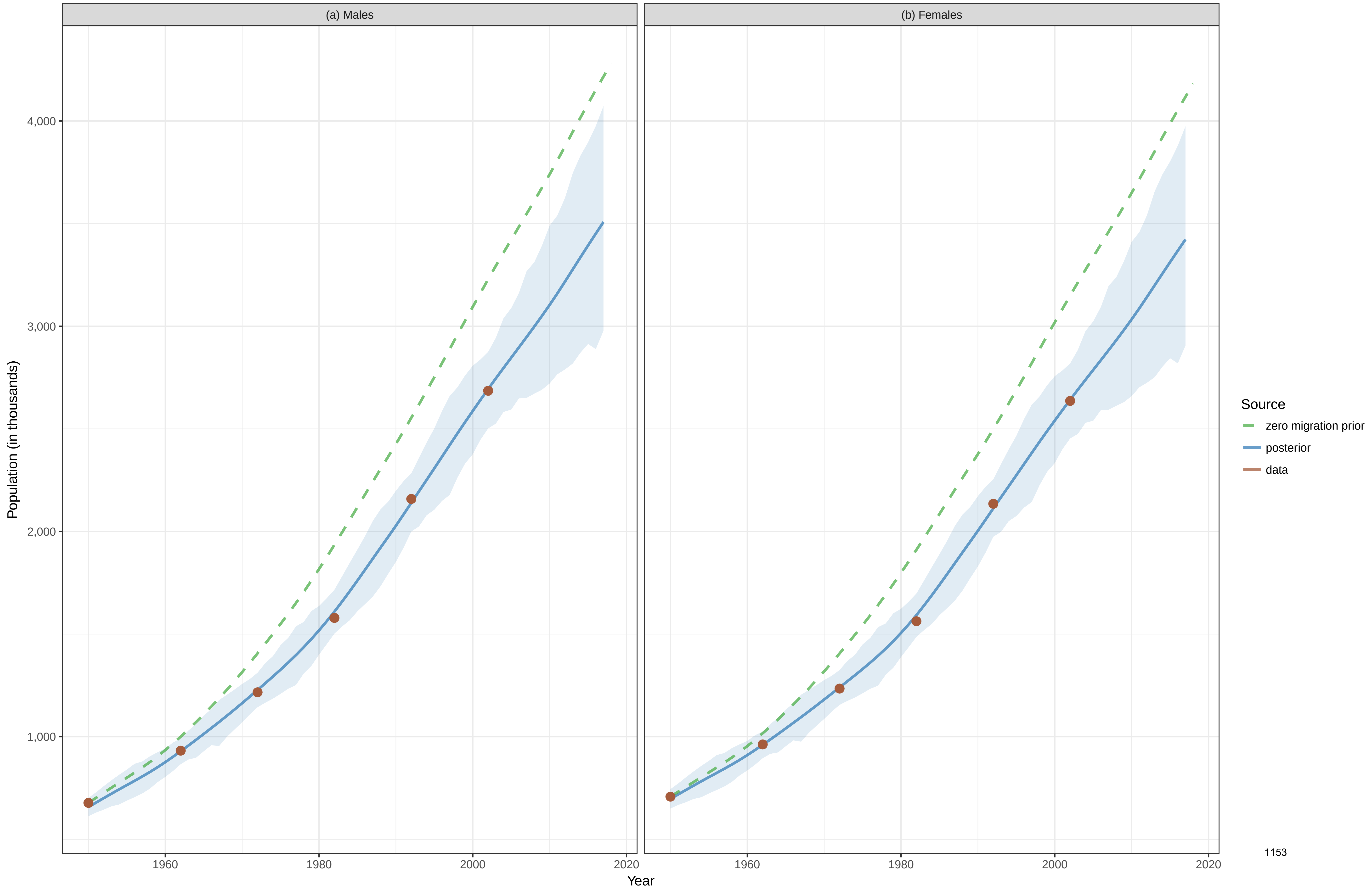

Supplemental Results Figure 4: GBD Bayesian Demographic Balancing Model fit in Peru for (a) total population of males, and (b) total population of females, 1950−2017.

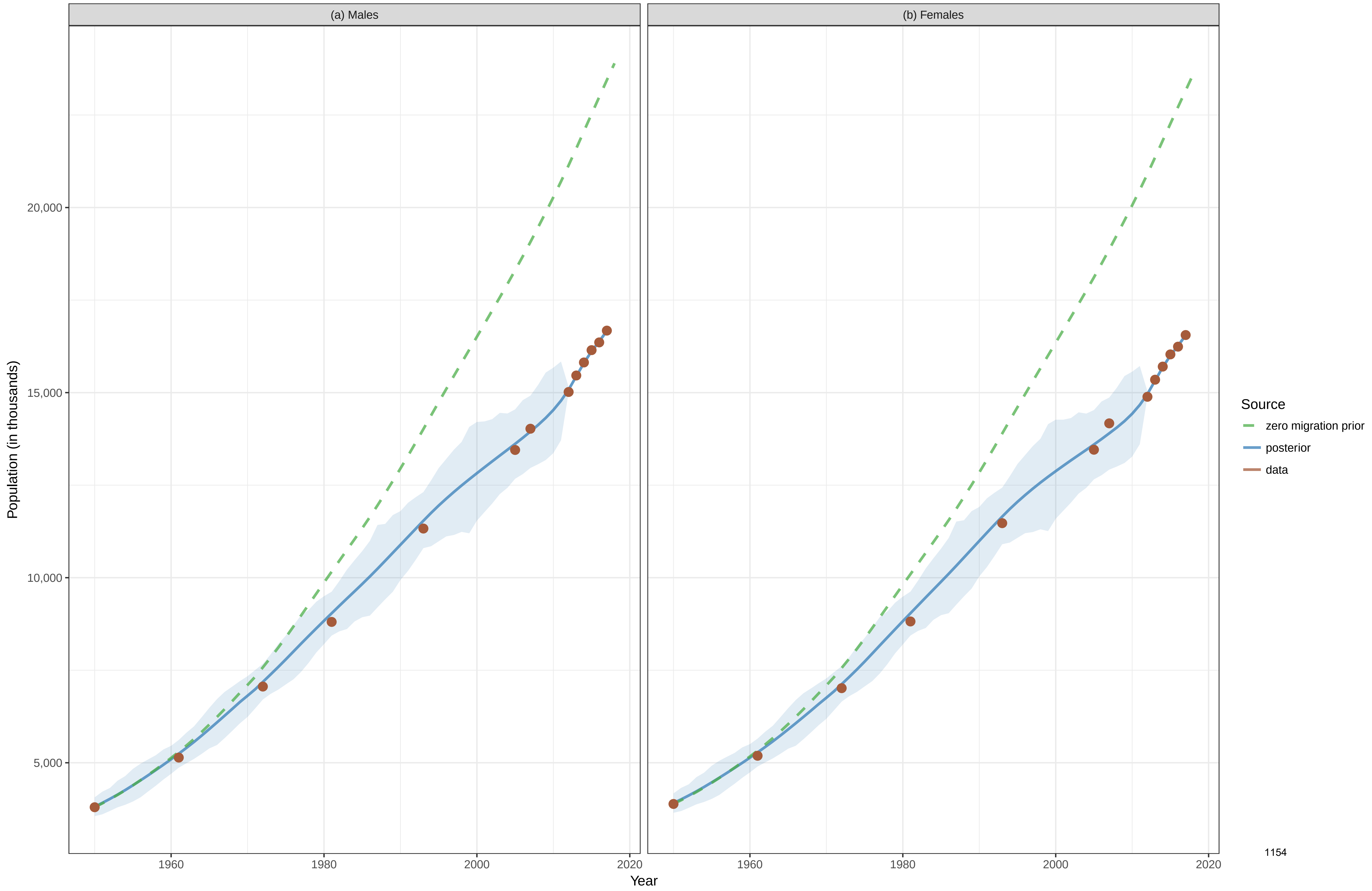

Supplemental Results Figure 4: GBD Bayesian Demographic Balancing Model fit in Philippines for (a) total population of males, and (b) total population of females, 1950−2017.

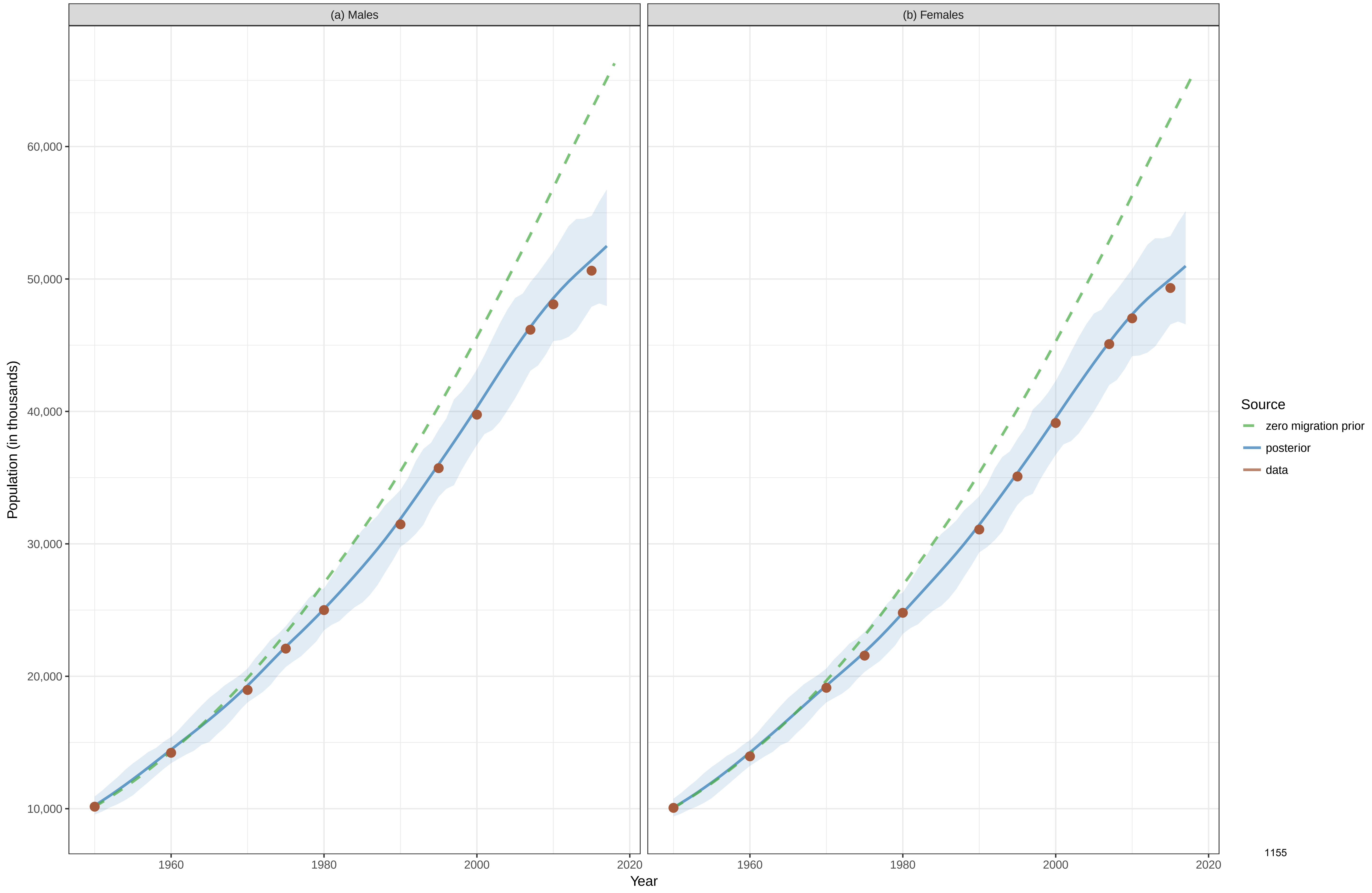

Supplemental Results Figure 4: GBD Bayesian Demographic Balancing Model fit in Poland for (a) total population of males, and (b) total population of females, 1950−2017.

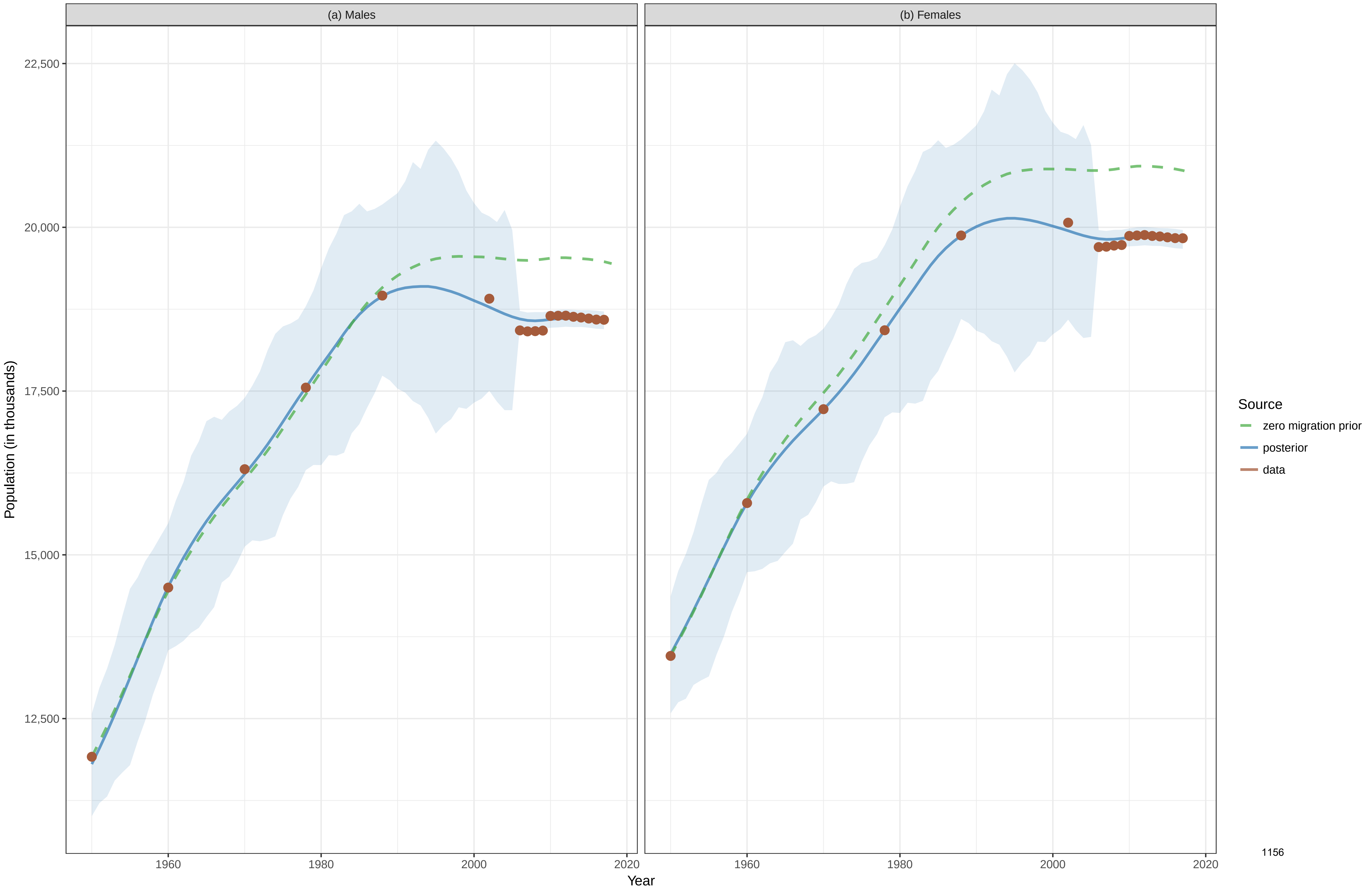

Supplemental Results Figure 4: GBD Bayesian Demographic Balancing Model fit in Portugal for (a) total population of males, and (b) total population of females, 1950−2017.

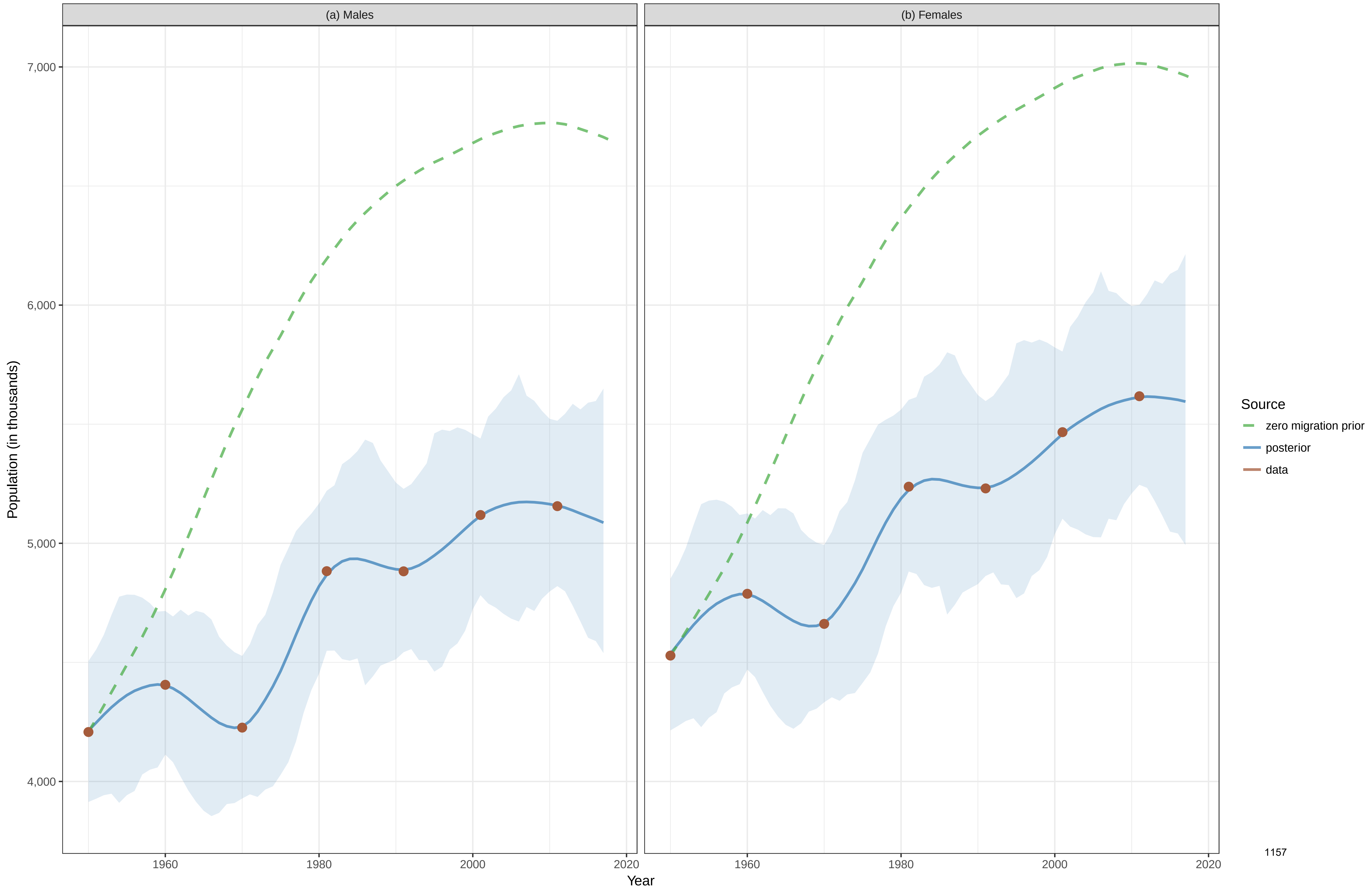

Supplemental Results Figure 4: GBD Bayesian Demographic Balancing Model fit in Puerto Rico for (a) total population of males, and (b) total population of females, 1950−2017.

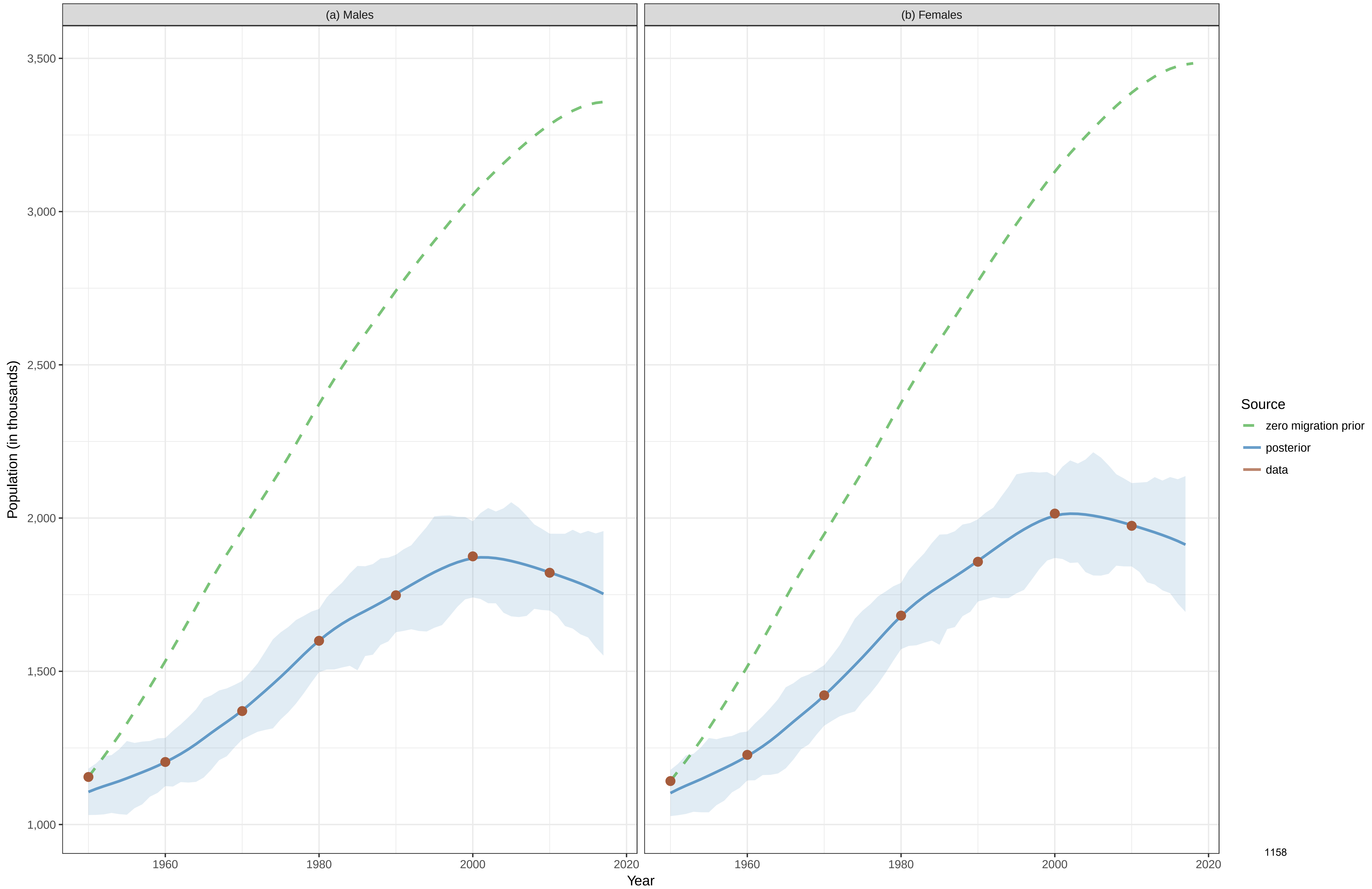

Supplemental Results Figure 4: GBD Bayesian Demographic Balancing Model fit in Qatar for (a) total population of males, and (b) total population of females, 1950−2017.

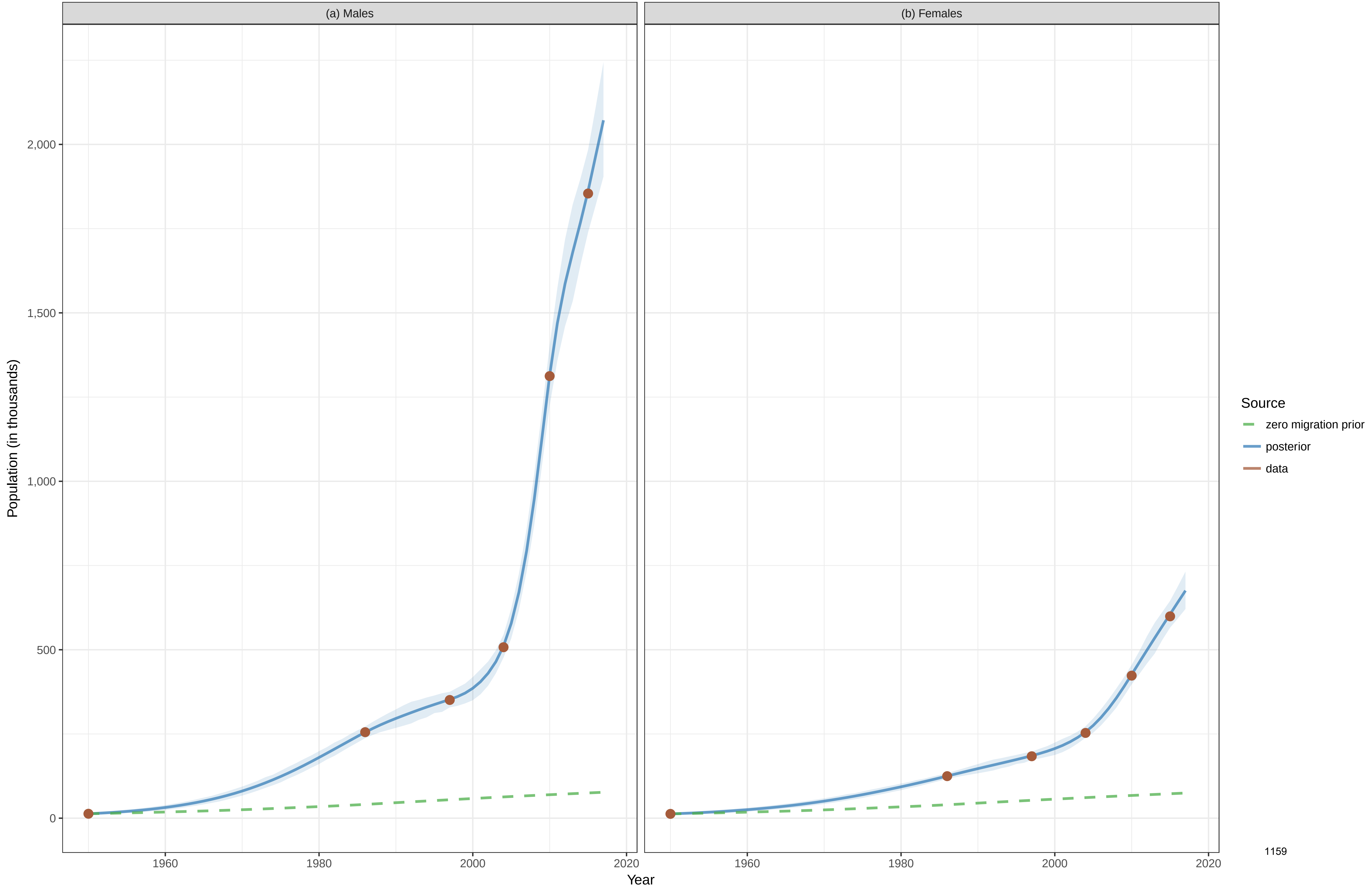

Supplemental Results Figure 4: GBD Bayesian Demographic Balancing Model fit in Romania for (a) total population of males, and (b) total population of females, 1950−2017.

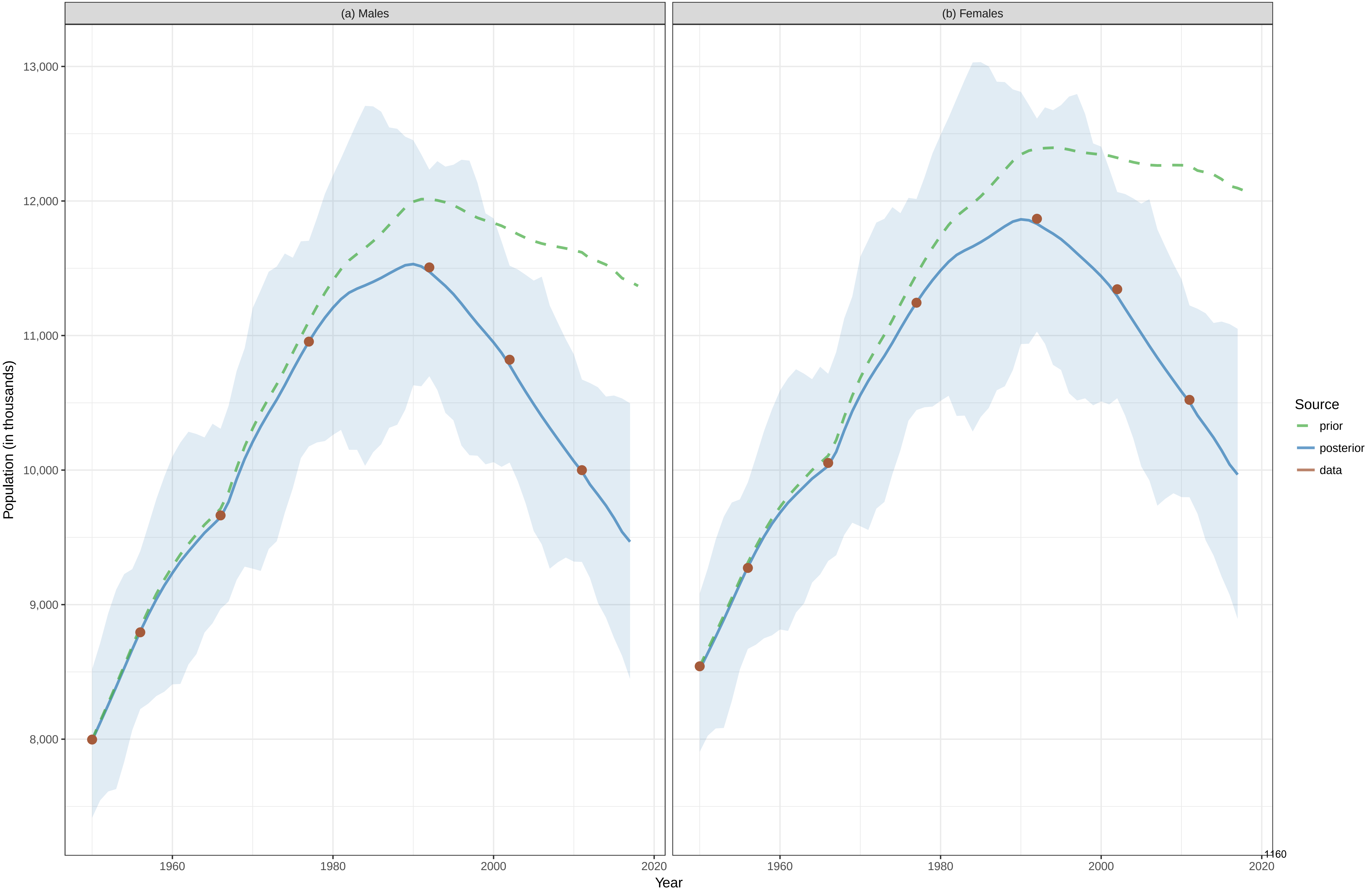

Supplemental Results Figure 4: GBD Bayesian Demographic Balancing Model fit in Russian Federation for (a) total population of males, and (b) total population of females, 1950−2017.

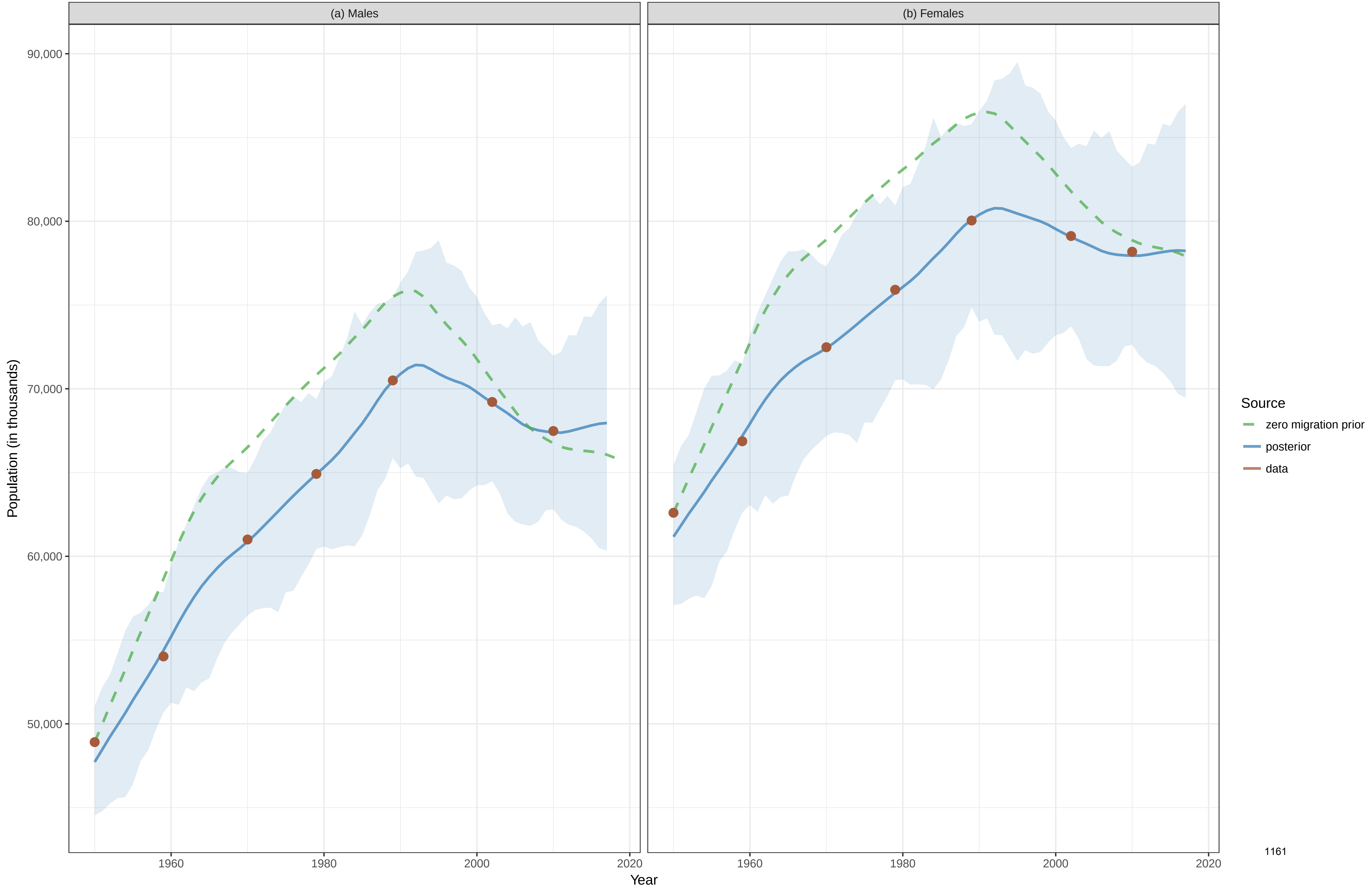

Supplemental Results Figure 4: GBD Bayesian Demographic Balancing Model fit in Rwanda for (a) total population of males, and (b) total population of females, 1950−2017.

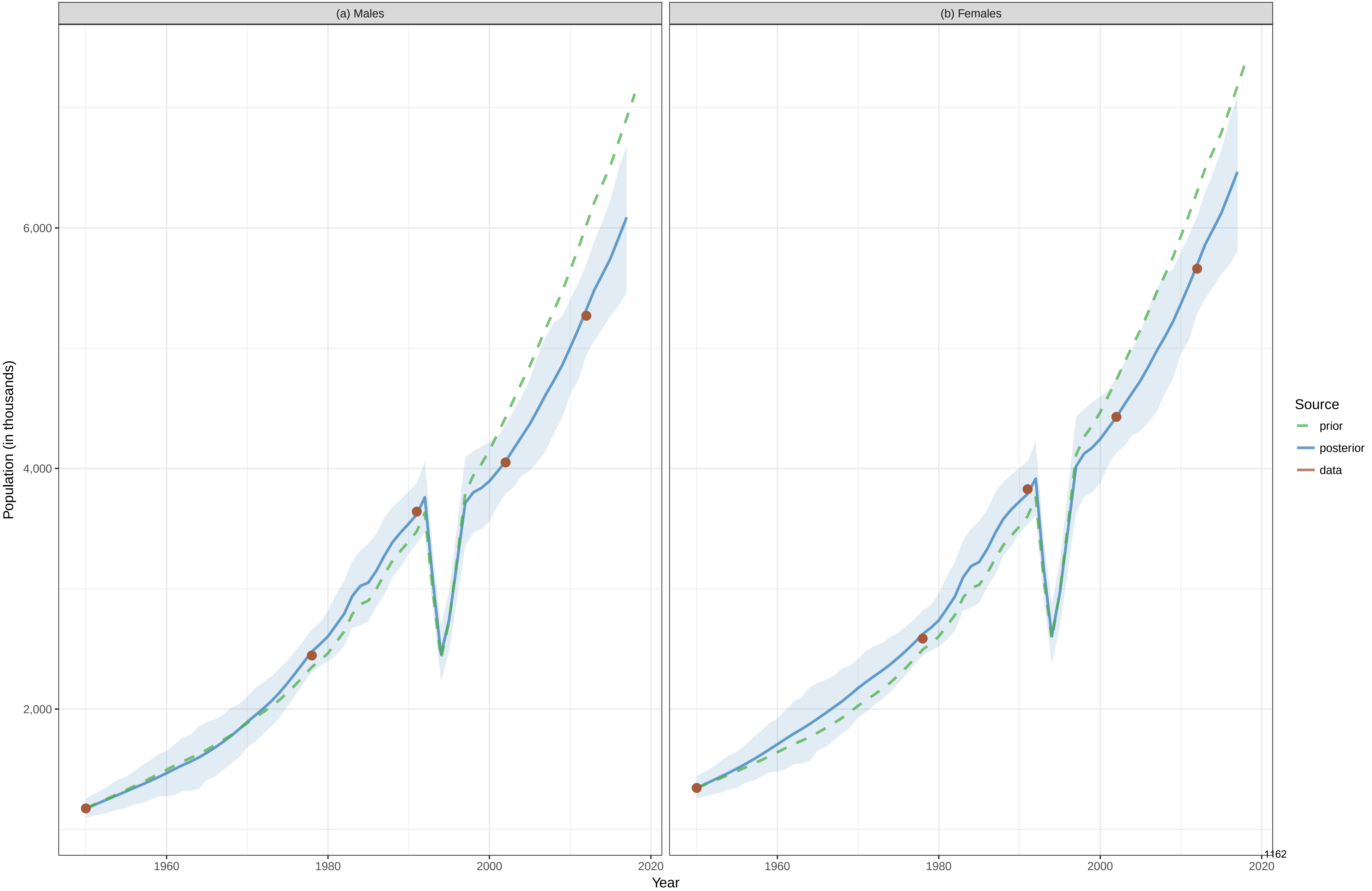

Supplemental Results Figure 4: GBD Bayesian Demographic Balancing Model fit in Saint Lucia for (a) total population of males, and (b) total population of females, 1950−2017.

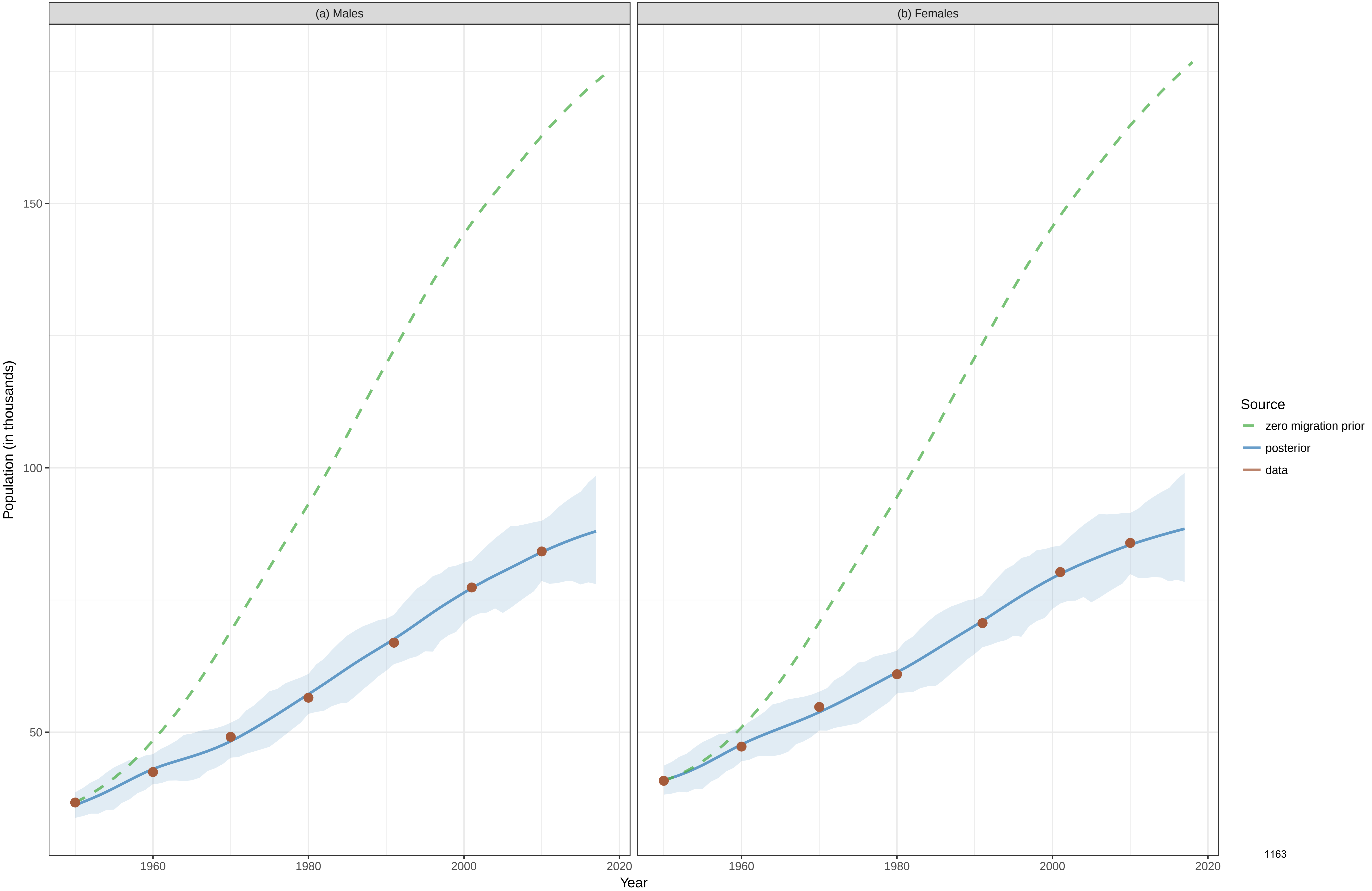

Supplemental Results Figure 4: GBD Bayesian Demographic Balancing Model fit in Saint Vincent and the Grenadines for (a) total population of males, and (b) total population of females, 1950 –2017.

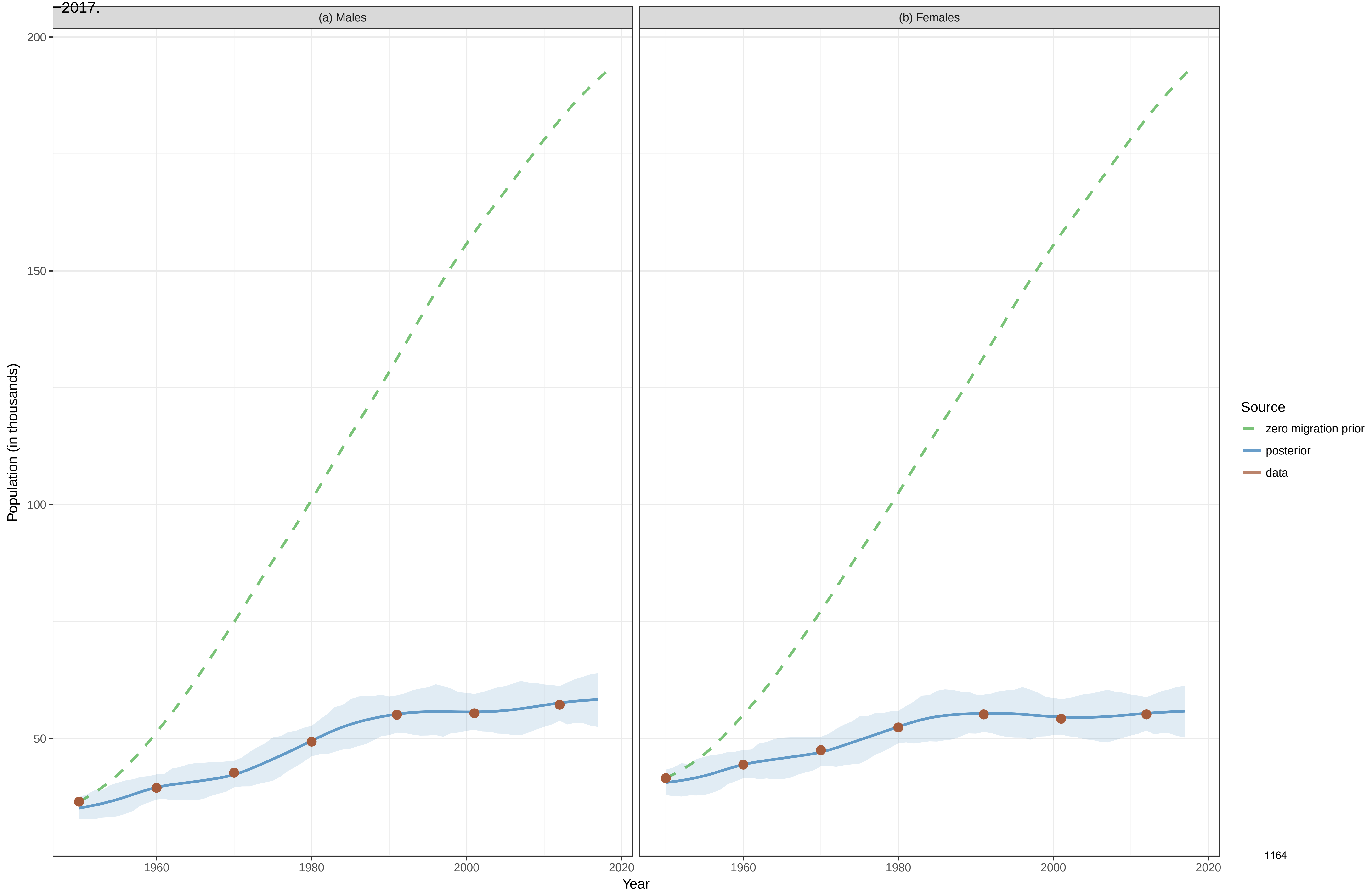

Supplemental Results Figure 4: GBD Bayesian Demographic Balancing Model fit in Samoa for (a) total population of males, and (b) total population of females, 1950−2017.

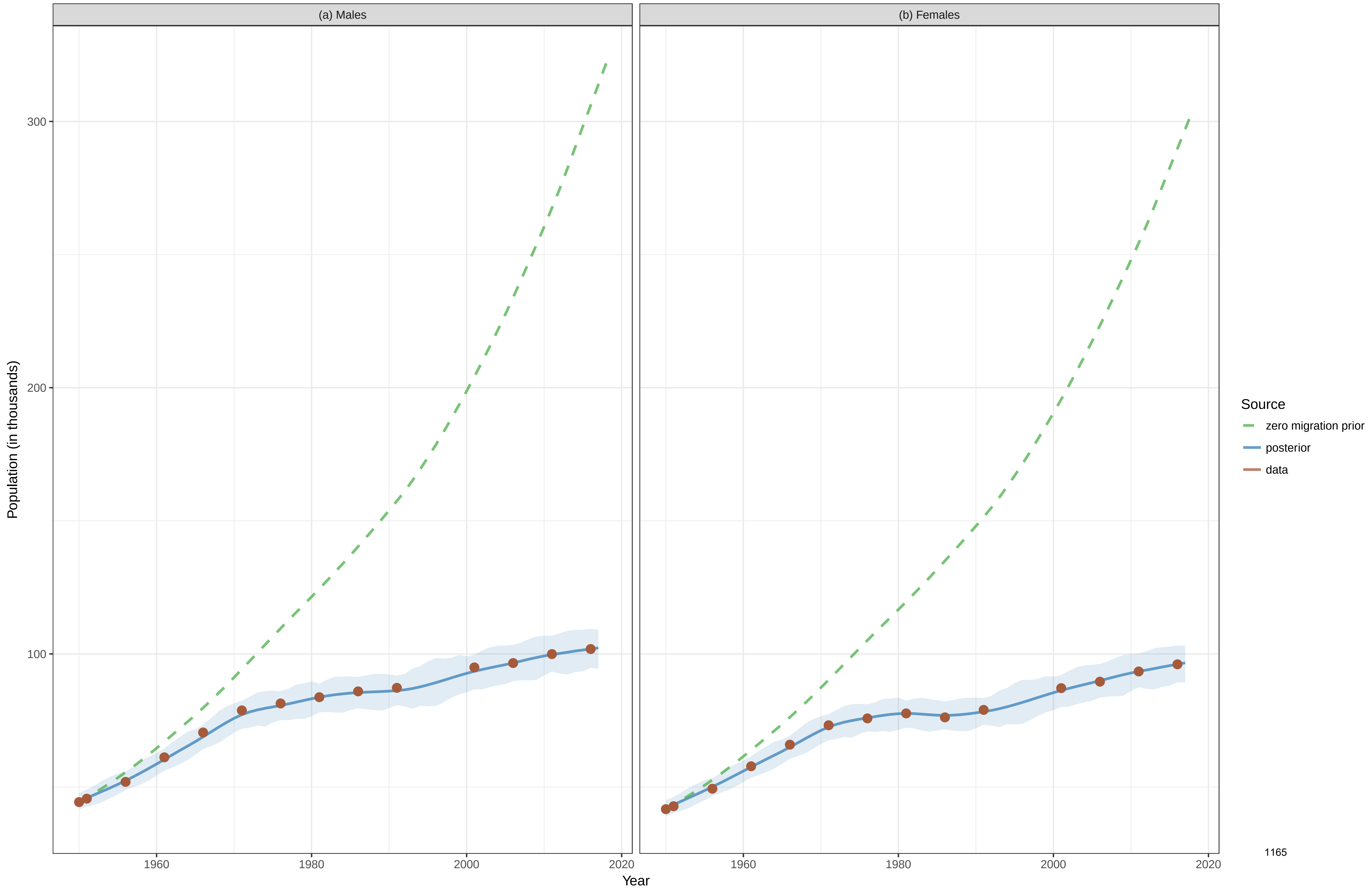

Supplemental Results Figure 4: GBD Bayesian Demographic Balancing Model fit in Sao Tome and Principe for (a) total population of males, and (b) total population of females, 1950−2017.

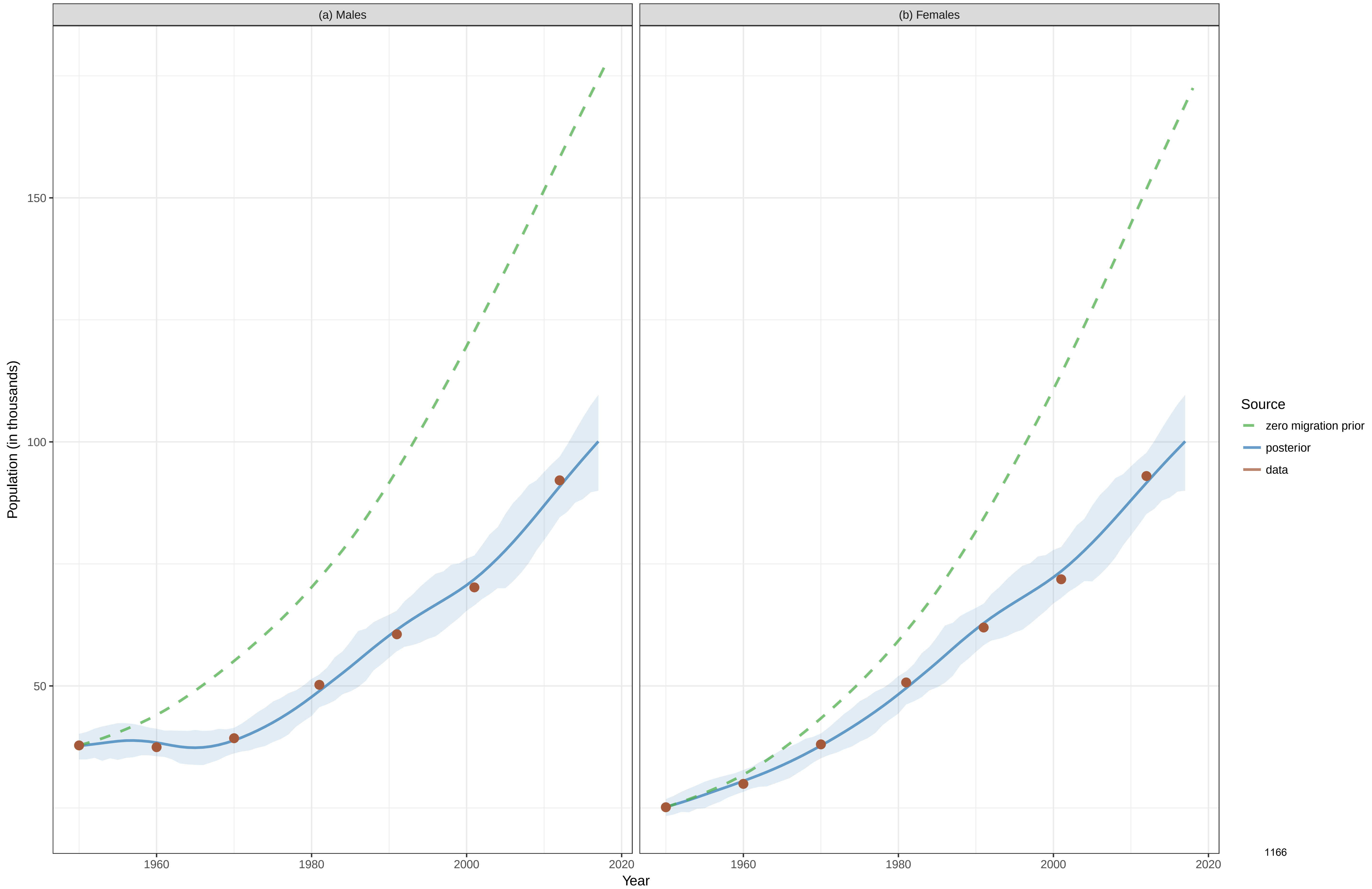

Supplemental Results Figure 4: GBD Bayesian Demographic Balancing Model fit in Saudi Arabia for (a) total population of males, and (b) total population of females, 1950−2017.

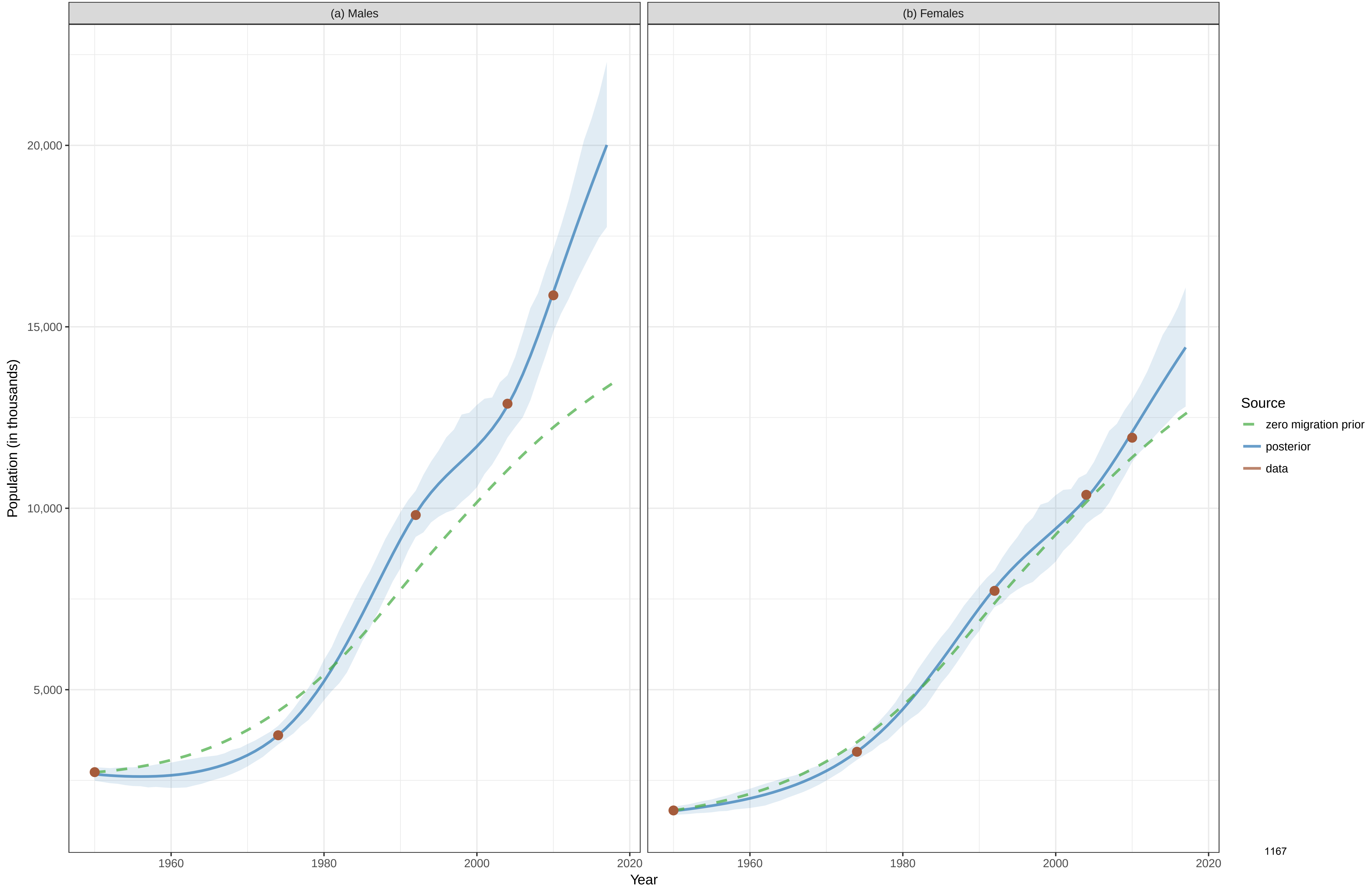

Supplemental Results Figure 4: GBD Bayesian Demographic Balancing Model fit in Scotland for (a) total population of males, and (b) total population of females, 1950−2017.

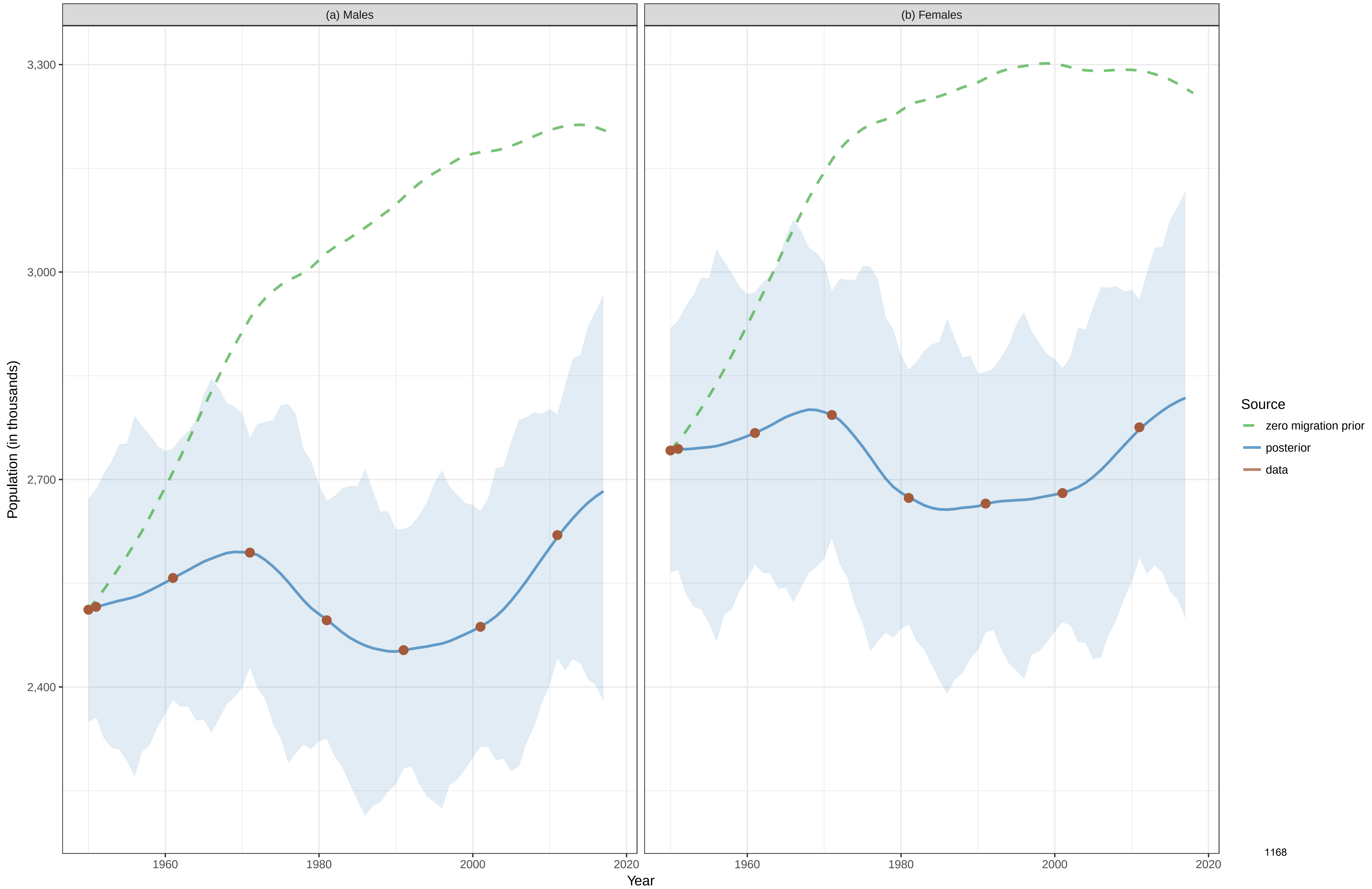

Supplemental Results Figure 4: GBD Bayesian Demographic Balancing Model fit in Senegal for (a) total population of males, and (b) total population of females, 1950−2017.

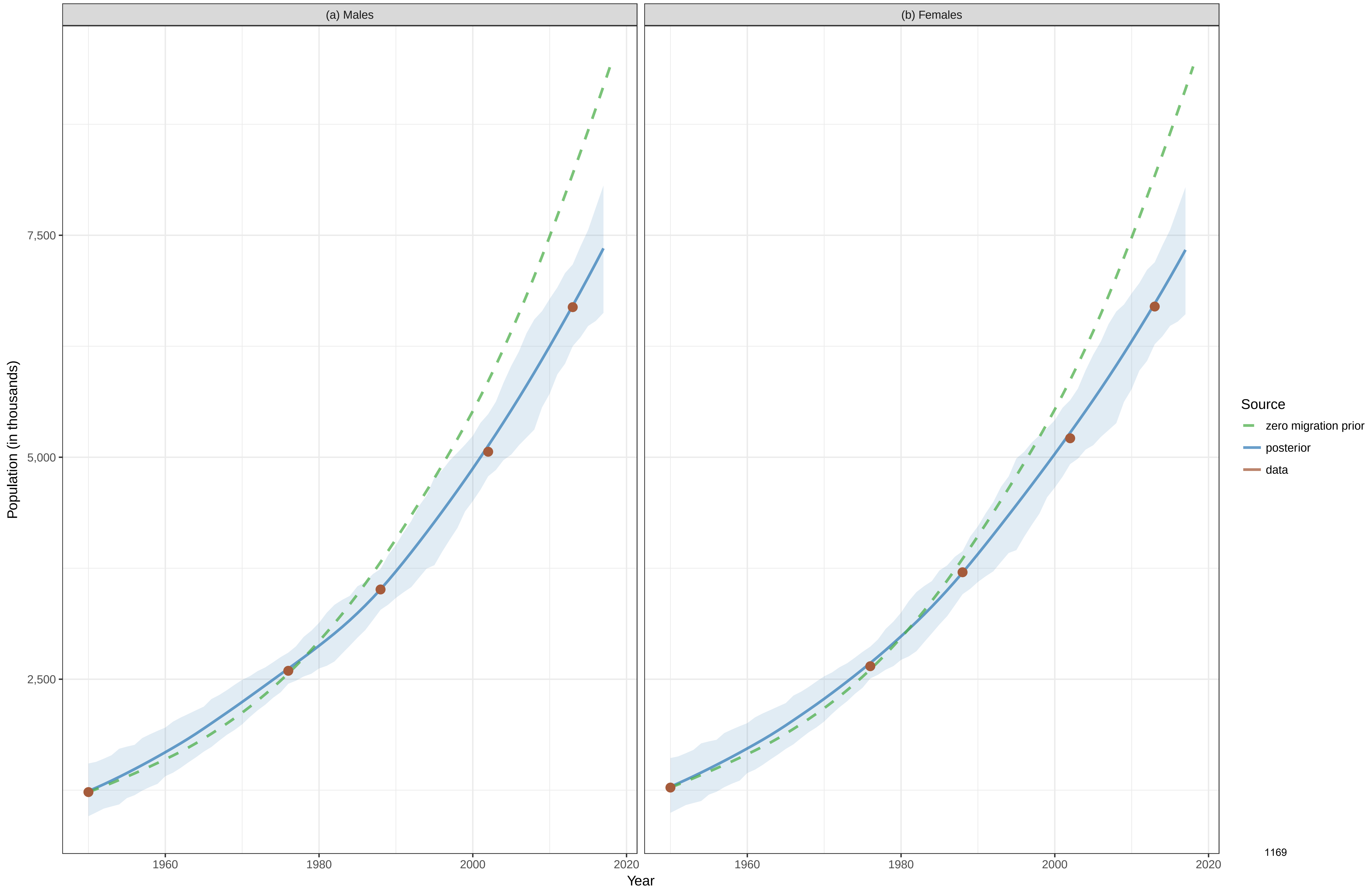

Supplemental Results Figure 4: GBD Bayesian Demographic Balancing Model fit in Serbia for (a) total population of males, and (b) total population of females, 1950−2017.

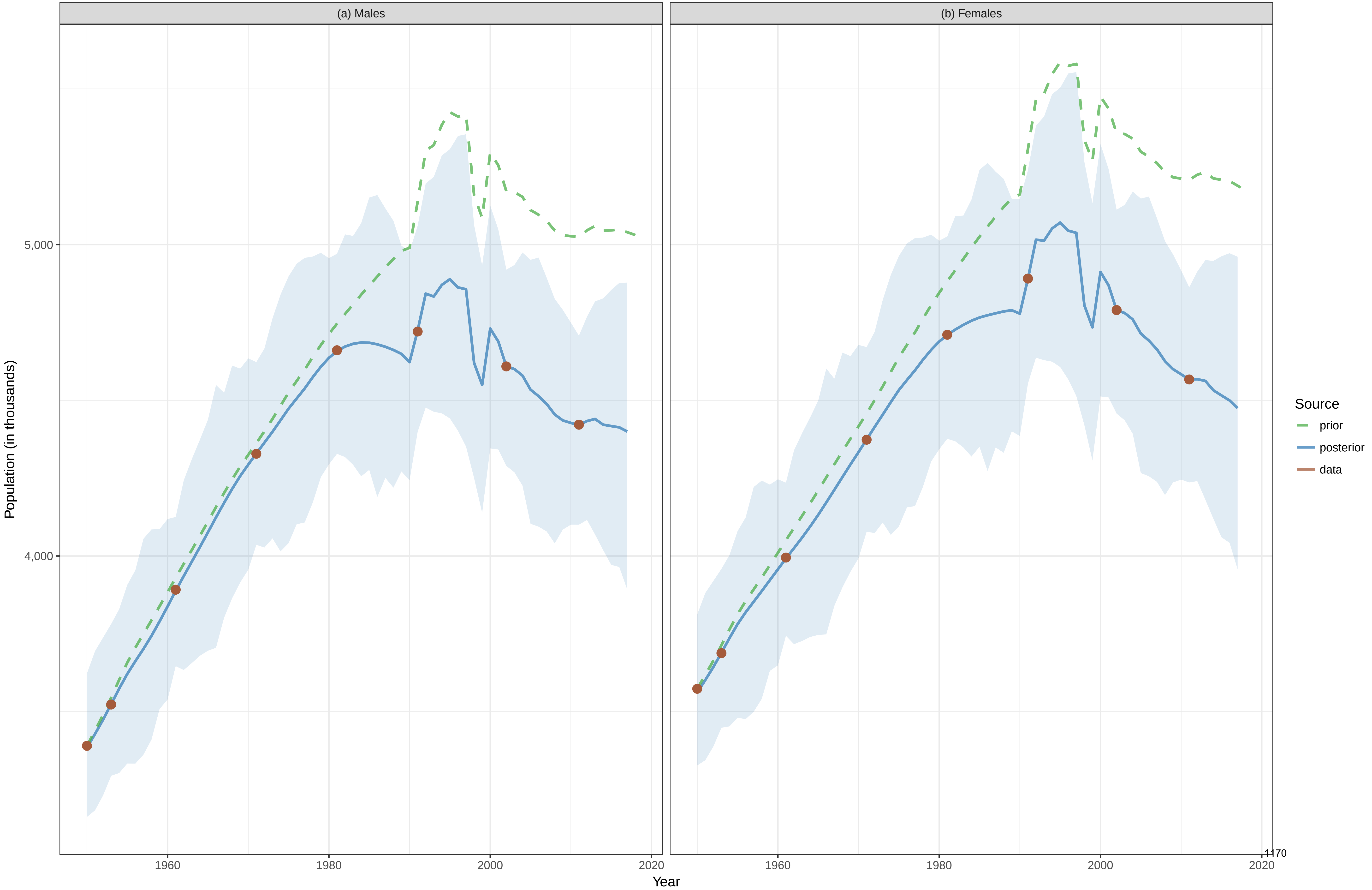

Supplemental Results Figure 4: GBD Bayesian Demographic Balancing Model fit in Seychelles for (a) total population of males, and (b) total population of females, 1950−2017.

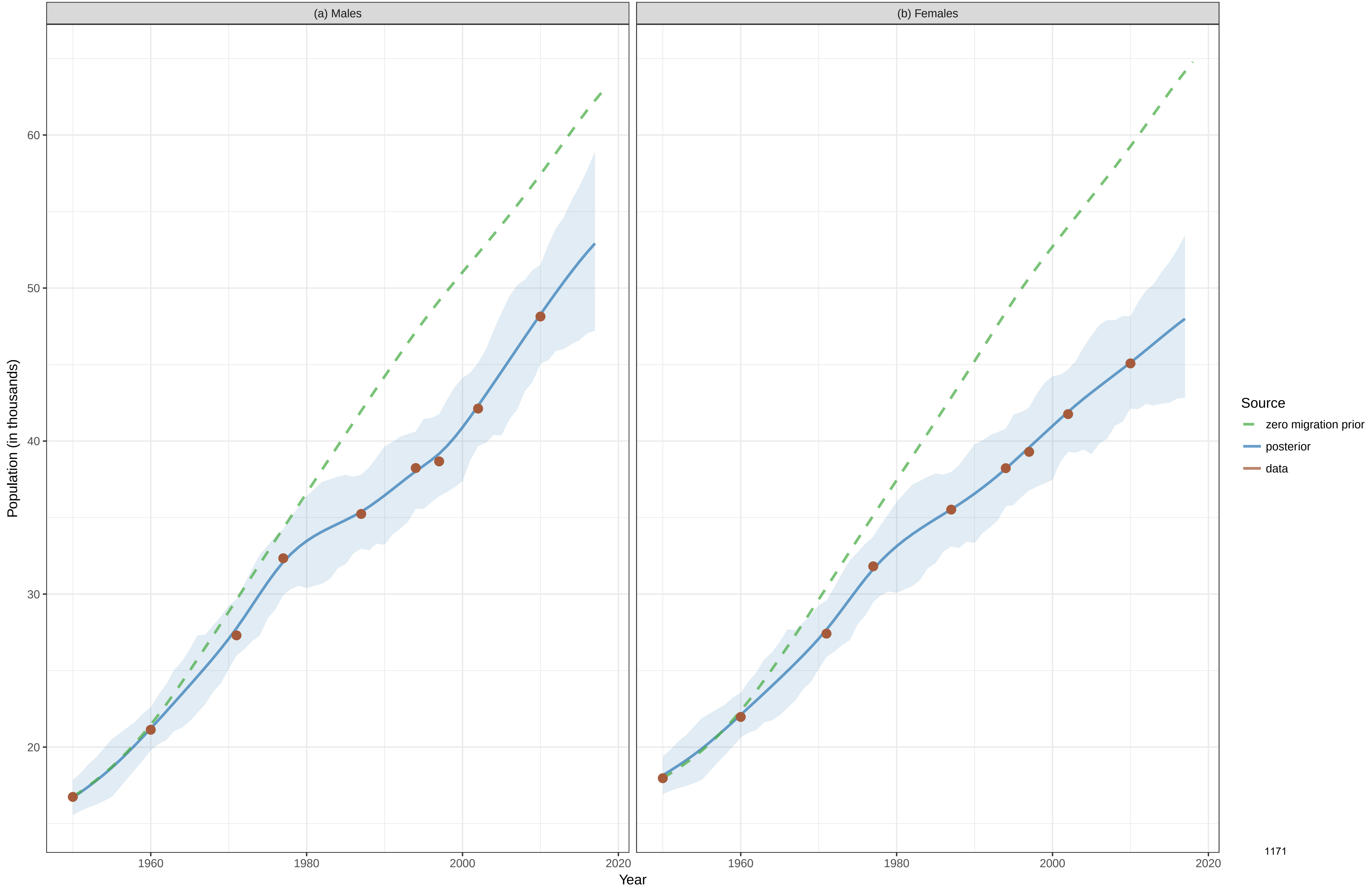

Supplemental Results Figure 4: GBD Bayesian Demographic Balancing Model fit in Sierra Leone for (a) total population of males, and (b) total population of females, 1950−2017.

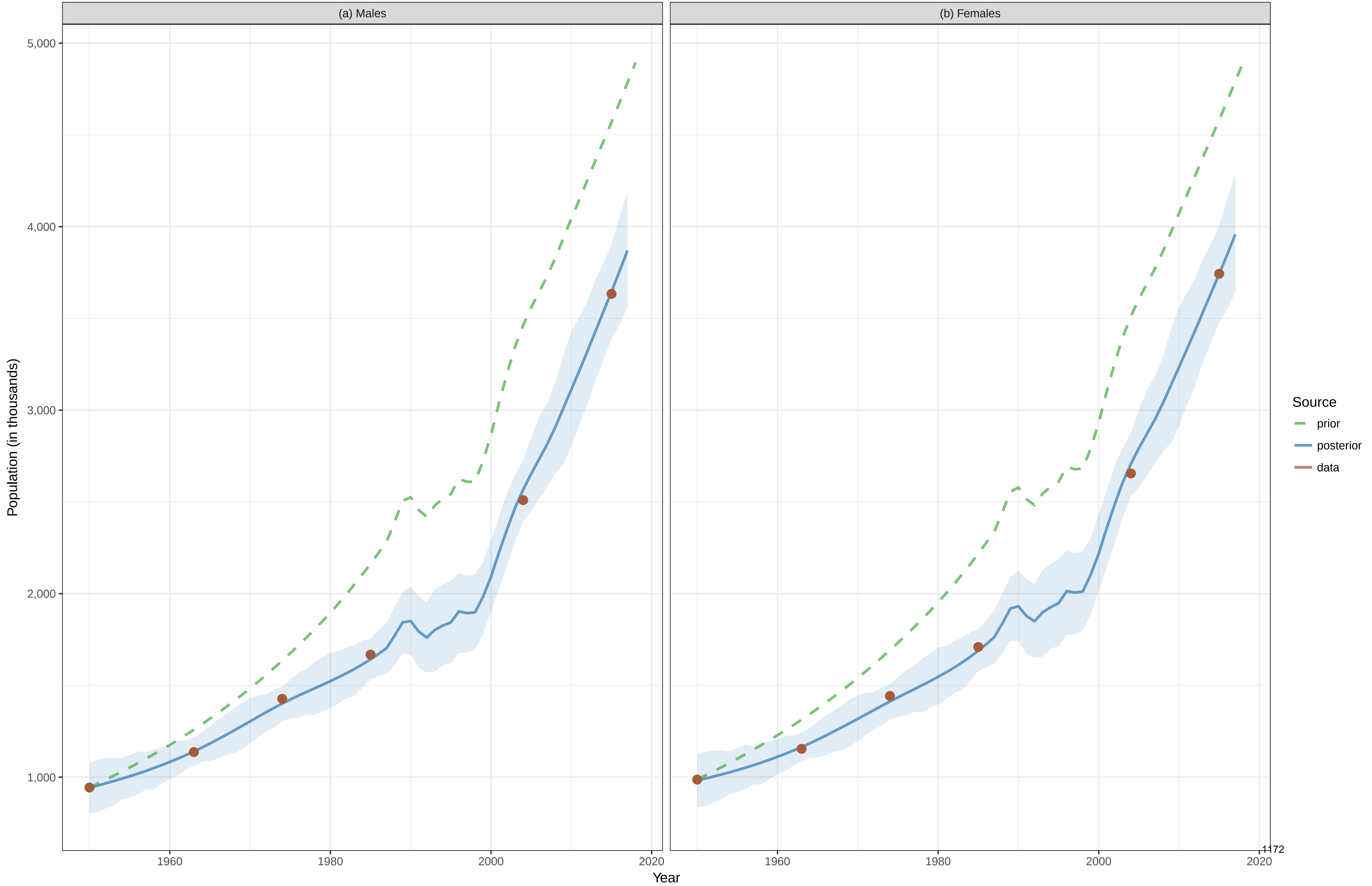

Supplemental Results Figure 4: GBD Bayesian Demographic Balancing Model fit in Singapore for (a) total population of males, and (b) total population of females, 1950−2017.

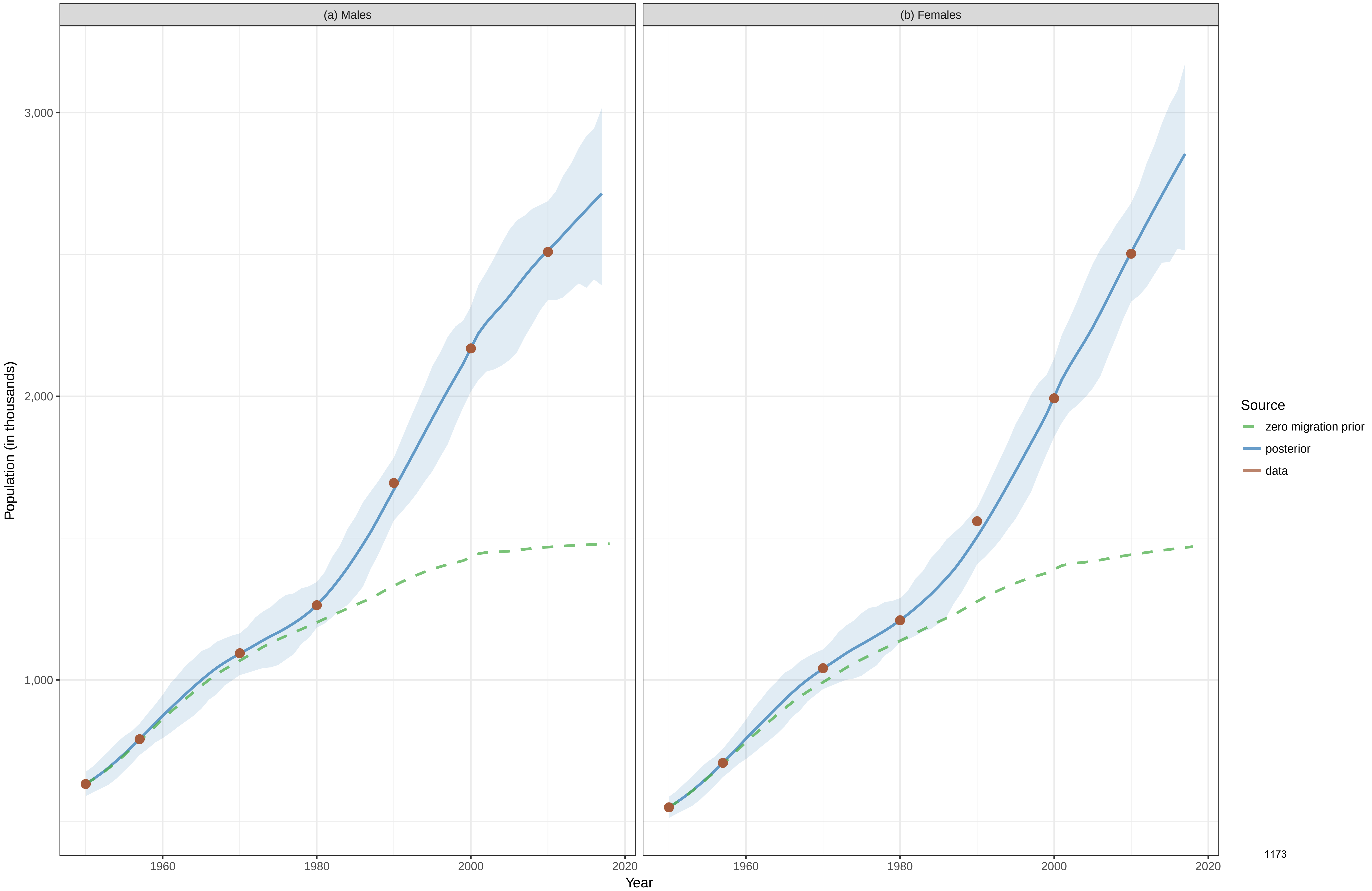

Supplemental Results Figure 4: GBD Bayesian Demographic Balancing Model fit in Slovakia for (a) total population of males, and (b) total population of females, 1950−2017.

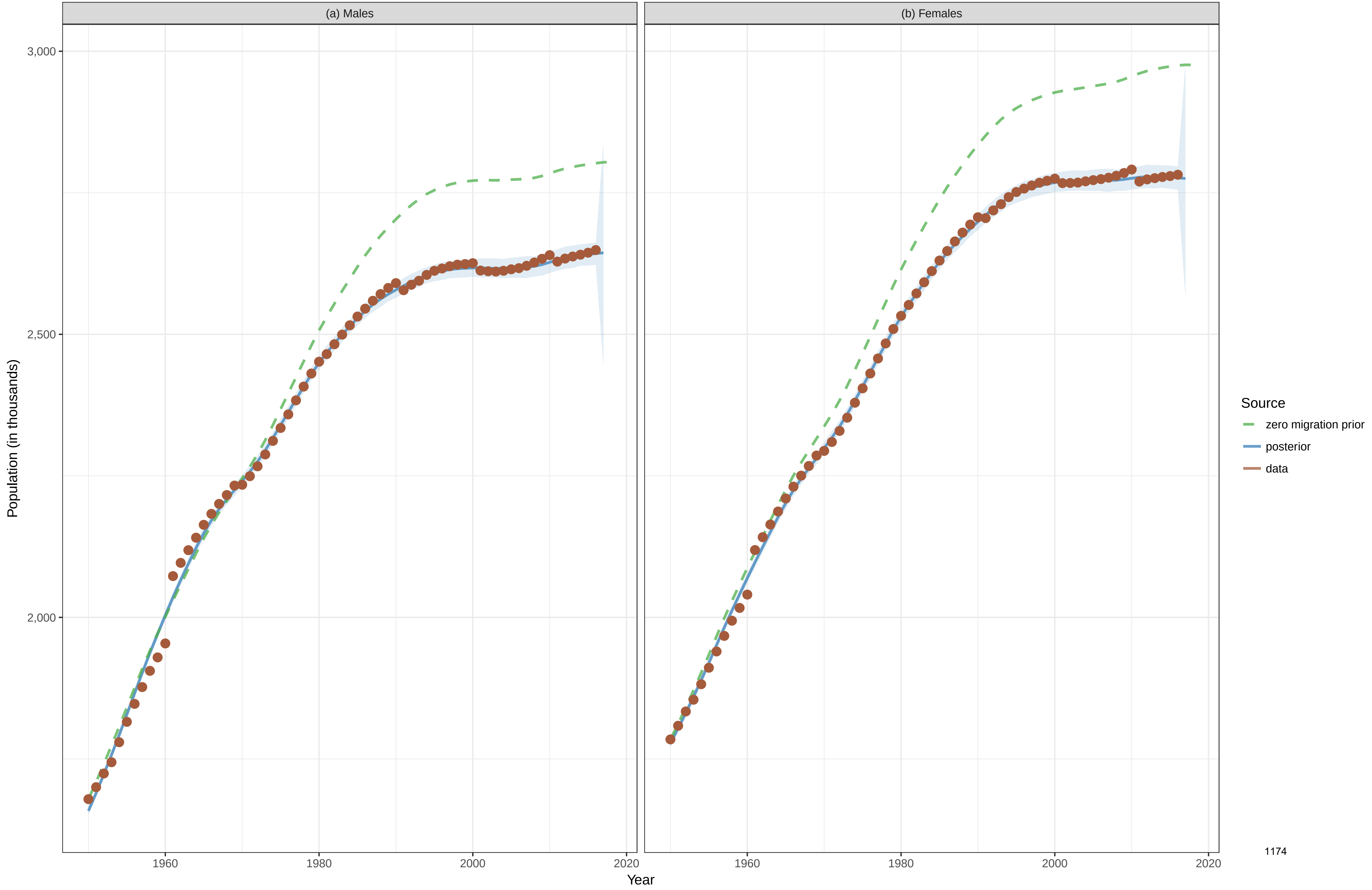

Supplemental Results Figure 4: GBD Bayesian Demographic Balancing Model fit in Slovenia for (a) total population of males, and (b) total population of females, 1950−2017.

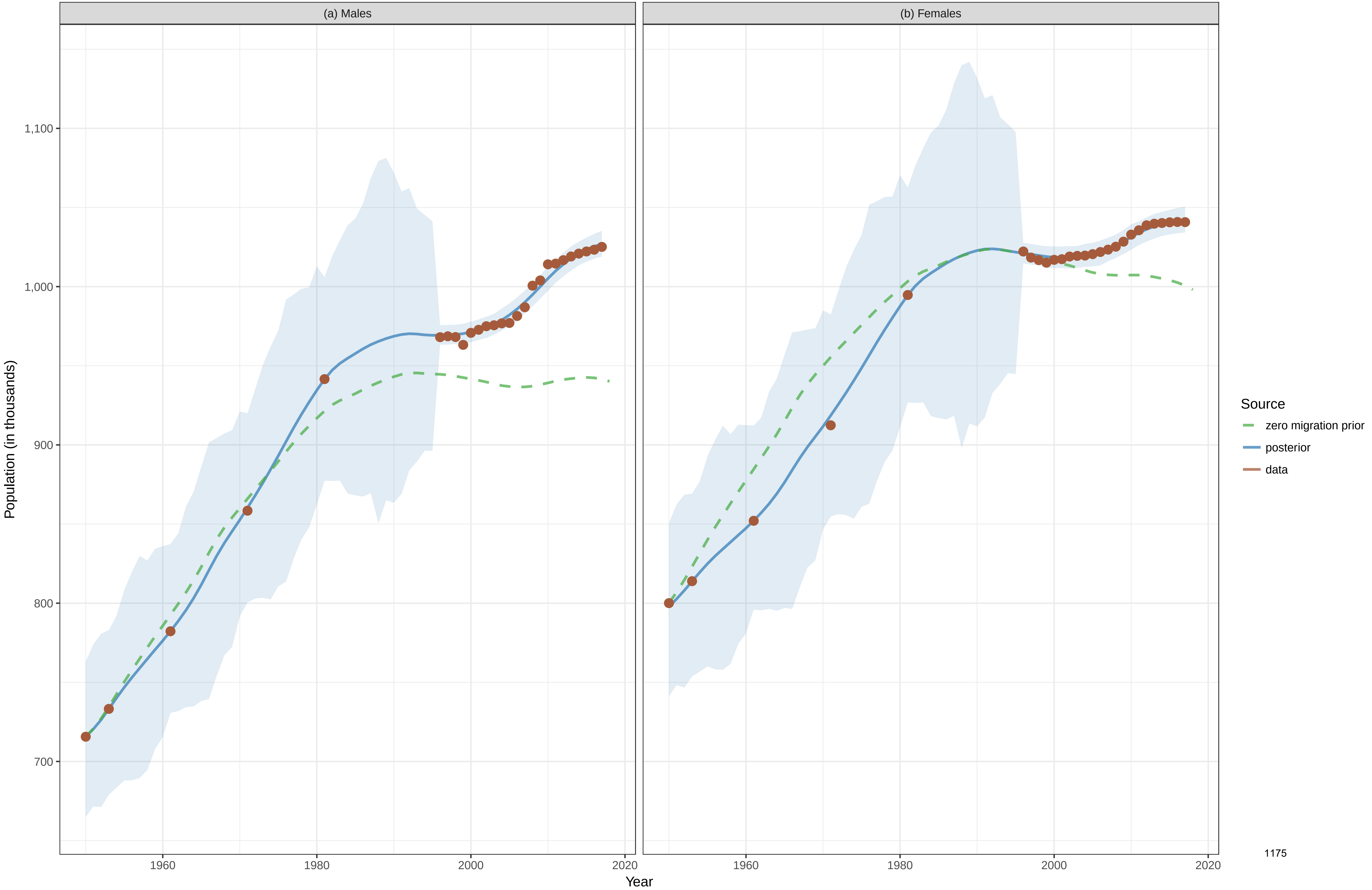

Supplemental Results Figure 4: GBD Bayesian Demographic Balancing Model fit in Solomon Islands for (a) total population of males, and (b) total population of females, 1950−2017.

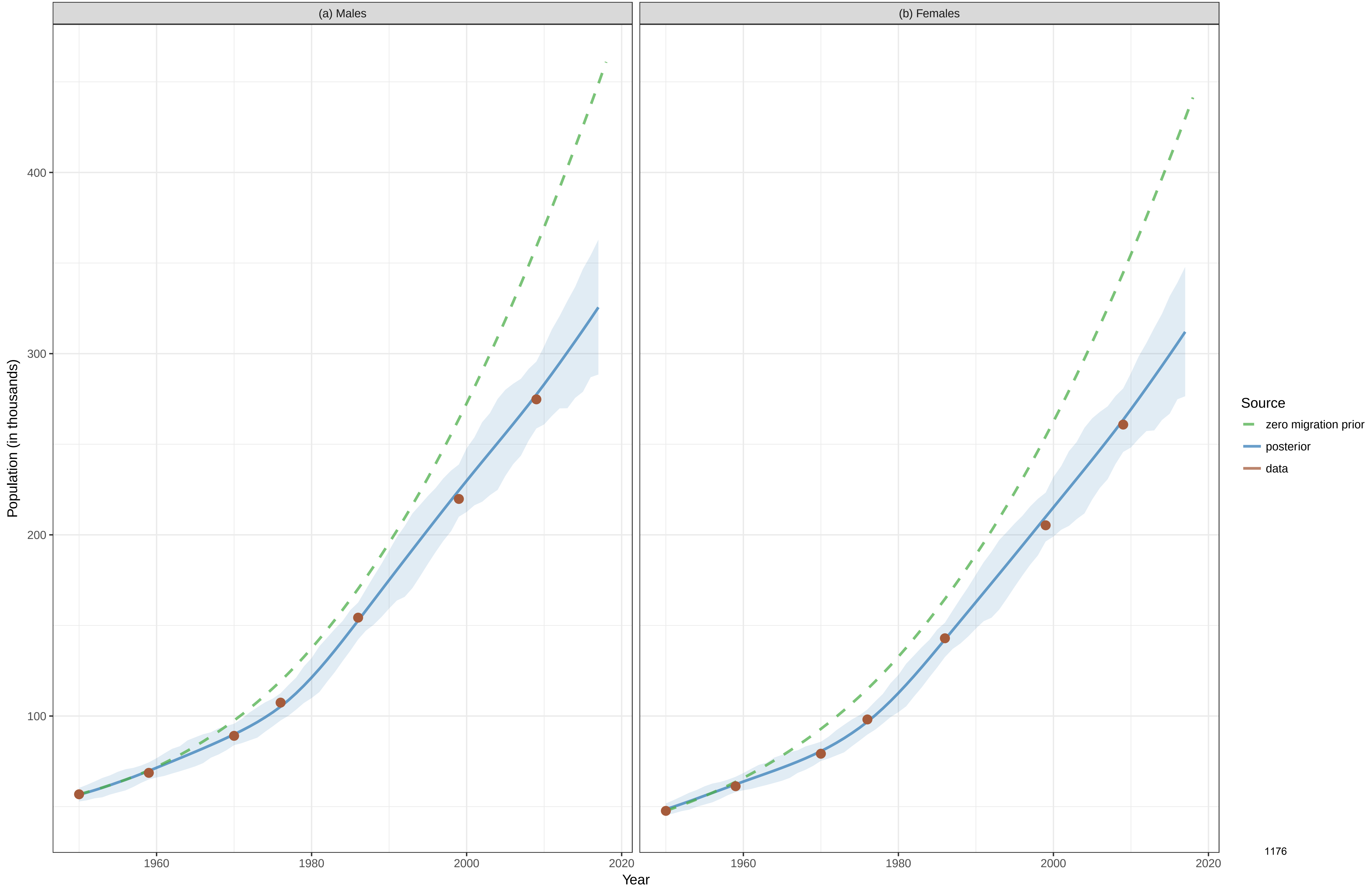

Supplemental Results Figure 4: GBD Bayesian Demographic Balancing Model fit in Somalia for (a) total population of males, and (b) total population of females, 1950−2017.

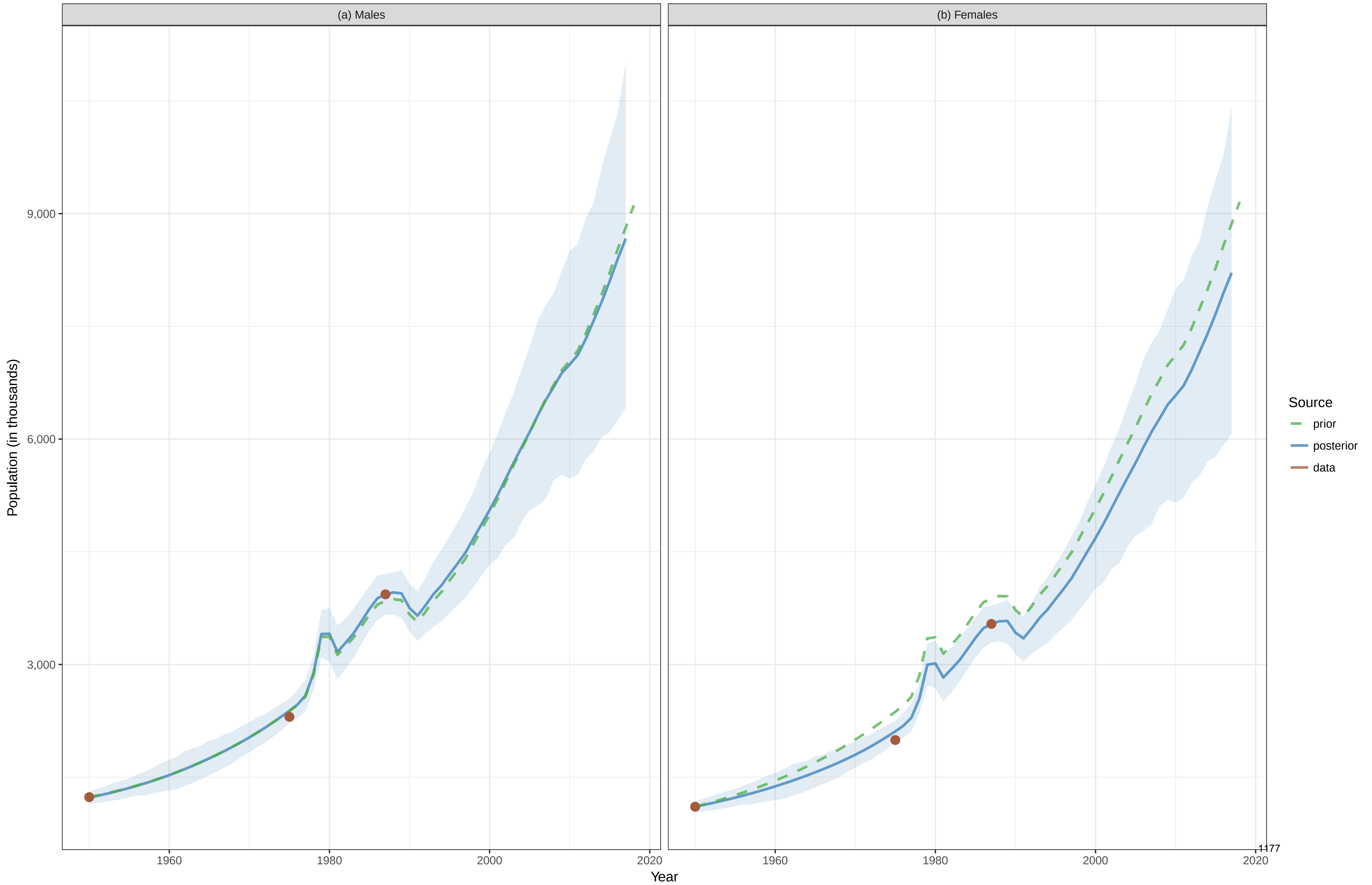

Supplemental Results Figure 4: GBD Bayesian Demographic Balancing Model fit in South Africa for (a) total population of males, and (b) total population of females, 1950−2017.

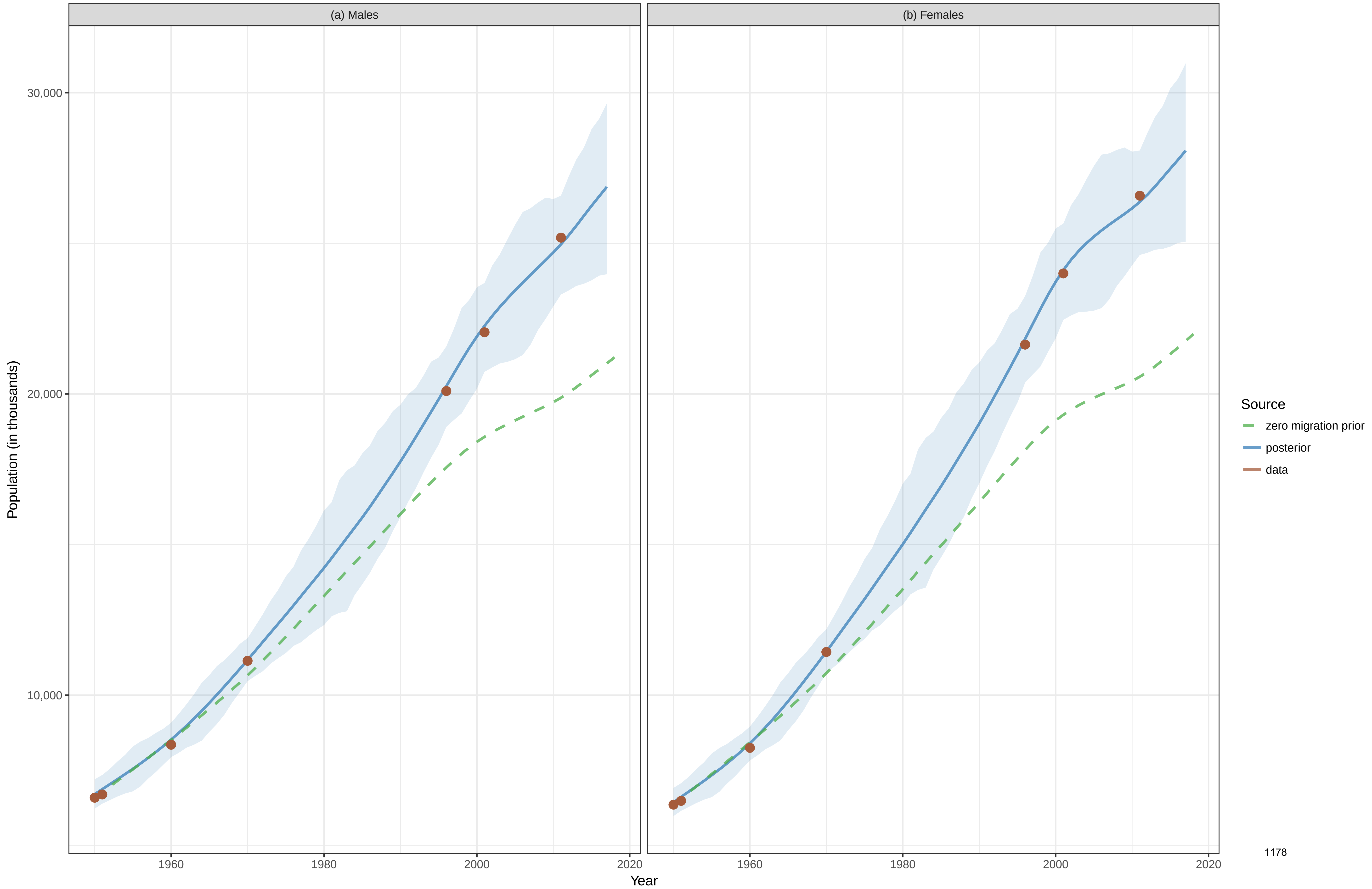

Supplemental Results Figure 4: GBD Bayesian Demographic Balancing Model fit in South Korea for (a) total population of males, and (b) total population of females, 1950−2017.

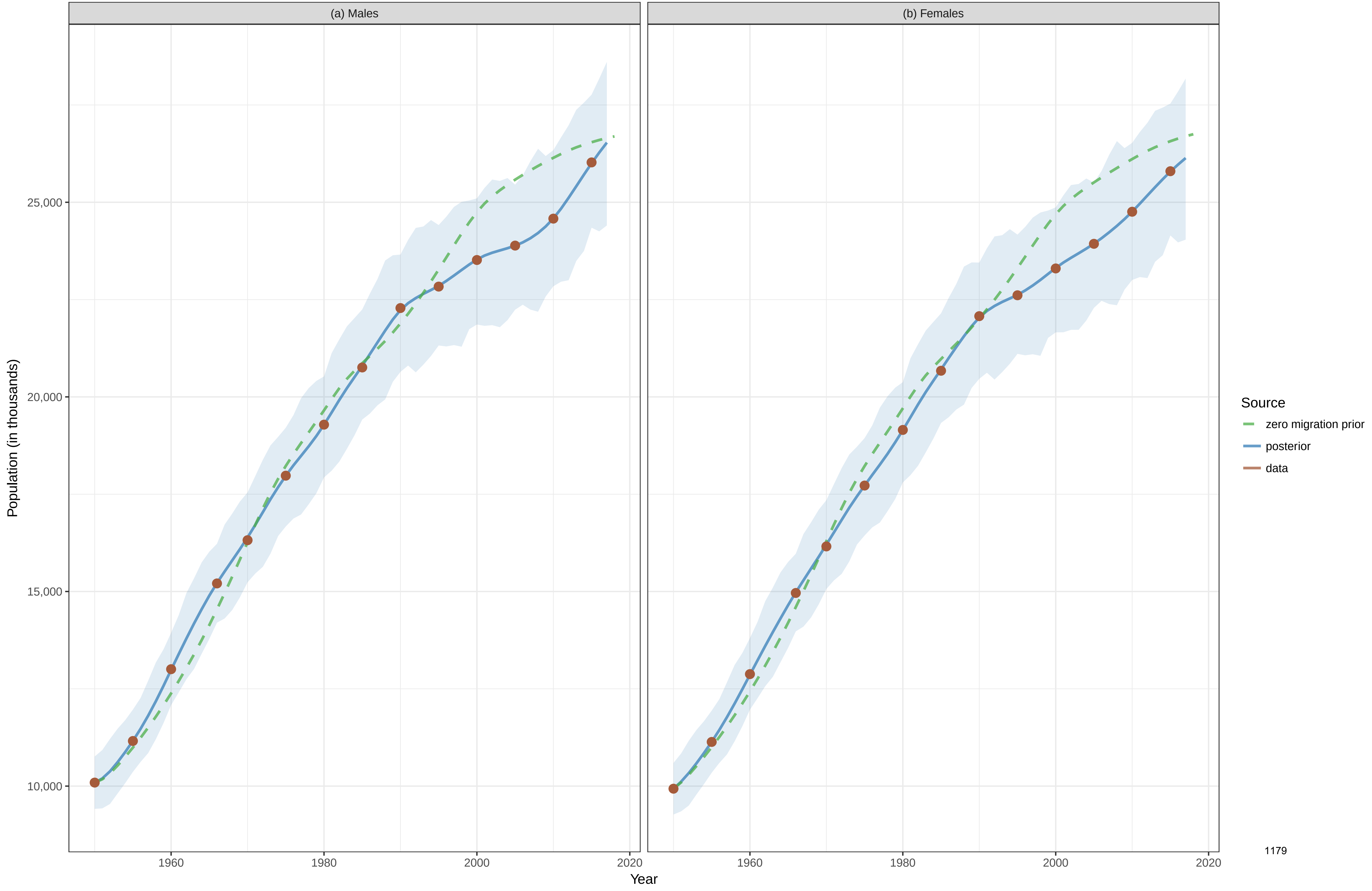

Supplemental Results Figure 4: GBD Bayesian Demographic Balancing Model fit in South Sudan for (a) total population of males, and (b) total population of females, 1950−2017.

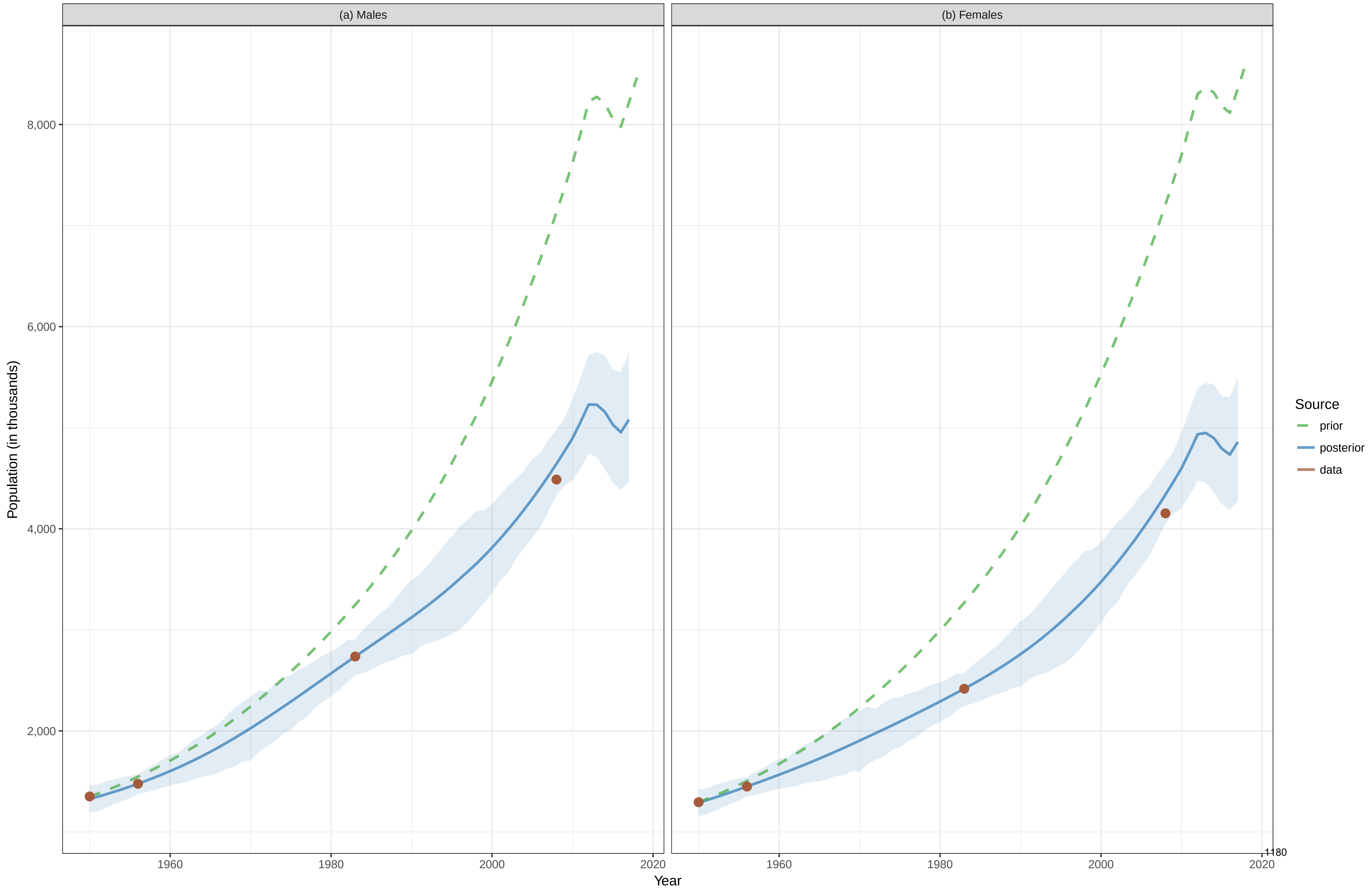

Supplemental Results Figure 4: GBD Bayesian Demographic Balancing Model fit in Spain for (a) total population of males, and (b) total population of females, 1950−2017.

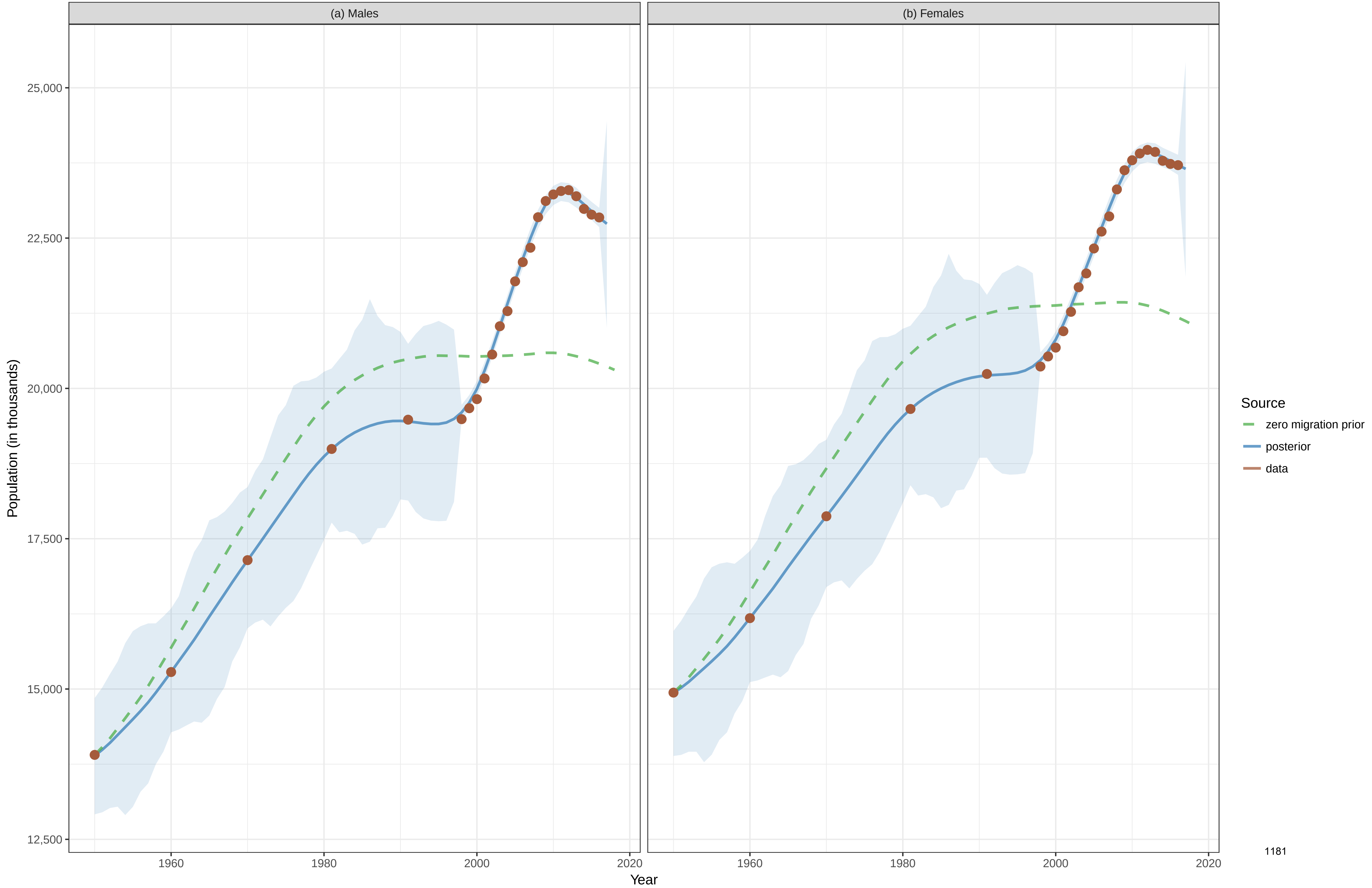

Supplemental Results Figure 4: GBD Bayesian Demographic Balancing Model fit in Sri Lanka for (a) total population of males, and (b) total population of females, 1950−2017.

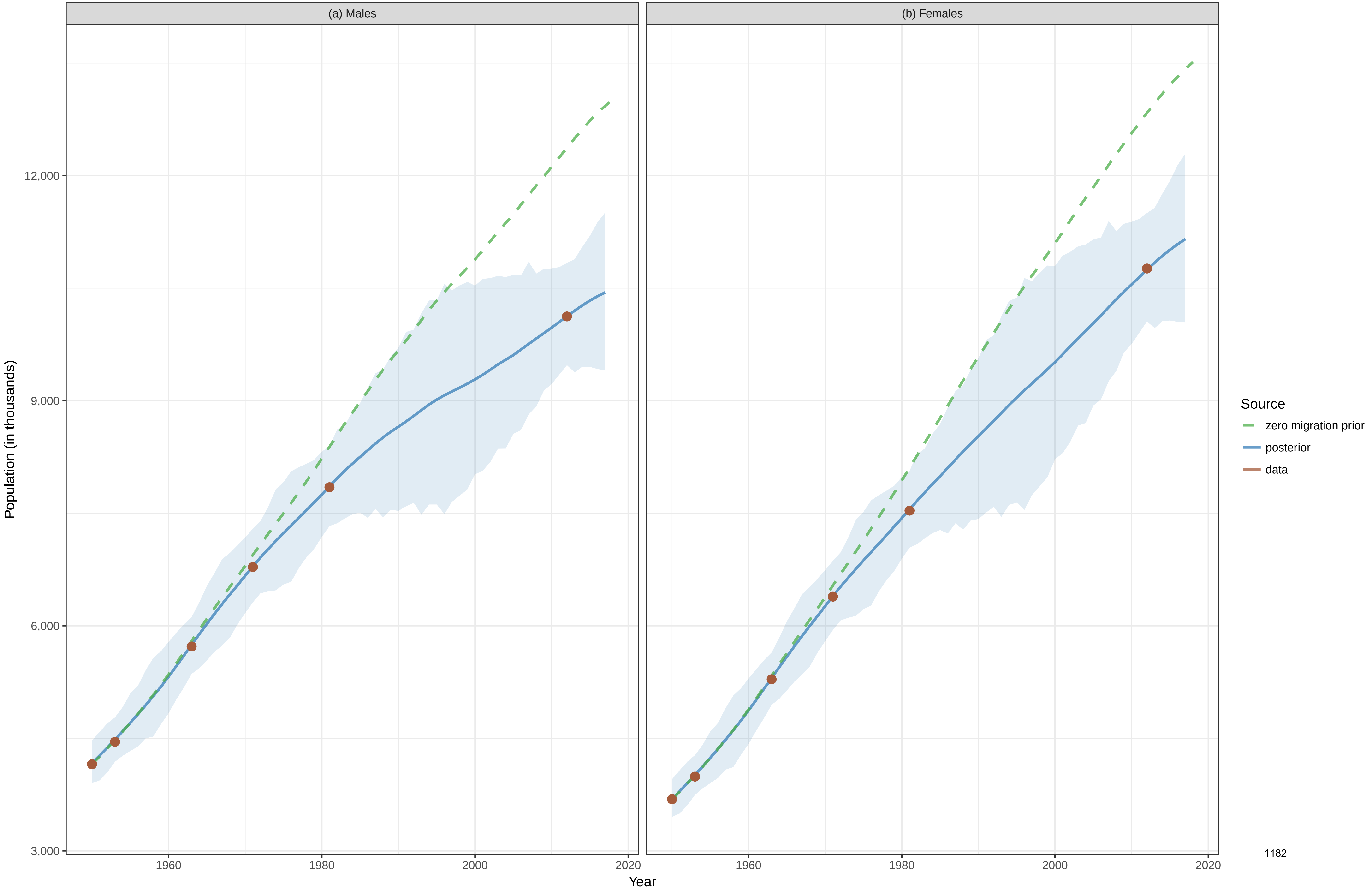

Supplemental Results Figure 4: GBD Bayesian Demographic Balancing Model fit in Sudan for (a) total population of males, and (b) total population of females, 1950−2017.

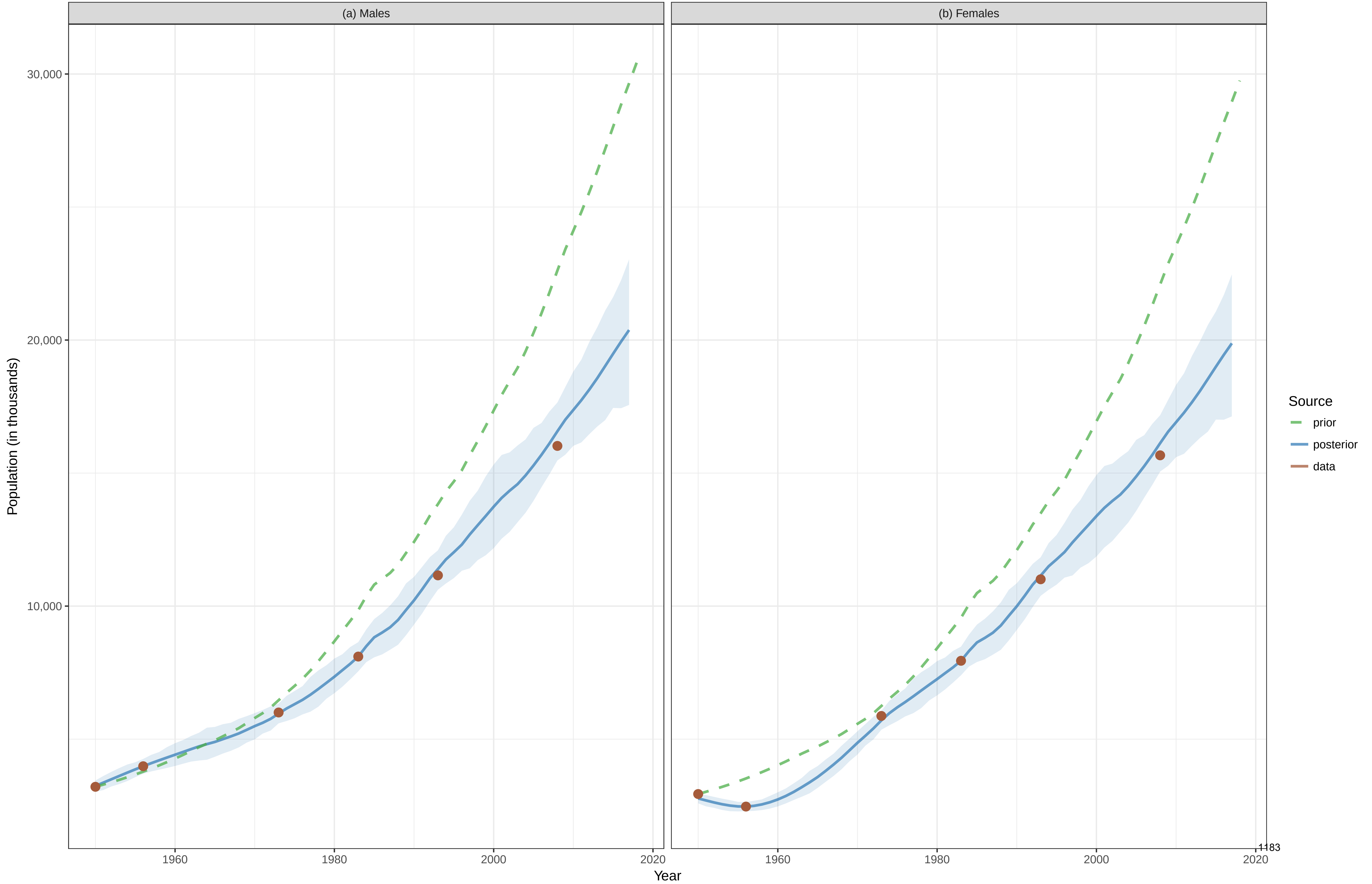

Supplemental Results Figure 4: GBD Bayesian Demographic Balancing Model fit in Suriname for (a) total population of males, and (b) total population of females, 1950−2017.

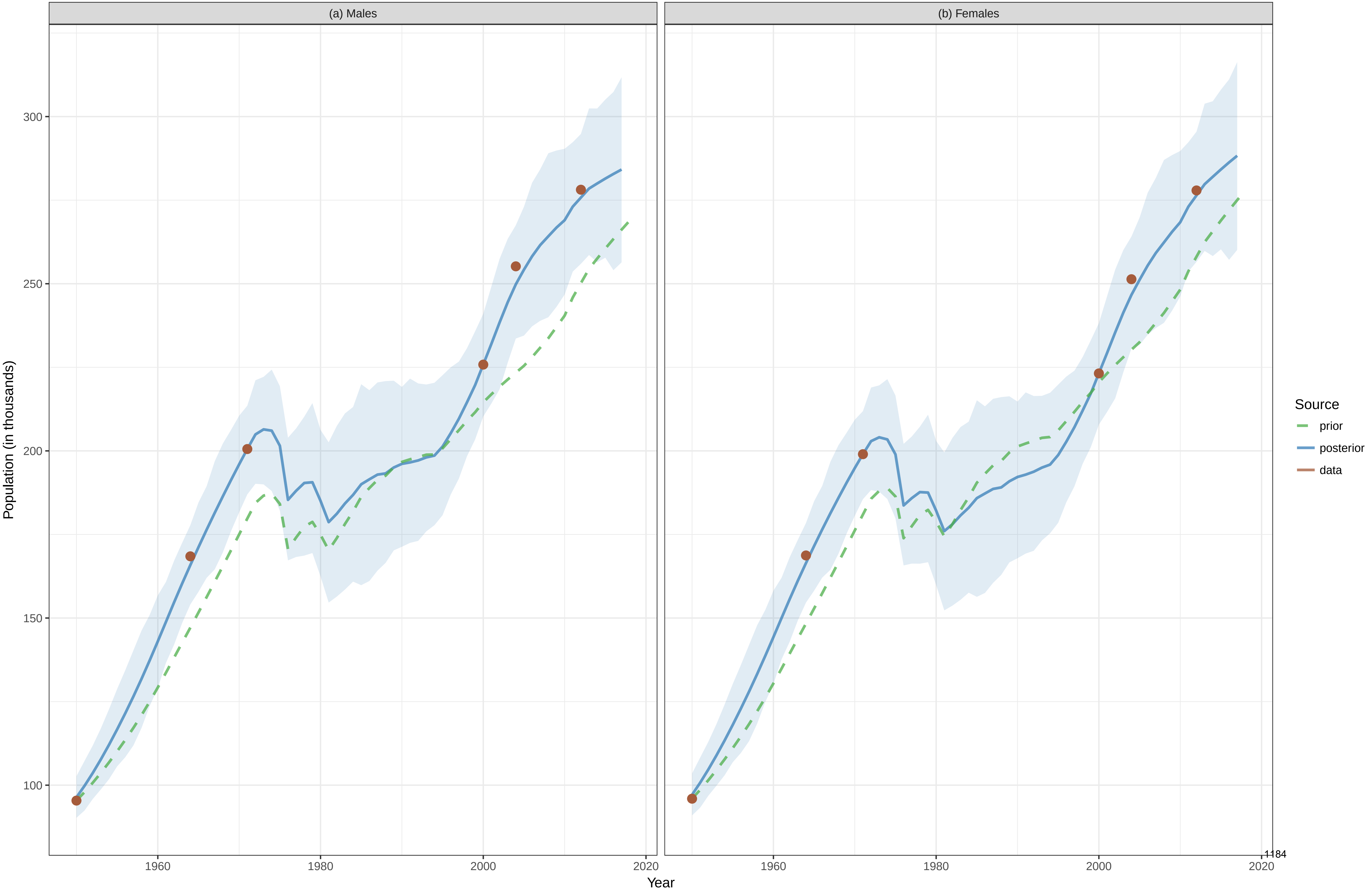

Supplemental Results Figure 4: GBD Bayesian Demographic Balancing Model fit in Swaziland for (a) total population of males, and (b) total population of females, 1950−2017.

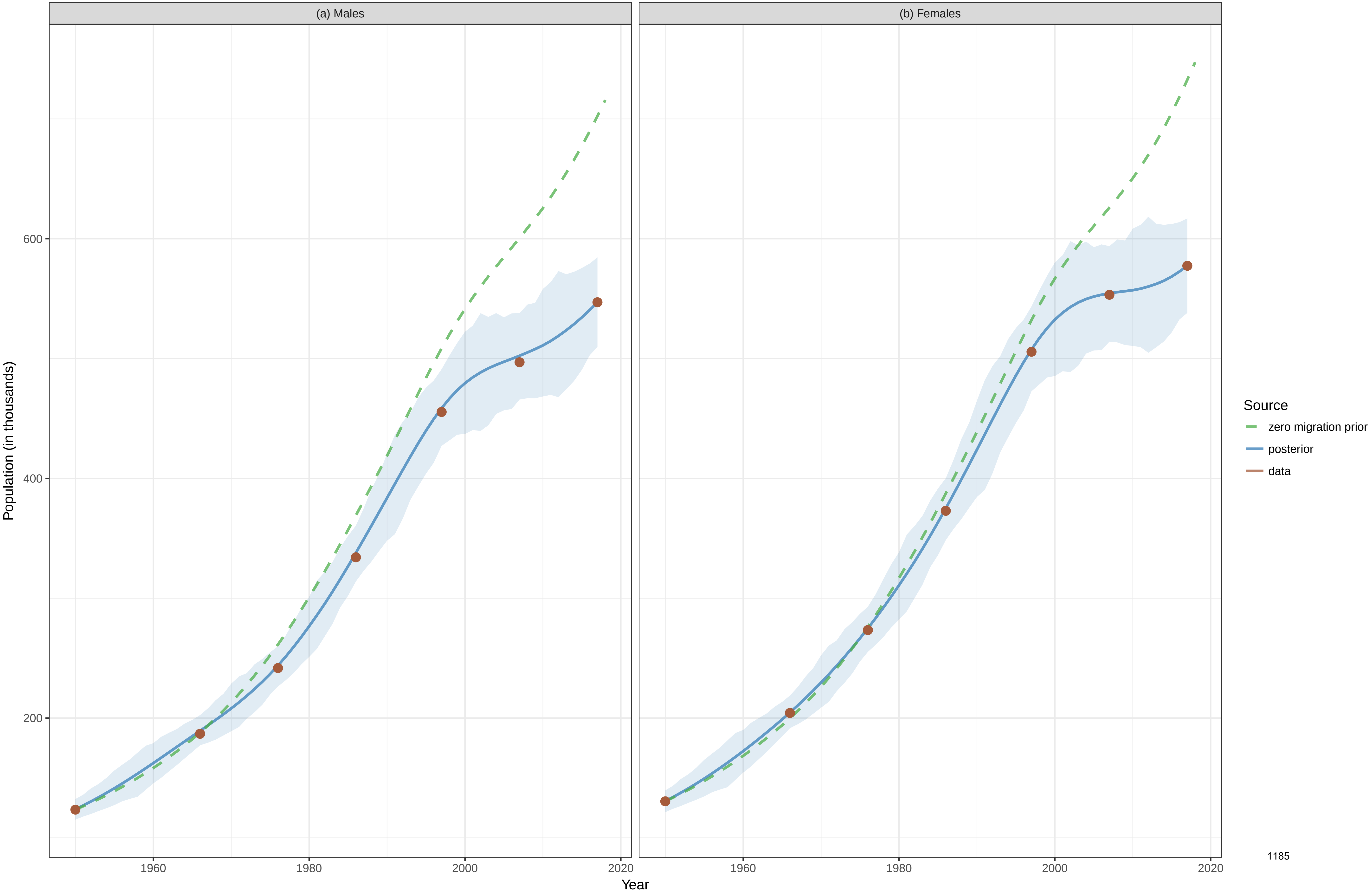

Supplemental Results Figure 4: GBD Bayesian Demographic Balancing Model fit in Sweden for (a) total population of males, and (b) total population of females, 1950−2017.

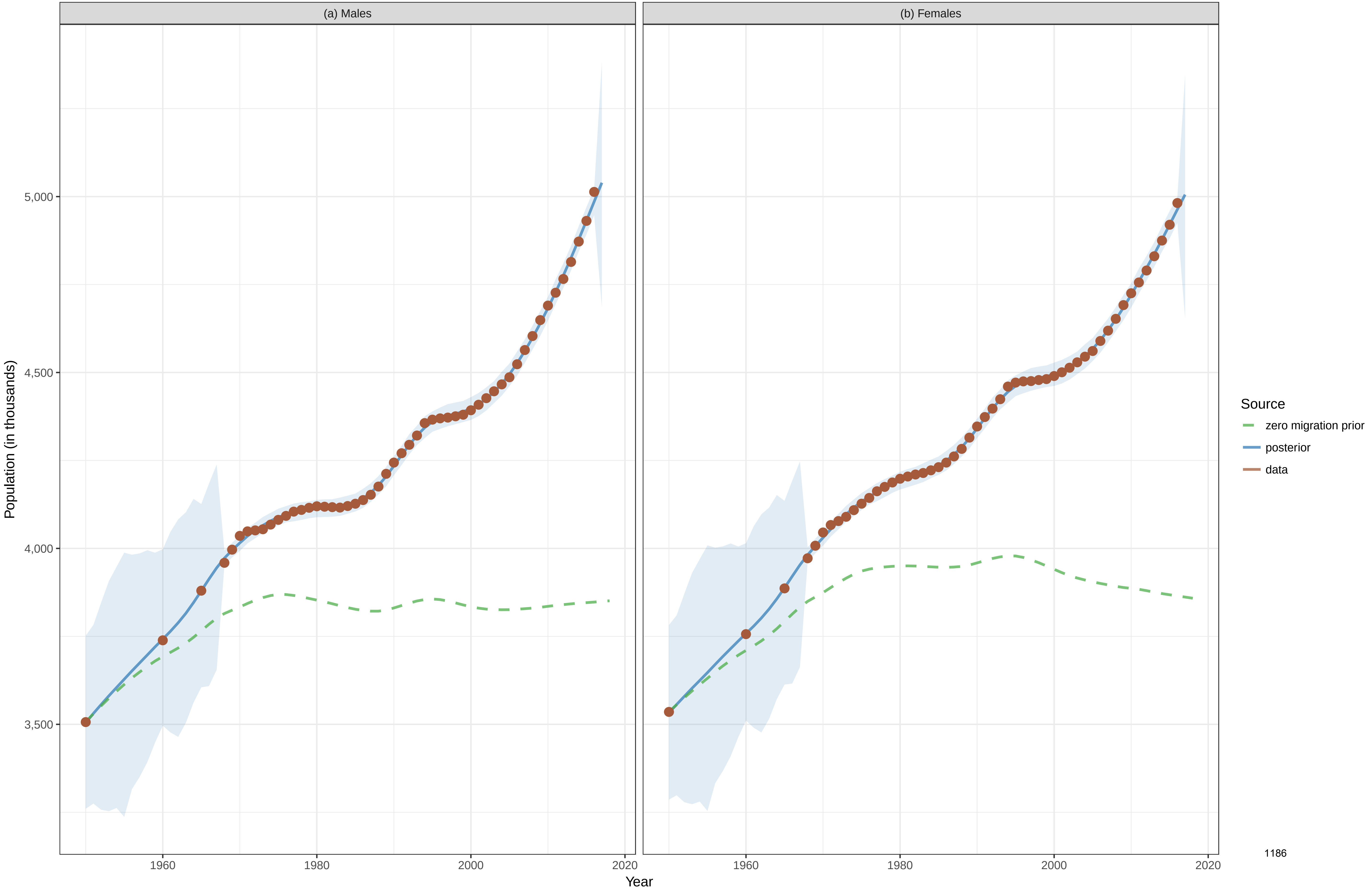

Supplemental Results Figure 4: GBD Bayesian Demographic Balancing Model fit in Switzerland for (a) total population of males, and (b) total population of females, 1950−2017.

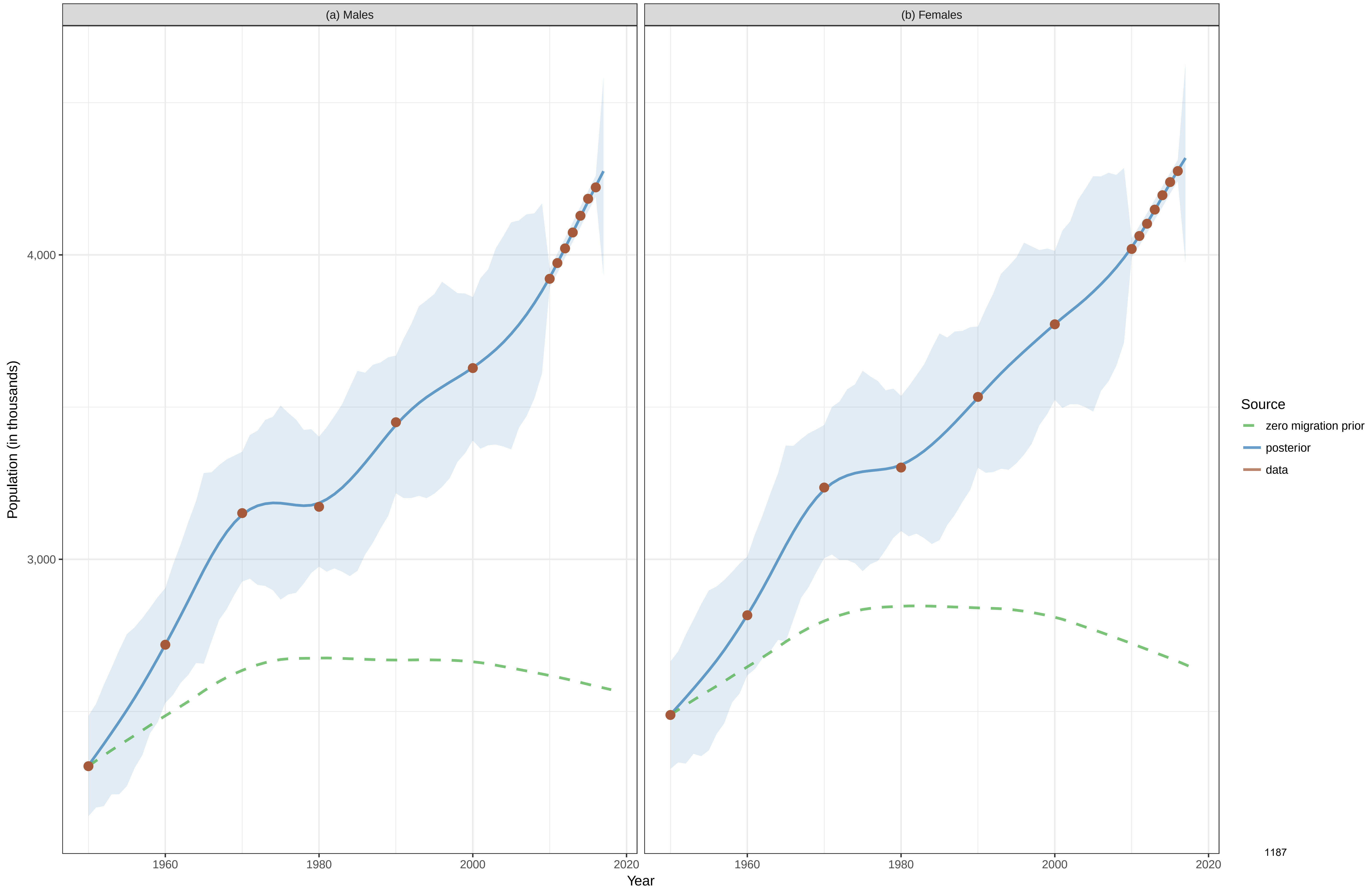

Supplemental Results Figure 4: GBD Bayesian Demographic Balancing Model fit in Syria for (a) total population of males, and (b) total population of females, 1950−2017.

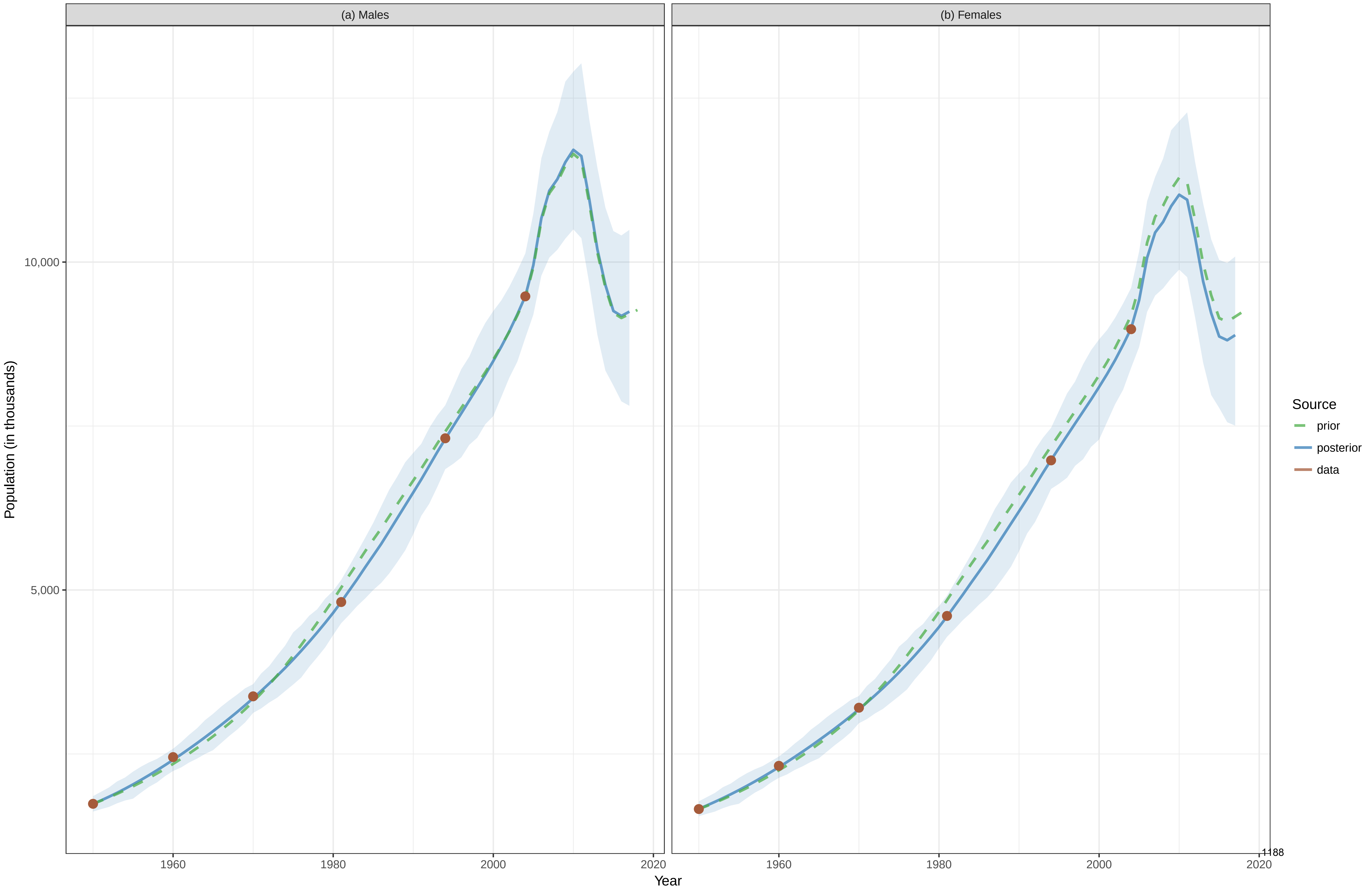

Supplemental Results Figure 4: GBD Bayesian Demographic Balancing Model fit in Taiwan for (a) total population of males, and (b) total population of females, 1950−2017.

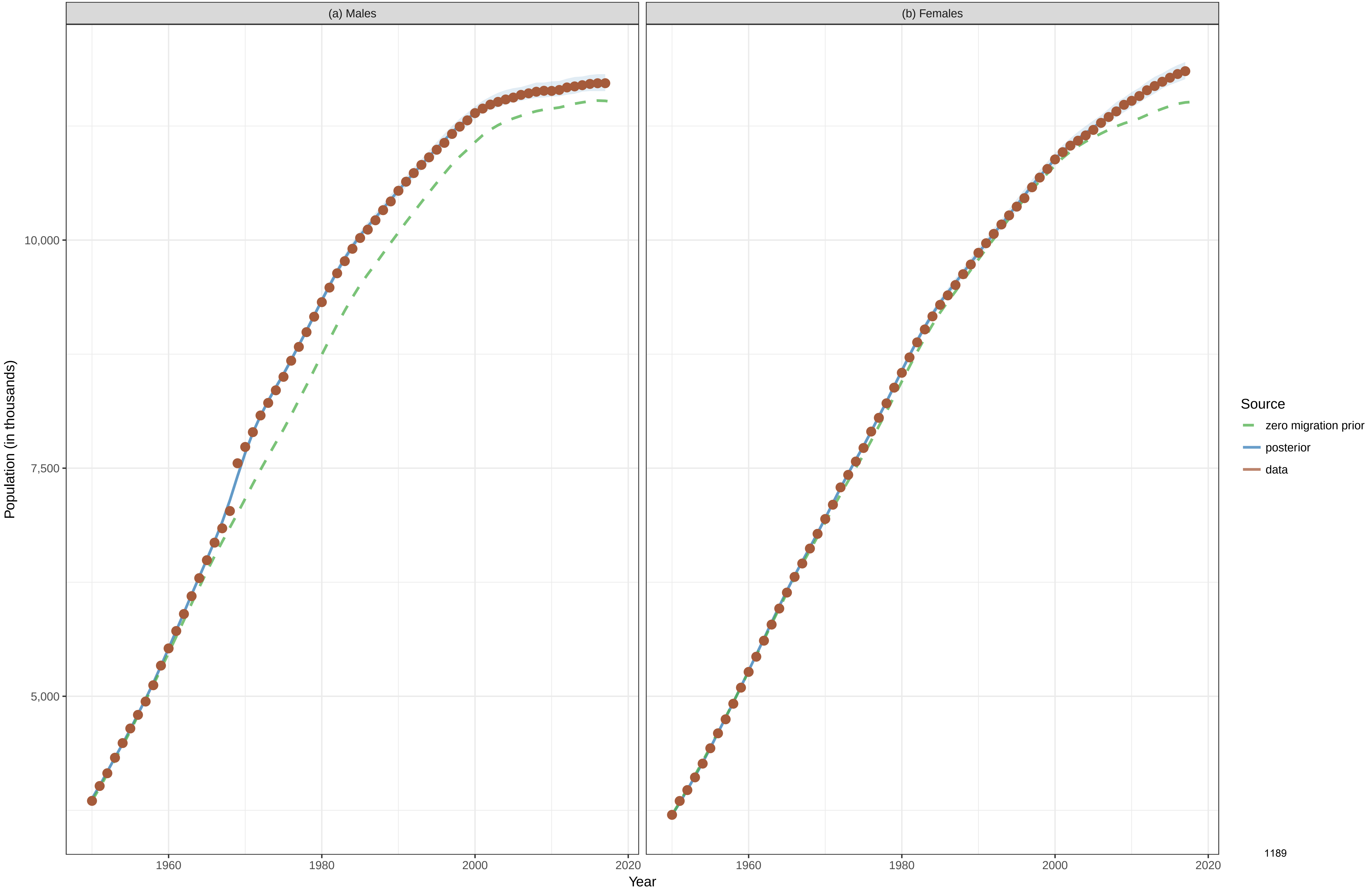

Supplemental Results Figure 4: GBD Bayesian Demographic Balancing Model fit in Tajikistan for (a) total population of males, and (b) total population of females, 1950−2017.

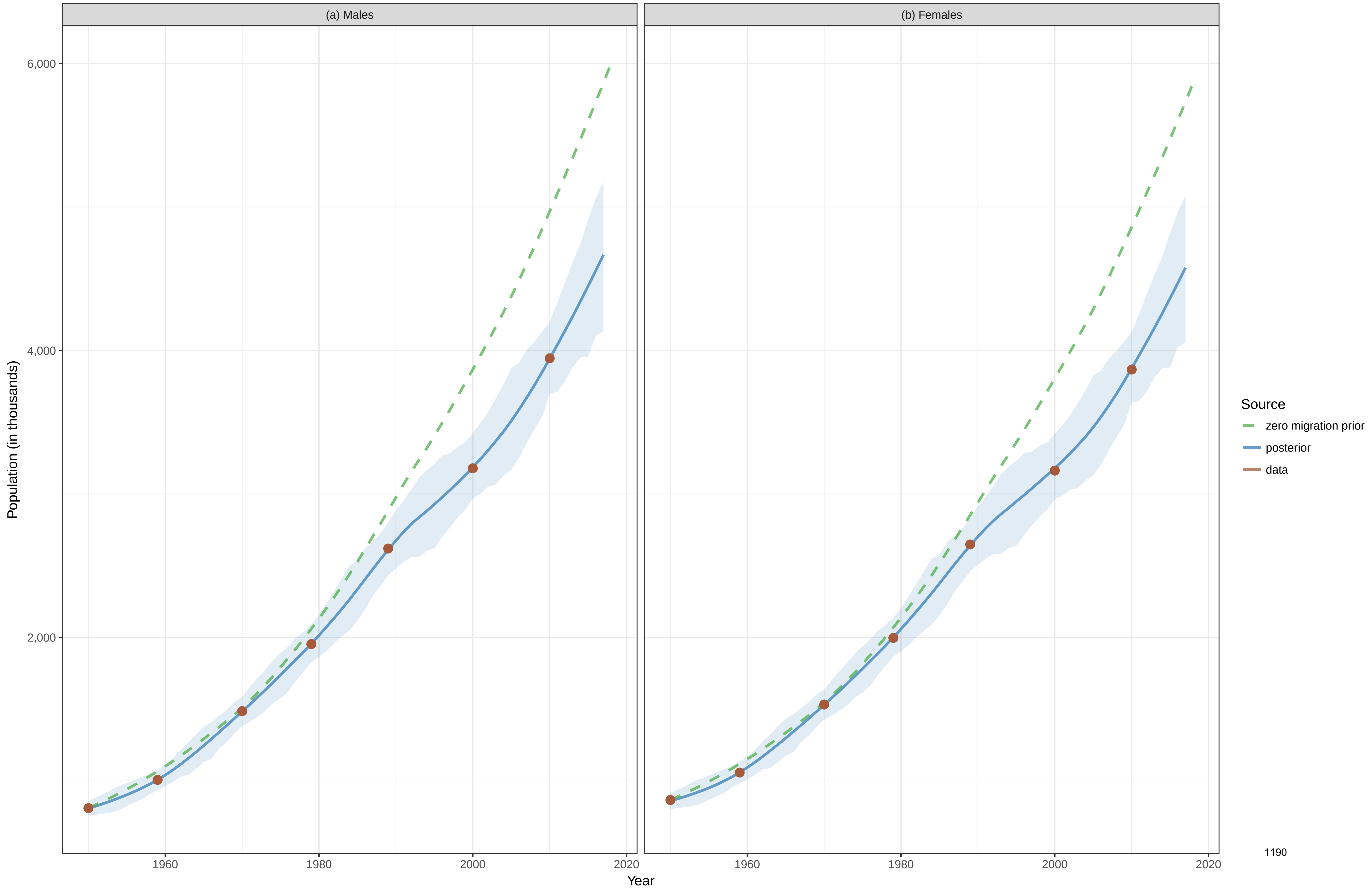

Supplemental Results Figure 4: GBD Bayesian Demographic Balancing Model fit in Tanzania for (a) total population of males, and (b) total population of females, 1950−2017.

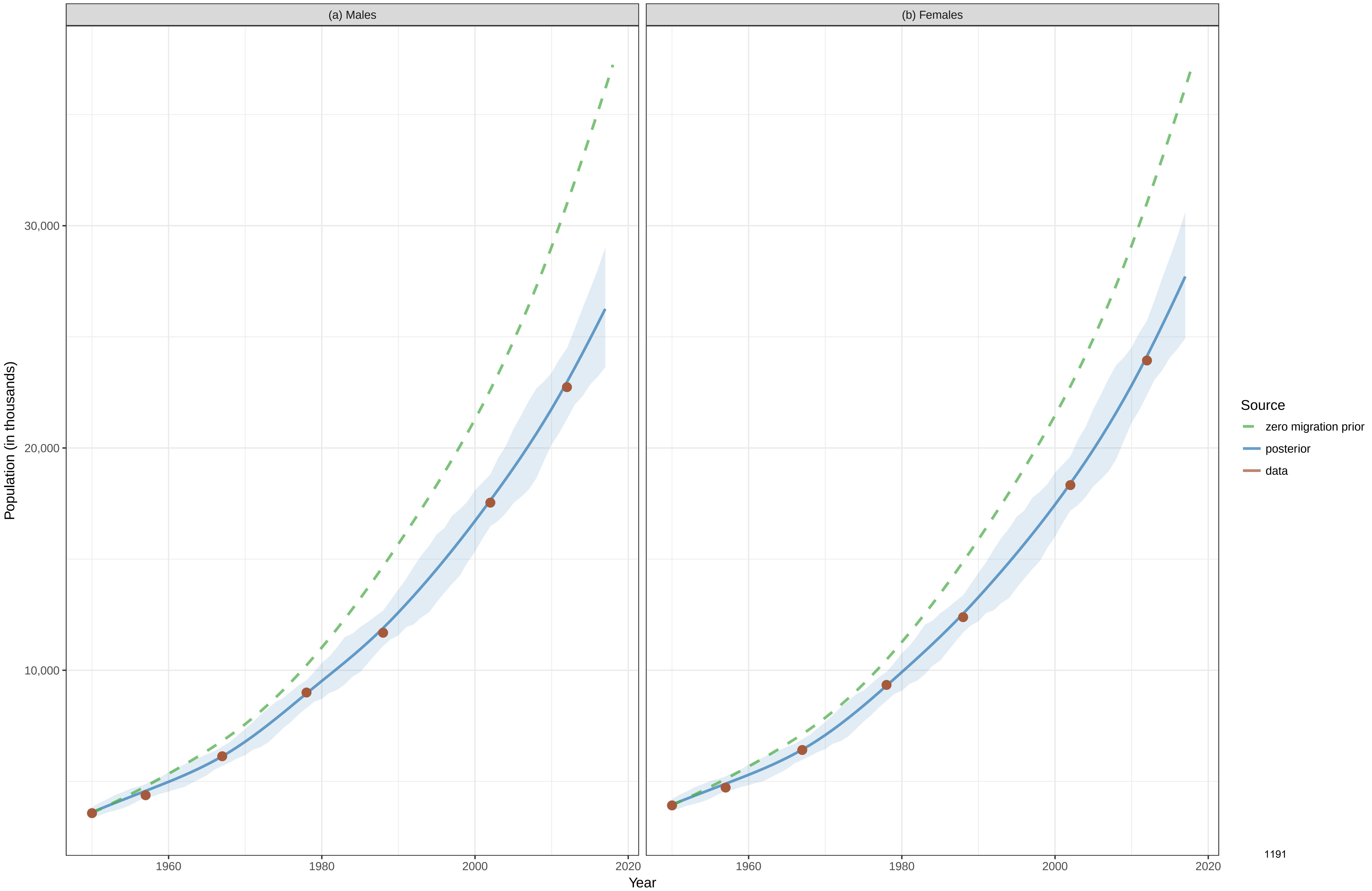

Supplemental Results Figure 4: GBD Bayesian Demographic Balancing Model fit in Thailand for (a) total population of males, and (b) total population of females, 1950−2017.

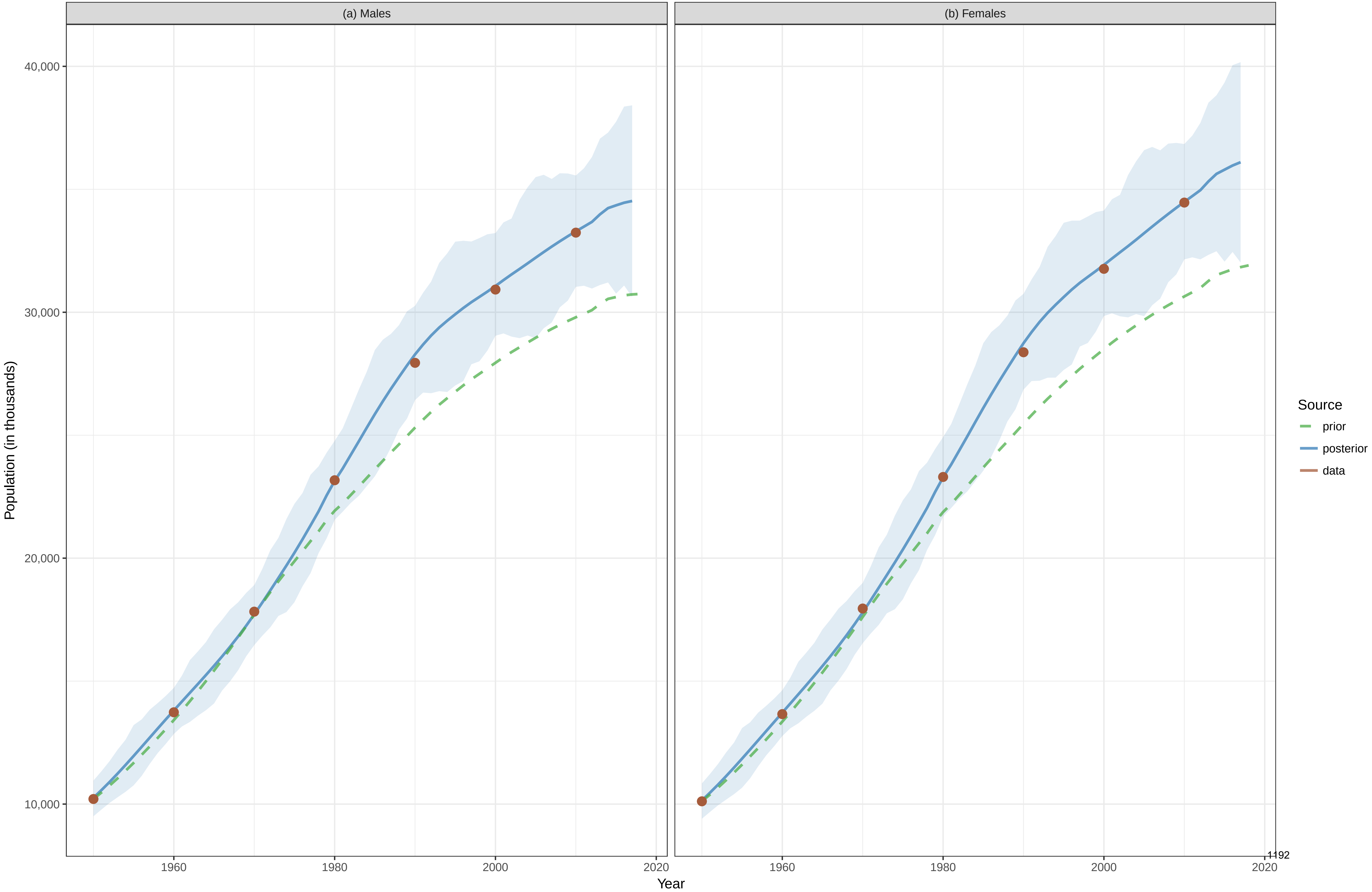

Supplemental Results Figure 4: GBD Bayesian Demographic Balancing Model fit in The Bahamas for (a) total population of males, and (b) total population of females, 1950−2017.

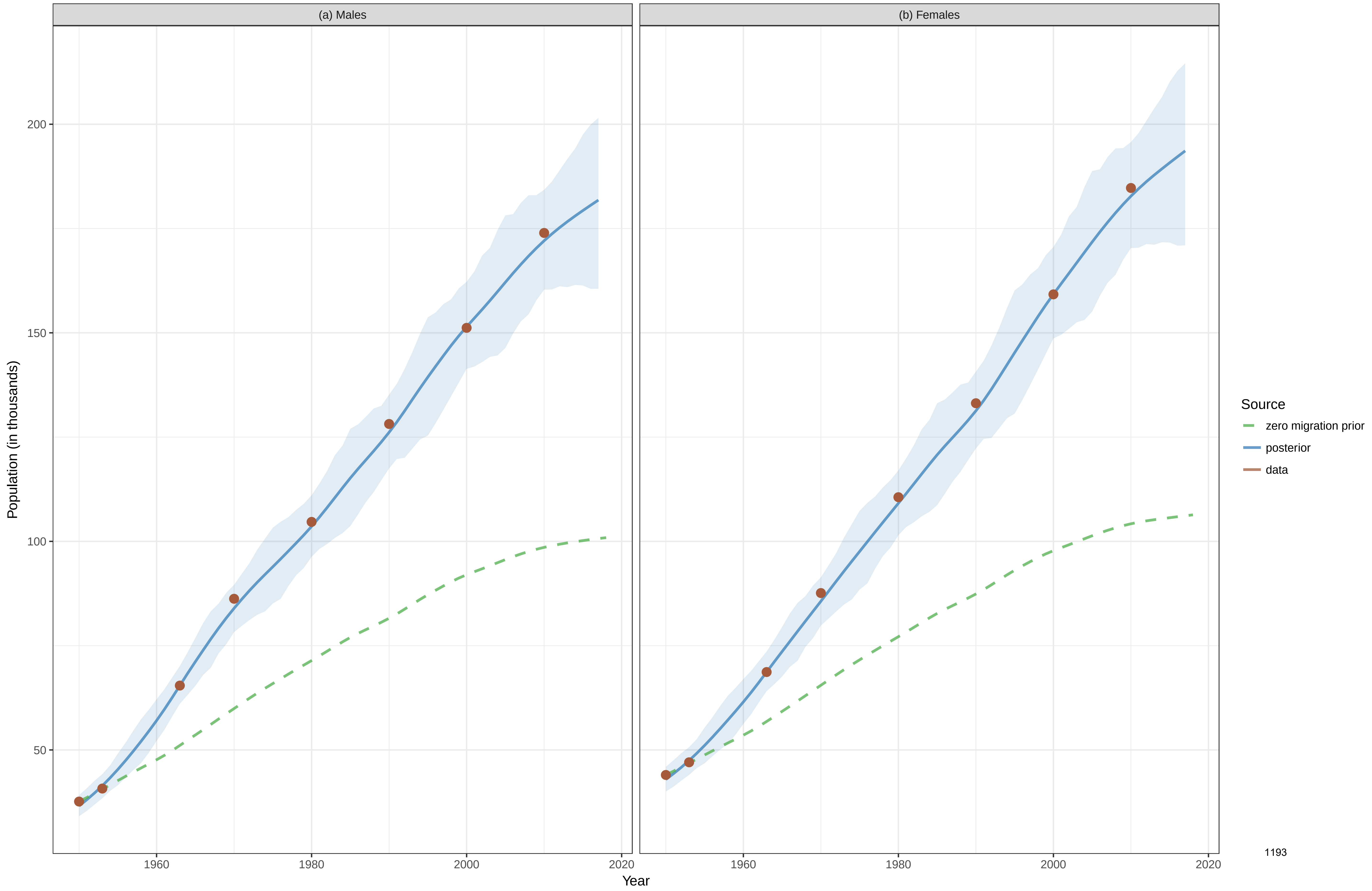

Supplemental Results Figure 4: GBD Bayesian Demographic Balancing Model fit in The Gambia for (a) total population of males, and (b) total population of females, 1950−2017.

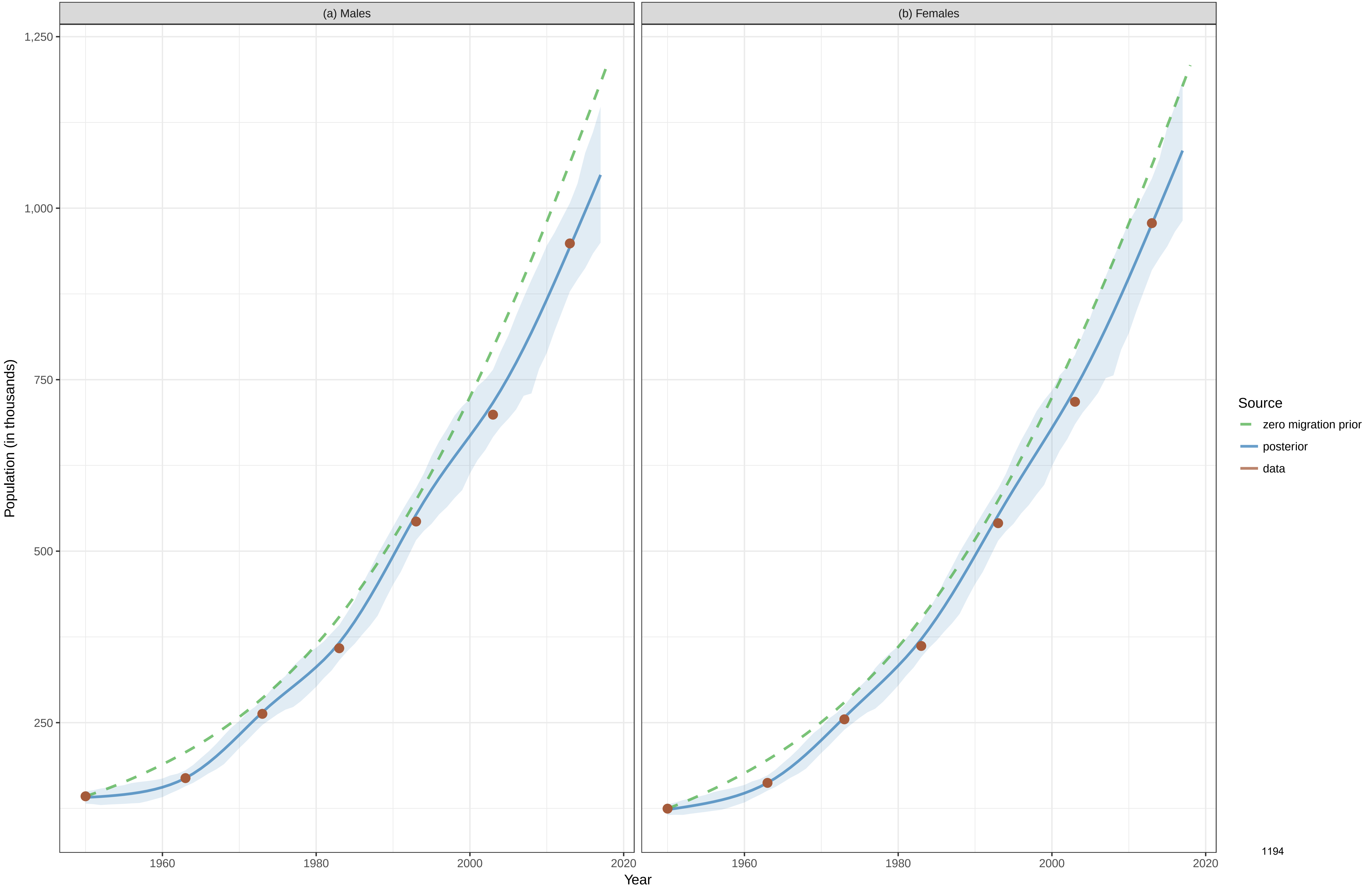

Supplemental Results Figure 4: GBD Bayesian Demographic Balancing Model fit in Timor−Leste for (a) total population of males, and (b) total population of females, 1950−2017.

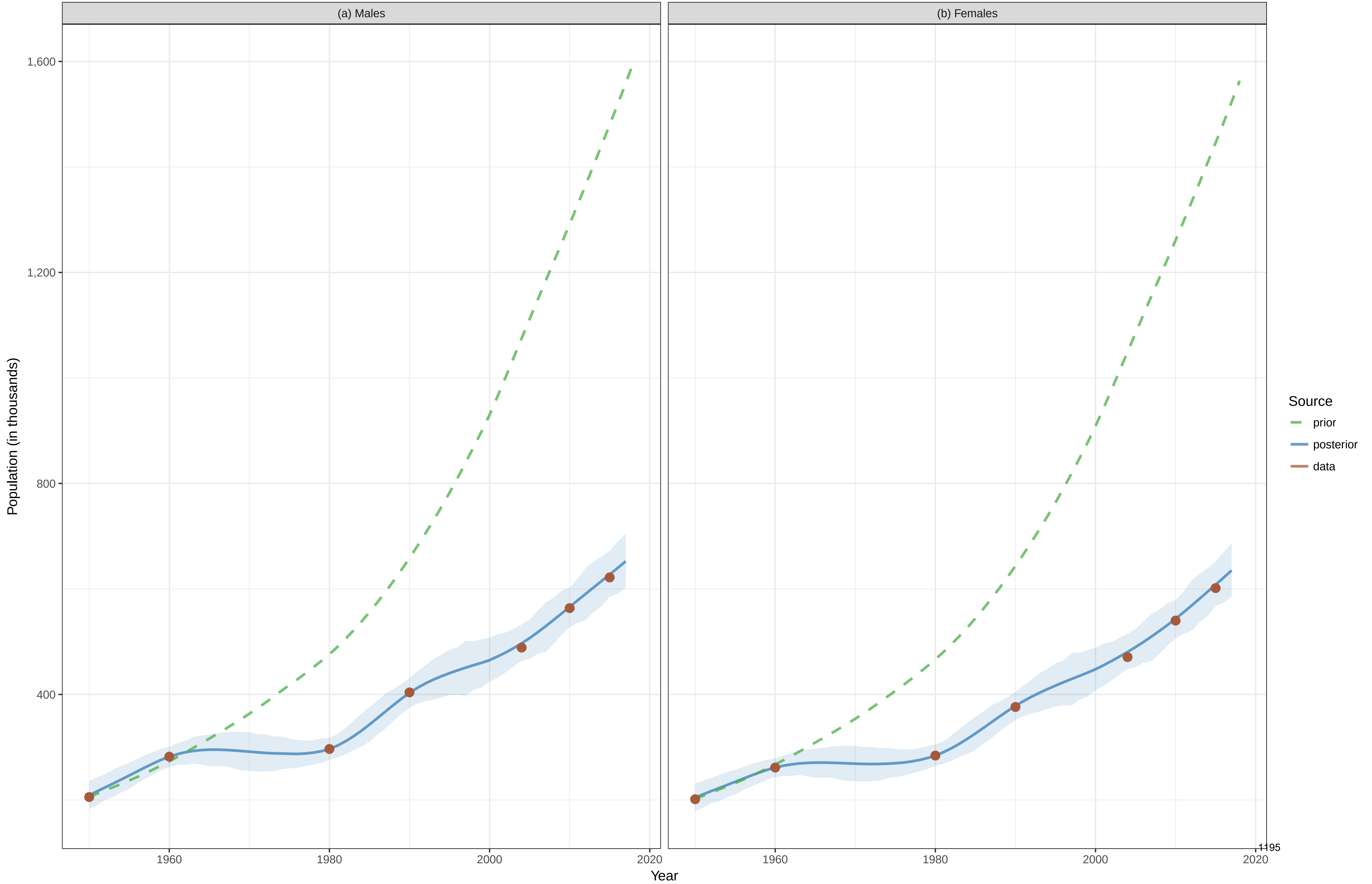

Supplemental Results Figure 4: GBD Bayesian Demographic Balancing Model fit in Togo for (a) total population of males, and (b) total population of females, 1950−2017.

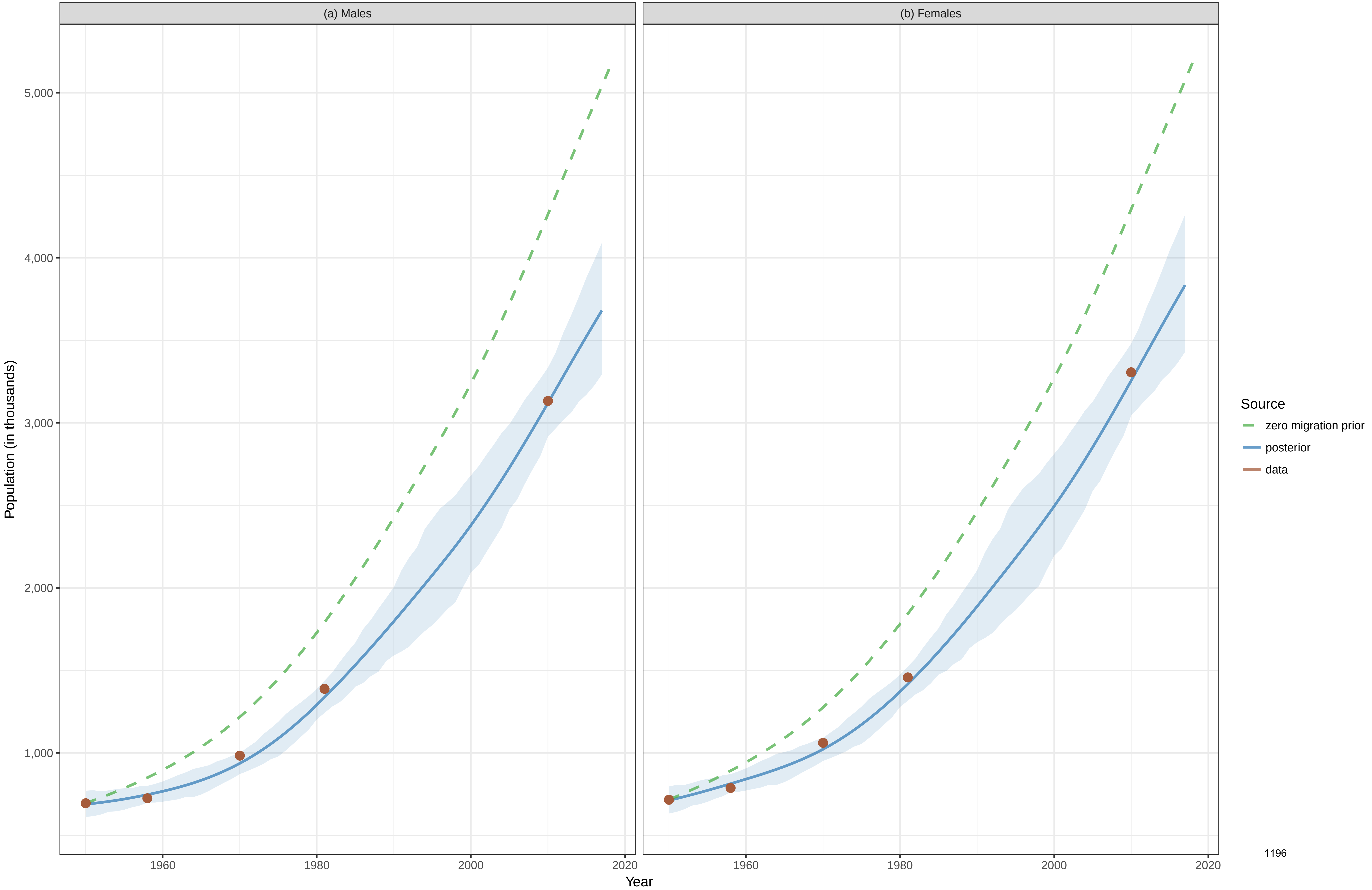

Supplemental Results Figure 4: GBD Bayesian Demographic Balancing Model fit in Tonga for (a) total population of males, and (b) total population of females, 1950−2017.

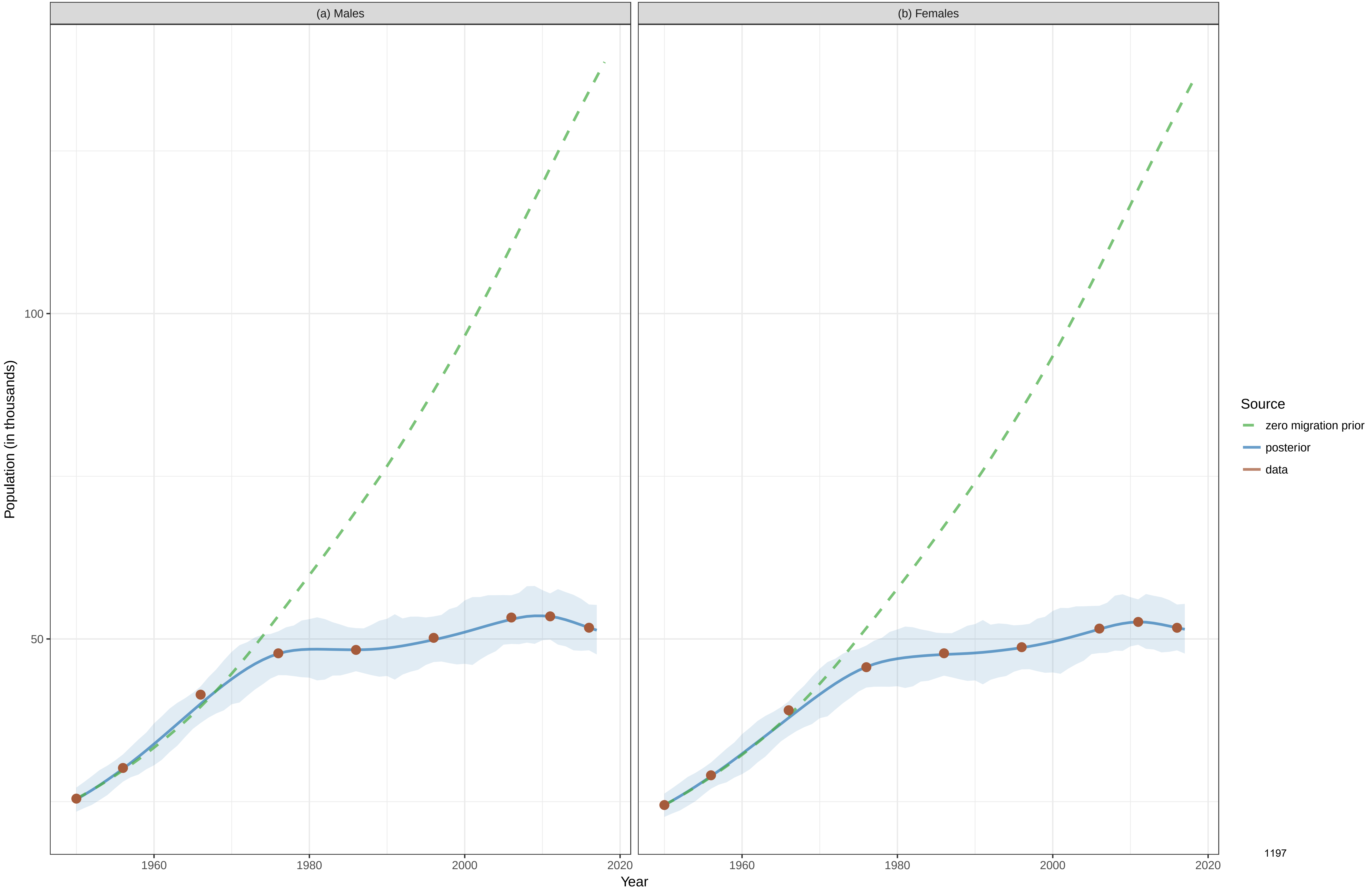

Supplemental Results Figure 4: GBD Bayesian Demographic Balancing Model fit in Trinidad and Tobago for (a) total population of males, and (b) total population of females, 1950−2017.

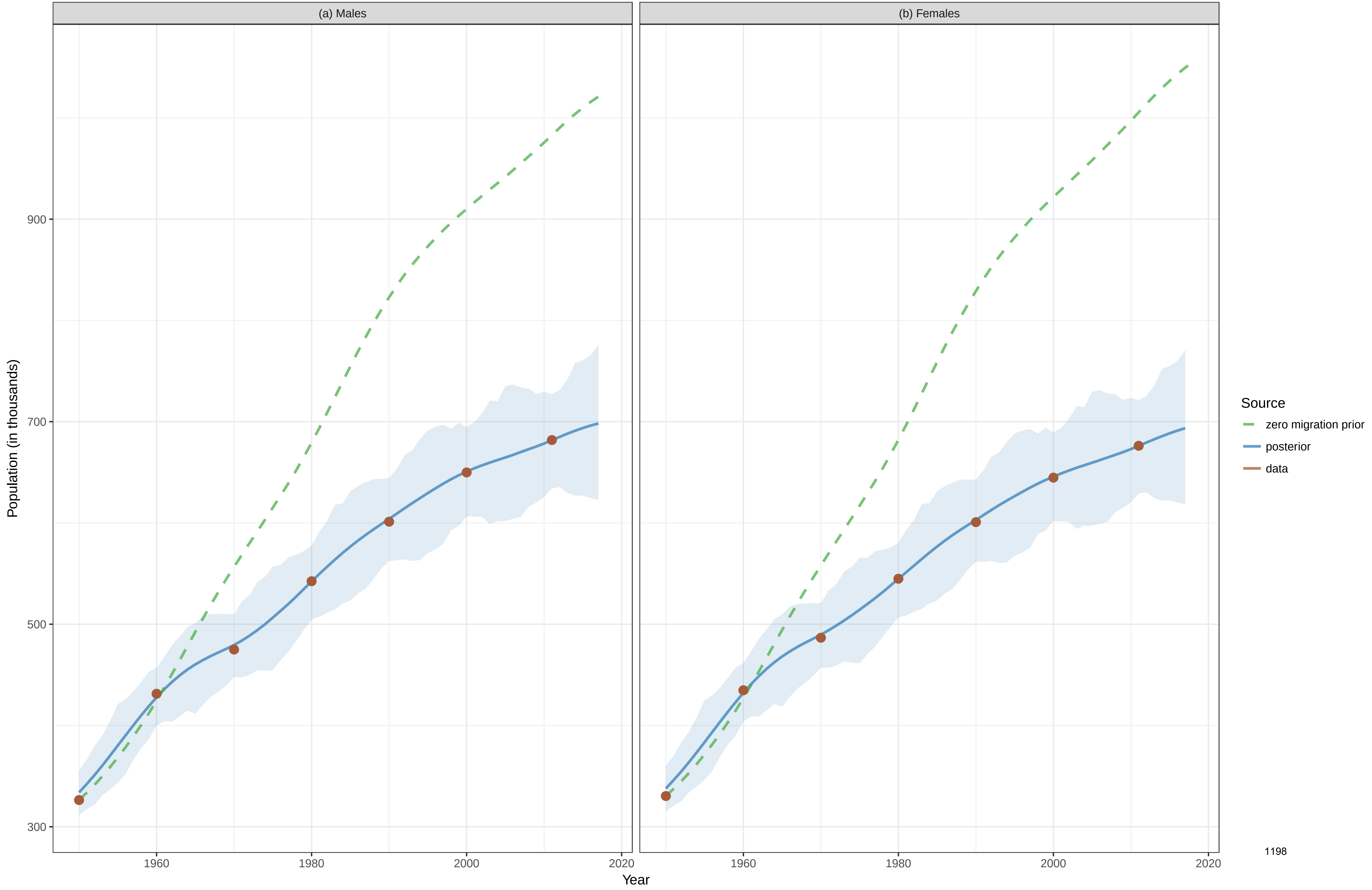

Supplemental Results Figure 4: GBD Bayesian Demographic Balancing Model fit in Tunisia for (a) total population of males, and (b) total population of females, 1950−2017.

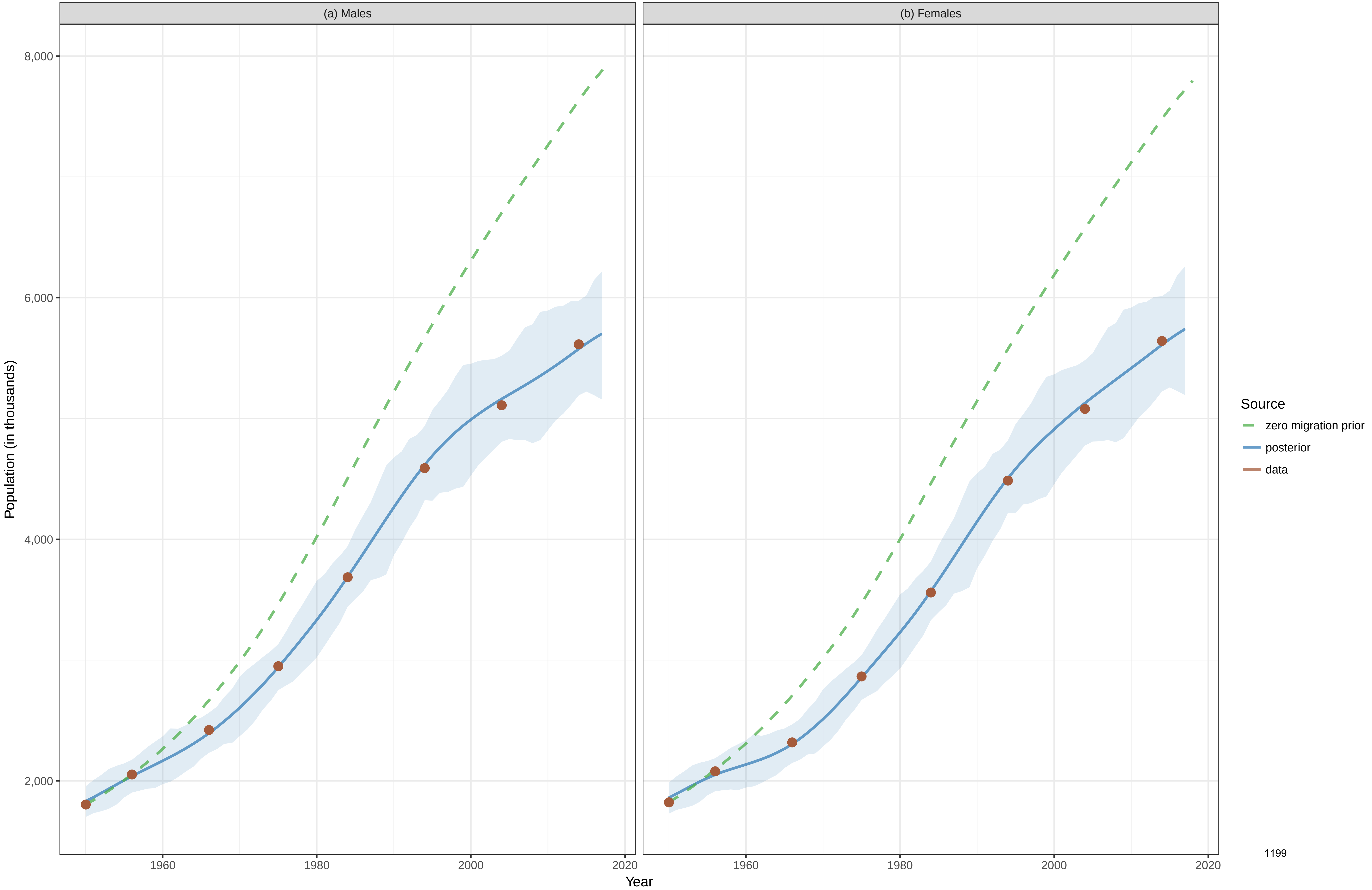

Supplemental Results Figure 4: GBD Bayesian Demographic Balancing Model fit in Turkey for (a) total population of males, and (b) total population of females, 1950−2017.

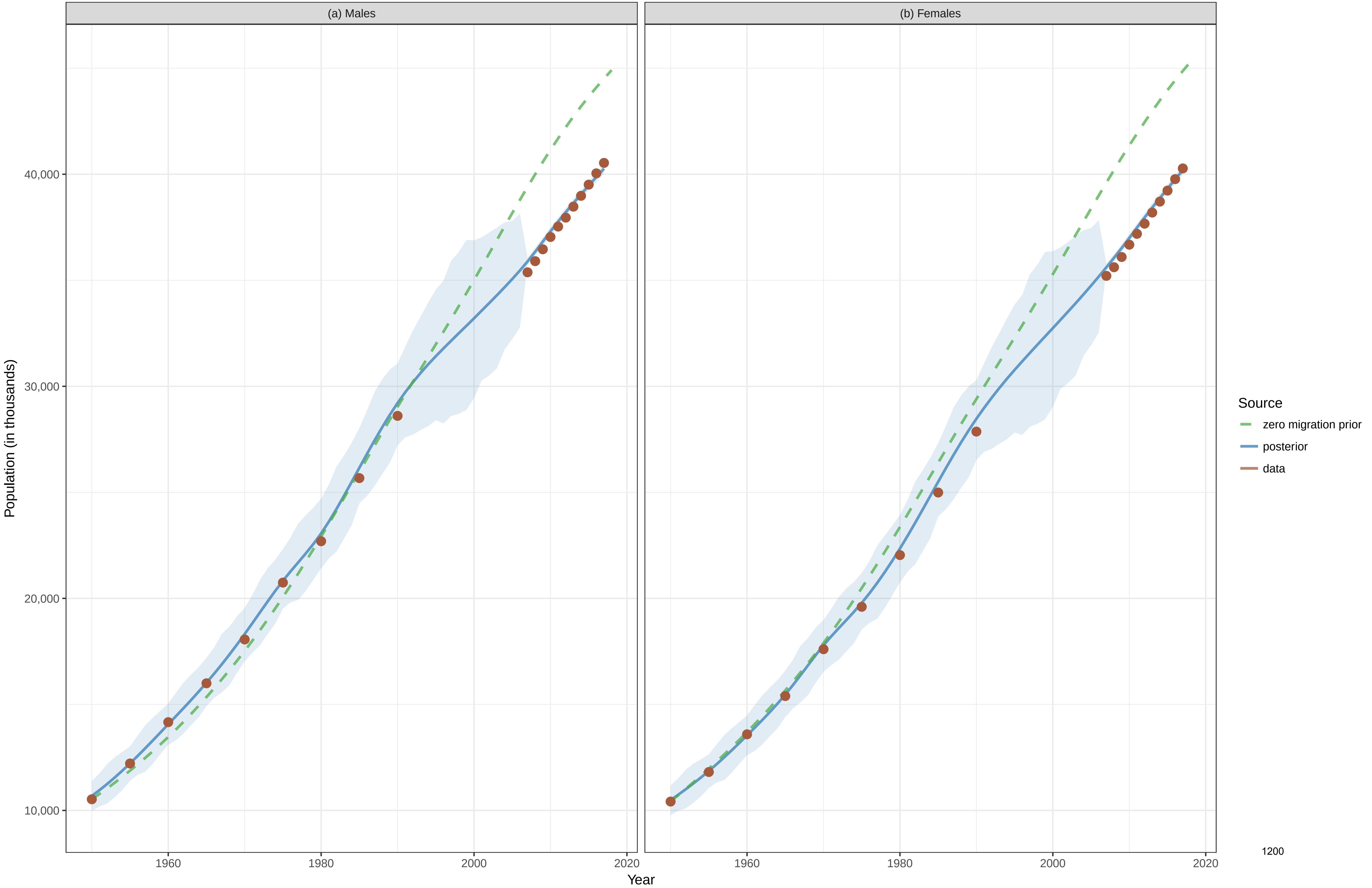

Supplemental Results Figure 4: GBD Bayesian Demographic Balancing Model fit in Turkmenistan for (a) total population of males, and (b) total population of females, 1950−2017.

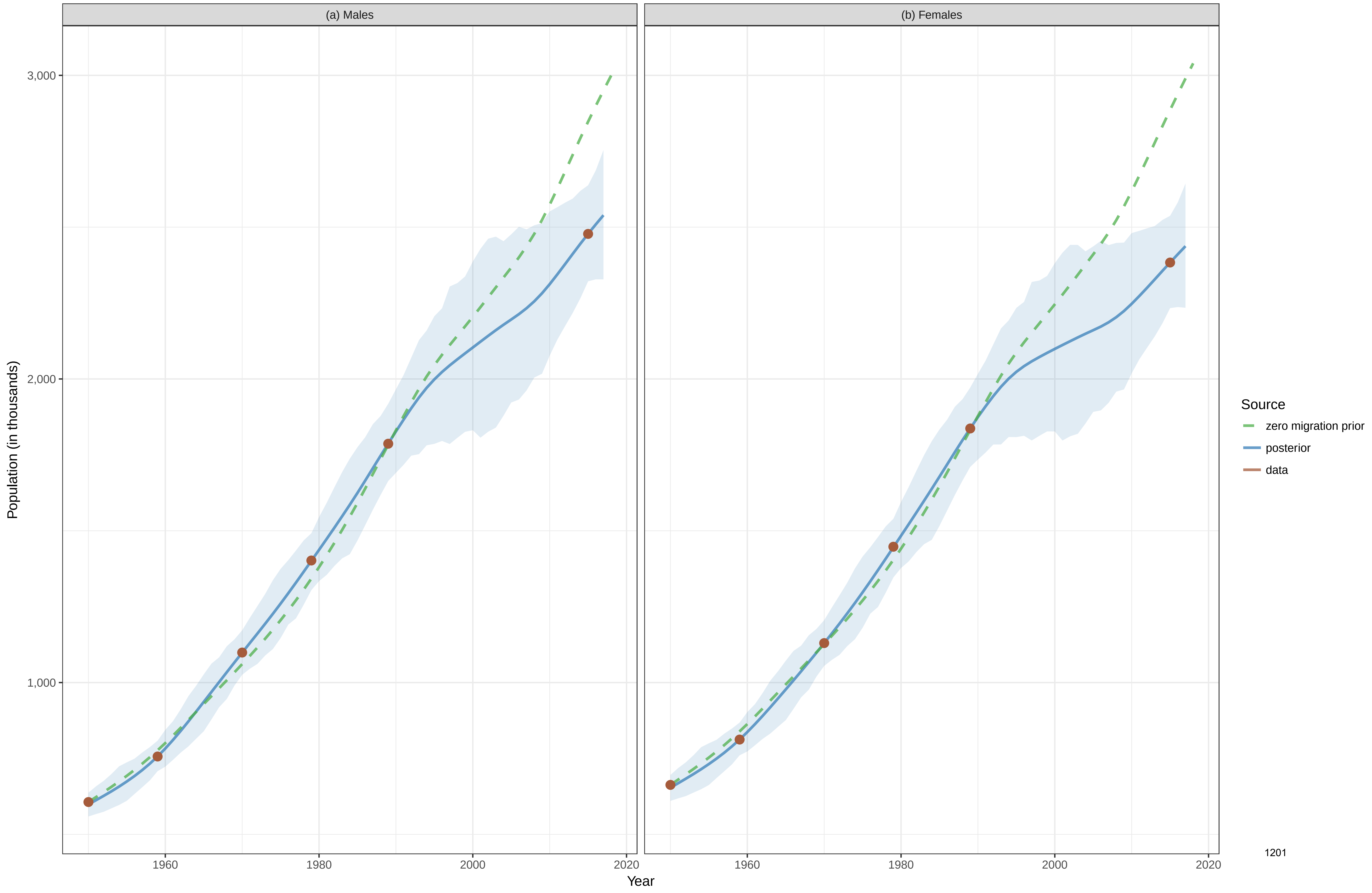

Supplemental Results Figure 4: GBD Bayesian Demographic Balancing Model fit in Uganda for (a) total population of males, and (b) total population of females, 1950−2017.

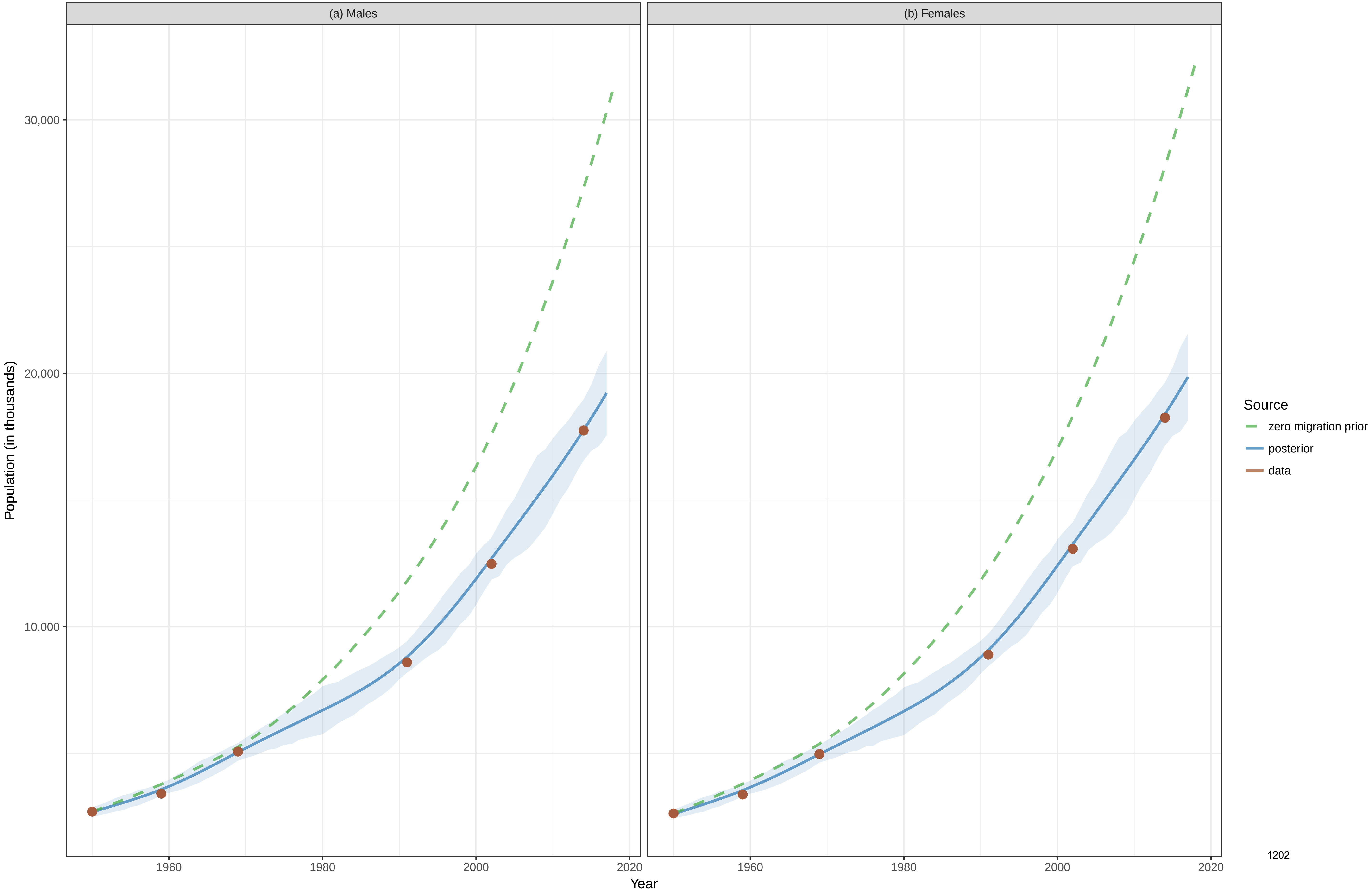

Supplemental Results Figure 4: GBD Bayesian Demographic Balancing Model fit in Ukraine for (a) total population of males, and (b) total population of females, 1950−2017.

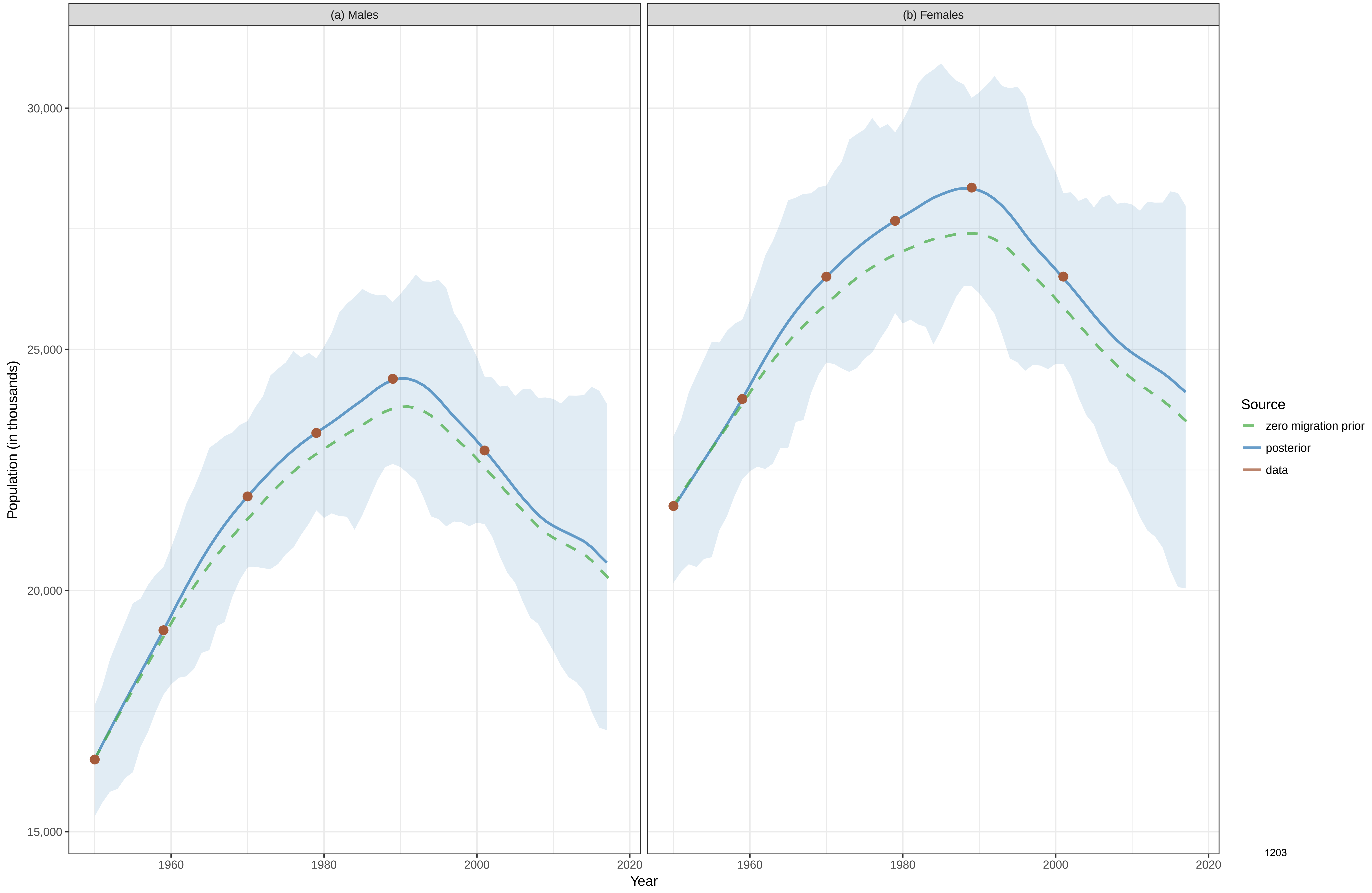

Supplemental Results Figure 4: GBD Bayesian Demographic Balancing Model fit in United Arab Emirates for (a) total population of males, and (b) total population of females, 1950−2017.

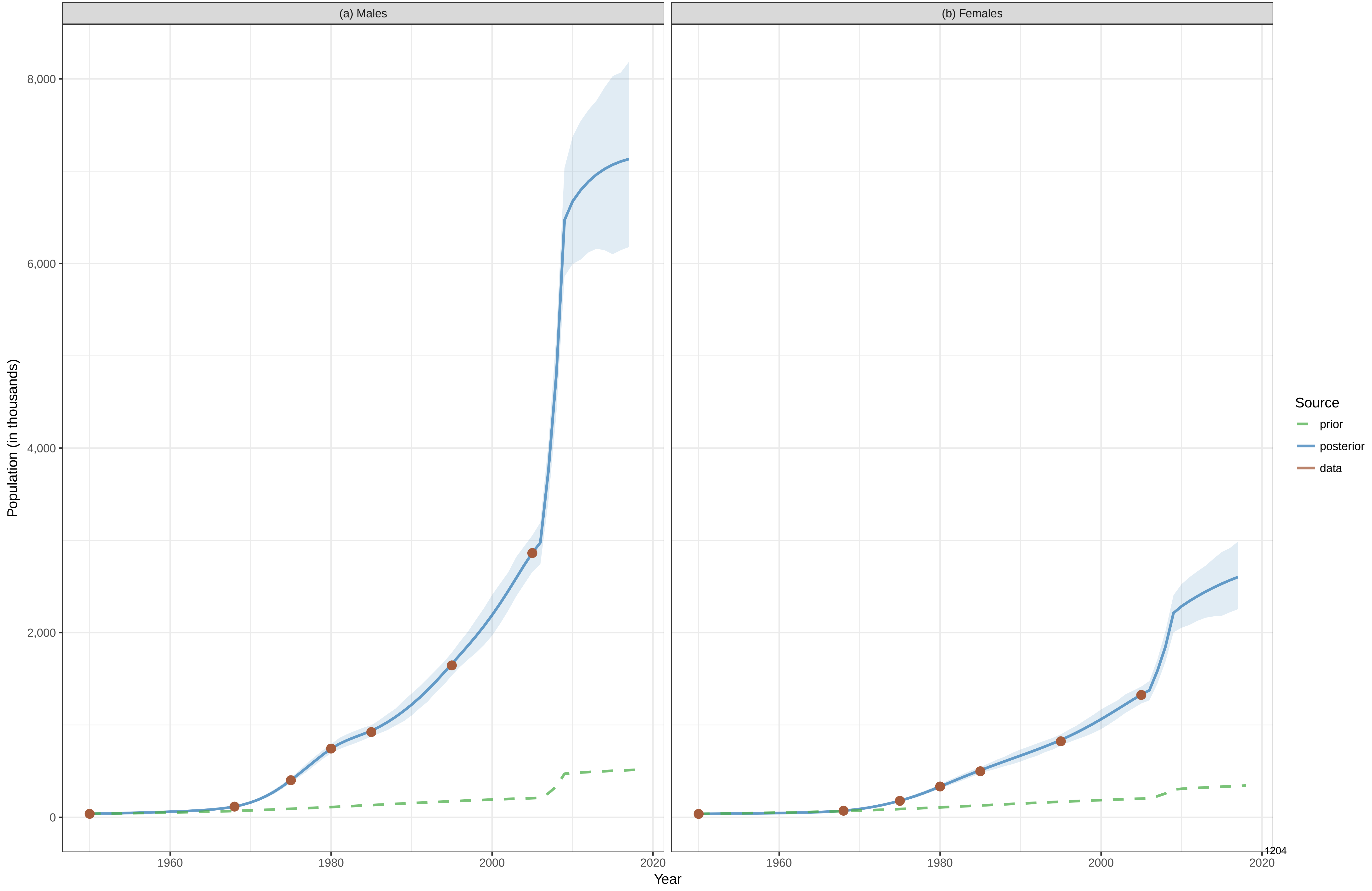

Supplemental Results Figure 4: GBD Bayesian Demographic Balancing Model fit in United States for (a) total population of males, and (b) total population of females, 1950−2017.

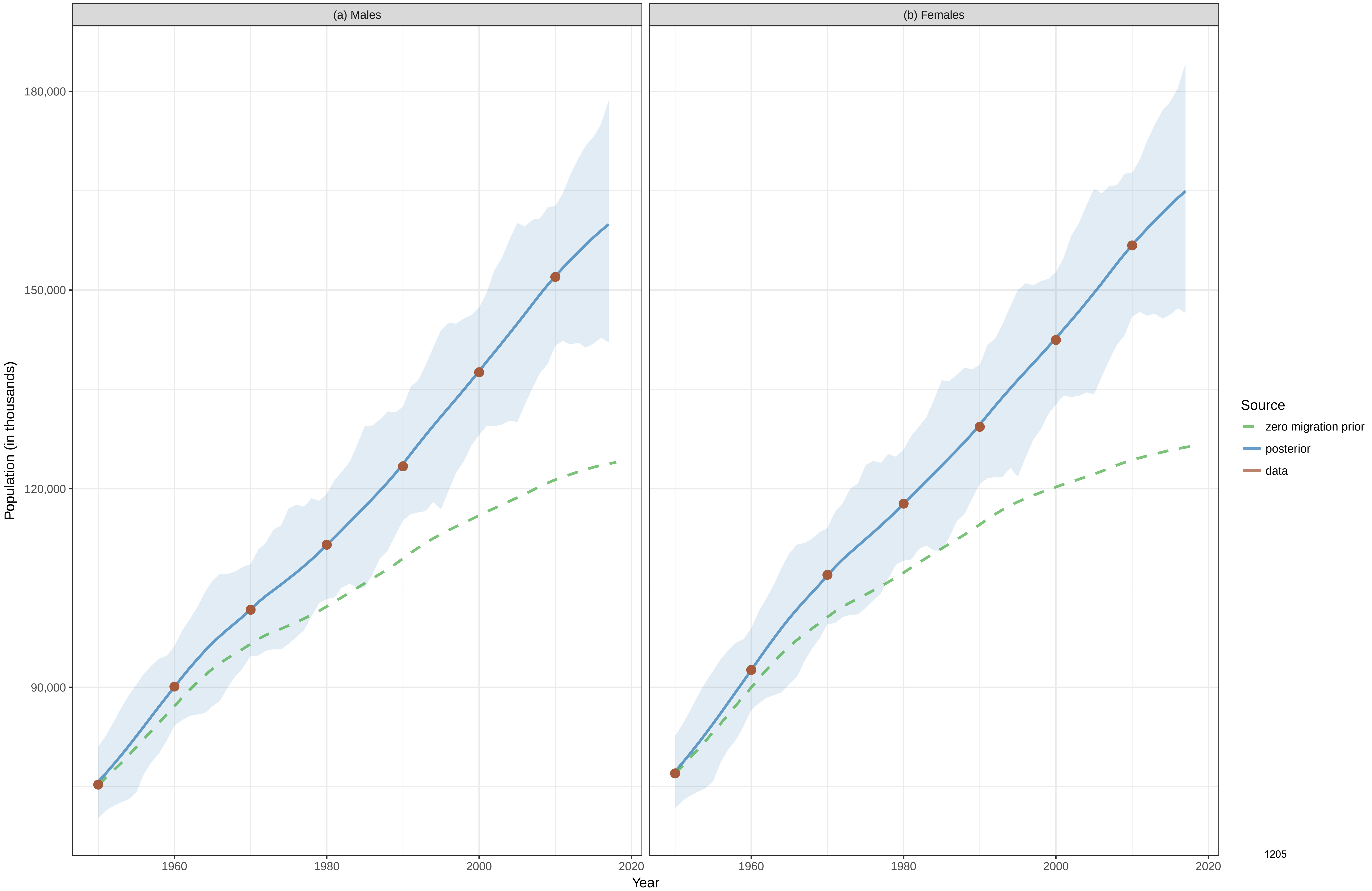

Supplemental Results Figure 4: GBD Bayesian Demographic Balancing Model fit in Uruguay for (a) total population of males, and (b) total population of females, 1950−2017.

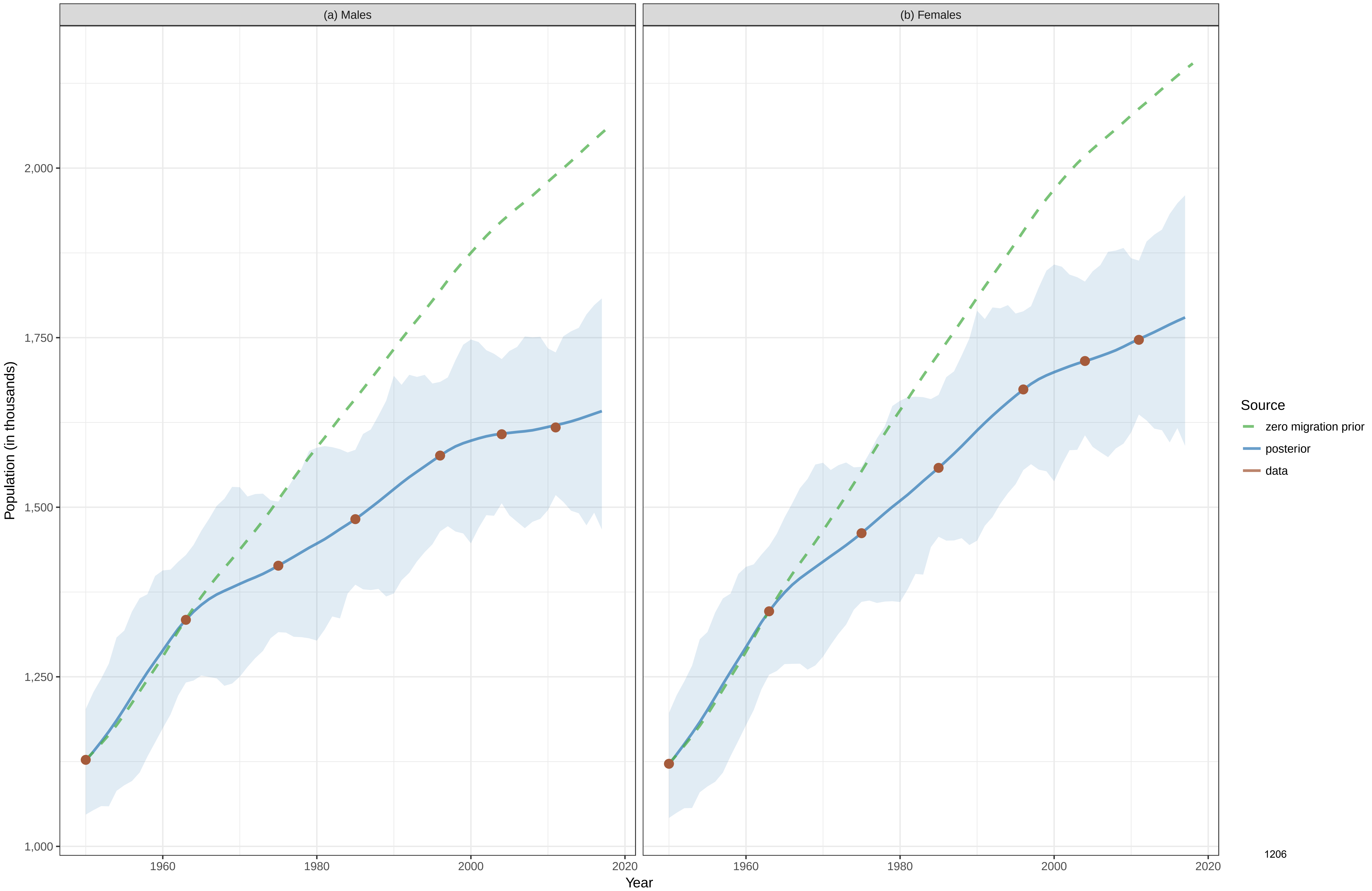

Supplemental Results Figure 4: GBD Bayesian Demographic Balancing Model fit in Uzbekistan for (a) total population of males, and (b) total population of females, 1950−2017.

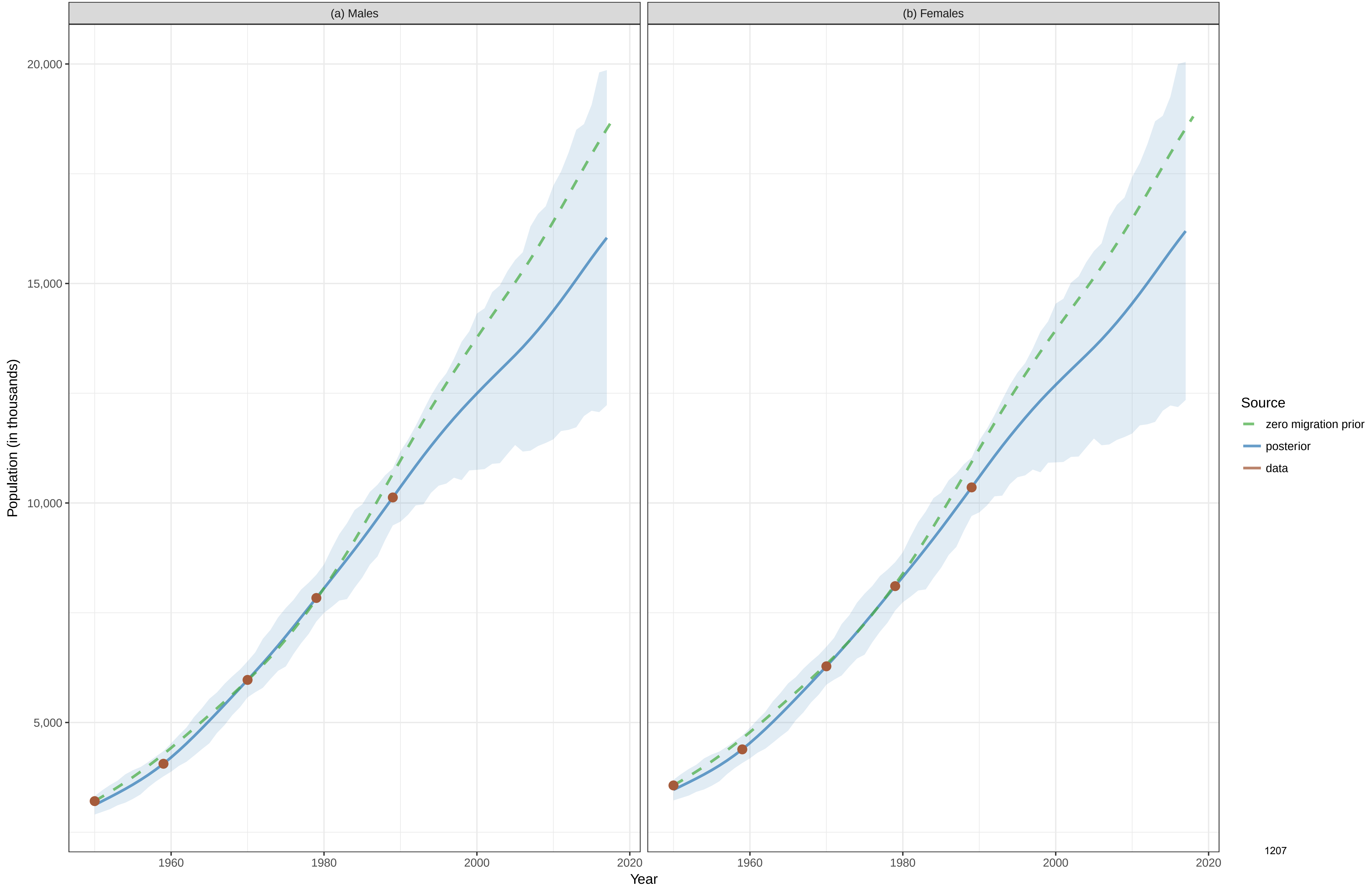

Supplemental Results Figure 4: GBD Bayesian Demographic Balancing Model fit in Vanuatu for (a) total population of males, and (b) total population of females, 1950−2017.

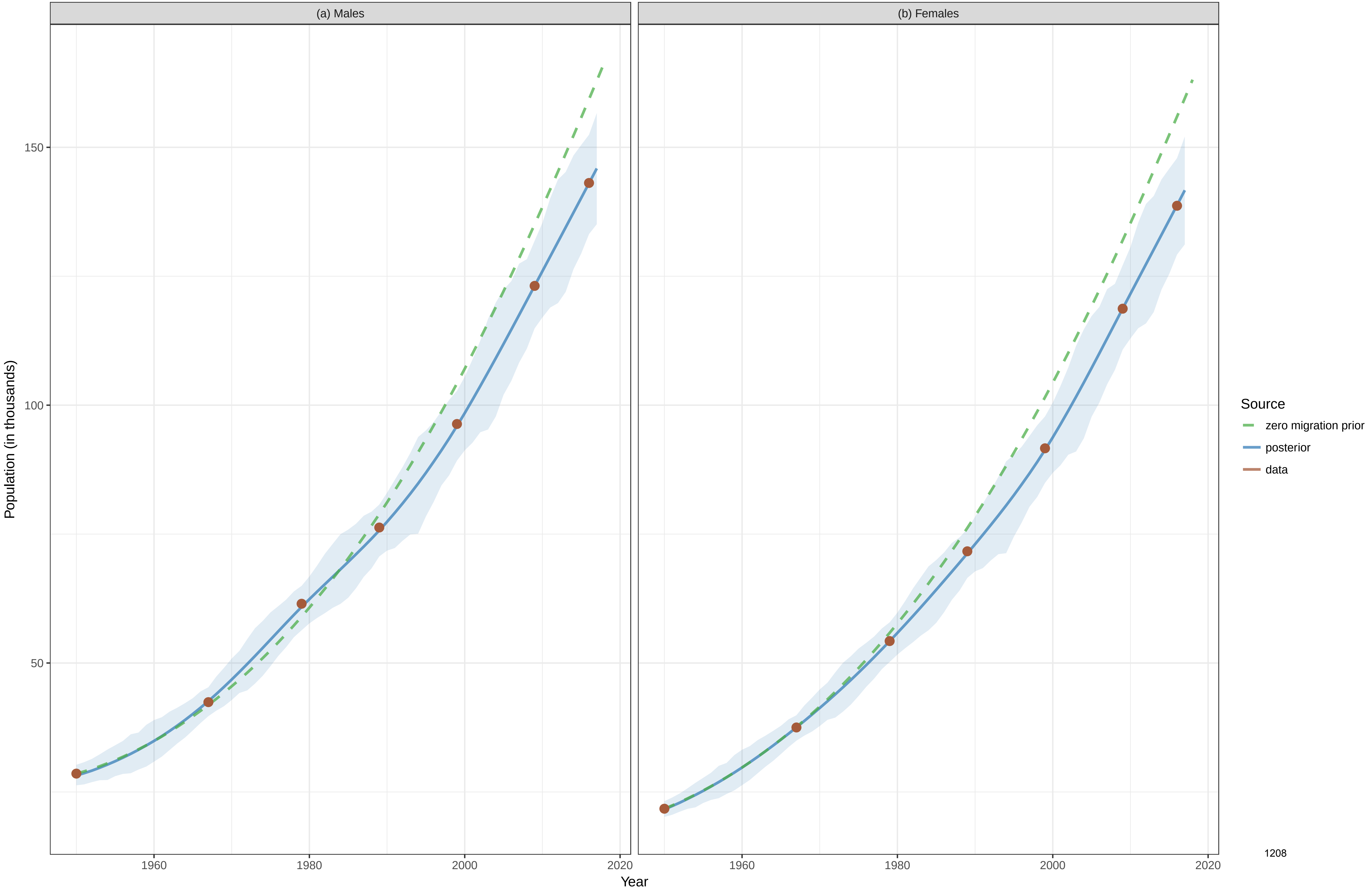

Supplemental Results Figure 4: GBD Bayesian Demographic Balancing Model fit in Venezuela for (a) total population of males, and (b) total population of females, 1950−2017.

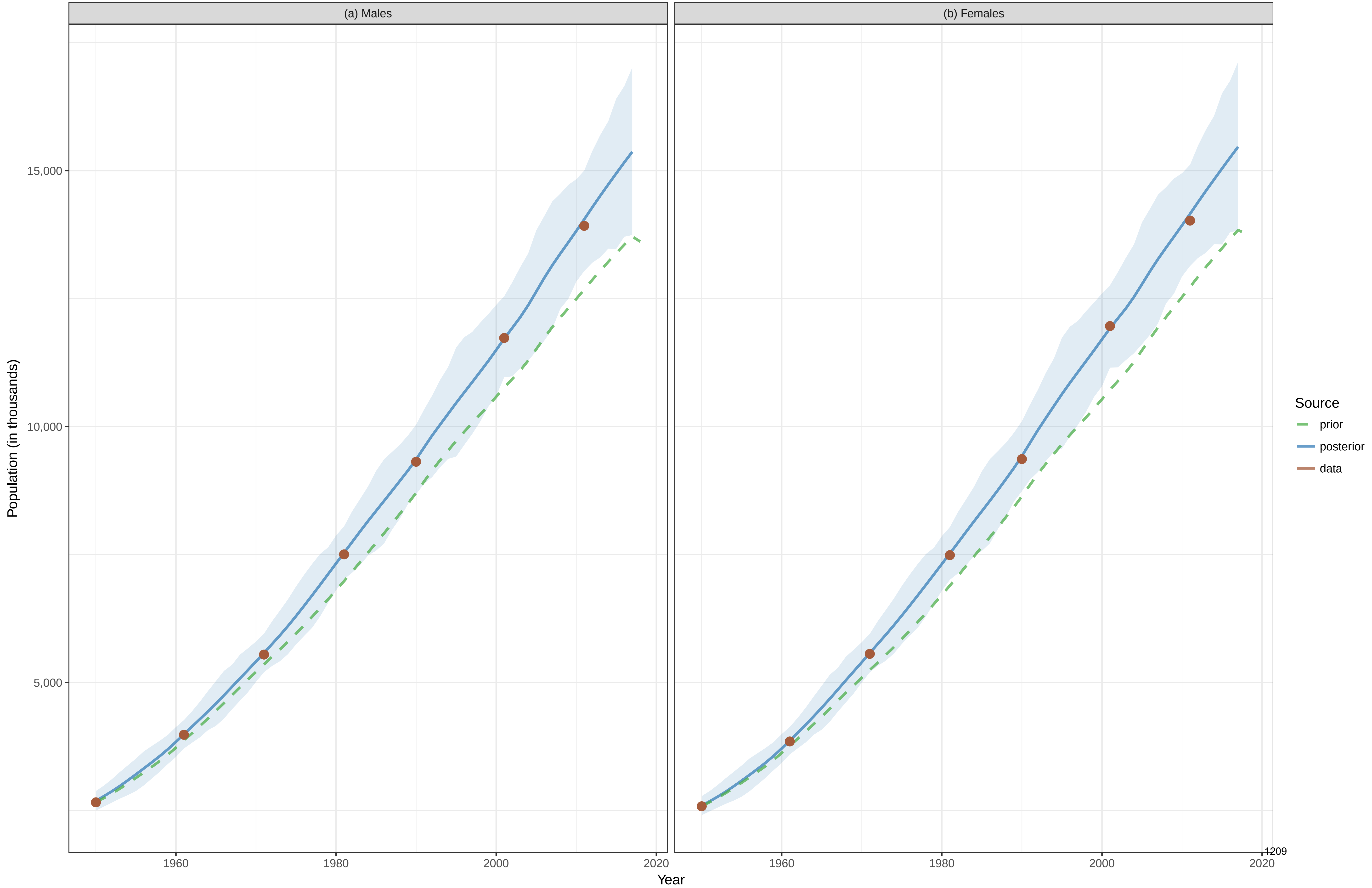

Supplemental Results Figure 4: GBD Bayesian Demographic Balancing Model fit in Vietnam for (a) total population of males, and (b) total population of females, 1950−2017.

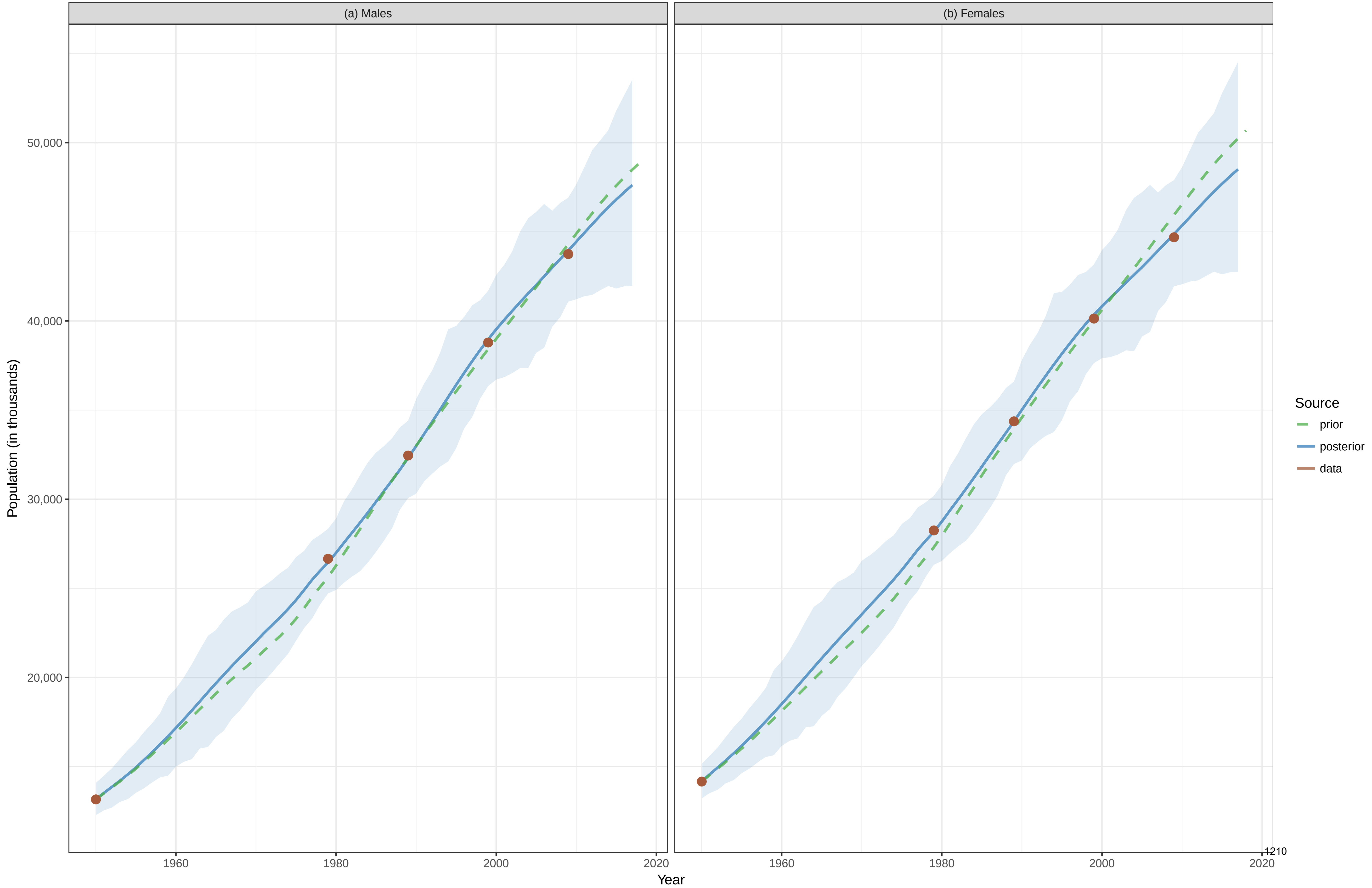

Supplemental Results Figure 4: GBD Bayesian Demographic Balancing Model fit in Virgin Islands, U.S. for (a) total population of males, and (b) total population of females, 1950−2017.

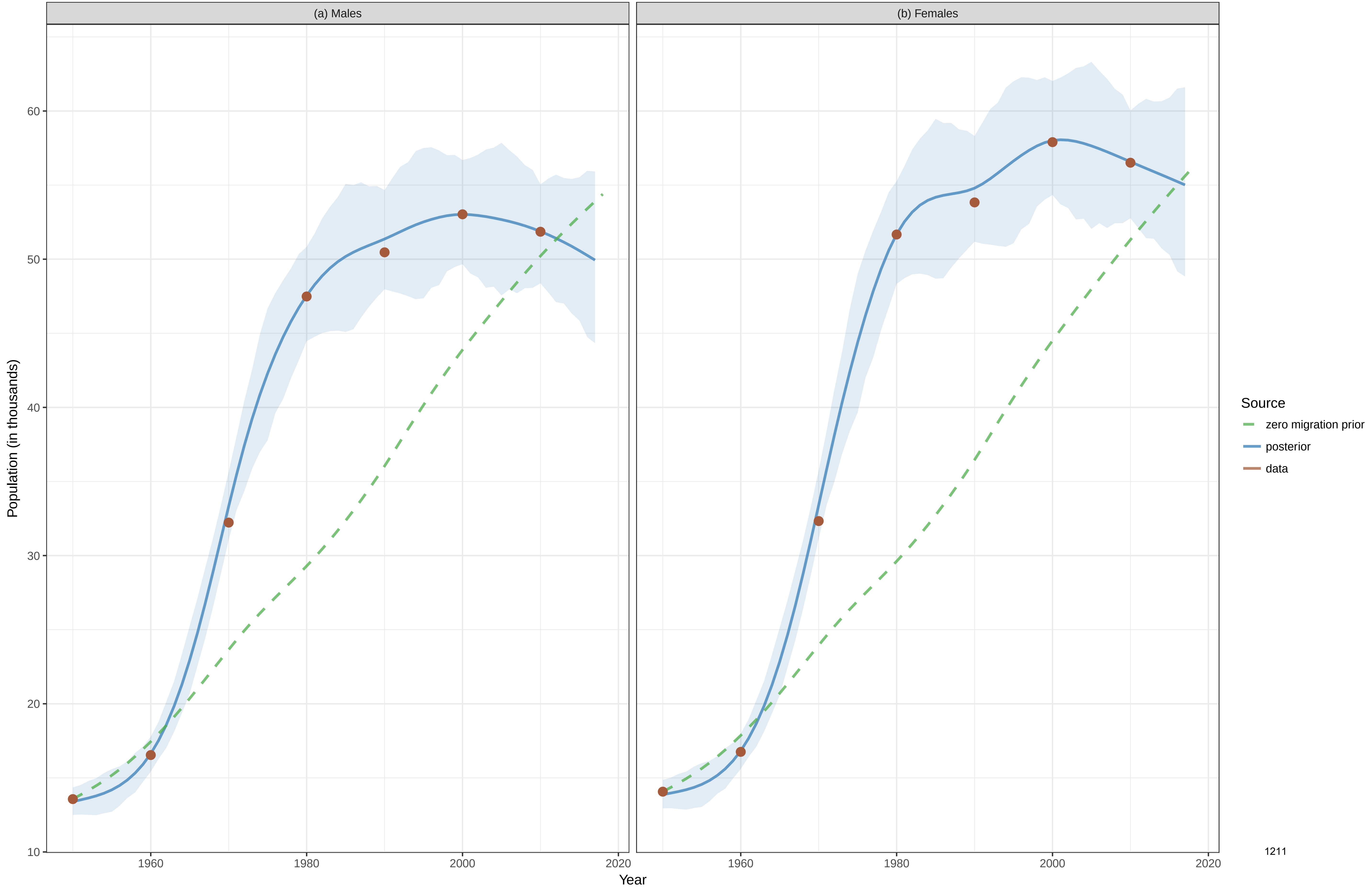

Supplemental Results Figure 4: GBD Bayesian Demographic Balancing Model fit in Wales for (a) total population of males, and (b) total population of females, 1950−2017.

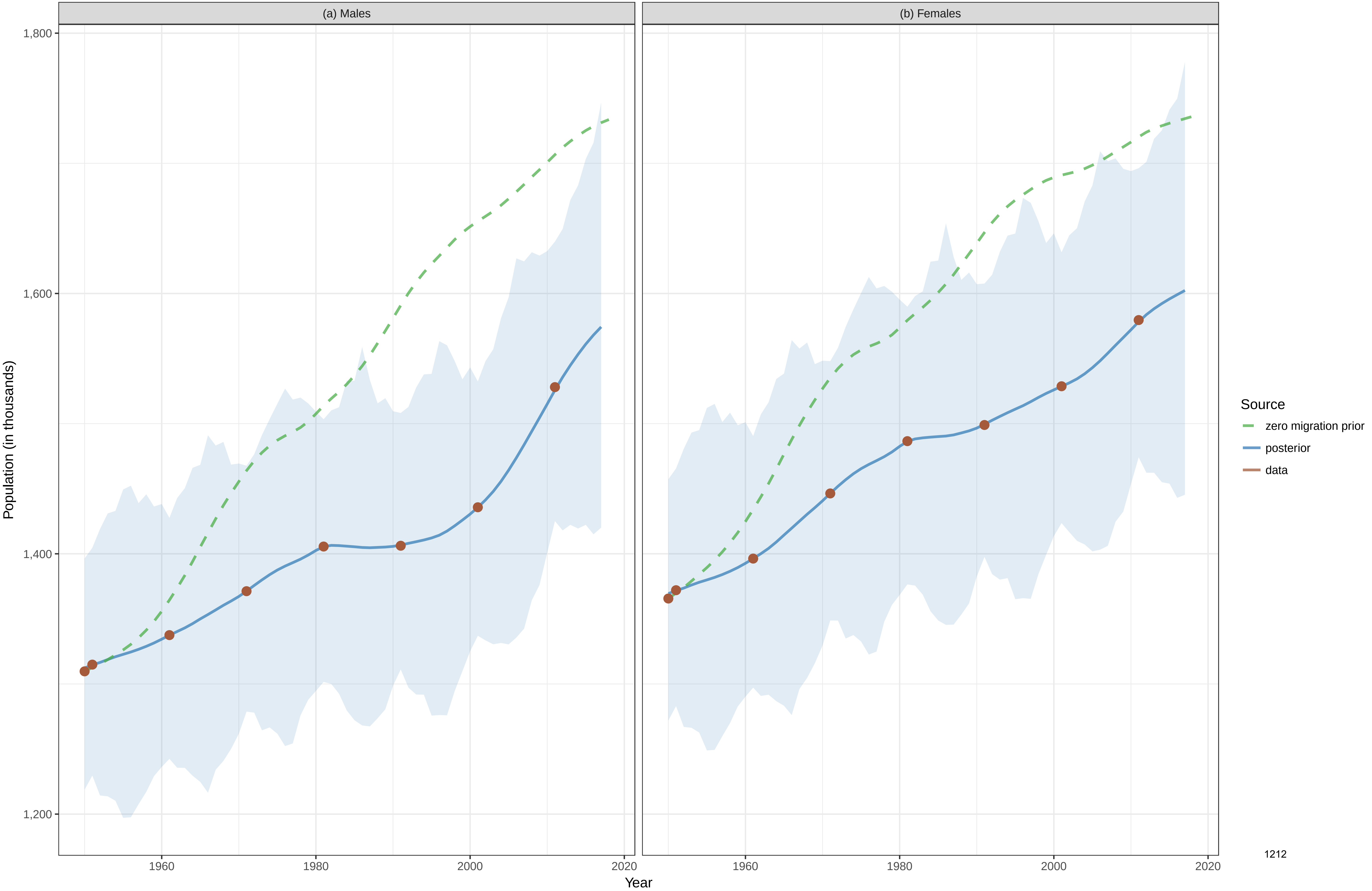

Supplemental Results Figure 4: GBD Bayesian Demographic Balancing Model fit in Yemen for (a) total population of males, and (b) total population of females, 1950−2017.

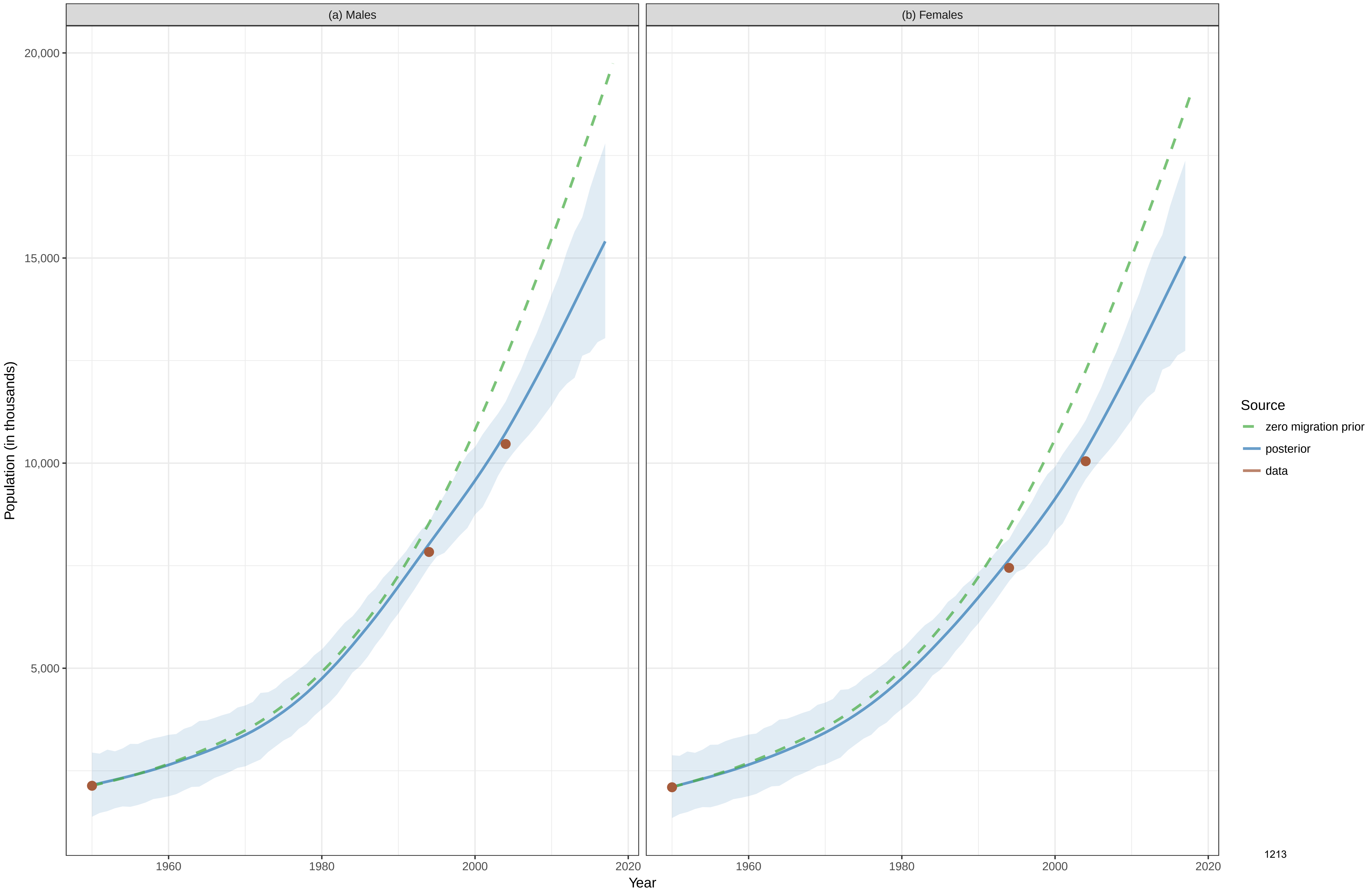

Supplemental Results Figure 4: GBD Bayesian Demographic Balancing Model fit in Zambia for (a) total population of males, and (b) total population of females, 1950−2017.

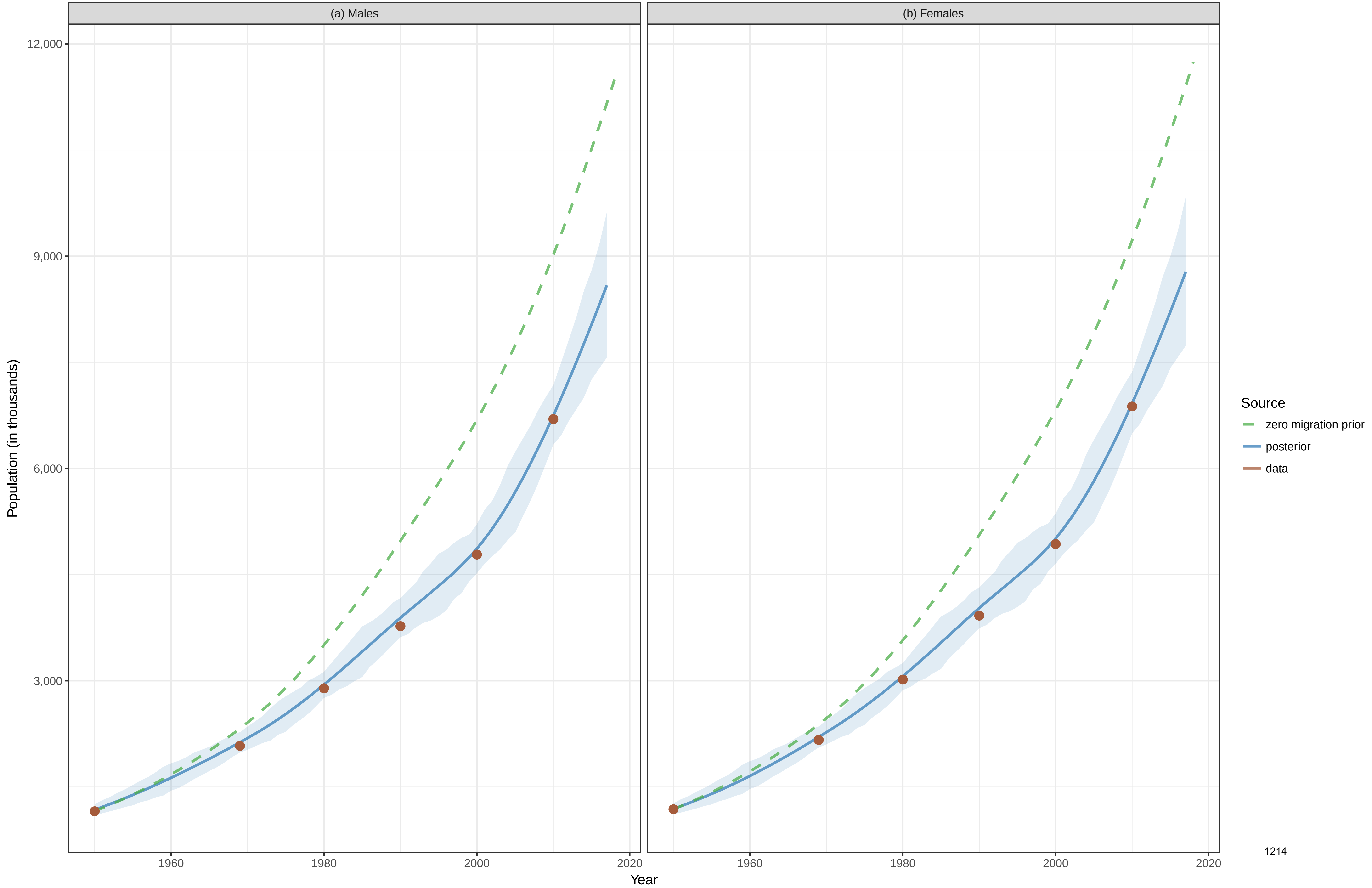

Supplemental Results Figure 4: GBD Bayesian Demographic Balancing Model fit in Zimbabwe for (a) total population of males, and (b) total population of females, 1950−2017.

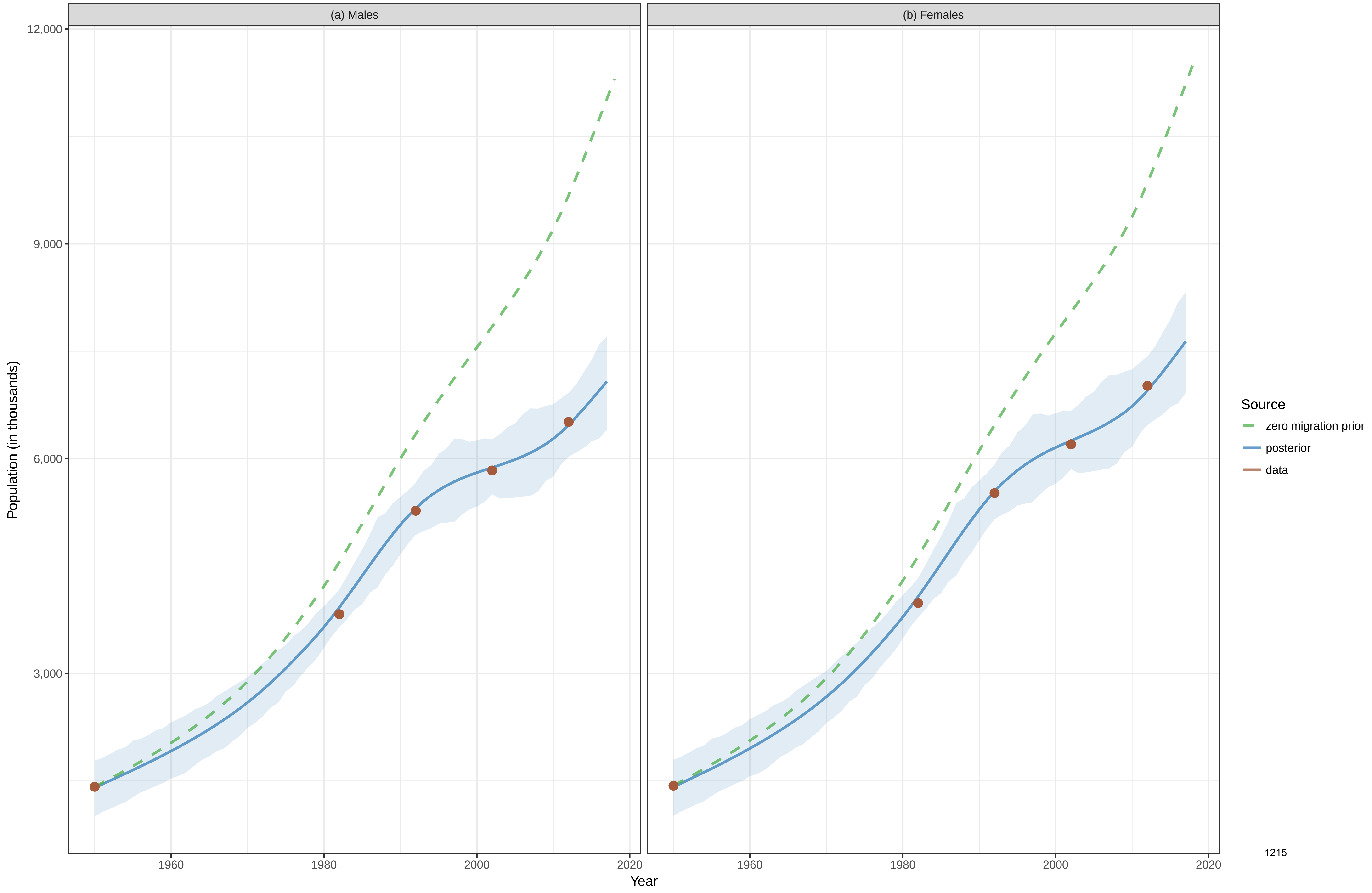

Supplemental Figure 5: Population pyramids for females and males, by age and by location, for 1950, 1975, 2000, and 2017. Horizontal lines show the median and mean population in females and males in each year.

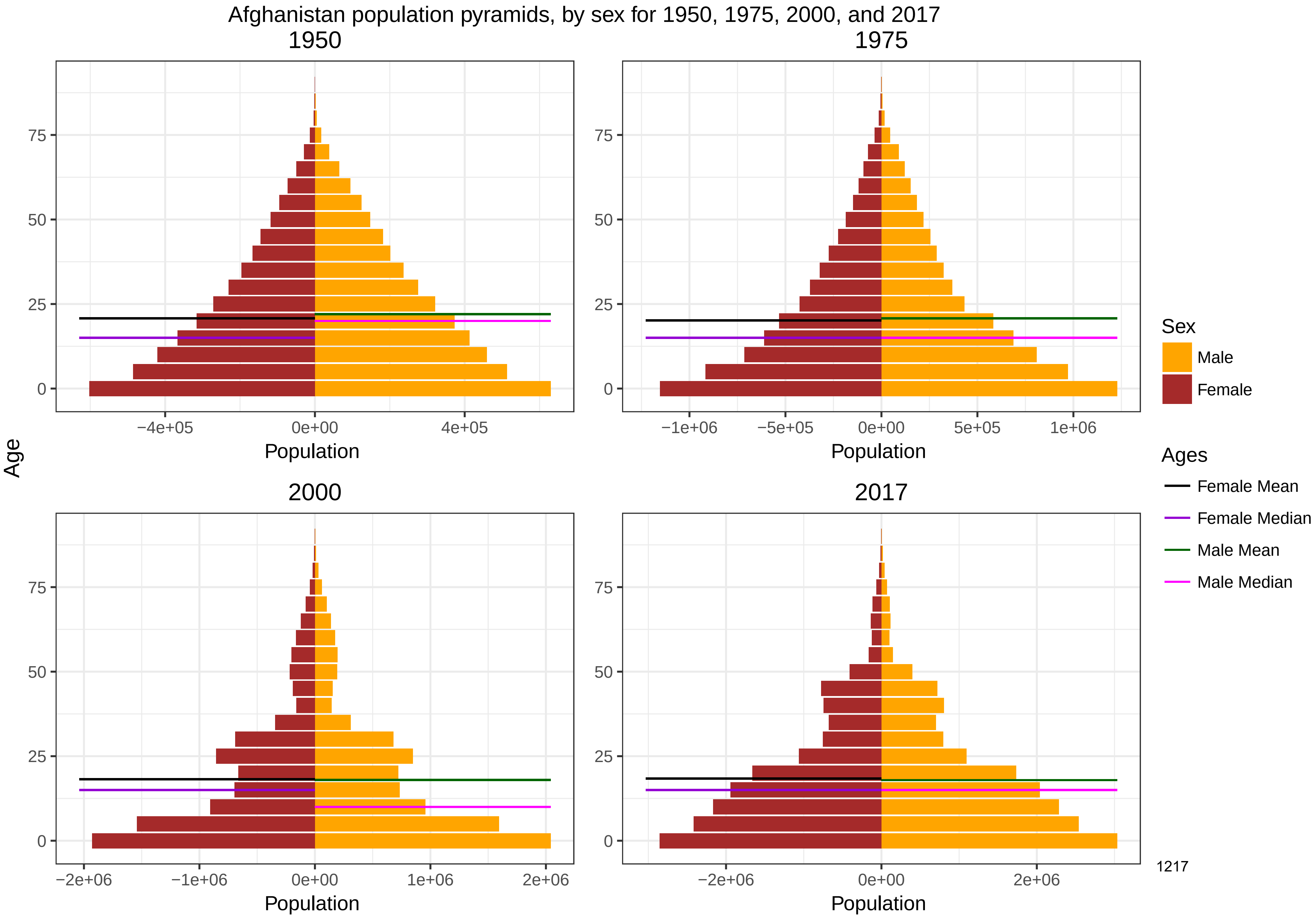
Afghanistan population pyramids, by sex for 1950, 1975, 2000, and 2017
1950
1975
2000
2017
Age
Population
0
25
50
75
−4e+05
0e+00
4e+05
−1e+06
−5e+05
5e+05
1e+06
−2e+06
2e+06
Sex
Male
Female
Ages
Female Mean
Female Median
Male Mean
Male Median

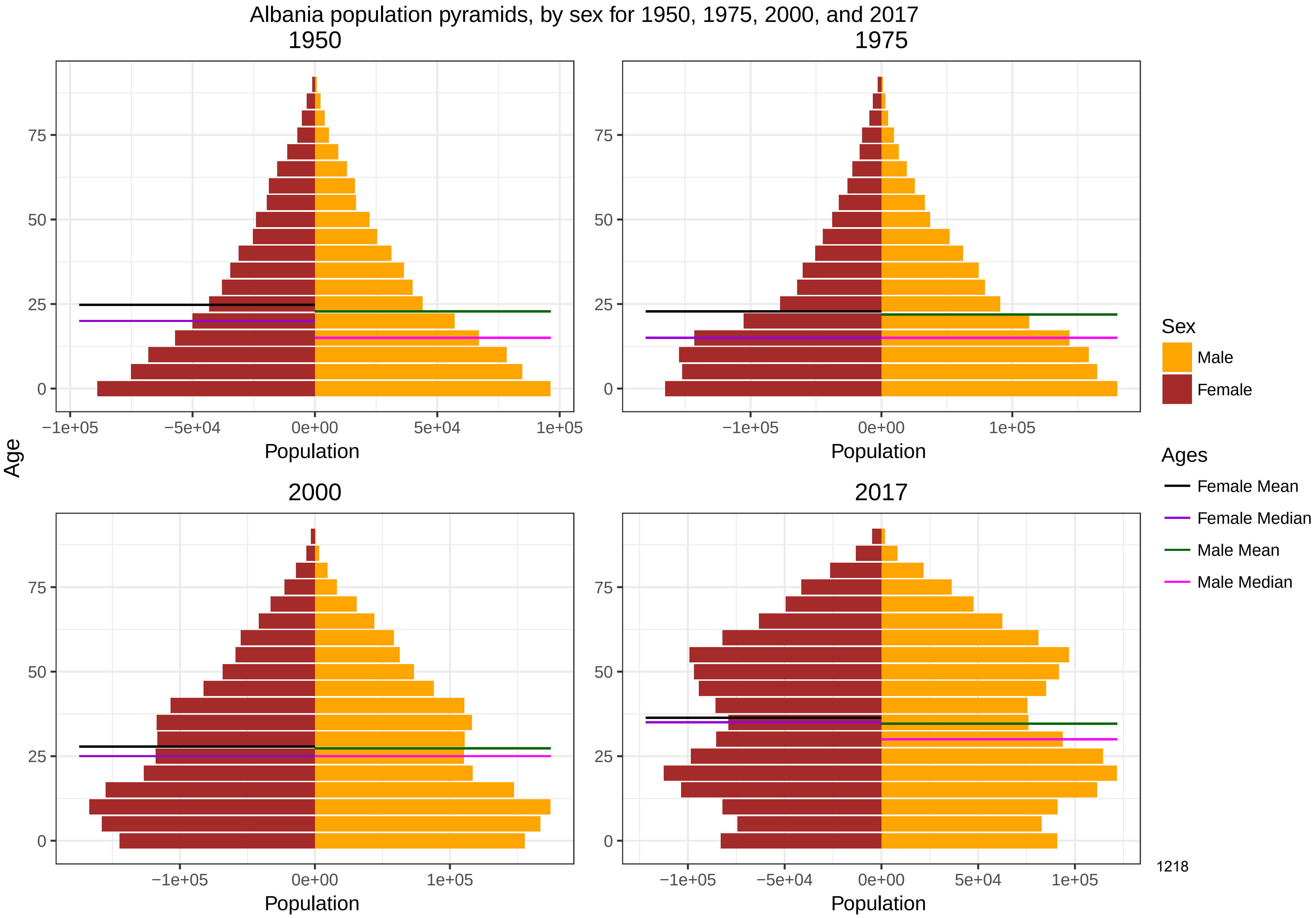
Albania population pyramids, by sex for 1950, 1975, 2000, and 2017
1950
1975
2000
2017
Age
Population
Sex
Male
Female
Ages
Female Mean
Female Median
Male Mean
Male Median

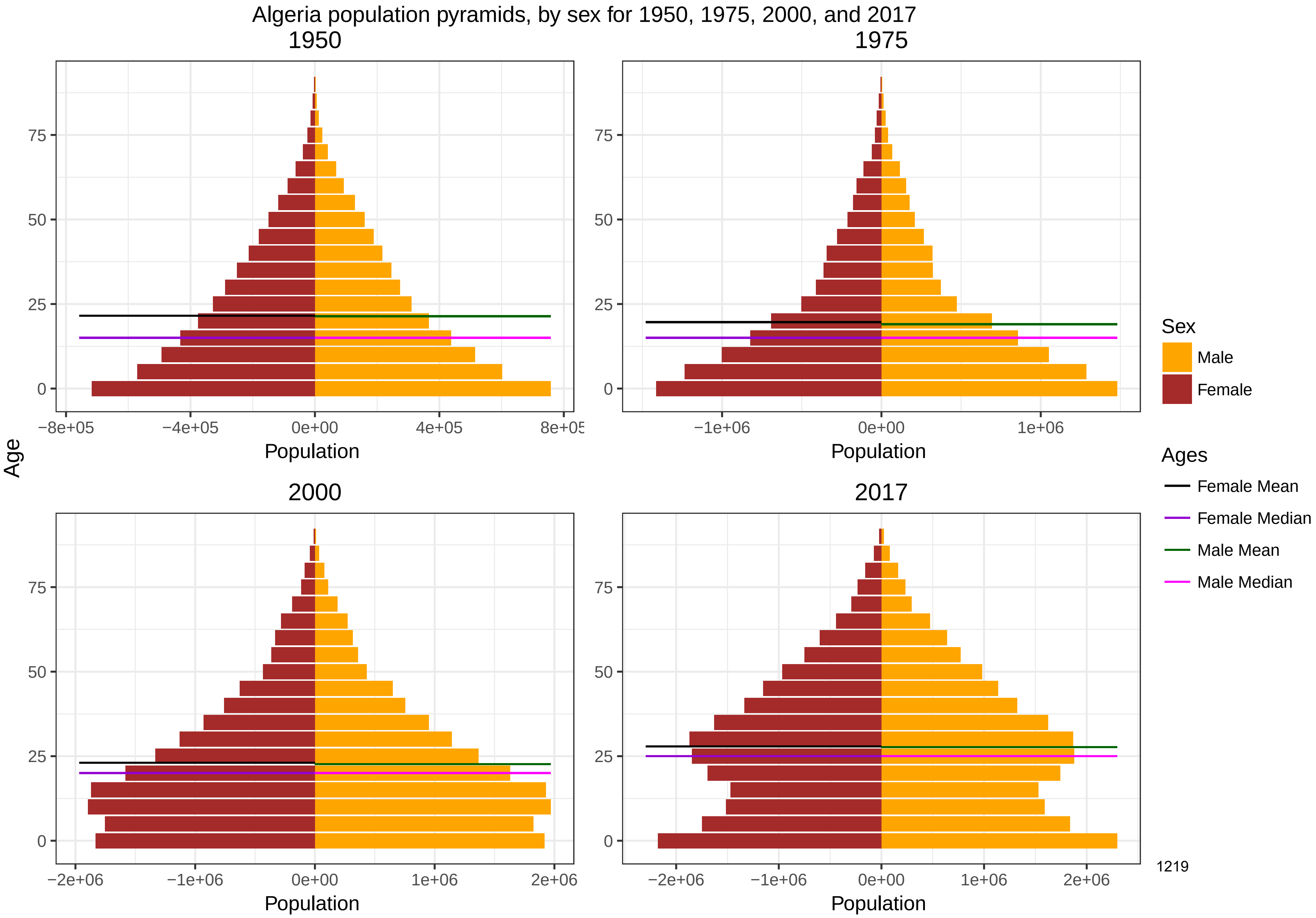

Algeria population pyramids, by sex for 1950, 1975, 2000, and 2017
1950
1975
2000
2017
Age
Population
0
25
50
75
−8e+05
−4e+05
0e+00
4e+05
8e+05
−1e+06
1e+06
−2e+06
2e+06
Sex
Male
Female
Ages
Female Mean
Female Median
Male Mean
Male Median

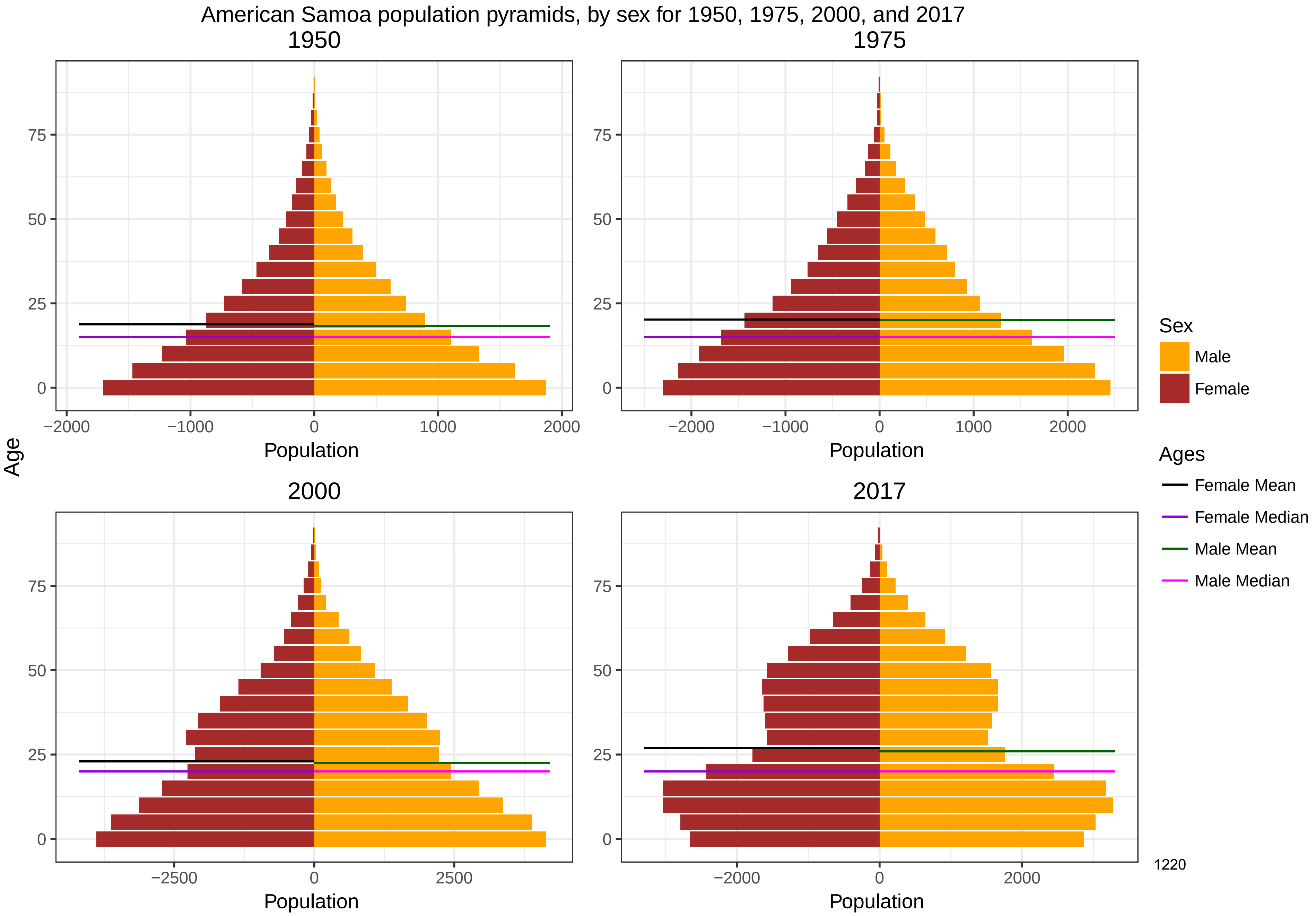
American Samoa population pyramids, by sex for 1950, 1975, 2000, and 2017
1950
1975
2000
2017
Age
Population
Sex
Male
Female
Ages
Female Mean
Female Median
Male Mean
Male Median

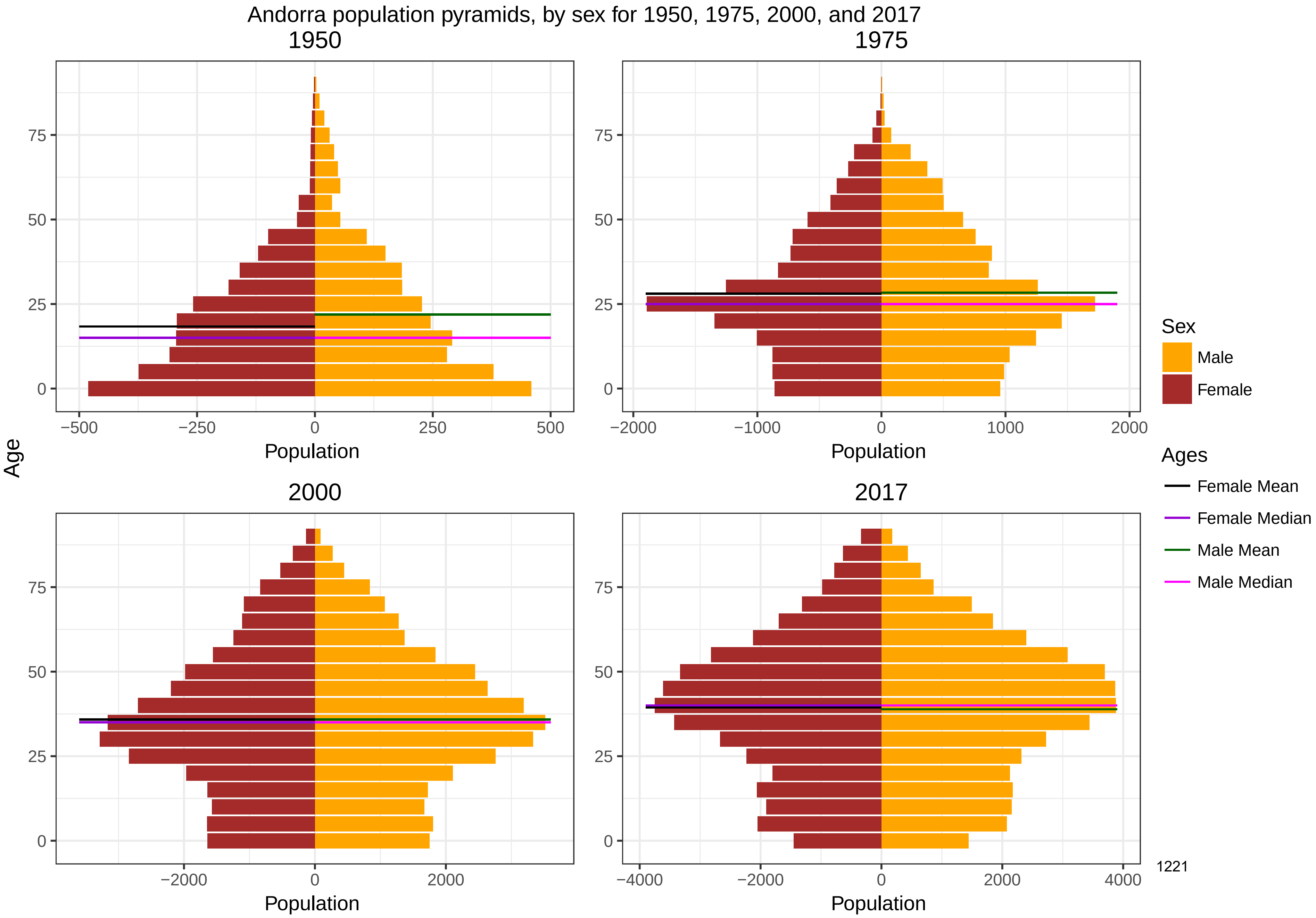
Andorra population pyramids, by sex for 1950, 1975, 2000, and 2017
1950
1975
2000
2017
Age
Population
Sex
Male
Female
Ages
Female Mean
Female Median
Male Mean
Male Median

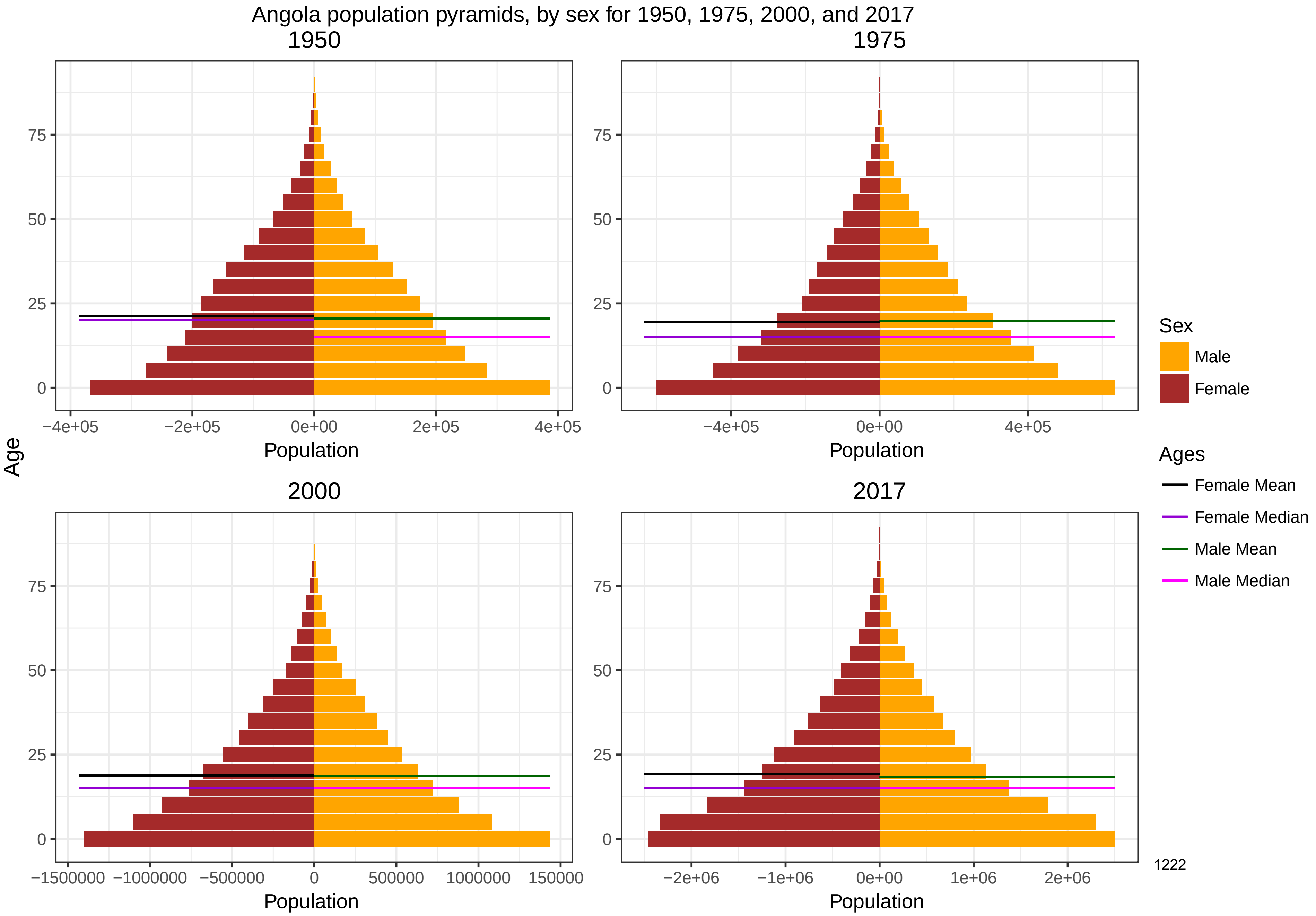
Angola population pyramids, by sex for 1950, 1975, 2000, and 2017
1950
1975
2000
2017
Age
Population
Sex
Male
Female
Ages
Female Mean
Female Median
Male Mean
Male Median
−4e+05
−2e+05
0e+00
2e+05
4e+05
1e+06
2e+06
−1e+06
−2e+06
−1500000
−1000000
−500000
0
500000
1000000
150000
75
50
25

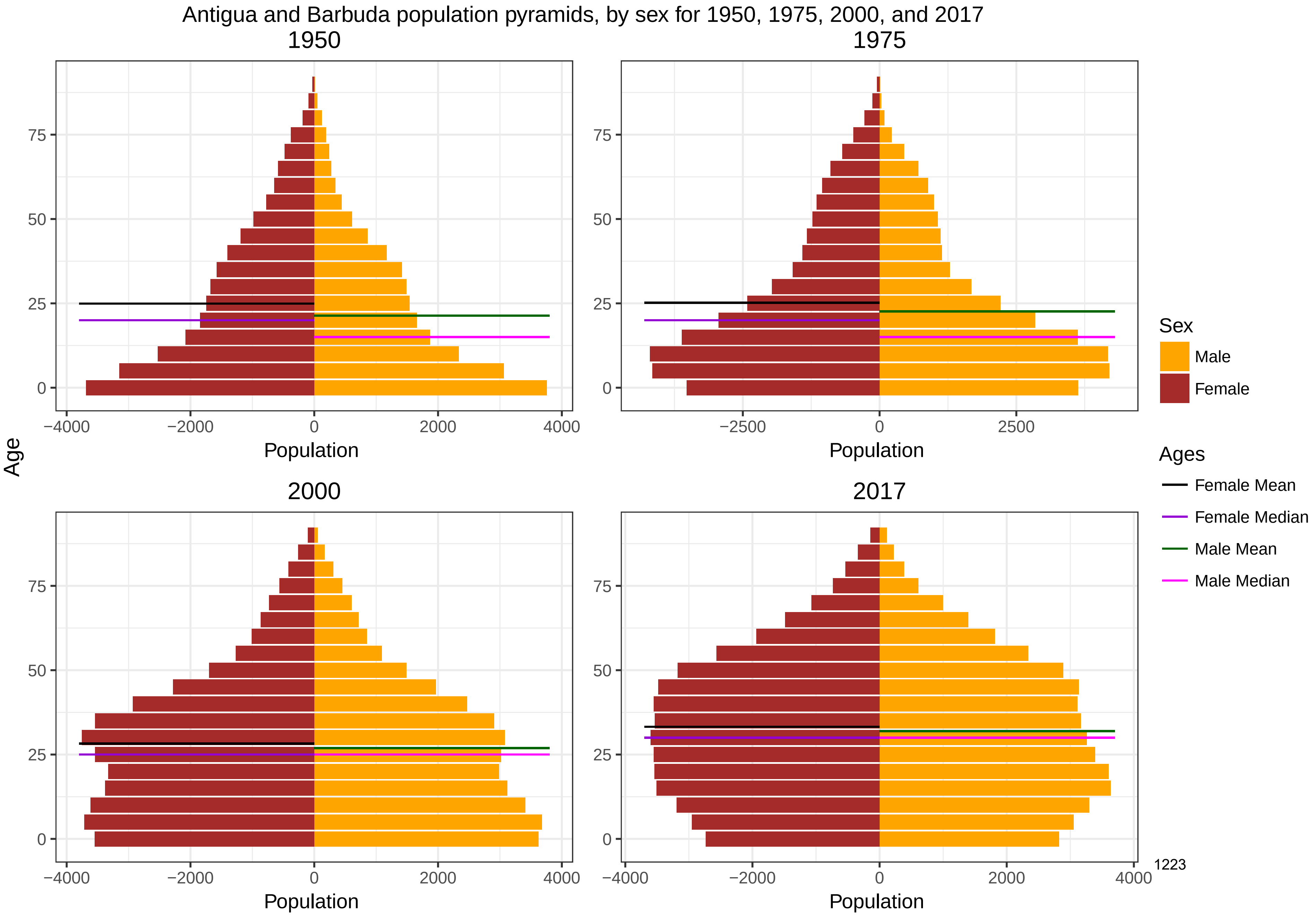

Antigua and Barbuda population pyramids, by sex for 1950, 1975, 2000, and 2017
1950
1975
2000
2017
Age
Population
Sex
Male
Female
Ages
Female Mean
Female Median
Male Mean
Male Median

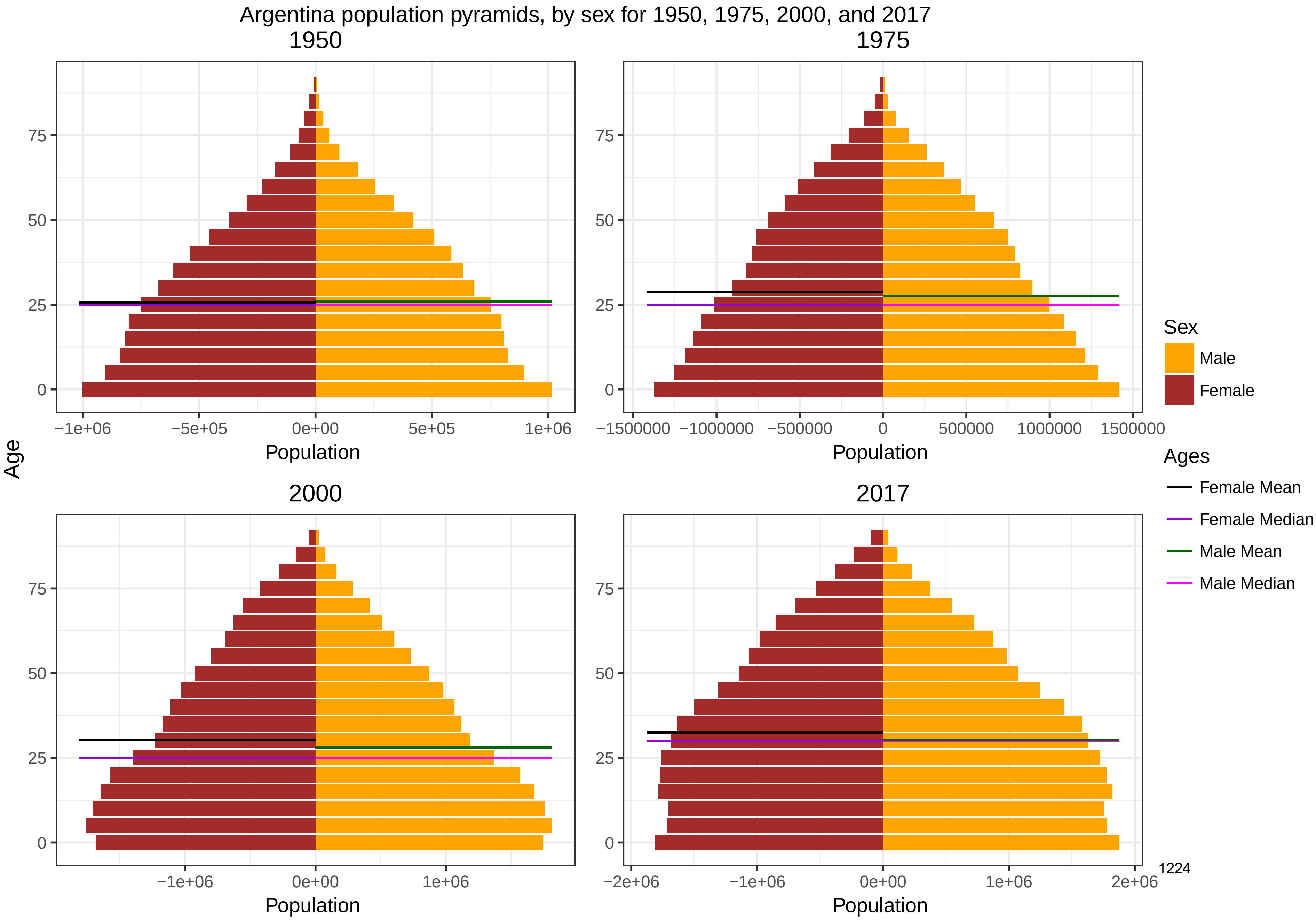

Argentina population pyramids, by sex for 1950, 1975, 2000, and 2017
1950
1975
2000
2017
Age
Population
Sex
Male
Female
Ages
Female Mean
Female Median
Male Mean
Male Median
0
25
50
75
−1e+06
−5e+05
0e+00
5e+05
1e+06
−1500000
−1000000
−500000
0
500000
1000000
1500000
−2e+06
2e+06

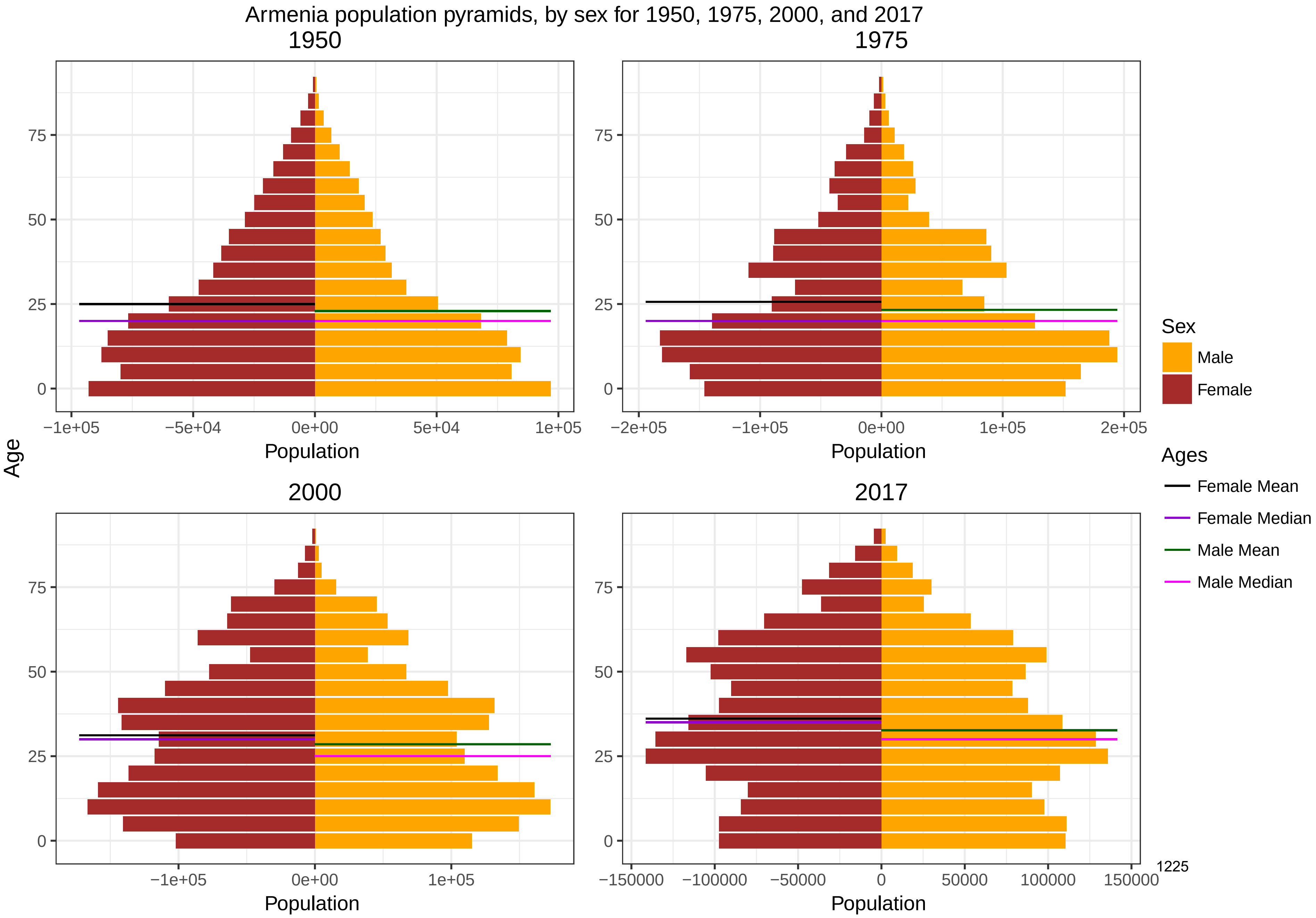

Armenia population pyramids, by sex for 1950, 1975, 2000, and 2017
1950
1975
2000
2017
Age
Population
0
25
50
75
−1e+05
−5e+04
0e+00
5e+04
1e+05
−2e+05
2e+05
−150000
−100000
−50000
50000
100000
150000
Sex
Male
Female
Ages
Female Mean
Female Median
Male Mean
Male Median

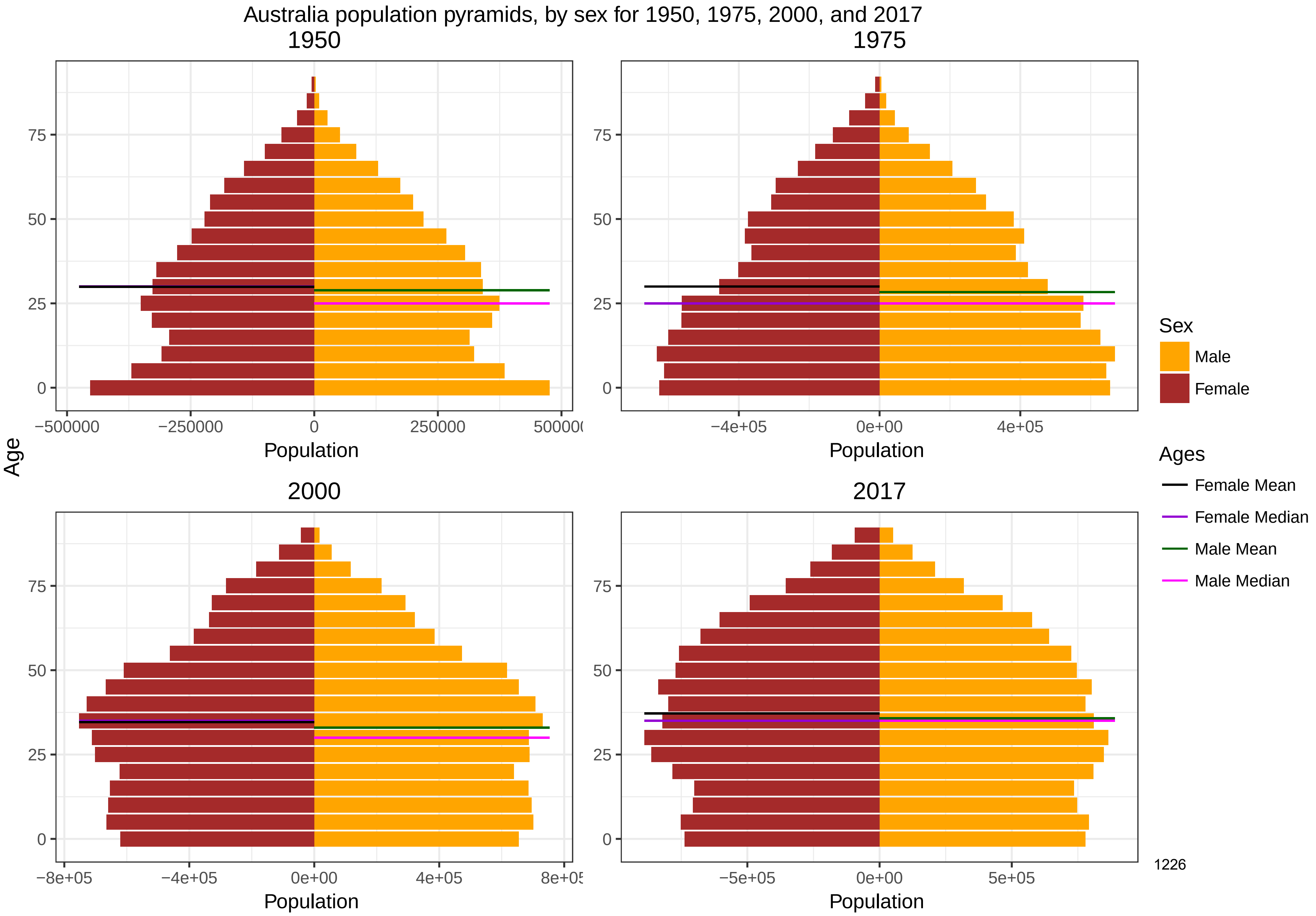
Australia population pyramids, by sex for 1950, 1975, 2000, and 2017
1950
1975
2000
2017
Age
Population
Sex
Male
Female
Ages
Female Mean
Female Median
Male Mean
Male Median
0
25
50
75
−500000
−250000
250000
−4e+05
0e+00
4e+05
−8e+05
−5e+05
5e+05

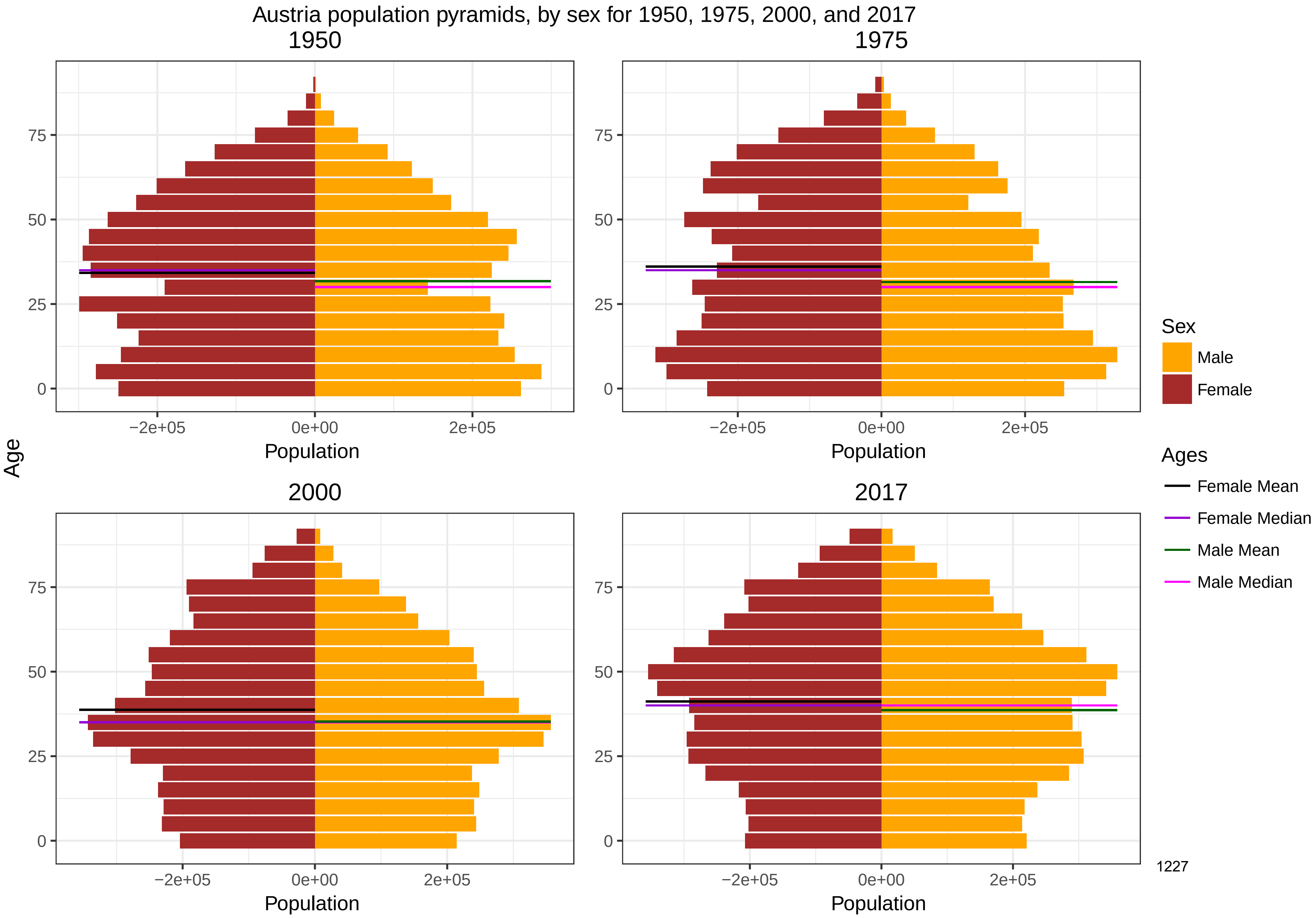

Austria population pyramids, by sex for 1950, 1975, 2000, and 2017
1950
1975
2000
2017
Age
Population
−2e+05
0e+00
2e+05
0
25
50
75
Sex
Male
Female
Ages
Female Mean
Female Median
Male Mean
Male Median

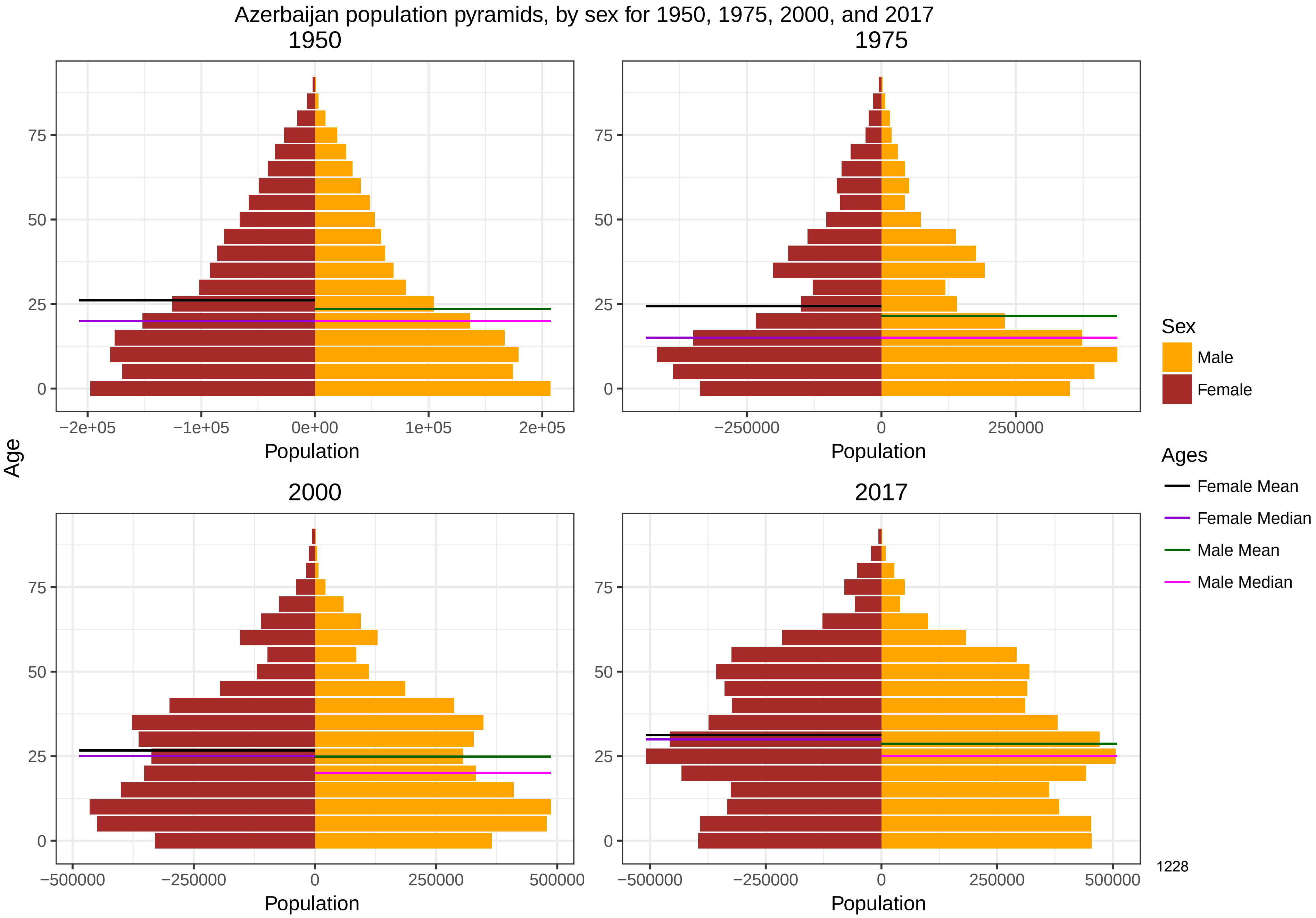

Azerbaijan population pyramids, by sex for 1950, 1975, 2000, and 2017
1950
1975
2000
2017
Age
Population
−2e+05
−1e+05
0e+00
1e+05
2e+05
−250000
0
250000
−500000
500000
0
25
50
75
Sex
Male
Female
Ages
Female Mean
Female Median
Male Mean
Male Median

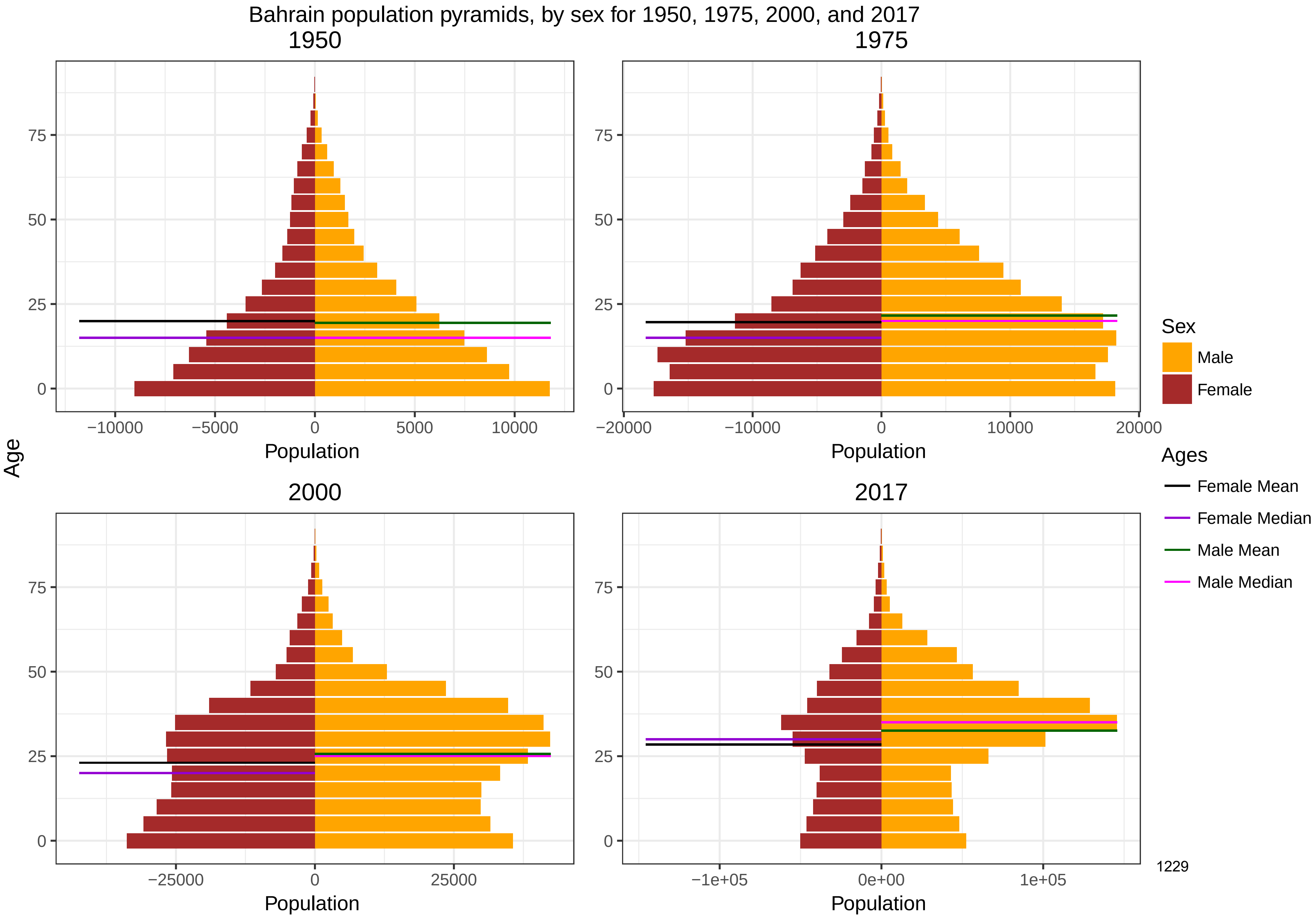
Bahrain population pyramids, by sex for 1950, 1975, 2000, and 2017
1950
1975
2000
2017
Age
Population
Sex
Male
Female
Ages
Female Mean
Female Median
Male Mean
Male Median

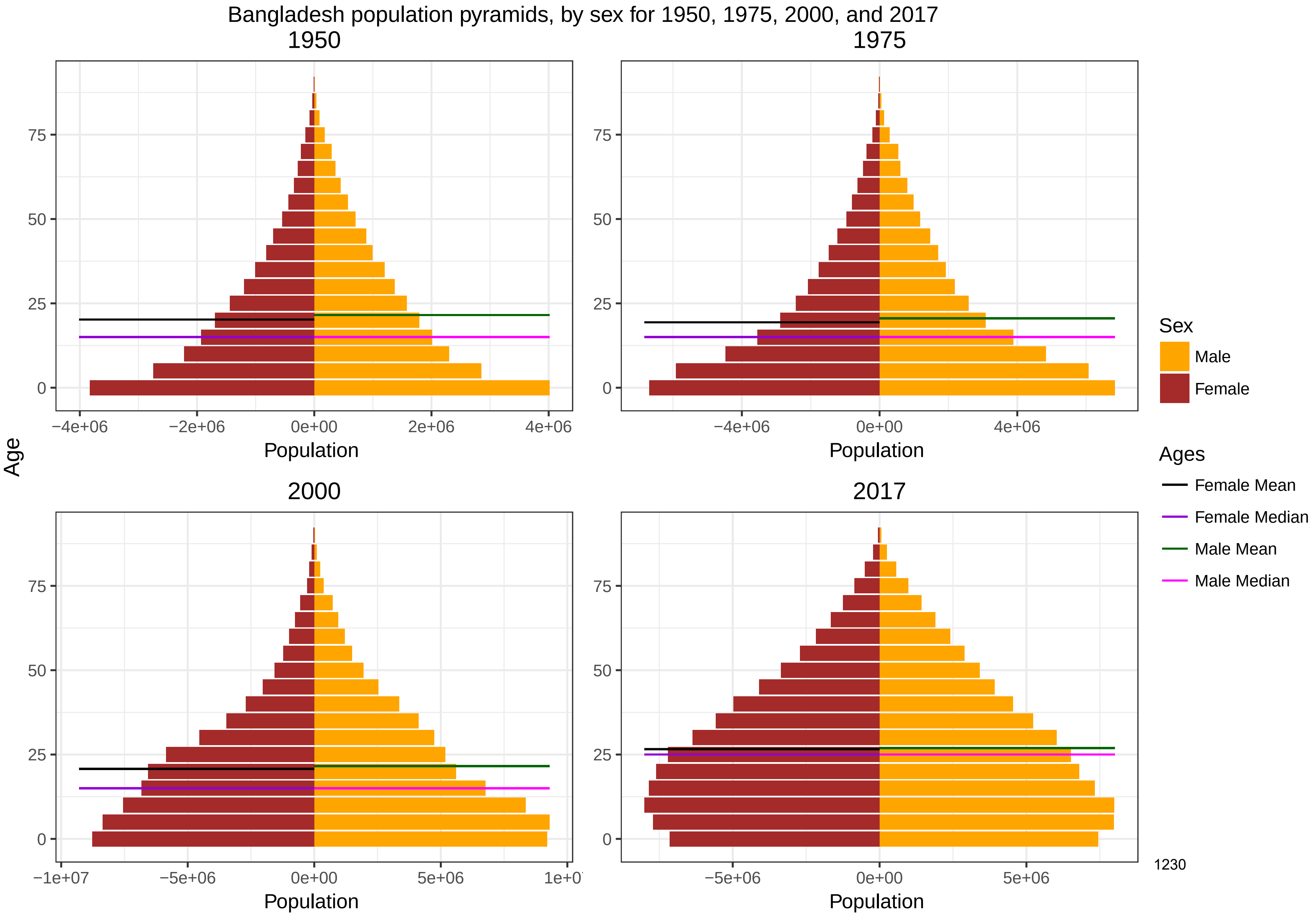
Bangladesh population pyramids, by sex for 1950, 1975, 2000, and 2017
1950
1975
2000
2017
Age
Population
Sex
Male
Female
Ages
Female Mean
Female Median
Male Mean
Male Median
0
25
50
75
−4e+06
−2e+06
0e+00
2e+06
4e+06
−1e+07
−5e+06
5e+06

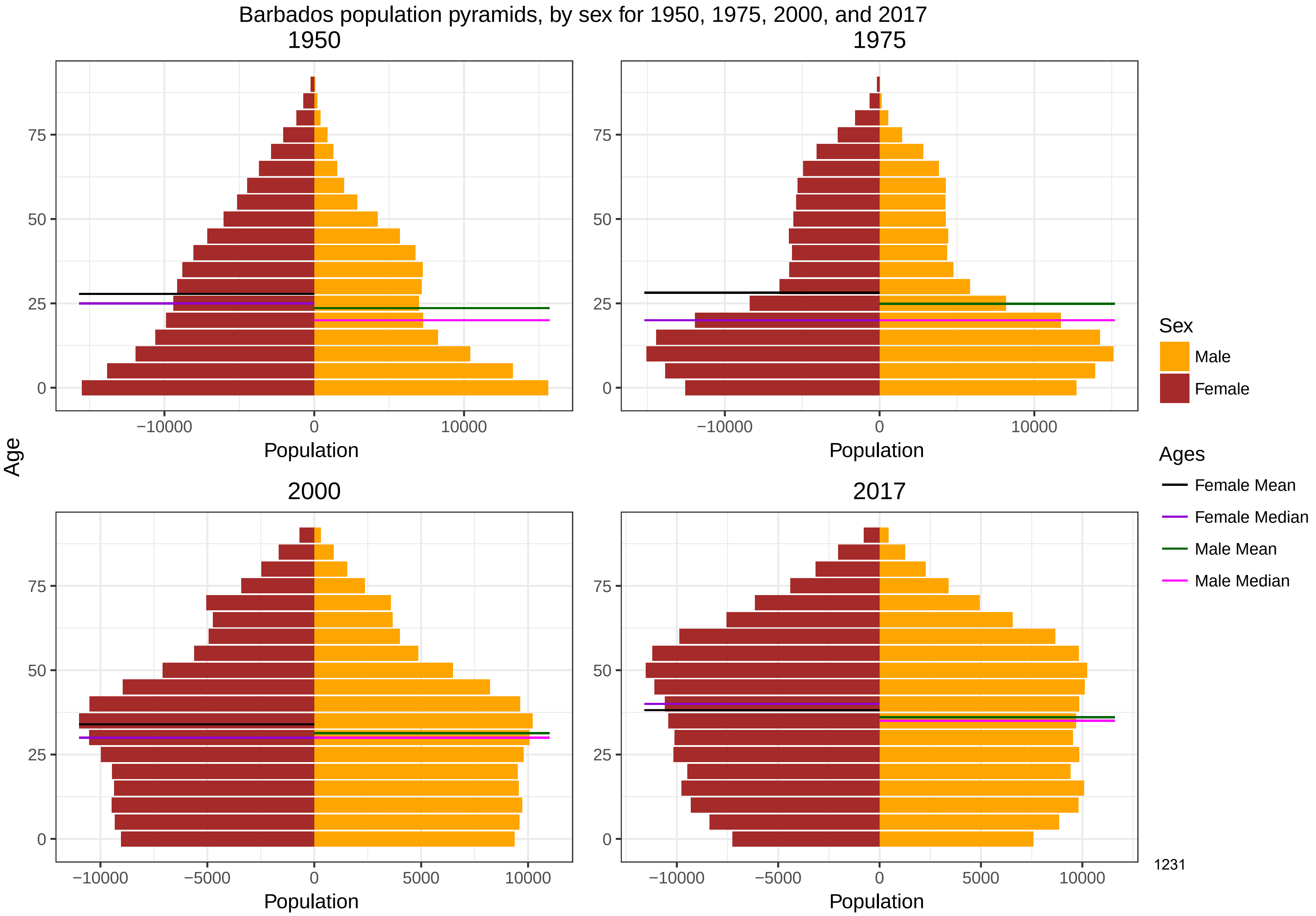
Barbados population pyramids, by sex for 1950, 1975, 2000, and 2017
1950
1975
2000
2017
Age
Population
Sex
Male
Female
Ages
Female Mean
Female Median
Male Mean
Male Median

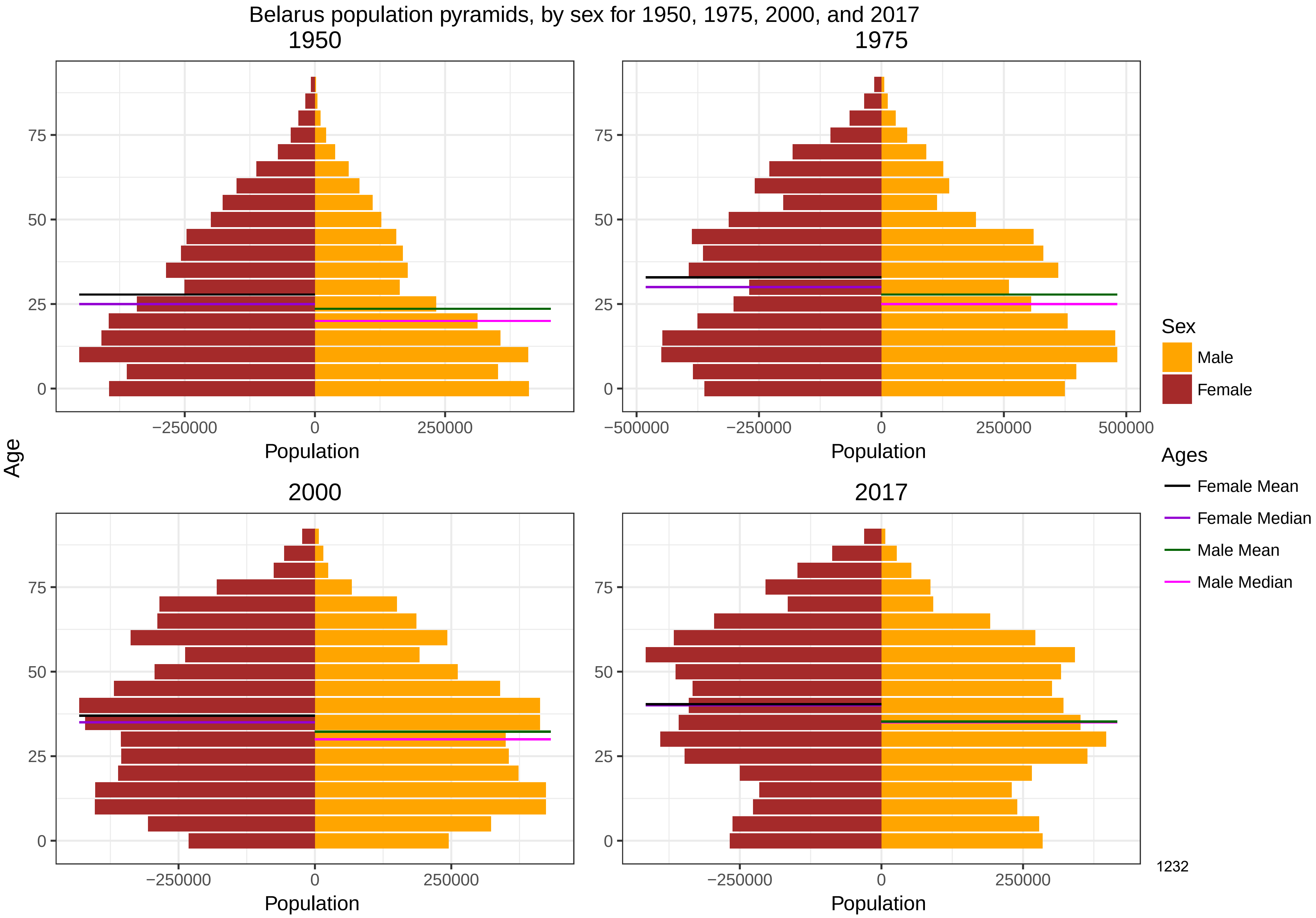

Belarus population pyramids, by sex for 1950, 1975, 2000, and 2017
1950
1975
2000
2017
Age
Population
−500000
−250000
0
250000
500000
75
50
25
0
Sex
Male
Female
Ages
Female Mean
Female Median
Male Mean
Male Median

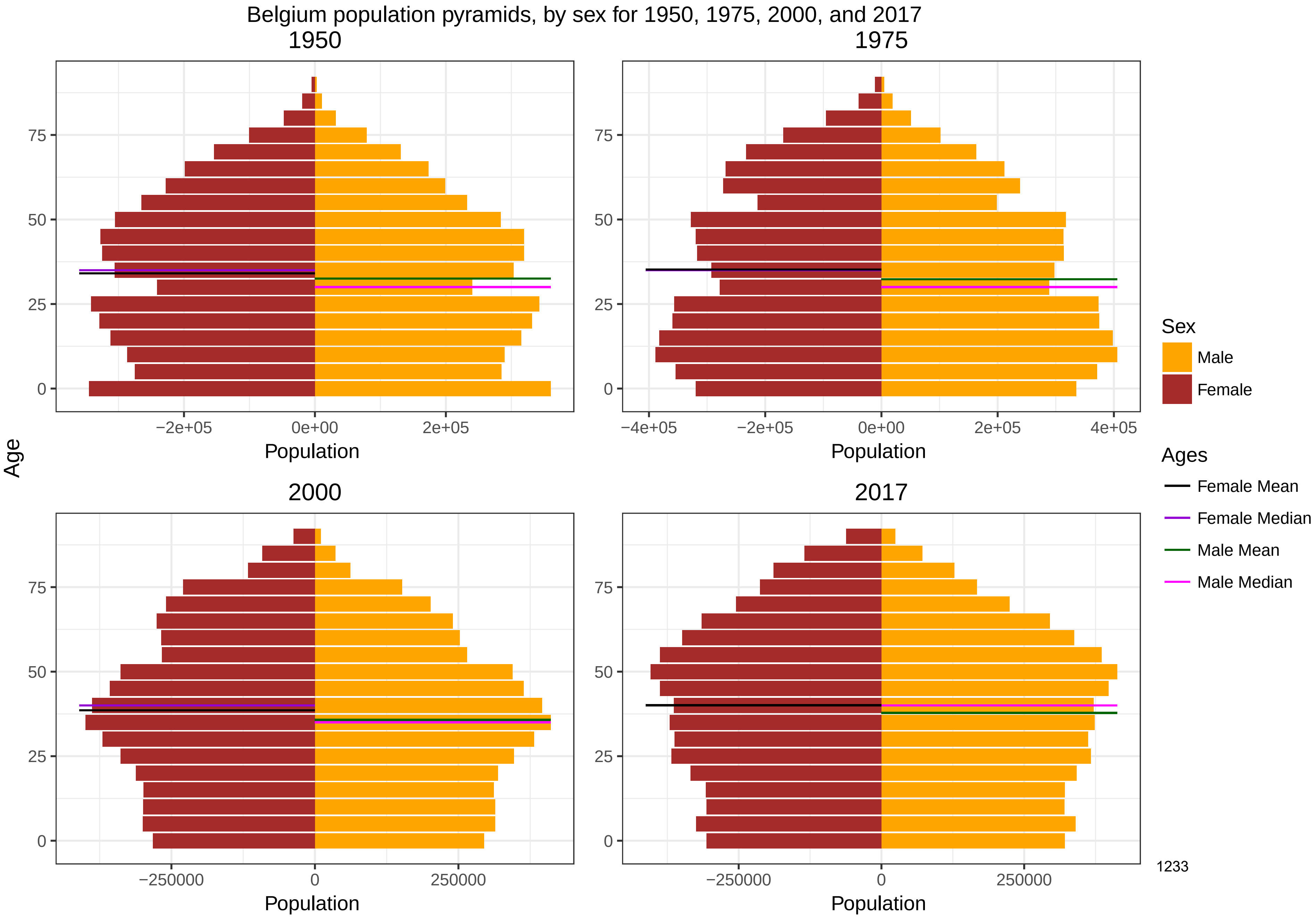
Belgium population pyramids, by sex for 1950, 1975, 2000, and 2017
1950
1975
2000
2017
Age
Population
Sex
Male
Female
Ages
Female Mean
Female Median
Male Mean
Male Median
−2e+05
0e+00
2e+05
−4e+05
4e+05
−250000
0
250000
0
25
50
75

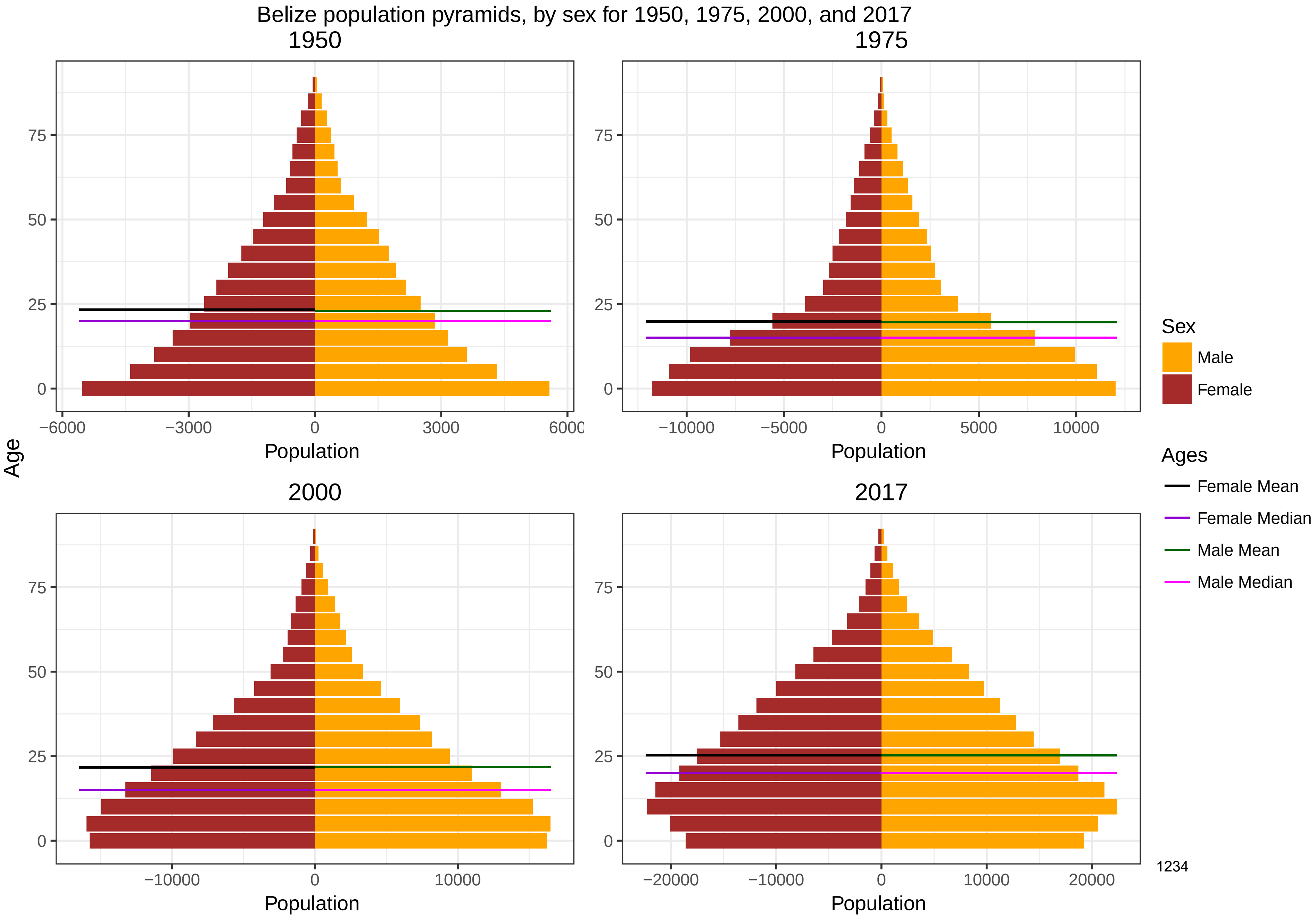
Belize population pyramids, by sex for 1950, 1975, 2000, and 2017
1950
1975
2000
2017
Age
Population
0
25
50
75
−6000
−3000
0
3000
6000
−10000
−5000
5000
10000
20000
−20000
Sex
Male
Female
Ages
Female Mean
Female Median
Male Mean
Male Median

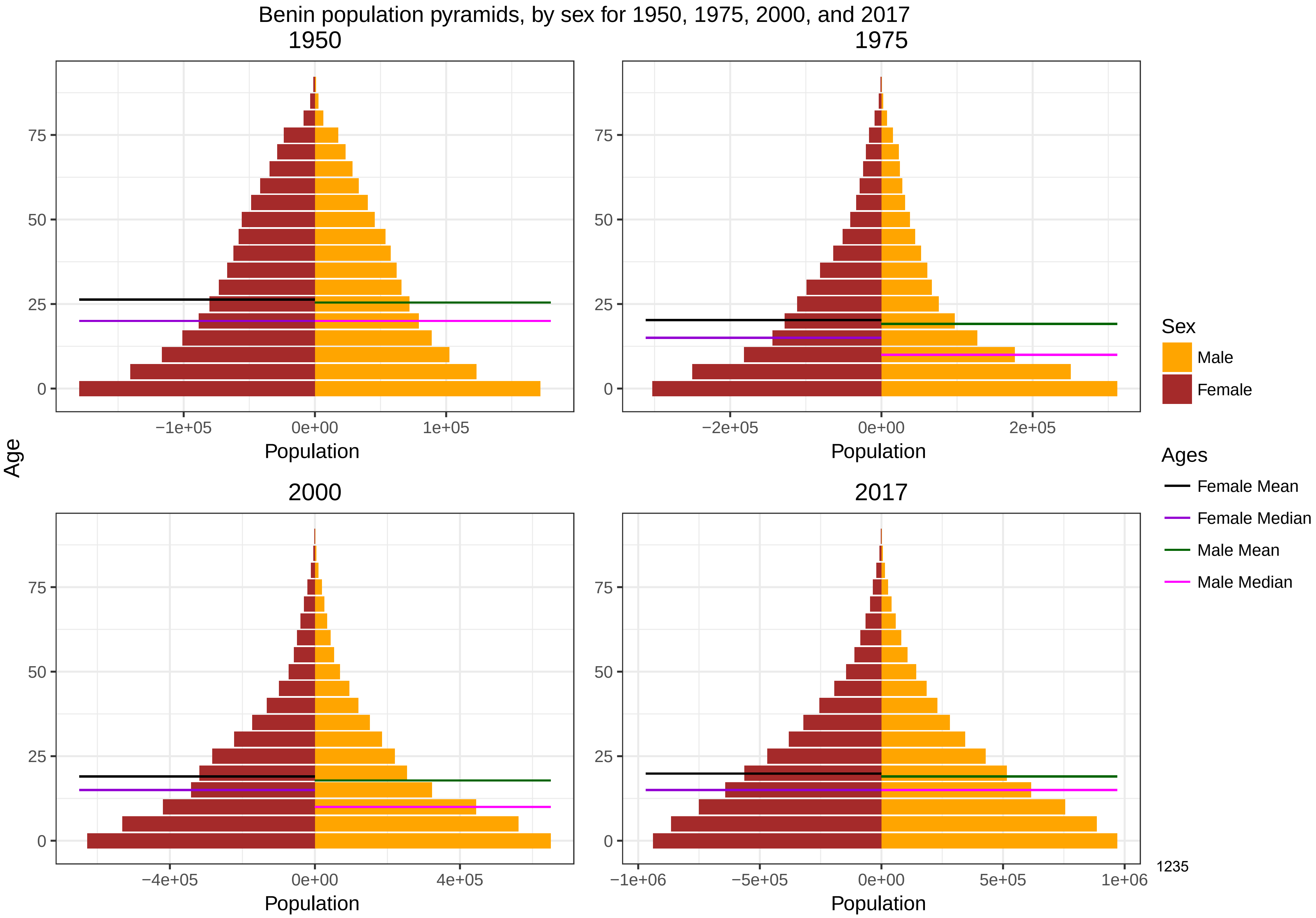

Benin population pyramids, by sex for 1950, 1975, 2000, and 2017
1950
1975
2000
2017
Age
Population
0
25
50
75
−1e+05
0e+00
1e+05
−2e+05
2e+05
−4e+05
4e+05
−1e+06
−5e+05
5e+05
1e+06
Sex
Male
Female
Ages
Female Mean
Female Median
Male Mean
Male Median

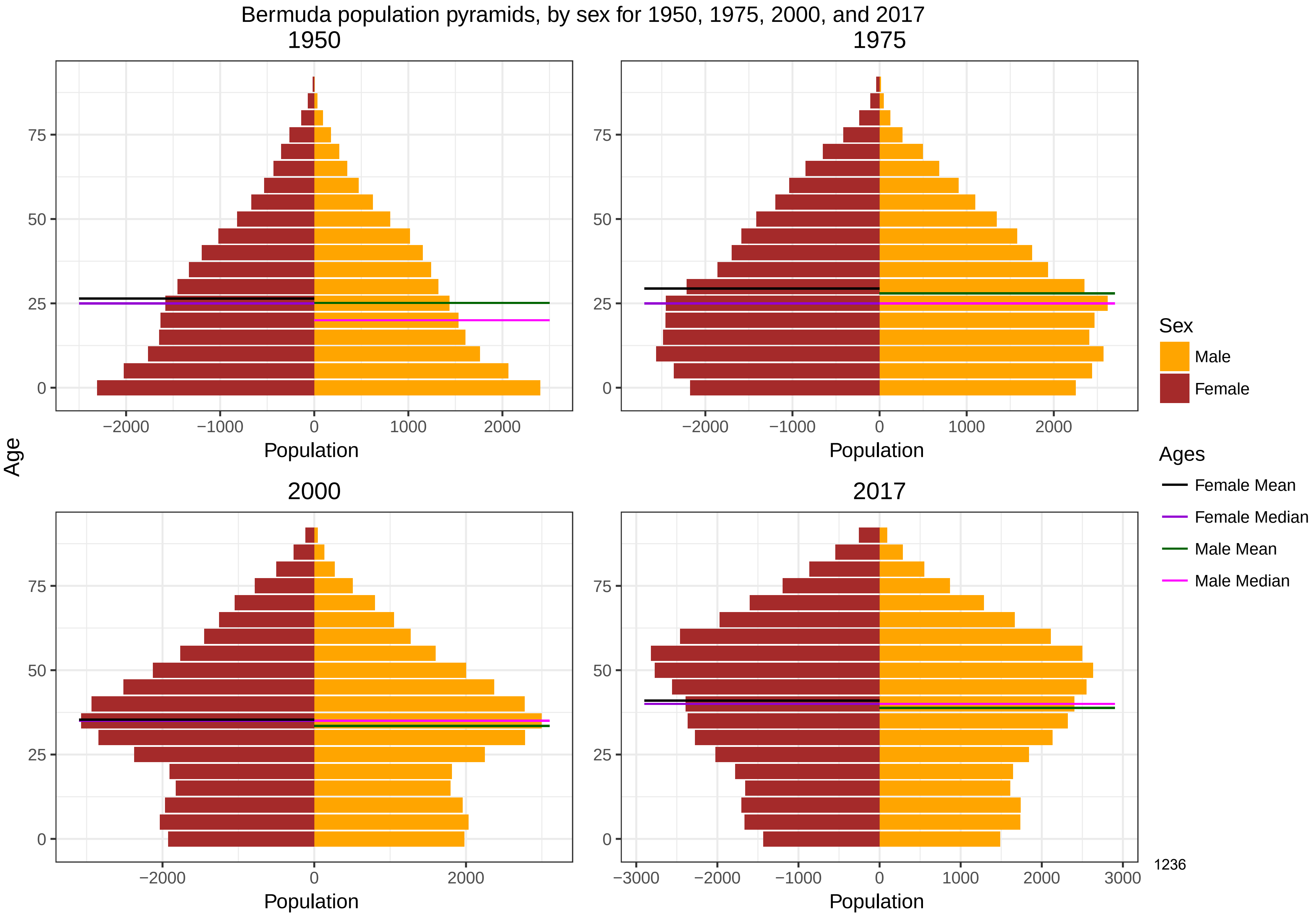

Bermuda population pyramids, by sex for 1950, 1975, 2000, and 2017
1950
1975
2000
2017
Age
Population
−3000
−2000
−1000
0
1000
2000
3000
75
50
25
0
Sex
Male
Female
Ages
Female Mean
Female Median
Male Mean
Male Median

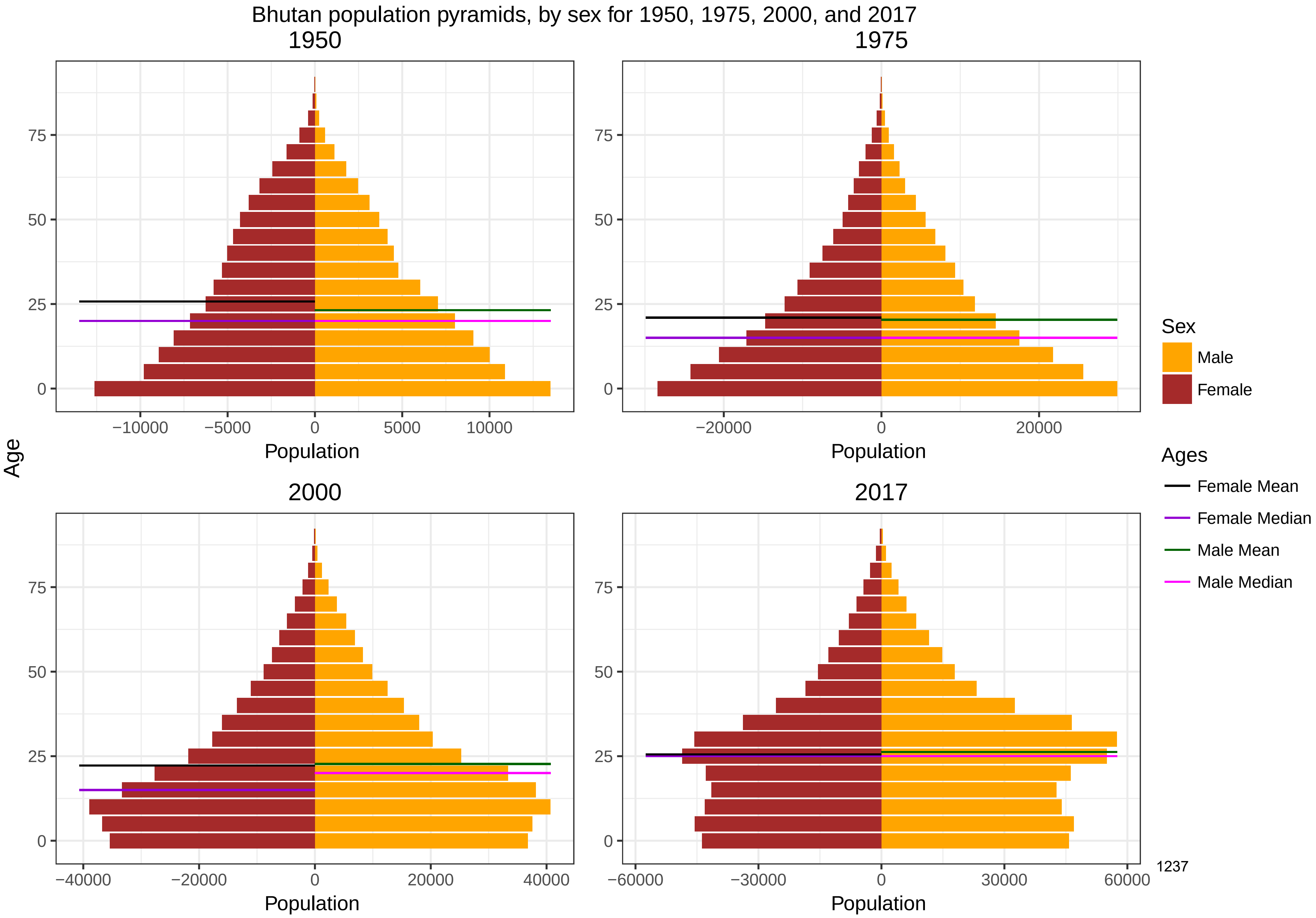
Bhutan population pyramids, by sex for 1950, 1975, 2000, and 2017
1950
1975
2000
2017
Age
Population
Sex
Male
Female
Ages
Female Mean
Female Median
Male Mean
Male Median

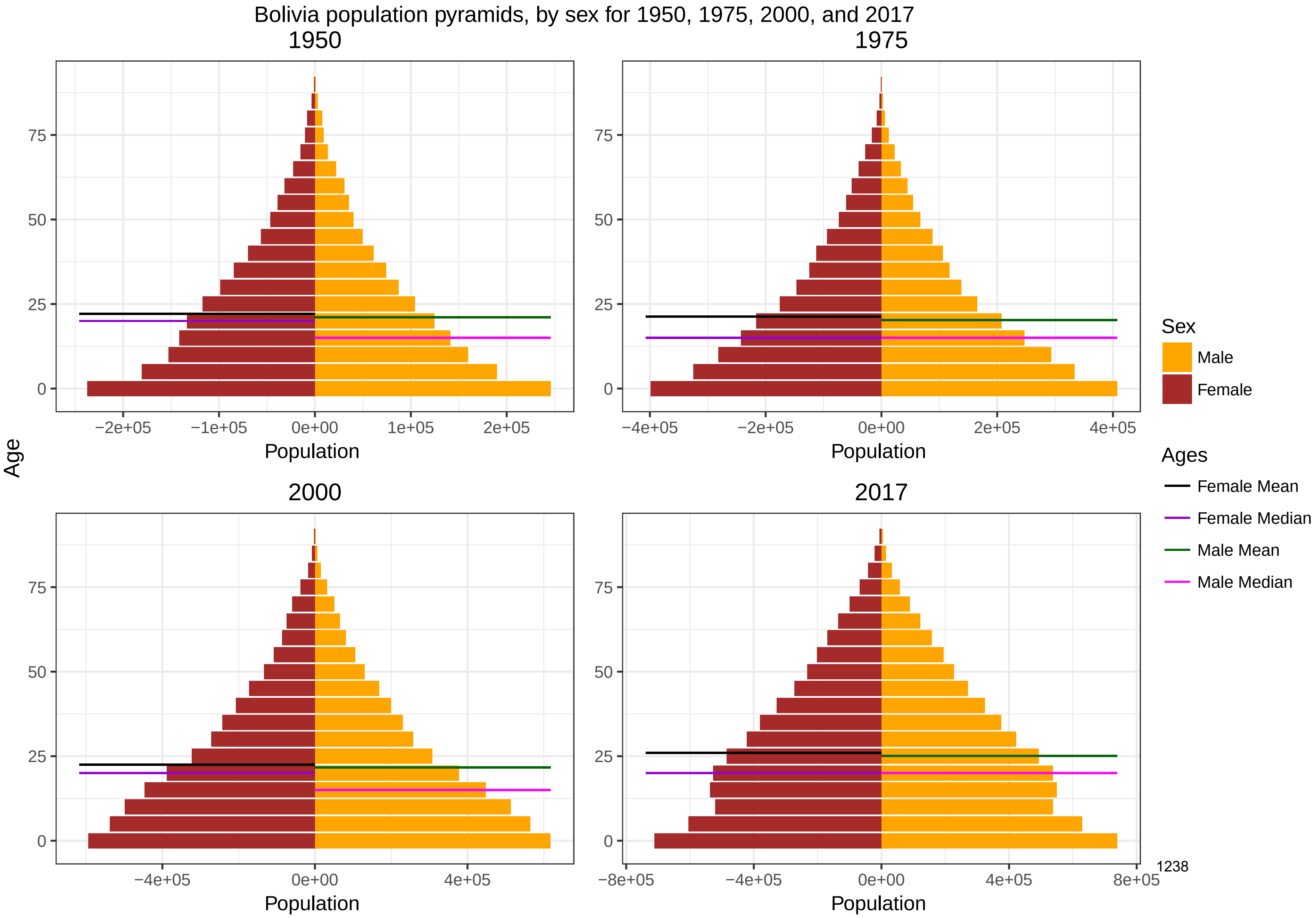

Bolivia population pyramids, by sex for 1950, 1975, 2000, and 2017
1950
1975
2000
2017
Age
Population
0
25
50
75
−2e+05
−1e+05
0e+00
1e+05
2e+05
−4e+05
−2e+05
2e+05
4e+05
8e+05
−8e+05
Sex
Male
Female
Ages
Female Mean
Female Median
Male Mean
Male Median

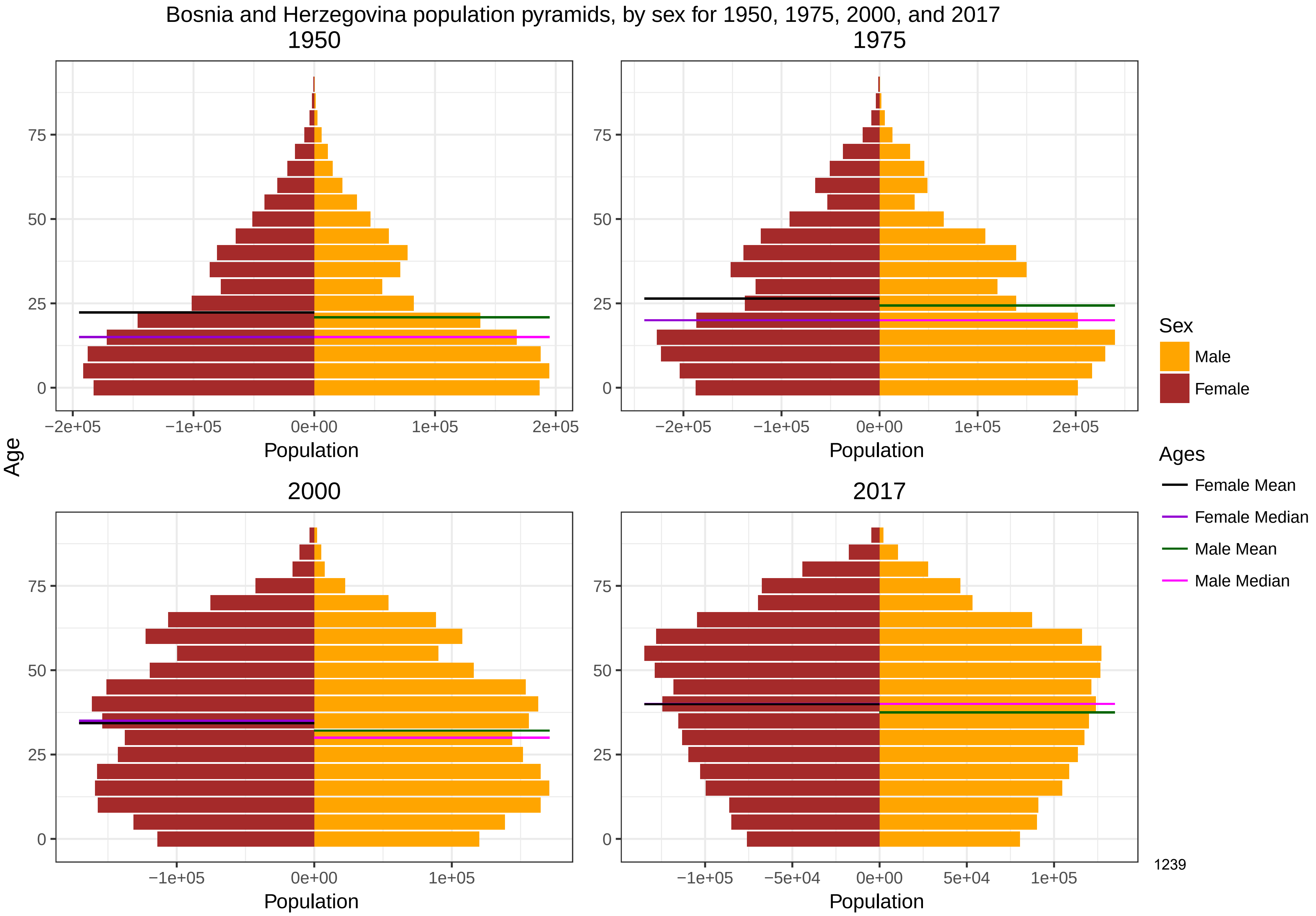
Bosnia and Herzegovina population pyramids, by sex for 1950, 1975, 2000, and 2017
1950
1975
2000
2017
Age
Population
Sex
Male
Female
Ages
Female Mean
Female Median
Male Mean
Male Median
−2e+05
−1e+05
0e+00
1e+05
2e+05
−5e+04
5e+04
0
25
50
75

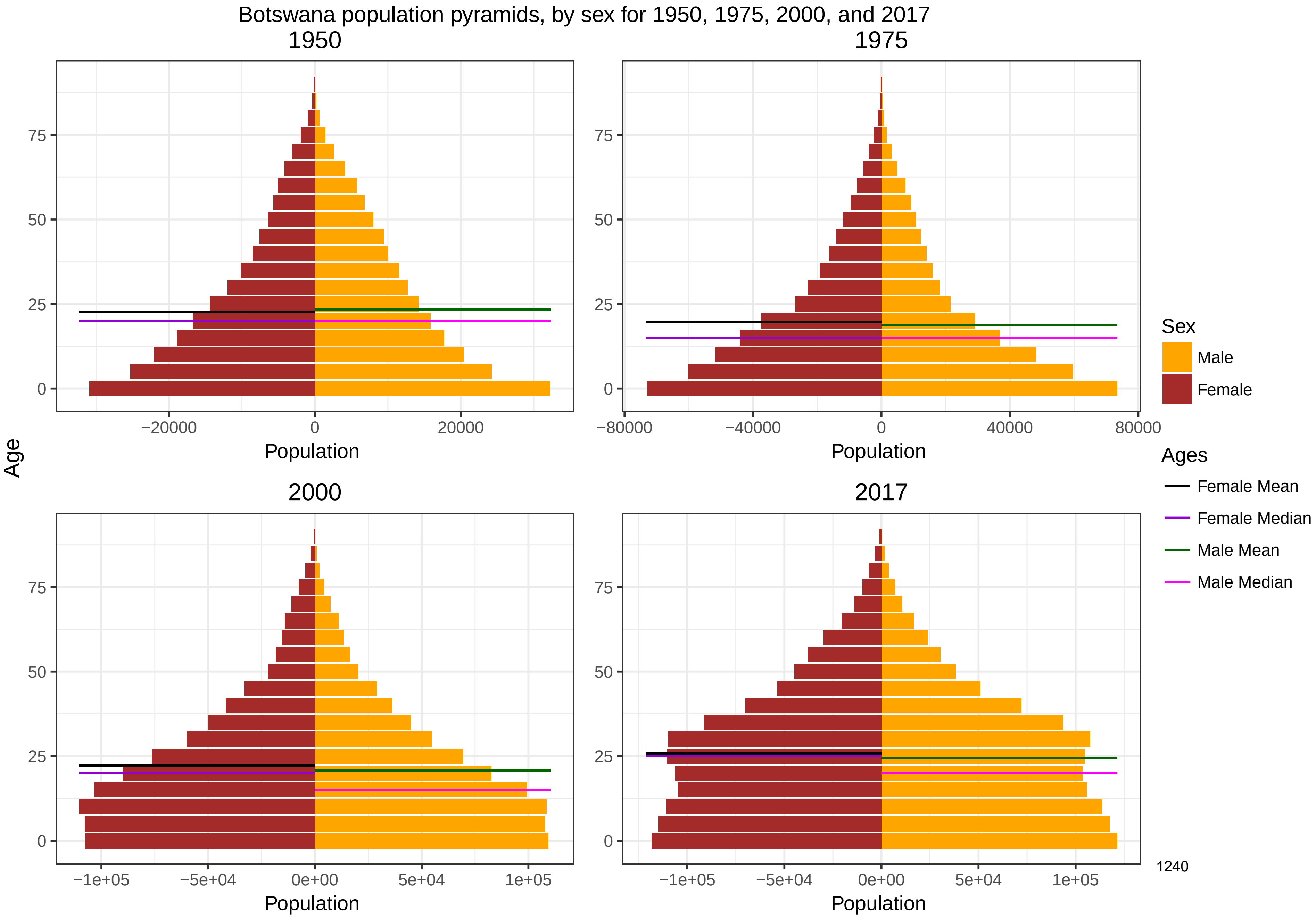
Botswana population pyramids, by sex for 1950, 1975, 2000, and 2017
1950
1975
2000
2017
Age
Population
−20000
0
20000
−80000
−40000
40000
80000
−1e+05
−5e+04
0e+00
5e+04
1e+05
75
50
25
Sex
Male
Female
Ages
Female Mean
Female Median
Male Mean
Male Median

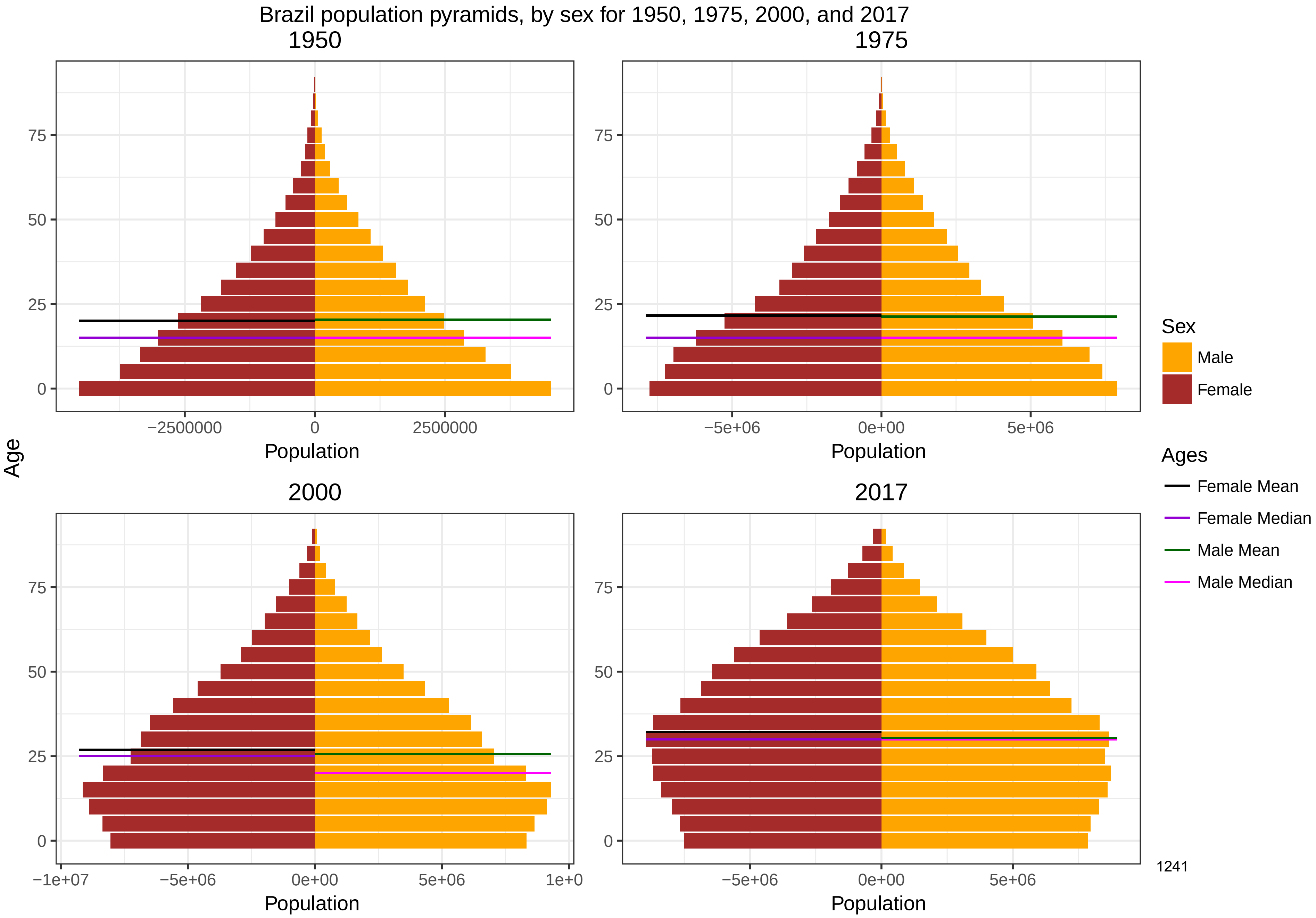
Brazil population pyramids, by sex for 1950, 1975, 2000, and 2017
1950
1975
2000
2017
Age
Population
−2500000
0
2500000
−5e+06
0e+00
5e+06
−1e+07
1e+07
0
25
50
75
Sex
Male
Female
Ages
Female Mean
Female Median
Male Mean
Male Median

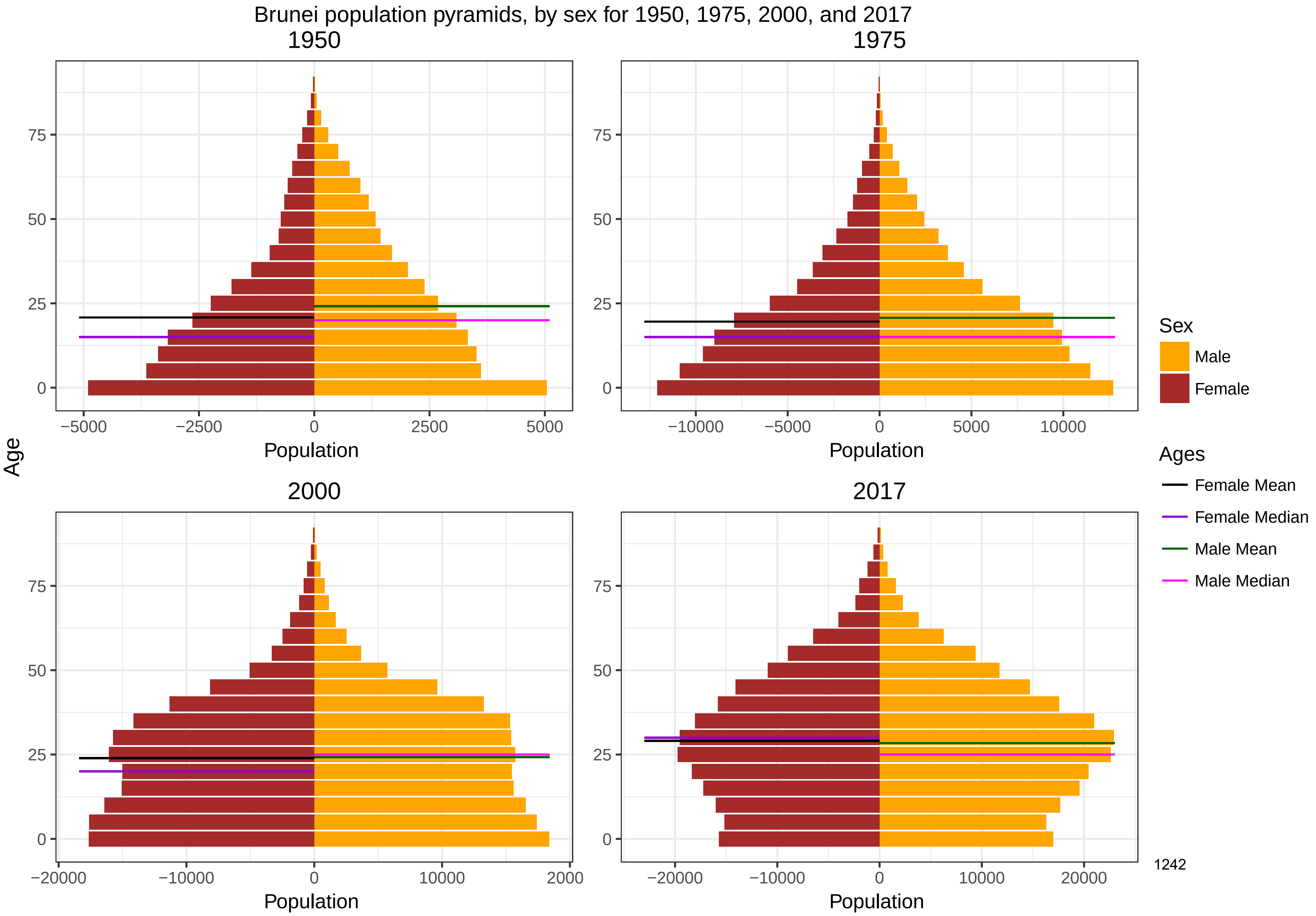
Brunei population pyramids, by sex for 1950, 1975, 2000, and 2017
1950
1975
2000
2017
Age
Population
−5000
−2500
0
2500
5000
−10000
−5000
0
5000
10000
−20000
−10000
0
10000
2000
−20000
−10000
0
10000
20000
75
50
25
0
Sex
Male
Female
Ages
Female Mean
Female Median
Male Mean
Male Median

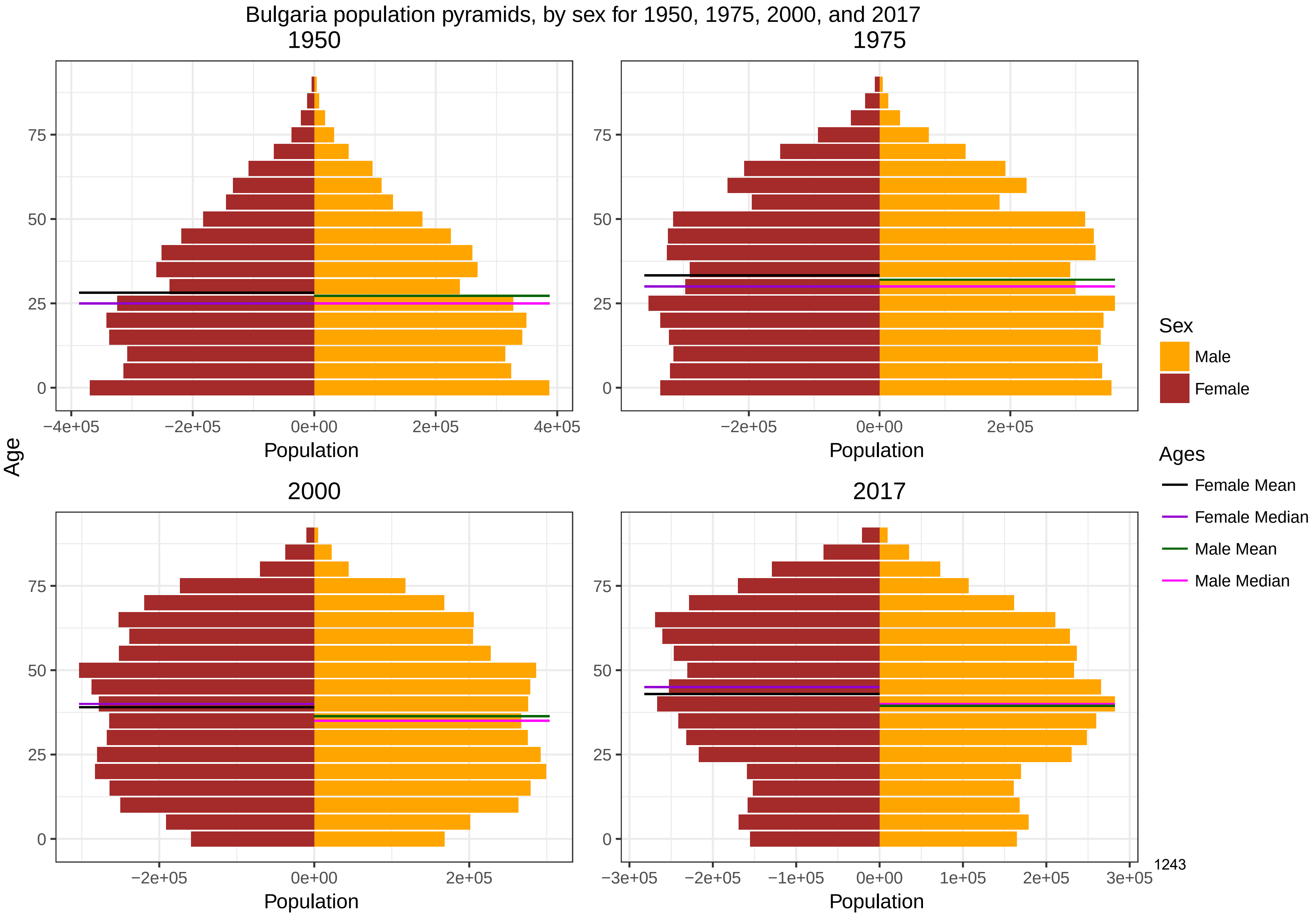

Bulgaria population pyramids, by sex for 1950, 1975, 2000, and 2017
1950
1975
2000
2017
Age
Population
Sex
Male
Female
Ages
Female Mean
Female Median
Male Mean
Male Median

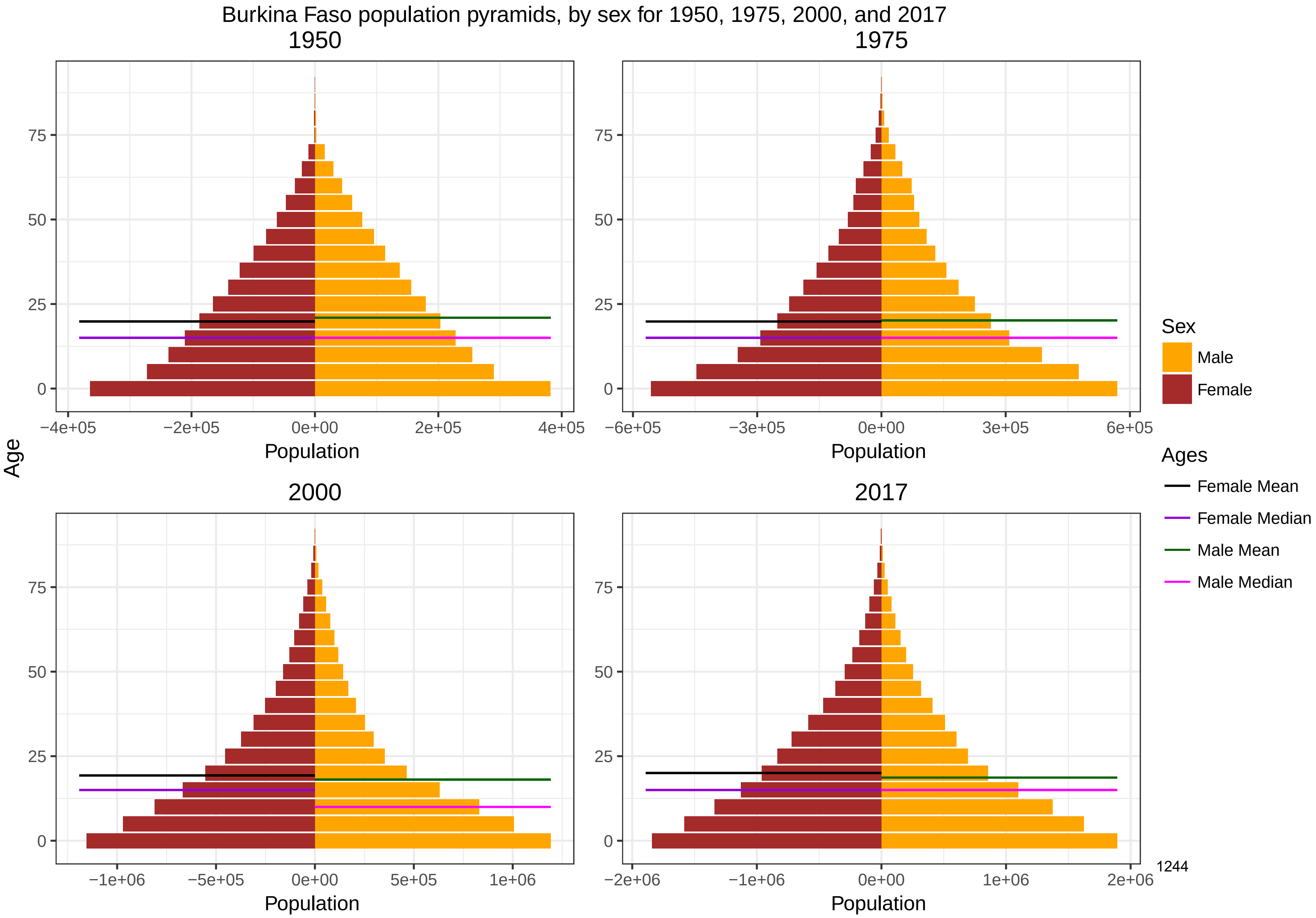
Burkina Faso population pyramids, by sex for 1950, 1975, 2000, and 2017
1950
1975
2000
2017
Age
Population
Sex
Male
Female
Ages
Female Mean
Female Median
Male Mean
Male Median

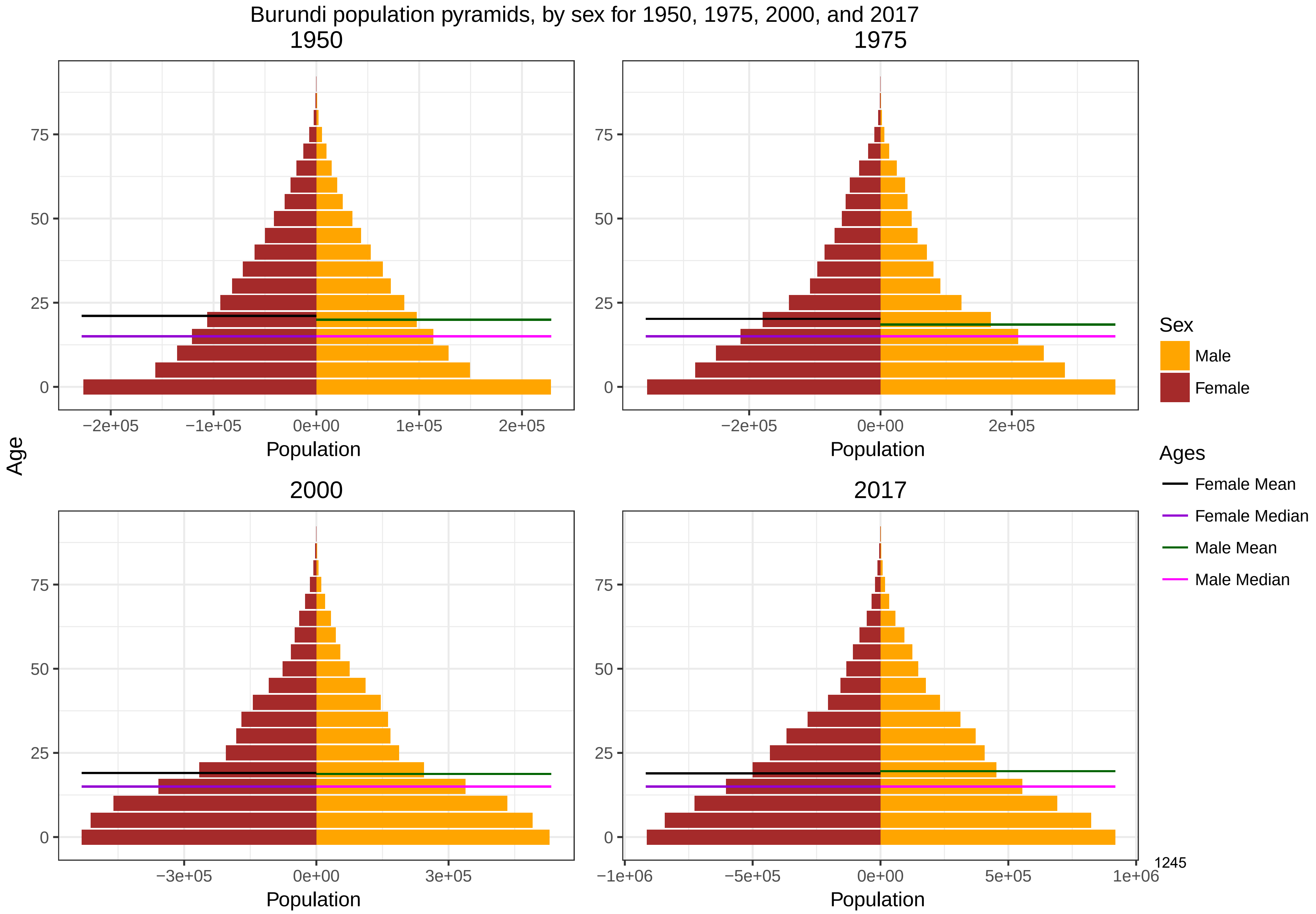
Burundi population pyramids, by sex for 1950, 1975, 2000, and 2017
1950
1975
2000
2017
Age
Population
75
50
25
0
−2e+05
−1e+05
0e+00
1e+05
2e+05
−3e+05
3e+05
−1e+06
−5e+05
5e+05
1e+06
Sex
Male
Female
Ages
Female Mean
Female Median
Male Mean
Male Median

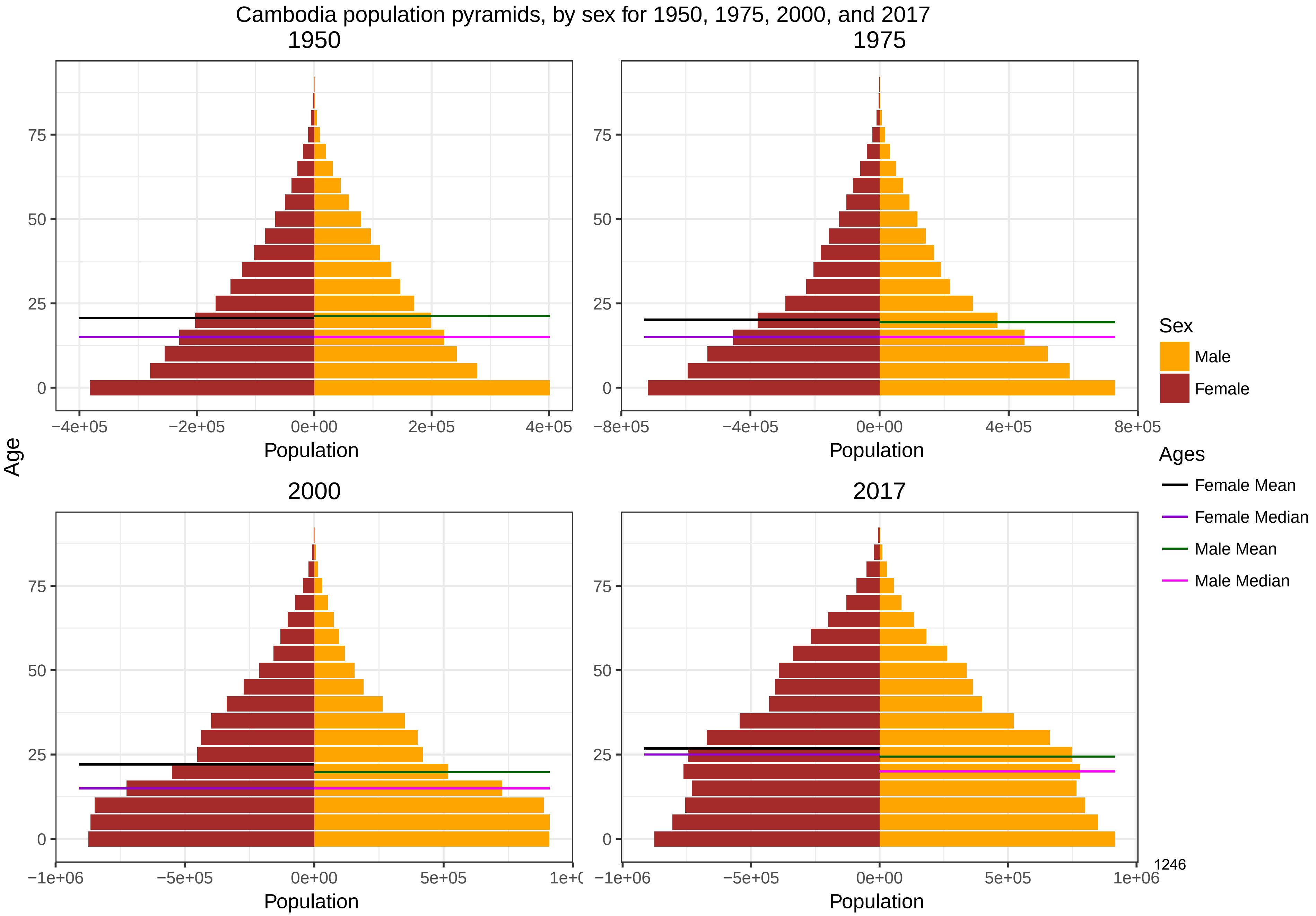
Cambodia population pyramids, by sex for 1950, 1975, 2000, and 2017
1950
1975
2000
2017
Age
Population
0
25
50
75
−4e+05
−2e+05
0e+00
2e+05
4e+05
−8e+05
−4e+05
4e+05
8e+05
−1e+06
−5e+05
5e+05
1e+06
Sex
Male
Female
Ages
Female Mean
Female Median
Male Mean
Male Median

1246

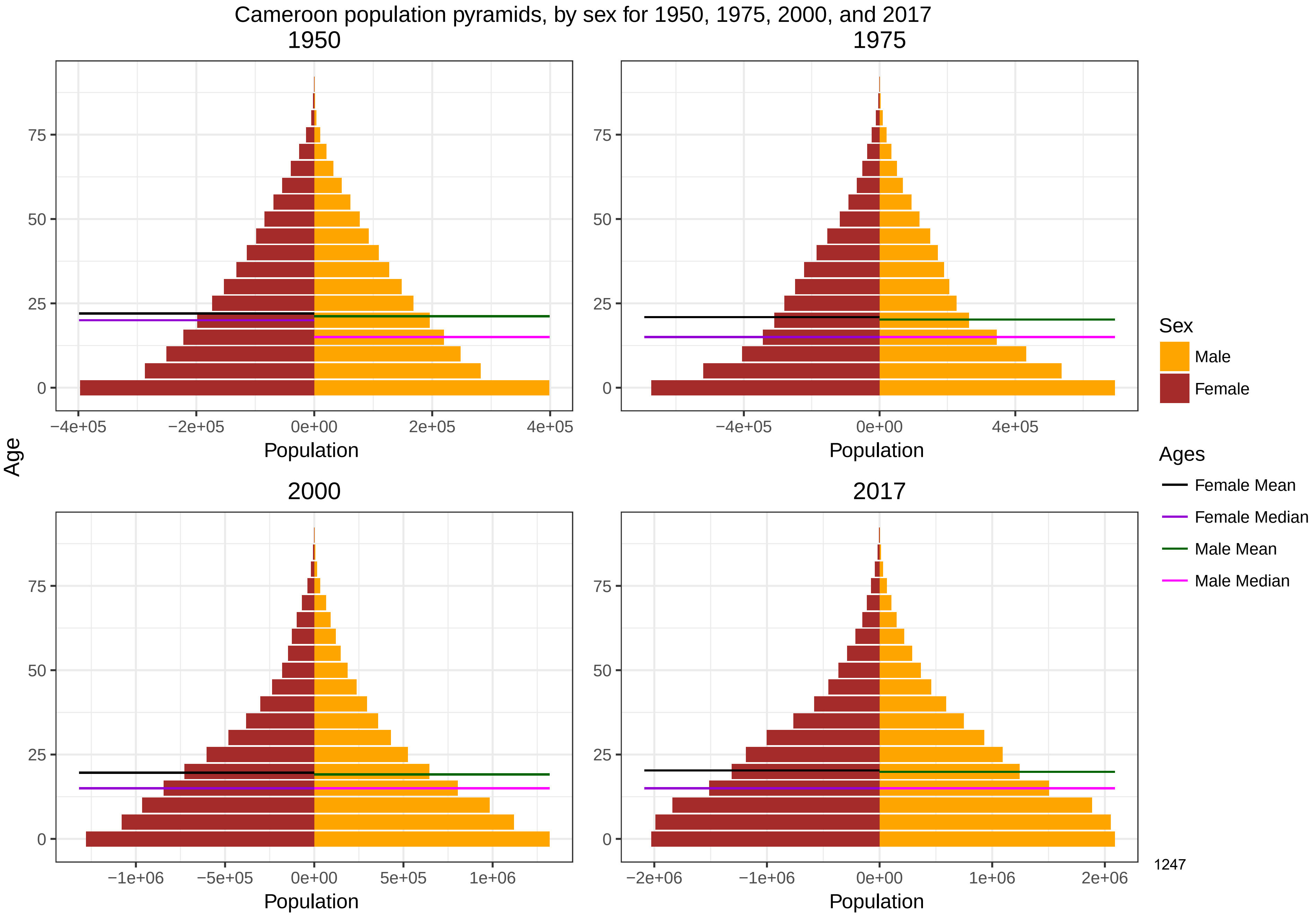

Cameroon population pyramids, by sex for 1950, 1975, 2000, and 2017
1950
1975
2000
2017
Age
Population
Sex
Male
Female
Ages
Female Mean
Female Median
Male Mean
Male Median

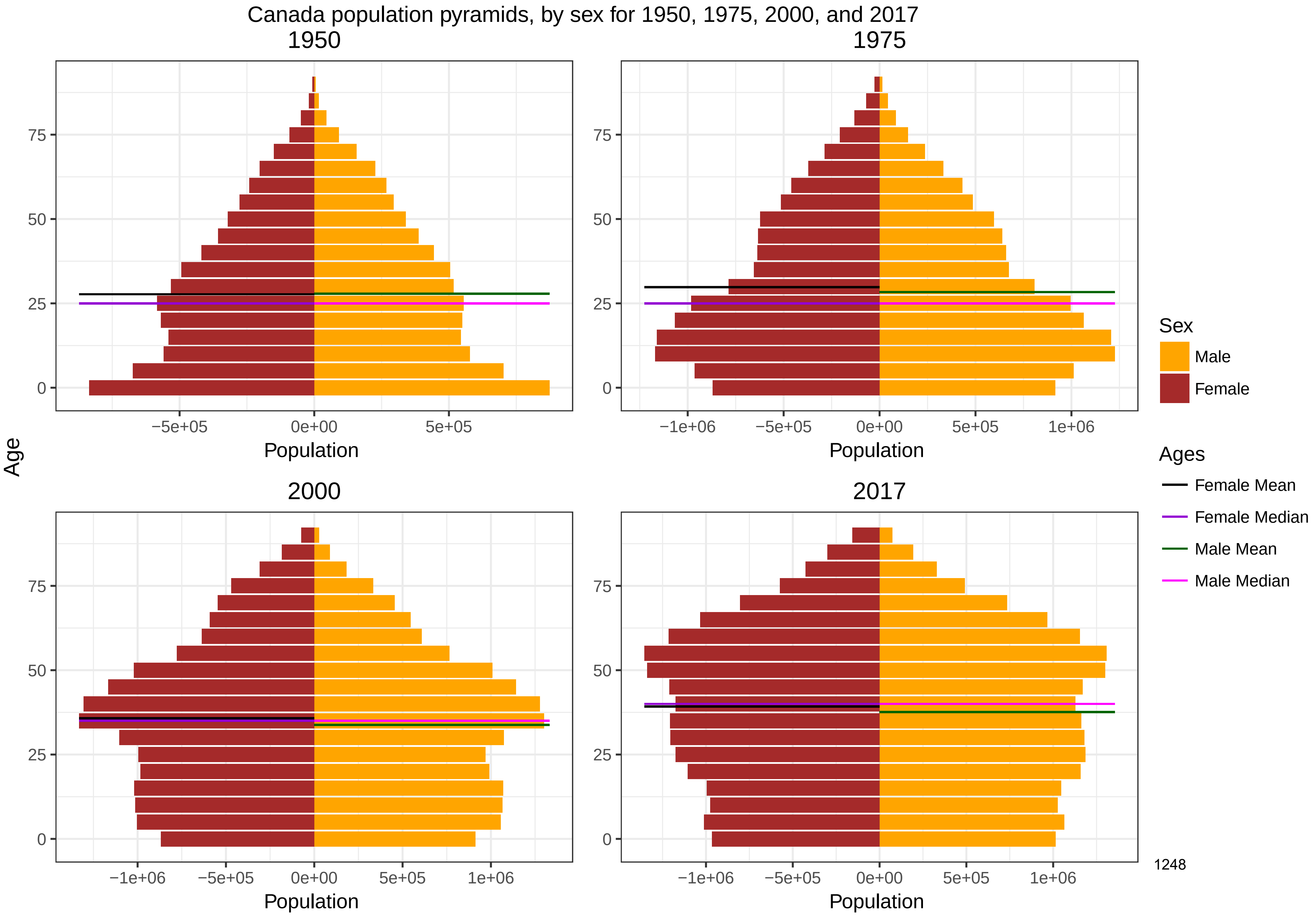
Canada population pyramids, by sex for 1950, 1975, 2000, and 2017
1950
1975
2000
2017
Age
Population
Sex
Male
Female
Ages
Female Mean
Female Median
Male Mean
Male Median

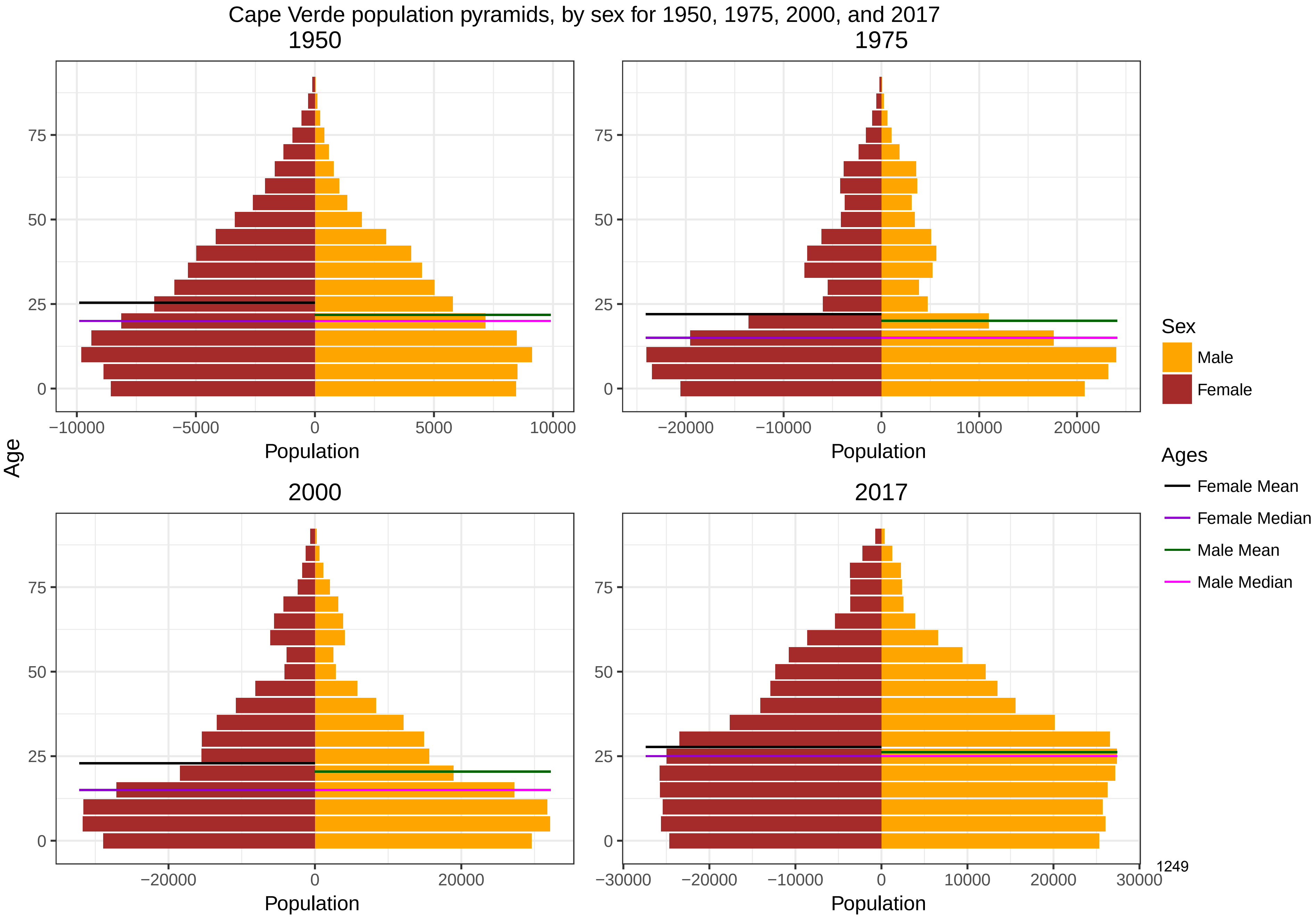

Cape Verde population pyramids, by sex for 1950, 1975, 2000, and 2017
1950
1975
2000
2017
Age
Population
0
25
50
75
−10000
−5000
0
5000
10000
−20000
−10000
0
10000
20000
−30000
30000
Sex
Male
Female
Ages
Female Mean
Female Median
Male Mean
Male Median

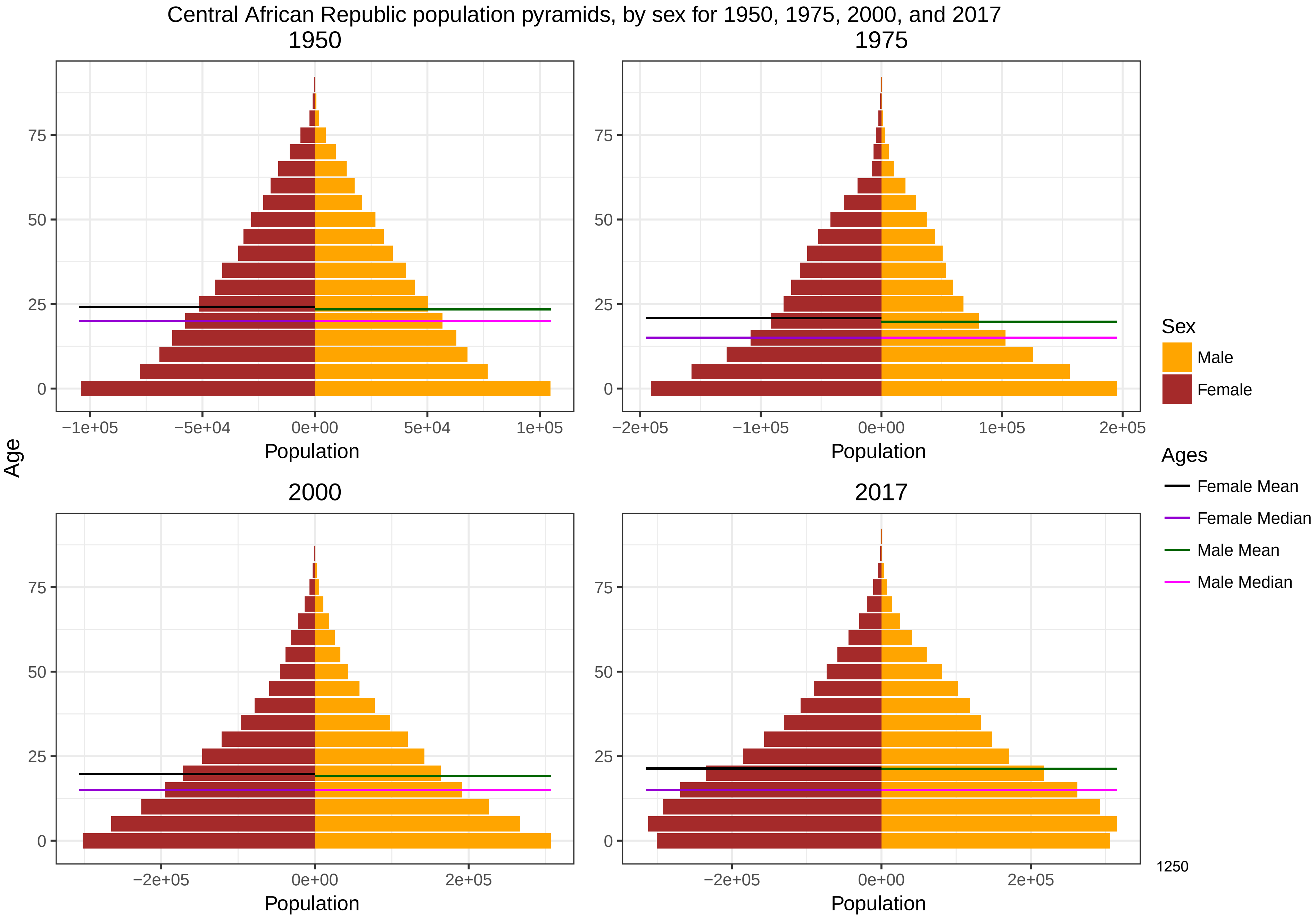
Central African Republic population pyramids, by sex for 1950, 1975, 2000, and 2017
1950
1975
2000
2017
Age
Population
−1e+05
−5e+04
0e+00
5e+04
1e+05
−2e+05
2e+05
0
25
50
75
Sex
Male
Female
Ages
Female Mean
Female Median
Male Mean
Male Median

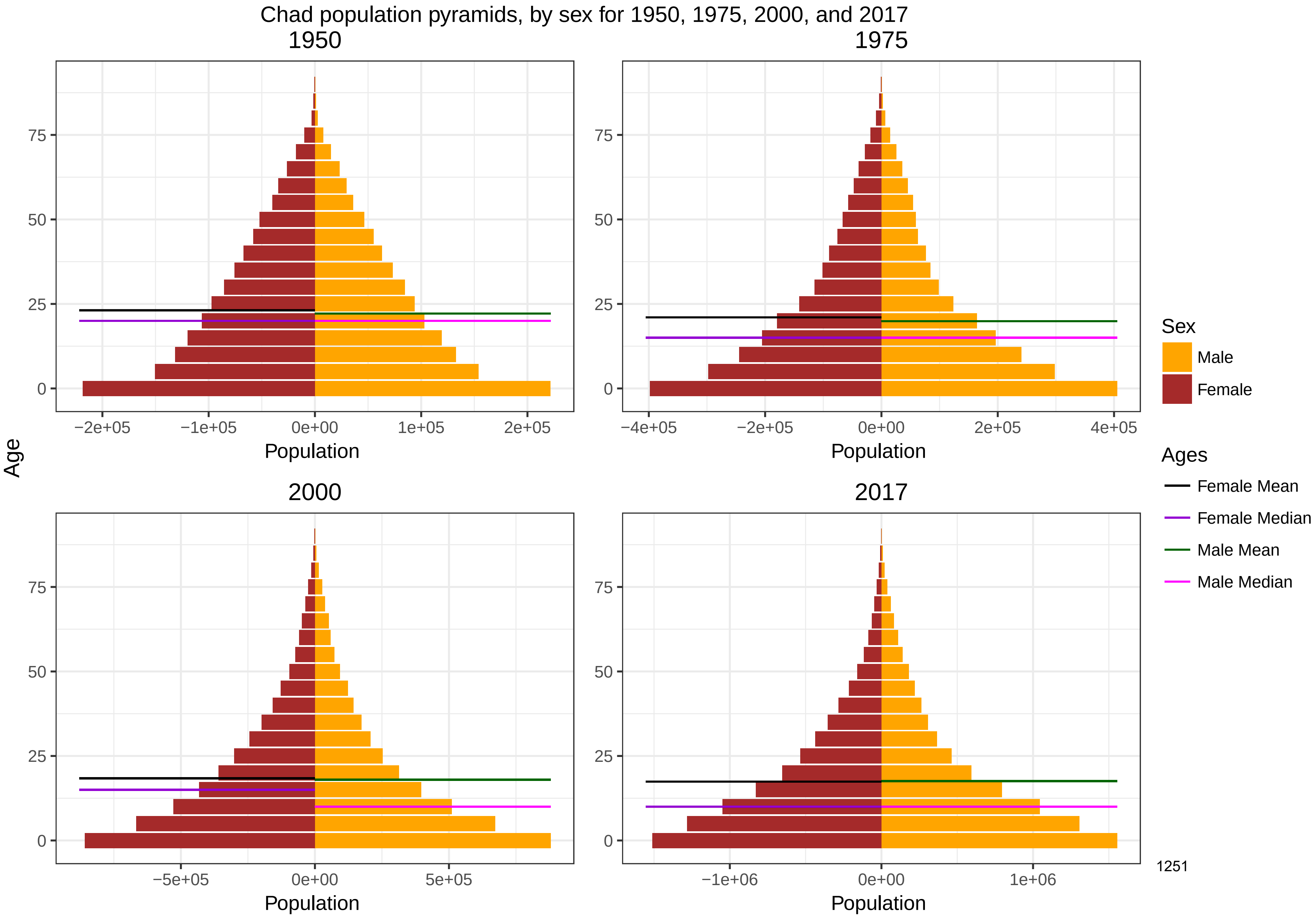
Chad population pyramids, by sex for 1950, 1975, 2000, and 2017
1950
1975
2000
2017
Age
Population
Sex
Male
Female
Ages
Female Mean
Female Median
Male Mean
Male Median

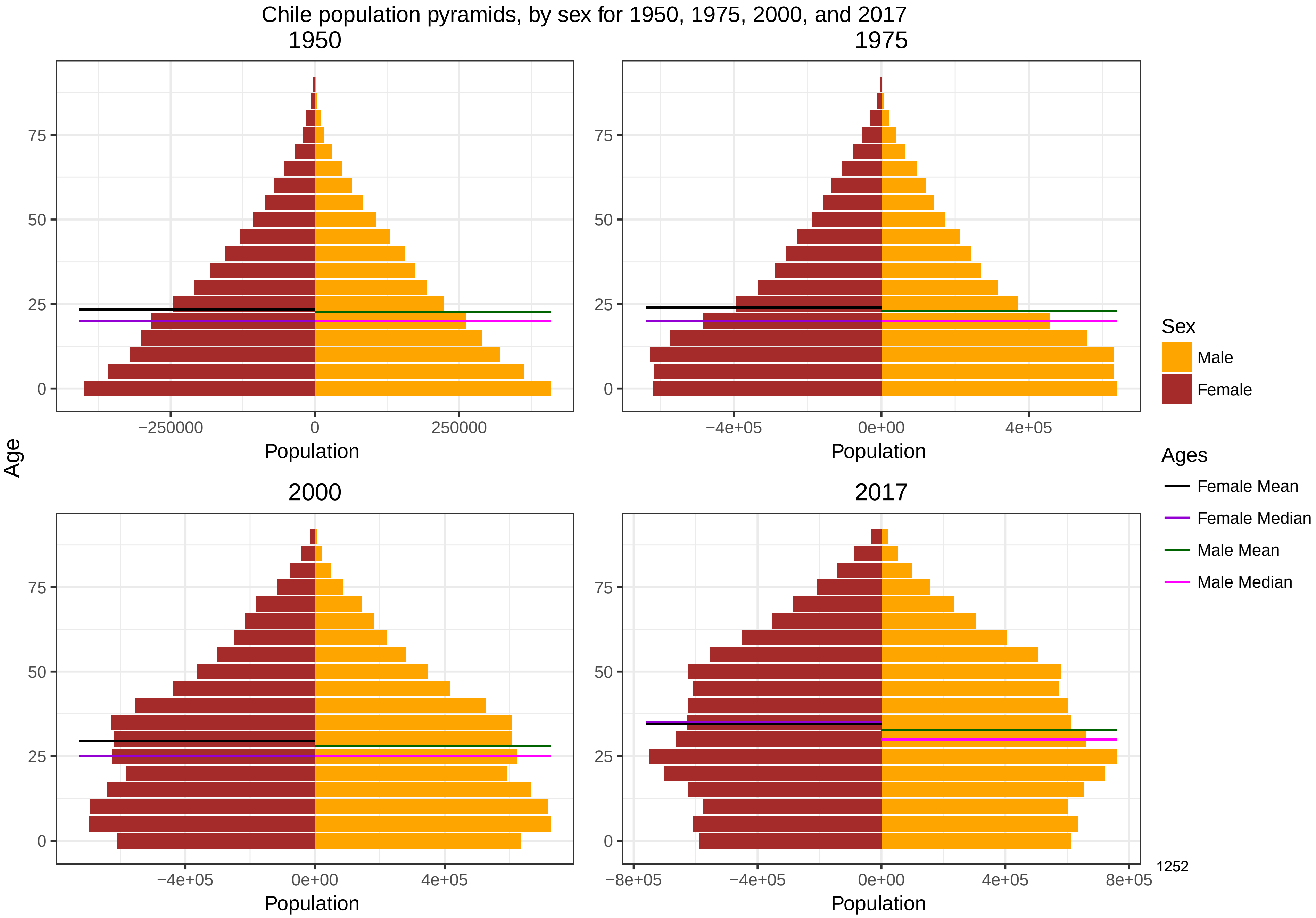

Chile population pyramids, by sex for 1950, 1975, 2000, and 2017
1950
1975
2000
2017
Age
Population
−250000
0
250000
−4e+05
0e+00
4e+05
−8e+05
8e+05
0
25
50
75
Sex
Male
Female
Ages
Female Mean
Female Median
Male Mean
Male Median

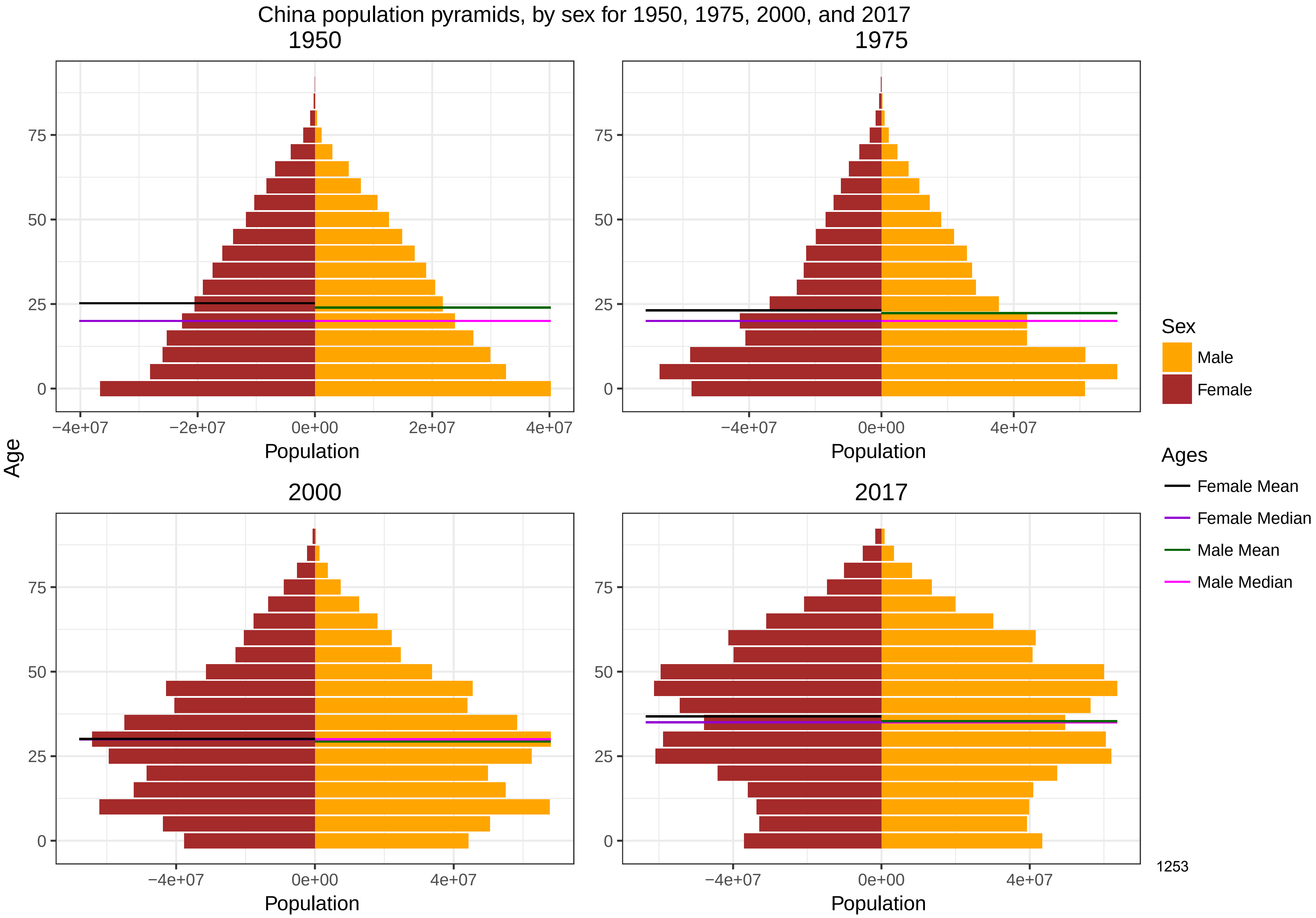

China population pyramids, by sex for 1950, 1975, 2000, and 2017
1950
1975
2000
2017
Age
Population
0
25
50
75
−4e+07
−2e+07
0e+00
2e+07
4e+07
Sex
Male
Female
Ages
Female Mean
Female Median
Male Mean
Male Median

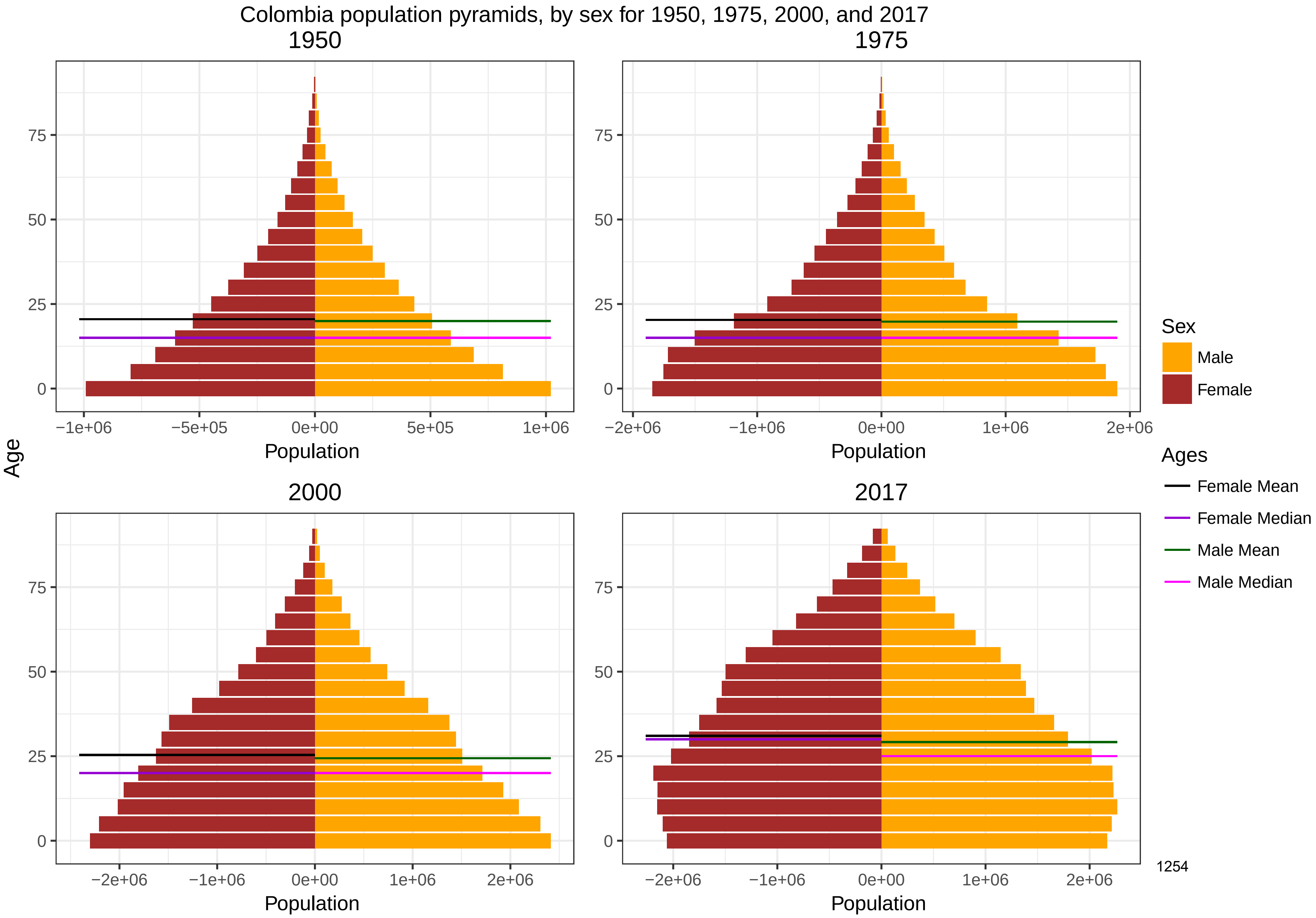
Colombia population pyramids, by sex for 1950, 1975, 2000, and 2017
1950
1975
2000
2017
Age
Population
0
25
50
75
−1e+06
−5e+05
0e+00
5e+05
1e+06
−2e+06
2e+06
Sex
Male
Female
Ages
Female Mean
Female Median
Male Mean
Male Median

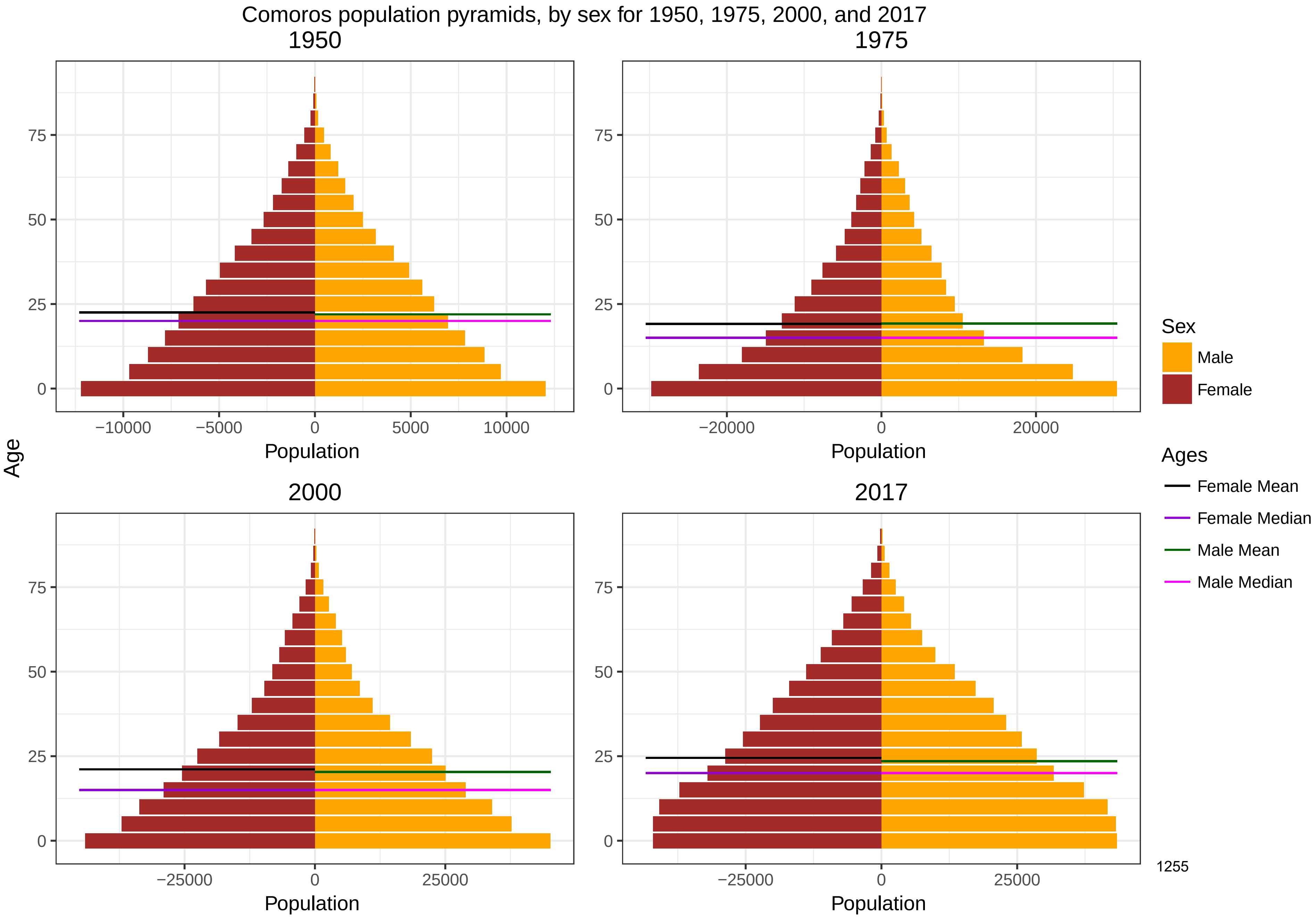
Comoros population pyramids, by sex for 1950, 1975, 2000, and 2017
1950
1975
2000
2017
Age
Population
Sex
Male
Female
Ages
Female Mean
Female Median
Male Mean
Male Median
0
25
50
75
−10000
−5000
0
5000
10000
−20000
20000
−25000
25000

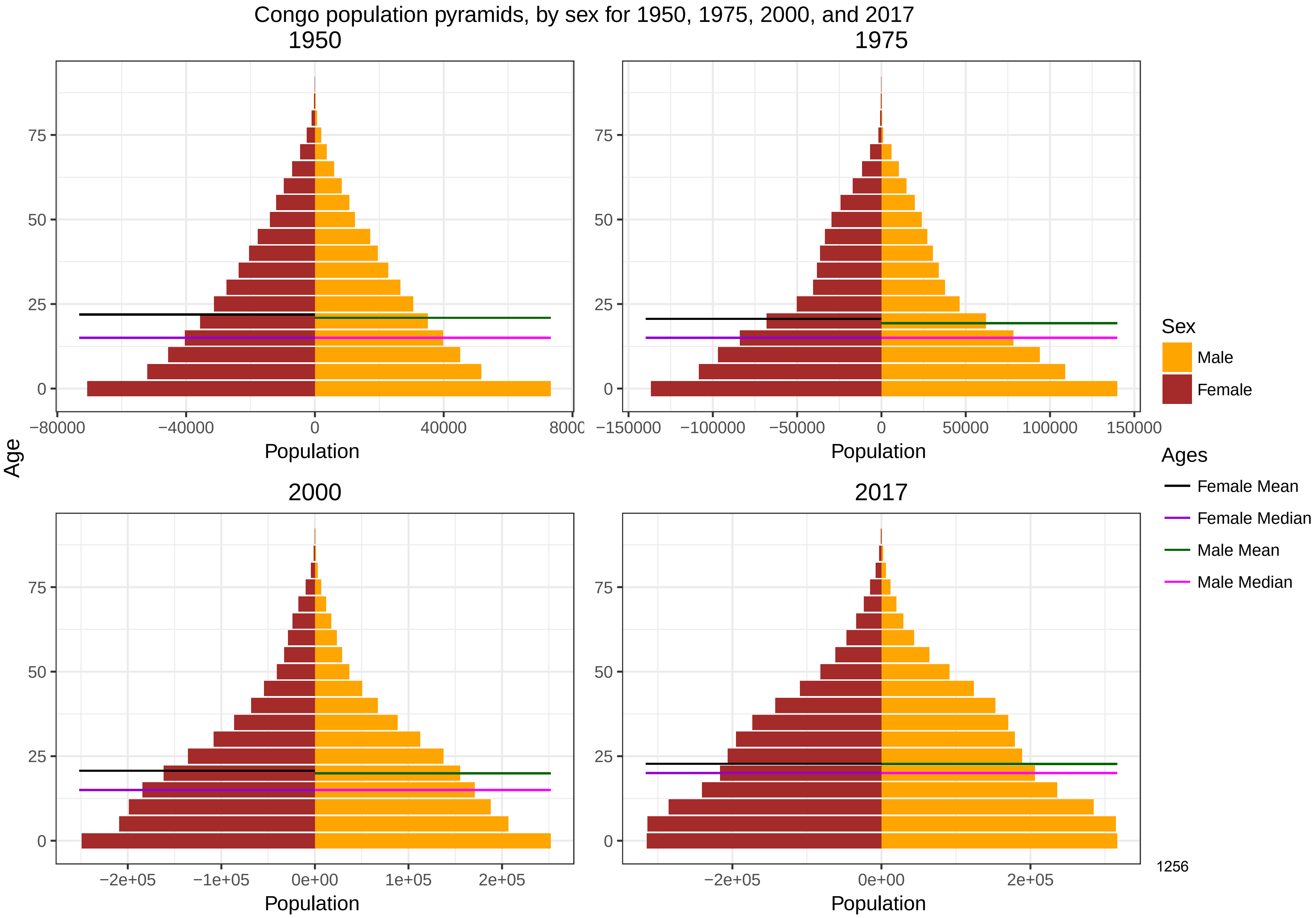
Congo population pyramids, by sex for 1950, 1975, 2000, and 2017
1950
1975
2000
2017
Age
Population
Sex
Male
Female
Ages
Female Mean
Female Median
Male Mean
Male Median
−80000
−40000
0
40000
8000
−150000
−100000
−50000
50000
100000
150000
−2e+05
−1e+05
0e+00
1e+05
2e+05
0
25
50
75

Costa Rica population pyramids, by sex for 1950, 1975, 2000, and 2017

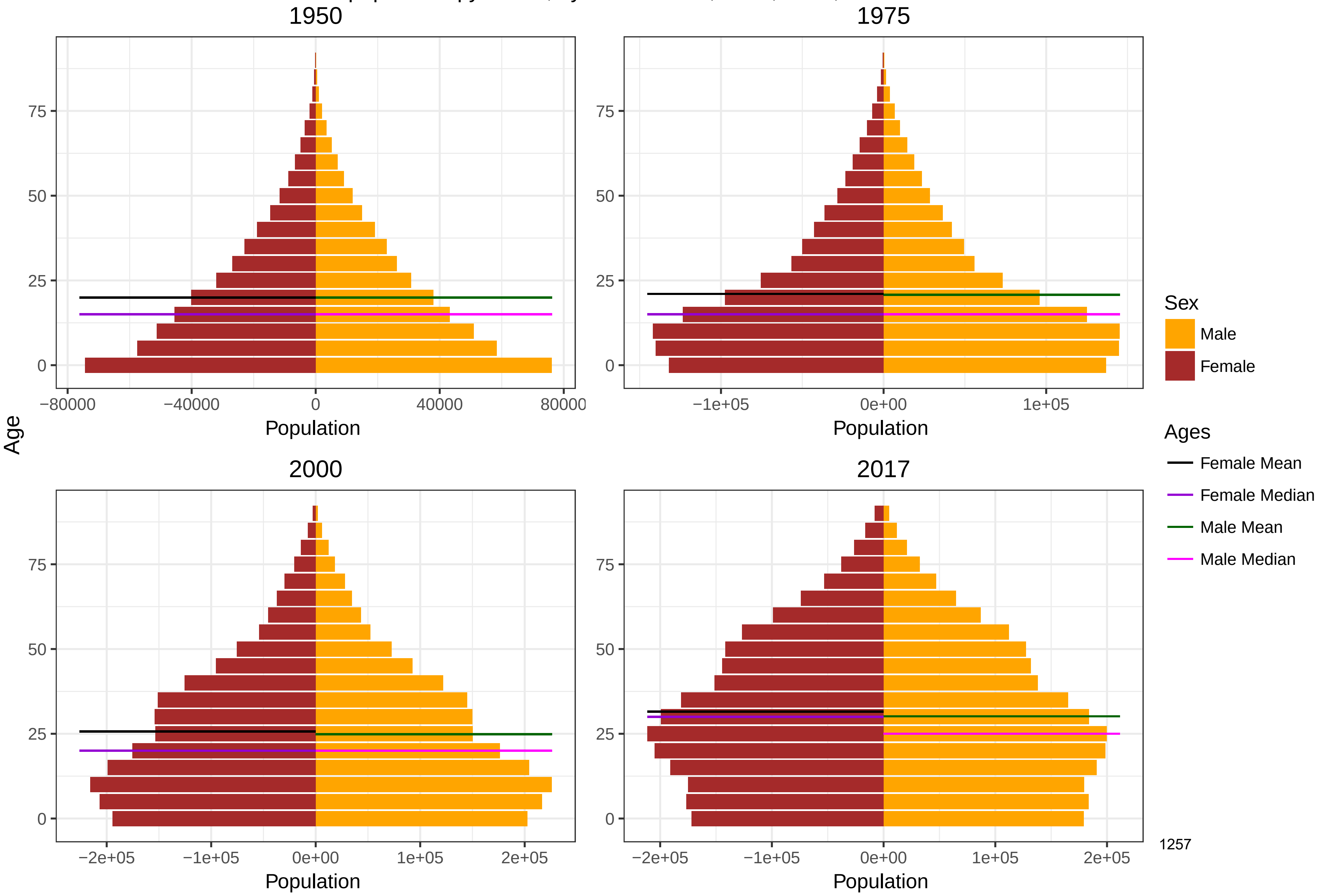

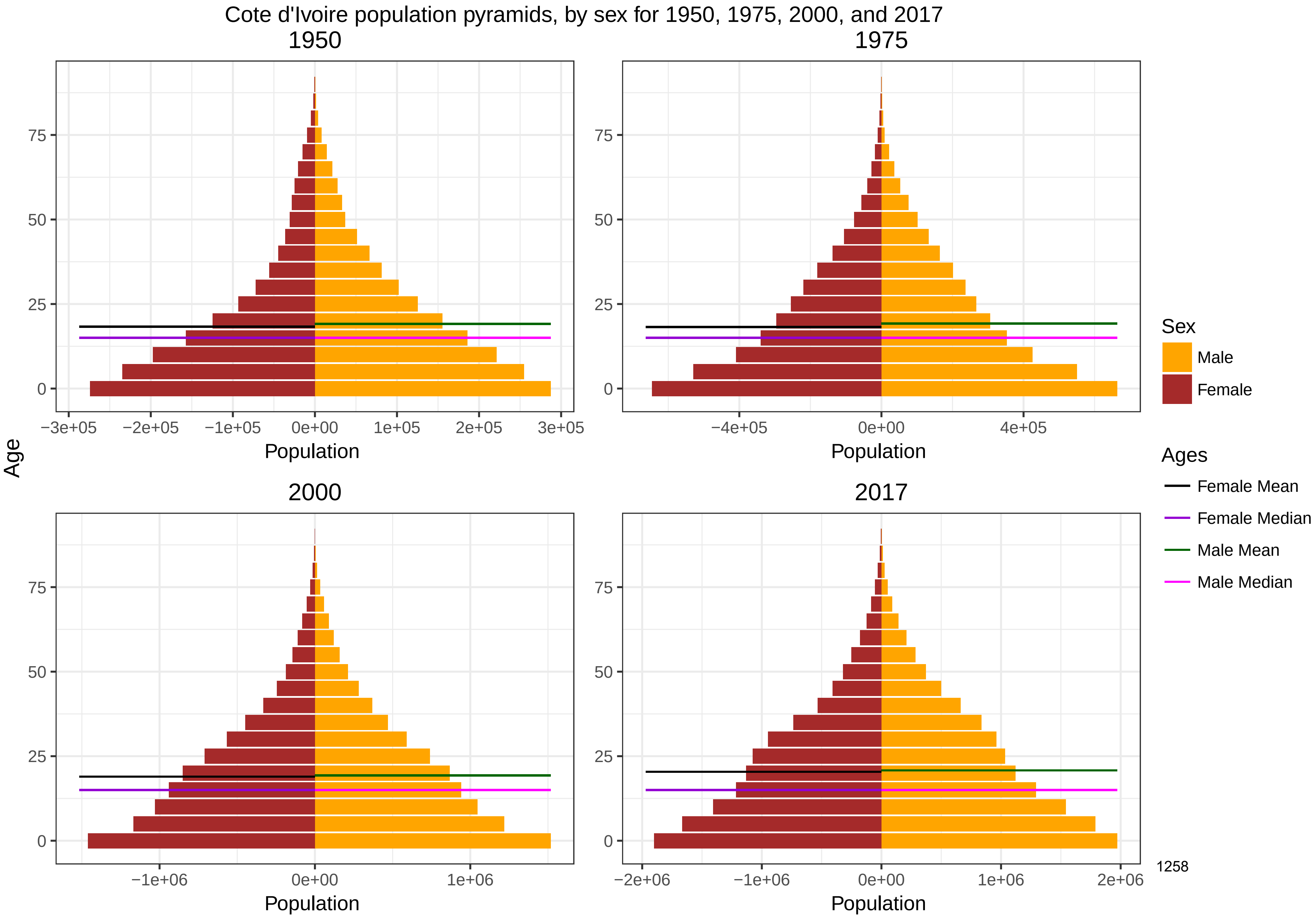

Cote d'Ivoire population pyramids, by sex for 1950, 1975, 2000, and 2017
1950
1975
2000
2017
Age
Population
0
25
50
75
−3e+05
−2e+05
−1e+05
0e+00
1e+05
2e+05
3e+05
−4e+05
4e+05
−1e+06
1e+06
−2e+06
2e+06
Sex
Male
Female
Ages
Female Mean
Female Median
Male Mean
Male Median

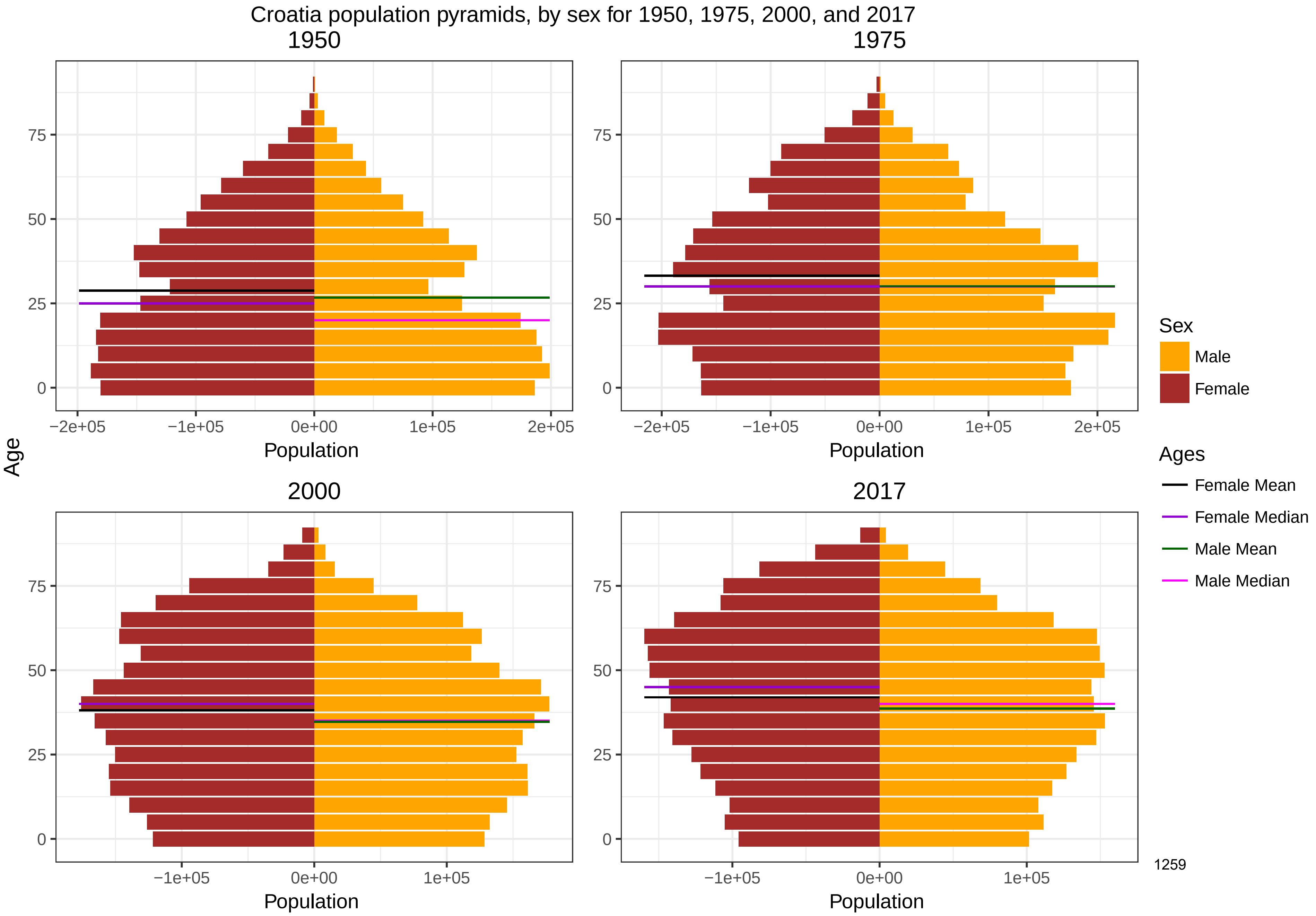
Croatia population pyramids, by sex for 1950, 1975, 2000, and 2017
1950
1975
2000
2017
Age
Population
0
25
50
75
−2e+05
−1e+05
0e+00
1e+05
2e+05
Sex
Male
Female
Ages
Female Mean
Female Median
Male Mean
Male Median

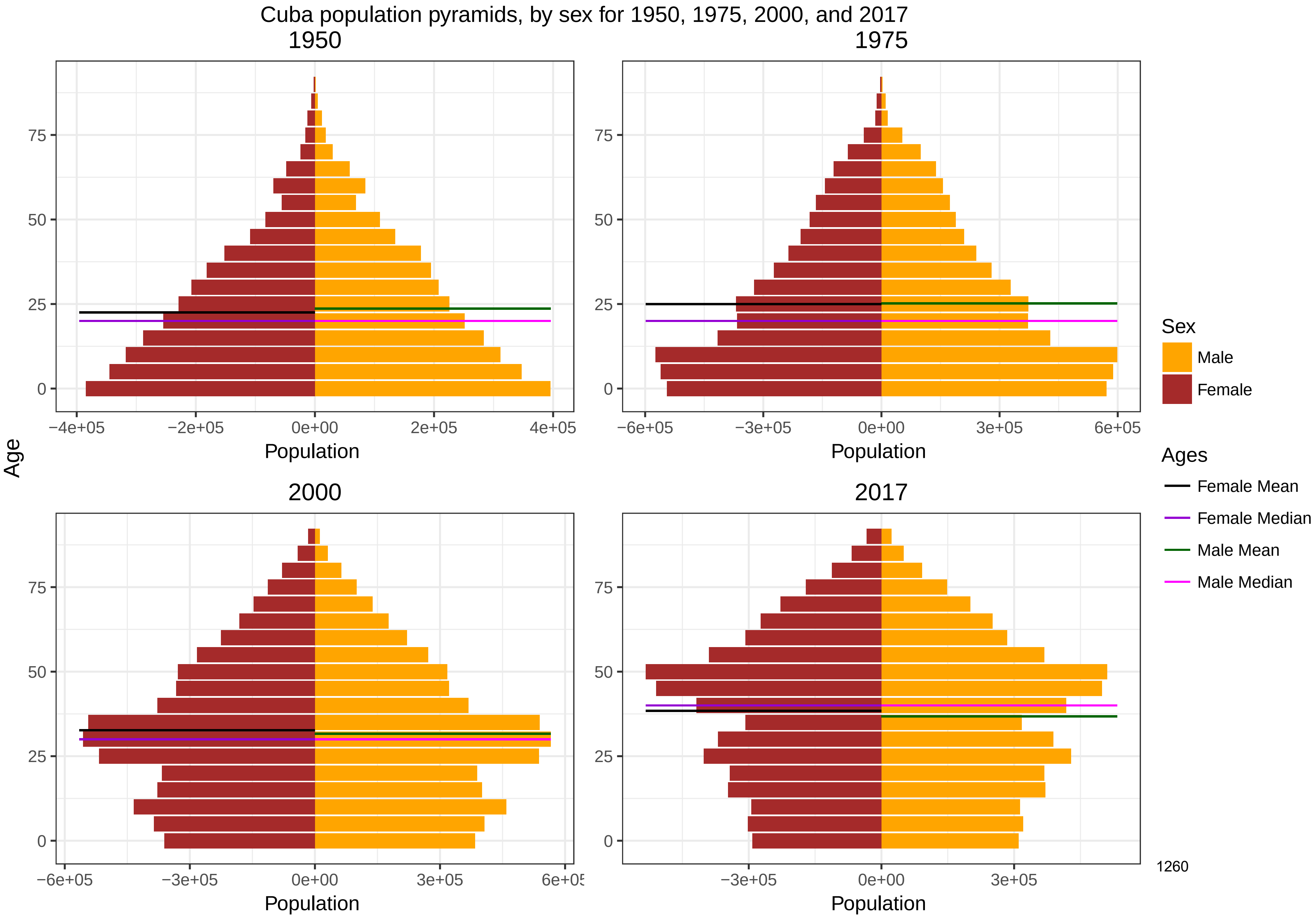
Cuba population pyramids, by sex for 1950, 1975, 2000, and 2017
1950
1975
2000
2017
Age
Population
Sex
Male
Female
Ages
Female Mean
Female Median
Male Mean
Male Median

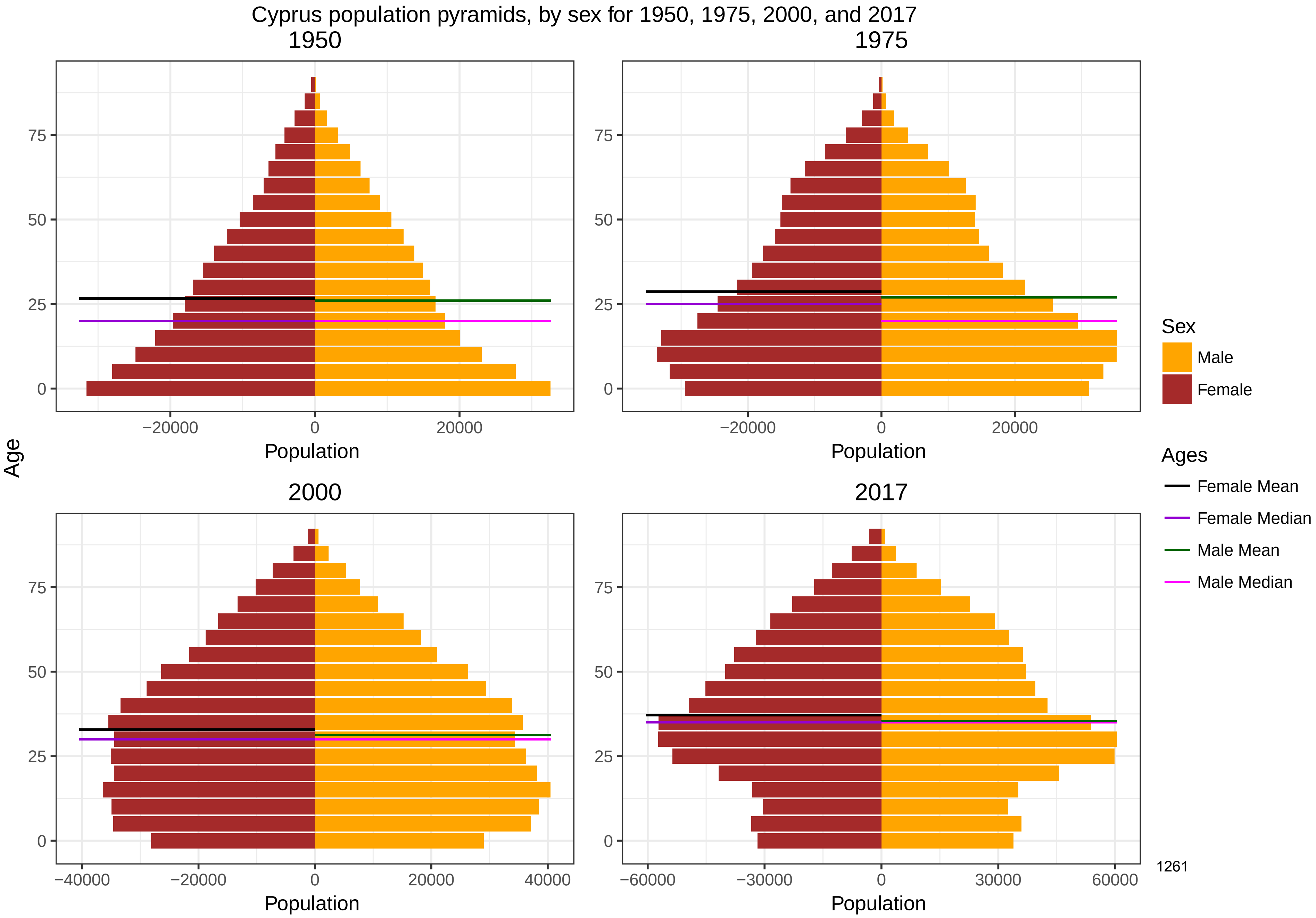
Cyprus population pyramids, by sex for 1950, 1975, 2000, and 2017
1950
1975
2000
2017
Age
Population
0
25
50
75
−20000
0
20000
−40000
40000
−60000
−30000
30000
60000
Sex
Male
Female
Ages
Female Mean
Female Median
Male Mean
Male Median

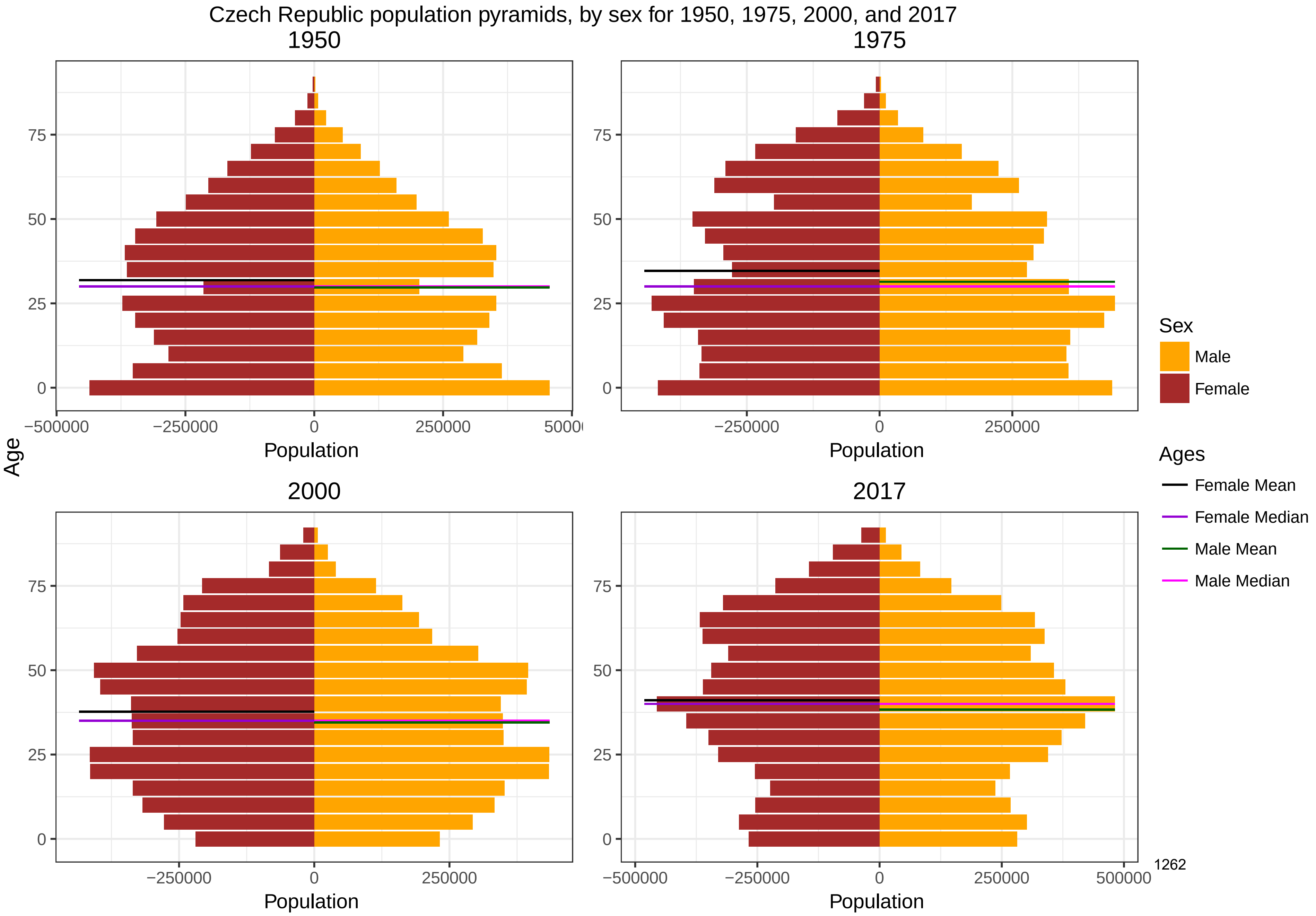

Czech Republic population pyramids, by sex for 1950, 1975, 2000, and 2017
1950
1975
2000
2017
Age
Population
Sex
Male
Female
Ages
Female Mean
Female Median
Male Mean
Male Median

1262

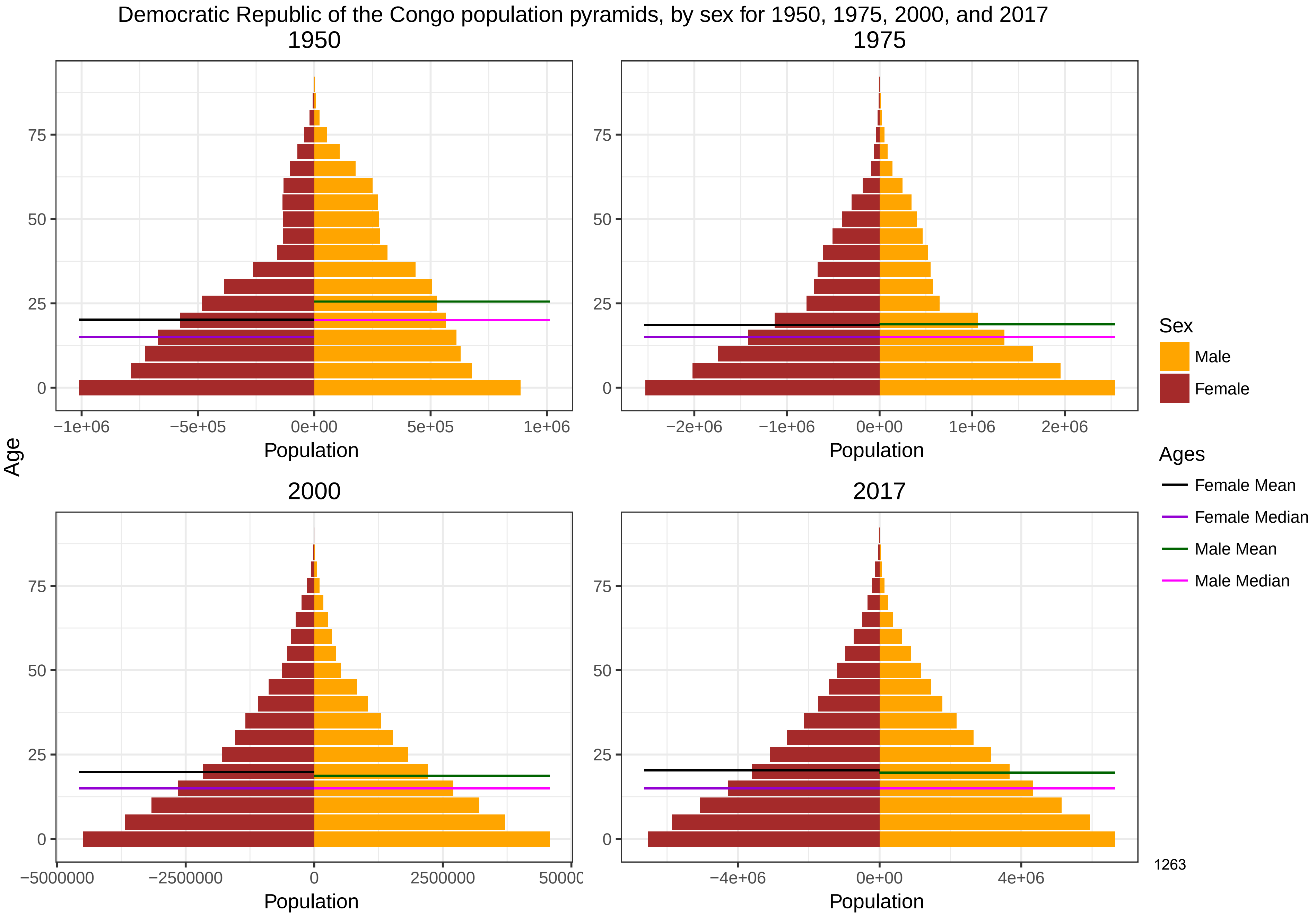
Democratic Republic of the Congo population pyramids, by sex for 1950, 1975, 2000, and 2017
1950
1975
2000
2017
Age
Population
Sex
Male
Female
Ages
Female Mean
Female Median
Male Mean
Male Median

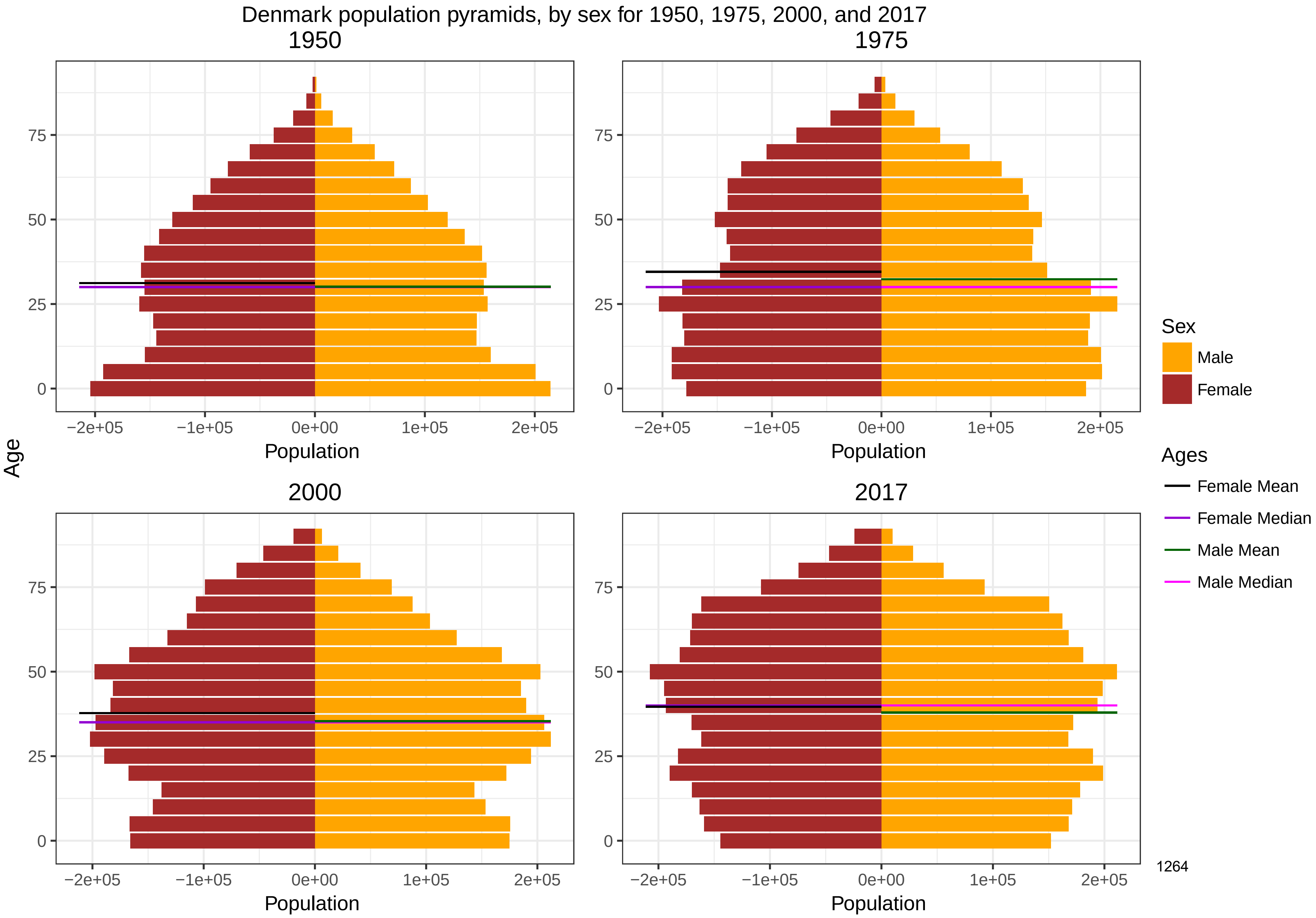

Denmark population pyramids, by sex for 1950, 1975, 2000, and 2017
1950
1975
2000
2017
Age
Population
0
25
50
75
−2e+05
−1e+05
0e+00
1e+05
2e+05
Sex
Male
Female
Ages
Female Mean
Female Median
Male Mean
Male Median

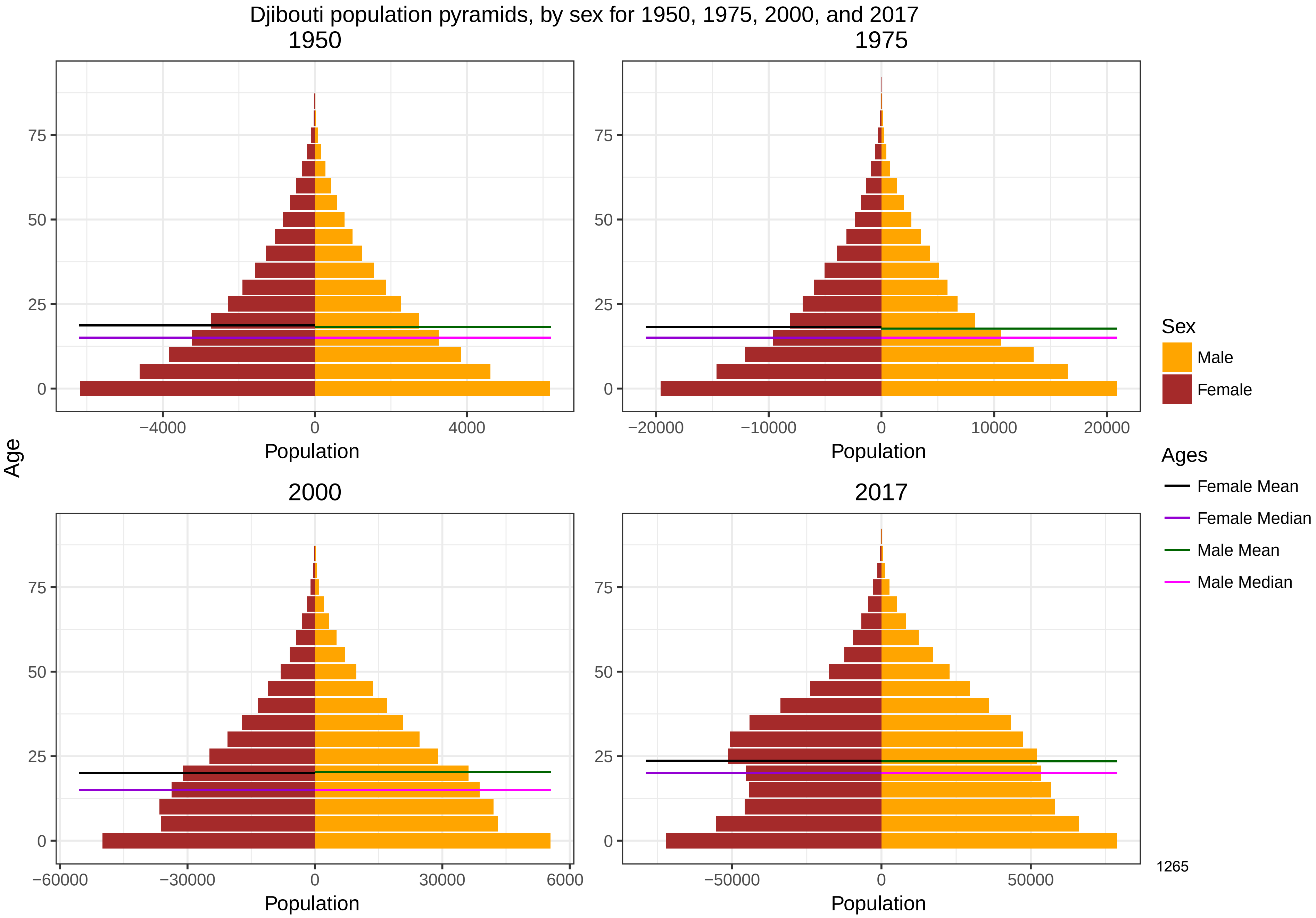
Djibouti population pyramids, by sex for 1950, 1975, 2000, and 2017
1950
1975
2000
2017
Age
Population
Sex
Male
Female
Ages
Female Mean
Female Median
Male Mean
Male Median
−4000
0
4000
−20000
−10000
10000
20000
−60000
−30000
30000
6000
−50000
50000
25
50
75

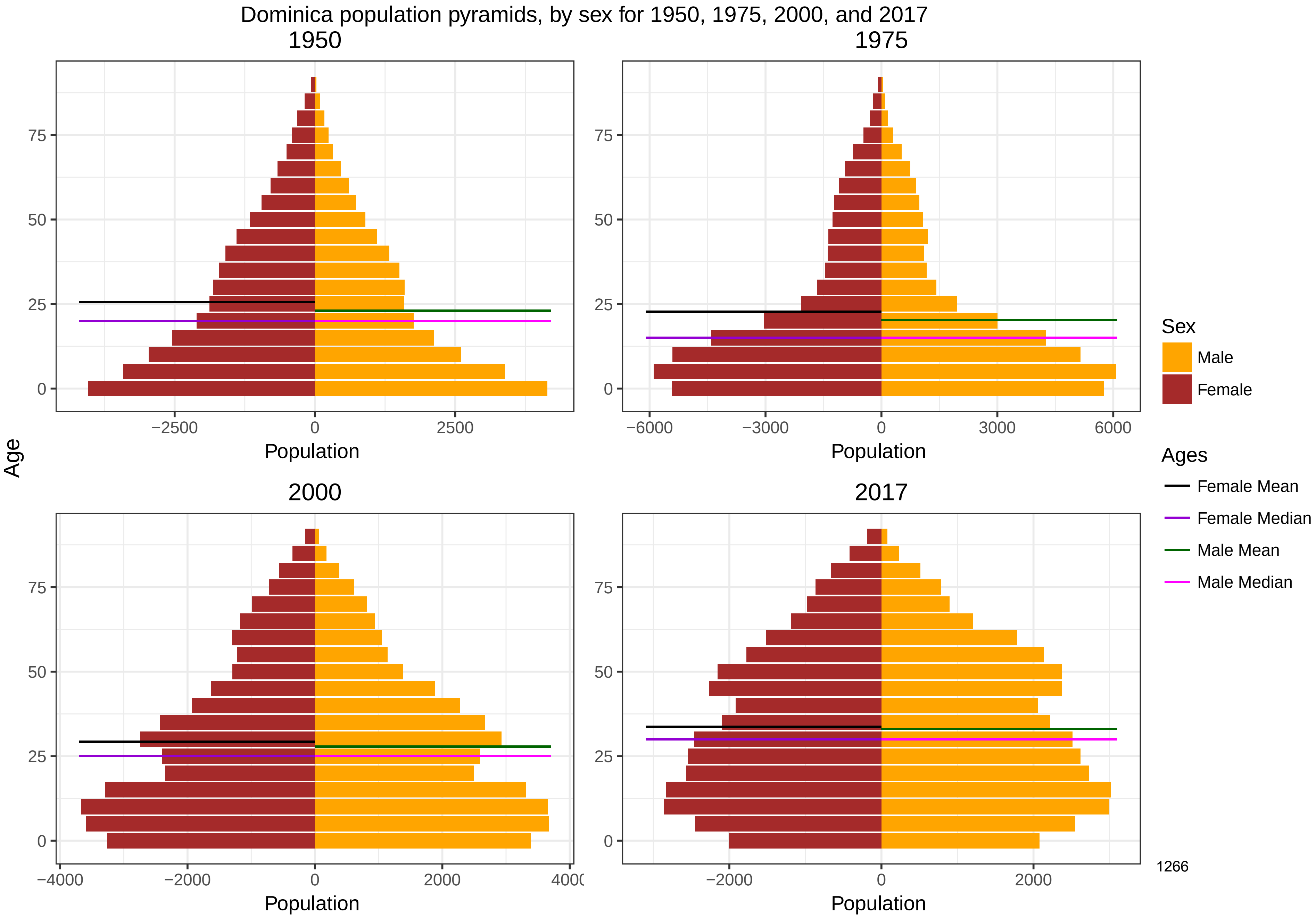

Dominica population pyramids, by sex for 1950, 1975, 2000, and 2017
1950
1975
2000
2017
Age
Population
Sex
Male
Female
Ages
Female Mean
Female Median
Male Mean
Male Median

1266

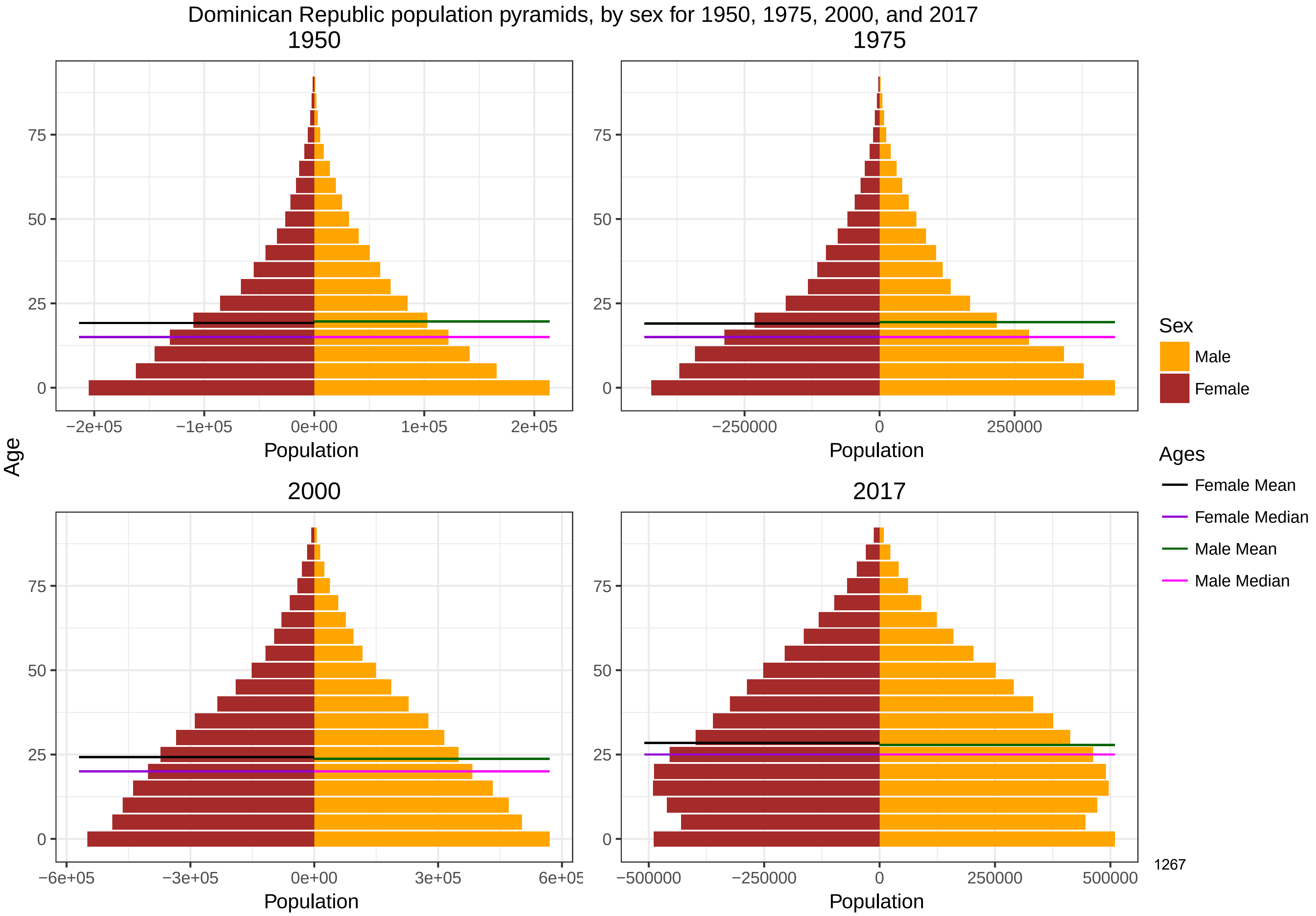

Dominican Republic population pyramids, by sex for 1950, 1975, 2000, and 2017
1950
1975
2000
2017
Age
Population
−2e+05
−1e+05
0e+00
1e+05
2e+05
−250000
0
250000
−6e+05
−3e+05
3e+05
6e+05
−500000
500000
0
25
50
75
Sex
Male
Female
Ages
Female Mean
Female Median
Male Mean
Male Median

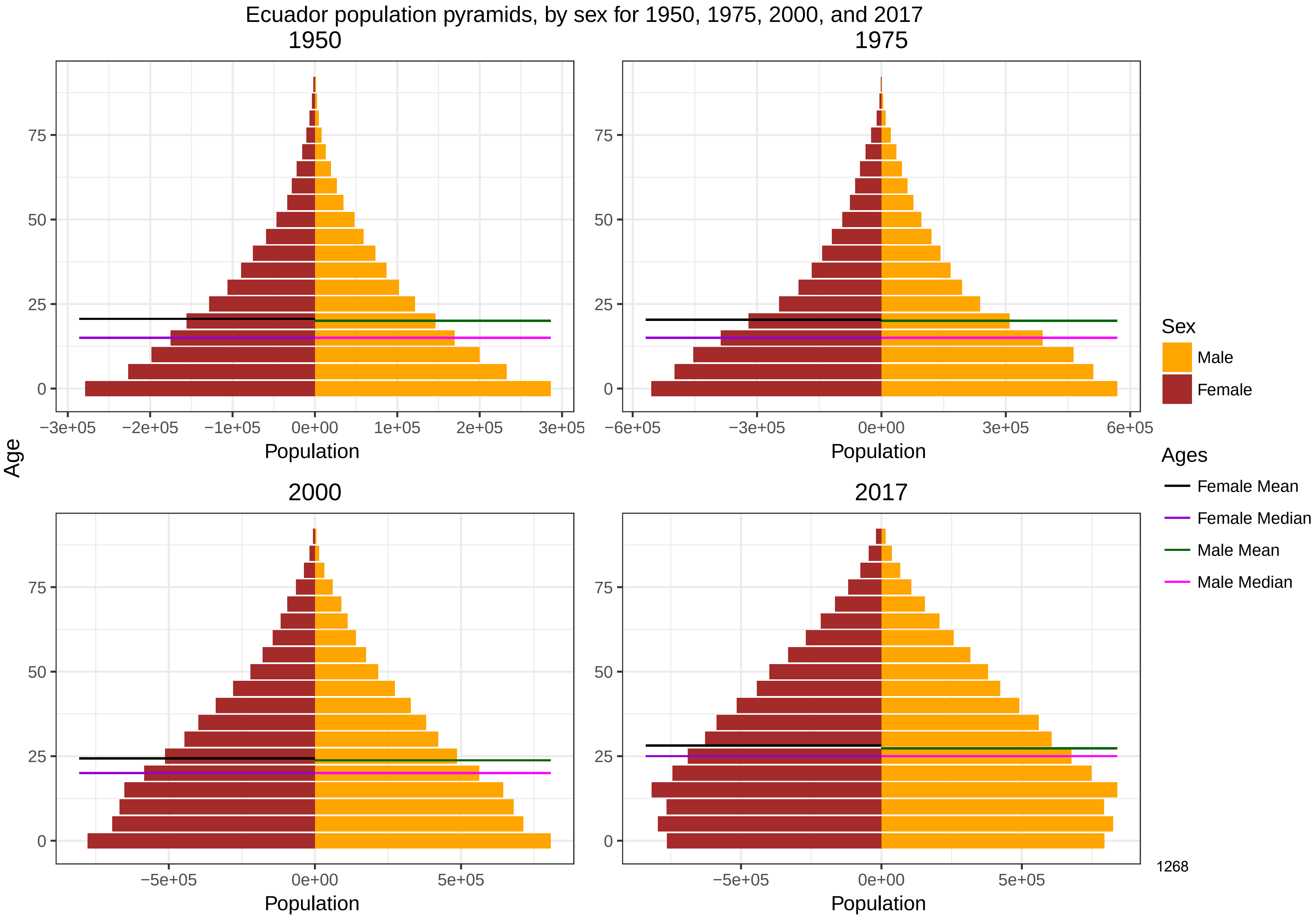
Ecuador population pyramids, by sex for 1950, 1975, 2000, and 2017
1950
1975
2000
2017
Age
Population
Sex
Male
Female
Ages
Female Mean
Female Median
Male Mean
Male Median

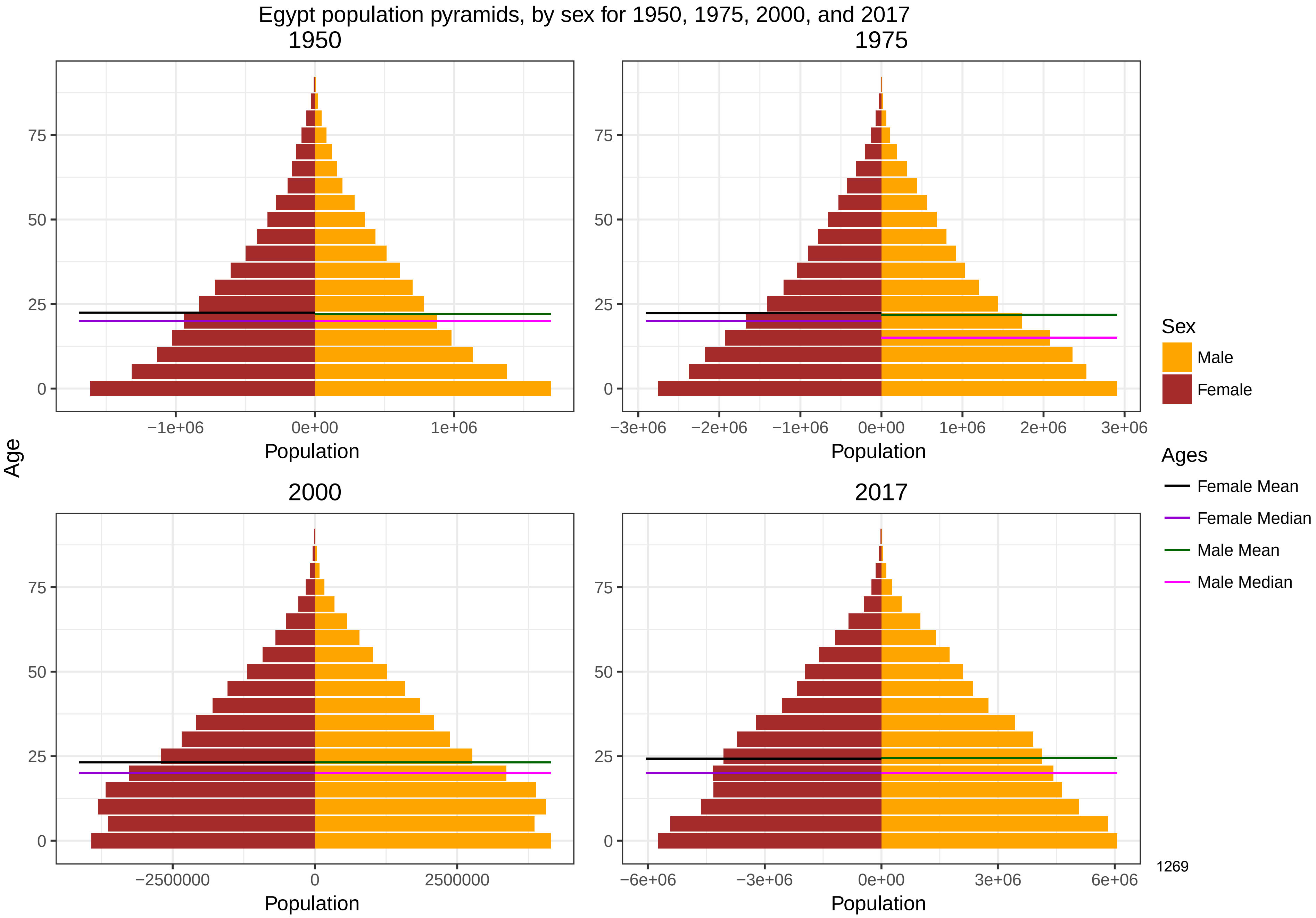

Egypt population pyramids, by sex for 1950, 1975, 2000, and 2017
1950
1975
2000
2017
Age
Population
0
25
50
75
−1e+06
0e+00
1e+06
−3e+06
−2e+06
−1e+06
2e+06
3e+06
−2500000
2500000
−6e+06
−3e+06
3e+06
6e+06
Sex
Male
Female
Ages
Female Mean
Female Median
Male Mean
Male Median

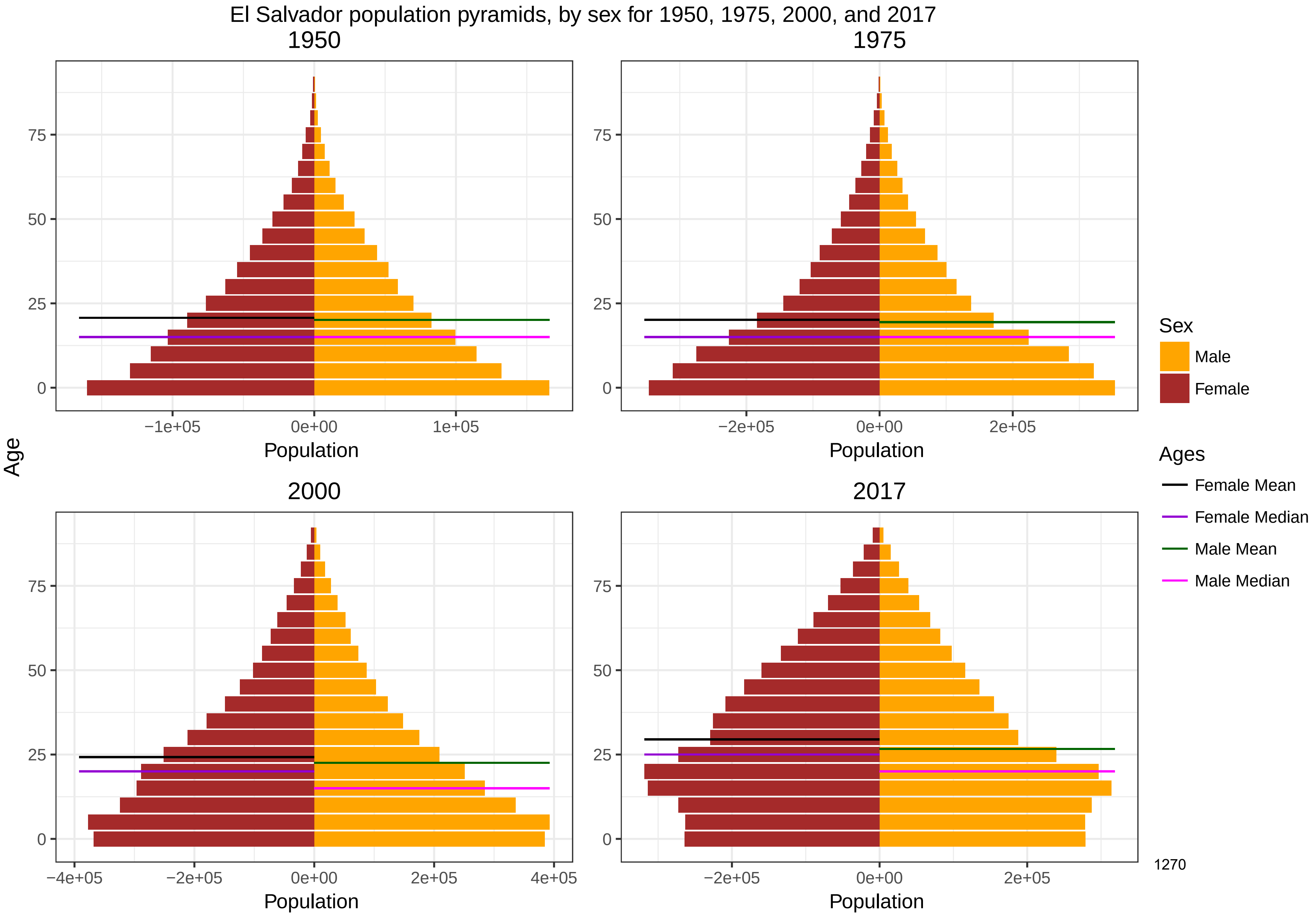
El Salvador population pyramids, by sex for 1950, 1975, 2000, and 2017
1950
1975
2000
2017
Age
Population
0
25
50
75
−1e+05
0e+00
1e+05
−2e+05
2e+05
−4e+05
4e+05
Sex
Male
Female
Ages
Female Mean
Female Median
Male Mean
Male Median

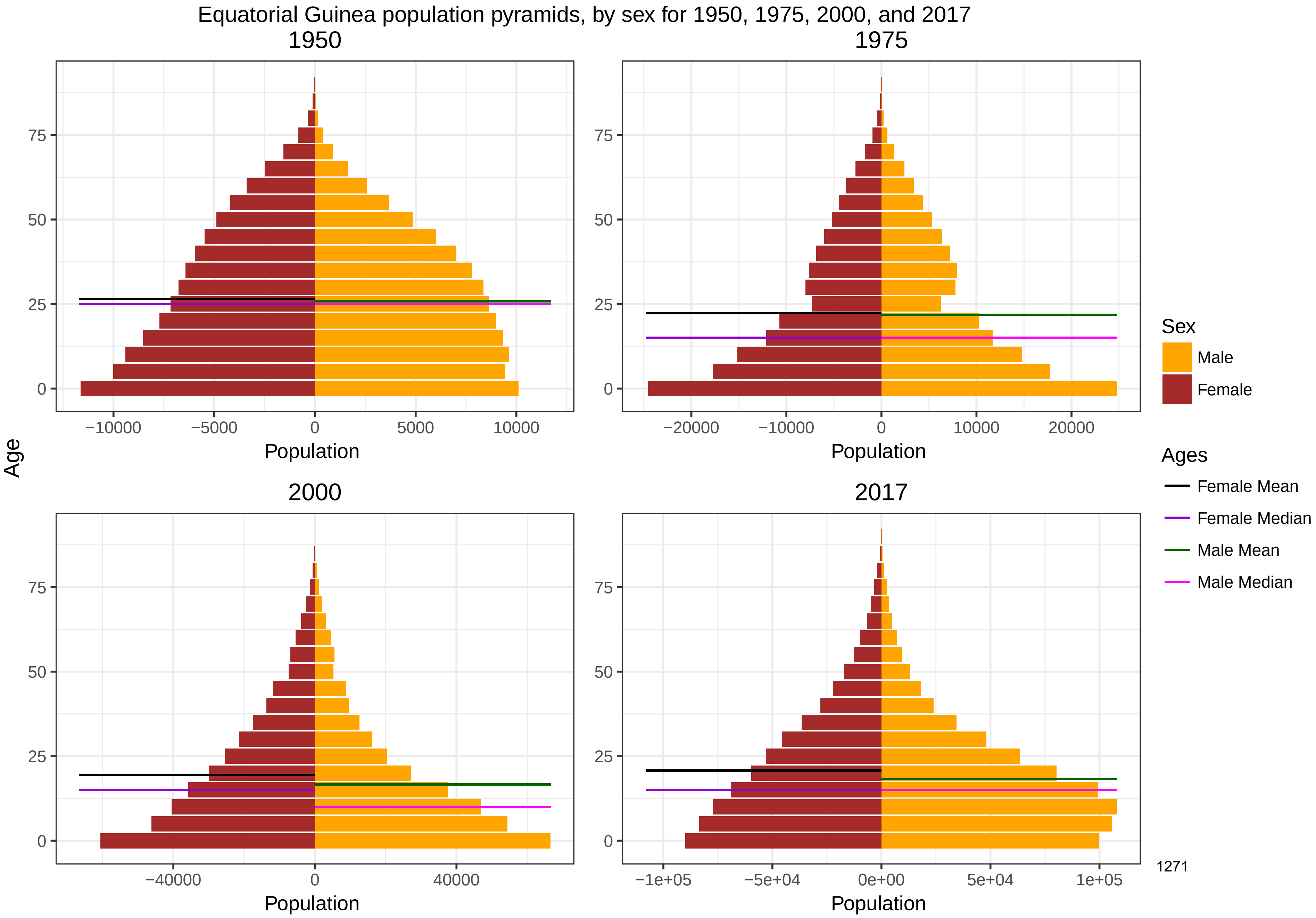
Equatorial Guinea population pyramids, by sex for 1950, 1975, 2000, and 2017
1950
1975
2000
2017
Age
Population
Sex
Male
Female
Ages
Female Mean
Female Median
Male Mean
Male Median
0
25
50
75
−10000
−5000
0
5000
10000
−20000
−10000
0
10000
20000
−40000
0
40000
−1e+05
−5e+04
0e+00
5e+04
1e+05

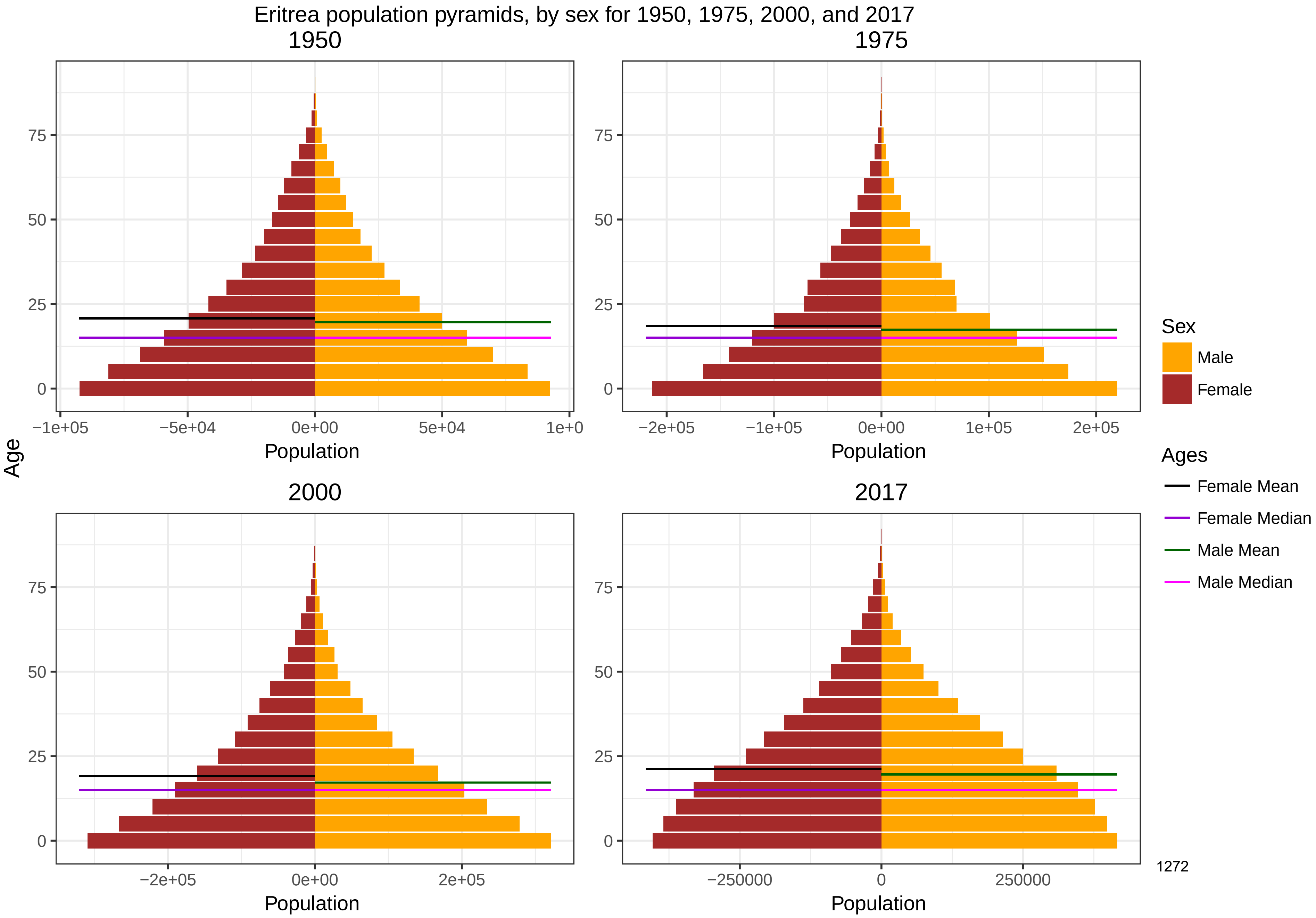

Eritrea population pyramids, by sex for 1950, 1975, 2000, and 2017
1950
1975
2000
2017
Age
Population
0
25
50
75
−1e+05
−5e+04
0e+00
5e+04
1e+0
−2e+05
−1e+05
1e+05
2e+05
−250000
250000
Sex
Male
Female
Ages
Female Mean
Female Median
Male Mean
Male Median

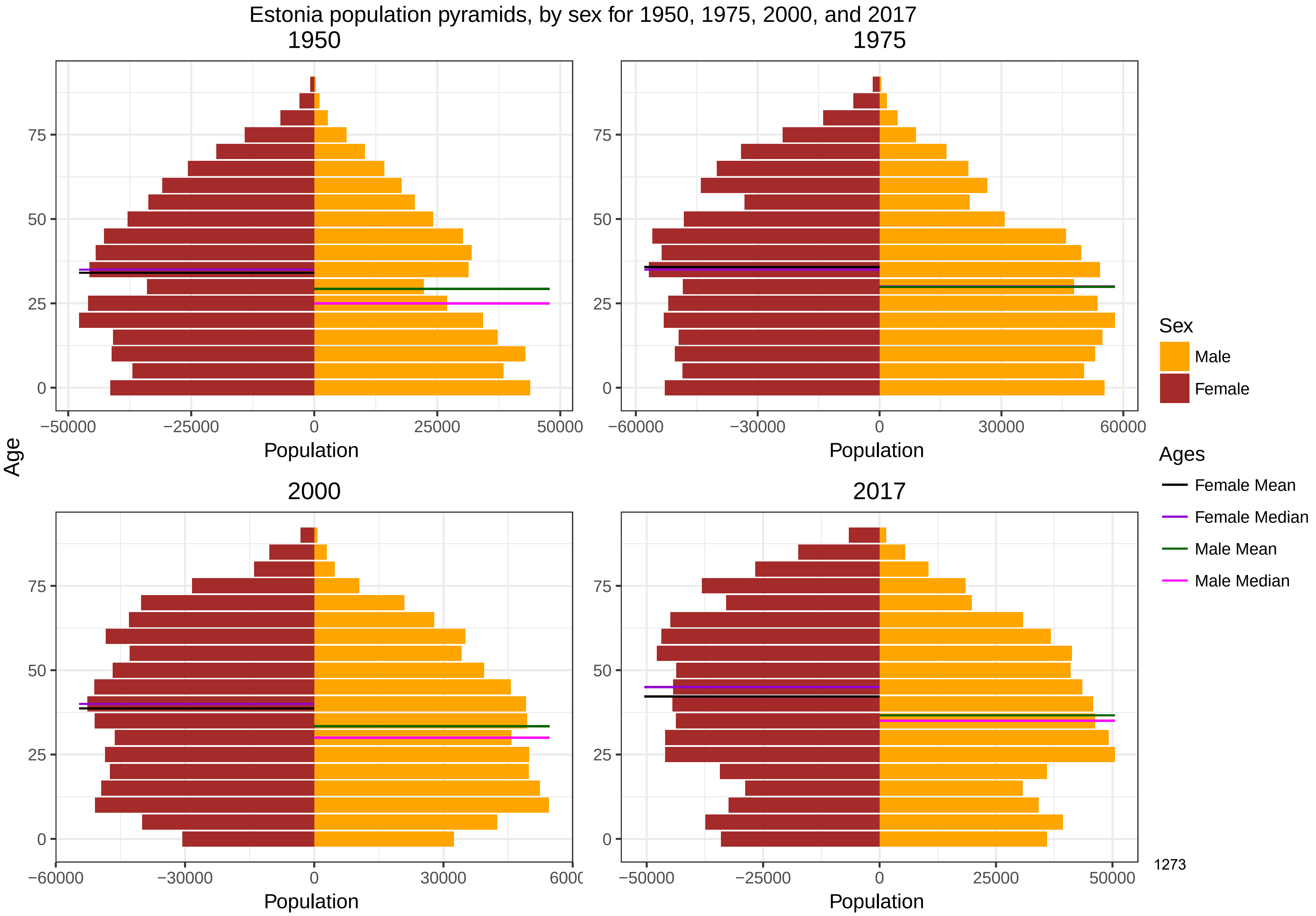
Estonia population pyramids, by sex for 1950, 1975, 2000, and 2017
1950
1975
2000
2017
Age
Population
Sex
Male
Female
Ages
Female Mean
Female Median
Male Mean
Male Median
−50000
−25000
0
25000
50000
−60000
−30000
30000
60000
75
50
25
0

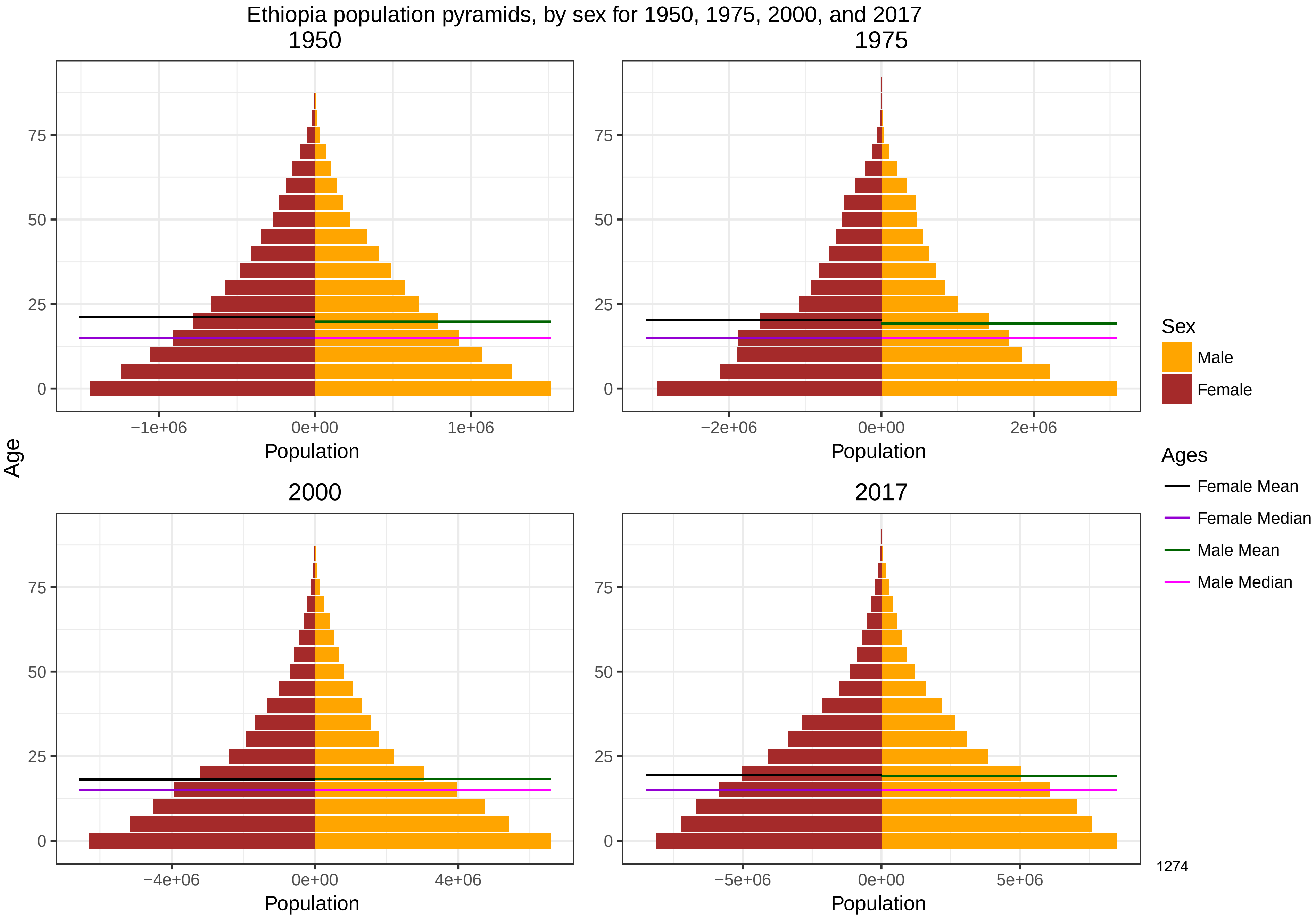
Ethiopia population pyramids, by sex for 1950, 1975, 2000, and 2017
1950
1975
2000
2017
Age
0
25
50
75
Population
−1e+06
0e+00
1e+06
−2e+06
2e+06
−4e+06
4e+06
−5e+06
5e+06
Sex
Male
Female
Ages
Female Mean
Female Median
Male Mean
Male Median

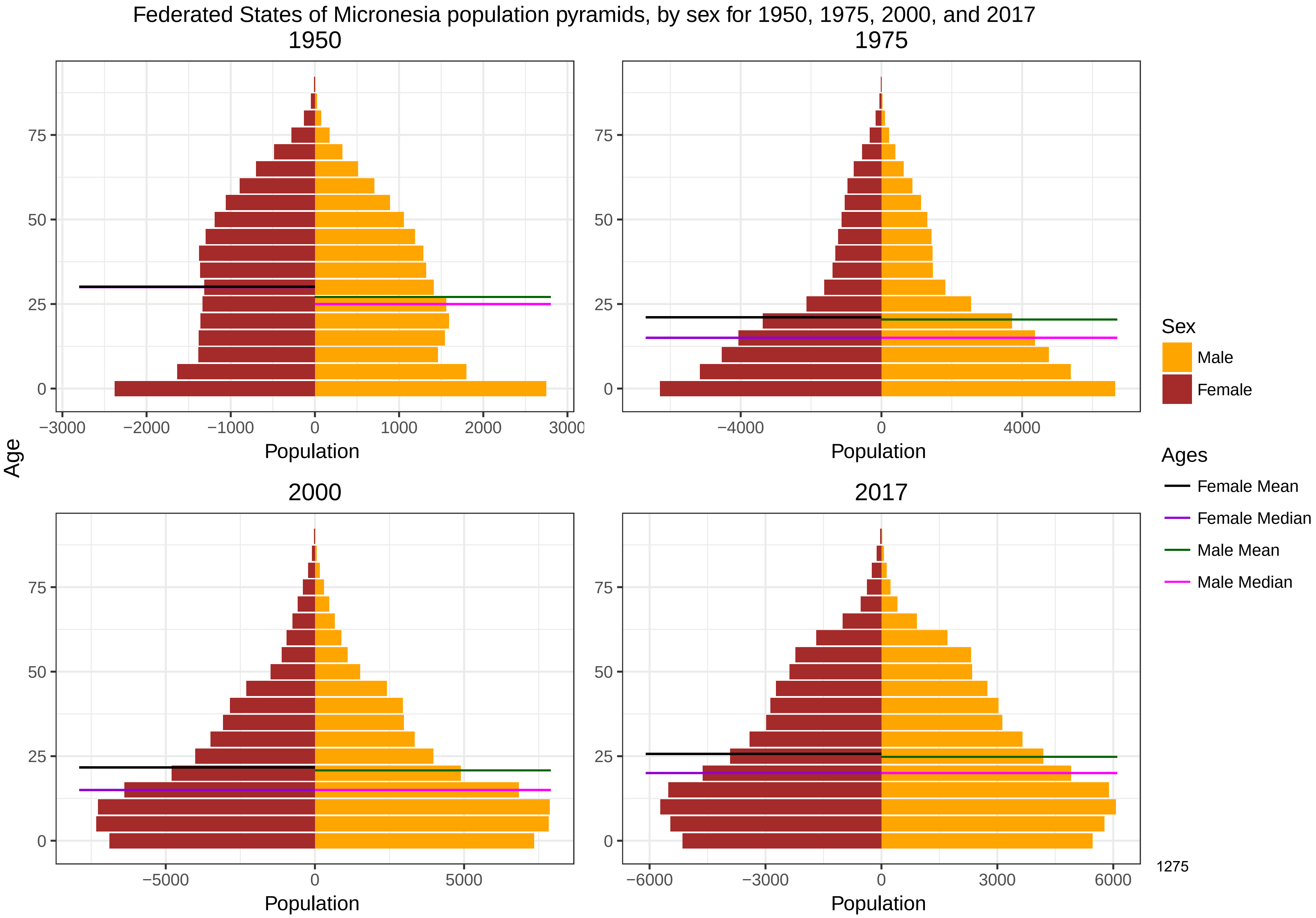
Federated States of Micronesia population pyramids, by sex for 1950, 1975, 2000, and 2017
1950
1975
2000
2017
Age
Population
75
50
25
0
−3000
−2000
−1000
0
1000
2000
3000
−4000
4000
−5000
5000
−6000
−3000
3000
6000
Sex
Male
Female
Ages
Female Mean
Female Median
Male Mean
Male Median

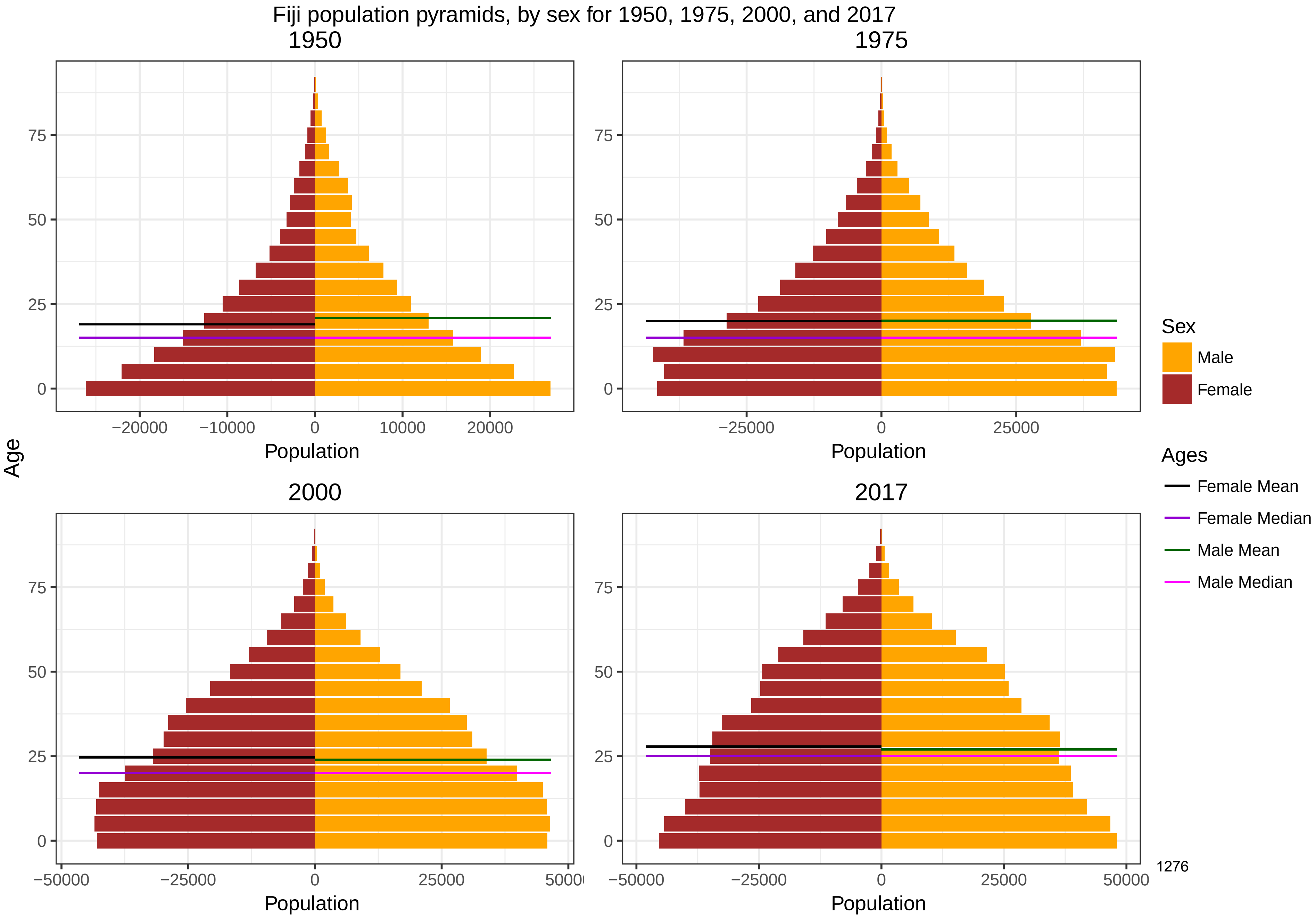
Fiji population pyramids, by sex for 1950, 1975, 2000, and 2017
1950
1975
2000
2017
Age
Population
Sex
Male
Female
Ages
Female Mean
Female Median
Male Mean
Male Median

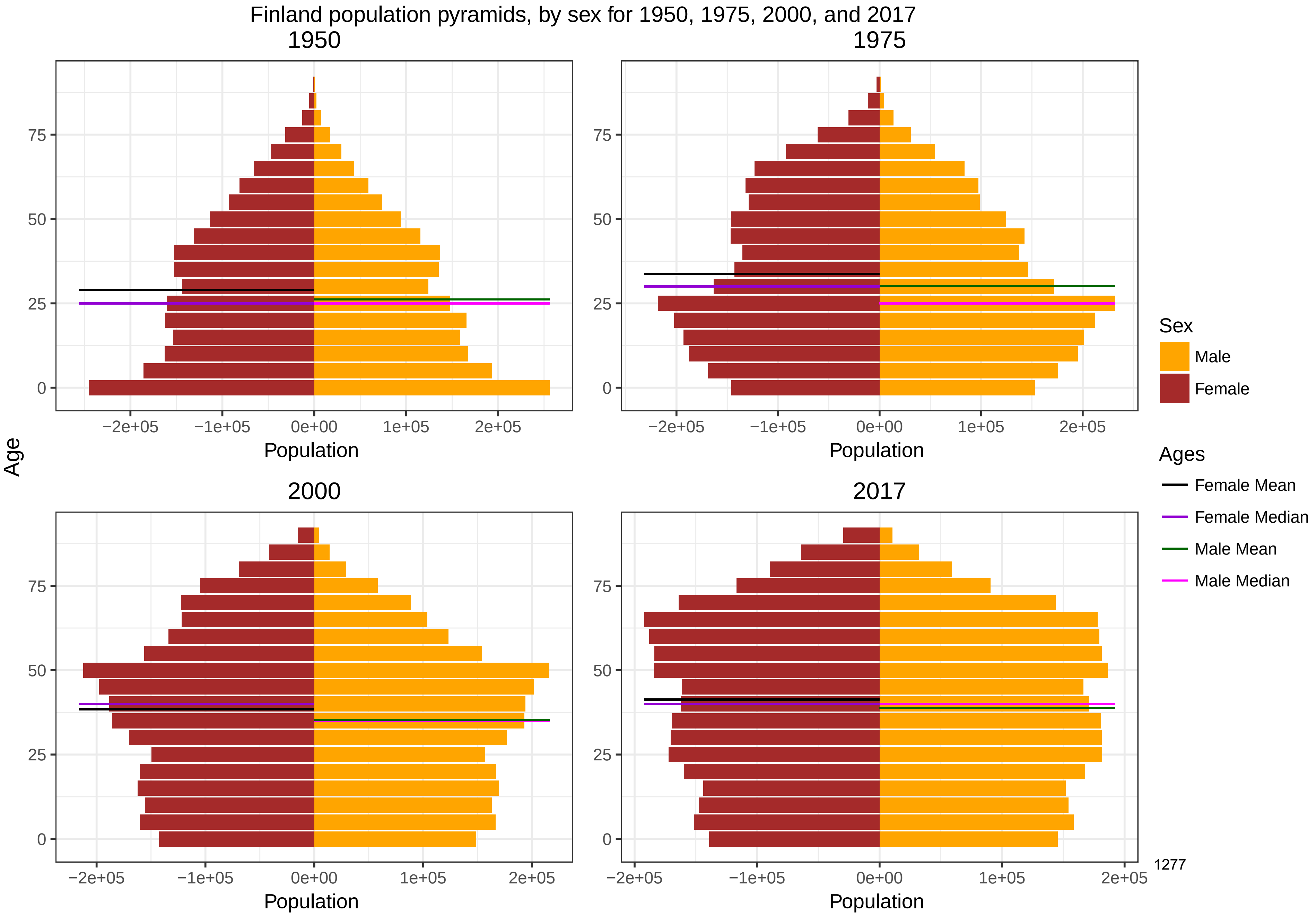
Finland population pyramids, by sex for 1950, 1975, 2000, and 2017
1950
1975
2000
2017
Age
Population
0
25
50
75
−2e+05
−1e+05
0e+00
1e+05
2e+05
Sex
Male
Female
Ages
Female Mean
Female Median
Male Mean
Male Median

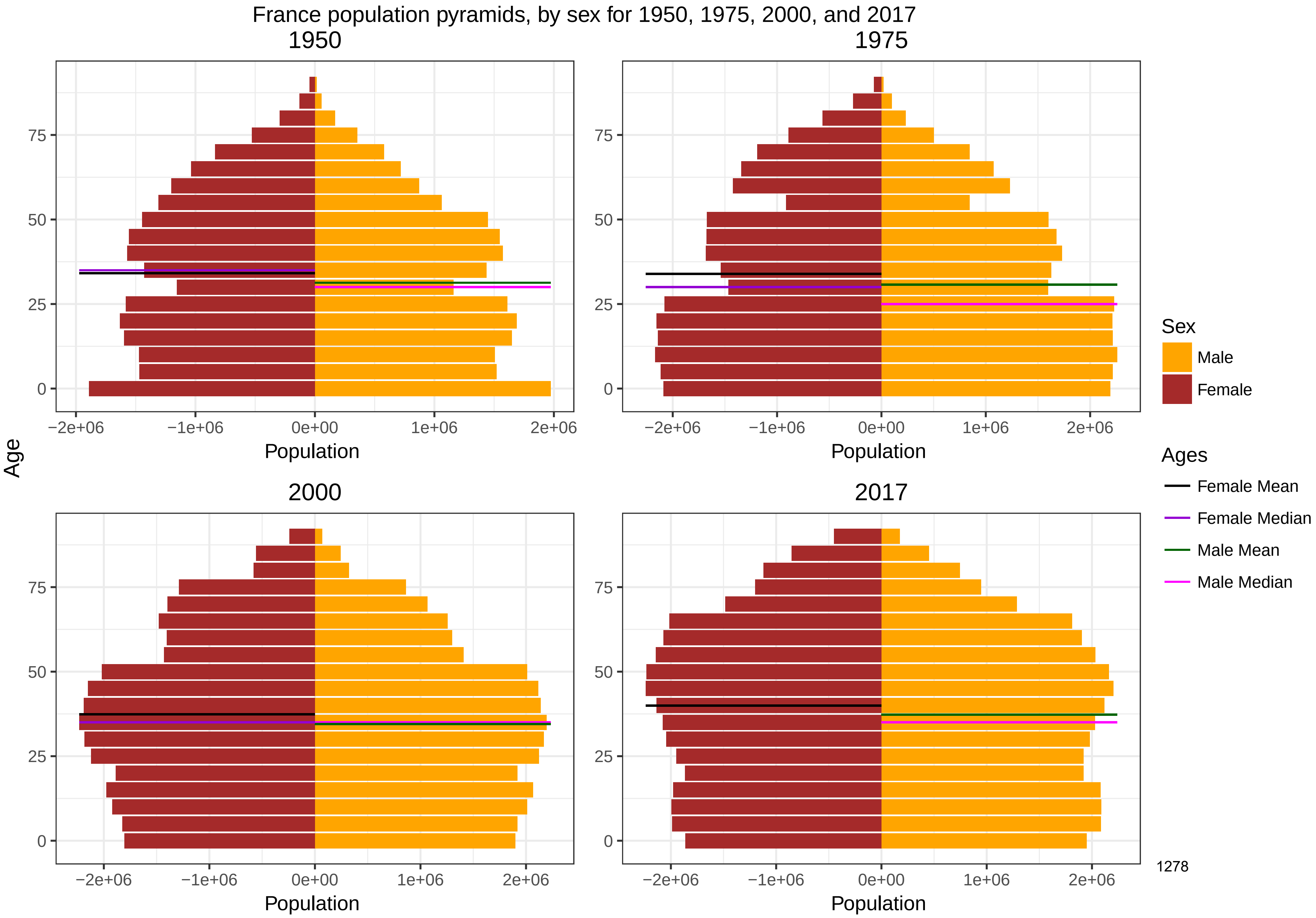

France population pyramids, by sex for 1950, 1975, 2000, and 2017
1950
1975
2000
2017
Age
Population
0
25
50
75
−2e+06
−1e+06
0e+00
1e+06
2e+06
Sex
Male
Female
Ages
Female Mean
Female Median
Male Mean
Male Median

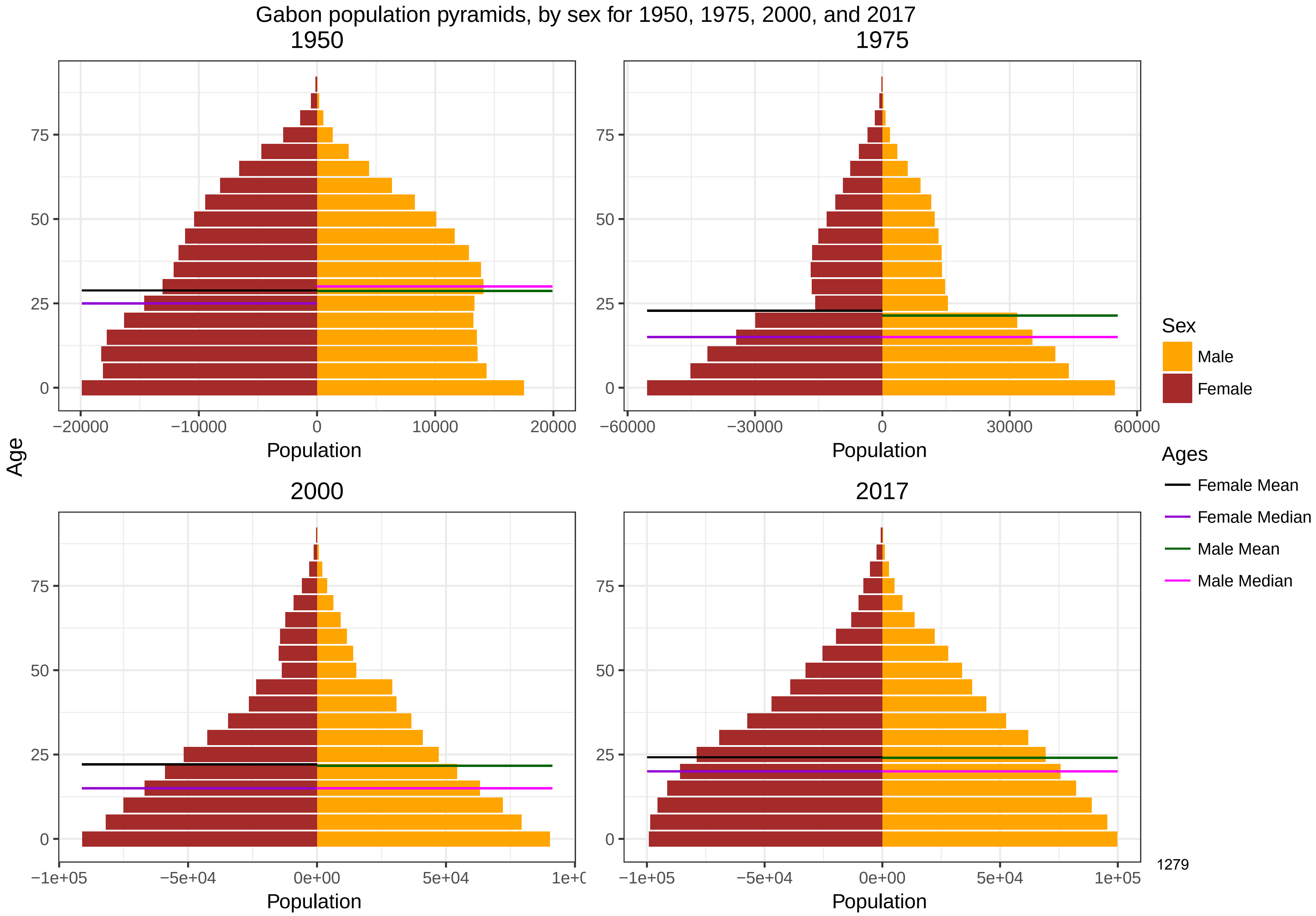
Gabon population pyramids, by sex for 1950, 1975, 2000, and 2017
1950
1975
2000
2017
Age
Population
0
25
50
75
−20000
−10000
0
10000
20000
−60000
−30000
0
30000
60000
−1e+05
−5e+04
0e+00
5e+04
1e+05
Sex
Male
Female
Ages
Female Mean
Female Median
Male Mean
Male Median

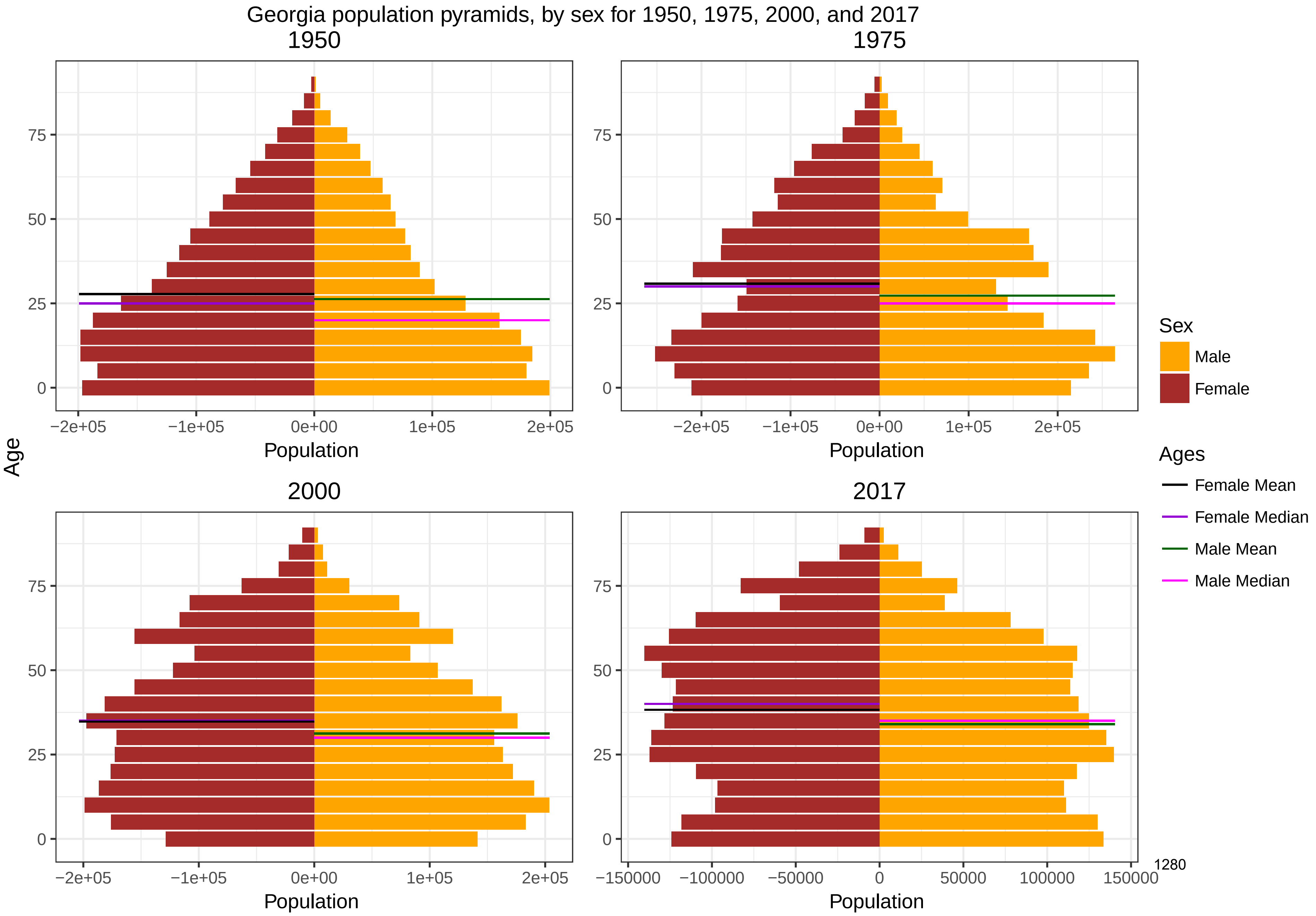
Georgia population pyramids, by sex for 1950, 1975, 2000, and 2017
1950
1975
2000
2017
Age
Population
−2e+05
−1e+05
0e+00
1e+05
2e+05
−150000
−100000
−50000
0
50000
100000
150000
0
25
50
75
Sex
Male
Female
Ages
Female Mean
Female Median
Male Mean
Male Median

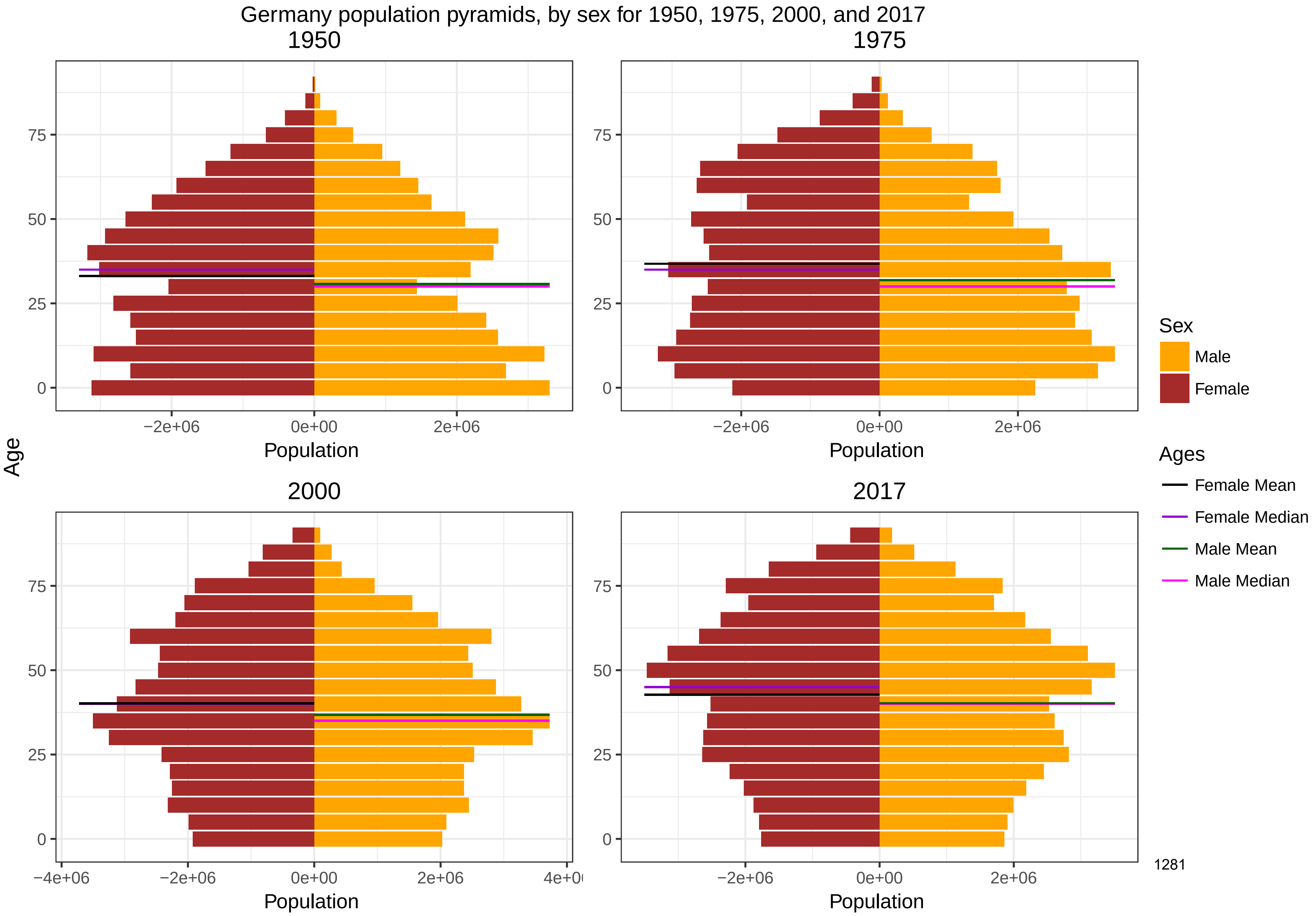

Germany population pyramids, by sex for 1950, 1975, 2000, and 2017
1950
1975
2000
2017
Age
Population
0
25
50
75
−4e+06
−2e+06
0e+00
2e+06
4e+06
Sex
Male
Female
Ages
Female Mean
Female Median
Male Mean
Male Median

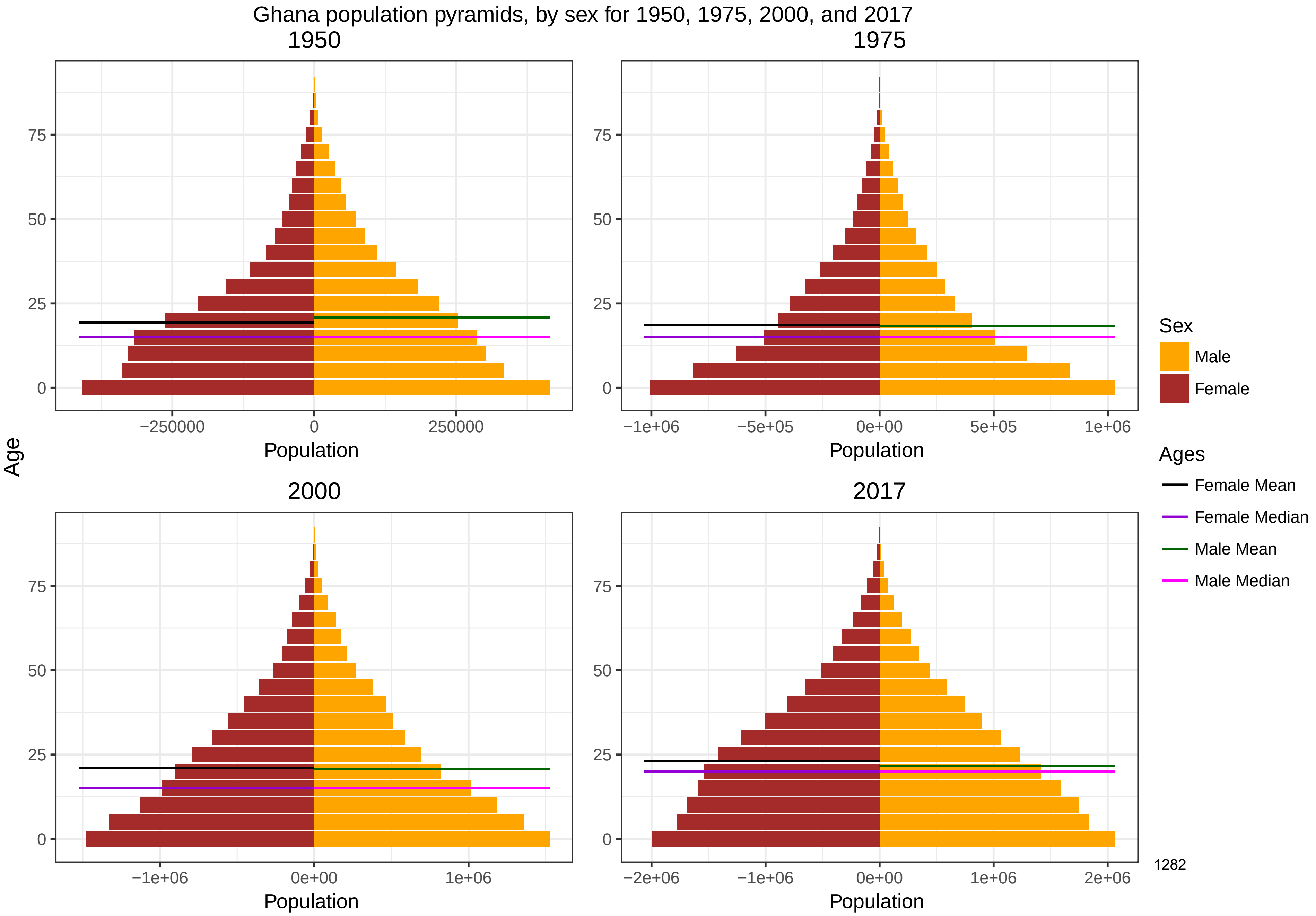

Ghana population pyramids, by sex for 1950, 1975, 2000, and 2017
1950
1975
2000
2017
Age
Population
75
50
25
0
−250000
0
250000
−1e+06
−5e+05
0e+00
5e+05
1e+06
−2e+06
2e+06
Sex
Male
Female
Ages
Female Mean
Female Median
Male Mean
Male Median

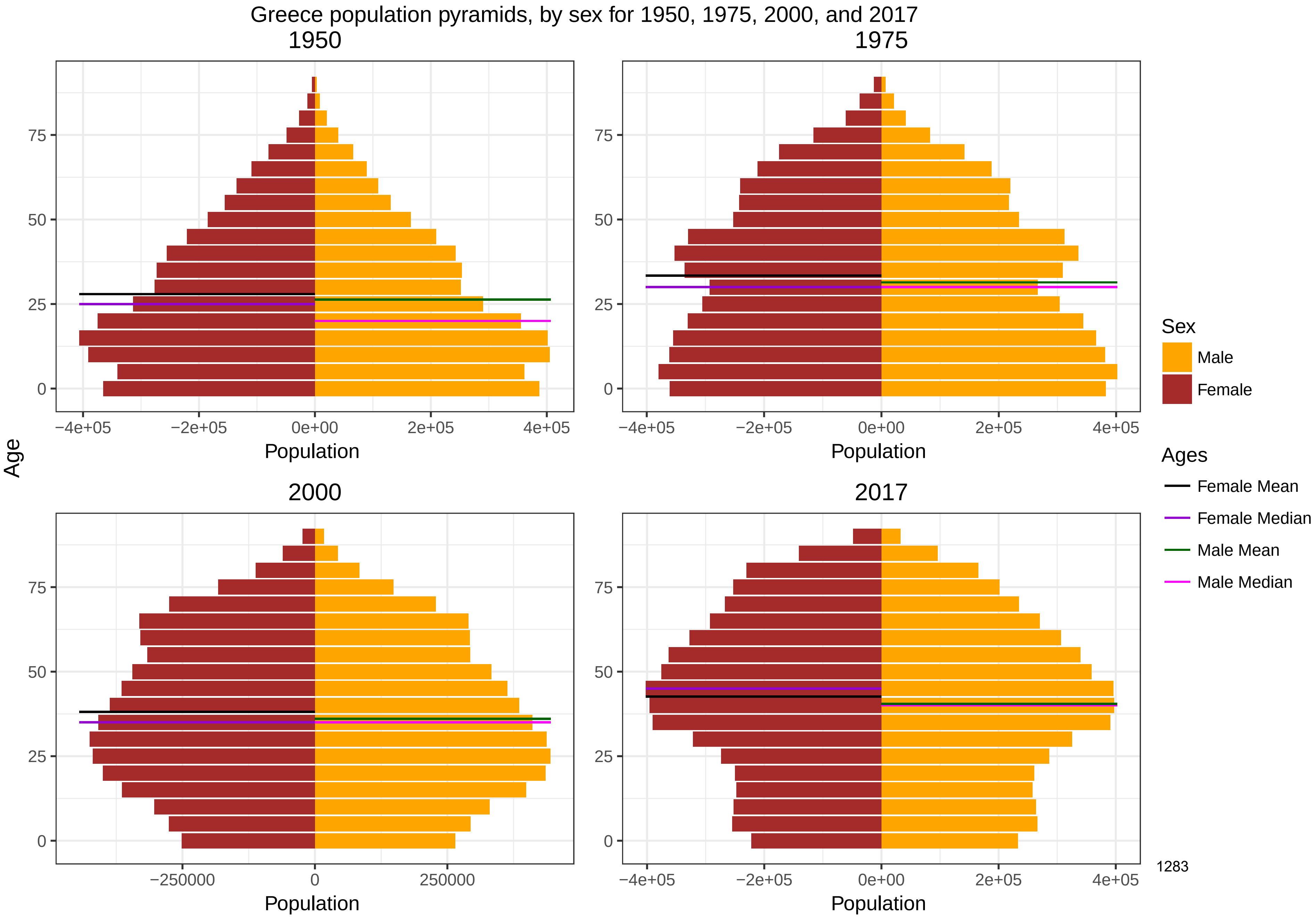
Greece population pyramids, by sex for 1950, 1975, 2000, and 2017
1950
1975
2000
2017
Age
Population
0
25
50
75
−4e+05
−2e+05
0e+00
2e+05
4e+05
−250000
0
250000
Sex
Male
Female
Ages
Female Mean
Female Median
Male Mean
Male Median

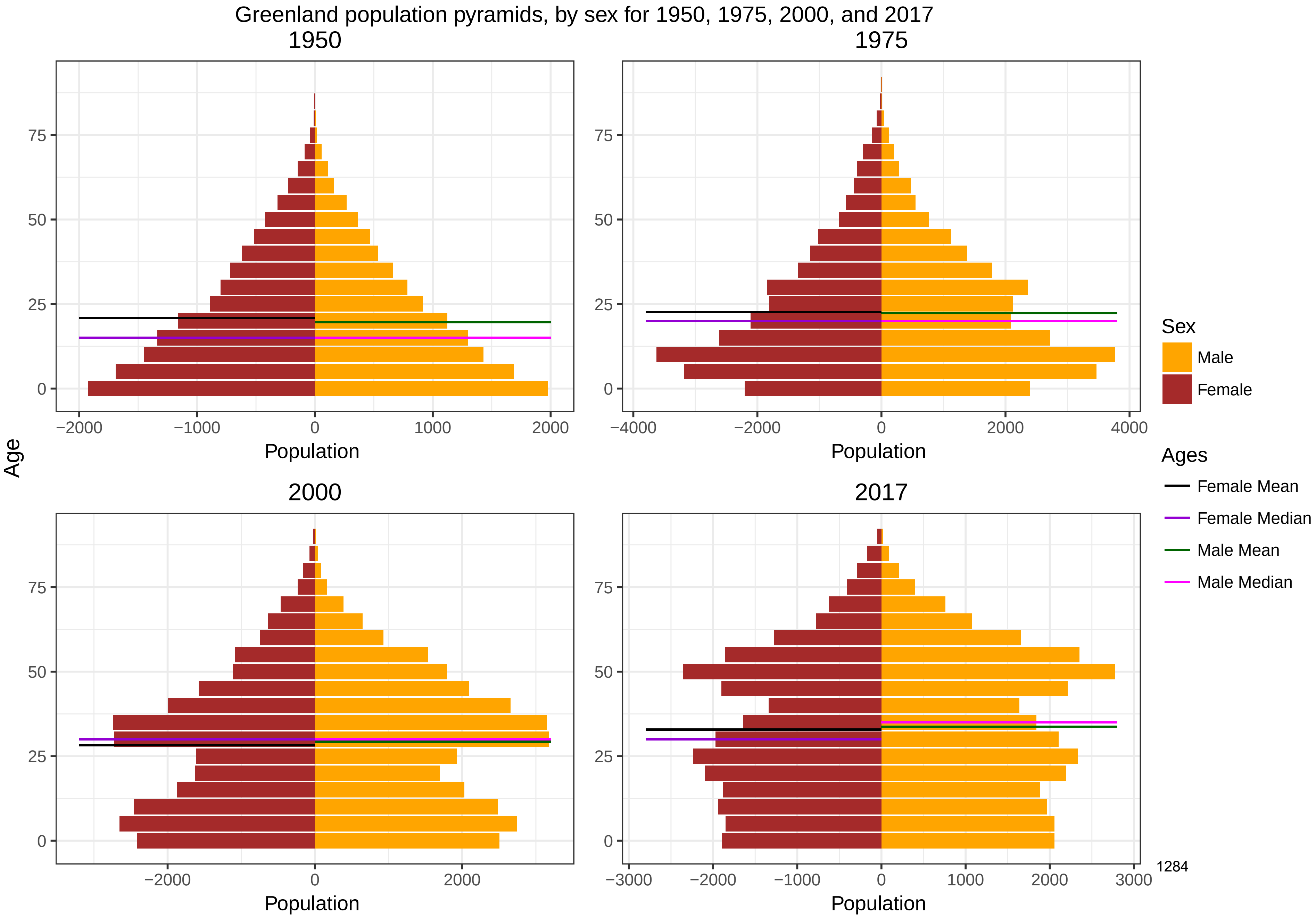

Greenland population pyramids, by sex for 1950, 1975, 2000, and 2017
1950
1975
2000
2017
Age
Population
Sex
Male
Female
Ages
Female Mean
Female Median
Male Mean
Male Median

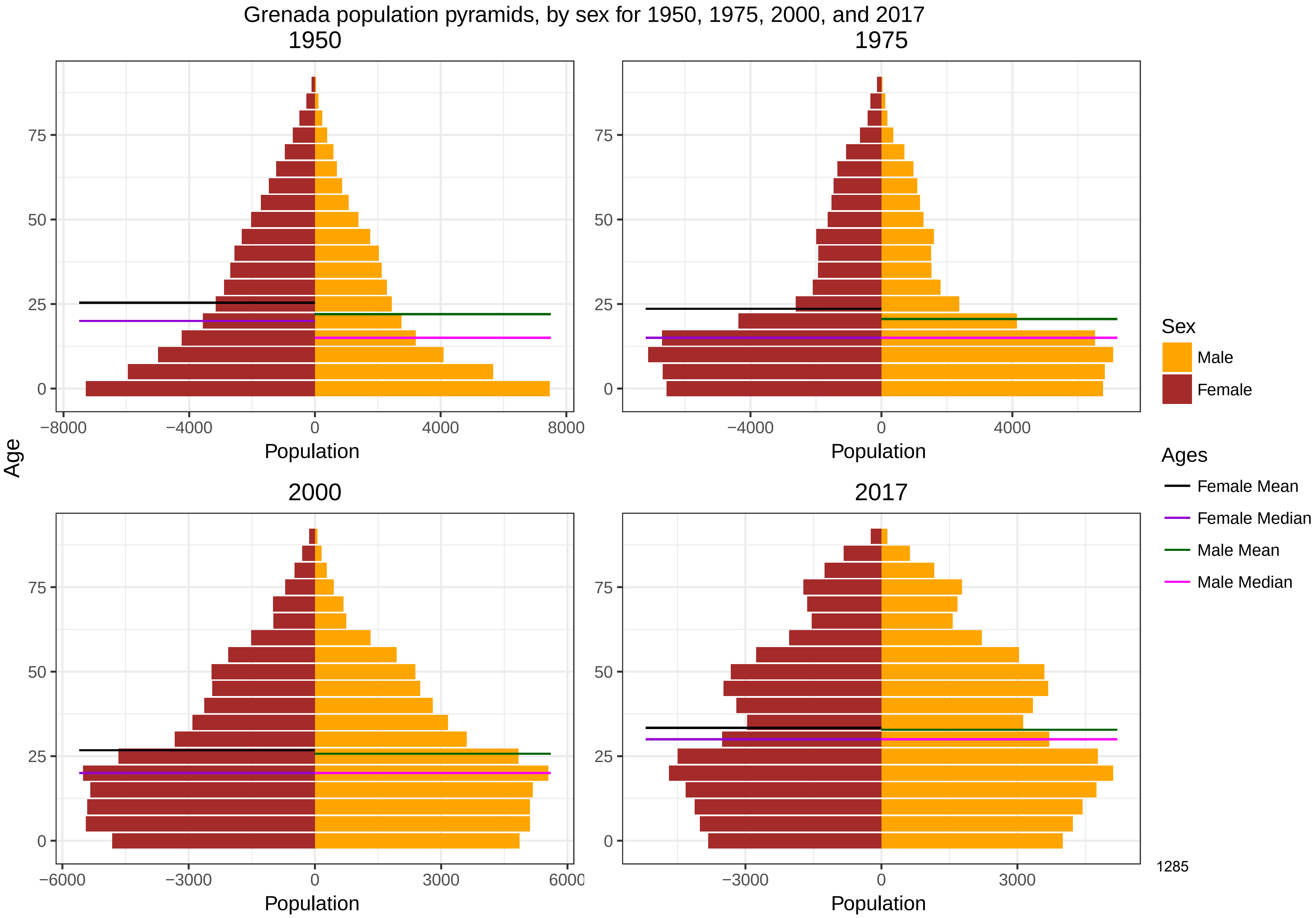

Grenada population pyramids, by sex for 1950, 1975, 2000, and 2017
1950
1975
2000
2017
Age
Population
75
50
25
0
−8000
−4000
0
4000
8000
−6000
−3000
3000
6000
Sex
Male
Female
Ages
Female Mean
Female Median
Male Mean
Male Median

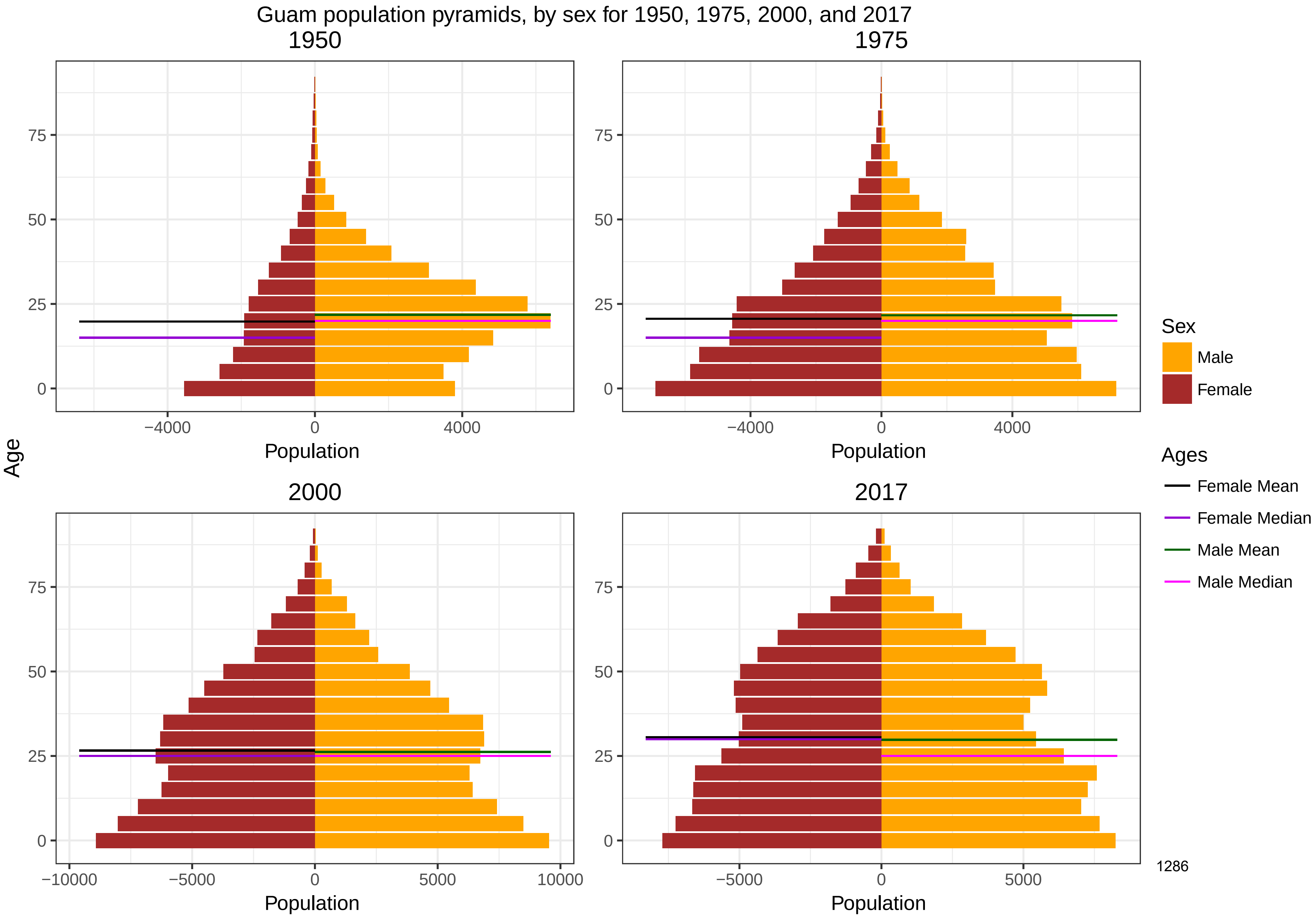

Guam population pyramids, by sex for 1950, 1975, 2000, and 2017
1950
1975
2000
2017
Age
Population
Sex
Male
Female
Ages
Female Mean
Female Median
Male Mean
Male Median

Guatemala population pyramids, by sex for 1950, 1975, 2000, and 2017

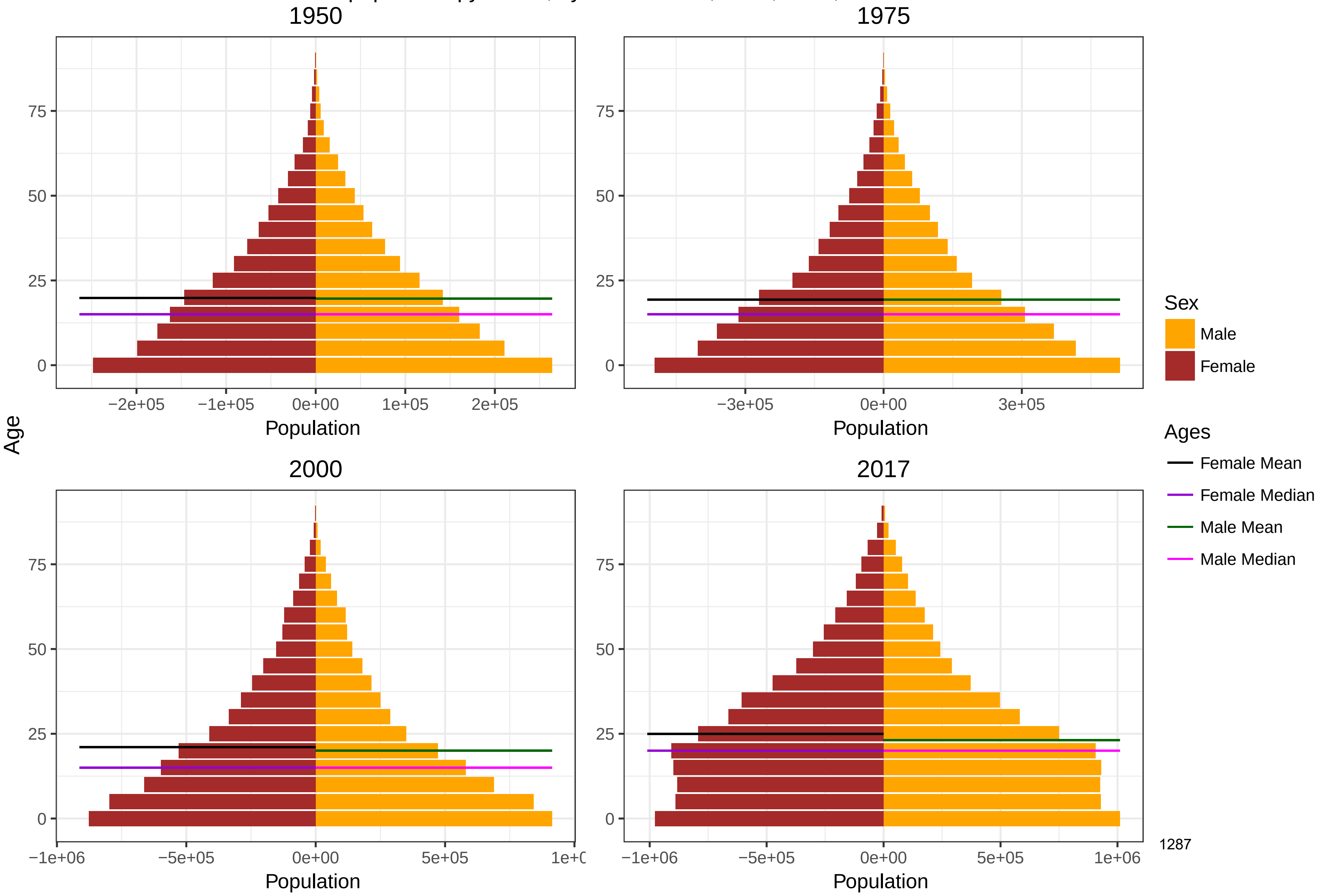

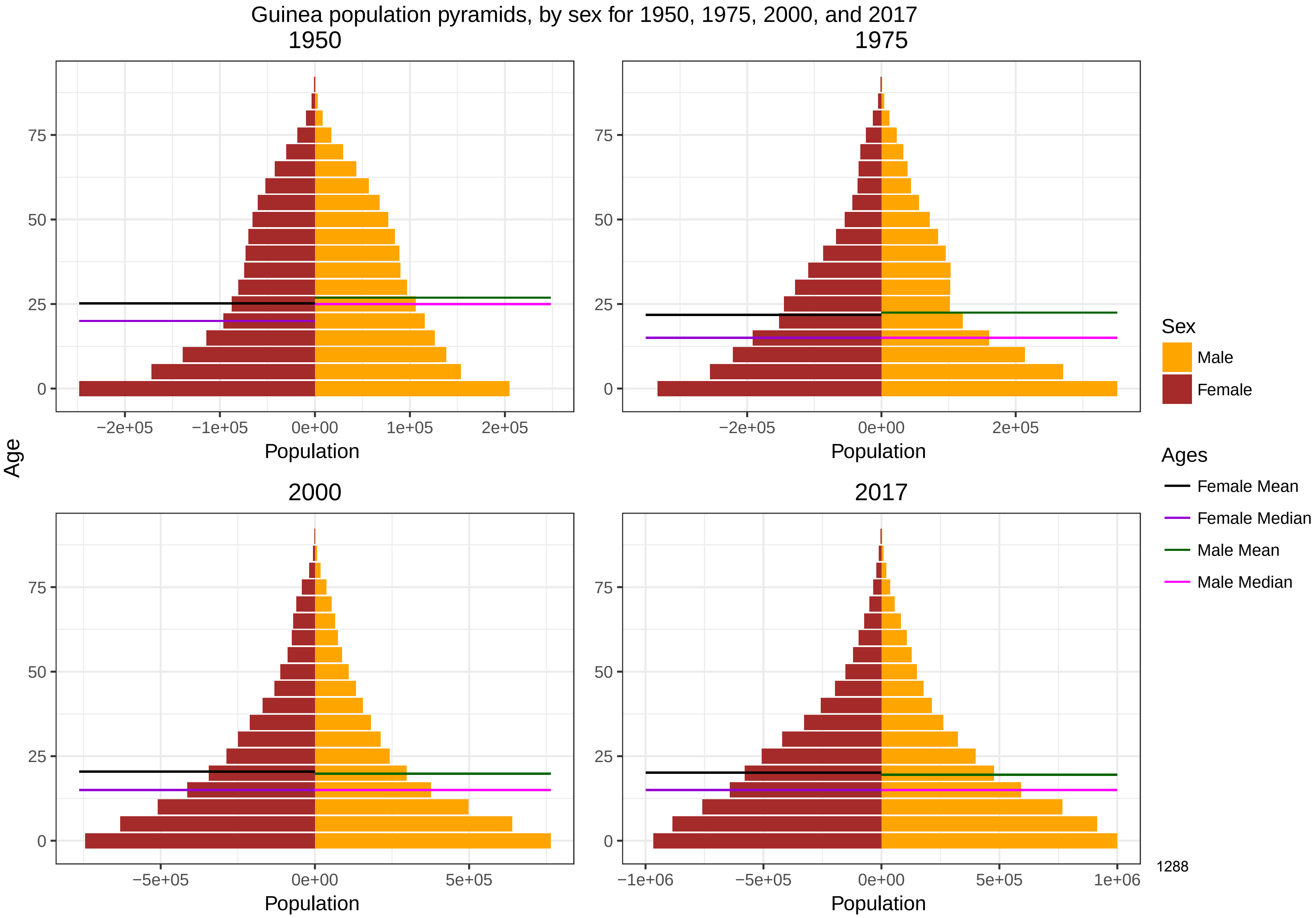
Guinea population pyramids, by sex for 1950, 1975, 2000, and 2017
1950
1975
2000
2017
Age
Population
0
25
50
75
−2e+05
−1e+05
0e+00
1e+05
2e+05
−5e+05
5e+05
−1e+06
1e+06
Sex
Male
Female
Ages
Female Mean
Female Median
Male Mean
Male Median

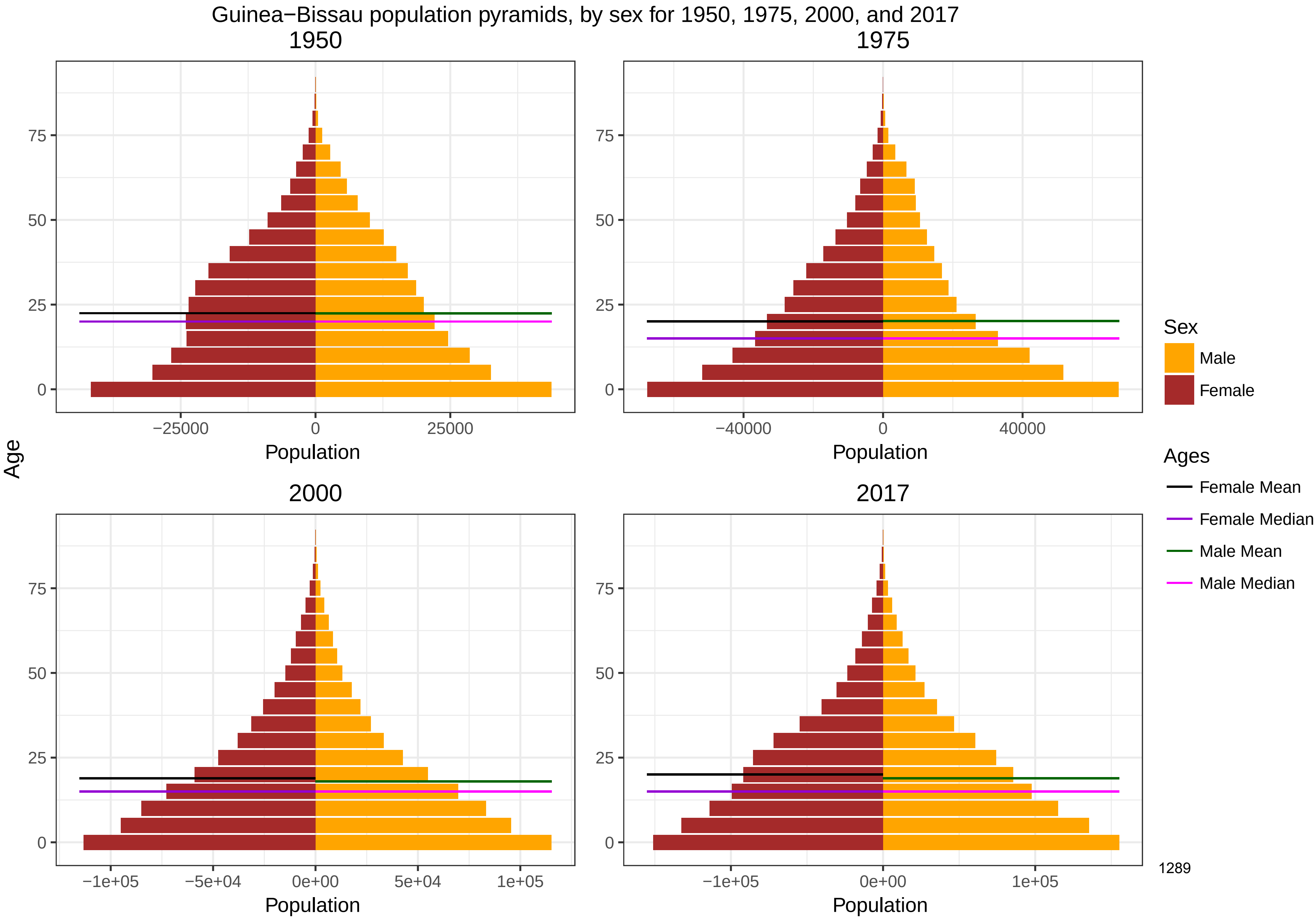

Guinea−Bissau population pyramids, by sex for 1950, 1975, 2000, and 2017
1950
1975
2000
2017
Age
Population
Sex
Male
Female
Ages
Female Mean
Female Median
Male Mean
Male Median
0
25
50
75
−25000
25000
−40000
40000
−1e+05
−5e+04
0e+00
5e+04
1e+05

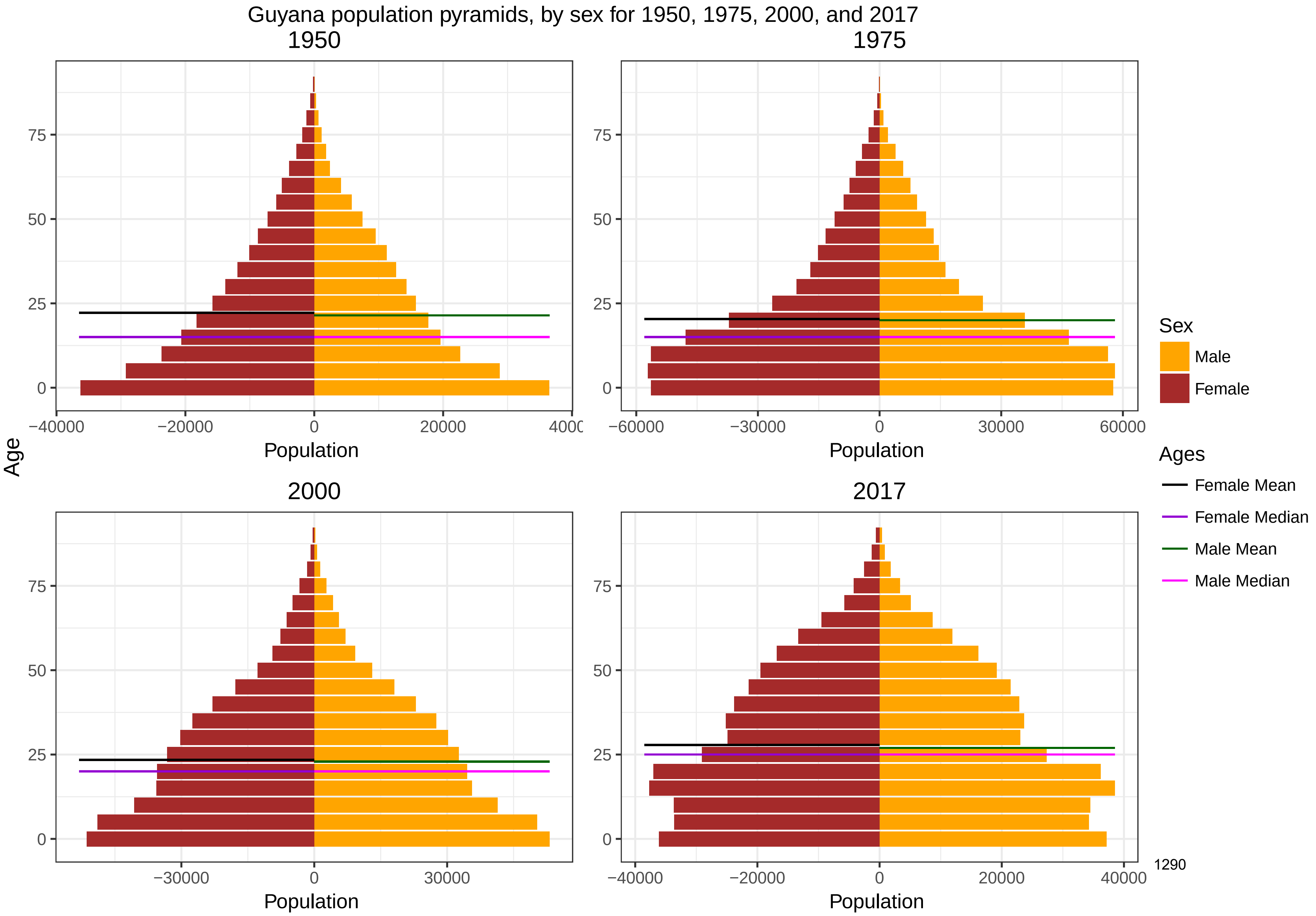

Guyana population pyramids, by sex for 1950, 1975, 2000, and 2017
1950
1975
2000
2017
Age
Population
Sex
Male
Female
Ages
Female Mean
Female Median
Male Mean
Male Median

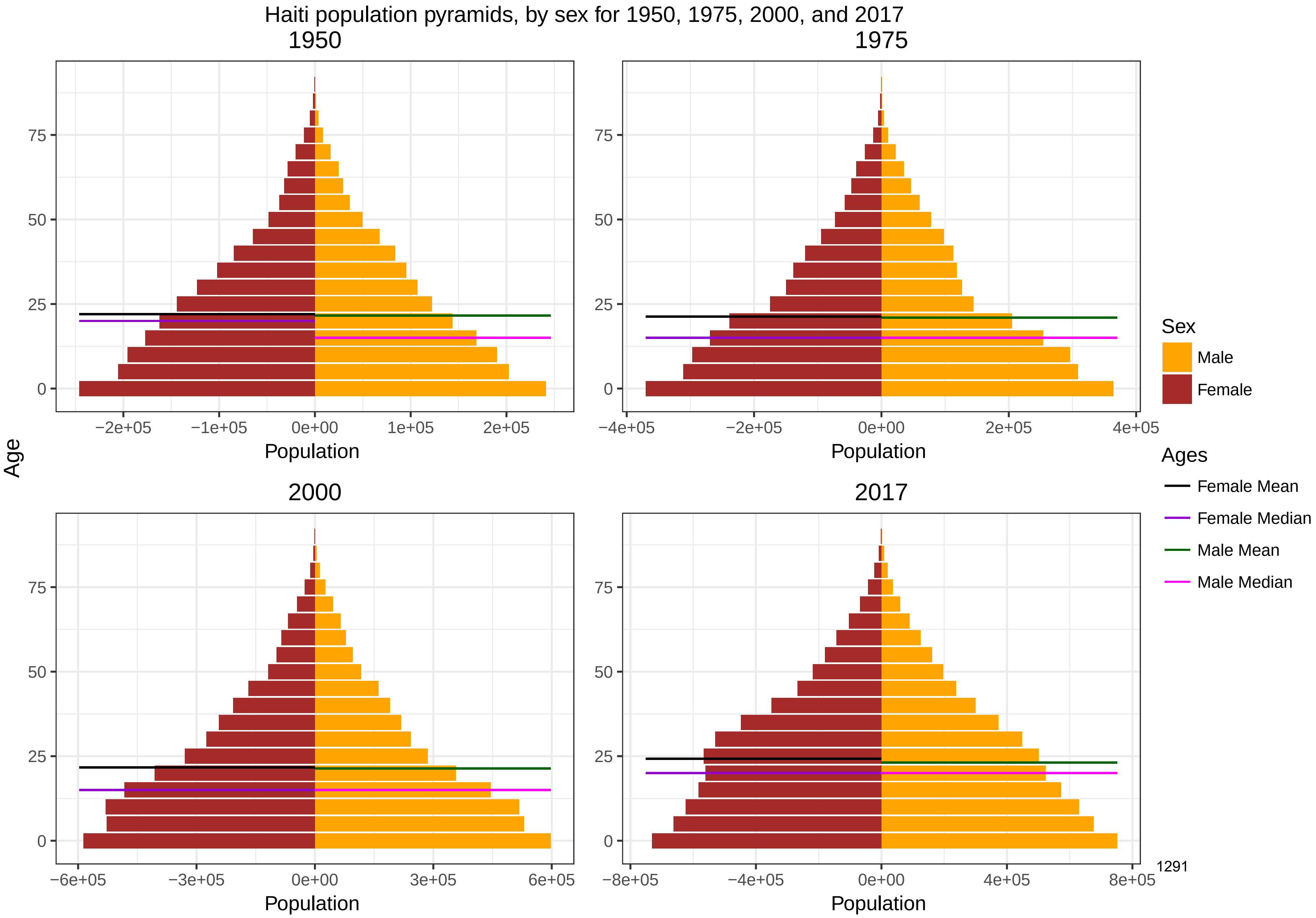
Haiti population pyramids, by sex for 1950, 1975, 2000, and 2017
1950
1975
2000
2017
Age
Population
Sex
Male
Female
Ages
Female Mean
Female Median
Male Mean
Male Median
0
25
50
75
−2e+05
−1e+05
0e+00
1e+05
2e+05
−4e+05
4e+05
−6e+05
−3e+05
3e+05
6e+05
−8e+05
8e+05

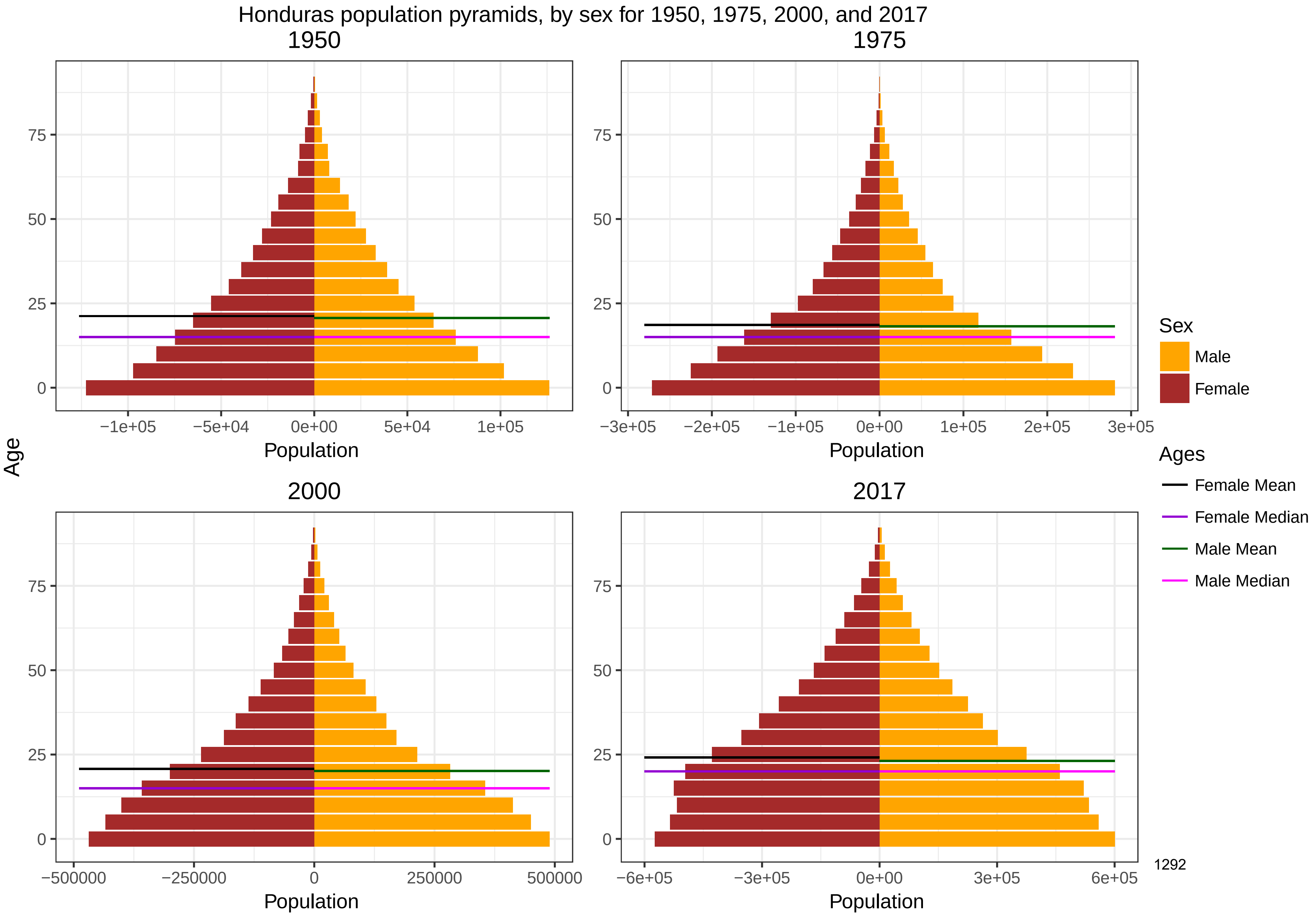

Honduras population pyramids, by sex for 1950, 1975, 2000, and 2017
1950
1975
2000
2017
Age
Population
Sex
Male
Female
Ages
Female Mean
Female Median
Male Mean
Male Median
−1e+05
−5e+04
0e+00
5e+04
1e+05
−3e+05
−2e+05
−1e+05
2e+05
3e+05
−500000
−250000
0
250000
500000
−6e+05
−3e+05
6e+05
75
50
25

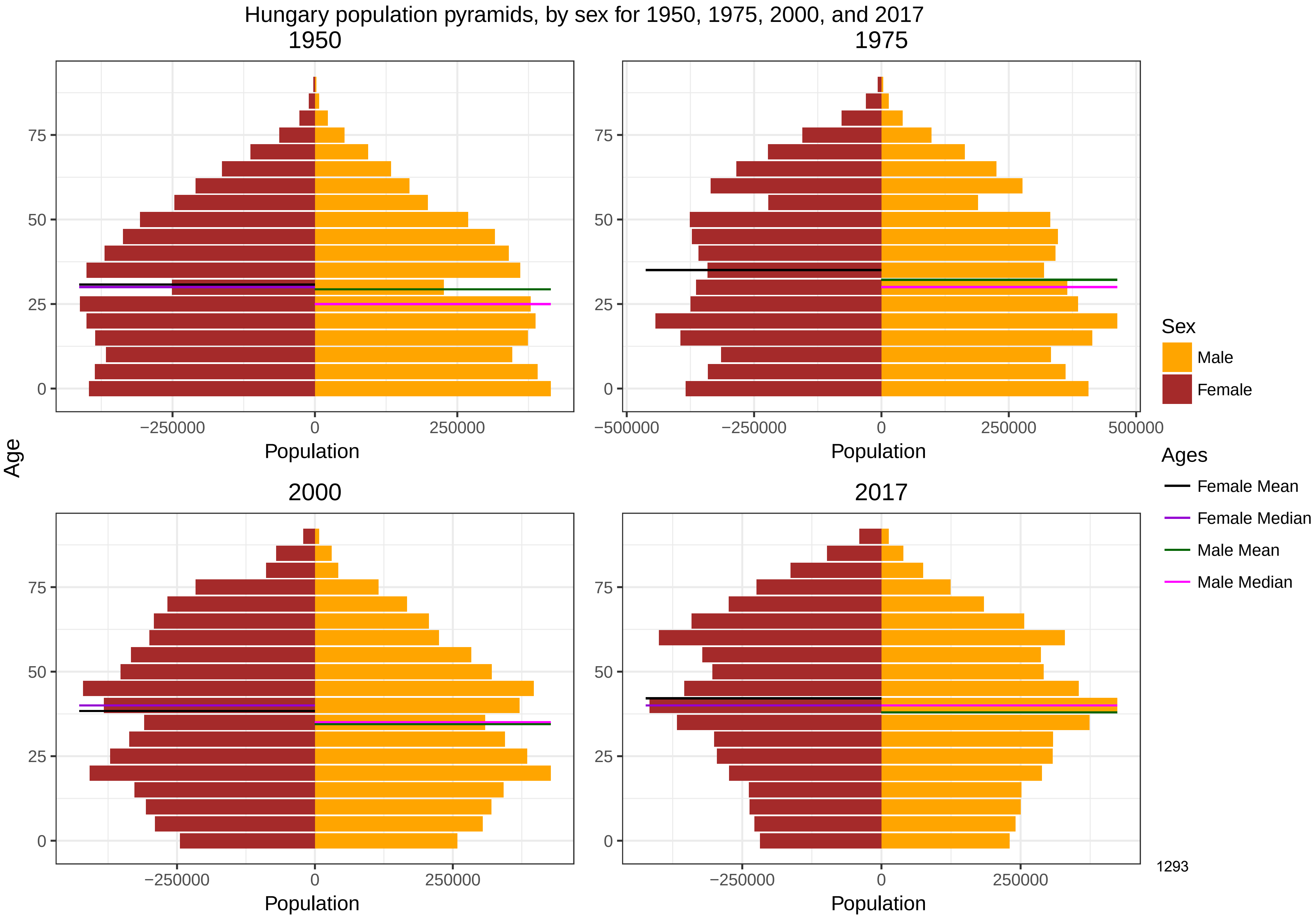

Hungary population pyramids, by sex for 1950, 1975, 2000, and 2017
1950
1975
2000
2017
Age
Population
0
25
50
75
−500000
−250000
0
250000
500000
Sex
Male
Female
Ages
Female Mean
Female Median
Male Mean
Male Median

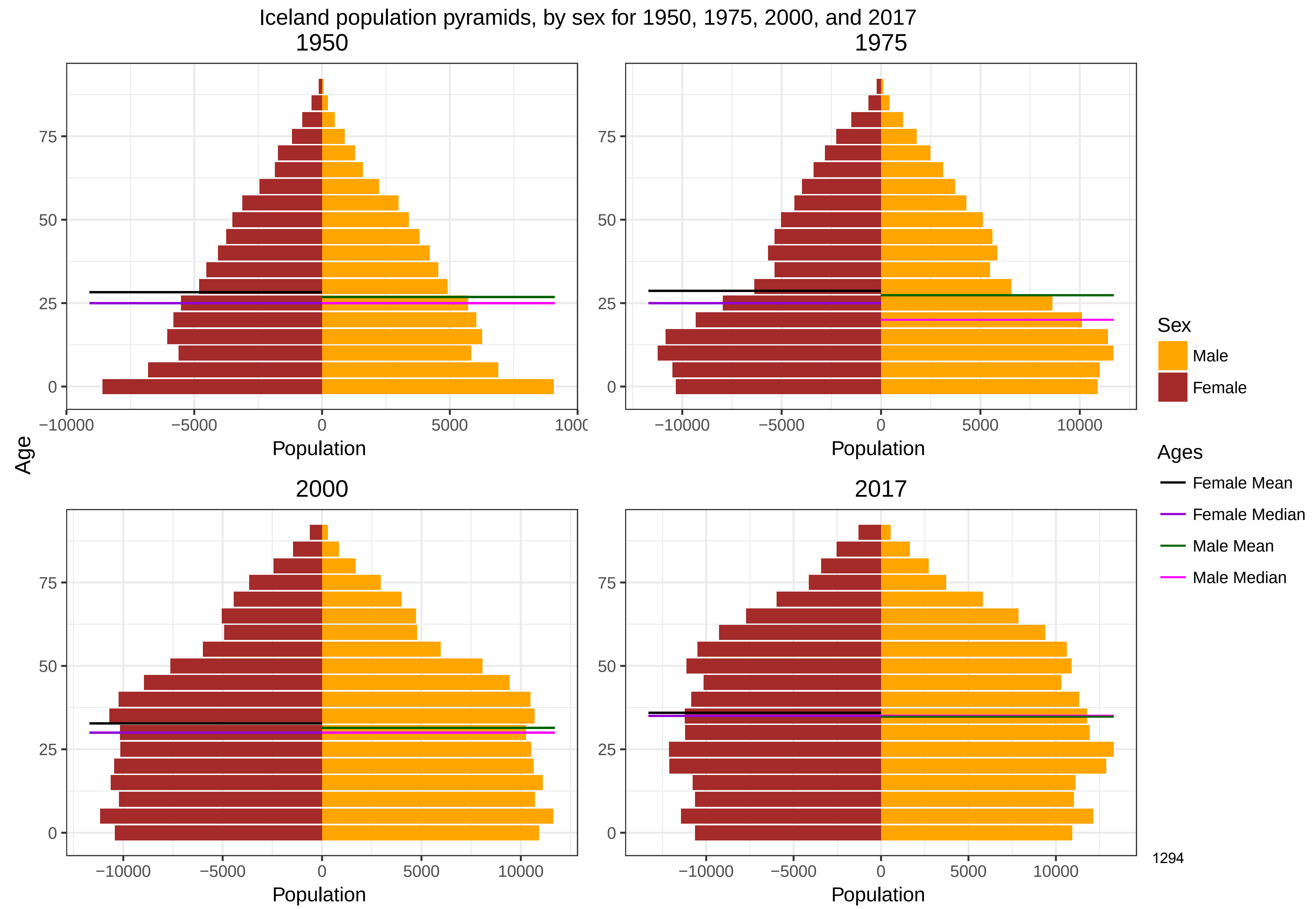
Iceland population pyramids, by sex for 1950, 1975, 2000, and 2017
1950
1975
2000
2017
Age
Population
Sex
Male
Female
Ages
Female Mean
Female Median
Male Mean
Male Median

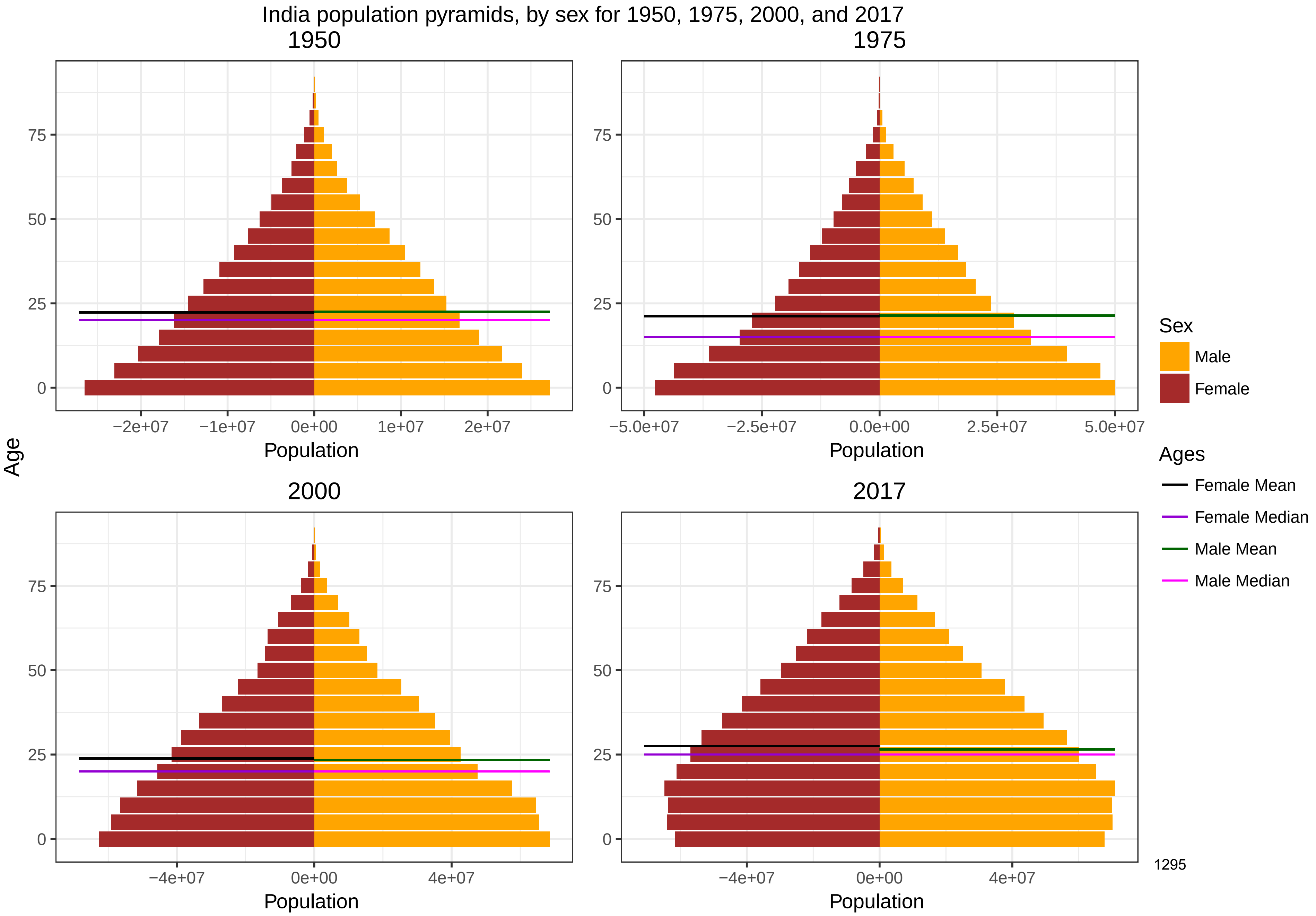
India population pyramids, by sex for 1950, 1975, 2000, and 2017
1950
1975
2000
2017
Age
Population
0
25
50
75
−2e+07
−1e+07
0e+00
1e+07
2e+07
−5.0e+07
−2.5e+07
0.0e+00
2.5e+07
5.0e+07
−4e+07
4e+07
Sex
Male
Female
Ages
Female Mean
Female Median
Male Mean
Male Median

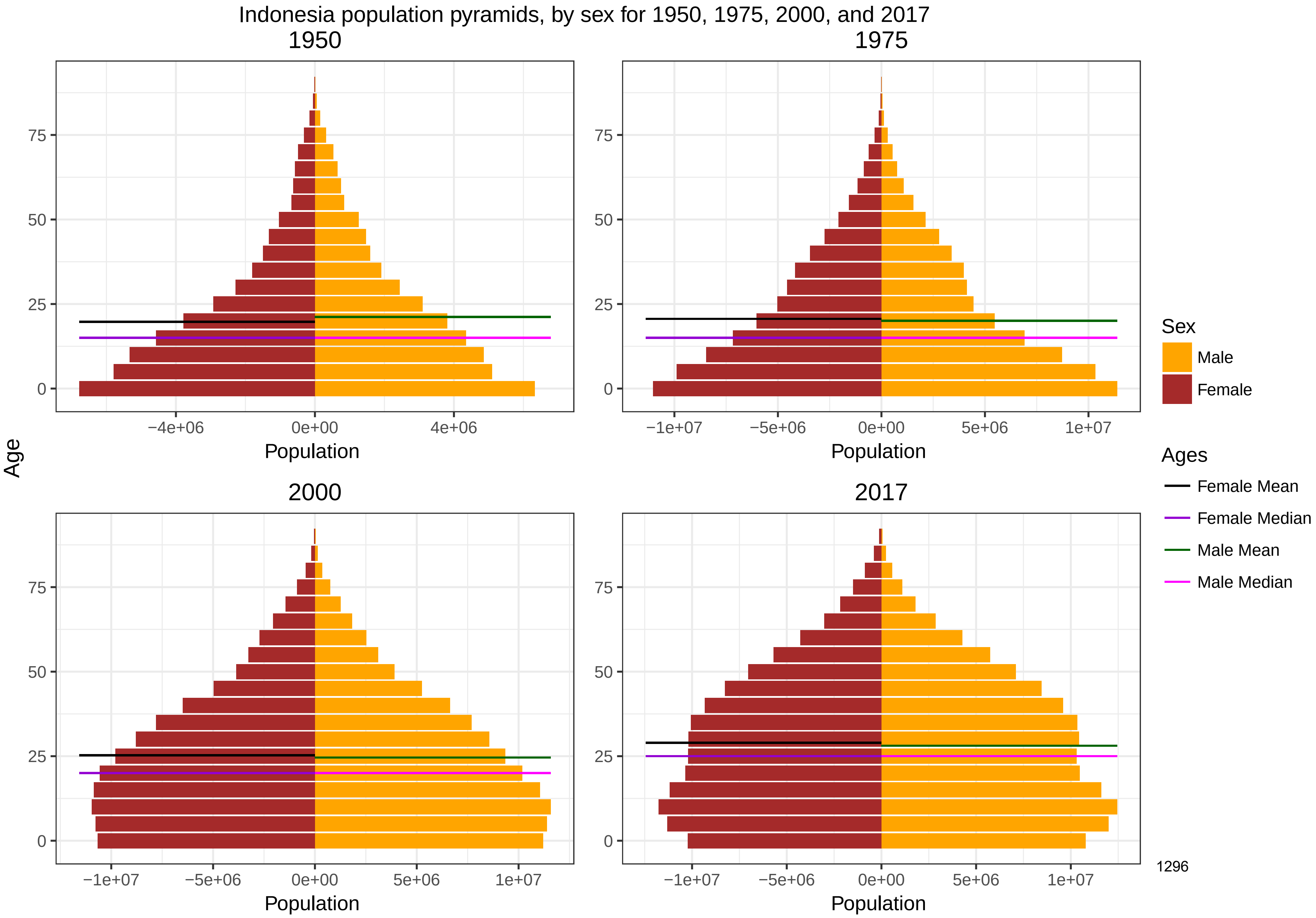
Indonesia population pyramids, by sex for 1950, 1975, 2000, and 2017
1950
1975
2000
2017
Age
Population
0
25
50
75
−4e+06
0e+00
4e+06
−1e+07
−5e+06
5e+06
1e+07
Sex
Male
Female
Ages
Female Mean
Female Median
Male Mean
Male Median

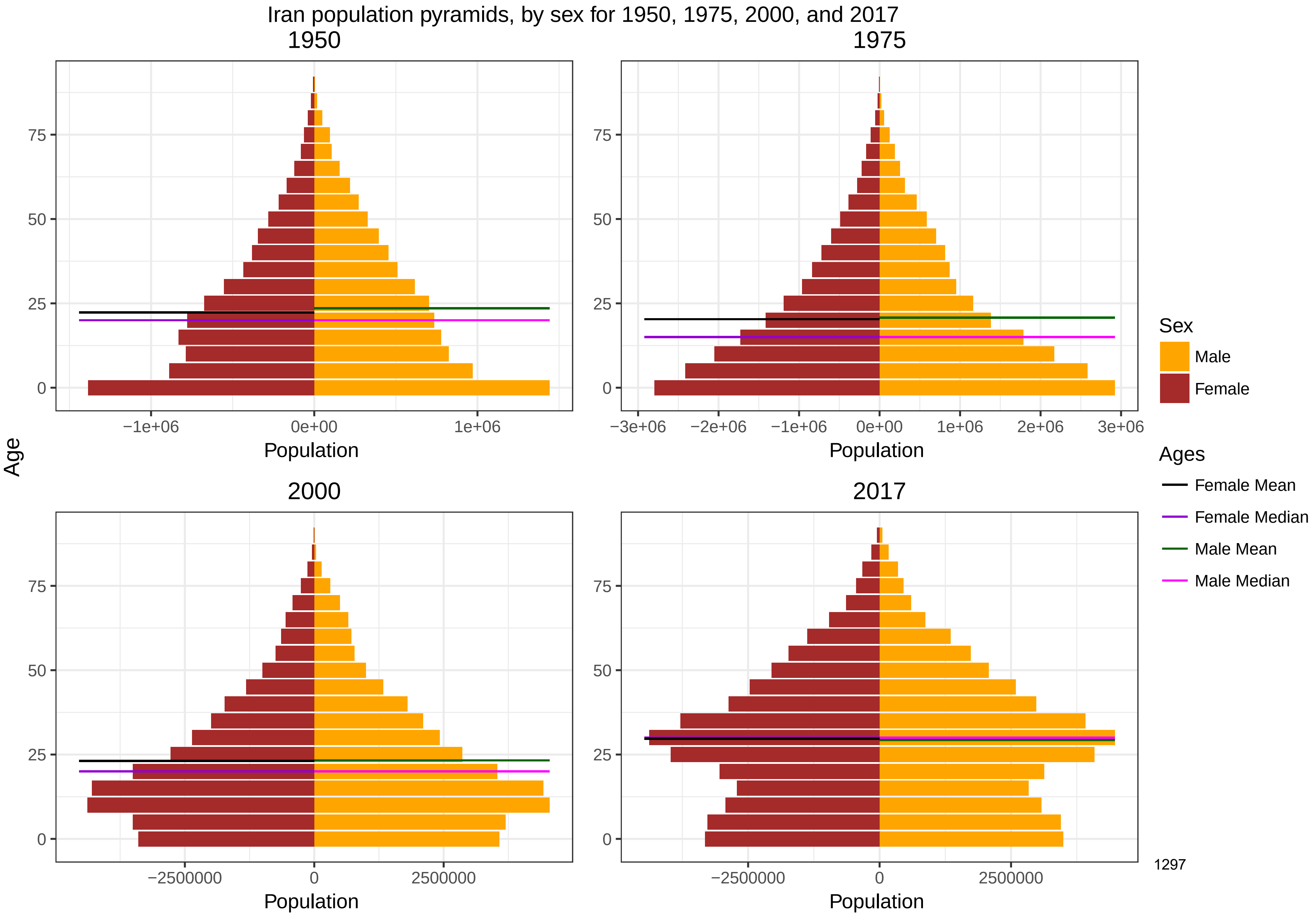

Iran population pyramids, by sex for 1950, 1975, 2000, and 2017
1950
1975
2000
2017
Age
Population
Sex
Male
Female
Ages
Female Mean
Female Median
Male Mean
Male Median

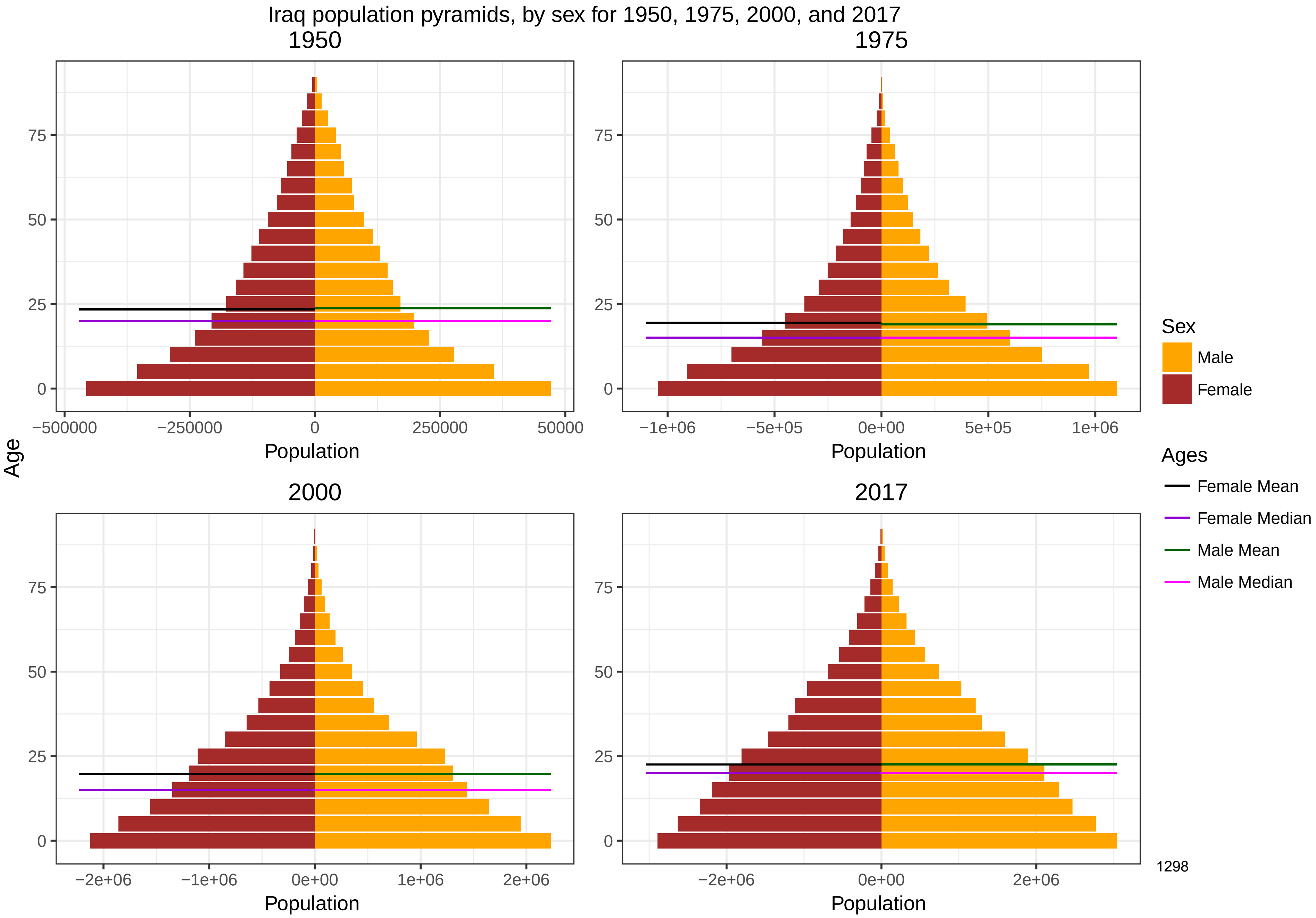

Iraq population pyramids, by sex for 1950, 1975, 2000, and 2017
1950
1975
2000
2017
Age
Population
−500000
−250000
0
250000
50000
−1e+06
−5e+05
0e+00
5e+05
1e+06
−2e+06
−1e+06
1e+06
2e+06
0
25
50
75
Sex
Male
Female
Ages
Female Mean
Female Median
Male Mean
Male Median

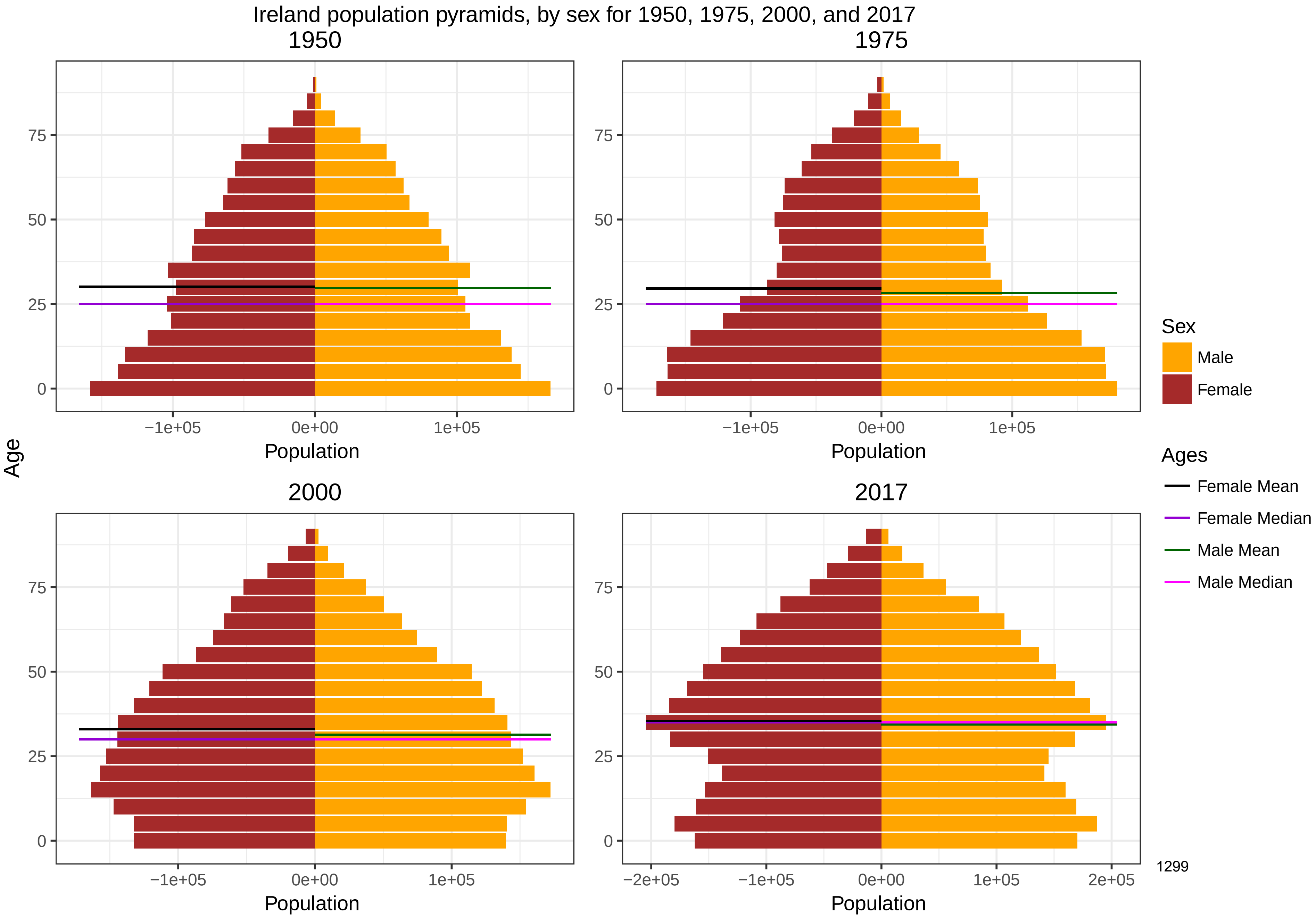
Ireland population pyramids, by sex for 1950, 1975, 2000, and 2017
1950
1975
2000
2017
Age
Population
−2e+05
−1e+05
0e+00
1e+05
2e+05
0
25
50
75
Sex
Male
Female
Ages
Female Mean
Female Median
Male Mean
Male Median

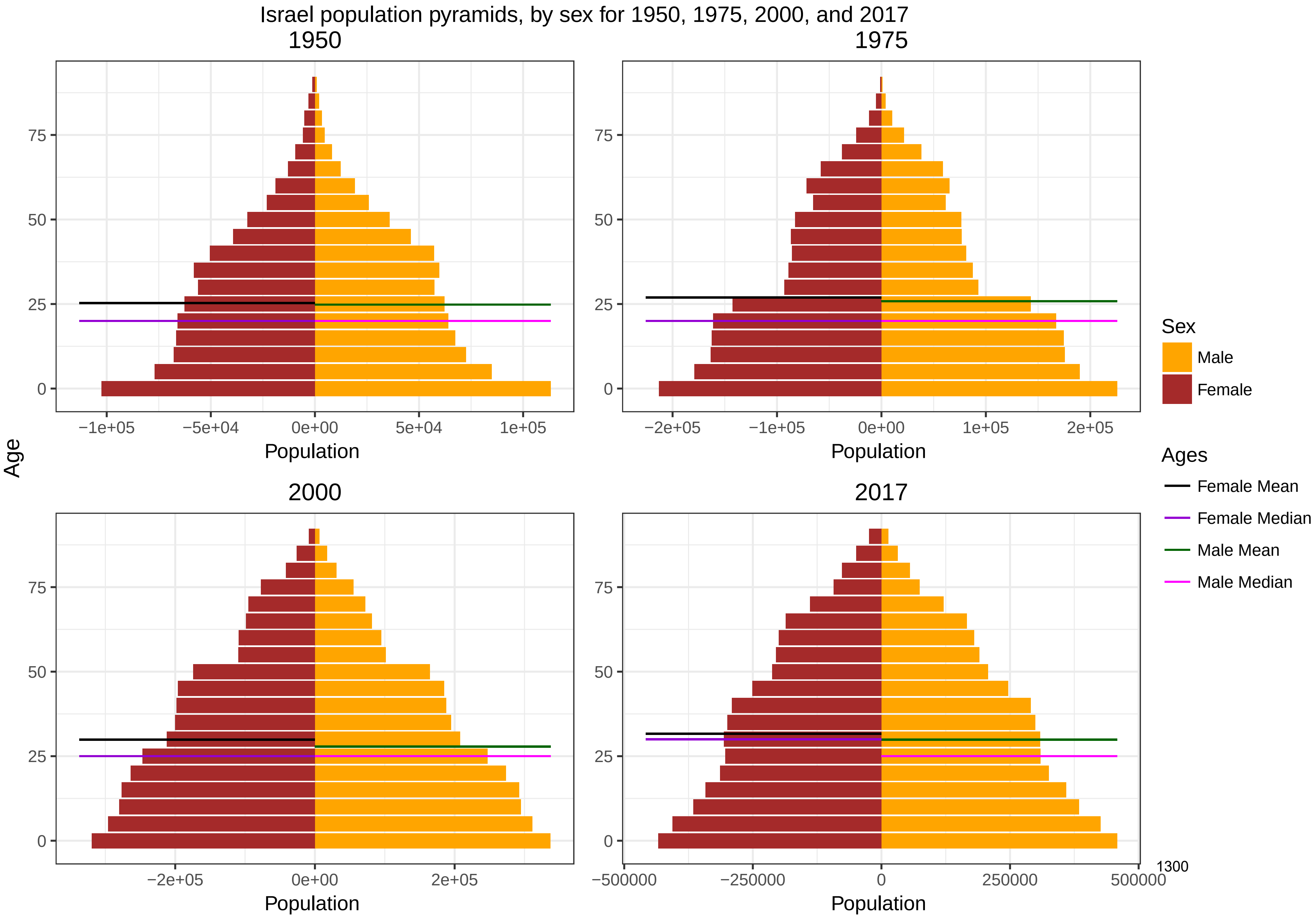
Israel population pyramids, by sex for 1950, 1975, 2000, and 2017
1950
1975
2000
2017
Age
Population
0
25
50
75
−1e+05
−5e+04
0e+00
5e+04
1e+05
−2e+05
2e+05
−500000
−250000
250000
500000
Sex
Male
Female
Ages
Female Mean
Female Median
Male Mean
Male Median

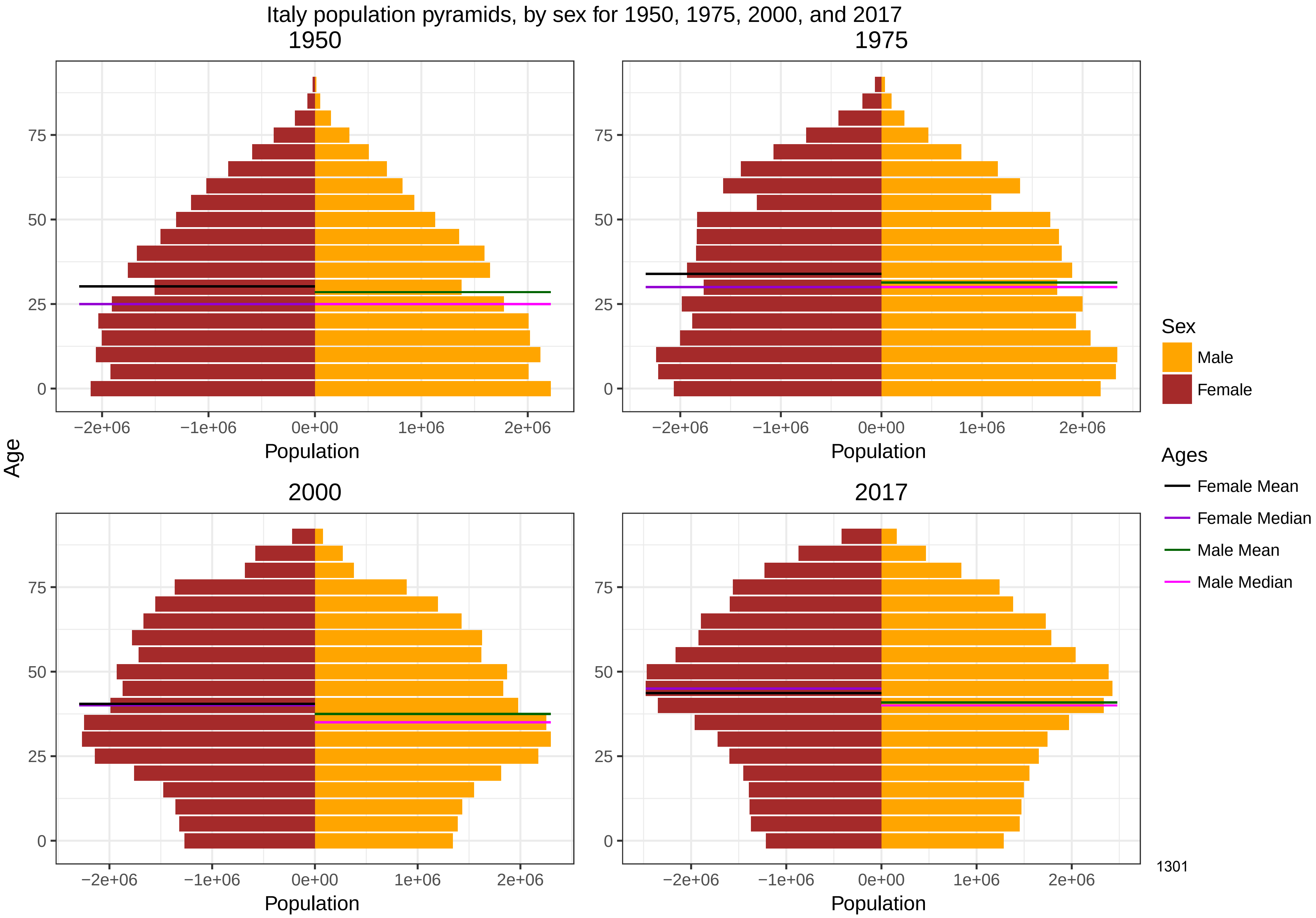
Italy population pyramids, by sex for 1950, 1975, 2000, and 2017
1950
1975
2000
2017
Age
Population
0
25
50
75
−2e+06
−1e+06
0e+00
1e+06
2e+06
Sex
Male
Female
Ages
Female Mean
Female Median
Male Mean
Male Median

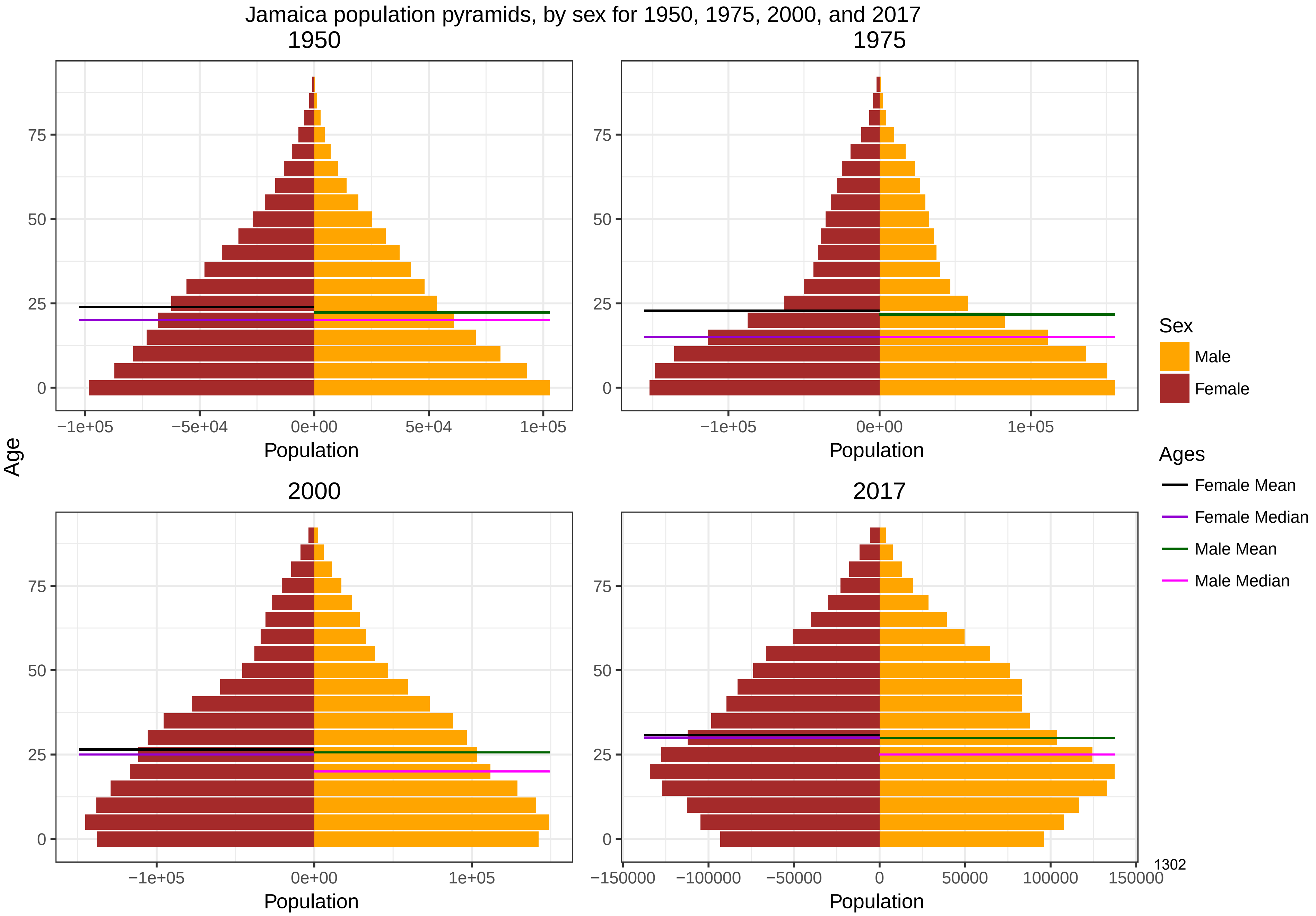

Jamaica population pyramids, by sex for 1950, 1975, 2000, and 2017
1950
1975
2000
2017
Age
Population
0
25
50
75
−1e+05
−5e+04
0e+00
5e+04
1e+05
−150000
−100000
−50000
0
50000
100000
150000
Sex
Male
Female
Ages
Female Mean
Female Median
Male Mean
Male Median

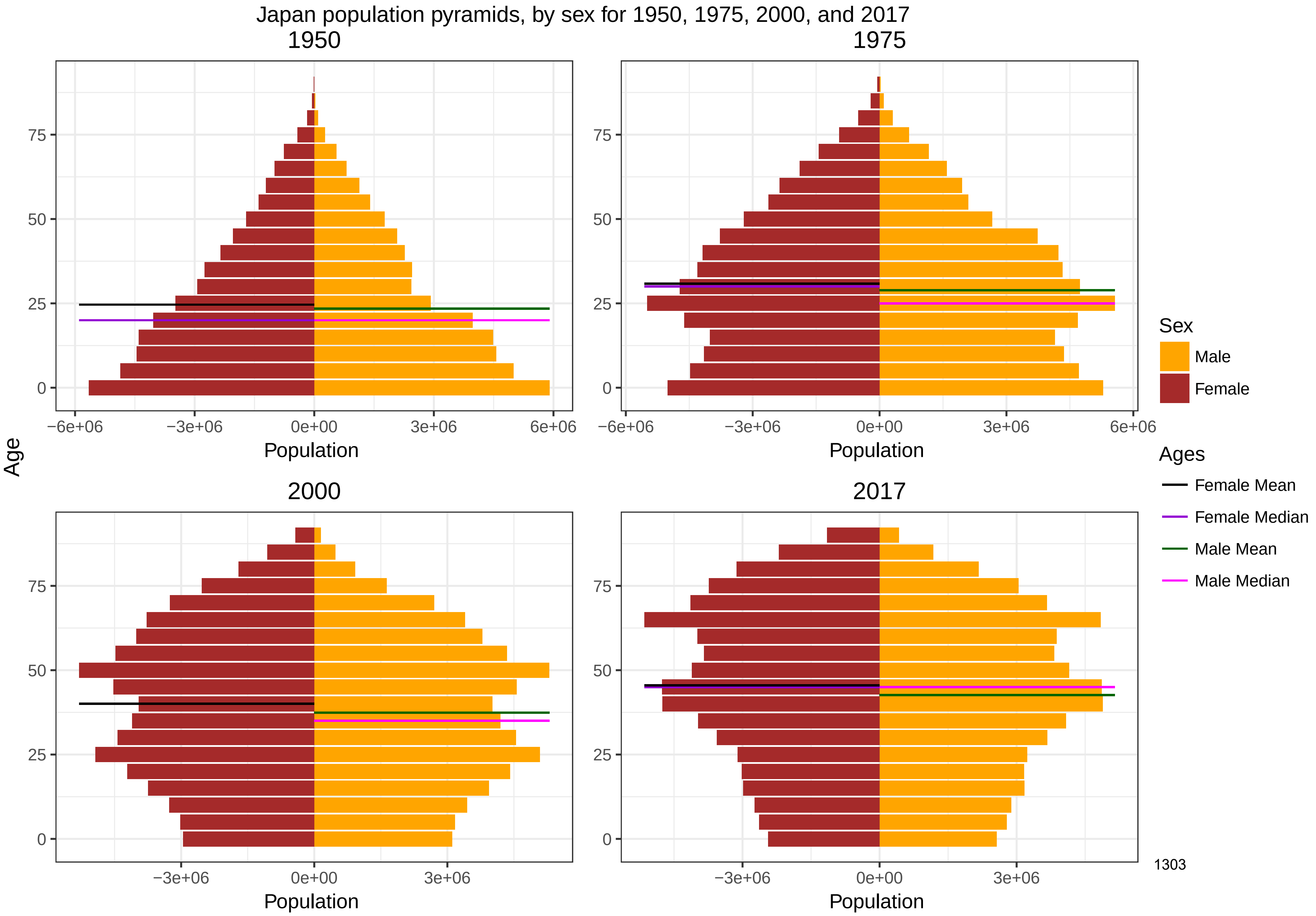

Japan population pyramids, by sex for 1950, 1975, 2000, and 2017
1950
1975
2000
2017
Age
Population
−6e+06
−3e+06
0e+00
3e+06
6e+06
0
25
50
75
Sex
Male
Female
Ages
Female Mean
Female Median
Male Mean
Male Median

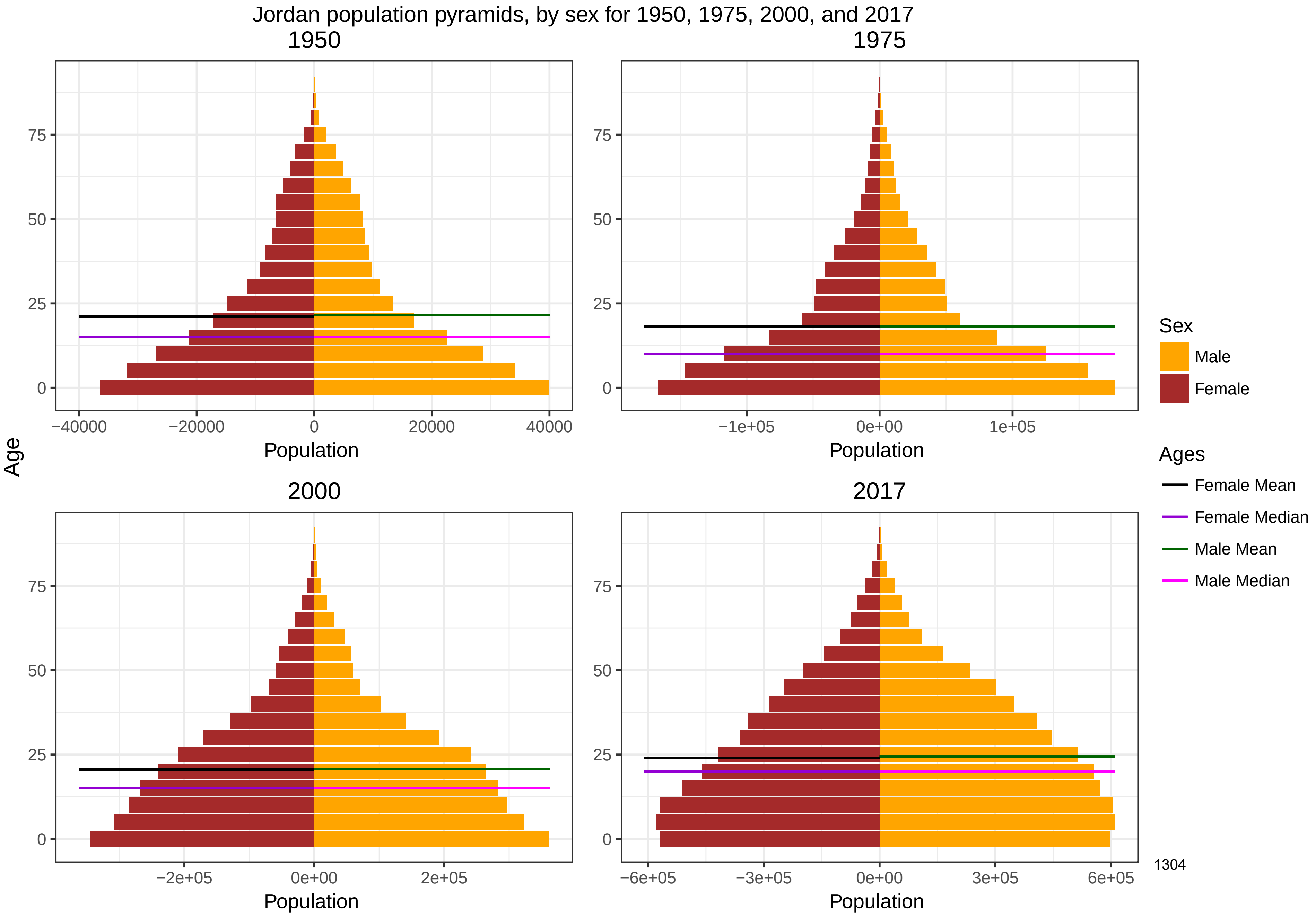
Jordan population pyramids, by sex for 1950, 1975, 2000, and 2017
1950
1975
2000
2017
Age
Population
−40000
−20000
0
20000
40000
−1e+05
0e+00
1e+05
−2e+05
2e+05
−6e+05
−3e+05
3e+05
6e+05
0
25
50
75
Sex
Male
Female
Ages
Female Mean
Female Median
Male Mean
Male Median

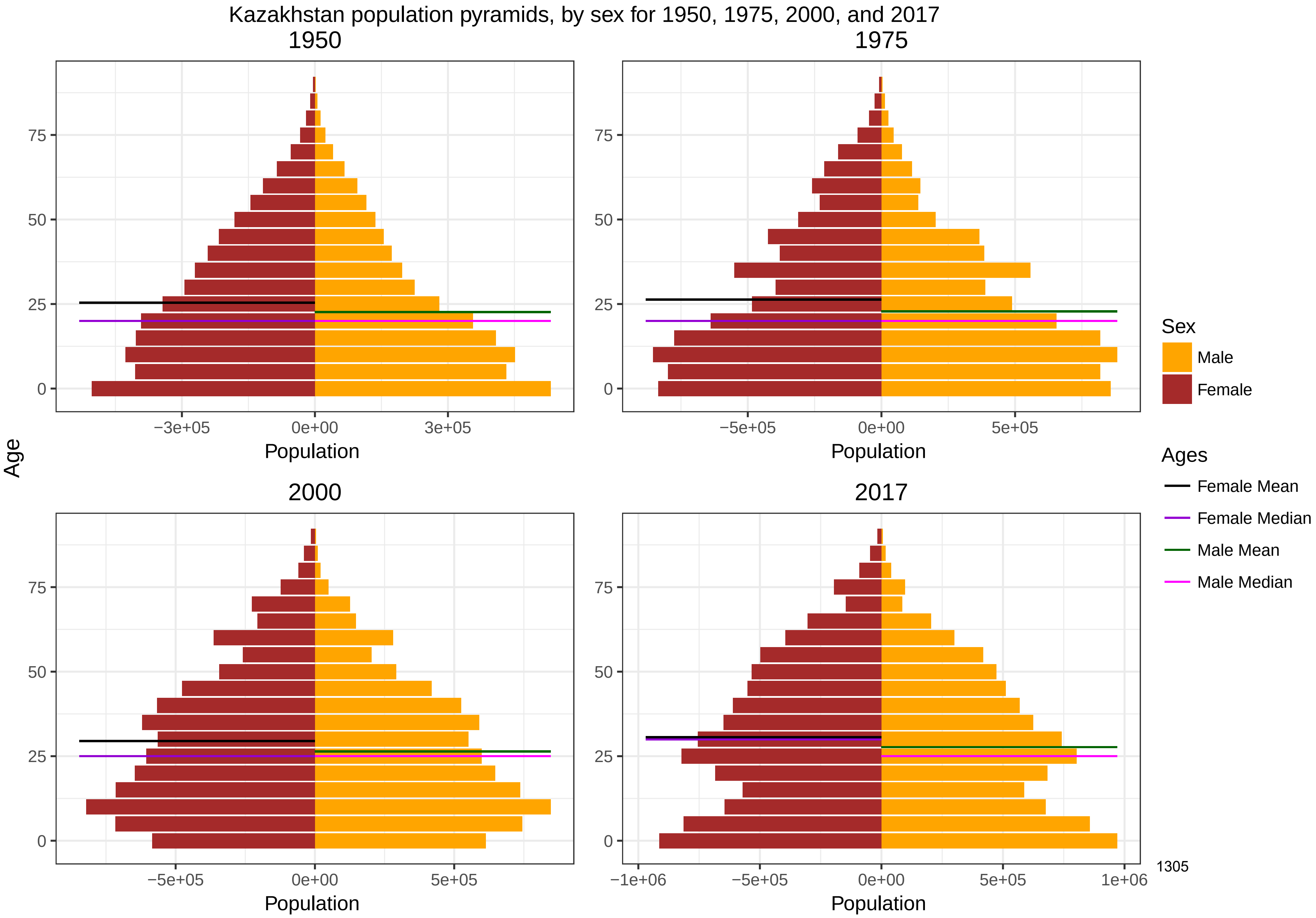
Kazakhstan population pyramids, by sex for 1950, 1975, 2000, and 2017
1950
1975
2000
2017
Age
Population
0
25
50
75
−3e+05
0e+00
3e+05
−5e+05
5e+05
−1e+06
1e+06
Sex
Male
Female
Ages
Female Mean
Female Median
Male Mean
Male Median

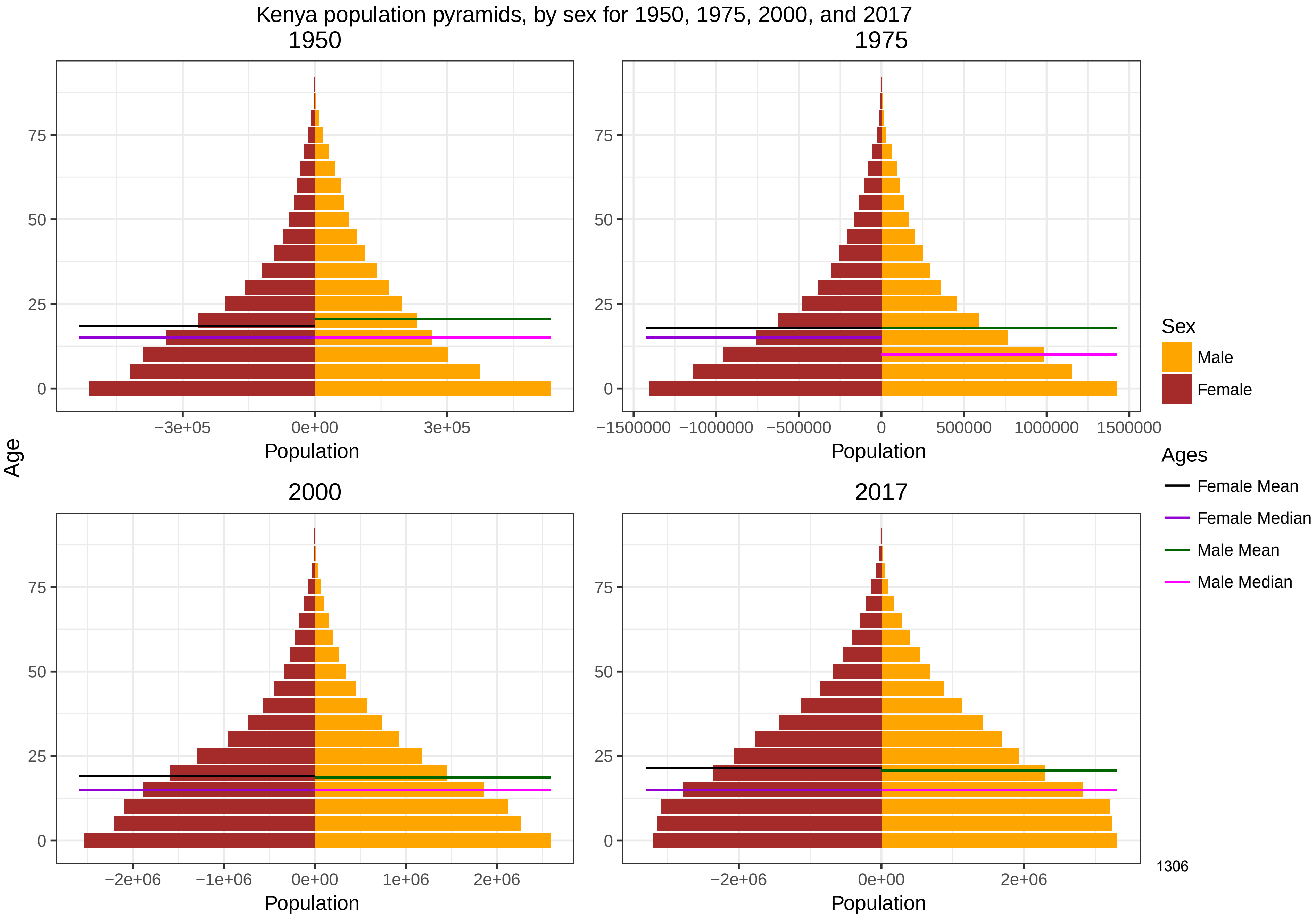
Kenya population pyramids, by sex for 1950, 1975, 2000, and 2017
1950
1975
2000
2017
Age
Population
−3e+05
0e+00
3e+05
−1500000
−1000000
−500000
0
500000
1000000
1500000
−2e+06
−1e+06
1e+06
2e+06
75
50
25
Sex
Male
Female
Ages
Female Mean
Female Median
Male Mean
Male Median

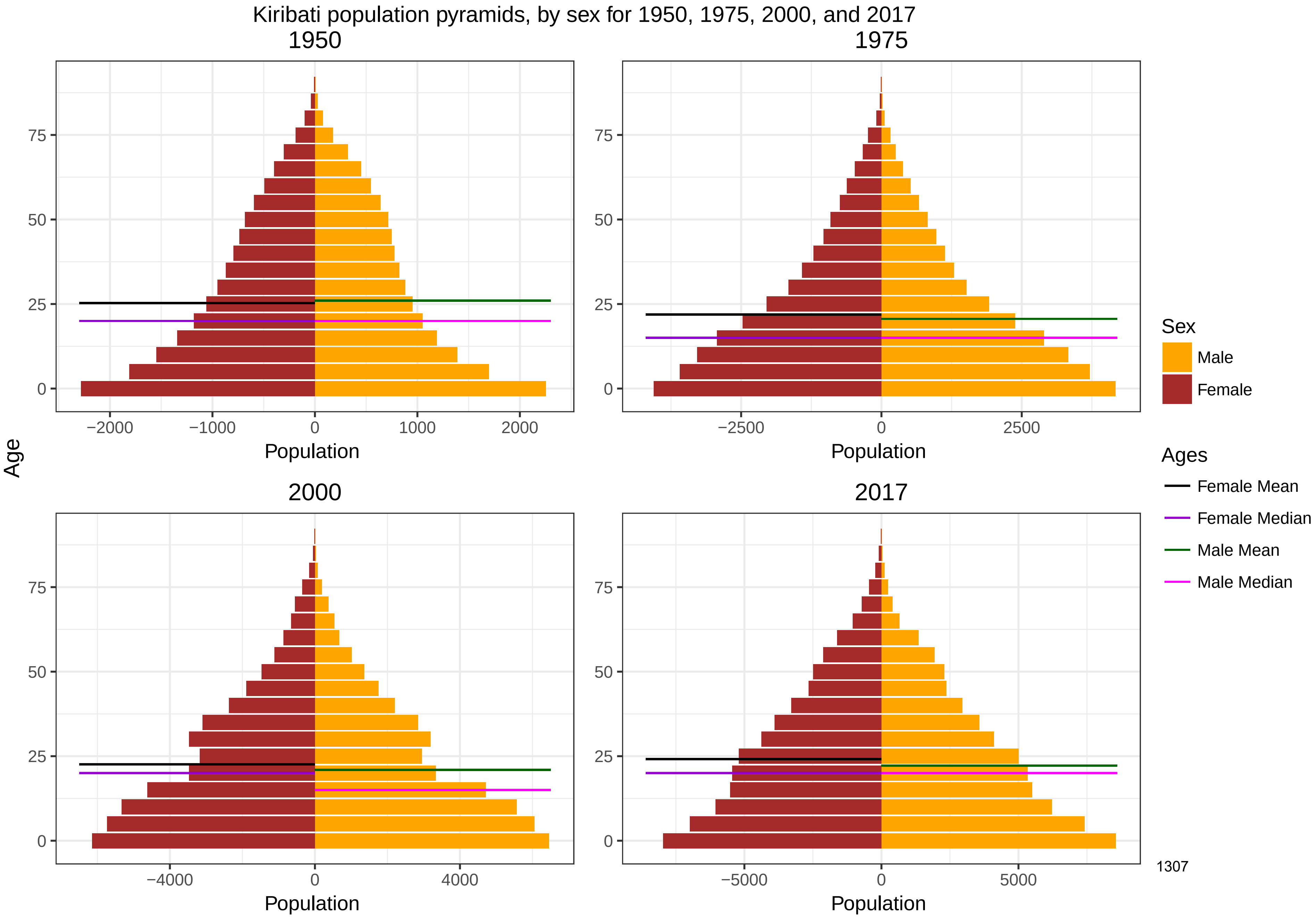

Kiribati population pyramids, by sex for 1950, 1975, 2000, and 2017
1950
1975
2000
2017
Age
Population
Sex
Male
Female
Ages
Female Mean
Female Median
Male Mean
Male Median

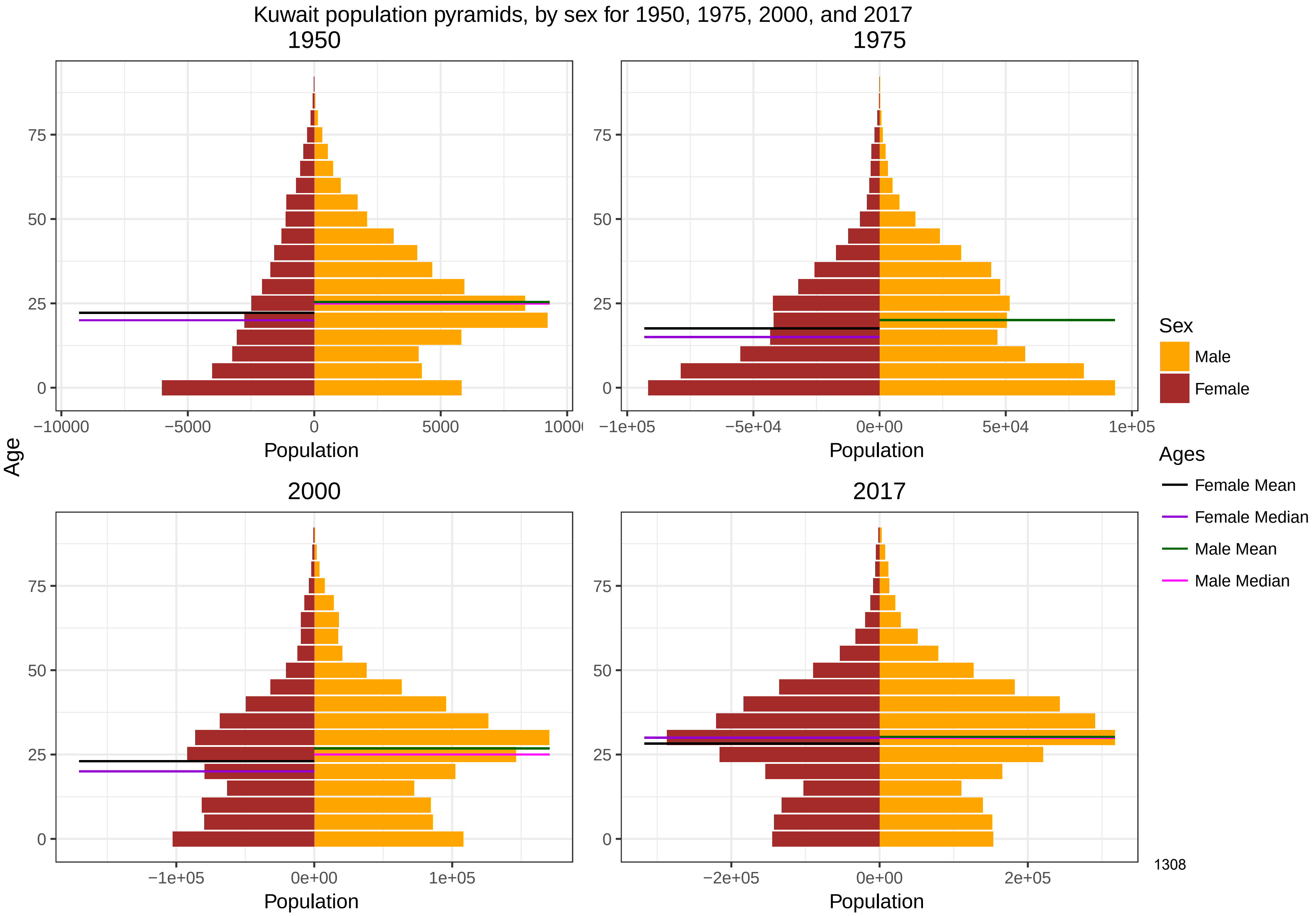
Kuwait population pyramids, by sex for 1950, 1975, 2000, and 2017
1950
1975
2000
2017
Age
Population
Sex
Male
Female
Ages
Female Mean
Female Median
Male Mean
Male Median
−10000
−5000
0
5000
10000
−1e+05
−5e+04
0e+00
5e+04
1e+05
2e+05
−2e+05
0
25
50
75

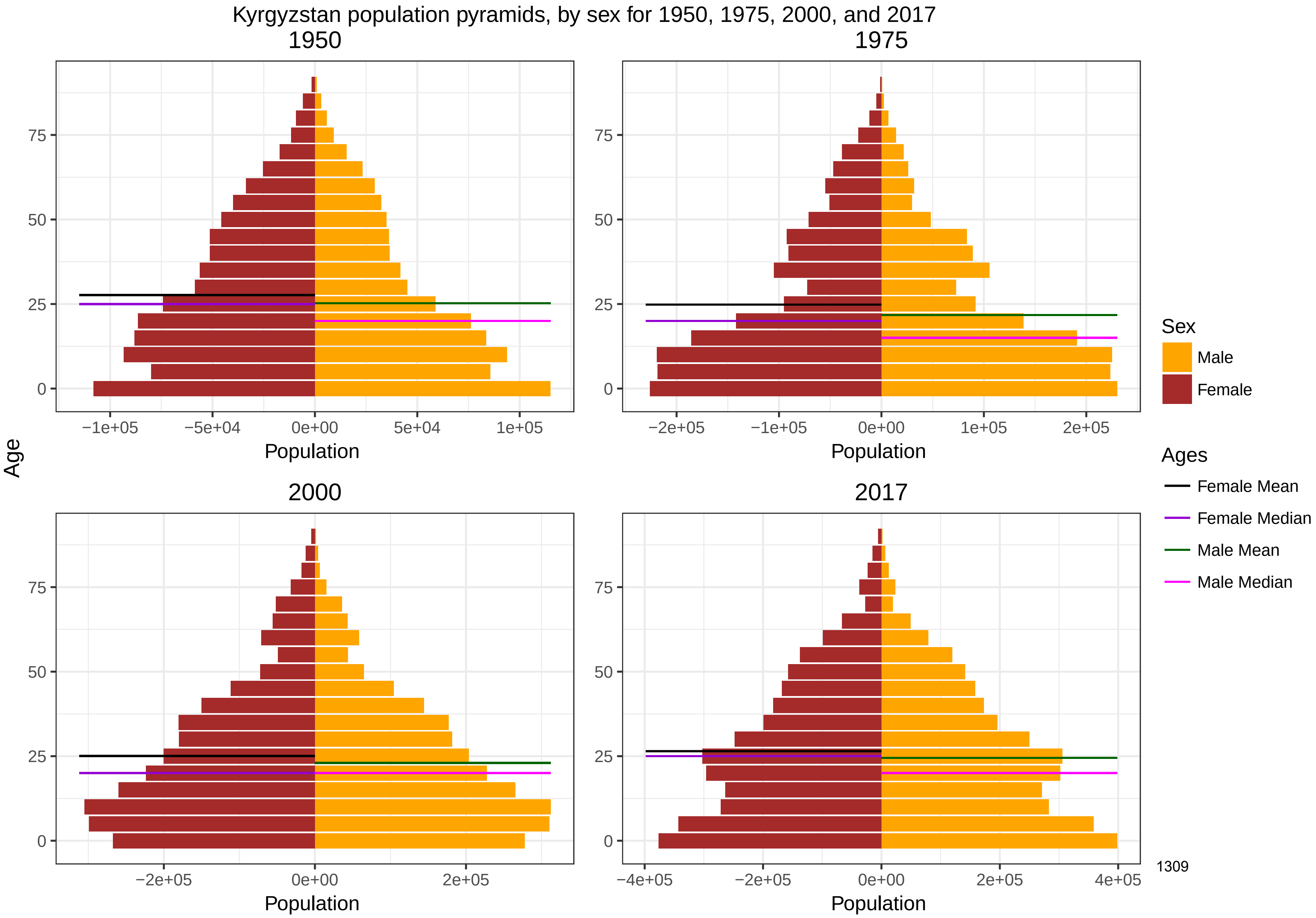

Kyrgyzstan population pyramids, by sex for 1950, 1975, 2000, and 2017
1950
1975
2000
2017
Age
Population
0
25
50
75
−1e+05
−5e+04
0e+00
5e+04
1e+05
−2e+05
2e+05
−4e+05
4e+05
Sex
Male
Female
Ages
Female Mean
Female Median
Male Mean
Male Median

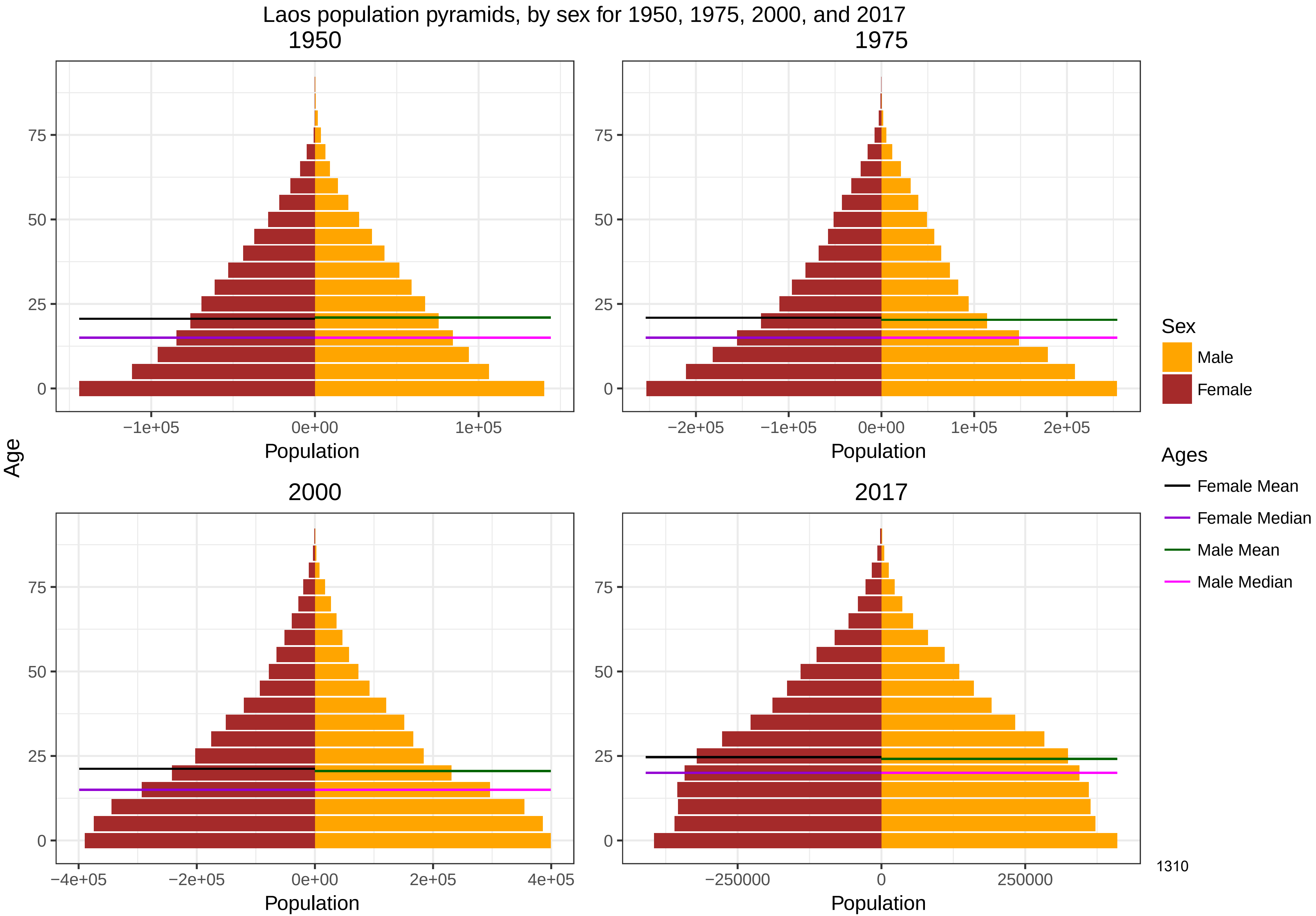

Laos population pyramids, by sex for 1950, 1975, 2000, and 2017
1950
1975
2000
2017
Age
Population
−1e+05
0e+00
1e+05
−2e+05
2e+05
−4e+05
4e+05
−250000
0
250000
75
50
25
Sex
Male
Female
Ages
Female Mean
Female Median
Male Mean
Male Median

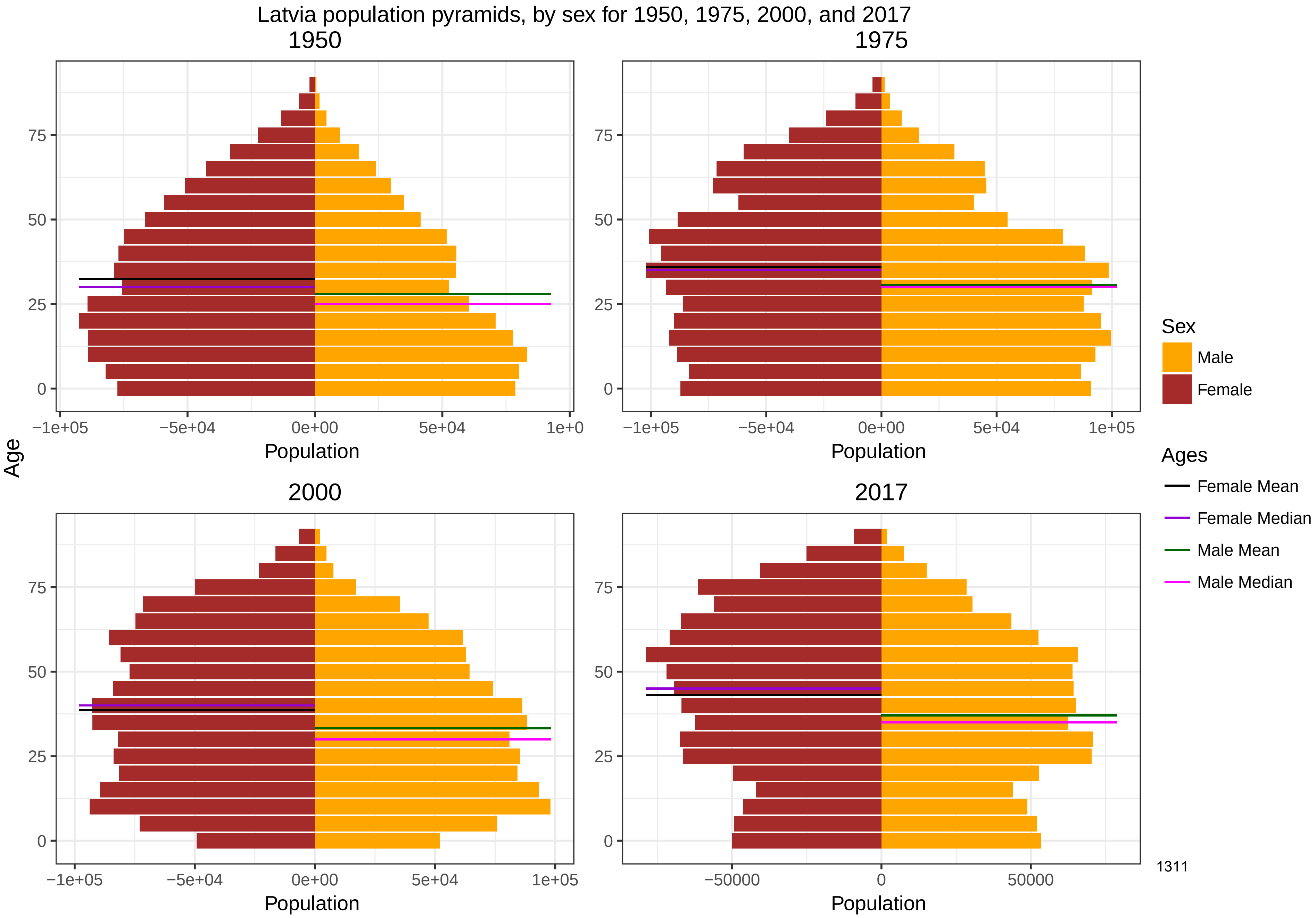
Latvia population pyramids, by sex for 1950, 1975, 2000, and 2017
1950
1975
2000
2017
Age
Population
Sex
Male
Female
Ages
Female Mean
Female Median
Male Mean
Male Median

Lebanon population pyramids, by sex for 1950, 1975, 2000, and 2017

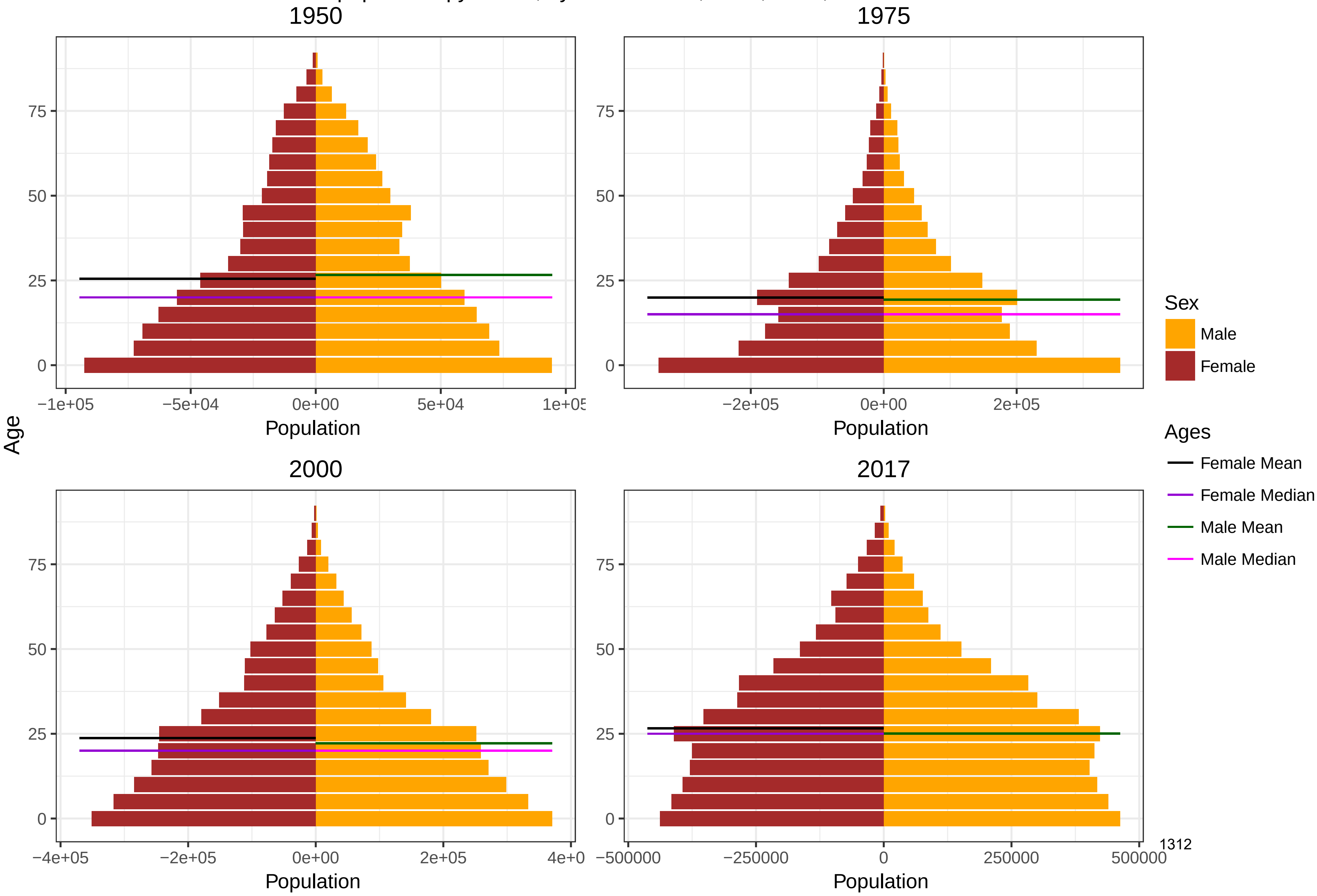

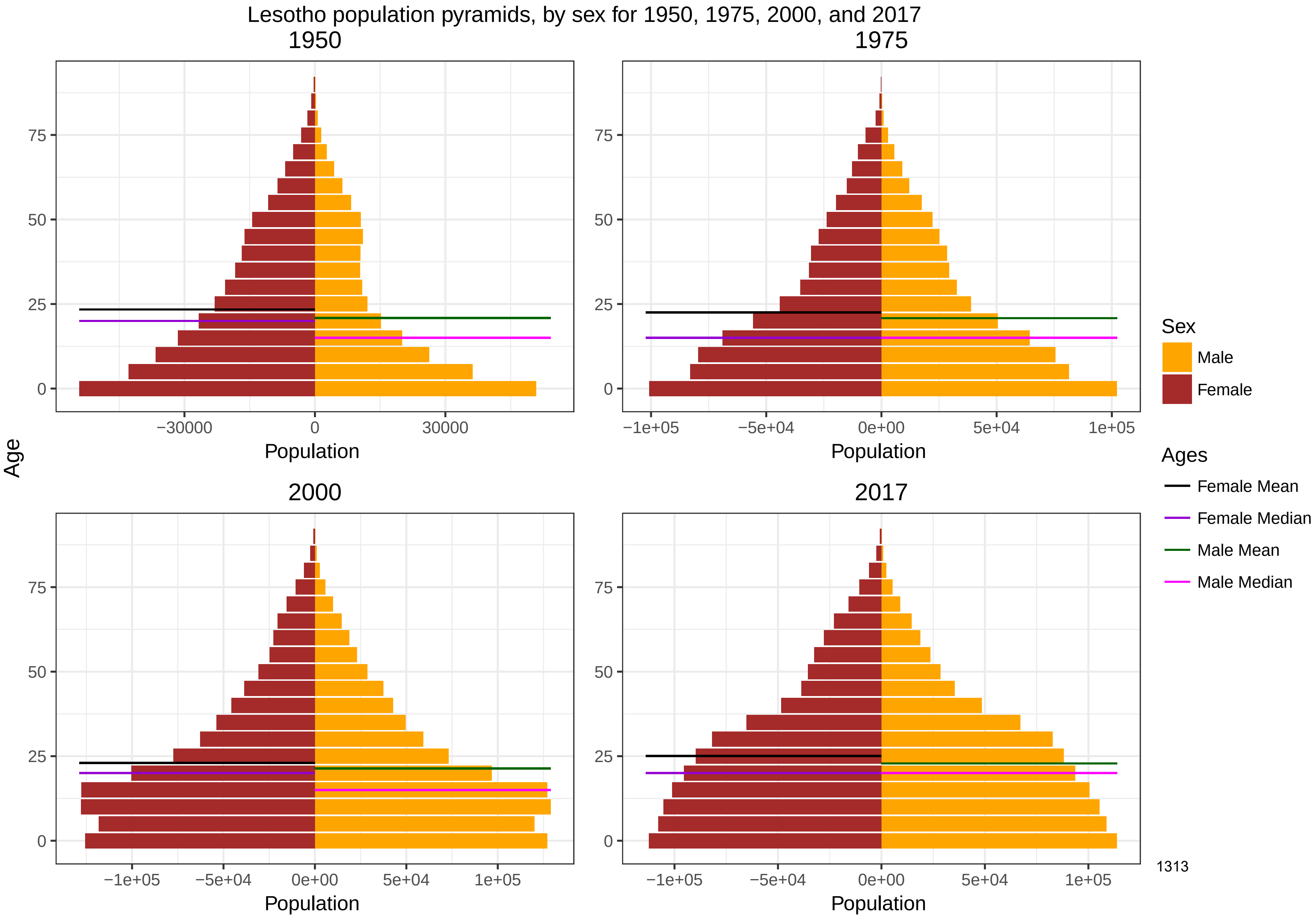

Lesotho population pyramids, by sex for 1950, 1975, 2000, and 2017
1950
1975
2000
2017
Age
Population
−30000
0
30000
−1e+05
−5e+04
0e+00
5e+04
1e+05
0
25
50
75
Sex
Male
Female
Ages
Female Mean
Female Median
Male Mean
Male Median

1313

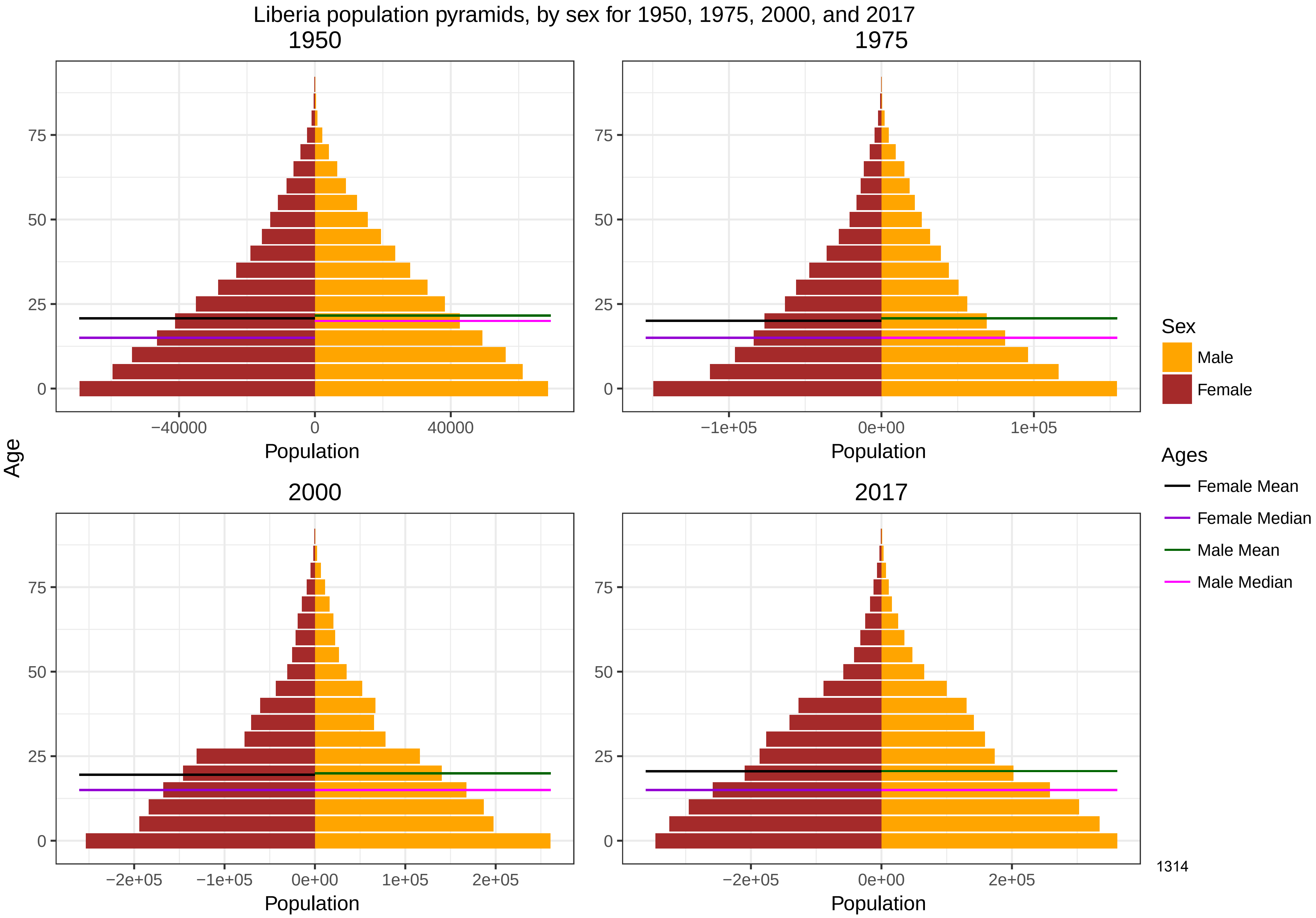
Liberia population pyramids, by sex for 1950, 1975, 2000, and 2017
1950
1975
2000
2017
Age
Population
Sex
Male
Female
Ages
Female Mean
Female Median
Male Mean
Male Median

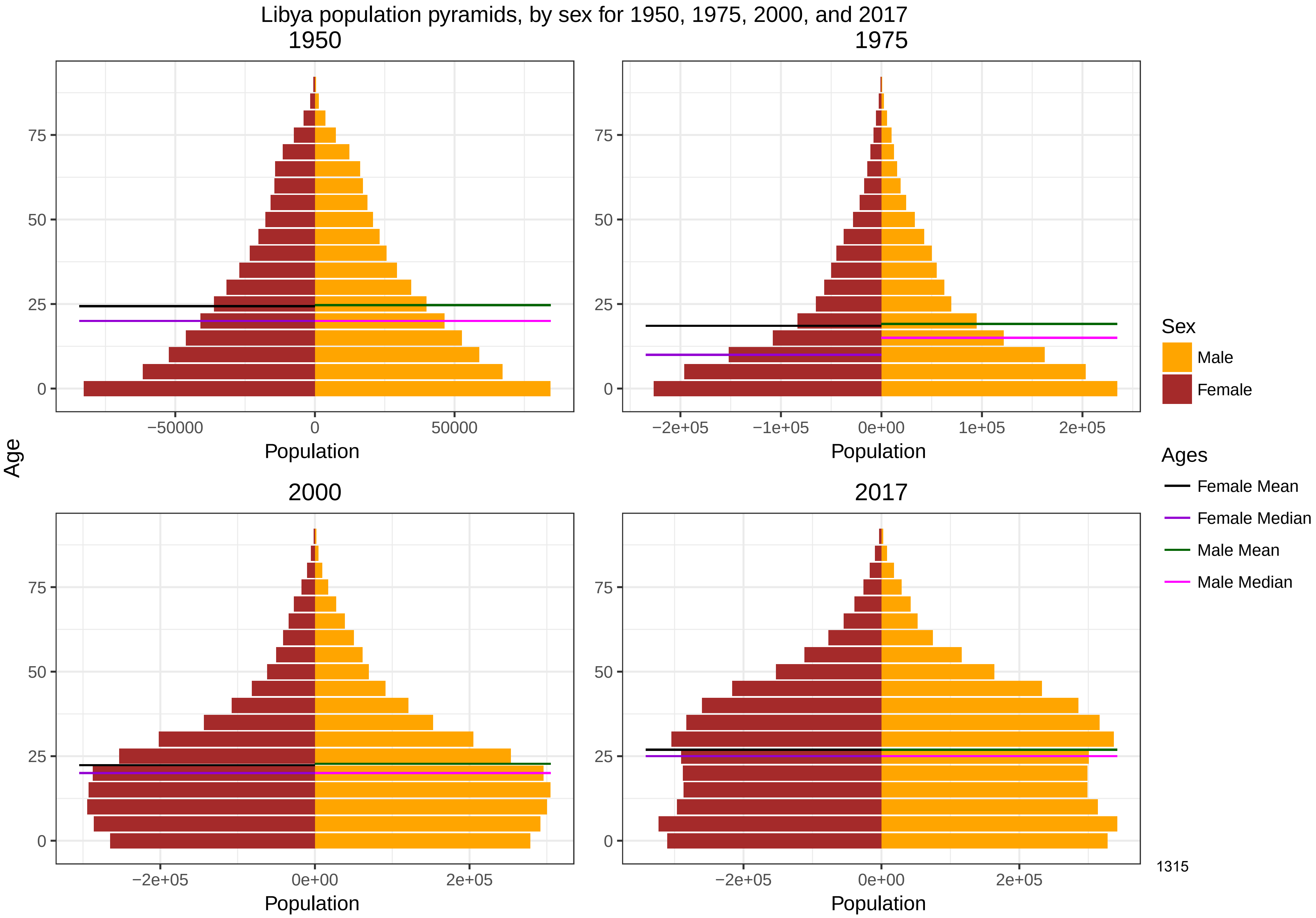
Libya population pyramids, by sex for 1950, 1975, 2000, and 2017
1950
1975
2000
2017
Age
Population
−50000
0
50000
−2e+05
−1e+05
0e+00
1e+05
2e+05
0
25
50
75
Sex
Male
Female
Ages
Female Mean
Female Median
Male Mean
Male Median

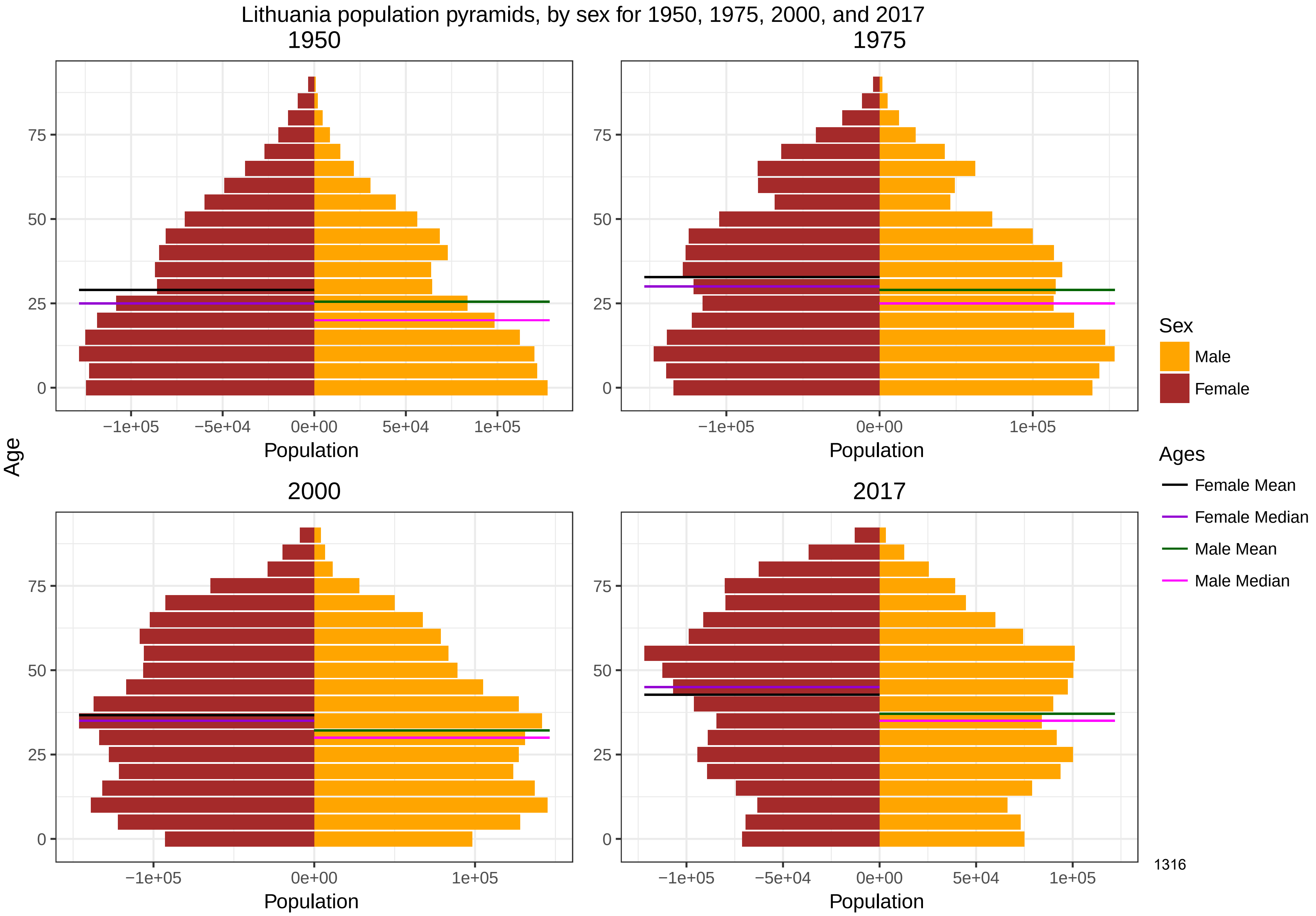

Lithuania population pyramids, by sex for 1950, 1975, 2000, and 2017
1950
1975
2000
2017
Age
Population
0
25
50
75
−1e+05
−5e+04
0e+00
5e+04
1e+05
Sex
Male
Female
Ages
Female Mean
Female Median
Male Mean
Male Median

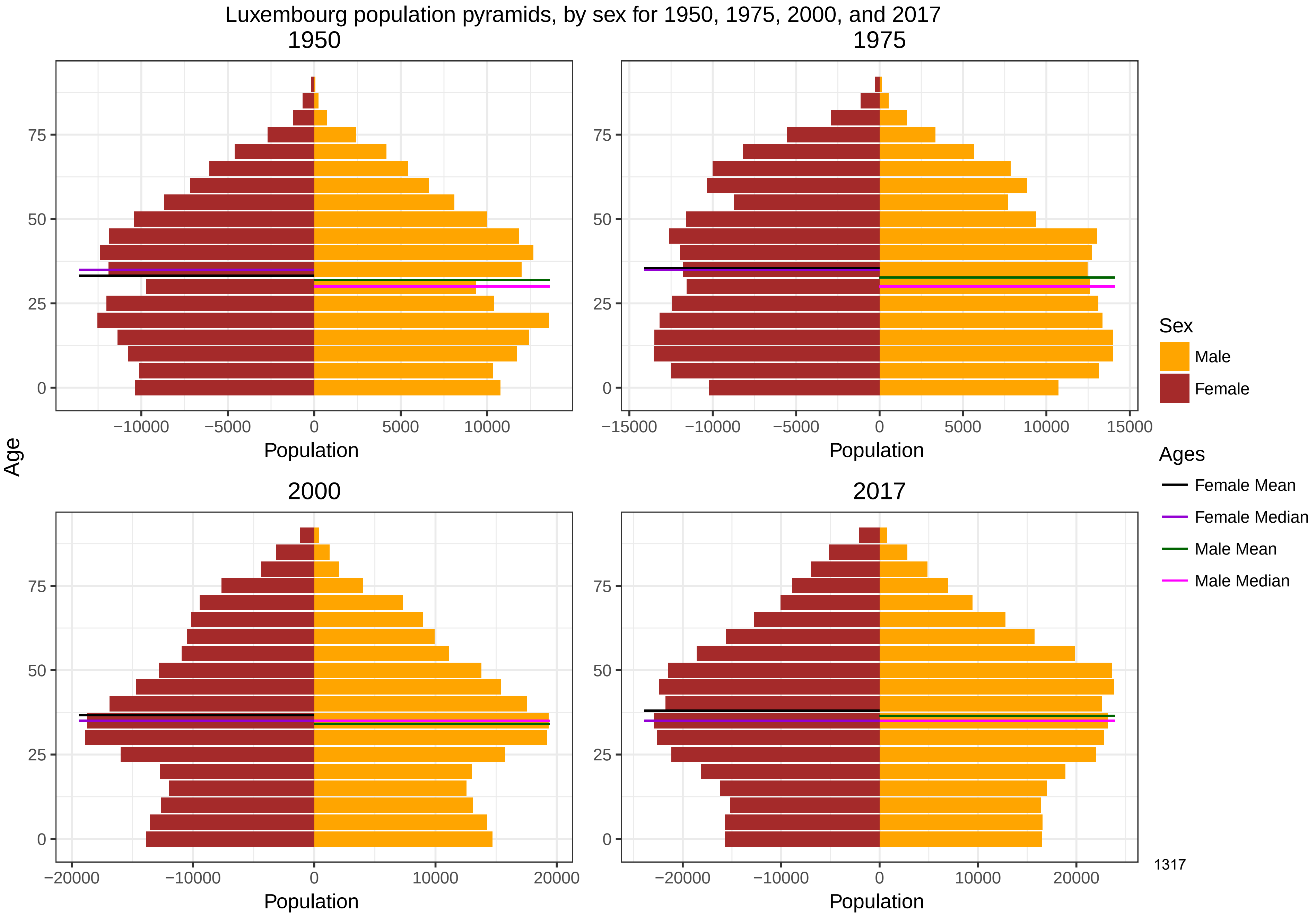
Luxembourg population pyramids, by sex for 1950, 1975, 2000, and 2017
1950
1975
2000
2017
Age
Population
Sex
Male
Female
Ages
Female Mean
Female Median
Male Mean
Male Median

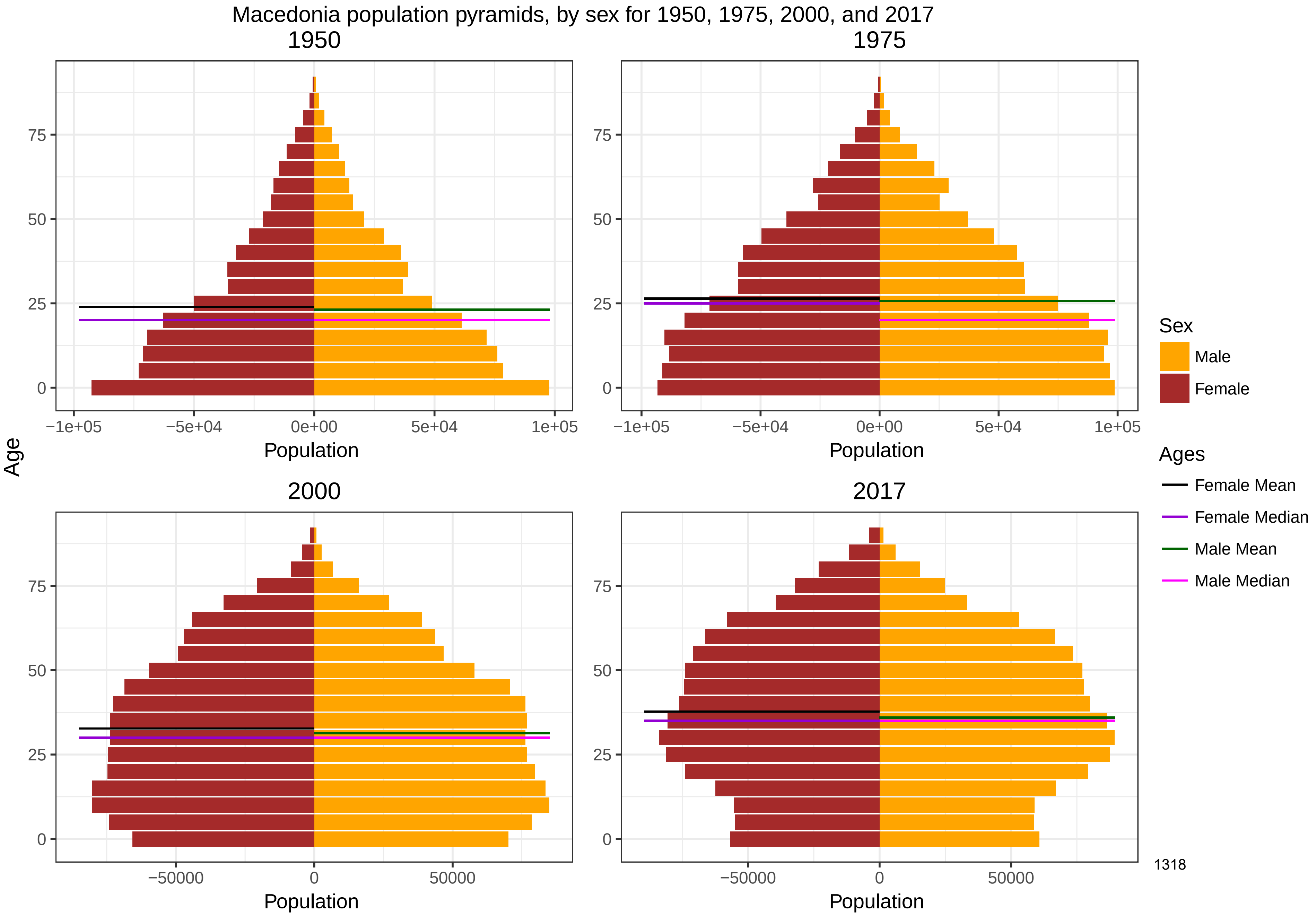

Macedonia population pyramids, by sex for 1950, 1975, 2000, and 2017
1950
1975
2000
2017
Age
Population
Sex
Male
Female
Ages
Female Mean
Female Median
Male Mean
Male Median

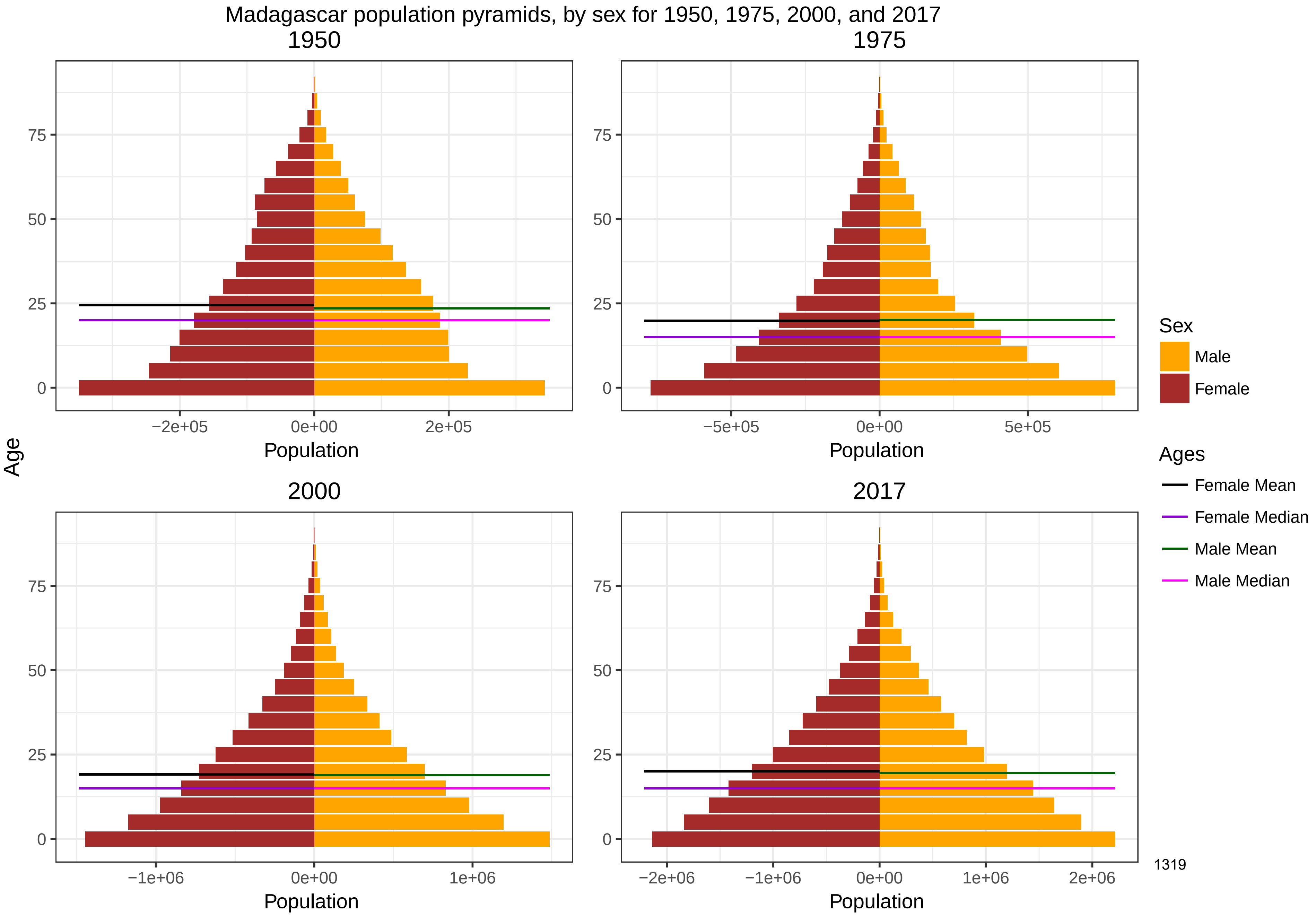

Madagascar population pyramids, by sex for 1950, 1975, 2000, and 2017
1950
1975
2000
2017
Age
Population
0
25
50
75
−2e+05
0e+00
2e+05
−5e+05
5e+05
−1e+06
1e+06
−2e+06
2e+06
Sex
Male
Female
Ages
Female Mean
Female Median
Male Mean
Male Median

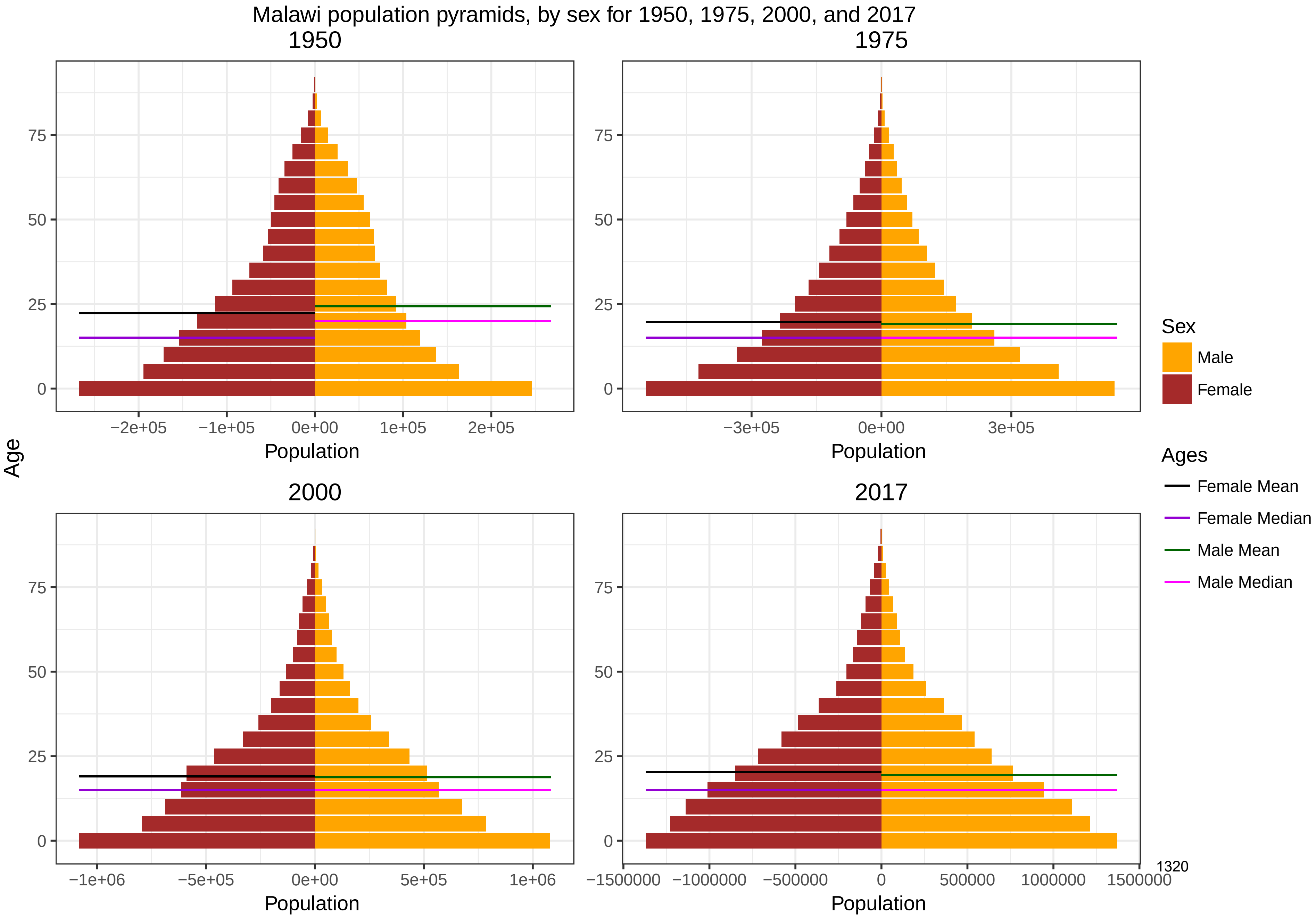

Malawi population pyramids, by sex for 1950, 1975, 2000, and 2017
1950
1975
2000
2017
Age
Population
−2e+05
−1e+05
0e+00
1e+05
2e+05
−3e+05
3e+05
−1e+06
−5e+05
5e+05
1e+06
−1500000
−1000000
−500000
0
500000
1000000
1500000
25
50
75
Sex
Male
Female
Ages
Female Mean
Female Median
Male Mean
Male Median

1320

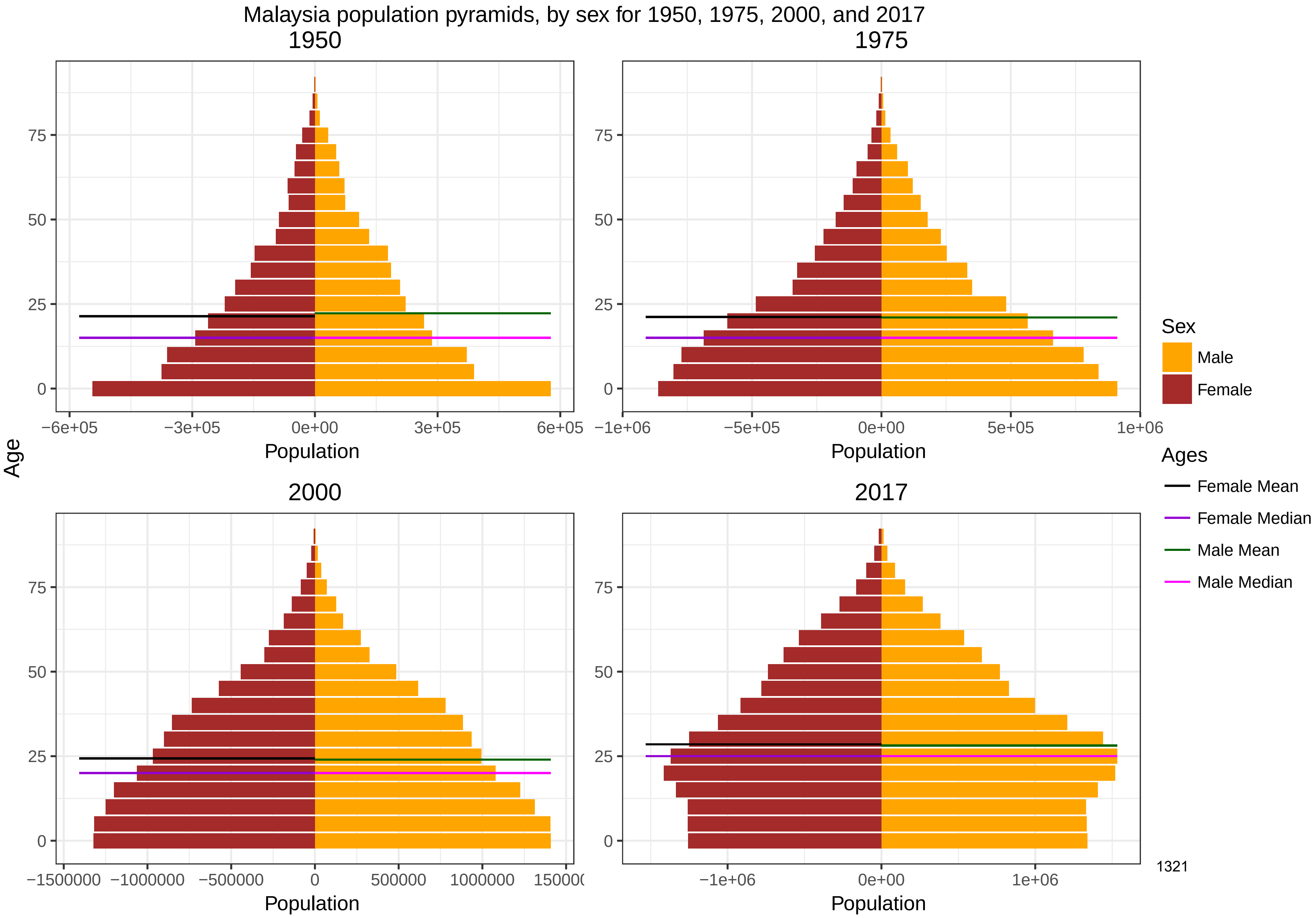
Malaysia population pyramids, by sex for 1950, 1975, 2000, and 2017
1950
1975
2000
2017
Age
Population
Sex
Male
Female
Ages
Female Mean
Female Median
Male Mean
Male Median

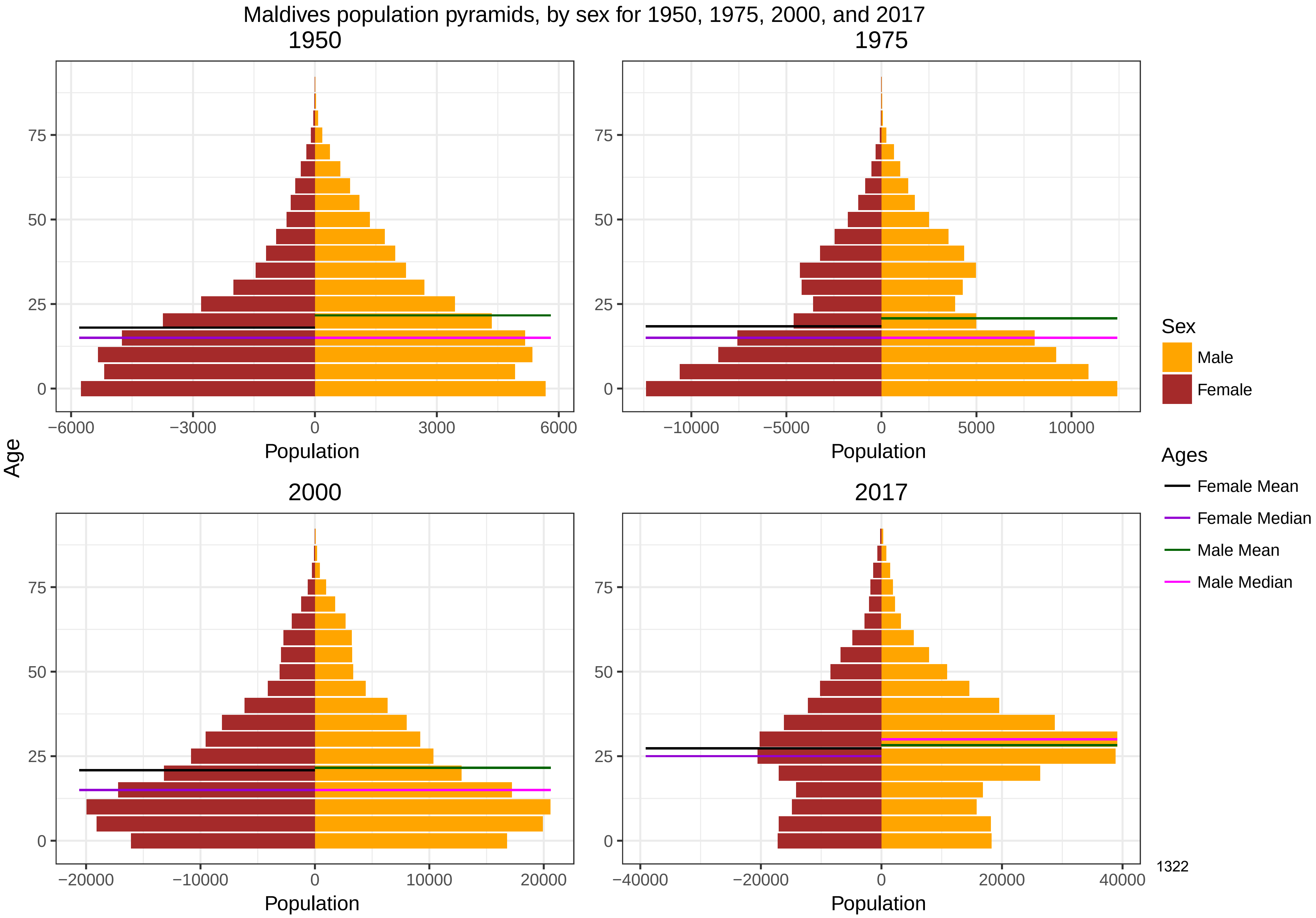

Maldives population pyramids, by sex for 1950, 1975, 2000, and 2017
1950
1975
2000
2017
Age
Population
75
50
25
0
−6000
−3000
0
3000
6000
−10000
−5000
0
5000
10000
−20000
−10000
0
10000
20000
−40000
−20000
0
20000
40000
Sex
Male
Female
Ages
Female Mean
Female Median
Male Mean
Male Median

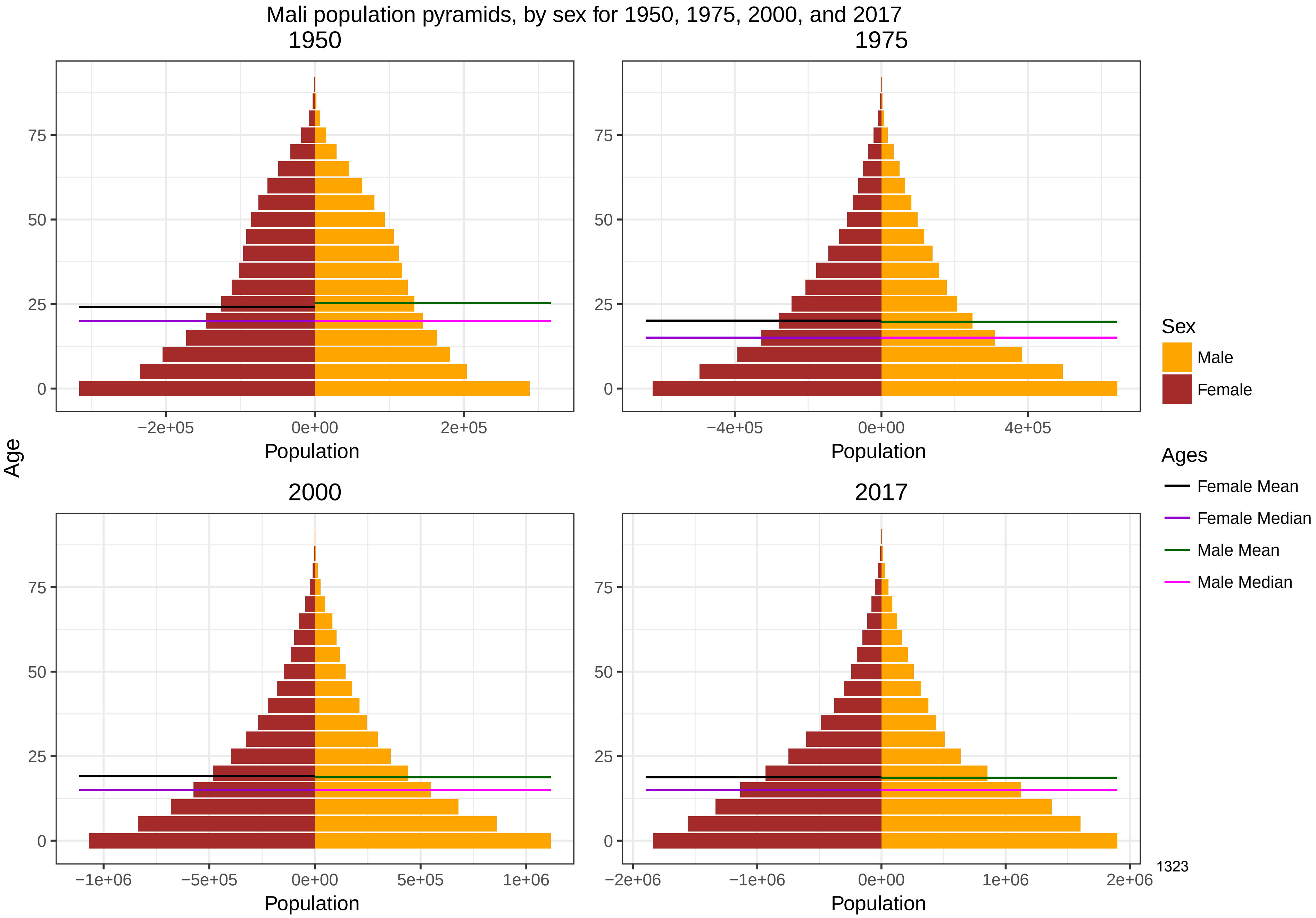
Mali population pyramids, by sex for 1950, 1975, 2000, and 2017
1950
1975
2000
2017
Age
Population
0
25
50
75
−2e+05
0e+00
2e+05
−4e+05
4e+05
−1e+06
−5e+05
5e+05
1e+06
−2e+06
2e+06
Sex
Male
Female
Ages
Female Mean
Female Median
Male Mean
Male Median

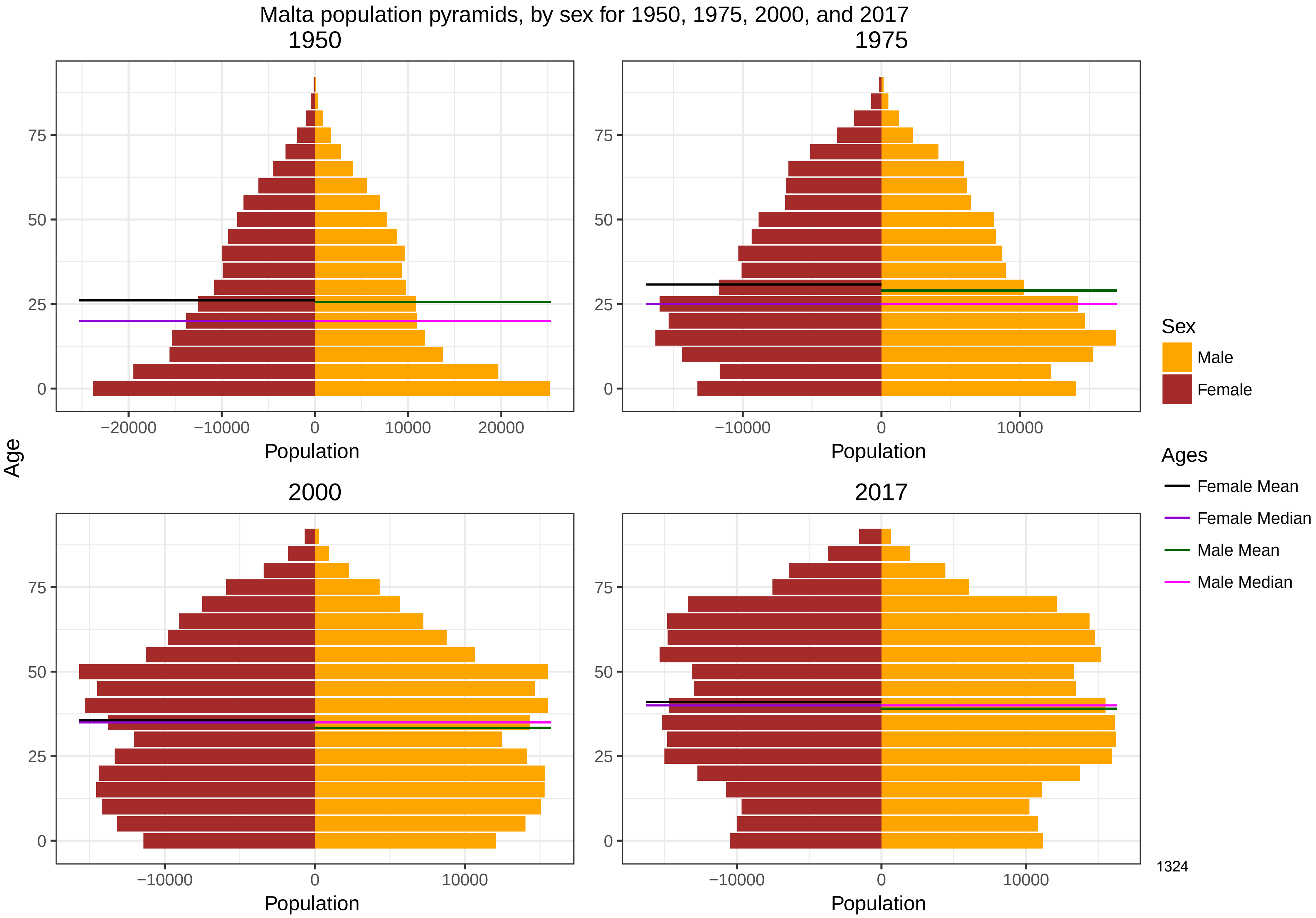
Malta population pyramids, by sex for 1950, 1975, 2000, and 2017
1950
1975
2000
2017
Age
Population
Sex
Male
Female
Ages
Female Mean
Female Median
Male Mean
Male Median

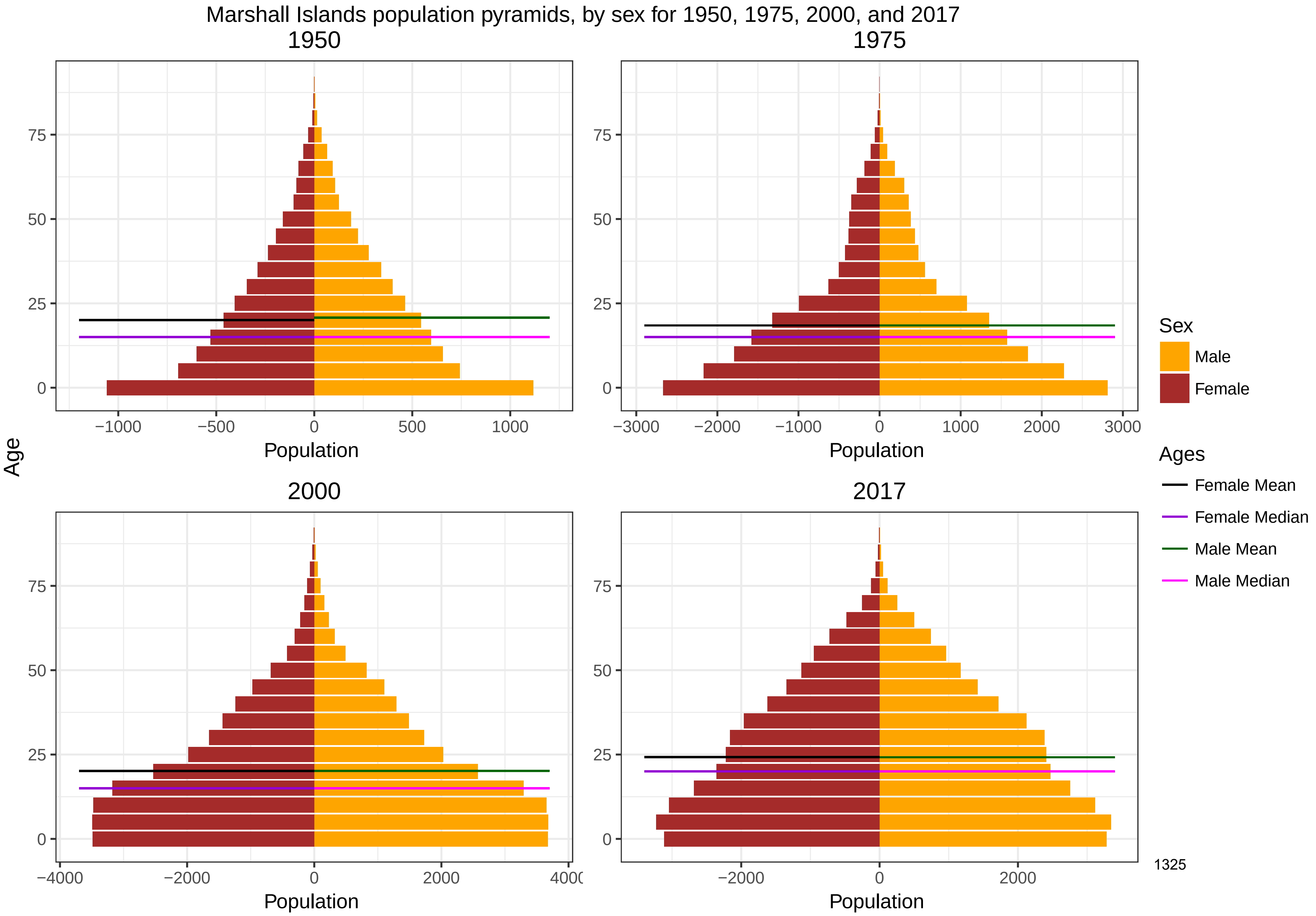
Marshall Islands population pyramids, by sex for 1950, 1975, 2000, and 2017
1950
1975
2000
2017
Age
Population
Sex
Male
Female
Ages
Female Mean
Female Median
Male Mean
Male Median

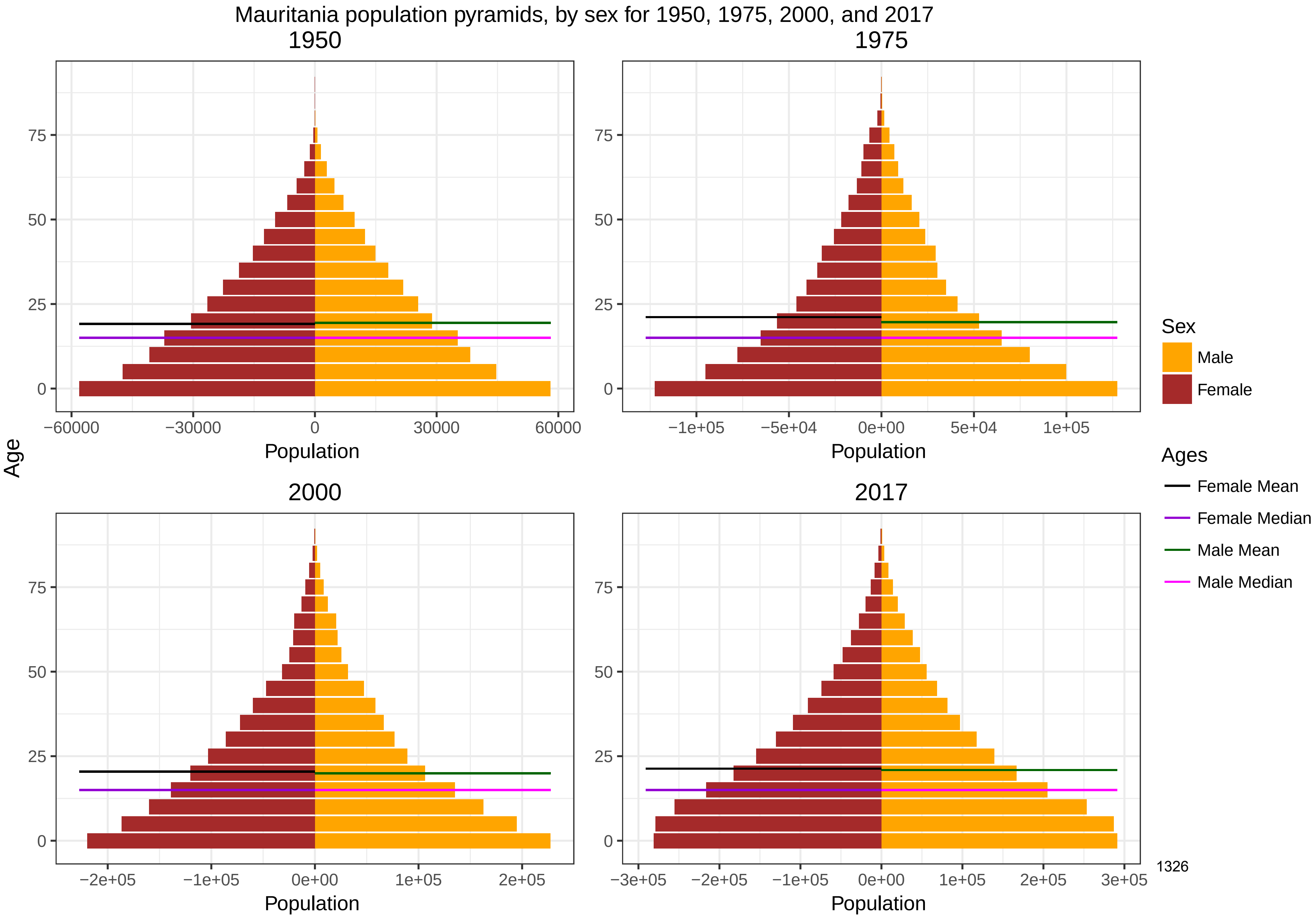

Mauritania population pyramids, by sex for 1950, 1975, 2000, and 2017
1950
1975
2000
2017
Age
Population
0
25
50
75
−60000
−30000
0
30000
60000
−1e+05
−5e+04
0e+00
5e+04
1e+05
−2e+05
−1e+05
2e+05
−3e+05
3e+05
Sex
Male
Female
Ages
Female Mean
Female Median
Male Mean
Male Median

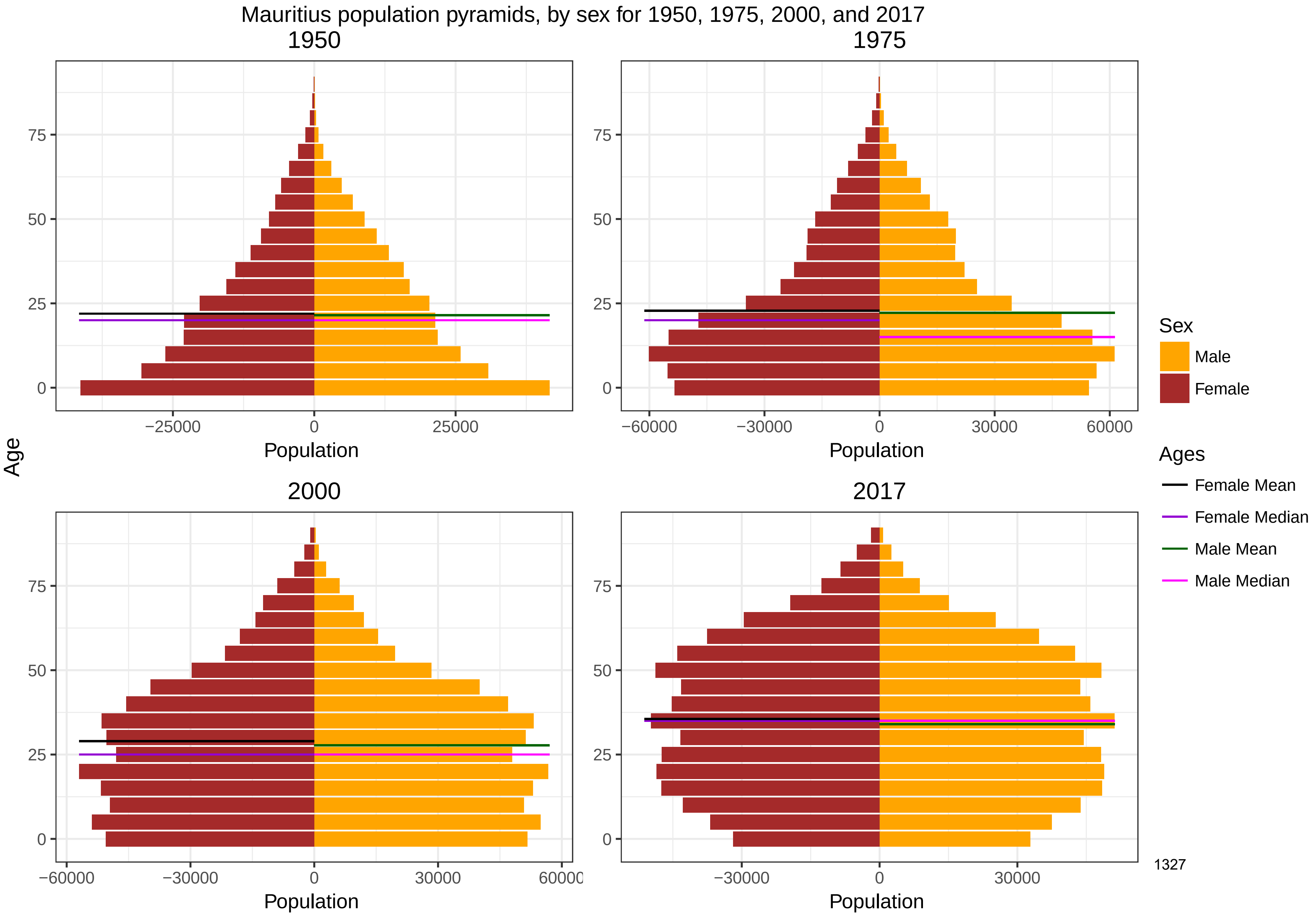

Mauritius population pyramids, by sex for 1950, 1975, 2000, and 2017
1950
1975
2000
2017
Age
Population
−25000
0
25000
−60000
−30000
30000
60000
75
50
25
Sex
Male
Female
Ages
Female Mean
Female Median
Male Mean
Male Median

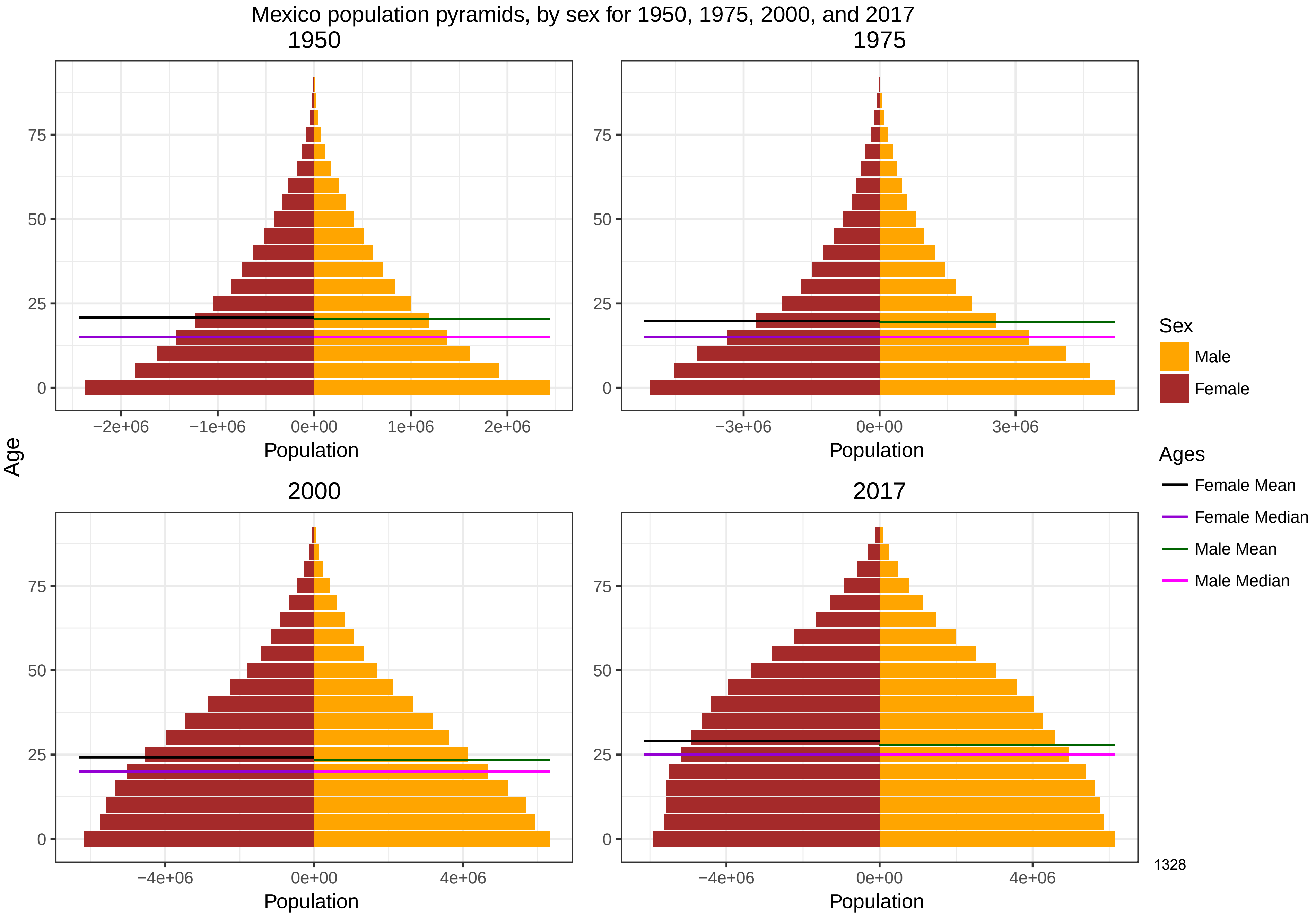
Mexico population pyramids, by sex for 1950, 1975, 2000, and 2017
1950
1975
2000
2017
Age
Population
Sex
Male
Female
Ages
Female Mean
Female Median
Male Mean
Male Median

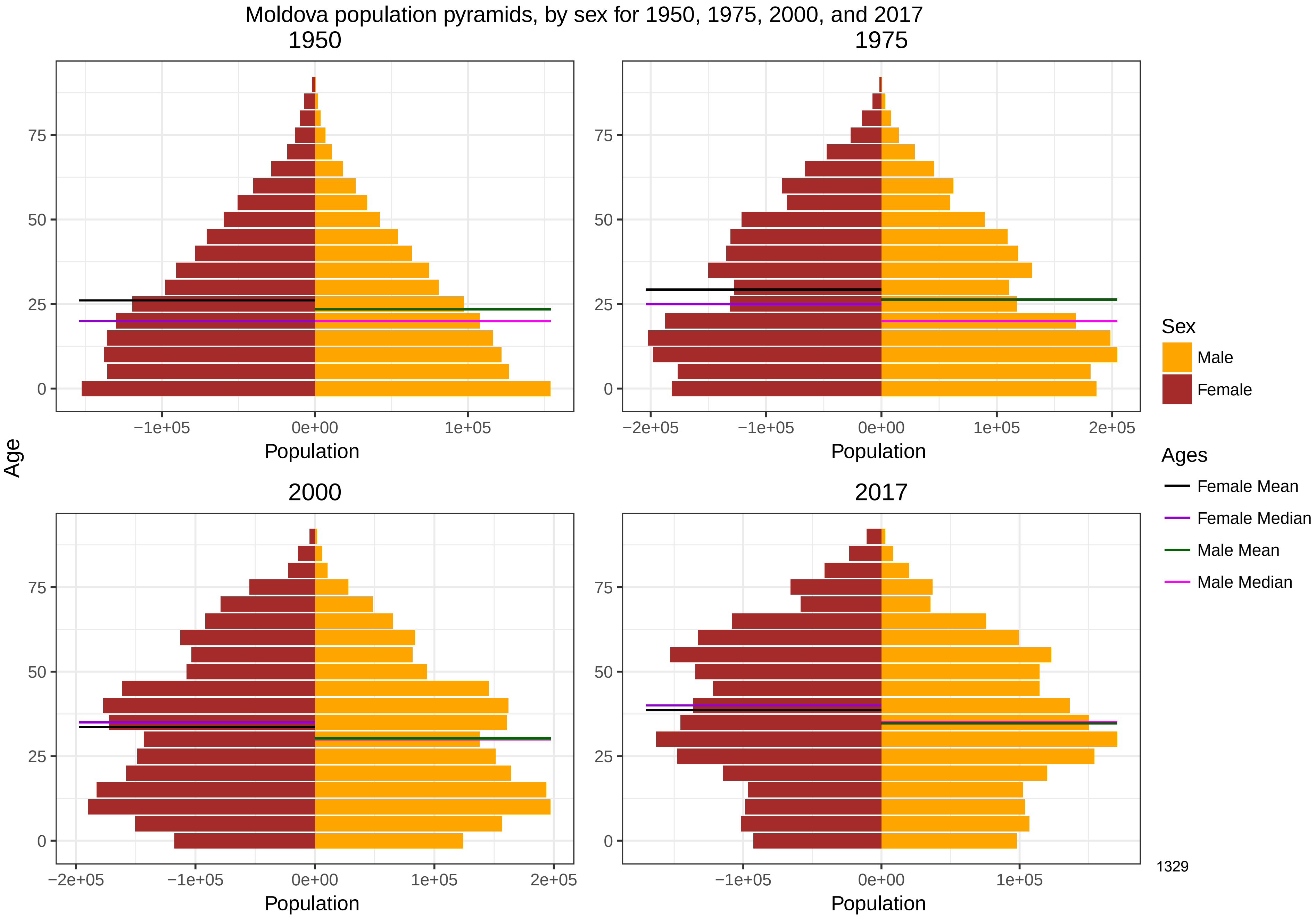

Moldova population pyramids, by sex for 1950, 1975, 2000, and 2017
1950
1975
2000
2017
Age
Population
75
50
25
0
−2e+05
−1e+05
0e+00
1e+05
2e+05
Sex
Male
Female
Ages
Female Mean
Female Median
Male Mean
Male Median

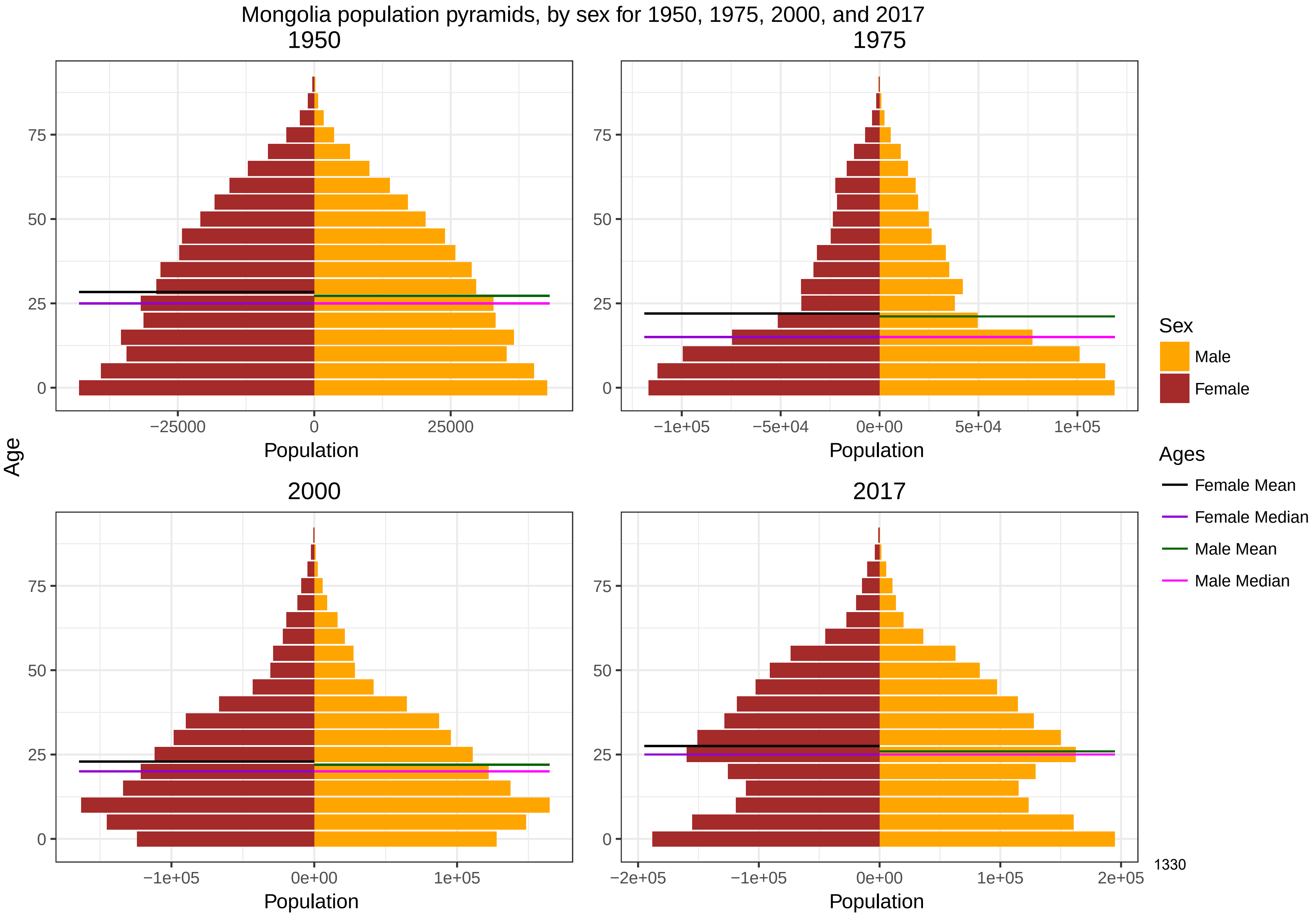
Mongolia population pyramids, by sex for 1950, 1975, 2000, and 2017
1950
1975
2000
2017
Age
Population
Sex
Male
Female
Ages
Female Mean
Female Median
Male Mean
Male Median

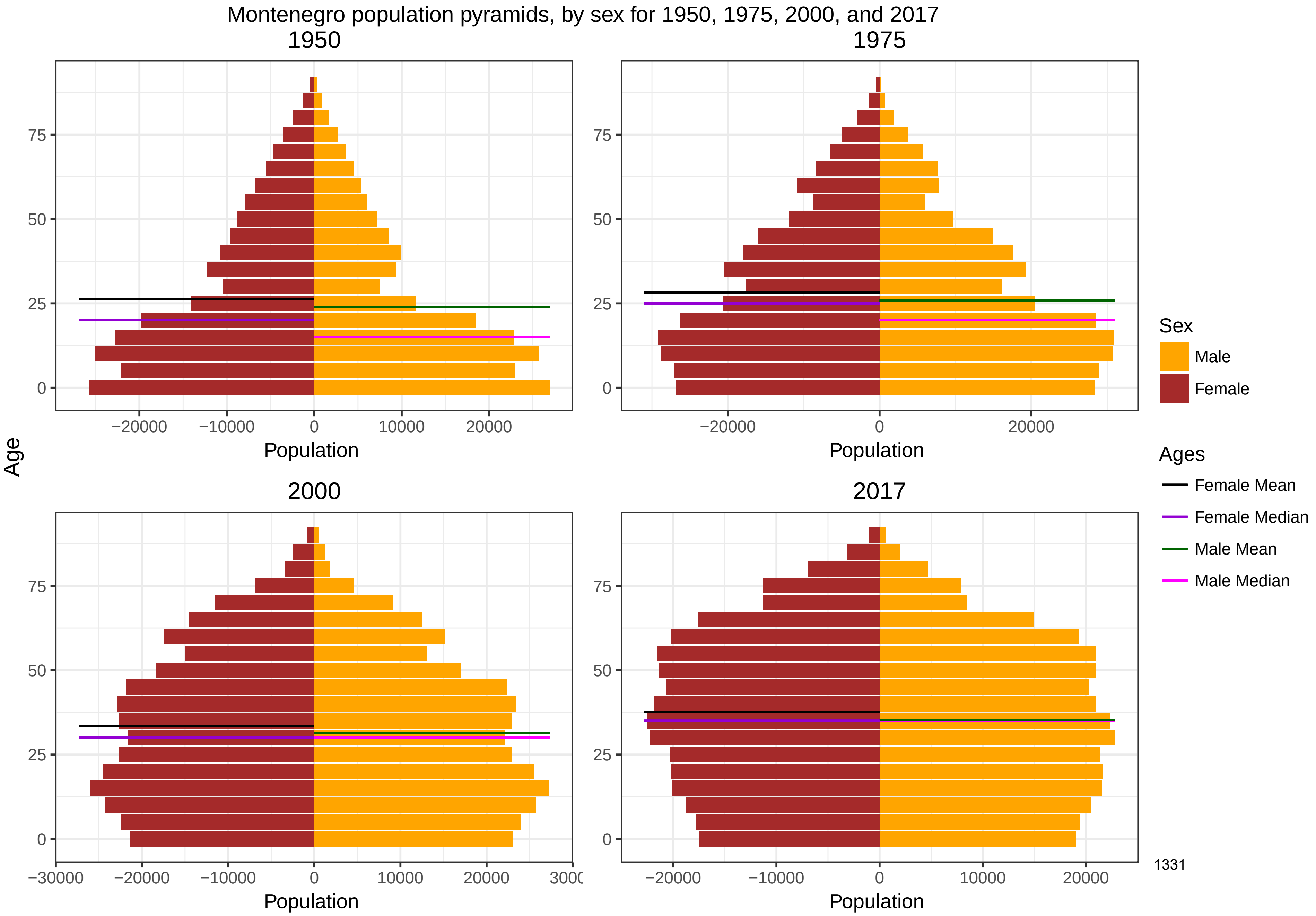
Montenegro population pyramids, by sex for 1950, 1975, 2000, and 2017
1950
1975
2000
2017
Age
Population
Sex
Male
Female
Ages
Female Mean
Female Median
Male Mean
Male Median

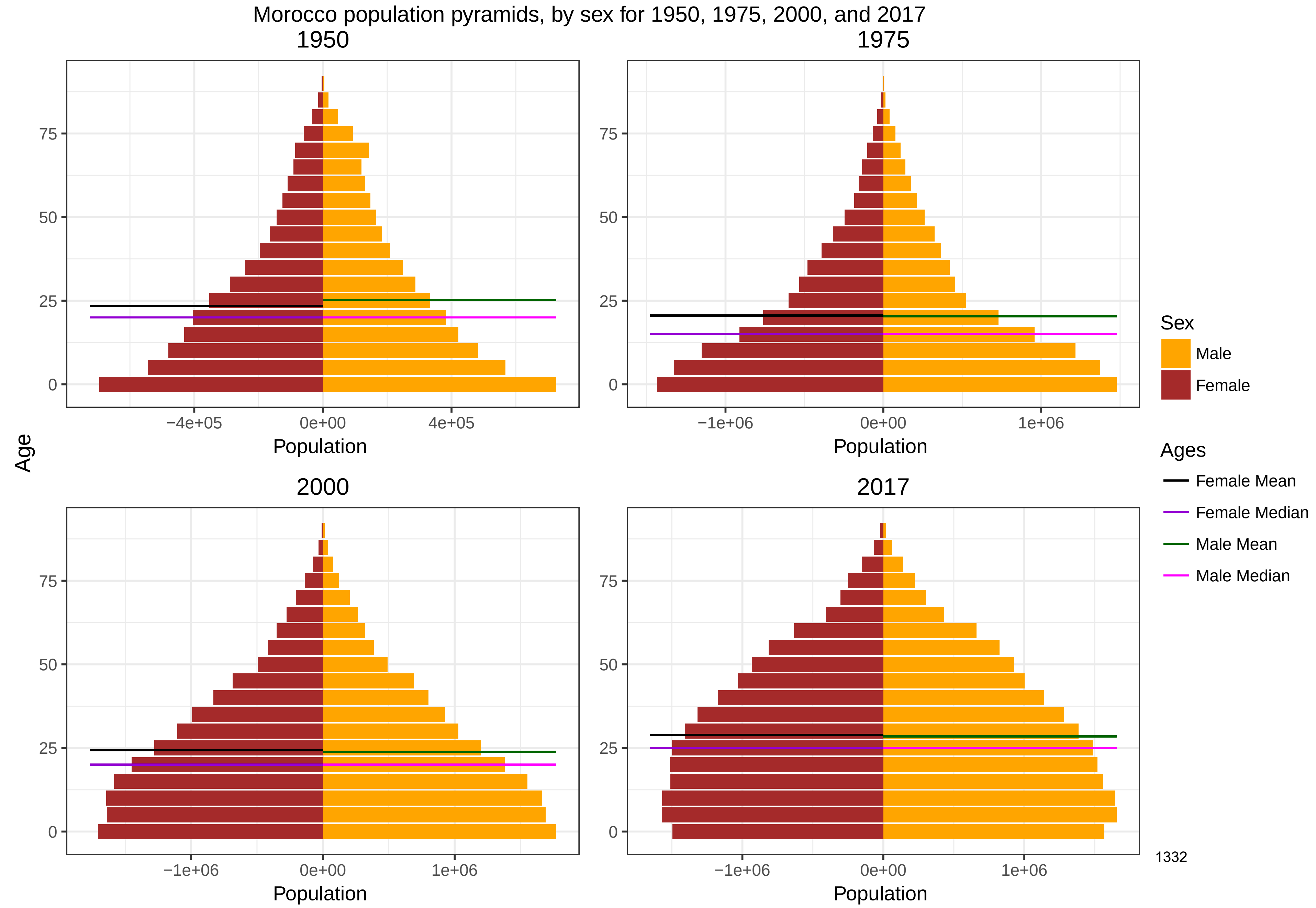
Morocco population pyramids, by sex for 1950, 1975, 2000, and 2017
1950
1975
2000
2017
Age
Population
0
25
50
75
−4e+05
0e+00
4e+05
−1e+06
1e+06
Sex
Male
Female
Ages
Female Mean
Female Median
Male Mean
Male Median

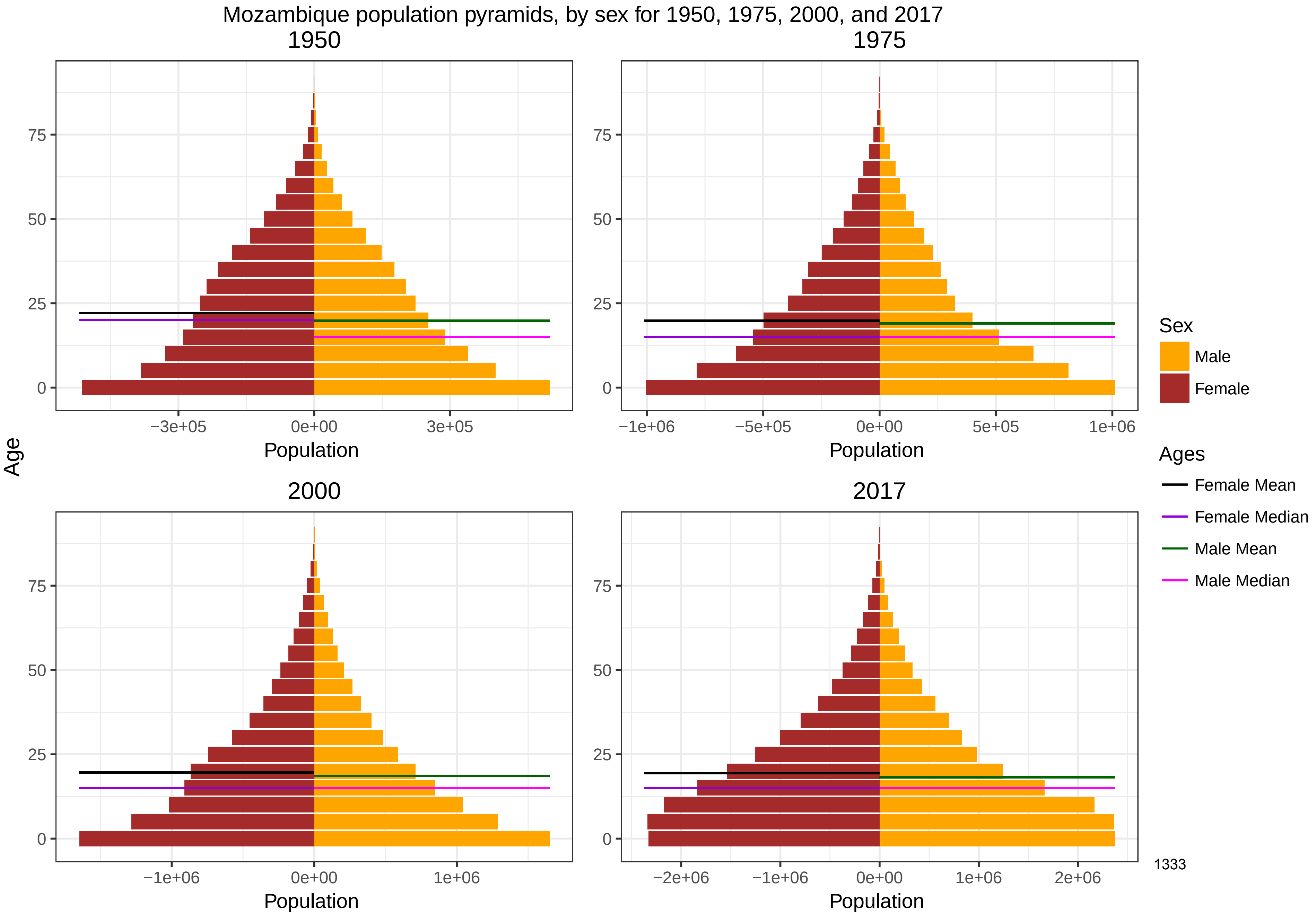
Mozambique population pyramids, by sex for 1950, 1975, 2000, and 2017
1950
1975
2000
2017
Age
Population
0
25
50
75
−3e+05
0e+00
3e+05
−1e+06
−5e+05
5e+05
1e+06
−2e+06
2e+06
Sex
Male
Female
Ages
Female Mean
Female Median
Male Mean
Male Median

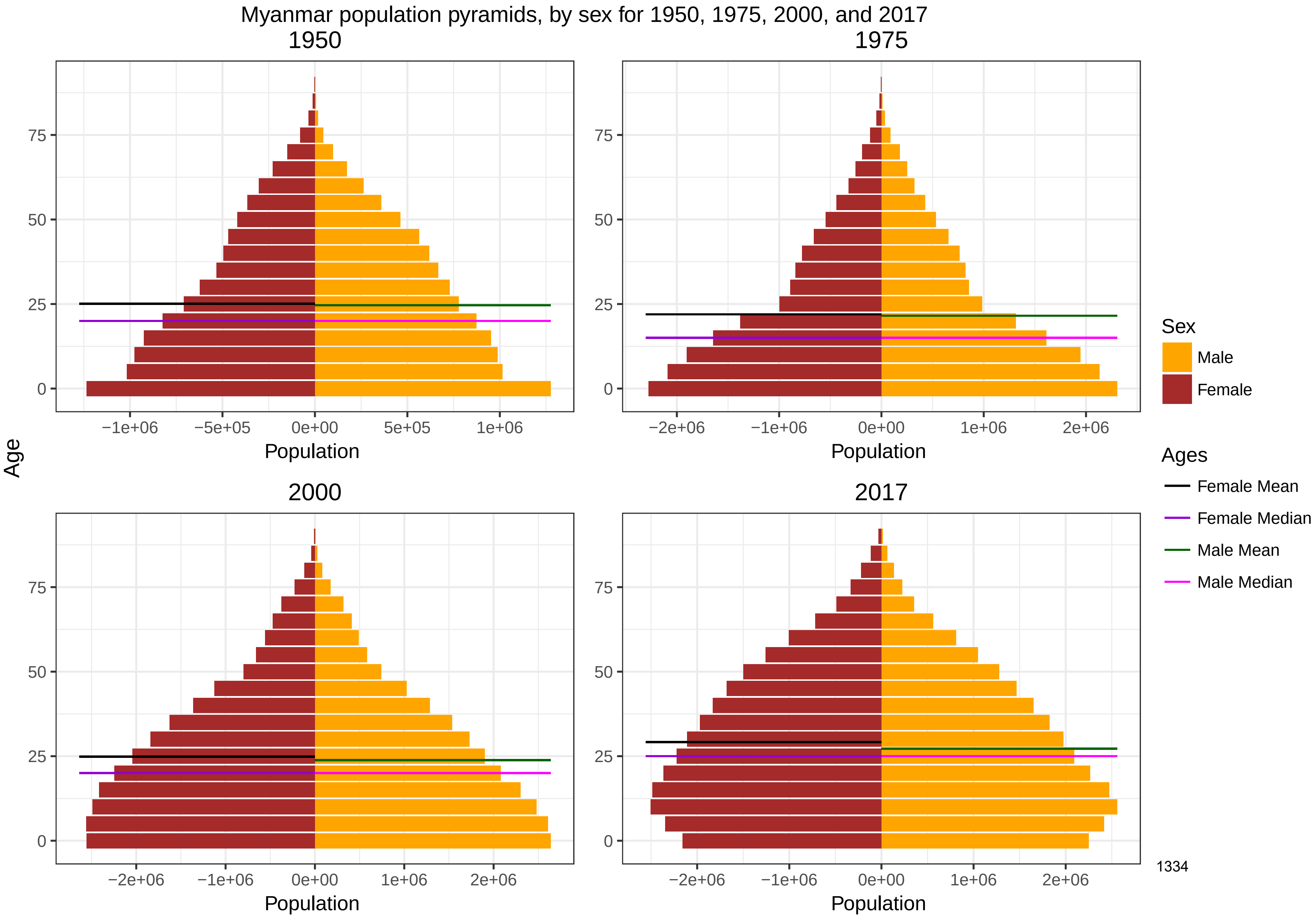

Myanmar population pyramids, by sex for 1950, 1975, 2000, and 2017
1950
1975
2000
2017
Age
Population
0
25
50
75
−1e+06
−5e+05
0e+00
5e+05
1e+06
−2e+06
2e+06
Sex
Male
Female
Ages
Female Mean
Female Median
Male Mean
Male Median

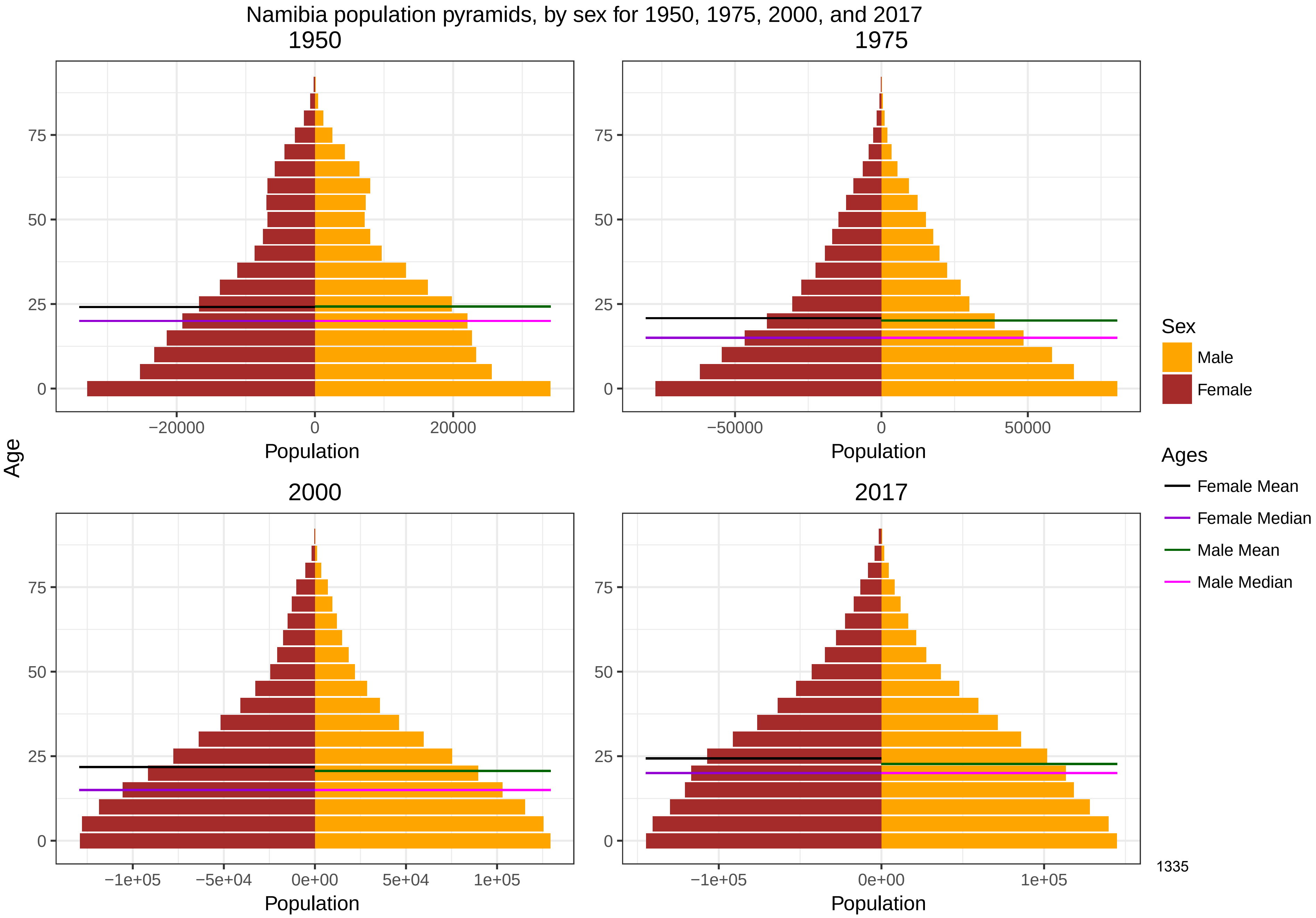

Namibia population pyramids, by sex for 1950, 1975, 2000, and 2017
1950
1975
2000
2017
Age
Population
0
25
50
75
−20000
0
20000
−50000
50000
−1e+05
−5e+04
0e+00
5e+04
1e+05
Sex
Male
Female
Ages
Female Mean
Female Median
Male Mean
Male Median

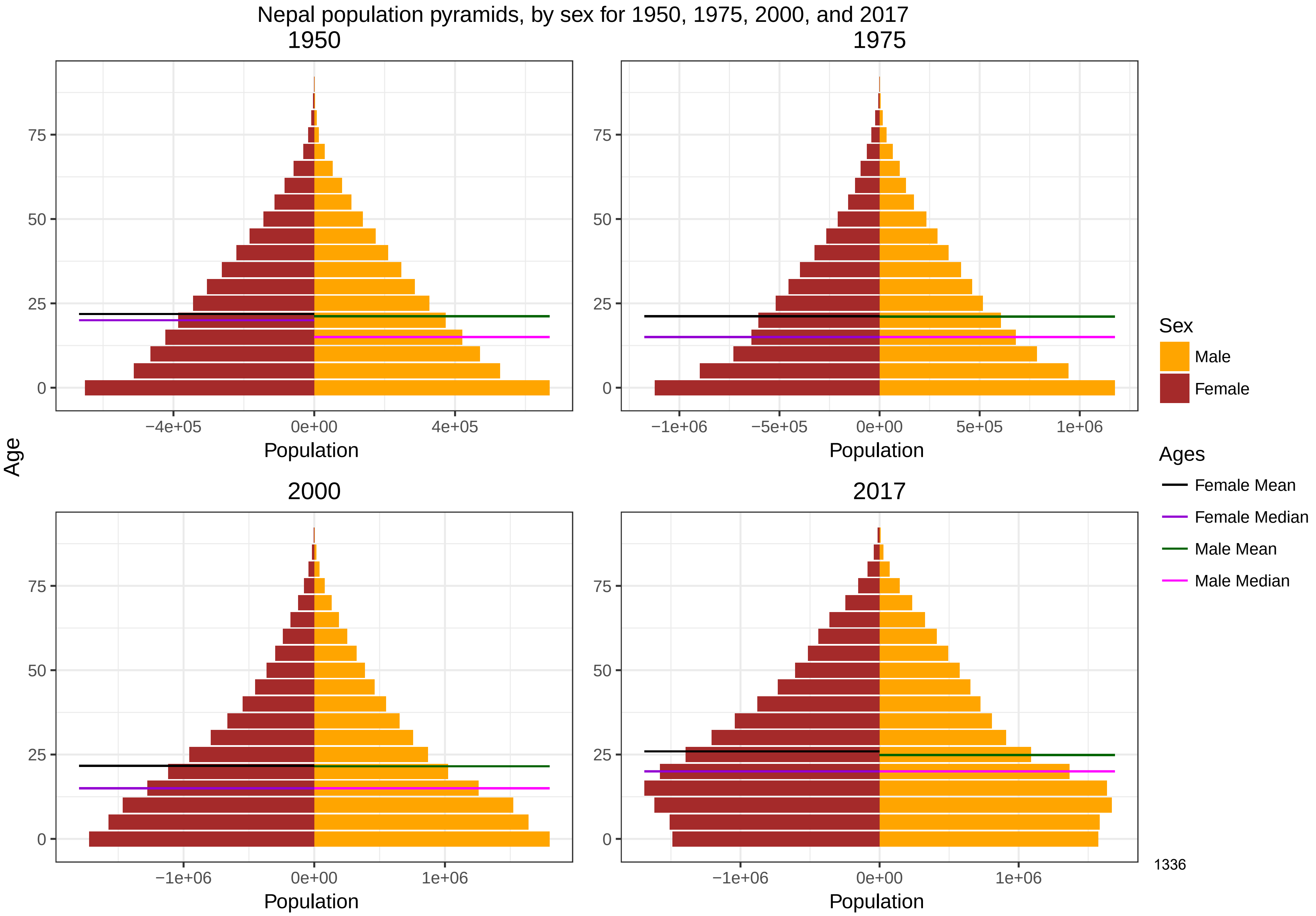
Nepal population pyramids, by sex for 1950, 1975, 2000, and 2017
1950
1975
2000
2017
Age
Population
−4e+05
0e+00
4e+05
−1e+06
−5e+05
5e+05
1e+06
0
25
50
75
Sex
Male
Female
Ages
Female Mean
Female Median
Male Mean
Male Median

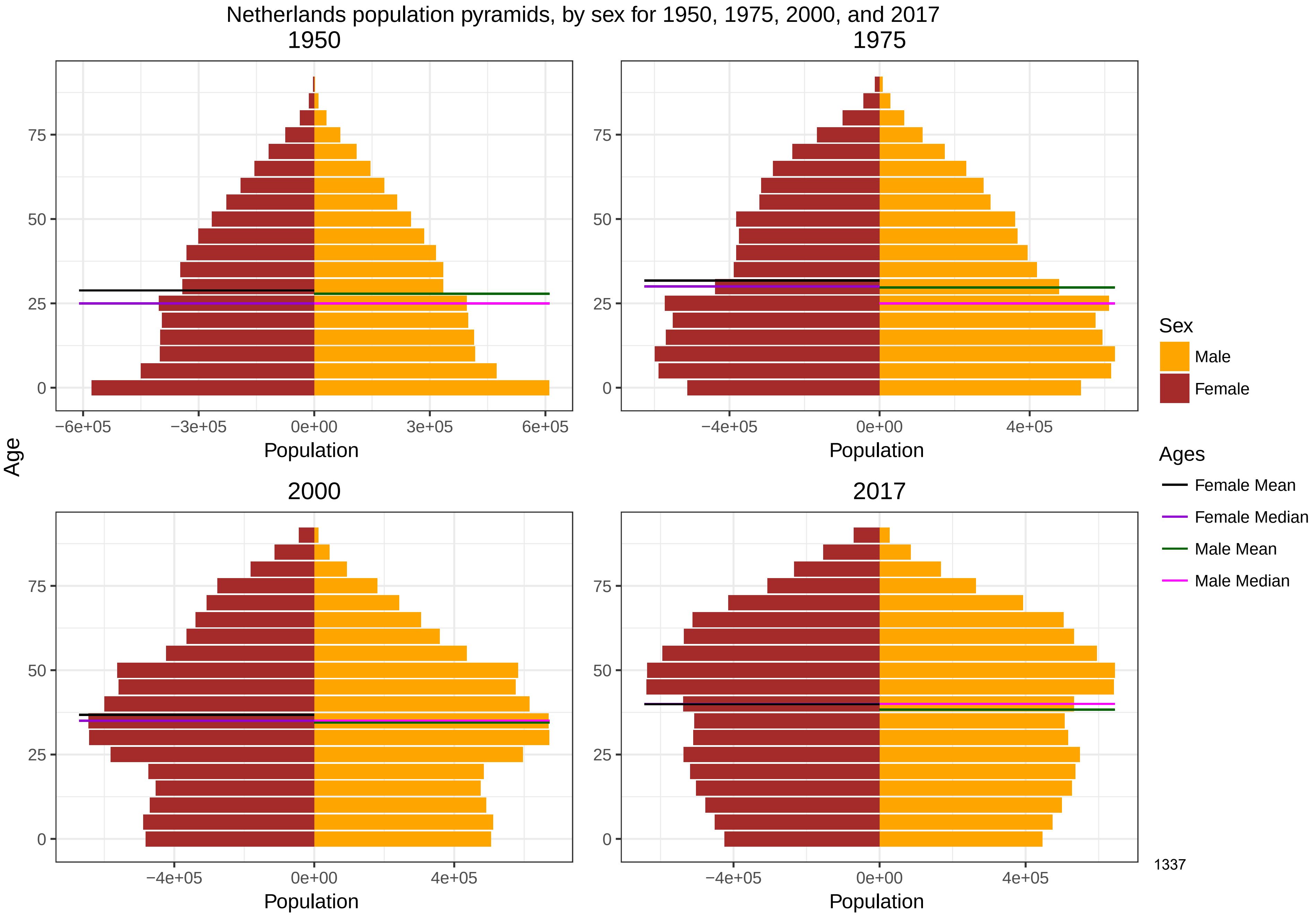

Netherlands population pyramids, by sex for 1950, 1975, 2000, and 2017
1950
1975
2000
2017
Age
Population
Sex
Male
Female
Ages
Female Mean
Female Median
Male Mean
Male Median

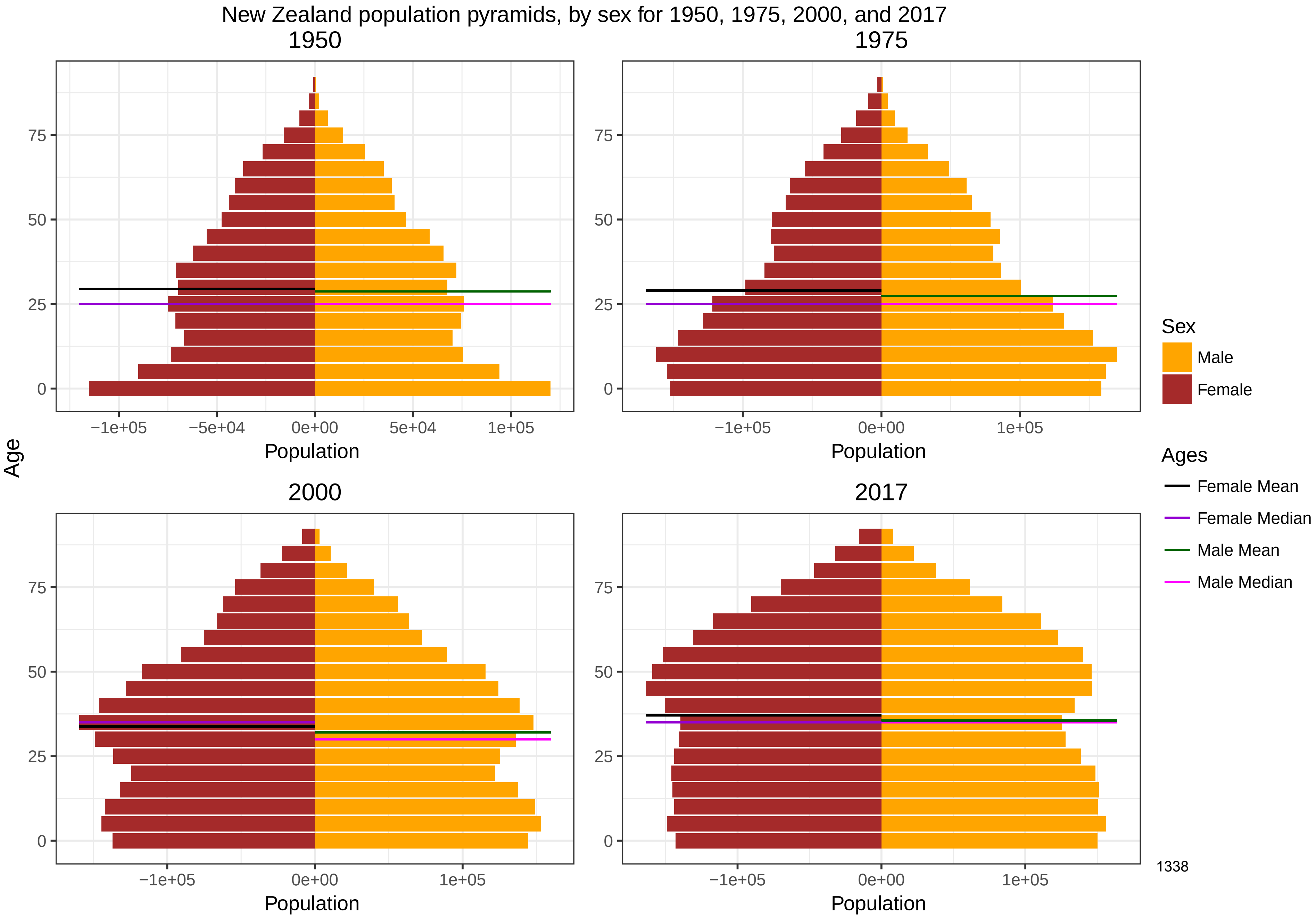
New Zealand population pyramids, by sex for 1950, 1975, 2000, and 2017
1950
1975
2000
2017
Age
Population
75
50
25
0
−1e+05
−5e+04
0e+00
5e+04
1e+05
Sex
Male
Female
Ages
Female Mean
Female Median
Male Mean
Male Median

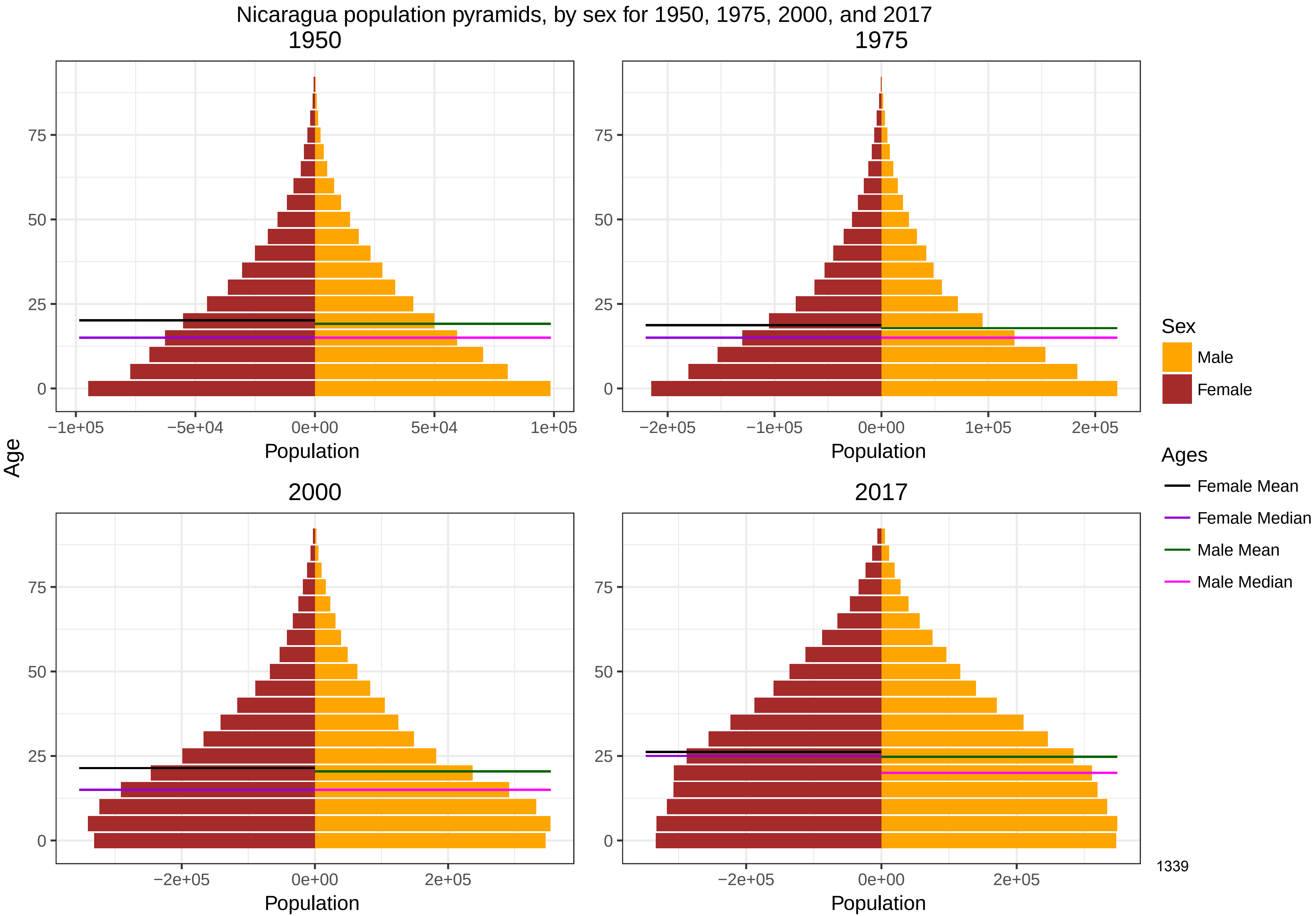

Nicaragua population pyramids, by sex for 1950, 1975, 2000, and 2017
1950
1975
2000
2017
Age
Population
Sex
Male
Female
Ages
Female Mean
Female Median
Male Mean
Male Median

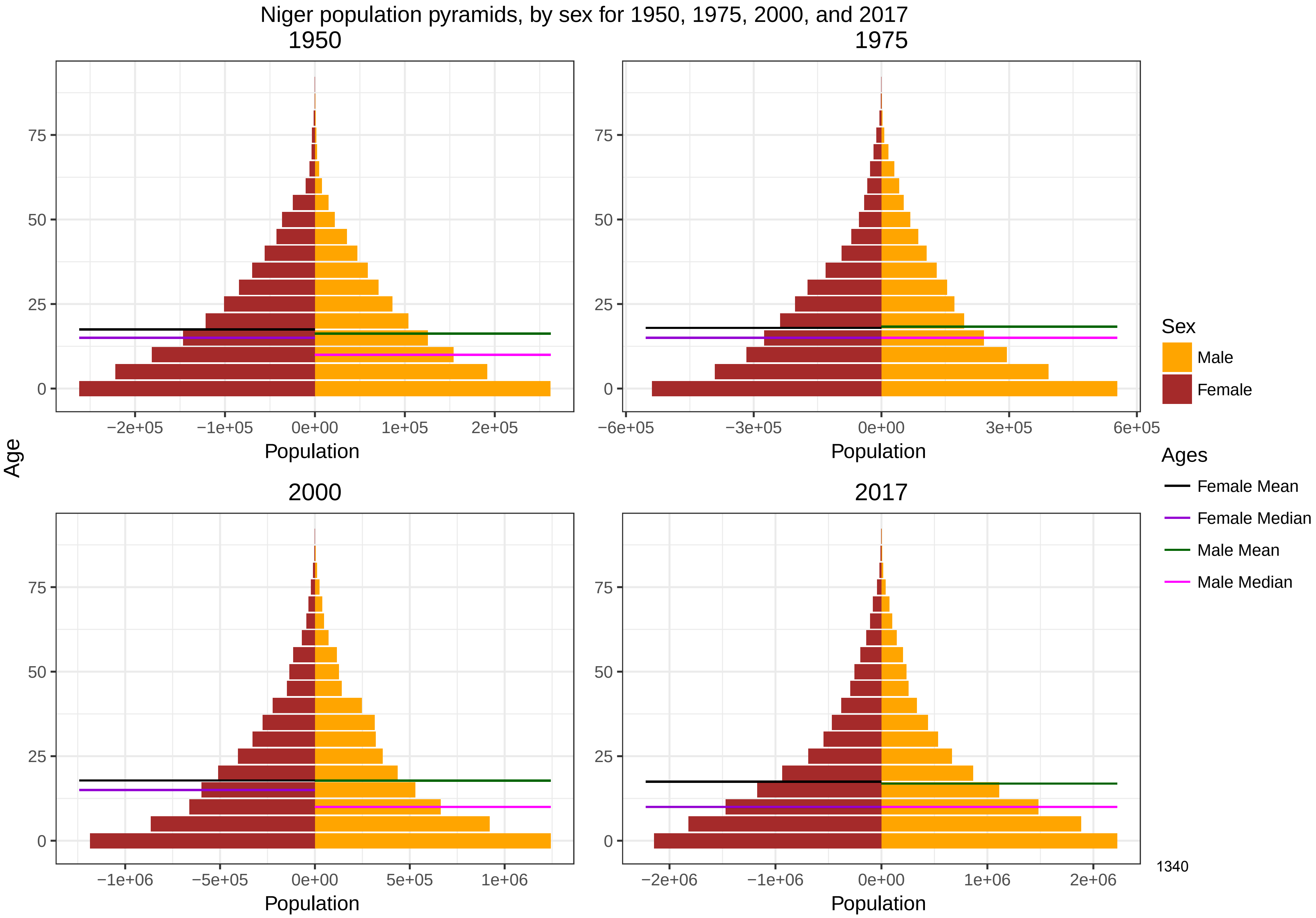

Niger population pyramids, by sex for 1950, 1975, 2000, and 2017
1950
1975
2000
2017
Age
Population
Sex
Male
Female
Ages
Female Mean
Female Median
Male Mean
Male Median
0
25
50
75
−2e+05
−1e+05
0e+00
1e+05
2e+05
−6e+05
−3e+05
3e+05
6e+05
−1e+06
−5e+05
5e+05
1e+06
−2e+06
2e+06

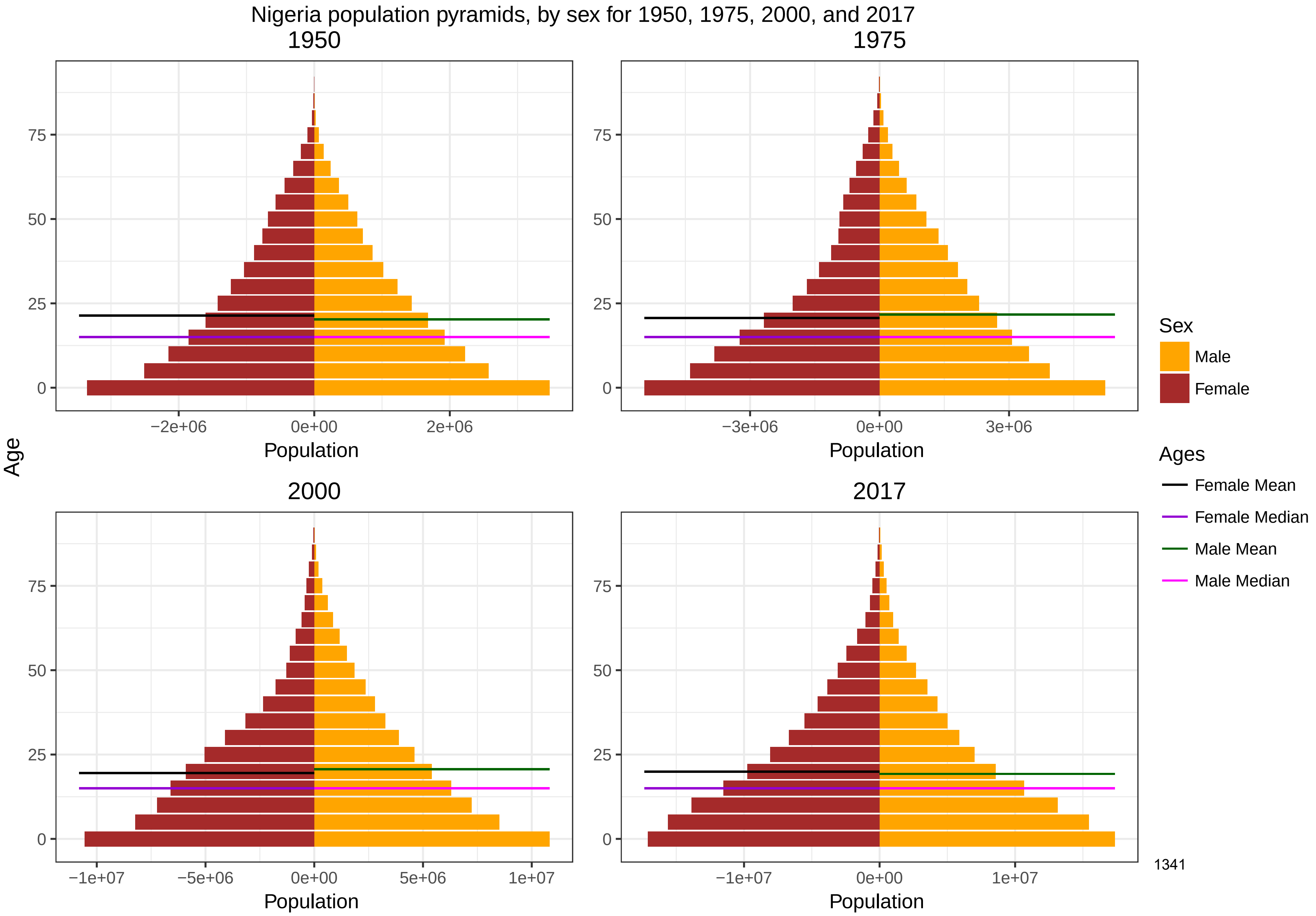

Nigeria population pyramids, by sex for 1950, 1975, 2000, and 2017
1950
1975
2000
2017
Age
Population
75
50
25
0
−2e+06
0e+00
2e+06
−3e+06
3e+06
−1e+07
−5e+06
5e+06
1e+07
Sex
Male
Female
Ages
Female Mean
Female Median
Male Mean
Male Median

North Korea population pyramids, by sex for 1950, 1975, 2000, and 2017

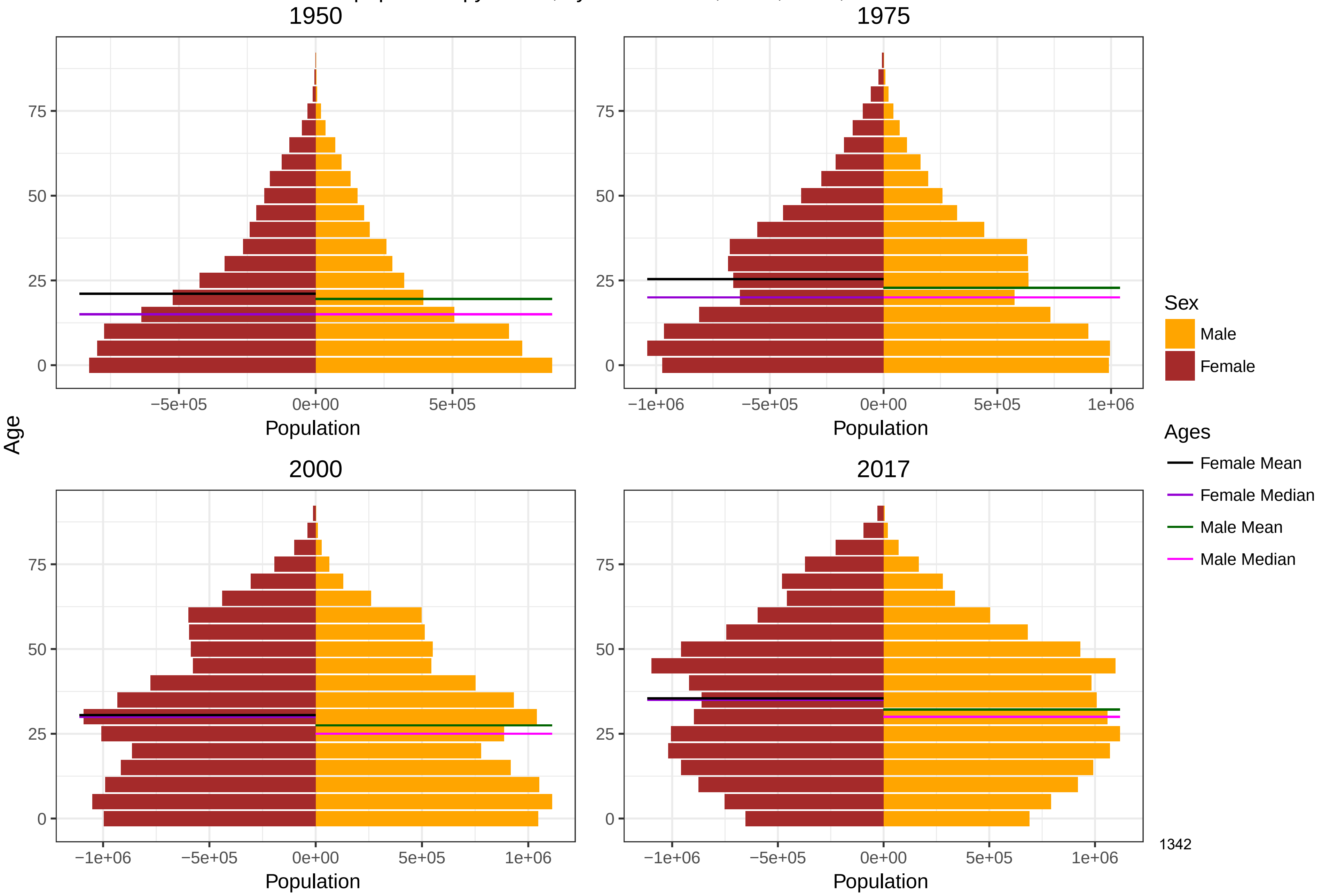

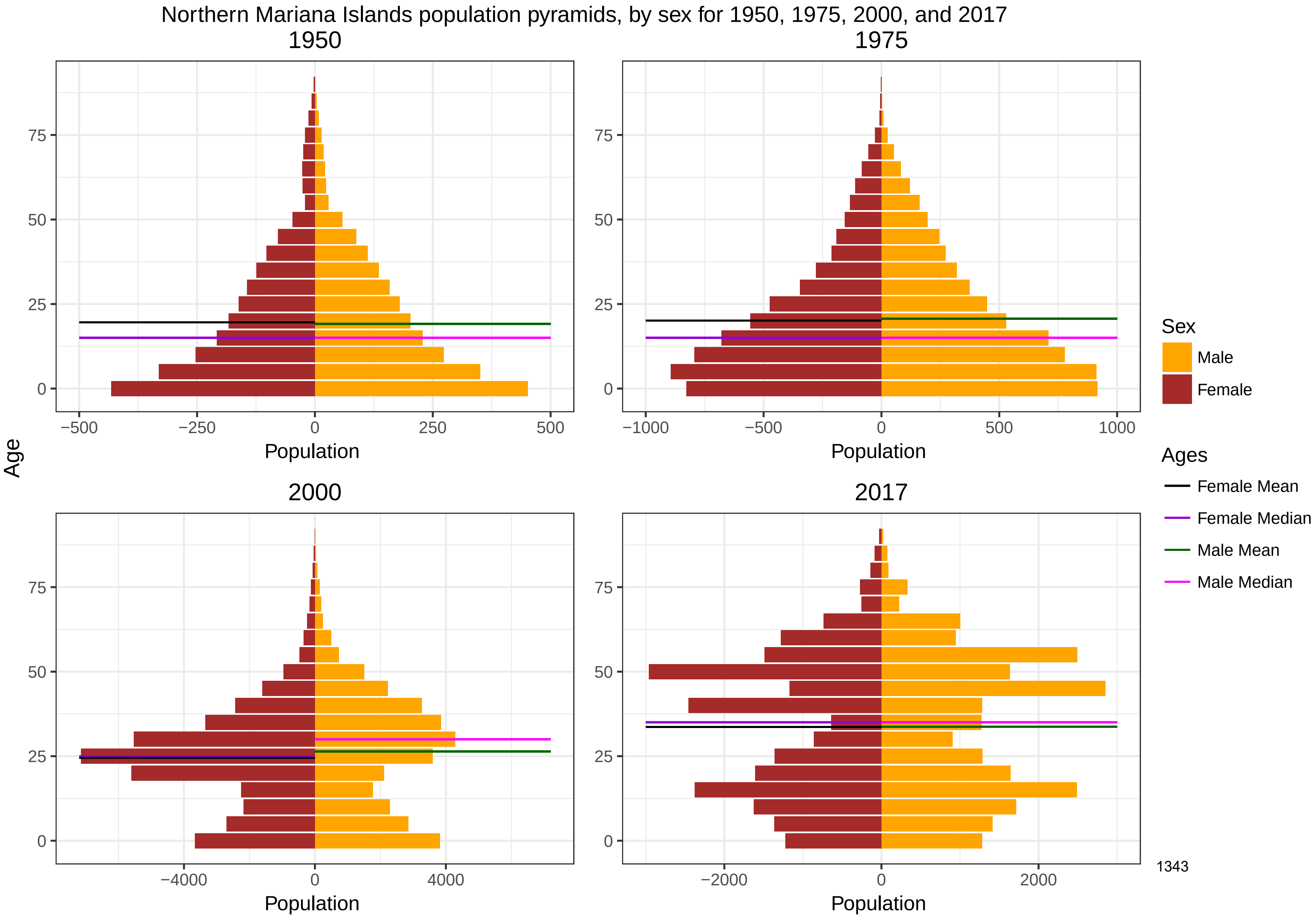
Northern Mariana Islands population pyramids, by sex for 1950, 1975, 2000, and 2017
1950
1975
2000
2017
Age
Population
Sex
Male
Female
Ages
Female Mean
Female Median
Male Mean
Male Median
−500
−250
0
250
500
−1000
−500
0
500
1000
−4000
0
4000
−2000
0
2000
0
25
50
75

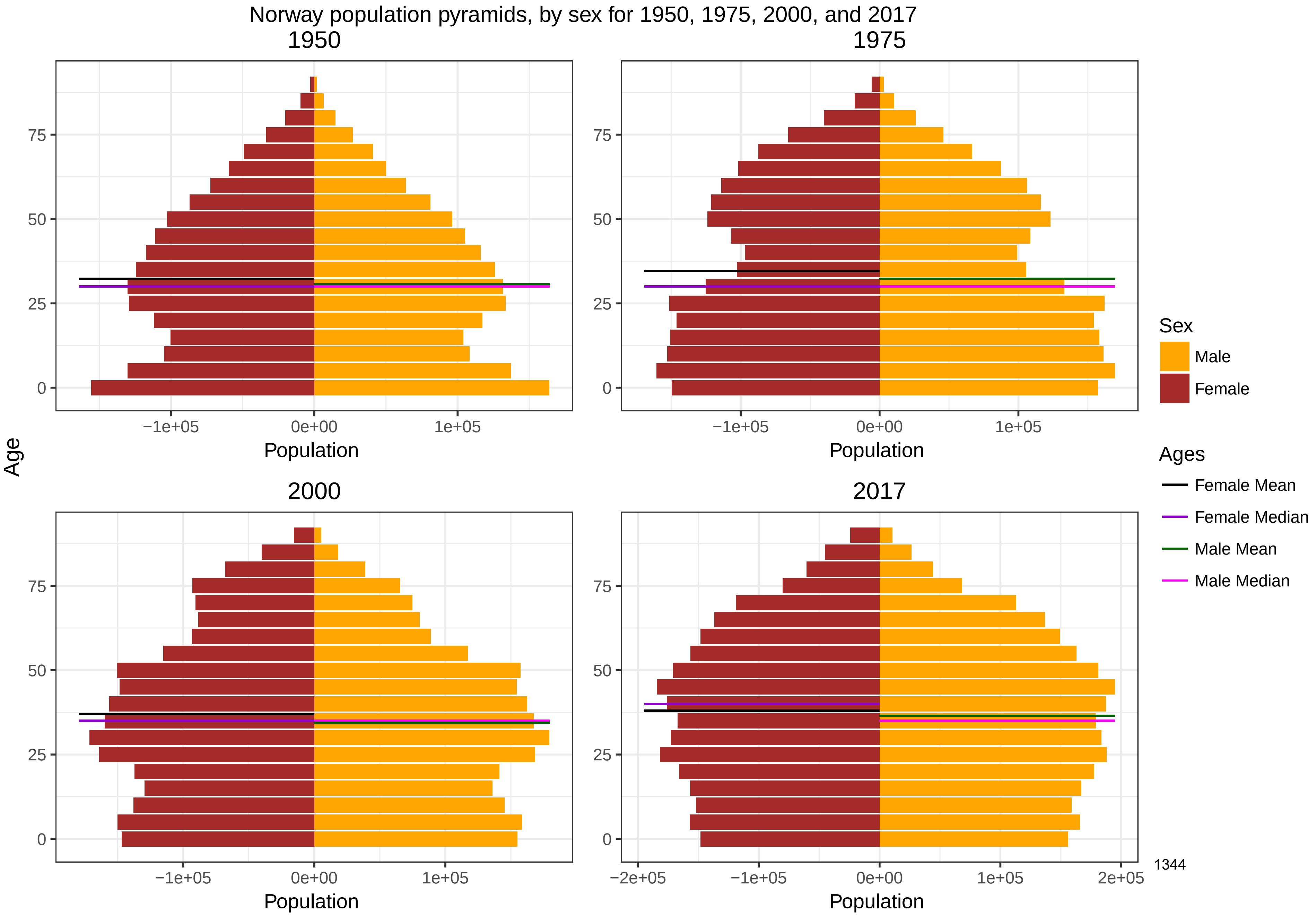

Norway population pyramids, by sex for 1950, 1975, 2000, and 2017
1950
1975
2000
2017
Age
Population
0
25
50
75
−2e+05
−1e+05
0e+00
1e+05
2e+05
Sex
Male
Female
Ages
Female Mean
Female Median
Male Mean
Male Median

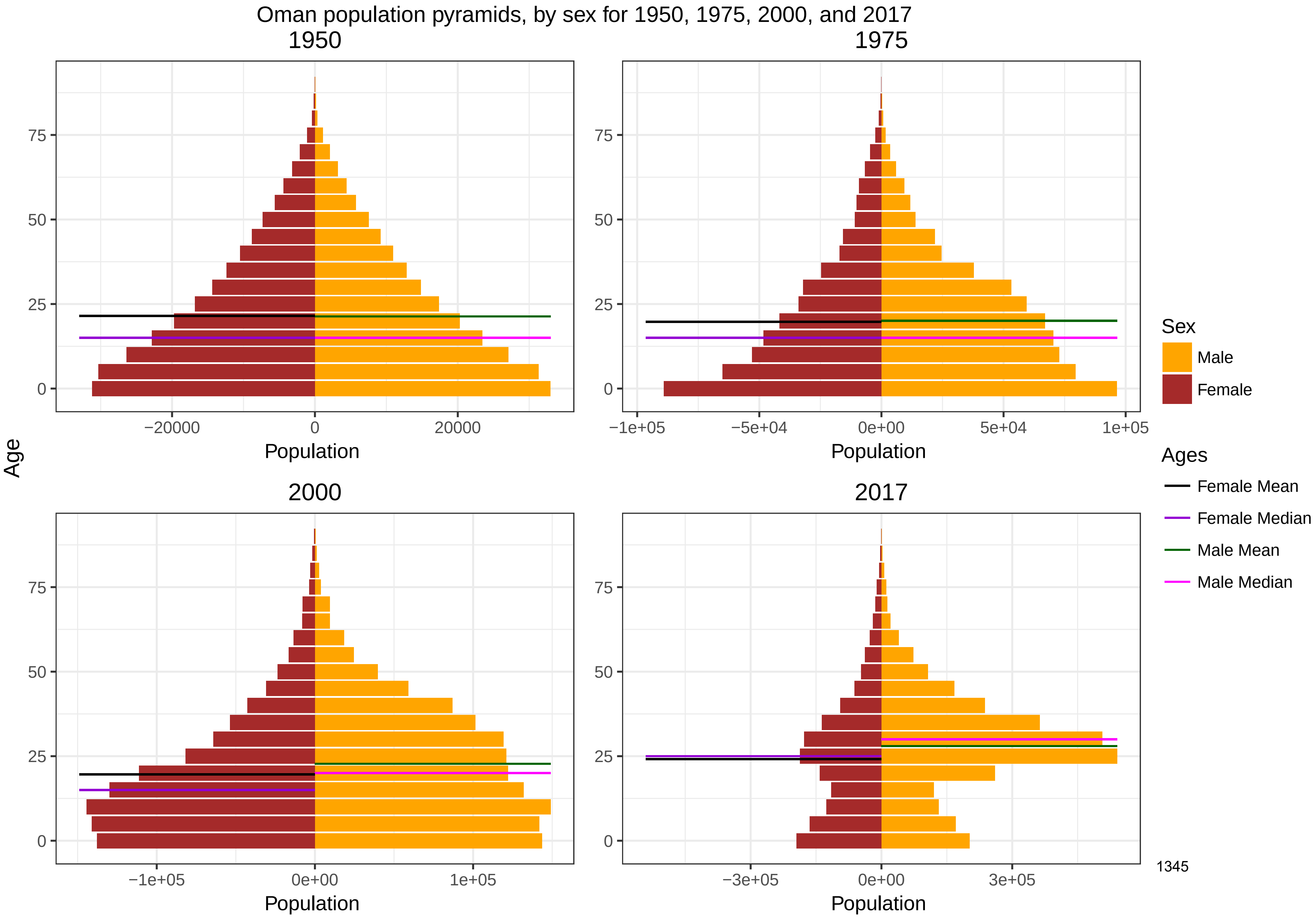

Oman population pyramids, by sex for 1950, 1975, 2000, and 2017
1950
1975
2000
2017
Age
Population
−20000
0
20000
−1e+05
−5e+04
0e+00
5e+04
1e+05
−3e+05
3e+05
0
25
50
75
Sex
Male
Female
Ages
Female Mean
Female Median
Male Mean
Male Median

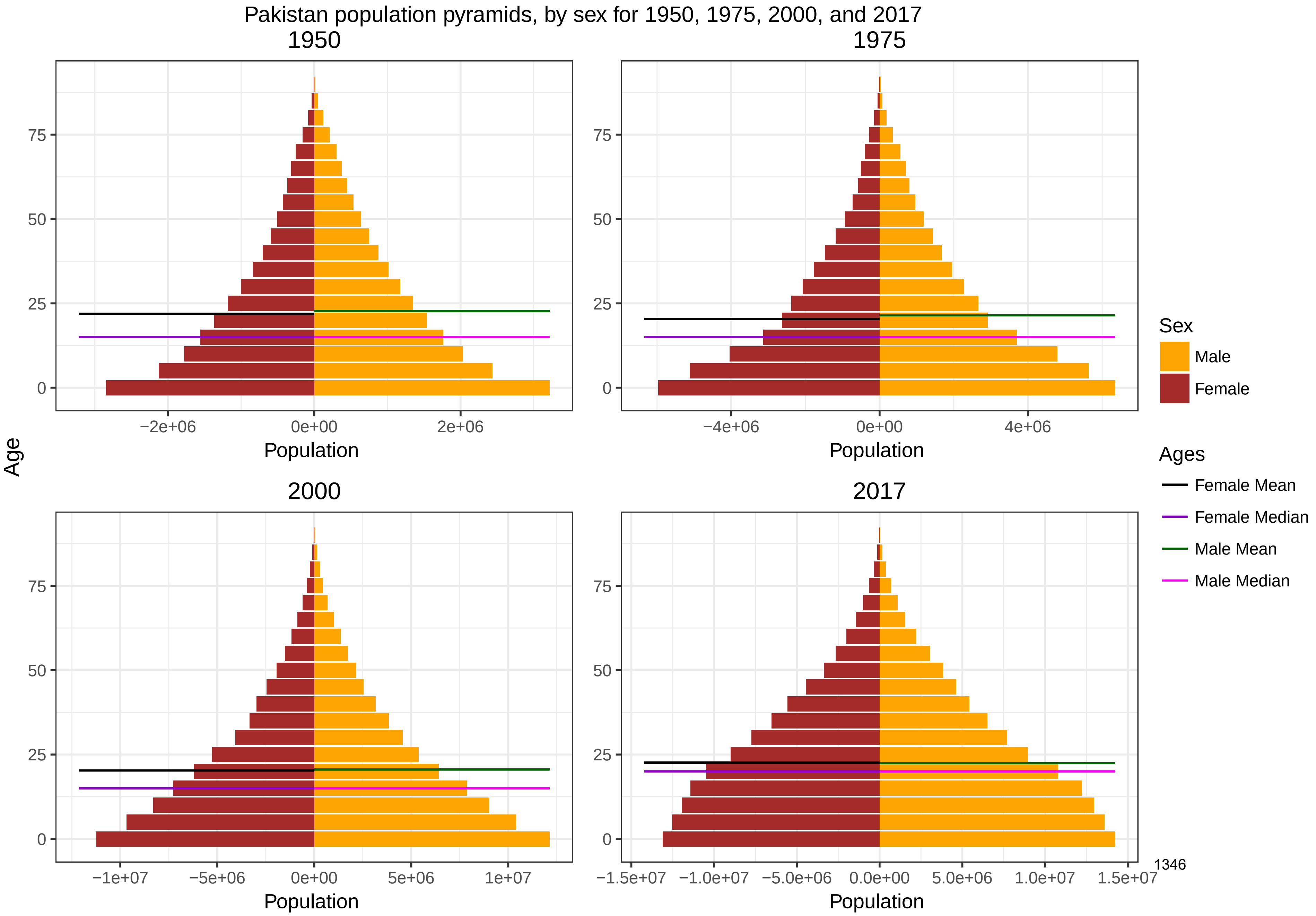

Pakistan population pyramids, by sex for 1950, 1975, 2000, and 2017
1950
1975
2000
2017
Age
Population
0
25
50
75
−2e+06
0e+00
2e+06
−4e+06
4e+06
−1e+07
−5e+06
5e+06
1e+07
−1.5e+07
−1.0e+07
−5.0e+06
0.0e+00
5.0e+06
1.0e+07
1.5e+07
Sex
Male
Female
Ages
Female Mean
Female Median
Male Mean
Male Median

1346

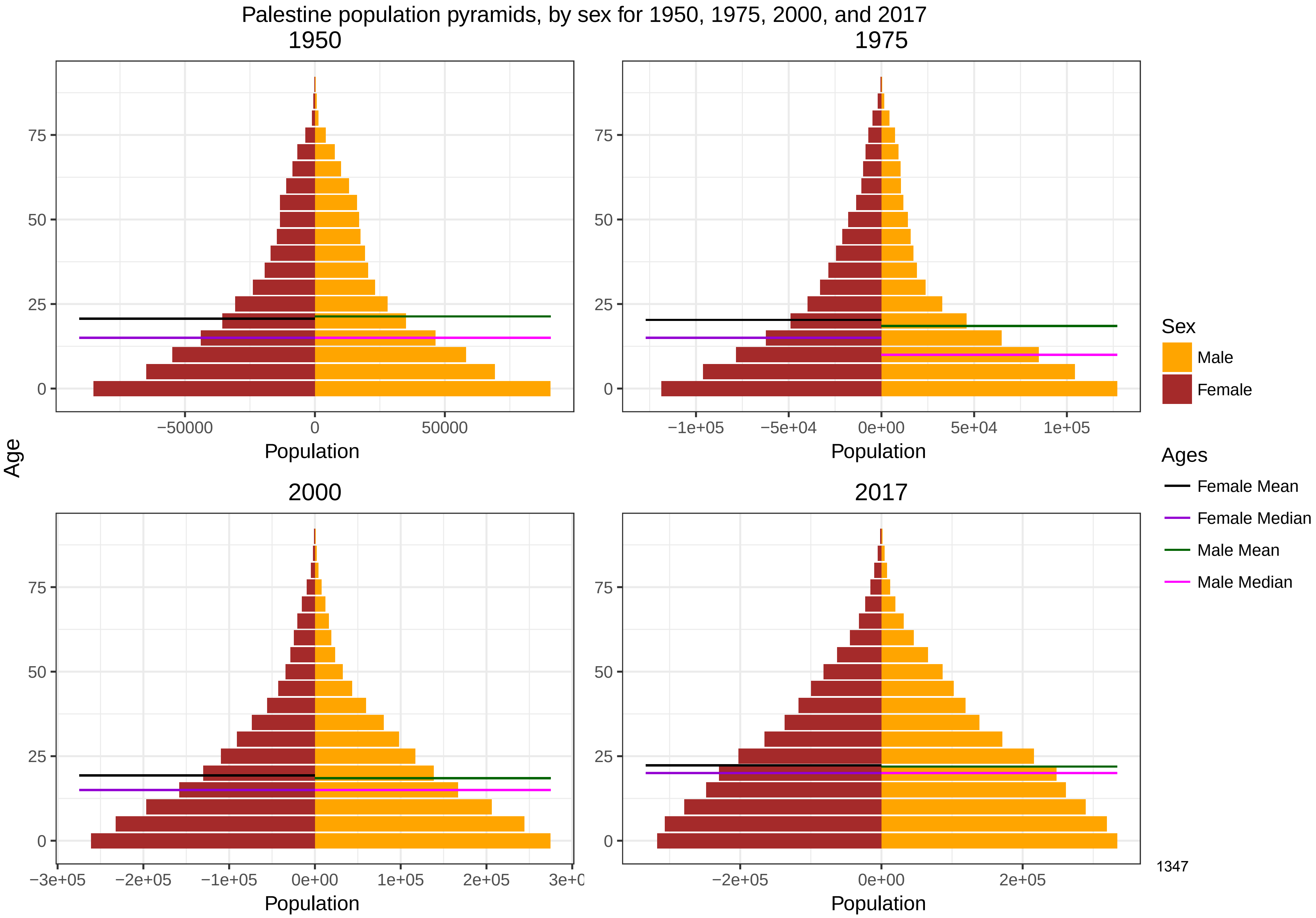

Palestine population pyramids, by sex for 1950, 1975, 2000, and 2017
1950
1975
2000
2017
Age
Population
0
25
50
75
−50000
0
50000
−1e+05
−5e+04
0e+00
5e+04
1e+05
−3e+05
−2e+05
−1e+05
1e+05
2e+05
Sex
Male
Female
Ages
Female Mean
Female Median
Male Mean
Male Median

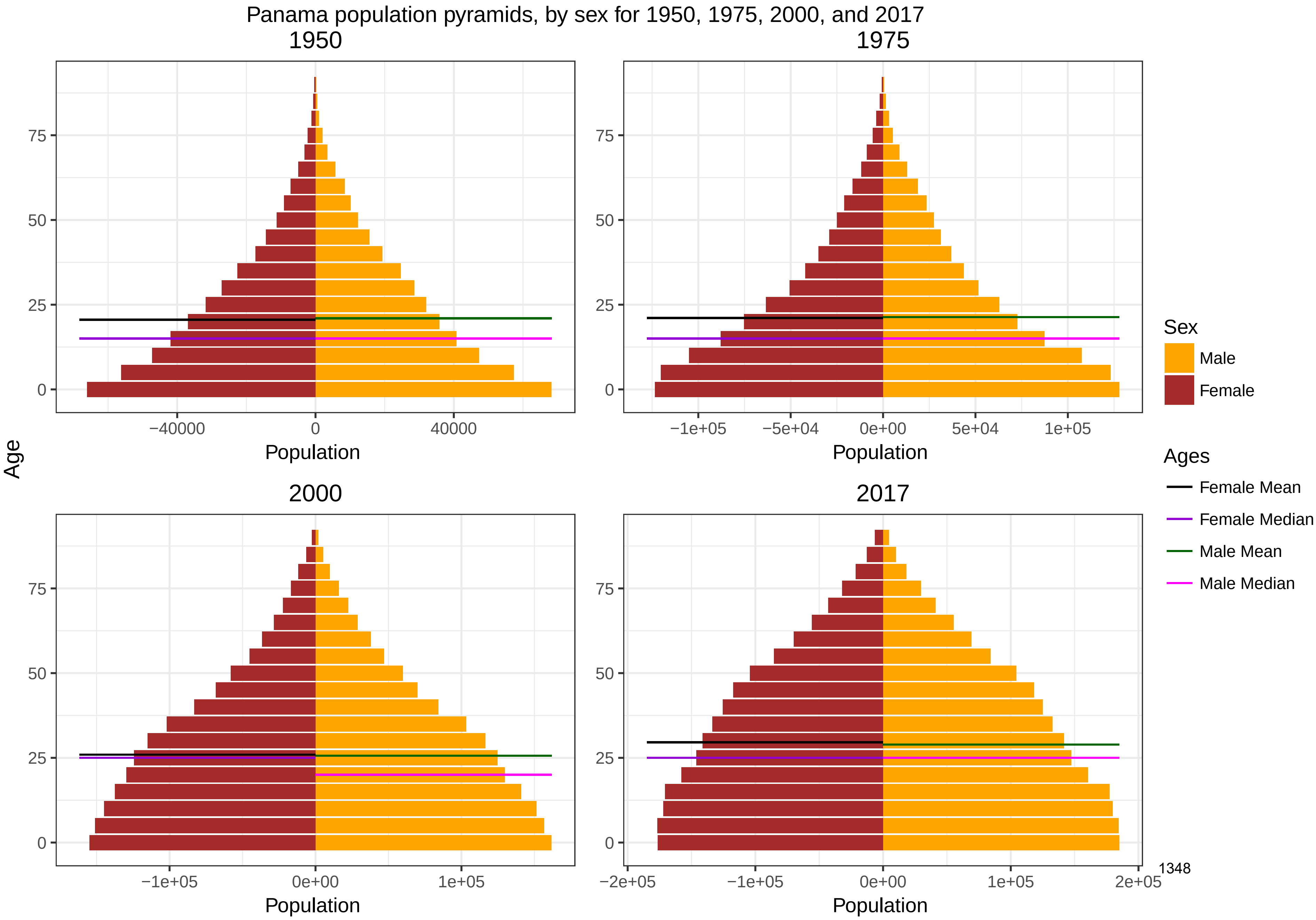
Panama population pyramids, by sex for 1950, 1975, 2000, and 2017
1950
1975
2000
2017
Age
Population
−40000
0
40000
−1e+05
−5e+04
0e+00
5e+04
1e+05
−2e+05
2e+05
0
25
50
75
Sex
Male
Female
Ages
Female Mean
Female Median
Male Mean
Male Median

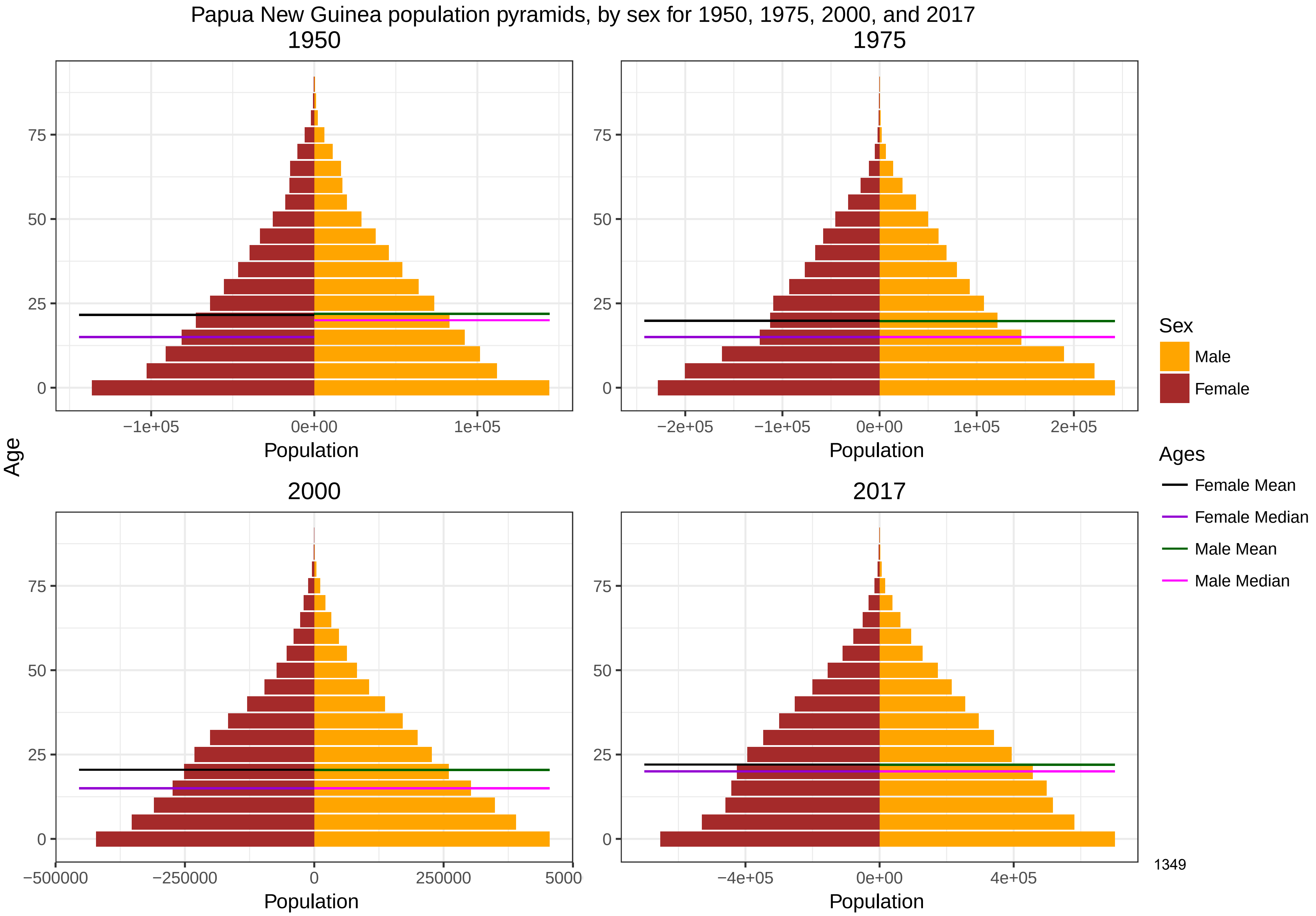

Papua New Guinea population pyramids, by sex for 1950, 1975, 2000, and 2017
1950
1975
2000
2017
Age
Population
0
25
50
75
−1e+05
0e+00
1e+05
−2e+05
2e+05
−500000
−250000
250000
5000
−4e+05
4e+05
Sex
Male
Female
Ages
Female Mean
Female Median
Male Mean
Male Median

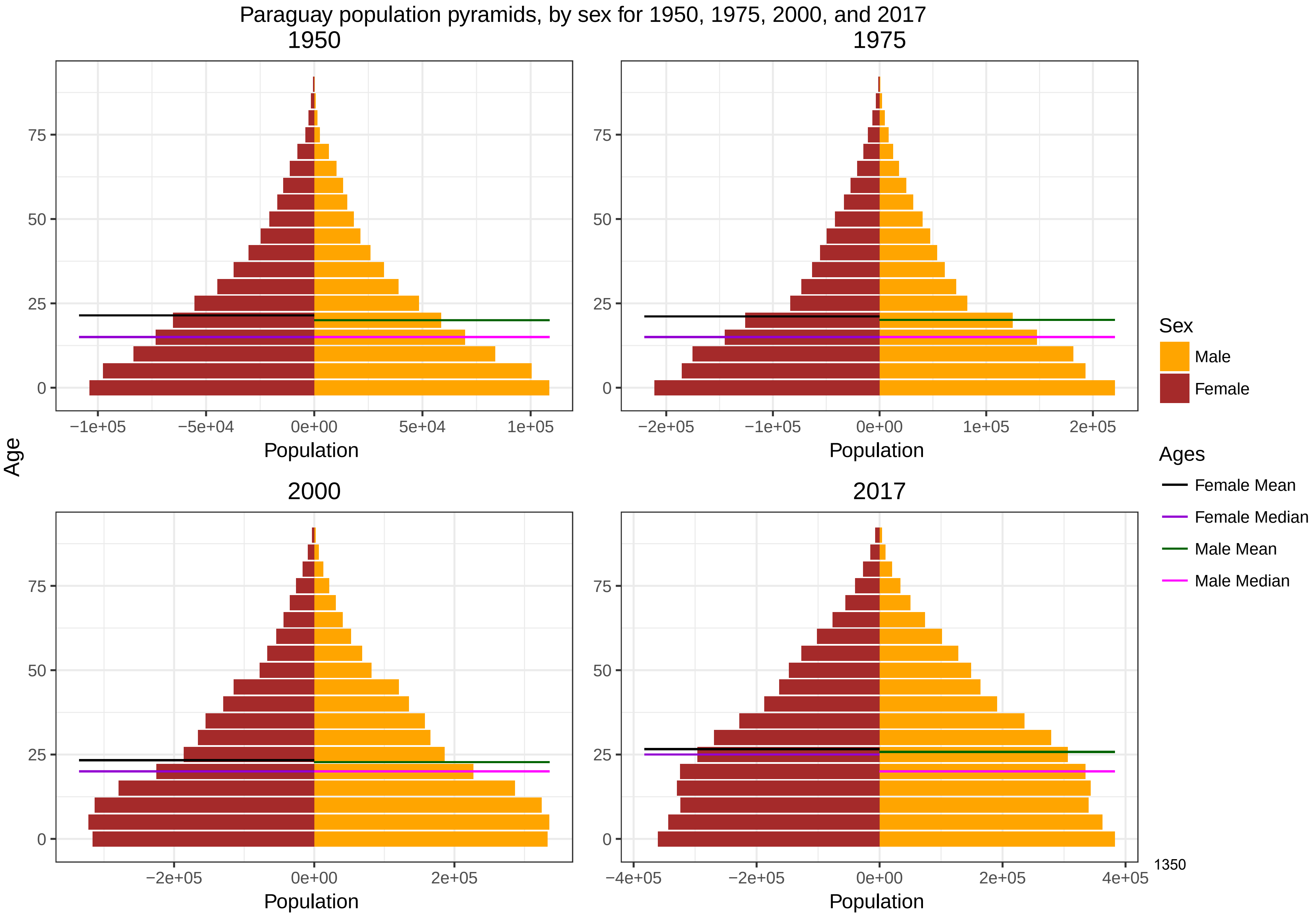
Paraguay population pyramids, by sex for 1950, 1975, 2000, and 2017
1950
1975
2000
2017
Age
Population
Sex
Male
Female
Ages
Female Mean
Female Median
Male Mean
Male Median
0
25
50
75
−1e+05
−5e+04
0e+00
5e+04
1e+05
−2e+05
−1e+05
2e+05
−4e+05
4e+05

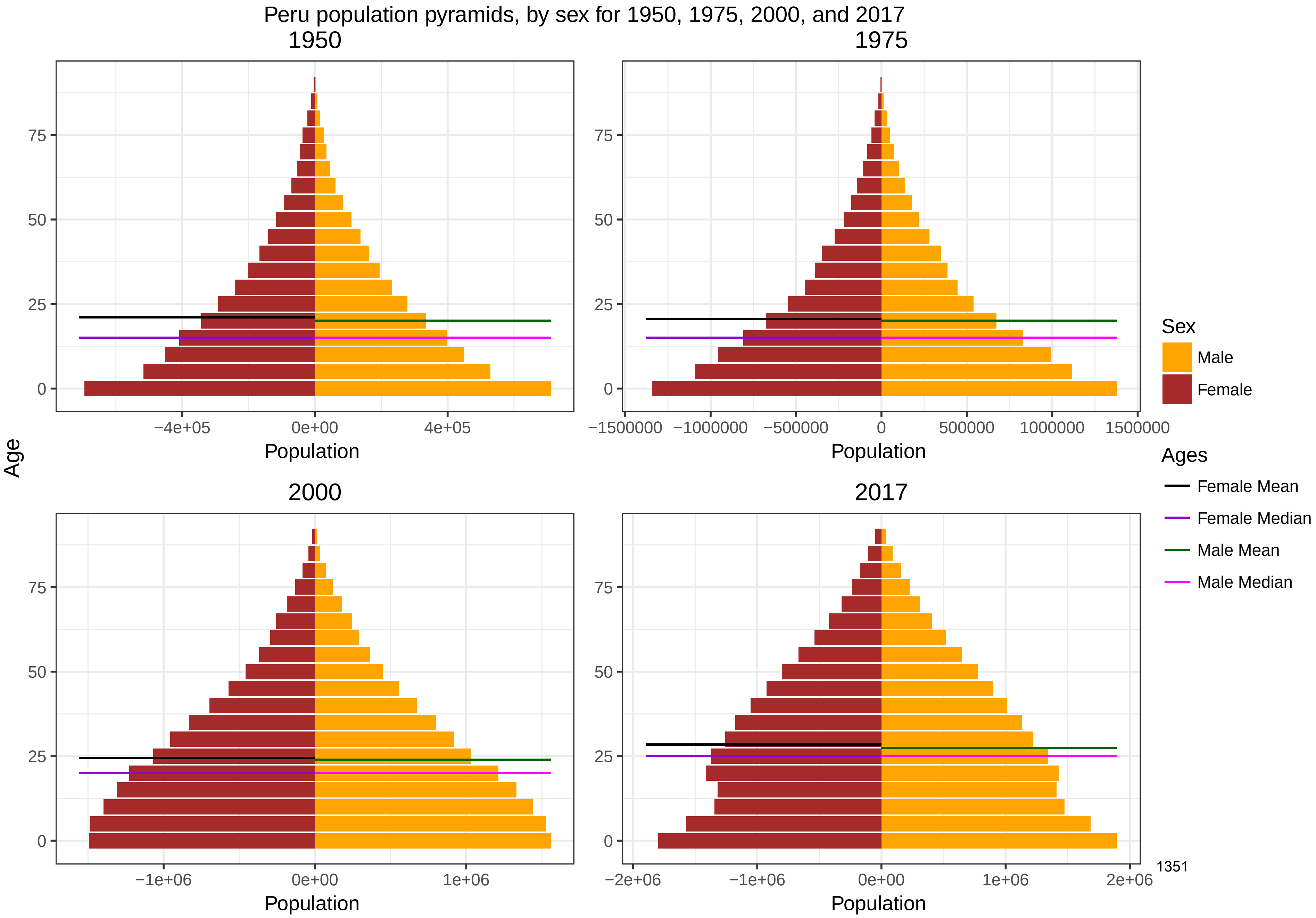

Peru population pyramids, by sex for 1950, 1975, 2000, and 2017
1950
1975
2000
2017
Age
Population
0
25
50
75
−4e+05
0e+00
4e+05
−1500000
−1000000
−500000
0
500000
1000000
1500000
−1e+06
1e+06
−2e+06
2e+06
Sex
Male
Female
Ages
Female Mean
Female Median
Male Mean
Male Median

1351

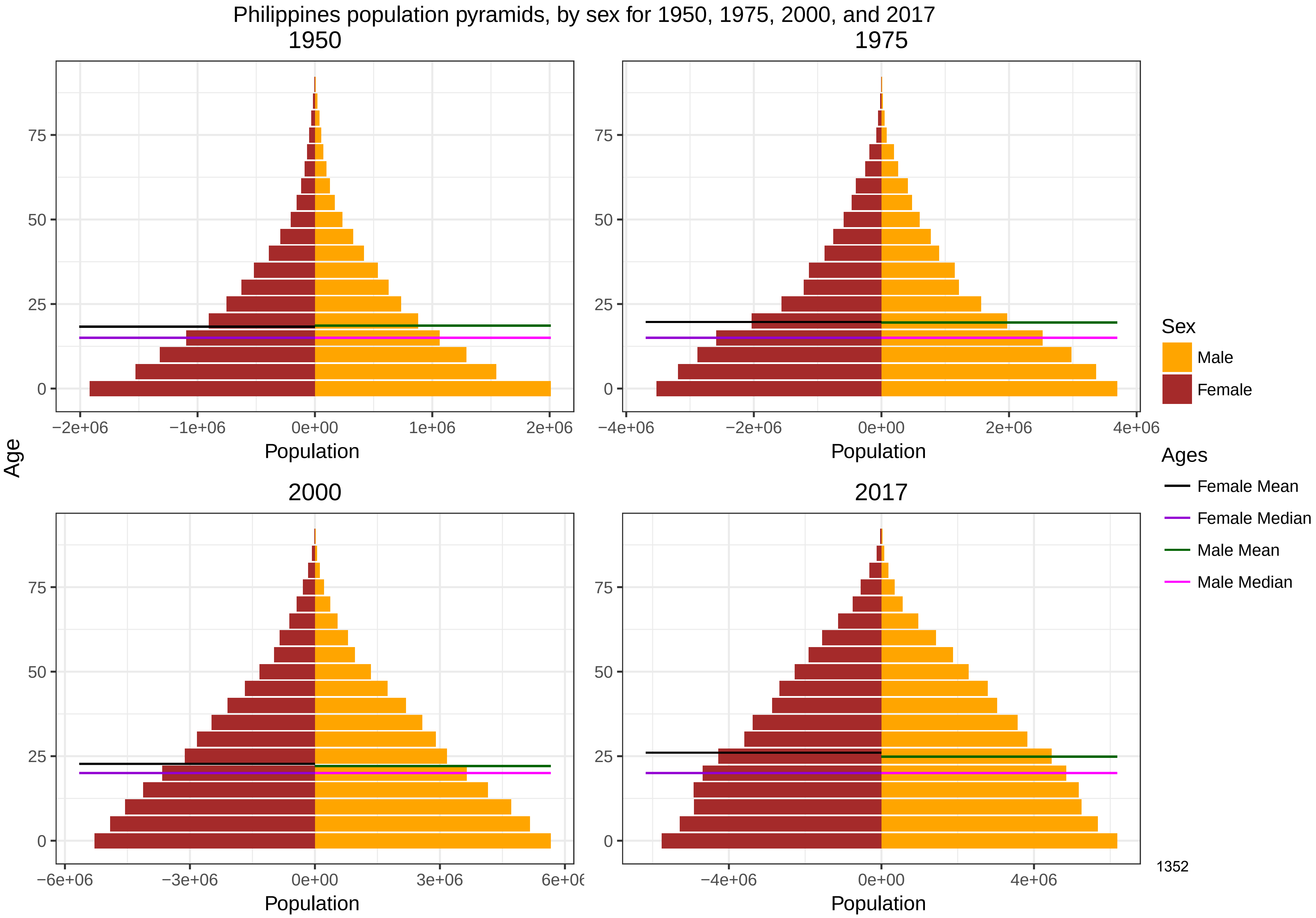
Philippines population pyramids, by sex for 1950, 1975, 2000, and 2017
1950
1975
2000
2017
Age
Population
Sex
Male
Female
Ages
Female Mean
Female Median
Male Mean
Male Median
0
25
50
75
−2e+06
−1e+06
0e+00
1e+06
2e+06
−4e+06
−2e+06
2e+06
4e+06
−6e+06
−3e+06
3e+06
6e+06
−4e+06
4e+06

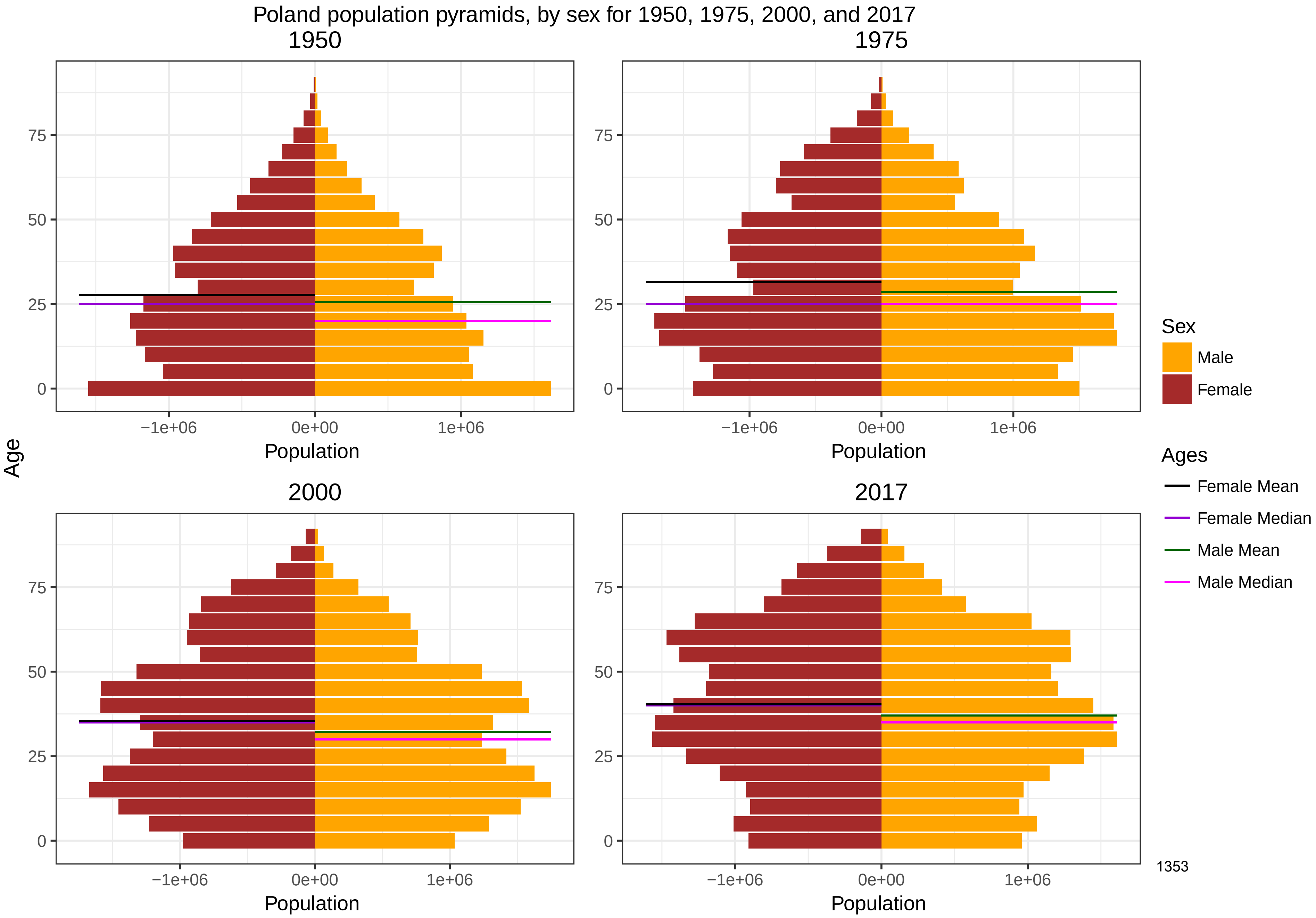

Poland population pyramids, by sex for 1950, 1975, 2000, and 2017
1950
1975
2000
2017
Age
0
25
50
75
Population
−1e+06
0e+00
1e+06
Sex
Male
Female
Ages
Female Mean
Female Median
Male Mean
Male Median

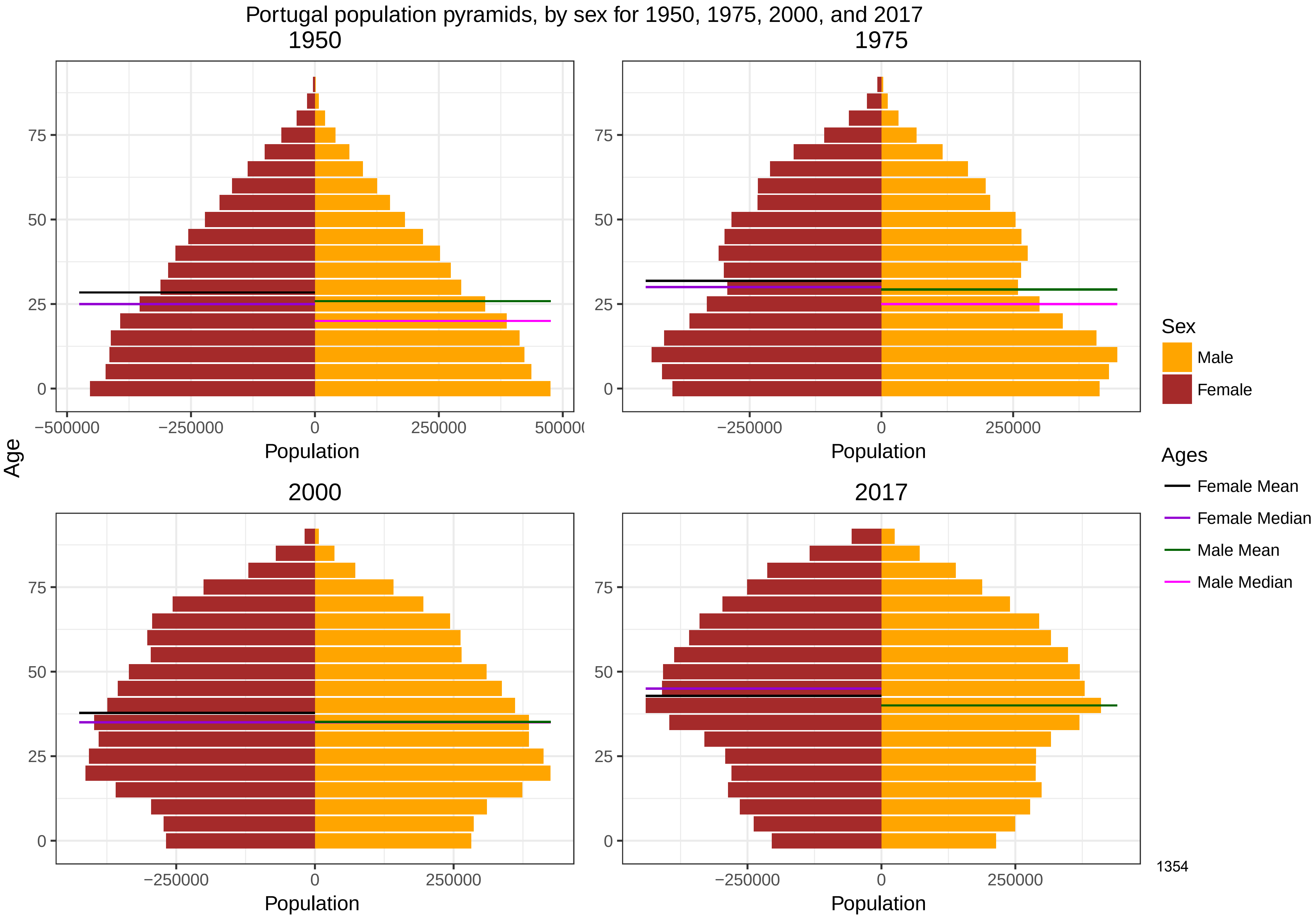

Portugal population pyramids, by sex for 1950, 1975, 2000, and 2017
1950
1975
2000
2017
Age
Population
75
50
25
0
−500000
−250000
0
250000
500000
Sex
Male
Female
Ages
Female Mean
Female Median
Male Mean
Male Median

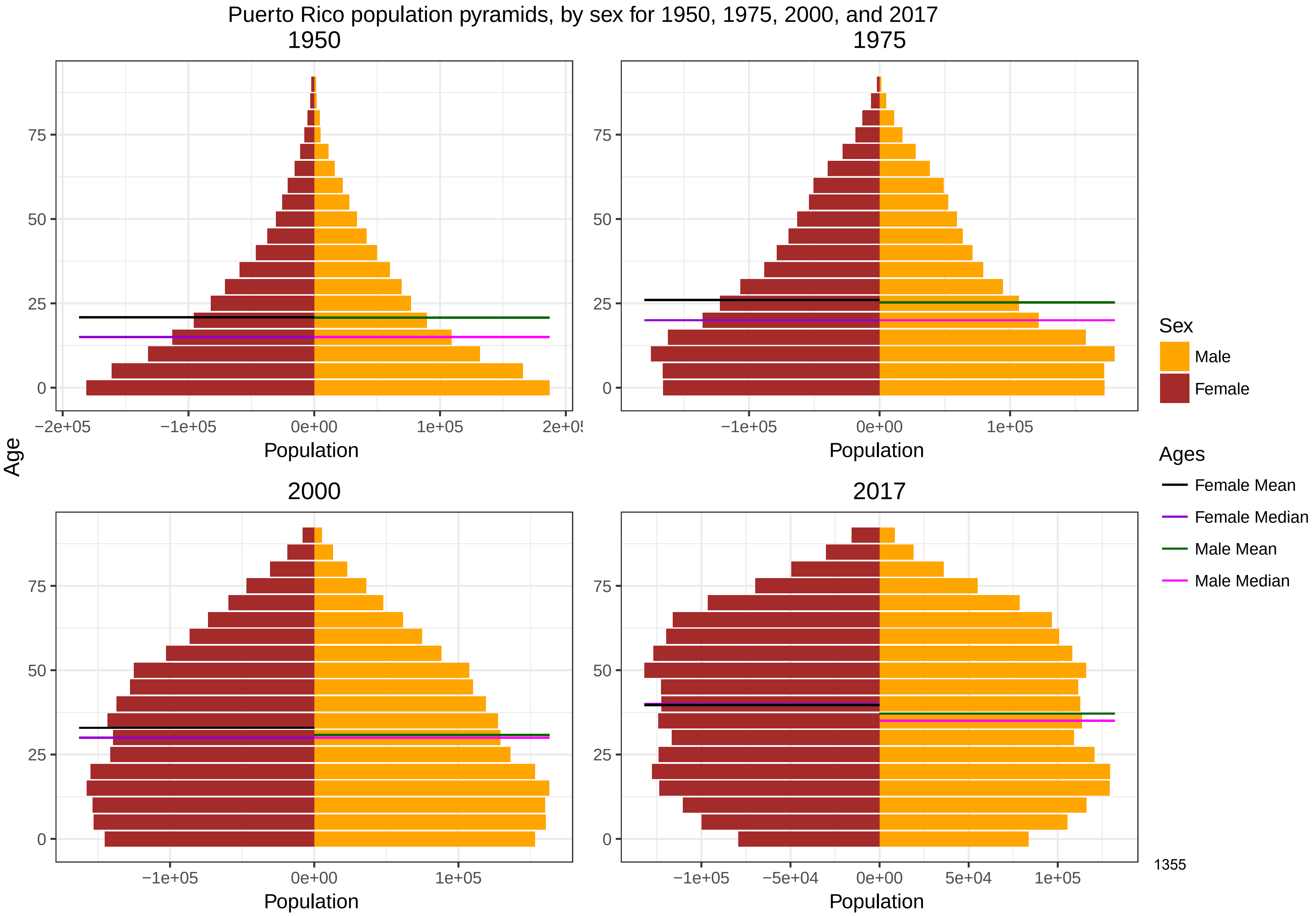
Puerto Rico population pyramids, by sex for 1950, 1975, 2000, and 2017
1950
1975
2000
2017
Age
Population
0
25
50
75
−2e+05
−1e+05
0e+00
1e+05
−5e+04
5e+04
Sex
Male
Female
Ages
Female Mean
Female Median
Male Mean
Male Median

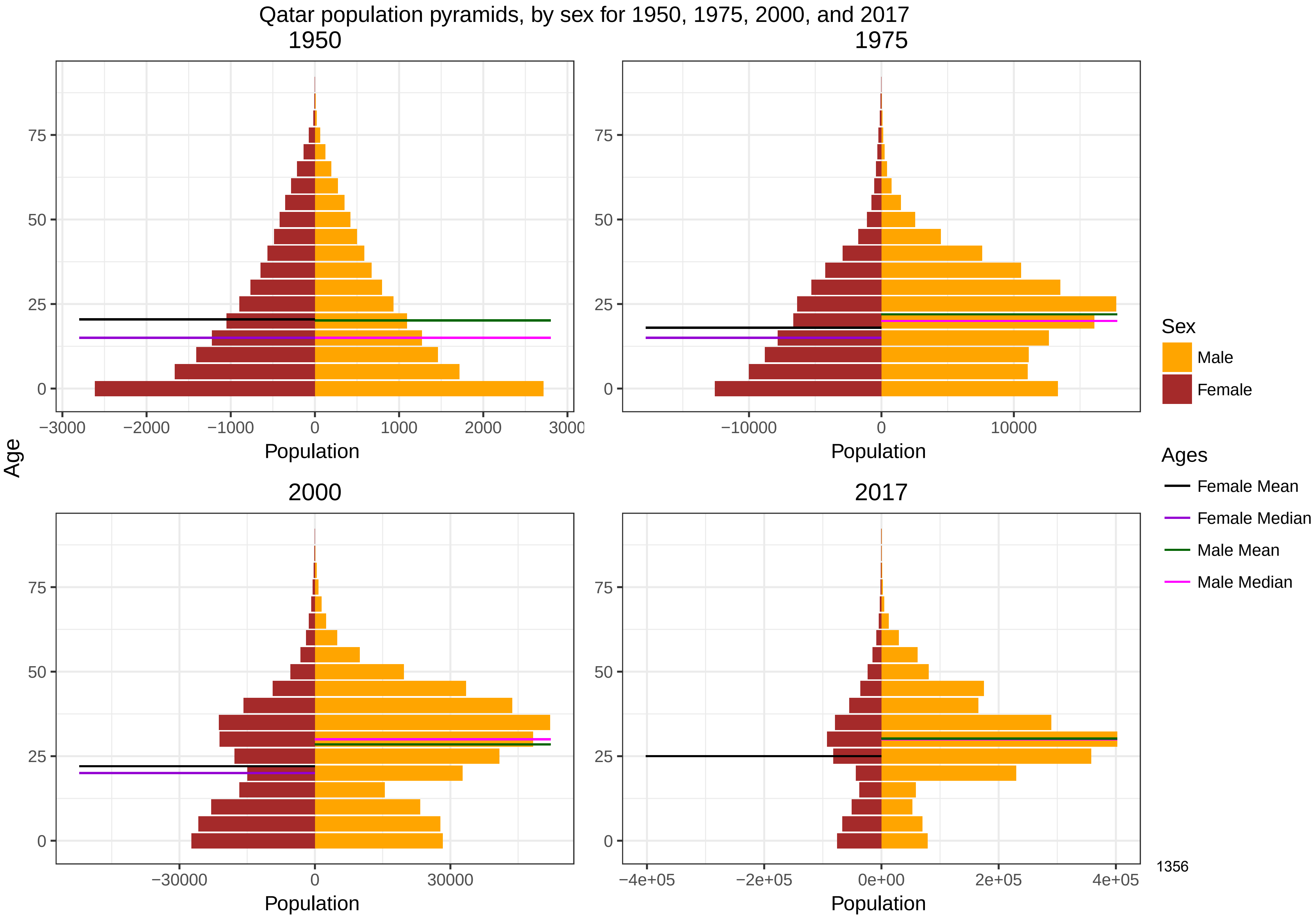

Qatar population pyramids, by sex for 1950, 1975, 2000, and 2017
1950
1975
2000
2017
Age
Population
0
25
50
75
−3000
−2000
−1000
0
1000
2000
3000
−10000
10000
−30000
30000
−4e+05
−2e+05
0e+00
2e+05
4e+05
Sex
Male
Female
Ages
Female Mean
Female Median
Male Mean
Male Median

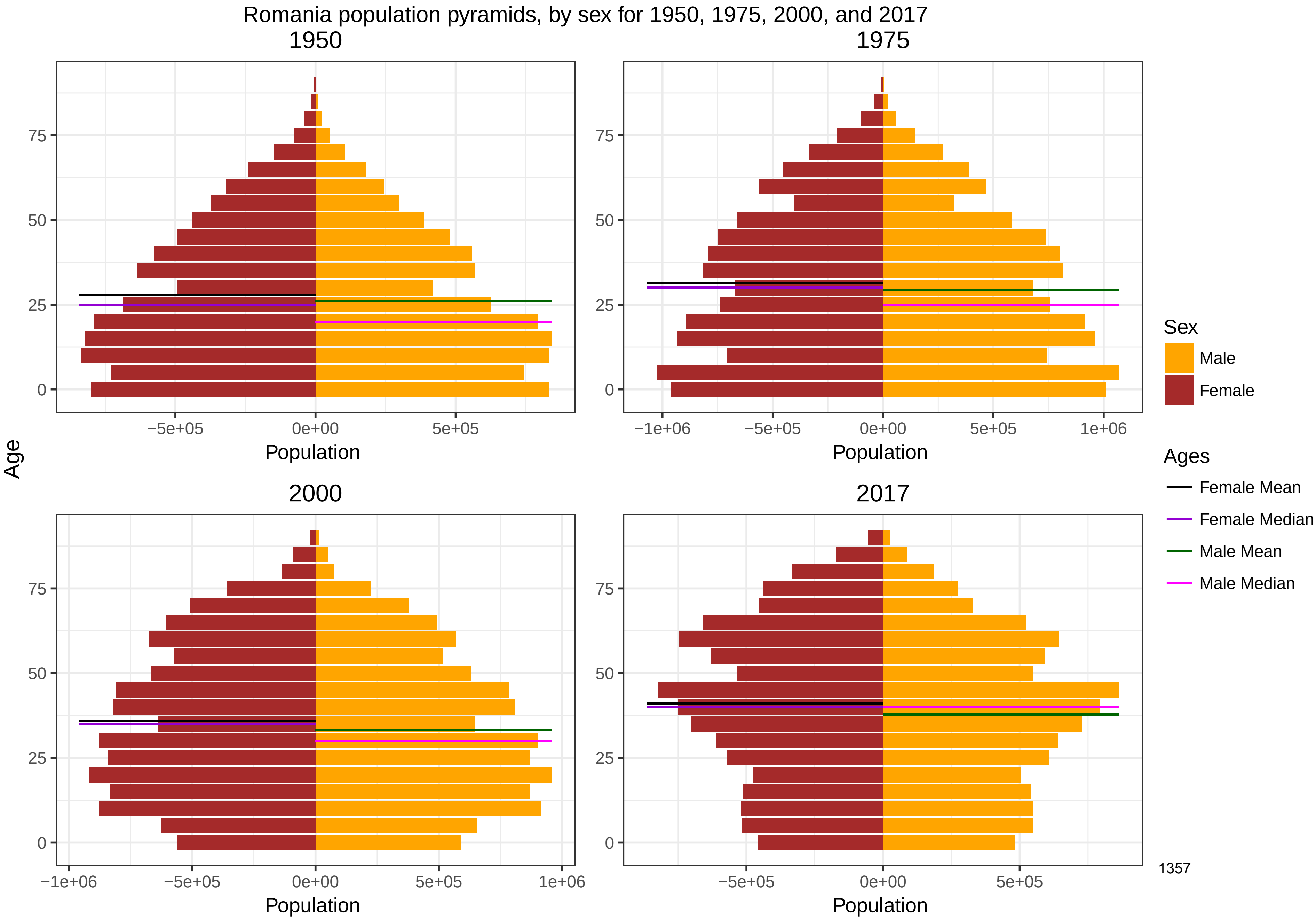

Romania population pyramids, by sex for 1950, 1975, 2000, and 2017
1950
1975
2000
2017
Age
Population
Sex
Male
Female
Ages
Female Mean
Female Median
Male Mean
Male Median

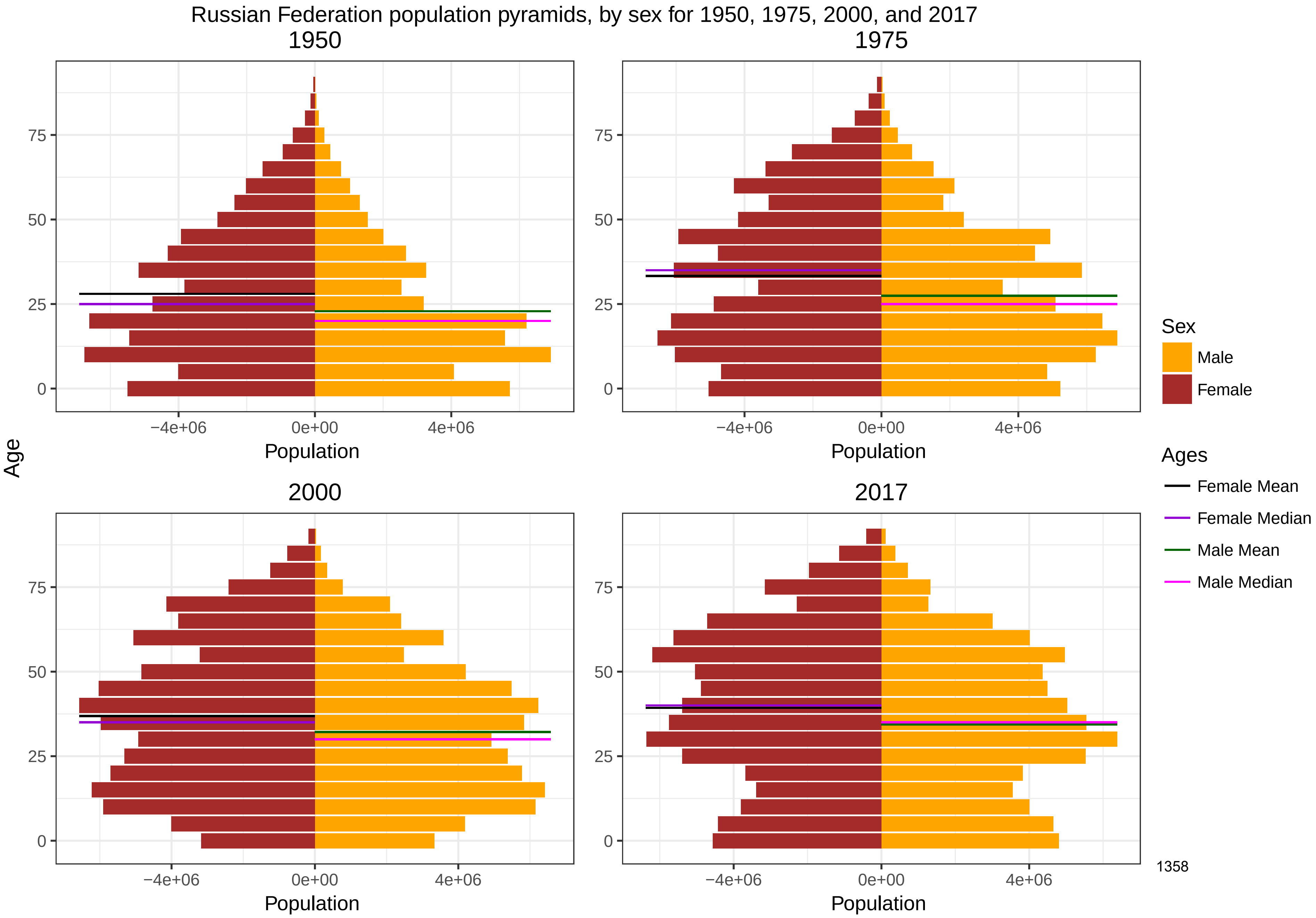
Russian Federation population pyramids, by sex for 1950, 1975, 2000, and 2017
1950
1975
2000
2017
Age
Population
−4e+06
0e+00
4e+06
0
25
50
75
Sex
Male
Female
Ages
Female Mean
Female Median
Male Mean
Male Median

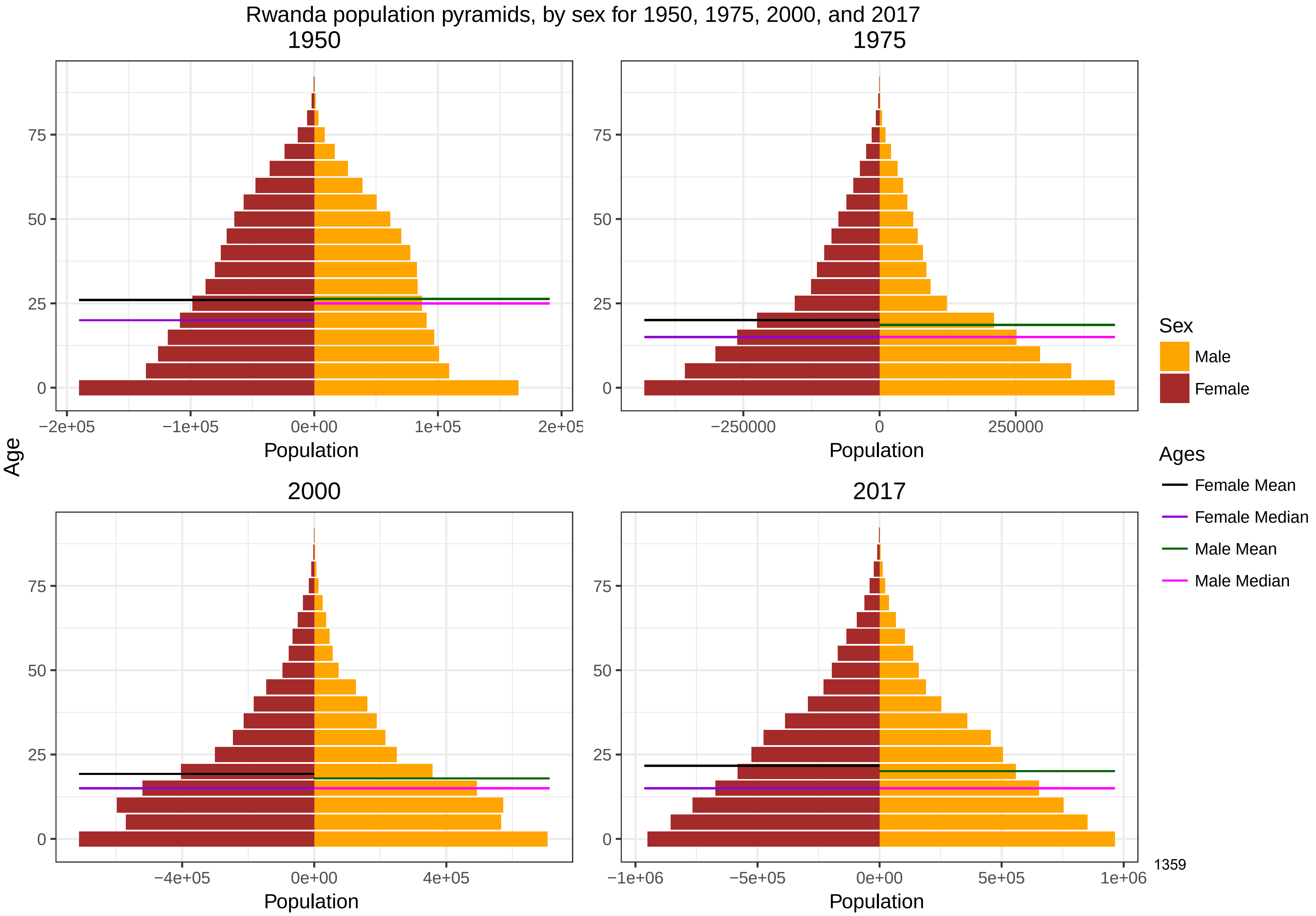

Rwanda population pyramids, by sex for 1950, 1975, 2000, and 2017
1950
1975
2000
2017
Age
Population
Sex
Male
Female
Ages
Female Mean
Female Median
Male Mean
Male Median
−2e+05
−1e+05
0e+00
1e+05
2e+05
−250000
0
250000
−4e+05
4e+05
−1e+06
−5e+05
5e+05
1e+06
0
25
50
75

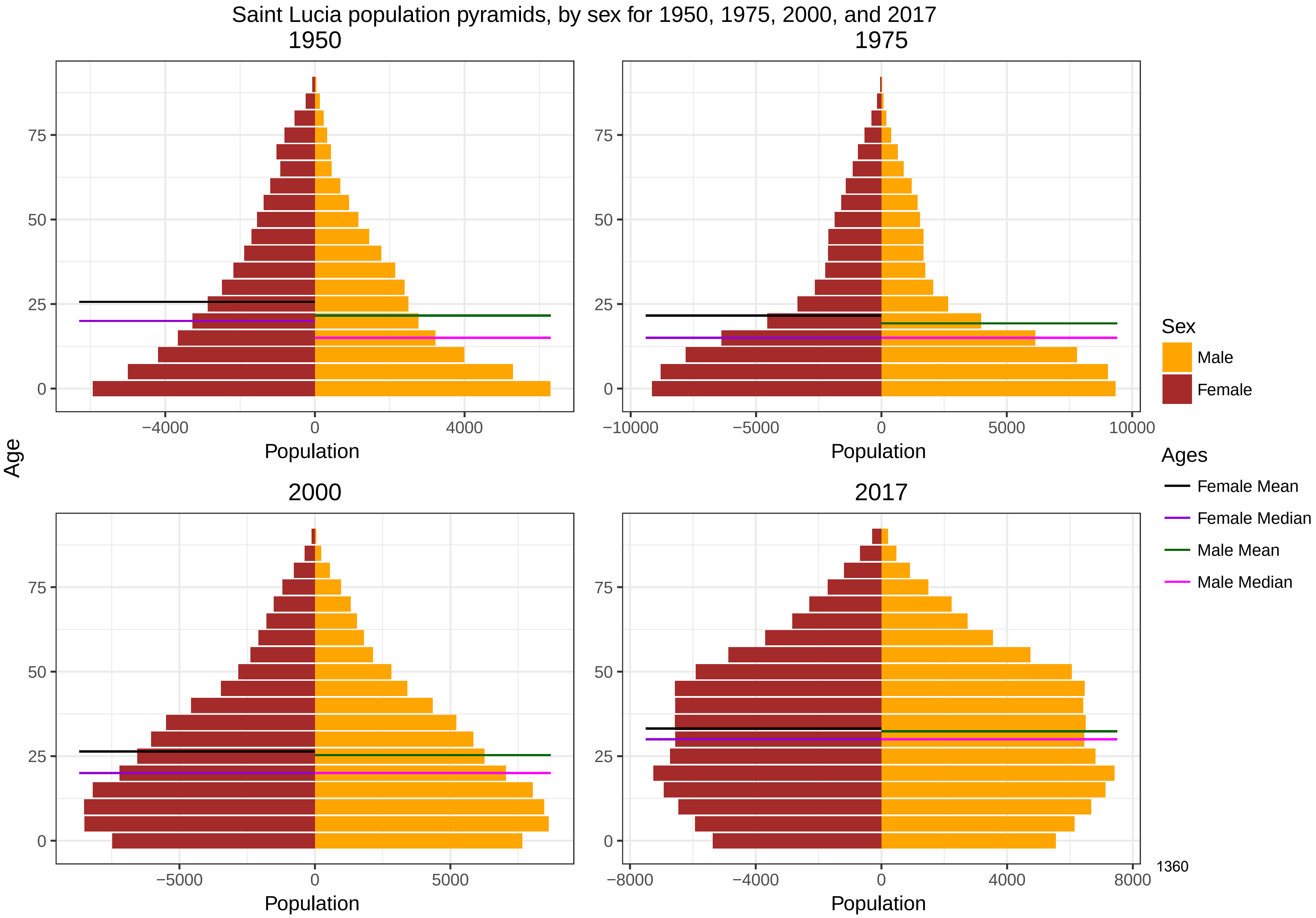

Saint Lucia population pyramids, by sex for 1950, 1975, 2000, and 2017
1950
1975
2000
2017
Age
Population
0
25
50
75
−4000
0
4000
−10000
−5000
5000
10000
−8000
8000
Sex
Male
Female
Ages
Female Mean
Female Median
Male Mean
Male Median

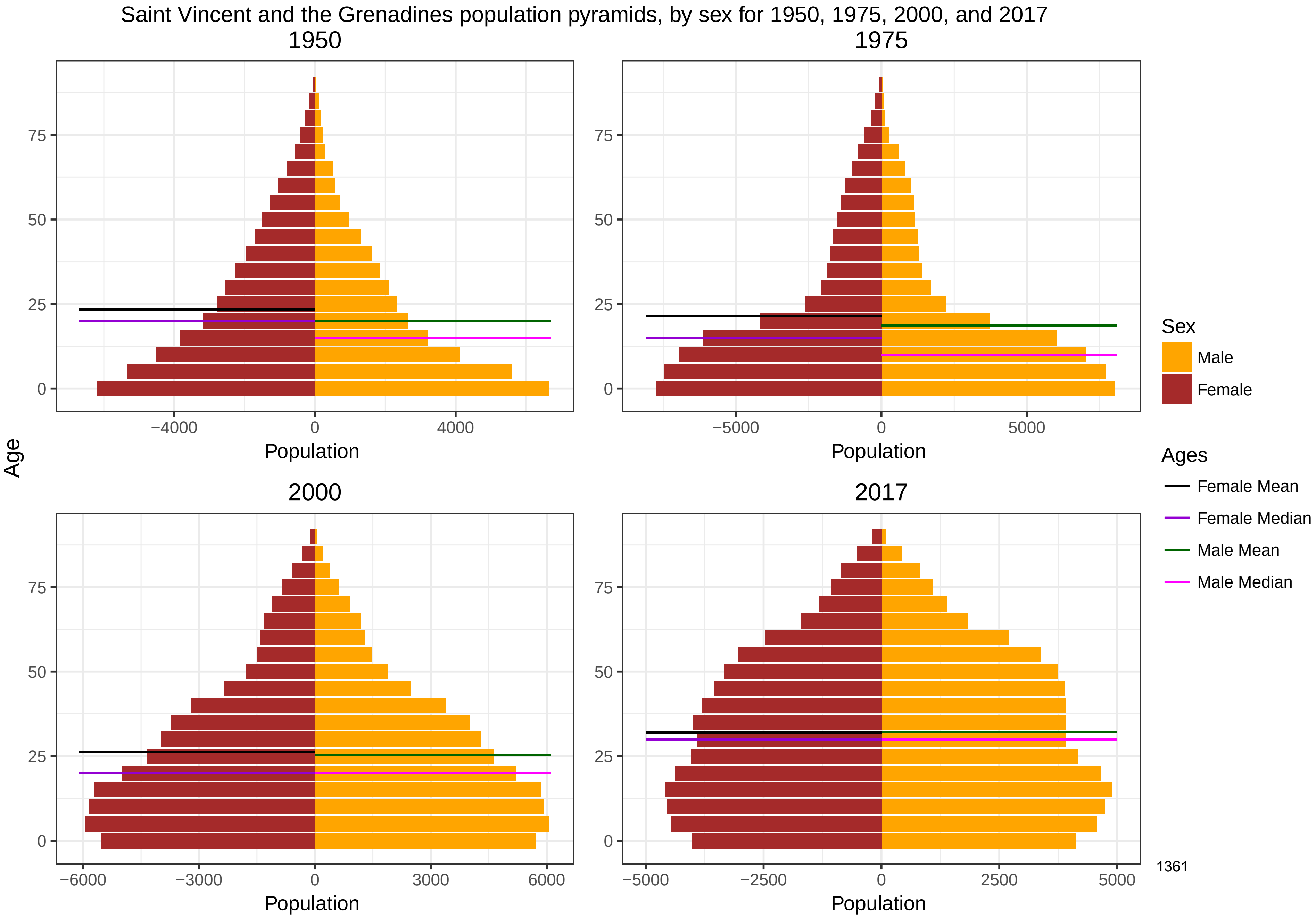

Saint Vincent and the Grenadines population pyramids, by sex for 1950, 1975, 2000, and 2017
1950
1975
2000
2017
Age
Population
0
25
50
75
−4000
0
4000
−5000
5000
−6000
−3000
3000
6000
−2500
2500
Sex
Male
Female
Ages
Female Mean
Female Median
Male Mean
Male Median

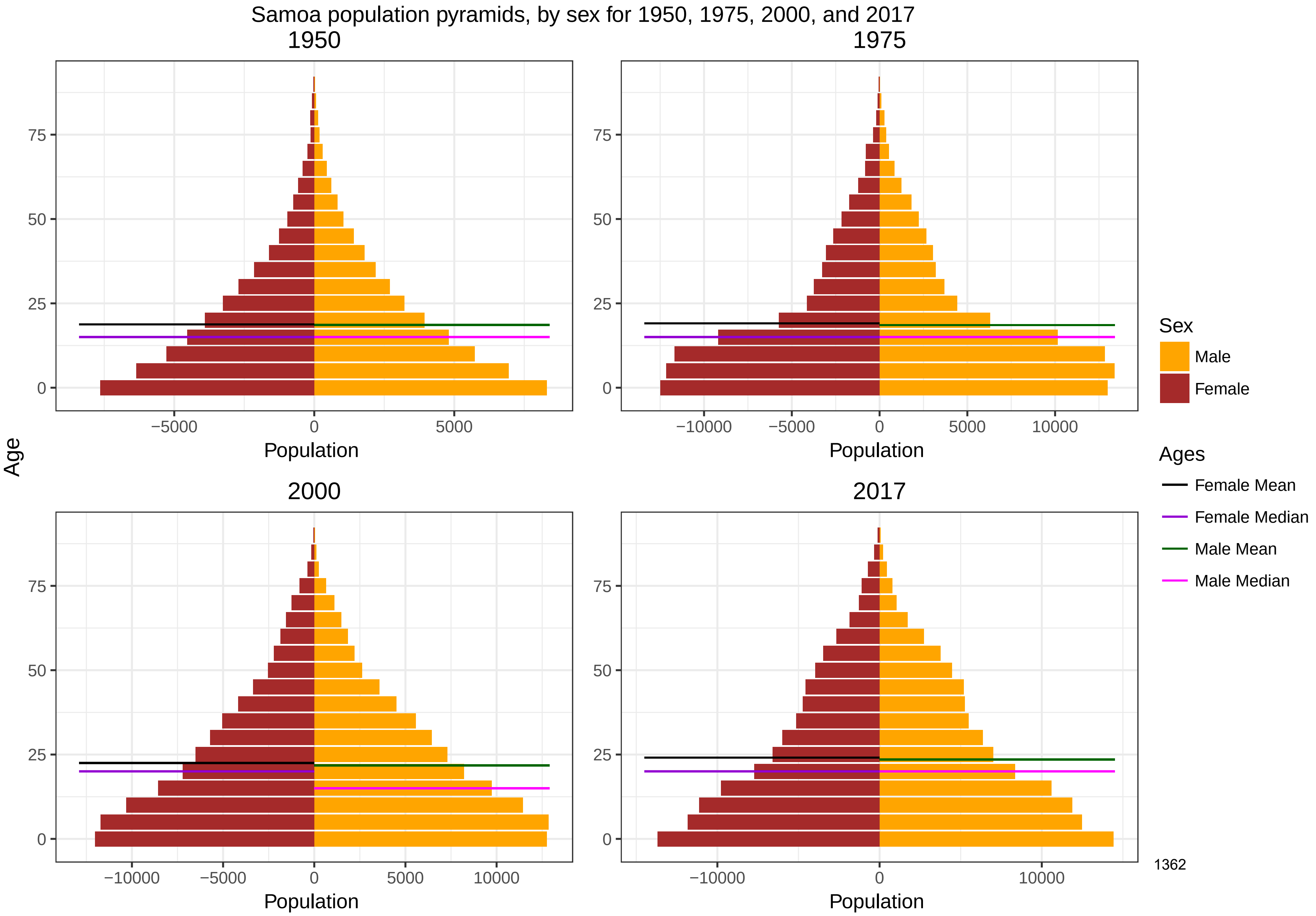

Samoa population pyramids, by sex for 1950, 1975, 2000, and 2017
1950
1975
2000
2017
Age
Population
75
50
25
0
−5000
0
5000
−10000
10000
Sex
Male
Female
Ages
Female Mean
Female Median
Male Mean
Male Median

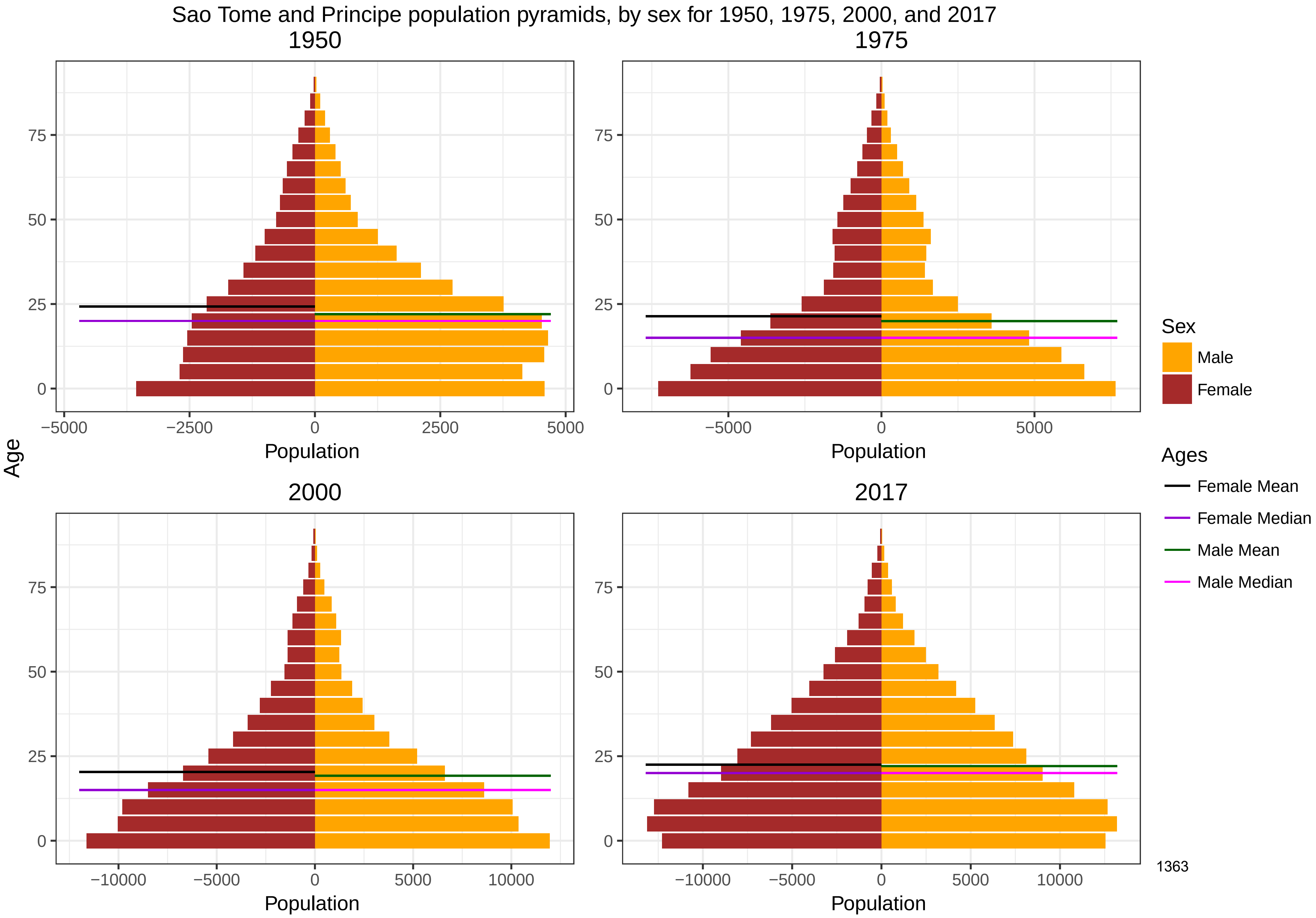

Sao Tome and Principe population pyramids, by sex for 1950, 1975, 2000, and 2017
1950
1975
2000
2017
Age
Population
Sex
Male
Female
Ages
Female Mean
Female Median
Male Mean
Male Median

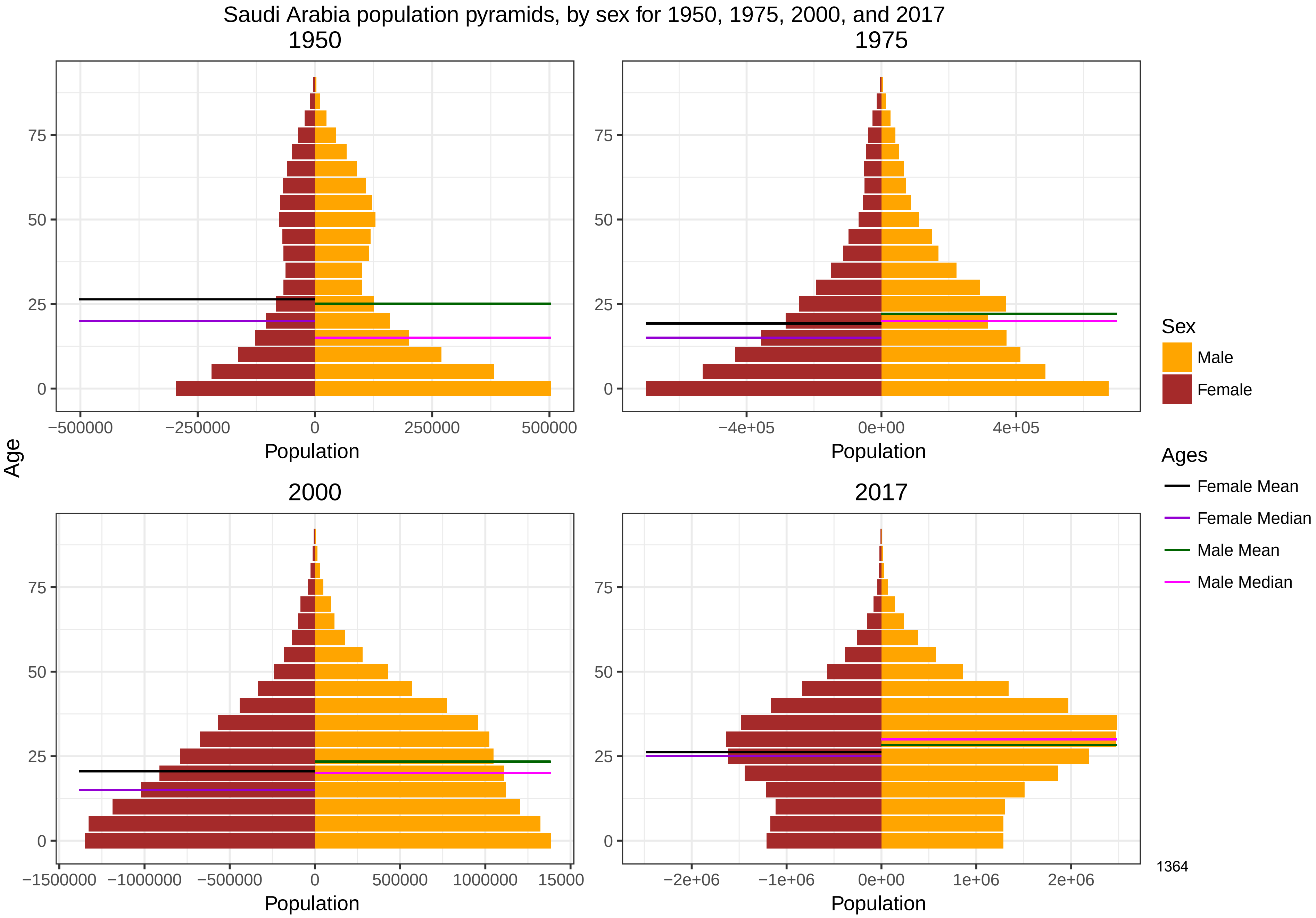
Saudi Arabia population pyramids, by sex for 1950, 1975, 2000, and 2017
1950
1975
2000
2017
Age
Population
Sex
Male
Female
Ages
Female Mean
Female Median
Male Mean
Male Median
−500000
−250000
0
250000
500000
−4e+05
0e+00
4e+05
−1500000
−1000000
−500000
1000000
15000
−2e+06
−1e+06
1e+06
2e+06
75
50
25

1364

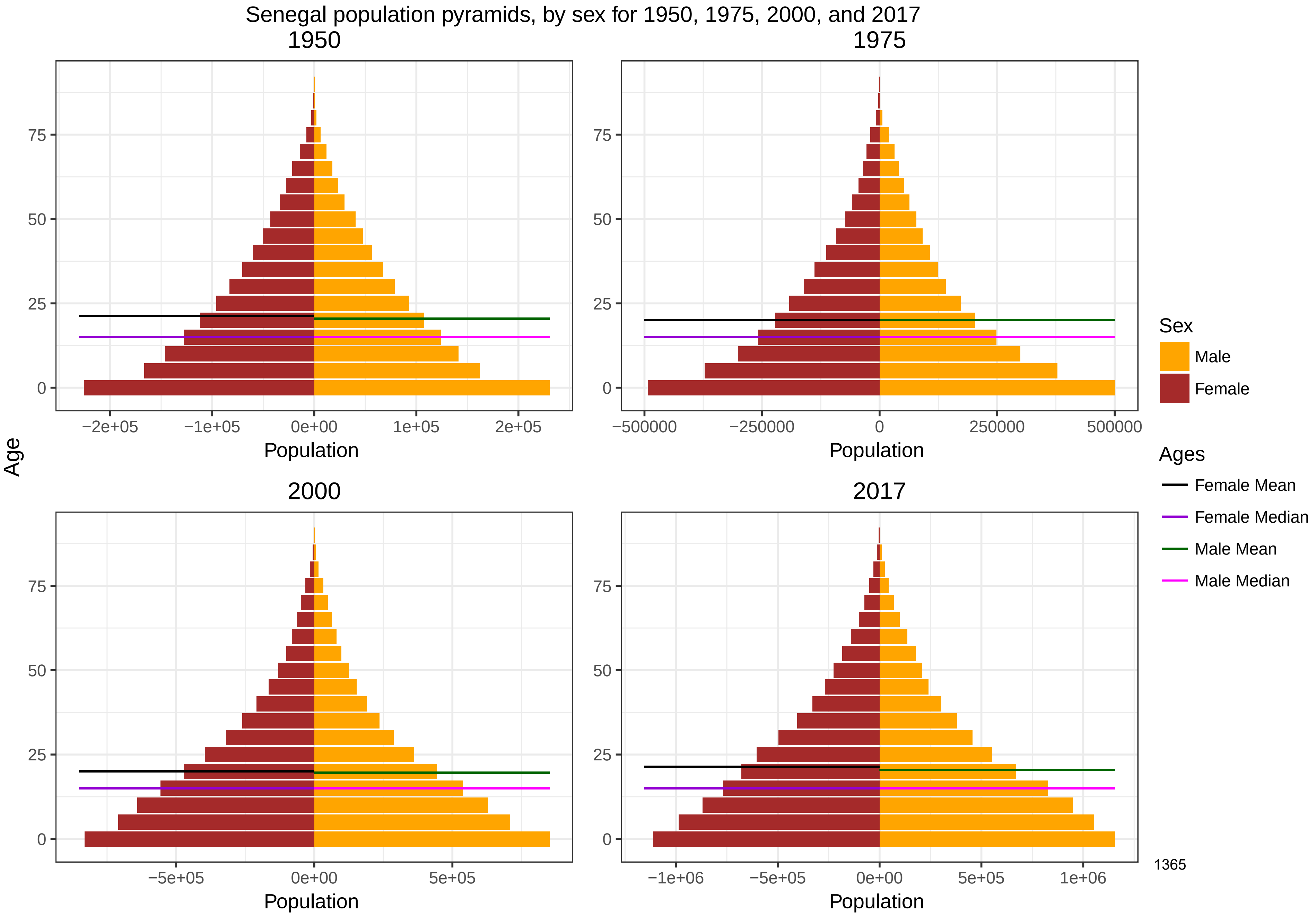
Senegal population pyramids, by sex for 1950, 1975, 2000, and 2017
1950
1975
2000
2017
Age
Population
0
25
50
75
−2e+05
−1e+05
0e+00
1e+05
2e+05
−500000
−250000
0
250000
500000
−5e+05
5e+05
−1e+06
1e+06
Sex
Male
Female
Ages
Female Mean
Female Median
Male Mean
Male Median

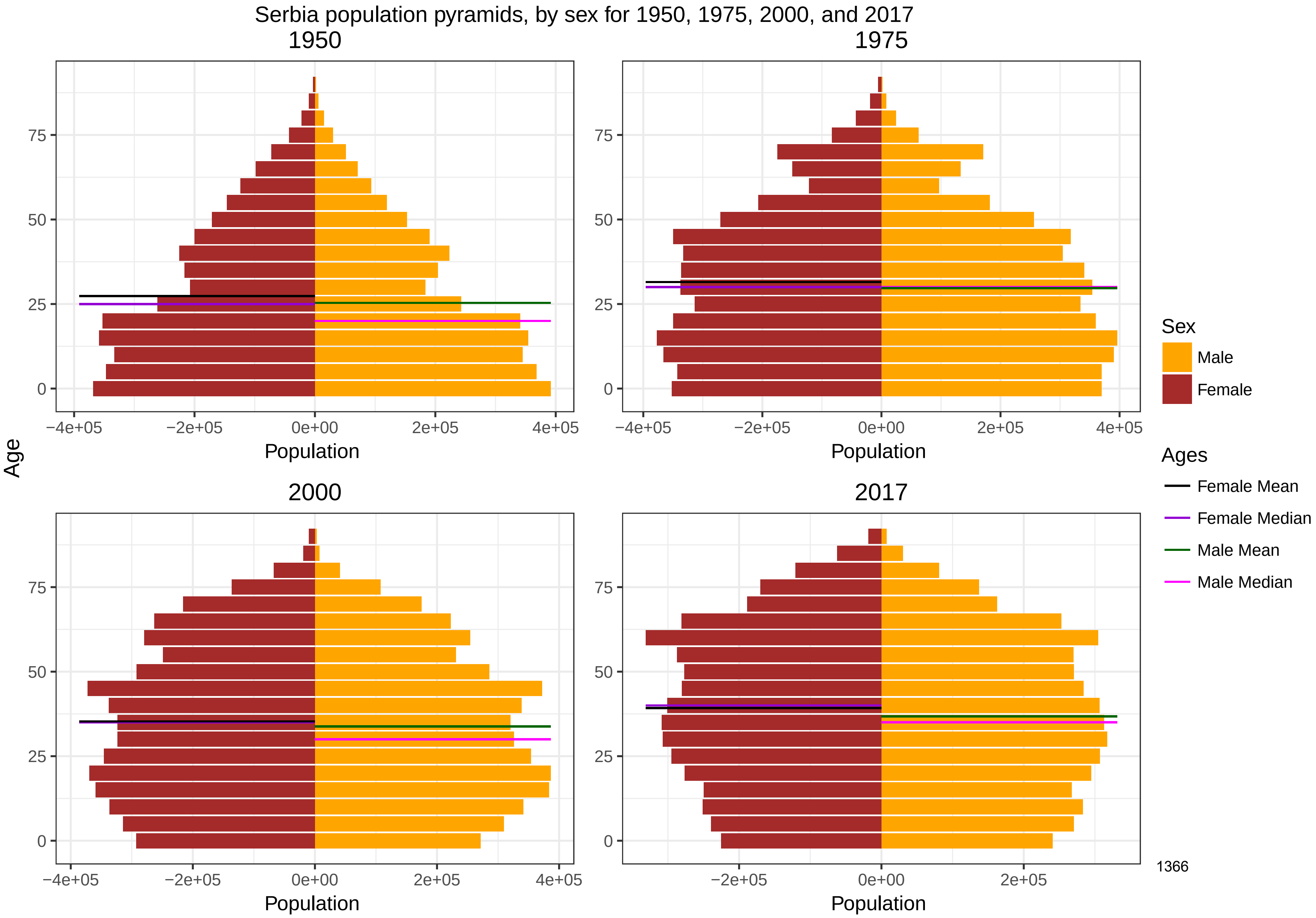
Serbia population pyramids, by sex for 1950, 1975, 2000, and 2017
1950
1975
2000
2017
Age
Population
Sex
Male
Female
Ages
Female Mean
Female Median
Male Mean
Male Median
0
25
50
75
−4e+05
−2e+05
0e+00
2e+05
4e+05

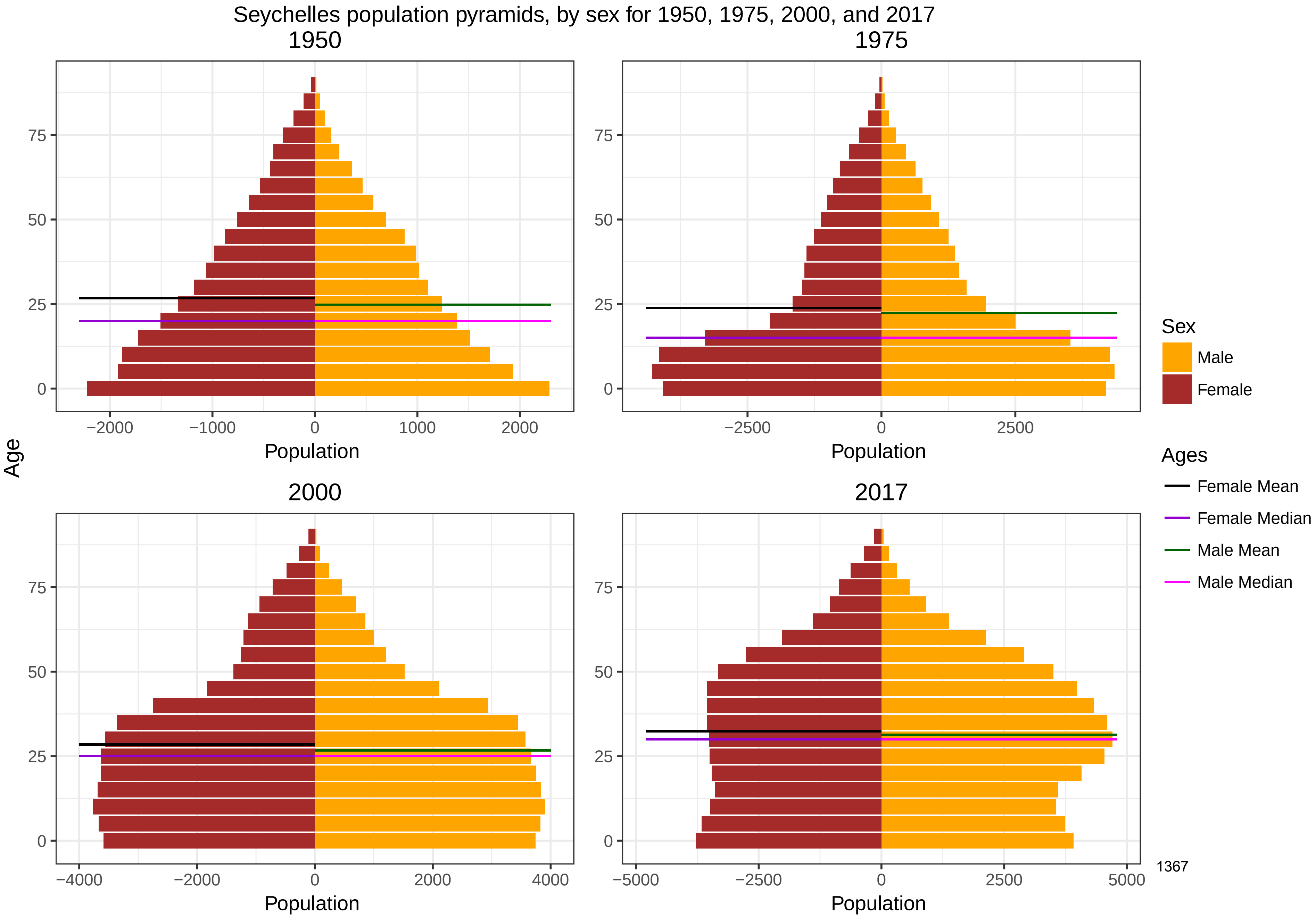
Seychelles population pyramids, by sex for 1950, 1975, 2000, and 2017
1950
1975
2000
2017
Age
Population
Sex
Male
Female
Ages
Female Mean
Female Median
Male Mean
Male Median

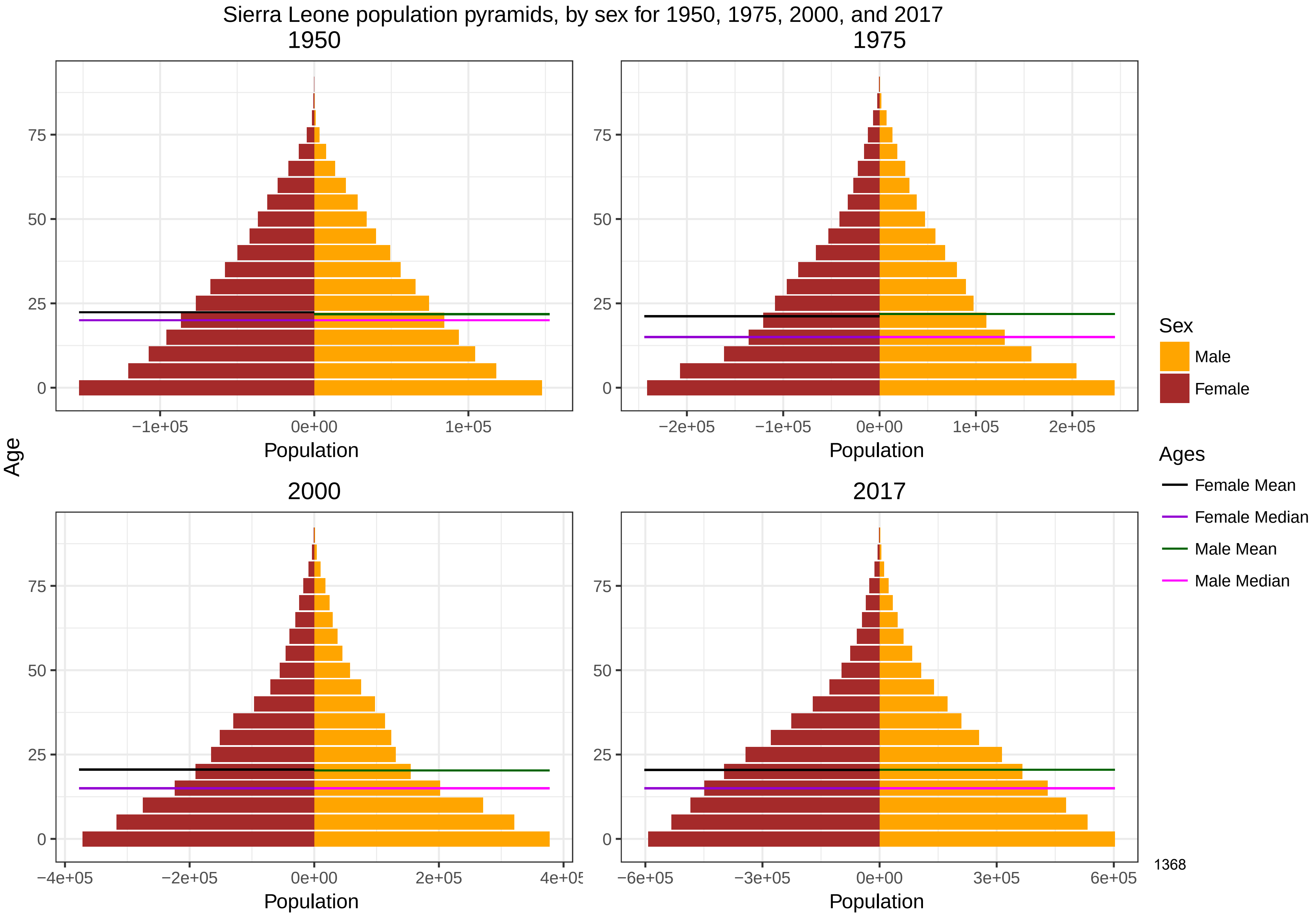

Sierra Leone population pyramids, by sex for 1950, 1975, 2000, and 2017
1950
1975
2000
2017
Age
Population
0
25
50
75
−1e+05
0e+00
1e+05
−2e+05
2e+05
−4e+05
4e+05
−6e+05
−3e+05
3e+05
6e+05
Sex
Male
Female
Ages
Female Mean
Female Median
Male Mean
Male Median

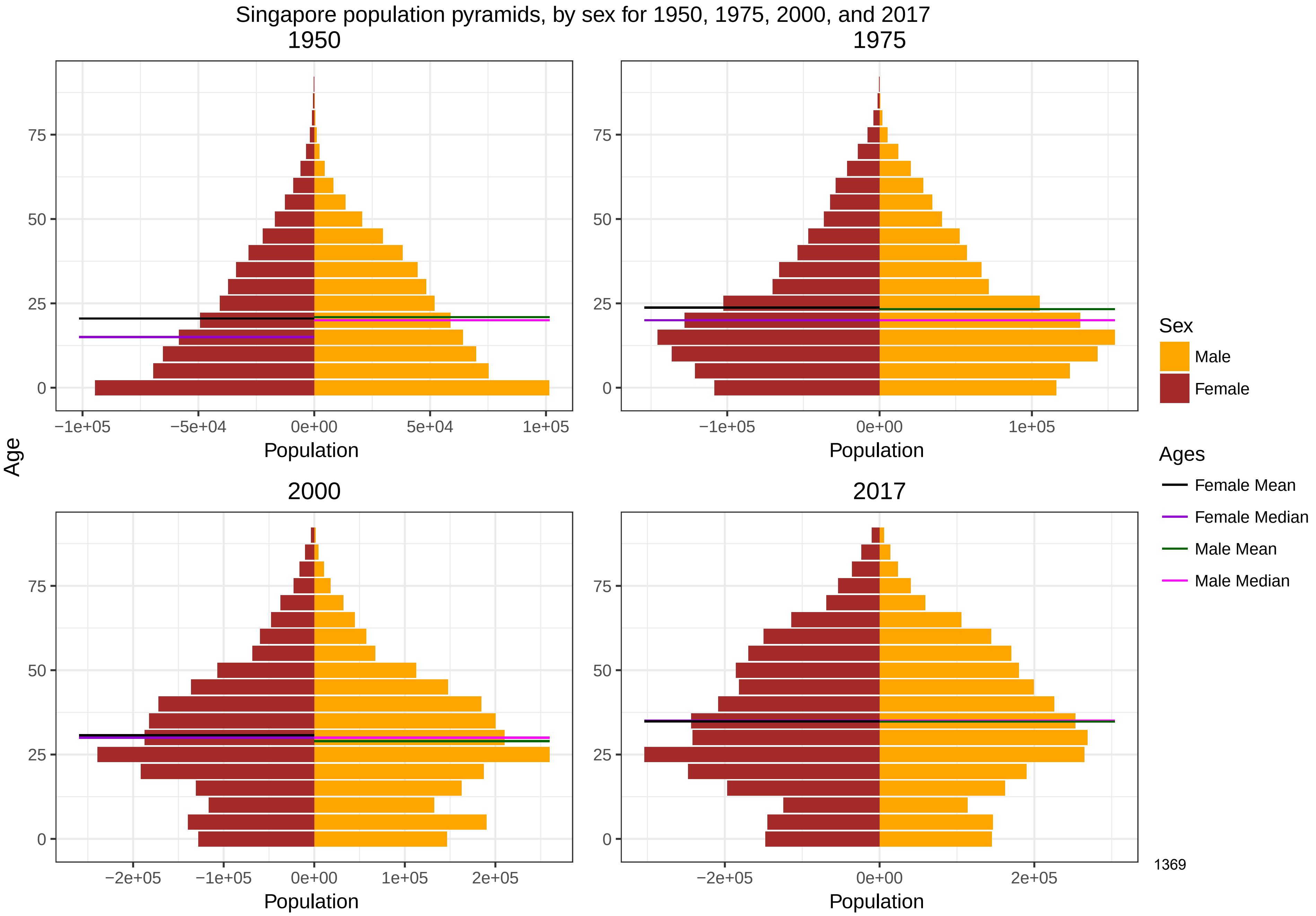

Singapore population pyramids, by sex for 1950, 1975, 2000, and 2017
1950
1975
2000
2017
Age
Population
−1e+05
−5e+04
0e+00
5e+04
1e+05
−2e+05
2e+05
0
25
50
75
Sex
Male
Female
Ages
Female Mean
Female Median
Male Mean
Male Median

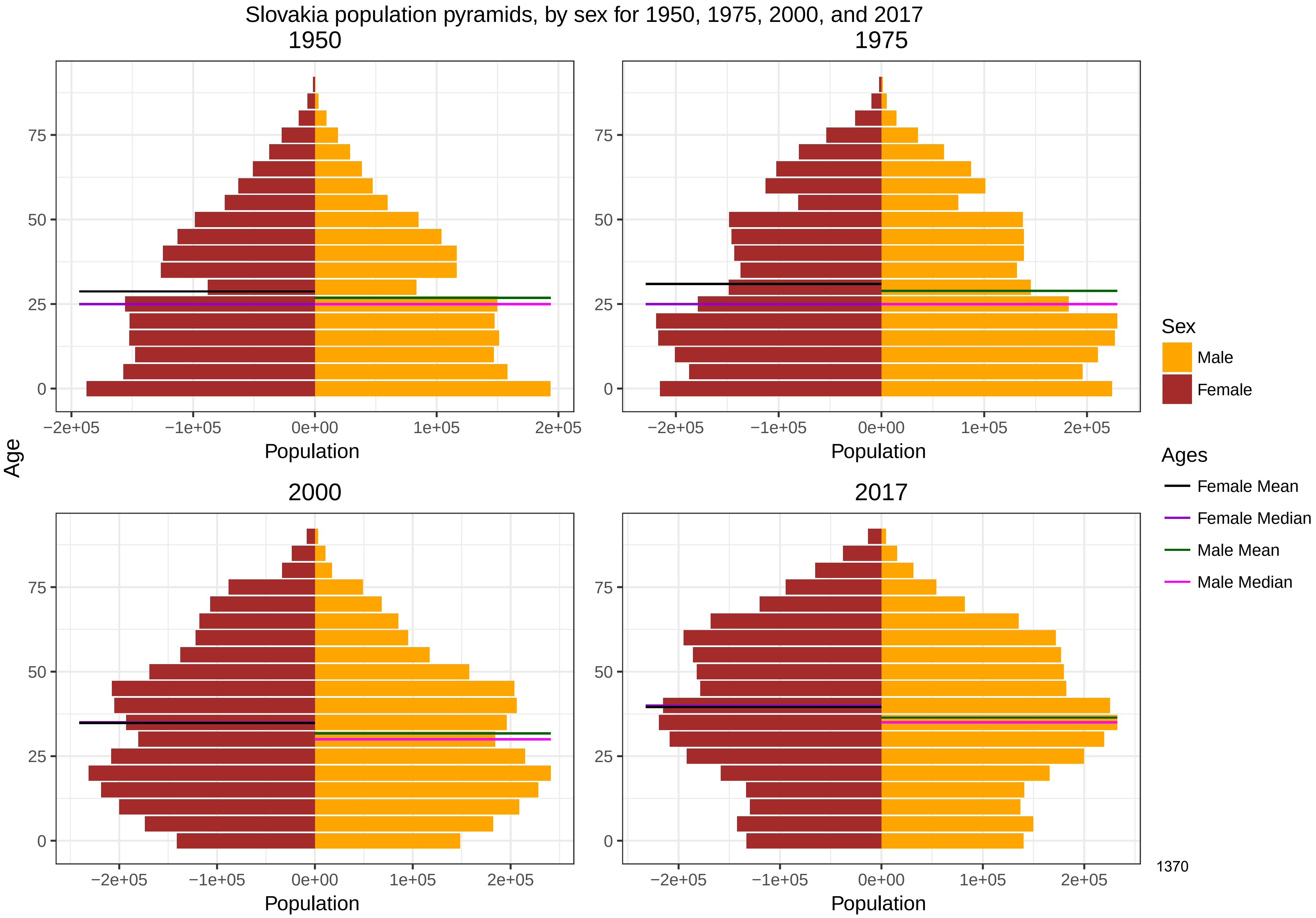

Slovakia population pyramids, by sex for 1950, 1975, 2000, and 2017
1950
1975
2000
2017
Age
Population
0
25
50
75
−2e+05
−1e+05
0e+00
1e+05
2e+05
Sex
Male
Female
Ages
Female Mean
Female Median
Male Mean
Male Median

Slovenia population pyramids, by sex for 1950, 1975, 2000, and 2017

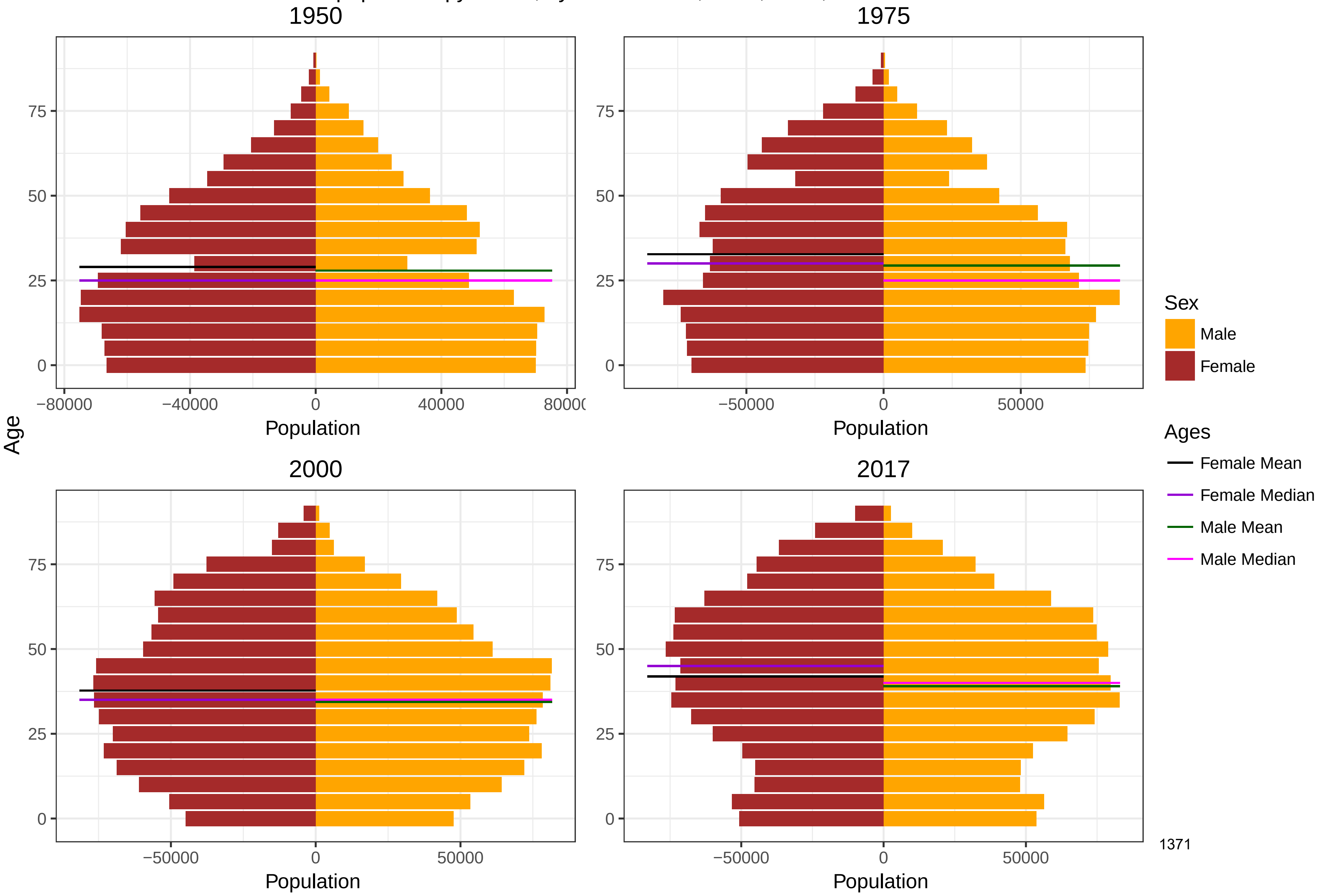

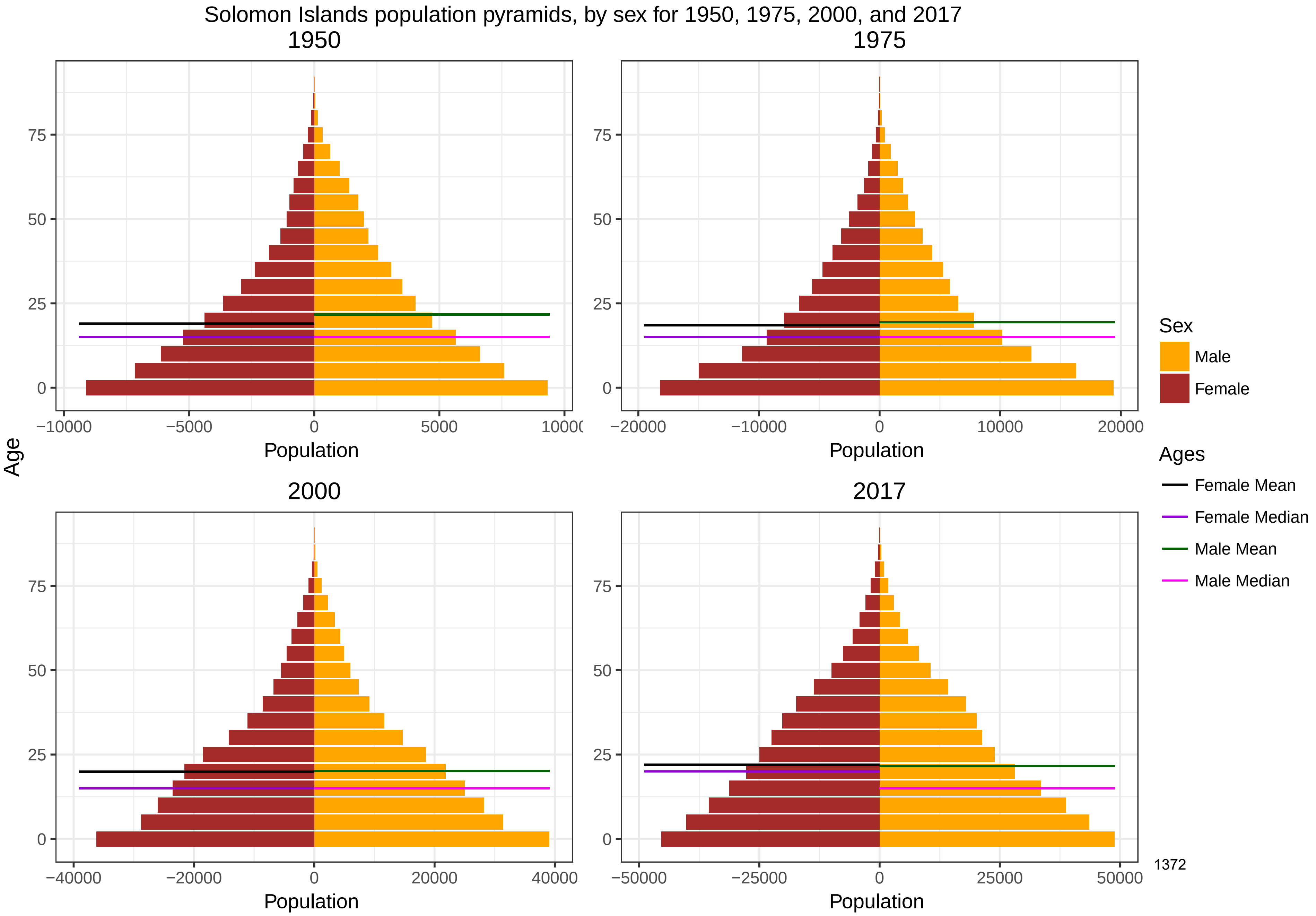

Solomon Islands population pyramids, by sex for 1950, 1975, 2000, and 2017
1950
1975
2000
2017
Age
Population
Sex
Male
Female
Ages
Female Mean
Female Median
Male Mean
Male Median

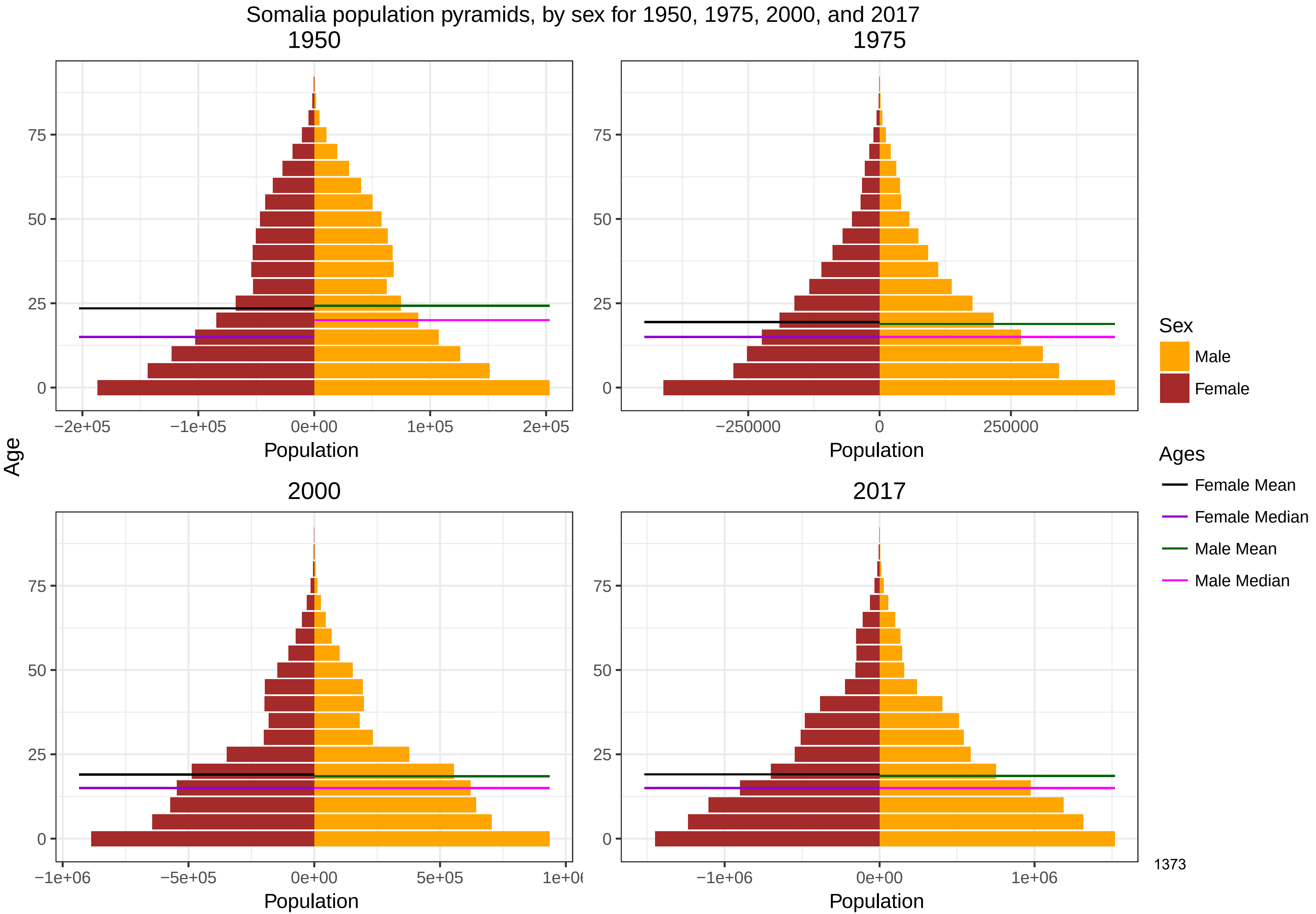
Somalia population pyramids, by sex for 1950, 1975, 2000, and 2017
1950
1975
2000
2017
Age
Population
Sex
Male
Female
Ages
Female Mean
Female Median
Male Mean
Male Median

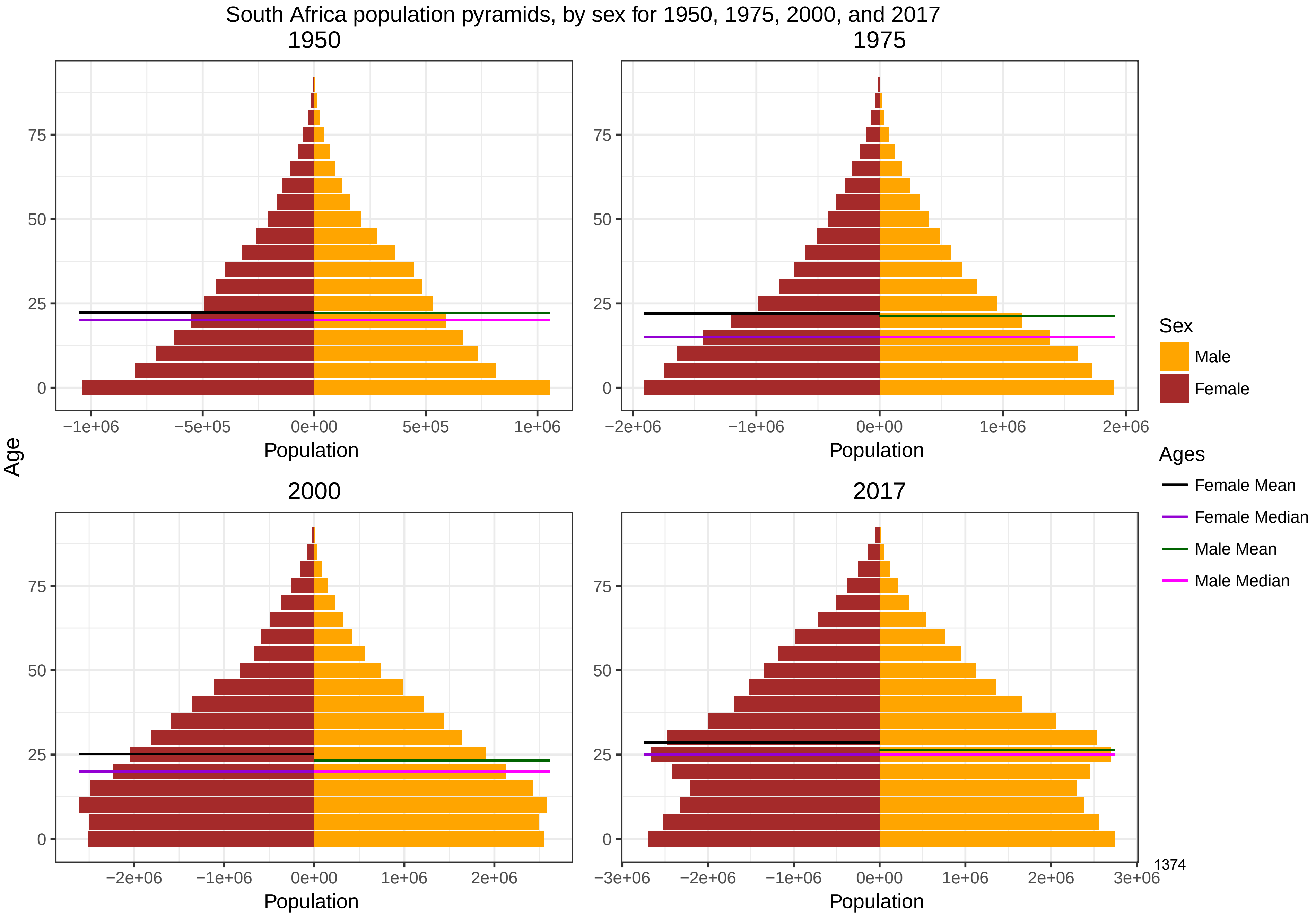
South Africa population pyramids, by sex for 1950, 1975, 2000, and 2017
1950
1975
2000
2017
Age
Population
75
50
25
0
−1e+06
−5e+05
0e+00
5e+05
1e+06
−2e+06
2e+06
−3e+06
3e+06
Sex
Male
Female
Ages
Female Mean
Female Median
Male Mean
Male Median

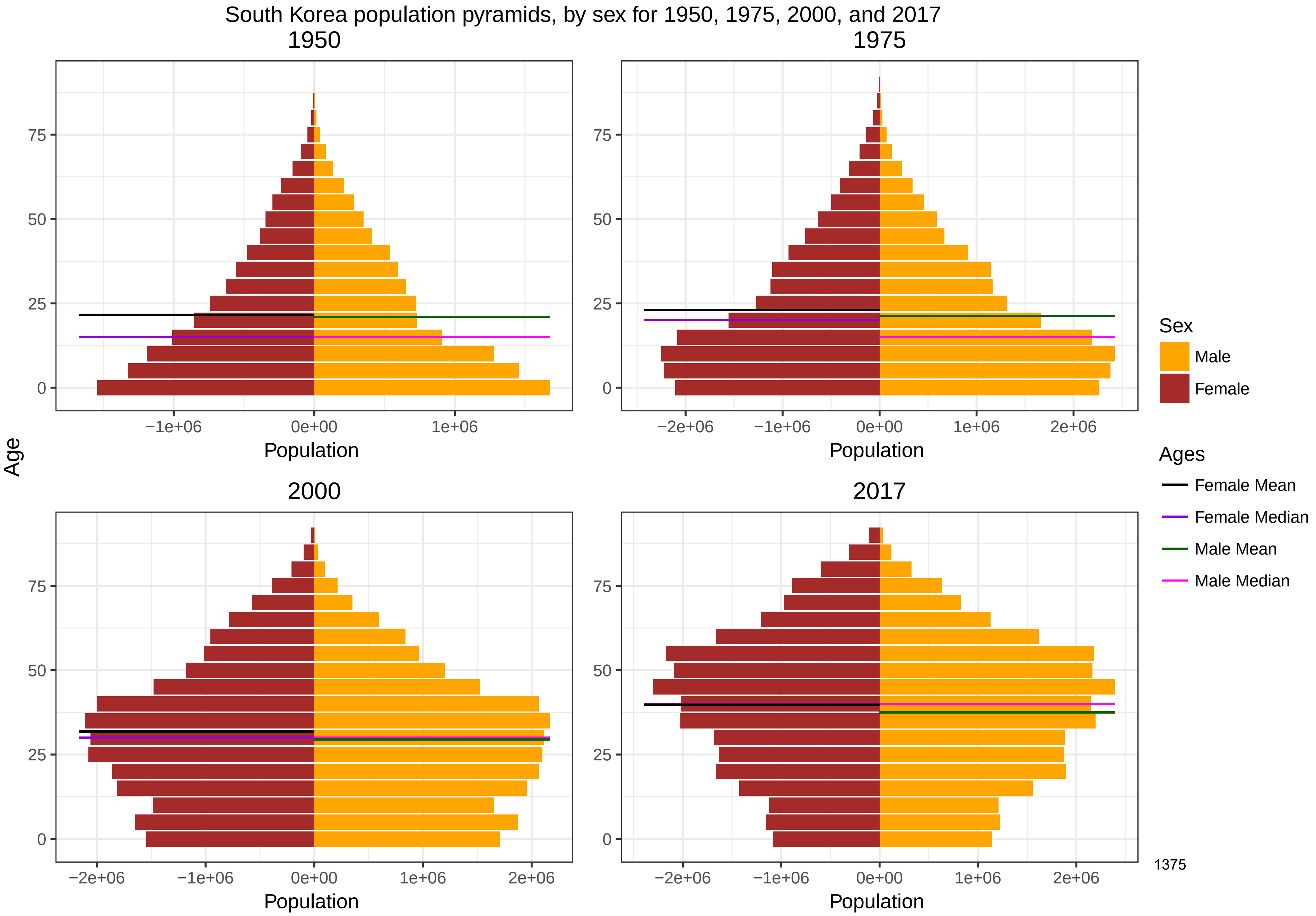

South Korea population pyramids, by sex for 1950, 1975, 2000, and 2017
1950
1975
2000
2017
Age
Population
0
25
50
75
−2e+06
−1e+06
0e+00
1e+06
2e+06
Sex
Male
Female
Ages
Female Mean
Female Median
Male Mean
Male Median

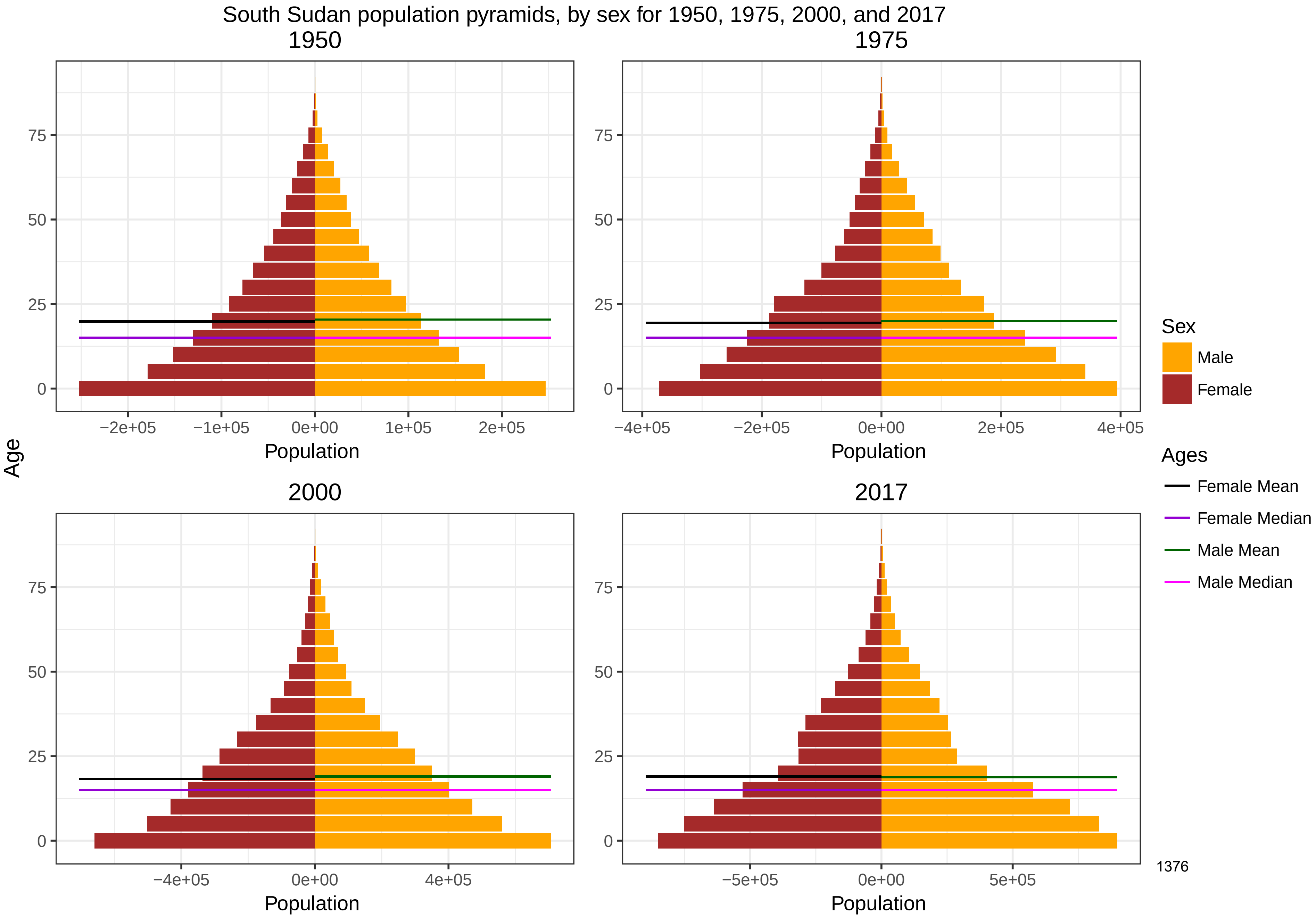

South Sudan population pyramids, by sex for 1950, 1975, 2000, and 2017
1950
1975
2000
2017
Age
Population
75
50
25
0
−2e+05
−1e+05
0e+00
1e+05
2e+05
−4e+05
4e+05
5e+05
−5e+05
Sex
Male
Female
Ages
Female Mean
Female Median
Male Mean
Male Median

1376

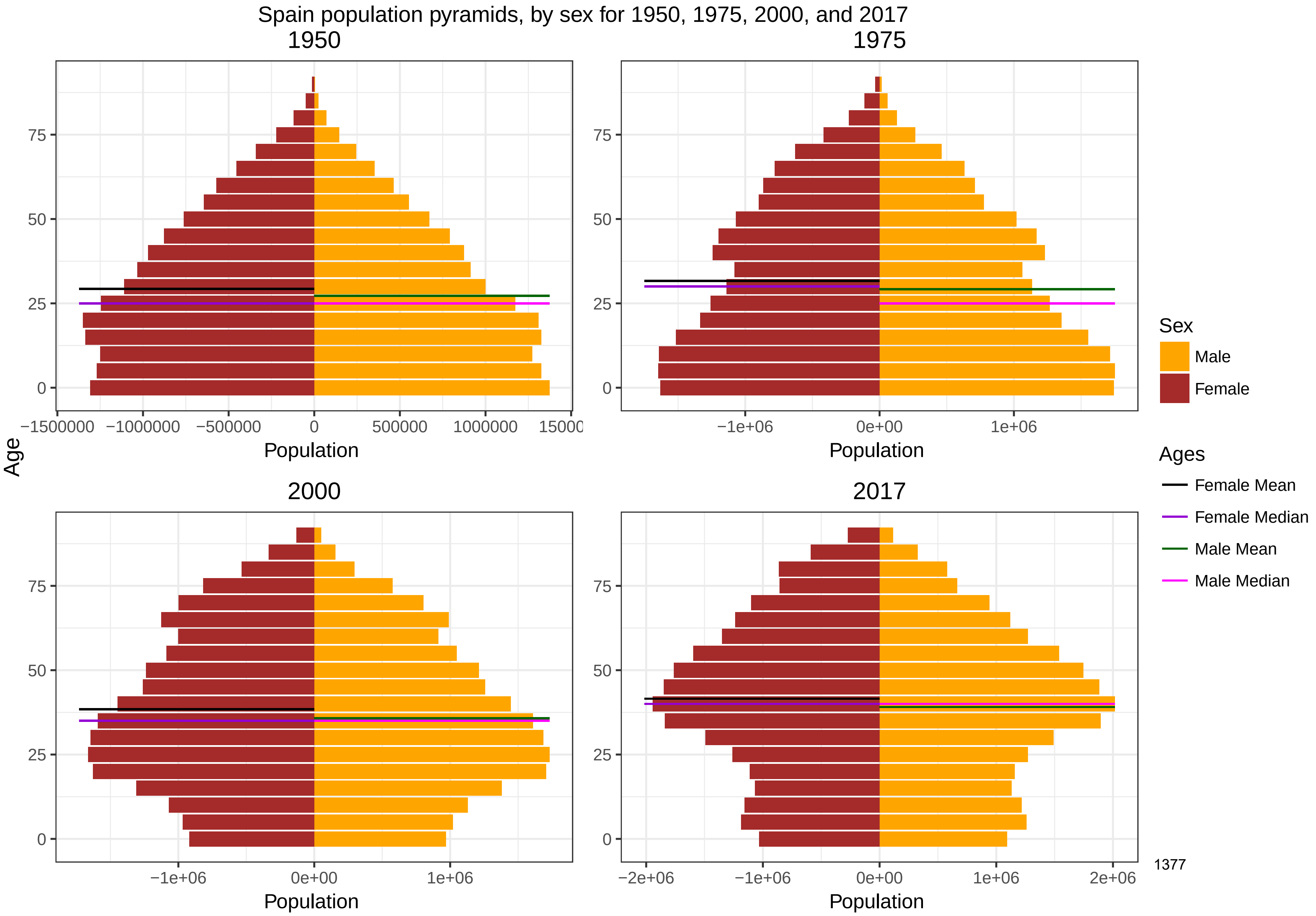
Spain population pyramids, by sex for 1950, 1975, 2000, and 2017
1950
1975
2000
2017
Age
Population
−1500000
−1000000
−500000
0
500000
1000000
15000
−2e+06
−1e+06
0e+00
1e+06
2e+06
0
25
50
75
Sex
Male
Female
Ages
Female Mean
Female Median
Male Mean
Male Median

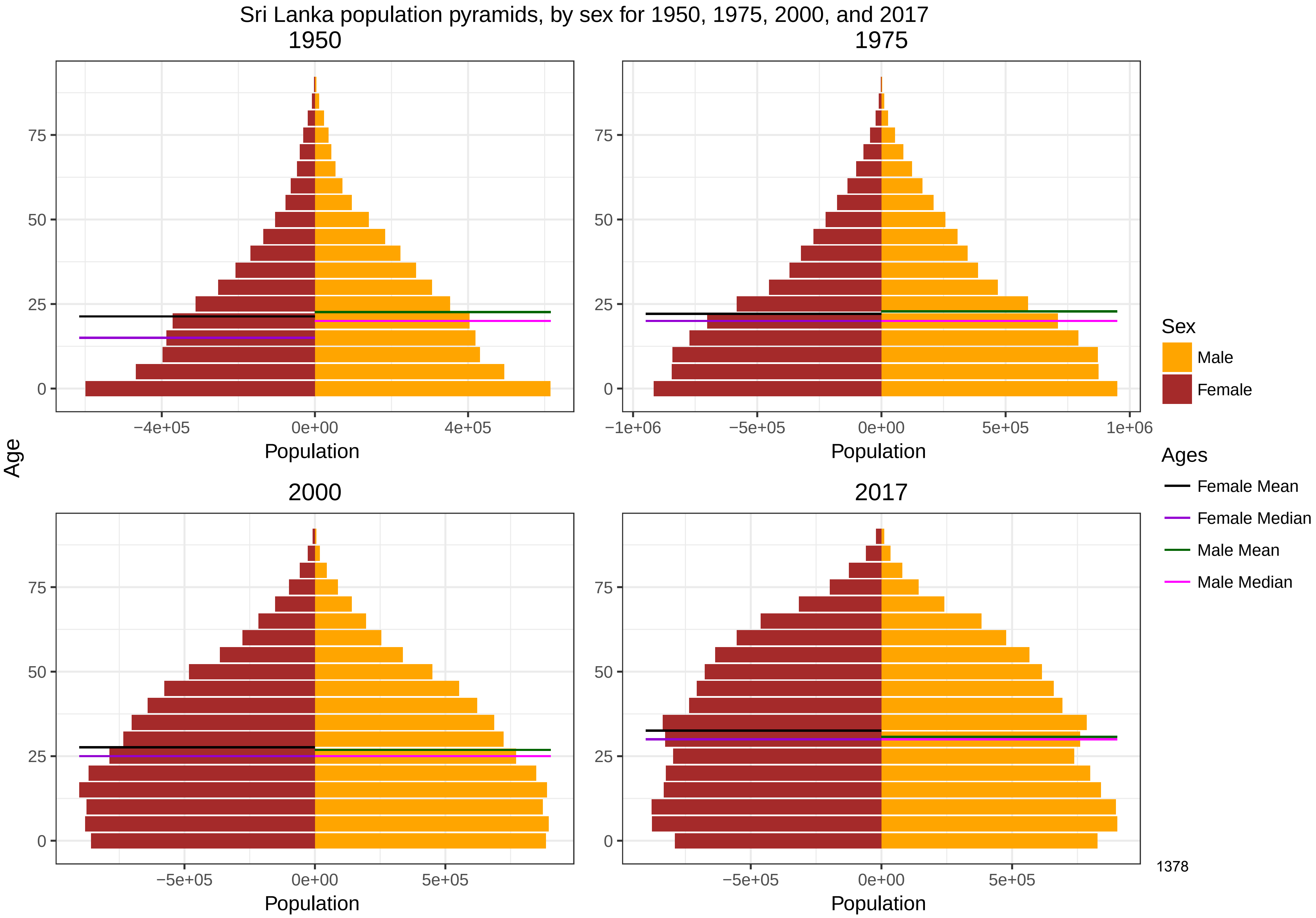
Sri Lanka population pyramids, by sex for 1950, 1975, 2000, and 2017
1950
1975
2000
2017
Age
Population
0
25
50
75
−4e+05
0e+00
4e+05
−1e+06
−5e+05
5e+05
1e+06
Sex
Male
Female
Ages
Female Mean
Female Median
Male Mean
Male Median

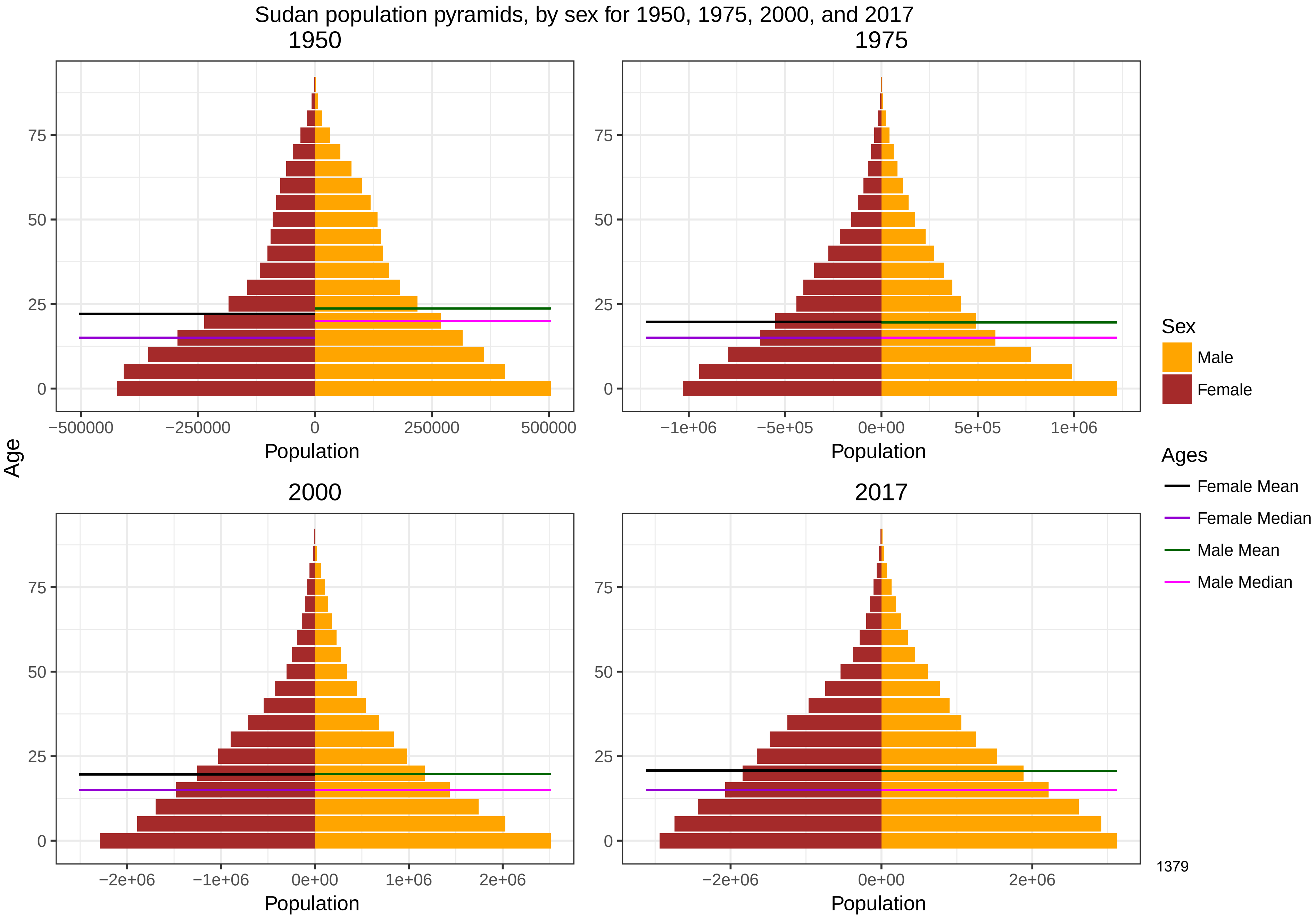
Sudan population pyramids, by sex for 1950, 1975, 2000, and 2017
1950
1975
2000
2017
Age
Population
Sex
Male
Female
Ages
Female Mean
Female Median
Male Mean
Male Median

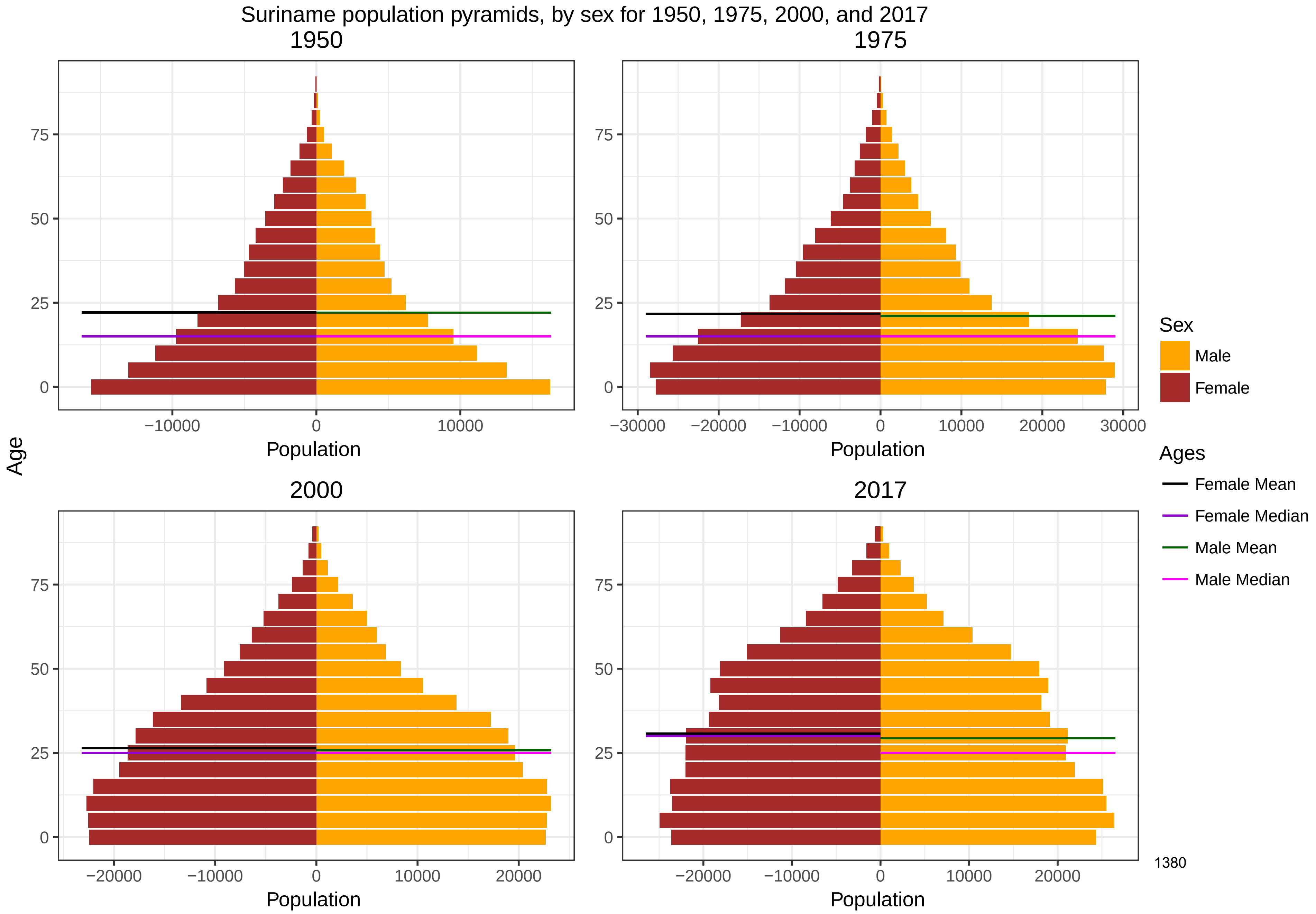
Suriname population pyramids, by sex for 1950, 1975, 2000, and 2017
1950
1975
2000
2017
Age
Population
Sex
Male
Female
Ages
Female Mean
Female Median
Male Mean
Male Median
0
25
50
75
−30000
−20000
−10000
0
10000
20000
30000

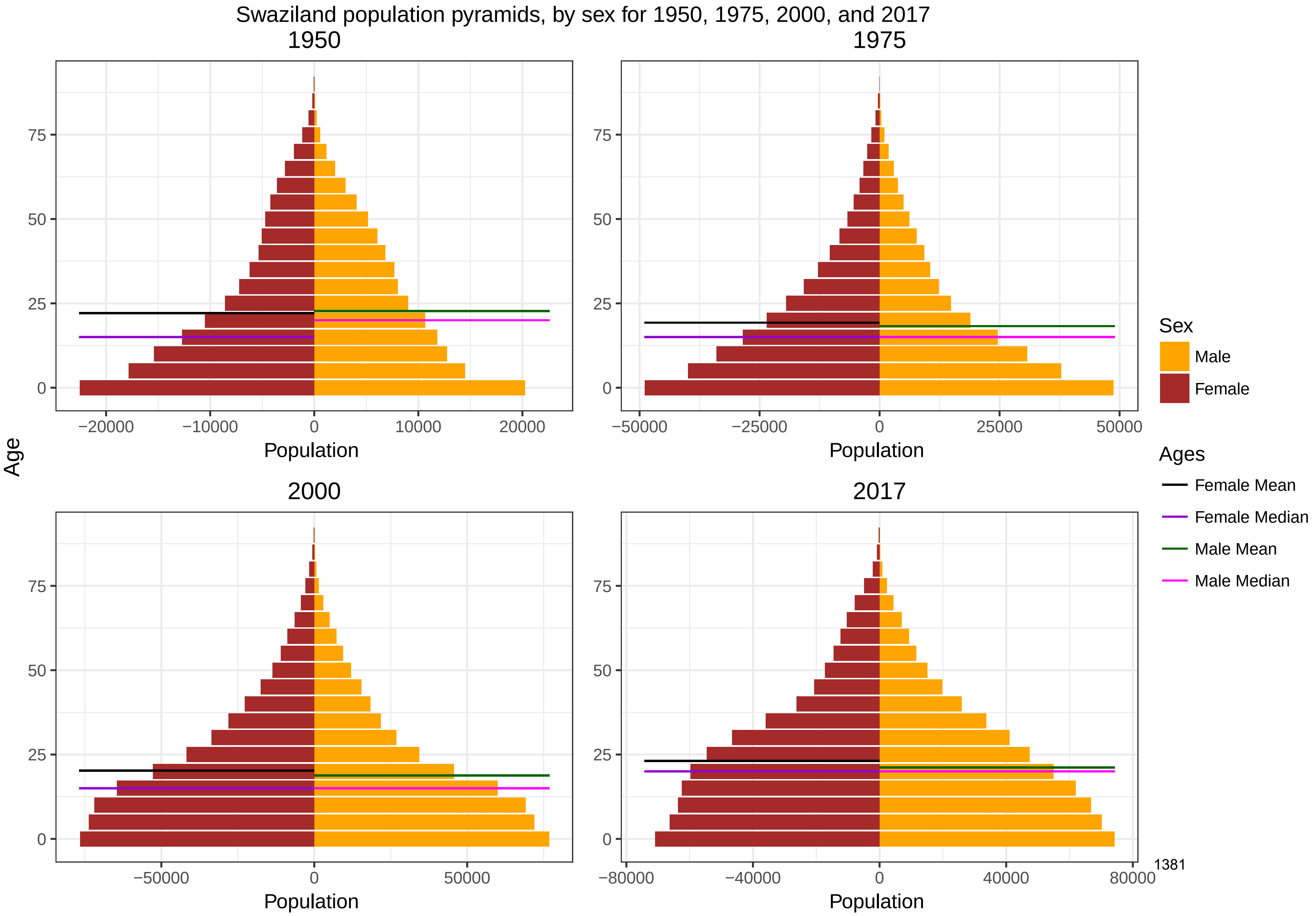

Swaziland population pyramids, by sex for 1950, 1975, 2000, and 2017
1950
1975
2000
2017
Age
Population
Sex
Male
Female
Ages
Female Mean
Female Median
Male Mean
Male Median

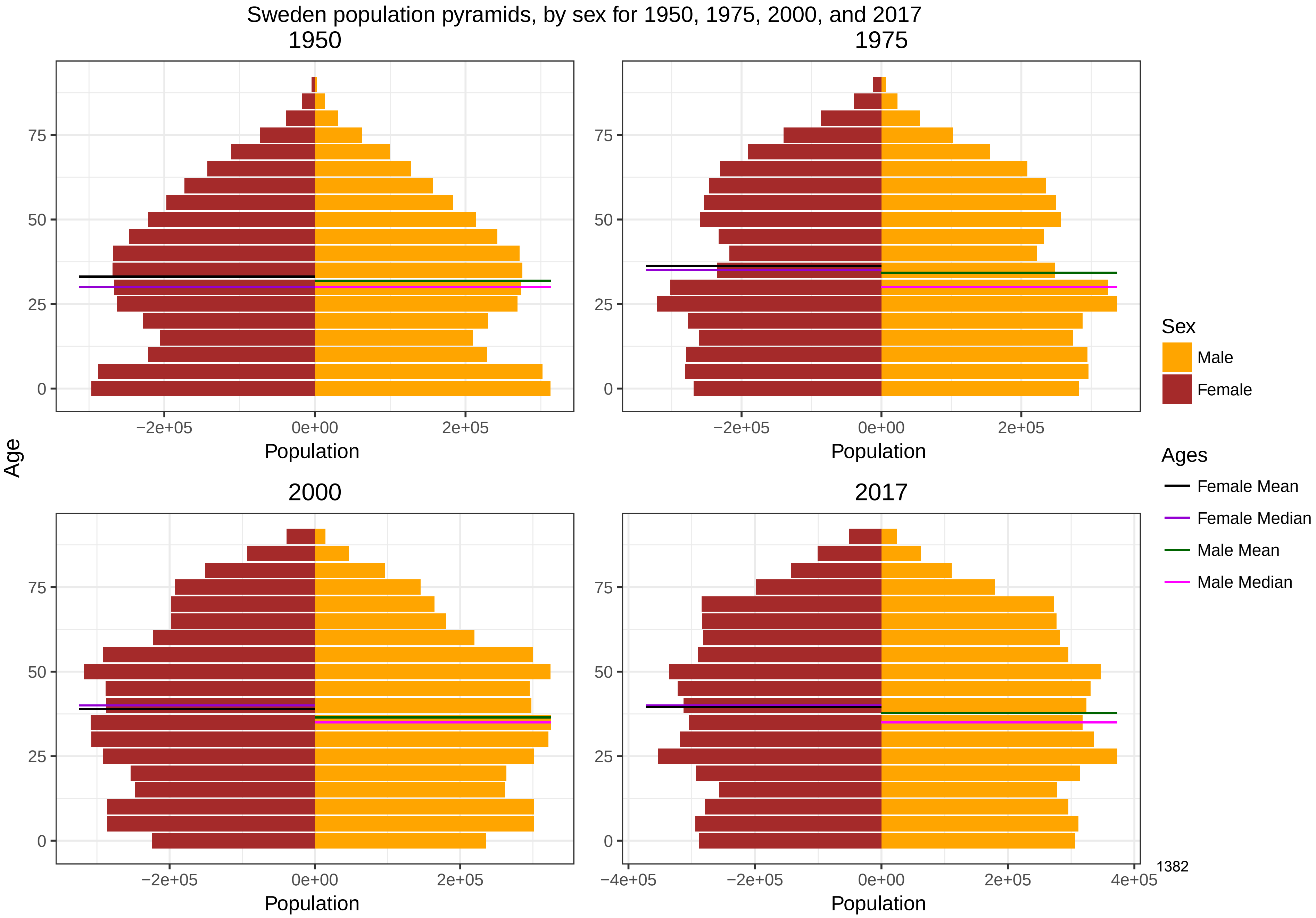

Sweden population pyramids, by sex for 1950, 1975, 2000, and 2017
1950
1975
2000
2017
Age
Population
0
25
50
75
−4e+05
−2e+05
0e+00
2e+05
4e+05
Sex
Male
Female
Ages
Female Mean
Female Median
Male Mean
Male Median

Switzerland population pyramids, by sex for 1950, 1975, 2000, and 2017

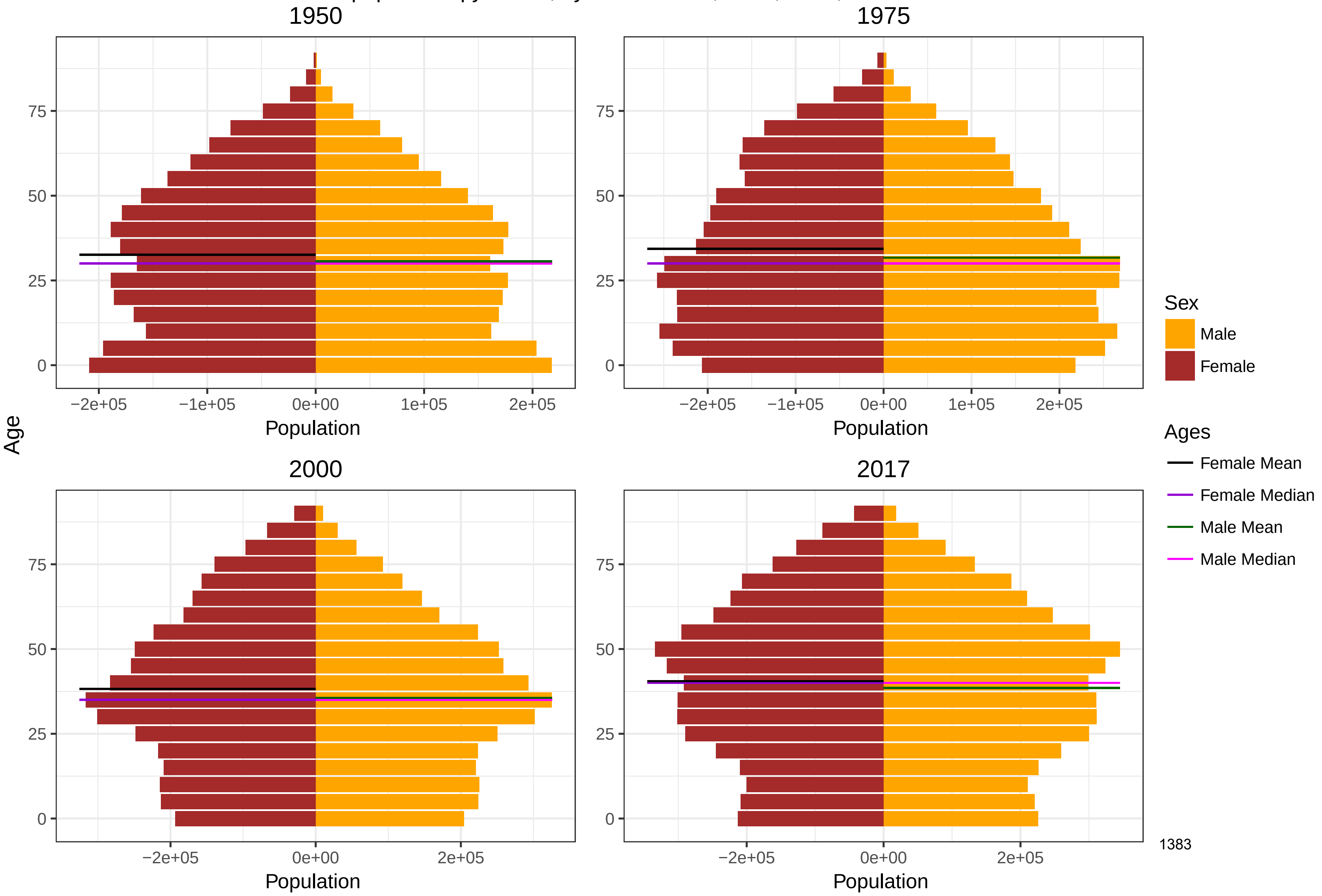

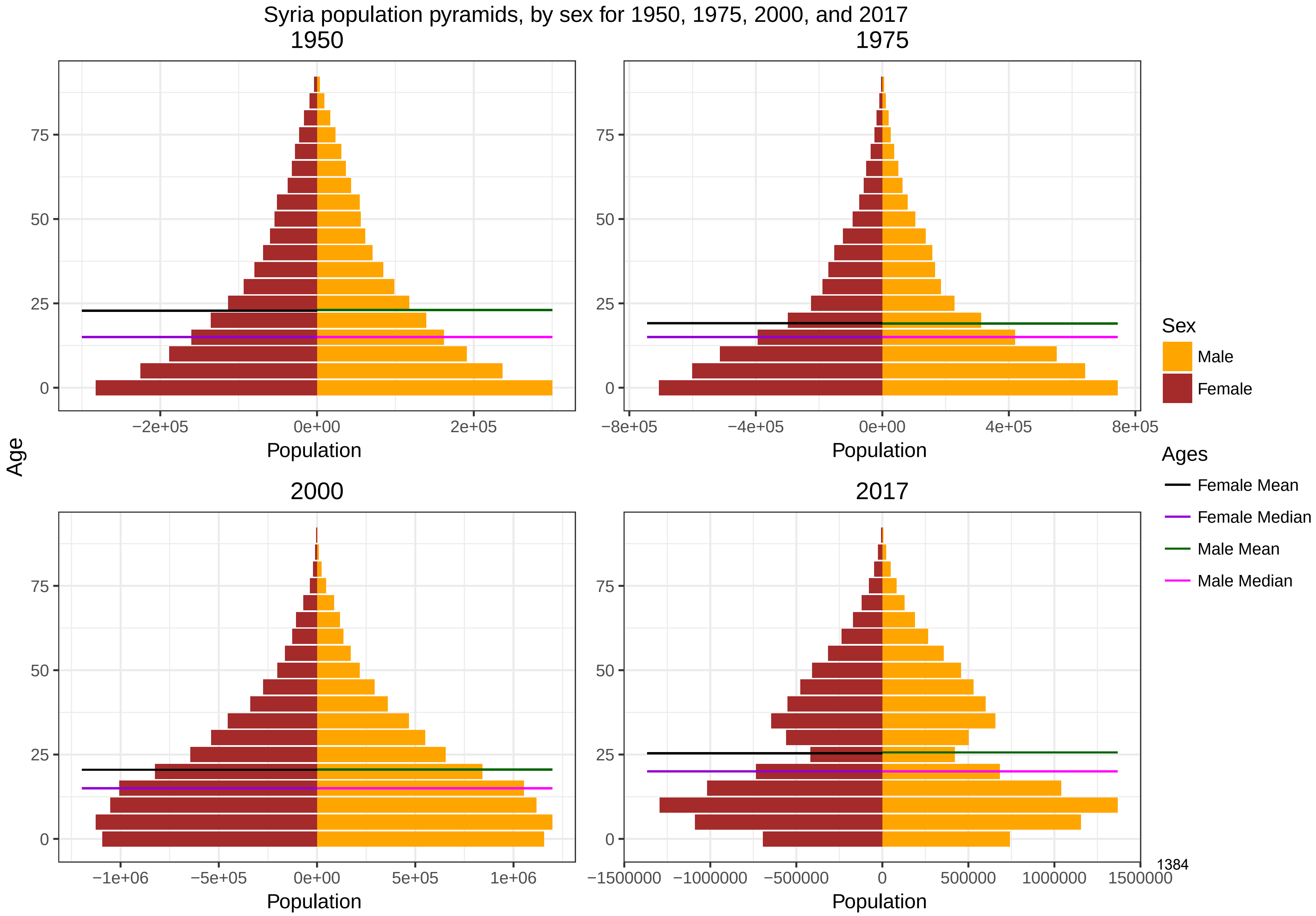
Syria population pyramids, by sex for 1950, 1975, 2000, and 2017
1950
1975
2000
2017
Age
Population
75
50
25
0
−2e+05
0e+00
2e+05
−8e+05
−4e+05
4e+05
8e+05
−1e+06
−5e+05
5e+05
1e+06
−1500000
−1000000
−500000
500000
1000000
1500000
Sex
Male
Female
Ages
Female Mean
Female Median
Male Mean
Male Median

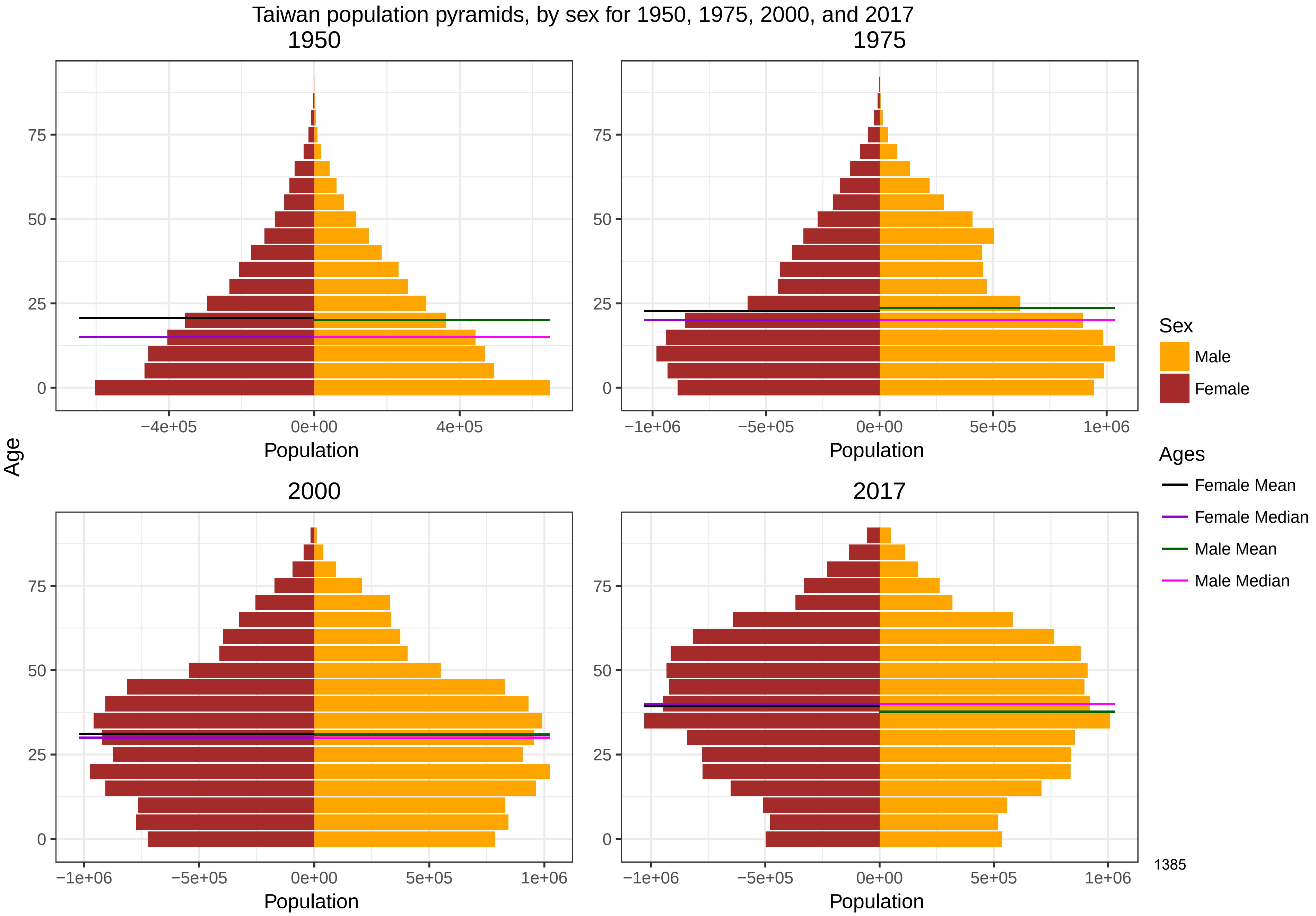

Taiwan population pyramids, by sex for 1950, 1975, 2000, and 2017
1950
1975
2000
2017
Age
Population
Sex
Male
Female
Ages
Female Mean
Female Median
Male Mean
Male Median

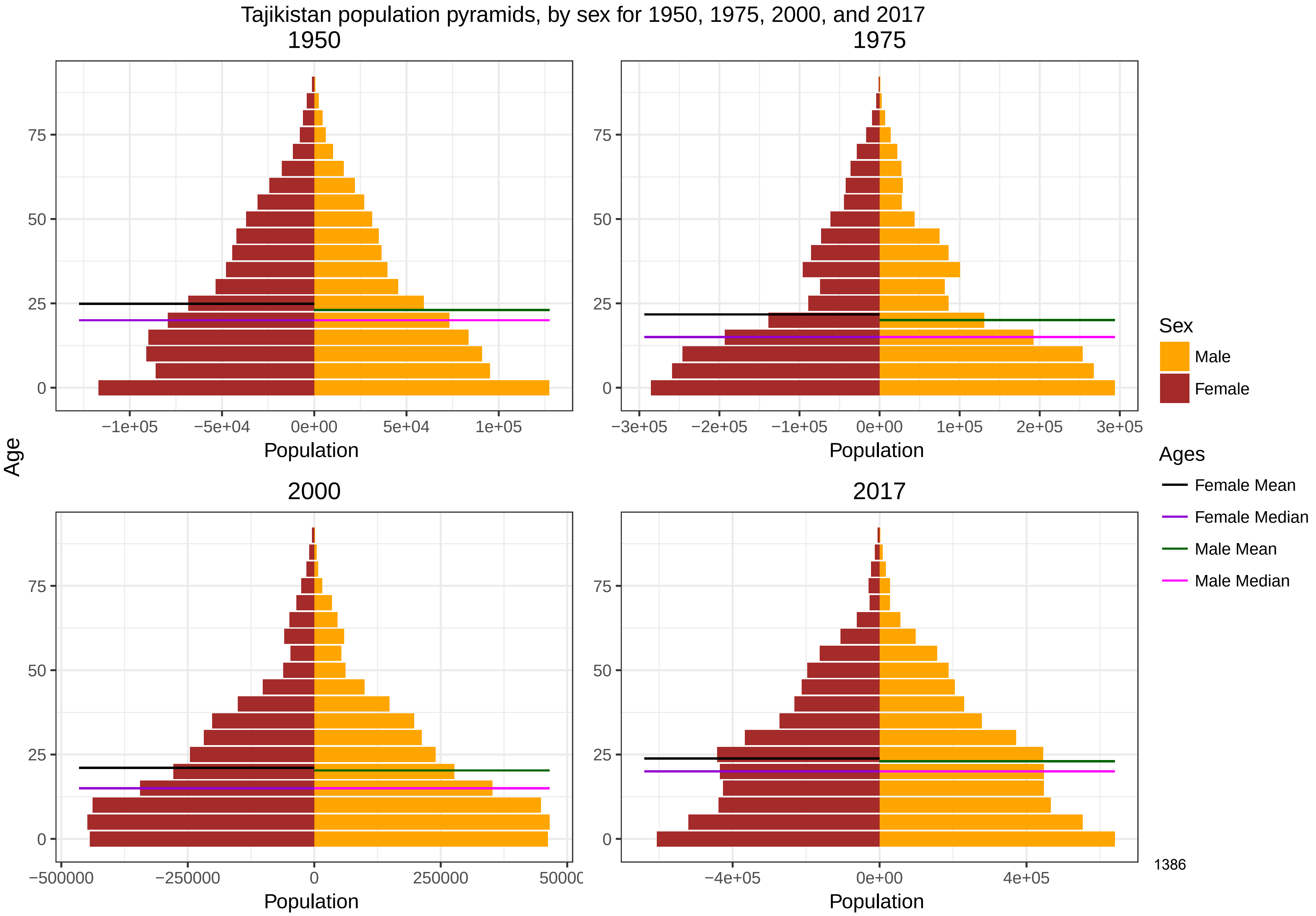
Tajikistan population pyramids, by sex for 1950, 1975, 2000, and 2017
1950
1975
2000
2017
Age
Population
0
25
50
75
−1e+05
−5e+04
0e+00
5e+04
1e+05
−3e+05
−2e+05
−1e+05
1e+05
2e+05
3e+05
−500000
−250000
250000
−4e+05
4e+05
Sex
Male
Female
Ages
Female Mean
Female Median
Male Mean
Male Median

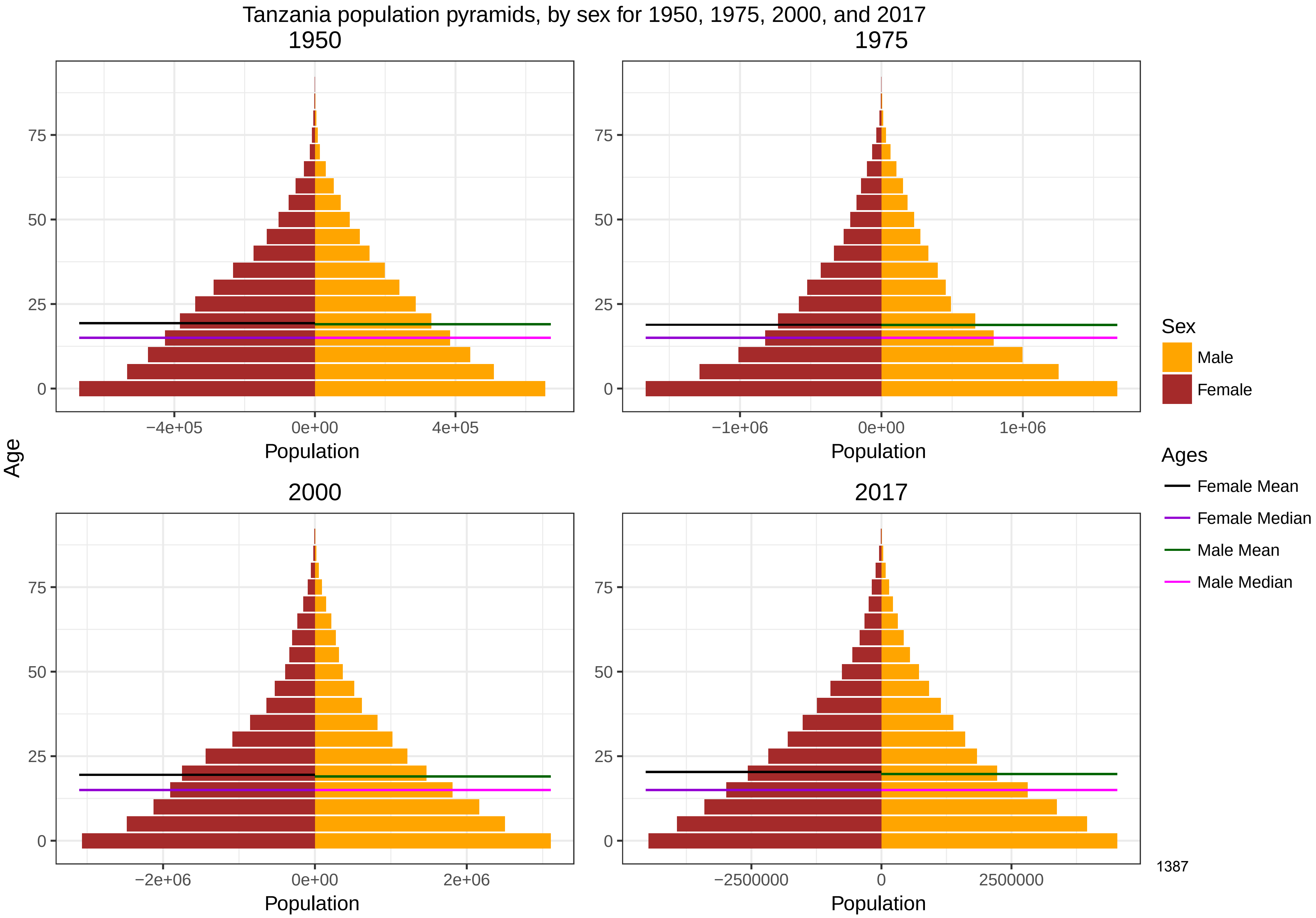

Tanzania population pyramids, by sex for 1950, 1975, 2000, and 2017
1950
1975
2000
2017
Age
Population
−4e+05
0e+00
4e+05
−1e+06
1e+06
−2e+06
2e+06
−2500000
0
2500000
75
50
25
0
Sex
Male
Female
Ages
Female Mean
Female Median
Male Mean
Male Median

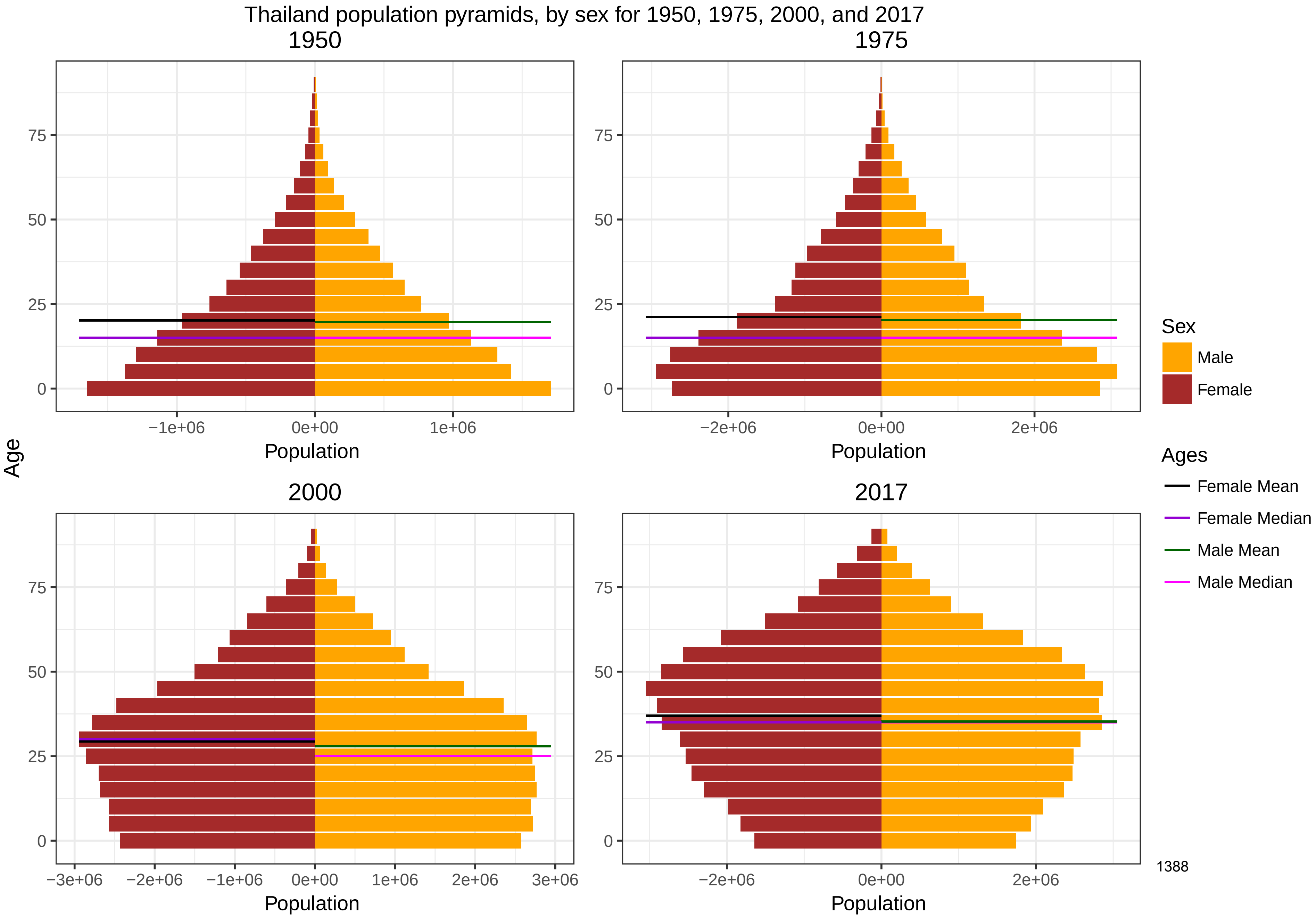
Thailand population pyramids, by sex for 1950, 1975, 2000, and 2017
1950
1975
2000
2017
Age
Population
0
25
50
75
−1e+06
0e+00
1e+06
−2e+06
2e+06
−3e+06
3e+06
Sex
Male
Female
Ages
Female Mean
Female Median
Male Mean
Male Median

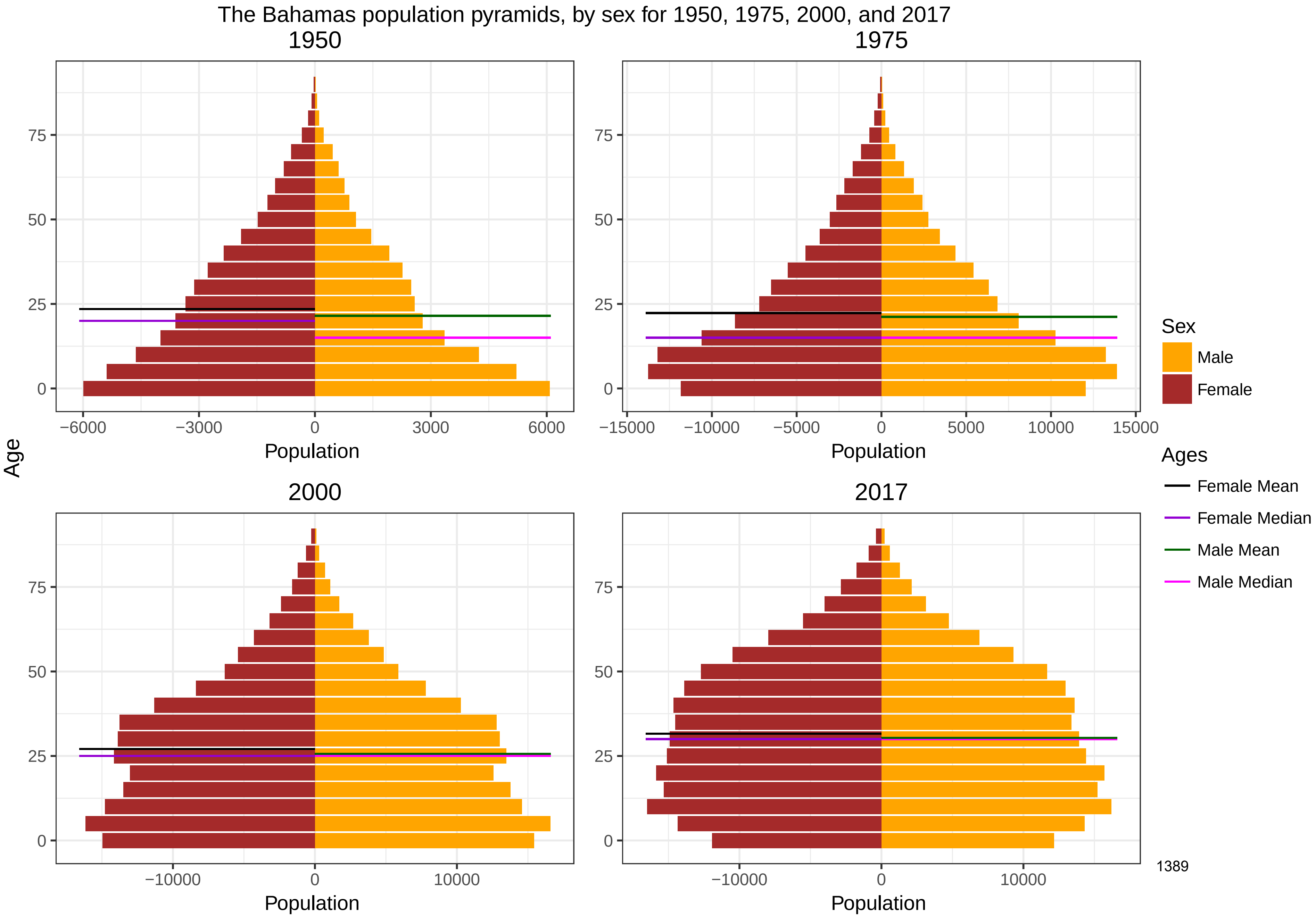
The Bahamas population pyramids, by sex for 1950, 1975, 2000, and 2017
1950
1975
2000
2017
Age
Population
Sex
Male
Female
Ages
Female Mean
Female Median
Male Mean
Male Median

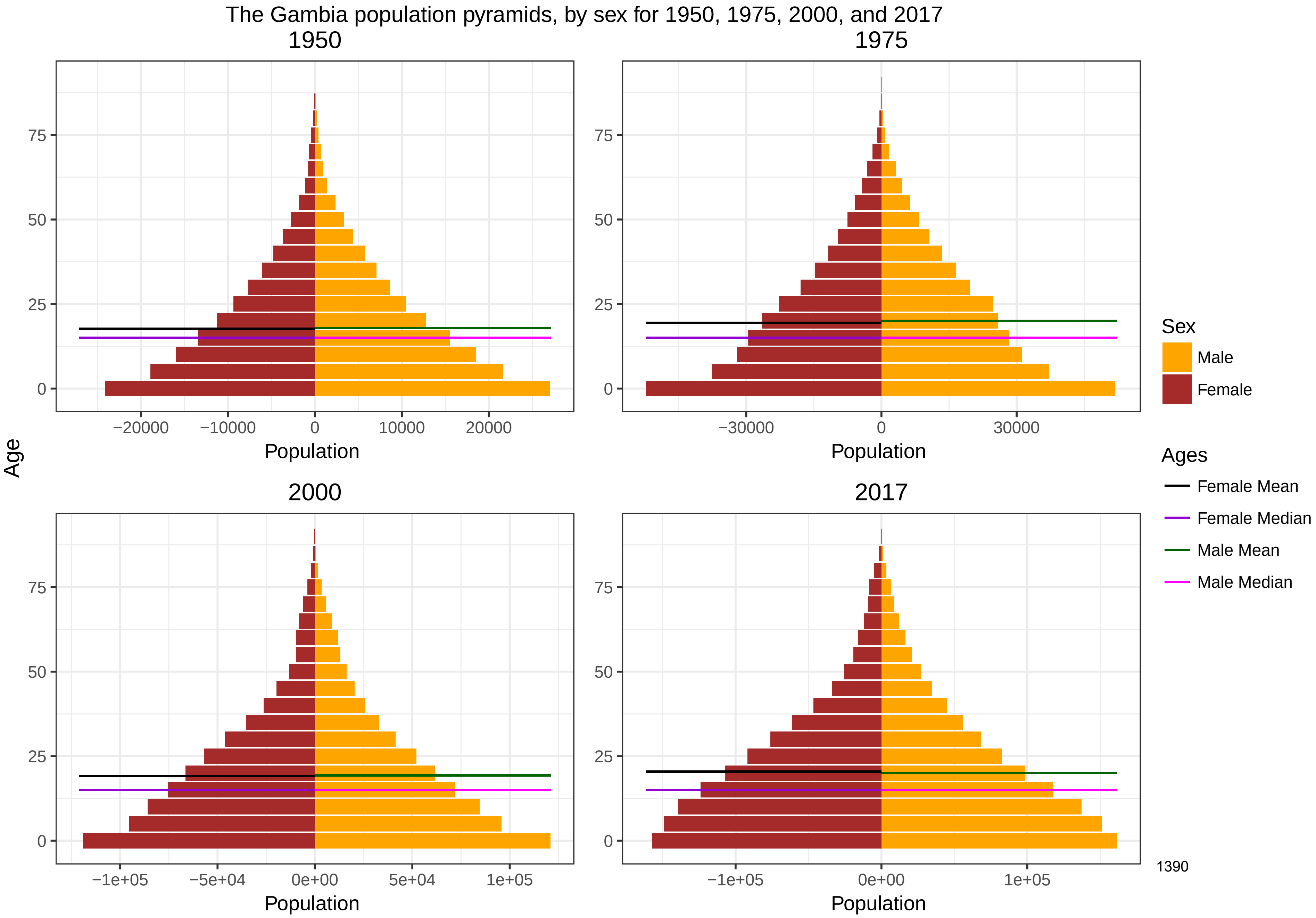
The Gambia population pyramids, by sex for 1950, 1975, 2000, and 2017
1950
1975
2000
2017
Age
Population
Sex
Male
Female
Ages
Female Mean
Female Median
Male Mean
Male Median
−20000
−10000
0
10000
20000
−30000
30000
−1e+05
−5e+04
0e+00
5e+04
1e+05
0
25
50
75

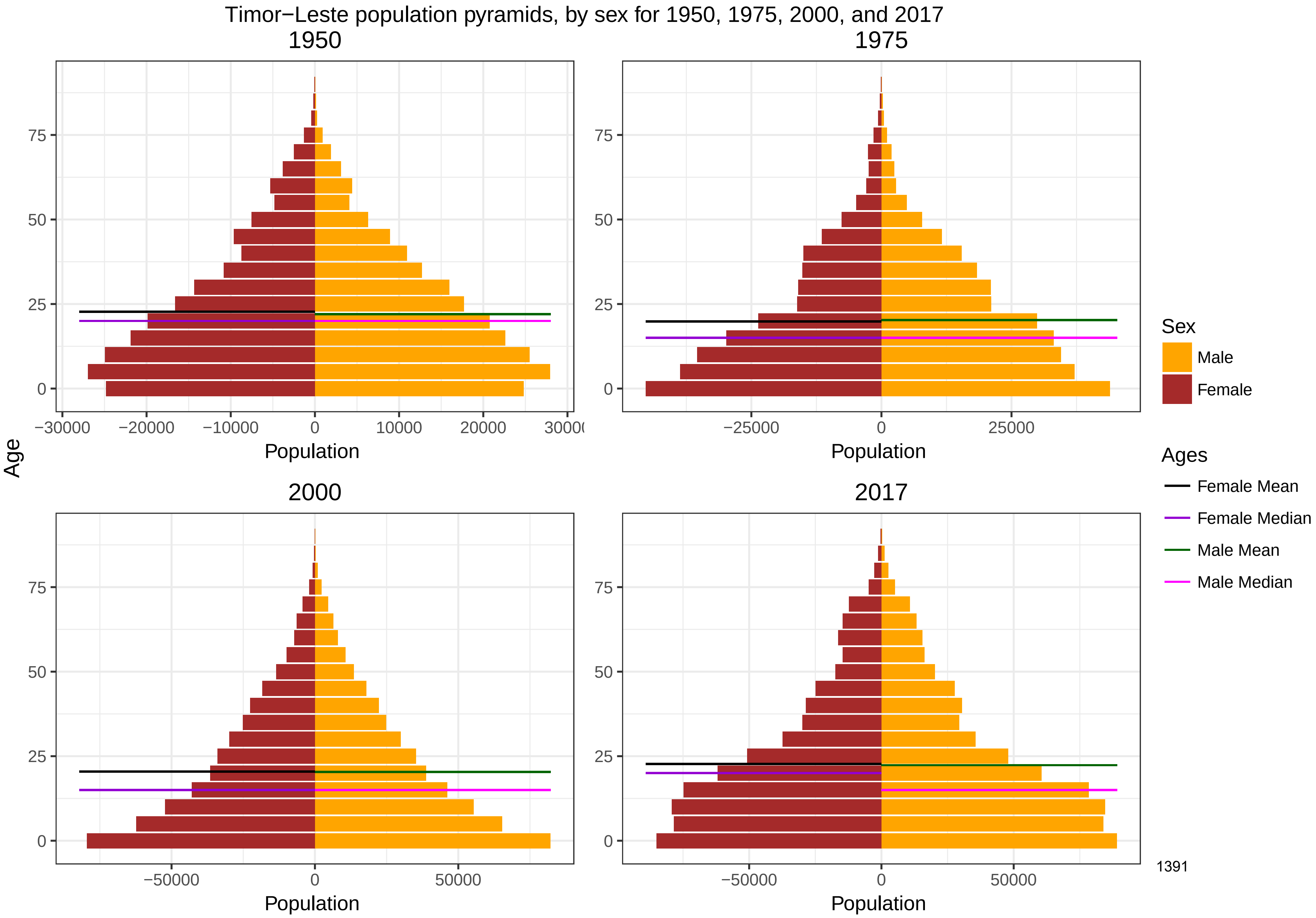

Timor−Leste population pyramids, by sex for 1950, 1975, 2000, and 2017
1950
1975
2000
2017
Age
Population
Sex
Male
Female
Ages
Female Mean
Female Median
Male Mean
Male Median

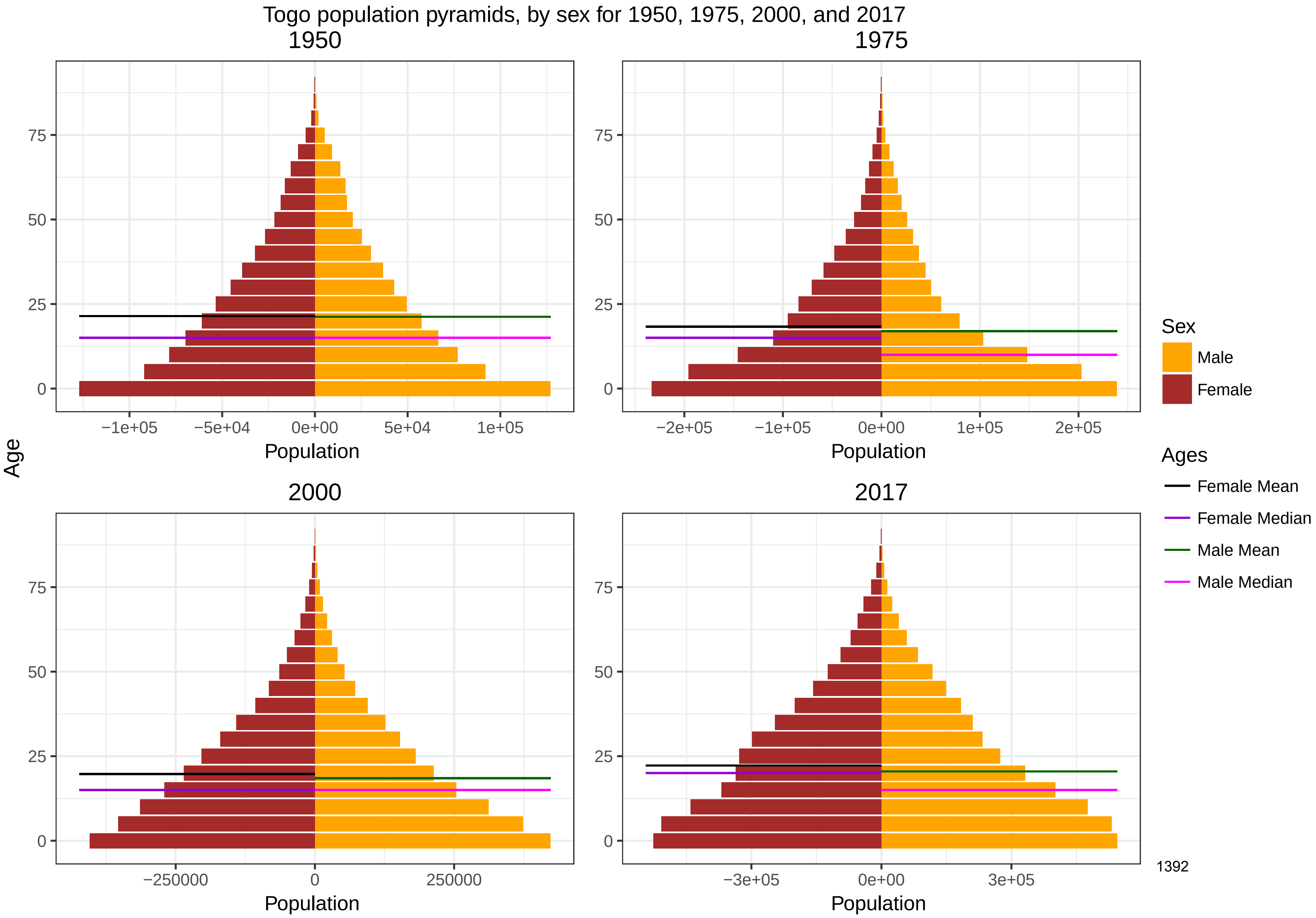

Togo population pyramids, by sex for 1950, 1975, 2000, and 2017
1950
1975
2000
2017
Age
Population
0
25
50
75
−1e+05
−5e+04
0e+00
5e+04
1e+05
−2e+05
2e+05
−250000
250000
−3e+05
3e+05
Sex
Male
Female
Ages
Female Mean
Female Median
Male Mean
Male Median

1392

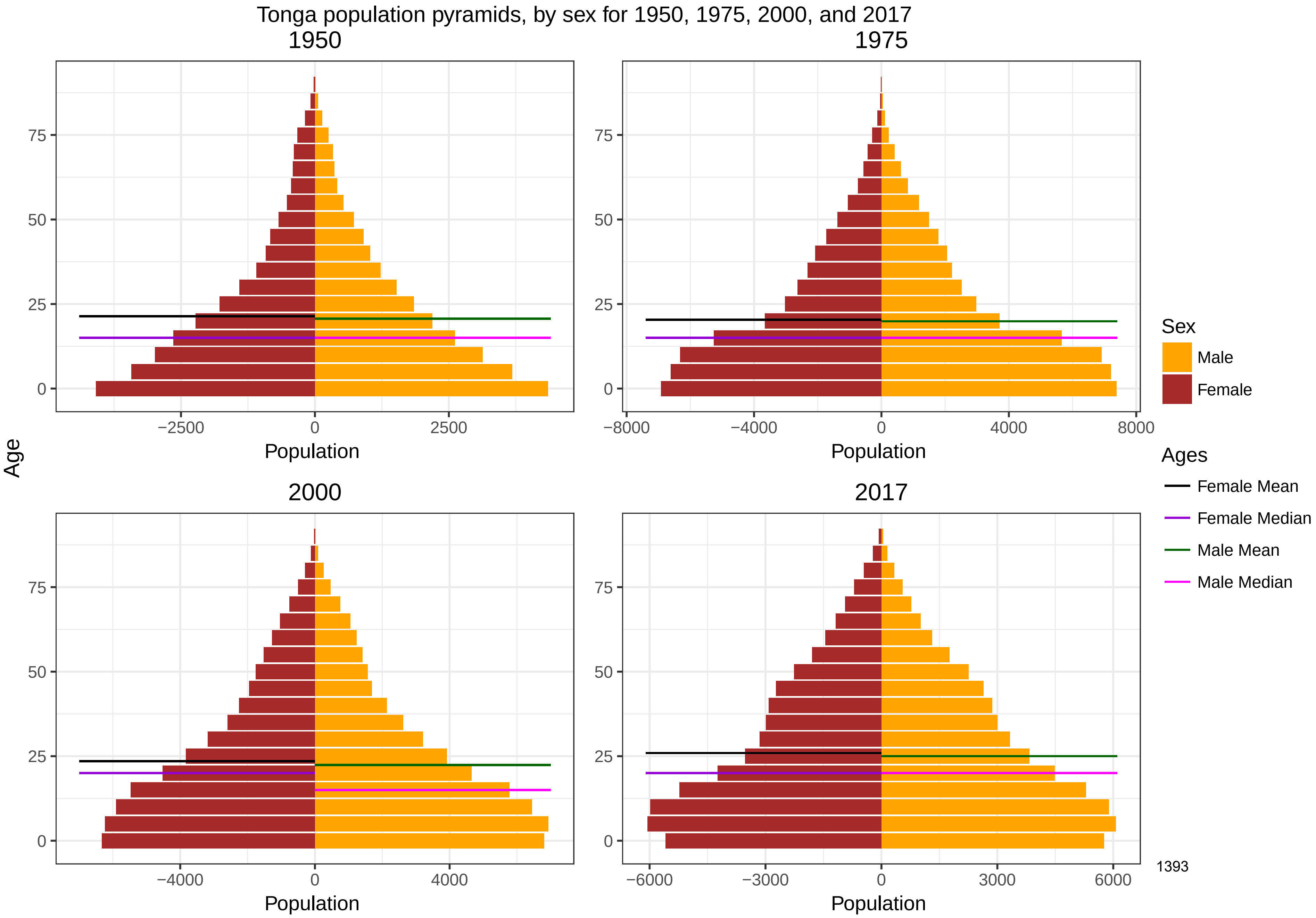
Tonga population pyramids, by sex for 1950, 1975, 2000, and 2017
1950
1975
2000
2017
Age
Population
Sex
Male
Female
Ages
Female Mean
Female Median
Male Mean
Male Median

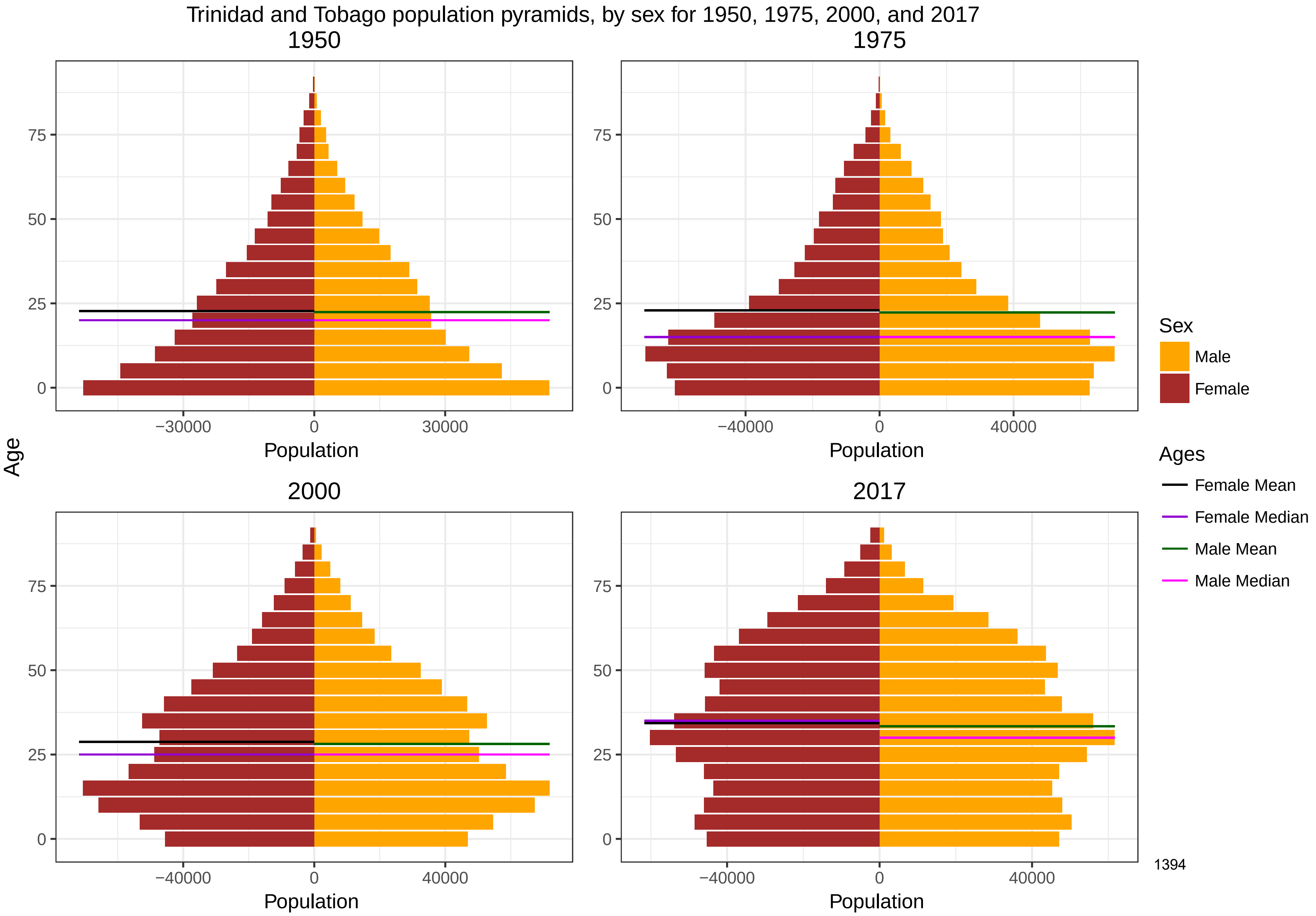
Trinidad and Tobago population pyramids, by sex for 1950, 1975, 2000, and 2017
1950
1975
2000
2017
Age
Population
−30000
0
30000
−40000
40000
0
25
50
75
Sex
Male
Female
Ages
Female Mean
Female Median
Male Mean
Male Median

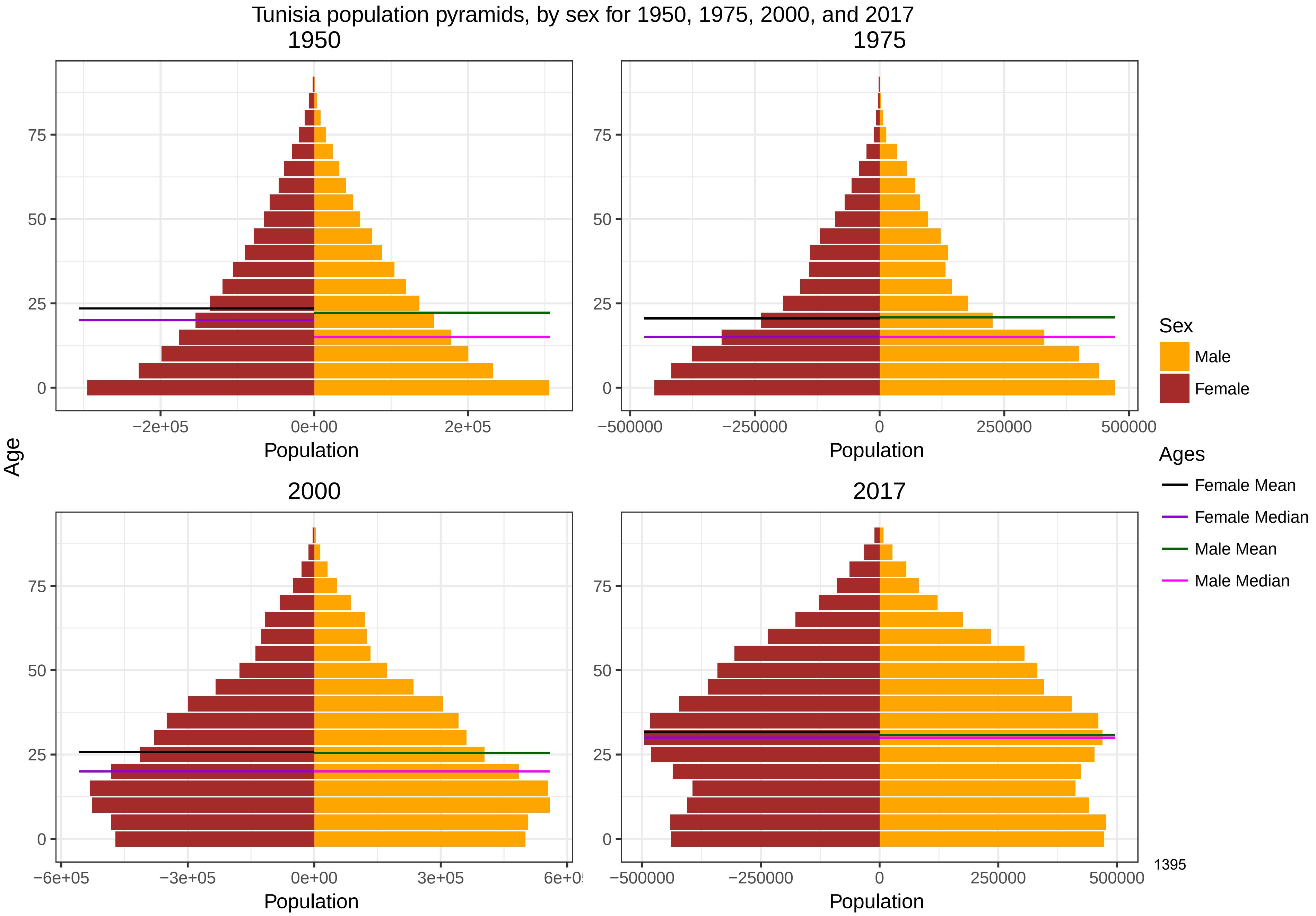

Tunisia population pyramids, by sex for 1950, 1975, 2000, and 2017
1950
1975
2000
2017
Age
Population
Sex
Male
Female
Ages
Female Mean
Female Median
Male Mean
Male Median

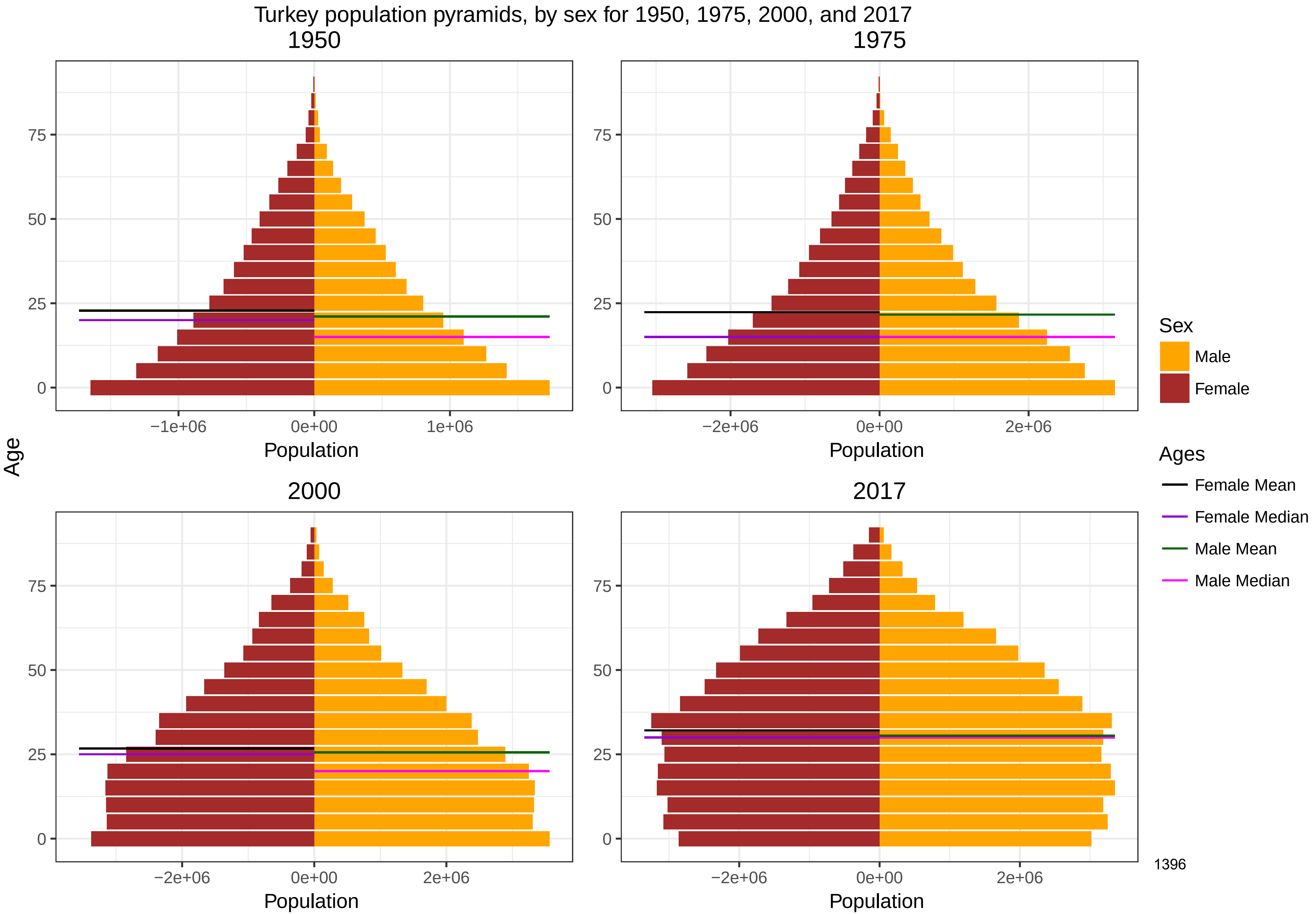
Turkey population pyramids, by sex for 1950, 1975, 2000, and 2017
1950
1975
2000
2017
Age
Population
−1e+06
0e+00
1e+06
−2e+06
2e+06
0
25
50
75
Sex
Male
Female
Ages
Female Mean
Female Median
Male Mean
Male Median

Turkmenistan population pyramids, by sex for 1950, 1975, 2000, and 2017

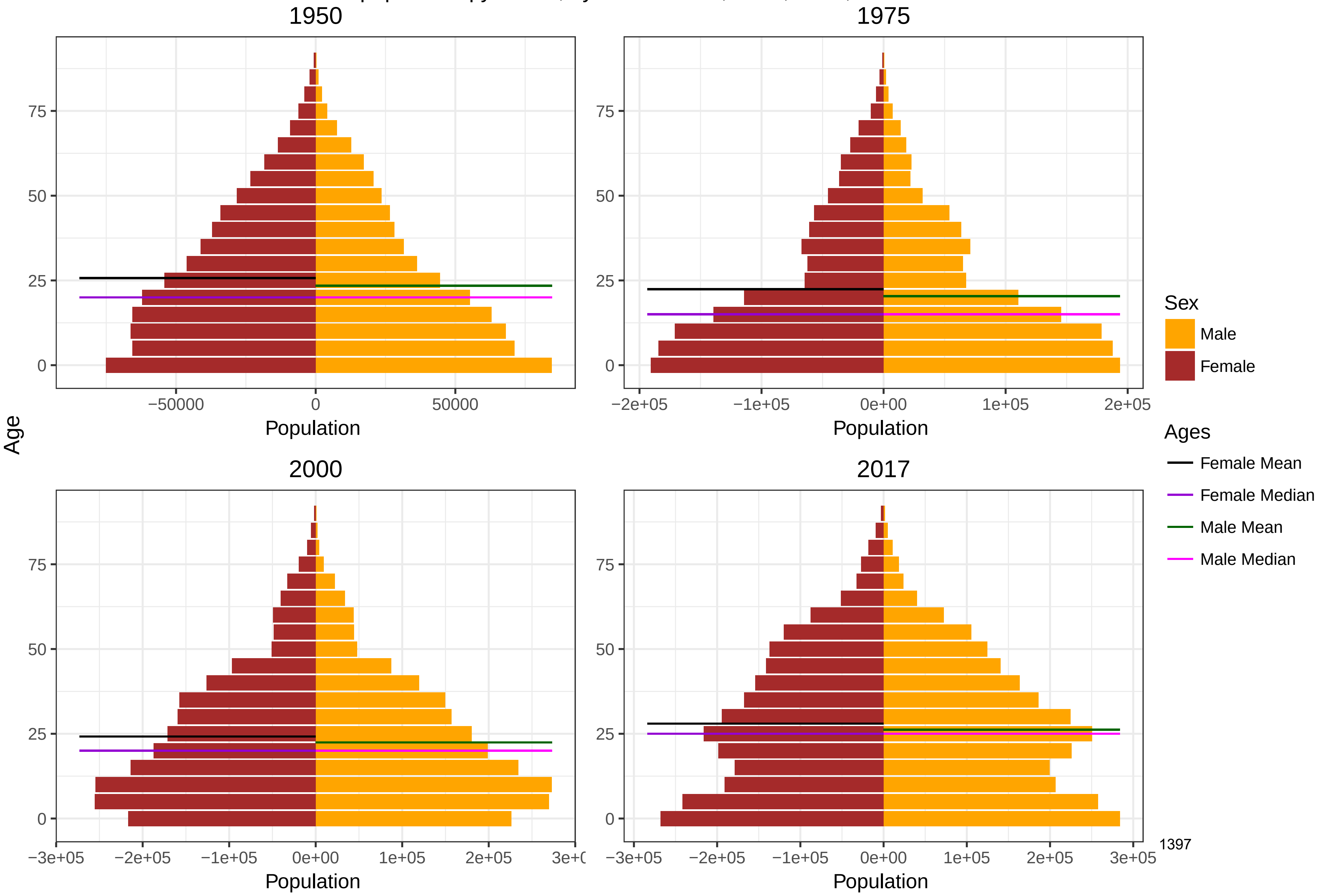

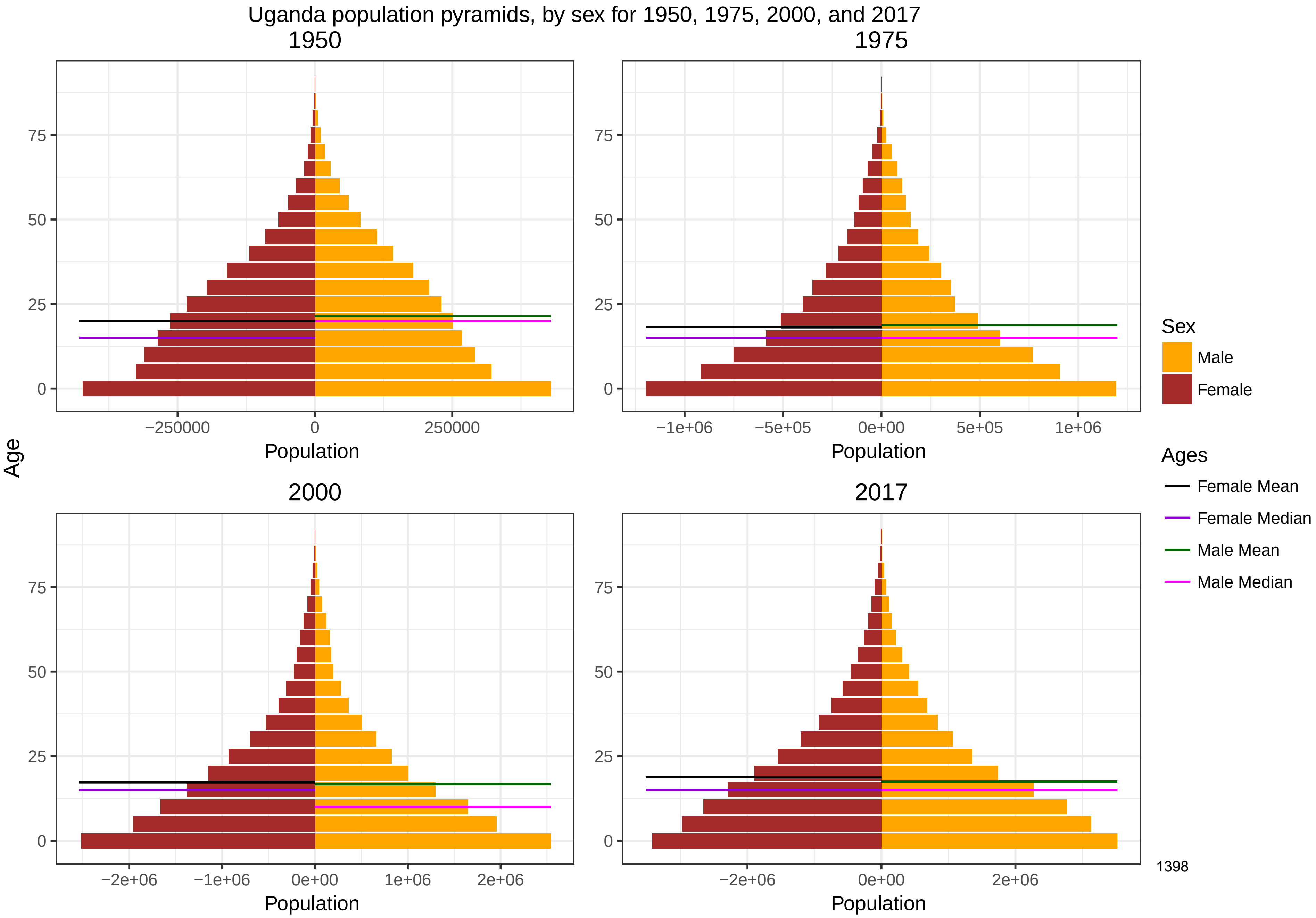
Uganda population pyramids, by sex for 1950, 1975, 2000, and 2017
1950
1975
2000
2017
Age
Population
0
25
50
75
−250000
0
250000
−1e+06
−5e+05
0e+00
5e+05
1e+06
−2e+06
−1e+06
0e+00
1e+06
2e+06
−2e+06
0e+00
2e+06
Sex
Male
Female
Ages
Female Mean
Female Median
Male Mean
Male Median

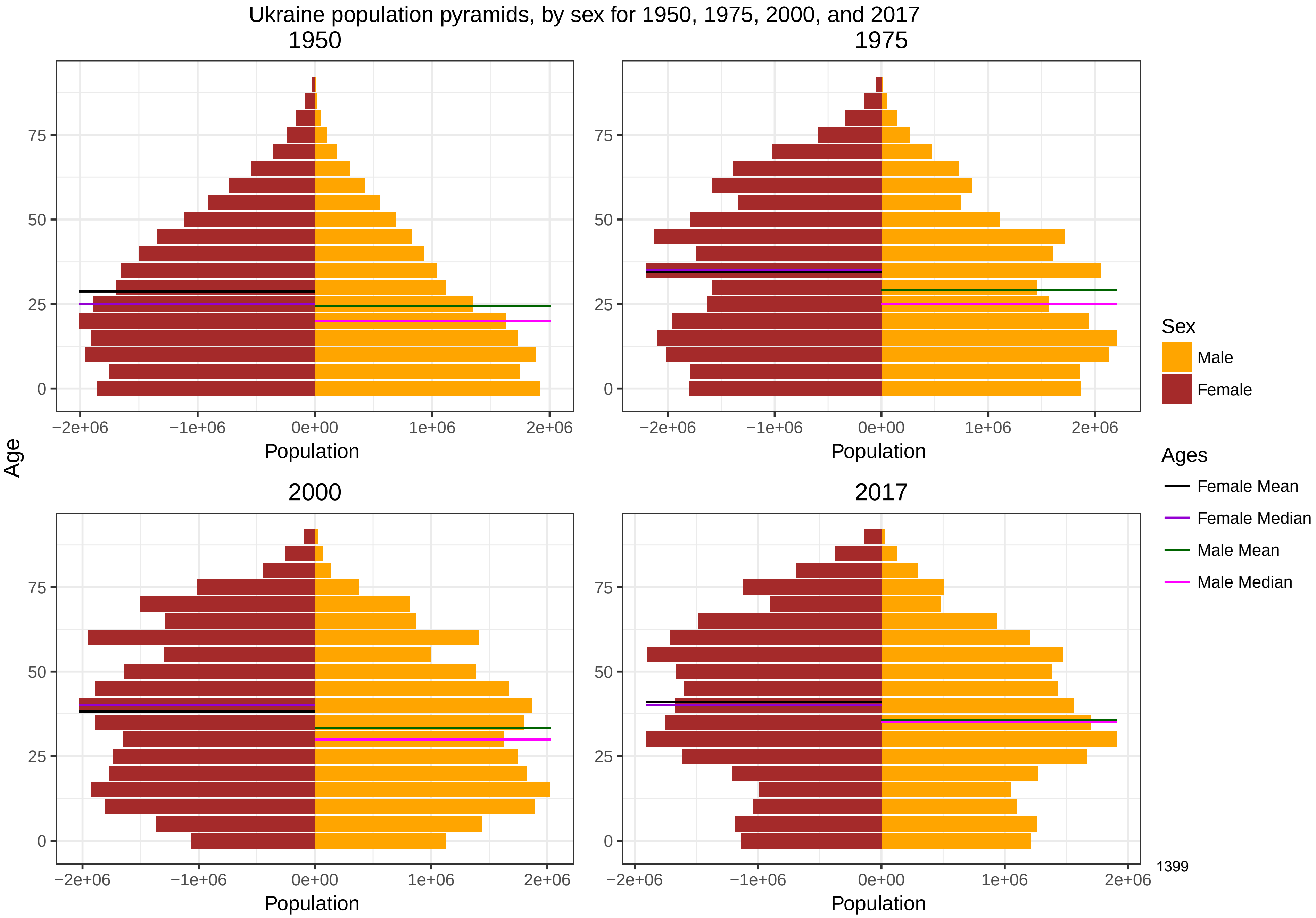
Ukraine population pyramids, by sex for 1950, 1975, 2000, and 2017
1950
1975
2000
2017
Age
Population
0
25
50
75
−2e+06
−1e+06
0e+00
1e+06
2e+06
Sex
Male
Female
Ages
Female Mean
Female Median
Male Mean
Male Median

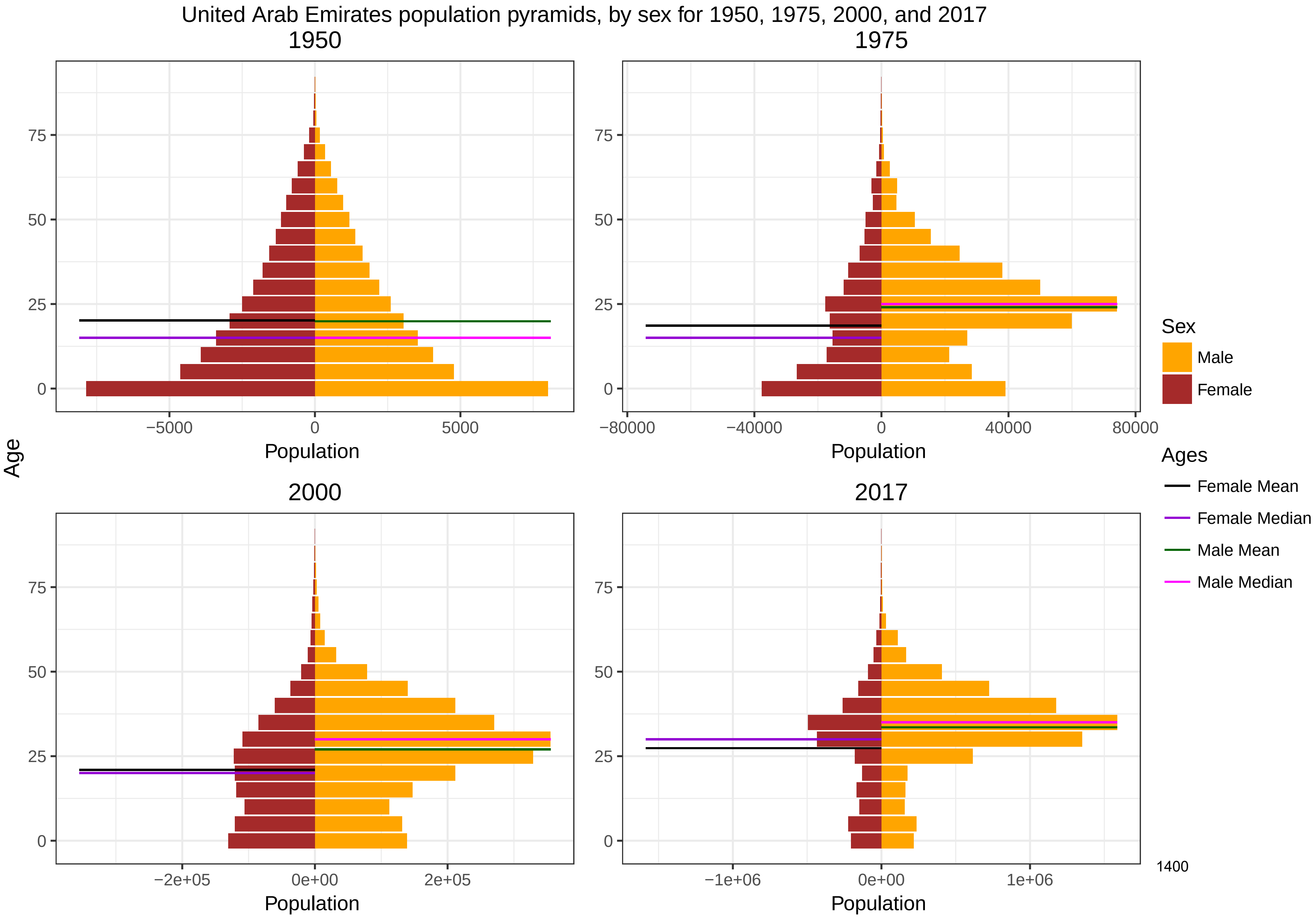
United Arab Emirates population pyramids, by sex for 1950, 1975, 2000, and 2017
1950
1975
2000
2017
Age
Population
75
50
25
0
−5000
0
5000
−80000
−40000
40000
80000
−2e+05
0e+00
2e+05
−1e+06
1e+06
Sex
Male
Female
Ages
Female Mean
Female Median
Male Mean
Male Median

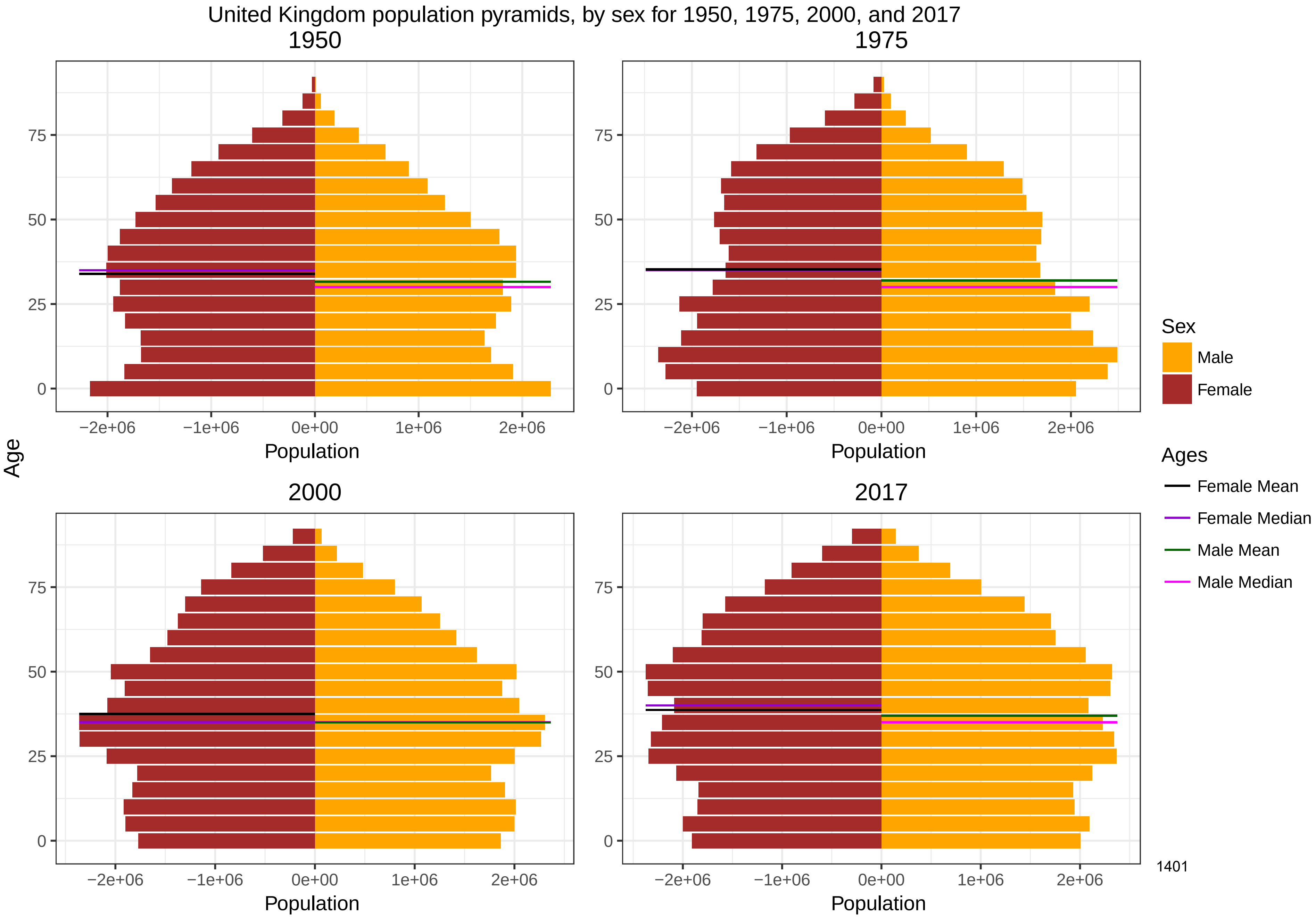

United Kingdom population pyramids, by sex for 1950, 1975, 2000, and 2017
1950
1975
2000
2017
Age
Population
0
25
50
75
−2e+06
−1e+06
0e+00
1e+06
2e+06
Sex
Male
Female
Ages
Female Mean
Female Median
Male Mean
Male Median

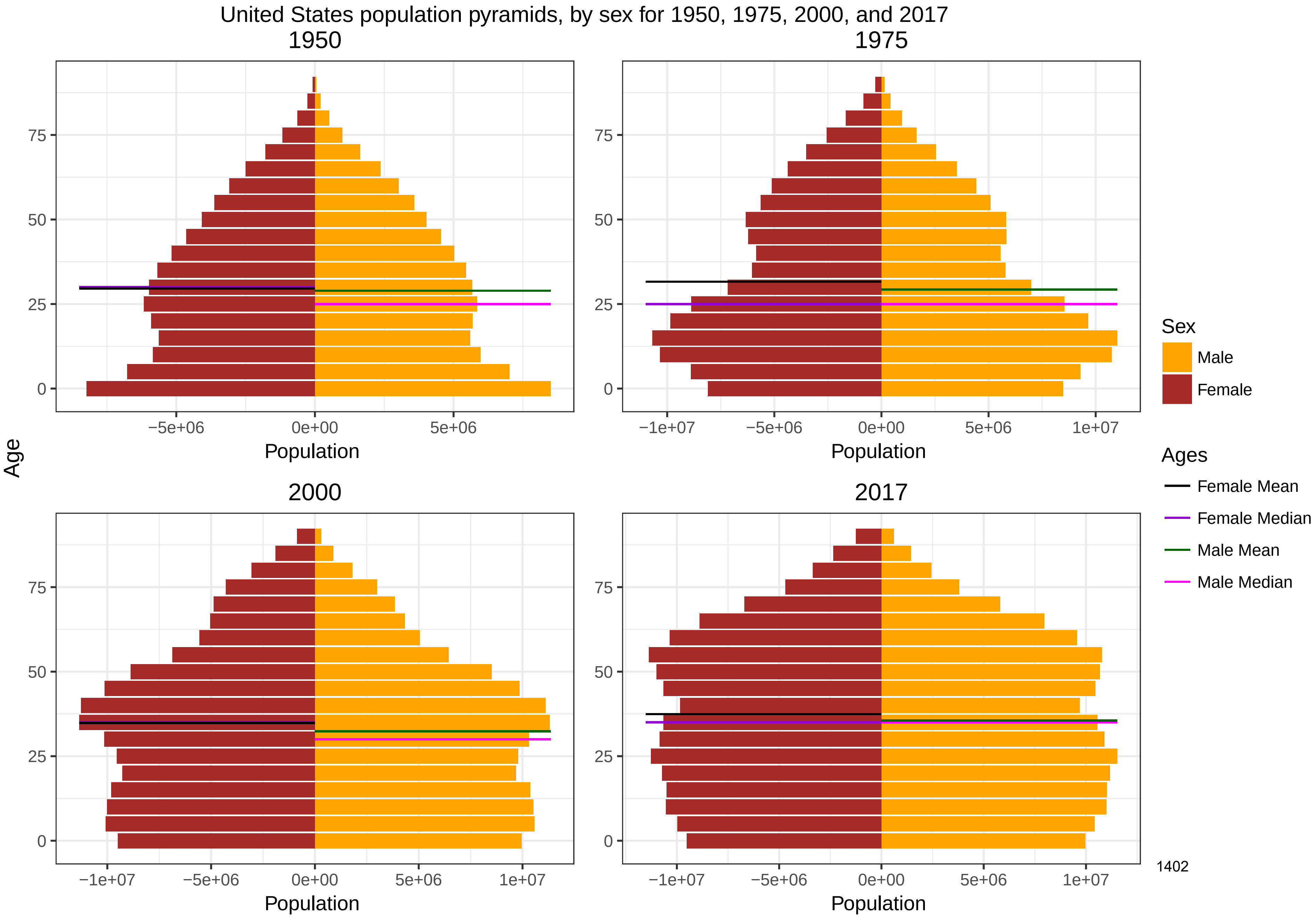
United States population pyramids, by sex for 1950, 1975, 2000, and 2017
1950
1975
2000
2017
Age
Population
−1e+07
−5e+06
0e+00
5e+06
1e+07
0
25
50
75
Sex
Male
Female
Ages
Female Mean
Female Median
Male Mean
Male Median

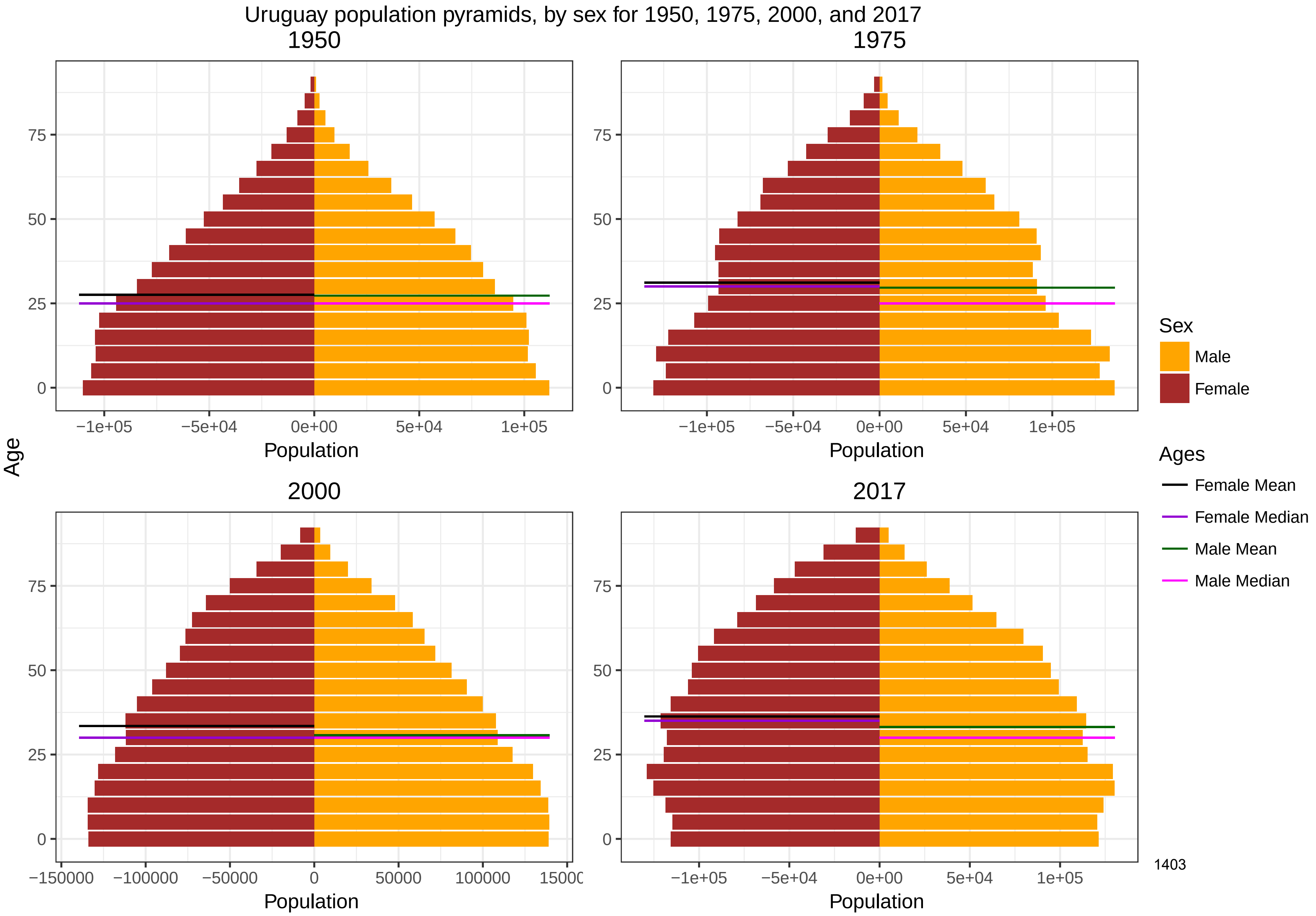
Uruguay population pyramids, by sex for 1950, 1975, 2000, and 2017
1950
1975
2000
2017
Age
Population
75
50
25
0
−1e+05
−5e+04
0e+00
5e+04
1e+05
−150000
−100000
−50000
0
50000
100000
Sex
Male
Female
Ages
Female Mean
Female Median
Male Mean
Male Median

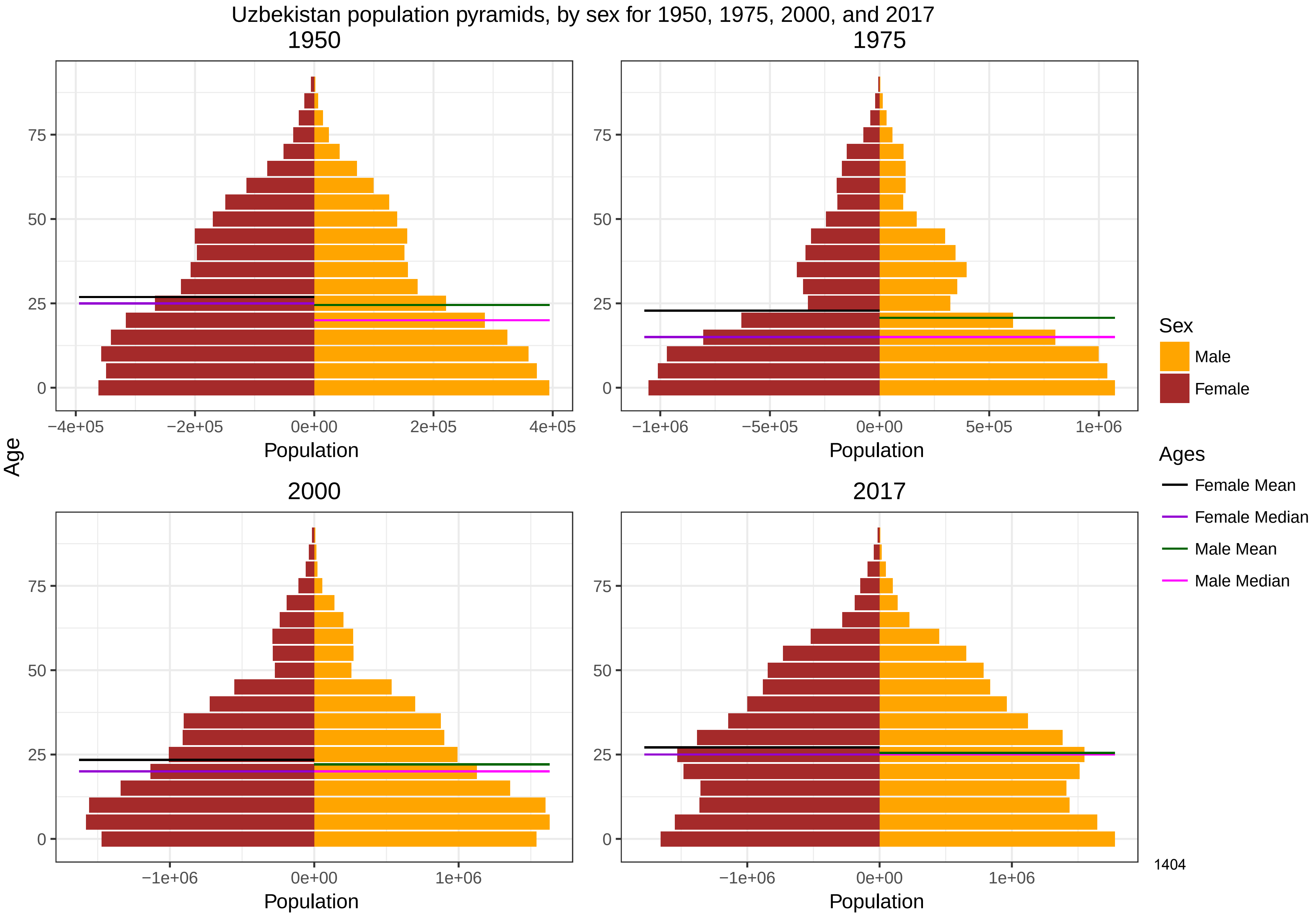
Uzbekistan population pyramids, by sex for 1950, 1975, 2000, and 2017
1950
1975
2000
2017
Age
Population
Sex
Male
Female
Ages
Female Mean
Female Median
Male Mean
Male Median

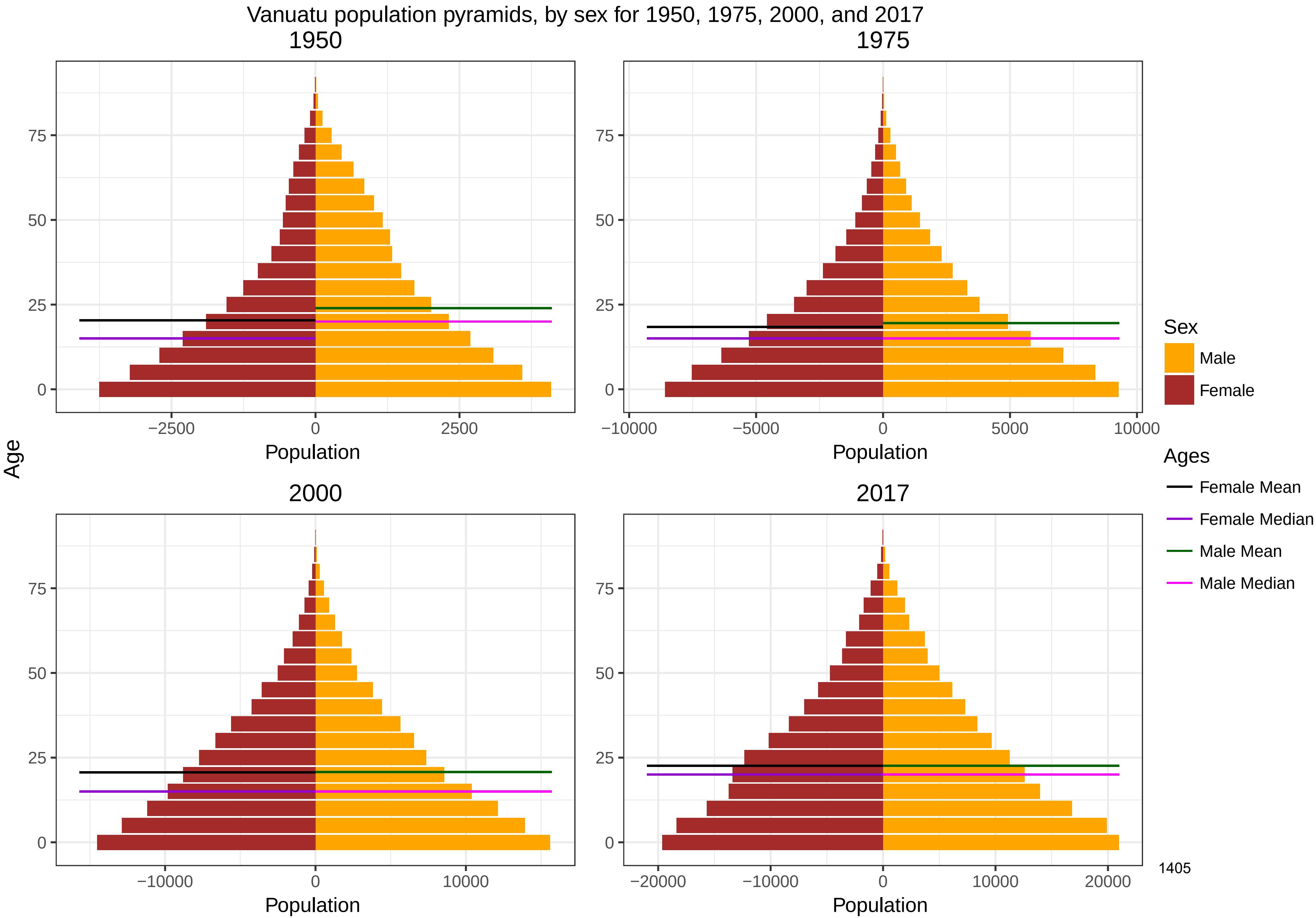
Vanuatu population pyramids, by sex for 1950, 1975, 2000, and 2017
1950
1975
2000
2017
Age
Population
Sex
Male
Female
Ages
Female Mean
Female Median
Male Mean
Male Median

1405

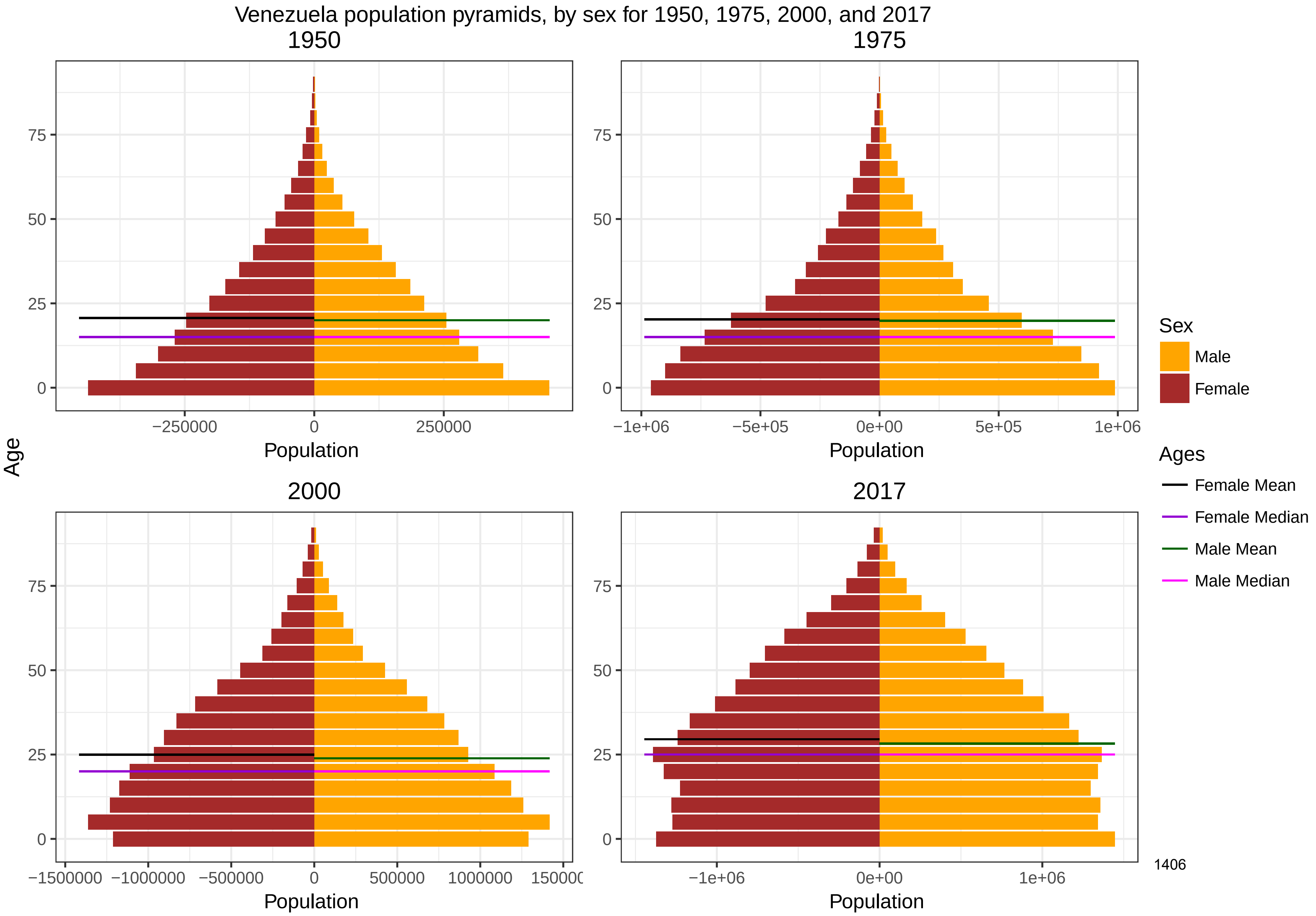

Venezuela population pyramids, by sex for 1950, 1975, 2000, and 2017
1950
1975
2000
2017
Age
Population
−250000
0
250000
−1e+06
−5e+05
0e+00
5e+05
1e+06
−1500000
−1000000
−500000
500000
1000000
150000
0
25
50
75
Sex
Male
Female
Ages
Female Mean
Female Median
Male Mean
Male Median

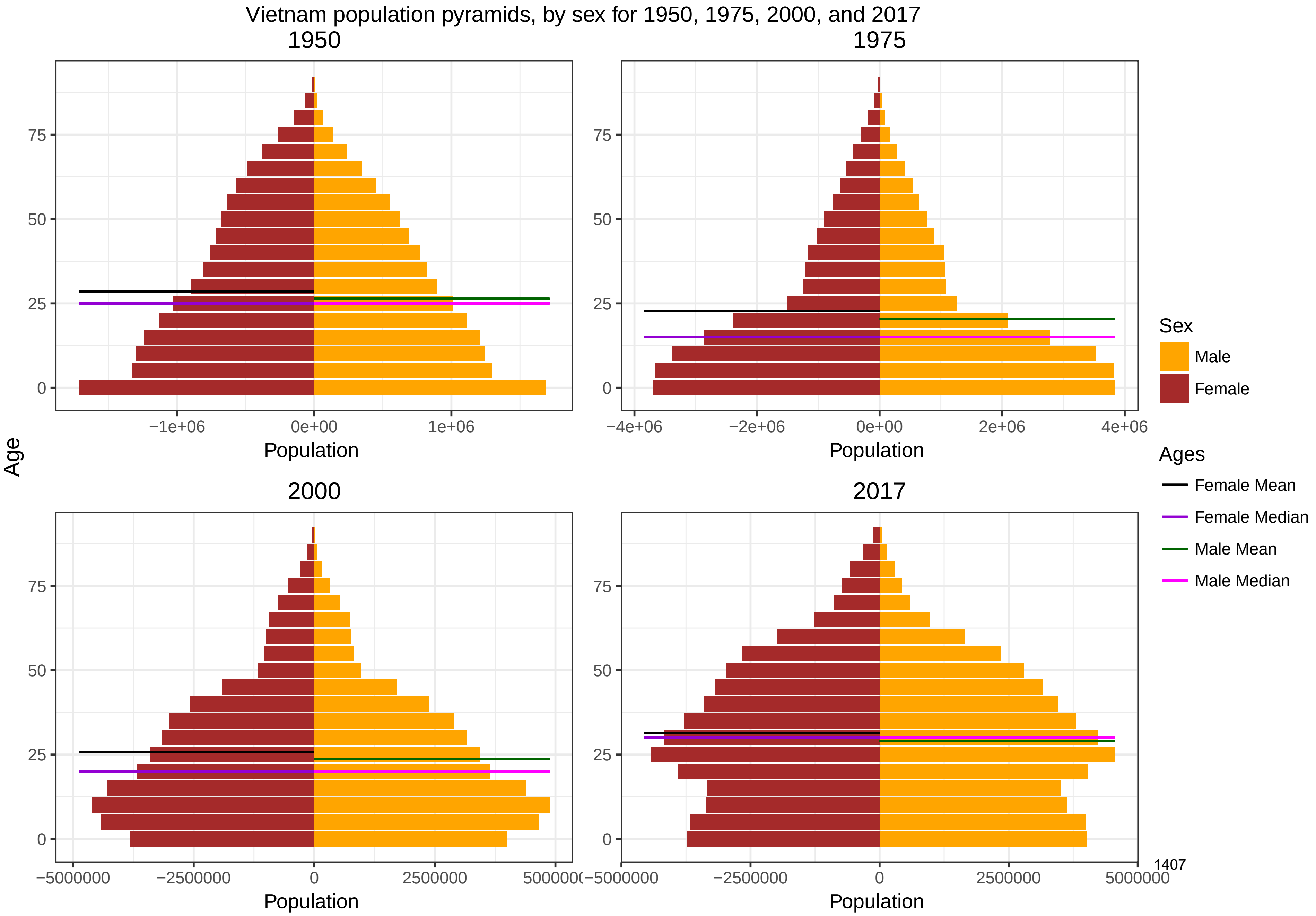
Vietnam population pyramids, by sex for 1950, 1975, 2000, and 2017
1950
1975
2000
2017
Age
Population
75
50
25
0
−1e+06
0e+00
1e+06
−4e+06
−2e+06
2e+06
4e+06
−5000000
−2500000
2500000
5000000
Sex
Male
Female
Ages
Female Mean
Female Median
Male Mean
Male Median

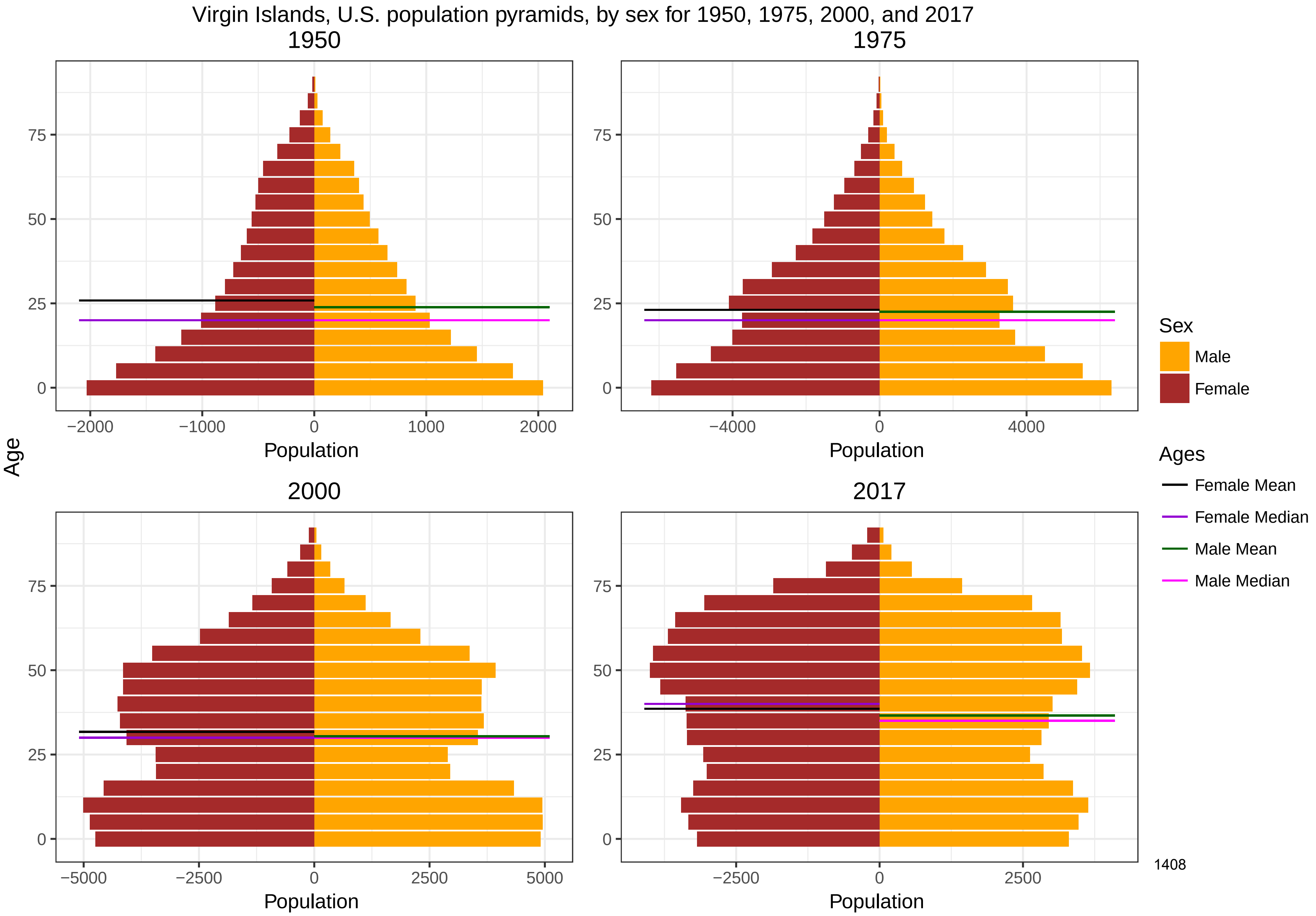

Virgin Islands, U.S. population pyramids, by sex for 1950, 1975, 2000, and 2017
1950
1975
2000
2017
Age
Population
Sex
Male
Female
Ages
Female Mean
Female Median
Male Mean
Male Median

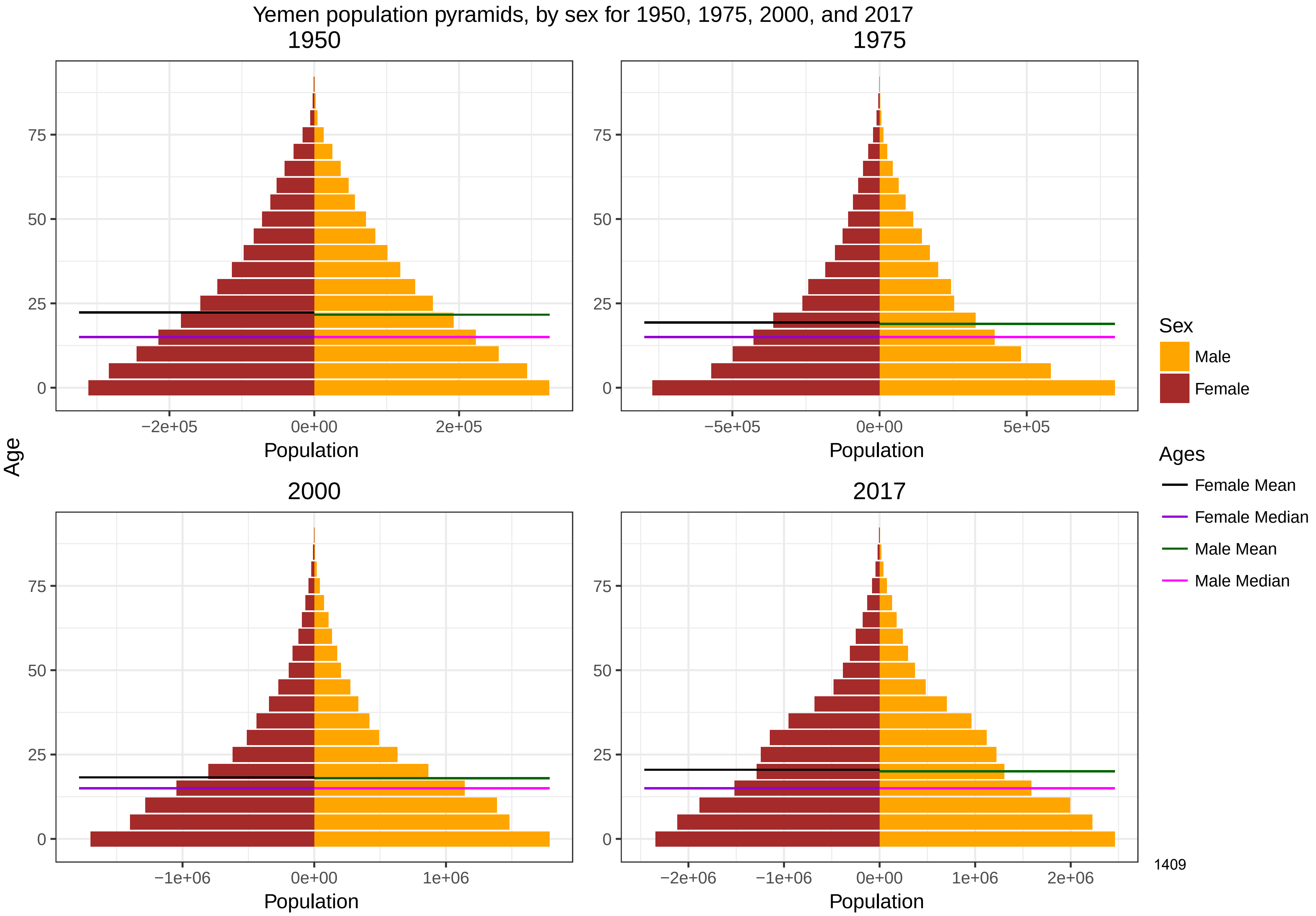
Yemen population pyramids, by sex for 1950, 1975, 2000, and 2017
1950
1975
2000
2017
Age
Population
−2e+05
0e+00
2e+05
−5e+05
5e+05
−1e+06
1e+06
−2e+06
2e+06
0
25
50
75
Sex
Male
Female
Ages
Female Mean
Female Median
Male Mean
Male Median

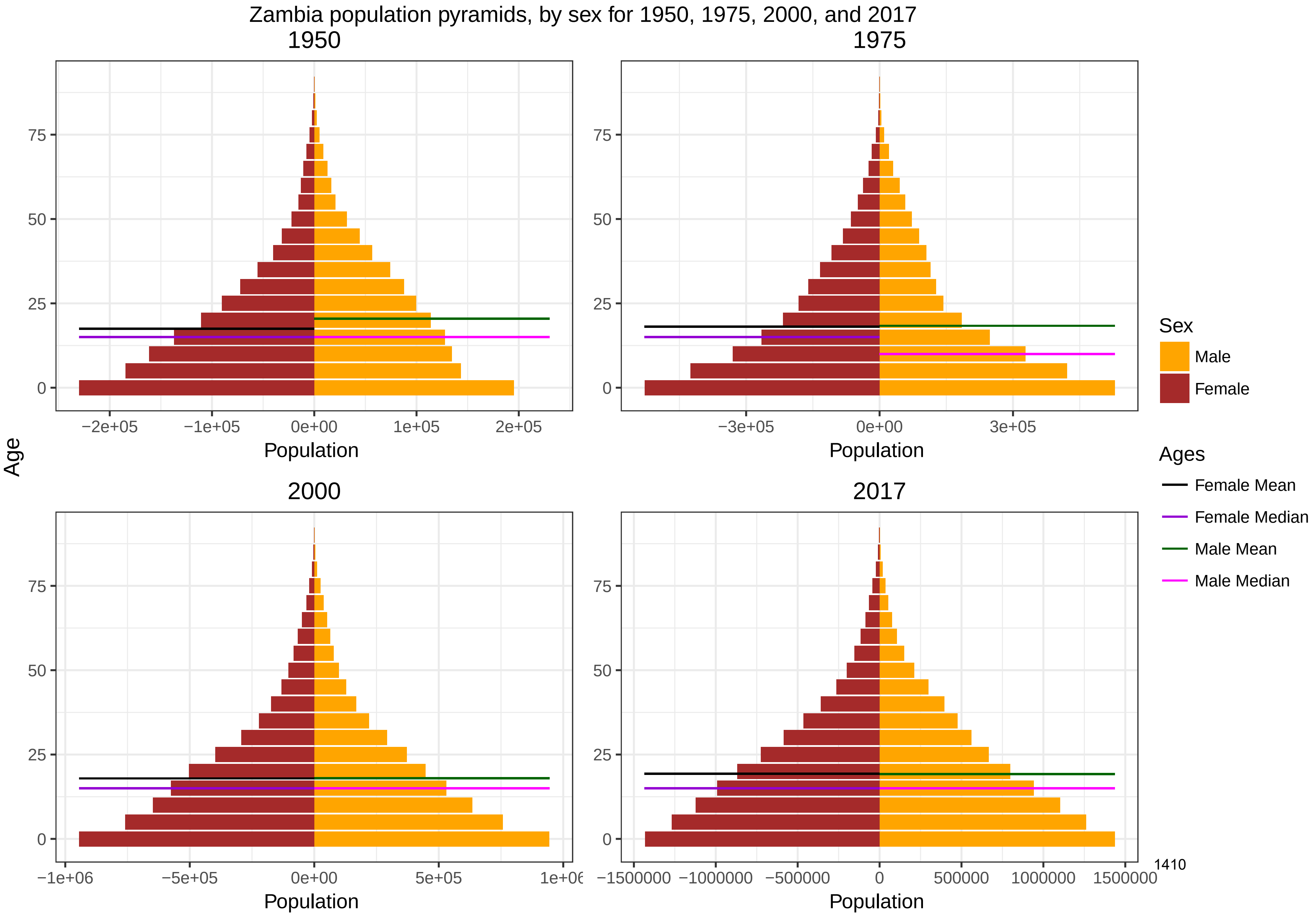
Zambia population pyramids, by sex for 1950, 1975, 2000, and 2017
1950
1975
2000
2017
Age
Population
Sex
Male
Female
Ages
Female Mean
Female Median
Male Mean
Male Median
−2e+05
−1e+05
0e+00
1e+05
2e+05
−3e+05
3e+05
−1e+06
−5e+05
5e+05
−1500000
−1000000
−500000
0
500000
1000000
1500000
25
50
75

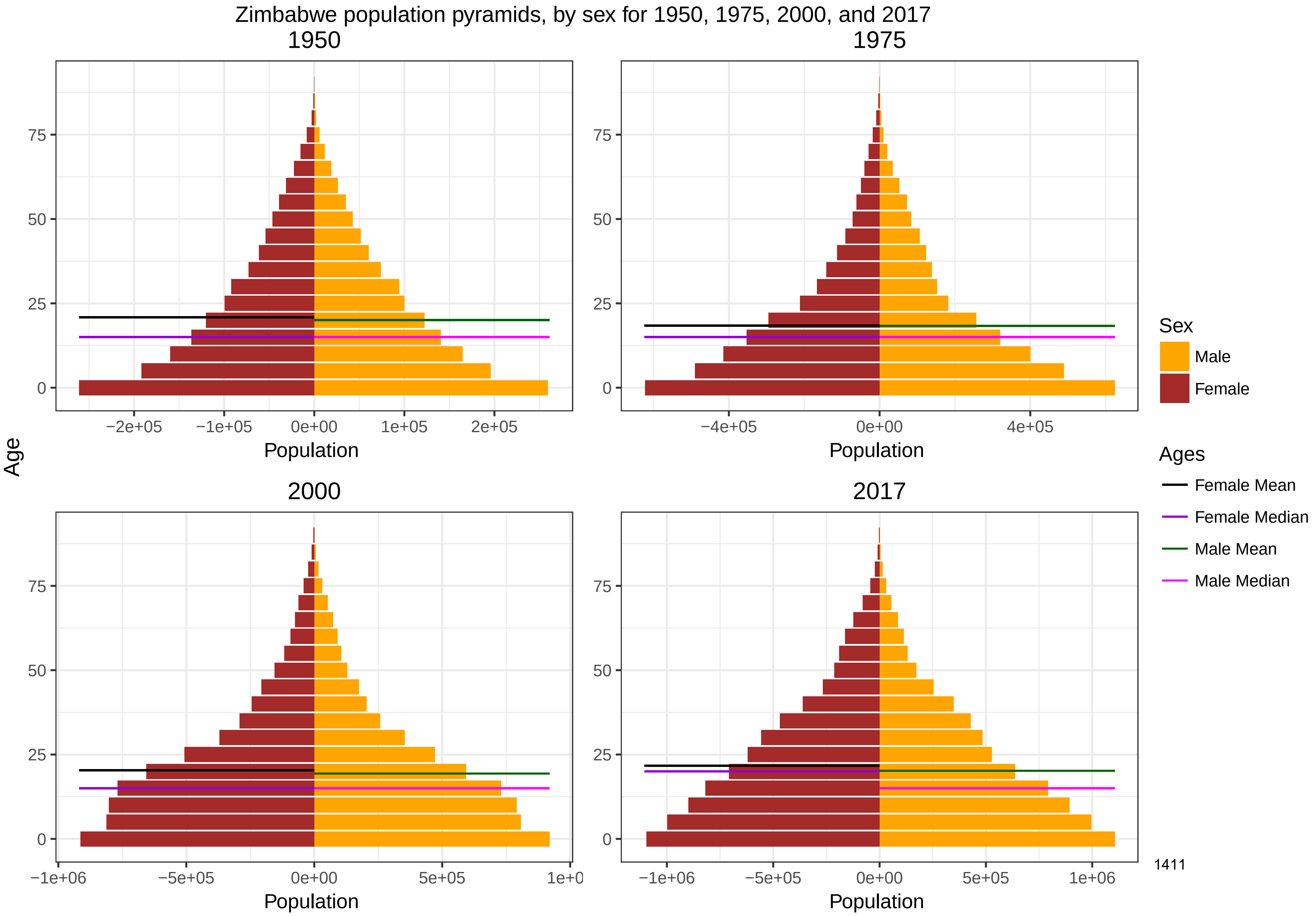

Zimbabwe population pyramids, by sex for 1950, 1975, 2000, and 2017
1950
1975
2000
2017
Age
Population
Sex
Male
Female
Ages
Female Mean
Female Median
Male Mean
Male Median

Supplemental Figure 6: Global net migrants, all ages and both sexes combined

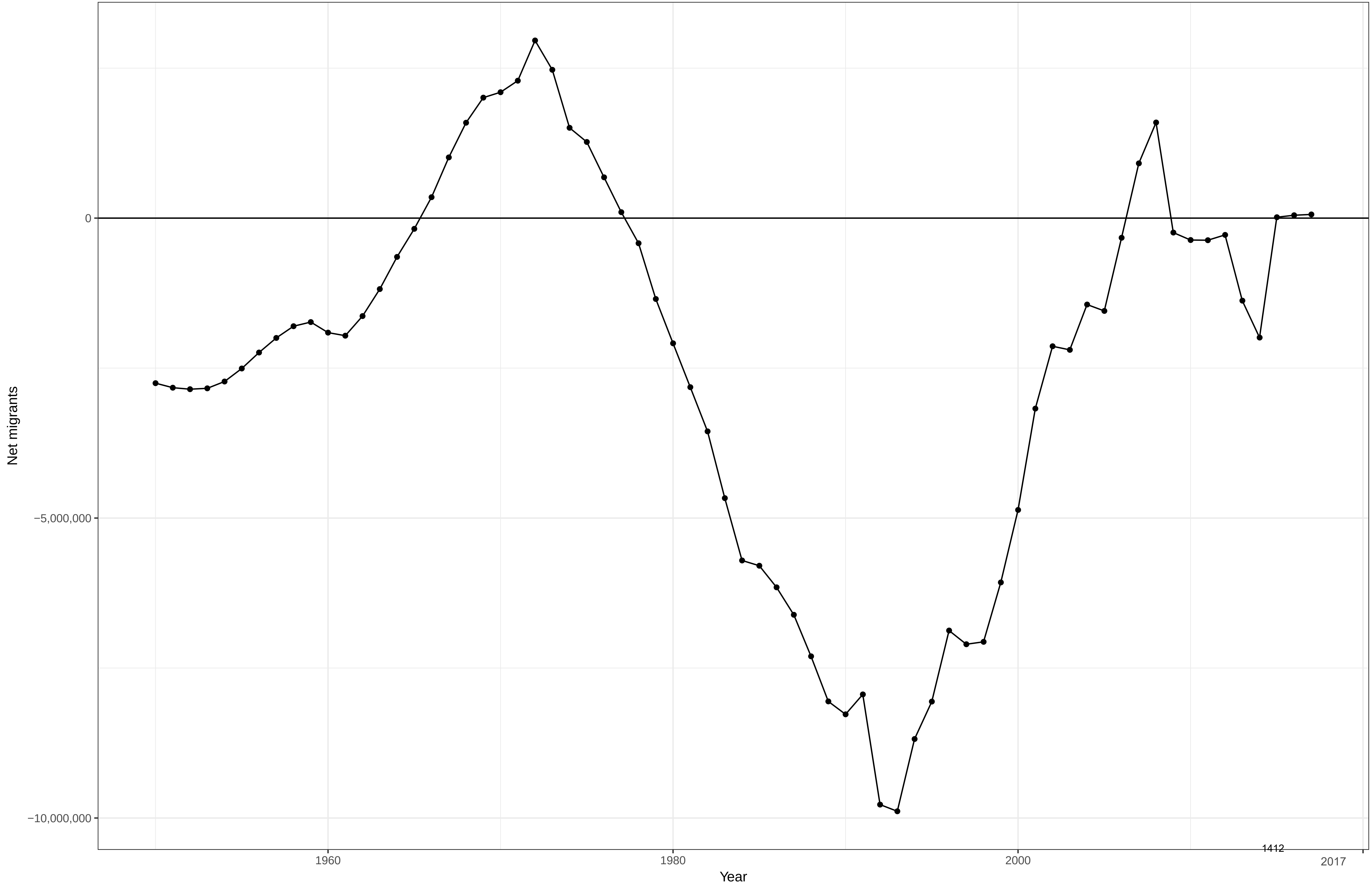

Supplemental Results Table 1: Correlation of age-specific fertility rates across 195 countries or territories by age of mother, 2017.									
	**10-14**	**15-19**	**20-24**	**25-29**	**30-34**	**35-39**	**40-44**	**45-49**	**50-54**
10-14	1	0.135	0.151	0.152	0.178	0.161	0.118	0.118	0.122
15-19	0.135	1	0.978	0.971	0.926	0.914	0.943	0.967	0.963
20-24	0.151	0.978	1	0.993	0.967	0.953	0.974	0.991	0.989
25-29	0.152	0.971	0.993	1	0.983	0.972	0.989	0.993	0.992
30-34	0.178	0.926	0.967	0.983	1	0.996	0.996	0.981	0.98
35-39	0.161	0.914	0.953	0.972	0.996	1	0.992	0.971	0.974
40-44	0.118	0.943	0.974	0.989	0.996	0.992	1	0.986	0.986
45-49	0.118	0.967	0.991	0.993	0.981	0.971	0.986	1	0.995
50-54	0.122	0.963	0.989	0.992	0.98	0.974	0.986	0.995	1

Supplemental Results Table 2: Live births (in thousands) by location, both sexes combined, 1950, 1960, 1970, 1980, 1990, 2000, 2010, 2017

Geography	1950	1960	1970	1980	1990	2000	2010	2017
Global	92,617 (88,864-96,400)	107,491 (105,117-109,765)	118,098 (116,158-119,938)	124,158 (121,805-126,472)	137,005 (134,022-139,942)	133,935 (130,721-137,079)	138,897 (134,050-144,108)	138,810 (129,960-149,058)
Central Europe, Eastern Europe, and Central Asia	7,370 (7,229-7,529)	7,426 (7,334-7,511)	6,303 (6,189-6,416)	7,146 (7,063-7,232)	6,623 (6,548-6,699)	4,600 (4,520-4,681)	5,634 (5,528-5,747)	5,224 (4,687-5,805)
Central Asia	1,021 (979-1,063)	1,413 (1,377-1,448)	1,395 (1,327-1,465)	1,763 (1,721-1,803)	1,997 (1,953-2,042)	1,496 (1,441-1,550)	1,898 (1,809-1,988)	1,910 (1,754-2,076)
Armenia	48 (45-51)	69 (67-72)	56 (53-60)	74 (71-77)	77 (75-80)	40 (38-41)	43 (41-45)	38 (34-41)
Azerbaijan	100 (93-108)	161 (155-167)	147 (138-156)	161 (153-169)	191 (184-200)	131 (124-138)	181 (169-195)	173 (153-196)
Georgia	86 (78-95)	101 (94-109)	87 (80-95)	88 (84-94)	81 (77-85)	50 (47-54)	59 (56-63)	50 (45-55)
Kazakhstan	254 (244-263)	359 (346-371)	355 (334-378)	389 (379-399)	379 (370-387)	241 (233-249)	350 (336-365)	347 (315-381)
Kyrgyzstan	59 (56-62)	77 (74-79)	89 (82-95)	110 (106-114)	126 (122-129)	101 (97-105)	147 (143-152)	151 (141-161)
Mongolia	33 (31-35)	38 (37-40)	43 (41-45)	59 (58-61)	67 (65-69)	49 (47-51)	70 (68-72)	75 (70-81)
Tajikistan	88 (85-90)	118 (115-121)	125 (120-129)	171 (164-178)	216 (208-224)	191 (182-201)	248 (231-265)	285 (259-310)
Turkmenistan	54 (52-56)	75 (73-76)	72 (68-77)	99 (96-102)	124 (120-127)	89 (84-94)	112 (106-119)	109 (98-123)
Uzbekistan	296 (284-307)	411 (400-423)	416 (395-437)	607 (584-629)	732 (707-760)	600 (564-637)	683 (623-741)	679 (619-740)
Central Europe	2,298 (2,248-2,348)	1,961 (1,935-1,989)	1,909 (1,886-1,933)	2,062 (2,044-2,080)	1,689 (1,678-1,701)	1,203 (1,192-1,214)	1,208 (1,193-1,222)	1,066 (960-1,187)
Albania	48 (47-49)	68 (66-70)	74 (71-78)	77 (74-80)	87 (84-89)	55 (53-56)	38 (36-39)	37 (32-42)
Bosnia and Herzegovina	77 (66-88)	88 (77-99)	73 (63-87)	74 (65-84)	66 (65-68)	38 (38-39)	34 (33-35)	27 (25-29)
Bulgaria	168 (166-170)	139 (137-140)	136 (134-138)	128 (126-129)	101 (99-103)	70 (69-71)	74 (73-76)	58 (51-66)
Croatia	86 (74-101)	81 (71-93)	65 (61-69)	70 (67-74)	53 (52-54)	43 (42-43)	42 (41-43)	36 (34-38)
Czech Republic	186 (184-188)	128 (126-129)	148 (146-150)	150 (149-152)	128 (126-129)	91 (89-92)	113 (111-115)	104 (95-114)
Hungary	192 (190-194)	143 (141-144)	150 (148-151)	147 (145-149)	126 (124-128)	96 (95-98)	90 (89-92)	86 (76-97)
Macedonia	36 (32-41)	36 (31-41)	35 (32-39)	41 (38-44)	35 (34-35)	26 (25-26)	24 (23-24)	23 (22-25)
Montenegro	12 (11-13)	12 (11-13)	10 (10-11)	10 (9-10)	9 (8-9)	8 (8-8)	7 (7-7)	7 (6-7)
Poland	757 (753-761)	643 (639-647)	545 (541-549)	687 (683-691)	548 (544-552)	371 (366-376)	402 (398-406)	355 (315-402)
Romania	411 (379-447)	338 (335-342)	411 (408-414)	393 (390-396)	292 (289-295)	223 (220-226)	208 (205-212)	177 (158-198)
Serbia	187 (186-189)	165 (163-167)	147 (145-149)	155 (153-156)	136 (134-138)	106 (104-108)	88 (80-98)	80 (71-91)
Slovakia	98 (97-100)	87 (85-88)	80 (79-82)	94 (93-95)	80 (79-81)	53 (52-54)	60 (58-61)	52 (46-59)
Slovenia	33 (31-36)	28 (27-29)	28 (27-28)	30 (29-31)	23 (22-23)	18 (17-18)	22 (21-22)	19 (17-21)
Eastern Europe	4,050 (3,969-4,146)	4,051 (4,004-4,093)	2,999 (2,953-3,045)	3,320 (3,285-3,357)	2,935 (2,906-2,964)	1,901 (1,874-1,928)	2,527 (2,496-2,560)	2,246 (1,958-2,577)
Belarus	185 (175-196)	192 (185-199)	148 (143-153)	161 (156-166)	137 (133-141)	93 (89-96)	113 (106-119)	101 (90-114)
Estonia	20 (19-20)	20 (19-20)	21 (21-22)	22 (22-23)	20 (20-21)	13 (12-13)	15 (14-15)	13 (11-15)
Latvia	33 (31-34)	35 (34-36)	34 (34-35)	35 (35-36)	36 (35-36)	20 (19-21)	20 (19-20)	19 (17-21)
Lithuania	58 (54-62)	61 (60-63)	55 (54-56)	51 (51-52)	57 (56-57)	33 (32-34)	34 (34-35)	29 (25-32)
Moldova	86 (81-91)	83 (79-87)	70 (63-79)	83 (80-87)	77 (74-79)	45 (43-47)	43 (41-45)	35 (31-40)
Russian Federation	2,815 (2,794-2,840)	2,814 (2,801-2,828)	1,951 (1,939-1,963)	2,213 (2,203-2,224)	1,977 (1,967-1,987)	1,286 (1,275-1,296)	1,793 (1,784-1,804)	1,622 (1,410-1,868)
Ukraine	851 (803-906)	842 (805-880)	715 (676-754)	751 (721-780)	628 (607-651)	408 (391-429)	506 (481-532)	424 (369-487)
High-income	13,496 (13,296-13,706)	14,440 (14,337-14,549)	13,954 (13,847-14,061)	12,547 (12,472-12,622)	12,573 (12,505-12,642)	11,882 (11,804-11,963)	11,908 (11,844-11,975)	11,470 (10,419-12,658)
Australasia	242 (239-245)	298 (295-301)	326 (324-329)	279 (276-281)	315 (312-319)	302 (297-308)	359 (355-363)	373 (338-413)
Australia	191 (190-193)	233 (231-235)	264 (261-266)	228 (226-231)	256 (253-259)	246 (242-251)	297 (294-300)	313 (278-353)
New Zealand	50 (48-52)	65 (63-66)	62 (61-63)	50 (49-51)	59 (57-60)	55 (54-57)	61 (60-63)	60 (54-66)
High-income Asia Pacific	2,941 (2,849-3,042)	2,550 (2,460-2,640)	3,095 (3,016-3,172)	2,505 (2,445-2,568)	2,000 (1,952-2,054)	1,860 (1,808-1,917)	1,607 (1,557-1,659)	1,427 (1,260-1,623)
Brunei	2 (2-2)	4 (3-4)	4 (4-4)	5 (5-6)	6 (6-7)	7 (6-7)	6 (6-7)	7 (6-7)
Japan	2,211 (2,134-2,292)	1,593 (1,526-1,659)	1,978 (1,902-2,056)	1,564 (1,503-1,627)	1,224 (1,176-1,278)	1,180 (1,129-1,233)	1,073 (1,024-1,128)	922 (767-1,106)
Singapore	46 (45-47)	58 (57-60)	45 (43-47)	41 (39-43)	49 (46-51)	74 (69-79)	56 (53-59)	64 (54-77)
South Korea	680 (619-745)	894 (835-958)	1,067 (1,044-1,090)	894 (890-897)	719 (710-728)	598 (593-604)	470 (467-474)	432 (412-453)
High-income North America	4,012 (3,995-4,028)	4,715 (4,701-4,732)	3,955 (3,938-3,974)	3,959 (3,940-3,978)	4,622 (4,602-4,644)	4,380 (4,363-4,399)	4,331 (4,312-4,349)	4,314 (3,982-4,683)
Canada	360 (358-363)	458 (455-461)	348 (345-351)	360 (356-364)	399 (395-402)	332 (328-336)	374 (371-378)	390 (334-455)
Greenland	1 (0-1)	1 (1-1)	1 (1-1)	0 (0-1)	1 (1-1)	0 (0-1)	0 (0-0)	0 (0-0)
United States	3,650 (3,633-3,666)	4,255 (4,242-4,271)	3,605 (3,589-3,623)	3,598 (3,581-3,616)	4,222 (4,202-4,243)	4,047 (4,030-4,065)	3,956 (3,938-3,972)	3,923 (3,646-4,226)
Southern Latin America	696 (665-728)	804 (798-812)	841 (833-850)	980 (974-985)	1,027 (1,020-1,034)	1,006 (995-1,018)	1,044 (1,035-1,052)	1,041 (958-1,130)

Supplemental Results Table 2: Live births (in thousands) by location, both sexes combined, 1950, 1960, 1970, 1980, 1990, 2000, 2010, 2017								
**Geography**	**1950**	**1960**	**1970**	**1980**	**1990**	**2000**	**2010**	**2017**
Argentina	452 (422-484)	470 (464-476)	539 (532-546)	684 (680-688)	684 (679-690)	706 (696-717)	748 (742-754)	747 (695-801)
Chile	198 (196-200)	273 (271-275)	246 (243-248)	242 (239-244)	287 (285-290)	247 (244-249)	248 (244-251)	245 (215-279)
Uruguay	44 (43-45)	60 (59-62)	56 (55-56)	54 (53-54)	55 (54-56)	52 (51-53)	47 (44-51)	48 (41-55)
Western Europe	5,604 (5,465-5,769)	6,070 (6,038-6,104)	5,734 (5,683-5,783)	4,822 (4,800-4,844)	4,606 (4,585-4,628)	4,331 (4,309-4,354)	4,566 (4,542-4,589)	4,313 (3,871-4,807)
Andorra	0 (0-0)	0 (0-0)	0 (0-0)	0 (0-0)	0 (0-0)	0 (0-0)	0 (0-0)	0 (0-0)
Austria	104 (101-107)	128 (127-130)	110 (108-111)	91 (90-93)	92 (90-94)	77 (76-79)	78 (76-79)	86 (79-94)
Belgium	141 (140-143)	157 (155-159)	142 (140-143)	123 (121-125)	124 (123-126)	115 (113-117)	128 (126-130)	121 (109-134)
Cyprus	13 (13-14)	14 (14-14)	13 (13-14)	12 (12-13)	11 (11-12)	10 (9-11)	11 (11-12)	10 (9-12)
Denmark	78 (76-79)	76 (74-77)	73 (72-74)	55 (54-56)	63 (62-65)	66 (65-67)	61 (60-62)	60 (54-67)
Finland	95 (94-96)	81 (80-82)	62 (60-63)	63 (62-64)	64 (63-65)	55 (54-56)	60 (59-61)	55 (49-61)
France	836 (831-841)	827 (821-832)	853 (848-858)	801 (795-806)	761 (755-766)	776 (770-781)	798 (793-804)	737 (664-819)
Germany	1,093 (1,012-1,191)	1,267 (1,260-1,273)	1,026 (1,018-1,034)	849 (842-855)	872 (866-878)	758 (752-765)	671 (664-677)	710 (633-798)
Greece	155 (152-158)	154 (152-156)	143 (141-145)	143 (141-145)	102 (100-103)	102 (100-103)	110 (108-113)	89 (79-100)
Iceland	3 (3-4)	4 (4-4)	4 (4-4)	4 (4-4)	4 (4-4)	4 (3-4)	4 (4-4)	4 (3-4)
Ireland	64 (61-68)	61 (60-62)	65 (64-66)	72 (71-73)	52 (51-53)	55 (54-56)	73 (72-74)	64 (57-72)
Israel	48 (45-52)	55 (54-56)	80 (79-82)	93 (91-94)	103 (102-104)	135 (133-137)	165 (163-166)	177 (161-194)
Italy	882 (877-887)	918 (913-924)	903 (897-909)	634 (629-640)	565 (560-570)	540 (534-546)	558 (553-563)	464 (410-526)
Luxembourg	4 (4-4)	5 (4-5)	4 (4-4)	4 (4-4)	4 (4-5)	5 (5-5)	5 (5-5)	6 (5-6)
Malta	9 (9-10)	8 (7-8)	5 (5-5)	5 (5-5)	5 (5-5)	4 (3-4)	4 (3-4)	4 (3-4)
Netherlands	228 (226-230)	243 (240-245)	230 (228-232)	177 (175-179)	196 (194-199)	205 (202-207)	182 (180-184)	172 (155-191)
Norway	61 (60-62)	62 (61-63)	65 (64-66)	51 (50-52)	60 (59-61)	57 (56-59)	60 (59-61)	60 (55-66)
Portugal	206 (203-208)	218 (215-220)	180 (178-182)	152 (150-154)	114 (112-117)	116 (114-118)	99 (96-101)	85 (74-97)
Spain	557 (553-561)	650 (645-654)	660 (656-665)	547 (543-552)	397 (392-402)	397 (393-401)	480 (476-485)	407 (370-447)
Sweden	113 (111-115)	102 (101-104)	111 (110-113)	95 (94-97)	122 (121-124)	91 (90-93)	112 (111-114)	118 (109-127)
Switzerland	82 (81-84)	96 (94-97)	97 (95-99)	74 (73-75)	84 (83-85)	76 (74-77)	80 (78-81)	87 (78-97)
United Kingdom	817 (775-870)	931 (908-958)	892 (851-936)	761 (755-766)	795 (789-800)	673 (668-679)	812 (806-817)	783 (703-873)
England	650 (611-698)	748 (728-770)	725 (690-761)	619 (613-625)	660 (655-666)	567 (562-573)	694 (689-699)	672 (604-749)
Northern Ireland	31 (29-32)	32 (31-34)	31 (29-32)	28 (27-29)	25 (24-26)	21 (20-22)	24 (23-24)	23 (20-26)
Scotland	91 (89-92)	101 (97-105)	89 (84-93)	71 (68-73)	66 (64-68)	52 (50-54)	57 (56-59)	53 (48-59)
Wales	44 (41-47)	49 (47-51)	47 (44-49)	42 (40-43)	42 (41-43)	31 (30-33)	35 (34-36)	33 (29-37)
Latin America and Caribbean	6,395 (6,026-6,756)	8,098 (7,766-8,425)	9,420 (9,149-9,690)	10,545 (10,231-10,880)	10,981 (10,601-11,368)	10,797 (10,377-11,222)	10,695 (10,128-11,262)	10,393 (9,469-11,430)
Andean Latin America	737 (706-766)	906 (877-933)	1,067 (1,046-1,088)	1,206 (1,169-1,244)	1,301 (1,256-1,346)	1,246 (1,188-1,308)	1,337 (1,264-1,406)	1,386 (1,260-1,526)
Bolivia	159 (153-165)	178 (171-184)	195 (190-201)	237 (229-244)	258 (246-270)	261 (245-276)	283 (264-302)	301 (271-334)
Ecuador	158 (152-165)	201 (200-203)	232 (231-234)	261 (254-269)	301 (287-317)	326 (303-349)	327 (301-352)	315 (268-370)
Peru	419 (394-442)	525 (502-548)	638 (622-656)	708 (675-739)	741 (705-777)	658 (621-699)	727 (684-768)	769 (687-866)
Caribbean	679 (653-705)	833 (808-856)	885 (872-898)	839 (823-856)	917 (897-938)	887 (859-913)	824 (785-864)	815 (746-889)
Antigua and Barbuda	1 (1-1)	1 (1-1)	1 (1-1)	1 (1-1)	1 (1-1)	1 (1-1)	1 (1-1)	1 (0-1)
The Bahamas	2 (2-3)	3 (3-3)	4 (4-4)	4 (4-5)	5 (5-5)	5 (4-5)	4 (4-4)	4 (3-5)
Barbados	6 (6-7)	7 (7-7)	5 (5-5)	4 (4-4)	4 (4-4)	3 (3-4)	3 (3-3)	2 (2-3)
Belize	2 (2-3)	4 (4-4)	4 (4-5)	5 (5-6)	6 (6-7)	6 (6-7)	7 (6-7)	7 (6-8)
Bermuda	1 (1-1)	1 (1-1)	0 (0-1)	0 (0-0)	0 (0-0)	0 (0-0)	0 (0-0)	0 (0-0)
Cuba	155 (146-163)	224 (214-234)	248 (245-251)	139 (137-141)	177 (175-180)	149 (146-151)	130 (128-132)	109 (103-116)
Dominica	2 (1-2)	2 (2-2)	2 (2-2)	1 (1-1)	1 (1-1)	1 (1-1)	0 (0-0)	0 (0-0)
Dominican Republic	113 (105-121)	147 (141-152)	189 (183-195)	228 (219-237)	238 (227-250)	244 (232-258)	201 (187-215)	216 (186-250)
Grenada	3 (3-3)	3 (3-3)	2 (2-2)	2 (2-2)	2 (2-2)	1 (1-2)	1 (1-2)	1 (1-1)
Guyana	18 (17-19)	25 (24-25)	24 (23-25)	25 (24-26)	26 (25-27)	20 (19-21)	15 (14-17)	15 (13-18)
Haiti	172 (162-182)	182 (171-192)	182 (176-188)	212 (205-219)	252 (244-260)	275 (263-288)	306 (285-329)	325 (290-365)
Jamaica	48 (45-52)	67 (66-69)	66 (64-68)	61 (59-64)	60 (58-62)	53 (49-58)	43 (39-47)	38 (33-43)

Supplemental Results Table 2: Live births (in thousands) by location, both sexes combined, 1950, 1960, 1970, 1980, 1990, 2000, 2010, 2017

Geography	1950	1960	1970	1980	1990	2000	2010	2017
Puerto Rico	85 (84-86)	75 (74-76)	68 (67-69)	71 (70-72)	65 (63-66)	57 (55-58)	42 (39-45)	29 (27-32)
Saint Lucia	2 (2-3)	4 (3-4)	4 (3-4)	3 (3-3)	3 (3-3)	2 (2-2)	2 (1-2)	2 (1-2)
Saint Vincent and the Grenadines	2 (2-3)	3 (3-4)	3 (3-3)	3 (3-3)	2 (2-2)	2 (1-2)	1 (1-1)	1 (1-1)
Suriname	7 (7-7)	12 (11-12)	13 (12-14)	10 (10-10)	9 (9-9)	10 (9-10)	10 (9-10)	9 (8-11)
Trinidad and Tobago	23 (22-25)	32 (31-33)	25 (25-26)	29 (29-30)	23 (22-24)	17 (17-18)	19 (17-20)	17 (15-19)
Virgin Islands, U.S.	0 (0-0)	1 (1-1)	2 (2-2)	2 (2-2)	2 (2-2)	1 (1-2)	1 (1-1)	1 (1-1)
Central Latin America	2,489 (2,388-2,593)	3,326 (3,200-3,442)	4,003 (3,880-4,124)	4,522 (4,365-4,679)	5,139 (4,929-5,361)	5,080 (4,860-5,299)	4,963 (4,693-5,219)	5,004 (4,502-5,583)
Colombia	492 (462-527)	730 (709-751)	825 (800-847)	885 (848-920)	981 (948-1,016)	979 (936-1,022)	909 (857-961)	851 (733-985)
Costa Rica	38 (36-39)	59 (58-60)	55 (55-56)	70 (69-71)	80 (79-82)	75 (72-77)	72 (71-74)	69 (64-76)
El Salvador	93 (88-97)	122 (119-124)	147 (144-150)	154 (150-158)	157 (151-163)	135 (130-140)	108 (102-113)	107 (94-122)
Guatemala	148 (147-149)	187 (186-188)	218 (216-219)	311 (309-313)	352 (350-354)	349 (340-359)	362 (355-368)	430 (372-496)
Honduras	75 (70-80)	90 (87-93)	114 (112-116)	152 (149-154)	180 (177-184)	192 (187-197)	209 (196-221)	244 (212-283)
Mexico	1,338 (1,249-1,428)	1,683 (1,586-1,775)	2,106 (2,001-2,210)	2,285 (2,140-2,422)	2,600 (2,387-2,807)	2,623 (2,448-2,815)	2,505 (2,284-2,735)	2,518 (2,153-2,972)
Nicaragua	54 (51-58)	74 (73-76)	92 (90-94)	127 (125-129)	145 (141-150)	136 (131-141)	138 (124-151)	137 (117-160)
Panama	28 (26-31)	41 (40-43)	52 (50-53)	51 (49-53)	57 (55-60)	61 (59-64)	68 (64-72)	69 (59-81)
Venezuela	219 (215-222)	336 (334-339)	391 (388-395)	484 (480-487)	581 (577-586)	526 (521-531)	589 (585-595)	575 (511-645)
Tropical Latin America	2,488 (2,272-2,709)	3,032 (2,846-3,213)	3,464 (3,314-3,622)	3,976 (3,836-4,127)	3,622 (3,468-3,781)	3,583 (3,427-3,740)	3,569 (3,359-3,806)	3,186 (2,886-3,554)
Brazil	2,426 (2,208-2,644)	2,962 (2,776-3,143)	3,375 (3,225-3,531)	3,861 (3,721-4,009)	3,501 (3,352-3,655)	3,456 (3,303-3,612)	3,428 (3,221-3,658)	3,040 (2,742-3,409)
Paraguay	61 (56-67)	69 (66-72)	89 (86-92)	115 (111-119)	120 (115-125)	127 (120-133)	141 (126-156)	145 (125-169)
North Africa and Middle East	5,551 (5,279-5,812)	6,717 (6,486-6,943)	8,195 (7,991-8,390)	10,433 (10,188-10,686)	11,226 (10,906-11,558)	12,217 (11,765-12,658)	13,318 (12,619-14,006)	13,008 (12,060-14,103)
North Africa and Middle East	5,551 (5,279-5,812)	6,717 (6,486-6,943)	8,195 (7,991-8,390)	10,433 (10,188-10,686)	11,226 (10,906-11,558)	12,217 (11,765-12,658)	13,318 (12,619-14,006)	13,008 (12,060-14,103)
Afghanistan	403 (380-425)	470 (448-490)	567 (544-590)	510 (492-528)	455 (438-471)	911 (886-935)	1,096 (1,057-1,134)	1,376 (1,303-1,448)
Algeria	445 (424-467)	513 (488-538)	597 (577-618)	782 (762-803)	835 (798-870)	873 (802-941)	938 (858-1,012)	963 (855-1,073)
Bahrain	4 (3-4)	6 (6-6)	6 (6-7)	10 (9-10)	13 (13-13)	13 (13-14)	19 (18-20)	19 (18-21)
Egypt	892 (842-943)	1,157 (1,106-1,205)	1,257 (1,207-1,304)	1,612 (1,551-1,669)	1,672 (1,601-1,742)	1,734 (1,648-1,822)	2,161 (2,019-2,306)	2,127 (1,940-2,330)
Iran	883 (827-939)	996 (951-1,039)	1,196 (1,148-1,243)	1,729 (1,669-1,786)	1,610 (1,510-1,716)	1,479 (1,366-1,591)	1,498 (1,390-1,605)	1,274 (1,085-1,494)
Iraq	259 (245-273)	328 (312-342)	419 (403-434)	570 (551-588)	668 (641-696)	946 (910-980)	1,075 (1,002-1,147)	1,255 (1,135-1,393)
Jordan	26 (25-26)	41 (40-42)	64 (63-65)	90 (88-91)	131 (126-136)	155 (147-162)	216 (203-229)	243 (223-266)
Kuwait	3 (2-3)	11 (10-11)	33 (33-33)	50 (49-50)	29 (28-30)	41 (39-42)	54 (52-55)	60 (55-67)
Lebanon	69 (66-72)	85 (82-89)	103 (98-107)	127 (118-136)	139 (125-151)	155 (138-171)	153 (135-172)	186 (160-217)
Libya	58 (57-60)	74 (72-77)	89 (87-91)	116 (113-118)	129 (119-138)	106 (104-108)	133 (119-149)	122 (102-146)
Morocco	423 (402-447)	527 (503-551)	618 (589-646)	706 (668-743)	760 (718-801)	742 (685-797)	679 (593-762)	601 (528-683)
Palestine	45 (43-47)	49 (47-51)	53 (51-55)	64 (61-67)	90 (86-94)	118 (112-122)	143 (135-152)	138 (125-152)
Oman	25 (24-26)	33 (32-34)	42 (40-44)	57 (54-59)	60 (56-64)	60 (54-65)	67 (62-73)	80 (72-88)
Qatar	1 (1-1)	2 (2-2)	4 (4-4)	7 (6-7)	10 (10-11)	11 (10-11)	21 (20-23)	30 (27-32)
Saudi Arabia	141 (136-146)	185 (180-190)	280 (272-288)	476 (462-490)	663 (626-701)	551 (506-598)	519 (465-579)	502 (444-565)
Sudan	290 (274-305)	324 (309-337)	540 (524-557)	790 (766-815)	1,026 (987-1,064)	1,255 (1,205-1,304)	1,379 (1,296-1,459)	1,336 (1,216-1,472)
Syria	153 (144-162)	202 (192-212)	279 (266-293)	378 (361-396)	412 (380-444)	448 (442-455)	533 (516-551)	276 (239-317)
Tunisia	167 (156-177)	188 (179-196)	195 (189-201)	225 (214-235)	229 (214-243)	205 (193-218)	193 (179-207)	167 (144-197)
Turkey	998 (942-1,053)	1,206 (1,153-1,257)	1,451 (1,399-1,504)	1,551 (1,489-1,607)	1,547 (1,473-1,620)	1,481 (1,403-1,553)	1,321 (1,227-1,420)	1,116 (1,004-1,245)
United Arab Emirates	3 (3-4)	4 (4-4)	9 (9-10)	36 (35-38)	48 (46-51)	53 (48-59)	93 (84-101)	71 (62-80)
Yemen	252 (241-264)	304 (293-315)	379 (368-391)	532 (518-546)	683 (661-704)	863 (824-902)	1,006 (943-1,073)	1,046 (953-1,151)
South Asia	19,911 (18,273-21,587)	23,437 (23,252-23,620)	28,001 (27,784-28,199)	30,696 (30,326-31,043)	35,905 (35,012-36,848)	37,791 (36,680-38,879)	37,425 (35,570-39,287)	33,968 (30,525-38,074)
South Asia	19,911 (18,273-21,587)	23,437 (23,252-23,620)	28,001 (27,784-28,199)	30,696 (30,326-31,043)	35,905 (35,012-36,848)	37,791 (36,680-38,879)	37,425 (35,570-39,287)	33,968 (30,525-38,074)
Bangladesh	2,106 (1,977-2,231)	2,409 (2,321-2,499)	3,013 (2,888-3,129)	3,173 (3,069-3,276)	3,988 (3,889-4,098)	3,606 (3,497-3,708)	3,177 (3,031-3,318)	2,858 (2,598-3,180)
Bhutan	8 (8-9)	10 (9-11)	13 (12-13)	16 (15-17)	20 (18-22)	15 (13-17)	17 (15-19)	17 (15-19)
India	15,905 (14,528-17,321)	18,973 (18,924-19,021)	22,063 (22,020-22,113)	23,531 (23,299-23,751)	26,797 (26,055-27,587)	28,319 (27,430-29,228)	28,065 (26,477-29,648)	24,568 (22,072-27,481)
Nepal	404 (376-431)	448 (427-470)	519 (498-537)	662 (636-689)	761 (731-790)	753 (713-794)	646 (596-702)	632 (560-717)

Supplemental Results Table 2: Live births (in thousands) by location, both sexes combined, 1950, 1960, 1970, 1980, 1990, 2000, 2010, 2017

Geography	1950	1960	1970	1980	1990	2000	2010	2017
Pakistan	1,486 (1,383-1,590)	1,596 (1,500-1,695)	2,392 (2,287-2,493)	3,313 (3,136-3,480)	4,337 (4,175-4,488)	5,096 (4,845-5,347)	5,518 (5,112-5,932)	5,891 (5,173-6,733)
Southeast Asia, East Asia, and Oceania	30,471 (29,236-31,776)	35,966 (34,722-37,264)	38,314 (37,242-39,409)	35,174 (33,876-36,483)	37,653 (36,142-39,311)	29,096 (27,978-30,180)	26,977 (25,628-28,295)	28,562 (27,037-30,297)
East Asia	21,835 (20,809-22,957)	25,255 (24,245-26,289)	26,664 (25,684-27,668)	22,699 (21,520-23,880)	24,946 (23,536-26,545)	16,654 (15,642-17,702)	14,483 (13,428-15,616)	17,180 (16,167-18,210)
China	20,873 (19,881-21,979)	24,018 (23,061-25,018)	25,392 (24,432-26,384)	21,519 (20,357-22,682)	23,756 (22,363-25,350)	15,686 (14,700-16,739)	13,748 (12,714-14,840)	16,469 (15,475-17,488)
North Korea	326 (291-365)	425 (380-472)	436 (392-482)	382 (344-427)	447 (398-499)	414 (359-473)	307 (269-351)	258 (228-295)
Taiwan	345 (343-347)	415 (413-417)	378 (376-381)	408 (405-410)	326 (323-329)	281 (278-284)	190 (186-193)	175 (154-200)
Oceania	116 (111-122)	138 (133-143)	156 (151-161)	176 (172-181)	222 (215-229)	285 (269-301)	355 (327-382)	398 (364-433)
American Samoa	0 (0-0)	0 (0-0)	1 (0-1)	1 (1-1)	1 (1-1)	1 (1-1)	1 (1-1)	1 (0-1)
Federated States of Micronesia	2 (1-2)	2 (2-2)	2 (2-2)	3 (3-3)	3 (3-3)	2 (2-2)	2 (2-2)	2 (1-2)
Fiji	11 (10-12)	16 (16-17)	17 (17-18)	19 (19-20)	19 (18-20)	18 (17-19)	19 (18-21)	18 (16-20)
Guam	1 (1-1)	2 (2-2)	2 (2-2)	2 (2-3)	3 (3-4)	3 (3-3)	3 (3-3)	3 (3-3)
Kiribati	1 (1-1)	1 (1-1)	1 (1-1)	2 (2-2)	2 (2-2)	2 (2-2)	3 (3-3)	3 (3-4)
Marshall Islands	0 (0-0)	0 (0-0)	1 (0-1)	1 (1-1)	1 (1-1)	1 (1-1)	1 (1-1)	1 (1-1)
Northern Mariana Islands	0 (0-0)	0 (0-0)	0 (0-0)	0 (0-0)	1 (1-1)	2 (2-2)	0 (0-0)	0 (0-0)
Papua New Guinea	77 (73-82)	88 (84-91)	99 (95-103)	112 (109-115)	148 (143-152)	202 (191-213)	265 (243-287)	309 (282-336)
Samoa	4 (3-4)	4 (4-5)	5 (4-5)	3 (3-4)	3 (3-4)	5 (4-5)	5 (5-6)	6 (5-6)
Solomon Islands	4 (4-5)	6 (6-6)	7 (7-7)	9 (9-10)	13 (12-14)	17 (16-18)	19 (18-21)	20 (18-22)
Tonga	1 (1-1)	2 (2-2)	2 (2-2)	2 (2-2)	2 (2-2)	2 (2-2)	2 (2-2)	2 (1-2)
Vanuatu	1 (1-2)	2 (2-2)	3 (3-3)	4 (4-4)	5 (5-5)	6 (6-7)	7 (7-8)	8 (7-9)
Southeast Asia	8,519 (7,957-9,064)	10,572 (10,127-11,007)	11,493 (11,151-11,826)	12,298 (11,909-12,650)	12,485 (12,120-12,844)	12,155 (11,735-12,568)	12,139 (11,440-12,871)	10,983 (9,949-12,233)
Cambodia	220 (204-235)	274 (259-289)	334 (320-348)	376 (364-387)	452 (442-461)	390 (377-402)	380 (354-406)	377 (343-419)
Indonesia	3,684 (3,468-3,899)	4,748 (4,524-4,957)	5,053 (4,894-5,200)	5,060 (4,912-5,207)	4,707 (4,531-4,878)	4,656 (4,481-4,829)	4,636 (4,280-5,014)	4,032 (3,491-4,705)
Laos	91 (84-97)	106 (100-112)	124 (118-130)	145 (140-150)	159 (154-163)	179 (174-184)	178 (168-188)	176 (161-194)
Malaysia	261 (240-283)	312 (310-314)	328 (326-330)	391 (388-394)	490 (487-493)	485 (481-489)	485 (481-489)	508 (457-566)
Maldives	3 (2-3)	4 (4-4)	5 (5-5)	6 (6-6)	7 (7-8)	5 (5-5)	7 (7-7)	6 (6-7)
Mauritius	23 (22-24)	25 (24-26)	21 (20-23)	24 (22-25)	22 (22-23)	20 (20-21)	14 (14-15)	12 (11-13)
Myanmar	851 (784-919)	997 (930-1,062)	1,059 (992-1,128)	1,158 (1,064-1,248)	1,158 (1,051-1,267)	1,093 (982-1,213)	919 (827-1,019)	876 (804-959)
Philippines	960 (902-1,018)	1,223 (1,161-1,286)	1,495 (1,425-1,561)	1,832 (1,757-1,903)	2,129 (2,049-2,219)	2,331 (2,232-2,432)	2,619 (2,460-2,772)	2,526 (2,304-2,800)
Sri Lanka	304 (282-330)	365 (351-378)	397 (390-404)	412 (392-435)	376 (352-403)	367 (340-394)	360 (324-398)	292 (248-344)
Seychelles	1 (1-1)	1 (1-1)	1 (1-1)	1 (1-1)	1 (1-1)	1 (1-1)	1 (1-1)	1 (1-1)
Thailand	848 (770-927)	1,035 (977-1,087)	1,146 (1,107-1,185)	1,158 (1,101-1,217)	1,139 (1,074-1,198)	978 (917-1,043)	783 (718-849)	613 (539-701)
Timor-Leste	25 (23-26)	28 (27-29)	25 (24-26)	28 (26-29)	37 (36-39)	42 (40-44)	36 (33-39)	38 (34-43)
Vietnam	1,231 (1,140-1,324)	1,432 (1,350-1,512)	1,481 (1,397-1,562)	1,686 (1,612-1,760)	1,784 (1,734-1,835)	1,586 (1,535-1,637)	1,699 (1,607-1,792)	1,504 (1,372-1,660)
Sub-Saharan Africa	9,421 (8,881-9,948)	11,403 (10,934-11,849)	13,907 (13,439-14,384)	17,615 (17,097-18,110)	22,042 (21,433-22,663)	27,549 (26,770-28,381)	32,937 (31,533-34,300)	36,181 (34,016-38,650)
Central Sub-Saharan Africa	1,092 (1,046-1,134)	1,408 (1,356-1,459)	1,679 (1,610-1,742)	2,071 (1,996-2,144)	2,589 (2,497-2,675)	3,284 (3,176-3,400)	4,098 (3,934-4,268)	4,318 (4,060-4,568)
Angola	253 (244-264)	287 (275-298)	310 (298-321)	382 (366-397)	519 (489-547)	718 (670-761)	977 (902-1,049)	1,052 (962-1,146)
Central African Republic	61 (57-66)	80 (75-85)	98 (91-105)	103 (96-109)	119 (111-127)	143 (131-156)	157 (143-171)	133 (119-149)
Congo	43 (40-46)	50 (47-53)	60 (57-62)	77 (73-81)	96 (91-100)	121 (116-126)	143 (133-152)	131 (119-145)
Democratic Republic of the Congo	702 (666-735)	956 (916-994)	1,173 (1,118-1,226)	1,462 (1,408-1,514)	1,796 (1,736-1,853)	2,230 (2,155-2,301)	2,741 (2,621-2,860)	2,920 (2,712-3,123)
Equatorial Guinea	10 (10-11)	11 (11-11)	12 (12-13)	15 (14-16)	21 (20-23)	30 (28-31)	37 (33-40)	39 (33-44)
Gabon	19 (18-21)	22 (20-23)	24 (22-25)	30 (28-32)	36 (32-39)	39 (35-44)	41 (37-46)	41 (36-47)
Eastern Sub-Saharan Africa	3,476 (3,281-3,669)	4,355 (4,189-4,512)	5,468 (5,317-5,620)	7,187 (7,022-7,341)	9,143 (8,943-9,333)	11,163 (10,871-11,461)	12,815 (12,242-13,372)	13,995 (13,041-15,084)
Burundi	135 (128-142)	155 (148-161)	172 (167-176)	221 (215-225)	249 (241-256)	245 (237-255)	350 (333-367)	406 (379-436)
Comoros	6 (5-6)	8 (8-9)	13 (12-13)	17 (16-17)	19 (18-20)	21 (19-23)	19 (17-22)	18 (15-20)
Djibouti	3 (3-3)	5 (4-5)	8 (7-8)	15 (14-15)	23 (22-24)	28 (26-30)	32 (29-35)	34 (30-39)
Eritrea	61 (57-64)	77 (74-81)	102 (96-107)	125 (118-131)	129 (121-137)	143 (131-156)	179 (160-200)	177 (155-203)
Ethiopia	933 (868-996)	1,119 (1,057-1,175)	1,427 (1,374-1,480)	1,822 (1,764-1,876)	2,563 (2,479-2,644)	3,158 (3,035-3,282)	3,370 (3,176-3,573)	3,714 (3,402-4,069)
Kenya	329 (314-343)	475 (460-489)	593 (572-613)	825 (798-852)	986 (948-1,022)	1,146 (1,088-1,200)	1,281 (1,183-1,379)	1,365 (1,208-1,535)

Supplemental Results Table 2: Live births (in thousands) by location, both sexes combined, 1950, 1960, 1970, 1980, 1990, 2000, 2010, 2017								
Geography	1950	1960	1970	1980	1990	2000	2010	2017
Madagascar	208<br>(198-219)	264<br>(254-274)	351<br>(339-362)	448<br>(434-461)	537<br>(514-559)	693<br>(659-724)	839<br>(789-890)	975<br>(871-1,083)
Malawi	166<br>(157-174)	215<br>(208-222)	260<br>(254-265)	333<br>(327-338)	500<br>(491-510)	551<br>(539-563)	579<br>(559-600)	612<br>(571-660)
Mozambique	334<br>(310-357)	373<br>(353-392)	445<br>(423-467)	578<br>(561-593)	664<br>(649-679)	808<br>(788-827)	893<br>(860-923)	988<br>(912-1,068)
Rwanda	132<br>(124-140)	155<br>(146-163)	193<br>(187-199)	264<br>(259-268)	326<br>(317-334)	333<br>(322-346)	370<br>(350-391)	423<br>(381-470)
Somalia	104<br>(98-109)	139<br>(133-145)	193<br>(184-201)	353<br>(337-369)	325<br>(307-340)	466<br>(440-490)	600<br>(564-633)	685<br>(638-737)
South Sudan	117<br>(106-127)	144<br>(134-156)	168<br>(157-178)	208<br>(194-222)	262<br>(242-281)	345<br>(321-367)	443<br>(413-473)	413<br>(387-444)
Tanzania	484<br>(458-508)	601<br>(575-624)	752<br>(734-769)	966<br>(942-991)	1,214<br>(1,185-1,243)	1,495<br>(1,449-1,544)	1,839<br>(1,752-1,929)	1,986<br>(1,828-2,163)
Uganda	326<br>(310-341)	428<br>(412-442)	555<br>(540-568)	711<br>(698-722)	951<br>(933-968)	1,264<br>(1,236-1,289)	1,447<br>(1,400-1,495)	1,550<br>(1,471-1,638)
Zambia	132<br>(124-139)	188<br>(179-197)	229<br>(217-241)	293<br>(279-306)	386<br>(365-406)	453<br>(430-477)	559<br>(515-603)	634<br>(568-710)
Southern Sub-Saharan Africa	820<br>(754-886)	952<br>(892-1,007)	1,183<br>(1,113-1,254)	1,426<br>(1,329-1,523)	1,552<br>(1,433-1,669)	1,700<br>(1,590-1,809)	1,782<br>(1,629-1,954)	1,748<br>(1,595-1,938)
Botswana	18<br>(17-19)	22<br>(21-23)	28<br>(27-29)	37<br>(36-38)	42<br>(41-43)	46<br>(44-47)	48<br>(46-50)	48<br>(45-52)
Lesotho	31<br>(29-33)	36<br>(34-38)	45<br>(43-47)	57<br>(55-59)	62<br>(60-64)	57<br>(55-59)	52<br>(50-55)	48<br>(44-53)
Namibia	22<br>(20-23)	27<br>(26-28)	33<br>(31-34)	40<br>(39-41)	50<br>(49-51)	54<br>(53-55)	60<br>(58-62)	59<br>(54-64)
South Africa	584<br>(533-636)	661<br>(611-708)	801<br>(742-861)	913<br>(823-1,005)	973<br>(866-1,082)	1,092<br>(987-1,197)	1,125<br>(983-1,286)	1,091<br>(976-1,238)
Swaziland	12<br>(11-12)	15<br>(15-16)	20<br>(19-21)	27<br>(26-28)	31<br>(30-33)	34<br>(32-35)	32<br>(29-34)	30<br>(26-35)
Zimbabwe	151<br>(142-159)	187<br>(179-195)	253<br>(243-261)	349<br>(342-357)	390<br>(382-399)	416<br>(405-426)	463<br>(453-473)	469<br>(438-501)
Western Sub-Saharan Africa	4,032<br>(3,799-4,254)	4,686<br>(4,474-4,889)	5,576<br>(5,368-5,778)	6,929<br>(6,684-7,156)	8,757<br>(8,480-9,028)	11,402<br>(11,031-11,754)	14,240<br>(13,632-14,856)	16,119<br>(15,142-17,204)
Benin	112<br>(104-119)	117<br>(111-122)	134<br>(130-138)	169<br>(164-173)	231<br>(225-237)	299<br>(289-308)	379<br>(362-397)	420<br>(387-457)
Burkina Faso	199<br>(183-214)	199<br>(186-211)	243<br>(233-254)	340<br>(329-350)	453<br>(444-463)	558<br>(541-574)	715<br>(682-748)	850<br>(792-913)
Cameroon	240<br>(222-256)	277<br>(259-295)	321<br>(307-336)	368<br>(355-382)	445<br>(426-463)	633<br>(607-659)	848<br>(791-906)	860<br>(767-970)
Cape Verde	7<br>(6-7)	9<br>(9-9)	8<br>(8-9)	10<br>(10-11)	11<br>(11-12)	11<br>(10-12)	10<br>(9-12)	9<br>(8-11)
Chad	145<br>(136-153)	160<br>(153-167)	192<br>(185-199)	247<br>(238-256)	323<br>(314-331)	437<br>(425-449)	594<br>(573-614)	716<br>(684-753)
Cote d'Ivoire	130<br>(121-139)	207<br>(195-217)	281<br>(270-291)	398<br>(382-415)	568<br>(537-597)	734<br>(687-779)	835<br>(769-902)	863<br>(785-951)
The Gambia	12<br>(11-13)	14<br>(14-15)	23<br>(21-24)	34<br>(33-36)	47<br>(44-50)	58<br>(54-62)	65<br>(59-71)	68<br>(60-78)
Ghana	202<br>(182-223)	328<br>(308-348)	439<br>(420-458)	537<br>(517-557)	579<br>(553-604)	678<br>(644-713)	766<br>(715-817)	876<br>(760-1,005)
Guinea	124<br>(118-130)	158<br>(150-166)	204<br>(195-213)	248<br>(239-257)	304<br>(294-313)	358<br>(344-370)	395<br>(376-414)	434<br>(409-461)
Guinea-Bissau	33<br>(31-34)	31<br>(29-33)	31<br>(29-33)	42<br>(40-44)	50<br>(46-52)	57<br>(53-61)	65<br>(59-70)	68<br>(62-75)
Liberia	45<br>(42-48)	52<br>(48-54)	72<br>(70-75)	99<br>(95-103)	86<br>(82-89)	133<br>(127-138)	156<br>(145-166)	154<br>(138-172)
Mali	187<br>(175-197)	247<br>(236-257)	317<br>(307-328)	387<br>(376-397)	450<br>(440-460)	539<br>(521-556)	718<br>(691-744)	877<br>(829-932)
Mauritania	35<br>(32-37)	42<br>(39-44)	53<br>(51-56)	73<br>(71-75)	89<br>(86-93)	103<br>(99-106)	116<br>(110-122)	118<br>(109-129)
Niger	155<br>(146-163)	202<br>(193-210)	266<br>(259-274)	349<br>(342-355)	441<br>(434-448)	621<br>(609-632)	812<br>(786-837)	1,005<br>(952-1,063)
Nigeria	2,103<br>(1,984-2,216)	2,278<br>(2,171-2,381)	2,533<br>(2,415-2,647)	3,063<br>(2,909-3,208)	3,987<br>(3,819-4,152)	5,391<br>(5,173-5,602)	6,802<br>(6,472-7,157)	7,798<br>(7,206-8,409)
Sao Tome and Principe	2<br>(2-2)	3<br>(3-3)	3<br>(3-3)	3<br>(3-3)	4<br>(4-4)	5<br>(5-5)	5<br>(4-5)	4<br>(4-5)
Senegal	130<br>(120-139)	163<br>(152-173)	228<br>(218-238)	289<br>(274-302)	341<br>(319-362)	397<br>(374-421)	468<br>(431-503)	496<br>(457-543)
Sierra Leone	93<br>(85-101)	106<br>(98-114)	118<br>(109-126)	134<br>(126-142)	178<br>(169-187)	188<br>(177-198)	249<br>(230-266)	269<br>(243-296)
Togo	71<br>(67-75)	86<br>(83-88)	98<br>(95-102)	129<br>(126-132)	160<br>(156-165)	195<br>(188-202)	234<br>(224-243)	223<br>(207-241)

Supplemental Results Table 3: Live births (in thousands) by location, males, 1950, 1960, 1970, 1980, 1990, 2000, 2010, 2017								
**Geography**	**1950**	**1960**	**1970**	**1980**	**1990**	**2000**	**2010**	**2017**
Global	47,326 (45,411-49,256)	54,965 (53,752-56,129)	60,579 (59,585-61,526)	63,822 (62,615-65,011)	70,784 (69,236-72,328)	69,441 (67,771-71,065)	71,959 (69,454-74,638)	71,871 (67,281-77,160)
Central Europe, Eastern Europe, and Central Asia	3,775 (3,703-3,856)	3,796 (3,749-3,840)	3,219 (3,161-3,277)	3,657 (3,615-3,701)	3,401 (3,362-3,440)	2,373 (2,332-2,415)	2,907 (2,852-2,965)	2,697 (2,420-2,997)
Central Asia	520 (498-541)	719 (701-737)	708 (674-744)	899 (878-920)	1,024 (1,001-1,047)	774 (746-802)	984 (938-1,031)	992 (910-1,078)
Armenia	24 (23-26)	35 (34-37)	29 (27-30)	38 (36-39)	40 (38-41)	21 (20-22)	23 (22-24)	20 (18-22)
Azerbaijan	51 (47-55)	82 (79-85)	75 (70-79)	83 (79-87)	99 (95-103)	70 (66-73)	97 (91-104)	92 (82-105)
Georgia	44 (39-48)	51 (48-55)	44 (41-48)	45 (43-48)	42 (40-44)	27 (25-28)	31 (29-33)	26 (23-28)
Kazakhstan	129 (124-134)	182 (176-189)	180 (169-192)	198 (193-203)	194 (190-199)	124 (120-128)	180 (173-187)	179 (162-196)
Kyrgyzstan	30 (28-31)	39 (37-40)	45 (41-48)	56 (54-58)	64 (62-66)	52 (50-54)	75 (73-78)	78 (72-83)
Mongolia	17 (16-18)	19 (18-20)	22 (21-22)	30 (29-31)	34 (33-35)	25 (24-26)	36 (34-37)	38 (35-41)
Tajikistan	44 (43-46)	60 (58-61)	64 (61-66)	87 (84-91)	111 (107-115)	99 (94-104)	128 (119-137)	147 (133-160)
Turkmenistan	27 (26-28)	38 (37-38)	37 (34-39)	50 (49-52)	63 (61-65)	45 (43-48)	58 (54-61)	56 (50-63)
Uzbekistan	150 (144-156)	209 (203-214)	210 (199-221)	308 (297-320)	373 (360-387)	309 (290-328)	353 (322-383)	353 (321-385)
Central Europe	1,183 (1,158-1,209)	1,010 (997-1,025)	981 (970-994)	1,059 (1,050-1,068)	868 (862-873)	619 (614-625)	621 (614-629)	548 (494-610)
Albania	24 (24-25)	35 (34-36)	38 (36-40)	40 (38-41)	45 (43-46)	28 (28-29)	20 (19-21)	19 (16-22)
Bosnia and Herzegovina	39 (34-45)	45 (39-51)	37 (32-44)	38 (33-43)	34 (33-35)	20 (19-20)	17 (17-18)	14 (13-15)
Bulgaria	86 (85-87)	71 (70-72)	70 (69-71)	65 (64-66)	52 (51-52)	36 (35-37)	38 (37-39)	30 (26-34)
Croatia	44 (38-52)	41 (36-48)	33 (31-35)	36 (34-38)	27 (27-27)	22 (21-22)	21 (21-22)	18 (17-19)
Czech Republic	95 (95-96)	65 (65-66)	76 (75-77)	77 (76-78)	65 (65-66)	46 (46-47)	58 (57-58)	53 (49-58)
Hungary	99 (98-100)	73 (73-74)	77 (76-78)	75 (74-76)	64 (63-65)	49 (48-50)	46 (45-47)	44 (39-50)
Macedonia	18 (16-21)	18 (16-21)	18 (16-20)	21 (20-23)	18 (18-18)	13 (13-13)	12 (12-12)	12 (11-13)
Montenegro	6 (5-6)	6 (6-6)	5 (5-5)	5 (5-5)	4 (4-5)	4 (4-4)	3 (3-4)	3 (3-3)
Poland	391 (389-393)	332 (330-334)	280 (278-283)	353 (351-355)	281 (279-283)	191 (188-193)	206 (204-209)	182 (162-206)
Romania	210 (194-229)	174 (172-176)	211 (209-213)	201 (200-203)	149 (148-151)	115 (113-116)	107 (105-109)	91 (81-101)
Serbia	96 (95-97)	85 (84-86)	75 (74-76)	79 (78-80)	70 (69-71)	54 (53-55)	45 (41-50)	41 (36-47)
Slovakia	50 (49-51)	44 (44-45)	41 (40-41)	48 (47-48)	41 (40-41)	27 (26-27)	30 (30-31)	26 (23-30)
Slovenia	17 (16-18)	14 (14-15)	14 (14-14)	15 (15-15)	11 (11-12)	9 (9-9)	11 (11-11)	9 (8-10)
Eastern Europe	2,071 (2,030-2,120)	2,066 (2,042-2,088)	1,528 (1,505-1,552)	1,698 (1,680-1,717)	1,508 (1,493-1,523)	979 (965-993)	1,300 (1,284-1,317)	1,156 (1,008-1,327)
Belarus	95 (89-100)	98 (94-102)	75 (73-78)	82 (79-85)	70 (68-72)	48 (46-49)	58 (54-61)	52 (46-59)
Estonia	10 (10-10)	10 (10-10)	11 (10-11)	11 (11-11)	10 (10-11)	6 (6-7)	7 (7-8)	6 (6-7)
Latvia	16 (16-17)	18 (17-18)	17 (17-18)	18 (17-18)	18 (18-18)	10 (10-10)	10 (9-10)	10 (8-11)
Lithuania	29 (27-31)	31 (31-32)	28 (27-29)	26 (26-27)	29 (28-29)	17 (16-17)	17 (17-18)	14 (13-16)
Moldova	44 (41-46)	42 (40-44)	36 (32-40)	42 (41-44)	39 (38-40)	23 (22-24)	22 (21-23)	18 (16-20)
Russian Federation	1,438 (1,427-1,451)	1,434 (1,427-1,441)	993 (987-999)	1,131 (1,125-1,136)	1,015 (1,010-1,020)	662 (656-667)	921 (916-927)	834 (725-960)
Ukraine	436 (412-464)	431 (412-450)	365 (345-385)	385 (370-400)	323 (313-335)	210 (202-221)	262 (249-275)	219 (191-251)
High-income	6,922 (6,819-7,030)	7,402 (7,349-7,457)	7,167 (7,111-7,222)	6,442 (6,403-6,481)	6,457 (6,422-6,492)	6,097 (6,057-6,139)	6,100 (6,067-6,134)	5,877 (5,338-6,486)
Australasia	124 (122-125)	153 (151-154)	167 (166-168)	143 (141-144)	162 (160-163)	155 (152-158)	184 (182-186)	191 (173-212)
Australia	98 (97-99)	119 (118-120)	135 (134-136)	117 (116-118)	131 (130-133)	126 (124-128)	152 (151-154)	161 (143-181)
New Zealand	25 (24-26)	33 (32-34)	32 (31-32)	25 (25-26)	30 (29-30)	28 (27-29)	31 (30-32)	30 (28-34)
High-income Asia Pacific	1,509 (1,462-1,561)	1,310 (1,263-1,356)	1,595 (1,554-1,634)	1,289 (1,259-1,321)	1,038 (1,013-1,066)	961 (935-991)	825 (800-852)	732 (646-832)
Brunei	1 (1-1)	2 (2-2)	2 (2-2)	2 (2-3)	3 (3-3)	3 (3-3)	3 (3-3)	3 (3-3)
Japan	1,133 (1,094-1,174)	818 (784-852)	1,020 (981-1,060)	804 (772-836)	628 (603-656)	605 (579-633)	550 (525-578)	472 (392-566)
Singapore	24 (23-24)	30 (29-31)	23 (22-24)	21 (20-22)	25 (24-26)	38 (36-40)	28 (27-30)	33 (27-39)
South Korea	350 (319-383)	459 (429-492)	549 (537-560)	461 (459-463)	380 (375-385)	313 (310-316)	242 (240-244)	222 (211-233)

Supplemental Results Table 3: Live births (in thousands) by location, males, 1950, 1960, 1970, 1980, 1990, 2000, 2010, 2017								
Geography	1950	1960	1970	1980	1990	2000	2010	2017
High-income North America	2,053 (2,045-2,062)	2,412 (2,405-2,420)	2,027 (2,018-2,037)	2,029 (2,019-2,039)	2,366 (2,356-2,377)	2,242 (2,233-2,251)	2,215 (2,205-2,224)	2,208 (2,038-2,397)
Canada	185 (183-186)	235 (234-236)	179 (177-180)	185 (182-187)	204 (202-206)	170 (168-172)	191 (190-193)	199 (171-232)
Greenland	0 (0-0)	0 (0-0)	0 (0-0)	0 (0-0)	0 (0-0)	0 (0-0)	0 (0-0)	0 (0-0)
United States	1,867 (1,859-1,876)	2,176 (2,169-2,184)	1,847 (1,839-1,856)	1,843 (1,835-1,852)	2,161 (2,150-2,172)	2,071 (2,062-2,080)	2,022 (2,013-2,031)	2,008 (1,866-2,163)
Southern Latin America	354 (338-371)	409 (406-413)	430 (426-434)	500 (497-502)	524 (520-527)	514 (508-520)	532 (528-536)	531 (488-576)
Argentina	231 (215-247)	239 (236-242)	275 (272-279)	348 (346-350)	349 (346-352)	360 (355-366)	381 (378-384)	381 (354-408)
Chile	100 (99-101)	139 (138-140)	125 (124-126)	123 (122-124)	146 (145-148)	126 (125-127)	126 (124-128)	125 (110-142)
Uruguay	22 (22-23)	31 (30-31)	28 (28-29)	27 (27-28)	28 (27-28)	26 (26-27)	24 (22-26)	24 (21-28)
Western Europe	2,880 (2,808-2,965)	3,116 (3,099-3,133)	2,946 (2,920-2,971)	2,479 (2,468-2,490)	2,365 (2,354-2,376)	2,223 (2,212-2,235)	2,342 (2,330-2,354)	2,213 (1,986-2,467)
Andorra	0 (0-0)	0 (0-0)	0 (0-0)	0 (0-0)	0 (0-0)	0 (0-0)	0 (0-0)	0 (0-0)
Austria	53 (52-54)	66 (65-66)	56 (55-57)	47 (46-47)	47 (46-48)	39 (39-40)	40 (39-41)	44 (40-48)
Belgium	72 (72-73)	81 (80-81)	73 (72-73)	63 (62-64)	64 (63-64)	59 (58-59)	65 (64-66)	62 (56-69)
Cyprus	7 (6-7)	7 (7-7)	7 (6-7)	6 (6-6)	6 (5-6)	5 (5-5)	6 (5-6)	5 (4-6)
Denmark	40 (39-40)	39 (38-39)	37 (37-38)	28 (27-28)	32 (32-33)	33 (33-34)	31 (30-31)	31 (28-34)
Finland	48 (48-49)	41 (40-42)	31 (31-32)	32 (32-33)	33 (32-33)	28 (27-28)	30 (30-31)	28 (25-31)
France	428 (425-431)	422 (420-425)	437 (434-440)	411 (408-413)	389 (387-392)	397 (394-400)	408 (405-411)	377 (339-419)
Germany	561 (520-612)	650 (647-653)	527 (523-531)	436 (432-439)	447 (444-451)	389 (386-392)	344 (340-347)	364 (324-409)
Greece	80 (78-81)	79 (78-80)	73 (72-75)	74 (73-75)	52 (51-53)	52 (51-53)	56 (55-58)	46 (41-51)
Iceland	2 (1-2)	2 (2-2)	2 (2-2)	2 (2-2)	2 (2-2)	2 (2-2)	2 (2-2)	2 (1-2)
Ireland	33 (31-35)	31 (30-31)	33 (32-34)	37 (36-37)	27 (26-27)	28 (28-29)	37 (37-38)	33 (29-37)
Israel	25 (23-26)	28 (28-28)	41 (40-42)	47 (47-48)	53 (52-53)	69 (68-70)	84 (83-85)	91 (82-100)
Italy	452 (450-455)	471 (468-474)	464 (461-467)	326 (323-329)	291 (288-293)	277 (274-281)	287 (284-290)	238 (210-270)
Luxembourg	2 (2-2)	2 (2-2)	2 (2-2)	2 (2-2)	2 (2-2)	2 (2-2)	2 (2-3)	3 (3-3)
Malta	5 (4-5)	4 (4-4)	2 (2-2)	2 (2-3)	2 (2-2)	2 (1-2)	2 (2-2)	2 (1-2)
Netherlands	117 (116-118)	124 (123-126)	118 (117-119)	91 (89-92)	100 (99-101)	105 (103-106)	93 (92-94)	88 (79-98)
Norway	31 (31-32)	32 (31-32)	33 (33-34)	26 (25-26)	31 (30-31)	29 (29-30)	30 (30-31)	30 (28-33)
Portugal	106 (105-107)	112 (111-113)	93 (92-94)	78 (77-79)	59 (58-60)	59 (58-60)	50 (49-51)	43 (38-50)
Spain	287 (285-289)	333 (331-335)	339 (337-341)	283 (281-286)	205 (202-207)	204 (202-206)	248 (245-250)	209 (190-230)
Sweden	58 (57-59)	52 (52-53)	57 (56-58)	49 (48-49)	63 (62-63)	47 (46-48)	57 (57-58)	60 (56-65)
Switzerland	42 (41-43)	49 (48-49)	50 (49-51)	38 (37-38)	43 (42-44)	39 (38-39)	41 (40-42)	44 (40-50)
United Kingdom	420 (398-447)	479 (467-493)	459 (438-481)	391 (388-394)	407 (404-410)	345 (342-348)	416 (413-419)	401 (360-448)
England	334 (314-359)	385 (375-396)	373 (355-391)	318 (315-321)	338 (335-341)	291 (288-293)	356 (353-358)	345 (310-384)
Northern Ireland	15 (15-16)	16 (16-17)	15 (15-16)	14 (14-15)	13 (12-13)	10 (10-11)	12 (12-12)	12 (10-13)
Scotland	46 (46-47)	52 (50-54)	45 (43-48)	36 (35-37)	34 (33-35)	27 (25-28)	29 (28-30)	27 (24-30)
Wales	22 (21-24)	25 (24-26)	24 (23-25)	21 (20-22)	21 (21-22)	16 (15-16)	18 (17-18)	17 (15-19)
Latin America and Caribbean	3,254 (3,066-3,438)	4,128 (3,959-4,295)	4,802 (4,664-4,940)	5,374 (5,214-5,544)	5,595 (5,402-5,792)	5,510 (5,296-5,727)	5,467 (5,177-5,757)	5,313 (4,841-5,843)
Andean Latin America	376 (360-391)	462 (447-476)	544 (533-554)	614 (595-633)	664 (641-686)	635 (605-667)	683 (645-718)	707 (643-779)
Bolivia	81 (78-84)	91 (87-94)	99 (96-102)	120 (117-124)	132 (125-138)	133 (125-141)	144 (135-154)	153 (138-170)
Ecuador	80 (77-83)	102 (102-103)	118 (117-118)	132 (129-136)	154 (147-162)	166 (155-178)	167 (154-180)	161 (137-189)
Peru	214 (201-226)	268 (256-280)	326 (317-335)	360 (344-377)	377 (359-395)	335 (316-356)	370 (349-391)	392 (350-442)
Caribbean	346 (333-359)	425 (412-437)	452 (445-458)	428 (420-437)	468 (457-479)	453 (438-466)	420 (401-441)	416 (381-454)
Antigua and Barbuda	0 (0-0)	0 (0-0)	0 (0-0)	0 (0-0)	0 (0-0)	0 (0-0)	0 (0-0)	0 (0-0)
The Bahamas	1 (1-1)	1 (1-1)	2 (2-2)	2 (2-2)	2 (2-2)	2 (2-2)	2 (2-2)	2 (1-2)

Supplemental Results Table 3: Live births (in thousands) by location, males, 1950, 1960, 1970, 1980, 1990, 2000, 2010, 2017								
**Geography**	**1950**	**1960**	**1970**	**1980**	**1990**	**2000**	**2010**	**2017**
Barbados	3 (3-3)	3 (3-3)	2 (2-2)	2 (2-2)	2 (2-2)	1 (1-2)	1 (1-1)	1 (1-1)
Belize	1 (1-1)	2 (2-2)	2 (2-2)	3 (2-3)	3 (3-3)	3 (3-3)	3 (3-3)	3 (3-4)
Bermuda	0 (0-0)	0 (0-0)	0 (0-0)	0 (0-0)	0 (0-0)	0 (0-0)	0 (0-0)	0 (0-0)
Cuba	79 (75-83)	115 (110-120)	127 (125-128)	71 (70-72)	91 (90-92)	77 (75-78)	67 (66-68)	56 (53-59)
Dominica	1 (0-1)	1 (1-1)	1 (1-1)	0 (0-0)	0 (0-0)	0 (0-0)	0 (0-0)	0 (0-0)
Dominican Republic	57 (54-62)	75 (72-78)	96 (94-99)	116 (112-121)	121 (115-127)	124 (118-131)	102 (95-110)	110 (95-128)
Grenada	1 (1-1)	1 (1-1)	1 (1-1)	1 (1-1)	1 (1-1)	0 (0-1)	0 (0-1)	0 (0-0)
Guyana	9 (9-10)	12 (12-13)	12 (12-12)	13 (12-13)	13 (12-14)	10 (10-10)	8 (7-8)	8 (6-9)
Haiti	87 (82-92)	92 (87-97)	92 (89-95)	108 (104-111)	128 (124-132)	139 (133-146)	156 (145-167)	165 (147-185)
Jamaica	24 (23-26)	34 (33-35)	33 (32-34)	31 (30-32)	30 (29-31)	27 (25-29)	22 (20-24)	19 (17-22)
Puerto Rico	43 (43-43)	38 (37-38)	35 (34-35)	36 (35-36)	33 (32-33)	29 (28-30)	21 (20-23)	15 (13-16)
Saint Lucia	1 (1-1)	2 (2-2)	2 (2-2)	1 (1-1)	1 (1-1)	1 (1-1)	1 (1-1)	1 (0-1)
Saint Vincent and the Grenadines	1 (1-1)	2 (1-2)	1 (1-1)	1 (1-1)	1 (1-1)	1 (1-1)	0 (0-0)	0 (0-0)
Suriname	3 (3-3)	6 (6-6)	6 (6-7)	5 (5-5)	4 (4-5)	5 (5-5)	5 (4-5)	4 (4-5)
Trinidad and Tobago	12 (11-13)	16 (16-16)	13 (12-13)	15 (14-15)	12 (11-12)	9 (8-9)	9 (8-10)	8 (7-10)
Virgin Islands, U.S.	0 (0-0)	0 (0-0)	1 (1-1)	1 (1-1)	1 (1-1)	1 (0-1)	0 (0-0)	0 (0-0)
Central Latin America	1,270 (1,218-1,323)	1,700 (1,635-1,759)	2,041 (1,978-2,103)	2,300 (2,220-2,379)	2,613 (2,507-2,726)	2,588 (2,477-2,699)	2,537 (2,399-2,668)	2,557 (2,300-2,852)
Colombia	251 (235-268)	371 (360-381)	419 (406-430)	450 (431-467)	501 (484-519)	503 (481-525)	467 (440-494)	436 (376-505)
Costa Rica	19 (18-20)	30 (30-30)	28 (28-28)	36 (35-36)	41 (40-41)	38 (37-39)	37 (36-37)	35 (32-39)
El Salvador	47 (45-49)	62 (60-63)	74 (73-76)	78 (76-80)	80 (77-83)	69 (67-72)	55 (53-58)	55 (48-63)
Guatemala	76 (75-76)	95 (95-96)	111 (110-112)	158 (157-160)	179 (178-180)	177 (172-182)	183 (180-187)	218 (188-251)
Honduras	38 (36-41)	46 (45-47)	58 (57-59)	77 (76-79)	92 (90-94)	98 (96-101)	107 (100-113)	125 (109-144)
Mexico	683 (637-728)	862 (813-910)	1,075 (1,021-1,128)	1,159 (1,086-1,229)	1,315 (1,207-1,420)	1,326 (1,237-1,423)	1,276 (1,164-1,394)	1,284 (1,098-1,516)
Nicaragua	28 (26-30)	38 (37-39)	47 (46-48)	65 (63-66)	74 (72-76)	69 (67-72)	70 (64-77)	70 (60-81)
Panama	14 (13-15)	21 (20-21)	26 (25-27)	26 (25-27)	29 (28-30)	31 (30-33)	34 (32-36)	35 (30-41)
Venezuela	111 (109-113)	171 (170-172)	199 (197-201)	247 (245-249)	298 (296-301)	273 (270-275)	303 (301-306)	295 (262-331)
Tropical Latin America	1,260 (1,151-1,372)	1,539 (1,445-1,631)	1,764 (1,688-1,845)	2,030 (1,959-2,108)	1,848 (1,770-1,929)	1,833 (1,753-1,914)	1,825 (1,718-1,946)	1,632 (1,478-1,820)
Brazil	1,228 (1,118-1,339)	1,504 (1,409-1,595)	1,718 (1,642-1,798)	1,971 (1,900-2,047)	1,786 (1,710-1,865)	1,767 (1,689-1,847)	1,752 (1,647-1,870)	1,556 (1,403-1,745)
Paraguay	31 (29-34)	35 (34-37)	45 (44-47)	59 (57-61)	62 (59-64)	65 (62-69)	72 (65-80)	75 (64-87)
North Africa and Middle East	2,843 (2,704-2,977)	3,442 (3,323-3,557)	4,200 (4,095-4,300)	5,348 (5,223-5,478)	5,764 (5,599-5,934)	6,282 (6,050-6,509)	6,843 (6,484-7,197)	6,684 (6,197-7,246)
North Africa and Middle East	2,843 (2,704-2,977)	3,442 (3,323-3,557)	4,200 (4,095-4,300)	5,348 (5,223-5,478)	5,764 (5,599-5,934)	6,282 (6,050-6,509)	6,843 (6,484-7,197)	6,684 (6,197-7,246)
Afghanistan	207 (196-218)	242 (230-252)	292 (280-304)	263 (253-272)	235 (226-243)	471 (458-484)	567 (547-587)	711 (674-749)
Algeria	228 (217-239)	263 (250-275)	306 (295-316)	401 (391-412)	428 (410-446)	448 (412-483)	482 (441-520)	495 (440-552)
Bahrain	2 (1-2)	3 (3-3)	3 (3-3)	5 (5-5)	6 (6-7)	6 (6-7)	9 (9-10)	10 (9-11)
Egypt	459 (433-485)	595 (569-620)	648 (622-673)	827 (796-856)	859 (822-895)	893 (848-938)	1,109 (1,036-1,184)	1,092 (996-1,196)
Iran	452 (423-480)	510 (487-532)	612 (587-636)	884 (853-913)	825 (774-880)	760 (702-818)	769 (714-824)	654 (557-767)
Iraq	133 (126-140)	168 (160-176)	215 (207-223)	293 (283-303)	343 (329-357)	486 (467-503)	551 (514-588)	644 (582-715)
Jordan	13 (13-13)	21 (21-21)	32 (32-33)	46 (45-47)	67 (64-70)	79 (75-83)	110 (104-117)	124 (114-136)
Kuwait	1 (1-1)	5 (5-5)	17 (16-17)	25 (25-25)	14 (14-15)	21 (20-21)	27 (27-28)	31 (28-34)
Lebanon	35 (33-37)	43 (42-45)	52 (50-55)	65 (60-69)	71 (64-77)	79 (71-87)	78 (69-88)	95 (82-111)
Libya	29 (29-30)	38 (36-39)	45 (44-46)	59 (58-60)	66 (61-70)	54 (53-55)	68 (61-76)	62 (52-75)
Morocco	216 (205-228)	270 (257-281)	316 (301-330)	361 (341-380)	388 (366-409)	379 (350-407)	347 (303-390)	307 (270-350)
Palestine	23 (22-24)	25 (24-26)	27 (26-28)	33 (31-34)	46 (44-48)	60 (57-63)	73 (69-78)	71 (64-78)

Supplemental Results Table 3: Live births (in thousands) by location, males, 1950, 1960, 1970, 1980, 1990, 2000, 2010, 2017								
Geography	1950	1960	1970	1980	1990	2000	2010	2017
Oman	13 (12-13)	17 (16-17)	21 (20-22)	29 (27-30)	30 (28-32)	30 (27-33)	34 (31-37)	40 (37-45)
Qatar	0 (0-0)	1 (1-1)	2 (2-2)	3 (3-3)	5 (5-5)	5 (5-6)	11 (10-11)	15 (14-16)
Saudi Arabia	72 (69-75)	95 (92-97)	143 (139-147)	244 (237-251)	340 (321-360)	283 (260-307)	266 (239-297)	258 (228-290)
Sudan	148 (140-156)	166 (158-173)	277 (268-285)	405 (393-418)	527 (506-546)	643 (618-669)	706 (664-747)	684 (623-754)
Syria	78 (74-83)	104 (98-109)	144 (136-150)	194 (186-204)	212 (196-228)	231 (228-234)	274 (265-284)	142 (123-163)
Tunisia	85 (79-90)	96 (91-100)	99 (96-102)	115 (110-120)	118 (111-126)	106 (100-113)	100 (93-107)	86 (74-102)
Turkey	508 (480-536)	614 (587-640)	738 (712-765)	793 (762-822)	794 (756-832)	763 (723-800)	679 (631-730)	573 (516-640)
United Arab Emirates	1 (1-2)	2 (2-2)	5 (4-5)	18 (18-19)	25 (23-26)	27 (25-30)	47 (43-52)	36 (32-41)
Yemen	129 (123-135)	156 (150-161)	194 (188-200)	273 (265-280)	350 (339-361)	443 (423-463)	516 (483-550)	536 (488-590)
South Asia	10,127 (9,294-10,979)	11,968 (11,874-12,062)	14,335 (14,224-14,437)	15,788 (15,597-15,966)	18,568 (18,106-19,056)	19,713 (19,133-20,281)	19,537 (18,567-20,510)	17,738 (15,939-19,882)
South Asia	10,127 (9,294-10,979)	11,968 (11,874-12,062)	14,335 (14,224-14,437)	15,788 (15,597-15,966)	18,568 (18,106-19,056)	19,713 (19,133-20,281)	19,537 (18,567-20,510)	17,738 (15,939-19,882)
Bangladesh	1,076 (1,010-1,140)	1,231 (1,186-1,277)	1,540 (1,476-1,599)	1,622 (1,569-1,675)	2,038 (1,987-2,094)	1,843 (1,787-1,895)	1,625 (1,550-1,697)	1,463 (1,330-1,628)
Bhutan	4 (4-4)	5 (5-5)	6 (6-7)	8 (7-9)	10 (9-11)	7 (6-8)	8 (7-9)	8 (7-10)
India	8,080 (7,380-8,799)	9,686 (9,662-9,711)	11,293 (11,271-11,319)	12,113 (11,994-12,227)	13,888 (13,503-14,297)	14,822 (14,356-15,298)	14,697 (13,866-15,527)	12,872 (11,564-14,399)
Nepal	207 (192-221)	229 (218-240)	266 (255-275)	339 (326-353)	390 (375-405)	386 (366-407)	332 (306-361)	325 (288-369)
Pakistan	759 (706-812)	815 (766-866)	1,228 (1,174-1,280)	1,703 (1,612-1,789)	2,240 (2,157-2,318)	2,653 (2,522-2,784)	2,872 (2,660-3,087)	3,067 (2,693-3,505)
Southeast Asia, East Asia, and Oceania	15,593 (14,961-16,261)	18,407 (17,770-19,071)	19,754 (19,200-20,321)	18,221 (17,546-18,903)	19,752 (18,952-20,632)	15,415 (14,818-15,996)	14,319 (13,599-15,030)	15,118 (14,316-16,018)
East Asia	11,178 (10,653-11,752)	12,938 (12,421-13,467)	13,803 (13,296-14,323)	11,836 (11,221-12,452)	13,229 (12,480-14,078)	9,016 (8,467-9,586)	7,875 (7,300-8,491)	9,250 (8,704-9,804)
China	10,685 (10,178-11,251)	12,302 (11,812-12,814)	13,146 (12,649-13,659)	11,224 (10,619-11,831)	12,608 (11,869-13,454)	8,508 (7,973-9,079)	7,489 (6,925-8,083)	8,877 (8,341-9,425)
North Korea	168 (149-188)	219 (196-243)	224 (202-248)	197 (177-220)	230 (205-257)	213 (185-243)	158 (138-181)	133 (117-152)
Taiwan	176 (175-177)	213 (212-214)	195 (194-197)	211 (209-212)	170 (168-171)	147 (145-148)	98 (97-100)	91 (79-103)
Oceania	59 (57-62)	71 (68-73)	80 (77-82)	91 (88-93)	114 (111-118)	147 (139-155)	183 (169-197)	206 (188-224)
American Samoa	0 (0-0)	0 (0-0)	0 (0-0)	0 (0-0)	0 (0-0)	0 (0-0)	0 (0-0)	0 (0-0)
Federated States of Micronesia	1 (1-1)	1 (1-1)	1 (1-1)	1 (1-1)	1 (1-1)	1 (1-1)	1 (1-1)	1 (0-1)
Fiji	5 (5-6)	8 (8-8)	9 (8-9)	10 (9-10)	10 (9-10)	9 (8-10)	10 (9-11)	9 (8-10)
Guam	0 (0-0)	1 (1-1)	1 (1-1)	1 (1-1)	1 (1-2)	1 (1-1)	1 (1-1)	1 (1-1)
Kiribati	0 (0-0)	0 (0-0)	0 (0-0)	1 (1-1)	1 (1-1)	1 (1-1)	1 (1-1)	1 (1-2)
Marshall Islands	0 (0-0)	0 (0-0)	0 (0-0)	0 (0-0)	0 (0-0)	0 (0-0)	0 (0-0)	0 (0-0)
Northern Mariana Islands	0 (0-0)	0 (0-0)	0 (0-0)	0 (0-0)	0 (0-0)	1 (1-1)	0 (0-0)	0 (0-0)
Papua New Guinea	40 (37-42)	45 (43-47)	51 (49-53)	57 (56-59)	76 (74-78)	104 (98-110)	137 (125-148)	159 (145-174)
Samoa	2 (2-2)	2 (2-2)	2 (2-2)	2 (1-2)	1 (1-2)	2 (2-2)	3 (2-3)	3 (2-3)
Solomon Islands	2 (2-2)	3 (3-3)	3 (3-4)	5 (4-5)	7 (6-7)	9 (8-9)	10 (9-11)	10 (9-11)
Tonga	0 (0-1)	1 (1-1)	1 (1-1)	1 (1-1)	1 (1-1)	1 (1-1)	1 (1-1)	1 (0-1)
Vanuatu	1 (0-1)	1 (1-1)	1 (1-1)	2 (2-2)	2 (2-3)	3 (3-3)	4 (3-4)	4 (3-4)
Southeast Asia	4,354 (4,067-4,633)	5,397 (5,170-5,619)	5,870 (5,695-6,040)	6,294 (6,095-6,474)	6,408 (6,220-6,592)	6,250 (6,035-6,463)	6,260 (5,900-6,637)	5,662 (5,129-6,306)
Cambodia	112 (104-120)	140 (132-148)	170 (163-178)	192 (186-198)	231 (226-236)	199 (193-206)	194 (181-207)	193 (175-214)
Indonesia	1,879 (1,769-1,989)	2,419 (2,305-2,525)	2,572 (2,490-2,646)	2,585 (2,510-2,660)	2,411 (2,321-2,499)	2,390 (2,300-2,479)	2,382 (2,198-2,576)	2,072 (1,794-2,418)
Laos	46 (43-49)	54 (51-57)	63 (60-66)	74 (71-76)	81 (79-83)	91 (89-94)	91 (86-96)	90 (82-99)
Malaysia	133 (123-144)	159 (158-160)	168 (167-169)	201 (199-202)	252 (250-253)	250 (248-252)	250 (248-252)	262 (236-292)
Maldives	1 (1-1)	2 (2-2)	2 (2-2)	3 (3-3)	4 (3-4)	2 (2-3)	3 (3-3)	3 (3-3)
Mauritius	11 (11-12)	12 (12-13)	11 (10-11)	12 (11-12)	11 (11-11)	10 (10-10)	7 (7-7)	6 (5-6)
Myanmar	435 (400-469)	509 (475-543)	541 (507-576)	592 (544-638)	592 (538-648)	560 (503-621)	471 (424-522)	449 (413-492)
Philippines	495 (466-526)	629 (597-661)	767 (732-801)	942 (904-979)	1,103 (1,061-1,149)	1,207 (1,155-1,259)	1,359 (1,276-1,439)	1,309 (1,194-1,451)

Supplemental Results Table 3: Live births (in thousands) by location, males, 1950, 1960, 1970, 1980, 1990, 2000, 2010, 2017								
**Geography**	**1950**	**1960**	**1970**	**1980**	**1990**	**2000**	**2010**	**2017**
Sri Lanka	154 (143-167)	185 (178-192)	202 (198-205)	210 (200-222)	192 (180-206)	187 (174-201)	184 (166-204)	150 (127-176)
Seychelles	0 (0-0)	0 (0-0)	0 (0-0)	0 (0-0)	0 (0-0)	0 (0-0)	0 (0-0)	0 (0-0)
Thailand	433 (394-474)	529 (500-556)	589 (568-609)	592 (563-623)	584 (551-615)	504 (472-537)	402 (369-436)	315 (277-360)
Timor-Leste	12 (12-13)	14 (13-14)	13 (12-13)	14 (13-14)	19 (18-20)	21 (20-22)	18 (17-20)	19 (17-22)
Vietnam	629 (582-676)	730 (689-771)	757 (714-798)	863 (825-901)	914 (888-940)	814 (788-841)	883 (835-931)	780 (711-860)
Sub-Saharan Africa	4,809 (4,533-5,078)	5,820 (5,581-6,048)	7,098 (6,860-7,342)	8,989 (8,725-9,242)	11,246 (10,935-11,563)	14,048 (13,649-14,472)	16,784 (16,069-17,478)	18,441 (17,338-19,699)
Central Sub-Saharan Africa	557 (533-579)	718 (692-744)	856 (822-889)	1,057 (1,018-1,094)	1,321 (1,274-1,364)	1,671 (1,616-1,731)	2,081 (1,998-2,168)	2,193 (2,062-2,320)
Angola	129 (124-135)	147 (140-152)	158 (152-164)	195 (187-203)	266 (250-280)	366 (342-389)	498 (460-535)	537 (491-585)
Central African Republic	31 (29-33)	40 (38-43)	50 (46-53)	52 (49-55)	60 (56-64)	73 (66-79)	79 (72-86)	67 (60-75)
Congo	22 (20-23)	25 (24-26)	30 (29-31)	39 (37-41)	48 (46-50)	61 (58-63)	72 (67-76)	65 (60-73)
Democratic Republic of the Congo	358 (340-375)	488 (467-507)	598 (571-626)	746 (719-773)	916 (886-945)	1,134 (1,096-1,170)	1,391 (1,330-1,451)	1,482 (1,376-1,584)
Equatorial Guinea	5 (5-5)	5 (5-6)	6 (6-6)	7 (7-8)	11 (10-11)	15 (14-16)	18 (17-20)	19 (17-22)
Gabon	10 (9-10)	11 (10-11)	12 (11-12)	15 (14-16)	18 (16-19)	20 (17-22)	21 (19-23)	20 (18-23)
Eastern Sub-Saharan Africa	1,770 (1,671-1,868)	2,218 (2,133-2,298)	2,786 (2,709-2,864)	3,660 (3,577-3,739)	4,654 (4,552-4,751)	5,679 (5,530-5,831)	6,519 (6,227-6,803)	7,122 (6,637-7,677)
Burundi	68 (65-72)	79 (75-82)	87 (84-89)	112 (109-114)	126 (122-130)	124 (120-129)	177 (168-186)	205 (192-221)
Comoros	3 (2-3)	4 (4-4)	6 (6-6)	8 (8-9)	10 (9-10)	10 (9-11)	10 (9-11)	9 (8-10)
Djibouti	1 (1-1)	2 (2-2)	4 (4-4)	7 (7-8)	11 (11-12)	14 (13-15)	16 (15-18)	17 (15-20)
Eritrea	31 (29-33)	39 (37-41)	52 (49-54)	63 (60-67)	65 (62-69)	73 (66-79)	91 (81-101)	90 (79-103)
Ethiopia	479 (446-511)	575 (543-604)	734 (706-761)	937 (907-965)	1,317 (1,274-1,359)	1,621 (1,558-1,685)	1,730 (1,631-1,834)	1,906 (1,746-2,089)
Kenya	167 (159-174)	241 (233-248)	302 (291-312)	418 (405-432)	499 (480-518)	582 (552-610)	652 (602-702)	696 (616-783)
Madagascar	106 (101-112)	135 (130-140)	179 (173-185)	229 (222-236)	274 (262-285)	353 (336-369)	428 (402-454)	498 (445-553)
Malawi	84 (79-88)	109 (105-112)	131 (128-134)	168 (165-171)	253 (248-258)	278 (272-284)	292 (282-302)	308 (287-332)
Mozambique	169 (157-181)	189 (179-199)	226 (214-237)	293 (284-301)	336 (328-344)	408 (398-418)	452 (435-467)	500 (462-540)
Rwanda	67 (63-71)	78 (74-82)	98 (95-101)	133 (131-136)	165 (160-169)	168 (163-175)	187 (177-198)	214 (193-237)
Somalia	53 (50-56)	71 (68-74)	99 (95-103)	182 (173-190)	167 (158-175)	239 (226-251)	307 (289-324)	351 (327-378)
South Sudan	59 (54-64)	73 (68-79)	85 (80-90)	105 (98-113)	133 (123-143)	175 (163-187)	226 (210-241)	210 (197-226)
Tanzania	245 (232-257)	304 (291-316)	381 (372-390)	489 (477-501)	614 (599-629)	755 (732-780)	928 (885-974)	1,003 (923-1,093)
Uganda	164 (156-172)	216 (208-223)	280 (273-287)	359 (353-365)	480 (472-489)	639 (624-651)	731 (707-756)	783 (744-828)
Zambia	66 (63-70)	95 (90-99)	115 (109-122)	148 (140-154)	194 (184-204)	228 (216-240)	281 (259-304)	320 (286-358)
Southern Sub-Saharan Africa	414 (381-448)	481 (451-509)	598 (562-634)	721 (671-770)	785 (725-844)	861 (805-916)	902 (824-988)	884 (807-980)
Botswana	9 (8-10)	11 (10-11)	14 (14-15)	18 (18-19)	21 (21-22)	23 (22-24)	24 (23-25)	24 (23-26)
Lesotho	16 (14-17)	18 (17-19)	23 (22-23)	29 (28-29)	31 (30-32)	29 (28-30)	26 (25-27)	24 (22-27)
Namibia	11 (10-11)	13 (13-14)	16 (16-17)	20 (19-20)	25 (25-26)	27 (26-27)	30 (29-31)	29 (27-32)
South Africa	295 (269-322)	334 (309-358)	405 (375-435)	461 (416-508)	492 (438-547)	553 (500-607)	569 (497-651)	552 (494-626)
Swaziland	6 (5-6)	8 (7-8)	10 (10-10)	13 (13-14)	16 (15-16)	17 (16-18)	16 (15-17)	15 (13-17)
Zimbabwe	76 (72-80)	94 (90-98)	127 (123-132)	176 (172-180)	197 (193-201)	210 (205-215)	234 (229-239)	237 (221-253)
Western Sub-Saharan Africa	2,066 (1,947-2,180)	2,401 (2,292-2,505)	2,857 (2,750-2,960)	3,549 (3,424-3,666)	4,485 (4,343-4,624)	5,835 (5,645-6,015)	7,280 (6,969-7,595)	8,240 (7,741-8,795)
Benin	57 (53-61)	60 (56-62)	68 (66-71)	86 (84-88)	118 (115-121)	153 (148-157)	194 (185-202)	215 (198-233)
Burkina Faso	101 (93-109)	101 (95-108)	124 (119-130)	173 (168-178)	231 (227-236)	284 (276-293)	365 (348-381)	433 (404-466)
Cameroon	122 (113-131)	141 (132-151)	164 (157-171)	188 (181-194)	227 (217-236)	322 (309-336)	432 (402-461)	438 (390-493)
Cape Verde	3 (3-3)	4 (4-4)	4 (4-4)	5 (5-5)	5 (5-6)	6 (5-6)	5 (4-6)	5 (4-5)
Chad	74 (69-78)	82 (78-85)	98 (95-102)	126 (122-131)	165 (160-169)	223 (217-229)	303 (292-313)	365 (349-384)
Cote d'Ivoire	66 (62-71)	105 (99-110)	143 (138-148)	203 (195-212)	289 (274-305)	374 (350-397)	426 (392-460)	440 (400-485)

Supplemental Results Table 3: Live births (in thousands) by location, males, 1950, 1960, 1970, 1980, 1990, 2000, 2010, 2017

Geography	1950	1960	1970	1980	1990	2000	2010	2017
The Gambia	6<br>(5-6)	7<br>(7-7)	11<br>(11-12)	17<br>(16-18)	24<br>(22-25)	29<br>(27-31)	33<br>(30-36)	35<br>(31-39)
Ghana	103<br>(93-114)	168<br>(157-178)	225<br>(215-234)	275<br>(264-285)	296<br>(283-309)	346<br>(329-365)	391<br>(365-417)	448<br>(388-513)
Guinea	63<br>(60-66)	81<br>(77-85)	104<br>(100-109)	127<br>(122-131)	155<br>(150-160)	183<br>(176-189)	201<br>(192-211)	221<br>(209-235)
Guinea-Bissau	16<br>(16-17)	16<br>(15-16)	15<br>(15-16)	21<br>(20-22)	25<br>(23-27)	29<br>(27-31)	33<br>(30-36)	34<br>(31-38)
Liberia	23<br>(21-24)	26<br>(25-28)	37<br>(36-38)	51<br>(49-53)	44<br>(42-45)	68<br>(65-71)	80<br>(74-85)	79<br>(71-88)
Mali	96<br>(90-101)	127<br>(121-132)	163<br>(158-168)	199<br>(193-204)	231<br>(226-236)	276<br>(268-285)	368<br>(354-381)	449<br>(424-477)
Mauritania	18<br>(16-19)	21<br>(20-22)	27<br>(26-28)	37<br>(36-38)	46<br>(44-47)	52<br>(50-54)	59<br>(56-62)	60<br>(56-66)
Niger	79<br>(74-83)	103<br>(99-107)	136<br>(132-140)	178<br>(175-181)	225<br>(221-229)	317<br>(311-323)	414<br>(401-428)	513<br>(486-543)
Nigeria	1,079<br>(1,018-1,137)	1,169<br>(1,114-1,222)	1,300<br>(1,239-1,359)	1,573<br>(1,493-1,647)	2,047<br>(1,961-2,131)	2,765<br>(2,653-2,873)	3,484<br>(3,315-3,665)	3,994<br>(3,691-4,307)
Sao Tome and Principe	1<br>(1-1)	1<br>(1-1)	1<br>(1-1)	1<br>(1-1)	2<br>(2-2)	2<br>(2-2)	2<br>(2-2)	2<br>(2-2)
Senegal	66<br>(61-71)	83<br>(77-88)	116<br>(111-121)	147<br>(139-154)	174<br>(162-184)	202<br>(190-214)	238<br>(219-256)	252<br>(232-276)
Sierra Leone	47<br>(43-51)	54<br>(50-58)	60<br>(56-64)	68<br>(64-72)	90<br>(86-95)	95<br>(90-101)	126<br>(117-135)	136<br>(123-150)
Togo	36<br>(34-38)	43<br>(42-45)	50<br>(48-52)	66<br>(64-67)	82<br>(79-84)	99<br>(96-103)	119<br>(114-124)	113<br>(105-123)

Supplemental Results Table 4: Live births (in thousands) by location, females, 1950, 1960, 1970, 1980, 1990, 2000, 2010, 2017

Geography	1950	1960	1970	1980	1990	2000	2010	2017
Global	45,291 (43,453-47,144)	52,525 (51,365-53,636)	57,519 (56,573-58,416)	60,335 (59,190-61,459)	66,220 (64,786-67,625)	64,494 (62,956-66,007)	66,937 (64,594-69,459)	66,939 (62,648-71,897)
Central Europe, Eastern Europe, and Central Asia	3,594 (3,526-3,672)	3,629 (3,584-3,671)	3,084 (3,028-3,139)	3,488 (3,448-3,530)	3,221 (3,185-3,258)	2,226 (2,187-2,265)	2,727 (2,676-2,781)	2,526 (2,267-2,807)
Central Asia	501 (480-521)	693 (676-711)	686 (652-720)	863 (842-883)	973 (951-995)	721 (695-748)	914 (870-956)	918 (843-998)
Armenia	23 (21-25)	34 (32-35)	27 (25-29)	36 (34-37)	37 (36-38)	18 (17-19)	20 (19-21)	17 (16-19)
Azerbaijan	49 (45-52)	79 (76-82)	72 (67-76)	78 (74-82)	92 (88-96)	61 (58-64)	84 (78-90)	80 (71-91)
Georgia	42 (38-46)	49 (46-53)	43 (39-46)	43 (41-45)	39 (37-41)	23 (22-25)	28 (26-30)	24 (22-26)
Kazakhstan	124 (120-129)	176 (170-182)	175 (164-186)	191 (186-196)	184 (180-188)	117 (113-120)	170 (163-177)	168 (152-185)
Kyrgyzstan	29 (27-30)	38 (36-39)	43 (40-46)	54 (52-56)	61 (59-63)	49 (47-51)	71 (69-73)	73 (68-77)
Mongolia	16 (15-17)	18 (18-19)	21 (20-22)	29 (28-30)	33 (32-33)	24 (23-24)	34 (33-35)	36 (34-39)
Tajikistan	43 (41-44)	57 (56-59)	61 (58-63)	83 (80-86)	105 (101-108)	92 (88-97)	120 (112-128)	138 (125-150)
Turkmenistan	26 (25-27)	36 (36-37)	35 (33-38)	48 (47-50)	60 (59-62)	43 (40-46)	54 (51-57)	53 (47-59)
Uzbekistan	145 (139-151)	202 (197-208)	205 (195-216)	298 (287-309)	359 (346-372)	291 (273-309)	329 (300-357)	326 (297-355)
Central Europe	1,114 (1,090-1,138)	951 (938-964)	927 (916-939)	1,002 (994-1,011)	821 (816-827)	583 (577-588)	586 (579-593)	518 (466-576)
Albania	23 (22-24)	33 (32-34)	36 (34-37)	37 (36-38)	42 (40-43)	26 (25-27)	18 (17-18)	17 (15-20)
Bosnia and Herzegovina	37 (32-43)	43 (37-48)	36 (30-42)	36 (32-40)	32 (31-33)	18 (18-19)	16 (16-16)	13 (12-14)
Bulgaria	81 (80-82)	67 (66-68)	66 (65-67)	62 (61-63)	49 (48-50)	34 (33-34)	36 (35-36)	28 (25-32)
Croatia	42 (36-48)	39 (34-45)	32 (30-34)	34 (32-36)	25 (25-26)	20 (20-21)	20 (20-21)	17 (16-18)
Czech Republic	90 (89-91)	62 (61-63)	72 (71-73)	73 (72-74)	62 (61-63)	44 (43-44)	55 (54-56)	50 (46-55)
Hungary	92 (91-93)	69 (68-70)	72 (71-73)	71 (70-72)	61 (60-62)	46 (46-47)	44 (43-45)	41 (37-47)
Macedonia	17 (15-20)	17 (15-20)	17 (15-19)	20 (18-21)	17 (16-17)	12 (12-12)	11 (11-12)	11 (10-12)
Montenegro	5 (5-6)	6 (5-6)	5 (4-5)	4 (4-5)	4 (4-4)	4 (3-4)	3 (3-3)	3 (3-3)
Poland	366 (364-368)	311 (309-312)	264 (262-266)	334 (332-336)	266 (264-268)	180 (178-182)	195 (193-197)	173 (153-195)
Romania	200 (184-217)	164 (162-165)	199 (198-201)	191 (189-192)	142 (140-144)	108 (106-109)	101 (99-102)	85 (76-96)
Serbia	91 (90-92)	80 (79-81)	71 (70-72)	75 (74-76)	66 (65-67)	51 (50-52)	42 (39-47)	39 (34-44)
Slovakia	48 (47-48)	42 (41-42)	39 (38-40)	46 (45-46)	39 (38-39)	26 (25-26)	29 (28-30)	25 (22-28)
Slovenia	16 (15-17)	13 (13-14)	13 (13-14)	14 (14-15)	11 (10-11)	8 (8-9)	10 (10-11)	9 (8-10)
Eastern Europe	1,979 (1,939-2,026)	1,984 (1,961-2,005)	1,470 (1,448-1,493)	1,622 (1,605-1,640)	1,426 (1,412-1,441)	921 (908-935)	1,226 (1,211-1,242)	1,089 (950-1,250)
Belarus	90 (85-96)	94 (90-97)	72 (70-75)	78 (76-81)	66 (64-68)	45 (43-46)	54 (51-57)	49 (43-55)
Estonia	9 (9-9)	9 (9-10)	10 (10-10)	11 (10-11)	10 (9-10)	6 (6-6)	7 (7-7)	6 (5-7)
Latvia	16 (15-16)	17 (17-17)	17 (16-17)	17 (17-17)	17 (17-17)	9 (9-10)	9 (9-10)	9 (8-10)
Lithuania	28 (26-30)	30 (29-30)	27 (26-27)	25 (24-25)	27 (27-28)	16 (15-16)	16 (16-17)	14 (12-15)
Moldova	42 (39-44)	40 (38-42)	34 (31-38)	40 (39-42)	37 (36-38)	22 (21-23)	21 (20-22)	17 (15-19)
Russian Federation	1,377 (1,366-1,389)	1,380 (1,374-1,387)	957 (951-963)	1,082 (1,077-1,088)	962 (957-967)	624 (618-628)	871 (867-876)	788 (684-907)
Ukraine	415 (391-441)	411 (393-430)	350 (330-369)	366 (351-380)	304 (294-315)	197 (189-207)	244 (232-257)	205 (178-235)
High-income	6,574 (6,476-6,676)	7,038 (6,988-7,091)	6,787 (6,735-6,839)	6,104 (6,068-6,141)	6,116 (6,083-6,149)	5,785 (5,747-5,824)	5,808 (5,776-5,840)	5,593 (5,080-6,172)
Australasia	117 (116-119)	145 (143-146)	159 (158-160)	135 (134-137)	153 (151-155)	147 (145-150)	174 (172-176)	181 (164-200)
Australia	93 (92-94)	113 (112-114)	128 (127-129)	111 (110-112)	124 (123-126)	120 (118-122)	144 (143-146)	152 (135-171)
New Zealand	24 (23-25)	31 (31-32)	30 (30-31)	24 (23-25)	28 (28-29)	27 (26-27)	30 (29-30)	29 (26-32)
High-income Asia Pacific	1,431 (1,387-1,481)	1,240 (1,196-1,284)	1,500 (1,461-1,537)	1,215 (1,186-1,246)	961 (938-988)	898 (873-926)	781 (757-807)	695 (614-790)
Brunei	1 (1-1)	1 (1-2)	2 (2-2)	2 (2-2)	3 (3-3)	3 (3-3)	3 (2-3)	3 (3-3)
Japan	1,077 (1,040-1,117)	774 (742-806)	958 (921-995)	760 (730-791)	596 (572-622)	574 (549-600)	523 (499-549)	449 (374-539)
Singapore	22 (22-23)	28 (27-29)	22 (21-22)	19 (18-20)	23 (22-24)	35 (33-38)	27 (25-28)	31 (26-37)
South Korea	330 (300-361)	435 (406-466)	518 (506-529)	432 (431-434)	338 (334-343)	285 (282-287)	228 (226-229)	210 (200-220)

Supplemental Results Table 4: Live births (in thousands) by location, females, 1950, 1960, 1970, 1980, 1990, 2000, 2010, 2017								
Geography	1950	1960	1970	1980	1990	2000	2010	2017
High-income North America	1,958<br>(1,950-1,966)	2,303<br>(2,296-2,311)	1,928<br>(1,919-1,937)	1,930<br>(1,920-1,939)	2,256<br>(2,246-2,267)	2,138<br>(2,130-2,147)	2,116<br>(2,106-2,124)	2,105<br>(1,943-2,285)
Canada	175<br>(174-176)	223<br>(221-224)	169<br>(168-170)	175<br>(173-177)	194<br>(192-196)	161<br>(159-163)	182<br>(180-184)	190<br>(163-222)
Greenland	0<br>(0-0)	0<br>(0-0)	0<br>(0-0)	0<br>(0-0)	0<br>(0-0)	0<br>(0-0)	0<br>(0-0)	0<br>(0-0)
United States	1,782<br>(1,774-1,790)	2,079<br>(2,072-2,087)	1,758<br>(1,750-1,766)	1,754<br>(1,746-1,763)	2,061<br>(2,051-2,071)	1,976<br>(1,967-1,985)	1,933<br>(1,924-1,941)	1,914<br>(1,779-2,063)
Southern Latin America	341<br>(326-357)	394<br>(391-398)	411<br>(407-415)	480<br>(477-482)	503<br>(499-506)	492<br>(487-498)	511<br>(507-515)	510<br>(469-554)
Argentina	221<br>(207-237)	230<br>(227-233)	263<br>(260-266)	335<br>(333-337)	335<br>(332-338)	345<br>(341-351)	367<br>(364-370)	366<br>(340-393)
Chile	98<br>(97-98)	134<br>(133-135)	120<br>(119-121)	118<br>(117-119)	141<br>(139-142)	120<br>(119-122)	121<br>(119-122)	120<br>(105-137)
Uruguay	21<br>(21-22)	29<br>(29-30)	27<br>(27-27)	26<br>(26-26)	26<br>(26-27)	25<br>(25-26)	23<br>(21-25)	23<br>(20-26)
Western Europe	2,724<br>(2,656-2,804)	2,954<br>(2,939-2,971)	2,787<br>(2,763-2,811)	2,342<br>(2,332-2,353)	2,241<br>(2,230-2,251)	2,107<br>(2,096-2,118)	2,223<br>(2,212-2,234)	2,099<br>(1,884-2,340)
Andorra	0<br>(0-0)	0<br>(0-0)	0<br>(0-0)	0<br>(0-0)	0<br>(0-0)	0<br>(0-0)	0<br>(0-0)	0<br>(0-0)
Austria	50<br>(49-52)	62<br>(61-63)	53<br>(52-54)	44<br>(44-45)	45<br>(44-45)	37<br>(37-38)	38<br>(37-38)	42<br>(38-45)
Belgium	68<br>(68-69)	76<br>(75-77)	69<br>(68-69)	60<br>(59-60)	60<br>(60-61)	56<br>(55-57)	62<br>(61-63)	59<br>(53-65)
Cyprus	6<br>(6-6)	7<br>(6-7)	6<br>(6-6)	6<br>(5-6)	5<br>(5-6)	5<br>(4-5)	5<br>(5-5)	5<br>(4-6)
Denmark	37<br>(37-38)	36<br>(36-37)	35<br>(35-36)	26<br>(26-27)	31<br>(30-31)	32<br>(31-32)	29<br>(29-30)	29<br>(26-32)
Finland	46<br>(45-47)	39<br>(39-40)	30<br>(29-30)	31<br>(30-31)	31<br>(31-32)	27<br>(26-27)	29<br>(28-30)	26<br>(24-30)
France	407<br>(405-410)	404<br>(401-406)	416<br>(413-418)	390<br>(387-392)	371<br>(368-373)	378<br>(375-381)	390<br>(387-393)	360<br>(324-400)
Germany	531<br>(492-578)	616<br>(613-619)	499<br>(495-503)	412<br>(409-416)	424<br>(421-427)	369<br>(366-372)	327<br>(323-330)	346<br>(308-389)
Greece	74<br>(73-76)	74<br>(73-75)	69<br>(68-70)	69<br>(68-70)	49<br>(48-50)	49<br>(48-50)	53<br>(52-54)	43<br>(38-48)
Iceland	1<br>(1-2)	2<br>(2-2)	2<br>(1-2)	2<br>(2-2)	2<br>(2-2)	2<br>(1-2)	2<br>(2-2)	2<br>(1-2)
Ireland	31<br>(29-33)	29<br>(29-30)	31<br>(31-32)	35<br>(34-35)	25<br>(24-25)	27<br>(26-27)	35<br>(35-36)	31<br>(28-35)
Israel	23<br>(22-25)	26<br>(26-27)	39<br>(38-39)	45<br>(44-45)	50<br>(49-51)	65<br>(65-66)	80<br>(79-81)	86<br>(78-94)
Italy	429<br>(426-431)	447<br>(444-449)	439<br>(436-442)	308<br>(305-310)	274<br>(271-276)	262<br>(259-265)	271<br>(268-273)	225<br>(199-255)
Luxembourg	2<br>(2-2)	2<br>(2-2)	2<br>(2-2)	2<br>(2-2)	2<br>(2-2)	2<br>(2-2)	2<br>(2-2)	3<br>(2-3)
Malta	4<br>(4-5)	3<br>(3-4)	2<br>(2-2)	2<br>(2-2)	2<br>(2-2)	1<br>(1-2)	1<br>(1-2)	2<br>(1-2)
Netherlands	110<br>(109-111)	118<br>(117-119)	112<br>(111-113)	86<br>(85-87)	96<br>(94-97)	100<br>(98-101)	89<br>(88-90)	84<br>(75-93)
Norway	29<br>(29-30)	30<br>(30-30)	31<br>(31-32)	24<br>(24-25)	29<br>(28-29)	28<br>(27-28)	29<br>(28-29)	29<br>(26-32)
Portugal	99<br>(98-100)	105<br>(104-106)	87<br>(86-88)	73<br>(73-74)	55<br>(54-56)	56<br>(55-57)	48<br>(47-49)	41<br>(36-47)
Spain	270<br>(268-272)	316<br>(314-318)	320<br>(318-323)	264<br>(261-266)	191<br>(189-194)	192<br>(190-194)	232<br>(230-235)	197<br>(179-217)
Sweden	54<br>(54-55)	49<br>(49-50)	54<br>(53-55)	46<br>(45-47)	59<br>(58-60)	44<br>(43-45)	54<br>(54-55)	57<br>(52-62)
Switzerland	40<br>(39-41)	46<br>(46-47)	47<br>(46-48)	36<br>(35-36)	41<br>(40-41)	37<br>(36-37)	38<br>(38-39)	42<br>(37-47)
United Kingdom	396<br>(376-422)	452<br>(440-465)	433<br>(413-454)	370<br>(367-372)	387<br>(384-390)	328<br>(325-331)	395<br>(393-398)	381<br>(342-425)
England	315<br>(296-339)	363<br>(353-373)	352<br>(335-369)	301<br>(298-303)	322<br>(319-324)	276<br>(274-279)	338<br>(336-341)	327<br>(294-364)
Northern Ireland	15<br>(14-15)	15<br>(15-16)	15<br>(14-15)	14<br>(13-14)	12<br>(12-12)	10<br>(9-10)	11<br>(11-12)	11<br>(10-13)
Scotland	44<br>(43-44)	49<br>(47-50)	43<br>(41-45)	34<br>(33-35)	32<br>(31-33)	25<br>(24-26)	28<br>(27-28)	26<br>(23-28)
Wales	21<br>(20-23)	23<br>(22-24)	22<br>(21-24)	20<br>(19-21)	20<br>(20-21)	15<br>(15-16)	17<br>(16-17)	16<br>(14-18)
Latin America and Caribbean	3,140<br>(2,959-3,318)	3,970<br>(3,807-4,130)	4,617<br>(4,484-4,750)	5,171<br>(5,017-5,336)	5,385<br>(5,199-5,576)	5,286<br>(5,080-5,495)	5,227<br>(4,950-5,505)	5,080<br>(4,627-5,587)
Andean Latin America	361<br>(345-375)	443<br>(429-456)	523<br>(513-533)	592<br>(573-610)	637<br>(615-659)	610<br>(582-641)	654<br>(618-688)	678<br>(616-747)
Bolivia	77<br>(74-80)	87<br>(84-90)	96<br>(93-98)	116<br>(112-119)	126<br>(120-132)	127<br>(119-135)	138<br>(129-147)	147<br>(132-163)
Ecuador	78<br>(75-81)	99<br>(98-99)	114<br>(114-115)	129<br>(125-132)	147<br>(140-154)	159<br>(148-171)	159<br>(147-172)	154<br>(131-180)
Peru	204<br>(192-216)	256<br>(245-268)	312<br>(304-321)	347<br>(331-362)	363<br>(346-381)	323<br>(304-343)	356<br>(335-376)	376<br>(336-424)
Caribbean	332<br>(320-345)	407<br>(395-419)	432<br>(426-439)	411<br>(403-419)	449<br>(439-459)	434<br>(420-447)	403<br>(384-422)	399<br>(365-435)
Antigua and Barbuda	0<br>(0-0)	0<br>(0-0)	0<br>(0-0)	0<br>(0-0)	0<br>(0-0)	0<br>(0-0)	0<br>(0-0)	0<br>(0-0)
The Bahamas	1<br>(1-1)	1<br>(1-1)	2<br>(1-2)	2<br>(2-2)	2<br>(2-2)	2<br>(2-2)	2<br>(2-2)	2<br>(1-2)

Supplemental Results Table 4: Live births (in thousands) by location, females, 1950, 1960, 1970, 1980, 1990, 2000, 2010, 2017								
Geography	1950	1960	1970	1980	1990	2000	2010	2017
Barbados	3<br>(3-3)	3<br>(3-3)	2<br>(2-2)	2<br>(2-2)	2<br>(1-2)	1<br>(1-1)	1<br>(1-1)	1<br>(1-1)
Belize	1<br>(1-1)	2<br>(2-2)	2<br>(2-2)	2<br>(2-2)	3<br>(3-3)	3<br>(3-3)	3<br>(3-3)	3<br>(3-4)
Bermuda	0<br>(0-0)	0<br>(0-0)	0<br>(0-0)	0<br>(0-0)	0<br>(0-0)	0<br>(0-0)	0<br>(0-0)	0<br>(0-0)
Cuba	75<br>(71-79)	109<br>(104-114)	120<br>(119-122)	67<br>(66-68)	86<br>(85-87)	72<br>(71-73)	63<br>(62-64)	53<br>(50-56)
Dominica	0<br>(0-1)	1<br>(1-1)	1<br>(1-1)	0<br>(0-0)	0<br>(0-0)	0<br>(0-0)	0<br>(0-0)	0<br>(0-0)
Dominican Republic	55<br>(51-59)	71<br>(69-74)	92<br>(89-95)	111<br>(107-116)	117<br>(111-122)	119<br>(113-126)	98<br>(91-105)	105<br>(91-122)
Grenada	1<br>(1-1)	1<br>(1-1)	1<br>(1-1)	1<br>(1-1)	1<br>(1-1)	0<br>(0-0)	0<br>(0-0)	0<br>(0-0)
Guyana	9<br>(8-9)	12<br>(11-12)	11<br>(11-12)	12<br>(12-13)	12<br>(12-13)	10<br>(9-10)	7<br>(7-8)	7<br>(6-8)
Haiti	84<br>(79-89)	89<br>(84-94)	89<br>(86-92)	104<br>(101-108)	124<br>(120-128)	135<br>(129-141)	150<br>(140-161)	159<br>(142-179)
Jamaica	24<br>(22-25)	33<br>(32-34)	32<br>(31-33)	30<br>(29-31)	29<br>(28-30)	26<br>(24-28)	21<br>(19-23)	18<br>(16-21)
Puerto Rico	41<br>(41-42)	36<br>(36-37)	33<br>(32-34)	34<br>(34-35)	31<br>(31-32)	27<br>(27-28)	20<br>(19-22)	14<br>(13-15)
Saint Lucia	1<br>(1-1)	1<br>(1-2)	1<br>(1-2)	1<br>(1-1)	1<br>(1-1)	1<br>(1-1)	1<br>(0-1)	1<br>(0-1)
Saint Vincent and the Grenadines	1<br>(1-1)	1<br>(1-1)	1<br>(1-1)	1<br>(1-1)	1<br>(1-1)	1<br>(0-1)	0<br>(0-0)	0<br>(0-0)
Suriname	3<br>(3-3)	5<br>(5-6)	6<br>(6-6)	5<br>(5-5)	4<br>(4-4)	4<br>(4-5)	4<br>(4-5)	4<br>(4-5)
Trinidad and Tobago	11<br>(11-12)	15<br>(15-16)	12<br>(12-13)	14<br>(14-15)	11<br>(11-12)	8<br>(8-9)	9<br>(8-10)	8<br>(7-9)
Virgin Islands, U.S.	0<br>(0-0)	0<br>(0-0)	1<br>(1-1)	1<br>(1-1)	1<br>(1-1)	0<br>(0-1)	0<br>(0-0)	0<br>(0-0)
Central Latin America	1,218<br>(1,169-1,269)	1,626<br>(1,564-1,682)	1,961<br>(1,901-2,021)	2,222<br>(2,144-2,299)	2,525<br>(2,421-2,634)	2,491<br>(2,383-2,599)	2,425<br>(2,293-2,551)	2,447<br>(2,202-2,730)
Colombia	241<br>(226-258)	359<br>(348-369)	405<br>(393-416)	435<br>(417-452)	480<br>(463-496)	476<br>(455-497)	442<br>(416-467)	414<br>(357-479)
Costa Rica	18<br>(17-19)	29<br>(28-29)	27<br>(26-27)	34<br>(33-34)	39<br>(38-40)	36<br>(35-38)	35<br>(34-36)	34<br>(31-37)
El Salvador	45<br>(43-47)	60<br>(58-61)	72<br>(71-73)	75<br>(73-77)	77<br>(74-80)	65<br>(63-67)	52<br>(49-54)	52<br>(45-59)
Guatemala	72<br>(71-73)	91<br>(90-91)	106<br>(105-107)	152<br>(151-153)	172<br>(171-173)	172<br>(167-176)	178<br>(175-181)	212<br>(183-244)
Honduras	36<br>(34-39)	44<br>(42-45)	55<br>(54-56)	74<br>(72-75)	88<br>(86-89)	93<br>(91-96)	102<br>(95-108)	119<br>(103-138)
Mexico	655<br>(612-699)	820<br>(773-865)	1,030<br>(979-1,081)	1,125<br>(1,054-1,193)	1,284<br>(1,179-1,387)	1,297<br>(1,210-1,392)	1,228<br>(1,120-1,341)	1,233<br>(1,055-1,456)
Nicaragua	26<br>(25-28)	36<br>(35-37)	44<br>(44-45)	62<br>(61-63)	71<br>(69-73)	66<br>(63-68)	67<br>(60-73)	67<br>(57-78)
Panama	14<br>(12-15)	20<br>(19-21)	25<br>(24-26)	25<br>(24-26)	28<br>(27-29)	30<br>(28-31)	33<br>(31-35)	33<br>(29-39)
Venezuela	107<br>(105-109)	165<br>(164-166)	192<br>(190-194)	237<br>(235-238)	283<br>(280-285)	253<br>(251-255)	286<br>(283-288)	279<br>(248-313)
Tropical Latin America	1,228<br>(1,121-1,337)	1,492<br>(1,401-1,581)	1,699<br>(1,626-1,777)	1,945<br>(1,877-2,019)	1,773<br>(1,698-1,851)	1,749<br>(1,673-1,826)	1,743<br>(1,641-1,859)	1,554<br>(1,408-1,734)
Brazil	1,198<br>(1,090-1,305)	1,458<br>(1,366-1,547)	1,656<br>(1,583-1,732)	1,889<br>(1,821-1,962)	1,715<br>(1,641-1,790)	1,688<br>(1,613-1,764)	1,675<br>(1,574-1,788)	1,484<br>(1,338-1,664)
Paraguay	30<br>(27-32)	33<br>(32-35)	43<br>(41-44)	55<br>(54-58)	58<br>(56-60)	61<br>(58-64)	68<br>(60-75)	70<br>(60-81)
North Africa and Middle East	2,707<br>(2,574-2,835)	3,275<br>(3,162-3,385)	3,995<br>(3,896-4,090)	5,084<br>(4,965-5,207)	5,462<br>(5,306-5,624)	5,934<br>(5,715-6,149)	6,474<br>(6,135-6,809)	6,324<br>(5,863-6,856)
North Africa and Middle East	2,707<br>(2,574-2,835)	3,275<br>(3,162-3,385)	3,995<br>(3,896-4,090)	5,084<br>(4,965-5,207)	5,462<br>(5,306-5,624)	5,934<br>(5,715-6,149)	6,474<br>(6,135-6,809)	6,324<br>(5,863-6,856)
Afghanistan	195<br>(184-206)	228<br>(217-238)	275<br>(264-286)	247<br>(238-256)	220<br>(212-227)	440<br>(427-451)	529<br>(510-547)	664<br>(629-699)
Algeria	217<br>(206-228)	250<br>(238-262)	291<br>(281-301)	381<br>(371-391)	406<br>(388-423)	424<br>(390-457)	455<br>(417-492)	467<br>(415-521)
Bahrain	1<br>(1-2)	3<br>(2-3)	3<br>(3-3)	4<br>(4-5)	6<br>(6-6)	6<br>(6-6)	9<br>(9-9)	9<br>(8-10)
Egypt	433<br>(409-458)	561<br>(536-584)	608<br>(584-631)	784<br>(754-812)	813<br>(779-847)	841<br>(799-884)	1,051<br>(982-1,122)	1,035<br>(944-1,134)
Iran	431<br>(403-458)	485<br>(464-507)	583<br>(560-606)	844<br>(815-872)	784<br>(736-836)	718<br>(663-773)	728<br>(676-780)	619<br>(527-726)
Iraq	126<br>(119-132)	159<br>(151-166)	203<br>(196-211)	276<br>(267-285)	324<br>(311-338)	460<br>(442-477)	523<br>(487-558)	611<br>(552-678)
Jordan	12<br>(12-13)	20<br>(19-20)	31<br>(30-31)	43<br>(43-44)	63<br>(61-66)	75<br>(71-79)	105<br>(99-111)	118<br>(109-130)
Kuwait	1<br>(1-1)	5<br>(5-5)	16<br>(16-16)	24<br>(24-24)	14<br>(13-14)	20<br>(19-20)	26<br>(25-26)	29<br>(26-32)
Lebanon	33<br>(32-35)	41<br>(40-43)	50<br>(48-52)	62<br>(57-66)	67<br>(61-73)	75<br>(67-83)	74<br>(65-84)	90<br>(78-105)
Libya	28<br>(28-29)	36<br>(35-37)	43<br>(42-44)	56<br>(55-57)	62<br>(58-67)	51<br>(50-52)	65<br>(58-72)	59<br>(49-71)
Morocco	207<br>(196-218)	257<br>(245-269)	302<br>(287-315)	344<br>(326-363)	372<br>(351-392)	363<br>(335-390)	331<br>(290-372)	293<br>(257-333)
Palestine	22<br>(21-23)	24<br>(23-24)	25<br>(24-27)	31<br>(29-32)	44<br>(42-45)	57<br>(54-59)	69<br>(65-73)	67<br>(60-73)

Supplemental Results Table 4: Live births (in thousands) by location, females, 1950, 1960, 1970, 1980, 1990, 2000, 2010, 2017								
Geography	1950	1960	1970	1980	1990	2000	2010	2017
Oman	12 (12-13)	16 (15-17)	20 (19-21)	27 (26-29)	29 (27-31)	29 (26-32)	33 (30-35)	39 (35-43)
Qatar	0 (0-0)	1 (1-1)	2 (2-2)	3 (3-3)	5 (4-5)	5 (5-5)	10 (10-11)	14 (13-16)
Saudi Arabia	68 (66-71)	90 (87-92)	136 (132-140)	231 (225-238)	323 (305-341)	268 (246-291)	252 (226-281)	244 (215-274)
Sudan	141 (133-148)	158 (150-164)	263 (255-271)	385 (373-397)	499 (480-518)	611 (587-635)	672 (632-711)	652 (593-718)
Syria	74 (69-79)	98 (93-103)	135 (129-142)	183 (175-192)	200 (184-215)	217 (214-220)	258 (250-267)	133 (116-153)
Tunisia	82 (76-87)	92 (87-96)	95 (92-98)	109 (104-114)	110 (103-117)	98 (93-105)	92 (86-99)	80 (69-95)
Turkey	489 (461-516)	592 (566-617)	713 (687-738)	758 (727-785)	752 (716-788)	717 (679-752)	641 (596-689)	542 (488-605)
United Arab Emirates	1 (1-1)	2 (2-2)	4 (4-4)	17 (17-18)	23 (22-25)	26 (23-28)	45 (40-49)	34 (30-39)
Yemen	123 (117-128)	148 (142-153)	185 (179-190)	259 (252-266)	332 (322-342)	420 (401-438)	490 (459-522)	509 (464-561)
South Asia	9,783 (8,978-10,607)	11,468 (11,378-11,557)	13,665 (13,559-13,762)	14,908 (14,728-15,076)	17,336 (16,906-17,792)	18,077 (17,546-18,597)	17,888 (17,002-18,776)	16,230 (14,585-18,192)
South Asia	9,783 (8,978-10,607)	11,468 (11,378-11,557)	13,665 (13,559-13,762)	14,908 (14,728-15,076)	17,336 (16,906-17,792)	18,077 (17,546-18,597)	17,888 (17,002-18,776)	16,230 (14,585-18,192)
Bangladesh	1,030 (967-1,091)	1,177 (1,135-1,222)	1,473 (1,411-1,529)	1,550 (1,499-1,600)	1,950 (1,901-2,004)	1,762 (1,709-1,812)	1,552 (1,480-1,621)	1,394 (1,267-1,552)
Bhutan	4 (3-4)	5 (4-5)	6 (6-6)	8 (7-8)	9 (8-10)	7 (6-8)	8 (7-9)	8 (7-9)
India	7,824 (7,147-8,521)	9,286 (9,262-9,310)	10,769 (10,748-10,794)	11,417 (11,304-11,524)	12,908 (12,551-13,289)	13,497 (13,073-13,930)	13,367 (12,611-14,121)	11,696 (10,507-13,082)
Nepal	197 (183-210)	218 (208-229)	253 (243-262)	322 (309-336)	370 (356-385)	366 (347-386)	313 (289-340)	307 (272-348)
Pakistan	727 (676-777)	780 (733-828)	1,163 (1,112-1,213)	1,609 (1,523-1,691)	2,096 (2,018-2,169)	2,443 (2,323-2,563)	2,646 (2,451-2,844)	2,824 (2,479-3,227)
Southeast Asia, East Asia, and Oceania	14,877 (14,275-15,515)	17,559 (16,952-18,192)	18,559 (18,042-19,087)	16,952 (16,330-17,579)	17,900 (17,185-18,678)	13,681 (13,162-14,180)	12,658 (12,029-13,269)	13,444 (12,719-14,275)
East Asia	10,656 (10,156-11,204)	12,317 (11,824-12,821)	12,860 (12,388-13,345)	10,862 (10,299-11,428)	11,716 (11,055-12,466)	7,638 (7,174-8,118)	6,607 (6,127-7,125)	7,930 (7,463-8,405)
China	10,187 (9,703-10,727)	11,716 (11,249-12,203)	12,246 (11,783-12,724)	10,294 (9,738-10,850)	11,147 (10,494-11,895)	7,178 (6,726-7,660)	6,259 (5,788-6,756)	7,592 (7,134-8,062)
North Korea	158 (141-177)	206 (184-229)	211 (190-233)	185 (166-206)	216 (192-241)	200 (174-229)	149 (130-170)	125 (110-143)
Taiwan	169 (168-170)	201 (200-202)	183 (182-184)	197 (195-198)	156 (155-158)	134 (133-136)	91 (89-92)	84 (74-96)
Oceania	56 (54-59)	67 (65-69)	75 (73-78)	85 (83-88)	107 (104-111)	138 (130-145)	171 (157-184)	192 (176-209)
American Samoa	0 (0-0)	0 (0-0)	0 (0-0)	0 (0-0)	0 (0-0)	0 (0-0)	0 (0-0)	0 (0-0)
Federated States of Micronesia	0 (0-1)	1 (0-1)	1 (1-1)	1 (1-1)	1 (1-1)	1 (1-1)	1 (1-1)	1 (0-1)
Fiji	5 (5-5)	8 (7-8)	8 (8-8)	9 (9-10)	9 (9-9)	8 (8-9)	9 (8-10)	8 (7-10)
Guam	0 (0-0)	1 (1-1)	1 (1-1)	1 (1-1)	1 (1-1)	1 (1-1)	1 (1-1)	1 (1-1)
Kiribati	0 (0-0)	0 (0-0)	0 (0-0)	1 (1-1)	1 (1-1)	1 (1-1)	1 (1-1)	1 (1-1)
Marshall Islands	0 (0-0)	0 (0-0)	0 (0-0)	0 (0-0)	0 (0-0)	0 (0-0)	0 (0-0)	0 (0-0)
Northern Mariana Islands	0 (0-0)	0 (0-0)	0 (0-0)	0 (0-0)	0 (0-0)	1 (1-1)	0 (0-0)	0 (0-0)
Papua New Guinea	37 (35-40)	42 (40-44)	48 (46-50)	54 (53-56)	71 (69-73)	97 (92-103)	128 (117-138)	149 (136-162)
Samoa	1 (1-2)	2 (2-2)	2 (2-2)	1 (1-2)	1 (1-2)	2 (2-2)	2 (2-3)	2 (2-3)
Solomon Islands	2 (2-2)	3 (2-3)	3 (3-3)	4 (4-4)	6 (6-7)	8 (7-9)	9 (8-10)	9 (8-10)
Tonga	0 (0-0)	1 (1-1)	1 (1-1)	1 (1-1)	1 (1-1)	1 (1-1)	1 (1-1)	1 (0-1)
Vanuatu	0 (0-1)	1 (1-1)	1 (1-1)	2 (2-2)	2 (2-2)	3 (2-3)	3 (3-4)	4 (3-4)
Southeast Asia	4,164 (3,889-4,430)	5,175 (4,957-5,387)	5,623 (5,455-5,785)	6,003 (5,814-6,176)	6,077 (5,899-6,252)	5,904 (5,700-6,105)	5,879 (5,540-6,233)	5,321 (4,820-5,927)
Cambodia	107 (100-115)	134 (126-141)	163 (156-170)	183 (178-189)	220 (216-225)	190 (184-196)	185 (173-198)	184 (167-204)
Indonesia	1,804 (1,698-1,909)	2,329 (2,219-2,432)	2,481 (2,403-2,553)	2,474 (2,402-2,546)	2,295 (2,209-2,378)	2,265 (2,180-2,350)	2,254 (2,081-2,438)	1,960 (1,697-2,287)
Laos	44 (41-47)	52 (49-54)	60 (57-63)	70 (68-73)	77 (75-79)	87 (85-90)	87 (82-92)	86 (79-95)
Malaysia	127 (117-138)	152 (151-153)	159 (158-161)	190 (188-191)	238 (236-239)	235 (233-237)	234 (233-236)	246 (221-273)
Maldives	1 (1-1)	2 (2-2)	2 (2-2)	3 (3-3)	3 (3-4)	2 (2-2)	3 (3-3)	3 (3-3)
Mauritius	11 (11-11)	12 (12-12)	10 (10-11)	11 (11-12)	11 (10-11)	10 (9-10)	7 (7-7)	6 (5-6)
Myanmar	416 (383-449)	487 (454-519)	517 (485-551)	566 (520-610)	565 (513-619)	533 (479-591)	447 (402-496)	426 (391-467)
Philippines	464 (436-492)	594 (564-625)	727 (693-759)	889 (853-924)	1,026 (988-1,069)	1,124 (1,076-1,173)	1,259 (1,183-1,333)	1,216 (1,109-1,348)

Supplemental Results Table 4: Live births (in thousands) by location, females, 1950, 1960, 1970, 1980, 1990, 2000, 2010, 2017

Geography	1950	1960	1970	1980	1990	2000	2010	2017
Sri Lanka	149 (138-162)	179 (172-185)	194 (191-198)	202 (192-213)	184 (172-197)	179 (166-192)	175 (158-194)	142 (120-167)
Seychelles	0 (0-0)	0 (0-0)	0 (0-0)	0 (0-0)	0 (0-0)	0 (0-0)	0 (0-0)	0 (0-0)
Thailand	414 (376-453)	505 (477-530)	557 (538-576)	565 (537-594)	554 (523-583)	474 (445-505)	380 (348-412)	297 (262-340)
Timor-Leste	12 (11-12)	13 (13-14)	12 (12-12)	13 (13-14)	18 (17-19)	20 (19-21)	17 (16-19)	18 (16-21)
Vietnam	602 (557-648)	701 (661-740)	723 (682-763)	823 (786-859)	870 (845-895)	771 (746-796)	815 (771-860)	724 (660-799)
Sub-Saharan Africa	4,612 (4,347-4,870)	5,583 (5,353-5,801)	6,808 (6,579-7,042)	8,625 (8,372-8,868)	10,796 (10,497-11,100)	13,501 (13,120-13,909)	16,152 (15,464-16,821)	17,739 (16,678-18,950)
Central Sub-Saharan Africa	534 (512-555)	689 (664-714)	822 (788-853)	1,014 (977-1,049)	1,268 (1,223-1,310)	1,612 (1,559-1,669)	2,016 (1,935-2,099)	2,124 (1,997-2,247)
Angola	124 (119-129)	140 (134-145)	151 (145-157)	186 (179-194)	253 (239-267)	351 (327-372)	478 (442-514)	515 (471-561)
Central African Republic	30 (28-32)	39 (37-42)	48 (45-51)	50 (47-53)	58 (54-62)	70 (64-76)	77 (70-84)	65 (58-73)
Congo	21 (20-23)	25 (23-26)	29 (28-31)	38 (36-40)	47 (45-49)	60 (57-62)	71 (66-75)	65 (59-71)
Democratic Republic of the Congo	343 (326-360)	468 (448-486)	574 (547-600)	715 (689-741)	879 (850-907)	1,095 (1,058-1,130)	1,350 (1,290-1,409)	1,438 (1,336-1,538)
Equatorial Guinea	5 (5-5)	5 (5-5)	6 (5-6)	7 (7-8)	10 (10-11)	14 (14-15)	18 (16-20)	19 (16-21)
Gabon	9 (9-10)	10 (10-11)	11 (11-12)	14 (13-15)	17 (16-19)	19 (17-21)	20 (18-22)	20 (17-23)
Eastern Sub-Saharan Africa	1,705 (1,610-1,800)	2,137 (2,055-2,213)	2,682 (2,607-2,756)	3,526 (3,445-3,601)	4,489 (4,390-4,581)	5,483 (5,340-5,629)	6,295 (6,014-6,569)	6,872 (6,404-7,407)
Burundi	66 (62-69)	76 (73-79)	84 (82-87)	108 (106-111)	122 (119-126)	121 (116-125)	172 (164-181)	200 (187-215)
Comoros	3 (2-3)	4 (4-4)	6 (6-6)	8 (7-8)	9 (9-10)	10 (9-11)	9 (8-10)	8 (7-10)
Djibouti	1 (1-1)	2 (2-2)	3 (3-4)	7 (7-7)	11 (10-11)	13 (12-14)	15 (14-17)	16 (14-19)
Eritrea	30 (28-31)	38 (36-40)	50 (47-52)	61 (58-64)	63 (59-67)	70 (64-76)	88 (78-98)	87 (76-99)
Ethiopia	453 (422-484)	543 (514-571)	693 (667-719)	884 (857-911)	1,245 (1,205-1,284)	1,536 (1,476-1,596)	1,639 (1,545-1,738)	1,807 (1,655-1,980)
Kenya	162 (154-169)	234 (226-241)	291 (281-301)	407 (393-419)	486 (467-504)	564 (535-590)	629 (581-677)	668 (591-751)
Madagascar	101 (97-107)	129 (124-133)	171 (165-177)	219 (211-225)	262 (251-273)	339 (323-355)	410 (386-435)	477 (426-530)
Malawi	82 (77-86)	106 (103-109)	128 (125-131)	164 (161-167)	247 (242-252)	273 (267-279)	287 (277-297)	304 (283-327)
Mozambique	164 (152-176)	184 (174-193)	219 (208-230)	284 (276-292)	327 (320-335)	399 (389-409)	441 (425-456)	487 (450-527)
Rwanda	65 (61-69)	76 (72-80)	95 (92-98)	130 (128-132)	160 (156-165)	164 (158-170)	183 (173-193)	209 (188-232)
Somalia	50 (47-53)	67 (64-70)	93 (89-97)	171 (163-179)	157 (149-165)	226 (214-238)	292 (274-308)	333 (310-359)
South Sudan	57 (52-62)	71 (66-76)	82 (77-87)	102 (95-109)	128 (118-138)	169 (157-180)	217 (202-232)	202 (190-217)
Tanzania	238 (226-250)	296 (284-308)	371 (362-379)	477 (465-489)	599 (585-613)	739 (716-763)	910 (867-954)	982 (904-1,070)
Uganda	161 (153-169)	211 (204-218)	274 (267-281)	351 (345-357)	470 (461-478)	625 (611-637)	715 (692-739)	766 (727-809)
Zambia	65 (61-68)	93 (88-97)	113 (107-119)	145 (138-151)	191 (181-201)	225 (213-236)	277 (255-299)	314 (281-352)
Southern Sub-Saharan Africa	405 (373-438)	470 (441-498)	584 (550-620)	705 (657-753)	767 (708-824)	839 (785-892)	880 (804-965)	863 (788-957)
Botswana	9 (8-9)	11 (10-11)	14 (13-14)	18 (17-19)	21 (20-21)	22 (22-23)	23 (22-24)	23 (22-25)
Lesotho	15 (14-16)	18 (17-19)	22 (21-23)	28 (27-29)	30 (29-31)	28 (27-29)	26 (24-27)	24 (22-26)
Namibia	10 (10-11)	13 (12-14)	16 (15-17)	20 (19-20)	25 (24-25)	26 (26-27)	30 (29-31)	29 (27-32)
South Africa	288 (263-314)	327 (302-350)	396 (367-425)	452 (407-497)	481 (427-534)	538 (486-590)	555 (485-635)	538 (481-611)
Swaziland	6 (5-6)	7 (7-8)	10 (9-10)	13 (13-14)	15 (15-16)	16 (16-17)	16 (14-17)	15 (13-17)
Zimbabwe	74 (70-78)	92 (88-96)	125 (120-129)	173 (169-176)	193 (189-197)	205 (200-210)	229 (224-234)	231 (216-247)
Western Sub-Saharan Africa	1,966 (1,852-2,074)	2,285 (2,181-2,383)	2,719 (2,618-2,817)	3,379 (3,260-3,490)	4,271 (4,136-4,403)	5,566 (5,385-5,738)	6,959 (6,662-7,260)	7,878 (7,401-8,409)
Benin	54 (50-58)	57 (54-59)	65 (63-67)	82 (80-84)	112 (109-116)	146 (141-150)	185 (177-194)	205 (189-223)
Burkina Faso	97 (89-105)	97 (91-103)	119 (114-124)	166 (161-171)	222 (217-226)	273 (265-281)	350 (334-366)	416 (388-447)
Cameroon	117 (109-125)	135 (126-144)	157 (150-164)	180 (173-187)	218 (208-227)	310 (297-323)	416 (388-445)	422 (376-476)
Cape Verde	3 (3-3)	4 (4-4)	4 (4-4)	5 (4-5)	5 (5-5)	5 (5-6)	5 (4-6)	4 (4-5)
Chad	70 (66-74)	78 (74-81)	93 (90-97)	120 (116-125)	157 (153-161)	213 (208-219)	290 (280-300)	350 (335-369)
Cote d'Ivoire	63 (59-68)	101 (95-106)	137 (132-142)	195 (187-203)	278 (263-292)	359 (336-382)	409 (376-442)	423 (385-466)

Supplemental Results Table 4: Live births (in thousands) by location, females, 1950, 1960, 1970, 1980, 1990, 2000, 2010, 2017								
**Geography**	**1950**	**1960**	**1970**	**1980**	**1990**	**2000**	**2010**	**2017**
The Gambia	6<br>(5-6)	7<br>(6-7)	11<br>(10-11)	17<br>(16-17)	23<br>(21-24)	28<br>(26-30)	31<br>(28-34)	33<br>(29-38)
Ghana	98<br>(89-108)	160<br>(150-170)	214<br>(205-223)	262<br>(252-271)	282<br>(269-295)	331<br>(314-348)	374<br>(350-399)	428<br>(371-491)
Guinea	60<br>(57-63)	77<br>(73-81)	99<br>(95-104)	121<br>(116-125)	148<br>(143-153)	175<br>(168-181)	193<br>(184-202)	212<br>(200-226)
Guinea-Bissau	16<br>(15-16)	15<br>(14-16)	15<br>(14-16)	20<br>(19-21)	24<br>(22-25)	28<br>(25-29)	31<br>(28-34)	33<br>(30-36)
Liberia	22<br>(20-23)	25<br>(23-26)	35<br>(34-36)	48<br>(46-50)	41<br>(40-43)	64<br>(62-67)	75<br>(70-81)	74<br>(67-83)
Mali	90<br>(85-96)	120<br>(114-124)	154<br>(149-159)	188<br>(182-193)	218<br>(213-223)	262<br>(253-270)	350<br>(337-363)	428<br>(404-455)
Mauritania	17<br>(16-18)	20<br>(19-21)	26<br>(25-27)	35<br>(34-36)	43<br>(42-45)	50<br>(48-52)	56<br>(53-59)	58<br>(53-63)
Niger	75<br>(71-80)	99<br>(94-102)	130<br>(126-133)	170<br>(167-173)	215<br>(212-219)	303<br>(298-309)	397<br>(384-409)	492<br>(466-520)
Nigeria	1,023<br>(965-1,078)	1,108<br>(1,057-1,158)	1,233<br>(1,175-1,288)	1,490<br>(1,415-1,561)	1,939<br>(1,858-2,020)	2,626<br>(2,520-2,729)	3,318<br>(3,157-3,491)	3,804<br>(3,515-4,102)
Sao Tome and Principe	1<br>(1-1)	1<br>(1-1)	1<br>(1-1)	1<br>(1-1)	2<br>(2-2)	2<br>(2-2)	2<br>(2-2)	2<br>(2-2)
Senegal	63<br>(59-68)	80<br>(75-84)	112<br>(107-116)	142<br>(134-148)	167<br>(156-177)	195<br>(184-206)	229<br>(211-247)	243<br>(224-266)
Sierra Leone	45<br>(41-49)	52<br>(48-55)	57<br>(53-61)	65<br>(62-69)	87<br>(82-91)	92<br>(87-97)	122<br>(113-131)	132<br>(119-145)
Togo	35<br>(32-37)	42<br>(40-43)	48<br>(46-50)	63<br>(61-65)	78<br>(76-80)	95<br>(92-98)	114<br>(110-119)	109<br>(101-118)

Supplemental Results Table 5: Age-specific fertility rates per 1000, cumulative total fertility up to maternal age 25 (TFU25), cumulative total fertility from maternal age 30 to age 55 (TFO30), and cumulative total fertility, Global, 1950-2017

Year	10-14	15-19	20-24	25-29	30-34	35-39	40-44	45-49	50-54	TFR	TFU25	TFO30
1950	0·8 (0·3–1·7)	75·7 (69·7–82·0)	255·7 (244·9–266·4)	263·7 (258·6–268·7)	178·3 (171·1–185·3)	111·7 (104·7–118·3)	42·4 (38·2–46·5)	9·9 (8·3–11·6)	0·2 (0·2–0·2)	4·69 (4·5–4·89)	1·66 (1·58–1·75)	1·71 (1·62–1·8)
1951	0·8 (0·3–1·7)	75·9 (70·2–82·1)	256·0 (245·3–266·2)	263·2 (258·2–267·9)	178·1 (171·2–184·7)	110·9 (104·3–117·6)	41·9 (38·1–45·7)	9·8 (8·2–11·5)	0·2 (0·2–0·2)	4·68 (4·5–4·87)	1·66 (1·58–1·74)	1·7 (1·62–1·79)
1952	0·8 (0·3–1·7)	75·8 (70·3–81·5)	255·7 (245·0–265·8)	262·9 (257·8–267·5)	177·2 (170·4–184·0)	110·4 (103·7–117·2)	41·6 (38·0–45·3)	9·7 (8·3–11·3)	0·2 (0·2–0·2)	4·67 (4·49–4·85)	1·66 (1·58–1·74)	1·7 (1·61–1·78)
1953	0·8 (0·4–1·7)	76·0 (70·7–81·3)	256·2 (244·7–267·0)	263·0 (258·3–267·3)	175·8 (169·6–182·1)	110·3 (103·9–117·0)	41·3 (37·9–44·7)	9·6 (8·3–11·1)	0·2 (0·2–0·2)	4·67 (4·49–4·84)	1·66 (1·58–1·74)	1·69 (1·61–1·77)
1954	0·8 (0·4–1·7)	76·3 (71·3–81·0)	256·9 (244·7–268·2)	263·3 (258·8–267·5)	174·2 (168·1–180·3)	110·0 (103·6–116·4)	40·8 (37·5–44·1)	9·5 (8·3–10·9)	0·2 (0·2–0·2)	4·66 (4·48–4·82)	1·67 (1·59–1·75)	1·67 (1·59–1·75)
1955	0·8 (0·4–1·8)	77·1 (72·6–81·6)	257·1 (246·4–267·6)	263·6 (259·3–267·8)	172·4 (166·3–178·4)	109·6 (103·4–115·6)	40·2 (36·9–43·6)	9·4 (8·3–10·6)	0·2 (0·2–0·2)	4·65 (4·49–4·81)	1·68 (1·6–1·74)	1·66 (1·58–1·73)
1956	0·8 (0·4–1·8)	78·7 (74·4–82·6)	258·4 (248·6–267·9)	264·9 (260·7–268·8)	171·9 (166·4–177·6)	109·2 (102·9–115·5)	39·9 (36·7–43·1)	9·3 (8·2–10·4)	0·2 (0·2–0·2)	4·67 (4·51–4·82)	1·69 (1·62–1·76)	1·65 (1·58–1·73)
1957	0·9 (0·4–1·8)	80·4 (76·3–84·4)	259·7 (250·0–268·8)	266·4 (262·6–270·3)	172·0 (166·1–177·7)	108·6 (102·3–115·2)	39·9 (36·5–43·3)	9·3 (8·3–10·4)	0·2 (0·2–0·2)	4·69 (4·54–4·84)	1·7 (1·64–1·77)	1·65 (1·57–1·73)
1958	0·9 (0·4–1·8)	82·4 (78·7–86·4)	261·0 (251·9–269·6)	267·5 (264·0–270·9)	172·1 (165·9–178·3)	107·5 (101·4–114·0)	40·2 (37·1–43·6)	9·3 (8·3–10·4)	0·2 (0·2–0·2)	4·7 (4·55–4·86)	1·72 (1·66–1·78)	1·65 (1·57–1·73)
1959	0·9 (0·4–1·8)	84·2 (80·8–87·7)	261·1 (254·0–268·2)	269·0 (266·1–271·8)	171·9 (166·2–177·4)	106·1 (100·9–111·2)	40·5 (37·7–43·5)	9·2 (8·3–10·2)	0·2 (0·2–0·2)	4·72 (4·59–4·84)	1·73 (1·68–1·78)	1·64 (1·57–1·71)
1960	0·8 (0·4–1·8)	85·3 (82·2–88·4)	261·1 (255·3–266·9)	269·8 (267·2–272·3)	171·9 (166·5–176·7)	104·9 (100·5–109·6)	40·8 (38·2–43·5)	9·2 (8·4–10·0)	0·2 (0·2–0·2)	4·72 (4·61–4·83)	1·74 (1·69–1·78)	1·63 (1·58–1·69)
1961	0·8 (0·4–1·8)	85·1 (82·0–88·3)	261·5 (256·0–266·7)	270·2 (267·5–272·6)	171·9 (166·8–176·9)	104·0 (99·9–108·2)	40·8 (38·4–43·3)	9·3 (8·5–10·2)	0·2 (0·2–0·2)	4·72 (4·62–4·81)	1·74 (1·7–1·78)	1·63 (1·58–1·68)
1962	0·8 (0·4–1·7)	83·6 (80·6–86·7)	261·6 (255·8–267·2)	270·7 (268·2–273·1)	172·1 (166·9–177·4)	103·3 (99·0–107·6)	40·5 (38·1–43·0)	9·4 (8·6–10·3)	0·2 (0·2–0·2)	4·71 (4·61–4·81)	1·73 (1·69–1·77)	1·63 (1·58–1·68)
1963	0·8 (0·4–1·7)	82·4 (79·5–85·3)	261·4 (255·6–266·6)	270·3 (267·9–272·7)	171·9 (166·8–176·6)	102·6 (98·8–107·0)	40·0 (37·6–42·4)	9·6 (8·8–10·5)	0·2 (0·2–0·2)	4·7 (4·6–4·79)	1·72 (1·68–1·76)	1·62 (1·57–1·67)
1964	0·8 (0·4–1·7)	81·3 (78·5–84·1)	260·2 (255·1–265·5)	267·9 (265·3–270·4)	171·2 (166·5–175·6)	101·4 (97·5–105·5)	39·4 (37·1–41·8)	9·7 (9·0–10·5)	0·2 (0·2–0·2)	4·66 (4·57–4·75)	1·71 (1·67–1·75)	1·61 (1·56–1·66)
1965	0·8 (0·4–1·7)	79·8 (77·5–82·4)	258·8 (254·1–263·6)	264·4 (261·8–266·8)	169·4 (165·2–173·5)	100·0 (96·3–104·0)	38·8 (36·7–41·1)	9·7 (8·9–10·6)	0·2 (0·2–0·2)	4·61 (4·53–4·69)	1·7 (1·66–1·73)	1·59 (1·55–1·64)
1966	0·8 (0·4–1·7)	78·9 (76·6–81·4)	259·2 (254·3–263·9)	263·9 (261·6–266·1)	168·9 (165·0–172·9)	99·2 (95·4–102·9)	38·3 (36·2–40·6)	9·7 (8·9–10·5)	0·2 (0·2–0·2)	4·59 (4·51–4·67)	1·69 (1·66–1·73)	1·58 (1·54–1·62)
1967	0·8 (0·4–1·7)	78·2 (75·9–80·5)	258·6 (253·4–263·4)	264·7 (262·3–266·8)	168·2 (164·3–172·1)	98·5 (95·0–102·0)	37·9 (35·9–40·0)	9·5 (8·7–10·3)	0·2 (0·2–0·2)	4·58 (4·5–4·66)	1·69 (1·65–1·72)	1·57 (1·53–1·61)
1968	0·8 (0·4–1·7)	77·2 (75·0–79·5)	255·2 (250·0–260·1)	263·2 (260·9–265·5)	165·4 (161·6–169·4)	97·1 (93·8–100·6)	37·5 (35·4–39·7)	9·3 (8·5–10·1)	0·2 (0·2–0·2)	4·53 (4·45–4·6)	1·67 (1·63–1·7)	1·55 (1·51–1·59)
1969	0·8 (0·4–1·7)	76·7 (74·7–78·9)	250·2 (245·2–254·8)	261·2 (258·8–263·4)	162·1 (158·4–166·2)	95·6 (92·3–98·9)	36·9 (34·8–38·8)	9·0 (8·2–9·7)	0·2 (0·2–0·2)	4·46 (4·39–4·53)	1·64 (1·61–1·67)	1·52 (1·48–1·56)
1970	0·8 (0·4–1·8)	77·1 (75·0–79·3)	244·7 (239·4–249·4)	257·7 (255·4–260·2)	158·1 (154·2–162·1)	93·8 (90·1–97·2)	36·1 (34·2–38·1)	8·6 (7·9–9·4)	0·2 (0·2–0·2)	4·39 (4·31–4·46)	1·61 (1·58–1·64)	1·48 (1·44–1·53)
1971	0·8 (0·4–1·8)	76·4 (74·4–78·7)	239·3 (233·7–244·6)	253·0 (250·5–255·5)	153·7 (149·7–157·7)	91·1 (87·4–94·8)	35·1 (33·2–37·0)	8·3 (7·6–9·0)	0·2 (0·2–0·2)	4·29 (4·21–4·37)	1·58 (1·55–1·62)	1·44 (1·4–1·48)
1972	0·8 (0·4–1·7)	75·6 (73·6–77·8)	234·2 (227·6–240·7)	246·6 (243·8–249·5)	149·0 (145·4–153·0)	87·7 (84·3–91·2)	34·1 (32·2–36·0)	8·0 (7·3–8·7)	0·2 (0·1–0·2)	4·18 (4·11–4·26)	1·55 (1·51–1·6)	1·39 (1·35–1·44)
1973	0·8 (0·4–1·7)	74·8 (72·8–77·0)	229·2 (222·1–236·3)	239·1 (236·1–242·1)	144·7 (141·0–148·7)	84·1 (81·2–87·5)	33·2 (31·3–35·0)	7·7 (7·1–8·4)	0·1 (0·1–0·2)	4·07 (3·99–4·15)	1·52 (1·48–1·57)	1·35 (1·31–1·4)
1974	0·8 (0·4–1·7)	74·0 (72·0–76·0)	222·6 (216·2–228·6)	230·6 (227·2–233·7)	141·3 (138·0–144·6)	81·3 (78·6–84·3)	32·4 (30·6–34·1)	7·5 (6·9–8·2)	0·1 (0·1–0·1)	3·95 (3·88–4·02)	1·49 (1·45–1·52)	1·31 (1·28–1·35)
1975	0·8 (0·3–1·7)	73·5 (71·5–75·5)	216·5 (211·0–222·4)	221·9 (218·5–225·1)	138·5 (135·3–141·5)	79·2 (76·7–81·6)	31·6 (30·0–33·3)	7·4 (6·8–8·1)	0·1 (0·1–0·1)	3·85 (3·78–3·91)	1·45 (1·42–1·49)	1·28 (1·25–1·32)
1976	0·8 (0·3–1·7)	73·6 (71·7–75·6)	212·2 (205·9–217·8)	216·0 (212·3–219·7)	135·5 (132·2–138·8)	77·1 (74·7–79·6)	30·8 (29·1–32·4)	7·4 (6·8–8·0)	0·1 (0·1–0·1)	3·77 (3·7–3·84)	1·43 (1·4–1·47)	1·25 (1·22–1·29)
1977	0·8 (0·3–1·7)	73·7 (71·7–75·8)	208·7 (202·7–214·7)	212·2 (208·4–215·6)	131·7 (128·7–135·1)	75·2 (73·0–77·5)	29·8 (28·2–31·4)	7·3 (6·7–7·9)	0·1 (0·1–0·1)	3·7 (3·63–3·77)	1·42 (1·38–1·45)	1·22 (1·19–1·25)
1978	0·8 (0·3–1·7)	72·9 (70·9–74·8)	206·6 (200·8–212·5)	209·4 (205·7–213·0)	129·5 (126·6–132·5)	74·1 (71·9–76·4)	29·0 (27·5–30·5)	7·2 (6·6–7·8)	0·1 (0·1–0·1)	3·65 (3·58–3·71)	1·4 (1·37–1·43)	1·2 (1·17–1·23)
1979	0·8 (0·3–1·7)	71·7 (69·8–73·7)	205·8 (200·7–210·9)	206·8 (203·1–210·5)	127·9 (125·0–131·0)	73·7 (71·7–76·0)	28·5 (27·0–29·9)	7·1 (6·6–7·7)	0·1 (0·1–0·1)	3·61 (3·55–3·67)	1·39 (1·36–1·42)	1·19 (1·16–1·22)
1980	0·8 (0·3–1·7)	70·2 (68·3–72·2)	204·7 (199·8–209·7)	204·8 (200·8–208·8)	125·9 (123·1–128·9)	73·8 (71·5–76·0)	28·1 (26·7–29·5)	7·1 (6·6–7·7)	0·1 (0·1–0·1)	3·58 (3·51–3·64)	1·38 (1·35–1·41)	1·18 (1·15–1·2)
1981	0·8 (0·3–1·7)	68·3 (66·0–70·7)	205·0 (199·4–210·7)	205·8 (201·5–209·9)	125·7 (122·4–129·3)	74·0 (71·4–76·7)	27·9 (26·1–29·7)	7·0 (6·4–7·6)	0·1 (0·1–0·1)	3·57 (3·49–3·65)	1·37 (1·33–1·41)	1·17 (1·14–1·21)
1982	0·8 (0·3–1·7)	66·7 (64·1–69·4)	207·0 (200·4–213·4)	208·7 (204·0–213·1)	126·1 (122·2–129·8)	73·4 (70·3–76·6)	27·6 (25·8–29·5)	6·8 (6·3–7·5)	0·1 (0·1–0·1)	3·59 (3·5–3·67)	1·37 (1·33–1·41)	1·17 (1·13–1·21)
1983	0·8 (0·3–1·7)	65·7 (62·9–68·7)	208·1 (202·1–214·5)	208·4 (203·6–212·6)	125·3 (121·5–129·1)	71·9 (68·9–75·2)	27·3 (25·4–29·4)	6·7 (6·1–7·3)	0·1 (0·1–0·1)	3·57 (3·49–3·65)	1·37 (1·33–1·41)	1·16 (1·12–1·2)
1984	0·8 (0·3–1·7)	64·8 (62·0–67·9)	208·2 (202·9–213·7)	205·3 (201·3–209·2)	124·0 (120·5–127·6)	69·6 (67·0–72·3)	27·0 (25·1–28·8)	6·5 (5·9–7·1)	0·1 (0·1–0·1)	3·53 (3·45–3·61)	1·37 (1·33–1·41)	1·14 (1·1–1·17)
1985	0·8 (0·3–1·7)	64·0 (61·1–67·0)	207·9 (202·0–213·7)	202·8 (199·1–206·5)	124·1 (120·6–127·5)	66·8 (64·4–69·2)	26·8 (25·0–28·6)	6·3 (5·8–7·0)	0·1 (0·1–0·1)	3·5 (3·42–3·57)	1·36 (1·32–1·4)	1·12 (1·09–1·15)
1986	0·8 (0·4–1·7)	63·1 (60·1–66·1)	207·0 (200·1–213·8)	199·7 (195·9–203·5)	123·2 (119·2–127·0)	64·8 (62·5–67·6)	26·3 (24·6–28·1)	6·2 (5·6–6·8)	0·1 (0·1–0·1)	3·46 (3·38–3·54)	1·35 (1·31–1·4)	1·1 (1·07–1·14)
1987	0·8 (0·4–1·7)	62·2 (59·2–65·3)	203·1 (195·7–211·0)	194·5 (190·8–198·3)	120·3 (116·1–124·6)	63·0 (60·5–65·6)	25·0 (23·5–26·7)	6·1 (5·4–6·7)	0·1 (0·1–0·1)	3·38 (3·29–3·46)	1·33 (1·28–1·38)	1·07 (1·04–1·11)
1988	0·8 (0·4–1·7)	61·8 (59·0–64·7)	197·9 (190·5–205·5)	188·3 (184·6–192·3)	117·0 (113·3–120·6)	61·2 (58·9–63·6)	23·6 (22·4–25·0)	6·0 (5·4–6·5)	0·1 (0·1–0·1)	3·28 (3·21–3·36)	1·3 (1·26–1·35)	1·04 (1·01–1·07)
1989	0·8 (0·4–1·8)	61·9 (59·2–64·8)	194·0 (188·1–200·2)	182·4 (178·7–186·3)	114·2 (111·2–117·1)	59·5 (57·6–61·5)	22·2 (21·1–23·4)	5·9 (5·4–6·4)	0·1 (0·1–0·1)	3·21 (3·14–3·27)	1·28 (1·25–1·32)	1·01 (0·98–1·04)
1990	0·8 (0·4–1·8)	61·9 (59·0–64·8)	189·1 (183·3–195·2)	177·2 (173·6–180·9)	111·3 (108·6–113·8)	57·9 (56·2–59·6)	20·7 (19·7–21·8)	5·8 (5·3–6·3)	0·1 (0·1–0·1)	3·12 (3·06–3·19)	1·26 (1·22–1·3)	0·98 (0·95–1·0)
1991	0·8 (0·4–1·8)	61·6 (58·7–64·4)	184·2 (177·9–190·6)	172·7 (168·6–176·7)	108·6 (106·1–111·0)	56·1 (54·3–57·8)	19·8 (18·8–20·7)	5·6 (5·2–6·1)	0·1 (0·1–0·1)	3·05 (2·98–3·12)	1·23 (1·19–1·27)	0·95 (0·93–0·97)
1992	0·8 (0·4–1·8)	60·8 (57·8–63·9)	180·5 (174·3–187·3)	168·9 (164·2–173·2)	106·6 (104·2–109·0)	54·8 (53·1–56·6)	19·2 (18·3–20·2)	5·4 (5·0–5·8)	0·1 (0·1–0·1)	2·99 (2·91–3·06)	1·21 (1·17–1·25)	0·93 (0·91–0·95)
1993	0·8 (0·4–1·8)	60·1 (56·9–63·3)	177·8 (171·7–184·0)	165·8 (161·5–170·2)	104·6 (102·5–107·0)	54·1 (52·6–55·7)	18·8 (17·8–19·8)	5·1 (4·7–5·6)	0·1 (0·1–0·1)	2·94 (2·87–3·01)	1·19 (1·15–1·23)	0·91 (0·89–0·94)
1994	0·8 (0·4–1·7)	59·6 (56·4–62·9)	175·7 (169·8–181·4)	162·7 (158·6–167·2)	102·3 (100·2–104·5)	53·7 (52·2–55·3)	18·4 (17·5–19·3)	4·9 (4·5–5·3)	0·1 (0·1–0·1)	2·89 (2·82–2·96)	1·18 (1·14–1·22)	0·9 (0·88–0·92)
1995	0·8 (0·4–1·7)	58·5 (55·5–61·9)	172·3 (167·2–177·2)	159·9 (156·0–163·9)	100·2 (98·1–102·3)	53·7 (52·1–55·3)	18·2 (17·4–19·0)	4·7 (4·3–5·1)	0·1 (0·1–0·1)	2·84 (2·78–2·91)	1·16 (1·12–1·2)	0·88 (0·86–0·91)
1996	0·8 (0·4–1·7)	57·6 (54·7–60·6)	168·7 (163·7–173·8)	158·2 (154·0–162·7)	99·2 (97·1–101·5)	53·9 (52·4–55·5)	17·9 (17·2–18·8)	4·5 (4·1–4·9)	0·1 (0·1–0·1)	2·81 (2·74–2·87)	1·14 (1·1–1·17)	0·88 (0·86–0·9)
1997	0·8 (0·3–1·7)	57·3 (54·4–60·2)	166·6 (161·6–171·7)	156·9 (153·0–161·1)	98·8 (96·7–100·9)	54·0 (52·5–55·6)	17·9 (17·1–18·8)	4·4 (4·0–4·7)	0·1 (0·1–0·1)	2·78 (2·72–2·85)	1·12 (1·09–1·16)	0·88 (0·85–0·9)
1998	0·8 (0·3–1·7)	57·0 (54·1–59·8)	164·9 (160·1–169·9)	155·3 (151·9–159·1)	98·5 (96·5–100·8)	53·5 (52·0–54·9)	18·0 (17·2–18·9)	4·3 (3·9–4·6)	0·1 (0·1–0·1)	2·76 (2·7–2·83)	1·11 (1·08–1·15)	0·87 (0·85–0·89)
1999	0·8 (0·3–1·7)	55·9 (53·3–58·8)	162·8 (158·1–167·6)	153·5 (150·0–156·9)	98·5 (96·2–100·9)	52·4 (51·0–53·9)	18·1 (17·3–19·0)	4·2 (3·9–4·5)	0·1 (0·1–0·1)	2·73 (2·67–2·79)	1·1 (1·06–1·13)	0·87 (0·85–0·89)
2000	0·8 (0·3–1·6)	54·4 (51·9–57·2)	160·2 (155·4–165·0)	151·4 (148·1–154·8)	98·3 (95·9–100·8)	51·3 (49·7–52·6)	18·1 (17·3–18·9)	4·1 (3·8–4·5)	0·1 (0·1–0·1)	2·69 (2·63–2·76)	1·08 (1·04–1·11)	0·86 (0·84–0·88)
2001	0·8 (0·3–1·6)	52·9 (50·4–55·6)	157·6 (152·9–162·3)	149·8 (146·4–153·3)	97·6 (95·2–100·2)	50·2 (48·6–51·8)	18·0 (17·1–18·8)	4·0 (3·7–4·3)	0·1 (0·1–0·1)	2·65 (2·59–2·72)	1·06 (1·02–1·09)	0·85 (0·83–0·87)
2002	0·8 (0·3–1·7)	51·5 (48·9–54·2)	156·2 (151·5–161·0)	149·2 (145·6–152·6)	97·6 (95·1–100·1)	49·5 (47·8–51·2)	17·8 (17·0–18·7)	4·0 (3·7–4·3)	0·1 (0·1–0·1)	2·63 (2·57–2·7)	1·04 (1·01–1·08)	0·84 (0·82–0·87)
2003	0·8 (0·3–1·7)	50·2 (47·7–53·0)	154·9 (149·4–160·3)	148·6 (145·0–152·3)	97·8 (95·3–100·3)	49·3 (47·7–51·0)	17·6 (16·7–18·4)	4·0 (3·6–4·3)	0·1 (0·1–0·1)	2·62 (2·55–2·69)	1·03 (0·99–1·07)	0·84 (0·82–0·87)
2004	0·8 (0·4–1·7)	49·0 (46·4–51·8)	152·8 (147·4–158·2)	147·4 (143·7–151·2)	97·7 (95·2–100·4)	49·5 (47·9–51·2)	17·3 (16·5–18·2)	3·9 (3·6–4·3)	0·1 (0·1–0·1)	2·59 (2·52–2·67)	1·01 (0·98–1·05)	0·84 (0·82–0·87)
2005	0·8 (0·4–1·7)	48·1 (45·5–50·7)	150·3 (144·6–156·2)	146·1 (142·2–150·2)	98·0 (95·4–101·0)	50·3 (48·5–52·0)	17·2 (16·4–18·1)	4·0 (3·7–4·3)	0·1 (0·1–0·1)	2·57 (2·5–2·65)	1·0 (0·96–1·04)	0·85 (0·82–0·87)
2006	0·8 (0·4–1·7)	47·7 (45·2–50·4)	147·7 (141·7–153·9)	145·3 (141·2–149·4)	98·8 (96·1–101·7)	51·3 (49·4–53·1)	17·2 (16·3–18·0)	4·0 (3·7–4·3)	0·1 (0·1–0·1)	2·56 (2·48–2·65)	0·98 (0·94–1·02)	0·86 (0·83–0·88)
2007	0·8 (0·4–1·7)	47·7 (45·1–50·4)	145·1 (138·6–151·5)	144·2 (139·9–148·3)	99·3 (96·4–102·2)	52·0 (49·9–53·9)	17·1 (16·1–18·0)	3·9 (3·6–4·3)	0·1 (0·1–0·1)	2·55 (2·47–2·64)	0·97 (0·92–1·01)	0·86 (0·83–0·89)
2008	0·8 (0·4–1·7)	47·7 (44·9–50·4)	142·6 (135·8–149·3)	142·5 (138·4–146·7)	99·3 (96·3–102·5)	52·0 (49·7–54·1)	17·0 (16·0–18·0)	3·8 (3·5–4·2)	0·1 (0·1–0·1)	2·53 (2·44–2·62)	0·96 (0·91–1·0)	0·86 (0·83–0·89)
2009	0·8 (0·4–1·7)	47·7 (45·0–50·6)	140·1 (133·1–146·9)	140·8 (136·7–145·0)	99·1 (95·7–102·5)	51·5 (49·3–53·7)	17·0 (16·0–17·9)	3·7 (3·4–4·0)	0·1 (0·1–0·1)	2·5 (2·41–2·59)	0·94 (0·9–0·99)	0·86 (0·82–0·89)
2010	0·8 (0·4–1·7)	48·0 (45·0–51·1)	138·0 (131·3–145·0)	139·7 (135·7–143·8)	98·8 (95·2–102·3)	51·1 (48·9–53·3)	16·9 (15·9–17·9)	3·5 (3·2–3·8)	0·1 (0·1–0·1)	2·48 (2·4–2·58)	0·93 (0·89–0·98)	0·85 (0·82–0·88)
2011	0·8 (0·4–1·8)	48·6 (45·5–52·3)	137·5 (130·9–144·2)	139·5 (135·0–143·7)	98·9 (95·5–102·5)	51·2 (48·9–53·7)	16·8 (15·8–17·9)	3·5 (3·1–3·8)	0·1 (0·1–0·1)	2·49 (2·39–2·58)	0·93 (0·89–0·98)	0·85 (0·82–0·88)
2012	0·8 (0·4–1·8)	48·6 (45·2–52·3)	137·6 (130·3–144·7)	139·3 (134·8–143·6)	99·5 (95·8–102·9)	52·0 (49·5–54·6)	16·9 (15·8–18·0)	3·4 (3·1–3·7)	0·1 (0·1–0·1)	2·49 (2·39–2·59)	0·94 (0·88–0·99)	0·86 (0·82–0·89)
2013	0·8 (0·4–1·7)	46·7 (43·2–50·7)	136·5 (129·3–144·0)	137·1 (132·8–141·3)	99·9 (96·1–103·5)	52·8 (50·1–55·6)	16·9 (15·8–18·1)	3·4 (3·1–3·7)	0·1 (0·1–0·1)	2·47 (2·36–2·58)	0·92 (0·87–0·98)	0·87 (0·83–0·9)
2014	0·8 (0·4–1·7)	44·4 (40·7–48·5)	132·9 (124·8–141·6)	132·5 (128·1–137·1)	99·0 (95·1–103·0)	53·1 (50·1–56·2)	17·0 (15·7–18·3)	3·4 (3·1–3·7)	0·1 (0·1–0·1)	2·41 (2·3–2·54)	0·89 (0·83–0·95)	0·86 (0·82–0·9)
2015	0·8 (0·4–1·7)	43·0 (39·0–47·5)	131·8 (122·3–142·4)	132·6 (127·6–138·1)	99·2 (94·5–104·2)	53·3 (49·6–57·2)	17·2 (15·7–18·8)	3·4 (3·1–3·8)	0·1 (0·1–0·1)	2·41 (2·28–2·55)	0·88 (0·81–0·95)	0·87 (0·82–0·92)
2016	0·8 (0·4–1·7)	42·6 (38·5–47·6)	131·9 (121·3–144·4)	134·3 (128·5–140·7)	99·2 (94·0–105·1)	53·2 (48·9–57·7)	17·3 (15·7–19·1)	3·4 (3·1–3·8)	0·1 (0·1–0·1)	2·41 (2·27–2·58)	0·88 (0·8–0·96)	0·87 (0·81–0·93)
2017	0·8 (0·4–1·7)	42·9 (38·6–48·0)	129·4 (117·8–142·7)	131·9 (125·9–138·7)	96·8 (91·4–102·9)	52·4 (47·7–57·5)	17·2 (15·4–19·2)	3·4 (3·1–3·8)	0·1 (0·1–0·1)	2·37 (2·22–2·55)	0·87 (0·78–0·96)	0·85 (0·79–0·92)

Supplemental Results Table 5: Age-specific fertility rates per 1000, cumulative total fertility up to maternal age 25 (TFU25), cumulative total fertility from maternal age 30 to age 55 (TFO30), and cumulative total fertility, Low SDI, 1950-2017

Year	10-14	15-19	20-24	25-29	30-34	35-39	40-44	45-49	50-54	TFR	TFU25	TFO30
1950	1·7 (0·8–3·7)	151·4 (137·2–166·6)	323·4 (302·1–343·8)	310·9 (300·2–321·5)	266·2 (253·0–278·9)	196·8 (180·9–211·6)	87·6 (75·6–99·6)	30·0 (24·6–35·4)	0·6 (0·6–0·6)	6·84 (6·38–7·3)	2·38 (2·2–2·56)	2·91 (2·67–3·13)
1951	1·7 (0·8–3·7)	151·4 (137·6–166·2)	323·1 (302·1–343·5)	310·7 (299·9–321·1)	266·3 (252·8–278·8)	196·8 (180·8–211·3)	87·6 (75·7–99·4)	30·0 (24·8–35·1)	0·6 (0·6–0·6)	6·84 (6·39–7·29)	2·38 (2·21–2·56)	2·91 (2·68–3·12)
1952	1·8 (0·8–3·7)	151·7 (138·4–165·8)	322·8 (301·0–342·0)	310·4 (299·7–320·4)	266·3 (253·3–278·7)	196·9 (181·5–211·5)	87·6 (76·7–99·0)	30·0 (24·9–35·0)	0·6 (0·6–0·6)	6·84 (6·42–7·26)	2·38 (2·21–2·55)	2·91 (2·69–3·11)
1953	1·8 (0·8–3·7)	151·9 (139·7–164·8)	322·4 (301·6–341·1)	310·2 (299·4–320·2)	266·4 (253·4–277·9)	196·9 (181·5–211·7)	87·6 (76·5–98·7)	30·0 (25·1–34·7)	0·6 (0·6–0·6)	6·84 (6·45–7·21)	2·38 (2·22–2·54)	2·91 (2·7–3·1)
1954	1·8 (0·8–3·8)	152·3 (141·2–163·8)	321·8 (300·9–340·1)	309·7 (299·1–319·5)	266·3 (254·7–277·6)	197·0 (182·7–211·2)	87·5 (76·8–98·8)	29·9 (25·3–34·3)	0·6 (0·6–0·6)	6·83 (6·48–7·18)	2·38 (2·23–2·52)	2·91 (2·71–3·1)
1955	1·8 (0·8–3·8)	153·3 (143·0–163·8)	321·2 (302·3–338·1)	309·1 (299·2–318·3)	266·1 (255·4–276·6)	196·8 (183·5–209·5)	87·5 (77·4–98·0)	29·9 (25·8–33·9)	0·6 (0·6–0·6)	6·83 (6·5–7·15)	2·38 (2·24–2·51)	2·9 (2·72–3·09)
1956	1·8 (0·8–3·8)	155·2 (145·8–165·0)	320·5 (303·0–335·3)	308·3 (300·0–316·2)	265·8 (256·5–275·0)	196·5 (184·6–207·7)	87·4 (77·9–96·7)	29·8 (26·2–33·4)	0·6 (0·6–0·6)	6·83 (6·54–7·11)	2·39 (2·26–2·51)	2·9 (2·75–3·05)
1957	1·8 (0·8–3·8)	158·1 (149·5–166·9)	319·5 (304·6–333·4)	307·3 (299·9–314·5)	265·4 (257·7–272·8)	196·3 (186·6–205·3)	87·3 (79·4–95·0)	29·7 (26·6–32·7)	0·6 (0·6–0·6)	6·83 (6·59–7·06)	2·4 (2·28–2·5)	2·9 (2·77–3·02)
1958	1·8 (0·8–3·9)	161·7 (153·7–169·7)	318·5 (306·3–329·7)	306·2 (300·2–312·1)	265·0 (259·0–270·9)	196·2 (188·7–202·6)	87·2 (81·2–92·9)	29·6 (27·2–32·0)	0·6 (0·6–0·6)	6·83 (6·63–7·03)	2·41 (2·31–2·5)	2·89 (2·8–2·98)
1959	1·8 (0·8–3·9)	164·8 (157·4–172·1)	317·6 (307·7–326·2)	305·4 (300·6–310·0)	264·9 (260·7–269·1)	196·3 (191·5–200·5)	87·3 (82·9–91·2)	29·5 (27·6–31·3)	0·6 (0·6–0·6)	6·84 (6·68–7·0)	2·42 (2·34–2·5)	2·89 (2·82–2·96)
1960	1·8 (0·8–3·9)	166·3 (159·4–173·4)	317·6 (309·0–325·0)	305·3 (300·7–309·7)	265·2 (261·4–268·9)	196·7 (192·3–200·6)	87·5 (83·6–91·0)	29·5 (27·8–31·0)	0·6 (0·6–0·6)	6·85 (6·7–6·99)	2·43 (2·36–2·5)	2·9 (2·83–2·95)
1961	1·8 (0·8–3·9)	166·8 (160·2–173·7)	318·1 (310·5–325·3)	306·0 (301·6–310·2)	266·0 (262·2–269·6)	197·3 (192·9–201·1)	87·9 (83·7–91·7)	29·5 (27·9–31·1)	0·6 (0·6–0·6)	6·87 (6·73–7·0)	2·43 (2·36–2·5)	2·91 (2·84–2·96)
1962	1·8 (0·8–3·9)	166·9 (160·6–173·7)	319·0 (312·0–325·5)	307·5 (303·3–311·5)	267·0 (263·3–270·5)	198·2 (193·7–202·0)	88·4 (84·2–92·3)	29·6 (27·9–31·2)	0·6 (0·6–0·6)	6·89 (6·76–7·02)	2·44 (2·37–2·5)	2·92 (2·86–2·98)
1963	1·8 (0·8–3·9)	167·2 (161·1–173·9)	320·4 (314·1–326·4)	309·1 (305·1–313·0)	267·9 (264·4–271·4)	199·0 (194·2–203·1)	88·9 (84·5–93·0)	29·7 (27·9–31·3)	0·6 (0·6–0·6)	6·92 (6·79–7·05)	2·45 (2·39–2·51)	2·93 (2·86–2·99)
1964	1·8 (0·8–3·9)	168·3 (162·3–174·9)	322·0 (315·8–327·7)	310·6 (306·8–314·4)	268·8 (265·5–272·2)	199·7 (194·8–204·0)	89·3 (85·0–93·5)	29·8 (28·1–31·5)	0·6 (0·6–0·6)	6·95 (6·83–7·08)	2·46 (2·4–2·52)	2·94 (2·88–3·0)
1965	1·8 (0·8–3·9)	168·8 (162·7–175·4)	322·8 (317·0–328·3)	311·7 (308·2–315·3)	269·9 (266·6–273·1)	200·3 (195·4–204·4)	89·8 (85·5–94·0)	29·9 (28·2–31·6)	0·6 (0·6–0·6)	6·98 (6·85–7·11)	2·47 (2·41–2·53)	2·95 (2·89–3·01)
1966	1·8 (0·8–3·9)	168·3 (162·3–174·6)	323·4 (317·5–328·8)	312·6 (309·2–315·8)	270·8 (267·5–274·0)	200·9 (196·1–205·0)	90·3 (86·1–94·4)	30·1 (28·3–31·8)	0·6 (0·6–0·6)	6·99 (6·86–7·12)	2·47 (2·41–2·52)	2·96 (2·9–3·02)
1967	1·8 (0·8–3·9)	168·0 (162·1–173·9)	324·2 (318·4–329·7)	313·2 (310·1–316·2)	271·4 (268·3–274·5)	201·3 (196·7–205·2)	90·9 (86·9–94·9)	30·1 (28·3–31·8)	0·6 (0·6–0·6)	7·01 (6·89–7·13)	2·47 (2·41–2·52)	2·97 (2·91–3·03)
1968	1·8 (0·8–3·9)	166·9 (161·0–172·7)	323·9 (318·0–329·5)	313·0 (309·8–316·0)	271·4 (268·4–274·5)	201·2 (196·8–205·0)	91·5 (87·4–95·2)	29·9 (28·2–31·5)	0·6 (0·6–0·6)	7·0 (6·88–7·12)	2·46 (2·41–2·52)	2·97 (2·91–3·03)
1969	1·8 (0·8–3·9)	165·4 (159·4–171·4)	322·7 (316·6–328·3)	312·0 (308·8–315·1)	270·6 (267·4–273·7)	200·2 (195·7–204·1)	91·4 (87·4–94·9)	29·4 (27·7–31·0)	0·6 (0·5–0·6)	6·97 (6·84–7·09)	2·45 (2·39–2·5)	2·96 (2·9–3·02)
1970	1·8 (0·8–3·9)	164·7 (158·6–170·7)	320·7 (314·3–326·3)	309·4 (306·0–312·6)	267·9 (264·7–271·2)	197·7 (193·3–201·4)	90·1 (86·2–93·7)	28·7 (27·0–30·2)	0·6 (0·5–0·6)	6·91 (6·78–7·02)	2·44 (2·38–2·49)	2·93 (2·87–2·98)
1971	1·8 (0·8–3·9)	164·4 (158·2–170·4)	318·4 (312·5–324·3)	305·4 (301·6–308·9)	263·7 (260·0–267·0)	193·9 (189·5–197·7)	88·2 (84·5–91·7)	27·8 (26·2–29·5)	0·5 (0·5–0·6)	6·82 (6·69–6·94)	2·42 (2·37–2·48)	2·87 (2·81–2·93)
1972	1·8 (0·8–3·9)	163·8 (157·7–169·7)	316·3 (310·1–321·8)	302·5 (298·6–306·0)	259·3 (255·7–262·7)	190·1 (185·9–193·8)	86·5 (82·9–89·9)	27·0 (25·5–28·8)	0·5 (0·5–0·5)	6·74 (6·62–6·86)	2·41 (2·35–2·46)	2·82 (2·76–2·87)
1973	1·8 (0·8–3·9)	162·1 (156·0–168·2)	314·3 (308·5–320·3)	301·1 (296·9–304·7)	256·1 (252·5–259·4)	187·3 (183·2–191·0)	85·5 (82·0–88·7)	26·4 (24·8–28·1)	0·5 (0·5–0·5)	6·68 (6·55–6·79)	2·39 (2·33–2·45)	2·78 (2·73–2·83)
1974	1·8 (0·8–3·9)	159·2 (152·9–165·3)	312·3 (306·4–317·7)	300·2 (295·9–303·9)	254·3 (250·6–257·5)	185·8 (182·0–189·4)	84·8 (81·6–87·7)	26·1 (24·4–27·6)	0·5 (0·5–0·5)	6·62 (6·5–6·74)	2·37 (2·31–2·42)	2·76 (2·7–2·81)
1975	1·8 (0·8–3·8)	157·5 (151·3–163·7)	309·6 (303·9–315·0)	297·9 (293·8–301·8)	251·5 (247·6–254·9)	184·0 (180·4–187·6)	83·6 (80·6–86·4)	25·9 (24·3–27·5)	0·5 (0·5–0·5)	6·56 (6·45–6·68)	2·34 (2·29–2·4)	2·73 (2·68–2·78)
1976	1·8 (0·8–3·8)	157·6 (151·4–163·5)	306·1 (300·5–311·3)	294·8 (290·6–298·7)	246·9 (243·1–250·5)	180·9 (177·5–184·6)	81·6 (78·8–84·1)	25·9 (24·5–27·4)	0·5 (0·5–0·5)	6·48 (6·37–6·6)	2·33 (2·27–2·38)	2·68 (2·63–2·73)
1977	1·8 (0·8–3·8)	157·7 (151·7–163·6)	302·3 (296·8–307·4)	291·1 (287·1–295·1)	242·3 (238·3–245·9)	176·7 (173·1–180·3)	79·0 (76·4–81·5)	25·9 (24·5–27·3)	0·5 (0·5–0·5)	6·39 (6·27–6·5)	2·31 (2·26–2·36)	2·62 (2·57–2·67)
1978	1·8 (0·8–3·8)	156·0 (150·4–161·5)	299·6 (294·4–304·5)	288·4 (284·6–292·4)	239·3 (235·3–243·0)	173·2 (169·6–176·7)	76·6 (74·1–78·9)	25·7 (24·4–27·0)	0·5 (0·5–0·5)	6·31 (6·2–6·42)	2·29 (2·23–2·34)	2·58 (2·53–2·63)
1979	1·8 (0·8–3·8)	154·0 (148·4–159·5)	297·3 (292·5–302·1)	285·9 (282·2–289·6)	236·8 (233·0–240·5)	170·5 (167·0–173·8)	74·8 (72·5–76·9)	25·6 (24·3–26·8)	0·5 (0·5–0·5)	6·24 (6·13–6·34)	2·27 (2·22–2·31)	2·54 (2·49–2·59)
1980	1·8 (0·8–3·8)	151·7 (146·2–157·2)	297·4 (292·9–301·6)	286·1 (282·4–289·5)	236·1 (232·5–239·5)	170·2 (166·7–173·8)	73·9 (71·3–76·3)	25·7 (24·4–27·0)	0·5 (0·5–0·5)	6·22 (6·11–6·32)	2·25 (2·21–2·3)	2·53 (2·48–2·58)
1981	1·8 (0·8–3·7)	147·6 (141·6–153·6)	300·7 (295·7–305·3)	290·8 (287·2–294·6)	239·0 (234·7–243·2)	173·1 (167·7–178·5)	73·9 (70·1–77·7)	25·8 (24·2–27·4)	0·5 (0·5–0·5)	6·27 (6·14–6·38)	2·25 (2·2–2·3)	2·56 (2·5–2·62)
1982	1·7 (0·8–3·7)	142·9 (136·1–149·6)	303·0 (297·0–308·8)	295·0 (290·9–299·0)	240·8 (236·0–245·6)	175·3 (168·1–182·9)	73·8 (69·0–78·7)	25·7 (23·8–27·5)	0·5 (0·5–0·5)	6·29 (6·16–6·42)	2·24 (2·19–2·29)	2·58 (2·51–2·65)
1983	1·7 (0·7–3·7)	139·2 (132·3–146·5)	303·7 (297·3–309·8)	296·5 (292·1–300·9)	241·6 (236·3–246·7)	175·3 (167·2–183·6)	73·4 (67·8–78·9)	25·7 (23·5–27·8)	0·5 (0·5–0·5)	6·29 (6·14–6·42)	2·22 (2·17–2·28)	2·58 (2·51–2·66)
1984	1·7 (0·7–3·7)	136·8 (129·9–143·8)	303·1 (296·9–309·6)	295·3 (291·1–299·7)	240·4 (235·1–245·6)	172·3 (164·7–179·6)	72·8 (67·5–78·2)	25·6 (23·4–28·0)	0·5 (0·5–0·5)	6·24 (6·1–6·38)	2·21 (2·16–2·26)	2·56 (2·48–2·63)
1985	1·7 (0·7–3·6)	134·7 (128·1–141·5)	301·5 (295·0–307·8)	292·2 (287·9–296·5)	237·0 (231·3–242·4)	166·5 (160·3–173·1)	72·2 (67·3–77·3)	25·5 (23·2–28·0)	0·5 (0·5–0·5)	6·16 (6·02–6·3)	2·19 (2·14–2·25)	2·51 (2·43–2·58)
1986	1·7 (0·7–3·6)	132·5 (125·8–139·1)	299·2 (292·8–305·6)	289·0 (284·7–293·2)	233·9 (228·1–239·5)	161·3 (155·9–167·1)	71·7 (66·7–76·6)	25·3 (22·8–27·9)	0·5 (0·5–0·5)	6·08 (5·95–6·2)	2·17 (2·12–2·22)	2·46 (2·4–2·53)
1987	1·7 (0·7–3·6)	130·9 (124·5–137·4)	296·7 (290·7–303·0)	286·4 (281·9–290·7)	232·7 (226·9–239·1)	158·6 (153·2–164·2)	71·4 (66·3–76·9)	25·0 (22·4–27·6)	0·5 (0·5–0·5)	6·02 (5·89–6·14)	2·15 (2·1–2·2)	2·44 (2·38–2·51)
1988	1·7 (0·7–3·6)	130·3 (124·1–136·8)	294·5 (288·5–300·6)	283·8 (279·2–288·1)	231·4 (225·5–237·3)	157·6 (152·3–162·9)	71·0 (65·7–76·6)	24·7 (22·2–27·3)	0·5 (0·5–0·5)	5·98 (5·85–6·09)	2·13 (2·08–2·18)	2·43 (2·36–2·49)
1989	1·7 (0·7–3·6)	129·7 (123·7–136·4)	292·6 (286·4–298·7)	281·0 (276·3–285·4)	228·5 (223·3–234·5)	156·8 (151·7–162·1)	70·1 (65·0–75·4)	24·3 (21·9–26·7)	0·5 (0·4–0·5)	5·93 (5·81–6·04)	2·12 (2·06–2·18)	2·4 (2·34–2·46)
1990	1·7 (0·7–3·6)	128·8 (122·6–135·4)	290·9 (285·4–296·3)	278·6 (274·3–282·7)	225·0 (220·0–230·1)	155·6 (151·2–160·6)	68·7 (64·5–73·1)	23·8 (21·8–25·9)	0·5 (0·4–0·5)	5·87 (5·76–5·98)	2·11 (2·05–2·16)	2·37 (2·31–2·42)
1991	1·7 (0·7–3·6)	127·6 (121·4–134·2)	289·1 (283·7–294·7)	276·3 (272·0–280·1)	222·2 (217·3–227·2)	154·5 (150·0–159·3)	67·1 (63·5–70·8)	23·3 (21·4–25·3)	0·5 (0·4–0·5)	5·81 (5·7–5·92)	2·09 (2·04–2·14)	2·34 (2·28–2·39)
1992	1·7 (0·7–3·6)	125·5 (119·5–132·3)	286·8 (281·0–293·0)	273·8 (269·8–277·9)	220·5 (215·5–225·8)	153·5 (148·4–159·4)	65·7 (62·3–69·1)	22·8 (21·0–24·6)	0·4 (0·4–0·5)	5·75 (5·64–5·86)	2·07 (2·02–2·12)	2·31 (2·25–2·37)
1993	1·7 (0·7–3·6)	123·3 (117·3–129·7)	284·5 (278·7–290·7)	271·6 (267·4–275·8)	219·3 (214·2–224·5)	151·9 (146·6–157·6)	64·7 (61·2–68·4)	22·2 (20·4–24·0)	0·4 (0·4–0·4)	5·7 (5·59–5·8)	2·05 (2·0–2·1)	2·29 (2·23–2·35)
1994	1·7 (0·7–3·5)	121·5 (115·4–127·7)	282·2 (276·6–288·0)	269·4 (265·0–273·8)	217·2 (212·1–222·1)	149·5 (144·2–154·5)	63·8 (60·4–67·7)	21·7 (19·9–23·3)	0·4 (0·4–0·4)	5·64 (5·53–5·75)	2·03 (1·98–2·07)	2·26 (2·2–2·32)
1995	1·7 (0·7–3·5)	119·3 (113·2–125·2)	279·6 (273·6–285·6)	266·6 (262·2–271·3)	213·9 (209·2–218·6)	146·6 (141·9–151·6)	63·2 (60·2–66·9)	21·2 (19·5–22·9)	0·4 (0·4–0·4)	5·56 (5·46–5·67)	2·0 (1·95–2·05)	2·23 (2·17–2·28)
1996	1·6 (0·7–3·5)	117·1 (110·9–123·2)	276·6 (270·9–282·4)	263·8 (259·4–268·2)	210·9 (206·5–215·6)	144·7 (140·4–149·1)	63·3 (60·2–66·6)	20·9 (19·4–22·5)	0·4 (0·4–0·4)	5·5 (5·4–5·6)	1·98 (1·93–2·03)	2·2 (2·15–2·25)
1997	1·6 (0·7–3·4)	115·6 (109·2–122·0)	274·2 (268·6–279·4)	261·1 (257·0–265·4)	208·9 (204·6–213·3)	144·2 (140·1–148·6)	64·0 (60·8–67·4)	21·0 (19·5–22·7)	0·4 (0·4–0·4)	5·46 (5·35–5·56)	1·96 (1·91–2·01)	2·19 (2·15–2·24)
1998	1·6 (0·7–3·4)	114·2 (107·8–121·0)	272·2 (266·8–277·7)	257·9 (253·5–262·2)	206·0 (201·2–210·5)	142·9 (138·6–147·0)	64·2 (61·1–67·5)	21·2 (19·5–22·9)	0·4 (0·4–0·4)	5·4 (5·3–5·51)	1·94 (1·89–1·99)	2·17 (2·13–2·22)
1999	1·6 (0·7–3·4)	112·1 (105·7–118·8)	269·5 (263·3–275·5)	254·1 (249·0–258·5)	201·8 (197·1–206·5)	139·8 (135·4–144·0)	63·6 (60·4–66·8)	21·2 (19·5–22·9)	0·4 (0·4–0·4)	5·32 (5·21–5·43)	1·92 (1·86–1·97)	2·13 (2·08–2·19)
2000	1·6 (0·7–3·4)	110·0 (104·1–116·4)	266·2 (259·7–272·5)	250·3 (245·0–254·9)	197·4 (192·6–202·3)	136·0 (131·5–140·6)	62·3 (59·2–65·6)	21·1 (19·4–22·8)	0·4 (0·4–0·4)	5·23 (5·11–5·34)	1·89 (1·84–1·94)	2·09 (2·03–2·15)
2001	1·6 (0·7–3·4)	108·3 (102·3–114·2)	263·1 (256·7–269·4)	246·9 (242·0–251·9)	193·4 (188·3–198·5)	132·2 (127·1–137·5)	60·9 (57·5–64·4)	21·0 (19·3–22·7)	0·4 (0·4–0·4)	5·14 (5·02–5·26)	1·86 (1·81–1·92)	2·04 (1·98–2·1)
2002	1·6 (0·7–3·3)	106·4 (100·5–112·3)	260·7 (254·1–267·4)	244·0 (238·8–249·2)	189·8 (185·0–195·2)	128·5 (123·3–133·8)	59·4 (56·1–62·9)	20·8 (19·2–22·5)	0·4 (0·4–0·4)	5·06 (4·93–5·18)	1·84 (1·79–1·9)	1·99 (1·94–2·06)
2003	1·6 (0·7–3·3)	103·9 (98·1–109·9)	258·0 (250·6–264·9)	240·9 (235·1–246·5)	186·0 (180·8–191·1)	124·8 (120·1–130·0)	58·2 (54·7–61·5)	20·3 (18·8–21·9)	0·4 (0·4–0·4)	4·97 (4·84–5·1)	1·82 (1·76–1·87)	1·95 (1·89–2·01)
2004	1·5 (0·7–3·3)	101·3 (95·8–107·2)	254·8 (246·7–262·8)	237·9 (231·9–243·8)	182·3 (177·2–187·7)	121·6 (116·6–126·5)	57·1 (53·8–60·5)	19·7 (18·1–21·2)	0·4 (0·4–0·4)	4·88 (4·74–5·02)	1·79 (1·72–1·85)	1·91 (1·84–1·97)
2005	1·5 (0·7–3·2)	99·0 (93·5–104·9)	251·9 (243·7–260·5)	235·5 (228·9–242·0)	178·9 (173·3–184·5)	118·9 (113·9–124·0)	56·0 (52·8–59·4)	19·0 (17·5–20·5)	0·4 (0·3–0·4)	4·81 (4·66–4·94)	1·76 (1·69–1·83)	1·87 (1·8–1·93)
2006	1·5 (0·7–3·2)	96·9 (91·7–102·9)	249·1 (239·9–258·3)	233·2 (226·4–240·1)	175·6 (169·8–181·4)	116·5 (111·5–121·5)	54·7 (51·4–58·1)	18·4 (17·0–19·8)	0·4 (0·3–0·4)	4·73 (4·57–4·88)	1·74 (1·67–1·81)	1·83 (1·76–1·89)
2007	1·5 (0·6–3·2)	94·7 (89·3–100·6)	246·1 (236·1–255·6)	230·2 (223·1–237·3)	172·1 (166·1–178·0)	113·8 (108·5–118·7)	53·2 (49·8–56·8)	17·9 (16·5–19·1)	0·3 (0·3–0·4)	4·65 (4·49–4·81)	1·71 (1·64–1·78)	1·79 (1·72–1·85)
2008	1·5 (0·6–3·1)	92·4 (87·0–98·1)	242·6 (232·5–252·4)	226·6 (219·5–233·6)	168·4 (162·1–174·3)	110·5 (105·1–115·6)	51·6 (48·1–55·1)	17·3 (16·0–18·6)	0·3 (0·3–0·3)	4·56 (4·39–4·71)	1·68 (1·61–1·76)	1·74 (1·67–1·81)
2009	1·5 (0·6–3·1)	90·4 (85·0–95·9)	239·3 (229·3–249·3)	223·9 (217·1–230·8)	165·2 (158·9–171·2)	107·4 (102·0–112·5)	50·3 (46·9–53·5)	16·8 (15·4–18·0)	0·3 (0·3–0·3)	4·47 (4·31–4·64)	1·66 (1·58–1·73)	1·7 (1·62–1·77)
2010	1·4 (0·6–3·1)	88·9 (83·2–94·8)	237·0 (227·2–247·2)	222·5 (216·2–229·2)	162·9 (156·5–169·3)	104·8 (99·1–110·1)	49·3 (46·0–52·3)	16·2 (15·0–17·5)	0·3 (0·3–0·3)	4·42 (4·25–4·58)	1·64 (1·56–1·71)	1·67 (1·59–1·74)
2011	1·4 (0·6–3·0)	88·2 (82·3–94·8)	235·2 (224·7–245·6)	221·4 (214·6–228·2)	160·8 (154·4–167·3)	102·4 (96·7–107·8)	48·3 (45·3–51·4)	15·8 (14·6–17·0)	0·3 (0·3–0·3)	4·37 (4·2–4·54)	1·62 (1·55–1·7)	1·64 (1·57–1·71)
2012	1·4 (0·6–3·0)	86·4 (80·1–93·3)	232·3 (220·7–243·8)	218·1 (211·0–225·3)	158·4 (151·7–165·0)	100·0 (94·1–105·4)	47·4 (44·2–50·5)	15·5 (14·3–16·8)	0·3 (0·3–0·3)	4·3 (4·12–4·48)	1·6 (1·51–1·69)	1·61 (1·54–1·68)
2013	1·4 (0·6–3·0)	81·3 (75·0–88·4)	226·3 (214·3–238·7)	210·5 (202·9–218·4)	155·4 (148·5–162·4)	97·5 (91·5–103·2)	46·6 (43·1–49·7)	15·4 (14·1–16·6)	0·3 (0·3–0·3)	4·17 (3·99–4·37)	1·54 (1·46–1·64)	1·58 (1·5–1·65)
2014	1·4 (0·6–2·9)	76·3 (69·8–83·4)	218·3 (204·2–233·4)	202·3 (193·7–211·3)	152·8 (145·4–160·6)	95·7 (89·1–101·9)	45·9 (42·3–49·5)	15·3 (14·0–16·6)	0·3 (0·3–0·3)	4·04 (3·84–4·27)	1·48 (1·38–1·59)	1·55 (1·47–1·64)
2015	1·4 (0·6–2·9)	73·3 (66·4–80·8)	210·9 (194·1–228·8)	195·8 (186·3–206·4)	150·5 (142·2–159·5)	94·5 (87·0–102·3)	45·3 (41·1–49·7)	15·3 (14·0–16·7)	0·3 (0·3–0·3)	3·94 (3·7–4·19)	1·43 (1·31–1·56)	1·53 (1·43–1·63)
2016	1·4 (0·6–2·9)	71·9 (65·0–80·0)	205·2 (186·5–226·7)	190·6 (180·4–202·4)	148·0 (138·7–158·4)	93·6 (84·6–102·8)	44·7 (40·0–49·6)	15·2 (13·8–16·7)	0·3 (0·3–0·3)	3·85 (3·61–4·14)	1·39 (1·27–1·54)	1·51 (1·4–1·63)
2017	1·4 (0·6–2·9)	71·9 (65·0–80·0)	202·6 (181·7–225·9)	188·4 (177·3–200·9)	147·0 (136·9–158·2)	93·4 (83·6–103·5)	44·6 (39·5–49·9)	15·1 (13·7–16·7)	0·3 (0·3–0·3)	3·82 (3·56–4·14)	1·38 (1·24–1·54)	1·5 (1·38–1·64)

Supplemental Results Table 5: Age-specific fertility rates per 1000, cumulative total fertility up to maternal age 25 (TFU25), cumulative total fertility from maternal age 30 to age 55 (TFO30), and cumulative total fertility, Low-middle SDI, 1950-2017

Year	10-14	15-19	20-24	25-29	30-34	35-39	40-44	45-49	50-54	TFR	TFU25	TFO30
1950	1·1 (0·5–2·3)	116·3 (104·7–128·7)	299·8 (279·4–319·9)	296·9 (286·7–307·3)	228·1 (214·8–241·8)	156·1 (140·7–171·7)	60·9 (52·1–70·4)	17·8 (14·0–22·1)	0·3 (0·3–0·4)	5·89 (5·47–6·32)	2·09 (1·92–2·25)	2·32 (2·11–2·53)
1951	1·1 (0·5–2·3)	116·2 (104·9–128·4)	299·1 (278·5–318·7)	296·2 (285·9–305·9)	227·7 (214·7–241·0)	155·8 (140·4–170·9)	60·8 (52·3–69·9)	17·7 (14·0–21·8)	0·3 (0·3–0·4)	5·87 (5·47–6·3)	2·08 (1·92–2·24)	2·31 (2·11–2·52)
1952	1·1 (0·5–2·3)	116·1 (105·7–127·6)	298·5 (277·5–317·8)	295·5 (285·3–305·4)	227·4 (215·4–239·7)	155·5 (140·7–169·2)	60·7 (52·6–69·1)	17·6 (14·2–21·5)	0·3 (0·3–0·4)	5·86 (5·48–6·25)	2·08 (1·92–2·23)	2·31 (2·13–2·5)
1953	1·1 (0·5–2·3)	116·1 (105·8–126·4)	297·8 (277·5–316·3)	294·9 (284·8–304·3)	227·2 (215·9–239·0)	155·2 (141·7–167·9)	60·6 (53·1–68·3)	17·5 (14·4–21·2)	0·3 (0·3–0·4)	5·85 (5·5–6·2)	2·08 (1·93–2·21)	2·3 (2·14–2·48)
1954	1·1 (0·5–2·3)	116·4 (106·8–126·0)	297·1 (277·3–314·6)	294·2 (284·3–303·2)	226·8 (215·8–238·1)	154·9 (142·3–167·2)	60·4 (53·3–67·6)	17·5 (14·5–20·8)	0·3 (0·3–0·4)	5·84 (5·51–6·16)	2·07 (1·94–2·2)	2·3 (2·14–2·47)
1955	1·1 (0·5–2·3)	117·3 (108·3–126·2)	296·3 (278·7–312·8)	293·5 (284·3–301·8)	226·4 (216·0–237·1)	154·5 (142·9–166·2)	60·3 (54·1–67·1)	17·4 (14·9–20·4)	0·3 (0·3–0·4)	5·84 (5·54–6·13)	2·07 (1·95–2·19)	2·3 (2·15–2·45)
1956	1·1 (0·5–2·3)	119·3 (110·8–127·3)	295·4 (280·0–310·2)	292·7 (284·7–300·0)	225·9 (216·4–235·5)	154·2 (143·3–164·8)	60·2 (54·5–66·1)	17·4 (15·0–20·0)	0·3 (0·3–0·3)	5·83 (5·56–6·1)	2·08 (1·97–2·18)	2·29 (2·15–2·43)
1957	1·1 (0·5–2·4)	122·2 (114·5–129·8)	294·6 (281·1–307·7)	291·9 (285·0–298·7)	225·4 (217·1–233·7)	153·9 (144·5–163·4)	60·1 (54·8–65·3)	17·4 (15·2–19·7)	0·3 (0·3–0·3)	5·83 (5·59–6·07)	2·09 (1·99–2·18)	2·29 (2·16–2·41)
1958	1·1 (0·5–2·4)	125·7 (118·8–132·6)	293·6 (282·6–304·2)	291·0 (285·1–296·6)	224·7 (217·2–232·1)	153·7 (145·7–161·8)	60·1 (55·5–64·6)	17·4 (15·4–19·4)	0·3 (0·3–0·3)	5·84 (5·63–6·05)	2·1 (2·01–2·18)	2·28 (2·17–2·39)
1959	1·1 (0·5–2·4)	128·3 (122·1–134·6)	292·8 (283·9–301·2)	290·3 (285·6–294·8)	224·2 (217·8–230·3)	153·5 (146·7–160·2)	60·1 (56·1–64·3)	17·4 (15·6–19·2)	0·3 (0·3–0·3)	5·84 (5·66–6·02)	2·11 (2·04–2·18)	2·28 (2·19–2·37)
1960	1·1 (0·5–2·4)	128·9 (123·0–134·8)	292·4 (284·3–300·5)	289·9 (285·7–294·0)	224·0 (218·0–229·4)	153·4 (147·1–159·5)	60·4 (56·6–64·2)	17·4 (15·7–19·2)	0·3 (0·3–0·3)	5·84 (5·67–6·0)	2·11 (2·05–2·18)	2·28 (2·19–2·36)
1961	1·1 (0·5–2·4)	128·6 (122·6–134·4)	292·5 (284·7–300·1)	290·2 (286·1–294·1)	224·0 (218·3–229·6)	153·4 (147·0–159·6)	60·8 (57·0–64·6)	17·4 (15·7–19·2)	0·3 (0·3–0·3)	5·84 (5·68–6·0)	2·11 (2·04–2·18)	2·28 (2·19–2·37)
1962	1·1 (0·5–2·4)	127·9 (122·0–133·6)	292·7 (285·0–300·0)	291·1 (287·2–295·0)	224·2 (218·6–229·6)	153·3 (147·0–159·5)	61·2 (57·4–65·1)	17·4 (15·6–19·2)	0·3 (0·3–0·3)	5·85 (5·68–6·01)	2·11 (2·04–2·17)	2·28 (2·2–2·37)
1963	1·1 (0·5–2·4)	127·1 (121·3–132·9)	293·2 (285·8–300·3)	292·0 (288·2–295·7)	224·1 (218·8–229·4)	153·0 (147·0–159·0)	61·7 (57·9–65·7)	17·4 (15·7–19·2)	0·3 (0·3–0·3)	5·85 (5·69–6·0)	2·11 (2·04–2·17)	2·28 (2·2–2·37)
1964	1·1 (0·5–2·3)	126·6 (121·0–132·3)	293·3 (286·5–300·1)	292·4 (288·7–296·1)	223·8 (218·7–228·7)	152·2 (146·5–157·9)	62·0 (57·9–66·0)	17·5 (15·8–19·1)	0·3 (0·3–0·3)	5·85 (5·7–6·0)	2·11 (2·04–2·16)	2·28 (2·2–2·36)
1965	1·1 (0·5–2·3)	125·9 (120·5–131·3)	292·8 (286·2–299·3)	292·8 (289·0–296·4)	223·5 (218·5–228·1)	151·1 (145·6–156·6)	62·2 (58·1–66·1)	17·4 (15·9–19·0)	0·3 (0·3–0·3)	5·84 (5·7–5·98)	2·1 (2·04–2·15)	2·27 (2·19–2·35)
1966	1·1 (0·5–2·3)	125·1 (119·9–130·3)	292·3 (285·8–298·7)	293·2 (289·6–296·7)	223·0 (218·2–227·8)	150·2 (144·6–155·9)	62·2 (58·1–66·2)	17·4 (15·9–19·0)	0·3 (0·3–0·3)	5·82 (5·69–5·96)	2·09 (2·04–2·14)	2·27 (2·19–2·34)
1967	1·1 (0·5–2·3)	124·3 (119·4–129·2)	292·1 (285·9–298·3)	293·5 (289·9–297·1)	222·4 (217·6–227·1)	149·3 (143·6–154·9)	62·2 (58·1–66·2)	17·4 (15·7–18·9)	0·3 (0·3–0·3)	5·81 (5·68–5·95)	2·09 (2·03–2·14)	2·26 (2·18–2·34)
1968	1·1 (0·5–2·3)	122·9 (118·3–127·6)	291·1 (285·2–297·1)	293·1 (289·5–296·6)	221·4 (216·5–226·1)	148·3 (142·6–154·0)	62·1 (58·0–66·1)	17·2 (15·6–18·8)	0·3 (0·3–0·3)	5·79 (5·66–5·92)	2·07 (2·02–2·12)	2·25 (2·17–2·32)
1969	1·1 (0·5–2·3)	121·2 (116·8–125·7)	288·9 (283·2–294·8)	291·8 (288·3–295·2)	220·0 (215·0–224·6)	147·0 (141·3–152·8)	61·7 (57·8–65·6)	17·0 (15·4–18·6)	0·3 (0·3–0·3)	5·74 (5·62–5·87)	2·06 (2·01–2·1)	2·23 (2·15–2·31)
1970	1·1 (0·5–2·3)	120·6 (116·1–124·9)	286·1 (280·6–291·8)	289·0 (285·7–292·4)	217·0 (212·1–221·6)	144·7 (139·1–150·4)	60·5 (56·6–64·4)	16·6 (15·0–18·2)	0·3 (0·3–0·3)	5·68 (5·55–5·81)	2·04 (1·99–2·09)	2·2 (2·12–2·27)
1971	1·1 (0·5–2·3)	120·3 (115·9–124·4)	283·5 (278·0–289·1)	285·2 (281·8–288·5)	212·7 (207·9–217·4)	141·3 (135·7–146·7)	58·8 (55·0–62·5)	16·1 (14·5–17·7)	0·3 (0·3–0·3)	5·6 (5·47–5·72)	2·02 (1·98–2·07)	2·15 (2·07–2·22)
1972	1·1 (0·5–2·3)	120·1 (116·1–123·9)	281·2 (275·8–286·4)	281·5 (278·2–284·8)	208·8 (204·2–213·4)	137·9 (132·2–143·2)	57·2 (53·6–60·9)	15·5 (14·0–17·1)	0·3 (0·3–0·3)	5·52 (5·4–5·64)	2·01 (1·97–2·06)	2·1 (2·02–2·17)
1973	1·1 (0·5–2·2)	118·6 (115·0–122·4)	278·9 (273·6–284·0)	278·4 (275·1–281·6)	206·0 (201·3–210·3)	135·1 (129·8–140·2)	56·1 (52·7–59·7)	15·1 (13·6–16·7)	0·3 (0·3–0·3)	5·45 (5·33–5·56)	1·99 (1·95–2·03)	2·06 (1·99–2·13)
1974	1·0 (0·5–2·2)	115·8 (112·3–119·4)	275·8 (270·8–280·6)	275·4 (272·2–278·6)	203·6 (199·2–207·9)	133·0 (128·0–137·6)	55·2 (51·8–58·6)	14·9 (13·4–16·4)	0·3 (0·3–0·3)	5·37 (5·26–5·48)	1·96 (1·92–2·0)	2·03 (1·96–2·1)
1975	1·0 (0·5–2·2)	114·7 (111·3–118·0)	272·0 (267·0–276·6)	271·1 (267·8–274·2)	200·0 (195·6–204·3)	130·3 (125·4–134·8)	54·1 (50·8–57·3)	14·6 (13·2–16·0)	0·3 (0·3–0·3)	5·29 (5·18–5·4)	1·94 (1·9–1·98)	2·0 (1·93–2·06)
1976	1·0 (0·5–2·2)	115·7 (112·7–118·9)	267·5 (262·6–272·0)	265·9 (262·8–268·9)	195·2 (190·7–199·4)	126·7 (122·2–131·1)	52·7 (49·6–55·8)	14·3 (12·9–15·7)	0·3 (0·3–0·3)	5·2 (5·09–5·3)	1·92 (1·88–1·96)	1·95 (1·88–2·01)
1977	1·0 (0·5–2·2)	116·8 (113·8–119·8)	263·2 (258·1–268·0)	260·9 (257·9–263·9)	190·2 (186·0–194·3)	123·1 (119·0–127·3)	51·2 (48·3–54·1)	14·0 (12·6–15·4)	0·3 (0·3–0·3)	5·1 (5·0–5·2)	1·9 (1·87–1·94)	1·89 (1·84–1·95)
1978	1·0 (0·4–2·2)	115·5 (112·6–118·4)	260·6 (255·3–265·7)	257·0 (254·0–259·9)	186·7 (182·4–190·7)	120·4 (116·5–124·3)	49·8 (47·1–52·6)	13·6 (12·3–15·0)	0·3 (0·3–0·3)	5·02 (4·92–5·12)	1·89 (1·85–1·92)	1·85 (1·8–1·91)
1979	1·0 (0·4–2·2)	113·6 (110·8–116·4)	258·2 (252·8–263·6)	253·1 (250·3–255·9)	183·6 (179·4–187·6)	118·0 (114·3–121·7)	48·6 (46·2–51·3)	13·3 (12·0–14·6)	0·3 (0·2–0·3)	4·95 (4·84–5·05)	1·86 (1·83–1·9)	1·82 (1·76–1·87)
1980	1·0 (0·4–2·2)	111·4 (108·2–114·2)	257·5 (251·9–263·2)	251·7 (248·5–254·8)	181·9 (177·7–185·9)	116·9 (113·1–120·3)	47·8 (45·1–50·4)	13·1 (11·8–14·4)	0·2 (0·2–0·3)	4·91 (4·79–5·01)	1·85 (1·81–1·89)	1·8 (1·74–1·85)
1981	1·0 (0·4–2·1)	107·2 (103·3–111·2)	259·5 (253·2–265·5)	254·5 (250·4–258·3)	182·8 (177·8–187·6)	117·6 (113·4–121·4)	47·1 (44·1–50·0)	12·8 (11·6–14·1)	0·2 (0·2–0·3)	4·91 (4·79–5·03)	1·84 (1·79–1·88)	1·8 (1·74–1·86)
1982	1·0 (0·4–2·1)	102·4 (97·8–107·3)	260·7 (253·9–267·5)	256·7 (251·9–261·4)	182·7 (177·2–188·1)	117·4 (112·8–122·1)	46·4 (43·1–49·7)	12·5 (11·3–13·8)	0·2 (0·2–0·2)	4·9 (4·77–5·03)	1·82 (1·77–1·87)	1·8 (1·73–1·86)
1983	1·0 (0·4–2·1)	98·9 (93·9–103·8)	260·5 (253·6–267·2)	256·2 (251·1–261·4)	181·3 (176·0–187·0)	115·7 (110·8–120·6)	45·4 (42·0–49·0)	12·3 (11·1–13·5)	0·2 (0·2–0·2)	4·86 (4·72–4·99)	1·8 (1·75–1·86)	1·77 (1·71–1·84)
1984	1·0 (0·4–2·1)	96·4 (91·4–101·7)	259·0 (252·4–265·1)	252·9 (247·9–257·8)	178·3 (173·3–183·3)	112·5 (108·0–117·1)	44·4 (41·2–47·7)	12·1 (10·9–13·3)	0·2 (0·2–0·2)	4·78 (4·65–4·92)	1·78 (1·73–1·84)	1·74 (1·68–1·8)
1985	1·0 (0·4–2·1)	94·1 (88·8–99·5)	256·5 (250·0–263·0)	248·1 (243·4–252·9)	174·1 (169·4–178·9)	108·3 (104·3–112·5)	43·4 (40·6–46·3)	11·8 (10·7–13·0)	0·2 (0·2–0·2)	4·69 (4·56–4·81)	1·76 (1·71–1·81)	1·69 (1·64–1·74)
1986	1·0 (0·4–2·0)	91·9 (86·6–97·5)	253·1 (246·7–259·7)	242·7 (238·1–247·2)	169·8 (165·1–174·3)	104·3 (100·6–108·3)	42·4 (40·0–44·9)	11·5 (10·4–12·7)	0·2 (0·2–0·2)	4·58 (4·46–4·7)	1·73 (1·68–1·79)	1·64 (1·59–1·7)
1987	1·0 (0·4–2·0)	90·0 (84·6–95·6)	248·8 (242·3–255·7)	237·4 (233·1–241·7)	166·1 (161·4–170·7)	101·1 (97·4–104·9)	41·4 (39·1–43·7)	11·2 (10·1–12·3)	0·2 (0·2–0·2)	4·49 (4·37–4·6)	1·7 (1·65–1·76)	1·6 (1·55–1·65)
1988	1·0 (0·4–2·0)	89·1 (83·7–94·7)	244·9 (238·3–251·7)	232·7 (228·6–236·9)	162·8 (158·2–167·6)	98·6 (94·8–102·4)	40·2 (37·9–42·7)	10·9 (9·9–12·0)	0·2 (0·2–0·2)	4·4 (4·29–4·51)	1·67 (1·62–1·73)	1·56 (1·51–1·61)
1989	1·0 (0·4–2·0)	88·9 (83·4–94·5)	242·1 (235·3–248·4)	228·5 (224·1–232·7)	159·3 (154·8–163·9)	96·2 (92·6–99·9)	39·0 (36·7–41·5)	10·7 (9·6–11·7)	0·2 (0·2–0·2)	4·33 (4·22–4·44)	1·66 (1·6–1·72)	1·53 (1·48–1·58)
1990	1·0 (0·4–2·0)	88·8 (83·5–94·2)	240·0 (233·9–246·3)	224·7 (220·6–228·7)	155·7 (151·4–160·0)	94·1 (90·8–97·6)	37·8 (35·4–40·2)	10·5 (9·5–11·5)	0·2 (0·2–0·2)	4·26 (4·16–4·37)	1·65 (1·59–1·7)	1·49 (1·44–1·54)
1991	1·0 (0·4–2·0)	88·0 (82·6–93·4)	237·8 (231·7–244·3)	220·7 (216·7–224·7)	152·3 (148·5–156·4)	92·0 (88·5–95·2)	36·7 (34·4–39·0)	10·3 (9·2–11·3)	0·2 (0·2–0·2)	4·19 (4·09–4·31)	1·63 (1·58–1·69)	1·46 (1·41–1·51)
1992	1·0 (0·4–2·0)	86·1 (80·0–91·7)	234·2 (227·7–240·8)	216·4 (211·9–220·7)	149·2 (145·4–153·1)	89·9 (86·3–93·4)	35·7 (33·5–38·0)	10·0 (9·0–11·1)	0·2 (0·2–0·2)	4·11 (4·0–4·23)	1·61 (1·55–1·66)	1·43 (1·38–1·47)
1993	1·0 (0·4–2·0)	84·2 (77·8–90·0)	230·3 (223·7–236·9)	212·5 (207·9–216·7)	146·4 (142·8–150·4)	88·1 (84·5–91·8)	34·8 (32·7–37·0)	9·8 (8·8–10·9)	0·2 (0·2–0·2)	4·04 (3·92–4·15)	1·58 (1·52–1·64)	1·4 (1·35–1·45)
1994	0·9 (0·4–2·0)	82·5 (76·1–88·8)	227·1 (220·7–233·4)	209·1 (204·9–213·6)	143·6 (139·9–147·5)	86·3 (82·7–90·1)	34·0 (32·1–35·9)	9·6 (8·6–10·7)	0·2 (0·2–0·2)	3·97 (3·85–4·09)	1·55 (1·5–1·61)	1·37 (1·32–1·41)
1995	0·9 (0·4–2·0)	80·6 (74·8–86·7)	223·9 (217·3–230·6)	205·9 (201·8–210·3)	140·4 (137·0–144·0)	84·5 (81·1–88·0)	33·3 (31·6–35·0)	9·4 (8·3–10·5)	0·2 (0·2–0·2)	3·9 (3·79–4·01)	1·53 (1·47–1·58)	1·34 (1·3–1·38)
1996	0·9 (0·4–1·9)	79·3 (74·0–84·6)	220·6 (213·6–227·4)	203·2 (198·7–207·9)	137·7 (134·1–141·3)	82·9 (79·7–86·2)	32·8 (31·1–34·5)	9·2 (8·2–10·3)	0·2 (0·2–0·2)	3·83 (3·72–3·95)	1·5 (1·45–1·56)	1·31 (1·27–1·36)
1997	0·9 (0·4–1·9)	79·0 (74·3–83·8)	218·5 (211·1–225·7)	201·4 (197·0–205·9)	136·1 (132·6–139·5)	81·9 (78·8–85·0)	32·5 (30·6–34·4)	9·0 (8·0–10·1)	0·2 (0·2–0·2)	3·8 (3·69–3·91)	1·49 (1·44–1·55)	1·3 (1·26–1·34)
1998	0·9 (0·4–1·9)	78·5 (74·4–83·0)	216·8 (209·5–224·2)	199·1 (194·8–203·6)	134·4 (130·6–137·9)	80·5 (77·5–83·7)	32·1 (30·1–34·1)	8·8 (7·9–9·8)	0·2 (0·2–0·2)	3·76 (3·64–3·87)	1·48 (1·43–1·54)	1·28 (1·24–1·32)
1999	0·9 (0·4–1·9)	76·8 (72·5–81·1)	213·9 (206·3–221·4)	195·9 (191·5–200·3)	131·9 (128·1–135·6)	78·3 (75·2–81·3)	31·4 (29·5–33·5)	8·6 (7·8–9·5)	0·2 (0·2–0·2)	3·69 (3·58–3·8)	1·46 (1·4–1·51)	1·25 (1·21–1·3)
2000	0·9 (0·4–1·9)	74·7 (70·4–79·0)	210·3 (202·7–217·8)	192·8 (188·4–197·3)	129·5 (125·9–133·4)	75·9 (72·7–79·0)	30·7 (28·8–32·8)	8·4 (7·6–9·3)	0·2 (0·2–0·2)	3·62 (3·5–3·73)	1·43 (1·37–1·49)	1·22 (1·18–1·27)
2001	0·9 (0·4–1·9)	73·1 (68·8–77·6)	207·1 (199·4–214·9)	190·4 (185·9–195·0)	127·7 (124·0–131·5)	73·8 (70·5–76·9)	30·1 (28·3–32·0)	8·2 (7·4–9·0)	0·2 (0·1–0·2)	3·56 (3·44–3·67)	1·41 (1·35–1·46)	1·2 (1·16–1·24)
2002	0·9 (0·4–1·9)	71·7 (67·0–76·3)	204·4 (196·3–212·4)	188·0 (183·4–192·9)	126·0 (122·3–129·9)	72·1 (68·9–75·1)	29·4 (27·8–31·2)	8·0 (7·2–8·8)	0·2 (0·1–0·2)	3·5 (3·39–3·62)	1·38 (1·32–1·44)	1·18 (1·14–1·22)
2003	0·9 (0·4–1·9)	69·7 (64·9–74·6)	201·3 (192·8–209·8)	185·5 (180·5–190·6)	124·5 (120·5–128·7)	70·7 (67·6–73·8)	28·8 (27·2–30·5)	7·8 (7·0–8·6)	0·1 (0·1–0·2)	3·45 (3·32–3·57)	1·36 (1·3–1·42)	1·16 (1·12–1·21)
2004	0·9 (0·4–1·9)	67·5 (62·7–72·4)	198·2 (189·6–206·6)	183·3 (178·1–188·7)	123·1 (118·7–127·6)	69·7 (66·7–72·8)	28·3 (26·7–30·0)	7·7 (6·9–8·5)	0·1 (0·1–0·1)	3·39 (3·27–3·52)	1·33 (1·27–1·4)	1·14 (1·1–1·19)
2005	0·9 (0·4–1·9)	65·6 (61·2–70·4)	195·5 (186·7–204·4)	181·4 (176·1–187·1)	122·0 (117·2–126·7)	69·1 (66·0–72·4)	27·8 (26·2–29·5)	7·5 (6·7–8·4)	0·1 (0·1–0·1)	3·35 (3·22–3·49)	1·31 (1·25–1·37)	1·13 (1·08–1·18)
2006	0·9 (0·4–1·9)	64·3 (59·9–68·9)	193·1 (183·7–202·7)	179·6 (174·0–185·7)	121·0 (116·0–125·9)	68·7 (65·4–72·2)	27·3 (25·5–29·1)	7·4 (6·6–8·2)	0·1 (0·1–0·1)	3·31 (3·18–3·46)	1·29 (1·23–1·36)	1·12 (1·07–1·17)
2007	0·9 (0·4–1·9)	63·3 (59·1–67·7)	190·8 (180·7–201·0)	177·5 (171·7–183·7)	120·0 (115·0–124·9)	68·3 (64·7–72·2)	26·8 (24·9–28·6)	7·3 (6·5–8·1)	0·1 (0·1–0·1)	3·28 (3·13–3·42)	1·28 (1·21–1·35)	1·11 (1·06–1·17)
2008	0·9 (0·4–1·9)	62·4 (58·0–66·8)	188·3 (177·4–199·1)	175·0 (168·7–181·4)	118·9 (113·7–123·9)	67·8 (64·0–71·8)	26·3 (24·3–28·2)	7·2 (6·4–8·0)	0·1 (0·1–0·1)	3·23 (3·09–3·38)	1·26 (1·18–1·33)	1·1 (1·05–1·16)
2009	0·9 (0·4–1·9)	61·7 (57·4–66·4)	185·8 (174·9–196·7)	172·8 (166·8–179·0)	117·9 (112·7–123·1)	67·0 (63·1–70·9)	26·0 (23·9–27·9)	7·0 (6·2–7·8)	0·1 (0·1–0·1)	3·2 (3·05–3·34)	1·24 (1·17–1·32)	1·09 (1·03–1·15)
2010	0·9 (0·4–1·9)	61·6 (57·3–66·5)	183·9 (173·3–195·1)	171·8 (165·7–177·9)	117·0 (111·5–122·6)	66·1 (62·1–70·1)	25·7 (23·6–27·7)	6·9 (6·1–7·7)	0·1 (0·1–0·1)	3·17 (3·03–3·32)	1·23 (1·16–1·31)	1·08 (1·02–1·13)
2011	0·9 (0·4–1·9)	62·3 (57·5–67·6)	182·8 (171·7–194·3)	171·4 (165·2–177·7)	116·4 (110·9–122·0)	65·5 (61·2–69·6)	25·5 (23·3–27·5)	6·8 (6·0–7·6)	0·1 (0·1–0·1)	3·16 (3·0–3·31)	1·23 (1·16–1·31)	1·07 (1·01–1·13)
2012	0·9 (0·4–1·9)	61·7 (56·6–67·6)	180·9 (169·0–192·6)	169·7 (163·5–176·0)	116·1 (110·5–121·8)	65·2 (60·9–69·7)	25·2 (22·9–27·4)	6·7 (5·9–7·5)	0·1 (0·1–0·1)	3·13 (2·96–3·3)	1·22 (1·14–1·3)	1·07 (1·01–1·13)
2013	0·9 (0·4–1·9)	58·3 (53·0–64·3)	176·3 (164·4–188·7)	165·3 (158·9–171·9)	115·9 (109·9–122·1)	65·2 (60·5–70·1)	24·9 (22·5–27·2)	6·6 (5·8–7·5)	0·1 (0·1–0·1)	3·07 (2·9–3·25)	1·18 (1·09–1·27)	1·06 (1·0–1·13)
2014	0·9 (0·4–1·8)	54·6 (49·2–61·0)	169·8 (157·4–182·9)	160·6 (153·7–168·0)	115·4 (109·0–122·2)	65·1 (60·1–70·8)	24·7 (22·1–27·4)	6·6 (5·8–7·6)	0·1 (0·1–0·1)	2·99 (2·8–3·19)	1·13 (1·04–1·22)	1·06 (0·99–1·14)
2015	0·9 (0·4–1·8)	52·4 (46·7–59·4)	163·8 (150·3–178·8)	156·9 (149·6–165·3)	114·5 (107·4–122·3)	64·8 (59·2–71·2)	24·5 (21·7–27·5)	6·5 (5·7–7·5)	0·1 (0·1–0·1)	2·92 (2·72–3·14)	1·09 (0·99–1·19)	1·05 (0·98–1·14)
2016	0·9 (0·4–1·8)	51·5 (45·5–58·9)	159·1 (144·3–175·9)	153·7 (145·8–162·9)	113·2 (105·4–122·3)	64·2 (57·8–71·6)	24·2 (21·3–27·5)	6·4 (5·5–7·5)	0·1 (0·1–0·1)	2·87 (2·65–3·11)	1·06 (0·96–1·18)	1·04 (0·95–1·14)
2017	0·9 (0·4–1·8)	51·7 (45·5–59·3)	156·6 (141·0–174·3)	152·0 (143·4–161·4)	112·4 (104·4–121·8)	63·8 (57·0–72·0)	24·1 (21·0–27·7)	6·4 (5·5–7·5)	0·1 (0·1–0·1)	2·84 (2·61–3·11)	1·05 (0·94–1·17)	1·03 (0·94–1·14)

Supplemental Results Table 5: Age-specific fertility rates per 1000, cumulative total fertility up to maternal age 25 (TFU25), cumulative total fertility from maternal age 30 to age 55 (TFO30), and cumulative total fertility, Middle SDI, 1950-2017												
**Year**	**10-14**	**15-19**	**20-24**	**25-29**	**30-34**	**35-39**	**40-44**	**45-49**	**50-54**	**TFR**	**TFU25**	**TFO30**
1950	0·9 (0·4–1·9)	85·7 (78·5–93·5)	309·3 (294·8–323·5)	325·6 (319·3–331·6)	219·5 (208·9–230·3)	146·8 (136·6–156·9)	61·5 (53·6–69·8)	15·9 (13·1–18·8)	0·3 (0·3–0·3)	5·83 (5·57–6·08)	1·98 (1·87–2·09)	2·22 (2·09–2·34)
1951	0·9 (0·4–1·9)	85·8 (79·1–93·2)	308·4 (294·3–321·9)	324·9 (318·3–330·6)	218·7 (208·5–228·8)	145·6 (136·2–155·7)	61·1 (53·9–68·7)	15·7 (12·9–18·8)	0·3 (0·3–0·3)	5·81 (5·56–6·04)	1·98 (1·87–2·08)	2·21 (2·09–2·33)
1952	0·9 (0·4–1·9)	85·6 (79·3–92·0)	307·6 (293·2–320·4)	324·3 (318·3–329·6)	218·1 (208·7–227·6)	144·6 (135·1–154·2)	60·9 (54·4–68·0)	15·5 (12·9–18·4)	0·3 (0·3–0·3)	5·79 (5·56–6·01)	1·97 (1·87–2·06)	2·2 (2·08–2·32)
1953	0·9 (0·4–1·9)	85·5 (79·6–91·5)	306·2 (291·4–320·0)	323·2 (317·6–328·3)	216·7 (207·7–225·5)	143·2 (133·8–153·1)	60·5 (54·2–67·8)	15·4 (12·8–18·3)	0·3 (0·3–0·3)	5·76 (5·54–5·97)	1·96 (1·86–2·06)	2·18 (2·07–2·29)
1954	0·9 (0·4–1·9)	85·8 (80·2–91·1)	303·9 (287·7–318·1)	321·5 (315·9–326·7)	214·1 (205·4–222·5)	141·2 (131·2–150·7)	60·0 (53·7–67·0)	15·2 (12·8–17·9)	0·3 (0·3–0·3)	5·71 (5·49–5·91)	1·95 (1·85–2·04)	2·15 (2·04–2·26)
1955	0·9 (0·4–1·9)	86·3 (81·1–91·3)	301·8 (287·5–315·2)	320·4 (315·2–325·6)	212·6 (203·4–221·5)	139·9 (130·1–149·7)	59·7 (53·6–66·5)	15·1 (12·7–17·6)	0·3 (0·3–0·3)	5·69 (5·49–5·88)	1·95 (1·85–2·03)	2·14 (2·03–2·24)
1956	0·9 (0·4–1·9)	87·2 (82·2–91·9)	301·2 (288·3–313·5)	320·6 (315·6–325·5)	213·1 (204·2–222·1)	140·1 (130·4–149·7)	59·9 (53·5–66·9)	15·0 (12·7–17·6)	0·3 (0·3–0·3)	5·69 (5·49–5·89)	1·95 (1·87–2·03)	2·14 (2·03–2·25)
1957	0·9 (0·4–1·9)	87·7 (83·0–92·4)	301·3 (289·0–313·1)	321·1 (316·2–325·8)	214·1 (205·0–223·3)	141·1 (130·8–151·9)	60·2 (53·2–67·6)	15·0 (12·6–17·9)	0·3 (0·3–0·3)	5·71 (5·49–5·92)	1·95 (1·87–2·02)	2·15 (2·02–2·28)
1958	0·9 (0·4–2·0)	88·6 (84·1–93·0)	301·5 (290·4–312·9)	321·2 (316·9–325·4)	214·9 (205·3–224·6)	141·7 (131·5–152·3)	60·4 (53·7–68·2)	15·0 (12·5–18·1)	0·3 (0·3–0·3)	5·72 (5·49–5·95)	1·96 (1·88–2·03)	2·16 (2·02–2·31)
1959	0·9 (0·4–2·0)	89·5 (85·6–93·5)	301·0 (291·9–309·9)	320·9 (317·1–324·4)	214·9 (205·9–224·0)	141·3 (132·2–149·9)	60·5 (54·0–67·6)	14·9 (12·6–17·6)	0·3 (0·3–0·3)	5·72 (5·53–5·9)	1·96 (1·9–2·02)	2·16 (2·04–2·27)
1960	0·9 (0·4–2·0)	89·9 (86·4–93·5)	300·2 (292·4–307·9)	319·3 (315·9–322·6)	215·0 (206·3–223·1)	141·1 (133·8–149·5)	60·8 (55·4–67·1)	14·9 (12·8–17·1)	0·3 (0·3–0·3)	5·71 (5·56–5·87)	1·95 (1·9–2·01)	2·16 (2·06–2·26)
1961	0·9 (0·4–2·0)	89·4 (86·0–93·0)	300·3 (292·8–307·6)	318·1 (314·8–321·4)	215·4 (206·4–224·0)	141·4 (134·1–149·2)	61·2 (55·8–67·1)	14·8 (13·0–17·1)	0·3 (0·3–0·3)	5·71 (5·57–5·84)	1·95 (1·9–2·0)	2·17 (2·07–2·25)
1962	0·9 (0·4–1·9)	88·2 (84·7–91·9)	301·0 (292·9–309·0)	318·0 (314·9–321·1)	215·5 (206·3–224·7)	141·1 (133·5–148·8)	61·2 (55·9–67·3)	14·7 (12·9–16·8)	0·3 (0·3–0·3)	5·7 (5·56–5·85)	1·95 (1·9–2·0)	2·16 (2·07–2·26)
1963	0·9 (0·4–1·9)	87·3 (83·7–90·9)	300·7 (291·9–308·9)	317·2 (314·3–320·1)	214·3 (205·3–222·9)	140·1 (133·2–148·0)	61·0 (55·4–67·2)	14·6 (13·0–16·4)	0·3 (0·3–0·3)	5·68 (5·54–5·82)	1·94 (1·89–2·0)	2·15 (2·06–2·24)
1964	0·9 (0·4–1·9)	86·3 (83·0–89·6)	299·5 (291·6–307·7)	314·9 (311·8–317·9)	212·3 (204·3–220·1)	138·4 (131·8–145·5)	60·4 (55·1–66·2)	14·5 (13·1–16·3)	0·3 (0·3–0·3)	5·64 (5·52–5·76)	1·93 (1·88–1·99)	2·13 (2·05–2·21)
1965	0·9 (0·4–1·9)	84·6 (81·7–87·7)	297·8 (290·1–306·0)	313·4 (310·4–316·3)	210·3 (203·3–217·2)	136·5 (130·3–143·5)	59·9 (55·3–65·4)	14·6 (13·0–16·4)	0·3 (0·3–0·3)	5·59 (5·48–5·7)	1·92 (1·87–1·97)	2·11 (2·04–2·18)
1966	0·9 (0·4–1·9)	82·9 (80·2–86·0)	297·4 (289·2–306·0)	313·2 (310·2–315·9)	208·8 (201·9–215·4)	134·9 (128·5–142·0)	59·5 (54·8–64·9)	14·5 (13·0–16·3)	0·3 (0·3–0·3)	5·56 (5·45–5·68)	1·91 (1·86–1·96)	2·09 (2·02–2·16)
1967	0·9 (0·4–1·9)	81·1 (78·4–84·0)	297·1 (288·3–305·8)	313·2 (310·4–315·9)	207·0 (200·8–213·8)	133·2 (127·0–139·5)	58·8 (54·4–63·5)	14·5 (13·0–16·2)	0·3 (0·3–0·3)	5·53 (5·42–5·64)	1·9 (1·84–1·95)	2·07 (2·01–2·13)
1968	0·9 (0·4–1·9)	79·0 (76·3–81·9)	293·5 (284·2–303·1)	312·3 (309·4–315·2)	203·6 (197·0–210·3)	130·5 (124·4–137·0)	57·6 (52·9–62·7)	14·3 (12·9–16·0)	0·3 (0·3–0·3)	5·46 (5·36–5·57)	1·87 (1·82–1·92)	2·03 (1·97–2·1)
1969	0·9 (0·4–1·9)	78·0 (75·2–80·9)	287·2 (278·7–295·9)	309·4 (306·2–312·5)	199·1 (192·7–206·0)	127·2 (121·4–132·8)	56·1 (51·9–60·7)	14·0 (12·5–15·7)	0·3 (0·3–0·3)	5·36 (5·26–5·46)	1·83 (1·78–1·88)	1·98 (1·92–2·05)
1970	0·9 (0·4–1·9)	78·2 (75·4–81·1)	281·2 (271·8–290·4)	303·4 (299·9–306·8)	193·9 (186·9–200·9)	123·6 (117·5–130·0)	54·5 (50·6–58·8)	13·6 (12·0–15·3)	0·3 (0·2–0·3)	5·25 (5·14–5·35)	1·8 (1·75–1·85)	1·93 (1·87–1·99)
1971	0·9 (0·4–1·9)	77·5 (74·5–80·5)	275·1 (264·7–284·7)	296·9 (293·3–300·2)	187·8 (180·4–195·6)	118·9 (112·1–126·0)	52·3 (48·7–56·5)	13·1 (11·7–14·8)	0·2 (0·2–0·3)	5·11 (4·99–5·22)	1·77 (1·71–1·82)	1·86 (1·8–1·93)
1972	0·9 (0·4–1·9)	76·4 (73·2–79·6)	268·1 (256·1–279·9)	289·6 (285·7–293·5)	180·1 (173·0–187·7)	112·9 (106·5–119·7)	50·0 (46·5–53·8)	12·6 (11·3–14·1)	0·2 (0·2–0·2)	4·95 (4·82–5·08)	1·73 (1·66–1·8)	1·78 (1·71–1·85)
1973	0·9 (0·4–1·9)	75·9 (72·8–79·0)	260·1 (246·1–273·6)	279·7 (275·2–283·9)	171·5 (164·8–178·5)	106·0 (100·8–111·6)	47·3 (43·8–51·3)	12·1 (10·9–13·4)	0·2 (0·2–0·2)	4·77 (4·63–4·9)	1·68 (1·61–1·76)	1·69 (1·61–1·76)
1974	0·9 (0·4–1·8)	75·6 (72·5–78·7)	250·1 (238·2–261·9)	268·0 (263·1–273·1)	163·6 (158·1–169·4)	100·1 (95·8–105·0)	44·8 (41·8–48·2)	11·6 (10·5–12·9)	0·2 (0·2–0·2)	4·58 (4·46–4·69)	1·63 (1·57–1·7)	1·6 (1·54–1·66)
1975	0·9 (0·4–1·8)	75·6 (72·5–78·6)	240·8 (231·3–250·9)	257·0 (251·2–262·3)	157·0 (151·6–162·4)	96·1 (92·1–100·2)	42·9 (40·0–46·0)	11·3 (10·2–12·5)	0·2 (0·2–0·2)	4·41 (4·31–4·51)	1·59 (1·53–1·64)	1·54 (1·48–1·59)
1976	0·8 (0·4–1·7)	75·4 (72·3–78·6)	234·1 (224·5–243·2)	247·8 (241·9–253·6)	150·6 (145·5–156·0)	92·5 (88·6–96·5)	41·1 (38·0–44·1)	10·9 (9·9–12·1)	0·2 (0·2–0·2)	4·27 (4·16–4·36)	1·55 (1·5–1·6)	1·48 (1·42–1·53)
1977	0·8 (0·4–1·7)	74·7 (71·6–77·9)	228·7 (219·7–237·7)	240·2 (234·1–245·6)	144·8 (139·8–150·0)	88·6 (84·8–92·4)	39·6 (36·7–42·6)	10·5 (9·6–11·6)	0·2 (0·2–0·2)	4·14 (4·04–4·24)	1·52 (1·47–1·57)	1·42 (1·37–1·47)
1978	0·8 (0·3–1·7)	72·9 (69·9–75·9)	225·1 (216·1–234·3)	234·2 (228·3–239·7)	141·4 (136·7–146·4)	85·8 (81·9–89·6)	38·4 (35·4–41·6)	10·1 (9·2–11·2)	0·2 (0·2–0·2)	4·04 (3·94–4·15)	1·49 (1·44–1·55)	1·38 (1·33–1·42)
1979	0·8 (0·3–1·7)	70·8 (67·9–73·7)	222·6 (214·6–230·9)	228·4 (222·7–234·3)	139·4 (134·7–144·7)	84·0 (80·6–88·0)	37·6 (34·7–40·7)	9·8 (8·8–10·7)	0·2 (0·2–0·2)	3·97 (3·87–4·07)	1·47 (1·42–1·52)	1·35 (1·31–1·4)
1980	0·8 (0·3–1·6)	68·5 (65·6–71·5)	220·1 (213·0–227·1)	223·5 (217·4–229·7)	137·5 (132·8–142·5)	82·1 (78·3–85·8)	37·0 (34·2–40·1)	9·4 (8·5–10·4)	0·2 (0·2–0·2)	3·9 (3·8–3·99)	1·45 (1·4–1·49)	1·33 (1·29–1·38)
1981	0·8 (0·3–1·6)	65·8 (62·8–69·0)	219·4 (212·0–226·8)	223·8 (217·1–229·9)	136·6 (131·2–142·5)	80·0 (75·7–84·4)	36·5 (33·4–40·0)	9·0 (8·1–10·0)	0·2 (0·2–0·2)	3·86 (3·75–3·97)	1·43 (1·38–1·48)	1·31 (1·26–1·36)
1982	0·8 (0·3–1·6)	63·7 (60·4–67·1)	220·5 (211·8–228·6)	226·7 (219·4–233·3)	135·7 (129·9–141·8)	78·4 (73·7–83·7)	35·5 (32·4–38·9)	8·5 (7·7–9·5)	0·2 (0·2–0·2)	3·85 (3·73–3·96)	1·42 (1·37–1·48)	1·29 (1·23–1·35)
1983	0·8 (0·3–1·6)	62·1 (58·7–65·8)	220·7 (212·3–229·5)	224·9 (218·2–231·0)	133·4 (127·7–139·7)	76·5 (71·9–81·4)	34·1 (31·0–37·9)	7·9 (7·2–8·8)	0·2 (0·1–0·2)	3·8 (3·69–3·92)	1·42 (1·37–1·47)	1·26 (1·2–1·32)
1984	0·8 (0·3–1·6)	60·2 (56·9–63·7)	219·5 (211·9–227·0)	218·8 (213·0–224·3)	130·3 (125·1–135·9)	73·8 (70·0–77·9)	32·7 (29·6–35·9)	7·4 (6·7–8·2)	0·1 (0·1–0·1)	3·72 (3·61–3·83)	1·4 (1·35–1·45)	1·22 (1·17–1·27)
1985	0·8 (0·3–1·6)	58·7 (55·4–62·1)	218·3 (210·3–226·5)	213·8 (208·5–218·8)	129·6 (124·5–134·5)	71·3 (67·5–75·4)	31·8 (28·6–35·0)	7·0 (6·2–7·8)	0·1 (0·1–0·1)	3·66 (3·56–3·76)	1·39 (1·34–1·44)	1·2 (1·15–1·24)
1986	0·8 (0·3–1·6)	57·3 (54·2–60·6)	216·9 (207·3–226·7)	208·5 (203·0–213·9)	127·5 (121·8–133·0)	68·6 (64·7–73·4)	30·3 (27·1–33·8)	6·6 (5·8–7·5)	0·1 (0·1–0·1)	3·58 (3·47–3·7)	1·37 (1·32–1·43)	1·17 (1·12–1·21)
1987	0·8 (0·3–1·6)	55·7 (52·5–59·0)	211·8 (201·1–223·3)	201·1 (195·6–206·7)	121·3 (115·5–127·3)	64·4 (60·7–68·6)	27·3 (24·7–30·3)	6·2 (5·5–7·1)	0·1 (0·1–0·1)	3·44 (3·33–3·57)	1·34 (1·27–1·41)	1·1 (1·05–1·15)
1988	0·8 (0·3–1·6)	54·5 (51·5–57·5)	205·2 (195·1–215·8)	193·8 (188·4–199·5)	115·3 (110·4–120·2)	60·5 (57·0–64·3)	24·4 (22·7–26·6)	5·8 (5·2–6·5)	0·1 (0·1–0·1)	3·3 (3·2–3·41)	1·3 (1·24–1·37)	1·03 (0·99–1·08)
1989	0·8 (0·3–1·6)	53·8 (51·0–56·6)	200·1 (192·1–209·1)	186·8 (181·5–192·6)	110·6 (106·7–114·4)	57·1 (54·4–60·0)	22·1 (20·7–23·7)	5·6 (5·1–6·2)	0·1 (0·1–0·1)	3·18 (3·09–3·28)	1·27 (1·22–1·33)	0·98 (0·94–1·01)
1990	0·8 (0·3–1·6)	53·0 (50·2–55·9)	193·8 (185·4–202·6)	180·2 (175·1–185·8)	106·5 (103·0–109·7)	54·0 (51·7–56·4)	19·9 (18·8–21·0)	5·3 (4·8–5·9)	0·1 (0·1–0·1)	3·07 (2·98–3·16)	1·24 (1·19–1·29)	0·93 (0·9–0·96)
1991	0·8 (0·3–1·6)	52·2 (49·3–55·2)	187·8 (179·1–197·3)	175·0 (169·0–181·1)	103·0 (99·6–106·4)	51·4 (49·1–53·8)	18·4 (17·3–19·6)	4·9 (4·4–5·4)	0·1 (0·1–0·1)	2·97 (2·88–3·06)	1·2 (1·15–1·25)	0·89 (0·86–0·92)
1992	0·8 (0·3–1·6)	51·3 (48·3–54·5)	183·1 (174·4–192·2)	170·6 (164·0–176·9)	100·0 (96·8–103·3)	49·5 (47·2–51·8)	17·5 (16·4–18·7)	4·6 (4·1–5·1)	0·1 (0·1–0·1)	2·89 (2·8–2·98)	1·18 (1·13–1·23)	0·86 (0·83–0·89)
1993	0·8 (0·3–1·6)	50·5 (47·3–53·8)	179·5 (170·6–188·6)	166·6 (160·1–173·1)	96·9 (93·9–100·1)	48·4 (46·4–50·5)	16·9 (15·8–18·2)	4·4 (3·9–4·9)	0·1 (0·1–0·1)	2·82 (2·73–2·91)	1·15 (1·1–1·2)	0·83 (0·8–0·86)
1994	0·8 (0·3–1·6)	50·0 (46·6–53·4)	176·7 (168·4–185·0)	162·2 (156·1–168·6)	93·3 (90·3–96·4)	48·0 (46·0–50·0)	16·3 (15·3–17·6)	4·2 (3·8–4·8)	0·1 (0·1–0·1)	2·76 (2·67–2·84)	1·14 (1·09–1·18)	0·81 (0·78–0·84)
1995	0·8 (0·3–1·6)	49·2 (45·9–52·5)	172·2 (164·3–180·0)	158·3 (152·6–163·9)	90·1 (87·4–92·8)	48·1 (45·9–50·1)	15·8 (14·8–16·9)	4·1 (3·6–4·6)	0·1 (0·1–0·1)	2·69 (2·61–2·77)	1·11 (1·07–1·16)	0·79 (0·76–0·82)
1996	0·7 (0·3–1·5)	48·7 (45·7–51·8)	167·4 (159·8–175·3)	156·2 (150·3–162·7)	88·4 (85·4–91·4)	48·1 (46·0–50·2)	15·3 (14·4–16·4)	3·7 (3·3–4·2)	0·1 (0·1–0·1)	2·64 (2·56–2·73)	1·08 (1·04–1·13)	0·78 (0·75–0·81)
1997	0·7 (0·3–1·5)	49·0 (46·0–52·0)	164·2 (156·2–172·0)	154·5 (148·9–160·5)	87·5 (84·7–90·6)	47·6 (45·7–49·7)	15·1 (14·1–16·2)	3·3 (3·0–3·7)	0·1 (0·1–0·1)	2·61 (2·53–2·69)	1·07 (1·02–1·12)	0·77 (0·74–0·8)
1998	0·7 (0·3–1·5)	49·5 (46·6–52·4)	161·8 (154·5–169·5)	152·3 (147·4–157·7)	87·1 (84·4–90·1)	46·5 (44·7–48·5)	15·1 (14·1–16·2)	3·0 (2·7–3·4)	0·1 (0·1–0·1)	2·58 (2·51–2·66)	1·06 (1·02–1·1)	0·76 (0·74–0·78)
1999	0·7 (0·3–1·5)	48·9 (46·2–51·8)	159·2 (152·5–166·1)	149·7 (145·0–154·6)	86·9 (83·9–90·1)	45·1 (43·3–46·9)	15·1 (14·1–16·1)	2·8 (2·6–3·1)	0·1 (0·1–0·1)	2·54 (2·47–2·61)	1·04 (1·01–1·08)	0·75 (0·72–0·77)
2000	0·7 (0·3–1·4)	47·1 (44·4–49·6)	155·8 (149·4–162·5)	146·8 (142·3–151·1)	86·3 (83·4–89·8)	43·3 (41·6–45·0)	14·9 (13·9–15·8)	2·7 (2·4–2·9)	0·1 (0·0–0·1)	2·49 (2·42–2·56)	1·02 (0·98–1·06)	0·74 (0·71–0·76)
2001	0·7 (0·3–1·4)	45·1 (42·5–47·8)	152·3 (146·2–158·5)	144·3 (139·7–149·2)	85·2 (82·0–88·5)	41·8 (39·8–43·7)	14·6 (13·7–15·5)	2·5 (2·2–2·7)	0·0 (0·0–0·0)	2·43 (2·36–2·5)	0·99 (0·95–1·03)	0·72 (0·69–0·74)
2002	0·7 (0·3–1·4)	43·8 (41·3–46·5)	150·4 (143·8–157·0)	143·0 (138·1–147·8)	84·6 (81·5–88·0)	40·8 (38·8–42·6)	14·4 (13·5–15·3)	2·4 (2·1–2·7)	0·0 (0·0–0·0)	2·4 (2·33–2·48)	0·97 (0·93–1·01)	0·71 (0·69–0·74)
2003	0·7 (0·3–1·4)	42·8 (40·4–45·5)	149·1 (141·8–156·3)	141·9 (137·3–146·6)	84·3 (81·2–87·5)	40·4 (38·4–42·3)	14·2 (13·3–15·1)	2·4 (2·1–2·6)	0·0 (0·0–0·0)	2·38 (2·31–2·45)	0·96 (0·92–1·0)	0·71 (0·68–0·73)
2004	0·7 (0·3–1·4)	41·7 (39·2–44·3)	146·8 (140·0–153·5)	140·0 (135·3–144·8)	84·0 (81·0–87·0)	40·5 (38·6–42·5)	14·0 (13·2–14·9)	2·4 (2·2–2·7)	0·0 (0·0–0·0)	2·35 (2·28–2·43)	0·95 (0·9–0·99)	0·71 (0·68–0·73)
2005	0·7 (0·3–1·4)	40·6 (38·1–43·2)	143·7 (136·6–150·9)	138·0 (133·3–143·0)	84·0 (80·8–87·3)	41·2 (39·2–43·2)	14·0 (13·1–15·0)	2·5 (2·2–2·9)	0·0 (0·0–0·0)	2·32 (2·24–2·4)	0·92 (0·88–0·97)	0·71 (0·68–0·74)
2006	0·7 (0·3–1·4)	40·0 (37·4–42·6)	140·5 (132·8–148·1)	136·3 (131·5–141·3)	84·2 (80·9–87·7)	42·0 (39·9–44·2)	14·0 (13·0–15·1)	2·6 (2·2–3·0)	0·1 (0·0–0·1)	2·3 (2·21–2·39)	0·91 (0·86–0·95)	0·71 (0·69–0·74)
2007	0·7 (0·3–1·4)	39·7 (37·0–42·3)	137·5 (129·8–145·5)	134·5 (129·6–139·4)	84·1 (80·8–87·8)	42·3 (40·0–44·6)	13·9 (12·8–15·3)	2·5 (2·2–3·0)	0·1 (0·0–0·1)	2·28 (2·19–2·37)	0·89 (0·84–0·94)	0·71 (0·69–0·75)
2008	0·7 (0·3–1·4)	39·5 (36·7–42·2)	134·8 (126·5–143·1)	132·5 (127·7–137·3)	83·7 (80·2–87·6)	41·8 (39·4–44·2)	13·7 (12·5–15·0)	2·4 (2·1–2·9)	0·0 (0·0–0·0)	2·25 (2·15–2·34)	0·87 (0·83–0·93)	0·71 (0·68–0·74)
2009	0·7 (0·3–1·4)	39·5 (36·7–42·5)	132·1 (123·5–140·4)	130·6 (125·8–135·4)	83·1 (79·1–87·3)	40·7 (38·4–43·1)	13·4 (12·3–14·6)	2·2 (1·9–2·6)	0·0 (0·0–0·0)	2·21 (2·12–2·3)	0·86 (0·81–0·91)	0·7 (0·67–0·73)
2010	0·7 (0·3–1·4)	39·8 (36·8–43·0)	129·4 (121·6–137·5)	129·3 (124·5–134·2)	82·4 (78·2–86·8)	39·4 (37·1–41·8)	12·9 (11·9–14·2)	2·0 (1·7–2·4)	0·0 (0·0–0·0)	2·18 (2·09–2·27)	0·85 (0·8–0·9)	0·68 (0·65–0·72)
2011	0·7 (0·3–1·4)	40·1 (37·1–43·2)	127·7 (120·2–135·3)	128·8 (123·6–133·7)	82·1 (78·3–86·1)	39·1 (36·6–41·5)	12·6 (11·5–13·8)	1·9 (1·6–2·3)	0·0 (0·0–0·0)	2·16 (2·06–2·26)	0·84 (0·79–0·89)	0·68 (0·65–0·71)
2012	0·7 (0·3–1·4)	39·8 (36·8–43·1)	126·4 (118·3–134·6)	128·7 (123·4–133·4)	82·7 (78·9–86·5)	39·6 (37·0–42·4)	12·3 (11·3–13·5)	1·8 (1·5–2·1)	0·0 (0·0–0·0)	2·16 (2·05–2·26)	0·83 (0·78–0·89)	0·68 (0·65–0·72)
2013	0·7 (0·3–1·4)	38·0 (35·0–41·2)	123·8 (115·8–132·1)	126·4 (121·5–131·4)	83·2 (79·3–87·2)	40·4 (37·6–43·5)	12·1 (11·1–13·2)	1·7 (1·5–1·9)	0·0 (0·0–0·0)	2·13 (2·02–2·24)	0·81 (0·76–0·87)	0·69 (0·65–0·72)
2014	0·6 (0·3–1·3)	35·6 (32·6–39·0)	118·3 (109·7–127·5)	121·2 (115·9–126·8)	82·0 (77·9–86·4)	40·3 (37·3–43·7)	11·8 (10·8–13·0)	1·6 (1·4–1·8)	0·0 (0·0–0·0)	2·06 (1·94–2·18)	0·77 (0·72–0·83)	0·68 (0·64–0·72)
2015	0·6 (0·3–1·3)	34·2 (31·0–37·8)	116·5 (107·0–127·3)	121·6 (115·8–128·3)	81·9 (77·3–86·9)	40·4 (36·8–44·4)	12·0 (10·8–13·4)	1·5 (1·3–1·7)	0·0 (0·0–0·0)	2·04 (1·92–2·19)	0·76 (0·69–0·83)	0·68 (0·64–0·73)
2016	0·6 (0·3–1·3)	33·7 (30·3–37·5)	116·5 (105·8–129·4)	123·6 (117·5–130·8)	81·9 (76·7–87·6)	40·2 (36·0–44·8)	12·1 (10·7–13·6)	1·5 (1·3–1·7)	0·0 (0·0–0·0)	2·05 (1·91–2·21)	0·75 (0·69–0·83)	0·68 (0·62–0·74)
2017	0·6 (0·3–1·3)	33·5 (30·1–37·6)	112·4 (100·6–125·9)	120·1 (113·3–127·9)	79·2 (73·7–85·3)	39·0 (34·4–44·2)	11·3 (9·9–13·1)	1·4 (1·1–1·6)	0·0 (0·0–0·0)	1·99 (1·84–2·17)	0·73 (0·66–0·82)	0·65 (0·6–0·72)

Supplemental Results Table 5: Age-specific fertility rates per 1000, cumulative total fertility up to maternal age 25 (TFU25), cumulative total fertility from maternal age 30 to age 55 (TFO30), and cumulative total fertility, High-middle SDI, 1950-2017

Year	10-14	15-19	20-24	25-29	30-34	35-39	40-44	45-49	50-54	TFR	TFU25	TFO30
1950	0·5 (0·2–1·0)	42·7 (38·8–47·3)	249·0 (237·8–260·5)	268·7 (264·8–272·3)	144·1 (135·3–153·9)	85·0 (78·1–92·2)	30·1 (27·4–33·1)	5·9 (5·1–7·0)	0·1 (0·1–0·1)	4·13 (3·98–4·29)	1·46 (1·39–1·54)	1·33 (1·25–1·4)
1951	0·5 (0·2–1·0)	42·5 (38·8–46·5)	248·8 (238·5–258·9)	268·3 (264·7–271·9)	143·8 (135·3–153·1)	83·1 (76·2–90·8)	29·8 (27·3–32·7)	5·8 (4·9–6·8)	0·1 (0·1–0·1)	4·11 (3·98–4·24)	1·46 (1·39–1·52)	1·31 (1·25–1·39)
1952	0·5 (0·2–1·0)	42·1 (38·7–45·9)	247·4 (236·5–257·3)	267·3 (263·7–270·8)	143·3 (135·3–152·5)	81·5 (74·4–89·3)	29·5 (27·1–32·3)	5·6 (4·8–6·5)	0·1 (0·1–0·1)	4·09 (3·97–4·21)	1·45 (1·39–1·51)	1·3 (1·23–1·37)
1953	0·5 (0·2–1·0)	42·1 (38·7–45·8)	247·7 (237·1–258·2)	267·5 (264·1–270·8)	142·5 (134·5–150·9)	80·5 (73·2–88·4)	29·0 (26·5–31·9)	5·5 (4·7–6·4)	0·1 (0·1–0·1)	4·08 (3·95–4·2)	1·45 (1·38–1·52)	1·29 (1·22–1·36)
1954	0·5 (0·2–1·1)	42·3 (39·2–45·8)	248·8 (237·0–260·4)	267·1 (263·6–270·4)	141·0 (133·1–148·8)	78·5 (71·6–86·0)	28·1 (25·6–30·8)	5·3 (4·6–6·2)	0·1 (0·1–0·1)	4·06 (3·92–4·18)	1·46 (1·39–1·53)	1·26 (1·2–1·33)
1955	0·5 (0·2–1·1)	43·1 (40·0–46·4)	248·0 (236·7–258·6)	265·8 (262·2–269·1)	138·9 (131·0–146·7)	77·1 (69·9–85·1)	26·6 (24·3–29·3)	5·2 (4·5–6·0)	0·1 (0·1–0·1)	4·03 (3·89–4·15)	1·46 (1·39–1·52)	1·24 (1·17–1·3)
1956	0·5 (0·2–1·1)	44·3 (41·3–47·7)	250·6 (238·9–261·6)	267·1 (263·3–270·3)	139·2 (131·4–147·0)	76·9 (69·0–85·2)	25·4 (22·9–28·5)	5·1 (4·4–5·9)	0·1 (0·1–0·1)	4·05 (3·92–4·18)	1·48 (1·41–1·54)	1·23 (1·17–1·31)
1957	0·5 (0·2–1·1)	45·5 (42·4–48·9)	253·9 (241·7–265·4)	269·4 (265·7–272·7)	140·3 (132·2–148·8)	77·0 (68·4–86·4)	24·9 (22·1–27·9)	5·0 (4·2–5·9)	0·1 (0·1–0·1)	4·08 (3·92–4·25)	1·5 (1·43–1·57)	1·24 (1·15–1·33)
1958	0·5 (0·2–1·1)	46·7 (43·5–50·2)	256·7 (244·9–268·1)	271·0 (267·6–274·3)	140·7 (131·8–150·2)	76·5 (67·7–86·7)	24·6 (21·8–28·1)	4·8 (4·0–5·9)	0·1 (0·1–0·1)	4·11 (3·92–4·32)	1·52 (1·44–1·59)	1·23 (1·13–1·35)
1959	0·5 (0·2–1·1)	48·0 (44·9–51·4)	256·7 (245·8–267·6)	274·8 (271·4–277·6)	140·1 (131·6–148·9)	75·5 (67·9–83·6)	24·4 (21·6–27·7)	4·7 (3·9–5·7)	0·1 (0·1–0·1)	4·12 (3·97–4·28)	1·53 (1·46–1·59)	1·22 (1·14–1·31)
1960	0·5 (0·2–1·1)	48·7 (45·8–51·9)	257·0 (248·6–266·2)	276·8 (273·8–279·4)	138·9 (130·4–146·6)	73·9 (67·3–81·5)	24·3 (21·9–27·3)	4·5 (3·8–5·4)	0·1 (0·1–0·1)	4·12 (3·99–4·25)	1·53 (1·48–1·59)	1·21 (1·14–1·28)
1961	0·5 (0·2–1·0)	48·0 (45·0–51·3)	257·4 (249·2–265·9)	277·5 (274·8–280·0)	137·5 (129·4–145·6)	72·6 (66·3–79·7)	24·2 (21·8–27·0)	4·5 (3·7–5·4)	0·1 (0·1–0·1)	4·11 (4·0–4·22)	1·53 (1·48–1·58)	1·19 (1·13–1·25)
1962	0·5 (0·2–1·0)	46·4 (43·3–50·1)	256·7 (247·9–265·1)	276·7 (274·0–279·3)	135·9 (128·0–144·2)	70·9 (64·4–77·8)	23·8 (21·4–26·4)	4·4 (3·6–5·4)	0·1 (0·1–0·1)	4·08 (3·96–4·19)	1·52 (1·47–1·57)	1·18 (1·11–1·24)
1963	0·5 (0·2–1·0)	45·3 (42·2–48·8)	256·1 (246·8–264·9)	273·5 (270·9–276·2)	132·8 (124·8–140·6)	68·5 (62·8–75·4)	23·3 (20·9–25·8)	4·4 (3·6–5·5)	0·1 (0·1–0·1)	4·02 (3·91–4·13)	1·51 (1·45–1·56)	1·14 (1·08–1·21)
1964	0·5 (0·2–1·0)	44·4 (41·4–47·4)	255·0 (245·3–264·0)	266·5 (263·6–269·6)	130·1 (122·0–138·5)	65·7 (60·2–72·2)	22·6 (20·3–25·3)	4·3 (3·6–5·3)	0·1 (0·1–0·1)	3·95 (3·85–4·05)	1·5 (1·45–1·55)	1·11 (1·06–1·17)
1965	0·5 (0·2–1·0)	43·4 (40·8–46·1)	255·2 (246·0–264·1)	262·4 (259·3–265·3)	128·4 (121·0–135·8)	63·5 (58·1–69·8)	21·9 (19·9–24·6)	4·3 (3·6–5·1)	0·1 (0·1–0·1)	3·9 (3·8–4·0)	1·49 (1·45–1·54)	1·09 (1·04–1·15)
1966	0·5 (0·2–1·0)	42·6 (40·3–45·3)	259·5 (249·7–269·0)	262·2 (259·3–264·9)	128·5 (121·6–135·8)	62·4 (56·9–68·5)	21·5 (19·3–24·1)	4·2 (3·5–5·1)	0·1 (0·1–0·1)	3·91 (3·81–4·01)	1·51 (1·46–1·56)	1·08 (1·03–1·14)
1967	0·5 (0·2–1·0)	41·8 (39·5–44·4)	263·0 (252·9–271·7)	262·2 (259·3–265·0)	127·5 (120·7–135·0)	61·4 (56·3–67·1)	21·0 (18·8–23·3)	4·0 (3·4–4·8)	0·1 (0·1–0·1)	3·91 (3·81–3·99)	1·53 (1·47–1·58)	1·07 (1·02–1·12)
1968	0·5 (0·2–1·0)	40·6 (38·4–43·1)	259·2 (249·2–268·3)	261·3 (258·1–264·3)	123·5 (116·8–130·6)	59·0 (54·1–64·2)	20·2 (18·1–22·7)	3·9 (3·3–4·6)	0·1 (0·1–0·1)	3·84 (3·75–3·93)	1·5 (1·44–1·55)	1·03 (0·98–1·09)
1969	0·5 (0·2–1·0)	40·1 (38·0–42·3)	250·4 (240·9–259·8)	258·6 (255·3–262·2)	117·5 (111·4–124·4)	56·4 (51·5–61·4)	19·3 (17·5–21·5)	3·6 (3·0–4·4)	0·1 (0·1–0·1)	3·73 (3·64–3·82)	1·45 (1·4–1·5)	0·98 (0·94–1·04)
1970	0·5 (0·2–1·0)	40·5 (38·3–42·8)	241·3 (230·6–251·6)	253·6 (249·5–257·7)	112·0 (105·6–118·6)	54·1 (49·3–59·4)	18·6 (16·8–20·6)	3·4 (2·8–4·0)	0·1 (0·1–0·1)	3·62 (3·52–3·71)	1·41 (1·35–1·46)	0·94 (0·89–0·99)
1971	0·5 (0·2–1·0)	39·5 (37·4–41·9)	233·4 (221·9–243·6)	249·4 (244·5–254·0)	107·4 (101·2–113·8)	52·2 (47·0–57·7)	17·8 (16·0–19·9)	3·1 (2·6–3·8)	0·1 (0·1–0·1)	3·52 (3·41–3·62)	1·37 (1·31–1·42)	0·9 (0·85–0·95)
1972	0·5 (0·2–1·0)	37·7 (35·5–39·9)	224·8 (212·8–236·6)	242·5 (237·1–247·9)	102·2 (96·3–108·7)	49·3 (44·5–54·6)	17·1 (15·5–19·0)	2·9 (2·4–3·5)	0·1 (0·1–0·1)	3·39 (3·28–3·49)	1·31 (1·25–1·38)	0·86 (0·81–0·91)
1973	0·5 (0·2–0·9)	36·8 (35·0–38·7)	214·8 (203·0–226·1)	230·3 (224·1–236·1)	97·2 (91·4–103·7)	45·8 (41·8–50·5)	16·2 (14·5–18·2)	2·7 (2·3–3·3)	0·1 (0·1–0·1)	3·22 (3·11–3·32)	1·26 (1·19–1·32)	0·81 (0·76–0·87)
1974	0·4 (0·2–0·9)	36·2 (34·6–37·8)	202·5 (190·9–213·7)	215·2 (208·6–221·9)	94·0 (88·9–99·6)	43·0 (39·8–46·9)	15·5 (14·0–17·1)	2·6 (2·2–3·0)	0·0 (0·0–0·1)	3·05 (2·95–3·14)	1·2 (1·13–1·25)	0·78 (0·73–0·82)
1975	0·4 (0·2–0·9)	35·6 (34·1–37·2)	192·0 (180·6–203·7)	201·8 (194·8–208·4)	92·0 (86·6–97·3)	41·5 (38·5–44·7)	14·9 (13·4–16·5)	2·4 (2·1–2·9)	0·0 (0·0–0·0)	2·9 (2·81–2·99)	1·14 (1·08–1·2)	0·75 (0·72–0·79)
1976	0·4 (0·2–0·9)	34·9 (33·4–36·6)	186·1 (174·2–198·5)	193·8 (186·5–200·7)	89·7 (84·4–95·3)	40·3 (37·5–43·5)	14·4 (12·8–16·0)	2·3 (2·0–2·8)	0·0 (0·0–0·0)	2·81 (2·71–2·9)	1·11 (1·04–1·17)	0·73 (0·69–0·78)
1977	0·4 (0·2–0·9)	34·5 (32·9–36·2)	182·7 (169·6–196·0)	189·2 (181·9–195·7)	87·3 (82·0–93·3)	39·2 (36·3–42·3)	13·9 (12·5–15·5)	2·3 (1·9–2·7)	0·0 (0·0–0·0)	2·75 (2·64–2·86)	1·09 (1·02–1·16)	0·71 (0·68–0·76)
1978	0·4 (0·2–0·8)	34·2 (32·7–35·7)	181·1 (168·6–194·5)	185·6 (178·3–192·1)	86·8 (81·7–92·4)	39·2 (36·1–42·5)	13·6 (12·3–15·0)	2·2 (1·9–2·6)	0·0 (0·0–0·0)	2·72 (2·62–2·82)	1·08 (1·01–1·14)	0·71 (0·67–0·75)
1979	0·4 (0·2–0·8)	34·0 (32·6–35·4)	180·8 (169·7–191·7)	182·3 (175·3–189·5)	87·8 (82·7–93·5)	40·3 (37·3–43·9)	13·4 (12·2–14·8)	2·2 (1·9–2·6)	0·0 (0·0–0·0)	2·71 (2·62–2·79)	1·08 (1·02–1·13)	0·72 (0·68–0·75)
1980	0·4 (0·2–0·9)	33·5 (32·2–35·1)	178·7 (168·5–189·6)	179·1 (171·7–186·8)	87·8 (82·7–92·9)	40·7 (37·8–44·1)	13·3 (12·1–14·6)	2·2 (1·9–2·5)	0·0 (0·0–0·0)	2·68 (2·59–2·77)	1·06 (1·01–1·12)	0·72 (0·69–0·76)
1981	0·4 (0·2–0·9)	33·5 (32·0–35·4)	177·4 (165·4–188·7)	179·5 (171·6–187·2)	87·4 (81·9–93·3)	39·8 (36·7–43·7)	13·1 (12·0–14·4)	2·1 (1·8–2·5)	0·0 (0·0–0·0)	2·67 (2·57–2·77)	1·06 (1·0–1·11)	0·71 (0·68–0·75)
1982	0·4 (0·2–0·9)	34·9 (32·8–37·1)	182·2 (169·5–195·0)	184·9 (176·6–193·0)	87·4 (81·5–93·6)	38·9 (35·3–43·7)	12·9 (11·7–14·2)	1·9 (1·7–2·3)	0·0 (0·0–0·0)	2·72 (2·62–2·82)	1·09 (1·02–1·15)	0·71 (0·66–0·75)
1983	0·4 (0·2–0·9)	36·2 (34·0–38·6)	187·2 (175·1–200·4)	185·6 (177·7–193·1)	86·0 (79·6–92·6)	38·0 (34·7–42·0)	12·8 (11·5–14·4)	1·8 (1·5–2·0)	0·0 (0·0–0·0)	2·74 (2·64–2·84)	1·12 (1·06–1·19)	0·69 (0·65–0·74)
1984	0·5 (0·2–0·9)	36·5 (34·2–39·0)	190·1 (178·4–202·2)	181·7 (174·4–188·5)	84·6 (79·6–90·5)	36·9 (34·2–40·1)	12·7 (11·4–14·0)	1·6 (1·4–1·9)	0·0 (0·0–0·0)	2·72 (2·63–2·82)	1·14 (1·08–1·2)	0·68 (0·65–0·72)
1985	0·5 (0·2–1·0)	36·3 (34·1–38·7)	192·5 (181·3–204·0)	180·4 (173·3–187·0)	87·2 (81·7–92·7)	36·6 (33·9–39·6)	12·7 (11·6–14·1)	1·5 (1·3–1·8)	0·0 (0·0–0·0)	2·74 (2·64–2·83)	1·15 (1·09–1·21)	0·69 (0·66–0·72)
1986	0·5 (0·2–1·0)	35·9 (33·6–38·2)	193·3 (179·7–206·5)	177·6 (170·5–184·5)	88·6 (81·9–94·9)	36·5 (33·3–40·2)	12·4 (11·2–13·7)	1·5 (1·2–1·7)	0·0 (0·0–0·0)	2·73 (2·64–2·83)	1·15 (1·08–1·22)	0·69 (0·65–0·73)
1987	0·5 (0·2–1·0)	34·7 (32·6–37·1)	187·1 (173·0–201·7)	168·1 (161·1–175·1)	84·1 (77·5–91·3)	34·7 (31·5–38·4)	11·1 (10·2–12·1)	1·4 (1·2–1·6)	0·0 (0·0–0·0)	2·61 (2·5–2·71)	1·11 (1·04–1·18)	0·66 (0·62–0·7)
1988	0·5 (0·2–1·0)	33·5 (31·6–35·6)	176·9 (162·7–191·5)	155·7 (149·1–163·0)	78·0 (72·0–83·6)	32·3 (29·6–35·7)	9·8 (9·1–10·6)	1·3 (1·1–1·5)	0·0 (0·0–0·0)	2·44 (2·35–2·54)	1·05 (0·98–1·13)	0·61 (0·57–0·65)
1989	0·5 (0·2–1·0)	32·8 (31·2–34·4)	168·5 (157·4–181·0)	144·2 (137·7–151·1)	72·4 (68·3–76·6)	29·9 (27·8–32·3)	8·5 (8·0–9·2)	1·3 (1·1–1·5)	0·0 (0·0–0·0)	2·29 (2·22–2·37)	1·01 (0·95–1·07)	0·56 (0·53–0·59)
1990	0·5 (0·2–1·0)	31·8 (30·4–33·2)	157·7 (146·6–169·8)	134·9 (128·7–141·7)	66·5 (62·5–70·3)	27·1 (25·3–28·9)	7·4 (7·0–7·9)	1·3 (1·1–1·4)	0·0 (0·0–0·0)	2·14 (2·06–2·22)	0·95 (0·89–1·01)	0·51 (0·49–0·53)
1991	0·5 (0·2–1·0)	30·6 (29·2–32·0)	147·7 (135·4–160·9)	128·1 (121·5–134·8)	61·2 (57·4–64·9)	24·6 (22·8–26·7)	6·7 (6·3–7·2)	1·2 (1·0–1·3)	0·0 (0·0–0·0)	2·0 (1·92–2·09)	0·89 (0·83–0·96)	0·47 (0·45–0·49)
1992	0·5 (0·2–1·0)	29·4 (28·2–30·8)	141·3 (129·2–154·2)	123·1 (116·3–129·9)	57·5 (54·2–60·9)	22·8 (21·3–24·6)	6·3 (6·0–6·8)	1·1 (0·9–1·2)	0·0 (0·0–0·0)	1·91 (1·83–2·0)	0·86 (0·8–0·92)	0·44 (0·42–0·46)
1993	0·5 (0·2–1·0)	28·7 (27·6–30·1)	137·3 (126·2–149·2)	119·1 (112·2–125·8)	55·0 (51·9–58·4)	21·7 (20·4–23·1)	6·0 (5·7–6·4)	1·0 (0·9–1·2)	0·0 (0·0–0·0)	1·85 (1·77–1·93)	0·83 (0·78–0·89)	0·42 (0·4–0·44)
1994	0·5 (0·2–0·9)	28·2 (27·1–29·4)	134·0 (123·9–144·2)	114·5 (108·1–121·3)	52·3 (49·7–55·4)	21·2 (20·1–22·4)	5·8 (5·4–6·1)	1·0 (0·8–1·2)	0·0 (0·0–0·0)	1·79 (1·71–1·86)	0·81 (0·76–0·87)	0·4 (0·38–0·42)
1995	0·4 (0·2–0·9)	27·2 (26·1–28·3)	127·3 (118·2–137·2)	110·5 (104·5–117·0)	50·6 (47·9–53·3)	21·4 (20·4–22·5)	5·6 (5·3–6·0)	0·9 (0·8–1·1)	0·0 (0·0–0·0)	1·72 (1·65–1·79)	0·77 (0·73–0·82)	0·39 (0·38–0·41)
1996	0·4 (0·2–0·9)	25·9 (24·8–26·9)	119·9 (111·1–130·1)	108·5 (102·5–114·9)	50·6 (47·7–53·9)	21·9 (20·8–23·1)	5·5 (5·2–5·9)	0·8 (0·7–1·0)	0·0 (0·0–0·0)	1·67 (1·61–1·73)	0·73 (0·69–0·78)	0·39 (0·38–0·41)
1997	0·4 (0·2–0·8)	24·7 (23·6–25·9)	114·5 (106·2–123·3)	106·5 (100·8–112·6)	51·0 (48·1–54·0)	22·0 (21·0–23·2)	5·4 (5·1–5·8)	0·7 (0·6–0·9)	0·0 (0·0–0·0)	1·63 (1·57–1·69)	0·7 (0·65–0·74)	0·4 (0·38–0·41)
1998	0·4 (0·2–0·8)	23·7 (22·6–24·8)	109·7 (102·4–117·9)	104·1 (98·5–110·0)	51·6 (48·8–54·6)	22·0 (20·9–23·1)	5·5 (5·2–5·9)	0·7 (0·6–0·8)	0·0 (0·0–0·0)	1·59 (1·53–1·65)	0·67 (0·63–0·71)	0·4 (0·38–0·42)
1999	0·4 (0·2–0·8)	22·7 (21·7–23·8)	105·8 (99·1–113·6)	102·1 (96·7–107·8)	52·6 (49·8–55·9)	21·7 (20·7–22·8)	5·6 (5·3–6·0)	0·6 (0·6–0·8)	0·0 (0·0–0·0)	1·56 (1·5–1·62)	0·64 (0·61–0·68)	0·4 (0·39–0·42)
2000	0·4 (0·2–0·8)	21·4 (20·4–22·4)	102·6 (96·0–110·1)	100·0 (94·9–105·2)	53·3 (50·4–56·7)	21·4 (20·4–22·5)	5·7 (5·4–6·2)	0·6 (0·6–0·7)	0·0 (0·0–0·0)	1·53 (1·48–1·58)	0·62 (0·59–0·66)	0·41 (0·39–0·42)
2001	0·4 (0·2–0·8)	20·1 (19·2–21·0)	99·9 (93·3–107·0)	98·6 (93·5–104·1)	53·6 (50·4–57·0)	21·2 (20·2–22·5)	5·8 (5·5–6·2)	0·6 (0·5–0·7)	0·0 (0·0–0·0)	1·5 (1·45–1·56)	0·6 (0·57–0·64)	0·41 (0·39–0·43)
2002	0·4 (0·2–0·8)	19·2 (18·4–20·1)	99·2 (92·1–107·1)	99·0 (93·7–104·5)	54·5 (51·4–58·0)	21·3 (20·1–22·6)	5·9 (5·5–6·3)	0·6 (0·5–0·7)	0·0 (0·0–0·0)	1·5 (1·44–1·57)	0·59 (0·56–0·63)	0·41 (0·39–0·43)
2003	0·4 (0·2–0·8)	18·8 (17·9–19·6)	99·2 (91·4–107·2)	99·3 (94·2–104·7)	55·6 (52·5–58·9)	21·6 (20·5–23·0)	5·9 (5·5–6·3)	0·6 (0·5–0·7)	0·0 (0·0–0·0)	1·51 (1·45–1·57)	0·59 (0·55–0·63)	0·42 (0·4–0·44)
2004	0·4 (0·2–0·9)	18·4 (17·5–19·2)	97·2 (90·1–104·4)	98·1 (93·1–103·3)	56·2 (53·4–59·3)	22·3 (21·2–23·5)	5·9 (5·5–6·3)	0·7 (0·6–0·8)	0·0 (0·0–0·0)	1·5 (1·44–1·55)	0·58 (0·54–0·62)	0·43 (0·41–0·45)
2005	0·4 (0·2–0·9)	18·0 (17·1–18·9)	93·4 (86·7–101·6)	96·7 (91·7–101·8)	57·4 (54·4–60·6)	23·7 (22·4–25·3)	6·1 (5·6–6·6)	0·8 (0·6–0·9)	0·0 (0·0–0·0)	1·48 (1·43–1·54)	0·56 (0·52–0·6)	0·44 (0·42–0·46)
2006	0·4 (0·2–0·9)	18·0 (17·0–18·9)	89·7 (82·5–97·7)	96·1 (91·3–101·3)	59·3 (55·9–62·7)	25·8 (24·1–27·7)	6·4 (5·8–7·0)	0·9 (0·7–1·1)	0·0 (0·0–0·0)	1·48 (1·43–1·54)	0·54 (0·5–0·58)	0·46 (0·44–0·48)
2007	0·4 (0·2–0·9)	18·4 (17·4–19·4)	86·8 (79·6–94·4)	96·1 (91·4–101·2)	61·0 (57·8–64·5)	27·5 (25·6–29·6)	6·7 (6·0–7·5)	0·9 (0·7–1·2)	0·0 (0·0–0·0)	1·49 (1·43–1·54)	0·53 (0·49–0·57)	0·48 (0·46–0·5)
2008	0·5 (0·2–0·9)	18·8 (17·9–19·9)	84·8 (77·4–92·0)	95·9 (91·0–100·9)	62·2 (58·9–65·9)	28·2 (26·2–30·7)	6·9 (6·2–7·8)	0·9 (0·7–1·1)	0·0 (0·0–0·0)	1·49 (1·43–1·55)	0·52 (0·48–0·56)	0·49 (0·47–0·51)
2009	0·5 (0·2–1·0)	19·1 (18·2–20·2)	82·4 (75·1–91·2)	94·9 (90·2–99·8)	62·9 (59·1–66·6)	28·1 (26·2–30·3)	7·0 (6·3–7·8)	0·8 (0·6–1·0)	0·0 (0·0–0·0)	1·48 (1·42–1·54)	0·51 (0·47–0·55)	0·49 (0·47–0·52)
2010	0·5 (0·2–1·0)	19·3 (18·3–20·4)	79·4 (72·4–87·2)	93·7 (88·9–98·5)	63·2 (59·6–67·3)	27·6 (25·8–29·5)	6·9 (6·2–7·7)	0·7 (0·6–0·9)	0·0 (0·0–0·0)	1·46 (1·4–1·51)	0·5 (0·46–0·54)	0·49 (0·47–0·52)
2011	0·5 (0·2–1·0)	19·7 (18·6–20·8)	78·5 (72·0–85·3)	94·9 (89·8–99·9)	64·5 (61·0–68·0)	28·3 (26·6–30·3)	6·9 (6·3–7·7)	0·7 (0·6–0·9)	0·0 (0·0–0·0)	1·47 (1·41–1·53)	0·49 (0·46–0·53)	0·5 (0·48–0·53)
2012	0·5 (0·2–1·0)	20·3 (19·2–21·6)	79·8 (73·6–86·3)	98·8 (94·1–103·9)	67·1 (63·7–70·7)	30·1 (28·2–32·1)	7·1 (6·5–7·8)	0·7 (0·6–0·8)	0·0 (0·0–0·0)	1·52 (1·46–1·58)	0·5 (0·47–0·54)	0·53 (0·5–0·55)
2013	0·5 (0·2–1·0)	20·8 (19·8–22·0)	81·8 (78·1–85·9)	100·9 (98·2–103·7)	69·4 (66·9–71·9)	32·1 (30·4–33·8)	7·3 (6·8–7·8)	0·7 (0·6–0·8)	0·0 (0·0–0·0)	1·57 (1·51–1·62)	0·52 (0·49–0·54)	0·55 (0·53–0·57)
2014	0·4 (0·2–0·9)	20·3 (19·2–21·5)	79·6 (76·6–82·7)	96·6 (94·7–98·7)	68·4 (66·2–70·5)	32·5 (31·0–34·1)	7·5 (7·0–8·0)	0·6 (0·6–0·7)	0·0 (0·0–0·0)	1·53 (1·49–1·57)	0·5 (0·48–0·52)	0·54 (0·53–0·56)
2015	0·4 (0·2–0·9)	20·0 (18·8–21·3)	84·9 (81·0–88·9)	102·8 (100·5–105·3)	70·8 (68·3–73·3)	33·3 (31·4–35·3)	7·9 (7·3–8·5)	0·7 (0·6–0·8)	0·0 (0·0–0·0)	1·6 (1·55–1·66)	0·53 (0·5–0·55)	0·56 (0·54–0·59)
2016	0·4 (0·2–0·9)	20·0 (18·6–21·6)	91·6 (87·4–96·2)	112·1 (109·5–114·9)	74·0 (71·0–77·0)	33·9 (31·5–36·4)	8·3 (7·6–9·0)	0·7 (0·6–0·8)	0·0 (0·0–0·0)	1·7 (1·64–1·77)	0·56 (0·53–0·59)	0·58 (0·55–0·62)
2017	0·4 (0·2–0·9)	19·8 (18·3–21·6)	84·2 (79·0–89·9)	107·0 (103·4–110·7)	69·4 (65·8–73·1)	32·1 (29·2–35·3)	8·0 (7·2–8·8)	0·6 (0·5–0·7)	0·0 (0·0–0·0)	1·61 (1·53–1·69)	0·52 (0·49–0·56)	0·55 (0·51–0·59)

Supplemental Results Table 5: Age-specific fertility rates per 1000, cumulative total fertility up to maternal age 25 (TFU25), cumulative total fertility from maternal age 30 to age 55 (TFO30), and cumulative total fertility, High SDI, 1950-2017

Year	10-14	15-19	20-24	25-29	30-34	35-39	40-44	45-49	50-54	TFR	TFU25	TFO30
1950	0·3 (0·1–0·7)	36·5 (35·8–37·3)	158·9 (155·9–162·3)	174·5 (172·8–176·1)	117·7 (115·7–119·5)	68·1 (66·4–69·7)	22·9 (22·3–23·6)	2·0 (1·8–2·2)	0·0 (0·0–0·0)	2·9 (2·87–2·95)	0·98 (0·96–1·0)	1·05 (1·03–1·07)
1951	0·3 (0·1–0·7)	36·3 (35·8–36·9)	159·0 (156·6–161·3)	173·4 (172·1–174·6)	117·9 (116·4–119·5)	65·7 (64·1–67·1)	22·1 (21·6–22·7)	1·9 (1·7–2·1)	0·0 (0·0–0·0)	2·88 (2·85–2·91)	0·98 (0·96–0·99)	1·04 (1·02–1·06)
1952	0·3 (0·1–0·7)	36·0 (35·5–36·5)	158·6 (156·2–161·1)	172·6 (171·3–173·9)	117·2 (115·7–118·7)	63·5 (62·2–64·9)	21·4 (20·9–22·0)	1·9 (1·7–2·1)	0·0 (0·0–0·0)	2·86 (2·83–2·88)	0·97 (0·96–0·99)	1·02 (1·01–1·03)
1953	0·3 (0·1–0·7)	36·2 (35·7–36·7)	159·4 (157·0–161·7)	172·1 (170·8–173·4)	116·2 (114·8–117·5)	61·9 (60·7–63·1)	20·9 (20·4–21·4)	1·8 (1·7–2·0)	0·0 (0·0–0·0)	2·84 (2·82–2·87)	0·98 (0·97–0·99)	1·0 (0·99–1·02)
1954	0·3 (0·2–0·7)	36·4 (35·8–36·9)	161·2 (158·9–163·5)	173·7 (172·4–175·0)	115·5 (114·0–117·0)	62·0 (60·7–63·2)	20·4 (19·9–20·8)	1·8 (1·6–2·0)	0·0 (0·0–0·0)	2·86 (2·83–2·88)	0·99 (0·98–1·0)	1·0 (0·99–1·01)
1955	0·4 (0·2–0·8)	37·1 (36·7–37·6)	163·6 (161·3–165·8)	175·7 (174·4–177·1)	113·2 (111·7–114·6)	62·0 (60·9–63·3)	19·5 (19·1–20·0)	1·7 (1·6–1·9)	0·0 (0·0–0·0)	2·87 (2·84–2·89)	1·01 (0·99–1·02)	0·98 (0·97–1·0)
1956	0·4 (0·2–0·8)	38·6 (38·0–39·1)	165·8 (163·4–168·2)	178·0 (176·7–179·4)	111·3 (109·9–112·7)	61·6 (60·4–62·7)	18·5 (18·1–19·0)	1·7 (1·5–1·9)	0·0 (0·0–0·0)	2·88 (2·86–2·9)	1·02 (1·01–1·04)	0·97 (0·95–0·98)
1957	0·4 (0·2–0·8)	39·3 (38·7–39·8)	167·2 (164·9–169·5)	180·2 (178·7–181·7)	109·9 (108·8–111·2)	60·1 (59·1–61·1)	17·7 (17·3–18·1)	1·6 (1·5–1·8)	0·0 (0·0–0·0)	2·88 (2·86–2·91)	1·03 (1·02–1·05)	0·95 (0·94–0·96)
1958	0·4 (0·2–0·8)	39·8 (39·3–40·3)	169·6 (167·3–172·0)	181·8 (180·3–183·3)	108·8 (107·6–110·2)	58·4 (57·4–59·4)	17·3 (16·9–17·8)	1·6 (1·4–1·7)	0·0 (0·0–0·0)	2·89 (2·86–2·91)	1·05 (1·04–1·06)	0·93 (0·92–0·94)
1959	0·4 (0·2–0·8)	40·4 (39·9–40·9)	171·3 (169·1–173·7)	183·1 (181·7–184·5)	107·9 (106·6–109·2)	56·5 (55·6–57·5)	17·3 (16·9–17·8)	1·5 (1·3–1·7)	0·0 (0·0–0·0)	2·89 (2·87–2·92)	1·06 (1·05–1·07)	0·92 (0·91–0·93)
1960	0·4 (0·2–0·8)	41·1 (40·6–41·6)	171·4 (169·4–173·7)	184·1 (182·6–185·5)	107·7 (106·3–109·0)	54·6 (53·7–55·5)	17·3 (16·9–17·8)	1·5 (1·3–1·7)	0·0 (0·0–0·0)	2·89 (2·87–2·91)	1·06 (1·05–1·08)	0·91 (0·89–0·92)
1961	0·4 (0·2–0·8)	40·8 (40·4–41·3)	171·7 (169·8–173·8)	184·1 (182·5–185·8)	107·1 (105·8–108·5)	52·8 (51·9–53·9)	17·2 (16·7–17·7)	1·5 (1·3–1·7)	0·0 (0·0–0·0)	2·88 (2·86–2·9)	1·06 (1·05–1·08)	0·89 (0·88–0·91)
1962	0·4 (0·2–0·8)	39·4 (38·9–39·9)	171·3 (169·2–173·6)	184·8 (183·1–186·4)	107·0 (105·8–108·3)	51·7 (50·9–52·6)	16·8 (16·4–17·3)	1·5 (1·3–1·7)	0·0 (0·0–0·0)	2·86 (2·84–2·89)	1·06 (1·04–1·07)	0·89 (0·88–0·9)
1963	0·4 (0·2–0·8)	38·4 (37·9–38·8)	169·5 (167·4–171·7)	185·6 (184·2–187·0)	108·1 (106·7–109·5)	51·8 (51·0–52·7)	16·4 (16·0–16·9)	1·4 (1·3–1·6)	0·0 (0·0–0·0)	2·86 (2·84–2·88)	1·04 (1·03–1·05)	0·89 (0·88–0·9)
1964	0·4 (0·2–0·8)	38·3 (37·8–38·7)	165·4 (163·4–167·6)	184·1 (182·8–185·5)	107·2 (105·9–108·7)	50·9 (50·2–51·8)	15·6 (15·2–16·1)	1·4 (1·2–1·5)	0·0 (0·0–0·0)	2·82 (2·8–2·84)	1·02 (1·01–1·03)	0·88 (0·87–0·89)
1965	0·4 (0·2–0·8)	38·7 (38·3–39·2)	159·0 (157·3–160·9)	173·2 (171·7–174·7)	100·8 (99·5–102·0)	48·2 (47·4–49·0)	14·6 (14·1–15·0)	1·3 (1·1–1·4)	0·0 (0·0–0·0)	2·68 (2·66–2·7)	0·99 (0·98–1·0)	0·82 (0·81–0·83)
1966	0·4 (0·2–0·8)	38·6 (38·2–39·0)	154·6 (152·7–156·6)	169·7 (168·3–171·0)	97·3 (96·1–98·6)	46·1 (45·3–46·9)	13·5 (13·1–14·0)	1·2 (1·0–1·3)	0·0 (0·0–0·0)	2·61 (2·59–2·63)	0·97 (0·96–0·98)	0·79 (0·78–0·8)
1967	0·4 (0·2–0·8)	38·3 (37·9–38·7)	150·5 (148·6–152·5)	172·4 (171·0–173·9)	96·5 (95·0–97·9)	44·2 (43·4–45·0)	12·7 (12·3–13·1)	1·1 (1·0–1·3)	0·0 (0·0–0·0)	2·58 (2·56–2·6)	0·95 (0·94–0·96)	0·77 (0·76–0·78)
1968	0·4 (0·2–0·8)	38·6 (38·2–38·9)	147·0 (145·2–148·9)	167·4 (166·0–168·9)	92·6 (91·3–94·0)	42·2 (41·4–42·9)	12·1 (11·7–12·5)	1·0 (0·9–1·2)	0·0 (0·0–0·0)	2·51 (2·49–2·52)	0·93 (0·92–0·94)	0·74 (0·73–0·75)
1969	0·4 (0·2–0·8)	39·6 (39·2–40·0)	145·6 (143·7–147·5)	165·9 (164·4–167·3)	90·2 (88·8–91·6)	40·5 (39·8–41·2)	11·5 (11·2–11·9)	1·0 (0·8–1·1)	0·0 (0·0–0·0)	2·47 (2·45–2·49)	0·93 (0·92–0·94)	0·72 (0·71–0·73)
1970	0·4 (0·2–0·8)	39·8 (39·4–40·1)	143·1 (141·3–145·1)	164·0 (162·5–165·4)	87·6 (86·3–88·8)	38·7 (38·0–39·5)	10·9 (10·6–11·2)	0·9 (0·8–1·0)	0·0 (0·0–0·0)	2·43 (2·41–2·44)	0·92 (0·91–0·93)	0·69 (0·68–0·7)
1971	0·4 (0·2–0·8)	38·9 (38·5–39·3)	137·8 (135·7–139·7)	157·8 (156·4–159·1)	83·6 (82·3–84·9)	36·1 (35·5–36·8)	10·1 (9·8–10·4)	0·8 (0·7–0·9)	0·0 (0·0–0·0)	2·33 (2·31–2·35)	0·89 (0·87–0·9)	0·65 (0·64–0·66)
1972	0·4 (0·2–0·8)	38·0 (37·7–38·4)	132·0 (130·1–134·0)	150·8 (149·5–152·1)	79·1 (77·8–80·4)	33·1 (32·5–33·8)	9·3 (9·0–9·6)	0·7 (0·6–0·8)	0·0 (0·0–0·0)	2·22 (2·2–2·23)	0·85 (0·84–0·86)	0·61 (0·6–0·62)
1973	0·4 (0·2–0·7)	37·0 (36·7–37·3)	128·5 (126·7–130·3)	147·2 (145·9–148·7)	75·2 (74·0–76·5)	30·3 (29·8–30·9)	8·5 (8·2–8·8)	0·7 (0·6–0·8)	0·0 (0·0–0·0)	2·14 (2·12–2·15)	0·83 (0·82–0·84)	0·57 (0·57–0·58)
1974	0·3 (0·2–0·7)	36·0 (35·7–36·4)	124·5 (122·9–126·2)	143·2 (141·7–144·7)	71·1 (70·0–72·3)	28·0 (27·4–28·6)	7·8 (7·5–8·0)	0·6 (0·5–0·7)	0·0 (0·0–0·0)	2·06 (2·04–2·07)	0·8 (0·8–0·81)	0·54 (0·53–0·55)
1975	0·3 (0·1–0·7)	34·7 (34·4–35·0)	121·2 (119·7–122·7)	138·8 (137·6–140·1)	68·3 (67·2–69·3)	26·1 (25·6–26·6)	7·2 (6·9–7·4)	0·6 (0·5–0·7)	0·0 (0·0–0·0)	1·99 (1·97–2·0)	0·78 (0·77–0·79)	0·51 (0·5–0·52)
1976	0·3 (0·1–0·7)	33·2 (32·9–33·5)	120·6 (119·2–122·1)	137·2 (135·9–138·5)	67·7 (66·5–68·8)	24·5 (24·0–25·0)	6·6 (6·3–6·9)	0·6 (0·5–0·6)	0·0 (0·0–0·0)	1·95 (1·94–1·97)	0·77 (0·76–0·78)	0·5 (0·49–0·5)
1977	0·3 (0·1–0·7)	32·2 (31·9–32·6)	118·4 (117·2–119·6)	136·7 (135·5–138·0)	68·0 (67·0–69·1)	23·4 (22·9–24·0)	6·0 (5·7–6·3)	0·5 (0·4–0·6)	0·0 (0·0–0·0)	1·93 (1·91–1·94)	0·75 (0·75–0·76)	0·49 (0·48–0·5)
1978	0·3 (0·1–0·6)	31·7 (31·3–32·1)	116·3 (115·2–117·5)	136·6 (135·4–137·7)	68·8 (67·7–70·0)	22·8 (22·3–23·4)	5·7 (5·4–6·0)	0·5 (0·4–0·6)	0·0 (0·0–0·0)	1·91 (1·9–1·93)	0·74 (0·74–0·75)	0·49 (0·48–0·5)
1979	0·3 (0·1–0·6)	31·4 (31·0–31·7)	117·1 (116·0–118·1)	137·0 (135·9–138·1)	69·3 (68·2–70·4)	22·6 (22·1–23·1)	5·5 (5·2–5·8)	0·5 (0·4–0·5)	0·0 (0·0–0·0)	1·92 (1·9–1·93)	0·74 (0·74–0·75)	0·49 (0·48–0·5)
1980	0·3 (0·1–0·6)	30·5 (30·1–30·8)	115·6 (114·6–116·7)	136·6 (135·5–137·6)	68·8 (67·7–69·9)	22·7 (22·2–23·3)	5·3 (5·0–5·5)	0·4 (0·4–0·5)	0·0 (0·0–0·0)	1·9 (1·89–1·91)	0·73 (0·73–0·74)	0·49 (0·48–0·49)
1981	0·3 (0·1–0·6)	29·6 (29·2–29·9)	112·9 (111·9–113·9)	135·2 (134·2–136·2)	68·3 (67·3–69·4)	23·2 (22·7–23·7)	5·0 (4·7–5·4)	0·4 (0·3–0·5)	0·0 (0·0–0·0)	1·87 (1·86–1·89)	0·71 (0·71–0·72)	0·48 (0·48–0·49)
1982	0·3 (0·1–0·6)	28·9 (28·5–29·2)	109·7 (108·7–110·7)	132·8 (131·8–133·8)	68·6 (67·5–69·7)	23·5 (22·9–24·1)	4·9 (4·6–5·2)	0·4 (0·3–0·5)	0·0 (0·0–0·0)	1·84 (1·83–1·86)	0·69 (0·69–0·7)	0·49 (0·48–0·49)
1983	0·3 (0·1–0·6)	28·0 (27·6–28·4)	105·3 (104·2–106·4)	130·2 (129·0–131·2)	69·2 (68·2–70·4)	23·7 (23·2–24·3)	4·8 (4·5–5·1)	0·4 (0·3–0·5)	0·0 (0·0–0·0)	1·81 (1·8–1·82)	0·67 (0·66–0·67)	0·49 (0·48–0·5)
1984	0·3 (0·1–0·6)	27·4 (27·0–27·8)	102·4 (101·4–103·4)	129·0 (128·1–130·0)	70·5 (69·4–71·5)	24·0 (23·5–24·6)	4·8 (4·5–5·1)	0·4 (0·3–0·5)	0·0 (0·0–0·0)	1·79 (1·78–1·8)	0·65 (0·64–0·66)	0·5 (0·49–0·51)
1985	0·3 (0·1–0·6)	26·9 (26·5–27·3)	99·6 (98·7–100·6)	127·9 (127·0–128·7)	71·6 (70·6–72·5)	24·1 (23·6–24·7)	4·9 (4·6–5·2)	0·4 (0·3–0·4)	0·0 (0·0–0·0)	1·78 (1·77–1·79)	0·63 (0·63–0·64)	0·5 (0·5–0·51)
1986	0·3 (0·1–0·6)	26·3 (25·9–26·8)	96·4 (95·5–97·3)	126·8 (125·9–127·6)	72·9 (71·9–73·9)	24·5 (24·0–25·1)	5·0 (4·8–5·3)	0·4 (0·3–0·4)	0·0 (0·0–0·0)	1·76 (1·75–1·77)	0·62 (0·61–0·62)	0·51 (0·51–0·52)
1987	0·3 (0·1–0·6)	26·3 (25·9–26·7)	93·9 (93·0–94·8)	127·0 (126·1–127·8)	74·8 (73·9–75·8)	25·6 (25·0–26·1)	5·2 (4·9–5·5)	0·4 (0·3–0·4)	0·0 (0·0–0·0)	1·77 (1·75–1·78)	0·6 (0·6–0·61)	0·53 (0·52–0·54)
1988	0·3 (0·1–0·6)	26·9 (26·5–27·4)	92·1 (91·2–93·0)	126·3 (125·5–127·2)	76·2 (75·4–77·1)	26·7 (26·2–27·2)	5·3 (5·1–5·6)	0·4 (0·3–0·4)	0·0 (0·0–0·0)	1·77 (1·76–1·78)	0·6 (0·59–0·6)	0·54 (0·54–0·55)
1989	0·3 (0·1–0·7)	27·7 (27·3–28·1)	91·3 (90·4–92·3)	126·1 (125·2–127·0)	77·9 (76·9–78·7)	27·8 (27·3–28·4)	5·4 (5·1–5·7)	0·4 (0·3–0·5)	0·0 (0·0–0·0)	1·78 (1·77–1·8)	0·6 (0·59–0·6)	0·56 (0·55–0·56)
1990	0·3 (0·1–0·7)	28·0 (27·6–28·5)	89·7 (88·8–90·6)	125·4 (124·6–126·2)	79·2 (78·3–79·9)	28·8 (28·3–29·3)	5·4 (5·2–5·7)	0·4 (0·3–0·5)	0·0 (0·0–0·0)	1·79 (1·77–1·8)	0·59 (0·58–0·6)	0·57 (0·56–0·57)
1991	0·3 (0·1–0·7)	27·6 (27·2–28·0)	86·2 (85·3–87·1)	122·9 (122·1–123·7)	79·6 (78·7–80·4)	29·4 (28·8–30·0)	5·5 (5·2–5·8)	0·4 (0·3–0·5)	0·0 (0·0–0·0)	1·76 (1·75–1·77)	0·57 (0·56–0·58)	0·57 (0·57–0·58)
1992	0·3 (0·1–0·7)	26·8 (26·4–27·3)	82·9 (81·9–84·0)	120·2 (119·3–121·0)	80·2 (79·3–81·1)	29·8 (29·3–30·3)	5·6 (5·4–5·9)	0·4 (0·3–0·5)	0·0 (0·0–0·0)	1·73 (1·72–1·74)	0·55 (0·54–0·56)	0·58 (0·57–0·59)
1993	0·3 (0·1–0·7)	26·3 (25·8–26·7)	79·8 (78·8–80·8)	117·7 (116·8–118·7)	81·0 (80·1–82·0)	30·3 (29·8–30·9)	5·8 (5·6–6·1)	0·4 (0·3–0·5)	0·0 (0·0–0·0)	1·71 (1·7–1·72)	0·53 (0·53–0·54)	0·59 (0·58–0·59)
1994	0·3 (0·1–0·7)	25·7 (25·3–26·2)	77·3 (76·3–78·5)	115·5 (114·5–116·4)	81·9 (81·0–82·9)	30·8 (30·3–31·4)	6·0 (5·7–6·3)	0·4 (0·3–0·5)	0·0 (0·0–0·0)	1·69 (1·68–1·7)	0·52 (0·51–0·52)	0·6 (0·59–0·6)
1995	0·3 (0·1–0·7)	24·9 (24·5–25·3)	74·7 (73·6–75·7)	113·1 (112·2–114·0)	83·2 (82·2–84·1)	31·5 (30·9–32·1)	6·1 (5·9–6·4)	0·4 (0·3–0·5)	0·0 (0·0–0·0)	1·67 (1·66–1·68)	0·5 (0·49–0·51)	0·61 (0·6–0·61)
1996	0·3 (0·1–0·7)	24·1 (23·7–24·6)	72·3 (71·2–73·5)	111·0 (110·0–112·0)	84·7 (83·8–85·7)	32·6 (32·1–33·2)	6·4 (6·1–6·7)	0·4 (0·3–0·5)	0·0 (0·0–0·0)	1·66 (1·65–1·67)	0·48 (0·48–0·49)	0·62 (0·61–0·63)
1997	0·3 (0·1–0·7)	23·7 (23·3–24·1)	71·5 (70·4–72·7)	109·4 (108·3–110·5)	85·5 (84·5–86·4)	33·4 (32·8–34·1)	6·5 (6·2–6·8)	0·4 (0·4–0·5)	0·0 (0·0–0·0)	1·65 (1·64–1·67)	0·48 (0·47–0·48)	0·63 (0·62–0·64)
1998	0·3 (0·1–0·7)	23·4 (23·0–23·8)	70·9 (69·8–72·1)	108·0 (107·1–108·9)	86·1 (85·2–87·1)	34·1 (33·5–34·8)	6·6 (6·3–6·9)	0·4 (0·4–0·5)	0·0 (0·0–0·0)	1·65 (1·64–1·66)	0·47 (0·47–0·48)	0·64 (0·63–0·64)
1999	0·3 (0·1–0·7)	23·0 (22·6–23·4)	70·6 (69·6–71·7)	107·2 (106·1–108·2)	88·1 (87·2–89·1)	35·4 (34·8–36·0)	6·8 (6·5–7·1)	0·4 (0·4–0·5)	0·0 (0·0–0·0)	1·66 (1·65–1·67)	0·47 (0·46–0·48)	0·65 (0·65–0·66)
2000	0·3 (0·1–0·7)	22·4 (22·0–22·7)	69·7 (68·6–70·8)	105·3 (104·4–106·2)	89·1 (88·1–90·2)	36·8 (36·2–37·5)	7·1 (6·8–7·4)	0·5 (0·4–0·5)	0·0 (0·0–0·0)	1·66 (1·64–1·67)	0·46 (0·46–0·47)	0·67 (0·66–0·68)
2001	0·3 (0·1–0·7)	21·4 (21·1–21·7)	67·6 (66·6–68·6)	102·2 (101·3–103·0)	88·6 (87·6–89·7)	37·7 (37·1–38·4)	7·3 (7·0–7·6)	0·5 (0·4–0·6)	0·0 (0·0–0·0)	1·63 (1·62–1·64)	0·45 (0·44–0·45)	0·67 (0·66–0·68)
2002	0·3 (0·1–0·7)	20·6 (20·3–21·0)	66·2 (65·2–67·1)	100·9 (99·9–101·9)	89·5 (88·5–90·6)	39·0 (38·3–39·7)	7·6 (7·3–7·9)	0·5 (0·4–0·6)	0·0 (0·0–0·0)	1·62 (1·61–1·64)	0·44 (0·43–0·44)	0·68 (0·68–0·69)
2003	0·3 (0·1–0·7)	20·2 (19·9–20·6)	65·2 (64·2–66·3)	100·3 (99·5–101·2)	91·0 (89·9–92·1)	40·6 (39·9–41·3)	7·8 (7·5–8·1)	0·5 (0·4–0·6)	0·0 (0·0–0·0)	1·63 (1·62–1·64)	0·43 (0·42–0·44)	0·7 (0·69–0·71)
2004	0·3 (0·1–0·7)	19·9 (19·6–20·3)	64·4 (63·5–65·4)	99·3 (98·4–100·1)	91·9 (90·9–93·0)	41·9 (41·1–42·7)	8·1 (7·8–8·4)	0·5 (0·4–0·6)	0·0 (0·0–0·0)	1·63 (1·62–1·65)	0·42 (0·42–0·43)	0·71 (0·7–0·72)
2005	0·3 (0·1–0·7)	20·0 (19·7–20·4)	64·6 (63·5–65·8)	99·2 (98·3–100·1)	93·3 (92·2–94·3)	43·4 (42·6–44·2)	8·4 (8·1–8·7)	0·5 (0·5–0·6)	0·0 (0·0–0·0)	1·65 (1·64–1·66)	0·42 (0·42–0·43)	0·73 (0·72–0·74)
2006	0·3 (0·1–0·7)	20·4 (20·0–20·7)	65·3 (64·3–66·3)	100·0 (99·1–100·8)	95·7 (94·6–96·8)	45·1 (44·4–46·0)	8·8 (8·5–9·1)	0·5 (0·5–0·6)	0·0 (0·0–0·0)	1·68 (1·67–1·69)	0·43 (0·42–0·44)	0·75 (0·74–0·76)
2007	0·3 (0·1–0·7)	20·3 (20·0–20·7)	64·9 (63·7–66·1)	99·9 (99·1–100·8)	97·2 (96·1–98·3)	46·3 (45·5–47·2)	9·1 (8·8–9·4)	0·5 (0·5–0·6)	0·0 (0·0–0·0)	1·69 (1·68–1·71)	0·43 (0·42–0·43)	0·77 (0·76–0·77)
2008	0·3 (0·1–0·6)	19·7 (19·4–20·0)	63·2 (62·1–64·3)	98·6 (97·8–99·3)	97·5 (96·4–98·5)	47·1 (46·2–48·0)	9·4 (9·1–9·7)	0·5 (0·5–0·6)	0·0 (0·0–0·0)	1·68 (1·67–1·7)	0·42 (0·41–0·42)	0·77 (0·76–0·78)
2009	0·3 (0·1–0·6)	18·4 (18·1–18·8)	60·4 (59·3–61·6)	96·7 (95·6–97·7)	97·3 (96·3–98·4)	47·8 (46·9–48·7)	9·8 (9·5–10·2)	0·6 (0·5–0·6)	0·0 (0·0–0·0)	1·66 (1·64–1·67)	0·4 (0·39–0·4)	0·78 (0·77–0·79)
2010	0·3 (0·1–0·6)	17·0 (16·7–17·4)	57·7 (56·8–58·6)	95·2 (94·5–96·0)	97·4 (96·4–98·5)	48·8 (47·9–49·8)	10·2 (9·9–10·5)	0·6 (0·5–0·7)	0·0 (0·0–0·0)	1·64 (1·62–1·65)	0·38 (0·37–0·38)	0·78 (0·78–0·79)
2011	0·3 (0·1–0·6)	15·9 (15·5–16·2)	55·9 (54·9–57·1)	94·2 (93·4–95·0)	97·7 (96·7–98·8)	49·8 (48·9–50·8)	10·5 (10·1–10·8)	0·6 (0·5–0·7)	0·0 (0·0–0·0)	1·62 (1·61–1·64)	0·36 (0·35–0·37)	0·79 (0·78–0·8)
2012	0·3 (0·1–0·6)	14·8 (14·4–15·2)	54·3 (53·4–55·1)	93·1 (92·1–94·1)	97·6 (96·6–98·6)	50·7 (49·7–51·7)	10·8 (10·4–11·1)	0·6 (0·5–0·7)	0·0 (0·0–0·0)	1·61 (1·6–1·63)	0·35 (0·34–0·35)	0·8 (0·79–0·81)
2013	0·3 (0·1–0·5)	13·7 (13·3–14·2)	52·8 (51·7–54·0)	92·3 (91·6–93·1)	98·1 (97·1–99·1)	51·7 (50·6–52·7)	11·1 (10·7–11·4)	0·6 (0·6–0·7)	0·0 (0·0–0·0)	1·6 (1·59–1·62)	0·33 (0·33–0·34)	0·81 (0·8–0·82)
2014	0·3 (0·1–0·5)	12·9 (12·5–13·4)	51·6 (50·7–52·5)	91·6 (90·9–92·4)	99·1 (98·2–100·1)	52·9 (51·9–53·9)	11·3 (10·9–11·7)	0·7 (0·6–0·7)	0·0 (0·0–0·0)	1·6 (1·59–1·62)	0·32 (0·32–0·33)	0·82 (0·81–0·83)
2015	0·2 (0·1–0·5)	12·3 (11·8–12·8)	50·4 (48·8–52·0)	91·1 (89·6–92·5)	99·6 (97·8–101·3)	53·0 (51·4–54·8)	11·4 (10·9–11·9)	0·7 (0·6–0·7)	0·0 (0·0–0·0)	1·59 (1·56–1·63)	0·31 (0·3–0·33)	0·82 (0·8–0·84)
2016	0·2 (0·1–0·5)	12·2 (11·4–13·1)	49·7 (46·7–53·1)	90·5 (87·4–93·7)	99·5 (95·2–104·0)	52·4 (48·8–56·4)	11·2 (10·2–12·3)	0·6 (0·6–0·7)	0·0 (0·0–0·0)	1·58 (1·5–1·67)	0·31 (0·29–0·33)	0·82 (0·78–0·87)
2017	0·2 (0·1–0·5)	12·5 (11·3–14·0)	49·6 (44·4–55·7)	89·5 (84·3–95·2)	98·6 (91·2–106·8)	51·8 (45·2–59·4)	11·1 (9·4–13·2)	0·6 (0·5–0·7)	0·0 (0·0–0·0)	1·57 (1·43–1·72)	0·31 (0·28–0·35)	0·81 (0·73–0·9)

Supplemental Results Table 5: Age-specific fertility rates per 1000, cumulative total fertility up to maternal age 25 (TFU25), cumulative total fertility from maternal age 30 to age 55 (TFO30), and cumulative total fertility, Central Europe, Eastern Europe, and Central Asia, 1950-2017

Year	10-14	15-19	20-24	25-29	30-34	35-39	40-44	45-49	50-54	TFR	TFU25	TFO30
1950	0·1 (0·0–0·2)	35·2 (32·9–37·9)	169·2 (165·8–173·1)	165·4 (163·2–168·0)	104·4 (102·5–106·4)	75·6 (74·0–77·3)	34·9 (34·1–35·7)	5·5 (5·2–5·9)	0·1 (0·1–0·1)	2·95 (2·9–3·01)	1·02 (0·99–1·06)	1·1 (1·08–1·12)
1951	0·1 (0·0–0·2)	34·2 (32·0–36·6)	168·1 (164·8–171·5)	167·7 (165·7–170·0)	105·7 (103·7–107·7)	71·4 (69·7–73·0)	34·5 (33·8–35·3)	5·2 (4·8–5·6)	0·1 (0·1–0·1)	2·94 (2·88–2·99)	1·01 (0·99–1·04)	1·08 (1·07–1·1)
1952	0·1 (0·0–0·2)	33·2 (31·1–35·6)	161·8 (158·9–165·0)	167·4 (165·6–169·3)	106·3 (104·5–108·2)	67·5 (65·9–69·1)	33·8 (33·1–34·5)	4·9 (4·5–5·2)	0·1 (0·1–0·1)	2·88 (2·83–2·93)	0·98 (0·95–1·0)	1·06 (1·04–1·08)
1953	0·1 (0·0–0·2)	33·2 (31·2–35·3)	161·8 (159·1–164·4)	170·1 (168·5–171·9)	109·1 (107·5–110·7)	66·3 (64·9–67·7)	32·8 (32·2–33·5)	4·6 (4·3–5·0)	0·1 (0·1–0·1)	2·89 (2·85–2·93)	0·98 (0·95–1·0)	1·06 (1·05–1·08)
1954	0·1 (0·1–0·2)	33·1 (31·5–35·0)	165·3 (162·7–167·8)	173·3 (171·7–174·8)	112·3 (110·9–113·7)	65·3 (64·1–66·5)	31·2 (30·5–31·8)	4·4 (4·1–4·7)	0·1 (0·1–0·1)	2·93 (2·89–2·96)	0·99 (0·97–1·01)	1·07 (1·05–1·08)
1955	0·1 (0·1–0·3)	33·2 (31·8–34·8)	164·5 (161·8–167·0)	168·7 (167·2–170·4)	110·9 (109·5–112·3)	63·6 (62·4–64·8)	28·0 (27·4–28·6)	4·1 (3·8–4·4)	0·1 (0·1–0·1)	2·87 (2·83–2·9)	0·99 (0·97–1·01)	1·03 (1·02–1·05)
1956	0·1 (0·1–0·3)	34·3 (33·0–35·7)	167·5 (164·9–170·2)	165·3 (163·8–166·9)	108·9 (107·6–110·3)	61·9 (60·7–63·0)	24·7 (24·1–25·3)	3·8 (3·5–4·1)	0·1 (0·1–0·1)	2·83 (2·8–2·87)	1·01 (0·99–1·03)	1·0 (0·98–1·01)
1957	0·1 (0·1–0·3)	35·9 (34·9–36·9)	172·6 (170·1–175·0)	160·8 (159·3–162·2)	107·4 (105·9–108·9)	58·9 (57·8–60·1)	22·7 (22·2–23·4)	3·5 (3·2–3·8)	0·1 (0·1–0·1)	2·81 (2·77–2·84)	1·04 (1·03–1·06)	0·96 (0·95–0·98)
1958	0·1 (0·1–0·3)	37·6 (36·8–38·5)	176·5 (174·0–178·7)	154·4 (152·8–155·7)	105·1 (103·6–106·6)	56·5 (55·5–57·6)	21·7 (21·1–22·3)	3·2 (2·9–3·5)	0·1 (0·1–0·1)	2·78 (2·74–2·81)	1·07 (1·05–1·09)	0·93 (0·92–0·95)
1959	0·1 (0·1–0·3)	39·5 (38·6–40·5)	177·5 (175·1–179·9)	150·6 (149·1–152·0)	103·2 (101·7–104·7)	55·4 (54·3–56·5)	21·0 (20·4–21·5)	2·9 (2·6–3·2)	0·1 (0·1–0·1)	2·75 (2·72–2·78)	1·09 (1·07–1·1)	0·91 (0·9–0·93)
1960	0·1 (0·1–0·3)	40·9 (39·9–41·8)	179·0 (176·6–181·4)	147·0 (145·5–148·5)	99·5 (97·9–101·0)	53·2 (52·1–54·3)	20·4 (19·8–20·9)	2·6 (2·3–2·9)	0·1 (0·0–0·1)	2·71 (2·68–2·75)	1·1 (1·08–1·12)	0·88 (0·86–0·89)
1961	0·1 (0·1–0·3)	39·6 (38·7–40·5)	176·5 (174·0–178·9)	143·7 (142·2–145·2)	94·9 (93·3–96·5)	50·7 (49·7–51·9)	19·5 (19·0–20·1)	2·4 (2·1–2·7)	0·0 (0·0–0·0)	2·64 (2·6–2·67)	1·08 (1·06–1·1)	0·84 (0·82–0·85)
1962	0·1 (0·1–0·2)	37·2 (36·3–38·1)	170·7 (168·3–173·2)	143·2 (141·6–144·7)	90·7 (89·2–92·3)	49·5 (48·4–50·5)	18·6 (18·0–19·1)	2·3 (2·0–2·6)	0·0 (0·0–0·0)	2·56 (2·53–2·6)	1·04 (1·02–1·06)	0·81 (0·79–0·82)
1963	0·1 (0·1–0·2)	35·5 (34·6–36·4)	166·5 (163·9–169·1)	142·0 (140·5–143·6)	86·5 (84·9–88·2)	47·5 (46·4–48·7)	17·7 (17·1–18·3)	2·2 (1·9–2·5)	0·0 (0·0–0·0)	2·49 (2·45–2·53)	1·01 (0·99–1·03)	0·77 (0·75–0·79)
1964	0·1 (0·1–0·2)	34·4 (33·5–35·3)	160·4 (157·4–163·3)	139·1 (137·6–140·7)	83·7 (82·0–85·5)	45·6 (44·3–47·0)	16·9 (16·2–17·5)	2·1 (1·8–2·4)	0·0 (0·0–0·0)	2·41 (2·37–2·45)	0·97 (0·96–0·99)	0·74 (0·72–0·76)
1965	0·1 (0·1–0·2)	34·7 (33·9–35·5)	158·2 (155·0–161·6)	137·7 (136·2–139·3)	83·4 (81·7–85·3)	44·5 (43·0–46·0)	16·1 (15·4–16·8)	2·0 (1·7–2·3)	0·0 (0·0–0·0)	2·38 (2·34–2·43)	0·97 (0·95–0·99)	0·73 (0·71–0·75)
1966	0·1 (0·1–0·2)	36·0 (35·2–36·9)	163·2 (159·8–166·6)	137·3 (135·6–138·9)	84·9 (83·1–86·9)	43·9 (42·3–45·6)	15·6 (14·8–16·4)	1·9 (1·6–2·2)	0·0 (0·0–0·0)	2·42 (2·37–2·46)	1·0 (0·98–1·02)	0·73 (0·71–0·75)
1967	0·1 (0·1–0·3)	37·3 (36·5–38·1)	169·3 (166·1–172·6)	134·6 (132·9–136·1)	86·2 (84·2–88·4)	43·1 (41·4–44·9)	15·0 (14·2–15·9)	1·8 (1·5–2·1)	0·0 (0·0–0·0)	2·44 (2·39–2·48)	1·03 (1·01–1·05)	0·73 (0·71–0·75)
1968	0·1 (0·1–0·2)	38·1 (37·3–39·0)	171·9 (168·8–174·9)	131·0 (129·4–132·6)	85·6 (83·5–87·7)	41·7 (40·0–43·6)	14·2 (13·3–15·1)	1·6 (1·4–1·9)	0·0 (0·0–0·0)	2·42 (2·38–2·47)	1·05 (1·03–1·07)	0·72 (0·69–0·74)
1969	0·1 (0·1–0·2)	38·9 (38·0–39·8)	174·6 (172·1–177·2)	128·7 (126·9–130·4)	83·7 (81·4–85·8)	40·5 (38·7–42·4)	13·6 (12·7–14·5)	1·5 (1·3–1·8)	0·0 (0·0–0·0)	2·41 (2·36–2·45)	1·07 (1·05–1·08)	0·7 (0·67–0·72)
1970	0·1 (0·1–0·2)	39·5 (38·6–40·4)	175·9 (173·7–178·1)	128·8 (127·1–130·5)	81·7 (79·5–83·9)	40·1 (38·2–42·1)	13·0 (12·1–13·9)	1·4 (1·2–1·7)	0·0 (0·0–0·0)	2·4 (2·36–2·45)	1·08 (1·06–1·09)	0·68 (0·66–0·71)
1971	0·1 (0·0–0·2)	39·7 (38·8–40·7)	176·1 (173·8–178·4)	131·4 (129·6–133·0)	79·7 (77·6–81·8)	39·9 (37·9–41·8)	12·5 (11·6–13·4)	1·3 (1·1–1·5)	0·0 (0·0–0·0)	2·4 (2·36–2·45)	1·08 (1·06–1·09)	0·67 (0·64–0·69)
1972	0·1 (0·0–0·2)	40·2 (39·3–41·2)	174·6 (172·2–177·1)	133·9 (132·3–135·5)	76·3 (74·3–78·2)	39·4 (37·6–41·2)	11·9 (11·0–12·8)	1·2 (1·0–1·4)	0·0 (0·0–0·0)	2·39 (2·35–2·43)	1·07 (1·06–1·09)	0·64 (0·62–0·67)
1973	0·1 (0·0–0·2)	41·4 (40·4–42·3)	175·4 (173·0–177·8)	136·5 (135·0–137·9)	73·5 (71·7–75·3)	38·5 (36·8–40·2)	11·6 (10·7–12·4)	1·1 (0·9–1·3)	0·0 (0·0–0·0)	2·39 (2·35–2·43)	1·08 (1·07–1·1)	0·62 (0·6–0·64)
1974	0·1 (0·0–0·2)	42·6 (41·7–43·5)	177·1 (174·7–179·4)	137·1 (135·7–138·6)	71·6 (69·9–73·3)	37·0 (35·5–38·6)	11·3 (10·5–12·1)	1·0 (0·9–1·3)	0·0 (0·0–0·0)	2·39 (2·35–2·43)	1·1 (1·08–1·11)	0·6 (0·58–0·62)
1975	0·1 (0·1–0·2)	43·5 (42·5–44·3)	177·5 (175·2–180·0)	134·6 (133·3–135·8)	70·6 (69·0–72·2)	34·7 (33·3–36·2)	11·0 (10·3–11·7)	1·0 (0·8–1·2)	0·0 (0·0–0·0)	2·36 (2·33–2·4)	1·11 (1·09–1·12)	0·59 (0·57–0·6)
1976	0·1 (0·1–0·2)	44·0 (43·1–45·0)	176·9 (174·5–179·4)	132·6 (131·2–133·9)	70·6 (69·1–72·1)	32·4 (31·0–33·7)	10·5 (9·9–11·2)	0·9 (0·7–1·1)	0·0 (0·0–0·0)	2·34 (2·31–2·38)	1·11 (1·09–1·12)	0·57 (0·56–0·59)
1977	0·1 (0·1–0·2)	44·9 (44·0–45·8)	175·9 (173·4–178·3)	130·6 (129·3–132·0)	70·5 (69·0–71·9)	30·0 (28·8–31·2)	10·0 (9·4–10·6)	0·9 (0·7–1·0)	0·0 (0·0–0·0)	2·31 (2·28–2·35)	1·1 (1·09–1·12)	0·56 (0·54–0·57)
1978	0·1 (0·1–0·3)	46·2 (45·3–47·1)	175·8 (173·4–177·9)	128·3 (127·0–129·8)	69·3 (68·0–70·6)	28·3 (27·3–29·4)	9·4 (8·9–9·9)	0·8 (0·7–1·0)	0·0 (0·0–0·0)	2·29 (2·26–2·32)	1·11 (1·1–1·12)	0·54 (0·53–0·55)
1979	0·1 (0·1–0·3)	47·1 (46·1–48·0)	175·8 (173·7–177·9)	126·9 (125·5–128·3)	67·3 (66·2–68·5)	27·1 (26·1–28·1)	8·7 (8·2–9·1)	0·8 (0·6–0·9)	0·0 (0·0–0·0)	2·27 (2·24–2·3)	1·12 (1·1–1·13)	0·52 (0·51–0·53)
1980	0·1 (0·1–0·3)	47·1 (46·2–48·1)	175·1 (173·1–177·0)	126·9 (125·5–128·3)	65·7 (64·6–66·7)	26·7 (25·7–27·7)	8·0 (7·6–8·4)	0·7 (0·6–0·9)	0·0 (0·0–0·0)	2·25 (2·23–2·28)	1·11 (1·1–1·12)	0·51 (0·49–0·52)
1981	0·1 (0·1–0·3)	47·1 (46·2–48·2)	175·4 (173·5–177·2)	127·6 (126·2–129·1)	66·2 (65·2–67·2)	27·4 (26·6–28·3)	7·5 (7·1–8·0)	0·7 (0·6–0·8)	0·0 (0·0–0·0)	2·26 (2·23–2·29)	1·11 (1·1–1·13)	0·51 (0·5–0·52)
1982	0·1 (0·1–0·3)	47·7 (46·7–48·6)	178·8 (176·9–180·6)	131·4 (130·0–132·8)	68·3 (67·3–69·4)	28·4 (27·6–29·2)	7·3 (6·9–7·7)	0·7 (0·6–0·8)	0·0 (0·0–0·0)	2·31 (2·29–2·34)	1·13 (1·12–1·14)	0·52 (0·51–0·53)
1983	0·1 (0·1–0·3)	48·4 (47·5–49·4)	180·4 (178·4–182·2)	133·4 (132·0–134·8)	69·3 (68·3–70·3)	28·5 (27·8–29·3)	7·2 (6·8–7·6)	0·6 (0·5–0·7)	0·0 (0·0–0·0)	2·34 (2·31–2·37)	1·14 (1·13–1·16)	0·53 (0·52–0·54)
1984	0·1 (0·1–0·3)	48·9 (48·0–49·8)	181·3 (179·6–183·3)	133·9 (132·6–135·3)	69·1 (68·1–70·1)	28·0 (27·3–28·8)	7·1 (6·8–7·5)	0·6 (0·5–0·7)	0·0 (0·0–0·0)	2·35 (2·32–2·37)	1·15 (1·14–1·17)	0·52 (0·52–0·53)
1985	0·1 (0·1–0·3)	49·0 (48·1–49·9)	181·9 (180·2–183·7)	135·8 (134·5–137·1)	70·5 (69·5–71·4)	28·1 (27·5–28·8)	7·2 (6·8–7·6)	0·5 (0·4–0·6)	0·0 (0·0–0·0)	2·37 (2·34–2·39)	1·16 (1·14–1·17)	0·53 (0·52–0·54)
1986	0·1 (0·1–0·3)	49·3 (48·3–50·3)	181·3 (179·4–183·2)	136·1 (134·7–137·5)	71·4 (70·5–72·4)	29·0 (28·3–29·7)	7·3 (6·9–7·7)	0·5 (0·4–0·6)	0·0 (0·0–0·0)	2·37 (2·35–2·4)	1·15 (1·14–1·17)	0·54 (0·53–0·55)
1987	0·1 (0·1–0·3)	49·4 (48·5–50·4)	180·4 (178·4–182·4)	133·6 (132·2–135·0)	69·7 (68·8–70·7)	28·4 (27·8–29·0)	7·1 (6·8–7·5)	0·5 (0·4–0·5)	0·0 (0·0–0·0)	2·35 (2·32–2·37)	1·15 (1·14–1·16)	0·53 (0·52–0·54)
1988	0·1 (0·1–0·3)	49·5 (48·6–50·4)	176·6 (174·6–178·6)	128·3 (126·9–129·7)	65·9 (65·1–66·8)	26·6 (25·9–27·2)	6·7 (6·4–7·0)	0·4 (0·4–0·5)	0·0 (0·0–0·0)	2·27 (2·25–2·3)	1·13 (1·12–1·14)	0·5 (0·49–0·51)
1989	0·1 (0·1–0·3)	50·2 (49·3–51·0)	172·8 (170·7–174·9)	123·2 (121·8–124·6)	62·0 (61·1–62·8)	24·7 (24·1–25·3)	6·1 (5·8–6·4)	0·4 (0·4–0·5)	0·0 (0·0–0·0)	2·2 (2·17–2·22)	1·12 (1·1–1·13)	0·47 (0·46–0·47)
1990	0·1 (0·1–0·2)	50·4 (49·5–51·2)	167·8 (165·7–169·9)	118·1 (116·7–119·5)	58·2 (57·3–59·1)	23·0 (22·4–23·5)	5·4 (5·2–5·7)	0·4 (0·3–0·4)	0·0 (0·0–0·0)	2·12 (2·09–2·14)	1·09 (1·08–1·1)	0·43 (0·43–0·44)
1991	0·1 (0·1–0·2)	49·1 (48·3–50·0)	159·7 (157·7–161·7)	111·5 (110·1–112·9)	54·0 (53·2–54·9)	21·1 (20·5–21·7)	5·0 (4·7–5·2)	0·3 (0·3–0·4)	0·0 (0·0–0·0)	2·0 (1·98–2·03)	1·04 (1·03–1·06)	0·4 (0·39–0·41)
1992	0·1 (0·0–0·2)	46·6 (45·7–47·5)	149·8 (148·0–151·8)	105·1 (103·7–106·5)	50·2 (49·4–51·1)	19·6 (19·1–20·2)	4·7 (4·4–4·9)	0·3 (0·2–0·3)	0·0 (0·0–0·0)	1·88 (1·86–1·91)	0·98 (0·97–1·0)	0·37 (0·37–0·38)
1993	0·1 (0·0–0·2)	44·4 (43·5–45·3)	142·7 (140·8–144·6)	101·4 (100·0–102·8)	48·3 (47·5–49·1)	18·6 (18·1–19·1)	4·4 (4·2–4·7)	0·2 (0·2–0·3)	0·0 (0·0–0·0)	1·8 (1·78–1·83)	0·94 (0·92–0·95)	0·36 (0·35–0·36)
1994	0·1 (0·0–0·2)	42·0 (41·1–43·0)	136·7 (134·7–138·6)	99·4 (98·0–100·8)	47·7 (46·9–48·5)	18·0 (17·6–18·5)	4·2 (4·0–4·5)	0·2 (0·2–0·3)	0·0 (0·0–0·0)	1·74 (1·72–1·77)	0·89 (0·88–0·91)	0·35 (0·34–0·36)
1995	0·1 (0·0–0·2)	39·1 (38·1–40·0)	128·1 (126·0–130·0)	96·1 (94·7–97·6)	47·4 (46·5–48·1)	17·6 (17·2–18·1)	4·1 (3·9–4·3)	0·2 (0·2–0·2)	0·0 (0·0–0·0)	1·66 (1·64–1·69)	0·84 (0·82–0·85)	0·35 (0·34–0·35)
1996	0·1 (0·0–0·2)	36·6 (35·6–37·6)	120·4 (118·3–122·4)	93·0 (91·5–94·4)	47·0 (46·2–47·8)	17·3 (16·8–17·8)	4·0 (3·7–4·2)	0·2 (0·2–0·2)	0·0 (0·0–0·0)	1·59 (1·57–1·62)	0·79 (0·77–0·8)	0·34 (0·34–0·35)
1997	0·1 (0·0–0·2)	34·2 (33·3–35·2)	115·9 (113·7–118·3)	91·2 (89·7–92·6)	47·1 (46·1–48·1)	17·1 (16·7–17·6)	3·9 (3·6–4·1)	0·2 (0·2–0·2)	0·0 (0·0–0·0)	1·55 (1·52–1·57)	0·75 (0·74–0·77)	0·34 (0·33–0·35)
1998	0·1 (0·0–0·2)	31·3 (30·4–32·2)	111·5 (109·4–113·7)	89·8 (88·4–91·2)	47·2 (46·3–48·0)	17·2 (16·7–17·6)	3·8 (3·6–4·1)	0·2 (0·2–0·2)	0·0 (0·0–0·0)	1·51 (1·48–1·53)	0·71 (0·7–0·73)	0·34 (0·33–0·35)
1999	0·1 (0·0–0·2)	28·8 (28·0–29·7)	107·6 (105·4–110·0)	89·1 (87·6–90·6)	47·6 (46·7–48·5)	17·4 (16·9–17·9)	3·8 (3·6–4·1)	0·2 (0·2–0·2)	0·0 (0·0–0·0)	1·47 (1·45–1·5)	0·68 (0·67–0·7)	0·34 (0·34–0·35)
2000	0·1 (0·0–0·2)	27·7 (26·9–28·5)	105·4 (103·3–107·5)	89·5 (87·9–91·0)	48·7 (47·7–49·7)	18·0 (17·5–18·4)	3·8 (3·6–4·2)	0·2 (0·2–0·2)	0·0 (0·0–0·0)	1·47 (1·44–1·49)	0·67 (0·65–0·68)	0·35 (0·35–0·36)
2001	0·1 (0·0–0·2)	27·1 (26·3–27·9)	103·6 (101·4–105·9)	90·4 (88·9–91·9)	50·3 (49·3–51·3)	18·8 (18·3–19·4)	3·9 (3·6–4·2)	0·2 (0·2–0·2)	0·0 (0·0–0·0)	1·47 (1·45–1·5)	0·65 (0·64–0·67)	0·37 (0·36–0·37)
2002	0·1 (0·0–0·2)	26·7 (25·9–27·5)	102·8 (100·4–105·1)	91·8 (90·1–93·3)	52·1 (51·1–53·1)	19·8 (19·3–20·3)	4·0 (3·7–4·3)	0·2 (0·2–0·3)	0·0 (0·0–0·0)	1·49 (1·46–1·51)	0·65 (0·63–0·66)	0·38 (0·37–0·39)
2003	0·1 (0·0–0·2)	26·4 (25·6–27·2)	101·8 (99·4–104·3)	93·3 (91·7–94·8)	54·2 (53·2–55·2)	20·9 (20·3–21·4)	4·1 (3·9–4·4)	0·2 (0·2–0·3)	0·0 (0·0–0·0)	1·5 (1·48–1·53)	0·64 (0·63–0·66)	0·4 (0·39–0·4)
2004	0·1 (0·0–0·2)	26·2 (25·4–27·0)	99·7 (97·3–102·2)	94·2 (92·5–95·9)	56·0 (55·1–57·0)	21·9 (21·3–22·5)	4·4 (4·1–4·6)	0·2 (0·2–0·3)	0·0 (0·0–0·0)	1·51 (1·49–1·54)	0·63 (0·61–0·65)	0·41 (0·4–0·42)
2005	0·1 (0·0–0·2)	26·4 (25·6–27·3)	98·4 (95·9–100·8)	95·1 (93·4–96·7)	58·1 (57·1–59·1)	22·9 (22·3–23·6)	4·6 (4·4–4·9)	0·2 (0·2–0·3)	0·0 (0·0–0·0)	1·53 (1·5–1·56)	0·62 (0·61–0·64)	0·43 (0·42–0·44)
2006	0·1 (0·0–0·2)	27·1 (26·1–28·0)	99·2 (96·6–101·7)	98·0 (96·5–99·6)	61·8 (60·7–62·8)	24·6 (23·9–25·4)	4·9 (4·7–5·2)	0·2 (0·2–0·3)	0·0 (0·0–0·0)	1·58 (1·55–1·61)	0·63 (0·61–0·65)	0·46 (0·45–0·47)
2007	0·1 (0·0–0·2)	27·9 (26·9–29·0)	101·0 (98·2–103·9)	102·0 (100·4–103·6)	66·4 (65·3–67·4)	26·8 (26·1–27·6)	5·3 (5·0–5·6)	0·2 (0·2–0·3)	0·0 (0·0–0·0)	1·65 (1·62–1·68)	0·65 (0·63–0·66)	0·49 (0·48–0·5)
2008	0·1 (0·0–0·2)	28·3 (27·3–29·4)	102·6 (99·7–105·7)	104·9 (103·4–106·5)	69·8 (68·7–70·9)	28·7 (28·0–29·4)	5·7 (5·4–6·0)	0·3 (0·2–0·3)	0·0 (0·0–0·0)	1·7 (1·67–1·73)	0·66 (0·64–0·68)	0·52 (0·51–0·53)
2009	0·1 (0·0–0·2)	27·9 (26·8–29·1)	102·6 (99·4–105·8)	106·8 (105·1–108·3)	72·1 (71·1–73·2)	30·3 (29·6–31·0)	6·1 (5·8–6·4)	0·3 (0·2–0·3)	0·0 (0·0–0·0)	1·73 (1·7–1·76)	0·65 (0·63–0·67)	0·54 (0·53–0·55)
2010	0·1 (0·0–0·2)	27·6 (26·4–28·8)	102·4 (99·0–105·8)	107·4 (105·8–109·0)	73·5 (72·3–74·7)	31·6 (30·9–32·3)	6·5 (6·1–6·8)	0·3 (0·2–0·3)	0·0 (0·0–0·0)	1·75 (1·71–1·78)	0·65 (0·63–0·67)	0·56 (0·55–0·57)
2011	0·1 (0·0–0·2)	27·6 (26·3–29·0)	104·2 (100·7–108·1)	108·8 (107·0–110·7)	75·2 (73·8–76·5)	32·9 (32·2–33·7)	6·8 (6·5–7·1)	0·3 (0·2–0·4)	0·0 (0·0–0·0)	1·78 (1·74–1·82)	0·66 (0·64–0·69)	0·58 (0·57–0·59)
2012	0·1 (0·0–0·2)	27·6 (26·2–29·2)	105·9 (102·1–110·0)	110·8 (108·8–112·9)	76·9 (75·5–78·3)	34·3 (33·5–35·1)	7·1 (6·8–7·4)	0·3 (0·3–0·4)	0·0 (0·0–0·0)	1·82 (1·77–1·86)	0·67 (0·64–0·7)	0·59 (0·58–0·61)
2013	0·1 (0·0–0·2)	27·6 (25·9–29·2)	105·9 (101·7–110·2)	112·4 (110·3–114·6)	78·9 (77·4–80·3)	35·8 (34·9–36·5)	7·4 (7·0–7·7)	0·3 (0·3–0·4)	0·0 (0·0–0·0)	1·84 (1·8–1·89)	0·67 (0·64–0·7)	0·61 (0·6–0·62)
2014	0·1 (0·0–0·2)	27·5 (25·6–29·6)	105·4 (100·1–111·3)	113·6 (110·7–116·5)	81·1 (78·9–83·4)	36·6 (35·2–38·0)	7·4 (6·9–7·9)	0·3 (0·3–0·4)	0·0 (0·0–0·0)	1·86 (1·79–1·93)	0·67 (0·63–0·7)	0·63 (0·61–0·65)
2015	0·1 (0·0–0·2)	27·3 (24·8–30·1)	104·5 (96·2–113·1)	112·7 (108·0–117·4)	80·8 (76·6–85·4)	35·8 (32·8–38·9)	7·0 (6·3–7·8)	0·3 (0·3–0·4)	0·0 (0·0–0·0)	1·84 (1·73–1·96)	0·66 (0·61–0·72)	0·62 (0·58–0·66)
2016	0·1 (0·0–0·2)	27·0 (23·9–30·7)	103·3 (92·1–115·8)	111·0 (104·7–117·6)	78·9 (72·9–85·3)	34·1 (30·0–38·8)	6·6 (5·7–7·7)	0·3 (0·2–0·4)	0·0 (0·0–0·0)	1·81 (1·65–1·97)	0·65 (0·58–0·73)	0·6 (0·54–0·66)
2017	0·1 (0·0–0·2)	27·0 (23·5–31·2)	102·9 (89·3–118·2)	110·0 (102·9–117·7)	77·0 (70·1–84·8)	32·6 (27·8–38·2)	6·3 (5·3–7·5)	0·3 (0·2–0·3)	0·0 (0·0–0·0)	1·78 (1·59–1·99)	0·65 (0·56–0·75)	0·58 (0·52–0·66)

Supplemental Results Table 5: Age-specific fertility rates per 1000, cumulative total fertility up to maternal age 25 (TFU25), cumulative total fertility from maternal age 30 to age 55 (TFO30), and cumulative total fertility, Central Asia, 1950-2017

Year	10-14	15-19	20-24	25-29	30-34	35-39	40-44	45-49	50-54	TFR	TFU25	TFO30
1950	0·1 (0·0–0·1)	44·4 (40·6–49·3)	252·7 (237·6–267·8)	229·3 (221·8–236·4)	159·7 (153·7–165·1)	120·7 (114·8–126·4)	66·6 (62·6–70·4)	12·2 (10·5–14·1)	0·2 (0·2–0·2)	4·43 (4·25–4·6)	1·49 (1·4–1·58)	1·8 (1·72–1·87)
1951	0·1 (0·0–0·1)	44·5 (40·8–48·9)	250·8 (236·8–264·3)	228·5 (221·3–234·9)	159·1 (153·6–164·3)	119·1 (113·9–124·4)	65·5 (61·8–68·8)	11·9 (10·3–13·7)	0·2 (0·2–0·2)	4·4 (4·24–4·56)	1·48 (1·4–1·56)	1·78 (1·72–1·84)
1952	0·1 (0·0–0·1)	44·9 (41·1–49·1)	250·4 (238·3–262·2)	229·2 (222·5–235·4)	159·4 (154·3–164·5)	118·4 (113·8–123·1)	64·9 (61·7–67·7)	11·6 (10·0–13·4)	0·2 (0·2–0·2)	4·4 (4·25–4·55)	1·48 (1·41–1·55)	1·77 (1·71–1·83)
1953	0·1 (0·0–0·1)	45·7 (42·1–49·6)	251·4 (240·9–261·7)	232·2 (226·2–238·3)	161·3 (156·6–166·0)	119·0 (114·9–123·3)	65·0 (62·2–67·6)	11·5 (9·9–13·2)	0·2 (0·2–0·2)	4·43 (4·29–4·57)	1·49 (1·42–1·55)	1·78 (1·73–1·84)
1954	0·1 (0·0–0·1)	46·6 (43·2–50·4)	254·3 (245·4–263·9)	236·2 (230·5–242·4)	164·2 (159·1–169·3)	120·4 (116·1–124·8)	65·6 (63·0–68·2)	11·3 (9·7–13·0)	0·2 (0·2–0·2)	4·49 (4·36–4·63)	1·5 (1·44–1·57)	1·81 (1·75–1·87)
1955	0·1 (0·0–0·1)	47·6 (44·2–51·3)	257·1 (248·2–265·7)	240·5 (235·0–246·0)	167·4 (161·9–173·1)	122·1 (117·5–126·7)	66·2 (63·6–68·9)	11·2 (9·6–12·9)	0·2 (0·2–0·2)	4·56 (4·43–4·69)	1·52 (1·47–1·58)	1·84 (1·77–1·9)
1956	0·1 (0·0–0·1)	48·7 (45·3–52·3)	260·1 (252·1–267·9)	244·7 (239·1–249·6)	170·7 (164·9–176·3)	124·1 (119·5–128·8)	66·9 (64·4–69·6)	11·1 (9·5–12·8)	0·2 (0·2–0·2)	4·63 (4·5–4·76)	1·54 (1·49–1·6)	1·86 (1·8–1·93)
1957	0·1 (0·0–0·1)	50·0 (46·6–53·5)	262·8 (254·8–270·5)	249·3 (243·8–254·3)	174·6 (168·9–180·1)	126·2 (121·3–131·0)	67·8 (65·2–70·6)	10·9 (9·3–12·9)	0·2 (0·2–0·2)	4·71 (4·58–4·84)	1·56 (1·51–1·62)	1·9 (1·84–1·96)
1958	0·1 (0·0–0·2)	51·4 (48·0–55·1)	266·2 (257·9–274·4)	253·8 (248·7–259·2)	178·7 (173·4–183·9)	128·6 (123·7–133·6)	68·9 (66·1–71·6)	10·8 (9·2–12·8)	0·2 (0·2–0·2)	4·79 (4·66–4·92)	1·59 (1·53–1·64)	1·94 (1·87–2·0)
1959	0·1 (0·0–0·2)	52·8 (49·1–56·6)	268·8 (259·8–277·8)	258·2 (252·6–263·5)	182·5 (177·7–187·4)	130·6 (125·7–135·9)	69·8 (67·1–72·5)	10·6 (9·0–12·6)	0·2 (0·2–0·2)	4·87 (4·74–4·99)	1·61 (1·55–1·67)	1·97 (1·91–2·03)
1960	0·1 (0·0–0·2)	53·6 (49·7–57·9)	269·9 (260·4–279·4)	259·9 (254·0–265·6)	185·2 (180·6–190·0)	131·5 (126·6–136·7)	70·2 (67·5–72·7)	10·4 (8·8–12·3)	0·2 (0·2–0·2)	4·9 (4·78–5·03)	1·62 (1·56–1·68)	1·99 (1·93–2·05)
1961	0·1 (0·0–0·2)	54·0 (50·0–58·6)	269·4 (259·8–279·7)	259·4 (253·3–265·2)	186·2 (181·2–191·2)	130·6 (125·9–135·7)	70·0 (67·4–72·5)	10·1 (8·5–11·9)	0·2 (0·2–0·2)	4·9 (4·77–5·03)	1·62 (1·56–1·68)	1·99 (1·93–2·04)
1962	0·1 (0·0–0·1)	54·4 (50·3–59·1)	268·4 (258·3–278·4)	257·7 (251·8–263·6)	185·0 (179·3–190·3)	128·9 (123·8–133·8)	68·7 (66·3–71·3)	9·7 (8·1–11·5)	0·2 (0·2–0·2)	4·87 (4·73–5·0)	1·61 (1·55–1·68)	1·96 (1·9–2·02)
1963	0·1 (0·0–0·1)	54·4 (50·2–59·0)	268·0 (257·2–279·0)	255·9 (249·8–261·9)	182·6 (176·5–188·9)	125·9 (120·0–131·6)	66·8 (63·9–69·5)	9·4 (7·8–11·2)	0·2 (0·2–0·2)	4·82 (4·67–4·96)	1·61 (1·54–1·68)	1·92 (1·85–2·0)
1964	0·1 (0·0–0·1)	54·0 (49·9–58·8)	267·9 (255·4–280·6)	253·8 (247·6–260·4)	180·4 (173·2–187·7)	122·6 (115·3–130·2)	64·5 (60·9–68·0)	9·0 (7·3–10·8)	0·2 (0·2–0·2)	4·76 (4·6–4·93)	1·61 (1·53–1·69)	1·88 (1·79–1·98)
1965	0·1 (0·0–0·1)	54·0 (50·0–58·6)	268·2 (253·3–283·4)	252·3 (245·4–259·3)	178·3 (170·3–187·0)	119·6 (111·1–128·6)	62·1 (57·8–66·5)	8·5 (6·9–10·4)	0·2 (0·2–0·2)	4·72 (4·53–4·91)	1·61 (1·52–1·71)	1·84 (1·74–1·95)
1966	0·1 (0·0–0·1)	54·5 (50·6–58·9)	268·4 (252·4–286·2)	252·0 (245·0–259·1)	175·8 (166·7–185·2)	117·1 (107·0–127·4)	59·5 (54·3–64·5)	8·1 (6·4–10·0)	0·2 (0·1–0·2)	4·68 (4·48–4·89)	1·61 (1·52–1·72)	1·8 (1·68–1·92)
1967	0·1 (0·0–0·1)	55·2 (51·2–59·4)	267·5 (251·4–284·9)	252·5 (245·4–259·4)	173·2 (162·9–184·1)	114·8 (103·4–125·7)	57·1 (51·2–62·8)	7·8 (6·0–9·8)	0·1 (0·1–0·2)	4·64 (4·42–4·86)	1·61 (1·52–1·72)	1·76 (1·62–1·9)
1968	0·1 (0·0–0·1)	55·9 (51·9–60·0)	265·3 (250·3–281·4)	253·9 (247·1–260·4)	170·5 (159·2–183·0)	112·9 (100·5–124·6)	54·6 (48·0–60·9)	7·4 (5·6–9·4)	0·1 (0·1–0·1)	4·6 (4·38–4·83)	1·61 (1·51–1·7)	1·73 (1·57–1·88)
1969	0·1 (0·0–0·1)	56·5 (52·5–60·5)	261·7 (248·8–275·3)	255·0 (248·4–261·6)	167·8 (156·0–180·3)	111·0 (98·2–123·5)	52·2 (45·4–59·1)	7·0 (5·2–9·1)	0·1 (0·1–0·1)	4·56 (4·32–4·79)	1·59 (1·51–1·68)	1·69 (1·53–1·86)
1970	0·1 (0·0–0·1)	56·6 (52·6–61·0)	258·5 (247·3–269·8)	255·6 (248·9–262·4)	164·8 (152·6–177·3)	108·6 (95·8–121·9)	49·9 (43·1–57·4)	6·6 (4·9–8·7)	0·1 (0·1–0·1)	4·5 (4·28–4·73)	1·58 (1·51–1·65)	1·65 (1·49–1·82)
1971	0·1 (0·0–0·1)	56·4 (52·3–60·9)	257·9 (246·7–268·2)	255·2 (247·9–262·0)	162·4 (150·9–173·7)	105·3 (92·6–118·8)	47·8 (41·0–55·3)	6·1 (4·4–8·3)	0·1 (0·1–0·1)	4·46 (4·25–4·68)	1·57 (1·5–1·64)	1·61 (1·45–1·78)
1972	0·1 (0·0–0·1)	56·3 (52·0–60·7)	257·8 (247·1–268·1)	253·4 (246·2–259·8)	160·4 (150·1–170·4)	101·8 (89·4–114·5)	45·7 (39·1–52·6)	5·7 (4·2–7·6)	0·1 (0·1–0·1)	4·41 (4·21–4·62)	1·57 (1·5–1·64)	1·57 (1·42–1·72)
1973	0·1 (0·0–0·1)	56·1 (51·9–60·5)	257·6 (247·5–267·4)	249·6 (243·3–255·7)	158·8 (149·6–167·6)	97·7 (86·8–109·3)	43·8 (38·1–49·9)	5·3 (4·0–6·9)	0·1 (0·1–0·1)	4·35 (4·17–4·53)	1·57 (1·51–1·63)	1·53 (1·4–1·67)
1974	0·1 (0·0–0·1)	55·9 (51·8–60·2)	257·4 (248·3–266·0)	244·2 (238·6–249·7)	156·4 (147·7–164·6)	93·5 (83·5–104·3)	42·1 (37·0–47·5)	4·9 (3·8–6·3)	0·1 (0·1–0·1)	4·27 (4·11–4·45)	1·57 (1·51–1·63)	1·48 (1·37–1·6)
1975	0·1 (0·0–0·1)	55·5 (51·6–59·6)	256·3 (247·6–265·1)	239·1 (234·2–244·0)	154·1 (146·4–161·9)	89·3 (80·4–98·9)	40·1 (35·6–44·7)	4·6 (3·5–5·7)	0·1 (0·1–0·1)	4·2 (4·04–4·37)	1·56 (1·5–1·62)	1·44 (1·34–1·55)
1976	0·1 (0·0–0·1)	55·0 (51·3–58·8)	253·6 (245·0–262·0)	236·5 (231·9–241·4)	151·7 (144·8–158·9)	85·9 (78·2–93·6)	37·8 (33·9–41·8)	4·3 (3·3–5·3)	0·1 (0·1–0·1)	4·12 (3·98–4·27)	1·54 (1·49–1·6)	1·4 (1·31–1·49)
1977	0·1 (0·0–0·1)	54·4 (50·9–58·0)	251·2 (243·1–259·1)	234·1 (229·4–238·9)	148·9 (142·8–154·9)	82·7 (76·5–89·2)	35·4 (31·9–38·8)	4·0 (3·2–4·9)	0·1 (0·1–0·1)	4·05 (3·93–4·19)	1·53 (1·47–1·58)	1·36 (1·28–1·43)
1978	0·1 (0·0–0·1)	54·0 (50·6–57·3)	249·2 (241·8–256·3)	231·9 (227·2–236·5)	145·0 (139·9–149·8)	79·9 (75·2–85·2)	33·0 (30·0–35·8)	3·8 (3·1–4·6)	0·1 (0·1–0·1)	3·98 (3·87–4·11)	1·52 (1·47–1·56)	1·31 (1·25–1·37)
1979	0·1 (0·0–0·1)	53·9 (50·7–57·1)	247·6 (240·6–253·8)	229·9 (225·3–234·5)	140·4 (136·6–144·6)	76·9 (73·0–81·0)	30·6 (28·4–32·9)	3·6 (3·0–4·2)	0·1 (0·1–0·1)	3·92 (3·82–4·02)	1·51 (1·46–1·55)	1·26 (1·21–1·31)
1980	0·1 (0·0–0·1)	53·8 (50·8–56·9)	246·6 (240·1–252·5)	227·9 (223·3–232·4)	136·7 (133·2–140·3)	74·6 (71·0–78·2)	28·5 (26·6–30·4)	3·3 (2·8–3·9)	0·1 (0·1–0·1)	3·86 (3·77–3·95)	1·5 (1·46–1·54)	1·22 (1·18–1·26)
1981	0·1 (0·0–0·1)	53·8 (50·9–57·0)	245·6 (239·4–251·4)	225·3 (220·7–229·6)	135·6 (132·2–139·2)	72·9 (69·2–76·5)	26·9 (25·2–28·7)	3·0 (2·6–3·5)	0·1 (0·1–0·1)	3·82 (3·73–3·91)	1·5 (1·46–1·54)	1·19 (1·15–1·23)
1982	0·1 (0·0–0·1)	54·1 (51·1–57·2)	245·0 (238·8–251·0)	224·6 (220·4–228·9)	136·0 (132·4–139·7)	71·7 (68·2–75·4)	25·8 (24·1–27·5)	2·8 (2·4–3·2)	0·1 (0·0–0·1)	3·8 (3·71–3·89)	1·5 (1·45–1·54)	1·18 (1·14–1·22)
1983	0·1 (0·0–0·1)	54·7 (51·8–57·7)	244·7 (238·8–250·9)	225·0 (220·7–229·2)	137·1 (133·8–140·7)	70·0 (66·4–73·6)	25·0 (23·5–26·6)	2·5 (2·2–2·9)	0·0 (0·0–0·0)	3·8 (3·71–3·89)	1·5 (1·46–1·54)	1·17 (1·14–1·21)
1984	0·1 (0·0–0·1)	55·5 (52·6–58·5)	244·9 (238·8–250·8)	225·1 (220·8–229·2)	137·4 (134·2–140·8)	68·0 (64·7–71·4)	24·3 (22·8–25·8)	2·3 (2·0–2·7)	0·0 (0·0–0·0)	3·79 (3·7–3·88)	1·5 (1·46–1·54)	1·16 (1·13–1·2)
1985	0·1 (0·0–0·1)	56·3 (53·2–59·4)	245·1 (239·0–250·8)	223·8 (219·7–228·1)	136·1 (132·9–139·4)	65·9 (62·8–69·0)	23·6 (22·1–25·1)	2·1 (1·8–2·4)	0·0 (0·0–0·0)	3·77 (3·68–3·85)	1·51 (1·46–1·55)	1·14 (1·11–1·17)
1986	0·1 (0·0–0·1)	56·6 (53·4–59·7)	244·1 (237·8–250·4)	220·2 (216·0–224·5)	132·9 (129·7–136·1)	63·9 (61·0–67·1)	22·6 (21·2–24·2)	1·9 (1·6–2·2)	0·0 (0·0–0·0)	3·71 (3·62–3·8)	1·5 (1·46–1·55)	1·11 (1·08–1·14)
1987	0·1 (0·0–0·1)	56·6 (53·4–59·7)	241·5 (235·0–248·3)	215·2 (211·2–219·3)	129·4 (126·4–132·5)	61·6 (58·7–64·7)	21·4 (20·1–22·8)	1·7 (1·5–2·0)	0·0 (0·0–0·0)	3·64 (3·55–3·72)	1·49 (1·45–1·54)	1·07 (1·04–1·1)
1988	0·1 (0·0–0·1)	56·8 (53·8–60·0)	238·5 (231·9–245·7)	210·2 (206·1–214·4)	125·8 (122·8–128·8)	59·4 (56·9–62·0)	19·8 (18·6–21·1)	1·6 (1·4–1·8)	0·0 (0·0–0·0)	3·56 (3·48–3·64)	1·48 (1·43–1·52)	1·03 (1·01–1·06)
1989	0·1 (0·0–0·1)	57·4 (54·5–60·4)	235·8 (228·9–243·1)	205·4 (201·1–209·7)	121·7 (118·9–124·7)	56·9 (54·9–59·0)	18·0 (17·0–19·1)	1·5 (1·3–1·7)	0·0 (0·0–0·0)	3·48 (3·41–3·56)	1·47 (1·42–1·51)	0·99 (0·97–1·01)
1990	0·1 (0·0–0·1)	57·7 (54·9–60·6)	233·1 (226·1–240·5)	199·9 (195·3–204·3)	117·1 (114·1–119·9)	53·8 (52·1–55·7)	16·3 (15·4–17·3)	1·4 (1·2–1·5)	0·0 (0·0–0·0)	3·4 (3·32–3·47)	1·45 (1·41–1·5)	0·94 (0·92–0·97)
1991	0·1 (0·0–0·1)	57·3 (54·6–60·2)	229·5 (222·1–237·4)	192·4 (187·9–196·9)	111·8 (108·7–114·6)	50·5 (48·9–52·2)	15·0 (14·1–15·9)	1·2 (1·0–1·4)	0·0 (0·0–0·0)	3·29 (3·22–3·36)	1·43 (1·39–1·48)	0·89 (0·87–0·92)
1992	0·1 (0·0–0·1)	55·9 (53·2–58·7)	224·2 (216·9–231·9)	182·5 (178·0–187·0)	106·7 (103·6–109·6)	47·5 (45·9–49·2)	13·8 (13·0–14·6)	1·1 (0·9–1·2)	0·0 (0·0–0·0)	3·16 (3·08–3·23)	1·4 (1·35–1·45)	0·85 (0·82–0·87)
1993	0·1 (0·0–0·1)	53·8 (51·0–56·5)	217·4 (209·8–224·9)	174·0 (169·5–178·5)	102·3 (99·4–105·0)	44·9 (43·5–46·6)	12·8 (12·0–13·5)	0·9 (0·8–1·1)	0·0 (0·0–0·0)	3·03 (2·95–3·11)	1·36 (1·31–1·4)	0·8 (0·78–0·83)
1994	0·1 (0·0–0·1)	51·1 (48·5–53·9)	210·4 (202·9–217·8)	168·1 (163·6–172·9)	98·3 (95·6–100·7)	42·5 (41·0–44·2)	11·9 (11·2–12·6)	0·8 (0·7–0·9)	0·0 (0·0–0·0)	2·92 (2·84–2·99)	1·31 (1·26–1·35)	0·77 (0·75–0·79)
1995	0·1 (0·0–0·1)	48·2 (45·6–50·9)	203·9 (196·1–211·2)	162·8 (158·0–167·5)	94·5 (91·9–96·9)	40·0 (38·6–41·8)	11·1 (10·5–11·7)	0·7 (0·6–0·8)	0·0 (0·0–0·0)	2·81 (2·73–2·88)	1·26 (1·21–1·31)	0·73 (0·71–0·75)
1996	0·1 (0·0–0·1)	45·2 (42·6–47·8)	197·9 (189·4–205·9)	157·5 (152·4–162·7)	91·1 (88·5–93·7)	37·9 (36·4–39·6)	10·5 (9·9–11·1)	0·6 (0·5–0·7)	0·0 (0·0–0·0)	2·7 (2·62–2·78)	1·22 (1·17–1·27)	0·7 (0·68–0·72)
1997	0·1 (0·0–0·1)	42·1 (39·7–44·9)	192·6 (184·1–201·0)	152·7 (147·3–158·0)	88·6 (85·8–91·2)	36·5 (35·0–38·0)	10·0 (9·4–10·6)	0·5 (0·5–0·7)	0·0 (0·0–0·0)	2·62 (2·53–2·7)	1·17 (1·12–1·23)	0·68 (0·66–0·7)
1998	0·1 (0·0–0·1)	39·1 (36·7–41·7)	187·7 (179·7–196·2)	148·8 (143·2–154·3)	86·9 (84·0–89·7)	35·7 (34·3–37·2)	9·6 (9·1–10·2)	0·5 (0·4–0·6)	0·0 (0·0–0·0)	2·54 (2·46–2·63)	1·13 (1·08–1·19)	0·66 (0·65–0·68)
1999	0·1 (0·0–0·1)	36·5 (34·3–38·9)	183·3 (175·1–192·4)	145·7 (140·3–151·2)	85·4 (82·5–88·1)	35·0 (33·7–36·5)	9·2 (8·7–9·8)	0·5 (0·4–0·6)	0·0 (0·0–0·0)	2·48 (2·39–2·56)	1·1 (1·05–1·16)	0·65 (0·63–0·67)
2000	0·1 (0·0–0·1)	34·6 (32·4–37·1)	179·9 (171·4–188·9)	143·2 (137·7–148·4)	84·0 (81·2–86·9)	34·6 (33·1–36·1)	8·9 (8·4–9·5)	0·5 (0·4–0·6)	0·0 (0·0–0·0)	2·43 (2·34–2·51)	1·07 (1·02–1·13)	0·64 (0·62–0·66)
2001	0·0 (0·0–0·1)	33·3 (31·0–35·7)	177·4 (168·2–187·1)	141·3 (135·8–146·6)	83·3 (80·3–86·3)	34·4 (32·9–36·1)	8·8 (8·3–9·4)	0·5 (0·4–0·5)	0·0 (0·0–0·0)	2·4 (2·31–2·48)	1·05 (1·0–1·11)	0·64 (0·61–0·66)
2002	0·0 (0·0–0·1)	32·5 (30·2–34·8)	175·7 (165·8–185·9)	140·4 (134·9–145·7)	83·6 (80·4–86·5)	34·7 (33·0–36·3)	8·7 (8·2–9·3)	0·5 (0·4–0·5)	0·0 (0·0–0·0)	2·38 (2·29–2·47)	1·04 (0·99–1·1)	0·64 (0·62–0·66)
2003	0·0 (0·0–0·1)	31·9 (29·7–34·3)	174·8 (164·8–185·6)	141·2 (135·4–146·7)	84·6 (81·5–87·5)	35·4 (33·8–37·1)	8·8 (8·3–9·4)	0·5 (0·4–0·6)	0·0 (0·0–0·0)	2·39 (2·3–2·48)	1·03 (0·98–1·1)	0·65 (0·63–0·67)
2004	0·0 (0·0–0·1)	31·8 (29·5–34·2)	174·9 (164·7–185·4)	143·3 (137·5–148·9)	86·4 (83·3–89·5)	36·5 (34·8–38·3)	9·1 (8·5–9·6)	0·5 (0·4–0·6)	0·0 (0·0–0·0)	2·41 (2·32–2·5)	1·03 (0·97–1·1)	0·66 (0·64–0·68)
2005	0·0 (0·0–0·1)	32·0 (29·7–34·5)	175·7 (165·2–186·0)	145·5 (139·6–151·1)	88·5 (85·4–91·9)	37·7 (35·8–39·8)	9·4 (8·8–10·0)	0·5 (0·4–0·6)	0·0 (0·0–0·0)	2·45 (2·35–2·54)	1·04 (0·98–1·1)	0·68 (0·66–0·7)
2006	0·0 (0·0–0·1)	32·6 (30·0–35·3)	176·5 (165·7–187·1)	147·1 (141·2–152·9)	91·0 (87·6–94·6)	38·9 (36·8–41·2)	9·7 (9·1–10·3)	0·5 (0·4–0·6)	0·0 (0·0–0·0)	2·48 (2·38–2·58)	1·05 (0·98–1·11)	0·7 (0·68–0·73)
2007	0·0 (0·0–0·1)	33·5 (30·8–36·3)	177·2 (165·8–188·2)	148·8 (143·0–154·5)	93·5 (89·8–97·2)	40·2 (37·9–42·7)	10·0 (9·3–10·7)	0·5 (0·4–0·6)	0·0 (0·0–0·0)	2·52 (2·41–2·62)	1·05 (0·98–1·12)	0·72 (0·69–0·75)
2008	0·0 (0·0–0·1)	34·3 (31·4–37·2)	177·7 (165·2–189·7)	150·2 (144·5–155·9)	95·3 (91·4–99·0)	41·4 (38·9–43·9)	10·3 (9·6–11·1)	0·5 (0·4–0·6)	0·0 (0·0–0·0)	2·55 (2·44–2·66)	1·06 (0·99–1·13)	0·74 (0·71–0·77)
2009	0·0 (0·0–0·1)	34·8 (31·8–37·9)	177·7 (165·7–189·9)	151·2 (145·5–156·6)	96·5 (92·4–100·8)	42·2 (39·8–44·8)	10·5 (9·7–11·2)	0·5 (0·4–0·6)	0·0 (0·0–0·0)	2·57 (2·45–2·68)	1·06 (0·99–1·14)	0·75 (0·72–0·78)
2010	0·0 (0·0–0·1)	35·2 (32·1–38·5)	177·9 (165·4–190·3)	151·9 (146·3–157·5)	97·7 (93·6–102·0)	42·8 (40·5–45·3)	10·6 (9·8–11·4)	0·5 (0·4–0·6)	0·0 (0·0–0·0)	2·58 (2·47–2·7)	1·07 (0·99–1·14)	0·76 (0·73–0·79)
2011	0·0 (0·0–0·1)	35·7 (32·4–39·4)	178·4 (165·8–191·1)	152·6 (146·6–158·9)	98·4 (94·0–103·0)	43·5 (41·1–46·0)	10·7 (9·8–11·7)	0·6 (0·5–0·7)	0·0 (0·0–0·0)	2·6 (2·49–2·72)	1·07 (0·99–1·15)	0·77 (0·73–0·8)
2012	0·0 (0·0–0·1)	36·2 (32·7–40·3)	179·0 (166·4–191·8)	153·4 (147·1–160·2)	98·9 (94·3–103·9)	44·2 (41·6–46·8)	10·9 (9·9–11·9)	0·6 (0·5–0·7)	0·0 (0·0–0·0)	2·62 (2·49–2·74)	1·08 (1·0–1·16)	0·77 (0·73–0·81)
2013	0·0 (0·0–0·1)	36·5 (32·7–40·9)	179·2 (166·7–192·4)	153·7 (147·1–160·9)	98·9 (93·8–104·1)	44·4 (41·5–47·6)	10·9 (9·9–11·9)	0·6 (0·5–0·7)	0·0 (0·0–0·0)	2·62 (2·49–2·76)	1·08 (1·0–1·17)	0·77 (0·73–0·82)
2014	0·0 (0·0–0·1)	36·4 (32·3–41·1)	178·4 (164·5–193·5)	152·2 (145·2–159·7)	97·6 (91·8–103·5)	43·5 (39·9–47·1)	10·6 (9·5–11·9)	0·6 (0·4–0·7)	0·0 (0·0–0·0)	2·6 (2·45–2·75)	1·07 (0·98–1·17)	0·76 (0·71–0·81)
2015	0·0 (0·0–0·1)	36·1 (31·5–41·2)	176·2 (159·6–193·5)	149·2 (141·5–157·3)	95·0 (88·1–102·5)	41·7 (37·2–46·6)	10·2 (8·9–11·8)	0·6 (0·4–0·8)	0·0 (0·0–0·0)	2·55 (2·38–2·72)	1·06 (0·95–1·17)	0·74 (0·67–0·81)
2016	0·0 (0·0–0·1)	35·8 (30·9–41·4)	173·6 (154·5–193·7)	146·6 (138·5–155·1)	92·4 (84·3–101·6)	40·2 (34·8–46·2)	9·8 (8·2–11·8)	0·5 (0·4–0·8)	0·0 (0·0–0·0)	2·49 (2·31–2·69)	1·05 (0·93–1·17)	0·71 (0·64–0·8)
2017	0·0 (0·0–0·1)	35·8 (30·7–41·8)	172·3 (151·6–195·2)	145·6 (137·2–154·7)	91·2 (82·3–102·1)	39·3 (33·4–47·0)	9·6 (7·8–12·0)	0·5 (0·4–0·8)	0·0 (0·0–0·0)	2·47 (2·26–2·69)	1·04 (0·91–1·18)	0·7 (0·62–0·81)

Supplemental Results Table 5: Age-specific fertility rates per 1000, cumulative total fertility up to maternal age 25 (TFU25), cumulative total fertility from maternal age 30 to age 55 (TFO30), and cumulative total fertility, Armenia, 1950-2017

Year	10-14	15-19	20-24	25-29	30-34	35-39	40-44	45-49	50-54	TFR	TFU25	TFO30
1950	0·1 (0·0–0·1)	39·1 (34·0–45·5)	220·9 (204·2–237·7)	209·4 (199·1–219·7)	161·3 (150·0–172·7)	122·0 (111·4–132·7)	58·2 (52·2–64·4)	11·9 (8·7–15·6)	0·2 (0·2–0·2)	4·11 (3·82–4·42)	1·3 (1·21–1·39)	1·77 (1·61–1·93)
1951	0·1 (0·0–0·1)	39·3 (34·3–45·2)	220·1 (204·0–235·7)	210·7 (200·3–220·5)	162·4 (151·9–173·1)	122·2 (112·1–132·3)	57·7 (52·2–63·2)	11·5 (8·5–15·0)	0·2 (0·2–0·2)	4·12 (3·86–4·39)	1·3 (1·21–1·38)	1·77 (1·63–1·91)
1952	0·1 (0·0–0·1)	39·5 (34·6–45·2)	220·0 (204·7–234·9)	213·1 (203·1–222·6)	164·2 (154·0–173·7)	123·0 (113·6–132·1)	57·4 (52·2–62·8)	11·2 (8·3–14·7)	0·2 (0·2–0·2)	4·14 (3·92–4·38)	1·3 (1·22–1·38)	1·78 (1·65–1·91)
1953	0·1 (0·0–0·1)	39·9 (35·0–45·5)	220·7 (206·2–235·1)	216·9 (207·1–226·0)	166·9 (157·1–176·4)	124·6 (115·4–133·3)	57·4 (52·2–62·7)	11·0 (8·2–14·4)	0·2 (0·2–0·2)	4·19 (3·98–4·39)	1·3 (1·23–1·38)	1·8 (1·69–1·91)
1954	0·1 (0·0–0·1)	40·4 (35·5–45·6)	221·9 (207·9–235·5)	221·1 (211·6–230·5)	170·0 (160·3–179·3)	126·4 (117·8–134·8)	57·6 (52·6–62·8)	10·8 (8·0–14·2)	0·2 (0·2–0·2)	4·24 (4·04–4·42)	1·31 (1·23–1·39)	1·82 (1·72–1·92)
1955	0·1 (0·0–0·1)	40·8 (35·7–46·1)	223·0 (208·8–236·3)	225·0 (215·8–233·8)	173·0 (163·4–182·4)	127·9 (119·0–136·2)	57·6 (52·6–62·8)	10·6 (7·9–13·8)	0·2 (0·2–0·2)	4·29 (4·11–4·46)	1·32 (1·23–1·4)	1·85 (1·75–1·93)
1956	0·1 (0·0–0·1)	41·2 (36·3–46·3)	223·9 (210·3–237·9)	228·9 (219·7–237·2)	175·9 (166·6–185·0)	129·1 (120·3–137·1)	57·4 (52·7–62·6)	10·4 (7·7–13·6)	0·2 (0·2–0·2)	4·34 (4·15–4·51)	1·33 (1·24–1·42)	1·87 (1·76–1·96)
1957	0·1 (0·0–0·1)	41·6 (36·7–46·8)	224·8 (210·7–238·7)	232·5 (223·4–240·7)	178·2 (168·5–187·5)	129·6 (120·9–137·8)	56·9 (52·1–62·0)	10·2 (7·5–13·3)	0·2 (0·2–0·2)	4·37 (4·18–4·55)	1·33 (1·24–1·42)	1·88 (1·77–1·97)
1958	0·1 (0·0–0·1)	42·1 (37·3–47·9)	226·0 (212·4–239·7)	234·4 (225·0–242·8)	179·0 (169·1–188·7)	128·2 (120·0–136·7)	55·7 (50·8–60·7)	9·8 (7·2–12·8)	0·2 (0·2–0·2)	4·38 (4·2–4·55)	1·34 (1·25–1·44)	1·86 (1·77–1·96)
1959	0·1 (0·0–0·1)	42·3 (37·4–48·0)	226·4 (212·6–240·3)	233·0 (223·6–241·5)	177·7 (168·3–187·7)	124·7 (116·4–133·0)	53·3 (48·7–58·1)	9·3 (6·9–12·2)	0·2 (0·2–0·2)	4·34 (4·16–4·5)	1·34 (1·25–1·44)	1·83 (1·73–1·92)
1960	0·1 (0·0–0·1)	42·0 (37·1–47·7)	224·9 (210·7–239·2)	228·6 (219·3–237·6)	174·7 (165·8–184·9)	119·4 (110·8–127·9)	50·2 (45·6–55·0)	8·8 (6·5–11·7)	0·2 (0·2–0·2)	4·24 (4·08–4·42)	1·33 (1·25–1·42)	1·77 (1·67–1·86)
1961	0·1 (0·0–0·1)	41·6 (36·5–47·4)	222·5 (208·2–236·8)	223·4 (214·1–232·3)	170·3 (161·0–180·6)	113·3 (104·8–121·8)	46·8 (42·2–51·6)	8·3 (6·1–11·1)	0·2 (0·2–0·2)	4·13 (3·98–4·3)	1·32 (1·23–1·41)	1·69 (1·6–1·8)
1962	0·1 (0·0–0·1)	41·7 (36·5–47·6)	220·6 (205·9–235·1)	219·5 (210·3–228·5)	164·9 (155·4–174·8)	107·2 (98·2–115·9)	43·6 (39·1–48·5)	7·7 (5·5–10·3)	0·1 (0·1–0·2)	4·03 (3·88–4·19)	1·31 (1·22–1·4)	1·62 (1·51–1·73)
1963	0·1 (0·0–0·1)	42·1 (36·7–48·2)	219·6 (204·5–235·2)	216·6 (207·1–226·0)	159·1 (149·3–169·3)	101·1 (92·2–110·0)	40·5 (36·0–45·1)	7·2 (5·2–9·7)	0·1 (0·1–0·1)	3·93 (3·78–4·09)	1·31 (1·22–1·4)	1·54 (1·43–1·66)
1964	0·1 (0·0–0·1)	42·4 (37·0–48·2)	218·8 (202·3–235·2)	213·2 (203·5–223·4)	153·0 (142·3–163·9)	95·2 (85·6–104·6)	37·5 (32·9–42·1)	6·7 (4·8–9·2)	0·1 (0·1–0·1)	3·83 (3·67–3·99)	1·31 (1·22–1·4)	1·46 (1·34–1·58)
1965	0·1 (0·0–0·1)	42·5 (37·3–48·3)	217·6 (200·5–235·7)	208·8 (198·4–219·2)	146·4 (135·6–157·5)	89·3 (79·5–99·0)	34·5 (29·8–39·2)	6·1 (4·3–8·4)	0·1 (0·1–0·1)	3·73 (3·55–3·9)	1·3 (1·21–1·4)	1·38 (1·26–1·5)
1966	0·1 (0·0–0·1)	42·6 (37·5–48·4)	216·4 (198·3–235·0)	203·9 (192·7–214·4)	139·6 (128·4–151·2)	83·6 (72·8–94·6)	31·5 (26·7–36·5)	5·5 (3·8–7·6)	0·1 (0·1–0·1)	3·62 (3·42–3·81)	1·3 (1·2–1·4)	1·3 (1·19–1·42)
1967	0·1 (0·0–0·1)	42·6 (37·6–48·4)	215·4 (197·2–236·0)	198·7 (187·2–210·3)	132·6 (120·7–145·2)	78·0 (67·2–89·8)	28·8 (24·1–34·2)	4·9 (3·4–7·0)	0·1 (0·1–0·1)	3·51 (3·3–3·72)	1·29 (1·2–1·4)	1·22 (1·11–1·33)
1968	0·1 (0·0–0·1)	42·5 (37·5–48·0)	214·2 (195·3–236·0)	193·1 (181·7–205·1)	125·6 (113·8–138·0)	72·5 (62·0–84·3)	26·1 (21·6–31·5)	4·4 (3·0–6·3)	0·1 (0·1–0·1)	3·39 (3·18–3·62)	1·28 (1·18–1·4)	1·14 (1·04–1·25)
1969	0·1 (0·0–0·1)	42·3 (37·3–47·4)	212·7 (192·8–234·2)	187·3 (175·5–199·2)	118·6 (107·0–131·2)	67·1 (57·0–78·6)	23·7 (19·4–28·9)	3·9 (2·7–5·7)	0·1 (0·1–0·1)	3·28 (3·07–3·5)	1·28 (1·17–1·39)	1·07 (0·97–1·17)
1970	0·1 (0·0–0·1)	42·3 (37·6–47·4)	211·0 (191·2–231·7)	182·0 (170·5–194·1)	111·6 (100·2–124·1)	61·6 (52·3–73·2)	21·3 (17·5–26·3)	3·4 (2·3–5·0)	0·1 (0·1–0·1)	3·17 (2·97–3·38)	1·27 (1·16–1·38)	0·99 (0·9–1·09)
1971	0·1 (0·0–0·1)	42·7 (38·0–47·5)	209·2 (190·8–228·8)	176·7 (165·6–188·7)	104·3 (93·1–116·2)	55·9 (47·4–66·4)	19·1 (15·7–23·3)	3·0 (1·9–4·4)	0·1 (0·1–0·1)	3·06 (2·87–3·25)	1·26 (1·15–1·37)	0·91 (0·82–1·0)
1972	0·1 (0·0–0·1)	43·0 (38·5–47·8)	207·2 (189·5–224·6)	170·8 (160·3–182·6)	97·1 (86·1–108·3)	50·4 (42·5–59·8)	17·1 (14·0–20·7)	2·6 (1·6–3·8)	0·0 (0·0–0·1)	2·94 (2·77–3·12)	1·25 (1·15–1·35)	0·84 (0·75–0·92)
1973	0·1 (0·0–0·1)	43·1 (38·6–47·8)	204·8 (189·1–219·7)	164·5 (154·5–175·8)	90·2 (79·9–100·5)	45·4 (38·5–53·5)	15·2 (12·5–18·3)	2·2 (1·4–3·3)	0·0 (0·0–0·0)	2·83 (2·67–2·99)	1·24 (1·14–1·34)	0·77 (0·69–0·85)
1974	0·1 (0·0–0·1)	43·3 (39·0–48·0)	202·0 (188·0–214·9)	158·7 (149·2–169·3)	84·1 (74·7–93·4)	40·8 (34·7–48·0)	13·5 (11·1–16·2)	2·0 (1·3–2·8)	0·0 (0·0–0·0)	2·72 (2·57–2·87)	1·23 (1·14–1·31)	0·7 (0·63–0·78)
1975	0·1 (0·0–0·1)	44·0 (39·8–48·5)	199·8 (187·5–211·5)	154·5 (145·3–164·4)	79·1 (70·5–87·9)	36·8 (31·6–42·8)	12·0 (9·9–14·2)	1·7 (1·1–2·5)	0·0 (0·0–0·0)	2·64 (2·5–2·78)	1·22 (1·14–1·3)	0·65 (0·58–0·72)
1976	0·1 (0·0–0·1)	45·2 (40·8–49·8)	198·9 (186·9–210·6)	151·6 (142·6–161·0)	74·8 (66·9–83·0)	33·2 (28·5–38·2)	10·6 (8·8–12·4)	1·5 (1·0–2·2)	0·0 (0·0–0·0)	2·58 (2·45–2·71)	1·22 (1·15–1·3)	0·6 (0·53–0·67)
1977	0·1 (0·0–0·1)	46·3 (41·9–51·0)	198·5 (186·7–210·1)	149·1 (140·9–157·9)	70·9 (63·8–78·3)	29·9 (25·7–34·5)	9·4 (7·9–11·0)	1·3 (0·9–1·9)	0·0 (0·0–0·0)	2·53 (2·41–2·65)	1·22 (1·15–1·3)	0·56 (0·49–0·63)
1978	0·1 (0·0–0·1)	47·6 (43·1–52·4)	198·0 (186·6–209·4)	147·5 (139·6–155·6)	67·6 (60·9–74·5)	27·2 (23·4–31·3)	8·3 (7·0–9·7)	1·2 (0·8–1·7)	0·0 (0·0–0·0)	2·49 (2·37–2·6)	1·23 (1·16–1·31)	0·52 (0·46–0·59)
1979	0·1 (0·0–0·1)	49·2 (44·6–54·2)	198·1 (186·7–209·7)	147·3 (139·9–154·8)	65·4 (58·9–72·0)	25·2 (21·6–28·7)	7·5 (6·4–8·7)	1·1 (0·7–1·5)	0·0 (0·0–0·0)	2·47 (2·36–2·58)	1·24 (1·16–1·31)	0·5 (0·44–0·55)
1980	0·1 (0·0–0·1)	51·1 (46·6–55·8)	199·3 (188·0–210·8)	147·7 (140·5–154·7)	64·7 (58·7–71·2)	23·9 (20·7–27·3)	6·9 (5·9–7·9)	0·9 (0·6–1·3)	0·0 (0·0–0·0)	2·47 (2·36–2·59)	1·25 (1·18–1·33)	0·48 (0·43–0·53)
1981	0·1 (0·0–0·1)	53·4 (48·8–58·1)	201·5 (190·2–212·4)	148·2 (141·3–155·0)	65·2 (59·3–71·5)	23·2 (20·3–26·5)	6·4 (5·6–7·4)	0·8 (0·6–1·2)	0·0 (0·0–0·0)	2·49 (2·38–2·61)	1·27 (1·2–1·35)	0·48 (0·44–0·52)
1982	0·1 (0·0–0·1)	56·0 (51·2–61·2)	204·6 (193·8–214·8)	149·9 (143·4–156·5)	66·6 (60·9–72·6)	22·9 (20·1–26·2)	6·1 (5·3–7·1)	0·8 (0·5–1·1)	0·0 (0·0–0·0)	2·53 (2·42–2·65)	1·3 (1·23–1·37)	0·48 (0·44–0·53)
1983	0·1 (0·0–0·1)	59·0 (54·0–64·5)	208·0 (197·7–217·6)	152·5 (146·3–159·2)	68·8 (63·5–74·7)	22·8 (20·1–26·0)	5·9 (5·1–6·8)	0·7 (0·5–1·0)	0·0 (0·0–0·0)	2·59 (2·49–2·69)	1·34 (1·27–1·4)	0·49 (0·45–0·54)
1984	0·1 (0·0–0·1)	62·1 (57·1–67·7)	211·1 (201·2–221·3)	155·5 (149·4–161·9)	71·4 (66·1–77·2)	23·1 (20·4–26·2)	5·7 (4·9–6·6)	0·6 (0·4–0·9)	0·0 (0·0–0·0)	2·65 (2·55–2·75)	1·37 (1·31–1·43)	0·5 (0·46–0·55)
1985	0·1 (0·0–0·1)	65·0 (59·6–70·4)	214·0 (204·6–223·7)	157·1 (151·0–163·1)	73·1 (67·9–78·5)	23·7 (20·7–26·6)	5·7 (4·9–6·5)	0·6 (0·4–0·8)	0·0 (0·0–0·0)	2·7 (2·61–2·79)	1·4 (1·34–1·46)	0·51 (0·48–0·56)
1986	0·1 (0·0–0·1)	67·3 (61·6–72·8)	216·0 (206·8–225·9)	156·1 (150·0–162·0)	73·2 (68·3–78·6)	24·3 (21·7–27·3)	5·6 (4·9–6·5)	0·5 (0·4–0·8)	0·0 (0·0–0·0)	2·72 (2·64–2·8)	1·42 (1·36–1·47)	0·52 (0·48–0·56)
1987	0·1 (0·0–0·1)	69·2 (63·4–74·8)	216·2 (206·5–225·8)	153·1 (147·1–159·0)	72·8 (68·0–78·1)	25·0 (22·2–28·1)	5·6 (4·9–6·5)	0·5 (0·4–0·7)	0·0 (0·0–0·0)	2·71 (2·63–2·8)	1·43 (1·37–1·49)	0·52 (0·48–0·56)
1988	0·1 (0·0–0·2)	71·3 (65·4–76·8)	214·8 (205·8–224·3)	149·7 (143·8–155·7)	72·4 (67·7–77·5)	26·0 (23·3–29·0)	5·6 (4·8–6·4)	0·5 (0·3–0·6)	0·0 (0·0–0·0)	2·7 (2·62–2·79)	1·43 (1·38–1·49)	0·52 (0·49–0·56)
1989	0·1 (0·0–0·2)	73·2 (67·6–79·2)	212·3 (203·3–221·9)	145·3 (139·3–151·1)	71·4 (66·9–76·3)	26·8 (24·2–29·6)	5·6 (4·8–6·4)	0·4 (0·3–0·6)	0·0 (0·0–0·0)	2·68 (2·59–2·77)	1·43 (1·37–1·48)	0·52 (0·49–0·56)
1990	0·1 (0·0–0·2)	74·5 (69·0–80·3)	208·5 (199·8–217·9)	138·1 (132·3–143·8)	69·0 (64·8–73·6)	26·9 (24·6–29·5)	5·5 (4·8–6·3)	0·4 (0·3–0·6)	0·0 (0·0–0·0)	2·61 (2·53–2·7)	1·42 (1·36–1·47)	0·51 (0·48–0·54)
1991	0·1 (0·0–0·2)	74·1 (68·9–79·8)	203·2 (194·2–212·4)	127·0 (121·7–132·2)	65·2 (61·1–69·3)	26·0 (23·8–28·5)	5·3 (4·6–6·0)	0·4 (0·3–0·5)	0·0 (0·0–0·0)	2·51 (2·43–2·59)	1·39 (1·34–1·44)	0·48 (0·46–0·52)
1992	0·1 (0·0–0·2)	71·4 (66·1–76·8)	195·6 (186·4–204·7)	112·1 (107·3–116·7)	60·5 (56·9–64·5)	24·3 (22·2–26·7)	4·9 (4·3–5·6)	0·4 (0·3–0·5)	0·0 (0·0–0·0)	2·35 (2·27–2·42)	1·33 (1·28–1·39)	0·45 (0·43–0·48)
1993	0·1 (0·0–0·1)	66·5 (61·5–71·9)	185·9 (177·2–195·0)	100·9 (96·7–105·2)	56·5 (53·0–60·3)	22·5 (20·4–24·7)	4·5 (4·0–5·1)	0·3 (0·2–0·5)	0·0 (0·0–0·0)	2·19 (2·12–2·26)	1·26 (1·21–1·31)	0·42 (0·4–0·44)
1994	0·1 (0·0–0·1)	60·8 (56·3–65·5)	176·0 (168·3–184·5)	97·4 (93·4–101·7)	53·3 (50·0–56·8)	20·8 (18·9–22·8)	4·1 (3·7–4·6)	0·3 (0·2–0·4)	0·0 (0·0–0·0)	2·06 (2·0–2·13)	1·18 (1·14–1·23)	0·39 (0·37–0·41)
1995	0·1 (0·0–0·1)	55·0 (50·9–59·5)	166·8 (159·2–174·9)	96·0 (91·8–100·3)	50·4 (47·4–53·7)	19·0 (17·4–20·6)	3·8 (3·4–4·2)	0·3 (0·2–0·4)	0·0 (0·0–0·0)	1·96 (1·89–2·02)	1·11 (1·06–1·15)	0·37 (0·35–0·39)
1996	0·1 (0·0–0·1)	49·2 (45·4–53·1)	158·2 (150·2–165·8)	93·9 (89·9–97·8)	47·4 (44·5–50·4)	17·1 (15·7–18·6)	3·4 (3·1–3·8)	0·3 (0·2–0·4)	0·0 (0·0–0·0)	1·85 (1·78–1·91)	1·04 (0·99–1·08)	0·34 (0·32–0·36)
1997	0·1 (0·0–0·1)	43·5 (40·0–47·0)	149·8 (142·3–157·9)	91·7 (87·5–95·8)	44·6 (41·7–47·4)	16·0 (14·7–17·5)	3·2 (2·9–3·6)	0·2 (0·2–0·3)	0·0 (0·0–0·0)	1·75 (1·68–1·81)	0·97 (0·93–1·01)	0·32 (0·3–0·34)
1998	0·1 (0·0–0·1)	38·1 (35·0–41·4)	141·8 (134·4–149·6)	90·0 (86·0–94·0)	42·3 (39·6–45·0)	15·9 (14·5–17·4)	3·2 (2·9–3·6)	0·2 (0·2–0·3)	0·0 (0·0–0·0)	1·66 (1·6–1·72)	0·9 (0·86–0·94)	0·31 (0·29–0·32)
1999	0·0 (0·0–0·1)	33·6 (30·8–36·6)	134·8 (127·7–142·6)	89·1 (85·1–93·0)	40·9 (38·3–43·5)	15·8 (14·5–17·2)	3·3 (2·9–3·6)	0·2 (0·1–0·3)	0·0 (0·0–0·0)	1·59 (1·53–1·65)	0·84 (0·81–0·88)	0·3 (0·29–0·32)
2000	0·0 (0·0–0·1)	30·5 (28·0–33·2)	129·9 (123·2–136·9)	88·8 (84·8–93·0)	40·1 (37·5–42·7)	15·4 (14·1–16·8)	3·2 (2·9–3·6)	0·2 (0·1–0·2)	0·0 (0·0–0·0)	1·54 (1·48–1·6)	0·8 (0·77–0·84)	0·29 (0·28–0·31)
2001	0·0 (0·0–0·1)	28·7 (26·2–31·4)	127·2 (119·8–134·0)	89·1 (84·8–93·3)	39·6 (37·0–42·4)	14·8 (13·6–16·1)	3·1 (2·8–3·5)	0·2 (0·1–0·2)	0·0 (0·0–0·0)	1·51 (1·46–1·57)	0·78 (0·74–0·81)	0·29 (0·27–0·31)
2002	0·0 (0·0–0·1)	27·4 (25·1–29·9)	125·8 (118·2–132·5)	90·2 (86·0–94·6)	39·6 (37·0–42·5)	14·3 (13·1–15·7)	3·0 (2·7–3·3)	0·1 (0·1–0·2)	0·0 (0·0–0·0)	1·5 (1·44–1·56)	0·77 (0·73–0·8)	0·29 (0·27–0·31)
2003	0·0 (0·0–0·1)	26·3 (24·2–28·8)	124·6 (117·2–131·9)	92·2 (87·9–96·7)	40·1 (37·4–42·9)	14·2 (13·0–15·6)	3·0 (2·7–3·3)	0·1 (0·1–0·2)	0·0 (0·0–0·0)	1·5 (1·45–1·56)	0·75 (0·72–0·79)	0·29 (0·27–0·31)
2004	0·0 (0·0–0·1)	25·5 (23·3–28·0)	123·5 (115·7–131·1)	94·3 (89·9–99·0)	40·9 (38·1–43·7)	14·3 (13·1–15·7)	2·9 (2·7–3·3)	0·1 (0·1–0·2)	0·0 (0·0–0·0)	1·51 (1·45–1·57)	0·75 (0·71–0·79)	0·29 (0·27–0·31)
2005	0·0 (0·0–0·1)	25·0 (22·7–27·5)	122·8 (115·2–130·6)	96·3 (91·8–101·2)	42·1 (39·2–45·0)	14·5 (13·2–15·9)	2·9 (2·6–3·2)	0·1 (0·1–0·2)	0·0 (0·0–0·0)	1·52 (1·46–1·58)	0·74 (0·7–0·78)	0·3 (0·28–0·31)
2006	0·0 (0·0–0·1)	24·8 (22·4–27·3)	122·3 (114·6–129·9)	98·2 (93·6–103·1)	43·5 (40·5–46·5)	14·8 (13·5–16·2)	2·9 (2·6–3·2)	0·1 (0·1–0·2)	0·0 (0·0–0·0)	1·53 (1·47–1·6)	0·74 (0·69–0·78)	0·31 (0·29–0·32)
2007	0·0 (0·0–0·1)	24·4 (22·0–26·9)	121·7 (113·6–130·1)	100·0 (95·3–105·3)	44·8 (41·8–47·9)	15·5 (14·0–17·0)	2·9 (2·6–3·3)	0·1 (0·1–0·2)	0·0 (0·0–0·0)	1·55 (1·48–1·61)	0·73 (0·69–0·78)	0·32 (0·3–0·34)
2008	0·0 (0·0–0·1)	23·7 (21·4–26·2)	120·3 (112·2–129·1)	101·5 (96·5–106·9)	46·2 (43·1–49·6)	16·5 (14·9–18·1)	3·1 (2·7–3·4)	0·1 (0·1–0·2)	0·0 (0·0–0·0)	1·56 (1·5–1·62)	0·72 (0·67–0·77)	0·33 (0·31–0·35)
2009	0·0 (0·0–0·1)	23·0 (20·6–25·5)	118·5 (110·2–127·4)	102·6 (97·3–108·3)	48·1 (44·8–51·6)	17·5 (15·9–19·3)	3·2 (2·8–3·5)	0·1 (0·1–0·2)	0·0 (0·0–0·0)	1·57 (1·51–1·63)	0·71 (0·66–0·76)	0·34 (0·32–0·37)
2010	0·0 (0·0–0·1)	22·7 (20·4–25·3)	117·3 (109·0–126·0)	103·5 (98·0–109·2)	49·9 (46·4–53·5)	18·4 (16·7–20·2)	3·3 (2·9–3·6)	0·2 (0·1–0·2)	0·0 (0·0–0·0)	1·58 (1·51–1·64)	0·7 (0·65–0·75)	0·36 (0·33–0·39)
2011	0·0 (0·0–0·1)	22·9 (20·6–25·5)	116·9 (108·3–126·2)	104·0 (98·3–110·1)	51·0 (47·3–55·1)	19·3 (17·5–21·3)	3·4 (3·0–3·7)	0·2 (0·1–0·2)	0·0 (0·0–0·0)	1·59 (1·52–1·66)	0·7 (0·65–0·76)	0·37 (0·34–0·4)
2012	0·0 (0·0–0·1)	23·3 (20·7–25·9)	116·9 (107·5–126·5)	104·5 (98·4–111·0)	51·7 (47·8–56·3)	20·0 (18·0–22·2)	3·5 (3·1–3·9)	0·2 (0·1–0·3)	0·0 (0·0–0·0)	1·6 (1·52–1·68)	0·7 (0·64–0·76)	0·38 (0·35–0·41)
2013	0·0 (0·0–0·1)	23·6 (20·8–26·6)	116·9 (106·9–127·1)	104·9 (98·9–111·4)	52·1 (47·6–56·9)	20·7 (18·6–23·2)	3·6 (3·1–4·0)	0·2 (0·1–0·3)	0·0 (0·0–0·0)	1·61 (1·52–1·7)	0·7 (0·64–0·77)	0·38 (0·35–0·42)
2014	0·0 (0·0–0·1)	23·9 (20·9–27·2)	116·6 (105·8–128·1)	104·9 (98·4–112·1)	52·1 (47·1–57·7)	21·0 (18·5–23·8)	3·6 (3·1–4·2)	0·2 (0·1–0·3)	0·0 (0·0–0·0)	1·61 (1·51–1·71)	0·7 (0·64–0·77)	0·38 (0·35–0·42)
2015	0·0 (0·0–0·1)	24·2 (21·0–27·7)	115·7 (103·8–128·8)	104·5 (97·2–112·6)	51·7 (46·2–58·0)	21·0 (18·2–24·3)	3·7 (3·1–4·3)	0·2 (0·1–0·3)	0·0 (0·0–0·0)	1·61 (1·49–1·72)	0·7 (0·63–0·78)	0·38 (0·35–0·42)
2016	0·0 (0·0–0·1)	24·5 (21·2–28·2)	114·3 (101·3–128·5)	103·9 (96·3–113·1)	51·0 (45·1–58·2)	20·8 (17·5–24·5)	3·7 (3·0–4·4)	0·2 (0·1–0·3)	0·0 (0·0–0·0)	1·59 (1·47–1·72)	0·69 (0·61–0·78)	0·38 (0·34–0·42)
2017	0·0 (0·0–0·1)	24·8 (21·3–28·8)	113·7 (99·3–129·9)	103·4 (95·0–113·6)	50·3 (44·0–58·1)	20·5 (16·9–24·9)	3·7 (2·9–4·6)	0·2 (0·1–0·3)	0·0 (0·0–0·0)	1·58 (1·44–1·72)	0·69 (0·6–0·79)	0·37 (0·33–0·42)

Supplemental Results Table 5: Age-specific fertility rates per 1000, cumulative total fertility up to maternal age 25 (TFU25), cumulative total fertility from maternal age 30 to age 55 (TFO30), and cumulative total fertility, Azerbaijan, 1950-2017

Year	10-14	15-19	20-24	25-29	30-34	35-39	40-44	45-49	50-54	TFR	TFU25	TFO30
1950	0·0 (0·0–0·0)	28·5 (24·0–34·3)	220·8 (200·6–241·2)	203·3 (192·4–214·4)	174·0 (163·2–184·9)	127·5 (118·2–136·9)	69·0 (63·5–74·5)	14·1 (10·6–18·1)	0·3 (0·3–0·3)	4·19 (3·88–4·51)	1·25 (1·14–1·36)	1·92 (1·78–2·07)
1951	0·0 (0·0–0·0)	28·6 (24·2–34·2)	219·4 (199·9–238·7)	203·1 (192·5–213·4)	174·6 (164·6–184·8)	127·1 (118·7–135·6)	68·5 (63·5–73·3)	13·7 (10·3–17·4)	0·3 (0·3–0·3)	4·18 (3·9–4·45)	1·24 (1·14–1·34)	1·92 (1·8–2·05)
1952	0·0 (0·0–0·0)	29·0 (24·6–34·8)	219·3 (200·6–238·2)	205·5 (195·4–215·4)	177·1 (167·5–187·0)	128·1 (120·4–136·1)	68·9 (64·4–73·5)	13·4 (10·1–17·0)	0·3 (0·2–0·3)	4·21 (3·96–4·45)	1·24 (1·14–1·34)	1·94 (1·83–2·05)
1953	0·0 (0·0–0·0)	29·6 (25·2–35·3)	220·8 (202·8–239·0)	210·9 (200·7–221·0)	181·7 (172·2–191·5)	130·8 (123·1–138·3)	70·1 (65·6–74·3)	13·3 (10·0–16·8)	0·3 (0·2–0·3)	4·29 (4·07–4·51)	1·25 (1·16–1·35)	1·98 (1·89–2·07)
1954	0·0 (0·0–0·0)	30·5 (26·0–36·2)	223·4 (205·3–241·4)	217·8 (208·2–227·9)	187·7 (178·5–196·8)	134·5 (126·6–141·8)	71·8 (67·6–76·1)	13·2 (10·1–16·6)	0·3 (0·2–0·3)	4·4 (4·21–4·59)	1·27 (1·17–1·37)	2·04 (1·95–2·12)
1955	0·0 (0·0–0·0)	31·3 (26·8–37·5)	226·4 (209·5–243·7)	225·0 (215·5–234·4)	194·2 (185·8–202·5)	138·4 (130·9–145·7)	73·6 (69·5–77·8)	13·1 (10·0–16·5)	0·3 (0·2–0·3)	4·51 (4·34–4·68)	1·29 (1·19–1·38)	2·1 (2·02–2·17)
1956	0·0 (0·0–0·0)	32·2 (27·4–38·5)	229·6 (211·5–246·0)	232·2 (223·0–241·4)	201·0 (192·8–209·1)	142·2 (135·1–149·9)	75·4 (71·1–79·6)	13·0 (9·9–16·2)	0·2 (0·2–0·3)	4·63 (4·46–4·79)	1·31 (1·22–1·4)	2·16 (2·08–2·23)
1957	0·0 (0·0–0·0)	33·2 (28·2–39·3)	232·9 (214·5–250·4)	239·2 (230·1–248·6)	207·8 (199·1–215·8)	145·9 (138·8–153·7)	77·1 (72·7–81·2)	12·8 (9·8–16·3)	0·2 (0·2–0·3)	4·75 (4·58–4·91)	1·33 (1·23–1·42)	2·22 (2·14–2·3)
1958	0·0 (0·0–0·0)	34·1 (28·9–40·1)	236·2 (217·5–254·1)	245·9 (237·0–254·8)	214·6 (206·3–222·6)	149·3 (142·5–156·9)	78·6 (74·2–82·7)	12·7 (9·5–16·1)	0·2 (0·2–0·3)	4·86 (4·69–5·02)	1·35 (1·25–1·44)	2·28 (2·2–2·36)
1959	0·0 (0·0–0·0)	34·9 (29·5–41·1)	239·1 (221·0–256·5)	251·2 (242·0–260·1)	220·6 (212·5–228·7)	152·1 (145·3–159·1)	79·7 (75·5–83·7)	12·5 (9·3–16·0)	0·2 (0·2–0·2)	4·95 (4·78–5·12)	1·37 (1·27–1·46)	2·33 (2·24–2·41)
1960	0·0 (0·0–0·0)	35·5 (30·0–42·0)	240·9 (222·4–258·6)	254·3 (245·0–263·5)	225·4 (217·2–233·3)	153·9 (146·8–160·6)	80·2 (75·9–84·1)	12·2 (9·1–15·7)	0·2 (0·2–0·2)	5·01 (4·83–5·19)	1·38 (1·28–1·48)	2·36 (2·27–2·45)
1961	0·0 (0·0–0·0)	35·9 (30·7–42·4)	241·8 (223·0–260·5)	256·3 (246·6–265·4)	228·5 (220·0–236·6)	154·9 (147·6–162·1)	79·9 (75·5–84·2)	11·8 (8·7–15·6)	0·2 (0·2–0·2)	5·05 (4·86–5·23)	1·39 (1·28–1·49)	2·38 (2·28–2·47)
1962	0·0 (0·0–0·0)	36·3 (30·8–42·6)	242·4 (223·1–262·1)	258·1 (248·0–267·7)	229·6 (220·7–238·3)	155·4 (147·5–163·4)	78·7 (74·0–83·4)	11·5 (8·3–15·2)	0·2 (0·2–0·2)	5·06 (4·86–5·26)	1·39 (1·29–1·5)	2·38 (2·27–2·48)
1963	0·0 (0·0–0·0)	36·5 (31·1–43·0)	242·3 (222·1–262·6)	258·4 (248·3–268·3)	227·9 (218·7–237·4)	154·8 (146·2–163·9)	76·4 (71·0–81·8)	11·1 (8·0–14·8)	0·2 (0·2–0·2)	5·04 (4·82–5·26)	1·39 (1·29–1·5)	2·35 (2·24–2·46)
1964	0·0 (0·0–0·0)	36·5 (30·9–43·0)	241·1 (221·1–262·4)	256·8 (246·3–267·0)	223·9 (213·8–234·4)	152·6 (142·8–162·7)	73·1 (67·1–79·0)	10·6 (7·5–14·3)	0·2 (0·2–0·2)	4·97 (4·72–5·24)	1·39 (1·28–1·51)	2·3 (2·18–2·42)
1965	0·0 (0·0–0·0)	36·5 (30·7–42·9)	239·5 (218·1–262·3)	254·7 (243·7–265·8)	219·1 (208·3–231·1)	149·9 (138·7–161·1)	69·4 (63·0–76·0)	10·1 (7·1–13·7)	0·2 (0·2–0·2)	4·9 (4·61–5·2)	1·38 (1·27–1·5)	2·24 (2·11–2·39)
1966	0·0 (0·0–0·0)	36·5 (30·8–42·9)	238·3 (216·9–262·2)	253·1 (242·4–265·3)	214·3 (202·4–227·0)	147·5 (135·8–159·7)	65·7 (58·8–73·0)	9·7 (6·9–13·2)	0·2 (0·2–0·2)	4·83 (4·52–5·16)	1·37 (1·26–1·5)	2·19 (2·04–2·35)
1967	0·0 (0·0–0·0)	36·7 (31·0–42·8)	237·3 (215·7–261·7)	251·5 (240·5–263·9)	209·5 (197·4–223·0)	145·1 (133·5–158·1)	62·0 (55·3–69·9)	9·2 (6·5–12·7)	0·2 (0·2–0·2)	4·76 (4·44–5·11)	1·37 (1·26–1·5)	2·13 (1·98–2·3)
1968	0·0 (0·0–0·0)	36·7 (31·2–42·8)	236·1 (214·6–260·2)	249·3 (237·8–262·1)	204·7 (192·8–218·4)	142·8 (131·7–155·6)	58·4 (52·1–66·1)	8·9 (6·3–12·1)	0·2 (0·2–0·2)	4·69 (4·38–5·04)	1·36 (1·25–1·49)	2·07 (1·93–2·25)
1969	0·0 (0·0–0·0)	36·3 (30·9–42·1)	234·6 (214·0–257·0)	246·6 (234·7–258·9)	199·5 (187·8–212·3)	140·0 (129·3–151·6)	55·1 (49·0–61·9)	8·4 (5·9–11·5)	0·2 (0·2–0·2)	4·6 (4·31–4·93)	1·35 (1·24–1·47)	2·02 (1·88–2·17)
1970	0·0 (0·0–0·0)	35·3 (30·1–40·9)	232·8 (213·3–252·5)	244·1 (232·1–256·7)	193·4 (181·4–206·0)	136·2 (125·9–147·2)	52·1 (46·6–58·1)	7·9 (5·6–10·7)	0·2 (0·1–0·2)	4·51 (4·24–4·79)	1·34 (1·23–1·45)	1·95 (1·82–2·09)
1971	0·0 (0·0–0·0)	34·3 (29·3–39·6)	230·8 (211·5–249·9)	242·0 (230·5–253·8)	186·9 (175·8–198·9)	131·5 (121·7–141·8)	49·4 (44·4–55·1)	7·4 (5·1–10·1)	0·1 (0·1–0·1)	4·41 (4·16–4·67)	1·33 (1·22–1·43)	1·88 (1·75–2·01)
1972	0·0 (0·0–0·0)	33·5 (28·6–38·8)	228·8 (210·8–247·3)	240·4 (229·2–251·7)	180·3 (169·0–191·7)	126·1 (116·6–136·4)	47·1 (42·1–52·1)	6·8 (4·8–9·3)	0·1 (0·1–0·1)	4·31 (4·09–4·55)	1·31 (1·22–1·4)	1·8 (1·68–1·94)
1973	0·0 (0·0–0·0)	32·9 (28·1–38·0)	226·7 (209·8–243·3)	238·7 (227·7–249·6)	173·5 (162·9–184·8)	119·8 (110·3–129·2)	44·8 (40·0–49·9)	6·2 (4·4–8·6)	0·1 (0·1–0·1)	4·21 (3·99–4·44)	1·3 (1·21–1·38)	1·72 (1·6–1·85)
1974	0·0 (0·0–0·0)	32·0 (27·4–37·0)	224·1 (207·4–240·2)	235·9 (225·2–246·1)	166·7 (156·2–177·2)	112·4 (103·1–121·6)	42·5 (37·9–47·3)	5·7 (4·0–7·7)	0·1 (0·1–0·1)	4·1 (3·89–4·31)	1·28 (1·19–1·36)	1·64 (1·51–1·76)
1975	0·0 (0·0–0·0)	30·7 (26·2–35·7)	220·4 (204·4–236·6)	231·7 (221·4–241·6)	159·9 (149·3–170·5)	104·1 (95·3–112·8)	40·0 (35·6–44·6)	5·2 (3·6–7·0)	0·1 (0·1–0·1)	3·96 (3·76–4·17)	1·26 (1·17–1·34)	1·55 (1·42–1·67)
1976	0·0 (0·0–0·0)	29·3 (25·2–33·9)	216·5 (200·6–232·0)	226·9 (216·5–236·7)	153·6 (142·7–163·7)	95·5 (87·2–103·8)	37·2 (33·2–41·5)	4·7 (3·3–6·3)	0·1 (0·1–0·1)	3·82 (3·62–4·02)	1·23 (1·15–1·31)	1·46 (1·34–1·57)
1977	0·0 (0·0–0·0)	28·2 (24·1–32·5)	212·6 (197·7–227·9)	221·8 (212·7–231·4)	147·7 (137·3–157·7)	87·2 (79·4–94·8)	34·3 (30·5–38·2)	4·2 (3·0–5·7)	0·1 (0·1–0·1)	3·68 (3·5–3·87)	1·2 (1·12–1·29)	1·37 (1·27–1·47)
1978	0·0 (0·0–0·0)	27·7 (23·7–31·9)	208·9 (193·6–224·4)	216·4 (208·0–225·7)	141·9 (131·9–151·6)	79·4 (72·6–86·4)	31·1 (27·7–34·6)	3·8 (2·6–5·1)	0·1 (0·1–0·1)	3·55 (3·38–3·73)	1·18 (1·1–1·26)	1·28 (1·19–1·37)
1979	0·0 (0·0–0·0)	27·6 (23·6–31·8)	205·5 (190·5–221·9)	211·1 (203·0–220·2)	136·5 (127·1–146·3)	72·3 (66·0–78·6)	27·9 (24·8–30·9)	3·4 (2·4–4·6)	0·1 (0·1–0·1)	3·42 (3·26–3·59)	1·17 (1·09–1·25)	1·2 (1·12–1·28)
1980	0·0 (0·0–0·0)	27·4 (23·5–31·6)	202·5 (188·2–218·2)	205·9 (197·6–214·8)	131·9 (123·2–141·0)	66·1 (60·4–72·0)	24·7 (22·1–27·5)	3·0 (2·1–4·1)	0·1 (0·1–0·1)	3·31 (3·15–3·47)	1·15 (1·07–1·23)	1·13 (1·06–1·19)
1981	0·0 (0·0–0·0)	27·2 (23·3–31·4)	200·0 (186·0–214·1)	201·1 (193·1–210·0)	128·0 (120·3–136·5)	60·7 (55·2–66·1)	21·8 (19·5–24·3)	2·6 (1·9–3·6)	0·1 (0·0–0·1)	3·21 (3·05–3·36)	1·14 (1·06–1·21)	1·07 (1·01–1·12)
1982	0·0 (0·0–0·0)	27·6 (23·8–31·7)	198·0 (184·0–212·1)	197·1 (189·0–205·6)	125·1 (117·6–132·9)	56·1 (51·2–61·3)	19·1 (17·1–21·3)	2·3 (1·7–3·2)	0·0 (0·0–0·0)	3·13 (2·98–3·28)	1·13 (1·06–1·2)	1·01 (0·96–1·07)
1983	0·0 (0·0–0·0)	28·8 (24·8–33·4)	196·6 (183·3–210·8)	194·2 (186·4–202·6)	122·7 (115·7–129·9)	52·4 (47·8–57·4)	16·9 (15·1–18·9)	2·1 (1·5–2·8)	0·0 (0·0–0·0)	3·07 (2·93–3·22)	1·13 (1·06–1·2)	0·97 (0·92–1·02)
1984	0·0 (0·0–0·0)	30·3 (26·1–35·1)	196·3 (183·4–210·5)	192·4 (184·5–201·0)	120·0 (113·1–127·1)	49·6 (45·5–54·2)	15·1 (13·4–16·9)	1·8 (1·3–2·5)	0·0 (0·0–0·0)	3·03 (2·9–3·17)	1·13 (1·06–1·21)	0·93 (0·88–0·99)
1985	0·0 (0·0–0·0)	31·7 (27·2–36·6)	196·4 (183·4–210·3)	190·5 (182·7–198·8)	116·2 (109·2–123·1)	47·4 (43·3–51·7)	13·6 (12·0–15·3)	1·6 (1·2–2·2)	0·0 (0·0–0·0)	2·99 (2·86–3·12)	1·14 (1·07–1·21)	0·89 (0·84–0·95)
1986	0·0 (0·0–0·0)	32·9 (28·3–38·0)	196·4 (182·8–210·4)	187·8 (180·3–196·4)	112·2 (105·8–118·7)	45·5 (41·9–49·6)	12·4 (10·9–13·9)	1·4 (1·0–2·0)	0·0 (0·0–0·0)	2·94 (2·82–3·07)	1·15 (1·08–1·22)	0·86 (0·81–0·91)
1987	0·0 (0·0–0·0)	34·3 (29·6–39·6)	196·0 (183·1–210·2)	184·7 (177·5–193·0)	108·8 (102·5–114·9)	44·2 (40·8–47·9)	11·3 (10·0–12·7)	1·3 (0·9–1·7)	0·0 (0·0–0·0)	2·9 (2·79–3·02)	1·15 (1·08–1·22)	0·83 (0·78–0·88)
1988	0·0 (0·0–0·0)	36·2 (31·5–41·5)	195·7 (182·3–209·3)	181·5 (174·5–189·3)	105·8 (99·9–112·1)	43·3 (40·0–46·8)	10·4 (9·2–11·7)	1·1 (0·8–1·6)	0·0 (0·0–0·0)	2·87 (2·77–2·98)	1·16 (1·09–1·23)	0·8 (0·76–0·85)
1989	0·0 (0·0–0·0)	38·4 (33·3–44·0)	195·4 (182·0–209·8)	178·0 (170·9–185·6)	102·7 (96·8–108·7)	42·3 (39·1–45·6)	9·7 (8·5–10·9)	1·0 (0·7–1·4)	0·0 (0·0–0·0)	2·84 (2·73–2·95)	1·17 (1·1–1·24)	0·78 (0·73–0·82)
1990	0·0 (0·0–0·0)	40·0 (34·8–45·6)	194·6 (181·9–209·1)	172·8 (165·9–180·2)	98·9 (93·1–104·6)	40·7 (37·6–43·9)	9·1 (8·0–10·1)	0·9 (0·7–1·3)	0·0 (0·0–0·0)	2·78 (2·68–2·9)	1·17 (1·1–1·25)	0·75 (0·71–0·79)
1991	0·0 (0·0–0·0)	40·9 (35·4–46·5)	192·3 (179·6–205·9)	163·5 (156·7–170·7)	94·0 (88·5–99·5)	38·5 (35·4–41·6)	8·5 (7·5–9·4)	0·9 (0·6–1·2)	0·0 (0·0–0·0)	2·69 (2·58–2·8)	1·17 (1·1–1·24)	0·71 (0·67–0·75)
1992	0·0 (0·0–0·0)	41·1 (35·5–46·5)	187·8 (175·3–200·6)	150·1 (143·5–157·0)	88·4 (83·1–93·5)	36·1 (33·2–39·1)	8·0 (7·1–8·8)	0·8 (0·6–1·1)	0·0 (0·0–0·0)	2·56 (2·46–2·67)	1·14 (1·08–1·21)	0·67 (0·63–0·71)
1993	0·0 (0·0–0·0)	40·6 (35·2–46·1)	181·2 (169·0–193·8)	139·6 (133·3–145·8)	82·6 (77·6–87·1)	33·8 (31·0–36·4)	7·5 (6·8–8·3)	0·7 (0·5–1·0)	0·0 (0·0–0·0)	2·43 (2·33–2·53)	1·11 (1·05–1·17)	0·62 (0·59–0·66)
1994	0·0 (0·0–0·0)	39·9 (34·7–45·3)	174·3 (162·1–186·8)	133·6 (127·6–139·7)	77·0 (72·5–81·3)	31·5 (29·1–34·1)	7·1 (6·4–7·8)	0·7 (0·5–0·9)	0·0 (0·0–0·0)	2·32 (2·23–2·42)	1·07 (1·01–1·14)	0·58 (0·55–0·61)
1995	0·0 (0·0–0·0)	38·8 (33·7–44·1)	167·5 (155·8–179·7)	128·0 (122·0–134·0)	71·7 (67·3–75·8)	29·2 (27·1–31·6)	6·7 (6·0–7·4)	0·6 (0·4–0·9)	0·0 (0·0–0·0)	2·21 (2·12–2·31)	1·03 (0·97–1·1)	0·54 (0·51–0·57)
1996	0·0 (0·0–0·0)	37·2 (32·4–42·6)	160·7 (148·2–173·1)	122·5 (116·6–128·6)	66·5 (62·7–70·5)	26·9 (24·8–29·1)	6·3 (5·7–7·0)	0·6 (0·4–0·8)	0·0 (0·0–0·0)	2·1 (2·0–2·2)	0·99 (0·92–1·06)	0·5 (0·47–0·53)
1997	0·0 (0·0–0·0)	35·6 (30·9–40·7)	154·2 (142·3–166·5)	117·7 (111·8–123·8)	62·3 (58·6–66·2)	24·7 (22·8–26·8)	5·9 (5·4–6·5)	0·5 (0·4–0·7)	0·0 (0·0–0·0)	2·0 (1·91–2·11)	0·95 (0·88–1·01)	0·47 (0·44–0·5)
1998	0·0 (0·0–0·0)	34·2 (29·6–39·2)	148·8 (137·2–160·6)	113·9 (108·1–119·6)	59·2 (55·8–63·1)	23·6 (21·8–25·5)	5·8 (5·2–6·4)	0·5 (0·4–0·7)	0·0 (0·0–0·0)	1·93 (1·84–2·03)	0·91 (0·85–0·98)	0·45 (0·42–0·47)
1999	0·0 (0·0–0·0)	33·5 (29·0–38·2)	144·9 (133·4–156·6)	111·1 (105·5–116·8)	57·1 (53·7–60·7)	23·3 (21·7–25·1)	5·8 (5·3–6·4)	0·5 (0·3–0·6)	0·0 (0·0–0·0)	1·88 (1·79–1·98)	0·89 (0·83–0·96)	0·43 (0·41–0·46)
2000	0·0 (0·0–0·0)	33·5 (29·1–38·5)	142·8 (131·2–154·9)	109·5 (103·9–115·6)	55·8 (52·5–59·2)	22·9 (21·2–24·7)	5·8 (5·2–6·4)	0·4 (0·3–0·6)	0·0 (0·0–0·0)	1·85 (1·76–1·95)	0·88 (0·82–0·95)	0·42 (0·4–0·45)
2001	0·0 (0·0–0·0)	34·0 (29·6–39·2)	142·3 (130·4–154·4)	108·9 (103·3–114·7)	55·2 (51·9–58·5)	22·4 (20·7–24·2)	5·7 (5·1–6·2)	0·4 (0·3–0·5)	0·0 (0·0–0·0)	1·84 (1·75–1·94)	0·88 (0·82–0·95)	0·42 (0·4–0·44)
2002	0·0 (0·0–0·0)	35·1 (30·4–40·4)	143·3 (131·5–155·6)	109·7 (103·9–115·6)	55·9 (52·4–59·7)	22·2 (20·4–23·8)	5·5 (5·0–6·1)	0·4 (0·3–0·5)	0·0 (0·0–0·0)	1·86 (1·77–1·96)	0·89 (0·83–0·96)	0·42 (0·4–0·44)
2003	0·0 (0·0–0·0)	36·8 (31·9–42·3)	145·8 (134·1–158·1)	111·8 (106·0–117·6)	57·8 (54·2–61·9)	22·4 (20·5–24·2)	5·5 (5·0–6·1)	0·4 (0·3–0·5)	0·0 (0·0–0·0)	1·9 (1·81–2·0)	0·91 (0·84–0·98)	0·43 (0·41–0·45)
2004	0·0 (0·0–0·0)	38·8 (33·6–44·4)	148·9 (136·2–161·4)	114·0 (107·9–120·2)	59·9 (55·9–64·2)	23·2 (21·2–25·2)	5·6 (5·1–6·2)	0·4 (0·3–0·5)	0·0 (0·0–0·0)	1·95 (1·86–2·05)	0·94 (0·87–1·01)	0·45 (0·42–0·47)
2005	0·0 (0·0–0·0)	40·9 (35·5–46·9)	151·4 (138·4–163·8)	115·6 (109·2–121·9)	61·3 (57·1–65·8)	24·0 (22·1–26·1)	5·8 (5·2–6·3)	0·4 (0·3–0·5)	0·0 (0·0–0·0)	2·0 (1·9–2·1)	0·96 (0·89–1·04)	0·46 (0·44–0·48)
2006	0·0 (0·0–0·0)	43·6 (37·8–50·1)	153·6 (139·9–166·1)	116·8 (109·9–123·3)	62·3 (57·8–66·9)	24·6 (22·6–26·8)	5·9 (5·3–6·5)	0·4 (0·3–0·6)	0·0 (0·0–0·0)	2·04 (1·93–2·15)	0·99 (0·91–1·06)	0·47 (0·44–0·49)
2007	0·0 (0·0–0·0)	47·2 (40·8–54·0)	156·3 (141·8–169·9)	118·0 (110·7–125·0)	63·2 (58·8–68·0)	25·2 (23·0–27·4)	6·0 (5·4–6·6)	0·4 (0·3–0·6)	0·0 (0·0–0·0)	2·08 (1·96–2·2)	1·02 (0·94–1·1)	0·47 (0·45–0·5)
2008	0·0 (0·0–0·0)	49·9 (43·1–57·3)	158·4 (143·6–172·7)	118·9 (111·8–125·9)	63·5 (58·9–68·4)	25·5 (23·2–27·8)	5·9 (5·4–6·6)	0·5 (0·3–0·6)	0·0 (0·0–0·0)	2·11 (1·99–2·24)	1·04 (0·96–1·12)	0·48 (0·45–0·5)
2009	0·0 (0·0–0·0)	49·5 (42·8–56·8)	158·6 (143·6–173·5)	119·4 (112·0–126·7)	62·9 (58·3–68·0)	25·3 (23·1–27·7)	5·8 (5·2–6·5)	0·5 (0·3–0·6)	0·0 (0·0–0·0)	2·11 (1·97–2·25)	1·04 (0·96–1·12)	0·47 (0·45–0·5)
2010	0·0 (0·0–0·0)	48·1 (41·5–55·3)	157·8 (142·8–173·7)	119·5 (112·0–127·2)	62·2 (57·6–67·1)	25·2 (23·0–27·5)	5·6 (5·0–6·2)	0·5 (0·3–0·6)	0·0 (0·0–0·0)	2·09 (1·96–2·25)	1·03 (0·95–1·12)	0·47 (0·44–0·5)
2011	0·0 (0·0–0·0)	47·8 (41·1–54·9)	157·4 (142·6–173·4)	119·6 (112·1–127·4)	61·5 (56·5–66·7)	24·9 (22·7–27·4)	5·4 (4·8–6·1)	0·5 (0·3–0·7)	0·0 (0·0–0·0)	2·09 (1·94–2·24)	1·03 (0·94–1·12)	0·46 (0·43–0·49)
2012	0·0 (0·0–0·0)	47·6 (41·1–55·0)	156·7 (141·4–173·5)	119·4 (111·6–127·5)	60·6 (55·5–65·8)	24·4 (22·0–27·0)	5·2 (4·6–5·9)	0·5 (0·3–0·7)	0·0 (0·0–0·0)	2·07 (1·92–2·23)	1·02 (0·93–1·12)	0·45 (0·42–0·49)
2013	0·0 (0·0–0·0)	47·3 (40·8–54·7)	155·7 (139·6–173·4)	119·0 (111·1–127·7)	59·5 (54·4–65·0)	23·7 (21·2–26·7)	5·0 (4·4–5·7)	0·5 (0·3–0·7)	0·0 (0·0–0·0)	2·05 (1·89–2·24)	1·01 (0·91–1·13)	0·44 (0·41–0·48)
2014	0·0 (0·0–0·0)	46·8 (40·2–54·2)	154·5 (138·2–172·7)	118·6 (110·5–127·6)	58·4 (52·9–64·3)	23·0 (20·3–26·1)	4·8 (4·2–5·6)	0·5 (0·3–0·7)	0·0 (0·0–0·0)	2·03 (1·86–2·24)	1·01 (0·9–1·13)	0·43 (0·39–0·48)
2015	0·0 (0·0–0·0)	45·9 (39·2–53·7)	152·6 (134·7–171·9)	118·3 (109·5–127·8)	57·0 (51·4–63·2)	22·1 (19·2–25·4)	4·7 (4·0–5·5)	0·5 (0·3–0·7)	0·0 (0·0–0·0)	2·01 (1·81–2·23)	0·99 (0·87–1·13)	0·42 (0·38–0·47)
2016	0·0 (0·0–0·0)	44·8 (38·0–52·5)	149·9 (131·5–170·3)	118·3 (109·2–128·3)	55·9 (49·6–62·6)	21·3 (18·2–24·9)	4·5 (3·8–5·4)	0·5 (0·3–0·7)	0·0 (0·0–0·0)	1·98 (1·75–2·22)	0·97 (0·85–1·11)	0·41 (0·36–0·47)
2017	0·0 (0·0–0·0)	44·1 (37·4–52·0)	148·3 (128·8–169·9)	118·6 (108·9–129·2)	55·6 (48·9–63·2)	21·0 (17·5–25·2)	4·5 (3·6–5·5)	0·5 (0·3–0·7)	0·0 (0·0–0·0)	1·96 (1·73–2·23)	0·96 (0·83–1·11)	0·41 (0·35–0·47)

Supplemental Results Table 5: Age-specific fertility rates per 1000, cumulative total fertility up to maternal age 25 (TFU25), cumulative total fertility from maternal age 30 to age 55 (TFO30), and cumulative total fertility, Georgia, 1950-2017

Year	10-14	15-19	20-24	25-29	30-34	35-39	40-44	45-49	50-54	TFR	TFU25	TFO30
1950	0·2 (0·1–0·4)	25·8 (21·7–30·5)	163·3 (145·2–182·1)	136·5 (127·1–146·2)	112·2 (102·8–122·1)	70·7 (62·3–79·8)	29·4 (25·7–33·4)	7·8 (6·4–9·3)	0·1 (0·1–0·2)	2·73 (2·48–2·99)	0·95 (0·84–1·06)	1·1 (1·0–1·2)
1951	0·2 (0·1–0·4)	25·2 (21·2–29·8)	160·1 (143·2–178·1)	137·5 (128·3–146·6)	110·0 (101·0–119·1)	67·5 (59·6–75·3)	27·6 (24·5–30·9)	7·4 (6·2–8·6)	0·1 (0·1–0·1)	2·68 (2·44–2·92)	0·93 (0·82–1·04)	1·06 (0·98–1·15)
1952	0·2 (0·1–0·4)	24·8 (20·8–29·2)	157·2 (139·7–174·7)	138·8 (130·3–147·6)	108·1 (99·9–116·8)	64·5 (57·5–71·7)	25·9 (23·2–28·8)	7·1 (6·1–8·3)	0·1 (0·1–0·1)	2·63 (2·42–2·85)	0·91 (0·8–1·02)	1·03 (0·95–1·11)
1953	0·2 (0·1–0·4)	24·4 (20·4–28·8)	155·0 (138·4–171·9)	141·0 (132·0–150·0)	106·7 (98·6–115·2)	61·9 (55·6–69·1)	24·4 (21·9–27·2)	6·8 (5·9–7·9)	0·1 (0·1–0·1)	2·6 (2·4–2·81)	0·9 (0·8–1·0)	1·0 (0·93–1·07)
1954	0·2 (0·1–0·4)	24·0 (20·2–28·5)	153·0 (136·2–169·8)	143·2 (134·6–151·9)	105·4 (97·8–114·3)	59·4 (53·5–66·2)	22·9 (20·5–25·6)	6·5 (5·6–7·5)	0·1 (0·1–0·1)	2·57 (2·38–2·77)	0·89 (0·79–0·98)	0·97 (0·9–1·04)
1955	0·2 (0·1–0·4)	23·7 (19·8–28·3)	150·9 (134·8–167·3)	145·1 (136·5–153·9)	104·1 (96·1–112·3)	57·0 (51·3–63·6)	21·6 (19·3–24·1)	6·2 (5·4–7·2)	0·1 (0·1–0·1)	2·54 (2·35–2·75)	0·87 (0·78–0·98)	0·95 (0·87–1·02)
1956	0·2 (0·1–0·4)	23·6 (19·7–28·0)	149·4 (133·7–165·2)	147·4 (138·4–156·7)	103·8 (95·8–112·2)	55·5 (49·7–61·9)	20·6 (18·4–23·1)	6·0 (5·1–6·9)	0·1 (0·1–0·1)	2·53 (2·32–2·75)	0·87 (0·77–0·97)	0·93 (0·86–1·01)
1957	0·2 (0·1–0·4)	24·0 (20·2–28·5)	149·3 (133·5–164·8)	149·8 (140·6–159·0)	104·8 (97·2–112·9)	55·3 (49·6–61·6)	20·2 (17·9–22·7)	5·8 (4·9–6·7)	0·1 (0·1–0·1)	2·55 (2·34–2·77)	0·87 (0·77–0·96)	0·93 (0·85–1·01)
1958	0·2 (0·1–0·4)	25·0 (21·0–29·4)	151·3 (135·3–167·5)	151·4 (142·3–160·8)	106·4 (98·7–114·6)	55·9 (50·1–62·4)	20·0 (17·8–22·4)	5·5 (4·7–6·4)	0·1 (0·1–0·1)	2·58 (2·38–2·8)	0·88 (0·79–0·98)	0·94 (0·86–1·03)
1959	0·2 (0·1–0·4)	25·9 (21·7–30·7)	153·8 (137·9–170·2)	152·5 (143·2–162·2)	107·6 (99·8–115·6)	56·3 (50·2–63·2)	19·8 (17·5–22·1)	5·2 (4·4–6·1)	0·1 (0·1–0·1)	2·61 (2·41–2·81)	0·9 (0·81–1·0)	0·95 (0·87–1·03)
1960	0·2 (0·1–0·4)	26·6 (22·3–31·6)	155·4 (139·1–172·0)	153·3 (143·9–162·5)	108·0 (100·3–116·2)	56·1 (50·1–62·7)	19·3 (17·1–21·5)	4·9 (4·1–5·8)	0·1 (0·1–0·1)	2·62 (2·43–2·81)	0·91 (0·82–1·01)	0·94 (0·87–1·02)
1961	0·2 (0·1–0·4)	27·3 (22·8–32·3)	156·3 (140·3–173·0)	153·6 (144·1–162·6)	107·6 (99·8–115·8)	55·5 (49·6–62·5)	18·6 (16·4–21·0)	4·6 (3·9–5·5)	0·1 (0·1–0·1)	2·62 (2·43–2·81)	0·92 (0·83–1·01)	0·93 (0·86–1·0)
1962	0·2 (0·1–0·4)	27·9 (23·4–33·1)	157·3 (140·7–174·0)	153·8 (144·4–163·1)	106·5 (98·1–114·9)	54·9 (48·7–61·9)	17·9 (15·7–20·3)	4·3 (3·5–5·2)	0·1 (0·1–0·1)	2·61 (2·42–2·81)	0·93 (0·83–1·02)	0·92 (0·85–0·99)
1963	0·2 (0·1–0·4)	28·6 (24·0–34·1)	158·5 (140·8–175·4)	153·8 (144·4–163·2)	105·0 (96·5–113·8)	54·2 (47·7–61·2)	17·2 (14·9–19·7)	4·0 (3·2–4·9)	0·1 (0·1–0·1)	2·61 (2·4–2·82)	0·94 (0·84–1·03)	0·9 (0·83–0·98)
1964	0·2 (0·1–0·4)	29·2 (24·6–35·0)	159·9 (142·5–177·7)	153·5 (143·9–163·6)	103·5 (94·5–112·6)	53·5 (46·6–61·0)	16·6 (14·1–19·0)	3·8 (3·0–4·7)	0·1 (0·1–0·1)	2·6 (2·39–2·82)	0·95 (0·85–1·05)	0·89 (0·81–0·97)
1965	0·2 (0·1–0·4)	29·9 (25·2–36·1)	161·2 (143·9–179·5)	153·1 (143·2–163·5)	102·0 (92·8–111·3)	52·6 (45·3–60·5)	15·9 (13·5–18·5)	3·6 (2·8–4·5)	0·1 (0·1–0·1)	2·59 (2·38–2·81)	0·96 (0·86–1·07)	0·87 (0·79–0·96)
1966	0·2 (0·1–0·4)	30·9 (26·0–37·0)	162·4 (144·2–182·6)	152·5 (142·5–163·2)	100·6 (90·7–110·6)	51·4 (44·2–59·2)	15·3 (12·9–18·2)	3·3 (2·6–4·1)	0·1 (0·1–0·1)	2·58 (2·38–2·79)	0·97 (0·86–1·09)	0·85 (0·77–0·95)
1967	0·2 (0·1–0·4)	32·3 (27·2–38·3)	164·3 (145·9–185·7)	152·1 (141·2–163·0)	99·8 (89·2–110·4)	50·7 (42·7–58·9)	14·9 (12·4–17·8)	3·0 (2·3–3·9)	0·1 (0·1–0·1)	2·59 (2·38–2·79)	0·98 (0·87–1·12)	0·84 (0·75–0·94)
1968	0·2 (0·1–0·5)	33·5 (28·2–39·6)	167·7 (147·7–190·3)	152·0 (140·8–163·5)	99·0 (88·4–110·0)	50·1 (41·9–58·6)	14·5 (11·9–17·4)	2·8 (2·1–3·6)	0·1 (0·1–0·1)	2·6 (2·4–2·81)	1·01 (0·89–1·14)	0·83 (0·73–0·94)
1969	0·2 (0·1–0·5)	34·1 (28·9–40·0)	169·9 (150·7–193·0)	151·1 (139·7–162·8)	96·8 (86·0–107·4)	48·3 (40·1–56·5)	13·8 (11·3–16·7)	2·5 (1·9–3·3)	0·0 (0·0–0·1)	2·58 (2·38–2·79)	1·02 (0·9–1·16)	0·81 (0·7–0·91)
1970	0·2 (0·1–0·5)	34·2 (28·9–40·2)	168·7 (148·2–193·0)	148·7 (137·3–160·4)	93·2 (82·7–103·8)	45·3 (37·3–53·5)	13·0 (10·5–15·8)	2·3 (1·8–3·0)	0·0 (0·0–0·0)	2·53 (2·33–2·74)	1·02 (0·89–1·16)	0·77 (0·67–0·88)
1971	0·2 (0·1–0·5)	34·3 (29·1–40·2)	166·8 (145·6–189·5)	145·8 (134·7–157·8)	89·4 (79·5–100·1)	42·2 (34·8–50·3)	12·2 (9·9–15·0)	2·1 (1·6–2·8)	0·0 (0·0–0·0)	2·47 (2·27–2·68)	1·01 (0·89–1·14)	0·73 (0·63–0·84)
1972	0·2 (0·1–0·5)	33·7 (28·9–39·4)	165·5 (143·9–187·6)	143·2 (131·9–154·5)	85·5 (75·8–96·4)	39·2 (32·3–47·3)	11·5 (9·3–14·1)	2·0 (1·5–2·6)	0·0 (0·0–0·0)	2·4 (2·21–2·61)	1·0 (0·88–1·13)	0·69 (0·59–0·8)
1973	0·2 (0·1–0·5)	33·6 (28·6–39·0)	163·4 (143·2–183·6)	141·0 (129·9–152·2)	81·8 (72·6–92·2)	36·4 (30·1–44·0)	10·8 (8·8–13·2)	1·8 (1·4–2·4)	0·0 (0·0–0·0)	2·35 (2·16–2·54)	0·99 (0·87–1·11)	0·65 (0·57–0·76)
1974	0·2 (0·1–0·5)	35·1 (30·0–40·7)	162·2 (142·8–181·6)	140·4 (129·8–151·5)	79·5 (70·9–89·3)	34·6 (28·8–41·6)	10·4 (8·5–12·6)	1·7 (1·3–2·2)	0·0 (0·0–0·0)	2·32 (2·15–2·51)	0·99 (0·88–1·1)	0·63 (0·55–0·73)
1975	0·2 (0·1–0·5)	36·8 (31·7–42·5)	162·9 (144·4–180·8)	139·6 (129·1–150·5)	77·6 (69·5–86·6)	33·2 (27·8–39·0)	10·0 (8·3–12·0)	1·6 (1·2–2·0)	0·0 (0·0–0·0)	2·31 (2·14–2·49)	1·0 (0·9–1·1)	0·61 (0·54–0·7)
1976	0·2 (0·1–0·5)	37·9 (32·6–43·9)	162·7 (145·1–179·3)	135·9 (125·8–146·2)	74·2 (66·9–82·1)	30·8 (25·9–36·0)	9·2 (7·8–11·0)	1·4 (1·1–1·7)	0·0 (0·0–0·0)	2·26 (2·1–2·43)	1·0 (0·91–1·1)	0·58 (0·51–0·65)
1977	0·2 (0·1–0·5)	38·6 (33·3–44·5)	160·9 (145·4–176·5)	131·7 (122·4–141·2)	70·5 (63·5–78·1)	28·2 (24·1–32·9)	8·4 (7·1–9·9)	1·2 (1·0–1·5)	0·0 (0·0–0·0)	2·2 (2·04–2·36)	1·0 (0·91–1·08)	0·54 (0·48–0·61)
1978	0·2 (0·1–0·5)	39·7 (34·2–45·9)	159·7 (145·1–174·5)	129·3 (120·5–138·3)	68·1 (61·7–75·3)	26·5 (22·9–30·8)	7·8 (6·7–9·2)	1·1 (0·9–1·3)	0·0 (0·0–0·0)	2·16 (2·02–2·31)	1·0 (0·92–1·08)	0·52 (0·46–0·58)
1979	0·2 (0·1–0·5)	42·0 (36·1–48·2)	160·8 (147·4–175·1)	127·6 (119·4–136·1)	67·2 (61·1–73·9)	26·0 (22·6–29·7)	7·5 (6·5–8·6)	1·0 (0·8–1·2)	0·0 (0·0–0·0)	2·16 (2·03–2·3)	1·02 (0·94–1·09)	0·51 (0·46–0·56)
1980	0·3 (0·1–0·5)	44·4 (38·4–51·1)	162·5 (150·0–176·9)	125·6 (118·3–133·6)	67·0 (61·0–73·5)	26·1 (22·9–29·6)	7·2 (6·2–8·1)	0·9 (0·8–1·1)	0·0 (0·0–0·0)	2·17 (2·05–2·3)	1·04 (0·96–1·11)	0·51 (0·46–0·56)
1981	0·3 (0·1–0·5)	46·2 (40·3–53·0)	162·9 (150·7–176·7)	124·0 (116·8–131·3)	66·6 (61·1–72·6)	26·1 (22·9–29·7)	6·8 (5·9–7·7)	0·9 (0·7–1·1)	0·0 (0·0–0·0)	2·17 (2·06–2·28)	1·05 (0·97–1·12)	0·5 (0·46–0·55)
1982	0·3 (0·1–0·5)	47·1 (41·1–54·0)	161·7 (149·4–174·7)	123·5 (116·8–130·4)	66·0 (60·7–71·7)	25·8 (22·8–29·2)	6·4 (5·6–7·3)	0·9 (0·7–1·0)	0·0 (0·0–0·0)	2·16 (2·06–2·26)	1·05 (0·97–1·12)	0·5 (0·45–0·54)
1983	0·3 (0·1–0·5)	48·3 (42·1–55·3)	159·5 (147·2–172·0)	123·8 (117·2–130·4)	65·9 (60·5–71·3)	25·5 (22·5–28·9)	6·0 (5·4–6·8)	0·8 (0·7–1·0)	0·0 (0·0–0·0)	2·15 (2·05–2·25)	1·04 (0·97–1·11)	0·49 (0·45–0·54)
1984	0·3 (0·1–0·6)	50·8 (44·4–58·2)	158·6 (146·2–170·5)	122·9 (116·3–129·7)	66·4 (61·5–71·7)	25·4 (22·5–28·7)	5·9 (5·2–6·7)	0·8 (0·6–0·9)	0·0 (0·0–0·0)	2·16 (2·06–2·26)	1·05 (0·98–1·12)	0·49 (0·45–0·54)
1985	0·3 (0·1–0·6)	53·6 (46·9–61·4)	158·2 (145·6–169·9)	119·7 (113·5–126·3)	66·1 (61·2–71·2)	25·0 (22·2–28·0)	5·8 (5·1–6·5)	0·7 (0·6–0·8)	0·0 (0·0–0·0)	2·15 (2·05–2·25)	1·06 (0·99–1·14)	0·49 (0·45–0·53)
1986	0·3 (0·1–0·6)	55·1 (48·1–62·9)	155·9 (143·2–168·0)	115·9 (110·0–122·1)	64·7 (60·0–69·3)	23·6 (21·0–26·4)	5·6 (4·9–6·3)	0·6 (0·5–0·7)	0·0 (0·0–0·0)	2·11 (2·0–2·21)	1·06 (0·98–1·13)	0·47 (0·44–0·51)
1987	0·3 (0·1–0·6)	55·2 (48·0–63·3)	151·4 (140·1–163·5)	112·9 (107·0–118·8)	62·6 (57·8–66·9)	22·0 (19·6–24·6)	5·3 (4·7–5·9)	0·5 (0·4–0·6)	0·0 (0·0–0·0)	2·05 (1·95–2·15)	1·03 (0·96–1·11)	0·45 (0·42–0·48)
1988	0·3 (0·1–0·6)	55·1 (47·9–62·9)	145·8 (134·6–157·7)	110·4 (104·8–116·1)	60·3 (56·1–64·3)	21·2 (19·0–23·7)	5·1 (4·5–5·7)	0·4 (0·4–0·5)	0·0 (0·0–0·0)	1·99 (1·89–2·09)	1·01 (0·93–1·08)	0·44 (0·41–0·46)
1989	0·3 (0·1–0·6)	55·7 (48·7–63·5)	141·1 (130·6–152·7)	107·1 (101·5–112·3)	57·4 (53·6–61·0)	20·6 (18·5–23·1)	5·0 (4·4–5·6)	0·4 (0·3–0·5)	0·0 (0·0–0·0)	1·94 (1·84–2·03)	0·99 (0·92–1·05)	0·42 (0·39–0·44)
1990	0·3 (0·1–0·6)	55·2 (48·0–63·1)	136·6 (126·0–147·8)	101·5 (95·8–106·7)	52·3 (48·7–55·9)	19·7 (17·6–21·9)	4·7 (4·2–5·3)	0·4 (0·3–0·4)	0·0 (0·0–0·0)	1·85 (1·76–1·94)	0·96 (0·9–1·03)	0·39 (0·36–0·41)
1991	0·3 (0·1–0·6)	52·5 (45·4–59·8)	129·3 (119·1–140·6)	94·8 (89·4–99·9)	45·1 (41·9–48·4)	18·8 (16·8–20·9)	4·5 (4·0–5·1)	0·3 (0·3–0·4)	0·0 (0·0–0·0)	1·73 (1·64–1·81)	0·91 (0·85–0·97)	0·34 (0·32–0·37)
1992	0·3 (0·1–0·6)	49·2 (42·5–56·1)	119·8 (109·7–130·9)	89·5 (84·5–94·4)	41·4 (38·4–44·3)	18·3 (16·4–20·2)	4·4 (3·9–4·9)	0·3 (0·3–0·4)	0·0 (0·0–0·0)	1·62 (1·53–1·69)	0·85 (0·79–0·91)	0·32 (0·3–0·35)
1993	0·3 (0·1–0·5)	46·6 (40·6–53·4)	111·3 (101·4–122·0)	86·3 (81·5–91·0)	41·7 (38·8–44·7)	18·1 (16·3–20·0)	4·3 (3·8–4·8)	0·4 (0·3–0·5)	0·0 (0·0–0·0)	1·54 (1·47–1·62)	0·79 (0·74–0·85)	0·32 (0·3–0·34)
1994	0·3 (0·1–0·5)	45·2 (39·2–51·9)	105·6 (96·3–116·0)	83·9 (79·0–88·5)	41·7 (38·8–44·7)	17·8 (16·1–19·7)	4·2 (3·7–4·7)	0·4 (0·4–0·5)	0·0 (0·0–0·0)	1·5 (1·43–1·56)	0·76 (0·7–0·81)	0·32 (0·3–0·34)
1995	0·3 (0·1–0·5)	44·0 (38·2–50·0)	102·1 (92·7–112·1)	81·6 (76·7–86·3)	41·1 (38·3–44·0)	17·4 (15·7–19·3)	4·1 (3·6–4·6)	0·5 (0·4–0·6)	0·0 (0·0–0·0)	1·46 (1·39–1·53)	0·73 (0·68–0·79)	0·32 (0·3–0·34)
1996	0·2 (0·1–0·5)	41·4 (35·8–47·1)	98·5 (89·5–108·5)	79·7 (75·0–84·4)	41·3 (38·4–44·2)	17·4 (15·7–19·3)	4·1 (3·7–4·6)	0·5 (0·4–0·6)	0·0 (0·0–0·0)	1·42 (1·35–1·49)	0·7 (0·64–0·76)	0·32 (0·3–0·34)
1997	0·2 (0·1–0·5)	37·9 (32·8–43·3)	94·1 (85·3–103·7)	78·6 (74·1–83·1)	42·6 (39·7–45·7)	17·9 (16·1–19·8)	4·2 (3·8–4·7)	0·5 (0·5–0·6)	0·0 (0·0–0·0)	1·38 (1·31–1·46)	0·66 (0·6–0·72)	0·33 (0·31–0·35)
1998	0·2 (0·1–0·5)	35·7 (30·8–40·9)	91·2 (82·7–100·0)	79·4 (74·9–84·1)	44·6 (41·6–47·6)	18·8 (17·0–20·7)	4·3 (3·9–4·8)	0·6 (0·5–0·7)	0·0 (0·0–0·0)	1·37 (1·3–1·46)	0·64 (0·58–0·7)	0·34 (0·32–0·36)
1999	0·2 (0·1–0·5)	35·5 (30·5–40·7)	91·4 (82·3–100·7)	81·1 (76·5–85·9)	46·0 (42·7–49·3)	19·8 (17·8–21·7)	4·6 (4·1–5·1)	0·6 (0·5–0·7)	0·0 (0·0–0·0)	1·4 (1·32–1·49)	0·64 (0·57–0·7)	0·35 (0·33–0·38)
2000	0·2 (0·1–0·5)	36·3 (31·2–42·0)	93·9 (85·1–103·3)	83·0 (78·2–88·0)	46·6 (43·4–50·3)	20·5 (18·4–22·6)	4·8 (4·3–5·4)	0·6 (0·5–0·7)	0·0 (0·0–0·0)	1·43 (1·34–1·53)	0·65 (0·59–0·72)	0·36 (0·34–0·39)
2001	0·2 (0·1–0·5)	38·3 (33·0–44·4)	97·6 (88·0–107·6)	86·9 (81·9–92·3)	47·8 (44·3–51·6)	21·4 (19·2–23·8)	5·2 (4·6–5·8)	0·5 (0·4–0·6)	0·0 (0·0–0·0)	1·49 (1·4–1·59)	0·68 (0·62–0·75)	0·37 (0·35–0·41)
2002	0·2 (0·1–0·5)	41·1 (35·4–47·6)	101·9 (92·2–112·8)	92·2 (86·8–98·1)	49·5 (45·8–53·7)	22·7 (20·3–25·3)	5·6 (4·9–6·3)	0·5 (0·4–0·6)	0·0 (0·0–0·0)	1·57 (1·47–1·67)	0·72 (0·65–0·79)	0·39 (0·36–0·43)
2003	0·3 (0·1–0·5)	44·5 (38·5–51·3)	106·4 (95·7–118·3)	98·2 (92·3–104·5)	51·9 (48·0–56·2)	24·4 (21·7–27·2)	6·1 (5·4–6·9)	0·5 (0·4–0·6)	0·0 (0·0–0·0)	1·66 (1·57–1·76)	0·76 (0·69–0·83)	0·41 (0·38–0·45)
2004	0·3 (0·1–0·5)	47·2 (40·5–54·4)	110·8 (99·0–122·5)	103·3 (97·0–109·9)	54·1 (49·8–58·7)	25·9 (23·1–28·9)	6·7 (6·0–7·6)	0·5 (0·4–0·6)	0·0 (0·0–0·0)	1·74 (1·63–1·85)	0·79 (0·72–0·87)	0·44 (0·4–0·47)
2005	0·3 (0·1–0·5)	47·5 (40·8–54·6)	114·8 (102·3–127·3)	105·9 (99·5–112·4)	54·5 (49·9–59·1)	26·6 (23·7–29·8)	7·1 (6·3–8·0)	0·5 (0·4–0·6)	0·0 (0·0–0·0)	1·78 (1·67–1·89)	0·81 (0·74–0·89)	0·44 (0·41–0·48)
2006	0·3 (0·1–0·5)	47·7 (41·0–54·8)	119·1 (105·9–132·2)	108·5 (101·8–115·8)	56·6 (51·9–61·6)	27·2 (24·1–30·6)	7·3 (6·5–8·3)	0·5 (0·4–0·6)	0·0 (0·0–0·0)	1·84 (1·72–1·95)	0·84 (0·76–0·91)	0·46 (0·42–0·5)
2007	0·3 (0·1–0·5)	48·3 (41·4–55·9)	124·2 (111·0–138·6)	112·1 (105·2–119·6)	61·7 (56·5–66·9)	28·4 (25·0–32·0)	7·7 (6·7–8·8)	0·5 (0·4–0·6)	0·0 (0·0–0·0)	1·92 (1·8–2·04)	0·86 (0·78–0·95)	0·49 (0·44–0·54)
2008	0·3 (0·1–0·6)	48·8 (41·7–56·7)	128·8 (115·5–143·1)	115·2 (107·9–122·8)	66·3 (60·6–72·1)	29·6 (26·0–33·5)	8·0 (7·0–9·1)	0·6 (0·5–0·7)	0·0 (0·0–0·0)	1·99 (1·86–2·12)	0·89 (0·81–0·97)	0·52 (0·47–0·58)
2009	0·3 (0·1–0·6)	49·2 (42·0–57·2)	131·4 (117·9–146·9)	116·9 (109·4–124·3)	69·5 (63·6–75·7)	30·7 (26·7–34·4)	8·1 (7·1–9·3)	0·6 (0·5–0·7)	0·0 (0·0–0·0)	2·03 (1·91–2·17)	0·9 (0·83–0·99)	0·54 (0·49–0·6)
2010	0·3 (0·1–0·6)	49·0 (42·1–56·9)	132·5 (119·2–147·9)	117·8 (110·3–125·4)	71·8 (65·5–77·9)	31·7 (27·6–35·6)	8·2 (7·2–9·3)	0·7 (0·5–0·8)	0·0 (0·0–0·0)	2·06 (1·94–2·2)	0·91 (0·83–1·0)	0·56 (0·51–0·61)
2011	0·3 (0·1–0·6)	48·9 (41·8–56·9)	133·4 (120·1–149·3)	119·0 (111·1–126·7)	74·0 (67·5–80·3)	32·7 (28·8–36·9)	8·4 (7·4–9·4)	0·7 (0·6–0·9)	0·0 (0·0–0·0)	2·09 (1·96–2·23)	0·91 (0·83–1·01)	0·58 (0·53–0·63)
2012	0·3 (0·1–0·6)	49·0 (42·3–57·1)	134·3 (120·2–149·9)	120·7 (112·4–128·7)	76·4 (69·9–83·0)	34·0 (29·9–38·2)	8·6 (7·6–9·7)	0·8 (0·7–1·0)	0·0 (0·0–0·0)	2·12 (1·98–2·27)	0·92 (0·83–1·01)	0·6 (0·55–0·65)
2013	0·3 (0·1–0·6)	49·1 (42·2–57·3)	134·9 (120·0–150·4)	122·3 (113·7–130·9)	78·3 (71·6–85·3)	35·3 (30·8–40·0)	8·9 (7·7–10·1)	0·9 (0·7–1·1)	0·0 (0·0–0·0)	2·15 (1·99–2·31)	0·92 (0·83–1·03)	0·62 (0·56–0·67)
2014	0·3 (0·1–0·6)	48·5 (41·5–56·5)	134·3 (118·6–150·0)	123·0 (114·5–132·5)	78·6 (71·8–86·3)	36·5 (31·9–41·9)	9·2 (7·9–10·6)	1·0 (0·8–1·2)	0·0 (0·0–0·0)	2·16 (2·0–2·33)	0·92 (0·81–1·03)	0·63 (0·57–0·69)
2015	0·3 (0·1–0·5)	47·3 (40·3–55·1)	132·1 (115·7–148·6)	122·2 (113·6–132·1)	76·5 (69·4–85·5)	37·1 (31·9–43·0)	9·4 (8·0–11·1)	1·0 (0·8–1·2)	0·0 (0·0–0·0)	2·13 (1·96–2·31)	0·9 (0·78–1·02)	0·62 (0·55–0·7)
2016	0·3 (0·1–0·5)	46·5 (39·4–54·7)	128·8 (112·1–146·5)	120·7 (111·6–131·3)	73·1 (65·5–82·6)	36·5 (31·1–43·3)	9·2 (7·8–11·1)	0·9 (0·7–1·2)	0·0 (0·0–0·0)	2·08 (1·9–2·27)	0·88 (0·76–1·01)	0·6 (0·53–0·69)
2017	0·3 (0·1–0·5)	46·3 (39·3–54·6)	126·8 (109·2–146·5)	119·8 (109·9–131·5)	71·1 (62·7–81·3)	35·8 (29·6–43·9)	9·0 (7·3–11·3)	0·9 (0·7–1·2)	0·0 (0·0–0·0)	2·05 (1·86–2·25)	0·87 (0·74–1·01)	0·58 (0·5–0·69)

Supplemental Results Table 5: Age-specific fertility rates per 1000, cumulative total fertility up to maternal age 25 (TFU25), cumulative total fertility from maternal age 30 to age 55 (TFO30), and cumulative total fertility, Kazakhstan, 1950-2017

Year	10-14	15-19	20-24	25-29	30-34	35-39	40-44	45-49	50-54	TFR	TFU25	TFO30
1950	0·1<br>(0·0–0·1)	44·7<br>(40·9–49·5)	216·6<br>(202·0–231·4)	187·6<br>(178·3–197·2)	136·9<br>(127·2–146·7)	110·9<br>(100·6–121·3)	59·3<br>(53·3–65·5)	9·1<br>(6·4–12·4)	0·2<br>(0·2–0·2)	3·83<br>(3·67–3·98)	1·31<br>(1·23–1·39)	1·58<br>(1·47–1·69)
1951	0·1<br>(0·0–0·1)	44·6<br>(41·0–48·8)	214·2<br>(199·9–228·3)	184·7<br>(176·5–193·4)	134·8<br>(126·1–143·8)	108·3<br>(98·7–118·5)	57·7<br>(51·9–63·2)	8·8<br>(6·3–11·8)	0·2<br>(0·2–0·2)	3·77<br>(3·63–3·91)	1·29<br>(1·23–1·37)	1·55<br>(1·45–1·65)
1952	0·1<br>(0·0–0·1)	45·1<br>(41·5–48·7)	213·9<br>(200·5–227·8)	185·0<br>(177·0–193·8)	134·3<br>(125·6–143·2)	107·1<br>(97·8–116·8)	56·6<br>(50·8–62·4)	8·6<br>(6·0–11·5)	0·2<br>(0·2–0·2)	3·75<br>(3·61–3·9)	1·3<br>(1·22–1·37)	1·53<br>(1·45–1·62)
1953	0·1<br>(0·0–0·1)	46·4<br>(43·0–49·8)	217·3<br>(204·5–230·6)	190·4<br>(182·1–199·1)	136·6<br>(127·5–145·7)	108·3<br>(98·9–117·5)	56·9<br>(51·2–62·7)	8·5<br>(5·9–11·4)	0·2<br>(0·2–0·2)	3·82<br>(3·69–3·96)	1·32<br>(1·25–1·39)	1·55<br>(1·47–1·64)
1954	0·1<br>(0·0–0·1)	48·2<br>(44·8–51·5)	223·4<br>(210·6–237·5)	198·4<br>(190·0–207·5)	140·6<br>(131·7–149·1)	111·0<br>(101·2–120·3)	57·9<br>(52·5–63·9)	8·5<br>(6·1–11·5)	0·2<br>(0·2–0·2)	3·94<br>(3·81–4·09)	1·36<br>(1·29–1·43)	1·59<br>(1·5–1·68)
1955	0·1<br>(0·0–0·1)	49·9<br>(46·6–53·4)	230·0<br>(216·7–243·3)	205·7<br>(197·3–214·3)	144·7<br>(135·9–153·4)	113·3<br>(104·1–122·4)	58·8<br>(53·3–64·6)	8·5<br>(6·0–11·5)	0·2<br>(0·2–0·2)	4·06<br>(3·91–4·21)	1·4<br>(1·33–1·47)	1·63<br>(1·54–1·73)
1956	0·1<br>(0·0–0·1)	51·5<br>(48·2–54·8)	236·2<br>(223·6–248·8)	211·9<br>(203·5–220·0)	148·3<br>(139·1–157·0)	114·9<br>(105·9–124·3)	59·2<br>(53·7–64·7)	8·5<br>(5·9–11·5)	0·2<br>(0·2–0·2)	4·15<br>(4·0–4·3)	1·44<br>(1·37–1·51)	1·66<br>(1·56–1·75)
1957	0·1<br>(0·0–0·1)	53·2<br>(49·8–56·6)	242·2<br>(229·7–255·8)	217·9<br>(209·5–226·7)	151·6<br>(142·2–160·5)	116·2<br>(106·4–126·2)	59·5<br>(54·0–65·0)	8·4<br>(5·9–11·3)	0·2<br>(0·2–0·2)	4·25<br>(4·08–4·41)	1·48<br>(1·41–1·55)	1·68<br>(1·59–1·76)
1958	0·1<br>(0·0–0·1)	54·7<br>(51·3–58·3)	248·2<br>(235·0–261·0)	223·9<br>(215·5–232·7)	154·8<br>(145·5–163·6)	117·4<br>(108·0–127·5)	59·8<br>(54·0–65·4)	8·3<br>(5·9–11·3)	0·2<br>(0·2–0·2)	4·34<br>(4·16–4·52)	1·52<br>(1·45–1·58)	1·7<br>(1·61–1·79)
1959	0·1<br>(0·0–0·1)	55·9<br>(52·3–59·6)	253·7<br>(240·8–266·9)	229·1<br>(220·9–236·9)	157·4<br>(148·1–166·7)	118·1<br>(108·7–127·8)	59·8<br>(54·3–65·2)	8·1<br>(5·6–11·4)	0·2<br>(0·1–0·2)	4·41<br>(4·24–4·59)	1·55<br>(1·48–1·62)	1·72<br>(1·62–1·81)
1960	0·1<br>(0·0–0·1)	56·2<br>(52·6–59·9)	257·3<br>(243·9–270·9)	230·5<br>(222·5–238·2)	157·9<br>(148·8–167·3)	116·8<br>(107·8–127·1)	58·5<br>(52·9–64·0)	7·8<br>(5·4–10·9)	0·2<br>(0·1–0·2)	4·43<br>(4·27–4·57)	1·57<br>(1·49–1·65)	1·71<br>(1·61–1·8)
1961	0·1<br>(0·0–0·1)	55·6<br>(51·9–59·3)	257·3<br>(244·1–270·4)	226·8<br>(218·8–234·9)	154·6<br>(145·9–163·8)	112·8<br>(103·1–122·7)	55·6<br>(50·1–61·4)	7·4<br>(5·1–10·3)	0·1<br>(0·1–0·1)	4·35<br>(4·21–4·49)	1·56<br>(1·48–1·65)	1·65<br>(1·57–1·75)
1962	0·1<br>(0·0–0·1)	54·3<br>(50·6–58·1)	253·9<br>(241·6–267·4)	219·9<br>(211·3–228·6)	148·1<br>(139·3–157·6)	107·0<br>(97·6–116·9)	52·0<br>(46·8–58·0)	6·8<br>(4·7–9·7)	0·1<br>(0·1–0·1)	4·21<br>(4·07–4·35)	1·54<br>(1·46–1·62)	1·57<br>(1·48–1·66)
1963	0·1<br>(0·0–0·1)	52·9<br>(49·2–56·5)	249·2<br>(236·2–261·4)	213·4<br>(204·0–222·9)	141·8<br>(132·3–152·4)	101·2<br>(91·1–111·4)	48·6<br>(42·7–54·8)	6·3<br>(4·2–9·0)	0·1<br>(0·1–0·1)	4·07<br>(3·91–4·22)	1·51<br>(1·44–1·58)	1·49<br>(1·39–1·59)
1964	0·1<br>(0·0–0·1)	51·9<br>(48·4–55·3)	244·7<br>(230·6–259·1)	210·8<br>(200·6–221·3)	138·3<br>(127·4–150·0)	96·9<br>(84·7–109·5)	45·9<br>(39·1–52·8)	5·9<br>(3·9–8·7)	0·1<br>(0·1–0·1)	3·97<br>(3·8–4·16)	1·48<br>(1·41–1·56)	1·44<br>(1·31–1·57)
1965	0·1<br>(0·0–0·1)	51·1<br>(47·7–54·4)	241·0<br>(223·8–260·1)	211·8<br>(200·5–223·5)	136·5<br>(123·7–150·2)	93·9<br>(80·3–109·2)	43·9<br>(35·6–52·1)	5·5<br>(3·6–8·1)	0·1<br>(0·1–0·1)	3·92<br>(3·72–4·14)	1·46<br>(1·37–1·56)	1·4<br>(1·25–1·57)
1966	0·1<br>(0·0–0·1)	50·4<br>(47·2–53·5)	238·2<br>(219·4–259·5)	214·0<br>(202·3–227·4)	135·5<br>(121·5–150·8)	91·4<br>(75·2–108·9)	42·0<br>(33·2–51·8)	5·2<br>(3·3–7·7)	0·1<br>(0·1–0·1)	3·88<br>(3·66–4·13)	1·44<br>(1·35–1·55)	1·37<br>(1·2–1·55)
1967	0·1<br>(0·0–0·1)	49·7<br>(46·7–53·0)	235·7<br>(217·7–255·0)	217·4<br>(205·7–229·6)	135·0<br>(119·8–153·4)	89·3<br>(71·7–108·5)	40·3<br>(31·0–50·8)	4·8<br>(3·0–7·1)	0·1<br>(0·1–0·1)	3·86<br>(3·64–4·11)	1·43<br>(1·33–1·53)	1·35<br>(1·15–1·56)
1968	0·1<br>(0·0–0·1)	49·3<br>(46·3–52·2)	234·0<br>(219·2–249·6)	220·6<br>(210·6–231·4)	135·4<br>(119·4–152·8)	87·6<br>(68·8–107·1)	38·7<br>(29·1–48·9)	4·5<br>(2·9–6·6)	0·1<br>(0·1–0·1)	3·85<br>(3·62–4·1)	1·42<br>(1·34–1·5)	1·33<br>(1·12–1·55)
1969	0·1<br>(0·0–0·1)	48·9<br>(45·8–51·8)	232·9<br>(220·9–245·6)	220·9<br>(210·9–231·5)	136·2<br>(120·6–152·8)	86·0<br>(67·6–105·4)	37·0<br>(28·0–47·2)	4·2<br>(2·7–6·2)	0·1<br>(0·1–0·1)	3·83<br>(3·61–4·08)	1·41<br>(1·34–1·48)	1·32<br>(1·11–1·54)
1970	0·1<br>(0·0–0·1)	48·4<br>(45·5–51·3)	231·4<br>(218·3–243·8)	218·5<br>(208·0–230·1)	136·8<br>(121·0–152·9)	83·8<br>(66·5–103·1)	35·1<br>(26·7–45·2)	3·9<br>(2·5–5·7)	0·1<br>(0·1–0·1)	3·79<br>(3·56–4·04)	1·4<br>(1·33–1·47)	1·3<br>(1·1–1·52)
1971	0·1<br>(0·0–0·1)	47·9<br>(45·1–50·8)	228·9<br>(214·7–241·6)	215·1<br>(203·9–226·8)	137·5<br>(122·9–152·4)	81·2<br>(64·6–100·4)	33·1<br>(25·0–42·8)	3·5<br>(2·3–5·3)	0·1<br>(0·1–0·1)	3·74<br>(3·52–3·96)	1·38<br>(1·3–1·46)	1·28<br>(1·09–1·48)
1972	0·1<br>(0·0–0·1)	47·3<br>(44·5–50·1)	226·1<br>(213·1–238·8)	211·4<br>(200·5–221·7)	138·9<br>(125·5–152·2)	78·6<br>(62·0–96·6)	30·9<br>(23·0–39·7)	3·3<br>(2·1–4·9)	0·1<br>(0·1–0·1)	3·68<br>(3·47–3·9)	1·37<br>(1·29–1·44)	1·26<br>(1·09–1·44)
1973	0·1<br>(0·0–0·1)	46·6<br>(44·0–49·3)	222·8<br>(211·7–233·7)	207·7<br>(198·0–216·6)	138·7<br>(127·1–150·4)	76·0<br>(60·1–92·2)	28·6<br>(21·5–36·9)	3·0<br>(1·9–4·4)	0·1<br>(0·1–0·1)	3·62<br>(3·43–3·82)	1·35<br>(1·28–1·41)	1·23<br>(1·08–1·39)
1974	0·1<br>(0·0–0·1)	46·0<br>(43·5–48·5)	219·3<br>(209·0–229·2)	204·0<br>(196·1–212·1)	134·2<br>(122·4–145·9)	73·7<br>(58·4–90·1)	26·5<br>(19·6–34·3)	2·7<br>(1·7–4·0)	0·1<br>(0·0–0·1)	3·53<br>(3·36–3·7)	1·33<br>(1·27–1·38)	1·19<br>(1·05–1·33)
1975	0·1<br>(0·0–0·1)	45·6<br>(43·2–48·1)	216·4<br>(204·7–227·4)	200·0<br>(192·3–207·6)	128·5<br>(116·7–140·4)	72·1<br>(58·0–87·4)	24·6<br>(18·6–31·6)	2·5<br>(1·5–3·6)	0·0<br>(0·0–0·0)	3·45<br>(3·28–3·61)	1·31<br>(1·25–1·37)	1·14<br>(1·01–1·27)
1976	0·1<br>(0·0–0·1)	45·4<br>(42·9–47·9)	214·4<br>(201·9–226·7)	195·8<br>(187·9–203·7)	124·0<br>(112·6–135·9)	71·1<br>(59·0–84·6)	23·0<br>(17·6–29·2)	2·2<br>(1·4–3·3)	0·0<br>(0·0–0·0)	3·38<br>(3·22–3·53)	1·3<br>(1·24–1·36)	1·1<br>(0·99–1·21)
1977	0·1<br>(0·0–0·1)	44·9<br>(42·7–47·1)	212·7<br>(200·7–225·8)	191·6<br>(183·7–199·5)	120·3<br>(110·1–130·0)	69·9<br>(59·6–81·3)	21·5<br>(17·0–26·8)	2·0<br>(1·3–3·0)	0·0<br>(0·0–0·0)	3·32<br>(3·17–3·45)	1·29<br>(1·23–1·35)	1·07<br>(0·98–1·15)
1978	0·1<br>(0·0–0·1)	44·3<br>(42·3–46·5)	210·6<br>(200·1–220·8)	186·9<br>(180·6–193·9)	117·4<br>(109·0–125·6)	67·0<br>(59·1–75·4)	20·1<br>(16·3–24·5)	1·9<br>(1·2–2·7)	0·0<br>(0·0–0·0)	3·24<br>(3·11–3·37)	1·27<br>(1·22–1·33)	1·03<br>(0·96–1·09)
1979	0·1<br>(0·0–0·1)	43·9<br>(41·9–45·8)	208·7<br>(199·8–217·2)	182·1<br>(176·2–187·9)	114·8<br>(108·2–121·7)	61·6<br>(55·1–68·2)	18·8<br>(15·8–22·1)	1·7<br>(1·1–2·5)	0·0<br>(0·0–0·0)	3·16<br>(3·06–3·26)	1·26<br>(1·22–1·3)	0·98<br>(0·93–1·04)
1980	0·1<br>(0·0–0·1)	43·6<br>(41·7–45·5)	207·7<br>(200·1–216·2)	177·0<br>(171·4–182·9)	111·8<br>(105·6–118·2)	56·3<br>(50·1–62·5)	18·0<br>(15·5–20·5)	1·6<br>(1·0–2·3)	0·0<br>(0·0–0·0)	3·08<br>(3·0–3·16)	1·26<br>(1·21–1·3)	0·94<br>(0·89–0·99)
1981	0·1<br>(0·0–0·1)	43·4<br>(41·5–45·2)	206·3<br>(197·7–215·5)	172·9<br>(166·7–179·1)	108·8<br>(102·5–115·4)	53·2<br>(47·5–59·2)	17·5<br>(15·1–20·0)	1·4<br>(0·9–2·1)	0·0<br>(0·0–0·0)	3·02<br>(2·94–3·11)	1·25<br>(1·21–1·3)	0·91<br>(0·86–0·95)
1982	0·1<br>(0·0–0·1)	43·7<br>(41·7–45·5)	205·9<br>(196·3–215·8)	173·1<br>(167·1–179·6)	108·5<br>(102·3–115·1)	53·2<br>(47·4–59·1)	17·7<br>(15·2–20·3)	1·3<br>(0·9–2·0)	0·0<br>(0·0–0·0)	3·02<br>(2·93–3·11)	1·25<br>(1·2–1·3)	0·9<br>(0·86–0·95)
1983	0·1<br>(0·0–0·1)	44·6<br>(42·6–46·5)	207·3<br>(198·4–215·8)	177·0<br>(171·8–182·6)	110·3<br>(105·1–115·8)	55·2<br>(50·1–60·7)	18·0<br>(15·8–20·4)	1·3<br>(0·8–1·8)	0·0<br>(0·0–0·0)	3·07<br>(3·0–3·14)	1·26<br>(1·22–1·3)	0·92<br>(0·89–0·96)
1984	0·1<br>(0·0–0·1)	45·8<br>(44·0–47·7)	209·4<br>(202·1–217·2)	180·4<br>(175·1–186·0)	110·3<br>(105·3–115·4)	56·9<br>(52·1–62·0)	17·4<br>(15·3–19·7)	1·2<br>(0·8–1·7)	0·0<br>(0·0–0·0)	3·11<br>(3·04–3·18)	1·28<br>(1·24–1·32)	0·93<br>(0·89–0·97)
1985	0·1<br>(0·0–0·1)	46·9<br>(45·1–48·8)	211·6<br>(203·9–219·1)	180·7<br>(175·3–186·3)	107·9<br>(102·6–113·6)	56·7<br>(52·1–61·7)	16·3<br>(14·4–18·6)	1·1<br>(0·7–1·6)	0·0<br>(0·0–0·0)	3·11<br>(3·04–3·18)	1·29<br>(1·25–1·33)	0·91<br>(0·87–0·95)
1986	0·1<br>(0·0–0·1)	47·7<br>(45·7–49·8)	211·2<br>(203·3–218·7)	176·3<br>(170·8–181·8)	104·5<br>(99·2–109·7)	54·3<br>(50·0–59·1)	15·4<br>(13·4–17·5)	1·0<br>(0·7–1·5)	0·0<br>(0·0–0·0)	3·05<br>(2·98–3·12)	1·29<br>(1·25–1·33)	0·88<br>(0·84–0·92)
1987	0·1<br>(0·0–0·1)	48·4<br>(46·2–50·8)	207·9<br>(199·7–215·9)	169·3<br>(163·9–174·6)	101·2<br>(96·5–106·6)	51·4<br>(47·2–55·9)	14·5<br>(12·8–16·3)	0·9<br>(0·6–1·3)	0·0<br>(0·0–0·0)	2·97<br>(2·9–3·05)	1·28<br>(1·24–1·32)	0·84<br>(0·8–0·88)
1988	0·1<br>(0·0–0·1)	49·3<br>(47·2–51·8)	204·2<br>(196·7–211·4)	162·3<br>(157·7–166·9)	97·7<br>(93·4–102·5)	49·2<br>(45·7–53·0)	13·6<br>(12·2–15·1)	0·8<br>(0·6–1·1)	0·0<br>(0·0–0·0)	2·89<br>(2·82–2·95)	1·27<br>(1·23–1·31)	0·81<br>(0·77–0·84)
1989	0·1<br>(0·0–0·1)	50·4<br>(48·1–52·7)	201·2<br>(194·4–208·0)	155·6<br>(151·2–160·0)	92·9<br>(89·0–97·1)	46·4<br>(43·3–49·9)	12·6<br>(11·4–13·9)	0·7<br>(0·5–1·0)	0·0<br>(0·0–0·0)	2·8<br>(2·74–2·86)	1·26<br>(1·22–1·29)	0·76<br>(0·73–0·79)
1990	0·1<br>(0·0–0·1)	51·4<br>(49·2–53·8)	198·6<br>(191·8–206·0)	149·9<br>(145·6–154·3)	87·6<br>(83·7–91·6)	43·1<br>(39·9–46·7)	11·5<br>(10·4–12·7)	0·6<br>(0·4–0·9)	0·0<br>(0·0–0·0)	2·71<br>(2·65–2·78)	1·25<br>(1·21–1·29)	0·71<br>(0·68–0·74)
1991	0·1<br>(0·0–0·1)	52·0<br>(49·8–54·4)	195·7<br>(188·1–203·6)	143·6<br>(138·9–148·5)	82·7<br>(78·7–87·0)	40·2<br>(37·0–43·5)	10·3<br>(9·2–11·4)	0·5<br>(0·4–0·7)	0·0<br>(0·0–0·0)	2·63<br>(2·56–2·69)	1·24<br>(1·2–1·28)	0·67<br>(0·64–0·7)
1992	0·1<br>(0·0–0·1)	51·2<br>(49·0–53·6)	190·5<br>(182·0–199·2)	133·4<br>(128·6–138·4)	78·8<br>(74·3–83·3)	38·0<br>(35·0–41·3)	9·3<br>(8·3–10·4)	0·4<br>(0·3–0·6)	0·0<br>(0·0–0·0)	2·51<br>(2·44–2·58)	1·21<br>(1·16–1·25)	0·63<br>(0·6–0·66)
1993	0·1<br>(0·0–0·1)	48·8<br>(46·6–51·1)	181·4<br>(173·7–189·2)	127·0<br>(122·6–131·6)	76·5<br>(72·5–80·8)	37·0<br>(34·3–40·1)	8·8<br>(7·9–9·7)	0·4<br>(0·3–0·6)	0·0<br>(0·0–0·0)	2·4<br>(2·34–2·47)	1·15<br>(1·11–1·19)	0·61<br>(0·59–0·64)
1994	0·1<br>(0·0–0·1)	45·2<br>(43·2–47·3)	170·4<br>(163·8–177·6)	125·0<br>(121·0–129·3)	74·0<br>(70·5–77·7)	35·5<br>(32·6–38·1)	8·3<br>(7·5–9·2)	0·4<br>(0·3–0·5)	0·0<br>(0·0–0·0)	2·29<br>(2·24–2·35)	1·08<br>(1·04–1·12)	0·59<br>(0·57–0·62)
1995	0·1<br>(0·0–0·1)	41·3<br>(39·3–43·3)	158·6<br>(151·3–166·2)	120·6<br>(116·5–125·2)	71·1<br>(67·4–74·7)	32·5<br>(29·8–35·3)	7·8<br>(7·0–8·7)	0·3<br>(0·2–0·5)	0·0<br>(0·0–0·0)	2·16<br>(2·11–2·21)	1·0<br>(0·96–1·04)	0·56<br>(0·53–0·59)
1996	0·1<br>(0·0–0·1)	38·0<br>(36·1–40·1)	149·6<br>(141·4–158·1)	115·8<br>(111·2–120·7)	69·0<br>(65·1–73·0)	30·2<br>(27·6–33·1)	7·6<br>(6·7–8·5)	0·3<br>(0·2–0·4)	0·0<br>(0·0–0·0)	2·05<br>(1·99–2·11)	0·94<br>(0·9–0·98)	0·54<br>(0·51–0·56)
1997	0·1<br>(0·0–0·1)	35·4<br>(33·6–37·5)	145·2<br>(136·5–154·7)	113·7<br>(108·8–118·9)	68·7<br>(64·6–73·4)	30·0<br>(27·2–33·3)	7·7<br>(6·8–8·8)	0·2<br>(0·2–0·3)	0·0<br>(0·0–0·0)	2·01<br>(1·94–2·07)	0·9<br>(0·86–0·95)	0·53<br>(0·51–0·56)
1998	0·1<br>(0·0–0·1)	33·0<br>(31·2–35·0)	142·5<br>(134·5–150·8)	114·4<br>(109·8–119·6)	69·9<br>(65·7–73·9)	30·8<br>(28·2–34·1)	7·8<br>(6·9–8·9)	0·3<br>(0·2–0·4)	0·0<br>(0·0–0·0)	1·99<br>(1·93–2·06)	0·88<br>(0·84–0·92)	0·54<br>(0·51–0·58)
1999	0·0<br>(0·0–0·1)	30·6<br>(28·9–32·4)	140·0<br>(132·5–147·7)	115·7<br>(111·0–120·5)	70·1<br>(66·1–74·3)	31·1<br>(28·6–34·0)	7·6<br>(6·7–8·5)	0·3<br>(0·2–0·4)	0·0<br>(0·0–0·0)	1·98<br>(1·91–2·04)	0·85<br>(0·81–0·89)	0·54<br>(0·52–0·57)
2000	0·0<br>(0·0–0·1)	28·3<br>(26·7–30·0)	137·0<br>(129·2–144·9)	116·0<br>(110·9–120·9)	69·1<br>(64·8–73·5)	31·2<br>(28·3–34·1)	7·3<br>(6·4–8·2)	0·3<br>(0·2–0·4)	0·0<br>(0·0–0·0)	1·95<br>(1·88–2·01)	0·83<br>(0·79–0·87)	0·54<br>(0·51–0·57)
2001	0·0<br>(0·0–0·1)	26·8<br>(25·2–28·4)	134·4<br>(125·9–143·4)	116·6<br>(111·2–122·3)	69·7<br>(65·0–74·6)	32·4<br>(29·0–35·6)	7·2<br>(6·3–8·3)	0·3<br>(0·2–0·5)	0·0<br>(0·0–0·0)	1·94<br>(1·87–2·01)	0·81<br>(0·76–0·85)	0·55<br>(0·52–0·58)
2002	0·0<br>(0·0–0·1)	26·1<br>(24·5–27·8)	134·0<br>(124·8–144·0)	120·1<br>(114·0–126·1)	73·0<br>(67·7–78·4)	34·6<br>(30·6–38·1)	7·6<br>(6·5–8·5)	0·4<br>(0·2–0·5)	0·0<br>(0·0–0·0)	1·98<br>(1·9–2·06)	0·8<br>(0·75–0·85)	0·58<br>(0·54–0·61)
2003	0·0<br>(0·0–0·1)	26·0<br>(24·4–27·6)	135·5<br>(126·3–145·4)	126·3<br>(120·5–132·1)	77·8<br>(72·4–83·5)	37·5<br>(33·3–41·9)	8·1<br>(7·1–9·2)	0·4<br>(0·3–0·6)	0·0<br>(0·0–0·0)	2·06<br>(1·99–2·13)	0·81<br>(0·76–0·86)	0·62<br>(0·58–0·65)
2004	0·0<br>(0·0–0·1)	26·1<br>(24·5–27·9)	136·8<br>(128·9–145·8)	133·2<br>(127·5–139·1)	82·8<br>(77·5–88·0)	40·6<br>(36·7–44·6)	8·9<br>(7·8–9·9)	0·4<br>(0·3–0·6)	0·0<br>(0·0–0·0)	2·14<br>(2·08–2·21)	0·82<br>(0·78–0·86)	0·66<br>(0·63–0·7)
2005	0·0<br>(0·0–0·1)	26·7<br>(24·9–28·6)	137·3<br>(129·9–145·6)	139·2<br>(133·8–145·0)	88·2<br>(82·7–93·8)	43·9<br>(39·9–47·7)	9·8<br>(8·8–10·9)	0·4<br>(0·3–0·6)	0·0<br>(0·0–0·0)	2·23<br>(2·17–2·29)	0·82<br>(0·78–0·86)	0·71<br>(0·68–0·74)
2006	0·0<br>(0·0–0·1)	27·5<br>(25·6–29·6)	138·9<br>(129·8–147·5)	144·4<br>(138·4–150·4)	94·7<br>(88·6–100·7)	47·7<br>(43·2–52·5)	10·9<br>(9·7–12·3)	0·5<br>(0·3–0·7)	0·0<br>(0·0–0·0)	2·32<br>(2·25–2·39)	0·83<br>(0·79–0·87)	0·77<br>(0·73–0·81)
2007	0·0<br>(0·0–0·1)	28·1<br>(26·0–30·3)	140·3<br>(130·3–150·1)	148·2<br>(141·4–154·6)	99·6<br>(93·0–106·1)	51·1<br>(46·1–56·8)	11·9<br>(10·5–13·6)	0·5<br>(0·3–0·7)	0·0<br>(0·0–0·0)	2·4<br>(2·31–2·48)	0·84<br>(0·79–0·89)	0·82<br>(0·77–0·86)
2008	0·0<br>(0·0–0·1)	28·4<br>(26·2–30·6)	139·3<br>(129·4–149·1)	149·9<br>(143·1–156·5)	101·4<br>(94·7–107·8)	53·0<br>(47·6–58·7)	12·6<br>(11·1–14·5)	0·5<br>(0·3–0·7)	0·0<br>(0·0–0·0)	2·43<br>(2·33–2·52)	0·84<br>(0·79–0·88)	0·84<br>(0·79–0·88)
2009	0·0<br>(0·0–0·1)	28·8<br>(26·6–31·2)	138·5<br>(129·2–148·0)	151·2<br>(144·3–157·8)	102·9<br>(96·5–109·4)	53·7<br>(48·5–59·3)	12·9<br>(11·3–14·7)	0·5<br>(0·3–0·7)	0·0<br>(0·0–0·0)	2·44<br>(2·35–2·53)	0·84<br>(0·79–0·89)	0·85<br>(0·8–0·9)
2010	0·0<br>(0·0–0·1)	30·0<br>(27·7–32·6)	141·3<br>(131·2–151·0)	153·0<br>(145·6–160·6)	105·7<br>(99·1–112·8)	54·7<br>(49·3–60·4)	13·3<br>(11·5–15·0)	0·5<br>(0·4–0·8)	0·0<br>(0·0–0·0)	2·49<br>(2·39–2·59)	0·86<br>(0·81–0·91)	0·87<br>(0·81–0·94)
2011	0·1<br>(0·0–0·1)	31·5<br>(29·0–34·2)	146·6<br>(135·2–157·4)	155·0<br>(147·3–163·1)	108·8<br>(101·1–116·4)	57·1<br>(51·2–63·6)	14·1<br>(12·2–16·2)	0·6<br>(0·4–0·8)	0·0<br>(0·0–0·0)	2·57<br>(2·45–2·67)	0·89<br>(0·83–0·95)	0·9<br>(0·83–0·98)
2012	0·1<br>(0·0–0·1)	32·9<br>(30·1–35·7)	151·9<br>(140·0–163·7)	156·7<br>(149·2–164·4)	111·1<br>(103·1–119·6)	60·9<br>(54·2–68·4)	15·1<br>(13·0–17·5)	0·6<br>(0·4–0·9)	0·0<br>(0·0–0·0)	2·65<br>(2·54–2·75)	0·92<br>(0·87–0·98)	0·94<br>(0·87–1·02)
2013	0·1<br>(0·0–0·1)	33·4<br>(30·4–36·4)	155·4<br>(143·1–168·4)	157·6<br>(149·1–166·1)	112·2<br>(103·6–120·9)	63·1<br>(55·9–71·4)	15·7<br>(13·4–18·3)	0·6<br>(0·4–1·0)	0·0<br>(0·0–0·0)	2·69<br>(2·58–2·8)	0·94<br>(0·88–1·01)	0·96<br>(0·88–1·05)
2014	0·1<br>(0·0–0·1)	32·9<br>(29·7–36·3)	155·0<br>(142·1–169·0)	156·6<br>(147·6–165·6)	110·0<br>(100·3–119·8)	61·4<br>(53·6–70·3)	15·1<br>(12·8–17·9)	0·6<br>(0·4–0·9)	0·0<br>(0·0–0·0)	2·66<br>(2·53–2·78)	0·94<br>(0·87–1·02)	0·94<br>(0·85–1·03)
2015	0·1<br>(0·0–0·1)	31·7<br>(28·4–35·3)	150·4<br>(135·0–167·1)	153·8<br>(143·9–164·2)	103·8<br>(93·4–115·8)	57·3<br>(48·2–67·7)	13·8<br>(11·2–17·2)	0·6<br>(0·4–0·9)	0·0<br>(0·0–0·0)	2·56<br>(2·4–2·72)	0·91<br>(0·82–1·01)	0·88<br>(0·78–0·99)
2016	0·0<br>(0·0–0·1)	30·6<br>(27·2–34·3)	144·3<br>(126·1–165·9)	150·8<br>(139·8–162·7)	97·1<br>(85·4–110·3)	54·3<br>(44·2–66·0)	12·9<br>(10·0–16·8)	0·5<br>(0·3–0·8)	0·0<br>(0·0–0·0)	2·45<br>(2·25–2·67)	0·87<br>(0·77–1·0)	0·82<br>(0·7–0·97)
2017	0·0<br>(0·0–0·1)	30·1<br>(26·5–34·4)	140·1<br>(120·0–162·7)	149·2<br>(137·1–162·3)	93·7<br>(81·5–108·8)	52·5<br>(41·6–66·6)	12·4<br>(9·3–16·8)	0·5<br>(0·3–0·8)	0·0<br>(0·0–0·0)	2·39<br>(2·16–2·63)	0·85<br>(0·73–0·99)	0·8<br>(0·66–0·97)

Supplemental Results Table 5: Age-specific fertility rates per 1000, cumulative total fertility up to maternal age 25 (TFU25), cumulative total fertility from maternal age 30 to age 55 (TFO30), and cumulative total fertility, Kyrgyzstan, 1950-2017

Year	10-14	15-19	20-24	25-29	30-34	35-39	40-44	45-49	50-54	TFR	TFU25	TFO30
1950	0·0 (0·0–0·0)	44·1 (37·8–52·1)	220·8 (201·7–240·0)	200·2 (188·7–211·9)	171·5 (161·0–182·0)	127·5 (117·9–137·3)	76·3 (70·8–81·8)	6·6 (4·5–9·3)	0·1 (0·1–0·1)	4·24 (4·05–4·43)	1·32 (1·22–1·42)	1·91 (1·81–2·01)
1951	0·0 (0·0–0·0)	44·0 (37·8–51·2)	218·6 (200·6–236·2)	198·2 (187·3–208·8)	170·0 (160·1–179·5)	125·5 (117·2–134·3)	75·0 (70·2–80·2)	6·4 (4·4–8·8)	0·1 (0·1–0·1)	4·19 (4·01–4·37)	1·31 (1·22–1·41)	1·88 (1·8–1·97)
1952	0·0 (0·0–0·0)	44·1 (38·0–51·4)	218·0 (200·5–235·5)	198·6 (187·7–209·5)	170·2 (160·5–179·7)	124·9 (116·6–133·5)	74·6 (69·7–79·6)	6·2 (4·2–8·6)	0·1 (0·1–0·1)	4·18 (3·99–4·36)	1·31 (1·22–1·41)	1·88 (1·8–1·97)
1953	0·0 (0·0–0·0)	44·4 (38·0–51·7)	218·6 (201·8–235·1)	199·9 (189·0–211·2)	171·5 (161·7–181·9)	125·1 (116·8–133·5)	74·7 (69·9–79·4)	6·0 (4·0–8·3)	0·1 (0·1–0·1)	4·2 (4·01–4·38)	1·32 (1·22–1·41)	1·89 (1·8–1·97)
1954	0·0 (0·0–0·0)	44·8 (38·5–52·1)	219·3 (203·7–236·4)	200·8 (189·8–211·5)	172·6 (163·5–183·2)	124·8 (116·5–132·9)	74·5 (69·7–79·1)	5·8 (3·9–8·1)	0·1 (0·1–0·1)	4·21 (4·03–4·4)	1·32 (1·23–1·41)	1·89 (1·81–1·98)
1955	0·0 (0·0–0·0)	45·3 (39·2–52·3)	220·2 (202·9–237·7)	202·0 (190·9–212·7)	174·0 (164·4–184·2)	124·0 (116·0–132·0)	74·1 (69·3–78·7)	5·6 (3·8–7·9)	0·1 (0·1–0·1)	4·23 (4·06–4·4)	1·33 (1·24–1·42)	1·89 (1·81–1·97)
1956	0·0 (0·0–0·0)	45·9 (39·8–52·8)	221·7 (203·5–238·3)	203·8 (192·3–213·9)	175·7 (165·9–185·6)	123·3 (115·0–131·0)	73·6 (68·7–78·4)	5·4 (3·7–7·6)	0·1 (0·1–0·1)	4·25 (4·08–4·42)	1·34 (1·24–1·43)	1·89 (1·82–1·96)
1957	0·0 (0·0–0·0)	46·6 (40·1–53·8)	223·3 (206·5–240·1)	206·2 (195·8–216·6)	178·0 (167·8–187·9)	122·7 (114·7–131·3)	73·2 (68·3–78·0)	5·3 (3·6–7·5)	0·1 (0·1–0·1)	4·28 (4·1–4·44)	1·35 (1·25–1·44)	1·9 (1·82–1·97)
1958	0·0 (0·0–0·0)	47·9 (41·6–55·0)	225·7 (207·2–242·0)	210·9 (200·3–221·2)	182·1 (171·6–192·0)	123·7 (115·6–132·7)	73·7 (69·1–78·3)	5·1 (3·4–7·3)	0·1 (0·1–0·1)	4·35 (4·18–4·5)	1·37 (1·27–1·46)	1·92 (1·86–1·99)
1959	0·0 (0·0–0·0)	49·3 (42·8–56·7)	228·5 (211·6–245·4)	216·2 (206·0–226·8)	187·6 (177·7–196·4)	126·0 (117·7–134·6)	74·9 (70·1–79·7)	5·0 (3·2–7·1)	0·1 (0·1–0·1)	4·44 (4·28–4·6)	1·39 (1·3–1·48)	1·97 (1·89–2·04)
1960	0·0 (0·0–0·0)	50·4 (43·8–57·9)	229·3 (211·8–245·0)	217·3 (207·2–228·0)	191·3 (181·7–200·4)	127·0 (118·3–136·1)	75·5 (70·8–80·3)	4·8 (3·0–6·9)	0·1 (0·1–0·1)	4·48 (4·33–4·62)	1·4 (1·31–1·49)	1·99 (1·91–2·07)
1961	0·0 (0·0–0·0)	51·5 (44·6–59·6)	227·3 (208·9–245·0)	216·1 (205·7–226·8)	192·3 (182·6–201·1)	126·2 (117·6–134·9)	74·9 (70·4–79·7)	4·5 (2·9–6·6)	0·1 (0·1–0·1)	4·46 (4·32–4·6)	1·39 (1·3–1·49)	1·99 (1·92–2·06)
1962	0·0 (0·0–0·0)	52·8 (45·4–61·1)	226·0 (209·1–244·1)	217·3 (206·5–228·3)	191·1 (181·4–200·3)	125·4 (116·9–133·9)	74·0 (69·2–78·8)	4·2 (2·8–6·3)	0·1 (0·1–0·1)	4·45 (4·3–4·6)	1·39 (1·3–1·49)	1·97 (1·9–2·05)
1963	0·0 (0·0–0·0)	53·8 (46·3–61·8)	226·9 (208·9–246·9)	221·0 (210·3–232·2)	188·8 (177·8–199·7)	124·0 (114·4–133·3)	72·5 (67·2–77·8)	4·1 (2·7–6·0)	0·1 (0·1–0·1)	4·46 (4·27–4·63)	1·4 (1·31–1·5)	1·95 (1·86–2·04)
1964	0·0 (0·0–0·0)	55·1 (47·4–63·5)	228·7 (208·7–250·5)	225·9 (214·0–238·3)	186·6 (173·6–199·2)	122·2 (110·4–133·8)	70·6 (63·8–77·3)	3·9 (2·5–6·0)	0·1 (0·1–0·1)	4·47 (4·23–4·69)	1·42 (1·31–1·53)	1·92 (1·8–2·04)
1965	0·0 (0·0–0·0)	57·0 (49·4–65·5)	230·7 (209·5–254·9)	231·5 (219·0–244·4)	185·1 (169·7–200·3)	120·7 (105·5–136·8)	69·1 (60·4–78·0)	3·7 (2·3–5·5)	0·1 (0·1–0·1)	4·49 (4·23–4·76)	1·44 (1·32–1·57)	1·89 (1·75–2·04)
1966	0·0 (0·0–0·0)	59·0 (50·9–67·7)	233·1 (211·9–256·7)	237·3 (223·9–250·9)	184·1 (167·3–200·9)	119·5 (102·1–138·8)	67·8 (57·9–78·8)	3·4 (2·2–5·1)	0·1 (0·1–0·1)	4·52 (4·23–4·83)	1·46 (1·35–1·59)	1·87 (1·71–2·04)
1967	0·0 (0·0–0·0)	60·4 (52·5–68·9)	235·4 (215·2–255·4)	243·3 (229·3–257·0)	183·4 (164·7–201·1)	118·4 (100·2–138·5)	66·5 (55·8–78·3)	3·2 (2·0–4·8)	0·1 (0·1–0·1)	4·55 (4·26–4·87)	1·48 (1·36–1·6)	1·86 (1·68–2·04)
1968	0·0 (0·0–0·0)	61·2 (53·0–69·6)	237·7 (217·4–257·9)	250·2 (236·6–264·2)	183·3 (164·2–201·7)	117·6 (99·3–137·1)	65·4 (53·8–77·1)	3·0 (1·9–4·6)	0·1 (0·1–0·1)	4·59 (4·27–4·92)	1·49 (1·38–1·61)	1·85 (1·65–2·05)
1969	0·0 (0·0–0·0)	62·0 (53·7–70·7)	240·9 (219·4–262·3)	258·1 (244·7–271·9)	183·9 (165·2–202·1)	117·3 (99·3–137·2)	64·6 (53·1–76·7)	2·9 (1·8–4·3)	0·1 (0·1–0·1)	4·65 (4·32–4·97)	1·51 (1·39–1·64)	1·84 (1·63–2·08)
1970	0·0 (0·0–0·0)	62·7 (54·8–71·1)	244·6 (224·2–265·2)	264·9 (251·7–278·2)	184·3 (166·5–202·9)	116·7 (97·2–136·9)	63·7 (52·2–75·7)	2·7 (1·8–4·1)	0·1 (0·1–0·1)	4·7 (4·38–5·03)	1·54 (1·42–1·66)	1·84 (1·59–2·09)
1971	0·0 (0·0–0·0)	62·8 (54·9–71·3)	246·7 (228·3–265·5)	267·6 (255·1–279·6)	183·3 (165·0–201·5)	114·2 (94·7–135·3)	61·9 (50·4–74·2)	2·5 (1·6–3·8)	0·0 (0·0–0·1)	4·7 (4·39–5·02)	1·55 (1·44–1·66)	1·81 (1·57–2·07)
1972	0·0 (0·0–0·0)	62·1 (54·5–70·4)	244·9 (227·3–262·2)	265·8 (254·1–277·3)	180·8 (162·0–198·5)	109·8 (90·5–130·6)	58·9 (47·7–71·2)	2·3 (1·4–3·5)	0·0 (0·0–0·0)	4·62 (4·33–4·93)	1·53 (1·43–1·65)	1·76 (1·53–2·0)
1973	0·0 (0·0–0·0)	61·1 (54·0–69·3)	241·2 (225·4–257·2)	263·1 (251·8–274·4)	178·3 (160·6–196·2)	105·1 (85·2–125·4)	55·9 (44·7–68·1)	2·1 (1·3–3·2)	0·0 (0·0–0·0)	4·53 (4·26–4·83)	1·51 (1·4–1·63)	1·71 (1·51–1·91)
1974	0·0 (0·0–0·0)	60·1 (52·8–68·1)	238·4 (221·9–255·4)	262·0 (250·4–273·8)	176·5 (160·0–194·6)	101·3 (82·3–121·7)	53·3 (41·4–65·3)	1·9 (1·2–2·9)	0·0 (0·0–0·0)	4·47 (4·21–4·74)	1·49 (1·38–1·6)	1·67 (1·48–1·85)
1975	0·0 (0·0–0·0)	59·5 (52·2–67·1)	236·3 (218·9–254·0)	262·3 (251·3–273·3)	175·2 (159·8–192·6)	97·6 (79·8–116·6)	51·0 (40·8–62·1)	1·7 (1·1–2·6)	0·0 (0·0–0·0)	4·42 (4·18–4·68)	1·48 (1·37–1·59)	1·63 (1·46–1·8)
1976	0·0 (0·0–0·0)	59·2 (52·1–66·8)	233·9 (217·6–251·4)	261·1 (251·0–271·9)	174·2 (160·8–189·7)	94·0 (77·6–110·6)	48·8 (39·5–58·3)	1·6 (1·0–2·3)	0·0 (0·0–0·0)	4·36 (4·15–4·59)	1·47 (1·37–1·57)	1·59 (1·45–1·74)
1977	0·0 (0·0–0·0)	58·6 (51·8–66·0)	231·6 (215·8–247·7)	255·5 (246·1–265·4)	171·5 (160·5–183·7)	90·7 (77·0–104·2)	46·8 (38·8–54·8)	1·4 (0·9–2·1)	0·0 (0·0–0·0)	4·28 (4·1–4·47)	1·45 (1·37–1·54)	1·55 (1·44–1·67)
1978	0·0 (0·0–0·0)	57·2 (50·7–64·1)	229·7 (216·0–242·7)	246·8 (238·1–255·3)	165·7 (156·3–175·3)	87·8 (77·7–98·1)	44·9 (39·2–50·8)	1·3 (0·8–2·0)	0·0 (0·0–0·0)	4·17 (4·02–4·31)	1·43 (1·36–1·51)	1·5 (1·41–1·6)
1979	0·0 (0·0–0·0)	55·9 (49·5–62·6)	229·0 (215·8–241·8)	240·1 (231·2–248·5)	160·9 (152·9–169·4)	86·2 (78·7–93·8)	43·2 (38·8–47·2)	1·2 (0·8–1·8)	0·0 (0·0–0·0)	4·08 (3·95–4·22)	1·42 (1·35–1·5)	1·46 (1·38–1·53)
1980	0·0 (0·0–0·0)	55·6 (49·0–62·4)	230·8 (217·8–243·0)	239·7 (231·1–248·0)	161·1 (152·6–169·3)	86·9 (80·5–93·7)	41·5 (37·7–45·4)	1·1 (0·7–1·7)	0·0 (0·0–0·0)	4·08 (3·94–4·22)	1·43 (1·36–1·5)	1·45 (1·38–1·52)
1981	0·0 (0·0–0·0)	56·1 (50·0–62·6)	233·8 (220·4–246·4)	243·5 (235·3–251·8)	164·8 (156·7–172·7)	90·1 (83·1–96·9)	40·0 (36·3–44·0)	1·0 (0·7–1·5)	0·0 (0·0–0·0)	4·15 (4·0–4·29)	1·45 (1·37–1·52)	1·48 (1·4–1·55)
1982	0·0 (0·0–0·0)	56·6 (50·8–63·1)	236·4 (222·6–248·7)	246·1 (238·3–253·9)	167·6 (160·0–175·0)	92·7 (86·6–98·7)	38·2 (34·5–42·1)	1·0 (0·6–1·4)	0·0 (0·0–0·0)	4·19 (4·07–4·33)	1·47 (1·39–1·53)	1·5 (1·44–1·56)
1983	0·0 (0·0–0·0)	56·5 (50·9–63·1)	236·2 (224·2–248·2)	244·2 (237·0–251·7)	166·3 (158·6–173·6)	90·6 (84·5–96·7)	35·9 (32·3–39·4)	0·9 (0·6–1·3)	0·0 (0·0–0·0)	4·15 (4·04–4·26)	1·46 (1·39–1·53)	1·47 (1·42–1·52)
1984	0·0 (0·0–0·0)	56·4 (50·7–62·8)	233·5 (221·5–244·6)	240·3 (233·0–247·8)	163·1 (155·9–170·6)	86·0 (80·1–92·4)	33·8 (30·6–37·0)	0·8 (0·5–1·2)	0·0 (0·0–0·0)	4·07 (3·97–4·17)	1·45 (1·38–1·51)	1·42 (1·37–1·47)
1985	0·0 (0·0–0·0)	56·9 (51·0–63·5)	231·3 (219·4–243·8)	237·9 (230·5–245·5)	161·1 (154·1–168·4)	83·0 (77·4–89·2)	33·2 (30·3–36·4)	0·7 (0·5–1·0)	0·0 (0·0–0·0)	4·02 (3·92–4·12)	1·44 (1·37–1·51)	1·39 (1·34–1·44)
1986	0·0 (0·0–0·0)	57·4 (51·3–63·9)	228·7 (216·6–240·6)	234·2 (226·8–241·8)	159·1 (152·5–166·1)	80·8 (75·5–86·2)	33·2 (30·3–36·0)	0·6 (0·4–0·9)	0·0 (0·0–0·0)	3·97 (3·88–4·06)	1·43 (1·36–1·5)	1·37 (1·33–1·41)
1987	0·0 (0·0–0·0)	57·7 (51·7–64·0)	223·9 (212·9–235·9)	227·5 (219·6–235·0)	154·4 (148·4–160·8)	77·2 (72·3–82·2)	32·2 (29·8–34·8)	0·5 (0·3–0·8)	0·0 (0·0–0·0)	3·87 (3·78–3·96)	1·41 (1·34–1·48)	1·32 (1·28–1·36)
1988	0·0 (0·0–0·0)	58·1 (51·7–64·6)	219·4 (209·2–229·5)	220·7 (213·5–228·1)	148·2 (141·9–154·2)	71·2 (66·9–75·7)	29·3 (27·1–31·7)	0·5 (0·3–0·7)	0·0 (0·0–0·0)	3·74 (3·65–3·82)	1·39 (1·33–1·45)	1·25 (1·21–1·28)
1989	0·0 (0·0–0·0)	57·9 (51·6–64·6)	216·9 (206·7–227·9)	214·8 (207·9–222·3)	141·9 (135·9–148·1)	64·5 (60·4–68·8)	25·6 (23·4–28·0)	0·4 (0·3–0·6)	0·0 (0·0–0·0)	3·61 (3·51–3·71)	1·37 (1·32–1·44)	1·16 (1·12–1·2)
1990	0·0 (0·0–0·0)	57·0 (51·0–63·2)	213·8 (202·8–225·7)	207·4 (200·3–214·8)	135·7 (129·4–142·5)	59·4 (55·3–63·4)	22·6 (20·6–24·7)	0·4 (0·2–0·5)	0·0 (0·0–0·0)	3·48 (3·38–3·58)	1·35 (1·29–1·42)	1·09 (1·05–1·14)
1991	0·0 (0·0–0·0)	54·3 (48·5–60·3)	207·6 (196·7–219·7)	196·6 (189·5–204·3)	129·5 (123·5–135·4)	55·8 (51·9–59·9)	20·6 (18·9–22·4)	0·3 (0·2–0·5)	0·0 (0·0–0·0)	3·32 (3·22–3·43)	1·31 (1·25–1·38)	1·03 (0·99–1·07)
1992	0·0 (0·0–0·0)	51·1 (45·8–56·5)	199·3 (188·4–209·9)	183·6 (177·1–190·9)	123·1 (117·6–128·5)	52·7 (49·2–56·3)	19·2 (17·7–20·7)	0·3 (0·2–0·4)	0·0 (0·0–0·0)	3·15 (3·05–3·25)	1·25 (1·19–1·31)	0·98 (0·94–1·01)
1993	0·0 (0·0–0·0)	49·9 (44·8–55·7)	190·8 (180·1–201·1)	172·5 (165·8–179·5)	117·1 (111·4–122·5)	49·0 (45·9–52·0)	17·4 (16·1–18·7)	0·2 (0·2–0·3)	0·0 (0·0–0·0)	2·98 (2·88–3·08)	1·2 (1·14–1·26)	0·92 (0·88–0·96)
1994	0·0 (0·0–0·0)	49·9 (44·7–55·7)	185·5 (174·7–196·1)	166·4 (159·2–173·4)	112·7 (107·0–118·2)	46·1 (42·8–49·4)	15·8 (14·4–17·3)	0·2 (0·1–0·3)	0·0 (0·0–0·0)	2·88 (2·78–2·99)	1·18 (1·11–1·24)	0·87 (0·82–0·92)
1995	0·0 (0·0–0·0)	48·6 (43·5–54·3)	183·7 (172·1–194·2)	162·1 (155·0–169·4)	109·6 (103·5–115·6)	44·9 (41·4–48·5)	14·9 (13·5–16·5)	0·2 (0·1–0·2)	0·0 (0·0–0·0)	2·82 (2·72–2·92)	1·16 (1·09–1·23)	0·85 (0·8–0·9)
1996	0·0 (0·0–0·0)	45·8 (40·7–51·0)	180·5 (168·1–192·4)	157·6 (150·3–165·3)	106·2 (100·0–112·4)	44·3 (40·3–48·2)	14·5 (13·1–16·0)	0·2 (0·1–0·2)	0·0 (0·0–0·0)	2·74 (2·65–2·83)	1·13 (1·06–1·2)	0·83 (0·78–0·87)
1997	0·0 (0·0–0·0)	42·9 (37·9–48·0)	174·8 (162·6–186·4)	153·7 (146·6–161·6)	103·3 (97·7–109·2)	43·8 (40·0–48·0)	14·2 (12·8–15·6)	0·2 (0·1–0·3)	0·0 (0·0–0·0)	2·66 (2·58–2·75)	1·09 (1·02–1·16)	0·81 (0·77–0·84)
1998	0·0 (0·0–0·0)	40·6 (36·1–45·4)	168·5 (158·0–179·3)	150·2 (143·3–157·1)	101·0 (95·8–106·8)	43·1 (39·8–46·7)	13·9 (12·6–15·2)	0·2 (0·1–0·3)	0·0 (0·0–0·0)	2·59 (2·5–2·68)	1·05 (0·97–1·11)	0·79 (0·76–0·83)
1999	0·0 (0·0–0·0)	38·7 (34·6–42·9)	162·9 (153·1–173·3)	146·9 (140·7–153·4)	98·5 (93·5–103·8)	42·3 (39·4–45·3)	13·5 (12·4–14·8)	0·2 (0·1–0·3)	0·0 (0·0–0·0)	2·51 (2·44–2·6)	1·01 (0·94–1·07)	0·77 (0·74–0·81)
2000	0·0 (0·0–0·0)	37·1 (33·2–41·3)	159·0 (148·0–170·0)	145·6 (138·7–151·8)	96·8 (91·2–102·2)	42·3 (39·2–45·9)	13·5 (12·3–14·9)	0·2 (0·1–0·3)	0·0 (0·0–0·0)	2·47 (2·39–2·56)	0·98 (0·91–1·05)	0·76 (0·73–0·8)
2001	0·0 (0·0–0·0)	35·6 (31·8–39·9)	156·5 (146·2–167·7)	146·8 (139·4–153·3)	97·3 (91·5–103·3)	43·7 (40·3–47·5)	14·0 (12·7–15·6)	0·2 (0·1–0·3)	0·0 (0·0–0·0)	2·47 (2·39–2·56)	0·96 (0·89–1·04)	0·78 (0·74–0·81)
2002	0·0 (0·0–0·0)	33·8 (30·1–38·0)	154·2 (144·0–165·5)	149·2 (142·4–155·9)	99·2 (93·8–104·6)	45·5 (41·9–49·2)	14·6 (13·2–16·1)	0·2 (0·1–0·3)	0·0 (0·0–0·0)	2·48 (2·39–2·57)	0·94 (0·87–1·01)	0·8 (0·76–0·83)
2003	0·0 (0·0–0·0)	31·8 (28·5–35·4)	152·4 (141·8–163·1)	152·6 (145·9–160·0)	101·7 (96·4–106·9)	47·7 (44·2–51·1)	15·0 (13·6–16·5)	0·2 (0·1–0·3)	0·0 (0·0–0·0)	2·51 (2·42–2·59)	0·92 (0·86–0·98)	0·82 (0·78–0·87)
2004	0·0 (0·0–0·0)	30·6 (27·3–34·1)	152·6 (142·4–162·7)	157·1 (150·0–164·5)	104·6 (99·3–109·6)	50·9 (47·5–54·4)	15·4 (14·1–16·8)	0·2 (0·2–0·3)	0·0 (0·0–0·0)	2·56 (2·47–2·64)	0·92 (0·86–0·97)	0·86 (0·82–0·9)
2005	0·0 (0·0–0·0)	31·1 (27·7–34·6)	154·2 (143·8–164·6)	161·8 (154·3–169·5)	108·3 (102·5–113·7)	54·1 (50·5–58·0)	16·2 (14·8–17·8)	0·2 (0·2–0·4)	0·0 (0·0–0·0)	2·63 (2·55–2·71)	0·93 (0·87–0·98)	0·89 (0·86–0·93)
2006	0·0 (0·0–0·0)	32·5 (28·7–36·0)	155·7 (145·0–167·0)	165·8 (158·0–173·9)	112·7 (107·1–118·8)	56·4 (52·5–60·5)	17·4 (15·9–19·0)	0·3 (0·2–0·4)	0·0 (0·0–0·0)	2·7 (2·62–2·79)	0·94 (0·88–1·0)	0·93 (0·89–0·98)
2007	0·0 (0·0–0·0)	33·7 (29·8–37·6)	157·7 (146·7–169·2)	169·9 (162·5–177·9)	117·8 (111·9–124·0)	58·5 (54·2–62·6)	18·6 (17·1–20·2)	0·3 (0·2–0·4)	0·0 (0·0–0·0)	2·78 (2·69–2·87)	0·96 (0·9–1·02)	0·98 (0·93–1·02)
2008	0·0 (0·0–0·0)	35·3 (31·2–39·5)	159·9 (149·3–171·2)	174·4 (167·3–182·2)	123·2 (117·1–129·2)	61·5 (57·4–65·5)	19·8 (18·3–21·4)	0·3 (0·2–0·4)	0·0 (0·0–0·0)	2·87 (2·78–2·96)	0·98 (0·92–1·04)	1·02 (0·98–1·06)
2009	0·0 (0·0–0·0)	37·3 (33·0–41·8)	161·8 (150·9–173·1)	178·7 (171·2–186·3)	128·1 (121·7–134·1)	64·8 (60·7–69·2)	21·3 (19·6–23·0)	0·3 (0·2–0·5)	0·0 (0·0–0·0)	2·96 (2·88–3·05)	1·0 (0·94–1·06)	1·07 (1·03–1·11)
2010	0·0 (0·0–0·0)	38·9 (34·3–43·8)	163·6 (152·2–175·7)	181·9 (174·1–190·4)	131·9 (125·4–138·7)	67·6 (63·1–72·4)	22·6 (20·7–24·7)	0·3 (0·2–0·5)	0·0 (0·0–0·0)	3·03 (2·95–3·12)	1·01 (0·95–1·07)	1·11 (1·07–1·16)
2011	0·0 (0·0–0·0)	39·5 (35·2–44·3)	163·8 (151·4–176·3)	183·1 (175·0–191·4)	133·5 (126·9–140·5)	68·5 (63·7–73·4)	23·1 (21·2–25·1)	0·3 (0·2–0·5)	0·0 (0·0–0·0)	3·06 (2·96–3·17)	1·02 (0·95–1·08)	1·13 (1·08–1·17)
2012	0·0 (0·0–0·0)	39·7 (35·3–44·7)	162·5 (150·5–175·7)	182·2 (174·0–190·1)	133·1 (126·4–139·9)	67·4 (62·7–72·2)	22·6 (20·7–24·6)	0·3 (0·2–0·5)	0·0 (0·0–0·0)	3·04 (2·91–3·17)	1·01 (0·94–1·08)	1·12 (1·06–1·17)
2013	0·0 (0·0–0·0)	39·8 (35·1–44·8)	160·9 (148·0–174·5)	180·6 (172·1–188·7)	132·1 (124·8–139·2)	65·8 (61·2–70·9)	21·6 (19·8–23·7)	0·3 (0·2–0·4)	0·0 (0·0–0·0)	3·01 (2·9–3·12)	1·0 (0·94–1·08)	1·1 (1·05–1·15)
2014	0·0 (0·0–0·0)	39·8 (35·1–45·0)	158·7 (145·6–172·7)	177·9 (168·7–186·7)	130·1 (122·7–137·7)	64·3 (59·6–69·6)	20·7 (18·8–22·8)	0·3 (0·2–0·4)	0·0 (0·0–0·0)	2·96 (2·86–3·06)	0·99 (0·92–1·07)	1·08 (1·03–1·13)
2015	0·0 (0·0–0·0)	39·4 (34·3–44·9)	155·7 (140·6–170·0)	174·9 (165·0–184·7)	126·3 (118·3–134·0)	61·9 (56·6–67·8)	19·7 (17·6–21·9)	0·3 (0·2–0·4)	0·0 (0·0–0·0)	2·89 (2·77–3·02)	0·98 (0·88–1·07)	1·04 (0·98–1·1)
2016	0·0 (0·0–0·0)	38·6 (33·2–44·6)	152·7 (135·6–171·1)	172·9 (162·2–184·3)	122·0 (112·3–132·1)	59·0 (52·2–66·0)	18·7 (16·1–21·6)	0·3 (0·2–0·4)	0·0 (0·0–0·0)	2·82 (2·66–2·98)	0·96 (0·84–1·08)	1·0 (0·91–1·09)
2017	0·0 (0·0–0·0)	38·2 (32·6–44·7)	151·2 (132·1–172·4)	171·9 (159·8–186·0)	119·1 (107·2–131·6)	57·0 (48·0–66·9)	18·0 (14·6–22·0)	0·3 (0·2–0·4)	0·0 (0·0–0·0)	2·78 (2·59–2·97)	0·95 (0·82–1·09)	0·97 (0·85–1·1)

Supplemental Results Table 5: Age-specific fertility rates per 1000, cumulative total fertility up to maternal age 25 (TFU25), cumulative total fertility from maternal age 30 to age 55 (TFO30), and cumulative total fertility, Mongolia, 1950-2017

Year	10-14	15-19	20-24	25-29	30-34	35-39	40-44	45-49	50-54	TFR	TFU25	TFO30
1950	0·3 (0·1–0·6)	45·6 (39·0–53·3)	275·9 (252·2–299·8)	289·9 (278·1–301·8)	210·1 (194·6–225·9)	176·9 (161·5–192·9)	105·6 (98·0–113·2)	33·2 (28·5–37·9)	0·6 (0·6–0·7)	5·69 (5·37–6·01)	1·61 (1·46–1·77)	2·63 (2·48–2·79)
1951	0·3 (0·1–0·6)	44·6 (38·2–51·6)	270·1 (247·0–292·3)	281·0 (268·7–292·8)	204·8 (189·8–220·2)	171·7 (157·3–187·5)	103·0 (95·2–110·7)	32·5 (27·7–37·3)	0·6 (0·6–0·7)	5·54 (5·24–5·86)	1·57 (1·43–1·72)	2·56 (2·41–2·72)
1952	0·3 (0·1–0·6)	43·4 (37·6–50·1)	263·9 (240·7–286·5)	270·6 (258·4–282·6)	198·8 (184·3–213·6)	165·8 (151·9–181·1)	100·0 (92·1–107·4)	31·8 (26·9–36·6)	0·6 (0·6–0·6)	5·38 (5·08–5·68)	1·54 (1·4–1·68)	2·48 (2·34–2·64)
1953	0·3 (0·1–0·6)	42·3 (36·9–48·5)	257·0 (235·1–278·3)	259·5 (247·8–272·0)	192·6 (178·5–207·0)	159·7 (146·0–172·9)	96·7 (89·3–103·6)	30·9 (26·1–35·6)	0·6 (0·6–0·6)	5·2 (4·9–5·49)	1·5 (1·37–1·63)	2·4 (2·26–2·55)
1954	0·3 (0·1–0·5)	42·0 (37·0–47·6)	251·3 (230·4–271·5)	253·1 (241·2–265·7)	188·9 (175·6–202·7)	156·0 (143·1–167·8)	94·7 (87·9–101·1)	30·2 (25·6–35·1)	0·6 (0·6–0·6)	5·08 (4·79–5·37)	1·47 (1·35–1·59)	2·35 (2·2–2·49)
1955	0·3 (0·1–0·6)	42·9 (38·1–48·3)	249·9 (229·9–268·9)	255·9 (243·6–267·8)	190·3 (177·4–202·9)	157·2 (145·3–167·9)	95·5 (88·9–101·3)	30·1 (25·5–35·1)	0·6 (0·6–0·6)	5·11 (4·82–5·39)	1·47 (1·35–1·57)	2·37 (2·21–2·51)
1956	0·3 (0·1–0·6)	44·9 (40·0–50·3)	252·2 (232·1–271·0)	265·2 (253·1–277·2)	195·6 (183·4–207·4)	162·2 (151·3–172·1)	98·5 (92·6–103·8)	30·5 (25·9–35·3)	0·6 (0·6–0·6)	5·25 (4·99–5·52)	1·49 (1·38–1·59)	2·44 (2·28–2·58)
1957	0·3 (0·1–0·6)	47·5 (42·1–52·8)	256·4 (237·1–274·7)	277·6 (266·4–288·4)	202·6 (190·3–215·0)	168·8 (158·3–178·1)	102·5 (96·7–107·5)	31·1 (26·4–35·7)	0·6 (0·6–0·6)	5·44 (5·18–5·69)	1·52 (1·42–1·62)	2·53 (2·38–2·67)
1958	0·3 (0·1–0·6)	50·1 (44·4–56·1)	261·8 (243·6–280·0)	291·0 (279·9–301·4)	210·4 (198·0–222·3)	176·0 (165·4–185·1)	106·7 (101·6–111·5)	31·8 (27·1–36·1)	0·6 (0·6–0·6)	5·64 (5·4–5·88)	1·56 (1·46–1·66)	2·63 (2·48–2·76)
1959	0·3 (0·1–0·6)	52·0 (46·0–58·6)	266·8 (248·0–284·5)	302·0 (291·5–311·6)	217·0 (204·5–228·9)	181·9 (172·1–191·3)	110·2 (105·1–114·5)	32·3 (27·6–36·5)	0·6 (0·6–0·6)	5·82 (5·58–6·05)	1·6 (1·49–1·7)	2·71 (2·56–2·85)
1960	0·3 (0·1–0·6)	53·2 (47·3–60·4)	270·0 (250·4–288·5)	310·0 (299·8–319·0)	221·5 (209·0–233·7)	185·5 (174·8–195·3)	112·3 (107·4–116·7)	32·4 (27·9–36·6)	0·6 (0·6–0·6)	5·93 (5·69–6·16)	1·62 (1·5–1·72)	2·76 (2·6–2·91)
1961	0·3 (0·1–0·6)	54·4 (48·4–61·8)	272·7 (252·5–291·0)	317·2 (307·3–326·0)	225·3 (212·8–238·0)	188·3 (177·3–198·7)	113·8 (108·9–118·2)	32·5 (27·9–36·8)	0·6 (0·6–0·6)	6·03 (5·78–6·27)	1·64 (1·52–1·76)	2·8 (2·65–2·95)
1962	0·3 (0·1–0·6)	55·6 (49·2–63·5)	275·6 (255·7–294·6)	324·4 (315·3–332·8)	229·2 (217·0–242·3)	191·1 (180·2–201·7)	115·4 (110·7–119·7)	32·6 (27·8–36·8)	0·6 (0·6–0·7)	6·12 (5·88–6·37)	1·66 (1·54–1·79)	2·84 (2·69–3·0)
1963	0·3 (0·1–0·6)	56·3 (50·0–63·9)	278·0 (258·8–297·9)	330·1 (321·9–338·6)	232·6 (219·9–245·8)	193·4 (181·5–203·6)	116·7 (111·9–120·8)	32·7 (27·9–36·9)	0·6 (0·6–0·7)	6·2 (5·95–6·46)	1·67 (1·55–1·81)	2·88 (2·73–3·02)
1964	0·3 (0·1–0·6)	56·2 (49·9–63·6)	278·4 (258·5–298·5)	333·2 (324·8–341·6)	234·1 (221·3–248·0)	193·9 (181·6–204·2)	117·3 (112·5–121·2)	32·6 (27·7–36·8)	0·6 (0·6–0·7)	6·23 (5·99–6·5)	1·67 (1·55–1·81)	2·89 (2·75–3·03)
1965	0·3 (0·1–0·6)	55·5 (49·5–62·5)	276·5 (256·7–298·6)	334·4 (325·9–342·6)	233·3 (220·2–247·8)	192·3 (179·3–203·6)	116·5 (111·4–120·8)	32·1 (27·4–36·4)	0·6 (0·6–0·6)	6·21 (5·95–6·48)	1·66 (1·54–1·8)	2·87 (2·73–3·01)
1966	0·3 (0·1–0·6)	54·8 (49·0–61·4)	273·9 (254·0–295·8)	335·5 (327·0–343·7)	231·9 (217·9–246·9)	189·9 (176·7–201·8)	115·3 (110·0–120·1)	31·5 (26·7–35·9)	0·6 (0·6–0·6)	6·17 (5·91–6·45)	1·64 (1·53–1·78)	2·85 (2·7–2·98)
1967	0·3 (0·1–0·6)	54·1 (48·6–60·3)	271·5 (252·2–293·3)	337·5 (329·4–346·1)	230·9 (217·1–246·5)	187·7 (174·4–200·5)	114·1 (108·3–119·4)	30·9 (26·1–35·5)	0·6 (0·6–0·6)	6·14 (5·88–6·4)	1·63 (1·51–1·75)	2·82 (2·67–2·95)
1968	0·3 (0·1–0·6)	53·3 (48·0–59·1)	269·1 (249·9–289·5)	338·6 (330·5–346·7)	230·1 (216·0–245·5)	185·7 (171·9–199·6)	113·0 (106·9–118·8)	30·3 (25·4–35·0)	0·6 (0·6–0·6)	6·11 (5·86–6·36)	1·61 (1·51–1·73)	2·8 (2·66–2·93)
1969	0·3 (0·1–0·6)	52·5 (47·2–57·9)	266·8 (247·9–285·8)	337·4 (329·6–345·5)	229·5 (215·2–245·0)	183·8 (169·8–198·0)	112·0 (105·4–117·9)	29·8 (24·7–34·7)	0·6 (0·6–0·6)	6·06 (5·82–6·3)	1·6 (1·49–1·7)	2·78 (2·63–2·92)
1970	0·3 (0·1–0·6)	51·6 (46·6–56·8)	264·7 (246·6–283·6)	335·7 (327·6–343·5)	229·1 (215·4–244·0)	182·0 (167·2–196·4)	111·0 (104·2–117·1)	29·2 (24·0–34·2)	0·6 (0·5–0·6)	6·02 (5·78–6·26)	1·58 (1·48–1·68)	2·76 (2·61–2·9)
1971	0·3 (0·1–0·6)	50·7 (46·0–55·7)	262·8 (245·0–280·4)	334·8 (326·6–342·6)	228·8 (215·0–243·2)	180·4 (165·4–194·4)	110·0 (103·2–116·2)	28·5 (23·5–33·5)	0·5 (0·5–0·6)	5·98 (5·76–6·21)	1·57 (1·47–1·66)	2·74 (2·59–2·88)
1972	0·3 (0·1–0·6)	49·9 (45·4–54·7)	260·8 (243·6–277·0)	334·8 (326·7–342·3)	228·9 (215·9–241·7)	179·1 (164·8–192·9)	109·2 (102·6–115·3)	27·8 (23·1–32·9)	0·5 (0·5–0·6)	5·96 (5·73–6·17)	1·55 (1·47–1·64)	2·73 (2·57–2·87)
1973	0·3 (0·1–0·6)	49·5 (45·0–54·0)	259·8 (243·4–275·4)	335·7 (328·0–342·9)	229·4 (216·7–241·9)	178·5 (164·5–192·0)	108·7 (102·0–114·8)	27·2 (22·4–32·1)	0·5 (0·5–0·5)	5·95 (5·71–6·18)	1·55 (1·46–1·63)	2·72 (2·57–2·86)
1974	0·3 (0·1–0·6)	49·1 (45·0–53·7)	260·0 (244·7–274·7)	336·2 (328·7–343·0)	229·6 (217·3–241·6)	178·3 (164·9–190·9)	108·3 (101·8–114·3)	26·5 (21·6–31·5)	0·5 (0·5–0·5)	5·94 (5·7–6·18)	1·55 (1·47–1·62)	2·72 (2·56–2·86)
1975	0·3 (0·1–0·6)	48·7 (44·7–52·9)	259·8 (244·9–274·2)	336·4 (329·2–343·2)	229·9 (217·8–240·8)	177·9 (165·7–189·8)	108·0 (101·8–113·6)	25·7 (21·1–30·5)	0·5 (0·5–0·5)	5·94 (5·69–6·17)	1·54 (1·46–1·62)	2·71 (2·56–2·85)
1976	0·3 (0·1–0·6)	48·2 (44·3–52·3)	259·3 (245·4–273·5)	337·2 (330·3–343·7)	230·7 (219·5–240·7)	177·7 (166·6–188·6)	107·7 (102·1–113·0)	24·9 (20·5–29·7)	0·5 (0·5–0·5)	5·93 (5·7–6·15)	1·54 (1·46–1·61)	2·71 (2·56–2·85)
1977	0·3 (0·1–0·6)	47·7 (43·9–51·5)	258·9 (245·9–272·8)	337·6 (331·1–344·0)	231·3 (221·3–240·9)	177·2 (167·6–186·7)	107·2 (102·2–112·1)	24·1 (19·6–28·8)	0·5 (0·4–0·5)	5·92 (5·71–6·13)	1·53 (1·46–1·6)	2·7 (2·56–2·83)
1978	0·3 (0·1–0·6)	47·4 (43·8–50·9)	258·6 (245·8–271·5)	337·0 (330·8–343·4)	231·1 (221·7–240·4)	175·6 (166·8–183·9)	106·2 (101·5–110·7)	23·5 (19·2–28·1)	0·5 (0·4–0·5)	5·9 (5·71–6·09)	1·53 (1·47–1·6)	2·68 (2·56–2·81)
1979	0·3 (0·1–0·6)	47·1 (43·8–50·5)	258·3 (246·2–270·1)	335·3 (329·1–341·7)	229·3 (220·6–238·2)	171·6 (163·0–180·1)	103·6 (98·9–107·9)	22·5 (18·1–26·9)	0·4 (0·4–0·4)	5·84 (5·66–6·02)	1·53 (1·47–1·59)	2·64 (2·51–2·75)
1980	0·3 (0·1–0·6)	46·5 (43·4–49·7)	256·6 (244·9–268·5)	330·9 (324·6–337·2)	224·5 (215·8–232·9)	163·9 (155·2–172·2)	98·0 (93·1–102·8)	20·4 (16·1–24·9)	0·4 (0·4–0·4)	5·71 (5·53–5·88)	1·52 (1·46–1·58)	2·54 (2·42–2·65)
1981	0·3 (0·1–0·6)	45·8 (42·9–48·8)	253·1 (241·7–264·9)	324·6 (318·4–330·9)	218·4 (209·9–226·8)	154·5 (145·7–163·0)	90·4 (85·3–95·3)	18·0 (14·0–22·4)	0·3 (0·3–0·4)	5·53 (5·36–5·7)	1·5 (1·44–1·56)	2·41 (2·29–2·52)
1982	0·3 (0·1–0·6)	45·2 (42·5–48·3)	249·6 (238·4–261·0)	318·6 (312·4–324·8)	212·8 (204·6–220·8)	146·0 (137·3–154·5)	83·1 (77·8–88·2)	16·1 (12·2–20·2)	0·3 (0·3–0·3)	5·36 (5·2–5·52)	1·48 (1·42–1·53)	2·29 (2·18–2·41)
1983	0·3 (0·1–0·6)	45·2 (42·5–48·2)	247·7 (236·8–258·2)	314·2 (307·7–320·1)	207·8 (199·9–215·8)	139·0 (130·5–147·6)	76·4 (71·0–81·6)	14·6 (11·2–18·5)	0·3 (0·3–0·3)	5·23 (5·08–5·38)	1·47 (1·41–1·52)	2·19 (2·08–2·3)
1984	0·3 (0·1–0·6)	45·4 (42·6–48·3)	247·0 (236·8–256·3)	310·5 (304·2–316·2)	203·2 (195·5–211·0)	132·6 (124·8–140·7)	69·9 (64·9–75·0)	13·4 (10·2–17·0)	0·3 (0·2–0·3)	5·11 (4·97–5·25)	1·46 (1·41–1·51)	2·1 (2·0–2·2)
1985	0·3 (0·1–0·6)	45·6 (42·9–48·2)	245·8 (236·1–255·0)	304·1 (297·8–310·2)	197·4 (190·3–204·7)	125·8 (118·4–133·6)	63·4 (59·0–68·2)	12·0 (9·1–15·3)	0·2 (0·2–0·2)	4·97 (4·84–5·1)	1·46 (1·4–1·51)	1·99 (1·92–2·08)
1986	0·3 (0·1–0·6)	45·6 (42·9–48·3)	242·7 (233·8–251·7)	294·0 (287·7–300·5)	189·8 (183·1–196·9)	118·9 (111·7–125·8)	57·1 (52·8–61·6)	10·7 (8·1–13·7)	0·2 (0·2–0·2)	4·8 (4·68–4·91)	1·44 (1·39–1·49)	1·88 (1·82–1·95)
1987	0·3 (0·1–0·6)	45·5 (42·8–48·2)	238·0 (229·1–246·9)	281·3 (274·7–288·3)	180·9 (174·4–187·7)	111·9 (105·0–118·3)	51·4 (47·3–55·9)	9·6 (7·3–12·5)	0·2 (0·2–0·2)	4·6 (4·49–4·71)	1·42 (1·37–1·47)	1·77 (1·71–1·83)
1988	0·3 (0·1–0·6)	45·5 (42·7–48·3)	232·4 (223·1–241·9)	267·6 (260·8–275·0)	171·3 (164·9–178·2)	104·7 (98·1–110·9)	46·5 (42·6–50·6)	8·8 (6·6–11·5)	0·2 (0·2–0·2)	4·39 (4·27–4·5)	1·39 (1·34–1·44)	1·66 (1·6–1·71)
1989	0·3 (0·1–0·6)	45·6 (42·9–48·3)	226·7 (217·8–236·4)	253·0 (246·3–260·2)	161·3 (154·9–168·2)	97·1 (90·6–103·3)	41·9 (38·2–45·9)	8·1 (6·0–10·6)	0·2 (0·1–0·2)	4·17 (4·07–4·29)	1·36 (1·31–1·42)	1·54 (1·49–1·59)
1990	0·3 (0·1–0·6)	45·2 (42·3–48·0)	219·3 (210·3–229·0)	234·0 (226·7–241·2)	149·4 (143·0–156·1)	88·3 (82·5–94·5)	37·2 (33·6–40·9)	7·2 (5·3–9·4)	0·1 (0·1–0·1)	3·91 (3·8–4·01)	1·32 (1·27–1·38)	1·41 (1·36–1·46)
1991	0·3 (0·1–0·6)	44·2 (41·5–46·8)	209·3 (199·8–218·8)	210·4 (202·9–217·7)	136·1 (129·9–142·2)	78·9 (73·4–84·7)	32·6 (29·2–35·9)	6·3 (4·6–8·3)	0·1 (0·1–0·1)	3·59 (3·49–3·69)	1·27 (1·22–1·32)	1·27 (1·22–1·32)
1992	0·3 (0·1–0·6)	43·1 (40·5–45·7)	198·8 (189·1–208·4)	186·5 (179·5–194·2)	123·7 (117·4–129·6)	70·1 (65·1–75·4)	28·6 (25·6–31·6)	5·3 (3·8–7·1)	0·1 (0·1–0·1)	3·28 (3·18–3·38)	1·21 (1·16–1·26)	1·14 (1·09–1·18)
1993	0·3 (0·1–0·5)	41·8 (39·3–44·3)	189·4 (179·5–198·9)	166·1 (158·9–173·1)	113·2 (107·6–119·0)	62·4 (57·9–67·3)	25·3 (22·6–28·0)	4·5 (3·2–6·0)	0·1 (0·1–0·1)	3·02 (2·92–3·12)	1·16 (1·1–1·21)	1·03 (0·99–1·07)
1994	0·3 (0·1–0·5)	40·1 (37·6–42·5)	180·5 (170·7–189·9)	154·7 (147·6–161·8)	104·6 (99·4–109·8)	56·0 (51·8–60·4)	22·5 (20·2–25·1)	3·7 (2·7–5·0)	0·1 (0·1–0·1)	2·81 (2·71–2·91)	1·1 (1·04–1·16)	0·93 (0·9–0·97)
1995	0·3 (0·1–0·5)	38·2 (35·8–40·6)	172·3 (163·0–181·2)	149·9 (142·8–156·8)	97·8 (93·0–102·8)	50·8 (47·0–54·7)	20·3 (18·2–22·4)	3·0 (2·1–4·0)	0·1 (0·1–0·1)	2·66 (2·57–2·76)	1·05 (1·0–1·11)	0·86 (0·83–0·89)
1996	0·2 (0·1–0·5)	36·5 (34·2–38·9)	165·0 (156·2–173·9)	145·5 (138·6–152·4)	92·6 (88·0–97·6)	46·7 (43·2–50·5)	18·4 (16·6–20·3)	2·5 (1·8–3·3)	0·0 (0·0–0·1)	2·54 (2·45–2·62)	1·01 (0·95–1·06)	0·8 (0·77–0·83)
1997	0·2 (0·1–0·5)	34·8 (32·7–37·1)	158·5 (149·6–167·1)	141·8 (135·3–148·3)	88·6 (84·4–93·2)	43·1 (40·2–46·3)	16·6 (15·0–18·4)	2·3 (1·7–3·1)	0·0 (0·0–0·0)	2·43 (2·35–2·51)	0·97 (0·92–1·02)	0·75 (0·72–0·79)
1998	0·2 (0·1–0·5)	33·1 (31·1–35·2)	153·1 (144·5–161·5)	139·1 (132·6–145·9)	85·7 (81·6–90·2)	40·1 (37·3–43·1)	15·1 (13·6–16·6)	2·3 (1·6–3·0)	0·0 (0·0–0·0)	2·34 (2·27–2·42)	0·93 (0·89–0·98)	0·72 (0·69–0·74)
1999	0·2 (0·1–0·5)	31·4 (29·4–33·5)	148·8 (140·6–157·3)	137·3 (130·7–143·8)	83·9 (79·9–88·4)	38·7 (36·2–41·5)	14·1 (12·7–15·5)	2·2 (1·6–2·9)	0·0 (0·0–0·0)	2·28 (2·21–2·36)	0·9 (0·86–0·95)	0·69 (0·67–0·72)
2000	0·2 (0·1–0·5)	29·6 (27·7–31·6)	145·2 (137·2–153·9)	136·1 (129·7–143·1)	82·7 (78·8–87·2)	39·0 (36·5–41·8)	13·8 (12·4–15·1)	2·0 (1·5–2·7)	0·0 (0·0–0·0)	2·24 (2·17–2·32)	0·87 (0·83–0·92)	0·69 (0·66–0·72)
2001	0·2 (0·1–0·4)	27·7 (25·8–29·6)	142·2 (134·3–150·7)	135·5 (129·2–142·4)	82·0 (78·1–86·1)	39·9 (37·3–42·8)	13·7 (12·3–15·0)	2·0 (1·4–2·6)	0·0 (0·0–0·0)	2·22 (2·14–2·3)	0·85 (0·81–0·89)	0·69 (0·66–0·72)
2002	0·2 (0·1–0·4)	25·9 (24·1–27·7)	139·8 (131·8–148·4)	135·5 (129·0–142·5)	82·1 (78·1–86·3)	41·2 (38·5–44·2)	13·6 (12·3–15·0)	1·9 (1·3–2·5)	0·0 (0·0–0·0)	2·2 (2·13–2·28)	0·83 (0·79–0·87)	0·69 (0·67–0·72)
2003	0·2 (0·1–0·4)	24·4 (22·8–26·1)	138·4 (130·3–147·0)	136·5 (129·7–143·4)	83·3 (79·2–87·6)	42·9 (40·1–46·1)	13·6 (12·3–15·1)	1·8 (1·3–2·4)	0·0 (0·0–0·0)	2·21 (2·13–2·29)	0·81 (0·77–0·86)	0·71 (0·68–0·74)
2004	0·2 (0·1–0·4)	23·4 (21·9–25·0)	137·9 (129·4–146·5)	138·4 (131·9–145·9)	85·7 (81·4–89·8)	44·9 (42·0–48·3)	13·8 (12·5–15·2)	1·7 (1·3–2·4)	0·0 (0·0–0·0)	2·23 (2·15–2·31)	0·81 (0·77–0·85)	0·73 (0·7–0·76)
2005	0·2 (0·1–0·4)	22·9 (21·4–24·6)	138·2 (129·6–147·2)	141·3 (134·5–148·7)	89·2 (84·5–93·7)	47·4 (44·3–50·8)	14·2 (12·8–15·4)	1·7 (1·2–2·4)	0·0 (0·0–0·0)	2·28 (2·2–2·36)	0·81 (0·76–0·85)	0·76 (0·73–0·79)
2006	0·2 (0·1–0·4)	22·5 (20·9–24·2)	139·0 (130·7–148·3)	144·8 (137·7–152·3)	93·8 (88·8–98·9)	50·7 (47·6–54·3)	14·8 (13·5–16·1)	1·7 (1·2–2·3)	0·0 (0·0–0·0)	2·34 (2·26–2·42)	0·81 (0·76–0·86)	0·81 (0·77–0·84)
2007	0·2 (0·1–0·4)	22·1 (20·6–23·8)	140·1 (131·6–149·5)	148·3 (141·0–156·4)	99·0 (93·9–104·4)	54·7 (51·2–58·4)	15·6 (14·4–16·9)	1·7 (1·3–2·3)	0·0 (0·0–0·0)	2·41 (2·33–2·49)	0·81 (0·77–0·86)	0·86 (0·83–0·89)
2008	0·2 (0·1–0·4)	21·7 (20·1–23·4)	141·2 (132·4–150·4)	151·5 (143·8–159·8)	103·7 (98·5–109·5)	58·6 (54·7–62·8)	16·5 (15·1–18·0)	1·7 (1·3–2·3)	0·0 (0·0–0·0)	2·48 (2·39–2·56)	0·82 (0·77–0·87)	0·9 (0·87–0·94)
2009	0·2 (0·1–0·4)	21·4 (19·8–23·1)	142·2 (132·9–151·8)	154·3 (146·5–162·8)	108·1 (102·6–114·0)	62·2 (57·9–66·9)	17·5 (16·0–19·1)	1·7 (1·3–2·3)	0·0 (0·0–0·0)	2·54 (2·45–2·62)	0·82 (0·77–0·87)	0·95 (0·91–0·99)
2010	0·2 (0·1–0·4)	21·4 (19·7–23·2)	143·8 (134·3–153·4)	157·1 (148·8–165·4)	112·5 (106·5–119·4)	65·5 (60·7–70·3)	18·3 (16·8–20·1)	1·7 (1·3–2·3)	0·0 (0·0–0·0)	2·6 (2·52–2·69)	0·83 (0·78–0·88)	0·99 (0·95–1·03)
2011	0·2 (0·1–0·4)	21·8 (20·1–23·6)	145·7 (135·9–155·5)	159·5 (150·8–168·2)	116·7 (110·5–123·4)	68·4 (63·4–73·4)	19·2 (17·5–21·0)	1·7 (1·2–2·2)	0·0 (0·0–0·0)	2·67 (2·58–2·75)	0·84 (0·79–0·89)	1·03 (0·99–1·07)
2012	0·2 (0·1–0·4)	22·7 (20·8–24·6)	147·3 (137·1–157·6)	161·0 (152·0–170·1)	119·8 (113·3–126·4)	71·2 (66·0–76·1)	20·1 (18·2–22·1)	1·7 (1·2–2·2)	0·0 (0·0–0·0)	2·72 (2·62–2·81)	0·85 (0·8–0·91)	1·06 (1·02–1·11)
2013	0·2 (0·1–0·4)	24·1 (22·0–26·3)	148·7 (137·5–160·2)	161·9 (152·1–171·5)	121·5 (114·4–128·7)	73·8 (67·9–79·3)	21·0 (18·9–23·3)	1·6 (1·2–2·2)	0·0 (0·0–0·0)	2·76 (2·65–2·87)	0·86 (0·8–0·93)	1·09 (1·03–1·14)
2014	0·2 (0·1–0·4)	25·5 (23·0–28·2)	149·5 (136·5–162·2)	161·9 (152·1–171·7)	121·3 (113·1–128·9)	74·7 (68·0–81·6)	21·3 (19·1–23·9)	1·5 (1·1–2·1)	0·0 (0·0–0·0)	2·78 (2·65–2·92)	0·88 (0·8–0·95)	1·09 (1·02–1·17)
2015	0·2 (0·1–0·4)	26·4 (23·7–29·4)	149·3 (135·5–163·6)	161·3 (151·0–171·5)	119·1 (110·3–127·7)	73·3 (66·0–80·9)	21·0 (18·2–23·9)	1·5 (1·1–2·1)	0·0 (0·0–0·0)	2·76 (2·59–2·92)	0·88 (0·8–0·96)	1·07 (0·99–1·16)
2016	0·2 (0·1–0·4)	26·8 (23·8–30·1)	148·0 (132·9–164·7)	160·4 (149·6–171·4)	116·4 (107·2–126·2)	71·2 (62·9–79·5)	20·5 (17·3–23·8)	1·5 (1·0–2·1)	0·0 (0·0–0·0)	2·73 (2·53–2·92)	0·88 (0·78–0·97)	1·05 (0·94–1·16)
2017	0·2 (0·1–0·4)	27·2 (23·9–30·8)	147·3 (130·5–165·0)	159·7 (148·4–171·4)	114·5 (103·9–125·6)	69·6 (60·3–79·7)	20·1 (16·6–24·1)	1·5 (1·0–2·1)	0·0 (0·0–0·0)	2·7 (2·49–2·91)	0·87 (0·77–0·98)	1·03 (0·91–1·16)

Supplemental Results Table 5: Age-specific fertility rates per 1000, cumulative total fertility up to maternal age 25 (TFU25), cumulative total fertility from maternal age 30 to age 55 (TFO30), and cumulative total fertility, Tajikistan, 1950-2017

Year	10-14	15-19	20-24	25-29	30-34	35-39	40-44	45-49	50-54	TFR	TFU25	TFO30
1950	0·1 (0·0–0·2)	88·8 (77·0–103·5)	361·6 (345·9–375·9)	347·6 (340·0–354·8)	231·0 (216·1–245·3)	195·5 (182·9–207·1)	104·5 (97·4–110·9)	32·2 (28·3–35·9)	0·6 (0·6–0·6)	6·81 (6·63–7·0)	2·25 (2·15–2·36)	2·82 (2·69–2·94)
1951	0·1 (0·0–0·2)	89·8 (78·0–103·6)	361·3 (344·9–375·3)	348·4 (341·6–355·3)	231·7 (217·5–245·8)	195·7 (183·5–206·3)	104·6 (97·9–110·8)	32·3 (28·5–35·6)	0·6 (0·6–0·6)	6·82 (6·64–7·01)	2·26 (2·16–2·36)	2·82 (2·71–2·94)
1952	0·1 (0·0–0·2)	90·7 (78·8–104·3)	361·0 (344·9–375·2)	349·1 (342·3–356·0)	232·3 (217·1–246·4)	195·8 (183·4–207·5)	104·8 (98·1–111·1)	32·3 (28·6–35·8)	0·6 (0·6–0·6)	6·83 (6·65–7·01)	2·26 (2·16–2·36)	2·83 (2·71–2·94)
1953	0·1 (0·0–0·2)	91·9 (79·8–105·1)	360·8 (343·8–374·8)	349·9 (342·9–356·3)	233·2 (218·3–247·7)	196·0 (184·5–207·4)	105·1 (98·2–111·2)	32·3 (28·8–35·9)	0·6 (0·6–0·6)	6·85 (6·67–7·03)	2·26 (2·16–2·36)	2·84 (2·72–2·95)
1954	0·1 (0·0–0·2)	93·1 (81·1–106·5)	360·8 (344·6–374·3)	350·7 (343·7–357·0)	234·0 (219·4–249·5)	196·3 (183·7–207·3)	105·5 (99·2–111·4)	32·3 (28·9–35·8)	0·6 (0·6–0·6)	6·87 (6·68–7·06)	2·27 (2·17–2·37)	2·84 (2·73–2·97)
1955	0·1 (0·0–0·2)	94·1 (81·9–107·5)	360·5 (344·2–374·6)	350·9 (343·8–356·9)	234·4 (220·3–249·1)	196·2 (183·6–207·2)	105·8 (99·4–111·5)	32·2 (28·6–35·5)	0·6 (0·6–0·6)	6·87 (6·67–7·08)	2·27 (2·17–2·38)	2·85 (2·71–2·97)
1956	0·1 (0·0–0·2)	95·0 (82·5–108·4)	359·4 (343·4–373·4)	350·2 (343·2–356·3)	234·0 (219·8–247·7)	195·5 (182·8–206·9)	105·8 (99·1–111·6)	31·9 (28·4–35·3)	0·6 (0·6–0·6)	6·86 (6·64–7·09)	2·27 (2·17–2·38)	2·84 (2·69–2·98)
1957	0·1 (0·0–0·2)	96·3 (84·0–109·6)	357·9 (342·3–372·4)	349·8 (342·5–356·3)	234·1 (219·5–247·5)	195·1 (182·2–206·5)	106·0 (99·5–112·2)	31·6 (27·8–35·1)	0·6 (0·6–0·6)	6·86 (6·63–7·09)	2·27 (2·17–2·37)	2·84 (2·67–2·99)
1958	0·1 (0·0–0·2)	98·3 (85·8–111·4)	357·5 (342·0–371·5)	351·0 (343·7–357·7)	236·8 (223·1–250·0)	196·6 (184·3–207·9)	107·4 (101·0–113·3)	31·6 (27·9–35·1)	0·6 (0·6–0·6)	6·9 (6·67–7·12)	2·28 (2·18–2·38)	2·87 (2·7–3·0)
1959	0·1 (0·0–0·2)	99·8 (86·8–112·8)	357·8 (342·0–371·6)	352·7 (345·5–359·3)	241·7 (228·7–254·5)	199·3 (186·9–210·3)	110·1 (103·6–115·4)	31·8 (27·8–35·3)	0·6 (0·6–0·6)	6·97 (6·75–7·18)	2·29 (2·19–2·39)	2·92 (2·78–3·04)
1960	0·1 (0·0–0·2)	100·6 (87·2–114·6)	356·7 (340·3–371·0)	353·2 (345·9–359·9)	248·0 (235·1–259·8)	202·7 (190·9–213·0)	113·6 (107·8–118·6)	31·9 (27·9–35·5)	0·6 (0·6–0·6)	7·04 (6·84–7·23)	2·29 (2·19–2·38)	2·98 (2·86–3·09)
1961	0·1 (0·0–0·2)	101·8 (89·0–116·4)	354·1 (337·8–368·6)	353·4 (346·1–360·1)	254·4 (242·2–266·1)	206·3 (194·6–215·4)	117·1 (111·8–121·1)	31·8 (27·8–35·3)	0·6 (0·6–0·6)	7·1 (6·92–7·28)	2·28 (2·18–2·38)	3·05 (2·94–3·15)
1962	0·1 (0·0–0·2)	104·0 (90·7–118·7)	353·4 (336·1–368·2)	355·2 (348·0–361·8)	257·4 (244·4–269·2)	208·5 (197·2–217·7)	118·6 (113·8–122·4)	31·8 (27·4–35·5)	0·6 (0·6–0·6)	7·15 (6·98–7·32)	2·29 (2·18–2·39)	3·08 (2·98–3·18)
1963	0·1 (0·0–0·2)	106·3 (93·0–120·9)	354·6 (336·9–370·1)	357·0 (349·9–363·8)	255·5 (242·4–267·3)	207·4 (195·5–217·2)	117·6 (112·5–121·9)	31·7 (27·2–35·8)	0·6 (0·6–0·6)	7·15 (6·97–7·33)	2·3 (2·2–2·41)	3·06 (2·95–3·16)
1964	0·1 (0·0–0·2)	107·3 (94·1–121·7)	354·8 (337·4–370·5)	356·9 (350·3–363·6)	251·7 (237·7–264·6)	204·4 (191·6–214·1)	115·5 (109·6–120·5)	31·1 (26·5–35·3)	0·6 (0·6–0·6)	7·11 (6·9–7·31)	2·31 (2·2–2·43)	3·02 (2·89–3·13)
1965	0·1 (0·0–0·2)	108·2 (95·1–122·7)	354·4 (337·1–371·0)	356·4 (349·9–363·3)	248·5 (234·0–261·9)	201·4 (187·2–212·7)	113·4 (106·6–119·3)	30·2 (25·5–34·7)	0·6 (0·6–0·6)	7·07 (6·85–7·29)	2·31 (2·21–2·43)	2·97 (2·81–3·11)
1966	0·1 (0·0–0·2)	109·4 (96·4–123·3)	354·7 (336·8–371·4)	356·7 (349·9–363·8)	246·0 (230·8–260·4)	198·8 (184·5–212·3)	111·4 (104·1–118·1)	29·4 (24·3–34·4)	0·6 (0·5–0·6)	7·04 (6·79–7·27)	2·32 (2·21–2·44)	2·93 (2·75–3·09)
1967	0·1 (0·0–0·2)	111·0 (98·4–124·8)	355·4 (338·2–372·8)	357·2 (350·5–363·9)	243·7 (228·6–258·0)	196·4 (180·8–210·5)	109·5 (101·6–116·7)	28·5 (23·0–33·7)	0·5 (0·5–0·6)	7·01 (6·75–7·27)	2·33 (2·23–2·45)	2·89 (2·7–3·07)
1968	0·1 (0·0–0·2)	112·8 (100·1–126·8)	356·2 (338·9–372·7)	357·9 (351·1–364·7)	241·6 (226·3–256·9)	194·3 (178·0–208·4)	107·7 (98·7–115·0)	27·7 (21·9–32·9)	0·5 (0·5–0·6)	6·99 (6·73–7·24)	2·35 (2·23–2·46)	2·86 (2·64–3·05)
1969	0·1 (0·0–0·2)	114·5 (101·8–128·5)	357·5 (340·3–373·4)	358·9 (352·5–365·5)	240·0 (224·8–255·3)	192·5 (175·3–207·1)	106·0 (96·8–113·9)	26·9 (21·1–32·0)	0·5 (0·5–0·5)	6·98 (6·71–7·23)	2·36 (2·25–2·47)	2·83 (2·61–3·03)
1970	0·1 (0·0–0·2)	115·2 (102·1–129·4)	360·1 (343·5–375·1)	360·3 (354·1–366·7)	238·8 (223·8–254·1)	191·1 (174·5–206·2)	104·5 (94·5–112·7)	26·1 (20·3–31·4)	0·5 (0·5–0·5)	6·98 (6·72–7·23)	2·38 (2·27–2·48)	2·81 (2·59–3·02)
1971	0·1 (0·0–0·2)	115·8 (102·7–129·6)	363·6 (347·1–377·4)	362·1 (356·3–368·0)	238·0 (222·2–253·3)	190·1 (174·0–205·2)	103·2 (94·0–111·7)	25·2 (19·9–30·8)	0·5 (0·5–0·5)	6·99 (6·74–7·23)	2·4 (2·3–2·49)	2·79 (2·58–2·98)
1972	0·1 (0·0–0·2)	117·4 (104·7–131·5)	368·3 (354·1–381·0)	364·2 (358·7–370·0)	237·6 (221·5–252·8)	189·6 (174·6–203·9)	102·2 (92·8–110·7)	24·4 (19·1–29·6)	0·5 (0·5–0·5)	7·02 (6·79–7·25)	2·43 (2·33–2·52)	2·77 (2·59–2·96)
1973	0·1 (0·0–0·2)	119·4 (106·5–133·2)	374·5 (361·4–386·4)	366·1 (360·5–371·8)	237·3 (221·9–251·5)	188·6 (172·7–203·4)	100·9 (91·2–109·4)	23·5 (18·1–28·8)	0·5 (0·4–0·5)	7·05 (6·84–7·27)	2·47 (2·37–2·55)	2·75 (2·58–2·91)
1974	0·1 (0·0–0·2)	119·0 (106·3–132·8)	376·7 (364·1–387·8)	365·7 (360·3–371·1)	235·4 (219·5–249·9)	184·6 (167·8–199·8)	97·9 (87·5–106·7)	22·3 (17·0–27·5)	0·4 (0·4–0·4)	7·01 (6·8–7·21)	2·48 (2·39–2·56)	2·7 (2·53–2·86)
1975	0·1 (0·0–0·2)	115·1 (102·6–128·0)	368·8 (354·9–381·2)	361·7 (355·6–367·6)	230·5 (215·1–245·9)	176·9 (159·7–193·0)	92·8 (82·2–102·3)	20·6 (15·6–25·7)	0·4 (0·4–0·4)	6·83 (6·6–7·04)	2·42 (2·33–2·51)	2·61 (2·43–2·77)
1976	0·1 (0·0–0·2)	110·7 (98·7–123·0)	355·9 (339·7–369·6)	356·6 (349·7–362·8)	224·8 (209·0–240·1)	168·6 (151·2–184·5)	87·3 (76·6–97·0)	18·9 (14·3–23·7)	0·4 (0·3–0·4)	6·62 (6·38–6·84)	2·33 (2·23–2·43)	2·5 (2·32–2·68)
1977	0·1 (0·0–0·2)	108·0 (96·4–120·2)	346·9 (329·5–360·5)	354·0 (347·2–360·2)	220·6 (205·3–235·4)	161·9 (145·0–178·3)	82·6 (71·4–93·2)	17·4 (13·1–22·2)	0·3 (0·3–0·3)	6·46 (6·23–6·67)	2·27 (2·17–2·38)	2·41 (2·24–2·58)
1978	0·1 (0·0–0·2)	106·6 (95·5–118·8)	342·1 (325·7–356·4)	350·9 (343·5–357·8)	217·0 (202·7–231·3)	156·3 (139·7–172·4)	78·7 (68·7–89·1)	16·0 (12·3–20·1)	0·3 (0·3–0·3)	6·34 (6·11–6·55)	2·24 (2·13–2·35)	2·34 (2·18–2·5)
1979	0·1 (0·0–0·2)	104·9 (94·3–116·8)	337·5 (320·0–352·3)	344·1 (335·8–351·9)	213·3 (198·3–226·7)	150·7 (133·6–166·1)	74·5 (65·5–84·4)	14·7 (11·3–18·4)	0·3 (0·3–0·3)	6·2 (5·95–6·43)	2·21 (2·11–2·32)	2·27 (2·1–2·42)
1980	0·1 (0·0–0·2)	102·7 (92·1–114·1)	331·0 (313·3–346·5)	337·5 (328·9–345·8)	210·3 (195·5–223·4)	145·4 (128·9–160·1)	70·4 (61·0–79·8)	13·5 (10·4–16·9)	0·3 (0·3–0·3)	6·06 (5·79–6·31)	2·17 (2·07–2·27)	2·2 (2·02–2·36)
1981	0·1 (0·0–0·2)	100·8 (90·9–112·6)	325·1 (307·8–341·2)	334·5 (325·8–343·3)	209·3 (195·6–223·1)	141·7 (126·4–156·4)	66·7 (57·9–75·2)	12·5 (9·5–15·6)	0·2 (0·2–0·3)	5·95 (5·67–6·23)	2·13 (2·03–2·23)	2·15 (1·96–2·34)
1982	0·1 (0·0–0·2)	99·5 (89·3–110·7)	320·9 (302·7–337·6)	334·6 (325·8–342·7)	210·1 (196·6–222·9)	139·2 (123·6–153·9)	63·5 (54·9–72·1)	11·7 (9·0–14·8)	0·2 (0·2–0·2)	5·9 (5·62–6·18)	2·1 (2·0–2·2)	2·12 (1·93–2·31)
1983	0·1 (0·0–0·2)	98·6 (88·5–109·8)	317·7 (299·6–335·3)	336·3 (328·1–344·0)	211·5 (198·8–224·3)	137·2 (121·9–152·5)	60·6 (52·1–69·4)	10·8 (8·3–13·6)	0·2 (0·2–0·2)	5·87 (5·59–6·14)	2·08 (1·98–2·18)	2·1 (1·93–2·28)
1984	0·1 (0·0–0·2)	98·2 (87·9–109·4)	315·6 (297·4–332·7)	336·2 (328·1–343·7)	211·5 (198·9–223·8)	135·5 (119·7–150·8)	58·3 (50·1–66·8)	10·1 (7·9–12·7)	0·2 (0·2–0·2)	5·83 (5·59–6·07)	2·07 (1·97–2·18)	2·08 (1·93–2·23)
1985	0·1 (0·0–0·2)	97·0 (86·7–107·6)	313·3 (294·7–331·0)	332·2 (324·1–339·8)	208·6 (196·0–221·2)	133·3 (118·0–148·1)	56·0 (48·4–64·3)	9·4 (7·3–12·0)	0·2 (0·2–0·2)	5·75 (5·54–5·98)	2·05 (1·95–2·15)	2·04 (1·91–2·17)
1986	0·1 (0·0–0·2)	94·3 (84·3–104·4)	308·7 (290·2–326·9)	325·2 (315·7–333·1)	203·5 (189·9–216·1)	129·6 (114·3–145·3)	53·0 (45·1–60·9)	8·7 (6·8–11·2)	0·2 (0·2–0·2)	5·62 (5·41–5·84)	2·02 (1·92–2·11)	1·98 (1·86–2·1)
1987	0·1 (0·0–0·2)	90·6 (80·7–100·5)	301·8 (282·4–320·6)	317·5 (307·5–326·2)	197·9 (184·3–210·3)	125·0 (111·0–140·2)	49·6 (42·0–57·3)	8·0 (6·2–10·2)	0·2 (0·1–0·2)	5·45 (5·24–5·65)	1·96 (1·87–2·06)	1·9 (1·78–2·02)
1988	0·1 (0·0–0·2)	88·1 (78·4–97·6)	295·2 (276·3–312·9)	311·8 (301·7–320·9)	193·6 (180·0–206·0)	121·2 (108·1–135·7)	46·7 (39·7–53·7)	7·4 (5·9–9·3)	0·1 (0·1–0·1)	5·32 (5·13–5·51)	1·92 (1·81–2·01)	1·85 (1·74–1·95)
1989	0·1 (0·0–0·2)	88·2 (78·3–97·7)	291·4 (272·5–310·0)	307·8 (297·4–317·2)	190·2 (176·6–202·3)	117·6 (105·1–130·8)	44·3 (37·6–51·0)	6·8 (5·4–8·5)	0·1 (0·1–0·1)	5·23 (5·05–5·42)	1·9 (1·79–2·0)	1·8 (1·69–1·91)
1990	0·1 (0·0–0·2)	87·8 (78·1–97·7)	288·0 (268·9–307·0)	301·0 (290·8–311·0)	184·4 (171·7–196·4)	112·0 (99·7–124·7)	41·1 (34·9–47·8)	6·2 (4·9–7·8)	0·1 (0·1–0·1)	5·1 (4·89–5·3)	1·88 (1·77–1·98)	1·72 (1·61–1·83)
1991	0·1 (0·0–0·2)	85·8 (76·5–95·8)	281·6 (262·9–301·1)	289·6 (279·0–300·9)	175·4 (162·5–186·5)	104·3 (92·1–117·3)	36·9 (31·3–43·2)	5·5 (4·3–6·9)	0·1 (0·1–0·1)	4·9 (4·68–5·12)	1·84 (1·73–1·95)	1·61 (1·49–1·73)
1992	0·1 (0·0–0·2)	83·3 (73·9–93·0)	274·0 (253·2–295·0)	276·9 (265·0–288·8)	166·4 (153·3–178·4)	96·9 (85·6–109·8)	33·1 (28·2–39·0)	4·9 (3·8–6·2)	0·1 (0·1–0·1)	4·68 (4·46–4·9)	1·79 (1·67–1·9)	1·51 (1·39–1·63)
1993	0·1 (0·0–0·2)	80·9 (71·8–90·8)	266·7 (245·3–286·9)	265·9 (254·0–278·5)	159·4 (147·0–171·0)	91·1 (79·5–103·0)	30·1 (25·5–35·4)	4·3 (3·3–5·6)	0·1 (0·1–0·1)	4·49 (4·3–4·69)	1·74 (1·62–1·85)	1·42 (1·31–1·55)
1994	0·1 (0·0–0·2)	79·0 (70·0–89·0)	261·3 (239·2–282·4)	258·9 (247·0–271·6)	154·7 (142·6–166·8)	87·0 (76·1–98·6)	27·9 (23·3–32·8)	3·8 (2·9–4·9)	0·1 (0·1–0·1)	4·36 (4·19–4·55)	1·7 (1·58–1·82)	1·37 (1·26–1·48)
1995	0·1 (0·0–0·2)	77·4 (68·7–86·7)	258·5 (236·3–279·3)	255·1 (242·6–267·8)	152·1 (139·5–164·5)	84·5 (72·8–95·9)	26·6 (22·0–31·2)	3·5 (2·6–4·6)	0·1 (0·1–0·1)	4·29 (4·11–4·46)	1·68 (1·56–1·79)	1·33 (1·23–1·44)
1996	0·1 (0·0–0·2)	75·7 (67·4–84·6)	256·5 (234·4–276·8)	251·3 (238·5–263·7)	150·1 (137·8–163·2)	82·5 (70·5–93·6)	25·5 (20·8–29·9)	3·3 (2·4–4·3)	0·1 (0·1–0·1)	4·23 (4·05–4·4)	1·66 (1·54–1·77)	1·31 (1·21–1·41)
1997	0·1 (0·0–0·2)	73·7 (65·6–82·3)	253·6 (230·9–273·6)	245·3 (231·9–258·2)	147·0 (134·8–159·8)	80·2 (68·4–91·6)	24·3 (20·0–28·5)	3·2 (2·4–4·1)	0·1 (0·1–0·1)	4·14 (3·96–4·3)	1·64 (1·51–1·74)	1·27 (1·18–1·37)
1998	0·1 (0·0–0·2)	70·5 (62·5–78·8)	248·9 (227·7–270·9)	236·7 (223·0–249·6)	143·3 (131·9–156·1)	77·5 (66·7–88·1)	23·0 (19·1–27·0)	3·0 (2·3–3·9)	0·1 (0·1–0·1)	4·02 (3·85–4·18)	1·6 (1·48–1·72)	1·23 (1·15–1·32)
1999	0·1 (0·0–0·1)	66·3 (58·7–74·1)	243·1 (220·0–264·7)	229·0 (215·6–242·3)	140·6 (129·2–152·7)	75·1 (65·2–86·1)	21·9 (18·4–26·1)	2·9 (2·2–3·7)	0·1 (0·1–0·1)	3·89 (3·73–4·06)	1·55 (1·42–1·66)	1·2 (1·11–1·29)
2000	0·1 (0·0–0·1)	62·6 (55·2–70·2)	239·0 (215·0–262·1)	224·5 (210·8–237·8)	139·1 (127·4–151·5)	73·6 (63·8–85·3)	21·2 (17·8–25·3)	2·8 (2·1–3·6)	0·1 (0·1–0·1)	3·81 (3·63–4·0)	1·51 (1·38–1·64)	1·18 (1·09–1·28)
2001	0·1 (0·0–0·1)	60·0 (52·7–67·1)	236·3 (213·5–259·3)	221·2 (207·6–234·6)	137·5 (124·8–151·3)	72·4 (62·0–84·2)	20·6 (16·8–24·6)	2·7 (2·1–3·5)	0·1 (0·1–0·1)	3·75 (3·56–3·93)	1·48 (1·36–1·61)	1·17 (1·07–1·28)
2002	0·1 (0·0–0·1)	57·4 (50·2–64·4)	233·7 (210·3–257·0)	216·2 (203·2–229·5)	134·9 (121·8–148·2)	70·6 (59·8–82·1)	20·0 (16·4–24·2)	2·7 (2·0–3·4)	0·1 (0·0–0·1)	3·68 (3·48–3·87)	1·46 (1·33–1·58)	1·14 (1·04–1·25)
2003	0·1 (0·0–0·1)	55·6 (48·3–62·9)	230·1 (207·1–254·7)	212·6 (198·7–225·9)	132·7 (119·1–145·8)	69·5 (58·4–81·3)	19·5 (16·0–23·9)	2·7 (2·0–3·5)	0·1 (0·0–0·1)	3·61 (3·43–3·8)	1·43 (1·3–1·57)	1·12 (1·02–1·23)
2004	0·1 (0·0–0·1)	57·2 (49·5–65·0)	231·2 (207·6–257·6)	216·7 (202·7–230·8)	132·8 (119·2–145·6)	70·8 (59·8–82·8)	19·9 (16·2–24·2)	2·9 (2·1–3·8)	0·1 (0·1–0·1)	3·66 (3·47–3·85)	1·44 (1·31–1·59)	1·13 (1·02–1·24)
2005	0·1 (0·0–0·1)	59·8 (51·8–68·3)	237·1 (212·6–262·2)	220·7 (205·8–234·7)	132·8 (119·6–146·3)	71·6 (59·9–84·5)	20·2 (16·3–24·5)	3·0 (2·2–4·0)	0·1 (0·1–0·1)	3·73 (3·54–3·92)	1·49 (1·34–1·63)	1·14 (1·03–1·24)
2006	0·1 (0·0–0·1)	58·4 (50·7–66·6)	236·8 (212·8–261·1)	218·3 (203·4–232·0)	132·2 (118·7–145·6)	70·8 (58·9–83·6)	19·9 (15·9–24·5)	2·9 (2·1–3·9)	0·1 (0·1–0·1)	3·7 (3·5–3·89)	1·48 (1·33–1·62)	1·13 (1·02–1·23)
2007	0·1 (0·0–0·1)	56·6 (48·9–64·7)	234·6 (210·5–259·1)	217·9 (203·8–232·2)	132·9 (118·8–146·5)	70·8 (58·4–83·5)	19·9 (15·7–24·6)	2·7 (1·9–3·8)	0·1 (0·1–0·1)	3·68 (3·47–3·89)	1·46 (1·3–1·61)	1·13 (1·03–1·24)
2008	0·1 (0·0–0·1)	56·2 (48·5–64·3)	235·7 (210·7–261·6)	219·9 (205·7–233·6)	134·2 (119·9–149·2)	71·4 (58·2–84·6)	20·0 (15·7–24·9)	2·6 (1·8–3·6)	0·1 (0·0–0·1)	3·7 (3·47–3·94)	1·46 (1·3–1·63)	1·14 (1·02–1·26)
2009	0·1 (0·0–0·1)	55·1 (47·3–63·4)	235·5 (209·6–261·0)	218·4 (204·8–232·3)	134·0 (119·6–149·2)	71·0 (57·7–85·3)	19·8 (15·4–24·9)	2·5 (1·8–3·4)	0·0 (0·0–0·1)	3·68 (3·44–3·94)	1·45 (1·29–1·61)	1·14 (1·02–1·26)
2010	0·1 (0·0–0·1)	53·4 (45·7–61·5)	233·0 (206·9–257·4)	213·9 (199·7–228·2)	131·8 (116·8–147·3)	69·7 (56·5–83·7)	19·4 (15·2–24·2)	2·4 (1·7–3·3)	0·0 (0·0–0·0)	3·62 (3·38–3·86)	1·43 (1·27–1·58)	1·12 (1·0–1·25)
2011	0·1 (0·0–0·1)	52·1 (44·4–60·3)	229·4 (203·5–255·0)	209·0 (195·5–223·5)	128·8 (113·9–144·4)	68·3 (55·4–81·4)	19·0 (14·7–23·7)	2·3 (1·6–3·1)	0·0 (0·0–0·0)	3·55 (3·3–3·8)	1·41 (1·26–1·57)	1·09 (0·96–1·23)
2012	0·1 (0·0–0·1)	52·2 (44·4–60·7)	226·7 (201·5–254·3)	207·0 (193·8–221·0)	126·8 (112·4–142·2)	67·4 (55·1–81·5)	18·8 (14·7–23·6)	2·1 (1·6–2·9)	0·0 (0·0–0·0)	3·51 (3·26–3·78)	1·39 (1·25–1·55)	1·08 (0·93–1·23)
2013	0·1 (0·0–0·1)	53·1 (45·1–61·8)	226·1 (200·3–252·2)	207·8 (194·0–222·1)	126·7 (111·4–142·1)	67·5 (54·5–81·4)	18·9 (14·6–23·7)	2·1 (1·5–2·8)	0·0 (0·0–0·0)	3·51 (3·23–3·79)	1·4 (1·24–1·55)	1·08 (0·92–1·24)
2014	0·1 (0·0–0·1)	53·9 (45·7–62·7)	226·5 (200·7–252·5)	208·2 (194·1–222·1)	127·5 (111·8–143·0)	67·8 (54·3–83·1)	19·0 (14·4–24·2)	2·1 (1·4–3·0)	0·0 (0·0–0·0)	3·53 (3·25–3·81)	1·4 (1·25–1·56)	1·08 (0·91–1·26)
2015	0·1 (0·0–0·1)	54·6 (46·5–63·9)	226·7 (200·7–254·5)	207·9 (193·4–222·3)	128·1 (112·1–145·2)	67·9 (53·9–84·1)	19·1 (14·2–24·9)	2·1 (1·4–3·1)	0·0 (0·0–0·0)	3·53 (3·25–3·82)	1·41 (1·24–1·58)	1·09 (0·91–1·27)
2016	0·1 (0·0–0·1)	55·2 (47·2–64·7)	226·6 (200·1–255·1)	208·0 (193·3–222·8)	128·6 (113·0–146·2)	68·1 (53·5–85·1)	19·1 (14·1–25·8)	2·1 (1·4–3·2)	0·0 (0·0–0·0)	3·54 (3·23–3·86)	1·41 (1·24–1·59)	1·09 (0·92–1·3)
2017	0·1 (0·0–0·1)	55·7 (47·3–65·4)	226·8 (199·4–255·5)	208·3 (193·6–223·8)	128·9 (112·4–148·6)	68·3 (53·5–87·5)	19·2 (14·0–26·4)	2·1 (1·4–3·4)	0·0 (0·0–0·0)	3·55 (3·22–3·87)	1·41 (1·23–1·6)	1·09 (0·91–1·33)

Supplemental Results Table 5: Age-specific fertility rates per 1000, cumulative total fertility up to maternal age 25 (TFU25), cumulative total fertility from maternal age 30 to age 55 (TFO30), and cumulative total fertility, Turkmenistan, 1950-2017

Year	10-14	15-19	20-24	25-29	30-34	35-39	40-44	45-49	50-54	TFR	TFU25	TFO30
1950	0·0 (0·0–0·1)	28·9 (25·6–32·6)	260·0 (242·2–277·5)	306·8 (296·3–317·0)	207·2 (198·4–216·1)	161·1 (153·6–168·4)	92·2 (87·9–96·5)	17·4 (13·7–21·3)	0·3 (0·3–0·3)	5·37 (5·18–5·55)	1·44 (1·34–1·55)	2·39 (2·3–2·48)
1951	0·0 (0·0–0·1)	28·8 (25·7–32·4)	258·9 (242·0–275·3)	306·5 (296·3–316·3)	206·6 (198·2–214·4)	159·9 (153·2–166·1)	91·6 (87·9–95·5)	17·0 (13·4–20·7)	0·3 (0·3–0·3)	5·35 (5·18–5·52)	1·44 (1·34–1·54)	2·38 (2·29–2·46)
1952	0·0 (0·0–0·1)	28·9 (25·8–32·1)	258·8 (242·6–274·1)	307·1 (297·0–316·9)	207·0 (198·9–215·1)	159·5 (153·0–165·8)	91·5 (87·8–95·0)	16·6 (13·3–20·3)	0·3 (0·3–0·3)	5·35 (5·19–5·51)	1·44 (1·34–1·53)	2·37 (2·29–2·46)
1953	0·0 (0·0–0·1)	29·1 (26·2–32·3)	259·8 (244·4–274·5)	309·2 (299·5–318·8)	209·0 (201·0–216·4)	160·1 (153·8–166·7)	92·1 (88·8–95·4)	16·4 (13·1–19·8)	0·3 (0·3–0·3)	5·38 (5·23–5·53)	1·44 (1·36–1·53)	2·39 (2·3–2·48)
1954	0·0 (0·0–0·1)	29·4 (26·5–32·5)	261·5 (245·9–276·1)	311·5 (302·2–321·3)	211·5 (203·3–218·9)	161·1 (154·6–167·8)	93·1 (89·6–96·2)	16·2 (12·8–19·8)	0·3 (0·3–0·3)	5·42 (5·28–5·56)	1·45 (1·37–1·54)	2·41 (2·33–2·5)
1955	0·0 (0·0–0·1)	29·7 (26·8–32·9)	263·4 (248·4–277·6)	314·1 (304·6–323·5)	214·4 (207·0–222·1)	162·4 (156·5–169·0)	94·2 (90·9–97·4)	16·0 (12·7–19·3)	0·3 (0·3–0·3)	5·47 (5·33–5·61)	1·47 (1·39–1·54)	2·44 (2·37–2·51)
1956	0·0 (0·0–0·1)	30·4 (27·5–33·6)	266·4 (251·7–280·5)	318·2 (309·2–327·0)	219·0 (211·6–226·0)	165·1 (159·1–171·2)	96·3 (93·1–99·3)	15·9 (12·7–19·1)	0·3 (0·3–0·3)	5·56 (5·41–5·7)	1·48 (1·41–1·56)	2·48 (2·42–2·55)
1957	0·0 (0·0–0·1)	31·3 (28·2–34·7)	270·6 (256·0–284·6)	323·4 (314·6–331·8)	225·1 (218·2–232·0)	169·1 (163·1–174·8)	99·2 (96·0–102·1)	15·9 (12·7–19·2)	0·3 (0·3–0·3)	5·67 (5·53–5·81)	1·51 (1·43–1·59)	2·55 (2·49–2·6)
1958	0·0 (0·0–0·1)	32·5 (29·3–36·1)	275·4 (260·9–289·1)	329·2 (320·5–337·0)	232·3 (225·8–238·9)	174·1 (168·3–179·6)	102·7 (99·8–105·5)	15·9 (12·9–19·1)	0·3 (0·3–0·3)	5·81 (5·68–5·94)	1·54 (1·46–1·62)	2·63 (2·57–2·68)
1959	0·0 (0·0–0·1)	33·7 (30·0–37·3)	280·5 (265·6–293·6)	334·3 (326·3–342·0)	239·6 (232·7–245·9)	179·3 (174·0–184·7)	106·4 (103·6–108·9)	15·9 (12·7–19·5)	0·3 (0·3–0·3)	5·95 (5·83–6·06)	1·57 (1·48–1·65)	2·71 (2·65–2·76)
1960	0·0 (0·0–0·1)	34·7 (30·7–38·7)	284·4 (269·5–297·9)	336·9 (329·3–344·8)	245·8 (238·9–252·2)	183·6 (178·3–188·7)	109·3 (106·8–111·6)	15·7 (12·6–19·3)	0·3 (0·3–0·3)	6·05 (5·94–6·17)	1·6 (1·51–1·68)	2·77 (2·72–2·83)
1961	0·1 (0·0–0·1)	35·5 (31·3–39·8)	286·9 (271·0–301·2)	337·7 (330·2–345·3)	250·3 (243·3–257·0)	187·0 (181·4–192·0)	111·4 (108·9–113·6)	15·4 (12·1–19·2)	0·3 (0·3–0·3)	6·12 (6·0–6·25)	1·61 (1·52–1·7)	2·82 (2·76–2·88)
1962	0·1 (0·0–0·1)	36·0 (31·5–40·5)	287·8 (271·3–302·7)	338·4 (330·5–345·4)	252·2 (245·0–259·3)	188·8 (182·6–194·0)	112·2 (109·4–114·6)	15·0 (11·6–18·9)	0·3 (0·3–0·3)	6·15 (6·02–6·29)	1·62 (1·52–1·71)	2·84 (2·77–2·91)
1963	0·1 (0·0–0·1)	36·0 (31·6–40·9)	286·5 (269·3–303·0)	338·1 (330·4–345·4)	251·0 (243·1–258·9)	187·6 (180·7–194·0)	111·1 (108·1–114·1)	14·5 (11·0–18·4)	0·3 (0·3–0·3)	6·13 (5·97–6·28)	1·61 (1·51–1·71)	2·82 (2·74–2·9)
1964	0·1 (0·0–0·1)	35·9 (31·4–41·2)	283·1 (265·0–301·3)	335·7 (327·7–343·3)	247·8 (238·4–256·9)	184·1 (175·9–192·1)	108·9 (104·8–112·7)	13·8 (10·4–18·0)	0·3 (0·3–0·3)	6·05 (5·86–6·24)	1·6 (1·49–1·7)	2·77 (2·66–2·88)
1965	0·1 (0·0–0·1)	35·8 (31·2–41·7)	279·2 (258·5–299·8)	332·5 (324·2–340·1)	244·4 (233·3–254·9)	180·2 (169·3–189·9)	106·1 (100·9–110·8)	13·1 (9·7–17·3)	0·3 (0·2–0·3)	5·96 (5·73–6·19)	1·58 (1·47–1·69)	2·72 (2·58–2·85)
1966	0·1 (0·0–0·1)	35·9 (31·1–41·7)	275·9 (254·4–297·2)	329·9 (321·5–338·1)	241·3 (228·7–252·7)	176·4 (163·4–188·0)	103·2 (96·5–109·0)	12·5 (9·2–16·8)	0·2 (0·2–0·2)	5·88 (5·6–6·15)	1·56 (1·45–1·67)	2·67 (2·5–2·82)
1967	0·1 (0·0–0·1)	36·0 (31·1–42·1)	273·0 (250·0–295·4)	327·6 (318·5–336·2)	238·3 (224·4–250·8)	172·5 (157·7–185·4)	100·1 (92·3–106·8)	12·0 (8·7–16·4)	0·2 (0·2–0·2)	5·8 (5·48–6·1)	1·55 (1·43–1·66)	2·62 (2·42–2·78)
1968	0·1 (0·0–0·1)	36·1 (31·3–42·1)	270·1 (247·2–293·0)	325·3 (316·1–334·4)	235·5 (220·7–249·2)	168·5 (153·6–182·7)	96·7 (88·1–104·4)	11·4 (8·1–15·8)	0·2 (0·2–0·2)	5·72 (5·38–6·05)	1·53 (1·42–1·65)	2·56 (2·36–2·75)
1969	0·1 (0·0–0·1)	36·1 (31·3–41·8)	267·3 (244·1–290·7)	323·2 (313·6–332·3)	232·8 (218·2–247·6)	164·6 (149·0–179·7)	93·1 (84·2–101·5)	11·0 (7·7–15·1)	0·2 (0·2–0·2)	5·64 (5·29–6·01)	1·52 (1·4–1·63)	2·51 (2·3–2·71)
1970	0·1 (0·0–0·1)	35·9 (31·1–41·4)	264·7 (241·5–288·4)	321·3 (311·8–331·1)	230·5 (215·8–245·1)	160·8 (145·0–177·3)	89·6 (80·2–98·8)	10·5 (7·3–14·5)	0·2 (0·2–0·2)	5·57 (5·19–5·95)	1·5 (1·38–1·62)	2·46 (2·25–2·67)
1971	0·1 (0·0–0·1)	35·7 (31·0–40·9)	262·4 (239·2–285·5)	319·7 (309·6–329·7)	228·4 (213·9–243·0)	157·0 (140·5–173·6)	86·0 (76·4–95·5)	10·0 (7·0–13·7)	0·2 (0·2–0·2)	5·5 (5·12–5·89)	1·49 (1·37–1·61)	2·41 (2·19–2·63)
1972	0·1 (0·0–0·1)	35·6 (30·9–40·4)	260·2 (238·3–282·4)	318·1 (308·0–328·1)	226·4 (212·0–240·3)	153·3 (136·8–169·0)	82·4 (73·0–91·7)	9·6 (6·8–13·2)	0·2 (0·2–0·2)	5·43 (5·08–5·8)	1·48 (1·37–1·59)	2·36 (2·15–2·57)
1973	0·1 (0·0–0·1)	35·5 (31·1–40·0)	258·0 (237·5–279·9)	316·2 (305·3–326·6)	224·4 (210·5–238·4)	149·5 (133·1–164·8)	78·8 (69·3–87·8)	9·2 (6·4–12·4)	0·2 (0·2–0·2)	5·36 (5·02–5·71)	1·47 (1·36–1·57)	2·31 (2·1–2·51)
1974	0·1 (0·0–0·1)	35·3 (31·1–39·7)	255·5 (235·7–275·1)	314·1 (302·8–325·0)	222·4 (208·7–235·5)	145·7 (130·1–160·6)	75·3 (66·1–84·1)	8·8 (6·1–11·9)	0·2 (0·2–0·2)	5·29 (4·97–5·61)	1·45 (1·36–1·56)	2·26 (2·06–2·45)
1975	0·1 (0·0–0·1)	35·1 (31·1–39·3)	253·2 (233·5–272·8)	312·2 (300·5–323·2)	220·6 (207·2–233·2)	142·4 (127·4–156·4)	72·3 (63·7–80·5)	8·4 (5·9–11·3)	0·2 (0·2–0·2)	5·22 (4·93–5·51)	1·44 (1·34–1·54)	2·22 (2·03–2·4)
1976	0·1 (0·0–0·1)	34·9 (31·1–38·7)	251·2 (232·6–270·5)	310·5 (299·2–321·1)	219·4 (206·8–231·4)	139·5 (126·4–152·6)	69·6 (61·9–77·4)	8·0 (5·7–10·8)	0·2 (0·1–0·2)	5·17 (4·92–5·43)	1·43 (1·34–1·53)	2·18 (2·01–2·35)
1977	0·0 (0·0–0·1)	34·6 (31·2–38·2)	249·2 (231·6–266·8)	308·5 (297·8–318·9)	218·1 (206·7–229·1)	136·7 (125·5–148·0)	67·2 (60·3–74·1)	7·6 (5·4–10·3)	0·1 (0·1–0·2)	5·11 (4·89–5·34)	1·42 (1·33–1·51)	2·15 (2·0–2·3)
1978	0·0 (0·0–0·1)	34·4 (31·1–37·8)	247·0 (231·0–263·6)	306·0 (295·5–316·6)	216·2 (206·2–226·3)	133·3 (123·3–143·2)	64·5 (58·8–70·3)	7·2 (5·3–9·7)	0·1 (0·1–0·1)	5·04 (4·85–5·23)	1·41 (1·32–1·49)	2·11 (1·98–2·23)
1979	0·0 (0·0–0·1)	34·2 (31·0–37·5)	244·6 (229·8–260·1)	303·4 (292·8–313·9)	213·5 (204·3–223·0)	129·3 (120·9–137·8)	61·0 (56·4–66·0)	6·8 (5·0–9·1)	0·1 (0·1–0·1)	4·97 (4·8–5·13)	1·39 (1·32–1·47)	2·05 (1·95–2·15)
1980	0·0 (0·0–0·1)	34·0 (31·0–37·3)	242·3 (228·6–257·1)	300·6 (290·4–310·8)	211·0 (201·9–219·9)	125·5 (118·1–132·6)	57·4 (53·2–61·5)	6·4 (4·7–8·6)	0·1 (0·1–0·1)	4·89 (4·74–5·04)	1·38 (1·31–1·46)	2·0 (1·92–2·08)
1981	0·0 (0·0–0·1)	33·9 (30·9–36·9)	240·4 (227·3–254·6)	298·9 (288·6–309·2)	209·5 (201·1–217·6)	122·3 (115·5–128·7)	54·2 (50·6–58·0)	6·1 (4·4–8·0)	0·1 (0·1–0·1)	4·83 (4·68–4·97)	1·37 (1·3–1·45)	1·96 (1·88–2·03)
1982	0·0 (0·0–0·1)	33·7 (30·9–36·6)	238·5 (225·2–252·4)	297·7 (287·0–307·8)	208·3 (200·7–215·8)	119·5 (112·9–125·8)	51·4 (47·9–55·2)	5·7 (4·3–7·5)	0·1 (0·1–0·1)	4·78 (4·63–4·92)	1·36 (1·29–1·43)	1·93 (1·86–1·99)
1983	0·0 (0·0–0·1)	33·4 (30·7–36·4)	236·3 (222·7–249·8)	296·5 (285·7–306·7)	207·4 (200·1–214·7)	116·8 (110·6–123·5)	48·9 (45·5–52·7)	5·4 (4·0–6·9)	0·1 (0·1–0·1)	4·72 (4·59–4·86)	1·35 (1·28–1·42)	1·89 (1·83–1·96)
1984	0·0 (0·0–0·1)	33·4 (30·7–36·3)	234·5 (221·1–248·0)	296·1 (285·5–306·0)	207·0 (200·0–214·2)	115·1 (108·9–121·4)	47·0 (43·6–50·6)	5·0 (3·8–6·6)	0·1 (0·1–0·1)	4·69 (4·56–4·82)	1·34 (1·27–1·41)	1·87 (1·81–1·94)
1985	0·0 (0·0–0·1)	33·4 (30·8–36·4)	233·4 (220·4–246·5)	294·5 (284·4–304·0)	206·0 (198·9–212·8)	113·7 (107·5–119·9)	45·4 (42·0–48·9)	4·7 (3·6–6·2)	0·1 (0·1–0·1)	4·66 (4·53–4·78)	1·33 (1·26–1·41)	1·85 (1·79–1·91)
1986	0·0 (0·0–0·1)	33·1 (30·4–36·0)	231·2 (217·7–244·1)	289·8 (279·3–299·3)	203·3 (196·4–209·8)	111·4 (105·3–117·5)	43·3 (40·3–46·7)	4·4 (3·3–5·7)	0·1 (0·1–0·1)	4·58 (4·46–4·7)	1·32 (1·25–1·4)	1·81 (1·75–1·88)
1987	0·0 (0·0–0·1)	32·5 (29·8–35·3)	227·3 (214·6–239·9)	282·6 (272·2–292·0)	199·5 (192·8–206·1)	108·2 (102·2–113·8)	40·9 (38·0–43·9)	4·0 (3·0–5·1)	0·1 (0·1–0·1)	4·48 (4·35–4·59)	1·3 (1·22–1·37)	1·76 (1·7–1·83)
1988	0·0 (0·0–0·1)	31·8 (29·2–34·6)	223·0 (210·6–235·9)	274·3 (263·7–284·4)	195·4 (188·7–201·5)	104·7 (99·0–109·9)	38·4 (35·6–41·3)	3·6 (2·7–4·6)	0·1 (0·1–0·1)	4·36 (4·23–4·47)	1·27 (1·2–1·35)	1·71 (1·65–1·76)
1989	0·0 (0·0–0·1)	31·2 (28·6–34·0)	218·9 (206·7–231·2)	266·1 (254·8–277·0)	190·6 (183·6–197·1)	100·4 (94·8–105·8)	36·0 (33·2–38·8)	3·1 (2·4–4·0)	0·1 (0·1–0·1)	4·23 (4·12–4·34)	1·25 (1·18–1·33)	1·65 (1·6–1·7)
1990	0·0 (0·0–0·1)	30·8 (28·4–33·5)	215·1 (202·8–227·5)	257·9 (246·3–269·2)	184·9 (178·2–191·3)	95·5 (90·6–101·0)	33·7 (31·2–36·5)	2·7 (2·1–3·5)	0·1 (0·1–0·1)	4·1 (3·99–4·21)	1·23 (1·16–1·3)	1·58 (1·54–1·63)
1991	0·0 (0·0–0·1)	30·3 (27·8–32·9)	211·0 (198·8–223·9)	248·5 (236·6–260·4)	178·8 (172·2–185·0)	90·8 (85·6–96·4)	31·6 (29·2–34·4)	2·3 (1·7–3·0)	0·0 (0·0–0·0)	3·97 (3·86–4·07)	1·21 (1·14–1·28)	1·52 (1·47–1·56)
1992	0·0 (0·0–0·1)	29·5 (27·1–32·1)	206·0 (193·8–218·6)	236·2 (223·8–248·5)	172·0 (165·3–178·3)	85·9 (80·8–91·3)	29·3 (26·9–31·8)	1·8 (1·4–2·5)	0·0 (0·0–0·0)	3·8 (3·7–3·91)	1·18 (1·11–1·24)	1·44 (1·4–1·49)
1993	0·0 (0·0–0·1)	28·0 (25·7–30·6)	199·0 (186·2–211·3)	218·3 (205·8–231·1)	162·9 (156·0–169·2)	79·6 (74·9–84·8)	26·3 (24·2–28·5)	1·4 (1·1–1·9)	0·0 (0·0–0·0)	3·58 (3·46–3·69)	1·14 (1·07–1·2)	1·35 (1·31–1·4)
1994	0·0 (0·0–0·1)	25·9 (23·7–28·3)	189·7 (176·7–202·4)	197·4 (184·9–210·1)	151·8 (145·3–157·9)	72·0 (67·7–76·4)	22·8 (21·0–25·0)	1·0 (0·7–1·3)	0·0 (0·0–0·0)	3·3 (3·18–3·42)	1·08 (1·01–1·14)	1·24 (1·19–1·28)
1995	0·0 (0·0–0·1)	23·7 (21·7–26·0)	180·2 (167·0–192·9)	184·1 (170·9–196·6)	140·5 (134·1–146·8)	64·3 (60·4–68·4)	19·7 (18·0–21·4)	0·7 (0·5–0·9)	0·0 (0·0–0·0)	3·07 (2·94–3·19)	1·02 (0·95–1·08)	1·13 (1·08–1·17)
1996	0·0 (0·0–0·1)	21·9 (20·0–24·1)	171·9 (158·8–184·9)	178·0 (164·8–190·5)	130·7 (124·0–137·0)	57·9 (53·9–62·1)	17·2 (15·7–18·8)	0·4 (0·3–0·6)	0·0 (0·0–0·0)	2·89 (2·75–3·02)	0·97 (0·9–1·04)	1·03 (0·98–1·08)
1997	0·0 (0·0–0·1)	20·4 (18·6–22·5)	165·4 (152·6–178·4)	174·2 (161·1–187·2)	124·2 (117·3–130·3)	53·7 (49·7–57·7)	15·4 (14·0–17·0)	0·3 (0·2–0·4)	0·0 (0·0–0·0)	2·77 (2·63–2·91)	0·93 (0·86–1·0)	0·97 (0·91–1·02)
1998	0·0 (0·0–0·1)	19·0 (17·2–20·9)	160·5 (147·3–173·9)	172·7 (160·0–186·0)	120·7 (113·7–127·1)	51·3 (46·9–55·3)	14·2 (12·8–15·7)	0·2 (0·1–0·2)	0·0 (0·0–0·0)	2·69 (2·55–2·84)	0·9 (0·82–0·97)	0·93 (0·88–0·98)
1999	0·0 (0·0–0·1)	17·7 (16·0–19·6)	155·8 (142·5–169·7)	171·4 (158·3–184·3)	118·2 (111·0–125·2)	49·6 (45·3–53·9)	13·2 (11·9–14·6)	0·1 (0·1–0·1)	0·0 (0·0–0·0)	2·63 (2·49–2·79)	0·87 (0·79–0·95)	0·91 (0·85–0·96)
2000	0·0 (0·0–0·1)	17·1 (15·5–19·1)	152·1 (139·1–166·8)	170·6 (157·6–183·3)	116·7 (109·5–123·7)	48·3 (43·9–52·8)	12·6 (11·3–14·2)	0·1 (0·1–0·1)	0·0 (0·0–0·0)	2·59 (2·44–2·75)	0·85 (0·77–0·93)	0·89 (0·83–0·94)
2001	0·0 (0·0–0·1)	17·0 (15·4–18·9)	150·3 (136·9–165·1)	170·7 (157·5–183·7)	115·9 (108·8–123·5)	47·3 (42·8–51·9)	12·2 (10·9–13·8)	0·1 (0·0–0·1)	0·0 (0·0–0·0)	2·57 (2·41–2·73)	0·84 (0·76–0·92)	0·88 (0·82–0·94)
2002	0·0 (0·0–0·1)	16·7 (15·0–18·5)	148·8 (135·3–163·7)	171·6 (157·7–184·7)	114·5 (107·1–122·1)	46·5 (41·6–51·5)	11·9 (10·5–13·5)	0·1 (0·0–0·1)	0·0 (0·0–0·0)	2·55 (2·39–2·73)	0·83 (0·75–0·91)	0·86 (0·8–0·93)
2003	0·0 (0·0–0·1)	16·1 (14·4–18·0)	146·4 (132·8–162·0)	172·4 (159·6–185·2)	112·2 (104·7–119·5)	46·4 (41·8–51·4)	11·9 (10·4–13·5)	0·0 (0·0–0·1)	0·0 (0·0–0·0)	2·53 (2·37–2·71)	0·81 (0·74–0·9)	0·85 (0·79–0·91)
2004	0·0 (0·0–0·1)	15·4 (13·7–17·2)	143·4 (129·3–159·2)	172·5 (159·4–185·4)	110·6 (103·3–117·7)	46·5 (42·0–51·3)	11·8 (10·4–13·5)	0·0 (0·0–0·0)	0·0 (0·0–0·0)	2·5 (2·35–2·67)	0·79 (0·72–0·88)	0·84 (0·79–0·9)
2005	0·0 (0·0–0·1)	15·2 (13·5–17·1)	142·7 (128·1–158·7)	173·6 (160·6–186·3)	112·2 (105·0–119·4)	45·9 (41·8–50·6)	11·6 (10·1–13·3)	0·0 (0·0–0·0)	0·0 (0·0–0·0)	2·51 (2·36–2·66)	0·79 (0·71–0·88)	0·85 (0·8–0·9)
2006	0·0 (0·0–0·1)	16·4 (14·7–18·4)	146·7 (131·3–162·3)	177·4 (164·2–190·3)	116·6 (109·1–124·0)	46·0 (41·8–50·5)	11·5 (10·0–13·1)	0·0 (0·0–0·0)	0·0 (0·0–0·0)	2·57 (2·43–2·73)	0·82 (0·73–0·9)	0·87 (0·82–0·92)
2007	0·0 (0·0–0·1)	18·2 (16·3–20·4)	152·0 (136·4–167·4)	182·0 (169·0–195·2)	121·6 (114·1–129·7)	48·0 (43·7–52·5)	11·9 (10·4–13·5)	0·0 (0·0–0·0)	0·0 (0·0–0·0)	2·67 (2·53–2·82)	0·85 (0·77–0·93)	0·91 (0·85–0·96)
2008	0·0 (0·0–0·1)	19·7 (17·6–22·1)	156·6 (140·7–173·4)	186·3 (173·2–199·4)	127·6 (119·7–135·8)	51·6 (46·9–56·5)	12·3 (10·7–13·9)	0·0 (0·0–0·0)	0·0 (0·0–0·0)	2·77 (2·62–2·92)	0·88 (0·8–0·97)	0·96 (0·89–1·02)
2009	0·0 (0·0–0·1)	20·9 (18·6–23·5)	161·2 (145·1–177·8)	190·7 (177·4–203·9)	133·7 (125·0–142·4)	55·6 (50·3–60·8)	12·5 (10·9–14·2)	0·0 (0·0–0·0)	0·0 (0·0–0·0)	2·87 (2·72–3·03)	0·91 (0·83–0·99)	1·01 (0·93–1·08)
2010	0·0 (0·0–0·1)	21·7 (19·2–24·4)	164·4 (147·5–181·1)	193·7 (180·0–207·2)	137·3 (128·2–146·1)	57·9 (52·2–63·3)	12·5 (11·0–14·2)	0·0 (0·0–0·0)	0·0 (0·0–0·0)	2·94 (2·78–3·11)	0·93 (0·85–1·02)	1·04 (0·96–1·12)
2011	0·0 (0·0–0·1)	21·7 (19·3–24·6)	165·5 (148·2–183·2)	194·5 (180·5–208·9)	138·1 (128·4–147·4)	58·3 (52·6–64·3)	12·4 (10·9–14·1)	0·0 (0·0–0·0)	0·0 (0·0–0·0)	2·95 (2·78–3·13)	0·94 (0·85–1·03)	1·04 (0·96–1·12)
2012	0·0 (0·0–0·1)	21·7 (19·3–24·5)	166·6 (149·0–185·8)	195·2 (180·9–210·3)	137·9 (128·3–147·4)	57·9 (52·2–63·9)	12·2 (10·6–13·8)	0·0 (0·0–0·0)	0·0 (0·0–0·0)	2·96 (2·78–3·15)	0·94 (0·85–1·04)	1·04 (0·96–1·12)
2013	0·0 (0·0–0·1)	20·8 (18·3–23·5)	166·8 (148·6–186·5)	195·6 (181·4–211·4)	136·1 (125·8–146·3)	56·3 (50·4–62·6)	11·6 (10·1–13·3)	0·0 (0·0–0·0)	0·0 (0·0–0·0)	2·94 (2·74–3·17)	0·94 (0·84–1·04)	1·02 (0·94–1·11)
2014	0·0 (0·0–0·1)	19·2 (16·8–21·9)	164·0 (145·4–184·8)	194·1 (179·6–210·9)	132·6 (121·9–143·4)	53·8 (47·5–60·5)	11·1 (9·4–12·9)	0·0 (0·0–0·0)	0·0 (0·0–0·0)	2·87 (2·64–3·13)	0·92 (0·82–1·02)	0·99 (0·9–1·08)
2015	0·0 (0·0–0·1)	18·7 (16·3–21·6)	160·4 (140·8–182·5)	192·3 (178·1–208·8)	129·3 (118·3–141·5)	51·5 (44·8–59·3)	10·7 (8·9–12·7)	0·0 (0·0–0·0)	0·0 (0·0–0·0)	2·82 (2·56–3·11)	0·9 (0·8–1·0)	0·96 (0·86–1·07)
2016	0·0 (0·0–0·1)	19·1 (16·3–22·1)	157·6 (137·3–181·2)	191·3 (177·3–207·2)	126·9 (115·2–140·4)	49·8 (42·3–58·8)	10·4 (8·5–12·9)	0·0 (0·0–0·0)	0·0 (0·0–0·0)	2·78 (2·5–3·09)	0·88 (0·78–1·0)	0·94 (0·83–1·06)
2017	0·0 (0·0–0·1)	19·2 (16·4–22·5)	156·1 (135·1–181·5)	190·9 (176·5–207·5)	125·8 (113·3–140·8)	49·1 (41·1–59·4)	10·4 (8·3–13·2)	0·0 (0·0–0·0)	0·0 (0·0–0·0)	2·76 (2·47–3·11)	0·88 (0·77–1·0)	0·93 (0·81–1·07)

Supplemental Results Table 5: Age-specific fertility rates per 1000, cumulative total fertility up to maternal age 25 (TFU25), cumulative total fertility from maternal age 30 to age 55 (TFO30), and cumulative total fertility, Uzbekistan, 1950-2017

Year	10-14	15-19	20-24	25-29	30-34	35-39	40-44	45-49	50-54	TFR	TFU25	TFO30
1950	0·0 (0·0–0·1)	55·8 (48·5–63·9)	351·3 (336·1–365·3)	311·1 (301·0–321·1)	176·1 (165·0–187·3)	125·7 (117·1–134·3)	76·9 (70·4–83·4)	11·0 (8·0–14·5)	0·2 (0·2–0·2)	5·54 (5·33–5·73)	2·04 (1·92–2·15)	1·95 (1·87–2·03)
1951	0·0 (0·0–0·1)	56·3 (49·0–64·3)	351·4 (336·5–364·8)	312·5 (302·4–322·2)	176·6 (165·9–186·8)	125·6 (117·9–133·1)	76·7 (70·7–82·8)	10·7 (7·9–14·0)	0·2 (0·2–0·2)	5·55 (5·35–5·74)	2·04 (1·93–2·14)	1·95 (1·88–2·02)
1952	0·0 (0·0–0·1)	57·1 (49·8–64·6)	351·9 (337·6–365·3)	314·3 (304·5–324·0)	178·2 (168·1–188·2)	126·5 (119·2–133·9)	77·0 (71·2–82·7)	10·5 (7·8–13·7)	0·2 (0·2–0·2)	5·58 (5·39–5·76)	2·05 (1·94–2·14)	1·96 (1·89–2·04)
1953	0·0 (0·0–0·1)	58·1 (50·8–65·8)	352·9 (338·5–366·2)	316·8 (307·5–326·2)	181·0 (171·1–191·0)	128·5 (121·6–135·7)	78·0 (72·2–83·4)	10·3 (7·7–13·3)	0·2 (0·2–0·2)	5·63 (5·45–5·8)	2·06 (1·95–2·15)	1·99 (1·92–2·06)
1954	0·0 (0·0–0·1)	59·4 (52·3–67·1)	354·3 (340·0–367·1)	319·6 (310·3–329·0)	184·9 (175·4–195·0)	131·3 (124·4–138·4)	79·5 (74·1–84·7)	10·2 (7·6–13·0)	0·2 (0·2–0·2)	5·7 (5·53–5·87)	2·07 (1·97–2·16)	2·03 (1·95–2·11)
1955	0·0 (0·0–0·1)	60·8 (53·4–68·5)	355·8 (342·5–368·3)	322·6 (313·7–331·6)	189·7 (180·1–199·4)	134·7 (127·8–141·8)	81·4 (75·8–86·7)	10·1 (7·5–12·9)	0·2 (0·2–0·2)	5·78 (5·6–5·94)	2·08 (1·99–2·17)	2·08 (1·98–2·17)
1956	0·0 (0·0–0·1)	62·2 (54·7–70·2)	357·3 (344·2–369·5)	325·6 (316·7–334·2)	195·0 (185·7–204·8)	138·6 (131·4–145·8)	83·6 (78·2–88·8)	9·9 (7·3–12·9)	0·2 (0·2–0·2)	5·86 (5·69–6·02)	2·1 (2·01–2·18)	2·14 (2·03–2·24)
1957	0·0 (0·0–0·1)	63·7 (55·8–71·6)	358·7 (345·9–370·6)	328·5 (319·8–336·9)	200·8 (192·1–210·0)	142·7 (135·6–149·8)	86·0 (80·7–91·3)	9·8 (7·3–12·9)	0·2 (0·2–0·2)	5·95 (5·78–6·11)	2·11 (2·03–2·19)	2·2 (2·09–2·31)
1958	0·0 (0·0–0·1)	65·1 (56·8–73·2)	359·8 (347·5–371·4)	330·9 (322·5–338·9)	206·8 (197·4–215·4)	147·0 (140·2–153·9)	88·4 (83·2–93·6)	9·6 (7·0–12·6)	0·2 (0·2–0·2)	6·04 (5·87–6·2)	2·13 (2·05–2·2)	2·26 (2·15–2·36)
1959	0·0 (0·0–0·1)	66·4 (58·1–74·9)	360·3 (347·7–372·1)	332·4 (324·3–340·1)	212·5 (203·6–220·9)	151·0 (144·2–157·9)	90·7 (85·6–95·7)	9·4 (6·9–12·5)	0·2 (0·2–0·2)	6·12 (5·94–6·28)	2·13 (2·05–2·21)	2·32 (2·22–2·41)
1960	0·0 (0·0–0·1)	67·3 (58·6–76·5)	359·6 (346·3–371·8)	332·4 (324·5–340·6)	217·6 (208·9–226·0)	154·2 (147·6–161·4)	92·8 (87·8–97·8)	9·2 (6·7–12·1)	0·2 (0·2–0·2)	6·17 (6·0–6·33)	2·13 (2·05–2·22)	2·37 (2·29–2·45)
1961	0·0 (0·0–0·1)	68·0 (59·2–77·8)	357·9 (343·6–370·2)	331·7 (323·3–339·7)	221·8 (212·9–230·3)	156·8 (149·8–164·1)	94·4 (89·2–99·6)	8·9 (6·5–11·9)	0·2 (0·2–0·2)	6·2 (6·02–6·37)	2·13 (2·04–2·22)	2·41 (2·33–2·49)
1962	0·0 (0·0–0·1)	68·9 (59·7–79·1)	356·6 (342·2–369·1)	331·6 (323·1–339·5)	223·7 (214·3–232·3)	158·0 (150·7–165·2)	94·7 (89·1–100·0)	8·6 (6·1–11·5)	0·2 (0·2–0·2)	6·21 (6·03–6·38)	2·13 (2·03–2·23)	2·43 (2·33–2·51)
1963	0·0 (0·0–0·1)	69·7 (60·3–80·5)	355·7 (341·0–368·9)	331·3 (322·7–339·4)	222·5 (212·6–231·7)	156·6 (147·9–164·8)	93·1 (86·6–99·0)	8·2 (5·9–11·2)	0·2 (0·2–0·2)	6·19 (6·0–6·36)	2·13 (2·02–2·24)	2·4 (2·31–2·49)
1964	0·0 (0·0–0·1)	70·4 (61·1–80·8)	354·0 (338·5–367·9)	329·7 (320·9–338·0)	219·9 (208·9–230·5)	153·5 (143·4–163·6)	90·5 (83·2–97·1)	7·9 (5·6–10·7)	0·2 (0·1–0·2)	6·13 (5·93–6·32)	2·12 (2·01–2·24)	2·36 (2·25–2·47)
1965	0·0 (0·0–0·1)	71·0 (62·0–82·2)	351·7 (335·5–366·5)	327·6 (318·7–336·2)	217·0 (205·2–229·1)	149·9 (137·4–162·0)	87·6 (79·5–94·9)	7·5 (5·3–10·3)	0·1 (0·1–0·1)	6·06 (5·86–6·27)	2·11 (1·99–2·24)	2·31 (2·19–2·44)
1966	0·0 (0·0–0·1)	71·6 (62·4–82·5)	349·6 (333·2–365·4)	325·7 (316·5–334·2)	214·1 (201·0–227·3)	146·1 (131·1–160·0)	84·4 (75·4–92·6)	7·1 (4·9–10·1)	0·1 (0·1–0·1)	5·99 (5·76–6·22)	2·11 (1·98–2·24)	2·26 (2·11–2·42)
1967	0·0 (0·0–0·1)	72·2 (63·2–82·3)	347·6 (330·7–364·2)	323·7 (314·7–332·7)	211·0 (197·1–225·3)	142·1 (125·5–157·1)	81·1 (71·2–89·9)	6·8 (4·5–9·7)	0·1 (0·1–0·1)	5·92 (5·68–6·17)	2·1 (1·98–2·23)	2·21 (2·03–2·38)
1968	0·0 (0·0–0·1)	73·0 (63·9–82·5)	345·4 (328·2–363·2)	321·5 (312·1–330·9)	207·8 (192·7–222·9)	138·0 (120·9–154·3)	77·5 (67·3–86·9)	6·5 (4·4–9·2)	0·1 (0·1–0·1)	5·85 (5·58–6·12)	2·09 (1·96–2·22)	2·15 (1·95–2·36)
1969	0·0 (0·0–0·1)	73·6 (64·6–82·8)	343·2 (325·7–360·4)	319·2 (309·3–328·9)	204·5 (189·6–220·4)	133·9 (116·5–151·0)	73·8 (63·3–84·0)	6·2 (4·1–8·8)	0·1 (0·1–0·1)	5·77 (5·48–6·06)	2·08 (1·96–2·21)	2·09 (1·88–2·32)
1970	0·0 (0·0–0·1)	73·7 (64·6–83·1)	341·2 (324·2–358·2)	317·2 (307·0–327·3)	201·5 (186·0–217·8)	130·0 (112·5–148·1)	70·1 (59·5–80·9)	5·9 (3·9–8·5)	0·1 (0·1–0·1)	5·7 (5·4–5·98)	2·07 (1·96–2·2)	2·04 (1·81–2·28)
1971	0·0 (0·0–0·1)	73·7 (64·6–82·7)	339·5 (322·1–356·8)	315·4 (305·0–325·6)	198·7 (182·7–215·3)	126·3 (109·1–144·5)	66·5 (56·0–77·7)	5·6 (3·7–8·2)	0·1 (0·1–0·1)	5·63 (5·34–5·93)	2·07 (1·95–2·18)	1·99 (1·76–2·23)
1972	0·0 (0·0–0·1)	73·7 (65·2–82·6)	338·1 (319·9–354·8)	313·7 (303·4–324·1)	196·1 (180·6–212·0)	122·7 (106·2–140·3)	63·0 (52·4–73·8)	5·3 (3·4–7·8)	0·1 (0·1–0·1)	5·56 (5·28–5·87)	2·06 (1·95–2·17)	1·94 (1·71–2·16)
1973	0·0 (0·0–0·1)	73·8 (65·6–82·1)	336·7 (319·0–353·3)	311·7 (301·2–322·0)	193·5 (177·8–209·1)	119·0 (103·3–136·3)	59·5 (49·4–70·4)	5·0 (3·2–7·3)	0·1 (0·1–0·1)	5·5 (5·22–5·79)	2·05 (1·95–2·15)	1·89 (1·67–2·1)
1974	0·0 (0·0–0·1)	73·6 (65·6–82·2)	335·0 (317·9–350·3)	309·0 (298·6–319·7)	190·7 (174·9–206·6)	115·2 (99·9–131·3)	56·0 (46·4–66·3)	4·7 (3·1–6·9)	0·1 (0·1–0·1)	5·42 (5·16–5·7)	2·04 (1·94–2·14)	1·83 (1·64–2·03)
1975	0·0 (0·0–0·1)	72·9 (65·0–81·4)	332·4 (315·8–347·5)	305·8 (295·6–317·1)	187·8 (172·4–202·9)	111·2 (97·2–125·7)	52·6 (43·6–62·3)	4·4 (2·9–6·4)	0·1 (0·1–0·1)	5·34 (5·08–5·62)	2·03 (1·93–2·11)	1·78 (1·61–1·96)
1976	0·0 (0·0–0·1)	71·7 (64·3–79·6)	329·5 (312·5–344·1)	302·8 (292·2–313·7)	185·2 (171·0–199·4)	107·4 (95·0–120·4)	49·4 (41·1–57·8)	4·1 (2·7–6·0)	0·1 (0·1–0·1)	5·25 (5·0–5·52)	2·01 (1·92–2·09)	1·73 (1·59–1·9)
1977	0·0 (0·0–0·1)	70·3 (63·3–77·7)	326·7 (310·5–340·8)	299·7 (289·1–310·2)	182·9 (170·0–195·7)	103·9 (92·7–115·2)	46·4 (39·0–54·0)	3·9 (2·6–5·5)	0·1 (0·1–0·1)	5·17 (4·93–5·42)	1·99 (1·9–2·07)	1·69 (1·56–1·83)
1978	0·0 (0·0–0·1)	69·1 (62·6–76·3)	324·0 (308·3–337·4)	296·7 (285·9–307·4)	180·7 (169·1–192·5)	100·4 (90·8–110·2)	43·7 (37·5–50·8)	3·6 (2·4–5·0)	0·1 (0·1–0·1)	5·09 (4·87–5·32)	1·97 (1·88–2·05)	1·64 (1·53–1·77)
1979	0·0 (0·0–0·1)	68·3 (61·8–75·6)	321·5 (306·7–334·1)	294·1 (282·9–304·4)	178·7 (167·9–189·6)	96·9 (89·0–105·4)	41·1 (35·7–47·5)	3·3 (2·3–4·7)	0·1 (0·1–0·1)	5·02 (4·82–5·23)	1·95 (1·86–2·03)	1·6 (1·51–1·7)
1980	0·0 (0·0–0·1)	67·6 (61·1–74·3)	319·1 (304·4–331·8)	291·2 (280·3–301·7)	177·1 (167·1–186·6)	93·7 (86·9–101·3)	38·7 (33·7–44·3)	3·1 (2·1–4·3)	0·1 (0·1–0·1)	4·95 (4·77–5·14)	1·93 (1·85–2·01)	1·56 (1·48–1·65)
1981	0·0 (0·0–0·1)	66·8 (60·5–73·4)	316·8 (302·0–329·4)	288·2 (277·7–298·4)	176·1 (167·0–185·1)	91·0 (84·6–97·7)	36·6 (32·2–41·4)	2·8 (2·0–3·9)	0·1 (0·1–0·1)	4·89 (4·71–5·07)	1·92 (1·83–2·0)	1·53 (1·46–1·61)
1982	0·0 (0·0–0·1)	66·4 (60·3–73·1)	314·6 (300·5–327·4)	285·3 (274·3–295·6)	175·3 (166·6–183·9)	88·4 (82·6–94·7)	34·7 (30·8–39·3)	2·6 (1·9–3·6)	0·1 (0·0–0·1)	4·84 (4·66–5·01)	1·91 (1·82–1·99)	1·51 (1·44–1·57)
1983	0·0 (0·0–0·1)	66·2 (60·3–72·7)	312·2 (299·4–325·6)	282·2 (271·2–292·5)	174·3 (166·6–182·1)	85·8 (79·7–91·8)	33·0 (29·2–37·1)	2·4 (1·7–3·3)	0·0 (0·0–0·0)	4·78 (4·6–4·96)	1·89 (1·8–1·98)	1·48 (1·41–1·54)
1984	0·0 (0·0–0·1)	66·2 (60·4–72·6)	309·8 (296·8–322·7)	279·1 (268·8–289·4)	172·6 (165·6–180·0)	83·3 (77·3–89·3)	31·4 (27·7–35·4)	2·2 (1·6–3·0)	0·0 (0·0–0·0)	4·72 (4·55–4·91)	1·88 (1·79–1·97)	1·45 (1·38–1·51)
1985	0·0 (0·0–0·1)	66·2 (60·1–72·6)	307·5 (293·6–321·0)	275·6 (265·1–285·9)	169·7 (162·6–176·7)	80·7 (75·2–86·6)	29·9 (26·4–33·6)	2·1 (1·5–2·8)	0·0 (0·0–0·0)	4·66 (4·48–4·84)	1·87 (1·77–1·96)	1·41 (1·35–1·47)
1986	0·0 (0·0–0·1)	65·7 (59·6–72·3)	304·9 (291·1–318·1)	271·0 (260·7–280·9)	165·6 (158·6–172·7)	77·8 (72·6–83·1)	28·3 (25·1–31·8)	1·9 (1·3–2·5)	0·0 (0·0–0·0)	4·58 (4·4–4·75)	1·85 (1·76–1·94)	1·37 (1·3–1·43)
1987	0·0 (0·0–0·1)	65·0 (59·1–71·7)	301·8 (288·2–315·4)	265·6 (255·8–275·3)	161·1 (154·0–168·1)	74·4 (69·9–79·4)	26·6 (23·6–29·9)	1·7 (1·2–2·3)	0·0 (0·0–0·0)	4·48 (4·32–4·65)	1·83 (1·74–1·93)	1·32 (1·26–1·38)
1988	0·0 (0·0–0·1)	64·6 (58·8–71·2)	298·5 (284·1–312·6)	259·6 (249·7–269·7)	156·1 (149·3–163·0)	70·5 (66·4–75·0)	24·9 (22·1–28·1)	1·6 (1·1–2·1)	0·0 (0·0–0·0)	4·38 (4·22–4·54)	1·82 (1·72–1·91)	1·27 (1·21–1·32)
1989	0·0 (0·0–0·1)	64·6 (58·6–71·2)	295·0 (280·3–309·4)	253·6 (243·0–264·1)	151·0 (144·9–157·6)	66·2 (62·5–70·1)	23·2 (20·5–25·8)	1·4 (1·0–1·9)	0·0 (0·0–0·0)	4·27 (4·14–4·42)	1·8 (1·7–1·9)	1·21 (1·16–1·26)
1990	0·0 (0·0–0·1)	64·5 (58·7–70·9)	291·5 (276·6–307·0)	247·7 (236·7–258·7)	145·9 (139·4–152·0)	62·0 (58·4–65·7)	21·4 (19·0–23·9)	1·2 (0·9–1·7)	0·0 (0·0–0·0)	4·17 (4·03–4·32)	1·78 (1·68–1·89)	1·15 (1·11–1·2)
1991	0·0 (0·0–0·1)	64·0 (58·2–70·5)	288·0 (273·7–303·5)	240·8 (229·5–252·1)	140·5 (134·3–146·3)	58·0 (54·3–61·6)	19·6 (17·3–21·9)	1·1 (0·8–1·5)	0·0 (0·0–0·0)	4·06 (3·93–4·21)	1·76 (1·66–1·87)	1·1 (1·05–1·14)
1992	0·0 (0·0–0·1)	62·8 (57·1–69·2)	283·8 (268·5–299·2)	232·2 (221·3–243·4)	134·3 (128·3–139·9)	54·1 (50·6–57·4)	17·7 (15·7–19·8)	1·0 (0·7–1·3)	0·0 (0·0–0·0)	3·93 (3·79–4·08)	1·73 (1·63–1·84)	1·04 (0·99–1·08)
1993	0·0 (0·0–0·1)	60·3 (54·6–66·6)	278·3 (262·9–293·8)	223·0 (212·1–234·8)	127·7 (121·6–133·2)	50·6 (47·5–53·7)	15·9 (14·2–17·9)	0·8 (0·6–1·2)	0·0 (0·0–0·0)	3·78 (3·64–3·93)	1·69 (1·59–1·8)	0·98 (0·93–1·02)
1994	0·0 (0·0–0·1)	56·8 (51·5–63·1)	272·1 (255·6–288·1)	214·5 (203·2–226·5)	121·7 (115·8–126·9)	47·8 (44·9–50·8)	14·5 (12·8–16·3)	0·7 (0·5–0·9)	0·0 (0·0–0·0)	3·64 (3·49–3·78)	1·64 (1·54–1·74)	0·92 (0·88–0·96)
1995	0·0 (0·0–0·1)	52·9 (47·7–58·5)	265·8 (249·6–281·8)	205·9 (194·3–217·8)	116·2 (110·6–121·7)	45·3 (42·7–48·2)	13·3 (11·8–15·0)	0·6 (0·4–0·8)	0·0 (0·0–0·0)	3·5 (3·35–3·65)	1·59 (1·49–1·69)	0·88 (0·84–0·91)
1996	0·0 (0·0–0·1)	48·9 (44·0–54·1)	259·3 (243·1–275·8)	196·7 (185·0–208·9)	111·0 (105·6–116·6)	42·8 (40·2–45·6)	12·2 (10·8–13·7)	0·5 (0·3–0·6)	0·0 (0·0–0·0)	3·36 (3·21–3·52)	1·54 (1·44–1·64)	0·83 (0·8–0·87)
1997	0·0 (0·0–0·1)	44·6 (40·1–49·6)	252·5 (235·5–269·6)	186·9 (174·9–199·3)	106·2 (100·6–111·6)	40·3 (37·8–43·2)	11·3 (10·0–12·6)	0·4 (0·3–0·5)	0·0 (0·0–0·0)	3·21 (3·05–3·38)	1·49 (1·38–1·6)	0·79 (0·76–0·82)
1998	0·0 (0·0–0·1)	40·2 (35·9–45·0)	245·5 (228·4–263·8)	177·8 (165·7–190·0)	102·1 (96·5–107·6)	38·1 (35·7–40·7)	10·4 (9·2–11·7)	0·3 (0·2–0·4)	0·0 (0·0–0·0)	3·07 (2·91–3·25)	1·43 (1·32–1·54)	0·75 (0·72–0·79)
1999	0·0 (0·0–0·1)	36·7 (32·9–41·1)	239·0 (221·5–257·5)	170·2 (158·4–183·0)	98·7 (93·2–104·2)	36·3 (33·9–39·0)	9·7 (8·6–10·9)	0·3 (0·2–0·3)	0·0 (0·0–0·0)	2·95 (2·79–3·13)	1·38 (1·27–1·49)	0·72 (0·69–0·76)
2000	0·0 (0·0–0·1)	34·2 (30·5–38·3)	233·2 (215·0–251·7)	163·4 (151·3–175·8)	95·7 (90·4–101·5)	34·7 (32·5–37·3)	9·1 (8·1–10·3)	0·2 (0·2–0·3)	0·0 (0·0–0·0)	2·85 (2·69–3·02)	1·34 (1·23–1·45)	0·7 (0·66–0·73)
2001	0·0 (0·0–0·1)	32·3 (28·7–36·3)	227·9 (209·4–247·7)	156·7 (145·3–168·9)	93·0 (87·6–98·9)	33·3 (30·9–35·8)	8·6 (7·6–9·7)	0·2 (0·1–0·3)	0·0 (0·0–0·0)	2·76 (2·61–2·92)	1·3 (1·19–1·42)	0·68 (0·64–0·71)
2002	0·0 (0·0–0·1)	30·8 (27·2–34·8)	223·0 (203·2–243·7)	151·0 (139·5–162·8)	90·8 (85·1–96·6)	32·1 (29·6–34·6)	8·1 (7·1–9·2)	0·2 (0·1–0·3)	0·0 (0·0–0·0)	2·68 (2·53–2·84)	1·27 (1·16–1·39)	0·66 (0·62–0·69)
2003	0·0 (0·0–0·1)	29·6 (26·2–33·7)	219·0 (198·3–239·6)	147·2 (135·4–158·8)	89·6 (83·5–95·7)	31·2 (28·5–33·9)	7·8 (6·8–8·9)	0·2 (0·1–0·3)	0·0 (0·0–0·0)	2·62 (2·46–2·79)	1·24 (1·13–1·36)	0·64 (0·61–0·68)
2004	0·0 (0·0–0·1)	28·1 (24·9–32·0)	216·2 (194·4–238·3)	145·4 (133·0–157·3)	89·4 (82·8–95·9)	30·8 (27·8–33·4)	7·6 (6·5–8·8)	0·2 (0·1–0·3)	0·0 (0·0–0·0)	2·59 (2·41–2·77)	1·22 (1·1–1·35)	0·64 (0·6–0·68)
2005	0·0 (0·0–0·1)	27·0 (23·6–30·8)	214·4 (191·8–237·4)	144·6 (132·5–157·2)	89·7 (82·5–97·0)	30·5 (27·4–33·6)	7·4 (6·2–8·6)	0·2 (0·1–0·3)	0·0 (0·0–0·0)	2·57 (2·38–2·76)	1·21 (1·08–1·34)	0·64 (0·6–0·68)
2006	0·0 (0·0–0·1)	27·3 (23·6–31·3)	213·1 (189·4–235·8)	144·0 (131·6–156·7)	90·0 (82·4–98·2)	30·4 (26·7–33·8)	7·2 (6·0–8·6)	0·2 (0·1–0·3)	0·0 (0·0–0·0)	2·56 (2·35–2·77)	1·2 (1·07–1·33)	0·64 (0·59–0·69)
2007	0·0 (0·0–0·1)	28·3 (24·4–32·4)	211·7 (187·7–234·9)	143·4 (131·0–155·8)	90·3 (82·0–99·5)	30·2 (26·3–34·0)	7·1 (5·8–8·5)	0·2 (0·1–0·3)	0·0 (0·0–0·0)	2·56 (2·34–2·78)	1·2 (1·06–1·34)	0·64 (0·59–0·7)
2008	0·0 (0·0–0·1)	29·2 (25·2–33·5)	210·4 (186·8–233·7)	143·1 (130·4–155·4)	90·5 (82·1–100·1)	30·0 (26·0–34·4)	6·9 (5·6–8·4)	0·2 (0·1–0·3)	0·0 (0·0–0·0)	2·55 (2·33–2·78)	1·2 (1·06–1·34)	0·64 (0·58–0·7)
2009	0·0 (0·0–0·1)	30·0 (25·9–34·7)	209·1 (185·8–233·1)	142·9 (130·1–155·0)	90·6 (81·8–100·0)	29·8 (25·7–34·3)	6·7 (5·4–8·2)	0·2 (0·1–0·3)	0·0 (0·0–0·0)	2·55 (2·32–2·77)	1·2 (1·06–1·34)	0·64 (0·58–0·7)
2010	0·0 (0·0–0·1)	30·9 (26·6–35·7)	207·6 (184·3–231·7)	143·0 (130·7–155·9)	90·7 (81·9–100·2)	29·7 (25·7–33·9)	6·5 (5·3–8·0)	0·2 (0·1–0·3)	0·0 (0·0–0·0)	2·54 (2·33–2·76)	1·19 (1·05–1·34)	0·64 (0·58–0·69)
2011	0·0 (0·0–0·1)	31·7 (27·4–36·7)	206·3 (183·1–230·6)	143·8 (131·8–156·9)	91·0 (82·3–100·0)	29·6 (25·9–33·3)	6·3 (5·1–7·7)	0·2 (0·1–0·3)	0·0 (0·0–0·0)	2·54 (2·33–2·76)	1·19 (1·05–1·34)	0·64 (0·58–0·7)
2012	0·0 (0·0–0·1)	32·3 (27·9–37·6)	205·3 (182·6–230·8)	144·8 (132·4–158·2)	91·3 (82·9–100·6)	29·4 (25·9–32·8)	6·1 (5·0–7·5)	0·2 (0·1–0·3)	0·0 (0·0–0·0)	2·55 (2·34–2·75)	1·19 (1·05–1·34)	0·63 (0·58–0·7)
2013	0·0 (0·0–0·1)	32·5 (28·1–37·9)	204·1 (181·7–229·5)	144·4 (131·8–158·4)	91·2 (82·4–100·3)	28·9 (25·6–32·4)	5·8 (4·8–7·2)	0·2 (0·1–0·3)	0·0 (0·0–0·0)	2·54 (2·34–2·73)	1·18 (1·05–1·34)	0·63 (0·57–0·69)
2014	0·0 (0·0–0·1)	32·3 (28·0–37·7)	202·0 (179·5–227·6)	140·8 (128·2–154·6)	90·0 (80·9–99·1)	28·0 (24·7–31·7)	5·5 (4·5–7·0)	0·2 (0·1–0·3)	0·0 (0·0–0·0)	2·49 (2·29–2·69)	1·17 (1·04–1·33)	0·62 (0·55–0·69)
2015	0·0 (0·0–0·1)	32·2 (27·6–37·5)	199·0 (175·3–223·9)	134·6 (122·2–148·7)	87·9 (79·0–98·0)	26·8 (23·2–31·1)	5·3 (4·3–6·7)	0·2 (0·1–0·3)	0·0 (0·0–0·0)	2·43 (2·23–2·63)	1·16 (1·01–1·31)	0·6 (0·53–0·68)
2016	0·0 (0·0–0·1)	32·1 (27·4–37·4)	195·8 (171·7–220·6)	129·5 (117·2–143·4)	85·9 (76·4–96·8)	25·7 (21·7–30·7)	5·1 (4·0–6·7)	0·2 (0·1–0·3)	0·0 (0·0–0·0)	2·37 (2·17–2·58)	1·14 (1·0–1·29)	0·58 (0·51–0·67)
2017	0·0 (0·0–0·1)	32·3 (27·4–38·0)	194·3 (169·4–221·0)	127·8 (115·6–142·5)	85·2 (74·9–97·8)	25·4 (20·8–31·5)	5·1 (3·9–6·7)	0·2 (0·1–0·3)	0·0 (0·0–0·0)	2·35 (2·14–2·57)	1·13 (0·98–1·3)	0·58 (0·5–0·68)

Supplemental Results Table 5: Age-specific fertility rates per 1000, cumulative total fertility up to maternal age 25 (TFU25), cumulative total fertility from maternal age 30 to age 55 (TFO30), and cumulative total fertility, Central Europe, 1950-2017

Year	10-14	15-19	20-24	25-29	30-34	35-39	40-44	45-49	50-54	TFR	TFU25	TFO30
1950	0·3 (0·1–0·6)	48·0 (45·6–50·8)	191·2 (186·3–196·3)	173·3 (171·0–175·8)	118·6 (116·1–121·2)	68·4 (66·4–70·6)	27·1 (26·1–28·1)	3·0 (2·7–3·4)	0·1 (0·1–0·1)	3·15 (3·08–3·22)	1·2 (1·16–1·23)	1·09 (1·06–1·11)
1951	0·3 (0·1–0·6)	47·0 (44·6–49·7)	189·1 (184·4–193·7)	171·1 (168·9–173·3)	116·3 (114·1–118·7)	65·7 (63·6–67·8)	26·5 (25·6–27·6)	3·0 (2·6–3·3)	0·1 (0·1–0·1)	3·09 (3·03–3·16)	1·18 (1·15–1·21)	1·06 (1·03–1·08)
1952	0·3 (0·1–0·6)	46·5 (44·4–48·7)	189·0 (184·8–193·1)	169·7 (167·5–171·9)	115·1 (113·0–117·2)	63·8 (61·9–65·9)	26·0 (25·0–27·0)	2·9 (2·6–3·2)	0·1 (0·1–0·1)	3·07 (3·0–3·13)	1·18 (1·15–1·21)	1·04 (1·02–1·06)
1953	0·3 (0·1–0·6)	46·8 (45·0–48·6)	190·9 (187·2–194·5)	169·5 (167·7–171·2)	114·1 (112·2–116·0)	64·0 (62·1–65·7)	25·3 (24·3–26·3)	2·8 (2·5–3·1)	0·1 (0·1–0·1)	3·07 (3·01–3·12)	1·19 (1·16–1·22)	1·03 (1·01–1·05)
1954	0·3 (0·1–0·6)	47·6 (46·2–48·9)	193·0 (190·2–195·9)	168·5 (167·0–170·0)	111·4 (110·0–112·9)	65·7 (64·2–67·2)	24·1 (23·2–25·0)	2·6 (2·4–2·9)	0·0 (0·0–0·1)	3·07 (3·02–3·11)	1·2 (1·18–1·22)	1·02 (1·0–1·04)
1955	0·3 (0·1–0·6)	48·4 (47·4–49·5)	192·3 (189·7–194·9)	163·7 (162·4–165·2)	106·0 (104·8–107·3)	66·2 (64·9–67·5)	22·2 (21·3–23·1)	2·5 (2·2–2·8)	0·0 (0·0–0·0)	3·01 (2·97–3·04)	1·21 (1·19–1·22)	0·98 (0·97–1·0)
1956	0·3 (0·1–0·6)	49·5 (48·5–50·5)	190·6 (187·9–193·2)	157·3 (156·0–158·7)	99·0 (97·7–100·2)	61·9 (60·6–63·2)	20·1 (19·3–20·9)	2·3 (2·1–2·6)	0·0 (0·0–0·0)	2·9 (2·87–2·94)	1·2 (1·19–1·22)	0·92 (0·9–0·93)
1957	0·3 (0·1–0·6)	50·8 (49·8–51·8)	188·9 (186·4–191·6)	151·1 (149·8–152·5)	92·1 (90·8–93·3)	54·9 (53·8–56·1)	18·2 (17·4–19·1)	2·2 (2·0–2·5)	0·0 (0·0–0·0)	2·79 (2·76–2·83)	1·2 (1·18–1·22)	0·84 (0·82–0·85)
1958	0·3 (0·1–0·6)	51·9 (50·9–53·1)	186·2 (183·5–188·8)	144·9 (143·6–146·3)	85·9 (84·6–87·2)	49·3 (48·2–50·4)	17·1 (16·4–18·0)	2·0 (1·8–2·3)	0·0 (0·0–0·0)	2·69 (2·65–2·72)	1·19 (1·17–1·21)	0·77 (0·76–0·79)
1959	0·3 (0·1–0·6)	52·5 (51·5–53·6)	183·3 (180·7–185·9)	139·2 (137·7–140·5)	80·5 (79·3–81·9)	44·1 (43·0–45·3)	16·6 (15·9–17·4)	1·9 (1·7–2·1)	0·0 (0·0–0·0)	2·59 (2·56–2·63)	1·18 (1·16–1·2)	0·72 (0·7–0·73)
1960	0·3 (0·1–0·6)	51·8 (50·8–52·7)	180·4 (177·7–183·3)	133·9 (132·5–135·3)	76·0 (74·8–77·4)	39·8 (38·8–40·9)	15·8 (15·0–16·6)	1·8 (1·5–2·0)	0·0 (0·0–0·0)	2·5 (2·47–2·54)	1·16 (1·15–1·18)	0·67 (0·65–0·68)
1961	0·3 (0·1–0·6)	49·9 (48·9–50·8)	177·3 (174·6–179·9)	130·1 (128·8–131·5)	73·1 (71·8–74·4)	37·0 (36·0–38·1)	14·8 (14·0–15·6)	1·7 (1·5–1·9)	0·0 (0·0–0·0)	2·42 (2·39–2·46)	1·14 (1·12–1·15)	0·63 (0·62–0·65)
1962	0·3 (0·1–0·6)	47·9 (47·0–48·8)	176·2 (173·3–179·0)	128·9 (127·4–130·3)	72·1 (70·9–73·5)	35·5 (34·5–36·6)	13·6 (12·9–14·4)	1·7 (1·5–2·0)	0·0 (0·0–0·0)	2·38 (2·35–2·42)	1·12 (1·1–1·14)	0·62 (0·6–0·63)
1963	0·3 (0·1–0·6)	46·4 (45·6–47·3)	176·0 (173·4–178·5)	128·5 (127·1–129·9)	71·4 (70·1–72·8)	34·5 (33·4–35·6)	12·4 (11·8–13·1)	1·8 (1·6–2·0)	0·0 (0·0–0·0)	2·36 (2·32–2·39)	1·11 (1·1–1·13)	0·6 (0·59–0·61)
1964	0·3 (0·1–0·6)	44·7 (43·8–45·6)	173·2 (170·8–175·7)	125·8 (124·4–127·2)	69·0 (67·6–70·5)	33·0 (32·0–34·1)	11·1 (10·5–11·7)	1·8 (1·5–2·0)	0·0 (0·0–0·0)	2·29 (2·26–2·33)	1·09 (1·08–1·11)	0·57 (0·56–0·59)
1965	0·3 (0·1–0·6)	44·4 (43·4–45·2)	169·1 (166·7–171·6)	123·3 (121·9–124·6)	68·2 (66·8–69·8)	32·3 (31·3–33·4)	10·5 (9·9–11·1)	1·7 (1·5–1·9)	0·0 (0·0–0·0)	2·25 (2·22–2·28)	1·07 (1·05–1·08)	0·56 (0·55–0·58)
1966	0·3 (0·1–0·6)	47·0 (46·1–47·9)	175·6 (173·3–177·6)	130·7 (129·3–132·0)	73·8 (72·4–75·2)	34·9 (33·8–36·0)	10·8 (10·3–11·5)	1·6 (1·3–1·8)	0·0 (0·0–0·0)	2·37 (2·34–2·41)	1·11 (1·1–1·13)	0·61 (0·59–0·62)
1967	0·3 (0·1–0·6)	49·4 (48·6–50·3)	183·4 (181·5–185·2)	138·9 (137·6–140·2)	79·6 (78·3–81·1)	37·8 (36·8–38·9)	11·2 (10·6–11·9)	1·4 (1·2–1·6)	0·0 (0·0–0·0)	2·51 (2·48–2·54)	1·17 (1·15–1·18)	0·65 (0·64–0·66)
1968	0·3 (0·1–0·6)	49·1 (48·4–49·8)	179·7 (178·0–181·5)	135·1 (133·9–136·4)	77·7 (76·3–79·2)	36·7 (35·8–37·7)	10·5 (9·9–11·2)	1·2 (1·0–1·4)	0·0 (0·0–0·0)	2·45 (2·42–2·48)	1·15 (1·13–1·16)	0·63 (0·62–0·65)
1969	0·3 (0·1–0·6)	47·8 (47·0–48·6)	175·1 (173·4–176·9)	129·9 (128·7–131·2)	73·5 (72·3–74·9)	34·4 (33·5–35·4)	10·0 (9·4–10·6)	1·0 (0·9–1·2)	0·0 (0·0–0·0)	2·36 (2·33–2·39)	1·12 (1·11–1·13)	0·6 (0·58–0·61)
1970	0·3 (0·1–0·6)	46·4 (45·6–47·2)	172·9 (171·3–174·6)	125·5 (124·2–126·8)	70·2 (69·0–71·5)	32·8 (31·9–33·9)	9·6 (9·1–10·2)	0·9 (0·8–1·1)	0·0 (0·0–0·0)	2·29 (2·27–2·32)	1·1 (1·09–1·11)	0·57 (0·56–0·58)
1971	0·3 (0·1–0·6)	45·5 (44·6–46·3)	172·1 (170·5–173·9)	123·1 (122·0–124·4)	67·6 (66·4–69·0)	31·6 (30·6–32·7)	9·3 (8·7–9·9)	0·9 (0·7–1·0)	0·0 (0·0–0·0)	2·25 (2·22–2·28)	1·09 (1·08–1·1)	0·55 (0·53–0·56)
1972	0·3 (0·1–0·6)	46·0 (45·1–46·9)	173·0 (171·3–174·8)	124·8 (123·6–126·1)	66·7 (65·5–67·9)	31·0 (29·9–32·0)	9·0 (8·4–9·7)	0·8 (0·7–1·0)	0·0 (0·0–0·0)	2·26 (2·23–2·29)	1·1 (1·09–1·11)	0·54 (0·53–0·55)
1973	0·3 (0·1–0·6)	48·2 (47·3–49·1)	178·5 (176·8–180·5)	129·7 (128·7–130·8)	67·5 (66·3–68·7)	31·1 (30·2–32·2)	8·9 (8·3–9·5)	0·8 (0·7–0·9)	0·0 (0·0–0·0)	2·33 (2·3–2·35)	1·14 (1·12–1·15)	0·54 (0·53–0·55)
1974	0·3 (0·1–0·6)	50·5 (49·6–51·4)	182·5 (180·8–184·2)	132·0 (130·9–133·2)	67·5 (66·4–68·7)	30·8 (29·8–31·8)	8·7 (8·1–9·2)	0·8 (0·6–0·9)	0·0 (0·0–0·0)	2·37 (2·34–2·39)	1·17 (1·16–1·18)	0·54 (0·53–0·55)
1975	0·3 (0·1–0·6)	51·6 (50·8–52·4)	182·8 (181·0–184·5)	131·2 (130·2–132·2)	66·0 (64·9–67·1)	29·6 (28·6–30·6)	8·2 (7·7–8·8)	0·7 (0·6–0·9)	0·0 (0·0–0·0)	2·35 (2·33–2·38)	1·17 (1·16–1·19)	0·52 (0·51–0·53)
1976	0·3 (0·1–0·6)	51·9 (51·1–52·8)	182·3 (180·5–184·2)	129·6 (128·5–130·6)	63·9 (62·7–64·9)	28·0 (27·1–29·0)	7·7 (7·2–8·3)	0·7 (0·6–0·9)	0·0 (0·0–0·0)	2·32 (2·3–2·35)	1·17 (1·16–1·19)	0·5 (0·49–0·51)
1977	0·3 (0·1–0·6)	52·0 (51·1–52·9)	180·7 (179·0–182·5)	127·3 (126·3–128·3)	61·9 (61·0–62·9)	26·6 (25·7–27·5)	7·3 (6·7–7·8)	0·7 (0·5–0·8)	0·0 (0·0–0·0)	2·28 (2·26–2·31)	1·16 (1·15–1·18)	0·48 (0·47–0·49)
1978	0·3 (0·1–0·7)	51·9 (51·1–52·8)	181·0 (179·4–182·7)	125·9 (125·0–126·8)	60·6 (59·7–61·5)	25·5 (24·7–26·4)	7·0 (6·4–7·5)	0·6 (0·5–0·8)	0·0 (0·0–0·0)	2·26 (2·24–2·29)	1·17 (1·16–1·18)	0·47 (0·46–0·48)
1979	0·3 (0·1–0·7)	51·2 (50·4–52·2)	180·8 (179·3–182·5)	124·6 (123·7–125·5)	59·1 (58·2–59·9)	24·3 (23·4–25·2)	6·6 (6·1–7·2)	0·6 (0·5–0·8)	0·0 (0·0–0·0)	2·24 (2·22–2·26)	1·16 (1·15–1·17)	0·45 (0·44–0·46)
1980	0·3 (0·1–0·7)	50·2 (49·4–50·9)	177·9 (176·4–179·3)	122·1 (121·2–123·1)	57·2 (56·3–58·2)	22·9 (22·0–23·8)	6·2 (5·7–6·7)	0·6 (0·4–0·7)	0·0 (0·0–0·0)	2·19 (2·17–2·21)	1·14 (1·13–1·15)	0·43 (0·43–0·44)
1981	0·3 (0·1–0·7)	49·2 (48·5–49·9)	176·4 (174·9–177·8)	120·1 (119·3–121·0)	56·2 (55·3–57·0)	22·2 (21·4–23·1)	5·9 (5·4–6·4)	0·5 (0·4–0·7)	0·0 (0·0–0·0)	2·15 (2·14–2·17)	1·13 (1·12–1·14)	0·42 (0·42–0·43)
1982	0·3 (0·1–0·7)	48·4 (47·7–49·2)	176·8 (175·4–178·2)	120·5 (119·7–121·3)	56·2 (55·4–57·0)	22·1 (21·3–22·9)	5·7 (5·2–6·2)	0·5 (0·4–0·7)	0·0 (0·0–0·0)	2·15 (2·13–2·17)	1·13 (1·12–1·14)	0·42 (0·41–0·43)
1983	0·3 (0·1–0·6)	47·9 (47·1–48·7)	177·5 (176·2–178·7)	122·3 (121·5–123·2)	56·8 (56·0–57·6)	22·1 (21·4–22·9)	5·6 (5·2–6·1)	0·5 (0·4–0·6)	0·0 (0·0–0·0)	2·17 (2·15–2·18)	1·13 (1·12–1·14)	0·43 (0·42–0·43)
1984	0·3 (0·1–0·6)	47·5 (46·7–48·3)	177·9 (176·7–179·0)	124·0 (123·2–124·7)	57·4 (56·6–58·1)	22·3 (21·7–22·9)	5·6 (5·1–6·1)	0·5 (0·4–0·6)	0·0 (0·0–0·0)	2·18 (2·16–2·19)	1·13 (1·12–1·14)	0·43 (0·42–0·44)
1985	0·3 (0·1–0·6)	47·3 (46·6–47·9)	175·8 (174·7–176·9)	124·1 (123·3–124·9)	57·6 (56·8–58·3)	22·4 (21·7–23·0)	5·5 (5·0–6·0)	0·4 (0·3–0·6)	0·0 (0·0–0·0)	2·17 (2·15–2·18)	1·12 (1·11–1·12)	0·43 (0·42–0·44)
1986	0·3 (0·1–0·6)	47·2 (46·5–47·8)	172·6 (171·5–173·7)	123·5 (122·7–124·3)	57·3 (56·6–58·1)	22·5 (21·9–23·2)	5·5 (5·0–6·0)	0·4 (0·3–0·6)	0·0 (0·0–0·0)	2·15 (2·13–2·16)	1·1 (1·09–1·11)	0·43 (0·42–0·44)
1987	0·3 (0·1–0·6)	46·3 (45·7–47·0)	170·1 (169·1–171·1)	122·9 (122·1–123·7)	56·8 (56·1–57·5)	22·5 (21·9–23·1)	5·4 (5·0–5·9)	0·4 (0·3–0·5)	0·0 (0·0–0·0)	2·12 (2·11–2·14)	1·08 (1·08–1·09)	0·43 (0·42–0·43)
1988	0·3 (0·1–0·6)	44·3 (43·7–45·0)	166·9 (165·8–168·0)	121·2 (120·4–122·0)	55·4 (54·7–56·1)	21·7 (21·0–22·3)	5·2 (4·8–5·6)	0·4 (0·3–0·5)	0·0 (0·0–0·0)	2·08 (2·06–2·09)	1·06 (1·05–1·06)	0·41 (0·41–0·42)
1989	0·3 (0·1–0·6)	42·3 (41·6–43·0)	160·5 (159·6–161·6)	117·6 (116·9–118·4)	53·2 (52·6–53·9)	20·5 (19·9–21·2)	4·9 (4·5–5·3)	0·3 (0·3–0·4)	0·0 (0·0–0·0)	2·0 (1·99–2·01)	1·02 (1·01–1·02)	0·4 (0·39–0·4)
1990	0·3 (0·1–0·6)	40·8 (40·1–41·5)	153·4 (152·5–154·3)	113·3 (112·6–114·0)	51·4 (50·8–52·1)	19·8 (19·2–20·5)	4·7 (4·3–5·1)	0·3 (0·2–0·4)	0·0 (0·0–0·0)	1·92 (1·91–1·93)	0·97 (0·97–0·98)	0·38 (0·38–0·39)
1991	0·3 (0·1–0·5)	39·2 (38·5–40·0)	145·1 (144·2–146·0)	109·4 (108·8–110·1)	50·0 (49·4–50·6)	19·2 (18·5–20·0)	4·5 (4·2–4·9)	0·3 (0·2–0·3)	0·0 (0·0–0·0)	1·84 (1·83–1·85)	0·92 (0·92–0·93)	0·37 (0·36–0·38)
1992	0·3 (0·1–0·5)	37·6 (37·0–38·4)	135·8 (134·9–136·6)	106·3 (105·6–107·0)	49·1 (48·5–49·8)	19·1 (18·4–19·7)	4·5 (4·2–4·9)	0·2 (0·2–0·3)	0·0 (0·0–0·0)	1·76 (1·75–1·78)	0·87 (0·86–0·87)	0·36 (0·36–0·37)
1993	0·2 (0·1–0·5)	35·6 (34·9–36·3)	128·0 (127·0–128·9)	103·6 (102·8–104·3)	48·9 (48·2–49·7)	19·2 (18·6–19·9)	4·5 (4·1–4·8)	0·2 (0·2–0·3)	0·0 (0·0–0·0)	1·7 (1·69–1·72)	0·82 (0·81–0·83)	0·36 (0·36–0·37)
1994	0·2 (0·1–0·5)	32·5 (31·8–33·2)	118·8 (117·9–119·8)	100·4 (99·7–101·1)	49·0 (48·3–49·7)	19·2 (18·6–19·8)	4·4 (4·1–4·8)	0·2 (0·2–0·3)	0·0 (0·0–0·0)	1·62 (1·61–1·64)	0·76 (0·75–0·76)	0·36 (0·36–0·37)
1995	0·2 (0·1–0·5)	29·9 (29·2–30·7)	110·4 (109·5–111·5)	97·2 (96·5–97·9)	49·0 (48·3–49·7)	18·9 (18·3–19·5)	4·3 (4·0–4·7)	0·2 (0·2–0·3)	0·0 (0·0–0·0)	1·55 (1·54–1·57)	0·7 (0·7–0·71)	0·36 (0·36–0·37)
1996	0·2 (0·1–0·4)	28·3 (27·4–29·2)	104·3 (103·4–105·1)	94·6 (93·9–95·3)	48·8 (48·0–49·6)	18·7 (18·1–19·3)	4·2 (3·9–4·6)	0·2 (0·2–0·3)	0·0 (0·0–0·0)	1·5 (1·48–1·51)	0·66 (0·66–0·67)	0·36 (0·35–0·37)
1997	0·2 (0·1–0·4)	27·0 (26·2–27·8)	98·5 (97·7–99·4)	92·6 (91·9–93·4)	48·3 (47·6–49·1)	18·4 (17·8–19·0)	4·1 (3·8–4·5)	0·2 (0·2–0·3)	0·0 (0·0–0·0)	1·45 (1·43–1·46)	0·63 (0·62–0·63)	0·36 (0·35–0·36)
1998	0·2 (0·1–0·4)	25·8 (25·1–26·5)	93·4 (92·3–94·5)	92·0 (91·3–92·7)	47·7 (47·0–48·5)	18·2 (17·6–18·8)	4·1 (3·7–4·5)	0·2 (0·2–0·3)	0·0 (0·0–0·0)	1·41 (1·39–1·42)	0·6 (0·59–0·6)	0·35 (0·34–0·36)
1999	0·2 (0·1–0·4)	24·6 (24·0–25·3)	88·3 (87·3–89·3)	91·7 (91·0–92·5)	47·8 (47·1–48·6)	18·3 (17·6–18·9)	4·0 (3·7–4·5)	0·2 (0·2–0·3)	0·0 (0·0–0·0)	1·38 (1·36–1·39)	0·57 (0·56–0·57)	0·35 (0·34–0·36)
2000	0·2 (0·1–0·4)	23·1 (22·5–23·7)	82·9 (82·2–83·7)	91·1 (90·4–91·9)	48·8 (48·0–49·4)	18·7 (18·1–19·3)	4·1 (3·7–4·5)	0·2 (0·2–0·3)	0·0 (0·0–0·0)	1·35 (1·33–1·36)	0·53 (0·53–0·54)	0·36 (0·35–0·37)
2001	0·2 (0·1–0·4)	21·7 (21·1–22·4)	77·4 (76·6–78·2)	90·2 (89·5–90·9)	49·9 (49·3–50·6)	19·2 (18·6–19·9)	4·2 (3·7–4·7)	0·2 (0·2–0·3)	0·0 (0·0–0·0)	1·32 (1·3–1·33)	0·5 (0·49–0·5)	0·37 (0·36–0·38)
2002	0·2 (0·1–0·4)	21·0 (20·4–21·8)	73·1 (72·2–74·1)	89·4 (88·7–90·0)	51·4 (50·7–52·1)	19·8 (19·1–20·5)	4·2 (3·8–4·7)	0·2 (0·2–0·3)	0·0 (0·0–0·0)	1·3 (1·28–1·31)	0·47 (0·47–0·48)	0·38 (0·37–0·39)
2003	0·2 (0·1–0·4)	20·7 (20·1–21·4)	70·1 (69·1–71·0)	89·5 (88·8–90·2)	54·1 (53·4–54·8)	20·5 (19·8–21·2)	4·3 (3·8–4·8)	0·2 (0·2–0·3)	0·0 (0·0–0·0)	1·3 (1·28–1·31)	0·45 (0·45–0·46)	0·4 (0·39–0·4)
2004	0·2 (0·1–0·4)	20·6 (19·9–21·2)	67·2 (66·3–68·1)	90·3 (89·4–91·2)	57·7 (57·1–58·4)	21·4 (20·7–22·1)	4·4 (3·9–5·0)	0·2 (0·2–0·3)	0·0 (0·0–0·0)	1·31 (1·3–1·32)	0·44 (0·43–0·45)	0·42 (0·41–0·43)
2005	0·2 (0·1–0·4)	20·7 (20·0–21·3)	65·0 (64·0–66·1)	91·3 (90·5–92·1)	61·7 (61·0–62·4)	22·5 (21·7–23·2)	4·5 (4·0–5·0)	0·2 (0·2–0·3)	0·0 (0·0–0·0)	1·33 (1·32–1·35)	0·43 (0·42–0·44)	0·44 (0·44–0·45)
2006	0·2 (0·1–0·4)	21·2 (20·6–21·9)	64·2 (63·3–65·1)	92·4 (91·7–93·1)	65·6 (64·9–66·4)	23·8 (23·1–24·6)	4·6 (4·2–5·1)	0·2 (0·2–0·3)	0·0 (0·0–0·0)	1·36 (1·35–1·38)	0·43 (0·42–0·43)	0·47 (0·46–0·48)
2007	0·2 (0·1–0·4)	22·0 (21·4–22·7)	64·8 (63·8–65·8)	94·3 (93·6–95·0)	69·4 (68·6–70·1)	25·5 (24·8–26·2)	4·8 (4·4–5·2)	0·2 (0·2–0·3)	0·0 (0·0–0·0)	1·41 (1·39–1·42)	0·43 (0·43–0·44)	0·5 (0·49–0·51)
2008	0·2 (0·1–0·4)	22·3 (21·5–23·0)	65·1 (64·2–66·0)	96·0 (95·2–96·8)	72·6 (71·8–73·4)	27·4 (26·6–28·2)	5·0 (4·6–5·4)	0·2 (0·2–0·3)	0·0 (0·0–0·0)	1·44 (1·43–1·46)	0·44 (0·43–0·45)	0·53 (0·52–0·53)
2009	0·2 (0·1–0·4)	21·5 (20·8–22·3)	63·4 (62·2–64·6)	95·6 (94·7–96·4)	74·3 (73·6–75·1)	29·1 (28·4–29·9)	5·3 (4·8–5·7)	0·2 (0·2–0·3)	0·0 (0·0–0·0)	1·45 (1·43–1·46)	0·43 (0·42–0·43)	0·54 (0·54–0·55)
2010	0·2 (0·1–0·4)	20·6 (19·8–21·5)	60·7 (59·4–62·0)	93·3 (92·4–94·2)	74·2 (73·5–75·0)	30·1 (29·5–30·8)	5·5 (5·0–6·0)	0·3 (0·2–0·3)	0·0 (0·0–0·0)	1·42 (1·41–1·44)	0·41 (0·4–0·42)	0·55 (0·54–0·56)
2011	0·2 (0·1–0·4)	20·1 (19·3–21·0)	59·0 (57·7–60·2)	91·3 (90·3–92·3)	73·8 (73·0–74·6)	30·7 (30·0–31·5)	5·7 (5·2–6·2)	0·3 (0·2–0·3)	0·0 (0·0–0·0)	1·41 (1·39–1·42)	0·4 (0·39–0·41)	0·55 (0·55–0·56)
2012	0·2 (0·1–0·4)	19·7 (18·9–20·6)	57·5 (56·2–59·0)	90·2 (89·1–91·2)	74·3 (73·5–75·1)	31·5 (30·8–32·2)	5·9 (5·4–6·4)	0·3 (0·2–0·3)	0·0 (0·0–0·0)	1·4 (1·38–1·42)	0·39 (0·38–0·4)	0·56 (0·55–0·57)
2013	0·2 (0·1–0·4)	19·5 (18·7–20·4)	56·3 (55·0–57·6)	90·1 (89·2–91·0)	76·3 (75·4–77·1)	32·8 (32·0–33·6)	6·1 (5·6–6·6)	0·3 (0·2–0·3)	0·0 (0·0–0·0)	1·41 (1·39–1·42)	0·38 (0·37–0·39)	0·58 (0·57–0·58)
2014	0·2 (0·1–0·4)	19·5 (18·5–20·6)	55·9 (54·7–57·2)	91·2 (90·3–92·3)	79·1 (78·3–79·9)	34·2 (33·4–35·2)	6·2 (5·7–6·7)	0·3 (0·2–0·3)	0·0 (0·0–0·0)	1·43 (1·41–1·45)	0·38 (0·37–0·39)	0·6 (0·59–0·61)
2015	0·2 (0·1–0·4)	19·5 (18·2–20·9)	56·2 (53·7–58·8)	92·4 (90·3–94·6)	80·3 (78·5–82·2)	34·5 (32·9–36·0)	6·0 (5·5–6·6)	0·3 (0·2–0·3)	0·0 (0·0–0·0)	1·45 (1·4–1·5)	0·38 (0·36–0·4)	0·61 (0·59–0·62)
2016	0·2 (0·1–0·4)	19·5 (17·8–21·3)	56·7 (51·8–62·4)	93·0 (88·8–97·5)	79·4 (74·9–84·3)	33·3 (30·0–37·0)	5·7 (5·1–6·4)	0·2 (0·2–0·3)	0·0 (0·0–0·0)	1·44 (1·34–1·55)	0·38 (0·35–0·42)	0·59 (0·55–0·64)
2017	0·2 (0·1–0·4)	19·5 (17·5–21·6)	57·2 (49·7–66·0)	93·1 (86·7–99·8)	78·0 (70·9–86·2)	31·9 (27·0–37·8)	5·5 (4·8–6·4)	0·2 (0·2–0·3)	0·0 (0·0–0·0)	1·43 (1·29–1·59)	0·38 (0·34–0·44)	0·58 (0·52–0·65)

Supplemental Results Table 5: Age-specific fertility rates per 1000, cumulative total fertility up to maternal age 25 (TFU25), cumulative total fertility from maternal age 30 to age 55 (TFO30), and cumulative total fertility, Albania, 1950-2017

Year	10-14	15-19	20-24	25-29	30-34	35-39	40-44	45-49	50-54	TFR	TFU25	TFO30
1950	0·1 (0·1–0·2)	60·0 (51·9–70·1)	256·3 (241·7–270·8)	286·7 (278·6–294·7)	253·6 (246·3–260·5)	181·8 (173·0–190·4)	101·6 (94·5–108·2)	33·9 (31·3–36·3)	0·7 (0·6–0·7)	5·87 (5·73–6·01)	1·58 (1·49–1·67)	2·86 (2·79–2·93)
1951	0·1 (0·1–0·3)	61·9 (54·2–71·2)	254·9 (242·3–267·4)	287·9 (280·7–295·2)	256·4 (250·1–262·1)	180·8 (173·4–188·5)	100·8 (93·9–107·1)	33·5 (31·0–35·8)	0·6 (0·6–0·7)	5·88 (5·76–6·01)	1·58 (1·51–1·66)	2·86 (2·8–2·92)
1952	0·1 (0·1–0·3)	64·8 (57·0–73·5)	258·3 (245·9–270·2)	296·0 (289·1–302·7)	263·0 (257·4–268·3)	186·0 (179·1–192·7)	102·0 (95·4–108·0)	33·5 (31·2–35·7)	0·6 (0·6–0·7)	6·02 (5·9–6·15)	1·62 (1·54–1·69)	2·93 (2·86–2·98)
1953	0·1 (0·1–0·3)	67·7 (59·6–76·7)	263·6 (250·8–276·4)	305·0 (298·1–311·6)	268·6 (263·3–273·7)	195·6 (189·0–201·6)	103·7 (97·5–109·5)	33·8 (31·6–35·9)	0·6 (0·6–0·7)	6·19 (6·08–6·31)	1·66 (1·58–1·74)	3·01 (2·95–3·07)
1954	0·1 (0·1–0·3)	69·7 (61·2–78·8)	267·3 (253·7–279·5)	310·5 (303·5–317·1)	269·9 (264·0–275·6)	205·1 (199·0–210·2)	104·8 (98·8–110·9)	34·0 (31·9–36·1)	0·7 (0·6–0·7)	6·31 (6·19–6·42)	1·69 (1·6–1·76)	3·07 (3·02–3·13)
1955	0·1 (0·1–0·3)	70·1 (62·0–78·3)	269·4 (255·0–282·6)	311·8 (304·2–318·7)	267·8 (261·1–274·3)	209·5 (203·3–214·9)	104·6 (98·1–110·6)	34·2 (31·9–36·1)	0·7 (0·6–0·7)	6·34 (6·22–6·46)	1·7 (1·61–1·78)	3·08 (3·02–3·15)
1956	0·1 (0·1–0·3)	70·1 (61·9–78·9)	270·7 (255·9–284·6)	311·7 (304·2–318·5)	264·8 (258·0–271·7)	210·2 (203·9–215·9)	104·8 (98·3–111·2)	34·2 (31·8–36·2)	0·7 (0·6–0·7)	6·34 (6·21–6·48)	1·7 (1·62–1·79)	3·07 (3·01–3·14)
1957	0·1 (0·1–0·3)	70·6 (62·4–79·5)	272·9 (258·1–286·8)	313·4 (306·1–320·5)	262·8 (256·0–269·5)	210·5 (204·1–216·1)	107·6 (101·4–113·5)	34·3 (31·8–36·4)	0·7 (0·6–0·7)	6·36 (6·23–6·5)	1·72 (1·64–1·8)	3·08 (3·01–3·15)
1958	0·1 (0·1–0·3)	70·8 (62·0–80·0)	276·4 (261·9–289·9)	315·1 (307·8–322·3)	261·6 (254·3–268·2)	209·3 (202·5–215·3)	113·2 (108·0–118·2)	34·6 (32·2–36·7)	0·7 (0·6–0·7)	6·41 (6·27–6·55)	1·74 (1·66–1·82)	3·1 (3·02–3·16)
1959	0·1 (0·1–0·3)	70·3 (61·3–79·5)	280·3 (266·9–293·7)	314·8 (307·6–321·7)	260·5 (253·5–267·3)	205·6 (198·4–211·8)	118·2 (113·7–122·1)	34·9 (32·6–37·0)	0·7 (0·6–0·7)	6·43 (6·28–6·58)	1·75 (1·68–1·83)	3·1 (3·01–3·17)
1960	0·1 (0·1–0·3)	69·0 (60·1–77·7)	283·9 (271·0–296·9)	312·8 (305·7–319·9)	258·6 (251·6–265·3)	200·2 (193·1–207·6)	119·1 (115·0–122·7)	34·7 (32·4–36·9)	0·7 (0·6–0·7)	6·4 (6·24–6·55)	1·77 (1·69–1·84)	3·07 (2·98–3·15)
1961	0·1 (0·1–0·3)	66·6 (58·5–75·5)	287·0 (274·1–300·4)	310·4 (302·8–317·3)	255·7 (248·6–262·6)	194·1 (186·5–201·5)	116·9 (112·4–120·9)	34·1 (31·8–36·4)	0·7 (0·6–0·7)	6·33 (6·18–6·48)	1·77 (1·69–1·84)	3·01 (2·91–3·1)
1962	0·1 (0·1–0·3)	63·9 (55·9–72·5)	288·7 (274·7–301·8)	307·7 (300·0–315·1)	251·7 (244·4–258·9)	187·2 (179·1–195·0)	113·1 (107·6–117·9)	33·2 (30·8–35·4)	0·6 (0·6–0·7)	6·23 (6·07–6·38)	1·76 (1·68–1·84)	2·93 (2·83–3·01)
1963	0·1 (0·1–0·3)	61·6 (53·7–69·8)	287·1 (273·4–300·9)	303·5 (295·3–311·5)	245·2 (237·5–252·9)	179·5 (170·0–188·0)	106·8 (100·4–112·7)	32·0 (29·5–34·2)	0·6 (0·6–0·6)	6·08 (5·93–6·24)	1·74 (1·65–1·83)	2·82 (2·73–2·9)
1964	0·1 (0·1–0·2)	60·2 (52·6–68·5)	282·6 (268·1–295·9)	299·5 (291·4–307·5)	236·6 (228·2–244·8)	171·7 (161·5–181·8)	98·9 (91·1–105·6)	30·5 (28·0–32·8)	0·6 (0·6–0·6)	5·9 (5·75–6·06)	1·71 (1·61–1·81)	2·69 (2·61–2·78)
1965	0·1 (0·1–0·2)	59·8 (52·1–67·8)	278·3 (263·4–292·0)	299·6 (291·5–307·5)	229·3 (220·9–237·6)	166·4 (156·0–176·4)	91·7 (83·6–99·0)	28·7 (26·0–31·2)	0·6 (0·5–0·6)	5·77 (5·61–5·92)	1·69 (1·58–1·79)	2·58 (2·5–2·67)
1966	0·1 (0·1–0·2)	60·3 (52·4–68·6)	277·4 (262·6–292·0)	304·4 (296·3–311·9)	225·4 (216·8–233·6)	164·0 (154·0–173·9)	86·8 (77·9–95·2)	26·5 (23·8–29·2)	0·5 (0·5–0·5)	5·73 (5·55–5·88)	1·69 (1·58–1·79)	2·52 (2·43–2·6)
1967	0·1 (0·1–0·3)	61·0 (53·4–69·4)	279·1 (264·3–293·7)	310·0 (302·3–317·2)	223·8 (215·0–232·4)	162·5 (152·4–172·6)	83·2 (74·1–91·8)	24·2 (21·4–27·0)	0·5 (0·4–0·5)	5·72 (5·55–5·89)	1·7 (1·6–1·81)	2·47 (2·38–2·56)
1968	0·1 (0·1–0·3)	61·2 (53·5–69·4)	280·7 (265·5–295·6)	313·0 (305·6–320·5)	222·3 (213·0–231·2)	159·9 (150·3–169·9)	79·7 (70·7–88·7)	21·9 (19·0–24·6)	0·4 (0·4–0·4)	5·7 (5·52–5·88)	1·71 (1·6–1·82)	2·42 (2·32–2·52)
1969	0·1 (0·1–0·2)	60·0 (52·9–68·2)	281·6 (266·1–295·9)	312·0 (304·1–319·8)	220·7 (210·8–230·3)	155·8 (145·1–166·2)	76·8 (67·6–86·3)	19·7 (16·7–22·6)	0·4 (0·4–0·4)	5·64 (5·43–5·85)	1·71 (1·6–1·81)	2·37 (2·24–2·49)
1970	0·1 (0·1–0·2)	57·8 (50·5–66·1)	280·1 (264·2–295·9)	307·5 (299·1–315·8)	218·3 (208·3–228·7)	150·8 (139·0–162·8)	74·0 (64·4–83·9)	17·5 (14·6–20·7)	0·3 (0·3–0·3)	5·53 (5·3–5·8)	1·69 (1·59–1·8)	2·3 (2·16–2·46)
1971	0·1 (0·1–0·2)	55·9 (48·5–64·1)	273·8 (256·7–291·9)	300·2 (290·9–309·9)	213·5 (201·8–226·1)	145·2 (131·5–160·0)	70·5 (60·6–80·8)	15·4 (12·5–18·8)	0·3 (0·3–0·3)	5·37 (5·08–5·69)	1·65 (1·55–1·75)	2·22 (2·05–2·41)
1972	0·1 (0·0–0·2)	54·5 (47·3–62·4)	264·6 (245·1–286·2)	291·1 (280·0–301·6)	206·5 (193·4–221·4)	139·0 (123·5–156·2)	66·3 (55·9–77·5)	13·3 (10·5–16·9)	0·3 (0·2–0·3)	5·18 (4·85–5·56)	1·6 (1·49–1·71)	2·13 (1·94–2·34)
1973	0·1 (0·0–0·2)	53·3 (46·3–60·9)	255·2 (235·9–276·6)	282·0 (270·3–294·5)	198·8 (184·1–215·2)	132·6 (115·3–151·0)	61·3 (50·1–73·0)	11·4 (8·8–14·7)	0·2 (0·2–0·2)	4·97 (4·59–5·38)	1·54 (1·43–1·66)	2·02 (1·79–2·27)
1974	0·1 (0·0–0·2)	51·9 (45·1–58·9)	246·5 (228·2–265·7)	273·5 (261·1–287·2)	190·8 (174·9–208·7)	126·5 (108·6–147·0)	56·2 (45·5–69·0)	9·7 (7·4–12·8)	0·2 (0·2–0·2)	4·78 (4·39–5·19)	1·49 (1·38–1·61)	1·92 (1·68–2·19)
1975	0·1 (0·0–0·2)	49·3 (42·9–55·9)	238·9 (220·7–257·6)	265·7 (254·1–278·7)	183·1 (167·6–200·2)	120·9 (103·3–139·7)	51·2 (40·8–63·4)	8·2 (6·3–10·8)	0·2 (0·2–0·2)	4·59 (4·27–4·94)	1·44 (1·34–1·56)	1·82 (1·6–2·07)
1976	0·1 (0·0–0·2)	46·0 (40·1–52·6)	232·1 (213·8–250·9)	257·4 (246·2–269·4)	175·9 (161·5–190·1)	115·4 (99·6–131·8)	46·2 (37·0–57·0)	7·0 (5·3–9·0)	0·1 (0·1–0·1)	4·4 (4·14–4·69)	1·39 (1·29–1·5)	1·72 (1·53–1·93)
1977	0·1 (0·0–0·2)	42·4 (36·9–48·3)	225·3 (208·6–242·2)	248·0 (236·9–259·3)	169·7 (157·0–181·9)	110·8 (96·9–124·5)	41·6 (33·2–50·8)	5·9 (4·6–7·6)	0·1 (0·1–0·1)	4·22 (4·0–4·44)	1·34 (1·25–1·43)	1·64 (1·47–1·81)
1978	0·1 (0·0–0·2)	39·5 (34·7–44·7)	218·3 (203·5–232·9)	237·7 (228·0–248·2)	165·0 (154·2–175·5)	107·1 (95·7–119·2)	38·2 (30·5–46·5)	5·1 (4·0–6·4)	0·1 (0·1–0·1)	4·06 (3·88–4·23)	1·29 (1·21–1·37)	1·58 (1·44–1·72)
1979	0·1 (0·0–0·2)	36·9 (32·6–41·8)	211·7 (199·4–224·4)	227·3 (218·1–237·0)	161·5 (151·7–171·1)	101·7 (91·5–112·4)	35·7 (28·8–43·4)	4·5 (3·5–5·6)	0·1 (0·1–0·1)	3·9 (3·75–4·05)	1·24 (1·17–1·31)	1·52 (1·41–1·64)
1980	0·1 (0·0–0·2)	33·9 (30·0–38·2)	204·9 (193·3–217·2)	219·2 (210·1–228·0)	158·4 (148·7–167·7)	94·5 (85·3–104·9)	33·6 (27·0–40·7)	3·9 (3·1–4·8)	0·1 (0·1–0·1)	3·74 (3·6–3·89)	1·19 (1·13–1·26)	1·45 (1·36–1·55)
1981	0·1 (0·0–0·2)	31·1 (27·5–34·9)	198·8 (186·6–211·0)	213·9 (204·3–222·6)	154·8 (145·4–163·6)	87·8 (78·7–97·8)	31·8 (25·5–38·7)	3·4 (2·7–4·2)	0·1 (0·1–0·1)	3·61 (3·46–3·75)	1·15 (1·08–1·21)	1·39 (1·31–1·47)
1982	0·1 (0·0–0·2)	29·6 (26·1–33·2)	195·3 (182·4–208·9)	211·9 (202·4–220·8)	149·8 (141·0–158·5)	83·2 (74·0–92·2)	30·3 (24·1–36·8)	3·0 (2·4–3·7)	0·1 (0·1–0·1)	3·52 (3·37–3·65)	1·13 (1·06–1·19)	1·33 (1·25–1·41)
1983	0·1 (0·0–0·2)	29·8 (26·5–33·5)	195·1 (182·4–207·4)	213·5 (204·7–222·1)	143·5 (135·2–151·6)	80·6 (71·7–89·8)	28·7 (22·7–35·3)	2·8 (2·3–3·4)	0·1 (0·1–0·1)	3·47 (3·35–3·6)	1·12 (1·06–1·19)	1·28 (1·2–1·35)
1984	0·1 (0·0–0·2)	30·2 (26·8–34·1)	195·9 (184·6–207·6)	213·7 (205·2–222·0)	136·2 (128·4–143·5)	77·4 (68·4–86·3)	26·3 (21·0–32·4)	2·6 (2·1–3·2)	0·1 (0·0–0·1)	3·41 (3·31–3·53)	1·13 (1·07–1·19)	1·21 (1·14–1·29)
1985	0·1 (0·0–0·2)	29·5 (26·3–33·0)	194·0 (183·3–204·5)	209·9 (201·4–217·7)	130·2 (122·7–137·8)	72·3 (63·9–81·4)	23·2 (18·4–28·5)	2·4 (2·0–3·0)	0·0 (0·0–0·0)	3·31 (3·21–3·42)	1·12 (1·06–1·17)	1·14 (1·07–1·22)
1986	0·1 (0·0–0·2)	28·5 (25·2–31·7)	190·5 (179·2–202·6)	205·7 (197·1–214·4)	126·4 (118·3–134·5)	66·6 (58·7–75·0)	20·5 (16·2–25·4)	2·3 (1·9–2·8)	0·0 (0·0–0·0)	3·2 (3·09–3·31)	1·09 (1·04–1·15)	1·08 (1·0–1·16)
1987	0·1 (0·0–0·2)	28·1 (24·8–31·6)	188·5 (177·0–201·1)	205·4 (197·2–213·9)	124·0 (115·8–132·0)	61·6 (54·2–69·0)	18·8 (14·8–23·4)	2·1 (1·7–2·6)	0·0 (0·0–0·0)	3·14 (3·02–3·27)	1·08 (1·02–1·15)	1·03 (0·95–1·11)
1988	0·1 (0·0–0·2)	29·4 (26·0–32·9)	190·3 (179·3–202·8)	209·3 (201·9–216·8)	122·2 (114·9–129·6)	57·9 (50·9–64·3)	18·1 (14·4–22·4)	2·0 (1·6–2·4)	0·0 (0·0–0·0)	3·15 (3·04–3·26)	1·1 (1·04–1·17)	1·0 (0·93–1·07)
1989	0·1 (0·0–0·2)	31·4 (27·9–35·1)	194·5 (184·0–205·4)	213·1 (205·7–220·1)	120·1 (114·1–126·2)	55·3 (49·3–61·1)	17·6 (14·2–21·8)	1·8 (1·5–2·3)	0·0 (0·0–0·0)	3·17 (3·07–3·26)	1·13 (1·08–1·19)	0·97 (0·92–1·02)
1990	0·1 (0·0–0·2)	32·7 (29·2–36·6)	197·7 (188·0–207·8)	213·3 (206·1–219·9)	117·2 (112·0–122·8)	52·9 (47·5–58·6)	16·9 (13·5–20·7)	1·7 (1·4–2·1)	0·0 (0·0–0·0)	3·16 (3·07–3·25)	1·15 (1·1–1·21)	0·94 (0·9–0·99)
1991	0·1 (0·0–0·2)	33·4 (29·8–37·5)	198·4 (187·8–209·9)	210·8 (203·7–218·3)	113·6 (107·8–119·7)	50·7 (45·1–56·6)	15·9 (12·6–19·5)	1·5 (1·2–1·9)	0·0 (0·0–0·0)	3·12 (3·02–3·23)	1·16 (1·11–1·22)	0·91 (0·87–0·95)
1992	0·1 (0·0–0·2)	34·3 (30·6–38·6)	197·8 (186·8–209·4)	207·6 (200·4–215·3)	110·0 (103·8–116·6)	48·9 (43·3–55·2)	14·9 (11·9–18·2)	1·4 (1·1–1·7)	0·0 (0·0–0·0)	3·08 (2·96–3·2)	1·16 (1·1–1·22)	0·88 (0·83–0·93)
1993	0·1 (0·0–0·2)	35·3 (31·4–39·8)	196·4 (184·8–207·9)	204·7 (196·8–212·5)	106·9 (100·6–113·2)	47·3 (41·8–53·1)	14·0 (11·1–17·3)	1·2 (1·0–1·5)	0·0 (0·0–0·0)	3·03 (2·93–3·15)	1·16 (1·1–1·22)	0·85 (0·8–0·89)
1994	0·1 (0·0–0·2)	35·8 (32·0–40·1)	194·1 (183·7–204·7)	201·0 (193·4–207·8)	103·9 (98·7–109·4)	45·2 (40·6–50·1)	13·1 (10·5–16·3)	1·1 (0·9–1·4)	0·0 (0·0–0·0)	2·97 (2·87–3·07)	1·15 (1·09–1·21)	0·82 (0·78–0·86)
1995	0·1 (0·0–0·2)	35·3 (31·8–39·2)	190·6 (180·4–200·8)	194·3 (187·3–201·3)	100·2 (95·0–105·3)	42·0 (38·0–46·5)	12·4 (10·0–15·3)	1·0 (0·8–1·2)	0·0 (0·0–0·0)	2·88 (2·79–2·97)	1·13 (1·08–1·19)	0·78 (0·74–0·81)
1996	0·1 (0·0–0·2)	34·2 (30·4–38·3)	185·8 (175·2–198·0)	185·3 (178·1–192·7)	95·5 (90·5–101·0)	38·0 (33·9–42·6)	11·6 (9·2–14·5)	0·8 (0·7–1·1)	0·0 (0·0–0·0)	2·76 (2·67–2·84)	1·1 (1·04–1·16)	0·73 (0·7–0·77)
1997	0·1 (0·0–0·2)	32·2 (28·8–36·0)	179·3 (167·8–191·0)	177·8 (170·4–185·4)	89·5 (84·4–94·7)	33·5 (29·8–37·7)	10·5 (8·5–13·0)	0·7 (0·6–0·9)	0·0 (0·0–0·0)	2·62 (2·53–2·71)	1·06 (1·0–1·12)	0·67 (0·64–0·71)
1998	0·1 (0·0–0·2)	29·4 (26·2–32·8)	170·0 (159·0–181·3)	171·6 (164·4–179·0)	83·4 (78·2–88·3)	29·5 (26·4–32·9)	8·9 (7·2–11·0)	0·6 (0·5–0·8)	0·0 (0·0–0·0)	2·47 (2·39–2·54)	1·0 (0·94–1·06)	0·61 (0·58–0·64)
1999	0·1 (0·0–0·2)	26·8 (24·0–29·9)	159·9 (149·6–170·0)	166·1 (159·5–173·3)	79·6 (75·0–84·5)	26·9 (24·3–29·8)	7·5 (6·2–9·2)	0·5 (0·4–0·7)	0·0 (0·0–0·0)	2·34 (2·27–2·4)	0·93 (0·88–0·99)	0·57 (0·55–0·6)
2000	0·1 (0·0–0·2)	25·0 (22·4–27·8)	152·1 (142·6–161·6)	162·4 (155·6–169·1)	78·1 (74·1–82·7)	26·0 (23·5–28·4)	6·7 (5·5–8·2)	0·5 (0·4–0·6)	0·0 (0·0–0·0)	2·25 (2·19–2·32)	0·89 (0·84–0·94)	0·56 (0·53–0·58)
2001	0·1 (0·0–0·1)	23·3 (20·9–26·1)	145·2 (135·1–155·1)	159·4 (152·6–166·2)	77·3 (72·9–81·9)	26·5 (23·7–29·3)	6·5 (5·2–7·8)	0·5 (0·4–0·6)	0·0 (0·0–0·0)	2·19 (2·12–2·27)	0·84 (0·79–0·89)	0·55 (0·52–0·58)
2002	0·1 (0·0–0·1)	21·8 (19·3–24·7)	137·9 (127·2–148·0)	156·3 (148·8–163·3)	75·9 (71·1–81·3)	27·4 (24·5–30·9)	6·4 (5·1–7·7)	0·4 (0·3–0·5)	0·0 (0·0–0·0)	2·13 (2·04–2·22)	0·8 (0·74–0·85)	0·55 (0·52–0·58)
2003	0·1 (0·0–0·1)	20·3 (17·8–22·9)	130·6 (120·3–140·1)	152·5 (144·8–159·4)	72·0 (67·2–77·0)	27·4 (24·6–30·8)	6·3 (5·0–7·5)	0·4 (0·3–0·4)	0·0 (0·0–0·0)	2·05 (1·95–2·14)	0·76 (0·7–0·8)	0·53 (0·5–0·57)
2004	0·1 (0·0–0·1)	18·9 (16·5–21·3)	123·5 (114·0–132·5)	148·1 (140·9–155·5)	66·8 (62·7–71·2)	26·5 (23·8–29·5)	6·0 (4·8–7·2)	0·3 (0·3–0·4)	0·0 (0·0–0·0)	1·95 (1·86–2·04)	0·71 (0·66–0·76)	0·5 (0·47–0·54)
2005	0·1 (0·0–0·1)	18·2 (15·9–20·5)	117·5 (109·2–126·3)	143·6 (136·8–151·1)	64·5 (60·6–68·3)	25·2 (22·8–27·9)	5·5 (4·5–6·8)	0·3 (0·3–0·4)	0·0 (0·0–0·0)	1·88 (1·8–1·95)	0·68 (0·64–0·72)	0·48 (0·45–0·51)
2006	0·1 (0·0–0·1)	17·8 (15·6–20·1)	112·9 (104·8–122·1)	139·8 (132·9–147·1)	64·7 (60·8–68·6)	24·2 (21·8–26·7)	5·2 (4·3–6·4)	0·3 (0·3–0·4)	0·0 (0·0–0·0)	1·83 (1·76–1·89)	0·65 (0·61–0·7)	0·47 (0·44–0·51)
2007	0·1 (0·0–0·1)	17·4 (15·3–19·8)	109·7 (100·7–119·2)	138·1 (130·7–145·2)	65·4 (60·9–70·1)	24·1 (21·4–27·0)	5·2 (4·2–6·4)	0·3 (0·3–0·4)	0·0 (0·0–0·0)	1·8 (1·72–1·87)	0·64 (0·59–0·68)	0·47 (0·44–0·51)
2008	0·1 (0·0–0·1)	17·2 (15·2–19·7)	108·2 (98·5–118·0)	138·5 (130·7–145·7)	66·6 (61·7–71·8)	24·8 (21·9–28·1)	5·3 (4·2–6·6)	0·3 (0·3–0·4)	0·0 (0·0–0·0)	1·81 (1·72–1·88)	0·63 (0·58–0·68)	0·49 (0·45–0·52)
2009	0·1 (0·0–0·1)	17·7 (15·4–20·3)	107·9 (98·1–117·5)	139·8 (132·1–147·4)	68·1 (63·3–73·4)	25·7 (22·7–29·1)	5·4 (4·3–6·8)	0·3 (0·2–0·4)	0·0 (0·0–0·0)	1·82 (1·75–1·9)	0·63 (0·58–0·68)	0·5 (0·46–0·53)
2010	0·1 (0·0–0·1)	18·5 (16·1–21·3)	108·4 (98·4–118·5)	141·6 (133·4–149·6)	69·6 (65·0–74·9)	26·4 (23·2–29·9)	5·5 (4·4–6·9)	0·3 (0·2–0·4)	0·0 (0·0–0·0)	1·85 (1·77–1·93)	0·63 (0·58–0·69)	0·51 (0·48–0·55)
2011	0·1 (0·0–0·1)	19·5 (16·9–22·4)	109·3 (99·1–120·7)	143·3 (135·1–151·6)	71·4 (66·3–76·9)	27·0 (23·7–30·9)	5·5 (4·4–6·8)	0·3 (0·2–0·4)	0·0 (0·0–0·0)	1·88 (1·79–1·98)	0·64 (0·59–0·7)	0·52 (0·49–0·56)
2012	0·1 (0·0–0·1)	20·4 (17·5–23·5)	110·2 (99·4–122·3)	144·4 (135·9–153·6)	72·9 (67·0–79·1)	27·4 (23·8–31·5)	5·5 (4·4–7·0)	0·3 (0·3–0·4)	0·0 (0·0–0·0)	1·91 (1·79–2·03)	0·65 (0·6–0·71)	0·53 (0·5–0·57)
2013	0·1 (0·0–0·1)	20·5 (17·6–23·5)	110·2 (99·0–122·7)	145·0 (136·2–154·8)	73·7 (67·2–80·5)	27·8 (24·1–32·2)	5·6 (4·4–7·2)	0·3 (0·3–0·4)	0·0 (0·0–0·0)	1·92 (1·79–2·06)	0·65 (0·6–0·72)	0·54 (0·49–0·58)
2014	0·1 (0·0–0·1)	20·0 (17·1–23·2)	109·1 (96·4–122·4)	145·0 (134·7–155·8)	73·9 (67·2–81·3)	28·1 (24·0–33·2)	5·7 (4·4–7·4)	0·3 (0·3–0·4)	0·0 (0·0–0·0)	1·91 (1·75–2·08)	0·65 (0·57–0·72)	0·54 (0·49–0·59)
2015	0·1 (0·0–0·1)	19·6 (16·5–23·0)	107·2 (92·8–122·4)	144·7 (134·1–156·0)	74·0 (65·7–82·9)	28·3 (23·3–34·5)	5·8 (4·3–7·7)	0·4 (0·3–0·5)	0·0 (0·0–0·0)	1·9 (1·71–2·1)	0·63 (0·55–0·72)	0·54 (0·48–0·62)
2016	0·1 (0·0–0·1)	19·2 (16·2–22·8)	105·2 (89·5–122·3)	144·8 (132·9–157·2)	74·1 (64·9–84·7)	28·4 (22·4–35·6)	5·9 (4·3–7·9)	0·4 (0·3–0·5)	0·0 (0·0–0·0)	1·89 (1·67–2·14)	0·62 (0·53–0·73)	0·54 (0·46–0·64)
2017	0·1 (0·0–0·1)	19·0 (15·9–22·7)	104·0 (86·8–123·8)	144·9 (132·5–158·3)	73·8 (63·5–85·5)	28·4 (21·9–36·5)	5·9 (4·2–8·1)	0·4 (0·3–0·5)	0·0 (0·0–0·0)	1·88 (1·63–2·18)	0·62 (0·51–0·73)	0·54 (0·45–0·65)

Supplemental Results Table 5: Age-specific fertility rates per 1000, cumulative total fertility up to maternal age 25 (TFU25), cumulative total fertility from maternal age 30 to age 55 (TFO30), and cumulative total fertility, Bosnia and Herzegovina, 1950-2017

Year	10-14	15-19	20-24	25-29	30-34	35-39	40-44	45-49	50-54	TFR	TFU25	TFO30
1950	0·1 (0·1–0·3)	57·6 (48·8–67·9)	216·0 (188·4–245·1)	160·1 (146·2–175·1)	126·3 (109·9–144·3)	80·7 (64·0–99·9)	33·7 (25·4–43·6)	6·3 (4·1–9·2)	0·1 (0·1–0·1)	3·4 (2·93–3·93)	1·37 (1·19–1·57)	1·24 (1·02–1·49)
1951	0·1 (0·1–0·3)	57·2 (48·3–66·9)	213·8 (186·3–242·0)	156·3 (142·3–170·7)	124·5 (109·0–142·6)	78·8 (62·6–97·1)	32·8 (24·6–42·4)	6·1 (4·0–9·0)	0·1 (0·1–0·1)	3·35 (2·9–3·85)	1·36 (1·18–1·54)	1·21 (1·01–1·46)
1952	0·1 (0·1–0·3)	56·8 (47·7–66·8)	211·6 (183·1–240·0)	152·7 (138·2–167·3)	122·8 (106·8–141·2)	77·0 (60·9–95·1)	31·9 (23·7–41·6)	6·0 (3·9–8·8)	0·1 (0·1–0·1)	3·3 (2·85–3·77)	1·34 (1·16–1·53)	1·19 (0·98–1·43)
1953	0·1 (0·1–0·3)	56·4 (47·5–66·2)	209·5 (181·0–237·6)	149·1 (134·7–163·8)	121·0 (104·6–138·6)	75·2 (58·5–93·0)	31·0 (23·0–40·2)	5·8 (3·8–8·7)	0·1 (0·1–0·1)	3·24 (2·81–3·7)	1·33 (1·15–1·51)	1·17 (0·97–1·39)
1954	0·1 (0·1–0·3)	56·0 (47·0–65·4)	207·5 (178·7–235·6)	145·5 (131·6–159·8)	119·1 (102·3–136·9)	73·3 (56·9–91·0)	30·1 (22·0–39·3)	5·7 (3·7–8·5)	0·1 (0·1–0·1)	3·19 (2·79–3·62)	1·32 (1·14–1·5)	1·14 (0·95–1·35)
1955	0·1 (0·1–0·3)	55·6 (47·2–64·9)	205·3 (175·7–232·8)	141·9 (128·4–155·9)	117·2 (100·1–134·5)	71·4 (55·0–88·7)	29·2 (21·3–38·2)	5·5 (3·6–8·2)	0·1 (0·1–0·1)	3·13 (2·73–3·54)	1·31 (1·12–1·48)	1·12 (0·93–1·31)
1956	0·1 (0·1–0·3)	55·3 (46·5–64·7)	203·2 (173·7–231·5)	138·3 (125·1–152·5)	115·2 (98·5–132·7)	69·5 (53·3–86·8)	28·2 (20·5–36·8)	5·4 (3·5–8·0)	0·1 (0·1–0·1)	3·08 (2·68–3·47)	1·29 (1·11–1·48)	1·09 (0·91–1·28)
1957	0·1 (0·1–0·3)	55·0 (46·2–64·4)	201·1 (172·3–229·9)	135·0 (121·8–148·6)	113·2 (96·5–130·5)	67·5 (52·1–84·6)	27·3 (20·1–35·7)	5·2 (3·4–7·8)	0·1 (0·1–0·1)	3·02 (2·63–3·42)	1·28 (1·1–1·46)	1·07 (0·89–1·25)
1958	0·1 (0·1–0·3)	54·7 (45·8–64·5)	199·1 (170·8–226·7)	131·7 (118·8–144·8)	111·3 (95·2–128·5)	65·6 (50·6–82·2)	26·4 (19·3–34·8)	5·1 (3·2–7·5)	0·1 (0·1–0·1)	2·97 (2·59–3·37)	1·27 (1·08–1·45)	1·04 (0·87–1·22)
1959	0·1 (0·1–0·3)	54·4 (45·9–63·8)	197·1 (169·5–224·6)	128·4 (115·7–141·5)	109·4 (94·2–125·9)	63·7 (49·2–79·7)	25·6 (18·9–33·6)	4·9 (3·2–7·3)	0·1 (0·1–0·1)	2·92 (2·53–3·31)	1·26 (1·08–1·44)	1·02 (0·86–1·19)
1960	0·1 (0·1–0·3)	54·1 (45·3–63·4)	195·0 (167·1–222·7)	125·1 (112·5–138·1)	107·5 (92·1–123·7)	61·8 (47·8–77·5)	24·7 (18·1–32·7)	4·7 (3·0–7·0)	0·1 (0·1–0·1)	2·87 (2·49–3·23)	1·25 (1·06–1·43)	0·99 (0·84–1·16)
1961	0·1 (0·1–0·3)	53·7 (44·9–62·6)	193·0 (165·8–221·1)	121·8 (109·3–134·7)	105·5 (90·6–122·5)	59·9 (45·6–75·2)	23·7 (17·4–31·7)	4·6 (2·9–6·7)	0·1 (0·1–0·1)	2·81 (2·46–3·18)	1·23 (1·06–1·41)	0·97 (0·82–1·14)
1962	0·1 (0·1–0·3)	53·4 (45·0–62·4)	190·9 (163·4–219·1)	118·7 (106·5–132·2)	103·6 (88·6–121·8)	58·0 (44·3–72·9)	22·8 (16·7–30·6)	4·4 (2·8–6·5)	0·1 (0·1–0·1)	2·76 (2·42–3·12)	1·22 (1·05–1·41)	0·94 (0·8–1·11)
1963	0·1 (0·1–0·3)	53·0 (45·1–62·4)	188·9 (161·7–216·6)	115·6 (103·3–129·4)	101·6 (86·7–118·9)	56·2 (43·3–70·5)	21·9 (16·0–29·4)	4·2 (2·7–6·4)	0·1 (0·1–0·1)	2·71 (2·36–3·09)	1·21 (1·04–1·39)	0·92 (0·78–1·09)
1964	0·1 (0·1–0·3)	52·8 (44·6–62·3)	186·8 (159·8–215·6)	112·6 (101·2–126·5)	99·7 (85·0–116·7)	54·3 (42·3–69·6)	21·0 (15·4–28·3)	4·1 (2·6–6·1)	0·1 (0·1–0·1)	2·66 (2·3–3·04)	1·2 (1·03–1·38)	0·9 (0·75–1·07)
1965	0·1 (0·1–0·3)	52·5 (44·3–62·1)	184·9 (157·9–213·9)	109·8 (98·3–123·2)	97·8 (83·3–114·1)	52·5 (40·4–67·5)	20·2 (14·5–27·2)	3·9 (2·5–5·9)	0·1 (0·1–0·1)	2·61 (2·24–3·02)	1·19 (1·01–1·38)	0·87 (0·72–1·06)
1966	0·1 (0·1–0·3)	52·2 (44·2–62·5)	183·0 (154·9–213·4)	108·1 (96·7–121·4)	95·9 (81·3–112·3)	50·8 (38·6–66·2)	19·4 (13·9–26·5)	3·8 (2·4–5·7)	0·1 (0·1–0·1)	2·57 (2·19–3·0)	1·18 (1·0–1·36)	0·85 (0·69–1·04)
1967	0·1 (0·1–0·3)	51·9 (43·8–62·5)	181·1 (153·4–212·0)	107·9 (96·3–121·0)	94·1 (79·3–110·7)	49·0 (37·1–64·9)	18·5 (13·4–25·6)	3·6 (2·3–5·5)	0·1 (0·1–0·1)	2·53 (2·15–2·99)	1·17 (0·99–1·36)	0·83 (0·67–1·02)
1968	0·1 (0·1–0·3)	51·6 (43·4–61·9)	179·2 (152·8–210·6)	108·0 (96·6–121·3)	92·2 (78·5–108·5)	47·2 (35·8–62·6)	17·7 (12·8–24·7)	3·4 (2·2–5·3)	0·1 (0·1–0·1)	2·5 (2·11–2·98)	1·15 (0·98–1·36)	0·8 (0·65–1·01)
1969	0·1 (0·1–0·2)	51·2 (43·1–61·6)	177·2 (151·1–207·8)	108·2 (96·8–121·9)	90·2 (76·6–107·1)	45·3 (34·8–59·7)	16·9 (12·2–23·3)	3·2 (2·1–5·0)	0·1 (0·1–0·1)	2·46 (2·09–2·93)	1·14 (0·97–1·35)	0·78 (0·63–0·97)
1970	0·1 (0·1–0·2)	50·8 (42·9–60·9)	175·2 (148·9–206·0)	108·3 (97·4–121·8)	88·1 (74·7–104·3)	43·4 (33·0–57·1)	16·0 (11·5–22·2)	3·1 (2·0–4·7)	0·1 (0·1–0·1)	2·43 (2·07–2·87)	1·13 (0·97–1·34)	0·75 (0·61–0·93)
1971	0·1 (0·1–0·2)	50·4 (42·5–60·2)	173·1 (147·3–204·6)	108·3 (97·7–122·3)	85·9 (72·3–101·6)	41·4 (31·5–54·2)	15·1 (10·8–20·9)	2·9 (1·9–4·4)	0·1 (0·1–0·1)	2·39 (2·05–2·82)	1·12 (0·96–1·32)	0·73 (0·59–0·9)
1972	0·1 (0·1–0·2)	50·0 (42·1–59·4)	170·9 (145·1–201·7)	108·4 (97·4–122·1)	83·6 (70·6–98·2)	39·5 (30·1–52·2)	14·3 (10·5–19·4)	2·7 (1·8–4·2)	0·1 (0·1–0·1)	2·35 (2·04–2·76)	1·11 (0·95–1·31)	0·7 (0·57–0·86)
1973	0·1 (0·1–0·2)	49·7 (41·8–58·6)	168·9 (143·3–198·0)	108·6 (97·8–122·2)	81·5 (68·8–96·1)	37·6 (28·4–49·6)	13·4 (9·6–18·1)	2·6 (1·7–3·9)	0·0 (0·0–0·1)	2·31 (2·0–2·7)	1·09 (0·93–1·28)	0·68 (0·56–0·82)
1974	0·1 (0·1–0·2)	49·3 (41·8–58·5)	166·8 (141·9–195·5)	108·8 (97·7–121·6)	79·3 (66·7–93·2)	35·7 (27·2–46·6)	12·6 (9·0–16·9)	2·4 (1·6–3·5)	0·0 (0·0–0·0)	2·28 (1·98–2·63)	1·08 (0·93–1·26)	0·65 (0·54–0·78)
1975	0·1 (0·1–0·2)	48·9 (41·3–57·9)	164·7 (140·4–192·6)	109·0 (97·8–122·1)	77·2 (64·9–90·0)	33·9 (25·9–44·6)	11·9 (8·4–15·9)	2·2 (1·5–3·2)	0·0 (0·0–0·0)	2·24 (1·96–2·57)	1·07 (0·92–1·24)	0·63 (0·53–0·75)
1976	0·1 (0·1–0·2)	48·5 (40·9–57·5)	162·6 (138·7–190·4)	109·2 (98·2–121·3)	75·0 (63·3–87·5)	32·2 (24·4–42·2)	11·1 (7·9–14·8)	2·1 (1·4–3·0)	0·0 (0·0–0·0)	2·2 (1·94–2·52)	1·06 (0·9–1·23)	0·6 (0·51–0·71)
1977	0·1 (0·1–0·2)	48·1 (40·9–57·0)	160·4 (136·4–186·5)	109·5 (98·6–121·9)	73·0 (61·7–84·9)	30·5 (23·1–39·6)	10·3 (7·5–13·7)	1·9 (1·3–2·8)	0·0 (0·0–0·0)	2·17 (1·91–2·48)	1·04 (0·89–1·22)	0·58 (0·49–0·67)
1978	0·1 (0·1–0·2)	47·8 (40·6–56·6)	158·3 (135·1–183·6)	109·8 (99·1–121·9)	71·0 (59·9–82·6)	28·9 (22·1–37·4)	9·6 (7·0–12·8)	1·8 (1·2–2·6)	0·0 (0·0–0·0)	2·14 (1·89–2·43)	1·03 (0·88–1·2)	0·56 (0·48–0·65)
1979	0·1 (0·1–0·2)	47·7 (40·5–56·1)	156·2 (133·6–180·2)	110·2 (99·4–122·1)	69·1 (58·6–80·2)	27·3 (20·9–35·0)	9·0 (6·7–11·7)	1·7 (1·1–2·3)	0·0 (0·0–0·0)	2·11 (1·86–2·38)	1·02 (0·87–1·18)	0·54 (0·46–0·62)
1980	0·1 (0·1–0·2)	47·3 (40·2–55·3)	154·2 (131·5–176·5)	110·7 (100·3–121·7)	67·3 (57·4–77·6)	25·8 (20·1–32·4)	8·4 (6·3–10·7)	1·5 (1·1–2·1)	0·0 (0·0–0·0)	2·08 (1·84–2·33)	1·01 (0·86–1·16)	0·52 (0·45–0·59)
1981	0·1 (0·1–0·2)	46·1 (39·5–53·6)	152·3 (131·3–172·6)	111·3 (100·7–122·0)	65·5 (56·3–74·9)	24·4 (19·2–30·1)	7·8 (6·0–9·9)	1·4 (1·0–1·9)	0·0 (0·0–0·0)	2·04 (1·82–2·28)	0·99 (0·85–1·13)	0·5 (0·44–0·56)
1982	0·1 (0·0–0·2)	44·8 (38·2–51·9)	150·5 (131·4–169·1)	111·9 (101·8–122·3)	63·8 (55·7–72·4)	23·0 (18·5–27·8)	7·2 (5·7–9·1)	1·3 (1·0–1·7)	0·0 (0·0–0·0)	2·01 (1·8–2·22)	0·98 (0·85–1·1)	0·48 (0·42–0·53)
1983	0·1 (0·0–0·2)	43·7 (37·4–50·5)	148·6 (131·9–165·6)	112·5 (103·3–121·7)	62·0 (54·6–70·4)	21·9 (18·1–26·2)	6·8 (5·5–8·3)	1·2 (0·9–1·6)	0·0 (0·0–0·0)	1·98 (1·79–2·18)	0·96 (0·85–1·07)	0·46 (0·41–0·51)
1984	0·1 (0·0–0·2)	42·8 (37·0–48·7)	146·6 (131·9–160·8)	113·0 (104·4–121·5)	60·4 (53·7–67·7)	21·3 (17·9–25·0)	6·5 (5·4–7·7)	1·1 (0·8–1·4)	0·0 (0·0–0·0)	1·96 (1·79–2·13)	0·95 (0·85–1·05)	0·45 (0·41–0·49)
1985	0·1 (0·0–0·2)	42·0 (36·8–47·6)	144·5 (132·0–157·1)	113·5 (105·6–121·2)	59·1 (52·8–65·5)	20·9 (18·1–23·9)	6·2 (5·4–7·2)	1·0 (0·8–1·3)	0·0 (0·0–0·0)	1·94 (1·8–2·08)	0·93 (0·85–1·02)	0·44 (0·4–0·47)
1986	0·1 (0·0–0·2)	41·0 (36·4–45·6)	142·6 (132·5–153·3)	113·8 (106·9–120·4)	58·0 (52·5–63·6)	20·5 (18·1–23·2)	5·9 (5·2–6·7)	0·9 (0·7–1·1)	0·0 (0·0–0·0)	1·91 (1·8–2·03)	0·92 (0·85–0·99)	0·43 (0·39–0·46)
1987	0·1 (0·0–0·2)	39·9 (35·9–44·1)	140·5 (132·3–148·7)	113·8 (107·9–119·2)	57·0 (52·6–61·8)	20·2 (18·0–22·5)	5·7 (5·0–6·4)	0·8 (0·6–1·0)	0·0 (0·0–0·0)	1·89 (1·8–1·99)	0·9 (0·85–0·96)	0·42 (0·39–0·45)
1988	0·1 (0·0–0·2)	39·0 (35·6–42·7)	138·1 (132·2–144·3)	113·5 (109·0–117·7)	56·0 (52·4–59·9)	20·0 (17·8–22·3)	5·4 (4·7–6·2)	0·7 (0·6–0·9)	0·0 (0·0–0·0)	1·86 (1·8–1·94)	0·89 (0·85–0·93)	0·41 (0·38–0·44)
1989	0·1 (0·0–0·2)	38·3 (35·4–41·4)	135·2 (131·4–139·2)	112·7 (109·4–115·8)	55·0 (52·0–58·2)	20·0 (17·8–22·5)	5·2 (4·4–6·0)	0·7 (0·5–0·9)	0·0 (0·0–0·0)	1·84 (1·78–1·89)	0·87 (0·84–0·9)	0·4 (0·38–0·43)
1990	0·1 (0·0–0·2)	37·3 (34·7–40·0)	131·3 (128·4–134·4)	111·2 (108·2–114·0)	53·9 (51·1–56·7)	20·5 (18·1–23·1)	5·1 (4·2–6·0)	0·6 (0·4–0·8)	0·0 (0·0–0·0)	1·8 (1·75–1·85)	0·84 (0·82–0·87)	0·4 (0·38–0·42)
1991	0·1 (0·0–0·2)	35·9 (33·2–38·5)	126·8 (122·8–130·8)	108·9 (105·9–111·9)	52·6 (49·6–55·6)	21·2 (18·8–24·0)	5·0 (4·1–6·1)	0·5 (0·4–0·7)	0·0 (0·0–0·0)	1·76 (1·7–1·8)	0·81 (0·78–0·84)	0·4 (0·37–0·42)
1992	0·1 (0·0–0·2)	34·6 (31·9–37·2)	122·8 (117·3–127·7)	106·5 (102·8–110·0)	51·7 (48·4–54·9)	22·1 (19·4–25·2)	4·9 (4·0–6·2)	0·5 (0·3–0·6)	0·0 (0·0–0·0)	1·72 (1·66–1·77)	0·79 (0·75–0·83)	0·4 (0·37–0·42)
1993	0·1 (0·0–0·2)	33·8 (31·1–36·5)	119·7 (114·0–125·2)	104·2 (100·3–107·8)	52·0 (48·8–55·3)	22·9 (20·1–25·8)	4·8 (3·8–6·1)	0·4 (0·3–0·6)	0·0 (0·0–0·0)	1·69 (1·64–1·74)	0·77 (0·73–0·81)	0·4 (0·38–0·42)
1994	0·1 (0·0–0·2)	33·2 (30·6–35·9)	117·4 (112·2–122·2)	101·9 (98·3–105·7)	53·3 (50·0–56·6)	23·5 (20·9–26·4)	4·7 (3·7–5·9)	0·4 (0·3–0·6)	0·0 (0·0–0·0)	1·67 (1·62–1·72)	0·75 (0·72–0·79)	0·41 (0·39–0·43)
1995	0·1 (0·0–0·2)	32·2 (29·8–34·7)	115·4 (111·2–119·4)	99·4 (96·0–102·7)	54·1 (51·1–57·0)	23·8 (21·5–26·5)	4·6 (3·6–5·8)	0·4 (0·3–0·5)	0·0 (0·0–0·0)	1·65 (1·61–1·69)	0·74 (0·71–0·77)	0·41 (0·4–0·44)
1996	0·1 (0·0–0·2)	30·0 (27·9–32·3)	112·2 (109·2–115·3)	96·2 (93·3–99·1)	54·1 (51·5–56·6)	23·6 (21·5–26·0)	4·5 (3·6–5·6)	0·4 (0·3–0·5)	0·0 (0·0–0·0)	1·61 (1·57–1·64)	0·71 (0·69–0·73)	0·41 (0·4–0·43)
1997	0·1 (0·0–0·2)	26·9 (25·2–29·0)	106·7 (104·0–109·4)	92·2 (89·8–94·7)	53·1 (50·8–55·2)	22·8 (21·0–25·1)	4·4 (3·5–5·4)	0·4 (0·3–0·5)	0·0 (0·0–0·0)	1·53 (1·5–1·56)	0·67 (0·65–0·69)	0·4 (0·39–0·42)
1998	0·1 (0·0–0·2)	24·0 (22·3–25·8)	100·0 (97·5–102·6)	88·0 (85·8–90·2)	51·1 (49·0–53·0)	21·9 (20·1–23·9)	4·3 (3·5–5·2)	0·4 (0·3–0·5)	0·0 (0·0–0·0)	1·45 (1·42–1·47)	0·62 (0·6–0·64)	0·39 (0·37–0·4)
1999	0·1 (0·0–0·2)	21·6 (20·0–23·3)	93·6 (91·1–96·1)	83·8 (81·8–86·0)	48·8 (46·6–50·7)	20·9 (19·2–22·9)	4·2 (3·4–5·0)	0·4 (0·3–0·5)	0·0 (0·0–0·0)	1·37 (1·34–1·39)	0·58 (0·56–0·59)	0·37 (0·36–0·38)
2000	0·1 (0·0–0·1)	19·7 (18·2–21·4)	87·8 (85·4–90·0)	80·1 (78·0–82·3)	46·9 (44·8–48·9)	20·3 (18·6–22·0)	4·2 (3·5–4·9)	0·4 (0·3–0·5)	0·0 (0·0–0·0)	1·3 (1·27–1·32)	0·54 (0·52–0·55)	0·36 (0·34–0·37)
2001	0·1 (0·0–0·1)	18·1 (16·7–19·8)	82·6 (80·3–85·0)	77·0 (74·9–79·1)	45·9 (43·8–48·0)	20·0 (18·3–21·8)	4·2 (3·6–4·9)	0·4 (0·3–0·5)	0·0 (0·0–0·0)	1·24 (1·22–1·27)	0·5 (0·49–0·52)	0·35 (0·34–0·37)
2002	0·1 (0·0–0·1)	16·9 (15·5–18·5)	78·2 (75·8–80·8)	75·1 (73·1–77·3)	45·8 (43·7–47·9)	20·0 (18·3–21·8)	4·2 (3·6–4·9)	0·4 (0·3–0·5)	0·0 (0·0–0·0)	1·2 (1·18–1·23)	0·48 (0·46–0·49)	0·35 (0·34–0·37)
2003	0·1 (0·0–0·1)	16·2 (14·8–17·8)	75·1 (72·8–77·6)	74·7 (72·8–76·9)	46·3 (44·4–48·5)	20·1 (18·5–21·9)	4·2 (3·7–4·9)	0·4 (0·2–0·5)	0·0 (0·0–0·0)	1·19 (1·16–1·21)	0·46 (0·44–0·47)	0·36 (0·34–0·37)
2004	0·1 (0·0–0·1)	15·8 (14·3–17·4)	73·1 (70·9–75·6)	75·7 (73·8–77·9)	47·7 (45·7–49·8)	20·4 (18·7–22·3)	4·3 (3·7–4·9)	0·3 (0·2–0·5)	0·0 (0·0–0·0)	1·19 (1·16–1·22)	0·44 (0·43–0·46)	0·36 (0·34–0·39)
2005	0·1 (0·0–0·1)	15·4 (13·9–17·1)	71·6 (69·3–74·0)	77·7 (75·7–79·9)	49·9 (47·9–52·1)	20·7 (19·0–22·6)	4·3 (3·8–5·0)	0·3 (0·2–0·5)	0·0 (0·0–0·0)	1·2 (1·17–1·23)	0·44 (0·42–0·45)	0·38 (0·36–0·4)
2006	0·1 (0·0–0·1)	14·9 (13·5–16·6)	70·1 (67·7–72·6)	80·1 (78·0–82·2)	52·6 (50·6–54·8)	21·2 (19·4–23·1)	4·4 (3·8–5·0)	0·3 (0·2–0·4)	0·0 (0·0–0·0)	1·22 (1·2–1·24)	0·43 (0·41–0·44)	0·39 (0·38–0·41)
2007	0·1 (0·0–0·1)	14·4 (13·0–16·0)	68·3 (65·9–70·9)	82·4 (80·4–84·5)	55·2 (53·1–57·3)	21·7 (19·9–23·7)	4·5 (3·9–5·2)	0·3 (0·2–0·4)	0·0 (0·0–0·0)	1·23 (1·21–1·26)	0·41 (0·4–0·43)	0·41 (0·39–0·42)
2008	0·1 (0·0–0·1)	13·6 (12·2–15·2)	66·0 (63·6–68·5)	84·5 (82·2–86·4)	57·7 (55·5–59·8)	22·5 (20·6–24·6)	4·7 (4·0–5·4)	0·3 (0·2–0·4)	0·0 (0·0–0·0)	1·25 (1·22–1·27)	0·4 (0·38–0·41)	0·43 (0·41–0·44)
2009	0·1 (0·0–0·1)	12·4 (11·2–13·9)	63·0 (60·6–65·4)	86·2 (83·9–88·4)	60·6 (58·2–63·0)	23·5 (21·6–25·8)	4·8 (4·2–5·6)	0·3 (0·2–0·4)	0·0 (0·0–0·0)	1·25 (1·22–1·29)	0·38 (0·36–0·39)	0·45 (0·43–0·47)
2010	0·1 (0·0–0·1)	11·4 (10·2–12·7)	59·5 (57·1–61·9)	87·9 (85·6–90·1)	63·9 (61·5–66·3)	24·7 (22·7–26·9)	5·0 (4·3–5·8)	0·3 (0·2–0·4)	0·0 (0·0–0·0)	1·26 (1·23–1·3)	0·35 (0·34–0·37)	0·47 (0·45–0·49)
2011	0·1 (0·0–0·1)	11·0 (9·7–12·3)	56·6 (54·2–59·1)	89·3 (86·9–91·6)	66·7 (64·2–69·2)	25·6 (23·5–27·8)	5·1 (4·4–5·9)	0·3 (0·2–0·4)	0·0 (0·0–0·0)	1·27 (1·24–1·31)	0·34 (0·32–0·35)	0·49 (0·47–0·51)
2012	0·1 (0·0–0·1)	10·7 (9·4–12·0)	53·9 (51·5–56·5)	89·9 (87·5–92·1)	69·2 (66·6–71·6)	26·4 (24·3–28·6)	5·1 (4·4–6·0)	0·3 (0·2–0·4)	0·0 (0·0–0·0)	1·28 (1·24–1·31)	0·32 (0·31–0·34)	0·51 (0·49–0·52)
2013	0·1 (0·0–0·1)	10·4 (9·1–11·8)	51·4 (48·7–54·2)	89·6 (87·2–92·1)	71·3 (68·8–73·9)	27·0 (24·8–29·4)	5·2 (4·4–6·1)	0·3 (0·2–0·4)	0·0 (0·0–0·0)	1·28 (1·24–1·31)	0·31 (0·29–0·33)	0·52 (0·5–0·54)
2014	0·1 (0·0–0·1)	10·1 (8·8–11·5)	49·5 (46·5–52·9)	88·9 (86·2–92·0)	72·8 (69·8–75·8)	27·4 (25·0–30·1)	5·1 (4·3–6·0)	0·3 (0·2–0·5)	0·0 (0·0–0·0)	1·27 (1·23–1·31)	0·3 (0·28–0·32)	0·53 (0·51–0·55)
2015	0·1 (0·0–0·1)	10·1 (8·7–11·6)	48·5 (44·9–52·7)	88·3 (84·8–92·2)	73·7 (69·6–77·6)	27·6 (24·8–30·7)	5·0 (4·2–6·0)	0·3 (0·2–0·5)	0·0 (0·0–0·0)	1·27 (1·21–1·33)	0·29 (0·27–0·32)	0·53 (0·51–0·56)
2016	0·1 (0·0–0·1)	10·1 (8·7–11·6)	48·2 (43·4–53·1)	87·7 (83·2–92·4)	73·9 (68·6–79·3)	27·4 (23·9–31·2)	4·9 (4·0–5·9)	0·3 (0·2–0·5)	0·0 (0·0–0·0)	1·26 (1·19–1·34)	0·29 (0·26–0·32)	0·53 (0·5–0·57)
2017	0·1 (0·0–0·1)	10·1 (8·6–11·7)	48·3 (42·7–54·6)	87·1 (81·7–92·8)	74·0 (67·7–80·7)	27·3 (23·2–31·9)	4·8 (3·9–5·9)	0·3 (0·2–0·5)	0·0 (0·0–0·0)	1·26 (1·17–1·36)	0·29 (0·26–0·33)	0·53 (0·49–0·58)

Supplemental Results Table 5: Age-specific fertility rates per 1000, cumulative total fertility up to maternal age 25 (TFU25), cumulative total fertility from maternal age 30 to age 55 (TFO30), and cumulative total fertility, Bulgaria, 1950-2017

Year	10-14	15-19	20-24	25-29	30-34	35-39	40-44	45-49	50-54	TFR	TFU25	TFO30
1950	0·9 (0·4–1·8)	53·9 (51·7–56·0)	188·7 (186·1–191·4)	155·7 (153·7–158·2)	80·2 (77·5–82·8)	44·3 (41·7–46·7)	14·5 (12·5–16·9)	2·6 (1·8–3·7)	0·1 (0·0–0·1)	2·7 (2·67–2·73)	1·22 (1·2–1·24)	0·71 (0·68–0·73)
1951	0·9 (0·4–1·8)	53·2 (51·2–55·4)	173·0 (170·5–175·4)	139·8 (137·5–142·0)	72·9 (70·5–75·4)	37·5 (35·1–40·0)	12·5 (10·7–14·4)	2·3 (1·6–3·2)	0·0 (0·0–0·0)	2·46 (2·43–2·49)	1·14 (1·12–1·15)	0·63 (0·6–0·65)
1952	0·9 (0·4–1·8)	54·9 (53·0–57·0)	171·1 (168·7–173·7)	134·9 (132·7–137·1)	70·2 (67·9–72·6)	33·4 (31·1–36·0)	11·5 (9·8–13·3)	2·1 (1·4–2·9)	0·0 (0·0–0·0)	2·4 (2·37–2·43)	1·13 (1·12–1·15)	0·59 (0·57–0·61)
1953	0·9 (0·4–1·9)	56·3 (54·3–58·5)	167·4 (164·8–170·0)	131·3 (129·2–133·4)	67·8 (65·6–70·3)	30·6 (28·4–33·0)	10·6 (9·1–12·4)	1·9 (1·3–2·6)	0·0 (0·0–0·0)	2·33 (2·31–2·36)	1·12 (1·1–1·14)	0·55 (0·54–0·57)
1954	0·9 (0·4–1·9)	57·8 (55·7–60·2)	169·3 (166·7–171·8)	128·9 (126·7–131·0)	65·4 (63·1–67·7)	29·7 (27·6–32·3)	10·3 (8·7–11·9)	1·8 (1·2–2·5)	0·0 (0·0–0·0)	2·32 (2·29–2·35)	1·14 (1·12–1·16)	0·54 (0·52–0·56)
1955	0·9 (0·4–1·9)	58·9 (56·9–61·2)	175·2 (172·8–177·7)	125·3 (123·1–127·7)	61·3 (59·1–63·5)	28·6 (26·3–31·1)	9·6 (8·0–11·4)	1·7 (1·1–2·4)	0·0 (0·0–0·0)	2·31 (2·28–2·34)	1·18 (1·16–1·19)	0·51 (0·49–0·53)
1956	0·9 (0·4–1·9)	59·7 (57·7–62·1)	176·1 (173·7–178·7)	119·7 (117·5–121·8)	55·9 (53·9–58·0)	26·0 (23·8–28·3)	8·5 (6·9–10·0)	1·5 (1·0–2·2)	0·0 (0·0–0·0)	2·24 (2·21–2·27)	1·18 (1·17–1·2)	0·46 (0·44–0·48)
1957	0·9 (0·4–2·0)	61·6 (59·4–63·7)	174·5 (172·0–177·1)	115·3 (113·1–117·5)	51·9 (49·8–54·0)	23·2 (21·2–25·5)	7·4 (6·0–8·9)	1·3 (0·9–2·0)	0·0 (0·0–0·0)	2·18 (2·15–2·21)	1·19 (1·17–1·2)	0·42 (0·4–0·44)
1958	1·0 (0·4–2·0)	65·6 (63·3–67·8)	175·0 (172·4–177·7)	114·0 (111·9–116·1)	50·0 (47·9–52·0)	21·4 (19·6–23·4)	6·9 (5·5–8·4)	1·2 (0·8–1·8)	0·0 (0·0–0·0)	2·18 (2·15–2·2)	1·21 (1·19–1·22)	0·4 (0·38–0·41)
1959	1·0 (0·4–2·1)	69·6 (67·4–71·8)	178·7 (176·1–181·3)	115·3 (113·3–117·6)	49·6 (47·6–51·8)	20·4 (18·5–22·2)	6·6 (5·3–8·1)	1·1 (0·7–1·7)	0·0 (0·0–0·0)	2·21 (2·19–2·24)	1·25 (1·23–1·26)	0·39 (0·37–0·4)
1960	1·0 (0·4–2·1)	69·8 (67·7–72·0)	180·8 (178·1–183·4)	116·6 (114·5–118·8)	49·7 (47·6–52·0)	19·4 (17·6–21·2)	6·3 (5·0–7·8)	1·0 (0·6–1·5)	0·0 (0·0–0·0)	2·22 (2·2–2·25)	1·26 (1·24–1·28)	0·38 (0·36–0·4)
1961	1·0 (0·4–2·1)	67·6 (65·4–69·7)	178·9 (176·2–181·7)	115·4 (113·2–117·7)	49·4 (47·5–51·4)	18·4 (16·7–20·3)	5·8 (4·6–7·2)	0·9 (0·6–1·4)	0·0 (0·0–0·0)	2·19 (2·16–2·21)	1·24 (1·22–1·25)	0·37 (0·36–0·39)
1962	1·0 (0·4–2·0)	66·5 (64·4–68·7)	177·0 (174·2–179·8)	112·9 (110·6–115·2)	48·9 (46·9–51·1)	17·9 (16·2–20·0)	5·5 (4·3–6·8)	0·8 (0·5–1·3)	0·0 (0·0–0·0)	2·15 (2·12–2·19)	1·22 (1·21–1·24)	0·37 (0·35–0·38)
1963	1·0 (0·4–2·1)	67·1 (64·9–69·2)	177·4 (174·8–180·2)	109·3 (107·1–111·5)	47·7 (45·8–49·9)	17·4 (15·5–19·3)	5·0 (3·9–6·3)	0·8 (0·5–1·2)	0·0 (0·0–0·0)	2·13 (2·1–2·16)	1·23 (1·21–1·25)	0·35 (0·34–0·37)
1964	1·0 (0·4–2·0)	66·2 (64·0–68·3)	173·7 (171·0–176·5)	104·5 (102·2–106·6)	44·7 (42·8–46·9)	16·0 (14·3–17·6)	4·3 (3·4–5·5)	0·7 (0·4–1·1)	0·0 (0·0–0·0)	2·06 (2·03–2·08)	1·2 (1·19–1·22)	0·33 (0·31–0·34)
1965	1·0 (0·4–2·0)	63·8 (61·8–65·9)	166·7 (164·2–169·3)	100·7 (98·5–102·8)	41·4 (39·4–43·5)	14·7 (13·1–16·3)	3·8 (2·9–4·9)	0·6 (0·4–1·0)	0·0 (0·0–0·0)	1·96 (1·94–1·99)	1·16 (1·14–1·17)	0·3 (0·29–0·32)
1966	1·0 (0·4–2·0)	64·5 (62·4–66·7)	166·5 (164·0–169·2)	101·0 (98·7–103·4)	41·0 (39·2–43·0)	14·7 (13·1–16·5)	3·8 (2·9–4·9)	0·6 (0·4–0·9)	0·0 (0·0–0·0)	1·97 (1·93–2·0)	1·16 (1·14–1·18)	0·3 (0·29–0·31)
1967	1·0 (0·4–2·1)	68·7 (66·7–70·6)	177·7 (175·2–180·2)	108·9 (106·5–111·2)	43·6 (41·5–45·6)	15·7 (14·0–17·5)	3·9 (3·0–5·2)	0·5 (0·3–0·9)	0·0 (0·0–0·0)	2·1 (2·07–2·13)	1·24 (1·22–1·25)	0·32 (0·3–0·33)
1968	1·0 (0·4–2·1)	71·1 (69·1–73·1)	185·8 (183·4–188·2)	114·6 (112·4–117·0)	45·5 (43·6–47·7)	15·6 (14·0–17·6)	3·8 (2·8–5·0)	0·5 (0·3–0·8)	0·0 (0·0–0·0)	2·19 (2·16–2·22)	1·29 (1·27–1·31)	0·33 (0·31–0·34)
1969	1·0 (0·4–2·1)	70·5 (68·5–72·7)	182·1 (179·4–184·7)	111·4 (109·2–113·5)	45·0 (43·1–47·2)	14·5 (12·9–16·2)	3·4 (2·5–4·4)	0·4 (0·3–0·7)	0·0 (0·0–0·0)	2·14 (2·12–2·17)	1·27 (1·25–1·29)	0·32 (0·3–0·33)
1970	1·0 (0·4–2·1)	68·8 (66·8–70·9)	177·8 (175·4–180·3)	105·2 (103·1–107·5)	42·4 (40·5–44·5)	13·3 (11·9–15·0)	3·0 (2·3–4·0)	0·4 (0·2–0·6)	0·0 (0·0–0·0)	2·06 (2·04–2·09)	1·24 (1·22–1·26)	0·3 (0·28–0·31)
1971	1·0 (0·4–2·1)	67·9 (65·8–69·8)	174·4 (171·8–176·8)	98·6 (96·5–100·8)	39·7 (37·6–41·8)	12·5 (11·0–14·3)	2·8 (2·1–3·7)	0·3 (0·2–0·5)	0·0 (0·0–0·0)	1·99 (1·96–2·01)	1·22 (1·2–1·23)	0·28 (0·26–0·29)
1972	1·0 (0·4–2·1)	69·9 (67·7–72·1)	178·9 (176·4–181·7)	100·9 (98·7–103·0)	39·8 (37·7–42·0)	13·2 (11·6–15·0)	3·0 (2·2–3·9)	0·3 (0·2–0·5)	0·0 (0·0–0·0)	2·03 (2·01–2·06)	1·25 (1·23–1·27)	0·28 (0·27–0·3)
1973	1·0 (0·4–2·2)	73·1 (71·0–75·2)	192·3 (189·6–194·8)	109·7 (107·4–111·9)	41·3 (39·2–43·4)	14·1 (12·5–15·9)	3·1 (2·3–4·2)	0·3 (0·2–0·4)	0·0 (0·0–0·0)	2·17 (2·15–2·2)	1·33 (1·31–1·35)	0·29 (0·28–0·31)
1974	1·0 (0·5–2·2)	74·6 (72·5–76·9)	197·1 (194·5–199·7)	111·7 (109·7–113·9)	41·4 (39·4–43·4)	14·1 (12·7–15·7)	3·0 (2·2–4·0)	0·3 (0·2–0·4)	0·0 (0·0–0·0)	2·22 (2·19–2·24)	1·36 (1·35–1·38)	0·29 (0·28–0·31)
1975	1·0 (0·5–2·2)	75·2 (73·0–77·4)	197·1 (194·6–199·4)	109·4 (107·3–111·5)	40·1 (38·2–42·2)	13·6 (12·0–15·3)	2·8 (2·1–3·8)	0·2 (0·2–0·4)	0·0 (0·0–0·0)	2·2 (2·17–2·22)	1·37 (1·35–1·38)	0·28 (0·27–0·3)
1976	1·1 (0·5–2·2)	75·9 (73·8–78·2)	199·0 (196·5–201·5)	105·4 (103·3–107·4)	38·1 (36·1–40·0)	12·7 (11·2–14·5)	2·7 (2·0–3·7)	0·2 (0·1–0·3)	0·0 (0·0–0·0)	2·18 (2·15–2·2)	1·38 (1·36–1·4)	0·27 (0·26–0·28)
1977	1·1 (0·5–2·2)	76·8 (74·8–79·1)	198·5 (196·0–201·1)	100·5 (98·4–102·6)	36·0 (34·1–37·9)	11·8 (10·2–13·4)	2·6 (1·8–3·5)	0·2 (0·1–0·3)	0·0 (0·0–0·0)	2·14 (2·11–2·16)	1·38 (1·37–1·4)	0·25 (0·24–0·27)
1978	1·1 (0·5–2·2)	78·2 (76·0–80·5)	197·4 (194·8–200·0)	97·1 (95·0–99·2)	34·5 (32·6–36·4)	10·8 (9·2–12·5)	2·4 (1·8–3·3)	0·2 (0·1–0·3)	0·0 (0·0–0·0)	2·11 (2·09–2·13)	1·38 (1·37–1·4)	0·24 (0·23–0·25)
1979	1·1 (0·5–2·3)	79·2 (76·7–81·4)	193·1 (190·6–195·8)	93·2 (91·2–95·5)	33·1 (31·4–34·9)	9·9 (8·3–11·6)	2·2 (1·6–3·1)	0·2 (0·1–0·3)	0·0 (0·0–0·0)	2·06 (2·03–2·09)	1·37 (1·35–1·38)	0·23 (0·21–0·24)
1980	1·1 (0·5–2·3)	80·0 (77·8–82·3)	187·1 (184·4–189·8)	90·0 (87·9–92·3)	32·2 (30·4–34·0)	9·3 (7·9–11·0)	2·1 (1·5–2·9)	0·2 (0·1–0·2)	0·0 (0·0–0·0)	2·01 (1·98–2·04)	1·34 (1·32–1·36)	0·22 (0·21–0·23)
1981	1·1 (0·5–2·3)	81·5 (79·3–83·8)	184·8 (182·1–187·4)	90·0 (87·9–92·1)	32·0 (30·3–33·9)	9·3 (7·9–11·1)	2·0 (1·4–2·8)	0·1 (0·1–0·2)	0·0 (0·0–0·0)	2·0 (1·98–2·03)	1·34 (1·32–1·36)	0·22 (0·2–0·23)
1982	1·1 (0·5–2·3)	81·5 (79·2–84·0)	184·4 (181·7–187·2)	90·9 (88·7–92·9)	32·1 (30·3–34·0)	9·6 (8·2–11·2)	2·0 (1·5–2·8)	0·1 (0·1–0·2)	0·0 (0·0–0·0)	2·01 (1·98–2·04)	1·34 (1·32–1·35)	0·22 (0·2–0·24)
1983	1·1 (0·5–2·3)	79·3 (76·9–81·7)	183·7 (181·1–186·4)	91·3 (89·1–93·2)	32·5 (30·6–34·4)	10·1 (8·7–11·7)	2·0 (1·5–2·8)	0·1 (0·1–0·2)	0·0 (0·0–0·0)	2·0 (1·97–2·03)	1·32 (1·3–1·34)	0·22 (0·21–0·24)
1984	1·1 (0·5–2·2)	76·9 (74·7–79·3)	181·0 (178·5–183·5)	92·0 (89·9–94·0)	33·6 (31·7–35·5)	10·9 (9·5–12·4)	2·1 (1·5–2·8)	0·1 (0·1–0·2)	0·0 (0·0–0·0)	1·99 (1·96–2·01)	1·29 (1·28–1·31)	0·23 (0·22–0·25)
1985	1·1 (0·5–2·2)	75·8 (73·5–78·2)	181·1 (178·6–183·9)	92·8 (90·7–95·0)	34·6 (32·7–36·5)	11·7 (10·3–13·2)	2·2 (1·6–2·9)	0·1 (0·1–0·2)	0·0 (0·0–0·0)	2·0 (1·97–2·02)	1·29 (1·27–1·31)	0·24 (0·23–0·26)
1986	1·1 (0·5–2·2)	75·9 (73·7–78·1)	181·5 (178·7–184·2)	93·2 (91·2–95·4)	34·7 (32·9–36·6)	12·1 (10·8–13·6)	2·3 (1·7–3·0)	0·1 (0·1–0·2)	0·0 (0·0–0·0)	2·0 (1·98–2·03)	1·29 (1·27–1·31)	0·25 (0·23–0·26)
1987	1·1 (0·5–2·2)	76·3 (74·0–78·5)	179·9 (177·2–182·7)	92·3 (90·1–94·4)	34·3 (32·4–36·1)	12·2 (10·8–13·8)	2·3 (1·8–3·0)	0·1 (0·1–0·2)	0·0 (0·0–0·0)	1·99 (1·97–2·02)	1·29 (1·27–1·31)	0·24 (0·23–0·26)
1988	1·0 (0·5–2·2)	75·1 (72·8–77·2)	176·6 (174·1–179·5)	88·8 (86·7–91·1)	33·3 (31·4–35·2)	11·7 (10·3–13·2)	2·3 (1·9–2·9)	0·1 (0·1–0·2)	0·0 (0·0–0·0)	1·95 (1·92–1·97)	1·26 (1·25–1·28)	0·24 (0·23–0·25)
1989	1·0 (0·4–2·1)	72·1 (69·8–74·4)	166·6 (164·0–169·1)	82·7 (80·7–85·0)	31·7 (29·8–33·6)	10·5 (9·2–11·9)	2·2 (1·8–2·6)	0·1 (0·1–0·2)	0·0 (0·0–0·0)	1·83 (1·81–1·86)	1·2 (1·18–1·22)	0·22 (0·21–0·23)
1990	1·0 (0·4–2·1)	69·3 (67·1–71·4)	152·9 (150·3–155·7)	75·1 (72·9–77·3)	29·4 (27·6–31·3)	9·7 (8·5–10·9)	2·0 (1·7–2·3)	0·1 (0·1–0·1)	0·0 (0·0–0·0)	1·7 (1·67–1·72)	1·12 (1·1–1·14)	0·21 (0·19–0·22)
1991	1·0 (0·4–2·1)	67·4 (65·4–69·7)	139·6 (136·9–142·4)	68·7 (66·5–70·9)	27·1 (25·3–29·0)	9·7 (8·5–10·9)	2·1 (1·8–2·4)	0·1 (0·1–0·1)	0·0 (0·0–0·0)	1·58 (1·55–1·6)	1·04 (1·02–1·06)	0·19 (0·18–0·21)
1992	1·0 (0·4–2·0)	65·3 (63·3–67·4)	128·5 (125·9–131·1)	64·9 (62·5–67·2)	24·5 (22·8–26·4)	9·8 (8·6–11·2)	2·1 (1·8–2·5)	0·1 (0·1–0·1)	0·0 (0·0–0·0)	1·48 (1·45–1·51)	0·97 (0·96–0·99)	0·18 (0·17–0·2)
1993	0·9 (0·4–1·9)	61·0 (58·9–63·2)	118·3 (115·6–120·6)	63·4 (61·3–65·6)	23·9 (22·2–26·0)	10·0 (8·7–11·5)	2·2 (1·8–2·6)	0·1 (0·1–0·1)	0·0 (0·0–0·0)	1·4 (1·38–1·42)	0·9 (0·88–0·92)	0·18 (0·17–0·19)
1994	0·9 (0·4–1·9)	55·6 (53·4–57·6)	107·9 (105·4–110·5)	63·3 (61·2–65·4)	25·3 (23·5–27·2)	10·4 (8·9–11·8)	2·2 (1·9–2·6)	0·1 (0·1–0·1)	0·0 (0·0–0·0)	1·33 (1·31–1·35)	0·82 (0·81–0·84)	0·19 (0·18–0·2)
1995	0·9 (0·4–1·8)	50·7 (48·7–52·7)	100·2 (97·8–102·8)	63·1 (61·1–65·2)	26·5 (24·6–28·4)	10·6 (9·3–12·2)	2·2 (2·0–2·6)	0·1 (0·1–0·1)	0·0 (0·0–0·0)	1·27 (1·25–1·3)	0·76 (0·74–0·78)	0·2 (0·19–0·21)
1996	0·8 (0·4–1·7)	46·6 (44·6–48·7)	93·0 (90·4–95·7)	62·5 (60·5–64·7)	27·8 (25·8–29·8)	10·7 (9·4–12·2)	2·3 (1·9–2·6)	0·1 (0·1–0·1)	0·0 (0·0–0·0)	1·22 (1·2–1·24)	0·7 (0·68–0·72)	0·2 (0·19–0·22)
1997	0·8 (0·4–1·7)	45·6 (43·6–47·8)	89·3 (86·8–91·7)	63·9 (61·7–66·1)	28·2 (26·2–30·2)	10·3 (9·0–11·9)	2·2 (1·8–2·5)	0·1 (0·1–0·1)	0·0 (0·0–0·0)	1·2 (1·18–1·23)	0·68 (0·66–0·7)	0·2 (0·19–0·22)
1998	0·8 (0·4–1·7)	47·4 (45·2–49·8)	93·0 (90·3–95·4)	68·4 (66·2–70·4)	28·2 (26·3–30·2)	9·8 (8·3–11·5)	2·0 (1·6–2·5)	0·1 (0·1–0·1)	0·0 (0·0–0·0)	1·25 (1·22–1·27)	0·71 (0·69–0·72)	0·2 (0·19–0·21)
1999	0·8 (0·4–1·7)	47·5 (45·4–49·6)	95·9 (93·4–98·5)	72·8 (70·7–74·8)	29·7 (27·7–31·7)	9·7 (8·2–11·5)	2·0 (1·5–2·6)	0·1 (0·1–0·1)	0·0 (0·0–0·0)	1·29 (1·27–1·32)	0·72 (0·7–0·74)	0·21 (0·19–0·22)
2000	0·8 (0·3–1·6)	44·7 (42·7–46·8)	91·9 (89·4–94·5)	73·8 (71·7–76·0)	32·1 (30·2–34·1)	10·1 (8·5–12·1)	2·1 (1·5–2·7)	0·1 (0·1–0·1)	0·0 (0·0–0·0)	1·28 (1·25–1·3)	0·69 (0·67–0·7)	0·22 (0·21–0·24)
2001	0·8 (0·3–1·6)	41·7 (39·6–43·7)	85·6 (83·1–88·3)	73·4 (71·3–75·5)	34·3 (32·4–36·5)	10·7 (8·9–12·7)	2·1 (1·5–2·9)	0·1 (0·1–0·1)	0·0 (0·0–0·0)	1·24 (1·21–1·27)	0·64 (0·62–0·66)	0·24 (0·22–0·25)
2002	0·8 (0·3–1·6)	40·3 (38·2–42·4)	82·1 (79·7–84·7)	74·2 (72·0–76·4)	36·5 (34·5–38·6)	11·4 (9·6–13·2)	2·2 (1·6–3·1)	0·1 (0·1–0·1)	0·0 (0·0–0·0)	1·24 (1·21–1·27)	0·62 (0·6–0·63)	0·25 (0·23–0·27)
2003	0·8 (0·3–1·5)	39·9 (37·8–42·0)	81·1 (78·6–83·6)	76·5 (74·3–78·6)	39·7 (37·6–41·8)	12·3 (10·7–14·2)	2·3 (1·6–3·2)	0·1 (0·1–0·1)	0·0 (0·0–0·0)	1·26 (1·24–1·29)	0·61 (0·59–0·63)	0·27 (0·26–0·29)
2004	0·7 (0·3–1·5)	39·8 (37·7–41·9)	80·4 (77·9–83·1)	79·5 (77·4–81·7)	44·1 (42·0–46·4)	13·7 (11·9–15·6)	2·4 (1·7–3·4)	0·1 (0·1–0·2)	0·0 (0·0–0·0)	1·3 (1·28–1·33)	0·6 (0·59–0·62)	0·3 (0·29–0·32)
2005	0·8 (0·3–1·6)	40·3 (38·3–42·4)	79·5 (76·9–82·1)	82·6 (80·5–85·0)	49·2 (46·9–51·4)	15·4 (13·5–17·4)	2·6 (1·9–3·5)	0·1 (0·1–0·2)	0·0 (0·0–0·0)	1·35 (1·32–1·38)	0·6 (0·59–0·62)	0·34 (0·32–0·35)
2006	0·8 (0·3–1·6)	41·8 (39·7–44·0)	79·2 (76·6–81·8)	85·6 (83·5–87·8)	54·1 (51·9–56·3)	17·3 (15·5–19·3)	2·8 (2·0–3·7)	0·1 (0·1–0·2)	0·0 (0·0–0·0)	1·41 (1·38–1·44)	0·61 (0·59–0·63)	0·37 (0·36–0·39)
2007	0·8 (0·3–1·6)	44·2 (41·9–46·6)	79·9 (77·3–82·5)	88·7 (86·5–91·1)	58·8 (56·5–61·0)	19·5 (17·6–21·7)	3·0 (2·2–4·0)	0·1 (0·1–0·2)	0·0 (0·0–0·0)	1·48 (1·45–1·5)	0·62 (0·61–0·64)	0·41 (0·39–0·42)
2008	0·8 (0·4–1·7)	45·9 (43·5–48·4)	80·5 (77·9–83·0)	91·5 (89·2–93·7)	63·3 (61·1–65·7)	22·1 (20·1–24·3)	3·3 (2·5–4·3)	0·1 (0·1–0·2)	0·0 (0·0–0·0)	1·54 (1·51–1·57)	0·64 (0·62–0·65)	0·44 (0·43–0·46)
2009	0·8 (0·4–1·7)	45·2 (42·9–47·6)	78·1 (75·6–80·7)	91·8 (89·6–94·0)	66·8 (64·5–68·9)	24·6 (22·5–26·7)	3·6 (2·7–4·7)	0·2 (0·1–0·3)	0·0 (0·0–0·0)	1·56 (1·53–1·59)	0·62 (0·6–0·64)	0·48 (0·46–0·49)
2010	0·8 (0·3–1·6)	43·6 (41·0–46·1)	73·7 (71·1–76·4)	90·1 (87·7–92·4)	67·8 (65·4–70·3)	26·3 (24·0–28·5)	4·0 (3·0–5·1)	0·2 (0·1–0·3)	0·0 (0·0–0·0)	1·53 (1·5–1·56)	0·59 (0·57–0·61)	0·49 (0·47–0·51)
2011	0·8 (0·3–1·6)	42·9 (40·5–45·6)	71·3 (68·7–74·0)	88·5 (86·3–90·7)	66·9 (64·5–69·6)	27·1 (24·8–29·4)	4·2 (3·2–5·4)	0·2 (0·1–0·3)	0·0 (0·0–0·0)	1·51 (1·48–1·54)	0·57 (0·55–0·6)	0·49 (0·48–0·51)
2012	0·8 (0·3–1·6)	42·7 (40·2–45·4)	71·3 (68·6–74·2)	87·7 (85·4–90·0)	66·3 (63·7–68·8)	27·8 (25·8–30·0)	4·4 (3·4–5·5)	0·2 (0·1–0·3)	0·0 (0·0–0·0)	1·51 (1·47–1·54)	0·57 (0·55–0·6)	0·49 (0·48–0·51)
2013	0·8 (0·3–1·6)	42·2 (39·6–44·9)	72·4 (69·7–75·4)	87·8 (85·5–90·4)	67·2 (64·6–69·7)	29·0 (26·8–31·2)	4·6 (3·6–5·7)	0·2 (0·2–0·3)	0·0 (0·0–0·0)	1·52 (1·49–1·56)	0·58 (0·55–0·6)	0·5 (0·49–0·52)
2014	0·8 (0·3–1·6)	41·3 (38·4–44·4)	73·1 (70·0–76·2)	88·4 (85·8–91·1)	68·8 (66·4–71·4)	29·8 (27·6–32·2)	4·6 (3·7–5·6)	0·2 (0·2–0·3)	0·0 (0·0–0·0)	1·54 (1·49–1·58)	0·58 (0·55–0·61)	0·52 (0·5–0·54)
2015	0·8 (0·3–1·6)	40·4 (36·8–44·3)	73·2 (68·5–78·4)	88·5 (84·7–92·5)	69·0 (65·2–73·1)	29·3 (26·3–32·5)	4·4 (3·6–5·3)	0·2 (0·2–0·3)	0·0 (0·0–0·0)	1·53 (1·45–1·61)	0·57 (0·53–0·62)	0·51 (0·48–0·55)
2016	0·7 (0·3–1·5)	39·7 (35·2–44·6)	72·6 (65·1–80·9)	87·8 (82·2–93·8)	67·4 (61·4–74·2)	27·4 (23·2–32·0)	4·1 (3·3–5·0)	0·2 (0·1–0·3)	0·0 (0·0–0·0)	1·5 (1·37–1·64)	0·57 (0·51–0·63)	0·5 (0·45–0·55)
2017	0·7 (0·3–1·5)	39·3 (34·2–45·4)	72·2 (61·9–84·0)	87·0 (79·8–94·9)	65·4 (57·6–74·1)	25·7 (20·8–31·7)	3·9 (3·0–4·9)	0·2 (0·1–0·3)	0·0 (0·0–0·0)	1·47 (1·29–1·67)	0·56 (0·48–0·65)	0·48 (0·41–0·55)

Supplemental Results Table 5: Age-specific fertility rates per 1000, cumulative total fertility up to maternal age 25 (TFU25), cumulative total fertility from maternal age 30 to age 55 (TFO30), and cumulative total fertility, Croatia, 1950-2017

Year	10-14	15-19	20-24	25-29	30-34	35-39	40-44	45-49	50-54	TFR	TFU25	TFO30
1950	0·1 (0·1–0·3)	49·4 (41·7–58·3)	198·2 (171·2–227·2)	137·9 (124·9–152·1)	93·9 (80·1–109·5)	47·9 (36·4–61·9)	17·7 (12·8–23·9)	1·8 (1·2–2·8)	0·0 (0·0–0·0)	2·73 (2·34–3·18)	1·24 (1·07–1·43)	0·81 (0·65–0·99)
1951	0·1 (0·1–0·3)	48·8 (41·2–57·5)	196·2 (169·2–225·6)	134·7 (121·8–148·4)	92·1 (78·5–107·3)	46·3 (35·2–60·0)	16·9 (12·2–22·7)	1·7 (1·1–2·7)	0·0 (0·0–0·0)	2·68 (2·3–3·12)	1·23 (1·05–1·42)	0·79 (0·63–0·96)
1952	0·1 (0·1–0·3)	48·3 (41·0–56·5)	194·2 (168·2–222·7)	131·5 (118·6–145·1)	90·3 (76·9–105·0)	44·8 (34·3–58·0)	16·1 (11·7–21·6)	1·7 (1·0–2·5)	0·0 (0·0–0·0)	2·63 (2·27–3·04)	1·21 (1·05–1·39)	0·76 (0·62–0·93)
1953	0·1 (0·1–0·3)	47·8 (40·8–56·1)	192·2 (166·3–219·9)	128·2 (115·4–141·1)	88·5 (75·0–103·2)	43·2 (33·1–55·3)	15·3 (11·1–20·6)	1·6 (1·0–2·4)	0·0 (0·0–0·0)	2·59 (2·25–2·95)	1·2 (1·04–1·37)	0·74 (0·61–0·9)
1954	0·1 (0·1–0·3)	47·4 (40·4–55·2)	190·2 (164·6–216·1)	124·9 (112·5–137·6)	86·7 (73·2–100·7)	41·7 (31·9–53·3)	14·6 (10·4–19·6)	1·5 (0·9–2·2)	0·0 (0·0–0·0)	2·54 (2·21–2·89)	1·19 (1·04–1·35)	0·72 (0·6–0·86)
1955	0·1 (0·1–0·3)	46·8 (39·9–54·5)	188·1 (162·7–213·8)	121·4 (109·2–134·0)	84·8 (71·7–98·6)	40·1 (30·6–51·2)	13·9 (9·8–18·5)	1·4 (0·9–2·1)	0·0 (0·0–0·0)	2·48 (2·18–2·82)	1·18 (1·03–1·33)	0·7 (0·58–0·83)
1956	0·1 (0·1–0·3)	46·3 (39·3–53·7)	185·9 (160·6–212·3)	117·9 (106·0–130·4)	83·0 (70·0–96·6)	38·6 (29·3–49·1)	13·2 (9·3–17·9)	1·3 (0·8–2·0)	0·0 (0·0–0·0)	2·43 (2·14–2·75)	1·16 (1·02–1·31)	0·68 (0·56–0·81)
1957	0·1 (0·1–0·3)	45·8 (38·9–53·0)	183·8 (158·4–211·0)	115·1 (103·1–126·9)	81·1 (68·8–94·5)	37·1 (28·1–47·7)	12·5 (8·8–17·3)	1·2 (0·8–1·8)	0·0 (0·0–0·0)	2·38 (2·09–2·71)	1·15 (1·01–1·3)	0·66 (0·54–0·79)
1958	0·1 (0·1–0·3)	45·2 (38·8–52·4)	181·6 (156·5–209·3)	114·0 (102·5–126·7)	79·3 (67·1–92·4)	35·6 (26·8–46·5)	11·9 (8·3–16·4)	1·2 (0·7–1·7)	0·0 (0·0–0·0)	2·34 (2·05–2·69)	1·13 (1·0–1·28)	0·64 (0·52–0·78)
1959	0·1 (0·1–0·3)	44·6 (38·6–51·4)	179·5 (155·5–206·2)	113·9 (102·8–126·8)	77·5 (65·5–90·9)	34·1 (25·6–45·1)	11·2 (7·9–15·6)	1·1 (0·7–1·6)	0·0 (0·0–0·0)	2·31 (2·02–2·65)	1·12 (0·99–1·26)	0·62 (0·5–0·77)
1960	0·1 (0·1–0·3)	44·0 (38·2–50·3)	177·2 (154·8–202·9)	113·8 (102·5–126·7)	75·7 (63·8–89·7)	32·7 (24·5–43·4)	10·6 (7·5–14·8)	1·0 (0·7–1·5)	0·0 (0·0–0·0)	2·28 (1·99–2·62)	1·11 (0·99–1·24)	0·6 (0·48–0·75)
1961	0·1 (0·1–0·3)	43·5 (37·9–49·5)	174·9 (153·3–197·9)	113·6 (102·3–126·9)	73·9 (62·2–87·9)	31·3 (23·4–41·9)	10·0 (7·2–14·1)	1·0 (0·6–1·4)	0·0 (0·0–0·0)	2·24 (1·96–2·57)	1·09 (0·98–1·22)	0·58 (0·47–0·73)
1962	0·1 (0·1–0·3)	43·1 (37·7–48·6)	172·5 (152·5–194·2)	113·5 (102·5–126·2)	72·1 (60·6–85·8)	30·0 (22·5–39·9)	9·4 (6·8–13·2)	0·9 (0·6–1·3)	0·0 (0·0–0·0)	2·21 (1·95–2·52)	1·08 (0·96–1·19)	0·56 (0·45–0·7)
1963	0·1 (0·1–0·3)	42·6 (37·5–48·0)	170·0 (151·7–189·6)	113·3 (102·2–125·2)	70·2 (59·0–83·4)	28·6 (21·6–38·3)	8·9 (6·4–12·4)	0·8 (0·5–1·3)	0·0 (0·0–0·0)	2·17 (1·94–2·46)	1·06 (0·96–1·17)	0·54 (0·44–0·68)
1964	0·1 (0·1–0·3)	42·2 (37·3–47·4)	167·4 (150·4–184·1)	113·1 (102·5–124·4)	68·3 (57·7–81·4)	27·4 (20·7–35·9)	8·3 (6·0–11·4)	0·8 (0·5–1·2)	0·0 (0·0–0·0)	2·14 (1·92–2·4)	1·05 (0·95–1·15)	0·52 (0·43–0·65)
1965	0·1 (0·1–0·3)	41·7 (37·2–46·8)	164·9 (149·7–179·6)	113·0 (102·8–124·1)	66·5 (56·0–79·6)	26·2 (20·0–34·6)	7·8 (5·8–10·6)	0·7 (0·5–1·1)	0·0 (0·0–0·0)	2·11 (1·91–2·34)	1·03 (0·94–1·13)	0·51 (0·41–0·62)
1966	0·1 (0·1–0·3)	41·3 (36·8–46·2)	162·6 (148·8–176·3)	113·0 (103·6–123·4)	64·8 (54·9–76·9)	25·2 (19·5–32·6)	7·4 (5·6–10·0)	0·7 (0·5–1·0)	0·0 (0·0–0·0)	2·07 (1·89–2·28)	1·02 (0·93–1·11)	0·49 (0·4–0·6)
1967	0·1 (0·1–0·3)	40·9 (36·5–45·4)	160·3 (147·2–173·7)	112·9 (104·2–123·3)	63·1 (54·1–74·0)	24·2 (19·1–31·2)	7·0 (5·3–9·3)	0·6 (0·4–0·9)	0·0 (0·0–0·0)	2·05 (1·88–2·23)	1·01 (0·92–1·09)	0·47 (0·4–0·57)
1968	0·1 (0·1–0·3)	40·5 (36·3–45·0)	158·0 (145·0–170·6)	113·0 (104·8–122·0)	61·5 (53·3–71·3)	23·3 (18·5–29·5)	6·6 (5·1–8·6)	0·6 (0·4–0·8)	0·0 (0·0–0·0)	2·02 (1·87–2·18)	0·99 (0·91–1·07)	0·46 (0·39–0·55)
1969	0·1 (0·1–0·3)	40·2 (36·2–44·4)	155·9 (143·7–167·8)	113·0 (106·0–121·1)	59·9 (52·2–69·1)	22·4 (18·0–27·8)	6·2 (4·9–8·0)	0·5 (0·4–0·7)	0·0 (0·0–0·0)	1·99 (1·86–2·14)	0·98 (0·91–1·05)	0·45 (0·38–0·53)
1970	0·1 (0·1–0·3)	39·8 (36·0–43·9)	153·7 (142·6–164·6)	113·0 (106·4–120·5)	58·4 (51·3–66·5)	21·7 (17·6–26·3)	5·9 (4·7–7·5)	0·5 (0·4–0·7)	0·0 (0·0–0·0)	1·97 (1·85–2·09)	0·97 (0·9–1·04)	0·43 (0·37–0·5)
1971	0·1 (0·1–0·3)	39·7 (36·1–43·5)	152·2 (142·2–162·3)	112·9 (106·5–119·6)	57·6 (51·0–64·9)	21·2 (17·5–25·4)	5·7 (4·6–7·0)	0·5 (0·3–0·6)	0·0 (0·0–0·0)	1·95 (1·84–2·07)	0·96 (0·9–1·02)	0·42 (0·37–0·49)
1972	0·1 (0·1–0·3)	39·9 (36·2–43·7)	152·8 (142·7–162·9)	113·0 (106·8–119·0)	57·5 (51·5–64·2)	20·8 (17·5–25·0)	5·5 (4·5–6·8)	0·4 (0·3–0·6)	0·0 (0·0–0·0)	1·95 (1·84–2·06)	0·96 (0·9–1·03)	0·42 (0·37–0·47)
1973	0·1 (0·1–0·3)	40·2 (36·5–44·1)	154·8 (144·6–165·3)	113·2 (107·0–119·3)	57·8 (51·8–64·1)	20·5 (17·1–24·6)	5·4 (4·4–6·6)	0·4 (0·3–0·5)	0·0 (0·0–0·0)	1·96 (1·85–2·07)	0·98 (0·91–1·05)	0·42 (0·38–0·47)
1974	0·1 (0·1–0·3)	40·5 (36·9–44·4)	157·0 (146·3–167·5)	113·4 (107·5–119·5)	57·9 (52·2–64·1)	20·2 (16·7–24·4)	5·2 (4·2–6·4)	0·4 (0·3–0·5)	0·0 (0·0–0·0)	1·97 (1·86–2·09)	0·99 (0·92–1·06)	0·42 (0·38–0·46)
1975	0·1 (0·1–0·3)	40·5 (36·8–44·5)	158·6 (148·0–168·3)	113·5 (107·3–119·5)	57·6 (51·9–63·7)	19·7 (16·3–23·6)	5·1 (4·0–6·3)	0·4 (0·3–0·5)	0·0 (0·0–0·0)	1·98 (1·87–2·09)	1·0 (0·92–1·06)	0·41 (0·37–0·46)
1976	0·1 (0·1–0·3)	40·2 (36·3–44·1)	158·9 (149·0–169·2)	113·0 (106·4–119·2)	57·3 (51·7–63·1)	18·9 (15·6–22·8)	4·8 (3·8–5·9)	0·3 (0·3–0·4)	0·0 (0·0–0·0)	1·97 (1·86–2·07)	1·0 (0·93–1·06)	0·41 (0·37–0·45)
1977	0·1 (0·1–0·3)	39·7 (36·1–43·6)	158·5 (148·4–168·9)	112·4 (106·1–118·1)	56·9 (51·8–62·6)	18·1 (15·1–21·6)	4·5 (3·7–5·6)	0·3 (0·3–0·4)	0·0 (0·0–0·0)	1·95 (1·85–2·06)	0·99 (0·93–1·05)	0·4 (0·37–0·44)
1978	0·1 (0·1–0·3)	39·3 (35·7–43·2)	158·0 (148·1–168·3)	111·7 (106·0–116·8)	56·4 (51·2–61·6)	17·4 (14·7–20·5)	4·3 (3·5–5·2)	0·3 (0·2–0·4)	0·0 (0·0–0·0)	1·94 (1·83–2·04)	0·99 (0·93–1·04)	0·39 (0·36–0·43)
1979	0·1 (0·1–0·3)	38·8 (35·7–42·5)	157·2 (148·1–166·5)	110·9 (105·8–115·9)	55·5 (50·9–60·7)	16·6 (14·2–19·3)	4·1 (3·4–4·8)	0·3 (0·2–0·4)	0·0 (0·0–0·0)	1·92 (1·82–2·01)	0·98 (0·93–1·03)	0·38 (0·35–0·42)
1980	0·1 (0·1–0·3)	38·1 (35·1–41·3)	155·7 (147·6–164·3)	109·7 (105·1–114·1)	54·3 (50·1–58·6)	15·7 (13·8–17·8)	3·8 (3·3–4·4)	0·3 (0·2–0·3)	0·0 (0·0–0·0)	1·89 (1·8–1·98)	0·97 (0·92–1·02)	0·37 (0·34–0·4)
1981	0·1 (0·1–0·2)	37·1 (34·4–40·0)	153·3 (146·2–161·0)	108·3 (104·1–112·4)	52·9 (49·1–56·7)	14·8 (13·2–16·5)	3·6 (3·1–4·1)	0·2 (0·2–0·3)	0·0 (0·0–0·0)	1·85 (1·78–1·93)	0·95 (0·91–1·0)	0·36 (0·33–0·38)
1982	0·1 (0·1–0·2)	36·2 (33·5–38·9)	150·5 (144·0–157·7)	106·9 (103·1–110·7)	51·6 (48·2–55·3)	14·0 (12·6–15·4)	3·4 (3·0–3·8)	0·2 (0·2–0·3)	0·0 (0·0–0·0)	1·81 (1·74–1·89)	0·93 (0·89–0·98)	0·35 (0·32–0·37)
1983	0·1 (0·1–0·2)	35·4 (32·9–38·1)	147·7 (141·1–154·7)	105·9 (102·3–109·6)	50·6 (47·5–54·1)	13·7 (12·5–15·0)	3·3 (3·0–3·6)	0·2 (0·2–0·3)	0·0 (0·0–0·0)	1·78 (1·71–1·86)	0·92 (0·87–0·96)	0·34 (0·32–0·36)
1984	0·1 (0·1–0·2)	34·6 (32·2–37·2)	145·0 (139·0–151·8)	105·1 (101·7–108·6)	49·8 (46·8–53·2)	13·8 (12·6–15·1)	3·3 (3·0–3·6)	0·2 (0·2–0·2)	0·0 (0·0–0·0)	1·76 (1·69–1·83)	0·9 (0·86–0·94)	0·34 (0·32–0·35)
1985	0·1 (0·1–0·2)	33·7 (31·6–36·0)	142·1 (136·9–148·0)	104·3 (101·1–107·7)	48·7 (45·9–51·6)	14·0 (12·7–15·2)	3·3 (3·0–3·7)	0·2 (0·2–0·2)	0·0 (0·0–0·0)	1·73 (1·68–1·79)	0·88 (0·85–0·91)	0·33 (0·32–0·35)
1986	0·1 (0·0–0·2)	32·4 (30·5–34·4)	138·4 (134·2–143·1)	103·4 (100·4–106·6)	47·2 (44·8–49·7)	14·2 (12·9–15·5)	3·3 (3·0–3·7)	0·2 (0·2–0·2)	0·0 (0·0–0·0)	1·7 (1·65–1·74)	0·85 (0·83–0·88)	0·32 (0·31–0·34)
1987	0·1 (0·0–0·2)	30·8 (28·9–32·7)	133·8 (130·3–137·2)	102·3 (99·6–104·9)	45·4 (43·2–47·8)	14·5 (13·3–15·9)	3·4 (3·0–3·8)	0·2 (0·1–0·2)	0·0 (0·0–0·0)	1·65 (1·62–1·69)	0·82 (0·8–0·84)	0·32 (0·3–0·33)
1988	0·1 (0·0–0·2)	28·9 (27·1–30·8)	128·5 (125·9–131·3)	101·3 (99·0–103·5)	43·4 (41·5–45·4)	15·0 (13·7–16·4)	3·5 (3·0–4·0)	0·2 (0·1–0·2)	0·0 (0·0–0·0)	1·6 (1·58–1·63)	0·79 (0·77–0·8)	0·31 (0·3–0·32)
1989	0·1 (0·0–0·2)	26·5 (25·0–28·2)	122·3 (119·7–124·9)	100·2 (98·1–102·3)	42·7 (41·0–44·6)	15·8 (14·4–17·2)	3·6 (3·1–4·2)	0·2 (0·1–0·2)	0·0 (0·0–0·0)	1·56 (1·53–1·58)	0·74 (0·73–0·76)	0·31 (0·3–0·32)
1990	0·1 (0·0–0·2)	24·0 (22·6–25·6)	114·8 (112·5–117·1)	98·8 (96·9–100·7)	45·0 (43·1–46·9)	16·7 (15·3–18·3)	3·9 (3·3–4·5)	0·2 (0·1–0·2)	0·0 (0·0–0·0)	1·52 (1·49–1·54)	0·69 (0·68–0·71)	0·33 (0·32–0·34)
1991	0·1 (0·0–0·2)	21·8 (20·4–23·3)	106·8 (104·4–109·2)	97·7 (95·7–99·8)	48·8 (46·9–50·9)	17·8 (16·3–19·5)	4·1 (3·5–4·8)	0·2 (0·1–0·2)	0·0 (0·0–0·0)	1·49 (1·46–1·51)	0·64 (0·63–0·66)	0·35 (0·34–0·37)
1992	0·1 (0·0–0·2)	20·3 (19·0–21·8)	100·6 (98·1–103·0)	98·3 (96·3–100·3)	52·6 (50·7–54·8)	19·0 (17·4–20·7)	4·3 (3·7–5·0)	0·2 (0·1–0·2)	0·0 (0·0–0·0)	1·48 (1·45–1·5)	0·61 (0·59–0·62)	0·38 (0·37–0·39)
1993	0·1 (0·0–0·2)	19·6 (18·2–21·2)	97·5 (95·0–100·0)	100·6 (98·4–102·9)	56·0 (53·9–58·0)	20·2 (18·5–21·9)	4·5 (3·8–5·2)	0·2 (0·1–0·2)	0·0 (0·0–0·0)	1·49 (1·47–1·52)	0·59 (0·57–0·6)	0·4 (0·39–0·42)
1994	0·1 (0·0–0·2)	19·4 (18·0–20·9)	97·0 (94·5–99·5)	104·0 (101·8–106·2)	58·9 (56·8–61·0)	21·5 (19·8–23·2)	4·6 (3·9–5·4)	0·2 (0·1–0·2)	0·0 (0·0–0·0)	1·53 (1·5–1·55)	0·58 (0·57–0·6)	0·43 (0·41–0·44)
1995	0·1 (0·0–0·2)	19·2 (17·7–20·8)	97·3 (94·9–99·8)	106·7 (104·6–108·9)	61·3 (59·4–63·4)	22·7 (21·1–24·5)	4·6 (3·9–5·4)	0·2 (0·1–0·2)	0·0 (0·0–0·0)	1·56 (1·53–1·59)	0·58 (0·57–0·6)	0·44 (0·43–0·46)
1996	0·1 (0·0–0·2)	18·5 (17·1–20·1)	96·2 (93·7–98·5)	107·1 (104·9–109·3)	63·0 (61·1–65·1)	23·9 (22·2–25·6)	4·8 (4·0–5·6)	0·2 (0·1–0·2)	0·0 (0·0–0·0)	1·57 (1·54–1·59)	0·57 (0·56–0·59)	0·46 (0·44–0·47)
1997	0·1 (0·0–0·2)	17·4 (16·0–18·8)	92·3 (89·8–94·7)	104·7 (102·3–106·6)	63·4 (61·3–65·5)	24·5 (22·8–26·2)	4·9 (4·1–5·8)	0·2 (0·1–0·3)	0·0 (0·0–0·0)	1·54 (1·51–1·56)	0·55 (0·53–0·56)	0·46 (0·45–0·48)
1998	0·1 (0·0–0·2)	16·1 (14·9–17·5)	86·9 (84·5–89·4)	100·9 (98·7–102·9)	62·7 (60·6–64·8)	24·8 (22·9–26·4)	5·0 (4·3–6·0)	0·2 (0·2–0·3)	0·0 (0·0–0·0)	1·48 (1·46–1·51)	0·52 (0·5–0·53)	0·46 (0·45–0·48)
1999	0·1 (0·0–0·2)	14·9 (13·7–16·2)	81·2 (79·0–83·8)	97·5 (95·3–99·6)	62·0 (59·9–64·1)	24·8 (23·0–26·7)	5·2 (4·4–6·1)	0·2 (0·2–0·3)	0·0 (0·0–0·0)	1·43 (1·41–1·45)	0·48 (0·47–0·5)	0·46 (0·45–0·47)
2000	0·1 (0·0–0·1)	13·9 (12·8–15·3)	75·9 (73·6–78·5)	95·1 (93·0–97·3)	62·0 (59·9–64·2)	24·7 (23·0–26·7)	5·3 (4·4–6·2)	0·2 (0·2–0·3)	0·0 (0·0–0·0)	1·39 (1·36–1·41)	0·45 (0·44–0·46)	0·46 (0·45–0·48)
2001	0·1 (0·0–0·1)	13·3 (12·2–14·7)	71·4 (69·0–74·0)	93·8 (91·7–96·2)	62·6 (60·5–64·9)	24·7 (22·9–26·7)	5·3 (4·5–6·3)	0·3 (0·2–0·3)	0·0 (0·0–0·0)	1·36 (1·34–1·38)	0·42 (0·41–0·44)	0·46 (0·45–0·48)
2002	0·1 (0·0–0·1)	13·1 (11·9–14·4)	68·3 (65·9–70·9)	93·5 (91·4–95·8)	64·0 (61·8–66·3)	24·8 (22·9–26·8)	5·4 (4·5–6·4)	0·3 (0·2–0·3)	0·0 (0·0–0·0)	1·35 (1·32–1·37)	0·41 (0·39–0·42)	0·47 (0·46–0·49)
2003	0·1 (0·0–0·1)	13·0 (11·8–14·3)	66·3 (63·8–68·6)	94·3 (92·2–96·5)	66·3 (64·0–68·5)	25·4 (23·4–27·5)	5·4 (4·4–6·5)	0·3 (0·2–0·4)	0·0 (0·0–0·0)	1·35 (1·33–1·38)	0·4 (0·38–0·41)	0·49 (0·47–0·5)
2004	0·1 (0·0–0·1)	13·1 (11·7–14·5)	65·0 (62·5–67·3)	95·8 (93·6–97·9)	69·3 (66·9–71·6)	26·4 (24·6–28·5)	5·4 (4·4–6·6)	0·3 (0·2–0·4)	0·0 (0·0–0·0)	1·38 (1·35–1·4)	0·39 (0·38–0·41)	0·51 (0·49–0·53)
2005	0·1 (0·0–0·1)	13·2 (11·8–14·7)	64·0 (61·6–66·1)	97·6 (95·5–99·7)	72·6 (70·4–75·1)	27·8 (25·8–30·0)	5·5 (4·5–6·7)	0·3 (0·2–0·4)	0·0 (0·0–0·0)	1·4 (1·38–1·43)	0·39 (0·37–0·4)	0·53 (0·51–0·55)
2006	0·1 (0·0–0·1)	13·3 (11·9–14·8)	63·1 (60·7–65·4)	99·5 (97·3–101·6)	76·1 (73·8–78·5)	29·5 (27·5–31·6)	5·6 (4·5–6·8)	0·3 (0·2–0·4)	0·0 (0·0–0·0)	1·44 (1·41–1·47)	0·38 (0·37–0·4)	0·56 (0·54–0·58)
2007	0·1 (0·0–0·1)	13·2 (11·8–14·7)	62·1 (59·6–64·5)	101·1 (99·0–103·3)	79·6 (77·3–82·0)	31·3 (29·4–33·6)	5·7 (4·6–7·0)	0·3 (0·2–0·4)	0·0 (0·0–0·0)	1·47 (1·44–1·49)	0·38 (0·36–0·39)	0·58 (0·57–0·6)
2008	0·1 (0·0–0·1)	12·9 (11·5–14·4)	60·5 (58·2–62·9)	101·9 (99·7–104·0)	82·9 (80·6–85·4)	33·3 (31·2–35·6)	5·9 (4·7–7·2)	0·3 (0·2–0·4)	0·0 (0·0–0·0)	1·49 (1·46–1·52)	0·37 (0·35–0·38)	0·61 (0·59–0·63)
2009	0·1 (0·0–0·1)	12·4 (11·1–13·9)	58·2 (55·9–60·7)	101·2 (99·1–103·4)	85·6 (83·4–88·0)	35·2 (33·0–37·4)	6·1 (4·9–7·4)	0·3 (0·2–0·4)	0·0 (0·0–0·0)	1·5 (1·47–1·52)	0·35 (0·34–0·37)	0·64 (0·62–0·65)
2010	0·1 (0·0–0·1)	11·8 (10·5–13·3)	55·5 (53·3–58·1)	99·3 (97·1–101·6)	87·3 (85·1–89·6)	36·9 (34·6–39·2)	6·3 (5·1–7·7)	0·3 (0·2–0·4)	0·0 (0·0–0·0)	1·49 (1·46–1·52)	0·34 (0·32–0·35)	0·65 (0·64–0·67)
2011	0·1 (0·0–0·1)	11·2 (10·0–12·7)	52·8 (50·5–55·4)	96·8 (94·6–99·1)	88·3 (86·1–90·6)	38·3 (36·0–40·7)	6·5 (5·3–7·9)	0·3 (0·2–0·4)	0·0 (0·0–0·0)	1·47 (1·44–1·5)	0·32 (0·31–0·34)	0·67 (0·65–0·69)
2012	0·1 (0·0–0·1)	10·7 (9·4–12·1)	50·3 (47·9–52·8)	94·0 (91·9–96·3)	88·8 (86·4–91·3)	39·5 (37·2–42·0)	6·7 (5·5–8·0)	0·3 (0·2–0·5)	0·0 (0·0–0·0)	1·45 (1·42–1·48)	0·3 (0·29–0·32)	0·68 (0·65–0·7)
2013	0·1 (0·0–0·1)	10·2 (9·0–11·6)	48·0 (45·5–50·4)	91·4 (89·2–93·8)	88·9 (86·2–91·6)	40·4 (37·9–42·9)	6·9 (5·7–8·1)	0·3 (0·2–0·5)	0·0 (0·0–0·0)	1·43 (1·4–1·46)	0·29 (0·28–0·31)	0·68 (0·66–0·71)
2014	0·1 (0·0–0·1)	9·9 (8·7–11·2)	46·3 (43·5–49·0)	89·4 (86·9–92·1)	88·3 (85·5–91·4)	40·7 (38·0–43·4)	6·9 (5·8–8·0)	0·3 (0·2–0·5)	0·0 (0·0–0·0)	1·41 (1·37–1·45)	0·28 (0·27–0·3)	0·68 (0·65–0·71)
2015	0·1 (0·0–0·1)	9·8 (8·6–11·2)	45·5 (42·2–49·0)	88·3 (85·1–91·7)	87·4 (83·7–91·3)	40·2 (37·0–43·6)	6·7 (5·6–7·9)	0·3 (0·2–0·5)	0·0 (0·0–0·0)	1·39 (1·35–1·44)	0·28 (0·26–0·3)	0·67 (0·64–0·71)
2016	0·1 (0·0–0·1)	9·8 (8·6–11·4)	45·4 (41·2–49·8)	87·9 (83·9–92·2)	86·4 (81·3–91·6)	39·2 (35·3–43·6)	6·5 (5·4–7·8)	0·3 (0·2–0·4)	0·0 (0·0–0·0)	1·38 (1·32–1·44)	0·28 (0·25–0·3)	0·66 (0·61–0·71)
2017	0·1 (0·0–0·1)	9·8 (8·5–11·6)	45·6 (40·5–51·3)	87·7 (82·7–93·0)	85·9 (79·4–92·6)	38·4 (33·5–43·8)	6·4 (5·2–7·7)	0·3 (0·2–0·4)	0·0 (0·0–0·0)	1·37 (1·3–1·45)	0·28 (0·25–0·31)	0·66 (0·59–0·72)

Supplemental Results Table 5: Age-specific fertility rates per 1000, cumulative total fertility up to maternal age 25 (TFU25), cumulative total fertility from maternal age 30 to age 55 (TFO30), and cumulative total fertility, Czech Republic, 1950-2017

Year	10-14	15-19	20-24	25-29	30-34	35-39	40-44	45-49	50-54	TFR	TFU25	TFO30
1950	0·1 (0·0–0·1)	50·0 (47·8–52·4)	189·3 (186·7–191·9)	159·2 (157·1–161·3)	99·3 (96·5–102·2)	50·1 (47·8–52·2)	16·2 (14·3–18·1)	0·9 (0·6–1·3)	0·0 (0·0–0·0)	2·83 (2·8–2·86)	1·2 (1·18–1·22)	0·83 (0·81–0·85)
1951	0·1 (0·0–0·1)	49·4 (47·3–51·6)	189·2 (186·7–191·9)	155·3 (153·1–157·3)	96·6 (93·9–99·0)	46·9 (44·8–49·3)	15·5 (13·8–17·3)	0·8 (0·5–1·2)	0·0 (0·0–0·0)	2·77 (2·74–2·8)	1·19 (1·18–1·21)	0·8 (0·78–0·82)
1952	0·1 (0·0–0·1)	47·2 (45·2–49·4)	186·8 (184·1–189·3)	149·3 (147·2–151·4)	92·1 (89·8–94·5)	43·8 (41·4–46·3)	14·4 (12·8–16·2)	0·8 (0·5–1·1)	0·0 (0·0–0·0)	2·67 (2·64–2·7)	1·17 (1·15–1·19)	0·76 (0·74–0·78)
1953	0·1 (0·0–0·1)	45·1 (43·0–47·1)	186·3 (183·7–188·9)	145·6 (143·4–147·8)	87·7 (85·5–90·0)	42·5 (39·9–45·1)	13·4 (11·9–15·1)	0·7 (0·4–1·1)	0·0 (0·0–0·0)	2·61 (2·58–2·63)	1·16 (1·14–1·17)	0·72 (0·7–0·74)
1954	0·1 (0·0–0·1)	44·2 (42·1–46·3)	188·6 (186·0–191·1)	144·7 (142·7–147·0)	84·1 (81·9–86·4)	42·9 (40·4–45·4)	12·4 (10·9–14·1)	0·7 (0·4–1·0)	0·0 (0·0–0·0)	2·59 (2·56–2·62)	1·16 (1·15–1·18)	0·7 (0·68–0·72)
1955	0·1 (0·0–0·1)	44·9 (42·8–47·0)	191·7 (189·1–194·4)	144·1 (141·9–146·2)	80·8 (78·7–83·1)	43·0 (40·7–45·5)	11·2 (9·6–12·8)	0·6 (0·4–0·9)	0·0 (0·0–0·0)	2·58 (2·55–2·62)	1·18 (1·17–1·2)	0·68 (0·66–0·7)
1956	0·1 (0·0–0·1)	45·7 (43·6–47·7)	193·2 (190·3–196·1)	139·6 (137·4–141·8)	75·7 (73·6–77·8)	40·0 (37·7–42·4)	9·4 (8·0–11·0)	0·5 (0·3–0·8)	0·0 (0·0–0·0)	2·52 (2·49–2·55)	1·19 (1·18–1·21)	0·63 (0·61–0·65)
1957	0·1 (0·0–0·1)	45·4 (43·5–47·5)	189·7 (186·8–192·5)	130·1 (127·9–132·4)	67·0 (64·8–69·1)	33·3 (31·2–35·4)	7·6 (6·2–9·1)	0·5 (0·3–0·7)	0·0 (0·0–0·0)	2·37 (2·34–2·4)	1·18 (1·16–1·19)	0·54 (0·53–0·56)
1958	0·1 (0·0–0·1)	44·6 (42·7–46·7)	184·1 (181·4–186·7)	120·1 (117·9–122·2)	57·5 (55·5–59·8)	26·2 (24·4–28·1)	6·3 (5·1–7·8)	0·4 (0·2–0·6)	0·0 (0·0–0·0)	2·2 (2·17–2·23)	1·14 (1·13–1·16)	0·45 (0·44–0·47)
1959	0·1 (0·0–0·1)	44·3 (42·4–46·1)	182·5 (179·8–185·0)	114·9 (112·7–117·2)	51·7 (49·7–53·8)	21·8 (20·0–23·8)	5·7 (4·5–7·2)	0·4 (0·2–0·6)	0·0 (0·0–0·0)	2·11 (2·08–2·13)	1·13 (1·12–1·15)	0·4 (0·38–0·41)
1960	0·1 (0·0–0·1)	44·2 (42·3–46·0)	185·3 (182·6–188·1)	113·7 (111·5–116·0)	49·7 (47·7–51·7)	19·7 (18·0–21·4)	5·4 (4·3–6·8)	0·3 (0·2–0·5)	0·0 (0·0–0·0)	2·09 (2·07–2·12)	1·15 (1·13–1·16)	0·38 (0·36–0·39)
1961	0·1 (0·0–0·1)	44·9 (43·0–46·9)	189·9 (187·2–192·5)	116·9 (114·7–119·2)	50·5 (48·5–52·6)	19·3 (17·6–21·1)	5·4 (4·2–6·8)	0·3 (0·2–0·5)	0·0 (0·0–0·0)	2·14 (2·11–2·16)	1·17 (1·16–1·19)	0·38 (0·36–0·39)
1962	0·1 (0·0–0·1)	46·9 (45·1–48·9)	197·7 (195·1–200·4)	126·9 (124·6–129·4)	54·4 (52·3–56·6)	20·4 (18·6–22·4)	5·5 (4·3–7·0)	0·3 (0·2–0·5)	0·0 (0·0–0·0)	2·26 (2·24–2·29)	1·22 (1·21–1·24)	0·4 (0·39–0·42)
1963	0·1 (0·0–0·1)	48·4 (46·6–50·3)	203·0 (200·4–205·5)	134·0 (131·7–136·2)	58·2 (56·1–60·4)	21·2 (19·3–23·2)	5·2 (4·1–6·6)	0·3 (0·2–0·5)	0·0 (0·0–0·0)	2·35 (2·32–2·38)	1·26 (1·24–1·27)	0·42 (0·41–0·44)
1964	0·1 (0·0–0·1)	48·2 (46·4–50·0)	196·5 (194·1–199·0)	127·4 (125·1–129·7)	57·0 (55·0–59·3)	20·3 (18·5–22·2)	4·5 (3·5–5·7)	0·3 (0·2–0·4)	0·0 (0·0–0·0)	2·27 (2·24–2·3)	1·22 (1·21–1·24)	0·41 (0·4–0·43)
1965	0·1 (0·0–0·1)	47·7 (45·8–49·5)	184·5 (182·2–187·2)	116·6 (114·4–118·9)	51·9 (49·8–54·1)	18·3 (16·6–20·2)	3·8 (2·9–4·9)	0·2 (0·1–0·4)	0·0 (0·0–0·0)	2·12 (2·09–2·14)	1·16 (1·15–1·18)	0·37 (0·36–0·39)
1966	0·1 (0·0–0·1)	47·3 (45·5–49·3)	175·3 (172·9–177·9)	108·7 (106·5–111·0)	46·3 (44·2–48·4)	16·4 (14·7–18·3)	3·3 (2·5–4·3)	0·2 (0·1–0·3)	0·0 (0·0–0·0)	1·99 (1·96–2·01)	1·11 (1·1–1·13)	0·33 (0·32–0·35)
1967	0·1 (0·0–0·1)	47·1 (45·4–48·9)	170·9 (168·5–173·4)	102·7 (100·5–104·9)	42·8 (40·6–45·0)	15·0 (13·4–16·8)	3·0 (2·3–4·0)	0·2 (0·1–0·3)	0·0 (0·0–0·0)	1·91 (1·89–1·94)	1·09 (1·08–1·1)	0·31 (0·29–0·32)
1968	0·1 (0·0–0·1)	47·6 (45·7–49·6)	171·4 (169·1–173·6)	101·0 (98·8–103·3)	41·7 (39·6–43·8)	14·3 (12·6–16·1)	2·9 (2·2–3·8)	0·2 (0·1–0·3)	0·0 (0·0–0·0)	1·9 (1·87–1·92)	1·1 (1·08–1·11)	0·3 (0·28–0·32)
1969	0·1 (0·0–0·1)	48·1 (46·2–50·0)	173·4 (171·1–175·6)	103·4 (101·3–105·5)	41·8 (39·7–44·1)	13·7 (12·0–15·5)	2·8 (2·0–3·7)	0·2 (0·1–0·3)	0·0 (0·0–0·0)	1·92 (1·89–1·95)	1·11 (1·09–1·12)	0·29 (0·27–0·31)
1970	0·1 (0·0–0·1)	48·0 (46·2–49·9)	174·9 (172·6–177·2)	105·9 (103·9–108·0)	43·2 (41·1–45·4)	13·4 (11·8–15·2)	2·7 (2·0–3·6)	0·2 (0·1–0·3)	0·0 (0·0–0·0)	1·94 (1·92–1·97)	1·11 (1·1–1·13)	0·3 (0·28–0·32)
1971	0·1 (0·0–0·1)	48·7 (46·8–50·6)	181·0 (178·6–183·4)	112·4 (110·5–114·4)	46·7 (44·7–49·0)	14·0 (12·4–15·8)	2·8 (2·1–3·8)	0·2 (0·1–0·3)	0·0 (0·0–0·0)	2·03 (2·01–2·05)	1·15 (1·13–1·16)	0·32 (0·3–0·34)
1972	0·1 (0·0–0·1)	51·4 (49·5–53·5)	194·5 (191·9–196·9)	126·4 (124·4–128·4)	52·2 (50·1–54·2)	15·7 (13·8–17·6)	3·1 (2·2–4·2)	0·2 (0·1–0·2)	0·0 (0·0–0·0)	2·22 (2·19–2·24)	1·23 (1·21–1·25)	0·36 (0·34–0·37)
1973	0·1 (0·0–0·1)	55·8 (53·8–57·9)	209·4 (206·9–211·8)	137·7 (135·6–139·8)	56·9 (54·8–58·9)	17·1 (15·1–19·2)	3·2 (2·4–4·4)	0·2 (0·1–0·2)	0·0 (0·0–0·0)	2·4 (2·38–2·43)	1·33 (1·31–1·34)	0·39 (0·37–0·4)
1974	0·1 (0·0–0·1)	60·0 (57·9–61·9)	216·0 (213·8–218·2)	137·6 (135·6–139·6)	57·7 (55·6–59·7)	17·3 (15·5–19·4)	3·1 (2·3–4·3)	0·2 (0·1–0·2)	0·0 (0·0–0·0)	2·46 (2·44–2·48)	1·38 (1·36–1·4)	0·39 (0·38–0·41)
1975	0·1 (0·0–0·1)	62·3 (60·2–64·4)	214·6 (212·4–216·9)	131·3 (129·3–133·3)	55·5 (53·5–57·5)	16·9 (15·1–18·9)	3·0 (2·2–3·9)	0·1 (0·1–0·2)	0·0 (0·0–0·0)	2·42 (2·4–2·44)	1·38 (1·37–1·4)	0·38 (0·36–0·39)
1976	0·1 (0·0–0·1)	62·4 (60·3–64·6)	212·9 (210·5–215·1)	126·9 (125·0–128·9)	53·6 (51·7–55·7)	16·7 (15·0–18·5)	2·8 (2·1–3·8)	0·1 (0·1–0·2)	0·0 (0·0–0·0)	2·38 (2·36–2·4)	1·38 (1·36–1·39)	0·37 (0·35–0·38)
1977	0·1 (0·0–0·1)	60·9 (58·6–63·0)	214·7 (212·4–217·2)	126·4 (124·3–128·3)	52·7 (50·8–54·6)	16·8 (15·0–18·7)	2·8 (2·1–3·8)	0·1 (0·1–0·2)	0·0 (0·0–0·0)	2·37 (2·35–2·4)	1·38 (1·36–1·39)	0·36 (0·35–0·38)
1978	0·1 (0·0–0·1)	58·0 (55·9–60·2)	216·0 (213·4–218·6)	123·5 (121·5–125·6)	50·2 (48·3–52·1)	15·9 (14·3–17·7)	2·7 (2·0–3·7)	0·1 (0·1–0·2)	0·0 (0·0–0·0)	2·33 (2·31–2·36)	1·37 (1·35–1·39)	0·34 (0·33–0·36)
1979	0·1 (0·0–0·1)	54·5 (52·3–56·5)	207·7 (205·1–210·3)	113·9 (111·9–115·9)	45·2 (43·5–47·1)	14·1 (12·5–15·7)	2·4 (1·8–3·3)	0·1 (0·1–0·2)	0·0 (0·0–0·0)	2·19 (2·17–2·21)	1·31 (1·29–1·33)	0·31 (0·3–0·32)
1980	0·1 (0·0–0·1)	52·6 (50·6–54·4)	196·9 (194·5–199·5)	106·5 (104·4–108·5)	41·0 (39·3–42·8)	12·7 (11·3–14·2)	2·2 (1·6–3·0)	0·1 (0·1–0·1)	0·0 (0·0–0·0)	2·06 (2·04–2·08)	1·25 (1·23–1·26)	0·28 (0·27–0·29)
1981	0·1 (0·0–0·1)	53·0 (51·0–54·9)	193·2 (190·7–195·8)	104·2 (102·3–106·2)	39·2 (37·5–41·0)	12·2 (10·7–13·7)	2·1 (1·5–2·8)	0·1 (0·1–0·1)	0·0 (0·0–0·0)	2·02 (2·0–2·04)	1·23 (1·21–1·25)	0·27 (0·26–0·28)
1982	0·1 (0·0–0·1)	54·4 (52·3–56·5)	190·5 (187·9–193·2)	102·5 (100·6–104·4)	38·2 (36·5–40·0)	11·7 (10·2–13·1)	1·9 (1·4–2·6)	0·1 (0·1–0·1)	0·0 (0·0–0·0)	2·0 (1·97–2·02)	1·23 (1·21–1·24)	0·26 (0·25–0·27)
1983	0·1 (0·0–0·1)	55·5 (53·6–57·6)	188·1 (185·7–190·6)	101·9 (99·9–104·1)	37·8 (36·1–39·5)	11·4 (10·0–12·9)	1·9 (1·4–2·5)	0·1 (0·1–0·1)	0·0 (0·0–0·0)	1·98 (1·96–2·01)	1·22 (1·2–1·24)	0·26 (0·24–0·27)
1984	0·1 (0·0–0·1)	54·9 (52·9–57·0)	188·1 (185·6–190·7)	102·5 (100·5–104·6)	37·6 (35·9–39·3)	11·2 (9·9–12·7)	1·8 (1·4–2·3)	0·1 (0·0–0·1)	0·0 (0·0–0·0)	1·98 (1·96–2·01)	1·22 (1·2–1·23)	0·25 (0·24–0·26)
1985	0·1 (0·0–0·1)	52·7 (50·8–54·6)	186·0 (183·6–188·6)	102·0 (100·0–104·1)	37·2 (35·5–39·0)	10·9 (9·7–12·3)	1·7 (1·4–2·1)	0·1 (0·0–0·1)	0·0 (0·0–0·0)	1·95 (1·93–1·98)	1·19 (1·18–1·21)	0·25 (0·24–0·26)
1986	0·1 (0·0–0·1)	50·4 (48·5–52·4)	182·4 (179·8–184·9)	103·1 (101·0–105·2)	37·2 (35·6–39·1)	10·9 (9·7–12·1)	1·7 (1·3–2·1)	0·1 (0·0–0·1)	0·0 (0·0–0·0)	1·93 (1·9–1·96)	1·16 (1·15–1·18)	0·25 (0·24–0·26)
1987	0·1 (0·0–0·1)	48·5 (46·6–50·5)	180·7 (178·0–183·3)	105·6 (103·5–107·7)	37·7 (35·9–39·5)	11·1 (9·9–12·4)	1·6 (1·3–2·1)	0·1 (0·0–0·1)	0·0 (0·0–0·0)	1·93 (1·9–1·96)	1·15 (1·13–1·16)	0·25 (0·24–0·27)
1988	0·1 (0·0–0·1)	46·9 (45·0–48·8)	178·9 (176·3–181·6)	106·0 (103·9–108·2)	37·8 (36·0–39·6)	11·2 (9·9–12·5)	1·6 (1·3–2·1)	0·1 (0·0–0·1)	0·0 (0·0–0·0)	1·91 (1·89–1·94)	1·13 (1·11–1·15)	0·25 (0·24–0·27)
1989	0·1 (0·0–0·1)	45·7 (43·9–47·7)	177·4 (174·8–180·0)	105·2 (103·0–107·2)	37·7 (35·9–39·5)	11·3 (10·0–12·6)	1·6 (1·3–2·1)	0·1 (0·0–0·1)	0·0 (0·0–0·0)	1·89 (1·87–1·92)	1·12 (1·1–1·13)	0·25 (0·24–0·27)
1990	0·1 (0·0–0·1)	45·2 (43·5–47·0)	174·8 (172·3–177·4)	102·7 (100·7–104·8)	37·3 (35·6–39·1)	10·9 (9·7–12·1)	1·6 (1·2–2·0)	0·1 (0·0–0·1)	0·0 (0·0–0·0)	1·86 (1·84–1·89)	1·1 (1·09–1·11)	0·25 (0·24–0·26)
1991	0·1 (0·0–0·1)	45·3 (43·5–47·0)	164·7 (162·2–167·2)	99·1 (97·0–101·0)	36·9 (35·0–38·8)	9·9 (8·8–11·0)	1·4 (1·1–1·7)	0·1 (0·0–0·1)	0·0 (0·0–0·0)	1·79 (1·76–1·81)	1·05 (1·03–1·07)	0·24 (0·23–0·25)
1992	0·1 (0·0–0·1)	43·6 (41·9–45·4)	151·4 (149·0–153·9)	95·7 (93·6–97·7)	36·1 (34·3–38·0)	9·6 (8·5–10·8)	1·3 (1·1–1·6)	0·1 (0·0–0·1)	0·0 (0·0–0·0)	1·69 (1·67–1·71)	0·98 (0·96–0·99)	0·24 (0·22–0·25)
1993	0·1 (0·0–0·1)	37·3 (35·6–39·0)	134·3 (131·8–136·8)	90·5 (88·4–92·6)	33·9 (32·0–35·8)	10·3 (9·1–11·6)	1·4 (1·2–1·8)	0·0 (0·0–0·1)	0·0 (0·0–0·0)	1·54 (1·52–1·56)	0·86 (0·84–0·87)	0·23 (0·21–0·24)
1994	0·0 (0·0–0·1)	29·4 (27·7–31·0)	113·2 (110·9–115·6)	84·7 (82·7–86·9)	34·2 (32·3–36·3)	11·0 (9·7–12·5)	1·6 (1·3–2·0)	0·1 (0·0–0·1)	0·0 (0·0–0·0)	1·37 (1·35–1·4)	0·71 (0·7–0·73)	0·23 (0·22–0·25)
1995	0·0 (0·0–0·1)	23·7 (22·2–25·3)	97·9 (95·6–100·2)	81·5 (79·5–83·9)	35·9 (34·0–37·8)	11·4 (10·0–13·0)	1·7 (1·4–2·1)	0·1 (0·0–0·1)	0·0 (0·0–0·0)	1·26 (1·24–1·29)	0·61 (0·59–0·62)	0·25 (0·23–0·26)
1996	0·0 (0·0–0·1)	20·2 (18·8–21·7)	89·7 (87·6–91·9)	81·6 (79·8–83·6)	36·1 (34·2–38·0)	11·7 (10·2–13·4)	1·8 (1·4–2·2)	0·1 (0·0–0·1)	0·0 (0·0–0·0)	1·21 (1·18–1·23)	0·55 (0·54–0·56)	0·25 (0·24–0·26)
1997	0·0 (0·0–0·1)	17·8 (16·4–19·4)	84·2 (82·0–86·4)	83·4 (81·4–85·5)	36·9 (35·0–38·9)	12·2 (10·7–14·1)	1·9 (1·5–2·4)	0·1 (0·0–0·1)	0·0 (0·0–0·0)	1·18 (1·16–1·21)	0·51 (0·5–0·52)	0·26 (0·24–0·27)
1998	0·0 (0·0–0·1)	15·7 (14·3–17·1)	78·2 (76·1–80·4)	85·5 (83·5–87·5)	38·9 (36·9–40·9)	12·9 (11·4–14·7)	2·0 (1·6–2·6)	0·1 (0·1–0·1)	0·0 (0·0–0·0)	1·17 (1·14–1·19)	0·47 (0·46–0·48)	0·27 (0·26–0·28)
1999	0·0 (0·0–0·1)	13·9 (12·6–15·3)	72·1 (70·1–74·2)	88·0 (86·2–89·9)	42·0 (40·0–44·0)	13·8 (12·3–15·6)	2·2 (1·8–2·8)	0·1 (0·1–0·1)	0·0 (0·0–0·0)	1·16 (1·14–1·18)	0·43 (0·42–0·44)	0·29 (0·28–0·3)
2000	0·0 (0·0–0·1)	12·8 (11·5–14·1)	67·2 (65·1–69·2)	91·0 (89·2–92·8)	45·8 (44·0–47·8)	14·8 (13·3–16·4)	2·4 (1·9–2·9)	0·1 (0·1–0·1)	0·0 (0·0–0·0)	1·17 (1·15–1·19)	0·4 (0·39–0·41)	0·32 (0·3–0·33)
2001	0·0 (0·0–0·1)	12·2 (11·0–13·6)	63·7 (61·6–65·8)	93·6 (91·8–95·6)	49·9 (47·8–52·0)	15·9 (14·4–17·6)	2·6 (2·1–3·2)	0·1 (0·1–0·2)	0·0 (0·0–0·0)	1·19 (1·17–1·22)	0·38 (0·37–0·39)	0·34 (0·33–0·36)
2002	0·0 (0·0–0·1)	11·7 (10·5–13·0)	59·2 (57·1–61·3)	94·9 (93·0–96·8)	54·4 (52·4–56·4)	17·5 (15·8–19·0)	3·0 (2·3–3·7)	0·1 (0·1–0·2)	0·0 (0·0–0·0)	1·2 (1·18–1·22)	0·35 (0·34–0·37)	0·37 (0·36–0·39)
2003	0·0 (0·0–0·1)	11·0 (9·8–12·3)	53·7 (51·6–55·9)	96·3 (94·3–98·2)	59·5 (57·5–61·4)	19·4 (17·5–21·0)	3·3 (2·6–4·2)	0·1 (0·1–0·2)	0·0 (0·0–0·0)	1·22 (1·19–1·24)	0·32 (0·31–0·34)	0·41 (0·4–0·43)
2004	0·0 (0·0–0·1)	10·7 (9·6–12·0)	49·9 (47·7–52·1)	99·1 (97·3–101·0)	65·8 (63·9–67·7)	21·6 (19·7–23·5)	3·7 (2·9–4·7)	0·2 (0·1–0·2)	0·0 (0·0–0·0)	1·26 (1·23–1·28)	0·3 (0·29–0·32)	0·46 (0·44–0·47)
2005	0·0 (0·0–0·1)	10·8 (9·6–12·2)	47·9 (45·7–50·1)	102·1 (100·3–104·1)	73·6 (71·6–75·7)	24·5 (22·7–26·5)	4·1 (3·1–5·2)	0·2 (0·1–0·3)	0·0 (0·0–0·0)	1·32 (1·29–1·34)	0·29 (0·28–0·31)	0·51 (0·5–0·53)
2006	0·0 (0·0–0·1)	11·2 (9·9–12·5)	47·6 (45·4–49·8)	105·3 (103·2–107·3)	82·8 (80·9–84·8)	27·9 (26·0–29·9)	4·4 (3·4–5·5)	0·2 (0·1–0·3)	0·0 (0·0–0·0)	1·4 (1·37–1·42)	0·29 (0·28–0·31)	0·58 (0·56–0·59)
2007	0·0 (0·0–0·1)	11·6 (10·3–13·0)	48·1 (46·1–50·2)	107·7 (105·6–109·9)	91·9 (89·9–93·8)	31·4 (29·4–33·4)	4·7 (3·7–5·8)	0·2 (0·1–0·3)	0·0 (0·0–0·0)	1·48 (1·46–1·5)	0·3 (0·29–0·31)	0·64 (0·63–0·66)
2008	0·0 (0·0–0·1)	11·8 (10·5–13·2)	48·1 (45·9–50·3)	107·1 (105·1–109·3)	97·5 (95·6–99·6)	34·3 (32·2–36·2)	5·1 (4·0–6·2)	0·2 (0·1–0·3)	0·0 (0·0–0·0)	1·52 (1·5–1·54)	0·3 (0·29–0·31)	0·69 (0·67–0·7)
2009	0·0 (0·0–0·1)	11·7 (10·4–13·1)	46·9 (44·8–49·0)	103·2 (101·1–105·4)	99·7 (97·6–101·7)	36·2 (34·2–38·2)	5·5 (4·3–6·7)	0·2 (0·1–0·3)	0·0 (0·0–0·0)	1·52 (1·49–1·54)	0·29 (0·28–0·31)	0·71 (0·69–0·72)
2010	0·0 (0·0–0·1)	11·4 (10·1–12·8)	44·7 (42·6–46·9)	97·9 (95·8–99·9)	99·1 (97·1–101·3)	36·9 (35·1–38·9)	5·9 (4·7–7·2)	0·2 (0·1–0·4)	0·0 (0·0–0·0)	1·48 (1·46–1·5)	0·28 (0·27–0·3)	0·71 (0·7–0·73)
2011	0·0 (0·0–0·1)	11·2 (9·9–12·7)	42·9 (40·8–45·2)	94·1 (92·0–96·1)	98·6 (96·6–100·7)	37·4 (35·5–39·3)	6·4 (5·1–7·7)	0·3 (0·2–0·4)	0·0 (0·0–0·0)	1·45 (1·43–1·48)	0·27 (0·26–0·29)	0·71 (0·7–0·73)
2012	0·0 (0·0–0·1)	11·3 (10·0–12·9)	42·4 (40·3–44·7)	93·3 (91·1–95·3)	100·2 (97·9–102·4)	39·0 (37·2–40·9)	6·8 (5·5–8·1)	0·3 (0·2–0·4)	0·0 (0·0–0·0)	1·47 (1·44–1·49)	0·27 (0·25–0·29)	0·73 (0·71–0·75)
2013	0·0 (0·0–0·1)	11·5 (10·2–13·1)	43·2 (41·1–45·8)	94·4 (92·3–96·5)	103·5 (101·4–105·8)	41·7 (39·9–43·7)	7·1 (6·0–8·5)	0·3 (0·2–0·4)	0·0 (0·0–0·0)	1·51 (1·48–1·53)	0·27 (0·26–0·29)	0·76 (0·75–0·78)
2014	0·0 (0·0–0·1)	11·9 (10·5–13·6)	45·6 (43·1–48·0)	96·6 (94·5–98·9)	106·9 (104·7–109·1)	44·9 (43·1–47·0)	7·3 (6·2–8·7)	0·2 (0·1–0·4)	0·0 (0·0–0·0)	1·57 (1·54–1·59)	0·29 (0·27–0·31)	0·8 (0·78–0·81)
2015	0·0 (0·0–0·1)	12·4 (10·7–14·1)	48·5 (46·0–51·0)	98·5 (96·3–100·7)	108·4 (105·9–110·7)	47·1 (45·1–49·5)	7·2 (5·9–8·6)	0·2 (0·1–0·3)	0·0 (0·0–0·0)	1·61 (1·58–1·64)	0·3 (0·29–0·32)	0·81 (0·8–0·83)
2016	0·0 (0·0–0·1)	12·6 (10·8–14·6)	50·5 (46·6–54·6)	99·4 (95·8–103·0)	106·6 (102·5–110·6)	46·1 (42·5–49·8)	6·7 (5·3–8·2)	0·2 (0·1–0·3)	0·0 (0·0–0·0)	1·61 (1·54–1·69)	0·32 (0·29–0·34)	0·8 (0·76–0·83)
2017	0·0 (0·0–0·1)	12·6 (10·7–14·8)	51·1 (44·9–58·2)	99·4 (93·5–105·9)	103·0 (95·5–111·2)	43·4 (37·6–50·0)	6·2 (4·7–7·9)	0·2 (0·1–0·3)	0·0 (0·0–0·0)	1·58 (1·45–1·73)	0·32 (0·28–0·37)	0·76 (0·7–0·84)

Supplemental Results Table 5: Age-specific fertility rates per 1000, cumulative total fertility up to maternal age 25 (TFU25), cumulative total fertility from maternal age 30 to age 55 (TFO30), and cumulative total fertility, Hungary, 1950-2017

Year	10-14	15-19	20-24	25-29	30-34	35-39	40-44	45-49	50-54	TFR	TFU25	TFO30
1950	0·4 (0·2–0·9)	49·9 (48·0–51·9)	168·3 (165·9–170·5)	139·1 (137·1–141·3)	91·9 (89·4–94·5)	45·8 (43·7–47·8)	17·2 (15·3–19·2)	1·1 (0·7–1·6)	0·0 (0·0–0·0)	2·57 (2·54–2·59)	1·09 (1·08–1·11)	0·78 (0·76–0·8)
1951	0·4 (0·2–0·9)	48·1 (46·1–50·0)	165·5 (163·1–168·0)	137·1 (135·1–139·1)	91·7 (89·3–94·0)	42·4 (40·1–44·6)	16·5 (14·9–18·4)	1·1 (0·7–1·6)	0·0 (0·0–0·0)	2·51 (2·49–2·54)	1·07 (1·06–1·09)	0·76 (0·74–0·78)
1952	0·4 (0·2–0·9)	48·7 (46·7–50·8)	171·1 (168·6–173·5)	143·7 (141·7–145·7)	98·5 (96·2–100·9)	44·7 (42·4–47·0)	17·3 (15·7–19·2)	1·1 (0·7–1·6)	0·0 (0·0–0·0)	2·63 (2·6–2·66)	1·1 (1·08–1·12)	0·81 (0·79–0·83)
1953	0·4 (0·2–0·9)	51·3 (49·3–53·5)	186·1 (183·6–188·5)	158·4 (156·3–160·5)	106·2 (104·0–108·5)	50·9 (48·6–53·6)	18·1 (16·4–20·0)	1·1 (0·7–1·6)	0·0 (0·0–0·0)	2·86 (2·83–2·89)	1·19 (1·17–1·2)	0·88 (0·86–0·9)
1954	0·4 (0·2–0·9)	53·5 (51·5–55·5)	192·5 (190·1–195·0)	159·1 (156·9–161·3)	102·5 (100·4–104·6)	54·2 (51·7–56·9)	16·6 (14·9–18·4)	1·0 (0·7–1·5)	0·0 (0·0–0·0)	2·9 (2·86–2·93)	1·23 (1·21–1·25)	0·87 (0·85–0·89)
1955	0·4 (0·2–0·9)	54·3 (52·3–56·3)	186·6 (184·4–188·9)	143·0 (141·1–145·1)	89·5 (87·3–91·6)	51·0 (48·5–53·6)	13·6 (11·8–15·4)	0·9 (0·6–1·3)	0·0 (0·0–0·0)	2·7 (2·67–2·73)	1·21 (1·19–1·22)	0·78 (0·76–0·79)
1956	0·4 (0·2–0·9)	54·5 (52·4–56·5)	176·3 (174·0–178·9)	125·8 (123·8–127·9)	74·7 (72·5–76·8)	43·2 (40·8–45·6)	10·8 (9·2–12·4)	0·8 (0·5–1·2)	0·0 (0·0–0·0)	2·43 (2·4–2·46)	1·16 (1·14–1·17)	0·65 (0·63–0·67)
1957	0·4 (0·2–0·9)	54·4 (52·4–56·5)	167·8 (165·4–170·3)	113·3 (111·1–115·2)	63·8 (61·7–65·7)	35·5 (33·3–37·7)	9·1 (7·6–10·6)	0·7 (0·4–1·0)	0·0 (0·0–0·0)	2·22 (2·19–2·25)	1·11 (1·1–1·13)	0·55 (0·53–0·57)
1958	0·4 (0·2–0·9)	53·8 (51·8–55·8)	162·5 (160·0–165·1)	106·9 (104·8–109·0)	57·8 (55·8–59·8)	30·1 (28·0–32·1)	8·3 (6·9–9·8)	0·6 (0·4–0·9)	0·0 (0·0–0·0)	2·1 (2·07–2·13)	1·08 (1·07–1·1)	0·48 (0·46–0·51)
1959	0·4 (0·2–0·9)	52·8 (51·1–54·8)	158·1 (155·6–160·6)	103·8 (101·7–105·8)	54·2 (52·3–56·2)	26·4 (24·7–28·4)	7·9 (6·4–9·3)	0·5 (0·3–0·8)	0·0 (0·0–0·0)	2·02 (1·99–2·05)	1·06 (1·04–1·07)	0·45 (0·43–0·46)
1960	0·4 (0·2–0·9)	51·3 (49·6–53·1)	154·2 (151·7–156·6)	100·6 (98·5–102·5)	51·1 (49·3–53·2)	23·4 (21·8–25·4)	7·3 (6·0–8·8)	0·5 (0·3–0·7)	0·0 (0·0–0·0)	1·94 (1·92–1·97)	1·03 (1·01–1·05)	0·41 (0·4–0·43)
1961	0·4 (0·2–0·9)	48·4 (46·6–50·2)	148·0 (145·7–150·4)	97·2 (95·2–99·4)	48·4 (46·5–50·3)	21·6 (20·0–23·5)	6·8 (5·6–8·1)	0·4 (0·3–0·6)	0·0 (0·0–0·0)	1·86 (1·83–1·88)	0·98 (0·97–1·0)	0·39 (0·37–0·4)
1962	0·4 (0·2–0·8)	45·5 (43·7–47·5)	144·1 (141·6–146·6)	97·1 (95·1–99·1)	47·4 (45·5–49·3)	20·8 (19·1–22·6)	6·4 (5·2–7·7)	0·4 (0·2–0·6)	0·0 (0·0–0·0)	1·81 (1·79–1·84)	0·95 (0·93–0·97)	0·37 (0·36–0·39)
1963	0·4 (0·2–0·8)	43·6 (41·9–45·6)	145·4 (143·1–148·0)	99·1 (97·0–101·1)	47·3 (45·4–49·2)	19·7 (18·0–21·4)	5·7 (4·6–6·9)	0·4 (0·2–0·6)	0·0 (0·0–0·0)	1·81 (1·78–1·83)	0·95 (0·93–0·96)	0·37 (0·35–0·38)
1964	0·4 (0·2–0·8)	43·3 (41·4–45·1)	146·6 (144·2–149·0)	100·7 (98·8–102·7)	46·9 (45·0–48·9)	18·6 (17·0–20·3)	5·2 (4·1–6·3)	0·4 (0·2–0·6)	0·0 (0·0–0·0)	1·81 (1·78–1·84)	0·95 (0·94–0·97)	0·36 (0·34–0·37)
1965	0·4 (0·2–0·8)	45·4 (43·7–47·3)	150·1 (147·6–152·7)	103·9 (101·8–106·0)	47·9 (46·0–49·9)	18·6 (16·9–20·3)	5·0 (4·0–6·2)	0·4 (0·2–0·6)	0·0 (0·0–0·0)	1·86 (1·83–1·88)	0·98 (0·96–1·0)	0·36 (0·34–0·38)
1966	0·4 (0·2–0·9)	49·2 (47·4–51·2)	158·8 (156·3–161·4)	110·1 (108·1–112·2)	50·8 (48·8–52·9)	19·6 (18·0–21·4)	5·2 (4·1–6·5)	0·4 (0·2–0·6)	0·0 (0·0–0·0)	1·97 (1·95–2·0)	1·04 (1·03–1·06)	0·38 (0·37–0·4)
1967	0·4 (0·2–0·9)	52·2 (50·2–54·0)	166·5 (164·1–169·1)	115·6 (113·5–117·6)	53·3 (51·2–55·5)	20·1 (18·4–22·0)	5·2 (4·0–6·5)	0·4 (0·2–0·6)	0·0 (0·0–0·0)	2·07 (2·04–2·09)	1·1 (1·08–1·11)	0·39 (0·38–0·41)
1968	0·4 (0·2–0·9)	52·5 (50·7–54·4)	167·1 (164·7–169·4)	116·3 (114·1–118·5)	53·8 (51·7–56·0)	19·4 (17·7–21·3)	4·8 (3·7–6·1)	0·3 (0·2–0·5)	0·0 (0·0–0·0)	2·07 (2·05–2·1)	1·1 (1·08–1·12)	0·39 (0·38–0·41)
1969	0·4 (0·2–0·9)	51·5 (49·7–53·3)	163·6 (161·2–166·0)	112·5 (110·4–114·4)	52·9 (51·0–54·9)	18·7 (17·0–20·5)	4·5 (3·4–5·7)	0·3 (0·2–0·5)	0·0 (0·0–0·0)	2·02 (2·0–2·05)	1·08 (1·06–1·09)	0·38 (0·37–0·4)
1970	0·4 (0·2–0·9)	51·0 (49·3–52·8)	162·4 (160·1–164·7)	107·7 (105·7–109·6)	51·1 (49·2–53·1)	18·0 (16·4–19·9)	4·2 (3·3–5·4)	0·3 (0·2–0·4)	0·0 (0·0–0·0)	1·98 (1·95–2·0)	1·07 (1·05–1·08)	0·37 (0·35–0·38)
1971	0·4 (0·2–0·9)	51·5 (49·7–53·3)	161·2 (158·8–163·6)	105·3 (103·3–107·4)	48·7 (46·6–50·6)	17·1 (15·5–18·8)	4·0 (3·1–5·1)	0·3 (0·2–0·4)	0·0 (0·0–0·0)	1·94 (1·92–1·97)	1·07 (1·05–1·08)	0·35 (0·34–0·36)
1972	0·4 (0·2–0·9)	54·8 (53·0–56·7)	160·3 (157·9–162·9)	108·4 (106·3–110·5)	49·7 (47·7–51·7)	17·6 (15·9–19·3)	4·1 (3·1–5·1)	0·3 (0·2–0·4)	0·0 (0·0–0·0)	1·98 (1·95–2·0)	1·08 (1·06–1·09)	0·36 (0·34–0·37)
1973	0·5 (0·2–1·0)	62·0 (60·2–63·9)	172·6 (170·2–174·9)	121·6 (119·4–123·7)	56·2 (54·2–58·2)	19·9 (18·1–21·7)	4·6 (3·5–5·9)	0·3 (0·2–0·4)	0·0 (0·0–0·0)	2·19 (2·16–2·21)	1·18 (1·16–1·19)	0·4 (0·39–0·42)
1974	0·5 (0·2–1·1)	68·7 (66·7–70·6)	185·3 (182·8–187·7)	133·8 (131·8–135·9)	61·0 (59·1–62·9)	21·1 (19·5–22·9)	4·8 (3·7–6·2)	0·3 (0·2–0·4)	0·0 (0·0–0·0)	2·38 (2·35–2·4)	1·27 (1·26–1·29)	0·44 (0·42–0·45)
1975	0·5 (0·2–1·1)	71·8 (69·7–73·8)	182·7 (180·5–185·0)	132·2 (130·1–134·3)	58·9 (56·9–60·9)	19·9 (18·3–21·7)	4·5 (3·5–5·8)	0·2 (0·1–0·4)	0·0 (0·0–0·0)	2·35 (2·33–2·38)	1·28 (1·26–1·29)	0·42 (0·4–0·43)
1976	0·5 (0·2–1·1)	72·8 (70·7–75·0)	175·8 (173·6–178·0)	123·6 (121·5–125·7)	54·3 (52·4–56·3)	18·3 (16·6–20·1)	4·2 (3·3–5·4)	0·2 (0·1–0·3)	0·0 (0·0–0·0)	2·25 (2·23–2·27)	1·25 (1·23–1·26)	0·38 (0·37–0·4)
1977	0·5 (0·2–1·1)	73·0 (70·9–75·1)	170·3 (168·1–172·7)	114·6 (112·7–116·5)	50·3 (48·4–52·4)	17·2 (15·6–19·0)	4·0 (3·1–5·1)	0·2 (0·1–0·3)	0·0 (0·0–0·0)	2·15 (2·13–2·18)	1·22 (1·2–1·23)	0·36 (0·34–0·38)
1978	0·5 (0·2–1·1)	72·0 (70·0–74·2)	165·7 (163·5–168·0)	108·4 (106·5–110·4)	46·6 (44·8–48·5)	16·3 (14·9–18·0)	3·8 (3·1–4·8)	0·2 (0·1–0·3)	0·0 (0·0–0·0)	2·07 (2·04–2·09)	1·19 (1·18–1·21)	0·33 (0·32–0·35)
1979	0·5 (0·2–1·1)	69·1 (67·0–71·4)	159·1 (156·6–161·5)	104·1 (102·2–106·0)	43·8 (41·9–45·6)	15·4 (14·1–16·8)	3·6 (2·9–4·4)	0·2 (0·1–0·3)	0·0 (0·0–0·0)	1·98 (1·96–2·0)	1·14 (1·13–1·16)	0·31 (0·3–0·33)
1980	0·5 (0·2–1·0)	65·2 (63·0–67·2)	153·7 (151·1–156·2)	101·0 (99·2–103·0)	42·0 (40·3–43·8)	14·3 (13·1–15·7)	3·4 (2·8–4·2)	0·2 (0·1–0·2)	0·0 (0·0–0·0)	1·9 (1·88–1·93)	1·1 (1·08–1·12)	0·3 (0·28–0·32)
1981	0·5 (0·2–1·0)	60·6 (58·4–62·7)	149·9 (147·4–152·4)	98·0 (96·0–99·9)	40·2 (38·4–42·1)	13·4 (11·9–15·1)	3·3 (2·6–4·1)	0·1 (0·1–0·2)	0·0 (0·0–0·0)	1·83 (1·81–1·85)	1·05 (1·04–1·07)	0·29 (0·27–0·3)
1982	0·5 (0·2–0·9)	56·1 (54·0–58·2)	145·4 (143·0–147·8)	94·9 (93·0–96·8)	38·9 (37·1–41·1)	13·2 (11·7–15·2)	3·3 (2·6–4·0)	0·1 (0·1–0·2)	0·0 (0·0–0·0)	1·76 (1·74–1·79)	1·01 (0·99–1·03)	0·28 (0·26–0·3)
1983	0·4 (0·2–0·9)	53·5 (51·4–55·5)	144·6 (142·2–147·1)	94·7 (92·7–96·8)	39·9 (38·1–41·8)	13·6 (12·1–15·3)	3·3 (2·7–4·0)	0·1 (0·1–0·2)	0·0 (0·0–0·0)	1·75 (1·72–1·78)	0·99 (0·97–1·02)	0·28 (0·27–0·3)
1984	0·4 (0·2–0·9)	52·3 (50·2–54·3)	148·1 (145·5–150·7)	98·6 (96·6–100·5)	42·6 (40·8–44·3)	14·5 (13·2–16·0)	3·4 (2·8–3·9)	0·1 (0·1–0·2)	0·0 (0·0–0·0)	1·8 (1·77–1·83)	1·0 (0·99–1·02)	0·3 (0·29–0·32)
1985	0·4 (0·2–0·9)	50·7 (48·7–52·9)	150·1 (147·6–152·5)	103·6 (101·6–105·5)	44·4 (42·7–46·1)	15·2 (13·8–16·5)	3·4 (2·9–4·0)	0·1 (0·1–0·2)	0·0 (0·0–0·0)	1·84 (1·81–1·86)	1·01 (0·99–1·02)	0·32 (0·3–0·33)
1986	0·4 (0·2–0·9)	48·5 (46·5–50·4)	148·4 (145·7–151·0)	106·5 (104·5–108·7)	44·5 (42·8–46·3)	15·2 (13·7–16·7)	3·3 (2·7–4·1)	0·1 (0·1–0·2)	0·0 (0·0–0·0)	1·83 (1·81–1·87)	0·99 (0·97–1·0)	0·32 (0·3–0·33)
1987	0·4 (0·2–0·8)	45·7 (43·9–47·8)	146·4 (143·9–149·1)	108·3 (106·2–110·5)	43·8 (42·1–45·7)	15·5 (13·9–17·2)	3·4 (2·7–4·2)	0·1 (0·1–0·2)	0·0 (0·0–0·0)	1·82 (1·79–1·85)	0·96 (0·95–0·98)	0·31 (0·3–0·33)
1988	0·4 (0·2–0·8)	42·6 (40·7–44·2)	144·8 (142·2–147·5)	110·2 (108·1–112·4)	43·8 (42·0–45·7)	16·2 (14·6–17·9)	3·5 (2·8–4·3)	0·1 (0·1–0·2)	0·0 (0·0–0·0)	1·81 (1·79–1·83)	0·94 (0·92–0·95)	0·32 (0·31–0·33)
1989	0·4 (0·2–0·8)	40·1 (38·4–42·0)	144·5 (142·0–146·9)	112·5 (110·3–114·8)	45·6 (43·8–47·5)	17·0 (15·5–18·6)	3·5 (3·0–4·2)	0·1 (0·1–0·2)	0·0 (0·0–0·0)	1·82 (1·8–1·84)	0·92 (0·91–0·94)	0·33 (0·32–0·34)
1990	0·4 (0·2–0·8)	38·9 (37·2–40·8)	145·1 (142·6–147·5)	114·4 (112·2–116·4)	47·9 (46·1–49·7)	17·3 (16·0–18·7)	3·5 (3·0–4·1)	0·1 (0·1–0·2)	0·0 (0·0–0·0)	1·84 (1·81–1·86)	0·92 (0·91–0·94)	0·34 (0·33–0·36)
1991	0·4 (0·2–0·8)	37·2 (35·5–39·2)	141·0 (138·4–143·5)	114·2 (112·2–116·4)	48·2 (46·3–50·2)	17·0 (15·6–18·5)	3·5 (2·9–4·2)	0·1 (0·1–0·2)	0·0 (0·0–0·0)	1·81 (1·78–1·84)	0·89 (0·88–0·91)	0·34 (0·33–0·36)
1992	0·4 (0·2–0·7)	35·1 (33·5–36·8)	130·3 (127·8–132·9)	112·8 (110·6–115·1)	46·4 (44·6–48·6)	16·6 (15·0–18·4)	3·6 (2·9–4·4)	0·1 (0·1–0·2)	0·0 (0·0–0·0)	1·73 (1·7–1·75)	0·83 (0·81–0·85)	0·33 (0·32–0·35)
1993	0·3 (0·2–0·7)	33·6 (31·9–35·3)	119·6 (117·1–122·3)	111·5 (109·4–113·7)	46·6 (44·6–48·7)	16·4 (14·8–18·2)	3·7 (2·9–4·6)	0·1 (0·1–0·2)	0·0 (0·0–0·0)	1·66 (1·64–1·68)	0·77 (0·75–0·78)	0·33 (0·32–0·35)
1994	0·3 (0·1–0·7)	32·0 (30·4–33·7)	110·1 (107·7–112·4)	109·1 (107·1–111·4)	48·5 (46·5–50·6)	16·6 (15·0–18·4)	3·8 (3·1–4·6)	0·1 (0·1–0·2)	0·0 (0·0–0·0)	1·6 (1·58–1·63)	0·71 (0·7–0·73)	0·35 (0·33–0·36)
1995	0·3 (0·1–0·7)	29·8 (28·2–31·6)	99·5 (97·1–101·6)	104·3 (102·2–106·4)	48·9 (46·8–50·9)	17·0 (15·5–18·7)	3·9 (3·3–4·7)	0·1 (0·1–0·2)	0·0 (0·0–0·0)	1·52 (1·5–1·54)	0·65 (0·63–0·66)	0·35 (0·34–0·36)
1996	0·3 (0·1–0·6)	27·7 (26·1–29·3)	89·2 (87·0–91·5)	98·9 (96·9–100·9)	48·6 (46·6–50·8)	17·2 (15·6–18·9)	4·0 (3·3–4·8)	0·1 (0·1–0·2)	0·0 (0·0–0·0)	1·43 (1·41–1·45)	0·59 (0·57–0·6)	0·35 (0·34–0·36)
1997	0·3 (0·1–0·6)	25·9 (24·2–27·5)	81·1 (79·0–83·4)	95·5 (93·5–97·4)	48·9 (46·8–51·1)	17·2 (15·5–19·1)	4·0 (3·2–4·9)	0·2 (0·1–0·2)	0·0 (0·0–0·0)	1·37 (1·34–1·39)	0·54 (0·52–0·55)	0·35 (0·34–0·37)
1998	0·3 (0·1–0·6)	24·6 (22·9–26·3)	75·2 (73·1–77·3)	94·0 (92·0–96·1)	50·2 (48·2–52·2)	17·6 (15·9–19·6)	4·1 (3·2–5·1)	0·2 (0·1–0·2)	0·0 (0·0–0·0)	1·33 (1·31–1·36)	0·5 (0·49–0·51)	0·36 (0·35–0·38)
1999	0·3 (0·1–0·6)	23·4 (21·8–25·0)	70·7 (68·6–72·8)	94·1 (92·0–96·2)	53·0 (50·9–55·1)	18·9 (16·9–21·0)	4·3 (3·4–5·3)	0·2 (0·1–0·2)	0·0 (0·0–0·0)	1·32 (1·3–1·35)	0·47 (0·46–0·48)	0·38 (0·37–0·4)
2000	0·3 (0·1–0·6)	22·2 (20·6–23·8)	66·1 (64·1–68·2)	93·9 (91·9–95·8)	56·6 (54·5–58·8)	20·7 (18·9–22·7)	4·5 (3·7–5·5)	0·2 (0·1–0·2)	0·0 (0·0–0·0)	1·32 (1·3–1·35)	0·44 (0·43–0·46)	0·41 (0·39–0·43)
2001	0·3 (0·1–0·6)	21·5 (19·9–23·1)	61·8 (59·8–64·0)	92·5 (90·6–94·5)	59·5 (57·4–61·6)	22·2 (20·4–24·1)	4·6 (3·8–5·6)	0·2 (0·1–0·3)	0·0 (0·0–0·0)	1·31 (1·29–1·34)	0·42 (0·41–0·43)	0·43 (0·42–0·45)
2002	0·3 (0·1–0·6)	21·1 (19·5–22·7)	58·5 (56·3–60·8)	90·7 (88·9–92·8)	61·9 (59·7–64·0)	23·3 (21·5–25·3)	4·7 (3·8–5·8)	0·2 (0·1–0·3)	0·0 (0·0–0·0)	1·3 (1·28–1·33)	0·4 (0·39–0·41)	0·45 (0·44–0·47)
2003	0·3 (0·1–0·5)	20·6 (19·0–22·3)	55·4 (53·3–57·6)	89·6 (87·7–91·6)	65·4 (63·2–67·5)	24·6 (22·8–26·7)	4·8 (3·8–6·0)	0·2 (0·1–0·3)	0·0 (0·0–0·0)	1·3 (1·28–1·33)	0·38 (0·37–0·4)	0·48 (0·46–0·49)
2004	0·3 (0·1–0·5)	20·2 (18·6–21·9)	52·9 (50·6–55·3)	90·0 (88·0–92·0)	70·3 (68·3–72·5)	26·3 (24·5–28·2)	4·9 (3·9–6·2)	0·2 (0·1–0·3)	0·0 (0·0–0·0)	1·33 (1·3–1·35)	0·37 (0·35–0·38)	0·51 (0·49–0·52)
2005	0·3 (0·1–0·5)	19·9 (18·4–21·7)	51·1 (48·9–53·4)	90·7 (88·9–92·7)	75·7 (73·5–77·8)	28·0 (26·1–30·2)	5·1 (4·0–6·4)	0·2 (0·1–0·3)	0·0 (0·0–0·0)	1·36 (1·33–1·38)	0·36 (0·34–0·37)	0·55 (0·53–0·56)
2006	0·3 (0·1–0·5)	19·7 (18·1–21·5)	49·5 (47·2–51·8)	90·7 (88·6–92·7)	79·2 (77·0–81·2)	29·6 (27·6–31·6)	5·3 (4·2–6·6)	0·2 (0·1–0·3)	0·0 (0·0–0·0)	1·37 (1·34–1·4)	0·35 (0·33–0·36)	0·57 (0·55–0·59)
2007	0·3 (0·1–0·5)	19·6 (18·0–21·4)	48·3 (46·2–50·6)	89·9 (87·9–92·0)	80·4 (78·3–82·6)	31·1 (29·0–33·2)	5·7 (4·5–6·9)	0·2 (0·1–0·3)	0·0 (0·0–0·0)	1·38 (1·35–1·4)	0·34 (0·33–0·35)	0·59 (0·57–0·6)
2008	0·3 (0·1–0·5)	19·1 (17·5–20·9)	46·8 (44·6–49·1)	87·6 (85·6–89·7)	80·5 (78·5–82·4)	32·9 (30·9–34·9)	6·1 (4·9–7·3)	0·2 (0·1–0·4)	0·0 (0·0–0·0)	1·37 (1·34–1·39)	0·33 (0·32–0·34)	0·6 (0·58–0·61)
2009	0·3 (0·1–0·5)	18·4 (16·8–20·2)	44·2 (42·1–46·4)	83·2 (81·1–85·4)	79·6 (77·5–81·7)	34·6 (32·6–36·8)	6·5 (5·3–7·9)	0·3 (0·2–0·4)	0·0 (0·0–0·0)	1·34 (1·31–1·36)	0·31 (0·3–0·33)	0·61 (0·59–0·62)
2010	0·3 (0·1–0·5)	18·2 (16·7–20·1)	42·1 (40·0–44·5)	79·0 (77·0–81·2)	79·0 (76·8–81·1)	36·3 (34·4–38·5)	7·0 (5·7–8·4)	0·3 (0·2–0·4)	0·0 (0·0–0·0)	1·31 (1·29–1·34)	0·3 (0·29–0·32)	0·61 (0·6–0·63)
2011	0·3 (0·1–0·5)	18·9 (17·3–20·8)	41·6 (39·5–43·8)	77·1 (74·9–79·3)	79·9 (77·8–82·1)	38·0 (36·0–40·0)	7·5 (6·2–8·9)	0·3 (0·2–0·4)	0·0 (0·0–0·0)	1·32 (1·29–1·35)	0·3 (0·29–0·32)	0·63 (0·61–0·65)
2012	0·3 (0·1–0·5)	20·2 (18·5–21·9)	42·4 (40·3–44·6)	76·7 (74·6–78·8)	81·7 (79·7–83·9)	39·7 (37·8–41·7)	7·9 (6·6–9·4)	0·3 (0·2–0·4)	0·0 (0·0–0·0)	1·35 (1·32–1·37)	0·31 (0·3–0·33)	0·65 (0·63–0·66)
2013	0·3 (0·1–0·6)	21·5 (19·7–23·3)	43·7 (41·5–46·0)	77·0 (74·8–79·2)	84·2 (81·9–86·5)	41·5 (39·6–43·6)	8·3 (7·0–9·8)	0·3 (0·2–0·4)	0·0 (0·0–0·0)	1·38 (1·36–1·41)	0·33 (0·31–0·35)	0·67 (0·65–0·69)
2014	0·3 (0·1–0·6)	22·4 (20·4–24·4)	45·2 (42·8–47·7)	78·2 (76·0–80·6)	87·0 (84·5–89·3)	43·3 (41·2–45·5)	8·5 (7·3–9·9)	0·3 (0·2–0·3)	0·0 (0·0–0·0)	1·43 (1·39–1·46)	0·34 (0·32–0·36)	0·7 (0·68–0·71)
2015	0·3 (0·1–0·6)	22·6 (20·3–25·0)	46·5 (43·2–50·2)	79·9 (76·6–83·4)	88·5 (85·0–92·0)	43·6 (40·6–46·7)	8·3 (7·1–9·6)	0·2 (0·2–0·3)	0·0 (0·0–0·0)	1·45 (1·39–1·51)	0·35 (0·32–0·37)	0·7 (0·68–0·73)
2016	0·3 (0·1–0·6)	22·2 (19·5–25·2)	47·0 (41·7–52·7)	81·4 (76·3–86·8)	87·8 (81·6–94·5)	41·9 (37·1–47·4)	7·7 (6·3–9·4)	0·2 (0·2–0·3)	0·0 (0·0–0·0)	1·44 (1·33–1·56)	0·35 (0·31–0·39)	0·69 (0·64–0·74)
2017	0·3 (0·1–0·6)	21·7 (18·7–25·3)	47·0 (40·0–55·3)	82·4 (75·7–89·7)	86·4 (77·5–96·4)	39·9 (33·2–47·7)	7·2 (5·6–9·5)	0·2 (0·2–0·3)	0·0 (0·0–0·0)	1·43 (1·26–1·61)	0·35 (0·29–0·4)	0·67 (0·59–0·76)

Supplemental Results Table 5: Age-specific fertility rates per 1000, cumulative total fertility up to maternal age 25 (TFU25), cumulative total fertility from maternal age 30 to age 55 (TFO30), and cumulative total fertility, Macedonia, 1950-2017

Year	10-14	15-19	20-24	25-29	30-34	35-39	40-44	45-49	50-54	TFR	TFU25	TFO30
1950	0·6 (0·2–1·2)	65·1 (55·3–76·4)	226·5 (198·9–255·5)	214·7 (199·9–230·1)	117·3 (101·5–134·8)	67·5 (52·6–85·0)	24·5 (18·1–32·5)	3·3 (2·1–5·0)	0·1 (0·1–0·1)	3·6 (3·14–4·1)	1·46 (1·27–1·66)	1·06 (0·87–1·29)
1951	0·6 (0·2–1·2)	64·5 (54·8–75·4)	224·6 (197·3–252·8)	211·3 (196·8–226·5)	115·8 (100·4–132·6)	65·8 (51·7–83·1)	23·7 (17·4–31·3)	3·2 (2·0–4·8)	0·1 (0·1–0·1)	3·55 (3·11–4·03)	1·45 (1·27–1·64)	1·04 (0·86–1·26)
1952	0·6 (0·2–1·1)	63·9 (54·0–74·7)	222·6 (195·1–250·3)	207·9 (192·8–222·9)	114·1 (98·1–130·6)	64·2 (50·3–81·3)	22·9 (16·7–30·5)	3·1 (1·9–4·6)	0·1 (0·1–0·1)	3·5 (3·07–3·96)	1·44 (1·25–1·62)	1·02 (0·85–1·23)
1953	0·5 (0·2–1·1)	63·4 (53·3–74·2)	220·7 (193·3–248·8)	204·5 (189·7–219·6)	112·4 (96·2–128·8)	62·5 (49·0–79·0)	22·1 (16·0–29·4)	2·9 (1·8–4·5)	0·1 (0·1–0·1)	3·45 (3·03–3·88)	1·42 (1·24–1·6)	1·0 (0·83–1·19)
1954	0·5 (0·2–1·1)	62·9 (53·2–73·8)	218·7 (190·9–245·9)	201·2 (185·8–216·1)	110·8 (94·6–127·4)	61·0 (47·5–76·3)	21·3 (15·3–28·6)	2·8 (1·8–4·3)	0·1 (0·1–0·1)	3·4 (3·0–3·84)	1·41 (1·23–1·59)	0·98 (0·82–1·15)
1955	0·5 (0·2–1·1)	62·4 (52·7–73·0)	216·9 (188·5–244·3)	198·0 (182·9–213·2)	109·2 (93·6–125·4)	59·5 (46·4–74·0)	20·6 (14·8–27·7)	2·7 (1·7–4·1)	0·1 (0·1–0·1)	3·35 (2·96–3·75)	1·4 (1·23–1·57)	0·96 (0·81–1·12)
1956	0·5 (0·2–1·1)	62·0 (52·3–72·6)	215·1 (186·5–242·8)	194·8 (179·9–209·4)	107·6 (92·3–123·4)	57·9 (45·0–72·2)	19·8 (14·3–26·5)	2·6 (1·6–3·9)	0·0 (0·0–0·1)	3·3 (2·93–3·69)	1·39 (1·22–1·56)	0·94 (0·8–1·1)
1957	0·5 (0·2–1·1)	61·5 (52·1–71·9)	213·3 (184·3–240·4)	191·6 (176·5–206·4)	106·1 (90·2–122·0)	56·4 (43·9–70·4)	19·1 (13·9–25·5)	2·5 (1·6–3·7)	0·0 (0·0–0·0)	3·25 (2·87–3·64)	1·38 (1·2–1·55)	0·92 (0·78–1·08)
1958	0·5 (0·2–1·1)	60·9 (51·3–71·4)	211·4 (182·7–238·5)	188·5 (172·9–203·8)	104·6 (88·9–121·3)	54·9 (42·1–68·6)	18·4 (13·2–24·8)	2·4 (1·5–3·6)	0·0 (0·0–0·0)	3·21 (2·81–3·62)	1·36 (1·19–1·54)	0·9 (0·76–1·07)
1959	0·5 (0·2–1·1)	60·4 (51·2–71·0)	209·7 (181·2–237·9)	185·4 (170·0–200·3)	103·2 (87·7–119·0)	53·4 (40·4–68·0)	17·8 (12·8–24·2)	2·3 (1·4–3·4)	0·0 (0·0–0·0)	3·16 (2·77–3·6)	1·35 (1·17–1·54)	0·88 (0·74–1·06)
1960	0·5 (0·2–1·1)	59·9 (50·7–71·0)	208·0 (179·5–237·7)	182·4 (167·1–197·9)	101·8 (86·8–118·4)	51·9 (39·5–67·6)	17·1 (12·3–23·5)	2·2 (1·4–3·3)	0·0 (0·0–0·0)	3·12 (2·72–3·57)	1·34 (1·16–1·54)	0·87 (0·71–1·04)
1961	0·5 (0·2–1·1)	59·3 (50·2–70·2)	206·2 (178·8–235·4)	179·3 (164·8–195·6)	100·4 (86·2–117·1)	50·4 (38·5–65·8)	16·5 (11·9–22·7)	2·1 (1·3–3·1)	0·0 (0·0–0·0)	3·07 (2·67–3·54)	1·33 (1·15–1·53)	0·85 (0·7–1·04)
1962	0·5 (0·2–1·1)	58·7 (49·8–69·5)	204·5 (177·1–234·8)	176·2 (162·2–192·4)	99·0 (85·3–116·4)	49·0 (37·3–63·8)	15·8 (11·4–21·8)	2·0 (1·2–3·0)	0·0 (0·0–0·0)	3·03 (2·64–3·5)	1·32 (1·14–1·52)	0·83 (0·68–1·02)
1963	0·5 (0·2–1·1)	58·1 (49·4–68·8)	202·7 (175·5–232·6)	172·9 (158·5–189·3)	97·7 (83·5–114·8)	47·5 (36·1–62·2)	15·2 (11·2–20·7)	1·9 (1·2–2·8)	0·0 (0·0–0·0)	2·98 (2·6–3·45)	1·31 (1·13–1·51)	0·81 (0·67–0·99)
1964	0·5 (0·2–1·1)	57·5 (48·9–67·9)	200·8 (174·2–230·6)	169·5 (155·2–185·7)	96·3 (82·7–113·3)	46·1 (35·2–59·9)	14·6 (10·7–19·6)	1·8 (1·1–2·7)	0·0 (0·0–0·0)	2·94 (2·56–3·38)	1·29 (1·13–1·49)	0·79 (0·65–0·96)
1965	0·5 (0·2–1·1)	57·0 (48·2–67·3)	198·8 (172·7–227·3)	166·1 (152·1–181·6)	95·1 (81·4–111·7)	44·7 (34·4–57·4)	14·0 (10·3–18·4)	1·7 (1·1–2·5)	0·0 (0·0–0·0)	2·89 (2·54–3·3)	1·28 (1·12–1·47)	0·78 (0·64–0·94)
1966	0·5 (0·2–1·1)	56·4 (47·8–66·3)	196·9 (171·3–224·2)	162·7 (149·2–177·6)	93·8 (80·3–109·2)	43·4 (33·3–54·8)	13·4 (9·9–17·6)	1·6 (1·1–2·4)	0·0 (0·0–0·0)	2·84 (2·53–3·23)	1·27 (1·11–1·44)	0·76 (0·63–0·91)
1967	0·5 (0·2–1·1)	55·7 (47·6–65·2)	194·9 (168·9–220·0)	159·2 (145·8–173·9)	92·5 (78·9–107·0)	42·1 (32·9–52·6)	12·8 (9·6–16·5)	1·5 (1·0–2·2)	0·0 (0·0–0·0)	2·8 (2·51–3·15)	1·26 (1·1–1·42)	0·74 (0·63–0·88)
1968	0·5 (0·2–1·1)	55·1 (47·2–64·5)	192·9 (168·1–218·4)	155·9 (142·3–169·6)	91·3 (78·0–104·7)	40·9 (32·4–50·7)	12·3 (9·3–15·7)	1·4 (1·0–2·1)	0·0 (0·0–0·0)	2·75 (2·48–3·08)	1·24 (1·09–1·4)	0·73 (0·63–0·85)
1969	0·5 (0·2–1·1)	54·6 (47·2–63·9)	191·0 (168·7–216·2)	152·6 (139·9–165·5)	90·2 (78·0–102·7)	39·8 (31·9–48·8)	11·8 (9·0–15·0)	1·4 (0·9–2·0)	0·0 (0·0–0·0)	2·71 (2·44–3·01)	1·23 (1·09–1·4)	0·72 (0·62–0·82)
1970	0·5 (0·2–1·1)	54·3 (46·8–62·8)	189·1 (166·6–212·8)	149·8 (137·4–162·6)	89·4 (77·7–102·0)	38·9 (31·5–46·6)	11·4 (8·9–14·2)	1·3 (0·9–1·8)	0·0 (0·0–0·0)	2·67 (2·41–2·96)	1·22 (1·08–1·38)	0·7 (0·62–0·8)
1971	0·5 (0·2–1·1)	54·4 (47·4–62·8)	187·7 (165·9–210·6)	148·7 (137·0–161·6)	88·6 (77·2–101·1)	38·1 (31·2–45·5)	11·0 (8·7–13·7)	1·2 (0·8–1·7)	0·0 (0·0–0·0)	2·65 (2·39–2·95)	1·21 (1·07–1·37)	0·69 (0·61–0·79)
1972	0·5 (0·2–1·1)	55·2 (47·9–63·8)	187·7 (166·0–211·0)	148·9 (137·7–161·6)	87·1 (76·2–99·2)	37·3 (30·4–44·6)	10·6 (8·4–13·3)	1·2 (0·8–1·7)	0·0 (0·0–0·0)	2·64 (2·36–2·95)	1·22 (1·07–1·37)	0·68 (0·59–0·78)
1973	0·5 (0·2–1·1)	56·5 (49·0–65·1)	188·3 (167·2–210·6)	149·5 (138·5–162·5)	85·4 (74·3–97·4)	36·3 (29·4–43·7)	10·3 (8·1–13·0)	1·1 (0·7–1·6)	0·0 (0·0–0·0)	2·64 (2·35–2·96)	1·23 (1·08–1·38)	0·67 (0·57–0·77)
1974	0·5 (0·2–1·1)	57·6 (50·0–66·1)	189·1 (167·7–211·1)	150·4 (139·3–163·1)	84·0 (73·5–95·9)	35·5 (28·6–43·2)	9·9 (7·7–12·6)	1·1 (0·7–1·5)	0·0 (0·0–0·0)	2·64 (2·35–2·96)	1·24 (1·09–1·39)	0·65 (0·56–0·76)
1975	0·5 (0·2–1·1)	57·5 (49·7–66·0)	189·7 (167·8–210·7)	151·2 (139·6–163·6)	82·7 (72·3–94·3)	34·7 (27·5–42·6)	9·6 (7·3–12·3)	1·0 (0·7–1·5)	0·0 (0·0–0·0)	2·64 (2·35–2·94)	1·24 (1·1–1·38)	0·64 (0·55–0·75)
1976	0·5 (0·2–1·1)	57·0 (49·7–65·3)	190·4 (169·4–210·8)	152·4 (140·6–164·1)	81·7 (71·3–92·5)	34·1 (27·2–42·2)	9·4 (7·0–12·0)	1·0 (0·6–1·4)	0·0 (0·0–0·0)	2·63 (2·36–2·92)	1·24 (1·1–1·37)	0·63 (0·54–0·73)
1977	0·5 (0·2–1·1)	57·3 (50·1–65·0)	191·5 (171·4–211·1)	154·5 (142·7–166·4)	81·1 (71·0–91·6)	33·7 (26·7–41·8)	9·2 (6·8–11·9)	0·9 (0·6–1·4)	0·0 (0·0–0·0)	2·64 (2·39–2·9)	1·25 (1·12–1·38)	0·62 (0·53–0·73)
1978	0·5 (0·2–1·1)	57·9 (50·6–65·5)	193·1 (174·0–212·4)	156·6 (144·5–168·3)	80·4 (70·5–90·5)	33·2 (26·3–41·4)	9·0 (6·6–11·9)	0·9 (0·6–1·3)	0·0 (0·0–0·0)	2·66 (2·42–2·9)	1·26 (1·13–1·39)	0·62 (0·52–0·72)
1979	0·5 (0·2–1·1)	57·7 (50·6–64·9)	193·8 (175·8–211·1)	156·5 (145·1–167·6)	78·8 (69·4–88·3)	32·2 (25·5–39·8)	8·7 (6·3–11·5)	0·8 (0·5–1·3)	0·0 (0·0–0·0)	2·64 (2·43–2·86)	1·26 (1·14–1·38)	0·6 (0·52–0·69)
1980	0·5 (0·2–1·1)	55·9 (49·4–62·5)	191·8 (175·2–208·3)	153·4 (142·8–163·2)	76·2 (67·5–85·1)	30·5 (24·5–37·8)	8·1 (6·0–10·9)	0·8 (0·5–1·2)	0·0 (0·0–0·0)	2·59 (2·4–2·77)	1·24 (1·13–1·36)	0·58 (0·5–0·66)
1981	0·5 (0·2–1·1)	54·4 (48·5–60·5)	189·0 (173·2–204·0)	150·1 (141·0–159·1)	73·6 (65·6–81·6)	28·9 (23·5–35·5)	7·7 (5·5–10·3)	0·7 (0·5–1·1)	0·0 (0·0–0·0)	2·52 (2·37–2·69)	1·22 (1·11–1·32)	0·55 (0·49–0·63)
1982	0·5 (0·2–1·0)	53·6 (48·3–59·5)	187·0 (173·9–200·6)	148·7 (140·7–157·0)	71·3 (64·2–78·4)	27·6 (22·9–33·5)	7·3 (5·3–9·7)	0·7 (0·4–1·1)	0·0 (0·0–0·0)	2·48 (2·35–2·62)	1·21 (1·12–1·3)	0·53 (0·48–0·6)
1983	0·5 (0·2–1·0)	53·1 (48·2–58·4)	185·5 (174·9–196·3)	147·0 (139·6–154·1)	69·1 (62·8–75·2)	26·3 (22·1–31·1)	6·9 (5·1–9·1)	0·6 (0·4–1·0)	0·0 (0·0–0·0)	2·45 (2·34–2·56)	1·2 (1·12–1·27)	0·51 (0·47–0·56)
1984	0·5 (0·2–1·0)	51·8 (47·6–56·4)	183·1 (175·0–191·3)	145·0 (139·2–150·8)	66·5 (61·2–71·7)	24·7 (21·1–28·5)	6·5 (4·8–8·6)	0·6 (0·4–0·9)	0·0 (0·0–0·0)	2·39 (2·3–2·49)	1·18 (1·13–1·23)	0·49 (0·45–0·53)
1985	0·5 (0·2–1·0)	49·7 (46·2–53·5)	179·4 (174·2–185·0)	143·6 (139·1–148·2)	64·0 (59·7–68·2)	23·1 (20·0–26·6)	6·1 (4·6–8·0)	0·6 (0·4–0·8)	0·0 (0·0–0·0)	2·34 (2·26–2·41)	1·15 (1·11–1·18)	0·47 (0·44–0·5)
1986	0·5 (0·2–1·0)	48·2 (45·2–51·5)	176·5 (172·8–180·5)	142·6 (139·0–146·3)	62·3 (58·9–66·0)	22·2 (19·5–25·1)	5·8 (4·5–7·5)	0·5 (0·3–0·8)	0·0 (0·0–0·0)	2·29 (2·24–2·35)	1·13 (1·1–1·15)	0·45 (0·43–0·49)
1987	0·5 (0·2–1·0)	47·5 (44·7–50·4)	174·9 (171·6–178·3)	142·2 (139·1–145·1)	61·5 (58·6–64·8)	21·8 (19·3–24·5)	5·7 (4·5–7·1)	0·5 (0·3–0·7)	0·0 (0·0–0·0)	2·27 (2·23–2·32)	1·11 (1·09–1·14)	0·45 (0·42–0·48)
1988	0·5 (0·2–1·0)	46·9 (44·5–49·6)	173·5 (170·2–176·8)	142·0 (139·3–145·0)	61·0 (58·2–64·0)	21·6 (19·2–24·2)	5·5 (4·4–6·7)	0·5 (0·3–0·7)	0·0 (0·0–0·0)	2·26 (2·22–2·3)	1·1 (1·08–1·13)	0·44 (0·42–0·47)
1989	0·5 (0·2–1·0)	45·9 (43·5–48·6)	171·0 (167·8–174·3)	141·6 (138·8–144·5)	60·3 (57·6–63·0)	21·1 (18·9–23·5)	5·2 (4·2–6·3)	0·4 (0·3–0·6)	0·0 (0·0–0·0)	2·23 (2·19–2·27)	1·09 (1·07–1·11)	0·43 (0·41–0·46)
1990	0·5 (0·2–0·9)	44·6 (42·2–47·0)	167·2 (163·9–170·6)	140·4 (137·6–143·3)	59·5 (56·8–62·1)	20·5 (18·5–22·8)	4·8 (3·9–5·9)	0·4 (0·3–0·6)	0·0 (0·0–0·0)	2·19 (2·16–2·22)	1·06 (1·04–1·08)	0·43 (0·41–0·45)
1991	0·5 (0·2–0·9)	43·8 (41·4–46·3)	163·8 (160·3–167·1)	139·5 (136·6–142·2)	59·3 (56·6–61·8)	20·1 (18·2–22·3)	4·6 (3·7–5·7)	0·4 (0·3–0·5)	0·0 (0·0–0·0)	2·16 (2·13–2·19)	1·04 (1·02–1·06)	0·42 (0·4–0·44)
1992	0·4 (0·2–0·9)	43·4 (41·0–46·0)	161·7 (158·2–165·3)	139·1 (136·3–142·1)	60·1 (57·4–62·7)	20·0 (18·1–22·1)	4·5 (3·7–5·5)	0·4 (0·3–0·5)	0·0 (0·0–0·0)	2·15 (2·12–2·18)	1·03 (1·01–1·05)	0·42 (0·41–0·44)
1993	0·4 (0·2–0·9)	43·0 (40·7–45·5)	160·3 (157·1–163·8)	138·7 (135·9–141·8)	61·4 (58·7–64·0)	20·0 (18·0–21·8)	4·4 (3·6–5·3)	0·3 (0·2–0·5)	0·0 (0·0–0·0)	2·14 (2·11–2·17)	1·02 (1·0–1·04)	0·43 (0·41–0·45)
1994	0·4 (0·2–0·9)	41·8 (39·5–44·4)	157·9 (154·7–161·4)	137·4 (134·3–140·2)	62·3 (59·5–65·0)	19·3 (17·5–21·1)	4·1 (3·4–5·0)	0·3 (0·2–0·4)	0·0 (0·0–0·0)	2·12 (2·09–2·15)	1·0 (0·98–1·02)	0·43 (0·41–0·45)
1995	0·4 (0·2–0·9)	39·7 (37·4–42·1)	153·4 (150·1–156·7)	134·5 (131·6–137·5)	61·9 (59·2–64·7)	18·2 (16·5–19·9)	3·8 (3·2–4·5)	0·3 (0·2–0·4)	0·0 (0·0–0·0)	2·06 (2·03–2·1)	0·97 (0·95–0·99)	0·42 (0·4–0·44)
1996	0·4 (0·2–0·9)	36·8 (34·5–39·3)	146·3 (143·2–149·8)	131·0 (128·1–133·9)	60·4 (57·6–63·2)	17·9 (16·2–19·7)	3·6 (3·1–4·3)	0·3 (0·2–0·4)	0·0 (0·0–0·0)	1·98 (1·95–2·02)	0·92 (0·9–0·94)	0·41 (0·39–0·43)
1997	0·4 (0·2–0·8)	33·7 (31·6–36·0)	137·6 (134·4–141·2)	127·3 (124·4–130·3)	58·3 (55·7–60·9)	18·4 (16·6–20·2)	3·7 (3·1–4·3)	0·2 (0·2–0·3)	0·0 (0·0–0·0)	1·9 (1·86–1·94)	0·86 (0·84–0·88)	0·4 (0·38–0·43)
1998	0·4 (0·2–0·8)	31·0 (28·9–33·2)	129·3 (126·2–132·7)	124·7 (121·9–127·6)	56·5 (53·9–59·1)	18·7 (17·0–20·5)	3·7 (3·2–4·3)	0·2 (0·1–0·3)	0·0 (0·0–0·0)	1·82 (1·79–1·86)	0·8 (0·78–0·82)	0·4 (0·37–0·42)
1999	0·4 (0·2–0·8)	29·2 (27·2–31·5)	123·6 (120·3–126·9)	124·2 (121·3–127·1)	55·6 (53·1–58·2)	18·8 (17·1–20·6)	3·7 (3·2–4·3)	0·2 (0·1–0·3)	0·0 (0·0–0·0)	1·78 (1·74–1·81)	0·77 (0·74–0·79)	0·39 (0·37–0·41)
2000	0·4 (0·2–0·7)	27·9 (25·9–30·2)	119·9 (116·5–123·1)	124·4 (121·6–127·2)	55·9 (53·4–58·5)	18·8 (17·1–20·5)	3·7 (3·2–4·3)	0·2 (0·1–0·2)	0·0 (0·0–0·0)	1·76 (1·72–1·79)	0·74 (0·72–0·76)	0·39 (0·37–0·41)
2001	0·3 (0·1–0·7)	26·3 (24·3–28·4)	115·2 (111·8–118·8)	123·3 (120·5–126·0)	57·5 (54·8–59·9)	18·9 (17·2–20·7)	3·7 (3·2–4·3)	0·2 (0·1–0·2)	0·0 (0·0–0·0)	1·73 (1·69–1·76)	0·71 (0·69–0·73)	0·4 (0·38–0·42)
2002	0·3 (0·1–0·7)	24·1 (22·2–26·0)	108·2 (104·9–111·7)	119·7 (116·9–122·4)	59·3 (56·6–61·9)	19·3 (17·7–21·1)	3·8 (3·3–4·3)	0·2 (0·1–0·3)	0·0 (0·0–0·0)	1·67 (1·64–1·71)	0·66 (0·64–0·68)	0·41 (0·39–0·43)
2003	0·3 (0·1–0·6)	21·6 (19·9–23·5)	99·1 (95·8–102·3)	114·3 (111·5–117·1)	60·9 (58·3–63·7)	19·9 (18·2–21·9)	3·9 (3·3–4·4)	0·2 (0·2–0·3)	0·0 (0·0–0·0)	1·6 (1·56–1·63)	0·6 (0·59–0·62)	0·42 (0·4–0·45)
2004	0·3 (0·1–0·6)	19·9 (18·2–21·8)	90·6 (87·5–93·7)	109·4 (106·7–112·3)	61·7 (59·0–64·6)	20·5 (18·7–22·5)	3·9 (3·4–4·5)	0·2 (0·2–0·3)	0·0 (0·0–0·0)	1·53 (1·5–1·57)	0·55 (0·54–0·57)	0·43 (0·41–0·46)
2005	0·3 (0·1–0·6)	19·4 (17·6–21·3)	85·5 (82·3–88·5)	106·8 (104·1–109·8)	61·8 (59·2–64·5)	20·8 (18·9–22·8)	3·9 (3·4–4·5)	0·2 (0·2–0·3)	0·0 (0·0–0·0)	1·49 (1·46–1·53)	0·53 (0·51–0·54)	0·43 (0·41–0·46)
2006	0·3 (0·1–0·6)	19·2 (17·4–21·2)	82·6 (79·6–85·9)	106·1 (103·4–109·1)	62·2 (59·5–64·9)	21·1 (19·1–23·2)	3·8 (3·3–4·4)	0·2 (0·2–0·3)	0·0 (0·0–0·0)	1·48 (1·45–1·51)	0·51 (0·49–0·53)	0·44 (0·42–0·46)
2007	0·3 (0·1–0·6)	19·1 (17·2–21·1)	80·6 (77·7–83·9)	106·5 (103·8–109·3)	63·3 (60·7–66·1)	21·6 (19·5–23·8)	3·8 (3·3–4·4)	0·2 (0·2–0·3)	0·0 (0·0–0·0)	1·48 (1·45–1·51)	0·5 (0·48–0·52)	0·44 (0·43–0·47)
2008	0·3 (0·1–0·6)	19·0 (17·1–21·0)	79·2 (76·4–82·6)	107·5 (104·8–110·2)	65·0 (62·4–67·9)	22·4 (20·2–24·5)	3·8 (3·2–4·5)	0·2 (0·2–0·3)	0·0 (0·0–0·0)	1·49 (1·45–1·52)	0·49 (0·48–0·51)	0·46 (0·44–0·48)
2009	0·3 (0·1–0·6)	18·8 (16·8–20·9)	78·0 (74·9–81·2)	108·6 (105·9–111·4)	67·1 (64·5–69·9)	23·2 (21·1–25·3)	3·8 (3·2–4·5)	0·2 (0·2–0·3)	0·0 (0·0–0·0)	1·5 (1·47–1·53)	0·49 (0·47–0·5)	0·47 (0·45–0·49)
2010	0·3 (0·1–0·6)	18·4 (16·6–20·6)	76·5 (73·4–79·8)	109·3 (106·5–112·0)	69·7 (67·0–72·6)	24·2 (22·1–26·5)	3·8 (3·2–4·5)	0·2 (0·2–0·3)	0·0 (0·0–0·0)	1·51 (1·48–1·55)	0·48 (0·46–0·5)	0·49 (0·47–0·51)
2011	0·3 (0·1–0·6)	17·9 (16·0–20·1)	74·3 (71·3–77·5)	109·2 (106·5–112·0)	72·7 (69·8–75·7)	25·4 (23·2–27·8)	3·9 (3·2–4·6)	0·2 (0·2–0·3)	0·0 (0·0–0·0)	1·52 (1·48–1·56)	0·46 (0·44–0·48)	0·51 (0·49–0·53)
2012	0·3 (0·1–0·6)	17·3 (15·4–19·6)	71·7 (68·8–74·9)	108·8 (105·9–111·7)	75·9 (72·8–79·1)	26·7 (24·4–29·2)	4·0 (3·3–4·8)	0·2 (0·2–0·3)	0·0 (0·0–0·0)	1·52 (1·49–1·56)	0·45 (0·43–0·47)	0·53 (0·51–0·56)
2013	0·3 (0·1–0·6)	16·8 (14·9–19·1)	69·3 (66·2–72·6)	108·0 (105·2–111·1)	78·7 (75·7–81·8)	28·0 (25·5–30·7)	4·1 (3·3–5·1)	0·3 (0·2–0·3)	0·0 (0·0–0·0)	1·53 (1·49–1·57)	0·43 (0·41–0·45)	0·56 (0·53–0·58)
2014	0·3 (0·1–0·5)	16·4 (14·3–18·7)	67·4 (64·2–70·9)	107·0 (103·9–110·3)	80·8 (77·7–84·1)	29·0 (26·4–32·1)	4·2 (3·3–5·4)	0·3 (0·2–0·4)	0·0 (0·0–0·0)	1·53 (1·48–1·58)	0·42 (0·4–0·44)	0·57 (0·55–0·6)
2015	0·3 (0·1–0·5)	16·1 (14·0–18·4)	66·0 (61·8–70·1)	105·9 (102·0–109·5)	81·9 (78·4–85·9)	29·6 (26·5–33·1)	4·3 (3·3–5·5)	0·3 (0·2–0·4)	0·0 (0·0–0·0)	1·52 (1·46–1·58)	0·41 (0·39–0·44)	0·58 (0·55–0·61)
2016	0·3 (0·1–0·5)	15·9 (13·8–18·4)	65·0 (60·0–70·1)	105·2 (100·5–109·7)	81·9 (77·1–87·1)	29·6 (25·8–33·6)	4·2 (3·2–5·5)	0·3 (0·2–0·4)	0·0 (0·0–0·0)	1·51 (1·44–1·59)	0·41 (0·38–0·44)	0·58 (0·55–0·61)
2017	0·3 (0·1–0·5)	15·9 (13·7–18·5)	64·9 (58·5–71·7)	104·8 (99·2–110·7)	81·5 (75·4–87·9)	29·4 (25·1–34·1)	4·2 (3·1–5·6)	0·3 (0·2–0·4)	0·0 (0·0–0·0)	1·51 (1·41–1·61)	0·41 (0·37–0·44)	0·58 (0·54–0·62)

Supplemental Results Table 5: Age-specific fertility rates per 1000, cumulative total fertility up to maternal age 25 (TFU25), cumulative total fertility from maternal age 30 to age 55 (TFO30), and cumulative total fertility, Montenegro, 1950-2017

Year	10-14	15-19	20-24	25-29	30-34	35-39	40-44	45-49	50-54	TFR	TFU25	TFO30
1950	0·3 (0·1–0·6)	52·1 (44·9–60·5)	212·9 (194·4–232·5)	227·1 (216·0–238·7)	165·9 (153·4–179·1)	109·7 (99·5–120·3)	48·6 (41·9–55·8)	4·7 (3·2–6·6)	0·1 (0·1–0·1)	4·11 (3·83–4·39)	1·33 (1·2–1·47)	1·64 (1·52–1·77)
1951	0·3 (0·1–0·5)	51·1 (44·1–59·1)	209·7 (192·5–228·1)	222·1 (211·5–233·0)	163·2 (151·1–175·5)	107·0 (97·1–117·5)	47·2 (40·6–54·1)	4·5 (3·1–6·3)	0·1 (0·1–0·1)	4·03 (3·75–4·29)	1·31 (1·18–1·43)	1·61 (1·49–1·73)
1952	0·3 (0·1–0·5)	49·9 (43·2–57·8)	206·0 (190·1–223·3)	216·7 (205·8–227·5)	160·1 (148·3–171·9)	104·2 (94·5–114·5)	45·6 (39·1–52·4)	4·3 (3·0–6·0)	0·1 (0·1–0·1)	3·94 (3·67–4·19)	1·28 (1·17–1·4)	1·57 (1·46–1·68)
1953	0·3 (0·1–0·5)	48·8 (42·3–56·3)	202·4 (186·7–219·1)	211·3 (200·5–222·0)	157·0 (145·3–168·9)	101·4 (91·9–111·5)	44·1 (37·5–50·7)	4·2 (2·9–5·7)	0·1 (0·1–0·1)	3·85 (3·59–4·09)	1·26 (1·15–1·37)	1·53 (1·42–1·64)
1954	0·3 (0·1–0·5)	47·7 (41·6–55·2)	198·8 (183·3–215·0)	206·0 (195·7–216·6)	153·8 (142·6–165·1)	98·7 (89·0–108·8)	42·5 (36·2–48·8)	4·0 (2·7–5·4)	0·1 (0·1–0·1)	3·76 (3·51–4·0)	1·23 (1·13–1·34)	1·5 (1·38–1·6)
1955	0·3 (0·1–0·5)	46·7 (40·8–53·6)	195·4 (180·7–210·0)	200·7 (190·5–210·7)	150·6 (140·2–161·4)	96·0 (86·7–106·0)	41·0 (35·1–47·2)	3·8 (2·6–5·3)	0·1 (0·1–0·1)	3·67 (3·43–3·91)	1·21 (1·12–1·31)	1·46 (1·34–1·57)
1956	0·2 (0·1–0·5)	45·7 (39·8–52·4)	192·0 (178·7–205·5)	195·5 (186·0–205·0)	147·5 (137·1–158·1)	93·2 (83·9–102·9)	39·5 (33·7–45·7)	3·7 (2·5–5·1)	0·1 (0·1–0·1)	3·59 (3·36–3·82)	1·19 (1·1–1·28)	1·42 (1·31–1·53)
1957	0·2 (0·1–0·5)	44·8 (38·9–51·2)	188·8 (176·8–200·9)	190·5 (181·0–199·7)	144·4 (134·2–154·5)	90·6 (81·8–100·0)	38·1 (32·6–44·1)	3·5 (2·4–4·9)	0·1 (0·1–0·1)	3·5 (3·29–3·72)	1·17 (1·09–1·24)	1·38 (1·28–1·49)
1958	0·2 (0·1–0·5)	43·9 (38·2–50·0)	185·7 (174·9–196·5)	185·9 (177·1–194·5)	141·5 (132·0–151·3)	88·1 (79·6–96·5)	36·9 (31·4–42·6)	3·4 (2·3–4·7)	0·1 (0·1–0·1)	3·43 (3·23–3·62)	1·15 (1·08–1·21)	1·35 (1·25–1·46)
1959	0·2 (0·1–0·5)	43·1 (37·6–49·1)	183·0 (173·2–193·1)	183·1 (174·6–191·7)	138·8 (129·6–147·9)	85·7 (77·6–93·4)	35·5 (30·4–41·2)	3·2 (2·2–4·4)	0·1 (0·1–0·1)	3·36 (3·18–3·55)	1·13 (1·07–1·19)	1·32 (1·22–1·41)
1960	0·2 (0·1–0·5)	42·2 (36·6–47·8)	180·5 (171·2–190·7)	181·5 (173·2–190·1)	136·0 (126·7–145·1)	82·9 (75·3–90·5)	33·8 (28·9–39·0)	3·1 (2·1–4·2)	0·1 (0·1–0·1)	3·3 (3·12–3·48)	1·11 (1·05–1·18)	1·28 (1·19–1·37)
1961	0·2 (0·1–0·5)	41·1 (35·7–46·5)	177·9 (167·9–188·7)	179·1 (170·6–187·6)	132·9 (123·6–142·3)	79·7 (72·2–87·1)	31·8 (27·2–36·9)	2·9 (2·0–4·0)	0·1 (0·1–0·1)	3·23 (3·06–3·41)	1·1 (1·03–1·16)	1·24 (1·15–1·33)
1962	0·2 (0·1–0·5)	40·0 (34·8–45·3)	175·3 (164·6–186·3)	176·1 (167·7–184·6)	129·8 (120·7–138·8)	76·4 (68·9–84·0)	29·9 (25·5–34·6)	2·7 (1·9–3·8)	0·1 (0·1–0·1)	3·15 (2·98–3·33)	1·08 (1·01–1·14)	1·19 (1·11–1·28)
1963	0·2 (0·1–0·5)	38·9 (33·8–44·3)	172·7 (161·5–183·9)	173·1 (164·5–181·3)	126·7 (117·5–135·3)	73·1 (65·8–80·9)	28·1 (23·8–32·7)	2·6 (1·8–3·6)	0·0 (0·0–0·1)	3·08 (2·91–3·25)	1·06 (0·99–1·13)	1·15 (1·07–1·24)
1964	0·2 (0·1–0·5)	37·9 (33·0–43·4)	170·2 (158·7–182·1)	170·0 (161·6–178·2)	123·6 (114·6–132·8)	69·9 (62·8–77·6)	26·3 (22·3–30·7)	2·5 (1·7–3·4)	0·0 (0·0–0·0)	3·0 (2·84–3·18)	1·04 (0·97–1·11)	1·11 (1·03–1·19)
1965	0·2 (0·1–0·5)	36·9 (32·1–42·2)	167·8 (156·6–179·6)	166·8 (158·5–175·3)	120·5 (111·9–129·6)	66·8 (59·8–74·3)	24·6 (20·8–28·8)	2·3 (1·6–3·2)	0·0 (0·0–0·0)	2·93 (2·76–3·1)	1·02 (0·95–1·1)	1·07 (1·0–1·15)
1966	0·2 (0·1–0·4)	36·0 (31·0–41·2)	165·4 (154·0–176·8)	163·7 (155·5–172·1)	117·6 (109·2–126·4)	63·8 (57·2–71·0)	23·0 (19·5–26·9)	2·2 (1·5–3·0)	0·0 (0·0–0·0)	2·86 (2·69–3·02)	1·01 (0·94–1·08)	1·03 (0·96–1·11)
1967	0·2 (0·1–0·4)	35·0 (30·2–40·2)	163·0 (152·4–173·5)	160·6 (152·9–168·9)	114·7 (106·5–123·3)	60·8 (54·9–67·8)	21·5 (18·3–25·0)	2·0 (1·4–2·8)	0·0 (0·0–0·0)	2·79 (2·63–2·95)	0·99 (0·92–1·06)	1·0 (0·93–1·07)
1968	0·2 (0·1–0·4)	34·1 (29·4–39·2)	160·6 (150·7–170·8)	157·8 (150·1–166·0)	111·9 (104·2–120·2)	58·0 (52·4–64·4)	20·1 (17·0–23·3)	1·9 (1·3–2·6)	0·0 (0·0–0·0)	2·72 (2·58–2·88)	0·97 (0·91–1·04)	0·96 (0·9–1·02)
1969	0·2 (0·1–0·4)	33·2 (28·8–37·9)	158·2 (148·9–168·0)	155·2 (147·9–163·3)	109·3 (101·9–116·6)	55·1 (50·0–60·8)	18·7 (15·9–21·5)	1·8 (1·3–2·5)	0·0 (0·0–0·0)	2·66 (2·52–2·81)	0·96 (0·9–1·02)	0·92 (0·87–0·98)
1970	0·2 (0·1–0·4)	32·4 (28·1–36·9)	155·5 (146·6–165·0)	152·7 (145·3–160·7)	106·8 (99·5–114·3)	52·1 (47·5–57·4)	17·4 (14·8–19·9)	1·7 (1·2–2·3)	0·0 (0·0–0·0)	2·59 (2·47–2·74)	0·94 (0·89–1·0)	0·89 (0·84–0·95)
1971	0·2 (0·1–0·4)	31·8 (27·6–36·2)	152·6 (143·4–162·4)	150·3 (143·1–157·9)	104·5 (97·2–111·7)	49·1 (44·6–53·7)	16·2 (13·8–18·7)	1·5 (1·1–2·1)	0·0 (0·0–0·0)	2·53 (2·41–2·66)	0·92 (0·87–0·98)	0·86 (0·81–0·91)
1972	0·2 (0·1–0·4)	31·1 (27·0–35·5)	149·9 (140·5–160·2)	148·2 (140·8–155·5)	102·1 (94·9–109·2)	46·4 (41·9–50·9)	15·2 (13·1–17·7)	1·4 (1·0–2·0)	0·0 (0·0–0·0)	2·47 (2·36–2·6)	0·91 (0·85–0·96)	0·83 (0·77–0·88)
1973	0·2 (0·1–0·4)	30·5 (26·5–34·9)	147·3 (137·3–158·1)	146·2 (138·7–153·6)	99·8 (92·5–107·3)	44·7 (40·1–49·6)	14·7 (12·5–17·2)	1·3 (0·9–1·9)	0·0 (0·0–0·0)	2·42 (2·31–2·54)	0·89 (0·83–0·95)	0·8 (0·75–0·86)
1974	0·2 (0·1–0·4)	29·8 (25·9–34·2)	144·7 (134·7–155·1)	144·1 (136·6–151·6)	97·5 (89·9–105·2)	43·8 (38·9–48·9)	14·4 (12·1–16·9)	1·2 (0·8–1·7)	0·0 (0·0–0·0)	2·38 (2·26–2·5)	0·87 (0·82–0·93)	0·78 (0·73–0·84)
1975	0·2 (0·1–0·4)	29·2 (25·4–33·5)	142·0 (132·2–152·4)	142·0 (134·8–149·5)	95·3 (87·9–103·0)	42·7 (37·7–48·2)	14·1 (11·7–16·6)	1·1 (0·8–1·6)	0·0 (0·0–0·0)	2·33 (2·22–2·46)	0·86 (0·8–0·91)	0·77 (0·71–0·83)
1976	0·2 (0·1–0·4)	28·5 (24·8–32·7)	139·4 (129·5–149·4)	140·0 (132·6–147·5)	93·2 (85·4–101·1)	41·7 (36·7–47·0)	13·8 (11·5–16·4)	1·0 (0·7–1·5)	0·0 (0·0–0·0)	2·29 (2·18–2·41)	0·84 (0·79–0·89)	0·75 (0·69–0·81)
1977	0·2 (0·1–0·4)	27·9 (24·3–32·1)	136·9 (127·3–146·8)	138·1 (130·6–145·3)	91·2 (83·4–99·0)	40·6 (35·5–46·1)	13·5 (11·2–16·0)	1·0 (0·7–1·3)	0·0 (0·0–0·0)	2·25 (2·14–2·36)	0·82 (0·77–0·88)	0·73 (0·67–0·79)
1978	0·2 (0·1–0·4)	27·3 (23·8–31·3)	134·5 (125·1–143·8)	136·3 (128·9–143·2)	89·4 (82·0–97·2)	39·6 (34·8–44·7)	13·2 (11·0–15·8)	0·9 (0·6–1·2)	0·0 (0·0–0·0)	2·21 (2·1–2·31)	0·81 (0·76–0·86)	0·72 (0·66–0·78)
1979	0·2 (0·1–0·4)	26·9 (23·4–30·7)	132·5 (123·6–141·9)	134·9 (127·6–141·8)	87·9 (80·8–95·3)	38·6 (34·0–43·5)	12·9 (10·8–15·4)	0·8 (0·5–1·1)	0·0 (0·0–0·0)	2·17 (2·08–2·28)	0·8 (0·75–0·85)	0·7 (0·65–0·76)
1980	0·2 (0·1–0·4)	27·1 (23·6–31·0)	131·0 (122·0–139·9)	133·6 (126·3–140·7)	86·5 (79·5–94·3)	37·8 (33·5–42·5)	12·4 (10·4–14·9)	0·7 (0·5–1·1)	0·0 (0·0–0·0)	2·15 (2·04–2·25)	0·79 (0·74–0·84)	0·69 (0·63–0·75)
1981	0·2 (0·1–0·4)	27·7 (24·1–31·6)	129·9 (121·0–138·8)	132·2 (125·2–139·8)	85·3 (78·4–92·9)	37·0 (32·9–41·9)	12·0 (10·0–14·4)	0·7 (0·5–1·0)	0·0 (0·0–0·0)	2·12 (2·02–2·23)	0·79 (0·74–0·84)	0·67 (0·62–0·73)
1982	0·2 (0·1–0·4)	28·2 (24·6–31·9)	128·9 (119·7–138·3)	130·6 (123·4–138·6)	84·0 (76·8–92·1)	36·3 (31·9–41·3)	11·5 (9·6–13·9)	0·6 (0·4–0·9)	0·0 (0·0–0·0)	2·1 (1·99–2·22)	0·79 (0·74–0·84)	0·66 (0·61–0·72)
1983	0·2 (0·1–0·4)	28·4 (24·8–32·3)	128·0 (117·7–137·8)	129·0 (121·6–137·5)	82·7 (75·3–90·9)	35·7 (31·1–40·8)	11·1 (9·1–13·4)	0·6 (0·4–0·9)	0·0 (0·0–0·0)	2·08 (1·96–2·19)	0·78 (0·73–0·83)	0·65 (0·6–0·71)
1984	0·2 (0·1–0·4)	28·5 (24·9–32·6)	127·0 (117·0–137·1)	127·4 (119·9–136·0)	81·3 (74·0–89·8)	35·1 (30·3–40·1)	10·7 (8·7–12·8)	0·6 (0·4–0·8)	0·0 (0·0–0·0)	2·05 (1·93–2·17)	0·78 (0·72–0·83)	0·64 (0·58–0·69)
1985	0·2 (0·1–0·4)	28·6 (25·0–32·6)	126·1 (116·2–136·3)	125·9 (118·5–134·6)	80·1 (72·7–89·0)	34·6 (29·8–39·8)	10·2 (8·3–12·4)	0·5 (0·4–0·8)	0·0 (0·0–0·0)	2·03 (1·91–2·16)	0·77 (0·72–0·83)	0·63 (0·57–0·68)
1986	0·2 (0·1–0·4)	28·7 (25·1–32·9)	125·2 (116·0–135·4)	124·5 (117·2–133·2)	78·8 (71·5–87·4)	34·2 (29·3–39·3)	9·9 (8·1–12·0)	0·5 (0·3–0·7)	0·0 (0·0–0·0)	2·01 (1·9–2·13)	0·77 (0·72–0·82)	0·62 (0·56–0·67)
1987	0·2 (0·1–0·4)	28·6 (25·1–32·6)	124·2 (115·4–133·4)	123·3 (116·4–131·6)	77·5 (70·3–85·8)	33·8 (29·1–39·0)	9·6 (7·8–11·6)	0·5 (0·3–0·7)	0·0 (0·0–0·0)	1·99 (1·88–2·11)	0·77 (0·72–0·81)	0·61 (0·55–0·66)
1988	0·2 (0·1–0·4)	28·3 (24·9–32·2)	122·9 (114·4–131·5)	122·2 (115·3–130·1)	76·1 (69·3–83·8)	33·6 (28·9–38·4)	9·3 (7·6–11·3)	0·4 (0·3–0·6)	0·0 (0·0–0·0)	1·96 (1·87–2·07)	0·76 (0·71–0·81)	0·6 (0·55–0·65)
1989	0·2 (0·1–0·4)	27·9 (24·5–31·7)	121·6 (113·5–129·6)	121·6 (114·6–128·7)	75·1 (68·5–82·2)	33·4 (28·9–37·9)	9·0 (7·4–11·0)	0·4 (0·3–0·6)	0·0 (0·0–0·0)	1·95 (1·86–2·05)	0·75 (0·71–0·79)	0·59 (0·54–0·64)
1990	0·2 (0·1–0·4)	27·7 (24·3–31·3)	120·7 (113·0–128·5)	121·7 (114·9–128·6)	74·4 (67·8–81·2)	33·5 (29·1–38·1)	8·9 (7·3–10·9)	0·4 (0·3–0·6)	0·0 (0·0–0·0)	1·94 (1·85–2·03)	0·74 (0·7–0·78)	0·59 (0·54–0·63)
1991	0·2 (0·1–0·4)	27·5 (24·2–31·0)	119·9 (112·0–127·6)	122·0 (115·5–128·8)	73·6 (67·3–80·2)	33·6 (29·5–38·3)	8·8 (7·2–10·8)	0·4 (0·2–0·5)	0·0 (0·0–0·0)	1·93 (1·84–2·02)	0·74 (0·69–0·78)	0·58 (0·54–0·63)
1992	0·2 (0·1–0·4)	27·1 (23·9–30·5)	118·9 (110·6–127·2)	122·1 (115·5–128·8)	73·5 (67·2–79·9)	33·7 (29·3–38·8)	8·8 (7·0–10·8)	0·4 (0·2–0·5)	0·0 (0·0–0·0)	1·92 (1·83–2·01)	0·73 (0·69–0·77)	0·58 (0·53–0·63)
1993	0·2 (0·1–0·4)	26·6 (23·5–30·0)	117·6 (108·9–126·2)	121·9 (115·4–128·3)	74·1 (67·8–80·5)	33·8 (29·3–39·2)	8·7 (6·9–10·8)	0·3 (0·2–0·5)	0·0 (0·0–0·0)	1·92 (1·82–2·01)	0·72 (0·68–0·77)	0·58 (0·54–0·63)
1994	0·2 (0·1–0·4)	26·1 (23·0–29·5)	116·0 (107·3–125·1)	121·6 (115·0–127·9)	74·7 (68·5–81·2)	34·0 (29·4–39·7)	8·6 (6·8–10·7)	0·3 (0·2–0·5)	0·0 (0·0–0·0)	1·91 (1·81–2·01)	0·71 (0·67–0·76)	0·59 (0·54–0·64)
1995	0·2 (0·1–0·4)	25·5 (22·5–28·8)	114·6 (106·0–123·4)	121·3 (114·8–127·5)	75·3 (69·2–81·8)	34·1 (29·4–40·1)	8·5 (6·6–10·8)	0·3 (0·2–0·5)	0·0 (0·0–0·0)	1·9 (1·81–2·0)	0·7 (0·66–0·75)	0·59 (0·55–0·64)
1996	0·2 (0·1–0·4)	25·0 (21·9–28·2)	113·1 (105·1–121·3)	121·0 (114·8–126·9)	76·0 (70·1–82·2)	34·2 (29·6–40·0)	8·5 (6·6–10·8)	0·3 (0·2–0·5)	0·0 (0·0–0·0)	1·89 (1·8–1·99)	0·69 (0·65–0·74)	0·6 (0·55–0·64)
1997	0·2 (0·1–0·4)	24·3 (21·4–27·5)	111·6 (104·2–119·5)	120·5 (114·7–126·1)	76·6 (71·0–82·4)	34·4 (29·8–40·2)	8·4 (6·5–10·6)	0·3 (0·2–0·5)	0·0 (0·0–0·0)	1·88 (1·8–1·97)	0·68 (0·64–0·72)	0·6 (0·56–0·64)
1998	0·2 (0·1–0·4)	23·6 (20·8–26·9)	109·7 (103·3–116·7)	119·6 (114·3–124·9)	77·2 (72·0–82·6)	34·6 (30·2–39·7)	8·4 (6·6–10·7)	0·3 (0·2–0·5)	0·0 (0·0–0·0)	1·87 (1·79–1·95)	0·67 (0·63–0·71)	0·6 (0·56–0·64)
1999	0·2 (0·1–0·4)	22·8 (20·2–26·0)	107·6 (101·8–113·7)	118·6 (113·6–123·3)	77·7 (72·8–82·8)	34·9 (30·6–39·4)	8·4 (6·5–10·6)	0·3 (0·2–0·5)	0·0 (0·0–0·0)	1·85 (1·78–1·92)	0·65 (0·62–0·69)	0·61 (0·57–0·65)
2000	0·2 (0·1–0·3)	21·9 (19·4–25·0)	105·2 (99·9–110·5)	117·6 (113·1–121·9)	78·2 (73·7–83·2)	35·2 (31·1–39·4)	8·4 (6·6–10·4)	0·3 (0·2–0·5)	0·0 (0·0–0·0)	1·83 (1·77–1·9)	0·64 (0·61–0·67)	0·61 (0·57–0·65)
2001	0·2 (0·1–0·3)	21·1 (18·7–23·8)	102·5 (97·5–107·2)	116·5 (112·4–120·7)	78·8 (74·7–83·4)	35·6 (32·1–39·6)	8·3 (6·6–10·3)	0·3 (0·2–0·5)	0·0 (0·0–0·0)	1·82 (1·75–1·88)	0·62 (0·59–0·64)	0·62 (0·58–0·65)
2002	0·2 (0·1–0·3)	20·2 (17·9–22·8)	99·3 (94·8–103·7)	115·5 (111·6–119·5)	79·6 (75·7–83·6)	36·1 (33·0–39·7)	8·3 (6·6–10·2)	0·3 (0·2–0·5)	0·0 (0·0–0·0)	1·8 (1·74–1·86)	0·6 (0·57–0·62)	0·62 (0·59–0·66)
2003	0·2 (0·1–0·3)	19·2 (17·0–21·6)	95·9 (91·6–100·2)	114·5 (110·8–118·4)	80·3 (76·4–84·3)	36·7 (33·6–40·2)	8·3 (6·7–10·1)	0·3 (0·2–0·5)	0·0 (0·0–0·0)	1·78 (1·72–1·84)	0·58 (0·55–0·6)	0·63 (0·6–0·66)
2004	0·2 (0·1–0·3)	18·3 (16·2–20·6)	92·4 (88·5–96·7)	113·8 (110·2–117·5)	81·1 (77·3–85·2)	37·3 (34·3–40·7)	8·3 (6·7–10·1)	0·3 (0·2–0·5)	0·0 (0·0–0·0)	1·76 (1·7–1·82)	0·55 (0·53–0·58)	0·64 (0·6–0·67)
2005	0·2 (0·1–0·3)	17·6 (15·6–19·8)	89·3 (85·2–93·6)	113·5 (110·0–117·2)	81·7 (77·9–85·7)	38·0 (34·8–41·1)	8·4 (6·8–10·1)	0·3 (0·2–0·5)	0·0 (0·0–0·0)	1·74 (1·69–1·8)	0·54 (0·51–0·56)	0·64 (0·61–0·68)
2006	0·1 (0·1–0·3)	16·9 (14·9–19·0)	86·6 (82·3–90·8)	113·6 (110·0–117·2)	82·3 (78·5–86·4)	38·7 (35·5–41·8)	8·4 (7·0–10·1)	0·4 (0·2–0·5)	0·0 (0·0–0·0)	1·73 (1·68–1·79)	0·52 (0·5–0·54)	0·65 (0·62–0·68)
2007	0·1 (0·1–0·3)	16·3 (14·3–18·3)	84·0 (80·0–88·4)	113·7 (110·0–117·3)	83·0 (79·2–87·1)	39·3 (36·1–42·7)	8·5 (7·1–10·2)	0·4 (0·2–0·5)	0·0 (0·0–0·0)	1·73 (1·67–1·79)	0·5 (0·48–0·53)	0·66 (0·62–0·69)
2008	0·1 (0·1–0·3)	15·5 (13·6–17·5)	81·4 (77·2–85·8)	113·6 (109·8–117·5)	84·0 (80·3–88·1)	40·0 (36·8–43·5)	8·6 (7·2–10·3)	0·4 (0·2–0·6)	0·0 (0·0–0·0)	1·72 (1·66–1·78)	0·49 (0·46–0·51)	0·66 (0·63–0·7)
2009	0·1 (0·1–0·3)	14·7 (12·8–16·7)	78·5 (74·4–82·6)	113·3 (109·6–117·3)	85·2 (81·4–89·2)	40·6 (37·3–44·3)	8·7 (7·3–10·4)	0·4 (0·3–0·6)	0·0 (0·0–0·0)	1·71 (1·65–1·77)	0·47 (0·44–0·49)	0·67 (0·64–0·72)
2010	0·1 (0·1–0·3)	13·8 (12·0–15·8)	75·6 (71·5–79·8)	113·0 (109·2–116·9)	86·7 (82·9–90·8)	41·2 (37·8–45·0)	8·9 (7·4–10·6)	0·4 (0·3–0·6)	0·0 (0·0–0·0)	1·7 (1·64–1·77)	0·45 (0·43–0·47)	0·69 (0·65–0·73)
2011	0·1 (0·1–0·3)	13·0 (11·2–14·9)	72·7 (68·9–77·2)	112·7 (109·0–116·6)	88·4 (84·5–92·5)	41·9 (38·6–45·7)	9·0 (7·4–10·7)	0·4 (0·3–0·6)	0·0 (0·0–0·0)	1·69 (1·63–1·76)	0·43 (0·41–0·46)	0·7 (0·66–0·74)
2012	0·1 (0·1–0·3)	12·3 (10·6–14·2)	70·1 (66·2–74·8)	112·6 (108·6–116·6)	90·3 (86·3–94·2)	42·6 (39·0–46·5)	9·1 (7·5–10·9)	0·4 (0·3–0·6)	0·0 (0·0–0·0)	1·69 (1·63–1·75)	0·41 (0·39–0·44)	0·71 (0·68–0·75)
2013	0·1 (0·1–0·2)	11·8 (10·1–13·7)	67·9 (63·5–73·0)	112·5 (108·4–116·7)	92·0 (87·9–96·3)	43·1 (39·3–47·2)	9·2 (7·5–11·0)	0·5 (0·3–0·7)	0·0 (0·0–0·0)	1·68 (1·62–1·75)	0·4 (0·37–0·43)	0·72 (0·69–0·76)
2014	0·1 (0·1–0·2)	11·5 (9·8–13·4)	66·0 (61·2–71·8)	112·5 (108·0–116·9)	93·4 (88·9–98·3)	43·4 (39·0–48·0)	9·2 (7·4–11·1)	0·5 (0·3–0·7)	0·0 (0·0–0·0)	1·68 (1·62–1·75)	0·39 (0·36–0·43)	0·73 (0·7–0·77)
2015	0·1 (0·1–0·2)	11·3 (9·6–13·4)	64·6 (59·0–71·3)	112·5 (107·4–117·5)	94·5 (89·4–100·1)	43·5 (38·6–48·8)	9·1 (7·3–11·3)	0·5 (0·3–0·7)	0·0 (0·0–0·0)	1·68 (1·61–1·75)	0·38 (0·35–0·42)	0·74 (0·7–0·78)
2016	0·1 (0·1–0·2)	11·2 (9·5–13·3)	63·7 (57·7–71·0)	112·5 (106·8–118·1)	95·0 (89·1–101·3)	43·2 (38·1–48·8)	8·9 (7·1–11·3)	0·5 (0·3–0·7)	0·0 (0·0–0·0)	1·68 (1·6–1·75)	0·38 (0·34–0·42)	0·74 (0·7–0·78)
2017	0·1 (0·1–0·2)	11·3 (9·6–13·5)	63·3 (56·4–71·9)	112·5 (106·2–119·1)	95·1 (88·3–102·2)	42·9 (37·2–49·4)	8·8 (6·9–11·4)	0·4 (0·3–0·7)	0·0 (0·0–0·0)	1·67 (1·59–1·76)	0·37 (0·33–0·43)	0·74 (0·69–0·79)

Supplemental Results Table 5: Age-specific fertility rates per 1000, cumulative total fertility up to maternal age 25 (TFU25), cumulative total fertility from maternal age 30 to age 55 (TFO30), and cumulative total fertility, Poland, 1950-2017

Year	10-14	15-19	20-24	25-29	30-34	35-39	40-44	45-49	50-54	TFR	TFU25	TFO30
1950	0·1 (0·0–0·2)	37·8 (36·6–39·1)	185·9 (184·2–187·6)	196·8 (195·4–198·1)	141·9 (140·2–143·6)	98·0 (96·3–99·6)	36·7 (35·3–38·1)	3·1 (2·5–3·7)	0·1 (0·1–0·1)	3·5 (3·48–3·52)	1·12 (1·11–1·13)	1·4 (1·38–1·41)
1951	0·1 (0·0–0·2)	37·2 (36·0–38·4)	187·9 (186·2–189·4)	197·4 (196·2–198·7)	140·5 (138·9–141·9)	96·0 (94·2–97·7)	37·1 (35·7–38·5)	3·1 (2·5–3·8)	0·1 (0·1–0·1)	3·5 (3·47–3·52)	1·13 (1·12–1·14)	1·38 (1·37–1·4)
1952	0·1 (0·0–0·2)	36·8 (35·7–38·1)	190·5 (189·0–192·0)	194·6 (193·3–195·9)	138·0 (136·6–139·5)	91·1 (89·2–92·9)	36·1 (34·7–37·5)	3·0 (2·4–3·8)	0·1 (0·1–0·1)	3·45 (3·43–3·48)	1·14 (1·13–1·15)	1·34 (1·33–1·36)
1953	0·1 (0·0–0·2)	37·9 (36·7–39·1)	194·8 (193·2–196·5)	192·3 (191·0–193·7)	135·9 (134·4–137·4)	86·7 (85·0–88·5)	34·4 (33·1–35·8)	2·8 (2·2–3·5)	0·1 (0·1–0·1)	3·43 (3·4–3·45)	1·16 (1·15–1·17)	1·3 (1·28–1·32)
1954	0·1 (0·0–0·2)	40·0 (38·8–41·2)	201·0 (199·3–202·6)	193·4 (192·0–194·7)	134·3 (132·9–135·9)	83·8 (82·0–85·5)	32·5 (31·3–34·0)	2·6 (2·1–3·1)	0·0 (0·0–0·1)	3·44 (3·42–3·46)	1·21 (1·2–1·22)	1·27 (1·25–1·28)
1955	0·1 (0·0–0·2)	42·0 (40·8–43·2)	205·1 (203·5–206·8)	193·0 (191·7–194·4)	131·0 (129·5–132·5)	79·8 (78·1–81·4)	30·6 (29·2–32·0)	2·4 (2·0–2·9)	0·0 (0·0–0·0)	3·42 (3·4–3·44)	1·24 (1·23–1·25)	1·22 (1·21–1·23)
1956	0·1 (0·0–0·2)	44·1 (42·8–45·4)	208·6 (206·9–210·2)	190·8 (189·4–192·1)	126·5 (125·0–127·9)	75·1 (73·5–76·7)	28·3 (26·9–29·7)	2·3 (1·8–2·9)	0·0 (0·0–0·0)	3·38 (3·36–3·4)	1·26 (1·25–1·27)	1·16 (1·15–1·17)
1957	0·1 (0·0–0·2)	46·2 (44·9–47·5)	211·5 (209·7–213·1)	187·8 (186·4–189·1)	121·7 (120·4–123·1)	71·0 (69·5–72·5)	25·7 (24·3–27·2)	2·2 (1·7–2·7)	0·0 (0·0–0·0)	3·33 (3·31–3·35)	1·29 (1·28–1·3)	1·1 (1·08–1·12)
1958	0·1 (0·0–0·2)	47·1 (45·9–48·4)	208·4 (206·8–210·0)	180·6 (179·3–181·8)	114·3 (112·9–115·7)	66·6 (65·2–68·0)	23·4 (22·0–25·0)	1·9 (1·5–2·4)	0·0 (0·0–0·0)	3·21 (3·19–3·23)	1·28 (1·27–1·29)	1·03 (1·02–1·04)
1959	0·1 (0·0–0·2)	45·5 (44·2–46·9)	202·3 (200·6–203·9)	169·4 (168·0–170·7)	104·5 (103·2–106·0)	59·9 (58·4–61·3)	21·2 (19·9–22·5)	1·7 (1·3–2·0)	0·0 (0·0–0·0)	3·02 (3·0–3·04)	1·24 (1·23–1·25)	0·94 (0·92–0·95)
1960	0·1 (0·0–0·2)	41·5 (40·2–42·8)	195·8 (194·2–197·6)	159·1 (157·7–160·4)	95·9 (94·5–97·4)	53·3 (51·9–54·8)	19·4 (18·2–20·7)	1·5 (1·2–1·9)	0·0 (0·0–0·0)	2·83 (2·82–2·85)	1·19 (1·18–1·2)	0·85 (0·84–0·86)
1961	0·1 (0·0–0·2)	36·7 (35·5–37·9)	193·4 (191·7–195·1)	153·4 (152·0–154·8)	91·0 (89·5–92·5)	49·3 (47·9–50·7)	18·2 (16·9–19·5)	1·5 (1·1–1·9)	0·0 (0·0–0·0)	2·72 (2·7–2·74)	1·15 (1·14–1·16)	0·8 (0·79–0·81)
1962	0·1 (0·0–0·2)	32·4 (31·4–33·6)	192·1 (190·3–193·8)	150·9 (149·5–152·3)	89·2 (87·8–90·6)	46·9 (45·5–48·2)	17·0 (15·8–18·3)	1·4 (1·1–1·9)	0·0 (0·0–0·0)	2·65 (2·63–2·67)	1·12 (1·11–1·13)	0·77 (0·76–0·78)
1963	0·1 (0·0–0·2)	29·9 (28·8–31·0)	187·8 (186·1–189·6)	148·4 (147·1–149·8)	87·5 (86·0–88·9)	45·2 (43·8–46·6)	15·8 (14·7–17·0)	1·4 (1·1–1·8)	0·0 (0·0–0·0)	2·58 (2·56–2·6)	1·09 (1·08–1·1)	0·75 (0·74–0·76)
1964	0·1 (0·0–0·2)	29·3 (28·3–30·4)	181·5 (179·8–183·3)	144·8 (143·4–146·1)	84·4 (83·0–85·9)	43·6 (42·2–45·0)	14·5 (13·5–15·6)	1·3 (1·1–1·6)	0·0 (0·0–0·0)	2·5 (2·48–2·51)	1·05 (1·04–1·06)	0·72 (0·71–0·73)
1965	0·1 (0·0–0·2)	29·4 (28·4–30·4)	173·1 (171·4–174·8)	141·2 (139·8–142·7)	81·1 (79·6–82·4)	41·9 (40·6–43·3)	13·3 (12·3–14·2)	1·2 (1·0–1·5)	0·0 (0·0–0·0)	2·41 (2·39–2·42)	1·01 (1·0–1·02)	0·69 (0·68–0·7)
1966	0·1 (0·0–0·2)	29·3 (28·3–30·4)	162·5 (160·9–164·3)	136·5 (135·0–138·0)	77·8 (76·4–79·2)	40·3 (39·0–41·6)	12·4 (11·5–13·5)	1·1 (0·8–1·4)	0·0 (0·0–0·0)	2·3 (2·28–2·32)	0·96 (0·95–0·97)	0·66 (0·65–0·67)
1967	0·1 (0·0–0·2)	29·1 (28·2–30·1)	157·2 (155·6–158·8)	132·2 (130·8–133·6)	74·9 (73·5–76·3)	39·0 (37·8–40·4)	11·9 (11·0–12·9)	1·0 (0·7–1·2)	0·0 (0·0–0·0)	2·23 (2·21–2·25)	0·93 (0·92–0·94)	0·63 (0·62–0·65)
1968	0·1 (0·0–0·2)	29·3 (28·4–30·2)	156·3 (154·7–157·9)	129·1 (127·6–130·4)	72·4 (71·0–73·8)	37·6 (36·3–38·9)	11·4 (10·5–12·4)	0·8 (0·6–1·1)	0·0 (0·0–0·0)	2·19 (2·17–2·2)	0·93 (0·92–0·94)	0·61 (0·6–0·62)
1969	0·1 (0·0–0·2)	29·7 (28·8–30·7)	156·4 (154·8–158·1)	126·8 (125·3–128·2)	71·1 (69·7–72·5)	36·0 (34·7–37·4)	10·9 (10·0–11·8)	0·8 (0·6–0·9)	0·0 (0·0–0·0)	2·16 (2·14–2·17)	0·93 (0·92–0·94)	0·59 (0·58–0·61)
1970	0·1 (0·0–0·2)	29·1 (28·2–30·2)	158·4 (156·9–159·9)	125·0 (123·6–126·5)	71·0 (69·7–72·4)	35·3 (33·9–36·6)	10·7 (9·8–11·7)	0·7 (0·6–0·9)	0·0 (0·0–0·0)	2·15 (2·14–2·17)	0·94 (0·93–0·95)	0·59 (0·58–0·6)
1971	0·1 (0·0–0·2)	28·0 (27·0–29·0)	160·1 (158·7–161·7)	124·2 (122·9–125·6)	70·7 (69·2–72·0)	34·7 (33·4–36·0)	10·5 (9·6–11·5)	0·7 (0·5–0·9)	0·0 (0·0–0·0)	2·14 (2·13–2·16)	0·94 (0·93–0·95)	0·58 (0·57–0·59)
1972	0·1 (0·0–0·2)	27·8 (26·8–28·8)	161·0 (159·5–162·5)	124·9 (123·7–126·3)	70·1 (68·6–71·4)	34·0 (32·7–35·4)	10·2 (9·3–11·0)	0·7 (0·5–0·9)	0·0 (0·0–0·0)	2·14 (2·13–2·16)	0·94 (0·94–0·95)	0·57 (0·56–0·58)
1973	0·1 (0·0–0·2)	28·8 (27·8–29·8)	163·1 (161·6–164·7)	125·7 (124·4–127·0)	69·6 (68·1–71·0)	33·7 (32·4–35·0)	9·9 (9·1–10·9)	0·7 (0·5–0·9)	0·0 (0·0–0·0)	2·16 (2·14–2·17)	0·96 (0·95–0·97)	0·57 (0·56–0·58)
1974	0·1 (0·0–0·2)	30·7 (29·8–31·8)	165·0 (163·6–166·6)	126·8 (125·5–128·1)	69·5 (68·1–70·9)	33·5 (32·2–34·9)	9·9 (8·9–10·9)	0·7 (0·5–0·9)	0·0 (0·0–0·0)	2·18 (2·17–2·2)	0·98 (0·97–0·99)	0·57 (0·56–0·58)
1975	0·1 (0·0–0·2)	32·8 (31·7–33·8)	169·0 (167·6–170·4)	128·9 (127·6–130·3)	69·1 (67·6–70·5)	33·0 (31·7–34·3)	9·6 (8·6–10·6)	0·7 (0·5–0·9)	0·0 (0·0–0·0)	2·22 (2·2–2·23)	1·01 (1·0–1·02)	0·56 (0·55–0·57)
1976	0·1 (0·0–0·2)	33·8 (32·7–34·9)	169·5 (168·0–171·0)	128·9 (127·7–130·1)	66·9 (65·5–68·3)	31·2 (29·9–32·6)	8·8 (7·9–9·8)	0·7 (0·4–0·9)	0·0 (0·0–0·0)	2·2 (2·19–2·21)	1·02 (1·01–1·03)	0·54 (0·53–0·55)
1977	0·1 (0·0–0·2)	34·0 (33·0–35·2)	166·8 (165·4–168·2)	127·6 (126·4–128·8)	64·9 (63·6–66·2)	29·4 (28·1–30·8)	8·1 (7·2–9·0)	0·6 (0·4–0·9)	0·0 (0·0–0·0)	2·16 (2·14–2·17)	1·0 (1·0–1·01)	0·52 (0·5–0·53)
1978	0·1 (0·0–0·2)	34·1 (33·1–35·2)	169·8 (168·4–171·3)	129·6 (128·4–130·9)	65·0 (63·8–66·3)	29·0 (27·7–30·3)	7·8 (7·0–8·8)	0·6 (0·4–0·9)	0·0 (0·0–0·0)	2·18 (2·17–2·2)	1·02 (1·01–1·03)	0·51 (0·5–0·52)
1979	0·1 (0·0–0·2)	33·3 (32·2–34·4)	174·9 (173·5–176·4)	132·4 (131·2–133·6)	64·8 (63·6–66·1)	28·5 (27·2–30·0)	7·6 (6·7–8·6)	0·6 (0·4–0·9)	0·0 (0·0–0·0)	2·21 (2·2–2·23)	1·04 (1·03–1·05)	0·51 (0·5–0·52)
1980	0·1 (0·0–0·2)	32·3 (31·3–33·5)	175·4 (174·0–176·9)	131·5 (130·3–132·7)	63·8 (62·6–65·1)	27·9 (26·6–29·2)	7·3 (6·4–8·4)	0·6 (0·4–0·8)	0·0 (0·0–0·0)	2·19 (2·18–2·21)	1·04 (1·03–1·05)	0·5 (0·49–0·51)
1981	0·1 (0·0–0·2)	32·9 (31·8–34·1)	178·7 (177·2–180·4)	132·2 (131·0–133·4)	65·2 (64·1–66·4)	28·8 (27·6–30·2)	7·4 (6·5–8·5)	0·6 (0·4–0·8)	0·0 (0·0–0·0)	2·23 (2·21–2·25)	1·06 (1·05–1·07)	0·51 (0·5–0·52)
1982	0·1 (0·0–0·2)	34·2 (33·0–35·3)	186·3 (184·7–188·0)	138·6 (137·5–139·9)	68·6 (67·5–69·8)	29·8 (28·5–31·0)	7·5 (6·6–8·6)	0·6 (0·4–0·8)	0·0 (0·0–0·0)	2·33 (2·31–2·35)	1·1 (1·09–1·11)	0·53 (0·52–0·54)
1983	0·1 (0·0–0·2)	34·8 (33·7–36·0)	187·3 (185·7–189·0)	141·9 (140·6–143·0)	69·9 (68·7–71·2)	29·5 (28·3–30·6)	7·3 (6·3–8·3)	0·5 (0·3–0·8)	0·0 (0·0–0·0)	2·36 (2·34–2·37)	1·11 (1·1–1·12)	0·54 (0·53–0·55)
1984	0·1 (0·0–0·2)	34·4 (33·3–35·6)	184·7 (183·1–186·3)	139·6 (138·4–140·9)	68·7 (67·5–69·8)	28·7 (27·6–30·0)	7·0 (6·1–8·0)	0·5 (0·3–0·8)	0·0 (0·0–0·0)	2·32 (2·3–2·33)	1·1 (1·09–1·11)	0·52 (0·52–0·53)
1985	0·1 (0·0–0·2)	33·0 (31·9–34·2)	179·2 (177·7–180·8)	135·6 (134·4–136·8)	65·9 (64·7–67·2)	27·6 (26·5–28·8)	6·7 (5·8–7·6)	0·5 (0·3–0·7)	0·0 (0·0–0·0)	2·24 (2·23–2·26)	1·06 (1·05–1·07)	0·5 (0·49–0·51)
1986	0·1 (0·0–0·2)	31·5 (30·5–32·6)	173·1 (171·5–174·7)	130·8 (129·6–132·0)	63·0 (61·8–64·2)	26·6 (25·5–27·8)	6·5 (5·6–7·5)	0·4 (0·3–0·7)	0·0 (0·0–0·0)	2·16 (2·14–2·18)	1·02 (1·01–1·03)	0·48 (0·47–0·49)
1987	0·1 (0·0–0·2)	30·6 (29·6–31·7)	169·9 (168·2–171·5)	128·4 (127·1–129·6)	61·4 (60·3–62·5)	25·7 (24·6–26·9)	6·4 (5·5–7·3)	0·4 (0·3–0·6)	0·0 (0·0–0·0)	2·11 (2·1–2·13)	1·0 (0·99–1·01)	0·47 (0·46–0·48)
1988	0·1 (0·0–0·2)	30·1 (29·1–31·2)	166·3 (164·8–168·1)	126·3 (125·0–127·6)	60·0 (58·9–61·1)	24·6 (23·5–25·6)	6·2 (5·4–7·1)	0·4 (0·2–0·6)	0·0 (0·0–0·0)	2·07 (2·05–2·08)	0·98 (0·97–0·99)	0·46 (0·45–0·47)
1989	0·1 (0·0–0·2)	30·4 (29·4–31·4)	162·9 (161·3–164·5)	123·1 (121·8–124·3)	58·5 (57·3–59·8)	24·1 (23·0–25·2)	6·1 (5·4–7·0)	0·4 (0·2–0·6)	0·0 (0·0–0·0)	2·03 (2·01–2·04)	0·97 (0·96–0·98)	0·45 (0·44–0·45)
1990	0·1 (0·0–0·2)	30·8 (29·8–31·9)	161·4 (159·8–162·9)	121·4 (120·2–122·7)	58·4 (57·3–59·6)	24·2 (23·2–25·3)	6·0 (5·2–6·8)	0·3 (0·2–0·6)	0·0 (0·0–0·0)	2·01 (2·0–2·03)	0·96 (0·95–0·97)	0·44 (0·44–0·45)
1991	0·1 (0·0–0·2)	29·6 (28·6–30·7)	154·3 (152·6–156·0)	119·2 (117·8–120·5)	58·0 (56·7–59·0)	24·1 (23·1–25·1)	5·7 (5·0–6·5)	0·3 (0·2–0·5)	0·0 (0·0–0·0)	1·96 (1·94–1·97)	0·92 (0·91–0·93)	0·44 (0·43–0·45)
1992	0·1 (0·0–0·2)	27·6 (26·6–28·6)	141·3 (139·7–142·9)	115·6 (114·3–116·9)	58·1 (56·9–59·4)	24·6 (23·6–25·7)	5·8 (5·0–6·6)	0·3 (0·2–0·4)	0·0 (0·0–0·0)	1·87 (1·85–1·88)	0·85 (0·84–0·85)	0·44 (0·43–0·45)
1993	0·1 (0·0–0·2)	25·5 (24·5–26·4)	131·5 (129·9–133·0)	112·6 (111·2–113·9)	58·1 (56·8–59·3)	25·2 (24·2–26·4)	5·9 (5·2–6·7)	0·2 (0·1–0·4)	0·0 (0·0–0·0)	1·8 (1·78–1·81)	0·79 (0·78–0·79)	0·45 (0·44–0·46)
1994	0·1 (0·0–0·2)	23·0 (22·1–24·0)	119·2 (117·6–120·7)	107·7 (106·4–109·0)	56·5 (55·2–57·9)	24·5 (23·5–25·6)	5·8 (5·1–6·6)	0·2 (0·1–0·3)	0·0 (0·0–0·0)	1·69 (1·67–1·7)	0·71 (0·7–0·72)	0·44 (0·42–0·45)
1995	0·1 (0·0–0·1)	21·2 (20·3–22·2)	109·1 (107·5–110·5)	103·3 (102·0–104·6)	54·8 (53·5–56·1)	23·2 (22·1–24·4)	5·6 (4·8–6·4)	0·2 (0·1–0·3)	0·0 (0·0–0·0)	1·59 (1·57–1·6)	0·65 (0·64–0·66)	0·42 (0·41–0·43)
1996	0·1 (0·0–0·1)	19·9 (19·0–20·8)	103·3 (101·9–104·8)	100·8 (99·5–102·1)	53·3 (52·1–54·5)	22·4 (21·4–23·5)	5·3 (4·6–6·0)	0·2 (0·1–0·3)	0·0 (0·0–0·0)	1·53 (1·51–1·54)	0·62 (0·61–0·63)	0·41 (0·4–0·42)
1997	0·1 (0·0–0·1)	18·6 (17·7–19·5)	95·8 (94·5–97·1)	97·4 (96·1–98·6)	52·3 (51·1–53·6)	21·8 (20·7–22·9)	5·1 (4·4–5·8)	0·2 (0·1–0·3)	0·0 (0·0–0·0)	1·46 (1·44–1·47)	0·57 (0·56–0·58)	0·4 (0·39–0·41)
1998	0·1 (0·0–0·1)	17·7 (16·8–18·6)	88·6 (87·3–89·9)	94·4 (93·2–95·7)	51·4 (50·2–52·7)	21·4 (20·3–22·5)	4·9 (4·2–5·6)	0·2 (0·1–0·3)	0·0 (0·0–0·0)	1·39 (1·38–1·41)	0·53 (0·52–0·54)	0·39 (0·38–0·4)
1999	0·1 (0·0–0·1)	17·0 (16·1–17·9)	83·6 (82·3–85·0)	93·1 (91·9–94·4)	50·9 (49·6–52·2)	21·1 (20·0–22·2)	4·8 (4·1–5·6)	0·2 (0·1–0·3)	0·0 (0·0–0·0)	1·35 (1·34–1·37)	0·5 (0·5–0·51)	0·39 (0·37–0·4)
2000	0·1 (0·0–0·1)	16·1 (15·3–17·0)	78·0 (76·7–79·3)	91·7 (90·5–92·9)	51·1 (49·8–52·4)	21·1 (20·0–22·3)	4·7 (4·0–5·6)	0·2 (0·1–0·4)	0·0 (0·0–0·0)	1·32 (1·3–1·33)	0·47 (0·46–0·48)	0·39 (0·37–0·4)
2001	0·1 (0·0–0·1)	15·1 (14·3–15·9)	70·6 (69·4–71·9)	89·0 (87·8–90·2)	51·2 (50·0–52·5)	21·1 (20·0–22·3)	4·8 (4·1–5·6)	0·3 (0·2–0·4)	0·0 (0·0–0·0)	1·26 (1·24–1·28)	0·43 (0·42–0·44)	0·39 (0·37–0·4)
2002	0·1 (0·0–0·1)	14·5 (13·6–15·3)	64·8 (63·6–66·1)	87·0 (85·9–88·3)	51·5 (50·2–52·7)	21·0 (19·8–22·2)	4·8 (4·1–5·7)	0·3 (0·2–0·4)	0·0 (0·0–0·0)	1·22 (1·2–1·24)	0·4 (0·39–0·4)	0·39 (0·38–0·4)
2003	0·1 (0·0–0·1)	13·9 (13·1–14·8)	61·5 (60·2–62·8)	87·2 (86·0–88·4)	53·2 (51·9–54·4)	21·2 (20·1–22·5)	4·9 (4·1–5·8)	0·3 (0·2–0·4)	0·0 (0·0–0·0)	1·21 (1·2–1·23)	0·38 (0·37–0·39)	0·4 (0·39–0·41)
2004	0·1 (0·0–0·1)	13·6 (12·7–14·5)	59·2 (58·0–60·5)	88·4 (87·3–89·6)	56·3 (55·1–57·5)	22·0 (20·9–23·2)	4·9 (4·2–5·9)	0·3 (0·2–0·4)	0·0 (0·0–0·0)	1·22 (1·21–1·24)	0·36 (0·36–0·37)	0·42 (0·41–0·43)
2005	0·1 (0·0–0·1)	13·7 (12·9–14·6)	57·9 (56·6–59·1)	89·4 (88·2–90·7)	60·0 (58·8–61·3)	23·2 (21·9–24·4)	5·0 (4·3–6·0)	0·3 (0·2–0·4)	0·0 (0·0–0·0)	1·25 (1·23–1·26)	0·36 (0·35–0·37)	0·44 (0·43–0·45)
2006	0·1 (0·0–0·1)	14·5 (13·6–15·4)	58·0 (56·8–59·2)	90·5 (89·3–91·6)	64·2 (62·9–65·5)	24·8 (23·5–26·0)	5·1 (4·3–6·1)	0·2 (0·1–0·4)	0·0 (0·0–0·0)	1·29 (1·27–1·3)	0·36 (0·36–0·37)	0·47 (0·46–0·48)
2007	0·1 (0·0–0·1)	15·6 (14·7–16·5)	59·9 (58·6–61·1)	93·0 (91·9–94·3)	68·9 (67·7–70·1)	26·7 (25·5–28·0)	5·3 (4·5–6·1)	0·2 (0·1–0·4)	0·0 (0·0–0·0)	1·35 (1·33–1·37)	0·38 (0·37–0·38)	0·51 (0·49–0·52)
2008	0·1 (0·0–0·1)	16·0 (15·1–16·9)	60·3 (59·0–61·6)	95·4 (94·3–96·5)	72·6 (71·3–73·9)	28·7 (27·5–30·0)	5·5 (4·7–6·4)	0·2 (0·1–0·4)	0·0 (0·0–0·0)	1·39 (1·38–1·41)	0·38 (0·37–0·39)	0·54 (0·53–0·55)
2009	0·1 (0·0–0·1)	15·5 (14·5–16·5)	57·6 (56·3–59·0)	94·9 (93·8–96·1)	73·9 (72·6–75·2)	30·2 (28·9–31·4)	5·8 (5·0–6·7)	0·2 (0·2–0·4)	0·0 (0·0–0·0)	1·39 (1·38–1·41)	0·37 (0·36–0·37)	0·55 (0·54–0·56)
2010	0·1 (0·0–0·1)	14·7 (13·7–15·6)	53·9 (52·6–55·1)	91·8 (90·7–93·0)	72·6 (71·4–73·9)	30·5 (29·3–31·7)	6·0 (5·2–7·0)	0·3 (0·2–0·4)	0·0 (0·0–0·0)	1·35 (1·33–1·36)	0·34 (0·33–0·35)	0·55 (0·54–0·56)
2011	0·1 (0·0–0·1)	14·1 (13·2–15·1)	51·1 (49·8–52·3)	89·0 (87·8–90·2)	70·7 (69·5–71·9)	30·2 (29·1–31·4)	6·2 (5·3–7·1)	0·3 (0·2–0·4)	0·0 (0·0–0·0)	1·31 (1·29–1·32)	0·33 (0·32–0·33)	0·54 (0·53–0·55)
2012	0·1 (0·0–0·1)	13·6 (12·7–14·6)	49·6 (48·3–50·9)	87·5 (86·3–88·6)	69·9 (68·8–71·1)	30·0 (28·9–31·1)	6·3 (5·4–7·2)	0·3 (0·2–0·4)	0·0 (0·0–0·0)	1·29 (1·27–1·3)	0·32 (0·31–0·32)	0·53 (0·52–0·54)
2013	0·1 (0·0–0·1)	13·1 (12·1–14·2)	48·2 (46·9–49·5)	87·0 (85·8–88·3)	71·2 (70·1–72·4)	30·5 (29·3–31·7)	6·4 (5·5–7·3)	0·3 (0·2–0·4)	0·0 (0·0–0·0)	1·28 (1·27–1·3)	0·31 (0·3–0·32)	0·54 (0·53–0·55)
2014	0·1 (0·0–0·1)	12·8 (11·7–14·0)	47·5 (46·2–48·9)	87·8 (86·5–89·0)	73·6 (72·5–74·9)	31·3 (30·1–32·6)	6·4 (5·5–7·2)	0·3 (0·2–0·4)	0·0 (0·0–0·0)	1·3 (1·28–1·32)	0·3 (0·29–0·31)	0·56 (0·55–0·57)
2015	0·1 (0·0–0·1)	12·7 (11·4–14·1)	47·8 (45·3–50·4)	88·5 (86·2–90·8)	74·4 (72·0–77·0)	31·3 (29·3–33·4)	6·2 (5·4–7·0)	0·3 (0·2–0·3)	0·0 (0·0–0·0)	1·31 (1·26–1·36)	0·3 (0·28–0·32)	0·56 (0·54–0·58)
2016	0·1 (0·0–0·1)	12·7 (11·1–14·5)	48·8 (43·7–54·2)	89·4 (84·7–94·3)	73·9 (68·6–79·5)	30·5 (26·7–34·5)	5·9 (4·9–7·0)	0·2 (0·2–0·3)	0·0 (0·0–0·0)	1·31 (1·2–1·42)	0·31 (0·28–0·34)	0·55 (0·5–0·61)
2017	0·1 (0·0–0·1)	12·7 (10·9–14·9)	49·8 (42·6–58·2)	89·9 (83·1–97·3)	73·5 (65·7–82·2)	29·7 (24·5–35·8)	5·7 (4·5–7·1)	0·2 (0·2–0·3)	0·0 (0·0–0·0)	1·31 (1·16–1·48)	0·31 (0·27–0·37)	0·55 (0·47–0·63)

Supplemental Results Table 5: Age-specific fertility rates per 1000, cumulative total fertility up to maternal age 25 (TFU25), cumulative total fertility from maternal age 30 to age 55 (TFO30), and cumulative total fertility, Romania, 1950-2017												
**Year**	**10-14**	**15-19**	**20-24**	**25-29**	**30-34**	**35-39**	**40-44**	**45-49**	**50-54**	**TFR**	**TFU25**	**TFO30**
1950	0·5 (0·2–1·0)	55·6 (47·9–64·4)	192·0 (173·3–213·9)	161·6 (151·8–173·2)	107·0 (98·1–117·8)	53·4 (46·2–62·4)	24·3 (21·0–27·8)	2·3 (2·0–2·8)	0·0 (0·0–0·0)	2·98 (2·76–3·25)	1·24 (1·14–1·35)	0·94 (0·86–1·04)
1951	0·5 (0·2–1·0)	53·0 (45·8–61·0)	188·6 (171·7–207·7)	160·6 (151·6–170·7)	107·3 (99·2–117·1)	50·8 (44·2–58·0)	23·8 (20·8–27·2)	2·2 (1·9–2·6)	0·0 (0·0–0·0)	2·93 (2·72–3·17)	1·21 (1·11–1·32)	0·92 (0·85–1·01)
1952	0·5 (0·2–1·0)	50·8 (44·4–57·5)	185·7 (171·4–201·4)	162·0 (154·2–170·5)	109·1 (101·8–117·4)	50·4 (44·7–57·2)	23·9 (21·1–27·1)	2·2 (1·9–2·6)	0·0 (0·0–0·0)	2·92 (2·73–3·14)	1·18 (1·1–1·28)	0·93 (0·86–1·0)
1953	0·5 (0·2–1·0)	49·4 (45·1–54·1)	183·9 (174·2–194·8)	164·6 (159·0–170·9)	111·7 (105·9–117·8)	53·7 (48·7–59·0)	24·4 (21·8–27·2)	2·2 (1·9–2·6)	0·0 (0·0–0·0)	2·95 (2·82–3·1)	1·17 (1·11–1·24)	0·96 (0·91–1·01)
1954	0·5 (0·2–0·9)	48·7 (46·1–51·2)	181·8 (177·8–186·1)	165·0 (162·3–168·0)	111·9 (109·0–115·0)	62·3 (59·0–65·6)	24·4 (22·4–26·6)	2·2 (1·8–2·7)	0·0 (0·0–0·0)	2·98 (2·93–3·04)	1·15 (1·13–1·18)	1·0 (0·98–1·03)
1955	0·5 (0·2–0·9)	49·0 (47·5–50·6)	177·5 (175·6–179·4)	158·9 (157·3–160·4)	105·9 (104·2–107·6)	75·6 (73·6–77·8)	22·8 (21·2–24·5)	2·1 (1·6–2·7)	0·0 (0·0–0·0)	2·96 (2·94–2·98)	1·14 (1·12–1·15)	1·03 (1·01–1·05)
1956	0·5 (0·2–1·0)	50·6 (49·2–52·1)	173·1 (171·2–174·9)	148·5 (146·9–150·1)	95·4 (93·8–97·1)	73·3 (71·2–75·4)	19·5 (18·0–21·1)	2·0 (1·5–2·6)	0·0 (0·0–0·0)	2·81 (2·79–2·84)	1·12 (1·11–1·13)	0·95 (0·93–0·97)
1957	0·5 (0·2–1·0)	52·6 (51·0–54·2)	169·9 (168·1–171·8)	138·8 (137·2–140·4)	84·4 (82·8–86·0)	57·0 (55·1–59·1)	16·0 (14·6–17·5)	1·8 (1·3–2·5)	0·0 (0·0–0·0)	2·6 (2·59–2·63)	1·11 (1·1–1·13)	0·8 (0·78–0·81)
1958	0·5 (0·2–1·0)	54·8 (53·4–56·4)	164·5 (162·7–166·2)	129·3 (127·8–130·8)	74·8 (73·2–76·4)	46·9 (45·2–48·6)	14·1 (12·7–15·6)	1·6 (1·1–2·2)	0·0 (0·0–0·0)	2·43 (2·41–2·45)	1·1 (1·09–1·11)	0·69 (0·67–0·7)
1959	0·5 (0·2–1·0)	57·0 (55·4–58·5)	159·1 (157·2–160·9)	120·6 (119·0–122·2)	67·2 (65·6–68·8)	40·7 (39·1–42·3)	13·3 (11·9–14·8)	1·3 (0·9–1·9)	0·0 (0·0–0·0)	2·3 (2·28–2·32)	1·08 (1·07–1·1)	0·61 (0·6–0·63)
1960	0·5 (0·2–1·0)	58·4 (56·9–59·9)	152·4 (150·6–154·2)	111·7 (110·1–113·2)	60·4 (58·8–62·0)	35·2 (33·6–36·9)	12·7 (11·3–14·3)	1·1 (0·7–1·7)	0·0 (0·0–0·0)	2·16 (2·14–2·19)	1·06 (1·04–1·07)	0·55 (0·53–0·56)
1961	0·5 (0·2–1·0)	59·0 (57·4–60·6)	143·6 (141·8–145·4)	103·4 (102·0–104·8)	55·0 (53·5–56·5)	30·9 (29·4–32·5)	12·2 (10·9–13·7)	1·0 (0·6–1·5)	0·0 (0·0–0·0)	2·03 (2·01–2·05)	1·02 (1·0–1·03)	0·5 (0·48–0·51)
1962	0·5 (0·2–1·1)	59·4 (57·8–60·9)	139·5 (137·5–141·4)	99·6 (98·1–101·0)	52·8 (51·3–54·4)	28·2 (26·6–29·7)	11·3 (10·1–12·7)	0·9 (0·6–1·3)	0·0 (0·0–0·0)	1·96 (1·94–1·98)	1·0 (0·98–1·01)	0·47 (0·45–0·48)
1963	0·5 (0·2–1·0)	58·1 (56·4–59·7)	139·8 (137·8–141·7)	99·0 (97·6–100·5)	51·5 (49·9–53·1)	26·0 (24·5–27·5)	9·8 (8·7–11·0)	0·8 (0·5–1·3)	0·0 (0·0–0·0)	1·93 (1·91–1·95)	0·99 (0·98–1·0)	0·44 (0·43–0·45)
1964	0·5 (0·2–1·0)	51·7 (50·3–53·3)	135·9 (134·0–137·8)	95·2 (93·6–96·8)	47·4 (46·1–48·8)	23·0 (21·7–24·4)	8·2 (7·1–9·2)	0·8 (0·5–1·2)	0·0 (0·0–0·0)	1·81 (1·79–1·83)	0·94 (0·93–0·95)	0·4 (0·39–0·41)
1965	0·5 (0·2–1·0)	51·8 (50·4–53·3)	134·3 (132·5–136·3)	94·5 (93·1–96·0)	53·9 (52·4–55·5)	25·0 (23·7–26·5)	8·7 (7·7–9·8)	0·9 (0·6–1·4)	0·0 (0·0–0·0)	1·85 (1·83–1·87)	0·93 (0·92–0·95)	0·44 (0·43–0·46)
1966	0·5 (0·2–1·1)	66·5 (64·9–68·2)	191·7 (189·7–193·7)	139·0 (137·4–140·7)	88·4 (86·8–90·1)	41·9 (40·3–43·5)	13·6 (12·3–15·1)	1·3 (0·8–1·9)	0·0 (0·0–0·0)	2·71 (2·69–2·74)	1·29 (1·28–1·31)	0·73 (0·71–0·74)
1967	0·6 (0·3–1·2)	78·0 (76·5–79·5)	245·4 (243·5–247·2)	184·5 (182·8–186·1)	120·0 (118·2–121·5)	59·8 (58·0–61·4)	16·8 (15·4–18·3)	1·3 (0·9–2·0)	0·0 (0·0–0·0)	3·53 (3·5–3·56)	1·62 (1·61–1·63)	0·99 (0·98–1·0)
1968	0·6 (0·2–1·2)	74·2 (72·7–75·6)	226·5 (224·6–228·5)	172·0 (170·4–173·6)	113·6 (111·8–115·3)	57·8 (56·1–59·5)	15·0 (13·7–16·4)	1·2 (0·8–1·7)	0·0 (0·0–0·0)	3·3 (3·28–3·33)	1·51 (1·49–1·52)	0·94 (0·92–0·95)
1969	0·5 (0·2–1·1)	67·0 (65·5–68·5)	205·8 (203·7–207·6)	154·1 (152·4–155·9)	97·1 (95·5–98·8)	51·1 (49·5–52·6)	13·7 (12·5–14·9)	1·0 (0·6–1·5)	0·0 (0·0–0·0)	2·95 (2·93–2·97)	1·37 (1·35–1·38)	0·81 (0·8–0·83)
1970	0·5 (0·2–1·1)	61·8 (60·4–63·2)	194·0 (192·0–196·0)	139·7 (138·1–141·4)	83·9 (82·3–85·5)	45·8 (44·1–47·4)	12·7 (11·6–14·0)	0·9 (0·6–1·3)	0·0 (0·0–0·0)	2·7 (2·68–2·72)	1·28 (1·27–1·29)	0·72 (0·7–0·73)
1971	0·5 (0·2–1·0)	58·8 (57·4–60·2)	186·0 (184·1–187·9)	128·9 (127·2–130·5)	74·6 (73·0–76·2)	41·1 (39·5–42·7)	11·5 (10·3–12·7)	0·8 (0·5–1·2)	0·0 (0·0–0·0)	2·51 (2·49–2·53)	1·23 (1·21–1·24)	0·64 (0·62–0·66)
1972	0·5 (0·2–1·0)	58·4 (57·0–59·9)	181·1 (179·3–183·1)	124·6 (122·9–126·3)	69·9 (68·4–71·6)	38·2 (36·6–39·8)	10·8 (9·6–11·9)	0·8 (0·5–1·2)	0·0 (0·0–0·0)	2·42 (2·4–2·44)	1·2 (1·19–1·21)	0·6 (0·58–0·61)
1973	0·5 (0·2–1·1)	61·8 (60·5–63·4)	189·5 (187·6–191·5)	132·6 (131·0–134·3)	70·5 (68·8–72·1)	38·1 (36·6–39·6)	10·7 (9·5–11·9)	0·8 (0·5–1·2)	0·0 (0·0–0·0)	2·52 (2·5–2·54)	1·26 (1·25–1·27)	0·6 (0·59–0·61)
1974	0·5 (0·2–1·1)	64·8 (63·3–66·3)	197·7 (196·0–199·4)	138·4 (136·8–140·1)	69·8 (68·2–71·5)	36·5 (35·1–38·1)	10·1 (9·0–11·4)	0·8 (0·5–1·2)	0·0 (0·0–0·0)	2·59 (2·57–2·61)	1·31 (1·3–1·33)	0·59 (0·57–0·6)
1975	0·5 (0·2–1·1)	65·1 (63·7–66·6)	194·5 (192·7–196·3)	136·3 (134·7–137·8)	67·0 (65·4–68·5)	33·5 (32·0–35·0)	9·2 (8·2–10·4)	0·7 (0·5–1·1)	0·0 (0·0–0·0)	2·53 (2·52–2·55)	1·3 (1·29–1·31)	0·55 (0·54–0·57)
1976	0·5 (0·2–1·1)	65·8 (64·3–67·3)	195·7 (194·0–197·5)	136·2 (134·5–137·8)	65·7 (64·1–67·4)	31·4 (30·0–32·9)	8·7 (7·7–9·9)	0·7 (0·5–1·1)	0·0 (0·0–0·0)	2·52 (2·5–2·54)	1·31 (1·3–1·32)	0·53 (0·52–0·55)
1977	0·5 (0·2–1·1)	66·9 (65·3–68·4)	195·9 (194·1–197·7)	133·2 (131·6–134·8)	63·9 (62·2–65·6)	29·6 (28·1–31·1)	8·2 (7·1–9·3)	0·7 (0·4–1·0)	0·0 (0·0–0·0)	2·49 (2·47–2·51)	1·32 (1·3–1·33)	0·51 (0·5–0·52)
1978	0·5 (0·2–1·1)	68·1 (66·5–69·8)	194·6 (192·8–196·3)	128·6 (127·1–130·1)	61·9 (60·3–63·4)	27·9 (26·3–29·5)	7·6 (6·5–8·7)	0·7 (0·4–1·0)	0·0 (0·0–0·0)	2·45 (2·43–2·47)	1·32 (1·3–1·33)	0·49 (0·48–0·5)
1979	0·5 (0·2–1·1)	69·6 (68·0–71·3)	193·5 (191·7–195·3)	126·7 (125·2–128·2)	60·1 (58·5–61·7)	26·1 (24·5–27·8)	7·0 (5·9–8·1)	0·6 (0·4–0·9)	0·0 (0·0–0·0)	2·42 (2·4–2·44)	1·32 (1·31–1·33)	0·47 (0·46–0·48)
1980	0·6 (0·2–1·2)	70·4 (68·9–72·0)	189·5 (187·7–191·5)	122·6 (121·1–124·0)	57·0 (55·5–58·6)	23·7 (22·3–25·2)	6·1 (5·2–7·1)	0·5 (0·3–0·8)	0·0 (0·0–0·0)	2·35 (2·33–2·37)	1·3 (1·29–1·32)	0·44 (0·42–0·45)
1981	0·5 (0·2–1·1)	67·8 (66·2–69·4)	182·0 (180·0–183·9)	112·6 (111·2–114·0)	50·8 (49·3–52·2)	20·5 (19·2–22·0)	5·2 (4·3–6·2)	0·5 (0·3–0·8)	0·0 (0·0–0·0)	2·2 (2·18–2·22)	1·25 (1·24–1·26)	0·39 (0·37–0·4)
1982	0·5 (0·2–1·1)	62·0 (60·5–63·6)	174·0 (172·2–175·8)	103·1 (101·6–104·5)	45·6 (44·2–47·0)	18·7 (17·3–20·0)	4·8 (3·9–5·7)	0·5 (0·3–0·7)	0·0 (0·0–0·0)	2·05 (2·03–2·06)	1·18 (1·17–1·2)	0·35 (0·33–0·36)
1983	0·5 (0·2–1·0)	57·6 (56·2–59·1)	179·0 (177·0–180·9)	105·2 (103·8–106·8)	46·5 (45·1–48·0)	19·5 (18·2–20·9)	5·2 (4·2–6·2)	0·4 (0·3–0·7)	0·0 (0·0–0·0)	2·07 (2·05–2·09)	1·19 (1·17–1·2)	0·36 (0·35–0·37)
1984	0·5 (0·2–1·0)	56·9 (55·5–58·3)	188·6 (186·7–190·7)	114·4 (113·0–116·0)	51·8 (50·3–53·3)	21·6 (20·1–23·1)	5·9 (4·9–7·0)	0·5 (0·3–0·7)	0·0 (0·0–0·0)	2·2 (2·18–2·22)	1·23 (1·22–1·24)	0·4 (0·39–0·41)
1985	0·5 (0·2–1·1)	59·5 (58·2–60·8)	191·8 (189·8–193·7)	120·2 (118·8–121·8)	58·2 (56·7–59·7)	24·3 (22·9–25·8)	6·7 (5·6–7·8)	0·4 (0·3–0·7)	0·0 (0·0–0·0)	2·31 (2·29–2·33)	1·26 (1·25–1·27)	0·45 (0·44–0·46)
1986	0·5 (0·2–1·1)	62·8 (61·5–64·1)	191·4 (189·5–193·6)	123·9 (122·4–125·5)	63·2 (61·7–64·7)	27·3 (25·9–28·7)	7·4 (6·5–8·5)	0·4 (0·3–0·7)	0·0 (0·0–0·0)	2·39 (2·37–2·41)	1·27 (1·26–1·29)	0·49 (0·48–0·5)
1987	0·5 (0·2–1·1)	62·9 (61·6–64·3)	188·4 (186·3–190·4)	123·5 (121·9–125·1)	63·9 (62·5–65·4)	29·0 (27·5–30·4)	7·7 (6·8–8·7)	0·4 (0·3–0·6)	0·0 (0·0–0·0)	2·38 (2·36–2·41)	1·26 (1·25–1·27)	0·51 (0·49–0·52)
1988	0·5 (0·2–1·1)	59·4 (58·0–60·8)	182·2 (180·4–184·1)	119·3 (117·6–121·0)	59·9 (58·4–61·4)	26·6 (25·2–28·0)	6·9 (5·9–7·8)	0·4 (0·3–0·6)	0·0 (0·0–0·0)	2·28 (2·25–2·3)	1·21 (1·2–1·22)	0·47 (0·46–0·48)
1989	0·5 (0·2–1·0)	53·8 (52·5–55·2)	162·8 (160·9–164·6)	107·4 (105·8–109·2)	50·1 (48·6–51·7)	21·1 (19·9–22·4)	5·4 (4·6–6·2)	0·3 (0·2–0·4)	0·0 (0·0–0·0)	2·01 (1·99–2·03)	1·09 (1·07–1·1)	0·38 (0·37–0·4)
1990	0·5 (0·2–1·0)	49·2 (47·9–50·6)	140·8 (138·9–142·5)	90·5 (89·0–92·1)	39·4 (38·1–40·8)	16·9 (15·7–18·1)	4·7 (4·0–5·4)	0·3 (0·2–0·3)	0·0 (0·0–0·0)	1·71 (1·69–1·73)	0·95 (0·94–0·96)	0·31 (0·29–0·32)
1991	0·4 (0·2–0·9)	46·7 (45·4–48·1)	129·4 (127·6–131·1)	79·9 (78·3–81·5)	32·5 (31·2–33·8)	14·3 (13·2–15·5)	4·4 (3·9–5·0)	0·2 (0·2–0·3)	0·0 (0·0–0·0)	1·54 (1·52–1·56)	0·88 (0·87–0·89)	0·26 (0·25–0·27)
1992	0·4 (0·2–0·9)	46·5 (45·2–47·9)	122·6 (121·0–124·4)	77·3 (75·7–78·9)	28·7 (27·5–30·0)	12·4 (11·4–13·6)	4·0 (3·5–4·6)	0·2 (0·2–0·2)	0·0 (0·0–0·0)	1·46 (1·44–1·48)	0·85 (0·84–0·86)	0·23 (0·22–0·24)
1993	0·4 (0·2–0·9)	46·2 (44·9–47·5)	118·2 (116·4–120·0)	78·2 (76·7–79·7)	27·5 (26·2–28·8)	11·4 (10·4–12·6)	3·7 (3·2–4·3)	0·2 (0·2–0·2)	0·0 (0·0–0·0)	1·43 (1·41–1·45)	0·82 (0·81–0·84)	0·21 (0·2–0·22)
1994	0·4 (0·2–0·9)	42·6 (41·3–43·8)	113·1 (111·4–114·9)	77·4 (76·0–78·9)	28·2 (26·8–29·6)	11·0 (9·9–12·2)	3·6 (3·0–4·2)	0·2 (0·2–0·3)	0·0 (0·0–0·0)	1·38 (1·37–1·4)	0·78 (0·77–0·79)	0·21 (0·2–0·23)
1995	0·4 (0·2–0·8)	39·7 (38·5–41·0)	106·2 (104·4–107·9)	75·1 (73·7–76·5)	29·9 (28·4–31·3)	11·0 (9·9–12·2)	3·5 (2·9–4·1)	0·2 (0·1–0·3)	0·0 (0·0–0·0)	1·33 (1·31–1·35)	0·73 (0·72–0·74)	0·22 (0·21–0·24)
1996	0·4 (0·2–0·8)	39·8 (38·5–41·1)	102·0 (100·2–103·7)	74·1 (72·7–75·4)	32·5 (31·0–33·9)	11·2 (10·1–12·4)	3·4 (2·8–4·0)	0·2 (0·1–0·3)	0·0 (0·0–0·0)	1·32 (1·3–1·33)	0·71 (0·7–0·72)	0·24 (0·22–0·25)
1997	0·4 (0·2–0·9)	40·2 (38·8–41·6)	100·5 (98·6–102·1)	75·5 (74·1–76·8)	35·3 (33·9–36·8)	11·5 (10·4–12·7)	3·3 (2·7–4·0)	0·2 (0·1–0·3)	0·0 (0·0–0·0)	1·33 (1·32–1·35)	0·71 (0·69–0·72)	0·25 (0·24–0·26)
1998	0·4 (0·2–0·8)	39·9 (38·6–41·3)	96·9 (95·2–98·6)	77·5 (76·1–79·0)	37·3 (36·0–38·8)	12·1 (11·0–13·4)	3·3 (2·6–4·0)	0·2 (0·1–0·3)	0·0 (0·0–0·0)	1·34 (1·32–1·35)	0·69 (0·67–0·7)	0·26 (0·25–0·28)
1999	0·4 (0·2–0·8)	39·0 (37·7–40·4)	91·9 (90·2–93·7)	78·4 (77·0–79·8)	38·2 (36·9–39·6)	12·9 (11·7–14·2)	3·3 (2·7–4·1)	0·2 (0·1–0·3)	0·0 (0·0–0·0)	1·32 (1·3–1·34)	0·66 (0·65–0·67)	0·27 (0·26–0·28)
2000	0·4 (0·2–0·8)	36·6 (35·3–38·0)	87·9 (86·4–89·6)	78·8 (77·4–80·2)	38·5 (37·1–39·8)	13·8 (12·5–15·1)	3·4 (2·8–4·3)	0·2 (0·2–0·3)	0·0 (0·0–0·0)	1·3 (1·28–1·32)	0·62 (0·62–0·64)	0·28 (0·27–0·29)
2001	0·4 (0·2–0·8)	34·0 (32·7–35·3)	83·0 (81·4–84·8)	78·4 (77·0–79·8)	38·9 (37·5–40·2)	14·9 (13·6–16·3)	3·6 (2·9–4·3)	0·2 (0·2–0·3)	0·0 (0·0–0·0)	1·27 (1·25–1·28)	0·59 (0·58–0·6)	0·29 (0·28–0·3)
2002	0·4 (0·2–0·8)	33·2 (31·9–34·5)	79·9 (78·2–81·5)	78·6 (77·1–80·1)	40·2 (38·9–41·6)	16·0 (14·8–17·4)	3·6 (3·0–4·4)	0·2 (0·2–0·3)	0·0 (0·0–0·0)	1·26 (1·24–1·28)	0·57 (0·56–0·58)	0·3 (0·29–0·31)
2003	0·4 (0·2–0·8)	33·3 (32·1–34·6)	78·2 (76·5–79·9)	80·0 (78·6–81·4)	43·7 (42·4–45·0)	17·2 (16·0–18·5)	3·7 (3·0–4·6)	0·2 (0·2–0·3)	0·0 (0·0–0·0)	1·28 (1·26–1·3)	0·56 (0·55–0·57)	0·32 (0·31–0·34)
2004	0·4 (0·2–0·8)	33·6 (32·3–35·0)	75·4 (73·7–77·1)	82·5 (81·0–84·0)	48·8 (47·3–50·1)	18·3 (16·9–19·7)	3·8 (3·0–4·8)	0·2 (0·1–0·3)	0·0 (0·0–0·0)	1·31 (1·3–1·33)	0·55 (0·54–0·56)	0·36 (0·35–0·37)
2005	0·4 (0·2–0·8)	34·7 (33·4–36·1)	73·8 (72·2–75·6)	85·6 (84·1–87·1)	52·9 (51·5–54·4)	19·1 (17·9–20·5)	3·9 (3·1–5·1)	0·2 (0·1–0·3)	0·0 (0·0–0·0)	1·35 (1·34–1·37)	0·54 (0·53–0·56)	0·38 (0·37–0·39)
2006	0·4 (0·2–0·8)	36·5 (35·1–38·0)	73·7 (71·9–75·3)	88·0 (86·5–89·6)	55·4 (54·0–57·0)	19·8 (18·6–21·1)	4·1 (3·2–5·1)	0·2 (0·1–0·3)	0·0 (0·0–0·0)	1·39 (1·37–1·41)	0·55 (0·54–0·57)	0·4 (0·39–0·41)
2007	0·4 (0·2–0·8)	38·9 (37·4–40·3)	74·8 (73·0–76·6)	90·6 (89·0–92·2)	57·8 (56·3–59·4)	20·7 (19·4–21·9)	4·2 (3·4–5·2)	0·2 (0·1–0·3)	0·0 (0·0–0·0)	1·44 (1·42–1·46)	0·57 (0·56–0·58)	0·41 (0·4–0·43)
2008	0·4 (0·2–0·9)	40·3 (38·8–41·8)	76·3 (74·6–78·0)	93·3 (91·7–94·9)	61·0 (59·4–62·6)	22·1 (20·7–23·5)	4·4 (3·6–5·4)	0·2 (0·1–0·3)	0·0 (0·0–0·0)	1·49 (1·47–1·51)	0·59 (0·57–0·6)	0·44 (0·43–0·45)
2009	0·4 (0·2–0·8)	39·2 (37·7–40·8)	74·7 (72·8–76·4)	93·2 (91·6–94·9)	63·5 (61·9–65·0)	23·8 (22·4–25·2)	4·6 (3·8–5·6)	0·2 (0·1–0·3)	0·0 (0·0–0·0)	1·5 (1·48–1·52)	0·57 (0·56–0·58)	0·46 (0·45–0·47)
2010	0·4 (0·2–0·8)	37·7 (36·2–39·2)	72·0 (70·3–73·8)	90·6 (89·0–92·3)	63·6 (62·0–65·1)	24·9 (23·5–26·3)	4·7 (3·9–5·6)	0·2 (0·1–0·3)	0·0 (0·0–0·0)	1·47 (1·45–1·5)	0·55 (0·54–0·56)	0·47 (0·46–0·48)
2011	0·4 (0·2–0·8)	36·6 (35·1–38·1)	72·1 (70·5–73·9)	88·6 (86·9–90·2)	63·7 (62·0–65·4)	25·7 (24·3–27·1)	4·7 (3·9–5·6)	0·2 (0·2–0·3)	0·0 (0·0–0·0)	1·46 (1·44–1·48)	0·55 (0·53–0·56)	0·47 (0·46–0·48)
2012	0·4 (0·2–0·8)	35·1 (33·7–36·7)	70·9 (69·1–72·6)	87·5 (85·9–89·0)	65·1 (63·5–66·8)	26·8 (25·3–28·3)	4·9 (4·1–5·9)	0·2 (0·2–0·4)	0·0 (0·0–0·0)	1·45 (1·43–1·48)	0·53 (0·52–0·54)	0·49 (0·47–0·5)
2013	0·4 (0·2–0·8)	34·7 (33·1–36·3)	69·4 (67·5–71·4)	88·8 (87·3–90·5)	68·2 (66·5–69·9)	28·3 (26·7–30·0)	5·1 (4·3–6·1)	0·2 (0·2–0·3)	0·0 (0·0–0·0)	1·48 (1·46–1·5)	0·52 (0·51–0·53)	0·51 (0·5–0·52)
2014	0·4 (0·2–0·8)	35·4 (33·7–37·1)	71·3 (69·3–73·3)	93·9 (92·3–95·7)	73·4 (71·7–75·2)	30·4 (28·9–32·0)	5·2 (4·3–6·2)	0·2 (0·2–0·3)	0·0 (0·0–0·0)	1·55 (1·53–1·57)	0·54 (0·52–0·55)	0·55 (0·53–0·56)
2015	0·4 (0·2–0·8)	35·6 (33·1–38·2)	72·8 (69·3–76·5)	97·9 (95·0–100·8)	76·8 (73·6–80·0)	31·4 (28·8–34·0)	5·1 (4·2–6·1)	0·2 (0·2–0·3)	0·0 (0·0–0·0)	1·6 (1·54–1·66)	0·54 (0·52–0·57)	0·57 (0·54–0·59)
2016	0·4 (0·2–0·8)	35·2 (31·5–39·5)	72·6 (65·4–80·4)	98·4 (93·0–104·1)	76·2 (70·3–82·5)	30·4 (26·2–35·1)	4·9 (4·0–6·0)	0·2 (0·2–0·3)	0·0 (0·0–0·0)	1·59 (1·48–1·71)	0·54 (0·5–0·58)	0·56 (0·51–0·61)
2017	0·4 (0·2–0·8)	34·9 (30·5–40·6)	71·9 (61·9–83·5)	97·4 (90·0–105·5)	73·7 (65·7–82·8)	28·9 (23·6–35·3)	4·7 (3·6–6·1)	0·2 (0·2–0·3)	0·0 (0·0–0·0)	1·56 (1·4–1·74)	0·54 (0·48–0·6)	0·54 (0·47–0·62)

Supplemental Results Table 5: Age-specific fertility rates per 1000, cumulative total fertility up to maternal age 25 (TFU25), cumulative total fertility from maternal age 30 to age 55 (TFO30), and cumulative total fertility, Serbia, 1950-2017

Year	10-14	15-19	20-24	25-29	30-34	35-39	40-44	45-49	50-54	TFR	TFU25	TFO30
1950	0·4 (0·2–0·7)	47·3 (45·2–49·3)	207·7 (205·0–210·3)	182·3 (179·9–184·7)	129·0 (126·1–131·8)	63·5 (60·6–66·5)	30·6 (28·0–33·3)	8·2 (6·5–10·3)	0·2 (0·2–0·2)	3·35 (3·31–3·38)	1·28 (1·26–1·29)	1·16 (1·13–1·19)
1951	0·4 (0·2–0·7)	46·5 (44·5–48·5)	203·9 (201·5–206·2)	178·6 (176·2–181·0)	118·2 (115·4–121·0)	60·9 (58·1–63·8)	28·4 (26·2–31·1)	7·7 (6·2–9·5)	0·1 (0·1–0·2)	3·22 (3·19–3·26)	1·25 (1·24–1·27)	1·08 (1·05–1·11)
1952	0·4 (0·2–0·7)	47·3 (45·3–49·2)	200·5 (198·1–203·0)	177·0 (174·7–179·3)	110·5 (107·8–113·2)	62·0 (59·2–64·9)	26·9 (24·7–29·3)	7·4 (5·9–9·2)	0·1 (0·1–0·1)	3·16 (3·13–3·2)	1·24 (1·22–1·26)	1·03 (1·01–1·06)
1953	0·4 (0·2–0·8)	49·2 (47·2–51·3)	200·3 (197·9–202·8)	172·0 (169·8–174·0)	101·7 (99·1–104·4)	65·2 (62·4–68·1)	25·4 (23·2–27·9)	7·2 (5·7–9·0)	0·1 (0·1–0·1)	3·11 (3·07–3·14)	1·25 (1·23–1·26)	1·0 (0·97–1·02)
1954	0·4 (0·2–0·8)	50·1 (48·1–52·1)	197·9 (195·4–200·3)	160·4 (158·1–162·6)	91·8 (89·4–94·4)	65·5 (62·6–68·5)	22·9 (20·6–25·3)	6·8 (5·3–8·5)	0·1 (0·1–0·1)	2·98 (2·95–3·02)	1·24 (1·23–1·26)	0·94 (0·91–0·96)
1955	0·4 (0·2–0·8)	49·8 (47·9–51·7)	185·0 (182·5–187·4)	147·4 (145·3–149·5)	81·4 (79·0–83·9)	59·5 (56·8–62·4)	20·0 (17·7–22·3)	6·2 (4·8–7·6)	0·1 (0·1–0·1)	2·75 (2·72–2·79)	1·18 (1·16–1·19)	0·84 (0·81–0·87)
1956	0·4 (0·2–0·8)	50·3 (48·2–52·2)	173·1 (170·6–175·4)	138·7 (136·6–140·8)	72·4 (70·2–74·6)	51·4 (48·7–54·2)	18·6 (16·5–20·7)	5·6 (4·2–7·0)	0·1 (0·1–0·1)	2·55 (2·52–2·59)	1·12 (1·1–1·13)	0·74 (0·72–0·76)
1957	0·4 (0·2–0·8)	52·7 (50·6–54·9)	168·9 (166·4–171·3)	132·5 (130·4–134·7)	66·2 (64·1–68·5)	45·2 (42·7–47·9)	19·1 (16·9–21·4)	5·2 (3·9–6·7)	0·1 (0·1–0·1)	2·45 (2·42–2·48)	1·11 (1·09–1·13)	0·68 (0·65–0·71)
1958	0·4 (0·2–0·8)	56·9 (54·9–59·1)	171·4 (168·9–173·9)	130·7 (128·6–132·9)	64·8 (62·8–67·1)	40·7 (38·2–43·3)	20·4 (18·2–23·0)	5·1 (3·8–6·6)	0·1 (0·1–0·1)	2·45 (2·42–2·49)	1·14 (1·13–1·16)	0·66 (0·63–0·68)
1959	0·4 (0·2–0·9)	62·2 (60·1–64·4)	175·9 (173·2–178·5)	132·2 (130·2–134·5)	66·9 (64·9–69·1)	36·8 (34·3–39·2)	21·3 (19·2–23·8)	5·1 (3·8–6·7)	0·1 (0·1–0·1)	2·5 (2·47–2·53)	1·19 (1·18–1·21)	0·65 (0·63–0·67)
1960	0·4 (0·2–0·9)	65·6 (63·3–67·8)	180·3 (177·7–182·9)	133·6 (131·4–135·8)	68·4 (66·2–70·5)	33·1 (31·0–35·3)	20·4 (18·2–22·7)	5·2 (3·8–6·8)	0·1 (0·1–0·1)	2·54 (2·51–2·57)	1·23 (1·21–1·25)	0·64 (0·61–0·66)
1961	0·4 (0·2–0·9)	64·8 (62·6–66·9)	179·7 (177·0–182·3)	132·4 (130·0–134·6)	67·6 (65·5–69·9)	30·3 (28·3–32·5)	18·0 (16·0–20·3)	5·3 (4·0–6·9)	0·1 (0·1–0·1)	2·49 (2·47–2·52)	1·22 (1·21–1·24)	0·61 (0·59–0·63)
1962	0·4 (0·2–0·9)	62·4 (60·4–64·6)	178·7 (176·1–181·4)	131·6 (129·4–133·8)	66·6 (64·5–68·8)	29·3 (27·4–31·4)	15·7 (13·9–17·8)	5·4 (4·0–6·9)	0·1 (0·1–0·1)	2·45 (2·42–2·48)	1·21 (1·19–1·22)	0·59 (0·57–0·61)
1963	0·4 (0·2–0·9)	61·7 (59·7–63·7)	180·5 (178·0–183·2)	132·8 (130·5–135·1)	66·4 (64·1–68·6)	29·6 (27·5–31·7)	14·1 (12·3–16·1)	5·4 (4·0–6·8)	0·1 (0·1–0·1)	2·45 (2·43–2·49)	1·21 (1·2–1·23)	0·58 (0·56–0·6)
1964	0·4 (0·2–0·9)	63·1 (60·9–65·3)	184·9 (182·1–187·5)	133·6 (131·4–135·8)	66·0 (63·8–68·2)	29·8 (27·9–31·8)	12·6 (10·9–14·7)	5·2 (3·9–6·7)	0·1 (0·1–0·1)	2·48 (2·45–2·51)	1·24 (1·22–1·26)	0·57 (0·55–0·59)
1965	0·4 (0·2–0·9)	64·6 (62·6–66·9)	183·9 (181·2–186·6)	131·0 (128·9–133·2)	65·0 (62·9–67·0)	29·3 (27·4–31·2)	11·1 (9·3–12·9)	4·6 (3·3–6·1)	0·1 (0·1–0·1)	2·45 (2·42–2·48)	1·24 (1·23–1·26)	0·55 (0·53–0·57)
1966	0·4 (0·2–0·9)	65·1 (63·1–67·2)	179·6 (176·9–182·4)	128·3 (126·1–130·6)	64·8 (62·8–67·0)	29·4 (27·6–31·4)	10·1 (8·5–11·8)	4·0 (2·8–5·4)	0·1 (0·1–0·1)	2·41 (2·38–2·44)	1·23 (1·21–1·24)	0·54 (0·52–0·56)
1967	0·4 (0·2–0·9)	64·9 (62·8–67·0)	178·9 (176·2–181·8)	126·9 (124·7–129·2)	66·0 (63·8–68·4)	30·2 (28·3–32·4)	9·8 (8·3–11·4)	3·4 (2·4–4·6)	0·1 (0·1–0·1)	2·4 (2·37–2·43)	1·22 (1·2–1·24)	0·55 (0·53–0·57)
1968	0·4 (0·2–0·9)	64·4 (62·3–66·4)	177·1 (174·5–179·7)	125·4 (123·2–127·7)	66·3 (64·2–68·5)	29·8 (27·8–31·8)	9·4 (8·0–11·0)	2·8 (2·0–3·8)	0·1 (0·1–0·1)	2·38 (2·35–2·4)	1·21 (1·19–1·22)	0·54 (0·52–0·56)
1969	0·4 (0·2–0·9)	63·6 (61·6–65·7)	170·8 (168·2–173·3)	123·2 (120·9–125·8)	65·0 (62·8–67·1)	28·8 (26·8–31·1)	9·2 (7·7–10·6)	2·3 (1·6–3·1)	0·0 (0·0–0·0)	2·32 (2·29–2·35)	1·17 (1·16–1·19)	0·53 (0·51–0·54)
1970	0·4 (0·2–0·9)	63·2 (61·2–65·3)	166·6 (163·8–169·2)	122·3 (120·1–124·6)	64·3 (62·2–66·6)	28·8 (26·8–31·0)	9·3 (8·0–10·6)	2·0 (1·4–2·6)	0·0 (0·0–0·0)	2·28 (2·26–2·31)	1·15 (1·13–1·17)	0·52 (0·51–0·54)
1971	0·4 (0·2–0·9)	63·2 (61·1–65·3)	166·9 (164·4–169·7)	123·5 (121·4–125·7)	64·5 (62·2–66·8)	29·3 (27·4–31·3)	9·6 (8·4–10·9)	1·7 (1·3–2·3)	0·0 (0·0–0·0)	2·3 (2·27–2·32)	1·15 (1·14–1·17)	0·53 (0·51–0·54)
1972	0·4 (0·2–0·9)	62·6 (60·7–64·7)	166·3 (163·8–168·9)	124·7 (122·4–126·7)	65·0 (62·7–67·2)	29·4 (27·5–31·4)	9·6 (8·3–10·9)	1·6 (1·2–2·1)	0·0 (0·0–0·0)	2·3 (2·27–2·33)	1·15 (1·13–1·16)	0·53 (0·51–0·55)
1973	0·4 (0·2–0·9)	62·1 (60·1–64·2)	165·6 (163·3–168·0)	125·1 (123·0–127·4)	66·5 (64·4–68·8)	29·7 (27·7–31·7)	9·4 (8·1–10·8)	1·5 (1·1–2·0)	0·0 (0·0–0·0)	2·3 (2·28–2·33)	1·14 (1·13–1·16)	0·54 (0·52–0·55)
1974	0·4 (0·2–0·9)	62·1 (60·0–64·1)	166·7 (164·4–169·2)	125·1 (123·0–127·3)	68·7 (66·5–71·1)	30·2 (28·3–32·3)	9·4 (8·0–10·9)	1·4 (1·0–2·0)	0·0 (0·0–0·0)	2·32 (2·29–2·35)	1·15 (1·13–1·16)	0·55 (0·53–0·57)
1975	0·4 (0·2–0·9)	61·5 (59·3–63·7)	168·2 (165·6–170·6)	125·5 (123·2–127·7)	69·9 (67·8–72·4)	30·4 (28·4–32·6)	9·3 (7·9–10·9)	1·4 (0·9–2·0)	0·0 (0·0–0·0)	2·33 (2·31–2·36)	1·15 (1·13–1·17)	0·56 (0·54–0·57)
1976	0·4 (0·2–0·8)	60·1 (58·0–62·2)	167·1 (164·6–169·5)	125·0 (122·9–127·4)	69·5 (67·3–71·8)	29·8 (27·9–31·8)	9·0 (7·6–10·7)	1·3 (0·9–1·9)	0·0 (0·0–0·0)	2·31 (2·29–2·34)	1·14 (1·12–1·15)	0·55 (0·53–0·56)
1977	0·4 (0·2–0·8)	58·9 (56·8–60·8)	165·4 (162·8–167·9)	123·9 (121·7–126·1)	68·0 (65·7–70·2)	29·1 (27·0–31·2)	8·7 (7·4–10·3)	1·3 (0·8–1·8)	0·0 (0·0–0·0)	2·28 (2·25–2·31)	1·12 (1·11–1·14)	0·54 (0·52–0·55)
1978	0·4 (0·2–0·8)	58·0 (55·9–60·2)	166·1 (163·6–168·5)	123·4 (121·3–125·6)	66·8 (64·5–68·9)	29·3 (27·4–31·5)	8·6 (7·3–10·3)	1·2 (0·8–1·8)	0·0 (0·0–0·0)	2·27 (2·24–2·29)	1·12 (1·11–1·14)	0·53 (0·51–0·55)
1979	0·4 (0·2–0·8)	55·8 (53·8–57·9)	167·8 (165·4–170·2)	123·0 (120·8–125·0)	65·5 (63·3–67·7)	29·5 (27·4–31·6)	8·4 (7·0–10·0)	1·2 (0·7–1·7)	0·0 (0·0–0·0)	2·26 (2·23–2·28)	1·12 (1·1–1·14)	0·52 (0·51–0·54)
1980	0·4 (0·2–0·8)	52·6 (50·6–54·7)	164·5 (161·9–166·9)	122·7 (120·5–124·9)	63·7 (61·5–65·9)	29·0 (27·1–31·2)	8·0 (6·7–9·5)	1·1 (0·7–1·6)	0·0 (0·0–0·0)	2·21 (2·19–2·23)	1·09 (1·07–1·1)	0·51 (0·49–0·53)
1981	0·4 (0·2–0·8)	50·9 (49·0–52·9)	163·9 (161·3–166·4)	125·2 (123·2–127·3)	63·5 (61·3–65·7)	29·2 (27·1–31·2)	8·0 (6·6–9·5)	1·0 (0·7–1·6)	0·0 (0·0–0·0)	2·21 (2·18–2·24)	1·08 (1·06–1·09)	0·51 (0·49–0·53)
1982	0·4 (0·2–0·8)	50·6 (48·7–52·6)	167·4 (165·0–170·0)	129·9 (127·7–132·0)	64·9 (62·9–67·2)	29·4 (27·2–31·4)	8·2 (6·9–9·7)	1·0 (0·6–1·5)	0·0 (0·0–0·0)	2·26 (2·23–2·28)	1·09 (1·08–1·11)	0·52 (0·5–0·53)
1983	0·4 (0·2–0·8)	50·4 (48·5–52·6)	168·5 (166·0–170·8)	133·5 (131·5–135·5)	65·9 (63·8–68·1)	28·8 (26·6–30·9)	8·2 (6·8–9·7)	1·0 (0·6–1·5)	0·0 (0·0–0·0)	2·28 (2·26–2·31)	1·1 (1·08–1·11)	0·52 (0·5–0·54)
1984	0·4 (0·2–0·8)	49·8 (47·6–51·8)	166·6 (164·0–169·0)	134·4 (132·3–136·6)	64·8 (62·7–67·0)	27·4 (25·4–29·5)	7·8 (6·4–9·4)	0·9 (0·6–1·5)	0·0 (0·0–0·0)	2·26 (2·24–2·29)	1·08 (1·07–1·1)	0·51 (0·49–0·52)
1985	0·4 (0·2–0·7)	48·6 (46·7–50·7)	162·5 (160·0–164·9)	134·2 (132·2–136·4)	63·5 (61·4–65·6)	26·1 (24·2–28·3)	7·5 (6·2–9·0)	0·9 (0·6–1·4)	0·0 (0·0–0·0)	2·22 (2·19–2·24)	1·06 (1·04–1·07)	0·49 (0·48–0·51)
1986	0·4 (0·2–0·7)	47·8 (45·9–49·9)	162·1 (159·6–164·6)	135·6 (133·7–137·6)	63·9 (61·9–66·1)	25·8 (23·7–28·0)	7·4 (6·1–8·9)	0·9 (0·5–1·3)	0·0 (0·0–0·0)	2·22 (2·19–2·25)	1·05 (1·03–1·07)	0·49 (0·47–0·51)
1987	0·4 (0·2–0·7)	46·7 (44·8–48·8)	162·5 (160·1–165·0)	135·7 (133·6–137·8)	64·0 (62·0–66·2)	25·0 (22·9–27·0)	7·0 (5·7–8·5)	0·8 (0·5–1·3)	0·0 (0·0–0·0)	2·21 (2·19–2·24)	1·05 (1·03–1·06)	0·48 (0·47–0·5)
1988	0·3 (0·2–0·7)	44·9 (43·0–46·8)	157·8 (155·4–160·4)	133·1 (131·0–135·3)	63·0 (61·0–65·1)	23·8 (21·9–25·8)	6·3 (5·0–7·8)	0·7 (0·5–1·2)	0·0 (0·0–0·0)	2·15 (2·12–2·18)	1·02 (1·0–1·03)	0·47 (0·45–0·48)
1989	0·3 (0·1–0·7)	43·2 (41·3–45·1)	152·9 (150·4–155·2)	131·5 (129·4–133·6)	63·5 (61·6–65·5)	22·8 (21·0–24·8)	5·7 (4·5–7·0)	0·7 (0·4–1·1)	0·0 (0·0–0·0)	2·1 (2·07–2·13)	0·98 (0·97–1·0)	0·46 (0·45–0·48)
1990	0·3 (0·1–0·7)	41·1 (39·3–42·9)	149·7 (147·3–152·4)	130·6 (128·3–132·9)	64·5 (62·6–66·7)	22·3 (20·5–24·3)	5·3 (4·2–6·7)	0·6 (0·4–1·0)	0·0 (0·0–0·0)	2·07 (2·05–2·1)	0·96 (0·94–0·97)	0·46 (0·45–0·48)
1991	0·3 (0·1–0·7)	38·4 (36·5–40·1)	141·6 (139·2–144·0)	126·3 (124·2–128·5)	63·6 (61·6–65·5)	22·2 (20·5–24·1)	5·1 (4·1–6·4)	0·5 (0·3–0·8)	0·0 (0·0–0·0)	1·99 (1·97–2·02)	0·9 (0·89–0·92)	0·46 (0·44–0·47)
1992	0·3 (0·1–0·6)	36·3 (34·6–38·2)	134·3 (131·9–136·7)	121·4 (119·1–123·4)	63·0 (60·9–65·0)	22·3 (20·6–24·1)	5·0 (3·9–6·2)	0·5 (0·3–0·7)	0·0 (0·0–0·0)	1·92 (1·89–1·94)	0·85 (0·84–0·87)	0·45 (0·44–0·47)
1993	0·3 (0·1–0·6)	34·9 (33·1–36·7)	130·0 (127·6–132·5)	118·9 (116·8–120·8)	64·0 (61·9–66·1)	22·6 (20·9–24·6)	4·8 (3·8–6·1)	0·4 (0·3–0·6)	0·0 (0·0–0·0)	1·88 (1·85–1·9)	0·83 (0·81–0·84)	0·46 (0·44–0·48)
1994	0·3 (0·1–0·6)	33·5 (31·9–35·4)	126·8 (124·3–129·2)	118·6 (116·7–120·8)	66·1 (64·0–68·3)	23·1 (21·2–24·9)	4·6 (3·6–5·9)	0·4 (0·2–0·6)	0·0 (0·0–0·0)	1·87 (1·84–1·89)	0·8 (0·79–0·82)	0·47 (0·46–0·49)
1995	0·3 (0·1–0·6)	32·0 (30·3–33·8)	123·6 (121·3–126·1)	118·2 (116·2–120·4)	66·4 (64·3–68·4)	23·4 (21·7–25·3)	4·5 (3·4–5·6)	0·3 (0·2–0·5)	0·0 (0·0–0·0)	1·84 (1·82–1·87)	0·78 (0·76–0·79)	0·47 (0·46–0·49)
1996	0·3 (0·1–0·6)	30·7 (29·0–32·4)	117·7 (115·1–120·2)	114·2 (112·1–116·3)	63·8 (61·7–65·8)	23·0 (21·1–24·9)	4·3 (3·3–5·4)	0·3 (0·2–0·5)	0·0 (0·0–0·0)	1·77 (1·75–1·8)	0·74 (0·73–0·76)	0·46 (0·44–0·47)
1997	0·3 (0·1–0·6)	29·7 (28·0–31·7)	111·0 (108·6–113·3)	106·7 (104·6–108·7)	58·6 (56·4–60·5)	21·4 (19·5–23·3)	4·1 (3·1–5·3)	0·3 (0·2–0·5)	0·0 (0·0–0·0)	1·66 (1·63–1·69)	0·71 (0·69–0·72)	0·42 (0·4–0·44)
1998	0·3 (0·1–0·6)	28·5 (26·8–30·5)	104·7 (102·3–107·2)	99·5 (97·2–101·7)	53·6 (51·4–55·8)	20·1 (18·4–22·0)	4·0 (3·0–5·1)	0·3 (0·2–0·4)	0·0 (0·0–0·0)	1·55 (1·52–1·59)	0·67 (0·65–0·68)	0·39 (0·37–0·41)
1999	0·3 (0·1–0·5)	27·4 (25·6–29·2)	99·3 (96·8–101·6)	96·9 (94·7–99·1)	52·6 (50·5–54·8)	19·8 (18·1–21·8)	3·9 (3·0–5·1)	0·3 (0·2–0·4)	0·0 (0·0–0·0)	1·5 (1·47–1·54)	0·63 (0·62–0·65)	0·38 (0·37–0·4)
2000	0·3 (0·1–0·5)	26·8 (25·1–28·5)	97·1 (94·9–99·5)	99·8 (97·6–101·9)	55·7 (53·6–57·8)	20·3 (18·5–22·3)	3·9 (2·9–5·0)	0·2 (0·2–0·4)	0·0 (0·0–0·0)	1·52 (1·49–1·55)	0·62 (0·61–0·64)	0·4 (0·39–0·42)
2001	0·3 (0·1–0·5)	26·3 (24·6–28·0)	96·3 (94·0–98·7)	104·0 (101·8–106·1)	60·6 (58·4–62·6)	21·2 (19·3–23·2)	3·9 (2·9–5·0)	0·2 (0·1–0·4)	0·0 (0·0–0·0)	1·56 (1·54–1·59)	0·61 (0·6–0·63)	0·43 (0·42–0·44)
2002	0·3 (0·1–0·5)	25·9 (24·2–27·6)	95·0 (92·7–97·5)	105·5 (103·4–107·6)	64·0 (61·8–66·3)	22·1 (20·3–24·1)	3·9 (2·9–4·9)	0·2 (0·1–0·3)	0·0 (0·0–0·0)	1·58 (1·56–1·61)	0·61 (0·59–0·62)	0·45 (0·44–0·47)
2003	0·3 (0·1–0·5)	25·0 (23·4–26·9)	92·4 (90·1–94·9)	103·8 (101·6–105·9)	65·5 (63·3–67·7)	22·8 (20·8–24·8)	3·8 (3·0–4·8)	0·2 (0·1–0·3)	0·0 (0·0–0·0)	1·57 (1·54–1·6)	0·59 (0·57–0·6)	0·46 (0·45–0·48)
2004	0·2 (0·1–0·5)	23·6 (22·0–25·4)	86·8 (84·3–89·4)	98·8 (96·7–101·1)	65·0 (62·7–67·3)	22·9 (20·9–25·0)	3·8 (3·1–4·6)	0·2 (0·2–0·3)	0·0 (0·0–0·0)	1·51 (1·47–1·54)	0·55 (0·54–0·57)	0·46 (0·44–0·48)
2005	0·2 (0·1–0·5)	22·3 (20·5–24·1)	80·4 (77·2–83·7)	93·4 (90·8–96·0)	63·9 (61·2–66·8)	22·6 (20·6–25·0)	3·8 (3·1–4·5)	0·2 (0·2–0·3)	0·0 (0·0–0·0)	1·43 (1·4–1·47)	0·51 (0·5–0·53)	0·45 (0·44–0·47)
2006	0·2 (0·1–0·5)	21·6 (19·8–23·4)	76·3 (73·0–79·6)	90·3 (87·6–93·0)	62·9 (60·2–65·7)	22·6 (20·6–25·0)	3·8 (3·2–4·4)	0·2 (0·2–0·3)	0·0 (0·0–0·0)	1·39 (1·35–1·43)	0·49 (0·47–0·51)	0·45 (0·43–0·47)
2007	0·2 (0·1–0·5)	21·5 (19·6–23·5)	75·9 (72·0–79·9)	90·7 (87·3–94·1)	61·3 (58·2–64·4)	22·7 (20·5–25·0)	3·8 (3·2–4·4)	0·3 (0·2–0·3)	0·0 (0·0–0·0)	1·38 (1·33–1·44)	0·49 (0·46–0·51)	0·44 (0·41–0·47)
2008	0·2 (0·1–0·5)	21·4 (19·4–23·5)	77·9 (70·9–85·1)	93·2 (88·1–98·8)	60·1 (55·8–64·5)	23·0 (20·6–25·4)	4·0 (3·4–4·5)	0·3 (0·2–0·3)	0·0 (0·0–0·0)	1·4 (1·32–1·48)	0·5 (0·46–0·54)	0·44 (0·4–0·47)
2009	0·2 (0·1–0·5)	21·0 (19·0–23·1)	79·3 (70·6–89·7)	95·5 (89·3–102·8)	60·0 (55·0–65·4)	23·4 (20·9–26·2)	4·2 (3·7–4·8)	0·3 (0·2–0·3)	0·0 (0·0–0·0)	1·42 (1·32–1·54)	0·5 (0·45–0·56)	0·44 (0·4–0·48)
2010	0·2 (0·1–0·5)	20·2 (18·1–22·5)	79·2 (70·1–91·1)	96·9 (90·2–105·0)	60·9 (55·7–67·2)	24·0 (21·5–27·1)	4·5 (3·9–5·2)	0·3 (0·3–0·4)	0·0 (0·0–0·0)	1·43 (1·31–1·58)	0·5 (0·45–0·56)	0·45 (0·41–0·5)
2011	0·2 (0·1–0·5)	19·3 (17·4–21·7)	77·4 (67·9–89·0)	97·4 (90·5–105·6)	63·0 (57·8–69·6)	24·9 (22·2–27·9)	4·8 (4·2–5·5)	0·4 (0·3–0·4)	0·0 (0·0–0·0)	1·44 (1·31–1·58)	0·48 (0·43–0·55)	0·47 (0·43–0·51)
2012	0·2 (0·1–0·4)	18·1 (16·3–20·3)	73·6 (64·8–83·6)	96·8 (90·2–104·1)	66·7 (61·4–72·8)	26·7 (23·8–29·7)	5·1 (4·5–5·8)	0·4 (0·3–0·5)	0·0 (0·0–0·0)	1·44 (1·33–1·56)	0·46 (0·41–0·52)	0·49 (0·46–0·54)
2013	0·2 (0·1–0·4)	16·7 (15·1–18·5)	68·1 (61·6–75·1)	95·1 (89·9–100·4)	72·8 (67·9–78·1)	29·6 (26·8–32·6)	5·3 (4·7–6·1)	0·4 (0·4–0·5)	0·0 (0·0–0·0)	1·44 (1·36–1·52)	0·42 (0·39–0·46)	0·54 (0·5–0·58)
2014	0·2 (0·1–0·4)	15·4 (13·8–17·1)	61·8 (57·7–65·9)	92·4 (88·9–95·9)	78·8 (75·1–82·6)	32·5 (29·9–35·2)	5·3 (4·6–6·1)	0·4 (0·3–0·5)	0·0 (0·0–0·0)	1·43 (1·38–1·48)	0·39 (0·36–0·41)	0·59 (0·56–0·61)
2015	0·2 (0·1–0·4)	14·8 (13·1–16·6)	57·9 (54·1–62·3)	90·2 (86·8–94·0)	79·5 (75·7–83·3)	32·4 (29·7–35·4)	4·9 (4·1–5·7)	0·4 (0·3–0·5)	0·0 (0·0–0·0)	1·4 (1·34–1·47)	0·36 (0·34–0·39)	0·59 (0·56–0·62)
2016	0·2 (0·1–0·4)	15·1 (13·1–17·4)	58·1 (51·9–66·0)	90·3 (85·1–96·1)	76·6 (70·1–83·5)	30·1 (26·1–34·5)	4·5 (3·6–5·5)	0·3 (0·3–0·5)	0·0 (0·0–0·0)	1·38 (1·27–1·51)	0·37 (0·33–0·42)	0·56 (0·51–0·61)
2017	0·2 (0·1–0·4)	15·4 (13·3–18·0)	60·1 (51·0–70·7)	90·8 (83·4–99·0)	74·7 (66·3–84·2)	28·5 (23·4–34·6)	4·4 (3·4–5·7)	0·3 (0·2–0·4)	0·0 (0·0–0·0)	1·37 (1·21–1·56)	0·38 (0·32–0·44)	0·54 (0·47–0·62)

Supplemental Results Table 5: Age-specific fertility rates per 1000, cumulative total fertility up to maternal age 25 (TFU25), cumulative total fertility from maternal age 30 to age 55 (TFO30), and cumulative total fertility, Slovakia, 1950-2017

Year	10-14	15-19	20-24	25-29	30-34	35-39	40-44	45-49	50-54	TFR	TFU25	TFO30
1950	0·2 (0·1–0·4)	52·8 (49·9–56·0)	212·4 (209·0–216·0)	199·1 (196·1–202·3)	137·9 (134·1–141·8)	85·2 (81·4–88·9)	33·1 (29·8–36·6)	2·6 (1·8–3·8)	0·1 (0·0–0·1)	3·62 (3·56–3·67)	1·33 (1·3–1·35)	1·29 (1·26–1·33)
1951	0·2 (0·1–0·4)	52·4 (49·9–55·3)	213·3 (210·1–216·5)	199·5 (196·7–202·5)	138·2 (134·8–141·5)	81·4 (77·6–85·1)	32·3 (29·4–35·4)	2·5 (1·7–3·6)	0·0 (0·0–0·1)	3·6 (3·55–3·65)	1·33 (1·31–1·35)	1·27 (1·24–1·3)
1952	0·2 (0·1–0·4)	50·4 (47·8–53·1)	212·4 (208·9–215·9)	195·7 (192·9–198·6)	136·3 (132·9–139·7)	77·0 (73·3–80·7)	30·9 (28·0–33·9)	2·4 (1·6–3·4)	0·0 (0·0–0·0)	3·53 (3·48–3·58)	1·32 (1·29–1·35)	1·23 (1·2–1·27)
1953	0·2 (0·1–0·4)	48·5 (45·8–51·3)	213·5 (210·2–217·0)	193·1 (190·1–196·0)	133·2 (130·1–136·3)	74·4 (70·7–78·0)	29·6 (26·9–32·6)	2·3 (1·5–3·3)	0·0 (0·0–0·0)	3·47 (3·43–3·52)	1·31 (1·29–1·34)	1·2 (1·17–1·23)
1954	0·2 (0·1–0·4)	47·9 (45·3–50·6)	217·6 (214·3–220·7)	193·1 (190·2–196·2)	130·3 (127·2–133·5)	74·8 (70·7–78·9)	28·2 (25·5–31·2)	2·2 (1·5–3·2)	0·0 (0·0–0·0)	3·47 (3·43–3·51)	1·33 (1·31–1·35)	1·18 (1·15–1·21)
1955	0·2 (0·1–0·4)	48·6 (45·8–51·3)	222·3 (218·9–225·8)	192·9 (189·9–195·8)	127·1 (123·7–130·1)	75·6 (71·5–79·6)	25·9 (23·3–28·8)	2·1 (1·4–3·0)	0·0 (0·0–0·0)	3·47 (3·43–3·51)	1·36 (1·34–1·38)	1·15 (1·13–1·18)
1956	0·2 (0·1–0·4)	49·1 (46·6–51·9)	224·1 (220·8–227·7)	187·9 (184·8–190·9)	122·0 (118·9–125·2)	73·4 (69·7–77·2)	22·9 (20·1–25·9)	1·9 (1·3–2·8)	0·0 (0·0–0·0)	3·41 (3·37–3·44)	1·37 (1·35–1·39)	1·1 (1·07–1·13)
1957	0·2 (0·1–0·4)	49·2 (46·7–51·9)	221·9 (218·6–225·7)	178·7 (175·5–181·6)	114·3 (111·2–117·5)	67·2 (63·9–70·8)	20·0 (17·3–23·1)	1·7 (1·1–2·5)	0·0 (0·0–0·0)	3·27 (3·23–3·3)	1·36 (1·34–1·38)	1·02 (0·99–1·04)
1958	0·2 (0·1–0·4)	49·5 (46·7–52·2)	220·0 (216·7–223·4)	170·7 (167·6–173·8)	105·6 (102·5–108·8)	59·7 (56·6–63·1)	18·4 (15·7–21·4)	1·6 (1·0–2·3)	0·0 (0·0–0·0)	3·13 (3·09–3·16)	1·35 (1·33–1·37)	0·93 (0·9–0·95)
1959	0·2 (0·1–0·4)	49·9 (47·3–52·9)	221·1 (217·5–225·0)	166·6 (163·6–169·5)	99·0 (96·1–102·1)	54·0 (51·0–57·2)	17·6 (14·8–20·3)	1·5 (0·9–2·1)	0·0 (0·0–0·0)	3·05 (3·01–3·09)	1·36 (1·34–1·38)	0·86 (0·84–0·89)
1960	0·2 (0·1–0·4)	49·5 (47·0–52·0)	222·0 (218·6–225·4)	162·7 (159·8–165·8)	94·6 (91·5–97·7)	49·9 (46·9–52·9)	16·7 (14·2–19·6)	1·3 (0·9–2·0)	0·0 (0·0–0·0)	2·98 (2·94–3·02)	1·36 (1·34–1·38)	0·81 (0·79–0·84)
1961	0·2 (0·1–0·4)	48·3 (45·8–50·8)	219·6 (216·2–222·8)	159·4 (156·5–162·5)	91·6 (88·2–94·7)	46·8 (44·1–49·8)	15·8 (13·5–18·5)	1·2 (0·8–1·8)	0·0 (0·0–0·0)	2·91 (2·87–2·96)	1·34 (1·32–1·36)	0·78 (0·75–0·81)
1962	0·2 (0·1–0·4)	47·1 (44·7–49·8)	218·6 (215·3–221·8)	160·9 (157·9–163·8)	91·5 (88·4–94·7)	46·0 (43·0–49·1)	15·1 (12·9–17·6)	1·1 (0·7–1·7)	0·0 (0·0–0·0)	2·9 (2·86–2·94)	1·33 (1·31–1·35)	0·77 (0·74–0·8)
1963	0·2 (0·1–0·4)	45·5 (43·1–48·0)	218·7 (215·2–222·1)	163·1 (160·1–166·2)	92·4 (89·2–95·3)	45·9 (43·0–49·0)	14·3 (12·1–16·8)	1·0 (0·6–1·6)	0·0 (0·0–0·0)	2·91 (2·87–2·94)	1·32 (1·3–1·34)	0·77 (0·74–0·8)
1964	0·2 (0·1–0·4)	43·3 (40·7–45·5)	215·7 (212·2–219·2)	161·4 (158·2–164·2)	90·4 (87·5–93·6)	44·4 (41·5–47·3)	13·2 (11·2–15·6)	1·0 (0·6–1·5)	0·0 (0·0–0·0)	2·85 (2·81–2·88)	1·3 (1·27–1·32)	0·75 (0·72–0·78)
1965	0·2 (0·1–0·4)	41·2 (38·8–43·5)	210·1 (206·9–213·6)	154·6 (151·7–157·7)	85·1 (82·2–88·1)	41·3 (38·6–44·2)	11·8 (9·8–14·1)	0·9 (0·5–1·4)	0·0 (0·0–0·0)	2·73 (2·69–2·76)	1·26 (1·24–1·28)	0·7 (0·66–0·73)
1966	0·2 (0·1–0·4)	39·7 (37·5–41·9)	201·9 (198·4–205·3)	144·0 (141·2–146·8)	78·3 (75·5–81·3)	37·3 (34·5–40·2)	10·3 (8·5–12·4)	0·8 (0·5–1·2)	0·0 (0·0–0·0)	2·56 (2·53–2·6)	1·21 (1·19–1·23)	0·63 (0·61–0·66)
1967	0·2 (0·1–0·4)	39·5 (37·2–41·7)	195·0 (191·6–198·5)	137·0 (134·1–140·1)	73·4 (70·5–76·6)	34·3 (31·6–37·3)	9·4 (7·6–11·2)	0·7 (0·4–1·1)	0·0 (0·0–0·0)	2·45 (2·41–2·48)	1·17 (1·15–1·19)	0·59 (0·57–0·61)
1968	0·2 (0·1–0·4)	40·5 (38·2–42·6)	194·3 (191·1–197·5)	136·9 (133·6–140·0)	71·3 (68·3–74·4)	32·7 (30·0–35·4)	8·9 (7·1–10·8)	0·6 (0·4–1·0)	0·0 (0·0–0·0)	2·43 (2·39–2·46)	1·17 (1·15–1·19)	0·57 (0·55–0·59)
1969	0·2 (0·1–0·4)	41·0 (38·9–43·1)	195·7 (192·4–199·0)	137·5 (134·5–140·5)	70·3 (67·5–73·5)	31·2 (28·4–33·6)	8·4 (6·7–10·2)	0·6 (0·4–0·9)	0·0 (0·0–0·0)	2·42 (2·39–2·47)	1·18 (1·17–1·2)	0·55 (0·53–0·58)
1970	0·2 (0·1–0·4)	40·3 (38·2–42·3)	195·4 (192·3–198·6)	137·6 (134·7–140·6)	69·8 (66·7–72·9)	29·8 (27·1–32·4)	7·9 (6·4–9·8)	0·5 (0·3–0·8)	0·0 (0·0–0·0)	2·41 (2·37–2·44)	1·18 (1·16–1·2)	0·54 (0·52–0·56)
1971	0·2 (0·1–0·4)	40·0 (37·9–42·1)	196·9 (193·9–200·1)	141·9 (138·9–144·9)	70·5 (67·4–73·8)	29·5 (26·9–32·2)	7·7 (6·1–9·6)	0·5 (0·3–0·8)	0·0 (0·0–0·0)	2·44 (2·4–2·47)	1·19 (1·17–1·2)	0·54 (0·52–0·56)
1972	0·2 (0·1–0·4)	41·3 (39·1–43·6)	202·9 (199·8–205·9)	150·4 (147·6–153·4)	72·0 (68·9–75·2)	29·8 (27·0–32·4)	7·7 (6·1–9·5)	0·5 (0·3–0·7)	0·0 (0·0–0·0)	2·52 (2·49–2·55)	1·22 (1·2–1·24)	0·55 (0·53–0·57)
1973	0·2 (0·1–0·4)	43·8 (41·5–46·1)	209·3 (206·3–212·3)	155·1 (152·3–158·0)	72·2 (69·3–75·2)	29·4 (26·6–32·3)	7·3 (5·9–9·2)	0·5 (0·3–0·7)	0·0 (0·0–0·0)	2·59 (2·55–2·63)	1·27 (1·25–1·29)	0·55 (0·53–0·57)
1974	0·2 (0·1–0·4)	46·4 (44·0–48·6)	210·3 (207·5–213·6)	153·4 (150·4–156·2)	70·4 (67·6–73·5)	27·9 (25·2–30·6)	6·7 (5·3–8·4)	0·4 (0·3–0·6)	0·0 (0·0–0·0)	2·58 (2·54–2·62)	1·28 (1·27–1·3)	0·53 (0·51–0·55)
1975	0·2 (0·1–0·4)	47·7 (45·4–49·8)	209·0 (205·9–211·9)	150·3 (147·5–153·2)	68·2 (65·2–71·4)	26·3 (23·8–29·2)	6·2 (4·8–7·9)	0·4 (0·2–0·6)	0·0 (0·0–0·0)	2·54 (2·51–2·58)	1·28 (1·26–1·31)	0·51 (0·49–0·53)
1976	0·2 (0·1–0·4)	48·1 (46·0–50·3)	209·1 (205·9–212·0)	147·6 (144·9–150·3)	66·2 (63·3–69·0)	25·0 (22·5–27·7)	5·8 (4·4–7·6)	0·4 (0·2–0·5)	0·0 (0·0–0·0)	2·51 (2·48–2·55)	1·29 (1·27–1·31)	0·49 (0·47–0·51)
1977	0·2 (0·1–0·4)	48·9 (46·6–51·2)	210·5 (207·5–213·4)	146·2 (143·5–148·9)	64·7 (61·8–67·5)	23·9 (21·4–26·8)	5·5 (4·1–7·3)	0·3 (0·2–0·5)	0·0 (0·0–0·0)	2·5 (2·47–2·53)	1·3 (1·28–1·32)	0·47 (0·45–0·49)
1978	0·2 (0·1–0·4)	49·7 (47·4–52·1)	212·5 (209·7–215·6)	143·7 (141·0–146·4)	62·6 (59·9–65·4)	22·5 (20·0–25·3)	5·1 (3·7–7·0)	0·3 (0·2–0·5)	0·0 (0·0–0·0)	2·48 (2·45–2·52)	1·31 (1·29–1·33)	0·45 (0·43–0·48)
1979	0·2 (0·1–0·4)	49·4 (47·2–51·8)	209·3 (206·5–212·3)	137·1 (134·5–139·8)	59·1 (56·5–61·9)	20·5 (18·0–23·1)	4·6 (3·3–6·2)	0·3 (0·2–0·4)	0·0 (0·0–0·0)	2·4 (2·37–2·43)	1·29 (1·28–1·31)	0·42 (0·4–0·44)
1980	0·2 (0·1–0·4)	48·5 (46·4–50·6)	203·7 (200·7–206·5)	131·5 (128·8–134·0)	56·4 (53·8–59·0)	19·1 (16·7–21·7)	4·1 (3·0–5·7)	0·3 (0·2–0·4)	0·0 (0·0–0·0)	2·32 (2·29–2·35)	1·26 (1·24–1·28)	0·4 (0·38–0·42)
1981	0·2 (0·1–0·4)	48·5 (46·2–50·9)	202·5 (199·5–205·3)	129·4 (126·9–132·0)	55·1 (52·6–57·7)	18·7 (16·4–21·1)	3·9 (2·8–5·2)	0·2 (0·1–0·4)	0·0 (0·0–0·0)	2·29 (2·26–2·32)	1·26 (1·24–1·27)	0·39 (0·37–0·41)
1982	0·2 (0·1–0·4)	49·7 (47·4–52·1)	203·2 (200·1–206·1)	127·7 (125·1–130·3)	54·2 (51·7–56·8)	18·5 (16·2–21·1)	3·7 (2·7–5·0)	0·2 (0·1–0·3)	0·0 (0·0–0·0)	2·29 (2·26–2·32)	1·27 (1·25–1·29)	0·38 (0·37–0·4)
1983	0·2 (0·1–0·4)	51·5 (49·1–54·0)	203·2 (200·2–206·2)	126·5 (123·8–129·1)	53·6 (51·2–56·0)	18·3 (16·0–20·8)	3·6 (2·6–4·8)	0·2 (0·1–0·3)	0·0 (0·0–0·0)	2·29 (2·26–2·31)	1·27 (1·26–1·29)	0·38 (0·36–0·4)
1984	0·2 (0·1–0·4)	52·7 (50·3–55·1)	203·1 (199·9–206·3)	125·8 (123·3–128·4)	53·0 (50·5–55·5)	18·0 (15·8–20·5)	3·5 (2·5–4·7)	0·2 (0·1–0·3)	0·0 (0·0–0·0)	2·28 (2·25–2·31)	1·28 (1·26–1·3)	0·37 (0·36–0·39)
1985	0·2 (0·1–0·4)	52·1 (49·7–54·5)	200·6 (197·5–203·7)	123·5 (121·2–126·1)	51·4 (49·0–53·7)	17·3 (15·2–19·8)	3·3 (2·4–4·5)	0·2 (0·1–0·3)	0·0 (0·0–0·0)	2·24 (2·21–2·27)	1·26 (1·25–1·28)	0·36 (0·34–0·38)
1986	0·2 (0·1–0·4)	50·5 (48·1–52·9)	195·7 (192·6–198·7)	120·9 (118·5–123·4)	49·3 (47·2–51·8)	16·6 (14·5–19·2)	3·2 (2·3–4·4)	0·2 (0·1–0·3)	0·0 (0·0–0·0)	2·18 (2·15–2·21)	1·23 (1·21–1·25)	0·35 (0·33–0·36)
1987	0·2 (0·1–0·4)	49·3 (47·0–51·9)	192·2 (189·0–195·4)	119·7 (117·1–122·4)	47·9 (45·4–50·3)	16·3 (14·3–18·6)	3·1 (2·3–4·2)	0·2 (0·1–0·2)	0·0 (0·0–0·0)	2·14 (2·11–2·18)	1·21 (1·19–1·23)	0·34 (0·32–0·35)
1988	0·2 (0·1–0·4)	48·9 (46·6–51·4)	189·4 (186·2–192·7)	119·0 (116·6–121·6)	47·2 (44·9–49·5)	16·4 (14·5–18·6)	3·2 (2·3–4·2)	0·2 (0·1–0·2)	0·0 (0·0–0·0)	2·12 (2·1–2·15)	1·19 (1·17–1·21)	0·33 (0·32–0·35)
1989	0·2 (0·1–0·4)	49·8 (47·7–52·4)	187·5 (184·3–190·7)	117·8 (115·3–120·4)	47·2 (44·8–49·6)	17·0 (15·2–18·9)	3·3 (2·3–4·4)	0·1 (0·1–0·2)	0·0 (0·0–0·0)	2·11 (2·09–2·14)	1·19 (1·17–1·21)	0·34 (0·32–0·36)
1990	0·2 (0·1–0·4)	50·4 (48·1–52·9)	185·0 (182·0–188·0)	114·2 (111·5–116·8)	46·9 (44·5–49·3)	17·1 (15·5–19·0)	3·3 (2·3–4·4)	0·1 (0·1–0·2)	0·0 (0·0–0·0)	2·09 (2·06–2·12)	1·18 (1·16–1·2)	0·34 (0·32–0·35)
1991	0·2 (0·1–0·4)	49·3 (47·0–51·7)	176·7 (173·6–179·8)	109·5 (107·0–111·9)	45·9 (43·6–48·1)	16·4 (14·7–18·2)	3·1 (2·3–4·0)	0·1 (0·1–0·2)	0·0 (0·0–0·0)	2·01 (1·97–2·04)	1·13 (1·1–1·16)	0·33 (0·31–0·35)
1992	0·2 (0·1–0·4)	46·5 (44·2–48·9)	164·7 (161·4–168·1)	105·0 (102·4–107·5)	44·9 (42·3–47·5)	14·8 (13·0–16·6)	2·7 (2·0–3·4)	0·1 (0·1–0·2)	0·0 (0·0–0·0)	1·89 (1·86–1·93)	1·06 (1·03–1·08)	0·31 (0·29–0·33)
1993	0·2 (0·1–0·4)	42·2 (40·0–44·6)	149·6 (146·4–153·0)	99·5 (96·9–101·9)	43·2 (40·9–45·7)	14·3 (12·6–16·1)	2·5 (2·0–3·2)	0·1 (0·1–0·2)	0·0 (0·0–0·0)	1·76 (1·72–1·79)	0·96 (0·93–0·99)	0·3 (0·28–0·32)
1994	0·2 (0·1–0·4)	37·8 (35·8–40·0)	132·6 (129·5–135·8)	94·8 (92·3–97·4)	40·6 (38·3–42·8)	15·3 (13·6–17·1)	2·7 (2·2–3·3)	0·1 (0·1–0·1)	0·0 (0·0–0·0)	1·62 (1·59–1·65)	0·85 (0·83–0·87)	0·29 (0·28–0·31)
1995	0·2 (0·1–0·3)	34·3 (32·4–36·1)	120·5 (117·7–123·5)	92·7 (90·1–95·1)	40·2 (37·9–42·5)	15·8 (14·2–17·7)	2·9 (2·4–3·3)	0·1 (0·1–0·1)	0·0 (0·0–0·0)	1·53 (1·51–1·56)	0·77 (0·76–0·79)	0·29 (0·28–0·31)
1996	0·2 (0·1–0·3)	31·2 (29·3–33·1)	112·2 (109·5–115·1)	92·1 (89·6–94·5)	41·3 (39·0–43·8)	15·8 (14·0–17·6)	2·9 (2·5–3·5)	0·1 (0·1–0·1)	0·0 (0·0–0·0)	1·48 (1·45–1·51)	0·72 (0·7–0·73)	0·3 (0·29–0·32)
1997	0·1 (0·1–0·3)	28·4 (26·5–30·4)	104·2 (101·4–107·1)	91·7 (89·2–94·4)	42·0 (39·6–44·8)	15·7 (13·8–17·8)	3·0 (2·5–3·7)	0·1 (0·1–0·1)	0·0 (0·0–0·0)	1·43 (1·4–1·46)	0·66 (0·65–0·68)	0·3 (0·29–0·32)
1998	0·1 (0·1–0·3)	25·9 (24·0–27·7)	95·6 (92·9–98·3)	91·3 (88·6–93·9)	42·7 (40·2–45·2)	16·0 (14·1–18·0)	3·2 (2·6–3·8)	0·1 (0·1–0·2)	0·0 (0·0–0·0)	1·37 (1·34–1·41)	0·61 (0·59–0·62)	0·31 (0·29–0·33)
1999	0·1 (0·1–0·3)	23·4 (21·7–25·2)	87·0 (84·2–89·7)	90·3 (87·8–92·8)	43·8 (41·3–46·5)	16·6 (14·8–18·5)	3·3 (2·8–4·0)	0·1 (0·1–0·2)	0·0 (0·0–0·0)	1·32 (1·3–1·35)	0·55 (0·54–0·57)	0·32 (0·3–0·34)
2000	0·1 (0·1–0·3)	21·2 (19·6–22·9)	78·8 (76·2–81·4)	88·0 (85·7–90·5)	45·3 (42·9–47·9)	17·1 (15·5–18·9)	3·5 (3·0–4·1)	0·1 (0·1–0·2)	0·0 (0·0–0·0)	1·27 (1·25–1·3)	0·5 (0·49–0·52)	0·33 (0·32–0·35)
2001	0·1 (0·1–0·3)	20·0 (18·4–21·7)	71·8 (69·2–74·6)	85·5 (83·4–87·8)	47·0 (44·5–49·5)	17·5 (15·6–19·5)	3·6 (3·0–4·3)	0·2 (0·1–0·2)	0·0 (0·0–0·0)	1·23 (1·2–1·26)	0·46 (0·45–0·48)	0·34 (0·32–0·36)
2002	0·1 (0·1–0·3)	19·9 (18·2–21·7)	67·3 (64·7–70·1)	85·1 (82·8–87·4)	49·1 (46·6–51·5)	17·7 (15·8–19·9)	3·7 (2·9–4·7)	0·2 (0·1–0·2)	0·0 (0·0–0·0)	1·22 (1·18–1·25)	0·44 (0·42–0·45)	0·35 (0·34–0·37)
2003	0·1 (0·1–0·3)	20·2 (18·5–22·0)	65·0 (62·3–67·8)	86·3 (83·9–88·5)	52·1 (49·6–54·7)	18·4 (16·3–20·6)	3·8 (2·9–4·9)	0·2 (0·1–0·2)	0·0 (0·0–0·0)	1·23 (1·2–1·26)	0·43 (0·41–0·44)	0·37 (0·36–0·39)
2004	0·1 (0·1–0·3)	20·2 (18·3–22·2)	62·6 (60·0–65·1)	87·0 (84·5–89·2)	56·1 (53·7–58·6)	19·6 (17·4–21·9)	3·9 (2·9–5·2)	0·2 (0·1–0·3)	0·0 (0·0–0·0)	1·25 (1·22–1·28)	0·41 (0·4–0·43)	0·4 (0·38–0·42)
2005	0·1 (0·1–0·3)	20·0 (18·2–22·0)	59·2 (56·6–61·8)	86·2 (83·7–88·6)	60·5 (58·1–63·3)	21·4 (19·1–23·9)	4·1 (3·1–5·4)	0·2 (0·1–0·3)	0·0 (0·0–0·0)	1·26 (1·23–1·29)	0·4 (0·38–0·41)	0·43 (0·42–0·45)
2006	0·1 (0·1–0·3)	20·2 (18·4–22·1)	56·4 (53·9–59·1)	85·5 (83·0–87·9)	64·9 (62·3–67·6)	23·6 (21·2–26·0)	4·4 (3·2–5·8)	0·2 (0·1–0·3)	0·0 (0·0–0·0)	1·28 (1·25–1·3)	0·38 (0·37–0·4)	0·47 (0·45–0·48)
2007	0·1 (0·1–0·3)	20·9 (19·0–22·8)	55·4 (53·0–58·0)	86·8 (84·4–89·3)	69·6 (67·0–72·2)	26·1 (23·9–28·6)	4·6 (3·5–6·0)	0·2 (0·1–0·3)	0·0 (0·0–0·0)	1·32 (1·29–1·35)	0·38 (0·36–0·4)	0·5 (0·48–0·52)
2008	0·1 (0·1–0·3)	21·7 (19·7–23·7)	55·7 (53·0–58·3)	89·3 (86·9–91·9)	75·1 (72·6–77·6)	28·9 (26·6–31·5)	4·9 (3·8–6·2)	0·2 (0·1–0·3)	0·0 (0·0–0·0)	1·38 (1·35–1·41)	0·39 (0·37–0·41)	0·55 (0·53–0·56)
2009	0·1 (0·1–0·3)	22·0 (20·1–24·0)	55·0 (52·1–57·5)	90·1 (87·7–92·5)	80·0 (77·4–82·6)	31·6 (29·1–34·1)	5·2 (4·0–6·5)	0·2 (0·2–0·3)	0·0 (0·0–0·0)	1·42 (1·39–1·45)	0·39 (0·37–0·4)	0·59 (0·57–0·6)
2010	0·1 (0·1–0·3)	21·8 (19·7–23·9)	53·0 (50·4–55·7)	88·5 (86·0–91·0)	82·1 (79·7–84·8)	33·4 (31·0–35·9)	5·5 (4·3–6·9)	0·2 (0·2–0·4)	0·0 (0·0–0·0)	1·42 (1·39–1·46)	0·37 (0·36–0·39)	0·61 (0·58–0·63)
2011	0·1 (0·1–0·3)	21·5 (19·5–23·8)	50·7 (48·0–53·6)	85·5 (83·2–87·9)	80·2 (77·8–82·8)	33·9 (31·6–36·4)	5·7 (4·4–7·2)	0·3 (0·2–0·4)	0·0 (0·0–0·0)	1·39 (1·36–1·42)	0·36 (0·34–0·38)	0·6 (0·58–0·62)
2012	0·1 (0·1–0·3)	21·6 (19·6–24·1)	49·3 (46·7–52·1)	82·8 (80·4–85·3)	77·4 (74·8–80·0)	34·0 (31·6–36·5)	5·9 (4·6–7·4)	0·3 (0·2–0·4)	0·0 (0·0–0·0)	1·36 (1·32–1·39)	0·36 (0·34–0·37)	0·59 (0·57–0·61)
2013	0·1 (0·1–0·3)	22·2 (20·1–24·5)	49·1 (46·4–51·9)	81·7 (79·4–84·4)	77·3 (74·8–79·9)	34·6 (32·0–37·2)	6·1 (4·8–7·4)	0·2 (0·2–0·4)	0·0 (0·0–0·0)	1·36 (1·32–1·39)	0·36 (0·34–0·38)	0·59 (0·57–0·61)
2014	0·1 (0·1–0·3)	23·0 (20·8–25·3)	50·0 (47·2–53·4)	82·6 (79·9–85·6)	78·9 (76·1–81·5)	35·1 (32·6–37·9)	6·0 (4·9–7·4)	0·2 (0·2–0·3)	0·0 (0·0–0·0)	1·38 (1·34–1·42)	0·37 (0·34–0·39)	0·6 (0·58–0·62)
2015	0·1 (0·1–0·3)	23·6 (21·3–26·0)	51·8 (47·9–56·3)	84·8 (81·0–88·7)	79·5 (75·3–83·8)	34·7 (31·5–38·1)	5·8 (4·8–7·0)	0·2 (0·2–0·3)	0·0 (0·0–0·0)	1·4 (1·34–1·47)	0·38 (0·35–0·41)	0·6 (0·57–0·63)
2016	0·1 (0·1–0·3)	23·5 (21·2–26·3)	53·5 (47·5–60·1)	86·4 (81·3–92·0)	78·2 (71·2–85·6)	33·1 (28·5–38·1)	5·5 (4·4–6·8)	0·2 (0·2–0·3)	0·0 (0·0–0·0)	1·4 (1·29–1·53)	0·39 (0·34–0·43)	0·59 (0·53–0·64)
2017	0·1 (0·1–0·3)	23·1 (20·2–26·5)	54·5 (46·4–63·9)	87·0 (79·9–94·8)	76·7 (68·2–86·3)	31·8 (26·1–38·6)	5·3 (4·1–6·7)	0·2 (0·1–0·3)	0·0 (0·0–0·0)	1·39 (1·23–1·58)	0·39 (0·33–0·45)	0·57 (0·5–0·65)

Supplemental Results Table 5: Age-specific fertility rates per 1000, cumulative total fertility up to maternal age 25 (TFU25), cumulative total fertility from maternal age 30 to age 55 (TFO30), and cumulative total fertility, Slovenia, 1950-2017												
**Year**	**10-14**	**15-19**	**20-24**	**25-29**	**30-34**	**35-39**	**40-44**	**45-49**	**50-54**	**TFR**	**TFU25**	**TFO30**
1950	0·1 (0·0–0·2)	29·3 (24·9–34·3)	146·8 (128·9–168·5)	147·1 (136·6–158·4)	118·2 (105·8–131·8)	65·2 (53·6–78·7)	25·4 (19·7–32·1)	1·9 (1·2–3·0)	0·0 (0·0–0·0)	2·67 (2·47–2·89)	0·88 (0·79–0·99)	1·05 (0·93–1·19)
1951	0·1 (0·0–0·2)	27·3 (23·2–31·8)	140·7 (128·7–155·7)	150·3 (142·1–159·2)	120·0 (109·7–130·4)	70·1 (60·2–81·1)	27·6 (22·1–33·3)	1·8 (1·1–2·8)	0·0 (0·0–0·0)	2·69 (2·53–2·86)	0·84 (0·77–0·92)	1·1 (1·0–1·2)
1952	0·1 (0·0–0·2)	25·6 (22·1–29·4)	134·7 (127·0–142·9)	151·3 (145·8–157·2)	120·0 (112·8–127·5)	73·9 (66·2–82·0)	29·3 (24·6–34·2)	1·7 (1·1–2·5)	0·0 (0·0–0·0)	2·68 (2·58–2·78)	0·8 (0·76–0·85)	1·12 (1·06–1·19)
1953	0·1 (0·0–0·2)	24·8 (21·8–28·1)	131·1 (126·4–135·8)	147·3 (143·1–151·4)	115·5 (110·5–120·3)	73·7 (68·2–79·7)	28·7 (24·9–32·8)	1·6 (1·0–2·4)	0·0 (0·0–0·0)	2·61 (2·56–2·68)	0·78 (0·75–0·81)	1·1 (1·06–1·14)
1954	0·1 (0·0–0·2)	24·9 (22·2–28·0)	131·3 (127·0–135·7)	141·8 (137·9–145·7)	108·7 (104·3–113·3)	71·0 (65·8–76·6)	26·2 (22·6–30·2)	1·5 (0·9–2·3)	0·0 (0·0–0·0)	2·53 (2·47–2·59)	0·78 (0·76–0·81)	1·04 (1·0–1·08)
1955	0·1 (0·0–0·2)	25·2 (22·6–28·2)	133·4 (128·8–137·7)	137·5 (133·5–141·7)	101·4 (96·8–105·7)	67·6 (62·3–73·0)	23·2 (20·0–27·0)	1·4 (0·9–2·2)	0·0 (0·0–0·0)	2·45 (2·39–2·51)	0·79 (0·77–0·82)	0·97 (0·92–1·02)
1956	0·1 (0·0–0·2)	25·2 (22·6–28·1)	133·4 (128·8–138·0)	133·6 (129·6–137·4)	93·4 (89·2–97·6)	63·0 (58·0–68·1)	20·7 (17·5–24·5)	1·3 (0·9–2·0)	0·0 (0·0–0·0)	2·35 (2·29–2·42)	0·79 (0·77–0·82)	0·89 (0·85–0·94)
1957	0·1 (0·0–0·2)	25·3 (22·6–28·2)	131·7 (126·9–136·5)	130·5 (126·8–134·3)	86·9 (82·9–91·1)	57·6 (53·1–62·2)	18·8 (15·7–22·5)	1·3 (0·8–1·9)	0·0 (0·0–0·0)	2·26 (2·21–2·32)	0·79 (0·76–0·81)	0·82 (0·79–0·86)
1958	0·1 (0·0–0·2)	26·2 (23·4–29·1)	131·6 (126·9–136·6)	129·0 (124·9–132·9)	82·8 (78·7–87·4)	52·7 (48·3–57·0)	17·4 (14·3–21·2)	1·2 (0·7–1·8)	0·0 (0·0–0·0)	2·2 (2·15–2·26)	0·79 (0·76–0·82)	0·77 (0·74–0·8)
1959	0·1 (0·0–0·2)	27·6 (24·8–30·8)	132·9 (128·1–137·7)	128·5 (124·5–132·3)	80·5 (76·8–84·9)	48·5 (44·6–52·5)	16·4 (13·4–20·2)	1·1 (0·7–1·7)	0·0 (0·0–0·0)	2·18 (2·12–2·24)	0·8 (0·78–0·83)	0·73 (0·7–0·77)
1960	0·1 (0·0–0·2)	30·0 (27·1–33·4)	136·1 (131·6–141·2)	128·4 (124·7–132·5)	79·3 (75·5–83·3)	44·9 (41·0–48·9)	15·5 (12·6–19·1)	1·1 (0·7–1·6)	0·0 (0·0–0·0)	2·18 (2·11–2·25)	0·83 (0·8–0·86)	0·7 (0·67–0·74)
1961	0·1 (0·0–0·2)	32·3 (29·3–35·6)	142·1 (137·3–146·8)	128·5 (125·0–132·6)	78·9 (75·3–83·1)	41·8 (38·3–46·0)	14·5 (11·6–17·9)	1·1 (0·7–1·6)	0·0 (0·0–0·0)	2·2 (2·14–2·26)	0·87 (0·85–0·9)	0·68 (0·65–0·72)
1962	0·1 (0·0–0·2)	34·0 (30·9–37·4)	149·2 (144·2–153·9)	129·2 (125·3–133·2)	79·0 (75·2–83·1)	41·2 (37·6–45·4)	14·3 (11·3–17·5)	1·0 (0·7–1·6)	0·0 (0·0–0·0)	2·24 (2·19–2·29)	0·92 (0·89–0·95)	0·68 (0·65–0·71)
1963	0·1 (0·0–0·2)	35·9 (32·6–39·3)	157·0 (152·2–161·6)	131·7 (127·9–135·6)	80·4 (76·5–84·6)	43·3 (39·6–47·1)	14·7 (11·7–17·9)	1·0 (0·7–1·5)	0·0 (0·0–0·0)	2·32 (2·28–2·37)	0·97 (0·94–0·99)	0·7 (0·67–0·73)
1964	0·1 (0·0–0·2)	38·4 (35·0–41·9)	165·5 (160·7–170·4)	135·8 (131·9–139·8)	82·8 (79·0–86·8)	44·8 (40·8–48·6)	14·8 (12·0–18·1)	1·0 (0·7–1·5)	0·0 (0·0–0·0)	2·42 (2·37–2·47)	1·02 (0·99–1·05)	0·72 (0·69–0·75)
1965	0·1 (0·0–0·2)	40·4 (37·1–44·0)	169·8 (164·8–174·4)	138·0 (133·7–142·6)	83·9 (80·0–88·0)	44·2 (40·3–48·3)	14·2 (11·5–17·3)	1·0 (0·6–1·4)	0·0 (0·0–0·0)	2·46 (2·4–2·51)	1·05 (1·02–1·08)	0·72 (0·69–0·75)
1966	0·1 (0·0–0·2)	41·0 (37·7–44·5)	166·7 (161·7–171·6)	135·6 (131·6–139·8)	81·6 (77·7–85·6)	41·6 (37·8–45·8)	12·8 (10·4–15·7)	0·9 (0·6–1·3)	0·0 (0·0–0·0)	2·4 (2·35–2·46)	1·04 (1·01–1·07)	0·68 (0·65–0·71)
1967	0·1 (0·0–0·2)	40·8 (37·7–44·3)	160·2 (154·9–165·1)	129·6 (125·3–133·7)	77·1 (73·1–81·1)	38·3 (34·5–42·4)	11·4 (9·2–14·0)	0·8 (0·5–1·2)	0·0 (0·0–0·0)	2·29 (2·24–2·35)	1·01 (0·98–1·03)	0·64 (0·61–0·67)
1968	0·1 (0·0–0·2)	41·3 (38·2–44·5)	155·0 (150·2–160·0)	122·9 (118·7–127·1)	73·2 (69·1–77·6)	35·6 (32·0–39·5)	10·5 (8·3–12·9)	0·7 (0·5–1·1)	0·0 (0·0–0·0)	2·2 (2·14–2·25)	0·98 (0·95–1·01)	0·6 (0·56–0·64)
1969	0·1 (0·0–0·2)	42·7 (39·5–46·0)	154·4 (149·5–159·3)	117·9 (114·1–121·9)	70·8 (66·6–74·8)	34·2 (30·6–37·8)	10·1 (7·9–12·4)	0·7 (0·4–1·0)	0·0 (0·0–0·0)	2·15 (2·1–2·21)	0·99 (0·96–1·01)	0·58 (0·54–0·62)
1970	0·1 (0·0–0·2)	44·7 (41·6–48·2)	156·4 (151·7–161·1)	115·8 (112·2–119·6)	68·7 (64·7–72·6)	33·0 (29·7–36·9)	9·7 (7·7–12·0)	0·6 (0·4–0·9)	0·0 (0·0–0·0)	2·15 (2·09–2·2)	1·01 (0·98–1·03)	0·56 (0·53–0·59)
1971	0·1 (0·0–0·2)	46·8 (43·8–50·0)	156·4 (152·0–160·6)	115·1 (111·5–118·9)	66·2 (62·4–70·1)	31·6 (28·4–35·5)	9·2 (7·4–11·2)	0·6 (0·4–0·9)	0·0 (0·0–0·0)	2·13 (2·08–2·18)	1·02 (0·99–1·05)	0·54 (0·51–0·57)
1972	0·1 (0·0–0·2)	49·0 (45·7–52·3)	155·0 (150·9–159·7)	114·2 (110·5–117·9)	63·2 (59·6–67·0)	30·1 (26·8–33·8)	8·5 (6·8–10·5)	0·6 (0·4–0·8)	0·0 (0·0–0·0)	2·1 (2·06–2·15)	1·02 (0·99–1·05)	0·51 (0·48–0·54)
1973	0·1 (0·0–0·2)	51·3 (47·8–54·7)	153·7 (149·4–158·3)	114·0 (110·0–118·1)	60·4 (56·8–64·5)	28·6 (25·3–32·3)	7·9 (6·2–10·1)	0·5 (0·3–0·8)	0·0 (0·0–0·0)	2·08 (2·03–2·14)	1·03 (1·0–1·05)	0·49 (0·46–0·52)
1974	0·1 (0·1–0·2)	53·5 (50·0–57·0)	154·4 (150·2–159·0)	115·3 (111·4–119·6)	59·0 (55·2–63·0)	27·6 (24·3–31·3)	7·5 (5·7–9·7)	0·5 (0·3–0·7)	0·0 (0·0–0·0)	2·09 (2·03–2·15)	1·04 (1·01–1·07)	0·47 (0·44–0·51)
1975	0·1 (0·1–0·2)	54·8 (51·2–58·2)	157·4 (152·9–161·9)	116·7 (112·8–120·6)	58·5 (54·6–62·4)	26·5 (23·1–30·1)	7·2 (5·4–9·4)	0·5 (0·3–0·7)	0·0 (0·0–0·0)	2·11 (2·06–2·16)	1·06 (1·03–1·09)	0·46 (0·43–0·5)
1976	0·1 (0·1–0·2)	54·7 (51·4–58·2)	159·4 (155·2–163·8)	115·9 (112·1–119·5)	57·3 (53·6–61·1)	25·2 (21·6–29·2)	6·9 (5·1–9·2)	0·5 (0·3–0·7)	0·0 (0·0–0·0)	2·1 (2·05–2·15)	1·07 (1·04–1·1)	0·45 (0·41–0·48)
1977	0·1 (0·1–0·2)	54·0 (50·6–57·6)	161·1 (156·8–165·3)	114·2 (110·3–117·7)	56·1 (52·3–60·0)	24·2 (20·7–28·0)	6·6 (4·9–8·7)	0·4 (0·3–0·7)	0·0 (0·0–0·0)	2·08 (2·04–2·13)	1·08 (1·05–1·11)	0·44 (0·4–0·47)
1978	0·1 (0·1–0·2)	53·3 (49·8–56·9)	162·9 (158·0–167·4)	112·6 (108·8–116·0)	55·6 (51·8–59·4)	23·5 (20·1–27·2)	6·3 (4·6–8·6)	0·4 (0·3–0·6)	0·0 (0·0–0·0)	2·07 (2·03–2·12)	1·08 (1·05–1·11)	0·43 (0·4–0·46)
1979	0·1 (0·0–0·2)	52·2 (48·5–55·9)	162·8 (158·2–167·4)	111·0 (107·2–114·8)	55·1 (51·4–58·9)	23·0 (19·6–27·0)	6·0 (4·3–8·4)	0·4 (0·2–0·6)	0·0 (0·0–0·0)	2·05 (2·01–2·1)	1·08 (1·05–1·1)	0·42 (0·4–0·45)
1980	0·1 (0·0–0·2)	50·2 (46·7–53·7)	161·1 (157·0–165·7)	109·5 (105·9–113·1)	54·3 (50·9–57·9)	22·7 (19·7–26·3)	5·7 (4·2–7·9)	0·4 (0·2–0·5)	0·0 (0·0–0·0)	2·02 (1·98–2·06)	1·06 (1·03–1·09)	0·41 (0·39–0·44)
1981	0·1 (0·0–0·2)	47·6 (44·3–50·8)	158·7 (154·3–163·3)	106·6 (103·2–110·2)	51·8 (48·5–55·5)	21·5 (18·5–25·0)	5·3 (3·9–7·2)	0·3 (0·2–0·5)	0·0 (0·0–0·0)	1·96 (1·92–2·0)	1·03 (1·0–1·06)	0·4 (0·37–0·42)
1982	0·1 (0·0–0·2)	44·6 (41·5–47·9)	154·5 (150·1–158·8)	102·4 (98·9–105·9)	48·1 (44·8–51·6)	19·5 (16·6–22·5)	4·9 (3·6–6·5)	0·3 (0·2–0·5)	0·0 (0·0–0·0)	1·87 (1·83–1·91)	1·0 (0·97–1·02)	0·36 (0·34–0·39)
1983	0·1 (0·0–0·2)	42·1 (38·9–45·3)	148·5 (144·3–152·8)	99·2 (95·7–102·6)	45·2 (41·8–48·8)	18·4 (15·6–21·6)	4·7 (3·5–6·2)	0·3 (0·2–0·4)	0·0 (0·0–0·0)	1·79 (1·75–1·83)	0·95 (0·93–0·98)	0·34 (0·32–0·37)
1984	0·1 (0·0–0·2)	40·4 (37·2–43·9)	143·9 (139·6–148·3)	98·6 (95·0–102·0)	44·3 (40·9–47·9)	18·6 (15·8–21·8)	4·9 (3·7–6·2)	0·3 (0·2–0·4)	0·0 (0·0–0·0)	1·76 (1·71–1·8)	0·92 (0·89–0·95)	0·34 (0·32–0·37)
1985	0·1 (0·0–0·2)	39·0 (35·8–42·3)	141·8 (137·6–146·2)	99·9 (96·4–103·5)	45·0 (41·8–48·4)	18·7 (16·1–21·7)	5·0 (3·9–6·2)	0·2 (0·2–0·3)	0·0 (0·0–0·0)	1·75 (1·71–1·79)	0·9 (0·88–0·93)	0·34 (0·32–0·37)
1986	0·1 (0·0–0·2)	36·8 (33·9–40·0)	140·0 (135·7–144·3)	101·5 (98·1–104·8)	45·1 (42·1–48·0)	18·1 (15·5–20·8)	4·9 (3·9–6·1)	0·2 (0·1–0·3)	0·0 (0·0–0·0)	1·73 (1·69–1·77)	0·88 (0·86–0·91)	0·34 (0·32–0·37)
1987	0·1 (0·0–0·2)	34·0 (31·1–37·2)	136·1 (131·8–140·5)	101·9 (98·4–105·6)	43·5 (40·3–46·5)	17·2 (14·7–20·1)	4·8 (3·7–6·0)	0·2 (0·1–0·3)	0·0 (0·0–0·0)	1·69 (1·65–1·73)	0·85 (0·82–0·88)	0·33 (0·3–0·35)
1988	0·1 (0·0–0·2)	31·3 (28·4–34·4)	130·0 (125·6–134·9)	100·9 (97·2–104·6)	41·2 (38·0–44·5)	16·7 (14·0–19·8)	4·7 (3·7–6·0)	0·2 (0·1–0·3)	0·0 (0·0–0·0)	1·63 (1·59–1·66)	0·81 (0·78–0·84)	0·31 (0·29–0·34)
1989	0·1 (0·0–0·2)	29·6 (26·8–32·7)	123·6 (119·3–128·1)	99·6 (96·2–103·4)	39·9 (36·8–43·1)	16·8 (14·1–19·6)	4·7 (3·7–6·0)	0·2 (0·1–0·2)	0·0 (0·0–0·0)	1·57 (1·53–1·61)	0·77 (0·74–0·79)	0·31 (0·29–0·33)
1990	0·1 (0·0–0·2)	27·5 (24·6–30·2)	117·3 (113·3–121·7)	98·7 (95·2–102·5)	40·9 (37·9–44·2)	17·2 (14·6–19·9)	4·7 (3·8–5·9)	0·2 (0·1–0·2)	0·0 (0·0–0·0)	1·53 (1·49–1·58)	0·72 (0·7–0·75)	0·32 (0·29–0·34)
1991	0·1 (0·0–0·2)	23·6 (21·0–26·2)	109·2 (105·2–113·5)	97·0 (93·6–100·4)	43·2 (40·2–46·4)	17·4 (14·9–20·0)	4·6 (3·6–5·8)	0·2 (0·1–0·3)	0·0 (0·0–0·0)	1·48 (1·44–1·52)	0·66 (0·64–0·69)	0·33 (0·31–0·35)
1992	0·1 (0·0–0·1)	19·6 (17·3–22·1)	100·2 (96·0–104·7)	95·6 (92·1–98·7)	44·7 (41·8–47·9)	17·0 (14·5–19·8)	4·4 (3·4–5·6)	0·2 (0·1–0·3)	0·0 (0·0–0·0)	1·41 (1·37–1·45)	0·6 (0·58–0·62)	0·33 (0·31–0·36)
1993	0·1 (0·0–0·1)	16·6 (14·5–19·0)	92·4 (88·2–96·8)	95·5 (91·7–99·0)	46·2 (43·0–49·6)	16·7 (14·1–19·6)	4·3 (3·3–5·5)	0·2 (0·1–0·3)	0·0 (0·0–0·0)	1·36 (1·32–1·4)	0·55 (0·52–0·57)	0·34 (0·31–0·36)
1994	0·1 (0·0–0·1)	14·3 (12·4–16·3)	85·9 (81·6–90·4)	96·2 (92·6–99·9)	47·9 (44·8–51·4)	16·8 (14·1–19·9)	4·3 (3·2–5·5)	0·2 (0·1–0·3)	0·0 (0·0–0·0)	1·33 (1·29–1·37)	0·5 (0·48–0·53)	0·35 (0·32–0·37)
1995	0·1 (0·0–0·1)	12·3 (10·7–14·2)	80·1 (76·0–84·4)	97·4 (94·0–101·1)	50·1 (46·8–53·6)	17·5 (14·6–20·7)	4·4 (3·4–5·6)	0·2 (0·1–0·3)	0·0 (0·0–0·0)	1·31 (1·27–1·35)	0·46 (0·44–0·48)	0·36 (0·34–0·39)
1996	0·0 (0·0–0·1)	10·6 (9·2–12·2)	74·3 (70·4–78·3)	98·5 (94·9–102·4)	52·3 (49·1–55·6)	18·4 (15·6–21·5)	4·6 (3·7–5·8)	0·2 (0·2–0·3)	0·0 (0·0–0·0)	1·3 (1·26–1·33)	0·42 (0·4–0·45)	0·38 (0·35–0·4)
1997	0·0 (0·0–0·1)	9·3 (8·0–10·8)	68·7 (65·0–72·8)	99·1 (95·3–103·0)	53·9 (50·3–57·2)	19·1 (16·4–21·9)	4·7 (3·8–6·0)	0·2 (0·2–0·4)	0·0 (0·0–0·0)	1·28 (1·23–1·31)	0·39 (0·37–0·41)	0·39 (0·36–0·41)
1998	0·0 (0·0–0·1)	8·2 (7·1–9·5)	63·7 (59·6–67·6)	99·5 (95·4–103·5)	55·3 (51·8–59·0)	19·6 (16·7–22·6)	4·8 (3·8–6·1)	0·3 (0·2–0·4)	0·0 (0·0–0·0)	1·26 (1·22–1·3)	0·36 (0·34–0·38)	0·4 (0·37–0·43)
1999	0·0 (0·0–0·1)	7·3 (6·2–8·5)	58·9 (55·1–62·9)	100·0 (96·3–103·8)	57·7 (54·3–61·5)	20·4 (17·5–23·6)	5·0 (3·8–6·4)	0·3 (0·2–0·4)	0·0 (0·0–0·0)	1·25 (1·21–1·29)	0·33 (0·31–0·35)	0·42 (0·39–0·44)
2000	0·0 (0·0–0·1)	6·3 (5·4–7·4)	53·9 (50·3–57·7)	99·8 (96·1–103·7)	61·8 (58·2–65·6)	21·8 (18·8–24·8)	5·3 (4·1–6·9)	0·3 (0·2–0·4)	0·0 (0·0–0·0)	1·25 (1·21–1·29)	0·3 (0·28–0·32)	0·45 (0·42–0·47)
2001	0·0 (0·0–0·1)	5·5 (4·7–6·5)	49·1 (45·5–52·9)	98·7 (94·9–102·4)	66·5 (63·0–70·3)	23·3 (20·3–26·6)	5·7 (4·5–7·2)	0·3 (0·2–0·5)	0·0 (0·0–0·0)	1·25 (1·21–1·29)	0·27 (0·26–0·29)	0·48 (0·45–0·51)
2002	0·0 (0·0–0·1)	5·2 (4·4–6·0)	45·6 (42·0–49·4)	97·5 (93·9–101·4)	70·4 (66·7–74·4)	24·3 (21·3–27·6)	5·8 (4·5–7·3)	0·4 (0·2–0·5)	0·0 (0·0–0·0)	1·25 (1·21–1·29)	0·25 (0·24–0·27)	0·5 (0·48–0·53)
2003	0·0 (0·0–0·1)	5·0 (4·2–5·9)	43·3 (39·7–47·1)	96·9 (93·4–100·8)	74·0 (70·2–78·3)	25·0 (21·9–28·5)	5·7 (4·3–7·3)	0·4 (0·2–0·5)	0·0 (0·0–0·0)	1·25 (1·21–1·29)	0·24 (0·22–0·26)	0·53 (0·5–0·55)
2004	0·0 (0·0–0·1)	4·9 (4·1–5·8)	41·6 (37·9–45·2)	97·1 (93·3–100·9)	78·6 (74·7–83·0)	26·3 (23·1–30·0)	5·7 (4·2–7·4)	0·4 (0·2–0·5)	0·0 (0·0–0·0)	1·27 (1·23–1·32)	0·23 (0·21–0·25)	0·56 (0·53–0·58)
2005	0·0 (0·0–0·1)	4·9 (4·1–5·7)	40·5 (36·9–44·2)	98·4 (95·0–102·1)	84·4 (80·4–88·5)	28·4 (25·0–32·1)	5·6 (4·2–7·5)	0·4 (0·2–0·5)	0·0 (0·0–0·0)	1·31 (1·27–1·36)	0·23 (0·21–0·25)	0·59 (0·56–0·62)
2006	0·0 (0·0–0·1)	4·9 (4·1–5·9)	40·3 (36·7–44·3)	101·2 (97·6–105·1)	91·2 (87·0–95·0)	31·2 (27·7–34·7)	5·7 (4·2–7·5)	0·4 (0·2–0·5)	0·0 (0·0–0·0)	1·37 (1·34–1·42)	0·23 (0·2–0·25)	0·64 (0·61–0·67)
2007	0·0 (0·0–0·1)	5·0 (4·2–6·1)	40·8 (36·9–44·7)	104·7 (101·1–108·5)	98·3 (94·1–102·5)	34·7 (31·0–38·2)	5·7 (4·2–7·5)	0·3 (0·2–0·5)	0·0 (0·0–0·0)	1·45 (1·41–1·49)	0·23 (0·21–0·25)	0·7 (0·67–0·72)
2008	0·0 (0·0–0·1)	5·1 (4·2–6·2)	41·5 (37·8–45·4)	107·6 (103·9–111·5)	104·1 (99·7–108·5)	38·1 (34·4–42·1)	5·7 (4·3–7·5)	0·3 (0·2–0·5)	0·0 (0·0–0·0)	1·51 (1·47–1·56)	0·23 (0·21–0·25)	0·74 (0·71–0·77)
2009	0·0 (0·0–0·1)	5·2 (4·3–6·3)	41·9 (38·3–45·8)	109·3 (105·6–113·1)	107·2 (103·1–111·4)	40·8 (37·2–44·9)	5·9 (4·3–7·6)	0·3 (0·2–0·5)	0·0 (0·0–0·0)	1·55 (1·51–1·59)	0·24 (0·22–0·25)	0·77 (0·74–0·8)
2010	0·0 (0·0–0·1)	5·2 (4·2–6·2)	42·3 (38·7–46·2)	109·8 (106·3–113·6)	107·7 (103·7–112·0)	42·6 (39·0–46·8)	6·1 (4·5–7·8)	0·3 (0·2–0·5)	0·0 (0·0–0·0)	1·57 (1·53–1·61)	0·24 (0·22–0·26)	0·78 (0·76–0·81)
2011	0·0 (0·0–0·1)	5·1 (4·3–6·2)	42·6 (38·9–46·6)	109·6 (105·9–113·4)	106·6 (102·6–110·7)	43·3 (39·4–47·5)	6·3 (4·7–8·1)	0·3 (0·2–0·4)	0·0 (0·0–0·0)	1·57 (1·53–1·61)	0·24 (0·22–0·26)	0·78 (0·76–0·81)
2012	0·0 (0·0–0·1)	5·1 (4·2–6·1)	42·6 (38·7–46·7)	109·1 (105·2–113·0)	105·1 (101·2–109·2)	43·6 (39·9–47·8)	6·5 (4·9–8·5)	0·3 (0·2–0·4)	0·0 (0·0–0·0)	1·56 (1·52–1·61)	0·24 (0·22–0·26)	0·78 (0·75–0·81)
2013	0·0 (0·0–0·1)	5·0 (4·1–6·0)	42·3 (38·3–46·6)	108·9 (105·0–112·8)	104·4 (100·4–108·3)	44·2 (40·4–48·3)	6·7 (5·0–8·8)	0·3 (0·2–0·4)	0·0 (0·0–0·0)	1·56 (1·51–1·6)	0·24 (0·22–0·26)	0·78 (0·75–0·81)
2014	0·0 (0·0–0·1)	4·9 (4·0–5·8)	41·7 (37·6–46·3)	108·9 (105·1–112·8)	105·0 (101·0–109·1)	44·6 (40·9–48·6)	6·8 (5·2–8·9)	0·3 (0·2–0·4)	0·0 (0·0–0·0)	1·56 (1·52–1·61)	0·23 (0·21–0·26)	0·78 (0·75–0·82)
2015	0·0 (0·0–0·1)	4·9 (4·0–5·9)	41·3 (36·5–46·3)	108·9 (104·1–113·4)	105·8 (101·4–110·5)	44·0 (39·9–48·7)	6·7 (5·1–8·8)	0·3 (0·2–0·4)	0·0 (0·0–0·0)	1·56 (1·5–1·62)	0·23 (0·2–0·26)	0·78 (0·75–0·82)
2016	0·0 (0·0–0·1)	4·9 (4·1–6·0)	41·3 (35·3–47·4)	108·7 (102·3–115·5)	104·7 (98·3–111·5)	41·8 (36·5–48·0)	6·2 (4·6–8·3)	0·2 (0·1–0·4)	0·0 (0·0–0·0)	1·54 (1·45–1·64)	0·23 (0·2–0·27)	0·76 (0·71–0·83)
2017	0·0 (0·0–0·1)	5·0 (4·1–6·0)	41·5 (34·9–49·4)	108·1 (100·3–117·2)	103·0 (93·7–114·0)	39·7 (33·0–48·5)	5·9 (4·3–8·0)	0·2 (0·1–0·4)	0·0 (0·0–0·0)	1·52 (1·39–1·66)	0·23 (0·2–0·28)	0·74 (0·66–0·84)

Supplemental Results Table 5: Age-specific fertility rates per 1000, cumulative total fertility up to maternal age 25 (TFU25), cumulative total fertility from maternal age 30 to age 55 (TFO30), and cumulative total fertility, Eastern Europe, 1950-2017

Year	10-14	15-19	20-24	25-29	30-34	35-39	40-44	45-49	50-54	TFR	TFU25	TFO30
1950	0·0 (0·0–0·1)	26·9 (23·9–30·6)	147·2 (144·3–150·5)	151·0 (148·5–153·7)	89·3 (87·3–91·5)	73·2 (71·5–75·1)	34·7 (33·7–35·7)	5·9 (5·4–6·4)	0·1 (0·1–0·1)	2·64 (2·59–2·7)	0·87 (0·84–0·91)	1·02 (0·99–1·04)
1951	0·0 (0·0–0·1)	25·7 (22·9–29·0)	146·2 (143·7–149·0)	156·5 (154·5–159·1)	91·8 (89·9–93·7)	67·8 (66·2–69·4)	34·6 (33·8–35·5)	5·4 (5·0–5·9)	0·1 (0·1–0·1)	2·64 (2·6–2·69)	0·86 (0·83–0·89)	1·0 (0·98–1·02)
1952	0·0 (0·0–0·1)	24·4 (22·0–27·2)	135·3 (132·9–137·7)	157·0 (155·1–159·1)	92·9 (91·1–95·0)	62·3 (60·9–63·8)	33·8 (33·0–34·6)	5·0 (4·6–5·4)	0·1 (0·1–0·1)	2·55 (2·52–2·6)	0·8 (0·78–0·82)	0·97 (0·95–0·99)
1953	0·0 (0·0–0·1)	24·5 (22·3–27·1)	133·0 (130·8–135·2)	161·0 (159·2–162·9)	97·9 (96·2–99·8)	59·7 (58·3–61·2)	32·5 (31·7–33·3)	4·7 (4·3–5·1)	0·1 (0·1–0·1)	2·57 (2·53–2·6)	0·79 (0·77–0·81)	0·97 (0·96–0·99)
1954	0·0 (0·0–0·1)	24·4 (22·4–26·6)	135·3 (133·0–137·6)	165·8 (164·2–167·4)	104·3 (102·5–106·1)	57·1 (55·8–58·5)	30·3 (29·6–31·0)	4·4 (4·0–4·8)	0·1 (0·1–0·1)	2·61 (2·58–2·64)	0·8 (0·78–0·82)	0·98 (0·97–1·0)
1955	0·0 (0·0–0·1)	24·4 (22·7–26·2)	132·1 (129·9–134·5)	159·8 (158·2–161·5)	104·3 (102·6–106·0)	53·3 (51·9–54·6)	26·0 (25·3–26·7)	4·0 (3·6–4·3)	0·1 (0·1–0·1)	2·52 (2·49–2·55)	0·78 (0·77–0·8)	0·94 (0·92–0·95)
1956	0·0 (0·0–0·1)	25·7 (24·2–27·0)	136·9 (134·4–139·5)	156·1 (154·6–157·9)	104·3 (102·5–106·1)	51·8 (50·4–53·2)	21·5 (20·9–22·2)	3·6 (3·3–4·0)	0·1 (0·1–0·1)	2·5 (2·47–2·53)	0·81 (0·8–0·83)	0·91 (0·89–0·92)
1957	0·0 (0·0–0·1)	27·4 (26·5–28·3)	146·3 (143·8–148·8)	150·3 (148·7–151·9)	104·5 (102·7–106·4)	50·0 (48·6–51·4)	18·9 (18·3–19·5)	3·2 (2·9–3·6)	0·1 (0·1–0·1)	2·5 (2·48–2·53)	0·87 (0·85–0·88)	0·88 (0·87–0·9)
1958	0·0 (0·0–0·1)	28·6 (27·8–29·4)	154·3 (151·8–156·8)	141·2 (139·6–142·8)	102·7 (100·9–104·6)	49·0 (47·7–50·3)	17·1 (16·5–17·7)	2·8 (2·5–3·2)	0·1 (0·1–0·1)	2·48 (2·45–2·51)	0·91 (0·9–0·93)	0·86 (0·85–0·87)
1959	0·0 (0·0–0·1)	30·1 (29·3–31·0)	157·9 (155·3–160·5)	135·9 (134·2–137·7)	101·1 (99·2–103·0)	49·7 (48·5–51·1)	15·7 (15·1–16·3)	2·4 (2·1–2·8)	0·0 (0·0–0·0)	2·46 (2·44–2·49)	0·94 (0·93–0·95)	0·85 (0·83–0·86)
1960	0·0 (0·0–0·1)	31·5 (30·6–32·4)	162·1 (159·4–164·6)	131·1 (129·3–132·9)	96·5 (94·5–98·3)	48·1 (46·8–49·4)	14·7 (14·1–15·3)	2·1 (1·8–2·5)	0·0 (0·0–0·0)	2·43 (2·4–2·46)	0·97 (0·95–0·98)	0·81 (0·79–0·82)
1961	0·0 (0·0–0·1)	29·1 (28·2–30·0)	159·6 (157·1–162·2)	126·7 (124·9–128·6)	89·7 (87·9–91·6)	45·6 (44·3–47·0)	13·8 (13·2–14·4)	1·8 (1·5–2·1)	0·0 (0·0–0·0)	2·33 (2·3–2·36)	0·94 (0·93–0·96)	0·75 (0·74–0·77)
1962	0·0 (0·0–0·1)	25·3 (24·5–26·2)	151·0 (148·6–153·5)	126·1 (124·2–127·9)	83·3 (81·7–85·1)	44·2 (43·0–45·5)	13·1 (12·5–13·6)	1·5 (1·3–1·9)	0·0 (0·0–0·0)	2·22 (2·2–2·25)	0·88 (0·87–0·89)	0·71 (0·7–0·72)
1963	0·0 (0·0–0·1)	23·4 (22·7–24·2)	143·0 (140·5–145·6)	124·9 (123·0–126·8)	76·5 (74·8–78·1)	41·7 (40·5–43·0)	12·9 (12·3–13·4)	1·3 (1·1–1·6)	0·0 (0·0–0·0)	2·12 (2·09–2·15)	0·83 (0·82–0·85)	0·66 (0·65–0·68)
1964	0·0 (0·0–0·1)	23·5 (22·8–24·2)	131·8 (129·1–134·5)	122·2 (120·3–124·0)	72·7 (71·2–74·3)	39·3 (38·2–40·6)	12·6 (12·0–13·2)	1·2 (1·0–1·5)	0·0 (0·0–0·0)	2·02 (1·99–2·04)	0·78 (0·76–0·79)	0·63 (0·62–0·64)
1965	0·0 (0·0–0·1)	25·0 (24·3–25·7)	127·0 (124·0–130·0)	121·8 (120·0–123·5)	72·2 (70·7–74·0)	38·0 (36·9–39·3)	12·0 (11·4–12·5)	1·1 (0·9–1·4)	0·0 (0·0–0·0)	1·99 (1·96–2·02)	0·76 (0·74–0·78)	0·62 (0·6–0·63)
1966	0·0 (0·0–0·1)	26·0 (25·3–26·7)	129·5 (126·3–132·8)	118·3 (116·6–120·1)	71·8 (70·3–73·6)	36·0 (34·8–37·3)	11·3 (10·7–11·8)	1·1 (0·9–1·3)	0·0 (0·0–0·0)	1·97 (1·94–2·0)	0·78 (0·76–0·79)	0·6 (0·59–0·62)
1967	0·0 (0·0–0·1)	26·6 (25·8–27·4)	135·1 (131·5–138·4)	110·4 (108·7–112·0)	71·1 (69·5–72·9)	33·5 (32·3–34·8)	10·4 (9·9–10·9)	1·0 (0·8–1·2)	0·0 (0·0–0·0)	1·94 (1·91–1·97)	0·81 (0·79–0·83)	0·58 (0·57–0·6)
1968	0·0 (0·0–0·1)	28·0 (27·3–28·9)	144·7 (141·3–148·0)	104·6 (102·8–106·2)	71·7 (70·2–73·4)	31·5 (30·4–32·8)	9·7 (9·2–10·2)	1·0 (0·8–1·2)	0·0 (0·0–0·0)	1·96 (1·93–1·99)	0·86 (0·85–0·88)	0·57 (0·56–0·58)
1969	0·0 (0·0–0·1)	29·8 (29·1–30·6)	155·6 (152·7–158·6)	101·0 (99·0–103·0)	71·2 (69·6–72·9)	30·4 (29·2–31·8)	9·2 (8·7–9·8)	0·9 (0·7–1·2)	0·0 (0·0–0·0)	1·99 (1·96–2·02)	0·93 (0·91–0·94)	0·56 (0·55–0·57)
1970	0·0 (0·0–0·1)	31·3 (30·6–32·1)	160·9 (158·2–163·6)	101·4 (99·5–103·4)	70·4 (68·8–71·9)	30·3 (29·2–31·7)	8·7 (8·2–9·1)	0·8 (0·7–1·1)	0·0 (0·0–0·0)	2·02 (1·99–2·05)	0·96 (0·95–0·98)	0·55 (0·54–0·56)
1971	0·0 (0·0–0·1)	32·1 (31·4–32·9)	161·3 (158·9–163·8)	106·8 (104·8–108·7)	68·8 (67·4–70·3)	30·8 (29·6–32·0)	8·1 (7·6–8·5)	0·7 (0·6–1·0)	0·0 (0·0–0·0)	2·04 (2·01–2·07)	0·97 (0·95–0·98)	0·54 (0·53–0·55)
1972	0·0 (0·0–0·1)	32·6 (31·8–33·4)	157·4 (155·1–159·9)	111·2 (109·4–113·0)	64·2 (62·8–65·6)	30·7 (29·6–31·8)	7·5 (7·1–8·0)	0·7 (0·5–0·9)	0·0 (0·0–0·0)	2·02 (1·99–2·05)	0·95 (0·94–0·96)	0·52 (0·5–0·53)
1973	0·0 (0·0–0·1)	33·4 (32·7–34·2)	154·9 (152·4–157·4)	114·8 (113·1–116·6)	59·1 (57·6–60·5)	29·9 (28·8–31·0)	7·2 (6·7–7·7)	0·6 (0·5–0·8)	0·0 (0·0–0·0)	2·0 (1·97–2·03)	0·94 (0·93–0·96)	0·48 (0·47–0·49)
1974	0·0 (0·0–0·1)	34·4 (33·7–35·2)	154·6 (152·2–156·9)	117·0 (115·4–118·7)	55·6 (54·2–57·1)	28·5 (27·6–29·6)	7·0 (6·5–7·5)	0·6 (0·4–0·7)	0·0 (0·0–0·0)	1·99 (1·96–2·01)	0·95 (0·93–0·96)	0·46 (0·45–0·47)
1975	0·0 (0·0–0·1)	35·5 (34·7–36·3)	154·4 (152·2–156·7)	114·9 (113·4–116·5)	54·3 (52·9–55·9)	26·4 (25·5–27·4)	6·8 (6·3–7·3)	0·5 (0·4–0·7)	0·0 (0·0–0·0)	1·96 (1·94–1·99)	0·95 (0·94–0·96)	0·44 (0·43–0·45)
1976	0·0 (0·0–0·1)	36·6 (35·8–37·3)	153·6 (151·4–155·9)	112·3 (110·8–113·9)	55·9 (54·4–57·6)	23·9 (23·0–24·9)	6·6 (6·1–7·0)	0·5 (0·3–0·6)	0·0 (0·0–0·0)	1·95 (1·92–1·97)	0·95 (0·94–0·96)	0·43 (0·42–0·45)
1977	0·0 (0·0–0·1)	38·3 (37·5–39·1)	152·5 (150·4–154·8)	109·8 (108·3–111·4)	58·0 (56·5–59·5)	21·4 (20·6–22·3)	6·2 (5·8–6·7)	0·4 (0·3–0·6)	0·0 (0·0–0·0)	1·93 (1·91–1·96)	0·95 (0·94–0·97)	0·43 (0·42–0·44)
1978	0·0 (0·0–0·1)	40·8 (40·0–41·6)	152·2 (150·1–154·3)	106·0 (104·6–107·5)	58·2 (56·9–59·7)	19·3 (18·5–20·1)	5·8 (5·4–6·2)	0·4 (0·3–0·5)	0·0 (0·0–0·0)	1·91 (1·89–1·94)	0·97 (0·95–0·98)	0·42 (0·41–0·43)
1979	0·0 (0·0–0·1)	42·6 (41·8–43·5)	152·1 (150·0–154·2)	103·3 (101·8–104·7)	57·2 (55·9–58·4)	18·1 (17·2–19·0)	5·3 (4·9–5·7)	0·4 (0·3–0·5)	0·0 (0·0–0·0)	1·89 (1·87–1·92)	0·97 (0·96–0·99)	0·4 (0·39–0·41)
1980	0·0 (0·0–0·1)	43·2 (42·4–44·0)	152·0 (150·0–154·2)	103·8 (102·4–105·2)	56·4 (55·2–57·6)	18·3 (17·4–19·3)	4·8 (4·5–5·2)	0·4 (0·3–0·5)	0·0 (0·0–0·0)	1·89 (1·87–1·92)	0·98 (0·97–0·99)	0·4 (0·39–0·41)
1981	0·0 (0·0–0·1)	43·5 (42·7–44·4)	153·0 (151·1–155·0)	106·2 (104·9–107·6)	57·5 (56·4–58·8)	20·4 (19·5–21·4)	4·5 (4·2–4·9)	0·3 (0·3–0·5)	0·0 (0·0–0·0)	1·93 (1·91–1·95)	0·98 (0·97–0·99)	0·41 (0·4–0·42)
1982	0·0 (0·0–0·1)	44·7 (43·9–45·5)	158·2 (156·0–160·2)	112·2 (110·7–113·6)	60·6 (59·4–61·9)	23·0 (22·2–24·0)	4·5 (4·1–4·8)	0·3 (0·2–0·4)	0·0 (0·0–0·0)	2·02 (2·0–2·04)	1·01 (1·0–1·03)	0·44 (0·43–0·45)
1983	0·0 (0·0–0·1)	46·1 (45·2–47·0)	160·1 (158·0–162·1)	113·9 (112·5–115·4)	61·0 (59·8–62·3)	23·9 (23·1–24·8)	4·4 (4·0–4·8)	0·3 (0·2–0·4)	0·0 (0·0–0·0)	2·05 (2·03–2·07)	1·03 (1·02–1·04)	0·45 (0·44–0·46)
1984	0·0 (0·0–0·1)	46·8 (45·9–47·8)	160·7 (158·7–162·8)	113·0 (111·6–114·4)	59·4 (58·2–60·6)	23·5 (22·7–24·3)	4·4 (4·1–4·9)	0·3 (0·2–0·4)	0·0 (0·0–0·0)	2·04 (2·02–2·06)	1·04 (1·03–1·05)	0·44 (0·43–0·45)
1985	0·0 (0·0–0·1)	46·9 (45·9–47·9)	161·7 (159·6–163·8)	115·5 (114·1–116·9)	61·3 (60·2–62·4)	24·1 (23·4–24·9)	4·7 (4·3–5·1)	0·3 (0·2–0·4)	0·0 (0·0–0·0)	2·07 (2·05–2·09)	1·04 (1·03–1·06)	0·45 (0·44–0·46)
1986	0·0 (0·0–0·1)	47·4 (46·4–48·5)	161·7 (159·5–163·7)	116·3 (115·0–117·7)	63·5 (62·5–64·5)	25·6 (24·9–26·3)	5·1 (4·7–5·5)	0·3 (0·2–0·4)	0·0 (0·0–0·0)	2·1 (2·08–2·12)	1·05 (1·03–1·06)	0·47 (0·46–0·48)
1987	0·0 (0·0–0·1)	48·3 (47·3–49·4)	161·5 (159·4–163·8)	112·8 (111·5–114·1)	61·1 (60·1–62·1)	24·7 (24·0–25·4)	5·2 (4·8–5·7)	0·3 (0·2–0·3)	0·0 (0·0–0·0)	2·07 (2·05–2·09)	1·05 (1·04–1·06)	0·46 (0·45–0·46)
1988	0·0 (0·0–0·1)	49·6 (48·5–50·7)	156·7 (154·7–158·9)	104·6 (103·2–105·9)	55·4 (54·5–56·4)	22·1 (21·4–22·7)	5·0 (4·7–5·4)	0·3 (0·2–0·3)	0·0 (0·0–0·0)	1·97 (1·95–1·99)	1·03 (1·02–1·04)	0·41 (0·41–0·42)
1989	0·0 (0·0–0·1)	51·9 (50·9–52·8)	153·8 (151·5–156·0)	97·6 (96·4–98·9)	50·0 (49·0–51·0)	19·6 (19·0–20·2)	4·5 (4·1–4·8)	0·2 (0·2–0·3)	0·0 (0·0–0·0)	1·89 (1·87–1·91)	1·03 (1·02–1·04)	0·37 (0·36–0·38)
1990	0·0 (0·0–0·1)	53·1 (52·2–54·1)	149·2 (147·0–151·4)	91·0 (89·8–92·3)	44·8 (43·9–45·8)	17·2 (16·7–17·8)	3·8 (3·5–4·2)	0·2 (0·2–0·3)	0·0 (0·0–0·0)	1·8 (1·78–1·82)	1·01 (1·0–1·03)	0·33 (0·32–0·34)
1991	0·0 (0·0–0·1)	51·8 (50·9–52·8)	139·6 (137·5–141·8)	82·3 (81·1–83·6)	39·0 (38·0–40·0)	15·0 (14·3–15·6)	3·3 (3·0–3·6)	0·2 (0·1–0·2)	0·0 (0·0–0·0)	1·66 (1·64–1·67)	0·96 (0·94–0·97)	0·29 (0·28–0·3)
1992	0·0 (0·0–0·1)	48·3 (47·4–49·3)	127·9 (125·8–129·9)	74·6 (73·4–75·8)	33·7 (32·8–34·7)	13·0 (12·3–13·7)	3·0 (2·6–3·3)	0·1 (0·1–0·2)	0·0 (0·0–0·0)	1·5 (1·48–1·52)	0·88 (0·87–0·89)	0·25 (0·24–0·26)
1993	0·0 (0·0–0·1)	46·0 (45·1–46·9)	121·3 (119·1–123·2)	71·6 (70·5–72·8)	31·1 (30·3–32·0)	11·6 (11·0–12·2)	2·6 (2·4–2·9)	0·1 (0·1–0·2)	0·0 (0·0–0·0)	1·42 (1·41–1·44)	0·84 (0·82–0·85)	0·23 (0·22–0·23)
1994	0·0 (0·0–0·1)	44·1 (43·2–45·0)	117·8 (115·8–119·7)	71·4 (70·2–72·6)	30·7 (29·9–31·5)	11·0 (10·5–11·5)	2·4 (2·2–2·8)	0·1 (0·1–0·2)	0·0 (0·0–0·0)	1·39 (1·37–1·4)	0·81 (0·8–0·82)	0·22 (0·22–0·23)
1995	0·0 (0·0–0·1)	40·8 (40·0–41·7)	108·4 (106·5–110·2)	68·9 (67·7–70·0)	30·5 (29·8–31·3)	11·0 (10·5–11·4)	2·4 (2·1–2·7)	0·1 (0·1–0·2)	0·0 (0·0–0·0)	1·31 (1·29–1·33)	0·75 (0·74–0·76)	0·22 (0·22–0·23)
1996	0·0 (0·0–0·1)	37·9 (37·2–38·8)	99·2 (97·6–100·9)	66·4 (65·2–67·5)	30·6 (29·9–31·3)	10·9 (10·4–11·4)	2·3 (2·1–2·7)	0·1 (0·1–0·2)	0·0 (0·0–0·0)	1·24 (1·22–1·25)	0·69 (0·68–0·7)	0·22 (0·21–0·22)
1997	0·0 (0·0–0·1)	35·1 (34·4–35·9)	96·0 (94·4–97·7)	66·2 (65·0–67·4)	31·3 (30·6–32·1)	11·0 (10·5–11·4)	2·3 (2·0–2·6)	0·1 (0·1–0·2)	0·0 (0·0–0·0)	1·21 (1·19–1·23)	0·66 (0·65–0·67)	0·22 (0·22–0·23)
1998	0·0 (0·0–0·1)	31·1 (30·4–31·8)	92·3 (90·6–94·1)	65·7 (64·7–66·8)	32·0 (31·3–32·7)	11·2 (10·7–11·6)	2·3 (2·0–2·7)	0·1 (0·1–0·2)	0·0 (0·0–0·0)	1·17 (1·16–1·19)	0·62 (0·61–0·63)	0·23 (0·22–0·23)
1999	0·0 (0·0–0·1)	27·9 (27·2–28·6)	89·2 (87·6–90·9)	65·9 (64·7–67·2)	33·1 (32·4–33·8)	11·5 (11·0–12·0)	2·4 (2·1–2·7)	0·1 (0·1–0·2)	0·0 (0·0–0·0)	1·15 (1·13–1·17)	0·59 (0·58–0·6)	0·24 (0·23–0·24)
2000	0·0 (0·0–0·1)	27·1 (26·5–27·8)	89·0 (87·5–90·6)	68·2 (67·1–69·4)	35·3 (34·5–36·2)	12·2 (11·8–12·7)	2·4 (2·2–2·7)	0·1 (0·1–0·2)	0·0 (0·0–0·0)	1·17 (1·16–1·19)	0·58 (0·57–0·59)	0·25 (0·24–0·26)
2001	0·0 (0·0–0·1)	27·1 (26·5–27·8)	89·1 (87·7–90·7)	71·2 (70·2–72·4)	38·1 (37·3–38·9)	13·3 (12·8–13·9)	2·5 (2·2–2·8)	0·1 (0·1–0·2)	0·0 (0·0–0·0)	1·21 (1·19–1·22)	0·58 (0·57–0·59)	0·27 (0·26–0·28)
2002	0·0 (0·0–0·1)	26·9 (26·3–27·5)	90·0 (88·6–91·5)	74·6 (73·6–75·7)	40·9 (39·9–41·8)	14·5 (14·0–15·1)	2·6 (2·3–2·9)	0·1 (0·1–0·2)	0·0 (0·0–0·0)	1·25 (1·23–1·26)	0·58 (0·58–0·59)	0·29 (0·28–0·3)
2003	0·0 (0·0–0·1)	26·6 (26·0–27·2)	89·5 (88·1–90·9)	77·1 (76·1–78·3)	43·0 (42·0–43·9)	15·7 (15·2–16·3)	2·7 (2·5–3·0)	0·1 (0·1–0·2)	0·0 (0·0–0·0)	1·27 (1·26–1·29)	0·58 (0·57–0·59)	0·31 (0·3–0·31)
2004	0·0 (0·0–0·1)	26·4 (25·7–27·0)	86·3 (85·0–87·7)	77·6 (76·4–78·8)	44·0 (43·0–45·0)	16·7 (16·1–17·3)	3·0 (2·7–3·3)	0·2 (0·1–0·2)	0·0 (0·0–0·0)	1·27 (1·26–1·28)	0·56 (0·56–0·57)	0·32 (0·31–0·33)
2005	0·0 (0·0–0·1)	26·5 (25·9–27·2)	83·4 (82·1–84·8)	77·7 (76·6–79·0)	45·0 (43·9–46·0)	17·6 (17·1–18·2)	3·2 (2·9–3·5)	0·2 (0·1–0·2)	0·0 (0·0–0·0)	1·27 (1·25–1·28)	0·55 (0·54–0·56)	0·33 (0·32–0·34)
2006	0·0 (0·0–0·1)	27·1 (26·3–27·9)	83·9 (82·4–85·3)	81·9 (80·8–83·0)	48·9 (47·8–49·9)	19·7 (19·1–20·4)	3·5 (3·2–3·8)	0·2 (0·1–0·2)	0·0 (0·0–0·0)	1·33 (1·31–1·34)	0·56 (0·55–0·56)	0·36 (0·35–0·37)
2007	0·0 (0·0–0·1)	27·8 (26·9–28·8)	85·6 (84·1–87·3)	87·8 (86·7–89·0)	54·6 (53·6–55·7)	22·6 (21·9–23·3)	3·9 (3·6–4·3)	0·2 (0·1–0·3)	0·0 (0·0–0·0)	1·41 (1·39–1·43)	0·57 (0·56–0·58)	0·41 (0·4–0·41)
2008	0·0 (0·0–0·1)	28·0 (27·1–28·9)	86·7 (85·2–88·3)	91·6 (90·4–92·8)	58·7 (57·6–59·9)	24·8 (24·0–25·5)	4·4 (4·1–4·8)	0·2 (0·1–0·3)	0·0 (0·0–0·0)	1·47 (1·45–1·49)	0·57 (0·56–0·58)	0·44 (0·43–0·45)
2009	0·0 (0·0–0·1)	27·2 (26·4–28·1)	85·8 (84·3–87·5)	94·6 (93·4–95·8)	61·8 (60·7–63·0)	26·6 (25·8–27·4)	5·0 (4·6–5·4)	0·2 (0·1–0·3)	0·0 (0·0–0·0)	1·51 (1·49–1·53)	0·57 (0·56–0·58)	0·47 (0·46–0·48)
2010	0·0 (0·0–0·1)	26·5 (25·6–27·4)	85·0 (83·3–86·6)	96·1 (94·9–97·5)	63·9 (62·7–65·1)	28·3 (27·5–29·2)	5·5 (5·1–5·9)	0·2 (0·2–0·3)	0·0 (0·0–0·0)	1·53 (1·51–1·55)	0·56 (0·55–0·57)	0·49 (0·48–0·5)
2011	0·0 (0·0–0·1)	26·5 (25·6–27·4)	87·5 (86·0–89·2)	98·8 (97·5–100·2)	67·0 (65·7–68·2)	30·3 (29·4–31·1)	5·9 (5·6–6·3)	0·2 (0·2–0·3)	0·0 (0·0–0·0)	1·58 (1·56–1·6)	0·57 (0·56–0·58)	0·52 (0·51–0·53)
2012	0·0 (0·0–0·1)	26·3 (25·4–27·3)	89·5 (87·8–91·3)	102·1 (100·7–103·7)	69·7 (68·4–71·1)	32·3 (31·4–33·1)	6·4 (6·0–6·7)	0·2 (0·2–0·3)	0·0 (0·0–0·0)	1·63 (1·61–1·66)	0·58 (0·57–0·59)	0·54 (0·53–0·55)
2013	0·0 (0·0–0·1)	26·0 (25·1–27·1)	88·1 (85·9–90·4)	104·2 (102·8–105·8)	72·4 (71·0–73·8)	34·2 (33·3–35·2)	6·8 (6·4–7·2)	0·2 (0·2–0·3)	0·0 (0·0–0·0)	1·66 (1·63–1·69)	0·57 (0·56–0·59)	0·57 (0·56–0·58)
2014	0·0 (0·0–0·1)	25·9 (24·6–27·4)	86·1 (82·9–89·3)	105·7 (103·2–108·1)	75·5 (73·2–77·9)	35·4 (33·7–37·2)	6·8 (6·2–7·6)	0·2 (0·2–0·3)	0·0 (0·0–0·0)	1·68 (1·63–1·73)	0·56 (0·54–0·58)	0·59 (0·57–0·61)
2015	0·0 (0·0–0·1)	25·6 (23·5–28·1)	83·8 (77·0–91·1)	104·1 (99·2–109·1)	75·4 (70·4–80·7)	34·3 (30·6–38·3)	6·5 (5·4–7·6)	0·2 (0·2–0·3)	0·0 (0·0–0·0)	1·65 (1·54–1·77)	0·55 (0·5–0·59)	0·58 (0·53–0·63)
2016	0·0 (0·0–0·1)	25·3 (22·3–28·8)	81·5 (71·6–92·4)	101·0 (94·1–108·5)	73·1 (65·8–81·2)	32·2 (26·9–38·4)	6·0 (4·8–7·5)	0·2 (0·2–0·3)	0·0 (0·0–0·0)	1·6 (1·43–1·78)	0·53 (0·47–0·61)	0·56 (0·49–0·64)
2017	0·0 (0·0–0·1)	25·3 (21·8–29·4)	80·1 (68·1–94·0)	98·6 (90·2–107·8)	70·6 (61·7–80·6)	30·4 (24·2–37·8)	5·6 (4·3–7·3)	0·2 (0·2–0·3)	0·0 (0·0–0·0)	1·55 (1·35–1·79)	0·53 (0·45–0·62)	0·53 (0·45–0·63)

Supplemental Results Table 5: Age-specific fertility rates per 1000, cumulative total fertility up to maternal age 25 (TFU25), cumulative total fertility from maternal age 30 to age 55 (TFO30), and cumulative total fertility, Belarus, 1950-2017

Year	10-14	15-19	20-24	25-29	30-34	35-39	40-44	45-49	50-54	TFR	TFU25	TFO30
1950	0·0 (0·0–0·0)	17·4 (15·1–20·2)	139·0 (130·1–149·5)	172·5 (164·2–182·0)	121·2 (113·3–130·5)	76·9 (70·1–84·2)	39·7 (35·2–44·5)	8·3 (6·7–10·0)	0·2 (0·2–0·2)	2·88 (2·72–3·05)	0·78 (0·73–0·85)	1·23 (1·16–1·31)
1951	0·0 (0·0–0·0)	16·6 (14·5–19·0)	134·0 (126·9–142·1)	171·8 (164·1–180·5)	119·1 (111·9–127·4)	76·1 (70·1–82·2)	39·2 (35·2–43·5)	7·8 (6·5–9·3)	0·2 (0·1–0·2)	2·82 (2·7–2·96)	0·75 (0·71–0·81)	1·21 (1·15–1·28)
1952	0·0 (0·0–0·0)	16·1 (14·1–18·2)	130·2 (123·2–137·4)	170·8 (163·7–178·4)	118·3 (111·7–125·3)	74·6 (69·1–80·1)	37·8 (34·1–41·8)	7·3 (6·1–8·7)	0·1 (0·1–0·1)	2·78 (2·66–2·9)	0·73 (0·69–0·78)	1·19 (1·14–1·25)
1953	0·0 (0·0–0·0)	15·9 (14·0–17·9)	128·5 (121·6–135·5)	170·3 (163·2–177·5)	118·9 (112·7–125·6)	72·9 (67·5–78·4)	35·8 (32·2–39·5)	6·7 (5·7–8·0)	0·1 (0·1–0·1)	2·75 (2·65–2·84)	0·72 (0·68–0·76)	1·17 (1·12–1·22)
1954	0·0 (0·0–0·0)	15·9 (14·2–17·9)	128·7 (121·8–135·5)	169·9 (163·0–176·9)	120·4 (114·0–126·9)	71·0 (65·8–76·5)	33·2 (29·9–36·6)	6·2 (5·3–7·3)	0·1 (0·1–0·1)	2·73 (2·64–2·82)	0·72 (0·68–0·76)	1·15 (1·1–1·21)
1955	0·0 (0·0–0·0)	16·1 (14·3–18·0)	130·2 (123·2–137·1)	169·0 (162·0–175·9)	121·7 (115·4–128·1)	69·2 (64·0–74·7)	30·4 (27·3–33·4)	5·7 (4·8–6·7)	0·1 (0·1–0·1)	2·71 (2·63–2·8)	0·73 (0·69–0·77)	1·14 (1·09–1·19)
1956	0·0 (0·0–0·0)	16·3 (14·5–18·2)	132·6 (125·5–139·8)	167·7 (160·5–174·3)	122·4 (115·9–129·4)	67·5 (62·3–73·0)	27·7 (24·9–30·7)	5·2 (4·3–6·1)	0·1 (0·1–0·1)	2·7 (2·62–2·78)	0·74 (0·7–0·78)	1·11 (1·06–1·17)
1957	0·0 (0·0–0·0)	16·5 (14·6–18·3)	135·2 (128·1–142·6)	165·4 (158·3–172·2)	121·9 (115·2–128·9)	65·8 (60·6–71·0)	25·4 (22·7–28·4)	4·7 (3·8–5·6)	0·1 (0·1–0·1)	2·67 (2·59–2·76)	0·76 (0·72–0·8)	1·09 (1·04–1·14)
1958	0·0 (0·0–0·0)	16·5 (14·7–18·3)	137·3 (130·0–144·7)	162·0 (155·0–169·2)	119·9 (113·0–126·7)	64·0 (58·7–69·4)	23·5 (21·0–26·4)	4·1 (3·4–5·0)	0·1 (0·1–0·1)	2·64 (2·55–2·73)	0·77 (0·73–0·81)	1·06 (1·0–1·11)
1959	0·0 (0·0–0·0)	16·4 (14·7–18·2)	138·6 (131·2–146·3)	157·8 (150·4–165·0)	116·3 (109·4–123·2)	62·1 (57·2–67·5)	22·0 (19·4–24·7)	3·7 (3·0–4·4)	0·1 (0·1–0·1)	2·58 (2·49–2·68)	0·78 (0·74–0·81)	1·02 (0·96–1·08)
1960	0·0 (0·0–0·0)	16·2 (14·5–18·0)	139·0 (131·1–146·7)	153·1 (146·0–160·6)	111·7 (105·1–118·2)	60·1 (55·0–65·6)	20·7 (18·5–23·3)	3·2 (2·6–3·9)	0·1 (0·1–0·1)	2·52 (2·42–2·62)	0·78 (0·74–0·82)	0·98 (0·92–1·04)
1961	0·0 (0·0–0·0)	15·9 (14·3–17·7)	139·1 (131·4–146·8)	148·2 (140·7–155·3)	106·7 (100·1–113·1)	57·8 (52·8–63·1)	19·6 (17·1–22·0)	2·9 (2·3–3·5)	0·1 (0·1–0·1)	2·45 (2·35–2·55)	0·78 (0·73–0·82)	0·94 (0·88–0·99)
1962	0·0 (0·0–0·0)	15·8 (14·1–17·5)	139·5 (131·8–147·1)	143·7 (135·9–151·0)	102·0 (95·3–108·7)	55·4 (50·3–60·5)	18·4 (16·1–20·9)	2·5 (2·0–3·1)	0·0 (0·0–0·1)	2·39 (2·29–2·49)	0·78 (0·73–0·82)	0·89 (0·84–0·95)
1963	0·0 (0·0–0·0)	15·8 (14·2–17·4)	140·4 (132·7–147·6)	139·6 (132·4–146·6)	97·7 (91·2–104·3)	52·8 (47·7–57·7)	17·3 (15·0–19·7)	2·3 (1·8–2·8)	0·0 (0·0–0·0)	2·33 (2·23–2·43)	0·78 (0·73–0·82)	0·85 (0·8–0·9)
1964	0·0 (0·0–0·0)	16·0 (14·4–17·7)	141·8 (134·0–149·8)	136·2 (129·2–143·2)	94·3 (88·0–101·0)	50·0 (45·0–55·0)	16·1 (13·9–18·6)	2·1 (1·6–2·6)	0·0 (0·0–0·0)	2·28 (2·18–2·38)	0·79 (0·74–0·83)	0·81 (0·76–0·87)
1965	0·0 (0·0–0·0)	16·5 (14·9–18·2)	144·3 (136·0–152·3)	133·7 (126·7–140·8)	91·9 (85·2–99·1)	46·9 (42·0–51·9)	14·8 (12·8–17·2)	1·9 (1·5–2·4)	0·0 (0·0–0·0)	2·25 (2·15–2·35)	0·8 (0·76–0·85)	0·78 (0·73–0·83)
1966	0·0 (0·0–0·0)	17·4 (15·7–19·1)	148·2 (139·9–156·7)	132·0 (124·9–139·0)	90·2 (83·4–97·2)	45·3 (40·6–50·4)	14·0 (12·0–16·2)	1·8 (1·4–2·2)	0·0 (0·0–0·0)	2·24 (2·14–2·35)	0·83 (0·78–0·87)	0·76 (0·71–0·81)
1967	0·0 (0·0–0·0)	18·5 (16·8–20·4)	152·9 (144·6–161·2)	131·2 (124·6–138·0)	88·9 (82·4–95·8)	45·2 (40·3–50·2)	13·7 (11·8–15·8)	1·6 (1·3–2·0)	0·0 (0·0–0·0)	2·26 (2·16–2·36)	0·86 (0·81–0·9)	0·75 (0·7–0·79)
1968	0·0 (0·0–0·0)	19·8 (18·1–21·7)	157·9 (149·5–166·3)	130·9 (124·4–137·7)	87·3 (80·9–94·0)	44·8 (40·0–50·2)	13·3 (11·4–15·4)	1·4 (1·1–1·8)	0·0 (0·0–0·0)	2·28 (2·19–2·37)	0·89 (0·85–0·93)	0·73 (0·69–0·78)
1969	0·0 (0·0–0·0)	21·2 (19·4–23·1)	162·4 (154·0–171·1)	130·5 (123·9–137·2)	85·2 (78·7–91·9)	43·9 (39·2–49·2)	12·8 (10·9–14·8)	1·3 (1·0–1·6)	0·0 (0·0–0·0)	2·29 (2·21–2·37)	0·92 (0·88–0·96)	0·72 (0·67–0·77)
1970	0·0 (0·0–0·0)	22·4 (20·5–24·3)	165·8 (157·2–174·9)	130·1 (123·7–136·6)	82·5 (76·1–88·9)	42·3 (37·8–47·9)	12·0 (10·3–14·0)	1·1 (0·9–1·4)	0·0 (0·0–0·0)	2·28 (2·21–2·36)	0·94 (0·9–0·99)	0·69 (0·65–0·74)
1971	0·0 (0·0–0·0)	23·4 (21·4–25·5)	167·7 (159·5–176·7)	129·9 (123·4–136·5)	79·2 (73·2–85·2)	40·2 (35·6–45·4)	11·1 (9·5–12·9)	1·0 (0·8–1·2)	0·0 (0·0–0·0)	2·26 (2·19–2·34)	0·96 (0·91–1·0)	0·66 (0·61–0·7)
1972	0·0 (0·0–0·0)	24·3 (22·2–26·5)	168·5 (160·5–176·9)	129·5 (123·1–135·9)	75·7 (70·1–81·5)	37·6 (33·4–42·3)	10·2 (8·7–12·0)	0·8 (0·6–1·0)	0·0 (0·0–0·0)	2·23 (2·16–2·31)	0·96 (0·92–1·01)	0·62 (0·58–0·67)
1973	0·0 (0·0–0·0)	25·2 (23·0–27·5)	168·7 (161·6–176·5)	128·7 (122·5–135·2)	72·4 (67·0–77·9)	34·9 (30·8–39·3)	9·3 (7·9–11·1)	0·7 (0·6–0·9)	0·0 (0·0–0·0)	2·2 (2·12–2·28)	0·97 (0·93–1·01)	0·59 (0·55–0·63)
1974	0·0 (0·0–0·0)	26·2 (24·1–28·6)	168·8 (161·5–176·1)	127·4 (121·0–133·7)	69·3 (64·2–74·5)	32·1 (28·5–36·2)	8·5 (7·2–10·1)	0·6 (0·5–0·8)	0·0 (0·0–0·0)	2·17 (2·08–2·25)	0·97 (0·93–1·02)	0·55 (0·51–0·6)
1975	0·0 (0·0–0·0)	27·2 (25·0–29·7)	169·1 (161·8–176·4)	125·8 (119·4–131·6)	66·8 (62·0–71·6)	29·6 (26·3–33·1)	7·9 (6·7–9·2)	0·6 (0·4–0·7)	0·0 (0·0–0·0)	2·13 (2·05–2·22)	0·98 (0·93–1·03)	0·52 (0·48–0·57)
1976	0·0 (0·0–0·0)	28·3 (26·0–30·7)	169·8 (162·4–177·3)	123·9 (117·8–129·8)	64·9 (60·4–69·8)	27·3 (24·5–30·5)	7·2 (6·1–8·5)	0·5 (0·4–0·7)	0·0 (0·0–0·0)	2·11 (2·02–2·2)	0·99 (0·94–1·04)	0·5 (0·46–0·54)
1977	0·0 (0·0–0·0)	29·5 (27·2–32·1)	170·9 (163·6–177·9)	122·2 (116·5–128·1)	63·5 (59·0–68·1)	25·4 (22·8–28·2)	6·7 (5·7–7·8)	0·4 (0·3–0·6)	0·0 (0·0–0·0)	2·09 (2·01–2·18)	1·0 (0·96–1·05)	0·48 (0·44–0·52)
1978	0·0 (0·0–0·0)	30·7 (28·3–33·3)	172·3 (165·6–179·3)	120·9 (115·5–126·5)	62·2 (57·8–66·7)	23·9 (21·5–26·6)	6·1 (5·2–7·1)	0·4 (0·3–0·5)	0·0 (0·0–0·0)	2·08 (2·0–2·17)	1·02 (0·97–1·06)	0·46 (0·43–0·5)
1979	0·0 (0·0–0·0)	31·8 (29·3–34·3)	173·9 (167·4–180·7)	119·9 (114·5–125·3)	61·0 (56·9–65·4)	22·8 (20·4–25·3)	5·6 (4·8–6·5)	0·4 (0·3–0·5)	0·0 (0·0–0·0)	2·08 (2·0–2·16)	1·03 (0·99–1·07)	0·45 (0·42–0·48)
1980	0·0 (0·0–0·0)	32·7 (30·0–35·3)	175·5 (168·6–182·6)	119·4 (114·0–124·7)	60·1 (55·9–64·6)	22·2 (19·9–24·7)	5·3 (4·5–6·1)	0·3 (0·3–0·4)	0·0 (0·0–0·0)	2·08 (2·01–2·15)	1·04 (1·0–1·08)	0·44 (0·41–0·47)
1981	0·0 (0·0–0·0)	33·8 (31·1–36·4)	177·5 (170·2–185·0)	119·5 (114·2–125·0)	59·5 (55·6–63·8)	22·3 (20·0–24·8)	5·0 (4·3–5·8)	0·3 (0·2–0·4)	0·0 (0·0–0·0)	2·09 (2·02–2·16)	1·06 (1·02–1·1)	0·44 (0·41–0·46)
1982	0·0 (0·0–0·0)	34·9 (32·1–37·5)	179·6 (172·4–186·9)	119·7 (114·6–124·7)	59·2 (55·2–63·3)	22·6 (20·2–25·1)	4·8 (4·2–5·6)	0·3 (0·2–0·3)	0·0 (0·0–0·0)	2·11 (2·04–2·17)	1·07 (1·04–1·11)	0·43 (0·41–0·46)
1983	0·0 (0·0–0·0)	36·0 (33·2–38·9)	181·4 (174·8–188·2)	119·1 (114·3–124·2)	58·6 (54·8–62·3)	22·7 (20·3–25·3)	4·7 (4·1–5·5)	0·2 (0·2–0·3)	0·0 (0·0–0·0)	2·11 (2·05–2·17)	1·09 (1·05–1·13)	0·43 (0·41–0·46)
1984	0·0 (0·0–0·0)	36·8 (34·0–39·8)	182·0 (175·3–188·5)	117·6 (112·6–122·6)	57·6 (53·9–61·4)	22·5 (20·3–25·2)	4·7 (4·0–5·4)	0·2 (0·2–0·3)	0·0 (0·0–0·0)	2·11 (2·05–2·16)	1·09 (1·05–1·13)	0·43 (0·4–0·45)
1985	0·0 (0·0–0·0)	37·4 (34·4–40·3)	181·3 (174·3–187·7)	115·2 (110·1–120·4)	56·2 (52·6–60·0)	22·0 (19·8–24·4)	4·6 (3·9–5·3)	0·2 (0·2–0·3)	0·0 (0·0–0·0)	2·08 (2·03–2·14)	1·09 (1·06–1·13)	0·41 (0·39–0·44)
1986	0·0 (0·0–0·0)	37·8 (34·7–40·8)	179·4 (172·1–186·0)	111·9 (106·9–116·8)	54·1 (50·5–57·8)	20·9 (18·9–23·2)	4·5 (3·8–5·1)	0·2 (0·2–0·2)	0·0 (0·0–0·0)	2·04 (1·99–2·1)	1·09 (1·05–1·12)	0·4 (0·38–0·42)
1987	0·0 (0·0–0·0)	38·1 (34·9–41·3)	176·3 (169·4–183·4)	107·8 (102·9–112·6)	51·5 (48·4–54·8)	19·4 (17·7–21·3)	4·2 (3·6–4·9)	0·2 (0·1–0·2)	0·0 (0·0–0·0)	1·99 (1·93–2·04)	1·07 (1·04–1·11)	0·38 (0·36–0·4)
1988	0·0 (0·0–0·0)	38·4 (35·2–41·8)	172·3 (165·6–179·0)	103·0 (98·3–107·7)	48·6 (45·6–51·5)	17·6 (16·1–19·3)	3·9 (3·4–4·5)	0·2 (0·1–0·2)	0·0 (0·0–0·0)	1·92 (1·86–1·97)	1·05 (1·02–1·09)	0·35 (0·33–0·37)
1989	0·0 (0·0–0·0)	39·1 (35·9–42·5)	167·6 (161·2–174·2)	98·0 (93·5–102·3)	45·5 (42·6–48·4)	15·8 (14·5–17·4)	3·5 (3·0–4·1)	0·2 (0·1–0·2)	0·0 (0·0–0·0)	1·85 (1·79–1·9)	1·03 (1·0–1·07)	0·32 (0·31–0·34)
1990	0·0 (0·0–0·1)	39·5 (36·2–43·1)	162·0 (155·1–168·8)	92·9 (88·6–97·1)	42·3 (39·6–45·2)	14·1 (12·8–15·6)	3·1 (2·7–3·6)	0·2 (0·1–0·2)	0·0 (0·0–0·0)	1·77 (1·72–1·83)	1·01 (0·97–1·05)	0·3 (0·28–0·31)
1991	0·0 (0·0–0·0)	39·2 (36·0–42·7)	155·5 (148·8–162·8)	88·3 (84·0–92·5)	39·3 (36·7–42·0)	12·3 (11·2–13·6)	2·7 (2·3–3·1)	0·2 (0·1–0·2)	0·0 (0·0–0·0)	1·69 (1·64–1·74)	0·97 (0·94–1·01)	0·27 (0·26–0·29)
1992	0·0 (0·0–0·0)	38·1 (34·9–41·7)	148·1 (141·2–155·0)	84·1 (79·7–88·2)	36·4 (34·0–39·0)	11·4 (10·3–12·6)	2·5 (2·1–2·8)	0·1 (0·1–0·2)	0·0 (0·0–0·0)	1·6 (1·55–1·66)	0·93 (0·89–0·97)	0·25 (0·24–0·27)
1993	0·0 (0·0–0·0)	36·5 (33·4–39·7)	140·5 (133·8–147·1)	80·6 (76·5–84·8)	33·8 (31·5–36·3)	11·3 (10·2–12·5)	2·4 (2·1–2·7)	0·1 (0·1–0·2)	0·0 (0·0–0·0)	1·53 (1·48–1·57)	0·88 (0·85–0·92)	0·24 (0·22–0·25)
1994	0·0 (0·0–0·0)	34·4 (31·6–37·4)	133·0 (126·8–139·2)	77·8 (74·1–82·1)	31·5 (29·4–33·9)	11·1 (10·1–12·2)	2·4 (2·0–2·7)	0·1 (0·1–0·2)	0·0 (0·0–0·0)	1·45 (1·41–1·5)	0·84 (0·8–0·87)	0·23 (0·21–0·24)
1995	0·0 (0·0–0·0)	32·0 (29·3–35·0)	126·2 (120·2–132·3)	75·6 (71·9–79·7)	29·7 (27·7–31·9)	11·0 (10·0–12·1)	2·3 (2·0–2·6)	0·1 (0·1–0·1)	0·0 (0·0–0·0)	1·38 (1·34–1·43)	0·79 (0·76–0·82)	0·22 (0·2–0·23)
1996	0·0 (0·0–0·0)	29·6 (27·1–32·3)	120·2 (114·1–126·2)	74·4 (70·7–78·2)	29·5 (27·5–31·7)	10·9 (9·9–12·0)	2·2 (2·0–2·6)	0·1 (0·1–0·1)	0·0 (0·0–0·0)	1·33 (1·29–1·38)	0·75 (0·71–0·78)	0·21 (0·2–0·23)
1997	0·0 (0·0–0·0)	27·2 (25·0–29·6)	115·3 (109·2–121·2)	74·0 (70·4–77·7)	31·1 (29·0–33·3)	11·0 (9·9–12·1)	2·2 (1·9–2·5)	0·1 (0·1–0·1)	0·0 (0·0–0·0)	1·3 (1·26–1·35)	0·71 (0·68–0·74)	0·22 (0·21–0·23)
1998	0·0 (0·0–0·0)	25·3 (23·2–27·6)	111·3 (105·3–117·1)	74·2 (70·6–78·0)	32·9 (30·7–35·4)	11·2 (10·1–12·2)	2·2 (1·9–2·5)	0·1 (0·1–0·1)	0·0 (0·0–0·0)	1·29 (1·24–1·33)	0·68 (0·65–0·71)	0·23 (0·22–0·25)
1999	0·0 (0·0–0·0)	24·0 (22·0–26·1)	108·1 (102·3–113·8)	74·9 (71·1–78·9)	34·8 (32·5–37·3)	11·4 (10·3–12·5)	2·2 (1·9–2·5)	0·1 (0·1–0·1)	0·0 (0·0–0·0)	1·28 (1·23–1·32)	0·66 (0·63–0·69)	0·24 (0·23–0·26)
2000	0·0 (0·0–0·0)	23·0 (21·2–25·1)	105·2 (99·5–111·0)	75·8 (71·8–80·2)	36·6 (34·0–39·4)	11·8 (10·6–13·0)	2·2 (2·0–2·5)	0·1 (0·1–0·1)	0·0 (0·0–0·0)	1·27 (1·23–1·32)	0·64 (0·61–0·67)	0·25 (0·24–0·27)
2001	0·0 (0·0–0·0)	22·1 (20·3–24·2)	102·6 (96·6–108·7)	77·0 (72·7–81·4)	38·5 (35·7–41·5)	12·3 (11·1–13·6)	2·3 (2·0–2·6)	0·1 (0·1–0·1)	0·0 (0·0–0·0)	1·27 (1·23–1·32)	0·62 (0·59–0·66)	0·27 (0·25–0·28)
2002	0·0 (0·0–0·0)	21·3 (19·4–23·4)	100·2 (94·1–106·3)	78·6 (74·1–83·2)	40·5 (37·5–43·8)	13·0 (11·7–14·5)	2·3 (2·0–2·7)	0·1 (0·1–0·1)	0·0 (0·0–0·0)	1·28 (1·23–1·33)	0·61 (0·58–0·64)	0·28 (0·26–0·3)
2003	0·0 (0·0–0·0)	20·6 (18·6–22·6)	98·3 (92·3–104·3)	80·7 (76·0–85·7)	42·7 (39·6–46·2)	13·9 (12·5–15·4)	2·5 (2·1–2·8)	0·1 (0·1–0·1)	0·0 (0·0–0·0)	1·29 (1·24–1·34)	0·59 (0·56–0·63)	0·3 (0·28–0·32)
2004	0·0 (0·0–0·0)	20·2 (18·2–22·3)	97·0 (91·0–103·1)	83·5 (78·6–88·7)	45·2 (41·7–49·0)	14·9 (13·4–16·6)	2·6 (2·2–3·0)	0·1 (0·1–0·1)	0·0 (0·0–0·0)	1·32 (1·26–1·37)	0·59 (0·55–0·62)	0·31 (0·29–0·34)
2005	0·0 (0·0–0·0)	20·2 (18·2–22·4)	96·2 (90·3–102·7)	86·5 (81·5–91·9)	47·9 (44·1–51·9)	16·2 (14·6–18·0)	2·7 (2·4–3·2)	0·1 (0·1–0·1)	0·0 (0·0–0·0)	1·35 (1·29–1·41)	0·58 (0·55–0·62)	0·33 (0·31–0·36)
2006	0·0 (0·0–0·0)	20·5 (18·4–22·6)	95·9 (89·9–102·2)	89·8 (84·4–95·3)	50·7 (46·6–55·0)	17·6 (15·9–19·7)	2·9 (2·6–3·4)	0·1 (0·1–0·1)	0·0 (0·0–0·0)	1·39 (1·32–1·45)	0·58 (0·55–0·62)	0·36 (0·33–0·38)
2007	0·0 (0·0–0·0)	20·6 (18·5–23·0)	95·6 (89·2–102·5)	92·9 (87·3–98·4)	53·7 (49·3–58·1)	19·2 (17·1–21·4)	3·2 (2·8–3·6)	0·1 (0·1–0·1)	0·0 (0·0–0·0)	1·43 (1·35–1·5)	0·58 (0·54–0·62)	0·38 (0·35–0·41)
2008	0·0 (0·0–0·0)	20·7 (18·6–23·1)	95·3 (88·5–102·1)	95·9 (89·9–101·7)	56·6 (52·0–61·3)	20·8 (18·5–23·2)	3·4 (2·9–4·0)	0·1 (0·1–0·2)	0·0 (0·0–0·0)	1·46 (1·38–1·54)	0·58 (0·54–0·62)	0·41 (0·37–0·44)
2009	0·0 (0·0–0·0)	20·8 (18·6–23·3)	95·1 (88·2–102·1)	98·8 (93·0–105·2)	59·5 (54·6–64·5)	22·4 (19·9–24·9)	3·7 (3·1–4·3)	0·1 (0·1–0·2)	0·0 (0·0–0·0)	1·5 (1·42–1·59)	0·58 (0·54–0·63)	0·43 (0·39–0·47)
2010	0·0 (0·0–0·0)	21·0 (18·7–23·5)	95·2 (88·8–102·1)	101·8 (96·0–108·1)	62·2 (57·4–67·1)	24·1 (21·5–26·9)	4·0 (3·4–4·6)	0·1 (0·1–0·2)	0·0 (0·0–0·0)	1·54 (1·45–1·63)	0·58 (0·54–0·63)	0·45 (0·41–0·49)
2011	0·0 (0·0–0·0)	21·0 (18·6–23·7)	95·4 (88·7–102·2)	104·5 (98·1–111·0)	64·9 (60·1–69·9)	25·8 (23·1–28·9)	4·3 (3·6–4·9)	0·1 (0·1–0·2)	0·0 (0·0–0·0)	1·58 (1·49–1·67)	0·58 (0·54–0·63)	0·48 (0·44–0·52)
2012	0·0 (0·0–0·0)	20·9 (18·4–23·7)	94·8 (88·1–101·6)	106·3 (99·4–113·3)	67·6 (62·7–72·8)	27·6 (24·7–31·0)	4·6 (3·9–5·3)	0·1 (0·1–0·2)	0·0 (0·0–0·0)	1·61 (1·51–1·7)	0·58 (0·53–0·62)	0·5 (0·46–0·54)
2013	0·0 (0·0–0·0)	20·6 (18·1–23·3)	93·6 (86·2–100·7)	107·4 (100·1–114·6)	70·0 (64·8–75·6)	29·1 (25·9–32·6)	4·8 (4·1–5·6)	0·1 (0·1–0·2)	0·0 (0·0–0·0)	1·63 (1·52–1·73)	0·57 (0·53–0·62)	0·52 (0·48–0·56)
2014	0·0 (0·0–0·0)	20·0 (17·6–22·6)	91·7 (83·8–99·9)	107·7 (100·1–115·4)	72·1 (66·2–78·5)	30·3 (26·5–34·3)	5·0 (4·2–6·0)	0·2 (0·1–0·2)	0·0 (0·0–0·0)	1·63 (1·51–1·76)	0·56 (0·51–0·61)	0·54 (0·49–0·59)
2015	0·0 (0·0–0·0)	19·5 (17·0–22·1)	89·3 (81·1–98·5)	107·0 (99·1–115·0)	73·4 (66·8–80·6)	30·7 (26·4–35·4)	5·1 (4·2–6·1)	0·2 (0·1–0·2)	0·0 (0·0–0·0)	1·63 (1·48–1·78)	0·54 (0·49–0·6)	0·55 (0·49–0·61)
2016	0·0 (0·0–0·0)	19·2 (16·6–22·0)	86·5 (77·2–96·7)	105·5 (97·3–114·0)	73·3 (65·9–81·7)	30·1 (25·5–35·4)	4·9 (4·0–6·1)	0·2 (0·1–0·2)	0·0 (0·0–0·0)	1·6 (1·44–1·78)	0·53 (0·47–0·59)	0·54 (0·48–0·62)
2017	0·0 (0·0–0·0)	19·2 (16·5–22·2)	84·7 (74·5–96·3)	104·3 (95·9–113·6)	72·3 (64·1–81·4)	29·3 (24·3–35·3)	4·8 (3·8–6·1)	0·2 (0·1–0·2)	0·0 (0·0–0·0)	1·57 (1·4–1·78)	0·52 (0·46–0·59)	0·53 (0·46–0·62)

Supplemental Results Table 5: Age-specific fertility rates per 1000, cumulative total fertility up to maternal age 25 (TFU25), cumulative total fertility from maternal age 30 to age 55 (TFO30), and cumulative total fertility, Estonia, 1950-2017

Year	10-14	15-19	20-24	25-29	30-34	35-39	40-44	45-49	50-54	TFR	TFU25	TFO30
1950	0·0 (0·0–0·1)	19·2 (16·8–22·0)	131·2 (126·0–136·7)	139·0 (134·6–143·6)	95·9 (90·8–101·0)	52·0 (48·0–56·3)	20·4 (17·9–23·2)	1·5 (1·1–1·9)	0·0 (0·0–0·0)	2·3 (2·24–2·35)	0·75 (0·72–0·78)	0·85 (0·81–0·88)
1951	0·0 (0·0–0·1)	18·6 (16·1–21·2)	129·1 (124·0–134·0)	137·7 (133·8–141·8)	97·5 (92·7–102·0)	51·0 (47·0–55·2)	19·8 (17·5–22·3)	1·3 (1·0–1·7)	0·0 (0·0–0·0)	2·28 (2·22–2·33)	0·74 (0·71–0·77)	0·85 (0·81–0·88)
1952	0·0 (0·0–0·1)	18·0 (15·6–20·6)	126·3 (121·2–131·2)	135·4 (131·3–139·6)	97·3 (92·5–101·9)	50·0 (45·6–54·6)	18·9 (16·4–21·7)	1·2 (0·9–1·6)	0·0 (0·0–0·0)	2·24 (2·18–2·3)	0·72 (0·69–0·75)	0·84 (0·79–0·88)
1953	0·0 (0·0–0·1)	17·7 (15·2–20·3)	123·8 (119·1–128·9)	132·7 (128·5–136·9)	96·0 (91·2–101·1)	49·6 (44·8–54·4)	17·8 (14·9–20·8)	1·1 (0·8–1·4)	0·0 (0·0–0·0)	2·19 (2·13–2·26)	0·71 (0·68–0·74)	0·82 (0·77–0·87)
1954	0·0 (0·0–0·1)	17·6 (15·3–20·2)	122·1 (117·0–127·4)	129·3 (124·6–133·7)	92·5 (87·9–97·2)	49·2 (44·5–54·4)	16·6 (13·7–19·8)	1·0 (0·7–1·3)	0·0 (0·0–0·0)	2·14 (2·08–2·21)	0·7 (0·67–0·73)	0·8 (0·75–0·85)
1955	0·0 (0·0–0·1)	17·7 (15·3–20·3)	120·2 (115·1–125·5)	124·9 (120·5–129·1)	86·4 (82·0–91·1)	48·6 (43·9–53·8)	15·3 (12·6–18·5)	0·9 (0·7–1·2)	0·0 (0·0–0·0)	2·07 (2·01–2·13)	0·69 (0·66–0·72)	0·76 (0·71–0·8)
1956	0·0 (0·0–0·1)	18·1 (15·9–20·8)	119·7 (114·5–124·7)	121·0 (116·8–125·5)	80·7 (76·6–85·4)	47·3 (42·5–52·3)	14·2 (11·8–17·1)	0·8 (0·6–1·1)	0·0 (0·0–0·0)	2·01 (1·95–2·07)	0·69 (0·66–0·72)	0·71 (0·68–0·76)
1957	0·0 (0·0–0·1)	19·1 (17·0–21·7)	121·4 (116·6–126·3)	118·8 (114·6–123·0)	76·9 (73·2–81·0)	45·6 (41·5–50·3)	13·3 (11·1–15·8)	0·8 (0·6–1·1)	0·0 (0·0–0·0)	1·98 (1·92–2·04)	0·7 (0·67–0·74)	0·68 (0·65–0·72)
1958	0·0 (0·0–0·1)	20·2 (18·0–22·5)	123·9 (118·9–129·1)	118·0 (113·8–122·1)	75·1 (70·7–79·5)	43·9 (40·0–48·3)	12·7 (10·5–15·2)	0·7 (0·5–1·0)	0·0 (0·0–0·0)	1·97 (1·91–2·03)	0·72 (0·69–0·75)	0·66 (0·63–0·7)
1959	0·0 (0·0–0·1)	21·4 (19·0–23·9)	126·5 (121·4–131·8)	118·4 (114·0–122·2)	74·7 (70·5–79·3)	42·1 (38·0–46·7)	12·2 (9·9–15·0)	0·7 (0·5–1·0)	0·0 (0·0–0·0)	1·98 (1·93–2·03)	0·74 (0·71–0·77)	0·65 (0·61–0·69)
1960	0·0 (0·0–0·1)	22·1 (19·6–24·9)	128·8 (124·0–133·7)	119·0 (114·7–123·3)	74·4 (70·2–78·9)	39·9 (35·8–44·6)	11·8 (9·4–14·6)	0·6 (0·5–0·9)	0·0 (0·0–0·0)	1·98 (1·94–2·03)	0·76 (0·73–0·78)	0·63 (0·6–0·67)
1961	0·0 (0·0–0·1)	22·1 (19·6–24·9)	129·0 (123·8–134·2)	119·0 (114·9–123·2)	73·0 (68·8–77·2)	37·8 (33·7–42·2)	11·5 (9·0–14·6)	0·6 (0·4–0·8)	0·0 (0·0–0·0)	1·97 (1·92–2·01)	0·76 (0·73–0·78)	0·61 (0·58–0·65)
1962	0·0 (0·0–0·1)	21·8 (19·2–24·4)	126·0 (120·8–131·5)	118·4 (114·1–122·5)	70·4 (66·4–74·7)	36·2 (32·4–40·5)	11·2 (8·7–14·1)	0·5 (0·4–0·7)	0·0 (0·0–0·0)	1·92 (1·88–1·97)	0·74 (0·71–0·77)	0·59 (0·56–0·62)
1963	0·0 (0·0–0·1)	21·8 (19·5–24·3)	123·0 (118·4–127·9)	117·8 (113·8–122·2)	68·4 (64·1–72·6)	34·6 (30·6–38·7)	10·8 (8·3–13·5)	0·5 (0·3–0·7)	0·0 (0·0–0·0)	1·88 (1·84–1·93)	0·72 (0·7–0·75)	0·57 (0·54–0·6)
1964	0·0 (0·0–0·1)	22·5 (20·0–25·2)	123·9 (119·2–129·0)	116·8 (112·6–121·0)	67·7 (63·7–71·8)	32·9 (29·3–37·0)	10·1 (7·8–12·7)	0·5 (0·3–0·7)	0·0 (0·0–0·0)	1·87 (1·82–1·92)	0·73 (0·71–0·76)	0·56 (0·52–0·59)
1965	0·0 (0·0–0·1)	23·4 (21·0–26·2)	127·2 (122·0–132·4)	115·7 (111·7–119·9)	67·8 (63·7–72·0)	31·2 (27·5–35·1)	9·3 (7·2–11·9)	0·4 (0·3–0·7)	0·0 (0·0–0·0)	1·88 (1·82–1·93)	0·75 (0·73–0·78)	0·54 (0·51–0·58)
1966	0·0 (0·0–0·1)	24·9 (22·2–27·8)	132·5 (127·0–137·9)	116·5 (112·4–120·8)	69·0 (64·8–73·2)	29·6 (25·8–33·5)	8·5 (6·4–10·9)	0·4 (0·3–0·6)	0·0 (0·0–0·0)	1·91 (1·85–1·97)	0·79 (0·76–0·82)	0·54 (0·51–0·57)
1967	0·0 (0·0–0·1)	26·8 (23·9–29·9)	140·4 (135·0–145·6)	120·0 (115·8–124·1)	71·0 (66·9–75·4)	28·1 (24·5–32·3)	7·6 (5·7–10·0)	0·4 (0·3–0·6)	0·0 (0·0–0·0)	1·97 (1·92–2·03)	0·84 (0·8–0·87)	0·54 (0·51–0·57)
1968	0·1 (0·0–0·1)	28·8 (25·8–31·8)	148·6 (143·5–154·1)	125·2 (121·1–129·4)	73·0 (68·8–77·5)	28·6 (25·0–32·6)	7·4 (5·6–9·8)	0·4 (0·3–0·7)	0·0 (0·0–0·0)	2·06 (2·01–2·12)	0·89 (0·86–0·92)	0·55 (0·52–0·58)
1969	0·1 (0·0–0·1)	30·9 (27·9–33·8)	156·7 (151·7–161·9)	128·9 (125·0–133·1)	74·5 (70·2–79·1)	30·8 (27·0–34·8)	7·7 (5·7–10·0)	0·4 (0·3–0·7)	0·0 (0·0–0·0)	2·15 (2·09–2·21)	0·94 (0·9–0·97)	0·57 (0·54–0·6)
1970	0·1 (0·0–0·1)	32·6 (29·6–35·7)	162·8 (157·5–168·1)	129·5 (125·3–134·3)	74·4 (70·0–79·0)	31·9 (28·2–36·5)	7·6 (5·8–10·0)	0·4 (0·2–0·6)	0·0 (0·0–0·0)	2·2 (2·14–2·25)	0·98 (0·94–1·01)	0·57 (0·54–0·61)
1971	0·1 (0·0–0·1)	33·3 (30·1–36·6)	163·7 (158·3–169·0)	127·9 (123·5–132·6)	72·1 (67·7–76·2)	31·6 (27·7–36·0)	7·2 (5·3–9·4)	0·4 (0·2–0·6)	0·0 (0·0–0·0)	2·18 (2·12–2·24)	0·99 (0·95–1·02)	0·56 (0·52–0·59)
1972	0·1 (0·0–0·1)	33·3 (30·0–36·5)	160·6 (155·4–165·7)	125·9 (121·3–130·2)	68·9 (64·8–72·7)	30·0 (26·4–33·9)	6·7 (5·0–8·8)	0·3 (0·2–0·5)	0·0 (0·0–0·0)	2·13 (2·07–2·19)	0·97 (0·93–1·01)	0·53 (0·5–0·56)
1973	0·1 (0·0–0·1)	33·6 (30·3–36·7)	157·3 (152·3–162·0)	124·1 (119·7–128·2)	66·6 (62·9–70·3)	28·4 (24·7–32·6)	6·3 (4·7–8·3)	0·3 (0·2–0·5)	0·0 (0·0–0·0)	2·08 (2·03–2·14)	0·95 (0·92–0·99)	0·51 (0·48–0·54)
1974	0·1 (0·0–0·1)	34·8 (31·9–38·1)	158·1 (153·3–162·9)	122·1 (118·1–126·2)	64·9 (61·0–69·0)	27·7 (24·0–31·6)	6·2 (4·6–8·2)	0·3 (0·2–0·5)	0·0 (0·0–0·0)	2·07 (2·03–2·12)	0·97 (0·94–0·99)	0·5 (0·47–0·53)
1975	0·1 (0·0–0·1)	36·8 (33·8–40·1)	162·4 (157·4–167·6)	119·7 (115·6–123·6)	63·6 (59·4–68·2)	27·5 (23·8–31·4)	6·3 (4·7–8·4)	0·3 (0·2–0·4)	0·0 (0·0–0·0)	2·08 (2·04–2·13)	1·0 (0·97–1·02)	0·49 (0·46–0·52)
1976	0·1 (0·0–0·1)	38·4 (35·1–42·1)	166·0 (160·6–171·1)	117·3 (113·3–121·6)	62·7 (58·5–67·0)	27·1 (23·6–31·2)	6·2 (4·6–8·5)	0·3 (0·2–0·4)	0·0 (0·0–0·0)	2·09 (2·04–2·13)	1·02 (0·99–1·05)	0·48 (0·45–0·51)
1977	0·1 (0·0–0·1)	39·2 (35·9–42·6)	165·6 (160·4–170·8)	115·3 (111·3–119·6)	61·5 (57·2–65·5)	26·3 (23·0–30·0)	5·9 (4·3–8·2)	0·2 (0·2–0·4)	0·0 (0·0–0·0)	2·07 (2·02–2·12)	1·02 (0·99–1·05)	0·47 (0·44–0·5)
1978	0·1 (0·0–0·1)	39·7 (36·7–42·7)	162·7 (157·8–167·9)	114·6 (110·9–118·3)	60·6 (56·9–64·4)	25·6 (22·6–28·8)	5·6 (4·1–7·7)	0·2 (0·2–0·4)	0·0 (0·0–0·0)	2·05 (2·0–2·1)	1·01 (0·98–1·04)	0·46 (0·43–0·49)
1979	0·1 (0·0–0·1)	40·8 (37·6–44·2)	163·4 (158·7–168·5)	114·8 (111·1–118·4)	60·2 (56·4–64·1)	25·7 (22·7–28·9)	5·7 (4·2–7·7)	0·2 (0·1–0·3)	0·0 (0·0–0·0)	2·05 (2·01–2·1)	1·02 (0·99–1·05)	0·46 (0·43–0·49)
1980	0·1 (0·0–0·1)	41·9 (38·6–45·7)	168·5 (163·6–173·6)	115·4 (111·5–119·4)	60·1 (56·3–64·1)	26·4 (23·1–29·8)	6·0 (4·4–7·9)	0·2 (0·1–0·3)	0·0 (0·0–0·0)	2·09 (2·05–2·14)	1·05 (1·02–1·08)	0·46 (0·44–0·49)
1981	0·1 (0·0–0·1)	42·1 (38·4–45·7)	173·2 (168·1–178·2)	116·8 (112·8–121·1)	61·1 (57·0–65·2)	27·3 (23·8–31·3)	6·3 (4·8–8·0)	0·2 (0·1–0·3)	0·0 (0·0–0·0)	2·14 (2·09–2·18)	1·08 (1·04–1·11)	0·47 (0·45–0·5)
1982	0·1 (0·0–0·1)	41·3 (37·7–44·6)	174·9 (170·0–179·8)	118·9 (114·9–123·0)	62·9 (58·8–67·1)	27·9 (24·6–31·5)	6·5 (5·3–8·0)	0·2 (0·1–0·3)	0·0 (0·0–0·0)	2·16 (2·12–2·21)	1·08 (1·05–1·11)	0·49 (0·46–0·52)
1983	0·1 (0·0–0·1)	40·5 (37·0–43·6)	173·2 (168·4–178·4)	120·8 (116·9–124·6)	64·4 (60·6–68·2)	27·9 (24·9–31·0)	6·6 (5·5–7·8)	0·2 (0·1–0·3)	0·0 (0·0–0·0)	2·17 (2·12–2·22)	1·07 (1·04–1·1)	0·5 (0·46–0·53)
1984	0·1 (0·0–0·1)	40·6 (37·5–43·9)	171·8 (166·5–176·6)	121·4 (117·8–125·3)	64·8 (61·2–68·8)	27·7 (24·9–30·8)	6·6 (5·5–7·8)	0·2 (0·1–0·3)	0·0 (0·0–0·0)	2·17 (2·12–2·22)	1·06 (1·03–1·09)	0·5 (0·47–0·53)
1985	0·1 (0·0–0·1)	42·1 (38·8–45·8)	175·1 (169·9–180·1)	121·9 (118·0–125·6)	65·4 (61·6–69·4)	28·4 (25·0–31·7)	6·9 (5·6–8·4)	0·2 (0·1–0·3)	0·0 (0·0–0·0)	2·2 (2·16–2·24)	1·09 (1·06–1·12)	0·5 (0·48–0·53)
1986	0·1 (0·0–0·1)	43·7 (40·2–47·5)	181·4 (176·5–186·6)	123·8 (119·9–127·6)	66·7 (62·7–70·7)	29·5 (25·9–33·3)	7·4 (5·9–9·1)	0·2 (0·1–0·3)	0·0 (0·0–0·0)	2·26 (2·22–2·31)	1·13 (1·09–1·16)	0·52 (0·49–0·55)
1987	0·1 (0·0–0·1)	43·5 (40·1–47·0)	183·2 (178·3–188·4)	124·2 (120·1–127·9)	67·3 (63·6–71·7)	29·5 (26·0–33·1)	7·3 (6·0–8·8)	0·2 (0·1–0·3)	0·0 (0·0–0·0)	2·28 (2·22–2·34)	1·13 (1·1–1·17)	0·52 (0·49–0·55)
1988	0·1 (0·0–0·1)	42·9 (39·9–46·2)	176·6 (171·6–181·6)	120·1 (116·4–124·0)	65·8 (62·2–69·8)	27·8 (24·9–31·0)	6·6 (5·6–7·7)	0·2 (0·1–0·2)	0·0 (0·0–0·0)	2·2 (2·14–2·26)	1·1 (1·07–1·13)	0·5 (0·48–0·53)
1989	0·1 (0·0–0·1)	44·5 (41·2–47·9)	166·7 (161·7–171·6)	111·7 (107·8–115·3)	61·4 (57·9–64·9)	24·7 (22·3–27·5)	5·6 (4·7–6·6)	0·2 (0·1–0·2)	0·0 (0·0–0·0)	2·07 (2·03–2·12)	1·06 (1·03–1·09)	0·46 (0·44–0·48)
1990	0·1 (0·0–0·1)	46·7 (43·2–50·3)	156·7 (152·3–161·6)	100·2 (96·2–104·1)	54·1 (50·5–57·7)	20·9 (18·6–23·4)	4·7 (3·7–5·7)	0·1 (0·1–0·2)	0·0 (0·0–0·0)	1·92 (1·87–1·96)	1·02 (0·99–1·05)	0·4 (0·38–0·42)
1991	0·1 (0·0–0·1)	46·4 (43·0–50·2)	144·3 (139·1–149·3)	89·8 (85·7–93·9)	45·5 (42·0–49·2)	18·7 (16·2–21·8)	4·2 (3·3–5·4)	0·1 (0·1–0·2)	0·0 (0·0–0·0)	1·75 (1·69–1·8)	0·95 (0·92–0·99)	0·34 (0·32–0·37)
1992	0·1 (0·0–0·1)	43·7 (40·5–47·5)	129·4 (124·5–134·7)	83·0 (78·9–87·2)	38·8 (35·3–42·4)	18·2 (15·4–21·4)	4·2 (3·3–5·4)	0·1 (0·1–0·2)	0·0 (0·0–0·0)	1·59 (1·53–1·65)	0·87 (0·84–0·9)	0·31 (0·28–0·34)
1993	0·1 (0·0–0·1)	40·0 (36·8–43·6)	116·0 (111·2–121·2)	79·9 (76·0–84·0)	37·7 (34·4–41·4)	17·9 (15·0–21·2)	4·3 (3·4–5·6)	0·1 (0·1–0·2)	0·0 (0·0–0·0)	1·48 (1·42–1·54)	0·78 (0·75–0·81)	0·3 (0·27–0·34)
1994	0·1 (0·0–0·1)	36·7 (33·6–40·1)	106·3 (101·4–111·7)	79·6 (75·8–83·5)	39·8 (36·3–43·4)	18·0 (15·2–21·0)	4·5 (3·6–5·6)	0·1 (0·1–0·2)	0·0 (0·0–0·0)	1·43 (1·37–1·49)	0·72 (0·68–0·75)	0·31 (0·28–0·34)
1995	0·1 (0·0–0·1)	33·9 (30·8–37·2)	100·1 (95·6–105·3)	80·0 (76·3–83·7)	41·0 (37·5–44·6)	18·0 (15·5–21·0)	4·6 (3·7–5·7)	0·1 (0·1–0·2)	0·0 (0·0–0·0)	1·39 (1·34–1·44)	0·67 (0·64–0·7)	0·32 (0·29–0·35)
1996	0·1 (0·0–0·1)	31·2 (27·9–34·6)	95·4 (90·6–100·7)	80·0 (75·9–84·2)	41·9 (38·2–46·1)	18·2 (15·5–21·5)	4·8 (3·8–5·9)	0·1 (0·1–0·2)	0·0 (0·0–0·0)	1·36 (1·3–1·43)	0·63 (0·6–0·67)	0·33 (0·29–0·36)
1997	0·1 (0·0–0·1)	28·4 (25·5–31·7)	91·4 (86·5–96·7)	80·1 (76·0–84·9)	43·8 (39·9–48·5)	18·8 (15·8–22·8)	4·9 (3·9–6·2)	0·1 (0·1–0·2)	0·0 (0·0–0·0)	1·34 (1·27–1·41)	0·6 (0·56–0·64)	0·34 (0·3–0·38)
1998	0·0 (0·0–0·1)	25·6 (22·9–28·6)	87·8 (82·9–93·0)	81·0 (77·1–85·6)	47·2 (43·2–51·5)	20·1 (17·0–23·8)	5·2 (4·1–6·4)	0·2 (0·1–0·2)	0·0 (0·0–0·0)	1·34 (1·28–1·4)	0·57 (0·54–0·6)	0·36 (0·33–0·41)
1999	0·0 (0·0–0·1)	23·4 (20·8–26·2)	83·7 (79·1–88·7)	82·7 (78·8–86·9)	51·7 (47·6–55·7)	21·7 (18·8–25·2)	5·5 (4·5–6·7)	0·2 (0·1–0·2)	0·0 (0·0–0·0)	1·34 (1·29–1·41)	0·54 (0·51–0·57)	0·4 (0·36–0·43)
2000	0·0 (0·0–0·1)	22·4 (19·9–25·1)	80·4 (76·0–85·0)	84·1 (80·2–88·0)	54·9 (51·0–58·9)	23·3 (20·1–26·8)	5·6 (4·6–6·9)	0·2 (0·1–0·2)	0·0 (0·0–0·0)	1·35 (1·29–1·42)	0·51 (0·49–0·55)	0·42 (0·39–0·45)
2001	0·0 (0·0–0·1)	22·1 (19·6–25·1)	78·4 (73·5–83·0)	85·4 (81·2–89·5)	57·0 (52·9–61·4)	24·6 (21·0–28·3)	5·7 (4·5–7·2)	0·2 (0·1–0·3)	0·0 (0·0–0·0)	1·37 (1·3–1·43)	0·5 (0·47–0·53)	0·44 (0·4–0·48)
2002	0·0 (0·0–0·1)	22·1 (19·4–25·0)	77·2 (72·5–82·1)	87·6 (83·4–91·9)	59·3 (55·2–63·9)	26·2 (22·5–30·3)	5·8 (4·3–7·4)	0·2 (0·1–0·3)	0·0 (0·0–0·0)	1·39 (1·33–1·46)	0·5 (0·47–0·52)	0·46 (0·42–0·5)
2003	0·0 (0·0–0·1)	22·3 (19·7–25·3)	76·7 (72·0–81·5)	91·0 (86·8–95·5)	62·4 (58·1–67·0)	28·3 (24·4–32·6)	5·9 (4·4–7·9)	0·2 (0·1–0·3)	0·0 (0·0–0·0)	1·43 (1·37–1·5)	0·5 (0·47–0·52)	0·48 (0·45–0·52)
2004	0·0 (0·0–0·1)	22·5 (19·9–25·5)	76·1 (71·4–81·2)	95·4 (91·3–100·0)	66·6 (62·4–71·4)	31·0 (26·9–35·3)	6·2 (4·6–8·2)	0·2 (0·1–0·3)	0·0 (0·0–0·0)	1·49 (1·43–1·55)	0·49 (0·46–0·53)	0·52 (0·49–0·55)
2005	0·0 (0·0–0·1)	22·8 (20·1–25·7)	75·7 (71·2–80·5)	100·4 (96·1–104·9)	71·3 (66·8–75·8)	34·0 (29·9–38·0)	6·6 (4·8–8·7)	0·2 (0·1–0·3)	0·0 (0·0–0·0)	1·55 (1·5–1·61)	0·49 (0·46–0·53)	0·56 (0·53–0·59)
2006	0·0 (0·0–0·1)	22·8 (20·2–25·7)	75·4 (70·7–80·3)	105·1 (100·7–109·6)	76·2 (71·5–81·0)	36·9 (32·5–41·7)	7·1 (5·3–9·2)	0·2 (0·1–0·3)	0·0 (0·0–0·0)	1·62 (1·56–1·68)	0·49 (0·46–0·53)	0·6 (0·57–0·64)
2007	0·0 (0·0–0·1)	22·1 (19·5–25·1)	73·9 (69·6–78·9)	108·6 (104·2–113·0)	81·0 (76·4–85·7)	39·9 (35·7–44·8)	7·6 (5·8–9·9)	0·2 (0·1–0·3)	0·0 (0·0–0·0)	1·67 (1·61–1·73)	0·48 (0·45–0·51)	0·64 (0·61–0·68)
2008	0·0 (0·0–0·1)	20·6 (17·8–23·6)	70·7 (66·1–75·5)	110·1 (105·7–115·0)	85·0 (80·5–89·8)	42·5 (38·2–47·1)	8·3 (6·3–10·7)	0·2 (0·1–0·3)	0·0 (0·0–0·0)	1·69 (1·63–1·75)	0·46 (0·43–0·48)	0·68 (0·65–0·72)
2009	0·0 (0·0–0·1)	18·8 (16·1–21·9)	66·3 (61·6–71·1)	109·0 (104·4–113·8)	88·0 (83·4–92·8)	44·4 (40·1–49·4)	9·0 (6·9–11·3)	0·2 (0·1–0·4)	0·0 (0·0–0·0)	1·68 (1·63–1·74)	0·43 (0·4–0·45)	0·71 (0·68–0·74)
2010	0·0 (0·0–0·1)	17·1 (14·5–20·1)	61·7 (56·9–66·5)	105·3 (100·9–109·8)	89·1 (84·8–94·3)	45·6 (41·4–50·8)	9·6 (7·3–12·3)	0·3 (0·2–0·4)	0·0 (0·0–0·0)	1·64 (1·59–1·7)	0·39 (0·37–0·42)	0·72 (0·69–0·76)
2011	0·0 (0·0–0·1)	15·9 (13·6–18·9)	57·8 (53·3–62·8)	101·0 (97·0–105·5)	88·0 (83·3–93·0)	46·3 (41·6–51·3)	10·1 (7·7–12·9)	0·3 (0·2–0·4)	0·0 (0·0–0·0)	1·6 (1·55–1·65)	0·37 (0·34–0·4)	0·72 (0·69–0·76)
2012	0·0 (0·0–0·1)	15·2 (12·9–18·0)	55·0 (50·4–59·8)	97·9 (93·7–102·2)	86·4 (81·7–91·3)	47·0 (42·5–51·9)	10·4 (8·1–13·5)	0·3 (0·2–0·5)	0·0 (0·0–0·0)	1·56 (1·51–1·62)	0·35 (0·32–0·38)	0·72 (0·69–0·76)
2013	0·0 (0·0–0·1)	14·5 (12·3–17·0)	53·2 (48·6–58·6)	96·7 (92·6–101·0)	86·4 (81·9–91·2)	47·9 (43·7–52·8)	10·8 (8·3–13·6)	0·3 (0·2–0·5)	0·0 (0·0–0·0)	1·55 (1·49–1·61)	0·34 (0·31–0·38)	0·73 (0·69–0·77)
2014	0·0 (0·0–0·1)	13·9 (11·7–16·4)	52·1 (47·3–57·8)	97·4 (93·4–102·0)	88·4 (83·7–93·2)	49·2 (44·7–54·0)	11·1 (8·5–14·0)	0·3 (0·2–0·5)	0·0 (0·0–0·0)	1·56 (1·5–1·63)	0·33 (0·3–0·37)	0·74 (0·71–0·78)
2015	0·0 (0·0–0·1)	13·6 (11·5–16·0)	51·7 (46·3–57·6)	98·6 (94·0–103·6)	90·7 (85·7–96·2)	49·7 (44·7–55·1)	11·0 (8·3–14·1)	0·3 (0·2–0·5)	0·0 (0·0–0·0)	1·58 (1·49–1·68)	0·33 (0·29–0·36)	0·76 (0·71–0·81)
2016	0·0 (0·0–0·1)	13·8 (11·8–16·5)	52·0 (45·5–59·4)	99·0 (93·2–105·3)	91·2 (84·7–98·7)	48·4 (42·0–55·7)	10·5 (7·9–13·7)	0·3 (0·2–0·5)	0·0 (0·0–0·0)	1·58 (1·44–1·74)	0·33 (0·29–0·38)	0·75 (0·68–0·83)
2017	0·0 (0·0–0·1)	14·1 (11·8–16·9)	52·8 (44·6–62·5)	98·6 (91·1–106·9)	90·7 (81·6–100·8)	46·9 (38·9–56·2)	10·1 (7·5–13·5)	0·3 (0·2–0·5)	0·0 (0·0–0·0)	1·57 (1·38–1·79)	0·33 (0·28–0·4)	0·74 (0·64–0·85)

Supplemental Results Table 5: Age-specific fertility rates per 1000, cumulative total fertility up to maternal age 25 (TFU25), cumulative total fertility from maternal age 30 to age 55 (TFO30), and cumulative total fertility, Latvia, 1950-2017

Year	10-14	15-19	20-24	25-29	30-34	35-39	40-44	45-49	50-54	TFR	TFU25	TFO30
1950	0·0 (0·0–0·1)	18·6 (16·3–21·1)	105·1 (98·7–111·7)	120·2 (115·4–125·0)	67·3 (62·6–72·2)	49·1 (44·6–53·9)	21·8 (19·5–24·4)	2·5 (2·1–3·0)	0·0 (0·0–0·1)	1·92 (1·84–2·01)	0·62 (0·58–0·65)	0·7 (0·67–0·74)
1951	0·0 (0·0–0·1)	17·5 (15·5–19·7)	99·0 (95·1–103·1)	118·4 (114·7–121·8)	71·4 (67·7–75·3)	47·0 (43·5–50·5)	21·3 (19·3–23·5)	2·5 (2·1–3·0)	0·0 (0·0–0·1)	1·89 (1·83–1·94)	0·58 (0·56–0·61)	0·71 (0·68–0·74)
1952	0·0 (0·0–0·1)	17·1 (15·1–19·2)	94·5 (90·6–98·0)	116·4 (113·1–119·7)	76·1 (72·6–79·6)	43·9 (40·6–47·5)	20·1 (18·2–22·2)	2·4 (2·0–2·8)	0·0 (0·0–0·0)	1·85 (1·81–1·9)	0·56 (0·54–0·58)	0·71 (0·68–0·74)
1953	0·0 (0·0–0·1)	17·5 (15·4–19·7)	93·9 (90·0–98·0)	115·7 (112·2–119·2)	79·5 (75·5–82·9)	41·2 (37·9–44·7)	18·4 (16·7–20·4)	2·2 (1·8–2·5)	0·0 (0·0–0·0)	1·84 (1·8–1·89)	0·56 (0·53–0·58)	0·71 (0·68–0·74)
1954	0·0 (0·0–0·1)	18·2 (16·2–20·5)	96·9 (91·1–102·8)	115·4 (111·2–119·6)	80·1 (75·6–84·7)	39·6 (36·0–43·4)	16·5 (14·7–18·4)	1·9 (1·6–2·2)	0·0 (0·0–0·0)	1·84 (1·79–1·91)	0·58 (0·54–0·61)	0·69 (0·66–0·72)
1955	0·0 (0·0–0·1)	19·0 (17·0–21·3)	100·7 (92·1–109·8)	114·6 (109·3–119·9)	78·7 (73·1–84·9)	39·0 (34·8–43·1)	14·8 (13·2–16·5)	1·7 (1·4–1·9)	0·0 (0·0–0·0)	1·84 (1·75–1·94)	0·6 (0·56–0·65)	0·67 (0·64–0·71)
1956	0·0 (0·0–0·1)	19·7 (17·7–22·1)	104·2 (95·2–115·2)	114·5 (109·0–120·9)	77·5 (71·6–84·4)	39·4 (35·2–43·7)	13·5 (12·0–15·2)	1·5 (1·3–1·7)	0·0 (0·0–0·0)	1·85 (1·74–1·98)	0·62 (0·57–0·68)	0·66 (0·62–0·7)
1957	0·0 (0·0–0·1)	20·3 (18·3–22·7)	107·1 (99·0–116·5)	115·2 (110·1–120·9)	77·2 (71·6–83·1)	40·2 (36·2–44·5)	12·6 (11·2–14·1)	1·3 (1·1–1·6)	0·0 (0·0–0·0)	1·87 (1·78–1·97)	0·64 (0·59–0·69)	0·66 (0·62–0·69)
1958	0·0 (0·0–0·1)	20·9 (18·8–23·1)	109·3 (103·8–115·3)	116·4 (112·2–120·9)	77·5 (73·2–82·2)	40·8 (37·1–44·6)	12·0 (10·6–13·4)	1·2 (1·0–1·4)	0·0 (0·0–0·0)	1·89 (1·83–1·96)	0·65 (0·61–0·69)	0·66 (0·63–0·69)
1959	0·0 (0·0–0·1)	21·3 (19·2–23·7)	111·6 (107·7–116·1)	117·1 (113·8–120·6)	77·3 (74·1–80·7)	40·4 (37·2–43·9)	11·4 (10·1–12·7)	1·1 (0·9–1·3)	0·0 (0·0–0·0)	1·9 (1·86–1·95)	0·66 (0·64–0·69)	0·65 (0·62–0·68)
1960	0·0 (0·0–0·1)	21·7 (19·4–24·2)	114·6 (110·8–118·3)	116·6 (113·3–119·7)	76·0 (72·8–79·4)	39·0 (35·9–42·5)	10·9 (9·7–12·3)	1·0 (0·8–1·2)	0·0 (0·0–0·0)	1·9 (1·86–1·94)	0·68 (0·66–0·71)	0·63 (0·6–0·67)
1961	0·0 (0·0–0·1)	21·4 (19·2–23·8)	116·2 (112·5–120·2)	114·3 (110·9–117·8)	74·1 (70·7–77·5)	37·0 (33·9–40·3)	10·7 (9·2–12·4)	0·9 (0·7–1·1)	0·0 (0·0–0·0)	1·87 (1·83–1·91)	0·69 (0·67–0·71)	0·61 (0·59–0·64)
1962	0·0 (0·0–0·1)	20·5 (18·4–22·8)	113·6 (109·8–117·7)	111·0 (107·3–114·6)	72·2 (68·4–75·7)	35·2 (32·0–38·7)	10·5 (8·6–12·5)	0·9 (0·7–1·1)	0·0 (0·0–0·0)	1·82 (1·78–1·86)	0·67 (0·65–0·69)	0·59 (0·57–0·62)
1963	0·0 (0·0–0·1)	19·6 (17·6–21·8)	109·5 (105·9–113·3)	108·0 (104·6–111·4)	70·0 (66·7–73·3)	33·6 (30·4–37·3)	10·2 (8·2–12·4)	0·9 (0·6–1·1)	0·0 (0·0–0·0)	1·76 (1·72–1·8)	0·65 (0·62–0·67)	0·57 (0·55–0·6)
1964	0·0 (0·0–0·1)	19·4 (17·4–21·5)	107·7 (104·0–111·6)	106·1 (102·9–109·6)	67·5 (64·1–71·0)	32·2 (29·0–35·8)	9·8 (7·8–12·0)	0·8 (0·6–1·1)	0·0 (0·0–0·0)	1·72 (1·67–1·76)	0·64 (0·61–0·66)	0·55 (0·52–0·58)
1965	0·0 (0·0–0·1)	20·1 (18·0–22·5)	109·6 (105·6–113·8)	106·0 (102·5–109·8)	64·7 (61·3–68·1)	31·1 (28·0–34·6)	9·2 (7·2–11·4)	0·7 (0·5–1·0)	0·0 (0·0–0·0)	1·71 (1·66–1·75)	0·65 (0·62–0·67)	0·53 (0·5–0·56)
1966	0·0 (0·0–0·1)	21·5 (19·3–24·1)	114·8 (110·4–119·1)	107·4 (104·0–110·8)	62·3 (58·9–65·3)	30·1 (27·2–33·4)	8·6 (6·7–10·7)	0·6 (0·4–0·8)	0·0 (0·0–0·0)	1·73 (1·69–1·77)	0·68 (0·66–0·71)	0·51 (0·48–0·53)
1967	0·0 (0·0–0·1)	23·2 (20·9–25·8)	121·2 (116·9–125·8)	109·1 (105·9–112·2)	62·4 (59·1–65·4)	29·5 (26·4–32·5)	8·0 (6·2–10·1)	0·6 (0·4–0·8)	0·0 (0·0–0·0)	1·77 (1·73–1·81)	0·72 (0·7–0·75)	0·5 (0·48–0·53)
1968	0·0 (0·0–0·1)	24·9 (22·6–27·5)	128·1 (123·8–132·5)	110·9 (107·7–114·1)	64·6 (61·3–67·9)	29·0 (26·0–32·2)	7·6 (5·9–9·5)	0·5 (0·3–0·8)	0·0 (0·0–0·0)	1·83 (1·79–1·87)	0·77 (0·74–0·79)	0·51 (0·49–0·53)
1969	0·0 (0·0–0·1)	26·4 (24·1–28·8)	136·4 (132·0–140·7)	111·6 (108·3–114·9)	66·9 (63·7–70·5)	29·0 (25·9–32·1)	7·2 (5·6–9·3)	0·5 (0·3–0·8)	0·0 (0·0–0·0)	1·89 (1·85–1·93)	0·81 (0·79–0·84)	0·52 (0·5–0·54)
1970	0·0 (0·0–0·1)	27·3 (24·8–30·0)	143·5 (139·3–147·9)	111·2 (108·0–114·9)	68·5 (65·1–72·0)	29·4 (26·3–32·8)	7·0 (5·4–9·0)	0·5 (0·3–0·8)	0·0 (0·0–0·0)	1·94 (1·9–1·98)	0·85 (0·83–0·88)	0·53 (0·5–0·55)
1971	0·0 (0·0–0·1)	27·5 (24·9–30·1)	145·7 (141·7–149·9)	111·0 (107·6–114·6)	68·0 (64·3–71·4)	29·9 (26·7–33·3)	6·9 (5·3–8·9)	0·5 (0·3–0·7)	0·0 (0·0–0·0)	1·95 (1·9–1·99)	0·87 (0·84–0·89)	0·53 (0·5–0·55)
1972	0·0 (0·0–0·1)	27·5 (25·1–29·8)	144·1 (140·1–148·4)	111·6 (108·3–115·2)	66·2 (62·8–69·6)	30·2 (27·2–33·5)	6·8 (5·2–8·9)	0·4 (0·3–0·7)	0·0 (0·0–0·0)	1·93 (1·89–1·98)	0·86 (0·84–0·88)	0·52 (0·49–0·54)
1973	0·0 (0·0–0·1)	27·9 (25·5–30·3)	142·2 (138·0–146·3)	113·3 (109·9–116·7)	64·3 (61·1–67·5)	30·1 (27·1–33·5)	6·7 (5·1–8·7)	0·4 (0·3–0·6)	0·0 (0·0–0·0)	1·92 (1·88–1·97)	0·85 (0·83–0·87)	0·51 (0·48–0·53)
1974	0·0 (0·0–0·1)	28·8 (26·4–31·3)	143·2 (139·1–147·4)	113·8 (110·2–117·0)	62·1 (58·7–65·3)	29·3 (26·3–32·6)	6·5 (4·8–8·5)	0·4 (0·2–0·6)	0·0 (0·0–0·0)	1·92 (1·88–1·97)	0·86 (0·84–0·88)	0·49 (0·47–0·52)
1975	0·0 (0·0–0·1)	29·9 (27·3–32·4)	146·0 (142·0–150·3)	111·7 (108·3–115·0)	59·3 (55·8–62·8)	28·0 (25·0–31·1)	6·2 (4·6–8·2)	0·4 (0·2–0·6)	0·0 (0·0–0·0)	1·91 (1·86–1·95)	0·88 (0·86–0·9)	0·47 (0·44–0·49)
1976	0·0 (0·0–0·1)	31·1 (28·4–33·6)	147·0 (142·9–151·2)	108·4 (105·2–111·8)	56·7 (53·5–60·1)	26·5 (23·6–29·4)	6·0 (4·5–7·9)	0·3 (0·2–0·5)	0·0 (0·0–0·0)	1·88 (1·84–1·92)	0·89 (0·87–0·92)	0·45 (0·42–0·47)
1977	0·0 (0·0–0·1)	32·6 (29·8–35·5)	146·2 (141·9–150·4)	105·6 (102·4–109·1)	55·4 (52·1–58·7)	24·9 (22·3–27·6)	5·8 (4·3–7·7)	0·3 (0·2–0·5)	0·0 (0·0–0·0)	1·85 (1·82–1·89)	0·89 (0·87–0·92)	0·43 (0·41–0·46)
1978	0·0 (0·0–0·1)	34·3 (31·5–37·0)	145·5 (141·6–149·6)	104·5 (101·0–107·7)	54·9 (51·8–58·2)	23·3 (21·1–25·8)	5·6 (4·2–7·3)	0·3 (0·2–0·4)	0·0 (0·0–0·0)	1·84 (1·81–1·88)	0·9 (0·87–0·92)	0·42 (0·4–0·44)
1979	0·0 (0·0–0·1)	35·7 (33·1–38·4)	146·2 (142·4–150·1)	104·0 (100·8–107·2)	54·4 (51·4–57·9)	21·9 (19·8–24·4)	5·3 (4·0–6·9)	0·3 (0·2–0·4)	0·0 (0·0–0·0)	1·84 (1·81–1·87)	0·91 (0·89–0·93)	0·41 (0·39–0·43)
1980	0·0 (0·0–0·1)	36·7 (34·0–39·5)	148·0 (144·1–151·9)	104·0 (100·9–107·4)	54·5 (51·2–57·9)	21·5 (19·1–24·3)	5·2 (3·9–6·7)	0·3 (0·2–0·4)	0·0 (0·0–0·0)	1·85 (1·82–1·89)	0·92 (0·9–0·95)	0·41 (0·38–0·43)
1981	0·0 (0·0–0·1)	37·7 (34·8–41·0)	151·7 (147·8–155·8)	106·7 (103·4–110·2)	56·3 (52·9–59·5)	22·7 (20·0–25·7)	5·3 (4·1–6·7)	0·3 (0·2–0·4)	0·0 (0·0–0·0)	1·9 (1·87–1·94)	0·95 (0·92–0·97)	0·42 (0·4–0·45)
1982	0·0 (0·0–0·1)	38·6 (35·6–41·8)	157·0 (153·0–160·9)	112·2 (109·1–115·7)	59·2 (56·2–62·5)	24·1 (21·5–27·0)	5·4 (4·5–6·6)	0·3 (0·2–0·4)	0·0 (0·0–0·0)	1·98 (1·95–2·02)	0·98 (0·95–1·0)	0·45 (0·42–0·47)
1983	0·0 (0·0–0·1)	39·0 (36·2–42·2)	158·9 (155·1–162·6)	117·0 (113·9–120·3)	61·5 (58·3–64·8)	24·6 (22·1–27·2)	5·3 (4·6–6·1)	0·2 (0·2–0·4)	0·0 (0·0–0·0)	2·03 (2·0–2·07)	0·99 (0·97–1·01)	0·46 (0·44–0·48)
1984	0·0 (0·0–0·1)	39·7 (36·8–42·8)	158·7 (154·8–162·4)	118·5 (115·4–121·7)	62·4 (59·1–65·7)	25·1 (22·6–27·9)	5·2 (4·5–6·1)	0·2 (0·2–0·3)	0·0 (0·0–0·0)	2·05 (2·01–2·08)	0·99 (0·97–1·02)	0·47 (0·44–0·49)
1985	0·0 (0·0–0·1)	40·8 (38·0–43·9)	161·7 (157·6–165·5)	118·1 (115·0–121·3)	63·2 (59·8–66·5)	26·4 (23·6–29·3)	5·4 (4·4–6·4)	0·2 (0·2–0·3)	0·0 (0·0–0·0)	2·08 (2·04–2·11)	1·01 (0·99–1·04)	0·48 (0·45–0·5)
1986	0·0 (0·0–0·1)	41·5 (38·7–44·7)	165·5 (161·2–169·6)	117·1 (114·0–120·2)	63·7 (60·5–67·2)	27·3 (24·3–30·3)	5·4 (4·4–6·6)	0·2 (0·2–0·3)	0·0 (0·0–0·0)	2·1 (2·07–2·14)	1·04 (1·01–1·06)	0·48 (0·46–0·51)
1987	0·0 (0·0–0·1)	41·0 (37·9–44·0)	166·2 (162·4–170·7)	114·9 (111·7–118·1)	63·3 (60·0–66·6)	26·7 (24·2–29·5)	5·2 (4·4–6·3)	0·2 (0·2–0·3)	0·0 (0·0–0·0)	2·09 (2·05–2·13)	1·04 (1·01–1·06)	0·48 (0·45–0·5)
1988	0·0 (0·0–0·1)	40·8 (37·9–43·5)	163·2 (159·3–167·3)	111·0 (107·8–114·2)	61·8 (58·8–65·1)	25·0 (22·6–27·4)	4·8 (4·2–5·6)	0·2 (0·2–0·3)	0·0 (0·0–0·0)	2·03 (1·99–2·08)	1·02 (1·0–1·04)	0·46 (0·43–0·49)
1989	0·0 (0·0–0·1)	43·3 (40·3–46·2)	159·1 (155·2–163·0)	105·0 (102·0–108·2)	59·0 (55·9–62·3)	22·6 (20·4–25·0)	4·4 (3·7–5·0)	0·2 (0·1–0·2)	0·0 (0·0–0·0)	1·97 (1·92–2·01)	1·01 (0·99–1·04)	0·43 (0·41–0·46)
1990	0·0 (0·0–0·1)	45·9 (42·6–49·0)	153·3 (149·2–157·6)	96·5 (93·4–99·7)	53·4 (50·4–56·6)	20·0 (17·9–22·4)	3·9 (3·2–4·8)	0·2 (0·1–0·2)	0·0 (0·0–0·0)	1·87 (1·83–1·91)	1·0 (0·97–1·02)	0·39 (0·37–0·41)
1991	0·0 (0·0–0·1)	45·1 (41·9–48·2)	142·4 (138·3–146·4)	87·3 (84·1–90·6)	44·9 (42·0–48·0)	18·3 (16·0–20·9)	3·8 (3·0–4·7)	0·2 (0·1–0·2)	0·0 (0·0–0·0)	1·71 (1·67–1·75)	0·94 (0·91–0·96)	0·34 (0·32–0·36)
1992	0·0 (0·0–0·1)	40·9 (37·9–43·9)	127·1 (123·0–131·2)	79·7 (76·4–83·2)	38·0 (35·4–40·9)	17·5 (15·0–20·2)	3·9 (3·0–4·8)	0·1 (0·1–0·2)	0·0 (0·0–0·0)	1·54 (1·5–1·57)	0·84 (0·81–0·87)	0·3 (0·28–0·32)
1993	0·0 (0·0–0·1)	35·0 (32·3–37·9)	110·6 (106·8–114·7)	74·7 (71·5–78·0)	36·3 (33·5–39·1)	16·9 (14·5–19·7)	4·0 (3·2–4·9)	0·2 (0·1–0·2)	0·0 (0·0–0·0)	1·39 (1·35–1·43)	0·73 (0·7–0·75)	0·29 (0·27–0·31)
1994	0·0 (0·0–0·1)	29·5 (27·0–32·1)	96·9 (93·2–100·8)	71·5 (68·4–74·8)	36·5 (33·8–39·7)	16·5 (14·3–19·1)	4·1 (3·4–4·9)	0·2 (0·1–0·3)	0·0 (0·0–0·0)	1·28 (1·24–1·32)	0·63 (0·61–0·66)	0·29 (0·26–0·31)
1995	0·0 (0·0–0·1)	25·2 (22·9–27·8)	87·1 (83·3–91·2)	68·7 (65·6–72·1)	36·0 (33·1–39·0)	16·2 (14·1–18·7)	4·2 (3·5–5·1)	0·2 (0·1–0·3)	0·0 (0·0–0·0)	1·19 (1·15–1·23)	0·56 (0·53–0·59)	0·28 (0·26–0·31)
1996	0·0 (0·0–0·1)	22·1 (19·9–24·6)	81·0 (77·2–85·0)	67·3 (63·9–70·9)	35·9 (32·9–39·1)	16·2 (13·9–19·0)	4·4 (3·6–5·3)	0·2 (0·2–0·3)	0·0 (0·0–0·0)	1·14 (1·09–1·18)	0·52 (0·49–0·55)	0·28 (0·26–0·31)
1997	0·0 (0·0–0·1)	19·9 (17·7–22·1)	77·6 (73·9–81·9)	68·3 (65·1–71·7)	37·1 (34·0–40·5)	16·6 (14·0–19·8)	4·5 (3·6–5·6)	0·2 (0·2–0·3)	0·0 (0·0–0·0)	1·12 (1·08–1·17)	0·49 (0·46–0·51)	0·29 (0·27–0·32)
1998	0·0 (0·0–0·1)	18·4 (16·3–20·6)	76·0 (72·1–79·9)	72·0 (68·8–75·3)	40·1 (36·8–43·2)	17·4 (15·0–20·5)	4·7 (3·8–5·7)	0·2 (0·2–0·3)	0·0 (0·0–0·0)	1·14 (1·1–1·19)	0·47 (0·45–0·49)	0·31 (0·29–0·34)
1999	0·0 (0·0–0·1)	17·3 (15·4–19·4)	75·2 (71·2–79·3)	76·4 (73·1–79·8)	44·0 (41·0–47·0)	18·7 (16·4–21·4)	4·8 (4·1–5·8)	0·2 (0·2–0·3)	0·0 (0·0–0·0)	1·18 (1·15–1·22)	0·46 (0·44–0·49)	0·34 (0·32–0·36)
2000	0·0 (0·0–0·1)	16·8 (14·9–18·9)	74·7 (70·4–78·7)	78·8 (75·5–82·1)	47·5 (44·2–50·8)	20·0 (17·6–22·4)	5·0 (4·1–6·0)	0·2 (0·2–0·3)	0·0 (0·0–0·0)	1·22 (1·18–1·25)	0·46 (0·43–0·48)	0·36 (0·34–0·39)
2001	0·0 (0·0–0·1)	16·7 (14·7–18·7)	74·4 (70·5–78·3)	80·0 (76·5–83·6)	50·2 (46·9–53·8)	21·1 (18·4–24·0)	5·2 (4·1–6·4)	0·2 (0·2–0·3)	0·0 (0·0–0·0)	1·24 (1·2–1·28)	0·46 (0·43–0·48)	0·38 (0·36–0·41)
2002	0·0 (0·0–0·1)	16·8 (14·7–19·0)	74·2 (70·2–78·0)	81·4 (78·1–84·8)	53·1 (49·6–56·7)	22·6 (19·6–25·9)	5·4 (4·2–6·9)	0·2 (0·2–0·3)	0·0 (0·0–0·0)	1·27 (1·23–1·31)	0·45 (0·43–0·48)	0·41 (0·38–0·43)
2003	0·0 (0·0–0·1)	17·0 (14·9–19·2)	73·5 (69·8–77·8)	83·2 (79·9–87·1)	56·3 (52·8–59·9)	24·7 (21·5–28·0)	5·7 (4·4–7·5)	0·2 (0·2–0·4)	0·0 (0·0–0·0)	1·3 (1·25–1·36)	0·45 (0·43–0·48)	0·43 (0·41–0·47)
2004	0·0 (0·0–0·1)	17·4 (15·3–19·8)	72·7 (68·7–76·8)	86·2 (82·8–90·1)	60·0 (56·5–63·7)	27·1 (23·8–30·6)	6·0 (4·6–7·9)	0·2 (0·2–0·4)	0·0 (0·0–0·0)	1·35 (1·3–1·41)	0·45 (0·43–0·48)	0·47 (0·44–0·5)
2005	0·0 (0·0–0·1)	18·4 (16·2–21·0)	72·6 (68·6–76·9)	90·6 (87·1–94·3)	63·2 (59·6–66·7)	29·5 (26·2–33·2)	6·3 (4·7–8·5)	0·2 (0·2–0·4)	0·0 (0·0–0·0)	1·4 (1·35–1·46)	0·46 (0·43–0·48)	0·5 (0·47–0·53)
2006	0·0 (0·0–0·1)	19·8 (17·4–22·5)	73·2 (69·1–77·4)	95·0 (91·3–98·7)	65·2 (61·8–68·9)	31·1 (27·8–34·7)	6·5 (4·8–8·6)	0·2 (0·2–0·4)	0·0 (0·0–0·0)	1·46 (1·41–1·5)	0·47 (0·44–0·49)	0·51 (0·49–0·54)
2007	0·0 (0·0–0·1)	20·5 (18·0–23·4)	72·8 (69·0–76·5)	97·0 (93·4–100·8)	66·3 (62·6–69·9)	32·0 (28·8–35·7)	6·7 (5·0–8·8)	0·3 (0·2–0·4)	0·0 (0·0–0·0)	1·48 (1·43–1·52)	0·47 (0·44–0·49)	0·53 (0·5–0·55)
2008	0·0 (0·0–0·1)	19·7 (17·3–22·4)	68·6 (64·9–72·4)	94·9 (91·4–98·5)	67·0 (63·4–71·2)	32·5 (29·3–36·1)	7·1 (5·4–9·1)	0·3 (0·2–0·4)	0·0 (0·0–0·0)	1·45 (1·41–1·5)	0·44 (0·42–0·46)	0·53 (0·51–0·56)
2009	0·0 (0·0–0·1)	18·2 (16·0–20·7)	62·2 (58·5–66·0)	90·6 (87·0–94·2)	67·6 (63·6–71·5)	32·9 (29·6–36·5)	7·6 (5·8–9·8)	0·3 (0·2–0·5)	0·0 (0·0–0·0)	1·4 (1·35–1·44)	0·4 (0·38–0·42)	0·54 (0·51–0·57)
2010	0·0 (0·0–0·1)	17·3 (15·0–19·8)	57·4 (53·8–61·3)	88·0 (84·4–91·6)	69·0 (65·1–72·8)	33·9 (30·4–37·9)	8·1 (6·2–10·4)	0·4 (0·2–0·5)	0·0 (0·0–0·0)	1·37 (1·32–1·42)	0·37 (0·35–0·4)	0·56 (0·53–0·59)
2011	0·0 (0·0–0·1)	17·0 (14·8–19·8)	55·6 (51·9–59·3)	88·8 (85·1–92·4)	72·4 (68·4–76·2)	36·4 (32·4–40·7)	8·6 (6·6–10·8)	0·4 (0·3–0·6)	0·0 (0·0–0·0)	1·4 (1·35–1·44)	0·36 (0·34–0·38)	0·59 (0·56–0·62)
2012	0·0 (0·0–0·1)	17·2 (14·9–20·1)	56·0 (52·2–59·9)	92·2 (88·7–95·7)	77·8 (73·8–81·7)	40·1 (36·2–44·2)	9·0 (7·1–11·4)	0·4 (0·3–0·5)	0·0 (0·0–0·0)	1·46 (1·42–1·51)	0·37 (0·34–0·39)	0·64 (0·61–0·67)
2013	0·0 (0·0–0·1)	17·9 (15·6–20·8)	58·0 (54·0–61·8)	96·9 (93·2–100·4)	83·8 (80·0–88·1)	43·7 (39·9–47·6)	9·3 (7·4–11·6)	0·4 (0·3–0·5)	0·0 (0·0–0·0)	1·55 (1·5–1·59)	0·38 (0·36–0·4)	0·69 (0·66–0·72)
2014	0·0 (0·0–0·1)	18·6 (16·2–21·4)	60·6 (56·2–65·2)	100·9 (97·1–104·9)	88·5 (84·1–92·9)	46·0 (42·2–50·5)	9·2 (7·5–11·1)	0·3 (0·2–0·5)	0·0 (0·0–0·0)	1·62 (1·57–1·67)	0·4 (0·37–0·43)	0·72 (0·69–0·75)
2015	0·0 (0·0–0·1)	18·9 (16·5–21·7)	62·5 (56·0–69·1)	102·7 (98·1–108·0)	89·9 (84·4–95·9)	46·1 (41·2–51·5)	8·9 (7·4–10·5)	0·3 (0·2–0·4)	0·0 (0·0–0·0)	1·65 (1·56–1·74)	0·41 (0·37–0·45)	0·73 (0·68–0·77)
2016	0·0 (0·0–0·1)	18·8 (16·3–21·9)	62·9 (54·7–72·0)	101·9 (96·0–109·0)	87·6 (80·0–95·6)	43·7 (37·4–50·6)	8·5 (7·0–10·2)	0·3 (0·2–0·4)	0·0 (0·0–0·0)	1·62 (1·48–1·77)	0·41 (0·36–0·47)	0·7 (0·64–0·77)
2017	0·0 (0·0–0·1)	18·7 (15·9–22·0)	62·8 (53·0–74·2)	100·2 (92·2–109·0)	84·4 (74·8–95·1)	41·2 (33·8–50·0)	8·2 (6·4–10·3)	0·3 (0·2–0·4)	0·0 (0·0–0·0)	1·58 (1·39–1·79)	0·41 (0·34–0·48)	0·67 (0·59–0·77)

Supplemental Results Table 5: Age-specific fertility rates per 1000, cumulative total fertility up to maternal age 25 (TFU25), cumulative total fertility from maternal age 30 to age 55 (TFO30), and cumulative total fertility, Lithuania, 1950-2017

Year	10-14	15-19	20-24	25-29	30-34	35-39	40-44	45-49	50-54	TFR	TFU25	TFO30
1950	0·0 (0·0–0·1)	22·4 (20·0–24·9)	160·4 (146·3–177·1)	133·1 (124·6–143·1)	136·3 (126·4–147·9)	79·2 (72·3–86·5)	35·0 (31·8–38·4)	5·8 (4·9–6·8)	0·1 (0·1–0·1)	2·86 (2·69–3·05)	0·91 (0·84–1·0)	1·28 (1·22–1·35)
1951	0·0 (0·0–0·1)	20·9 (18·9–23·2)	152·4 (138·8–166·3)	131·6 (123·4–140·5)	129·4 (120·5–139·2)	70·6 (64·2–76·8)	30·5 (27·8–33·3)	5·2 (4·5–6·0)	0·1 (0·1–0·1)	2·7 (2·54–2·87)	0·87 (0·8–0·94)	1·18 (1·12–1·24)
1952	0·0 (0·0–0·1)	19·4 (17·7–21·4)	144·0 (131·9–156·8)	131·1 (123·3–139·4)	122·9 (114·4–131·9)	65·2 (59·5–71·4)	27·3 (25·0–29·9)	4·7 (4·1–5·4)	0·1 (0·1–0·1)	2·57 (2·42–2·73)	0·82 (0·76–0·88)	1·1 (1·05–1·16)
1953	0·0 (0·0–0·1)	18·0 (16·3–19·8)	136·1 (124·8–149·2)	132·1 (124·3–140·3)	118·0 (109·3–127·1)	63·2 (57·5–68·9)	25·7 (23·4–28·2)	4·2 (3·6–4·9)	0·1 (0·1–0·1)	2·49 (2·35–2·63)	0·77 (0·71–0·84)	1·06 (1·01–1·11)
1954	0·0 (0·0–0·1)	16·7 (15·1–18·3)	128·9 (117·9–140·8)	134·9 (127·4–142·8)	114·6 (106·6–123·1)	63·6 (58·1–69·2)	25·0 (22·7–27·3)	3·8 (3·3–4·4)	0·1 (0·1–0·1)	2·44 (2·3–2·58)	0·73 (0·67–0·79)	1·04 (0·98–1·09)
1955	0·0 (0·0–0·1)	15·6 (14·1–17·1)	122·4 (112·4–133·1)	139·2 (132·2–146·4)	113·7 (106·2–121·2)	66·1 (61·1–72·0)	24·9 (22·7–27·3)	3·5 (3·0–4·1)	0·1 (0·1–0·1)	2·43 (2·3–2·55)	0·69 (0·64–0·74)	1·04 (0·99–1·1)
1956	0·0 (0·0–0·1)	15·0 (13·5–16·6)	118·1 (109·8–126·4)	145·1 (139·0–151·2)	117·0 (110·4–123·6)	69·6 (64·8–75·2)	25·2 (22·8–27·8)	3·4 (2·8–4·0)	0·1 (0·1–0·1)	2·47 (2·38–2·56)	0·67 (0·62–0·71)	1·08 (1·02–1·13)
1957	0·0 (0·0–0·1)	15·1 (13·5–16·7)	116·8 (111·6–122·5)	151·6 (147·3–156·0)	120·4 (115·5–125·1)	72·6 (68·3–77·2)	25·3 (22·6–28·2)	3·2 (2·6–3·9)	0·1 (0·1–0·1)	2·53 (2·46–2·59)	0·66 (0·63–0·69)	1·11 (1·07–1·14)
1958	0·0 (0·0–0·1)	15·6 (14·0–17·4)	118·5 (115·0–122·3)	155·7 (152·5–159·0)	120·9 (117·4–124·5)	73·4 (69·5–77·6)	25·2 (22·1–28·5)	3·0 (2·4–3·8)	0·1 (0·1–0·1)	2·56 (2·52–2·61)	0·67 (0·65–0·69)	1·11 (1·08–1·14)
1959	0·0 (0·0–0·1)	16·4 (14·6–18·4)	120·8 (117·0–124·5)	156·3 (153·1–159·5)	120·2 (116·7–123·8)	71·6 (68·1–75·3)	25·0 (21·8–28·6)	2·8 (2·1–3·6)	0·1 (0·1–0·1)	2·57 (2·51–2·62)	0·69 (0·66–0·71)	1·1 (1·06–1·13)
1960	0·0 (0·0–0·1)	16·9 (15·1–19·1)	123·2 (119·5–126·9)	153·0 (149·8–156·2)	118·0 (114·6–121·6)	67·9 (64·5–71·5)	24·8 (21·6–28·4)	2·7 (1·9–3·5)	0·1 (0·0–0·1)	2·53 (2·49–2·58)	0·7 (0·68–0·72)	1·07 (1·03–1·1)
1961	0·0 (0·0–0·1)	16·8 (14·7–19·0)	123·5 (120·1–127·4)	145·7 (142·6–148·7)	113·2 (110·0–116·5)	64·1 (60·7–67·9)	24·6 (21·6–28·2)	2·4 (1·7–3·3)	0·0 (0·0–0·0)	2·45 (2·41–2·5)	0·7 (0·68–0·72)	1·02 (0·99–1·05)
1962	0·0 (0·0–0·1)	16·2 (14·2–18·3)	120·1 (116·6–123·6)	136·6 (133·3–139·8)	109·0 (105·8–112·3)	61·1 (57·6–65·0)	23·9 (20·7–27·3)	2·2 (1·5–3·1)	0·0 (0·0–0·0)	2·35 (2·3–2·39)	0·68 (0·66–0·7)	0·98 (0·95–1·02)
1963	0·0 (0·0–0·1)	15·8 (14·0–17·9)	117·3 (113·9–120·7)	129·3 (126·2–132·2)	106·2 (103·0–109·6)	58·4 (55·1–61·8)	22·6 (19·7–25·7)	2·1 (1·4–2·9)	0·0 (0·0–0·0)	2·26 (2·21–2·3)	0·67 (0·65–0·69)	0·95 (0·91–0·98)
1964	0·0 (0·0–0·1)	16·0 (14·1–18·1)	118·3 (114·8–122·1)	125·9 (122·9–128·7)	101·4 (98·2–104·7)	56·0 (52·7–59·3)	21·0 (18·3–24·0)	1·9 (1·3–2·7)	0·0 (0·0–0·0)	2·2 (2·16–2·25)	0·67 (0·65–0·69)	0·9 (0·87–0·93)
1965	0·0 (0·0–0·1)	16·5 (14·5–18·6)	122·4 (118·7–126·6)	126·7 (123·7–130·0)	95·7 (92·5–99·0)	54·2 (50·9–57·7)	19·5 (16·7–22·2)	1·8 (1·2–2·6)	0·0 (0·0–0·0)	2·18 (2·14–2·23)	0·69 (0·67–0·72)	0·86 (0·83–0·89)
1966	0·0 (0·0–0·1)	17·4 (15·3–19·7)	127·6 (124·0–131·6)	129·7 (126·6–132·9)	90·9 (87·7–94·2)	52·7 (49·5–56·0)	18·0 (15·6–20·6)	1·7 (1·1–2·5)	0·0 (0·0–0·0)	2·19 (2·15–2·23)	0·72 (0·7–0·75)	0·82 (0·79–0·85)
1967	0·0 (0·0–0·1)	18·8 (16·7–21·1)	134·5 (130·5–138·4)	132·9 (129·8–136·2)	87·5 (84·1–90·7)	50·1 (46·9–53·3)	16·4 (14·0–19·1)	1·6 (1·0–2·4)	0·0 (0·0–0·0)	2·21 (2·17–2·25)	0·77 (0·74–0·79)	0·78 (0·75–0·81)
1968	0·0 (0·0–0·1)	20·5 (18·3–22·7)	143·4 (139·4–147·4)	135·3 (132·1–138·5)	85·6 (82·4–88·8)	47·8 (44·6–51·2)	15·4 (13·2–18·0)	1·6 (1·0–2·3)	0·0 (0·0–0·0)	2·25 (2·21–2·29)	0·82 (0·8–0·84)	0·75 (0·72–0·78)
1969	0·0 (0·0–0·1)	22·0 (19·9–24·2)	151·8 (148·1–155·9)	136·5 (133·2–139·9)	85·0 (81·9–88·4)	47·5 (44·2–50·8)	15·4 (13·3–18·0)	1·5 (1·0–2·2)	0·0 (0·0–0·0)	2·3 (2·26–2·34)	0·87 (0·85–0·89)	0·75 (0·72–0·78)
1970	0·0 (0·0–0·1)	22·8 (20·7–25·0)	157·6 (154·0–161·4)	135·6 (132·5–138·7)	85·0 (81·7–88·4)	47·2 (44·0–50·5)	15·5 (13·2–18·0)	1·4 (0·9–2·1)	0·0 (0·0–0·0)	2·33 (2·28–2·37)	0·9 (0·88–0·93)	0·75 (0·72–0·78)
1971	0·0 (0·0–0·1)	22·7 (20·8–24·8)	158·3 (154·7–162·2)	132·5 (129·2–135·9)	83·3 (80·1–86·5)	45·0 (41·9–48·3)	14·5 (12·3–16·9)	1·2 (0·8–1·9)	0·0 (0·0–0·0)	2·29 (2·25–2·33)	0·91 (0·88–0·93)	0·72 (0·69–0·75)
1972	0·0 (0·0–0·1)	22·3 (20·4–24·4)	153·8 (150·2–157·8)	129·6 (126·3–132·9)	79·5 (76·2–82·5)	42·0 (38·9–45·3)	13·1 (11·0–15·5)	1·1 (0·7–1·7)	0·0 (0·0–0·0)	2·21 (2·17–2·25)	0·88 (0·86–0·9)	0·68 (0·65–0·71)
1973	0·0 (0·0–0·1)	22·3 (20·2–24·4)	150·2 (146·5–153·8)	128·9 (125·7–132·2)	76·0 (72·9–78·9)	40·0 (36·9–43·2)	12·1 (10·1–14·4)	1·0 (0·6–1·5)	0·0 (0·0–0·0)	2·15 (2·11–2·2)	0·86 (0·84–0·88)	0·65 (0·62–0·67)
1974	0·0 (0·0–0·1)	22·7 (20·5–24·8)	150·4 (146·4–154·5)	129·4 (126·1–132·9)	73·5 (70·5–76·8)	38·8 (35·6–42·0)	11·6 (9·6–13·8)	0·9 (0·6–1·4)	0·0 (0·0–0·0)	2·14 (2·1–2·18)	0·87 (0·84–0·89)	0·62 (0·59–0·66)
1975	0·0 (0·0–0·1)	23·3 (21·2–25·5)	151·9 (148·2–155·6)	129·3 (126·1–132·6)	71·2 (68·0–74·6)	37·4 (34·5–40·5)	11·2 (9·0–13·3)	0·8 (0·5–1·3)	0·0 (0·0–0·0)	2·13 (2·09–2·16)	0·88 (0·86–0·9)	0·6 (0·58–0·63)
1976	0·0 (0·0–0·1)	24·3 (22·3–26·5)	152·9 (149·1–156·6)	127·3 (124·0–130·7)	68·6 (65·6–71·8)	35·7 (33·0–38·7)	10·7 (8·7–12·9)	0·8 (0·5–1·2)	0·0 (0·0–0·0)	2·1 (2·06–2·15)	0·89 (0·86–0·91)	0·58 (0·55–0·61)
1977	0·0 (0·0–0·1)	25·5 (23·4–27·8)	153·1 (149·4–156·7)	123·1 (119·9–126·2)	66·7 (63·7–69·8)	33·8 (30·8–36·9)	10·3 (8·3–12·5)	0·7 (0·5–1·1)	0·0 (0·0–0·0)	2·07 (2·03–2·11)	0·89 (0·87–0·91)	0·56 (0·53–0·59)
1978	0·0 (0·0–0·1)	26·5 (24·3–28·8)	153·7 (150·3–157·5)	118·6 (115·4–121·6)	65·3 (62·1–68·5)	32·0 (29·3–35·0)	9·9 (8·0–12·1)	0·7 (0·4–1·0)	0·0 (0·0–0·0)	2·03 (1·99–2·08)	0·9 (0·88–0·92)	0·54 (0·51–0·57)
1979	0·0 (0·0–0·1)	26·6 (24·2–29·1)	153·4 (149·9–157·1)	116·2 (113·3–119·3)	63·8 (60·8–67·1)	30·0 (27·2–32·9)	9·3 (7·4–11·4)	0·6 (0·4–0·9)	0·0 (0·0–0·0)	2·0 (1·96–2·04)	0·9 (0·88–0·92)	0·52 (0·5–0·54)
1980	0·0 (0·0–0·1)	26·0 (23·6–28·8)	149·3 (146·0–152·8)	117·4 (114·6–120·5)	63·0 (59·9–66·2)	28·7 (26·0–31·8)	8·8 (7·1–10·9)	0·6 (0·4–0·9)	0·0 (0·0–0·0)	1·97 (1·94–2·01)	0·88 (0·86–0·9)	0·51 (0·48–0·53)
1981	0·0 (0·0–0·1)	25·9 (23·1–29·3)	146·1 (142·7–149·5)	122·0 (118·7–125·1)	64·0 (61·1–67·1)	28·8 (26·0–31·9)	8·5 (6·8–10·6)	0·5 (0·3–0·8)	0·0 (0·0–0·0)	1·98 (1·95–2·01)	0·86 (0·84–0·88)	0·51 (0·49–0·53)
1982	0·0 (0·0–0·1)	26·5 (23·2–30·3)	147·5 (144·0–150·7)	128·5 (125·5–131·7)	65·9 (62·9–69·0)	29·4 (26·7–32·5)	8·3 (6·6–10·3)	0·5 (0·3–0·8)	0·0 (0·0–0·0)	2·03 (1·99–2·07)	0·87 (0·85–0·9)	0·52 (0·5–0·55)
1983	0·0 (0·0–0·1)	26·9 (23·3–31·4)	150·1 (146·7–153·5)	133·6 (130·6–136·6)	66·8 (63·7–69·9)	30·2 (27·4–33·2)	8·0 (6·3–10·1)	0·5 (0·3–0·7)	0·0 (0·0–0·0)	2·08 (2·04–2·12)	0·88 (0·86–0·91)	0·53 (0·5–0·55)
1984	0·0 (0·0–0·1)	26·7 (23·3–30·9)	152·1 (148·6–155·8)	135·2 (132·3–138·3)	67·3 (64·3–70·4)	30·2 (27·4–33·3)	7·7 (6·0–9·7)	0·4 (0·3–0·7)	0·0 (0·0–0·0)	2·1 (2·06–2·14)	0·89 (0·87–0·92)	0·53 (0·5–0·55)
1985	0·0 (0·0–0·1)	26·4 (23·4–29·9)	153·3 (150·0–156·5)	134·3 (131·3–137·5)	67·9 (65·0–71·2)	29·8 (26·9–32·7)	7·3 (5·6–9·2)	0·4 (0·3–0·6)	0·0 (0·0–0·0)	2·1 (2·06–2·14)	0·9 (0·88–0·92)	0·53 (0·5–0·55)
1986	0·0 (0·0–0·1)	27·3 (24·6–30·2)	154·6 (151·1–158·2)	129·2 (126·3–132·1)	66·6 (63·6–69·5)	28·7 (25·9–31·6)	6·9 (5·3–8·7)	0·4 (0·2–0·6)	0·0 (0·0–0·0)	2·07 (2·03–2·1)	0·91 (0·89–0·93)	0·51 (0·49–0·54)
1987	0·0 (0·0–0·1)	29·9 (27·5–32·7)	157·0 (153·4–160·6)	120·7 (117·8–123·6)	62·8 (59·8–66·2)	26·8 (23·9–29·8)	6·4 (5·0–8·2)	0·4 (0·2–0·5)	0·0 (0·0–0·0)	2·02 (1·98–2·06)	0·94 (0·91–0·96)	0·48 (0·46–0·51)
1988	0·1 (0·0–0·1)	33·8 (31·2–36·5)	159·5 (156·0–162·9)	113·2 (110·4–116·0)	59·1 (56·0–61·9)	25·0 (22·2–27·9)	6·1 (4·6–8·0)	0·3 (0·2–0·5)	0·0 (0·0–0·0)	1·99 (1·95–2·03)	0·97 (0·95–0·99)	0·45 (0·43–0·48)
1989	0·1 (0·0–0·1)	38·3 (35·7–41·0)	161·7 (157·9–165·3)	109·3 (106·5–112·2)	57·7 (54·9–60·5)	24·2 (21·6–27·2)	5·8 (4·4–7·8)	0·3 (0·2–0·5)	0·0 (0·0–0·0)	1·99 (1·95–2·02)	1·0 (0·98–1·02)	0·44 (0·42–0·46)
1990	0·1 (0·0–0·1)	42·4 (39·7–45·0)	162·2 (158·8–166·0)	106·5 (103·7–109·3)	56·9 (54·0–59·6)	23·4 (20·9–26·0)	5·4 (4·1–7·4)	0·3 (0·2–0·4)	0·0 (0·0–0·0)	1·99 (1·95–2·02)	1·02 (1·0–1·05)	0·43 (0·41–0·45)
1991	0·1 (0·0–0·1)	44·2 (41·5–46·8)	157·8 (154·4–161·3)	101·3 (98·5–103·8)	52·9 (50·2–55·7)	20·8 (18·6–23·3)	4·7 (3·6–6·4)	0·3 (0·2–0·4)	0·0 (0·0–0·0)	1·91 (1·88–1·94)	1·01 (0·99–1·03)	0·39 (0·37–0·42)
1992	0·1 (0·0–0·1)	42·8 (39·9–45·6)	144·7 (141·1–148·5)	94·0 (91·1–96·5)	46·1 (43·6–48·8)	19·1 (16·7–21·7)	4·4 (3·3–5·8)	0·2 (0·1–0·3)	0·0 (0·0–0·0)	1·76 (1·73–1·79)	0·94 (0·91–0·96)	0·35 (0·33–0·37)
1993	0·1 (0·0–0·1)	40·5 (37·7–43·2)	129·7 (125·9–133·3)	87·6 (84·7–90·3)	40·3 (37·8–42·9)	18·7 (16·3–21·8)	4·5 (3·5–5·8)	0·2 (0·1–0·3)	0·0 (0·0–0·0)	1·61 (1·57–1·64)	0·85 (0·83–0·87)	0·32 (0·3–0·34)
1994	0·1 (0·0–0·1)	38·2 (35·7–41·0)	119·7 (116·2–123·2)	84·5 (81·7–87·3)	39·2 (36·8–41·7)	18·5 (16·1–21·2)	4·6 (3·8–5·7)	0·2 (0·1–0·3)	0·0 (0·0–0·0)	1·53 (1·49–1·56)	0·79 (0·77–0·81)	0·31 (0·29–0·33)
1995	0·1 (0·0–0·1)	35·5 (33·1–38·0)	112·5 (109·3–115·9)	83·8 (81·2–86·7)	41·3 (38·8–44·2)	18·7 (16·5–21·1)	4·8 (4·0–5·7)	0·2 (0·1–0·3)	0·0 (0·0–0·0)	1·48 (1·45–1·52)	0·74 (0·72–0·76)	0·33 (0·31–0·34)
1996	0·1 (0·0–0·1)	32·6 (30·3–35·0)	106·9 (103·4–110·5)	83·9 (81·1–86·8)	42·5 (39·8–45·3)	18·7 (16·5–21·1)	4·9 (4·1–5·7)	0·2 (0·1–0·3)	0·0 (0·0–0·0)	1·45 (1·41–1·48)	0·7 (0·68–0·72)	0·33 (0·31–0·35)
1997	0·0 (0·0–0·1)	29·7 (27·3–32·1)	103·1 (99·7–106·6)	84·3 (81·5–87·3)	43·0 (40·4–45·8)	18·5 (16·2–21·0)	4·9 (4·0–6·0)	0·2 (0·1–0·3)	0·0 (0·0–0·0)	1·42 (1·39–1·45)	0·66 (0·64–0·69)	0·33 (0·31–0·35)
1998	0·0 (0·0–0·1)	26·4 (24·3–28·8)	100·0 (96·6–103·4)	84·6 (81·8–87·6)	43·9 (41·2–46·6)	18·7 (16·3–21·2)	5·0 (4·0–6·2)	0·2 (0·1–0·3)	0·0 (0·0–0·0)	1·39 (1·36–1·43)	0·63 (0·61–0·66)	0·34 (0·32–0·36)
1999	0·0 (0·0–0·1)	22·6 (20·7–24·7)	94·7 (91·3–98·3)	83·8 (80·9–86·8)	45·6 (42·9–48·3)	19·4 (17·0–21·9)	5·2 (4·3–6·3)	0·2 (0·1–0·3)	0·0 (0·0–0·0)	1·36 (1·32–1·4)	0·59 (0·56–0·61)	0·35 (0·33–0·37)
2000	0·0 (0·0–0·1)	19·5 (17·9–21·3)	87·5 (84·0–91·2)	82·6 (79·8–85·6)	47·0 (44·0–49·7)	20·3 (17·9–22·8)	5·4 (4·5–6·3)	0·2 (0·2–0·3)	0·0 (0·0–0·0)	1·31 (1·27–1·35)	0·54 (0·51–0·56)	0·36 (0·34–0·39)
2001	0·0 (0·0–0·1)	18·5 (16·9–20·4)	82·3 (79·0–85·9)	82·1 (79·2–85·1)	46·6 (43·7–49·5)	20·3 (18·1–22·8)	5·3 (4·5–6·3)	0·2 (0·2–0·3)	0·0 (0·0–0·0)	1·28 (1·24–1·32)	0·5 (0·49–0·53)	0·36 (0·34–0·38)
2002	0·0 (0·0–0·1)	18·7 (16·9–20·6)	79·8 (76·1–83·5)	83·4 (80·3–86·5)	46·3 (43·5–49·4)	20·0 (17·7–22·9)	5·2 (4·2–6·5)	0·2 (0·2–0·3)	0·0 (0·0–0·0)	1·27 (1·23–1·31)	0·49 (0·47–0·51)	0·36 (0·34–0·38)
2003	0·0 (0·0–0·1)	18·8 (16·9–20·7)	77·1 (73·6–80·5)	86·2 (83·2–89·6)	47·9 (45·0–50·9)	20·2 (17·7–23·0)	5·1 (3·9–6·5)	0·2 (0·2–0·3)	0·0 (0·0–0·0)	1·28 (1·24–1·32)	0·48 (0·46–0·5)	0·37 (0·35–0·39)
2004	0·0 (0·0–0·1)	18·8 (16·9–20·8)	74·1 (70·7–77·9)	90·1 (87·0–93·3)	51·3 (48·2–54·4)	21·1 (18·4–24·0)	5·1 (3·8–6·6)	0·2 (0·2–0·3)	0·0 (0·0–0·0)	1·3 (1·26–1·35)	0·46 (0·44–0·49)	0·39 (0·36–0·41)
2005	0·0 (0·0–0·1)	19·1 (17·0–21·2)	72·8 (69·4–76·4)	96·2 (93·1–99·3)	55·8 (52·8–59·1)	22·5 (19·7–25·2)	5·0 (3·7–6·7)	0·2 (0·1–0·3)	0·0 (0·0–0·0)	1·36 (1·31–1·41)	0·46 (0·44–0·48)	0·42 (0·39–0·44)
2006	0·0 (0·0–0·1)	19·4 (17·3–21·6)	73·5 (70·0–76·9)	105·3 (102·0–108·7)	62·0 (58·9–65·2)	24·3 (21·7–27·0)	5·1 (3·8–6·7)	0·2 (0·1–0·3)	0·0 (0·0–0·0)	1·45 (1·41–1·49)	0·46 (0·44–0·49)	0·46 (0·44–0·48)
2007	0·0 (0·0–0·1)	19·3 (17·2–21·6)	74·5 (71·2–77·8)	116·5 (113·0–120·0)	70·4 (66·9–73·8)	26·8 (23·9–29·8)	5·2 (3·9–6·8)	0·2 (0·1–0·3)	0·0 (0·0–0·0)	1·57 (1·52–1·61)	0·47 (0·45–0·49)	0·51 (0·49–0·54)
2008	0·0 (0·0–0·1)	18·3 (16·3–20·3)	73·4 (69·9–76·8)	127·2 (123·9–130·7)	80·2 (76·6–83·6)	29·8 (26·9–32·8)	5·4 (4·0–7·1)	0·2 (0·1–0·3)	0·0 (0·0–0·0)	1·67 (1·63–1·71)	0·46 (0·44–0·48)	0·58 (0·55–0·61)
2009	0·0 (0·0–0·1)	16·7 (14·7–18·8)	69·1 (65·8–72·4)	133·0 (129·6–136·7)	88·1 (84·4–91·6)	32·5 (29·5–35·4)	5·7 (4·3–7·5)	0·2 (0·1–0·3)	0·0 (0·0–0·0)	1·73 (1·69–1·76)	0·43 (0·41–0·45)	0·63 (0·61–0·66)
2010	0·0 (0·0–0·1)	15·2 (13·2–17·4)	63·5 (60·1–66·9)	131·1 (128·0–134·7)	91·0 (87·2–94·6)	34·2 (31·2–37·3)	6·1 (4·6–8·0)	0·2 (0·2–0·4)	0·0 (0·0–0·0)	1·71 (1·67–1·74)	0·39 (0·38–0·41)	0·66 (0·63–0·68)
2011	0·0 (0·0–0·1)	14·0 (12·1–16·0)	58·2 (54·8–61·5)	123·3 (119·9–126·7)	90·4 (86·8–94·2)	35·3 (32·1–38·5)	6·5 (5·0–8·5)	0·3 (0·2–0·4)	0·0 (0·0–0·0)	1·64 (1·6–1·68)	0·36 (0·34–0·38)	0·66 (0·64–0·69)
2012	0·0 (0·0–0·1)	13·3 (11·6–15·4)	54·3 (50·9–57·8)	115·7 (112·3–119·0)	90·6 (87·0–94·6)	36·6 (33·4–40·1)	6·9 (5·4–8·7)	0·3 (0·2–0·4)	0·0 (0·0–0·0)	1·59 (1·55–1·63)	0·34 (0·32–0·36)	0·67 (0·65–0·7)
2013	0·0 (0·0–0·1)	13·3 (11·5–15·3)	52·6 (49·2–56·2)	112·7 (109·1–116·1)	93·9 (89·8–97·6)	38·4 (35·2–42·1)	7·0 (5·6–8·9)	0·3 (0·2–0·4)	0·0 (0·0–0·0)	1·59 (1·55–1·63)	0·33 (0·31–0·35)	0·7 (0·67–0·73)
2014	0·0 (0·0–0·1)	13·8 (12·0–15·7)	53·2 (49·5–56·9)	113·4 (109·7–117·2)	97·5 (93·6–101·4)	40·1 (36·6–43·8)	7·0 (5·8–8·5)	0·3 (0·2–0·4)	0·0 (0·0–0·0)	1·63 (1·58–1·67)	0·33 (0·31–0·36)	0·72 (0·69–0·76)
2015	0·0 (0·0–0·1)	14·7 (12·9–16·7)	55·5 (50·9–60·8)	115·0 (109·9–120·0)	97·2 (91·5–102·8)	39·7 (35·6–43·8)	6·7 (5·7–7·9)	0·3 (0·2–0·3)	0·0 (0·0–0·0)	1·65 (1·57–1·73)	0·35 (0·32–0·38)	0·72 (0·68–0·76)
2016	0·0 (0·0–0·1)	15·5 (13·6–17·9)	58·7 (51·7–66·5)	115·3 (107·8–123·1)	93·1 (85·1–101·2)	37·7 (32·4–43·2)	6·4 (5·4–7·6)	0·2 (0·2–0·3)	0·0 (0·0–0·0)	1·63 (1·5–1·78)	0·37 (0·33–0·42)	0·69 (0·62–0·75)
2017	0·0 (0·0–0·1)	15·8 (13·6–18·5)	61·1 (51·8–72·0)	114·5 (105·7–124·2)	89·8 (80·0–100·8)	36·2 (29·7–43·8)	6·3 (5·0–7·7)	0·2 (0·2–0·3)	0·0 (0·0–0·0)	1·62 (1·44–1·82)	0·39 (0·33–0·45)	0·66 (0·58–0·75)

Supplemental Results Table 5: Age-specific fertility rates per 1000, cumulative total fertility up to maternal age 25 (TFU25), cumulative total fertility from maternal age 30 to age 55 (TFO30), and cumulative total fertility, Moldova, 1950-2017											
Year	10-14	15-19	20-24	25-29	30-34	35-39	40-44	45-49	50-54	TFR	TFU25	TFO30
1950	0·1 (0·0–0·1)	46·5 (40·9–52·6)	219·9 (203·9–236·1)	186·3 (176·1–196·7)	144·9 (134·8–155·2)	110·5 (101·5–119·8)	58·1 (53·5–62·8)	6·9 (4·9–9·5)	0·1 (0·1–0·1)	3·87 (3·66–4·07)	1·33 (1·22–1·44)	1·6 (1·51–1·69)
1951	0·1 (0·0–0·1)	45·4 (40·1–51·2)	215·5 (200·5–231·0)	178·3 (168·7–188·1)	140·4 (131·2–149·7)	105·0 (96·9–113·2)	54·9 (50·9–59·0)	6·5 (4·7–8·9)	0·1 (0·1–0·1)	3·73 (3·54–3·9)	1·3 (1·21–1·41)	1·53 (1·46–1·61)
1952	0·1 (0·0–0·1)	44·6 (39·6–50·1)	211·5 (197·0–226·4)	172·0 (162·6–181·6)	136·7 (128·0–145·3)	100·3 (93·0–108·3)	52·2 (48·4–56·0)	6·2 (4·4–8·4)	0·1 (0·1–0·1)	3·62 (3·45–3·79)	1·28 (1·19–1·38)	1·48 (1·41–1·55)
1953	0·1 (0·0–0·1)	44·1 (39·3–49·2)	208·4 (194·7–222·1)	167·6 (158·3–176·9)	134·1 (125·7–142·9)	96·7 (89·5–104·3)	50·1 (46·4–53·9)	5·9 (4·2–7·9)	0·1 (0·1–0·1)	3·54 (3·38–3·7)	1·26 (1·18–1·35)	1·43 (1·37–1·5)
1954	0·1 (0·0–0·1)	43·9 (39·3–49·1)	205·8 (192·7–219·1)	164·7 (156·0–173·4)	132·3 (124·2–140·5)	94·0 (86·9–101·3)	48·5 (45·0–52·1)	5·6 (4·0–7·6)	0·1 (0·1–0·1)	3·47 (3·32–3·64)	1·25 (1·17–1·33)	1·4 (1·34–1·47)
1955	0·1 (0·0–0·1)	43·9 (39·6–48·8)	203·7 (190·7–216·4)	163·4 (154·8–172·0)	131·3 (123·5–139·4)	92·3 (85·0–99·5)	47·5 (44·0–51·1)	5·3 (3·8–7·2)	0·1 (0·1–0·1)	3·44 (3·27–3·62)	1·24 (1·17–1·31)	1·38 (1·31–1·46)
1956	0·1 (0·0–0·1)	44·2 (39·7–49·1)	202·2 (189·0–215·0)	163·4 (154·7–171·7)	131·1 (123·3–139·2)	91·3 (84·1–98·4)	47·0 (43·5–50·8)	5·0 (3·6–6·9)	0·1 (0·1–0·1)	3·42 (3·24–3·61)	1·23 (1·16–1·3)	1·37 (1·29–1·46)
1957	0·1 (0·0–0·1)	44·5 (39·9–49·4)	200·6 (187·7–213·3)	162·9 (154·6–171·1)	130·7 (122·9–138·5)	90·2 (83·4–97·1)	46·4 (43·0–50·1)	4·8 (3·4–6·5)	0·1 (0·1–0·1)	3·4 (3·21–3·6)	1·23 (1·15–1·29)	1·36 (1·27–1·45)
1958	0·1 (0·0–0·1)	44·4 (39·8–49·3)	198·1 (184·7–211·2)	160·4 (152·0–169·3)	129·4 (121·7–137·0)	88·2 (81·3–95·1)	45·4 (42·0–48·8)	4·5 (3·1–6·2)	0·1 (0·1–0·1)	3·35 (3·17–3·55)	1·21 (1·14–1·28)	1·34 (1·25–1·42)
1959	0·1 (0·0–0·1)	43·7 (39·0–48·7)	194·2 (180·8–207·3)	155·1 (146·5–164·1)	126·6 (118·8–134·3)	84·9 (78·0–91·8)	43·7 (40·3–47·0)	4·2 (2·9–5·9)	0·1 (0·1–0·1)	3·26 (3·09–3·44)	1·19 (1·12–1·26)	1·3 (1·22–1·37)
1960	0·1 (0·0–0·1)	42·7 (37·9–47·7)	189·0 (175·6–202·7)	147·2 (139·0–156·1)	122·5 (114·6–130·0)	80·6 (73·9–87·3)	41·3 (38·1–44·8)	3·8 (2·7–5·4)	0·1 (0·1–0·1)	3·14 (2·98–3·3)	1·16 (1·08–1·23)	1·24 (1·17–1·31)
1961	0·1 (0·0–0·1)	41·9 (37·0–46·9)	183·2 (170·1–196·6)	142·2 (133·6–151·1)	118·0 (110·2–125·9)	76·0 (69·1–82·9)	38·6 (35·3–42·2)	3·5 (2·4–4·8)	0·1 (0·1–0·1)	3·02 (2·87–3·16)	1·13 (1·06–1·2)	1·18 (1·11–1·25)
1962	0·1 (0·0–0·1)	41·5 (36·8–46·8)	178·7 (165·6–191·8)	140·9 (132·1–149·8)	113·8 (105·8–122·2)	71·8 (65·1–79·2)	35·9 (32·6–39·5)	3·2 (2·2–4·5)	0·1 (0·1–0·1)	2·93 (2·78–3·08)	1·1 (1·03–1·17)	1·12 (1·06–1·19)
1963	0·1 (0·0–0·1)	41·6 (36·5–47·1)	176·1 (162·3–189·6)	139·4 (130·1–148·9)	110·3 (101·8–119·5)	68·2 (60·6–76·3)	33·4 (29·9–37·2)	3·0 (2·1–4·2)	0·1 (0·1–0·1)	2·86 (2·7–3·02)	1·09 (1·02–1·16)	1·07 (1·01–1·15)
1964	0·1 (0·0–0·1)	41·9 (36·8–47·7)	174·9 (160·4–189·4)	137·6 (128·3–146·9)	107·3 (98·6–117·3)	64·9 (56·6–73·7)	31·0 (27·2–35·0)	2·8 (1·9–3·9)	0·1 (0·1–0·1)	2·8 (2·63–2·99)	1·08 (1·01–1·16)	1·03 (0·95–1·12)
1965	0·1 (0·0–0·1)	42·3 (37·2–48·5)	174·2 (157·9–190·5)	135·7 (125·6–145·9)	104·5 (95·0–115·2)	61·7 (52·1–71·8)	28·7 (24·3–33·3)	2·6 (1·8–3·7)	0·0 (0·0–0·1)	2·75 (2·55–2·95)	1·08 (1·0–1·17)	0·99 (0·9–1·09)
1966	0·1 (0·0–0·1)	42·8 (37·6–49·5)	173·6 (156·2–191·3)	133·7 (123·2–143·7)	101·8 (91·5–113·0)	58·5 (47·9–69·6)	26·5 (21·8–31·7)	2·4 (1·6–3·5)	0·0 (0·0–0·0)	2·7 (2·48–2·92)	1·08 (0·99–1·18)	0·95 (0·84–1·06)
1967	0·1 (0·0–0·1)	43·4 (37·9–50·4)	173·0 (155·5–191·1)	131·5 (121·0–141·9)	99·0 (88·0–110·6)	55·3 (44·8–66·8)	24·2 (19·5–29·6)	2·2 (1·5–3·3)	0·0 (0·0–0·0)	2·64 (2·41–2·9)	1·08 (0·99–1·18)	0·9 (0·79–1·03)
1968	0·1 (0·0–0·1)	44·0 (38·4–50·9)	172·6 (154·3–191·0)	129·6 (118·8–139·8)	96·4 (84·9–108·5)	52·4 (41·4–64·2)	22·2 (17·6–27·6)	2·0 (1·3–3·0)	0·0 (0·0–0·0)	2·6 (2·34–2·87)	1·08 (0·99–1·18)	0·87 (0·74–1·01)
1969	0·1 (0·0–0·1)	44·4 (39·0–51·1)	173·0 (155·0–192·6)	128·1 (117·6–138·8)	94·3 (83·0–106·8)	49·9 (39·6–61·8)	20·4 (15·9–25·8)	1·9 (1·2–2·8)	0·0 (0·0–0·0)	2·56 (2·3–2·85)	1·09 (0·99–1·19)	0·83 (0·7–0·98)
1970	0·1 (0·0–0·1)	44·6 (39·3–50·9)	173·9 (155·8–193·5)	126·6 (116·8–137·4)	92·3 (81·3–104·8)	47·5 (37·5–59·3)	18·8 (14·4–23·9)	1·7 (1·1–2·6)	0·0 (0·0–0·0)	2·53 (2·26–2·83)	1·09 (1·0–1·19)	0·8 (0·67–0·95)
1971	0·1 (0·0–0·1)	44·8 (39·4–50·9)	174·5 (156·9–194·5)	125·0 (114·9–136·0)	90·2 (79·4–102·3)	45·0 (35·4–56·5)	17·1 (12·9–22·3)	1·6 (1·0–2·4)	0·0 (0·0–0·0)	2·49 (2·23–2·78)	1·1 (1·0–1·2)	0·77 (0·65–0·91)
1972	0·1 (0·0–0·1)	45·0 (39·6–50·8)	174·6 (156·6–193·3)	123·2 (113·1–134·2)	88·1 (77·5–99·6)	42·3 (33·2–52·6)	15·4 (11·5–20·1)	1·5 (0·9–2·2)	0·0 (0·0–0·0)	2·45 (2·2–2·73)	1·1 (1·01–1·19)	0·74 (0·62–0·87)
1973	0·1 (0·0–0·1)	45·0 (39·6–50·6)	174·3 (156·8–192·0)	121·8 (111·9–132·3)	86·0 (75·4–96·9)	39·8 (31·5–49·3)	13·9 (10·2–18·3)	1·3 (0·9–2·0)	0·0 (0·0–0·0)	2·41 (2·19–2·66)	1·1 (1·01–1·19)	0·7 (0·61–0·82)
1974	0·1 (0·0–0·1)	44·5 (39·4–49·9)	174·3 (158·2–190·6)	121·0 (111·6–131·0)	83·4 (73·2–94·0)	37·5 (29·8–45·9)	12·5 (9·2–16·5)	1·2 (0·8–1·8)	0·0 (0·0–0·0)	2·37 (2·18–2·59)	1·09 (1·01–1·18)	0·67 (0·58–0·77)
1975	0·1 (0·0–0·1)	44·0 (39·1–49·2)	174·1 (158·8–189·6)	120·5 (111·3–130·7)	80·7 (71·1–91·1)	35·5 (28·6–43·2)	11·4 (8·4–14·7)	1·1 (0·7–1·6)	0·0 (0·0–0·0)	2·34 (2·17–2·52)	1·09 (1·02–1·17)	0·64 (0·56–0·73)
1976	0·1 (0·0–0·1)	43·9 (38·9–48·9)	174·3 (160·5–189·2)	120·2 (111·2–130·0)	79·0 (70·3–88·6)	34·1 (28·0–41·1)	10·5 (8·0–13·4)	1·0 (0·7–1·5)	0·0 (0·0–0·0)	2·32 (2·16–2·48)	1·09 (1·02–1·17)	0·62 (0·55–0·7)
1977	0·1 (0·0–0·1)	44·1 (39·4–48·9)	176·1 (163·4–190·1)	120·3 (111·9–129·4)	78·3 (70·2–86·9)	33·5 (28·0–39·6)	9·9 (7·8–12·2)	0·9 (0·6–1·4)	0·0 (0·0–0·0)	2·32 (2·18–2·47)	1·1 (1·04–1·17)	0·61 (0·55–0·68)
1978	0·1 (0·0–0·1)	44·2 (39·7–49·0)	178·6 (166·7–191·8)	120·8 (112·8–129·4)	78·1 (70·6–86·1)	33·0 (27·8–38·4)	9·5 (7·7–11·4)	0·9 (0·6–1·3)	0·0 (0·0–0·0)	2·33 (2·2–2·46)	1·11 (1·05–1·18)	0·61 (0·55–0·67)
1979	0·1 (0·0–0·1)	43·8 (39·5–48·4)	181·2 (169·8–193·2)	123·3 (115·5–131·3)	78·4 (71·3–85·8)	32·6 (28·0–37·2)	9·1 (7·7–10·7)	0·8 (0·5–1·2)	0·0 (0·0–0·0)	2·35 (2·24–2·46)	1·13 (1·06–1·19)	0·6 (0·55–0·66)
1980	0·1 (0·0–0·1)	43·7 (39·4–48·0)	184·3 (173·2–196·0)	128·8 (121·5–136·8)	79·2 (72·5–85·5)	32·6 (28·7–37·0)	8·9 (7·7–10·1)	0·8 (0·5–1·1)	0·0 (0·0–0·0)	2·39 (2·29–2·5)	1·14 (1·08–1·2)	0·61 (0·55–0·66)
1981	0·1 (0·0–0·1)	44·5 (40·4–48·7)	187·8 (177·0–199·3)	136·1 (129·0–143·6)	80·4 (74·1–86·6)	33·3 (29·8–37·4)	8·6 (7·7–9·7)	0·7 (0·5–1·0)	0·0 (0·0–0·0)	2·46 (2·36–2·57)	1·16 (1·11–1·22)	0·62 (0·56–0·67)
1982	0·1 (0·0–0·1)	45·9 (41·8–50·2)	191·7 (181·8–202·4)	142·7 (135·7–150·0)	81·4 (75·5–87·6)	34·4 (31·0–38·3)	8·4 (7·5–9·4)	0·6 (0·4–0·9)	0·0 (0·0–0·0)	2·53 (2·42–2·64)	1·19 (1·13–1·25)	0·62 (0·57–0·68)
1983	0·1 (0·0–0·1)	47·6 (43·4–52·1)	194·4 (184·2–204·8)	146·7 (139·8–153·8)	81·8 (76·0–88·2)	35·2 (31·9–39·0)	8·1 (7·3–9·0)	0·6 (0·4–0·8)	0·0 (0·0–0·0)	2·57 (2·47–2·68)	1·21 (1·16–1·27)	0·63 (0·58–0·68)
1984	0·1 (0·0–0·1)	49·2 (45·1–53·9)	195·5 (184·9–205·5)	148·3 (141·5–155·4)	81·6 (76·2–87·5)	35·7 (32·5–39·5)	7·8 (7·0–8·7)	0·5 (0·4–0·7)	0·0 (0·0–0·0)	2·59 (2·49–2·71)	1·22 (1·17–1·28)	0·63 (0·59–0·67)
1985	0·1 (0·0–0·1)	50·8 (46·4–55·8)	195·5 (185·2–205·7)	147·5 (140·7–154·2)	80·5 (75·4–86·3)	35·6 (32·5–39·3)	7·6 (6·8–8·4)	0·5 (0·3–0·7)	0·0 (0·0–0·0)	2·59 (2·49–2·7)	1·23 (1·18–1·29)	0·62 (0·58–0·66)
1986	0·1 (0·0–0·2)	52·7 (48·1–57·5)	194·6 (185·1–204·9)	144·0 (137·6–150·6)	78·5 (73·6–84·0)	34·8 (31·4–38·2)	7·5 (6·7–8·2)	0·4 (0·3–0·6)	0·0 (0·0–0·0)	2·56 (2·47–2·67)	1·24 (1·18–1·29)	0·61 (0·57–0·64)
1987	0·1 (0·0–0·2)	54·5 (49·8–59·4)	193·2 (183·6–203·2)	138·4 (132·2–144·7)	75·5 (70·9–80·5)	33·1 (30·0–36·4)	7·2 (6·5–8·0)	0·4 (0·3–0·5)	0·0 (0·0–0·0)	2·51 (2·42–2·6)	1·24 (1·18–1·3)	0·58 (0·55–0·62)
1988	0·1 (0·0–0·2)	56·6 (52·0–61·8)	191·1 (180·9–200·9)	130·6 (124·6–136·4)	71·6 (67·0–76·4)	30·9 (28·0–33·8)	6·9 (6·3–7·6)	0·4 (0·3–0·5)	0·0 (0·0–0·0)	2·44 (2·35–2·53)	1·24 (1·18–1·29)	0·55 (0·52–0·58)
1989	0·1 (0·0–0·2)	59·1 (54·4–64·2)	187·8 (178·2–197·2)	120·5 (114·8–125·8)	67·2 (63·0–71·9)	28·3 (25·5–31·0)	6·4 (5·8–7·1)	0·3 (0·2–0·4)	0·0 (0·0–0·0)	2·35 (2·26–2·43)	1·24 (1·18–1·29)	0·51 (0·49–0·54)
1990	0·1 (0·0–0·2)	61·0 (56·3–66·3)	182·9 (173·6–191·9)	112·8 (107·2–118·0)	62·7 (58·8–66·8)	25·5 (23·1–28·0)	5·7 (5·2–6·4)	0·3 (0·2–0·4)	0·0 (0·0–0·0)	2·26 (2·18–2·33)	1·22 (1·17–1·27)	0·47 (0·45–0·5)
1991	0·1 (0·0–0·2)	61·7 (57·0–67·1)	176·3 (167·3–185·8)	108·6 (103·2–113·7)	58·3 (54·5–62·2)	22·6 (20·5–24·8)	5·0 (4·5–5·5)	0·3 (0·2–0·3)	0·0 (0·0–0·0)	2·16 (2·08–2·24)	1·19 (1·14–1·24)	0·43 (0·41–0·45)
1992	0·1 (0·0–0·2)	61·1 (56·4–66·4)	168·5 (159·4–177·8)	104·8 (99·7–109·8)	54·1 (50·5–57·9)	19·7 (17·8–21·7)	4·3 (3·8–4·7)	0·2 (0·2–0·3)	0·0 (0·0–0·0)	2·06 (1·98–2·14)	1·15 (1·1–1·2)	0·39 (0·37–0·41)
1993	0·1 (0·0–0·2)	59·0 (54·4–64·2)	159·4 (150·6–168·3)	100·8 (95·9–105·8)	50·0 (46·7–53·4)	16·8 (15·2–18·4)	3·5 (3·2–3·9)	0·2 (0·1–0·3)	0·0 (0·0–0·0)	1·95 (1·87–2·03)	1·09 (1·04–1·14)	0·35 (0·33–0·37)
1994	0·1 (0·0–0·2)	55·8 (51·3–60·5)	149·6 (140·7–158·0)	96·7 (91·8–101·5)	46·3 (43·2–49·2)	15·2 (13·9–16·7)	3·2 (2·9–3·5)	0·2 (0·1–0·2)	0·0 (0·0–0·0)	1·84 (1·76–1·91)	1·03 (0·98–1·08)	0·32 (0·31–0·34)
1995	0·1 (0·0–0·2)	52·1 (47·7–56·7)	140·4 (132·0–148·7)	92·8 (88·0–97·6)	43·2 (40·4–45·9)	15·1 (13·8–16·6)	3·2 (2·9–3·5)	0·2 (0·1–0·2)	0·0 (0·0–0·0)	1·73 (1·67–1·8)	0·96 (0·92–1·01)	0·31 (0·29–0·32)
1996	0·1 (0·0–0·1)	48·5 (44·5–52·6)	132·4 (124·5–140·5)	89·5 (84·9–94·3)	40·5 (38·0–43·2)	15·1 (13·9–16·5)	3·2 (2·9–3·5)	0·1 (0·1–0·2)	0·0 (0·0–0·0)	1·65 (1·58–1·71)	0·91 (0·86–0·95)	0·29 (0·28–0·31)
1997	0·1 (0·0–0·1)	45·3 (41·6–49·0)	125·5 (117·7–133·3)	86·6 (82·2–91·2)	38·1 (35·6–40·6)	15·0 (13·7–16·4)	3·2 (2·9–3·5)	0·1 (0·1–0·2)	0·0 (0·0–0·0)	1·57 (1·51–1·63)	0·85 (0·81–0·9)	0·28 (0·27–0·3)
1998	0·1 (0·0–0·1)	42·4 (38·8–45·9)	119·6 (112·3–127·8)	84·2 (79·8–88·8)	37·6 (35·2–40·2)	14·8 (13·5–16·2)	3·1 (2·9–3·4)	0·1 (0·1–0·2)	0·0 (0·0–0·0)	1·51 (1·45–1·57)	0·81 (0·77–0·85)	0·28 (0·26–0·29)
1999	0·1 (0·0–0·1)	39·9 (36·6–43·3)	114·9 (107·7–122·9)	82·5 (78·3–87·2)	39·1 (36·8–41·8)	14·7 (13·5–16·2)	3·1 (2·8–3·4)	0·1 (0·1–0·1)	0·0 (0·0–0·0)	1·47 (1·41–1·54)	0·77 (0·73–0·82)	0·29 (0·27–0·3)
2000	0·1 (0·0–0·1)	37·9 (34·8–41·1)	111·0 (103·3–119·0)	81·5 (77·2–85·9)	40·7 (38·2–43·4)	14·9 (13·6–16·3)	3·0 (2·8–3·4)	0·1 (0·1–0·1)	0·0 (0·0–0·0)	1·45 (1·38–1·51)	0·74 (0·7–0·79)	0·29 (0·28–0·31)
2001	0·1 (0·0–0·1)	36·0 (32·9–39·3)	107·7 (100·6–115·6)	81·1 (76·8–85·5)	42·3 (39·7–45·1)	15·3 (14·0–16·7)	3·0 (2·7–3·3)	0·1 (0·1–0·1)	0·0 (0·0–0·0)	1·43 (1·37–1·49)	0·72 (0·68–0·76)	0·3 (0·29–0·32)
2002	0·1 (0·0–0·1)	34·3 (31·3–37·5)	105·0 (97·9–113·0)	81·2 (76·9–85·8)	43·7 (41·1–46·8)	15·9 (14·6–17·3)	3·0 (2·7–3·3)	0·1 (0·1–0·1)	0·0 (0·0–0·0)	1·42 (1·35–1·49)	0·7 (0·66–0·74)	0·31 (0·3–0·33)
2003	0·1 (0·0–0·1)	32·6 (29·7–35·6)	102·4 (95·5–110·2)	81·6 (77·1–86·4)	45·0 (42·2–48·1)	16·7 (15·3–18·2)	3·0 (2·8–3·3)	0·1 (0·1–0·1)	0·0 (0·0–0·0)	1·41 (1·35–1·48)	0·68 (0·64–0·72)	0·32 (0·31–0·34)
2004	0·1 (0·0–0·1)	31·1 (28·4–33·9)	99·8 (92·8–107·4)	82·2 (77·4–87·1)	46·0 (42·9–49·0)	17·6 (16·1–19·2)	3·1 (2·8–3·4)	0·1 (0·1–0·1)	0·0 (0·0–0·0)	1·4 (1·34–1·47)	0·66 (0·62–0·7)	0·33 (0·32–0·35)
2005	0·1 (0·0–0·1)	30·2 (27·5–32·9)	98·0 (91·2–105·5)	83·2 (78·5–88·2)	46·8 (43·8–49·9)	18·6 (17·0–20·3)	3·2 (2·9–3·5)	0·1 (0·1–0·1)	0·0 (0·0–0·0)	1·4 (1·34–1·46)	0·64 (0·61–0·68)	0·34 (0·33–0·36)
2006	0·1 (0·0–0·1)	29·6 (26·7–32·1)	96·6 (89·9–103·7)	84·6 (79·8–89·7)	47·8 (44·7–51·1)	19·7 (18·0–21·3)	3·3 (3·1–3·6)	0·1 (0·1–0·1)	0·0 (0·0–0·0)	1·41 (1·35–1·47)	0·63 (0·59–0·67)	0·35 (0·34–0·37)
2007	0·1 (0·0–0·1)	28·9 (26·0–31·6)	95·2 (88·4–102·8)	85·7 (80·7–90·9)	48·9 (45·6–52·3)	20·6 (18·8–22·4)	3·5 (3·2–3·8)	0·1 (0·1–0·1)	0·0 (0·0–0·0)	1·41 (1·35–1·48)	0·62 (0·58–0·66)	0·37 (0·35–0·39)
2008	0·1 (0·0–0·1)	28·2 (25·4–30·9)	93·6 (86·6–101·1)	86·6 (81·4–91·8)	50·0 (46·4–53·8)	21·5 (19·6–23·5)	3·6 (3·3–3·9)	0·1 (0·1–0·1)	0·0 (0·0–0·0)	1·42 (1·35–1·48)	0·61 (0·57–0·65)	0·38 (0·35–0·4)
2009	0·1 (0·0–0·1)	27·3 (24·6–30·2)	91·8 (84·3–99·5)	87·1 (81·7–92·5)	51·2 (47·7–55·1)	22·2 (20·1–24·4)	3·8 (3·4–4·2)	0·1 (0·1–0·1)	0·0 (0·0–0·0)	1·42 (1·35–1·49)	0·6 (0·56–0·63)	0·39 (0·36–0·41)
2010	0·1 (0·0–0·1)	26·2 (23·6–29·3)	89·0 (81·4–96·8)	86·8 (81·6–92·4)	52·1 (48·6–56·0)	22·6 (20·5–25·0)	3·9 (3·6–4·3)	0·1 (0·1–0·1)	0·0 (0·0–0·0)	1·4 (1·34–1·48)	0·58 (0·54–0·62)	0·39 (0·37–0·42)
2011	0·0 (0·0–0·1)	25·2 (22·7–28·0)	86·1 (78·9–94·1)	86·1 (80·5–91·9)	52·7 (49·2–56·6)	22·7 (20·7–25·0)	4·1 (3·7–4·5)	0·1 (0·1–0·1)	0·0 (0·0–0·0)	1·39 (1·32–1·46)	0·56 (0·52–0·6)	0·4 (0·38–0·42)
2012	0·0 (0·0–0·1)	24·7 (22·2–27·5)	83·6 (76·4–91·4)	85·0 (79·3–91·0)	52·8 (49·3–57·0)	22·7 (20·5–24·9)	4·2 (3·7–4·5)	0·1 (0·1–0·2)	0·0 (0·0–0·0)	1·37 (1·3–1·44)	0·54 (0·5–0·58)	0·4 (0·38–0·43)
2013	0·0 (0·0–0·1)	24·4 (21·7–27·5)	81·6 (74·1–89·7)	83·9 (78·1–89·9)	52·7 (48·8–57·4)	22·5 (20·1–25·0)	4·2 (3·8–4·6)	0·1 (0·1–0·2)	0·0 (0·0–0·0)	1·35 (1·27–1·43)	0·53 (0·49–0·57)	0·4 (0·37–0·43)
2014	0·0 (0·0–0·1)	24·1 (21·4–27·4)	79·8 (72·0–88·5)	83·2 (77·0–89·5)	52·9 (48·5–58·3)	22·3 (19·7–25·2)	4·2 (3·8–4·8)	0·1 (0·1–0·2)	0·0 (0·0–0·0)	1·33 (1·24–1·44)	0·52 (0·48–0·57)	0·4 (0·36–0·44)
2015	0·0 (0·0–0·1)	23·8 (20·9–27·2)	78·2 (69·2–88·4)	82·7 (76·1–89·8)	53·3 (48·1–59·5)	22·1 (19·1–25·7)	4·2 (3·7–4·9)	0·1 (0·1–0·2)	0·0 (0·0–0·0)	1·32 (1·21–1·45)	0·51 (0·46–0·56)	0·4 (0·36–0·45)
2016	0·0 (0·0–0·1)	23·5 (20·5–27·0)	76·5 (66·9–88·1)	82·4 (75·4–90·5)	53·6 (47·8–60·4)	21·9 (18·6–25·9)	4·2 (3·6–5·0)	0·1 (0·1–0·2)	0·0 (0·0–0·0)	1·31 (1·18–1·47)	0·5 (0·45–0·56)	0·4 (0·35–0·46)
2017	0·0 (0·0–0·1)	23·5 (20·3–27·3)	75·7 (65·1–89·2)	82·2 (74·9–91·0)	53·7 (47·3–61·6)	21·6 (17·9–26·5)	4·1 (3·4–5·2)	0·1 (0·1–0·2)	0·0 (0·0–0·0)	1·31 (1·16–1·49)	0·5 (0·44–0·56)	0·4 (0·34–0·47)

Supplemental Results Table 5: Age-specific fertility rates per 1000, cumulative total fertility up to maternal age 25 (TFU25), cumulative total fertility from maternal age 30 to age 55 (TFO30), and cumulative total fertility, Russian Federation, 1950-2017

Year	10-14	15-19	20-24	25-29	30-34	35-39	40-44	45-49	50-54	TFR	TFU25	TFO30
1950	0·0 (0·0–0·1)	27·2 (23·9–31·3)	149·8 (148·6–151·0)	155·6 (154·8–156·5)	88·3 (87·4–89·1)	79·7 (78·7–80·7)	37·6 (36·8–38·3)	6·4 (5·8–7·1)	0·1 (0·1–0·1)	2·72 (2·7–2·75)	0·89 (0·87–0·91)	1·06 (1·05–1·07)
1951	0·0 (0·0–0·1)	25·7 (22·7–29·4)	150·2 (149·1–151·3)	164·1 (163·3–165·0)	93·2 (92·3–94·1)	72·8 (71·8–73·8)	37·9 (37·1–38·6)	6·0 (5·4–6·6)	0·1 (0·1–0·1)	2·75 (2·73–2·77)	0·88 (0·86–0·9)	1·05 (1·04–1·06)
1952	0·0 (0·0–0·1)	24·0 (21·3–27·1)	136·0 (135·0–137·2)	164·7 (163·9–165·4)	95·4 (94·5–96·3)	65·8 (64·9–66·7)	37·4 (36·5–38·2)	5·4 (4·9–6·0)	0·1 (0·1–0·1)	2·64 (2·63–2·66)	0·8 (0·79–0·82)	1·02 (1·01–1·03)
1953	0·0 (0·0–0·1)	24·2 (21·5–27·2)	133·5 (132·5–134·6)	170·7 (169·9–171·5)	102·8 (102·0–103·8)	63·4 (62·6–64·3)	36·3 (35·6–37·1)	5·2 (4·7–5·8)	0·1 (0·1–0·1)	2·68 (2·66–2·7)	0·79 (0·77–0·8)	1·04 (1·03–1·05)
1954	0·0 (0·0–0·1)	23·9 (21·4–26·7)	137·0 (135·9–138·0)	177·6 (176·8–178·4)	112·6 (111·7–113·6)	60·5 (59·6–61·4)	34·2 (33·4–35·0)	4·9 (4·4–5·5)	0·1 (0·1–0·1)	2·75 (2·74–2·77)	0·8 (0·79–0·82)	1·06 (1·05–1·07)
1955	0·0 (0·0–0·1)	23·7 (21·4–26·1)	131·5 (130·4–132·6)	169·4 (168·6–170·2)	111·4 (110·6–112·3)	55·7 (54·8–56·7)	28·8 (28·0–29·5)	4·5 (4·0–5·0)	0·1 (0·1–0·1)	2·63 (2·61–2·64)	0·78 (0·76–0·79)	1·0 (0·99–1·01)
1956	0·0 (0·0–0·1)	25·2 (23·3–27·1)	137·5 (136·5–138·6)	164·4 (163·7–165·2)	110·3 (109·5–111·2)	54·2 (53·3–55·2)	23·2 (22·5–24·0)	4·1 (3·6–4·6)	0·1 (0·1–0·1)	2·6 (2·58–2·61)	0·81 (0·8–0·82)	0·96 (0·95–0·97)
1957	0·0 (0·0–0·1)	27·2 (26·1–28·3)	150·0 (148·9–151·1)	156·8 (156·1–157·6)	109·8 (108·9–110·7)	52·2 (51·3–53·1)	20·1 (19·4–20·8)	3·7 (3·2–4·2)	0·1 (0·1–0·1)	2·6 (2·59–2·61)	0·89 (0·88–0·89)	0·93 (0·92–0·94)
1958	0·0 (0·0–0·1)	28·8 (28·1–29·4)	160·5 (159·4–161·6)	144·3 (143·5–145·2)	107·2 (106·4–108·1)	50·9 (50·0–51·8)	18·2 (17·5–18·9)	3·2 (2·7–3·7)	0·1 (0·1–0·1)	2·57 (2·55–2·58)	0·95 (0·94–0·95)	0·9 (0·89–0·9)
1959	0·0 (0·0–0·1)	30·9 (30·2–31·6)	164·6 (163·4–165·7)	137·7 (136·9–138·5)	105·5 (104·7–106·3)	52·8 (51·9–53·7)	16·6 (15·9–17·3)	2·7 (2·3–3·2)	0·1 (0·1–0·1)	2·55 (2·54–2·56)	0·98 (0·97–0·98)	0·89 (0·88–0·9)
1960	0·0 (0·0–0·1)	33·2 (32·4–33·9)	170·1 (169·1–171·3)	131·8 (131·0–132·6)	100·0 (99·2–100·8)	50·8 (50·0–51·7)	15·5 (14·8–16·2)	2·3 (1·9–2·8)	0·0 (0·0–0·0)	2·52 (2·51–2·53)	1·02 (1·01–1·02)	0·84 (0·83–0·85)
1961	0·0 (0·0–0·1)	29·7 (29·0–30·4)	166·9 (165·7–168·1)	126·5 (125·7–127·2)	91·9 (91·1–92·8)	47·6 (46·7–48·4)	14·3 (13·6–15·1)	1·9 (1·6–2·4)	0·0 (0·0–0·0)	2·39 (2·38–2·41)	0·98 (0·98–0·99)	0·78 (0·77–0·79)
1962	0·0 (0·0–0·1)	23·9 (23·2–24·7)	155·3 (154·1–156·5)	126·8 (126·1–127·6)	84·4 (83·6–85·2)	46·1 (45·3–46·9)	13·5 (12·8–14·1)	1·7 (1·3–2·1)	0·0 (0·0–0·0)	2·26 (2·25–2·27)	0·9 (0·89–0·9)	0·73 (0·72–0·74)
1963	0·0 (0·0–0·1)	21·5 (20·9–22·2)	144·7 (143·6–145·9)	126·3 (125·5–127·1)	75·9 (75·1–76·7)	43·0 (42·2–43·8)	13·3 (12·7–14·0)	1·5 (1·1–1·9)	0·0 (0·0–0·0)	2·13 (2·12–2·14)	0·83 (0·82–0·84)	0·67 (0·66–0·68)
1964	0·0 (0·0–0·1)	22·0 (21·4–22·7)	128·3 (127·2–129·5)	122·9 (122·1–123·6)	71·4 (70·6–72·2)	40·2 (39·4–40·9)	13·2 (12·5–13·9)	1·3 (1·0–1·7)	0·0 (0·0–0·0)	2·0 (1·99–2·01)	0·75 (0·75–0·76)	0·63 (0·62–0·64)
1965	0·0 (0·0–0·1)	24·3 (23·6–24·9)	120·0 (119·0–121·1)	122·8 (122·0–123·5)	71·4 (70·6–72·2)	38·9 (38·2–39·7)	12·5 (11·9–13·2)	1·2 (0·9–1·5)	0·0 (0·0–0·0)	1·96 (1·95–1·97)	0·72 (0·72–0·73)	0·62 (0·61–0·63)
1966	0·0 (0·0–0·1)	25·3 (24·7–26·0)	122·3 (121·2–123·4)	118·0 (117·2–118·7)	71·2 (70·4–71·9)	36·6 (35·9–37·3)	11·7 (11·1–12·3)	1·1 (0·8–1·5)	0·0 (0·0–0·0)	1·93 (1·92–1·94)	0·74 (0·73–0·75)	0·6 (0·6–0·61)
1967	0·0 (0·0–0·1)	25·6 (25·0–26·1)	129·8 (128·6–131·0)	106·6 (105·9–107·4)	70·1 (69·3–70·9)	33·4 (32·8–34·2)	10·7 (10·1–11·4)	1·1 (0·8–1·4)	0·0 (0·0–0·0)	1·89 (1·88–1·9)	0·78 (0·77–0·78)	0·58 (0·57–0·58)
1968	0·0 (0·0–0·1)	26·9 (26·3–27·5)	143·3 (142·2–144·5)	97·7 (96·9–98·5)	71·0 (70·2–71·8)	31·0 (30·2–31·8)	9·9 (9·4–10·5)	1·1 (0·8–1·5)	0·0 (0·0–0·0)	1·91 (1·9–1·92)	0·85 (0·84–0·86)	0·57 (0·56–0·57)
1969	0·0 (0·0–0·1)	28·8 (28·2–29·4)	157·8 (156·6–158·9)	91·6 (90·8–92·4)	70·2 (69·4–71·0)	29·5 (28·7–30·2)	9·4 (8·8–10·0)	1·0 (0·7–1·4)	0·0 (0·0–0·0)	1·94 (1·93–1·95)	0·93 (0·93–0·94)	0·55 (0·54–0·56)
1970	0·0 (0·0–0·1)	30·3 (29·7–30·9)	163·7 (162·5–164·8)	91·5 (90·6–92·3)	69·3 (68·6–70·0)	29·3 (28·5–30·1)	8·7 (8·1–9·2)	0·9 (0·6–1·2)	0·0 (0·0–0·0)	1·97 (1·96–1·98)	0·97 (0·96–0·98)	0·54 (0·53–0·55)
1971	0·0 (0·0–0·1)	31·0 (30·4–31·6)	163·4 (162·3–164·6)	99·7 (98·7–100·6)	67·9 (67·0–68·7)	30·4 (29·6–31·1)	8·0 (7·4–8·5)	0·8 (0·6–1·1)	0·0 (0·0–0·0)	2·01 (2·0–2·02)	0·97 (0·97–0·98)	0·53 (0·53–0·54)
1972	0·0 (0·0–0·1)	31·3 (30·7–32·0)	158·0 (156·8–159·1)	107·1 (106·2–108·0)	62·2 (61·4–62·9)	30·8 (30·1–31·6)	7·4 (6·9–8·0)	0·7 (0·5–1·0)	0·0 (0·0–0·0)	1·99 (1·98–2·0)	0·95 (0·94–0·95)	0·51 (0·5–0·51)
1973	0·0 (0·0–0·1)	32·3 (31·7–32·9)	154·8 (153·7–155·9)	113·3 (112·4–114·1)	55·6 (54·8–56·4)	30·4 (29·8–31·2)	7·1 (6·6–7·6)	0·6 (0·4–0·9)	0·0 (0·0–0·0)	1·97 (1·96–1·98)	0·94 (0·93–0·94)	0·47 (0·46–0·47)
1974	0·0 (0·0–0·1)	33·4 (32·8–34·0)	154·7 (153·6–155·7)	116·8 (116·0–117·7)	51·1 (50·3–51·9)	29·1 (28·4–29·8)	6·9 (6·4–7·5)	0·6 (0·4–0·8)	0·0 (0·0–0·0)	1·96 (1·95–1·97)	0·94 (0·93–0·95)	0·44 (0·43–0·44)
1975	0·0 (0·0–0·1)	34·5 (33·9–35·1)	154·6 (153·5–155·7)	114·1 (113·3–114·8)	49·8 (49·0–50·7)	26·5 (25·9–27·2)	6·9 (6·3–7·5)	0·5 (0·4–0·7)	0·0 (0·0–0·0)	1·93 (1·92–1·94)	0·95 (0·94–0·95)	0·42 (0·41–0·43)
1976	0·0 (0·0–0·1)	35·6 (35·0–36·2)	153·5 (152·4–154·6)	110·8 (110·0–111·6)	53·1 (52·2–54·0)	23·7 (23·0–24·3)	6·7 (6·2–7·3)	0·5 (0·3–0·7)	0·0 (0·0–0·0)	1·92 (1·91–1·93)	0·95 (0·94–0·95)	0·42 (0·41–0·43)
1977	0·0 (0·0–0·1)	37·6 (37·0–38·2)	152·0 (150·9–153·0)	107·5 (106·7–108·3)	57·1 (56·2–58·0)	20·5 (19·8–21·2)	6·4 (5·9–6·9)	0·4 (0·3–0·6)	0·0 (0·0–0·0)	1·91 (1·9–1·92)	0·95 (0·94–0·95)	0·42 (0·42–0·43)
1978	0·0 (0·0–0·1)	40·8 (40·2–41·5)	151·6 (150·6–152·7)	102·3 (101·6–103·0)	58·0 (57·1–58·8)	17·7 (17·1–18·4)	5·9 (5·4–6·4)	0·4 (0·3–0·6)	0·0 (0·0–0·0)	1·88 (1·87–1·89)	0·96 (0·96–0·97)	0·41 (0·4–0·42)
1979	0·0 (0·0–0·1)	43·1 (42·5–43·8)	151·4 (150·2–152·5)	98·4 (97·6–99·1)	56·5 (55·7–57·4)	16·1 (15·4–16·8)	5·3 (4·8–5·8)	0·4 (0·3–0·6)	0·0 (0·0–0·0)	1·86 (1·85–1·87)	0·97 (0·97–0·98)	0·39 (0·39–0·4)
1980	0·0 (0·0–0·1)	43·5 (42·9–44·2)	150·8 (149·9–151·9)	99·0 (98·3–99·7)	55·4 (54·6–56·2)	16·4 (15·6–17·1)	4·7 (4·2–5·1)	0·4 (0·2–0·5)	0·0 (0·0–0·0)	1·85 (1·84–1·86)	0·97 (0·97–0·98)	0·38 (0·38–0·39)
1981	0·0 (0·0–0·1)	43·5 (42·8–44·2)	151·6 (150·7–152·6)	101·9 (101·2–102·7)	56·8 (56·0–57·5)	19·5 (18·8–20·3)	4·4 (4·0–4·8)	0·4 (0·2–0·5)	0·0 (0·0–0·0)	1·89 (1·88–1·9)	0·98 (0·97–0·98)	0·41 (0·4–0·41)
1982	0·0 (0·0–0·1)	44·7 (44·0–45·4)	158·5 (157·4–159·6)	109·9 (109·1–110·6)	61·0 (60·2–61·7)	23·5 (22·7–24·4)	4·3 (3·8–4·8)	0·3 (0·2–0·5)	0·0 (0·0–0·0)	2·01 (2·0–2·02)	1·02 (1·01–1·02)	0·45 (0·44–0·45)
1983	0·0 (0·0–0·1)	46·4 (45·7–47·1)	160·9 (159·8–162·0)	111·9 (111·2–112·6)	61·5 (60·8–62·3)	24·8 (24·1–25·6)	4·3 (3·8–4·8)	0·3 (0·2–0·5)	0·0 (0·0–0·0)	2·05 (2·04–2·06)	1·04 (1·03–1·04)	0·45 (0·45–0·46)
1984	0·0 (0·0–0·1)	47·2 (46·5–48·1)	162·1 (161·0–163·1)	110·6 (109·9–111·3)	59·3 (58·6–60·1)	24·1 (23·4–24·9)	4·4 (3·9–5·0)	0·3 (0·2–0·5)	0·0 (0·0–0·0)	2·04 (2·03–2·05)	1·05 (1·04–1·05)	0·44 (0·44–0·45)
1985	0·0 (0·0–0·1)	47·1 (46·4–47·8)	163·9 (162·7–164·9)	114·8 (114·1–115·6)	62·5 (61·8–63·3)	25·2 (24·5–25·9)	4·8 (4·2–5·4)	0·3 (0·2–0·4)	0·0 (0·0–0·0)	2·09 (2·08–2·1)	1·06 (1·05–1·06)	0·46 (0·46–0·47)
1986	0·0 (0·0–0·1)	47·7 (47·0–48·4)	164·5 (163·4–165·7)	117·2 (116·4–118·0)	66·4 (65·7–67·1)	27·6 (26·9–28·2)	5·5 (4·8–6·1)	0·3 (0·2–0·4)	0·0 (0·0–0·0)	2·15 (2·14–2·16)	1·06 (1·06–1·07)	0·5 (0·49–0·5)
1987	0·0 (0·0–0·1)	48·8 (48·1–49·5)	165·2 (164·1–166·4)	113·7 (113·0–114·5)	64·0 (63·2–64·7)	26·8 (26·1–27·5)	5·8 (5·3–6·4)	0·3 (0·2–0·4)	0·0 (0·0–0·0)	2·12 (2·11–2·13)	1·07 (1·06–1·08)	0·48 (0·48–0·49)
1988	0·0 (0·0–0·1)	50·5 (49·7–51·3)	159·6 (158·4–160·7)	103·7 (103·0–104·4)	57·0 (56·3–57·7)	23·6 (23·0–24·3)	5·5 (5·0–6·1)	0·3 (0·2–0·4)	0·0 (0·0–0·0)	2·0 (1·99–2·01)	1·05 (1·04–1·06)	0·43 (0·43–0·44)
1989	0·1 (0·0–0·1)	53·4 (52·6–54·1)	157·1 (156·0–158·3)	95·8 (95·0–96·5)	50·6 (49·9–51·2)	20·6 (19·9–21·3)	4·8 (4·3–5·3)	0·3 (0·2–0·3)	0·0 (0·0–0·0)	1·91 (1·9–1·92)	1·05 (1·05–1·06)	0·38 (0·38–0·39)
1990	0·1 (0·0–0·1)	54·6 (53·9–55·3)	152·5 (151·4–153·6)	88·3 (87·6–89·1)	44·7 (44·1–45·4)	17·8 (17·3–18·4)	3·9 (3·5–4·4)	0·2 (0·2–0·3)	0·0 (0·0–0·0)	1·81 (1·8–1·82)	1·04 (1·03–1·04)	0·33 (0·33–0·34)
1991	0·0 (0·0–0·1)	52·4 (51·7–53·2)	140·6 (139·5–141·7)	77·7 (77·0–78·4)	38·1 (37·5–38·8)	15·2 (14·6–15·8)	3·4 (3·0–3·8)	0·2 (0·1–0·3)	0·0 (0·0–0·0)	1·64 (1·63–1·65)	0·97 (0·96–0·97)	0·28 (0·28–0·29)
1992	0·0 (0·0–0·1)	47·6 (46·9–48·3)	126·2 (125·1–127·2)	68·5 (67·8–69·2)	32·4 (31·8–33·0)	12·8 (12·2–13·3)	3·0 (2·5–3·4)	0·2 (0·1–0·2)	0·0 (0·0–0·0)	1·45 (1·44–1·46)	0·87 (0·86–0·88)	0·24 (0·24–0·25)
1993	0·0 (0·0–0·1)	45·0 (44·4–45·7)	119·7 (118·7–120·8)	66·0 (65·3–66·7)	29·7 (29·1–30·4)	11·1 (10·5–11·6)	2·6 (2·2–3·0)	0·1 (0·1–0·2)	0·0 (0·0–0·0)	1·37 (1·36–1·38)	0·82 (0·82–0·83)	0·22 (0·21–0·22)
1994	0·0 (0·0–0·1)	43·7 (43·0–44·3)	118·1 (117·0–119·1)	67·2 (66·4–67·9)	29·5 (28·9–30·1)	10·5 (10·0–11·0)	2·4 (2·0–2·8)	0·1 (0·1–0·2)	0·0 (0·0–0·0)	1·36 (1·35–1·37)	0·81 (0·8–0·82)	0·21 (0·21–0·22)
1995	0·0 (0·0–0·1)	40·4 (39·7–41·0)	107·3 (106·3–108·3)	64·8 (64·1–65·5)	29·5 (28·9–30·1)	10·5 (10·0–11·1)	2·3 (1·9–2·7)	0·1 (0·1–0·2)	0·0 (0·0–0·0)	1·28 (1·27–1·28)	0·74 (0·73–0·74)	0·21 (0·21–0·22)
1996	0·0 (0·0–0·1)	37·6 (36·9–38·3)	96·6 (95·7–97·5)	62·1 (61·4–62·9)	29·8 (29·1–30·4)	10·6 (10·1–11·1)	2·3 (1·9–2·7)	0·1 (0·1–0·2)	0·0 (0·0–0·0)	1·2 (1·19–1·21)	0·67 (0·67–0·68)	0·21 (0·21–0·22)
1997	0·0 (0·0–0·1)	34·8 (34·2–35·5)	94·1 (93·2–95·1)	62·8 (62·1–63·4)	30·8 (30·1–31·4)	10·8 (10·2–11·3)	2·3 (1·9–2·7)	0·1 (0·1–0·2)	0·0 (0·0–0·0)	1·18 (1·17–1·19)	0·64 (0·64–0·65)	0·22 (0·21–0·23)
1998	0·0 (0·0–0·1)	30·2 (29·6–30·8)	90·6 (89·7–91·6)	62·5 (61·8–63·2)	31·4 (30·8–32·1)	11·0 (10·5–11·6)	2·3 (1·9–2·7)	0·1 (0·1–0·2)	0·0 (0·0–0·0)	1·14 (1·13–1·15)	0·6 (0·6–0·61)	0·22 (0·22–0·23)
1999	0·0 (0·0–0·1)	26·8 (26·2–27·4)	87·9 (87·0–88·9)	62·9 (62·2–63·6)	32·4 (31·7–33·0)	11·4 (10·8–12·0)	2·3 (2·0–2·8)	0·1 (0·1–0·2)	0·0 (0·0–0·0)	1·12 (1·11–1·13)	0·57 (0·57–0·58)	0·23 (0·23–0·24)
2000	0·0 (0·0–0·1)	26·6 (26·0–27·1)	89·1 (88·2–90·0)	66·0 (65·3–66·7)	35·0 (34·3–35·7)	12·2 (11·7–12·8)	2·4 (2·1–2·8)	0·1 (0·1–0·2)	0·0 (0·0–0·0)	1·16 (1·15–1·17)	0·58 (0·57–0·58)	0·25 (0·24–0·25)
2001	0·0 (0·0–0·1)	27·1 (26·5–27·7)	90·2 (89·3–91·2)	70·1 (69·4–70·8)	38·5 (37·8–39·2)	13·6 (13·0–14·2)	2·5 (2·1–2·9)	0·1 (0·1–0·2)	0·0 (0·0–0·0)	1·21 (1·2–1·22)	0·59 (0·58–0·59)	0·27 (0·27–0·28)
2002	0·0 (0·0–0·1)	27·0 (26·5–27·7)	92·2 (91·3–93·1)	74·5 (73·8–75·2)	41·7 (41·0–42·4)	15·1 (14·5–15·8)	2·6 (2·2–3·0)	0·1 (0·1–0·2)	0·0 (0·0–0·0)	1·27 (1·26–1·28)	0·6 (0·59–0·6)	0·3 (0·29–0·3)
2003	0·0 (0·0–0·1)	26·9 (26·3–27·4)	92·1 (91·3–93·1)	77·4 (76·6–78·1)	43·7 (42·9–44·4)	16·4 (15·8–17·1)	2·8 (2·4–3·2)	0·1 (0·1–0·2)	0·0 (0·0–0·0)	1·3 (1·29–1·31)	0·6 (0·59–0·6)	0·32 (0·31–0·32)
2004	0·0 (0·0–0·1)	26·6 (26·1–27·2)	88·1 (87·3–89·0)	76·9 (76·2–77·7)	43·9 (43·2–44·6)	17·2 (16·5–17·8)	3·0 (2·6–3·4)	0·2 (0·1–0·3)	0·0 (0·0–0·0)	1·28 (1·27–1·29)	0·57 (0·57–0·58)	0·32 (0·32–0·33)
2005	0·0 (0·0–0·1)	26·8 (26·2–27·4)	84·0 (83·2–84·9)	75·7 (75·1–76·5)	44·1 (43·4–44·8)	17·9 (17·2–18·6)	3·3 (2·9–3·7)	0·2 (0·1–0·3)	0·0 (0·0–0·0)	1·26 (1·25–1·27)	0·55 (0·55–0·56)	0·33 (0·32–0·33)
2006	0·0 (0·0–0·1)	27·5 (26·9–28·2)	84·4 (83·5–85·3)	80·2 (79·4–80·9)	48·5 (47·8–49·2)	20·2 (19·5–20·8)	3·6 (3·2–4·1)	0·2 (0·1–0·3)	0·0 (0·0–0·0)	1·32 (1·31–1·33)	0·56 (0·55–0·57)	0·36 (0·36–0·37)
2007	0·0 (0·0–0·1)	28·4 (27·8–29·1)	86·5 (85·7–87·4)	87·2 (86·5–88·0)	55·7 (54·9–56·4)	23·6 (22·9–24·4)	4·1 (3·7–4·6)	0·2 (0·1–0·3)	0·0 (0·0–0·0)	1·43 (1·42–1·44)	0·57 (0·57–0·58)	0·42 (0·41–0·42)
2008	0·0 (0·0–0·1)	28·7 (28·0–29·4)	88·0 (87·0–88·9)	91·7 (90·9–92·4)	60·4 (59·6–61·2)	26·1 (25·4–26·9)	4·7 (4·2–5·2)	0·2 (0·1–0·3)	0·0 (0·0–0·0)	1·5 (1·49–1·51)	0·58 (0·58–0·59)	0·46 (0·45–0·46)
2009	0·0 (0·0–0·1)	27·7 (27·0–28·3)	86·8 (86·0–87·8)	95·1 (94·3–95·8)	63·6 (62·9–64·5)	28·0 (27·3–28·7)	5·4 (4·9–5·9)	0·2 (0·1–0·3)	0·0 (0·0–0·0)	1·53 (1·53–1·54)	0·57 (0·57–0·58)	0·49 (0·48–0·49)
2010	0·0 (0·0–0·1)	26·8 (26·1–27·5)	85·8 (84·9–86·7)	96·7 (95·9–97·4)	65·8 (65·0–66·6)	29·9 (29·1–30·6)	6·0 (5·5–6·5)	0·2 (0·1–0·3)	0·0 (0·0–0·0)	1·56 (1·55–1·57)	0·56 (0·56–0·57)	0·51 (0·5–0·51)
2011	0·0 (0·0–0·1)	26·9 (26·2–27·7)	89·7 (88·8–90·7)	100·2 (99·5–101·0)	69·4 (68·7–70·1)	32·2 (31·5–32·9)	6·4 (5·9–7·0)	0·2 (0·1–0·3)	0·0 (0·0–0·0)	1·63 (1·62–1·64)	0·58 (0·58–0·59)	0·54 (0·54–0·55)
2012	0·0 (0·0–0·1)	26·8 (26·0–27·5)	92·9 (91·9–93·8)	104·8 (104·1–105·5)	73·0 (72·2–73·8)	34·7 (34·0–35·4)	7·0 (6·5–7·6)	0·2 (0·2–0·3)	0·0 (0·0–0·0)	1·7 (1·69–1·71)	0·6 (0·59–0·6)	0·57 (0·57–0·58)
2013	0·0 (0·0–0·1)	26·4 (25·7–27·2)	91·1 (90·1–92·1)	107·9 (107·1–108·6)	76·5 (75·7–77·4)	37·2 (36·5–38·0)	7·5 (7·0–8·2)	0·2 (0·2–0·3)	0·0 (0·0–0·0)	1·74 (1·72–1·75)	0·59 (0·58–0·59)	0·61 (0·6–0·61)
2014	0·0 (0·0–0·1)	26·4 (25·2–27·8)	88·7 (86·3–91·1)	110·1 (108·4–111·9)	80·8 (78·9–82·7)	38·8 (37·2–40·4)	7·6 (6·8–8·5)	0·2 (0·2–0·3)	0·0 (0·0–0·0)	1·76 (1·73–1·8)	0·58 (0·56–0·59)	0·64 (0·62–0·66)
2015	0·0 (0·0–0·1)	26·1 (23·9–28·5)	86·0 (79·0–93·3)	108·1 (103·2–113·1)	80·5 (75·1–86·1)	37·4 (33·2–41·9)	7·1 (5·9–8·4)	0·2 (0·2–0·3)	0·0 (0·0–0·0)	1·73 (1·61–1·85)	0·56 (0·52–0·6)	0·63 (0·57–0·68)
2016	0·0 (0·0–0·1)	25·7 (22·9–29·2)	83·3 (72·9–95·0)	104·2 (97·1–112·0)	77·5 (69·6–86·2)	34·6 (28·8–41·3)	6·4 (5·1–8·2)	0·2 (0·2–0·3)	0·0 (0·0–0·0)	1·66 (1·49–1·85)	0·55 (0·48–0·62)	0·59 (0·52–0·68)
2017	0·0 (0·0–0·1)	25·6 (22·0–29·9)	81·7 (68·9–96·4)	101·1 (92·5–110·7)	74·1 (64·7–84·8)	32·3 (25·6–40·5)	6·0 (4·4–7·9)	0·2 (0·2–0·3)	0·0 (0·0–0·0)	1·61 (1·39–1·85)	0·54 (0·46–0·63)	0·56 (0·48–0·67)

Supplemental Results Table 5: Age-specific fertility rates per 1000, cumulative total fertility up to maternal age 25 (TFU25), cumulative total fertility from maternal age 30 to age 55 (TFO30), and cumulative total fertility, Ukraine, 1950-2017												
**Year**	**10-14**	**15-19**	**20-24**	**25-29**	**30-34**	**35-39**	**40-44**	**45-49**	**50-54**	**TFR**	**TFU25**	**TFO30**
1950	0·0 (0·0–0·1)	27·7 (24·8–31·4)	136·9 (126·1–149·8)	135·9 (128·5–144·6)	82·1 (75·4–89·1)	51·7 (45·8–58·0)	25·5 (22·5–28·7)	4·1 (3·4–4·9)	0·1 (0·1–0·1)	2·32 (2·19–2·47)	0·82 (0·75–0·91)	0·82 (0·75–0·89)
1951	0·0 (0·0–0·1)	27·1 (24·3–30·3)	133·1 (123·2–144·3)	135·6 (128·9–143·8)	80·7 (74·7–87·2)	50·3 (45·1–56·1)	24·5 (21·9–27·2)	3·8 (3·2–4·5)	0·1 (0·1–0·1)	2·28 (2·16–2·4)	0·8 (0·74–0·87)	0·8 (0·74–0·86)
1952	0·0 (0·0–0·1)	26·7 (24·1–29·7)	130·4 (120·8–139·9)	134·9 (128·6–142·1)	80·3 (74·9–86·4)	48·5 (43·7–53·6)	23·0 (20·7–25·4)	3·4 (2·9–4·0)	0·1 (0·1–0·1)	2·24 (2·14–2·34)	0·79 (0·73–0·84)	0·78 (0·73–0·83)
1953	0·0 (0·0–0·1)	26·7 (24·1–29·5)	129·1 (120·1–137·9)	134·1 (127·5–140·7)	80·8 (75·6–86·6)	46·6 (42·2–51·4)	21·1 (19·1–23·3)	3·1 (2·6–3·6)	0·1 (0·1–0·1)	2·21 (2·12–2·3)	0·78 (0·73–0·83)	0·76 (0·71–0·8)
1954	0·0 (0·0–0·1)	27·1 (24·6–29·9)	129·4 (120·4–138·4)	133·0 (126·8–139·5)	81·8 (76·6–87·8)	44·7 (40·5–49·0)	18·9 (17·1–20·8)	2·7 (2·4–3·2)	0·1 (0·1–0·1)	2·19 (2·1–2·28)	0·78 (0·73–0·83)	0·74 (0·7–0·78)
1955	0·0 (0·0–0·1)	28·0 (25·5–30·6)	131·4 (122·3–140·3)	131·8 (125·5–138·5)	83·4 (78·0–89·2)	42·9 (38·8–46·9)	16·6 (15·0–18·5)	2·4 (2·1–2·9)	0·0 (0·0–0·0)	2·18 (2·09–2·28)	0·8 (0·75–0·85)	0·73 (0·69–0·76)
1956	0·0 (0·0–0·1)	29·1 (26·6–31·7)	134·8 (125·5–144·5)	130·5 (124·2–137·6)	85·1 (79·4–90·9)	41·3 (37·2–45·5)	14·5 (13·0–16·1)	2·2 (1·8–2·5)	0·0 (0·0–0·0)	2·19 (2·09–2·29)	0·82 (0·77–0·87)	0·72 (0·68–0·75)
1957	0·0 (0·0–0·1)	30·2 (27·7–32·8)	138·9 (129·2–149·1)	128·9 (122·4–135·5)	86·1 (79·9–92·2)	39·9 (35·8–44·2)	12·8 (11·4–14·3)	1·9 (1·6–2·2)	0·0 (0·0–0·0)	2·19 (2·1–2·29)	0·85 (0·79–0·9)	0·7 (0·67–0·74)
1958	0·0 (0·0–0·1)	31·1 (28·5–33·6)	142·5 (132·7–153·1)	127·0 (120·6–133·4)	86·1 (79·6–92·4)	38·7 (34·8–42·9)	11·5 (10·2–13·0)	1·6 (1·4–1·9)	0·0 (0·0–0·0)	2·19 (2·1–2·28)	0·87 (0·82–0·92)	0·69 (0·65–0·73)
1959	0·0 (0·0–0·1)	31·5 (29·0–34·1)	144·9 (134·3–155·2)	124·9 (118·5–131·6)	84·5 (77·8–90·9)	37·7 (33·6–42·0)	10·7 (9·3–12·1)	1·4 (1·2–1·7)	0·0 (0·0–0·0)	2·18 (2·09–2·27)	0·88 (0·83–0·93)	0·67 (0·63–0·71)
1960	0·0 (0·0–0·1)	31·4 (29·0–34·0)	145·6 (134·9–156·6)	123·0 (116·4–129·9)	81·7 (75·1–88·1)	36·7 (32·3–41·1)	10·1 (8·8–11·5)	1·2 (1·0–1·4)	0·0 (0·0–0·0)	2·15 (2·06–2·24)	0·89 (0·83–0·94)	0·65 (0·61–0·69)
1961	0·0 (0·0–0·1)	30·8 (28·5–33·3)	144·6 (134·4–155·3)	121·3 (114·5–128·2)	77·9 (71·2–84·4)	35·8 (31·4–40·4)	9·8 (8·5–11·1)	1·1 (0·9–1·3)	0·0 (0·0–0·0)	2·11 (2·01–2·2)	0·88 (0·82–0·93)	0·62 (0·58–0·67)
1962	0·0 (0·0–0·1)	29·8 (27·4–32·3)	142·4 (132·4–152·7)	119·7 (113·0–126·7)	73·7 (67·2–80·1)	34·9 (30·6–39·4)	9·6 (8·4–10·9)	0·9 (0·8–1·1)	0·0 (0·0–0·0)	2·06 (1·96–2·15)	0·86 (0·81–0·91)	0·6 (0·56–0·64)
1963	0·0 (0·0–0·1)	28·8 (26·6–31·3)	139·7 (130·7–149·8)	118·2 (111·6–125·2)	70·1 (63·8–76·5)	33·9 (29·6–38·5)	9·4 (8·2–10·7)	0·9 (0·7–1·0)	0·0 (0·0–0·0)	2·0 (1·91–2·1)	0·84 (0·79–0·9)	0·57 (0·53–0·61)
1964	0·0 (0·0–0·1)	28·2 (26·1–30·5)	137·9 (128·7–148·0)	117·0 (110·3–123·8)	67·7 (61·5–74·4)	32·9 (28·7–37·2)	9·2 (8·0–10·5)	0·8 (0·7–1·0)	0·0 (0·0–0·0)	1·97 (1·88–2·06)	0·83 (0·78–0·88)	0·55 (0·51–0·6)
1965	0·0 (0·0–0·1)	28·4 (26·2–30·7)	137·7 (127·8–147·9)	116·1 (109·5–123·3)	66·7 (60·9–73·8)	31·7 (27·7–36·1)	8·9 (7·7–10·2)	0·8 (0·6–0·9)	0·0 (0·0–0·0)	1·95 (1·86–2·04)	0·83 (0·78–0·88)	0·54 (0·5–0·6)
1966	0·0 (0·0–0·1)	29·2 (26·9–31·5)	138·9 (128·8–149·2)	115·4 (109·2–122·2)	66·7 (61·2–73·3)	30·4 (26·4–34·9)	8·5 (7·3–9·8)	0·7 (0·6–0·9)	0·0 (0·0–0·0)	1·95 (1·86–2·04)	0·84 (0·79–0·89)	0·53 (0·49–0·59)
1967	0·0 (0·0–0·1)	30·5 (28·1–32·9)	141·2 (130·2–151·3)	114·9 (108·7–121·5)	67·3 (61·9–73·8)	29·1 (25·4–33·7)	8·0 (6·9–9·3)	0·7 (0·6–0·9)	0·0 (0·0–0·0)	1·96 (1·87–2·05)	0·86 (0·8–0·91)	0·53 (0·48–0·58)
1968	0·0 (0·0–0·1)	32·2 (29·8–34·7)	144·5 (133·6–155·2)	114·5 (108·5–120·7)	68·3 (62·3–74·7)	27·9 (24·4–32·4)	7·5 (6·5–8·6)	0·7 (0·5–0·8)	0·0 (0·0–0·0)	1·98 (1·88–2·07)	0·88 (0·83–0·93)	0·52 (0·48–0·57)
1969	0·0 (0·0–0·1)	34·0 (31·5–36·7)	148·3 (137·5–159·0)	113·9 (107·7–119·7)	69·0 (62·8–75·3)	27·8 (24·4–32·0)	7·2 (6·2–8·3)	0·6 (0·5–0·8)	0·0 (0·0–0·0)	2·0 (1·9–2·11)	0·91 (0·86–0·96)	0·52 (0·48–0·57)
1970	0·0 (0·0–0·1)	35·6 (33·0–38·4)	151·5 (141·2–162·2)	113·5 (107·4–119·7)	68·8 (63·0–74·8)	28·2 (24·7–32·3)	7·1 (6·2–8·2)	0·6 (0·5–0·7)	0·0 (0·0–0·0)	2·03 (1·92–2·14)	0·94 (0·88–0·99)	0·52 (0·48–0·57)
1971	0·0 (0·0–0·1)	36·8 (34·2–39·8)	153·0 (142·7–163·2)	113·5 (107·4–119·6)	67·5 (61·8–73·2)	28·1 (24·6–32·1)	6·9 (5·9–7·9)	0·5 (0·4–0·7)	0·0 (0·0–0·0)	2·03 (1·92–2·14)	0·95 (0·9–1·0)	0·52 (0·48–0·55)
1972	0·0 (0·0–0·1)	37·7 (35·0–40·6)	152·7 (142·7–162·7)	114·0 (108·0–120·1)	65·3 (59·9–70·8)	27·4 (24·1–31·0)	6·6 (5·7–7·6)	0·5 (0·4–0·6)	0·0 (0·0–0·0)	2·02 (1·92–2·13)	0·95 (0·9–1·01)	0·5 (0·46–0·53)
1973	0·0 (0·0–0·1)	38·6 (35·9–41·3)	151·6 (141·3–162·0)	114·4 (108·2–120·9)	62·8 (57·7–68·1)	26·4 (23·2–30·0)	6·2 (5·4–7·2)	0·5 (0·4–0·5)	0·0 (0·0–0·0)	2·0 (1·91–2·1)	0·95 (0·9–1·01)	0·48 (0·45–0·51)
1974	0·0 (0·0–0·1)	39·5 (36·8–42·2)	150·5 (140·4–160·4)	114·5 (108·1–120·8)	60·4 (55·6–65·4)	25·2 (22·1–28·5)	6·0 (5·2–7·0)	0·4 (0·3–0·5)	0·0 (0·0–0·0)	1·98 (1·89–2·07)	0·95 (0·9–1·0)	0·46 (0·43–0·49)
1975	0·0 (0·0–0·1)	40·5 (37·7–43·4)	149·4 (140·0–159·0)	114·1 (108·0–120·1)	58·5 (53·9–63·4)	23·9 (21·1–27·1)	5·7 (5·0–6·7)	0·4 (0·3–0·5)	0·0 (0·0–0·0)	1·96 (1·88–2·05)	0·95 (0·9–1·0)	0·44 (0·41–0·47)
1976	0·0 (0·0–0·1)	41·6 (38·9–44·6)	148·8 (139·6–158·6)	113·4 (107·4–119·4)	57·3 (52·8–62·1)	22·6 (20·0–25·6)	5·5 (4·7–6·3)	0·4 (0·3–0·4)	0·0 (0·0–0·0)	1·95 (1·86–2·03)	0·95 (0·91–1·0)	0·43 (0·4–0·46)
1977	0·0 (0·0–0·1)	42·7 (40·0–45·9)	148·2 (139·0–157·6)	112·8 (106·7–118·9)	56·6 (52·1–61·1)	21·5 (19·0–24·2)	5·3 (4·5–6·0)	0·3 (0·3–0·4)	0·0 (0·0–0·0)	1·94 (1·85–2·03)	0·96 (0·91–1·01)	0·42 (0·39–0·45)
1978	0·0 (0·0–0·1)	43·8 (41·0–46·8)	147·5 (138·1–156·3)	112·3 (106·4–118·1)	56·2 (51·8–60·7)	20·6 (18·2–23·3)	5·0 (4·3–5·8)	0·3 (0·3–0·4)	0·0 (0·0–0·0)	1·93 (1·84–2·02)	0·96 (0·91–1·0)	0·41 (0·38–0·45)
1979	0·0 (0·0–0·1)	44·8 (42·0–47·8)	147·3 (138·4–155·6)	112·1 (106·4–117·9)	56·0 (51·5–60·5)	19·9 (17·5–22·5)	4·7 (4·1–5·4)	0·3 (0·3–0·4)	0·0 (0·0–0·0)	1·93 (1·85–2·01)	0·96 (0·91–1·0)	0·4 (0·37–0·44)
1980	0·0 (0·0–0·1)	45·8 (43·0–49·0)	147·8 (139·5–156·3)	112·2 (106·8–118·0)	56·3 (51·9–61·0)	19·6 (17·3–22·1)	4·5 (3·9–5·1)	0·3 (0·2–0·4)	0·0 (0·0–0·0)	1·93 (1·86–2·01)	0·97 (0·92–1·01)	0·4 (0·37–0·44)
1981	0·0 (0·0–0·1)	47·0 (44·1–50·0)	148·9 (140·7–158·1)	112·8 (107·3–118·5)	57·0 (52·5–61·4)	20·0 (17·6–22·6)	4·3 (3·8–4·9)	0·3 (0·2–0·4)	0·0 (0·0–0·0)	1·95 (1·88–2·02)	0·98 (0·94–1·02)	0·41 (0·38–0·44)
1982	0·0 (0·0–0·1)	48·2 (45·3–51·3)	150·1 (141·3–159·2)	113·8 (108·3–119·5)	57·6 (52·7–62·2)	20·5 (18·1–23·1)	4·2 (3·7–4·8)	0·3 (0·2–0·3)	0·0 (0·0–0·0)	1·97 (1·91–2·04)	0·99 (0·94–1·04)	0·41 (0·38–0·44)
1983	0·0 (0·0–0·1)	49·1 (46·1–52·2)	150·1 (141·7–158·6)	114·5 (109·0–120·0)	57·5 (52·7–62·1)	20·6 (18·1–23·0)	4·1 (3·6–4·6)	0·2 (0·2–0·3)	0·0 (0·0–0·0)	1·98 (1·92–2·05)	1·0 (0·95–1·04)	0·41 (0·39–0·44)
1984	0·0 (0·0–0·1)	49·6 (46·6–52·9)	149·3 (140·4–157·7)	114·3 (109·1–119·7)	56·9 (52·5–61·3)	20·2 (17·7–22·7)	3·9 (3·5–4·5)	0·2 (0·2–0·3)	0·0 (0·0–0·0)	1·97 (1·91–2·04)	0·99 (0·95–1·04)	0·41 (0·38–0·43)
1985	0·0 (0·0–0·1)	50·0 (47·0–53·2)	147·9 (139·3–156·3)	113·0 (107·9–118·3)	55·9 (51·8–60·2)	19·7 (17·2–22·2)	3·9 (3·4–4·4)	0·2 (0·2–0·3)	0·0 (0·0–0·0)	1·95 (1·89–2·02)	0·99 (0·94–1·04)	0·4 (0·37–0·42)
1986	0·0 (0·0–0·1)	50·4 (47·3–53·8)	146·2 (137·7–154·5)	110·6 (105·4–116·0)	54·4 (50·5–58·6)	19·0 (16·6–21·4)	3·9 (3·4–4·4)	0·2 (0·2–0·2)	0·0 (0·0–0·0)	1·92 (1·86–1·99)	0·98 (0·94–1·03)	0·39 (0·36–0·41)
1987	0·0 (0·0–0·1)	50·5 (47·3–53·9)	144·0 (135·8–152·1)	107·3 (102·4–112·3)	52·3 (48·6–56·2)	18·1 (16·0–20·3)	3·8 (3·3–4·3)	0·2 (0·2–0·2)	0·0 (0·0–0·0)	1·88 (1·81–1·95)	0·97 (0·93–1·02)	0·37 (0·35–0·4)
1988	0·0 (0·0–0·1)	50·6 (47·4–54·0)	140·7 (133·0–149·2)	103·6 (99·2–108·3)	49·5 (45·8–53·2)	17·0 (15·1–19·2)	3·7 (3·2–4·1)	0·2 (0·1–0·2)	0·0 (0·0–0·0)	1·83 (1·76–1·89)	0·96 (0·91–1·0)	0·35 (0·33–0·38)
1989	0·0 (0·0–0·1)	51·4 (48·2–54·6)	136·8 (129·1–145·1)	99·4 (94·9–104·0)	46·1 (42·7–49·5)	15·7 (14·0–17·7)	3·5 (3·1–3·9)	0·2 (0·1–0·2)	0·0 (0·0–0·0)	1·77 (1·71–1·83)	0·94 (0·9–0·99)	0·33 (0·3–0·36)
1990	0·0 (0·0–0·1)	52·3 (49·1–55·6)	132·6 (124·7–140·8)	95·1 (90·7–100·0)	42·2 (39·1–45·6)	14·6 (13·0–16·4)	3·3 (2·8–3·7)	0·1 (0·1–0·2)	0·0 (0·0–0·0)	1·7 (1·64–1·76)	0·92 (0·88–0·97)	0·3 (0·28–0·33)
1991	0·0 (0·0–0·1)	52·6 (49·3–56·0)	128·2 (120·8–136·2)	90·5 (86·5–95·1)	38·1 (35·3–41·2)	13·5 (12·0–15·1)	3·0 (2·6–3·4)	0·1 (0·1–0·2)	0·0 (0·0–0·0)	1·63 (1·57–1·69)	0·9 (0·86–0·95)	0·27 (0·25–0·3)
1992	0·0 (0·0–0·1)	52·2 (48·9–55·8)	123·8 (116·2–131·6)	86·0 (81·8–90·6)	34·0 (31·4–36·9)	12·6 (11·1–14·1)	2·8 (2·4–3·1)	0·1 (0·1–0·1)	0·0 (0·0–0·0)	1·56 (1·51–1·61)	0·88 (0·84–0·92)	0·25 (0·23–0·27)
1993	0·0 (0·0–0·1)	50·5 (47·3–53·8)	118·5 (111·1–126·0)	82·0 (78·0–86·2)	31·9 (29·4–34·6)	11·9 (10·6–13·3)	2·5 (2·2–2·9)	0·1 (0·1–0·1)	0·0 (0·0–0·0)	1·49 (1·43–1·54)	0·85 (0·8–0·89)	0·23 (0·22–0·25)
1994	0·0 (0·0–0·1)	47·5 (44·5–50·7)	112·2 (104·8–119·6)	78·6 (74·7–82·5)	31·6 (29·2–34·2)	11·3 (10·1–12·7)	2·4 (2·1–2·7)	0·1 (0·1–0·1)	0·0 (0·0–0·0)	1·42 (1·37–1·47)	0·8 (0·76–0·84)	0·23 (0·21–0·24)
1995	0·0 (0·0–0·1)	44·2 (41·4–47·2)	106·0 (98·9–112·8)	75·7 (71·9–79·5)	31·3 (29·0–33·9)	10·9 (9·8–12·3)	2·3 (2·0–2·6)	0·1 (0·1–0·1)	0·0 (0·0–0·0)	1·35 (1·3–1·41)	0·75 (0·71–0·79)	0·22 (0·21–0·24)
1996	0·0 (0·0–0·1)	41·1 (38·5–43·7)	100·5 (94·0–107·1)	73·2 (69·6–76·9)	31·0 (28·8–33·6)	10·6 (9·4–11·9)	2·1 (1·9–2·4)	0·1 (0·1–0·1)	0·0 (0·0–0·0)	1·29 (1·24–1·35)	0·71 (0·67–0·74)	0·22 (0·21–0·23)
1997	0·0 (0·0–0·1)	38·0 (35·6–40·5)	95·8 (89·6–102·3)	71·1 (67·4–74·6)	30·9 (28·7–33·2)	10·3 (9·2–11·5)	2·1 (1·8–2·3)	0·1 (0·1–0·1)	0·0 (0·0–0·0)	1·24 (1·18–1·3)	0·67 (0·63–0·71)	0·22 (0·2–0·23)
1998	0·0 (0·0–0·1)	35·0 (32·6–37·4)	91·5 (85·6–97·8)	69·8 (66·2–73·5)	31·3 (29·1–33·7)	10·2 (9·1–11·4)	2·1 (1·8–2·3)	0·1 (0·1–0·1)	0·0 (0·0–0·0)	1·2 (1·14–1·26)	0·63 (0·6–0·67)	0·22 (0·2–0·23)
1999	0·0 (0·0–0·1)	32·2 (30·1–34·4)	87·6 (81·9–94·0)	69·6 (66·0–73·5)	32·2 (29·8–34·5)	10·3 (9·3–11·5)	2·1 (1·8–2·3)	0·1 (0·1–0·1)	0·0 (0·0–0·0)	1·17 (1·12–1·23)	0·6 (0·57–0·64)	0·22 (0·21–0·24)
2000	0·0 (0·0–0·1)	30·0 (27·9–32·2)	84·5 (79·0–90·8)	70·2 (66·7–74·2)	33·5 (31·1–36·0)	10·6 (9·5–11·8)	2·1 (1·9–2·3)	0·1 (0·1–0·1)	0·0 (0·0–0·0)	1·16 (1·11–1·21)	0·57 (0·54–0·61)	0·23 (0·21–0·25)
2001	0·0 (0·0–0·1)	28·8 (26·8–31·0)	82·4 (77·1–88·5)	71·2 (67·8–75·0)	34·9 (32·4–37·5)	11·1 (10·0–12·2)	2·1 (1·9–2·4)	0·1 (0·1–0·1)	0·0 (0·0–0·0)	1·15 (1·11–1·21)	0·56 (0·52–0·59)	0·24 (0·22–0·26)
2002	0·0 (0·0–0·1)	28·1 (26·1–30·2)	81·2 (75·7–86·8)	72·4 (68·7–76·2)	36·8 (34·2–39·6)	11·8 (10·6–13·0)	2·2 (2·0–2·5)	0·1 (0·1–0·1)	0·0 (0·0–0·0)	1·16 (1·12–1·21)	0·55 (0·51–0·58)	0·25 (0·24–0·27)
2003	0·0 (0·0–0·1)	27·6 (25·5–29·6)	79·7 (73·9–85·4)	74·0 (70·2–78·0)	39·2 (36·4–42·1)	12·8 (11·6–14·2)	2·3 (2·1–2·6)	0·1 (0·1–0·1)	0·0 (0·0–0·0)	1·18 (1·14–1·22)	0·54 (0·51–0·57)	0·27 (0·25–0·29)
2004	0·0 (0·0–0·1)	27·3 (25·3–29·4)	78·6 (73·1–84·4)	76·4 (72·5–80·5)	42·0 (38·8–45·1)	14·2 (12·7–15·8)	2·5 (2·2–2·8)	0·1 (0·1–0·2)	0·0 (0·0–0·0)	1·21 (1·16–1·25)	0·53 (0·5–0·56)	0·29 (0·27–0·31)
2005	0·0 (0·0–0·1)	27·6 (25·3–29·9)	78·7 (73·0–84·7)	79·5 (75·8–83·9)	44·7 (41·3–48·0)	15·6 (14·1–17·4)	2·7 (2·4–3·1)	0·1 (0·1–0·2)	0·0 (0·0–0·0)	1·25 (1·2–1·29)	0·53 (0·5–0·56)	0·32 (0·3–0·34)
2006	0·0 (0·0–0·1)	28·1 (25·7–30·6)	79·7 (73·8–85·5)	83·1 (78·9–87·5)	47·4 (43·7–50·9)	17·3 (15·5–19·2)	3·0 (2·7–3·3)	0·1 (0·1–0·2)	0·0 (0·0–0·0)	1·29 (1·23–1·36)	0·54 (0·5–0·58)	0·34 (0·32–0·36)
2007	0·0 (0·0–0·1)	28·6 (26·0–31·1)	80·9 (74·9–87·4)	86·1 (81·5–91·0)	49·9 (46·0–53·8)	18·9 (16·8–21·2)	3·3 (2·9–3·7)	0·2 (0·1–0·2)	0·0 (0·0–0·0)	1·34 (1·27–1·41)	0·55 (0·51–0·59)	0·36 (0·34–0·39)
2008	0·0 (0·0–0·1)	28·8 (26·2–31·5)	82·0 (75·5–88·7)	88·6 (83·9–93·6)	52·2 (48·0–56·4)	20·5 (18·1–23·1)	3·6 (3·2–4·0)	0·2 (0·1–0·2)	0·0 (0·0–0·0)	1·38 (1·31–1·45)	0·55 (0·52–0·59)	0·38 (0·35–0·41)
2009	0·0 (0·0–0·1)	28·7 (26·1–31·3)	82·6 (76·1–89·4)	90·5 (85·5–95·7)	54·5 (50·2–59·4)	22·0 (19·5–24·8)	3·9 (3·5–4·4)	0·2 (0·1–0·2)	0·0 (0·0–0·0)	1·41 (1·34–1·49)	0·56 (0·52–0·59)	0·4 (0·37–0·44)
2010	0·0 (0·0–0·1)	28·4 (25·8–31·3)	82·7 (76·2–89·6)	92·2 (87·2–97·5)	56·8 (52·4–61·8)	23·5 (20·8–26·5)	4·2 (3·7–4·8)	0·2 (0·2–0·2)	0·0 (0·0–0·0)	1·44 (1·37–1·52)	0·56 (0·52–0·59)	0·42 (0·39–0·46)
2011	0·0 (0·0–0·1)	28·2 (25·5–31·2)	82·5 (75·7–89·8)	93·4 (88·2–98·7)	58·5 (53·5–63·6)	24·7 (21·8–27·8)	4·5 (3·9–5·1)	0·2 (0·2–0·3)	0·0 (0·0–0·0)	1·46 (1·38–1·54)	0·55 (0·51–0·6)	0·44 (0·4–0·48)
2012	0·0 (0·0–0·1)	28·0 (25·1–31·1)	82·6 (75·5–89·6)	93·7 (88·2–99·5)	59·2 (54·2–64·6)	25·3 (22·3–28·8)	4·7 (4·1–5·4)	0·2 (0·2–0·3)	0·0 (0·0–0·0)	1·47 (1·37–1·57)	0·55 (0·51–0·6)	0·45 (0·41–0·49)
2013	0·0 (0·0–0·1)	27·8 (24·7–30·8)	82·4 (74·7–90·7)	93·3 (87·4–99·5)	59·3 (53·9–65·3)	25·6 (22·2–29·2)	4·8 (4·1–5·6)	0·2 (0·2–0·3)	0·0 (0·0–0·0)	1·47 (1·35–1·59)	0·55 (0·5–0·61)	0·45 (0·41–0·5)
2014	0·0 (0·0–0·1)	27·5 (24·3–30·9)	81·7 (72·7–91·1)	92·6 (86·2–99·4)	59·2 (53·2–65·9)	25·4 (21·7–29·4)	4·9 (4·1–5·8)	0·2 (0·2–0·3)	0·0 (0·0–0·0)	1·46 (1·32–1·6)	0·55 (0·49–0·61)	0·45 (0·4–0·5)
2015	0·0 (0·0–0·1)	27·1 (23·8–30·7)	80·3 (70·5–91·2)	91·4 (84·3–99·2)	59·0 (52·4–66·2)	25·0 (20·8–29·7)	4·8 (3·9–5·9)	0·3 (0·2–0·3)	0·0 (0·0–0·0)	1·44 (1·29–1·61)	0·54 (0·47–0·61)	0·45 (0·39–0·51)
2016	0·0 (0·0–0·1)	26·9 (23·4–30·8)	78·7 (68·1–90·6)	90·2 (82·8–98·3)	58·6 (51·5–66·7)	24·3 (19·9–29·5)	4·7 (3·7–5·9)	0·2 (0·2–0·3)	0·0 (0·0–0·0)	1·42 (1·25–1·61)	0·53 (0·46–0·61)	0·44 (0·38–0·51)
2017	0·0 (0·0–0·1)	26·8 (23·3–30·9)	77·7 (66·2–90·9)	89·4 (81·5–98·1)	57·8 (50·3–66·5)	23·7 (19·0–29·5)	4·6 (3·5–5·8)	0·2 (0·2–0·3)	0·0 (0·0–0·0)	1·4 (1·22–1·61)	0·52 (0·45–0·61)	0·43 (0·37–0·51)

Supplemental Results Table 5: Age-specific fertility rates per 1000, cumulative total fertility up to maternal age 25 (TFU25), cumulative total fertility from maternal age 30 to age 55 (TFO30), and cumulative total fertility, High-income, 1950-2017

Year	10-14	15-19	20-24	25-29	30-34	35-39	40-44	45-49	50-54	TFR	TFU25	TFO30
1950	0·4 (0·2–0·8)	36·2 (35·5–37·0)	153·8 (150·5–157·3)	168·6 (166·7–170·4)	114·0 (112·0–116·0)	65·8 (64·1–67·6)	21·8 (21·2–22·5)	1·9 (1·7–2·1)	0·0 (0·0–0·0)	2·81 (2·77–2·86)	0·95 (0·93–0·97)	1·02 (1·0–1·04)
1951	0·4 (0·2–0·8)	36·1 (35·5–36·7)	153·4 (150·7–156·0)	167·0 (165·6–168·3)	113·9 (112·2–115·5)	63·5 (61·9–65·0)	20·9 (20·3–21·5)	1·8 (1·7–2·0)	0·0 (0·0–0·0)	2·78 (2·75–2·82)	0·95 (0·93–0·96)	1·0 (0·98–1·02)
1952	0·4 (0·2–0·8)	35·9 (35·4–36·4)	153·2 (150·7–155·9)	166·4 (165·0–167·9)	113·1 (111·6–114·7)	61·6 (60·3–63·1)	20·2 (19·7–20·8)	1·8 (1·6–2·0)	0·0 (0·0–0·0)	2·76 (2·74–2·79)	0·95 (0·93–0·96)	0·98 (0·97–1·0)
1953	0·4 (0·2–0·9)	36·2 (35·8–36·6)	153·9 (151·4–156·4)	166·0 (164·6–167·4)	112·4 (110·9–113·7)	60·2 (58·9–61·4)	19·8 (19·3–20·3)	1·8 (1·6–1·9)	0·0 (0·0–0·0)	2·75 (2·73–2·78)	0·95 (0·94–0·97)	0·97 (0·96–0·98)
1954	0·4 (0·2–0·9)	36·4 (36·0–36·9)	155·2 (152·9–157·6)	167·6 (166·2–169·0)	111·9 (110·4–113·4)	60·4 (59·1–61·7)	19·4 (18·9–19·9)	1·7 (1·6–1·9)	0·0 (0·0–0·0)	2·77 (2·74–2·79)	0·96 (0·95–0·97)	0·97 (0·96–0·98)
1955	0·4 (0·2–0·9)	37·2 (36·8–37·6)	157·6 (155·2–160·0)	170·1 (168·8–171·5)	110·0 (108·6–111·5)	60·7 (59·6–61·9)	18·9 (18·4–19·4)	1·7 (1·5–1·9)	0·0 (0·0–0·0)	2·78 (2·76–2·81)	0·98 (0·96–0·99)	0·96 (0·95–0·97)
1956	0·5 (0·2–0·9)	38·6 (38·2–39·1)	159·8 (157·3–162·5)	172·9 (171·6–174·3)	108·9 (107·5–110·3)	60·6 (59·5–61·7)	18·2 (17·7–18·7)	1·7 (1·5–1·8)	0·0 (0·0–0·0)	2·81 (2·78–2·83)	0·99 (0·98–1·01)	0·95 (0·94–0·96)
1957	0·5 (0·2–1·0)	39·3 (38·9–39·7)	161·2 (159·0–163·6)	175·6 (174·2–177·2)	108·4 (107·2–109·7)	59·6 (58·6–60·6)	17·6 (17·2–18·1)	1·6 (1·5–1·8)	0·0 (0·0–0·0)	2·82 (2·79–2·85)	1·0 (0·99–1·02)	0·94 (0·93–0·95)
1958	0·5 (0·2–0·9)	39·7 (39·3–40·1)	163·9 (161·7–166·3)	178·1 (176·5–179·6)	108·0 (106·7–109·5)	58·3 (57·3–59·3)	17·5 (17·0–18·0)	1·6 (1·4–1·8)	0·0 (0·0–0·0)	2·84 (2·81–2·86)	1·02 (1·01–1·03)	0·93 (0·92–0·94)
1959	0·5 (0·2–0·9)	40·4 (40·0–40·8)	166·2 (164·0–168·5)	180·2 (178·8–181·7)	108·0 (106·8–109·3)	57·0 (56·1–57·9)	17·6 (17·2–18·1)	1·6 (1·4–1·8)	0·0 (0·0–0·0)	2·86 (2·84–2·88)	1·04 (1·02–1·05)	0·92 (0·91–0·93)
1960	0·5 (0·2–1·0)	41·3 (40·9–41·7)	166·9 (164·9–169·1)	181·9 (180·4–183·3)	108·6 (107·3–110·1)	55·7 (54·9–56·6)	17·7 (17·3–18·2)	1·6 (1·4–1·8)	0·0 (0·0–0·0)	2·87 (2·85–2·89)	1·04 (1·03–1·05)	0·92 (0·91–0·93)
1961	0·5 (0·2–1·0)	41·4 (41·0–41·8)	167·5 (165·5–169·7)	182·2 (180·5–183·9)	108·6 (107·3–110·0)	54·3 (53·3–55·2)	17·7 (17·3–18·1)	1·6 (1·4–1·8)	0·0 (0·0–0·0)	2·87 (2·85–2·89)	1·05 (1·04–1·06)	0·91 (0·9–0·92)
1962	0·5 (0·2–0·9)	40·2 (39·9–40·6)	167·5 (165·2–169·8)	182·8 (181·1–184·4)	108·6 (107·2–109·8)	53·3 (52·4–54·1)	17·4 (17·0–17·8)	1·6 (1·4–1·8)	0·0 (0·0–0·0)	2·86 (2·84–2·88)	1·04 (1·03–1·05)	0·9 (0·89–0·91)
1963	0·5 (0·2–0·9)	39·5 (39·1–39·9)	166·1 (163·9–168·4)	183·8 (182·4–185·3)	109·6 (108·3–111·0)	53·6 (52·7–54·4)	17·1 (16·7–17·6)	1·6 (1·4–1·7)	0·0 (0·0–0·0)	2·86 (2·84–2·88)	1·03 (1·02–1·04)	0·91 (0·9–0·92)
1964	0·5 (0·2–0·9)	39·5 (39·1–39·9)	162·4 (160·4–164·7)	182·7 (181·3–184·2)	108·7 (107·4–110·2)	52·9 (52·1–53·7)	16·4 (16·0–16·8)	1·5 (1·3–1·6)	0·0 (0·0–0·0)	2·82 (2·8–2·84)	1·01 (1·0–1·02)	0·9 (0·89–0·91)
1965	0·5 (0·2–1·0)	39·9 (39·5–40·4)	156·4 (154·5–158·4)	171·8 (170·4–173·3)	102·1 (100·8–103·3)	50·2 (49·4–51·0)	15·4 (14·9–15·8)	1·4 (1·3–1·6)	0·0 (0·0–0·0)	2·69 (2·67–2·71)	0·98 (0·97–0·99)	0·85 (0·84–0·86)
1966	0·5 (0·2–1·0)	39·8 (39·4–40·2)	152·4 (150·5–154·4)	168·6 (167·2–169·9)	98·6 (97·4–100·0)	48·2 (47·4–49·0)	14·4 (13·9–14·8)	1·3 (1·2–1·5)	0·0 (0·0–0·0)	2·62 (2·6–2·64)	0·96 (0·95–0·98)	0·81 (0·8–0·82)
1967	0·5 (0·2–1·0)	39·4 (39·1–39·8)	148·5 (146·5–150·4)	171·8 (170·4–173·3)	98·1 (96·6–99·6)	46·4 (45·6–47·2)	13·7 (13·2–14·0)	1·3 (1·1–1·4)	0·0 (0·0–0·0)	2·6 (2·58–2·62)	0·94 (0·93–0·95)	0·8 (0·79–0·81)
1968	0·5 (0·2–1·0)	39·9 (39·6–40·2)	145·1 (143·2–147·0)	167·0 (165·5–168·5)	94·6 (93·3–96·1)	44·6 (43·8–45·4)	13·1 (12·7–13·5)	1·2 (1·1–1·4)	0·0 (0·0–0·0)	2·53 (2·51–2·55)	0·93 (0·92–0·94)	0·77 (0·76–0·78)
1969	0·5 (0·2–1·0)	41·1 (40·7–41·4)	143·4 (141·5–145·4)	165·4 (163·9–167·0)	92·5 (91·0–93·9)	42·9 (42·2–43·7)	12·6 (12·2–13·0)	1·2 (1·0–1·3)	0·0 (0·0–0·0)	2·5 (2·48–2·52)	0·92 (0·91–0·94)	0·75 (0·74–0·76)
1970	0·5 (0·2–1·0)	41·4 (41·1–41·8)	140·2 (138·3–142·3)	163·4 (161·9–164·9)	89·6 (88·3–90·9)	40·8 (40·1–41·6)	11·8 (11·5–12·2)	1·1 (0·9–1·2)	0·0 (0·0–0·0)	2·44 (2·43–2·46)	0·91 (0·9–0·92)	0·72 (0·71–0·73)
1971	0·5 (0·2–1·0)	40·7 (40·3–41·2)	134·3 (132·2–136·4)	157·1 (155·6–158·4)	85·3 (84·1–86·7)	38·0 (37·3–38·7)	11·0 (10·7–11·3)	1·0 (0·8–1·1)	0·0 (0·0–0·0)	2·34 (2·32–2·36)	0·88 (0·87–0·89)	0·68 (0·67–0·69)
1972	0·5 (0·2–0·9)	40·0 (39·5–40·4)	127·8 (125·8–130·0)	149·9 (148·5–151·3)	80·6 (79·1–81·9)	34·7 (34·0–35·4)	10·1 (9·8–10·4)	0·9 (0·8–1·0)	0·0 (0·0–0·0)	2·22 (2·2–2·24)	0·84 (0·83–0·85)	0·63 (0·62–0·64)
1973	0·4 (0·2–0·9)	38·8 (38·5–39·2)	123·7 (121·8–125·5)	146·3 (145·0–147·8)	76·5 (75·2–77·9)	31·7 (31·1–32·3)	9·2 (8·9–9·6)	0·8 (0·7–0·9)	0·0 (0·0–0·0)	2·14 (2·12–2·15)	0·81 (0·81–0·82)	0·59 (0·58–0·6)
1974	0·4 (0·2–0·9)	37·7 (37·3–38·1)	119·4 (117·7–121·2)	142·4 (141·0–143·8)	72·4 (71·3–73·7)	29·3 (28·7–29·9)	8·4 (8·1–8·7)	0·7 (0·6–0·8)	0·0 (0·0–0·0)	2·05 (2·04–2·07)	0·79 (0·78–0·8)	0·55 (0·55–0·56)
1975	0·4 (0·2–0·9)	36·1 (35·7–36·4)	115·5 (113·9–117·0)	137·9 (136·6–139·3)	69·5 (68·4–70·5)	27·3 (26·8–27·7)	7·7 (7·4–8·0)	0·7 (0·6–0·8)	0·0 (0·0–0·0)	1·98 (1·96–1·99)	0·76 (0·75–0·77)	0·53 (0·52–0·53)
1976	0·4 (0·2–0·9)	34·4 (34·1–34·8)	114·6 (113·2–116·3)	136·2 (134·8–137·6)	68·9 (67·8–70·0)	25·5 (25·0–26·1)	7·1 (6·8–7·4)	0·6 (0·6–0·7)	0·0 (0·0–0·0)	1·94 (1·92–1·95)	0·75 (0·74–0·76)	0·51 (0·5–0·52)
1977	0·4 (0·2–0·8)	33·3 (33·0–33·6)	112·6 (111·2–114·0)	135·7 (134·4–137·1)	69·3 (68·2–70·5)	24·4 (23·9–25·0)	6·4 (6·1–6·7)	0·6 (0·5–0·7)	0·0 (0·0–0·0)	1·91 (1·9–1·93)	0·73 (0·72–0·74)	0·5 (0·5–0·51)
1978	0·4 (0·2–0·8)	32·6 (32·3–33·0)	110·2 (109·1–111·3)	135·6 (134·4–136·7)	70·3 (69·2–71·4)	23·8 (23·2–24·4)	6·0 (5·7–6·4)	0·5 (0·5–0·6)	0·0 (0·0–0·0)	1·9 (1·88–1·91)	0·72 (0·71–0·72)	0·5 (0·5–0·51)
1979	0·4 (0·2–0·8)	32·2 (32·0–32·5)	111·0 (110·0–112·1)	136·1 (134·9–137·2)	70·8 (69·7–72·0)	23·4 (23·0–24·0)	5·8 (5·5–6·1)	0·5 (0·4–0·6)	0·0 (0·0–0·0)	1·9 (1·89–1·91)	0·72 (0·71–0·72)	0·5 (0·5–0·51)
1980	0·4 (0·2–0·8)	31·2 (30·9–31·4)	109·7 (108·7–110·7)	135·4 (134·3–136·6)	70·3 (69·1–71·4)	23·5 (22·9–24·1)	5·5 (5·3–5·8)	0·4 (0·4–0·5)	0·0 (0·0–0·0)	1·88 (1·87–1·89)	0·71 (0·7–0·71)	0·5 (0·49–0·51)
1981	0·4 (0·2–0·8)	29·9 (29·7–30·2)	106·5 (105·6–107·5)	133·5 (132·5–134·5)	69·6 (68·5–70·7)	23·8 (23·3–24·2)	5·2 (4·9–5·5)	0·4 (0·3–0·5)	0·0 (0·0–0·0)	1·85 (1·84–1·86)	0·68 (0·68–0·69)	0·49 (0·49–0·5)
1982	0·4 (0·2–0·8)	28·8 (28·5–29·1)	102·5 (101·5–103·5)	130·2 (129·2–131·3)	69·4 (68·3–70·6)	23·9 (23·3–24·6)	5·0 (4·7–5·3)	0·4 (0·3–0·4)	0·0 (0·0–0·0)	1·8 (1·79–1·81)	0·66 (0·65–0·66)	0·49 (0·49–0·5)
1983	0·4 (0·2–0·8)	27·6 (27·3–27·9)	97·8 (96·7–98·9)	126·9 (125·8–128·0)	69·9 (68·9–71·1)	24·0 (23·5–24·6)	4·8 (4·5–5·1)	0·3 (0·3–0·4)	0·0 (0·0–0·0)	1·76 (1·75–1·77)	0·63 (0·62–0·63)	0·49 (0·49–0·5)
1984	0·4 (0·2–0·8)	26·9 (26·7–27·2)	94·9 (94·0–95·9)	125·9 (125·0–126·9)	71·3 (70·3–72·4)	24·2 (23·6–24·7)	4·7 (4·5–5·0)	0·3 (0·3–0·4)	0·0 (0·0–0·0)	1·74 (1·73–1·75)	0·61 (0·61–0·62)	0·5 (0·5–0·51)
1985	0·4 (0·2–0·8)	26·5 (26·3–26·8)	92·7 (91·8–93·6)	125·3 (124·3–126·1)	72·6 (71·6–73·6)	24·3 (23·8–24·9)	4·8 (4·5–5·1)	0·3 (0·3–0·4)	0·0 (0·0–0·0)	1·73 (1·73–1·74)	0·6 (0·59–0·6)	0·51 (0·5–0·52)
1986	0·4 (0·2–0·8)	26·1 (25·8–26·3)	89·8 (89·0–90·6)	124·5 (123·6–125·3)	74·0 (73·0–75·0)	24·7 (24·2–25·3)	5·0 (4·7–5·2)	0·3 (0·3–0·4)	0·0 (0·0–0·0)	1·72 (1·72–1·73)	0·58 (0·58–0·59)	0·52 (0·51–0·53)
1987	0·4 (0·2–0·8)	26·1 (25·9–26·4)	87·5 (86·7–88·4)	124·8 (123·9–125·7)	76·2 (75·3–77·1)	25·9 (25·3–26·5)	5·1 (4·8–5·3)	0·3 (0·3–0·4)	0·0 (0·0–0·0)	1·73 (1·72–1·74)	0·57 (0·57–0·58)	0·54 (0·53–0·54)
1988	0·4 (0·2–0·9)	26·9 (26·6–27·2)	86·1 (85·3–86·9)	124·6 (123·8–125·5)	77·9 (77·1–78·8)	27·2 (26·7–27·7)	5·2 (4·9–5·4)	0·3 (0·3–0·4)	0·0 (0·0–0·0)	1·74 (1·73–1·75)	0·57 (0·56–0·57)	0·55 (0·55–0·56)
1989	0·4 (0·2–0·9)	27·8 (27·5–28·1)	85·6 (84·8–86·4)	124·9 (124·1–125·8)	79·9 (79·0–80·8)	28·5 (28·0–29·1)	5·3 (5·0–5·5)	0·3 (0·3–0·4)	0·0 (0·0–0·0)	1·76 (1·75–1·77)	0·57 (0·56–0·57)	0·57 (0·56–0·58)
1990	0·4 (0·2–0·9)	28·3 (28·1–28·6)	84·3 (83·5–85·1)	124·7 (123·8–125·5)	81·6 (80·7–82·4)	29·7 (29·2–30·2)	5·4 (5·1–5·6)	0·4 (0·3–0·4)	0·0 (0·0–0·0)	1·77 (1·76–1·78)	0·57 (0·56–0·57)	0·58 (0·58–0·59)
1991	0·5 (0·2–0·9)	28·1 (27·8–28·3)	81·4 (80·6–82·2)	122·4 (121·6–123·2)	82·2 (81·3–83·1)	30·4 (29·9–31·0)	5·5 (5·2–5·7)	0·4 (0·3–0·4)	0·0 (0·0–0·0)	1·75 (1·74–1·76)	0·55 (0·54–0·55)	0·59 (0·59–0·6)
1992	0·5 (0·2–0·9)	27·6 (27·3–27·8)	78·6 (77·7–79·6)	119·8 (118·9–120·6)	82·9 (82·0–83·8)	30·9 (30·4–31·5)	5·6 (5·4–5·9)	0·3 (0·3–0·4)	0·0 (0·0–0·0)	1·73 (1·72–1·74)	0·53 (0·53–0·54)	0·6 (0·59–0·61)
1993	0·5 (0·2–1·0)	27·5 (27·2–27·7)	76·0 (75·2–76·9)	117·3 (116·3–118·2)	83·9 (82·9–84·9)	31·6 (31·1–32·1)	5·8 (5·6–6·1)	0·3 (0·3–0·4)	0·0 (0·0–0·0)	1·71 (1·7–1·72)	0·52 (0·52–0·53)	0·61 (0·6–0·61)
1994	0·5 (0·2–1·0)	27·4 (27·1–27·7)	74·2 (73·3–75·1)	115·0 (114·1–115·9)	84·9 (83·9–85·9)	32·2 (31·7–32·8)	6·0 (5·8–6·3)	0·3 (0·3–0·4)	0·0 (0·0–0·0)	1·7 (1·69–1·71)	0·51 (0·5–0·52)	0·62 (0·61–0·62)
1995	0·5 (0·2–1·0)	27·0 (26·7–27·4)	72·1 (71·3–72·9)	112·7 (111·8–113·5)	86·2 (85·2–87·1)	33·0 (32·5–33·6)	6·2 (6·0–6·5)	0·3 (0·3–0·4)	0·0 (0·0–0·0)	1·69 (1·68–1·7)	0·5 (0·49–0·5)	0·63 (0·62–0·63)
1996	0·5 (0·2–1·0)	26·7 (26·4–27·1)	70·3 (69·5–71·2)	110·8 (109·9–111·6)	87·8 (86·9–88·7)	34·3 (33·7–34·9)	6·5 (6·2–6·8)	0·3 (0·3–0·4)	0·0 (0·0–0·0)	1·69 (1·68–1·7)	0·49 (0·48–0·49)	0·64 (0·64–0·65)
1997	0·5 (0·2–1·0)	26·7 (26·4–27·1)	70·3 (69·5–71·2)	109·7 (108·5–110·9)	88·7 (87·8–89·6)	35·2 (34·6–35·9)	6·7 (6·4–7·0)	0·4 (0·3–0·4)	0·0 (0·0–0·0)	1·69 (1·68–1·7)	0·49 (0·48–0·49)	0·65 (0·65–0·66)
1998	0·5 (0·2–1·0)	26·7 (26·4–27·0)	70·5 (69·8–71·2)	108·6 (107·7–109·5)	89·3 (88·4–90·3)	36·0 (35·4–36·6)	6·8 (6·6–7·1)	0·4 (0·3–0·4)	0·0 (0·0–0·0)	1·69 (1·68–1·7)	0·49 (0·48–0·49)	0·66 (0·66–0·67)
1999	0·5 (0·2–1·0)	26·5 (26·2–26·8)	70·7 (69·9–71·5)	107·7 (106·7–108·7)	91·2 (90·2–92·2)	37·4 (36·8–37·9)	7·1 (6·9–7·4)	0·4 (0·4–0·5)	0·0 (0·0–0·0)	1·71 (1·7–1·72)	0·49 (0·48–0·49)	0·68 (0·67–0·69)
2000	0·5 (0·2–0·9)	26·0 (25·7–26·4)	70·4 (69·5–71·3)	105·9 (105·1–106·8)	92·4 (91·4–93·3)	38·9 (38·2–39·6)	7·4 (7·2–7·7)	0·4 (0·4–0·5)	0·0 (0·0–0·0)	1·71 (1·7–1·72)	0·48 (0·48–0·49)	0·7 (0·69–0·7)
2001	0·5 (0·2–0·9)	25·2 (24·8–25·6)	69·0 (68·2–69·8)	103·2 (102·4–104·1)	92·2 (91·1–93·2)	40·0 (39·4–40·6)	7·8 (7·5–8·0)	0·5 (0·4–0·5)	0·0 (0·0–0·0)	1·69 (1·68–1·7)	0·47 (0·47–0·48)	0·7 (0·69–0·71)
2002	0·4 (0·2–0·9)	24·5 (24·1–24·9)	68·0 (67·1–69·0)	102·2 (101·3–103·2)	93·3 (92·4–94·4)	41·2 (40·7–41·9)	8·1 (7·9–8·4)	0·5 (0·4–0·5)	0·0 (0·0–0·0)	1·69 (1·68–1·7)	0·46 (0·46–0·47)	0·72 (0·71–0·72)
2003	0·4 (0·2–0·9)	24·1 (23·8–24·5)	67·4 (66·7–68·2)	101·8 (100·9–102·7)	95·0 (94·0–96·0)	42·9 (42·3–43·6)	8·4 (8·1–8·7)	0·5 (0·4–0·6)	0·0 (0·0–0·0)	1·7 (1·69–1·71)	0·46 (0·46–0·47)	0·73 (0·73–0·74)
2004	0·4 (0·2–0·9)	23·9 (23·6–24·2)	66·8 (66·2–67·5)	100·7 (100·0–101·4)	95·9 (94·8–97·0)	44·2 (43·6–44·9)	8·6 (8·3–8·9)	0·5 (0·4–0·6)	0·0 (0·0–0·0)	1·71 (1·7–1·71)	0·46 (0·45–0·46)	0·75 (0·74–0·75)
2005	0·4 (0·2–0·9)	24·0 (23·8–24·3)	67·3 (66·6–68·1)	100·5 (99·7–101·3)	97·2 (96·2–98·2)	45·7 (45·0–46·5)	8·9 (8·6–9·2)	0·5 (0·4–0·6)	0·0 (0·0–0·0)	1·72 (1·71–1·73)	0·46 (0·45–0·46)	0·76 (0·75–0·77)
2006	0·4 (0·2–0·9)	24·6 (24·3–24·9)	68·4 (67·8–69·0)	101·2 (100·5–102·1)	99·4 (98·4–100·5)	47·4 (46·7–48·3)	9·2 (8·9–9·5)	0·5 (0·4–0·6)	0·0 (0·0–0·0)	1·76 (1·75–1·77)	0·47 (0·46–0·47)	0·78 (0·78–0·79)
2007	0·4 (0·2–0·9)	24·6 (24·4–24·9)	68·4 (67·7–69·2)	101·2 (100·6–102·0)	100·8 (99·8–101·9)	48·6 (47·8–49·4)	9·5 (9·2–9·8)	0·5 (0·5–0·6)	0·0 (0·0–0·0)	1·77 (1·76–1·78)	0·47 (0·46–0·47)	0·8 (0·79–0·81)
2008	0·4 (0·2–0·9)	24·1 (23·8–24·4)	67·2 (66·6–67·8)	100·1 (99·4–100·7)	101·2 (100·2–102·3)	49·4 (48·6–50·3)	9·9 (9·6–10·1)	0·5 (0·5–0·6)	0·0 (0·0–0·0)	1·76 (1·75–1·77)	0·46 (0·45–0·46)	0·81 (0·8–0·81)
2009	0·4 (0·2–0·9)	22·9 (22·6–23·2)	64·8 (64·1–65·4)	98·5 (97·8–99·1)	101·4 (100·4–102·5)	50·3 (49·4–51·2)	10·3 (10·0–10·6)	0·6 (0·5–0·6)	0·0 (0·0–0·0)	1·75 (1·73–1·76)	0·44 (0·44–0·45)	0·81 (0·81–0·82)
2010	0·4 (0·2–0·9)	21·5 (21·2–21·8)	62·3 (61·8–62·9)	97·2 (96·6–97·8)	101·7 (100·7–102·8)	51·4 (50·5–52·3)	10·6 (10·3–10·9)	0·6 (0·5–0·7)	0·0 (0·0–0·0)	1·73 (1·72–1·74)	0·42 (0·42–0·42)	0·82 (0·81–0·83)
2011	0·4 (0·2–0·8)	20·2 (19·9–20·6)	60·5 (59·9–61·2)	95·9 (95·3–96·6)	101·8 (100·9–102·8)	52·4 (51·5–53·4)	10·9 (10·6–11·2)	0·6 (0·5–0·7)	0·0 (0·0–0·0)	1·71 (1·7–1·72)	0·41 (0·4–0·41)	0·83 (0·82–0·84)
2012	0·4 (0·2–0·8)	19·0 (18·7–19·3)	58·7 (58·2–59·2)	94·6 (94·0–95·3)	101·6 (100·7–102·6)	53·3 (52·4–54·2)	11·2 (10·9–11·5)	0·6 (0·6–0·7)	0·0 (0·0–0·0)	1·7 (1·69–1·71)	0·39 (0·39–0·39)	0·83 (0·83–0·84)
2013	0·4 (0·2–0·8)	17·9 (17·6–18·2)	57·2 (56·5–57·9)	94·0 (93·4–94·6)	102·3 (101·4–103·2)	54·5 (53·6–55·4)	11·5 (11·2–11·8)	0·7 (0·6–0·8)	0·0 (0·0–0·0)	1·69 (1·68–1·7)	0·38 (0·37–0·38)	0·84 (0·84–0·85)
2014	0·4 (0·2–0·8)	16·9 (16·5–17·3)	56·1 (55·5–56·7)	93·8 (93·1–94·4)	103·6 (102·7–104·6)	56·0 (55·0–56·9)	11·8 (11·4–12·2)	0·7 (0·6–0·8)	0·0 (0·0–0·0)	1·7 (1·68–1·71)	0·37 (0·36–0·37)	0·86 (0·85–0·87)
2015	0·4 (0·2–0·7)	16·1 (15·5–16·6)	54·6 (53·3–55·9)	92·8 (91·4–94·2)	104·3 (102·7–105·9)	56·6 (55·1–58·2)	11·9 (11·4–12·4)	0·7 (0·6–0·8)	0·0 (0·0–0·0)	1·69 (1·65–1·72)	0·35 (0·35–0·36)	0·87 (0·85–0·88)
2016	0·4 (0·2–0·7)	15·9 (14·9–16·9)	53·5 (50·4–56·8)	91·7 (88·5–95·1)	104·5 (100·0–109·4)	56·3 (52·3–60·7)	11·7 (10·6–13·0)	0·7 (0·6–0·8)	0·0 (0·0–0·0)	1·67 (1·59–1·76)	0·35 (0·33–0·37)	0·87 (0·82–0·92)
2017	0·4 (0·2–0·7)	16·2 (14·8–17·8)	53·5 (47·9–60·1)	91·0 (85·6–96·9)	104·4 (95·9–113·6)	56·1 (48·6–64·8)	11·6 (9·6–14·1)	0·7 (0·6–0·7)	0·0 (0·0–0·0)	1·67 (1·52–1·84)	0·35 (0·32–0·39)	0·86 (0·77–0·97)

Supplemental Results Table 5: Age-specific fertility rates per 1000, cumulative total fertility up to maternal age 25 (TFU25), cumulative total fertility from maternal age 30 to age 55 (TFO30), and cumulative total fertility, Australasia, 1950-2017

Year	10-14	15-19	20-24	25-29	30-34	35-39	40-44	45-49	50-54	TFR	TFU25	TFO30
1950	0·4 (0·2–0·8)	39·9 (38·0–42·1)	177·7 (174·6–180·8)	182·9 (180·6–185·2)	115·6 (113·2–118·0)	65·9 (63·6–68·1)	20·0 (18·2–21·7)	1·4 (1·0–1·8)	0·0 (0·0–0·0)	3·02 (2·98–3·05)	1·09 (1·07–1·11)	1·01 (0·99–1·03)
1951	0·4 (0·2–0·9)	41·1 (39·3–43·2)	186·0 (183·0–189·1)	187·5 (185·1–190·0)	118·1 (115·8–120·5)	66·1 (64·0–68·3)	20·7 (19·0–22·4)	1·5 (1·1–2·0)	0·0 (0·0–0·0)	3·11 (3·07–3·15)	1·14 (1·12–1·16)	1·03 (1·01–1·05)
1952	0·4 (0·2–0·9)	42·1 (40·3–43·9)	193·6 (190·6–196·6)	191·0 (188·8–193·1)	120·0 (117·7–122·3)	65·8 (63·7–68·0)	20·7 (19·2–22·5)	1·6 (1·2–2·1)	0·0 (0·0–0·0)	3·18 (3·14–3·21)	1·18 (1·16–1·2)	1·04 (1·02–1·06)
1953	0·4 (0·2–0·9)	43·0 (41·2–44·8)	197·3 (194·5–200·1)	193·0 (190·7–195·1)	120·2 (117·7–122·6)	65·1 (63·0–67·3)	20·5 (19·0–22·2)	1·6 (1·2–2·2)	0·0 (0·0–0·0)	3·21 (3·17–3·24)	1·2 (1·19–1·22)	1·04 (1·02–1·06)
1954	0·4 (0·2–0·9)	44·5 (42·5–46·5)	201·6 (198·7–204·5)	196·5 (194·3–198·7)	120·6 (118·3–123·0)	64·8 (62·7–66·9)	20·5 (18·9–22·1)	1·6 (1·2–2·2)	0·0 (0·0–0·0)	3·25 (3·22–3·28)	1·23 (1·21–1·25)	1·04 (1·02–1·06)
1955	0·4 (0·2–0·9)	46·2 (44·1–48·5)	207·2 (204·4–210·0)	201·8 (199·6–203·9)	122·4 (120·3–124·7)	65·1 (63·1–67·3)	20·6 (19·1–22·2)	1·7 (1·2–2·3)	0·0 (0·0–0·0)	3·33 (3·29–3·36)	1·27 (1·25–1·29)	1·05 (1·03–1·07)
1956	0·4 (0·2–0·9)	47·7 (45·4–50·2)	213·1 (210·2–215·8)	207·7 (205·2–209·7)	125·0 (122·9–127·5)	65·8 (63·6–68·1)	20·4 (18·9–22·0)	1·8 (1·3–2·4)	0·0 (0·0–0·0)	3·41 (3·38–3·45)	1·31 (1·29–1·33)	1·07 (1·05–1·08)
1957	0·4 (0·2–0·9)	48·4 (46·3–50·8)	217·1 (214·2–219·8)	212·3 (210·0–214·3)	126·5 (124·3–128·8)	66·1 (63·9–68·4)	20·1 (18·6–21·6)	1·8 (1·3–2·4)	0·0 (0·0–0·0)	3·46 (3·43–3·49)	1·33 (1·31–1·35)	1·07 (1·05–1·09)
1958	0·4 (0·2–0·9)	48·8 (46·9–50·6)	219·7 (216·9–222·5)	215·0 (212·8–217·2)	126·7 (124·6–129·0)	65·7 (63·5–67·8)	19·7 (18·2–21·4)	1·8 (1·3–2·5)	0·0 (0·0–0·0)	3·49 (3·46–3·52)	1·34 (1·33–1·36)	1·07 (1·05–1·09)
1959	0·4 (0·2–0·9)	49·2 (47·4–51·0)	222·1 (219·4–224·8)	217·5 (215·4–219·7)	128·1 (125·8–130·4)	65·2 (63·1–67·3)	19·7 (18·1–21·4)	1·8 (1·4–2·5)	0·0 (0·0–0·0)	3·52 (3·49–3·55)	1·36 (1·34–1·38)	1·07 (1·06–1·09)
1960	0·4 (0·2–0·9)	49·8 (48·0–51·5)	224·5 (221·5–227·4)	220·4 (218·4–222·5)	130·6 (128·4–132·9)	65·1 (63·1–67·2)	19·9 (18·3–21·6)	1·8 (1·3–2·5)	0·0 (0·0–0·0)	3·56 (3·53–3·59)	1·37 (1·36–1·39)	1·09 (1·07–1·11)
1961	0·4 (0·2–0·9)	49·9 (48·4–51·6)	222·1 (219·9–224·2)	220·4 (218·3–222·8)	130·5 (128·4–132·7)	63·7 (61·8–65·6)	19·6 (18·1–21·2)	1·7 (1·2–2·3)	0·0 (0·0–0·0)	3·54 (3·52–3·57)	1·36 (1·35–1·38)	1·08 (1·06–1·09)
1962	0·5 (0·2–0·9)	50·1 (48·5–51·7)	210·9 (208·8–213·1)	215·0 (212·9–217·1)	126·8 (124·7–128·8)	61·0 (59·0–63·1)	18·5 (17·0–20·1)	1·4 (1·0–1·9)	0·0 (0·0–0·0)	3·42 (3·39–3·45)	1·31 (1·29–1·32)	1·04 (1·02–1·06)
1963	0·5 (0·2–0·9)	50·6 (49·1–52·1)	196·0 (193·8–198·2)	205·1 (203·3–207·0)	121·2 (119·2–123·2)	58·3 (56·4–60·3)	17·1 (15·6–18·7)	1·2 (0·8–1·6)	0·0 (0·0–0·0)	3·25 (3·22–3·28)	1·24 (1·22–1·25)	0·99 (0·97–1·01)
1964	0·4 (0·2–0·9)	50·5 (49·0–52·0)	185·1 (183·0–187·2)	194·4 (192·6–196·3)	114·0 (111·9–116·0)	55·4 (53·6–57·4)	15·8 (14·5–17·3)	1·0 (0·7–1·4)	0·0 (0·0–0·0)	3·08 (3·06–3·11)	1·18 (1·17–1·19)	0·93 (0·91–0·95)
1965	0·4 (0·2–0·9)	50·2 (48·7–51·7)	179·1 (177·0–181·1)	187·1 (185·4–188·9)	106·9 (105·0–108·9)	51·9 (50·0–53·7)	14·8 (13·4–16·3)	1·0 (0·7–1·4)	0·0 (0·0–0·0)	2·96 (2·93–2·98)	1·15 (1·14–1·16)	0·87 (0·86–0·89)
1966	0·4 (0·2–0·9)	50·1 (48·7–51·6)	175·9 (173·9–177·9)	184·5 (182·7–186·2)	102·9 (100·9–104·7)	48·8 (47·0–50·6)	14·0 (12·6–15·5)	1·0 (0·7–1·4)	0·0 (0·0–0·0)	2·89 (2·87–2·91)	1·13 (1·12–1·14)	0·83 (0·82–0·85)
1967	0·4 (0·2–0·9)	50·3 (48·8–51·7)	176·4 (174·5–178·5)	185·3 (183·5–187·1)	101·7 (99·9–103·6)	46·6 (44·8–48·6)	13·3 (12·0–14·8)	1·0 (0·7–1·4)	0·0 (0·0–0·0)	2·88 (2·86–2·9)	1·14 (1·12–1·15)	0·81 (0·8–0·83)
1968	0·4 (0·2–0·9)	50·5 (49·1–51·9)	177·0 (175·0–179·0)	186·6 (184·9–188·2)	101·4 (99·5–103·3)	44·6 (42·7–46·7)	12·6 (11·3–14·0)	1·0 (0·6–1·4)	0·0 (0·0–0·0)	2·87 (2·85–2·89)	1·14 (1·13–1·15)	0·8 (0·78–0·81)
1969	0·4 (0·2–0·9)	52·2 (50·5–53·9)	175·9 (174·0–177·8)	187·5 (185·8–189·2)	101·0 (99·0–102·9)	43·7 (41·8–45·8)	12·1 (10·8–13·6)	1·0 (0·6–1·4)	0·0 (0·0–0·0)	2·87 (2·85–2·89)	1·14 (1·13–1·16)	0·79 (0·77–0·8)
1970	0·5 (0·2–1·0)	55·1 (53·4–56·9)	179·1 (177·3–181·0)	189·2 (187·5–190·8)	100·5 (98·7–102·4)	43·4 (41·5–45·3)	11·8 (10·5–13·2)	1·0 (0·6–1·4)	0·0 (0·0–0·0)	2·9 (2·88–2·93)	1·17 (1·16–1·19)	0·78 (0·77–0·8)
1971	0·5 (0·2–1·0)	55·6 (54·0–57·2)	176·9 (174·7–179·2)	184·4 (182·8–186·1)	95·6 (93·6–97·6)	39·9 (38·1–41·7)	10·6 (9·4–12·1)	0·8 (0·5–1·2)	0·0 (0·0–0·0)	2·82 (2·8–2·85)	1·17 (1·15–1·18)	0·74 (0·72–0·75)
1972	0·4 (0·2–0·9)	52·9 (51·4–54·2)	164·1 (162·3–166·1)	172·3 (170·7–174·0)	87·2 (85·4–89·1)	34·8 (33·0–36·4)	9·1 (8·0–10·4)	0·7 (0·4–1·0)	0·0 (0·0–0·0)	2·61 (2·59–2·63)	1·09 (1·08–1·1)	0·66 (0·64–0·67)
1973	0·4 (0·2–0·9)	48·8 (47·5–50·4)	154·6 (152·8–156·3)	162·9 (161·3–164·6)	80·5 (78·6–82·4)	30·8 (29·2–32·6)	8·0 (6·9–9·3)	0·6 (0·4–0·8)	0·0 (0·0–0·0)	2·43 (2·41–2·46)	1·02 (1·01–1·03)	0·6 (0·58–0·62)
1974	0·4 (0·2–0·9)	44·6 (43·2–46·1)	146·5 (144·7–148·3)	156·0 (154·4–157·5)	76·2 (74·5–78·0)	27·5 (26·0–29·1)	7·1 (5·9–8·2)	0·5 (0·3–0·7)	0·0 (0·0–0·0)	2·29 (2·27–2·32)	0·96 (0·95–0·97)	0·56 (0·54–0·57)
1975	0·4 (0·2–0·8)	40·4 (39·1–41·9)	137·5 (135·8–139·4)	150·5 (149·1–151·9)	74·2 (72·5–75·8)	25·1 (23·9–26·3)	6·3 (5·2–7·5)	0·4 (0·3–0·6)	0·0 (0·0–0·0)	2·17 (2·15–2·19)	0·89 (0·88–0·9)	0·53 (0·52–0·54)
1976	0·4 (0·2–0·8)	36·5 (35·2–37·8)	130·3 (128·5–132·1)	147·4 (145·9–148·8)	73·5 (71·8–75·1)	23·3 (22·1–24·6)	5·7 (4·6–6·9)	0·4 (0·2–0·5)	0·0 (0·0–0·0)	2·09 (2·07–2·11)	0·84 (0·82–0·85)	0·51 (0·5–0·53)
1977	0·4 (0·2–0·7)	33·3 (31·9–34·7)	123·0 (121·3–124·8)	145·2 (143·6–146·7)	72·9 (71·3–74·5)	22·5 (21·2–24·2)	5·1 (4·2–6·4)	0·3 (0·2–0·5)	0·0 (0·0–0·0)	2·01 (1·99–2·03)	0·78 (0·77–0·79)	0·5 (0·49–0·52)
1978	0·3 (0·2–0·7)	31·2 (29·7–32·9)	116·2 (114·6–118·0)	143·3 (141·8–144·8)	72·6 (70·9–74·3)	22·9 (21·4–24·6)	4·8 (3·9–5·9)	0·3 (0·2–0·4)	0·0 (0·0–0·0)	1·96 (1·94–1·98)	0·74 (0·73–0·75)	0·5 (0·49–0·52)
1979	0·3 (0·1–0·7)	30·0 (28·7–31·5)	112·0 (110·3–113·6)	142·3 (140·8–143·8)	73·3 (71·4–75·2)	23·3 (21·8–24·9)	4·7 (3·9–5·6)	0·3 (0·2–0·4)	0·0 (0·0–0·0)	1·93 (1·91–1·95)	0·71 (0·7–0·72)	0·51 (0·49–0·52)
1980	0·3 (0·1–0·7)	29·4 (28·1–30·6)	110·2 (108·4–112·0)	143·4 (141·8–145·0)	75·5 (73·7–77·4)	23·8 (22·5–25·2)	4·7 (4·2–5·4)	0·3 (0·2–0·4)	0·0 (0·0–0·0)	1·94 (1·92–1·96)	0·7 (0·69–0·71)	0·52 (0·51–0·53)
1981	0·3 (0·1–0·7)	28·8 (27·6–30·1)	108·6 (107·0–110·3)	145·4 (143·9–146·9)	78·2 (76·6–79·9)	24·3 (22·9–25·7)	4·7 (4·2–5·3)	0·3 (0·2–0·4)	0·0 (0·0–0·0)	1·95 (1·93–1·97)	0·69 (0·68–0·7)	0·54 (0·53–0·55)
1982	0·3 (0·1–0·6)	27·5 (26·3–28·7)	106·1 (104·4–107·8)	145·8 (144·4–147·4)	79·8 (78·3–81·3)	24·5 (22·8–26·2)	4·6 (4·0–5·3)	0·2 (0·2–0·3)	0·0 (0·0–0·0)	1·94 (1·93–1·96)	0·67 (0·66–0·68)	0·55 (0·53–0·56)
1983	0·3 (0·1–0·6)	25·6 (24·5–26·9)	101·3 (99·7–103·0)	144·3 (142·9–145·8)	81·0 (79·4–82·6)	24·8 (23·1–26·7)	4·3 (3·7–5·1)	0·2 (0·2–0·3)	0·0 (0·0–0·0)	1·91 (1·89–1·93)	0·64 (0·63–0·65)	0·55 (0·54–0·57)
1984	0·3 (0·1–0·6)	24·1 (22·9–25·3)	97·7 (96·0–99·3)	144·2 (142·7–145·8)	84·2 (82·6–85·8)	25·4 (23·8–27·1)	4·3 (3·7–5·0)	0·2 (0·2–0·3)	0·0 (0·0–0·0)	1·9 (1·88–1·92)	0·61 (0·6–0·62)	0·57 (0·56–0·58)
1985	0·3 (0·1–0·6)	22·9 (21·8–24·0)	95·0 (93·3–96·6)	144·0 (142·5–145·5)	87·8 (86·1–89·6)	26·5 (25·0–27·9)	4·5 (3·9–5·1)	0·2 (0·2–0·3)	0·0 (0·0–0·0)	1·91 (1·88–1·93)	0·59 (0·58–0·6)	0·59 (0·58–0·61)
1986	0·3 (0·1–0·6)	22·1 (21·0–23·3)	91·0 (89·4–92·6)	142·0 (140·5–143·6)	89·9 (87·9–92·0)	27·9 (26·5–29·2)	4·8 (4·1–5·5)	0·2 (0·2–0·3)	0·0 (0·0–0·0)	1·89 (1·87–1·91)	0·57 (0·56–0·58)	0·61 (0·6–0·63)
1987	0·3 (0·1–0·6)	22·0 (20·7–23·1)	87·6 (86·0–89·4)	140·5 (139·0–142·0)	92·5 (90·5–94·3)	29·5 (28·1–31·0)	5·0 (4·2–5·9)	0·2 (0·2–0·3)	0·0 (0·0–0·0)	1·89 (1·87–1·91)	0·55 (0·54–0·56)	0·64 (0·62–0·65)
1988	0·3 (0·1–0·6)	22·3 (21·0–23·8)	85·2 (83·6–86·9)	140·1 (138·6–141·6)	96·0 (94·1–98·0)	31·5 (29·9–33·0)	5·1 (4·2–6·3)	0·3 (0·2–0·3)	0·0 (0·0–0·0)	1·9 (1·88–1·92)	0·54 (0·53–0·55)	0·66 (0·65–0·68)
1989	0·3 (0·1–0·6)	22·9 (21·5–24·3)	83·9 (82·2–85·5)	140·5 (138·9–142·0)	100·0 (98·3–101·7)	33·5 (32·1–35·0)	5·3 (4·3–6·4)	0·3 (0·2–0·4)	0·0 (0·0–0·0)	1·93 (1·91–1·95)	0·54 (0·52–0·55)	0·7 (0·68–0·71)
1990	0·3 (0·1–0·6)	23·3 (22·1–24·5)	81·6 (80·0–83·4)	137·7 (136·2–139·2)	102·1 (100·5–103·7)	35·6 (34·1–37·0)	5·5 (4·5–6·6)	0·3 (0·2–0·4)	0·0 (0·0–0·0)	1·93 (1·91–1·95)	0·53 (0·51–0·54)	0·72 (0·7–0·73)
1991	0·3 (0·1–0·6)	23·3 (22·2–24·5)	78·6 (77·1–80·3)	133·9 (132·3–135·5)	103·5 (101·9–105·2)	37·4 (36·0–38·9)	5·8 (4·8–6·9)	0·3 (0·2–0·4)	0·0 (0·0–0·0)	1·92 (1·89–1·94)	0·51 (0·5–0·52)	0·73 (0·72–0·75)
1992	0·3 (0·1–0·6)	23·0 (21·9–24·2)	76·3 (74·8–77·8)	131·7 (130·2–133·2)	105·5 (103·9–107·1)	38·8 (37·5–40·3)	6·1 (5·1–7·2)	0·3 (0·2–0·4)	0·0 (0·0–0·0)	1·91 (1·89–1·93)	0·5 (0·49–0·51)	0·75 (0·74–0·77)
1993	0·3 (0·1–0·6)	22·6 (21·4–23·9)	74·0 (72·1–75·8)	128·8 (127·1–130·5)	105·8 (104·2–107·5)	40·2 (38·7–41·8)	6·5 (5·4–7·6)	0·3 (0·2–0·4)	0·0 (0·0–0·0)	1·89 (1·87–1·91)	0·48 (0·47–0·5)	0·76 (0·75–0·78)
1994	0·3 (0·1–0·6)	22·2 (20·9–23·5)	72·0 (69·8–74·3)	125·0 (123·2–126·9)	105·8 (103·7–107·8)	41·7 (40·0–43·4)	6·9 (5·9–7·9)	0·3 (0·2–0·4)	0·0 (0·0–0·0)	1·87 (1·84–1·9)	0·47 (0·46–0·49)	0·77 (0·76–0·79)
1995	0·3 (0·1–0·6)	21·8 (20·4–23·2)	69·9 (67·4–72·4)	121·0 (118·9–123·3)	106·1 (103·6–108·7)	43·1 (41·1–45·3)	7·3 (6·3–8·2)	0·3 (0·3–0·4)	0·0 (0·0–0·0)	1·85 (1·81–1·89)	0·46 (0·44–0·48)	0·78 (0·76–0·81)
1996	0·3 (0·1–0·6)	21·3 (19·7–22·8)	67·8 (65·1–70·6)	117·3 (115·2–119·5)	106·6 (104·3–109·2)	44·4 (42·4–46·4)	7·7 (6·8–8·5)	0·4 (0·3–0·4)	0·0 (0·0–0·0)	1·83 (1·78–1·88)	0·45 (0·43–0·47)	0·8 (0·77–0·82)
1997	0·3 (0·1–0·5)	20·7 (19·2–22·5)	66·1 (63·6–68·7)	114·3 (112·2–116·3)	107·5 (105·2–109·8)	45·6 (43·7–47·5)	8·1 (7·3–8·9)	0·4 (0·3–0·5)	0·0 (0·0–0·0)	1·81 (1·77–1·86)	0·44 (0·42–0·45)	0·81 (0·79–0·83)
1998	0·3 (0·1–0·5)	20·2 (18·7–21·7)	64·7 (62·8–66·9)	111·7 (109·9–113·6)	108·5 (106·5–110·6)	46·8 (45·0–48·7)	8·5 (7·7–9·4)	0·4 (0·3–0·5)	0·0 (0·0–0·0)	1·8 (1·78–1·84)	0·43 (0·41–0·44)	0·82 (0·8–0·84)
1999	0·3 (0·1–0·5)	19·6 (18·3–21·0)	63·4 (61·4–65·4)	109·7 (108·0–111·3)	109·6 (108·0–111·3)	48·4 (46·8–50·3)	8·9 (8·0–9·9)	0·4 (0·3–0·5)	0·0 (0·0–0·0)	1·8 (1·78–1·83)	0·42 (0·4–0·43)	0·84 (0·82–0·85)
2000	0·3 (0·1–0·5)	19·2 (17·9–20·6)	62·2 (59·8–64·5)	108·8 (107·0–110·8)	110·8 (108·6–112·9)	50·2 (48·5–52·3)	9·3 (8·3–10·3)	0·4 (0·4–0·5)	0·0 (0·0–0·0)	1·81 (1·78–1·84)	0·41 (0·39–0·42)	0·85 (0·84–0·87)
2001	0·3 (0·1–0·5)	18·8 (17·5–20·1)	61·1 (59·1–63·2)	108·6 (106·7–110·4)	111·9 (109·8–114·0)	52·1 (50·2–53·9)	9·7 (8·8–10·8)	0·5 (0·4–0·6)	0·0 (0·0–0·0)	1·81 (1·79–1·84)	0·4 (0·39–0·41)	0·87 (0·85–0·89)
2002	0·3 (0·1–0·5)	18·4 (17·1–19·7)	59·8 (58·2–61·6)	108·0 (106·4–109·5)	113·1 (111·7–114·7)	54·1 (52·7–55·6)	10·2 (9·2–11·3)	0·5 (0·4–0·6)	0·0 (0·0–0·0)	1·82 (1·8–1·84)	0·39 (0·38–0·4)	0·89 (0·88–0·9)
2003	0·3 (0·1–0·5)	18·0 (16·7–19·4)	58·4 (56·4–60·3)	107·2 (105·3–108·9)	115·0 (113·0–116·9)	56·5 (54·6–58·6)	10·8 (9·8–11·8)	0·5 (0·4–0·6)	0·0 (0·0–0·0)	1·83 (1·8–1·86)	0·38 (0·37–0·4)	0·91 (0·9–0·93)
2004	0·3 (0·1–0·5)	17·9 (16·7–19·3)	57·6 (55·7–59·7)	107·1 (105·2–109·0)	117·4 (115·3–119·3)	59·2 (57·0–61·1)	11·3 (10·3–12·4)	0·6 (0·5–0·7)	0·0 (0·0–0·0)	1·86 (1·83–1·89)	0·38 (0·36–0·4)	0·94 (0·92–0·96)
2005	0·3 (0·1–0·5)	18·3 (17·1–19·6)	58·4 (56·5–60·4)	108·6 (106·6–110·6)	121·0 (119·1–123·2)	62·2 (59·7–64·8)	11·8 (10·7–12·8)	0·6 (0·5–0·7)	0·0 (0·0–0·0)	1·91 (1·88–1·93)	0·38 (0·37–0·4)	0·98 (0·96–1·0)
2006	0·3 (0·1–0·5)	19·1 (17·9–20·2)	60·7 (58·8–62·7)	111·3 (109·5–113·0)	126·0 (123·9–128·2)	66·1 (64·0–68·1)	12·3 (11·3–13·4)	0·6 (0·4–0·7)	0·0 (0·0–0·0)	1·98 (1·96–2·01)	0·4 (0·39–0·41)	1·03 (1·01–1·04)
2007	0·3 (0·1–0·6)	19·6 (18·5–20·6)	62·9 (61·4–64·5)	113·2 (111·4–115·0)	129·8 (128·3–131·3)	69·5 (68·0–71·0)	13·1 (12·0–14·2)	0·6 (0·4–0·7)	0·0 (0·0–0·0)	2·04 (2·03–2·06)	0·41 (0·4–0·42)	1·06 (1·05–1·08)
2008	0·3 (0·1–0·5)	19·5 (18·3–20·6)	63·2 (61·8–64·7)	112·6 (110·8–114·4)	130·1 (128·5–131·6)	70·4 (68·8–71·9)	13·9 (12·8–15·0)	0·6 (0·4–0·8)	0·0 (0·0–0·0)	2·05 (2·03–2·07)	0·41 (0·41–0·42)	1·07 (1·06–1·09)
2009	0·3 (0·1–0·5)	18·9 (17·7–20·0)	61·9 (60·4–63·4)	110·8 (109·3–112·4)	128·9 (127·3–130·5)	70·0 (68·4–71·6)	14·5 (13·4–15·7)	0·6 (0·4–0·9)	0·0 (0·0–0·0)	2·03 (2·01–2·05)	0·41 (0·4–0·41)	1·07 (1·06–1·09)
2010	0·3 (0·1–0·5)	18·3 (17·3–19·4)	60·8 (59·2–62·3)	109·7 (108·3–111·2)	128·2 (126·6–129·8)	70·4 (68·8–72·0)	14·8 (13·5–16·1)	0·7 (0·4–1·0)	0·0 (0·0–0·0)	2·02 (2·0–2·04)	0·4 (0·39–0·41)	1·07 (1·06–1·08)
2011	0·3 (0·1–0·5)	17·5 (16·5–18·7)	60·1 (58·7–61·6)	109·4 (108·1–110·8)	128·5 (126·9–130·0)	70·9 (69·3–72·5)	14·9 (13·7–16·2)	0·7 (0·4–1·0)	0·0 (0·0–0·0)	2·01 (1·99–2·03)	0·39 (0·38–0·4)	1·08 (1·06–1·09)
2012	0·2 (0·1–0·5)	16·4 (15·3–17·6)	58·6 (57·2–60·1)	107·8 (106·4–109·2)	127·8 (126·3–129·2)	70·9 (69·4–72·5)	14·9 (13·8–16·1)	0·7 (0·5–1·1)	0·0 (0·0–0·0)	1·99 (1·97–2·01)	0·38 (0·37–0·39)	1·07 (1·06–1·09)
2013	0·2 (0·1–0·5)	15·2 (13·9–16·5)	55·8 (54·2–57·3)	104·7 (103·1–106·3)	124·6 (123·0–126·2)	69·8 (68·2–71·3)	14·9 (13·8–16·0)	0·8 (0·5–1·2)	0·0 (0·0–0·0)	1·93 (1·91–1·95)	0·36 (0·35–0·37)	1·05 (1·04–1·06)
2014	0·2 (0·1–0·5)	14·4 (13·0–15·8)	53·8 (52·3–55·2)	102·7 (101·1–104·4)	123·1 (121·6–124·6)	69·0 (67·5–70·5)	14·6 (13·4–15·8)	0·8 (0·5–1·2)	0·0 (0·0–0·0)	1·89 (1·87–1·91)	0·34 (0·33–0·35)	1·04 (1·03–1·05)
2015	0·2 (0·1–0·4)	14·2 (12·8–15·6)	52·9 (50·3–55·5)	101·8 (99·3–104·6)	124·2 (121·3–127·2)	69·4 (66·6–72·4)	14·4 (12·7–16·2)	0·8 (0·5–1·3)	0·0 (0·0–0·0)	1·89 (1·84–1·94)	0·34 (0·32–0·35)	1·04 (1·01–1·07)
2016	0·2 (0·1–0·4)	14·2 (12·7–16·0)	52·2 (47·3–57·5)	101·3 (96·4–106·5)	125·3 (118·4–132·5)	69·7 (63·5–76·5)	14·4 (12·3–17·0)	0·8 (0·5–1·3)	0·0 (0·0–0·0)	1·89 (1·77–2·03)	0·33 (0·3–0·37)	1·05 (0·98–1·13)
2017	0·2 (0·1–0·4)	14·5 (12·8–16·5)	51·9 (45·4–59·2)	101·1 (94·5–108·1)	125·9 (116·1–136·3)	69·8 (60·6–80·0)	14·4 (11·7–17·8)	0·8 (0·5–1·3)	0·0 (0·0–0·0)	1·89 (1·71–2·09)	0·33 (0·29–0·38)	1·05 (0·94–1·18)

Supplemental Results Table 5: Age-specific fertility rates per 1000, cumulative total fertility up to maternal age 25 (TFU25), cumulative total fertility from maternal age 30 to age 55 (TFO30), and cumulative total fertility, Australia, 1950-2017												
**Year**	**10-14**	**15-19**	**20-24**	**25-29**	**30-34**	**35-39**	**40-44**	**45-49**	**50-54**	**TFR**	**TFU25**	**TFO30**
1950	0·2 (0·1–0·5)	34·8 (32·7–37·0)	167·8 (165·1–170·5)	177·7 (175·5–179·9)	115·4 (113·1–117·9)	64·8 (62·5–67·2)	19·4 (17·3–21·5)	1·1 (0·7–1·7)	0·0 (0·0–0·0)	2·91 (2·88–2·94)	1·01 (1·0–1·03)	1·0 (0·98–1·02)
1951	0·2 (0·1–0·5)	36·2 (34·3–38·3)	177·7 (174·8–180·4)	182·9 (180·8–185·2)	118·1 (115·7–120·4)	64·9 (62·5–67·3)	20·1 (18·2–22·2)	1·3 (0·8–1·9)	0·0 (0·0–0·0)	3·01 (2·98–3·04)	1·07 (1·05–1·09)	1·02 (1·0–1·04)
1952	0·3 (0·1–0·5)	37·1 (35·2–39·2)	186·4 (183·7–189·1)	186·5 (184·3–188·6)	119·9 (117·5–122·0)	64·2 (61·9–66·7)	20·1 (18·3–22·1)	1·3 (0·9–2·0)	0·0 (0·0–0·0)	3·08 (3·05–3·11)	1·12 (1·1–1·14)	1·03 (1·01–1·05)
1953	0·3 (0·1–0·5)	37·9 (36·1–39·9)	190·1 (187·7–192·8)	187·9 (185·7–190·0)	119·5 (117·3–121·6)	63·0 (60·7–65·4)	19·7 (17·9–21·7)	1·4 (0·9–2·0)	0·0 (0·0–0·0)	3·1 (3·07–3·13)	1·14 (1·13–1·16)	1·02 (1·0–1·04)
1954	0·3 (0·1–0·5)	39·3 (37·3–41·3)	194·6 (192·0–197·2)	191·1 (189·0–193·3)	119·2 (117·1–121·4)	62·2 (59·9–64·4)	19·6 (17·7–21·4)	1·4 (0·9–2·1)	0·0 (0·0–0·0)	3·14 (3·11–3·17)	1·17 (1·15–1·19)	1·01 (0·99–1·03)
1955	0·3 (0·1–0·5)	41·1 (39·2–43·0)	200·8 (198·1–203·5)	196·6 (194·5–198·8)	120·7 (118·4–123·1)	62·3 (59·9–64·5)	19·6 (17·8–21·5)	1·5 (1·0–2·3)	0·0 (0·0–0·0)	3·21 (3·18–3·25)	1·21 (1·19–1·23)	1·02 (1·0–1·04)
1956	0·3 (0·1–0·6)	42·4 (40·6–44·5)	207·5 (204·8–210·2)	202·6 (200·5–204·7)	123·1 (120·9–125·5)	62·8 (60·4–65·2)	19·4 (17·6–21·2)	1·6 (1·1–2·3)	0·0 (0·0–0·0)	3·3 (3·27–3·33)	1·25 (1·23–1·27)	1·03 (1·02–1·05)
1957	0·3 (0·1–0·6)	43·1 (41·1–45·2)	211·7 (209·0–214·3)	207·1 (205·0–209·3)	124·1 (121·8–126·5)	63·0 (60·7–65·4)	18·8 (17·1–20·7)	1·6 (1·1–2·4)	0·0 (0·0–0·0)	3·35 (3·32–3·38)	1·28 (1·26–1·29)	1·04 (1·02–1·06)
1958	0·3 (0·1–0·6)	43·4 (41·5–45·4)	214·0 (211·4–216·6)	208·9 (206·8–211·1)	123·5 (121·4–125·6)	62·4 (60·2–64·7)	18·4 (16·7–20·4)	1·7 (1·1–2·4)	0·0 (0·0–0·0)	3·36 (3·33–3·39)	1·29 (1·27–1·3)	1·03 (1·01–1·05)
1959	0·3 (0·1–0·6)	43·9 (42·1–45·7)	215·7 (213·1–218·3)	210·4 (208·2–212·7)	124·2 (121·9–126·5)	61·7 (59·4–64·0)	18·4 (16·6–20·5)	1·7 (1·1–2·5)	0·0 (0·0–0·0)	3·38 (3·35–3·41)	1·3 (1·28–1·32)	1·03 (1·01–1·05)
1960	0·3 (0·1–0·6)	44·5 (42·6–46·4)	217·4 (214·8–219·9)	212·6 (210·4–214·9)	126·4 (124·3–128·5)	61·4 (59·2–63·7)	18·7 (16·8–20·8)	1·7 (1·1–2·5)	0·0 (0·0–0·0)	3·42 (3·39–3·44)	1·31 (1·29–1·33)	1·04 (1·02–1·06)
1961	0·3 (0·1–0·6)	44·7 (42·7–46·5)	214·7 (212·2–217·2)	212·7 (210·6–215·1)	126·4 (124·0–128·7)	60·6 (58·4–62·8)	18·7 (17·0–20·6)	1·6 (1·0–2·3)	0·0 (0·0–0·0)	3·4 (3·37–3·43)	1·3 (1·28–1·31)	1·04 (1·02–1·05)
1962	0·3 (0·1–0·6)	44·6 (42·8–46·4)	205·5 (203·1–208·0)	208·3 (206·1–210·5)	123·1 (120·9–125·5)	59·0 (56·8–61·2)	18·0 (16·4–20·0)	1·4 (0·9–2·0)	0·0 (0·0–0·0)	3·3 (3·27–3·33)	1·25 (1·24–1·27)	1·01 (0·99–1·03)
1963	0·3 (0·1–0·6)	44·9 (43·2–46·7)	192·6 (190·1–195·0)	199·2 (197·1–201·3)	118·3 (115·9–120·6)	56·9 (54·7–59·2)	16·7 (15·1–18·5)	1·1 (0·7–1·7)	0·0 (0·0–0·0)	3·15 (3·12–3·18)	1·19 (1·17–1·21)	0·97 (0·94–0·99)
1964	0·3 (0·1–0·6)	45·6 (43·7–47·3)	179·2 (176·7–181·6)	188·6 (186·5–190·8)	111·6 (109·3–113·9)	53·8 (51·7–56·1)	15·2 (13·6–16·9)	1·0 (0·6–1·4)	0·0 (0·0–0·0)	2·98 (2·95–3·01)	1·13 (1·11–1·14)	0·91 (0·89–0·93)
1965	0·3 (0·1–0·6)	46·3 (44·6–48·1)	170·1 (167·6–172·6)	181·6 (179·6–183·5)	105·0 (102·8–107·1)	50·3 (48·2–52·4)	14·0 (12·5–15·7)	0·9 (0·6–1·3)	0·0 (0·0–0·0)	2·84 (2·82–2·87)	1·08 (1·07–1·1)	0·85 (0·83–0·87)
1966	0·3 (0·1–0·6)	47·0 (45·2–48·7)	166·5 (164·1–168·6)	179·5 (177·6–181·4)	101·5 (99·3–103·7)	47·8 (45·7–49·7)	13·3 (11·8–15·0)	0·9 (0·6–1·3)	0·0 (0·0–0·0)	2·78 (2·76–2·81)	1·07 (1·05–1·08)	0·82 (0·8–0·83)
1967	0·3 (0·1–0·6)	47·4 (45·8–49·1)	168·3 (166·2–170·5)	181·1 (179·1–183·1)	100·8 (98·7–102·9)	46·1 (43·9–48·1)	12·9 (11·4–14·6)	0·9 (0·6–1·4)	0·0 (0·0–0·0)	2·79 (2·76–2·81)	1·08 (1·07–1·09)	0·8 (0·78–0·82)
1968	0·3 (0·1–0·6)	47·8 (46·2–49·5)	170·1 (167·9–172·3)	183·1 (181·3–185·0)	100·7 (98·5–102·8)	44·3 (42·2–46·5)	12·2 (10·7–13·9)	0·9 (0·6–1·4)	0·0 (0·0–0·0)	2·8 (2·77–2·82)	1·09 (1·08–1·1)	0·79 (0·77–0·81)
1969	0·3 (0·1–0·6)	49·6 (47·9–51·4)	169·7 (167·5–171·9)	185·0 (183·1–187·1)	100·7 (98·5–102·9)	43·5 (41·5–45·6)	11·8 (10·4–13·5)	0·9 (0·6–1·4)	0·0 (0·0–0·0)	2·81 (2·79–2·83)	1·1 (1·08–1·11)	0·79 (0·77–0·8)
1970	0·3 (0·1–0·6)	52·8 (51·1–54·6)	173·9 (171·8–176·0)	187·8 (185·9–189·6)	100·8 (98·7–103·0)	43·5 (41·4–45·6)	11·6 (10·1–13·4)	0·9 (0·6–1·4)	0·0 (0·0–0·0)	2·86 (2·83–2·88)	1·13 (1·12–1·15)	0·78 (0·77–0·8)
1971	0·3 (0·1–0·6)	53·4 (51·7–55·1)	172·3 (170·2–174·5)	183·3 (181·4–185·1)	96·0 (94·0–98·0)	40·0 (37·9–42·1)	10·4 (9·0–12·0)	0·8 (0·5–1·2)	0·0 (0·0–0·0)	2·78 (2·76–2·81)	1·13 (1·11–1·15)	0·74 (0·72–0·75)
1972	0·3 (0·1–0·6)	50·5 (48·9–52·1)	159·3 (157·2–161·6)	170·8 (169·0–172·7)	87·3 (85·3–89·4)	34·8 (32·9–36·7)	8·9 (7·6–10·5)	0·7 (0·4–1·0)	0·0 (0·0–0·0)	2·56 (2·54–2·59)	1·05 (1·04–1·06)	0·66 (0·64–0·67)
1973	0·3 (0·1–0·6)	46·5 (44·9–48·1)	150·3 (148·3–152·2)	161·5 (159·8–163·4)	80·7 (78·8–82·8)	31·0 (29·0–32·9)	7·9 (6·6–9·3)	0·6 (0·3–0·8)	0·0 (0·0–0·0)	2·39 (2·37–2·42)	0·99 (0·97–1·0)	0·6 (0·58–0·62)
1974	0·3 (0·1–0·6)	42·3 (40·8–44·0)	142·7 (140·6–144·8)	155·1 (153·2–156·8)	76·7 (74·8–78·7)	27·9 (26·1–29·8)	6·9 (5·7–8·3)	0·5 (0·3–0·7)	0·0 (0·0–0·0)	2·26 (2·23–2·29)	0·93 (0·91–0·94)	0·56 (0·54–0·58)
1975	0·3 (0·1–0·5)	38·3 (36·8–39·9)	133·6 (131·5–135·7)	149·8 (148·1–151·5)	75·0 (73·2–76·8)	25·7 (24·3–27·2)	6·1 (4·9–7·5)	0·4 (0·3–0·6)	0·0 (0·0–0·0)	2·15 (2·12–2·17)	0·86 (0·85–0·87)	0·54 (0·52–0·55)
1976	0·2 (0·1–0·5)	34·3 (32·9–35·8)	126·7 (124·6–128·9)	147·3 (145·6–149·0)	74·7 (72·7–76·4)	23·9 (22·6–25·4)	5·5 (4·4–6·9)	0·4 (0·2–0·5)	0·0 (0·0–0·0)	2·07 (2·05–2·09)	0·81 (0·79–0·82)	0·52 (0·51–0·54)
1977	0·2 (0·1–0·5)	31·1 (29·8–32·6)	120·3 (118·2–122·4)	145·5 (143·8–147·3)	74·0 (72·3–75·8)	23·2 (21·6–25·1)	5·1 (4·0–6·4)	0·3 (0·2–0·5)	0·0 (0·0–0·0)	2·0 (1·98–2·02)	0·76 (0·75–0·77)	0·51 (0·5–0·53)
1978	0·2 (0·1–0·5)	29·2 (27·8–30·7)	113·6 (111·7–115·6)	143·5 (141·7–145·2)	73·6 (71·8–75·3)	23·6 (21·9–25·7)	4·7 (3·8–5·8)	0·3 (0·2–0·4)	0·0 (0·0–0·0)	1·94 (1·92–1·97)	0·71 (0·7–0·73)	0·51 (0·5–0·53)
1979	0·2 (0·1–0·4)	28·2 (26·9–29·6)	109·3 (107·4–111·2)	142·3 (140·6–144·0)	74·4 (72·6–76·2)	24·0 (22·4–25·8)	4·6 (3·9–5·5)	0·3 (0·2–0·4)	0·0 (0·0–0·0)	1·92 (1·9–1·94)	0·69 (0·68–0·7)	0·52 (0·5–0·53)
1980	0·2 (0·1–0·4)	27·9 (26·5–29·3)	108·1 (106·1–110·1)	143·8 (142·1–145·5)	77·0 (75·3–78·8)	24·6 (23·1–26·2)	4·7 (4·2–5·4)	0·3 (0·2–0·4)	0·0 (0·0–0·0)	1·93 (1·91–1·95)	0·68 (0·67–0·69)	0·53 (0·52–0·55)
1981	0·2 (0·1–0·4)	27·7 (26·3–29·2)	107·3 (105·5–109·3)	146·1 (144·4–147·8)	80·0 (78·3–81·8)	25·0 (23·5–26·6)	4·8 (4·2–5·4)	0·3 (0·2–0·4)	0·0 (0·0–0·0)	1·96 (1·94–1·98)	0·68 (0·66–0·69)	0·55 (0·54–0·56)
1982	0·2 (0·1–0·4)	26·5 (25·2–28·0)	105·1 (103·2–107·0)	146·3 (144·6–148·1)	81·5 (79·8–83·4)	25·2 (23·5–26·8)	4·6 (3·9–5·4)	0·2 (0·1–0·3)	0·0 (0·0–0·0)	1·95 (1·93–1·97)	0·66 (0·65–0·67)	0·56 (0·55–0·57)
1983	0·2 (0·1–0·4)	24·6 (23·2–25·9)	100·0 (98·1–101·9)	144·2 (142·5–146·0)	82·3 (80·5–84·2)	25·4 (23·8–27·1)	4·4 (3·6–5·3)	0·2 (0·1–0·3)	0·0 (0·0–0·0)	1·91 (1·89–1·93)	0·62 (0·61–0·64)	0·56 (0·55–0·58)
1984	0·2 (0·1–0·4)	22·8 (21·5–24·1)	96·2 (94·3–98·1)	143·9 (142·3–145·7)	85·3 (83·6–87·2)	26·0 (24·4–27·6)	4·3 (3·6–5·1)	0·2 (0·1–0·3)	0·0 (0·0–0·0)	1·89 (1·87–1·92)	0·6 (0·58–0·61)	0·58 (0·57–0·59)
1985	0·2 (0·1–0·4)	21·3 (20·0–22·7)	93·3 (91·4–95·2)	143·9 (142·1–145·5)	88·8 (87·0–90·6)	27·0 (25·5–28·5)	4·5 (3·9–5·2)	0·2 (0·2–0·3)	0·0 (0·0–0·0)	1·9 (1·88–1·92)	0·57 (0·56–0·59)	0·6 (0·59–0·62)
1986	0·2 (0·1–0·4)	20·4 (19·1–21·7)	88·4 (86·6–90·3)	141·4 (139·6–143·1)	90·1 (88·3–91·9)	28·3 (26·8–29·8)	4·8 (4·2–5·6)	0·2 (0·2–0·3)	0·0 (0·0–0·0)	1·87 (1·85–1·89)	0·54 (0·53–0·56)	0·62 (0·6–0·63)
1987	0·2 (0·1–0·4)	20·0 (18·8–21·2)	84·2 (82·4–86·2)	139·2 (137·6–140·9)	92·0 (90·2–93·8)	29·8 (28·3–31·4)	5·0 (4·2–5·9)	0·2 (0·2–0·3)	0·0 (0·0–0·0)	1·85 (1·83–1·88)	0·52 (0·51–0·53)	0·64 (0·62–0·65)
1988	0·2 (0·1–0·4)	20·3 (19·0–21·6)	81·3 (79·5–83·2)	138·7 (137·0–140·4)	95·3 (93·5–97·0)	31·6 (30·0–33·3)	5·2 (4·3–6·2)	0·2 (0·2–0·4)	0·0 (0·0–0·0)	1·86 (1·84–1·88)	0·51 (0·5–0·52)	0·66 (0·65–0·67)
1989	0·2 (0·1–0·4)	20·9 (19·6–22·2)	80·0 (78·2–81·9)	139·2 (137·5–140·9)	99·2 (97·4–101·0)	33·6 (32·0–35·3)	5·3 (4·4–6·4)	0·3 (0·2–0·4)	0·0 (0·0–0·0)	1·89 (1·87–1·92)	0·51 (0·49–0·52)	0·69 (0·68–0·71)
1990	0·2 (0·1–0·4)	21·3 (20·1–22·6)	77·8 (76·1–79·7)	136·5 (134·8–138·1)	101·3 (99·6–103·0)	35·5 (33·9–37·1)	5·6 (4·6–6·7)	0·3 (0·2–0·4)	0·0 (0·0–0·0)	1·89 (1·87–1·91)	0·5 (0·49–0·51)	0·71 (0·7–0·73)
1991	0·2 (0·1–0·4)	21·5 (20·2–22·7)	75·1 (73·4–76·8)	132·7 (131·0–134·4)	102·7 (100·9–104·5)	37·2 (35·5–39·0)	5·9 (4·9–7·0)	0·3 (0·2–0·4)	0·0 (0·0–0·0)	1·88 (1·86–1·9)	0·48 (0·47–0·49)	0·73 (0·72–0·74)
1992	0·2 (0·1–0·4)	21·2 (20·0–22·5)	73·0 (71·2–74·5)	130·7 (129·1–132·5)	104·8 (103·1–106·6)	38·6 (37·0–40·3)	6·2 (5·2–7·3)	0·3 (0·2–0·4)	0·0 (0·0–0·0)	1·88 (1·86–1·9)	0·47 (0·46–0·48)	0·75 (0·74–0·76)
1993	0·2 (0·1–0·4)	20·8 (19·5–22·1)	70·6 (68·8–72·2)	127·8 (126·1–129·5)	105·4 (103·7–107·2)	40·1 (38·5–41·8)	6·6 (5·6–7·6)	0·3 (0·2–0·4)	0·0 (0·0–0·0)	1·86 (1·84–1·88)	0·46 (0·45–0·47)	0·76 (0·75–0·77)
1994	0·2 (0·1–0·4)	20·4 (19·1–21·7)	68·4 (66·7–70·3)	123·8 (122·1–125·3)	105·5 (103·8–107·2)	41·7 (40·0–43·4)	7·0 (6·0–8·0)	0·3 (0·2–0·4)	0·0 (0·0–0·0)	1·84 (1·82–1·86)	0·44 (0·43–0·46)	0·77 (0·76–0·79)
1995	0·2 (0·1–0·4)	20·0 (18·6–21·4)	66·1 (64·4–67·9)	119·4 (117·8–120·9)	105·8 (104·1–107·6)	43·1 (41·4–44·7)	7·4 (6·4–8·4)	0·3 (0·2–0·4)	0·0 (0·0–0·0)	1·81 (1·79–1·83)	0·43 (0·42–0·44)	0·78 (0·77–0·8)
1996	0·2 (0·1–0·4)	19·4 (17·9–21·3)	63·9 (62·2–65·6)	115·5 (113·8–117·1)	106·2 (104·5–107·9)	44·3 (42·6–46·1)	7·8 (6·9–8·6)	0·4 (0·3–0·4)	0·0 (0·0–0·0)	1·79 (1·77–1·81)	0·42 (0·41–0·43)	0·79 (0·78–0·81)
1997	0·2 (0·1–0·4)	18·9 (17·2–20·9)	62·2 (60·5–63·9)	112·5 (110·8–114·1)	106·8 (105·2–108·5)	45·3 (43·7–46·8)	8·1 (7·3–9·0)	0·4 (0·3–0·5)	0·0 (0·0–0·0)	1·77 (1·75–1·79)	0·41 (0·39–0·42)	0·8 (0·79–0·82)
1998	0·2 (0·1–0·4)	18·3 (16·8–20·1)	61·0 (59·3–62·9)	110·0 (108·4–111·6)	107·5 (105·8–109·2)	46·3 (44·7–48·0)	8·4 (7·6–9·4)	0·4 (0·3–0·5)	0·0 (0·0–0·0)	1·76 (1·74–1·78)	0·4 (0·39–0·41)	0·81 (0·8–0·83)
1999	0·2 (0·1–0·3)	17·9 (16·5–19·3)	60·0 (58·2–61·9)	108·3 (106·7–109·8)	108·7 (107·0–110·6)	47·8 (46·2–49·6)	8·7 (7·8–9·8)	0·4 (0·3–0·5)	0·0 (0·0–0·0)	1·76 (1·74–1·78)	0·39 (0·38–0·4)	0·83 (0·82–0·84)
2000	0·2 (0·1–0·3)	17·5 (16·2–19·0)	59·1 (56·6–61·7)	108·0 (106·1–110·2)	110·2 (107·9–112·6)	49·6 (47·6–51·7)	9·0 (8·0–10·2)	0·4 (0·4–0·6)	0·0 (0·0–0·0)	1·77 (1·74–1·8)	0·38 (0·37–0·4)	0·85 (0·83–0·86)
2001	0·2 (0·1–0·3)	17·1 (15·8–18·5)	58·3 (55·9–60·7)	108·3 (106·2–110·5)	111·7 (109·3–114·1)	51·4 (49·3–53·6)	9·4 (8·4–10·5)	0·5 (0·4–0·6)	0·0 (0·0–0·0)	1·78 (1·76–1·81)	0·38 (0·36–0·39)	0·87 (0·85–0·88)
2002	0·2 (0·1–0·3)	16·6 (15·2–17·9)	57·0 (55·2–58·9)	107·7 (106·0–109·4)	113·1 (111·5–114·7)	53·4 (51·9–55·2)	9·9 (8·9–11·0)	0·5 (0·4–0·6)	0·0 (0·0–0·0)	1·79 (1·77–1·81)	0·37 (0·36–0·38)	0·88 (0·87–0·9)
2003	0·2 (0·1–0·3)	16·0 (14·6–17·5)	55·2 (52·9–57·5)	106·5 (104·4–108·6)	114·6 (112·3–116·9)	55·9 (53·7–58·2)	10·5 (9·4–11·7)	0·5 (0·4–0·7)	0·0 (0·0–0·0)	1·8 (1·77–1·83)	0·36 (0·34–0·37)	0·91 (0·89–0·92)
2004	0·2 (0·1–0·3)	15·8 (14·4–17·2)	54·2 (52·0–56·5)	106·4 (104·2–108·5)	117·0 (114·6–119·2)	58·6 (56·4–60·7)	11·0 (9·9–12·2)	0·5 (0·4–0·7)	0·0 (0·0–0·0)	1·82 (1·79–1·85)	0·35 (0·33–0·37)	0·94 (0·92–0·95)
2005	0·2 (0·1–0·3)	16·0 (14·6–17·4)	55·0 (52·8–57·3)	107·8 (105·6–109·9)	120·9 (118·6–123·3)	61·7 (59·4–64·1)	11·5 (10·4–12·8)	0·5 (0·4–0·7)	0·0 (0·0–0·0)	1·87 (1·84–1·9)	0·36 (0·34–0·37)	0·97 (0·96–0·99)
2006	0·2 (0·1–0·3)	16·5 (15·1–17·9)	57·4 (55·2–59·6)	110·4 (108·3–112·4)	126·3 (123·8–128·8)	65·7 (63·5–68·1)	12·1 (11·0–13·4)	0·6 (0·4–0·7)	0·0 (0·0–0·0)	1·95 (1·92–1·98)	0·37 (0·36–0·38)	1·02 (1·01–1·04)
2007	0·2 (0·1–0·3)	17·0 (15·8–18·1)	59·5 (57·9–61·3)	112·3 (110·6–114·0)	130·5 (128·7–132·2)	69·3 (67·6–70·9)	13·0 (11·8–14·2)	0·6 (0·4–0·8)	0·0 (0·0–0·0)	2·01 (1·99–2·03)	0·38 (0·37–0·39)	1·07 (1·05–1·08)
2008	0·2 (0·1–0·3)	16·9 (15·8–18·1)	59·7 (58·0–61·3)	111·8 (110·2–113·5)	130·8 (129·0–132·5)	70·3 (68·6–71·9)	14·0 (12·7–15·3)	0·6 (0·4–0·9)	0·0 (0·0–0·0)	2·02 (2·0–2·04)	0·38 (0·37–0·39)	1·08 (1·06–1·09)
2009	0·2 (0·1–0·3)	16·6 (15·4–17·8)	58·3 (56·7–59·9)	109·9 (108·4–111·6)	129·5 (127·7–131·2)	70·0 (68·3–71·7)	14·7 (13·4–16·1)	0·6 (0·4–0·9)	0·0 (0·0–0·0)	2·0 (1·98–2·02)	0·38 (0·37–0·39)	1·07 (1·06–1·09)
2010	0·2 (0·1–0·3)	16·3 (15·1–17·5)	57·3 (55·6–58·9)	108·9 (107·4–110·5)	128·6 (126·9–130·4)	70·4 (68·8–72·1)	15·0 (13·7–16·3)	0·7 (0·4–1·0)	0·0 (0·0–0·0)	1·99 (1·97–2·01)	0·37 (0·36–0·38)	1·07 (1·06–1·09)
2011	0·2 (0·1–0·3)	15·8 (14·7–17·1)	56·9 (55·2–58·5)	108·7 (107·2–110·3)	129·0 (127·2–130·8)	71·2 (69·4–72·9)	15·0 (13·8–16·3)	0·7 (0·5–1·1)	0·0 (0·0–0·0)	1·99 (1·97–2·01)	0·36 (0·35–0·37)	1·08 (1·07–1·09)
2012	0·2 (0·1–0·3)	15·0 (13·8–16·2)	55·7 (54·0–57·2)	107·0 (105·5–108·6)	128·4 (126·7–130·0)	71·2 (69·5–73·0)	15·0 (13·8–16·2)	0·8 (0·5–1·2)	0·0 (0·0–0·0)	1·97 (1·95–1·99)	0·35 (0·34–0·36)	1·08 (1·06–1·09)
2013	0·1 (0·1–0·3)	13·9 (12·7–15·0)	53·0 (51·3–54·7)	103·5 (101·9–105·1)	124·7 (122·9–126·4)	69·8 (68·0–71·7)	14·9 (13·6–16·1)	0·8 (0·5–1·3)	0·0 (0·0–0·0)	1·9 (1·88–1·92)	0·33 (0·32–0·34)	1·05 (1·04–1·06)
2014	0·1 (0·1–0·3)	13·2 (12·0–14·5)	50·9 (49·3–52·6)	100·8 (99·3–102·4)	122·3 (120·5–123·9)	68·8 (67·1–70·4)	14·5 (13·2–15·9)	0·8 (0·5–1·3)	0·0 (0·0–0·0)	1·86 (1·84–1·88)	0·32 (0·31–0·33)	1·03 (1·02–1·05)
2015	0·1 (0·1–0·3)	13·0 (11·6–14·6)	50·1 (47·1–53·2)	99·6 (96·8–102·6)	123·1 (119·8–126·3)	69·0 (65·8–72·4)	14·3 (12·4–16·4)	0·8 (0·5–1·3)	0·0 (0·0–0·0)	1·85 (1·79–1·91)	0·32 (0·3–0·34)	1·04 (1·0–1·07)
2016	0·1 (0·1–0·3)	13·1 (11·4–15·1)	49·5 (44·0–55·3)	99·1 (93·6–104·6)	124·2 (116·5–132·0)	69·2 (61·9–77·2)	14·3 (11·8–17·3)	0·8 (0·5–1·3)	0·0 (0·0–0·0)	1·85 (1·71–2·01)	0·31 (0·28–0·35)	1·04 (0·96–1·13)
2017	0·1 (0·1–0·3)	13·3 (11·3–15·6)	49·2 (41·8–57·8)	98·9 (91·5–107·1)	125·0 (113·8–137·1)	69·4 (58·9–81·4)	14·4 (11·1–18·4)	0·8 (0·5–1·3)	0·0 (0·0–0·0)	1·86 (1·65–2·09)	0·31 (0·27–0·37)	1·05 (0·92–1·19)

Supplemental Results Table 5: Age-specific fertility rates per 1000, cumulative total fertility up to maternal age 25 (TFU25), cumulative total fertility from maternal age 30 to age 55 (TFO30), and cumulative total fertility, New Zealand, 1950-2017

Year	10-14	15-19	20-24	25-29	30-34	35-39	40-44	45-49	50-54	TFR	TFU25	TFO30
1950	1·1 (0·5–2·2)	62·1 (57·2–67·3)	223·4 (211·7–235·2)	207·2 (199·2–215·3)	116·5 (108·5–124·8)	70·7 (64·9–76·4)	22·7 (20·6–24·8)	2·4 (2·0–2·8)	0·0 (0·0–0·0)	3·53 (3·38–3·67)	1·43 (1·37–1·5)	1·06 (1·01–1·11)
1951	1·1 (0·5–2·2)	62·6 (58·1–67·5)	223·8 (212·7–234·4)	209·1 (201·8–216·0)	118·1 (110·9–125·9)	71·8 (66·6–77·3)	23·0 (21·1–25·0)	2·4 (2·1–2·8)	0·0 (0·0–0·0)	3·56 (3·44–3·68)	1·44 (1·38–1·49)	1·08 (1·03–1·13)
1952	1·1 (0·5–2·2)	63·4 (58·8–68·5)	225·6 (214·7–235·8)	212·4 (204·8–219·2)	120·6 (112·9–127·6)	73·1 (68·1–78·7)	23·4 (21·6–25·6)	2·5 (2·2–2·8)	0·0 (0·0–0·0)	3·61 (3·5–3·72)	1·45 (1·39–1·51)	1·1 (1·05–1·15)
1953	1·1 (0·5–2·3)	64·7 (60·2–69·7)	228·4 (217·9–239·0)	216·6 (209·3–223·4)	123·7 (116·0–130·7)	74·9 (69·8–80·5)	24·0 (22·0–26·1)	2·5 (2·2–2·9)	0·0 (0·0–0·1)	3·68 (3·58–3·78)	1·47 (1·41–1·53)	1·13 (1·08–1·17)
1954	1·1 (0·5–2·3)	66·1 (61·8–70·8)	231·4 (221·6–241·5)	221·1 (214·2–227·9)	127·1 (119·0–134·5)	76·6 (71·7–82·3)	24·6 (22·5–26·6)	2·6 (2·3–2·9)	0·0 (0·0–0·1)	3·75 (3·66–3·85)	1·49 (1·44–1·55)	1·15 (1·11–1·2)
1955	1·1 (0·5–2·3)	67·5 (63·5–72·1)	234·2 (223·4–244·2)	225·4 (218·3–232·5)	130·5 (122·8–138·3)	78·2 (72·8–84·0)	25·0 (23·0–27·1)	2·5 (2·2–2·9)	0·0 (0·0–0·1)	3·82 (3·72–3·92)	1·51 (1·45–1·57)	1·18 (1·14–1·23)
1956	1·1 (0·5–2·3)	68·6 (64·6–73·2)	237·0 (227·0–247·3)	230·1 (223·2–237·3)	134·1 (126·7–142·0)	79·4 (73·9–85·1)	25·4 (23·3–27·5)	2·5 (2·2–2·9)	0·0 (0·0–0·1)	3·89 (3·79–3·99)	1·53 (1·47–1·6)	1·21 (1·16–1·26)
1957	1·1 (0·5–2·3)	69·4 (64·9–74·2)	240·3 (230·2–250·5)	235·5 (228·7–242·2)	137·9 (130·5–146·1)	80·2 (74·4–85·2)	25·5 (23·5–27·7)	2·5 (2·2–2·9)	0·0 (0·0–0·1)	3·96 (3·87–4·06)	1·55 (1·5–1·61)	1·23 (1·18–1·28)
1958	1·1 (0·5–2·4)	70·0 (65·3–75·2)	244·6 (234·8–254·8)	241·5 (234·6–248·1)	141·9 (134·4–149·7)	80·6 (75·3–86·0)	25·5 (23·5–27·6)	2·5 (2·2–2·9)	0·0 (0·0–0·1)	4·04 (3·94–4·13)	1·58 (1·52–1·63)	1·25 (1·2–1·3)
1959	1·1 (0·5–2·4)	71·0 (66·2–76·2)	250·2 (241·2–259·3)	248·3 (241·8–254·7)	146·2 (138·8–153·8)	81·4 (76·2–86·8)	25·5 (23·5–27·7)	2·5 (2·2–3·0)	0·0 (0·0–0·1)	4·13 (4·04–4·22)	1·61 (1·56–1·66)	1·28 (1·23–1·33)
1960	1·1 (0·5–2·4)	72·0 (67·9–76·4)	255·7 (247·6–263·4)	254·3 (248·3–260·0)	150·0 (143·1–156·9)	82·2 (77·0–87·6)	25·4 (23·2–27·5)	2·5 (2·1–2·9)	0·0 (0·0–0·0)	4·22 (4·13–4·31)	1·64 (1·6–1·69)	1·3 (1·25–1·35)
1961	1·2 (0·5–2·4)	72·6 (68·9–76·4)	253·6 (248·2–258·9)	254·3 (249·8–259·0)	149·6 (144·1–154·6)	78·7 (74·2–83·6)	23·7 (21·3–26·2)	2·1 (1·7–2·5)	0·0 (0·0–0·0)	4·18 (4·11–4·25)	1·64 (1·6–1·67)	1·27 (1·23–1·31)
1962	1·2 (0·5–2·4)	74·2 (70·9–77·8)	233·2 (229·1–237·1)	245·1 (241·2–248·9)	143·4 (139·1–147·6)	70·7 (66·8–75·0)	20·5 (17·9–23·3)	1·5 (1·2–1·9)	0·0 (0·0–0·0)	3·95 (3·9–4·0)	1·54 (1·52–1·57)	1·18 (1·15–1·22)
1963	1·2 (0·5–2·5)	75·0 (71·9–78·3)	210·6 (206·5–215·0)	232·1 (228·5–235·7)	134·2 (130·1–138·1)	65·2 (61·4–69·2)	18·7 (16·2–21·4)	1·3 (0·9–1·6)	0·0 (0·0–0·0)	3·69 (3·64–3·74)	1·43 (1·41–1·46)	1·1 (1·07–1·13)
1964	1·1 (0·5–2·4)	71·5 (68·5–74·6)	210·3 (206·3–214·4)	221·1 (217·5–225·0)	124·6 (120·5–128·4)	63·1 (59·3–67·1)	18·9 (16·2–22·0)	1·3 (1·0–1·8)	0·0 (0·0–0·0)	3·56 (3·51–3·61)	1·41 (1·39–1·44)	1·04 (1·0–1·08)
1965	1·1 (0·5–2·3)	66·5 (63·6–69·4)	218·3 (214·3–222·1)	212·2 (208·8–215·7)	115·5 (111·5–119·4)	59·1 (55·3–62·9)	18·6 (15·7–21·7)	1·4 (1·0–1·9)	0·0 (0·0–0·0)	3·46 (3·41–3·51)	1·43 (1·4–1·45)	0·97 (0·93–1·01)
1966	1·1 (0·5–2·2)	63·5 (60·7–66·2)	218·1 (214·0–222·0)	206·5 (203·0–209·9)	109·1 (105·1–113·2)	53·5 (49·7–57·6)	17·2 (14·4–20·3)	1·4 (1·0–1·9)	0·0 (0·0–0·0)	3·35 (3·31–3·4)	1·41 (1·39–1·44)	0·91 (0·87–0·94)
1967	1·1 (0·5–2·2)	62·4 (59·3–65·2)	214·1 (210·0–218·1)	203·9 (200·6–207·3)	106·1 (102·0–109·8)	49·1 (45·5–53·1)	15·7 (13·0–18·7)	1·3 (0·9–1·9)	0·0 (0·0–0·0)	3·27 (3·23–3·31)	1·39 (1·36–1·41)	0·86 (0·83–0·89)
1968	1·1 (0·5–2·2)	62·2 (59·1–65·2)	209·0 (205·0–213·1)	201·7 (198·2–205·4)	104·7 (100·8–108·8)	46·2 (42·7–49·9)	14·4 (11·8–17·1)	1·2 (0·8–1·8)	0·0 (0·0–0·0)	3·2 (3·16–3·24)	1·36 (1·34–1·39)	0·83 (0·8–0·86)
1969	1·1 (0·5–2·2)	63·1 (60·3–66·2)	204·8 (201·1–208·4)	199·1 (195·6–202·6)	102·6 (98·8–106·6)	44·4 (40·8–48·3)	13·5 (11·0–16·3)	1·2 (0·8–1·7)	0·0 (0·0–0·0)	3·15 (3·11–3·19)	1·35 (1·32–1·37)	0·81 (0·78–0·84)
1970	1·1 (0·5–2·3)	64·9 (62·3–67·8)	203·5 (199·8–206·9)	196·0 (192·5–199·5)	99·1 (95·7–102·6)	42·9 (39·1–46·9)	12·8 (10·3–15·7)	1·1 (0·7–1·7)	0·0 (0·0–0·0)	3·11 (3·06–3·15)	1·35 (1·32–1·37)	0·78 (0·75–0·81)
1971	1·1 (0·5–2·3)	65·0 (62·4–67·8)	198·2 (194·6–201·6)	189·7 (186·4–193·0)	93·7 (90·1–97·2)	39·5 (35·9–43·4)	11·6 (9·3–14·0)	1·0 (0·6–1·6)	0·0 (0·0–0·0)	3·0 (2·96–3·04)	1·32 (1·3–1·35)	0·73 (0·7–0·76)
1972	1·1 (0·5–2·2)	62·6 (59·7–65·5)	186·2 (182·7–190·0)	179·9 (176·3–183·3)	86·7 (83·3–90·4)	34·8 (31·5–38·5)	10·0 (7·9–12·4)	0·8 (0·5–1·3)	0·0 (0·0–0·0)	2·81 (2·77–2·85)	1·25 (1·23–1·27)	0·66 (0·63–0·69)
1973	1·0 (0·5–2·1)	58·6 (55·8–61·6)	173·9 (170·5–177·8)	169·3 (166·0–172·8)	79·5 (76·2–83·1)	30·0 (26·9–33·6)	8·7 (6·8–11·0)	0·7 (0·4–1·1)	0·0 (0·0–0·0)	2·61 (2·57–2·65)	1·17 (1·15–1·19)	0·59 (0·57–0·62)
1974	1·0 (0·4–2·0)	54·0 (51·2–56·7)	163·4 (159·6–167·2)	160·5 (157·4–163·8)	73·7 (70·4–77·3)	25·8 (23·1–28·6)	7·7 (5·9–9·9)	0·6 (0·4–0·9)	0·0 (0·0–0·0)	2·43 (2·39–2·47)	1·09 (1·07–1·12)	0·54 (0·52–0·57)
1975	0·9 (0·4–2·0)	49·3 (46·8–51·9)	155·0 (151·4–158·8)	153·8 (150·9–156·8)	70·1 (67·0–73·4)	22·5 (20·5–24·7)	7·0 (5·2–9·0)	0·5 (0·3–0·8)	0·0 (0·0–0·0)	2·3 (2·26–2·33)	1·03 (1·01–1·05)	0·5 (0·48–0·52)
1976	0·9 (0·4–1·9)	45·1 (42·8–47·6)	145·8 (142·3–149·5)	147·9 (145·0–150·8)	68·2 (65·0–71·4)	20·4 (18·5–22·4)	6·2 (4·6–8·1)	0·4 (0·3–0·7)	0·0 (0·0–0·0)	2·17 (2·14–2·21)	0·96 (0·94–0·98)	0·48 (0·46–0·5)
1977	0·9 (0·4–1·8)	41·9 (39·5–44·4)	134·9 (131·3–138·5)	143·6 (140·6–146·5)	67·3 (64·1–70·4)	19·6 (17·4–21·9)	5·5 (4·1–7·2)	0·4 (0·2–0·6)	0·0 (0·0–0·0)	2·07 (2·04–2·11)	0·89 (0·87–0·91)	0·46 (0·44–0·48)
1978	0·8 (0·4–1·7)	39·5 (37·2–42·0)	127·9 (124·7–131·4)	142·5 (139·2–145·8)	67·5 (64·4–70·7)	19·8 (17·1–22·6)	5·1 (3·9–6·8)	0·4 (0·2–0·5)	0·0 (0·0–0·0)	2·02 (1·98–2·05)	0·84 (0·82–0·86)	0·46 (0·44–0·49)
1979	0·8 (0·4–1·7)	37·4 (35·1–39·8)	124·1 (120·5–127·8)	142·3 (139·2–145·5)	68·0 (64·8–71·5)	19·7 (16·9–22·7)	4·9 (3·7–6·5)	0·3 (0·2–0·5)	0·0 (0·0–0·0)	1·99 (1·95–2·03)	0·81 (0·79–0·84)	0·46 (0·44–0·49)
1980	0·8 (0·3–1·6)	35·3 (32·8–38·0)	119·6 (115·2–124·2)	141·6 (137·9–145·6)	68·4 (64·7–72·1)	19·8 (17·0–23·1)	4·7 (3·5–6·4)	0·3 (0·2–0·5)	0·0 (0·0–0·0)	1·95 (1·91–2·0)	0·78 (0·75–0·81)	0·47 (0·44–0·49)
1981	0·8 (0·3–1·6)	33·2 (30·6–36·1)	114·5 (110·3–119·0)	141·8 (138·0–145·5)	69·3 (65·6–73·0)	20·6 (17·4–23·9)	4·5 (3·2–6·3)	0·3 (0·2–0·5)	0·0 (0·0–0·0)	1·92 (1·88–1·97)	0·74 (0·72–0·77)	0·47 (0·45–0·5)
1982	0·7 (0·3–1·5)	31·5 (29·2–34·1)	110·5 (107·1–114·1)	143·3 (139·9–146·5)	71·3 (68·1–74·8)	21·1 (18·1–24·2)	4·3 (3·1–5·9)	0·3 (0·2–0·4)	0·0 (0·0–0·0)	1·92 (1·87–1·96)	0·71 (0·69–0·73)	0·49 (0·46–0·51)
1983	0·7 (0·3–1·5)	30·2 (28·0–32·9)	107·4 (104·1–110·7)	145·0 (142·0–148·2)	74·6 (71·2–77·8)	21·7 (19·0–24·7)	4·2 (3·0–5·7)	0·3 (0·2–0·4)	0·0 (0·0–0·0)	1·92 (1·88–1·96)	0·69 (0·67–0·71)	0·5 (0·48–0·53)
1984	0·7 (0·3–1·5)	29·5 (27·1–31·9)	104·4 (101·1–107·8)	145·3 (142·2–148·6)	78·4 (75·2–81·6)	22·6 (19·7–25·5)	4·1 (3·0–5·6)	0·3 (0·2–0·4)	0·0 (0·0–0·0)	1·93 (1·89–1·97)	0·67 (0·65–0·69)	0·53 (0·5–0·55)
1985	0·7 (0·3–1·5)	29·4 (27·1–31·8)	102·8 (99·2–106·2)	144·7 (141·5–147·9)	83·0 (79·8–86·1)	23·9 (21·2–26·9)	4·2 (3·1–5·8)	0·2 (0·2–0·4)	0·0 (0·0–0·0)	1·95 (1·9–2·0)	0·66 (0·64–0·69)	0·56 (0·54–0·58)
1986	0·7 (0·3–1·5)	30·0 (27·5–32·4)	102·7 (99·3–106·2)	145·3 (142·2–148·3)	88·7 (85·3–92·1)	25·7 (23·0–28·6)	4·5 (3·3–6·1)	0·3 (0·2–0·4)	0·0 (0·0–0·0)	1·99 (1·94–2·03)	0·67 (0·65–0·69)	0·6 (0·57–0·62)
1987	0·7 (0·3–1·5)	30·7 (28·4–33·3)	103·4 (99·9–106·7)	146·6 (143·5–149·6)	94·7 (91·4–98·1)	28·1 (25·3–30·9)	4·8 (3·5–6·5)	0·3 (0·2–0·4)	0·0 (0·0–0·0)	2·05 (2·01–2·08)	0·67 (0·65–0·7)	0·64 (0·62–0·66)
1988	0·7 (0·3–1·5)	31·6 (29·3–34·2)	103·2 (99·9–106·7)	147·1 (144·2–150·2)	100·0 (96·9–103·3)	30·6 (27·8–33·9)	5·0 (3·7–6·7)	0·3 (0·2–0·4)	0·0 (0·0–0·0)	2·09 (2·06–2·13)	0·68 (0·65–0·7)	0·68 (0·66–0·7)
1989	0·8 (0·3–1·5)	32·1 (29·6–34·7)	102·0 (98·5–105·4)	146·3 (143·3–149·6)	104·0 (100·8–107·3)	33·3 (30·2–36·4)	5·1 (3·8–6·9)	0·3 (0·2–0·4)	0·0 (0·0–0·0)	2·12 (2·08–2·16)	0·67 (0·65–0·7)	0·71 (0·69–0·74)
1990	0·8 (0·3–1·5)	32·2 (29·5–34·9)	99·6 (95·0–104·1)	143·7 (140·0–147·3)	106·0 (102·3–110·0)	36·0 (32·5–39·5)	5·1 (3·7–6·8)	0·3 (0·2–0·4)	0·0 (0·0–0·0)	2·12 (2·07–2·16)	0·66 (0·64–0·69)	0·74 (0·71–0·77)
1991	0·7 (0·3–1·5)	31·9 (29·2–34·9)	96·0 (91·7–100·4)	139·8 (136·0–143·2)	107·6 (103·6–111·4)	38·4 (34·7–42·2)	5·4 (3·9–7·1)	0·3 (0·2–0·4)	0·0 (0·0–0·0)	2·1 (2·05–2·15)	0·64 (0·62–0·67)	0·76 (0·73–0·79)
1992	0·7 (0·3–1·5)	31·4 (28·6–34·4)	92·8 (89·3–96·4)	136·3 (133·1–139·7)	108·5 (105·2–112·0)	39·9 (36·6–43·6)	5·8 (4·2–7·7)	0·3 (0·2–0·4)	0·0 (0·0–0·0)	2·08 (2·03–2·13)	0·62 (0·6–0·65)	0·77 (0·75–0·8)
1993	0·7 (0·3–1·5)	30·9 (27·6–34·4)	90·8 (85·4–96·5)	133·5 (128·9–138·5)	107·7 (102·6–113·0)	40·7 (36·3–45·7)	6·1 (4·4–8·2)	0·3 (0·2–0·5)	0·0 (0·0–0·0)	2·05 (1·97–2·14)	0·61 (0·58–0·65)	0·77 (0·73–0·82)
1994	0·7 (0·3–1·5)	30·5 (26·5–34·7)	89·4 (80·2–99·1)	131·0 (124·3–139·3)	107·0 (98·4–115·3)	41·8 (35·5–49·1)	6·4 (4·7–8·7)	0·3 (0·2–0·5)	0·0 (0·0–0·0)	2·04 (1·89–2·2)	0·6 (0·54–0·67)	0·78 (0·71–0·85)
1995	0·7 (0·3–1·5)	30·2 (26·1–35·0)	88·1 (76·7–101·0)	128·7 (119·4–138·3)	107·4 (97·0–118·4)	43·2 (35·5–52·8)	6·8 (4·9–9·3)	0·3 (0·2–0·5)	0·0 (0·0–0·0)	2·03 (1·82–2·27)	0·6 (0·52–0·68)	0·79 (0·69–0·9)
1996	0·7 (0·3–1·5)	29·9 (25·6–34·9)	86·8 (74·3–101·0)	126·3 (116·8–136·5)	108·8 (97·5–121·2)	45·0 (36·5–55·1)	7·4 (5·4–9·9)	0·4 (0·2–0·6)	0·0 (0·0–0·0)	2·03 (1·78–2·29)	0·59 (0·5–0·68)	0·81 (0·7–0·93)
1997	0·7 (0·3–1·5)	29·4 (25·3–34·1)	85·0 (73·4–97·5)	123·4 (114·5–132·7)	110·8 (100·2–122·4)	47·1 (38·7–56·8)	8·1 (5·9–10·8)	0·4 (0·2–0·6)	0·0 (0·0–0·0)	2·02 (1·82–2·25)	0·58 (0·5–0·66)	0·83 (0·73–0·94)
1998	0·7 (0·3–1·5)	28·9 (25·1–33·2)	82·8 (73·8–92·5)	120·2 (112·8–127·3)	112·9 (105·1–122·5)	49·3 (42·1–56·8)	8·9 (6·7–11·6)	0·4 (0·3–0·6)	0·0 (0·0–0·0)	2·02 (1·87–2·18)	0·56 (0·5–0·62)	0·86 (0·79–0·94)
1999	0·7 (0·3–1·4)	28·4 (24·9–32·1)	80·3 (74·3–86·0)	116·5 (111·7–121·5)	114·1 (108·7–119·7)	51·5 (46·4–57·0)	9·7 (7·5–12·4)	0·5 (0·3–0·7)	0·0 (0·0–0·0)	2·01 (1·92–2·09)	0·55 (0·51–0·59)	0·88 (0·83–0·92)
2000	0·7 (0·3–1·4)	27·6 (24·7–31·0)	77·7 (73·2–82·0)	112·7 (108·7–116·4)	113·6 (109·5–118·0)	53·4 (49·6–57·6)	10·5 (8·3–13·3)	0·5 (0·3–0·7)	0·0 (0·0–0·0)	1·98 (1·93–2·04)	0·53 (0·5–0·56)	0·89 (0·86–0·92)
2001	0·7 (0·3–1·4)	27·1 (24·2–29·9)	75·4 (71·2–79·4)	109·7 (106·0–113·2)	112·7 (108·8–116·8)	55·2 (51·5–59·4)	11·2 (8·9–13·8)	0·5 (0·3–0·8)	0·0 (0·0–0·0)	1·96 (1·91–2·01)	0·52 (0·49–0·54)	0·9 (0·87–0·93)
2002	0·7 (0·3–1·4)	27·1 (24·7–29·5)	74·2 (70·5–77·8)	109·2 (105·8–112·3)	113·6 (110·3–116·9)	57·4 (54·2–60·8)	11·7 (9·5–14·0)	0·6 (0·3–0·8)	0·0 (0·0–0·0)	1·97 (1·93–2·01)	0·51 (0·49–0·53)	0·92 (0·89–0·94)
2003	0·7 (0·3–1·4)	27·5 (25·0–30·0)	74·3 (70·7–77·8)	110·3 (107·0–113·4)	116·5 (113·3–119·8)	59·7 (56·4–63·0)	12·1 (10·0–14·5)	0·6 (0·4–0·9)	0·0 (0·0–0·0)	2·01 (1·96–2·06)	0·51 (0·49–0·54)	0·94 (0·92–0·97)
2004	0·7 (0·3–1·4)	28·1 (25·5–30·8)	74·8 (70·7–78·9)	111·2 (107·4–114·8)	119·5 (115·8–123·5)	62·0 (58·0–65·9)	12·5 (10·4–14·9)	0·6 (0·4–0·9)	0·0 (0·0–0·0)	2·05 (1·98–2·11)	0·52 (0·49–0·54)	0·97 (0·94–1·0)
2005	0·7 (0·3–1·5)	29·1 (26·6–31·8)	75·4 (71·2–79·3)	112·7 (108·9–116·5)	121·8 (117·7–126·0)	64·6 (60·9–68·6)	12·9 (10·7–15·1)	0·6 (0·4–0·9)	0·0 (0·0–0·0)	2·09 (2·03–2·15)	0·53 (0·5–0·55)	1·0 (0·97–1·03)
2006	0·7 (0·3–1·5)	30·5 (28·1–33·0)	77·4 (74·0–81·0)	115·8 (112·4–119·4)	124·6 (121·3–128·0)	67·9 (64·7–71·0)	13·1 (11·1–15·3)	0·6 (0·4–0·9)	0·0 (0·0–0·0)	2·15 (2·1–2·2)	0·54 (0·52–0·57)	1·03 (1·0–1·06)
2007	0·7 (0·3–1·5)	31·3 (28·9–33·8)	79·8 (76·4–83·2)	117·9 (114·8–121·2)	126·5 (123·4–129·8)	70·6 (67·6–73·7)	13·4 (11·4–15·7)	0·6 (0·3–0·9)	0·0 (0·0–0·0)	2·2 (2·16–2·25)	0·56 (0·54–0·58)	1·06 (1·03–1·08)
2008	0·7 (0·3–1·5)	30·7 (28·3–33·1)	80·8 (77·4–84·5)	117·1 (113·8–120·4)	126·5 (123·2–129·8)	71·0 (67·7–74·3)	13·6 (11·6–16·0)	0·6 (0·3–0·9)	0·0 (0·0–0·0)	2·2 (2·16–2·25)	0·56 (0·54–0·58)	1·06 (1·03–1·09)
2009	0·7 (0·3–1·5)	29·2 (26·8–31·8)	80·1 (76·0–84·6)	115·6 (112·2–119·4)	126·0 (122·0–130·2)	70·3 (66·3–74·4)	13·8 (11·7–16·4)	0·6 (0·4–0·9)	0·0 (0·0–0·0)	2·18 (2·13–2·24)	0·55 (0·53–0·57)	1·05 (1·02–1·08)
2010	0·7 (0·3–1·4)	27·4 (25·0–29·9)	78·6 (74·7–82·8)	114·4 (110·9–118·2)	126·0 (122·0–130·3)	70·0 (65·8–74·1)	14·1 (11·9–16·7)	0·6 (0·4–1·0)	0·0 (0·0–0·0)	2·16 (2·1–2·22)	0·53 (0·51–0·56)	1·05 (1·02–1·09)
2011	0·7 (0·3–1·4)	25·4 (23·1–27·8)	76·4 (73·1–79·7)	113·4 (110·5–116·4)	125·6 (122·5–129·1)	69·7 (66·3–72·9)	14·5 (12·4–16·8)	0·6 (0·4–1·0)	0·0 (0·0–0·0)	2·13 (2·09–2·18)	0·51 (0·49–0·53)	1·05 (1·03–1·08)
2012	0·6 (0·3–1·3)	23·2 (21·1–25·5)	73·2 (70·1–76·4)	112·2 (109·4–115·3)	124·2 (120·8–127·7)	69·4 (66·5–72·7)	14·7 (12·7–17·2)	0·7 (0·4–1·1)	0·0 (0·0–0·0)	2·09 (2·05–2·14)	0·49 (0·46–0·5)	1·05 (1·02–1·07)
2013	0·6 (0·3–1·2)	21·4 (19·3–23·6)	70·1 (66·9–73·2)	112·0 (109·1–115·1)	124·2 (120·9–127·7)	69·4 (66·2–72·8)	14·8 (12·8–17·2)	0·7 (0·4–1·1)	0·0 (0·0–0·0)	2·07 (2·02–2·11)	0·46 (0·44–0·48)	1·05 (1·02–1·07)
2014	0·6 (0·3–1·2)	20·2 (18·0–22·6)	68·6 (65·2–71·6)	113·8 (111·0–116·8)	127·7 (124·5–130·9)	70·4 (67·1–73·9)	14·7 (12·5–17·1)	0·7 (0·4–1·1)	0·0 (0·0–0·0)	2·08 (2·04–2·12)	0·45 (0·43–0·47)	1·07 (1·04–1·1)
2015	0·6 (0·3–1·2)	19·6 (17·2–22·2)	67·6 (64·2–71·0)	115·1 (112·1–118·5)	131·2 (127·7–134·6)	71·8 (68·4–75·3)	14·8 (12·4–17·4)	0·7 (0·5–1·2)	0·0 (0·0–0·0)	2·11 (2·06–2·15)	0·44 (0·42–0·46)	1·09 (1·06–1·12)
2016	0·6 (0·3–1·2)	19·7 (17·0–22·8)	66·6 (61·6–72·5)	114·8 (110·2–119·7)	132·1 (126·7–138·3)	72·1 (66·6–78·5)	14·8 (12·1–18·0)	0·7 (0·4–1·2)	0·0 (0·0–0·0)	2·11 (2·0–2·23)	0·43 (0·4–0·48)	1·1 (1·04–1·16)
2017	0·6 (0·3–1·2)	20·1 (17·2–23·9)	66·4 (58·4–76·4)	114·1 (106·9–122·5)	131·7 (122·3–142·7)	72·0 (63·0–83·0)	14·9 (11·7–18·6)	0·7 (0·5–1·2)	0·0 (0·0–0·0)	2·1 (1·92–2·32)	0·44 (0·38–0·5)	1·1 (1·0–1·21)

Supplemental Results Table 5: Age-specific fertility rates per 1000, cumulative total fertility up to maternal age 25 (TFU25), cumulative total fertility from maternal age 30 to age 55 (TFO30), and cumulative total fertility, High-income Asia Pacific, 1950-2017

Year	10-14	15-19	20-24	25-29	30-34	35-39	40-44	45-49	50-54	TFR	TFU25	TFO30
1950	0·0 (0·0–0·1)	14·8 (13·3–16·1)	167·6 (156·5–179·3)	236·5 (230·0–242·9)	166·7 (158·2–175·1)	97·2 (88·3–105·9)	32·0 (28·3–36·2)	1·7 (1·3–2·3)	0·0 (0·0–0·0)	3·58 (3·47–3·71)	0·91 (0·86–0·97)	1·49 (1·42–1·57)
1951	0·0 (0·0–0·1)	13·0 (11·9–14·3)	155·2 (144·5–165·3)	223·5 (217·1–229·7)	154·9 (146·1–163·3)	85·3 (76·8–93·3)	26·4 (22·9–29·9)	1·4 (1·0–1·9)	0·0 (0·0–0·0)	3·3 (3·17–3·43)	0·84 (0·79–0·89)	1·34 (1·24–1·44)
1952	0·0 (0·0–0·1)	12·0 (10·9–13·2)	147·8 (137·5–158·4)	215·0 (208·4–221·7)	144·2 (135·9–152·5)	76·0 (68·7–83·8)	22·4 (19·5–25·4)	1·2 (0·9–1·6)	0·0 (0·0–0·0)	3·09 (2·99–3·21)	0·8 (0·74–0·86)	1·22 (1·15–1·29)
1953	0·0 (0·0–0·1)	11·4 (10·3–12·5)	142·0 (131·8–152·3)	205·8 (199·1–212·5)	133·3 (125·5–141·2)	67·9 (60·8–75·0)	19·2 (16·7–22·0)	1·1 (0·8–1·5)	0·0 (0·0–0·0)	2·9 (2·8–3·01)	0·77 (0·71–0·82)	1·11 (1·05–1·17)
1954	0·0 (0·0–0·1)	11·0 (9·9–12·1)	139·1 (129·8–148·9)	202·0 (195·8–208·5)	126·1 (118·6–133·5)	62·5 (55·9–69·7)	17·1 (14·6–19·8)	1·0 (0·8–1·4)	0·0 (0·0–0·0)	2·79 (2·69–2·89)	0·75 (0·7–0·8)	1·03 (0·97–1·1)
1955	0·0 (0·0–0·0)	10·5 (9·5–11·6)	136·8 (127·6–146·5)	201·4 (194·4–208·2)	119·9 (112·5–127·7)	57·1 (50·7–63·8)	15·5 (13·3–18·0)	1·0 (0·7–1·3)	0·0 (0·0–0·0)	2·71 (2·61–2·81)	0·74 (0·69–0·79)	0·97 (0·91–1·03)
1956	0·0 (0·0–0·1)	10·1 (9·0–11·3)	131·8 (122·4–141·6)	197·1 (190·7–203·7)	110·8 (103·9–117·8)	51·7 (45·7–57·7)	13·8 (11·8–16·1)	0·9 (0·7–1·1)	0·0 (0·0–0·0)	2·58 (2·48–2·68)	0·71 (0·66–0·76)	0·89 (0·83–0·94)
1957	0·0 (0·0–0·0)	9·9 (8·9–11·0)	132·6 (123·6–142·2)	200·4 (194·2–206·9)	106·7 (100·1–113·7)	47·9 (42·5–52·8)	12·7 (10·9–14·8)	0·9 (0·7–1·2)	0·0 (0·0–0·0)	2·56 (2·46–2·66)	0·71 (0·67–0·76)	0·84 (0·79–0·89)
1958	0·0 (0·0–0·0)	9·8 (8·8–10·9)	136·9 (127·8–147·1)	205·1 (198·5–211·7)	105·1 (98·9–111·8)	45·4 (40·5–50·5)	12·1 (10·3–14·3)	0·9 (0·6–1·1)	0·0 (0·0–0·0)	2·58 (2·48–2·68)	0·73 (0·69–0·78)	0·82 (0·77–0·87)
1959	0·0 (0·0–0·0)	9·7 (8·7–10·8)	137·0 (127·7–146·8)	204·7 (198·5–211·5)	101·5 (95·2–108·3)	41·9 (37·6–47·0)	11·3 (9·5–13·3)	0·8 (0·6–1·1)	0·0 (0·0–0·0)	2·53 (2·44–2·64)	0·73 (0·68–0·78)	0·78 (0·73–0·83)
1960	0·0 (0·0–0·0)	9·5 (8·6–10·5)	133·1 (124·0–142·6)	205·5 (199·2–212·2)	100·2 (94·2–106·4)	39·2 (34·7–43·8)	10·5 (8·9–12·4)	0·7 (0·5–1·0)	0·0 (0·0–0·0)	2·49 (2·4–2·59)	0·71 (0·67–0·76)	0·75 (0·71–0·8)
1961	0·0 (0·0–0·0)	9·3 (8·4–10·4)	130·0 (121·6–138·9)	207·2 (200·9–214·3)	100·1 (94·1–106·5)	37·0 (32·9–41·5)	10·1 (8·5–12·1)	0·7 (0·5–1·0)	0·0 (0·0–0·0)	2·47 (2·39–2·57)	0·7 (0·65–0·74)	0·74 (0·69–0·79)
1962	0·0 (0·0–0·0)	9·1 (8·1–10·2)	128·9 (119·9–137·8)	211·6 (205·3–217·9)	102·8 (97·1–108·7)	36·2 (32·5–40·4)	9·8 (8·2–11·7)	0·7 (0·5–1·0)	0·0 (0·0–0·0)	2·5 (2·42–2·58)	0·69 (0·65–0·74)	0·75 (0·7–0·79)
1963	0·0 (0·0–0·1)	9·0 (8·1–10·0)	128·2 (119·5–137·9)	214·0 (208·0–220·0)	104·6 (98·6–110·6)	36·5 (32·7–40·9)	10·0 (8·3–11·9)	0·7 (0·5–1·0)	0·0 (0·0–0·0)	2·52 (2·44–2·6)	0·69 (0·64–0·73)	0·76 (0·71–0·81)
1964	0·0 (0·0–0·1)	9·1 (8·2–10·1)	134·1 (125·4–143·7)	224·5 (218·5–230·8)	109·5 (103·6–116·4)	37·9 (34·1–41·7)	10·1 (8·5–11·9)	0·8 (0·5–1·0)	0·0 (0·0–0·0)	2·63 (2·56–2·71)	0·72 (0·67–0·76)	0·79 (0·75–0·84)
1965	0·0 (0·0–0·1)	9·1 (8·2–10·1)	127·7 (119·7–136·5)	200·7 (194·6–207·1)	99·5 (93·8–105·1)	36·9 (33·4–40·6)	10·0 (8·5–11·9)	0·7 (0·5–1·0)	0·0 (0·0–0·0)	2·42 (2·35–2·5)	0·68 (0·64–0·73)	0·74 (0·7–0·78)
1966	0·0 (0·0–0·1)	9·0 (8·1–9·8)	126·3 (117·6–135·2)	204·5 (198·6–210·5)	101·3 (95·9–107·0)	37·7 (34·4–41·3)	10·0 (8·4–11·8)	0·8 (0·5–1·0)	0·0 (0·0–0·0)	2·45 (2·38–2·52)	0·68 (0·63–0·72)	0·75 (0·71–0·79)
1967	0·0 (0·0–0·1)	9·0 (8·2–9·9)	129·9 (121·1–139·1)	233·6 (227·5–239·7)	114·1 (108·0–120·6)	39·7 (36·2–43·3)	10·1 (8·6–11·6)	0·8 (0·5–1·0)	0·0 (0·0–0·0)	2·69 (2·6–2·77)	0·69 (0·65–0·74)	0·82 (0·78–0·87)
1968	0·0 (0·0–0·1)	9·1 (8·2–9·9)	120·3 (112·2–128·8)	223·5 (217·2–229·5)	110·1 (103·9–116·3)	39·3 (36·0–42·5)	10·2 (8·8–11·6)	0·8 (0·5–1·0)	0·0 (0·0–0·0)	2·57 (2·5–2·63)	0·65 (0·61–0·69)	0·8 (0·76–0·84)
1969	0·0 (0·0–0·1)	9·5 (8·7–10·3)	117·8 (109·8–126·0)	223·8 (217·3–230·1)	110·4 (104·6–115·9)	39·0 (36·0–42·3)	10·2 (9·1–11·4)	0·8 (0·6–1·0)	0·0 (0·0–0·0)	2·56 (2·49–2·62)	0·64 (0·59–0·68)	0·8 (0·77–0·84)
1970	0·0 (0·0–0·1)	9·5 (8·8–10·3)	121·5 (113·3–130·6)	226·1 (219·8–232·2)	109·8 (104·4–115·4)	38·1 (35·4–41·2)	9·9 (8·9–10·9)	0·7 (0·5–1·0)	0·0 (0·0–0·0)	2·58 (2·51–2·64)	0·66 (0·61–0·7)	0·79 (0·76–0·82)
1971	0·0 (0·0–0·1)	9·4 (8·7–10·1)	126·3 (116·9–135·5)	223·5 (216·9–229·7)	108·4 (103·0–114·3)	36·2 (33·8–39·0)	9·3 (8·5–10·2)	0·7 (0·5–0·9)	0·0 (0·0–0·0)	2·57 (2·5–2·64)	0·68 (0·63–0·72)	0·77 (0·74–0·81)
1972	0·0 (0·0–0·1)	9·1 (8·5–9·7)	128·3 (120·0–137·3)	219·5 (213·9–225·6)	105·9 (100·1–111·7)	33·3 (31·0–35·7)	8·4 (7·7–9·2)	0·6 (0·5–0·8)	0·0 (0·0–0·0)	2·53 (2·46–2·59)	0·69 (0·65–0·73)	0·74 (0·71–0·77)
1973	0·0 (0·0–0·1)	8·6 (8·1–9·2)	125·4 (116·9–134·0)	215·5 (209·0–222·1)	98·9 (93·8–104·7)	29·9 (27·7–32·4)	7·1 (6·5–7·8)	0·5 (0·4–0·6)	0·0 (0·0–0·0)	2·43 (2·37–2·49)	0·67 (0·63–0·71)	0·68 (0·65–0·71)
1974	0·0 (0·0–0·1)	8·0 (7·5–8·5)	119·5 (111·7–128·2)	205·1 (198·6–211·4)	87·4 (82·3–93·0)	25·9 (23·9–28·0)	5·9 (5·4–6·5)	0·4 (0·3–0·5)	0·0 (0·0–0·0)	2·26 (2·21–2·32)	0·64 (0·6–0·68)	0·6 (0·57–0·63)
1975	0·0 (0·0–0·1)	7·5 (7·1–8·0)	114·1 (106·3–122·1)	196·0 (189·7–202·1)	78·5 (73·6–83·3)	22·5 (20·8–24·4)	4·9 (4·5–5·4)	0·3 (0·2–0·4)	0·0 (0·0–0·0)	2·12 (2·06–2·18)	0·61 (0·57–0·65)	0·53 (0·5–0·56)
1976	0·0 (0·0–0·1)	7·2 (6·7–7·7)	109·9 (103·3–117·4)	191·3 (184·9–197·5)	75·3 (70·9–79·8)	20·1 (18·3–22·0)	4·1 (3·6–4·7)	0·3 (0·2–0·3)	0·0 (0·0–0·0)	2·04 (1·99–2·1)	0·59 (0·55–0·62)	0·5 (0·47–0·52)
1977	0·0 (0·0–0·0)	6·9 (6·5–7·4)	105·9 (100·0–113·3)	191·3 (185·3–197·4)	75·7 (70·7–80·7)	18·3 (16·4–20·3)	3·6 (3·2–4·0)	0·2 (0·2–0·3)	0·0 (0·0–0·0)	2·01 (1·95–2·07)	0·56 (0·53–0·6)	0·49 (0·45–0·52)
1978	0·0 (0·0–0·0)	6·8 (6·3–7·4)	104·5 (98·9–110·8)	192·5 (186·8–198·1)	77·7 (72·7–82·9)	17·0 (15·4–18·8)	3·2 (2·8–3·7)	0·2 (0·1–0·2)	0·0 (0·0–0·0)	2·01 (1·96–2·06)	0·56 (0·53–0·59)	0·49 (0·46–0·52)
1979	0·0 (0·0–0·0)	6·6 (6·2–7·1)	103·4 (98·2–108·8)	192·9 (187·3–198·3)	77·1 (72·2–82·5)	16·1 (14·4–18·0)	3·0 (2·6–3·6)	0·2 (0·1–0·2)	0·0 (0·0–0·0)	2·0 (1·95–2·05)	0·55 (0·52–0·58)	0·48 (0·46–0·51)
1980	0·0 (0·0–0·0)	6·4 (5·9–7·0)	102·5 (97·4–107·7)	192·2 (186·6–198·2)	74·3 (69·3–79·2)	15·6 (14·0–17·5)	2·8 (2·4–3·3)	0·1 (0·1–0·2)	0·0 (0·0–0·0)	1·97 (1·92–2·02)	0·54 (0·52–0·57)	0·46 (0·44–0·49)
1981	0·0 (0·0–0·0)	6·2 (5·7–6·9)	100·9 (96·3–106·3)	191·0 (185·6–196·3)	72·3 (67·4–77·1)	15·5 (13·8–17·4)	2·5 (2·1–3·0)	0·1 (0·1–0·1)	0·0 (0·0–0·0)	1·94 (1·9–1·98)	0·54 (0·51–0·56)	0·45 (0·43–0·48)
1982	0·0 (0·0–0·0)	6·1 (5·6–6·6)	95·5 (90·5–100·7)	185·9 (180·9–191·3)	72·1 (67·3–77·4)	15·7 (13·9–17·7)	2·3 (1·9–2·8)	0·1 (0·0–0·1)	0·0 (0·0–0·0)	1·89 (1·85–1·93)	0·51 (0·48–0·53)	0·45 (0·43–0·48)
1983	0·0 (0·0–0·0)	6·0 (5·5–6·5)	89·8 (85·1–94·9)	178·2 (172·8–183·2)	72·9 (68·0–78·7)	16·2 (14·1–18·6)	2·1 (1·8–2·6)	0·1 (0·0–0·1)	0·0 (0·0–0·0)	1·83 (1·78–1·88)	0·48 (0·46–0·5)	0·46 (0·43–0·49)
1984	0·0 (0·0–0·0)	5·7 (5·3–6·2)	83·2 (78·7–88·4)	170·8 (166·0–175·9)	73·5 (68·3–78·9)	16·1 (14·1–18·4)	2·0 (1·6–2·5)	0·1 (0·0–0·1)	0·0 (0·0–0·0)	1·76 (1·71–1·8)	0·44 (0·42–0·47)	0·46 (0·43–0·49)
1985	0·0 (0·0–0·0)	5·4 (4·9–5·8)	76·7 (72·3–81·3)	165·1 (160·0–169·5)	72·7 (67·6–77·7)	15·8 (13·7–18·0)	2·0 (1·6–2·5)	0·1 (0·0–0·1)	0·0 (0·0–0·0)	1·69 (1·65–1·73)	0·41 (0·39–0·43)	0·45 (0·42–0·48)
1986	0·0 (0·0–0·0)	5·0 (4·6–5·4)	71·6 (67·3–76·1)	162·2 (157·6–167·1)	73·1 (67·8–78·0)	15·9 (13·9–18·1)	2·0 (1·5–2·5)	0·1 (0·0–0·1)	0·0 (0·0–0·0)	1·65 (1·61–1·69)	0·38 (0·36–0·41)	0·46 (0·43–0·48)
1987	0·0 (0·0–0·0)	4·8 (4·3–5·2)	66·3 (62·3–70·6)	161·1 (156·2–165·8)	75·0 (70·3–80·4)	16·4 (14·2–18·7)	2·1 (1·7–2·7)	0·1 (0·0–0·1)	0·0 (0·0–0·0)	1·63 (1·58–1·67)	0·36 (0·34–0·38)	0·47 (0·44–0·49)
1988	0·0 (0·0–0·0)	4·6 (4·1–5·1)	62·1 (58·3–66·3)	157·3 (153·0–162·0)	75·4 (70·7–80·5)	17·0 (15·0–19·3)	2·3 (1·8–2·8)	0·1 (0·0–0·1)	0·0 (0·0–0·0)	1·59 (1·55–1·64)	0·33 (0·31–0·35)	0·47 (0·45–0·5)
1989	0·0 (0·0–0·0)	4·5 (4·1–5·0)	58·2 (54·5–62·3)	152·7 (148·6–157·4)	75·4 (70·7–80·1)	17·6 (15·3–20·3)	2·4 (1·9–3·0)	0·1 (0·0–0·1)	0·0 (0·0–0·0)	1·55 (1·51–1·6)	0·31 (0·3–0·33)	0·48 (0·45–0·5)
1990	0·0 (0·0–0·0)	4·4 (4·0–4·9)	55·4 (52·0–58·7)	151·5 (147·1–155·7)	77·2 (72·6–81·6)	18·2 (15·7–20·8)	2·4 (1·9–2·9)	0·1 (0·0–0·1)	0·0 (0·0–0·0)	1·55 (1·51–1·59)	0·3 (0·28–0·32)	0·49 (0·46–0·51)
1991	0·0 (0·0–0·0)	4·4 (4·0–4·9)	53·3 (49·7–57·0)	149·8 (145·5–154·3)	78·6 (73·8–83·5)	18·9 (16·7–21·6)	2·4 (1·9–3·0)	0·1 (0·0–0·1)	0·0 (0·0–0·0)	1·54 (1·5–1·58)	0·29 (0·27–0·31)	0·5 (0·47–0·53)
1992	0·0 (0·0–0·0)	4·4 (3·9–4·8)	50·8 (47·3–54·9)	145·0 (140·6–149·4)	79·6 (75·1–84·4)	19·5 (17·1–22·0)	2·5 (2·0–3·1)	0·1 (0·0–0·1)	0·0 (0·0–0·0)	1·51 (1·47–1·55)	0·28 (0·26–0·3)	0·51 (0·48–0·54)
1993	0·0 (0·0–0·0)	4·3 (3·9–4·9)	49·1 (45·5–52·9)	143·4 (138·6–148·3)	83·4 (78·7–88·4)	20·6 (18·3–23·5)	2·6 (2·1–3·3)	0·1 (0·0–0·1)	0·0 (0·0–0·0)	1·52 (1·47–1·57)	0·27 (0·25–0·29)	0·53 (0·51–0·56)
1994	0·0 (0·0–0·0)	4·3 (3·9–4·8)	47·3 (43·8–51·1)	140·5 (136·0–145·0)	86·0 (81·0–91·1)	21·6 (19·1–24·3)	2·7 (2·2–3·4)	0·1 (0·1–0·1)	0·0 (0·0–0·0)	1·51 (1·46–1·57)	0·26 (0·24–0·28)	0·55 (0·52–0·58)
1995	0·0 (0·0–0·0)	4·3 (3·8–4·7)	45·2 (41·6–48·9)	135·3 (130·9–139·7)	87·7 (82·3–93·1)	22·3 (20·0–25·1)	2·9 (2·3–3·5)	0·1 (0·1–0·1)	0·0 (0·0–0·0)	1·49 (1·44–1·53)	0·25 (0·23–0·27)	0·56 (0·53–0·6)
1996	0·0 (0·0–0·0)	4·3 (3·9–4·8)	43·5 (39·9–47·0)	131·3 (127·3–135·4)	89·6 (84·9–94·7)	23·3 (20·4–26·3)	2·9 (2·4–3·5)	0·1 (0·1–0·1)	0·0 (0·0–0·0)	1·48 (1·43–1·52)	0·24 (0·22–0·26)	0·58 (0·55–0·61)
1997	0·0 (0·0–0·0)	4·4 (4·0–4·9)	42·1 (38·8–45·6)	127·4 (122·2–132·2)	89·8 (84·8–94·6)	23·8 (21·2–26·7)	3·1 (2·5–3·8)	0·1 (0·1–0·1)	0·0 (0·0–0·0)	1·45 (1·41–1·5)	0·23 (0·22–0·25)	0·58 (0·56–0·61)
1998	0·0 (0·0–0·0)	4·6 (4·1–5·1)	40·8 (37·7–44·4)	122·5 (118·8–126·2)	89·9 (85·0–95·3)	24·7 (21·8–27·8)	3·2 (2·6–3·8)	0·1 (0·1–0·1)	0·0 (0·0–0·0)	1·43 (1·39–1·47)	0·23 (0·21–0·25)	0·59 (0·56–0·62)
1999	0·0 (0·0–0·0)	4·7 (4·3–5·3)	39·6 (36·3–43·2)	117·1 (113·3–120·8)	90·8 (86·1–96·0)	26·2 (23·3–29·2)	3·4 (2·8–4·1)	0·1 (0·1–0·1)	0·0 (0·0–0·0)	1·41 (1·37–1·45)	0·22 (0·21–0·24)	0·6 (0·57–0·63)
2000	0·0 (0·0–0·0)	4·8 (4·3–5·4)	38·0 (34·8–41·2)	109·8 (106·1–113·8)	89·0 (84·5–94·2)	27·8 (25·0–30·7)	3·6 (3·0–4·4)	0·1 (0·1–0·1)	0·0 (0·0–0·0)	1·37 (1·33–1·41)	0·21 (0·2–0·23)	0·6 (0·57–0·63)
2001	0·0 (0·0–0·0)	4·8 (4·3–5·4)	35·9 (33·1–39·1)	101·4 (97·9–105·4)	84·7 (79·8–89·6)	27·4 (24·5–30·8)	3·8 (3·2–4·5)	0·1 (0·1–0·1)	0·0 (0·0–0·0)	1·29 (1·25–1·33)	0·2 (0·19–0·22)	0·58 (0·55–0·61)
2002	0·0 (0·0–0·0)	4·7 (4·1–5·2)	33·8 (30·7–37·2)	95·6 (91·9–99·2)	83·8 (79·1–89·0)	27·9 (25·0–31·1)	4·1 (3·4–4·8)	0·1 (0·1–0·2)	0·0 (0·0–0·0)	1·25 (1·21–1·29)	0·19 (0·18–0·21)	0·58 (0·55–0·61)
2003	0·0 (0·0–0·0)	4·5 (3·9–5·0)	31·8 (29·0–35·1)	91·6 (88·3–95·3)	84·2 (79·8–88·9)	29·2 (25·9–32·7)	4·3 (3·6–5·0)	0·1 (0·1–0·2)	0·0 (0·0–0·0)	1·23 (1·19–1·27)	0·18 (0·17–0·2)	0·59 (0·56–0·62)
2004	0·0 (0·0–0·0)	4·3 (3·8–4·8)	30·4 (27·7–33·6)	87·8 (84·4–91·5)	84·8 (80·3–89·5)	30·2 (26·9–33·9)	4·5 (3·8–5·2)	0·1 (0·1–0·2)	0·0 (0·0–0·0)	1·21 (1·18–1·25)	0·17 (0·16–0·19)	0·6 (0·57–0·63)
2005	0·0 (0·0–0·0)	4·2 (3·7–4·7)	30·2 (27·3–33·3)	86·2 (82·9–89·7)	86·9 (82·2–91·7)	31·4 (28·1–35·0)	4·6 (4·0–5·5)	0·2 (0·1–0·2)	0·0 (0·0–0·0)	1·22 (1·18–1·26)	0·17 (0·16–0·19)	0·62 (0·59–0·65)
2006	0·0 (0·0–0·0)	4·1 (3·6–4·6)	30·4 (27·6–33·3)	85·5 (82·1–89·2)	90·4 (85·5–95·5)	33·4 (29·9–37·6)	5·0 (4·3–5·8)	0·2 (0·1–0·2)	0·0 (0·0–0·0)	1·25 (1·21–1·28)	0·17 (0·16–0·19)	0·64 (0·61–0·68)
2007	0·0 (0·0–0·0)	4·0 (3·6–4·6)	30·4 (27·5–33·5)	84·2 (81·0–87·8)	92·1 (87·2–97·3)	35·6 (32·4–39·8)	5·3 (4·6–6·2)	0·2 (0·1–0·2)	0·0 (0·0–0·0)	1·26 (1·22–1·3)	0·17 (0·16–0·19)	0·67 (0·63–0·7)
2008	0·0 (0·0–0·0)	3·9 (3·5–4·4)	29·9 (27·0–33·1)	83·1 (79·8–86·3)	93·7 (88·9–98·3)	37·6 (33·8–41·6)	5·8 (4·9–6·7)	0·2 (0·1–0·2)	0·0 (0·0–0·0)	1·27 (1·23–1·31)	0·17 (0·15–0·19)	0·69 (0·66–0·72)
2009	0·0 (0·0–0·0)	3·8 (3·3–4·2)	28·9 (26·1–31·8)	82·2 (79·0–85·4)	95·5 (90·6–100·8)	39·8 (35·8–44·1)	6·3 (5·4–7·4)	0·2 (0·1–0·3)	0·0 (0·0–0·0)	1·28 (1·24–1·33)	0·16 (0·15–0·18)	0·71 (0·68–0·74)
2010	0·0 (0·0–0·0)	3·6 (3·1–4·1)	27·9 (25·1–30·7)	81·9 (78·8–85·0)	97·9 (93·0–103·4)	41·8 (37·9–46·1)	6·7 (5·7–7·8)	0·2 (0·1–0·3)	0·0 (0·0–0·0)	1·3 (1·26–1·34)	0·16 (0·14–0·17)	0·73 (0·7–0·77)
2011	0·0 (0·0–0·0)	3·5 (3·1–4·0)	26·4 (24·0–29·0)	80·7 (77·3–84·2)	99·5 (94·8–104·6)	43·7 (39·4–48·3)	7·1 (6·1–8·3)	0·2 (0·1–0·3)	0·0 (0·0–0·0)	1·31 (1·27–1·34)	0·15 (0·14–0·16)	0·75 (0·72–0·79)
2012	0·0 (0·0–0·0)	3·4 (3·0–3·9)	24·9 (22·7–27·6)	78·6 (75·5–81·8)	100·5 (95·6–105·7)	46·3 (41·9–50·7)	7·8 (6·7–9·0)	0·2 (0·2–0·3)	0·0 (0·0–0·0)	1·31 (1·27–1·35)	0·14 (0·13–0·15)	0·77 (0·74–0·81)
2013	0·0 (0·0–0·0)	3·3 (2·9–3·8)	23·9 (21·5–26·3)	75·8 (72·5–79·4)	100·4 (95·8–105·1)	48·1 (43·7–52·7)	8·3 (7·2–9·7)	0·2 (0·2–0·3)	0·0 (0·0–0·0)	1·3 (1·26–1·34)	0·14 (0·12–0·15)	0·79 (0·75–0·82)
2014	0·0 (0·0–0·0)	3·3 (2·9–3·7)	23·0 (20·9–25·1)	74·1 (70·9–77·3)	101·8 (97·4–106·6)	50·4 (46·1–55·2)	8·8 (7·7–10·2)	0·2 (0·2–0·3)	0·0 (0·0–0·0)	1·31 (1·27–1·35)	0·13 (0·12–0·14)	0·81 (0·77–0·85)
2015	0·0 (0·0–0·0)	3·3 (2·8–3·9)	23·1 (20·0–26·7)	74·2 (69·9–78·8)	103·9 (97·7–110·7)	50·1 (44·6–56·5)	8·4 (7·1–10·1)	0·2 (0·2–0·3)	0·0 (0·0–0·0)	1·32 (1·22–1·43)	0·13 (0·11–0·15)	0·81 (0·75–0·88)
2016	0·0 (0·0–0·0)	3·4 (2·8–4·1)	23·7 (19·8–28·7)	74·5 (68·9–81·0)	104·0 (95·4–114·0)	47·9 (40·7–56·8)	7·6 (6·1–9·6)	0·2 (0·2–0·3)	0·0 (0·0–0·0)	1·31 (1·17–1·46)	0·14 (0·11–0·16)	0·8 (0·72–0·89)
2017	0·0 (0·0–0·0)	3·4 (2·9–4·0)	23·8 (19·5–29·1)	74·1 (67·8–80·8)	103·3 (93·5–114·1)	47·4 (39·7–56·5)	7·5 (5·8–9·8)	0·2 (0·2–0·3)	0·0 (0·0–0·0)	1·3 (1·15–1·47)	0·14 (0·11–0·17)	0·79 (0·69–0·9)

Supplemental Results Table 5: Age-specific fertility rates per 1000, cumulative total fertility up to maternal age 25 (TFU25), cumulative total fertility from maternal age 30 to age 55 (TFO30), and cumulative total fertility, Brunei, 1950-2017

Year	10-14	15-19	20-24	25-29	30-34	35-39	40-44	45-49	50-54	TFR	TFU25	TFO30
1950	0·7 (0·3–1·4)	66·6 (57·0–77·2)	337·5 (321·1–352·8)	330·2 (321·2–339·2)	269·1 (257·9–279·9)	220·4 (211·8–227·9)	98·2 (91·5–104·0)	19·9 (15·3–24·9)	0·4 (0·4–0·4)	6·71 (6·5–6·9)	2·02 (1·89–2·16)	3·04 (2·94–3·13)
1951	0·7 (0·3–1·4)	67·8 (57·9–78·9)	340·7 (325·2–354·6)	334·7 (325·8–343·2)	273·2 (262·5–283·2)	223·7 (215·7–230·0)	100·2 (94·0–105·8)	20·6 (15·6–25·6)	0·4 (0·4–0·4)	6·81 (6·61–6·99)	2·05 (1·92–2·17)	3·09 (3·0–3·17)
1952	0·7 (0·3–1·5)	69·7 (59·3–81·1)	346·3 (331·9–360·0)	341·0 (332·7–348·7)	279·5 (269·9–288·4)	228·4 (221·6–233·8)	103·3 (97·5–108·3)	21·6 (16·5–26·7)	0·4 (0·4–0·4)	6·95 (6·78–7·13)	2·08 (1·97–2·2)	3·17 (3·07–3·24)
1953	0·7 (0·3–1·5)	72·5 (62·1–84·3)	354·8 (341·4–366·8)	349·5 (341·8–356·4)	287·9 (278·8–296·4)	234·3 (228·8–238·4)	107·5 (102·4–112·1)	22·8 (17·6–27·9)	0·4 (0·4–0·5)	7·15 (6·97–7·33)	2·14 (2·04–2·25)	3·26 (3·18–3·33)
1954	0·7 (0·3–1·5)	75·6 (64·5–86·9)	363·7 (350·9–375·4)	358·3 (351·5–364·7)	296·5 (288·8–303·7)	237·9 (233·4–241·6)	111·7 (107·3–115·9)	23·8 (18·5–28·8)	0·5 (0·4–0·5)	7·34 (7·17–7·52)	2·2 (2·09–2·3)	3·35 (3·28–3·42)
1955	0·7 (0·3–1·6)	78·5 (67·4–90·2)	369·1 (356·9–380·6)	363·9 (358·3–369·3)	301·2 (294·2–308·0)	238·0 (233·8–241·9)	114·3 (110·4–117·8)	24·4 (19·0–29·5)	0·5 (0·5–0·5)	7·45 (7·29–7·62)	2·24 (2·13–2·34)	3·39 (3·33–3·45)
1956	0·8 (0·3–1·6)	81·6 (70·5–93·9)	371·0 (359·5–382·4)	367·7 (362·7–372·6)	300·9 (293·7–308·2)	237·2 (232·6–241·6)	115·5 (111·5–118·7)	24·7 (19·3–29·6)	0·5 (0·5–0·5)	7·5 (7·34–7·66)	2·27 (2·17–2·37)	3·39 (3·33–3·45)
1957	0·8 (0·3–1·6)	85·4 (74·0–97·6)	371·7 (359·5–382·6)	370·3 (365·5–375·3)	301·0 (293·6–308·2)	237·9 (233·1–242·1)	116·2 (112·3–119·5)	24·6 (19·3–29·6)	0·5 (0·5–0·5)	7·54 (7·4–7·69)	2·29 (2·19–2·39)	3·4 (3·34–3·46)
1958	0·8 (0·3–1·7)	90·2 (78·1–102·8)	368·1 (356·4–378·7)	366·8 (361·6–372·5)	301·8 (294·7–309·1)	239·0 (234·5–242·9)	115·4 (111·6–119·0)	23·8 (18·6–28·9)	0·5 (0·4–0·5)	7·53 (7·4–7·66)	2·3 (2·21–2·39)	3·4 (3·35–3·46)
1959	0·8 (0·4–1·7)	92·5 (80·7–105·4)	359·6 (346·7–371·4)	359·1 (353·1–365·2)	299·9 (292·7–306·9)	239·4 (234·8–243·1)	112·6 (108·6–116·6)	22·7 (17·6–27·7)	0·4 (0·4–0·5)	7·43 (7·31–7·57)	2·26 (2·18–2·35)	3·37 (3·32–3·43)
1960	0·8 (0·3–1·6)	87·4 (76·1–99·3)	348·6 (334·3–361·8)	351·0 (344·5–358·1)	295·4 (287·6–302·7)	237·5 (232·7–241·5)	108·4 (104·1–112·8)	21·1 (16·1–26·3)	0·4 (0·4–0·4)	7·25 (7·12–7·39)	2·18 (2·09–2·28)	3·31 (3·25–3·37)
1961	0·7 (0·3–1·6)	79·9 (69·2–91·4)	334·8 (318·8–348·8)	341·0 (332·9–349·5)	288·1 (279·3–295·9)	231·8 (226·2–236·9)	102·9 (97·7–108·1)	19·4 (14·5–24·5)	0·4 (0·4–0·4)	6·99 (6·85–7·14)	2·08 (1·98–2·18)	3·21 (3·13–3·28)
1962	0·7 (0·3–1·5)	75·0 (65·3–86·4)	320·8 (303·2–336·4)	331·3 (322·1–340·5)	280·7 (271·2–289·3)	224·6 (218·0–231·0)	97·5 (91·8–102·9)	17·9 (13·3–23·2)	0·3 (0·3–0·4)	6·74 (6·57–6·92)	1·98 (1·87–2·09)	3·11 (3·02–3·18)
1963	0·7 (0·3–1·5)	72·0 (62·4–82·6)	310·1 (292·2–326·9)	324·7 (315·1–333·5)	276·1 (266·4–285·2)	219·2 (211·5–226·6)	93·8 (87·9–100·1)	16·8 (12·3–21·9)	0·3 (0·3–0·3)	6·57 (6·35–6·78)	1·91 (1·78–2·04)	3·03 (2·94–3·12)
1964	0·7 (0·3–1·5)	69·8 (60·3–80·2)	300·9 (282·3–318·3)	318·8 (308·8–328·5)	272·4 (262·4–281·7)	214·3 (205·7–222·0)	91·0 (84·5–97·2)	15·8 (11·4–20·8)	0·3 (0·3–0·3)	6·42 (6·17–6·66)	1·86 (1·72–1·99)	2·97 (2·87–3·06)
1965	0·7 (0·3–1·4)	68·2 (59·0–78·1)	291·9 (274·1–309·7)	312·9 (302·6–322·9)	268·9 (258·9–278·6)	209·3 (200·0–217·8)	88·6 (81·7–95·1)	14·9 (10·7–19·7)	0·3 (0·3–0·3)	6·28 (6·01–6·53)	1·8 (1·67–1·93)	2·91 (2·81–3·01)
1966	0·7 (0·3–1·4)	67·4 (58·4–77·9)	284·4 (264·9–302·8)	308·6 (298·5–318·3)	267·1 (256·4–277·0)	205·5 (195·1–214·9)	87·0 (80·3–93·5)	14·2 (10·2–18·9)	0·3 (0·3–0·3)	6·18 (5·93–6·42)	1·76 (1·64–1·88)	2·87 (2·77–2·97)
1967	0·7 (0·3–1·4)	66·8 (57·8–76·6)	277·2 (258·7–294·4)	304·1 (294·0–313·3)	265·1 (254·8–274·7)	201·6 (191·0–211·3)	85·3 (78·7–91·8)	13·4 (9·5–18·0)	0·3 (0·2–0·3)	6·07 (5·85–6·27)	1·72 (1·62–1·82)	2·83 (2·74–2·92)
1968	0·7 (0·3–1·4)	66·2 (57·7–75·6)	268·3 (250·4–284·3)	297·8 (287·1–306·9)	261·3 (251·0–271·3)	196·0 (184·6–206·1)	82·8 (75·5–90·0)	12·4 (8·9–16·7)	0·2 (0·2–0·2)	5·93 (5·74–6·11)	1·68 (1·58–1·77)	2·76 (2·67–2·85)
1969	0·7 (0·3–1·4)	65·7 (58·1–74·4)	259·7 (245·4–273·2)	290·8 (280·3–299·8)	257·1 (247·3–267·0)	190·2 (178·2–200·8)	80·4 (72·0–88·1)	11·5 (8·1–15·5)	0·2 (0·2–0·2)	5·78 (5·62–5·94)	1·63 (1·55–1·71)	2·7 (2·6–2·79)
1970	0·7 (0·3–1·4)	64·2 (56·7–71·9)	251·3 (240·0–263·3)	282·6 (272·9–291·4)	252·7 (242·5–262·4)	184·5 (172·8–195·2)	78·1 (69·3–86·3)	10·4 (7·3–14·0)	0·2 (0·2–0·2)	5·62 (5·49–5·77)	1·58 (1·51–1·65)	2·63 (2·53–2·72)
1971	0·6 (0·3–1·3)	60·3 (53·5–67·6)	239·3 (228·5–250·5)	271·8 (262·2–280·7)	246·9 (236·7–256·3)	177·4 (166·2–188·7)	75·2 (66·0–83·8)	8·8 (6·1–12·0)	0·2 (0·2–0·2)	5·4 (5·27–5·54)	1·5 (1·44–1·57)	2·54 (2·44–2·64)
1972	0·6 (0·3–1·3)	55·1 (49·0–62·2)	222·6 (212·5–233·7)	259·5 (250·1–268·5)	239·7 (228·7–249·2)	169·5 (157·8–181·2)	71·3 (62·3–80·2)	7·1 (4·8–9·8)	0·1 (0·1–0·1)	5·13 (5·0–5·27)	1·39 (1·33–1·46)	2·44 (2·35–2·53)
1973	0·6 (0·3–1·2)	49·2 (43·3–55·8)	205·1 (194·3–217·0)	246·9 (237·5–255·7)	230·8 (219·6–240·7)	159·9 (147·0–172·4)	65·0 (56·1–73·3)	5·5 (3·6–7·7)	0·1 (0·1–0·1)	4·82 (4·67–4·96)	1·27 (1·21–1·35)	2·31 (2·21–2·41)
1974	0·5 (0·2–1·1)	41·2 (35·8–46·9)	185·5 (173·7–197·9)	235·8 (226·3–245·5)	222·1 (210·6–233·2)	150·6 (137·4–163·5)	56·6 (48·4–64·7)	4·1 (2·6–5·8)	0·1 (0·1–0·1)	4·48 (4·33–4·64)	1·14 (1·06–1·21)	2·17 (2·06–2·27)
1975	0·5 (0·2–1·0)	36·5 (31·4–41·8)	169·3 (157·0–182·9)	229·3 (219·3–238·9)	214·4 (202·7–225·4)	145·0 (131·2–157·8)	51·0 (43·4–58·7)	3·4 (2·2–5·0)	0·1 (0·1–0·1)	4·25 (4·09–4·42)	1·03 (0·96–1·11)	2·07 (1·95–2·19)
1976	0·5 (0·2–1·1)	39·4 (34·0–45·1)	168·0 (155·7–181·1)	228·7 (218·2–238·9)	204·3 (192·0–216·2)	140·5 (126·4–154·0)	50·2 (42·7–57·7)	3·6 (2·3–5·2)	0·1 (0·1–0·1)	4·18 (4·01–4·35)	1·04 (0·97–1·11)	1·99 (1·87–2·11)
1977	0·5 (0·2–1·1)	44·3 (38·6–50·8)	175·9 (163·2–189·3)	229·8 (219·6–240·2)	193·2 (180·5–204·3)	135·4 (121·5–149·1)	50·8 (43·4–58·7)	3·9 (2·5–5·7)	0·1 (0·1–0·1)	4·17 (4·01–4·33)	1·1 (1·03–1·18)	1·92 (1·8–2·03)
1978	0·6 (0·2–1·2)	46·1 (40·4–52·8)	181·8 (168·9–195·6)	228·9 (219·0–239·2)	184·2 (171·9–195·4)	128·9 (114·4–142·9)	50·5 (43·4–58·0)	4·0 (2·6–5·8)	0·1 (0·1–0·1)	4·13 (3·96–4·31)	1·14 (1·06–1·23)	1·84 (1·72–1·96)
1979	0·6 (0·2–1·2)	46·5 (40·6–53·4)	183·8 (169·8–197·8)	225·5 (216·1–235·6)	175·9 (164·4–187·9)	120·2 (106·0–133·9)	49·7 (42·7–57·1)	3·8 (2·5–5·6)	0·1 (0·1–0·1)	4·03 (3·86–4·22)	1·15 (1·06–1·25)	1·75 (1·62–1·88)
1980	0·6 (0·2–1·2)	46·1 (40·6–53·1)	182·4 (170·0–194·5)	220·2 (210·9–229·5)	167·4 (156·0–178·3)	110·4 (97·7–123·7)	48·6 (41·5–55·9)	3·5 (2·3–5·0)	0·1 (0·1–0·1)	3·9 (3·73–4·07)	1·15 (1·07–1·23)	1·65 (1·52–1·78)
1981	0·6 (0·2–1·1)	44·7 (39·4–51·4)	177·2 (167·0–187·2)	214·2 (206·4–222·7)	160·8 (150·7–170·6)	102·0 (90·0–114·6)	46·1 (38·7–54·0)	2·9 (1·9–4·3)	0·1 (0·1–0·1)	3·74 (3·6–3·9)	1·11 (1·05–1·17)	1·56 (1·45–1·66)
1982	0·5 (0·2–1·1)	42·8 (37·4–48·7)	169·8 (160·1–179·4)	210·5 (203·3–218·5)	157·6 (147·8–167·0)	97·1 (86·1–108·1)	42·9 (35·2–51·5)	2·4 (1·6–3·6)	0·0 (0·0–0·0)	3·62 (3·49–3·74)	1·07 (1·01–1·12)	1·5 (1·41–1·59)
1983	0·5 (0·2–1·1)	41·4 (36·3–47·0)	163·9 (154·3–173·5)	210·3 (202·7–218·0)	158·2 (149·0–167·6)	95·6 (85·5–107·0)	40·3 (33·1–48·6)	2·1 (1·3–3·2)	0·0 (0·0–0·0)	3·56 (3·46–3·67)	1·03 (0·98–1·08)	1·48 (1·4–1·57)
1984	0·5 (0·2–1·1)	40·8 (35·6–46·2)	160·5 (151·8–169·3)	210·5 (202·8–218·5)	160·6 (151·4–169·8)	96·2 (86·5–107·0)	38·3 (31·2–46·8)	1·9 (1·2–2·9)	0·0 (0·0–0·0)	3·55 (3·44–3·66)	1·01 (0·96–1·06)	1·48 (1·4–1·57)
1985	0·5 (0·2–1·1)	40·2 (35·2–45·7)	157·3 (148·2–166·3)	207·9 (200·4–216·0)	162·1 (153·4–171·4)	96·5 (86·0–106·8)	36·8 (29·6–44·9)	1·7 (1·0–2·5)	0·0 (0·0–0·0)	3·52 (3·41–3·63)	0·99 (0·94–1·04)	1·49 (1·41–1·57)
1986	0·5 (0·2–1·1)	39·1 (34·0–44·3)	152·2 (143·5–161·2)	202·1 (195·1–209·8)	161·3 (153·3–170·4)	95·2 (85·4–105·0)	35·3 (28·0–43·1)	1·5 (0·9–2·2)	0·0 (0·0–0·0)	3·44 (3·32–3·56)	0·96 (0·91–1·01)	1·47 (1·39–1·55)
1987	0·5 (0·2–1·0)	37·5 (32·7–42·9)	146·6 (137·7–155·3)	194·0 (186·3–200·6)	158·9 (150·8–167·6)	92·6 (83·5–102·9)	33·4 (26·7–41·2)	1·3 (0·8–1·9)	0·0 (0·0–0·0)	3·32 (3·2–3·46)	0·92 (0·87–0·98)	1·43 (1·35–1·52)
1988	0·5 (0·2–1·0)	36·1 (31·4–41·4)	142·1 (133·4–151·2)	186·0 (179·0–192·6)	155·7 (147·8–164·3)	89·7 (80·9–99·7)	31·8 (25·6–39·2)	1·1 (0·7–1·7)	0·0 (0·0–0·0)	3·22 (3·09–3·35)	0·89 (0·84–0·94)	1·39 (1·3–1·48)
1989	0·5 (0·2–1·0)	35·2 (30·5–40·2)	139·3 (130·6–148·2)	180·5 (173·3–187·6)	151·8 (143·8–159·4)	87·0 (78·0–96·0)	30·5 (24·4–37·7)	1·1 (0·7–1·6)	0·0 (0·0–0·0)	3·13 (3·0–3·25)	0·87 (0·83–0·92)	1·35 (1·26–1·44)
1990	0·5 (0·2–1·0)	34·7 (29·9–39·7)	137·0 (128·3–145·7)	176·9 (169·6–183·8)	147·0 (138·9–155·0)	84·7 (75·5–93·7)	29·2 (23·4–35·7)	1·0 (0·6–1·5)	0·0 (0·0–0·0)	3·06 (2·93–3·18)	0·86 (0·81–0·91)	1·31 (1·22–1·39)
1991	0·5 (0·2–1·0)	34·1 (29·3–39·1)	134·4 (125·9–143·7)	174·2 (166·5–182·0)	142·8 (134·0–151·0)	81·8 (72·0–91·6)	27·6 (21·8–33·8)	0·9 (0·6–1·4)	0·0 (0·0–0·0)	2·98 (2·86–3·1)	0·85 (0·79–0·9)	1·27 (1·18–1·35)
1992	0·5 (0·2–1·0)	33·0 (28·3–38·0)	130·9 (118·6–142·0)	171·9 (162·7–181·7)	139·9 (129·7–150·3)	77·3 (65·9–88·5)	25·8 (20·7–31·6)	0·8 (0·5–1·3)	0·0 (0·0–0·0)	2·9 (2·76–3·05)	0·82 (0·74–0·9)	1·22 (1·14–1·31)
1993	0·5 (0·2–0·9)	31·8 (27·1–37·0)	126·6 (112·4–142·2)	169·6 (159·2–180·9)	137·6 (125·6–150·6)	72·2 (60·5–84·9)	24·2 (19·3–30·1)	0·7 (0·5–1·2)	0·0 (0·0–0·0)	2·82 (2·65–2·99)	0·79 (0·7–0·9)	1·17 (1·07–1·29)
1994	0·5 (0·2–0·9)	30·8 (25·7–36·0)	122·4 (106·1–139·9)	167·0 (155·2–179·5)	135·6 (121·1–149·7)	68·1 (55·6–82·1)	23·0 (18·1–28·7)	0·7 (0·4–1·1)	0·0 (0·0–0·0)	2·74 (2·56–2·93)	0·77 (0·67–0·88)	1·14 (1·01–1·26)
1995	0·4 (0·2–0·9)	29·8 (24·9–35·5)	118·6 (101·1–138·3)	163·7 (151·3–176·5)	133·3 (118·5–148·5)	67·1 (54·7–81·4)	21·8 (17·1–27·1)	0·6 (0·4–0·9)	0·0 (0·0–0·0)	2·68 (2·48–2·89)	0·74 (0·63–0·87)	1·11 (0·98–1·26)
1996	0·4 (0·2–0·9)	28·7 (24·1–34·7)	114·2 (97·2–135·3)	159·3 (146·8–172·3)	130·0 (115·8–144·9)	67·7 (55·0–81·9)	20·8 (16·1–26·3)	0·5 (0·3–0·8)	0·0 (0·0–0·0)	2·61 (2·41–2·84)	0·72 (0·61–0·85)	1·1 (0·96–1·25)
1997	0·4 (0·2–0·9)	27·6 (23·1–33·1)	109·7 (93·3–127·3)	154·3 (142·3–166·3)	126·2 (113·4–140·0)	68·0 (55·8–81·6)	20·1 (15·4–26·2)	0·5 (0·3–0·7)	0·0 (0·0–0·0)	2·53 (2·36–2·75)	0·69 (0·59–0·81)	1·07 (0·95–1·21)
1998	0·4 (0·2–0·9)	26·8 (22·6–31·9)	105·7 (91·7–120·7)	148·9 (138·2–160·1)	122·9 (110·8–135·5)	68·8 (57·6–81·7)	19·9 (15·2–26·2)	0·4 (0·3–0·7)	0·0 (0·0–0·0)	2·47 (2·32–2·64)	0·67 (0·58–0·76)	1·06 (0·96–1·18)
1999	0·4 (0·2–0·9)	26·1 (22·2–30·8)	102·5 (91·1–115·2)	144·1 (135·4–153·8)	120·1 (110·3–130·7)	69·5 (59·6–80·8)	19·6 (14·9–26·1)	0·4 (0·2–0·6)	0·0 (0·0–0·0)	2·41 (2·29–2·55)	0·65 (0·58–0·72)	1·05 (0·95–1·16)
2000	0·4 (0·2–0·8)	25·1 (21·3–29·5)	99·8 (90·0–111·0)	140·4 (132·2–148·8)	117·4 (108·1–127·0)	69·1 (59·6–79·3)	18·7 (14·1–24·4)	0·3 (0·2–0·5)	0·0 (0·0–0·0)	2·36 (2·23–2·48)	0·63 (0·57–0·69)	1·03 (0·93–1·13)
2001	0·4 (0·2–0·8)	23·7 (20·0–27·6)	97·0 (88·4–108·0)	136·8 (128·2–145·3)	114·8 (106·0–123·9)	68·2 (59·2–78·5)	17·6 (13·2–22·7)	0·3 (0·2–0·5)	0·0 (0·0–0·0)	2·29 (2·18–2·43)	0·61 (0·56–0·67)	1·0 (0·91–1·11)
2002	0·4 (0·2–0·8)	22·1 (18·4–25·9)	92·6 (82·4–103·6)	133·6 (124·3–143·1)	112·7 (103·0–123·2)	66·9 (56·9–77·3)	16·5 (12·4–20·9)	0·3 (0·2–0·5)	0·0 (0·0–0·0)	2·23 (2·09–2·37)	0·58 (0·52–0·64)	0·98 (0·88–1·1)
2003	0·4 (0·2–0·7)	20·3 (16·9–24·1)	87·1 (75·8–99·4)	130·3 (119·9–140·6)	110·5 (99·8–121·6)	64·9 (53·7–76·0)	15·8 (11·9–20·2)	0·4 (0·2–0·5)	0·0 (0·0–0·0)	2·15 (1·97–2·33)	0·54 (0·48–0·61)	0·96 (0·84–1·07)
2004	0·3 (0·2–0·7)	18·7 (15·7–22·2)	81·6 (69·7–94·3)	126·4 (115·4–137·4)	107·9 (96·2–120·0)	62·9 (51·8–75·0)	15·3 (11·6–19·6)	0·4 (0·2–0·6)	0·0 (0·0–0·0)	2·07 (1·87–2·26)	0·5 (0·44–0·57)	0·93 (0·81–1·06)
2005	0·3 (0·1–0·7)	17·4 (14·6–20·6)	76·5 (64·9–88·4)	122·6 (112·3–133·3)	105·4 (93·5–117·6)	61·2 (50·7–73·7)	15·0 (11·7–18·8)	0·4 (0·2–0·6)	0·0 (0·0–0·0)	1·99 (1·82–2·17)	0·47 (0·41–0·53)	0·91 (0·8–1·03)
2006	0·3 (0·1–0·7)	16·5 (13·8–19·4)	72·4 (62·3–83·2)	119·8 (110·4–130·2)	103·5 (92·6–114·6)	59·8 (50·0–71·3)	14·9 (12·2–18·4)	0·4 (0·3–0·6)	0·0 (0·0–0·0)	1·94 (1·8–2·09)	0·45 (0·39–0·5)	0·89 (0·8–1·0)
2007	0·3 (0·1–0·6)	15·8 (13·1–18·7)	69·2 (60·4–79·0)	118·1 (109·4–127·7)	102·5 (92·8–112·9)	59·0 (49·5–69·5)	15·0 (12·4–18·3)	0·4 (0·3–0·7)	0·0 (0·0–0·0)	1·9 (1·79–2·03)	0·43 (0·38–0·48)	0·89 (0·81–0·97)
2008	0·3 (0·1–0·6)	15·1 (12·6–17·9)	66·7 (58·4–76·1)	116·6 (108·2–125·4)	102·4 (93·0–113·1)	59·1 (50·1–69·0)	15·2 (12·5–18·5)	0·5 (0·3–0·7)	0·0 (0·0–0·0)	1·88 (1·77–2·0)	0·41 (0·37–0·46)	0·89 (0·8–0·98)
2009	0·3 (0·1–0·6)	14·6 (12·1–17·2)	64·7 (56·1–73·5)	115·5 (107·2–124·2)	103·0 (93·3–113·9)	59·6 (50·8–70·4)	15·4 (12·6–18·5)	0·5 (0·3–0·7)	0·0 (0·0–0·0)	1·87 (1·74–2·02)	0·4 (0·35–0·45)	0·89 (0·8–1·0)
2010	0·3 (0·1–0·6)	14·1 (11·8–16·6)	63·1 (54·3–72·2)	114·9 (105·9–123·9)	103·9 (93·9–114·8)	60·5 (51·0–71·4)	15·8 (12·9–18·9)	0·5 (0·3–0·7)	0·0 (0·0–0·0)	1·86 (1·72–2·02)	0·39 (0·34–0·43)	0·9 (0·8–1·02)
2011	0·3 (0·1–0·6)	13·7 (11·5–16·3)	61·5 (53·3–70·4)	113·8 (105·5–122·3)	104·7 (95·3–115·4)	61·3 (52·4–72·1)	16·2 (13·2–19·7)	0·5 (0·3–0·7)	0·0 (0·0–0·0)	1·86 (1·73–2·01)	0·38 (0·34–0·42)	0·91 (0·82–1·03)
2012	0·3 (0·1–0·6)	13·2 (11·1–15·8)	60·0 (52·5–68·2)	112·4 (105·2–120·0)	105·4 (96·8–114·5)	62·2 (53·7–71·5)	16·8 (13·6–20·7)	0·5 (0·3–0·8)	0·0 (0·0–0·0)	1·85 (1·74–1·98)	0·37 (0·33–0·41)	0·92 (0·84–1·02)
2013	0·3 (0·1–0·6)	12·8 (10·7–15·1)	58·4 (51·7–65·6)	111·3 (104·7–118·0)	106·2 (98·6–114·2)	63·0 (55·5–71·1)	17·3 (13·9–21·6)	0·5 (0·3–0·8)	0·0 (0·0–0·0)	1·85 (1·75–1·96)	0·36 (0·32–0·4)	0·94 (0·86–1·02)
2014	0·3 (0·1–0·6)	12·5 (10·4–14·8)	56·8 (49·8–64·2)	110·3 (103·8–116·8)	107·1 (99·4–115·1)	64·2 (56·7–72·9)	17·9 (14·0–23·0)	0·5 (0·3–0·8)	0·0 (0·0–0·0)	1·85 (1·74–1·95)	0·35 (0·31–0·39)	0·95 (0·87–1·04)
2015	0·3 (0·1–0·6)	12·4 (10·3–14·8)	55·4 (47·2–64·4)	109·0 (101·1–116·2)	108·6 (98·9–118·2)	66·3 (57·3–77·1)	18·7 (14·6–24·3)	0·5 (0·3–0·8)	0·0 (0·0–0·0)	1·86 (1·74–1·98)	0·34 (0·29–0·39)	0·97 (0·89–1·06)
2016	0·3 (0·1–0·6)	12·4 (10·3–14·9)	54·3 (45·2–65·0)	107·9 (98·5–116·9)	110·5 (99·9–122·4)	68·7 (57·4–83·1)	19·5 (14·6–25·9)	0·5 (0·3–0·8)	0·0 (0·0–0·0)	1·87 (1·73–2·01)	0·33 (0·28–0·4)	1·0 (0·9–1·09)
2017	0·3 (0·1–0·6)	12·4 (10·3–14·9)	53·7 (43·8–65·4)	107·4 (97·8–117·5)	111·8 (98·6–125·8)	70·1 (56·8–87·0)	19·8 (14·6–26·3)	0·6 (0·3–0·9)	0·0 (0·0–0·0)	1·88 (1·74–2·03)	0·33 (0·27–0·4)	1·01 (0·91–1·12)

Supplemental Results Table 5: Age-specific fertility rates per 1000, cumulative total fertility up to maternal age 25 (TFU25), cumulative total fertility from maternal age 30 to age 55 (TFO30), and cumulative total fertility, Japan, 1950-2017

Year	10-14	15-19	20-24	25-29	30-34	35-39	40-44	45-49	50-54	TFR	TFU25	TFO30
1950	0·0 (0·0–0·0)	11·2 (9·9–12·5)	145·8 (133·2–159·6)	218·6 (210·7–226·6)	162·7 (153·1–172·5)	93·6 (83·2–104·0)	31·1 (26·9–35·7)	1·4 (0·9–2·0)	0·0 (0·0–0·0)	3·32 (3·21–3·44)	0·79 (0·72–0·85)	1·44 (1·37–1·52)
1951	0·0 (0·0–0·0)	9·0 (8·0–10·1)	130·6 (118·7–142·9)	202·9 (195·5–210·4)	148·1 (138·4–157·1)	79·2 (69·8–88·3)	24·3 (20·6–28·4)	1·0 (0·7–1·5)	0·0 (0·0–0·0)	2·98 (2·84–3·1)	0·7 (0·64–0·76)	1·26 (1·15–1·37)
1952	0·0 (0·0–0·0)	7·6 (6·7–8·6)	121·3 (110·3–133·7)	192·6 (184·7–200·2)	134·7 (125·7–144·5)	67·9 (59·8–76·6)	19·4 (16·4–22·5)	0·8 (0·5–1·2)	0·0 (0·0–0·0)	2·72 (2·62–2·82)	0·64 (0·59–0·71)	1·11 (1·05–1·18)
1953	0·0 (0·0–0·0)	6·7 (5·9–7·5)	114·2 (103·5–125·7)	181·3 (173·7–189·2)	120·9 (111·9–129·9)	57·9 (50·7–65·1)	15·5 (13·2–18·3)	0·7 (0·4–1·0)	0·0 (0·0–0·0)	2·49 (2·4–2·59)	0·6 (0·55–0·66)	0·98 (0·92–1·04)
1954	0·0 (0·0–0·0)	6·1 (5·3–6·9)	110·3 (99·0–120·9)	176·2 (169·0–184·1)	111·9 (103·7–120·1)	51·1 (44·5–58·6)	12·9 (10·6–15·5)	0·6 (0·3–0·8)	0·0 (0·0–0·0)	2·35 (2·26–2·43)	0·58 (0·53–0·64)	0·88 (0·83–0·94)
1955	0·0 (0·0–0·0)	5·5 (4·8–6·3)	107·2 (97·2–118·6)	174·9 (166·6–182·8)	103·9 (95·8–112·7)	44·7 (38·7–51·5)	11·0 (9·0–13·3)	0·4 (0·3–0·7)	0·0 (0·0–0·0)	2·24 (2·15–2·32)	0·56 (0·51–0·62)	0·8 (0·75–0·85)
1956	0·0 (0·0–0·0)	4·9 (4·2–5·7)	100·5 (90·2–111·5)	168·8 (161·7–176·2)	92·5 (85·1–100·0)	37·9 (32·8–43·7)	8·9 (7·3–10·9)	0·4 (0·2–0·6)	0·0 (0·0–0·0)	2·07 (1·99–2·15)	0·53 (0·47–0·58)	0·7 (0·65–0·75)
1957	0·0 (0·0–0·0)	4·7 (4·0–5·5)	101·2 (91·0–112·5)	172·0 (164·6–179·9)	87·1 (79·9–95·2)	33·1 (28·0–38·0)	7·5 (6·0–9·1)	0·4 (0·2–0·6)	0·0 (0·0–0·0)	2·03 (1·93–2·13)	0·53 (0·48–0·59)	0·64 (0·6–0·69)
1958	0·0 (0·0–0·0)	4·7 (4·1–5·5)	105·9 (95·4–117·5)	177·4 (169·4–185·3)	84·8 (77·9–91·8)	29·7 (25·5–34·1)	6·7 (5·4–8·2)	0·4 (0·2–0·6)	0·0 (0·0–0·0)	2·05 (1·97–2·13)	0·55 (0·5–0·61)	0·61 (0·57–0·65)
1959	0·0 (0·0–0·0)	4·9 (4·2–5·7)	105·5 (95·1–116·8)	176·1 (168·7–184·2)	79·8 (72·8–87·0)	25·3 (21·5–29·4)	5·6 (4·4–7·0)	0·3 (0·2–0·5)	0·0 (0·0–0·0)	1·99 (1·91–2·07)	0·55 (0·5–0·61)	0·55 (0·51–0·6)
1960	0·0 (0·0–0·0)	4·8 (4·2–5·5)	101·1 (90·8–112·0)	176·7 (169·4–184·5)	77·3 (70·9–84·0)	21·9 (18·5–25·1)	4·7 (3·7–5·8)	0·3 (0·2–0·4)	0·0 (0·0–0·0)	1·93 (1·86–2·01)	0·53 (0·48–0·58)	0·52 (0·48–0·56)
1961	0·0 (0·0–0·0)	4·5 (3·8–5·2)	97·8 (88·2–107·4)	178·4 (170·5–186·4)	76·0 (69·5–83·0)	19·5 (16·6–23·0)	4·1 (3·1–5·2)	0·2 (0·1–0·3)	0·0 (0·0–0·0)	1·9 (1·83–1·98)	0·51 (0·46–0·56)	0·5 (0·46–0·54)
1962	0·0 (0·0–0·0)	4·3 (3·6–5·0)	97·2 (86·6–108·2)	183·5 (176·1–191·0)	77·8 (71·5–84·1)	18·5 (15·7–21·5)	3·6 (2·8–4·7)	0·2 (0·1–0·3)	0·0 (0·0–0·0)	1·93 (1·85–2·0)	0·51 (0·45–0·56)	0·5 (0·46–0·54)
1963	0·0 (0·0–0·0)	4·2 (3·6–4·9)	97·4 (87·5–107·9)	185·5 (177·9–193·1)	78·6 (72·0–85·8)	18·4 (15·6–21·7)	3·4 (2·6–4·5)	0·2 (0·1–0·3)	0·0 (0·0–0·0)	1·94 (1·87–2·02)	0·51 (0·46–0·56)	0·5 (0·47–0·54)
1964	0·0 (0·0–0·0)	4·2 (3·6–4·8)	106·2 (96·6–117·4)	197·4 (189·6–205·4)	84·1 (77·2–91·7)	19·1 (16·1–22·2)	3·2 (2·5–4·2)	0·2 (0·1–0·3)	0·0 (0·0–0·0)	2·07 (2·0–2·15)	0·55 (0·5–0·61)	0·53 (0·5–0·57)
1965	0·0 (0·0–0·0)	4·1 (3·5–4·7)	97·8 (88·2–108·5)	166·6 (158·9–174·8)	71·0 (65·0–77·3)	17·0 (14·4–20·0)	2·9 (2·2–3·7)	0·2 (0·1–0·2)	0·0 (0·0–0·0)	1·8 (1·73–1·87)	0·51 (0·46–0·56)	0·46 (0·42–0·49)
1966	0·0 (0·0–0·0)	4·3 (3·7–5·0)	95·7 (86·4–105·6)	171·4 (163·6–178·8)	72·6 (66·0–79·4)	17·6 (14·9–20·6)	2·8 (2·1–3·7)	0·2 (0·1–0·2)	0·0 (0·0–0·0)	1·82 (1·75–1·9)	0·5 (0·45–0·55)	0·47 (0·43–0·5)
1967	0·0 (0·0–0·0)	4·6 (3·9–5·3)	102·4 (92·5–112·8)	208·9 (201·2–216·5)	88·7 (81·6–96·3)	19·9 (17·0–23·2)	2·8 (2·1–3·6)	0·2 (0·1–0·2)	0·0 (0·0–0·0)	2·14 (2·04–2·23)	0·53 (0·48–0·59)	0·56 (0·52–0·6)
1968	0·0 (0·0–0·0)	4·7 (4·1–5·4)	92·1 (82·7–102·9)	197·0 (188·9–205·0)	83·2 (76·0–90·3)	19·4 (16·5–22·5)	2·6 (2·0–3·5)	0·1 (0·1–0·2)	0·0 (0·0–0·0)	2·0 (1·92–2·07)	0·48 (0·44–0·54)	0·53 (0·49–0·57)
1969	0·0 (0·0–0·0)	5·1 (4·4–5·9)	90·9 (81·1–101·0)	199·3 (191·4–207·0)	83·0 (76·2–89·9)	19·2 (16·4–22·6)	2·6 (2·0–3·4)	0·1 (0·1–0·2)	0·0 (0·0–0·0)	2·0 (1·93–2·08)	0·48 (0·43–0·53)	0·52 (0·49–0·56)
1970	0·0 (0·0–0·0)	5·2 (4·5–6·0)	98·1 (87·6–109·7)	203·0 (195·2–210·8)	83·7 (77·3–90·7)	19·1 (16·2–22·3)	2·6 (2·0–3·3)	0·1 (0·1–0·2)	0·0 (0·0–0·0)	2·06 (1·98–2·14)	0·52 (0·47–0·57)	0·53 (0·49–0·57)
1971	0·0 (0·0–0·0)	5·1 (4·3–5·8)	107·7 (96·3–118·6)	200·5 (192·6–208·6)	84·1 (77·1–91·5)	18·6 (15·8–21·9)	2·7 (2·0–3·5)	0·1 (0·1–0·2)	0·0 (0·0–0·0)	2·09 (2·01–2·18)	0·56 (0·51–0·62)	0·53 (0·49–0·57)
1972	0·0 (0·0–0·0)	5·1 (4·4–5·9)	112·7 (102·1–124·2)	199·0 (191·9–206·5)	84·3 (76·7–91·6)	18·1 (15·4–21·1)	2·7 (2·0–3·5)	0·1 (0·1–0·2)	0·0 (0·0–0·0)	2·11 (2·03–2·19)	0·59 (0·54–0·65)	0·53 (0·49–0·57)
1973	0·0 (0·0–0·0)	5·0 (4·3–5·8)	112·9 (102·1–123·8)	198·6 (190·5–206·9)	80·7 (74·2–87·8)	17·3 (14·6–20·5)	2·5 (1·8–3·2)	0·1 (0·1–0·2)	0·0 (0·0–0·0)	2·09 (2·01–2·16)	0·59 (0·54–0·64)	0·5 (0·47–0·54)
1974	0·0 (0·0–0·0)	4·6 (4·0–5·3)	107·5 (97·1–118·7)	190·1 (181·9–198·0)	72·3 (65·8–79·1)	15·4 (13·0–18·1)	2·2 (1·6–2·8)	0·1 (0·1–0·2)	0·0 (0·0–0·0)	1·96 (1·89–2·04)	0·56 (0·51–0·62)	0·45 (0·41–0·49)
1975	0·0 (0·0–0·0)	4·2 (3·6–4·9)	99·4 (89·0–110·2)	182·1 (174·2–189·7)	65·8 (59·8–71·7)	14·1 (12·0–16·6)	1·9 (1·4–2·5)	0·1 (0·0–0·1)	0·0 (0·0–0·0)	1·84 (1·76–1·91)	0·52 (0·47–0·57)	0·41 (0·38–0·44)
1976	0·0 (0·0–0·0)	3·9 (3·3–4·6)	92·6 (83·0–103·3)	178·9 (170·9–186·8)	64·9 (59·4–70·8)	13·8 (11·7–16·2)	1·8 (1·3–2·4)	0·1 (0·0–0·1)	0·0 (0·0–0·0)	1·78 (1·71–1·86)	0·48 (0·44–0·54)	0·4 (0·37–0·44)
1977	0·0 (0·0–0·0)	3·8 (3·2–4·4)	86·2 (77·5–97·1)	179·0 (171·4–186·6)	67·8 (61·5–74·1)	13·6 (11·3–16·0)	1·8 (1·3–2·4)	0·1 (0·0–0·1)	0·0 (0·0–0·0)	1·76 (1·69–1·84)	0·45 (0·41–0·5)	0·42 (0·37–0·46)
1978	0·0 (0·0–0·0)	3·7 (3·2–4·4)	81·1 (72·7–90·6)	179·3 (171·9–186·4)	71·6 (65·2–78·1)	13·3 (11·3–15·6)	1·7 (1·3–2·3)	0·1 (0·0–0·1)	0·0 (0·0–0·0)	1·75 (1·69–1·82)	0·42 (0·38–0·47)	0·43 (0·4–0·47)
1979	0·0 (0·0–0·0)	3·8 (3·2–4·4)	76·6 (68·7–84·8)	177·5 (170·1–184·7)	71·9 (65·8–78·6)	12·9 (10·8–15·3)	1·7 (1·3–2·5)	0·1 (0·0–0·1)	0·0 (0·0–0·0)	1·72 (1·66–1·79)	0·4 (0·36–0·44)	0·43 (0·4–0·47)
1980	0·0 (0·0–0·0)	3·8 (3·2–4·4)	72·6 (64·7–80·8)	175·3 (167·5–183·4)	70·5 (64·3–76·7)	12·8 (10·7–15·1)	1·8 (1·3–2·3)	0·1 (0·0–0·1)	0·0 (0·0–0·0)	1·68 (1·62–1·75)	0·38 (0·34–0·42)	0·43 (0·39–0·46)
1981	0·0 (0·0–0·0)	4·0 (3·4–4·6)	70·3 (62·9–79·0)	176·4 (168·9–183·9)	71·3 (65·3–77·5)	13·4 (11·4–15·8)	1·8 (1·3–2·4)	0·1 (0·0–0·1)	0·0 (0·0–0·0)	1·69 (1·63–1·74)	0·37 (0·33–0·41)	0·43 (0·4–0·46)
1982	0·0 (0·0–0·0)	4·1 (3·5–4·8)	69·1 (61·0–77·4)	178·2 (170·8–186·3)	74·8 (68·8–81·3)	15·1 (12·7–17·6)	1·8 (1·3–2·4)	0·1 (0·0–0·1)	0·0 (0·0–0·0)	1·72 (1·66–1·78)	0·37 (0·33–0·41)	0·46 (0·43–0·5)
1983	0·0 (0·0–0·0)	4·2 (3·6–4·9)	67·3 (60·0–75·6)	178·4 (170·3–186·1)	79·0 (72·7–86·4)	16·7 (14·0–19·6)	1·8 (1·3–2·4)	0·1 (0·0–0·1)	0·0 (0·0–0·0)	1·74 (1·68–1·81)	0·36 (0·32–0·4)	0·49 (0·45–0·53)
1984	0·0 (0·0–0·0)	4·1 (3·5–4·8)	62·7 (55·6–70·8)	175·2 (167·9–183·2)	82·2 (75·4–89·5)	17·2 (14·7–20·1)	1·8 (1·4–2·4)	0·1 (0·0–0·1)	0·0 (0·0–0·0)	1·72 (1·66–1·78)	0·33 (0·3–0·38)	0·51 (0·47–0·55)
1985	0·0 (0·0–0·0)	3·9 (3·4–4·6)	58·9 (51·9–66·1)	168·7 (160·8–175·6)	83·8 (76·6–90·7)	17·1 (14·5–20·0)	1·9 (1·4–2·5)	0·1 (0·0–0·1)	0·0 (0·0–0·0)	1·67 (1·62–1·73)	0·31 (0·28–0·35)	0·51 (0·48–0·55)
1986	0·0 (0·0–0·0)	3·8 (3·3–4·4)	56·2 (49·5–63·0)	162·8 (155·5–170·5)	86·1 (78·8–92·7)	17·2 (14·6–20·1)	1·9 (1·4–2·6)	0·1 (0·0–0·1)	0·0 (0·0–0·0)	1·64 (1·59–1·7)	0·3 (0·27–0·33)	0·53 (0·49–0·56)
1987	0·0 (0·0–0·0)	3·7 (3·1–4·3)	51·9 (46·1–58·4)	157·4 (149·9–164·8)	88·7 (82·1–96·6)	17·8 (15·2–20·9)	2·2 (1·6–2·8)	0·1 (0·0–0·1)	0·0 (0·0–0·0)	1·61 (1·54–1·68)	0·28 (0·25–0·31)	0·54 (0·51–0·58)
1988	0·0 (0·0–0·0)	3·6 (3·0–4·1)	48·2 (42·6–54·5)	147·6 (140·6–155·0)	89·6 (82·3–97·3)	18·8 (16·1–21·7)	2·3 (1·7–3·1)	0·1 (0·0–0·1)	0·0 (0·0–0·0)	1·55 (1·49–1·61)	0·26 (0·23–0·29)	0·55 (0·51–0·6)
1989	0·0 (0·0–0·0)	3·6 (3·0–4·2)	44·7 (39·1–50·8)	137·5 (130·9–144·9)	89·2 (81·9–96·5)	19·8 (16·8–23·3)	2·4 (1·8–3·2)	0·1 (0·0–0·1)	0·0 (0·0–0·0)	1·49 (1·42–1·54)	0·24 (0·21–0·27)	0·56 (0·52–0·6)
1990	0·0 (0·0–0·0)	3·7 (3·1–4·2)	43·1 (37·9–48·1)	134·0 (127·1–140·6)	90·6 (83·5–97·9)	20·7 (17·5–24·1)	2·4 (1·8–3·1)	0·1 (0·0–0·1)	0·0 (0·0–0·0)	1·47 (1·41–1·54)	0·23 (0·21–0·26)	0·57 (0·53–0·61)
1991	0·0 (0·0–0·0)	3·8 (3·2–4·4)	43·0 (38·1–48·1)	130·2 (123·6–137·3)	91·1 (83·8–98·9)	21·8 (18·7–25·6)	2·4 (1·8–3·2)	0·1 (0·0–0·1)	0·0 (0·0–0·0)	1·46 (1·41–1·52)	0·23 (0·21–0·26)	0·58 (0·54–0·62)
1992	0·0 (0·0–0·0)	3·8 (3·3–4·5)	41·5 (36·8–46·5)	122·4 (115·9–129·0)	89·8 (82·8–97·6)	22·5 (19·1–26·2)	2·5 (1·9–3·3)	0·1 (0·0–0·1)	0·0 (0·0–0·0)	1·41 (1·36–1·47)	0·23 (0·2–0·25)	0·57 (0·54–0·62)
1993	0·0 (0·0–0·0)	3·9 (3·3–4·6)	40·9 (36·1–46·2)	120·2 (113·6–127·3)	92·5 (85·3–100·0)	24·2 (20·7–28·2)	2·7 (2·0–3·5)	0·1 (0·1–0·1)	0·0 (0·0–0·0)	1·42 (1·36–1·48)	0·22 (0·2–0·25)	0·6 (0·56–0·64)
1994	0·0 (0·0–0·0)	3·9 (3·3–4·6)	40·2 (35·6–45·4)	116·0 (109·6–122·2)	92·7 (85·3–100·6)	25·4 (21·5–29·5)	2·8 (2·1–3·7)	0·1 (0·1–0·1)	0·0 (0·0–0·0)	1·41 (1·34–1·47)	0·22 (0·2–0·25)	0·6 (0·56–0·65)
1995	0·0 (0·0–0·0)	3·9 (3·3–4·5)	39·1 (34·6–43·9)	109·3 (103·1–115·2)	91·8 (84·2–99·2)	26·2 (22·5–30·2)	3·0 (2·3–3·8)	0·1 (0·1–0·1)	0·0 (0·0–0·0)	1·37 (1·31–1·42)	0·22 (0·19–0·24)	0·61 (0·56–0·65)
1996	0·0 (0·0–0·0)	4·1 (3·5–4·8)	38·8 (33·8–43·5)	106·1 (100·2–112·0)	92·1 (85·0–100·1)	27·4 (23·2–32·1)	3·1 (2·4–3·9)	0·1 (0·1–0·1)	0·0 (0·0–0·0)	1·36 (1·3–1·41)	0·21 (0·19–0·24)	0·61 (0·57–0·66)
1997	0·0 (0·0–0·0)	4·3 (3·7–5·0)	38·7 (34·0–43·4)	102·8 (97·0–108·5)	90·7 (83·7–98·2)	27·9 (23·8–32·5)	3·3 (2·6–4·3)	0·1 (0·1–0·1)	0·0 (0·0–0·0)	1·34 (1·29–1·4)	0·21 (0·19–0·24)	0·61 (0·57–0·66)
1998	0·0 (0·0–0·0)	4·6 (3·9–5·3)	38·5 (34·2–43·5)	99·1 (93·6–104·6)	89·8 (82·7–98·1)	29·1 (24·6–34·0)	3·5 (2·7–4·4)	0·1 (0·1–0·2)	0·0 (0·0–0·0)	1·32 (1·27–1·38)	0·22 (0·19–0·24)	0·61 (0·57–0·66)
1999	0·0 (0·0–0·0)	4·9 (4·2–5·6)	38·3 (33·8–43·2)	96·2 (90·4–101·6)	89·3 (82·3–97·1)	29·9 (25·4–34·6)	3·7 (2·8–4·7)	0·1 (0·1–0·2)	0·0 (0·0–0·0)	1·31 (1·25–1·37)	0·22 (0·19–0·24)	0·61 (0·57–0·66)
2000	0·0 (0·0–0·0)	5·2 (4·5–6·1)	38·6 (34·0–43·3)	94·3 (88·9–100·1)	87·8 (81·2–95·2)	31·4 (26·9–35·8)	3·9 (3·0–4·9)	0·1 (0·1–0·2)	0·0 (0·0–0·0)	1·31 (1·25–1·37)	0·22 (0·2–0·24)	0·62 (0·57–0·66)
2001	0·0 (0·0–0·0)	5·5 (4·7–6·4)	38·8 (34·5–43·7)	91·6 (86·6–97·5)	85·1 (77·9–92·5)	31·5 (26·8–36·6)	4·0 (3·2–5·1)	0·1 (0·1–0·2)	0·0 (0·0–0·0)	1·28 (1·23–1·34)	0·22 (0·2–0·25)	0·6 (0·56–0·65)
2002	0·0 (0·0–0·0)	5·4 (4·7–6·3)	38·0 (33·3–43·2)	89·1 (83·7–94·6)	84·8 (78·0–92·5)	32·1 (27·6–37·1)	4·3 (3·3–5·5)	0·1 (0·1–0·2)	0·0 (0·0–0·0)	1·27 (1·21–1·33)	0·22 (0·19–0·24)	0·61 (0·56–0·65)
2003	0·0 (0·0–0·0)	5·2 (4·5–6·1)	36·6 (32·3–41·5)	86·8 (81·6–91·9)	84·3 (77·7–91·3)	33·6 (28·8–38·9)	4·6 (3·6–5·7)	0·1 (0·1–0·2)	0·0 (0·0–0·0)	1·26 (1·2–1·32)	0·21 (0·19–0·23)	0·61 (0·57–0·66)
2004	0·0 (0·0–0·0)	5·0 (4·3–5·8)	35·5 (31·3–40·5)	83·3 (78·3–88·6)	83·1 (76·6–90·0)	34·5 (29·6–40·1)	4·8 (3·8–5·9)	0·1 (0·1–0·2)	0·0 (0·0–0·0)	1·23 (1·18–1·28)	0·2 (0·18–0·23)	0·61 (0·57–0·66)
2005	0·0 (0·0–0·0)	4·9 (4·2–5·7)	35·3 (31·0–40·0)	82·3 (77·3–87·7)	83·9 (76·9–91·0)	35·6 (30·6–41·1)	5·1 (4·1–6·3)	0·1 (0·1–0·2)	0·0 (0·0–0·0)	1·24 (1·18–1·29)	0·2 (0·18–0·22)	0·62 (0·58–0·67)
2006	0·0 (0·0–0·0)	4·9 (4·2–5·6)	35·9 (31·6–40·2)	83·2 (78·1–88·5)	87·0 (80·0–94·5)	37·6 (32·5–43·9)	5·6 (4·6–6·8)	0·1 (0·1–0·2)	0·0 (0·0–0·0)	1·27 (1·22–1·33)	0·2 (0·18–0·23)	0·65 (0·61–0·7)
2007	0·0 (0·0–0·0)	4·9 (4·2–5·6)	35·9 (31·7–40·4)	82·9 (78·1–88·5)	87·8 (80·4–95·4)	39·9 (34·8–46·2)	6·1 (4·9–7·4)	0·1 (0·1–0·2)	0·0 (0·0–0·0)	1·29 (1·23–1·34)	0·2 (0·18–0·23)	0·67 (0·62–0·72)
2008	0·0 (0·0–0·0)	4·8 (4·1–5·5)	35·5 (31·2–40·2)	82·6 (77·5–87·8)	88·8 (81·8–95·7)	41·6 (36·0–47·5)	6·6 (5·4–8·0)	0·2 (0·1–0·2)	0·0 (0·0–0·0)	1·3 (1·25–1·36)	0·2 (0·18–0·23)	0·69 (0·64–0·73)
2009	0·0 (0·0–0·0)	4·6 (3·9–5·4)	34·5 (30·4–38·9)	82·5 (77·6–87·6)	89·9 (82·7–97·8)	43·3 (37·4–49·6)	7·3 (5·9–8·9)	0·2 (0·1–0·3)	0·0 (0·0–0·0)	1·31 (1·24–1·38)	0·2 (0·17–0·22)	0·7 (0·66–0·76)
2010	0·0 (0·0–0·0)	4·4 (3·8–5·2)	33·5 (29·2–37·8)	83·1 (78·2–88·0)	91·5 (84·1–99·8)	44·7 (38·9–50·9)	7·8 (6·3–9·4)	0·2 (0·1–0·3)	0·0 (0·0–0·0)	1·33 (1·27–1·39)	0·19 (0·17–0·21)	0·72 (0·67–0·77)
2011	0·0 (0·0–0·0)	4·3 (3·6–5·0)	31·9 (28·2–36·0)	82·7 (77·5–88·1)	92·1 (84·9–99·9)	46·1 (39·7–52·8)	8·2 (6·7–10·0)	0·2 (0·1–0·3)	0·0 (0·0–0·0)	1·33 (1·27–1·38)	0·18 (0·16–0·2)	0·73 (0·69–0·78)
2012	0·0 (0·0–0·0)	4·2 (3·6–4·9)	30·4 (26·9–34·4)	82·1 (77·3–87·1)	94·0 (86·4–102·3)	48·7 (42·4–55·1)	9·0 (7·4–10·9)	0·2 (0·1–0·3)	0·0 (0·0–0·0)	1·34 (1·29–1·4)	0·17 (0·16–0·19)	0·76 (0·71–0·82)
2013	0·0 (0·0–0·0)	4·1 (3·5–4·8)	29·3 (25·7–33·2)	80·7 (75·7–85·9)	94·8 (87·3–102·5)	50·3 (43·9–57·1)	9·6 (8·0–11·8)	0·2 (0·1–0·3)	0·0 (0·0–0·0)	1·35 (1·28–1·41)	0·17 (0·15–0·19)	0·77 (0·72–0·83)
2014	0·0 (0·0–0·0)	4·0 (3·4–4·7)	28·2 (24·6–32·0)	80·0 (75·1–85·3)	96·2 (88·9–104·2)	52·6 (46·0–60·0)	10·2 (8·6–12·2)	0·2 (0·1–0·4)	0·0 (0·0–0·0)	1·36 (1·29–1·43)	0·16 (0·14–0·18)	0·8 (0·74–0·86)
2015	0·0 (0·0–0·0)	4·0 (3·4–4·8)	28·6 (24·2–33·8)	81·3 (75·0–88·2)	98·7 (89·4–108·8)	51·4 (43·1–60·8)	9·5 (7·6–11·8)	0·2 (0·1–0·3)	0·0 (0·0–0·0)	1·37 (1·22–1·53)	0·16 (0·14–0·19)	0·8 (0·71–0·9)
2016	0·0 (0·0–0·0)	4·1 (3·4–5·0)	29·7 (23·6–37·6)	82·2 (73·7–92·4)	98·2 (85·6–113·1)	47·5 (37·1–60·7)	8·2 (6·1–10·9)	0·2 (0·1–0·3)	0·0 (0·0–0·0)	1·35 (1·15–1·58)	0·17 (0·13–0·21)	0·77 (0·65–0·91)
2017	0·0 (0·0–0·0)	4·0 (3·3–4·9)	29·6 (23·1–37·6)	81·9 (72·7–92·2)	96·7 (82·6–112·5)	46·3 (35·2–59·9)	8·0 (5·6–11·1)	0·2 (0·1–0·3)	0·0 (0·0–0·0)	1·33 (1·11–1·59)	0·17 (0·13–0·21)	0·76 (0·62–0·92)

Supplemental Results Table 5: Age-specific fertility rates per 1000, cumulative total fertility up to maternal age 25 (TFU25), cumulative total fertility from maternal age 30 to age 55 (TFO30), and cumulative total fertility, Aichi, 1950-2017

Year	10-14	15-19	20-24	25-29	30-34	35-39	40-44	45-49	50-54	TFR	TFU25	TFO30
1950	0·0 (0·0–0·0)	11·9 (10·5–13·3)	153·1 (139·5–167·7)	229·9 (221·3–238·6)	155·7 (146·5–165·0)	84·4 (74·9–93·7)	30·7 (26·5–35·2)	1·7 (1·1–2·4)	0·0 (0·0–0·0)	3·34 (3·22–3·45)	0·83 (0·76–0·9)	1·36 (1·29–1·43)
1951	0·0 (0·0–0·0)	9·5 (8·5–10·7)	137·3 (124·5–150·6)	214·0 (205·8–222·0)	140·7 (131·3–149·8)	68·1 (59·4–76·9)	23·7 (20·1–27·9)	1·2 (0·8–1·8)	0·0 (0·0–0·0)	2·97 (2·85–3·1)	0·73 (0·67–0·8)	1·17 (1·07–1·27)
1952	0·0 (0·0–0·0)	8·0 (7·1–9·1)	127·8 (115·6–142·0)	203·6 (194·9–212·3)	127·2 (117·0–137·3)	54·5 (46·9–63·6)	18·8 (15·5–22·2)	0·9 (0·6–1·4)	0·0 (0·0–0·0)	2·7 (2·6–2·82)	0·68 (0·62–0·75)	1·01 (0·94–1·08)
1953	0·0 (0·0–0·0)	7·0 (6·1–8·1)	120·7 (107·6–135·7)	191·9 (183·4–200·4)	113·5 (103·5–124·0)	44·7 (37·5–52·6)	14·9 (12·0–18·0)	0·8 (0·5–1·2)	0·0 (0·0–0·0)	2·47 (2·35–2·59)	0·64 (0·57–0·71)	0·87 (0·8–0·94)
1954	0·0 (0·0–0·0)	6·5 (5·5–7·4)	117·0 (103·5–130·8)	186·8 (179·0–194·9)	104·5 (95·7–114·5)	39·9 (33·4–47·1)	12·3 (9·9–15·2)	0·6 (0·4–0·9)	0·0 (0·0–0·0)	2·34 (2·22–2·45)	0·62 (0·55–0·69)	0·79 (0·72–0·86)
1955	0·0 (0·0–0·0)	5·8 (5·0–6·7)	114·2 (99·7–128·9)	185·7 (177·3–194·2)	96·0 (86·9–105·5)	35·9 (30·2–41·9)	10·3 (7·9–12·9)	0·5 (0·3–0·8)	0·0 (0·0–0·0)	2·24 (2·12–2·36)	0·6 (0·53–0·68)	0·71 (0·65–0·78)
1956	0·0 (0·0–0·0)	5·2 (4·5–6·1)	107·6 (95·0–120·6)	179·6 (171·8–187·9)	85·0 (76·7–94·0)	30·8 (25·9–36·2)	8·2 (6·4–10·5)	0·4 (0·3–0·6)	0·0 (0·0–0·0)	2·08 (1·98–2·19)	0·56 (0·5–0·63)	0·62 (0·57–0·68)
1957	0·0 (0·0–0·0)	5·0 (4·3–5·9)	108·8 (96·7–123·0)	183·1 (175·0–191·6)	80·5 (72·9–88·8)	26·4 (21·7–30·9)	7·0 (5·4–8·9)	0·4 (0·3–0·7)	0·0 (0·0–0·0)	2·06 (1·94–2·17)	0·57 (0·51–0·64)	0·57 (0·52–0·62)
1958	0·0 (0·0–0·0)	5·0 (4·3–5·8)	114·4 (100·3–128·8)	189·1 (180·2–198·1)	78·7 (71·5–86·8)	23·2 (18·9–27·7)	6·4 (5·0–8·0)	0·4 (0·2–0·7)	0·0 (0·0–0·0)	2·09 (1·98–2·19)	0·6 (0·53–0·67)	0·54 (0·5–0·59)
1959	0·0 (0·0–0·0)	5·2 (4·5–6·1)	114·7 (101·8–129·5)	188·2 (179·7–197·2)	73·6 (65·6–81·6)	20·0 (16·1–24·5)	5·3 (4·1–6·9)	0·4 (0·2–0·6)	0·0 (0·0–0·0)	2·04 (1·94–2·14)	0·6 (0·53–0·67)	0·5 (0·45–0·54)
1960	0·0 (0·0–0·0)	5·1 (4·4–6·0)	110·6 (98·0–124·1)	189·3 (180·8–198·1)	70·7 (63·3–78·2)	17·6 (14·4–21·0)	4·4 (3·3–5·6)	0·3 (0·2–0·4)	0·0 (0·0–0·0)	1·99 (1·9–2·08)	0·58 (0·52–0·64)	0·46 (0·42–0·51)
1961	0·0 (0·0–0·0)	4·8 (4·1–5·6)	107·8 (97·0–119·2)	191·6 (183·4–200·3)	69·1 (63·6–75·3)	15·8 (13·3–18·7)	3·8 (2·8–5·0)	0·2 (0·1–0·4)	0·0 (0·0–0·0)	1·97 (1·89–2·05)	0·56 (0·51–0·62)	0·44 (0·41–0·48)
1962	0·0 (0·0–0·0)	4·6 (3·9–5·4)	108·0 (96·0–120·2)	197·5 (189·2–205·6)	70·6 (64·8–76·7)	14·9 (12·1–18·0)	3·4 (2·5–4·4)	0·2 (0·1–0·4)	0·0 (0·0–0·0)	2·0 (1·91–2·08)	0·56 (0·5–0·62)	0·45 (0·41–0·48)
1963	0·0 (0·0–0·0)	4·5 (3·8–5·3)	109·0 (97·9–120·9)	199·6 (191·4–208·1)	72·1 (65·3–79·1)	14·4 (12·2–16·9)	3·4 (2·5–4·5)	0·2 (0·1–0·4)	0·0 (0·0–0·0)	2·02 (1·94–2·1)	0·57 (0·51–0·63)	0·45 (0·41–0·49)
1964	0·0 (0·0–0·0)	4·5 (3·8–5·3)	119·6 (107·5–133·1)	212·8 (203·8–221·5)	76·9 (69·8–84·3)	14·9 (12·4–17·4)	3·2 (2·4–4·2)	0·2 (0·1–0·4)	0·0 (0·0–0·0)	2·16 (2·07–2·25)	0·62 (0·56–0·69)	0·48 (0·44–0·52)
1965	0·0 (0·0–0·0)	4·4 (3·8–5·1)	110·8 (97·9–123·5)	179·9 (171·2–188·7)	63·9 (58·0–69·5)	13·5 (11·3–16·2)	2·8 (2·1–3·6)	0·2 (0·1–0·3)	0·0 (0·0–0·0)	1·88 (1·79–1·97)	0·58 (0·51–0·64)	0·4 (0·37–0·43)
1966	0·0 (0·0–0·0)	4·7 (4·0–5·4)	108·9 (96·5–122·5)	185·2 (176·6–193·3)	65·4 (59·2–72·6)	13·7 (11·3–16·7)	2·8 (2·1–3·7)	0·2 (0·1–0·3)	0·0 (0·0–0·0)	1·9 (1·82–2·0)	0·57 (0·51–0·63)	0·41 (0·37–0·45)
1967	0·0 (0·0–0·0)	4·9 (4·2–5·7)	116·9 (105·5–130·0)	225·9 (217·8–234·2)	78·8 (71·4–86·8)	15·5 (12·6–19·1)	2·7 (2·0–3·6)	0·2 (0·1–0·3)	0·0 (0·0–0·0)	2·22 (2·12–2·33)	0·61 (0·55–0·67)	0·49 (0·45–0·53)
1968	0·0 (0·0–0·0)	5·1 (4·4–5·8)	105·5 (93·9–118·1)	213·5 (204·5–222·1)	71·7 (64·8–78·8)	15·4 (12·6–18·7)	2·5 (1·8–3·3)	0·2 (0·1–0·2)	0·0 (0·0–0·0)	2·07 (1·98–2·16)	0·55 (0·5–0·61)	0·45 (0·41–0·49)
1969	0·0 (0·0–0·0)	5·5 (4·7–6·5)	104·5 (93·0–116·5)	215·8 (207·0–224·4)	71·9 (65·5–79·0)	15·1 (12·5–18·3)	2·5 (1·8–3·4)	0·1 (0·1–0·2)	0·0 (0·0–0·0)	2·08 (1·99–2·17)	0·55 (0·49–0·61)	0·45 (0·41–0·49)
1970	0·0 (0·0–0·0)	5·6 (4·6–6·7)	113·2 (100·2–127·2)	219·1 (211·0–227·1)	74·1 (67·0–81·1)	15·4 (12·6–18·6)	2·7 (1·9–3·7)	0·2 (0·1–0·3)	0·0 (0·0–0·0)	2·15 (2·06–2·24)	0·59 (0·53–0·66)	0·46 (0·42–0·5)
1971	0·0 (0·0–0·0)	5·5 (4·5–6·6)	124·6 (110·2–138·5)	215·7 (206·0–226·0)	75·2 (68·3–82·1)	16·2 (13·3–19·7)	3·0 (2·1–4·1)	0·2 (0·1–0·3)	0·0 (0·0–0·0)	2·2 (2·1–2·3)	0·65 (0·58–0·72)	0·47 (0·43–0·51)
1972	0·0 (0·0–0·0)	5·5 (4·5–6·6)	130·8 (117·6–145·4)	213·3 (205·5–222·0)	75·4 (68·2–82·5)	16·6 (13·8–19·5)	3·0 (2·1–4·1)	0·2 (0·1–0·3)	0·0 (0·0–0·0)	2·22 (2·14–2·32)	0·68 (0·61–0·75)	0·48 (0·43–0·51)
1973	0·0 (0·0–0·0)	5·3 (4·3–6·4)	131·0 (118·0–144·5)	212·1 (202·8–221·0)	71·6 (65·7–78·1)	15·0 (12·1–18·5)	2·7 (1·9–3·7)	0·2 (0·1–0·3)	0·0 (0·0–0·0)	2·19 (2·1–2·28)	0·68 (0·62–0·75)	0·45 (0·41–0·48)
1974	0·0 (0·0–0·0)	4·9 (4·0–5·9)	124·3 (111·7–137·7)	202·0 (192·9–210·8)	63·3 (57·5–69·6)	12·2 (10·0–15·0)	2·2 (1·6–3·2)	0·1 (0·1–0·2)	0·0 (0·0–0·0)	2·05 (1·97–2·13)	0·65 (0·58–0·71)	0·39 (0·36–0·43)
1975	0·0 (0·0–0·0)	4·4 (3·6–5·3)	113·9 (101·8–126·7)	192·6 (184·2–200·8)	56·7 (51·0–62·1)	10·9 (8·9–13·0)	1·8 (1·4–2·3)	0·1 (0·1–0·1)	0·0 (0·0–0·0)	1·9 (1·82–1·99)	0·59 (0·53–0·65)	0·35 (0·32–0·38)
1976	0·0 (0·0–0·0)	4·1 (3·3–5·0)	105·0 (94·0–116·8)	188·5 (180·3–196·7)	54·7 (49·8–59·9)	10·8 (9·2–12·5)	1·5 (1·1–2·1)	0·1 (0·0–0·1)	0·0 (0·0–0·0)	1·82 (1·75–1·91)	0·55 (0·49–0·61)	0·33 (0·31–0·36)
1977	0·0 (0·0–0·0)	3·9 (2·9–5·0)	96·8 (86·7–108·5)	189·0 (181·0–197·9)	56·9 (51·5–62·9)	10·7 (8·9–12·7)	1·3 (1·0–1·8)	0·1 (0·0–0·1)	0·0 (0·0–0·0)	1·79 (1·72–1·87)	0·5 (0·45–0·56)	0·34 (0·31–0·38)
1978	0·0 (0·0–0·0)	3·8 (3·1–4·7)	90·4 (81·6–100·5)	190·5 (182·9–198·1)	61·2 (55·4–67·2)	10·5 (8·5–12·8)	1·3 (0·9–1·8)	0·1 (0·0–0·1)	0·0 (0·0–0·0)	1·79 (1·72–1·86)	0·47 (0·43–0·52)	0·37 (0·34–0·4)
1979	0·0 (0·0–0·0)	3·9 (3·3–4·4)	84·9 (75·4–95·6)	189·9 (180·3–198·8)	62·3 (57·2–68·2)	10·2 (8·3–12·4)	1·4 (1·0–1·9)	0·1 (0·0–0·1)	0·0 (0·0–0·0)	1·76 (1·69–1·84)	0·44 (0·4–0·5)	0·37 (0·34–0·4)
1980	0·0 (0·0–0·0)	3·9 (3·3–4·7)	80·3 (71·3–89·5)	188·4 (180·3–196·9)	61·4 (55·8–67·2)	10·1 (8·2–12·1)	1·4 (1·0–1·9)	0·1 (0·0–0·1)	0·0 (0·0–0·0)	1·73 (1·66–1·8)	0·42 (0·38–0·47)	0·36 (0·34–0·4)
1981	0·0 (0·0–0·0)	4·1 (3·5–4·8)	77·8 (69·3–87·1)	190·6 (182·1–198·9)	62·2 (56·5–68·1)	10·5 (8·5–12·6)	1·4 (0·9–2·0)	0·1 (0·0–0·1)	0·0 (0·0–0·0)	1·73 (1·68–1·79)	0·41 (0·37–0·46)	0·37 (0·34–0·4)
1982	0·0 (0·0–0·0)	4·3 (3·5–5·1)	76·7 (67·6–86·2)	194·0 (185·8–202·8)	65·8 (60·0–71·7)	11·8 (9·7–14·3)	1·4 (1·0–1·9)	0·1 (0·0–0·1)	0·0 (0·0–0·0)	1·77 (1·71–1·83)	0·4 (0·36–0·45)	0·4 (0·37–0·43)
1983	0·0 (0·0–0·0)	4·4 (3·7–5·2)	75·0 (66·4–84·6)	196·0 (187·1–204·9)	70·5 (64·5–77·8)	13·0 (10·6–15·6)	1·4 (1·0–1·8)	0·1 (0·0–0·1)	0·0 (0·0–0·0)	1·8 (1·73–1·88)	0·4 (0·35–0·45)	0·42 (0·39–0·46)
1984	0·0 (0·0–0·0)	4·3 (3·6–5·1)	69·7 (62·5–77·8)	194·0 (185·4–203·2)	74·3 (68·0–81·2)	13·4 (10·9–16·5)	1·4 (1·0–1·9)	0·1 (0·0–0·1)	0·0 (0·0–0·0)	1·79 (1·72–1·85)	0·37 (0·33–0·41)	0·45 (0·41–0·48)
1985	0·0 (0·0–0·0)	4·1 (3·4–4·9)	65·3 (57·3–73·6)	187·5 (179·0–195·5)	76·5 (69·5–83·3)	13·3 (11·1–16·2)	1·5 (1·0–2·1)	0·1 (0·0–0·1)	0·0 (0·0–0·0)	1·74 (1·68–1·81)	0·35 (0·31–0·39)	0·46 (0·42–0·49)
1986	0·0 (0·0–0·0)	3·9 (3·3–4·6)	61·8 (54·5–69·6)	181·5 (172·6–190·4)	79·3 (72·7–85·8)	13·3 (11·1–15·8)	1·5 (1·1–2·1)	0·1 (0·0–0·1)	0·0 (0·0–0·0)	1·71 (1·65–1·77)	0·33 (0·29–0·37)	0·47 (0·44–0·51)
1987	0·0 (0·0–0·0)	3·8 (3·2–4·5)	56·4 (49·4–63·9)	175·6 (166·9–184·2)	82·1 (75·6–89·2)	13·9 (11·5–16·8)	1·7 (1·2–2·4)	0·1 (0·0–0·1)	0·0 (0·0–0·0)	1·67 (1·6–1·74)	0·3 (0·27–0·34)	0·49 (0·45–0·52)
1988	0·0 (0·0–0·0)	3·6 (3·0–4·3)	51·4 (45·2–58·1)	164·3 (156·8–172·6)	83·0 (75·8–90·9)	14·7 (12·4–17·4)	1·9 (1·3–2·6)	0·1 (0·0–0·1)	0·0 (0·0–0·0)	1·6 (1·53–1·66)	0·28 (0·24–0·31)	0·5 (0·46–0·54)
1989	0·0 (0·0–0·0)	3·6 (3·0–4·4)	46·8 (40·6–53·3)	152·8 (145·2–161·2)	82·7 (75·7–89·2)	15·5 (12·9–18·5)	2·0 (1·4–2·8)	0·1 (0·0–0·1)	0·0 (0·0–0·0)	1·52 (1·45–1·58)	0·25 (0·22–0·28)	0·5 (0·47–0·54)
1990	0·0 (0·0–0·0)	3·7 (3·1–4·5)	44·6 (39·4–49·8)	148·9 (141·2–156·6)	83·9 (77·0–90·9)	16·3 (13·6–19·2)	2·0 (1·4–2·7)	0·1 (0·0–0·1)	0·0 (0·0–0·0)	1·5 (1·44–1·57)	0·24 (0·21–0·27)	0·51 (0·47–0·55)
1991	0·0 (0·0–0·0)	3·8 (3·1–4·7)	44·0 (39·0–49·5)	145·1 (137·8–152·4)	84·8 (77·8–92·5)	17·2 (14·6–20·4)	2·0 (1·4–2·7)	0·1 (0·0–0·1)	0·0 (0·0–0·0)	1·49 (1·43–1·54)	0·24 (0·21–0·27)	0·52 (0·48–0·56)
1992	0·0 (0·0–0·0)	3·9 (3·2–4·8)	42·3 (36·6–49·2)	137·4 (129·8–145·5)	84·8 (78·5–91·5)	17·9 (14·9–21·2)	2·1 (1·6–2·8)	0·1 (0·0–0·1)	0·0 (0·0–0·0)	1·44 (1·38–1·5)	0·23 (0·2–0·27)	0·52 (0·49–0·56)
1993	0·0 (0·0–0·0)	3·9 (3·2–4·8)	41·6 (36·5–46·8)	137·2 (129·6–145·1)	90·0 (82·4–97·5)	19·6 (16·3–23·1)	2·2 (1·7–3·0)	0·1 (0·0–0·1)	0·0 (0·0–0·0)	1·47 (1·41–1·53)	0·23 (0·2–0·25)	0·56 (0·52–0·6)
1994	0·0 (0·0–0·0)	4·0 (3·3–4·8)	40·6 (35·9–46·1)	134·6 (126·0–143·1)	93·5 (85·8–101·9)	21·0 (17·6–25·0)	2·4 (1·7–3·1)	0·1 (0·1–0·1)	0·0 (0·0–0·0)	1·48 (1·41–1·55)	0·22 (0·2–0·25)	0·58 (0·54–0·63)
1995	0·0 (0·0–0·0)	4·0 (3·3–4·9)	39·2 (34·4–44·2)	126·8 (119·6–134·0)	94·8 (86·3–102·8)	22·2 (18·4–26·6)	2·5 (1·9–3·3)	0·1 (0·1–0·1)	0·0 (0·0–0·0)	1·45 (1·39–1·51)	0·22 (0·19–0·24)	0·6 (0·55–0·64)
1996	0·0 (0·0–0·0)	4·2 (3·4–5·1)	38·5 (33·7–43·6)	122·7 (115·5–130·0)	96·6 (88·7–105·0)	23·6 (19·9–27·9)	2·6 (1·9–3·4)	0·1 (0·1–0·1)	0·0 (0·0–0·0)	1·44 (1·37–1·51)	0·21 (0·19–0·24)	0·61 (0·57–0·66)
1997	0·0 (0·0–0·0)	4·4 (3·6–5·3)	38·2 (33·3–43·4)	119·1 (112·5–125·7)	96·8 (89·0–105·6)	24·5 (20·0–29·3)	2·8 (2·0–3·8)	0·1 (0·1–0·1)	0·0 (0·0–0·0)	1·43 (1·37–1·5)	0·21 (0·19–0·24)	0·62 (0·58–0·67)
1998	0·0 (0·0–0·0)	4·7 (3·9–5·7)	38·0 (33·4–43·5)	114·8 (108·2–121·2)	97·4 (89·8–106·2)	25·9 (21·6–30·4)	2·9 (2·2–3·9)	0·1 (0·1–0·1)	0·0 (0·0–0·0)	1·42 (1·36–1·48)	0·21 (0·19–0·24)	0·63 (0·59–0·68)
1999	0·0 (0·0–0·0)	5·1 (4·2–6·1)	37·8 (33·0–42·9)	110·9 (104·0–117·2)	97·9 (89·7–106·8)	26·9 (22·7–31·8)	3·1 (2·3–4·2)	0·1 (0·1–0·1)	0·0 (0·0–0·0)	1·41 (1·34–1·48)	0·21 (0·19–0·24)	0·64 (0·59–0·69)
2000	0·0 (0·0–0·0)	5·5 (4·5–6·5)	38·0 (33·9–42·5)	107·8 (101·7–114·7)	97·1 (89·6–105·0)	28·6 (24·2–33·1)	3·3 (2·4–4·4)	0·1 (0·1–0·1)	0·0 (0·0–0·0)	1·4 (1·34–1·47)	0·22 (0·19–0·24)	0·65 (0·6–0·69)
2001	0·0 (0·0–0·0)	5·8 (4·8–6·8)	38·3 (33·1–43·8)	104·0 (98·1–110·8)	94·9 (86·3–103·3)	29·0 (24·5–34·2)	3·5 (2·6–4·6)	0·1 (0·1–0·1)	0·0 (0·0–0·0)	1·38 (1·31–1·44)	0·22 (0·19–0·25)	0·64 (0·59–0·69)
2002	0·0 (0·0–0·0)	5·7 (4·8–6·8)	37·5 (32·5–43·1)	100·7 (94·3–107·2)	94·8 (86·7–103·2)	30·0 (25·3–34·8)	3·8 (2·7–5·0)	0·1 (0·1–0·2)	0·0 (0·0–0·0)	1·36 (1·3–1·43)	0·22 (0·19–0·25)	0·64 (0·6–0·69)
2003	0·0 (0·0–0·0)	5·6 (4·6–6·6)	36·3 (31·9–41·2)	97·9 (92·6–103·7)	94·3 (87·0–102·4)	31·9 (26·8–37·3)	4·1 (2·9–5·4)	0·1 (0·1–0·2)	0·0 (0·0–0·0)	1·35 (1·29–1·41)	0·21 (0·19–0·23)	0·65 (0·6–0·7)
2004	0·0 (0·0–0·0)	5·4 (4·5–6·3)	35·4 (30·7–41·0)	93·8 (87·8–100·2)	93·1 (85·6–100·4)	33·5 (28·3–39·3)	4·3 (3·1–5·7)	0·1 (0·1–0·2)	0·0 (0·0–0·0)	1·33 (1·27–1·39)	0·2 (0·18–0·23)	0·65 (0·61–0·7)
2005	0·0 (0·0–0·0)	5·3 (4·5–6·2)	35·6 (31·0–40·5)	92·6 (86·5–98·8)	94·3 (86·0–102·4)	35·4 (30·0–41·2)	4·5 (3·3–5·9)	0·1 (0·1–0·2)	0·0 (0·0–0·0)	1·34 (1·28–1·4)	0·2 (0·18–0·23)	0·67 (0·62–0·72)
2006	0·0 (0·0–0·0)	5·3 (4·5–6·2)	36·5 (31·7–41·7)	93·6 (87·7–99·5)	98·5 (89·9–107·0)	38·3 (33·1–44·2)	5·0 (3·8–6·4)	0·1 (0·1–0·2)	0·0 (0·0–0·0)	1·39 (1·33–1·45)	0·21 (0·18–0·24)	0·71 (0·66–0·77)
2007	0·0 (0·0–0·0)	5·3 (4·4–6·3)	36·7 (32·4–41·2)	93·3 (87·6–99·9)	100·1 (92·3–108·4)	41·4 (35·6–47·8)	5·4 (4·1–7·0)	0·1 (0·1–0·2)	0·0 (0·0–0·0)	1·41 (1·35–1·47)	0·21 (0·19–0·23)	0·74 (0·69–0·79)
2008	0·0 (0·0–0·0)	5·2 (4·4–6·1)	36·3 (31·3–42·1)	93·1 (86·9–99·2)	101·7 (92·8–110·5)	43·4 (37·4–49·9)	6·0 (4·7–7·5)	0·1 (0·1–0·2)	0·0 (0·0–0·0)	1·43 (1·36–1·5)	0·21 (0·18–0·24)	0·76 (0·7–0·81)
2009	0·0 (0·0–0·0)	4·9 (4·1–5·8)	35·1 (31·1–38·8)	93·1 (87·6–98·5)	102·9 (94·7–111·9)	45·1 (38·8–52·0)	6·7 (5·3–8·6)	0·1 (0·1–0·2)	0·0 (0·0–0·0)	1·44 (1·37–1·52)	0·2 (0·18–0·22)	0·77 (0·72–0·83)
2010	0·0 (0·0–0·0)	4·7 (4·0–5·5)	33·9 (28·9–39·2)	93·8 (87·5–100·2)	104·1 (95·7–113·7)	46·3 (39·8–52·9)	7·2 (5·8–9·0)	0·2 (0·1–0·3)	0·0 (0·0–0·0)	1·45 (1·38–1·53)	0·19 (0·17–0·22)	0·79 (0·73–0·85)
2011	0·0 (0·0–0·0)	4·5 (3·8–5·3)	31·9 (27·8–36·3)	93·0 (86·5–99·3)	103·8 (95·6–112·5)	47·2 (40·2–54·4)	7·7 (6·1–9·7)	0·2 (0·1–0·3)	0·0 (0·0–0·0)	1·44 (1·38–1·51)	0·18 (0·16–0·2)	0·79 (0·74–0·85)
2012	0·0 (0·0–0·0)	4·4 (3·6–5·2)	30·0 (26·0–34·8)	91·8 (86·7–97·1)	104·6 (96·0–113·4)	49·6 (43·1–56·0)	8·5 (6·8–10·7)	0·2 (0·1–0·3)	0·0 (0·0–0·0)	1·45 (1·39–1·51)	0·17 (0·15–0·19)	0·81 (0·76–0·87)
2013	0·0 (0·0–0·0)	4·2 (3·5–5·0)	28·6 (24·9–32·7)	89·8 (83·9–96·1)	104·3 (95·3–113·6)	51·1 (44·4–58·5)	9·1 (7·1–11·4)	0·2 (0·1–0·3)	0·0 (0·0–0·0)	1·44 (1·36–1·51)	0·16 (0·15–0·18)	0·82 (0·76–0·89)
2014	0·0 (0·0–0·0)	4·1 (3·5–4·9)	27·4 (23·5–31·4)	88·6 (82·6–94·9)	105·5 (96·8–114·7)	53·5 (46·6–61·1)	9·7 (7·7–12·1)	0·2 (0·1–0·3)	0·0 (0·0–0·0)	1·45 (1·37–1·53)	0·16 (0·14–0·18)	0·84 (0·78–0·91)
2015	0·0 (0·0–0·0)	4·1 (3·4–4·9)	27·7 (23·6–32·2)	89·9 (82·7–97·5)	108·4 (97·8–119·2)	52·3 (43·9–62·8)	9·0 (6·9–11·7)	0·2 (0·1–0·3)	0·0 (0·0–0·0)	1·46 (1·32–1·63)	0·16 (0·14–0·19)	0·85 (0·76–0·96)
2016	0·0 (0·0–0·0)	4·1 (3·4–5·1)	28·8 (22·6–37·1)	90·9 (81·5–102·2)	108·2 (93·8–124·5)	48·4 (37·6–62·6)	7·7 (5·5–10·6)	0·2 (0·1–0·3)	0·0 (0·0–0·0)	1·44 (1·23–1·69)	0·16 (0·13–0·21)	0·82 (0·7–0·97)
2017	0·0 (0·0–0·0)	4·1 (3·4–5·0)	28·7 (22·5–36·7)	90·6 (80·4–101·4)	106·8 (91·3–123·8)	47·2 (35·6–61·2)	7·5 (5·0–10·8)	0·2 (0·1–0·3)	0·0 (0·0–0·0)	1·43 (1·19–1·69)	0·16 (0·13–0·21)	0·81 (0·66–0·98)

Supplemental Results Table 5: Age-specific fertility rates per 1000, cumulative total fertility up to maternal age 25 (TFU25), cumulative total fertility from maternal age 30 to age 55 (TFO30), and cumulative total fertility, Akita, 1950-2017

Year	10-14	15-19	20-24	25-29	30-34	35-39	40-44	45-49	50-54	TFR	TFU25	TFO30
1950	0·0 (0·0–0·0)	7·9 (7·0–8·8)	164·5 (150·1–179·8)	215·6 (207·9–223·4)	153·8 (144·7–162·9)	86·0 (76·4–95·5)	23·7 (20·5–27·2)	1·1 (0·8–1·7)	0·0 (0·0–0·0)	3·26 (3·15–3·38)	0·86 (0·79–0·94)	1·32 (1·25–1·39)
1951	0·0 (0·0–0·0)	6·4 (5·6–7·1)	147·8 (133·7–162·1)	200·1 (191·7–208·6)	138·9 (129·4–148·5)	69·4 (60·5–78·1)	18·4 (15·3–21·7)	0·8 (0·5–1·2)	0·0 (0·0–0·0)	2·91 (2·78–3·04)	0·77 (0·7–0·84)	1·14 (1·04–1·24)
1952	0·0 (0·0–0·0)	5·4 (4·7–6·2)	137·7 (123·6–153·6)	190·0 (180·8–199·4)	125·5 (115·5–135·7)	55·5 (48·1–64·2)	14·5 (11·7–17·6)	0·7 (0·4–1·0)	0·0 (0·0–0·0)	2·65 (2·53–2·76)	0·72 (0·64–0·79)	0·98 (0·92–1·05)
1953	0·0 (0·0–0·0)	4·7 (4·0–5·4)	130·3 (115·5–146·8)	179·0 (169·2–190·3)	112·1 (101·7–122·2)	44·2 (37·4–52·1)	11·6 (9·0–14·7)	0·5 (0·3–0·8)	0·0 (0·0–0·0)	2·41 (2·3–2·53)	0·67 (0·6–0·76)	0·84 (0·77–0·91)
1954	0·0 (0·0–0·0)	4·3 (3·7–5·0)	126·4 (109·3–142·5)	174·2 (162·5–186·1)	103·2 (94·2–112·5)	36·8 (30·6–44·2)	9·5 (7·1–12·7)	0·4 (0·3–0·7)	0·0 (0·0–0·0)	2·27 (2·15–2·39)	0·65 (0·57–0·73)	0·75 (0·69–0·82)
1955	0·0 (0·0–0·0)	3·9 (3·2–4·6)	123·5 (107·9–141·9)	173·1 (161·1–186·6)	94·8 (86·0–104·3)	30·0 (25·0–35·5)	7·9 (5·7–10·8)	0·3 (0·2–0·5)	0·0 (0·0–0·0)	2·17 (2·05–2·29)	0·64 (0·56–0·73)	0·67 (0·61–0·72)
1956	0·0 (0·0–0·0)	3·5 (2·9–4·1)	116·5 (100·5–133·3)	167·2 (155·1–180·6)	83·9 (75·7–92·8)	24·2 (20·0–28·8)	6·4 (4·5–8·9)	0·3 (0·2–0·4)	0·0 (0·0–0·0)	2·01 (1·89–2·13)	0·6 (0·52–0·68)	0·57 (0·52–0·63)
1957	0·0 (0·0–0·0)	3·3 (2·7–4·0)	117·9 (101·9–136·8)	170·6 (159·0–184·0)	79·4 (71·5–87·9)	22·0 (18·2–25·7)	5·4 (3·8–7·4)	0·3 (0·2–0·5)	0·0 (0·0–0·0)	1·99 (1·87–2·14)	0·61 (0·53–0·7)	0·54 (0·49–0·59)
1958	0·0 (0·0–0·0)	3·4 (2·8–4·0)	124·0 (107·7–141·6)	176·2 (164·6–188·0)	77·6 (69·3–86·1)	20·6 (16·8–24·9)	4·9 (3·3–7·0)	0·3 (0·2–0·5)	0·0 (0·0–0·0)	2·03 (1·92–2·15)	0·64 (0·55–0·73)	0·52 (0·46–0·57)
1959	0·0 (0·0–0·0)	3·5 (2·8–4·2)	124·2 (107·4–142·8)	175·0 (165·3–185·6)	72·4 (64·0–80·6)	16·7 (13·4–20·4)	4·1 (2·7–5·8)	0·2 (0·1–0·4)	0·0 (0·0–0·0)	1·98 (1·88–2·1)	0·64 (0·56–0·73)	0·47 (0·42–0·51)
1960	0·0 (0·0–0·0)	3·4 (2·7–4·1)	119·5 (105·9–134·9)	175·4 (165·5–184·7)	69·3 (62·1–76·5)	13·7 (11·0–16·8)	3·3 (2·2–4·7)	0·2 (0·1–0·3)	0·0 (0·0–0·0)	1·92 (1·84–2·02)	0·61 (0·55–0·69)	0·43 (0·39–0·47)
1961	0·0 (0·0–0·0)	3·2 (2·5–3·9)	116·1 (104·1–128·4)	176·9 (168·5–186·0)	67·5 (61·1–74·9)	12·2 (9·8–15·2)	2·9 (1·9–4·1)	0·2 (0·1–0·3)	0·0 (0·0–0·0)	1·89 (1·81–1·98)	0·6 (0·54–0·66)	0·41 (0·38–0·45)
1962	0·0 (0·0–0·0)	3·0 (2·3–3·9)	115·9 (102·8–128·8)	181·6 (174·1–188·9)	68·8 (62·7–75·2)	11·5 (9·1–14·0)	2·5 (1·7–3·6)	0·1 (0·1–0·2)	0·0 (0·0–0·0)	1·92 (1·83–2·0)	0·59 (0·53–0·66)	0·41 (0·38–0·45)
1963	0·0 (0·0–0·0)	3·0 (2·3–3·8)	116·5 (103·8–131·1)	183·3 (175·6–191·0)	70·0 (64·0–76·5)	12·3 (10·0–14·8)	2·5 (1·7–3·6)	0·2 (0·1–0·3)	0·0 (0·0–0·0)	1·94 (1·85–2·03)	0·6 (0·53–0·67)	0·42 (0·39–0·46)
1964	0·0 (0·0–0·0)	3·0 (2·2–3·9)	127·4 (113·4–142·9)	194·8 (186·5–203·1)	74·4 (68·0–81·4)	12·6 (10·4–14·9)	2·3 (1·5–3·3)	0·2 (0·1–0·2)	0·0 (0·0–0·0)	2·07 (1·98–2·17)	0·65 (0·58–0·73)	0·45 (0·41–0·48)
1965	0·0 (0·0–0·0)	2·9 (2·2–3·9)	117·6 (102·0–135·4)	163·8 (155·3–172·4)	61·5 (55·1–68·0)	10·3 (8·7–12·2)	2·0 (1·3–2·9)	0·1 (0·1–0·2)	0·0 (0·0–0·0)	1·79 (1·69–1·9)	0·6 (0·53–0·69)	0·37 (0·34–0·4)
1966	0·0 (0·0–0·0)	3·1 (2·3–4·1)	115·2 (96·8–134·9)	168·2 (159·5–176·5)	62·6 (56·0–69·2)	10·9 (9·1–13·0)	2·0 (1·4–2·7)	0·1 (0·1–0·2)	0·0 (0·0–0·0)	1·81 (1·71–1·91)	0·59 (0·5–0·69)	0·38 (0·34–0·41)
1967	0·0 (0·0–0·0)	3·2 (2·4–4·3)	123·1 (102·9–144·9)	204·1 (195·2–213·1)	74·9 (67·2–83·7)	12·3 (10·1–14·7)	1·9 (1·4–2·5)	0·1 (0·1–0·2)	0·0 (0·0–0·0)	2·1 (1·97–2·23)	0·63 (0·53–0·74)	0·45 (0·4–0·5)
1968	0·0 (0·0–0·0)	3·3 (2·4–4·4)	110·5 (91·9–131·1)	191·5 (183·2–199·4)	67·7 (60·5–75·6)	11·5 (9·1–14·1)	1·7 (1·2–2·2)	0·1 (0·1–0·1)	0·0 (0·0–0·0)	1·93 (1·84–2·03)	0·57 (0·48–0·67)	0·41 (0·37–0·44)
1969	0·0 (0·0–0·0)	3·6 (2·7–4·7)	109·0 (90·5–129·2)	192·9 (184·9–200·7)	67·5 (60·7–75·0)	11·1 (8·9–13·9)	1·7 (1·2–2·2)	0·1 (0·1–0·1)	0·0 (0·0–0·0)	1·93 (1·84–2·03)	0·56 (0·47–0·66)	0·4 (0·37–0·44)
1970	0·0 (0·0–0·0)	3·7 (2·7–4·8)	117·7 (99·2–137·9)	195·9 (187·1–204·0)	69·2 (62·2–76·8)	11·9 (9·3–14·9)	1·8 (1·3–2·4)	0·1 (0·1–0·2)	0·0 (0·0–0·0)	2·0 (1·9–2·1)	0·61 (0·52–0·7)	0·41 (0·38–0·46)
1971	0·0 (0·0–0·0)	3·6 (2·7–4·7)	129·6 (111·1–150·5)	193·1 (184·6–201·9)	69·9 (62·6–77·7)	13·2 (10·4–16·8)	2·0 (1·4–2·7)	0·1 (0·1–0·2)	0·0 (0·0–0·0)	2·06 (1·95–2·17)	0·67 (0·58–0·77)	0·43 (0·38–0·47)
1972	0·0 (0·0–0·0)	3·7 (2·7–4·7)	136·6 (119·2–156·1)	191·2 (182·3–200·1)	69·9 (62·0–78·2)	13·5 (10·5–16·7)	2·0 (1·4–2·7)	0·1 (0·1–0·2)	0·0 (0·0–0·0)	2·08 (1·97–2·2)	0·7 (0·62–0·8)	0·43 (0·38–0·47)
1973	0·0 (0·0–0·0)	3·6 (2·7–4·6)	138·1 (122·6–154·0)	190·7 (181·1–200·0)	66·3 (60·0–72·9)	12·5 (9·8–15·6)	1·8 (1·3–2·5)	0·1 (0·1–0·2)	0·0 (0·0–0·0)	2·07 (1·96–2·17)	0·71 (0·63–0·79)	0·4 (0·36–0·44)
1974	0·0 (0·0–0·0)	3·4 (2·6–4·4)	133·6 (119·3–149·6)	182·9 (174·4–191·3)	58·9 (52·7–65·4)	9·8 (7·5–12·7)	1·6 (1·0–2·5)	0·1 (0·1–0·1)	0·0 (0·0–0·0)	1·95 (1·86–2·05)	0·68 (0·62–0·76)	0·35 (0·32–0·39)
1975	0·0 (0·0–0·0)	3·1 (2·4–4·0)	126·1 (112·4–140·0)	176·5 (168·3–185·2)	53·6 (47·8–58·9)	7·8 (5·9–10·2)	1·4 (0·8–2·1)	0·1 (0·0–0·1)	0·0 (0·0–0·0)	1·84 (1·75–1·93)	0·65 (0·58–0·71)	0·31 (0·28–0·35)
1976	0·0 (0·0–0·0)	3·0 (2·3–3·7)	119·8 (107·1–133·8)	174·9 (165·7–184·0)	53·1 (47·8–58·8)	7·1 (5·5–9·1)	1·2 (0·8–1·7)	0·1 (0·0–0·1)	0·0 (0·0–0·0)	1·8 (1·71–1·89)	0·61 (0·55–0·68)	0·31 (0·28–0·34)
1977	0·0 (0·0–0·0)	2·9 (2·3–3·5)	113·0 (100·9–127·4)	176·5 (168·3–185·1)	55·2 (49·5–61·3)	7·2 (5·5–9·6)	1·1 (0·7–1·6)	0·0 (0·0–0·1)	0·0 (0·0–0·0)	1·78 (1·7–1·87)	0·58 (0·52–0·65)	0·32 (0·28–0·36)
1978	0·0 (0·0–0·0)	2·8 (2·3–3·5)	107·0 (96·1–118·8)	178·2 (170·3–186·2)	57·7 (51·9–64·0)	7·3 (5·3–9·7)	0·9 (0·6–1·4)	0·0 (0·0–0·1)	0·0 (0·0–0·0)	1·77 (1·7–1·85)	0·55 (0·49–0·61)	0·33 (0·3–0·36)
1979	0·0 (0·0–0·0)	2·8 (2·3–3·3)	100·7 (89·3–113·1)	177·7 (170·0–185·4)	57·0 (51·6–62·9)	7·3 (5·7–9·4)	0·8 (0·5–1·3)	0·0 (0·0–0·0)	0·0 (0·0–0·0)	1·73 (1·66–1·81)	0·52 (0·46–0·58)	0·33 (0·3–0·36)
1980	0·0 (0·0–0·0)	2·8 (2·2–3·5)	94·4 (83·2–105·7)	176·3 (167·7–184·8)	56·1 (50·1–61·8)	7·6 (5·8–9·6)	0·8 (0·5–1·2)	0·0 (0·0–0·0)	0·0 (0·0–0·0)	1·69 (1·61–1·77)	0·49 (0·43–0·54)	0·32 (0·29–0·35)
1981	0·0 (0·0–0·0)	2·9 (2·3–3·6)	89·9 (80·6–101·1)	177·9 (169·6–186·3)	57·6 (51·9–63·4)	8·3 (6·4–10·4)	0·9 (0·6–1·2)	0·0 (0·0–0·0)	0·0 (0·0–0·0)	1·69 (1·62–1·76)	0·46 (0·42–0·52)	0·33 (0·3–0·36)
1982	0·0 (0·0–0·0)	2·9 (2·3–3·7)	86·8 (76·4–98·3)	180·4 (172·9–188·4)	61·1 (55·1–67·3)	9·6 (7·5–12·1)	0·9 (0·6–1·3)	0·0 (0·0–0·0)	0·0 (0·0–0·0)	1·71 (1·64–1·78)	0·45 (0·4–0·51)	0·36 (0·33–0·39)
1983	0·0 (0·0–0·0)	3·0 (2·3–3·7)	83·1 (73·1–94·3)	181·5 (172·3–190·1)	65·1 (58·8–72·0)	10·9 (8·5–13·8)	1·0 (0·7–1·3)	0·0 (0·0–0·0)	0·0 (0·0–0·0)	1·72 (1·64–1·8)	0·43 (0·38–0·49)	0·38 (0·35–0·42)
1984	0·0 (0·0–0·0)	2·8 (2·2–3·5)	76·1 (66·9–85·8)	179·6 (172·0–188·0)	68·5 (62·1–75·5)	11·6 (9·1–14·5)	1·1 (0·7–1·6)	0·0 (0·0–0·0)	0·0 (0·0–0·0)	1·7 (1·64–1·77)	0·39 (0·35–0·44)	0·41 (0·37–0·44)
1985	0·0 (0·0–0·0)	2·7 (2·1–3·3)	70·7 (61·7–81·1)	174·9 (163·9–185·1)	70·9 (63·9–78·1)	11·9 (9·1–14·8)	1·1 (0·8–1·5)	0·0 (0·0–0·1)	0·0 (0·0–0·0)	1·66 (1·59–1·74)	0·37 (0·32–0·42)	0·42 (0·38–0·45)
1986	0·0 (0·0–0·0)	2·6 (2·1–3·3)	67·0 (57·9–77·2)	171·2 (163·8–179·3)	74·4 (68·0–80·5)	12·3 (9·6–15·3)	1·3 (0·9–1·7)	0·0 (0·0–0·1)	0·0 (0·0–0·0)	1·64 (1·57–1·73)	0·35 (0·3–0·4)	0·44 (0·4–0·48)
1987	0·0 (0·0–0·0)	2·6 (2·1–3·2)	61·9 (54·7–70·3)	168·2 (158·7–177·6)	78·7 (72·3–86·5)	13·1 (10·3–16·4)	1·5 (1·0–2·1)	0·0 (0·0–0·1)	0·0 (0·0–0·0)	1·63 (1·55–1·71)	0·32 (0·29–0·37)	0·47 (0·43–0·51)
1988	0·0 (0·0–0·0)	2·6 (2·1–3·1)	58·0 (49·6–66·6)	160·2 (152·4–168·6)	81·9 (74·5–89·9)	14·1 (11·2–17·6)	1·6 (1·1–2·3)	0·0 (0·0–0·1)	0·0 (0·0–0·0)	1·59 (1·52–1·67)	0·3 (0·26–0·35)	0·49 (0·45–0·53)
1989	0·0 (0·0–0·0)	2·6 (2·1–3·2)	54·4 (46·4–63·4)	151·1 (142·9–159·9)	84·0 (76·5–91·5)	15·2 (11·9–19·3)	1·8 (1·2–2·5)	0·0 (0·0–0·1)	0·0 (0·0–0·0)	1·55 (1·47–1·62)	0·29 (0·25–0·33)	0·51 (0·46–0·55)
1990	0·0 (0·0–0·0)	2·7 (2·2–3·2)	53·3 (46·2–60·6)	148·6 (141·0–156·4)	87·8 (79·6–96·0)	16·3 (13·0–20·3)	1·7 (1·2–2·4)	0·0 (0·0–0·1)	0·0 (0·0–0·0)	1·55 (1·47–1·63)	0·28 (0·24–0·32)	0·53 (0·48–0·57)
1991	0·0 (0·0–0·0)	2·8 (2·3–3·4)	53·9 (46·2–62·2)	145·5 (134·6–157·3)	90·4 (81·9–98·8)	17·5 (13·8–22·0)	1·8 (1·2–2·4)	0·0 (0·0–0·1)	0·0 (0·0–0·0)	1·56 (1·49–1·64)	0·28 (0·24–0·33)	0·55 (0·5–0·6)
1992	0·0 (0·0–0·0)	2·9 (2·4–3·4)	52·7 (45·6–61·1)	138·4 (130·9–145·9)	90·8 (83·0–99·2)	18·4 (15·0–22·5)	1·9 (1·3–2·6)	0·0 (0·0–0·1)	0·0 (0·0–0·0)	1·53 (1·46–1·59)	0·28 (0·24–0·32)	0·56 (0·51–0·6)
1993	0·0 (0·0–0·0)	2·9 (2·4–3·5)	52·8 (45·3–61·5)	138·6 (129·1–148·3)	95·1 (86·9–104·0)	20·3 (16·6–24·9)	2·0 (1·4–2·7)	0·0 (0·0–0·1)	0·0 (0·0–0·0)	1·56 (1·48–1·64)	0·28 (0·24–0·32)	0·59 (0·54–0·64)
1994	0·0 (0·0–0·0)	2·9 (2·4–3·5)	52·4 (45·2–60·5)	136·2 (128·3–144·3)	96·3 (87·7–106·1)	21·8 (17·6–26·9)	2·1 (1·5–3·0)	0·0 (0·0–0·1)	0·0 (0·0–0·0)	1·56 (1·48–1·64)	0·28 (0·24–0·32)	0·6 (0·55–0·66)
1995	0·0 (0·0–0·0)	3·0 (2·4–3·6)	51·2 (44·0–59·2)	128·4 (120·2–136·5)	94·9 (85·7–103·5)	23·1 (18·9–27·7)	2·3 (1·6–3·1)	0·1 (0·0–0·1)	0·0 (0·0–0·0)	1·51 (1·44–1·59)	0·27 (0·23–0·31)	0·6 (0·55–0·65)
1996	0·0 (0·0–0·0)	3·1 (2·5–3·8)	50·8 (43·6–58·5)	124·1 (116·1–132·9)	94·3 (86·0–103·7)	24·6 (20·1–29·9)	2·4 (1·7–3·2)	0·1 (0·0–0·1)	0·0 (0·0–0·0)	1·5 (1·41–1·58)	0·27 (0·23–0·31)	0·61 (0·56–0·66)
1997	0·0 (0·0–0·0)	3·3 (2·6–3·9)	50·8 (42·9–59·2)	120·3 (112·0–129·0)	92·3 (84·1–101·5)	25·6 (21·1–30·7)	2·5 (1·8–3·5)	0·1 (0·0–0·1)	0·0 (0·0–0·0)	1·47 (1·4–1·56)	0·27 (0·23–0·31)	0·6 (0·56–0·66)
1998	0·0 (0·0–0·0)	3·5 (2·9–4·3)	50·7 (43·7–58·4)	115·9 (108·9–123·3)	91·1 (82·6–100·6)	26·9 (22·2–32·7)	2·7 (1·9–3·6)	0·1 (0·0–0·1)	0·0 (0·0–0·0)	1·45 (1·38–1·53)	0·27 (0·24–0·31)	0·6 (0·55–0·66)
1999	0·0 (0·0–0·0)	3·7 (3·0–4·5)	50·7 (43·5–58·8)	112·2 (104·5–120·2)	90·1 (81·8–98·7)	27·7 (22·7–33·6)	2·8 (2·0–3·8)	0·1 (0·0–0·1)	0·0 (0·0–0·0)	1·44 (1·36–1·51)	0·27 (0·24–0·31)	0·6 (0·55–0·66)
2000	0·0 (0·0–0·0)	4·0 (3·2–4·8)	51·2 (45·2–57·6)	109·6 (102·4–117·3)	88·3 (80·3–97·2)	29·1 (23·6–34·8)	3·0 (2·1–4·0)	0·1 (0·0–0·1)	0·0 (0·0–0·0)	1·43 (1·34–1·51)	0·28 (0·25–0·31)	0·6 (0·55–0·65)
2001	0·0 (0·0–0·0)	4·2 (3·4–5·0)	51·7 (44·5–59·5)	106·3 (99·2–114·1)	85·4 (77·1–93·7)	29·1 (23·8–34·6)	3·1 (2·2–4·2)	0·1 (0·0–0·1)	0·0 (0·0–0·0)	1·4 (1·33–1·47)	0·28 (0·24–0·32)	0·59 (0·54–0·64)
2002	0·0 (0·0–0·0)	4·1 (3·3–5·1)	50·6 (43·5–58·2)	103·1 (96·1–110·6)	84·9 (76·6–93·8)	29·7 (24·2–36·2)	3·3 (2·2–4·6)	0·1 (0·0–0·1)	0·0 (0·0–0·0)	1·38 (1·3–1·46)	0·27 (0·24–0·31)	0·59 (0·53–0·65)
2003	0·0 (0·0–0·0)	3·9 (3·1–4·8)	48·8 (42·5–55·9)	100·3 (93·6–107·4)	84·3 (76·1–92·8)	31·0 (25·6–37·1)	3·5 (2·4–4·7)	0·1 (0·0–0·1)	0·0 (0·0–0·0)	1·36 (1·28–1·44)	0·26 (0·23–0·3)	0·59 (0·54–0·65)
2004	0·0 (0·0–0·0)	3·7 (3·0–4·6)	47·3 (40·5–55·2)	96·0 (88·6–103·4)	82·7 (75·2–90·4)	31·7 (26·2–38·1)	3·6 (2·6–4·9)	0·1 (0·0–0·1)	0·0 (0·0–0·0)	1·33 (1·26–1·4)	0·25 (0·22–0·29)	0·59 (0·54–0·65)
2005	0·0 (0·0–0·0)	3·5 (2·8–4·3)	46·9 (40·6–54·0)	94·3 (88·1–100·6)	82·7 (74·9–90·5)	32·4 (26·6–39·1)	3·8 (2·7–5·3)	0·1 (0·0–0·1)	0·0 (0·0–0·0)	1·32 (1·25–1·4)	0·25 (0·22–0·29)	0·6 (0·54–0·65)
2006	0·0 (0·0–0·0)	3·4 (2·6–4·2)	47·3 (41·4–54·0)	94·7 (85·4–104·8)	84·7 (76·9–93·2)	33·8 (28·1–40·3)	4·1 (2·9–5·6)	0·1 (0·1–0·1)	0·0 (0·0–0·0)	1·34 (1·27–1·41)	0·25 (0·22–0·29)	0·61 (0·56–0·67)
2007	0·0 (0·0–0·0)	3·3 (2·5–4·1)	47·0 (39·0–56·3)	93·5 (87·4–100·1)	84·1 (75·9–92·7)	35·3 (29·5–41·9)	4·4 (3·1–5·9)	0·1 (0·1–0·1)	0·0 (0·0–0·0)	1·34 (1·26–1·42)	0·25 (0·21–0·3)	0·62 (0·57–0·67)
2008	0·0 (0·0–0·0)	3·2 (2·5–4·1)	45·9 (39·9–52·3)	92·3 (86·1–98·6)	83·7 (75·6–92·1)	36·2 (30·3–42·7)	4·8 (3·4–6·5)	0·1 (0·1–0·2)	0·0 (0·0–0·0)	1·33 (1·26–1·4)	0·25 (0·22–0·28)	0·62 (0·58–0·67)
2009	0·0 (0·0–0·0)	3·0 (2·3–3·8)	44·2 (36·5–52·7)	91·4 (82·5–99·6)	83·6 (75·7–92·3)	37·3 (31·1–44·1)	5·3 (3·7–7·1)	0·1 (0·1–0·2)	0·0 (0·0–0·0)	1·32 (1·23–1·42)	0·24 (0·2–0·28)	0·63 (0·58–0·69)
2010	0·0 (0·0–0·0)	2·9 (2·1–3·9)	42·6 (37·1–48·4)	91·3 (85·5–97·0)	84·4 (76·7–92·9)	38·2 (31·8–45·0)	5·5 (3·9–7·5)	0·1 (0·1–0·2)	0·0 (0·0–0·0)	1·32 (1·26–1·39)	0·23 (0·2–0·26)	0·64 (0·59–0·69)
2011	0·0 (0·0–0·0)	2·8 (2·1–3·6)	40·4 (35·2–46·5)	90·2 (83·5–97·4)	84·4 (76·2–93·1)	38·8 (32·6–45·8)	5·8 (4·1–8·0)	0·1 (0·1–0·2)	0·0 (0·0–0·0)	1·31 (1·24–1·39)	0·22 (0·19–0·25)	0·65 (0·59–0·71)
2012	0·0 (0·0–0·0)	2·7 (2·1–3·4)	38·3 (32·5–44·9)	89·1 (82·8–95·6)	85·9 (77·6–95·1)	40·4 (33·8–48·3)	6·2 (4·5–8·3)	0·1 (0·1–0·3)	0·0 (0·0–0·0)	1·31 (1·25–1·39)	0·2 (0·18–0·24)	0·66 (0·61–0·72)
2013	0·0 (0·0–0·0)	2·6 (1·9–3·4)	36·8 (31·2–42·9)	87·2 (80·1–94·4)	86·5 (78·7–94·7)	41·0 (34·3–48·0)	6·6 (4·7–9·1)	0·1 (0·1–0·3)	0·0 (0·0–0·0)	1·3 (1·23–1·38)	0·2 (0·17–0·23)	0·67 (0·62–0·73)
2014	0·0 (0·0–0·0)	2·5 (1·9–3·3)	35·4 (29·9–41·6)	86·6 (79·6–93·9)	87·7 (79·6–96·9)	41·9 (35·3–49·5)	6·9 (5·0–9·5)	0·1 (0·1–0·3)	0·0 (0·0–0·0)	1·31 (1·24–1·38)	0·19 (0·16–0·22)	0·68 (0·63–0·74)
2015	0·0 (0·0–0·0)	2·5 (2·0–3·3)	36·2 (26·6–47·7)	88·4 (79·3–97·7)	89·9 (80·5–100·2)	40·0 (32·2–48·7)	6·4 (4·4–9·0)	0·1 (0·1–0·3)	0·0 (0·0–0·0)	1·32 (1·16–1·5)	0·19 (0·14–0·25)	0·68 (0·6–0·77)
2016	0·0 (0·0–0·0)	2·6 (1·7–4·0)	37·4 (28·6–49·2)	89·9 (78·3–102·8)	89·7 (77·6–103·7)	36·6 (27·9–47·8)	5·6 (3·6–8·2)	0·1 (0·1–0·3)	0·0 (0·0–0·0)	1·31 (1·12–1·54)	0·2 (0·15–0·26)	0·66 (0·55–0·78)
2017	0·0 (0·0–0·0)	2·6 (1·9–3·5)	37·4 (27·3–50·4)	90·0 (77·4–103·7)	88·5 (75·4–103·6)	35·5 (25·9–47·2)	5·5 (3·4–8·2)	0·1 (0·1–0·3)	0·0 (0·0–0·0)	1·3 (1·08–1·55)	0·2 (0·15–0·27)	0·65 (0·53–0·79)

Supplemental Results Table 5: Age-specific fertility rates per 1000, cumulative total fertility up to maternal age 25 (TFU25), cumulative total fertility from maternal age 30 to age 55 (TFO30), and cumulative total fertility, Aomori, 1950-2017

Year	10-14	15-19	20-24	25-29	30-34	35-39	40-44	45-49	50-54	TFR	TFU25	TFO30
1950	0·0 (0·0–0·0)	14·3 (12·6–15·9)	172·6 (157·6–188·8)	206·8 (199·3–214·2)	173·8 (163·3–184·5)	145·2 (128·8–161·8)	44·6 (38·5–51·3)	2·4 (1·6–3·5)	0·0 (0·0–0·0)	3·8 (3·66–3·94)	0·93 (0·86–1·01)	1·83 (1·73–1·93)
1951	0·0 (0·0–0·0)	11·5 (10·2–12·9)	155·2 (140·9–169·5)	191·6 (183·7–199·6)	157·4 (145·9–168·4)	118·0 (102·3–133·2)	34·6 (28·6–41·1)	1·8 (1·2–2·6)	0·0 (0·0–0·0)	3·35 (3·18–3·52)	0·83 (0·76–0·9)	1·56 (1·41–1·7)
1952	0·0 (0·0–0·0)	9·7 (8·5–11·0)	144·8 (129·9–161·3)	181·8 (172·6–190·7)	142·5 (129·6–155·9)	95·1 (82·1–109·4)	27·5 (22·4–33·1)	1·4 (0·9–2·0)	0·0 (0·0–0·0)	3·01 (2·87–3·15)	0·77 (0·7–0·86)	1·33 (1·24–1·42)
1953	0·0 (0·0–0·0)	8·5 (7·4–9·8)	137·1 (121·8–154·0)	171·2 (161·7–181·7)	127·4 (113·4–141·8)	76·1 (64·3–88·3)	21·9 (17·3–27·2)	1·1 (0·7–1·7)	0·0 (0·0–0·0)	2·72 (2·58–2·87)	0·73 (0·65–0·81)	1·13 (1·05–1·22)
1954	0·0 (0·0–0·0)	7·8 (6·6–9·0)	133·3 (116·7–150·6)	166·6 (155·3–178·2)	117·5 (103·2–132·5)	63·6 (53·0–74·6)	18·1 (13·7–23·4)	0·9 (0·6–1·4)	0·0 (0·0–0·0)	2·54 (2·38–2·71)	0·71 (0·62–0·79)	1·0 (0·92–1·08)
1955	0·0 (0·0–0·0)	7·1 (6·1–8·3)	130·4 (114·0–149·0)	165·5 (154·1–178·8)	108·2 (93·9–122·4)	50·1 (42·3–59·3)	15·1 (10·9–20·5)	0·7 (0·4–1·2)	0·0 (0·0–0·0)	2·39 (2·23–2·55)	0·69 (0·6–0·78)	0·87 (0·79–0·95)
1956	0·0 (0·0–0·0)	6·3 (5·4–7·5)	123·1 (106·4–140·5)	159·9 (148·3–172·9)	95·9 (83·2–110·0)	39·8 (33·3–46·7)	12·2 (8·6–16·9)	0·6 (0·4–1·0)	0·0 (0·0–0·0)	2·19 (2·04–2·36)	0·65 (0·56–0·73)	0·74 (0·68–0·81)
1957	0·0 (0·0–0·0)	6·0 (5·2–7·1)	124·7 (107·9–144·8)	163·4 (152·3–176·6)	90·9 (78·7–104·5)	37·6 (31·3–43·5)	10·4 (7·3–14·2)	0·6 (0·4–1·0)	0·0 (0·0–0·0)	2·17 (2·01–2·34)	0·65 (0·57–0·75)	0·7 (0·63–0·76)
1958	0·0 (0·0–0·0)	6·1 (5·2–7·1)	131·2 (115·2–149·0)	168·9 (157·4–181·1)	88·8 (77·0–101·7)	36·0 (30·0–42·6)	9·4 (6·5–13·2)	0·6 (0·4–1·0)	0·0 (0·0–0·0)	2·2 (2·07–2·35)	0·69 (0·61–0·78)	0·67 (0·62–0·74)
1959	0·0 (0·0–0·0)	6·4 (5·4–7·4)	131·5 (115·2–150·0)	167·8 (158·3–177·4)	83·0 (72·6–94·6)	29·2 (23·7–35·6)	7·9 (5·3–10·9)	0·5 (0·3–0·9)	0·0 (0·0–0·0)	2·13 (2·0–2·27)	0·69 (0·61–0·78)	0·6 (0·55–0·66)
1960	0·0 (0·0–0·0)	6·3 (5·3–7·3)	126·7 (112·2–142·5)	168·2 (158·7–177·8)	79·5 (70·8–88·9)	23·0 (19·4–26·9)	6·4 (4·3–8·9)	0·4 (0·3–0·7)	0·0 (0·0–0·0)	2·05 (1·95–2·16)	0·66 (0·59–0·74)	0·55 (0·5–0·6)
1961	0·0 (0·0–0·0)	5·8 (4·9–6·9)	123·4 (111·4–136·2)	169·8 (161·6–178·2)	77·7 (68·1–88·4)	19·8 (16·0–23·9)	5·5 (3·7–7·9)	0·4 (0·2–0·6)	0·0 (0·0–0·0)	2·01 (1·92–2·12)	0·65 (0·58–0·71)	0·52 (0·47–0·57)
1962	0·0 (0·0–0·0)	5·6 (4·7–6·6)	123·5 (109·6–137·5)	174·7 (167·4–181·8)	79·1 (72·5–85·7)	18·8 (15·4–22·5)	4·9 (3·4–7·1)	0·3 (0·2–0·5)	0·0 (0·0–0·0)	2·04 (1·95–2·12)	0·65 (0·58–0·72)	0·52 (0·48–0·55)
1963	0·0 (0·0–0·0)	5·5 (4·6–6·6)	124·5 (111·0–139·6)	176·8 (169·6–184·5)	80·7 (73·9–88·2)	21·2 (17·9–24·8)	4·8 (3·4–6·8)	0·4 (0·2–0·6)	0·0 (0·0–0·0)	2·07 (1·98–2·17)	0·65 (0·58–0·73)	0·54 (0·49–0·58)
1964	0·0 (0·0–0·0)	5·5 (4·7–6·3)	136·4 (121·8–152·8)	188·2 (180·1–196·6)	85·9 (77·9–95·0)	22·2 (18·8–25·7)	4·6 (3·3–6·5)	0·3 (0·2–0·5)	0·0 (0·0–0·0)	2·22 (2·11–2·32)	0·71 (0·64–0·79)	0·56 (0·52–0·61)
1965	0·0 (0·0–0·0)	5·4 (4·6–6·2)	126·1 (110·1–144·9)	158·3 (149·9–167·0)	71·1 (62·4–79·8)	17·3 (14·2–21·0)	3·9 (2·8–5·4)	0·3 (0·2–0·4)	0·0 (0·0–0·0)	1·91 (1·8–2·04)	0·66 (0·58–0·75)	0·46 (0·41–0·51)
1966	0·0 (0·0–0·0)	5·7 (4·9–6·6)	123·8 (105·1–143·4)	162·8 (154·4–171·1)	72·6 (63·1–83·0)	18·8 (15·7–22·5)	3·9 (2·8–5·3)	0·3 (0·2–0·4)	0·0 (0·0–0·0)	1·94 (1·82–2·08)	0·65 (0·55–0·75)	0·48 (0·43–0·53)
1967	0·0 (0·0–0·0)	6·1 (5·1–7·1)	132·5 (111·5–155·7)	197·8 (189·3–206·0)	87·1 (74·7–100·7)	19·9 (16·2–24·0)	3·8 (2·8–4·9)	0·3 (0·2–0·4)	0·0 (0·0–0·0)	2·24 (2·07–2·4)	0·69 (0·59–0·81)	0·56 (0·49–0·62)
1968	0·0 (0·0–0·0)	6·2 (5·2–7·3)	119·1 (99·3–141·2)	185·7 (177·8–193·5)	79·0 (67·3–91·0)	16·5 (13·3–20·1)	3·4 (2·5–4·4)	0·2 (0·1–0·3)	0·0 (0·0–0·0)	2·05 (1·91–2·2)	0·63 (0·53–0·74)	0·5 (0·43–0·56)
1969	0·0 (0·0–0·0)	6·8 (5·6–8·1)	117·6 (98·2–139·3)	187·4 (179·6–195·0)	78·8 (67·1–91·8)	17·6 (14·2–21·5)	3·4 (2·5–4·5)	0·2 (0·1–0·3)	0·0 (0·0–0·0)	2·06 (1·93–2·21)	0·62 (0·53–0·73)	0·5 (0·44–0·56)
1970	0·0 (0·0–0·0)	6·9 (5·7–8·3)	127·0 (107·3–147·6)	190·8 (182·5–199·0)	80·7 (69·5–93·5)	21·4 (17·3–26·0)	3·6 (2·7–4·9)	0·2 (0·1–0·4)	0·0 (0·0–0·0)	2·15 (2·02–2·29)	0·67 (0·57–0·77)	0·53 (0·48–0·59)
1971	0·0 (0·0–0·0)	6·8 (5·5–8·3)	139·8 (120·7–161·7)	188·5 (180·1–197·1)	81·6 (70·3–95·0)	24·3 (19·8–29·5)	3·9 (2·8–5·3)	0·3 (0·2–0·4)	0·0 (0·0–0·0)	2·23 (2·09–2·37)	0·73 (0·64–0·84)	0·55 (0·49–0·61)
1972	0·0 (0·0–0·0)	6·9 (5·6–8·4)	147·1 (129·0–168·1)	186·7 (178·1–195·2)	81·4 (71·4–91·4)	24·8 (19·8–29·9)	3·9 (2·8–5·4)	0·3 (0·2–0·4)	0·0 (0·0–0·0)	2·26 (2·13–2·38)	0·77 (0·68–0·87)	0·55 (0·51–0·6)
1973	0·0 (0·0–0·0)	6·8 (5·5–8·3)	148·1 (131·7–164·7)	186·0 (176·7–195·0)	77·1 (68·9–86·1)	22·7 (18·5–27·5)	3·6 (2·5–5·1)	0·3 (0·2–0·4)	0·0 (0·0–0·0)	2·22 (2·11–2·33)	0·77 (0·69–0·85)	0·52 (0·48–0·56)
1974	0·0 (0·0–0·0)	6·4 (5·2–7·8)	142·3 (127·4–158·6)	177·9 (169·9–185·8)	68·3 (61·3–75·3)	17·8 (14·1–22·2)	3·1 (2·0–4·6)	0·2 (0·1–0·3)	0·0 (0·0–0·0)	2·08 (1·99–2·17)	0·74 (0·67–0·82)	0·45 (0·41–0·48)
1975	0·0 (0·0–0·0)	5·9 (4·7–7·3)	133·4 (119·2–147·4)	171·0 (163·0–178·9)	61·8 (55·7–67·8)	14·0 (11·1–17·5)	2·6 (1·5–4·1)	0·1 (0·1–0·2)	0·0 (0·0–0·0)	1·94 (1·85–2·03)	0·7 (0·63–0·77)	0·39 (0·36–0·43)
1976	0·0 (0·0–0·0)	5·5 (4·4–6·8)	126·0 (112·7–140·8)	168·6 (160·6–177·0)	60·6 (54·8–66·6)	11·9 (9·6–14·4)	2·2 (1·5–3·3)	0·1 (0·1–0·2)	0·0 (0·0–0·0)	1·87 (1·79–1·97)	0·66 (0·59–0·73)	0·37 (0·34–0·41)
1977	0·0 (0·0–0·0)	5·3 (4·4–6·4)	118·5 (105·8–133·6)	169·2 (161·2–176·9)	62·7 (56·5–69·8)	11·2 (9·0–13·8)	2·0 (1·4–2·9)	0·1 (0·1–0·2)	0·0 (0·0–0·0)	1·85 (1·76–1·93)	0·62 (0·56–0·7)	0·38 (0·34–0·42)
1978	0·0 (0·0–0·0)	5·2 (4·1–6·3)	112·4 (101·3–124·0)	170·1 (162·5–177·8)	65·7 (59·1–72·4)	11·4 (8·7–14·8)	1·8 (1·2–2·5)	0·1 (0·0–0·1)	0·0 (0·0–0·0)	1·83 (1·75–1·93)	0·59 (0·53–0·65)	0·39 (0·36–0·43)
1979	0·0 (0·0–0·0)	5·1 (4·2–6·1)	106·8 (94·8–120·0)	169·1 (161·7–176·6)	65·5 (59·6–72·3)	11·3 (9·4–13·6)	1·6 (1·1–2·4)	0·1 (0·0–0·1)	0·0 (0·0–0·0)	1·8 (1·72–1·88)	0·56 (0·5–0·63)	0·39 (0·36–0·43)
1980	0·0 (0·0–0·0)	5·1 (4·1–6·3)	101·6 (91·0–112·1)	167·1 (159·4–175·6)	64·1 (57·8–70·7)	11·4 (9·3–14·1)	1·5 (1·0–2·0)	0·1 (0·0–0·1)	0·0 (0·0–0·0)	1·75 (1·68–1·83)	0·53 (0·48–0·59)	0·38 (0·35–0·42)
1981	0·0 (0·0–0·0)	5·2 (4·1–6·5)	98·6 (86·5–113·1)	168·3 (160·5–176·3)	65·0 (58·8–71·4)	12·1 (9·7–14·7)	1·4 (1·0–2·1)	0·1 (0·0–0·1)	0·0 (0·0–0·0)	1·75 (1·68–1·83)	0·52 (0·46–0·59)	0·39 (0·36–0·43)
1982	0·0 (0·0–0·0)	5·3 (4·1–6·7)	96·9 (85·9–108·8)	170·5 (163·0–178·5)	68·0 (61·9–74·6)	13·6 (11·2–16·3)	1·4 (1·0–2·0)	0·0 (0·0–0·1)	0·0 (0·0–0·0)	1·78 (1·71–1·86)	0·51 (0·46–0·57)	0·42 (0·38–0·45)
1983	0·0 (0·0–0·0)	5·4 (4·2–6·7)	94·2 (84·3–104·9)	172·0 (163·1–180·2)	71·5 (65·0–79·0)	15·1 (12·2–18·4)	1·5 (1·1–1·9)	0·0 (0·0–0·1)	0·0 (0·0–0·0)	1·8 (1·73–1·88)	0·5 (0·45–0·55)	0·44 (0·4–0·48)
1984	0·0 (0·0–0·0)	5·1 (4·0–6·4)	87·5 (75·0–101·8)	170·7 (163·2–179·4)	74·4 (67·1–82·4)	15·7 (12·8–19·4)	1·6 (1·1–2·1)	0·0 (0·0–0·1)	0·0 (0·0–0·0)	1·78 (1·68–1·88)	0·46 (0·4–0·53)	0·46 (0·42–0·5)
1985	0·0 (0·0–0·0)	4·8 (3·8–6·0)	81·8 (72·4–92·3)	166·4 (158·7–173·7)	75·6 (69·5–82·0)	15·7 (12·7–19·1)	1·6 (1·2–2·2)	0·1 (0·0–0·1)	0·0 (0·0–0·0)	1·73 (1·66–1·8)	0·43 (0·39–0·48)	0·47 (0·43–0·5)
1986	0·0 (0·0–0·0)	4·6 (3·6–5·7)	77·6 (68·1–87·5)	162·6 (154·5–171·2)	77·9 (68·9–86·5)	15·8 (12·8–19·0)	1·8 (1·2–2·4)	0·1 (0·0–0·1)	0·0 (0·0–0·0)	1·7 (1·63–1·78)	0·41 (0·36–0·47)	0·48 (0·43–0·53)
1987	0·0 (0·0–0·0)	4·3 (3·4–5·4)	71·5 (62·7–81·2)	159·1 (151·4–166·5)	80·8 (74·7–87·4)	16·6 (13·4–20·2)	2·0 (1·4–2·7)	0·1 (0·0–0·1)	0·0 (0·0–0·0)	1·67 (1·59–1·75)	0·38 (0·33–0·43)	0·5 (0·46–0·54)
1988	0·0 (0·0–0·0)	4·2 (3·3–5·1)	66·3 (58·0–76·4)	151·0 (142·2–160·7)	82·6 (74·8–90·6)	17·6 (14·3–21·2)	2·2 (1·5–2·9)	0·1 (0·0–0·1)	0·0 (0·0–0·0)	1·62 (1·54–1·71)	0·35 (0·31–0·4)	0·51 (0·47–0·56)
1989	0·0 (0·0–0·0)	4·1 (3·4–5·1)	61·7 (53·3–70·9)	142·5 (135·3–150·2)	83·5 (75·8–91·0)	18·6 (15·2–22·3)	2·3 (1·7–3·1)	0·1 (0·0–0·1)	0·0 (0·0–0·0)	1·56 (1·49–1·64)	0·33 (0·29–0·37)	0·52 (0·48–0·56)
1990	0·0 (0·0–0·0)	4·3 (3·5–5·1)	60·2 (52·6–67·4)	140·6 (132·3–148·2)	86·1 (78·5–93·9)	19·6 (16·0–24·0)	2·3 (1·6–3·1)	0·1 (0·0–0·1)	0·0 (0·0–0·0)	1·57 (1·5–1·64)	0·32 (0·28–0·36)	0·54 (0·5–0·58)
1991	0·0 (0·0–0·0)	4·4 (3·7–5·4)	60·6 (52·6–69·3)	138·1 (130·9–146·1)	87·8 (79·7–96·2)	20·8 (17·5–25·1)	2·3 (1·6–3·1)	0·1 (0·0–0·1)	0·0 (0·0–0·0)	1·57 (1·5–1·64)	0·33 (0·29–0·37)	0·55 (0·51–0·6)
1992	0·0 (0·0–0·0)	4·6 (3·8–5·4)	59·1 (52·5–66·4)	131·8 (124·6–139·7)	87·6 (80·5–95·3)	21·6 (17·8–26·3)	2·4 (1·7–3·2)	0·1 (0·0–0·1)	0·0 (0·0–0·0)	1·54 (1·48–1·59)	0·32 (0·28–0·36)	0·56 (0·52–0·6)
1993	0·0 (0·0–0·0)	4·7 (3·8–5·7)	59·2 (50·8–67·8)	132·8 (123·5–142·3)	91·7 (83·9–99·8)	23·5 (19·6–28·0)	2·5 (1·8–3·4)	0·1 (0·0–0·1)	0·0 (0·0–0·0)	1·57 (1·49–1·65)	0·32 (0·28–0·36)	0·59 (0·54–0·64)
1994	0·0 (0·0–0·0)	4·7 (3·9–5·8)	58·7 (51·5–66·6)	131·3 (123·9–138·3)	93·3 (83·6–104·0)	25·0 (20·6–30·6)	2·6 (1·9–3·5)	0·1 (0·0–0·1)	0·0 (0·0–0·0)	1·58 (1·49–1·67)	0·32 (0·28–0·36)	0·6 (0·55–0·66)
1995	0·0 (0·0–0·0)	4·8 (4·0–5·7)	57·6 (50·3–65·3)	124·6 (116·8–132·4)	92·3 (83·7–100·8)	26·0 (21·8–30·9)	2·7 (2·1–3·6)	0·1 (0·0–0·1)	0·0 (0·0–0·0)	1·54 (1·45–1·63)	0·31 (0·28–0·35)	0·61 (0·56–0·66)
1996	0·0 (0·0–0·0)	5·0 (4·0–6·0)	57·4 (49·4–65·2)	121·0 (113·4–129·3)	92·1 (84·5–100·6)	27·3 (22·6–32·5)	2·9 (2·1–3·7)	0·1 (0·0–0·1)	0·0 (0·0–0·0)	1·53 (1·44–1·63)	0·31 (0·27–0·35)	0·61 (0·56–0·67)
1997	0·0 (0·0–0·0)	5·3 (4·3–6·3)	57·6 (50·5–65·2)	117·6 (110·2–125·0)	90·7 (83·2–99·5)	28·0 (23·3–33·6)	3·0 (2·2–4·0)	0·1 (0·0–0·1)	0·0 (0·0–0·0)	1·51 (1·43–1·6)	0·31 (0·28–0·35)	0·61 (0·56–0·66)
1998	0·0 (0·0–0·0)	5·6 (4·5–6·7)	57·5 (50·5–65·8)	113·3 (106·2–120·6)	90·0 (81·6–99·3)	29·1 (24·0–35·3)	3·2 (2·3–4·2)	0·1 (0·0–0·1)	0·0 (0·0–0·0)	1·49 (1·42–1·58)	0·32 (0·28–0·36)	0·61 (0·57–0·67)
1999	0·0 (0·0–0·0)	5·9 (4·9–7·1)	57·4 (50·2–64·9)	109·8 (102·5–117·2)	89·5 (82·6–97·1)	29·8 (24·9–35·3)	3·3 (2·4–4·5)	0·1 (0·0–0·1)	0·0 (0·0–0·0)	1·48 (1·41–1·55)	0·32 (0·28–0·35)	0·61 (0·57–0·66)
2000	0·0 (0·0–0·0)	6·2 (5·1–7·5)	57·7 (48·8–67·0)	107·4 (100·6–114·5)	88·0 (79·8–96·6)	31·1 (25·7–37·2)	3·5 (2·5–4·8)	0·1 (0·0–0·1)	0·0 (0·0–0·0)	1·47 (1·38–1·56)	0·32 (0·27–0·37)	0·61 (0·56–0·67)
2001	0·0 (0·0–0·0)	6·5 (5·3–7·8)	57·6 (51·1–65·0)	103·9 (97·2–111·3)	85·2 (77·7–93·0)	30·9 (25·5–36·9)	3·6 (2·6–5·0)	0·1 (0·0–0·1)	0·0 (0·0–0·0)	1·44 (1·37–1·51)	0·32 (0·29–0·36)	0·6 (0·55–0·65)
2002	0·0 (0·0–0·0)	6·3 (5·2–7·6)	55·8 (48·6–64·1)	100·4 (93·7–107·2)	84·4 (76·7–93·3)	31·3 (25·9–36·8)	3·9 (2·7–5·5)	0·1 (0·0–0·1)	0·0 (0·0–0·0)	1·41 (1·34–1·49)	0·31 (0·27–0·35)	0·6 (0·55–0·65)
2003	0·0 (0·0–0·0)	5·9 (4·7–7·3)	53·1 (46·3–60·8)	97·0 (90·9–103·9)	83·1 (75·8–90·4)	32·5 (27·1–38·5)	4·2 (2·9–5·8)	0·1 (0·1–0·1)	0·0 (0·0–0·0)	1·38 (1·31–1·45)	0·3 (0·26–0·34)	0·6 (0·55–0·66)
2004	0·0 (0·0–0·0)	5·6 (4·5–6·8)	50·7 (44·2–58·5)	92·5 (85·7–99·6)	80·7 (73·7–87·9)	32·9 (27·3–39·1)	4·4 (3·1–5·9)	0·1 (0·1–0·1)	0·0 (0·0–0·0)	1·33 (1·26–1·41)	0·28 (0·25–0·32)	0·59 (0·54–0·64)
2005	0·0 (0·0–0·0)	5·3 (4·3–6·5)	49·9 (40·4–60·2)	90·9 (85·2–97·0)	79·8 (72·4–87·6)	33·2 (27·7–39·4)	4·6 (3·3–6·2)	0·1 (0·1–0·1)	0·0 (0·0–0·0)	1·32 (1·24–1·41)	0·28 (0·23–0·33)	0·59 (0·54–0·64)
2006	0·0 (0·0–0·0)	5·2 (4·1–6·4)	49·9 (43·4–56·5)	91·5 (82·6–101·2)	81·1 (73·7–88·5)	34·4 (28·7–41·3)	5·1 (3·7–6·8)	0·1 (0·1–0·1)	0·0 (0·0–0·0)	1·34 (1·25–1·42)	0·28 (0·24–0·31)	0·6 (0·56–0·66)
2007	0·0 (0·0–0·0)	5·1 (4·0–6·3)	49·5 (43·2–56·6)	91·0 (85·2–97·1)	80·3 (72·7–87·8)	35·7 (30·0–42·4)	5·4 (4·0–7·1)	0·1 (0·1–0·2)	0·0 (0·0–0·0)	1·34 (1·27–1·4)	0·27 (0·24–0·31)	0·61 (0·56–0·66)
2008	0·0 (0·0–0·0)	5·0 (4·0–6·1)	48·7 (42·5–55·9)	90·8 (84·6–97·3)	80·1 (72·4–87·6)	36·6 (30·8–43·2)	5·8 (4·3–7·6)	0·1 (0·1–0·2)	0·0 (0·0–0·0)	1·34 (1·27–1·41)	0·27 (0·24–0·31)	0·61 (0·56–0·67)
2009	0·0 (0·0–0·0)	4·8 (3·8–5·8)	47·3 (39·6–55·5)	91·1 (83·0–99·3)	80·6 (72·7–89·5)	37·7 (31·6–44·3)	6·2 (4·7–8·4)	0·1 (0·1–0·2)	0·0 (0·0–0·0)	1·34 (1·25–1·43)	0·26 (0·22–0·3)	0·62 (0·58–0·68)
2010	0·0 (0·0–0·0)	4·6 (3·8–5·4)	46·0 (40·3–52·2)	92·1 (86·4–97·8)	82·0 (74·6–90·4)	38·8 (33·1–45·0)	6·5 (4·8–8·4)	0·1 (0·1–0·2)	0·0 (0·0–0·0)	1·35 (1·29–1·42)	0·25 (0·22–0·28)	0·64 (0·59–0·69)
2011	0·0 (0·0–0·0)	4·5 (3·7–5·4)	43·9 (38·5–50·4)	91·9 (85·4–98·9)	82·9 (74·8–91·4)	39·8 (33·5–46·8)	6·7 (5·0–8·7)	0·1 (0·1–0·2)	0·0 (0·0–0·0)	1·35 (1·29–1·42)	0·24 (0·21–0·27)	0·65 (0·6–0·7)
2012	0·0 (0·0–0·0)	4·4 (3·8–5·1)	42·0 (35·7–48·9)	91·7 (85·9–98·1)	85·0 (77·4–93·7)	41·9 (35·5–49·2)	7·1 (5·3–9·2)	0·1 (0·1–0·2)	0·0 (0·0–0·0)	1·36 (1·29–1·43)	0·23 (0·2–0·27)	0·67 (0·62–0·72)
2013	0·0 (0·0–0·0)	4·4 (3·6–5·2)	40·7 (34·9–46·9)	90·6 (84·0–97·9)	86·0 (78·8–94·2)	43·0 (36·5–49·6)	7·4 (5·6–9·8)	0·2 (0·1–0·2)	0·0 (0·0–0·0)	1·36 (1·29–1·43)	0·23 (0·2–0·26)	0·68 (0·63–0·74)
2014	0·0 (0·0–0·0)	4·3 (3·6–5·1)	39·4 (33·9–45·9)	90·6 (83·7–97·8)	87·4 (79·7–95·7)	44·4 (37·9–51·7)	7·7 (5·6–10·3)	0·2 (0·1–0·2)	0·0 (0·0–0·0)	1·37 (1·3–1·44)	0·22 (0·19–0·25)	0·7 (0·64–0·76)
2015	0·0 (0·0–0·0)	4·3 (3·5–5·2)	40·4 (30·7–52·1)	92·8 (83·7–103·1)	89·7 (80·5–99·5)	43·0 (35·4–51·7)	7·1 (5·0–9·9)	0·2 (0·1–0·2)	0·0 (0·0–0·0)	1·39 (1·22–1·58)	0·22 (0·17–0·29)	0·7 (0·62–0·79)
2016	0·0 (0·0–0·0)	4·4 (3·6–5·3)	41·8 (32·2–54·6)	94·2 (82·5–107·5)	89·6 (77·4–103·4)	39·6 (30·0–51·6)	6·2 (4·1–8·8)	0·1 (0·1–0·2)	0·0 (0·0–0·0)	1·38 (1·18–1·63)	0·23 (0·18–0·3)	0·68 (0·57–0·81)
2017	0·0 (0·0–0·0)	4·3 (3·6–5·3)	41·8 (30·9–55·8)	94·1 (81·4–108·0)	88·4 (75·3–103·2)	38·6 (28·5–50·9)	6·1 (3·9–9·2)	0·1 (0·1–0·2)	0·0 (0·0–0·0)	1·37 (1·14–1·63)	0·23 (0·17–0·3)	0·67 (0·55–0·81)

Supplemental Results Table 5: Age-specific fertility rates per 1000, cumulative total fertility up to maternal age 25 (TFU25), cumulative total fertility from maternal age 30 to age 55 (TFO30), and cumulative total fertility, Chiba, 1950-2017

Year	10-14	15-19	20-24	25-29	30-34	35-39	40-44	45-49	50-54	TFR	TFU25	TFO30
1950	0·0 (0·0–0·0)	12·3 (10·9–13·7)	142·6 (130·2–155·9)	219·8 (211·8–227·8)	171·1 (160·8–181·5)	87·6 (77·8–97·2)	31·0 (26·8–35·6)	1·5 (1·0–2·1)	0·0 (0·0–0·0)	3·33 (3·21–3·45)	0·77 (0·71–0·84)	1·46 (1·38–1·53)
1951	0·0 (0·0–0·0)	9·9 (8·8–11·1)	127·8 (115·8–140·1)	204·1 (196·5–211·6)	154·9 (144·0–165·4)	71·2 (62·7–79·8)	24·0 (20·4–28·2)	1·1 (0·7–1·6)	0·0 (0·0–0·0)	2·96 (2·84–3·1)	0·69 (0·63–0·75)	1·26 (1·15–1·37)
1952	0·0 (0·0–0·0)	8·3 (7·3–9·4)	118·7 (106·6–132·0)	193·8 (185·5–202·3)	140·2 (129·5–151·6)	62·9 (54·5–72·3)	19·0 (15·6–22·4)	0·8 (0·5–1·2)	0·0 (0·0–0·0)	2·72 (2·61–2·83)	0·64 (0·57–0·7)	1·11 (1·04–1·19)
1953	0·0 (0·0–0·0)	7·3 (6·3–8·4)	112·0 (99·5–126·2)	182·6 (174·6–190·8)	125·5 (114·3–136·9)	57·9 (49·1–67·1)	15·1 (12·1–18·2)	0·7 (0·4–1·0)	0·0 (0·0–0·0)	2·5 (2·39–2·62)	0·6 (0·53–0·67)	1·0 (0·92–1·07)
1954	0·0 (0·0–0·0)	6·7 (5·7–7·7)	108·5 (95·3–122·2)	177·5 (170·3–185·3)	115·7 (105·9–126·7)	52·7 (44·8–61·7)	12·4 (10·0–15·3)	0·6 (0·3–0·8)	0·0 (0·0–0·0)	2·37 (2·26–2·49)	0·58 (0·51–0·64)	0·91 (0·83–0·99)
1955	0·0 (0·0–0·0)	6·0 (5·2–7·0)	105·8 (92·4–119·9)	176·4 (167·8–184·7)	106·5 (96·5–117·0)	47·6 (39·9–55·8)	10·4 (8·0–13·1)	0·4 (0·3–0·7)	0·0 (0·0–0·0)	2·27 (2·14–2·38)	0·56 (0·49–0·63)	0·82 (0·76–0·9)
1956	0·0 (0·0–0·0)	5·4 (4·6–6·3)	99·5 (87·8–111·6)	170·4 (162·9–178·3)	94·5 (85·4–104·4)	40·9 (34·4–48·0)	8·3 (6·4–10·4)	0·4 (0·2–0·6)	0·0 (0·0–0·0)	2·1 (1·99–2·2)	0·52 (0·47–0·58)	0·72 (0·66–0·79)
1957	0·0 (0·0–0·0)	5·1 (4·4–6·1)	100·5 (89·5–114·0)	173·7 (166·2–181·7)	89·5 (81·5–97·9)	35·1 (29·1–41·0)	7·0 (5·5–8·9)	0·4 (0·2–0·6)	0·0 (0·0–0·0)	2·06 (1·95–2·17)	0·53 (0·47–0·6)	0·66 (0·6–0·72)
1958	0·0 (0·0–0·0)	5·2 (4·4–6·0)	105·6 (93·0–119·9)	179·4 (170·6–188·0)	87·7 (79·9–96·5)	31·1 (25·7–36·9)	6·4 (5·0–8·0)	0·4 (0·2–0·6)	0·0 (0·0–0·0)	2·08 (1·98–2·19)	0·55 (0·49–0·63)	0·63 (0·57–0·69)
1959	0·0 (0·0–0·0)	5·4 (4·6–6·3)	105·7 (94·2–119·6)	178·4 (170·3–186·9)	82·3 (73·5–91·1)	26·8 (22·2–32·2)	5·3 (4·1–6·9)	0·3 (0·2–0·5)	0·0 (0·0–0·0)	2·02 (1·93–2·12)	0·56 (0·5–0·62)	0·57 (0·52–0·62)
1960	0·0 (0·0–0·0)	5·3 (4·5–6·1)	101·8 (90·9–113·8)	179·3 (171·1–187·7)	79·3 (71·8–86·9)	23·7 (19·3–28·1)	4·4 (3·3–5·6)	0·2 (0·1–0·4)	0·0 (0·0–0·0)	1·97 (1·88–2·06)	0·54 (0·48–0·6)	0·54 (0·49–0·58)
1961	0·0 (0·0–0·0)	4·9 (4·2–5·7)	99·1 (88·6–109·7)	181·3 (172·9–189·9)	77·9 (71·8–84·2)	21·3 (17·8–25·5)	3·8 (2·9–5·0)	0·2 (0·1–0·3)	0·0 (0·0–0·0)	1·94 (1·87–2·02)	0·52 (0·47–0·57)	0·52 (0·48–0·55)
1962	0·0 (0·0–0·0)	4·7 (4·0–5·5)	99·0 (88·3–110·2)	186·7 (179·1–194·3)	80·0 (73·5–86·6)	20·2 (17·1–23·5)	3·4 (2·5–4·4)	0·2 (0·1–0·3)	0·0 (0·0–0·0)	1·97 (1·89–2·05)	0·52 (0·46–0·57)	0·52 (0·48–0·56)
1963	0·0 (0·0–0·0)	4·6 (3·9–5·4)	99·8 (89·2–110·9)	188·8 (181·0–196·6)	82·0 (74·8–89·6)	19·5 (16·6–22·9)	3·3 (2·5–4·5)	0·2 (0·1–0·3)	0·0 (0·0–0·0)	1·99 (1·91–2·07)	0·52 (0·47–0·58)	0·53 (0·48–0·57)
1964	0·0 (0·0–0·0)	4·6 (3·9–5·4)	109·3 (98·2–121·5)	201·1 (192·6–209·4)	88·1 (80·0–96·4)	20·2 (16·9–23·9)	3·2 (2·3–4·1)	0·2 (0·1–0·3)	0·0 (0·0–0·0)	2·13 (2·05–2·22)	0·57 (0·51–0·63)	0·56 (0·51–0·6)
1965	0·0 (0·0–0·0)	4·5 (3·9–5·2)	101·0 (90·6–113·2)	169·8 (161·7–178·1)	73·8 (67·1–80·3)	18·6 (15·5–22·0)	2·7 (2·0–3·6)	0·2 (0·1–0·2)	0·0 (0·0–0·0)	1·85 (1·77–1·94)	0·53 (0·48–0·59)	0·48 (0·44–0·51)
1966	0·0 (0·0–0·0)	4·8 (4·1–5·5)	99·2 (88·6–110·3)	174·8 (166·9–182·7)	76·2 (69·0–84·5)	19·1 (15·6–23·0)	2·7 (2·0–3·6)	0·2 (0·1–0·2)	0·0 (0·0–0·0)	1·88 (1·8–1·97)	0·52 (0·47–0·58)	0·49 (0·45–0·54)
1967	0·0 (0·0–0·0)	5·0 (4·3–5·9)	106·3 (95·4–117·8)	213·2 (205·3–221·2)	92·7 (84·4–102·1)	21·7 (17·9–26·3)	2·7 (2·0–3·5)	0·2 (0·1–0·2)	0·0 (0·0–0·0)	2·21 (2·1–2·32)	0·56 (0·5–0·61)	0·59 (0·54–0·64)
1968	0·0 (0·0–0·0)	5·1 (4·4–5·9)	95·7 (85·0–107·2)	201·4 (192·9–209·4)	85·7 (77·6–94·0)	21·7 (17·9–25·9)	2·4 (1·8–3·3)	0·1 (0·1–0·2)	0·0 (0·0–0·0)	2·06 (1·97–2·15)	0·5 (0·45–0·56)	0·55 (0·5–0·6)
1969	0·0 (0·0–0·0)	5·6 (4·8–6·6)	94·7 (84·6–105·4)	203·8 (195·6–211·9)	86·9 (79·2–95·1)	21·3 (17·6–25·7)	2·5 (1·8–3·3)	0·1 (0·1–0·2)	0·0 (0·0–0·0)	2·07 (1·99–2·16)	0·5 (0·45–0·56)	0·55 (0·51–0·6)
1970	0·0 (0·0–0·0)	5·7 (4·7–6·8)	102·5 (90·6–114·7)	207·6 (199·8–215·2)	90·0 (82·1–98·7)	20·4 (16·7–24·6)	2·7 (1·9–3·6)	0·1 (0·1–0·2)	0·0 (0·0–0·0)	2·14 (2·05–2·24)	0·54 (0·48–0·6)	0·57 (0·52–0·62)
1971	0·0 (0·0–0·0)	5·6 (4·5–6·8)	112·8 (99·8–124·6)	205·2 (195·8–215·2)	92·0 (83·9–100·6)	18·6 (15·4–22·4)	2·9 (2·1–4·0)	0·2 (0·1–0·3)	0·0 (0·0–0·0)	2·19 (2·09–2·28)	0·59 (0·53–0·65)	0·57 (0·52–0·62)
1972	0·0 (0·0–0·0)	5·6 (4·6–6·7)	118·5 (105·8–132·2)	203·8 (195·8–212·1)	93·0 (84·2–101·4)	17·6 (14·4–21·0)	2·9 (2·1–4·0)	0·2 (0·1–0·3)	0·0 (0·0–0·0)	2·21 (2·12–2·31)	0·62 (0·56–0·69)	0·57 (0·52–0·61)
1973	0·0 (0·0–0·0)	5·4 (4·4–6·6)	118·9 (106·2–131·6)	203·7 (195·1–212·2)	89·0 (81·7–97·2)	17·4 (14·3–21·3)	2·7 (1·9–3·7)	0·1 (0·1–0·2)	0·0 (0·0–0·0)	2·19 (2·1–2·28)	0·62 (0·56–0·69)	0·55 (0·51–0·59)
1974	0·0 (0·0–0·0)	5·0 (4·1–6·0)	113·1 (101·8–124·6)	195·1 (186·3–203·5)	78·9 (71·4–86·1)	16·4 (13·6–19·6)	2·3 (1·6–3·0)	0·1 (0·1–0·2)	0·0 (0·0–0·0)	2·05 (1·98–2·14)	0·59 (0·54–0·65)	0·49 (0·45–0·53)
1975	0·0 (0·0–0·0)	4·5 (3·7–5·5)	104·1 (91·1–117·6)	187·1 (178·8–195·1)	69·7 (63·2–76·1)	15·5 (12·7–18·4)	1·8 (1·4–2·3)	0·1 (0·0–0·1)	0·0 (0·0–0·0)	1·91 (1·83–2·0)	0·54 (0·48–0·61)	0·44 (0·4–0·47)
1976	0·0 (0·0–0·0)	4·2 (3·4–5·2)	96·0 (86·3–107·2)	183·4 (175·2–191·5)	68·0 (62·3–74·3)	15·2 (13·0–17·6)	1·7 (1·2–2·3)	0·1 (0·0–0·1)	0·0 (0·0–0·0)	1·84 (1·77–1·92)	0·5 (0·45–0·56)	0·43 (0·39–0·46)
1977	0·0 (0·0–0·0)	4·1 (3·1–5·3)	88·3 (79·0–99·0)	183·1 (175·5–191·5)	71·9 (65·0–78·9)	14·9 (12·2–18·0)	1·8 (1·3–2·4)	0·1 (0·0–0·1)	0·0 (0·0–0·0)	1·82 (1·74–1·91)	0·46 (0·42–0·52)	0·44 (0·4–0·49)
1978	0·0 (0·0–0·0)	4·0 (3·3–4·9)	82·3 (72·9–92·9)	182·7 (175·0–190·4)	76·4 (69·6–83·5)	14·5 (12·1–17·1)	1·8 (1·3–2·4)	0·1 (0·0–0·1)	0·0 (0·0–0·0)	1·81 (1·73–1·89)	0·43 (0·38–0·49)	0·46 (0·43–0·5)
1979	0·0 (0·0–0·0)	4·0 (3·5–4·6)	77·0 (69·0–85·4)	180·2 (172·7–187·4)	77·0 (70·5–84·3)	14·0 (11·5–17·0)	1·8 (1·3–2·6)	0·1 (0·0–0·1)	0·0 (0·0–0·0)	1·77 (1·7–1·84)	0·41 (0·36–0·45)	0·46 (0·43–0·5)
1980	0·0 (0·0–0·0)	4·1 (3·4–4·9)	72·6 (64·3–80·8)	177·7 (169·8–186·0)	75·7 (69·0–82·8)	13·8 (11·3–16·6)	1·8 (1·3–2·5)	0·1 (0·0–0·1)	0·0 (0·0–0·0)	1·73 (1·66–1·8)	0·38 (0·34–0·42)	0·46 (0·42–0·5)
1981	0·0 (0·0–0·0)	4·3 (3·6–5·0)	70·0 (62·0–78·9)	178·8 (170·6–187·0)	76·7 (70·1–83·8)	14·4 (11·9–17·2)	1·8 (1·3–2·7)	0·1 (0·0–0·1)	0·0 (0·0–0·0)	1·73 (1·67–1·79)	0·37 (0·33–0·42)	0·47 (0·43–0·5)
1982	0·0 (0·0–0·0)	4·4 (3·7–5·3)	68·5 (60·7–76·7)	180·1 (172·5–188·4)	80·3 (73·5–88·0)	16·0 (13·5–18·9)	1·8 (1·3–2·5)	0·1 (0·0–0·1)	0·0 (0·0–0·0)	1·76 (1·69–1·82)	0·36 (0·33–0·41)	0·49 (0·45–0·53)
1983	0·0 (0·0–0·0)	4·5 (3·8–5·4)	66·3 (58·7–74·7)	179·2 (170·9–187·2)	84·4 (77·0–92·5)	17·6 (14·4–21·4)	1·9 (1·4–2·5)	0·1 (0·0–0·1)	0·0 (0·0–0·0)	1·77 (1·71–1·84)	0·35 (0·31–0·39)	0·52 (0·48–0·57)
1984	0·0 (0·0–0·0)	4·3 (3·6–5·1)	61·1 (54·6–68·5)	173·9 (166·4–182·0)	87·2 (79·8–95·1)	18·2 (15·2–21·6)	1·9 (1·4–2·6)	0·1 (0·0–0·1)	0·0 (0·0–0·0)	1·73 (1·66–1·8)	0·33 (0·29–0·36)	0·54 (0·5–0·58)
1985	0·0 (0·0–0·0)	4·1 (3·4–4·9)	56·8 (49·6–64·2)	164·7 (157·4–171·5)	88·2 (80·6–96·4)	18·0 (15·0–21·3)	2·0 (1·3–2·8)	0·1 (0·0–0·1)	0·0 (0·0–0·0)	1·67 (1·62–1·73)	0·3 (0·27–0·34)	0·54 (0·5–0·58)
1986	0·0 (0·0–0·0)	3·9 (3·3–4·6)	53·5 (47·3–60·3)	156·5 (149·1–164·4)	90·0 (82·9–96·8)	18·1 (15·0–21·4)	2·1 (1·2–3·3)	0·1 (0·0–0·1)	0·0 (0·0–0·0)	1·62 (1·57–1·68)	0·29 (0·26–0·32)	0·55 (0·51–0·59)
1987	0·0 (0·0–0·0)	3·7 (3·1–4·4)	48·9 (42·1–56·1)	149·0 (141·6–156·5)	92·3 (84·9–101·0)	18·8 (15·8–22·9)	2·3 (1·5–3·3)	0·1 (0·0–0·1)	0·0 (0·0–0·0)	1·58 (1·5–1·66)	0·26 (0·23–0·3)	0·57 (0·53–0·61)
1988	0·0 (0·0–0·0)	3·6 (3·0–4·3)	44·7 (39·5–50·4)	137·9 (131·6–145·0)	92·5 (84·9–101·0)	19·9 (16·6–23·5)	2·5 (1·8–3·4)	0·1 (0·0–0·1)	0·0 (0·0–0·0)	1·51 (1·45–1·57)	0·24 (0·22–0·27)	0·57 (0·53–0·62)
1989	0·0 (0·0–0·0)	3·5 (2·9–4·3)	41·0 (35·6–46·5)	127·3 (120·6–134·2)	91·1 (83·5–98·5)	20·9 (17·3–24·8)	2·6 (1·9–3·6)	0·1 (0·1–0·1)	0·0 (0·0–0·0)	1·43 (1·37–1·5)	0·22 (0·19–0·25)	0·57 (0·53–0·62)
1990	0·0 (0·0–0·0)	3·6 (3·0–4·4)	39·3 (33·5–45·0)	123·3 (116·6–130·0)	91·4 (84·2–99·2)	21·9 (18·3–25·9)	2·6 (1·9–3·4)	0·1 (0·1–0·1)	0·0 (0·0–0·0)	1·41 (1·35–1·48)	0·21 (0·18–0·25)	0·58 (0·54–0·62)
1991	0·0 (0·0–0·0)	3·7 (3·0–4·5)	38·8 (34·2–43·8)	119·2 (113·4–125·2)	91·1 (83·8–98·4)	23·1 (19·6–27·1)	2·6 (1·9–3·5)	0·1 (0·1–0·1)	0·0 (0·0–0·0)	1·39 (1·34–1·45)	0·21 (0·19–0·24)	0·58 (0·54–0·63)
1992	0·0 (0·0–0·0)	3·7 (3·0–4·6)	37·3 (33·3–42·0)	112·3 (106·0–118·8)	89·7 (81·1–98·7)	23·8 (19·9–28·2)	2·8 (2·0–3·6)	0·1 (0·1–0·1)	0·0 (0·0–0·0)	1·35 (1·29–1·41)	0·21 (0·18–0·23)	0·58 (0·53–0·64)
1993	0·0 (0·0–0·0)	3·7 (3·0–4·6)	36·8 (31·6–41·7)	112·0 (105·8–118·7)	93·6 (86·4–101·5)	25·7 (21·7–30·5)	2·9 (2·2–3·9)	0·1 (0·1–0·2)	0·0 (0·0–0·0)	1·37 (1·31–1·44)	0·2 (0·18–0·23)	0·61 (0·57–0·66)
1994	0·0 (0·0–0·0)	3·7 (3·1–4·5)	35·9 (31·8–40·6)	109·6 (103·5–115·7)	95·4 (87·6–104·0)	27·1 (22·8–32·3)	3·1 (2·3–4·1)	0·1 (0·1–0·2)	0·0 (0·0–0·0)	1·37 (1·31–1·43)	0·2 (0·18–0·22)	0·63 (0·58–0·68)
1995	0·0 (0·0–0·0)	3·7 (3·0–4·6)	34·4 (30·4–39·1)	102·9 (96·9–108·7)	94·8 (86·9–103·3)	28·2 (23·6–33·4)	3·3 (2·4–4·4)	0·1 (0·1–0·2)	0·0 (0·0–0·0)	1·34 (1·28–1·4)	0·19 (0·17–0·21)	0·63 (0·58–0·68)
1996	0·0 (0·0–0·0)	3·9 (3·1–4·7)	33·6 (29·1–37·9)	99·0 (93·1–105·1)	94·6 (87·0–103·3)	29·6 (24·9–35·4)	3·5 (2·5–4·7)	0·1 (0·1–0·2)	0·0 (0·0–0·0)	1·32 (1·26–1·38)	0·19 (0·16–0·21)	0·64 (0·6–0·69)
1997	0·0 (0·0–0·0)	4·1 (3·3–4·9)	33·2 (29·1–37·6)	95·7 (90·2–101·4)	92·9 (85·7–100·8)	30·3 (25·5–35·5)	3·8 (2·7–5·1)	0·1 (0·1–0·2)	0·0 (0·0–0·0)	1·3 (1·25–1·36)	0·19 (0·17–0·21)	0·64 (0·59–0·68)
1998	0·0 (0·0–0·0)	4·3 (3·6–5·2)	33·0 (28·8–37·9)	92·2 (87·2–97·5)	92·1 (84·8–100·5)	31·5 (26·5–36·9)	3·9 (2·9–5·2)	0·1 (0·1–0·2)	0·0 (0·0–0·0)	1·29 (1·23–1·35)	0·19 (0·16–0·21)	0·64 (0·59–0·68)
1999	0·0 (0·0–0·0)	4·7 (3·9–5·7)	33·1 (28·7–37·8)	89·4 (83·9–95·0)	91·8 (84·3–99·9)	32·3 (27·3–37·8)	4·1 (3·0–5·6)	0·1 (0·1–0·2)	0·0 (0·0–0·0)	1·28 (1·22–1·34)	0·19 (0·17–0·21)	0·64 (0·59–0·69)
2000	0·0 (0·0–0·0)	5·1 (4·2–6·2)	33·8 (29·9–37·6)	87·6 (82·6–93·2)	90·5 (83·2–98·5)	33·9 (28·9–38·9)	4·3 (3·1–5·7)	0·1 (0·1–0·2)	0·0 (0·0–0·0)	1·28 (1·22–1·34)	0·19 (0·17–0·22)	0·64 (0·6–0·69)
2001	0·0 (0·0–0·0)	5·5 (4·6–6·5)	34·5 (30·0–39·2)	85·2 (80·2–90·7)	88·0 (80·1–95·8)	33·8 (28·8–39·1)	4·4 (3·3–5·9)	0·1 (0·1–0·2)	0·0 (0·0–0·0)	1·26 (1·2–1·32)	0·2 (0·18–0·22)	0·63 (0·58–0·68)
2002	0·0 (0·0–0·0)	5·5 (4·6–6·5)	34·2 (29·8–39·1)	83·0 (77·6–88·7)	87·7 (80·4–95·9)	34·4 (29·4–40·0)	4·7 (3·4–6·2)	0·1 (0·1–0·2)	0·0 (0·0–0·0)	1·25 (1·18–1·31)	0·2 (0·18–0·22)	0·63 (0·59–0·68)
2003	0·0 (0·0–0·0)	5·3 (4·4–6·3)	33·3 (29·5–37·9)	81·0 (76·5–85·6)	87·0 (80·3–93·9)	35·9 (30·5–42·1)	5·0 (3·5–6·6)	0·1 (0·1–0·2)	0·0 (0·0–0·0)	1·24 (1·18–1·3)	0·19 (0·17–0·22)	0·64 (0·59–0·69)
2004	0·0 (0·0–0·0)	5·1 (4·3–6·0)	32·6 (28·3–37·5)	78·0 (72·9–83·2)	85·3 (77·2–93·4)	36·8 (31·3–42·6)	5·2 (3·8–6·8)	0·1 (0·1–0·2)	0·0 (0·0–0·0)	1·22 (1·16–1·27)	0·19 (0·17–0·21)	0·64 (0·59–0·69)
2005	0·0 (0·0–0·0)	5·0 (4·2–5·9)	32·6 (28·6–37·1)	77·4 (72·3–82·5)	85·5 (78·2–92·9)	37·9 (32·3–44·0)	5·4 (4·1–7·1)	0·2 (0·1–0·2)	0·0 (0·0–0·0)	1·22 (1·16–1·28)	0·19 (0·17–0·21)	0·65 (0·6–0·69)
2006	0·0 (0·0–0·0)	5·0 (4·2–5·8)	33·3 (28·9–38·3)	78·6 (73·5–83·7)	88·4 (80·9–96·1)	40·2 (34·4–47·2)	5·9 (4·6–7·6)	0·2 (0·1–0·3)	0·0 (0·0–0·0)	1·26 (1·2–1·32)	0·19 (0·17–0·22)	0·67 (0·63–0·72)
2007	0·0 (0·0–0·0)	4·9 (4·1–5·8)	33·3 (29·2–37·7)	78·7 (73·9–84·7)	89·0 (82·0–96·7)	42·5 (36·6–49·2)	6·4 (4·9–8·2)	0·2 (0·1–0·3)	0·0 (0·0–0·0)	1·28 (1·22–1·33)	0·19 (0·17–0·21)	0·69 (0·64–0·74)
2008	0·0 (0·0–0·0)	4·8 (4·1–5·7)	33·0 (28·3–38·3)	78·7 (73·4–83·6)	90·0 (82·5–97·4)	44·1 (38·2–50·9)	7·0 (5·5–8·7)	0·2 (0·1–0·3)	0·0 (0·0–0·0)	1·29 (1·23–1·35)	0·19 (0·17–0·22)	0·71 (0·66–0·76)
2009	0·0 (0·0–0·0)	4·6 (3·8–5·4)	31·8 (28·6–35·1)	78·5 (73·6–83·1)	90·8 (83·2–98·7)	45·5 (39·0–52·5)	7·7 (6·1–9·9)	0·2 (0·1–0·3)	0·0 (0·0–0·0)	1·3 (1·23–1·36)	0·18 (0·16–0·2)	0·72 (0·67–0·77)
2010	0·0 (0·0–0·0)	4·3 (3·7–5·1)	30·7 (26·0–35·5)	78·8 (73·7–84·3)	91·6 (84·1–100·1)	46·4 (39·9–53·3)	8·2 (6·5–10·2)	0·2 (0·1–0·3)	0·0 (0·0–0·0)	1·3 (1·24–1·37)	0·18 (0·15–0·2)	0·73 (0·68–0·79)
2011	0·0 (0·0–0·0)	4·1 (3·5–4·9)	28·8 (25·1–33·2)	77·7 (72·4–83·2)	91·0 (83·9–98·7)	47·0 (40·6–54·5)	8·7 (6·8–10·8)	0·2 (0·1–0·4)	0·0 (0·0–0·0)	1·29 (1·23–1·35)	0·16 (0·15–0·19)	0·73 (0·69–0·79)
2012	0·0 (0·0–0·0)	4·0 (3·3–4·8)	27·1 (23·4–31·5)	76·5 (71·9–81·2)	91·7 (83·7–99·9)	48·9 (42·4–55·6)	9·5 (7·4–11·9)	0·2 (0·1–0·4)	0·0 (0·0–0·0)	1·29 (1·23–1·35)	0·16 (0·14–0·18)	0·75 (0·7–0·81)
2013	0·0 (0·0–0·0)	3·9 (3·2–4·6)	25·9 (22·3–29·6)	74·6 (69·7–79·9)	91·5 (84·0–99·3)	50·0 (43·5–56·8)	10·0 (7·8–12·7)	0·3 (0·2–0·4)	0·0 (0·0–0·0)	1·28 (1·22–1·34)	0·15 (0·13–0·17)	0·76 (0·7–0·81)
2014	0·0 (0·0–0·0)	3·8 (3·2–4·5)	24·8 (21·1–28·7)	73·6 (68·7–79·1)	92·5 (85·1–100·5)	51·9 (45·1–59·3)	10·6 (8·4–13·2)	0·3 (0·2–0·4)	0·0 (0·0–0·0)	1·29 (1·22–1·36)	0·14 (0·12–0·16)	0·78 (0·72–0·84)
2015	0·0 (0·0–0·0)	3·8 (3·1–4·5)	25·1 (21·5–29·7)	74·7 (68·9–80·9)	95·0 (85·5–104·9)	50·6 (42·5–59·7)	9·9 (7·6–12·7)	0·3 (0·2–0·4)	0·0 (0·0–0·0)	1·3 (1·16–1·45)	0·14 (0·12–0·17)	0·78 (0·69–0·87)
2016	0·0 (0·0–0·0)	3·8 (3·1–4·7)	26·2 (20·5–33·5)	75·7 (67·8–85·1)	94·9 (82·6–109·3)	47·0 (36·6–60·7)	8·5 (6·1–11·5)	0·2 (0·2–0·4)	0·0 (0·0–0·0)	1·28 (1·1–1·51)	0·15 (0·12–0·19)	0·75 (0·64–0·89)
2017	0·0 (0·0–0·0)	3·8 (3·1–4·6)	26·2 (20·5–33·5)	75·5 (67·0–84·8)	93·9 (80·3–108·6)	46·0 (34·4–60·0)	8·3 (5·7–12·1)	0·2 (0·2–0·4)	0·0 (0·0–0·0)	1·27 (1·06–1·51)	0·15 (0·12–0·19)	0·74 (0·61–0·9)

Supplemental Results Table 5: Age-specific fertility rates per 1000, cumulative total fertility up to maternal age 25 (TFU25), cumulative total fertility from maternal age 30 to age 55 (TFO30), and cumulative total fertility, Ehime, 1950-2017

Year	10-14	15-19	20-24	25-29	30-34	35-39	40-44	45-49	50-54	TFR	TFU25	TFO30
1950	0·0 (0·0–0·0)	11·8 (10·4–13·2)	159·7 (145·8–174·5)	221·3 (213·2–229·5)	166·3 (156·4–176·3)	104·9 (93·2–116·5)	36·0 (31·1–41·3)	2·1 (1·4–3·0)	0·0 (0·0–0·0)	3·51 (3·39–3·64)	0·86 (0·79–0·93)	1·55 (1·47–1·63)
1951	0·0 (0·0–0·0)	9·5 (8·4–10·8)	143·3 (130·4–156·2)	205·6 (197·7–213·4)	150·5 (139·5–160·9)	84·8 (73·3–95·7)	27·8 (23·2–32·8)	1·5 (1·0–2·2)	0·0 (0·0–0·0)	3·12 (2·97–3·26)	0·76 (0·7–0·83)	1·32 (1·2–1·44)
1952	0·0 (0·0–0·0)	8·0 (6·9–9·3)	133·5 (119·3–149·1)	195·4 (186·9–203·9)	136·1 (124·1–148·0)	67·9 (58·7–78·2)	22·0 (18·0–26·5)	1·2 (0·7–1·7)	0·0 (0·0–0·0)	2·82 (2·71–2·93)	0·71 (0·64–0·78)	1·14 (1·06–1·21)
1953	0·0 (0·0–0·0)	7·0 (6·0–8·3)	126·1 (112·4–141·4)	184·1 (176·0–192·8)	121·6 (108·6–134·9)	54·1 (45·8–62·9)	17·5 (14·0–21·7)	1·0 (0·6–1·5)	0·0 (0·0–0·0)	2·56 (2·45–2·69)	0·67 (0·6–0·74)	0·97 (0·9–1·05)
1954	0·0 (0·0–0·0)	6·4 (5·3–7·7)	122·3 (107·6–137·1)	179·1 (171·1–187·6)	112·1 (98·9–126·8)	45·0 (37·5–53·1)	14·4 (11·0–18·8)	0·8 (0·5–1·2)	0·0 (0·0–0·0)	2·4 (2·28–2·52)	0·64 (0·57–0·72)	0·86 (0·79–0·93)
1955	0·0 (0·0–0·0)	5·8 (4·7–7·1)	119·4 (105·5–135·5)	178·0 (169·4–186·5)	103·2 (89·5–117·3)	38·8 (32·8–46·0)	12·0 (8·9–16·3)	0·6 (0·4–1·0)	0·0 (0·0–0·0)	2·29 (2·18–2·4)	0·63 (0·55–0·71)	0·77 (0·7–0·84)
1956	0·0 (0·0–0·0)	5·2 (4·2–6·5)	112·5 (98·1–127·4)	172·0 (164·5–179·9)	91·4 (79·3–105·0)	33·1 (27·7–39·2)	9·6 (6·8–13·2)	0·5 (0·3–0·8)	0·0 (0·0–0·0)	2·12 (2·02–2·23)	0·59 (0·52–0·66)	0·67 (0·61–0·74)
1957	0·0 (0·0–0·0)	5·0 (3·9–6·2)	113·7 (99·3–130·7)	175·5 (167·9–183·5)	86·6 (75·2–100·2)	28·4 (23·6–32·9)	8·2 (5·7–11·1)	0·5 (0·3–0·8)	0·0 (0·0–0·0)	2·09 (1·96–2·21)	0·59 (0·52–0·68)	0·62 (0·56–0·68)
1958	0·0 (0·0–0·0)	5·0 (3·9–6·3)	119·4 (105·5–134·1)	181·3 (172·4–190·2)	84·6 (72·9–97·8)	25·1 (20·9–29·9)	7·4 (5·1–10·4)	0·5 (0·3–0·8)	0·0 (0·0–0·0)	2·12 (2·01–2·22)	0·62 (0·55–0·7)	0·59 (0·53–0·65)
1959	0·0 (0·0–0·0)	5·2 (4·0–6·5)	119·4 (105·7–134·6)	180·2 (171·7–189·2)	79·2 (69·3–90·7)	21·6 (17·6–26·6)	6·2 (4·1–8·6)	0·4 (0·3–0·7)	0·0 (0·0–0·0)	2·06 (1·96–2·17)	0·62 (0·55–0·7)	0·54 (0·49–0·59)
1960	0·0 (0·0–0·0)	5·1 (4·0–6·4)	114·8 (101·9–128·5)	180·9 (172·1–189·6)	75·9 (67·6–84·9)	18·9 (15·9–22·3)	5·0 (3·4–6·9)	0·3 (0·2–0·5)	0·0 (0·0–0·0)	2·01 (1·92–2·1)	0·6 (0·54–0·67)	0·5 (0·46–0·55)
1961	0·0 (0·0–0·0)	4·7 (3·7–5·8)	111·5 (100·1–122·8)	182·8 (174·4–191·7)	74·2 (65·2–84·8)	17·0 (13·8–20·6)	4·3 (2·9–6·1)	0·3 (0·2–0·5)	0·0 (0·0–0·0)	1·97 (1·88–2·07)	0·58 (0·52–0·64)	0·48 (0·43–0·53)
1962	0·0 (0·0–0·0)	4·5 (3·6–5·6)	111·2 (98·8–123·8)	188·0 (180·4–195·8)	75·7 (69·1–81·9)	16·1 (13·2–19·2)	3·8 (2·7–5·3)	0·3 (0·2–0·4)	0·0 (0·0–0·0)	2·0 (1·91–2·08)	0·58 (0·52–0·64)	0·48 (0·45–0·51)
1963	0·0 (0·0–0·0)	4·4 (3·6–5·4)	111·8 (99·8–124·2)	190·0 (182·4–197·8)	77·2 (70·8–84·4)	16·1 (13·6–18·9)	3·7 (2·6–5·4)	0·3 (0·2–0·5)	0·0 (0·0–0·0)	2·02 (1·94–2·1)	0·58 (0·52–0·64)	0·49 (0·45–0·52)
1964	0·0 (0·0–0·0)	4·4 (3·6–5·3)	122·2 (110·1–136·1)	202·3 (193·4–211·5)	82·4 (74·8–91·0)	16·6 (14·1–19·3)	3·5 (2·5–4·8)	0·3 (0·2–0·4)	0·0 (0·0–0·0)	2·16 (2·07–2·25)	0·63 (0·57–0·7)	0·51 (0·47–0·56)
1965	0·0 (0·0–0·0)	4·3 (3·6–5·1)	112·6 (100·9–126·3)	170·6 (161·6–179·7)	68·5 (60·3–76·8)	14·6 (12·4–17·1)	3·0 (2·1–4·0)	0·2 (0·1–0·3)	0·0 (0·0–0·0)	1·87 (1·78–1·96)	0·58 (0·52–0·65)	0·43 (0·39–0·48)
1966	0·0 (0·0–0·0)	4·6 (3·8–5·3)	110·1 (98·1–122·6)	175·3 (166·4–184·2)	70·0 (60·8–80·1)	14·8 (12·3–17·8)	3·0 (2·2–3·9)	0·2 (0·1–0·4)	0·0 (0·0–0·0)	1·89 (1·81–1·98)	0·57 (0·51–0·64)	0·44 (0·4–0·49)
1967	0·0 (0·0–0·0)	4·8 (4·1–5·6)	117·4 (105·4–130·0)	213·4 (204·5–221·6)	84·3 (72·5–97·9)	16·8 (13·8–20·2)	2·9 (2·1–3·7)	0·2 (0·1–0·3)	0·0 (0·0–0·0)	2·2 (2·09–2·31)	0·61 (0·55–0·67)	0·52 (0·46–0·59)
1968	0·0 (0·0–0·0)	4·8 (4·2–5·6)	105·2 (93·5–117·8)	200·9 (192·7–208·9)	76·7 (65·2–88·5)	16·7 (13·4–20·2)	2·5 (1·9–3·3)	0·2 (0·1–0·3)	0·0 (0·0–0·0)	2·04 (1·95–2·12)	0·55 (0·49–0·61)	0·48 (0·42–0·54)
1969	0·0 (0·0–0·0)	5·3 (4·5–6·2)	103·4 (91·5–115·3)	202·9 (194·8–211·1)	76·9 (64·9–90·0)	16·3 (13·3–20·0)	2·5 (1·9–3·4)	0·2 (0·1–0·2)	0·0 (0·0–0·0)	2·04 (1·96–2·12)	0·54 (0·48–0·6)	0·48 (0·43–0·54)
1970	0·0 (0·0–0·0)	5·3 (4·5–6·3)	111·4 (98·7–125·4)	206·2 (197·4–214·8)	79·1 (68·1–91·6)	16·0 (12·8–19·9)	2·7 (2·0–3·6)	0·2 (0·1–0·3)	0·0 (0·0–0·0)	2·1 (2·01–2·2)	0·58 (0·52–0·65)	0·49 (0·44–0·55)
1971	0·0 (0·0–0·0)	5·2 (4·2–6·3)	122·0 (107·7–135·9)	203·4 (194·5–212·5)	80·2 (69·0–93·5)	16·2 (13·2–19·5)	2·9 (2·1–4·0)	0·2 (0·1–0·3)	0·0 (0·0–0·0)	2·15 (2·05–2·26)	0·64 (0·57–0·71)	0·5 (0·44–0·56)
1972	0·0 (0·0–0·0)	5·2 (4·3–6·4)	127·7 (112·9–143·8)	201·6 (192·6–210·3)	80·2 (70·8–90·3)	16·4 (13·1–20·0)	2·9 (2·0–4·0)	0·2 (0·1–0·3)	0·0 (0·0–0·0)	2·17 (2·06–2·27)	0·66 (0·59–0·74)	0·5 (0·45–0·55)
1973	0·0 (0·0–0·0)	5·1 (4·0–6·3)	127·8 (114·3–142·1)	201·2 (191·3–210·8)	76·2 (68·2–85·1)	15·0 (11·8–18·4)	2·7 (1·9–3·7)	0·2 (0·1–0·3)	0·0 (0·0–0·0)	2·14 (2·05–2·24)	0·66 (0·6–0·74)	0·47 (0·43–0·51)
1974	0·0 (0·0–0·0)	4·7 (3·8–5·9)	122·1 (109·7–136·3)	193·0 (184·5–201·7)	67·4 (60·4–74·3)	12·5 (9·8–15·7)	2·3 (1·4–3·4)	0·1 (0·1–0·2)	0·0 (0·0–0·0)	2·01 (1·93–2·1)	0·63 (0·57–0·71)	0·41 (0·38–0·45)
1975	0·0 (0·0–0·0)	4·4 (3·4–5·6)	113·5 (100·8–127·0)	185·7 (177·2–194·3)	60·3 (54·1–66·4)	11·4 (9·0–14·4)	1·9 (1·1–3·0)	0·1 (0·1–0·2)	0·0 (0·0–0·0)	1·89 (1·8–1·97)	0·59 (0·53–0·66)	0·37 (0·34–0·4)
1976	0·0 (0·0–0·0)	4·1 (2·9–5·6)	106·6 (94·5–119·3)	182·9 (173·9–192·0)	58·2 (52·6–64·1)	11·3 (9·1–13·7)	1·6 (1·0–2·3)	0·1 (0·0–0·1)	0·0 (0·0–0·0)	1·82 (1·74–1·91)	0·55 (0·49–0·62)	0·36 (0·32–0·39)
1977	0·0 (0·0–0·0)	4·0 (3·1–5·1)	100·1 (88·9–113·3)	183·2 (174·6–191·5)	59·1 (53·1–65·7)	11·2 (8·8–14·2)	1·4 (0·9–2·0)	0·1 (0·0–0·1)	0·0 (0·0–0·0)	1·8 (1·72–1·88)	0·52 (0·46–0·59)	0·36 (0·32–0·4)
1978	0·0 (0·0–0·0)	3·9 (3·1–4·9)	94·9 (85·4–105·8)	183·6 (175·6–191·7)	62·3 (56·2–68·9)	11·1 (8·3–14·5)	1·3 (0·9–1·8)	0·1 (0·0–0·1)	0·0 (0·0–0·0)	1·79 (1·71–1·87)	0·49 (0·45–0·55)	0·37 (0·34–0·41)
1979	0·0 (0·0–0·0)	3·9 (3·3–4·7)	90·2 (79·8–101·7)	182·1 (174·8–189·5)	63·8 (57·9–70·2)	10·8 (9·0–13·0)	1·4 (0·9–2·1)	0·1 (0·0–0·1)	0·0 (0·0–0·0)	1·76 (1·69–1·83)	0·47 (0·42–0·53)	0·38 (0·35–0·41)
1980	0·0 (0·0–0·0)	4·0 (3·2–4·9)	85·8 (76·5–95·6)	180·0 (171·6–188·9)	63·5 (57·0–69·8)	10·8 (8·7–13·3)	1·4 (0·9–1·9)	0·1 (0·0–0·1)	0·0 (0·0–0·0)	1·73 (1·65–1·8)	0·45 (0·4–0·5)	0·38 (0·34–0·41)
1981	0·0 (0·0–0·0)	4·2 (3·2–5·4)	83·0 (73·5–94·6)	181·3 (172·9–189·8)	64·4 (58·7–70·4)	11·4 (9·2–13·9)	1·4 (0·9–2·1)	0·1 (0·0–0·1)	0·0 (0·0–0·0)	1·73 (1·66–1·8)	0·44 (0·39–0·49)	0·39 (0·35–0·42)
1982	0·0 (0·0–0·0)	4·4 (3·5–5·5)	81·6 (72·1–92·4)	183·5 (176·0–191·9)	67·3 (60·9–74·4)	12·7 (10·4–15·4)	1·4 (1·0–2·0)	0·1 (0·0–0·1)	0·0 (0·0–0·0)	1·76 (1·69–1·83)	0·43 (0·38–0·48)	0·41 (0·37–0·44)
1983	0·0 (0·0–0·0)	4·5 (3·6–5·6)	79·9 (70·1–90·8)	184·6 (175·7–192·9)	70·7 (64·0–78·3)	13·9 (11·0–17·1)	1·5 (1·1–1·9)	0·1 (0·0–0·1)	0·0 (0·0–0·0)	1·78 (1·7–1·86)	0·42 (0·37–0·48)	0·43 (0·39–0·47)
1984	0·0 (0·0–0·0)	4·4 (3·7–5·3)	75·2 (66·5–84·7)	182·5 (174·9–191·0)	73·2 (66·6–80·6)	14·3 (11·6–17·7)	1·5 (1·1–2·0)	0·1 (0·0–0·1)	0·0 (0·0–0·0)	1·76 (1·69–1·83)	0·4 (0·35–0·45)	0·45 (0·41–0·48)
1985	0·0 (0·0–0·0)	4·3 (3·5–5·3)	71·8 (62·9–81·8)	177·1 (166·3–187·2)	74·5 (67·0–81·5)	14·1 (11·2–17·2)	1·5 (1·1–2·0)	0·1 (0·0–0·1)	0·0 (0·0–0·0)	1·72 (1·65–1·79)	0·38 (0·33–0·43)	0·45 (0·41–0·49)
1986	0·0 (0·0–0·0)	4·2 (3·4–5·1)	69·6 (60·9–78·6)	172·4 (164·8–180·7)	76·9 (69·9–83·4)	14·1 (11·4–17·0)	1·6 (1·1–2·3)	0·1 (0·0–0·1)	0·0 (0·0–0·0)	1·69 (1·63–1·77)	0·37 (0·33–0·41)	0·46 (0·43–0·5)
1987	0·0 (0·0–0·0)	4·2 (3·4–5·0)	65·4 (58·3–73·4)	168·5 (159·2–177·5)	79·9 (73·4–87·4)	14·6 (11·7–17·9)	1·8 (1·1–2·7)	0·1 (0·0–0·1)	0·0 (0·0–0·0)	1·67 (1·6–1·74)	0·35 (0·31–0·39)	0·48 (0·45–0·53)
1988	0·0 (0·0–0·0)	4·1 (3·3–5·0)	61·8 (53·5–71·0)	159·8 (150·9–169·6)	81·6 (74·5–89·7)	15·3 (12·3–18·7)	1·9 (1·3–2·6)	0·1 (0·0–0·1)	0·0 (0·0–0·0)	1·62 (1·54–1·7)	0·33 (0·29–0·38)	0·49 (0·45–0·54)
1989	0·0 (0·0–0·0)	4·2 (3·3–5·1)	58·0 (50·3–67·3)	150·1 (142·7–158·0)	82·4 (75·2–89·3)	16·1 (12·9–20·1)	2·0 (1·4–2·7)	0·1 (0·0–0·1)	0·0 (0·0–0·0)	1·56 (1·49–1·64)	0·31 (0·27–0·36)	0·5 (0·46–0·54)
1990	0·0 (0·0–0·0)	4·3 (3·5–5·2)	56·6 (49·3–63·8)	147·1 (138·8–155·1)	84·6 (77·4–91·9)	16·9 (13·6–20·7)	2·0 (1·4–2·7)	0·1 (0·0–0·1)	0·0 (0·0–0·0)	1·56 (1·48–1·64)	0·3 (0·27–0·34)	0·52 (0·48–0·56)
1991	0·0 (0·0–0·0)	4·5 (3·7–5·5)	56·8 (49·3–64·9)	143·4 (136·0–151·7)	85·9 (77·8–94·2)	17·9 (14·3–22·0)	2·0 (1·4–2·7)	0·1 (0·0–0·1)	0·0 (0·0–0·0)	1·55 (1·49–1·62)	0·31 (0·27–0·35)	0·53 (0·48–0·58)
1992	0·0 (0·0–0·0)	4·6 (3·7–5·6)	55·1 (48·6–62·5)	135·6 (127·9–143·3)	85·5 (78·5–93·2)	18·6 (14·9–23·0)	2·1 (1·5–2·9)	0·1 (0·0–0·1)	0·0 (0·0–0·0)	1·51 (1·45–1·56)	0·3 (0·27–0·33)	0·53 (0·49–0·58)
1993	0·0 (0·0–0·0)	4·7 (3·8–5·6)	54·8 (47·2–63·2)	135·3 (126·6–143·8)	89·3 (81·8–98·0)	20·2 (16·3–24·7)	2·2 (1·5–2·9)	0·1 (0·0–0·1)	0·0 (0·0–0·0)	1·53 (1·46–1·6)	0·3 (0·26–0·34)	0·56 (0·52–0·6)
1994	0·0 (0·0–0·0)	4·7 (3·8–5·7)	54·2 (47·8–62·0)	132·6 (124·6–140·0)	91·0 (82·3–99·9)	21·5 (17·4–25·9)	2·3 (1·6–3·1)	0·1 (0·0–0·1)	0·0 (0·0–0·0)	1·53 (1·45–1·61)	0·29 (0·26–0·33)	0·57 (0·52–0·63)
1995	0·0 (0·0–0·0)	4·7 (3·9–5·7)	52·9 (45·6–60·7)	125·0 (117·4–133·3)	90·5 (82·1–99·2)	22·4 (18·6–27·0)	2·4 (1·8–3·3)	0·1 (0·0–0·1)	0·0 (0·0–0·0)	1·49 (1·41–1·57)	0·29 (0·25–0·33)	0·58 (0·53–0·63)
1996	0·0 (0·0–0·0)	4·9 (4·1–5·9)	52·4 (45·4–60·0)	120·6 (112·4–129·0)	91·0 (83·4–99·4)	23·7 (19·5–28·7)	2·5 (1·9–3·3)	0·1 (0·0–0·1)	0·0 (0·0–0·0)	1·48 (1·4–1·56)	0·29 (0·25–0·32)	0·59 (0·54–0·64)
1997	0·0 (0·0–0·0)	5·2 (4·2–6·2)	52·2 (45·3–59·6)	116·7 (109·3–123·7)	90·1 (82·8–98·6)	24·6 (20·3–29·4)	2·7 (2·0–3·6)	0·1 (0·0–0·1)	0·0 (0·0–0·0)	1·46 (1·39–1·53)	0·29 (0·25–0·32)	0·59 (0·54–0·64)
1998	0·0 (0·0–0·0)	5·5 (4·5–6·5)	51·8 (45·3–59·6)	112·3 (104·9–119·5)	89·9 (81·8–99·4)	26·0 (21·3–31·3)	2·8 (2·1–3·7)	0·1 (0·0–0·1)	0·0 (0·0–0·0)	1·44 (1·37–1·52)	0·29 (0·25–0·33)	0·59 (0·54–0·65)
1999	0·0 (0·0–0·0)	5·8 (4·8–6·9)	51·4 (44·9–58·2)	109·0 (102·0–116·2)	89·8 (82·0–98·5)	27·0 (22·5–31·8)	3·0 (2·2–4·0)	0·1 (0·0–0·1)	0·0 (0·0–0·0)	1·43 (1·36–1·5)	0·29 (0·25–0·32)	0·6 (0·55–0·65)
2000	0·0 (0·0–0·0)	6·2 (5·1–7·5)	51·8 (43·1–60·3)	106·9 (100·3–113·8)	88·5 (80·7–96·8)	28·7 (24·0–34·5)	3·2 (2·3–4·3)	0·1 (0·0–0·1)	0·0 (0·0–0·0)	1·43 (1·35–1·51)	0·29 (0·25–0·33)	0·6 (0·55–0·66)
2001	0·0 (0·0–0·0)	6·6 (5·4–8·0)	52·0 (46·0–58·8)	104·4 (97·7–111·6)	85·9 (77·9–93·9)	29·0 (23·9–35·0)	3·3 (2·4–4·4)	0·1 (0·0–0·2)	0·0 (0·0–0·0)	1·41 (1·34–1·48)	0·29 (0·26–0·33)	0·59 (0·54–0·64)
2002	0·0 (0·0–0·0)	6·6 (5·4–8·1)	51·1 (44·4–59·2)	102·2 (95·9–108·6)	85·6 (77·8–94·3)	29·6 (24·8–35·4)	3·5 (2·5–4·9)	0·1 (0·0–0·2)	0·0 (0·0–0·0)	1·39 (1·32–1·47)	0·29 (0·25–0·33)	0·59 (0·55–0·65)
2003	0·0 (0·0–0·0)	6·4 (5·1–8·0)	49·6 (43·3–56·4)	100·5 (92·3–109·2)	85·1 (77·4–93·1)	30·9 (25·8–36·7)	3·8 (2·6–5·1)	0·1 (0·0–0·2)	0·0 (0·0–0·0)	1·38 (1·3–1·47)	0·28 (0·25–0·31)	0·6 (0·55–0·65)
2004	0·0 (0·0–0·0)	6·2 (4·9–7·7)	48·4 (41·6–56·5)	97·4 (91·6–103·6)	83·7 (75·9–91·7)	31·6 (26·3–37·5)	3·9 (2·7–5·3)	0·1 (0·1–0·2)	0·0 (0·0–0·0)	1·36 (1·29–1·43)	0·27 (0·24–0·31)	0·6 (0·55–0·65)
2005	0·0 (0·0–0·0)	6·1 (4·8–7·5)	48·7 (42·7–55·6)	97·1 (90·3–104·3)	84·2 (76·8–92·3)	32·4 (26·9–38·7)	4·1 (2·9–5·5)	0·1 (0·1–0·2)	0·0 (0·0–0·0)	1·36 (1·3–1·43)	0·27 (0·24–0·31)	0·6 (0·56–0·66)
2006	0·0 (0·0–0·0)	6·1 (4·9–7·7)	49·9 (43·9–56·5)	98·8 (92·7–105·5)	87·2 (79·5–95·7)	34·0 (28·2–40·7)	4·4 (3·2–6·0)	0·1 (0·1–0·2)	0·0 (0·0–0·0)	1·4 (1·34–1·47)	0·28 (0·25–0·32)	0·63 (0·58–0·69)
2007	0·0 (0·0–0·0)	6·2 (4·9–7·7)	50·5 (42·0–60·3)	98·8 (92·3–106·1)	88·0 (80·0–96·4)	35·8 (30·0–42·9)	4·8 (3·5–6·3)	0·1 (0·1–0·2)	0·0 (0·0–0·0)	1·42 (1·34–1·5)	0·28 (0·24–0·34)	0·64 (0·59–0·7)
2008	0·0 (0·0–0·0)	6·2 (4·9–7·6)	50·3 (44·0–57·3)	98·8 (92·3–105·8)	89·3 (81·5–97·2)	37·2 (31·3–43·7)	5·1 (3·8–6·8)	0·1 (0·1–0·2)	0·0 (0·0–0·0)	1·44 (1·37–1·5)	0·28 (0·25–0·32)	0·66 (0·61–0·71)
2009	0·0 (0·0–0·0)	6·0 (4·8–7·4)	49·4 (42·2–57·3)	99·1 (90·3–107·5)	90·8 (82·7–99·4)	38·6 (32·4–45·4)	5·6 (4·2–7·6)	0·1 (0·1–0·2)	0·0 (0·0–0·0)	1·45 (1·36–1·55)	0·28 (0·24–0·31)	0·68 (0·62–0·73)
2010	0·0 (0·0–0·0)	5·8 (4·7–7·3)	48·6 (42·4–55·4)	100·2 (94·2–106·5)	92·4 (83·6–101·6)	39·8 (33·8–46·2)	5·9 (4·4–7·7)	0·1 (0·1–0·2)	0·0 (0·0–0·0)	1·46 (1·4–1·54)	0·27 (0·24–0·31)	0·69 (0·64–0·75)
2011	0·0 (0·0–0·0)	5·7 (4·6–7·0)	46·8 (40·9–53·4)	100·0 (93·2–107·4)	92·7 (84·5–101·8)	40·8 (34·5–47·4)	6·2 (4·6–8·1)	0·1 (0·1–0·2)	0·0 (0·0–0·0)	1·46 (1·4–1·53)	0·26 (0·23–0·3)	0·7 (0·65–0·75)
2012	0·0 (0·0–0·0)	5·7 (4·8–6·7)	45·1 (39·6–51·2)	99·6 (92·5–106·9)	93·9 (85·2–103·0)	42·6 (36·3–49·7)	6·6 (5·0–8·6)	0·1 (0·1–0·2)	0·0 (0·0–0·0)	1·47 (1·39–1·55)	0·25 (0·23–0·29)	0·72 (0·66–0·78)
2013	0·0 (0·0–0·0)	5·6 (4·6–6·7)	44·0 (36·6–51·8)	98·0 (91·3–104·8)	93·8 (86·1–102·0)	43·4 (37·1–50·2)	6·9 (5·3–9·1)	0·1 (0·1–0·2)	0·0 (0·0–0·0)	1·46 (1·39–1·54)	0·25 (0·21–0·29)	0·72 (0·67–0·78)
2014	0·0 (0·0–0·0)	5·5 (4·6–6·5)	42·6 (36·9–48·6)	97·3 (90·4–104·6)	94·3 (86·3–103·6)	44·6 (38·6–51·6)	7·3 (5·4–9·7)	0·1 (0·1–0·2)	0·0 (0·0–0·0)	1·46 (1·38–1·55)	0·24 (0·21–0·27)	0·73 (0·68–0·8)
2015	0·0 (0·0–0·0)	5·5 (4·5–6·6)	43·3 (36·0–51·7)	99·1 (90·9–108·3)	96·0 (85·7–106·5)	42·7 (34·9–51·2)	6·8 (4·8–9·3)	0·1 (0·1–0·2)	0·0 (0·0–0·0)	1·47 (1·31–1·64)	0·24 (0·2–0·29)	0·73 (0·64–0·82)
2016	0·0 (0·0–0·0)	5·5 (4·5–6·8)	44·9 (33·8–58·6)	100·5 (89·8–113·0)	95·1 (82·2–109·7)	38·9 (29·7–51·1)	5·9 (3·9–8·3)	0·1 (0·1–0·2)	0·0 (0·0–0·0)	1·45 (1·24–1·72)	0·25 (0·19–0·33)	0·7 (0·59–0·83)
2017	0·0 (0·0–0·0)	5·5 (4·5–6·6)	44·6 (33·2–58·5)	100·3 (88·8–112·8)	93·3 (79·5–108·7)	37·6 (27·8–49·6)	5·7 (3·7–8·6)	0·1 (0·1–0·2)	0·0 (0·0–0·0)	1·44 (1·21–1·7)	0·25 (0·19–0·32)	0·68 (0·56–0·83)

Supplemental Results Table 5: Age-specific fertility rates per 1000, cumulative total fertility up to maternal age 25 (TFU25), cumulative total fertility from maternal age 30 to age 55 (TFO30), and cumulative total fertility, Fukui, 1950-2017

Year	10-14	15-19	20-24	25-29	30-34	35-39	40-44	45-49	50-54	TFR	TFU25	TFO30
1950	0·0 (0·0–0·0)	7·5 (6·6–8·4)	172·9 (157·9–189·1)	233·3 (224·3–241·9)	159·6 (150·1–169·1)	107·0 (95·1–118·8)	33·5 (29·0–38·5)	1·4 (0·9–2·0)	0·0 (0·0–0·0)	3·58 (3·45–3·71)	0·9 (0·83–0·98)	1·51 (1·43–1·59)
1951	0·0 (0·0–0·0)	6·0 (5·3–6·7)	155·5 (140·4–171·5)	217·2 (207·6–226·6)	144·2 (134·1–154·2)	86·5 (75·9–96·9)	26·0 (21·9–30·5)	1·0 (0·7–1·5)	0·0 (0·0–0·0)	3·18 (3·03–3·34)	0·81 (0·73–0·89)	1·29 (1·17–1·4)
1952	0·0 (0·0–0·0)	5·1 (4·4–5·8)	144·9 (129·1–161·6)	206·7 (196·9–216·1)	130·3 (120·2–140·6)	69·4 (59·8–80·1)	20·6 (17·0–24·4)	0·8 (0·5–1·2)	0·0 (0·0–0·0)	2·89 (2·76–3·02)	0·75 (0·67–0·83)	1·11 (1·03–1·18)
1953	0·0 (0·0–0·0)	4·4 (3·8–5·1)	137·2 (121·3–153·3)	195·1 (182·2–209·5)	116·4 (106·0–126·4)	55·3 (46·6–65·1)	16·3 (13·2–20·1)	0·6 (0·4–1·0)	0·0 (0·0–0·0)	2·63 (2·5–2·76)	0·71 (0·63–0·79)	0·94 (0·87–1·02)
1954	0·0 (0·0–0·0)	4·1 (3·4–4·7)	133·2 (116·8–150·1)	189·9 (176·7–202·9)	107·2 (98·0–117·0)	46·1 (38·4–55·5)	13·5 (10·4–17·2)	0·5 (0·3–0·8)	0·0 (0·0–0·0)	2·47 (2·35–2·6)	0·69 (0·6–0·77)	0·84 (0·77–0·91)
1955	0·0 (0·0–0·0)	3·7 (3·1–4·3)	130·3 (113·9–148·4)	189·0 (175·4–203·9)	98·6 (89·3–108·2)	36·3 (30·5–42·7)	11·2 (8·5–14·3)	0·4 (0·3–0·7)	0·0 (0·0–0·0)	2·35 (2·23–2·46)	0·67 (0·59–0·76)	0·73 (0·67–0·79)
1956	0·0 (0·0–0·0)	3·3 (2·8–3·9)	123·0 (106·4–140·4)	182·8 (169·4–197·6)	87·3 (78·9–96·5)	28·8 (23·6–34·6)	9·0 (6·9–11·6)	0·3 (0·2–0·6)	0·0 (0·0–0·0)	2·17 (2·06–2·3)	0·63 (0·55–0·72)	0·63 (0·57–0·69)
1957	0·0 (0·0–0·0)	3·1 (2·6–3·7)	124·6 (108·5–143·7)	186·4 (172·3–201·6)	82·7 (74·7–91·3)	27·2 (22·6–31·9)	7·6 (5·7–10·0)	0·4 (0·2–0·6)	0·0 (0·0–0·0)	2·16 (2·03–2·3)	0·64 (0·56–0·73)	0·59 (0·53–0·64)
1958	0·0 (0·0–0·0)	3·2 (2·6–3·8)	131·1 (114·5–148·9)	192·4 (177·6–207·6)	80·9 (72·5–90·0)	26·1 (21·4–31·1)	6·9 (5·2–9·3)	0·4 (0·2–0·6)	0·0 (0·0–0·0)	2·2 (2·09–2·33)	0·67 (0·59–0·76)	0·57 (0·52–0·63)
1959	0·0 (0·0–0·0)	3·3 (2·8–3·9)	131·4 (114·4–149·9)	191·3 (178·8–203·8)	75·7 (67·1–84·6)	21·1 (17·0–26·0)	5·8 (4·3–7·7)	0·3 (0·2–0·5)	0·0 (0·0–0·0)	2·14 (2·03–2·26)	0·67 (0·59–0·77)	0·51 (0·46–0·57)
1960	0·0 (0·0–0·0)	3·2 (2·7–3·8)	126·5 (112·2–142·9)	192·2 (180·5–204·2)	72·5 (65·1–80·5)	16·7 (13·3–20·5)	4·7 (3·4–6·3)	0·2 (0·1–0·4)	0·0 (0·0–0·0)	2·08 (1·98–2·18)	0·65 (0·58–0·73)	0·47 (0·43–0·52)
1961	0·0 (0·0–0·0)	3·0 (2·5–3·5)	123·1 (110·6–136·4)	194·2 (184·4–204·6)	70·7 (64·1–78·1)	14·3 (11·4–17·7)	4·0 (2·9–5·6)	0·2 (0·1–0·3)	0·0 (0·0–0·0)	2·05 (1·96–2·14)	0·63 (0·57–0·7)	0·45 (0·41–0·49)
1962	0·0 (0·0–0·0)	2·9 (2·4–3·4)	123·2 (109·2–137·1)	199·7 (190·9–208·6)	72·1 (65·6–79·0)	13·6 (10·8–16·6)	3·6 (2·5–5·0)	0·2 (0·1–0·3)	0·0 (0·0–0·0)	2·08 (1·99–2·17)	0·63 (0·56–0·7)	0·45 (0·41–0·49)
1963	0·0 (0·0–0·0)	2·8 (2·3–3·4)	124·1 (110·6–138·9)	201·7 (193·4–210·1)	73·6 (67·4–80·5)	15·3 (12·6–18·5)	3·6 (2·4–5·1)	0·2 (0·1–0·3)	0·0 (0·0–0·0)	2·11 (2·02–2·2)	0·63 (0·57–0·71)	0·46 (0·43–0·5)
1964	0·0 (0·0–0·0)	2·8 (2·3–3·4)	136·0 (120·9–153·7)	214·6 (204·9–224·6)	78·4 (71·7–85·6)	16·1 (13·3–18·9)	3·4 (2·3–4·9)	0·2 (0·1–0·3)	0·0 (0·0–0·0)	2·26 (2·15–2·37)	0·69 (0·62–0·78)	0·49 (0·45–0·53)
1965	0·0 (0·0–0·0)	2·8 (2·3–3·4)	125·8 (109·6–145·2)	181·2 (168·8–194·6)	64·9 (58·2–71·8)	12·5 (10·6–14·8)	2·9 (1·9–4·3)	0·1 (0·1–0·2)	0·0 (0·0–0·0)	1·95 (1·83–2·08)	0·64 (0·56–0·74)	0·4 (0·37–0·44)
1966	0·0 (0·0–0·0)	2·9 (2·4–3·5)	123·5 (104·6–144·6)	186·1 (173·3–199·3)	66·3 (59·6–73·6)	13·7 (11·3–16·5)	2·9 (1·8–4·3)	0·1 (0·1–0·2)	0·0 (0·0–0·0)	1·98 (1·85–2·11)	0·63 (0·54–0·74)	0·42 (0·38–0·45)
1967	0·0 (0·0–0·0)	3·1 (2·6–3·7)	132·3 (110·6–156·5)	226·5 (209·3–244·8)	79·6 (71·6–88·2)	14·5 (12·1–17·5)	2·8 (1·8–4·0)	0·1 (0·1–0·2)	0·0 (0·0–0·0)	2·29 (2·12–2·48)	0·68 (0·57–0·8)	0·49 (0·44–0·53)
1968	0·0 (0·0–0·0)	3·2 (2·7–3·7)	119·0 (99·0–141·6)	213·3 (194·7–232·2)	72·1 (64·2–80·5)	12·1 (9·5–15·2)	2·5 (1·6–3·7)	0·1 (0·1–0·2)	0·0 (0·0–0·0)	2·11 (1·96–2·27)	0·61 (0·51–0·72)	0·43 (0·39–0·48)
1969	0·0 (0·0–0·0)	3·4 (2·9–4·1)	117·6 (99·0–138·6)	214·9 (197·7–233·3)	72·0 (65·1–79·6)	13·0 (10·1–16·4)	2·5 (1·7–3·6)	0·1 (0·1–0·2)	0·0 (0·0–0·0)	2·12 (1·98–2·27)	0·61 (0·51–0·71)	0·44 (0·4–0·48)
1970	0·0 (0·0–0·0)	3·5 (3·0–4·1)	127·2 (107·5–147·9)	217·7 (202·3–234·7)	73·9 (66·4–82·0)	15·9 (12·4–20·6)	2·7 (1·8–3·9)	0·1 (0·1–0·2)	0·0 (0·0–0·0)	2·2 (2·07–2·35)	0·65 (0·55–0·76)	0·46 (0·42–0·51)
1971	0·0 (0·0–0·0)	3·4 (2·8–4·1)	140·1 (119·8–162·8)	214·0 (199·9–229·5)	74·6 (66·7–82·7)	18·0 (14·2–23·0)	2·9 (2·0–4·2)	0·1 (0·1–0·2)	0·0 (0·0–0·0)	2·27 (2·14–2·41)	0·72 (0·62–0·83)	0·48 (0·43–0·53)
1972	0·0 (0·0–0·0)	3·5 (2·9–4·2)	147·5 (127·6–168·4)	211·5 (198·4–224·0)	74·3 (65·7–83·3)	18·5 (14·3–23·0)	2·9 (2·0–4·2)	0·1 (0·1–0·2)	0·0 (0·0–0·0)	2·29 (2·17–2·41)	0·76 (0·66–0·86)	0·48 (0·43–0·53)
1973	0·0 (0·0–0·0)	3·4 (2·8–4·1)	148·8 (131·5–167·5)	210·4 (198·4–221·9)	70·1 (62·2–78·2)	16·9 (13·3–21·7)	2·7 (1·8–3·9)	0·1 (0·1–0·2)	0·0 (0·0–0·0)	2·26 (2·15–2·39)	0·76 (0·67–0·86)	0·45 (0·4–0·5)
1974	0·0 (0·0–0·0)	3·2 (2·6–3·8)	143·1 (126·8–160·3)	201·0 (191·2–211·2)	61·8 (54·8–68·8)	13·2 (10·0–17·0)	2·3 (1·5–3·3)	0·1 (0·1–0·1)	0·0 (0·0–0·0)	2·12 (2·04–2·22)	0·73 (0·65–0·82)	0·39 (0·35–0·43)
1975	0·0 (0·0–0·0)	2·9 (2·4–3·5)	133·7 (118·3–148·6)	193·3 (184·0–203·3)	55·6 (49·2–61·4)	10·3 (7·8–13·5)	1·9 (1·3–2·9)	0·1 (0·0–0·1)	0·0 (0·0–0·0)	1·99 (1·9–2·08)	0·68 (0·61–0·76)	0·34 (0·3–0·38)
1976	0·0 (0·0–0·0)	2·7 (2·2–3·2)	126·0 (112·7–141·8)	191·1 (181·5–201·0)	54·2 (48·5–60·5)	8·6 (6·6–11·0)	1·6 (1·1–2·4)	0·1 (0·0–0·1)	0·0 (0·0–0·0)	1·92 (1·83–2·02)	0·64 (0·58–0·72)	0·32 (0·29–0·36)
1977	0·0 (0·0–0·0)	2·5 (2·1–3·0)	118·7 (106·9–133·4)	192·9 (184·0–202·3)	55·8 (49·6–62·0)	7·9 (5·9–10·5)	1·5 (0·9–2·3)	0·1 (0·0–0·1)	0·0 (0·0–0·0)	1·9 (1·81–1·99)	0·61 (0·55–0·68)	0·33 (0·29–0·36)
1978	0·0 (0·0–0·0)	2·5 (2·0–3·1)	112·8 (98·1–130·4)	194·9 (185·8–203·6)	58·3 (52·0–65·2)	7·9 (6·0–10·4)	1·3 (0·8–2·0)	0·0 (0·0–0·1)	0·0 (0·0–0·0)	1·89 (1·8–1·99)	0·58 (0·5–0·66)	0·34 (0·3–0·37)
1979	0·0 (0·0–0·0)	2·6 (1·9–3·3)	106·6 (94·7–118·6)	194·7 (185·6–204·1)	57·8 (52·0–64·1)	7·9 (6·0–10·1)	1·1 (0·6–2·1)	0·0 (0·0–0·1)	0·0 (0·0–0·0)	1·85 (1·77–1·94)	0·55 (0·49–0·61)	0·33 (0·31–0·37)
1980	0·0 (0·0–0·0)	2·6 (2·1–3·2)	100·4 (88·7–112·4)	194·1 (183·8–203·7)	56·0 (49·9–62·4)	8·0 (6·0–10·6)	1·0 (0·6–1·6)	0·0 (0·0–0·1)	0·0 (0·0–0·0)	1·81 (1·72–1·9)	0·52 (0·46–0·57)	0·33 (0·29–0·36)
1981	0·0 (0·0–0·0)	2·8 (2·3–3·4)	96·4 (85·3–108·3)	197·5 (187·8–207·0)	56·9 (51·3–63·2)	8·6 (6·5–11·1)	0·9 (0·6–1·3)	0·0 (0·0–0·0)	0·0 (0·0–0·0)	1·82 (1·75–1·89)	0·5 (0·44–0·56)	0·33 (0·3–0·36)
1982	0·0 (0·0–0·0)	3·0 (2·4–3·7)	93·9 (82·2–107·1)	202·3 (193·7–211·9)	60·6 (54·6–67·1)	9·7 (7·6–12·3)	0·9 (0·6–1·4)	0·0 (0·0–0·0)	0·0 (0·0–0·0)	1·85 (1·78–1·92)	0·48 (0·43–0·55)	0·36 (0·32–0·4)
1983	0·0 (0·0–0·0)	3·1 (2·5–3·8)	90·6 (80·0–101·7)	206·1 (195·4–216·1)	65·1 (58·6–72·4)	10·9 (8·4–13·9)	1·0 (0·6–1·5)	0·0 (0·0–0·0)	0·0 (0·0–0·0)	1·88 (1·8–1·97)	0·47 (0·42–0·52)	0·39 (0·35–0·43)
1984	0·0 (0·0–0·0)	3·1 (2·5–3·8)	83·8 (72·2–96·7)	206·3 (196·5–216·4)	68·9 (62·1–76·3)	11·4 (8·7–14·5)	1·1 (0·7–1·7)	0·0 (0·0–0·0)	0·0 (0·0–0·0)	1·87 (1·8–1·95)	0·43 (0·38–0·5)	0·41 (0·37–0·45)
1985	0·0 (0·0–0·0)	3·0 (2·5–3·6)	78·6 (68·4–90·2)	202·5 (193·2–211·9)	71·2 (64·0–78·7)	11·4 (8·7–14·6)	1·2 (0·8–1·8)	0·0 (0·0–0·1)	0·0 (0·0–0·0)	1·84 (1·77–1·92)	0·41 (0·36–0·46)	0·42 (0·38–0·46)
1986	0·0 (0·0–0·0)	3·0 (2·5–3·6)	75·0 (64·9–86·3)	199·5 (189·2–210·5)	74·4 (67·5–82·1)	11·6 (8·9–15·0)	1·3 (0·8–1·9)	0·0 (0·0–0·1)	0·0 (0·0–0·0)	1·82 (1·74–1·91)	0·39 (0·34–0·45)	0·44 (0·4–0·48)
1987	0·0 (0·0–0·0)	2·9 (2·5–3·5)	68·9 (61·2–78·2)	197·0 (187·4–207·3)	78·4 (71·0–87·0)	12·4 (9·6–15·9)	1·5 (0·9–2·2)	0·0 (0·0–0·1)	0·0 (0·0–0·0)	1·81 (1·72–1·9)	0·36 (0·32–0·4)	0·46 (0·42–0·51)
1988	0·0 (0·0–0·0)	2·9 (2·4–3·4)	63·4 (55·1–72·2)	188·5 (177·5–198·6)	81·2 (73·7–89·6)	13·4 (10·2–16·9)	1·6 (1·1–2·5)	0·0 (0·0–0·1)	0·0 (0·0–0·0)	1·76 (1·67–1·84)	0·33 (0·29–0·38)	0·48 (0·44–0·53)
1989	0·0 (0·0–0·0)	2·9 (2·4–3·5)	58·2 (50·1–68·3)	178·3 (168·2–188·3)	83·2 (75·2–91·5)	14·4 (11·1–18·3)	1·8 (1·1–2·8)	0·0 (0·0–0·1)	0·0 (0·0–0·0)	1·69 (1·61–1·78)	0·31 (0·26–0·36)	0·5 (0·46–0·54)
1990	0·0 (0·0–0·0)	3·0 (2·4–3·6)	55·6 (48·2–63·5)	175·2 (166·2–184·9)	86·5 (79·2–94·4)	15·4 (11·9–19·4)	1·8 (1·2–2·8)	0·0 (0·0–0·1)	0·0 (0·0–0·0)	1·69 (1·61–1·77)	0·29 (0·25–0·33)	0·52 (0·48–0·56)
1991	0·0 (0·0–0·0)	3·0 (2·5–3·7)	54·8 (47·2–63·0)	171·2 (158·9–184·3)	88·9 (80·9–98·3)	16·6 (13·0–20·9)	1·9 (1·2–2·8)	0·0 (0·0–0·1)	0·0 (0·0–0·0)	1·68 (1·61–1·77)	0·29 (0·25–0·33)	0·54 (0·49–0·58)
1992	0·0 (0·0–0·0)	3·1 (2·5–3·7)	52·3 (45·8–59·8)	162·7 (153·3–171·9)	89·6 (82·2–97·8)	17·5 (13·7–22·4)	2·0 (1·3–3·2)	0·0 (0·0–0·1)	0·0 (0·0–0·0)	1·64 (1·56–1·71)	0·28 (0·25–0·31)	0·55 (0·5–0·59)
1993	0·0 (0·0–0·0)	3·1 (2·5–3·7)	51·4 (43·9–59·5)	162·8 (152·0–173·9)	95·0 (86·1–104·2)	19·2 (15·0–24·1)	2·2 (1·3–3·3)	0·1 (0·0–0·1)	0·0 (0·0–0·0)	1·67 (1·59–1·75)	0·27 (0·23–0·31)	0·58 (0·53–0·64)
1994	0·0 (0·0–0·0)	3·0 (2·4–3·7)	50·3 (43·5–58·2)	160·0 (150·9–169·4)	98·2 (89·1–107·9)	20·7 (16·2–26·3)	2·3 (1·4–3·6)	0·1 (0·0–0·1)	0·0 (0·0–0·0)	1·67 (1·59–1·76)	0·27 (0·23–0·31)	0·61 (0·55–0·66)
1995	0·0 (0·0–0·0)	3·0 (2·4–3·6)	48·7 (41·5–57·0)	150·9 (141·1–160·6)	98·9 (89·2–108·1)	21·8 (17·2–27·4)	2·4 (1·5–3·7)	0·1 (0·0–0·1)	0·0 (0·0–0·0)	1·63 (1·55–1·71)	0·26 (0·22–0·3)	0·62 (0·56–0·66)
1996	0·0 (0·0–0·0)	3·1 (2·5–3·7)	48·0 (40·8–55·7)	145·2 (136·1–155·2)	100·8 (90·6–112·0)	23·4 (18·3–29·1)	2·6 (1·6–3·9)	0·1 (0·0–0·1)	0·0 (0·0–0·0)	1·62 (1·53–1·71)	0·26 (0·22–0·29)	0·63 (0·57–0·7)
1997	0·0 (0·0–0·0)	3·2 (2·5–3·9)	47·5 (40·4–55·4)	139·9 (131·1–148·6)	101·3 (92·6–110·8)	24·4 (19·7–30·2)	2·7 (1·7–4·1)	0·1 (0·0–0·1)	0·0 (0·0–0·0)	1·6 (1·53–1·67)	0·25 (0·22–0·29)	0·64 (0·59–0·7)
1998	0·0 (0·0–0·0)	3·4 (2·7–4·2)	46·8 (40·4–54·4)	133·8 (125·5–142·4)	102·6 (93·5–112·6)	26·0 (20·7–31·8)	2·9 (1·8–4·3)	0·1 (0·0–0·1)	0·0 (0·0–0·0)	1·58 (1·5–1·66)	0·25 (0·22–0·29)	0·66 (0·6–0·72)
1999	0·0 (0·0–0·0)	3·6 (2·8–4·5)	46·3 (39·4–54·2)	128·6 (119·9–137·2)	103·8 (94·8–114·2)	27·2 (22·0–33·7)	3·1 (2·0–4·6)	0·1 (0·0–0·1)	0·0 (0·0–0·0)	1·56 (1·48–1·65)	0·25 (0·22–0·29)	0·67 (0·62–0·74)
2000	0·0 (0·0–0·0)	3·8 (3·1–4·8)	46·4 (40·1–52·9)	124·8 (116·7–133·1)	103·4 (93·8–113·3)	29·1 (23·3–35·9)	3·3 (2·1–4·9)	0·1 (0·0–0·1)	0·0 (0·0–0·0)	1·55 (1·47–1·65)	0·25 (0·22–0·28)	0·68 (0·62–0·74)
2001	0·0 (0·0–0·0)	4·0 (3·3–4·9)	46·6 (39·5–54·0)	120·2 (112·4–128·9)	101·4 (91·5–111·4)	29·6 (24·0–36·2)	3·4 (2·3–4·9)	0·1 (0·0–0·1)	0·0 (0·0–0·0)	1·53 (1·44–1·61)	0·25 (0·22–0·29)	0·67 (0·62–0·73)
2002	0·0 (0·0–0·0)	4·0 (3·2–4·9)	45·6 (38·8–53·3)	116·2 (108·4–124·2)	101·9 (92·4–112·2)	30·7 (25·5–37·6)	3·6 (2·4–5·1)	0·1 (0·0–0·1)	0·0 (0·0–0·0)	1·51 (1·44–1·59)	0·25 (0·21–0·29)	0·68 (0·63–0·74)
2003	0·0 (0·0–0·0)	3·9 (3·1–4·7)	44·0 (37·8–51·2)	112·7 (105·2–120·8)	102·0 (93·3–111·4)	32·6 (26·3–39·3)	3·9 (2·6–5·3)	0·1 (0·0–0·2)	0·0 (0·0–0·0)	1·5 (1·41–1·59)	0·24 (0·21–0·28)	0·69 (0·64–0·75)
2004	0·0 (0·0–0·0)	3·8 (3·1–4·5)	42·7 (36·2–50·2)	108·1 (99·9–116·4)	101·0 (89·7–113·1)	33·7 (27·7–40·2)	4·0 (2·8–5·6)	0·1 (0·0–0·2)	0·0 (0·0–0·0)	1·47 (1·39–1·55)	0·23 (0·2–0·27)	0·69 (0·63–0·76)
2005	0·0 (0·0–0·0)	3·7 (3·0–4·4)	42·5 (36·4–49·2)	106·7 (99·9–113·8)	101·7 (92·9–110·9)	35·0 (29·0–42·2)	4·2 (2·9–5·9)	0·1 (0·0–0·2)	0·0 (0·0–0·0)	1·47 (1·4–1·55)	0·23 (0·2–0·26)	0·71 (0·65–0·76)
2006	0·0 (0·0–0·0)	3·6 (3·0–4·3)	42·9 (36·8–49·7)	107·7 (97·2–118·7)	105·0 (95·6–115·2)	37·3 (31·0–44·8)	4·6 (3·2–6·4)	0·1 (0·0–0·2)	0·0 (0·0–0·0)	1·51 (1·43–1·59)	0·23 (0·2–0·27)	0·74 (0·68–0·8)
2007	0·0 (0·0–0·0)	3·6 (3·0–4·3)	42·8 (35·6–50·4)	106·8 (99·8–114·7)	105·4 (96·1–115·4)	39·8 (32·8–47·3)	5·0 (3·4–6·8)	0·1 (0·0–0·2)	0·0 (0·0–0·0)	1·52 (1·44–1·6)	0·23 (0·2–0·27)	0·75 (0·69–0·82)
2008	0·0 (0·0–0·0)	3·6 (3·0–4·3)	42·0 (36·5–48·3)	105·9 (98·7–113·1)	106·1 (96·5–115·8)	41·7 (35·1–49·0)	5·4 (3·8–7·4)	0·1 (0·1–0·2)	0·0 (0·0–0·0)	1·52 (1·45–1·61)	0·23 (0·2–0·26)	0·77 (0·71–0·83)
2009	0·0 (0·0–0·0)	3·4 (2·8–4·1)	40·7 (33·1–49·5)	105·1 (94·7–114·5)	107·0 (97·1–117·8)	43·7 (36·8–51·4)	5·9 (4·1–8·0)	0·1 (0·1–0·2)	0·0 (0·0–0·0)	1·53 (1·41–1·64)	0·22 (0·18–0·27)	0·78 (0·72–0·85)
2010	0·0 (0·0–0·0)	3·4 (2·8–4·0)	39·4 (34·1–44·6)	104·9 (98·1–111·6)	108·3 (99·1–118·9)	45·5 (38·3–53·3)	6·1 (4·4–8·2)	0·1 (0·1–0·2)	0·0 (0·0–0·0)	1·54 (1·46–1·62)	0·21 (0·19–0·24)	0·8 (0·74–0·86)
2011	0·0 (0·0–0·0)	3·3 (2·7–4·1)	37·6 (32·4–43·6)	103·4 (95·8–111·3)	108·3 (98·4–119·2)	46·9 (39·6–55·5)	6·3 (4·5–8·6)	0·1 (0·1–0·2)	0·0 (0·0–0·0)	1·53 (1·45–1·61)	0·2 (0·18–0·23)	0·81 (0·75–0·88)
2012	0·0 (0·0–0·0)	3·3 (2·6–4·0)	35·9 (30·6–42·3)	101·6 (94·9–109·0)	109·8 (99·6–120·5)	49·2 (41·8–58·3)	6·8 (4·8–9·1)	0·1 (0·1–0·3)	0·0 (0·0–0·0)	1·53 (1·45–1·62)	0·2 (0·17–0·23)	0·83 (0·77–0·9)
2013	0·0 (0·0–0·0)	3·2 (2·6–3·8)	34·8 (29·2–40·4)	99·0 (91·1–106·9)	109·7 (100·2–119·8)	50·2 (42·5–58·2)	7·1 (5·0–9·4)	0·2 (0·1–0·3)	0·0 (0·0–0·0)	1·52 (1·44–1·62)	0·19 (0·16–0·22)	0·84 (0·77–0·9)
2014	0·0 (0·0–0·0)	3·1 (2·6–3·7)	33·8 (28·6–39·8)	98·2 (90·4–106·5)	110·2 (100·5–120·3)	51·3 (42·9–59·7)	7·3 (5·2–10·0)	0·2 (0·1–0·3)	0·0 (0·0–0·0)	1·52 (1·44–1·6)	0·18 (0·16–0·21)	0·84 (0·78–0·92)
2015	0·0 (0·0–0·0)	3·1 (2·6–3·8)	34·8 (25·6–46·3)	100·3 (90·3–110·7)	112·3 (100·8–124·7)	48·8 (39·3–60·0)	6·7 (4·7–9·6)	0·2 (0·1–0·3)	0·0 (0·0–0·0)	1·53 (1·35–1·74)	0·19 (0·14–0·25)	0·84 (0·74–0·95)
2016	0·0 (0·0–0·0)	3·2 (2·6–3·9)	36·2 (27·3–48·0)	101·9 (89·0–116·4)	111·2 (96·3–129·2)	44·2 (33·1–57·7)	5·8 (3·7–8·6)	0·1 (0·1–0·3)	0·0 (0·0–0·0)	1·51 (1·29–1·77)	0·2 (0·15–0·26)	0·81 (0·68–0·96)
2017	0·0 (0·0–0·0)	3·1 (2·6–3·8)	36·2 (26·4–48·7)	102·0 (88·0–117·3)	109·1 (92·9–127·0)	42·5 (31·1–56·6)	5·7 (3·5–8·5)	0·1 (0·1–0·3)	0·0 (0·0–0·0)	1·49 (1·25–1·77)	0·2 (0·14–0·26)	0·79 (0·65–0·95)

Supplemental Results Table 5: Age-specific fertility rates per 1000, cumulative total fertility up to maternal age 25 (TFU25), cumulative total fertility from maternal age 30 to age 55 (TFO30), and cumulative total fertility, Fukuoka, 1950-2017											
**Year**	**10-14**	**15-19**	**20-24**	**25-29**	**30-34**	**35-39**	**40-44**	**45-49**	**50-54**	**TFR**	**TFU25**	**TFO30**
1950	0·0 (0·0–0·0)	14·1 (12·5–15·7)	136·1 (124·4–148·8)	213·4 (205·7–221·1)	162·8 (153·1–172·5)	75·4 (67·0–83·7)	27·1 (23·5–31·2)	1·2 (0·8–1·8)	0·0 (0·0–0·0)	3·15 (3·04–3·26)	0·75 (0·69–0·81)	1·33 (1·26–1·4)
1951	0·0 (0·0–0·0)	11·3 (10·0–12·7)	121·9 (110·0–134·0)	197·9 (190·5–205·2)	147·2 (137·0–157·2)	66·1 (58·2–74·1)	21·0 (17·7–24·7)	0·9 (0·6–1·3)	0·0 (0·0–0·0)	2·83 (2·71–2·96)	0·67 (0·61–0·72)	1·18 (1·08–1·28)
1952	0·0 (0·0–0·0)	9·5 (8·4–10·8)	113·2 (102·2–125·8)	187·8 (179·8–196·0)	133·1 (122·8–143·7)	63·0 (54·7–72·3)	16·6 (13·6–19·8)	0·7 (0·4–1·1)	0·0 (0·0–0·0)	2·62 (2·51–2·74)	0·61 (0·56–0·68)	1·07 (1·0–1·15)
1953	0·0 (0·0–0·0)	8·3 (7·3–9·6)	106·7 (95·2–119·9)	176·7 (168·8–184·6)	118·9 (108·4–129·8)	57·9 (49·2–67·1)	13·1 (10·5–16·1)	0·6 (0·3–0·9)	0·0 (0·0–0·0)	2·41 (2·29–2·54)	0·58 (0·51–0·64)	0·95 (0·88–1·03)
1954	0·0 (0·0–0·0)	7·6 (6·5–8·8)	103·2 (91·0–115·9)	171·7 (164·7–179·3)	109·5 (100·4–120·2)	52·8 (44·8–61·8)	10·8 (8·5–13·7)	0·5 (0·3–0·7)	0·0 (0·0–0·0)	2·28 (2·16–2·4)	0·55 (0·49–0·62)	0·87 (0·79–0·95)
1955	0·0 (0·0–0·0)	6·9 (5·9–8·0)	100·5 (87·6–114·1)	170·3 (162·1–178·5)	101·2 (91·6–111·1)	47·6 (39·9–55·9)	9·1 (7·0–11·4)	0·4 (0·2–0·6)	0·0 (0·0–0·0)	2·18 (2·06–2·3)	0·54 (0·47–0·61)	0·79 (0·72–0·86)
1956	0·0 (0·0–0·0)	6·1 (5·2–7·2)	94·4 (83·1–105·8)	164·4 (157·1–172·0)	89·6 (81·0–99·1)	40·9 (34·4–48·0)	7·3 (5·7–9·1)	0·3 (0·2–0·5)	0·0 (0·0–0·0)	2·02 (1·92–2·12)	0·5 (0·45–0·56)	0·69 (0·63–0·76)
1957	0·0 (0·0–0·0)	5·8 (5·0–6·9)	95·2 (84·5–107·9)	167·5 (160·1–175·2)	84·4 (77·0–92·4)	35·1 (29·2–40·9)	6·0 (4·7–7·7)	0·3 (0·2–0·5)	0·0 (0·0–0·0)	1·97 (1·86–2·08)	0·51 (0·45–0·57)	0·63 (0·58–0·68)
1958	0·0 (0·0–0·0)	5·9 (5·0–6·8)	99·8 (88·0–113·3)	172·9 (164·4–181·2)	82·4 (75·0–90·7)	31·0 (25·6–36·8)	5·4 (4·2–6·8)	0·3 (0·2–0·5)	0·0 (0·0–0·0)	1·99 (1·89–2·1)	0·53 (0·47–0·6)	0·6 (0·54–0·66)
1959	0·0 (0·0–0·0)	6·1 (5·2–7·1)	99·6 (88·5–111·9)	171·7 (163·6–179·9)	77·2 (68·8–85·5)	26·8 (22·1–32·1)	4·5 (3·5–5·8)	0·3 (0·1–0·4)	0·0 (0·0–0·0)	1·93 (1·84–2·03)	0·53 (0·47–0·59)	0·54 (0·5–0·59)
1960	0·0 (0·0–0·0)	6·0 (5·1–7·0)	95·6 (85·2–107·5)	172·2 (164·2–180·1)	75·1 (67·9–82·3)	23·6 (19·3–28·0)	3·9 (2·9–5·0)	0·2 (0·1–0·4)	0·0 (0·0–0·0)	1·88 (1·8–1·97)	0·51 (0·46–0·57)	0·51 (0·47–0·56)
1961	0·0 (0·0–0·0)	5·6 (4·7–6·6)	92·7 (83·2–102·0)	173·8 (165·5–182·0)	74·6 (68·8–80·9)	21·2 (17·7–25·4)	3·5 (2·6–4·6)	0·2 (0·1–0·3)	0·0 (0·0–0·0)	1·86 (1·79–1·93)	0·49 (0·44–0·54)	0·5 (0·46–0·53)
1962	0·0 (0·0–0·0)	5·3 (4·3–6·3)	92·4 (82·4–102·6)	178·6 (171·2–185·7)	76·4 (70·2–82·8)	20·1 (16·9–23·6)	3·1 (2·2–4·1)	0·2 (0·1–0·3)	0·0 (0·0–0·0)	1·88 (1·81–1·95)	0·49 (0·44–0·54)	0·5 (0·46–0·54)
1963	0·0 (0·0–0·0)	5·2 (4·3–6·2)	92·6 (82·6–102·8)	180·3 (172·9–187·8)	76·8 (70·1–83·8)	19·3 (16·4–22·7)	2·8 (2·1–3·8)	0·2 (0·1–0·3)	0·0 (0·0–0·0)	1·89 (1·82–1·97)	0·49 (0·44–0·54)	0·5 (0·46–0·54)
1964	0·0 (0·0–0·0)	5·1 (4·3–6·0)	100·9 (90·4–112·8)	191·6 (183·5–199·3)	82·0 (74·3–89·8)	20·0 (16·7–23·6)	2·7 (2·0–3·5)	0·2 (0·1–0·3)	0·0 (0·0–0·0)	2·01 (1·93–2·1)	0·53 (0·48–0·59)	0·52 (0·48–0·57)
1965	0·0 (0·0–0·0)	5·0 (4·3–5·7)	92·7 (82·4–103·8)	161·1 (153·4–169·0)	70·0 (63·6–76·2)	18·4 (15·4–21·8)	2·5 (1·9–3·3)	0·1 (0·1–0·2)	0·0 (0·0–0·0)	1·75 (1·67–1·82)	0·49 (0·44–0·54)	0·46 (0·42–0·49)
1966	0·0 (0·0–0·0)	5·3 (4·5–6·1)	90·3 (80·3–101·5)	165·3 (157·7–172·7)	70·7 (64·1–78·5)	18·8 (15·4–22·6)	2·3 (1·7–3·1)	0·1 (0·1–0·2)	0·0 (0·0–0·0)	1·76 (1·69–1·85)	0·48 (0·43–0·53)	0·46 (0·42–0·5)
1967	0·0 (0·0–0·0)	5·5 (4·7–6·4)	95·9 (85·9–106·9)	200·7 (193·0–208·5)	87·6 (79·6–96·7)	21·3 (17·4–26·0)	2·4 (1·8–3·2)	0·1 (0·1–0·2)	0·0 (0·0–0·0)	2·07 (1·97–2·17)	0·51 (0·46–0·56)	0·56 (0·51–0·61)
1968	0·0 (0·0–0·0)	5·6 (4·8–6·5)	85·6 (76·0–95·9)	188·4 (180·5–196·0)	85·2 (77·1–93·4)	21·2 (17·3–25·6)	2·7 (2·0–3·7)	0·1 (0·1–0·2)	0·0 (0·0–0·0)	1·94 (1·86–2·03)	0·46 (0·41–0·51)	0·55 (0·5–0·6)
1969	0·0 (0·0–0·0)	6·1 (5·2–7·2)	83·9 (73·4–94·8)	189·9 (182·2–197·5)	83·5 (76·1–91·5)	20·8 (17·1–25·2)	2·4 (1·8–3·3)	0·1 (0·1–0·2)	0·0 (0·0–0·0)	1·93 (1·85–2·02)	0·45 (0·4–0·5)	0·53 (0·49–0·58)
1970	0·0 (0·0–0·0)	6·1 (5·1–7·2)	89·9 (80·1–100·4)	192·8 (185·5–200·0)	80·6 (73·5–88·5)	19·9 (16·3–24·1)	2·1 (1·5–2·8)	0·1 (0·1–0·2)	0·0 (0·0–0·0)	1·96 (1·87–2·04)	0·48 (0·43–0·53)	0·51 (0·47–0·56)
1971	0·0 (0·0–0·0)	5·9 (4·9–7·0)	98·0 (86·8–109·5)	190·0 (180·9–199·7)	79·4 (72·3–86·6)	18·0 (14·9–21·9)	2·0 (1·4–2·8)	0·1 (0·1–0·2)	0·0 (0·0–0·0)	1·97 (1·88–2·06)	0·52 (0·46–0·58)	0·5 (0·45–0·54)
1972	0·0 (0·0–0·0)	5·9 (4·9–7·0)	102·1 (91·2–113·9)	188·1 (180·8–196·0)	79·6 (71·9–87·1)	17·0 (14·2–20·0)	2·0 (1·4–2·7)	0·1 (0·1–0·2)	0·0 (0·0–0·0)	1·97 (1·89–2·07)	0·54 (0·49–0·6)	0·49 (0·45–0·53)
1973	0·0 (0·0–0·0)	5·7 (4·7–6·8)	101·6 (91·0–112·6)	187·4 (179·6–195·4)	75·7 (69·2–82·7)	16·7 (13·5–20·2)	1·8 (1·2–2·5)	0·1 (0·0–0·1)	0·0 (0·0–0·0)	1·94 (1·86–2·03)	0·54 (0·48–0·59)	0·47 (0·44–0·51)
1974	0·0 (0·0–0·0)	5·3 (4·3–6·4)	96·4 (86·4–106·5)	179·4 (171·1–187·1)	69·1 (62·7–75·8)	15·6 (13·0–18·7)	1·7 (1·2–2·2)	0·1 (0·0–0·1)	0·0 (0·0–0·0)	1·84 (1·77–1·91)	0·51 (0·46–0·56)	0·43 (0·4–0·47)
1975	0·0 (0·0–0·0)	4·9 (3·9–6·1)	89·4 (79·8–99·4)	172·6 (164·9–180·2)	65·6 (59·6–71·9)	14·7 (12·1–17·5)	1·7 (1·3–2·2)	0·1 (0·0–0·1)	0·0 (0·0–0·0)	1·75 (1·68–1·81)	0·47 (0·42–0·52)	0·41 (0·38–0·45)
1976	0·0 (0·0–0·0)	4·6 (3·7–5·8)	83·9 (75·2–93·9)	170·7 (163·1–178·3)	66·8 (61·1–73·0)	14·5 (12·4–16·9)	1·8 (1·3–2·5)	0·1 (0·0–0·1)	0·0 (0·0–0·0)	1·71 (1·64–1·79)	0·44 (0·4–0·49)	0·42 (0·38–0·45)
1977	0·0 (0·0–0·0)	4·6 (3·4–5·9)	79·1 (70·7–89·2)	172·3 (165·1–180·2)	71·0 (64·2–77·8)	14·4 (11·8–17·3)	1·9 (1·3–2·5)	0·1 (0·0–0·1)	0·0 (0·0–0·0)	1·72 (1·64–1·79)	0·42 (0·38–0·47)	0·44 (0·39–0·48)
1978	0·0 (0·0–0·0)	4·6 (3·7–5·6)	75·4 (67·9–83·6)	173·7 (166·5–181·1)	75·7 (68·9–82·9)	14·2 (11·8–16·9)	1·8 (1·3–2·4)	0·1 (0·0–0·1)	0·0 (0·0–0·0)	1·73 (1·66–1·8)	0·4 (0·36–0·44)	0·46 (0·42–0·5)
1979	0·0 (0·0–0·0)	4·7 (4·1–5·4)	72·2 (64·0–80·6)	172·9 (165·6–179·8)	76·4 (70·1–83·7)	14·0 (11·5–17·2)	1·9 (1·3–2·6)	0·1 (0·0–0·1)	0·0 (0·0–0·0)	1·71 (1·64–1·78)	0·38 (0·34–0·43)	0·46 (0·43–0·5)
1980	0·0 (0·0–0·0)	4·8 (4·0–5·7)	69·2 (61·2–77·7)	171·3 (163·8–179·4)	74·8 (67·8–81·7)	14·0 (11·4–16·9)	1·9 (1·3–2·6)	0·1 (0·0–0·1)	0·0 (0·0–0·0)	1·68 (1·61–1·75)	0·37 (0·33–0·41)	0·45 (0·42–0·49)
1981	0·0 (0·0–0·0)	5·0 (4·3–5·9)	67·9 (60·7–76·1)	173·0 (165·2–181·1)	75·2 (68·8–81·5)	14·7 (12·1–17·6)	1·9 (1·3–2·8)	0·1 (0·0–0·1)	0·0 (0·0–0·0)	1·69 (1·63–1·75)	0·36 (0·33–0·41)	0·46 (0·43–0·49)
1982	0·0 (0·0–0·0)	5·2 (4·3–6·2)	67·7 (59·9–76·4)	175·1 (167·8–183·2)	78·3 (70·4–86·8)	16·5 (13·8–19·6)	1·9 (1·4–2·6)	0·1 (0·0–0·1)	0·0 (0·0–0·0)	1·72 (1·66–1·79)	0·36 (0·32–0·41)	0·48 (0·44–0·53)
1983	0·0 (0·0–0·0)	5·3 (4·5–6·3)	66·6 (59·8–74·1)	175·3 (167·2–183·2)	82·2 (75·5–90·5)	18·4 (15·0–22·2)	1·9 (1·4–2·6)	0·1 (0·0–0·1)	0·0 (0·0–0·0)	1·75 (1·69–1·82)	0·36 (0·32–0·4)	0·51 (0·47–0·56)
1984	0·0 (0·0–0·0)	5·1 (4·3–6·0)	62·4 (53·8–71·5)	171·6 (164·3–179·7)	85·2 (78·1–93·1)	19·1 (15·8–23·1)	2·0 (1·4–2·8)	0·1 (0·0–0·1)	0·0 (0·0–0·0)	1·73 (1·66–1·8)	0·34 (0·29–0·38)	0·53 (0·49–0·58)
1985	0·0 (0·0–0·0)	4·8 (4·0–5·7)	58·6 (51·7–66·3)	164·2 (156·9–170·9)	86·6 (79·3–94·0)	19·0 (15·8–22·5)	2·1 (1·4–3·0)	0·1 (0·0–0·1)	0·0 (0·0–0·0)	1·68 (1·62–1·73)	0·32 (0·28–0·36)	0·54 (0·5–0·58)
1986	0·0 (0·0–0·0)	4·5 (3·8–5·4)	55·6 (49·3–62·5)	157·2 (149·8–165·2)	88·8 (79·6–97·2)	19·0 (15·9–22·8)	2·2 (1·3–3·5)	0·1 (0·0–0·1)	0·0 (0·0–0·0)	1·64 (1·58–1·7)	0·3 (0·27–0·33)	0·55 (0·5–0·6)
1987	0·0 (0·0–0·0)	4·4 (3·6–5·2)	51·2 (44·0–59·2)	151·3 (143·7–158·9)	91·2 (84·4–98·8)	19·8 (16·4–23·6)	2·5 (1·7–3·6)	0·1 (0·0–0·1)	0·0 (0·0–0·0)	1·6 (1·52–1·68)	0·28 (0·24–0·32)	0·57 (0·53–0·61)
1988	0·0 (0·0–0·0)	4·2 (3·5–5·1)	47·3 (41·7–53·7)	141·8 (135·4–149·3)	91·9 (84·0–100·4)	20·7 (17·5–24·5)	2·7 (1·9–3·7)	0·1 (0·1–0·1)	0·0 (0·0–0·0)	1·54 (1·48–1·61)	0·26 (0·23–0·29)	0·58 (0·53–0·62)
1989	0·0 (0·0–0·0)	4·2 (3·4–5·1)	44·0 (38·6–49·7)	132·6 (125·8–139·8)	91·2 (83·7–98·7)	21·7 (18·0–25·9)	2·8 (2·1–3·9)	0·1 (0·1–0·1)	0·0 (0·0–0·0)	1·48 (1·42–1·54)	0·24 (0·21–0·27)	0·58 (0·54–0·62)
1990	0·0 (0·0–0·0)	4·3 (3·6–5·3)	42·9 (36·2–49·6)	129·9 (122·9–137·0)	92·2 (84·6–99·7)	22·5 (18·8–26·7)	2·8 (2·0–3·7)	0·1 (0·1–0·1)	0·0 (0·0–0·0)	1·47 (1·41–1·54)	0·24 (0·2–0·27)	0·59 (0·55–0·63)
1991	0·0 (0·0–0·0)	4·5 (3·7–5·6)	43·1 (38·0–48·5)	126·6 (120·5–133·1)	92·3 (84·2–100·6)	23·6 (19·8–28·2)	2·8 (2·0–3·7)	0·1 (0·1–0·1)	0·0 (0·0–0·0)	1·47 (1·41–1·52)	0·24 (0·21–0·27)	0·59 (0·55–0·64)
1992	0·0 (0·0–0·0)	4·6 (3·8–5·7)	42·2 (37·6–47·3)	120·0 (113·4–127·1)	90·9 (83·5–98·2)	24·1 (20·3–28·8)	2·9 (2·1–3·8)	0·1 (0·1–0·1)	0·0 (0·0–0·0)	1·42 (1·37–1·48)	0·23 (0·21–0·26)	0·59 (0·55–0·64)
1993	0·0 (0·0–0·0)	4·7 (3·8–5·8)	42·4 (36·6–48·4)	120·2 (113·6–127·6)	94·6 (86·9–102·8)	25·8 (21·9–30·1)	3·0 (2·2–3·9)	0·1 (0·1–0·2)	0·0 (0·0–0·0)	1·45 (1·39–1·52)	0·24 (0·21–0·27)	0·62 (0·57–0·66)
1994	0·0 (0·0–0·0)	4·8 (3·9–5·8)	42·1 (37·2–47·7)	118·1 (111·4–124·6)	96·2 (88·2–104·8)	27·0 (22·8–32·1)	3·1 (2·2–4·1)	0·1 (0·1–0·2)	0·0 (0·0–0·0)	1·46 (1·39–1·52)	0·23 (0·21–0·26)	0·63 (0·58–0·68)
1995	0·0 (0·0–0·0)	4·8 (4·0–5·8)	41·1 (35·6–46·8)	111·3 (104·9–118·0)	95·3 (87·5–103·9)	27·9 (23·3–33·1)	3·2 (2·3–4·3)	0·1 (0·1–0·2)	0·0 (0·0–0·0)	1·42 (1·35–1·49)	0·23 (0·2–0·26)	0·63 (0·59–0·68)
1996	0·0 (0·0–0·0)	5·0 (4·2–6·0)	40·7 (35·0–46·4)	107·4 (101·1–113·9)	95·2 (87·4–103·8)	29·1 (24·3–35·0)	3·4 (2·5–4·4)	0·1 (0·1–0·2)	0·0 (0·0–0·0)	1·4 (1·34–1·47)	0·23 (0·2–0·26)	0·64 (0·6–0·69)
1997	0·0 (0·0–0·0)	5·2 (4·3–6·2)	40·4 (35·0–46·0)	104·0 (98·3–109·9)	93·6 (86·4–101·7)	29·7 (25·5–34·5)	3·6 (2·6–4·8)	0·1 (0·1–0·2)	0·0 (0·0–0·0)	1·38 (1·32–1·45)	0·23 (0·2–0·26)	0·64 (0·59–0·69)
1998	0·0 (0·0–0·0)	5·5 (4·4–6·6)	39·9 (34·6–45·4)	100·2 (94·4–106·2)	92·7 (84·7–101·6)	31·1 (25·8–36·6)	3·7 (2·7–5·1)	0·1 (0·1–0·2)	0·0 (0·0–0·0)	1·37 (1·3–1·43)	0·23 (0·2–0·26)	0·64 (0·59–0·69)
1999	0·0 (0·0–0·0)	5·8 (4·8–6·9)	39·3 (34·2–45·1)	97·2 (91·3–102·7)	92·2 (84·8–100·6)	32·0 (27·0–37·5)	4·0 (2·9–5·5)	0·1 (0·1–0·2)	0·0 (0·0–0·0)	1·35 (1·29–1·42)	0·23 (0·2–0·25)	0·64 (0·59–0·69)
2000	0·0 (0·0–0·0)	6·1 (5·1–7·4)	39·1 (34·9–43·6)	95·2 (89·5–101·6)	90·7 (83·1–99·1)	33·8 (28·8–38·9)	4·3 (3·1–5·6)	0·1 (0·1–0·2)	0·0 (0·0–0·0)	1·35 (1·29–1·41)	0·23 (0·2–0·25)	0·64 (0·6–0·7)
2001	0·0 (0·0–0·0)	6·4 (5·3–7·7)	39·1 (34·1–44·6)	92·5 (87·1–98·5)	88·0 (80·2–96·2)	33·9 (28·9–39·3)	4·5 (3·3–6·0)	0·1 (0·1–0·2)	0·0 (0·0–0·0)	1·32 (1·26–1·38)	0·23 (0·2–0·26)	0·63 (0·59–0·68)
2002	0·0 (0·0–0·0)	6·4 (5·3–7·6)	38·1 (33·1–43·6)	90·0 (84·3–96·2)	87·7 (80·1–95·6)	34·5 (28·7–41·1)	4·8 (3·5–6·4)	0·1 (0·1–0·2)	0·0 (0·0–0·0)	1·31 (1·24–1·37)	0·22 (0·2–0·25)	0·64 (0·59–0·69)
2003	0·0 (0·0–0·0)	6·2 (5·1–7·3)	36·8 (32·6–41·6)	87·9 (83·1–92·8)	87·3 (80·0–95·2)	36·0 (30·6–42·4)	5·2 (3·7–6·9)	0·1 (0·1–0·2)	0·0 (0·0–0·0)	1·3 (1·24–1·36)	0·21 (0·19–0·24)	0·64 (0·59–0·69)
2004	0·0 (0·0–0·0)	6·0 (5·0–7·0)	36·0 (31·4–41·3)	84·7 (79·2–90·4)	86·3 (78·6–94·3)	36·9 (31·5–42·9)	5·4 (4·0–7·2)	0·1 (0·1–0·2)	0·0 (0·0–0·0)	1·28 (1·22–1·34)	0·21 (0·19–0·24)	0·64 (0·6–0·7)
2005	0·0 (0·0–0·0)	5·9 (5·0–7·0)	36·4 (31·7–41·2)	84·3 (78·8–89·9)	87·4 (80·3–94·8)	38·1 (32·6–44·2)	5·6 (4·2–7·3)	0·1 (0·1–0·2)	0·0 (0·0–0·0)	1·29 (1·23–1·35)	0·21 (0·19–0·24)	0·66 (0·61–0·7)
2006	0·0 (0·0–0·0)	6·0 (5·0–7·0)	37·8 (32·5–43·4)	85·9 (80·4–91·6)	91·2 (83·6–98·7)	40·3 (34·5–47·4)	6·1 (4·7–7·8)	0·1 (0·1–0·2)	0·0 (0·0–0·0)	1·34 (1·28–1·4)	0·22 (0·19–0·25)	0·69 (0·64–0·74)
2007	0·0 (0·0–0·0)	6·1 (5·1–7·2)	38·6 (33·9–43·8)	86·3 (80·7–92·5)	92·4 (84·8–100·4)	42·6 (36·8–49·3)	6·6 (5·0–8·4)	0·1 (0·1–0·2)	0·0 (0·0–0·0)	1·36 (1·3–1·42)	0·22 (0·2–0·25)	0·71 (0·66–0·76)
2008	0·0 (0·0–0·0)	6·1 (5·2–7·2)	39·0 (33·5–45·3)	86·6 (80·7–92·4)	93·6 (86·0–101·1)	44·2 (37·8–50·9)	7·1 (5·6–8·9)	0·1 (0·1–0·2)	0·0 (0·0–0·0)	1·38 (1·32–1·45)	0·23 (0·2–0·26)	0·73 (0·68–0·77)
2009	0·0 (0·0–0·0)	5·9 (4·9–7·0)	38·5 (34·1–42·6)	87·1 (81·8–92·5)	94·7 (86·8–103·2)	45·8 (39·2–52·5)	7·8 (6·2–10·1)	0·2 (0·1–0·2)	0·0 (0·0–0·0)	1·4 (1·33–1·47)	0·22 (0·2–0·25)	0·74 (0·69–0·8)
2010	0·0 (0·0–0·0)	5·8 (5·0–6·6)	37·9 (32·3–44·1)	88·5 (82·8–94·5)	95·9 (88·0–104·8)	47·0 (40·1–53·5)	8·2 (6·5–10·2)	0·2 (0·1–0·3)	0·0 (0·0–0·0)	1·42 (1·34–1·5)	0·22 (0·19–0·25)	0·76 (0·7–0·81)
2011	0·0 (0·0–0·0)	5·7 (4·8–6·7)	36·4 (31·8–41·8)	88·4 (82·4–94·7)	95·9 (88·2–104·0)	48·1 (41·4–55·6)	8·6 (6·6–10·7)	0·2 (0·1–0·3)	0·0 (0·0–0·0)	1·42 (1·35–1·48)	0·21 (0·19–0·24)	0·76 (0·71–0·82)
2012	0·0 (0·0–0·0)	5·6 (4·6–6·7)	34·9 (30·3–40·1)	88·1 (83·3–93·6)	97·2 (89·2–105·4)	50·4 (43·9–57·6)	9·3 (7·3–11·6)	0·2 (0·1–0·3)	0·0 (0·0–0·0)	1·43 (1·37–1·49)	0·2 (0·18–0·23)	0·79 (0·73–0·84)
2013	0·0 (0·0–0·0)	5·5 (4·6–6·5)	33·8 (29·4–38·6)	86·6 (80·6–93·1)	97·4 (88·8–106·5)	51·9 (45·2–58·9)	9·8 (7·9–12·4)	0·2 (0·1–0·3)	0·0 (0·0–0·0)	1·43 (1·36–1·5)	0·2 (0·17–0·22)	0·8 (0·74–0·86)
2014	0·0 (0·0–0·0)	5·4 (4·6–6·5)	32·8 (28·0–37·8)	85·7 (79·8–91·7)	98·6 (90·4–107·2)	54·2 (46·8–61·8)	10·4 (8·1–12·8)	0·2 (0·1–0·3)	0·0 (0·0–0·0)	1·44 (1·35–1·52)	0·19 (0·17–0·22)	0·82 (0·75–0·88)
2015	0·0 (0·0–0·0)	5·4 (4·6–6·5)	33·3 (28·5–38·7)	86·8 (79·8–94·3)	101·1 (91·1–111·3)	53·0 (44·1–62·7)	9·6 (7·3–12·6)	0·2 (0·1–0·3)	0·0 (0·0–0·0)	1·45 (1·3–1·61)	0·19 (0·17–0·23)	0·82 (0·73–0·92)
2016	0·0 (0·0–0·0)	5·5 (4·5–6·7)	34·5 (27·1–44·1)	87·6 (78·4–98·7)	100·6 (87·6–115·9)	49·0 (38·2–63·1)	8·3 (6·0–11·4)	0·2 (0·1–0·3)	0·0 (0·0–0·0)	1·43 (1·22–1·69)	0·2 (0·16–0·25)	0·79 (0·67–0·94)
2017	0·0 (0·0–0·0)	5·4 (4·5–6·6)	34·3 (26·9–43·8)	87·2 (77·4–97·8)	99·0 (84·7–114·6)	47·8 (35·7–62·3)	8·0 (5·5–11·8)	0·2 (0·1–0·3)	0·0 (0·0–0·0)	1·41 (1·18–1·67)	0·2 (0·16–0·25)	0·78 (0·63–0·94)

Supplemental Results Table 5: Age-specific fertility rates per 1000, cumulative total fertility up to maternal age 25 (TFU25), cumulative total fertility from maternal age 30 to age 55 (TFO30), and cumulative total fertility, Fukushima, 1950-2017

Year	10-14	15-19	20-24	25-29	30-34	35-39	40-44	45-49	50-54	TFR	TFU25	TFO30
1950	0·0 (0·0–0·0)	12·1 (10·7–13·5)	173·1 (158·0–189·3)	227·7 (219·2–236·2)	199·6 (187·1–211·8)	159·3 (141·3–177·6)	50·5 (43·5–58·0)	1·9 (1·3–2·8)	0·0 (0·0–0·0)	4·12 (3·96–4·28)	0·93 (0·85–1·01)	2·06 (1·94–2·18)
1951	0·0 (0·0–0·0)	9·7 (8·7–11·0)	155·5 (140·5–171·1)	211·7 (203·5–219·8)	181·0 (168·0–193·6)	129·5 (112·6–146·0)	39·1 (32·6–45·7)	1·4 (0·9–2·1)	0·0 (0·0–0·0)	3·64 (3·44–3·83)	0·83 (0·75–0·91)	1·76 (1·58–1·92)
1952	0·0 (0·0–0·0)	8·2 (7·2–9·4)	144·9 (131·0–160·6)	201·4 (192·4–210·4)	164·1 (149·0–178·9)	104·1 (88·1–122·9)	30·9 (25·0–37·3)	1·1 (0·7–1·6)	0·0 (0·0–0·0)	3·27 (3·12–3·43)	0·77 (0·7–0·84)	1·5 (1·38–1·63)
1953	0·0 (0·0–0·0)	7·2 (6·2–8·3)	137·0 (121·7–153·7)	189·8 (181·2–199·4)	146·9 (131·4–163·5)	83·1 (67·3–100·0)	24·5 (19·3–31·4)	0·9 (0·5–1·4)	0·0 (0·0–0·0)	2·95 (2·78–3·1)	0·72 (0·64–0·81)	1·28 (1·15–1·42)
1954	0·0 (0·0–0·0)	6·6 (5·6–7·7)	132·8 (117·8–147·6)	184·8 (176·3–194·2)	135·4 (119·8–152·6)	69·3 (53·9–86·1)	20·3 (14·9–26·8)	0·7 (0·4–1·1)	0·0 (0·0–0·0)	2·75 (2·59–2·92)	0·7 (0·62–0·77)	1·13 (0·98–1·27)
1955	0·0 (0·0–0·0)	6·0 (5·0–6·9)	129·7 (114·4–145·7)	183·7 (174·8–192·1)	124·7 (108·6–141·9)	54·5 (42·0–70·3)	16·9 (12·1–23·0)	0·6 (0·3–0·9)	0·0 (0·0–0·0)	2·58 (2·44–2·73)	0·68 (0·6–0·76)	0·98 (0·85–1·12)
1956	0·0 (0·0–0·0)	5·3 (4·5–6·3)	122·2 (108·0–136·7)	177·6 (170·0–185·8)	110·5 (95·7–126·3)	43·3 (32·1–57·3)	13·5 (9·5–18·7)	0·5 (0·3–0·8)	0·0 (0·0–0·0)	2·36 (2·23–2·52)	0·64 (0·57–0·71)	0·84 (0·72–0·98)
1957	0·0 (0·0–0·0)	5·1 (4·3–6·0)	123·6 (108·9–139·9)	181·2 (173·4–189·9)	104·5 (90·5–119·9)	40·8 (28·8–53·6)	11·5 (7·9–15·7)	0·5 (0·3–0·8)	0·0 (0·0–0·0)	2·34 (2·17–2·51)	0·64 (0·57–0·73)	0·79 (0·66–0·91)
1958	0·0 (0·0–0·0)	5·1 (4·4–6·0)	129·9 (115·0–147·2)	187·3 (178·4–196·0)	102·1 (88·8–116·7)	38·9 (29·2–51·4)	10·4 (7·3–14·6)	0·5 (0·3–0·8)	0·0 (0·0–0·0)	2·37 (2·24–2·51)	0·68 (0·6–0·76)	0·76 (0·65–0·88)
1959	0·0 (0·0–0·0)	5·3 (4·5–6·2)	130·1 (115·9–145·2)	186·3 (177·9–195·1)	95·5 (84·0–108·9)	31·5 (23·7–40·6)	8·6 (5·8–12·1)	0·4 (0·2–0·7)	0·0 (0·0–0·0)	2·29 (2·17–2·41)	0·68 (0·6–0·75)	0·68 (0·59–0·78)
1960	0·0 (0·0–0·0)	5·2 (4·4–6·1)	125·2 (111·2–139·6)	187·3 (178·8–196·4)	91·6 (82·1–102·2)	24·8 (19·4–31·3)	7·0 (4·8–9·9)	0·3 (0·2–0·6)	0·0 (0·0–0·0)	2·21 (2·09–2·32)	0·65 (0·58–0·72)	0·62 (0·55–0·69)
1961	0·0 (0·0–0·0)	4·9 (4·1–5·7)	121·7 (109·0–134·7)	189·3 (181·3–197·8)	89·5 (78·9–100·9)	21·2 (16·7–26·4)	6·0 (4·1–8·5)	0·3 (0·1–0·5)	0·0 (0·0–0·0)	2·16 (2·07–2·27)	0·63 (0·57–0·7)	0·58 (0·52–0·65)
1962	0·0 (0·0–0·0)	4·6 (3·8–5·5)	121·4 (107·6–135·1)	194·8 (186·7–202·9)	91·1 (83·3–98·9)	20·0 (16·3–24·5)	5·3 (3·7–7·5)	0·3 (0·1–0·4)	0·0 (0·0–0·0)	2·19 (2·09–2·28)	0·63 (0·56–0·7)	0·58 (0·54–0·63)
1963	0·0 (0·0–0·0)	4·6 (3·9–5·4)	121·9 (109·2–135·6)	196·8 (188·4–205·6)	92·6 (84·8–101·0)	22·3 (18·9–26·4)	5·2 (3·7–7·2)	0·3 (0·1–0·5)	0·0 (0·0–0·0)	2·22 (2·13–2·31)	0·63 (0·57–0·7)	0·6 (0·56–0·65)
1964	0·0 (0·0–0·0)	4·5 (3·8–5·2)	133·1 (119·5–149·4)	209·4 (200·7–219·0)	98·5 (89·2–108·7)	23·2 (19·6–26·9)	4·9 (3·5–6·5)	0·3 (0·1–0·5)	0·0 (0·0–0·0)	2·37 (2·27–2·47)	0·69 (0·62–0·77)	0·63 (0·58–0·69)
1965	0·0 (0·0–0·0)	4·4 (3·7–5·1)	122·5 (109·2–136·9)	176·7 (167·4–186·5)	81·6 (72·3–91·6)	17·9 (14·9–21·4)	4·1 (3·0–5·4)	0·2 (0·1–0·4)	0·0 (0·0–0·0)	2·04 (1·94–2·14)	0·63 (0·57–0·71)	0·52 (0·47–0·57)
1966	0·0 (0·0–0·0)	4·7 (4·0–5·3)	119·7 (106·2–133·3)	181·6 (172·3–191·7)	83·2 (72·8–94·4)	19·4 (15·7–23·6)	4·0 (3·0–5·3)	0·2 (0·1–0·4)	0·0 (0·0–0·0)	2·06 (1·97–2·16)	0·62 (0·55–0·69)	0·53 (0·47–0·6)
1967	0·0 (0·0–0·0)	4·9 (4·2–5·7)	127·4 (114·6–141·2)	221·2 (211·4–230·0)	99·8 (86·0–114·3)	21·8 (17·1–27·2)	3·9 (2·9–5·0)	0·2 (0·1–0·4)	0·0 (0·0–0·0)	2·4 (2·26–2·53)	0·66 (0·6–0·73)	0·63 (0·54–0·72)
1968	0·0 (0·0–0·0)	5·0 (4·3–5·8)	114·0 (101·5–127·6)	208·5 (199·8–217·0)	90·6 (77·7–104·2)	19·5 (14·5–25·0)	3·4 (2·6–4·6)	0·2 (0·1–0·3)	0·0 (0·0–0·0)	2·21 (2·1–2·31)	0·59 (0·53–0·66)	0·57 (0·48–0·66)
1969	0·0 (0·0–0·0)	5·4 (4·6–6·4)	112·0 (98·6–126·3)	210·6 (202·3–219·0)	90·4 (77·0–105·2)	19·3 (14·6–25·2)	3·4 (2·5–4·6)	0·1 (0·1–0·3)	0·0 (0·0–0·0)	2·21 (2·11–2·31)	0·59 (0·52–0·66)	0·57 (0·48–0·66)
1970	0·0 (0·0–0·0)	5·5 (4·6–6·5)	120·4 (107·3–134·4)	214·2 (204·3–223·4)	92·4 (80·3–106·3)	21·5 (16·2–28·2)	3·6 (2·6–4·9)	0·2 (0·1–0·3)	0·0 (0·0–0·0)	2·29 (2·18–2·4)	0·63 (0·56–0·7)	0·59 (0·51–0·68)
1971	0·0 (0·0–0·0)	5·3 (4·4–6·4)	131·9 (116·6–147·1)	211·5 (201·6–221·6)	93·1 (81·8–106·1)	24·2 (18·1–32·0)	3·9 (2·7–5·3)	0·2 (0·1–0·3)	0·0 (0·0–0·0)	2·35 (2·24–2·46)	0·69 (0·61–0·76)	0·61 (0·52–0·7)
1972	0·0 (0·0–0·0)	5·4 (4·4–6·6)	138·2 (123·4–154·1)	210·0 (200·9–220·0)	92·8 (82·1–104·0)	24·5 (18·4–32·1)	3·8 (2·7–5·2)	0·2 (0·1–0·4)	0·0 (0·0–0·0)	2·37 (2·27–2·48)	0·72 (0·65–0·8)	0·61 (0·53–0·68)
1973	0·0 (0·0–0·0)	5·2 (4·3–6·4)	138·6 (123·8–153·6)	210·3 (200·5–220·3)	88·0 (79·5–97·3)	22·3 (16·8–28·7)	3·5 (2·3–4·9)	0·2 (0·1–0·3)	0·0 (0·0–0·0)	2·34 (2·24–2·44)	0·72 (0·65–0·79)	0·57 (0·51–0·63)
1974	0·0 (0·0–0·0)	4·9 (4·0–6·0)	132·8 (119·3–146·5)	202·8 (193·5–212·1)	78·2 (70·4–85·9)	17·4 (13·4–22·8)	2·9 (2·0–4·1)	0·1 (0·1–0·2)	0·0 (0·0–0·0)	2·2 (2·11–2·29)	0·69 (0·62–0·76)	0·49 (0·45–0·54)
1975	0·0 (0·0–0·0)	4·6 (3·7–5·6)	124·7 (111·9–138·1)	196·3 (187·7–204·7)	70·8 (64·3–77·6)	13·8 (10·7–17·3)	2·5 (1·5–4·0)	0·1 (0·0–0·2)	0·0 (0·0–0·0)	2·06 (1·97–2·15)	0·65 (0·58–0·71)	0·44 (0·4–0·48)
1976	0·0 (0·0–0·0)	4·3 (3·5–5·4)	118·4 (105·8–132·6)	194·1 (185·1–202·6)	69·3 (63·3–75·9)	12·8 (10·2–15·6)	2·2 (1·5–3·2)	0·1 (0·0–0·2)	0·0 (0·0–0·0)	2·01 (1·92–2·1)	0·61 (0·55–0·68)	0·42 (0·38–0·46)
1977	0·0 (0·0–0·0)	4·2 (3·3–5·3)	111·9 (100·8–125·4)	194·6 (185·8–203·0)	71·9 (65·0–78·7)	12·8 (10·4–15·7)	2·0 (1·4–2·8)	0·1 (0·0–0·1)	0·0 (0·0–0·0)	1·99 (1·9–2·08)	0·58 (0·52–0·65)	0·43 (0·39–0·48)
1978	0·0 (0·0–0·0)	4·2 (3·3–5·1)	107·0 (94·4–121·6)	194·6 (186·1–202·6)	75·6 (68·5–83·5)	12·7 (9·7–16·1)	1·8 (1·2–2·5)	0·1 (0·0–0·1)	0·0 (0·0–0·0)	1·98 (1·89–2·07)	0·56 (0·49–0·63)	0·45 (0·41–0·49)
1979	0·0 (0·0–0·0)	4·2 (3·6–4·8)	102·3 (91·4–113·1)	192·0 (183·7–200·0)	75·5 (68·9–83·2)	12·4 (10·4–14·9)	1·6 (1·1–2·4)	0·0 (0·0–0·1)	0·0 (0·0–0·0)	1·94 (1·86–2·02)	0·53 (0·48–0·59)	0·45 (0·41–0·49)
1980	0·0 (0·0–0·0)	4·2 (3·4–5·1)	98·1 (87·0–109·7)	188·8 (180·0–197·7)	73·9 (66·7–80·9)	12·4 (10·0–15·3)	1·6 (1·1–2·1)	0·0 (0·0–0·1)	0·0 (0·0–0·0)	1·89 (1·81–1·97)	0·51 (0·46–0·57)	0·44 (0·4–0·48)
1981	0·0 (0·0–0·0)	4·4 (3·5–5·6)	96·1 (85·9–107·7)	189·6 (181·5–197·9)	74·9 (68·7–81·2)	13·0 (10·5–15·5)	1·6 (1·0–2·3)	0·0 (0·0–0·1)	0·0 (0·0–0·0)	1·9 (1·83–1·97)	0·5 (0·45–0·56)	0·45 (0·41–0·48)
1982	0·0 (0·0–0·0)	4·6 (3·8–5·6)	95·7 (84·4–107·7)	192·0 (183·5–201·4)	78·7 (70·0–88·6)	14·5 (12·0–17·3)	1·6 (1·1–2·2)	0·0 (0·0–0·1)	0·0 (0·0–0·0)	1·94 (1·86–2·02)	0·5 (0·45–0·56)	0·47 (0·43–0·53)
1983	0·0 (0·0–0·0)	4·8 (3·9–5·8)	94·5 (84·6–105·1)	193·7 (184·2–202·7)	82·7 (75·7–90·6)	16·0 (13·0–19·6)	1·6 (1·2–2·1)	0·0 (0·0–0·1)	0·0 (0·0–0·0)	1·97 (1·89–2·05)	0·5 (0·45–0·55)	0·5 (0·46–0·54)
1984	0·0 (0·0–0·0)	4·7 (3·9–5·6)	89·0 (76·6–102·8)	192·2 (183·9–201·6)	85·8 (78·3–94·0)	16·6 (13·6–20·0)	1·7 (1·2–2·3)	0·0 (0·0–0·1)	0·0 (0·0–0·0)	1·95 (1·87–2·04)	0·47 (0·41–0·54)	0·52 (0·48–0·57)
1985	0·0 (0·0–0·0)	4·5 (3·6–5·5)	84·3 (74·4–94·8)	187·1 (178·6–194·8)	87·2 (79·5–95·9)	16·6 (13·6–20·0)	1·8 (1·2–2·4)	0·0 (0·0–0·1)	0·0 (0·0–0·0)	1·91 (1·84–1·98)	0·44 (0·39–0·5)	0·53 (0·49–0·57)
1986	0·0 (0·0–0·0)	4·4 (3·5–5·4)	80·7 (70·6–91·2)	182·8 (173·9–191·9)	89·8 (82·6–97·7)	16·9 (14·0–20·0)	1·9 (1·2–2·8)	0·0 (0·0–0·1)	0·0 (0·0–0·0)	1·88 (1·81–1·96)	0·43 (0·37–0·48)	0·54 (0·5–0·58)
1987	0·0 (0·0–0·0)	4·3 (3·5–5·3)	74·9 (66·1–84·7)	179·3 (170·6–187·8)	93·1 (85·6–101·4)	17·7 (14·5–21·2)	2·1 (1·4–3·0)	0·0 (0·0–0·1)	0·0 (0·0–0·0)	1·86 (1·77–1·95)	0·4 (0·35–0·45)	0·56 (0·52–0·61)
1988	0·0 (0·0–0·0)	4·2 (3·4–5·2)	70·2 (61·5–80·1)	170·6 (162·2–179·5)	94·6 (86·6–103·2)	18·8 (15·5–22·7)	2·3 (1·7–3·1)	0·0 (0·0–0·1)	0·0 (0·0–0·0)	1·8 (1·72–1·88)	0·37 (0·33–0·42)	0·58 (0·53–0·63)
1989	0·0 (0·0–0·0)	4·3 (3·4–5·3)	66·1 (57·3–75·7)	161·2 (153·4–170·0)	94·9 (87·0–102·9)	19·9 (16·5–23·6)	2·4 (1·7–3·3)	0·0 (0·0–0·1)	0·0 (0·0–0·0)	1·74 (1·67–1·82)	0·35 (0·31–0·4)	0·59 (0·54–0·63)
1990	0·0 (0·0–0·0)	4·5 (3·6–5·5)	64·9 (57·4–73·4)	158·7 (149·6–167·1)	97·1 (88·9–105·2)	20·9 (17·1–24·9)	2·4 (1·7–3·2)	0·0 (0·0–0·1)	0·0 (0·0–0·0)	1·74 (1·67–1·82)	0·35 (0·31–0·39)	0·6 (0·56–0·65)
1991	0·0 (0·0–0·0)	4·7 (3·9–5·8)	65·8 (57·1–74·7)	155·1 (147·7–163·4)	98·2 (89·4–107·6)	22·2 (18·7–26·2)	2·4 (1·7–3·2)	0·0 (0·0–0·1)	0·0 (0·0–0·0)	1·74 (1·67–1·81)	0·35 (0·31–0·4)	0·61 (0·56–0·67)
1992	0·0 (0·0–0·0)	4·9 (4·0–6·0)	64·4 (57·4–72·2)	147·3 (138·8–155·8)	97·3 (89·7–105·4)	23·1 (19·4–27·7)	2·6 (1·9–3·4)	0·1 (0·0–0·1)	0·0 (0·0–0·0)	1·7 (1·64–1·76)	0·35 (0·31–0·38)	0·61 (0·57–0·66)
1993	0·0 (0·0–0·0)	5·0 (4·2–5·9)	64·8 (56·5–73·3)	147·4 (138·5–156·7)	101·3 (92·5–110·4)	25·1 (21·0–29·6)	2·7 (2·0–3·6)	0·1 (0·0–0·1)	0·0 (0·0–0·0)	1·73 (1·65–1·82)	0·35 (0·31–0·39)	0·65 (0·59–0·7)
1994	0·0 (0·0–0·0)	5·1 (4·3–6·1)	64·8 (56·0–74·5)	144·9 (136·6–153·3)	102·8 (94·3–112·1)	26·6 (21·9–31·5)	2·8 (2·0–3·7)	0·1 (0·0–0·1)	0·0 (0·0–0·0)	1·74 (1·64–1·82)	0·35 (0·31–0·4)	0·66 (0·61–0·72)
1995	0·0 (0·0–0·0)	5·2 (4·3–6·2)	63·9 (56·2–72·5)	136·8 (128·8–145·0)	101·7 (92·2–110·7)	27·7 (23·3–32·6)	2·9 (2·2–3·8)	0·1 (0·0–0·1)	0·0 (0·0–0·0)	1·69 (1·61–1·78)	0·35 (0·31–0·39)	0·66 (0·61–0·72)
1996	0·0 (0·0–0·0)	5·5 (4·6–6·5)	64·3 (55·6–72·5)	132·3 (124·1–140·9)	101·3 (93·1–110·3)	29·1 (24·2–34·6)	3·0 (2·2–3·9)	0·1 (0·0–0·1)	0·0 (0·0–0·0)	1·68 (1·58–1·77)	0·35 (0·31–0·39)	0·67 (0·62–0·73)
1997	0·0 (0·0–0·0)	5·8 (4·8–7·0)	65·1 (56·9–74·1)	128·3 (120·6–135·7)	99·5 (91·0–109·1)	29·7 (24·7–35·4)	3·1 (2·3–4·2)	0·1 (0·0–0·1)	0·0 (0·0–0·0)	1·66 (1·58–1·74)	0·35 (0·32–0·4)	0·66 (0·61–0·72)
1998	0·0 (0·0–0·0)	6·2 (5·1–7·5)	65·7 (57·7–75·2)	123·9 (116·5–132·4)	98·3 (89·8–107·6)	30·9 (25·5–36·9)	3·2 (2·4–4·3)	0·1 (0·0–0·1)	0·0 (0·0–0·0)	1·64 (1·56–1·73)	0·36 (0·32–0·41)	0·66 (0·61–0·72)
1999	0·0 (0·0–0·0)	6·6 (5·5–8·0)	66·3 (57·7–75·8)	120·4 (112·6–127·9)	97·3 (89·3–106·0)	31·6 (26·1–37·5)	3·4 (2·4–4·6)	0·1 (0·0–0·1)	0·0 (0·0–0·0)	1·63 (1·55–1·71)	0·36 (0·32–0·41)	0·66 (0·61–0·72)
2000	0·0 (0·0–0·0)	7·1 (5·9–8·5)	67·2 (59·7–74·7)	118·2 (111·1–126·1)	94·8 (86·8–103·3)	32·9 (27·4–38·9)	3·5 (2·6–4·8)	0·1 (0·0–0·1)	0·0 (0·0–0·0)	1·62 (1·55–1·69)	0·37 (0·33–0·41)	0·66 (0·61–0·71)
2001	0·0 (0·0–0·0)	7·4 (6·1–8·9)	67·8 (58·5–77·6)	115·1 (108·0–123·1)	91·3 (83·1–99·3)	32·7 (27·2–38·9)	3·6 (2·6–5·0)	0·1 (0·0–0·1)	0·0 (0·0–0·0)	1·59 (1·51–1·67)	0·38 (0·33–0·42)	0·64 (0·59–0·69)
2002	0·0 (0·0–0·0)	7·3 (6·1–8·7)	66·0 (57·5–75·6)	112·0 (104·6–119·4)	90·2 (82·4–98·9)	33·1 (27·3–39·2)	3·9 (2·7–5·4)	0·1 (0·0–0·1)	0·0 (0·0–0·0)	1·56 (1·49–1·64)	0·37 (0·32–0·41)	0·64 (0·59–0·69)
2003	0·0 (0·0–0·0)	6·9 (5·7–8·4)	63·0 (54·5–72·2)	108·8 (102·5–115·3)	89·1 (81·6–97·2)	34·2 (28·3–40·4)	4·2 (2·9–5·7)	0·1 (0·0–0·1)	0·0 (0·0–0·0)	1·53 (1·46–1·61)	0·35 (0·31–0·39)	0·64 (0·59–0·69)
2004	0·0 (0·0–0·0)	6·5 (5·4–7·6)	60·3 (52·5–69·8)	104·1 (97·4–111·3)	87·1 (79·5–95·2)	34·7 (29·0–41·1)	4·4 (3·1–5·8)	0·1 (0·0–0·2)	0·0 (0·0–0·0)	1·49 (1·41–1·56)	0·33 (0·3–0·38)	0·63 (0·58–0·68)
2005	0·0 (0·0–0·0)	6·2 (5·3–7·4)	59·2 (52·2–66·9)	102·3 (95·2–109·4)	86·8 (78·8–94·7)	35·1 (29·4–41·5)	4·6 (3·3–6·1)	0·1 (0·0–0·2)	0·0 (0·0–0·0)	1·47 (1·4–1·54)	0·33 (0·29–0·37)	0·63 (0·58–0·68)
2006	0·0 (0·0–0·0)	6·1 (5·1–7·1)	59·4 (51·5–67·6)	102·6 (95·4–109·7)	88·5 (80·6–96·4)	36·2 (30·5–43·2)	5·0 (3·7–6·7)	0·1 (0·0–0·2)	0·0 (0·0–0·0)	1·49 (1·42–1·57)	0·33 (0·29–0·37)	0·65 (0·6–0·71)
2007	0·0 (0·0–0·0)	6·0 (5·1–7·1)	58·9 (52·6–66·3)	101·3 (94·5–108·7)	87·8 (79·9–95·9)	37·5 (31·4–43·9)	5·4 (4·0–6·9)	0·1 (0·0–0·2)	0·0 (0·0–0·0)	1·48 (1·42–1·55)	0·32 (0·29–0·36)	0·65 (0·6–0·71)
2008	0·0 (0·0–0·0)	5·9 (5·0–6·9)	58·0 (50·1–66·5)	99·9 (92·6–106·9)	87·4 (78·9–96·3)	38·2 (32·2–44·6)	5·7 (4·3–7·4)	0·1 (0·0–0·2)	0·0 (0·0–0·0)	1·48 (1·4–1·55)	0·32 (0·28–0·36)	0·66 (0·61–0·71)
2009	0·0 (0·0–0·0)	5·6 (4·7–6·7)	56·0 (50·2–61·6)	98·8 (92·6–105·0)	87·0 (79·5–95·1)	38·9 (33·0–45·0)	6·2 (4·6–8·0)	0·1 (0·1–0·2)	0·0 (0·0–0·0)	1·46 (1·39–1·54)	0·31 (0·28–0·34)	0·66 (0·62–0·71)
2010	0·0 (0·0–0·0)	5·4 (4·6–6·4)	54·6 (45·9–63·4)	99·0 (92·4–105·9)	87·2 (79·4–96·1)	39·6 (34·0–45·6)	6·4 (4·9–8·1)	0·1 (0·1–0·2)	0·0 (0·0–0·0)	1·46 (1·38–1·55)	0·3 (0·26–0·35)	0·67 (0·62–0·72)
2011	0·0 (0·0–0·0)	5·3 (4·4–6·3)	52·2 (45·0–59·8)	98·5 (91·7–105·5)	86·8 (79·1–94·7)	40·2 (34·0–46·7)	6·5 (4·9–8·4)	0·1 (0·1–0·2)	0·0 (0·0–0·0)	1·45 (1·38–1·52)	0·29 (0·25–0·33)	0·67 (0·62–0·72)
2012	0·0 (0·0–0·0)	5·3 (4·2–6·4)	50·2 (43·2–58·2)	98·8 (92·4–105·4)	88·4 (80·4–96·9)	42·1 (35·9–49·4)	7·0 (5·2–9·0)	0·1 (0·1–0·3)	0·0 (0·0–0·0)	1·46 (1·39–1·53)	0·28 (0·25–0·31)	0·69 (0·64–0·74)
2013	0·0 (0·0–0·0)	5·2 (4·4–6·2)	48·9 (42·3–55·7)	98·5 (91·8–105·3)	89·6 (82·3–98·4)	43·3 (37·1–49·4)	7·2 (5·4–9·6)	0·1 (0·1–0·3)	0·0 (0·0–0·0)	1·46 (1·4–1·54)	0·27 (0·24–0·3)	0·7 (0·65–0·76)
2014	0·0 (0·0–0·0)	5·2 (4·4–6·1)	47·6 (40·4–55·9)	99·2 (92·0–106·4)	91·7 (83·9–100·6)	45·0 (38·9–52·2)	7·6 (5·5–10·0)	0·1 (0·1–0·3)	0·0 (0·0–0·0)	1·48 (1·4–1·57)	0·26 (0·23–0·3)	0·72 (0·66–0·79)
2015	0·0 (0·0–0·0)	5·2 (4·3–6·2)	48·5 (41·5–57·0)	102·0 (94·1–110·8)	94·9 (85·7–104·9)	43·7 (35·8–52·4)	7·0 (4·9–9·7)	0·1 (0·1–0·3)	0·0 (0·0–0·0)	1·51 (1·35–1·68)	0·27 (0·23–0·32)	0·73 (0·65–0·82)
2016	0·0 (0·0–0·0)	5·2 (4·3–6·4)	50·5 (39·7–64·3)	104·1 (93·0–117·5)	95·2 (82·2–110·2)	40·3 (30·9–52·8)	6·0 (4·0–8·7)	0·1 (0·1–0·2)	0·0 (0·0–0·0)	1·51 (1·28–1·78)	0·28 (0·22–0·35)	0·71 (0·59–0·84)
2017	0·0 (0·0–0·0)	5·2 (4·3–6·3)	50·3 (39·4–64·3)	104·3 (92·6–117·1)	94·3 (80·3–109·4)	39·3 (29·1–51·6)	5·9 (3·8–8·9)	0·1 (0·1–0·3)	0·0 (0·0–0·0)	1·5 (1·25–1·79)	0·28 (0·22–0·35)	0·7 (0·57–0·85)

Supplemental Results Table 5: Age-specific fertility rates per 1000, cumulative total fertility up to maternal age 25 (TFU25), cumulative total fertility from maternal age 30 to age 55 (TFO30), and cumulative total fertility, Gifu, 1950-2017

Year	10-14	15-19	20-24	25-29	30-34	35-39	40-44	45-49	50-54	TFR	TFU25	TFO30
1950	0·0 (0·0–0·0)	7·9 (7·0–8·9)	151·4 (138·1–165·7)	223·6 (215·4–231·9)	138·0 (129·7–146·0)	72·0 (63·9–79·8)	24·4 (21·1–28·0)	1·4 (0·9–2·0)	0·0 (0·0–0·0)	3·09 (2·98–3·2)	0·8 (0·73–0·87)	1·18 (1·12–1·24)
1951	0·0 (0·0–0·0)	6·4 (5·7–7·1)	135·8 (123·4–148·2)	207·9 (199·9–215·6)	124·6 (115·3–133·5)	58·0 (50·4–65·7)	18·9 (15·8–22·2)	1·0 (0·7–1·5)	0·0 (0·0–0·0)	2·76 (2·65–2·88)	0·71 (0·65–0·77)	1·01 (0·92–1·1)
1952	0·0 (0·0–0·0)	5·4 (4·7–6·1)	126·5 (113·0–140·8)	197·7 (189·1–206·6)	112·6 (101·9–123·9)	46·4 (40·1–53·2)	15·0 (12·4–18·0)	0·8 (0·5–1·2)	0·0 (0·0–0·0)	2·52 (2·41–2·63)	0·66 (0·59–0·73)	0·87 (0·81–0·94)
1953	0·0 (0·0–0·0)	4·7 (4·1–5·4)	119·6 (105·9–134·5)	186·5 (178·0–195·9)	100·7 (88·8–112·3)	38·7 (32·7–45·2)	11·9 (9·7–14·4)	0·6 (0·4–1·0)	0·0 (0·0–0·0)	2·31 (2·21–2·43)	0·62 (0·55–0·7)	0·76 (0·7–0·82)
1954	0·0 (0·0–0·0)	4·3 (3·7–5·0)	116·0 (101·4–131·3)	181·5 (173·2–190·7)	92·8 (81·5–104·9)	35·0 (29·4–41·6)	9·8 (7·8–12·3)	0·5 (0·3–0·8)	0·0 (0·0–0·0)	2·2 (2·08–2·32)	0·6 (0·53–0·68)	0·69 (0·63–0·75)
1955	0·0 (0·0–0·0)	3·9 (3·3–4·6)	113·2 (98·6–130·0)	180·6 (171·7–188·7)	85·4 (73·6–97·3)	31·5 (26·6–37·5)	8·2 (6·3–10·3)	0·4 (0·3–0·7)	0·0 (0·0–0·0)	2·12 (2·01–2·22)	0·59 (0·51–0·67)	0·63 (0·57–0·69)
1956	0·0 (0·0–0·0)	3·5 (2·9–4·1)	106·7 (91·8–122·0)	174·6 (167·1–182·8)	75·7 (65·3–87·3)	27·0 (22·5–31·6)	6·6 (5·0–8·3)	0·3 (0·2–0·6)	0·0 (0·0–0·0)	1·97 (1·87–2·09)	0·55 (0·48–0·63)	0·55 (0·5–0·6)
1957	0·0 (0·0–0·0)	3·3 (2·8–3·9)	107·9 (92·9–125·4)	178·2 (170·7–186·6)	71·8 (62·2–83·4)	23·1 (19·1–26·8)	5·6 (4·3–7·1)	0·4 (0·2–0·6)	0·0 (0·0–0·0)	1·95 (1·83–2·08)	0·56 (0·48–0·64)	0·5 (0·45–0·55)
1958	0·0 (0·0–0·0)	3·4 (2·8–3·9)	113·3 (98·4–129·9)	184·1 (175·5–192·3)	70·3 (60·4–81·7)	20·4 (16·7–24·1)	5·1 (3·9–6·4)	0·3 (0·2–0·6)	0·0 (0·0–0·0)	1·98 (1·88–2·09)	0·58 (0·51–0·67)	0·48 (0·43–0·53)
1959	0·0 (0·0–0·0)	3·5 (3·0–4·1)	113·5 (98·9–129·9)	183·2 (174·7–192·3)	65·9 (57·8–75·5)	17·6 (14·2–21·5)	4·2 (3·2–5·5)	0·3 (0·2–0·5)	0·0 (0·0–0·0)	1·94 (1·84–2·04)	0·58 (0·51–0·67)	0·44 (0·4–0·48)
1960	0·0 (0·0–0·0)	3·4 (2·9–4·0)	109·2 (96·5–123·2)	184·2 (175·7–193·3)	63·2 (56·4–70·8)	15·4 (12·6–18·6)	3·5 (2·6–4·4)	0·2 (0·1–0·4)	0·0 (0·0–0·0)	1·9 (1·8–1·99)	0·56 (0·5–0·63)	0·41 (0·38–0·45)
1961	0·0 (0·0–0·0)	3·2 (2·6–3·8)	106·2 (95·6–117·8)	186·4 (178·4–195·1)	61·9 (54·2–70·6)	13·8 (11·3–16·7)	3·0 (2·2–4·0)	0·2 (0·1–0·3)	0·0 (0·0–0·0)	1·87 (1·79–1·96)	0·55 (0·5–0·6)	0·39 (0·35–0·44)
1962	0·0 (0·0–0·0)	3·0 (2·5–3·6)	106·2 (94·5–118·3)	192·1 (184·2–200·2)	63·3 (57·9–68·7)	13·1 (10·7–15·7)	2·7 (1·9–3·6)	0·2 (0·1–0·3)	0·0 (0·0–0·0)	1·9 (1·83–1·98)	0·55 (0·49–0·61)	0·4 (0·37–0·43)
1963	0·0 (0·0–0·0)	3·0 (2·5–3·6)	106·9 (95·1–120·3)	194·4 (186·4–202·8)	64·8 (59·3–70·9)	12·5 (10·5–14·9)	2·6 (1·9–3·6)	0·2 (0·1–0·3)	0·0 (0·0–0·0)	1·92 (1·84–2·0)	0·55 (0·49–0·62)	0·4 (0·37–0·43)
1964	0·0 (0·0–0·0)	3·0 (2·5–3·5)	117·1 (103·5–132·5)	207·4 (198·8–216·9)	69·5 (62·9–76·8)	12·9 (10·9–15·0)	2·5 (1·7–3·4)	0·2 (0·1–0·3)	0·0 (0·0–0·0)	2·06 (1·97–2·16)	0·6 (0·53–0·68)	0·43 (0·39–0·46)
1965	0·0 (0·0–0·0)	2·9 (2·5–3·4)	108·3 (94·3–124·4)	175·5 (166·1–185·0)	57·9 (51·0–64·9)	11·8 (9·6–14·3)	2·1 (1·5–2·9)	0·1 (0·1–0·2)	0·0 (0·0–0·0)	1·79 (1·68–1·91)	0·56 (0·48–0·64)	0·36 (0·32–0·4)
1966	0·0 (0·0–0·0)	3·1 (2·6–3·6)	106·3 (89·6–124·7)	180·8 (171·5–190·8)	59·6 (52·2–67·8)	11·9 (10·0–14·2)	2·1 (1·5–2·9)	0·2 (0·1–0·2)	0·0 (0·0–0·0)	1·82 (1·71–1·94)	0·55 (0·46–0·64)	0·37 (0·33–0·41)
1967	0·0 (0·0–0·0)	3·3 (2·8–3·8)	113·9 (95·5–134·5)	220·9 (211·4–229·7)	72·1 (62·0–83·0)	13·4 (11·0–16·3)	2·1 (1·6–2·8)	0·1 (0·1–0·2)	0·0 (0·0–0·0)	2·13 (1·97–2·29)	0·59 (0·5–0·69)	0·44 (0·39–0·49)
1968	0·0 (0·0–0·0)	3·3 (2·9–3·9)	102·5 (85·3–122·0)	208·9 (199·9–217·7)	66·0 (55·6–76·7)	13·3 (10·7–16·2)	1·9 (1·4–2·5)	0·1 (0·1–0·2)	0·0 (0·0–0·0)	1·98 (1·85–2·12)	0·53 (0·44–0·63)	0·41 (0·36–0·46)
1969	0·0 (0·0–0·0)	3·6 (3·2–4·3)	101·3 (84·2–120·3)	211·5 (203·1–219·9)	66·6 (56·6–77·8)	13·0 (10·6–15·7)	1·9 (1·4–2·5)	0·1 (0·1–0·2)	0·0 (0·0–0·0)	1·99 (1·88–2·13)	0·52 (0·44–0·62)	0·41 (0·36–0·46)
1970	0·0 (0·0–0·0)	3·7 (3·2–4·3)	109·5 (92·7–128·1)	215·4 (205·8–223·7)	69·0 (59·3–79·9)	12·5 (10·0–15·3)	2·0 (1·5–2·7)	0·1 (0·1–0·2)	0·0 (0·0–0·0)	2·06 (1·93–2·19)	0·57 (0·48–0·66)	0·42 (0·37–0·47)
1971	0·0 (0·0–0·0)	3·6 (3·0–4·2)	120·4 (103·2–140·5)	212·8 (202·9–222·7)	70·5 (60·6–82·1)	12·4 (10·1–15·1)	2·2 (1·5–3·1)	0·2 (0·1–0·2)	0·0 (0·0–0·0)	2·11 (1·98–2·25)	0·62 (0·54–0·72)	0·43 (0·38–0·48)
1972	0·0 (0·0–0·0)	3·7 (3·1–4·4)	126·6 (110·7–144·7)	211·3 (201·9–221·0)	71·1 (62·6–79·8)	12·7 (10·1–15·5)	2·2 (1·6–3·1)	0·2 (0·1–0·2)	0·0 (0·0–0·0)	2·14 (2·02–2·26)	0·65 (0·57–0·74)	0·43 (0·39–0·47)
1973	0·0 (0·0–0·0)	3·6 (2·9–4·3)	127·2 (112·9–141·7)	211·4 (201·6–221·4)	68·1 (60·9–75·8)	11·7 (9·2–14·4)	2·1 (1·4–2·9)	0·1 (0·1–0·2)	0·0 (0·0–0·0)	2·12 (2·02–2·22)	0·65 (0·58–0·73)	0·41 (0·38–0·45)
1974	0·0 (0·0–0·0)	3·3 (2·7–4·0)	121·6 (108·9–135·6)	202·8 (193·5–212·4)	60·6 (54·4–66·8)	9·8 (7·7–12·4)	1·7 (1·2–2·4)	0·1 (0·1–0·2)	0·0 (0·0–0·0)	2·0 (1·92–2·09)	0·62 (0·56–0·7)	0·36 (0·33–0·39)
1975	0·0 (0·0–0·0)	3·0 (2·4–3·7)	112·9 (101·0–125·1)	194·8 (185·9–203·3)	54·4 (49·0–59·8)	9·1 (7·1–11·6)	1·4 (1·0–2·0)	0·1 (0·0–0·1)	0·0 (0·0–0·0)	1·88 (1·79–1·96)	0·58 (0·52–0·64)	0·32 (0·3–0·35)
1976	0·0 (0·0–0·0)	2·8 (2·3–3·5)	105·5 (94·1–117·5)	191·8 (183·1–200·7)	52·4 (47·4–58·0)	9·0 (7·3–10·9)	1·2 (0·8–1·8)	0·1 (0·0–0·1)	0·0 (0·0–0·0)	1·81 (1·74–1·9)	0·54 (0·48–0·6)	0·31 (0·28–0·35)
1977	0·0 (0·0–0·0)	2·7 (2·2–3·3)	98·4 (87·8–111·3)	192·5 (184·1–201·3)	53·7 (48·5–59·7)	9·0 (7·0–11·4)	1·1 (0·7–1·6)	0·1 (0·0–0·1)	0·0 (0·0–0·0)	1·79 (1·71–1·87)	0·51 (0·45–0·57)	0·32 (0·29–0·36)
1978	0·0 (0·0–0·0)	2·7 (2·2–3·3)	92·7 (83·6–102·5)	193·5 (185·0–201·4)	57·0 (51·4–62·9)	8·9 (6·6–11·7)	1·1 (0·7–1·6)	0·1 (0·0–0·1)	0·0 (0·0–0·0)	1·78 (1·7–1·85)	0·48 (0·43–0·53)	0·34 (0·3–0·37)
1979	0·0 (0·0–0·0)	2·7 (2·3–3·2)	87·3 (77·4–98·3)	192·7 (184·6–201·2)	58·3 (53·0–63·9)	8·6 (7·1–10·5)	1·1 (0·7–1·7)	0·1 (0·0–0·1)	0·0 (0·0–0·0)	1·75 (1·68–1·83)	0·45 (0·4–0·51)	0·34 (0·31–0·37)
1980	0·0 (0·0–0·0)	2·7 (2·2–3·4)	82·1 (73·4–90·7)	191·8 (182·8–200·9)	57·8 (52·0–63·9)	8·5 (6·9–10·6)	1·1 (0·7–1·6)	0·1 (0·0–0·1)	0·0 (0·0–0·0)	1·72 (1·64–1·79)	0·42 (0·38–0·47)	0·34 (0·31–0·37)
1981	0·0 (0·0–0·0)	2·8 (2·2–3·5)	78·9 (69·0–90·7)	194·6 (185·8–203·2)	59·0 (53·6–64·4)	8·9 (7·1–11·1)	1·1 (0·8–1·6)	0·0 (0·0–0·1)	0·0 (0·0–0·0)	1·73 (1·66–1·8)	0·41 (0·36–0·47)	0·35 (0·32–0·37)
1982	0·0 (0·0–0·0)	2·9 (2·3–3·6)	76·7 (67·8–86·6)	198·0 (190·0–207·1)	62·4 (56·6–68·6)	10·0 (8·1–12·1)	1·1 (0·8–1·6)	0·0 (0·0–0·1)	0·0 (0·0–0·0)	1·76 (1·69–1·83)	0·4 (0·35–0·45)	0·37 (0·34–0·4)
1983	0·0 (0·0–0·0)	3·0 (2·4–3·7)	73·9 (66·0–82·4)	200·0 (189·9–209·5)	66·3 (60·3–73·4)	11·0 (8·8–13·7)	1·2 (0·9–1·5)	0·0 (0·0–0·1)	0·0 (0·0–0·0)	1·78 (1·71–1·84)	0·38 (0·35–0·43)	0·39 (0·36–0·43)
1984	0·0 (0·0–0·0)	2·9 (2·3–3·5)	68·1 (58·4–79·3)	198·1 (188·9–208·0)	69·2 (62·8–76·0)	11·4 (9·0–14·3)	1·2 (0·9–1·7)	0·1 (0·0–0·1)	0·0 (0·0–0·0)	1·76 (1·69–1·83)	0·36 (0·31–0·41)	0·41 (0·38–0·45)
1985	0·0 (0·0–0·0)	2·8 (2·2–3·4)	63·4 (55·7–71·4)	192·1 (183·5–200·3)	71·0 (63·9–77·5)	11·4 (9·1–14·2)	1·2 (0·9–1·6)	0·1 (0·0–0·1)	0·0 (0·0–0·0)	1·71 (1·64–1·77)	0·33 (0·29–0·37)	0·42 (0·38–0·45)
1986	0·0 (0·0–0·0)	2·6 (2·1–3·3)	59·8 (52·5–68·1)	187·1 (178·3–196·6)	73·8 (67·6–79·7)	11·5 (9·1–14·2)	1·3 (0·9–1·7)	0·1 (0·0–0·1)	0·0 (0·0–0·0)	1·68 (1·62–1·75)	0·31 (0·28–0·35)	0·43 (0·4–0·47)
1987	0·0 (0·0–0·0)	2·6 (2·0–3·2)	54·6 (48·5–61·7)	183·0 (174·3–192·0)	77·6 (71·0–85·1)	12·1 (9·7–14·7)	1·5 (1·0–2·1)	0·1 (0·0–0·1)	0·0 (0·0–0·0)	1·66 (1·59–1·73)	0·29 (0·25–0·32)	0·46 (0·42–0·5)
1988	0·0 (0·0–0·0)	2·5 (1·9–3·1)	50·1 (43·3–58·0)	173·7 (165·5–182·6)	80·6 (73·7–88·4)	12·9 (10·2–15·9)	1·6 (1·1–2·2)	0·1 (0·0–0·1)	0·0 (0·0–0·0)	1·61 (1·54–1·68)	0·26 (0·23–0·3)	0·48 (0·44–0·52)
1989	0·0 (0·0–0·0)	2·5 (2·0–3·0)	45·7 (40·1–52·2)	163·8 (155·3–173·1)	82·8 (75·7–90·3)	13·9 (11·1–17·4)	1·7 (1·2–2·4)	0·1 (0·0–0·1)	0·0 (0·0–0·0)	1·55 (1·49–1·62)	0·24 (0·21–0·27)	0·49 (0·46–0·53)
1990	0·0 (0·0–0·0)	2·5 (2·1–3·0)	43·6 (37·6–49·5)	161·3 (152·1–170·1)	86·5 (78·9–93·9)	14·7 (11·8–17·8)	1·7 (1·2–2·3)	0·1 (0·0–0·1)	0·0 (0·0–0·0)	1·55 (1·49–1·62)	0·23 (0·2–0·26)	0·51 (0·48–0·56)
1991	0·0 (0·0–0·0)	2·6 (2·1–3·1)	43·0 (37·2–49·1)	156·9 (149·0–165·1)	88·4 (80·3–96·6)	15·8 (12·9–19·4)	1·8 (1·3–2·4)	0·1 (0·0–0·1)	0·0 (0·0–0·0)	1·54 (1·48–1·6)	0·23 (0·2–0·26)	0·53 (0·49–0·57)
1992	0·0 (0·0–0·0)	2·7 (2·2–3·2)	41·1 (36·1–46·6)	144·7 (135·8–153·3)	86·0 (78·7–93·6)	16·6 (13·7–20·4)	1·9 (1·3–2·6)	0·1 (0·0–0·1)	0·0 (0·0–0·0)	1·46 (1·41–1·51)	0·22 (0·19–0·25)	0·52 (0·49–0·56)
1993	0·0 (0·0–0·0)	2·7 (2·2–3·3)	40·5 (34·7–46·8)	130·5 (122·7–138·6)	80·9 (73·7–88·7)	18·2 (14·9–22·1)	2·0 (1·4–2·7)	0·1 (0·0–0·1)	0·0 (0·0–0·0)	1·37 (1·31–1·43)	0·22 (0·19–0·25)	0·51 (0·46–0·55)
1994	0·0 (0·0–0·0)	2·8 (2·3–3·4)	39·8 (34·7–45·4)	117·7 (110·2–124·8)	76·0 (69·5–82·8)	19·6 (16·1–23·6)	2·1 (1·5–2·8)	0·1 (0·0–0·1)	0·0 (0·0–0·0)	1·29 (1·23–1·34)	0·21 (0·19–0·24)	0·49 (0·45–0·53)
1995	0·0 (0·0–0·0)	2·9 (2·3–3·5)	38·7 (33·1–44·9)	119·7 (112·3–127·6)	82·9 (75·3–90·7)	20·7 (17·2–24·8)	2·2 (1·6–3·0)	0·1 (0·0–0·1)	0·0 (0·0–0·0)	1·34 (1·28–1·39)	0·21 (0·18–0·24)	0·53 (0·49–0·57)
1996	0·0 (0·0–0·0)	3·0 (2·5–3·7)	38·4 (33·2–44·1)	128·0 (121·0–135·2)	94·0 (86·2–102·3)	22·0 (18·2–26·6)	2·3 (1·7–3·0)	0·1 (0·0–0·1)	0·0 (0·0–0·0)	1·44 (1·38–1·5)	0·21 (0·18–0·24)	0·59 (0·55–0·64)
1997	0·0 (0·0–0·0)	3·3 (2·7–4·0)	38·4 (33·2–43·4)	128·5 (118·3–138·5)	97·9 (89·1–106·3)	22·8 (18·9–27·4)	2·5 (1·8–3·3)	0·1 (0·0–0·1)	0·0 (0·0–0·0)	1·47 (1·4–1·54)	0·21 (0·18–0·23)	0·62 (0·57–0·66)
1998	0·0 (0·0–0·0)	3·5 (2·9–4·4)	38·4 (33·3–44·1)	124·2 (117·2–131·5)	99·5 (90·6–108·8)	24·0 (19·7–29·0)	2·6 (1·9–3·4)	0·1 (0·0–0·1)	0·0 (0·0–0·0)	1·46 (1·4–1·53)	0·21 (0·19–0·24)	0·63 (0·58–0·68)
1999	0·0 (0·0–0·0)	3·8 (3·2–4·7)	38·5 (33·8–43·8)	118·7 (111·2–126·1)	99·7 (91·7–109·4)	25·0 (20·9–29·8)	2·8 (2·0–3·8)	0·1 (0·0–0·1)	0·0 (0·0–0·0)	1·44 (1·38–1·51)	0·21 (0·19–0·24)	0·64 (0·59–0·68)
2000	0·0 (0·0–0·0)	4·2 (3·4–5·1)	39·1 (32·7–45·6)	113·9 (107·0–121·5)	98·5 (90·7–107·3)	26·7 (22·1–31·7)	2·9 (2·1–3·9)	0·1 (0·1–0·1)	0·0 (0·0–0·0)	1·43 (1·35–1·5)	0·22 (0·18–0·25)	0·64 (0·59–0·69)
2001	0·0 (0·0–0·0)	4·4 (3·6–5·4)	39·5 (34·9–44·7)	108·7 (102·1–116·2)	96·1 (87·2–104·9)	27·2 (22·6–32·4)	3·0 (2·2–4·1)	0·1 (0·1–0·1)	0·0 (0·0–0·0)	1·4 (1·33–1·46)	0·22 (0·2–0·25)	0·63 (0·58–0·68)
2002	0·0 (0·0–0·0)	4·4 (3·6–5·4)	38·8 (33·6–44·8)	104·4 (97·6–111·3)	95·9 (87·6–105·0)	28·3 (23·6–33·1)	3·2 (2·3–4·4)	0·1 (0·1–0·1)	0·0 (0·0–0·0)	1·38 (1·31–1·45)	0·22 (0·19–0·25)	0·64 (0·59–0·69)
2003	0·0 (0·0–0·0)	4·3 (3·4–5·2)	37·5 (32·9–42·5)	100·8 (94·0–107·7)	95·4 (87·3–104·1)	30·3 (25·4–35·9)	3·5 (2·5–4·7)	0·1 (0·1–0·2)	0·0 (0·0–0·0)	1·36 (1·29–1·43)	0·21 (0·19–0·23)	0·65 (0·6–0·7)
2004	0·0 (0·0–0·0)	4·1 (3·3–5·0)	36·4 (31·2–42·2)	96·3 (90·1–102·5)	93·7 (85·7–101·8)	31·7 (26·7–37·2)	3·6 (2·6–4·9)	0·1 (0·1–0·2)	0·0 (0·0–0·0)	1·33 (1·27–1·39)	0·2 (0·18–0·23)	0·65 (0·6–0·69)
2005	0·0 (0·0–0·0)	4·0 (3·2–4·9)	36·2 (31·8–41·6)	94·9 (88·5–101·6)	93·8 (85·2–102·2)	33·2 (28·0–39·2)	3·9 (2·7–5·3)	0·1 (0·1–0·2)	0·0 (0·0–0·0)	1·33 (1·27–1·4)	0·2 (0·18–0·23)	0·65 (0·61–0·71)
2006	0·0 (0·0–0·0)	4·0 (3·2–5·0)	36·7 (32·2–41·7)	95·9 (89·6–102·9)	96·3 (88·0–105·4)	35·5 (30·0–41·9)	4·3 (3·1–5·8)	0·1 (0·1–0·2)	0·0 (0·0–0·0)	1·36 (1·3–1·43)	0·2 (0·18–0·23)	0·68 (0·63–0·74)
2007	0·0 (0·0–0·0)	4·0 (3·2–5·0)	36·7 (30·4–44·2)	95·4 (89·5–102·2)	96·3 (87·7–105·4)	38·0 (32·3–44·4)	4·6 (3·4–6·3)	0·1 (0·1–0·2)	0·0 (0·0–0·0)	1·38 (1·3–1·45)	0·2 (0·17–0·24)	0·7 (0·64–0·75)
2008	0·0 (0·0–0·0)	3·9 (3·1–4·9)	36·0 (31·3–41·0)	95·0 (88·8–101·8)	96·8 (88·7–105·0)	39·9 (34·0–46·4)	5·1 (3·7–6·9)	0·1 (0·1–0·2)	0·0 (0·0–0·0)	1·38 (1·32–1·45)	0·2 (0·18–0·23)	0·71 (0·66–0·76)
2009	0·0 (0·0–0·0)	3·7 (2·9–4·8)	34·7 (29·4–40·5)	94·9 (88·8–100·5)	97·6 (88·8–107·1)	41·8 (35·6–48·5)	5·6 (4·2–7·4)	0·1 (0·1–0·2)	0·0 (0·0–0·0)	1·39 (1·31–1·48)	0·19 (0·17–0·22)	0·73 (0·67–0·79)
2010	0·0 (0·0–0·0)	3·6 (2·7–4·8)	33·4 (29·0–38·1)	95·6 (89·2–102·6)	99·1 (91·1–108·1)	43·4 (37·2–50·1)	6·0 (4·5–7·9)	0·1 (0·1–0·2)	0·0 (0·0–0·0)	1·41 (1·34–1·48)	0·19 (0·16–0·21)	0·74 (0·69–0·8)
2011	0·0 (0·0–0·0)	3·5 (2·8–4·4)	31·5 (27·4–36·0)	95·1 (88·5–101·9)	99·6 (90·0–109·6)	44·6 (38·3–51·6)	6·3 (4·8–8·3)	0·2 (0·1–0·3)	0·0 (0·0–0·0)	1·4 (1·34–1·47)	0·18 (0·15–0·2)	0·75 (0·7–0·82)
2012	0·0 (0·0–0·0)	3·4 (2·9–4·1)	29·9 (25·9–34·4)	94·3 (88·1–100·7)	101·4 (92·6–110·6)	46·6 (40·0–53·9)	6·9 (5·3–8·9)	0·2 (0·1–0·3)	0·0 (0·0–0·0)	1·41 (1·35–1·48)	0·17 (0·15–0·19)	0·78 (0·72–0·83)
2013	0·0 (0·0–0·0)	3·4 (2·8–4·1)	28·8 (24·7–33·1)	92·5 (86·2–98·9)	101·7 (93·4–111·0)	47·4 (40·9–54·4)	7·3 (5·5–9·5)	0·2 (0·1–0·3)	0·0 (0·0–0·0)	1·41 (1·34–1·47)	0·16 (0·14–0·18)	0·78 (0·73–0·84)
2014	0·0 (0·0–0·0)	3·3 (2·7–4·0)	27·9 (24·1–32·4)	91·6 (85·1–98·3)	102·7 (94·5–111·5)	48·6 (41·7–56·3)	7·7 (5·9–10·1)	0·2 (0·1–0·3)	0·0 (0·0–0·0)	1·41 (1·34–1·49)	0·16 (0·14–0·18)	0·8 (0·74–0·86)
2015	0·0 (0·0–0·0)	3·3 (2·7–4·0)	28·6 (21·6–36·8)	92·7 (85·5–101·0)	104·6 (93·8–115·8)	46·6 (38·4–56·0)	7·1 (5·1–9·5)	0·2 (0·1–0·3)	0·0 (0·0–0·0)	1·42 (1·26–1·6)	0·16 (0·12–0·2)	0·79 (0·7–0·89)
2016	0·0 (0·0–0·0)	3·4 (2·8–4·1)	29·7 (22·6–38·8)	93·1 (83·3–105·3)	103·2 (89·7–118·4)	42·5 (32·6–54·7)	6·1 (4·2–8·4)	0·2 (0·1–0·2)	0·0 (0·0–0·0)	1·39 (1·19–1·63)	0·17 (0·13–0·21)	0·76 (0·64–0·9)
2017	0·0 (0·0–0·0)	3·3 (2·8–4·1)	29·7 (21·8–39·7)	92·2 (81·8–103·6)	100·8 (85·9–117·0)	41·1 (30·4–54·2)	5·9 (3·8–8·9)	0·2 (0·1–0·3)	0·0 (0·0–0·0)	1·37 (1·15–1·62)	0·17 (0·12–0·22)	0·74 (0·61–0·9)

Supplemental Results Table 5: Age-specific fertility rates per 1000, cumulative total fertility up to maternal age 25 (TFU25), cumulative total fertility from maternal age 30 to age 55 (TFO30), and cumulative total fertility, Gunma, 1950-2017

Year	10-14	15-19	20-24	25-29	30-34	35-39	40-44	45-49	50-54	TFR	TFU25	TFO30
1950	0·0 (0·0–0·0)	11·9 (10·5–13·2)	148·5 (135·7–162·5)	214·3 (206·6–222·0)	173·0 (162·5–183·5)	98·6 (87·6–109·4)	34·2 (29·6–39·3)	1·7 (1·1–2·4)	0·0 (0·0–0·0)	3·41 (3·29–3·53)	0·8 (0·74–0·87)	1·54 (1·46–1·62)
1951	0·0 (0·0–0·0)	9·5 (8·5–10·7)	133·2 (120·6–146·1)	198·9 (191·4–206·1)	156·8 (145·1–167·6)	79·6 (69·4–90·1)	26·5 (22·3–31·0)	1·2 (0·8–1·8)	0·0 (0·0–0·0)	3·03 (2·88–3·17)	0·71 (0·65–0·78)	1·32 (1·2–1·44)
1952	0·0 (0·0–0·0)	8·0 (7·0–9·1)	123·8 (111·1–137·5)	188·9 (180·7–197·2)	142·0 (129·3–154·9)	65·8 (55·4–78·1)	20·9 (17·2–25·1)	0·9 (0·6–1·4)	0·0 (0·0–0·0)	2·75 (2·62–2·87)	0·66 (0·59–0·73)	1·15 (1·06–1·24)
1953	0·0 (0·0–0·0)	7·0 (6·1–8·0)	116·9 (104·7–130·5)	177·9 (170·3–186·3)	127·1 (113·7–141·4)	56·9 (45·7–69·9)	16·6 (13·5–20·1)	0·8 (0·5–1·2)	0·0 (0·0–0·0)	2·52 (2·39–2·67)	0·62 (0·56–0·69)	1·01 (0·91–1·11)
1954	0·0 (0·0–0·0)	6·4 (5·5–7·4)	113·2 (99·2–127·5)	173·0 (164·9–181·8)	117·3 (102·6–132·1)	52·0 (40·4–65·7)	13·7 (10·9–17·0)	0·6 (0·4–1·0)	0·0 (0·0–0·0)	2·38 (2·23–2·54)	0·6 (0·53–0·67)	0·92 (0·8–1·04)
1955	0·0 (0·0–0·0)	5·8 (4·9–6·7)	110·3 (97·3–125·0)	171·9 (163·6–180·1)	108·2 (93·6–122·5)	46·9 (36·0–60·9)	11·4 (8·7–14·2)	0·5 (0·3–0·8)	0·0 (0·0–0·0)	2·27 (2·13–2·43)	0·58 (0·51–0·66)	0·83 (0·71–0·96)
1956	0·0 (0·0–0·0)	5·2 (4·3–6·1)	103·8 (90·3–117·9)	166·2 (158·9–173·8)	96·0 (83·3–110·1)	40·3 (29·9–53·1)	9·0 (6·9–11·6)	0·4 (0·2–0·7)	0·0 (0·0–0·0)	2·1 (1·96–2·26)	0·54 (0·48–0·61)	0·73 (0·62–0·86)
1957	0·0 (0·0–0·0)	4·9 (4·2–5·8)	104·7 (91·9–119·2)	169·7 (162·4–177·5)	91·1 (78·6–105·0)	34·5 (24·3–45·4)	7·7 (6·0–9·7)	0·4 (0·2–0·7)	0·0 (0·0–0·0)	2·06 (1·91–2·22)	0·55 (0·48–0·62)	0·67 (0·56–0·78)
1958	0·0 (0·0–0·0)	5·0 (4·2–5·8)	109·8 (96·2–123·8)	175·4 (167·0–183·6)	89·2 (77·1–102·4)	30·5 (23·0–40·3)	6·9 (5·4–8·8)	0·4 (0·2–0·7)	0·0 (0·0–0·0)	2·09 (1·96–2·22)	0·57 (0·51–0·64)	0·63 (0·55–0·74)
1959	0·0 (0·0–0·0)	5·2 (4·4–6·1)	109·6 (96·9–123·9)	174·4 (166·3–182·6)	83·7 (73·3–95·7)	26·2 (19·5–34·1)	5·7 (4·4–7·4)	0·3 (0·2–0·6)	0·0 (0·0–0·0)	2·03 (1·91–2·14)	0·57 (0·51–0·65)	0·58 (0·51–0·67)
1960	0·0 (0·0–0·0)	5·0 (4·3–5·9)	105·2 (92·6–118·3)	175·2 (167·0–183·3)	80·5 (72·1–90·1)	23·2 (17·6–30·0)	4·6 (3·5–5·9)	0·3 (0·2–0·5)	0·0 (0·0–0·0)	1·97 (1·87–2·07)	0·55 (0·49–0·62)	0·54 (0·48–0·61)
1961	0·0 (0·0–0·0)	4·7 (3·9–5·5)	101·9 (91·1–112·2)	177·0 (169·1–185·0)	79·0 (69·6–89·6)	20·8 (16·6–25·8)	4·0 (2·9–5·4)	0·2 (0·1–0·4)	0·0 (0·0–0·0)	1·94 (1·85–2·03)	0·53 (0·48–0·59)	0·52 (0·47–0·58)
1962	0·0 (0·0–0·0)	4·4 (3·7–5·3)	101·4 (90·4–112·9)	182·3 (174·8–189·7)	80·9 (73·9–87·9)	19·7 (16·1–24·1)	3·5 (2·6–4·6)	0·2 (0·1–0·3)	0·0 (0·0–0·0)	1·96 (1·88–2·04)	0·53 (0·47–0·59)	0·52 (0·48–0·56)
1963	0·0 (0·0–0·0)	4·4 (3·7–5·3)	101·7 (91·0–113·1)	184·5 (176·7–192·6)	82·9 (75·9–90·5)	19·0 (16·0–22·5)	3·4 (2·5–4·6)	0·2 (0·1–0·4)	0·0 (0·0–0·0)	1·98 (1·9–2·06)	0·53 (0·48–0·59)	0·53 (0·49–0·57)
1964	0·0 (0·0–0·0)	4·3 (3·6–5·1)	110·7 (99·5–124·1)	196·7 (188·2–204·9)	89·0 (80·4–98·1)	19·7 (16·6–22·8)	3·2 (2·3–4·3)	0·2 (0·1–0·3)	0·0 (0·0–0·0)	2·12 (2·04–2·21)	0·58 (0·52–0·64)	0·56 (0·52–0·61)
1965	0·0 (0·0–0·0)	4·2 (3·5–4·9)	101·7 (90·7–113·7)	166·1 (157·6–175·2)	74·5 (65·6–83·7)	18·1 (15·3–21·4)	2·7 (2·0–3·6)	0·2 (0·1–0·3)	0·0 (0·0–0·0)	1·84 (1·76–1·93)	0·53 (0·48–0·59)	0·48 (0·43–0·53)
1966	0·0 (0·0–0·0)	4·4 (3·8–5·1)	99·1 (87·7–110·4)	171·1 (162·5–179·8)	76·8 (67·2–87·8)	18·4 (15·0–22·3)	2·7 (1·9–3·5)	0·2 (0·1–0·3)	0·0 (0·0–0·0)	1·86 (1·78–1·95)	0·52 (0·46–0·58)	0·49 (0·43–0·55)
1967	0·0 (0·0–0·0)	4·7 (4·0–5·4)	105·3 (94·3–116·9)	208·9 (200·1–217·5)	94·1 (80·6–108·5)	21·1 (15·8–28·2)	2·6 (1·9–3·4)	0·2 (0·1–0·2)	0·0 (0·0–0·0)	2·18 (2·07–2·3)	0·55 (0·5–0·61)	0·59 (0·51–0·68)
1968	0·0 (0·0–0·0)	4·7 (4·1–5·5)	94·0 (83·4–105·2)	197·4 (189·2–205·5)	88·1 (75·3–101·8)	21·0 (15·8–27·0)	2·4 (1·8–3·2)	0·1 (0·1–0·2)	0·0 (0·0–0·0)	2·04 (1·95–2·13)	0·49 (0·44–0·55)	0·56 (0·48–0·65)
1969	0·0 (0·0–0·0)	5·1 (4·4–6·1)	92·0 (82·5–102·7)	200·2 (192·2–208·3)	88·6 (75·6–103·1)	20·6 (15·6–27·0)	2·4 (1·8–3·1)	0·1 (0·1–0·2)	0·0 (0·0–0·0)	2·05 (1·96–2·14)	0·49 (0·44–0·54)	0·56 (0·48–0·66)
1970	0·0 (0·0–0·0)	5·2 (4·3–6·1)	98·7 (86·8–111·8)	204·6 (195·3–213·2)	90·3 (78·8–104·1)	19·6 (14·7–25·9)	2·4 (1·7–3·1)	0·1 (0·1–0·2)	0·0 (0·0–0·0)	2·1 (2·01–2·21)	0·52 (0·46–0·59)	0·56 (0·48–0·65)
1971	0·0 (0·0–0·0)	5·0 (4·1–6·0)	107·5 (94·6–120·5)	202·9 (193·8–212·0)	92·4 (79·9–106·6)	17·9 (13·4–23·7)	2·5 (1·8–3·4)	0·1 (0·1–0·2)	0·0 (0·0–0·0)	2·14 (2·04–2·25)	0·56 (0·5–0·63)	0·56 (0·48–0·66)
1972	0·0 (0·0–0·0)	5·0 (4·1–6·0)	112·0 (98·7–126·6)	202·3 (193·5–211·0)	93·2 (82·6–103·9)	16·9 (12·6–21·9)	2·5 (1·7–3·4)	0·1 (0·1–0·2)	0·0 (0·0–0·0)	2·16 (2·07–2·25)	0·59 (0·52–0·66)	0·56 (0·5–0·63)
1973	0·0 (0·0–0·0)	4·8 (4·0–5·8)	111·6 (99·1–125·0)	203·4 (194·3–213·3)	89·5 (81·0–98·8)	16·6 (12·4–21·5)	2·2 (1·6–3·0)	0·1 (0·1–0·2)	0·0 (0·0–0·0)	2·14 (2·05–2·23)	0·58 (0·52–0·65)	0·54 (0·49–0·6)
1974	0·0 (0·0–0·0)	4·5 (3·6–5·5)	105·9 (94·9–119·1)	196·7 (186·0–206·8)	79·6 (71·9–87·5)	15·5 (11·7–20·1)	1·9 (1·3–2·6)	0·1 (0·0–0·2)	0·0 (0·0–0·0)	2·02 (1·93–2·12)	0·55 (0·5–0·62)	0·49 (0·44–0·53)
1975	0·0 (0·0–0·0)	4·1 (3·4–5·0)	98·3 (87·2–110·3)	190·4 (181·6–199·0)	72·0 (64·8–79·0)	14·7 (11·5–18·7)	1·7 (1·0–2·6)	0·1 (0·0–0·1)	0·0 (0·0–0·0)	1·91 (1·83–1·99)	0·51 (0·46–0·57)	0·44 (0·4–0·48)
1976	0·0 (0·0–0·0)	3·9 (3·2–4·8)	92·4 (82·4–104·0)	187·9 (179·2–196·7)	71·4 (65·0–78·7)	14·5 (11·7–17·8)	1·7 (1·1–2·4)	0·1 (0·0–0·1)	0·0 (0·0–0·0)	1·86 (1·78–1·94)	0·48 (0·43–0·54)	0·44 (0·4–0·48)
1977	0·0 (0·0–0·0)	3·8 (3·2–4·5)	87·1 (77·3–98·5)	187·9 (179·6–195·9)	74·7 (66·5–83·4)	14·3 (11·5–17·3)	1·7 (1·2–2·4)	0·1 (0·0–0·1)	0·0 (0·0–0·0)	1·85 (1·77–1·93)	0·45 (0·41–0·51)	0·45 (0·4–0·51)
1978	0·0 (0·0–0·0)	3·9 (3·0–4·9)	83·2 (74·4–93·0)	187·3 (179·1–195·0)	78·3 (70·9–85·9)	13·9 (10·7–17·6)	1·7 (1·2–2·5)	0·1 (0·0–0·1)	0·0 (0·0–0·0)	1·84 (1·76–1·92)	0·44 (0·39–0·49)	0·47 (0·43–0·51)
1979	0·0 (0·0–0·0)	4·0 (3·0–5·1)	79·7 (70·6–89·4)	184·0 (176·3–191·9)	78·3 (71·5–85·8)	13·4 (11·3–16·0)	1·8 (1·2–2·6)	0·1 (0·0–0·1)	0·0 (0·0–0·0)	1·81 (1·73–1·88)	0·42 (0·37–0·47)	0·47 (0·43–0·51)
1980	0·0 (0·0–0·0)	4·0 (3·2–5·0)	76·4 (67·8–85·1)	180·2 (172·0–188·8)	76·6 (69·2–83·8)	13·2 (10·6–16·2)	1·8 (1·2–2·5)	0·1 (0·0–0·1)	0·0 (0·0–0·0)	1·76 (1·69–1·84)	0·4 (0·36–0·45)	0·46 (0·42–0·5)
1981	0·0 (0·0–0·0)	4·2 (3·5–5·1)	74·9 (67·0–84·1)	180·5 (172·3–188·6)	77·1 (70·2–84·2)	13·8 (11·3–16·4)	1·8 (1·2–2·5)	0·1 (0·0–0·1)	0·0 (0·0–0·0)	1·76 (1·68–1·84)	0·4 (0·36–0·44)	0·46 (0·43–0·5)
1982	0·0 (0·0–0·0)	4·4 (3·6–5·4)	74·5 (65·6–84·2)	182·1 (174·6–190·5)	80·1 (73·2–87·8)	15·3 (12·6–18·3)	1·8 (1·2–2·5)	0·1 (0·0–0·1)	0·0 (0·0–0·0)	1·79 (1·72–1·87)	0·39 (0·35–0·44)	0·49 (0·45–0·53)
1983	0·0 (0·0–0·0)	4·5 (3·7–5·4)	73·6 (65·2–83·0)	182·4 (173·1–191·1)	83·7 (76·8–91·5)	16·7 (13·6–20·4)	1·8 (1·3–2·3)	0·1 (0·0–0·1)	0·0 (0·0–0·0)	1·81 (1·74–1·9)	0·39 (0·35–0·44)	0·51 (0·47–0·56)
1984	0·0 (0·0–0·0)	4·4 (3·7–5·3)	69·6 (61·1–78·0)	179·4 (171·1–188·4)	86·5 (78·2–95·1)	17·2 (14·2–21·1)	1·8 (1·3–2·5)	0·1 (0·0–0·1)	0·0 (0·0–0·0)	1·8 (1·72–1·88)	0·37 (0·33–0·41)	0·53 (0·48–0·58)
1985	0·0 (0·0–0·0)	4·2 (3·5–5·0)	66·6 (58·1–75·5)	173·4 (165·6–181·0)	87·8 (79·7–95·7)	17·1 (14·0–20·6)	1·8 (1·3–2·4)	0·1 (0·0–0·1)	0·0 (0·0–0·0)	1·76 (1·68–1·83)	0·35 (0·31–0·4)	0·53 (0·49–0·58)
1986	0·0 (0·0–0·0)	4·1 (3·5–4·8)	64·6 (56·5–73·6)	168·4 (160·1–177·3)	90·5 (82·8–98·1)	17·2 (14·2–20·5)	1·9 (1·3–2·7)	0·1 (0·0–0·1)	0·0 (0·0–0·0)	1·73 (1·67–1·81)	0·34 (0·3–0·39)	0·55 (0·51–0·59)
1987	0·0 (0·0–0·0)	4·0 (3·4–4·8)	60·8 (54·1–67·8)	164·3 (156·6–172·1)	94·0 (85·5–103·4)	18·0 (14·9–21·6)	2·1 (1·4–3·0)	0·1 (0·0–0·1)	0·0 (0·0–0·0)	1·72 (1·64–1·8)	0·32 (0·29–0·36)	0·57 (0·52–0·62)
1988	0·0 (0·0–0·0)	3·9 (3·3–4·7)	57·4 (50·3–65·1)	155·6 (147·6–164·6)	96·0 (87·9–105·2)	19·1 (15·8–22·9)	2·3 (1·6–3·1)	0·1 (0·0–0·1)	0·0 (0·0–0·0)	1·67 (1·59–1·75)	0·31 (0·27–0·35)	0·59 (0·54–0·63)
1989	0·0 (0·0–0·0)	3·9 (3·3–4·7)	54·0 (47·0–62·0)	146·7 (139·7–154·1)	96·9 (88·5–105·4)	20·3 (16·5–24·5)	2·4 (1·7–3·3)	0·1 (0·0–0·1)	0·0 (0·0–0·0)	1·62 (1·55–1·69)	0·29 (0·25–0·33)	0·6 (0·55–0·64)
1990	0·0 (0·0–0·0)	4·1 (3·4–4·7)	52·8 (45·8–59·9)	144·6 (136·3–152·1)	99·3 (90·8–107·7)	21·3 (17·3–25·7)	2·4 (1·7–3·2)	0·1 (0·0–0·1)	0·0 (0·0–0·0)	1·62 (1·55–1·69)	0·28 (0·25–0·32)	0·62 (0·57–0·66)
1991	0·0 (0·0–0·0)	4·2 (3·5–5·0)	53·1 (46·1–60·3)	140·6 (133·5–148·7)	99·5 (90·5–108·6)	22·6 (19·0–27·3)	2·4 (1·7–3·1)	0·1 (0·0–0·1)	0·0 (0·0–0·0)	1·61 (1·54–1·69)	0·29 (0·25–0·32)	0·62 (0·57–0·67)
1992	0·0 (0·0–0·0)	4·2 (3·5–5·1)	51·6 (45·7–58·2)	129·4 (121·8–137·2)	95·0 (86·8–103·2)	23·4 (19·3–28·3)	2·5 (1·7–3·3)	0·1 (0·0–0·1)	0·0 (0·0–0·0)	1·53 (1·47–1·59)	0·28 (0·25–0·31)	0·6 (0·56–0·65)
1993	0·0 (0·0–0·0)	4·3 (3·4–5·2)	51·4 (44·7–59·2)	116·0 (108·9–123·7)	88·1 (80·6–96·2)	25·3 (21·2–30·1)	2·6 (1·9–3·5)	0·1 (0·0–0·1)	0·0 (0·0–0·0)	1·44 (1·38–1·51)	0·28 (0·24–0·32)	0·58 (0·54–0·62)
1994	0·0 (0·0–0·0)	4·3 (3·4–5·2)	50·7 (44·4–57·8)	104·3 (98·1–110·4)	81·5 (74·0–89·6)	26·7 (22·3–31·6)	2·7 (2·0–3·6)	0·1 (0·0–0·1)	0·0 (0·0–0·0)	1·35 (1·29–1·42)	0·27 (0·24–0·31)	0·56 (0·51–0·6)
1995	0·0 (0·0–0·0)	4·3 (3·3–5·2)	49·4 (42·7–56·7)	106·6 (99·6–113·6)	87·1 (79·2–94·7)	27·7 (23·3–32·6)	2·9 (2·1–3·8)	0·1 (0·0–0·1)	0·0 (0·0–0·0)	1·39 (1·33–1·46)	0·27 (0·23–0·3)	0·59 (0·54–0·64)
1996	0·0 (0·0–0·0)	4·4 (3·5–5·4)	49·0 (42·1–56·4)	115·1 (107·9–122·2)	96·7 (88·8–105·5)	29·0 (23·9–34·3)	3·0 (2·2–3·9)	0·1 (0·0–0·1)	0·0 (0·0–0·0)	1·49 (1·41–1·56)	0·27 (0·23–0·3)	0·64 (0·59–0·7)
1997	0·0 (0·0–0·0)	4·7 (3·7–5·7)	48·9 (42·2–55·9)	116·5 (109·6–123·2)	98·8 (90·3–107·8)	29·4 (24·4–34·8)	3·1 (2·3–4·2)	0·1 (0·0–0·1)	0·0 (0·0–0·0)	1·51 (1·44–1·58)	0·27 (0·23–0·3)	0·66 (0·61–0·71)
1998	0·0 (0·0–0·0)	4·9 (4·0–6·1)	49·0 (42·2–56·0)	113·9 (107·1–121·2)	98·6 (90·7–107·9)	30·4 (24·8–36·6)	3·2 (2·4–4·3)	0·1 (0·0–0·1)	0·0 (0·0–0·0)	1·5 (1·43–1·57)	0·27 (0·24–0·31)	0·66 (0·61–0·71)
1999	0·0 (0·0–0·0)	5·3 (4·3–6·5)	49·2 (42·9–56·3)	110·3 (103·7–117·0)	97·4 (88·4–107·9)	30·9 (25·5–36·7)	3·4 (2·5–4·5)	0·1 (0·0–0·1)	0·0 (0·0–0·0)	1·48 (1·41–1·55)	0·27 (0·24–0·31)	0·66 (0·6–0·72)
2000	0·0 (0·0–0·0)	5·7 (4·6–6·9)	49·8 (42·2–57·7)	107·3 (100·0–115·0)	94·9 (87·4–103·6)	32·2 (27·3–38·0)	3·6 (2·6–4·7)	0·1 (0·0–0·1)	0·0 (0·0–0·0)	1·47 (1·4–1·55)	0·28 (0·24–0·32)	0·65 (0·6–0·71)
2001	0·0 (0·0–0·0)	5·9 (4·8–7·1)	50·0 (44·4–56·4)	103·6 (97·0–110·4)	91·7 (83·5–100·1)	32·2 (27·0–38·3)	3·7 (2·7–4·9)	0·1 (0·1–0·1)	0·0 (0·0–0·0)	1·44 (1·37–1·51)	0·28 (0·25–0·31)	0·64 (0·59–0·69)
2002	0·0 (0·0–0·0)	5·8 (4·8–6·9)	48·6 (42·2–55·9)	100·3 (93·9–107·1)	91·0 (82·9–100·1)	32·9 (28·1–38·6)	3·9 (2·8–5·5)	0·1 (0·1–0·1)	0·0 (0·0–0·0)	1·41 (1·34–1·49)	0·27 (0·24–0·31)	0·64 (0·59–0·69)
2003	0·0 (0·0–0·0)	5·5 (4·5–6·6)	46·4 (41·0–52·5)	97·5 (91·1–104·6)	90·2 (82·6–98·5)	34·5 (28·9–40·5)	4·2 (3·0–5·7)	0·1 (0·1–0·2)	0·0 (0·0–0·0)	1·39 (1·32–1·47)	0·26 (0·23–0·29)	0·65 (0·6–0·69)
2004	0·0 (0·0–0·0)	5·2 (4·4–6·2)	44·4 (38·2–51·7)	93·5 (87·4–99·5)	88·6 (81·0–96·5)	35·4 (30·1–41·4)	4·5 (3·2–5·9)	0·1 (0·1–0·2)	0·0 (0·0–0·0)	1·36 (1·3–1·42)	0·25 (0·22–0·28)	0·64 (0·6–0·69)
2005	0·0 (0·0–0·0)	5·0 (4·2–6·1)	43·6 (38·2–50·0)	92·2 (86·0–99·1)	88·9 (81·2–96·8)	36·5 (31·1–42·8)	4·7 (3·4–6·3)	0·1 (0·1–0·2)	0·0 (0·0–0·0)	1·36 (1·29–1·42)	0·24 (0·22–0·27)	0·65 (0·6–0·7)
2006	0·0 (0·0–0·0)	4·9 (4·1–5·9)	43·5 (38·3–49·3)	93·0 (87·3–99·7)	91·5 (82·9–100·0)	38·4 (32·8–44·9)	5·2 (3·9–6·9)	0·1 (0·1–0·2)	0·0 (0·0–0·0)	1·38 (1·31–1·45)	0·24 (0·22–0·27)	0·68 (0·62–0·74)
2007	0·0 (0·0–0·0)	4·9 (4·1–5·8)	42·9 (35·8–51·4)	92·3 (86·3–99·2)	91·7 (83·9–100·6)	40·4 (34·4–47·3)	5·7 (4·2–7·4)	0·1 (0·1–0·2)	0·0 (0·0–0·0)	1·39 (1·31–1·47)	0·24 (0·2–0·28)	0·69 (0·64–0·75)
2008	0·0 (0·0–0·0)	4·8 (4·0–5·6)	41·6 (36·5–47·5)	91·5 (85·7–97·7)	92·1 (83·9–100·4)	41·8 (35·4–48·5)	6·2 (4·7–7·9)	0·1 (0·1–0·2)	0·0 (0·0–0·0)	1·39 (1·33–1·46)	0·23 (0·21–0·26)	0·7 (0·65–0·76)
2009	0·0 (0·0–0·0)	4·5 (3·8–5·4)	39·9 (34·0–46·4)	90·8 (84·0–97·8)	92·4 (84·5–100·9)	43·1 (36·8–50·2)	6·9 (5·2–8·8)	0·1 (0·1–0·2)	0·0 (0·0–0·0)	1·39 (1·31–1·48)	0·22 (0·19–0·26)	0·71 (0·66–0·77)
2010	0·0 (0·0–0·0)	4·4 (3·7–5·2)	38·3 (33·4–43·7)	90·9 (85·4–96·4)	93·0 (84·9–101·9)	44·2 (38·0–50·8)	7·2 (5·7–9·0)	0·2 (0·1–0·2)	0·0 (0·0–0·0)	1·39 (1·33–1·46)	0·21 (0·19–0·24)	0·72 (0·67–0·78)
2011	0·0 (0·0–0·0)	4·2 (3·5–5·1)	36·2 (31·5–41·3)	89·8 (83·5–96·4)	92·5 (84·4–101·0)	45·0 (38·5–52·2)	7·5 (5·8–9·6)	0·2 (0·1–0·3)	0·0 (0·0–0·0)	1·38 (1·31–1·44)	0·2 (0·18–0·23)	0·73 (0·68–0·78)
2012	0·0 (0·0–0·0)	4·2 (3·4–5·0)	34·4 (30·2–38·9)	88·8 (83·0–95·4)	93·5 (85·2–102·8)	46·8 (40·3–53·8)	8·1 (6·3–10·2)	0·2 (0·1–0·3)	0·0 (0·0–0·0)	1·38 (1·31–1·45)	0·19 (0·17–0·22)	0·74 (0·69–0·8)
2013	0·0 (0·0–0·0)	4·1 (3·4–4·8)	33·4 (27·6–39·6)	87·0 (81·2–92·8)	93·4 (85·7–102·0)	47·4 (40·7–54·3)	8·4 (6·3–10·7)	0·2 (0·1–0·3)	0·0 (0·0–0·0)	1·37 (1·3–1·45)	0·19 (0·16–0·22)	0·75 (0·69–0·8)
2014	0·0 (0·0–0·0)	4·0 (3·4–4·7)	32·3 (28·0–36·9)	85·9 (80·0–92·1)	94·4 (86·0–103·1)	48·5 (42·0–56·1)	8·7 (6·7–11·4)	0·2 (0·1–0·3)	0·0 (0·0–0·0)	1·37 (1·29–1·46)	0·18 (0·16–0·2)	0·76 (0·7–0·82)
2015	0·0 (0·0–0·0)	4·0 (3·3–4·8)	33·1 (27·4–39·8)	86·8 (79·7–94·2)	96·4 (86·8–107·0)	46·5 (38·4–54·8)	8·0 (5·9–10·9)	0·2 (0·1–0·3)	0·0 (0·0–0·0)	1·38 (1·23–1·54)	0·19 (0·15–0·22)	0·76 (0·67–0·85)
2016	0·0 (0·0–0·0)	4·1 (3·3–5·0)	34·7 (26·2–45·2)	87·3 (78·0–98·5)	95·4 (83·0–109·6)	42·5 (32·7–55·3)	6·9 (4·7–9·6)	0·2 (0·1–0·2)	0·0 (0·0–0·0)	1·36 (1·15–1·6)	0·19 (0·15–0·25)	0·73 (0·61–0·86)
2017	0·0 (0·0–0·0)	4·1 (3·4–4·9)	34·8 (25·9–45·7)	86·4 (76·5–97·2)	93·7 (79·8–108·7)	41·3 (30·7–54·1)	6·7 (4·3–10·1)	0·2 (0·1–0·3)	0·0 (0·0–0·0)	1·34 (1·12–1·59)	0·19 (0·15–0·25)	0·71 (0·58–0·86)

Supplemental Results Table 5: Age-specific fertility rates per 1000, cumulative total fertility up to maternal age 25 (TFU25), cumulative total fertility from maternal age 30 to age 55 (TFO30), and cumulative total fertility, Hiroshima, 1950-2017

Year	10-14	15-19	20-24	25-29	30-34	35-39	40-44	45-49	50-54	TFR	TFU25	TFO30
1950	0·0<br>(0·0–0·0)	11·4<br>(10·1–12·8)	157·3<br>(143·4–172·1)	228·6<br>(220·0–237·1)	162·7<br>(153·1–172·4)	99·3<br>(88·3–110·3)	32·1<br>(27·8–36·9)	1·6<br>(1·1–2·3)	0·0<br>(0·0–0·0)	3·47<br>(3·34–3·59)	0·84<br>(0·78–0·92)	1·48<br>(1·4–1·56)
1951	0·0<br>(0·0–0·0)	9·2<br>(8·2–10·3)	141·1<br>(128·2–155·4)	212·6<br>(204·6–220·4)	147·1<br>(136·8–157·1)	80·3<br>(69·4–90·4)	24·9<br>(20·8–29·1)	1·2<br>(0·8–1·8)	0·0<br>(0·0–0·0)	3·08<br>(2·94–3·23)	0·75<br>(0·69–0·82)	1·27<br>(1·15–1·38)
1952	0·0<br>(0·0–0·0)	7·7<br>(6·7–8·8)	131·4<br>(119·2–145·3)	202·2<br>(193·6–211·1)	133·1<br>(122·9–144·0)	64·4<br>(54·0–75·1)	19·7<br>(16·2–23·6)	0·9<br>(0·6–1·3)	0·0<br>(0·0–0·0)	2·8<br>(2·69–2·9)	0·7<br>(0·63–0·76)	1·09<br>(1·02–1·17)
1953	0·0<br>(0·0–0·0)	6·8<br>(5·9–7·8)	124·2<br>(110·6–138·8)	190·7<br>(182·2–199·2)	118·9<br>(108·9–129·1)	51·4<br>(41·8–62·9)	15·6<br>(12·6–18·9)	0·7<br>(0·5–1·1)	0·0<br>(0·0–0·0)	2·54<br>(2·44–2·65)	0·65<br>(0·59–0·73)	0·93<br>(0·87–1·0)
1954	0·0<br>(0·0–0·0)	6·2<br>(5·4–7·2)	120·4<br>(106·4–134·2)	185·6<br>(178·2–193·8)	109·5<br>(99·8–119·4)	43·8<br>(34·2–55·2)	12·9<br>(10·3–16·0)	0·6<br>(0·4–0·9)	0·0<br>(0·0–0·0)	2·4<br>(2·29–2·49)	0·63<br>(0·56–0·7)	0·83<br>(0·77–0·9)
1955	0·0<br>(0·0–0·0)	5·6<br>(4·8–6·5)	117·5<br>(103·3–132·7)	184·6<br>(175·7–192·8)	100·7<br>(91·2–110·7)	38·8<br>(29·9–50·0)	10·7<br>(8·3–13·4)	0·5<br>(0·3–0·8)	0·0<br>(0·0–0·0)	2·29<br>(2·2–2·39)	0·62<br>(0·54–0·69)	0·75<br>(0·7–0·81)
1956	0·0<br>(0·0–0·0)	5·0<br>(4·2–5·9)	110·8<br>(98·0–124·0)	178·5<br>(170·8–186·8)	89·2<br>(80·6–98·4)	33·3<br>(24·5–43·8)	8·6<br>(6·7–11·0)	0·4<br>(0·2–0·6)	0·0<br>(0·0–0·0)	2·13<br>(2·04–2·22)	0·58<br>(0·51–0·65)	0·66<br>(0·61–0·71)
1957	0·0<br>(0·0–0·0)	4·8<br>(4·1–5·7)	112·2<br>(99·4–126·8)	182·0<br>(174·4–190·6)	84·5<br>(76·2–93·1)	28·4<br>(20·0–37·4)	7·3<br>(5·7–9·3)	0·4<br>(0·2–0·6)	0·0<br>(0·0–0·0)	2·1<br>(1·99–2·21)	0·58<br>(0·52–0·66)	0·6<br>(0·56–0·65)
1958	0·0<br>(0·0–0·0)	4·8<br>(4·1–5·6)	118·0<br>(103·7–134·1)	188·0<br>(179·1–196·6)	82·6<br>(75·5–90·1)	25·0<br>(18·6–33·0)	6·7<br>(5·2–8·5)	0·4<br>(0·2–0·7)	0·0<br>(0·0–0·0)	2·13<br>(2·04–2·22)	0·61<br>(0·54–0·69)	0·57<br>(0·53–0·61)
1959	0·0<br>(0·0–0·0)	5·1<br>(4·3–5·9)	118·3<br>(105·6–133·1)	187·2<br>(178·8–196·6)	77·4<br>(69·0–85·7)	21·5<br>(16·3–27·8)	5·6<br>(4·3–7·2)	0·3<br>(0·2–0·6)	0·0<br>(0·0–0·0)	2·08<br>(1·99–2·17)	0·62<br>(0·55–0·69)	0·52<br>(0·48–0·57)
1960	0·0<br>(0·0–0·0)	4·9<br>(4·2–5·8)	114·2<br>(101·3–127·6)	188·3<br>(179·8–197·7)	74·4<br>(66·4–82·3)	19·0<br>(13·5–25·7)	4·6<br>(3·4–5·8)	0·3<br>(0·2–0·4)	0·0<br>(0·0–0·0)	2·03<br>(1·93–2·13)	0·6<br>(0·53–0·66)	0·49<br>(0·44–0·54)
1961	0·0<br>(0·0–0·0)	4·6<br>(3·9–5·5)	111·2<br>(99·5–123·1)	190·6<br>(182·1–199·3)	72·7<br>(67·5–78·0)	16·9<br>(13·2–21·2)	3·9<br>(2·9–5·3)	0·2<br>(0·1–0·4)	0·0<br>(0·0–0·0)	2·0<br>(1·92–2·08)	0·58<br>(0·52–0·64)	0·47<br>(0·44–0·5)
1962	0·0<br>(0·0–0·0)	4·4<br>(3·6–5·2)	111·4<br>(98·9–123·9)	196·2<br>(188·1–204·3)	74·3<br>(68·2–80·6)	15·9<br>(13·1–19·1)	3·5<br>(2·6–4·7)	0·2<br>(0·1–0·3)	0·0<br>(0·0–0·0)	2·03<br>(1·95–2·11)	0·58<br>(0·52–0·64)	0·47<br>(0·43–0·51)
1963	0·0<br>(0·0–0·0)	4·3<br>(3·7–5·2)	112·3<br>(100·4–124·6)	198·4<br>(190·2–207·1)	75·8<br>(69·2–82·5)	16·0<br>(13·5–19·1)	3·5<br>(2·5–4·7)	0·2<br>(0·1–0·4)	0·0<br>(0·0–0·0)	2·05<br>(1·97–2·14)	0·58<br>(0·52–0·65)	0·48<br>(0·44–0·52)
1964	0·0<br>(0·0–0·0)	4·3<br>(3·7–5·0)	123·3<br>(110·8–138·2)	211·4<br>(202·5–220·2)	81·0<br>(73·2–89·6)	16·5<br>(13·9–19·2)	3·3<br>(2·4–4·4)	0·2<br>(0·1–0·4)	0·0<br>(0·0–0·0)	2·2<br>(2·12–2·3)	0·64<br>(0·58–0·71)	0·51<br>(0·46–0·55)
1965	0·0<br>(0·0–0·0)	4·2<br>(3·5–5·0)	114·1<br>(101·8–127·7)	178·7<br>(170·0–187·7)	67·3<br>(60·4–74·1)	14·3<br>(11·9–17·0)	2·9<br>(2·1–3·8)	0·2<br>(0·1–0·3)	0·0<br>(0·0–0·0)	1·91<br>(1·82–2·0)	0·59<br>(0·53–0·66)	0·42<br>(0·39–0·46)
1966	0·0<br>(0·0–0·0)	4·5<br>(3·8–5·2)	112·2<br>(99·8–125·4)	184·0<br>(175·1–192·7)	68·9<br>(61·9–76·8)	14·7<br>(11·8–17·8)	2·9<br>(2·1–3·8)	0·2<br>(0·1–0·3)	0·0<br>(0·0–0·0)	1·94<br>(1·85–2·03)	0·58<br>(0·52–0·65)	0·43<br>(0·39–0·47)
1967	0·0<br>(0·0–0·0)	4·8<br>(4·1–5·5)	120·4<br>(108·1–133·4)	224·5<br>(216·0–232·9)	83·1<br>(74·2–93·3)	16·5<br>(13·0–20·9)	2·8<br>(2·1–3·6)	0·2<br>(0·1–0·3)	0·0<br>(0·0–0·0)	2·26<br>(2·15–2·37)	0·63<br>(0·56–0·69)	0·51<br>(0·46–0·56)
1968	0·0<br>(0·0–0·0)	4·9<br>(4·2–5·7)	108·5<br>(96·5–121·4)	212·1<br>(203·1–220·6)	75·7<br>(67·9–83·4)	16·1<br>(12·3–20·6)	2·5<br>(1·9–3·3)	0·1<br>(0·1–0·2)	0·0<br>(0·0–0·0)	2·1<br>(2·01–2·18)	0·57<br>(0·51–0·63)	0·47<br>(0·44–0·51)
1969	0·0<br>(0·0–0·0)	5·3<br>(4·5–6·2)	107·4<br>(94·6–121·7)	214·4<br>(205·8–222·9)	75·9<br>(69·0–83·9)	15·7<br>(11·4–21·1)	2·5<br>(1·9–3·3)	0·1<br>(0·1–0·2)	0·0<br>(0·0–0·0)	2·11<br>(2·02–2·19)	0·56<br>(0·5–0·63)	0·47<br>(0·44–0·51)
1970	0·0<br>(0·0–0·0)	5·4<br>(4·5–6·4)	116·2<br>(104·0–129·7)	217·8<br>(209·9–225·7)	78·1<br>(70·6–85·4)	16·6<br>(12·6–21·7)	2·8<br>(2·0–3·6)	0·2<br>(0·1–0·3)	0·0<br>(0·0–0·0)	2·19<br>(2·1–2·27)	0·61<br>(0·55–0·68)	0·49<br>(0·45–0·52)
1971	0·0<br>(0·0–0·0)	5·3<br>(4·3–6·4)	127·8<br>(112·7–143·8)	214·7<br>(205·0–225·3)	79·1<br>(71·5–87·0)	18·2<br>(13·7–24·2)	3·0<br>(2·1–4·0)	0·2<br>(0·1–0·3)	0·0<br>(0·0–0·0)	2·24<br>(2·15–2·33)	0·67<br>(0·59–0·74)	0·5<br>(0·46–0·55)
1972	0·0<br>(0·0–0·0)	5·3<br>(4·3–6·4)	134·1<br>(118·5–150·4)	212·6<br>(204·4–221·4)	79·0<br>(71·3–86·4)	18·6<br>(14·0–24·4)	3·0<br>(2·1–4·0)	0·2<br>(0·1–0·3)	0·0<br>(0·0–0·0)	2·26<br>(2·18–2·36)	0·7<br>(0·62–0·78)	0·5<br>(0·46–0·55)
1973	0·0<br>(0·0–0·0)	5·1<br>(4·2–6·2)	134·1<br>(120·1–149·0)	211·8<br>(202·6–221·2)	74·8<br>(68·3–82·0)	16·8<br>(12·7–21·8)	2·7<br>(1·8–3·8)	0·2<br>(0·1–0·2)	0·0<br>(0·0–0·0)	2·23<br>(2·13–2·32)	0·7<br>(0·63–0·77)	0·47<br>(0·43–0·51)
1974	0·0<br>(0·0–0·0)	4·7<br>(3·8–5·7)	127·1<br>(114·4–141·7)	202·3<br>(192·9–211·3)	65·8<br>(60·0–72·3)	13·1<br>(10·1–16·7)	2·2<br>(1·6–3·1)	0·1<br>(0·1–0·2)	0·0<br>(0·0–0·0)	2·08<br>(2·0–2·17)	0·66<br>(0·59–0·73)	0·41<br>(0·37–0·44)
1975	0·0<br>(0·0–0·0)	4·2<br>(3·4–5·2)	116·6<br>(104·0–129·2)	193·5<br>(184·5–202·0)	58·6<br>(53·0–64·2)	11·4<br>(9·1–14·2)	1·8<br>(1·1–2·8)	0·1<br>(0·1–0·1)	0·0<br>(0·0–0·0)	1·93<br>(1·85–2·01)	0·6<br>(0·54–0·67)	0·36<br>(0·33–0·39)
1976	0·0<br>(0·0–0·0)	3·9<br>(3·2–4·9)	107·7<br>(96·9–120·6)	189·9<br>(181·3–198·0)	56·3<br>(51·0–61·8)	11·4<br>(9·3–13·8)	1·5<br>(1·0–2·1)	0·1<br>(0·0–0·1)	0·0<br>(0·0–0·0)	1·85<br>(1·77–1·94)	0·56<br>(0·5–0·62)	0·35<br>(0·31–0·38)
1977	0·0<br>(0·0–0·0)	3·8<br>(2·9–4·8)	99·6<br>(89·6–111·4)	190·2<br>(180·7–199·3)	57·5<br>(51·8–63·5)	11·4<br>(9·2–13·7)	1·3<br>(0·9–1·8)	0·1<br>(0·0–0·1)	0·0<br>(0·0–0·0)	1·82<br>(1·74–1·9)	0·52<br>(0·47–0·58)	0·35<br>(0·32–0·39)
1978	0·0<br>(0·0–0·0)	3·8<br>(3·0–4·7)	93·5<br>(82·6–106·6)	190·7<br>(182·7–198·5)	61·3<br>(55·2–67·5)	11·2<br>(8·7–14·3)	1·2<br>(0·8–1·7)	0·1<br>(0·0–0·1)	0·0<br>(0·0–0·0)	1·81<br>(1·73–1·9)	0·49<br>(0·43–0·55)	0·37<br>(0·34–0·4)
1979	0·0<br>(0·0–0·0)	3·8<br>(3·3–4·4)	88·4<br>(79·1–97·6)	189·5<br>(181·4–197·4)	63·3<br>(57·6–69·7)	10·9<br>(9·1–13·0)	1·3<br>(0·9–2·0)	0·1<br>(0·0–0·1)	0·0<br>(0·0–0·0)	1·79<br>(1·72–1·86)	0·46<br>(0·41–0·51)	0·38<br>(0·35–0·41)
1980	0·0<br>(0·0–0·0)	3·9<br>(3·2–4·6)	83·9<br>(74·4–93·7)	188·1<br>(179·5–197·0)	63·4<br>(57·4–69·5)	10·8<br>(8·6–13·2)	1·3<br>(0·9–1·9)	0·1<br>(0·0–0·1)	0·0<br>(0·0–0·0)	1·76<br>(1·68–1·83)	0·44<br>(0·39–0·49)	0·38<br>(0·35–0·41)
1981	0·0<br>(0·0–0·0)	4·1<br>(3·3–4·9)	81·3<br>(72·7–91·2)	190·5<br>(181·7–198·4)	64·9<br>(59·1–70·7)	11·3<br>(9·2–13·5)	1·4<br>(0·9–2·0)	0·1<br>(0·0–0·1)	0·0<br>(0·0–0·0)	1·77<br>(1·7–1·84)	0·43<br>(0·38–0·48)	0·39<br>(0·36–0·42)
1982	0·0<br>(0·0–0·0)	4·3<br>(3·5–5·2)	79·9<br>(70·5–89·8)	193·8<br>(184·6–204·3)	68·4<br>(62·5–75·2)	12·5<br>(10·4–15·0)	1·4<br>(1·0–2·0)	0·0<br>(0·0–0·1)	0·0<br>(0·0–0·0)	1·8<br>(1·74–1·87)	0·42<br>(0·37–0·47)	0·41<br>(0·38–0·45)
1983	0·0<br>(0·0–0·0)	4·4<br>(3·6–5·4)	77·9<br>(69·9–86·8)	195·6<br>(186·5–204·3)	72·3<br>(66·0–80·0)	13·7<br>(11·2–17·0)	1·4<br>(1·1–1·9)	0·0<br>(0·0–0·1)	0·0<br>(0·0–0·0)	1·83<br>(1·76–1·91)	0·41<br>(0·37–0·46)	0·44<br>(0·4–0·48)
1984	0·0<br>(0·0–0·0)	4·4<br>(3·5–5·3)	72·7<br>(62·7–84·1)	193·6<br>(185·0–202·6)	75·4<br>(68·6–82·6)	14·1<br>(11·5–17·0)	1·5<br>(1·1–2·0)	0·1<br>(0·0–0·1)	0·0<br>(0·0–0·0)	1·81<br>(1·73–1·89)	0·39<br>(0·33–0·44)	0·45<br>(0·42–0·49)
1985	0·0<br>(0·0–0·0)	4·2<br>(3·4–5·2)	68·6<br>(60·3–77·0)	187·2<br>(178·9–195·0)	77·0<br>(69·9–84·4)	13·9<br>(11·4–16·8)	1·5<br>(1·1–2·1)	0·1<br>(0·0–0·1)	0·0<br>(0·0–0·0)	1·76<br>(1·7–1·82)	0·36<br>(0·32–0·41)	0·46<br>(0·42–0·5)
1986	0·0<br>(0·0–0·0)	4·1<br>(3·4–5·0)	65·5<br>(58·3–73·5)	181·0<br>(172·4–189·6)	79·7<br>(73·7–85·9)	14·0<br>(11·5–16·7)	1·6<br>(1·1–2·4)	0·1<br>(0·0–0·1)	0·0<br>(0·0–0·0)	1·73<br>(1·68–1·79)	0·35<br>(0·31–0·39)	0·48<br>(0·44–0·51)
1987	0·0<br>(0·0–0·0)	4·1<br>(3·3–4·9)	60·7<br>(52·0–70·5)	175·4<br>(166·7–184·2)	82·8<br>(75·7–90·7)	14·6<br>(12·0–17·2)	1·8<br>(1·3–2·5)	0·1<br>(0·0–0·1)	0·0<br>(0·0–0·0)	1·7<br>(1·61–1·78)	0·32<br>(0·28–0·37)	0·5<br>(0·46–0·54)
1988	0·0<br>(0·0–0·0)	4·0<br>(3·2–4·9)	56·5<br>(49·9–63·8)	165·1<br>(157·5–173·8)	84·2<br>(76·9–92·1)	15·4<br>(12·7–18·5)	2·0<br>(1·4–2·6)	0·1<br>(0·0–0·1)	0·0<br>(0·0–0·0)	1·64<br>(1·57–1·71)	0·3<br>(0·27–0·34)	0·51<br>(0·47–0·55)
1989	0·0<br>(0·0–0·0)	4·1<br>(3·2–5·0)	52·7<br>(45·6–60·7)	154·5<br>(146·4–163·5)	84·5<br>(76·9–91·7)	16·2<br>(13·4–19·4)	2·0<br>(1·5–2·8)	0·1<br>(0·0–0·1)	0·0<br>(0·0–0·0)	1·57<br>(1·51–1·63)	0·28<br>(0·25–0·32)	0·51<br>(0·47–0·55)
1990	0·0<br>(0·0–0·0)	4·2<br>(3·3–5·2)	51·2<br>(45·2–57·7)	150·6<br>(142·7–159·1)	86·3<br>(79·0–93·5)	17·0<br>(14·0–20·2)	2·0<br>(1·5–2·7)	0·1<br>(0·0–0·1)	0·0<br>(0·0–0·0)	1·56<br>(1·48–1·64)	0·28<br>(0·24–0·31)	0·53<br>(0·49–0·57)
1991	0·0<br>(0·0–0·0)	4·4<br>(3·6–5·4)	51·1<br>(44·4–58·1)	145·9<br>(138·4–154·2)	87·4<br>(79·3–95·6)	18·1<br>(15·0–21·5)	2·0<br>(1·4–2·7)	0·1<br>(0·0–0·1)	0·0<br>(0·0–0·0)	1·55<br>(1·49–1·61)	0·28<br>(0·24–0·31)	0·54<br>(0·49–0·58)
1992	0·0<br>(0·0–0·0)	4·5<br>(3·6–5·4)	49·4<br>(43·8–55·7)	137·2<br>(128·9–145·4)	87·2<br>(80·2–94·8)	18·8<br>(15·5–22·6)	2·1<br>(1·5–2·8)	0·1<br>(0·0–0·1)	0·0<br>(0·0–0·0)	1·5<br>(1·44–1·55)	0·27<br>(0·24–0·3)	0·54<br>(0·5–0·58)
1993	0·0<br>(0·0–0·0)	4·5<br>(3·6–5·5)	48·7<br>(42·5–55·2)	135·9<br>(127·6–144·5)	91·6<br>(84·1–100·0)	20·4<br>(17·3–23·5)	2·3<br>(1·7–3·0)	0·1<br>(0·0–0·1)	0·0<br>(0·0–0·0)	1·52<br>(1·45–1·59)	0·27<br>(0·24–0·3)	0·57<br>(0·53–0·62)
1994	0·0<br>(0·0–0·0)	4·5<br>(3·7–5·4)	47·7<br>(41·3–55·0)	132·2<br>(124·1–139·9)	94·0<br>(85·6–102·9)	21·8<br>(18·1–26·2)	2·4<br>(1·7–3·3)	0·1<br>(0·0–0·1)	0·0<br>(0·0–0·0)	1·51<br>(1·44–1·58)	0·26<br>(0·23–0·3)	0·59<br>(0·55–0·64)
1995	0·0<br>(0·0–0·0)	4·5<br>(3·7–5·4)	46·0<br>(40·5–51·9)	123·8<br>(116·2–131·6)	93·7<br>(85·4–102·3)	22·9<br>(19·1–27·1)	2·6<br>(1·9–3·4)	0·1<br>(0·0–0·1)	0·0<br>(0·0–0·0)	1·47<br>(1·4–1·53)	0·25<br>(0·23–0·28)	0·6<br>(0·55–0·64)
1996	0·0<br>(0·0–0·0)	4·7<br>(3·9–5·5)	45·1<br>(39·0–51·0)	119·0<br>(111·8–126·2)	93·9<br>(86·1–102·1)	24·3<br>(19·9–28·7)	2·7<br>(2·0–3·5)	0·1<br>(0·0–0·1)	0·0<br>(0·0–0·0)	1·45<br>(1·38–1·51)	0·25<br>(0·22–0·28)	0·6<br>(0·56–0·65)
1997	0·0<br>(0·0–0·0)	4·9<br>(4·0–5·9)	44·5<br>(39·1–50·2)	115·1<br>(108·3–121·8)	92·5<br>(84·3–100·7)	25·1<br>(20·8–30·0)	2·9<br>(2·2–4·0)	0·1<br>(0·0–0·1)	0·0<br>(0·0–0·0)	1·43<br>(1·36–1·49)	0·25<br>(0·22–0·28)	0·6<br>(0·56–0·65)
1998	0·0<br>(0·0–0·0)	5·2<br>(4·2–6·3)	44·0<br>(38·7–50·5)	111·1<br>(104·4–118·1)	91·7<br>(83·7–100·0)	26·5<br>(22·0–31·6)	3·1<br>(2·3–4·1)	0·1<br>(0·0–0·1)	0·0<br>(0·0–0·0)	1·41<br>(1·34–1·47)	0·25<br>(0·22–0·28)	0·61<br>(0·56–0·66)
1999	0·0<br>(0·0–0·0)	5·5<br>(4·5–6·7)	43·6<br>(38·4–49·2)	108·1<br>(101·6–114·3)	91·2<br>(83·9–99·8)	27·5<br>(22·7–32·6)	3·3<br>(2·4–4·4)	0·1<br>(0·1–0·1)	0·0<br>(0·0–0·0)	1·4<br>(1·33–1·46)	0·25<br>(0·22–0·27)	0·61<br>(0·56–0·66)
2000	0·0<br>(0·0–0·0)	6·0<br>(4·9–7·2)	43·8<br>(37·3–50·7)	106·4<br>(99·6–114·0)	90·0<br>(82·8–98·1)	29·2<br>(24·7–34·4)	3·5<br>(2·5–4·6)	0·1<br>(0·1–0·1)	0·0<br>(0·0–0·0)	1·39<br>(1·33–1·47)	0·25<br>(0·22–0·28)	0·61<br>(0·57–0·66)
2001	0·0<br>(0·0–0·0)	6·3<br>(5·3–7·6)	44·1<br>(39·2–49·8)	103·9<br>(97·3–110·7)	87·9<br>(79·9–96·2)	29·6<br>(24·8–35·0)	3·7<br>(2·7–4·9)	0·1<br>(0·1–0·1)	0·0<br>(0·0–0·0)	1·38<br>(1·31–1·44)	0·25<br>(0·23–0·28)	0·61<br>(0·56–0·66)
2002	0·0<br>(0·0–0·0)	6·3<br>(5·3–7·7)	43·4<br>(37·9–49·9)	101·9<br>(95·4–108·4)	88·2<br>(80·6–96·4)	30·5<br>(25·8–35·9)	3·9<br>(2·8–5·2)	0·1<br>(0·1–0·1)	0·0<br>(0·0–0·0)	1·37<br>(1·3–1·44)	0·25<br>(0·22–0·28)	0·61<br>(0·57–0·66)
2003	0·0<br>(0·0–0·0)	6·2<br>(5·1–7·5)	42·3<br>(35·8–49·3)	100·0<br>(94·3–105·7)	88·5<br>(81·3–96·2)	32·2<br>(26·9–38·0)	4·2<br>(2·9–5·7)	0·1<br>(0·1–0·2)	0·0<br>(0·0–0·0)	1·37<br>(1·31–1·43)	0·24<br>(0·21–0·28)	0·62<br>(0·58–0·67)
2004	0·0<br>(0·0–0·0)	6·0<br>(5·1–7·1)	41·4<br>(36·4–47·4)	96·8<br>(90·6–103·2)	88·0<br>(80·6–95·6)	33·3<br>(28·0–39·1)	4·4<br>(3·2–6·1)	0·1<br>(0·1–0·2)	0·0<br>(0·0–0·0)	1·35<br>(1·29–1·41)	0·24<br>(0·21–0·27)	0·63<br>(0·58–0·68)
2005	0·0<br>(0·0–0·0)	6·0<br>(5·1–7·1)	41·8<br>(36·7–47·4)	96·6<br>(90·1–103·2)	89·5<br>(81·6–97·2)	34·6<br>(29·2–40·6)	4·6<br>(3·3–6·3)	0·1<br>(0·1–0·2)	0·0<br>(0·0–0·0)	1·37<br>(1·31–1·43)	0·24<br>(0·21–0·27)	0·64<br>(0·6–0·69)
2006	0·0<br>(0·0–0·0)	6·1<br>(5·2–7·2)	43·2<br>(37·2–49·6)	98·8<br>(92·3–105·6)	93·5<br>(85·3–102·2)	36·8<br>(31·3–43·9)	5·1<br>(3·7–6·7)	0·1<br>(0·1–0·2)	0·0<br>(0·0–0·0)	1·42<br>(1·35–1·49)	0·25<br>(0·22–0·28)	0·68<br>(0·62–0·74)
2007	0·0<br>(0·0–0·0)	6·2<br>(5·2–7·3)	43·8<br>(39·1–49·1)	99·8<br>(93·2–107·1)	94·9<br>(86·4–103·5)	39·1<br>(33·4–45·4)	5·4<br>(3·9–7·3)	0·1<br>(0·1–0·2)	0·0<br>(0·0–0·0)	1·45<br>(1·38–1·51)	0·25<br>(0·23–0·28)	0·7<br>(0·65–0·75)
2008	0·0<br>(0·0–0·0)	6·2<br>(5·2–7·3)	44·0<br>(38·5–50·2)	100·7<br>(93·7–107·5)	96·6<br>(88·7–105·0)	40·8<br>(35·1–47·4)	5·9<br>(4·5–7·7)	0·1<br>(0·1–0·2)	0·0<br>(0·0–0·0)	1·47<br>(1·41–1·54)	0·25<br>(0·22–0·28)	0·72<br>(0·67–0·77)
2009	0·0<br>(0·0–0·0)	6·0<br>(4·9–7·2)	43·1<br>(37·6–49·6)	101·4<br>(95·5–107·6)	98·6<br>(90·2–107·4)	42·6<br>(36·5–49·4)	6·5<br>(5·0–8·3)	0·1<br>(0·1–0·2)	0·0<br>(0·0–0·0)	1·49<br>(1·41–1·58)	0·25<br>(0·22–0·28)	0·74<br>(0·69–0·8)
2010	0·0<br>(0·0–0·0)	5·8<br>(4·8–6·8)	42·0<br>(36·6–47·9)	102·6<br>(95·9–109·5)	100·9<br>(92·6–110·7)	44·2<br>(38·1–50·6)	6·9<br>(5·4–8·5)	0·1<br>(0·1–0·2)	0·0<br>(0·0–0·0)	1·51<br>(1·44–1·59)	0·24<br>(0·21–0·27)	0·76<br>(0·7–0·82)
2011	0·0<br>(0·0–0·0)	5·6<br>(4·6–6·8)	39·9<br>(35·0–45·6)	101·9<br>(95·1–109·1)	101·8<br>(92·1–112·4)	45·6<br>(39·1–52·9)	7·2<br>(5·6–9·2)	0·1<br>(0·1–0·2)	0·0<br>(0·0–0·0)	1·51<br>(1·44–1·58)	0·23<br>(0·2–0·26)	0·77<br>(0·72–0·84)
2012	0·0<br>(0·0–0·0)	5·5<br>(4·4–6·7)	37·9<br>(33·2–43·0)	100·7<br>(94·7–107·6)	103·5<br>(95·5–112·5)	48·1<br>(41·6–55·3)	7·9<br>(6·2–9·9)	0·2<br>(0·1–0·3)	0·0<br>(0·0–0·0)	1·52<br>(1·45–1·59)	0·22<br>(0·19–0·24)	0·8<br>(0·74–0·86)
2013	0·0<br>(0·0–0·0)	5·3<br>(4·4–6·4)	36·3<br>(31·8–40·9)	98·3<br>(91·6–106·0)	103·5<br>(95·1–112·9)	49·5<br>(43·3–56·6)	8·4<br>(6·5–10·6)	0·2<br>(0·1–0·3)	0·0<br>(0·0–0·0)	1·51<br>(1·44–1·58)	0·21<br>(0·19–0·23)	0·81<br>(0·75–0·87)
2014	0·0<br>(0·0–0·0)	5·2<br>(4·3–6·2)	34·8<br>(29·8–40·0)	96·7<br>(90·1–103·7)	104·2<br>(95·7–113·2)	51·4<br>(44·6–59·0)	8·9<br>(7·0–11·5)	0·2<br>(0·1–0·3)	0·0<br>(0·0–0·0)	1·51<br>(1·43–1·59)	0·2<br>(0·17–0·23)	0·82<br>(0·76–0·89)
2015	0·0<br>(0·0–0·0)	5·1<br>(4·3–6·2)	35·0<br>(29·8–41·0)	97·5<br>(89·8–105·7)	106·1<br>(96·1–117·3)	49·7<br>(41·3–59·3)	8·3<br>(5·8–11·4)	0·2<br>(0·1–0·3)	0·0<br>(0·0–0·0)	1·51<br>(1·35–1·69)	0·2<br>(0·17–0·24)	0·82<br>(0·73–0·92)
2016	0·0<br>(0·0–0·0)	5·2<br>(4·3–6·3)	36·2<br>(28·6–46·4)	98·2<br>(87·7–110·9)	104·9<br>(91·1–120·8)	45·5<br>(35·4–58·5)	7·3<br>(4·2–11·7)	0·2<br>(0·1–0·2)	0·0<br>(0·0–0·0)	1·49<br>(1·27–1·74)	0·21<br>(0·16–0·26)	0·79<br>(0·67–0·93)
2017	0·0<br>(0·0–0·0)	5·1<br>(4·2–6·2)	36·0<br>(28·2–46·1)	97·6<br>(86·6–109·7)	102·9<br>(87·8–119·3)	44·0<br>(32·9–57·7)	6·9<br>(4·4–10·3)	0·2<br>(0·1–0·2)	0·0<br>(0·0–0·0)	1·46<br>(1·23–1·74)	0·21<br>(0·16–0·26)	0·77<br>(0·63–0·93)

Supplemental Results Table 5: Age-specific fertility rates per 1000, cumulative total fertility up to maternal age 25 (TFU25), cumulative total fertility from maternal age 30 to age 55 (TFO30), and cumulative total fertility, Hokkaido, 1950-2017

Year	10-14	15-19	20-24	25-29	30-34	35-39	40-44	45-49	50-54	TFR	TFU25	TFO30
1950	0·0 (0·0–0·0)	14·2 (12·6–15·9)	144·2 (131·7–157·6)	203·1 (195·7–210·3)	153·1 (144·0–162·2)	78·7 (69·8–87·3)	28·0 (24·2–32·2)	1·4 (0·9–2·1)	0·0 (0·0–0·0)	3·11 (3·01–3·22)	0·79 (0·73–0·86)	1·31 (1·24–1·37)
1951	0·0 (0·0–0·0)	11·4 (10·0–12·9)	129·4 (117·4–141·7)	188·0 (181·0–194·8)	138·4 (128·6–147·6)	63·6 (56·0–71·3)	21·8 (18·3–25·6)	1·0 (0·7–1·5)	0·0 (0·0–0·0)	2·77 (2·64–2·89)	0·7 (0·64–0·76)	1·12 (1·03–1·22)
1952	0·0 (0·0–0·0)	9·7 (8·4–11·1)	120·4 (108·5–133·2)	178·2 (170·4–185·9)	125·1 (115·1–134·7)	54·9 (47·6–63·1)	17·2 (14·1–20·6)	0·8 (0·5–1·2)	0·0 (0·0–0·0)	2·53 (2·43–2·64)	0·65 (0·59–0·72)	0·99 (0·92–1·06)
1953	0·0 (0·0–0·0)	8·5 (7·2–9·9)	113·7 (101·1–126·7)	167·6 (160·0–175·2)	111·8 (101·9–122·0)	49·6 (42·1–57·6)	13·7 (11·0–16·8)	0·7 (0·4–1·0)	0·0 (0·0–0·0)	2·33 (2·22–2·43)	0·61 (0·55–0·68)	0·88 (0·81–0·95)
1954	0·0 (0·0–0·0)	7·8 (6·5–9·3)	110·1 (97·3–122·7)	162·8 (155·8–170·1)	102·9 (94·2–113·2)	45·2 (38·5–52·7)	11·3 (8·8–14·3)	0·5 (0·3–0·8)	0·0 (0·0–0·0)	2·2 (2·09–2·31)	0·59 (0·53–0·66)	0·8 (0·73–0·87)
1955	0·0 (0·0–0·0)	7·0 (5·7–8·6)	107·4 (93·9–121·1)	161·4 (153·6–168·9)	94·6 (85·6–104·2)	40·6 (34·5–47·4)	9·4 (7·2–11·7)	0·4 (0·3–0·7)	0·0 (0·0–0·0)	2·1 (1·99–2·21)	0·57 (0·5–0·65)	0·73 (0·66–0·79)
1956	0·0 (0·0–0·0)	6·3 (5·1–7·8)	101·1 (89·2–113·5)	155·8 (148·6–163·4)	83·8 (75·6–92·6)	34·9 (29·3–40·9)	7·5 (5·8–9·6)	0·4 (0·2–0·5)	0·0 (0·0–0·0)	1·95 (1·85–2·05)	0·54 (0·48–0·6)	0·63 (0·58–0·69)
1957	0·0 (0·0–0·0)	6·0 (4·8–7·5)	102·2 (90·5–116·1)	159·0 (152·1–166·4)	79·3 (72·0–87·0)	29·8 (24·9–34·7)	6·4 (5·0–8·0)	0·4 (0·2–0·6)	0·0 (0·0–0·0)	1·92 (1·8–2·03)	0·54 (0·48–0·61)	0·58 (0·53–0·63)
1958	0·0 (0·0–0·0)	6·1 (4·8–7·6)	107·5 (94·7–122·3)	164·3 (156·4–172·6)	77·6 (70·6–85·6)	26·3 (21·6–31·3)	5·8 (4·5–7·3)	0·4 (0·2–0·6)	0·0 (0·0–0·0)	1·94 (1·84–2·05)	0·57 (0·5–0·64)	0·55 (0·5–0·61)
1959	0·0 (0·0–0·0)	6·3 (5·0–7·9)	107·6 (95·7–120·5)	163·2 (155·9–171·4)	72·7 (64·8–80·6)	22·6 (18·7–27·2)	4·8 (3·7–6·2)	0·3 (0·2–0·5)	0·0 (0·0–0·0)	1·89 (1·8–1·99)	0·57 (0·51–0·64)	0·5 (0·46–0·55)
1960	0·0 (0·0–0·0)	6·2 (5·1–7·6)	103·8 (92·5–116·0)	163·7 (156·2–171·4)	69·9 (63·2–76·6)	19·8 (16·2–23·5)	4·0 (3·0–5·1)	0·2 (0·1–0·4)	0·0 (0·0–0·0)	1·84 (1·75–1·92)	0·55 (0·49–0·61)	0·47 (0·43–0·51)
1961	0·0 (0·0–0·0)	5·8 (4·7–6·9)	101·1 (90·6–111·7)	165·4 (157·8–173·0)	68·4 (63·0–74·2)	17·7 (14·8–21·3)	3·4 (2·6–4·6)	0·2 (0·1–0·3)	0·0 (0·0–0·0)	1·81 (1·74–1·89)	0·53 (0·48–0·59)	0·45 (0·42–0·48)
1962	0·0 (0·0–0·0)	5·5 (4·6–6·6)	101·2 (90·2–112·7)	170·2 (163·0–177·1)	70·0 (64·2–76·0)	16·7 (14·2–19·4)	3·1 (2·3–4·0)	0·2 (0·1–0·3)	0·0 (0·0–0·0)	1·83 (1·76–1·91)	0·53 (0·48–0·59)	0·45 (0·42–0·48)
1963	0·0 (0·0–0·0)	5·4 (4·5–6·5)	102·1 (91·7–113·0)	172·3 (165·1–179·4)	71·6 (65·1–78·3)	16·0 (13·6–18·7)	3·0 (2·2–4·0)	0·2 (0·1–0·3)	0·0 (0·0–0·0)	1·85 (1·78–1·93)	0·54 (0·49–0·59)	0·45 (0·42–0·49)
1964	0·0 (0·0–0·0)	5·4 (4·5–6·4)	111·9 (100·7–124·6)	183·3 (176·1–190·9)	76·5 (69·4–84·0)	16·4 (13·7–19·5)	2·9 (2·2–3·8)	0·2 (0·1–0·3)	0·0 (0·0–0·0)	1·98 (1·9–2·07)	0·59 (0·53–0·65)	0·48 (0·44–0·52)
1965	0·0 (0·0–0·0)	5·3 (4·5–6·1)	103·5 (92·9–115·9)	154·3 (146·7–161·8)	63·7 (57·8–69·3)	15·0 (12·4–17·7)	2·5 (1·9–3·2)	0·2 (0·1–0·2)	0·0 (0·0–0·0)	1·72 (1·65–1·8)	0·54 (0·49–0·61)	0·41 (0·37–0·44)
1966	0·0 (0·0–0·0)	5·6 (4·8–6·5)	101·7 (90·6–113·1)	158·7 (151·3–165·9)	65·3 (59·0–72·6)	15·1 (12·4–18·2)	2·5 (1·8–3·3)	0·2 (0·1–0·3)	0·0 (0·0–0·0)	1·75 (1·67–1·83)	0·54 (0·48–0·59)	0·42 (0·38–0·46)
1967	0·0 (0·0–0·0)	5·9 (5·0–7·0)	109·0 (97·6–120·5)	192·9 (185·3–200·7)	78·9 (71·6–87·0)	17·0 (14·0–20·6)	2·4 (1·8–3·1)	0·2 (0·1–0·2)	0·0 (0·0–0·0)	2·03 (1·93–2·14)	0·57 (0·52–0·63)	0·49 (0·45–0·54)
1968	0·0 (0·0–0·0)	6·1 (5·2–7·1)	98·2 (87·4–109·9)	181·3 (173·7–188·5)	72·0 (65·1–79·1)	16·8 (13·9–20·1)	2·2 (1·6–2·9)	0·1 (0·1–0·2)	0·0 (0·0–0·0)	1·88 (1·8–1·97)	0·52 (0·47–0·58)	0·46 (0·42–0·5)
1969	0·0 (0·0–0·0)	6·7 (5·6–7·9)	97·1 (86·4–108·0)	183·1 (176·1–190·2)	72·4 (65·9–79·7)	16·3 (13·5–19·6)	2·2 (1·6–3·0)	0·1 (0·1–0·2)	0·0 (0·0–0·0)	1·89 (1·81–1·98)	0·52 (0·46–0·58)	0·46 (0·41–0·5)
1970	0·0 (0·0–0·0)	6·8 (5·6–8·1)	105·1 (93·0–118·1)	186·7 (178·8–194·3)	74·7 (67·7–81·7)	15·4 (12·6–18·6)	2·4 (1·7–3·3)	0·1 (0·1–0·2)	0·0 (0·0–0·0)	1·96 (1·86–2·05)	0·56 (0·5–0·62)	0·46 (0·42–0·51)
1971	0·0 (0·0–0·0)	6·6 (5·5–7·9)	115·5 (101·8–128·5)	184·6 (176·9–192·4)	76·0 (69·3–83·0)	14·2 (11·7–17·0)	2·6 (1·7–3·7)	0·2 (0·1–0·2)	0·0 (0·0–0·0)	2·0 (1·91–2·09)	0·61 (0·54–0·68)	0·46 (0·42–0·51)
1972	0·0 (0·0–0·0)	6·7 (5·4–8·1)	121·1 (108·7–134·7)	183·0 (175·9–190·7)	76·3 (68·9–83·5)	13·6 (11·0–16·3)	2·6 (1·7–3·6)	0·2 (0·1–0·3)	0·0 (0·0–0·0)	2·02 (1·93–2·11)	0·64 (0·58–0·71)	0·46 (0·42–0·5)
1973	0·0 (0·0–0·0)	6·5 (5·3–7·8)	121·2 (109·1–133·7)	182·2 (174·6–189·7)	72·6 (66·6–79·2)	13·0 (10·5–15·8)	2·3 (1·5–3·4)	0·1 (0·1–0·2)	0·0 (0·0–0·0)	1·99 (1·91–2·08)	0·64 (0·58–0·7)	0·44 (0·41–0·48)
1974	0·0 (0·0–0·0)	6·0 (4·8–7·3)	114·9 (103·3–127·0)	173·9 (166·1–181·4)	64·1 (58·0–70·4)	11·9 (9·9–14·4)	1·9 (1·4–2·6)	0·1 (0·1–0·2)	0·0 (0·0–0·0)	1·86 (1·79–1·95)	0·6 (0·55–0·67)	0·39 (0·36–0·42)
1975	0·0 (0·0–0·0)	5·4 (4·4–6·6)	105·3 (94·2–116·6)	166·5 (159·1–173·7)	57·1 (51·9–62·2)	11·4 (9·3–13·7)	1·5 (1·2–2·0)	0·1 (0·0–0·1)	0·0 (0·0–0·0)	1·74 (1·66–1·81)	0·55 (0·5–0·61)	0·35 (0·32–0·38)
1976	0·0 (0·0–0·0)	5·0 (4·1–6·0)	97·0 (87·2–108·1)	163·5 (156·3–170·7)	56·4 (51·6–61·6)	11·5 (9·9–13·4)	1·4 (1·0–2·0)	0·1 (0·0–0·1)	0·0 (0·0–0·0)	1·67 (1·6–1·75)	0·51 (0·46–0·57)	0·35 (0·32–0·38)
1977	0·0 (0·0–0·0)	4·8 (4·1–5·5)	89·3 (79·8–100·1)	163·6 (156·8–171·1)	60·5 (54·8–66·4)	11·7 (9·7–14·0)	1·5 (1·0–2·0)	0·1 (0·0–0·1)	0·0 (0·0–0·0)	1·66 (1·59–1·73)	0·47 (0·42–0·52)	0·37 (0·33–0·41)
1978	0·0 (0·0–0·0)	4·7 (3·8–5·7)	83·2 (74·9–93·1)	163·5 (157·0–170·2)	65·4 (59·1–71·8)	11·7 (9·6–14·2)	1·5 (1·0–2·0)	0·1 (0·0–0·1)	0·0 (0·0–0·0)	1·65 (1·58–1·72)	0·44 (0·4–0·49)	0·39 (0·36–0·43)
1979	0·0 (0·0–0·0)	4·6 (3·6–5·8)	78·0 (69·6–87·1)	161·2 (152·9–168·8)	66·8 (61·2–72·8)	11·7 (9·7–14·1)	1·6 (1·1–2·2)	0·1 (0·0–0·1)	0·0 (0·0–0·0)	1·62 (1·55–1·69)	0·41 (0·37–0·46)	0·4 (0·37–0·43)
1980	0·0 (0·0–0·0)	4·6 (3·7–5·7)	73·6 (64·8–82·7)	158·3 (151·5–165·9)	66·3 (60·4–72·6)	11·9 (9·8–14·3)	1·6 (1·1–2·2)	0·1 (0·0–0·1)	0·0 (0·0–0·0)	1·58 (1·52–1·65)	0·39 (0·35–0·44)	0·4 (0·37–0·43)
1981	0·0 (0·0–0·0)	4·8 (3·8–6·1)	71·0 (63·6–79·7)	159·1 (152·0–166·1)	67·5 (61·5–73·5)	12·7 (10·6–15·1)	1·6 (1·1–2·4)	0·1 (0·0–0·1)	0·0 (0·0–0·0)	1·58 (1·52–1·65)	0·38 (0·34–0·42)	0·41 (0·38–0·44)
1982	0·0 (0·0–0·0)	4·9 (3·9–6·1)	69·9 (61·9–78·3)	161·0 (153·6–169·1)	70·8 (64·7–77·5)	14·5 (12·2–17·1)	1·6 (1·2–2·2)	0·1 (0·0–0·1)	0·0 (0·0–0·0)	1·61 (1·55–1·68)	0·37 (0·33–0·42)	0·43 (0·4–0·47)
1983	0·0 (0·0–0·0)	5·0 (4·0–6·1)	68·3 (60·5–77·2)	161·3 (154·2–168·5)	74·3 (68·0–82·0)	16·2 (13·4–19·6)	1·7 (1·3–2·3)	0·1 (0·0–0·1)	0·0 (0·0–0·0)	1·63 (1·57–1·71)	0·37 (0·33–0·41)	0·46 (0·43–0·5)
1984	0·0 (0·0–0·0)	4·8 (3·9–6·0)	63·9 (57·1–71·4)	158·2 (150·9–165·9)	76·7 (70·3–83·7)	16·8 (14·0–20·3)	1·8 (1·2–2·4)	0·1 (0·0–0·1)	0·0 (0·0–0·0)	1·61 (1·55–1·67)	0·34 (0·31–0·38)	0·48 (0·44–0·52)
1985	0·0 (0·0–0·0)	4·6 (3·6–5·6)	60·5 (52·8–68·1)	152·0 (145·0–158·3)	77·6 (70·9–84·4)	16·7 (14·0–19·7)	1·8 (1·2–2·6)	0·1 (0·0–0·1)	0·0 (0·0–0·0)	1·57 (1·51–1·63)	0·33 (0·29–0·37)	0·48 (0·44–0·52)
1986	0·0 (0·0–0·0)	4·4 (3·5–5·5)	58·1 (51·4–65·3)	146·3 (139·3–153·4)	79·3 (72·9–85·3)	16·7 (13·8–19·9)	1·9 (1·4–2·6)	0·1 (0·0–0·1)	0·0 (0·0–0·0)	1·53 (1·48–1·59)	0·31 (0·28–0·35)	0·49 (0·45–0·52)
1987	0·0 (0·0–0·0)	4·2 (3·4–5·2)	54·2 (46·9–62·2)	141·1 (134·4–148·1)	81·5 (74·9–88·7)	17·3 (14·3–20·8)	2·1 (1·5–2·9)	0·1 (0·0–0·1)	0·0 (0·0–0·0)	1·5 (1·43–1·58)	0·29 (0·26–0·33)	0·5 (0·47–0·54)
1988	0·0 (0·0–0·0)	4·1 (3·3–5·0)	50·7 (44·9–57·2)	132·3 (126·3–139·2)	82·2 (75·3–89·5)	18·1 (15·2–21·4)	2·3 (1·6–3·2)	0·1 (0·0–0·1)	0·0 (0·0–0·0)	1·45 (1·39–1·5)	0·27 (0·24–0·31)	0·51 (0·48–0·55)
1989	0·0 (0·0–0·0)	4·1 (3·3–5·0)	47·4 (41·0–54·0)	123·7 (117·4–130·5)	81·9 (75·1–88·7)	19·0 (15·7–22·6)	2·4 (1·6–3·4)	0·1 (0·0–0·1)	0·0 (0·0–0·0)	1·39 (1·33–1·46)	0·26 (0·23–0·29)	0·52 (0·47–0·56)
1990	0·0 (0·0–0·0)	4·2 (3·4–5·0)	46·1 (40·9–51·4)	121·2 (114·8–127·9)	83·1 (76·5–89·8)	19·8 (16·5–23·5)	2·3 (1·6–3·2)	0·1 (0·0–0·1)	0·0 (0·0–0·0)	1·38 (1·33–1·45)	0·25 (0·22–0·28)	0·53 (0·49–0·56)
1991	0·0 (0·0–0·0)	4·3 (3·5–5·2)	46·2 (40·7–52·2)	118·1 (112·2–124·0)	83·4 (76·3–91·0)	20·8 (17·4–24·8)	2·3 (1·7–3·1)	0·1 (0·0–0·1)	0·0 (0·0–0·0)	1·38 (1·32–1·43)	0·25 (0·23–0·28)	0·53 (0·49–0·57)
1992	0·0 (0·0–0·0)	4·4 (3·6–5·2)	44·8 (38·6–52·3)	111·6 (105·3–118·1)	82·0 (75·5–88·7)	21·5 (18·1–25·5)	2·4 (1·8–3·2)	0·1 (0·0–0·1)	0·0 (0·0–0·0)	1·33 (1·28–1·39)	0·25 (0·21–0·28)	0·53 (0·49–0·57)
1993	0·0 (0·0–0·0)	4·4 (3·6–5·3)	44·5 (39·1–50·3)	111·3 (105·5–118·0)	84·8 (77·9–91·8)	23·2 (19·6–27·4)	2·5 (1·9–3·3)	0·1 (0·0–0·1)	0·0 (0·0–0·0)	1·35 (1·29–1·41)	0·24 (0·22–0·27)	0·55 (0·51–0·59)
1994	0·0 (0·0–0·0)	4·4 (3·6–5·4)	43·9 (39·0–49·6)	109·1 (101·5–116·5)	85·5 (78·0–93·0)	24·4 (20·3–29·3)	2·6 (1·9–3·5)	0·1 (0·1–0·1)	0·0 (0·0–0·0)	1·35 (1·29–1·41)	0·24 (0·22–0·27)	0·56 (0·52–0·61)
1995	0·0 (0·0–0·0)	4·4 (3·6–5·3)	42·9 (37·7–48·6)	102·9 (97·1–108·7)	83·9 (76·9–91·5)	25·1 (21·0–29·8)	2·7 (2·0–3·6)	0·1 (0·1–0·1)	0·0 (0·0–0·0)	1·31 (1·25–1·37)	0·24 (0·21–0·27)	0·56 (0·52–0·6)
1996	0·0 (0·0–0·0)	4·6 (3·8–5·6)	42·8 (37·3–48·4)	99·6 (93·4–105·7)	83·1 (76·1–90·6)	26·0 (21·8–31·0)	2·8 (2·1–3·7)	0·1 (0·1–0·1)	0·0 (0·0–0·0)	1·29 (1·24–1·35)	0·24 (0·21–0·27)	0·56 (0·52–0·6)
1997	0·0 (0·0–0·0)	4·9 (4·0–5·9)	43·0 (37·7–48·7)	96·7 (91·0–102·5)	81·0 (74·2–87·9)	26·3 (21·9–31·1)	3·0 (2·2–4·0)	0·1 (0·0–0·1)	0·0 (0·0–0·0)	1·27 (1·21–1·34)	0·24 (0·21–0·27)	0·55 (0·51–0·59)
1998	0·0 (0·0–0·0)	5·2 (4·2–6·2)	43·2 (38·1–49·5)	93·4 (88·0–98·9)	79·7 (72·9–87·1)	27·0 (22·4–31·8)	3·1 (2·2–4·2)	0·1 (0·1–0·1)	0·0 (0·0–0·0)	1·26 (1·2–1·32)	0·24 (0·22–0·27)	0·55 (0·51–0·59)
1999	0·0 (0·0–0·0)	5·5 (4·5–6·6)	43·4 (38·0–49·1)	90·9 (85·5–96·4)	78·9 (72·6–85·8)	27·4 (23·0–32·2)	3·2 (2·3–4·3)	0·1 (0·1–0·1)	0·0 (0·0–0·0)	1·25 (1·19–1·31)	0·24 (0·22–0·28)	0·55 (0·51–0·59)
2000	0·0 (0·0–0·0)	5·9 (4·8–7·1)	43·8 (39·0–48·9)	89·7 (84·6–95·6)	77·6 (71·3–84·5)	28·5 (24·2–32·9)	3·4 (2·4–4·5)	0·1 (0·1–0·1)	0·0 (0·0–0·0)	1·25 (1·19–1·31)	0·25 (0·22–0·27)	0·55 (0·51–0·59)
2001	0·0 (0·0–0·0)	6·2 (5·1–7·5)	44·1 (38·4–50·5)	88·0 (82·9–94·0)	75·6 (68·6–82·9)	28·4 (24·1–33·1)	3·5 (2·6–4·7)	0·1 (0·1–0·1)	0·0 (0·0–0·0)	1·23 (1·16–1·3)	0·25 (0·22–0·28)	0·54 (0·49–0·58)
2002	0·0 (0·0–0·0)	6·1 (5·0–7·3)	42·9 (37·3–49·0)	86·3 (80·8–92·2)	75·6 (69·3–82·5)	28·8 (24·4–33·5)	3·8 (2·7–5·0)	0·1 (0·1–0·1)	0·0 (0·0–0·0)	1·22 (1·15–1·28)	0·24 (0·22–0·27)	0·54 (0·5–0·59)
2003	0·0 (0·0–0·0)	5·8 (4·7–6·9)	41·0 (36·2–46·6)	84·4 (79·9–89·3)	75·3 (69·2–81·8)	29·9 (25·2–35·2)	4·1 (2·9–5·4)	0·1 (0·1–0·2)	0·0 (0·0–0·0)	1·2 (1·15–1·26)	0·23 (0·21–0·26)	0·55 (0·5–0·59)
2004	0·0 (0·0–0·0)	5·5 (4·6–6·4)	39·4 (34·3–45·2)	81·2 (75·9–86·7)	74·2 (68·2–80·7)	30·4 (25·9–35·5)	4·3 (3·1–5·7)	0·1 (0·1–0·2)	0·0 (0·0–0·0)	1·17 (1·12–1·23)	0·22 (0·2–0·25)	0·54 (0·51–0·59)
2005	0·0 (0·0–0·0)	5·3 (4·4–6·3)	39·0 (34·3–44·1)	80·1 (74·8–85·6)	74·6 (68·3–81·2)	31·1 (26·6–35·9)	4·5 (3·3–5·8)	0·1 (0·1–0·2)	0·0 (0·0–0·0)	1·17 (1·12–1·23)	0·22 (0·2–0·25)	0·55 (0·51–0·59)
2006	0·0 (0·0–0·0)	5·2 (4·3–6·1)	39·5 (34·3–44·9)	80·7 (75·3–85·9)	77·2 (70·7–84·2)	32·7 (27·8–38·5)	4·9 (3·6–6·2)	0·1 (0·1–0·2)	0·0 (0·0–0·0)	1·2 (1·15–1·26)	0·22 (0·2–0·25)	0·57 (0·53–0·62)
2007	0·0 (0·0–0·0)	5·1 (4·3–6·1)	39·4 (35·1–44·3)	80·1 (74·7–85·9)	77·8 (71·3–84·9)	34·5 (29·7–40·2)	5·2 (3·9–6·8)	0·1 (0·1–0·2)	0·0 (0·0–0·0)	1·21 (1·16–1·26)	0·22 (0·2–0·25)	0·59 (0·55–0·63)
2008	0·0 (0·0–0·0)	5·0 (4·2–5·9)	39·1 (33·9–45·0)	79·7 (74·2–85·0)	78·5 (72·2–85·3)	35·9 (31·0–41·5)	5·6 (4·4–7·1)	0·1 (0·1–0·2)	0·0 (0·0–0·0)	1·22 (1·17–1·28)	0·22 (0·19–0·25)	0·6 (0·56–0·64)
2009	0·0 (0·0–0·0)	4·8 (4·0–5·6)	38·0 (34·3–41·9)	79·7 (75·0–84·6)	79·4 (72·8–86·6)	37·3 (32·0–43·1)	6·1 (4·9–7·8)	0·1 (0·1–0·2)	0·0 (0·0–0·0)	1·23 (1·16–1·3)	0·21 (0·19–0·23)	0·61 (0·57–0·66)
2010	0·0 (0·0–0·0)	4·6 (4·0–5·3)	37·1 (31·5–43·3)	80·5 (75·2–85·9)	80·6 (73·9–87·9)	38·6 (33·2–43·8)	6·5 (5·2–8·1)	0·1 (0·1–0·2)	0·0 (0·0–0·0)	1·24 (1·18–1·31)	0·21 (0·18–0·24)	0·63 (0·58–0·68)
2011	0·0 (0·0–0·0)	4·4 (3·7–5·3)	35·3 (30·7–40·6)	80·2 (74·7–86·1)	80·9 (74·2–87·9)	39·7 (34·0–45·7)	6·8 (5·4–8·5)	0·2 (0·1–0·3)	0·0 (0·0–0·0)	1·24 (1·18–1·29)	0·2 (0·18–0·22)	0·64 (0·6–0·68)
2012	0·0 (0·0–0·0)	4·3 (3·5–5·2)	33·7 (29·1–38·9)	79·7 (75·0–84·7)	82·4 (75·4–90·0)	41·8 (36·2–47·8)	7·4 (5·9–9·2)	0·2 (0·1–0·3)	0·0 (0·0–0·0)	1·25 (1·19–1·31)	0·19 (0·17–0·21)	0·66 (0·61–0·71)
2013	0·0 (0·0–0·0)	4·2 (3·5–5·0)	32·5 (28·3–37·2)	78·3 (73·2–83·9)	82·8 (75·8–90·1)	42·9 (37·6–49·2)	7·8 (6·2–9·8)	0·2 (0·1–0·3)	0·0 (0·0–0·0)	1·24 (1·19–1·3)	0·18 (0·16–0·21)	0·67 (0·62–0·72)
2014	0·0 (0·0–0·0)	4·2 (3·6–5·0)	31·5 (27·0–36·7)	77·6 (72·3–83·3)	84·0 (77·5–91·3)	44·5 (38·6–51·0)	8·3 (6·4–10·4)	0·2 (0·1–0·3)	0·0 (0·0–0·0)	1·25 (1·19–1·32)	0·18 (0·16–0·2)	0·68 (0·63–0·74)
2015	0·0 (0·0–0·0)	4·2 (3·5–5·0)	32·0 (27·4–37·2)	78·8 (72·7–85·3)	86·2 (77·9–95·4)	43·0 (35·7–51·0)	7·7 (5·6–10·3)	0·2 (0·1–0·3)	0·0 (0·0–0·0)	1·26 (1·13–1·41)	0·18 (0·15–0·21)	0·69 (0·61–0·77)
2016	0·0 (0·0–0·0)	4·2 (3·5–5·2)	33·2 (26·2–42·2)	79·9 (71·3–89·7)	85·9 (74·7–99·6)	39·6 (30·7–50·8)	6·7 (4·0–10·5)	0·2 (0·1–0·3)	0·0 (0·0–0·0)	1·25 (1·06–1·47)	0·19 (0·15–0·24)	0·66 (0·56–0·78)
2017	0·0 (0·0–0·0)	4·2 (3·5–5·1)	33·2 (26·0–42·4)	79·7 (70·7–89·5)	84·8 (72·4–98·3)	38·5 (28·8–50·4)	6·4 (4·2–9·5)	0·2 (0·1–0·3)	0·0 (0·0–0·0)	1·24 (1·03–1·47)	0·19 (0·15–0·24)	0·65 (0·53–0·78)

Supplemental Results Table 5: Age-specific fertility rates per 1000, cumulative total fertility up to maternal age 25 (TFU25), cumulative total fertility from maternal age 30 to age 55 (TFO30), and cumulative total fertility, Hyogo, 1950-2017

Year	10-14	15-19	20-24	25-29	30-34	35-39	40-44	45-49	50-54	TFR	TFU25	TFO30
1950	0·0 (0·0–0·0)	11·8 (10·4–13·1)	139·4 (127·3–152·2)	223·3 (215·0–231·6)	156·2 (147·0–165·5)	71·6 (63·6–79·4)	25·6 (22·1–29·4)	1·1 (0·7–1·6)	0·0 (0·0–0·0)	3·14 (3·04–3·26)	0·76 (0·7–0·82)	1·27 (1·21–1·34)
1951	0·0 (0·0–0·0)	9·5 (8·4–10·6)	124·9 (113·0–137·0)	207·5 (199·8–215·2)	141·2 (131·5–150·9)	60·3 (53·0–67·9)	19·8 (16·7–23·2)	0·8 (0·5–1·2)	0·0 (0·0–0·0)	2·82 (2·7–2·94)	0·67 (0·61–0·73)	1·11 (1·02–1·21)
1952	0·0 (0·0–0·0)	8·0 (7·0–9·0)	116·1 (104·3–128·5)	197·2 (188·8–205·9)	127·8 (117·9–137·9)	55·3 (47·6–63·6)	15·7 (13·0–18·6)	0·6 (0·4–0·9)	0·0 (0·0–0·0)	2·6 (2·5–2·71)	0·62 (0·56–0·68)	1·0 (0·93–1·07)
1953	0·0 (0·0–0·0)	7·0 (6·1–8·0)	109·5 (97·4–122·1)	185·8 (177·6–194·1)	114·2 (104·2–124·7)	50·9 (43·2–59·7)	12·4 (10·2–15·0)	0·5 (0·3–0·8)	0·0 (0·0–0·0)	2·4 (2·3–2·51)	0·58 (0·52–0·65)	0·89 (0·82–0·96)
1954	0·0 (0·0–0·0)	6·4 (5·5–7·4)	106·0 (93·8–118·2)	180·6 (173·3–188·4)	105·2 (96·5–115·9)	46·4 (39·3–54·1)	10·2 (8·2–12·6)	0·4 (0·3–0·6)	0·0 (0·0–0·0)	2·28 (2·17–2·39)	0·56 (0·5–0·62)	0·81 (0·74–0·88)
1955	0·0 (0·0–0·0)	5·7 (4·8–6·7)	103·2 (90·5–116·9)	179·4 (170·7–187·9)	96·8 (87·7–106·3)	41·9 (35·3–49·0)	8·5 (6·6–10·7)	0·3 (0·2–0·5)	0·0 (0·0–0·0)	2·18 (2·07–2·29)	0·54 (0·48–0·62)	0·74 (0·67–0·8)
1956	0·0 (0·0–0·0)	5·1 (4·4–6·0)	97·0 (85·5–108·8)	173·3 (165·7–181·3)	85·7 (77·4–94·8)	36·0 (30·2–42·2)	6·8 (5·2–8·5)	0·3 (0·2–0·4)	0·0 (0·0–0·0)	2·02 (1·92–2·12)	0·51 (0·45–0·57)	0·64 (0·59–0·7)
1957	0·0 (0·0–0·0)	4·9 (4·2–5·7)	97·9 (87·1–111·0)	176·6 (168·9–184·8)	81·3 (74·1–89·1)	30·8 (25·5–36·0)	5·7 (4·5–7·3)	0·3 (0·2–0·4)	0·0 (0·0–0·0)	1·99 (1·88–2·1)	0·51 (0·46–0·58)	0·59 (0·54–0·64)
1958	0·0 (0·0–0·0)	4·9 (4·2–5·7)	102·8 (90·6–117·2)	182·3 (173·3–191·0)	79·6 (72·4–87·6)	27·3 (22·2–32·5)	5·2 (4·1–6·5)	0·3 (0·2–0·4)	0·0 (0·0–0·0)	2·01 (1·92–2·11)	0·54 (0·48–0·61)	0·56 (0·51–0·61)
1959	0·0 (0·0–0·0)	5·1 (4·4–6·0)	102·8 (91·3–115·3)	181·3 (173·1–189·9)	74·7 (66·6–82·7)	23·5 (18·9–28·9)	4·3 (3·3–5·6)	0·2 (0·1–0·4)	0·0 (0·0–0·0)	1·96 (1·86–2·07)	0·54 (0·48–0·6)	0·51 (0·46–0·56)
1960	0·0 (0·0–0·0)	5·0 (4·3–5·8)	99·0 (88·1–110·6)	182·2 (173·9–190·6)	71·9 (64·9–78·8)	20·7 (16·9–24·7)	3·5 (2·6–4·5)	0·2 (0·1–0·3)	0·0 (0·0–0·0)	1·91 (1·82–2·0)	0·52 (0·47–0·58)	0·48 (0·44–0·52)
1961	0·0 (0·0–0·0)	4·7 (4·0–5·4)	96·3 (86·0–106·6)	184·2 (175·8–192·9)	70·6 (65·1–76·6)	18·6 (15·6–22·0)	3·1 (2·3–4·1)	0·2 (0·1–0·2)	0·0 (0·0–0·0)	1·89 (1·81–1·96)	0·5 (0·45–0·55)	0·46 (0·43–0·5)
1962	0·0 (0·0–0·0)	4·5 (3·8–5·2)	96·2 (85·7–106·8)	189·6 (181·8–197·4)	72·4 (66·5–78·6)	17·7 (14·3–21·3)	2·7 (2·0–3·6)	0·1 (0·1–0·2)	0·0 (0·0–0·0)	1·92 (1·84–1·99)	0·5 (0·45–0·56)	0·46 (0·43–0·5)
1963	0·0 (0·0–0·0)	4·4 (3·7–5·1)	96·8 (86·4–107·4)	191·5 (183·6–199·6)	74·3 (67·8–81·2)	17·0 (14·4–19·9)	2·7 (2·0–3·6)	0·2 (0·1–0·2)	0·0 (0·0–0·0)	1·93 (1·86–2·02)	0·51 (0·45–0·56)	0·47 (0·43–0·51)
1964	0·0 (0·0–0·0)	4·3 (3·7–5·1)	105·9 (95·4–117·9)	203·9 (195·4–212·3)	79·8 (72·4–87·5)	17·6 (14·8–20·6)	2·5 (1·9–3·4)	0·1 (0·1–0·2)	0·0 (0·0–0·0)	2·07 (1·99–2·16)	0·55 (0·5–0·61)	0·5 (0·46–0·54)
1965	0·0 (0·0–0·0)	4·2 (3·7–5·0)	97·8 (87·5–109·6)	172·2 (163·9–180·6)	66·8 (60·6–72·7)	16·1 (13·4–19·3)	2·2 (1·6–2·9)	0·1 (0·1–0·2)	0·0 (0·0–0·0)	1·8 (1·72–1·88)	0·51 (0·46–0·57)	0·43 (0·39–0·46)
1966	0·0 (0·0–0·0)	4·5 (3·9–5·2)	95·8 (85·9–106·5)	177·1 (169·0–184·9)	68·9 (62·4–76·4)	16·4 (13·5–20·0)	2·2 (1·6–2·9)	0·1 (0·1–0·2)	0·0 (0·0–0·0)	1·83 (1·75–1·91)	0·5 (0·45–0·55)	0·44 (0·4–0·48)
1967	0·0 (0·0–0·0)	4·7 (4·0–5·5)	102·5 (91·8–113·6)	215·8 (207·7–223·7)	85·1 (77·3–93·8)	18·7 (15·2–22·9)	2·3 (1·7–2·9)	0·1 (0·1–0·2)	0·0 (0·0–0·0)	2·15 (2·04–2·25)	0·54 (0·48–0·59)	0·53 (0·48–0·58)
1968	0·0 (0·0–0·0)	4·8 (4·1–5·6)	92·1 (81·8–103·2)	203·5 (194·9–211·6)	80·1 (72·5–88·0)	18·6 (15·1–22·5)	2·2 (1·6–3·0)	0·1 (0·1–0·2)	0·0 (0·0–0·0)	2·01 (1·92–2·1)	0·48 (0·43–0·54)	0·51 (0·46–0·55)
1969	0·0 (0·0–0·0)	5·3 (4·4–6·3)	90·9 (80·8–101·3)	205·6 (197·4–213·8)	79·7 (72·6–87·3)	18·2 (15·0–22·0)	2·1 (1·4–3·1)	0·1 (0·1–0·1)	0·0 (0·0–0·0)	2·01 (1·93–2·09)	0·48 (0·43–0·54)	0·5 (0·46–0·54)
1970	0·0 (0·0–0·0)	5·3 (4·4–6·3)	98·1 (86·6–110·2)	208·9 (201·1–216·6)	80·7 (73·6–88·6)	17·4 (14·2–21·0)	2·1 (1·5–2·8)	0·1 (0·1–0·2)	0·0 (0·0–0·0)	2·06 (1·97–2·15)	0·52 (0·46–0·58)	0·5 (0·46–0·55)
1971	0·0 (0·0–0·0)	5·2 (4·2–6·2)	107·8 (95·2–120·2)	206·1 (196·7–216·1)	82·4 (74·9–89·9)	15·8 (13·0–19·1)	2·3 (1·6–3·2)	0·1 (0·1–0·2)	0·0 (0·0–0·0)	2·1 (2·0–2·2)	0·56 (0·5–0·63)	0·5 (0·46–0·55)
1972	0·0 (0·0–0·0)	5·2 (4·3–6·2)	113·0 (100·9–126·0)	204·2 (196·3–212·5)	82·9 (75·0–90·6)	14·8 (12·4–17·5)	2·3 (1·6–3·2)	0·1 (0·1–0·2)	0·0 (0·0–0·0)	2·11 (2·03–2·2)	0·59 (0·53–0·65)	0·5 (0·46–0·54)
1973	0·0 (0·0–0·0)	5·0 (4·1–6·0)	113·1 (101·0–125·4)	203·6 (194·8–212·1)	78·9 (72·4–86·2)	14·6 (11·8–17·9)	2·1 (1·5–2·9)	0·1 (0·1–0·2)	0·0 (0·0–0·0)	2·09 (2·0–2·18)	0·59 (0·53–0·65)	0·48 (0·44–0·52)
1974	0·0 (0·0–0·0)	4·6 (3·8–5·6)	107·3 (96·1–119·2)	194·5 (185·9–202·6)	69·3 (62·8–75·9)	13·8 (11·5–16·6)	1·8 (1·2–2·5)	0·1 (0·0–0·1)	0·0 (0·0–0·0)	1·96 (1·88–2·04)	0·56 (0·5–0·62)	0·42 (0·39–0·46)
1975	0·0 (0·0–0·0)	4·2 (3·4–5·1)	98·6 (88·2–109·6)	186·1 (177·9–194·2)	62·1 (56·4–68·0)	13·2 (10·8–15·7)	1·5 (1·1–2·0)	0·1 (0·0–0·1)	0·0 (0·0–0·0)	1·83 (1·75–1·9)	0·51 (0·46–0·57)	0·38 (0·35–0·42)
1976	0·0 (0·0–0·0)	3·9 (3·1–4·9)	90·9 (81·2–101·4)	182·7 (174·3–190·7)	61·8 (56·5–67·5)	13·1 (11·2–15·1)	1·5 (1·1–2·1)	0·1 (0·0–0·1)	0·0 (0·0–0·0)	1·77 (1·7–1·85)	0·47 (0·42–0·53)	0·38 (0·35–0·42)
1977	0·0 (0·0–0·0)	3·8 (2·8–4·8)	83·5 (74·5–93·8)	182·9 (175·0–191·2)	65·3 (59·0–71·4)	13·0 (10·6–15·6)	1·6 (1·1–2·2)	0·1 (0·0–0·1)	0·0 (0·0–0·0)	1·75 (1·68–1·83)	0·44 (0·39–0·49)	0·4 (0·36–0·44)
1978	0·0 (0·0–0·0)	3·7 (3·0–4·5)	77·5 (70·0–85·7)	183·5 (175·6–191·6)	69·3 (63·1–76·3)	12·8 (10·5–15·4)	1·6 (1·1–2·2)	0·1 (0·0–0·1)	0·0 (0·0–0·0)	1·74 (1·68–1·81)	0·41 (0·37–0·45)	0·42 (0·38–0·46)
1979	0·0 (0·0–0·0)	3·7 (3·2–4·3)	72·6 (64·7–81·0)	182·3 (174·5–189·6)	70·0 (64·1–76·6)	12·4 (10·3–15·0)	1·7 (1·2–2·3)	0·1 (0·0–0·1)	0·0 (0·0–0·0)	1·71 (1·64–1·79)	0·38 (0·34–0·42)	0·42 (0·39–0·46)
1980	0·0 (0·0–0·0)	3·8 (3·1–4·7)	68·5 (60·8–77·0)	180·7 (172·6–189·1)	69·0 (62·9–75·4)	12·3 (10·2–14·9)	1·7 (1·2–2·3)	0·1 (0·0–0·1)	0·0 (0·0–0·0)	1·68 (1·61–1·75)	0·36 (0·32–0·4)	0·42 (0·38–0·45)
1981	0·0 (0·0–0·0)	4·0 (3·3–4·9)	66·4 (59·1–74·4)	182·5 (174·4–190·6)	70·0 (63·8–76·2)	12·9 (10·7–15·4)	1·7 (1·1–2·4)	0·1 (0·0–0·1)	0·0 (0·0–0·0)	1·69 (1·63–1·75)	0·35 (0·32–0·39)	0·42 (0·39–0·46)
1982	0·0 (0·0–0·0)	4·3 (3·5–5·2)	65·6 (57·7–73·8)	184·5 (176·0–194·1)	73·5 (67·5–80·2)	14·4 (12·1–17·2)	1·7 (1·2–2·3)	0·1 (0·0–0·1)	0·0 (0·0–0·0)	1·72 (1·66–1·78)	0·35 (0·31–0·39)	0·45 (0·41–0·48)
1983	0·0 (0·0–0·0)	4·4 (3·6–5·3)	64·3 (56·7–72·7)	184·3 (176·0–192·2)	77·5 (71·1–84·6)	15·9 (13·0–19·2)	1·7 (1·2–2·2)	0·1 (0·0–0·1)	0·0 (0·0–0·0)	1·74 (1·67–1·81)	0·34 (0·3–0·39)	0·48 (0·44–0·52)
1984	0·0 (0·0–0·0)	4·3 (3·7–5·2)	60·1 (53·4–67·3)	180·1 (171·8–188·8)	80·4 (73·9–87·4)	16·2 (13·5–19·5)	1·7 (1·2–2·4)	0·1 (0·0–0·1)	0·0 (0·0–0·0)	1·71 (1·66–1·78)	0·32 (0·29–0·36)	0·49 (0·45–0·53)
1985	0·0 (0·0–0·0)	4·2 (3·4–4·9)	56·7 (49·6–64·1)	172·1 (164·4–179·0)	81·5 (74·5–89·0)	16·0 (12·8–19·3)	1·7 (1·1–2·5)	0·1 (0·0–0·1)	0·0 (0·0–0·0)	1·66 (1·6–1·72)	0·3 (0·27–0·34)	0·5 (0·46–0·54)
1986	0·0 (0·0–0·0)	4·0 (3·4–4·7)	54·2 (48·0–61·1)	164·8 (157·1–173·0)	83·3 (76·6–89·3)	15·9 (13·5–18·8)	1·8 (1·1–2·9)	0·1 (0·0–0·1)	0·0 (0·0–0·0)	1·62 (1·57–1·68)	0·29 (0·26–0·33)	0·51 (0·47–0·54)
1987	0·0 (0·0–0·0)	3·9 (3·3–4·6)	50·0 (43·0–57·5)	158·3 (150·5–166·1)	85·5 (78·8–93·3)	16·5 (13·5–19·8)	2·0 (1·4–2·9)	0·1 (0·0–0·1)	0·0 (0·0–0·0)	1·58 (1·5–1·66)	0·27 (0·23–0·31)	0·52 (0·48–0·56)
1988	0·0 (0·0–0·0)	3·8 (3·2–4·5)	46·4 (41·1–52·3)	147·9 (141·3–155·5)	86·2 (79·2–94·0)	17·3 (14·5–20·4)	2·2 (1·6–2·9)	0·1 (0·0–0·1)	0·0 (0·0–0·0)	1·52 (1·46–1·58)	0·25 (0·22–0·28)	0·53 (0·49–0·57)
1989	0·0 (0·0–0·0)	3·8 (3·1–4·6)	43·1 (37·2–49·0)	138·1 (130·9–145·6)	85·9 (79·0–93·2)	18·2 (15·0–21·5)	2·3 (1·7–3·0)	0·1 (0·0–0·1)	0·0 (0·0–0·0)	1·46 (1·39–1·52)	0·23 (0·21–0·26)	0·53 (0·49–0·57)
1990	0·0 (0·0–0·0)	3·9 (3·2–4·7)	41·7 (36·8–46·5)	135·2 (128·0–142·6)	87·3 (80·2–94·3)	19·0 (15·8–22·6)	2·3 (1·6–3·0)	0·1 (0·0–0·1)	0·0 (0·0–0·0)	1·45 (1·39–1·51)	0·23 (0·2–0·26)	0·54 (0·51–0·58)
1991	0·0 (0·0–0·0)	4·0 (3·3–5·0)	41·8 (36·8–46·9)	132·3 (125·9–138·9)	87·9 (80·4–95·7)	19·9 (16·8–23·7)	2·3 (1·6–3·0)	0·1 (0·0–0·1)	0·0 (0·0–0·0)	1·44 (1·39–1·5)	0·23 (0·2–0·26)	0·55 (0·51–0·59)
1992	0·0 (0·0–0·0)	4·1 (3·3–5·0)	40·6 (34·9–47·5)	125·8 (118·8–132·8)	87·3 (80·4–94·4)	20·5 (17·1–24·2)	2·3 (1·8–3·0)	0·1 (0·0–0·1)	0·0 (0·0–0·0)	1·4 (1·34–1·47)	0·22 (0·19–0·26)	0·55 (0·51–0·6)
1993	0·0 (0·0–0·0)	4·2 (3·4–5·1)	40·4 (35·4–45·6)	126·0 (118·9–133·7)	91·7 (84·4–99·3)	22·2 (18·8–26·3)	2·4 (1·8–3·2)	0·1 (0·0–0·1)	0·0 (0·0–0·0)	1·43 (1·37–1·5)	0·22 (0·2–0·25)	0·58 (0·54–0·63)
1994	0·0 (0·0–0·0)	4·2 (3·4–5·0)	39·9 (35·4–44·9)	123·9 (116·8–130·7)	94·1 (86·3–102·3)	23·5 (19·6–27·8)	2·6 (1·9–3·4)	0·1 (0·1–0·1)	0·0 (0·0–0·0)	1·44 (1·38–1·51)	0·22 (0·2–0·25)	0·6 (0·56–0·65)
1995	0·0 (0·0–0·0)	4·2 (3·4–5·1)	38·9 (34·0–43·9)	116·8 (110·2–123·3)	94·1 (86·3–102·5)	24·4 (20·7–28·6)	2·7 (2·0–3·6)	0·1 (0·0–0·1)	0·0 (0·0–0·0)	1·41 (1·34–1·47)	0·22 (0·19–0·24)	0·61 (0·56–0·65)
1996	0·0 (0·0–0·0)	4·3 (3·6–5·1)	38·4 (33·6–43·5)	112·9 (106·2–119·8)	94·7 (86·9–103·3)	25·8 (21·3–30·4)	2·8 (2·0–3·7)	0·1 (0·1–0·1)	0·0 (0·0–0·0)	1·4 (1·33–1·46)	0·21 (0·19–0·24)	0·62 (0·58–0·67)
1997	0·0 (0·0–0·0)	4·5 (3·8–5·5)	38·2 (33·4–43·1)	109·5 (103·4–115·9)	94·1 (86·9–102·3)	26·5 (22·5–30·8)	3·0 (2·2–4·0)	0·1 (0·1–0·1)	0·0 (0·0–0·0)	1·38 (1·32–1·44)	0·21 (0·19–0·24)	0·62 (0·58–0·67)
1998	0·0 (0·0–0·0)	4·8 (3·9–5·7)	37·6 (33·0–43·0)	105·5 (99·8–111·4)	94·0 (86·3–102·7)	28·0 (23·4–32·9)	3·1 (2·3–4·2)	0·1 (0·1–0·1)	0·0 (0·0–0·0)	1·37 (1·3–1·43)	0·21 (0·19–0·24)	0·63 (0·58–0·67)
1999	0·0 (0·0–0·0)	5·0 (4·0–6·1)	36·9 (32·1–42·1)	101·8 (95·7–107·8)	94·2 (86·3–103·1)	29·1 (24·6–34·0)	3·4 (2·5–4·6)	0·1 (0·1–0·1)	0·0 (0·0–0·0)	1·35 (1·29–1·42)	0·21 (0·18–0·24)	0·63 (0·59–0·69)
2000	0·0 (0·0–0·0)	5·3 (4·4–6·4)	36·5 (32·2–41·2)	99·1 (93·5–105·3)	93·1 (85·6–100·5)	31·1 (26·5–35·9)	3·7 (2·7–4·9)	0·1 (0·1–0·1)	0·0 (0·0–0·0)	1·34 (1·28–1·4)	0·21 (0·19–0·23)	0·64 (0·59–0·69)
2001	0·0 (0·0–0·0)	5·5 (4·6–6·6)	36·2 (31·4–41·4)	95·6 (90·0–101·7)	90·6 (82·2–99·0)	31·5 (26·7–37·0)	3·9 (2·9–5·2)	0·1 (0·1–0·2)	0·0 (0·0–0·0)	1·32 (1·25–1·38)	0·21 (0·18–0·23)	0·63 (0·58–0·68)
2002	0·0 (0·0–0·0)	5·4 (4·5–6·5)	35·0 (30·5–39·8)	92·4 (86·5–98·7)	90·3 (82·6–98·4)	32·4 (27·5–37·9)	4·2 (3·0–5·6)	0·1 (0·1–0·2)	0·0 (0·0–0·0)	1·3 (1·24–1·36)	0·2 (0·18–0·23)	0·64 (0·59–0·68)
2003	0·0 (0·0–0·0)	5·2 (4·3–6·3)	33·5 (28·8–38·9)	89·5 (84·7–94·6)	89·8 (82·6–97·1)	34·1 (28·8–39·9)	4·6 (3·3–6·1)	0·1 (0·1–0·2)	0·0 (0·0–0·0)	1·28 (1·23–1·34)	0·19 (0·17–0·22)	0·64 (0·59–0·69)
2004	0·0 (0·0–0·0)	5·0 (4·2–5·9)	32·5 (28·5–37·1)	85·5 (80·1–91·3)	88·5 (80·2–97·0)	35·2 (29·9–41·1)	4·8 (3·5–6·5)	0·1 (0·1–0·2)	0·0 (0·0–0·0)	1·26 (1·2–1·32)	0·19 (0·17–0·21)	0·64 (0·59–0·69)
2005	0·0 (0·0–0·0)	4·9 (4·2–5·8)	32·6 (28·6–36·6)	84·4 (78·9–89·9)	89·4 (81·9–96·7)	36·4 (30·8–42·2)	5·1 (3·8–6·7)	0·1 (0·1–0·2)	0·0 (0·0–0·0)	1·26 (1·21–1·32)	0·19 (0·17–0·21)	0·65 (0·61–0·7)
2006	0·0 (0·0–0·0)	4·9 (4·2–5·8)	33·4 (28·9–38·3)	85·3 (79·8–90·7)	92·8 (85·1–100·8)	38·6 (33·2–45·2)	5·6 (4·3–7·2)	0·1 (0·1–0·2)	0·0 (0·0–0·0)	1·3 (1·25–1·37)	0·19 (0·17–0·22)	0·69 (0·64–0·74)
2007	0·0 (0·0–0·0)	5·0 (4·2–5·9)	33·8 (30·1–38·1)	85·2 (80·1–91·6)	93·8 (86·4–102·2)	41·1 (35·6–47·4)	6·0 (4·6–7·7)	0·1 (0·1–0·2)	0·0 (0·0–0·0)	1·33 (1·27–1·38)	0·19 (0·18–0·22)	0·71 (0·66–0·76)
2008	0·0 (0·0–0·0)	4·9 (4·2–5·9)	33·9 (29·3–39·3)	85·2 (79·5–90·6)	94·9 (87·4–102·6)	42·9 (36·6–49·3)	6·5 (5·2–8·2)	0·1 (0·1–0·2)	0·0 (0·0–0·0)	1·34 (1·29–1·41)	0·19 (0·17–0·22)	0·72 (0·67–0·77)
2009	0·0 (0·0–0·0)	4·7 (3·9–5·7)	33·2 (29·7–36·5)	85·5 (80·5–90·6)	96·2 (88·1–104·5)	44·5 (38·3–51·2)	7·2 (5·7–9·4)	0·2 (0·1–0·3)	0·0 (0·0–0·0)	1·36 (1·29–1·42)	0·19 (0·17–0·21)	0·74 (0·69–0·8)
2010	0·0 (0·0–0·0)	4·6 (3·8–5·4)	32·6 (27·6–38·2)	86·5 (81·0–92·3)	97·6 (89·7–106·7)	46·0 (39·4–53·0)	7·7 (6·0–9·6)	0·2 (0·1–0·3)	0·0 (0·0–0·0)	1·38 (1·31–1·45)	0·19 (0·16–0·21)	0·76 (0·7–0·81)
2011	0·0 (0·0–0·0)	4·4 (3·6–5·4)	31·1 (27·0–35·9)	86·3 (80·4–92·6)	97·8 (90·0–106·0)	47·2 (40·8–54·7)	8·1 (6·3–10·0)	0·2 (0·1–0·3)	0·0 (0·0–0·0)	1·38 (1·32–1·44)	0·18 (0·16–0·2)	0·77 (0·72–0·82)
2012	0·0 (0·0–0·0)	4·3 (3·5–5·3)	29·7 (25·6–34·4)	85·8 (80·8–91·1)	99·1 (90·4–107·7)	49·7 (43·4–56·8)	8·8 (6·9–11·1)	0·2 (0·1–0·3)	0·0 (0·0–0·0)	1·39 (1·33–1·45)	0·17 (0·15–0·19)	0·79 (0·73–0·85)
2013	0·0 (0·0–0·0)	4·2 (3·5–5·0)	28·6 (24·7–32·5)	84·4 (78·7–90·2)	99·3 (91·8–107·8)	51·3 (44·5–58·5)	9·4 (7·6–11·8)	0·2 (0·1–0·3)	0·0 (0·0–0·0)	1·39 (1·32–1·45)	0·16 (0·14–0·18)	0·8 (0·74–0·86)
2014	0·0 (0·0–0·0)	4·1 (3·4–4·9)	27·5 (23·6–32·0)	83·8 (78·2–89·9)	100·6 (92·3–109·5)	53·6 (46·3–61·1)	10·0 (7·9–12·3)	0·2 (0·1–0·3)	0·0 (0·0–0·0)	1·4 (1·32–1·48)	0·16 (0·14–0·18)	0·82 (0·76–0·89)
2015	0·0 (0·0–0·0)	4·1 (3·4–5·0)	27·9 (23·8–32·8)	85·4 (78·8–92·5)	103·3 (93·5–114·2)	52·4 (43·7–62·0)	9·3 (7·0–12·1)	0·2 (0·1–0·3)	0·0 (0·0–0·0)	1·41 (1·27–1·58)	0·16 (0·14–0·19)	0·83 (0·74–0·93)
2016	0·0 (0·0–0·0)	4·2 (3·4–5·1)	29·0 (22·8–37·0)	86·7 (77·5–97·6)	103·1 (89·9–119·5)	48·6 (37·8–62·5)	8·0 (5·7–11·0)	0·2 (0·1–0·3)	0·0 (0·0–0·0)	1·4 (1·2–1·65)	0·17 (0·13–0·21)	0·8 (0·68–0·95)
2017	0·0 (0·0–0·0)	4·1 (3·4–5·0)	28·9 (22·6–36·9)	86·6 (76·9–97·1)	101·7 (87·0–117·9)	47·5 (35·5–61·8)	7·8 (5·3–11·4)	0·2 (0·1–0·3)	0·0 (0·0–0·0)	1·38 (1·16–1·64)	0·17 (0·13–0·21)	0·79 (0·64–0·95)

Supplemental Results Table 5: Age-specific fertility rates per 1000, cumulative total fertility up to maternal age 25 (TFU25), cumulative total fertility from maternal age 30 to age 55 (TFO30), and cumulative total fertility, Ibaraki, 1950-2017

Year	10-14	15-19	20-24	25-29	30-34	35-39	40-44	45-49	50-54	TFR	TFU25	TFO30
1950	0·0 (0·0–0·0)	14·6 (12·9–16·3)	157·1 (143·2–171·8)	227·4 (218·9–235·9)	184·7 (173·4–196·1)	112·6 (100·1–125·1)	41·4 (35·8–47·6)	2·1 (1·4–3·1)	0·0 (0·0–0·0)	3·7 (3·57–3·83)	0·86 (0·79–0·93)	1·7 (1·61–1·8)
1951	0·0 (0·0–0·0)	11·7 (10·4–13·2)	140·9 (127·5–155·0)	211·5 (203·2–219·5)	167·4 (155·3–178·4)	91·0 (79·4–102·7)	32·1 (26·8–37·4)	1·6 (1·0–2·3)	0·0 (0·0–0·0)	3·28 (3·12–3·44)	0·76 (0·7–0·83)	1·46 (1·33–1·6)
1952	0·0 (0·0–0·0)	9·9 (8·7–11·2)	131·1 (118·2–145·6)	201·2 (192·6–209·8)	151·7 (140·3–163·7)	72·9 (63·0–84·5)	25·4 (20·8–30·6)	1·2 (0·8–1·8)	0·0 (0·0–0·0)	2·97 (2·84–3·09)	0·7 (0·64–0·78)	1·26 (1·17–1·34)
1953	0·0 (0·0–0·0)	8·6 (7·5–10·0)	123·8 (110·1–138·8)	189·7 (181·1–198·5)	135·7 (124·2–147·1)	58·0 (49·6–67·5)	20·1 (16·2–25·2)	1·0 (0·6–1·5)	0·0 (0·0–0·0)	2·68 (2·56–2·81)	0·66 (0·59–0·74)	1·07 (1·0–1·16)
1954	0·0 (0·0–0·0)	7·9 (6·8–9·1)	119·9 (106·7–133·3)	184·6 (177·1–192·8)	125·1 (114·3–136·4)	49·7 (41·9–58·6)	16·6 (12·8–21·4)	0·8 (0·5–1·2)	0·0 (0·0–0·0)	2·52 (2·41–2·64)	0·64 (0·57–0·71)	0·96 (0·88–1·04)
1955	0·0 (0·0–0·0)	7·1 (6·1–8·4)	116·9 (102·9–131·5)	183·5 (174·7–192·0)	115·1 (104·3–126·2)	44·3 (37·3–51·8)	13·8 (10·4–18·0)	0·6 (0·4–1·0)	0·0 (0·0–0·0)	2·41 (2·28–2·53)	0·62 (0·55–0·7)	0·87 (0·8–0·94)
1956	0·0 (0·0–0·0)	6·4 (5·5–7·4)	110·0 (97·3–123·1)	177·4 (169·7–185·6)	102·0 (92·6–112·4)	38·0 (32·0–44·7)	11·1 (8·4–14·2)	0·5 (0·3–0·8)	0·0 (0·0–0·0)	2·23 (2·12–2·34)	0·58 (0·52–0·65)	0·76 (0·69–0·83)
1957	0·0 (0·0–0·0)	6·1 (5·2–7·2)	111·2 (98·1–126·4)	180·9 (173·0–189·4)	96·6 (87·4–106·6)	32·5 (27·1–37·9)	9·4 (7·2–12·0)	0·5 (0·3–0·9)	0·0 (0·0–0·0)	2·19 (2·07–2·31)	0·59 (0·52–0·66)	0·7 (0·64–0·76)
1958	0·0 (0·0–0·0)	6·2 (5·2–7·2)	116·8 (103·1–132·1)	186·8 (178·0–195·4)	94·5 (86·6–103·2)	28·7 (24·0–34·0)	8·5 (6·6–10·8)	0·5 (0·3–0·9)	0·0 (0·0–0·0)	2·21 (2·11–2·32)	0·61 (0·55–0·69)	0·66 (0·61–0·72)
1959	0·0 (0·0–0·0)	6·4 (5·5–7·5)	116·8 (104·1–130·4)	186·0 (177·5–195·0)	88·7 (79·2–98·3)	24·7 (20·4–29·6)	7·0 (5·5–9·1)	0·4 (0·2–0·7)	0·0 (0·0–0·0)	2·15 (2·05–2·25)	0·62 (0·55–0·68)	0·6 (0·55–0·66)
1960	0·0 (0·0–0·0)	6·3 (5·4–7·2)	112·4 (99·5–125·6)	187·1 (178·7–196·0)	85·3 (76·2–95·0)	21·8 (18·2–25·9)	5·8 (4·3–7·4)	0·4 (0·2–0·6)	0·0 (0·0–0·0)	2·1 (2·0–2·19)	0·59 (0·53–0·66)	0·57 (0·52–0·62)
1961	0·0 (0·0–0·0)	5·8 (4·9–6·8)	109·2 (97·5–120·4)	189·3 (180·7–197·8)	83·5 (77·7–89·7)	19·6 (16·4–23·8)	4·9 (3·7–6·7)	0·3 (0·2–0·5)	0·0 (0·0–0·0)	2·06 (1·98–2·15)	0·58 (0·52–0·63)	0·54 (0·51–0·58)
1962	0·0 (0·0–0·0)	5·6 (4·7–6·5)	108·9 (96·8–121·2)	194·9 (186·8–203·0)	85·4 (78·5–92·7)	18·5 (15·9–21·2)	4·4 (3·2–5·9)	0·3 (0·2–0·4)	0·0 (0·0–0·0)	2·09 (2·0–2·17)	0·57 (0·51–0·63)	0·54 (0·5–0·59)
1963	0·0 (0·0–0·0)	5·5 (4·6–6·4)	109·4 (97·8–121·5)	197·0 (188·9–205·5)	87·3 (79·4–94·9)	18·0 (15·3–21·1)	4·3 (3·1–5·7)	0·3 (0·2–0·5)	0·0 (0·0–0·0)	2·11 (2·03–2·19)	0·57 (0·52–0·63)	0·55 (0·51–0·59)
1964	0·0 (0·0–0·0)	5·4 (4·6–6·4)	119·5 (107·4–134·2)	209·9 (201·1–218·6)	93·3 (84·7–103·2)	18·6 (15·6–21·9)	4·0 (3·0–5·3)	0·3 (0·2–0·5)	0·0 (0·0–0·0)	2·26 (2·17–2·35)	0·62 (0·56–0·7)	0·58 (0·53–0·63)
1965	0·0 (0·0–0·0)	5·3 (4·5–6·1)	110·1 (98·0–123·0)	177·4 (168·9–186·2)	77·8 (70·0–85·4)	17·0 (14·2–20·1)	3·5 (2·5–4·6)	0·2 (0·1–0·4)	0·0 (0·0–0·0)	1·96 (1·87–2·04)	0·58 (0·52–0·64)	0·49 (0·45–0·54)
1966	0·0 (0·0–0·0)	5·6 (4·8–6·5)	107·6 (95·7–120·2)	182·6 (173·8–191·3)	79·9 (71·6–89·2)	17·4 (14·1–21·1)	3·4 (2·5–4·6)	0·2 (0·1–0·4)	0·0 (0·0–0·0)	1·98 (1·89–2·08)	0·57 (0·5–0·63)	0·5 (0·46–0·56)
1967	0·0 (0·0–0·0)	5·9 (5·0–6·9)	114·6 (103·3–127·3)	222·8 (214·3–231·2)	96·6 (87·2–106·5)	19·7 (16·2–23·6)	3·3 (2·5–4·2)	0·2 (0·1–0·3)	0·0 (0·0–0·0)	2·32 (2·2–2·43)	0·6 (0·54–0·66)	0·6 (0·55–0·66)
1968	0·0 (0·0–0·0)	6·0 (5·1–7·0)	102·7 (91·3–114·9)	210·6 (201·7–219·0)	88·5 (79·8–97·4)	19·7 (16·3–23·6)	2·9 (2·2–3·9)	0·2 (0·1–0·3)	0·0 (0·0–0·0)	2·15 (2·06–2·24)	0·54 (0·49–0·6)	0·56 (0·51–0·61)
1969	0·0 (0·0–0·0)	6·5 (5·5–7·7)	100·9 (88·4–114·0)	213·0 (204·5–221·5)	89·0 (81·1–98·0)	19·3 (15·8–23·2)	2·9 (2·2–3·9)	0·2 (0·1–0·2)	0·0 (0·0–0·0)	2·16 (2·06–2·26)	0·54 (0·47–0·6)	0·56 (0·51–0·61)
1970	0·0 (0·0–0·0)	6·6 (5·5–7·9)	108·5 (96·5–121·2)	216·6 (208·6–224·3)	91·7 (83·1–100·4)	18·4 (15·0–22·3)	3·2 (2·3–4·3)	0·2 (0·1–0·3)	0·0 (0·0–0·0)	2·23 (2·13–2·32)	0·58 (0·51–0·64)	0·57 (0·52–0·62)
1971	0·0 (0·0–0·0)	6·4 (5·4–7·6)	118·7 (104·9–132·3)	213·8 (204·2–224·3)	93·1 (84·1–102·1)	17·8 (14·7–21·3)	3·4 (2·4–4·6)	0·2 (0·1–0·3)	0·0 (0·0–0·0)	2·27 (2·16–2·38)	0·63 (0·56–0·69)	0·57 (0·52–0·63)
1972	0·0 (0·0–0·0)	6·5 (5·3–7·8)	123·9 (110·7–138·2)	212·1 (203·9–220·9)	93·4 (84·3–102·2)	17·9 (14·4–21·8)	3·3 (2·3–4·6)	0·2 (0·1–0·3)	0·0 (0·0–0·0)	2·29 (2·19–2·39)	0·65 (0·59–0·72)	0·57 (0·52–0·62)
1973	0·0 (0·0–0·0)	6·3 (5·2–7·6)	123·6 (110·4–137·2)	212·0 (202·8–221·3)	89·0 (81·4–97·4)	16·6 (13·1–20·3)	3·1 (2·1–4·2)	0·2 (0·1–0·3)	0·0 (0·0–0·0)	2·25 (2·15–2·36)	0·65 (0·58–0·72)	0·54 (0·5–0·59)
1974	0·0 (0·0–0·0)	5·8 (4·7–7·0)	117·4 (105·4–129·5)	203·4 (194·0–212·5)	78·9 (71·8–86·5)	14·7 (12·0–17·7)	2·6 (1·8–3·6)	0·1 (0·1–0·2)	0·0 (0·0–0·0)	2·11 (2·03–2·2)	0·62 (0·56–0·68)	0·48 (0·44–0·52)
1975	0·0 (0·0–0·0)	5·3 (4·4–6·5)	108·8 (97·6–120·5)	195·3 (186·2–203·8)	70·4 (63·9–77·0)	13·9 (11·3–16·9)	2·1 (1·3–3·3)	0·1 (0·1–0·2)	0·0 (0·0–0·0)	1·98 (1·9–2·06)	0·57 (0·51–0·63)	0·43 (0·4–0·47)
1976	0·0 (0·0–0·0)	5·0 (4·1–6·1)	102·0 (91·3–114·2)	191·9 (183·0–200·0)	67·3 (61·5–73·8)	13·6 (11·5–16·0)	1·8 (1·2–2·6)	0·1 (0·0–0·1)	0·0 (0·0–0·0)	1·91 (1·82–2·0)	0·54 (0·48–0·6)	0·41 (0·38–0·45)
1977	0·0 (0·0–0·0)	4·9 (4·2–5·7)	96·0 (86·4–107·7)	191·9 (182·5–201·6)	68·8 (62·3–75·5)	13·4 (11·1–16·1)	1·7 (1·2–2·3)	0·1 (0·0–0·1)	0·0 (0·0–0·0)	1·88 (1·81–1·97)	0·5 (0·46–0·56)	0·42 (0·38–0·47)
1978	0·0 (0·0–0·0)	5·0 (4·0–6·1)	91·8 (80·7–104·5)	192·0 (183·8–199·8)	72·7 (65·7–79·8)	13·1 (10·2–16·4)	1·6 (1·1–2·3)	0·1 (0·0–0·1)	0·0 (0·0–0·0)	1·88 (1·8–1·97)	0·48 (0·43–0·55)	0·44 (0·4–0·48)
1979	0·0 (0·0–0·0)	5·1 (3·8–6·5)	88·0 (78·7–97·4)	189·9 (181·8–197·7)	73·3 (67·0–80·4)	12·6 (10·4–15·0)	1·6 (1·1–2·4)	0·1 (0·0–0·1)	0·0 (0·0–0·0)	1·85 (1·78–1·92)	0·47 (0·42–0·51)	0·44 (0·41–0·47)
1980	0·0 (0·0–0·0)	5·1 (4·1–6·3)	84·7 (75·1–94·8)	187·5 (179·0–196·2)	71·9 (65·2–78·9)	12·3 (10·1–14·9)	1·6 (1·1–2·3)	0·1 (0·0–0·1)	0·0 (0·0–0·0)	1·82 (1·74–1·89)	0·45 (0·4–0·5)	0·43 (0·4–0·47)
1981	0·0 (0·0–0·0)	5·4 (4·3–6·9)	83·1 (74·3–93·2)	188·8 (180·1–197·7)	72·6 (66·0–79·4)	12·8 (10·4–15·3)	1·7 (1·1–2·4)	0·1 (0·0–0·1)	0·0 (0·0–0·0)	1·82 (1·75–1·89)	0·44 (0·4–0·49)	0·44 (0·4–0·47)
1982	0·0 (0·0–0·0)	5·6 (4·5–6·8)	82·6 (72·8–93·0)	190·9 (182·6–199·6)	75·9 (69·4–82·6)	14·0 (11·8–16·7)	1·6 (1·2–2·3)	0·1 (0·0–0·1)	0·0 (0·0–0·0)	1·85 (1·79–1·92)	0·44 (0·39–0·49)	0·46 (0·42–0·5)
1983	0·0 (0·0–0·0)	5·6 (4·7–6·8)	81·1 (72·6–90·3)	191·3 (182·4–200·0)	79·9 (72·9–88·2)	15·3 (12·5–18·6)	1·7 (1·2–2·1)	0·1 (0·0–0·1)	0·0 (0·0–0·0)	1·87 (1·8–1·96)	0·43 (0·39–0·48)	0·48 (0·44–0·53)
1984	0·0 (0·0–0·0)	5·4 (4·6–6·4)	76·0 (65·4–87·9)	188·0 (179·7–196·7)	83·1 (76·0–91·0)	15·6 (12·9–18·7)	1·7 (1·2–2·3)	0·1 (0·0–0·1)	0·0 (0·0–0·0)	1·85 (1·76–1·94)	0·41 (0·35–0·47)	0·5 (0·47–0·54)
1985	0·0 (0·0–0·0)	5·1 (4·3–6·1)	71·6 (63·2–80·6)	180·9 (172·9–188·6)	84·8 (77·8–93·0)	15·5 (12·7–18·7)	1·7 (1·2–2·3)	0·1 (0·0–0·1)	0·0 (0·0–0·0)	1·8 (1·74–1·86)	0·38 (0·34–0·43)	0·51 (0·47–0·55)
1986	0·0 (0·0–0·0)	4·9 (4·1–5·7)	68·4 (59·8–77·3)	174·6 (166·3–183·0)	87·4 (80·1–94·5)	15·6 (13·1–18·6)	1·8 (1·1–2·7)	0·1 (0·0–0·1)	0·0 (0·0–0·0)	1·76 (1·7–1·83)	0·37 (0·32–0·41)	0·52 (0·49–0·56)
1987	0·0 (0·0–0·0)	4·7 (3·9–5·5)	63·2 (55·7–71·5)	169·1 (160·7–177·5)	90·3 (83·1–98·4)	16·4 (12·8–20·6)	2·0 (1·4–2·8)	0·1 (0·0–0·1)	0·0 (0·0–0·0)	1·73 (1·64–1·82)	0·34 (0·3–0·38)	0·54 (0·5–0·59)
1988	0·0 (0·0–0·0)	4·5 (3·7–5·3)	58·8 (51·5–67·1)	159·1 (151·7–167·4)	91·4 (83·8–99·8)	17·3 (14·4–20·5)	2·2 (1·6–2·9)	0·1 (0·0–0·1)	0·0 (0·0–0·0)	1·67 (1·6–1·74)	0·32 (0·28–0·36)	0·55 (0·51–0·6)
1989	0·0 (0·0–0·0)	4·4 (3·7–5·3)	54·7 (47·5–62·7)	148·9 (141·0–157·5)	91·3 (83·4–99·0)	18·3 (15·2–21·9)	2·3 (1·7–3·1)	0·1 (0·0–0·1)	0·0 (0·0–0·0)	1·6 (1·53–1·67)	0·3 (0·26–0·33)	0·56 (0·52–0·6)
1990	0·0 (0·0–0·0)	4·5 (3·7–5·3)	53·1 (47·0–60·0)	145·7 (138·1–154·0)	92·7 (85·0–100·4)	19·3 (15·8–23·0)	2·3 (1·6–3·0)	0·1 (0·0–0·1)	0·0 (0·0–0·0)	1·59 (1·52–1·66)	0·29 (0·25–0·33)	0·57 (0·53–0·62)
1991	0·0 (0·0–0·0)	4·7 (3·9–5·6)	53·2 (46·2–60·3)	141·9 (134·6–149·8)	93·0 (85·4–101·2)	20·5 (17·1–24·3)	2·3 (1·6–3·0)	0·1 (0·0–0·1)	0·0 (0·0–0·0)	1·58 (1·52–1·64)	0·29 (0·25–0·33)	0·58 (0·54–0·63)
1992	0·0 (0·0–0·0)	4·7 (3·9–5·6)	51·4 (45·8–57·6)	134·1 (126·0–142·1)	91·7 (84·5–99·5)	21·3 (18·0–25·2)	2·4 (1·7–3·1)	0·1 (0·0–0·1)	0·0 (0·0–0·0)	1·53 (1·47–1·59)	0·28 (0·25–0·31)	0·58 (0·54–0·62)
1993	0·0 (0·0–0·0)	4·7 (3·9–5·7)	50·9 (44·4–57·6)	133·6 (125·5–141·9)	95·3 (87·1–103·6)	23·3 (18·7–28·9)	2·5 (1·9–3·4)	0·1 (0·0–0·1)	0·0 (0·0–0·0)	1·55 (1·48–1·63)	0·28 (0·25–0·31)	0·61 (0·56–0·66)
1994	0·0 (0·0–0·0)	4·7 (3·8–5·8)	50·0 (43·2–57·4)	130·5 (122·7–138·4)	96·9 (88·7–105·8)	24·7 (20·8–29·3)	2·7 (1·9–3·5)	0·1 (0·0–0·1)	0·0 (0·0–0·0)	1·55 (1·48–1·61)	0·27 (0·24–0·31)	0·62 (0·58–0·67)
1995	0·0 (0·0–0·0)	4·7 (3·8–5·7)	48·4 (42·5–54·9)	122·6 (115·1–130·8)	96·3 (88·0–105·1)	25·8 (21·8–30·3)	2·8 (2·1–3·7)	0·1 (0·0–0·1)	0·0 (0·0–0·0)	1·5 (1·44–1·57)	0·27 (0·24–0·3)	0·63 (0·58–0·67)
1996	0·0 (0·0–0·0)	4·9 (4·0–6·0)	47·7 (41·4–53·9)	118·1 (111·6–124·8)	96·7 (88·9–105·4)	27·1 (22·8–32·0)	3·0 (2·2–3·8)	0·1 (0·0–0·1)	0·0 (0·0–0·0)	1·49 (1·42–1·55)	0·26 (0·23–0·29)	0·63 (0·59–0·68)
1997	0·0 (0·0–0·0)	5·2 (4·2–6·3)	47·5 (41·5–54·1)	114·2 (105·3–123·0)	95·8 (87·2–103·8)	27·9 (22·3–34·3)	3·1 (2·3–4·2)	0·1 (0·1–0·1)	0·0 (0·0–0·0)	1·47 (1·4–1·54)	0·26 (0·23–0·3)	0·63 (0·59–0·68)
1998	0·0 (0·0–0·0)	5·5 (4·4–6·8)	47·4 (41·6–54·2)	109·9 (103·7–115·9)	95·4 (87·4–104·4)	29·0 (24·4–34·3)	3·3 (2·4–4·4)	0·1 (0·1–0·1)	0·0 (0·0–0·0)	1·45 (1·39–1·52)	0·26 (0·23–0·3)	0·64 (0·59–0·69)
1999	0·0 (0·0–0·0)	5·9 (4·8–7·2)	47·4 (41·2–54·3)	106·4 (99·9–113·1)	95·2 (86·9–103·9)	29·9 (24·9–35·5)	3·5 (2·5–4·7)	0·1 (0·1–0·1)	0·0 (0·0–0·0)	1·44 (1·37–1·51)	0·27 (0·24–0·3)	0·64 (0·59–0·69)
2000	0·0 (0·0–0·0)	6·4 (5·2–7·8)	47·9 (42·5–53·4)	104·0 (97·8–111·0)	93·7 (86·2–101·8)	31·5 (26·6–36·8)	3·7 (2·6–5·1)	0·1 (0·1–0·2)	0·0 (0·0–0·0)	1·44 (1·37–1·51)	0·27 (0·25–0·3)	0·64 (0·6–0·69)
2001	0·0 (0·0–0·0)	6·7 (5·5–8·2)	48·4 (41·7–55·4)	100·8 (94·3–107·5)	90·9 (82·7–99·1)	31·6 (26·8–37·3)	3·8 (2·7–5·2)	0·1 (0·1–0·2)	0·0 (0·0–0·0)	1·41 (1·34–1·48)	0·28 (0·24–0·31)	0·63 (0·58–0·68)
2002	0·0 (0·0–0·0)	6·6 (5·4–8·0)	47·4 (41·2–54·3)	97·8 (91·6–104·1)	90·3 (82·4–98·9)	32·4 (27·5–37·8)	4·0 (2·9–5·6)	0·1 (0·1–0·2)	0·0 (0·0–0·0)	1·39 (1·33–1·46)	0·27 (0·24–0·3)	0·63 (0·59–0·68)
2003	0·0 (0·0–0·0)	6·4 (5·2–7·6)	45·8 (39·6–52·6)	95·0 (89·4–100·7)	89·3 (81·7–97·2)	33·9 (28·5–39·6)	4·3 (3·1–5·8)	0·1 (0·1–0·2)	0·0 (0·0–0·0)	1·37 (1·31–1·44)	0·26 (0·23–0·3)	0·64 (0·59–0·69)
2004	0·0 (0·0–0·0)	6·0 (5·0–7·1)	44·4 (38·6–51·4)	90·9 (84·9–97·2)	87·3 (80·0–94·5)	34·7 (29·4–40·6)	4·5 (3·3–5·9)	0·1 (0·1–0·2)	0·0 (0·0–0·0)	1·34 (1·28–1·4)	0·25 (0·22–0·29)	0·63 (0·59–0·68)
2005	0·0 (0·0–0·0)	5·9 (4·9–7·0)	44·1 (38·9–49·9)	89·6 (83·5–95·7)	87·1 (79·3–94·7)	35·7 (30·2–41·5)	4·7 (3·5–6·2)	0·1 (0·1–0·2)	0·0 (0·0–0·0)	1·34 (1·27–1·4)	0·25 (0·22–0·28)	0·64 (0·59–0·69)
2006	0·0 (0·0–0·0)	5·8 (4·9–6·8)	44·7 (38·7–51·0)	90·2 (84·3–96·4)	89·2 (81·7–97·4)	37·6 (32·0–44·1)	5·2 (3·9–6·8)	0·1 (0·1–0·2)	0·0 (0·0–0·0)	1·36 (1·3–1·43)	0·25 (0·22–0·29)	0·66 (0·61–0·72)
2007	0·0 (0·0–0·0)	5·8 (4·9–6·9)	44·5 (39·6–50·2)	89·5 (83·9–96·0)	89·0 (81·5–97·8)	39·6 (33·8–46·7)	5·6 (4·4–7·3)	0·1 (0·1–0·2)	0·0 (0·0–0·0)	1·37 (1·31–1·44)	0·25 (0·23–0·28)	0·67 (0·62–0·73)
2008	0·0 (0·0–0·0)	5·7 (4·8–6·7)	43·8 (37·7–50·5)	88·8 (82·6–95·3)	89·2 (81·6–97·1)	41·0 (35·2–47·3)	6·1 (4·8–7·7)	0·1 (0·1–0·2)	0·0 (0·0–0·0)	1·37 (1·31–1·45)	0·25 (0·22–0·28)	0·68 (0·63–0·73)
2009	0·0 (0·0–0·0)	5·4 (4·5–6·4)	42·3 (37·9–46·6)	88·4 (82·8–93·7)	89·6 (81·9–98·1)	42·4 (36·0–49·2)	6·8 (5·3–8·6)	0·1 (0·1–0·2)	0·0 (0·0–0·0)	1·38 (1·3–1·45)	0·24 (0·22–0·26)	0·69 (0·64–0·75)
2010	0·0 (0·0–0·0)	5·2 (4·5–5·9)	41·2 (34·6–47·9)	88·9 (82·6–95·1)	90·4 (82·7–98·9)	43·4 (37·3–49·9)	7·2 (5·7–8·8)	0·2 (0·1–0·2)	0·0 (0·0–0·0)	1·38 (1·31–1·46)	0·23 (0·2–0·27)	0·71 (0·65–0·76)
2011	0·0 (0·0–0·0)	5·1 (4·3–6·0)	39·3 (33·9–45·1)	88·3 (82·3–94·8)	90·3 (82·6–98·5)	44·1 (37·7–51·1)	7·5 (5·7–9·4)	0·2 (0·1–0·3)	0·0 (0·0–0·0)	1·37 (1·31–1·44)	0·22 (0·2–0·25)	0·71 (0·66–0·76)
2012	0·0 (0·0–0·0)	5·0 (4·0–6·0)	37·7 (32·4–43·7)	87·7 (82·1–93·4)	91·6 (83·3–99·9)	45·8 (39·3–52·6)	8·0 (6·3–10·2)	0·2 (0·1–0·3)	0·0 (0·0–0·0)	1·38 (1·32–1·45)	0·21 (0·19–0·24)	0·73 (0·68–0·78)
2013	0·0 (0·0–0·0)	4·9 (4·1–5·8)	36·7 (31·7–41·8)	86·4 (80·4–92·1)	91·7 (83·8–99·9)	46·6 (40·1–53·3)	8·3 (6·4–10·6)	0·2 (0·1–0·3)	0·0 (0·0–0·0)	1·37 (1·31–1·44)	0·21 (0·18–0·23)	0·73 (0·68–0·79)
2014	0·0 (0·0–0·0)	4·8 (4·0–5·7)	35·7 (30·3–42·0)	85·8 (79·8–92·2)	92·9 (85·6–100·8)	48·0 (41·4–55·1)	8·7 (6·8–11·2)	0·2 (0·1–0·3)	0·0 (0·0–0·0)	1·38 (1·3–1·47)	0·2 (0·18–0·23)	0·75 (0·69–0·81)
2015	0·0 (0·0–0·0)	4·8 (3·9–5·8)	36·4 (31·1–42·8)	87·5 (80·4–95·3)	95·4 (85·8–105·8)	46·4 (38·5–55·2)	8·0 (5·8–10·7)	0·2 (0·1–0·3)	0·0 (0·0–0·0)	1·39 (1·24–1·56)	0·21 (0·18–0·24)	0·75 (0·66–0·84)
2016	0·0 (0·0–0·0)	4·9 (4·0–6·0)	38·0 (29·9–48·4)	88·9 (79·5–99·9)	95·3 (82·9–109·7)	42·7 (33·1–55·0)	6·8 (4·7–9·5)	0·2 (0·1–0·2)	0·0 (0·0–0·0)	1·38 (1·18–1·63)	0·21 (0·17–0·27)	0·72 (0·61–0·86)
2017	0·0 (0·0–0·0)	4·8 (4·0–5·9)	38·0 (29·7–48·6)	88·9 (78·9–99·9)	94·1 (80·2–109·2)	41·6 (31·0–54·4)	6·7 (4·3–10·0)	0·2 (0·1–0·3)	0·0 (0·0–0·0)	1·37 (1·15–1·63)	0·21 (0·17–0·27)	0·71 (0·58–0·86)

Supplemental Results Table 5: Age-specific fertility rates per 1000, cumulative total fertility up to maternal age 25 (TFU25), cumulative total fertility from maternal age 30 to age 55 (TFO30), and cumulative total fertility, Ishikawa, 1950-2017

Year	10-14	15-19	20-24	25-29	30-34	35-39	40-44	45-49	50-54	TFR	TFU25	TFO30
1950	0·0 (0·0–0·0)	8·4 (7·5–9·4)	173·8 (158·7–190·1)	227·5 (219·0–236·0)	152·3 (143·3–161·4)	102·2 (90·8–113·4)	28·6 (24·7–32·9)	1·4 (0·9–2·0)	0·0 (0·0–0·0)	3·47 (3·35–3·6)	0·91 (0·84–0·99)	1·42 (1·35–1·5)
1951	0·0 (0·0–0·0)	6·8 (6·0–7·6)	156·3 (142·3–170·4)	211·6 (202·7–220·4)	137·6 (128·0–147·0)	82·6 (71·0–93·5)	22·2 (18·4–26·2)	1·0 (0·6–1·5)	0·0 (0·0–0·0)	3·09 (2·94–3·23)	0·82 (0·74–0·89)	1·22 (1·1–1·33)
1952	0·0 (0·0–0·0)	5·7 (5·0–6·6)	145·7 (131·1–162·5)	201·3 (191·7–211·2)	124·3 (114·7–133·8)	66·2 (56·9–76·0)	17·6 (14·5–21·4)	0·8 (0·5–1·1)	0·0 (0·0–0·0)	2·81 (2·7–2·93)	0·76 (0·68–0·84)	1·04 (0·98–1·12)
1953	0·0 (0·0–0·0)	5·0 (4·3–5·8)	137·9 (122·6–155·0)	189·9 (179·7–201·7)	111·0 (100·7–121·2)	52·9 (44·4–61·6)	14·0 (11·0–17·4)	0·6 (0·4–1·0)	0·0 (0·0–0·0)	2·56 (2·43–2·69)	0·71 (0·64–0·8)	0·89 (0·82–0·96)
1954	0·0 (0·0–0·0)	4·6 (3·9–5·3)	134·0 (117·2–151·5)	184·9 (172·9–197·7)	102·2 (93·7–111·7)	44·1 (36·5–52·6)	11·6 (8·8–14·9)	0·5 (0·3–0·8)	0·0 (0·0–0·0)	2·41 (2·28–2·54)	0·69 (0·61–0·78)	0·79 (0·73–0·86)
1955	0·0 (0·0–0·0)	4·1 (3·5–4·9)	131·0 (114·8–149·6)	184·0 (171·5–198·3)	94·0 (85·2–103·8)	34·7 (28·8–41·4)	9·6 (7·2–12·4)	0·4 (0·2–0·7)	0·0 (0·0–0·0)	2·29 (2·16–2·42)	0·68 (0·59–0·77)	0·69 (0·63–0·75)
1956	0·0 (0·0–0·0)	3·7 (3·1–4·4)	123·7 (106·9–141·2)	178·0 (165·2–191·9)	83·2 (75·1–92·1)	27·5 (23·0–32·8)	7·7 (5·8–10·1)	0·3 (0·2–0·6)	0·0 (0·0–0·0)	2·12 (1·99–2·25)	0·64 (0·55–0·72)	0·59 (0·54–0·65)
1957	0·0 (0·0–0·0)	3·5 (2·9–4·2)	125·3 (108·9–145·0)	181·6 (169·4–195·6)	78·9 (71·3–87·4)	26·0 (21·6–30·5)	6·6 (5·0–8·5)	0·4 (0·2–0·6)	0·0 (0·0–0·0)	2·11 (1·98–2·26)	0·64 (0·56–0·74)	0·56 (0·51–0·61)
1958	0·0 (0·0–0·0)	3·6 (3·0–4·2)	131·9 (115·2–150·1)	187·6 (175·4–199·8)	77·1 (69·2–85·4)	25·0 (20·5–29·8)	6·0 (4·5–7·9)	0·4 (0·2–0·6)	0·0 (0·0–0·0)	2·16 (2·03–2·28)	0·68 (0·59–0·77)	0·54 (0·49–0·6)
1959	0·0 (0·0–0·0)	3·8 (3·2–4·4)	132·3 (116·2–150·8)	186·5 (176·3–197·4)	72·2 (63·6–80·3)	20·3 (16·2–25·1)	5·0 (3·7–6·5)	0·3 (0·2–0·5)	0·0 (0·0–0·0)	2·1 (1·99–2·22)	0·68 (0·6–0·77)	0·49 (0·44–0·54)
1960	0·0 (0·0–0·0)	3·7 (3·1–4·3)	127·6 (112·6–144·5)	187·4 (177·1–197·5)	69·2 (62·0–76·3)	16·0 (13·1–19·2)	4·1 (3·0–5·3)	0·2 (0·1–0·4)	0·0 (0·0–0·0)	2·04 (1·95–2·14)	0·66 (0·58–0·74)	0·45 (0·41–0·49)
1961	0·0 (0·0–0·0)	3·4 (2·9–4·0)	124·2 (111·5–137·6)	189·3 (180·5–198·8)	67·5 (61·1–74·7)	13·7 (11·0–17·1)	3·5 (2·5–4·8)	0·2 (0·1–0·4)	0·0 (0·0–0·0)	2·01 (1·92–2·1)	0·64 (0·57–0·71)	0·42 (0·39–0·46)
1962	0·0 (0·0–0·0)	3·3 (2·7–3·9)	124·3 (110·3–138·4)	194·8 (186·7–202·9)	68·9 (62·9–75·6)	13·1 (10·5–15·9)	3·1 (2·2–4·3)	0·2 (0·1–0·3)	0·0 (0·0–0·0)	2·04 (1·95–2·13)	0·64 (0·57–0·71)	0·43 (0·39–0·46)
1963	0·0 (0·0–0·0)	3·2 (2·7–4·0)	125·4 (111·7–140·2)	196·8 (189·0–205·2)	70·4 (64·4–76·9)	14·8 (12·2–18·0)	3·1 (2·2–4·3)	0·2 (0·1–0·4)	0·0 (0·0–0·0)	2·07 (1·98–2·16)	0·64 (0·57–0·72)	0·44 (0·41–0·48)
1964	0·0 (0·0–0·0)	3·2 (2·6–3·9)	137·6 (122·4–154·0)	209·6 (200·1–218·9)	75·0 (68·6–82·2)	15·6 (12·8–18·4)	3·0 (2·0–4·1)	0·2 (0·1–0·4)	0·0 (0·0–0·0)	2·22 (2·12–2·33)	0·7 (0·63–0·79)	0·47 (0·43–0·51)
1965	0·0 (0·0–0·0)	3·1 (2·6–3·7)	127·4 (110·4–146·4)	177·0 (167·5–186·4)	62·2 (55·7–68·9)	12·1 (10·3–14·3)	2·5 (1·8–3·6)	0·2 (0·1–0·3)	0·0 (0·0–0·0)	1·92 (1·82–2·04)	0·65 (0·57–0·75)	0·39 (0·35–0·42)
1966	0·0 (0·0–0·0)	3·4 (2·8–4·0)	125·4 (105·8–146·5)	182·1 (172·7–191·3)	63·6 (56·9–70·5)	13·3 (11·2–15·8)	2·5 (1·8–3·5)	0·2 (0·1–0·3)	0·0 (0·0–0·0)	1·95 (1·84–2·06)	0·64 (0·55–0·75)	0·4 (0·36–0·43)
1967	0·0 (0·0–0·0)	3·6 (3·0–4·2)	134·6 (113·1–158·1)	221·8 (212·6–230·2)	76·4 (68·8–84·9)	14·2 (11·5–17·2)	2·5 (1·8–3·3)	0·2 (0·1–0·3)	0·0 (0·0–0·0)	2·27 (2·13–2·42)	0·69 (0·58–0·81)	0·47 (0·42–0·51)
1968	0·0 (0·0–0·0)	3·6 (3·1–4·2)	121·4 (101·2–143·9)	209·0 (200·5–217·4)	69·3 (61·9–77·0)	11·6 (9·2–14·3)	2·2 (1·6–3·0)	0·1 (0·1–0·2)	0·0 (0·0–0·0)	2·09 (1·99–2·2)	0·63 (0·53–0·74)	0·42 (0·38–0·46)
1969	0·0 (0·0–0·0)	4·0 (3·4–4·7)	120·2 (100·1–142·1)	211·0 (202·6–219·4)	69·3 (62·3–77·0)	12·5 (10·0–15·7)	2·2 (1·7–3·0)	0·1 (0·1–0·2)	0·0 (0·0–0·0)	2·1 (2·0–2·21)	0·62 (0·52–0·73)	0·42 (0·38–0·46)
1970	0·0 (0·0–0·0)	4·1 (3·5–4·8)	130·3 (109·8–152·4)	214·3 (205·2–223·1)	71·3 (64·2–79·0)	15·7 (12·3–19·5)	2·4 (1·8–3·2)	0·1 (0·1–0·3)	0·0 (0·0–0·0)	2·19 (2·08–2·3)	0·67 (0·57–0·78)	0·45 (0·4–0·5)
1971	0·0 (0·0–0·0)	4·0 (3·3–4·8)	143·9 (123·5–166·8)	211·2 (202·0–220·4)	72·2 (64·6–80·1)	18·0 (14·3–22·5)	2·7 (1·9–3·6)	0·2 (0·1–0·3)	0·0 (0·0–0·0)	2·26 (2·14–2·39)	0·74 (0·64–0·85)	0·47 (0·42–0·52)
1972	0·0 (0·0–0·0)	4·1 (3·4–4·9)	151·9 (133·0–173·6)	209·1 (199·8–218·6)	72·2 (64·0–80·6)	18·5 (14·4–22·8)	2·7 (2·0–3·6)	0·2 (0·1–0·3)	0·0 (0·0–0·0)	2·29 (2·17–2·42)	0·78 (0·69–0·89)	0·47 (0·42–0·52)
1973	0·0 (0·0–0·0)	4·0 (3·3–4·8)	153·5 (136·3–170·4)	208·4 (198·1–218·3)	68·3 (61·0–76·1)	17·0 (13·3–21·3)	2·5 (1·7–3·3)	0·1 (0·1–0·3)	0·0 (0·0–0·0)	2·27 (2·15–2·38)	0·79 (0·7–0·87)	0·44 (0·39–0·49)
1974	0·0 (0·0–0·0)	3·7 (3·0–4·6)	147·8 (132·4–165·2)	199·1 (190·3–208·1)	60·3 (53·9–67·3)	13·3 (10·2–16·9)	2·1 (1·4–2·9)	0·1 (0·1–0·2)	0·0 (0·0–0·0)	2·13 (2·04–2·23)	0·76 (0·68–0·84)	0·38 (0·34–0·42)
1975	0·0 (0·0–0·0)	3·4 (2·7–4·3)	138·1 (123·0–154·5)	191·0 (181·9–200·1)	54·3 (48·4–59·5)	10·4 (7·9–13·6)	1·7 (1·2–2·4)	0·1 (0·0–0·2)	0·0 (0·0–0·0)	1·99 (1·9–2·09)	0·71 (0·63–0·79)	0·33 (0·3–0·37)
1976	0·0 (0·0–0·0)	3·1 (2·5–3·9)	129·9 (115·7–145·2)	188·0 (178·7–197·5)	52·9 (47·6–58·7)	8·6 (6·6–11·0)	1·5 (0·9–2·5)	0·1 (0·0–0·1)	0·0 (0·0–0·0)	1·92 (1·83–2·02)	0·67 (0·59–0·74)	0·32 (0·28–0·35)
1977	0·0 (0·0–0·0)	3·0 (2·5–3·7)	121·8 (109·8–136·1)	189·0 (180·5–197·9)	54·4 (48·4–60·5)	7·7 (5·8–10·4)	1·4 (0·9–2·0)	0·1 (0·0–0·1)	0·0 (0·0–0·0)	1·89 (1·8–1·98)	0·62 (0·56–0·7)	0·32 (0·28–0·36)
1978	0·0 (0·0–0·0)	3·0 (2·4–3·6)	115·2 (100·4–132·9)	190·2 (182·0–198·5)	56·7 (50·9–63·4)	7·5 (5·6–10·0)	1·2 (0·7–1·8)	0·0 (0·0–0·1)	0·0 (0·0–0·0)	1·87 (1·77–1·98)	0·59 (0·52–0·68)	0·33 (0·29–0·36)
1979	0·0 (0·0–0·0)	3·0 (2·5–3·5)	108·5 (96·8–120·3)	189·2 (181·0–197·5)	55·9 (50·5–62·0)	7·4 (5·8–9·4)	1·1 (0·6–1·7)	0·0 (0·0–0·1)	0·0 (0·0–0·0)	1·83 (1·75–1·91)	0·56 (0·5–0·62)	0·32 (0·29–0·35)
1980	0·0 (0·0–0·0)	3·0 (2·4–3·8)	102·0 (90·4–113·9)	187·5 (178·6–197·3)	53·9 (48·3–59·8)	7·6 (5·8–9·7)	1·0 (0·6–1·4)	0·0 (0·0–0·1)	0·0 (0·0–0·0)	1·78 (1·69–1·86)	0·53 (0·47–0·58)	0·31 (0·28–0·34)
1981	0·0 (0·0–0·0)	3·2 (2·5–3·9)	97·5 (87·7–109·5)	189·5 (180·9–198·5)	54·1 (48·9–59·9)	8·1 (6·3–10·3)	0·9 (0·6–1·3)	0·0 (0·0–0·0)	0·0 (0·0–0·0)	1·77 (1·7–1·84)	0·5 (0·45–0·57)	0·32 (0·29–0·34)
1982	0·0 (0·0–0·0)	3·3 (2·5–4·1)	94·2 (83·2–106·7)	192·4 (183·8–201·0)	57·2 (51·6–63·3)	9·2 (7·3–11·7)	0·9 (0·6–1·3)	0·0 (0·0–0·0)	0·0 (0·0–0·0)	1·79 (1·71–1·87)	0·49 (0·43–0·55)	0·34 (0·3–0·37)
1983	0·0 (0·0–0·0)	3·3 (2·6–4·1)	90·0 (79·3–101·7)	194·1 (184·9–203·1)	61·1 (55·4–67·8)	10·4 (8·1–13·1)	1·0 (0·7–1·3)	0·0 (0·0–0·0)	0·0 (0·0–0·0)	1·8 (1·72–1·89)	0·47 (0·41–0·53)	0·36 (0·33–0·4)
1984	0·0 (0·0–0·0)	3·2 (2·5–4·0)	82·3 (72·8–92·7)	192·8 (184·6–201·7)	64·1 (58·1–70·7)	10·9 (8·4–13·8)	1·0 (0·7–1·4)	0·0 (0·0–0·0)	0·0 (0·0–0·0)	1·77 (1·71–1·84)	0·43 (0·38–0·48)	0·38 (0·35–0·42)
1985	0·0 (0·0–0·0)	3·0 (2·3–3·8)	76·3 (66·5–87·1)	188·2 (176·5–199·3)	65·8 (58·6–72·8)	10·9 (8·5–13·5)	1·1 (0·8–1·4)	0·0 (0·0–0·0)	0·0 (0·0–0·0)	1·73 (1·65–1·81)	0·4 (0·35–0·45)	0·39 (0·35–0·43)
1986	0·0 (0·0–0·0)	2·9 (2·3–3·6)	71·7 (62·6–81·0)	184·3 (176·4–193·1)	68·4 (62·4–74·2)	11·2 (8·7–14·2)	1·2 (0·9–1·5)	0·0 (0·0–0·1)	0·0 (0·0–0·0)	1·7 (1·63–1·78)	0·37 (0·33–0·42)	0·4 (0·37–0·44)
1987	0·0 (0·0–0·0)	2·8 (2·2–3·5)	65·1 (57·9–73·2)	181·0 (171·3–190·7)	72·0 (65·2–79·4)	11·9 (9·2–15·1)	1·3 (0·9–1·9)	0·0 (0·0–0·1)	0·0 (0·0–0·0)	1·67 (1·6–1·74)	0·34 (0·3–0·38)	0·43 (0·39–0·47)
1988	0·0 (0·0–0·0)	2·7 (2·2–3·4)	59·3 (51·4–68·7)	172·0 (163·6–181·0)	74·7 (67·7–82·1)	12·9 (10·2–16·0)	1·5 (1·0–2·1)	0·0 (0·0–0·1)	0·0 (0·0–0·0)	1·62 (1·54–1·69)	0·31 (0·27–0·36)	0·45 (0·41–0·49)
1989	0·0 (0·0–0·0)	2·7 (2·2–3·4)	53·7 (46·4–62·0)	161·8 (152·9–170·8)	76·7 (69·5–84·0)	14·0 (11·1–17·4)	1·6 (1·1–2·4)	0·0 (0·0–0·1)	0·0 (0·0–0·0)	1·55 (1·48–1·63)	0·28 (0·24–0·32)	0·46 (0·42–0·5)
1990	0·0 (0·0–0·0)	2·7 (2·2–3·3)	50·6 (44·0–57·3)	158·3 (150·4–166·7)	80·2 (73·0–87·6)	15·1 (11·7–18·9)	1·7 (1·1–2·4)	0·0 (0·0–0·1)	0·0 (0·0–0·0)	1·54 (1·47–1·62)	0·27 (0·23–0·3)	0·48 (0·44–0·53)
1991	0·0 (0·0–0·0)	2·8 (2·3–3·4)	49·3 (42·6–56·5)	154·1 (143·0–166·0)	83·0 (74·9–91·1)	16·2 (12·7–20·2)	1·8 (1·2–2·4)	0·0 (0·0–0·1)	0·0 (0·0–0·0)	1·54 (1·47–1·61)	0·26 (0·23–0·3)	0·5 (0·46–0·55)
1992	0·0 (0·0–0·0)	2·8 (2·3–3·4)	46·8 (41·1–53·4)	145·7 (137·7–153·5)	84·1 (76·8–91·7)	17·0 (13·1–21·7)	1·9 (1·3–2·7)	0·0 (0·0–0·1)	0·0 (0·0–0·0)	1·49 (1·43–1·55)	0·25 (0·22–0·28)	0·51 (0·48–0·56)
1993	0·0 (0·0–0·0)	2·9 (2·3–3·6)	45·9 (39·4–52·7)	145·3 (135·3–155·1)	89·4 (81·5–97·7)	18·5 (14·6–23·0)	2·0 (1·4–2·7)	0·0 (0·0–0·1)	0·0 (0·0–0·0)	1·52 (1·45–1·59)	0·24 (0·21–0·28)	0·55 (0·5–0·6)
1994	0·0 (0·0–0·0)	2·9 (2·4–3·6)	45·0 (39·3–51·6)	142·5 (134·9–150·5)	92·3 (83·9–101·6)	19·8 (15·8–24·8)	2·2 (1·5–3·1)	0·0 (0·0–0·1)	0·0 (0·0–0·0)	1·52 (1·45–1·6)	0·24 (0·21–0·27)	0·57 (0·53–0·62)
1995	0·0 (0·0–0·0)	3·0 (2·4–3·6)	43·7 (37·9–49·9)	134·5 (125·8–142·8)	92·7 (83·7–101·1)	20·7 (16·6–25·2)	2·3 (1·6–3·3)	0·0 (0·0–0·1)	0·0 (0·0–0·0)	1·48 (1·41–1·56)	0·23 (0·2–0·26)	0·58 (0·53–0·62)
1996	0·0 (0·0–0·0)	3·1 (2·5–3·8)	43·2 (37·0–49·3)	130·1 (122·0–138·9)	93·8 (85·6–103·0)	21·9 (17·7–26·9)	2·4 (1·7–3·3)	0·0 (0·0–0·1)	0·0 (0·0–0·0)	1·47 (1·4–1·55)	0·23 (0·2–0·26)	0·59 (0·55–0·64)
1997	0·0 (0·0–0·0)	3·3 (2·7–3·9)	43·1 (37·5–49·0)	126·2 (118·3–134·0)	93·6 (85·8–102·7)	22·8 (18·6–27·8)	2·6 (1·9–3·5)	0·0 (0·0–0·1)	0·0 (0·0–0·0)	1·46 (1·39–1·53)	0·23 (0·2–0·26)	0·6 (0·55–0·65)
1998	0·0 (0·0–0·0)	3·5 (2·9–4·3)	42·7 (37·1–49·6)	121·7 (114·0–129·4)	94·2 (86·1–103·2)	24·2 (19·8–29·2)	2·8 (2·0–3·7)	0·1 (0·0–0·1)	0·0 (0·0–0·0)	1·45 (1·37–1·53)	0·23 (0·2–0·27)	0·61 (0·56–0·66)
1999	0·0 (0·0–0·0)	3·7 (3·1–4·6)	42·3 (37·2–48·2)	117·9 (110·0–125·7)	95·2 (86·5–104·5)	25·4 (20·9–30·3)	3·0 (2·1–4·0)	0·1 (0·0–0·1)	0·0 (0·0–0·0)	1·44 (1·36–1·51)	0·23 (0·2–0·26)	0·62 (0·57–0·67)
2000	0·0 (0·0–0·0)	4·0 (3·3–4·9)	42·4 (35·0–50·0)	115·1 (107·8–122·5)	95·0 (86·7–104·1)	27·4 (22·9–32·8)	3·2 (2·3–4·4)	0·1 (0·0–0·1)	0·0 (0·0–0·0)	1·44 (1·36–1·51)	0·23 (0·2–0·27)	0·63 (0·58–0·68)
2001	0·0 (0·0–0·0)	4·2 (3·5–5·2)	42·2 (37·0–47·8)	111·3 (104·2–119·3)	93·4 (85·5–101·3)	28·4 (23·3–34·4)	3·4 (2·4–4·6)	0·1 (0·0–0·1)	0·0 (0·0–0·0)	1·42 (1·35–1·49)	0·23 (0·21–0·26)	0·63 (0·58–0·68)
2002	0·0 (0·0–0·0)	4·2 (3·4–5·1)	41·0 (35·3–47·9)	107·9 (100·7–115·3)	94·1 (85·5–103·5)	29·9 (24·9–35·5)	3·7 (2·5–5·1)	0·1 (0·0–0·1)	0·0 (0·0–0·0)	1·4 (1·33–1·48)	0·23 (0·2–0·26)	0·64 (0·59–0·69)
2003	0·0 (0·0–0·0)	4·0 (3·2–5·0)	39·3 (33·8–45·6)	104·5 (98·1–111·6)	94·2 (86·8–101·8)	32·2 (26·6–38·4)	4·0 (2·8–5·6)	0·1 (0·0–0·1)	0·0 (0·0–0·0)	1·39 (1·32–1·47)	0·22 (0·19–0·25)	0·65 (0·6–0·71)
2004	0·0 (0·0–0·0)	3·9 (3·1–4·8)	37·7 (32·7–43·4)	99·7 (92·5–107·3)	93·2 (83·4–104·4)	33·9 (28·5–40·2)	4·3 (3·0–5·8)	0·1 (0·0–0·1)	0·0 (0·0–0·0)	1·36 (1·29–1·44)	0·21 (0·18–0·24)	0·66 (0·6–0·72)
2005	0·0 (0·0–0·0)	3·8 (3·0–4·6)	37·4 (29·7–46·4)	97·7 (91·6–104·2)	93·8 (85·6–102·3)	35·6 (29·7–42·4)	4·5 (3·1–6·2)	0·1 (0·0–0·1)	0·0 (0·0–0·0)	1·36 (1·29–1·45)	0·21 (0·17–0·25)	0·67 (0·62–0·73)
2006	0·0 (0·0–0·0)	3·8 (3·0–4·7)	37·7 (32·7–43·1)	97·8 (88·5–107·8)	96·8 (88·2–106·3)	38·1 (32·2–45·6)	5·0 (3·6–6·8)	0·1 (0·0–0·1)	0·0 (0·0–0·0)	1·4 (1·31–1·48)	0·21 (0·18–0·23)	0·7 (0·65–0·76)
2007	0·0 (0·0–0·0)	3·8 (3·0–4·7)	37·6 (32·5–43·5)	96·5 (90·5–103·6)	97·0 (88·6–106·0)	40·7 (34·3–48·4)	5·4 (3·9–7·5)	0·1 (0·0–0·1)	0·0 (0·0–0·0)	1·41 (1·34–1·49)	0·21 (0·18–0·24)	0·72 (0·66–0·78)
2008	0·0 (0·0–0·0)	3·7 (3·0–4·7)	37·2 (32·2–42·6)	95·3 (89·0–101·8)	97·4 (89·0–105·6)	42·5 (35·5–49·8)	5·9 (4·3–8·2)	0·1 (0·0–0·2)	0·0 (0·0–0·0)	1·41 (1·34–1·48)	0·2 (0·18–0·23)	0·73 (0·67–0·79)
2009	0·0 (0·0–0·0)	3·6 (2·8–4·6)	36·3 (29·8–43·3)	94·3 (85·9–102·4)	98·2 (90·1–106·8)	44·3 (37·2–51·6)	6·5 (4·5–8·9)	0·1 (0·0–0·2)	0·0 (0·0–0·0)	1·42 (1·32–1·51)	0·2 (0·17–0·23)	0·75 (0·69–0·81)
2010	0·0 (0·0–0·0)	3·5 (2·6–4·6)	35·4 (30·8–40·2)	94·2 (88·2–100·3)	99·8 (91·0–109·8)	45·8 (39·0–53·3)	6·8 (4·9–9·3)	0·1 (0·1–0·2)	0·0 (0·0–0·0)	1·43 (1·36–1·5)	0·19 (0·17–0·22)	0·76 (0·7–0·82)
2011	0·0 (0·0–0·0)	3·4 (2·7–4·3)	33·9 (29·6–39·1)	93·2 (86·5–100·3)	100·5 (92·3–109·8)	47·2 (40·0–55·1)	7·1 (5·1–9·5)	0·1 (0·1–0·2)	0·0 (0·0–0·0)	1·43 (1·35–1·51)	0·19 (0·16–0·21)	0·77 (0·71–0·84)
2012	0·0 (0·0–0·0)	3·4 (2·8–4·0)	32·6 (27·5–38·0)	92·0 (85·9–98·5)	102·5 (93·3–112·3)	49·7 (42·3–58·1)	7·7 (5·6–10·2)	0·1 (0·1–0·2)	0·0 (0·0–0·0)	1·44 (1·37–1·52)	0·18 (0·15–0·21)	0·8 (0·74–0·86)
2013	0·0 (0·0–0·0)	3·3 (2·7–4·0)	31·6 (26·9–36·6)	90·2 (82·8–97·1)	103·0 (94·1–112·4)	51·1 (43·7–58·9)	8·1 (5·8–10·7)	0·1 (0·1–0·2)	0·0 (0·0–0·0)	1·44 (1·35–1·52)	0·17 (0·15–0·2)	0·81 (0·75–0·88)
2014	0·0 (0·0–0·0)	3·2 (2·7–3·9)	30·7 (26·0–36·0)	89·6 (82·7–97·0)	104·1 (95·6–113·5)	52·8 (45·2–61·8)	8·4 (6·2–11·2)	0·1 (0·1–0·3)	0·0 (0·0–0·0)	1·45 (1·37–1·53)	0·17 (0·15–0·2)	0·83 (0·77–0·9)
2015	0·0 (0·0–0·0)	3·2 (2·6–3·9)	31·6 (23·5–41·5)	91·5 (82·4–100·9)	106·5 (95·4–118·5)	50·8 (41·4–61·6)	7·8 (5·5–10·6)	0·1 (0·1–0·2)	0·0 (0·0–0·0)	1·46 (1·29–1·65)	0·17 (0·13–0·23)	0·83 (0·73–0·93)
2016	0·0 (0·0–0·0)	3·2 (2·7–4·0)	32·8 (25·2–43·2)	93·1 (81·3–106·3)	105·6 (91·6–122·3)	46·5 (35·2–60·4)	6·7 (4·5–9·3)	0·1 (0·1–0·2)	0·0 (0·0–0·0)	1·44 (1·23–1·69)	0·18 (0·14–0·24)	0·79 (0·67–0·94)
2017	0·0 (0·0–0·0)	3·2 (2·7–3·9)	32·9 (24·0–44·3)	93·2 (80·6–107·1)	103·8 (88·4–120·6)	44·9 (33·1–59·2)	6·5 (4·1–9·7)	0·1 (0·1–0·2)	0·0 (0·0–0·0)	1·42 (1·19–1·69)	0·18 (0·13–0·24)	0·78 (0·64–0·94)

Supplemental Results Table 5: Age-specific fertility rates per 1000, cumulative total fertility up to maternal age 25 (TFU25), cumulative total fertility from maternal age 30 to age 55 (TFO30), and cumulative total fertility, Iwate, 1950-2017

Year	10-14	15-19	20-24	25-29	30-34	35-39	40-44	45-49	50-54	TFR	TFU25	TFO30
1950	0·0 (0·0–0·0)	12·6 (11·1–14·0)	173·3 (158·2–189·5)	216·9 (209·1–224·8)	193·0 (180·9–204·8)	163·6 (145·1–182·3)	50·4 (43·5–57·9)	2·2 (1·5–3·2)	0·0 (0·0–0·0)	4·06 (3·9–4·22)	0·93 (0·85–1·01)	2·05 (1·93–2·17)
1951	0·0 (0·0–0·0)	10·1 (9·0–11·3)	155·8 (142·0–169·7)	201·3 (193·4–208·8)	175·1 (162·1–188·1)	133·3 (114·9–150·6)	39·1 (32·2–46·4)	1·6 (1·0–2·4)	0·0 (0·0–0·0)	3·58 (3·38–3·77)	0·83 (0·76–0·9)	1·75 (1·57–1·92)
1952	0·0 (0·0–0·0)	8·5 (7·4–9·7)	145·3 (130·5–161·9)	191·3 (182·8–199·8)	158·8 (144·1–172·9)	107·5 (90·6–125·3)	31·0 (25·2–37·4)	1·3 (0·8–1·9)	0·0 (0·0–0·0)	3·22 (3·05–3·38)	0·77 (0·69–0·85)	1·49 (1·36–1·62)
1953	0·0 (0·0–0·0)	7·5 (6·5–8·6)	137·6 (122·3–154·0)	180·2 (171·9–188·7)	142·1 (126·9–158·5)	86·2 (69·8–103·7)	24·6 (19·4–30·7)	1·0 (0·6–1·6)	0·0 (0·0–0·0)	2·9 (2·73–3·08)	0·73 (0·65–0·81)	1·27 (1·13–1·42)
1954	0·0 (0·0–0·0)	6·9 (5·9–7·9)	133·6 (117·6–149·7)	175·4 (167·8–183·5)	131·1 (114·6–147·7)	72·1 (55·6–90·6)	20·4 (15·4–26·3)	0·9 (0·5–1·3)	0·0 (0·0–0·0)	2·7 (2·52–2·91)	0·7 (0·62–0·78)	1·12 (0·97–1·28)
1955	0·0 (0·0–0·0)	6·2 (5·3–7·2)	130·7 (115·7–148·5)	174·4 (166·3–182·5)	120·8 (104·8–136·9)	56·9 (43·3–73·0)	17·0 (12·3–23·0)	0·7 (0·4–1·1)	0·0 (0·0–0·0)	2·53 (2·36–2·71)	0·68 (0·61–0·78)	0·98 (0·83–1·12)
1956	0·0 (0·0–0·0)	5·6 (4·7–6·6)	123·3 (107·4–140·0)	168·6 (161·2–176·4)	107·1 (92·8–122·5)	45·3 (33·8–59·6)	13·7 (9·7–19·0)	0·6 (0·3–0·9)	0·0 (0·0–0·0)	2·32 (2·16–2·51)	0·64 (0·57–0·73)	0·83 (0·71–0·98)
1957	0·0 (0·0–0·0)	5·3 (4·5–6·3)	124·8 (110·0–141·7)	172·2 (164·6–180·1)	101·4 (88·1–116·3)	42·8 (30·2–56·2)	11·7 (8·2–15·9)	0·6 (0·3–1·0)	0·0 (0·0–0·0)	2·29 (2·12–2·47)	0·65 (0·58–0·73)	0·78 (0·66–0·91)
1958	0·0 (0·0–0·0)	5·4 (4·6–6·3)	131·3 (114·7–148·2)	178·0 (169·3–186·9)	99·1 (86·2–113·7)	40·9 (30·7–54·2)	10·6 (7·3–14·7)	0·6 (0·3–1·0)	0·0 (0·0–0·0)	2·33 (2·18–2·48)	0·68 (0·6–0·77)	0·76 (0·65–0·88)
1959	0·0 (0·0–0·0)	5·6 (4·8–6·6)	131·5 (115·9–148·8)	177·1 (168·7–186·1)	92·7 (81·3–105·4)	33·2 (25·1–42·6)	8·8 (5·9–12·2)	0·5 (0·3–0·8)	0·0 (0·0–0·0)	2·25 (2·11–2·4)	0·69 (0·61–0·77)	0·68 (0·59–0·78)
1960	0·0 (0·0–0·0)	5·5 (4·6–6·4)	126·7 (112·5–142·6)	177·9 (169·0–186·5)	89·0 (79·5–99·4)	26·2 (20·5–33·2)	7·2 (4·8–10·0)	0·4 (0·2–0·7)	0·0 (0·0–0·0)	2·16 (2·05–2·28)	0·66 (0·59–0·74)	0·61 (0·54–0·69)
1961	0·0 (0·0–0·0)	5·1 (4·3–6·1)	123·4 (111·2–136·5)	179·9 (171·6–188·5)	87·0 (76·5–98·7)	22·4 (17·6–28·0)	6·2 (4·2–8·8)	0·3 (0·2–0·6)	0·0 (0·0–0·0)	2·12 (2·02–2·23)	0·64 (0·58–0·71)	0·58 (0·52–0·65)
1962	0·0 (0·0–0·0)	4·9 (4·1–5·8)	123·4 (109·5–137·4)	185·2 (177·7–192·8)	88·7 (81·1–96·2)	21·4 (17·3–26·1)	5·5 (3·8–7·9)	0·3 (0·2–0·5)	0·0 (0·0–0·0)	2·15 (2·06–2·24)	0·64 (0·57–0·71)	0·58 (0·53–0·63)
1963	0·0 (0·0–0·0)	4·8 (4·1–5·9)	124·3 (111·1–138·3)	187·5 (179·7–195·1)	90·4 (82·9–98·6)	24·0 (20·1–28·3)	5·4 (3·8–7·6)	0·3 (0·2–0·6)	0·0 (0·0–0·0)	2·18 (2·1–2·28)	0·65 (0·58–0·72)	0·6 (0·55–0·65)
1964	0·0 (0·0–0·0)	4·8 (4·1–5·7)	136·1 (123·0–151·7)	199·7 (190·9–208·9)	96·4 (87·3–106·6)	25·1 (21·2–29·1)	5·1 (3·6–7·2)	0·3 (0·2–0·6)	0·0 (0·0–0·0)	2·34 (2·24–2·44)	0·7 (0·64–0·78)	0·63 (0·58–0·69)
1965	0·0 (0·0–0·0)	4·7 (4·0–5·5)	125·7 (112·1–141·2)	168·5 (159·7–177·7)	80·0 (70·2–89·6)	19·6 (16·0–24·3)	4·4 (3·1–6·0)	0·2 (0·1–0·4)	0·0 (0·0–0·0)	2·02 (1·92–2·11)	0·65 (0·58–0·73)	0·52 (0·47–0·58)
1966	0·0 (0·0–0·0)	5·0 (4·3–5·8)	123·1 (109·8–138·0)	173·4 (164·6–182·2)	81·7 (71·4–93·4)	21·3 (17·4–25·8)	4·3 (3·1–5·9)	0·3 (0·1–0·5)	0·0 (0·0–0·0)	2·05 (1·95–2·14)	0·64 (0·57–0·72)	0·54 (0·48–0·6)
1967	0·0 (0·0–0·0)	5·3 (4·5–6·1)	131·6 (118·2–145·4)	211·3 (202·4–219·4)	98·3 (84·6–113·3)	22·8 (17·9–28·8)	4·2 (3·1–5·4)	0·2 (0·1–0·4)	0·0 (0·0–0·0)	2·37 (2·24–2·5)	0·68 (0·62–0·75)	0·63 (0·54–0·72)
1968	0·0 (0·0–0·0)	5·4 (4·7–6·3)	118·2 (105·3–132·2)	199·1 (190·9–206·9)	89·4 (76·2–103·1)	19·3 (14·4–25·3)	3·7 (2·8–4·9)	0·2 (0·1–0·4)	0·0 (0·0–0·0)	2·18 (2·08–2·28)	0·62 (0·55–0·69)	0·56 (0·48–0·65)
1969	0·0 (0·0–0·0)	5·9 (5·1–6·9)	116·6 (103·6–130·0)	201·4 (193·3–209·7)	89·4 (76·2–104·1)	20·5 (15·4–26·6)	3·7 (2·8–4·9)	0·2 (0·1–0·4)	0·0 (0·0–0·0)	2·19 (2·09–2·29)	0·61 (0·55–0·68)	0·57 (0·48–0·66)
1970	0·0 (0·0–0·0)	6·0 (5·0–7·1)	126·0 (111·6–141·8)	205·3 (196·5–213·8)	91·6 (79·1–105·8)	24·6 (18·2–32·2)	4·0 (2·9–5·3)	0·2 (0·1–0·4)	0·0 (0·0–0·0)	2·29 (2·17–2·4)	0·66 (0·59–0·74)	0·6 (0·52–0·7)
1971	0·0 (0·0–0·0)	5·9 (4·8–7·1)	138·4 (121·3–155·9)	203·0 (194·0–211·9)	92·6 (80·5–106·1)	27·9 (20·9–36·7)	4·3 (3·1–5·8)	0·2 (0·1–0·4)	0·0 (0·0–0·0)	2·36 (2·24–2·49)	0·72 (0·64–0·81)	0·63 (0·54–0·73)
1972	0·0 (0·0–0·0)	6·0 (4·9–7·3)	145·3 (128·9–163·1)	201·5 (192·5–210·5)	92·4 (81·1–104·5)	28·4 (21·4–37·4)	4·3 (3·0–5·9)	0·2 (0·1–0·5)	0·0 (0·0–0·0)	2·39 (2·27–2·52)	0·76 (0·67–0·84)	0·63 (0·55–0·71)
1973	0·0 (0·0–0·0)	5·9 (4·8–7·1)	146·0 (130·8–162·2)	201·4 (191·5–210·9)	87·6 (79·2–97·2)	26·0 (19·5–33·5)	3·9 (2·7–5·4)	0·2 (0·1–0·4)	0·0 (0·0–0·0)	2·36 (2·25–2·46)	0·76 (0·68–0·84)	0·59 (0·52–0·66)
1974	0·0 (0·0–0·0)	5·5 (4·5–6·7)	139·8 (125·8–156·4)	193·4 (185·0–201·9)	77·5 (69·6–85·5)	20·3 (15·8–26·2)	3·3 (2·1–5·0)	0·2 (0·1–0·3)	0·0 (0·0–0·0)	2·2 (2·11–2·3)	0·73 (0·66–0·81)	0·51 (0·46–0·56)
1975	0·0 (0·0–0·0)	5·1 (4·1–6·2)	130·6 (116·6–144·6)	186·3 (177·7–195·2)	69·9 (63·0–76·7)	15·8 (12·4–20·0)	2·8 (1·7–4·4)	0·1 (0·1–0·2)	0·0 (0·0–0·0)	2·05 (1·96–2·14)	0·68 (0·61–0·75)	0·44 (0·4–0·48)
1976	0·0 (0·0–0·0)	4·7 (3·9–5·7)	123·5 (110·5–138·1)	183·6 (175·0–192·5)	68·2 (62·0–75·4)	13·6 (10·9–16·7)	2·5 (1·6–3·6)	0·1 (0·0–0·2)	0·0 (0·0–0·0)	1·98 (1·89–2·08)	0·64 (0·58–0·71)	0·42 (0·38–0·46)
1977	0·0 (0·0–0·0)	4·5 (3·8–5·3)	116·8 (104·8–131·8)	183·9 (175·7–192·2)	70·5 (63·5–78·2)	12·8 (10·2–15·8)	2·2 (1·5–3·2)	0·1 (0·0–0·2)	0·0 (0·0–0·0)	1·95 (1·87–2·05)	0·61 (0·55–0·68)	0·43 (0·38–0·48)
1978	0·0 (0·0–0·0)	4·4 (3·5–5·4)	111·5 (100·6–123·6)	183·7 (175·8–191·5)	74·2 (67·1–81·6)	12·7 (9·7–16·2)	2·0 (1·3–2·8)	0·1 (0·0–0·1)	0·0 (0·0–0·0)	1·94 (1·86–2·02)	0·58 (0·53–0·64)	0·44 (0·41–0·49)
1979	0·0 (0·0–0·0)	4·4 (3·3–5·7)	106·6 (94·3–119·4)	180·8 (173·5–188·1)	74·3 (67·7–81·7)	12·4 (10·2–14·8)	1·8 (1·2–2·8)	0·1 (0·0–0·1)	0·0 (0·0–0·0)	1·9 (1·82–1·99)	0·55 (0·49–0·62)	0·44 (0·41–0·48)
1980	0·0 (0·0–0·0)	4·4 (3·5–5·4)	101·6 (89·7–114·0)	177·1 (168·6–185·7)	72·7 (66·0–80·0)	12·3 (9·8–15·4)	1·6 (1·1–2·3)	0·0 (0·0–0·1)	0·0 (0·0–0·0)	1·85 (1·76–1·93)	0·53 (0·47–0·59)	0·43 (0·4–0·47)
1981	0·0 (0·0–0·0)	4·5 (3·7–5·4)	98·3 (87·7–110·3)	177·2 (168·8–185·5)	73·4 (66·4–80·4)	13·0 (10·5–15·7)	1·6 (1·1–2·3)	0·0 (0·0–0·1)	0·0 (0·0–0·0)	1·84 (1·77–1·91)	0·51 (0·46–0·57)	0·44 (0·4–0·48)
1982	0·0 (0·0–0·0)	4·6 (3·8–5·6)	96·2 (84·5–109·0)	178·8 (171·7–186·4)	76·6 (69·3–84·4)	14·6 (11·9–17·6)	1·6 (1·1–2·2)	0·0 (0·0–0·1)	0·0 (0·0–0·0)	1·86 (1·79–1·95)	0·5 (0·44–0·57)	0·46 (0·43–0·51)
1983	0·0 (0·0–0·0)	4·6 (3·9–5·5)	92·9 (83·1–103·8)	179·6 (170·6–188·1)	80·5 (73·3–88·7)	16·3 (13·1–19·9)	1·6 (1·2–2·1)	0·0 (0·0–0·1)	0·0 (0·0–0·0)	1·88 (1·8–1·96)	0·49 (0·44–0·54)	0·49 (0·45–0·54)
1984	0·0 (0·0–0·0)	4·4 (3·7–5·3)	85·8 (74·6–98·6)	177·6 (170·3–185·6)	83·2 (75·6–91·1)	17·0 (13·9–21·1)	1·7 (1·2–2·3)	0·0 (0·0–0·1)	0·0 (0·0–0·0)	1·85 (1·76–1·94)	0·45 (0·39–0·51)	0·51 (0·46–0·56)
1985	0·0 (0·0–0·0)	4·2 (3·5–5·0)	80·3 (70·2–91·0)	172·7 (162·0–182·6)	84·1 (76·2–92·6)	17·2 (14·0–21·0)	1·8 (1·3–2·4)	0·0 (0·0–0·1)	0·0 (0·0–0·0)	1·8 (1·73–1·88)	0·42 (0·37–0·48)	0·52 (0·47–0·56)
1986	0·0 (0·0–0·0)	4·1 (3·4–4·8)	76·6 (66·9–86·4)	168·6 (161·3–176·7)	86·2 (78·9–93·5)	17·4 (14·2–20·9)	1·9 (1·4–2·7)	0·0 (0·0–0·1)	0·0 (0·0–0·0)	1·77 (1·7–1·85)	0·4 (0·36–0·45)	0·53 (0·49–0·57)
1987	0·0 (0·0–0·0)	4·0 (3·4–4·7)	71·3 (63·2–80·4)	165·7 (156·3–174·9)	89·3 (81·7–97·9)	18·2 (14·8–22·2)	2·2 (1·6–3·0)	0·0 (0·0–0·1)	0·0 (0·0–0·0)	1·75 (1·68–1·83)	0·38 (0·33–0·42)	0·55 (0·51–0·6)
1988	0·0 (0·0–0·0)	3·9 (3·3–4·6)	67·2 (58·1–77·3)	158·1 (150·5–166·4)	91·2 (82·9–99·5)	19·3 (15·5–23·3)	2·4 (1·7–3·2)	0·0 (0·0–0·1)	0·0 (0·0–0·0)	1·71 (1·64–1·79)	0·36 (0·31–0·41)	0·56 (0·52–0·61)
1989	0·0 (0·0–0·0)	3·9 (3·3–4·7)	63·6 (55·0–73·2)	149·8 (141·7–158·4)	92·2 (83·9–100·2)	20·3 (16·5–24·3)	2·5 (1·8–3·4)	0·0 (0·0–0·1)	0·0 (0·0–0·0)	1·66 (1·58–1·74)	0·34 (0·29–0·39)	0·57 (0·53–0·62)
1990	0·0 (0·0–0·0)	4·0 (3·4–4·7)	62·7 (54·8–70·4)	147·7 (140·1–155·5)	94·9 (86·5–103·2)	21·3 (17·4–25·8)	2·4 (1·8–3·3)	0·0 (0·0–0·1)	0·0 (0·0–0·0)	1·67 (1·6–1·74)	0·33 (0·29–0·37)	0·59 (0·55–0·64)
1991	0·0 (0·0–0·0)	4·1 (3·5–5·0)	63·6 (55·0–72·6)	144·3 (133·8–155·8)	96·6 (88·0–106·0)	22·6 (19·0–27·3)	2·4 (1·7–3·2)	0·0 (0·0–0·1)	0·0 (0·0–0·0)	1·67 (1·59–1·75)	0·34 (0·3–0·38)	0·61 (0·56–0·66)
1992	0·0 (0·0–0·0)	4·2 (3·5–5·0)	62·2 (54·9–70·4)	136·3 (128·9–143·6)	96·0 (88·5–104·2)	23·6 (19·3–28·1)	2·5 (1·8–3·4)	0·1 (0·0–0·1)	0·0 (0·0–0·0)	1·62 (1·56–1·69)	0·33 (0·3–0·37)	0·61 (0·57–0·66)
1993	0·0 (0·0–0·0)	4·3 (3·4–5·2)	62·3 (53·6–72·1)	135·5 (126·5–144·2)	100·1 (91·2–109·4)	25·8 (21·5–30·9)	2·6 (1·9–3·5)	0·1 (0·0–0·1)	0·0 (0·0–0·0)	1·65 (1·58–1·73)	0·33 (0·29–0·38)	0·64 (0·59–0·69)
1994	0·0 (0·0–0·0)	4·3 (3·5–5·2)	61·8 (54·4–70·7)	132·3 (124·3–139·8)	101·3 (92·6–110·7)	27·5 (22·8–33·3)	2·7 (2·0–3·6)	0·1 (0·0–0·1)	0·0 (0·0–0·0)	1·65 (1·57–1·73)	0·33 (0·29–0·38)	0·66 (0·6–0·72)
1995	0·0 (0·0–0·0)	4·3 (3·5–5·3)	60·6 (52·4–69·9)	124·4 (116·5–132·9)	99·8 (90·6–108·9)	28·8 (24·1–34·1)	2·9 (2·2–3·8)	0·1 (0·0–0·1)	0·0 (0·0–0·0)	1·6 (1·52–1·68)	0·32 (0·28–0·37)	0·66 (0·6–0·72)
1996	0·0 (0·0–0·0)	4·5 (3·6–5·5)	60·3 (52·3–68·9)	120·2 (111·9–128·7)	99·2 (91·0–108·3)	30·3 (25·2–35·9)	3·0 (2·3–4·0)	0·1 (0·0–0·1)	0·0 (0·0–0·0)	1·59 (1·5–1·67)	0·32 (0·28–0·37)	0·66 (0·61–0·72)
1997	0·0 (0·0–0·0)	4·8 (3·8–5·8)	60·6 (52·5–68·9)	116·8 (109·3–124·0)	97·1 (88·6–107·2)	31·1 (25·8–37·5)	3·2 (2·4–4·3)	0·1 (0·0–0·1)	0·0 (0·0–0·0)	1·57 (1·5–1·65)	0·33 (0·28–0·37)	0·66 (0·6–0·72)
1998	0·0 (0·0–0·0)	5·0 (4·0–6·2)	60·8 (52·9–69·5)	113·2 (105·5–120·6)	95·6 (87·1–105·4)	32·3 (26·7–39·1)	3·4 (2·5–4·4)	0·1 (0·0–0·1)	0·0 (0·0–0·0)	1·55 (1·48–1·64)	0·33 (0·29–0·37)	0·66 (0·61–0·71)
1999	0·0 (0·0–0·0)	5·4 (4·3–6·6)	60·9 (52·9–69·0)	110·7 (103·4–118·1)	94·3 (86·1–103·4)	32·8 (27·4–39·0)	3·5 (2·6–4·8)	0·1 (0·0–0·1)	0·0 (0·0–0·0)	1·54 (1·47–1·61)	0·33 (0·29–0·37)	0·65 (0·6–0·71)
2000	0·0 (0·0–0·0)	5·7 (4·6–7·1)	61·7 (52·6–71·3)	109·6 (102·7–116·8)	91·8 (83·6–100·7)	34·0 (28·4–40·6)	3·7 (2·7–5·0)	0·1 (0·0–0·1)	0·0 (0·0–0·0)	1·53 (1·45–1·62)	0·34 (0·29–0·38)	0·65 (0·6–0·7)
2001	0·0 (0·0–0·0)	6·0 (4·8–7·3)	62·1 (54·9–70·4)	107·5 (100·6–115·0)	88·0 (79·7–96·7)	33·5 (27·7–40·1)	3·8 (2·7–5·2)	0·1 (0·0–0·1)	0·0 (0·0–0·0)	1·5 (1·43–1·58)	0·34 (0·3–0·38)	0·63 (0·58–0·68)
2002	0·0 (0·0–0·0)	5·8 (4·8–7·0)	60·7 (53·0–69·6)	105·5 (98·9–112·2)	86·6 (78·9–95·4)	33·6 (28·3–39·9)	4·0 (2·8–5·7)	0·1 (0·0–0·1)	0·0 (0·0–0·0)	1·48 (1·4–1·56)	0·33 (0·29–0·38)	0·62 (0·57–0·68)
2003	0·0 (0·0–0·0)	5·5 (4·5–6·6)	58·3 (49·8–66·8)	103·4 (95·1–112·4)	85·3 (77·5–93·4)	34·5 (28·8–40·8)	4·3 (3·0–5·9)	0·1 (0·0–0·1)	0·0 (0·0–0·0)	1·46 (1·38–1·54)	0·32 (0·28–0·36)	0·62 (0·57–0·68)
2004	0·0 (0·0–0·0)	5·2 (4·3–6·1)	55·8 (48·6–64·4)	99·5 (93·7–105·7)	83·4 (76·0–90·9)	34·8 (29·0–41·4)	4·5 (3·1–6·0)	0·1 (0·0–0·2)	0·0 (0·0–0·0)	1·42 (1·34–1·49)	0·31 (0·27–0·35)	0·61 (0·56–0·67)
2005	0·0 (0·0–0·0)	4·9 (4·1–5·9)	54·8 (48·1–62·1)	98·3 (91·4–105·7)	83·1 (75·4–91·6)	35·1 (29·5–41·7)	4·8 (3·4–6·4)	0·1 (0·0–0·2)	0·0 (0·0–0·0)	1·41 (1·34–1·48)	0·3 (0·26–0·33)	0·62 (0·56–0·67)
2006	0·0 (0·0–0·0)	4·8 (4·0–5·7)	54·8 (46·9–63·3)	99·1 (92·7–105·8)	85·1 (77·4–92·9)	36·5 (30·5–43·4)	5·2 (3·8–7·1)	0·1 (0·0–0·2)	0·0 (0·0–0·0)	1·43 (1·36–1·5)	0·3 (0·26–0·34)	0·63 (0·58–0·69)
2007	0·0 (0·0–0·0)	4·7 (3·9–5·6)	53·9 (47·9–61·3)	98·2 (91·6–105·7)	84·7 (76·3–93·2)	38·1 (32·2–45·2)	5·6 (4·2–7·4)	0·1 (0·0–0·2)	0·0 (0·0–0·0)	1·43 (1·36–1·5)	0·29 (0·26–0·33)	0·64 (0·59–0·7)
2008	0·0 (0·0–0·0)	4·5 (3·8–5·4)	52·6 (45·8–60·4)	97·2 (91·1–104·0)	84·6 (76·8–92·3)	39·3 (32·9–46·3)	6·1 (4·5–8·0)	0·1 (0·1–0·2)	0·0 (0·0–0·0)	1·42 (1·36–1·49)	0·29 (0·25–0·32)	0·65 (0·6–0·7)
2009	0·0 (0·0–0·0)	4·3 (3·5–5·1)	50·5 (43·4–58·3)	96·5 (88·2–104·8)	84·9 (76·8–93·3)	40·5 (34·1–48·0)	6·6 (4·9–8·8)	0·1 (0·1–0·2)	0·0 (0·0–0·0)	1·42 (1·32–1·52)	0·27 (0·24–0·31)	0·66 (0·61–0·72)
2010	0·0 (0·0–0·0)	4·1 (3·4–4·8)	48·7 (42·0–55·6)	96·6 (90·8–102·5)	85·6 (78·3–93·9)	41·5 (35·4–48·3)	6·9 (5·1–8·9)	0·1 (0·1–0·2)	0·0 (0·0–0·0)	1·42 (1·35–1·49)	0·26 (0·23–0·3)	0·67 (0·62–0·73)
2011	0·0 (0·0–0·0)	4·0 (3·3–4·8)	46·3 (40·5–53·0)	95·7 (89·0–102·7)	85·7 (77·4–93·8)	42·3 (35·4–50·1)	7·1 (5·2–9·2)	0·1 (0·1–0·3)	0·0 (0·0–0·0)	1·41 (1·34–1·47)	0·25 (0·22–0·28)	0·68 (0·62–0·73)
2012	0·0 (0·0–0·0)	3·9 (3·2–4·8)	44·3 (38·9–50·1)	94·8 (87·8–102·1)	87·0 (78·9–95·7)	43·9 (37·4–51·2)	7·5 (5·6–9·7)	0·1 (0·1–0·3)	0·0 (0·0–0·0)	1·41 (1·34–1·49)	0·24 (0·21–0·27)	0·69 (0·64–0·75)
2013	0·0 (0·0–0·0)	3·9 (3·2–4·6)	43·0 (35·5–51·0)	93·2 (86·8–99·7)	87·3 (79·9–96·2)	44·3 (37·6–51·4)	7·8 (5·9–10·3)	0·2 (0·1–0·3)	0·0 (0·0–0·0)	1·4 (1·32–1·48)	0·23 (0·2–0·27)	0·7 (0·65–0·76)
2014	0·0 (0·0–0·0)	3·8 (3·1–4·5)	41·6 (36·0–47·5)	92·6 (85·8–99·9)	88·3 (80·7–97·0)	45·2 (38·8–52·4)	8·1 (5·9–10·8)	0·2 (0·1–0·3)	0·0 (0·0–0·0)	1·4 (1·32–1·49)	0·23 (0·2–0·26)	0·71 (0·65–0·78)
2015	0·0 (0·0–0·0)	3·8 (3·1–4·6)	42·4 (35·1–50·8)	94·3 (86·3–103·1)	90·4 (81·4–100·3)	43·1 (35·2–52·2)	7·5 (5·3–10·4)	0·2 (0·1–0·3)	0·0 (0·0–0·0)	1·41 (1·25–1·58)	0·23 (0·19–0·28)	0·71 (0·62–0·8)
2016	0·0 (0·0–0·0)	3·9 (3·2–4·7)	44·2 (33·5–57·8)	95·8 (85·6–107·7)	90·1 (77·7–104·5)	39·5 (30·3–51·3)	6·5 (4·3–9·2)	0·1 (0·1–0·3)	0·0 (0·0–0·0)	1·4 (1·19–1·66)	0·24 (0·18–0·31)	0·68 (0·57–0·81)
2017	0·0 (0·0–0·0)	3·8 (3·2–4·7)	44·1 (32·8–58·0)	95·6 (84·8–107·8)	89·0 (75·8–103·8)	38·3 (28·3–50·7)	6·4 (4·1–9·6)	0·1 (0·1–0·3)	0·0 (0·0–0·0)	1·39 (1·16–1·65)	0·24 (0·18–0·31)	0·67 (0·55–0·82)

Supplemental Results Table 5: Age-specific fertility rates per 1000, cumulative total fertility up to maternal age 25 (TFU25), cumulative total fertility from maternal age 30 to age 55 (TFO30), and cumulative total fertility, Kagawa, 1950-2017

Year	10-14	15-19	20-24	25-29	30-34	35-39	40-44	45-49	50-54	TFR	TFU25	TFO30
1950	0·0 (0·0–0·0)	11·7 (10·3–13·0)	168·1 (153·3–183·7)	224·1 (215·8–232·4)	156·2 (147·0–165·5)	107·4 (95·5–119·3)	33·6 (29·0–38·6)	1·2 (0·8–1·7)	0·0 (0·0–0·0)	3·51 (3·39–3·64)	0·9 (0·83–0·98)	1·49 (1·41–1·57)
1951	0·0 (0·0–0·0)	9·4 (8·3–10·6)	151·0 (136·3–166·2)	208·3 (199·5–216·8)	141·1 (131·3–151·0)	86·7 (75·5–98·3)	26·1 (21·6–30·5)	0·8 (0·5–1·3)	0·0 (0·0–0·0)	3·12 (2·97–3·27)	0·8 (0·73–0·88)	1·27 (1·16–1·39)
1952	0·0 (0·0–0·0)	7·9 (6·9–9·1)	140·8 (126·8–158·0)	198·0 (188·2–208·0)	127·6 (117·6–137·6)	69·5 (59·9–80·6)	20·6 (17·0–25·1)	0·7 (0·4–1·0)	0·0 (0·0–0·0)	2·83 (2·71–2·94)	0·74 (0·67–0·83)	1·09 (1·02–1·17)
1953	0·0 (0·0–0·0)	6·9 (5·9–8·1)	133·1 (118·2–149·2)	186·7 (175·7–198·1)	113·9 (103·7–123·7)	55·4 (47·2–64·7)	16·4 (12·9–20·6)	0·5 (0·3–0·8)	0·0 (0·0–0·0)	2·56 (2·45–2·69)	0·7 (0·63–0·78)	0·93 (0·86–1·0)
1954	0·0 (0·0–0·0)	6·4 (5·4–7·4)	129·2 (113·0–146·7)	181·7 (169·3–194·9)	104·9 (95·8–114·6)	46·1 (38·4–54·9)	13·5 (10·4–17·4)	0·4 (0·3–0·7)	0·0 (0·0–0·0)	2·41 (2·29–2·53)	0·68 (0·6–0·77)	0·82 (0·76–0·9)
1955	0·0 (0·0–0·0)	5·7 (4·8–6·7)	126·1 (110·0–143·8)	180·6 (167·8–194·4)	96·4 (87·4–105·7)	36·3 (30·3–42·9)	11·2 (8·4–14·4)	0·3 (0·2–0·6)	0·0 (0·0–0·0)	2·28 (2·18–2·4)	0·66 (0·58–0·75)	0·72 (0·66–0·78)
1956	0·0 (0·0–0·0)	5·1 (4·3–6·1)	118·9 (102·6–136·1)	174·6 (161·9–189·0)	85·3 (77·0–94·4)	28·7 (23·7–34·1)	9·0 (6·7–11·7)	0·3 (0·2–0·4)	0·0 (0·0–0·0)	2·11 (2·0–2·23)	0·62 (0·54–0·71)	0·62 (0·56–0·68)
1957	0·0 (0·0–0·0)	4·9 (4·1–5·8)	120·2 (104·6–138·8)	178·1 (164·3–193·0)	80·8 (73·0–89·2)	27·0 (22·3–31·5)	7·6 (5·8–9·8)	0·3 (0·2–0·4)	0·0 (0·0–0·0)	2·09 (1·97–2·23)	0·63 (0·55–0·72)	0·58 (0·52–0·63)
1958	0·0 (0·0–0·0)	4·9 (4·1–5·8)	126·4 (110·2–143·8)	184·0 (169·7–199·3)	79·0 (71·0–87·9)	25·8 (21·2–30·7)	6·9 (5·2–9·1)	0·3 (0·2–0·5)	0·0 (0·0–0·0)	2·14 (2·02–2·26)	0·66 (0·58–0·74)	0·56 (0·5–0·62)
1959	0·0 (0·0–0·0)	5·1 (4·3–6·0)	126·5 (110·5–144·6)	182·9 (170·4–195·3)	73·9 (65·5–82·6)	20·8 (16·8–25·5)	5·7 (4·3–7·5)	0·2 (0·1–0·4)	0·0 (0·0–0·0)	2·08 (1·97–2·19)	0·66 (0·58–0·75)	0·5 (0·45–0·56)
1960	0·0 (0·0–0·0)	5·0 (4·3–5·9)	121·7 (107·1–137·1)	183·7 (172·3–195·6)	70·8 (63·5–78·7)	16·6 (13·5–20·1)	4·7 (3·5–6·1)	0·2 (0·1–0·3)	0·0 (0·0–0·0)	2·01 (1·91–2·12)	0·63 (0·56–0·71)	0·46 (0·42–0·51)
1961	0·0 (0·0–0·0)	4·7 (3·9–5·5)	118·3 (106·3–130·9)	185·6 (176·4–196·4)	69·1 (62·6–76·2)	14·4 (11·5–17·9)	4·0 (2·9–5·5)	0·2 (0·1–0·3)	0·0 (0·0–0·0)	1·98 (1·9–2·07)	0·61 (0·56–0·68)	0·44 (0·4–0·48)
1962	0·0 (0·0–0·0)	4·4 (3·7–5·3)	118·1 (104·8–131·2)	191·0 (182·5–199·5)	70·5 (63·9–77·2)	13·5 (11·2–15·9)	3·6 (2·5–4·9)	0·1 (0·1–0·2)	0·0 (0·0–0·0)	2·01 (1·92–2·09)	0·61 (0·55–0·68)	0·44 (0·4–0·48)
1963	0·0 (0·0–0·0)	4·4 (3·6–5·2)	118·8 (105·7–132·3)	193·0 (185·2–200·8)	71·8 (65·8–78·5)	15·0 (12·0–18·8)	3·5 (2·5–4·8)	0·2 (0·1–0·3)	0·0 (0·0–0·0)	2·03 (1·94–2·12)	0·62 (0·55–0·68)	0·45 (0·42–0·49)
1964	0·0 (0·0–0·0)	4·3 (3·6–5·1)	129·9 (115·5–145·7)	205·5 (195·6–215·3)	76·5 (69·7–83·5)	15·5 (13·0–18·1)	3·3 (2·2–4·6)	0·1 (0·1–0·2)	0·0 (0·0–0·0)	2·18 (2·07–2·28)	0·67 (0·6–0·75)	0·48 (0·44–0·52)
1965	0·0 (0·0–0·0)	4·2 (3·6–5·0)	119·8 (104·8–137·1)	173·4 (160·3–186·1)	63·4 (56·9–70·1)	12·3 (10·4–14·5)	2·8 (2·0–4·0)	0·1 (0·1–0·2)	0·0 (0·0–0·0)	1·88 (1·76–2·01)	0·62 (0·54–0·71)	0·39 (0·36–0·43)
1966	0·0 (0·0–0·0)	4·5 (3·8–5·3)	117·3 (99·5–137·0)	178·3 (166·1–192·0)	64·7 (58·1–71·4)	13·4 (11·2–16·1)	2·8 (1·9–3·9)	0·1 (0·1–0·2)	0·0 (0·0–0·0)	1·91 (1·78–2·04)	0·61 (0·52–0·71)	0·4 (0·37–0·44)
1967	0·0 (0·0–0·0)	4·7 (4·0–5·5)	125·3 (104·6–147·3)	217·1 (200·7–235·2)	77·7 (69·4–86·4)	15·1 (12·2–18·2)	2·7 (1·9–3·6)	0·1 (0·1–0·2)	0·0 (0·0–0·0)	2·21 (2·03–2·4)	0·65 (0·55–0·76)	0·48 (0·43–0·53)
1968	0·0 (0·0–0·0)	4·8 (4·2–5·6)	112·5 (93·5–134·4)	204·5 (187·1–222·7)	70·5 (62·9–78·8)	13·7 (11·0–16·7)	2·4 (1·7–3·2)	0·1 (0·1–0·1)	0·0 (0·0–0·0)	2·04 (1·89–2·21)	0·59 (0·49–0·69)	0·43 (0·39–0·47)
1969	0·0 (0·0–0·0)	5·2 (4·5–6·2)	110·8 (93·1–130·8)	206·4 (190·5–224·1)	70·4 (63·6–77·7)	13·3 (10·6–16·5)	2·4 (1·8–3·1)	0·1 (0·1–0·1)	0·0 (0·0–0·0)	2·04 (1·9–2·2)	0·58 (0·49–0·68)	0·43 (0·39–0·48)
1970	0·0 (0·0–0·0)	5·3 (4·5–6·2)	119·4 (101·4–139·8)	209·6 (194·0–226·5)	72·3 (65·0–80·2)	14·6 (11·6–18·4)	2·5 (1·9–3·3)	0·1 (0·1–0·1)	0·0 (0·0–0·0)	2·12 (1·98–2·27)	0·62 (0·53–0·73)	0·45 (0·41–0·49)
1971	0·0 (0·0–0·0)	5·2 (4·3–6·2)	130·9 (111·2–152·4)	206·5 (193·2–220·4)	73·0 (65·1–81·2)	16·5 (13·2–20·6)	2·7 (2·0–3·7)	0·1 (0·1–0·2)	0·0 (0·0–0·0)	2·17 (2·03–2·32)	0·68 (0·58–0·79)	0·46 (0·41–0·51)
1972	0·0 (0·0–0·0)	5·2 (4·3–6·3)	137·2 (119·3–157·2)	204·3 (191·8–216·1)	72·8 (64·4–81·5)	16·6 (12·9–20·7)	2·7 (2·0–3·6)	0·1 (0·1–0·2)	0·0 (0·0–0·0)	2·2 (2·08–2·32)	0·71 (0·62–0·82)	0·46 (0·41–0·52)
1973	0·0 (0·0–0·0)	5·0 (4·0–6·2)	137·6 (120·9–154·1)	203·6 (192·2–215·1)	68·8 (62·1–75·9)	15·1 (11·9–18·8)	2·4 (1·7–3·3)	0·1 (0·1–0·1)	0·0 (0·0–0·0)	2·16 (2·06–2·27)	0·71 (0·63–0·8)	0·43 (0·39–0·48)
1974	0·0 (0·0–0·0)	4·7 (3·8–5·8)	131·5 (117·6–147·4)	194·7 (185·8–204·2)	60·6 (54·1–67·8)	11·6 (8·9–14·8)	2·0 (1·4–2·8)	0·1 (0·0–0·1)	0·0 (0·0–0·0)	2·03 (1·94–2·12)	0·68 (0·61–0·76)	0·37 (0·34–0·41)
1975	0·0 (0·0–0·0)	4·3 (3·5–5·4)	122·4 (107·8–137·6)	186·8 (178·2–196·0)	54·4 (48·4–59·9)	9·5 (7·2–12·3)	1·7 (1·2–2·4)	0·1 (0·0–0·1)	0·0 (0·0–0·0)	1·9 (1·81–1·99)	0·63 (0·56–0·71)	0·33 (0·29–0·36)
1976	0·0 (0·0–0·0)	4·1 (3·2–5·0)	114·9 (102·4–129·1)	183·5 (174·6–192·3)	52·9 (47·5–58·9)	9·2 (7·1–11·6)	1·5 (0·9–2·4)	0·0 (0·0–0·1)	0·0 (0·0–0·0)	1·83 (1·74–1·93)	0·6 (0·53–0·67)	0·32 (0·28–0·36)
1977	0·0 (0·0–0·0)	4·0 (3·2–4·9)	108·0 (97·4–120·7)	184·1 (175·6–192·6)	54·2 (48·3–60·3)	9·3 (7·1–12·2)	1·4 (0·9–2·0)	0·0 (0·0–0·1)	0·0 (0·0–0·0)	1·81 (1·73–1·89)	0·56 (0·51–0·62)	0·32 (0·29–0·36)
1978	0·0 (0·0–0·0)	4·0 (3·2–4·9)	102·8 (89·6–118·4)	185·3 (176·7–193·6)	56·4 (50·5–63·0)	9·3 (7·0–12·4)	1·2 (0·7–1·8)	0·0 (0·0–0·1)	0·0 (0·0–0·0)	1·8 (1·71–1·9)	0·53 (0·47–0·61)	0·33 (0·3–0·37)
1979	0·0 (0·0–0·0)	4·1 (3·4–4·8)	98·0 (87·0–108·9)	184·6 (176·7–192·5)	56·2 (50·8–62·0)	9·2 (7·2–11·6)	1·1 (0·7–1·8)	0·0 (0·0–0·0)	0·0 (0·0–0·0)	1·77 (1·69–1·84)	0·51 (0·45–0·56)	0·33 (0·3–0·36)
1980	0·0 (0·0–0·0)	4·2 (3·3–5·2)	93·4 (82·2–104·3)	183·4 (173·8–192·8)	55·7 (49·9–61·8)	9·3 (7·2–11·9)	1·1 (0·7–1·6)	0·0 (0·0–0·0)	0·0 (0·0–0·0)	1·74 (1·66–1·82)	0·49 (0·43–0·54)	0·33 (0·3–0·36)
1981	0·0 (0·0–0·0)	4·4 (3·5–5·3)	90·6 (81·1–101·8)	185·9 (177·1–195·3)	57·1 (51·6–63·3)	9·9 (7·7–12·4)	1·1 (0·8–1·6)	0·0 (0·0–0·0)	0·0 (0·0–0·0)	1·75 (1·68–1·81)	0·47 (0·43–0·53)	0·34 (0·31–0·37)
1982	0·0 (0·0–0·0)	4·6 (3·6–5·6)	89·0 (78·3–100·6)	189·4 (181·4–198·1)	60·3 (54·5–66·6)	11·1 (8·8–13·9)	1·2 (0·8–1·7)	0·0 (0·0–0·0)	0·0 (0·0–0·0)	1·78 (1·71–1·85)	0·47 (0·41–0·53)	0·36 (0·33–0·4)
1983	0·0 (0·0–0·0)	4·7 (3·8–5·8)	86·7 (76·7–97·9)	191·7 (181·9–200·8)	63·8 (57·8–70·9)	12·3 (9·7–15·3)	1·3 (0·9–1·7)	0·0 (0·0–0·0)	0·0 (0·0–0·0)	1·8 (1·72–1·88)	0·46 (0·41–0·51)	0·39 (0·35–0·43)
1984	0·0 (0·0–0·0)	4·5 (3·7–5·4)	80·8 (71·3–90·7)	190·8 (181·7–199·8)	66·5 (59·9–73·6)	12·7 (9·9–15·9)	1·3 (0·9–1·8)	0·0 (0·0–0·0)	0·0 (0·0–0·0)	1·78 (1·72–1·85)	0·43 (0·38–0·48)	0·4 (0·36–0·44)
1985	0·0 (0·0–0·0)	4·4 (3·5–5·3)	76·6 (67·1–87·4)	186·0 (177·1–195·5)	67·9 (60·9–74·9)	12·6 (9·6–15·6)	1·4 (1·0–1·8)	0·0 (0·0–0·0)	0·0 (0·0–0·0)	1·74 (1·67–1·83)	0·4 (0·36–0·46)	0·41 (0·37–0·45)
1986	0·0 (0·0–0·0)	4·2 (3·4–5·2)	73·7 (64·4–83·4)	181·5 (172·0–191·7)	70·2 (63·9–77·7)	12·7 (9·9–15·9)	1·4 (1·1–1·8)	0·0 (0·0–0·0)	0·0 (0·0–0·0)	1·72 (1·65–1·8)	0·39 (0·34–0·44)	0·42 (0·38–0·46)
1987	0·0 (0·0–0·0)	4·2 (3·4–5·0)	68·8 (61·0–77·4)	177·1 (168·9–185·5)	73·2 (66·4–81·2)	13·2 (10·4–16·9)	1·6 (1·1–2·3)	0·0 (0·0–0·1)	0·0 (0·0–0·0)	1·69 (1·61–1·77)	0·36 (0·33–0·41)	0·44 (0·4–0·49)
1988	0·0 (0·0–0·0)	4·1 (3·3–5·0)	64·4 (55·7–74·4)	167·2 (156·8–178·0)	74·9 (67·8–82·5)	14·0 (10·9–17·3)	1·8 (1·2–2·5)	0·0 (0·0–0·1)	0·0 (0·0–0·0)	1·63 (1·54–1·72)	0·34 (0·3–0·39)	0·45 (0·41–0·5)
1989	0·0 (0·0–0·0)	4·1 (3·3–5·0)	59·9 (51·8–68·8)	156·1 (147·4–165·1)	75·7 (68·6–83·2)	14·8 (11·7–18·6)	1·9 (1·3–2·7)	0·0 (0·0–0·1)	0·0 (0·0–0·0)	1·56 (1·48–1·65)	0·32 (0·28–0·36)	0·46 (0·42–0·5)
1990	0·0 (0·0–0·0)	4·2 (3·4–5·2)	57·9 (50·6–66·0)	151·9 (143·4–160·3)	78·0 (71·3–85·3)	15·7 (12·2–19·6)	1·9 (1·3–2·7)	0·0 (0·0–0·1)	0·0 (0·0–0·0)	1·55 (1·48–1·63)	0·31 (0·27–0·35)	0·48 (0·44–0·52)
1991	0·0 (0·0–0·0)	4·4 (3·6–5·4)	57·4 (49·6–65·7)	147·0 (139·0–155·9)	79·7 (72·4–87·9)	16·7 (13·0–20·9)	2·0 (1·3–2·7)	0·0 (0·0–0·1)	0·0 (0·0–0·0)	1·54 (1·47–1·61)	0·31 (0·27–0·35)	0·49 (0·45–0·54)
1992	0·0 (0·0–0·0)	4·5 (3·6–5·6)	55·3 (48·9–63·2)	138·5 (129·5–147·2)	80·0 (73·1–87·6)	17·5 (13·7–22·4)	2·1 (1·4–2·9)	0·0 (0·0–0·1)	0·0 (0·0–0·0)	1·49 (1·42–1·56)	0·3 (0·27–0·34)	0·5 (0·46–0·54)
1993	0·0 (0·0–0·0)	4·6 (3·7–5·7)	54·8 (46·6–63·0)	138·0 (129·7–146·5)	84·4 (76·4–92·7)	19·1 (14·9–23·8)	2·2 (1·5–3·1)	0·0 (0·0–0·1)	0·0 (0·0–0·0)	1·52 (1·43–1·6)	0·3 (0·26–0·34)	0·53 (0·48–0·58)
1994	0·0 (0·0–0·0)	4·7 (3·8–5·8)	54·1 (47·2–62·1)	135·6 (125·9–145·1)	87·0 (78·6–95·6)	20·5 (16·1–25·8)	2·3 (1·6–3·3)	0·0 (0·0–0·1)	0·0 (0·0–0·0)	1·52 (1·43–1·61)	0·29 (0·26–0·33)	0·55 (0·5–0·6)
1995	0·0 (0·0–0·0)	4·9 (4·0–5·9)	52·9 (45·4–61·2)	128·4 (120·4–137·5)	87·4 (78·7–95·6)	21·4 (17·0–26·3)	2·4 (1·7–3·5)	0·0 (0·0–0·1)	0·0 (0·0–0·0)	1·49 (1·4–1·58)	0·29 (0·25–0·33)	0·56 (0·51–0·6)
1996	0·0 (0·0–0·0)	5·1 (4·2–6·3)	52·8 (45·6–60·7)	125·1 (116·4–134·1)	88·7 (80·6–97·7)	22·8 (18·2–28·2)	2·5 (1·8–3·5)	0·0 (0·0–0·1)	0·0 (0·0–0·0)	1·49 (1·4–1·58)	0·29 (0·25–0·33)	0·57 (0·52–0·62)
1997	0·0 (0·0–0·0)	5·5 (4·5–6·6)	53·2 (46·0–61·2)	122·3 (114·4–129·7)	89·0 (80·8–97·7)	23·6 (19·0–29·1)	2·7 (1·9–3·7)	0·0 (0·0–0·1)	0·0 (0·0–0·0)	1·48 (1·4–1·57)	0·29 (0·26–0·33)	0·58 (0·53–0·63)
1998	0·0 (0·0–0·0)	5·9 (4·9–7·0)	53·3 (46·3–61·6)	118·9 (111·1–127·0)	90·0 (81·3–99·4)	24·9 (20·4–30·5)	2·8 (2·0–3·8)	0·0 (0·0–0·1)	0·0 (0·0–0·0)	1·48 (1·39–1·56)	0·3 (0·26–0·34)	0·59 (0·54–0·64)
1999	0·0 (0·0–0·0)	6·3 (5·2–7·5)	53·4 (46·6–60·7)	116·0 (108·4–123·7)	91·3 (83·1–100·1)	26·0 (21·3–31·4)	2·9 (2·1–4·0)	0·0 (0·0–0·1)	0·0 (0·0–0·0)	1·48 (1·4–1·56)	0·3 (0·26–0·34)	0·6 (0·55–0·65)
2000	0·0 (0·0–0·0)	6·7 (5·6–8·0)	54·0 (45·2–63·3)	114·1 (106·9–122·6)	91·3 (82·8–100·7)	27·7 (23·0–33·5)	3·1 (2·2–4·3)	0·1 (0·0–0·1)	0·0 (0·0–0·0)	1·49 (1·41–1·57)	0·3 (0·26–0·35)	0·61 (0·56–0·67)
2001	0·0 (0·0–0·0)	7·1 (5·8–8·6)	54·4 (48·0–61·3)	111·2 (103·8–119·3)	90·0 (81·5–98·7)	28·3 (23·1–34·2)	3·3 (2·3–4·5)	0·1 (0·0–0·1)	0·0 (0·0–0·0)	1·47 (1·4–1·54)	0·31 (0·27–0·34)	0·61 (0·56–0·66)
2002	0·0 (0·0–0·0)	7·0 (5·8–8·6)	53·3 (46·1–61·8)	108·6 (101·6–115·6)	90·8 (82·1–99·7)	29·3 (24·2–35·0)	3·5 (2·5–4·9)	0·1 (0·0–0·1)	0·0 (0·0–0·0)	1·46 (1·39–1·54)	0·3 (0·27–0·34)	0·62 (0·57–0·67)
2003	0·0 (0·0–0·0)	6·8 (5·4–8·4)	51·4 (44·9–59·3)	106·1 (97·2–115·3)	91·4 (84·0–98·8)	31·2 (25·5–37·3)	3·8 (2·6–5·3)	0·1 (0·0–0·1)	0·0 (0·0–0·0)	1·45 (1·36–1·55)	0·29 (0·26–0·33)	0·63 (0·58–0·69)
2004	0·0 (0·0–0·0)	6·5 (5·2–8·0)	49·8 (43·1–58·4)	102·0 (95·9–108·6)	90·8 (81·2–101·0)	32·4 (27·1–38·7)	4·0 (2·8–5·5)	0·1 (0·0–0·1)	0·0 (0·0–0·0)	1·43 (1·35–1·51)	0·28 (0·24–0·32)	0·64 (0·58–0·7)
2005	0·0 (0·0–0·0)	6·4 (5·1–7·9)	49·7 (43·2–57·2)	101·1 (93·9–108·8)	91·5 (82·9–100·2)	33·6 (28·1–40·2)	4·2 (2·9–5·8)	0·1 (0·0–0·1)	0·0 (0·0–0·0)	1·43 (1·36–1·52)	0·28 (0·25–0·32)	0·65 (0·59–0·71)
2006	0·0 (0·0–0·0)	6·4 (5·1–8·1)	50·7 (44·2–58·0)	102·5 (95·9–109·7)	94·4 (85·5–103·5)	35·6 (29·6–42·7)	4·6 (3·2–6·2)	0·1 (0·0–0·1)	0·0 (0·0–0·0)	1·47 (1·4–1·55)	0·29 (0·25–0·32)	0·67 (0·62–0·74)
2007	0·0 (0·0–0·0)	6·5 (5·1–8·0)	51·2 (42·7–60·8)	102·4 (95·5–110·0)	94·8 (86·8–103·8)	37·8 (31·4–44·6)	4·9 (3·5–6·8)	0·1 (0·0–0·1)	0·0 (0·0–0·0)	1·49 (1·4–1·58)	0·29 (0·24–0·34)	0·69 (0·63–0·75)
2008	0·0 (0·0–0·0)	6·4 (5·1–7·9)	50·9 (44·2–57·7)	102·3 (96·0–109·6)	95·5 (86·9–104·8)	39·6 (33·1–46·6)	5·3 (3·8–7·6)	0·1 (0·0–0·1)	0·0 (0·0–0·0)	1·5 (1·43–1·58)	0·29 (0·25–0·32)	0·7 (0·64–0·77)
2009	0·0 (0·0–0·0)	6·2 (5·0–7·6)	49·9 (41·5–59·0)	102·4 (93·4–111·2)	96·7 (88·2–105·8)	41·6 (35·2–48·7)	5·8 (4·0–8·0)	0·1 (0·0–0·2)	0·0 (0·0–0·0)	1·51 (1·41–1·62)	0·28 (0·24–0·32)	0·72 (0·66–0·78)
2010	0·0 (0·0–0·0)	6·0 (4·9–7·5)	48·9 (42·7–55·5)	103·1 (96·9–109·6)	98·7 (90·1–108·3)	43·5 (36·9–50·7)	6·1 (4·4–8·4)	0·1 (0·1–0·2)	0·0 (0·0–0·0)	1·53 (1·46–1·61)	0·27 (0·24–0·31)	0·74 (0·68–0·8)
2011	0·0 (0·0–0·0)	5·9 (4·7–7·3)	47·0 (41·1–53·8)	102·7 (95·4–110·2)	99·7 (91·3–108·9)	45·2 (38·2–53·3)	6·4 (4·6–8·7)	0·1 (0·1–0·2)	0·0 (0·0–0·0)	1·53 (1·46–1·62)	0·26 (0·23–0·3)	0·76 (0·69–0·82)
2012	0·0 (0·0–0·0)	5·8 (4·9–6·8)	45·1 (39·2–51·9)	102·1 (94·5–110·2)	101·9 (92·7–111·7)	47·6 (40·3–55·9)	6·9 (5·0–9·2)	0·1 (0·1–0·2)	0·0 (0·0–0·0)	1·55 (1·47–1·63)	0·25 (0·22–0·29)	0·78 (0·72–0·85)
2013	0·0 (0·0–0·0)	5·7 (4·6–6·8)	43·8 (37·4–50·4)	100·6 (93·6–107·8)	102·6 (93·4–112·3)	48·6 (41·3–56·3)	7·2 (5·1–9·7)	0·1 (0·1–0·2)	0·0 (0·0–0·0)	1·54 (1·46–1·63)	0·25 (0·22–0·28)	0·79 (0·73–0·86)
2014	0·0 (0·0–0·0)	5·6 (4·6–6·7)	42·5 (36·4–49·6)	100·2 (93·1–108·0)	103·9 (95·2–113·5)	49·8 (42·2–58·3)	7·6 (5·6–10·3)	0·1 (0·1–0·2)	0·0 (0·0–0·0)	1·55 (1·47–1·64)	0·24 (0·21–0·28)	0·81 (0·75–0·88)
2015	0·0 (0·0–0·0)	5·6 (4·6–6·8)	43·4 (33·1–56·3)	102·2 (93·8–111·3)	106·1 (95·1–118·2)	47·6 (38·5–58·2)	7·0 (4·9–9·6)	0·1 (0·1–0·2)	0·0 (0·0–0·0)	1·56 (1·38–1·77)	0·25 (0·19–0·31)	0·8 (0·71–0·91)
2016	0·0 (0·0–0·0)	5·7 (4·7–6·9)	44·9 (34·5–58·9)	103·6 (92·2–116·5)	105·2 (91·1–122·0)	43·3 (32·8–56·5)	6·0 (4·0–8·5)	0·1 (0·1–0·2)	0·0 (0·0–0·0)	1·54 (1·31–1·81)	0·25 (0·2–0·33)	0·77 (0·65–0·92)
2017	0·0 (0·0–0·0)	5·6 (4·6–6·8)	44·7 (32·9–59·7)	103·4 (91·7–116·6)	103·1 (87·8–120·1)	41·7 (30·5–55·2)	5·9 (3·7–8·8)	0·1 (0·1–0·2)	0·0 (0·0–0·0)	1·52 (1·27–1·81)	0·25 (0·19–0·33)	0·75 (0·62–0·91)

Supplemental Results Table 5: Age-specific fertility rates per 1000, cumulative total fertility up to maternal age 25 (TFU25), cumulative total fertility from maternal age 30 to age 55 (TFO30), and cumulative total fertility, Kagoshima, 1950-2017

Year	10-14	15-19	20-24	25-29	30-34	35-39	40-44	45-49	50-54	TFR	TFU25	TFO30
1950	0·0 (0·0–0·0)	9·7 (8·6–10·9)	150·0 (136·9–164·1)	229·6 (221·0–238·2)	198·9 (186·4–211·0)	124·6 (110·7–138·6)	51·4 (44·3–59·0)	2·2 (1·5–3·2)	0·0 (0·0–0·0)	3·83 (3·69–3·97)	0·8 (0·73–0·87)	1·88 (1·78–1·99)
1951	0·0 (0·0–0·0)	7·8 (6·9–8·8)	134·5 (122·3–146·5)	213·6 (205·5–221·9)	180·5 (167·7–193·4)	101·1 (88·5–115·0)	39·9 (33·2–46·9)	1·6 (1·0–2·4)	0·0 (0·0–0·0)	3·39 (3·22–3·56)	0·71 (0·65–0·77)	1·62 (1·47–1·77)
1952	0·0 (0·0–0·0)	6·6 (5·8–7·5)	125·1 (111·9–139·2)	203·3 (194·5–212·0)	163·7 (148·8–178·5)	82·2 (71·0–95·2)	31·6 (25·5–38·5)	1·2 (0·8–1·8)	0·0 (0·0–0·0)	3·07 (2·95–3·19)	0·66 (0·59–0·73)	1·39 (1·31–1·48)
1953	0·0 (0·0–0·0)	5·8 (5·0–6·7)	118·2 (104·9–132·6)	191·7 (183·3–201·0)	146·7 (130·9–162·5)	69·8 (58·6–81·8)	25·1 (19·4–32·5)	1·0 (0·6–1·6)	0·0 (0·0–0·0)	2·79 (2·67–2·93)	0·62 (0·55–0·69)	1·21 (1·13–1·3)
1954	0·0 (0·0–0·0)	5·3 (4·5–6·1)	114·6 (100·8–128·5)	186·7 (178·4–195·4)	135·5 (118·7–152·0)	63·6 (53·1–74·9)	20·8 (15·3–28·0)	0·8 (0·5–1·3)	0·0 (0·0–0·0)	2·64 (2·51–2·76)	0·6 (0·53–0·67)	1·1 (1·02–1·19)
1955	0·0 (0·0–0·0)	4·8 (4·1–5·6)	111·8 (98·8–127·2)	185·7 (176·7–194·5)	124·9 (108·4–141·2)	57·2 (48·3–66·8)	17·4 (12·3–23·7)	0·7 (0·4–1·0)	0·0 (0·0–0·0)	2·51 (2·4–2·63)	0·58 (0·52–0·66)	1·0 (0·92–1·08)
1956	0·0 (0·0–0·0)	4·3 (3·6–5·0)	105·2 (91·6–119·5)	179·5 (171·8–187·8)	110·8 (96·0–126·7)	49·1 (41·3–57·6)	13·9 (9·2–20·0)	0·5 (0·3–0·8)	0·0 (0·0–0·0)	2·32 (2·21–2·44)	0·55 (0·48–0·62)	0·87 (0·79–0·95)
1957	0·0 (0·0–0·0)	4·0 (3·4–4·8)	106·1 (92·8–121·4)	182·9 (174·4–191·9)	104·9 (91·2–120·4)	42·1 (35·1–49·3)	11·8 (7·8–16·9)	0·5 (0·3–0·8)	0·0 (0·0–0·0)	2·26 (2·13–2·4)	0·55 (0·48–0·63)	0·8 (0·72–0·87)
1958	0·0 (0·0–0·0)	4·1 (3·4–4·8)	111·3 (98·1–125·6)	188·8 (180·1–197·6)	102·5 (88·8–118·3)	37·2 (31·0–44·4)	10·6 (7·0–15·1)	0·5 (0·3–0·9)	0·0 (0·0–0·0)	2·28 (2·16–2·39)	0·58 (0·51–0·65)	0·75 (0·68–0·83)
1959	0·0 (0·0–0·0)	4·3 (3·6–5·0)	111·2 (98·1–125·1)	187·9 (179·3–196·9)	96·1 (84·5–109·5)	32·1 (26·4–38·6)	8·8 (5·7–12·9)	0·5 (0·3–0·7)	0·0 (0·0–0·0)	2·2 (2·1–2·31)	0·58 (0·51–0·65)	0·69 (0·63–0·76)
1960	0·0 (0·0–0·0)	4·2 (3·5–4·8)	106·7 (95·3–119·7)	188·9 (179·8–198·3)	92·4 (82·4–103·6)	28·3 (23·5–33·6)	7·2 (4·6–10·4)	0·4 (0·2–0·6)	0·0 (0·0–0·0)	2·14 (2·04–2·24)	0·55 (0·5–0·62)	0·64 (0·58–0·7)
1961	0·0 (0·0–0·0)	3·9 (3·2–4·6)	103·5 (93·4–114·3)	190·9 (182·8–199·8)	90·5 (80·3–102·7)	25·5 (21·5–30·6)	6·1 (4·0–8·6)	0·3 (0·2–0·5)	0·0 (0·0–0·0)	2·1 (2·01–2·21)	0·54 (0·49–0·59)	0·61 (0·55–0·68)
1962	0·0 (0·0–0·0)	3·7 (3·0–4·4)	103·1 (91·8–114·8)	196·4 (188·2–204·5)	92·5 (84·1–101·6)	24·3 (20·1–28·6)	5·4 (3·8–7·6)	0·3 (0·2–0·4)	0·0 (0·0–0·0)	2·13 (2·04–2·21)	0·53 (0·48–0·59)	0·61 (0·57–0·66)
1963	0·0 (0·0–0·0)	3·6 (3·0–4·3)	103·3 (92·2–115·1)	198·2 (189·8–206·7)	94·4 (86·4–103·0)	23·5 (19·9–27·5)	5·3 (3·6–7·3)	0·3 (0·2–0·5)	0·0 (0·0–0·0)	2·14 (2·06–2·23)	0·53 (0·48–0·59)	0·62 (0·57–0·66)
1964	0·0 (0·0–0·0)	3·6 (3·0–4·2)	112·6 (101·4–125·6)	211·0 (201·8–220·3)	101·0 (91·4–111·1)	24·4 (20·4–28·6)	4·9 (3·6–6·7)	0·3 (0·2–0·4)	0·0 (0·0–0·0)	2·29 (2·2–2·39)	0·58 (0·53–0·65)	0·65 (0·6–0·71)
1965	0·0 (0·0–0·0)	3·5 (2·9–4·1)	103·4 (92·4–115·9)	178·2 (168·6–187·2)	84·3 (74·8–94·5)	22·5 (18·6–26·9)	4·2 (3·1–5·6)	0·2 (0·1–0·3)	0·0 (0·0–0·0)	1·98 (1·89–2·08)	0·53 (0·48–0·6)	0·56 (0·51–0·61)
1966	0·0 (0·0–0·0)	3·7 (3·1–4·3)	100·7 (89·9–113·0)	183·2 (173·0–192·6)	86·5 (75·7–97·9)	23·1 (18·7–28·1)	4·1 (3·1–5·4)	0·2 (0·1–0·3)	0·0 (0·0–0·0)	2·01 (1·92–2·1)	0·52 (0·47–0·58)	0·57 (0·52–0·62)
1967	0·0 (0·0–0·0)	3·8 (3·3–4·5)	107·0 (96·0–118·8)	223·2 (213·9–232·0)	104·7 (90·2–120·1)	26·4 (21·5–32·8)	3·9 (3·0–5·1)	0·2 (0·1–0·3)	0·0 (0·0–0·0)	2·35 (2·23–2·46)	0·55 (0·5–0·61)	0·68 (0·6–0·75)
1968	0·0 (0·0–0·0)	3·9 (3·4–4·5)	95·5 (84·7–106·9)	210·7 (201·6–220·0)	96·9 (82·9–111·3)	26·4 (21·1–32·6)	3·6 (2·6–4·9)	0·2 (0·1–0·3)	0·0 (0·0–0·0)	2·19 (2·1–2·28)	0·5 (0·44–0·56)	0·64 (0·56–0·71)
1969	0·0 (0·0–0·0)	4·2 (3·6–5·0)	93·5 (82·6–104·5)	213·0 (204·5–221·4)	97·6 (83·4–113·4)	26·0 (21·5–31·2)	3·6 (2·6–4·9)	0·2 (0·1–0·2)	0·0 (0·0–0·0)	2·19 (2·11–2·27)	0·49 (0·43–0·54)	0·64 (0·58–0·7)
1970	0·0 (0·0–0·0)	4·3 (3·6–5·0)	100·3 (89·2–113·7)	216·4 (207·5–225·2)	99·7 (86·8–114·5)	24·9 (20·4–30·2)	3·7 (2·6–5·1)	0·2 (0·1–0·3)	0·0 (0·0–0·0)	2·25 (2·16–2·34)	0·52 (0·47–0·59)	0·64 (0·58–0·71)
1971	0·0 (0·0–0·0)	4·1 (3·4–4·9)	109·4 (95·3–124·2)	213·5 (204·3–223·2)	101·4 (88·8–115·7)	22·8 (18·8–27·6)	4·0 (2·7–5·7)	0·2 (0·1–0·3)	0·0 (0·0–0·0)	2·28 (2·18–2·38)	0·57 (0·5–0·64)	0·64 (0·58–0·71)
1972	0·0 (0·0–0·0)	4·2 (3·4–5·1)	114·1 (100·8–128·5)	211·9 (202·5–221·6)	102·0 (90·1–114·2)	21·7 (17·4–26·1)	4·0 (2·7–5·6)	0·2 (0·1–0·3)	0·0 (0·0–0·0)	2·29 (2·2–2·39)	0·59 (0·52–0·66)	0·64 (0·58–0·7)
1973	0·0 (0·0–0·0)	4·0 (3·3–4·9)	113·9 (101·2–126·6)	212·1 (202·1–223·1)	97·6 (88·3–107·8)	21·4 (17·4–26·4)	3·7 (2·4–5·4)	0·2 (0·1–0·2)	0·0 (0·0–0·0)	2·26 (2·17–2·36)	0·59 (0·53–0·65)	0·61 (0·56–0·67)
1974	0·0 (0·0–0·0)	3·8 (3·0–4·7)	108·6 (97·1–121·6)	204·3 (194·9–214·7)	87·3 (78·7–95·5)	20·1 (16·2–24·3)	3·1 (2·1–4·5)	0·1 (0·1–0·2)	0·0 (0·0–0·0)	2·14 (2·05–2·23)	0·56 (0·51–0·63)	0·55 (0·51–0·6)
1975	0·0 (0·0–0·0)	3·5 (2·8–4·4)	101·5 (90·8–113·5)	197·6 (188·4–206·8)	78·7 (71·0–86·3)	18·9 (15·5–22·9)	2·6 (1·8–3·6)	0·1 (0·1–0·1)	0·0 (0·0–0·0)	2·01 (1·93–2·1)	0·53 (0·47–0·58)	0·5 (0·45–0·55)
1976	0·0 (0·0–0·0)	3·4 (2·7–4·1)	96·5 (85·3–107·4)	195·5 (186·6–204·9)	77·2 (70·0–84·6)	18·6 (13·8–24·3)	2·4 (1·6–3·5)	0·1 (0·0–0·1)	0·0 (0·0–0·0)	1·97 (1·88–2·06)	0·5 (0·45–0·55)	0·49 (0·45–0·54)
1977	0·0 (0·0–0·0)	3·3 (2·7–4·0)	92·2 (82·3–104·5)	196·2 (187·7–204·6)	80·6 (72·6–88·9)	18·0 (14·3–22·2)	2·4 (1·6–3·6)	0·1 (0·1–0·1)	0·0 (0·0–0·0)	1·96 (1·88–2·06)	0·48 (0·43–0·54)	0·51 (0·45–0·57)
1978	0·0 (0·0–0·0)	3·4 (2·7–4·1)	89·3 (80·4–98·9)	196·2 (187·7–204·6)	84·8 (76·6–93·4)	17·3 (14·8–20·2)	2·4 (1·4–3·6)	0·1 (0·0–0·1)	0·0 (0·0–0·0)	1·97 (1·88–2·04)	0·46 (0·42–0·51)	0·52 (0·48–0·57)
1979	0·0 (0·0–0·0)	3·4 (2·8–4·0)	86·3 (76·8–97·0)	193·3 (184·7–201·7)	85·0 (77·5–93·1)	16·6 (13·0–20·7)	2·3 (1·6–3·3)	0·1 (0·0–0·1)	0·0 (0·0–0·0)	1·93 (1·85–2·02)	0·45 (0·4–0·5)	0·52 (0·48–0·56)
1980	0·0 (0·0–0·0)	3·4 (2·8–4·2)	83·0 (74·3–91·8)	189·4 (180·3–198·5)	83·0 (75·4–91·4)	16·1 (12·7–19·9)	2·3 (1·7–3·3)	0·1 (0·0–0·1)	0·0 (0·0–0·0)	1·89 (1·81–1·97)	0·43 (0·39–0·48)	0·51 (0·47–0·55)
1981	0·0 (0·0–0·0)	3·5 (2·8–4·3)	81·0 (70·8–93·1)	189·3 (180·0–198·2)	83·3 (75·7–91·1)	16·7 (13·3–20·4)	2·4 (1·6–3·4)	0·1 (0·0–0·1)	0·0 (0·0–0·0)	1·88 (1·8–1·95)	0·42 (0·37–0·48)	0·51 (0·47–0·55)
1982	0·0 (0·0–0·0)	3·6 (2·9–4·5)	79·4 (70·4–89·5)	190·2 (182·2–198·3)	86·1 (78·5–95·0)	18·4 (15·3–22·1)	2·3 (1·7–3·3)	0·1 (0·0–0·1)	0·0 (0·0–0·0)	1·9 (1·83–1·98)	0·42 (0·37–0·47)	0·53 (0·49–0·58)
1983	0·0 (0·0–0·0)	3·7 (2·9–4·5)	77·0 (68·9–85·8)	190·6 (181·4–199·9)	89·2 (81·2–97·7)	20·1 (16·2–24·6)	2·4 (1·8–3·1)	0·1 (0·0–0·1)	0·0 (0·0–0·0)	1·92 (1·84–2·0)	0·4 (0·36–0·45)	0·56 (0·51–0·61)
1984	0·0 (0·0–0·0)	3·5 (2·8–4·3)	71·4 (61·1–83·5)	188·2 (179·3–198·3)	91·0 (83·0–99·2)	20·5 (16·9–24·7)	2·5 (1·8–3·3)	0·1 (0·0–0·1)	0·0 (0·0–0·0)	1·89 (1·81–1·97)	0·37 (0·32–0·44)	0·57 (0·53–0·62)
1985	0·0 (0·0–0·0)	3·4 (2·7–4·1)	66·9 (58·8–75·4)	183·2 (174·8–191·0)	91·4 (82·8–99·8)	20·1 (16·4–24·2)	2·5 (1·8–3·3)	0·1 (0·0–0·1)	0·0 (0·0–0·0)	1·84 (1·77–1·9)	0·35 (0·31–0·39)	0·57 (0·52–0·62)
1986	0·0 (0·0–0·0)	3·3 (2·6–4·0)	64·0 (55·7–72·4)	180·0 (171·3–189·4)	93·4 (85·3–101·2)	19·9 (16·7–23·8)	2·6 (1·7–3·7)	0·1 (0·0–0·1)	0·0 (0·0–0·0)	1·82 (1·75–1·88)	0·34 (0·3–0·38)	0·58 (0·53–0·62)
1987	0·0 (0·0–0·0)	3·1 (2·5–3·8)	59·6 (52·7–67·2)	177·5 (169·0–186·0)	96·3 (88·4–105·3)	20·4 (16·7–24·6)	2·8 (1·9–4·0)	0·1 (0·0–0·1)	0·0 (0·0–0·0)	1·8 (1·72–1·88)	0·31 (0·28–0·35)	0·6 (0·55–0·65)
1988	0·0 (0·0–0·0)	3·0 (2·5–3·7)	55·7 (48·6–63·4)	168·9 (159·9–179·3)	97·9 (89·3–107·3)	21·2 (17·5–25·5)	3·0 (2·2–4·1)	0·1 (0·0–0·1)	0·0 (0·0–0·0)	1·75 (1·67–1·84)	0·29 (0·26–0·33)	0·61 (0·56–0·66)
1989	0·0 (0·0–0·0)	3·1 (2·5–3·8)	52·0 (44·7–60·4)	158·7 (150·9–167·2)	98·8 (90·3–107·5)	22·2 (18·3–26·4)	3·2 (2·3–4·3)	0·1 (0·1–0·1)	0·0 (0·0–0·0)	1·69 (1·62–1·76)	0·28 (0·24–0·32)	0·62 (0·57–0·67)
1990	0·0 (0·0–0·0)	3·2 (2·6–3·8)	50·5 (44·0–57·1)	154·9 (146·0–163·1)	101·8 (93·3–110·5)	23·1 (19·0–27·5)	3·2 (2·3–4·2)	0·1 (0·1–0·1)	0·0 (0·0–0·0)	1·68 (1·61–1·75)	0·27 (0·23–0·3)	0·64 (0·59–0·69)
1991	0·0 (0·0–0·0)	3·3 (2·7–4·0)	50·5 (43·4–58·0)	150·3 (142·7–158·7)	103·9 (94·9–114·2)	24·5 (19·5–30·2)	3·2 (2·3–4·2)	0·1 (0·1–0·1)	0·0 (0·0–0·0)	1·68 (1·61–1·75)	0·27 (0·23–0·31)	0·66 (0·6–0·72)
1992	0·0 (0·0–0·0)	3·4 (2·8–4·1)	49·0 (42·9–56·0)	141·8 (133·6–150·2)	103·7 (95·8–112·8)	25·3 (21·4–30·0)	3·3 (2·4–4·4)	0·1 (0·1–0·1)	0·0 (0·0–0·0)	1·63 (1·58–1·69)	0·26 (0·23–0·3)	0·66 (0·62–0·71)
1993	0·0 (0·0–0·0)	3·5 (2·8–4·2)	49·0 (42·2–55·9)	141·2 (132·5–150·2)	108·5 (99·7–118·8)	27·5 (23·3–32·6)	3·4 (2·5–4·6)	0·1 (0·1–0·1)	0·0 (0·0–0·0)	1·67 (1·59–1·75)	0·26 (0·23–0·3)	0·7 (0·64–0·76)
1994	0·0 (0·0–0·0)	3·6 (2·9–4·4)	48·7 (42·5–55·7)	137·9 (130·3–145·3)	110·1 (100·3–120·6)	29·2 (24·2–34·5)	3·6 (2·6–4·8)	0·1 (0·1–0·1)	0·0 (0·0–0·0)	1·67 (1·58–1·75)	0·26 (0·23–0·3)	0·71 (0·66–0·77)
1995	0·0 (0·0–0·0)	3·7 (3·0–4·5)	47·9 (40·9–54·8)	129·7 (121·5–138·0)	108·4 (98·8–118·3)	30·3 (25·6–35·7)	3·8 (2·8–4·9)	0·1 (0·1–0·1)	0·0 (0·0–0·0)	1·62 (1·54–1·7)	0·26 (0·22–0·29)	0·71 (0·66–0·77)
1996	0·0 (0·0–0·0)	3·9 (3·2–4·7)	47·9 (40·8–55·1)	125·0 (117·0–133·4)	107·5 (98·7–117·0)	31·9 (26·3–37·7)	3·9 (2·9–5·1)	0·1 (0·1–0·1)	0·0 (0·0–0·0)	1·6 (1·52–1·68)	0·26 (0·22–0·3)	0·72 (0·67–0·77)
1997	0·0 (0·0–0·0)	4·2 (3·5–5·1)	48·3 (40·9–55·9)	121·2 (113·9–129·0)	104·9 (96·4–114·5)	32·7 (27·1–38·7)	4·1 (3·1–5·6)	0·1 (0·1–0·1)	0·0 (0·0–0·0)	1·58 (1·51–1·66)	0·26 (0·23–0·3)	0·71 (0·66–0·77)
1998	0·0 (0·0–0·0)	4·5 (3·7–5·4)	48·6 (41·8–55·8)	117·5 (109·5–125·0)	103·2 (94·2–112·9)	34·0 (28·1–40·6)	4·3 (3·2–5·7)	0·1 (0·1–0·2)	0·0 (0·0–0·0)	1·56 (1·49–1·64)	0·27 (0·23–0·3)	0·71 (0·66–0·76)
1999	0·0 (0·0–0·0)	4·9 (4·1–5·9)	49·2 (41·8–57·2)	114·8 (107·2–122·0)	102·2 (93·5–111·7)	34·7 (28·7–41·2)	4·5 (3·2–6·2)	0·1 (0·1–0·1)	0·0 (0·0–0·0)	1·55 (1·47–1·64)	0·27 (0·23–0·31)	0·71 (0·64–0·77)
2000	0·0 (0·0–0·0)	5·3 (4·4–6·3)	50·1 (44·7–56·1)	113·4 (106·4–120·9)	100·4 (91·8–109·5)	36·3 (30·8–42·5)	4·7 (3·3–6·4)	0·1 (0·1–0·1)	0·0 (0·0–0·0)	1·55 (1·48–1·63)	0·28 (0·25–0·31)	0·71 (0·66–0·76)
2001	0·0 (0·0–0·0)	5·6 (4·6–6·8)	51·0 (43·9–58·5)	111·2 (104·1–118·6)	97·6 (89·2–106·4)	36·3 (30·5–43·0)	4·8 (3·4–6·7)	0·1 (0·1–0·1)	0·0 (0·0–0·0)	1·53 (1·45–1·62)	0·28 (0·25–0·32)	0·69 (0·64–0·75)
2002	0·0 (0·0–0·0)	5·6 (4·7–6·9)	50·5 (43·8–57·6)	109·2 (102·0–116·8)	97·2 (88·7–106·6)	36·8 (31·2–43·0)	5·0 (3·6–6·9)	0·1 (0·1–0·2)	0·0 (0·0–0·0)	1·52 (1·44–1·61)	0·28 (0·25–0·32)	0·7 (0·64–0·75)
2003	0·0 (0·0–0·0)	5·5 (4·4–6·7)	49·4 (41·8–57·8)	107·3 (100·9–114·4)	96·6 (88·3–105·6)	38·2 (31·9–44·9)	5·3 (3·7–7·3)	0·1 (0·1–0·2)	0·0 (0·0–0·0)	1·51 (1·43–1·61)	0·27 (0·24–0·32)	0·7 (0·64–0·77)
2004	0·0 (0·0–0·0)	5·3 (4·3–6·5)	48·5 (42·7–55·6)	103·7 (96·6–111·5)	95·1 (86·8–103·5)	38·9 (32·7–45·9)	5·5 (4·0–7·3)	0·1 (0·1–0·2)	0·0 (0·0–0·0)	1·49 (1·41–1·57)	0·27 (0·24–0·31)	0·7 (0·65–0·76)
2005	0·0 (0·0–0·0)	5·2 (4·2–6·4)	48·9 (42·8–55·3)	103·0 (95·7–110·4)	95·7 (86·8–105·0)	39·6 (33·3–47·1)	5·7 (4·1–7·6)	0·1 (0·1–0·2)	0·0 (0·0–0·0)	1·49 (1·42–1·56)	0·27 (0·24–0·3)	0·71 (0·65–0·77)
2006	0·0 (0·0–0·0)	5·2 (4·2–6·5)	50·2 (43·4–57·7)	104·5 (97·4–112·0)	99·1 (90·6–108·5)	41·5 (35·0–49·1)	6·2 (4·6–8·3)	0·1 (0·1–0·2)	0·0 (0·0–0·0)	1·53 (1·47–1·61)	0·28 (0·24–0·31)	0·73 (0·68–0·8)
2007	0·0 (0·0–0·0)	5·3 (4·2–6·6)	50·6 (44·9–57·2)	104·5 (97·4–112·2)	99·9 (91·0–109·2)	43·6 (36·9–51·3)	6·6 (5·0–8·7)	0·1 (0·1–0·2)	0·0 (0·0–0·0)	1·55 (1·48–1·63)	0·28 (0·25–0·31)	0·75 (0·69–0·81)
2008	0·0 (0·0–0·0)	5·2 (4·2–6·4)	50·5 (43·6–57·9)	104·6 (97·4–111·4)	100·8 (92·3–109·4)	45·2 (38·1–52·9)	7·1 (5·3–9·3)	0·1 (0·1–0·2)	0·0 (0·0–0·0)	1·57 (1·5–1·65)	0·28 (0·24–0·32)	0·77 (0·71–0·82)
2009	0·0 (0·0–0·0)	5·1 (4·0–6·2)	49·4 (44·2–54·8)	105·2 (98·8–111·2)	101·7 (93·4–111·4)	46·9 (39·8–54·6)	7·7 (5·9–9·9)	0·1 (0·1–0·2)	0·0 (0·0–0·0)	1·58 (1·49–1·67)	0·27 (0·25–0·3)	0·78 (0·73–0·85)
2010	0·0 (0·0–0·0)	4·9 (4·0–6·1)	48·5 (41·1–56·6)	106·8 (99·6–114·5)	103·0 (94·1–112·8)	48·4 (41·4–56·0)	8·0 (6·1–10·5)	0·1 (0·1–0·2)	0·0 (0·0–0·0)	1·6 (1·52–1·69)	0·27 (0·23–0·31)	0·8 (0·74–0·86)
2011	0·0 (0·0–0·0)	4·8 (3·9–5·9)	46·4 (40·1–53·5)	106·9 (99·5–114·2)	103·3 (94·8–112·7)	49·6 (42·2–57·4)	8·3 (6·3–10·7)	0·1 (0·1–0·2)	0·0 (0·0–0·0)	1·6 (1·52–1·67)	0·26 (0·22–0·29)	0·81 (0·75–0·87)
2012	0·0 (0·0–0·0)	4·8 (4·0–5·7)	44·4 (38·2–51·7)	106·8 (99·9–114·6)	104·7 (95·4–114·1)	51·7 (44·4–60·3)	8·9 (6·8–11·3)	0·2 (0·1–0·3)	0·0 (0·0–0·0)	1·61 (1·53–1·68)	0·25 (0·22–0·28)	0·83 (0·77–0·89)
2013	0·0 (0·0–0·0)	4·7 (3·9–5·7)	43·1 (35·7–51·0)	105·4 (98·1–113·7)	104·8 (95·5–114·1)	52·6 (45·0–60·6)	9·4 (7·1–12·0)	0·2 (0·1–0·3)	0·0 (0·0–0·0)	1·6 (1·52–1·68)	0·24 (0·2–0·28)	0·83 (0·77–0·9)
2014	0·0 (0·0–0·0)	4·6 (3·8–5·6)	41·5 (35·3–48·2)	104·6 (97·6–112·3)	105·9 (97·3–115·3)	54·1 (46·4–62·5)	9·9 (7·3–13·0)	0·2 (0·1–0·3)	0·0 (0·0–0·0)	1·6 (1·51–1·7)	0·23 (0·2–0·26)	0·85 (0·79–0·92)
2015	0·0 (0·0–0·0)	4·6 (3·8–5·6)	42·0 (34·8–50·5)	106·3 (97·7–115·4)	108·4 (98·2–120·2)	52·0 (43·0–62·6)	9·2 (6·6–12·2)	0·2 (0·1–0·2)	0·0 (0·0–0·0)	1·61 (1·45–1·81)	0·23 (0·19–0·28)	0·85 (0·75–0·96)
2016	0·0 (0·0–0·0)	4·6 (3·8–5·7)	43·3 (33·1–56·6)	107·5 (95·9–121·1)	107·7 (93·4–125·1)	47·6 (36·5–61·0)	7·9 (5·5–11·1)	0·2 (0·1–0·2)	0·0 (0·0–0·0)	1·59 (1·36–1·88)	0·24 (0·19–0·31)	0·82 (0·69–0·97)
2017	0·0 (0·0–0·0)	4·6 (3·8–5·6)	43·0 (32·1–56·3)	107·1 (95·1–120·3)	105·9 (90·2–122·9)	46·1 (34·2–61·4)	7·8 (5·1–11·6)	0·2 (0·1–0·2)	0·0 (0·0–0·0)	1·57 (1·32–1·86)	0·24 (0·18–0·31)	0·8 (0·65–0·98)

Supplemental Results Table 5: Age-specific fertility rates per 1000, cumulative total fertility up to maternal age 25 (TFU25), cumulative total fertility from maternal age 30 to age 55 (TFO30), and cumulative total fertility, Kanagawa, 1950-2017

Year	10-14	15-19	20-24	25-29	30-34	35-39	40-44	45-49	50-54	TFR	TFU25	TFO30
1950	0·0 (0·0–0·0)	11·6 (10·2–12·9)	127·3 (116·2–139·2)	217·8 (210·0–225·7)	158·7 (149·3–168·1)	67·0 (59·5–74·4)	21·1 (18·3–24·3)	0·9 (0·6–1·4)	0·0 (0·0–0·0)	3·02 (2·92–3·13)	0·69 (0·64–0·76)	1·24 (1·18–1·3)
1951	0·0 (0·0–0·0)	9·3 (8·2–10·5)	114·0 (102·9–124·6)	202·3 (194·6–209·5)	143·6 (133·8–153·1)	65·6 (56·6–73·8)	16·4 (13·7–19·5)	0·7 (0·4–1·0)	0·0 (0·0–0·0)	2·76 (2·64–2·87)	0·62 (0·56–0·67)	1·13 (1·03–1·23)
1952	0·0 (0·0–0·0)	7·8 (6·9–8·9)	105·8 (95·1–117·1)	192·0 (183·9–200·2)	130·0 (119·9–140·4)	65·0 (54·7–76·0)	13·0 (10·6–15·7)	0·5 (0·3–0·8)	0·0 (0·0–0·0)	2·57 (2·48–2·66)	0·57 (0·51–0·63)	1·04 (0·98–1·11)
1953	0·0 (0·0–0·0)	6·8 (6·0–7·9)	99·7 (88·3–111·4)	180·7 (172·8–188·9)	116·2 (106·0–126·9)	60·0 (48·6–73·5)	10·3 (8·1–12·9)	0·4 (0·3–0·6)	0·0 (0·0–0·0)	2·37 (2·28–2·47)	0·53 (0·48–0·59)	0·93 (0·87–1·0)
1954	0·0 (0·0–0·0)	6·3 (5·3–7·2)	96·3 (84·9–107·8)	175·6 (168·4–183·8)	108·0 (98·7–118·7)	54·9 (42·8–69·2)	8·7 (6·7–10·9)	0·4 (0·2–0·6)	0·0 (0·0–0·0)	2·25 (2·16–2·34)	0·51 (0·46–0·57)	0·86 (0·8–0·93)
1955	0·0 (0·0–0·0)	5·6 (4·9–6·6)	93·8 (81·9–106·3)	174·3 (166·2–182·4)	103·4 (93·7–113·7)	49·6 (38·4–63·8)	8·3 (6·3–10·6)	0·3 (0·2–0·5)	0·0 (0·0–0·0)	2·18 (2·08–2·27)	0·5 (0·43–0·56)	0·81 (0·75–0·87)
1956	0·0 (0·0–0·0)	5·0 (4·3–5·9)	88·1 (77·2–99·0)	168·3 (160·9–176·1)	94·1 (85·1–104·0)	42·8 (31·7–56·1)	7·2 (5·6–9·1)	0·3 (0·2–0·5)	0·0 (0·0–0·0)	2·03 (1·95–2·12)	0·47 (0·41–0·52)	0·72 (0·67–0·78)
1957	0·0 (0·0–0·0)	4·8 (4·1–5·7)	88·8 (78·5–101·6)	171·4 (164·0–179·3)	86·9 (78·8–95·5)	36·8 (26·6–48·1)	5·6 (4·3–7·0)	0·3 (0·2–0·4)	0·0 (0·0–0·0)	1·97 (1·87–2·07)	0·47 (0·42–0·53)	0·65 (0·6–0·7)
1958	0·0 (0·0–0·0)	4·8 (4·1–5·6)	93·2 (81·9–106·3)	176·9 (168·7–185·6)	83·2 (75·7–91·5)	32·5 (24·8–42·7)	4·6 (3·6–5·9)	0·2 (0·2–0·4)	0·0 (0·0–0·0)	1·98 (1·9–2·07)	0·49 (0·43–0·56)	0·6 (0·56–0·65)
1959	0·0 (0·0–0·0)	5·0 (4·3–5·9)	93·1 (82·4–104·8)	175·9 (167·7–184·3)	79·8 (71·2–88·4)	28·1 (21·9–35·7)	4·1 (3·1–5·3)	0·2 (0·1–0·4)	0·0 (0·0–0·0)	1·93 (1·85–2·02)	0·49 (0·44–0·55)	0·56 (0·52–0·61)
1960	0·0 (0·0–0·0)	4·9 (4·2–5·7)	89·6 (79·9–100·7)	176·6 (168·5–184·6)	79·6 (71·7–87·3)	24·8 (19·3–31·4)	3·8 (2·9–4·9)	0·2 (0·1–0·3)	0·0 (0·0–0·0)	1·9 (1·82–1·97)	0·47 (0·42–0·53)	0·54 (0·5–0·58)
1961	0·0 (0·0–0·0)	4·6 (3·9–5·3)	87·1 (78·0–96·5)	178·6 (170·6–186·7)	79·4 (73·1–86·1)	22·3 (18·2–27·3)	3·4 (2·6–4·6)	0·2 (0·1–0·3)	0·0 (0·0–0·0)	1·88 (1·8–1·96)	0·46 (0·41–0·51)	0·53 (0·48–0·57)
1962	0·0 (0·0–0·0)	4·4 (3·7–5·1)	87·1 (77·7–96·9)	183·8 (176·3–191·3)	81·5 (74·9–88·4)	21·1 (17·6–25·1)	3·0 (2·3–4·0)	0·2 (0·1–0·3)	0·0 (0·0–0·0)	1·91 (1·83–1·98)	0·46 (0·41–0·51)	0·53 (0·49–0·57)
1963	0·0 (0·0–0·0)	4·3 (3·6–5·1)	87·8 (78·5–97·8)	185·9 (178·3–193·6)	80·4 (73·3–88·3)	20·4 (17·4–24·0)	2·6 (1·9–3·3)	0·2 (0·1–0·2)	0·0 (0·0–0·0)	1·91 (1·83–1·99)	0·46 (0·41–0·51)	0·52 (0·48–0·56)
1964	0·0 (0·0–0·0)	4·3 (3·6–5·0)	96·1 (86·4–107·2)	198·0 (190·3–206·1)	86·2 (78·3–94·6)	21·2 (17·9–24·9)	2·4 (1·8–3·1)	0·1 (0·1–0·2)	0·0 (0·0–0·0)	2·04 (1·96–2·12)	0·5 (0·45–0·56)	0·55 (0·51–0·59)
1965	0·0 (0·0–0·0)	4·2 (3·6–4·9)	88·8 (79·6–99·3)	167·1 (159·1–175·2)	75·8 (68·9–82·4)	19·6 (15·4–24·8)	2·4 (1·8–3·2)	0·1 (0·1–0·2)	0·0 (0·0–0·0)	1·79 (1·71–1·87)	0·47 (0·42–0·52)	0·49 (0·45–0·53)
1966	0·0 (0·0–0·0)	4·5 (3·8–5·2)	87·2 (77·4–97·4)	172·0 (164·0–179·6)	76·8 (69·5–85·0)	20·0 (16·0–24·6)	2·2 (1·6–3·0)	0·1 (0·1–0·2)	0·0 (0·0–0·0)	1·81 (1·74–1·89)	0·46 (0·41–0·51)	0·5 (0·46–0·54)
1967	0·0 (0·0–0·0)	4·7 (4·0–5·5)	93·4 (83·8–103·7)	209·7 (201·7–217·6)	95·5 (87·1–105·3)	22·9 (17·7–29·0)	2·3 (1·6–3·1)	0·1 (0·1–0·2)	0·0 (0·0–0·0)	2·14 (2·05–2·24)	0·49 (0·44–0·54)	0·6 (0·56–0·65)
1968	0·0 (0·0–0·0)	4·8 (4·2–5·6)	84·2 (74·7–94·3)	197·9 (189·6–205·8)	93·7 (84·8–102·5)	22·9 (17·3–29·5)	2·6 (1·8–3·5)	0·1 (0·1–0·2)	0·0 (0·0–0·0)	2·03 (1·95–2·11)	0·44 (0·4–0·5)	0·6 (0·55–0·64)
1969	0·0 (0·0–0·0)	5·3 (4·5–6·2)	83·3 (73·9–93·5)	200·3 (192·2–208·2)	92·3 (84·3–101·1)	22·6 (17·0–29·0)	2·3 (1·7–3·2)	0·1 (0·1–0·2)	0·0 (0·0–0·0)	2·03 (1·96–2·1)	0·44 (0·4–0·5)	0·59 (0·54–0·63)
1970	0·0 (0·0–0·0)	5·3 (4·4–6·3)	90·1 (79·7–101·9)	203·9 (195·6–212·1)	90·1 (81·9–98·7)	21·6 (16·4–28·1)	2·0 (1·4–2·7)	0·1 (0·1–0·2)	0·0 (0·0–0·0)	2·07 (1·99–2·15)	0·48 (0·43–0·54)	0·57 (0·53–0·61)
1971	0·0 (0·0–0·0)	5·2 (4·3–6·3)	99·0 (87·2–111·3)	201·3 (193·3–209·8)	89·8 (81·7–98·0)	19·8 (15·2–25·8)	2·0 (1·4–2·7)	0·1 (0·1–0·2)	0·0 (0·0–0·0)	2·09 (2·01–2·17)	0·52 (0·46–0·58)	0·56 (0·52–0·61)
1972	0·0 (0·0–0·0)	5·2 (4·3–6·2)	103·7 (91·7–116·2)	199·7 (192·1–207·8)	90·6 (82·1–98·8)	18·8 (14·5–24·0)	2·0 (1·4–2·7)	0·1 (0·1–0·2)	0·0 (0·0–0·0)	2·1 (2·02–2·18)	0·54 (0·48–0·61)	0·56 (0·51–0·6)
1973	0·0 (0·0–0·0)	5·1 (4·1–6·1)	103·7 (92·9–114·7)	199·2 (190·7–207·8)	86·2 (79·3–93·8)	18·5 (14·5–23·3)	1·8 (1·3–2·5)	0·1 (0·1–0·1)	0·0 (0·0–0·0)	2·07 (1·99–2·16)	0·54 (0·49–0·6)	0·53 (0·49–0·57)
1974	0·0 (0·0–0·0)	4·6 (3·8–5·6)	98·2 (88·2–109·4)	190·4 (181·3–198·6)	77·7 (70·0–85·1)	17·4 (13·8–21·7)	1·7 (1·2–2·2)	0·1 (0·0–0·1)	0·0 (0·0–0·0)	1·95 (1·87–2·03)	0·51 (0·47–0·57)	0·48 (0·44–0·52)
1975	0·0 (0·0–0·0)	4·2 (3·5–5·1)	89·6 (79·3–100·2)	182·0 (173·8–190·0)	72·2 (65·4–78·9)	16·5 (13·7–19·7)	1·7 (1·3–2·1)	0·1 (0·0–0·1)	0·0 (0·0–0·0)	1·83 (1·75–1·91)	0·47 (0·42–0·52)	0·45 (0·41–0·49)
1976	0·0 (0·0–0·0)	3·9 (3·2–4·6)	81·7 (73·6–90·9)	178·2 (170·2–186·0)	72·4 (66·3–79·0)	16·2 (12·6–20·2)	1·8 (1·3–2·5)	0·1 (0·0–0·1)	0·0 (0·0–0·0)	1·77 (1·71–1·84)	0·43 (0·39–0·47)	0·45 (0·42–0·49)
1977	0·0 (0·0–0·0)	3·7 (3·1–4·2)	74·3 (66·4–83·9)	177·7 (169·8–185·3)	76·1 (68·8–83·6)	15·9 (12·6–19·5)	1·9 (1·3–2·7)	0·1 (0·1–0·1)	0·0 (0·0–0·0)	1·75 (1·68–1·82)	0·39 (0·35–0·44)	0·47 (0·42–0·52)
1978	0·0 (0·0–0·0)	3·6 (2·9–4·4)	68·4 (61·5–76·0)	177·5 (170·1–184·9)	80·3 (73·1–87·6)	15·3 (13·4–17·3)	2·0 (1·4–2·7)	0·1 (0·1–0·1)	0·0 (0·0–0·0)	1·74 (1·67–1·8)	0·36 (0·33–0·4)	0·49 (0·45–0·53)
1979	0·0 (0·0–0·0)	3·6 (2·8–4·6)	63·4 (56·4–70·8)	175·4 (167·9–182·6)	80·3 (73·6–87·6)	14·9 (11·8–18·3)	2·0 (1·4–2·8)	0·1 (0·1–0·1)	0·0 (0·0–0·0)	1·7 (1·63–1·77)	0·34 (0·3–0·37)	0·49 (0·45–0·53)
1980	0·0 (0·0–0·0)	3·6 (2·9–4·4)	59·4 (52·7–66·7)	172·7 (165·0–181·0)	78·2 (71·2–85·4)	14·7 (12·1–17·6)	2·1 (1·3–3·1)	0·1 (0·1–0·1)	0·0 (0·0–0·0)	1·65 (1·59–1·72)	0·31 (0·28–0·35)	0·48 (0·44–0·52)
1981	0·0 (0·0–0·0)	3·7 (3·1–4·4)	56·9 (50·5–64·3)	173·2 (165·4–180·9)	78·8 (72·1–85·7)	15·6 (12·7–18·8)	2·0 (1·4–2·8)	0·1 (0·0–0·1)	0·0 (0·0–0·0)	1·65 (1·59–1·71)	0·3 (0·27–0·34)	0·48 (0·45–0·52)
1982	0·0 (0·0–0·0)	3·8 (3·1–4·6)	55·6 (49·4–62·4)	173·7 (166·5–181·4)	82·7 (75·8–90·0)	17·6 (14·1–21·6)	2·0 (1·4–2·8)	0·1 (0·0–0·1)	0·0 (0·0–0·0)	1·68 (1·61–1·74)	0·3 (0·27–0·33)	0·51 (0·47–0·55)
1983	0·0 (0·0–0·0)	3·8 (3·2–4·6)	54·1 (47·8–61·0)	171·8 (163·4–179·7)	87·5 (79·7–96·6)	19·6 (16·0–23·5)	2·1 (1·5–2·7)	0·1 (0·0–0·1)	0·0 (0·0–0·0)	1·69 (1·63–1·77)	0·29 (0·26–0·32)	0·55 (0·5–0·6)
1984	0·0 (0·0–0·0)	3·7 (3·0–4·4)	50·2 (44·8–56·5)	166·0 (158·9–174·1)	91·5 (83·8–99·6)	20·3 (17·0–24·2)	2·1 (1·4–3·0)	0·1 (0·0–0·1)	0·0 (0·0–0·0)	1·67 (1·61–1·73)	0·27 (0·24–0·3)	0·57 (0·53–0·62)
1985	0·0 (0·0–0·0)	3·5 (2·9–4·2)	47·2 (41·4–53·1)	157·2 (150·3–163·6)	93·6 (85·7–101·3)	20·1 (16·7–24·0)	2·1 (1·2–3·5)	0·1 (0·0–0·1)	0·0 (0·0–0·0)	1·62 (1·56–1·68)	0·25 (0·22–0·28)	0·58 (0·54–0·62)
1986	0·0 (0·0–0·0)	3·3 (2·8–4·0)	44·9 (39·6–50·7)	149·7 (142·6–157·1)	96·3 (87·3–105·0)	20·2 (16·7–24·0)	2·2 (1·5–3·0)	0·1 (0·0–0·1)	0·0 (0·0–0·0)	1·58 (1·53–1·64)	0·24 (0·21–0·27)	0·59 (0·55–0·64)
1987	0·0 (0·0–0·0)	3·2 (2·7–3·8)	41·3 (36·8–46·3)	143·2 (136·3–150·0)	98·7 (91·1–107·3)	21·0 (17·5–24·7)	2·4 (1·7–3·4)	0·1 (0·1–0·1)	0·0 (0·0–0·0)	1·55 (1·48–1·62)	0·22 (0·2–0·25)	0·61 (0·57–0·66)
1988	0·0 (0·0–0·0)	3·1 (2·6–3·7)	38·1 (33·3–43·4)	132·9 (126·8–139·1)	98·3 (90·0–106·9)	22·1 (18·6–25·9)	2·6 (1·8–3·8)	0·1 (0·1–0·1)	0·0 (0·0–0·0)	1·49 (1·42–1·55)	0·21 (0·18–0·23)	0·62 (0·57–0·66)
1989	0·0 (0·0–0·0)	3·1 (2·6–3·7)	35·2 (30·5–40·2)	122·9 (116·6–129·6)	96·1 (88·4–104·2)	23·2 (19·4–27·5)	2·8 (1·9–3·8)	0·1 (0·1–0·1)	0·0 (0·0–0·0)	1·42 (1·36–1·48)	0·19 (0·17–0·22)	0·61 (0·57–0·65)
1990	0·0 (0·0–0·0)	3·2 (2·7–3·8)	34·0 (29·2–38·9)	119·3 (112·5–125·6)	95·7 (87·9–103·7)	24·3 (20·4–28·9)	2·7 (1·9–3·7)	0·1 (0·1–0·1)	0·0 (0·0–0·0)	1·4 (1·34–1·46)	0·19 (0·16–0·21)	0·61 (0·57–0·66)
1991	0·0 (0·0–0·0)	3·3 (2·8–4·0)	33·9 (29·8–38·1)	115·5 (109·9–121·6)	94·7 (87·0–102·8)	25·7 (21·4–30·4)	2·7 (1·9–3·9)	0·1 (0·1–0·1)	0·0 (0·0–0·0)	1·38 (1·33–1·44)	0·19 (0·17–0·21)	0·62 (0·57–0·66)
1992	0·0 (0·0–0·0)	3·3 (2·8–4·0)	32·7 (29·0–36·9)	108·8 (102·9–115·0)	92·8 (85·3–100·6)	26·4 (21·9–31·7)	2·9 (2·0–4·1)	0·1 (0·1–0·2)	0·0 (0·0–0·0)	1·34 (1·29–1·39)	0·18 (0·16–0·2)	0·61 (0·57–0·66)
1993	0·0 (0·0–0·0)	3·4 (2·7–4·1)	32·3 (28·0–36·8)	108·2 (102·2–114·7)	96·7 (88·7–104·7)	28·4 (23·8–33·5)	3·0 (2·0–4·3)	0·1 (0·1–0·2)	0·0 (0·0–0·0)	1·36 (1·3–1·42)	0·18 (0·16–0·2)	0·64 (0·59–0·69)
1994	0·0 (0·0–0·0)	3·4 (2·7–4·1)	31·5 (27·9–35·7)	105·7 (99·8–111·5)	98·7 (90·8–107·4)	29·8 (24·8–35·3)	3·2 (2·2–4·7)	0·1 (0·1–0·2)	0·0 (0·0–0·0)	1·36 (1·29–1·43)	0·17 (0·16–0·2)	0·66 (0·61–0·71)
1995	0·0 (0·0–0·0)	3·4 (2·7–4·1)	30·2 (26·3–34·2)	99·2 (93·5–104·7)	98·1 (90·0–106·6)	30·8 (25·5–36·4)	3·4 (2·3–4·9)	0·1 (0·1–0·2)	0·0 (0·0–0·0)	1·33 (1·26–1·39)	0·17 (0·15–0·19)	0·66 (0·61–0·71)
1996	0·0 (0·0–0·0)	3·5 (2·8–4·3)	29·4 (25·7–33·3)	95·5 (89·9–101·3)	98·1 (90·2–107·0)	32·2 (26·4–38·3)	3·7 (2·4–5·1)	0·1 (0·1–0·2)	0·0 (0·0–0·0)	1·31 (1·25–1·37)	0·16 (0·15–0·18)	0·67 (0·62–0·72)
1997	0·0 (0·0–0·0)	3·7 (3·0–4·5)	28·9 (25·3–32·7)	92·4 (87·5–97·5)	96·5 (88·6–105·4)	32·9 (27·7–38·7)	4·0 (2·7–5·7)	0·1 (0·1–0·2)	0·0 (0·0–0·0)	1·29 (1·23–1·36)	0·16 (0·14–0·18)	0·67 (0·62–0·72)
1998	0·0 (0·0–0·0)	3·9 (3·2–4·7)	28·5 (24·8–32·5)	89·0 (83·8–94·3)	95·5 (87·8–104·4)	34·4 (28·3–40·4)	4·2 (2·9–5·9)	0·1 (0·1–0·2)	0·0 (0·0–0·0)	1·28 (1·22–1·34)	0·16 (0·14–0·18)	0·67 (0·63–0·72)
1999	0·0 (0·0–0·0)	4·2 (3·5–5·1)	28·4 (24·9–32·2)	86·4 (80·9–91·7)	94·8 (87·2–103·3)	35·3 (29·6–41·2)	4·4 (3·1–6·1)	0·1 (0·1–0·2)	0·0 (0·0–0·0)	1·27 (1·21–1·33)	0·16 (0·15–0·18)	0·67 (0·63–0·73)
2000	0·0 (0·0–0·0)	4·6 (3·7–5·5)	28·8 (25·3–32·5)	84·8 (79·8–90·3)	92·9 (85·1–100·8)	37·1 (31·5–43·1)	4·6 (3·3–6·2)	0·1 (0·1–0·2)	0·0 (0·0–0·0)	1·26 (1·21–1·32)	0·17 (0·15–0·19)	0·67 (0·62–0·72)
2001	0·0 (0·0–0·0)	4·9 (4·0–5·8)	29·3 (25·4–33·2)	82·5 (77·8–87·7)	89·8 (81·8–97·5)	37·1 (31·6–43·5)	4·8 (3·4–6·5)	0·1 (0·1–0·2)	0·0 (0·0–0·0)	1·24 (1·18–1·3)	0·17 (0·15–0·19)	0·66 (0·61–0·71)
2002	0·0 (0·0–0·0)	4·8 (4·0–5·7)	28·8 (25·1–32·9)	80·3 (75·2–85·8)	89·0 (81·5–96·9)	37·6 (32·2–43·8)	5·1 (3·7–6·8)	0·1 (0·1–0·2)	0·0 (0·0–0·0)	1·23 (1·17–1·29)	0·17 (0·15–0·19)	0·66 (0·61–0·71)
2003	0·0 (0·0–0·0)	4·7 (3·9–5·5)	27·9 (24·4–32·1)	78·2 (74·0–82·9)	88·1 (81·0–95·5)	39·1 (33·2–45·6)	5·4 (4·0–7·2)	0·2 (0·1–0·2)	0·0 (0·0–0·0)	1·22 (1·16–1·29)	0·16 (0·15–0·18)	0·66 (0·61–0·72)
2004	0·0 (0·0–0·0)	4·5 (3·7–5·2)	27·0 (23·8–30·8)	75·0 (70·2–80·1)	86·5 (79·0–94·5)	40·0 (33·7–46·8)	5·7 (4·2–7·5)	0·2 (0·1–0·3)	0·0 (0·0–0·0)	1·19 (1·14–1·25)	0·16 (0·14–0·18)	0·66 (0·61–0·71)
2005	0·0 (0·0–0·0)	4·3 (3·7–5·2)	26·9 (23·6–30·3)	74·1 (69·2–79·0)	87·2 (80·1–94·6)	41·2 (35·4–47·5)	6·1 (4·5–8·0)	0·2 (0·1–0·3)	0·0 (0·0–0·0)	1·2 (1·15–1·25)	0·16 (0·14–0·17)	0·67 (0·63–0·72)
2006	0·0 (0·0–0·0)	4·3 (3·6–5·0)	27·2 (23·5–31·1)	74·7 (69·8–79·4)	90·7 (83·2–98·3)	43·6 (37·6–51·2)	6·7 (5·1–8·7)	0·2 (0·1–0·3)	0·0 (0·0–0·0)	1·24 (1·18–1·3)	0·16 (0·14–0·18)	0·71 (0·66–0·76)
2007	0·0 (0·0–0·0)	4·2 (3·5–5·0)	27·0 (24·1–30·4)	74·0 (69·4–79·1)	91·9 (84·2–100·6)	46·2 (40·1–53·6)	7·3 (5·5–9·5)	0·2 (0·1–0·3)	0·0 (0·0–0·0)	1·25 (1·2–1·31)	0·16 (0·14–0·17)	0·73 (0·67–0·78)
2008	0·0 (0·0–0·0)	4·1 (3·5–4·8)	26·5 (23·0–30·5)	73·3 (68·5–78·2)	92·9 (85·8–100·3)	47·9 (41·1–55·2)	8·0 (6·1–10·3)	0·2 (0·1–0·3)	0·0 (0·0–0·0)	1·26 (1·21–1·32)	0·15 (0·13–0·17)	0·74 (0·69–0·8)
2009	0·0 (0·0–0·0)	3·9 (3·2–4·6)	25·4 (22·2–28·8)	72·6 (68·3–76·8)	93·6 (85·9–102·4)	49·3 (42·7–56·8)	8·9 (6·8–11·6)	0·2 (0·1–0·4)	0·0 (0·0–0·0)	1·27 (1·2–1·34)	0·15 (0·13–0·16)	0·76 (0·71–0·82)
2010	0·0 (0·0–0·0)	3·7 (3·1–4·3)	24·4 (21·1–27·8)	72·7 (67·8–77·4)	94·3 (86·7–102·5)	50·3 (43·1–57·4)	9·5 (7·3–12·0)	0·2 (0·1–0·4)	0·0 (0·0–0·0)	1·28 (1·21–1·34)	0·14 (0·12–0·16)	0·77 (0·72–0·83)
2011	0·0 (0·0–0·0)	3·5 (3·0–4·2)	22·9 (20·1–26·1)	71·9 (66·9–77·0)	93·8 (86·0–101·3)	51·0 (43·8–59·1)	10·1 (7·8–12·7)	0·3 (0·2–0·4)	0·0 (0·0–0·0)	1·27 (1·21–1·33)	0·13 (0·12–0·15)	0·78 (0·73–0·83)
2012	0·0 (0·0–0·0)	3·4 (2·8–4·1)	21·6 (19·0–24·6)	71·0 (66·9–75·3)	94·9 (86·6–102·9)	53·3 (46·5–60·6)	11·1 (8·8–13·7)	0·3 (0·2–0·5)	0·0 (0·0–0·0)	1·28 (1·22–1·34)	0·13 (0·11–0·14)	0·8 (0·74–0·85)
2013	0·0 (0·0–0·0)	3·3 (2·7–3·9)	20·7 (18·2–23·3)	69·5 (64·9–74·4)	95·3 (87·9–103·4)	54·8 (47·4–62·4)	11·7 (9·4–14·6)	0·3 (0·2–0·5)	0·0 (0·0–0·0)	1·28 (1·22–1·34)	0·12 (0·11–0·13)	0·81 (0·75–0·87)
2014	0·0 (0·0–0·0)	3·2 (2·7–3·8)	19·9 (16·9–23·0)	68·7 (64·1–73·7)	97·0 (89·1–105·3)	57·4 (50·1–65·5)	12·4 (9·9–15·5)	0·3 (0·2–0·5)	0·0 (0·0–0·0)	1·29 (1·22–1·37)	0·12 (0·1–0·13)	0·84 (0·77–0·91)
2015	0·0 (0·0–0·0)	3·2 (2·7–3·9)	20·2 (17·2–23·6)	69·9 (64·3–75·9)	100·0 (90·5–110·5)	56·5 (47·4–67·3)	11·5 (9·1–14·5)	0·3 (0·2–0·5)	0·0 (0·0–0·0)	1·31 (1·17–1·46)	0·12 (0·1–0·14)	0·84 (0·75–0·95)
2016	0·0 (0·0–0·0)	3·3 (2·7–4·0)	21·2 (16·5–27·0)	70·9 (63·5–79·6)	100·0 (87·1–115·4)	52·7 (40·7–67·7)	9·9 (7·2–13·2)	0·3 (0·2–0·4)	0·0 (0·0–0·0)	1·29 (1·1–1·51)	0·12 (0·1–0·15)	0·81 (0·69–0·96)
2017	0·0 (0·0–0·0)	3·3 (2·7–4·0)	21·3 (16·7–27·3)	70·7 (62·7–79·3)	99·0 (84·6–114·7)	51·9 (38·8–68·0)	9·6 (6·5–13·7)	0·3 (0·2–0·4)	0·0 (0·0–0·0)	1·28 (1·06–1·52)	0·12 (0·1–0·16)	0·8 (0·65–0·97)

Supplemental Results Table 5: Age-specific fertility rates per 1000, cumulative total fertility up to maternal age 25 (TFU25), cumulative total fertility from maternal age 30 to age 55 (TFO30), and cumulative total fertility, Kochi, 1950-2017												
**Year**	**10-14**	**15-19**	**20-24**	**25-29**	**30-34**	**35-39**	**40-44**	**45-49**	**50-54**	**TFR**	**TFU25**	**TFO30**
1950	0·0 (0·0–0·1)	16·6 (14·7–18·6)	164·7 (150·3–180·0)	206·8 (199·3–214·2)	172·9 (162·5–183·4)	137·7 (122·2–153·4)	46·0 (39·7–52·8)	2·3 (1·5–3·3)	0·0 (0·0–0·0)	3·73 (3·6–3·88)	0·91 (0·84–0·98)	1·79 (1·7–1·9)
1951	0·0 (0·0–0·1)	13·4 (11·9–15·0)	147·9 (133·8–163·1)	191·5 (183·6–198·6)	156·4 (145·8–167·0)	111·7 (96·9–126·0)	35·7 (29·4–42·3)	1·7 (1·1–2·4)	0·0 (0·0–0·0)	3·29 (3·14–3·46)	0·81 (0·74–0·88)	1·53 (1·38–1·67)
1952	0·0 (0·0–0·1)	11·3 (9·8–12·8)	137·7 (122·8–153·9)	181·6 (174·2–188·7)	141·5 (130·6–152·7)	89·8 (77·5–103·2)	28·3 (22·9–34·4)	1·3 (0·8–2·0)	0·0 (0·0–0·0)	2·96 (2·84–3·09)	0·75 (0·67–0·83)	1·3 (1·22–1·4)
1953	0·0 (0·0–0·1)	9·9 (8·6–11·3)	130·3 (115·3–145·3)	171·0 (160·4–181·6)	126·5 (114·8–137·9)	71·7 (60·6–83·2)	22·4 (17·5–28·0)	1·0 (0·7–1·6)	0·0 (0·0–0·0)	2·66 (2·54–2·8)	0·7 (0·63–0·78)	1·11 (1·02–1·19)
1954	0·0 (0·0–0·1)	9·1 (7·8–10·5)	126·4 (110·3–142·9)	166·1 (159·3–173·4)	116·6 (106·7–127·1)	59·7 (49·4–70·8)	18·5 (13·8–24·1)	0·9 (0·5–1·3)	0·0 (0·0–0·0)	2·49 (2·38–2·6)	0·68 (0·6–0·76)	0·98 (0·9–1·06)
1955	0·0 (0·0–0·0)	8·2 (6·9–9·6)	123·4 (108·0–140·6)	164·9 (156·9–172·9)	107·2 (97·0–117·6)	48·7 (41·2–57·4)	15·4 (11·1–20·9)	0·7 (0·4–1·1)	0·0 (0·0–0·0)	2·34 (2·24–2·44)	0·66 (0·58–0·75)	0·86 (0·79–0·93)
1956	0·0 (0·0–0·0)	7·3 (6·2–8·6)	116·3 (100·3–133·0)	159·2 (151·8–167·2)	95·0 (86·0–104·9)	39·4 (33·2–46·4)	12·3 (8·7–17·3)	0·6 (0·3–0·9)	0·0 (0·0–0·0)	2·15 (2·05–2·25)	0·62 (0·54–0·7)	0·74 (0·67–0·81)
1957	0·0 (0·0–0·0)	7·0 (5·9–8·3)	117·6 (102·9–135·7)	162·6 (154·8–170·8)	89·9 (81·5–99·3)	35·7 (29·7–41·3)	10·5 (7·1–14·6)	0·6 (0·3–1·0)	0·0 (0·0–0·0)	2·12 (2·01–2·23)	0·62 (0·55–0·71)	0·68 (0·62–0·75)
1958	0·0 (0·0–0·0)	7·1 (6·0–8·3)	123·6 (108·6–140·8)	168·0 (159·5–177·5)	87·8 (78·9–98·1)	33·4 (27·6–39·5)	9·5 (6·5–13·4)	0·6 (0·3–0·9)	0·0 (0·0–0·0)	2·15 (2·06–2·25)	0·65 (0·58–0·74)	0·66 (0·6–0·72)
1959	0·0 (0·0–0·0)	7·4 (6·2–8·6)	123·6 (107·7–140·0)	166·8 (157·8–176·3)	82·1 (73·2–91·5)	27·1 (21·9–32·9)	7·9 (5·3–11·1)	0·5 (0·3–0·8)	0·0 (0·0–0·0)	2·08 (1·97–2·18)	0·65 (0·57–0·74)	0·59 (0·53–0·65)
1960	0·0 (0·0–0·0)	7·2 (6·1–8·4)	118·8 (105·4–133·7)	167·3 (158·4–176·4)	78·7 (70·8–87·5)	22·5 (18·9–26·4)	6·4 (4·4–9·0)	0·4 (0·2–0·7)	0·0 (0·0–0·0)	2·01 (1·92–2·1)	0·63 (0·56–0·7)	0·54 (0·49–0·59)
1961	0·0 (0·0–0·0)	6·7 (5·7–7·9)	115·3 (104·2–127·0)	168·9 (160·5–177·8)	76·9 (69·6–84·6)	20·2 (16·4–24·5)	5·5 (3·6–8·1)	0·3 (0·2–0·5)	0·0 (0·0–0·0)	1·97 (1·88–2·06)	0·61 (0·55–0·67)	0·51 (0·47–0·56)
1962	0·0 (0·0–0·0)	6·4 (5·3–7·6)	115·1 (102·2–128·0)	173·7 (165·6–181·4)	78·4 (71·4–85·4)	19·0 (15·6–22·7)	4·9 (3·3–6·9)	0·3 (0·2–0·5)	0·0 (0·0–0·0)	1·99 (1·9–2·08)	0·61 (0·54–0·67)	0·51 (0·47–0·56)
1963	0·0 (0·0–0·0)	6·3 (5·3–7·6)	115·6 (102·8–129·4)	175·8 (168·8–183·0)	79·9 (73·2–87·3)	20·1 (17·0–23·6)	4·8 (3·3–6·8)	0·3 (0·2–0·5)	0·0 (0·0–0·0)	2·01 (1·93–2·1)	0·61 (0·55–0·68)	0·53 (0·49–0·57)
1964	0·0 (0·0–0·0)	6·2 (5·2–7·3)	126·2 (112·5–141·0)	187·1 (178·6–195·7)	85·1 (77·9–93·0)	20·7 (17·5–24·0)	4·5 (3·1–6·2)	0·3 (0·2–0·5)	0·0 (0·0–0·0)	2·15 (2·06–2·25)	0·66 (0·59–0·74)	0·55 (0·51–0·6)
1965	0·0 (0·0–0·0)	6·1 (5·1–7·3)	116·3 (101·1–134·1)	157·6 (145·9–169·3)	70·6 (63·5–78·1)	17·3 (14·7–20·3)	3·8 (2·6–5·4)	0·2 (0·1–0·4)	0·0 (0·0–0·0)	1·86 (1·75–1·98)	0·61 (0·54–0·7)	0·46 (0·42–0·5)
1966	0·0 (0·0–0·0)	6·5 (5·5–7·5)	113·8 (96·1–132·7)	162·0 (151·1–173·6)	72·1 (64·7–80·0)	18·0 (14·9–21·7)	3·7 (2·6–5·1)	0·2 (0·1–0·4)	0·0 (0·0–0·0)	1·88 (1·78–2·0)	0·6 (0·51–0·7)	0·47 (0·43–0·51)
1967	0·0 (0·0–0·0)	6·8 (5·8–7·9)	121·3 (101·6–142·4)	196·9 (182·0–212·5)	86·5 (78·2–96·0)	20·4 (16·7–24·6)	3·6 (2·6–4·7)	0·2 (0·1–0·3)	0·0 (0·0–0·0)	2·18 (2·03–2·33)	0·64 (0·54–0·75)	0·55 (0·51–0·61)
1968	0·0 (0·0–0·0)	7·0 (6·0–8·0)	108·6 (90·1–129·8)	184·9 (169·4–201·0)	78·5 (70·6–86·9)	19·6 (15·6–23·7)	3·2 (2·4–4·2)	0·2 (0·1–0·3)	0·0 (0·0–0·0)	2·01 (1·88–2·14)	0·58 (0·48–0·68)	0·51 (0·46–0·56)
1969	0·0 (0·0–0·0)	7·6 (6·4–8·9)	106·8 (89·6–126·4)	186·6 (171·9–202·5)	78·5 (70·7–87·2)	19·1 (15·5–23·6)	3·2 (2·4–4·2)	0·2 (0·1–0·3)	0·0 (0·0–0·0)	2·01 (1·89–2·13)	0·57 (0·49–0·67)	0·5 (0·46–0·55)
1970	0·0 (0·0–0·0)	7·7 (6·5–9·1)	114·9 (96·6–135·5)	189·9 (175·8–204·3)	80·5 (72·6–89·1)	19·7 (15·7–24·5)	3·4 (2·6–4·4)	0·2 (0·1–0·3)	0·0 (0·0–0·0)	2·08 (1·96–2·2)	0·61 (0·52–0·71)	0·52 (0·47–0·57)
1971	0·0 (0·0–0·0)	7·5 (6·2–9·0)	125·9 (106·2–146·7)	187·5 (174·4–200·6)	81·5 (73·1–90·2)	21·0 (17·1–25·2)	3·6 (2·6–5·0)	0·2 (0·1–0·3)	0·0 (0·0–0·0)	2·14 (2·02–2·26)	0·67 (0·57–0·77)	0·53 (0·48–0·59)
1972	0·0 (0·0–0·0)	7·6 (6·3–9·0)	132·1 (113·2–152·0)	185·7 (173·6–197·5)	81·4 (72·4–90·5)	21·4 (17·0–26·1)	3·6 (2·5–4·9)	0·2 (0·1–0·4)	0·0 (0·0–0·0)	2·16 (2·04–2·28)	0·7 (0·61–0·8)	0·53 (0·48–0·59)
1973	0·0 (0·0–0·0)	7·5 (6·1–9·0)	132·8 (116·0–148·9)	185·0 (174·5–195·5)	77·4 (69·3–86·3)	19·7 (15·4–24·3)	3·3 (2·2–4·6)	0·2 (0·1–0·3)	0·0 (0·0–0·0)	2·13 (2·03–2·23)	0·7 (0·62–0·78)	0·5 (0·45–0·56)
1974	0·0 (0·0–0·0)	7·0 (5·8–8·5)	127·5 (113·5–144·4)	176·8 (167·9–186·0)	68·7 (61·2–76·6)	15·4 (12·0–19·7)	2·8 (1·8–4·3)	0·1 (0·1–0·3)	0·0 (0·0–0·0)	1·99 (1·91–2·09)	0·67 (0·6–0·76)	0·44 (0·39–0·48)
1975	0·0 (0·0–0·0)	6·5 (5·4–7·9)	119·5 (107·0–133·0)	169·6 (160·9–178·5)	62·2 (55·4–68·3)	13·1 (10·3–16·7)	2·4 (1·4–3·8)	0·1 (0·1–0·2)	0·0 (0·0–0·0)	1·87 (1·79–1·95)	0·63 (0·57–0·7)	0·39 (0·35–0·43)
1976	0·0 (0·0–0·0)	6·2 (5·0–7·4)	113·1 (99·5–126·9)	166·5 (158·2–175·3)	60·7 (54·6–67·5)	13·0 (10·4–15·9)	2·1 (1·4–3·1)	0·1 (0·1–0·1)	0·0 (0·0–0·0)	1·81 (1·72–1·9)	0·6 (0·53–0·67)	0·38 (0·34–0·42)
1977	0·0 (0·0–0·0)	5·9 (5·0–6·9)	106·8 (94·9–120·7)	166·3 (158·6–175·0)	62·4 (56·1–69·4)	13·1 (10·2–16·5)	1·9 (1·3–2·8)	0·1 (0·0–0·1)	0·0 (0·0–0·0)	1·78 (1·7–1·87)	0·56 (0·5–0·63)	0·39 (0·35–0·43)
1978	0·0 (0·0–0·0)	5·8 (4·6–7·2)	101·3 (90·5–113·2)	166·1 (158·5–173·7)	64·7 (58·0–72·0)	13·2 (9·8–17·4)	1·7 (1·1–2·4)	0·1 (0·0–0·1)	0·0 (0·0–0·0)	1·76 (1·68–1·86)	0·54 (0·48–0·6)	0·4 (0·36–0·44)
1979	0·0 (0·0–0·0)	5·8 (4·3–7·5)	96·1 (84·7–107·2)	164·5 (156·7–172·3)	65·0 (58·4–72·2)	13·0 (10·7–15·8)	1·6 (1·0–2·5)	0·1 (0·0–0·1)	0·0 (0·0–0·0)	1·73 (1·65–1·81)	0·51 (0·45–0·57)	0·4 (0·36–0·43)
1980	0·0 (0·0–0·0)	5·8 (4·5–7·2)	91·4 (80·4–102·2)	163·3 (154·9–172·0)	65·4 (58·5–72·4)	13·0 (10·4–16·2)	1·6 (1·1–2·3)	0·1 (0·0–0·1)	0·0 (0·0–0·0)	1·7 (1·62–1·78)	0·49 (0·43–0·54)	0·4 (0·36–0·44)
1981	0·0 (0·0–0·0)	5·9 (4·8–7·2)	88·5 (78·8–100·0)	165·3 (156·8–173·3)	67·4 (60·5–74·2)	13·8 (11·1–17·0)	1·7 (1·2–2·5)	0·1 (0·0–0·1)	0·0 (0·0–0·0)	1·71 (1·64–1·79)	0·47 (0·42–0·53)	0·41 (0·38–0·45)
1982	0·0 (0·0–0·0)	6·1 (5·0–7·4)	86·8 (76·2–98·5)	168·1 (160·1–176·6)	71·2 (64·4–78·8)	15·5 (12·3–19·7)	1·8 (1·2–2·5)	0·1 (0·0–0·1)	0·0 (0·0–0·0)	1·75 (1·66–1·84)	0·46 (0·41–0·52)	0·44 (0·41–0·48)
1983	0·0 (0·0–0·0)	6·1 (5·0–7·4)	84·6 (73·6–97·2)	169·6 (160·7–178·2)	75·0 (68·3–83·3)	17·2 (13·7–21·1)	1·9 (1·4–2·5)	0·1 (0·0–0·1)	0·0 (0·0–0·0)	1·77 (1·68–1·88)	0·45 (0·4–0·52)	0·47 (0·43–0·52)
1984	0·0 (0·0–0·0)	5·8 (4·8–7·0)	78·8 (69·5–88·8)	168·0 (159·5–176·7)	77·7 (70·6–86·3)	17·9 (14·7–21·5)	2·0 (1·3–2·7)	0·1 (0·0–0·1)	0·0 (0·0–0·0)	1·75 (1·67–1·84)	0·42 (0·38–0·47)	0·49 (0·45–0·53)
1985	0·0 (0·0–0·0)	5·5 (4·5–6·6)	74·4 (64·5–85·1)	163·1 (155·2–171·2)	78·8 (71·0–87·1)	17·9 (14·3–22·0)	2·0 (1·5–2·6)	0·1 (0·0–0·1)	0·0 (0·0–0·0)	1·71 (1·63–1·8)	0·4 (0·35–0·45)	0·49 (0·45–0·53)
1986	0·0 (0·0–0·0)	5·2 (4·4–6·2)	71·3 (62·3–81·5)	158·5 (149·8–167·3)	80·9 (74·2–88·7)	18·0 (14·4–22·0)	2·1 (1·5–2·9)	0·1 (0·0–0·1)	0·0 (0·0–0·0)	1·68 (1·6–1·76)	0·38 (0·34–0·43)	0·51 (0·47–0·55)
1987	0·0 (0·0–0·0)	5·0 (4·2–5·9)	66·6 (58·7–75·4)	154·5 (147·0–162·4)	83·7 (75·9–92·4)	18·6 (14·8–22·8)	2·4 (1·6–3·5)	0·1 (0·0–0·1)	0·0 (0·0–0·0)	1·65 (1·57–1·74)	0·36 (0·32–0·4)	0·52 (0·48–0·57)
1988	0·0 (0·0–0·0)	4·9 (4·1–5·7)	62·9 (54·1–72·3)	146·3 (137·1–156·1)	84·9 (77·1–93·2)	19·3 (15·6–23·6)	2·6 (1·8–3·6)	0·1 (0·0–0·1)	0·0 (0·0–0·0)	1·6 (1·51–1·69)	0·34 (0·29–0·39)	0·53 (0·49–0·58)
1989	0·0 (0·0–0·0)	4·9 (4·1–5·8)	59·4 (50·8–68·9)	137·9 (129·6–146·5)	84·9 (76·7–93·1)	20·2 (16·0–25·0)	2·7 (1·9–3·7)	0·1 (0·0–0·1)	0·0 (0·0–0·0)	1·55 (1·47–1·63)	0·32 (0·28–0·37)	0·54 (0·49–0·58)
1990	0·0 (0·0–0·0)	5·0 (4·2–5·8)	58·5 (50·9–67·5)	136·1 (127·8–144·6)	86·4 (78·3–94·7)	20·9 (16·7–25·7)	2·6 (1·9–3·6)	0·1 (0·0–0·1)	0·0 (0·0–0·0)	1·55 (1·47–1·64)	0·32 (0·28–0·37)	0·55 (0·5–0·6)
1991	0·0 (0·0–0·0)	5·2 (4·2–6·2)	59·3 (51·1–68·2)	134·0 (126·5–141·9)	87·1 (78·7–96·1)	21·8 (17·6–26·9)	2·6 (1·8–3·6)	0·1 (0·0–0·1)	0·0 (0·0–0·0)	1·55 (1·47–1·64)	0·32 (0·28–0·37)	0·56 (0·51–0·61)
1992	0·0 (0·0–0·0)	5·2 (4·3–6·3)	58·1 (51·0–66·4)	128·2 (119·5–137·4)	86·1 (78·4–94·4)	22·4 (17·7–27·6)	2·7 (1·9–3·8)	0·1 (0·0–0·1)	0·0 (0·0–0·0)	1·51 (1·44–1·59)	0·32 (0·28–0·36)	0·56 (0·51–0·61)
1993	0·0 (0·0–0·0)	5·2 (4·2–6·3)	58·0 (49·5–66·7)	128·9 (120·4–138·1)	89·3 (81·5–97·6)	24·0 (19·2–29·6)	2·9 (2·0–3·9)	0·1 (0·0–0·1)	0·0 (0·0–0·0)	1·54 (1·45–1·63)	0·32 (0·27–0·36)	0·58 (0·53–0·64)
1994	0·0 (0·0–0·0)	5·2 (4·2–6·4)	57·3 (50·5–65·2)	127·1 (118·3–135·8)	90·4 (81·6–99·3)	25·4 (20·0–31·4)	3·0 (2·1–4·1)	0·1 (0·0–0·1)	0·0 (0·0–0·0)	1·54 (1·46–1·62)	0·31 (0·28–0·35)	0·59 (0·54–0·65)
1995	0·0 (0·0–0·0)	5·2 (4·1–6·2)	55·6 (48·2–63·5)	120·5 (112·5–129·0)	89·2 (80·3–97·4)	26·3 (20·7–32·4)	3·1 (2·2–4·2)	0·1 (0·0–0·1)	0·0 (0·0–0·0)	1·5 (1·41–1·59)	0·3 (0·27–0·34)	0·59 (0·53–0·65)
1996	0·0 (0·0–0·0)	5·2 (4·3–6·3)	54·6 (46·8–62·3)	116·9 (108·5–125·8)	89·0 (80·6–98·2)	27·6 (21·9–34·2)	3·2 (2·4–4·2)	0·1 (0·0–0·1)	0·0 (0·0–0·0)	1·48 (1·39–1·58)	0·3 (0·26–0·34)	0·6 (0·54–0·66)
1997	0·0 (0·0–0·0)	5·4 (4·3–6·5)	53·9 (46·0–63·0)	113·6 (106·1–121·0)	87·7 (79·1–96·6)	28·3 (22·6–34·7)	3·5 (2·5–4·7)	0·1 (0·0–0·1)	0·0 (0·0–0·0)	1·46 (1·38–1·55)	0·3 (0·26–0·34)	0·6 (0·54–0·66)
1998	0·0 (0·0–0·0)	5·6 (4·4–6·8)	52·9 (45·9–60·6)	109·8 (102·2–117·7)	87·4 (79·1–97·0)	29·6 (23·2–36·6)	3·7 (2·7–4·8)	0·1 (0·0–0·1)	0·0 (0·0–0·0)	1·44 (1·35–1·54)	0·29 (0·26–0·33)	0·6 (0·54–0·67)
1999	0·0 (0·0–0·0)	5·8 (4·7–7·3)	51·9 (44·6–60·7)	106·6 (99·3–114·3)	87·7 (79·6–97·1)	30·4 (24·6–37·2)	3·9 (2·8–5·3)	0·1 (0·0–0·1)	0·0 (0·0–0·0)	1·43 (1·35–1·53)	0·29 (0·25–0·33)	0·61 (0·55–0·68)
2000	0·0 (0·0–0·0)	6·1 (5·0–7·6)	51·4 (44·8–58·4)	104·3 (97·2–112·5)	87·0 (78·5–95·6)	32·2 (25·9–39·5)	4·2 (3·0–5·6)	0·1 (0·0–0·1)	0·0 (0·0–0·0)	1·43 (1·34–1·52)	0·29 (0·25–0·32)	0·62 (0·56–0·68)
2001	0·0 (0·0–0·0)	6·3 (5·2–7·8)	50·8 (43·8–58·9)	101·1 (93·8–108·8)	85·0 (77·1–93·5)	32·5 (26·6–39·3)	4·3 (3·1–5·8)	0·1 (0·1–0·1)	0·0 (0·0–0·0)	1·4 (1·32–1·48)	0·29 (0·25–0·33)	0·61 (0·55–0·67)
2002	0·0 (0·0–0·0)	6·2 (5·0–7·6)	49·0 (41·7–57·0)	98·3 (91·9–104·5)	84·9 (75·8–94·2)	33·4 (27·3–40·4)	4·7 (3·3–6·5)	0·1 (0·1–0·2)	0·0 (0·0–0·0)	1·38 (1·31–1·46)	0·28 (0·24–0·32)	0·62 (0·55–0·68)
2003	0·0 (0·0–0·0)	5·9 (4·8–7·2)	46·8 (40·2–54·1)	95·9 (86·9–105·3)	84·2 (76·5–92·9)	35·1 (28·6–42·6)	5·0 (3·5–6·8)	0·1 (0·1–0·2)	0·0 (0·0–0·0)	1·37 (1·28–1·47)	0·26 (0·23–0·3)	0·62 (0·56–0·69)
2004	0·0 (0·0–0·0)	5·6 (4·6–6·7)	45·0 (38·9–51·7)	92·1 (86·4–98·2)	82·7 (74·7–91·4)	35·9 (29·5–43·1)	5·2 (3·7–6·9)	0·1 (0·1–0·2)	0·0 (0·0–0·0)	1·33 (1·27–1·4)	0·25 (0·22–0·29)	0·62 (0·56–0·68)
2005	0·0 (0·0–0·0)	5·5 (4·6–6·6)	44·8 (35·5–56·1)	90·9 (84·3–98·4)	82·7 (74·8–91·2)	36·6 (29·8–44·2)	5·4 (3·9–7·3)	0·1 (0·1–0·2)	0·0 (0·0–0·0)	1·33 (1·26–1·41)	0·25 (0·2–0·31)	0·62 (0·57–0·68)
2006	0·0 (0·0–0·0)	5·4 (4·5–6·4)	45·1 (38·5–52·3)	91·4 (84·9–98·4)	85·1 (76·7–93·9)	38·2 (31·9–46·0)	5·9 (4·3–8·0)	0·1 (0·1–0·2)	0·0 (0·0–0·0)	1·36 (1·28–1·43)	0·25 (0·22–0·29)	0·65 (0·59–0·71)
2007	0·0 (0·0–0·0)	5·4 (4·5–6·4)	45·1 (38·7–52·1)	90·7 (84·1–98·1)	85·2 (77·5–94·9)	39·8 (33·0–47·7)	6·3 (4·5–8·4)	0·1 (0·1–0·2)	0·0 (0·0–0·0)	1·36 (1·29–1·44)	0·25 (0·22–0·29)	0·66 (0·6–0·71)
2008	0·0 (0·0–0·0)	5·3 (4·5–6·3)	44·6 (38·5–51·4)	90·1 (83·9–96·8)	85·6 (77·4–94·4)	40·7 (33·7–48·3)	6·8 (4·8–9·1)	0·1 (0·1–0·2)	0·0 (0·0–0·0)	1·37 (1·3–1·44)	0·25 (0·22–0·29)	0·67 (0·61–0·73)
2009	0·0 (0·0–0·0)	5·1 (4·2–6·1)	43·6 (35·3–53·0)	89·7 (80·4–98·9)	86·6 (78·3–95·3)	41·8 (34·3–49·9)	7·4 (5·2–9·9)	0·1 (0·1–0·2)	0·0 (0·0–0·0)	1·37 (1·27–1·48)	0·24 (0·2–0·29)	0·68 (0·63–0·74)
2010	0·0 (0·0–0·0)	5·0 (4·1–5·9)	42·5 (36·8–48·2)	90·0 (84·2–96·2)	88·1 (80·1–97·5)	42·9 (35·9–51·4)	7·7 (5·4–10·4)	0·1 (0·1–0·3)	0·0 (0·0–0·0)	1·38 (1·31–1·46)	0·24 (0·21–0·27)	0·69 (0·64–0·76)
2011	0·0 (0·0–0·0)	4·9 (4·0–5·9)	40·9 (35·2–47·3)	89·5 (82·4–96·9)	88·9 (80·1–98·0)	43·7 (36·1–51·6)	7·9 (5·7–10·8)	0·2 (0·1–0·3)	0·0 (0·0–0·0)	1·38 (1·31–1·45)	0·23 (0·2–0·26)	0·7 (0·65–0·76)
2012	0·0 (0·0–0·0)	4·8 (3·9–5·9)	39·4 (33·3–46·4)	89·0 (82·9–96·0)	90·7 (82·1–100·2)	45·6 (38·6–53·5)	8·4 (6·1–11·1)	0·2 (0·1–0·3)	0·0 (0·0–0·0)	1·39 (1·31–1·48)	0·22 (0·19–0·26)	0·72 (0·66–0·79)
2013	0·0 (0·0–0·0)	4·8 (4·0–5·7)	38·5 (32·5–45·3)	87·7 (80·3–95·8)	91·1 (83·2–100·2)	46·4 (38·4–54·5)	8·7 (6·5–11·8)	0·2 (0·1–0·3)	0·0 (0·0–0·0)	1·39 (1·31–1·47)	0·22 (0·19–0·25)	0·73 (0·68–0·79)
2014	0·0 (0·0–0·0)	4·7 (3·9–5·7)	37·7 (32·0–44·7)	87·4 (80·7–94·4)	92·1 (83·2–101·7)	47·4 (39·8–55·4)	9·1 (6·5–12·3)	0·2 (0·1–0·3)	0·0 (0·0–0·0)	1·39 (1·32–1·47)	0·21 (0·18–0·25)	0·74 (0·68–0·81)
2015	0·0 (0·0–0·0)	4·8 (3·9–5·8)	38·8 (29·0–51·5)	89·3 (76·8–103·2)	94·0 (83·9–105·1)	45·4 (36·7–55·1)	8·4 (5·9–11·6)	0·2 (0·1–0·3)	0·0 (0·0–0·0)	1·4 (1·21–1·62)	0·22 (0·16–0·29)	0·74 (0·65–0·84)
2016	0·0 (0·0–0·0)	4·8 (4·0–5·9)	40·3 (30·8–53·7)	90·5 (79·5–104·1)	93·3 (80·6–108·0)	41·6 (31·5–54·2)	7·2 (4·7–10·3)	0·2 (0·1–0·3)	0·0 (0·0–0·0)	1·39 (1·18–1·64)	0·23 (0·17–0·3)	0·71 (0·6–0·84)
2017	0·0 (0·0–0·0)	4·8 (4·0–5·8)	40·3 (29·4–54·2)	90·5 (77·2–104·9)	91·6 (78·2–107·4)	40·4 (29·5–53·6)	7·0 (4·5–10·6)	0·2 (0·1–0·3)	0·0 (0·0–0·0)	1·37 (1·13–1·65)	0·23 (0·17–0·3)	0·7 (0·57–0·85)

Supplemental Results Table 5: Age-specific fertility rates per 1000, cumulative total fertility up to maternal age 25 (TFU25), cumulative total fertility from maternal age 30 to age 55 (TFO30), and cumulative total fertility, Kumamoto, 1950-2017

Year	10-14	15-19	20-24	25-29	30-34	35-39	40-44	45-49	50-54	TFR	TFU25	TFO30
1950	0·0 (0·0–0·0)	11·5 (10·2–12·9)	157·9 (144·0–172·7)	219·1 (211·2–227·1)	179·4 (168·5–190·4)	110·9 (98·6–123·2)	37·9 (32·7–43·5)	2·0 (1·3–2·8)	0·0 (0·0–0·0)	3·59 (3·46–3·72)	0·85 (0·78–0·92)	1·65 (1·56–1·74)
1951	0·0 (0·0–0·0)	9·2 (8·2–10·5)	141·6 (128·8–154·6)	203·4 (195·5–211·0)	162·4 (150·6–173·8)	89·5 (78·6–101·3)	29·3 (24·5–34·3)	1·4 (0·9–2·1)	0·0 (0·0–0·0)	3·18 (3·03–3·33)	0·75 (0·69–0·82)	1·41 (1·28–1·53)
1952	0·0 (0·0–0·0)	7·8 (6·8–8·9)	131·7 (118·7–146·0)	193·2 (184·9–201·5)	147·1 (133·8–160·4)	71·6 (61·5–82·4)	23·1 (19·1–27·8)	1·1 (0·7–1·7)	0·0 (0·0–0·0)	2·88 (2·75–3·0)	0·7 (0·63–0·77)	1·21 (1·13–1·3)
1953	0·0 (0·0–0·0)	6·8 (5·9–7·9)	124·4 (110·0–139·0)	182·0 (174·1–190·7)	131·5 (117·4–146·5)	57·0 (47·6–67·1)	18·4 (14·9–22·3)	0·9 (0·6–1·4)	0·0 (0·0–0·0)	2·61 (2·5–2·73)	0·66 (0·59–0·73)	1·04 (0·95–1·12)
1954	0·0 (0·0–0·0)	6·3 (5·3–7·3)	120·6 (107·2–134·6)	177·0 (169·1–185·1)	121·2 (107·0–137·3)	48·3 (40·4–57·8)	15·1 (12·1–18·8)	0·7 (0·5–1·1)	0·0 (0·0–0·0)	2·45 (2·33–2·56)	0·63 (0·57–0·7)	0·93 (0·85–1·01)
1955	0·0 (0·0–0·0)	5·6 (4·7–6·6)	117·6 (103·5–132·3)	175·8 (167·3–184·4)	111·5 (96·6–126·4)	42·6 (35·9–50·6)	12·6 (9·7–15·7)	0·6 (0·3–0·9)	0·0 (0·0–0·0)	2·33 (2·23–2·43)	0·62 (0·54–0·69)	0·84 (0·76–0·91)
1956	0·0 (0·0–0·0)	5·1 (4·2–5·9)	110·7 (98·0–123·9)	169·9 (162·4–177·7)	98·7 (85·7–113·1)	36·5 (30·7–43·2)	10·0 (7·8–12·8)	0·5 (0·3–0·8)	0·0 (0·0–0·0)	2·16 (2·07–2·26)	0·58 (0·52–0·65)	0·73 (0·66–0·8)
1957	0·0 (0·0–0·0)	4·8 (4·1–5·7)	111·9 (99·0–126·5)	173·3 (165·8–181·3)	93·4 (80·9–107·6)	31·2 (26·0–36·2)	8·5 (6·6–10·8)	0·5 (0·3–0·8)	0·0 (0·0–0·0)	2·12 (2·01–2·24)	0·58 (0·52–0·66)	0·67 (0·61–0·73)
1958	0·0 (0·0–0·0)	4·9 (4·1–5·7)	117·5 (103·4–133·2)	179·1 (170·4–187·5)	91·2 (78·9–105·3)	27·5 (22·9–32·6)	7·7 (6·0–9·8)	0·5 (0·3–0·8)	0·0 (0·0–0·0)	2·14 (2·04–2·25)	0·61 (0·54–0·69)	0·63 (0·57–0·7)
1959	0·0 (0·0–0·0)	5·1 (4·3–5·9)	117·5 (104·7–131·6)	178·0 (169·9–186·7)	85·3 (75·0–97·5)	23·7 (19·5–29·0)	6·4 (4·9–8·3)	0·4 (0·2–0·7)	0·0 (0·0–0·0)	2·08 (1·99–2·18)	0·61 (0·55–0·68)	0·58 (0·53–0·64)
1960	0·0 (0·0–0·0)	4·9 (4·2–5·8)	113·0 (100·4–126·2)	178·8 (170·5–187·2)	81·8 (72·9–91·4)	20·8 (17·2–25·3)	5·2 (3·9–6·6)	0·3 (0·2–0·6)	0·0 (0·0–0·0)	2·02 (1·93–2·12)	0·59 (0·53–0·66)	0·54 (0·49–0·59)
1961	0·0 (0·0–0·0)	4·6 (3·9–5·5)	109·8 (98·7–121·5)	180·6 (172·6–188·9)	79·9 (70·0–91·0)	18·7 (15·3–22·6)	4·5 (3·3–6·0)	0·3 (0·2–0·5)	0·0 (0·0–0·0)	1·99 (1·9–2·09)	0·57 (0·52–0·63)	0·52 (0·47–0·57)
1962	0·0 (0·0–0·0)	4·4 (3·6–5·2)	109·4 (97·2–121·8)	185·8 (178·2–193·4)	81·3 (74·3–88·3)	17·6 (14·3–21·0)	3·9 (2·9–5·2)	0·3 (0·1–0·4)	0·0 (0·0–0·0)	2·01 (1·93–2·1)	0·57 (0·51–0·63)	0·52 (0·48–0·55)
1963	0·0 (0·0–0·0)	4·3 (3·6–5·2)	109·8 (98·6–122·6)	187·8 (179·7–195·8)	82·8 (75·8–90·4)	17·1 (14·4–20·2)	3·9 (2·8–5·2)	0·3 (0·2–0·4)	0·0 (0·0–0·0)	2·03 (1·95–2·11)	0·57 (0·51–0·63)	0·52 (0·48–0·56)
1964	0·0 (0·0–0·0)	4·3 (3·6–5·0)	119·8 (107·6–133·8)	199·7 (191·4–209·0)	88·2 (79·8–97·2)	17·6 (14·9–20·5)	3·6 (2·6–4·8)	0·3 (0·1–0·4)	0·0 (0·0–0·0)	2·17 (2·08–2·26)	0·62 (0·56–0·69)	0·55 (0·5–0·6)
1965	0·0 (0·0–0·0)	4·2 (3·4–5·0)	110·1 (98·3–123·6)	168·3 (159·3–177·9)	73·1 (64·5–81·7)	16·1 (13·2–19·5)	3·1 (2·2–4·1)	0·2 (0·1–0·3)	0·0 (0·0–0·0)	1·88 (1·79–1·97)	0·57 (0·51–0·64)	0·46 (0·42–0·51)
1966	0·0 (0·0–0·0)	4·4 (3·8–5·1)	107·4 (96·1–119·6)	172·9 (164·0–182·4)	74·6 (65·7–84·8)	16·3 (13·8–19·4)	3·0 (2·2–4·0)	0·2 (0·1–0·3)	0·0 (0·0–0·0)	1·89 (1·82–1·98)	0·56 (0·5–0·62)	0·47 (0·42–0·52)
1967	0·0 (0·0–0·0)	4·6 (3·9–5·4)	114·3 (102·5–126·9)	210·3 (201·1–218·8)	89·6 (77·2–102·9)	18·4 (15·0–22·4)	2·9 (2·1–3·7)	0·2 (0·1–0·3)	0·0 (0·0–0·0)	2·2 (2·09–2·3)	0·59 (0·54–0·66)	0·56 (0·49–0·62)
1968	0·0 (0·0–0·0)	4·7 (4·0–5·4)	102·1 (90·7–114·4)	197·9 (189·4–205·9)	81·4 (69·1–93·9)	18·3 (14·7–22·2)	2·6 (1·9–3·4)	0·1 (0·1–0·2)	0·0 (0·0–0·0)	2·04 (1·95–2·12)	0·53 (0·48–0·6)	0·51 (0·45–0·57)
1969	0·0 (0·0–0·0)	5·1 (4·3–6·0)	100·2 (87·9–113·0)	199·7 (191·7–207·8)	81·2 (69·3–94·9)	17·8 (14·5–21·6)	2·5 (1·9–3·3)	0·1 (0·1–0·2)	0·0 (0·0–0·0)	2·03 (1·95–2·12)	0·53 (0·46–0·59)	0·51 (0·45–0·57)
1970	0·0 (0·0–0·0)	5·2 (4·3–6·1)	107·5 (95·6–120·0)	202·9 (193·3–211·5)	83·1 (71·6–96·2)	16·9 (13·6–20·3)	2·7 (1·9–3·5)	0·2 (0·1–0·2)	0·0 (0·0–0·0)	2·09 (2·01–2·18)	0·56 (0·5–0·63)	0·51 (0·46–0·57)
1971	0·0 (0·0–0·0)	5·0 (4·1–6·0)	117·5 (103·8–131·3)	200·1 (190·6–210·1)	84·0 (72·4–97·5)	15·9 (13·1–19·4)	2·9 (2·1–3·9)	0·2 (0·1–0·3)	0·0 (0·0–0·0)	2·13 (2·04–2·22)	0·61 (0·54–0·68)	0·52 (0·46–0·58)
1972	0·0 (0·0–0·0)	5·1 (4·1–6·1)	122·7 (109·7–136·9)	198·1 (188·8–208·0)	83·9 (73·8–94·1)	15·7 (12·5–19·0)	2·8 (2·0–3·9)	0·2 (0·1–0·3)	0·0 (0·0–0·0)	2·14 (2·05–2·24)	0·64 (0·57–0·71)	0·51 (0·47–0·56)
1973	0·0 (0·0–0·0)	4·9 (4·0–6·1)	122·6 (109·4–136·4)	197·5 (188·1–207·2)	79·6 (71·4–88·6)	14·6 (11·7–18·0)	2·6 (1·8–3·6)	0·2 (0·1–0·2)	0·0 (0·0–0·0)	2·11 (2·03–2·2)	0·64 (0·57–0·71)	0·48 (0·44–0·53)
1974	0·0 (0·0–0·0)	4·6 (3·7–5·7)	117·0 (104·8–129·6)	189·2 (179·4–199·4)	70·7 (63·5–78·0)	13·0 (10·3–16·2)	2·2 (1·5–3·1)	0·1 (0·1–0·2)	0·0 (0·0–0·0)	1·98 (1·91–2·07)	0·61 (0·55–0·67)	0·43 (0·4–0·47)
1975	0·0 (0·0–0·0)	4·3 (3·3–5·3)	109·3 (97·3–121·9)	182·0 (174·0–190·0)	64·1 (57·8–70·1)	12·3 (9·7–15·2)	1·9 (1·1–2·9)	0·1 (0·0–0·2)	0·0 (0·0–0·0)	1·87 (1·79–1·95)	0·57 (0·51–0·63)	0·39 (0·36–0·42)
1976	0·0 (0·0–0·0)	4·0 (3·2–5·0)	103·5 (91·7–114·9)	179·5 (171·1–187·9)	62·7 (57·0–68·9)	12·1 (9·9–14·7)	1·6 (1·1–2·4)	0·1 (0·0–0·1)	0·0 (0·0–0·0)	1·82 (1·73–1·9)	0·54 (0·48–0·59)	0·38 (0·35–0·42)
1977	0·0 (0·0–0·0)	3·9 (3·2–4·7)	98·3 (87·9–111·3)	180·3 (172·1–188·2)	64·7 (58·4–71·3)	12·1 (9·7–14·8)	1·5 (1·0–2·1)	0·1 (0·0–0·1)	0·0 (0·0–0·0)	1·8 (1·72–1·89)	0·51 (0·46–0·58)	0·39 (0·35–0·43)
1978	0·0 (0·0–0·0)	3·9 (3·2–4·7)	94·6 (85·2–104·7)	181·3 (173·0–188·8)	68·8 (62·2–76·1)	12·0 (9·0–15·5)	1·4 (0·9–2·0)	0·1 (0·0–0·1)	0·0 (0·0–0·0)	1·81 (1·74–1·89)	0·49 (0·45–0·54)	0·41 (0·37–0·45)
1979	0·0 (0·0–0·0)	4·0 (3·3–4·7)	91·1 (80·9–102·2)	180·1 (171·9–187·8)	70·4 (64·1–77·4)	11·7 (9·7–14·0)	1·4 (0·9–2·1)	0·1 (0·0–0·1)	0·0 (0·0–0·0)	1·79 (1·72–1·87)	0·48 (0·42–0·53)	0·42 (0·38–0·45)
1980	0·0 (0·0–0·0)	4·0 (3·2–5·0)	87·4 (78·1–96·8)	178·1 (169·7–186·5)	70·0 (63·4–76·7)	11·7 (9·5–14·4)	1·4 (1·0–2·0)	0·1 (0·0–0·1)	0·0 (0·0–0·0)	1·76 (1·69–1·84)	0·46 (0·41–0·5)	0·42 (0·38–0·45)
1981	0·0 (0·0–0·0)	4·1 (3·3–5·0)	85·5 (74·7–98·1)	179·5 (170·9–187·5)	71·2 (64·2–77·9)	12·4 (10·0–15·0)	1·5 (1·0–2·1)	0·1 (0·0–0·1)	0·0 (0·0–0·0)	1·77 (1·7–1·84)	0·45 (0·4–0·51)	0·43 (0·39–0·46)
1982	0·0 (0·0–0·0)	4·3 (3·4–5·2)	84·6 (74·8–95·3)	182·0 (173·8–190·3)	74·5 (68·0–81·5)	13·9 (11·4–16·6)	1·5 (1·1–2·1)	0·1 (0·0–0·1)	0·0 (0·0–0·0)	1·8 (1·74–1·88)	0·44 (0·39–0·5)	0·45 (0·42–0·49)
1983	0·0 (0·0–0·0)	4·3 (3·4–5·3)	83·1 (74·3–92·6)	183·8 (175·0–192·6)	78·4 (71·3–86·5)	15·5 (12·5–19·0)	1·6 (1·2–2·0)	0·1 (0·0–0·1)	0·0 (0·0–0·0)	1·83 (1·76–1·91)	0·44 (0·39–0·49)	0·48 (0·44–0·52)
1984	0·0 (0·0–0·0)	4·2 (3·3–5·1)	77·7 (66·6–90·2)	182·3 (174·4–191·3)	81·4 (74·3–89·2)	16·1 (13·2–19·8)	1·7 (1·2–2·2)	0·1 (0·0–0·1)	0·0 (0·0–0·0)	1·82 (1·74–1·91)	0·41 (0·35–0·47)	0·5 (0·46–0·54)
1985	0·0 (0·0–0·0)	4·0 (3·1–4·8)	73·1 (64·4–82·4)	177·2 (169·2–184·9)	82·9 (75·8–90·0)	16·2 (13·2–19·7)	1·7 (1·2–2·3)	0·1 (0·0–0·1)	0·0 (0·0–0·0)	1·78 (1·7–1·85)	0·39 (0·34–0·43)	0·5 (0·46–0·54)
1986	0·0 (0·0–0·0)	3·8 (3·0–4·7)	69·8 (61·2–79·5)	172·7 (164·4–181·6)	85·4 (76·7–94·1)	16·4 (13·5–19·6)	1·9 (1·2–2·7)	0·1 (0·0–0·1)	0·0 (0·0–0·0)	1·75 (1·68–1·83)	0·37 (0·33–0·41)	0·52 (0·47–0·57)
1987	0·0 (0·0–0·0)	3·7 (2·9–4·4)	64·8 (57·6–73·1)	168·8 (160·6–177·0)	88·5 (81·7–96·0)	17·2 (14·1–20·7)	2·1 (1·4–2·9)	0·1 (0·0–0·1)	0·0 (0·0–0·0)	1·73 (1·65–1·8)	0·34 (0·31–0·38)	0·54 (0·5–0·58)
1988	0·0 (0·0–0·0)	3·6 (2·9–4·4)	60·8 (52·6–70·2)	160·2 (151·6–169·6)	90·2 (82·1–98·6)	18·1 (14·9–22·0)	2·3 (1·6–3·0)	0·1 (0·0–0·1)	0·0 (0·0–0·0)	1·68 (1·61–1·75)	0·32 (0·28–0·37)	0·55 (0·51–0·6)
1989	0·0 (0·0–0·0)	3·6 (3·0–4·4)	57·0 (50·0–64·9)	151·0 (143·6–158·9)	90·7 (82·7–98·9)	19·0 (15·8–23·1)	2·4 (1·8–3·3)	0·1 (0·0–0·1)	0·0 (0·0–0·0)	1·62 (1·55–1·69)	0·3 (0·27–0·34)	0·56 (0·52–0·61)
1990	0·0 (0·0–0·0)	3·7 (3·1–4·4)	55·8 (48·3–63·4)	148·5 (140·0–156·3)	93·0 (84·9–100·8)	20·0 (16·3–24·0)	2·4 (1·8–3·2)	0·1 (0·0–0·1)	0·0 (0·0–0·0)	1·62 (1·55–1·69)	0·3 (0·26–0·34)	0·58 (0·53–0·62)
1991	0·0 (0·0–0·0)	3·9 (3·2–4·7)	56·1 (48·8–64·0)	145·4 (138·1–153·6)	94·5 (85·6–103·3)	21·2 (17·6–25·5)	2·5 (1·8–3·3)	0·1 (0·0–0·1)	0·0 (0·0–0·0)	1·62 (1·55–1·69)	0·3 (0·26–0·34)	0·59 (0·55–0·64)
1992	0·0 (0·0–0·0)	4·1 (3·3–4·9)	54·6 (48·4–61·9)	138·2 (130·4–146·4)	94·0 (86·3–101·7)	22·0 (18·2–26·6)	2·6 (1·9–3·4)	0·1 (0·0–0·1)	0·0 (0·0–0·0)	1·58 (1·52–1·64)	0·29 (0·26–0·33)	0·59 (0·55–0·64)
1993	0·0 (0·0–0·0)	4·2 (3·4–5·1)	54·8 (47·5–62·6)	138·6 (129·5–147·6)	98·2 (89·4–107·1)	24·0 (20·1–28·4)	2·8 (2·0–3·6)	0·1 (0·0–0·1)	0·0 (0·0–0·0)	1·61 (1·54–1·69)	0·3 (0·26–0·34)	0·62 (0·58–0·67)
1994	0·0 (0·0–0·0)	4·4 (3·6–5·3)	54·7 (47·7–62·5)	136·6 (128·7–144·5)	99·8 (90·6–108·9)	25·6 (21·2–30·8)	2·9 (2·1–3·9)	0·1 (0·0–0·1)	0·0 (0·0–0·0)	1·62 (1·54–1·69)	0·3 (0·26–0·33)	0·64 (0·59–0·69)
1995	0·0 (0·0–0·0)	4·5 (3·6–5·5)	53·8 (47·2–61·3)	129·3 (121·1–137·5)	98·8 (89·7–108·5)	26·9 (22·6–31·8)	3·0 (2·3–4·0)	0·1 (0·0–0·1)	0·0 (0·0–0·0)	1·58 (1·51–1·65)	0·29 (0·26–0·33)	0·64 (0·59–0·7)
1996	0·0 (0·0–0·0)	4·8 (3·9–5·7)	53·9 (46·5–61·1)	125·3 (117·0–133·9)	98·8 (90·5–107·7)	28·5 (23·7–33·8)	3·2 (2·3–4·1)	0·1 (0·0–0·1)	0·0 (0·0–0·0)	1·57 (1·5–1·64)	0·29 (0·25–0·33)	0·65 (0·61–0·7)
1997	0·0 (0·0–0·0)	5·1 (4·1–6·1)	54·3 (47·6–61·6)	121·7 (114·3–128·7)	97·5 (88·3–105·8)	29·5 (24·7–34·9)	3·3 (2·4–4·5)	0·1 (0·0–0·1)	0·0 (0·0–0·0)	1·56 (1·49–1·63)	0·3 (0·26–0·33)	0·65 (0·61–0·7)
1998	0·0 (0·0–0·0)	5·4 (4·4–6·6)	54·6 (47·8–62·8)	117·8 (110·1–125·5)	96·9 (88·6–106·0)	31·0 (25·6–37·1)	3·5 (2·6–4·7)	0·1 (0·0–0·1)	0·0 (0·0–0·0)	1·55 (1·48–1·62)	0·3 (0·27–0·34)	0·66 (0·61–0·71)
1999	0·0 (0·0–0·0)	5·8 (4·7–7·0)	54·7 (48·1–61·8)	115·0 (107·8–122·3)	96·4 (87·9–105·3)	32·1 (27·0–37·8)	3·7 (2·7–5·0)	0·1 (0·0–0·1)	0·0 (0·0–0·0)	1·54 (1·47–1·61)	0·3 (0·27–0·34)	0·66 (0·61–0·71)
2000	0·0 (0·0–0·0)	6·2 (5·1–7·5)	55·3 (46·8–63·9)	113·5 (106·4–120·9)	94·8 (86·7–103·8)	34·0 (28·2–40·2)	4·0 (2·9–5·3)	0·1 (0·0–0·1)	0·0 (0·0–0·0)	1·54 (1·46–1·63)	0·31 (0·27–0·35)	0·66 (0·61–0·72)
2001	0·0 (0·0–0·0)	6·5 (5·4–7·8)	55·5 (49·3–62·7)	111·1 (104·1–118·3)	92·3 (83·8–101·1)	34·4 (28·5–40·6)	4·2 (3·1–5·6)	0·1 (0·1–0·1)	0·0 (0·0–0·0)	1·52 (1·45–1·59)	0·31 (0·28–0·35)	0·65 (0·61–0·71)
2002	0·0 (0·0–0·0)	6·4 (5·2–7·8)	54·1 (47·2–62·1)	108·9 (101·8–116·2)	92·4 (83·8–101·7)	35·3 (29·8–41·7)	4·5 (3·3–6·4)	0·1 (0·1–0·1)	0·0 (0·0–0·0)	1·51 (1·43–1·58)	0·3 (0·27–0·34)	0·66 (0·61–0·72)
2003	0·0 (0·0–0·0)	6·2 (4·9–7·5)	52·2 (44·8–60·6)	106·7 (100·1–113·6)	92·5 (84·7–101·2)	37·1 (30·8–43·9)	4·9 (3·4–6·6)	0·1 (0·1–0·2)	0·0 (0·0–0·0)	1·5 (1·43–1·58)	0·29 (0·26–0·33)	0·67 (0·62–0·73)
2004	0·0 (0·0–0·0)	5·9 (4·8–7·2)	50·6 (44·4–58·0)	103·1 (96·1–110·7)	91·8 (84·2–100·3)	38·0 (32·0–44·6)	5·2 (3·7–6·8)	0·1 (0·1–0·2)	0·0 (0·0–0·0)	1·47 (1·4–1·54)	0·28 (0·25–0·32)	0·68 (0·62–0·73)
2005	0·0 (0·0–0·0)	5·8 (4·6–7·1)	50·7 (44·6–57·5)	102·5 (95·3–110·1)	93·0 (85·2–101·6)	38·9 (32·7–45·7)	5·4 (4·0–7·1)	0·1 (0·1–0·2)	0·0 (0·0–0·0)	1·48 (1·42–1·55)	0·28 (0·25–0·32)	0·69 (0·64–0·74)
2006	0·0 (0·0–0·0)	5·8 (4·6–7·3)	51·9 (44·9–59·6)	104·1 (97·2–111·4)	96·6 (88·2–105·8)	40·8 (34·6–48·5)	5·9 (4·4–7·8)	0·1 (0·1–0·2)	0·0 (0·0–0·0)	1·53 (1·46–1·6)	0·29 (0·25–0·33)	0·72 (0·67–0·78)
2007	0·0 (0·0–0·0)	5·8 (4·6–7·2)	52·5 (46·5–59·2)	104·2 (96·9–111·8)	97·3 (88·8–106·3)	42·8 (36·6–50·2)	6·4 (4·8–8·2)	0·1 (0·1–0·2)	0·0 (0·0–0·0)	1·55 (1·47–1·62)	0·29 (0·26–0·32)	0·73 (0·68–0·79)
2008	0·0 (0·0–0·0)	5·8 (4·5–7·2)	52·7 (45·1–61·0)	104·3 (97·1–111·0)	98·2 (90·7–106·2)	44·3 (37·2–51·7)	6·8 (5·3–8·7)	0·1 (0·1–0·2)	0·0 (0·0–0·0)	1·56 (1·49–1·64)	0·29 (0·25–0·34)	0·75 (0·7–0·8)
2009	0·0 (0·0–0·0)	5·5 (4·3–7·1)	51·6 (45·8–57·3)	104·6 (98·2–110·6)	99·2 (90·8–109·0)	45·8 (38·8–53·3)	7·5 (5·8–9·4)	0·1 (0·1–0·2)	0·0 (0·0–0·0)	1·57 (1·48–1·67)	0·29 (0·26–0·31)	0·76 (0·71–0·83)
2010	0·0 (0·0–0·0)	5·4 (4·0–7·1)	50·8 (43·2–59·1)	105·9 (98·6–113·6)	100·6 (91·5–110·2)	47·1 (40·2–54·6)	7·9 (6·2–9·8)	0·1 (0·1–0·2)	0·0 (0·0–0·0)	1·59 (1·51–1·68)	0·28 (0·24–0·33)	0·78 (0·72–0·84)
2011	0·0 (0·0–0·0)	5·3 (4·2–6·6)	48·6 (42·0–56·2)	105·7 (98·4–113·0)	101·1 (92·5–109·8)	48·3 (41·0–56·0)	8·3 (6·4–10·5)	0·1 (0·1–0·2)	0·0 (0·0–0·0)	1·59 (1·5–1·67)	0·27 (0·23–0·31)	0·79 (0·73–0·85)
2012	0·0 (0·0–0·0)	5·2 (4·3–6·1)	46·4 (39·9–53·7)	105·2 (98·5–112·9)	102·9 (93·6–112·5)	50·6 (43·6–58·5)	9·0 (7·1–11·5)	0·2 (0·1–0·3)	0·0 (0·0–0·0)	1·6 (1·53–1·67)	0·26 (0·23–0·29)	0·81 (0·76–0·87)
2013	0·0 (0·0–0·0)	5·1 (4·2–6·1)	44·7 (38·4–51·3)	103·5 (96·5–111·5)	103·3 (94·8–112·5)	51·7 (44·7–59·6)	9·6 (7·3–12·3)	0·2 (0·1–0·3)	0·0 (0·0–0·0)	1·59 (1·52–1·66)	0·25 (0·22–0·28)	0·82 (0·76–0·89)
2014	0·0 (0·0–0·0)	5·0 (4·1–5·9)	42·9 (36·8–49·4)	102·5 (95·6–109·9)	104·5 (96·0–113·7)	53·4 (45·7–61·8)	10·3 (8·0–13·4)	0·2 (0·1–0·3)	0·0 (0·0–0·0)	1·59 (1·51–1·68)	0·24 (0·21–0·27)	0·84 (0·77–0·91)
2015	0·0 (0·0–0·0)	4·9 (4·1–6·0)	43·3 (37·0–50·9)	103·8 (95·4–112·7)	106·8 (96·4–118·5)	51·4 (42·7–61·3)	9·6 (7·0–12·8)	0·2 (0·1–0·3)	0·0 (0·0–0·0)	1·6 (1·43–1·79)	0·24 (0·21–0·28)	0·84 (0·74–0·94)
2016	0·0 (0·0–0·0)	5·0 (4·0–6·1)	44·7 (35·0–57·4)	104·8 (93·5–118·1)	106·0 (92·0–122·7)	47·1 (36·4–61·0)	8·3 (5·7–11·6)	0·2 (0·1–0·2)	0·0 (0·0–0·0)	1·58 (1·35–1·86)	0·25 (0·2–0·32)	0·81 (0·68–0·96)
2017	0·0 (0·0–0·0)	4·9 (4·1–6·0)	44·3 (34·7–56·8)	104·3 (92·6–117·1)	104·0 (88·7–120·7)	45·5 (34·0–59·7)	8·1 (5·2–12·1)	0·2 (0·1–0·3)	0·0 (0·0–0·0)	1·56 (1·3–1·85)	0·25 (0·19–0·31)	0·79 (0·65–0·95)

Supplemental Results Table 5: Age-specific fertility rates per 1000, cumulative total fertility up to maternal age 25 (TFU25), cumulative total fertility from maternal age 30 to age 55 (TFO30), and cumulative total fertility, Kyoto, 1950-2017

Year	10-14	15-19	20-24	25-29	30-34	35-39	40-44	45-49	50-54	TFR	TFU25	TFO30
1950	0·0 (0·0–0·0)	9·4 (8·4–10·5)	118·0 (107·6–129·3)	220·9 (212·8–229·0)	136·9 (128·8–144·9)	52·6 (46·6–58·4)	13·7 (11·9–15·8)	0·6 (0·4–0·8)	0·0 (0·0–0·0)	2·76 (2·66–2·86)	0·64 (0·58–0·69)	1·02 (0·97–1·07)
1951	0·0 (0·0–0·0)	7·6 (6·8–8·5)	105·4 (95·8–114·6)	205·2 (197·2–212·9)	123·6 (114·7–132·4)	57·2 (49·9–64·8)	10·6 (8·9–12·5)	0·4 (0·3–0·6)	0·0 (0·0–0·0)	2·55 (2·45–2·65)	0·57 (0·52–0·61)	0·96 (0·88–1·04)
1952	0·0 (0·0–0·0)	6·4 (5·6–7·2)	97·8 (86·8–110·2)	194·8 (186·5–203·4)	113·6 (104·5–123·1)	56·7 (49·1–65·5)	8·9 (7·2–10·7)	0·3 (0·2–0·5)	0·0 (0·0–0·0)	2·39 (2·31–2·48)	0·52 (0·47–0·58)	0·9 (0·84–0·96)
1953	0·0 (0·0–0·0)	5·6 (4·8–6·5)	92·0 (80·9–103·7)	183·4 (174·5–192·4)	105·0 (96·1–114·3)	52·2 (44·7–60·9)	8·0 (6·4–9·9)	0·3 (0·2–0·5)	0·0 (0·0–0·0)	2·23 (2·14–2·32)	0·49 (0·43–0·55)	0·83 (0·76–0·89)
1954	0·0 (0·0–0·0)	5·1 (4·3–5·9)	88·8 (77·3–101·1)	178·2 (170·7–186·3)	99·7 (91·0–109·1)	47·7 (40·1–56·5)	7·3 (5·7–9·3)	0·3 (0·2–0·5)	0·0 (0·0–0·0)	2·14 (2·05–2·22)	0·47 (0·41–0·53)	0·77 (0·71–0·84)
1955	0·0 (0·0–0·0)	4·6 (3·9–5·4)	86·4 (74·8–99·8)	176·9 (168·6–185·2)	95·8 (86·8–105·2)	43·0 (36·0–50·2)	7·1 (5·3–9·0)	0·3 (0·2–0·5)	0·0 (0·0–0·0)	2·07 (1·98–2·16)	0·45 (0·4–0·53)	0·73 (0·67–0·8)
1956	0·0 (0·0–0·0)	4·1 (3·5–4·8)	81·0 (69·4–93·8)	170·7 (163·3–178·7)	87·2 (78·8–96·2)	37·0 (31·1–43·4)	6·2 (4·8–7·9)	0·3 (0·1–0·4)	0·0 (0·0–0·0)	1·93 (1·85–2·02)	0·43 (0·37–0·49)	0·65 (0·6–0·72)
1957	0·0 (0·0–0·0)	3·9 (3·3–4·6)	81·6 (69·3–95·7)	173·7 (166·2–181·9)	80·4 (72·5–88·9)	31·8 (26·4–37·0)	4·8 (3·7–6·0)	0·2 (0·1–0·4)	0·0 (0·0–0·0)	1·88 (1·79–1·99)	0·43 (0·37–0·5)	0·59 (0·53–0·64)
1958	0·0 (0·0–0·0)	3·9 (3·3–4·6)	85·3 (73·8–97·9)	179·1 (170·9–187·7)	77·0 (70·4–84·2)	28·2 (23·7–33·4)	4·0 (3·1–5·1)	0·2 (0·1–0·4)	0·0 (0·0–0·0)	1·89 (1·8–1·96)	0·45 (0·39–0·51)	0·55 (0·5–0·6)
1959	0·0 (0·0–0·0)	4·0 (3·4–4·8)	84·9 (73·6–97·8)	177·8 (169·4–186·1)	73·8 (65·6–81·7)	24·4 (20·1–29·1)	3·6 (2·7–4·6)	0·2 (0·1–0·3)	0·0 (0·0–0·0)	1·84 (1·76–1·92)	0·44 (0·39–0·51)	0·51 (0·46–0·56)
1960	0·0 (0·0–0·0)	3·9 (3·3–4·6)	81·3 (71·1–92·2)	178·3 (169·8–187·0)	73·5 (65·6–82·0)	21·5 (18·0–25·7)	3·3 (2·5–4·3)	0·2 (0·1–0·3)	0·0 (0·0–0·0)	1·81 (1·73–1·89)	0·43 (0·38–0·48)	0·49 (0·45–0·54)
1961	0·0 (0·0–0·0)	3·6 (3·0–4·3)	78·5 (70·3–87·2)	179·8 (171·5–187·9)	73·3 (68·1–78·9)	19·5 (16·3–23·6)	3·0 (2·2–4·1)	0·2 (0·1–0·3)	0·0 (0·0–0·0)	1·79 (1·72–1·86)	0·41 (0·37–0·45)	0·48 (0·45–0·51)
1962	0·0 (0·0–0·0)	3·5 (2·8–4·1)	78·0 (69·5–86·9)	184·5 (176·9–192·0)	75·1 (69·0–81·5)	18·5 (15·9–21·2)	2·7 (2·0–3·6)	0·2 (0·1–0·3)	0·0 (0·0–0·0)	1·81 (1·74–1·88)	0·41 (0·37–0·45)	0·48 (0·45–0·52)
1963	0·0 (0·0–0·0)	3·4 (2·8–4·0)	78·0 (69·3–87·8)	186·0 (178·4–194·1)	73·6 (67·1–80·2)	17·9 (15·2–21·0)	2·3 (1·7–3·0)	0·1 (0·1–0·3)	0·0 (0·0–0·0)	1·81 (1·74–1·88)	0·41 (0·36–0·46)	0·47 (0·43–0·51)
1964	0·0 (0·0–0·0)	3·3 (2·8–4·0)	84·8 (75·0–95·9)	197·6 (189·2–206·3)	78·7 (71·0–87·1)	18·6 (15·5–21·9)	2·1 (1·5–2·8)	0·1 (0·1–0·2)	0·0 (0·0–0·0)	1·93 (1·85–2·01)	0·44 (0·39–0·5)	0·5 (0·46–0·54)
1965	0·0 (0·0–0·0)	3·2 (2·7–3·8)	77·9 (67·0–90·2)	166·5 (158·2–175·0)	69·3 (62·1–76·2)	17·1 (14·2–20·2)	2·2 (1·6–2·9)	0·1 (0·1–0·2)	0·0 (0·0–0·0)	1·68 (1·61–1·76)	0·41 (0·35–0·47)	0·44 (0·4–0·48)
1966	0·0 (0·0–0·0)	3·4 (2·9–4·0)	75·9 (63·0–89·6)	170·9 (162·7–178·7)	69·9 (62·8–77·9)	17·5 (14·2–21·2)	2·0 (1·5–2·7)	0·1 (0·1–0·2)	0·0 (0·0–0·0)	1·7 (1·62–1·78)	0·4 (0·33–0·47)	0·45 (0·41–0·49)
1967	0·0 (0·0–0·0)	3·6 (3·0–4·2)	80·6 (66·5–96·4)	207·8 (199·8–215·9)	86·8 (77·6–97·5)	19·8 (16·4–23·9)	2·1 (1·6–2·7)	0·1 (0·1–0·2)	0·0 (0·0–0·0)	2·0 (1·9–2·1)	0·42 (0·35–0·5)	0·54 (0·49–0·6)
1968	0·0 (0·0–0·0)	3·6 (3·2–4·2)	72·0 (59·0–86·5)	195·6 (187·4–203·4)	84·8 (76·5–93·2)	19·7 (16·2–23·8)	2·4 (1·8–3·1)	0·1 (0·1–0·3)	0·0 (0·0–0·0)	1·89 (1·82–1·97)	0·38 (0·31–0·45)	0·54 (0·49–0·58)
1969	0·0 (0·0–0·0)	3·9 (3·4–4·6)	70·5 (57·8–84·6)	197·2 (189·2–205·2)	83·4 (76·0–92·0)	19·4 (16·1–23·4)	2·2 (1·6–3·0)	0·1 (0·0–0·2)	0·0 (0·0–0·0)	1·88 (1·81–1·96)	0·37 (0·31–0·44)	0·53 (0·48–0·58)
1970	0·0 (0·0–0·0)	3·9 (3·4–4·6)	75·5 (63·1–89·3)	200·2 (192·1–208·6)	79·7 (72·2–87·1)	18·6 (15·1–22·5)	1·8 (1·3–2·4)	0·1 (0·0–0·2)	0·0 (0·0–0·0)	1·9 (1·82–1·97)	0·4 (0·34–0·47)	0·5 (0·45–0·55)
1971	0·0 (0·0–0·0)	3·8 (3·2–4·5)	82·3 (70·1–96·0)	197·3 (188·7–205·4)	76·0 (68·8–83·6)	16·9 (13·9–20·4)	1·5 (1·1–2·1)	0·1 (0·0–0·1)	0·0 (0·0–0·0)	1·89 (1·82–1·97)	0·43 (0·37–0·5)	0·47 (0·43–0·52)
1972	0·0 (0·0–0·0)	3·8 (3·2–4·5)	85·9 (74·3–98·9)	195·5 (187·8–203·8)	74·8 (67·5–81·9)	16·0 (12·8–19·4)	1·4 (1·0–2·0)	0·1 (0·0–0·1)	0·0 (0·0–0·0)	1·89 (1·81–1·96)	0·45 (0·39–0·51)	0·46 (0·42–0·5)
1973	0·0 (0·0–0·0)	3·7 (3·1–4·4)	85·6 (75·3–95·9)	195·3 (186·7–203·9)	73·2 (66·9–80·2)	15·7 (12·6–19·1)	1·4 (1·0–2·1)	0·1 (0·0–0·1)	0·0 (0·0–0·0)	1·87 (1·8–1·95)	0·45 (0·4–0·5)	0·45 (0·41–0·49)
1974	0·0 (0·0–0·0)	3·4 (2·8–4·1)	81·4 (72·7–91·3)	187·4 (179·1–195·6)	68·8 (62·7–75·5)	14·7 (11·8–17·8)	1·5 (1·1–2·2)	0·1 (0·0–0·1)	0·0 (0·0–0·0)	1·79 (1·72–1·85)	0·42 (0·38–0·47)	0·43 (0·39–0·46)
1975	0·0 (0·0–0·0)	3·1 (2·6–3·9)	75·2 (66·9–84·4)	180·3 (172·1–188·2)	65·5 (59·4–71·5)	13·9 (11·4–16·7)	1·6 (1·1–2·1)	0·1 (0·0–0·1)	0·0 (0·0–0·0)	1·7 (1·63–1·77)	0·39 (0·35–0·44)	0·4 (0·37–0·44)
1976	0·0 (0·0–0·0)	3·0 (2·4–3·6)	70·0 (62·1–78·2)	177·5 (169·6–185·2)	66·3 (60·7–72·6)	13·8 (11·7–16·1)	1·6 (1·1–2·3)	0·1 (0·0–0·1)	0·0 (0·0–0·0)	1·66 (1·59–1·74)	0·36 (0·33–0·41)	0·41 (0·37–0·45)
1977	0·0 (0·0–0·0)	2·9 (2·4–3·4)	64·8 (58·2–72·6)	177·7 (169·7–185·6)	70·1 (63·5–76·9)	13·8 (11·5–16·6)	1·7 (1·2–2·4)	0·1 (0·0–0·1)	0·0 (0·0–0·0)	1·65 (1·59–1·72)	0·34 (0·31–0·38)	0·43 (0·38–0·47)
1978	0·0 (0·0–0·0)	2·9 (2·3–3·6)	60·6 (52·4–70·5)	177·6 (170·1–185·4)	74·2 (67·3–81·9)	13·7 (10·8–17·1)	1·7 (1·2–2·4)	0·1 (0·0–0·1)	0·0 (0·0–0·0)	1·65 (1·58–1·74)	0·32 (0·28–0·37)	0·45 (0·41–0·49)
1979	0·0 (0·0–0·0)	2·9 (2·2–3·8)	56·8 (50·7–62·9)	175·5 (167·9–182·6)	74·5 (67·9–81·5)	13·5 (11·1–16·2)	1·7 (1·2–2·5)	0·1 (0·0–0·1)	0·0 (0·0–0·0)	1·63 (1·57–1·69)	0·3 (0·27–0·33)	0·45 (0·41–0·49)
1980	0·0 (0·0–0·0)	3·0 (2·4–3·7)	53·6 (47·3–59·9)	173·2 (165·4–181·2)	73·0 (66·2–80·3)	13·5 (11·1–16·4)	1·7 (1·3–2·4)	0·1 (0·0–0·2)	0·0 (0·0–0·0)	1·59 (1·52–1·66)	0·28 (0·25–0·31)	0·44 (0·41–0·48)
1981	0·0 (0·0–0·0)	3·1 (2·6–3·8)	51·9 (46·6–58·4)	174·2 (166·3–182·5)	73·9 (67·3–80·7)	14·3 (11·8–17·2)	1·8 (1·2–2·5)	0·1 (0·0–0·1)	0·0 (0·0–0·0)	1·6 (1·54–1·65)	0·28 (0·25–0·31)	0·45 (0·41–0·49)
1982	0·0 (0·0–0·0)	3·4 (2·8–4·1)	51·3 (45·1–58·1)	175·8 (168·9–183·9)	77·8 (71·0–85·2)	16·1 (12·4–21·0)	1·7 (1·2–2·4)	0·1 (0·0–0·1)	0·0 (0·0–0·0)	1·63 (1·57–1·69)	0·27 (0·24–0·31)	0·48 (0·44–0·52)
1983	0·0 (0·0–0·0)	3·5 (2·9–4·2)	50·4 (44·2–57·1)	175·7 (166·9–183·9)	82·5 (75·1–90·9)	17·7 (14·6–21·0)	1·8 (1·4–2·2)	0·1 (0·0–0·1)	0·0 (0·0–0·0)	1·66 (1·59–1·72)	0·27 (0·24–0·3)	0·51 (0·47–0·56)
1984	0·0 (0·0–0·0)	3·4 (2·8–4·1)	47·4 (41·9–53·1)	172·2 (164·7–180·4)	86·0 (78·7–94·4)	18·1 (14·9–22·1)	1·8 (1·3–2·4)	0·1 (0·0–0·1)	0·0 (0·0–0·0)	1·64 (1·58–1·71)	0·25 (0·23–0·28)	0·53 (0·49–0·57)
1985	0·0 (0·0–0·0)	3·3 (2·7–3·9)	45·1 (39·4–51·3)	165·4 (157·9–172·3)	87·5 (80·2–95·2)	17·8 (14·9–21·7)	1·8 (1·3–2·5)	0·1 (0·0–0·1)	0·0 (0·0–0·0)	1·6 (1·54–1·67)	0·24 (0·21–0·27)	0·54 (0·49–0·57)
1986	0·0 (0·0–0·0)	3·2 (2·7–3·8)	43·2 (37·8–48·6)	159·0 (151·3–167·3)	89·8 (80·0–98·9)	17·7 (14·7–21·1)	1·9 (1·3–2·9)	0·1 (0·0–0·1)	0·0 (0·0–0·0)	1·57 (1·52–1·64)	0·23 (0·21–0·26)	0·55 (0·49–0·6)
1987	0·0 (0·0–0·0)	3·0 (2·6–3·6)	39·9 (35·3–45·1)	153·0 (145·7–160·4)	92·2 (85·0–100·0)	18·4 (14·3–23·2)	2·2 (1·5–3·1)	0·1 (0·0–0·1)	0·0 (0·0–0·0)	1·54 (1·48–1·61)	0·21 (0·19–0·24)	0·56 (0·52–0·61)
1988	0·0 (0·0–0·0)	2·9 (2·5–3·4)	36·9 (31·9–42·5)	142·7 (135·6–150·3)	92·7 (84·9–101·2)	19·3 (16·2–22·9)	2·4 (1·7–3·2)	0·1 (0·0–0·1)	0·0 (0·0–0·0)	1·48 (1·42–1·55)	0·2 (0·17–0·23)	0·57 (0·53–0·62)
1989	0·0 (0·0–0·0)	2·9 (2·4–3·5)	33·9 (29·3–39·4)	132·1 (125·7–139·3)	91·6 (84·0–99·8)	20·3 (16·8–24·1)	2·5 (1·8–3·4)	0·1 (0·0–0·1)	0·0 (0·0–0·0)	1·42 (1·35–1·48)	0·18 (0·16–0·21)	0·57 (0·53–0·61)
1990	0·0 (0·0–0·0)	3·0 (2·5–3·5)	32·4 (28·4–36·6)	127·9 (120·6–135·1)	92·1 (84·4–99·9)	21·2 (17·5–25·1)	2·5 (1·8–3·3)	0·1 (0·0–0·1)	0·0 (0·0–0·0)	1·4 (1·34–1·45)	0·18 (0·15–0·2)	0·58 (0·54–0·62)
1991	0·0 (0·0–0·0)	3·0 (2·5–3·6)	32·0 (27·7–36·7)	123·5 (117·2–130·3)	91·8 (84·6–99·2)	22·5 (18·1–27·8)	2·5 (1·9–3·4)	0·1 (0·0–0·2)	0·0 (0·0–0·0)	1·38 (1·32–1·44)	0·17 (0·15–0·2)	0·58 (0·54–0·63)
1992	0·0 (0·0–0·0)	3·1 (2·6–3·7)	30·6 (26·7–34·7)	116·1 (109·4–122·9)	90·2 (81·4–99·9)	23·1 (19·4–27·5)	2·7 (1·9–3·5)	0·1 (0·0–0·2)	0·0 (0·0–0·0)	1·33 (1·28–1·38)	0·17 (0·15–0·19)	0·58 (0·54–0·63)
1993	0·0 (0·0–0·0)	3·1 (2·5–3·8)	30·1 (25·9–34·5)	115·4 (108·5–122·8)	93·7 (85·9–102·1)	24·9 (20·7–29·9)	2·8 (2·1–3·7)	0·1 (0·0–0·2)	0·0 (0·0–0·0)	1·35 (1·28–1·42)	0·17 (0·14–0·19)	0·61 (0·56–0·66)
1994	0·0 (0·0–0·0)	3·2 (2·5–3·9)	29·5 (25·9–33·8)	113·0 (106·2–119·5)	95·2 (87·1–104·1)	26·1 (21·2–31·4)	3·0 (2·2–3·9)	0·1 (0·0–0·2)	0·0 (0·0–0·0)	1·35 (1·29–1·41)	0·16 (0·15–0·18)	0·62 (0·57–0·67)
1995	0·0 (0·0–0·0)	3·2 (2·6–3·9)	28·7 (24·9–32·8)	106·5 (100·1–112·8)	94·6 (86·2–103·2)	26·9 (21·8–32·5)	3·1 (2·3–4·1)	0·1 (0·0–0·2)	0·0 (0·0–0·0)	1·31 (1·25–1·38)	0·16 (0·14–0·18)	0·62 (0·57–0·67)
1996	0·0 (0·0–0·0)	3·3 (2·7–4·0)	28·3 (24·2–32·2)	102·9 (96·5–109·2)	94·8 (86·9–103·1)	28·0 (22·8–33·8)	3·3 (2·4–4·2)	0·1 (0·0–0·2)	0·0 (0·0–0·0)	1·3 (1·24–1·36)	0·16 (0·14–0·18)	0·63 (0·59–0·68)
1997	0·0 (0·0–0·0)	3·5 (2·8–4·2)	28·0 (24·4–31·7)	99·7 (93·7–105·5)	93·8 (85·5–102·0)	28·7 (23·5–34·3)	3·5 (2·5–4·6)	0·1 (0·0–0·2)	0·0 (0·0–0·0)	1·29 (1·23–1·35)	0·16 (0·14–0·18)	0·63 (0·58–0·68)
1998	0·0 (0·0–0·0)	3·6 (2·9–4·5)	27·6 (24·2–32·1)	95·9 (89·9–102·3)	93·3 (85·1–102·0)	30·0 (24·1–36·0)	3·7 (2·7–4·9)	0·1 (0·1–0·2)	0·0 (0·0–0·0)	1·27 (1·21–1·33)	0·16 (0·14–0·18)	0·64 (0·59–0·69)
1999	0·0 (0·0–0·0)	3·9 (3·1–4·8)	27·2 (23·9–30·9)	92·5 (87·0–98·2)	92·9 (85·4–101·8)	31·1 (25·8–36·7)	3·9 (2·9–5·2)	0·1 (0·1–0·2)	0·0 (0·0–0·0)	1·26 (1·2–1·32)	0·16 (0·14–0·17)	0·64 (0·59–0·69)
2000	0·0 (0·0–0·0)	4·1 (3·3–5·1)	27·1 (22·5–32·0)	89·9 (83·9–96·2)	91·4 (84·4–99·8)	33·0 (27·6–38·9)	4·2 (3·0–5·7)	0·1 (0·1–0·2)	0·0 (0·0–0·0)	1·25 (1·19–1·31)	0·16 (0·13–0·18)	0·64 (0·6–0·69)
2001	0·0 (0·0–0·0)	4·3 (3·5–5·2)	26·8 (23·6–30·4)	86·4 (81·1–92·0)	88·9 (80·5–97·0)	33·4 (28·2–39·6)	4·4 (3·2–5·9)	0·1 (0·1–0·2)	0·0 (0·0–0·0)	1·22 (1·16–1·28)	0·16 (0·14–0·17)	0·63 (0·59–0·69)
2002	0·0 (0·0–0·0)	4·3 (3·5–5·2)	26·0 (22·3–30·2)	83·3 (77·9–88·9)	88·6 (80·9–97·4)	34·3 (29·2–40·1)	4·8 (3·4–6·6)	0·1 (0·1–0·3)	0·0 (0·0–0·0)	1·21 (1·14–1·27)	0·15 (0·13–0·17)	0·64 (0·59–0·69)
2003	0·0 (0·0–0·0)	4·1 (3·4–5·0)	25·0 (21·5–28·8)	80·4 (75·0–86·4)	88·4 (81·4–95·9)	36·2 (30·2–42·5)	5·2 (3·7–6·9)	0·2 (0·1–0·3)	0·0 (0·0–0·0)	1·2 (1·14–1·26)	0·15 (0·13–0·16)	0·65 (0·6–0·7)
2004	0·0 (0·0–0·0)	4·0 (3·3–4·7)	24·1 (21·0–27·8)	76·8 (71·8–81·9)	87·3 (79·9–94·7)	37·2 (31·7–43·5)	5·4 (4·0–7·1)	0·2 (0·1–0·3)	0·0 (0·0–0·0)	1·17 (1·12–1·23)	0·14 (0·12–0·16)	0·65 (0·6–0·7)
2005	0·0 (0·0–0·0)	3·9 (3·3–4·6)	24·1 (19·1–29·6)	75·5 (70·3–81·0)	88·0 (79·9–96·0)	38·5 (33·0–44·7)	5·7 (4·3–7·4)	0·2 (0·1–0·3)	0·0 (0·0–0·0)	1·18 (1·12–1·24)	0·14 (0·12–0·17)	0·66 (0·61–0·71)
2006	0·0 (0·0–0·0)	3·9 (3·2–4·6)	24·4 (21·2–27·8)	75·9 (71·0–81·5)	90·9 (83·3–99·1)	40·8 (34·8–47·6)	6·3 (4·8–8·1)	0·2 (0·1–0·3)	0·0 (0·0–0·0)	1·21 (1·15–1·28)	0·14 (0·12–0·16)	0·69 (0·64–0·75)
2007	0·0 (0·0–0·0)	3·9 (3·2–4·6)	24·5 (21·2–28·3)	75·3 (70·6–80·7)	91·1 (82·8–99·5)	43·1 (37·0–50·4)	6·9 (5·3–8·8)	0·2 (0·1–0·3)	0·0 (0·0–0·0)	1·22 (1·16–1·29)	0·14 (0·13–0·16)	0·71 (0·66–0·76)
2008	0·0 (0·0–0·0)	3·8 (3·2–4·5)	24·3 (21·0–27·7)	74·7 (69·6–80·1)	91·3 (83·7–99·1)	44·7 (38·3–51·7)	7·4 (5·8–9·4)	0·2 (0·1–0·3)	0·0 (0·0–0·0)	1·23 (1·17–1·29)	0·14 (0·12–0·16)	0·72 (0·67–0·77)
2009	0·0 (0·0–0·0)	3·7 (3·0–4·5)	23·7 (19·5–28·1)	74·1 (69·3–78·6)	91·7 (83·4–100·8)	46·3 (39·8–53·7)	8·2 (6·5–10·3)	0·2 (0·1–0·4)	0·0 (0·0–0·0)	1·24 (1·17–1·31)	0·14 (0·11–0·16)	0·73 (0·68–0·79)
2010	0·0 (0·0–0·0)	3·6 (2·7–4·6)	23·0 (20·1–26·1)	74·0 (68·7–79·3)	92·5 (85·0–100·9)	47·5 (41·1–54·7)	8·7 (6·8–10·6)	0·2 (0·1–0·4)	0·0 (0·0–0·0)	1·25 (1·19–1·31)	0·13 (0·12–0·15)	0·74 (0·69–0·8)
2011	0·0 (0·0–0·0)	3·4 (2·7–4·3)	22·0 (19·2–25·3)	73·0 (67·9–78·6)	92·4 (83·4–101·8)	48·6 (41·7–56·4)	9·0 (7·1–11·2)	0·2 (0·1–0·5)	0·0 (0·0–0·0)	1·24 (1·18–1·31)	0·13 (0·11–0·14)	0·75 (0·7–0·81)
2012	0·0 (0·0–0·0)	3·4 (2·7–4·2)	21·1 (18·1–24·3)	71·9 (67·1–77·1)	93·6 (85·0–102·7)	51·0 (44·1–58·5)	9·7 (7·7–12·3)	0·3 (0·1–0·5)	0·0 (0·0–0·0)	1·25 (1·2–1·32)	0·12 (0·11–0·14)	0·77 (0·72–0·83)
2013	0·0 (0·0–0·0)	3·3 (2·7–4·0)	20·4 (17·4–23·6)	70·3 (65·4–75·2)	93·6 (86·2–101·8)	52·5 (45·5–60·2)	10·2 (8·1–12·8)	0·3 (0·1–0·5)	0·0 (0·0–0·0)	1·25 (1·19–1·32)	0·12 (0·1–0·14)	0·78 (0·73–0·84)
2014	0·0 (0·0–0·0)	3·3 (2·7–3·9)	19·8 (16·8–23·1)	69·7 (64·7–75·1)	94·5 (86·5–102·7)	54·6 (47·4–62·7)	10·7 (8·4–13·8)	0·3 (0·1–0·5)	0·0 (0·0–0·0)	1·26 (1·2–1·34)	0·12 (0·1–0·13)	0·8 (0·74–0·87)
2015	0·0 (0·0–0·0)	3·3 (2·7–4·0)	20·3 (15·1–26·7)	71·2 (65·3–77·8)	96·6 (86·8–107·0)	53·1 (44·0–63·3)	9·9 (7·3–13·0)	0·3 (0·1–0·5)	0·0 (0·0–0·0)	1·27 (1·13–1·44)	0·12 (0·09–0·15)	0·8 (0·71–0·9)
2016	0·0 (0·0–0·0)	3·4 (2·8–4·1)	21·2 (16·2–27·8)	72·6 (64·9–81·7)	96·1 (83·5–111·0)	49·1 (38·1–63·4)	8·5 (6·1–11·7)	0·3 (0·1–0·5)	0·0 (0·0–0·0)	1·26 (1·07–1·48)	0·12 (0·09–0·16)	0·77 (0·65–0·91)
2017	0·0 (0·0–0·0)	3·4 (2·8–4·1)	21·2 (15·5–28·6)	72·7 (64·4–81·9)	94·9 (80·8–110·0)	47·9 (35·8–62·8)	8·2 (5·6–12·2)	0·3 (0·1–0·5)	0·0 (0·0–0·0)	1·24 (1·04–1·49)	0·12 (0·09–0·16)	0·76 (0·61–0·92)

Supplemental Results Table 5: Age-specific fertility rates per 1000, cumulative total fertility up to maternal age 25 (TFU25), cumulative total fertility from maternal age 30 to age 55 (TFO30), and cumulative total fertility, Mie, 1950-2017

Year	10-14	15-19	20-24	25-29	30-34	35-39	40-44	45-49	50-54	TFR	TFU25	TFO30
1950	0·0 (0·0–0·0)	11·2 (9·9–12·5)	164·8 (150·4–180·1)	225·6 (217·3–233·9)	153·0 (143·9–162·0)	95·0 (84·4–105·5)	28·5 (24·6–32·7)	1·4 (0·9–2·0)	0·0 (0·0–0·0)	3·4 (3·28–3·52)	0·88 (0·81–0·96)	1·39 (1·32–1·46)
1951	0·0 (0·0–0·0)	9·0 (8·0–10·1)	148·0 (134·5–161·5)	209·7 (201·8–217·7)	138·1 (127·7–147·6)	76·7 (66·7–86·9)	22·1 (18·4–25·9)	1·0 (0·7–1·5)	0·0 (0·0–0·0)	3·02 (2·89–3·15)	0·78 (0·72–0·85)	1·19 (1·08–1·3)
1952	0·0 (0·0–0·0)	7·6 (6·6–8·6)	137·9 (123·6–152·8)	199·4 (190·7–208·1)	124·9 (112·7–137·5)	61·5 (53·0–70·4)	17·5 (14·2–21·1)	0·8 (0·5–1·2)	0·0 (0·0–0·0)	2·75 (2·63–2·87)	0·73 (0·66–0·8)	1·02 (0·94–1·11)
1953	0·0 (0·0–0·0)	6·7 (5·8–7·6)	130·4 (116·1–146·1)	188·0 (180·0–197·0)	111·6 (98·4–124·5)	49·0 (41·4–57·2)	13·9 (11·2–17·1)	0·6 (0·4–1·0)	0·0 (0·0–0·0)	2·5 (2·38–2·64)	0·69 (0·61–0·76)	0·88 (0·8–0·96)
1954	0·0 (0·0–0·0)	6·1 (5·3–7·1)	126·6 (111·7–142·0)	183·0 (174·4–192·1)	102·8 (89·9–116·0)	40·8 (34·2–48·5)	11·4 (8·9–14·8)	0·5 (0·3–0·8)	0·0 (0·0–0·0)	2·36 (2·23–2·49)	0·66 (0·59–0·74)	0·78 (0·71–0·85)
1955	0·0 (0·0–0·0)	5·5 (4·7–6·4)	123·7 (109·5–140·2)	182·0 (173·3–190·8)	94·5 (81·5–107·4)	33·2 (28·1–39·5)	9·5 (7·1–12·3)	0·4 (0·2–0·7)	0·0 (0·0–0·0)	2·24 (2·13–2·37)	0·65 (0·57–0·73)	0·69 (0·62–0·75)
1956	0·0 (0·0–0·0)	4·9 (4·1–5·8)	116·6 (101·7–132·1)	176·0 (168·4–184·1)	83·7 (72·4–96·1)	26·8 (22·3–31·6)	7·6 (5·8–9·9)	0·3 (0·2–0·6)	0·0 (0·0–0·0)	2·08 (1·97–2·19)	0·61 (0·53–0·68)	0·59 (0·54–0·65)
1957	0·0 (0·0–0·0)	4·7 (4·0–5·6)	117·9 (103·5–134·5)	179·4 (171·7–187·5)	79·2 (68·7–91·7)	24·4 (20·2–28·3)	6·5 (4·9–8·2)	0·4 (0·2–0·6)	0·0 (0·0–0·0)	2·06 (1·94–2·19)	0·61 (0·54–0·7)	0·55 (0·5–0·61)
1958	0·0 (0·0–0·0)	4·8 (4·1–5·5)	123·9 (108·8–139·5)	185·3 (176·5–194·2)	77·4 (66·6–89·4)	23·0 (19·0–27·1)	5·9 (4·5–7·5)	0·4 (0·2–0·6)	0·0 (0·0–0·0)	2·1 (2·0–2·21)	0·64 (0·57–0·72)	0·53 (0·48–0·59)
1959	0·0 (0·0–0·0)	5·0 (4·2–5·8)	124·1 (109·7–139·2)	184·3 (175·6–193·8)	72·4 (63·4–82·4)	18·6 (15·2–22·6)	4·9 (3·7–6·4)	0·3 (0·2–0·5)	0·0 (0·0–0·0)	2·05 (1·94–2·16)	0·65 (0·57–0·72)	0·48 (0·44–0·53)
1960	0·0 (0·0–0·0)	4·8 (4·1–5·7)	119·5 (106·4–133·8)	185·2 (176·3–194·3)	69·3 (61·6–77·6)	15·3 (12·5–18·5)	4·0 (3·0–5·1)	0·2 (0·1–0·4)	0·0 (0·0–0·0)	1·99 (1·9–2·08)	0·62 (0·56–0·69)	0·44 (0·41–0·49)
1961	0·0 (0·0–0·0)	4·5 (3·8–5·3)	116·3 (104·7–128·4)	187·3 (179·3–195·4)	67·7 (59·2–77·3)	13·6 (11·1–16·5)	3·4 (2·5–4·7)	0·2 (0·1–0·4)	0·0 (0·0–0·0)	1·97 (1·88–2·06)	0·6 (0·55–0·66)	0·42 (0·38–0·48)
1962	0·0 (0·0–0·0)	4·3 (3·6–5·1)	116·3 (103·2–129·2)	192·8 (184·9–200·9)	68·9 (63·1–74·7)	12·8 (10·5–15·3)	3·1 (2·2–4·2)	0·2 (0·1–0·3)	0·0 (0·0–0·0)	1·99 (1·91–2·08)	0·6 (0·54–0·67)	0·43 (0·39–0·46)
1963	0·0 (0·0–0·0)	4·2 (3·6–5·1)	117·1 (104·6–130·1)	194·9 (187·0–203·3)	70·3 (64·3–76·9)	13·9 (11·7–16·5)	3·0 (2·1–4·1)	0·2 (0·1–0·4)	0·0 (0·0–0·0)	2·02 (1·93–2·1)	0·61 (0·54–0·67)	0·44 (0·4–0·47)
1964	0·0 (0·0–0·0)	4·2 (3·6–4·9)	128·2 (115·1–143·0)	207·5 (198·9–216·6)	74·9 (67·7–82·8)	14·3 (12·1–16·6)	2·8 (2·0–4·0)	0·2 (0·1–0·4)	0·0 (0·0–0·0)	2·16 (2·08–2·25)	0·66 (0·6–0·73)	0·46 (0·42–0·5)
1965	0·0 (0·0–0·0)	4·1 (3·5–4·8)	118·4 (105·8–133·0)	175·1 (165·9–185·0)	62·0 (54·4–69·8)	11·6 (9·5–14·0)	2·4 (1·7–3·3)	0·2 (0·1–0·3)	0·0 (0·0–0·0)	1·87 (1·78–1·96)	0·61 (0·55–0·69)	0·38 (0·34–0·42)
1966	0·0 (0·0–0·0)	4·4 (3·7–5·0)	116·0 (103·8–129·2)	180·1 (170·8–189·2)	63·3 (55·1–72·0)	12·5 (10·5–14·9)	2·4 (1·7–3·3)	0·2 (0·1–0·3)	0·0 (0·0–0·0)	1·89 (1·81–1·98)	0·6 (0·54–0·67)	0·39 (0·35–0·44)
1967	0·0 (0·0–0·0)	4·6 (3·9–5·4)	124·0 (111·1–137·1)	219·3 (210·0–228·3)	76·0 (64·4–88·4)	14·1 (11·6–17·2)	2·3 (1·7–3·0)	0·1 (0·1–0·3)	0·0 (0·0–0·0)	2·2 (2·09–2·31)	0·64 (0·58–0·71)	0·46 (0·41–0·52)
1968	0·0 (0·0–0·0)	4·7 (4·0–5·5)	111·4 (99·1–124·6)	206·7 (198·2–215·0)	68·8 (58·5–78·9)	13·1 (10·5–15·9)	2·1 (1·6–2·7)	0·1 (0·1–0·2)	0·0 (0·0–0·0)	2·03 (1·95–2·12)	0·58 (0·52–0·65)	0·42 (0·37–0·47)
1969	0·0 (0·0–0·0)	5·1 (4·3–6·1)	109·9 (97·2–122·2)	208·7 (200·4–217·0)	68·7 (57·8–81·3)	12·7 (10·4–15·4)	2·1 (1·6–2·7)	0·1 (0·1–0·2)	0·0 (0·0–0·0)	2·04 (1·95–2·12)	0·58 (0·51–0·64)	0·42 (0·36–0·48)
1970	0·0 (0·0–0·0)	5·2 (4·3–6·2)	118·6 (105·5–133·3)	212·0 (202·9–220·6)	70·4 (60·5–81·7)	13·9 (11·0–17·1)	2·2 (1·7–2·9)	0·1 (0·1–0·3)	0·0 (0·0–0·0)	2·11 (2·01–2·21)	0·62 (0·56–0·69)	0·43 (0·39–0·49)
1971	0·0 (0·0–0·0)	5·1 (4·2–6·2)	130·2 (115·4–145·1)	208·9 (199·9–218·5)	71·2 (61·1–83·1)	15·6 (12·6–18·9)	2·4 (1·7–3·4)	0·2 (0·1–0·3)	0·0 (0·0–0·0)	2·17 (2·07–2·27)	0·68 (0·6–0·75)	0·45 (0·4–0·5)
1972	0·0 (0·0–0·0)	5·1 (4·2–6·3)	136·6 (121·4–153·5)	206·8 (197·6–215·7)	70·9 (62·3–80·0)	15·9 (12·6–19·4)	2·4 (1·7–3·3)	0·2 (0·1–0·3)	0·0 (0·0–0·0)	2·19 (2·08–2·29)	0·71 (0·63–0·79)	0·45 (0·41–0·49)
1973	0·0 (0·0–0·0)	5·0 (4·1–6·1)	137·1 (122·5–152·4)	206·0 (196·7–216·0)	66·9 (59·7–75·0)	14·5 (11·4–18·0)	2·2 (1·5–3·1)	0·1 (0·1–0·2)	0·0 (0·0–0·0)	2·16 (2·07–2·25)	0·71 (0·64–0·79)	0·42 (0·38–0·46)
1974	0·0 (0·0–0·0)	4·6 (3·8–5·6)	130·9 (117·7–146·0)	196·9 (187·1–206·1)	58·9 (52·9–65·0)	11·2 (8·7–14·1)	1·8 (1·2–2·8)	0·1 (0·1–0·2)	0·0 (0·0–0·0)	2·02 (1·94–2·11)	0·68 (0·61–0·76)	0·36 (0·33–0·39)
1975	0·0 (0·0–0·0)	4·2 (3·5–5·2)	121·7 (109·0–134·7)	188·7 (179·7–197·6)	52·8 (47·3–58·3)	9·1 (7·1–11·7)	1·5 (1·1–2·1)	0·1 (0·0–0·1)	0·0 (0·0–0·0)	1·89 (1·81–1·97)	0·63 (0·57–0·7)	0·32 (0·29–0·34)
1976	0·0 (0·0–0·0)	4·0 (3·3–4·8)	114·1 (101·7–127·4)	185·5 (176·6–194·6)	51·3 (46·3–56·7)	8·8 (7·1–10·7)	1·3 (0·9–1·9)	0·1 (0·0–0·1)	0·0 (0·0–0·0)	1·83 (1·74–1·92)	0·59 (0·53–0·66)	0·31 (0·28–0·34)
1977	0·0 (0·0–0·0)	3·9 (3·3–4·6)	107·0 (95·3–120·8)	185·9 (177·5–194·4)	52·7 (47·3–58·7)	8·7 (6·8–11·2)	1·2 (0·8–1·7)	0·1 (0·0–0·1)	0·0 (0·0–0·0)	1·8 (1·72–1·88)	0·55 (0·5–0·62)	0·31 (0·28–0·35)
1978	0·0 (0·0–0·0)	3·9 (3·1–4·7)	101·4 (91·4–112·8)	186·7 (178·5–195·3)	54·8 (50·0–60·2)	8·6 (6·4–11·5)	1·0 (0·6–1·5)	0·0 (0·0–0·1)	0·0 (0·0–0·0)	1·78 (1·71–1·86)	0·53 (0·48–0·58)	0·32 (0·3–0·35)
1979	0·0 (0·0–0·0)	3·9 (3·0–5·1)	96·5 (85·4–108·6)	185·9 (177·8–194·2)	55·3 (48·2–63·4)	8·4 (6·8–10·2)	1·0 (0·6–1·5)	0·0 (0·0–0·1)	0·0 (0·0–0·0)	1·76 (1·67–1·84)	0·5 (0·45–0·56)	0·32 (0·29–0·37)
1980	0·0 (0·0–0·0)	4·0 (3·2–5·0)	91·8 (81·5–102·0)	184·9 (176·4–194·0)	55·4 (50·5–60·7)	8·4 (6·7–10·5)	1·0 (0·7–1·4)	0·0 (0·0–0·1)	0·0 (0·0–0·0)	1·73 (1·65–1·8)	0·48 (0·43–0·53)	0·32 (0·3–0·35)
1981	0·0 (0·0–0·0)	4·2 (3·5–5·1)	89·3 (78·9–101·3)	187·8 (179·2–196·5)	57·0 (51·9–63·0)	8·8 (7·0–10·9)	1·0 (0·7–1·5)	0·0 (0·0–0·1)	0·0 (0·0–0·0)	1·74 (1·67–1·81)	0·47 (0·41–0·53)	0·33 (0·31–0·36)
1982	0·0 (0·0–0·0)	4·4 (3·6–5·4)	88·3 (77·9–99·8)	191·6 (183·5–200·7)	60·3 (54·7–66·1)	9·8 (7·8–12·1)	1·0 (0·7–1·5)	0·0 (0·0–0·1)	0·0 (0·0–0·0)	1·78 (1·71–1·85)	0·46 (0·41–0·52)	0·36 (0·33–0·39)
1983	0·0 (0·0–0·0)	4·5 (3·7–5·5)	86·8 (77·7–97·4)	193·8 (184·1–202·6)	64·0 (58·3–70·7)	10·8 (8·5–13·4)	1·1 (0·8–1·4)	0·0 (0·0–0·1)	0·0 (0·0–0·0)	1·8 (1·73–1·88)	0·46 (0·41–0·51)	0·38 (0·34–0·42)
1984	0·0 (0·0–0·0)	4·4 (3·5–5·3)	81·6 (70·3–93·9)	192·3 (184·2–201·2)	66·7 (60·6–73·4)	11·0 (8·7–13·9)	1·1 (0·8–1·6)	0·0 (0·0–0·0)	0·0 (0·0–0·0)	1·79 (1·71–1·86)	0·43 (0·37–0·49)	0·39 (0·36–0·43)
1985	0·0 (0·0–0·0)	4·2 (3·4–5·1)	77·4 (68·3–87·5)	187·1 (176·1–197·0)	68·2 (61·8–74·7)	10·9 (8·7–13·6)	1·1 (0·8–1·5)	0·0 (0·0–0·1)	0·0 (0·0–0·0)	1·74 (1·67–1·82)	0·41 (0·36–0·46)	0·4 (0·37–0·44)
1986	0·0 (0·0–0·0)	4·0 (3·3–5·0)	74·2 (65·0–84·2)	182·5 (174·4–191·0)	70·6 (64·0–76·6)	11·1 (8·7–13·8)	1·2 (0·9–1·6)	0·0 (0·0–0·1)	0·0 (0·0–0·0)	1·72 (1·65–1·8)	0·39 (0·35–0·44)	0·41 (0·38–0·45)
1987	0·0 (0·0–0·0)	3·9 (3·2–4·8)	68·3 (60·0–77·3)	178·5 (169·1–188·3)	73·8 (67·1–81·4)	11·7 (9·4–14·4)	1·4 (0·9–1·9)	0·0 (0·0–0·1)	0·0 (0·0–0·0)	1·69 (1·61–1·77)	0·36 (0·32–0·41)	0·43 (0·4–0·48)
1988	0·0 (0·0–0·0)	3·8 (3·0–4·6)	62·7 (55·0–71·8)	169·4 (160·3–179·2)	75·6 (69·5–82·6)	12·6 (9·9–15·6)	1·5 (1·1–2·1)	0·0 (0·0–0·1)	0·0 (0·0–0·0)	1·63 (1·56–1·7)	0·33 (0·29–0·38)	0·45 (0·42–0·49)
1989	0·0 (0·0–0·0)	3·7 (3·0–4·5)	57·2 (49·7–65·6)	159·4 (151·4–168·3)	76·4 (69·0–84·1)	13·6 (10·7–17·2)	1·7 (1·2–2·3)	0·0 (0·0–0·1)	0·0 (0·0–0·0)	1·56 (1·49–1·63)	0·3 (0·27–0·35)	0·46 (0·42–0·5)
1990	0·0 (0·0–0·0)	3·7 (3·1–4·5)	54·3 (47·6–60·6)	156·3 (147·7–164·5)	78·4 (71·7–85·2)	14·5 (11·6–17·8)	1·7 (1·2–2·3)	0·0 (0·0–0·1)	0·0 (0·0–0·0)	1·54 (1·48–1·61)	0·29 (0·25–0·33)	0·47 (0·43–0·51)
1991	0·0 (0·0–0·0)	3·8 (3·1–4·6)	53·0 (46·0–60·5)	152·4 (144·9–161·0)	79·9 (73·3–86·6)	15·6 (12·7–19·2)	1·7 (1·2–2·3)	0·0 (0·0–0·1)	0·0 (0·0–0·0)	1·53 (1·47–1·6)	0·28 (0·25–0·32)	0·49 (0·45–0·52)
1992	0·0 (0·0–0·0)	3·8 (3·1–4·5)	50·3 (44·4–56·8)	144·4 (136·2–152·9)	80·2 (71·8–89·5)	16·4 (13·4–20·2)	1·9 (1·3–2·5)	0·1 (0·0–0·1)	0·0 (0·0–0·0)	1·48 (1·43–1·54)	0·27 (0·24–0·3)	0·49 (0·45–0·54)
1993	0·0 (0·0–0·0)	3·8 (3·1–4·6)	49·2 (42·3–56·6)	144·1 (135·4–153·5)	84·8 (77·5–92·5)	18·0 (14·6–21·7)	2·0 (1·4–2·7)	0·1 (0·0–0·1)	0·0 (0·0–0·0)	1·51 (1·44–1·58)	0·27 (0·23–0·3)	0·52 (0·48–0·57)
1994	0·0 (0·0–0·0)	3·9 (3·1–4·7)	48·0 (42·1–54·8)	141·2 (133·1–149·5)	87·8 (79·9–96·1)	19·4 (15·8–23·5)	2·1 (1·5–2·9)	0·1 (0·0–0·1)	0·0 (0·0–0·0)	1·51 (1·44–1·58)	0·26 (0·23–0·29)	0·55 (0·5–0·6)
1995	0·0 (0·0–0·0)	3·9 (3·2–4·7)	46·5 (40·0–53·5)	133·0 (124·9–141·3)	88·7 (80·6–96·6)	20·4 (16·9–24·5)	2·3 (1·7–3·1)	0·1 (0·0–0·1)	0·0 (0·0–0·0)	1·47 (1·41–1·54)	0·25 (0·22–0·29)	0·56 (0·51–0·61)
1996	0·0 (0·0–0·0)	4·1 (3·3–5·0)	45·9 (39·8–52·6)	128·2 (120·3–136·0)	90·3 (83·2–98·5)	21·6 (17·7–25·9)	2·4 (1·7–3·2)	0·1 (0·0–0·1)	0·0 (0·0–0·0)	1·46 (1·4–1·53)	0·25 (0·22–0·28)	0·57 (0·53–0·62)
1997	0·0 (0·0–0·0)	4·4 (3·5–5·3)	45·9 (39·9–52·1)	123·9 (116·7–131·3)	90·6 (82·8–98·6)	22·4 (18·4–26·8)	2·6 (1·9–3·4)	0·1 (0·0–0·1)	0·0 (0·0–0·0)	1·45 (1·39–1·51)	0·25 (0·22–0·28)	0·58 (0·53–0·63)
1998	0·0 (0·0–0·0)	4·7 (3·7–5·8)	45·7 (39·9–52·8)	119·1 (111·8–127·0)	91·4 (83·0–100·2)	23·7 (19·3–28·7)	2·7 (2·0–3·5)	0·1 (0·0–0·1)	0·0 (0·0–0·0)	1·44 (1·37–1·51)	0·25 (0·22–0·29)	0·59 (0·55–0·64)
1999	0·0 (0·0–0·0)	5·1 (4·1–6·3)	45·5 (39·6–51·7)	115·2 (108·4–122·4)	92·4 (84·6–101·1)	24·7 (20·6–29·3)	2·9 (2·1–3·9)	0·1 (0·0–0·1)	0·0 (0·0–0·0)	1·43 (1·36–1·5)	0·25 (0·22–0·29)	0·6 (0·55–0·65)
2000	0·0 (0·0–0·0)	5·5 (4·4–6·8)	45·8 (38·6–53·7)	112·4 (104·9–120·7)	92·0 (84·6–100·3)	26·5 (21·8–31·6)	3·0 (2·2–4·1)	0·1 (0·0–0·1)	0·0 (0·0–0·0)	1·43 (1·35–1·51)	0·26 (0·22–0·29)	0·61 (0·56–0·66)
2001	0·0 (0·0–0·0)	5·8 (4·7–7·0)	45·9 (40·5–51·7)	108·8 (102·2–115·7)	90·3 (81·9–98·5)	27·1 (22·3–32·4)	3·2 (2·3–4·3)	0·1 (0·0–0·1)	0·0 (0·0–0·0)	1·41 (1·34–1·47)	0·26 (0·23–0·29)	0·6 (0·56–0·65)
2002	0·0 (0·0–0·0)	5·8 (4·7–7·1)	44·8 (38·9–51·7)	105·5 (98·8–112·6)	90·6 (82·2–99·7)	28·2 (23·7–33·3)	3·4 (2·4–4·7)	0·1 (0·0–0·1)	0·0 (0·0–0·0)	1·39 (1·32–1·46)	0·25 (0·22–0·29)	0·61 (0·56–0·66)
2003	0·0 (0·0–0·0)	5·6 (4·6–6·7)	43·2 (37·7–49·6)	102·5 (95·4–109·9)	90·7 (82·9–99·1)	30·1 (25·1–35·6)	3·7 (2·6–5·1)	0·1 (0·0–0·2)	0·0 (0·0–0·0)	1·38 (1·31–1·46)	0·24 (0·22–0·27)	0·62 (0·57–0·67)
2004	0·0 (0·0–0·0)	5·3 (4·4–6·3)	41·9 (36·1–49·1)	98·0 (91·7–104·4)	89·7 (82·3–97·1)	31·3 (26·2–37·0)	3·9 (2·8–5·3)	0·1 (0·0–0·2)	0·0 (0·0–0·0)	1·35 (1·29–1·42)	0·24 (0·21–0·27)	0·62 (0·57–0·68)
2005	0·0 (0·0–0·0)	5·2 (4·4–6·2)	41·8 (36·6–48·0)	96·5 (89·9–103·6)	90·5 (82·3–98·7)	32·8 (27·9–38·6)	4·1 (2·9–5·6)	0·1 (0·0–0·2)	0·0 (0·0–0·0)	1·35 (1·29–1·42)	0·24 (0·21–0·27)	0·64 (0·59–0·69)
2006	0·0 (0·0–0·0)	5·2 (4·3–6·2)	42·6 (37·4–48·4)	97·4 (91·2–104·4)	93·7 (85·7–102·2)	35·0 (29·8–40·9)	4·5 (3·2–6·1)	0·1 (0·0–0·2)	0·0 (0·0–0·0)	1·39 (1·33–1·46)	0·24 (0·21–0·27)	0·67 (0·62–0·72)
2007	0·0 (0·0–0·0)	5·2 (4·3–6·2)	42·9 (35·9–50·7)	96·9 (90·6–104·1)	94·3 (85·7–103·5)	37·4 (31·8–44·1)	4·9 (3·5–6·6)	0·1 (0·0–0·2)	0·0 (0·0–0·0)	1·41 (1·33–1·49)	0·24 (0·21–0·28)	0·68 (0·63–0·74)
2008	0·0 (0·0–0·0)	5·1 (4·3–6·2)	42·5 (37·1–48·8)	96·5 (90·3–103·1)	95·0 (87·0–103·3)	39·4 (33·4–45·9)	5·3 (3·9–7·1)	0·1 (0·1–0·2)	0·0 (0·0–0·0)	1·42 (1·35–1·49)	0·24 (0·21–0·27)	0·7 (0·64–0·76)
2009	0·0 (0·0–0·0)	4·9 (4·0–6·0)	41·4 (35·2–48·4)	96·5 (89·2–103·8)	95·9 (87·4–105·4)	41·2 (35·2–48·2)	5·8 (4·4–7·6)	0·1 (0·1–0·2)	0·0 (0·0–0·0)	1·43 (1·34–1·52)	0·23 (0·2–0·27)	0·72 (0·66–0·78)
2010	0·0 (0·0–0·0)	4·7 (3·9–5·6)	40·3 (34·9–45·9)	97·1 (91·3–102·8)	97·3 (89·3–106·7)	42·8 (36·4–49·2)	6·2 (4·7–8·1)	0·1 (0·1–0·2)	0·0 (0·0–0·0)	1·44 (1·37–1·52)	0·23 (0·2–0·25)	0·73 (0·67–0·79)
2011	0·0 (0·0–0·0)	4·6 (3·8–5·6)	38·4 (33·6–43·8)	96·3 (89·4–103·2)	97·6 (88·8–107·2)	44·1 (37·6–51·1)	6·5 (5·0–8·5)	0·1 (0·1–0·2)	0·0 (0·0–0·0)	1·44 (1·37–1·51)	0·21 (0·19–0·24)	0·74 (0·69–0·8)
2012	0·0 (0·0–0·0)	4·5 (3·7–5·5)	36·5 (32·1–41·3)	95·4 (89·2–102·3)	99·1 (90·4–108·4)	46·2 (39·5–53·2)	7·2 (5·5–9·2)	0·1 (0·1–0·3)	0·0 (0·0–0·0)	1·45 (1·38–1·52)	0·21 (0·18–0·23)	0·76 (0·71–0·82)
2013	0·0 (0·0–0·0)	4·4 (3·7–5·3)	35·2 (29·0–41·8)	93·4 (87·1–99·6)	99·4 (91·1–108·2)	47·1 (40·5–53·7)	7·6 (5·7–9·8)	0·1 (0·1–0·3)	0·0 (0·0–0·0)	1·44 (1·36–1·51)	0·2 (0·17–0·23)	0·77 (0·71–0·83)
2014	0·0 (0·0–0·0)	4·3 (3·6–5·2)	33·8 (29·1–38·5)	92·3 (86·0–98·9)	100·6 (92·5–109·5)	48·5 (41·7–56·2)	8·0 (6·1–10·6)	0·2 (0·1–0·3)	0·0 (0·0–0·0)	1·44 (1·36–1·53)	0·19 (0·17–0·21)	0·79 (0·72–0·85)
2015	0·0 (0·0–0·0)	4·3 (3·6–5·3)	34·2 (28·2–40·8)	93·9 (86·2–101·8)	103·0 (92·5–114·0)	46·4 (38·5–56·0)	7·4 (5·4–9·8)	0·1 (0·1–0·3)	0·0 (0·0–0·0)	1·45 (1·29–1·61)	0·19 (0·16–0·23)	0·78 (0·7–0·88)
2016	0·0 (0·0–0·0)	4·4 (3·6–5·4)	35·6 (27·1–46·4)	95·2 (85·0–107·5)	102·2 (88·7–118·4)	42·1 (31·8–54·9)	6·3 (4·4–8·7)	0·1 (0·1–0·3)	0·0 (0·0–0·0)	1·43 (1·22–1·69)	0·2 (0·15–0·26)	0·75 (0·63–0·9)
2017	0·0 (0·0–0·0)	4·3 (3·6–5·3)	35·5 (26·4–46·6)	95·0 (84·3–106·9)	100·5 (85·6–116·6)	40·6 (30·0–53·5)	6·2 (3·9–9·2)	0·1 (0·1–0·3)	0·0 (0·0–0·0)	1·41 (1·19–1·67)	0·2 (0·15–0·26)	0·74 (0·61–0·89)

Supplemental Results Table 5: Age-specific fertility rates per 1000, cumulative total fertility up to maternal age 25 (TFU25), cumulative total fertility from maternal age 30 to age 55 (TFO30), and cumulative total fertility, Miyagi, 1950-2017

Year	10-14	15-19	20-24	25-29	30-34	35-39	40-44	45-49	50-54	TFR	TFU25	TFO30
1950	0·0 (0·0–0·0)	10·8 (9·6–12·1)	153·8 (140·1–168·5)	210·3 (202·7–217·9)	165·4 (155·6–175·3)	102·8 (91·4–114·2)	33·5 (28·9–38·4)	1·5 (1·0–2·2)	0·0 (0·0–0·0)	3·39 (3·27–3·51)	0·82 (0·76–0·89)	1·52 (1·44–1·6)
1951	0·0 (0·0–0·0)	8·7 (7·7–9·9)	138·0 (124·9–151·9)	195·1 (187·7–202·0)	149·7 (138·5–160·1)	83·1 (72·6–93·6)	25·9 (21·7–30·4)	1·1 (0·7–1·7)	0·0 (0·0–0·0)	3·01 (2·87–3·15)	0·73 (0·67–0·8)	1·3 (1·18–1·41)
1952	0·0 (0·0–0·0)	7·3 (6·4–8·4)	128·5 (115·1–142·2)	185·1 (177·1–193·3)	135·5 (123·1–148·0)	66·7 (56·8–77·3)	20·5 (16·9–24·6)	0·9 (0·6–1·3)	0·0 (0·0–0·0)	2·72 (2·61–2·83)	0·68 (0·61–0·75)	1·12 (1·05–1·2)
1953	0·0 (0·0–0·0)	6·4 (5·5–7·5)	121·4 (108·9–135·4)	174·4 (166·4–182·9)	121·3 (108·4–134·9)	54·2 (45·1–63·8)	16·3 (13·2–19·8)	0·7 (0·4–1·1)	0·0 (0·0–0·0)	2·47 (2·36–2·59)	0·64 (0·58–0·71)	0·96 (0·89–1·04)
1954	0·0 (0·0–0·0)	5·9 (5·0–6·9)	117·7 (103·2–132·4)	169·6 (161·7–178·3)	111·9 (97·8–125·9)	47·9 (40·2–57·2)	13·4 (10·7–16·7)	0·6 (0·4–0·9)	0·0 (0·0–0·0)	2·34 (2·22–2·45)	0·62 (0·55–0·69)	0·87 (0·8–0·94)
1955	0·0 (0·0–0·0)	5·3 (4·5–6·2)	114·8 (101·2–130·3)	168·5 (159·9–176·1)	103·0 (88·9–116·9)	43·1 (35·6–51·9)	11·2 (8·7–14·0)	0·5 (0·3–0·7)	0·0 (0·0–0·0)	2·23 (2·13–2·34)	0·6 (0·53–0·68)	0·79 (0·72–0·86)
1956	0·0 (0·0–0·0)	4·8 (4·0–5·6)	108·1 (94·3–122·7)	162·8 (155·6–170·4)	91·3 (79·2–104·9)	36·9 (31·0–44·0)	8·9 (6·9–11·4)	0·4 (0·2–0·6)	0·0 (0·0–0·0)	2·07 (1·97–2·18)	0·56 (0·49–0·64)	0·69 (0·63–0·75)
1957	0·0 (0·0–0·0)	4·5 (3·8–5·4)	109·2 (95·2–125·2)	166·2 (158·9–174·1)	86·5 (75·0–100·0)	31·6 (26·2–36·6)	7·6 (5·9–9·5)	0·4 (0·2–0·6)	0·0 (0·0–0·0)	2·03 (1·91–2·15)	0·57 (0·5–0·65)	0·63 (0·57–0·69)
1958	0·0 (0·0–0·0)	4·5 (3·9–5·3)	114·5 (100·0–130·1)	171·8 (163·7–179·9)	84·6 (73·1–97·7)	27·8 (22·9–32·9)	6·8 (5·3–8·7)	0·4 (0·2–0·6)	0·0 (0·0–0·0)	2·05 (1·95–2·16)	0·6 (0·52–0·67)	0·6 (0·54–0·66)
1959	0·0 (0·0–0·0)	4·7 (4·0–5·6)	114·4 (101·1–129·8)	170·9 (163·0–179·2)	79·2 (69·4–90·7)	24·0 (19·5–29·2)	5·6 (4·3–7·3)	0·3 (0·2–0·5)	0·0 (0·0–0·0)	2·0 (1·9–2·1)	0·6 (0·53–0·67)	0·55 (0·5–0·6)
1960	0·0 (0·0–0·0)	4·6 (3·9–5·4)	110·0 (97·3–123·2)	171·6 (163·9–179·8)	76·1 (67·9–85·3)	21·1 (17·3–25·4)	4·6 (3·5–5·8)	0·3 (0·1–0·4)	0·0 (0·0–0·0)	1·94 (1·85–2·03)	0·57 (0·51–0·64)	0·51 (0·47–0·56)
1961	0·0 (0·0–0·0)	4·3 (3·6–5·1)	106·7 (96·1–118·1)	173·4 (165·9–181·5)	74·5 (65·6–84·4)	19·0 (15·4–23·1)	3·9 (2·9–5·3)	0·2 (0·1–0·4)	0·0 (0·0–0·0)	1·91 (1·82–2·0)	0·56 (0·5–0·61)	0·49 (0·44–0·54)
1962	0·0 (0·0–0·0)	4·1 (3·4–4·9)	106·3 (94·6–118·4)	178·5 (171·1–185·7)	76·0 (69·1–83·1)	18·0 (15·0–21·3)	3·5 (2·6–4·6)	0·2 (0·1–0·3)	0·0 (0·0–0·0)	1·93 (1·85–2·01)	0·55 (0·49–0·61)	0·49 (0·45–0·53)
1963	0·0 (0·0–0·0)	4·0 (3·4–4·8)	106·7 (95·3–119·0)	180·7 (172·8–188·2)	77·6 (71·1–84·8)	17·3 (14·5–20·6)	3·4 (2·5–4·6)	0·2 (0·1–0·3)	0·0 (0·0–0·0)	1·95 (1·87–2·03)	0·55 (0·5–0·62)	0·49 (0·45–0·53)
1964	0·0 (0·0–0·0)	4·0 (3·3–4·7)	116·3 (104·7–130·0)	192·5 (184·7–201·0)	82·9 (74·9–91·6)	17·9 (15·1–20·8)	3·2 (2·3–4·3)	0·2 (0·1–0·3)	0·0 (0·0–0·0)	2·08 (2·0–2·17)	0·6 (0·54–0·67)	0·52 (0·48–0·57)
1965	0·0 (0·0–0·0)	3·9 (3·3–4·5)	107·0 (95·4–119·8)	162·3 (153·5–171·6)	69·0 (60·5–77·3)	16·4 (14·0–19·3)	2·7 (2·0–3·6)	0·2 (0·1–0·3)	0·0 (0·0–0·0)	1·81 (1·72–1·91)	0·55 (0·5–0·62)	0·44 (0·4–0·48)
1966	0·0 (0·0–0·0)	4·1 (3·5–4·7)	104·5 (92·5–116·4)	167·2 (158·6–176·4)	70·7 (61·7–81·1)	16·7 (13·8–20·1)	2·7 (2·0–3·5)	0·2 (0·1–0·3)	0·0 (0·0–0·0)	1·83 (1·75–1·91)	0·54 (0·48–0·6)	0·45 (0·41–0·5)
1967	0·0 (0·0–0·0)	4·3 (3·7–5·0)	111·2 (99·7–123·3)	203·8 (194·6–212·5)	85·5 (73·3–98·9)	18·9 (15·4–22·9)	2·6 (1·9–3·3)	0·1 (0·1–0·2)	0·0 (0·0–0·0)	2·13 (2·02–2·24)	0·58 (0·52–0·64)	0·54 (0·47–0·6)
1968	0·0 (0·0–0·0)	4·4 (3·7–5·0)	99·5 (88·3–111·4)	192·1 (184·4–199·5)	78·1 (66·8–90·4)	18·7 (15·6–22·2)	2·3 (1·7–3·0)	0·1 (0·1–0·2)	0·0 (0·0–0·0)	1·98 (1·9–2·05)	0·52 (0·46–0·58)	0·5 (0·44–0·55)
1969	0·0 (0·0–0·0)	4·7 (4·1–5·5)	97·7 (86·1–110·8)	194·6 (186·7–202·5)	78·7 (66·8–91·9)	18·4 (14·4–23·3)	2·3 (1·7–2·9)	0·1 (0·1–0·2)	0·0 (0·0–0·0)	1·98 (1·9–2·07)	0·51 (0·45–0·58)	0·5 (0·44–0·56)
1970	0·0 (0·0–0·0)	4·8 (4·1–5·6)	105·1 (94·0–117·4)	198·6 (189·6–206·6)	81·3 (70·4–93·8)	17·4 (14·3–20·9)	2·4 (1·7–3·2)	0·1 (0·1–0·2)	0·0 (0·0–0·0)	2·05 (1·96–2·14)	0·55 (0·49–0·61)	0·51 (0·45–0·56)
1971	0·0 (0·0–0·0)	4·7 (3·9–5·5)	115·2 (101·3–129·8)	196·9 (188·1–206·3)	83·0 (72·6–94·8)	16·0 (13·2–19·4)	2·6 (1·9–3·5)	0·1 (0·1–0·2)	0·0 (0·0–0·0)	2·09 (2·0–2·19)	0·6 (0·53–0·67)	0·51 (0·46–0·57)
1972	0·0 (0·0–0·0)	4·7 (3·9–5·6)	120·8 (106·9–136·1)	196·1 (186·8–204·7)	83·9 (73·9–93·8)	15·4 (12·4–18·6)	2·6 (1·8–3·5)	0·1 (0·1–0·2)	0·0 (0·0–0·0)	2·12 (2·02–2·22)	0·63 (0·56–0·7)	0·51 (0·46–0·56)
1973	0·0 (0·0–0·0)	4·6 (3·8–5·5)	121·4 (108·0–135·9)	196·8 (187·9–206·6)	80·9 (72·9–89·5)	14·7 (11·8–18·0)	2·4 (1·6–3·3)	0·1 (0·1–0·2)	0·0 (0·0–0·0)	2·1 (2·01–2·2)	0·63 (0·56–0·7)	0·49 (0·45–0·54)
1974	0·0 (0·0–0·0)	4·2 (3·4–5·1)	116·6 (104·9–129·9)	189·8 (180·4–198·8)	72·9 (65·8–80·4)	13·2 (10·8–16·1)	2·0 (1·4–2·9)	0·1 (0·1–0·2)	0·0 (0·0–0·0)	1·99 (1·91–2·09)	0·6 (0·55–0·67)	0·44 (0·41–0·48)
1975	0·0 (0·0–0·0)	4·0 (3·3–4·8)	109·7 (98·2–121·1)	183·5 (174·6–192·2)	66·6 (60·3–73·1)	12·5 (10·1–15·3)	1·8 (1·1–2·7)	0·1 (0·0–0·1)	0·0 (0·0–0·0)	1·89 (1·81–1·96)	0·57 (0·51–0·63)	0·4 (0·37–0·44)
1976	0·0 (0·0–0·0)	3·8 (3·1–4·5)	104·3 (93·4–116·2)	181·7 (173·3–189·5)	65·3 (59·3–71·6)	12·3 (10·2–14·8)	1·6 (1·1–2·3)	0·1 (0·0–0·1)	0·0 (0·0–0·0)	1·84 (1·76–1·93)	0·54 (0·49–0·6)	0·4 (0·36–0·43)
1977	0·0 (0·0–0·0)	3·8 (3·2–4·4)	98·7 (89·0–110·7)	183·0 (174·7–191·3)	67·4 (60·7–74·3)	12·2 (10·0–14·9)	1·4 (1·0–2·0)	0·1 (0·0–0·1)	0·0 (0·0–0·0)	1·83 (1·76–1·92)	0·51 (0·46–0·57)	0·41 (0·37–0·45)
1978	0·0 (0·0–0·0)	3·8 (3·0–4·7)	94·2 (82·9–107·2)	184·2 (176·0–192·1)	71·5 (64·5–78·9)	12·2 (9·4–15·6)	1·4 (0·9–1·9)	0·1 (0·0–0·1)	0·0 (0·0–0·0)	1·84 (1·76–1·92)	0·49 (0·43–0·55)	0·43 (0·39–0·47)
1979	0·0 (0·0–0·0)	3·9 (3·0–5·0)	89·7 (80·2–99·1)	183·4 (175·4–191·0)	72·9 (66·3–79·8)	12·0 (10·0–14·2)	1·4 (0·9–2·1)	0·1 (0·0–0·1)	0·0 (0·0–0·0)	1·82 (1·74–1·89)	0·47 (0·42–0·52)	0·43 (0·4–0·47)
1980	0·0 (0·0–0·0)	4·0 (3·2–4·8)	85·3 (75·6–95·4)	181·8 (173·3–190·4)	72·2 (65·5–78·8)	12·0 (9·8–14·4)	1·5 (1·0–2·0)	0·1 (0·0–0·1)	0·0 (0·0–0·0)	1·78 (1·71–1·85)	0·45 (0·4–0·5)	0·43 (0·39–0·46)
1981	0·0 (0·0–0·0)	4·1 (3·4–4·9)	82·6 (73·9–92·7)	183·2 (175·3–191·1)	73·3 (66·6–80·0)	12·7 (10·3–15·3)	1·5 (1·0–2·2)	0·1 (0·0–0·1)	0·0 (0·0–0·0)	1·79 (1·72–1·86)	0·43 (0·39–0·48)	0·44 (0·4–0·47)
1982	0·0 (0·0–0·0)	4·2 (3·5–5·1)	81·0 (71·4–91·2)	184·7 (175·8–195·0)	76·5 (70·0–83·6)	14·2 (11·8–16·9)	1·5 (1·1–2·1)	0·1 (0·0–0·1)	0·0 (0·0–0·0)	1·81 (1·75–1·88)	0·43 (0·38–0·48)	0·46 (0·43–0·5)
1983	0·0 (0·0–0·0)	4·3 (3·5–5·2)	78·5 (70·2–87·5)	184·0 (175·2–192·8)	80·0 (73·2–88·0)	15·8 (12·9–19·2)	1·6 (1·2–2·1)	0·1 (0·0–0·1)	0·0 (0·0–0·0)	1·82 (1·75–1·89)	0·41 (0·37–0·46)	0·49 (0·45–0·53)
1984	0·0 (0·0–0·0)	4·1 (3·3–5·0)	72·7 (62·5–84·3)	179·8 (171·9–188·7)	82·6 (75·3–90·2)	16·4 (13·5–19·7)	1·7 (1·2–2·2)	0·1 (0·0–0·1)	0·0 (0·0–0·0)	1·79 (1·72–1·86)	0·38 (0·33–0·44)	0·5 (0·47–0·55)
1985	0·0 (0·0–0·0)	3·9 (3·1–4·8)	67·8 (59·9–76·4)	172·6 (164·6–179·7)	83·8 (76·9–91·7)	16·4 (13·6–19·6)	1·7 (1·2–2·3)	0·1 (0·0–0·1)	0·0 (0·0–0·0)	1·73 (1·66–1·8)	0·36 (0·32–0·4)	0·51 (0·47–0·55)
1986	0·0 (0·0–0·0)	3·7 (3·0–4·5)	64·1 (56·2–72·8)	166·6 (158·4–174·9)	86·2 (78·8–93·6)	16·6 (13·9–20·0)	1·9 (1·2–2·7)	0·1 (0·0–0·1)	0·0 (0·0–0·0)	1·7 (1·63–1·77)	0·34 (0·3–0·38)	0·52 (0·48–0·56)
1987	0·0 (0·0–0·0)	3·6 (2·9–4·3)	58·8 (52·2–66·0)	161·9 (154·0–169·7)	89·2 (82·2–97·3)	17·5 (14·2–20·9)	2·1 (1·5–2·9)	0·1 (0·0–0·1)	0·0 (0·0–0·0)	1·67 (1·59–1·74)	0·31 (0·28–0·35)	0·54 (0·5–0·59)
1988	0·0 (0·0–0·0)	3·4 (2·7–4·2)	54·3 (47·4–62·3)	153·4 (145·7–161·5)	90·8 (82·9–99·4)	18·5 (15·3–22·3)	2·3 (1·7–3·1)	0·1 (0·0–0·1)	0·0 (0·0–0·0)	1·61 (1·55–1·68)	0·29 (0·25–0·33)	0·56 (0·52–0·6)
1989	0·0 (0·0–0·0)	3·4 (2·8–4·2)	50·1 (43·7–57·1)	144·7 (137·4–152·7)	91·4 (83·8–99·6)	19·7 (16·3–23·4)	2·4 (1·8–3·3)	0·1 (0·0–0·1)	0·0 (0·0–0·0)	1·56 (1·49–1·62)	0·27 (0·24–0·3)	0·57 (0·52–0·61)
1990	0·0 (0·0–0·0)	3·5 (2·9–4·2)	48·2 (41·8–54·7)	142·1 (134·2–150·1)	93·7 (85·2–102·6)	20·7 (17·1–24·5)	2·4 (1·8–3·2)	0·1 (0·0–0·1)	0·0 (0·0–0·0)	1·55 (1·49–1·62)	0·26 (0·22–0·29)	0·58 (0·54–0·63)
1991	0·0 (0·0–0·0)	3·6 (3·0–4·4)	47·7 (41·3–54·5)	137·4 (130·5–144·8)	94·3 (86·5–103·6)	22·0 (18·2–26·1)	2·5 (1·8–3·3)	0·1 (0·0–0·1)	0·0 (0·0–0·0)	1·54 (1·48–1·6)	0·26 (0·23–0·29)	0·59 (0·55–0·65)
1992	0·0 (0·0–0·0)	3·7 (3·1–4·5)	45·8 (40·6–51·8)	125·2 (118·0–132·4)	90·6 (83·8–98·3)	22·7 (19·0–27·4)	2·6 (1·9–3·4)	0·1 (0·0–0·1)	0·0 (0·0–0·0)	1·45 (1·39–1·52)	0·25 (0·22–0·28)	0·58 (0·54–0·62)
1993	0·0 (0·0–0·0)	3·8 (3·1–4·6)	45·3 (39·2–52·0)	111·1 (104·1–118·2)	84·4 (77·4–92·0)	24·5 (20·6–29·0)	2·7 (2·0–3·6)	0·1 (0·0–0·1)	0·0 (0·0–0·0)	1·36 (1·3–1·43)	0·25 (0·21–0·28)	0·56 (0·52–0·6)
1994	0·0 (0·0–0·0)	3·9 (3·1–4·8)	44·6 (39·1–50·9)	99·0 (93·1–105·0)	78·3 (71·5–85·5)	25·9 (21·5–30·9)	2·9 (2·1–3·9)	0·1 (0·1–0·1)	0·0 (0·0–0·0)	1·27 (1·2–1·34)	0·24 (0·21–0·27)	0·54 (0·49–0·58)
1995	0·0 (0·0–0·0)	4·0 (3·2–5·0)	43·6 (38·1–49·9)	100·9 (94·7–106·8)	83·9 (76·5–91·8)	26·8 (22·6–31·6)	3·0 (2·2–3·9)	0·1 (0·1–0·1)	0·0 (0·0–0·0)	1·31 (1·24–1·39)	0·24 (0·21–0·27)	0·57 (0·52–0·62)
1996	0·0 (0·0–0·0)	4·2 (3·4–5·3)	43·6 (37·6–49·4)	108·9 (102·1–116·1)	93·1 (85·6–101·4)	28·1 (23·3–33·3)	3·1 (2·3–4·0)	0·1 (0·0–0·1)	0·0 (0·0–0·0)	1·41 (1·33–1·49)	0·24 (0·21–0·27)	0·62 (0·58–0·67)
1997	0·0 (0·0–0·0)	4·6 (3·6–5·7)	44·0 (38·6–49·9)	109·8 (103·0–116·3)	94·8 (87·3–103·2)	28·6 (23·9–34·0)	3·2 (2·4–4·4)	0·1 (0·0–0·1)	0·0 (0·0–0·0)	1·43 (1·35–1·5)	0·24 (0·22–0·27)	0·63 (0·59–0·69)
1998	0·0 (0·0–0·0)	4·9 (3·9–6·2)	44·5 (38·9–51·0)	106·7 (100·2–113·7)	94·2 (86·3–103·0)	29·6 (24·4–35·6)	3·3 (2·5–4·4)	0·1 (0·1–0·1)	0·0 (0·0–0·0)	1·42 (1·35–1·49)	0·25 (0·22–0·28)	0·64 (0·59–0·69)
1999	0·0 (0·0–0·0)	5·3 (4·2–6·6)	44·9 (39·4–51·1)	102·7 (95·9–109·3)	92·3 (84·5–101·0)	30·2 (24·8–35·8)	3·5 (2·5–4·7)	0·1 (0·1–0·1)	0·0 (0·0–0·0)	1·39 (1·32–1·46)	0·25 (0·22–0·28)	0·63 (0·57–0·69)
2000	0·0 (0·0–0·0)	5·7 (4·5–7·1)	45·7 (39·1–52·2)	99·3 (93·1–105·8)	89·2 (81·7–96·8)	31·5 (26·7–36·9)	3·6 (2·6–4·9)	0·1 (0·0–0·2)	0·0 (0·0–0·0)	1·38 (1·31–1·45)	0·26 (0·22–0·29)	0·62 (0·57–0·67)
2001	0·0 (0·0–0·0)	6·0 (4·8–7·4)	46·0 (40·4–52·4)	95·4 (89·4–102·0)	85·3 (77·6–93·1)	31·4 (26·4–37·1)	3·7 (2·8–5·0)	0·1 (0·1–0·1)	0·0 (0·0–0·0)	1·34 (1·27–1·4)	0·26 (0·23–0·29)	0·6 (0·56–0·65)
2002	0·0 (0·0–0·0)	5·8 (4·8–7·2)	44·7 (39·0–51·2)	92·1 (86·0–98·4)	84·0 (76·6–92·6)	31·9 (27·1–37·3)	4·0 (2·9–5·7)	0·1 (0·1–0·2)	0·0 (0·0–0·0)	1·31 (1·25–1·38)	0·25 (0·22–0·29)	0·6 (0·55–0·65)
2003	0·0 (0·0–0·0)	5·5 (4·5–6·7)	42·7 (36·6–48·8)	89·2 (84·1–94·8)	82·9 (75·5–90·2)	33·2 (27·7–38·6)	4·3 (3·0–5·8)	0·1 (0·1–0·2)	0·0 (0·0–0·0)	1·29 (1·23–1·35)	0·24 (0·21–0·27)	0·6 (0·55–0·65)
2004	0·0 (0·0–0·0)	5·1 (4·3–6·1)	40·7 (35·4–46·8)	85·2 (79·5–91·2)	81·1 (73·9–88·2)	34·0 (28·9–39·7)	4·6 (3·3–6·0)	0·1 (0·1–0·2)	0·0 (0·0–0·0)	1·25 (1·2–1·31)	0·23 (0·2–0·26)	0·6 (0·56–0·64)
2005	0·0 (0·0–0·0)	4·9 (4·1–5·8)	39·7 (34·8–45·3)	84·0 (78·3–90·0)	81·0 (73·8–88·5)	34·9 (29·6–41·1)	4·9 (3·6–6·4)	0·1 (0·1–0·2)	0·0 (0·0–0·0)	1·25 (1·19–1·31)	0·22 (0·2–0·25)	0·6 (0·56–0·65)
2006	0·0 (0·0–0·0)	4·7 (4·0–5·6)	39·6 (33·9–45·6)	84·7 (79·2–90·9)	83·2 (75·7–91·3)	36·8 (31·4–43·0)	5·4 (4·0–7·1)	0·1 (0·1–0·2)	0·0 (0·0–0·0)	1·27 (1·21–1·34)	0·22 (0·19–0·25)	0·63 (0·58–0·68)
2007	0·0 (0·0–0·0)	4·6 (3·9–5·5)	39·0 (34·8–44·2)	84·1 (78·8–90·2)	83·0 (76·0–90·6)	38·6 (32·8–45·4)	5·9 (4·5–7·6)	0·1 (0·1–0·2)	0·0 (0·0–0·0)	1·28 (1·22–1·33)	0·22 (0·2–0·24)	0·64 (0·59–0·69)
2008	0·0 (0·0–0·0)	4·5 (3·7–5·3)	38·0 (32·9–43·6)	83·4 (77·7–89·4)	83·1 (76·1–90·2)	39·8 (33·7–46·2)	6·4 (5·0–8·1)	0·1 (0·1–0·2)	0·0 (0·0–0·0)	1·28 (1·22–1·34)	0·21 (0·19–0·24)	0·65 (0·6–0·69)
2009	0·0 (0·0–0·0)	4·2 (3·3–5·2)	36·4 (31·3–41·9)	83·0 (77·7–88·2)	83·5 (76·5–91·2)	40·9 (34·9–47·0)	7·1 (5·5–8·9)	0·2 (0·1–0·3)	0·0 (0·0–0·0)	1·28 (1·21–1·35)	0·2 (0·18–0·23)	0·66 (0·61–0·71)
2010	0·0 (0·0–0·0)	4·0 (3·1–5·2)	35·0 (30·2–40·1)	83·4 (77·5–89·3)	84·6 (77·0–93·0)	42·0 (35·9–48·6)	7·5 (5·9–9·3)	0·2 (0·1–0·3)	0·0 (0·0–0·0)	1·28 (1·22–1·34)	0·2 (0·17–0·22)	0·67 (0·63–0·72)
2011	0·0 (0·0–0·0)	3·9 (3·1–4·9)	33·0 (28·8–37·6)	82·9 (77·2–89·0)	85·3 (78·3–93·2)	43·0 (36·7–50·1)	7·9 (6·1–10·1)	0·2 (0·1–0·3)	0·0 (0·0–0·0)	1·28 (1·22–1·35)	0·18 (0·16–0·21)	0·68 (0·63–0·74)
2012	0·0 (0·0–0·0)	3·8 (3·1–4·7)	31·2 (27·4–35·4)	82·4 (77·1–88·0)	87·4 (79·4–95·8)	45·0 (38·6–52·1)	8·5 (6·7–10·8)	0·2 (0·1–0·3)	0·0 (0·0–0·0)	1·29 (1·24–1·35)	0·18 (0·15–0·2)	0·71 (0·66–0·76)
2013	0·0 (0·0–0·0)	3·7 (3·1–4·5)	30·1 (25·1–35·5)	80·9 (75·4–86·5)	88·4 (80·4–96·4)	45·9 (39·7–52·6)	9·0 (6·8–11·5)	0·2 (0·1–0·3)	0·0 (0·0–0·0)	1·29 (1·23–1·36)	0·17 (0·14–0·2)	0·72 (0·66–0·77)
2014	0·0 (0·0–0·0)	3·7 (3·0–4·4)	29·1 (24·9–33·7)	79·9 (73·9–85·7)	89·9 (82·6–97·9)	47·3 (40·6–54·5)	9·4 (7·3–12·3)	0·2 (0·1–0·4)	0·0 (0·0–0·0)	1·3 (1·23–1·37)	0·16 (0·14–0·19)	0·73 (0·68–0·8)
2015	0·0 (0·0–0·0)	3·7 (3·0–4·5)	29·6 (24·5–35·5)	80·8 (74·4–87·9)	92·1 (83·2–101·7)	45·6 (37·8–54·2)	8·6 (6·3–11·6)	0·2 (0·1–0·3)	0·0 (0·0–0·0)	1·3 (1·17–1·46)	0·17 (0·14–0·2)	0·73 (0·65–0·82)
2016	0·0 (0·0–0·0)	3·7 (3·1–4·6)	31·0 (23·5–40·6)	81·2 (72·5–91·7)	91·3 (79·0–105·7)	41·8 (32·0–54·1)	7·4 (5·1–10·4)	0·2 (0·1–0·3)	0·0 (0·0–0·0)	1·28 (1·09–1·51)	0·17 (0·13–0·23)	0·7 (0·59–0·84)
2017	0·0 (0·0–0·0)	3·7 (3·1–4·5)	31·1 (23·1–40·8)	80·3 (71·1–90·3)	89·7 (76·4–104·0)	40·7 (30·4–53·4)	7·2 (4·7–10·9)	0·2 (0·1–0·3)	0·0 (0·0–0·0)	1·26 (1·06–1·5)	0·17 (0·13–0·23)	0·69 (0·56–0·84)

Supplemental Results Table 5: Age-specific fertility rates per 1000, cumulative total fertility up to maternal age 25 (TFU25), cumulative total fertility from maternal age 30 to age 55 (TFO30), and cumulative total fertility, Miyazaki, 1950-2017

Year	10-14	15-19	20-24	25-29	30-34	35-39	40-44	45-49	50-54	TFR	TFU25	TFO30
1950	0·0 (0·0–0·0)	12·3 (10·9–13·7)	170·0 (155·0–185·9)	225·8 (217·4–234·1)	190·2 (178·3–201·9)	150·1 (133·1–167·3)	48·3 (41·6–55·5)	2·5 (1·6–3·6)	0·0 (0·0–0·0)	4·0 (3·84–4·15)	0·91 (0·84–0·99)	1·96 (1·84–2·07)
1951	0·0 (0·0–0·0)	9·9 (8·8–11·2)	152·7 (138·9–166·5)	209·9 (201·6–217·8)	172·3 (160·8–183·9)	122·0 (106·2–136·8)	37·4 (31·3–44·1)	1·8 (1·2–2·7)	0·0 (0·0–0·0)	3·53 (3·35–3·7)	0·81 (0·74–0·88)	1·67 (1·51–1·82)
1952	0·0 (0·0–0·0)	8·4 (7·3–9·5)	142·3 (127·6–158·8)	199·6 (190·9–208·3)	156·1 (143·9–168·7)	98·2 (84·6–112·5)	29·6 (24·2–35·7)	1·4 (0·9–2·0)	0·0 (0·0–0·0)	3·18 (3·06–3·31)	0·75 (0·68–0·83)	1·43 (1·33–1·53)
1953	0·0 (0·0–0·0)	7·3 (6·3–8·5)	134·6 (119·8–150·8)	188·2 (179·6–197·4)	139·6 (126·8–152·5)	78·4 (66·3–91·1)	23·5 (18·8–29·1)	1·1 (0·7–1·8)	0·0 (0·0–0·0)	2·86 (2·74–3·0)	0·71 (0·64–0·79)	1·21 (1·12–1·31)
1954	0·0 (0·0–0·0)	6·7 (5·7–7·9)	130·7 (115·1–146·8)	183·2 (175·4–191·6)	128·7 (118·1–140·9)	65·3 (54·5–77·6)	19·5 (14·9–25·3)	1·0 (0·6–1·4)	0·0 (0·0–0·0)	2·68 (2·56–2·79)	0·69 (0·61–0·77)	1·07 (0·99–1·17)
1955	0·0 (0·0–0·0)	6·1 (5·1–7·1)	127·7 (113·1–144·9)	182·3 (173·8–190·8)	118·5 (107·7–130·0)	51·3 (43·2–60·4)	16·2 (12·0–21·9)	0·7 (0·5–1·2)	0·0 (0·0–0·0)	2·51 (2·4–2·63)	0·67 (0·59–0·76)	0·93 (0·86–1·01)
1956	0·0 (0·0–0·0)	5·4 (4·6–6·4)	120·4 (105·0–136·8)	176·3 (168·7–184·4)	105·1 (95·2–115·9)	40·7 (34·3–47·9)	13·0 (9·2–17·9)	0·6 (0·4–1·0)	0·0 (0·0–0·0)	2·31 (2·2–2·41)	0·63 (0·55–0·71)	0·8 (0·73–0·87)
1957	0·0 (0·0–0·0)	5·2 (4·4–6·2)	121·8 (106·7–139·7)	179·9 (171·4–188·9)	99·5 (90·7–109·2)	38·4 (32·0–44·4)	11·1 (7·7–15·0)	0·6 (0·4–1·0)	0·0 (0·0–0·0)	2·28 (2·16–2·41)	0·64 (0·56–0·72)	0·75 (0·68–0·81)
1958	0·0 (0·0–0·0)	5·2 (4·4–6·1)	128·1 (113·1–143·9)	185·9 (176·7–194·4)	97·4 (85·6–111·0)	36·7 (30·7–43·5)	10·0 (6·9–14·1)	0·6 (0·4–1·0)	0·0 (0·0–0·0)	2·32 (2·21–2·43)	0·67 (0·59–0·75)	0·72 (0·65–0·81)
1959	0·0 (0·0–0·0)	5·5 (4·6–6·4)	128·2 (113·3–144·4)	185·0 (176·2–194·4)	91·1 (82·2–100·6)	29·7 (24·1–36·7)	8·3 (5·6–11·6)	0·5 (0·3–0·9)	0·0 (0·0–0·0)	2·24 (2·13–2·35)	0·67 (0·59–0·75)	0·65 (0·59–0·71)
1960	0·0 (0·0–0·0)	5·3 (4·5–6·2)	123·3 (109·8–138·0)	185·9 (176·9–194·6)	87·4 (78·2–97·4)	23·4 (19·7–27·3)	6·8 (4·6–9·3)	0·4 (0·3–0·7)	0·0 (0·0–0·0)	2·16 (2·06–2·26)	0·64 (0·57–0·72)	0·59 (0·54–0·64)
1961	0·0 (0·0–0·0)	5·0 (4·2–5·9)	119·9 (107·6–132·0)	187·9 (179·7–196·8)	85·3 (77·7–94·1)	20·0 (16·2–24·4)	5·8 (3·9–8·2)	0·4 (0·2–0·6)	0·0 (0·0–0·0)	2·12 (2·03–2·22)	0·62 (0·56–0·68)	0·56 (0·51–0·61)
1962	0·0 (0·0–0·0)	4·7 (3·9–5·7)	119·7 (106·1–133·0)	193·4 (185·4–201·5)	87·0 (79·5–94·6)	18·9 (16·1–22·0)	5·1 (3·6–7·2)	0·3 (0·2–0·5)	0·0 (0·0–0·0)	2·15 (2·06–2·24)	0·62 (0·55–0·69)	0·56 (0·51–0·6)
1963	0·0 (0·0–0·0)	4·7 (3·9–5·6)	120·3 (107·5–133·8)	195·5 (187·3–203·9)	88·6 (81·2–96·6)	21·2 (17·7–24·9)	5·1 (3·6–7·3)	0·4 (0·2–0·6)	0·0 (0·0–0·0)	2·18 (2·09–2·27)	0·63 (0·56–0·69)	0·58 (0·53–0·62)
1964	0·0 (0·0–0·0)	4·6 (3·9–5·5)	131·5 (118·9–146·6)	208·2 (199·2–217·5)	94·4 (86·2–103·3)	22·1 (18·7–25·7)	4·8 (3·4–6·5)	0·3 (0·2–0·5)	0·0 (0·0–0·0)	2·33 (2·24–2·43)	0·68 (0·62–0·76)	0·61 (0·56–0·66)
1965	0·0 (0·0–0·0)	4·5 (3·7–5·4)	121·2 (108·3–136·1)	175·8 (167·0–184·8)	78·3 (70·3–86·0)	17·3 (14·6–20·2)	4·1 (2·9–5·4)	0·3 (0·2–0·4)	0·0 (0·0–0·0)	2·01 (1·91–2·11)	0·63 (0·56–0·7)	0·5 (0·46–0·54)
1966	0·0 (0·0–0·0)	4·8 (4·1–5·6)	118·5 (105·9–132·8)	180·8 (171·7–189·9)	79·9 (71·3–89·1)	18·8 (15·5–22·6)	4·0 (2·9–5·3)	0·3 (0·2–0·4)	0·0 (0·0–0·0)	2·04 (1·94–2·13)	0·62 (0·55–0·69)	0·51 (0·47–0·56)
1967	0·0 (0·0–0·0)	5·1 (4·3–5·9)	126·4 (113·5–139·6)	220·4 (211·2–229·5)	96·0 (86·5–105·5)	21·2 (17·4–25·2)	3·8 (2·9–5·0)	0·2 (0·2–0·4)	0·0 (0·0–0·0)	2·37 (2·25–2·48)	0·66 (0·59–0·72)	0·61 (0·55–0·66)
1968	0·0 (0·0–0·0)	5·2 (4·5–6·0)	113·3 (100·7–126·7)	207·9 (199·0–216·5)	87·3 (78·4–96·6)	19·1 (15·6–23·1)	3·4 (2·6–4·5)	0·2 (0·1–0·3)	0·0 (0·0–0·0)	2·18 (2·09–2·29)	0·59 (0·53–0·66)	0·55 (0·5–0·6)
1969	0·0 (0·0–0·0)	5·7 (4·8–6·7)	111·5 (98·8–124·3)	210·2 (201·9–218·7)	87·3 (78·8–96·3)	18·8 (15·2–22·9)	3·4 (2·6–4·5)	0·2 (0·1–0·3)	0·0 (0·0–0·0)	2·19 (2·08–2·29)	0·59 (0·52–0·65)	0·55 (0·49–0·61)
1970	0·0 (0·0–0·0)	5·8 (4·8–6·8)	120·2 (106·5–135·6)	213·9 (204·6–222·5)	89·6 (81·0–98·4)	20·9 (16·8–25·6)	3·6 (2·7–4·9)	0·2 (0·1–0·3)	0·0 (0·0–0·0)	2·27 (2·16–2·38)	0·63 (0·56–0·71)	0·57 (0·52–0·63)
1971	0·0 (0·0–0·0)	5·7 (4·6–6·8)	131·9 (115·6–148·8)	211·1 (202·3–220·3)	90·6 (81·6–100·0)	23·6 (19·3–28·8)	3·9 (2·8–5·3)	0·3 (0·2–0·4)	0·0 (0·0–0·0)	2·34 (2·22–2·45)	0·69 (0·61–0·77)	0·59 (0·53–0·65)
1972	0·0 (0·0–0·0)	5·7 (4·7–6·9)	138·3 (122·5–155·5)	209·5 (200·0–219·0)	90·6 (81·1–100·3)	24·0 (19·1–29·1)	3·9 (2·7–5·4)	0·3 (0·2–0·4)	0·0 (0·0–0·0)	2·36 (2·24–2·49)	0·72 (0·64–0·8)	0·59 (0·54–0·65)
1973	0·0 (0·0–0·0)	5·6 (4·6–7·0)	138·9 (124·0–154·3)	209·4 (199·1–219·2)	86·1 (77·7–94·8)	21·9 (17·7–26·9)	3·5 (2·5–4·9)	0·2 (0·1–0·3)	0·0 (0·0–0·0)	2·33 (2·22–2·44)	0·72 (0·65–0·8)	0·56 (0·51–0·61)
1974	0·0 (0·0–0·0)	5·3 (4·2–6·6)	133·2 (119·6–148·9)	201·1 (191·9–210·9)	76·5 (69·2–84·7)	17·2 (13·7–21·6)	3·0 (1·9–4·6)	0·2 (0·1–0·3)	0·0 (0·0–0·0)	2·18 (2·09–2·28)	0·69 (0·62–0·77)	0·48 (0·44–0·53)
1975	0·0 (0·0–0·0)	4·9 (3·8–6·1)	124·8 (111·0–138·4)	193·8 (185·0–203·2)	69·3 (62·0–75·9)	13·7 (10·8–17·2)	2·6 (1·5–4·1)	0·1 (0·1–0·2)	0·0 (0·0–0·0)	2·05 (1·95–2·14)	0·65 (0·58–0·72)	0·43 (0·39–0·47)
1976	0·0 (0·0–0·0)	4·6 (3·6–5·6)	118·3 (105·8–132·5)	190·9 (181·3–200·3)	67·8 (61·4–74·8)	12·7 (10·2–15·6)	2·3 (1·5–3·3)	0·1 (0·1–0·2)	0·0 (0·0–0·0)	1·98 (1·89–2·08)	0·61 (0·55–0·69)	0·41 (0·38–0·46)
1977	0·0 (0·0–0·0)	4·4 (3·5–5·3)	112·2 (100·8–125·6)	191·1 (182·8–200·8)	70·1 (62·4–78·3)	12·7 (10·2–15·7)	2·1 (1·4–2·9)	0·1 (0·1–0·2)	0·0 (0·0–0·0)	1·96 (1·88–2·06)	0·58 (0·53–0·65)	0·42 (0·38–0·48)
1978	0·0 (0·0–0·0)	4·3 (3·5–5·2)	107·6 (93·7–124·5)	191·2 (182·2–200·2)	73·2 (66·8–80·0)	12·7 (9·7–16·5)	1·8 (1·2–2·6)	0·1 (0·0–0·1)	0·0 (0·0–0·0)	1·95 (1·86–2·06)	0·56 (0·49–0·64)	0·44 (0·4–0·48)
1979	0·0 (0·0–0·0)	4·3 (3·6–5·0)	102·9 (91·7–114·4)	189·0 (180·9–197·2)	72·6 (63·3–82·9)	12·4 (10·3–14·9)	1·7 (1·1–2·6)	0·1 (0·0–0·1)	0·0 (0·0–0·0)	1·91 (1·83–2·01)	0·54 (0·48–0·59)	0·43 (0·39–0·49)
1980	0·0 (0·0–0·0)	4·2 (3·4–5·3)	98·4 (87·2–110·2)	186·2 (177·6–195·5)	70·1 (64·1–77·0)	12·4 (10·0–15·3)	1·5 (1·1–2·1)	0·1 (0·0–0·1)	0·0 (0·0–0·0)	1·86 (1·78–1·95)	0·51 (0·46–0·57)	0·42 (0·39–0·46)
1981	0·0 (0·0–0·0)	4·3 (3·4–5·3)	95·3 (85·6–107·2)	187·2 (178·7–196·0)	70·7 (63·8–77·7)	13·0 (10·4–15·9)	1·5 (1·0–2·2)	0·1 (0·0–0·1)	0·0 (0·0–0·0)	1·86 (1·79–1·94)	0·5 (0·45–0·56)	0·43 (0·39–0·46)
1982	0·0 (0·0–0·0)	4·4 (3·5–5·4)	93·0 (82·1–105·4)	189·2 (180·7–198·3)	74·4 (67·7–81·6)	14·6 (11·9–17·6)	1·6 (1·1–2·2)	0·1 (0·0–0·1)	0·0 (0·0–0·0)	1·89 (1·81–1·98)	0·49 (0·43–0·55)	0·45 (0·42–0·49)
1983	0·0 (0·0–0·0)	4·4 (3·5–5·4)	90·0 (79·4–102·3)	190·3 (181·2–199·2)	78·4 (70·6–87·0)	16·1 (12·9–19·8)	1·7 (1·3–2·2)	0·1 (0·0–0·1)	0·0 (0·0–0·0)	1·9 (1·83–1·99)	0·47 (0·42–0·53)	0·48 (0·44–0·53)
1984	0·0 (0·0–0·0)	4·2 (3·4–5·1)	83·3 (73·7–93·4)	188·6 (180·6–197·6)	81·4 (73·8–89·8)	16·7 (13·6–20·7)	1·8 (1·3–2·4)	0·1 (0·0–0·1)	0·0 (0·0–0·0)	1·88 (1·8–1·96)	0·44 (0·39–0·49)	0·5 (0·46–0·54)
1985	0·0 (0·0–0·0)	4·0 (3·2–4·9)	78·0 (67·6–89·4)	183·6 (172·4–193·9)	82·9 (75·0–91·2)	16·6 (13·4–20·4)	1·8 (1·3–2·4)	0·1 (0·0–0·1)	0·0 (0·0–0·0)	1·84 (1·76–1·91)	0·41 (0·36–0·46)	0·51 (0·47–0·55)
1986	0·0 (0·0–0·0)	3·8 (3·0–4·6)	74·1 (64·5–83·8)	179·3 (171·6–188·0)	85·8 (78·1–94·0)	16·8 (13·7–20·2)	2·0 (1·3–2·7)	0·1 (0·0–0·1)	0·0 (0·0–0·0)	1·81 (1·73–1·9)	0·39 (0·34–0·44)	0·52 (0·48–0·57)
1987	0·0 (0·0–0·0)	3·7 (2·9–4·4)	68·4 (60·7–76·8)	175·8 (166·4–185·2)	89·4 (81·9–98·0)	17·7 (14·3–21·6)	2·2 (1·4–3·3)	0·1 (0·0–0·1)	0·0 (0·0–0·0)	1·79 (1·71–1·86)	0·36 (0·32–0·4)	0·55 (0·5–0·59)
1988	0·0 (0·0–0·0)	3·6 (2·9–4·4)	63·8 (55·0–73·4)	167·2 (159·0–176·1)	91·6 (83·3–100·6)	18·7 (15·1–22·7)	2·4 (1·7–3·4)	0·1 (0·0–0·1)	0·0 (0·0–0·0)	1·74 (1·65–1·82)	0·34 (0·29–0·39)	0·56 (0·52–0·61)
1989	0·0 (0·0–0·0)	3·6 (2·9–4·4)	59·5 (51·3–68·9)	157·7 (149·2–166·7)	92·8 (84·4–100·7)	19·9 (16·0–24·0)	2·6 (1·8–3·6)	0·1 (0·0–0·1)	0·0 (0·0–0·0)	1·68 (1·6–1·76)	0·32 (0·28–0·36)	0·58 (0·53–0·62)
1990	0·0 (0·0–0·0)	3·8 (3·1–4·5)	58·3 (50·9–65·8)	155·3 (147·2–163·6)	95·7 (87·5–104·4)	21·0 (17·1–25·5)	2·6 (1·8–3·5)	0·1 (0·0–0·1)	0·0 (0·0–0·0)	1·68 (1·6–1·77)	0·31 (0·27–0·35)	0·6 (0·55–0·64)
1991	0·0 (0·0–0·0)	4·0 (3·2–4·8)	59·2 (51·6–67·8)	152·2 (141·1–164·2)	97·6 (88·4–106·6)	22·3 (18·6–27·1)	2·6 (1·8–3·5)	0·1 (0·0–0·1)	0·0 (0·0–0·0)	1·69 (1·61–1·77)	0·32 (0·28–0·36)	0·61 (0·56–0·66)
1992	0·0 (0·0–0·0)	4·1 (3·3–5·0)	58·5 (51·8–67·1)	144·7 (136·9–152·8)	97·6 (89·7–106·5)	23·1 (18·8–27·6)	2·7 (1·9–3·7)	0·1 (0·0–0·1)	0·0 (0·0–0·0)	1·65 (1·58–1·73)	0·31 (0·28–0·36)	0·62 (0·57–0·66)
1993	0·0 (0·0–0·0)	4·3 (3·5–5·2)	59·5 (51·0–68·0)	145·1 (135·9–154·9)	102·2 (93·3–111·9)	25·0 (20·7–30·0)	2·8 (2·0–3·7)	0·1 (0·0–0·1)	0·0 (0·0–0·0)	1·7 (1·62–1·77)	0·32 (0·28–0·36)	0·65 (0·6–0·7)
1994	0·0 (0·0–0·0)	4·5 (3·6–5·5)	60·1 (52·6–68·6)	143·0 (134·4–151·4)	104·1 (95·1–114·1)	26·6 (21·9–32·3)	2·9 (2·1–3·9)	0·1 (0·0–0·1)	0·0 (0·0–0·0)	1·71 (1·62–1·79)	0·32 (0·29–0·37)	0·67 (0·62–0·72)
1995	0·0 (0·0–0·0)	4·6 (3·8–5·5)	59·8 (51·4–68·9)	135·4 (126·9–144·3)	103·0 (93·4–112·8)	27·6 (23·1–32·9)	3·0 (2·2–4·1)	0·1 (0·0–0·1)	0·0 (0·0–0·0)	1·67 (1·58–1·75)	0·32 (0·28–0·37)	0·67 (0·62–0·72)
1996	0·0 (0·0–0·0)	4·8 (4·0–5·8)	60·1 (52·0–68·7)	131·0 (122·4–140·0)	103·0 (94·2–113·2)	29·1 (24·1–34·4)	3·1 (2·3–4·1)	0·1 (0·0–0·1)	0·0 (0·0–0·0)	1·66 (1·56–1·75)	0·32 (0·28–0·37)	0·68 (0·63–0·73)
1997	0·0 (0·0–0·0)	5·1 (4·2–6·1)	60·5 (52·8–69·0)	127·1 (118·8–136·0)	101·5 (92·2–111·3)	30·0 (24·8–36·3)	3·3 (2·5–4·5)	0·1 (0·0–0·1)	0·0 (0·0–0·0)	1·64 (1·56–1·72)	0·33 (0·29–0·37)	0·67 (0·62–0·73)
1998	0·0 (0·0–0·0)	5·4 (4·5–6·5)	60·5 (53·1–69·6)	122·7 (114·7–130·7)	100·6 (91·9–110·8)	31·5 (26·0–38·2)	3·5 (2·6–4·6)	0·1 (0·0–0·1)	0·0 (0·0–0·0)	1·62 (1·54–1·71)	0·33 (0·29–0·38)	0·68 (0·63–0·73)
1999	0·0 (0·0–0·0)	5·8 (4·8–7·0)	60·4 (53·3–68·1)	119·5 (111·1–127·7)	99·9 (90·9–109·9)	32·4 (26·7–38·5)	3·7 (2·7–4·9)	0·1 (0·0–0·1)	0·0 (0·0–0·0)	1·61 (1·53–1·69)	0·33 (0·29–0·37)	0·68 (0·63–0·74)
2000	0·0 (0·0–0·0)	6·2 (5·1–7·5)	61·1 (51·2–71·5)	117·8 (109·7–125·7)	98·0 (88·6–106·9)	34·0 (28·2–40·3)	3·9 (2·8–5·2)	0·1 (0·1–0·1)	0·0 (0·0–0·0)	1·61 (1·5–1·71)	0·34 (0·29–0·39)	0·68 (0·62–0·74)
2001	0·0 (0·0–0·0)	6·6 (5·4–8·0)	61·4 (54·4–69·6)	115·2 (107·7–123·6)	94·8 (86·0–103·8)	34·0 (28·2–40·8)	4·0 (3·0–5·4)	0·1 (0·1–0·1)	0·0 (0·0–0·0)	1·58 (1·5–1·66)	0·34 (0·3–0·38)	0·66 (0·61–0·72)
2002	0·0 (0·0–0·0)	6·5 (5·4–8·0)	60·1 (52·0–68·4)	113·1 (105·9–120·7)	94·1 (85·7–103·7)	34·4 (28·7–41·4)	4·3 (3·0–5·9)	0·1 (0·1–0·1)	0·0 (0·0–0·0)	1·56 (1·47–1·65)	0·33 (0·29–0·38)	0·66 (0·61–0·72)
2003	0·0 (0·0–0·0)	6·4 (5·0–7·9)	58·4 (49·1–68·4)	111·4 (102·9–120·5)	93·3 (84·9–102·1)	35·7 (29·4–43·2)	4·5 (3·2–6·1)	0·1 (0·1–0·1)	0·0 (0·0–0·0)	1·55 (1·45–1·65)	0·32 (0·28–0·37)	0·67 (0·61–0·72)
2004	0·0 (0·0–0·0)	6·1 (4·9–7·6)	56·9 (49·7–65·6)	108·2 (101·6–115·3)	91·7 (83·6–100·2)	36·3 (29·9–43·1)	4·7 (3·3–6·3)	0·1 (0·1–0·2)	0·0 (0·0–0·0)	1·52 (1·44–1·6)	0·32 (0·28–0·36)	0·66 (0·61–0·72)
2005	0·0 (0·0–0·0)	6·0 (4·8–7·4)	57·1 (50·2–64·4)	108·4 (101·0–116·5)	92·2 (83·7–101·3)	36·8 (30·5–44·0)	4·9 (3·5–6·5)	0·1 (0·1–0·2)	0·0 (0·0–0·0)	1·53 (1·46–1·6)	0·32 (0·28–0·35)	0·67 (0·62–0·73)
2006	0·0 (0·0–0·0)	6·0 (4·8–7·5)	58·6 (50·3–67·6)	110·9 (103·5–119·3)	95·4 (87·2–104·5)	38·2 (32·0–46·0)	5·3 (3·8–7·1)	0·1 (0·1–0·2)	0·0 (0·0–0·0)	1·57 (1·5–1·65)	0·32 (0·28–0·37)	0·69 (0·64–0·76)
2007	0·0 (0·0–0·0)	6·1 (4·8–7·6)	59·1 (52·4–66·9)	111·7 (104·6–120·1)	96·2 (87·3–106·0)	39·9 (33·6–47·4)	5·6 (4·1–7·4)	0·1 (0·1–0·2)	0·0 (0·0–0·0)	1·59 (1·52–1·68)	0·33 (0·29–0·37)	0·71 (0·65–0·77)
2008	0·0 (0·0–0·0)	6·0 (4·9–7·4)	59·1 (51·4–67·5)	112·4 (104·3–120·7)	97·3 (89·0–106·1)	41·0 (34·3–48·2)	6·0 (4·4–7·9)	0·1 (0·1–0·2)	0·0 (0·0–0·0)	1·61 (1·53–1·69)	0·33 (0·29–0·37)	0·72 (0·66–0·79)
2009	0·0 (0·0–0·0)	5·9 (4·6–7·3)	57·9 (49·9–67·3)	112·8 (105·4–120·2)	98·9 (89·9–108·7)	42·4 (35·7–50·3)	6·5 (4·8–8·7)	0·1 (0·1–0·2)	0·0 (0·0–0·0)	1·62 (1·53–1·73)	0·32 (0·28–0·36)	0·74 (0·68–0·81)
2010	0·0 (0·0–0·0)	5·7 (4·7–7·1)	56·7 (49·4–64·5)	113·7 (105·8–122·2)	101·1 (91·9–110·9)	43·9 (37·4–51·1)	6·7 (5·0–8·8)	0·1 (0·1–0·2)	0·0 (0·0–0·0)	1·64 (1·56–1·72)	0·31 (0·28–0·35)	0·76 (0·7–0·83)
2011	0·0 (0·0–0·0)	5·6 (4·6–6·9)	54·3 (47·6–61·8)	113·0 (105·3–121·3)	102·1 (93·5–111·6)	45·3 (38·0–53·7)	6·9 (5·2–9·1)	0·1 (0·1–0·2)	0·0 (0·0–0·0)	1·64 (1·56–1·71)	0·3 (0·26–0·34)	0·77 (0·71–0·84)
2012	0·0 (0·0–0·0)	5·6 (4·7–6·6)	51·9 (45·6–58·9)	112·1 (104·4–120·2)	104·1 (94·9–114·3)	47·6 (40·7–55·5)	7·4 (5·6–9·6)	0·1 (0·1–0·2)	0·0 (0·0–0·0)	1·64 (1·56–1·73)	0·29 (0·26–0·32)	0·8 (0·74–0·87)
2013	0·0 (0·0–0·0)	5·5 (4·5–6·6)	50·3 (41·9–59·4)	110·1 (102·3–117·8)	104·4 (95·8–113·7)	48·7 (41·4–56·3)	7·8 (5·9–10·3)	0·1 (0·1–0·2)	0·0 (0·0–0·0)	1·63 (1·55–1·72)	0·28 (0·24–0·32)	0·81 (0·74–0·87)
2014	0·0 (0·0–0·0)	5·4 (4·5–6·5)	48·4 (42·0–55·8)	109·0 (101·1–117·1)	105·4 (96·1–115·0)	50·0 (43·1–58·1)	8·2 (6·0–10·9)	0·2 (0·1–0·2)	0·0 (0·0–0·0)	1·63 (1·54–1·73)	0·27 (0·24–0·31)	0·82 (0·76–0·9)
2015	0·0 (0·0–0·0)	5·4 (4·5–6·6)	49·0 (40·4–58·5)	110·7 (101·8–120·8)	107·4 (96·9–119·1)	47·8 (39·3–58·1)	7·6 (5·4–10·5)	0·1 (0·1–0·2)	0·0 (0·0–0·0)	1·64 (1·46–1·83)	0·27 (0·23–0·32)	0·81 (0·72–0·92)
2016	0·0 (0·0–0·0)	5·4 (4·5–6·7)	50·6 (38·6–66·1)	111·9 (100·0–126·2)	106·4 (92·3–122·7)	43·6 (33·6–56·9)	6·6 (4·4–9·4)	0·1 (0·1–0·2)	0·0 (0·0–0·0)	1·62 (1·38–1·91)	0·28 (0·22–0·36)	0·78 (0·66–0·93)
2017	0·0 (0·0–0·0)	5·4 (4·4–6·5)	50·2 (37·4–65·8)	111·5 (98·9–125·4)	104·3 (88·8–121·2)	42·1 (31·2–55·5)	6·5 (4·1–9·8)	0·1 (0·1–0·2)	0·0 (0·0–0·0)	1·6 (1·35–1·89)	0·28 (0·21–0·36)	0·77 (0·63–0·93)

Supplemental Results Table 5: Age-specific fertility rates per 1000, cumulative total fertility up to maternal age 25 (TFU25), cumulative total fertility from maternal age 30 to age 55 (TFO30), and cumulative total fertility, Nagano, 1950-2017												
**Year**	**10-14**	**15-19**	**20-24**	**25-29**	**30-34**	**35-39**	**40-44**	**45-49**	**50-54**	**TFR**	**TFU25**	**TFO30**
1950	0·0 (0·0–0·0)	5·1 (4·5–5·7)	123·3 (112·6–134·8)	239·6 (230·4–248·5)	173·9 (163·4–184·6)	62·4 (55·4–69·3)	15·7 (13·5–18·0)	0·7 (0·4–1·0)	0·0 (0·0–0·0)	3·1 (2·99–3·22)	0·64 (0·59–0·7)	1·26 (1·2–1·33)
1951	0·0 (0·0–0·0)	4·1 (3·6–4·7)	110·4 (99·1–121·7)	223·5 (214·6–232·3)	157·7 (147·1–167·8)	64·2 (55·9–72·4)	12·1 (10·2–14·2)	0·5 (0·3–0·7)	0·0 (0·0–0·0)	2·86 (2·74–2·99)	0·57 (0·52–0·63)	1·17 (1·08–1·27)
1952	0·0 (0·0–0·0)	3·5 (3·0–4·0)	102·4 (91·9–115·3)	212·9 (203·6–222·4)	142·9 (131·7–154·0)	63·6 (54·9–73·7)	9·6 (7·9–11·5)	0·4 (0·2–0·6)	0·0 (0·0–0·0)	2·68 (2·57–2·78)	0·53 (0·48–0·59)	1·08 (1·01–1·16)
1953	0·0 (0·0–0·0)	3·0 (2·6–3·6)	96·5 (85·1–108·5)	200·9 (191·7–211·1)	129·1 (118·3–140·1)	58·5 (50·0–68·1)	7·9 (6·4–9·5)	0·3 (0·2–0·5)	0·0 (0·0–0·0)	2·48 (2·38–2·58)	0·5 (0·44–0·56)	0·98 (0·91–1·06)
1954	0·0 (0·0–0·0)	2·8 (2·3–3·3)	93·2 (81·1–105·9)	195·5 (186·5–205·3)	121·8 (111·0–133·1)	53·4 (45·0–62·9)	7·0 (5·6–8·8)	0·3 (0·2–0·4)	0·0 (0·0–0·0)	2·37 (2·27–2·47)	0·48 (0·42–0·54)	0·91 (0·84–0·99)
1955	0·0 (0·0–0·0)	2·5 (2·0–3·1)	90·6 (79·5–103·5)	194·5 (185·2–203·3)	117·0 (106·0–128·3)	48·1 (40·4–56·2)	6·8 (5·3–8·6)	0·3 (0·2–0·4)	0·0 (0·0–0·0)	2·3 (2·2–2·4)	0·47 (0·41–0·53)	0·86 (0·79–0·94)
1956	0·0 (0·0–0·0)	2·2 (1·8–2·8)	85·0 (73·7–97·3)	188·0 (180·0–196·6)	106·5 (96·7–117·2)	41·4 (34·8–48·5)	6·0 (4·6–7·7)	0·3 (0·2–0·4)	0·0 (0·0–0·0)	2·15 (2·05–2·24)	0·44 (0·38–0·5)	0·77 (0·7–0·84)
1957	0·0 (0·0–0·0)	2·1 (1·6–2·7)	85·6 (74·9–98·0)	191·3 (183·0–200·5)	98·3 (89·5–107·8)	35·5 (29·6–41·4)	4·6 (3·6–5·8)	0·2 (0·1–0·4)	0·0 (0·0–0·0)	2·09 (1·98–2·21)	0·44 (0·38–0·5)	0·69 (0·63–0·75)
1958	0·0 (0·0–0·0)	2·1 (1·6–2·7)	89·5 (78·4–101·6)	197·2 (188·2–206·2)	94·3 (86·2–102·8)	31·5 (26·4–37·3)	3·8 (3·0–4·9)	0·2 (0·1–0·3)	0·0 (0·0–0·0)	2·09 (2·0–2·18)	0·46 (0·4–0·52)	0·65 (0·6–0·7)
1959	0·0 (0·0–0·0)	2·2 (1·7–2·8)	89·2 (78·4–101·2)	196·1 (187·1–205·5)	90·4 (80·8–100·1)	27·2 (22·5–32·5)	3·4 (2·6–4·5)	0·2 (0·1–0·3)	0·0 (0·0–0·0)	2·04 (1·96–2·14)	0·46 (0·4–0·52)	0·61 (0·55–0·66)
1960	0·0 (0·0–0·0)	2·1 (1·7–2·7)	85·3 (75·4–96·0)	197·1 (187·7–206·7)	90·2 (81·4–99·1)	24·0 (20·1–28·5)	3·2 (2·4–4·1)	0·2 (0·1–0·3)	0·0 (0·0–0·0)	2·01 (1·92–2·1)	0·44 (0·39–0·49)	0·59 (0·54–0·64)
1961	0·0 (0·0–0·0)	2·0 (1·5–2·5)	82·5 (74·1–91·5)	199·2 (190·7–208·2)	90·1 (83·7–96·5)	21·7 (18·1–26·4)	2·9 (2·2–3·9)	0·2 (0·1–0·3)	0·0 (0·0–0·0)	1·99 (1·92–2·07)	0·42 (0·38–0·47)	0·57 (0·54–0·62)
1962	0·0 (0·0–0·0)	1·9 (1·5–2·4)	81·9 (73·0–91·2)	204·8 (196·2–213·4)	92·6 (85·1–100·6)	20·6 (17·7–23·6)	2·6 (1·9–3·4)	0·1 (0·1–0·2)	0·0 (0·0–0·0)	2·02 (1·94–2·1)	0·42 (0·37–0·47)	0·58 (0·54–0·62)
1963	0·0 (0·0–0·0)	1·8 (1·4–2·3)	81·9 (73·3–91·4)	206·5 (197·8–215·6)	91·0 (83·1–99·2)	20·0 (17·0–23·4)	2·2 (1·6–2·9)	0·1 (0·1–0·2)	0·0 (0·0–0·0)	2·02 (1·94–2·09)	0·42 (0·38–0·46)	0·57 (0·52–0·61)
1964	0·0 (0·0–0·0)	1·8 (1·4–2·2)	89·1 (79·8–100·3)	219·8 (210·5–229·3)	97·7 (88·4–107·7)	20·7 (17·4–24·5)	2·0 (1·5–2·7)	0·1 (0·1–0·2)	0·0 (0·0–0·0)	2·16 (2·07–2·25)	0·45 (0·41–0·51)	0·6 (0·55–0·66)
1965	0·0 (0·0–0·0)	1·8 (1·4–2·2)	81·7 (72·3–91·7)	185·9 (176·7–195·9)	86·3 (77·5–94·5)	19·1 (16·0–22·5)	2·1 (1·5–2·8)	0·1 (0·1–0·2)	0·0 (0·0–0·0)	1·88 (1·79–1·98)	0·42 (0·37–0·47)	0·54 (0·49–0·58)
1966	0·0 (0·0–0·0)	1·9 (1·5–2·2)	79·4 (70·4–89·5)	191·0 (181·1–200·5)	87·6 (78·4–97·5)	19·5 (15·9–23·7)	1·9 (1·4–2·6)	0·1 (0·1–0·2)	0·0 (0·0–0·0)	1·91 (1·82–2·0)	0·41 (0·36–0·46)	0·55 (0·5–0·6)
1967	0·0 (0·0–0·0)	1·9 (1·6–2·3)	84·2 (74·5–94·6)	233·0 (223·5–242·5)	109·2 (98·8–120·6)	22·2 (18·3–26·6)	2·1 (1·5–2·7)	0·1 (0·1–0·2)	0·0 (0·0–0·0)	2·26 (2·15–2·38)	0·43 (0·38–0·48)	0·67 (0·61–0·73)
1968	0·0 (0·0–0·0)	2·0 (1·7–2·3)	75·0 (66·1–84·3)	220·5 (211·4–230·1)	107·1 (96·3–117·8)	22·2 (18·4–26·6)	2·3 (1·7–3·0)	0·1 (0·1–0·2)	0·0 (0·0–0·0)	2·15 (2·05–2·24)	0·38 (0·34–0·43)	0·66 (0·6–0·72)
1969	0·0 (0·0–0·0)	2·1 (1·8–2·5)	73·3 (64·7–82·6)	222·9 (214·1–231·6)	105·9 (96·7–116·7)	21·8 (17·9–26·1)	2·1 (1·6–2·8)	0·1 (0·1–0·2)	0·0 (0·0–0·0)	2·14 (2·05–2·24)	0·38 (0·33–0·42)	0·65 (0·59–0·71)
1970	0·0 (0·0–0·0)	2·1 (1·8–2·5)	78·4 (69·1–88·8)	226·4 (217·2–235·0)	101·9 (92·8–111·3)	20·9 (17·0–25·3)	1·7 (1·2–2·2)	0·1 (0·1–0·1)	0·0 (0·0–0·0)	2·16 (2·05–2·26)	0·4 (0·36–0·46)	0·62 (0·57–0·68)
1971	0·0 (0·0–0·0)	2·1 (1·7–2·4)	85·4 (74·5–96·4)	223·4 (213·8–233·6)	97·8 (88·7–107·5)	19·0 (15·6–22·7)	1·5 (1·1–2·0)	0·1 (0·0–0·1)	0·0 (0·0–0·0)	2·15 (2·04–2·25)	0·44 (0·38–0·49)	0·59 (0·54–0·65)
1972	0·0 (0·0–0·0)	2·1 (1·7–2·5)	88·9 (78·0–100·4)	222·2 (212·7–231·6)	96·7 (87·3–105·7)	18·0 (14·4–21·9)	1·4 (1·0–1·8)	0·1 (0·0–0·1)	0·0 (0·0–0·0)	2·15 (2·05–2·24)	0·45 (0·4–0·51)	0·58 (0·53–0·63)
1973	0·0 (0·0–0·0)	2·0 (1·6–2·4)	88·7 (78·0–99·7)	223·5 (213·9–234·0)	94·6 (86·5–103·5)	17·6 (14·0–21·5)	1·4 (0·9–1·9)	0·1 (0·0–0·1)	0·0 (0·0–0·0)	2·14 (2·04–2·24)	0·45 (0·4–0·51)	0·57 (0·52–0·62)
1974	0·0 (0·0–0·0)	1·8 (1·5–2·3)	84·4 (75·2–94·5)	216·5 (206·1–226·4)	88·6 (80·7–97·4)	16·4 (13·5–19·7)	1·4 (1·0–2·0)	0·1 (0·0–0·1)	0·0 (0·0–0·0)	2·05 (1·98–2·12)	0·43 (0·39–0·48)	0·53 (0·49–0·58)
1975	0·0 (0·0–0·0)	1·7 (1·3–2·2)	78·3 (69·2–87·6)	210·1 (200·4–219·4)	83·3 (75·3–90·9)	15·5 (12·6–18·8)	1·5 (1·1–2·0)	0·1 (0·0–0·1)	0·0 (0·0–0·0)	1·95 (1·87–2·04)	0·4 (0·35–0·45)	0·5 (0·46–0·54)
1976	0·0 (0·0–0·0)	1·6 (1·1–2·1)	73·3 (65·0–81·9)	208·0 (199·0–217·6)	82·9 (75·6–90·6)	15·2 (12·9–17·9)	1·5 (1·0–2·2)	0·1 (0·0–0·1)	0·0 (0·0–0·0)	1·91 (1·83–1·99)	0·37 (0·33–0·42)	0·5 (0·45–0·54)
1977	0·0 (0·0–0·0)	1·5 (1·2–1·9)	68·5 (60·9–77·5)	208·1 (198·9–217·0)	86·3 (77·8–95·3)	15·1 (12·6–18·2)	1·6 (1·1–2·2)	0·1 (0·0–0·1)	0·0 (0·0–0·0)	1·91 (1·83–1·99)	0·35 (0·31–0·4)	0·52 (0·46–0·57)
1978	0·0 (0·0–0·0)	1·5 (1·2–1·9)	64·5 (57·3–72·2)	206·5 (197·4–214·9)	90·6 (82·2–99·0)	14·9 (11·7–18·5)	1·6 (1·1–2·3)	0·1 (0·0–0·1)	0·0 (0·0–0·0)	1·9 (1·82–1·97)	0·33 (0·29–0·37)	0·54 (0·49–0·58)
1979	0·0 (0·0–0·0)	1·5 (1·3–1·8)	60·6 (53·5–67·9)	201·1 (192·7–209·9)	90·7 (82·9–99·8)	14·5 (12·1–17·4)	1·6 (1·1–2·4)	0·1 (0·0–0·1)	0·0 (0·0–0·0)	1·85 (1·78–1·92)	0·31 (0·28–0·35)	0·53 (0·49–0·58)
1980	0·0 (0·0–0·0)	1·6 (1·2–1·9)	57·0 (50·2–64·1)	194·5 (185·4–203·7)	88·7 (80·6–97·5)	14·4 (11·7–17·6)	1·7 (1·1–2·3)	0·1 (0·0–0·1)	0·0 (0·0–0·0)	1·79 (1·72–1·86)	0·29 (0·26–0·33)	0·52 (0·48–0·57)
1981	0·0 (0·0–0·0)	1·7 (1·3–2·1)	54·9 (49·0–62·1)	192·2 (183·7–200·7)	89·2 (81·1–97·3)	15·0 (12·3–17·9)	1·7 (1·1–2·5)	0·1 (0·0–0·1)	0·0 (0·0–0·0)	1·77 (1·71–1·84)	0·28 (0·25–0·32)	0·53 (0·49–0·57)
1982	0·0 (0·0–0·0)	1·8 (1·4–2·2)	54·0 (47·0–62·0)	192·1 (184·4–201·0)	92·8 (84·7–101·3)	16·7 (14·1–19·8)	1·7 (1·2–2·4)	0·1 (0·0–0·1)	0·0 (0·0–0·0)	1·8 (1·74–1·86)	0·28 (0·24–0·32)	0·56 (0·51–0·6)
1983	0·0 (0·0–0·0)	1·9 (1·5–2·3)	53·2 (46·8–60·9)	192·0 (182·2–201·2)	97·1 (88·4–106·3)	18·3 (15·0–22·4)	1·8 (1·3–2·3)	0·1 (0·0–0·1)	0·0 (0·0–0·0)	1·82 (1·75–1·9)	0·28 (0·24–0·31)	0·59 (0·54–0·64)
1984	0·0 (0·0–0·0)	1·9 (1·5–2·3)	50·4 (44·6–56·9)	189·5 (180·7–198·9)	100·3 (91·6–109·4)	18·9 (15·5–22·8)	1·8 (1·3–2·4)	0·1 (0·0–0·1)	0·0 (0·0–0·0)	1·81 (1·75–1·89)	0·26 (0·23–0·29)	0·61 (0·56–0·65)
1985	0·0 (0·0–0·0)	1·9 (1·5–2·3)	48·4 (42·1–55·6)	183·7 (175·3–191·8)	101·8 (92·8–110·7)	18·7 (15·5–22·4)	1·8 (1·3–2·5)	0·1 (0·0–0·1)	0·0 (0·0–0·0)	1·78 (1·72–1·84)	0·25 (0·22–0·29)	0·61 (0·57–0·66)
1986	0·0 (0·0–0·0)	1·9 (1·5–2·3)	47·1 (41·1–53·7)	178·7 (170·2–187·7)	104·5 (95·7–112·8)	18·9 (15·7–22·6)	2·0 (1·3–2·8)	0·1 (0·0–0·1)	0·0 (0·0–0·0)	1·77 (1·71–1·83)	0·24 (0·22–0·28)	0·63 (0·58–0·67)
1987	0·0 (0·0–0·0)	1·9 (1·5–2·3)	44·4 (39·1–50·2)	174·6 (166·3–183·0)	107·7 (98·7–117·5)	19·8 (16·3–23·9)	2·2 (1·5–3·0)	0·1 (0·0–0·1)	0·0 (0·0–0·0)	1·75 (1·67–1·83)	0·23 (0·21–0·26)	0·65 (0·6–0·7)
1988	0·0 (0·0–0·0)	1·9 (1·5–2·3)	42·0 (36·6–48·1)	165·3 (157·0–174·5)	108·9 (99·7–118·5)	21·1 (17·6–25·0)	2·4 (1·7–3·2)	0·1 (0·1–0·1)	0·0 (0·0–0·0)	1·71 (1·64–1·78)	0·22 (0·19–0·25)	0·66 (0·61–0·72)
1989	0·0 (0·0–0·0)	2·0 (1·6–2·4)	39·7 (34·2–45·9)	155·1 (147·2–163·6)	109·0 (99·4–118·5)	22·5 (18·5–26·9)	2·5 (1·8–3·4)	0·1 (0·1–0·1)	0·0 (0·0–0·0)	1·65 (1·58–1·72)	0·21 (0·18–0·24)	0·67 (0·62–0·72)
1990	0·0 (0·0–0·0)	2·1 (1·7–2·5)	39·0 (33·9–44·6)	151·4 (142·7–159·6)	111·1 (101·8–120·2)	23·9 (19·4–28·6)	2·6 (1·9–3·4)	0·1 (0·1–0·1)	0·0 (0·0–0·0)	1·65 (1·58–1·73)	0·21 (0·18–0·24)	0·69 (0·63–0·74)
1991	0·0 (0·0–0·0)	2·2 (1·8–2·7)	39·5 (34·2–45·3)	146·9 (139·5–154·8)	112·1 (102·8–122·6)	25·6 (21·3–30·4)	2·6 (1·9–3·5)	0·1 (0·1–0·1)	0·0 (0·0–0·0)	1·64 (1·58–1·71)	0·21 (0·18–0·24)	0·7 (0·65–0·76)
1992	0·0 (0·0–0·0)	2·3 (1·9–2·8)	38·8 (34·1–44·3)	138·3 (130·5–146·3)	110·9 (102·7–120·0)	26·7 (22·3–32·0)	2·8 (2·0–3·6)	0·1 (0·1–0·1)	0·0 (0·0–0·0)	1·6 (1·53–1·66)	0·21 (0·18–0·23)	0·7 (0·65–0·76)
1993	0·0 (0·0–0·0)	2·4 (2·0–2·9)	39·2 (33·6–45·0)	137·3 (129·1–146·3)	115·3 (105·3–125·1)	29·1 (24·2–34·3)	2·9 (2·2–3·9)	0·1 (0·1–0·1)	0·0 (0·0–0·0)	1·63 (1·55–1·71)	0·21 (0·18–0·24)	0·74 (0·68–0·8)
1994	0·0 (0·0–0·0)	2·5 (2·0–3·1)	39·4 (34·4–45·3)	133·9 (125·7–141·9)	116·9 (107·1–127·5)	30·8 (26·1–36·5)	3·1 (2·2–4·2)	0·1 (0·1–0·1)	0·0 (0·0–0·0)	1·63 (1·55–1·71)	0·21 (0·18–0·24)	0·75 (0·7–0·82)
1995	0·0 (0·0–0·0)	2·6 (2·1–3·1)	39·2 (33·6–45·4)	125·6 (118·0–133·6)	115·3 (105·1–125·6)	31·9 (26·4–38·4)	3·3 (2·4–4·3)	0·1 (0·1–0·1)	0·0 (0·0–0·0)	1·59 (1·5–1·68)	0·21 (0·18–0·24)	0·75 (0·69–0·82)
1996	0·0 (0·0–0·0)	2·8 (2·3–3·4)	39·6 (34·2–45·2)	121·0 (113·5–128·4)	114·7 (105·4–124·4)	33·3 (27·6–40·2)	3·4 (2·5–4·4)	0·1 (0·1–0·1)	0·0 (0·0–0·0)	1·57 (1·5–1·66)	0·21 (0·19–0·24)	0·76 (0·7–0·82)
1997	0·0 (0·0–0·0)	3·1 (2·5–3·7)	40·5 (33·6–47·3)	117·1 (110·1–124·1)	112·2 (102·2–121·6)	34·0 (28·5–40·2)	3·6 (2·6–4·8)	0·1 (0·1–0·1)	0·0 (0·0–0·0)	1·55 (1·48–1·63)	0·22 (0·18–0·25)	0·75 (0·69–0·81)
1998	0·0 (0·0–0·0)	3·3 (2·8–4·0)	41·0 (35·8–47·0)	113·0 (106·1–120·5)	110·5 (101·2–120·9)	35·4 (29·4–41·8)	3·8 (2·8–5·0)	0·1 (0·1–0·1)	0·0 (0·0–0·0)	1·54 (1·46–1·62)	0·22 (0·2–0·25)	0·75 (0·69–0·81)
1999	0·0 (0·0–0·0)	3·7 (3·0–4·4)	41·5 (35·4–47·9)	110·0 (103·3–116·3)	108·8 (99·5–118·6)	36·4 (29·9–43·8)	4·0 (2·9–5·6)	0·1 (0·1–0·1)	0·0 (0·0–0·0)	1·52 (1·45–1·6)	0·23 (0·2–0·26)	0·75 (0·69–0·81)
2000	0·0 (0·0–0·0)	4·0 (3·3–4·8)	42·3 (37·3–47·5)	108·2 (101·0–116·2)	105·9 (97·4–115·2)	38·2 (32·2–44·9)	4·3 (3·1–5·8)	0·1 (0·1–0·1)	0·0 (0·0–0·0)	1·52 (1·44–1·59)	0·23 (0·21–0·26)	0·74 (0·69–0·8)
2001	0·0 (0·0–0·0)	4·3 (3·5–5·2)	42·9 (36·9–49·1)	105·6 (99·1–112·7)	101·8 (92·4–111·1)	38·2 (32·4–45·0)	4·4 (3·2–6·1)	0·1 (0·1–0·1)	0·0 (0·0–0·0)	1·49 (1·41–1·56)	0·24 (0·21–0·27)	0·72 (0·67–0·78)
2002	0·0 (0·0–0·0)	4·3 (3·5–5·3)	42·2 (36·3–48·5)	103·1 (96·5–109·9)	100·4 (92·0–110·0)	38·8 (33·1–45·3)	4·8 (3·4–6·6)	0·1 (0·1–0·2)	0·0 (0·0–0·0)	1·47 (1·4–1·54)	0·23 (0·2–0·26)	0·72 (0·67–0·78)
2003	0·0 (0·0–0·0)	4·2 (3·3–5·2)	40·9 (35·7–46·9)	100·7 (93·9–107·5)	99·1 (91·0–107·9)	40·3 (33·9–47·1)	5·1 (3·6–6·9)	0·1 (0·1–0·2)	0·0 (0·0–0·0)	1·45 (1·37–1·54)	0·23 (0·2–0·26)	0·72 (0·67–0·79)
2004	0·0 (0·0–0·0)	4·0 (3·2–5·0)	39·6 (34·3–46·4)	96·8 (90·6–103·0)	97·2 (89·0–105·4)	41·0 (34·6–48·1)	5·4 (4·0–7·1)	0·1 (0·1–0·2)	0·0 (0·0–0·0)	1·42 (1·35–1·49)	0·22 (0·19–0·25)	0·72 (0·67–0·78)
2005	0·0 (0·0–0·0)	4·0 (3·1–4·9)	39·4 (34·4–45·2)	95·5 (88·8–102·3)	97·5 (88·6–106·5)	41·7 (35·5–48·6)	5·7 (4·1–7·7)	0·1 (0·1–0·2)	0·0 (0·0–0·0)	1·42 (1·35–1·49)	0·22 (0·19–0·25)	0·73 (0·67–0·78)
2006	0·0 (0·0–0·0)	3·9 (3·1–4·9)	39·8 (34·8–45·3)	96·2 (90·2–102·9)	100·5 (91·9–109·5)	43·4 (37·2–50·7)	6·3 (4·8–8·4)	0·1 (0·1–0·2)	0·0 (0·0–0·0)	1·45 (1·38–1·52)	0·22 (0·19–0·25)	0·75 (0·7–0·81)
2007	0·0 (0·0–0·0)	3·9 (3·1–4·8)	39·7 (33·1–47·1)	95·3 (89·1–102·3)	100·5 (91·9–109·5)	45·2 (38·6–53·2)	6·9 (5·2–8·9)	0·1 (0·1–0·2)	0·0 (0·0–0·0)	1·46 (1·38–1·54)	0·22 (0·18–0·26)	0·76 (0·71–0·83)
2008	0·0 (0·0–0·0)	3·8 (3·1–4·7)	39·0 (33·8–44·7)	94·3 (88·4–100·8)	100·7 (92·5–109·7)	46·5 (39·5–53·9)	7·5 (5·7–9·8)	0·1 (0·1–0·2)	0·0 (0·0–0·0)	1·46 (1·39–1·53)	0·21 (0·19–0·24)	0·77 (0·72–0·83)
2009	0·0 (0·0–0·0)	3·7 (2·9–4·6)	37·7 (31·7–44·2)	93·8 (86·7–100·9)	101·3 (92·4–110·7)	48·0 (40·5–56·3)	8·2 (6·3–10·5)	0·2 (0·1–0·3)	0·0 (0·0–0·0)	1·46 (1·37–1·56)	0·21 (0·18–0·24)	0·79 (0·73–0·85)
2010	0·0 (0·0–0·0)	3·6 (2·9–4·5)	36·6 (31·9–41·4)	94·2 (88·6–99·8)	102·5 (94·0–112·4)	49·3 (42·1–56·8)	8·6 (6·7–10·9)	0·2 (0·1–0·3)	0·0 (0·0–0·0)	1·47 (1·41–1·54)	0·2 (0·18–0·23)	0·8 (0·75–0·86)
2011	0·0 (0·0–0·0)	3·5 (2·8–4·4)	35·0 (30·5–39·9)	93·7 (87·0–100·8)	102·9 (94·0–112·2)	50·4 (42·9–58·3)	8·8 (6·9–11·3)	0·2 (0·1–0·3)	0·0 (0·0–0·0)	1·47 (1·4–1·55)	0·19 (0·17–0·22)	0·81 (0·75–0·88)
2012	0·0 (0·0–0·0)	3·5 (2·9–4·1)	33·6 (29·4–38·4)	93·1 (87·2–99·9)	104·8 (95·3–114·9)	52·5 (45·1–60·5)	9·4 (7·3–12·1)	0·2 (0·1–0·3)	0·0 (0·0–0·0)	1·49 (1·42–1·56)	0·19 (0·16–0·21)	0·83 (0·78–0·9)
2013	0·0 (0·0–0·0)	3·5 (2·9–4·2)	32·7 (27·0–38·8)	91·7 (85·3–97·8)	105·4 (96·7–114·8)	53·4 (46·0–60·8)	9·8 (7·5–12·6)	0·2 (0·1–0·3)	0·0 (0·0–0·0)	1·48 (1·41–1·56)	0·18 (0·15–0·21)	0·84 (0·78–0·91)
2014	0·0 (0·0–0·0)	3·4 (2·8–4·1)	31·8 (27·3–36·8)	91·1 (84·7–97·7)	107·0 (97·9–116·3)	55·0 (47·2–63·5)	10·2 (8·0–13·2)	0·2 (0·1–0·3)	0·0 (0·0–0·0)	1·49 (1·41–1·58)	0·18 (0·15–0·2)	0·86 (0·79–0·93)
2015	0·0 (0·0–0·0)	3·4 (2·8–4·2)	32·4 (26·7–38·8)	92·9 (85·3–100·7)	110·0 (99·1–121·4)	53·0 (44·1–63·1)	9·4 (7·0–12·6)	0·2 (0·1–0·3)	0·0 (0·0–0·0)	1·51 (1·34–1·68)	0·18 (0·15–0·21)	0·86 (0·77–0·97)
2016	0·0 (0·0–0·0)	3·4 (2·8–4·2)	33·8 (25·7–44·0)	94·5 (84·3–106·6)	109·7 (95·5–126·4)	48·6 (37·8–62·2)	8·1 (5·6–11·1)	0·2 (0·1–0·3)	0·0 (0·0–0·0)	1·49 (1·28–1·75)	0·19 (0·14–0·24)	0·83 (0·71–0·99)
2017	0·0 (0·0–0·0)	3·4 (2·8–4·1)	33·7 (25·0–44·3)	94·4 (83·8–106·2)	108·2 (92·2–125·5)	47·2 (35·3–61·8)	7·8 (5·1–11·7)	0·2 (0·1–0·3)	0·0 (0·0–0·0)	1·47 (1·24–1·75)	0·19 (0·14–0·24)	0·82 (0·67–0·99)

Supplemental Results Table 5: Age-specific fertility rates per 1000, cumulative total fertility up to maternal age 25 (TFU25), cumulative total fertility from maternal age 30 to age 55 (TFO30), and cumulative total fertility, Nagasaki, 1950-2017

Year	10-14	15-19	20-24	25-29	30-34	35-39	40-44	45-49	50-54	TFR	TFU25	TFO30
1950	0·0 (0·0–0·0)	10·8 (9·5–12·0)	149·5 (136·5–163·6)	222·4 (214·3–230·6)	200·1 (187·6–212·4)	125·3 (111·3–139·4)	53·2 (45·9–61·0)	2·3 (1·5–3·3)	0·0 (0·0–0·0)	3·82 (3·68–3·96)	0·8 (0·74–0·87)	1·9 (1·8–2·01)
1951	0·0 (0·0–0·0)	8·7 (7·7–9·7)	134·1 (121·9–145·9)	206·7 (198·7–214·6)	181·7 (168·6–194·8)	101·7 (88·8–115·9)	41·4 (34·4–48·9)	1·7 (1·1–2·5)	0·0 (0·0–0·0)	3·38 (3·2–3·55)	0·71 (0·65–0·77)	1·63 (1·48–1·78)
1952	0·0 (0·0–0·0)	7·3 (6·4–8·3)	124·8 (111·1–139·5)	196·5 (187·9–204·8)	164·9 (150·3–180·5)	83·2 (71·9–96·7)	32·8 (26·6–40·0)	1·3 (0·8–1·9)	0·0 (0·0–0·0)	3·05 (2·93–3·18)	0·66 (0·6–0·73)	1·41 (1·32–1·5)
1953	0·0 (0·0–0·0)	6·4 (5·5–7·3)	117·8 (104·6–132·1)	185·2 (177·2–194·1)	147·8 (131·9–164·8)	71·0 (59·4–83·3)	26·1 (20·2–33·5)	1·1 (0·7–1·6)	0·0 (0·0–0·0)	2·78 (2·65–2·93)	0·62 (0·55–0·69)	1·23 (1·14–1·32)
1954	0·0 (0·0–0·0)	5·9 (5·0–6·8)	114·2 (100·3–128·1)	180·2 (171·9–188·8)	136·5 (120·6–153·9)	64·6 (54·2–76·4)	21·6 (15·8–29·1)	0·9 (0·5–1·3)	0·0 (0·0–0·0)	2·62 (2·49–2·75)	0·6 (0·53–0·67)	1·12 (1·04–1·21)
1955	0·0 (0·0–0·0)	5·3 (4·5–6·1)	111·4 (98·3–126·6)	179·2 (170·4–187·8)	126·0 (109·1–143·0)	58·1 (49·1–68·3)	18·0 (12·8–24·5)	0·7 (0·4–1·1)	0·0 (0·0–0·0)	2·49 (2·38–2·61)	0·58 (0·52–0·66)	1·01 (0·93–1·1)
1956	0·0 (0·0–0·0)	4·7 (4·0–5·6)	104·9 (91·4–119·2)	173·2 (165·7–181·2)	111·8 (97·0–127·9)	49·9 (42·0–58·5)	14·4 (9·7–20·7)	0·6 (0·3–0·9)	0·0 (0·0–0·0)	2·3 (2·18–2·42)	0·55 (0·48–0·62)	0·88 (0·8–0·97)
1957	0·0 (0·0–0·0)	4·5 (3·8–5·3)	105·9 (92·6–121·8)	176·6 (169·2–184·6)	106·0 (91·9–121·5)	42·7 (35·7–50·0)	12·3 (8·0–17·5)	0·6 (0·3–0·9)	0·0 (0·0–0·0)	2·24 (2·11–2·37)	0·55 (0·49–0·63)	0·81 (0·73–0·89)
1958	0·0 (0·0–0·0)	4·5 (3·9–5·3)	111·2 (98·1–125·0)	182·4 (173·5–191·3)	103·7 (89·8–118·8)	37·8 (31·1–45·1)	11·1 (7·2–15·7)	0·6 (0·3–0·9)	0·0 (0·0–0·0)	2·26 (2·15–2·37)	0·58 (0·51–0·65)	0·77 (0·69–0·85)
1959	0·0 (0·0–0·0)	4·7 (4·0–5·5)	111·1 (98·1–125·5)	181·4 (172·5–190·2)	97·3 (85·6–110·9)	32·6 (26·9–39·6)	9·2 (6·0–13·3)	0·5 (0·3–0·8)	0·0 (0·0–0·0)	2·18 (2·08–2·3)	0·58 (0·51–0·65)	0·7 (0·63–0·78)
1960	0·0 (0·0–0·0)	4·6 (3·9–5·4)	106·7 (94·5–119·5)	182·2 (173·7–191·0)	93·7 (83·9–104·3)	28·8 (23·9–34·4)	7·5 (4·9–10·7)	0·4 (0·2–0·6)	0·0 (0·0–0·0)	2·12 (2·03–2·21)	0·56 (0·49–0·62)	0·65 (0·6–0·71)
1961	0·0 (0·0–0·0)	4·3 (3·6–5·1)	103·4 (92·7–114·0)	184·2 (175·9–192·9)	91·9 (81·0–104·1)	25·9 (21·6–31·0)	6·4 (4·2–9·1)	0·3 (0·2–0·5)	0·0 (0·0–0·0)	2·08 (1·99–2·19)	0·54 (0·49–0·59)	0·62 (0·56–0·69)
1962	0·0 (0·0–0·0)	4·1 (3·4–4·9)	103·1 (91·8–114·8)	189·6 (181·9–197·5)	94·1 (85·7–102·8)	24·6 (20·6–28·8)	5·7 (3·9–7·9)	0·3 (0·2–0·5)	0·0 (0·0–0·0)	2·11 (2·03–2·19)	0·54 (0·48–0·59)	0·62 (0·57–0·67)
1963	0·0 (0·0–0·0)	4·0 (3·4–4·9)	103·4 (92·5–114·9)	191·6 (183·9–199·6)	96·3 (88·0–105·0)	23·8 (20·2–28·0)	5·5 (3·8–7·6)	0·3 (0·2–0·5)	0·0 (0·0–0·0)	2·13 (2·04–2·21)	0·54 (0·48–0·59)	0·63 (0·58–0·68)
1964	0·0 (0·0–0·0)	4·0 (3·3–4·7)	112·8 (101·8–125·5)	204·1 (195·4–213·4)	103·2 (93·4–113·8)	24·8 (20·7–29·1)	5·2 (3·8–7·1)	0·3 (0·2–0·5)	0·0 (0·0–0·0)	2·27 (2·18–2·37)	0·58 (0·53–0·65)	0·67 (0·62–0·72)
1965	0·0 (0·0–0·0)	3·9 (3·3–4·5)	103·7 (92·7–116·2)	172·3 (163·2–181·3)	86·4 (76·7–97·5)	22·8 (19·5–27·0)	4·4 (3·4–5·9)	0·2 (0·1–0·3)	0·0 (0·0–0·0)	1·97 (1·88–2·06)	0·54 (0·48–0·6)	0·57 (0·52–0·62)
1966	0·0 (0·0–0·0)	4·1 (3·5–4·7)	101·1 (90·0–113·4)	177·2 (168·2–186·2)	88·9 (78·2–101·0)	23·4 (18·7–28·8)	4·4 (3·3–5·8)	0·2 (0·1–0·4)	0·0 (0·0–0·0)	2·0 (1·91–2·1)	0·53 (0·47–0·59)	0·58 (0·52–0·65)
1967	0·0 (0·0–0·0)	4·3 (3·7–5·0)	107·5 (96·4–119·1)	216·0 (207·2–224·6)	108·0 (93·1–123·7)	26·6 (22·2–31·9)	4·2 (3·0–5·6)	0·2 (0·1–0·3)	0·0 (0·0–0·0)	2·33 (2·22–2·44)	0·56 (0·5–0·62)	0·7 (0·62–0·77)
1968	0·0 (0·0–0·0)	4·4 (3·8–5·1)	96·1 (85·3–107·6)	203·8 (195·1–212·0)	100·0 (85·4–115·2)	26·7 (21·7–32·5)	3·8 (2·7–5·2)	0·2 (0·1–0·3)	0·0 (0·0–0·0)	2·17 (2·09–2·26)	0·5 (0·45–0·56)	0·65 (0·58–0·73)
1969	0·0 (0·0–0·0)	4·8 (4·1–5·6)	94·3 (83·1–105·6)	205·9 (197·7–214·2)	101·1 (86·5–117·3)	26·3 (21·8–31·4)	3·8 (2·7–5·2)	0·2 (0·1–0·3)	0·0 (0·0–0·0)	2·18 (2·09–2·27)	0·5 (0·44–0·55)	0·66 (0·59–0·73)
1970	0·0 (0·0–0·0)	4·8 (4·1–5·6)	101·2 (89·5–114·6)	209·4 (200·6–218·2)	103·9 (90·7–119·3)	25·2 (20·7–30·5)	4·0 (2·8–5·7)	0·2 (0·1–0·3)	0·0 (0·0–0·0)	2·24 (2·15–2·34)	0·53 (0·47–0·6)	0·67 (0·6–0·73)
1971	0·0 (0·0–0·0)	4·7 (3·9–5·5)	110·5 (96·3–125·3)	206·6 (197·6–215·8)	105·9 (91·6–121·6)	23·1 (19·1–27·7)	4·3 (2·9–6·1)	0·2 (0·1–0·3)	0·0 (0·0–0·0)	2·28 (2·17–2·38)	0·58 (0·51–0·65)	0·67 (0·59–0·75)
1972	0·0 (0·0–0·0)	4·7 (3·9–5·6)	115·3 (101·9–129·9)	204·9 (195·8–213·7)	106·5 (94·5–118·4)	21·9 (17·6–26·4)	4·3 (2·9–6·2)	0·2 (0·1–0·3)	0·0 (0·0–0·0)	2·29 (2·19–2·38)	0·6 (0·53–0·67)	0·66 (0·61–0·73)
1973	0·0 (0·0–0·0)	4·6 (3·8–5·5)	115·1 (102·6–128·5)	204·6 (194·6–214·2)	101·9 (92·4–112·5)	21·7 (17·4–26·2)	4·0 (2·6–5·8)	0·2 (0·1–0·3)	0·0 (0·0–0·0)	2·26 (2·17–2·35)	0·6 (0·53–0·67)	0·64 (0·59–0·69)
1974	0·0 (0·0–0·0)	4·3 (3·5–5·2)	109·6 (98·3–122·5)	196·2 (187·7–204·9)	90·6 (81·8–99·3)	20·3 (16·3–24·9)	3·4 (2·2–4·8)	0·1 (0·1–0·2)	0·0 (0·0–0·0)	2·12 (2·04–2·21)	0·57 (0·51–0·64)	0·57 (0·52–0·62)
1975	0·0 (0·0–0·0)	3·9 (3·2–4·8)	101·9 (90·7–113·0)	188·7 (180·0–197·4)	80·7 (72·5–88·6)	19·1 (15·4–23·4)	2·8 (1·9–3·9)	0·1 (0·1–0·1)	0·0 (0·0–0·0)	1·99 (1·9–2·07)	0·53 (0·47–0·58)	0·51 (0·47–0·56)
1976	0·0 (0·0–0·0)	3·7 (3·0–4·5)	96·4 (86·0–108·1)	185·8 (177·2–195·1)	78·2 (70·8–85·9)	18·6 (13·9–23·9)	2·6 (1·7–3·9)	0·1 (0·1–0·1)	0·0 (0·0–0·0)	1·93 (1·84–2·02)	0·5 (0·45–0·56)	0·5 (0·45–0·55)
1977	0·0 (0·0–0·0)	3·6 (3·0–4·3)	91·6 (82·5–102·7)	186·2 (177·1–194·3)	81·0 (72·9–89·6)	18·0 (14·3–22·2)	2·6 (1·7–3·8)	0·1 (0·1–0·1)	0·0 (0·0–0·0)	1·92 (1·84–2·0)	0·48 (0·43–0·53)	0·51 (0·45–0·57)
1978	0·0 (0·0–0·0)	3·6 (2·9–4·4)	88·1 (76·5–102·1)	185·9 (178·0–194·4)	84·8 (76·7–93·2)	17·3 (14·7–20·3)	2·5 (1·7–3·7)	0·1 (0·1–0·1)	0·0 (0·0–0·0)	1·91 (1·82–2·01)	0·46 (0·4–0·53)	0·52 (0·48–0·57)
1979	0·0 (0·0–0·0)	3·6 (2·7–4·7)	84·2 (74·9–93·1)	183·0 (174·9–191·1)	84·8 (77·3–93·4)	16·8 (13·0–21·1)	2·5 (1·7–3·6)	0·1 (0·1–0·1)	0·0 (0·0–0·0)	1·88 (1·8–1·95)	0·44 (0·39–0·49)	0·52 (0·48–0·57)
1980	0·0 (0·0–0·0)	3·7 (2·9–4·5)	80·4 (70·9–89·5)	179·7 (171·2–188·5)	82·8 (75·5–90·9)	16·5 (12·7–20·3)	2·5 (1·7–3·5)	0·1 (0·1–0·1)	0·0 (0·0–0·0)	1·83 (1·75–1·91)	0·42 (0·37–0·47)	0·51 (0·47–0·56)
1981	0·0 (0·0–0·0)	3·8 (3·1–4·6)	78·1 (70·0–87·9)	180·2 (171·4–188·8)	83·1 (75·5–91·0)	17·2 (13·5–20·9)	2·4 (1·6–3·6)	0·1 (0·0–0·1)	0·0 (0·0–0·0)	1·82 (1·76–1·89)	0·41 (0·37–0·46)	0·51 (0·47–0·56)
1982	0·0 (0·0–0·0)	3·9 (3·2–4·7)	76·9 (67·9–87·2)	181·9 (173·6–190·2)	86·2 (78·6–94·4)	19·1 (14·8–24·2)	2·4 (1·7–3·4)	0·1 (0·0–0·1)	0·0 (0·0–0·0)	1·85 (1·78–1·93)	0·4 (0·36–0·46)	0·54 (0·49–0·59)
1983	0·0 (0·0–0·0)	4·0 (3·3–4·7)	75·1 (66·3–85·1)	182·6 (173·5–191·4)	90·0 (82·3–98·6)	21·0 (17·2–25·3)	2·4 (1·8–3·2)	0·1 (0·0–0·1)	0·0 (0·0–0·0)	1·88 (1·81–1·95)	0·4 (0·35–0·45)	0·57 (0·52–0·62)
1984	0·0 (0·0–0·0)	3·9 (3·2–4·6)	70·0 (62·0–78·5)	180·5 (172·4–189·7)	92·8 (83·4–102·3)	21·6 (17·7–26·4)	2·5 (1·8–3·3)	0·1 (0·1–0·1)	0·0 (0·0–0·0)	1·86 (1·78–1·94)	0·37 (0·33–0·41)	0·58 (0·53–0·64)
1985	0·0 (0·0–0·0)	3·7 (3·1–4·4)	66·2 (57·4–75·6)	175·5 (167·4–182·9)	93·7 (85·6–102·1)	21·3 (17·5–26·3)	2·5 (1·8–3·3)	0·1 (0·0–0·1)	0·0 (0·0–0·0)	1·81 (1·75–1·88)	0·35 (0·31–0·4)	0·59 (0·54–0·63)
1986	0·0 (0·0–0·0)	3·6 (3·0–4·2)	63·7 (55·5–72·0)	171·2 (162·7–180·1)	95·8 (87·4–103·8)	21·3 (17·6–25·5)	2·6 (1·8–3·6)	0·1 (0·0–0·1)	0·0 (0·0–0·0)	1·79 (1·73–1·86)	0·34 (0·3–0·38)	0·6 (0·55–0·64)
1987	0·0 (0·0–0·0)	3·5 (2·9–4·1)	59·6 (53·2–67·0)	167·5 (159·5–175·4)	98·6 (90·4–107·8)	21·9 (18·0–26·4)	2·8 (2·0–3·8)	0·1 (0·0–0·1)	0·0 (0·0–0·0)	1·77 (1·68–1·86)	0·32 (0·28–0·35)	0·62 (0·56–0·67)
1988	0·0 (0·0–0·0)	3·4 (2·9–4·0)	56·4 (48·6–65·1)	159·2 (150·2–169·3)	99·9 (91·3–108·9)	22·8 (18·9–27·3)	3·0 (2·1–4·1)	0·1 (0·1–0·1)	0·0 (0·0–0·0)	1·72 (1·65–1·8)	0·3 (0·26–0·34)	0·63 (0·58–0·68)
1989	0·0 (0·0–0·0)	3·4 (2·8–4·1)	53·3 (46·0–61·5)	150·3 (142·7–158·5)	100·1 (91·1–109·3)	23·6 (19·5–28·5)	3·2 (2·3–4·4)	0·1 (0·1–0·1)	0·0 (0·0–0·0)	1·67 (1·59–1·75)	0·28 (0·25–0·32)	0·63 (0·59–0·68)
1990	0·0 (0·0–0·0)	3·5 (3·0–4·1)	52·6 (45·8–59·1)	148·3 (139·7–156·3)	102·3 (93·2–111·2)	24·4 (20·0–29·4)	3·1 (2·2–4·1)	0·1 (0·1–0·1)	0·0 (0·0–0·0)	1·67 (1·6–1·76)	0·28 (0·24–0·31)	0·65 (0·6–0·7)
1991	0·0 (0·0–0·0)	3·7 (3·1–4·4)	53·3 (46·3–61·0)	145·7 (138·2–154·1)	103·4 (93·8–113·2)	25·5 (21·1–30·7)	3·0 (2·2–4·1)	0·1 (0·1–0·1)	0·0 (0·0–0·0)	1·67 (1·6–1·75)	0·28 (0·25–0·32)	0·66 (0·61–0·71)
1992	0·0 (0·0–0·0)	3·8 (3·1–4·5)	52·3 (46·2–59·4)	138·9 (131·1–146·7)	102·4 (94·3–111·4)	26·1 (21·4–31·3)	3·1 (2·3–4·2)	0·1 (0·1–0·1)	0·0 (0·0–0·0)	1·63 (1·58–1·69)	0·28 (0·25–0·32)	0·66 (0·61–0·71)
1993	0·0 (0·0–0·0)	3·8 (3·1–4·7)	52·6 (45·2–61·0)	139·5 (129·9–148·8)	106·6 (98·1–116·0)	28·0 (23·0–33·8)	3·2 (2·4–4·4)	0·1 (0·1–0·1)	0·0 (0·0–0·0)	1·67 (1·59–1·75)	0·28 (0·25–0·32)	0·69 (0·64–0·74)
1994	0·0 (0·0–0·0)	3·9 (3·2–4·8)	52·4 (46·4–60·4)	137·6 (129·2–145·2)	108·1 (98·8–118·0)	29·4 (24·0–35·4)	3·4 (2·5–4·4)	0·1 (0·1–0·1)	0·0 (0·0–0·0)	1·67 (1·59–1·76)	0·28 (0·25–0·32)	0·7 (0·65–0·76)
1995	0·0 (0·0–0·0)	3·9 (3·2–4·8)	51·6 (44·5–59·5)	130·4 (122·4–139·2)	106·8 (97·6–116·0)	30·3 (24·5–36·7)	3·5 (2·6–4·6)	0·1 (0·1–0·1)	0·0 (0·0–0·0)	1·63 (1·54–1·72)	0·28 (0·24–0·32)	0·7 (0·65–0·76)
1996	0·0 (0·0–0·0)	4·1 (3·3–5·0)	51·5 (44·5–59·0)	126·7 (118·1–135·5)	106·3 (97·7–115·8)	31·6 (25·7–38·3)	3·6 (2·7–4·7)	0·1 (0·1–0·1)	0·0 (0·0–0·0)	1·62 (1·53–1·72)	0·28 (0·24–0·31)	0·71 (0·66–0·77)
1997	0·0 (0·0–0·0)	4·3 (3·4–5·2)	51·7 (44·7–58·9)	123·4 (115·7–130·8)	104·2 (95·7–113·2)	32·1 (26·4–38·8)	3·8 (2·8–5·1)	0·1 (0·1–0·1)	0·0 (0·0–0·0)	1·6 (1·52–1·68)	0·28 (0·24–0·32)	0·7 (0·65–0·76)
1998	0·0 (0·0–0·0)	4·5 (3·5–5·6)	51·7 (44·8–59·3)	119·4 (111·4–127·1)	102·9 (93·9–112·4)	33·4 (27·1–40·1)	4·0 (3·0–5·2)	0·1 (0·1–0·2)	0·0 (0·0–0·0)	1·58 (1·5–1·66)	0·28 (0·25–0·32)	0·7 (0·65–0·76)
1999	0·0 (0·0–0·0)	4·8 (3·8–5·9)	51·5 (45·0–58·7)	116·3 (108·9–123·9)	101·9 (93·3–111·2)	34·2 (28·3–40·6)	4·2 (3·0–5·7)	0·1 (0·1–0·1)	0·0 (0·0–0·0)	1·56 (1·48–1·65)	0·28 (0·25–0·32)	0·7 (0·64–0·76)
2000	0·0 (0·0–0·0)	5·2 (4·2–6·4)	51·9 (43·6–60·4)	114·3 (107·3–121·8)	99·7 (91·2–109·2)	35·8 (30·0–42·2)	4·4 (3·1–5·9)	0·1 (0·1–0·1)	0·0 (0·0–0·0)	1·56 (1·47–1·64)	0·29 (0·24–0·33)	0·7 (0·64–0·77)
2001	0·0 (0·0–0·0)	5·4 (4·4–6·6)	52·2 (46·1–59·1)	111·5 (104·4–119·3)	96·4 (87·1–105·6)	35·7 (30·0–42·6)	4·5 (3·2–6·3)	0·1 (0·1–0·2)	0·0 (0·0–0·0)	1·53 (1·45–1·61)	0·29 (0·26–0·32)	0·68 (0·62–0·75)
2002	0·0 (0·0–0·0)	5·4 (4·4–6·6)	51·2 (44·7–58·7)	109·0 (102·2–116·0)	95·7 (87·4–105·2)	36·0 (30·2–42·3)	4·7 (3·3–6·6)	0·1 (0·1–0·2)	0·0 (0·0–0·0)	1·51 (1·43–1·59)	0·28 (0·25–0·32)	0·68 (0·62–0·75)
2003	0·0 (0·0–0·0)	5·3 (4·3–6·3)	49·8 (42·2–58·3)	106·7 (98·1–115·8)	95·0 (87·0–103·8)	37·3 (31·2–43·9)	5·0 (3·4–6·9)	0·1 (0·1–0·2)	0·0 (0·0–0·0)	1·5 (1·41–1·59)	0·28 (0·24–0·32)	0·69 (0·62–0·75)
2004	0·0 (0·0–0·0)	5·1 (4·2–6·0)	48·4 (42·2–55·8)	102·7 (96·5–109·0)	93·4 (85·2–102·2)	37·9 (31·6–45·3)	5·1 (3·7–7·0)	0·1 (0·1–0·2)	0·0 (0·0–0·0)	1·46 (1·39–1·54)	0·27 (0·24–0·31)	0·68 (0·63–0·74)
2005	0·0 (0·0–0·0)	5·0 (4·2–5·9)	48·5 (42·7–55·1)	101·8 (94·8–109·3)	93·7 (85·2–102·8)	38·6 (32·4–45·6)	5·3 (3·7–7·4)	0·1 (0·1–0·2)	0·0 (0·0–0·0)	1·47 (1·4–1·53)	0·27 (0·24–0·3)	0·69 (0·64–0·75)
2006	0·0 (0·0–0·0)	5·0 (4·1–5·9)	49·7 (42·6–57·4)	103·2 (96·7–110·5)	96·4 (87·9–105·0)	40·3 (33·9–47·8)	5·8 (4·2–7·9)	0·1 (0·1–0·2)	0·0 (0·0–0·0)	1·5 (1·44–1·57)	0·27 (0·24–0·31)	0·71 (0·66–0·77)
2007	0·0 (0·0–0·0)	5·0 (4·1–6·0)	50·1 (44·2–56·8)	103·3 (96·6–110·9)	96·7 (87·4–106·1)	42·2 (35·5–49·9)	6·1 (4·5–8·3)	0·1 (0·1–0·2)	0·0 (0·0–0·0)	1·52 (1·44–1·6)	0·28 (0·25–0·31)	0·73 (0·67–0·79)
2008	0·0 (0·0–0·0)	4·9 (4·1–5·9)	50·1 (43·6–57·2)	103·7 (96·8–110·9)	97·4 (88·6–106·0)	43·6 (36·9–51·0)	6·5 (4·9–8·8)	0·1 (0·1–0·2)	0·0 (0·0–0·0)	1·53 (1·46–1·6)	0·28 (0·24–0·31)	0·74 (0·69–0·8)
2009	0·0 (0·0–0·0)	4·7 (3·9–5·6)	49·2 (42·3–57·4)	104·4 (95·8–113·1)	98·7 (89·7–108·0)	45·2 (38·4–53·1)	7·0 (5·2–9·3)	0·1 (0·1–0·2)	0·0 (0·0–0·0)	1·55 (1·45–1·65)	0·27 (0·23–0·31)	0·76 (0·7–0·82)
2010	0·0 (0·0–0·0)	4·5 (3·9–5·2)	48·2 (42·0–55·0)	106·1 (99·9–112·7)	100·7 (92·1–110·4)	46·9 (40·0–54·4)	7·3 (5·4–9·6)	0·1 (0·1–0·2)	0·0 (0·0–0·0)	1·57 (1·5–1·65)	0·26 (0·23–0·3)	0·77 (0·71–0·83)
2011	0·0 (0·0–0·0)	4·4 (3·7–5·3)	46·2 (40·5–52·6)	106·4 (99·3–114·3)	101·7 (92·6–110·9)	48·4 (41·1–56·4)	7·5 (5·6–9·8)	0·2 (0·1–0·2)	0·0 (0·0–0·0)	1·57 (1·5–1·64)	0·25 (0·23–0·28)	0·79 (0·73–0·85)
2012	0·0 (0·0–0·0)	4·4 (3·5–5·2)	44·2 (38·8–50·1)	106·5 (99·5–114·0)	104·0 (94·6–113·9)	50·8 (43·8–59·2)	8·1 (6·0–10·3)	0·2 (0·1–0·3)	0·0 (0·0–0·0)	1·59 (1·51–1·67)	0·24 (0·21–0·27)	0·82 (0·76–0·88)
2013	0·0 (0·0–0·0)	4·3 (3·6–5·1)	42·8 (35·5–50·4)	105·2 (98·3–112·7)	105·0 (96·4–115·0)	52·0 (44·7–59·7)	8·5 (6·3–11·1)	0·2 (0·1–0·3)	0·0 (0·0–0·0)	1·59 (1·51–1·68)	0·24 (0·2–0·27)	0·83 (0·77–0·89)
2014	0·0 (0·0–0·0)	4·2 (3·6–5·1)	41·2 (35·7–47·5)	104·5 (97·4–112·1)	106·9 (98·1–117·2)	53·6 (45·8–61·8)	8·9 (6·6–11·7)	0·2 (0·1–0·3)	0·0 (0·0–0·0)	1·6 (1·51–1·69)	0·23 (0·2–0·26)	0·85 (0·78–0·93)
2015	0·0 (0·0–0·0)	4·2 (3·5–5·1)	41·8 (34·4–49·9)	106·2 (97·6–115·8)	109·9 (99·4–121·8)	51·5 (42·3–62·1)	8·3 (6·0–11·1)	0·2 (0·1–0·3)	0·0 (0·0–0·0)	1·61 (1·44–1·8)	0·23 (0·19–0·27)	0·85 (0·75–0·96)
2016	0·0 (0·0–0·0)	4·3 (3·5–5·3)	43·2 (32·8–56·5)	107·3 (96·2–120·5)	109·6 (94·9–127·4)	47·1 (36·2–60·7)	7·2 (4·9–10·1)	0·2 (0·1–0·2)	0·0 (0·0–0·0)	1·59 (1·36–1·88)	0·24 (0·18–0·31)	0·82 (0·69–0·98)
2017	0·0 (0·0–0·0)	4·2 (3·5–5·1)	42·9 (31·9–56·3)	106·9 (94·8–120·2)	107·8 (91·8–125·1)	45·5 (33·7–60·7)	7·0 (4·5–10·5)	0·2 (0·1–0·2)	0·0 (0·0–0·0)	1·57 (1·32–1·87)	0·24 (0·18–0·31)	0·8 (0·66–0·98)

Supplemental Results Table 5: Age-specific fertility rates per 1000, cumulative total fertility up to maternal age 25 (TFU25), cumulative total fertility from maternal age 30 to age 55 (TFO30), and cumulative total fertility, Nara, 1950-2017											
Year	10-14	15-19	20-24	25-29	30-34	35-39	40-44	45-49	50-54	TFR	TFU25	TFO30
1950	0·0 (0·0–0·0)	9·9 (8·8–11·1)	130·5 (119·2–142·5)	227·7 (219·2–236·2)	139·2 (130·9–147·3)	50·7 (44·9–56·4)	16·1 (13·9–18·5)	0·4 (0·3–0·6)	0·0 (0·0–0·0)	2·87 (2·77–2·97)	0·7 (0·65–0·76)	1·03 (0·98–1·09)
1951	0·0 (0·0–0·0)	8·0 (7·1–9·0)	116·8 (105·7–128·1)	211·9 (202·9–220·7)	125·6 (116·9–134·5)	47·5 (41·3–53·9)	12·5 (10·4–14·7)	0·3 (0·2–0·5)	0·0 (0·0–0·0)	2·61 (2·5–2·72)	0·62 (0·57–0·68)	0·93 (0·85–1·01)
1952	0·0 (0·0–0·0)	6·7 (5·9–7·6)	108·5 (95·9–121·4)	201·5 (191·9–211·5)	113·5 (103·8–123·1)	47·0 (40·6–54·2)	9·8 (8·0–11·9)	0·2 (0·2–0·4)	0·0 (0·0–0·0)	2·44 (2·35–2·54)	0·58 (0·51–0·64)	0·85 (0·79–0·91)
1953	0·0 (0·0–0·0)	5·9 (5·1–6·8)	102·3 (90·8–115·4)	190·0 (179·8–201·8)	101·3 (91·9–110·7)	43·3 (36·4–50·2)	7·8 (6·3–9·5)	0·2 (0·1–0·3)	0·0 (0·0–0·0)	2·25 (2·14–2·37)	0·54 (0·48–0·61)	0·76 (0·7–0·82)
1954	0·0 (0·0–0·0)	5·4 (4·6–6·2)	98·9 (86·1–112·8)	184·8 (172·8–197·4)	93·2 (84·7–102·1)	39·4 (32·8–46·8)	6·4 (5·0–8·1)	0·2 (0·1–0·3)	0·0 (0·0–0·0)	2·14 (2·03–2·25)	0·52 (0·46–0·59)	0·7 (0·64–0·76)
1955	0·0 (0·0–0·0)	4·8 (4·2–5·7)	96·3 (83·6–110·7)	183·8 (171·5–198·2)	87·8 (79·0–96·4)	35·5 (29·9–41·7)	5·8 (4·4–7·4)	0·1 (0·1–0·2)	0·0 (0·0–0·0)	2·07 (1·96–2·19)	0·51 (0·44–0·58)	0·65 (0·59–0·7)
1956	0·0 (0·0–0·0)	4·3 (3·7–5·1)	90·5 (77·7–103·9)	177·6 (164·9–191·5)	79·4 (71·6–87·9)	30·4 (25·2–36·0)	5·0 (3·8–6·4)	0·1 (0·1–0·2)	0·0 (0·0–0·0)	1·94 (1·83–2·05)	0·47 (0·41–0·54)	0·58 (0·52–0·63)
1957	0·0 (0·0–0·0)	4·1 (3·5–4·9)	91·3 (78·5–106·0)	180·9 (169·0–195·2)	73·3 (66·1–81·0)	26·1 (21·7–30·3)	3·8 (2·9–4·9)	0·1 (0·1–0·2)	0·0 (0·0–0·0)	1·9 (1·78–2·02)	0·48 (0·41–0·55)	0·52 (0·47–0·57)
1958	0·0 (0·0–0·0)	4·1 (3·5–4·9)	95·6 (83·5–109·5)	186·5 (174·0–199·0)	70·1 (62·8–78·0)	23·1 (19·1–27·4)	3·2 (2·4–4·1)	0·1 (0·1–0·2)	0·0 (0·0–0·0)	1·91 (1·81–2·02)	0·5 (0·44–0·57)	0·48 (0·44–0·53)
1959	0·0 (0·0–0·0)	4·3 (3·7–5·1)	95·4 (83·0–109·7)	185·2 (175·3–196·1)	66·9 (59·3–75·9)	19·9 (16·2–24·1)	2·9 (2·2–3·7)	0·1 (0·0–0·2)	0·0 (0·0–0·0)	1·87 (1·77–1·98)	0·5 (0·44–0·57)	0·45 (0·4–0·5)
1960	0·0 (0·0–0·0)	4·2 (3·5–4·9)	91·4 (81·0–103·8)	185·9 (175·7–196·3)	66·6 (60·1–73·2)	17·6 (14·4–21·1)	2·7 (2·0–3·4)	0·1 (0·0–0·2)	0·0 (0·0–0·0)	1·84 (1·77–1·92)	0·48 (0·43–0·54)	0·43 (0·4–0·47)
1961	0·0 (0·0–0·0)	3·9 (3·3–4·6)	88·5 (79·3–97·6)	187·6 (178·7–196·9)	66·4 (58·1–75·6)	15·8 (13·0–19·1)	2·4 (1·8–3·4)	0·1 (0·0–0·1)	0·0 (0·0–0·0)	1·82 (1·74–1·9)	0·46 (0·42–0·51)	0·42 (0·38–0·47)
1962	0·0 (0·0–0·0)	3·7 (3·1–4·4)	88·1 (78·6–98·0)	192·8 (184·9–200·9)	67·8 (62·1–73·6)	14·9 (12·6–17·3)	2·1 (1·5–3·0)	0·1 (0·0–0·1)	0·0 (0·0–0·0)	1·85 (1·77–1·92)	0·46 (0·41–0·51)	0·42 (0·39–0·46)
1963	0·0 (0·0–0·0)	3·6 (3·0–4·3)	88·2 (79·3–98·6)	194·5 (186·6–202·5)	66·6 (60·8–72·8)	14·4 (12·2–17·0)	1·8 (1·3–2·5)	0·1 (0·0–0·1)	0·0 (0·0–0·0)	1·85 (1·77–1·92)	0·46 (0·41–0·51)	0·41 (0·38–0·45)
1964	0·0 (0·0–0·0)	3·6 (3·1–4·2)	96·2 (85·3–108·8)	206·9 (197·4–215·9)	71·0 (64·8–77·9)	14·9 (12·6–17·3)	1·7 (1·2–2·4)	0·1 (0·0–0·1)	0·0 (0·0–0·0)	1·97 (1·89–2·06)	0·5 (0·44–0·56)	0·44 (0·4–0·47)
1965	0·0 (0·0–0·0)	3·5 (3·0–4·1)	88·5 (76·7–101·7)	174·6 (165·4–184·0)	62·1 (56·1–68·6)	13·6 (11·5–16·0)	1·7 (1·2–2·4)	0·1 (0·0–0·1)	0·0 (0·0–0·0)	1·72 (1·64–1·81)	0·46 (0·4–0·53)	0·39 (0·36–0·42)
1966	0·0 (0·0–0·0)	3·7 (3·1–4·3)	86·4 (72·3–101·5)	179·5 (170·2–188·5)	62·5 (56·2–69·6)	13·9 (11·5–16·7)	1·6 (1·1–2·2)	0·1 (0·0–0·1)	0·0 (0·0–0·0)	1·74 (1·65–1·83)	0·45 (0·38–0·53)	0·39 (0·35–0·43)
1967	0·0 (0·0–0·0)	3·9 (3·3–4·6)	92·0 (76·0–109·7)	218·5 (209·5–226·9)	77·3 (69·6–85·8)	15·7 (12·7–18·8)	1·7 (1·2–2·2)	0·1 (0·0–0·1)	0·0 (0·0–0·0)	2·05 (1·93–2·16)	0·48 (0·4–0·57)	0·47 (0·43–0·53)
1968	0·0 (0·0–0·0)	4·0 (3·4–4·6)	82·3 (67·7–99·0)	206·0 (197·7–214·2)	75·3 (68·2–82·1)	15·5 (12·6–18·8)	1·9 (1·4–2·5)	0·1 (0·0–0·1)	0·0 (0·0–0·0)	1·93 (1·85–2·0)	0·43 (0·36–0·51)	0·46 (0·42–0·51)
1969	0·0 (0·0–0·0)	4·3 (3·7–5·1)	80·8 (67·2–96·1)	207·8 (199·5–216·2)	73·7 (65·8–82·7)	15·1 (12·3–18·5)	1·7 (1·3–2·2)	0·0 (0·0–0·1)	0·0 (0·0–0·0)	1·92 (1·84–2·01)	0·43 (0·36–0·5)	0·45 (0·4–0·51)
1970	0·0 (0·0–0·0)	4·3 (3·7–5·1)	86·8 (72·0–102·3)	210·8 (201·8–219·6)	70·0 (63·0–77·5)	14·3 (11·5–17·6)	1·4 (1·0–1·8)	0·0 (0·0–0·1)	0·0 (0·0–0·0)	1·94 (1·86–2·02)	0·46 (0·38–0·53)	0·43 (0·39–0·47)
1971	0·0 (0·0–0·0)	4·2 (3·5–5·1)	94·9 (79·5–111·3)	207·4 (198·6–216·5)	67·8 (60·1–76·0)	12·9 (10·5–16·0)	1·3 (0·9–1·7)	0·0 (0·0–0·1)	0·0 (0·0–0·0)	1·94 (1·86–2·03)	0·5 (0·42–0·58)	0·41 (0·37–0·46)
1972	0·0 (0·0–0·0)	4·3 (3·5–5·2)	99·2 (84·6–115·1)	205·3 (196·1–214·5)	67·8 (60·8–75·1)	12·1 (9·6–14·9)	1·2 (0·9–1·7)	0·0 (0·0–0·1)	0·0 (0·0–0·0)	1·95 (1·87–2·03)	0·52 (0·45–0·6)	0·41 (0·36–0·45)
1973	0·0 (0·0–0·0)	4·1 (3·4–5·0)	99·4 (86·6–111·8)	204·8 (194·7–214·6)	64·7 (58·3–71·7)	11·9 (9·4–14·6)	1·2 (0·8–1·6)	0·0 (0·0–0·1)	0·0 (0·0–0·0)	1·93 (1·85–2·01)	0·52 (0·45–0·58)	0·39 (0·35–0·43)
1974	0·0 (0·0–0·0)	3·8 (3·1–4·6)	94·8 (84·1–108·0)	195·9 (187·3–204·6)	59·5 (53·6–65·8)	11·1 (8·6–14·1)	1·1 (0·7–1·7)	0·0 (0·0–0·0)	0·0 (0·0–0·0)	1·83 (1·76–1·91)	0·49 (0·44–0·56)	0·36 (0·33–0·39)
1975	0·0 (0·0–0·0)	3·5 (2·9–4·3)	87·8 (78·5–97·8)	187·9 (179·2–196·9)	56·6 (50·8–62·2)	10·6 (8·3–13·3)	1·2 (0·9–1·6)	0·0 (0·0–0·0)	0·0 (0·0–0·0)	1·74 (1·67–1·81)	0·46 (0·41–0·51)	0·34 (0·31–0·37)
1976	0·0 (0·0–0·0)	3·3 (2·7–4·0)	82·1 (72·1–92·0)	184·6 (175·9–193·5)	57·3 (51·8–63·2)	10·6 (8·4–13·0)	1·2 (0·8–1·7)	0·0 (0·0–0·0)	0·0 (0·0–0·0)	1·7 (1·62–1·77)	0·43 (0·38–0·48)	0·35 (0·31–0·38)
1977	0·0 (0·0–0·0)	3·2 (2·7–3·7)	76·6 (68·4–87·1)	184·8 (176·6–193·2)	60·7 (54·1–68·0)	10·6 (8·4–13·2)	1·3 (0·8–1·8)	0·0 (0·0–0·1)	0·0 (0·0–0·0)	1·69 (1·61–1·76)	0·4 (0·36–0·45)	0·36 (0·32–0·41)
1978	0·0 (0·0–0·0)	3·2 (2·5–3·9)	72·2 (64·6–81·1)	185·7 (177·7–193·6)	64·7 (59·2–70·9)	10·6 (8·0–13·8)	1·3 (0·8–1·9)	0·0 (0·0–0·1)	0·0 (0·0–0·0)	1·69 (1·62–1·75)	0·38 (0·34–0·42)	0·38 (0·35–0·42)
1979	0·0 (0·0–0·0)	3·2 (2·4–4·2)	68·2 (60·0–76·9)	185·6 (178·2–193·2)	66·1 (57·6–75·5)	10·5 (8·6–12·7)	1·3 (0·8–2·0)	0·0 (0·0–0·0)	0·0 (0·0–0·0)	1·67 (1·6–1·75)	0·36 (0·31–0·4)	0·39 (0·35–0·44)
1980	0·0 (0·0–0·0)	3·3 (2·6–4·1)	64·6 (56·9–73·1)	185·7 (176·6–194·9)	65·7 (60·1–71·9)	10·5 (8·4–13·0)	1·3 (0·9–1·9)	0·0 (0·0–0·1)	0·0 (0·0–0·0)	1·66 (1·59–1·72)	0·34 (0·3–0·38)	0·39 (0·36–0·42)
1981	0·0 (0·0–0·0)	3·4 (2·8–4·2)	62·4 (55·4–70·8)	188·8 (179·9–197·7)	67·3 (61·2–74·1)	11·2 (8·9–13·7)	1·3 (0·9–2·0)	0·0 (0·0–0·0)	0·0 (0·0–0·0)	1·67 (1·61–1·73)	0·33 (0·29–0·37)	0·4 (0·37–0·43)
1982	0·0 (0·0–0·0)	3·6 (2·9–4·4)	61·0 (53·3–69·7)	191·5 (184·1–199·9)	70·9 (64·5–77·7)	12·7 (10·0–15·9)	1·4 (0·9–1·9)	0·0 (0·0–0·0)	0·0 (0·0–0·0)	1·71 (1·64–1·77)	0·32 (0·28–0·37)	0·42 (0·39–0·47)
1983	0·0 (0·0–0·0)	3·7 (3·0–4·6)	59·0 (51·7–67·1)	191·5 (181·9–200·7)	74·8 (68·0–82·5)	14·2 (11·4–17·3)	1·4 (1·0–1·9)	0·0 (0·0–0·0)	0·0 (0·0–0·0)	1·72 (1·66–1·8)	0·31 (0·28–0·35)	0·45 (0·41–0·5)
1984	0·0 (0·0–0·0)	3·6 (2·8–4·3)	54·6 (48·3–61·7)	187·1 (179·4–195·5)	77·7 (70·8–85·0)	14·8 (11·8–18·3)	1·5 (1·0–2·0)	0·0 (0·0–0·0)	0·0 (0·0–0·0)	1·7 (1·63–1·77)	0·29 (0·26–0·33)	0·47 (0·43–0·51)
1985	0·0 (0·0–0·0)	3·4 (2·7–4·1)	51·2 (44·4–58·7)	178·8 (167·9–189·0)	79·0 (71·7–86·4)	14·7 (11·8–18·1)	1·5 (1·1–1·9)	0·0 (0·0–0·0)	0·0 (0·0–0·0)	1·64 (1·58–1·71)	0·27 (0·24–0·31)	0·48 (0·44–0·52)
1986	0·0 (0·0–0·0)	3·2 (2·6–4·0)	48·7 (42·8–55·4)	171·5 (164·1–179·7)	81·3 (74·0–88·0)	14·9 (12·0–18·1)	1·5 (1·1–2·1)	0·0 (0·0–0·0)	0·0 (0·0–0·0)	1·61 (1·54–1·67)	0·26 (0·23–0·29)	0·49 (0·45–0·52)
1987	0·0 (0·0–0·0)	3·1 (2·5–3·8)	45·0 (39·4–51·3)	165·4 (156·5–174·2)	84·1 (76·5–92·5)	15·6 (12·5–19·0)	1·7 (1·2–2·6)	0·0 (0·0–0·1)	0·0 (0·0–0·0)	1·57 (1·51–1·64)	0·24 (0·21–0·27)	0·51 (0·47–0·55)
1988	0·0 (0·0–0·0)	3·0 (2·4–3·7)	41·7 (35·9–48·0)	155·0 (147·4–163·1)	85·3 (78·0–93·2)	16·4 (13·3–20·1)	1·9 (1·3–2·7)	0·0 (0·0–0·1)	0·0 (0·0–0·0)	1·52 (1·45–1·59)	0·22 (0·19–0·26)	0·52 (0·48–0·56)
1989	0·0 (0·0–0·0)	2·9 (2·4–3·6)	38·4 (32·9–44·8)	144·2 (136·4–152·4)	85·4 (76·8–95·0)	17·4 (14·0–21·2)	2·0 (1·4–2·8)	0·0 (0·0–0·1)	0·0 (0·0–0·0)	1·45 (1·39–1·52)	0·21 (0·18–0·24)	0·52 (0·48–0·57)
1990	0·0 (0·0–0·0)	2·9 (2·4–3·5)	36·8 (31·9–42·5)	140·1 (132·9–147·6)	87·2 (79·6–94·7)	18·2 (14·5–22·2)	2·0 (1·4–2·8)	0·0 (0·0–0·1)	0·0 (0·0–0·0)	1·44 (1·37–1·5)	0·2 (0·17–0·23)	0·54 (0·5–0·58)
1991	0·0 (0·0–0·0)	2·9 (2·4–3·6)	36·1 (31·1–41·9)	135·6 (125·8–146·2)	88·6 (80·4–97·3)	19·3 (15·6–23·4)	2·1 (1·5–2·8)	0·0 (0·0–0·1)	0·0 (0·0–0·0)	1·42 (1·36–1·49)	0·2 (0·17–0·22)	0·55 (0·5–0·6)
1992	0·0 (0·0–0·0)	2·9 (2·4–3·5)	34·3 (30·1–39·5)	127·4 (120·5–134·3)	88·5 (81·2–96·8)	20·0 (16·0–24·4)	2·2 (1·5–3·1)	0·0 (0·0–0·1)	0·0 (0·0–0·0)	1·38 (1·32–1·44)	0·19 (0·16–0·21)	0·55 (0·51–0·6)
1993	0·0 (0·0–0·0)	3·0 (2·3–3·6)	33·6 (28·6–38·8)	126·2 (117·6–134·8)	93·2 (85·1–102·0)	21·6 (17·5–25·9)	2·4 (1·6–3·2)	0·0 (0·0–0·1)	0·0 (0·0–0·0)	1·4 (1·33–1·47)	0·18 (0·16–0·21)	0·59 (0·54–0·63)
1994	0·0 (0·0–0·0)	3·0 (2·4–3·6)	32·6 (28·4–37·2)	123·1 (116·4–129·9)	95·6 (87·1–104·9)	22·9 (18·2–28·2)	2·5 (1·7–3·4)	0·1 (0·0–0·1)	0·0 (0·0–0·0)	1·4 (1·33–1·47)	0·18 (0·16–0·2)	0·61 (0·56–0·65)
1995	0·0 (0·0–0·0)	3·0 (2·4–3·7)	31·5 (26·9–36·2)	115·6 (108·1–122·8)	95·7 (87·0–104·3)	23·9 (19·1–29·2)	2·7 (1·9–3·6)	0·1 (0·0–0·1)	0·0 (0·0–0·0)	1·36 (1·29–1·43)	0·17 (0·15–0·2)	0·61 (0·57–0·66)
1996	0·0 (0·0–0·0)	3·1 (2·4–3·8)	30·9 (26·4–35·5)	111·2 (104·2–118·9)	96·5 (87·4–106·5)	25·1 (19·8–31·0)	2·8 (2·0–3·6)	0·1 (0·0–0·1)	0·0 (0·0–0·0)	1·35 (1·28–1·42)	0·17 (0·15–0·19)	0·62 (0·58–0·67)
1997	0·0 (0·0–0·0)	3·3 (2·6–4·0)	30·6 (26·4–35·2)	107·3 (100·5–113·9)	95·7 (88·0–104·1)	25·8 (20·8–31·3)	3·0 (2·2–4·0)	0·1 (0·0–0·1)	0·0 (0·0–0·0)	1·33 (1·27–1·39)	0·17 (0·15–0·19)	0·62 (0·58–0·67)
1998	0·0 (0·0–0·0)	3·5 (2·7–4·3)	30·3 (26·3–35·2)	102·6 (95·9–109·2)	95·4 (87·3–104·2)	27·2 (21·7–33·2)	3·1 (2·4–4·2)	0·1 (0·0–0·1)	0·0 (0·0–0·0)	1·31 (1·25–1·38)	0·17 (0·15–0·19)	0·63 (0·58–0·68)
1999	0·0 (0·0–0·0)	3·7 (3·0–4·6)	30·0 (25·5–34·8)	98·5 (91·9–105·2)	95·3 (87·1–104·4)	28·4 (23·3–34·4)	3·4 (2·4–4·5)	0·1 (0·0–0·1)	0·0 (0·0–0·0)	1·3 (1·23–1·36)	0·17 (0·15–0·19)	0·64 (0·58–0·69)
2000	0·0 (0·0–0·0)	4·0 (3·2–4·9)	30·0 (25·9–34·2)	95·6 (89·4–101·8)	93·9 (85·9–102·4)	30·3 (24·9–36·4)	3·6 (2·6–4·8)	0·1 (0·0–0·1)	0·0 (0·0–0·0)	1·29 (1·22–1·36)	0·17 (0·15–0·19)	0·64 (0·59–0·69)
2001	0·0 (0·0–0·0)	4·2 (3·4–5·0)	30·1 (25·4–35·2)	92·1 (86·1–98·7)	91·3 (83·0–99·8)	30·7 (25·7–36·8)	3·7 (2·7–5·0)	0·1 (0·0–0·2)	0·0 (0·0–0·0)	1·26 (1·2–1·32)	0·17 (0·15–0·2)	0·63 (0·58–0·68)
2002	0·0 (0·0–0·0)	4·2 (3·4–5·0)	29·5 (25·1–34·8)	88·9 (83·0–95·2)	90·9 (83·0–99·5)	31·6 (26·4–38·2)	4·0 (2·8–5·6)	0·1 (0·0–0·2)	0·0 (0·0–0·0)	1·25 (1·18–1·31)	0·17 (0·15–0·19)	0·63 (0·59–0·68)
2003	0·0 (0·0–0·0)	4·1 (3·3–4·9)	28·5 (24·3–33·5)	86·0 (80·6–92·0)	90·4 (82·4–98·7)	33·3 (28·0–39·9)	4·2 (3·0–5·7)	0·1 (0·0–0·2)	0·0 (0·0–0·0)	1·23 (1·17–1·3)	0·16 (0·14–0·19)	0·64 (0·59–0·69)
2004	0·0 (0·0–0·0)	3·9 (3·3–4·7)	27·7 (23·9–32·3)	82·2 (76·1–88·8)	88·8 (81·2–97·2)	34·2 (28·5–41·0)	4·4 (3·2–6·0)	0·1 (0·0–0·2)	0·0 (0·0–0·0)	1·21 (1·15–1·27)	0·16 (0·14–0·18)	0·64 (0·59–0·69)
2005	0·0 (0·0–0·0)	3·9 (3·3–4·7)	27·8 (22·1–34·6)	80·8 (75·6–86·2)	88·9 (80·6–97·8)	35·3 (29·6–42·0)	4·6 (3·3–6·4)	0·1 (0·1–0·2)	0·0 (0·0–0·0)	1·21 (1·14–1·27)	0·16 (0·13–0·19)	0·64 (0·59–0·7)
2006	0·0 (0·0–0·0)	3·9 (3·2–4·6)	28·3 (24·1–33·1)	81·1 (73·2–89·5)	91·3 (83·2–99·9)	37·4 (31·4–44·4)	5·1 (3·6–6·9)	0·1 (0·1–0·2)	0·0 (0·0–0·0)	1·24 (1·16–1·31)	0·16 (0·14–0·19)	0·67 (0·62–0·73)
2007	0·0 (0·0–0·0)	3·9 (3·2–4·6)	28·4 (24·4–32·9)	80·1 (74·9–85·8)	91·3 (83·1–100·1)	39·5 (33·2–46·8)	5·4 (3·9–7·5)	0·1 (0·1–0·2)	0·0 (0·0–0·0)	1·24 (1·18–1·31)	0·16 (0·14–0·18)	0·68 (0·63–0·74)
2008	0·0 (0·0–0·0)	3·8 (3·2–4·5)	28·2 (24·3–32·3)	79·4 (73·9–85·0)	91·7 (83·8–99·7)	41·0 (34·7–47·7)	5·9 (4·3–8·2)	0·1 (0·1–0·3)	0·0 (0·0–0·0)	1·25 (1·19–1·31)	0·16 (0·14–0·18)	0·69 (0·64–0·75)
2009	0·0 (0·0–0·0)	3·7 (3·0–4·4)	27·5 (22·2–33·5)	79·0 (71·4–85·9)	92·6 (84·4–101·4)	42·7 (36·1–49·9)	6·4 (4·5–8·7)	0·1 (0·1–0·3)	0·0 (0·0–0·0)	1·26 (1·18–1·35)	0·16 (0·13–0·19)	0·71 (0·65–0·77)
2010	0·0 (0·0–0·0)	3·6 (3·0–4·2)	26·8 (23·2–30·4)	79·3 (74·3–84·4)	94·1 (84·9–103·8)	44·3 (37·7–51·3)	6·7 (4·9–9·1)	0·2 (0·1–0·3)	0·0 (0·0–0·0)	1·27 (1·21–1·34)	0·15 (0·13–0·17)	0·73 (0·67–0·79)
2011	0·0 (0·0–0·0)	3·5 (2·9–4·2)	25·6 (22·1–30·0)	78·7 (72·9–84·8)	94·7 (87·0–103·7)	45·7 (38·6–52·7)	7·0 (5·2–9·4)	0·2 (0·1–0·3)	0·0 (0·0–0·0)	1·28 (1·21–1·34)	0·15 (0·13–0·17)	0·74 (0·68–0·8)
2012	0·0 (0·0–0·0)	3·4 (2·8–4·1)	24·5 (20·8–28·9)	78·0 (72·7–83·5)	96·7 (88·0–105·9)	48·1 (41·3–56·1)	7·6 (5·5–10·0)	0·2 (0·1–0·4)	0·0 (0·0–0·0)	1·29 (1·22–1·37)	0·14 (0·12–0·16)	0·76 (0·7–0·83)
2013	0·0 (0·0–0·0)	3·4 (2·8–4·0)	23·7 (20·0–27·8)	76·7 (70·5–82·8)	97·5 (88·5–106·5)	49·3 (42·4–56·5)	8·0 (5·8–10·7)	0·2 (0·1–0·3)	0·0 (0·0–0·0)	1·29 (1·22–1·37)	0·14 (0·12–0·16)	0·77 (0·72–0·83)
2014	0·0 (0·0–0·0)	3·3 (2·8–4·0)	23·0 (19·4–27·2)	76·4 (70·4–82·9)	99·0 (91·1–108·3)	50·7 (43·5–58·7)	8·3 (6·1–11·0)	0·2 (0·1–0·4)	0·0 (0·0–0·0)	1·3 (1·23–1·38)	0·13 (0·11–0·15)	0·79 (0·73–0·86)
2015	0·0 (0·0–0·0)	3·4 (2·8–4·1)	23·6 (17·3–31·5)	78·2 (70·5–86·1)	101·7 (91·5–112·9)	48·6 (40·2–58·1)	7·7 (5·5–10·3)	0·2 (0·1–0·3)	0·0 (0·0–0·0)	1·32 (1·16–1·49)	0·14 (0·1–0·18)	0·79 (0·7–0·89)
2016	0·0 (0·0–0·0)	3·4 (2·8–4·2)	24·6 (18·7–32·5)	79·8 (69·6–91·1)	101·3 (87·8–117·3)	44·4 (34·2–57·7)	6·6 (4·5–9·2)	0·2 (0·1–0·3)	0·0 (0·0–0·0)	1·3 (1·11–1·53)	0·14 (0·11–0·18)	0·76 (0·64–0·91)
2017	0·0 (0·0–0·0)	3·4 (2·8–4·1)	24·6 (17·8–33·2)	79·9 (68·9–92·0)	99·8 (85·0–116·0)	42·9 (31·8–56·4)	6·4 (4·1–9·6)	0·2 (0·1–0·3)	0·0 (0·0–0·0)	1·29 (1·07–1·54)	0·14 (0·1–0·19)	0·75 (0·61–0·91)

Supplemental Results Table 5: Age-specific fertility rates per 1000, cumulative total fertility up to maternal age 25 (TFU25), cumulative total fertility from maternal age 30 to age 55 (TFO30), and cumulative total fertility, Niigata, 1950-2017

Year	10-14	15-19	20-24	25-29	30-34	35-39	40-44	45-49	50-54	TFR	TFU25	TFO30
1950	0·0 (0·0–0·0)	7·3 (6·4–8·1)	156·9 (143·0–171·7)	229·3 (220·7–237·9)	185·2 (173·8–196·6)	102·8 (91·3–114·1)	28·2 (24·4–32·4)	0·9 (0·6–1·3)	0·0 (0·0–0·0)	3·55 (3·43–3·68)	0·82 (0·75–0·89)	1·59 (1·5–1·67)
1951	0·0 (0·0–0·0)	5·9 (5·2–6·6)	140·8 (127·4–154·9)	213·4 (205·3–221·8)	167·8 (155·3–179·4)	83·1 (72·7–93·8)	21·8 (18·3–25·5)	0·7 (0·4–1·0)	0·0 (0·0–0·0)	3·17 (3·02–3·32)	0·73 (0·67–0·81)	1·37 (1·25–1·48)
1952	0·0 (0·0–0·0)	4·9 (4·3–5·6)	131·1 (117·7–145·5)	203·1 (194·3–212·1)	152·0 (138·3–165·7)	66·6 (57·0–77·0)	17·2 (14·2–20·7)	0·5 (0·3–0·7)	0·0 (0·0–0·0)	2·88 (2·75–3·0)	0·68 (0·61–0·75)	1·18 (1·1–1·27)
1953	0·0 (0·0–0·0)	4·3 (3·7–5·1)	123·9 (110·8–138·7)	191·5 (182·5–200·9)	136·0 (121·5–151·0)	53·0 (44·5–62·6)	13·7 (11·1–16·6)	0·4 (0·3–0·6)	0·0 (0·0–0·0)	2·61 (2·49–2·75)	0·64 (0·57–0·71)	1·02 (0·94–1·1)
1954	0·0 (0·0–0·0)	4·0 (3·3–4·7)	120·2 (105·0–136·1)	186·4 (178·4–195·1)	125·4 (109·9–141·1)	45·2 (38·2–53·9)	11·3 (9·0–14·0)	0·3 (0·2–0·5)	0·0 (0·0–0·0)	2·46 (2·33–2·6)	0·62 (0·54–0·7)	0·91 (0·83–0·99)
1955	0·0 (0·0–0·0)	3·6 (2·9–4·4)	117·3 (102·4–134·3)	185·3 (176·7–194·2)	115·5 (99·8–130·8)	40·1 (33·7–47·4)	9·4 (7·3–11·8)	0·3 (0·2–0·4)	0·0 (0·0–0·0)	2·36 (2·24–2·49)	0·6 (0·53–0·69)	0·83 (0·75–0·91)
1956	0·0 (0·0–0·0)	3·2 (2·6–4·0)	110·5 (95·2–126·6)	179·2 (171·4–187·5)	102·4 (88·7–117·3)	34·4 (28·6–40·7)	7·5 (5·8–9·6)	0·2 (0·1–0·3)	0·0 (0·0–0·0)	2·19 (2·07–2·32)	0·57 (0·49–0·65)	0·72 (0·66–0·79)
1957	0·0 (0·0–0·0)	3·1 (2·4–3·8)	111·7 (96·2–130·0)	182·6 (174·7–190·9)	96·9 (83·9–111·5)	29·4 (24·3–34·1)	6·4 (5·0–8·0)	0·2 (0·1–0·3)	0·0 (0·0–0·0)	2·15 (2·02–2·29)	0·57 (0·49–0·66)	0·66 (0·6–0·73)
1958	0·0 (0·0–0·0)	3·1 (2·4–3·9)	117·3 (102·3–133·9)	188·5 (179·7–197·4)	94·6 (81·7–108·5)	25·9 (21·1–30·8)	5·8 (4·5–7·3)	0·2 (0·1–0·4)	0·0 (0·0–0·0)	2·18 (2·05–2·3)	0·6 (0·53–0·68)	0·63 (0·57–0·69)
1959	0·0 (0·0–0·0)	3·2 (2·5–4·0)	117·3 (102·4–133·9)	187·5 (178·6–196·3)	88·5 (77·6–100·9)	22·3 (18·4–26·8)	4·8 (3·6–6·2)	0·2 (0·1–0·3)	0·0 (0·0–0·0)	2·12 (2·01–2·24)	0·6 (0·53–0·69)	0·58 (0·53–0·64)
1960	0·0 (0·0–0·0)	3·1 (2·4–3·9)	112·8 (99·6–126·6)	188·4 (179·4–197·3)	84·8 (75·7–94·6)	19·7 (15·1–24·9)	3·9 (2·9–4·9)	0·1 (0·1–0·2)	0·0 (0·0–0·0)	2·06 (1·96–2·17)	0·58 (0·51–0·65)	0·54 (0·49–0·6)
1961	0·0 (0·0–0·0)	2·9 (2·3–3·7)	109·5 (98·7–121·5)	190·4 (182·0–198·7)	82·9 (72·8–93·5)	17·7 (14·7–21·3)	3·3 (2·5–4·5)	0·1 (0·1–0·2)	0·0 (0·0–0·0)	2·03 (1·94–2·13)	0·56 (0·51–0·62)	0·52 (0·47–0·57)
1962	0·0 (0·0–0·0)	2·8 (2·2–3·5)	109·3 (97·1–121·6)	195·8 (187·6–203·9)	84·5 (77·0–92·2)	16·7 (13·8–19·8)	2·9 (2·2–3·9)	0·1 (0·1–0·2)	0·0 (0·0–0·0)	2·06 (1·97–2·14)	0·56 (0·5–0·62)	0·52 (0·48–0·56)
1963	0·0 (0·0–0·0)	2·7 (2·2–3·4)	109·8 (97·7–123·4)	197·6 (189·7–205·9)	86·1 (78·8–93·9)	16·2 (13·6–19·2)	2·9 (2·1–3·9)	0·1 (0·1–0·2)	0·0 (0·0–0·0)	2·08 (1·99–2·16)	0·56 (0·5–0·63)	0·53 (0·48–0·57)
1964	0·0 (0·0–0·0)	2·7 (2·2–3·3)	119·9 (106·0–135·1)	210·3 (201·4–219·3)	91·7 (83·2–101·3)	16·7 (14·1–19·4)	2·7 (2·0–3·6)	0·1 (0·1–0·2)	0·0 (0·0–0·0)	2·22 (2·12–2·33)	0·61 (0·54–0·69)	0·56 (0·51–0·61)
1965	0·0 (0·0–0·0)	2·6 (2·1–3·2)	110·6 (96·4–126·0)	177·5 (168·7–186·5)	76·2 (67·3–85·7)	15·1 (12·9–17·6)	2·3 (1·7–3·1)	0·1 (0·1–0·1)	0·0 (0·0–0·0)	1·92 (1·81–2·04)	0·57 (0·49–0·64)	0·47 (0·42–0·51)
1966	0·0 (0·0–0·0)	2·8 (2·4–3·3)	108·2 (90·8–126·4)	182·4 (173·7–190·8)	78·0 (68·0–88·3)	15·3 (12·7–18·4)	2·3 (1·7–3·1)	0·1 (0·1–0·1)	0·0 (0·0–0·0)	1·95 (1·82–2·07)	0·56 (0·47–0·65)	0·48 (0·43–0·53)
1967	0·0 (0·0–0·0)	2·9 (2·5–3·4)	115·5 (96·2–135·7)	222·3 (213·8–230·8)	93·9 (80·7–108·2)	17·4 (13·3–22·5)	2·2 (1·6–2·9)	0·1 (0·1–0·1)	0·0 (0·0–0·0)	2·27 (2·1–2·43)	0·59 (0·5–0·69)	0·57 (0·5–0·64)
1968	0·0 (0·0–0·0)	3·0 (2·6–3·5)	103·6 (86·2–123·1)	209·8 (200·9–218·1)	85·5 (73·5–98·4)	17·1 (14·2–20·6)	2·0 (1·5–2·6)	0·1 (0·0–0·1)	0·0 (0·0–0·0)	2·11 (1·97–2·25)	0·53 (0·45–0·63)	0·52 (0·47–0·58)
1969	0·0 (0·0–0·0)	3·2 (2·8–3·8)	102·1 (85·1–120·5)	211·9 (203·6–219·8)	85·7 (73·0–99·8)	16·7 (13·7–20·5)	2·0 (1·5–2·6)	0·1 (0·0–0·1)	0·0 (0·0–0·0)	2·11 (1·99–2·25)	0·53 (0·44–0·62)	0·52 (0·47–0·59)
1970	0·0 (0·0–0·0)	3·3 (2·7–3·9)	110·0 (93·6–127·9)	215·3 (206·5–224·1)	88·1 (76·3–101·4)	16·1 (13·0–19·4)	2·1 (1·5–2·8)	0·1 (0·0–0·1)	0·0 (0·0–0·0)	2·17 (2·04–2·31)	0·57 (0·48–0·65)	0·53 (0·48–0·59)
1971	0·0 (0·0–0·0)	3·2 (2·6–3·9)	120·7 (103·3–140·7)	212·4 (204·0–221·5)	89·3 (78·2–101·5)	15·9 (13·1–19·3)	2·3 (1·6–3·1)	0·1 (0·1–0·1)	0·0 (0·0–0·0)	2·22 (2·1–2·35)	0·62 (0·53–0·72)	0·54 (0·48–0·6)
1972	0·0 (0·0–0·0)	3·2 (2·6–4·0)	126·6 (110·1–144·6)	210·9 (202·7–219·7)	89·5 (79·2–100·0)	16·2 (13·0–19·5)	2·3 (1·6–3·1)	0·1 (0·0–0·1)	0·0 (0·0–0·0)	2·24 (2·13–2·36)	0·65 (0·57–0·74)	0·54 (0·49–0·59)
1973	0·0 (0·0–0·0)	3·1 (2·5–3·9)	127·0 (112·5–141·7)	211·1 (201·9–220·2)	85·3 (77·2–94·3)	14·8 (11·9–18·2)	2·1 (1·4–2·9)	0·1 (0·0–0·1)	0·0 (0·0–0·0)	2·22 (2·12–2·32)	0·65 (0·58–0·72)	0·51 (0·47–0·55)
1974	0·0 (0·0–0·0)	2·9 (2·3–3·6)	121·6 (108·6–135·8)	203·3 (194·0–212·4)	76·2 (68·6–83·6)	12·4 (10·1–15·1)	1·7 (1·2–2·5)	0·1 (0·0–0·1)	0·0 (0·0–0·0)	2·09 (2·01–2·19)	0·62 (0·56–0·69)	0·45 (0·41–0·49)
1975	0·0 (0·0–0·0)	2·7 (2·1–3·5)	113·5 (101·1–125·8)	196·4 (186·9–205·6)	69·0 (62·6–75·5)	11·4 (9·1–14·1)	1·5 (0·9–2·3)	0·0 (0·0–0·1)	0·0 (0·0–0·0)	1·97 (1·89–2·05)	0·58 (0·52–0·64)	0·41 (0·38–0·44)
1976	0·0 (0·0–0·0)	2·6 (1·8–3·5)	106·8 (95·8–119·2)	194·0 (185·1–202·1)	67·2 (61·2–73·5)	11·3 (9·2–13·6)	1·3 (0·9–1·9)	0·0 (0·0–0·1)	0·0 (0·0–0·0)	1·92 (1·84–2·0)	0·55 (0·49–0·61)	0·4 (0·36–0·44)
1977	0·0 (0·0–0·0)	2·4 (1·9–3·2)	100·2 (90·2–112·4)	194·2 (185·9–202·3)	68·8 (62·4–75·5)	11·2 (9·2–13·7)	1·1 (0·8–1·6)	0·0 (0·0–0·0)	0·0 (0·0–0·0)	1·89 (1·81–1·97)	0·51 (0·46–0·57)	0·41 (0·36–0·45)
1978	0·0 (0·0–0·0)	2·4 (1·9–3·0)	94·8 (83·6–108·0)	194·0 (185·0–202·5)	72·9 (66·0–80·5)	11·1 (8·5–14·2)	1·1 (0·7–1·5)	0·0 (0·0–0·0)	0·0 (0·0–0·0)	1·88 (1·8–1·97)	0·49 (0·43–0·55)	0·43 (0·39–0·47)
1979	0·0 (0·0–0·0)	2·4 (2·0–2·8)	89·6 (80·1–99·1)	191·2 (182·9–199·2)	74·5 (67·9–82·0)	11·0 (9·2–13·1)	1·1 (0·7–1·7)	0·0 (0·0–0·0)	0·0 (0·0–0·0)	1·85 (1·78–1·92)	0·46 (0·41–0·51)	0·43 (0·4–0·47)
1980	0·0 (0·0–0·0)	2·5 (2·0–3·0)	84·7 (75·0–94·6)	188·1 (179·4–197·0)	73·8 (66·9–80·8)	11·0 (9·0–13·3)	1·2 (0·8–1·6)	0·0 (0·0–0·0)	0·0 (0·0–0·0)	1·81 (1·73–1·88)	0·44 (0·39–0·48)	0·43 (0·39–0·47)
1981	0·0 (0·0–0·0)	2·6 (2·1–3·1)	81·8 (72·9–91·7)	189·0 (180·9–197·0)	74·7 (67·4–81·7)	11·7 (9·5–13·9)	1·2 (0·8–1·7)	0·0 (0·0–0·0)	0·0 (0·0–0·0)	1·8 (1·74–1·88)	0·42 (0·38–0·47)	0·44 (0·4–0·47)
1982	0·0 (0·0–0·0)	2·7 (2·2–3·3)	80·2 (70·8–90·3)	191·7 (182·2–202·1)	78·1 (71·3–85·9)	13·1 (10·8–15·7)	1·2 (0·9–1·7)	0·0 (0·0–0·0)	0·0 (0·0–0·0)	1·84 (1·77–1·91)	0·41 (0·37–0·47)	0·46 (0·43–0·5)
1983	0·0 (0·0–0·0)	2·8 (2·3–3·5)	78·1 (69·8–87·1)	193·7 (184·8–202·7)	82·2 (74·9–90·2)	14·6 (11·8–17·9)	1·3 (1·0–1·7)	0·0 (0·0–0·1)	0·0 (0·0–0·0)	1·86 (1·79–1·94)	0·4 (0·36–0·45)	0·49 (0·45–0·53)
1984	0·0 (0·0–0·0)	2·8 (2·3–3·4)	72·8 (62·5–84·6)	192·4 (184·0–201·4)	85·6 (78·1–93·2)	15·2 (12·5–18·3)	1·4 (1·0–1·8)	0·0 (0·0–0·0)	0·0 (0·0–0·0)	1·85 (1·76–1·94)	0·38 (0·33–0·44)	0·51 (0·47–0·55)
1985	0·0 (0·0–0·0)	2·7 (2·2–3·3)	68·4 (60·3–77·0)	187·1 (178·8–195·1)	87·5 (79·7–96·3)	15·4 (12·7–18·5)	1·4 (1·0–2·0)	0·0 (0·0–0·1)	0·0 (0·0–0·0)	1·81 (1·75–1·88)	0·36 (0·31–0·4)	0·52 (0·48–0·57)
1986	0·0 (0·0–0·0)	2·7 (2·2–3·2)	65·3 (57·3–74·2)	182·3 (173·5–191·5)	90·5 (82·9–97·9)	15·8 (12·9–18·8)	1·6 (1·0–2·2)	0·0 (0·0–0·1)	0·0 (0·0–0·0)	1·79 (1·72–1·87)	0·34 (0·3–0·38)	0·54 (0·5–0·58)
1987	0·0 (0·0–0·0)	2·6 (2·1–3·2)	60·8 (54·1–68·4)	177·9 (168·9–186·1)	94·2 (86·7–102·6)	16·6 (13·7–19·8)	1·7 (1·2–2·4)	0·0 (0·0–0·1)	0·0 (0·0–0·0)	1·77 (1·7–1·84)	0·32 (0·28–0·35)	0·56 (0·52–0·61)
1988	0·0 (0·0–0·0)	2·6 (2·2–3·1)	57·3 (49·9–65·7)	168·1 (159·9–176·8)	96·1 (87·9–104·8)	17·8 (14·6–21·4)	1·9 (1·4–2·5)	0·0 (0·0–0·1)	0·0 (0·0–0·0)	1·72 (1·65–1·79)	0·3 (0·26–0·34)	0·58 (0·54–0·62)
1989	0·0 (0·0–0·0)	2·7 (2·2–3·2)	54·0 (47·1–61·7)	157·1 (149·3–165·7)	96·6 (88·5–105·1)	18·9 (15·6–22·8)	2·0 (1·5–2·7)	0·0 (0·0–0·1)	0·0 (0·0–0·0)	1·66 (1·59–1·72)	0·28 (0·25–0·32)	0·59 (0·54–0·63)
1990	0·0 (0·0–0·0)	2·8 (2·3–3·3)	53·0 (46·0–60·1)	153·0 (144·3–161·2)	98·8 (90·5–107·5)	20·1 (16·4–24·1)	2·0 (1·5–2·7)	0·0 (0·0–0·1)	0·0 (0·0–0·0)	1·65 (1·58–1·72)	0·28 (0·24–0·32)	0·6 (0·56–0·65)
1991	0·0 (0·0–0·0)	2·9 (2·4–3·6)	53·5 (46·5–60·8)	148·4 (141·1–156·7)	100·0 (91·4–109·4)	21·5 (17·8–25·8)	2·1 (1·5–2·8)	0·0 (0·0–0·1)	0·0 (0·0–0·0)	1·64 (1·57–1·71)	0·28 (0·25–0·32)	0·62 (0·57–0·67)
1992	0·0 (0·0–0·0)	3·0 (2·5–3·7)	52·3 (46·5–59·0)	140·3 (132·4–148·5)	99·1 (91·5–107·4)	22·4 (18·3–27·2)	2·2 (1·5–2·8)	0·1 (0·0–0·1)	0·0 (0·0–0·0)	1·6 (1·53–1·67)	0·28 (0·25–0·31)	0·62 (0·58–0·67)
1993	0·0 (0·0–0·0)	3·1 (2·5–3·8)	52·5 (45·5–60·4)	140·1 (131·5–149·1)	103·0 (94·3–111·9)	24·3 (20·3–29·0)	2·3 (1·7–3·0)	0·1 (0·0–0·1)	0·0 (0·0–0·0)	1·63 (1·56–1·7)	0·28 (0·24–0·32)	0·65 (0·6–0·7)
1994	0·0 (0·0–0·0)	3·2 (2·5–3·9)	52·1 (45·6–59·3)	137·5 (129·3–145·6)	104·1 (95·3–113·8)	25·9 (21·2–30·8)	2·4 (1·7–3·2)	0·1 (0·0–0·1)	0·0 (0·0–0·0)	1·63 (1·55–1·7)	0·28 (0·25–0·31)	0·66 (0·61–0·72)
1995	0·0 (0·0–0·0)	3·2 (2·6–3·9)	50·9 (44·2–58·3)	129·6 (121·6–137·7)	102·4 (93·1–111·5)	27·0 (22·8–31·8)	2·6 (1·9–3·4)	0·1 (0·0–0·1)	0·0 (0·0–0·0)	1·58 (1·51–1·66)	0·27 (0·24–0·31)	0·66 (0·61–0·72)
1996	0·0 (0·0–0·0)	3·4 (2·8–4·1)	50·5 (43·4–57·9)	125·0 (117·4–132·6)	101·8 (93·5–110·7)	28·4 (23·6–33·8)	2·7 (2·0–3·5)	0·1 (0·0–0·1)	0·0 (0·0–0·0)	1·56 (1·48–1·63)	0·27 (0·23–0·31)	0·66 (0·61–0·72)
1997	0·0 (0·0–0·0)	3·5 (2·9–4·3)	50·2 (43·6–57·6)	120·8 (113·5–128·0)	99·8 (92·0–108·3)	29·2 (24·4–34·7)	2·9 (2·1–3·9)	0·1 (0·0–0·1)	0·0 (0·0–0·0)	1·53 (1·47–1·6)	0·27 (0·24–0·3)	0·66 (0·61–0·71)
1998	0·0 (0·0–0·0)	3·7 (3·0–4·6)	49·7 (42·9–56·7)	116·1 (108·6–123·8)	98·8 (90·3–108·6)	30·4 (25·1–36·4)	3·1 (2·3–4·1)	0·1 (0·0–0·1)	0·0 (0·0–0·0)	1·51 (1·44–1·59)	0·27 (0·23–0·3)	0·66 (0·61–0·72)
1999	0·0 (0·0–0·0)	3·9 (3·2–4·7)	48·9 (42·8–56·1)	112·2 (105·4–118·7)	97·9 (89·8–106·7)	31·2 (25·9–36·8)	3·3 (2·4–4·4)	0·1 (0·0–0·1)	0·0 (0·0–0·0)	1·49 (1·42–1·56)	0·26 (0·23–0·3)	0·66 (0·61–0·72)
2000	0·0 (0·0–0·0)	4·1 (3·3–5·0)	48·5 (41·1–56·0)	109·5 (102·0–117·5)	95·9 (88·1–104·3)	32·6 (27·1–38·5)	3·5 (2·6–4·8)	0·1 (0·0–0·1)	0·0 (0·0–0·0)	1·47 (1·4–1·54)	0·26 (0·23–0·3)	0·66 (0·61–0·71)
2001	0·0 (0·0–0·0)	4·2 (3·4–5·1)	47·7 (42·3–53·9)	106·0 (99·5–112·7)	92·6 (84·0–101·1)	32·7 (27·3–38·8)	3·7 (2·7–5·0)	0·1 (0·0–0·1)	0·0 (0·0–0·0)	1·43 (1·37–1·5)	0·26 (0·23–0·29)	0·65 (0·59–0·7)
2002	0·0 (0·0–0·0)	4·1 (3·4–5·0)	45·8 (40·1–52·8)	103·0 (96·4–109·8)	91·6 (83·7–100·4)	33·3 (27·9–39·1)	4·0 (2·9–5·7)	0·1 (0·0–0·1)	0·0 (0·0–0·0)	1·41 (1·34–1·47)	0·25 (0·22–0·28)	0·65 (0·6–0·7)
2003	0·0 (0·0–0·0)	3·8 (3·1–4·7)	43·5 (38·2–49·4)	100·2 (93·8–107·3)	90·7 (83·1–98·7)	34·8 (29·1–40·6)	4·3 (3·0–5·8)	0·1 (0·1–0·1)	0·0 (0·0–0·0)	1·39 (1·32–1·46)	0·24 (0·21–0·27)	0·65 (0·6–0·7)
2004	0·0 (0·0–0·0)	3·6 (2·9–4·4)	41·6 (36·4–47·7)	96·2 (90·2–102·4)	88·8 (81·4–96·5)	35·5 (29·8–41·8)	4·5 (3·3–6·0)	0·1 (0·1–0·2)	0·0 (0·0–0·0)	1·35 (1·29–1·42)	0·23 (0·2–0·26)	0·65 (0·6–0·7)
2005	0·0 (0·0–0·0)	3·5 (2·8–4·3)	41·1 (33·7–49·5)	94·9 (88·4–101·6)	89·0 (80·7–97·4)	36·5 (30·8–42·6)	4·8 (3·5–6·3)	0·1 (0·1–0·2)	0·0 (0·0–0·0)	1·35 (1·28–1·42)	0·22 (0·19–0·26)	0·65 (0·6–0·7)
2006	0·0 (0·0–0·0)	3·4 (2·7–4·3)	41·1 (35·8–46·4)	95·6 (89·6–102·3)	91·5 (83·4–99·7)	38·3 (32·6–45·4)	5·3 (3·9–6·9)	0·1 (0·1–0·2)	0·0 (0·0–0·0)	1·38 (1·31–1·45)	0·22 (0·2–0·25)	0·68 (0·63–0·73)
2007	0·0 (0·0–0·0)	3·4 (2·6–4·1)	40·7 (35·6–46·3)	94·9 (89·1–101·6)	91·4 (83·5–99·6)	40·3 (34·3–47·5)	5·7 (4·3–7·3)	0·1 (0·1–0·2)	0·0 (0·0–0·0)	1·38 (1·32–1·45)	0·22 (0·2–0·25)	0·69 (0·63–0·74)
2008	0·0 (0·0–0·0)	3·3 (2·6–4·2)	39·8 (34·3–45·7)	94·2 (87·9–101·2)	91·6 (84·0–99·9)	41·6 (35·0–48·5)	6·2 (4·8–7·8)	0·1 (0·1–0·2)	0·0 (0·0–0·0)	1·38 (1·32–1·45)	0·22 (0·19–0·24)	0·7 (0·65–0·75)
2009	0·0 (0·0–0·0)	3·2 (2·4–4·1)	38·3 (32·9–44·5)	93·6 (87·6–99·3)	92·2 (84·2–100·8)	43·0 (36·7–50·0)	6·8 (5·3–8·6)	0·1 (0·1–0·2)	0·0 (0·0–0·0)	1·39 (1·31–1·47)	0·21 (0·18–0·24)	0·71 (0·66–0·77)
2010	0·0 (0·0–0·0)	3·1 (2·2–4·0)	36·8 (32·1–42·0)	93·7 (87·2–100·5)	93·4 (85·6–102·2)	44·3 (38·0–51·3)	7·2 (5·7–9·0)	0·1 (0·1–0·2)	0·0 (0·0–0·0)	1·39 (1·33–1·46)	0·2 (0·18–0·22)	0·73 (0·68–0·78)
2011	0·0 (0·0–0·0)	3·0 (2·3–3·7)	34·8 (30·6–39·7)	92·5 (86·3–99·3)	93·7 (85·8–102·1)	45·4 (38·7–52·8)	7·6 (5·9–9·7)	0·2 (0·1–0·3)	0·0 (0·0–0·0)	1·38 (1·31–1·46)	0·19 (0·17–0·21)	0·73 (0·67–0·8)
2012	0·0 (0·0–0·0)	2·9 (2·4–3·4)	32·8 (28·5–37·6)	91·1 (85·1–97·2)	95·4 (87·0–104·2)	47·5 (40·6–55·0)	8·3 (6·5–10·5)	0·2 (0·1–0·3)	0·0 (0·0–0·0)	1·39 (1·33–1·46)	0·18 (0·16–0·2)	0·76 (0·7–0·81)
2013	0·0 (0·0–0·0)	2·8 (2·3–3·4)	31·4 (27·1–36·0)	88·7 (82·6–94·9)	95·8 (87·5–104·3)	48·5 (41·6–55·6)	8·8 (6·7–11·3)	0·2 (0·1–0·3)	0·0 (0·0–0·0)	1·38 (1·31–1·45)	0·17 (0·15–0·19)	0·77 (0·71–0·82)
2014	0·0 (0·0–0·0)	2·8 (2·3–3·3)	30·2 (26·1–35·1)	87·5 (81·2–94·1)	96·9 (89·1–105·4)	49·9 (43·1–57·2)	9·2 (7·2–12·1)	0·2 (0·1–0·4)	0·0 (0·0–0·0)	1·38 (1·31–1·46)	0·16 (0·14–0·19)	0·78 (0·72–0·85)
2015	0·0 (0·0–0·0)	2·8 (2·3–3·4)	30·8 (23·4–39·7)	88·9 (81·8–96·7)	99·2 (89·2–110·0)	48·0 (39·9–57·0)	8·5 (6·2–11·4)	0·2 (0·1–0·3)	0·0 (0·0–0·0)	1·39 (1·23–1·57)	0·17 (0·13–0·22)	0·78 (0·69–0·88)
2016	0·0 (0·0–0·0)	2·8 (2·3–3·5)	32·0 (24·7–41·8)	90·1 (80·4–101·9)	98·8 (85·6–114·0)	44·0 (34·0–57·3)	7·3 (5·0–10·3)	0·2 (0·1–0·3)	0·0 (0·0–0·0)	1·38 (1·18–1·61)	0·17 (0·14–0·23)	0·75 (0·64–0·89)
2017	0·0 (0·0–0·0)	2·8 (2·3–3·4)	32·1 (23·7–42·9)	89·9 (79·7–101·1)	97·5 (83·1–113·2)	42·8 (31·9–56·1)	7·2 (4·6–10·7)	0·2 (0·1–0·3)	0·0 (0·0–0·0)	1·36 (1·14–1·62)	0·17 (0·13–0·23)	0·74 (0·6–0·89)

Supplemental Results Table 5: Age-specific fertility rates per 1000, cumulative total fertility up to maternal age 25 (TFU25), cumulative total fertility from maternal age 30 to age 55 (TFO30), and cumulative total fertility, Oita, 1950-2017

Year	10-14	15-19	20-24	25-29	30-34	35-39	40-44	45-49	50-54	TFR	TFU25	TFO30
1950	0·0 (0·0–0·0)	9·5 (8·4–10·6)	147·9 (135·2–161·8)	210·4 (202·8–218·0)	153·0 (143·9–162·1)	79·9 (70·9–88·6)	30·7 (26·5–35·2)	1·3 (0·9–2·0)	0·0 (0·0–0·0)	3·16 (3·05–3·28)	0·79 (0·72–0·86)	1·32 (1·26–1·39)
1951	0·0 (0·0–0·0)	7·7 (6·8–8·6)	132·7 (119·7–145·7)	195·2 (187·4–202·4)	138·3 (128·7–147·8)	64·6 (56·1–72·9)	23·8 (19·9–28·1)	1·0 (0·6–1·5)	0·0 (0·0–0·0)	2·82 (2·69–2·94)	0·7 (0·64–0·77)	1·14 (1·04–1·24)
1952	0·0 (0·0–0·0)	6·5 (5·6–7·3)	123·5 (111·1–138·4)	185·3 (176·9–193·5)	125·1 (115·6–135·1)	53·6 (46·0–61·5)	18·9 (15·3–22·8)	0·8 (0·5–1·1)	0·0 (0·0–0·0)	2·57 (2·47–2·68)	0·65 (0·59–0·72)	0·99 (0·93–1·06)
1953	0·0 (0·0–0·0)	5·7 (4·9–6·5)	116·7 (103·5–130·8)	174·5 (166·4–182·7)	111·8 (101·4–121·9)	46·5 (39·2–54·1)	15·0 (12·1–18·4)	0·6 (0·4–1·0)	0·0 (0·0–0·0)	2·35 (2·25–2·47)	0·61 (0·55–0·68)	0·87 (0·8–0·94)
1954	0·0 (0·0–0·0)	5·2 (4·4–6·0)	113·1 (98·5–128·8)	169·7 (162·3–177·4)	103·0 (94·3–112·8)	42·3 (35·3–49·9)	12·3 (9·6–15·6)	0·5 (0·3–0·8)	0·0 (0·0–0·0)	2·23 (2·13–2·34)	0·59 (0·52–0·67)	0·79 (0·73–0·86)
1955	0·0 (0·0–0·0)	4·7 (4·0–5·5)	110·3 (96·3–126·2)	168·6 (160·7–176·6)	94·8 (85·7–104·3)	38·1 (32·2–45·2)	10·2 (7·8–13·0)	0·4 (0·2–0·6)	0·0 (0·0–0·0)	2·14 (2·04–2·23)	0·57 (0·5–0·66)	0·72 (0·66–0·78)
1956	0·0 (0·0–0·0)	4·2 (3·5–4·9)	103·8 (89·5–118·6)	162·9 (155·7–170·5)	84·0 (75·8–92·9)	32·6 (27·3–38·6)	8·2 (6·2–10·4)	0·3 (0·2–0·5)	0·0 (0·0–0·0)	1·98 (1·89–2·07)	0·54 (0·47–0·61)	0·63 (0·57–0·68)
1957	0·0 (0·0–0·0)	4·0 (3·4–4·7)	104·9 (90·6–121·6)	166·4 (158·9–173·9)	79·7 (72·1–87·9)	27·9 (23·2–32·4)	6·9 (5·3–8·8)	0·3 (0·2–0·5)	0·0 (0·0–0·0)	1·95 (1·85–2·06)	0·54 (0·47–0·63)	0·57 (0·53–0·63)
1958	0·0 (0·0–0·0)	4·0 (3·4–4·7)	110·1 (95·8–125·6)	172·0 (163·8–180·3)	78·1 (68·3–88·8)	24·7 (20·5–29·2)	6·3 (4·8–8·0)	0·3 (0·2–0·5)	0·0 (0·0–0·0)	1·98 (1·88–2·08)	0·57 (0·5–0·65)	0·55 (0·49–0·61)
1959	0·0 (0·0–0·0)	4·2 (3·6–4·9)	110·1 (95·9–126·2)	171·0 (163·0–179·7)	73·1 (65·6–81·0)	21·3 (17·2–26·0)	5·2 (3·9–6·8)	0·3 (0·2–0·4)	0·0 (0·0–0·0)	1·93 (1·83–2·02)	0·57 (0·5–0·65)	0·5 (0·45–0·55)
1960	0·0 (0·0–0·0)	4·1 (3·4–4·8)	105·8 (93·5–119·1)	171·7 (163·1–180·0)	70·2 (62·6–78·3)	18·7 (15·7–22·0)	4·3 (3·1–5·5)	0·2 (0·1–0·3)	0·0 (0·0–0·0)	1·87 (1·79–1·96)	0·55 (0·49–0·62)	0·47 (0·42–0·51)
1961	0·0 (0·0–0·0)	3·8 (3·2–4·5)	102·6 (92·2–114·0)	173·6 (165·6–182·2)	68·6 (62·5–76·0)	16·8 (13·7–20·5)	3·6 (2·7–5·1)	0·2 (0·1–0·3)	0·0 (0·0–0·0)	1·85 (1·77–1·93)	0·53 (0·48–0·59)	0·45 (0·41–0·49)
1962	0·0 (0·0–0·0)	3·6 (3·0–4·3)	102·3 (91·2–113·9)	178·7 (171·3–185·9)	70·2 (63·9–76·5)	15·9 (13·5–18·4)	3·2 (2·3–4·5)	0·2 (0·1–0·3)	0·0 (0·0–0·0)	1·87 (1·79–1·95)	0·53 (0·47–0·59)	0·45 (0·41–0·49)
1963	0·0 (0·0–0·0)	3·6 (2·9–4·3)	102·8 (91·5–115·2)	180·9 (173·6–188·4)	71·7 (65·7–78·4)	15·3 (12·8–18·2)	3·2 (2·2–4·4)	0·2 (0·1–0·3)	0·0 (0·0–0·0)	1·89 (1·81–1·97)	0·53 (0·48–0·59)	0·45 (0·42–0·49)
1964	0·0 (0·0–0·0)	3·5 (3·0–4·2)	112·2 (100·0–126·5)	192·9 (184·5–201·7)	76·7 (70·0–84·0)	15·8 (13·4–18·4)	3·0 (2·1–4·1)	0·2 (0·1–0·3)	0·0 (0·0–0·0)	2·02 (1·94–2·11)	0·58 (0·52–0·65)	0·48 (0·44–0·52)
1965	0·0 (0·0–0·0)	3·4 (2·9–4·1)	103·5 (90·6–118·9)	162·9 (154·2–172·0)	63·9 (57·2–70·5)	14·5 (12·3–16·9)	2·5 (1·8–3·5)	0·1 (0·1–0·2)	0·0 (0·0–0·0)	1·75 (1·67–1·85)	0·53 (0·47–0·61)	0·41 (0·37–0·44)
1966	0·0 (0·0–0·0)	3·7 (3·1–4·2)	101·3 (85·5–118·3)	167·9 (159·2–176·5)	65·6 (58·6–73·0)	14·7 (12·2–17·8)	2·5 (1·8–3·5)	0·1 (0·1–0·2)	0·0 (0·0–0·0)	1·78 (1·69–1·88)	0·52 (0·45–0·61)	0·42 (0·38–0·46)
1967	0·0 (0·0–0·0)	3·8 (3·3–4·5)	108·1 (90·1–127·4)	205·0 (196·3–213·1)	79·4 (71·5–87·5)	16·7 (13·6–20·0)	2·4 (1·8–3·2)	0·1 (0·1–0·2)	0·0 (0·0–0·0)	2·08 (1·95–2·2)	0·56 (0·47–0·66)	0·49 (0·45–0·54)
1968	0·0 (0·0–0·0)	3·9 (3·4–4·5)	97·0 (80·5–116·0)	193·6 (185·6–201·4)	72·6 (64·8–80·5)	16·5 (13·5–20·0)	2·2 (1·6–2·9)	0·1 (0·1–0·2)	0·0 (0·0–0·0)	1·93 (1·84–2·02)	0·5 (0·42–0·6)	0·46 (0·42–0·5)
1969	0·0 (0·0–0·0)	4·3 (3·7–5·0)	95·5 (79·9–113·3)	196·3 (188·3–204·3)	73·2 (66·0–81·4)	16·1 (13·2–19·4)	2·2 (1·6–2·9)	0·1 (0·1–0·2)	0·0 (0·0–0·0)	1·94 (1·86–2·03)	0·5 (0·42–0·59)	0·46 (0·41–0·51)
1970	0·0 (0·0–0·0)	4·3 (3·7–5·1)	103·0 (86·5–121·3)	200·6 (191·8–209·0)	75·9 (68·3–83·8)	15·3 (12·3–19·0)	2·3 (1·8–3·0)	0·1 (0·1–0·2)	0·0 (0·0–0·0)	2·01 (1·93–2·1)	0·54 (0·45–0·63)	0·47 (0·43–0·52)
1971	0·0 (0·0–0·0)	4·2 (3·6–5·0)	113·1 (95·2–131·5)	199·2 (190·5–208·2)	77·6 (69·1–86·6)	13·8 (11·2–16·8)	2·5 (1·8–3·4)	0·1 (0·1–0·2)	0·0 (0·0–0·0)	2·05 (1·96–2·15)	0·59 (0·5–0·68)	0·47 (0·42–0·52)
1972	0·0 (0·0–0·0)	4·3 (3·6–5·1)	119·0 (103·4–136·5)	198·8 (189·8–207·7)	78·5 (70·4–86·9)	13·0 (10·3–16·0)	2·5 (1·8–3·5)	0·1 (0·1–0·2)	0·0 (0·0–0·0)	2·08 (1·99–2·18)	0·62 (0·54–0·7)	0·47 (0·43–0·52)
1973	0·0 (0·0–0·0)	4·2 (3·5–5·0)	120·0 (105·3–134·8)	200·1 (190·1–209·6)	75·6 (68·2–83·5)	12·7 (10·1–15·6)	2·4 (1·6–3·3)	0·1 (0·1–0·2)	0·0 (0·0–0·0)	2·08 (1·98–2·17)	0·62 (0·55–0·69)	0·45 (0·41–0·5)
1974	0·0 (0·0–0·0)	3·9 (3·2–4·8)	115·6 (103·2–130·3)	193·7 (185·0–202·4)	67·8 (61·2–75·0)	11·7 (9·2–14·6)	2·0 (1·3–3·0)	0·1 (0·1–0·1)	0·0 (0·0–0·0)	1·97 (1·89–2·06)	0·6 (0·54–0·67)	0·41 (0·37–0·45)
1975	0·0 (0·0–0·0)	3·7 (2·9–4·5)	108·7 (97·1–120·5)	187·8 (178·9–196·7)	61·4 (55·0–67·4)	11·2 (8·8–14·2)	1·7 (1·2–2·4)	0·1 (0·0–0·1)	0·0 (0·0–0·0)	1·87 (1·79–1·96)	0·56 (0·5–0·62)	0·37 (0·34–0·41)
1976	0·0 (0·0–0·0)	3·5 (2·8–4·3)	103·1 (91·6–115·1)	185·8 (176·6–195·2)	59·7 (54·0–65·7)	11·1 (8·9–13·7)	1·5 (1·0–2·2)	0·1 (0·0–0·1)	0·0 (0·0–0·0)	1·82 (1·73–1·91)	0·53 (0·48–0·59)	0·36 (0·33–0·4)
1977	0·0 (0·0–0·0)	3·4 (2·8–4·1)	97·7 (87·1–110·3)	186·4 (178·0–195·2)	61·5 (55·3–68·3)	11·1 (8·7–14·1)	1·4 (0·9–2·0)	0·1 (0·0–0·1)	0·0 (0·0–0·0)	1·81 (1·73–1·89)	0·51 (0·45–0·57)	0·37 (0·33–0·41)
1978	0·0 (0·0–0·0)	3·4 (2·7–4·1)	93·3 (83·8–103·6)	186·5 (178·5–194·7)	65·1 (59·3–71·3)	11·0 (8·2–14·4)	1·4 (0·9–2·0)	0·0 (0·0–0·1)	0·0 (0·0–0·0)	1·8 (1·73–1·88)	0·48 (0·43–0·54)	0·39 (0·36–0·42)
1979	0·0 (0·0–0·0)	3·4 (2·9–4·1)	89·2 (78·8–100·1)	184·0 (176·0–192·1)	66·2 (57·6–75·7)	10·8 (8·8–13·1)	1·4 (0·9–2·1)	0·0 (0·0–0·1)	0·0 (0·0–0·0)	1·78 (1·7–1·86)	0·46 (0·41–0·52)	0·39 (0·35–0·44)
1980	0·0 (0·0–0·0)	3·5 (2·8–4·3)	84·9 (75·5–94·9)	180·6 (172·0–189·9)	65·2 (59·4–71·3)	10·8 (8·6–13·4)	1·4 (0·9–2·0)	0·0 (0·0–0·1)	0·0 (0·0–0·0)	1·73 (1·66–1·81)	0·44 (0·39–0·49)	0·39 (0·36–0·42)
1981	0·0 (0·0–0·0)	3·6 (2·8–4·4)	82·1 (73·6–92·5)	181·0 (172·8–189·6)	66·0 (60·0–72·8)	11·4 (9·2–14·0)	1·5 (1·0–2·1)	0·0 (0·0–0·1)	0·0 (0·0–0·0)	1·73 (1·67–1·8)	0·43 (0·39–0·48)	0·39 (0·36–0·43)
1982	0·0 (0·0–0·0)	3·7 (2·9–4·6)	80·4 (70·6–91·2)	182·7 (174·6–190·7)	69·0 (62·6–76·3)	12·8 (10·4–15·7)	1·5 (1·0–2·1)	0·0 (0·0–0·1)	0·0 (0·0–0·0)	1·75 (1·68–1·83)	0·42 (0·37–0·47)	0·42 (0·38–0·46)
1983	0·0 (0·0–0·0)	3·7 (3·0–4·6)	77·9 (68·9–88·1)	183·3 (174·7–191·7)	72·5 (65·3–80·0)	14·2 (11·4–17·6)	1·5 (1·1–2·0)	0·0 (0·0–0·1)	0·0 (0·0–0·0)	1·77 (1·7–1·84)	0·41 (0·36–0·46)	0·44 (0·4–0·48)
1984	0·0 (0·0–0·0)	3·6 (2·9–4·5)	72·2 (64·1–81·2)	181·3 (173·5–189·7)	75·4 (68·1–82·7)	14·7 (11·9–18·2)	1·6 (1·1–2·2)	0·0 (0·0–0·1)	0·0 (0·0–0·0)	1·74 (1·68–1·81)	0·38 (0·34–0·43)	0·46 (0·42–0·5)
1985	0·0 (0·0–0·0)	3·5 (2·8–4·4)	68·0 (59·0–77·5)	176·5 (165·4–186·9)	77·1 (69·4–85·1)	14·7 (11·9–18·0)	1·7 (1·2–2·2)	0·0 (0·0–0·1)	0·0 (0·0–0·0)	1·71 (1·64–1·79)	0·36 (0·31–0·41)	0·47 (0·43–0·51)
1986	0·0 (0·0–0·0)	3·4 (2·7–4·2)	65·3 (57·4–74·1)	172·9 (165·5–181·2)	80·1 (72·8–87·2)	14·8 (11·9–17·8)	1·7 (1·2–2·3)	0·0 (0·0–0·1)	0·0 (0·0–0·0)	1·69 (1·62–1·77)	0·34 (0·3–0·39)	0·48 (0·44–0·52)
1987	0·0 (0·0–0·0)	3·3 (2·7–4·1)	61·3 (54·5–69·2)	170·5 (160·9–179·9)	83·9 (76·3–91·8)	15·4 (12·4–18·7)	1·9 (1·3–2·9)	0·0 (0·0–0·1)	0·0 (0·0–0·0)	1·68 (1·61–1·76)	0·32 (0·29–0·36)	0·51 (0·46–0·55)
1988	0·0 (0·0–0·0)	3·3 (2·6–4·1)	58·1 (50·3–66·9)	163·6 (155·6–172·2)	86·4 (78·6–94·9)	16·1 (12·8–19·8)	2·1 (1·4–2·9)	0·1 (0·0–0·1)	0·0 (0·0–0·0)	1·65 (1·58–1·72)	0·31 (0·27–0·35)	0·52 (0·48–0·57)
1989	0·0 (0·0–0·0)	3·3 (2·7–4·1)	54·8 (47·2–63·5)	155·9 (147·3–164·7)	88·2 (79·9–95·9)	16·8 (13·1–21·4)	2·1 (1·5–3·0)	0·1 (0·0–0·1)	0·0 (0·0–0·0)	1·61 (1·54–1·67)	0·29 (0·25–0·33)	0·54 (0·49–0·58)
1990	0·0 (0·0–0·0)	3·4 (2·8–4·1)	53·5 (46·6–61·4)	154·3 (146·3–162·5)	91·3 (83·4–99·2)	17·3 (13·9–21·1)	2·1 (1·5–2·9)	0·1 (0·0–0·1)	0·0 (0·0–0·0)	1·61 (1·53–1·7)	0·28 (0·25–0·33)	0·55 (0·51–0·6)
1991	0·0 (0·0–0·0)	3·5 (2·9–4·2)	52·7 (45·5–60·3)	148·9 (137·9–160·9)	93·0 (84·2–101·8)	18·0 (14·5–22·0)	2·2 (1·6–3·0)	0·1 (0·0–0·1)	0·0 (0·0–0·0)	1·59 (1·52–1·67)	0·28 (0·25–0·32)	0·57 (0·52–0·62)
1992	0·0 (0·0–0·0)	3·3 (2·8–4·0)	48·5 (42·8–55·6)	131·2 (124·1–138·4)	92·0 (84·4–100·5)	18·9 (15·3–23·1)	2·6 (1·8–3·7)	0·1 (0·0–0·1)	0·0 (0·0–0·0)	1·48 (1·42–1·55)	0·26 (0·23–0·29)	0·57 (0·53–0·62)
1993	0·0 (0·0–0·0)	3·1 (2·5–3·8)	41·9 (35·9–48·6)	110·5 (102·9–118·0)	89·8 (82·1–98·1)	19·6 (16·2–23·4)	3·3 (2·3–4·4)	0·1 (0·0–0·1)	0·0 (0·0–0·0)	1·34 (1·27–1·41)	0·22 (0·19–0·26)	0·56 (0·52–0·61)
1994	0·0 (0·0–0·0)	3·1 (2·5–3·7)	37·2 (32·7–42·7)	98·7 (92·6–104·5)	84·3 (76·8–92·7)	19·0 (15·5–23·3)	3·5 (2·5–4·8)	0·1 (0·0–0·1)	0·0 (0·0–0·0)	1·23 (1·17–1·3)	0·2 (0·18–0·23)	0·53 (0·49–0·58)
1995	0·0 (0·0–0·0)	3·4 (2·8–4·2)	40·1 (34·6–46·5)	107·0 (100·3–114·6)	87·3 (78·4–95·3)	20·1 (16·6–24·1)	3·2 (2·3–4·4)	0·1 (0·0–0·1)	0·0 (0·0–0·0)	1·31 (1·23–1·38)	0·22 (0·19–0·25)	0·55 (0·51–0·6)
1996	0·0 (0·0–0·0)	3·9 (3·1–4·7)	45·7 (39·4–52·6)	120·8 (112·5–129·4)	94·8 (86·5–104·2)	23·2 (19·0–27·8)	3·0 (2·2–3·9)	0·1 (0·0–0·1)	0·0 (0·0–0·0)	1·46 (1·37–1·55)	0·25 (0·22–0·28)	0·61 (0·56–0·66)
1997	0·0 (0·0–0·0)	4·3 (3·5–5·2)	48·7 (41·8–56·0)	124·3 (116·2–131·8)	97·0 (88·3–106·1)	25·4 (21·1–30·7)	3·1 (2·3–4·2)	0·1 (0·0–0·1)	0·0 (0·0–0·0)	1·51 (1·43–1·6)	0·27 (0·23–0·3)	0·63 (0·58–0·68)
1998	0·0 (0·0–0·0)	4·6 (3·8–5·7)	50·0 (43·1–57·4)	121·8 (113·6–129·7)	98·0 (89·6–108·4)	28·0 (22·9–33·9)	3·2 (2·4–4·2)	0·1 (0·0–0·1)	0·0 (0·0–0·0)	1·53 (1·45–1·62)	0·27 (0·24–0·31)	0·65 (0·6–0·7)
1999	0·0 (0·0–0·0)	4·9 (4·1–6·1)	50·1 (43·5–57·1)	117·4 (109·6–125·1)	98·0 (89·2–107·5)	29·9 (24·5–36·1)	3·4 (2·4–4·5)	0·1 (0·0–0·1)	0·0 (0·0–0·0)	1·52 (1·44–1·6)	0·28 (0·24–0·31)	0·66 (0·6–0·72)
2000	0·0 (0·0–0·0)	5·2 (4·3–6·4)	50·4 (42·0–58·9)	113·4 (106·3–120·9)	96·5 (88·0–105·3)	32·4 (26·8–38·5)	3·6 (2·6–4·8)	0·1 (0·0–0·1)	0·0 (0·0–0·0)	1·51 (1·43–1·59)	0·28 (0·24–0·32)	0·66 (0·61–0·72)
2001	0·0 (0·0–0·0)	5·5 (4·5–6·6)	50·1 (44·2–56·6)	108·6 (101·7–116·3)	93·6 (85·1–102·2)	33·1 (27·4–39·7)	3·8 (2·7–5·1)	0·1 (0·0–0·1)	0·0 (0·0–0·0)	1·47 (1·4–1·54)	0·28 (0·25–0·31)	0·65 (0·6–0·71)
2002	0·0 (0·0–0·0)	5·4 (4·4–6·6)	48·7 (42·2–56·3)	104·8 (98·3–111·6)	93·0 (84·5–102·5)	34·2 (28·6–41·1)	4·1 (2·9–5·8)	0·1 (0·0–0·1)	0·0 (0·0–0·0)	1·45 (1·38–1·53)	0·27 (0·24–0·31)	0·66 (0·6–0·72)
2003	0·0 (0·0–0·0)	5·3 (4·2–6·5)	46·8 (40·7–53·6)	101·9 (93·6–110·8)	92·4 (84·1–101·0)	36·0 (29·5–43·8)	4·4 (3·1–6·0)	0·1 (0·0–0·1)	0·0 (0·0–0·0)	1·43 (1·34–1·53)	0·26 (0·23–0·29)	0·66 (0·6–0·72)
2004	0·0 (0·0–0·0)	5·1 (4·1–6·3)	45·4 (39·0–53·0)	97·9 (92·2–104·1)	91·1 (82·8–99·9)	36·9 (30·6–44·0)	4·7 (3·3–6·4)	0·1 (0·1–0·1)	0·0 (0·0–0·0)	1·41 (1·33–1·48)	0·25 (0·22–0·29)	0·66 (0·6–0·73)
2005	0·0 (0·0–0·0)	5·0 (4·0–6·1)	45·3 (39·4–52·2)	97·2 (90·4–104·5)	91·8 (83·9–100·6)	37·9 (31·7–44·9)	4·9 (3·5–6·8)	0·1 (0·1–0·1)	0·0 (0·0–0·0)	1·41 (1·34–1·49)	0·25 (0·22–0·29)	0·67 (0·62–0·73)
2006	0·0 (0·0–0·0)	5·0 (4·0–6·2)	46·3 (40·3–52·7)	98·9 (92·6–105·6)	95·0 (86·5–104·2)	39·6 (33·1–47·6)	5·4 (3·9–7·3)	0·1 (0·1–0·1)	0·0 (0·0–0·0)	1·45 (1·39–1·52)	0·26 (0·23–0·29)	0·7 (0·65–0·76)
2007	0·0 (0·0–0·0)	5·0 (4·0–6·2)	46·7 (38·9–55·8)	99·1 (92·5–106·9)	95·6 (86·9–104·8)	41·5 (34·6–49·4)	5·8 (4·2–8·1)	0·1 (0·1–0·2)	0·0 (0·0–0·0)	1·47 (1·39–1·56)	0·26 (0·22–0·3)	0·72 (0·66–0·77)
2008	0·0 (0·0–0·0)	4·9 (3·9–6·2)	46·5 (40·3–53·2)	99·3 (93·1–106·4)	96·3 (88·2–104·8)	42·8 (36·0–50·3)	6·3 (4·5–8·7)	0·1 (0·1–0·2)	0·0 (0·0–0·0)	1·48 (1·41–1·56)	0·26 (0·23–0·29)	0·73 (0·67–0·79)
2009	0·0 (0·0–0·0)	4·7 (3·7–6·1)	45·5 (38·2–53·1)	99·7 (91·2–108·5)	97·1 (88·8–106·6)	44·3 (37·2–52·3)	6·8 (4·9–9·2)	0·1 (0·1–0·2)	0·0 (0·0–0·0)	1·49 (1·39–1·6)	0·25 (0·22–0·29)	0·74 (0·69–0·81)
2010	0·0 (0·0–0·0)	4·6 (3·4–6·1)	44·4 (38·6–50·4)	100·8 (94·7–106·7)	98·2 (89·7–108·0)	45·7 (38·8–53·6)	7·2 (5·2–9·7)	0·1 (0·1–0·2)	0·0 (0·0–0·0)	1·51 (1·43–1·58)	0·25 (0·22–0·28)	0·76 (0·7–0·82)
2011	0·0 (0·0–0·0)	4·5 (3·6–5·7)	42·4 (37·1–48·6)	100·7 (93·7–108·1)	98·2 (90·2–107·1)	46·8 (39·5–54·5)	7·5 (5·5–10·0)	0·1 (0·1–0·2)	0·0 (0·0–0·0)	1·5 (1·43–1·57)	0·23 (0·21–0·27)	0·76 (0·71–0·82)
2012	0·0 (0·0–0·0)	4·4 (3·7–5·2)	40·6 (35·2–46·6)	100·6 (93·5–108·1)	99·3 (90·4–108·8)	48·8 (41·7–56·9)	8·0 (5·8–10·6)	0·1 (0·1–0·3)	0·0 (0·0–0·0)	1·51 (1·43–1·6)	0·22 (0·2–0·26)	0·78 (0·72–0·85)
2013	0·0 (0·0–0·0)	4·3 (3·5–5·3)	39·3 (33·6–45·1)	99·3 (92·5–106·3)	99·1 (90·2–108·5)	49·2 (41·9–56·5)	8·4 (6·1–11·2)	0·1 (0·1–0·3)	0·0 (0·0–0·0)	1·5 (1·43–1·58)	0·22 (0·19–0·25)	0·78 (0·73–0·85)
2014	0·0 (0·0–0·0)	4·3 (3·5–5·1)	38·2 (32·7–44·5)	98·9 (92·1–106·5)	99·7 (91·6–109·5)	49·9 (42·6–58·0)	8·7 (6·4–11·6)	0·2 (0·1–0·3)	0·0 (0·0–0·0)	1·5 (1·42–1·59)	0·21 (0·19–0·24)	0·79 (0·73–0·86)
2015	0·0 (0·0–0·0)	4·2 (3·5–5·2)	38·9 (29·9–50·4)	100·1 (92·0–109·2)	101·5 (91·2–112·7)	47·5 (38·9–56·9)	8·1 (5·8–11·0)	0·1 (0·1–0·3)	0·0 (0·0–0·0)	1·5 (1·32–1·7)	0·22 (0·17–0·28)	0·79 (0·69–0·89)
2016	0·0 (0·0–0·0)	4·3 (3·5–5·2)	39·9 (30·7–52·2)	100·2 (89·4–112·6)	100·3 (86·8–115·7)	43·1 (32·9–55·6)	7·1 (4·8–9·9)	0·1 (0·1–0·3)	0·0 (0·0–0·0)	1·47 (1·26–1·74)	0·22 (0·17–0·29)	0·75 (0·64–0·9)
2017	0·0 (0·0–0·0)	4·2 (3·5–5·1)	39·4 (29·1–52·7)	99·0 (87·7–111·4)	98·1 (83·6–114·2)	41·4 (30·6–54·5)	6·9 (4·4–10·4)	0·1 (0·1–0·3)	0·0 (0·0–0·0)	1·45 (1·21–1·72)	0·22 (0·16–0·29)	0·73 (0·6–0·89)

Supplemental Results Table 5: Age-specific fertility rates per 1000, cumulative total fertility up to maternal age 25 (TFU25), cumulative total fertility from maternal age 30 to age 55 (TFO30), and cumulative total fertility, Okayama, 1950-2017

Year	10-14	15-19	20-24	25-29	30-34	35-39	40-44	45-49	50-54	TFR	TFU25	TFO30
1950	0·0 (0·0–0·0)	11·7 (10·3–13·0)	165·8 (151·3–181·2)	229·9 (221·2–238·5)	163·1 (153·5–172·9)	104·5 (92·9–116·0)	34·4 (29·7–39·5)	1·8 (1·2–2·6)	0·0 (0·0–0·0)	3·56 (3·43–3·68)	0·89 (0·82–0·96)	1·52 (1·44–1·6)
1951	0·0 (0·0–0·0)	9·4 (8·3–10·6)	148·9 (134·9–163·9)	213·9 (205·6–222·1)	147·4 (136·4–158·2)	84·3 (73·5–95·4)	26·6 (22·3–31·2)	1·3 (0·8–2·0)	0·0 (0·0–0·0)	3·16 (3·01–3·3)	0·79 (0·72–0·87)	1·3 (1·17–1·41)
1952	0·0 (0·0–0·0)	7·9 (6·9–9·0)	138·7 (124·8–153·2)	203·5 (194·7–212·9)	133·3 (121·1–145·7)	67·6 (57·7–78·9)	21·1 (17·3–25·3)	1·0 (0·6–1·5)	0·0 (0·0–0·0)	2·87 (2·73–2·99)	0·73 (0·66–0·81)	1·12 (1·04–1·2)
1953	0·0 (0·0–0·0)	6·9 (5·9–8·0)	131·2 (117·7–146·2)	192·0 (183·2–201·6)	119·2 (105·4–132·6)	53·8 (44·8–63·9)	16·7 (13·6–20·3)	0·8 (0·5–1·3)	0·0 (0·0–0·0)	2·6 (2·48–2·73)	0·69 (0·62–0·77)	0·95 (0·88–1·03)
1954	0·0 (0·0–0·0)	6·4 (5·4–7·4)	127·3 (111·9–143·0)	186·9 (178·7–195·7)	109·8 (96·9–124·0)	44·8 (37·1–52·8)	13·8 (11·0–17·2)	0·7 (0·4–1·0)	0·0 (0·0–0·0)	2·45 (2·32–2·58)	0·67 (0·59–0·75)	0·85 (0·77–0·92)
1955	0·0 (0·0–0·0)	5·7 (4·8–6·7)	124·4 (110·0–141·3)	185·9 (176·6–194·6)	101·0 (87·5–115·1)	36·1 (30·2–43·2)	11·5 (8·9–14·4)	0·5 (0·3–0·9)	0·0 (0·0–0·0)	2·33 (2·21–2·44)	0·65 (0·58–0·74)	0·75 (0·68–0·81)
1956	0·0 (0·0–0·0)	5·1 (4·3–6·0)	117·3 (102·3–133·0)	179·9 (172·1–188·2)	89·4 (77·5–102·7)	28·8 (24·2–34·2)	9·2 (7·2–11·8)	0·4 (0·3–0·7)	0·0 (0·0–0·0)	2·15 (2·04–2·27)	0·61 (0·54–0·69)	0·64 (0·58–0·71)
1957	0·0 (0·0–0·0)	4·9 (4·1–5·8)	118·6 (103·8–135·3)	183·5 (175·5–192·0)	84·7 (73·5–97·7)	26·5 (22·0–30·7)	7·8 (6·1–9·9)	0·5 (0·3–0·7)	0·0 (0·0–0·0)	2·13 (2·01–2·27)	0·62 (0·54–0·7)	0·6 (0·54–0·66)
1958	0·0 (0·0–0·0)	4·9 (4·2–5·8)	124·7 (109·8–140·0)	189·5 (180·6–198·3)	82·7 (71·4–95·6)	25·2 (20·6–29·8)	7·1 (5·5–9·0)	0·4 (0·3–0·7)	0·0 (0·0–0·0)	2·17 (2·06–2·28)	0·65 (0·57–0·73)	0·58 (0·52–0·64)
1959	0·0 (0·0–0·0)	5·1 (4·3–6·0)	124·9 (110·5–140·6)	188·7 (180·1–197·6)	77·3 (67·8–87·9)	20·4 (16·8–24·4)	5·9 (4·5–7·7)	0·4 (0·2–0·6)	0·0 (0·0–0·0)	2·11 (2·01–2·22)	0·65 (0·58–0·73)	0·52 (0·47–0·57)
1960	0·0 (0·0–0·0)	5·0 (4·3–5·9)	120·4 (106·9–134·3)	189·7 (181·0–198·7)	74·1 (65·9–82·9)	16·7 (12·8–21·0)	4·8 (3·6–6·2)	0·3 (0·2–0·5)	0·0 (0·0–0·0)	2·06 (1·96–2·16)	0·63 (0·56–0·7)	0·48 (0·44–0·53)
1961	0·0 (0·0–0·0)	4·7 (3·9–5·5)	117·1 (105·4–129·3)	192·0 (184·0–200·9)	72·3 (63·5–82·5)	14·6 (12·2–17·4)	4·2 (3·1–5·6)	0·3 (0·2–0·4)	0·0 (0·0–0·0)	2·03 (1·93–2·12)	0·61 (0·55–0·67)	0·46 (0·41–0·51)
1962	0·0 (0·0–0·0)	4·5 (3·7–5·3)	117·1 (103·9–130·2)	197·8 (189·5–205·9)	73·7 (67·4–79·7)	13·7 (11·2–16·3)	3·7 (2·7–5·0)	0·2 (0·1–0·4)	0·0 (0·0–0·0)	2·05 (1·97–2·14)	0·61 (0·54–0·67)	0·46 (0·42–0·49)
1963	0·0 (0·0–0·0)	4·4 (3·7–5·3)	118·0 (105·4–131·4)	199·9 (191·4–208·5)	75·1 (68·8–82·1)	15·0 (12·6–17·7)	3·7 (2·7–5·0)	0·3 (0·2–0·4)	0·0 (0·0–0·0)	2·08 (2·0–2·17)	0·61 (0·55–0·68)	0·47 (0·43–0·51)
1964	0·0 (0·0–0·0)	4·4 (3·7–5·2)	129·2 (116·2–144·4)	213·1 (204·0–222·7)	80·0 (72·6–88·3)	15·4 (13·1–17·9)	3·5 (2·5–4·6)	0·2 (0·1–0·4)	0·0 (0·0–0·0)	2·23 (2·14–2·32)	0·67 (0·6–0·74)	0·5 (0·45–0·54)
1965	0·0 (0·0–0·0)	4·3 (3·5–5·1)	119·5 (106·7–133·8)	180·2 (170·7–190·0)	66·3 (58·3–74·4)	12·3 (10·1–14·9)	3·0 (2·2–4·0)	0·2 (0·1–0·3)	0·0 (0·0–0·0)	1·93 (1·83–2·03)	0·62 (0·55–0·69)	0·41 (0·37–0·45)
1966	0·0 (0·0–0·0)	4·5 (3·9–5·3)	117·2 (103·6–130·5)	185·6 (176·1–195·8)	67·7 (58·8–77·4)	13·4 (11·3–16·1)	3·0 (2·2–3·9)	0·2 (0·1–0·3)	0·0 (0·0–0·0)	1·96 (1·87–2·05)	0·61 (0·54–0·68)	0·42 (0·38–0·47)
1967	0·0 (0·0–0·0)	4·8 (4·1–5·6)	125·5 (113·0–138·9)	226·4 (216·5–235·5)	81·2 (69·8–94·3)	15·1 (12·3–18·3)	2·9 (2·1–3·7)	0·2 (0·1–0·3)	0·0 (0·0–0·0)	2·28 (2·16–2·4)	0·65 (0·59–0·72)	0·5 (0·44–0·56)
1968	0·0 (0·0–0·0)	4·9 (4·2–5·7)	112·9 (100·5–126·4)	213·9 (204·9–222·7)	73·7 (62·6–84·9)	13·7 (11·0–16·6)	2·6 (1·9–3·4)	0·2 (0·1–0·2)	0·0 (0·0–0·0)	2·11 (2·02–2·2)	0·59 (0·53–0·65)	0·45 (0·39–0·51)
1969	0·0 (0·0–0·0)	5·3 (4·5–6·2)	111·5 (97·9–126·1)	216·2 (207·7–224·7)	73·6 (62·2–86·2)	13·4 (10·8–16·3)	2·6 (1·9–3·4)	0·1 (0·1–0·2)	0·0 (0·0–0·0)	2·11 (2·02–2·21)	0·58 (0·52–0·66)	0·45 (0·39–0·51)
1970	0·0 (0·0–0·0)	5·4 (4·5–6·4)	120·4 (107·8–134·0)	219·6 (210·2–228·5)	75·3 (64·8–87·2)	15·0 (11·8–18·4)	2·8 (2·0–3·7)	0·2 (0·1–0·3)	0·0 (0·0–0·0)	2·19 (2·1–2·29)	0·63 (0·57–0·7)	0·47 (0·42–0·52)
1971	0·0 (0·0–0·0)	5·2 (4·3–6·3)	132·2 (115·9–148·8)	216·4 (207·1–226·5)	76·1 (65·4–88·7)	16·9 (13·7–20·7)	3·0 (2·1–4·1)	0·2 (0·1–0·3)	0·0 (0·0–0·0)	2·25 (2·15–2·36)	0·69 (0·61–0·77)	0·48 (0·43–0·54)
1972	0·0 (0·0–0·0)	5·3 (4·3–6·4)	138·6 (122·8–155·8)	214·1 (204·3–223·5)	75·6 (66·7–85·0)	17·1 (13·5–20·8)	3·0 (2·1–4·1)	0·2 (0·1–0·3)	0·0 (0·0–0·0)	2·27 (2·16–2·38)	0·72 (0·64–0·81)	0·48 (0·44–0·52)
1973	0·0 (0·0–0·0)	5·1 (4·2–6·1)	138·7 (124·2–154·4)	213·1 (203·7–223·6)	71·2 (63·8–79·5)	15·3 (12·3–18·9)	2·7 (1·8–3·9)	0·2 (0·1–0·3)	0·0 (0·0–0·0)	2·23 (2·13–2·33)	0·72 (0·65–0·8)	0·45 (0·41–0·49)
1974	0·0 (0·0–0·0)	4·7 (3·8–5·7)	131·9 (118·9–146·9)	203·1 (193·2–212·9)	62·4 (56·1–68·8)	11·6 (9·2–14·7)	2·2 (1·5–3·4)	0·1 (0·1–0·2)	0·0 (0·0–0·0)	2·08 (2·0–2·18)	0·68 (0·62–0·76)	0·38 (0·35–0·42)
1975	0·0 (0·0–0·0)	4·3 (3·5–5·2)	121·6 (108·6–135·1)	193·8 (184·8–202·7)	55·7 (50·2–61·4)	9·5 (7·5–12·0)	1·8 (1·1–2·9)	0·1 (0·1–0·1)	0·0 (0·0–0·0)	1·93 (1·85–2·02)	0·63 (0·56–0·7)	0·34 (0·31–0·36)
1976	0·0 (0·0–0·0)	4·0 (3·2–4·9)	113·0 (100·3–125·6)	189·5 (181·2–198·2)	54·0 (48·8–59·4)	9·4 (7·6–11·4)	1·6 (1·0–2·3)	0·1 (0·0–0·1)	0·0 (0·0–0·0)	1·86 (1·77–1·95)	0·58 (0·52–0·65)	0·32 (0·29–0·36)
1977	0·0 (0·0–0·0)	3·9 (3·0–4·9)	105·2 (94·1–119·1)	189·4 (181·0–197·5)	55·4 (50·0–61·4)	9·5 (7·6–11·9)	1·4 (0·9–1·9)	0·1 (0·0–0·1)	0·0 (0·0–0·0)	1·82 (1·74–1·91)	0·55 (0·49–0·61)	0·33 (0·3–0·37)
1978	0·0 (0·0–0·0)	3·9 (3·1–4·8)	99·6 (89·9–110·3)	190·4 (182·3–198·5)	58·0 (52·4–64·2)	9·6 (7·1–12·5)	1·2 (0·8–1·7)	0·0 (0·0–0·1)	0·0 (0·0–0·0)	1·81 (1·74–1·89)	0·52 (0·47–0·57)	0·34 (0·31–0·38)
1979	0·0 (0·0–0·0)	4·0 (3·4–4·6)	95·2 (84·6–106·9)	190·0 (182·0–198·0)	59·1 (53·7–64·9)	9·4 (7·8–11·4)	1·1 (0·7–1·8)	0·0 (0·0–0·1)	0·0 (0·0–0·0)	1·79 (1·72–1·88)	0·5 (0·44–0·56)	0·35 (0·32–0·38)
1980	0·0 (0·0–0·0)	4·1 (3·4–4·9)	91·2 (81·5–101·0)	189·6 (180·8–198·8)	59·7 (53·8–65·9)	9·5 (7·7–11·7)	1·2 (0·8–1·6)	0·0 (0·0–0·1)	0·0 (0·0–0·0)	1·78 (1·7–1·85)	0·48 (0·43–0·53)	0·35 (0·32–0·38)
1981	0·0 (0·0–0·0)	4·4 (3·6–5·3)	89·3 (78·2–102·4)	193·0 (184·2–201·9)	61·6 (56·1–67·4)	10·1 (8·0–12·5)	1·2 (0·8–1·7)	0·0 (0·0–0·1)	0·0 (0·0–0·0)	1·8 (1·72–1·88)	0·47 (0·41–0·53)	0·36 (0·33–0·39)
1982	0·0 (0·0–0·0)	4·6 (3·8–5·6)	88·3 (78·2–99·3)	197·0 (189·1–206·0)	65·5 (59·5–72·0)	11·3 (9·2–13·7)	1·2 (0·8–1·7)	0·0 (0·0–0·1)	0·0 (0·0–0·0)	1·84 (1·77–1·92)	0·46 (0·41–0·52)	0·39 (0·36–0·43)
1983	0·0 (0·0–0·0)	4·8 (4·0–5·9)	86·4 (77·2–96·3)	199·7 (189·9–208·7)	69·7 (63·5–77·4)	12·6 (10·0–15·6)	1·3 (0·9–1·6)	0·0 (0·0–0·1)	0·0 (0·0–0·0)	1·87 (1·8–1·94)	0·46 (0·41–0·51)	0·42 (0·38–0·46)
1984	0·0 (0·0–0·0)	4·7 (4·0–5·6)	80·7 (69·3–93·6)	198·5 (189·4–208·2)	72·8 (66·1–79·9)	13·1 (10·4–16·2)	1·3 (0·9–1·7)	0·0 (0·0–0·1)	0·0 (0·0–0·0)	1·86 (1·78–1·93)	0·43 (0·37–0·49)	0·44 (0·4–0·47)
1985	0·0 (0·0–0·0)	4·6 (3·8–5·4)	76·1 (67·1–85·8)	192·9 (184·4–201·2)	74·5 (67·0–81·4)	13·1 (10·5–16·2)	1·3 (1·0–1·8)	0·0 (0·0–0·1)	0·0 (0·0–0·0)	1·81 (1·75–1·88)	0·4 (0·36–0·45)	0·44 (0·41–0·48)
1986	0·0 (0·0–0·0)	4·4 (3·7–5·2)	72·7 (64·0–82·7)	187·6 (178·5–197·4)	77·0 (70·5–83·2)	13·2 (10·6–16·3)	1·4 (1·0–2·0)	0·0 (0·0–0·1)	0·0 (0·0–0·0)	1·78 (1·72–1·86)	0·39 (0·34–0·43)	0·46 (0·42–0·49)
1987	0·0 (0·0–0·0)	4·3 (3·7–5·1)	67·1 (59·7–75·3)	182·5 (173·1–192·1)	80·1 (73·2–87·9)	13·9 (11·0–17·0)	1·6 (1·1–2·3)	0·0 (0·0–0·1)	0·0 (0·0–0·0)	1·75 (1·67–1·82)	0·36 (0·32–0·4)	0·48 (0·44–0·52)
1988	0·0 (0·0–0·0)	4·2 (3·5–5·0)	62·2 (54·2–71·5)	171·9 (162·8–181·6)	81·9 (75·0–90·0)	14·8 (11·9–18·1)	1·8 (1·3–2·4)	0·1 (0·0–0·1)	0·0 (0·0–0·0)	1·68 (1·61–1·76)	0·33 (0·29–0·38)	0·49 (0·45–0·54)
1989	0·0 (0·0–0·0)	4·2 (3·5–5·0)	57·5 (50·1–65·6)	160·3 (152·4–168·9)	82·8 (75·5–90·1)	15·8 (12·6–19·6)	1·9 (1·4–2·6)	0·1 (0·0–0·1)	0·0 (0·0–0·0)	1·61 (1·55–1·68)	0·31 (0·27–0·35)	0·5 (0·47–0·54)
1990	0·0 (0·0–0·0)	4·3 (3·6–5·2)	55·3 (48·1–62·8)	155·7 (147·0–163·8)	85·1 (77·7–92·5)	16·7 (13·5–20·1)	1·9 (1·4–2·6)	0·1 (0·0–0·1)	0·0 (0·0–0·0)	1·6 (1·52–1·67)	0·3 (0·26–0·34)	0·52 (0·48–0·56)
1991	0·0 (0·0–0·0)	4·5 (3·7–5·4)	54·8 (47·7–62·3)	150·6 (143·1–159·0)	86·6 (78·9–94·8)	17·8 (14·6–21·7)	2·0 (1·4–2·6)	0·1 (0·0–0·1)	0·0 (0·0–0·0)	1·58 (1·51–1·65)	0·3 (0·26–0·33)	0·53 (0·49–0·58)
1992	0·0 (0·0–0·0)	4·5 (3·7–5·6)	52·8 (46·8–59·5)	141·8 (133·6–150·3)	86·4 (79·2–94·4)	18·6 (15·4–22·7)	2·1 (1·5–2·8)	0·1 (0·0–0·1)	0·0 (0·0–0·0)	1·53 (1·47–1·59)	0·29 (0·26–0·32)	0·54 (0·5–0·58)
1993	0·0 (0·0–0·0)	4·6 (3·8–5·6)	52·3 (45·4–60·3)	141·3 (132·8–150·3)	90·8 (83·4–99·2)	20·3 (16·6–24·3)	2·2 (1·6–3·0)	0·1 (0·0–0·1)	0·0 (0·0–0·0)	1·56 (1·49–1·64)	0·28 (0·25–0·32)	0·57 (0·52–0·61)
1994	0·0 (0·0–0·0)	4·6 (3·8–5·5)	51·6 (45·5–58·8)	138·8 (130·8–147·0)	93·3 (85·0–102·1)	21·6 (17·8–26·3)	2·3 (1·7–3·1)	0·1 (0·0–0·1)	0·0 (0·0–0·0)	1·56 (1·49–1·64)	0·28 (0·25–0·32)	0·59 (0·54–0·64)
1995	0·0 (0·0–0·0)	4·7 (3·9–5·6)	50·4 (43·6–57·8)	131·3 (123·2–139·4)	93·6 (85·1–101·8)	22·6 (18·8–27·0)	2·5 (1·8–3·3)	0·1 (0·0–0·1)	0·0 (0·0–0·0)	1·53 (1·46–1·6)	0·28 (0·24–0·31)	0·59 (0·55–0·64)
1996	0·0 (0·0–0·0)	4·9 (4·1–5·8)	50·0 (43·1–57·5)	127·3 (119·5–135·0)	94·7 (87·0–103·2)	24·0 (19·9–29·2)	2·6 (1·9–3·4)	0·1 (0·0–0·1)	0·0 (0·0–0·0)	1·52 (1·44–1·59)	0·27 (0·24–0·31)	0·61 (0·56–0·66)
1997	0·0 (0·0–0·0)	5·1 (4·2–6·1)	49·8 (42·9–57·0)	123·8 (116·5–130·8)	94·5 (86·5–102·6)	24·9 (20·7–29·8)	2·7 (2·0–3·7)	0·1 (0·0–0·1)	0·0 (0·0–0·0)	1·5 (1·44–1·58)	0·27 (0·24–0·31)	0·61 (0·57–0·66)
1998	0·0 (0·0–0·0)	5·4 (4·4–6·5)	49·4 (42·9–57·2)	119·7 (112·7–127·5)	94·8 (86·2–104·2)	26·3 (21·7–31·5)	2·9 (2·2–3·8)	0·1 (0·0–0·1)	0·0 (0·0–0·0)	1·49 (1·43–1·56)	0·27 (0·24–0·31)	0·62 (0·58–0·67)
1999	0·0 (0·0–0·0)	5·7 (4·7–6·8)	48·9 (42·7–55·8)	116·6 (109·6–123·4)	95·2 (87·3–104·4)	27·5 (22·9–32·5)	3·1 (2·2–4·1)	0·1 (0·0–0·1)	0·0 (0·0–0·0)	1·49 (1·42–1·55)	0·27 (0·24–0·31)	0·63 (0·58–0·68)
2000	0·0 (0·0–0·0)	6·0 (5·0–7·3)	48·8 (41·1–56·7)	114·4 (106·8–122·7)	94·4 (86·8–102·9)	29·3 (24·3–34·8)	3·3 (2·4–4·4)	0·1 (0·1–0·1)	0·0 (0·0–0·0)	1·48 (1·4–1·57)	0·27 (0·23–0·32)	0·64 (0·59–0·69)
2001	0·0 (0·0–0·0)	6·3 (5·2–7·5)	48·5 (43·0–54·7)	111·1 (104·6–118·6)	92·1 (83·7–100·9)	29·8 (24·8–35·8)	3·5 (2·5–4·7)	0·1 (0·1–0·1)	0·0 (0·0–0·0)	1·46 (1·39–1·53)	0·27 (0·25–0·31)	0·63 (0·58–0·68)
2002	0·0 (0·0–0·0)	6·2 (5·2–7·4)	46·8 (40·8–53·7)	107·9 (101·0–115·0)	92·2 (84·2–100·6)	30·8 (25·7–36·4)	3·8 (2·7–5·3)	0·1 (0·1–0·1)	0·0 (0·0–0·0)	1·44 (1·37–1·51)	0·27 (0·23–0·3)	0·63 (0·59–0·68)
2003	0·0 (0·0–0·0)	6·0 (4·9–7·2)	44·7 (39·3–50·4)	104·8 (97·8–112·0)	91·9 (84·2–100·5)	32·5 (26·9–38·4)	4·1 (2·9–5·5)	0·1 (0·1–0·2)	0·0 (0·0–0·0)	1·42 (1·35–1·49)	0·25 (0·23–0·28)	0·64 (0·59–0·7)
2004	0·0 (0·0–0·0)	5·7 (4·8–6·7)	43·0 (37·1–49·9)	100·1 (93·9–106·4)	90·7 (83·0–98·5)	33·6 (28·2–39·5)	4·3 (3·1–5·7)	0·1 (0·1–0·2)	0·0 (0·0–0·0)	1·39 (1·33–1·45)	0·24 (0·21–0·28)	0·64 (0·6–0·69)
2005	0·0 (0·0–0·0)	5·6 (4·7–6·6)	42·6 (37·3–48·7)	98·3 (91·4–105·4)	91·3 (82·7–99·6)	34·7 (29·2–40·9)	4·6 (3·3–6·1)	0·1 (0·1–0·2)	0·0 (0·0–0·0)	1·39 (1·32–1·45)	0·24 (0·21–0·27)	0·65 (0·6–0·71)
2006	0·0 (0·0–0·0)	5·5 (4·7–6·6)	43·0 (37·9–48·6)	98·9 (92·7–106·3)	94·3 (86·3–102·7)	36·7 (31·0–43·5)	5·0 (3·7–6·7)	0·1 (0·1–0·2)	0·0 (0·0–0·0)	1·42 (1·35–1·49)	0·24 (0·22–0·27)	0·68 (0·63–0·74)
2007	0·0 (0·0–0·0)	5·5 (4·6–6·5)	43·0 (35·9–51·3)	98·0 (92·0–105·0)	94·6 (86·1–103·8)	38·9 (33·0–45·5)	5·5 (4·1–7·1)	0·1 (0·1–0·2)	0·0 (0·0–0·0)	1·43 (1·35–1·5)	0·24 (0·21–0·29)	0·7 (0·64–0·75)
2008	0·0 (0·0–0·0)	5·4 (4·6–6·4)	42·2 (36·8–48·1)	97·2 (90·8–104·0)	95·3 (87·5–103·5)	40·6 (34·8–47·0)	6·0 (4·6–7·7)	0·1 (0·1–0·2)	0·0 (0·0–0·0)	1·43 (1·37–1·5)	0·24 (0·21–0·27)	0·71 (0·66–0·76)
2009	0·0 (0·0–0·0)	5·1 (4·3–6·1)	40·9 (35·1–47·3)	96·9 (90·8–102·6)	96·3 (87·9–105·7)	42·4 (36·0–49·3)	6·6 (5·0–8·5)	0·1 (0·1–0·2)	0·0 (0·0–0·0)	1·44 (1·36–1·53)	0·23 (0·2–0·26)	0·73 (0·67–0·79)
2010	0·0 (0·0–0·0)	4·9 (4·2–5·8)	39·6 (34·5–45·0)	97·6 (91·0–104·7)	97·8 (89·7–107·2)	44·0 (37·7–50·8)	7·0 (5·6–8·7)	0·2 (0·1–0·3)	0·0 (0·0–0·0)	1·46 (1·39–1·53)	0·22 (0·2–0·25)	0·74 (0·69–0·8)
2011	0·0 (0·0–0·0)	4·8 (4·0–5·7)	37·6 (32·9–42·9)	97·0 (90·4–103·9)	98·2 (89·3–107·6)	45·4 (38·8–52·5)	7·4 (5·7–9·5)	0·2 (0·1–0·3)	0·0 (0·0–0·0)	1·45 (1·39–1·52)	0·21 (0·19–0·24)	0·76 (0·7–0·81)
2012	0·0 (0·0–0·0)	4·7 (3·8–5·7)	35·8 (31·5–40·7)	96·4 (90·2–102·9)	99·6 (90·9–108·9)	47·7 (41·2–54·8)	8·0 (6·3–10·2)	0·2 (0·1–0·3)	0·0 (0·0–0·0)	1·46 (1·4–1·53)	0·2 (0·18–0·23)	0·78 (0·72–0·84)
2013	0·0 (0·0–0·0)	4·6 (3·9–5·5)	34·6 (28·9–40·9)	94·9 (88·5–101·5)	99·6 (91·2–108·4)	48·9 (42·4–56·1)	8·5 (6·5–10·7)	0·2 (0·1–0·4)	0·0 (0·0–0·0)	1·46 (1·39–1·53)	0·2 (0·17–0·23)	0·79 (0·73–0·84)
2014	0·0 (0·0–0·0)	4·5 (3·8–5·4)	33·4 (29·0–38·0)	94·4 (87·7–101·1)	100·2 (92·1–109·1)	50·5 (43·4–58·5)	8·9 (6·9–11·7)	0·2 (0·1–0·4)	0·0 (0·0–0·0)	1·46 (1·38–1·54)	0·19 (0·17–0·21)	0·8 (0·74–0·86)
2015	0·0 (0·0–0·0)	4·5 (3·8–5·5)	34·0 (28·3–40·6)	96·3 (88·8–104·9)	102·1 (91·7–113·0)	48·4 (40·0–58·5)	8·3 (6·0–11·1)	0·2 (0·1–0·4)	0·0 (0·0–0·0)	1·47 (1·32–1·64)	0·19 (0·16–0·23)	0·79 (0·71–0·89)
2016	0·0 (0·0–0·0)	4·6 (3·8–5·7)	35·5 (27·0–46·3)	97·9 (87·5–110·6)	101·2 (87·9–117·1)	44·2 (34·0–56·8)	7·1 (4·9–9·8)	0·2 (0·1–0·3)	0·0 (0·0–0·0)	1·45 (1·24–1·71)	0·2 (0·15–0·26)	0·76 (0·65–0·91)
2017	0·0 (0·0–0·0)	4·6 (3·8–5·5)	35·5 (26·4–46·6)	97·7 (86·7–109·8)	99·4 (84·7–115·4)	42·7 (31·5–56·3)	6·9 (4·5–10·4)	0·2 (0·1–0·3)	0·0 (0·0–0·0)	1·44 (1·21–1·7)	0·2 (0·15–0·26)	0·75 (0·61–0·91)

Supplemental Results Table 5: Age-specific fertility rates per 1000, cumulative total fertility up to maternal age 25 (TFU25), cumulative total fertility from maternal age 30 to age 55 (TFO30), and cumulative total fertility, Okinawa, 1950-2017

Year	10-14	15-19	20-24	25-29	30-34	35-39	40-44	45-49	50-54	TFR	TFU25	TFO30
1950	0·0 (0·0–0·0)	30·3 (26·8–33·9)	176·1 (160·7–192·5)	199·1 (191·9–206·1)	256·1 (239·0–272·3)	407·9 (354·8–461·2)	175·2 (150·6–201·8)	6·6 (4·4–9·6)	0·1 (0·1–0·1)	6·26 (5·89–6·64)	1·03 (0·95–1·12)	4·23 (3·89–4·59)
1951	0·0 (0·0–0·0)	24·5 (21·5–27·8)	158·4 (143·1–174·4)	184·3 (176·1–192·3)	234·0 (216·3–251·8)	342·7 (296·7–386·7)	138·4 (115·4–163·0)	4·9 (3·1–7·2)	0·1 (0·1–0·1)	5·44 (5·02–5·86)	0·91 (0·84–1·0)	3·6 (3·2–4·0)
1952	0·0 (0·0–0·0)	20·7 (17·8–23·8)	147·7 (133·6–163·4)	174·8 (165·9–184·0)	213·4 (194·1–232·6)	281·9 (238·3–329·3)	110·9 (90·7–132·7)	3·8 (2·4–5·7)	0·1 (0·1–0·1)	4·77 (4·46–5·08)	0·84 (0·77–0·93)	3·05 (2·79–3·35)
1953	0·0 (0·0–0·0)	18·2 (15·4–21·4)	139·9 (124·4–157·0)	164·7 (154·0–175·4)	192·1 (172·7–213·9)	228·5 (188·1–274·6)	88·8 (71·0–112·2)	3·1 (1·9–4·7)	0·1 (0·1–0·1)	4·18 (3·86–4·5)	0·79 (0·71–0·88)	2·56 (2·28–2·86)
1954	0·0 (0·0–0·0)	16·8 (13·9–19·9)	135·8 (120·3–151·0)	160·3 (148·0–173·4)	178·0 (157·6–199·8)	192·7 (154·2–236·9)	73·7 (55·4–95·5)	2·6 (1·6–3·9)	0·0 (0·0–0·1)	3·8 (3·47–4·16)	0·76 (0·68–0·84)	2·24 (1·95–2·56)
1955	0·0 (0·0–0·0)	15·2 (12·2–18·6)	132·8 (117·2–149·8)	159·3 (146·7–171·9)	164·9 (144·1–185·6)	154·0 (120·1–194·3)	61·8 (44·7–82·1)	2·0 (1·2–3·1)	0·0 (0·0–0·0)	3·45 (3·13–3·77)	0·74 (0·65–0·84)	1·91 (1·63–2·2)
1956	0·0 (0·0–0·0)	13·6 (11·0–16·9)	125·3 (110·6–140·2)	154·2 (142·6–167·6)	147·0 (128·0–167·6)	123·5 (93·5–159·6)	49·8 (35·7–68·3)	1·7 (1·0–2·7)	0·0 (0·0–0·0)	3·08 (2·8–3·39)	0·69 (0·62–0·77)	1·61 (1·38–1·9)
1957	0·0 (0·0–0·0)	13·0 (10·6–16·2)	126·9 (112·0–143·5)	158·0 (144·4–171·8)	139·6 (121·7–159·2)	116·3 (84·7–149·9)	42·4 (29·9–55·9)	1·7 (1·0–2·8)	0·0 (0·0–0·0)	2·99 (2·7–3·3)	0·7 (0·62–0·78)	1·5 (1·27–1·74)
1958	0·0 (0·0–0·0)	13·2 (10·6–16·3)	133·6 (118·7–150·7)	163·9 (150·4–178·5)	136·9 (119·5–156·4)	111·1 (85·1–142·4)	38·4 (27·3–52·7)	1·7 (0·9–2·8)	0·0 (0·0–0·0)	2·99 (2·75–3·26)	0·73 (0·66–0·82)	1·44 (1·24–1·66)
1959	0·0 (0·0–0·0)	13·9 (11·0–17·1)	133·9 (119·4–149·8)	163·5 (152·0–175·5)	129·2 (113·8–146·4)	91·0 (70·3–115·8)	32·1 (22·5–44·4)	1·5 (0·8–2·4)	0·0 (0·0–0·0)	2·83 (2·62–3·05)	0·74 (0·66–0·82)	1·27 (1·1–1·45)
1960	0·0 (0·0–0·0)	13·6 (11·1–16·7)	129·2 (114·2–144·2)	164·6 (153·3–176·0)	125·3 (112·4–139·6)	73·1 (57·1–90·8)	26·5 (18·6–35·1)	1·2 (0·7–2·1)	0·0 (0·0–0·0)	2·67 (2·5–2·85)	0·71 (0·64–0·79)	1·13 (1·0–1·27)
1961	0·0 (0·0–0·0)	12·7 (10·2–15·5)	125·9 (113·3–139·5)	167·0 (158·2–176·5)	123·8 (109·7–139·9)	63·2 (50·8–76·7)	22·9 (16·3–31·4)	1·0 (0·6–1·6)	0·0 (0·0–0·0)	2·58 (2·44–2·73)	0·69 (0·63–0·76)	1·05 (0·95–1·17)
1962	0·0 (0·0–0·0)	12·2 (10·0–14·8)	126·0 (111·9–140·2)	172·7 (164·9–180·4)	127·5 (115·4–139·8)	60·5 (50·4–72·3)	20·3 (15·0–27·3)	0·9 (0·5–1·5)	0·0 (0·0–0·0)	2·6 (2·47–2·73)	0·69 (0·62–0·76)	1·05 (0·96–1·14)
1963	0·0 (0·0–0·0)	12·1 (10·0–14·4)	126·9 (113·5–141·8)	175·8 (168·8–183·0)	130·9 (119·5–143·3)	67·7 (57·0–79·5)	20·0 (14·7–26·7)	1·0 (0·6–1·6)	0·0 (0·0–0·0)	2·67 (2·55–2·79)	0·69 (0·63–0·77)	1·1 (1·01–1·19)
1964	0·0 (0·0–0·0)	12·0 (10·3–14·1)	138·9 (125·1–155·4)	188·3 (178·7–197·6)	141·3 (128·0–155·6)	71·5 (59·0–85·2)	18·9 (13·8–24·7)	1·0 (0·5–1·6)	0·0 (0·0–0·0)	2·86 (2·72–3·01)	0·75 (0·68–0·84)	1·16 (1·06–1·27)
1965	0·0 (0·0–0·0)	11·8 (10·0–13·9)	128·3 (114·3–144·1)	159·7 (148·9–170·7)	119·5 (106·3–133·7)	56·8 (47·8–67·4)	16·3 (12·1–21·2)	0·7 (0·4–1·3)	0·0 (0·0–0·0)	2·47 (2·33–2·6)	0·7 (0·63–0·78)	0·97 (0·88–1·06)
1966	0·0 (0·0–0·0)	12·6 (10·6–14·8)	125·7 (111·9–139·7)	165·6 (154·1–178·4)	124·0 (108·8–139·4)	62·5 (49·7–77·7)	16·1 (11·9–21·6)	0·8 (0·5–1·2)	0·0 (0·0–0·0)	2·54 (2·38–2·71)	0·69 (0·62–0·76)	1·02 (0·9–1·14)
1967	0·0 (0·0–0·0)	13·4 (11·0–16·0)	134·4 (121·0–148·7)	203·4 (187·0–220·2)	152·3 (132·7–173·2)	67·3 (52·8–85·6)	15·7 (11·2–21·1)	0·7 (0·4–1·1)	0·0 (0·0–0·0)	2·94 (2·7–3·19)	0·74 (0·67–0·81)	1·18 (1·02–1·36)
1968	0·0 (0·0–0·0)	13·8 (11·2–16·5)	120·7 (107·6–134·9)	193·3 (176·6–210·6)	142·3 (123·0–161·8)	56·6 (43·8–72·5)	14·2 (9·8–19·3)	0·6 (0·4–0·9)	0·0 (0·0–0·0)	2·71 (2·5–2·92)	0·67 (0·61–0·74)	1·07 (0·93–1·22)
1969	0·0 (0·0–0·0)	15·2 (12·3–18·2)	119·2 (104·7–134·0)	197·5 (182·1–214·5)	145·1 (126·0–166·7)	61·7 (46·5–79·7)	14·2 (10·0–19·9)	0·6 (0·3–0·9)	0·0 (0·0–0·0)	2·77 (2·56–3·0)	0·67 (0·59–0·75)	1·11 (0·95–1·28)
1970	0·0 (0·0–0·0)	15·6 (12·4–19·1)	128·7 (114·5–143·5)	203·4 (188·0–219·7)	151·0 (133·2–171·6)	76·5 (58·6–97·7)	15·3 (10·8–21·5)	0·7 (0·4–1·0)	0·0 (0·0–0·0)	2·96 (2·74–3·19)	0·72 (0·65–0·8)	1·22 (1·06–1·4)
1971	0·0 (0·0–0·0)	15·5 (12·2–18·9)	141·7 (124·9–158·7)	203·2 (190·9–216·7)	155·0 (136·6–174·7)	86·9 (66·4–111·4)	16·5 (11·3–22·5)	0·8 (0·5–1·2)	0·0 (0·0–0·0)	3·1 (2·88–3·33)	0·79 (0·7–0·87)	1·3 (1·13–1·49)
1972	0·0 (0·0–0·0)	15·9 (12·7–19·6)	149·2 (133·4–166·4)	203·7 (192·5–215·5)	157·7 (140·6–175·3)	90·2 (71·0–114·3)	16·6 (11·6–23·1)	0·8 (0·4–1·2)	0·0 (0·0–0·0)	3·17 (2·98–3·37)	0·83 (0·74–0·91)	1·33 (1·17–1·5)
1973	0·0 (0·0–0·0)	15·8 (12·7–19·7)	150·6 (134·6–167·2)	205·6 (194·8–216·0)	153·3 (139·7–168·6)	85·9 (67·3–107·9)	15·5 (10·7–21·2)	0·7 (0·4–1·1)	0·0 (0·0–0·0)	3·14 (2·96–3·31)	0·83 (0·75–0·92)	1·28 (1·15–1·41)
1974	0·0 (0·0–0·0)	15·1 (12·2–18·5)	145·1 (130·0–160·7)	198·7 (188·6–208·6)	138·5 (125·2–151·9)	71·0 (56·5–89·3)	13·4 (9·9–18·4)	0·6 (0·3–1·0)	0·0 (0·0–0·0)	2·91 (2·78–3·05)	0·8 (0·72–0·88)	1·12 (1·01–1·23)
1975	0·0 (0·0–0·0)	14·1 (11·3–17·4)	136·9 (122·2–152·6)	190·9 (181·5–200·3)	124·6 (112·2–136·7)	58·9 (48·2–71·7)	11·5 (8·7–14·7)	0·4 (0·2–0·7)	0·0 (0·0–0·0)	2·69 (2·56–2·81)	0·75 (0·68–0·83)	0·98 (0·89–1·08)
1976	0·0 (0·0–0·0)	13·2 (10·4–16·3)	131·0 (116·1–145·8)	185·8 (176·5–194·8)	118·7 (108·0–129·8)	52·1 (42·5–62·1)	10·4 (7·5–14·4)	0·4 (0·2–0·6)	0·0 (0·0–0·0)	2·56 (2·45–2·68)	0·72 (0·65–0·8)	0·91 (0·84–0·98)
1977	0·0 (0·0–0·0)	12·5 (10·5–14·7)	125·5 (112·3–142·0)	182·6 (174·0–191·3)	118·1 (106·8–130·0)	46·5 (36·7–57·3)	9·7 (6·5–13·7)	0·3 (0·2–0·5)	0·0 (0·0–0·0)	2·48 (2·35–2·61)	0·69 (0·62–0·77)	0·87 (0·77–0·98)
1978	0·0 (0·0–0·0)	12·1 (10·1–14·7)	121·5 (109·6–134·4)	178·9 (170·8–187·2)	119·1 (109·1–130·1)	41·9 (36·9–47·3)	8·8 (5·9–12·6)	0·3 (0·2–0·5)	0·0 (0·0–0·0)	2·41 (2·31–2·51)	0·67 (0·61–0·73)	0·85 (0·79–0·92)
1979	0·0 (0·0–0·0)	11·8 (9·9–14·0)	117·5 (104·4–131·9)	173·2 (165·2–181·2)	115·0 (102·4–129·6)	39·2 (31·1–48·0)	8·1 (5·4–11·8)	0·2 (0·1–0·4)	0·0 (0·0–0·0)	2·32 (2·21–2·45)	0·65 (0·58–0·72)	0·81 (0·73–0·91)
1980	0·0 (0·0–0·0)	11·5 (9·3–14·0)	113·1 (101·0–124·9)	167·8 (160·0–176·3)	109·4 (100·1–119·5)	37·7 (30·7–45·0)	7·5 (5·0–10·5)	0·2 (0·1–0·3)	0·0 (0·0–0·0)	2·24 (2·14–2·34)	0·62 (0·56–0·68)	0·77 (0·71–0·85)
1981	0·0 (0·0–0·0)	11·6 (9·3–14·3)	110·7 (97·0–126·5)	166·9 (159·0–174·7)	108·8 (98·7–119·1)	38·7 (31·2–46·6)	7·3 (5·3–9·5)	0·2 (0·1–0·3)	0·0 (0·0–0·0)	2·22 (2·12–2·31)	0·61 (0·54–0·69)	0·77 (0·71–0·84)
1982	0·0 (0·0–0·0)	11·7 (9·4–14·3)	109·4 (96·8–122·7)	168·2 (160·6–176·4)	112·4 (103·0–123·3)	42·1 (35·0–50·3)	7·2 (4·8–10·2)	0·2 (0·1–0·3)	0·0 (0·0–0·0)	2·26 (2·16–2·36)	0·61 (0·54–0·67)	0·81 (0·75–0·88)
1983	0·0 (0·0–0·0)	11·6 (9·3–14·4)	107·4 (94·7–121·2)	169·6 (161·4–177·7)	114·2 (104·1–125·1)	45·4 (36·8–54·4)	6·7 (4·5–9·5)	0·1 (0·1–0·2)	0·0 (0·0–0·0)	2·28 (2·17–2·39)	0·6 (0·53–0·66)	0·83 (0·76–0·9)
1984	0·0 (0·0–0·0)	11·2 (9·0–13·6)	100·9 (89·4–112·7)	168·9 (160·7–177·3)	112·6 (102·3–123·4)	46·1 (38·3–55·8)	5·8 (3·8–8·5)	0·1 (0·1–0·2)	0·0 (0·0–0·0)	2·23 (2·13–2·33)	0·56 (0·5–0·62)	0·82 (0·75–0·9)
1985	0·0 (0·0–0·0)	10·6 (8·4–13·1)	95·7 (83·5–108·3)	165·6 (158·2–173·5)	110·9 (101·5–120·7)	45·1 (37·8–53·8)	5·5 (3·1–9·0)	0·1 (0·1–0·2)	0·0 (0·0–0·0)	2·17 (2·08–2·26)	0·53 (0·47–0·59)	0·81 (0·75–0·87)
1986	0·0 (0·0–0·0)	10·1 (7·8–12·9)	91·7 (81·0–104·0)	163·1 (154·5–172·1)	112·4 (103·2–121·5)	44·5 (37·1–53·1)	5·7 (4·0–7·9)	0·1 (0·1–0·2)	0·0 (0·0–0·0)	2·14 (2·05–2·23)	0·51 (0·46–0·57)	0·81 (0·76–0·87)
1987	0·0 (0·0–0·0)	9·8 (7·8–11·9)	85·4 (75·9–95·7)	161·4 (153·4–169·7)	114·1 (105·0–124·5)	44·9 (37·2–53·9)	6·4 (4·4–9·1)	0·1 (0·1–0·2)	0·0 (0·0–0·0)	2·11 (2·0–2·22)	0·48 (0·43–0·53)	0·83 (0·76–0·9)
1988	0·0 (0·0–0·0)	9·5 (7·8–11·5)	80·2 (70·0–92·2)	155·7 (145·8–165·5)	113·8 (104·3–123·9)	45·2 (38·0–52·8)	6·9 (4·7–9·8)	0·1 (0·1–0·2)	0·0 (0·0–0·0)	2·06 (1·97–2·16)	0·45 (0·4–0·51)	0·83 (0·77–0·89)
1989	0·0 (0·0–0·0)	9·6 (7·9–11·7)	75·5 (65·9–86·0)	149·2 (141·2–157·1)	112·2 (102·8–121·7)	45·2 (37·5–53·6)	7·1 (4·8–10·0)	0·1 (0·1–0·2)	0·0 (0·0–0·0)	1·99 (1·9–2·09)	0·43 (0·38–0·48)	0·82 (0·76–0·89)
1990	0·0 (0·0–0·0)	9·9 (8·2–11·7)	74·0 (64·2–83·9)	148·9 (141·0–157·1)	113·0 (103·7–122·2)	44·9 (37·6–53·3)	6·9 (4·7–9·8)	0·1 (0·1–0·2)	0·0 (0·0–0·0)	1·99 (1·9–2·08)	0·42 (0·36–0·47)	0·82 (0·76–0·89)
1991	0·0 (0·0–0·0)	10·1 (8·4–12·2)	73·9 (64·2–84·3)	144·9 (134·3–156·4)	112·3 (103·4–121·6)	45·0 (38·0–53·2)	7·0 (4·6–10·0)	0·1 (0·1–0·2)	0·0 (0·0–0·0)	1·97 (1·86–2·08)	0·42 (0·37–0·47)	0·82 (0·76–0·89)
1992	0·0 (0·0–0·0)	9·7 (8·1–11·6)	69·1 (61·4–78·0)	128·3 (121·3–135·2)	109·1 (97·5–121·6)	44·9 (37·5–52·9)	7·9 (5·1–11·5)	0·1 (0·1–0·2)	0·0 (0·0–0·0)	1·85 (1·75–1·94)	0·39 (0·35–0·44)	0·81 (0·74–0·88)
1993	0·0 (0·0–0·0)	9·0 (7·4–10·9)	60·5 (52·7–69·2)	107·9 (100·6–115·0)	104·8 (96·4–113·7)	44·6 (38·0–52·7)	9·3 (6·3–13·6)	0·2 (0·1–0·3)	0·0 (0·0–0·0)	1·68 (1·6–1·76)	0·35 (0·31–0·39)	0·79 (0·74–0·85)
1994	0·0 (0·0–0·0)	9·1 (7·4–11·0)	54·2 (47·6–61·8)	96·3 (90·4–102·0)	97·4 (88·6–106·5)	41·5 (34·0–49·4)	9·6 (6·4–14·0)	0·2 (0·1–0·3)	0·0 (0·0–0·0)	1·54 (1·47–1·62)	0·32 (0·28–0·36)	0·74 (0·69–0·8)
1995	0·0 (0·0–0·0)	10·0 (8·1–12·2)	58·3 (51·0–66·4)	105·2 (98·5–112·6)	100·0 (91·2–109·2)	42·1 (34·6–50·3)	8·6 (5·9–12·6)	0·2 (0·1–0·2)	0·0 (0·0–0·0)	1·62 (1·54–1·71)	0·34 (0·31–0·38)	0·75 (0·69–0·82)
1996	0·0 (0·0–0·0)	11·1 (9·0–13·5)	66·4 (57·6–75·0)	119·6 (111·4–127·9)	107·9 (99·2–117·6)	46·0 (37·8–54·8)	7·8 (5·3–11·2)	0·1 (0·1–0·2)	0·0 (0·0–0·0)	1·8 (1·71–1·88)	0·39 (0·34–0·43)	0·81 (0·75–0·87)
1997	0·0 (0·0–0·0)	11·9 (9·8–14·3)	70·5 (61·5–80·4)	123·5 (115·5–131·0)	109·9 (100·9–119·3)	47·8 (39·8–56·2)	7·7 (5·2–11·1)	0·1 (0·1–0·2)	0·0 (0·0–0·0)	1·86 (1·78–1·95)	0·41 (0·37–0·46)	0·83 (0·77–0·9)
1998	0·0 (0·0–0·0)	12·6 (10·3–15·2)	72·1 (63·4–82·6)	121·6 (113·9–130·1)	110·6 (101·0–120·7)	49·9 (41·1–59·3)	7·8 (5·3–11·2)	0·1 (0·1–0·2)	0·0 (0·0–0·0)	1·87 (1·78–1·97)	0·42 (0·38–0·48)	0·84 (0·78–0·91)
1999	0·0 (0·0–0·0)	13·1 (10·9–15·8)	72·4 (62·4–83·1)	117·9 (110·3–125·6)	110·3 (101·1–120·5)	50·6 (42·3–59·2)	8·0 (5·4–11·5)	0·1 (0·1–0·2)	0·0 (0·0–0·0)	1·86 (1·76–1·97)	0·43 (0·38–0·49)	0·85 (0·77–0·92)
2000	0·0 (0·0–0·0)	13·7 (11·4–16·5)	72·4 (64·6–80·5)	114·9 (107·9–122·6)	108·4 (99·2–118·6)	52·3 (44·1–61·2)	8·4 (5·7–11·6)	0·1 (0·1–0·2)	0·0 (0·0–0·0)	1·85 (1·76–1·96)	0·43 (0·39–0·47)	0·85 (0·78–0·92)
2001	0·0 (0·0–0·0)	14·0 (11·5–16·9)	72·1 (62·4–82·4)	111·2 (104·0–118·5)	104·9 (95·1–114·8)	51·4 (43·6–60·4)	8·5 (6·1–11·8)	0·1 (0·1–0·2)	0·0 (0·0–0·0)	1·81 (1·7–1·92)	0·43 (0·38–0·48)	0·82 (0·75–0·9)
2002	0·0 (0·0–0·0)	13·5 (11·2–16·6)	69·7 (60·7–79·9)	108·3 (101·4–115·8)	103·9 (95·1–114·0)	51·5 (43·9–60·1)	9·0 (6·4–12·2)	0·2 (0·1–0·2)	0·0 (0·0–0·0)	1·78 (1·69–1·88)	0·42 (0·37–0·46)	0·82 (0·76–0·89)
2003	0·0 (0·0–0·0)	12·8 (10·2–15·5)	66·6 (57·6–76·7)	106·2 (99·8–113·2)	102·9 (94·2–112·2)	53·0 (44·5–62·3)	9·4 (6·7–12·7)	0·2 (0·1–0·3)	0·0 (0·0–0·0)	1·76 (1·66–1·86)	0·4 (0·35–0·45)	0·83 (0·75–0·9)
2004	0·0 (0·0–0·0)	12·0 (9·8–14·5)	63·9 (55·6–74·0)	102·9 (95·8–110·9)	101·2 (92·7–110·2)	53·7 (45·3–62·6)	9·8 (7·2–12·7)	0·2 (0·1–0·3)	0·0 (0·0–0·0)	1·72 (1·63–1·81)	0·38 (0·34–0·43)	0·82 (0·76–0·89)
2005	0·0 (0·0–0·0)	11·5 (9·4–14·1)	63·1 (55·4–71·3)	102·9 (95·5–110·5)	101·6 (92·6–111·1)	54·9 (46·7–63·9)	10·2 (7·6–13·6)	0·2 (0·1–0·3)	0·0 (0·0–0·0)	1·72 (1·65–1·81)	0·37 (0·33–0·42)	0·83 (0·77–0·91)
2006	0·0 (0·0–0·0)	11·3 (9·2–13·9)	63·8 (55·0–72·9)	105·0 (97·9–112·3)	105·0 (95·5–114·7)	57·6 (49·0–67·6)	11·1 (8·2–14·4)	0·2 (0·1–0·3)	0·0 (0·0–0·0)	1·77 (1·68–1·86)	0·38 (0·33–0·42)	0·87 (0·8–0·95)
2007	0·0 (0·0–0·0)	11·3 (9·1–13·8)	63·6 (56·9–71·6)	105·5 (98·3–113·4)	105·6 (96·8–116·2)	60·5 (51·8–70·2)	11·8 (8·9–15·6)	0·2 (0·1–0·3)	0·0 (0·0–0·0)	1·79 (1·71–1·87)	0·37 (0·34–0·41)	0·89 (0·82–0·96)
2008	0·0 (0·0–0·0)	11·2 (9·1–13·6)	62·9 (54·7–72·1)	105·9 (98·5–113·6)	106·7 (97·7–115·8)	62·6 (53·7–72·6)	12·7 (9·5–16·8)	0·2 (0·1–0·3)	0·0 (0·0–0·0)	1·81 (1·72–1·91)	0·37 (0·32–0·42)	0·91 (0·84–0·98)
2009	0·0 (0·0–0·0)	10·8 (8·8–13·3)	61·1 (54·6–67·6)	106·5 (99·9–113·2)	108·1 (99·0–117·8)	64·6 (55·4–74·8)	13·9 (10·4–18·2)	0·2 (0·1–0·4)	0·0 (0·0–0·0)	1·83 (1·74–1·92)	0·36 (0·33–0·39)	0·93 (0·87–1·0)
2010	0·0 (0·0–0·0)	10·6 (8·7–12·7)	59·8 (50·5–69·7)	108·1 (100·6–116·0)	110·0 (100·6–120·9)	66·1 (57·0–76·1)	14·4 (10·9–18·5)	0·2 (0·1–0·4)	0·0 (0·0–0·0)	1·85 (1·74–1·95)	0·35 (0·3–0·41)	0·95 (0·88–1·03)
2011	0·0 (0·0–0·0)	10·5 (8·5–12·7)	57·3 (49·8–65·7)	108·2 (100·4–116·2)	110·5 (100·5–120·6)	67·0 (57·7–77·8)	14·8 (11·4–19·0)	0·2 (0·1–0·4)	0·0 (0·0–0·0)	1·84 (1·76–1·92)	0·34 (0·3–0·39)	0·96 (0·9–1·04)
2012	0·0 (0·0–0·0)	10·4 (8·8–12·1)	55·2 (47·7–63·9)	108·0 (100·8–115·2)	112·4 (102·7–122·6)	69·1 (59·6–79·5)	15·7 (12·2–19·9)	0·3 (0·2–0·4)	0·0 (0·0–0·0)	1·86 (1·77–1·94)	0·33 (0·29–0·37)	0·99 (0·92–1·07)
2013	0·0 (0·0–0·0)	10·3 (8·5–12·4)	53·9 (46·8–61·5)	106·5 (99·4–114·3)	112·8 (103·6–122·0)	69·5 (60·5–79·4)	16·2 (12·8–20·7)	0·3 (0·2–0·4)	0·0 (0·0–0·0)	1·85 (1·76–1·93)	0·32 (0·28–0·36)	0·99 (0·92–1·07)
2014	0·0 (0·0–0·0)	10·1 (8·5–12·1)	52·6 (44·6–60·9)	105·3 (98·3–113·2)	114·4 (105·0–124·5)	70·9 (61·0–81·1)	16·9 (13·4–21·6)	0·3 (0·2–0·5)	0·0 (0·0–0·0)	1·85 (1·74–1·97)	0·31 (0·27–0·36)	1·01 (0·93–1·1)
2015	0·0 (0·0–0·0)	10·1 (8·5–12·2)	53·5 (45·9–62·7)	105·8 (97·7–115·1)	117·4 (105·8–130·3)	68·0 (56·4–81·4)	15·7 (11·6–21·0)	0·3 (0·2–0·4)	0·0 (0·0–0·0)	1·85 (1·66–2·08)	0·32 (0·27–0·38)	1·01 (0·9–1·14)
2016	0·0 (0·0–0·0)	10·2 (8·4–12·5)	55·2 (43·3–71·2)	105·4 (94·1–118·9)	116·5 (101·3–134·8)	62·4 (47·9–79·9)	13·9 (8·3–21·7)	0·3 (0·2–0·4)	0·0 (0·0–0·0)	1·82 (1·54–2·16)	0·33 (0·26–0·42)	0·97 (0·81–1·15)
2017	0·0 (0·0–0·0)	10·0 (8·3–12·1)	54·5 (42·7–69·5)	103·7 (92·0–116·5)	114·1 (97·3–132·4)	60·3 (45·0–79·4)	13·4 (8·8–19·7)	0·3 (0·2–0·4)	0·0 (0·0–0·0)	1·78 (1·48–2·13)	0·32 (0·25–0·41)	0·94 (0·76–1·15)

Supplemental Results Table 5: Age-specific fertility rates per 1000, cumulative total fertility up to maternal age 25 (TFU25), cumulative total fertility from maternal age 30 to age 55 (TFO30), and cumulative total fertility, Osaka, 1950-2017

Year	10-14	15-19	20-24	25-29	30-34	35-39	40-44	45-49	50-54	TFR	TFU25	TFO30
1950	0·0<br>(0·0–0·0)	13·5<br>(11·9–15·1)	134·3<br>(122·8–146·8)	213·0<br>(205·3–220·6)	148·1<br>(139·3–156·8)	65·2<br>(57·9–72·4)	24·6<br>(21·2–28·2)	1·1<br>(0·8–1·7)	0·0<br>(0·0–0·0)	3·0<br>(2·89–3·11)	0·74<br>(0·68–0·8)	1·19<br>(1·13–1·26)
1951	0·0<br>(0·0–0·0)	10·9<br>(9·6–12·3)	120·3<br>(109·1–132·5)	197·5<br>(190·0–204·7)	133·8<br>(124·9–142·5)	58·4<br>(50·7–66·0)	19·0<br>(15·9–22·6)	0·8<br>(0·5–1·2)	0·0<br>(0·0–0·0)	2·7<br>(2·59–2·82)	0·66<br>(0·6–0·72)	1·06<br>(0·97–1·15)
1952	0·0<br>(0·0–0·0)	9·2<br>(7·9–10·5)	111·7<br>(100·6–124·3)	187·5<br>(179·6–195·7)	121·0<br>(111·4–131·0)	57·1<br>(47·8–67·1)	15·1<br>(12·2–18·1)	0·6<br>(0·4–0·9)	0·0<br>(0·0–0·0)	2·51<br>(2·41–2·61)	0·6<br>(0·55–0·67)	0·97<br>(0·91–1·03)
1953	0·0<br>(0·0–0·0)	8·0<br>(6·9–9·5)	105·4<br>(93·9–118·3)	176·4<br>(168·6–184·3)	108·1<br>(98·6–118·1)	52·6<br>(42·5–63·4)	12·0<br>(9·2–15·0)	0·5<br>(0·3–0·8)	0·0<br>(0·0–0·0)	2·31<br>(2·21–2·43)	0·57<br>(0·51–0·63)	0·87<br>(0·81–0·93)
1954	0·0<br>(0·0–0·0)	7·3<br>(6·1–8·8)	101·9<br>(89·7–114·3)	171·4<br>(164·5–179·2)	99·6<br>(91·0–109·2)	48·0<br>(37·5–59·5)	9·8<br>(7·5–12·4)	0·4<br>(0·3–0·7)	0·0<br>(0·0–0·0)	2·19<br>(2·1–2·28)	0·55<br>(0·49–0·61)	0·79<br>(0·74–0·85)
1955	0·0<br>(0·0–0·0)	6·6<br>(5·3–8·2)	99·3<br>(86·6–112·1)	170·2<br>(162·1–178·1)	92·7<br>(83·9–101·8)	43·4<br>(33·6–55·4)	8·5<br>(6·4–11·0)	0·4<br>(0·2–0·6)	0·0<br>(0·0–0·0)	2·11<br>(2·01–2·2)	0·53<br>(0·46–0·6)	0·72<br>(0·67–0·78)
1956	0·0<br>(0·0–0·0)	5·9<br>(4·7–7·4)	93·4<br>(82·1–104·6)	164·3<br>(157·0–172·0)	82·7<br>(74·6–91·4)	37·4<br>(27·7–49·1)	6·9<br>(5·3–8·8)	0·3<br>(0·2–0·5)	0·0<br>(0·0–0·0)	1·95<br>(1·88–2·04)	0·5<br>(0·44–0·55)	0·64<br>(0·59–0·69)
1957	0·0<br>(0·0–0·0)	5·6<br>(4·4–7·1)	94·3<br>(83·4–107·2)	167·6<br>(160·4–175·5)	77·6<br>(70·4–85·4)	32·1<br>(23·1–42·3)	5·6<br>(4·4–7·1)	0·3<br>(0·2–0·4)	0·0<br>(0·0–0·0)	1·92<br>(1·82–2·02)	0·5<br>(0·44–0·57)	0·58<br>(0·53–0·63)
1958	0·0<br>(0·0–0·0)	5·7<br>(4·4–7·2)	99·0<br>(87·3–112·4)	173·1<br>(165·0–181·5)	75·7<br>(68·8–83·3)	28·3<br>(21·5–36·8)	5·0<br>(3·9–6·3)	0·3<br>(0·2–0·4)	0·0<br>(0·0–0·0)	1·94<br>(1·85–2·02)	0·52<br>(0·46–0·59)	0·55<br>(0·51–0·59)
1959	0·0<br>(0·0–0·0)	5·9<br>(4·7–7·3)	99·1<br>(87·3–111·2)	172·1<br>(164·0–180·3)	71·1<br>(63·4–78·9)	24·4<br>(19·0–30·8)	4·2<br>(3·2–5·3)	0·2<br>(0·1–0·4)	0·0<br>(0·0–0·0)	1·89<br>(1·8–1·98)	0·52<br>(0·47–0·59)	0·5<br>(0·46–0·54)
1960	0·0<br>(0·0–0·0)	5·8<br>(4·7–7·1)	95·4<br>(85·0–106·8)	172·9<br>(165·0–180·7)	69·6<br>(62·3–76·9)	21·5<br>(16·7–27·3)	3·6<br>(2·7–4·6)	0·2<br>(0·1–0·3)	0·0<br>(0·0–0·0)	1·84<br>(1·76–1·92)	0·51<br>(0·45–0·56)	0·47<br>(0·43–0·51)
1961	0·0<br>(0·0–0·0)	5·4<br>(4·3–6·5)	92·8<br>(83·2–102·3)	174·9<br>(166·9–182·7)	69·3<br>(63·9–75·5)	19·4<br>(15·9–23·7)	3·3<br>(2·4–4·3)	0·2<br>(0·1–0·3)	0·0<br>(0·0–0·0)	1·83<br>(1·75–1·9)	0·49<br>(0·44–0·54)	0·46<br>(0·42–0·5)
1962	0·0<br>(0·0–0·0)	5·1<br>(4·2–6·2)	92·8<br>(82·8–103·1)	180·2<br>(172·7–187·4)	71·1<br>(65·3–77·2)	18·3<br>(15·5–21·6)	2·9<br>(2·1–3·8)	0·2<br>(0·1–0·3)	0·0<br>(0·0–0·0)	1·85<br>(1·78–1·93)	0·49<br>(0·44–0·54)	0·46<br>(0·43–0·5)
1963	0·0<br>(0·0–0·0)	5·1<br>(4·3–6·1)	93·5<br>(83·8–104·3)	182·4<br>(175·1–190·2)	72·2<br>(65·8–79·1)	17·7<br>(15·0–20·7)	2·7<br>(2·0–3·6)	0·2<br>(0·1–0·3)	0·0<br>(0·0–0·0)	1·87<br>(1·8–1·95)	0·49<br>(0·44–0·55)	0·46<br>(0·43–0·5)
1964	0·0<br>(0·0–0·0)	5·0<br>(4·3–5·9)	102·5<br>(92·1–114·3)	194·4<br>(186·4–202·5)	77·3<br>(70·2–84·7)	18·3<br>(15·3–21·5)	2·5<br>(1·9–3·3)	0·2<br>(0·1–0·2)	0·0<br>(0·0–0·0)	2·0<br>(1·92–2·08)	0·54<br>(0·49–0·6)	0·49<br>(0·45–0·53)
1965	0·0<br>(0·0–0·0)	4·9<br>(4·2–5·7)	94·8<br>(83·8–105·8)	164·1<br>(156·2–172·0)	65·7<br>(59·6–71·5)	16·9<br>(13·8–20·4)	2·3<br>(1·7–3·0)	0·1<br>(0·1–0·2)	0·0<br>(0·0–0·0)	1·74<br>(1·67–1·82)	0·5<br>(0·44–0·55)	0·42<br>(0·39–0·46)
1966	0·0<br>(0·0–0·0)	5·2<br>(4·5–6·0)	93·1<br>(82·4–104·3)	169·0<br>(161·6–176·6)	67·9<br>(61·2–75·4)	17·2<br>(13·7–21·7)	2·3<br>(1·6–3·1)	0·1<br>(0·1–0·2)	0·0<br>(0·0–0·0)	1·77<br>(1·7–1·86)	0·49<br>(0·44–0·55)	0·44<br>(0·4–0·48)
1967	0·0<br>(0·0–0·0)	5·5<br>(4·6–6·4)	99·7<br>(89·9–110·8)	206·1<br>(198·3–213·9)	84·3<br>(76·7–93·0)	19·6<br>(15·2–24·6)	2·3<br>(1·7–3·2)	0·1<br>(0·1–0·2)	0·0<br>(0·0–0·0)	2·09<br>(2·0–2·18)	0·53<br>(0·48–0·58)	0·53<br>(0·49–0·57)
1968	0·0<br>(0·0–0·0)	5·6<br>(4·7–6·5)	89·9<br>(79·8–100·7)	194·5<br>(186·4–202·2)	80·7<br>(73·0–88·4)	19·6<br>(14·9–25·6)	2·4<br>(1·7–3·3)	0·1<br>(0·1–0·2)	0·0<br>(0·0–0·0)	1·96<br>(1·89–2·03)	0·48<br>(0·43–0·53)	0·51<br>(0·48–0·55)
1969	0·0<br>(0·0–0·0)	6·1<br>(5·1–7·2)	88·8<br>(78·8–99·2)	196·8<br>(188·7–204·6)	79·6<br>(72·6–87·1)	19·2<br>(14·5–24·8)	2·2<br>(1·6–3·0)	0·1<br>(0·1–0·2)	0·0<br>(0·0–0·0)	1·96<br>(1·89–2·04)	0·47<br>(0·43–0·53)	0·51<br>(0·47–0·54)
1970	0·0<br>(0·0–0·0)	6·2<br>(5·1–7·4)	96·0<br>(84·7–107·9)	200·3<br>(192·3–207·9)	79·9<br>(72·8–87·7)	18·4<br>(13·9–23·9)	2·1<br>(1·5–2·9)	0·1<br>(0·1–0·2)	0·0<br>(0·0–0·0)	2·01<br>(1·93–2·1)	0·51<br>(0·46–0·57)	0·5<br>(0·47–0·54)
1971	0·0<br>(0·0–0·0)	6·0<br>(4·9–7·2)	105·4<br>(92·9–117·3)	197·6<br>(189·7–205·9)	81·6<br>(74·5–88·8)	16·7<br>(12·8–21·9)	2·3<br>(1·6–3·2)	0·1<br>(0·1–0·2)	0·0<br>(0·0–0·0)	2·05<br>(1·96–2·14)	0·56<br>(0·49–0·62)	0·5<br>(0·46–0·55)
1972	0·0<br>(0·0–0·0)	6·0<br>(5·0–7·2)	110·3<br>(99·1–122·6)	195·4<br>(188·3–203·3)	81·9<br>(74·2–89·4)	15·8<br>(12·1–20·2)	2·3<br>(1·6–3·3)	0·1<br>(0·1–0·2)	0·0<br>(0·0–0·0)	2·06<br>(1·98–2·15)	0·58<br>(0·53–0·64)	0·5<br>(0·46–0·54)
1973	0·0<br>(0·0–0·0)	5·8<br>(4·8–6·9)	109·9<br>(99·0–121·4)	194·0<br>(185·7–202·7)	77·5<br>(71·1–84·5)	15·6<br>(12·4–19·6)	2·1<br>(1·5–2·9)	0·1<br>(0·1–0·2)	0·0<br>(0·0–0·0)	2·03<br>(1·95–2·11)	0·58<br>(0·52–0·63)	0·48<br>(0·44–0·51)
1974	0·0<br>(0·0–0·0)	5·3<br>(4·3–6·4)	103·6<br>(93·4–114·5)	184·0<br>(174·9–192·8)	67·7<br>(61·3–74·3)	14·7<br>(11·7–18·4)	1·8<br>(1·3–2·4)	0·1<br>(0·0–0·1)	0·0<br>(0·0–0·0)	1·89<br>(1·81–1·97)	0·54<br>(0·49–0·6)	0·42<br>(0·39–0·46)
1975	0·0<br>(0·0–0·0)	4·7<br>(3·9–5·8)	94·2<br>(84·2–104·6)	174·5<br>(166·6–181·9)	61·4<br>(55·6–67·0)	14·0<br>(11·5–17·0)	1·6<br>(1·2–2·2)	0·1<br>(0·0–0·1)	0·0<br>(0·0–0·0)	1·75<br>(1·68–1·83)	0·49<br>(0·45–0·55)	0·39<br>(0·35–0·42)
1976	0·0<br>(0·0–0·0)	4·4<br>(3·5–5·5)	85·7<br>(76·7–95·4)	169·8<br>(162·3–177·3)	61·6<br>(56·3–67·2)	13·8<br>(10·8–17·4)	1·7<br>(1·2–2·4)	0·1<br>(0·0–0·1)	0·0<br>(0·0–0·0)	1·69<br>(1·62–1·75)	0·45<br>(0·41–0·5)	0·39<br>(0·36–0·41)
1977	0·0<br>(0·0–0·0)	4·2<br>(3·2–5·3)	77·9<br>(69·9–87·4)	169·1<br>(161·6–176·9)	64·8<br>(58·7–70·8)	13·6<br>(10·8–16·8)	1·9<br>(1·3–2·6)	0·1<br>(0·1–0·1)	0·0<br>(0·0–0·0)	1·66<br>(1·59–1·73)	0·41<br>(0·37–0·46)	0·4<br>(0·36–0·44)
1978	0·0<br>(0·0–0·0)	4·1<br>(3·3–4·9)	71·7<br>(64·6–79·3)	169·1<br>(161·8–176·4)	68·5<br>(62·1–74·9)	13·2<br>(11·6–14·9)	1·9<br>(1·3–2·5)	0·1<br>(0·1–0·1)	0·0<br>(0·0–0·0)	1·64<br>(1·58–1·7)	0·38<br>(0·34–0·42)	0·42<br>(0·39–0·45)
1979	0·0<br>(0·0–0·0)	4·1<br>(3·5–4·7)	66·8<br>(59·3–74·8)	167·8<br>(160·3–174·8)	68·6<br>(63·1–75·1)	12·9<br>(10·2–15·8)	1·9<br>(1·4–2·7)	0·1<br>(0·1–0·1)	0·0<br>(0·0–0·0)	1·61<br>(1·55–1·68)	0·35<br>(0·32–0·4)	0·42<br>(0·39–0·45)
1980	0·0<br>(0·0–0·0)	4·2<br>(3·4–5·1)	63·0<br>(55·8–70·5)	166·0<br>(158·7–173·7)	67·2<br>(61·0–73·5)	12·8<br>(10·5–15·3)	1·9<br>(1·4–2·6)	0·1<br>(0·1–0·1)	0·0<br>(0·0–0·0)	1·58<br>(1·51–1·64)	0·34<br>(0·3–0·37)	0·41<br>(0·38–0·45)
1981	0·0<br>(0·0–0·0)	4·4<br>(3·5–5·6)	61·2<br>(54·9–68·4)	167·6<br>(160·0–175·2)	68·0<br>(62·0–74·0)	13·5<br>(11·0–16·3)	1·9<br>(1·3–2·6)	0·1<br>(0·0–0·1)	0·0<br>(0·0–0·0)	1·58<br>(1·53–1·64)	0·33<br>(0·3–0·36)	0·42<br>(0·39–0·45)
1982	0·0<br>(0·0–0·0)	4·7<br>(3·8–5·8)	60·7<br>(53·5–68·2)	169·7<br>(162·6–177·0)	71·6<br>(65·7–78·2)	15·3<br>(12·4–18·9)	1·9<br>(1·3–2·6)	0·1<br>(0·0–0·1)	0·0<br>(0·0–0·0)	1·62<br>(1·57–1·68)	0·33<br>(0·29–0·36)	0·44<br>(0·41–0·48)
1983	0·0<br>(0·0–0·0)	4·9<br>(4·1–5·9)	60·0<br>(52·9–67·8)	170·1<br>(162·0–178·2)	76·2<br>(69·6–83·6)	16·9<br>(13·9–20·3)	1·9<br>(1·2–3·0)	0·1<br>(0·0–0·1)	0·0<br>(0·0–0·0)	1·65<br>(1·59–1·72)	0·32<br>(0·29–0·36)	0·48<br>(0·44–0·52)
1984	0·0<br>(0·0–0·0)	4·9<br>(4·0–5·9)	56·7<br>(50·8–63·3)	167·2<br>(159·7–174·9)	79·7<br>(72·1–87·6)	17·4<br>(14·6–20·8)	2·0<br>(1·3–2·8)	0·1<br>(0·0–0·1)	0·0<br>(0·0–0·0)	1·64<br>(1·57–1·71)	0·31<br>(0·28–0·34)	0·5<br>(0·46–0·54)
1985	0·0<br>(0·0–0·0)	4·7<br>(3·9–5·7)	54·1<br>(47·4–60·7)	160·8<br>(153·7–167·3)	81·4<br>(74·4–88·6)	17·1<br>(14·5–20·2)	1·9<br>(1·3–2·8)	0·1<br>(0·0–0·1)	0·0<br>(0·0–0·0)	1·6<br>(1·55–1·65)	0·29<br>(0·26–0·33)	0·5<br>(0·47–0·54)
1986	0·0<br>(0·0–0·0)	4·6<br>(3·8–5·5)	52·2<br>(46·0–58·8)	154·6<br>(147·1–162·2)	83·6<br>(77·1–90·2)	16·9<br>(14·0–20·0)	2·0<br>(1·4–2·7)	0·1<br>(0·0–0·1)	0·0<br>(0·0–0·0)	1·57<br>(1·52–1·63)	0·28<br>(0·25–0·32)	0·51<br>(0·48–0·55)
1987	0·0<br>(0·0–0·0)	4·5<br>(3·6–5·4)	48·5<br>(42·6–54·8)	148·4<br>(141·4–155·8)	85·8<br>(78·8–93·9)	17·4<br>(14·4–20·8)	2·2<br>(1·5–3·0)	0·1<br>(0·0–0·1)	0·0<br>(0·0–0·0)	1·53<br>(1·47–1·6)	0·26<br>(0·23–0·3)	0·53<br>(0·49–0·57)
1988	0·0<br>(0·0–0·0)	4·3<br>(3·5–5·2)	45·1<br>(39·9–50·8)	138·0<br>(131·5–144·8)	85·8<br>(78·8–93·7)	18·1<br>(15·2–21·4)	2·4<br>(1·6–3·4)	0·1<br>(0·0–0·1)	0·0<br>(0·0–0·0)	1·47<br>(1·41–1·54)	0·25<br>(0·22–0·28)	0·53<br>(0·49–0·57)
1989	0·0<br>(0·0–0·0)	4·3<br>(3·6–5·3)	41·8<br>(36·3–47·6)	127·4<br>(120·9–134·4)	84·3<br>(77·3–91·3)	18·9<br>(15·7–22·5)	2·5<br>(1·7–3·4)	0·1<br>(0·0–0·1)	0·0<br>(0·0–0·0)	1·4<br>(1·34–1·46)	0·23<br>(0·2–0·26)	0·53<br>(0·49–0·57)
1990	0·0<br>(0·0–0·0)	4·5<br>(3·6–5·4)	40·5<br>(35·6–45·2)	123·4<br>(116·7–129·8)	84·2<br>(77·4–91·3)	19·6<br>(16·5–23·4)	2·4<br>(1·7–3·3)	0·1<br>(0·1–0·1)	0·0<br>(0·0–0·0)	1·37<br>(1·32–1·43)	0·22<br>(0·2–0·25)	0·53<br>(0·49–0·57)
1991	0·0<br>(0·0–0·0)	4·6<br>(3·7–5·7)	40·4<br>(35·7–46·0)	119·3<br>(113·3–125·4)	83·7<br>(77·1–90·5)	20·5<br>(17·3–24·2)	2·4<br>(1·7–3·3)	0·1<br>(0·1–0·1)	0·0<br>(0·0–0·0)	1·36<br>(1·3–1·41)	0·22<br>(0·2–0·25)	0·53<br>(0·5–0·57)
1992	0·0<br>(0·0–0·0)	4·7<br>(3·8–5·7)	39·2<br>(34·0–45·2)	112·4<br>(106·1–118·9)	82·3<br>(74·4–90·6)	21·1<br>(17·7–24·7)	2·5<br>(1·8–3·3)	0·1<br>(0·1–0·1)	0·0<br>(0·0–0·0)	1·31<br>(1·26–1·36)	0·22<br>(0·19–0·25)	0·53<br>(0·49–0·57)
1993	0·0<br>(0·0–0·0)	4·7<br>(3·8–5·8)	38·9<br>(34·4–43·6)	112·3<br>(106·1–119·1)	86·0<br>(79·1–93·2)	22·7<br>(18·4–27·7)	2·6<br>(1·9–3·4)	0·1<br>(0·1–0·1)	0·0<br>(0·0–0·0)	1·34<br>(1·28–1·4)	0·22<br>(0·19–0·24)	0·56<br>(0·51–0·6)
1994	0·0<br>(0·0–0·0)	4·8<br>(4·0–5·8)	38·4<br>(33·2–44·6)	111·0<br>(105·2–117·1)	88·2<br>(80·9–96·2)	23·9<br>(20·1–28·1)	2·7<br>(1·9–3·6)	0·1<br>(0·1–0·2)	0·0<br>(0·0–0·0)	1·35<br>(1·29–1·41)	0·22<br>(0·19–0·25)	0·57<br>(0·53–0·62)
1995	0·0<br>(0·0–0·0)	4·8<br>(4·0–5·8)	37·3<br>(33·1–41·9)	106·0<br>(99·5–112·7)	88·4<br>(81·1–96·1)	24·8<br>(20·8–29·4)	2·8<br>(2·1–3·7)	0·1<br>(0·1–0·2)	0·0<br>(0·0–0·0)	1·32<br>(1·26–1·38)	0·21<br>(0·19–0·23)	0·58<br>(0·54–0·62)
1996	0·0<br>(0·0–0·0)	5·0<br>(4·2–6·0)	36·9<br>(31·9–42·2)	103·8<br>(97·8–110·2)	89·1<br>(82·0–97·1)	26·2<br>(22·0–31·1)	2·9<br>(2·2–3·9)	0·1<br>(0·1–0·2)	0·0<br>(0·0–0·0)	1·32<br>(1·26–1·38)	0·21<br>(0·19–0·24)	0·59<br>(0·55–0·64)
1997	0·0<br>(0·0–0·0)	5·3<br>(4·4–6·3)	36·6<br>(31·7–41·6)	101·9<br>(96·1–107·6)	88·3<br>(81·5–95·9)	27·0<br>(22·7–31·6)	3·1<br>(2·3–4·4)	0·1<br>(0·1–0·2)	0·0<br>(0·0–0·0)	1·31<br>(1·26–1·37)	0·21<br>(0·19–0·23)	0·59<br>(0·55–0·64)
1998	0·0<br>(0·0–0·0)	5·6<br>(4·6–6·7)	36·1<br>(31·5–41·4)	98·8<br>(92·8–104·8)	88·3<br>(81·3–96·3)	28·4<br>(23·8–33·2)	3·3<br>(2·4–4·6)	0·1<br>(0·1–0·2)	0·0<br>(0·0–0·0)	1·3<br>(1·24–1·37)	0·21<br>(0·19–0·24)	0·6<br>(0·56–0·64)
1999	0·0<br>(0·0–0·0)	5·9<br>(4·9–7·0)	35·5<br>(30·8–40·9)	95·7<br>(89·6–101·3)	88·6<br>(81·6–96·5)	29·5<br>(24·9–34·5)	3·6<br>(2·5–4·8)	0·1<br>(0·1–0·2)	0·0<br>(0·0–0·0)	1·29<br>(1·23–1·36)	0·21<br>(0·18–0·24)	0·61<br>(0·57–0·66)
2000	0·0<br>(0·0–0·0)	6·3<br>(5·2–7·5)	35·3<br>(31·7–39·1)	93·2<br>(87·8–99·3)	87·7<br>(81·0–95·4)	31·1<br>(26·6–35·9)	3·9<br>(2·7–5·2)	0·1<br>(0·1–0·2)	0·0<br>(0·0–0·0)	1·29<br>(1·23–1·35)	0·21<br>(0·19–0·23)	0·61<br>(0·57–0·66)
2001	0·0<br>(0·0–0·0)	6·6<br>(5·4–8·0)	35·1<br>(30·4–40·3)	89·9<br>(84·7–95·7)	85·6<br>(77·9–93·3)	31·2<br>(26·4–36·3)	4·0<br>(2·9–5·5)	0·1<br>(0·1–0·2)	0·0<br>(0·0–0·0)	1·26<br>(1·2–1·32)	0·21<br>(0·18–0·24)	0·6<br>(0·56–0·65)
2002	0·0<br>(0·0–0·0)	6·5<br>(5·4–8·0)	34·1<br>(29·7–38·8)	86·7<br>(81·2–92·3)	85·5<br>(78·3–93·3)	31·8<br>(27·3–37·3)	4·4<br>(3·2–5·9)	0·1<br>(0·1–0·2)	0·0<br>(0·0–0·0)	1·25<br>(1·19–1·31)	0·2<br>(0·18–0·23)	0·61<br>(0·56–0·66)
2003	0·0<br>(0·0–0·0)	6·3<br>(5·1–7·9)	32·8<br>(29·1–37·2)	83·8<br>(79·2–88·9)	85·1<br>(78·5–91·9)	33·2<br>(28·0–38·7)	4·7<br>(3·5–6·2)	0·1<br>(0·1–0·2)	0·0<br>(0·0–0·0)	1·23<br>(1·18–1·29)	0·2<br>(0·17–0·22)	0·62<br>(0·57–0·67)
2004	0·0<br>(0·0–0·0)	6·1<br>(4·8–7·5)	32·0<br>(27·8–36·7)	79·9<br>(74·9–85·3)	83·9<br>(76·8–91·6)	34·1<br>(29·2–39·9)	4·9<br>(3·8–6·3)	0·2<br>(0·1–0·2)	0·0<br>(0·0–0·0)	1·21<br>(1·15–1·26)	0·19<br>(0·17–0·21)	0·62<br>(0·57–0·66)
2005	0·0<br>(0·0–0·0)	6·1<br>(4·8–7·4)	32·2<br>(28·1–36·6)	78·6<br>(73·5–84·0)	84·6<br>(77·2–92·0)	35·3<br>(30·2–40·6)	5·2<br>(4·0–6·7)	0·2<br>(0·1–0·2)	0·0<br>(0·0–0·0)	1·21<br>(1·16–1·27)	0·19<br>(0·17–0·22)	0·63<br>(0·58–0·67)
2006	0·0<br>(0·0–0·0)	6·1<br>(4·8–7·6)	33·2<br>(28·6–38·0)	79·1<br>(74·1–84·3)	87·7<br>(80·4–95·3)	37·7<br>(32·5–43·7)	5·6<br>(4·4–7·0)	0·2<br>(0·1–0·3)	0·0<br>(0·0–0·0)	1·25<br>(1·19–1·31)	0·2<br>(0·17–0·22)	0·66<br>(0·61–0·71)
2007	0·0<br>(0·0–0·0)	6·2<br>(4·9–7·7)	33·7<br>(29·7–37·9)	78·5<br>(73·5–84·1)	88·4<br>(81·2–96·2)	40·2<br>(34·7–46·4)	6·0<br>(4·7–7·5)	0·2<br>(0·1–0·3)	0·0<br>(0·0–0·0)	1·27<br>(1·21–1·32)	0·2<br>(0·18–0·22)	0·67<br>(0·63–0·72)
2008	0·0<br>(0·0–0·0)	6·1<br>(4·9–7·6)	33·9<br>(29·2–39·2)	78·0<br>(72·8–83·0)	89·1<br>(81·9–95·8)	41·9<br>(36·2–48·3)	6·5<br>(5·2–8·1)	0·2<br>(0·1–0·3)	0·0<br>(0·0–0·0)	1·28<br>(1·22–1·34)	0·2<br>(0·17–0·23)	0·69<br>(0·64–0·74)
2009	0·0<br>(0·0–0·0)	5·9<br>(4·7–7·3)	33·2<br>(29·9–36·6)	77·7<br>(73·1–82·2)	89·8<br>(82·3–97·8)	43·3<br>(37·3–50·3)	7·2<br>(5·7–9·1)	0·2<br>(0·1–0·3)	0·0<br>(0·0–0·0)	1·29<br>(1·23–1·35)	0·2<br>(0·18–0·21)	0·7<br>(0·66–0·75)
2010	0·0<br>(0·0–0·0)	5·6<br>(4·6–6·7)	32·5<br>(27·6–37·7)	78·1<br>(72·8–83·3)	90·8<br>(83·4–99·2)	44·3<br>(38·2–50·8)	7·6<br>(6·1–9·6)	0·2<br>(0·1–0·3)	0·0<br>(0·0–0·0)	1·3<br>(1·23–1·37)	0·19<br>(0·16–0·22)	0·71<br>(0·66–0·77)
2011	0·0<br>(0·0–0·0)	5·4<br>(4·4–6·6)	30·9<br>(26·7–35·4)	77·2<br>(72·0–82·8)	90·8<br>(83·7–98·2)	45·3<br>(39·2–52·0)	8·1<br>(6·3–10·0)	0·2<br>(0·1–0·3)	0·0<br>(0·0–0·0)	1·29<br>(1·23–1·35)	0·18<br>(0·16–0·2)	0·72<br>(0·68–0·77)
2012	0·0<br>(0·0–0·0)	5·3<br>(4·5–6·0)	29·3<br>(25·5–33·8)	76·1<br>(71·8–80·8)	92·1<br>(84·1–100·0)	47·6<br>(41·2–54·0)	8·9<br>(7·0–11·2)	0·2<br>(0·1–0·4)	0·0<br>(0·0–0·0)	1·3<br>(1·24–1·35)	0·17<br>(0·15–0·19)	0·74<br>(0·69–0·8)
2013	0·0<br>(0·0–0·0)	5·1<br>(4·3–6·1)	28·1<br>(24·5–32·1)	74·2<br>(69·3–79·3)	92·1<br>(84·6–99·8)	49·3<br>(42·7–55·7)	9·5<br>(7·7–11·9)	0·2<br>(0·1–0·4)	0·0<br>(0·0–0·0)	1·29<br>(1·23–1·36)	0·17<br>(0·15–0·19)	0·76<br>(0·7–0·81)
2014	0·0<br>(0·0–0·0)	5·0<br>(4·2–6·0)	27·0<br>(23·2–31·2)	73·0<br>(68·2–78·1)	93·2<br>(85·8–101·1)	51·9<br>(45·0–59·2)	10·2<br>(8·2–12·8)	0·2<br>(0·2–0·4)	0·0<br>(0·0–0·0)	1·3<br>(1·23–1·38)	0·16<br>(0·14–0·18)	0·78<br>(0·72–0·84)
2015	0·0<br>(0·0–0·0)	5·0<br>(4·2–6·0)	27·4<br>(23·4–31·9)	74·1<br>(68·5–80·4)	95·5<br>(86·0–105·7)	51·1<br>(42·6–60·8)	9·5<br>(7·3–12·2)	0·2<br>(0·2–0·4)	0·0<br>(0·0–0·0)	1·31<br>(1·18–1·47)	0·16<br>(0·14–0·19)	0·78<br>(0·7–0·88)
2016	0·0<br>(0·0–0·0)	5·0<br>(4·1–6·2)	28·4<br>(22·5–36·5)	75·1<br>(67·2–84·4)	95·1<br>(82·5–109·6)	47·6<br>(37·0–60·9)	8·2<br>(5·9–11·1)	0·2<br>(0·2–0·3)	0·0<br>(0·0–0·0)	1·3<br>(1·11–1·52)	0·17<br>(0·13–0·21)	0·76<br>(0·64–0·89)
2017	0·0<br>(0·0–0·0)	5·0<br>(4·1–6·0)	28·4<br>(22·2–36·3)	75·0<br>(66·6–84·1)	93·8<br>(80·2–108·7)	46·7<br>(35·0–61·3)	8·0<br>(5·3–11·4)	0·2<br>(0·1–0·3)	0·0<br>(0·0–0·0)	1·29<br>(1·07–1·53)	0·17<br>(0·13–0·21)	0·74<br>(0·61–0·9)

Supplemental Results Table 5: Age-specific fertility rates per 1000, cumulative total fertility up to maternal age 25 (TFU25), cumulative total fertility from maternal age 30 to age 55 (TFO30), and cumulative total fertility, Saga, 1950-2017

Year	10-14	15-19	20-24	25-29	30-34	35-39	40-44	45-49	50-54	TFR	TFU25	TFO30
1950	0·0 (0·0–0·0)	10·8 (9·5–12·0)	151·7 (138·3–166·1)	227·8 (219·3–236·3)	192·9 (180·8–204·8)	108·1 (96·1–120·0)	42·0 (36·2–48·2)	2·0 (1·3–2·9)	0·0 (0·0–0·0)	3·68 (3·55–3·81)	0·81 (0·75–0·88)	1·72 (1·64–1·82)
1951	0·0 (0·0–0·0)	8·7 (7·7–9·8)	136·1 (122·7–149·2)	212·0 (202·8–220·8)	175·0 (163·2–186·9)	87·5 (76·2–98·6)	32·6 (27·0–38·6)	1·5 (0·9–2·2)	0·0 (0·0–0·0)	3·27 (3·11–3·42)	0·72 (0·66–0·79)	1·48 (1·35–1·62)
1952	0·0 (0·0–0·0)	7·3 (6·4–8·3)	126·7 (114·1–142·2)	201·7 (192·1–212·0)	158·6 (146·2–171·0)	70·1 (60·2–80·5)	25·8 (21·0–31·1)	1·1 (0·7–1·7)	0·0 (0·0–0·0)	2·96 (2·84–3·08)	0·67 (0·61–0·75)	1·28 (1·2–1·36)
1953	0·0 (0·0–0·0)	6·4 (5·6–7·4)	119·7 (106·3–134·3)	190·2 (178·8–201·9)	141·9 (129·0–154·8)	58·3 (49·2–67·9)	20·5 (16·2–25·5)	0·9 (0·6–1·4)	0·0 (0·0–0·0)	2·69 (2·57–2·82)	0·63 (0·56–0·7)	1·11 (1·02–1·19)
1954	0·0 (0·0–0·0)	5·9 (5·0–6·7)	116·0 (101·2–131·0)	185·2 (172·2–199·0)	130·8 (119·7–143·0)	52·7 (44·1–62·1)	16·9 (12·7–21·8)	0·8 (0·5–1·1)	0·0 (0·0–0·0)	2·54 (2·43–2·66)	0·61 (0·53–0·68)	1·01 (0·93–1·09)
1955	0·0 (0·0–0·0)	5·3 (4·4–6·2)	113·0 (99·4–128·2)	184·2 (171·2–198·1)	120·5 (109·6–132·0)	47·4 (40·1–56·2)	14·1 (10·2–19·0)	0·6 (0·3–0·9)	0·0 (0·0–0·0)	2·43 (2·32–2·53)	0·59 (0·52–0·67)	0·91 (0·84–0·99)
1956	0·0 (0·0–0·0)	4·7 (3·9–5·6)	106·4 (92·7–120·6)	178·1 (165·1–192·6)	106·8 (96·8–117·8)	40·6 (34·0–47·9)	11·2 (8·0–15·5)	0·5 (0·3–0·8)	0·0 (0·0–0·0)	2·24 (2·14–2·34)	0·56 (0·49–0·63)	0·8 (0·73–0·87)
1957	0·0 (0·0–0·0)	4·5 (3·8–5·3)	107·4 (94·5–122·8)	181·5 (167·1–196·3)	101·1 (92·3–110·9)	34·7 (28·9–40·2)	9·5 (6·7–13·0)	0·5 (0·3–0·8)	0·0 (0·0–0·0)	2·2 (2·08–2·31)	0·56 (0·49–0·64)	0·73 (0·67–0·79)
1958	0·0 (0·0–0·0)	4·5 (3·8–5·4)	112·6 (99·3–127·4)	187·3 (173·2–202·9)	98·9 (87·0–112·7)	30·7 (25·6–36·4)	8·6 (6·0–12·0)	0·5 (0·3–0·8)	0·0 (0·0–0·0)	2·22 (2·1–2·33)	0·59 (0·52–0·66)	0·69 (0·62–0·77)
1959	0·0 (0·0–0·0)	4·7 (4·0–5·6)	112·4 (99·0–127·2)	186·3 (173·3–199·0)	92·6 (83·2–102·0)	26·5 (21·5–32·2)	7·1 (4·8–9·9)	0·4 (0·2–0·7)	0·0 (0·0–0·0)	2·15 (2·05–2·26)	0·59 (0·52–0·66)	0·63 (0·57–0·69)
1960	0·0 (0·0–0·0)	4·6 (3·9–5·4)	107·9 (96·6–121·5)	187·1 (175·1–199·1)	88·9 (79·7–99·3)	23·3 (19·5–27·3)	5·8 (3·9–8·1)	0·3 (0·2–0·6)	0·0 (0·0–0·0)	2·09 (1·99–2·19)	0·56 (0·5–0·63)	0·59 (0·54–0·65)
1961	0·0 (0·0–0·0)	4·3 (3·5–5·0)	104·5 (93·4–115·4)	189·1 (179·3–199·0)	86·9 (79·2–96·0)	21·0 (17·0–25·5)	4·9 (3·3–7·1)	0·3 (0·2–0·4)	0·0 (0·0–0·0)	2·05 (1·97–2·15)	0·54 (0·49–0·6)	0·57 (0·52–0·62)
1962	0·0 (0·0–0·0)	4·0 (3·3–4·9)	103·9 (92·6–115·8)	194·5 (186·0–202·9)	88·7 (81·0–96·4)	19·8 (17·0–22·9)	4·3 (3·0–6·2)	0·2 (0·1–0·4)	0·0 (0·0–0·0)	2·08 (2·0–2·16)	0·54 (0·48–0·6)	0·57 (0·52–0·61)
1963	0·0 (0·0–0·0)	4·0 (3·2–4·8)	104·1 (93·2–115·8)	196·4 (188·3–204·4)	90·4 (82·8–98·5)	19·1 (16·0–22·6)	4·2 (3·0–5·9)	0·3 (0·1–0·4)	0·0 (0·0–0·0)	2·09 (2·01–2·17)	0·54 (0·49–0·6)	0·57 (0·53–0·61)
1964	0·0 (0·0–0·0)	3·9 (3·2–4·7)	113·2 (101·7–126·7)	209·0 (198·8–219·1)	96·4 (88·1–105·4)	19·8 (16·8–23·0)	3·9 (2·8–5·6)	0·2 (0·1–0·4)	0·0 (0·0–0·0)	2·23 (2·14–2·33)	0·59 (0·53–0·65)	0·6 (0·56–0·65)
1965	0·0 (0·0–0·0)	3·8 (3·1–4·5)	103·9 (92·2–116·8)	176·5 (164·9–188·2)	80·2 (72·1–88·3)	18·2 (15·5–21·2)	3·3 (2·4–4·5)	0·2 (0·1–0·3)	0·0 (0·0–0·0)	1·93 (1·84–2·03)	0·54 (0·48–0·6)	0·51 (0·46–0·56)
1966	0·0 (0·0–0·0)	4·0 (3·3–4·7)	101·1 (88·8–113·2)	181·4 (168·5–194·9)	82·0 (73·3–90·8)	18·6 (15·4–22·3)	3·3 (2·3–4·4)	0·2 (0·1–0·3)	0·0 (0·0–0·0)	1·95 (1·86–2·06)	0·53 (0·47–0·59)	0·52 (0·47–0·57)
1967	0·0 (0·0–0·0)	4·2 (3·5–4·9)	107·2 (95·0–120·6)	221·0 (204·5–239·4)	98·9 (88·9–109·5)	21·1 (17·3–25·4)	3·1 (2·3–4·0)	0·2 (0·1–0·3)	0·0 (0·0–0·0)	2·28 (2·15–2·4)	0·56 (0·5–0·62)	0·62 (0·56–0·68)
1968	0·0 (0·0–0·0)	4·3 (3·7–5·0)	95·5 (84·6–106·4)	208·4 (191·3–226·6)	91·2 (82·2–101·0)	21·0 (17·2–25·4)	2·8 (2·1–3·7)	0·1 (0·1–0·2)	0·0 (0·0–0·0)	2·12 (2·03–2·21)	0·5 (0·44–0·55)	0·58 (0·53–0·62)
1969	0·0 (0·0–0·0)	4·6 (4·0–5·4)	93·3 (82·6–104·8)	210·3 (194·2–228·3)	91·4 (82·9–101·0)	20·6 (16·8–25·1)	2·8 (2·1–3·7)	0·1 (0·1–0·2)	0·0 (0·0–0·0)	2·12 (2·03–2·21)	0·49 (0·44–0·55)	0·57 (0·52–0·64)
1970	0·0 (0·0–0·0)	4·6 (3·9–5·5)	99·8 (88·3–113·7)	213·5 (197·2–230·4)	92·9 (84·0–102·1)	19·6 (15·8–24·1)	2·9 (2·1–3·9)	0·1 (0·1–0·2)	0·0 (0·0–0·0)	2·17 (2·08–2·26)	0·52 (0·47–0·59)	0·58 (0·53–0·63)
1971	0·0 (0·0–0·0)	4·5 (3·7–5·3)	108·7 (95·8–122·4)	210·5 (196·8–224·9)	94·2 (85·0–103·9)	17·7 (14·4–21·9)	3·0 (2·2–4·1)	0·2 (0·1–0·2)	0·0 (0·0–0·0)	2·19 (2·1–2·29)	0·57 (0·5–0·63)	0·58 (0·52–0·64)
1972	0·0 (0·0–0·0)	4·5 (3·7–5·5)	113·3 (98·3–129·7)	208·7 (196·0–221·0)	94·4 (84·0–105·2)	16·7 (13·2–20·4)	3·0 (2·1–4·1)	0·2 (0·1–0·2)	0·0 (0·0–0·0)	2·2 (2·09–2·31)	0·59 (0·51–0·67)	0·57 (0·51–0·63)
1973	0·0 (0·0–0·0)	4·4 (3·5–5·4)	113·1 (100·0–127·6)	208·8 (196·9–220·7)	90·0 (81·8–98·3)	16·3 (12·9–20·1)	2·8 (1·9–3·9)	0·1 (0·1–0·2)	0·0 (0·0–0·0)	2·18 (2·09–2·27)	0·59 (0·52–0·66)	0·55 (0·5–0·6)
1974	0·0 (0·0–0·0)	4·0 (3·3–5·0)	108·0 (95·0–121·3)	201·0 (191·9–210·7)	80·1 (72·1–88·3)	15·1 (11·9–19·0)	2·4 (1·5–3·6)	0·1 (0·1–0·2)	0·0 (0·0–0·0)	2·05 (1·97–2·14)	0·56 (0·5–0·63)	0·49 (0·44–0·54)
1975	0·0 (0·0–0·0)	3·8 (3·0–4·8)	101·2 (88·8–113·1)	194·2 (185·2–203·7)	72·2 (64·8–78·9)	14·2 (11·2–17·9)	2·0 (1·2–3·2)	0·1 (0·0–0·1)	0·0 (0·0–0·0)	1·94 (1·85–2·02)	0·52 (0·46–0·58)	0·44 (0·4–0·48)
1976	0·0 (0·0–0·0)	3·7 (2·9–4·5)	96·5 (85·4–108·2)	192·0 (182·3–201·2)	71·0 (64·3–78·4)	13·9 (11·1–17·3)	1·9 (1·2–2·7)	0·1 (0·0–0·1)	0·0 (0·0–0·0)	1·89 (1·81–1·98)	0·5 (0·45–0·56)	0·43 (0·39–0·48)
1977	0·0 (0·0–0·0)	3·7 (3·0–4·4)	92·3 (82·4–104·5)	192·8 (183·8–202·0)	74·6 (67·1–82·4)	13·7 (10·7–17·6)	1·9 (1·2–2·6)	0·1 (0·0–0·1)	0·0 (0·0–0·0)	1·9 (1·82–1·98)	0·48 (0·43–0·54)	0·45 (0·4–0·5)
1978	0·0 (0·0–0·0)	3·7 (2·9–4·5)	89·3 (79·8–100·0)	193·4 (184·4–202·8)	79·3 (71·3–87·9)	13·4 (10·0–17·5)	1·8 (1·2–2·5)	0·1 (0·0–0·1)	0·0 (0·0–0·0)	1·9 (1·82–1·99)	0·47 (0·42–0·52)	0·47 (0·43–0·52)
1979	0·0 (0·0–0·0)	3·8 (3·2–4·5)	86·2 (76·3–97·3)	191·4 (183·0–200·0)	80·1 (72·9–88·0)	13·1 (10·6–16·3)	1·8 (1·2–2·7)	0·1 (0·0–0·1)	0·0 (0·0–0·0)	1·88 (1·8–1·96)	0·45 (0·4–0·51)	0·48 (0·44–0·52)
1980	0·0 (0·0–0·0)	3·8 (3·0–4·8)	82·9 (72·9–93·4)	188·6 (178·7–197·8)	79·0 (70·9–86·8)	13·0 (10·4–16·3)	1·8 (1·2–2·5)	0·1 (0·0–0·1)	0·0 (0·0–0·0)	1·85 (1·76–1·93)	0·43 (0·38–0·49)	0·47 (0·43–0·51)
1981	0·0 (0·0–0·0)	4·0 (3·2–5·0)	80·9 (71·9–91·6)	189·6 (180·7–199·1)	79·7 (71·9–87·1)	13·8 (10·9–17·1)	1·8 (1·2–2·6)	0·1 (0·0–0·1)	0·0 (0·0–0·0)	1·85 (1·78–1·92)	0·42 (0·38–0·48)	0·48 (0·44–0·52)
1982	0·0 (0·0–0·0)	4·2 (3·3–5·3)	79·9 (69·8–91·1)	191·8 (183·5–201·0)	82·9 (75·5–91·7)	15·4 (12·6–18·8)	1·8 (1·3–2·5)	0·1 (0·0–0·1)	0·0 (0·0–0·0)	1·88 (1·81–1·96)	0·42 (0·37–0·48)	0·5 (0·46–0·55)
1983	0·0 (0·0–0·0)	4·3 (3·4–5·4)	78·4 (68·7–89·1)	192·8 (182·7–201·9)	86·6 (78·7–95·9)	17·0 (13·6–21·2)	1·9 (1·4–2·4)	0·1 (0·0–0·1)	0·0 (0·0–0·0)	1·91 (1·82–2·0)	0·41 (0·36–0·47)	0·53 (0·48–0·58)
1984	0·0 (0·0–0·0)	4·2 (3·3–5·2)	73·5 (65·0–82·9)	190·5 (181·3–199·7)	89·5 (81·6–97·9)	17·7 (14·4–21·9)	2·0 (1·4–2·6)	0·1 (0·0–0·1)	0·0 (0·0–0·0)	1·89 (1·81–1·96)	0·39 (0·35–0·44)	0·55 (0·5–0·59)
1985	0·0 (0·0–0·0)	4·0 (3·2–4·9)	69·7 (60·4–79·9)	184·4 (175·4–193·7)	90·7 (83·3–98·5)	17·7 (14·2–21·6)	2·0 (1·5–2·7)	0·1 (0·0–0·1)	0·0 (0·0–0·0)	1·84 (1·76–1·93)	0·37 (0·32–0·42)	0·55 (0·51–0·59)
1986	0·0 (0·0–0·0)	3·9 (3·1–4·8)	67·0 (58·4–75·9)	178·8 (169·3–189·2)	93·2 (82·0–103·8)	17·9 (14·5–21·7)	2·1 (1·5–3·0)	0·1 (0·0–0·1)	0·0 (0·0–0·0)	1·81 (1·74–1·9)	0·35 (0·31–0·4)	0·57 (0·51–0·62)
1987	0·0 (0·0–0·0)	3·8 (3·0–4·6)	62·7 (54·6–71·1)	174·1 (165·6–183·1)	96·2 (88·7–104·7)	18·7 (15·1–22·9)	2·4 (1·5–3·6)	0·1 (0·0–0·1)	0·0 (0·0–0·0)	1·79 (1·71–1·87)	0·33 (0·29–0·38)	0·59 (0·54–0·64)
1988	0·0 (0·0–0·0)	3·7 (3·0–4·5)	59·2 (51·3–68·0)	164·8 (154·4–175·5)	97·8 (88·9–106·7)	19·9 (15·9–24·4)	2·6 (1·8–3·5)	0·1 (0·0–0·1)	0·0 (0·0–0·0)	1·74 (1·66–1·83)	0·31 (0·28–0·36)	0·6 (0·55–0·65)
1989	0·0 (0·0–0·0)	3·7 (2·9–4·6)	55·9 (48·0–64·7)	155·1 (146·7–163·4)	98·4 (90·0–107·4)	21·1 (16·6–26·8)	2·7 (1·9–3·7)	0·1 (0·0–0·1)	0·0 (0·0–0·0)	1·68 (1·61–1·77)	0·3 (0·26–0·34)	0·61 (0·56–0·66)
1990	0·0 (0·0–0·0)	3·8 (3·1–4·5)	54·8 (47·8–62·8)	152·6 (144·6–161·0)	100·8 (91·9–109·6)	22·3 (18·0–27·6)	2·7 (1·9–3·6)	0·1 (0·0–0·1)	0·0 (0·0–0·0)	1·69 (1·61–1·77)	0·29 (0·26–0·33)	0·63 (0·58–0·68)
1991	0·0 (0·0–0·0)	3·9 (3·2–4·7)	55·4 (47·9–63·8)	149·3 (138·1–161·6)	101·9 (93·3–110·5)	23·8 (19·1–29·3)	2·7 (1·9–3·7)	0·1 (0·0–0·1)	0·0 (0·0–0·0)	1·69 (1·59–1·78)	0·3 (0·26–0·34)	0·64 (0·59–0·7)
1992	0·0 (0·0–0·0)	4·0 (3·4–4·8)	54·3 (47·4–62·4)	141·9 (134·2–149·4)	101·1 (90·5–113·1)	24·9 (20·2–30·5)	2·8 (2·0–3·9)	0·1 (0·0–0·1)	0·0 (0·0–0·0)	1·65 (1·56–1·73)	0·29 (0·26–0·33)	0·64 (0·59–0·71)
1993	0·0 (0·0–0·0)	4·2 (3·4–5·0)	54·6 (46·7–63·9)	142·2 (132·6–151·8)	105·4 (96·5–114·8)	27·0 (22·1–32·5)	3·0 (2·2–4·0)	0·1 (0·0–0·1)	0·0 (0·0–0·0)	1·68 (1·59–1·78)	0·29 (0·25–0·34)	0·68 (0·62–0·74)
1994	0·0 (0·0–0·0)	4·3 (3·4–5·1)	54·6 (47·4–62·8)	140·2 (131·0–148·8)	107·2 (97·2–117·8)	28·8 (23·6–34·9)	3·1 (2·2–4·2)	0·1 (0·0–0·1)	0·0 (0·0–0·0)	1·69 (1·6–1·79)	0·29 (0·26–0·33)	0·7 (0·64–0·75)
1995	0·0 (0·0–0·0)	4·4 (3·5–5·3)	54·1 (46·3–62·8)	133·0 (124·4–142·4)	106·2 (96·0–116·1)	29·9 (24·8–35·8)	3·2 (2·4–4·4)	0·1 (0·0–0·1)	0·0 (0·0–0·0)	1·65 (1·55–1·75)	0·29 (0·25–0·33)	0·7 (0·64–0·76)
1996	0·0 (0·0–0·0)	4·6 (3·7–5·6)	54·4 (46·8–62·4)	129·4 (120·3–138·8)	106·3 (97·0–116·9)	31·4 (25·7–37·9)	3·3 (2·5–4·4)	0·1 (0·0–0·1)	0·0 (0·0–0·0)	1·65 (1·55–1·76)	0·3 (0·26–0·34)	0·71 (0·65–0·77)
1997	0·0 (0·0–0·0)	5·0 (4·0–6·0)	55·2 (46·9–63·0)	126·3 (117·4–134·9)	104·9 (95·5–115·0)	32·0 (26·5–38·5)	3·5 (2·6–4·7)	0·1 (0·0–0·1)	0·0 (0·0–0·0)	1·63 (1·55–1·73)	0·3 (0·26–0·34)	0·7 (0·65–0·76)
1998	0·0 (0·0–0·0)	5·3 (4·4–6·5)	55·8 (48·0–64·1)	122·7 (114·6–130·5)	104·3 (94·8–114·9)	33·3 (27·0–40·2)	3·6 (2·7–4·7)	0·1 (0·0–0·1)	0·0 (0·0–0·0)	1·63 (1·54–1·72)	0·31 (0·27–0·35)	0·71 (0·65–0·77)
1999	0·0 (0·0–0·0)	5·7 (4·7–7·0)	56·2 (49·2–63·9)	119·7 (111·8–128·1)	103·8 (94·6–113·6)	34·0 (28·3–40·5)	3·8 (2·7–5·1)	0·1 (0·0–0·1)	0·0 (0·0–0·0)	1·62 (1·54–1·7)	0·31 (0·27–0·35)	0·71 (0·65–0·77)
2000	0·0 (0·0–0·0)	6·1 (5·0–7·4)	57·1 (47·5–67·2)	117·6 (110·0–126·1)	101·9 (93·2–111·7)	35·7 (29·5–42·4)	4·0 (2·9–5·4)	0·1 (0·0–0·1)	0·0 (0·0–0·0)	1·61 (1·51–1·72)	0·32 (0·27–0·36)	0·71 (0·65–0·77)
2001	0·0 (0·0–0·0)	6·4 (5·2–7·7)	57·4 (50·4–64·9)	114·3 (106·8–122·6)	98·6 (89·1–108·4)	35·7 (29·4–42·9)	4·1 (2·9–5·7)	0·1 (0·1–0·1)	0·0 (0·0–0·0)	1·58 (1·5–1·67)	0·32 (0·28–0·36)	0·69 (0·63–0·75)
2002	0·0 (0·0–0·0)	6·3 (5·1–7·7)	56·1 (48·5–65·1)	111·3 (104·2–118·5)	97·7 (88·3–107·5)	36·4 (30·3–43·9)	4·4 (3·0–6·1)	0·1 (0·1–0·1)	0·0 (0·0–0·0)	1·56 (1·48–1·64)	0·31 (0·27–0·36)	0·69 (0·63–0·76)
2003	0·0 (0·0–0·0)	6·0 (4·8–7·3)	54·1 (47·4–62·2)	108·5 (99·7–118·3)	96·7 (88·0–105·8)	38·0 (31·1–46·0)	4·7 (3·2–6·5)	0·1 (0·1–0·1)	0·0 (0·0–0·0)	1·54 (1·43–1·66)	0·3 (0·27–0·34)	0·7 (0·63–0·77)
2004	0·0 (0·0–0·0)	5·7 (4·6–7·0)	52·5 (45·3–61·4)	104·3 (98·1–110·9)	94·7 (85·9–104·3)	38·7 (32·1–46·2)	4·9 (3·4–6·5)	0·1 (0·1–0·2)	0·0 (0·0–0·0)	1·5 (1·43–1·58)	0·29 (0·25–0·34)	0·69 (0·63–0·76)
2005	0·0 (0·0–0·0)	5·5 (4·5–6·8)	52·2 (45·5–59·9)	103·3 (95·8–111·3)	94·7 (85·7–103·7)	39·5 (32·3–47·1)	5·1 (3·6–6·9)	0·1 (0·1–0·2)	0·0 (0·0–0·0)	1·5 (1·43–1·58)	0·29 (0·26–0·33)	0·7 (0·63–0·76)
2006	0·0 (0·0–0·0)	5·5 (4·4–6·9)	53·0 (46·4–60·3)	104·6 (97·8–112·0)	97·3 (88·1–107·1)	41·2 (34·5–49·2)	5·6 (4·0–7·7)	0·1 (0·1–0·2)	0·0 (0·0–0·0)	1·54 (1·46–1·61)	0·29 (0·26–0·33)	0·72 (0·66–0·79)
2007	0·0 (0·0–0·0)	5·5 (4·3–6·9)	53·1 (44·5–63·1)	104·4 (97·4–112·5)	97·2 (88·3–106·3)	43·3 (36·2–51·4)	5·9 (4·2–7·8)	0·1 (0·1–0·2)	0·0 (0·0–0·0)	1·55 (1·46–1·64)	0·29 (0·25–0·34)	0·73 (0·67–0·79)
2008	0·0 (0·0–0·0)	5·4 (4·3–6·8)	52·6 (45·6–59·7)	104·1 (97·7–111·4)	97·5 (88·1–106·8)	44·9 (37·8–52·9)	6·3 (4·7–8·4)	0·1 (0·1–0·2)	0·0 (0·0–0·0)	1·55 (1·48–1·63)	0·29 (0·26–0·33)	0·74 (0·69–0·8)
2009	0·0 (0·0–0·0)	5·3 (4·1–6·6)	51·4 (42·9–60·2)	104·0 (94·8–113·2)	98·1 (88·5–107·8)	46·6 (38·9–54·9)	6·9 (5·1–9·1)	0·1 (0·1–0·2)	0·0 (0·0–0·0)	1·56 (1·46–1·66)	0·28 (0·24–0·33)	0·76 (0·7–0·83)
2010	0·0 (0·0–0·0)	5·1 (3·7–6·8)	50·3 (43·8–57·0)	104·7 (98·2–111·5)	99·2 (90·1–108·7)	48·0 (40·3–56·1)	7·2 (5·4–9·4)	0·1 (0·1–0·2)	0·0 (0·0–0·0)	1·57 (1·5–1·65)	0·28 (0·24–0·31)	0·77 (0·71–0·84)
2011	0·0 (0·0–0·0)	5·0 (4·0–6·3)	48·3 (42·1–55·2)	104·1 (96·7–111·7)	99·2 (90·0–109·1)	49·0 (41·1–57·6)	7·4 (5·5–9·7)	0·1 (0·1–0·2)	0·0 (0·0–0·0)	1·57 (1·49–1·64)	0·27 (0·23–0·3)	0·78 (0·72–0·84)
2012	0·0 (0·0–0·0)	5·0 (4·1–5·9)	46·5 (40·7–52·8)	103·5 (95·7–111·6)	100·5 (90·9–111·3)	50·8 (43·2–59·7)	7·9 (5·9–10·3)	0·1 (0·1–0·3)	0·0 (0·0–0·0)	1·57 (1·49–1·67)	0·26 (0·23–0·29)	0·8 (0·73–0·87)
2013	0·0 (0·0–0·0)	4·9 (4·0–6·0)	45·4 (37·0–54·5)	101·8 (94·7–109·1)	100·4 (91·8–109·9)	51·3 (43·8–59·6)	8·3 (6·2–10·9)	0·2 (0·1–0·3)	0·0 (0·0–0·0)	1·56 (1·47–1·65)	0·25 (0·21–0·3)	0·8 (0·73–0·87)
2014	0·0 (0·0–0·0)	4·9 (4·0–5·8)	44·0 (37·7–50·7)	101·0 (93·8–108·9)	101·2 (92·1–111·1)	52·2 (44·8–61·0)	8·7 (6·3–11·5)	0·2 (0·1–0·3)	0·0 (0·0–0·0)	1·56 (1·47–1·66)	0·24 (0·21–0·28)	0·81 (0·75–0·88)
2015	0·0 (0·0–0·0)	4·9 (4·0–5·9)	44·7 (36·5–53·8)	102·8 (94·3–112·2)	103·3 (92·7–115·0)	49·8 (40·6–60·3)	8·1 (5·7–11·3)	0·2 (0·1–0·3)	0·0 (0·0–0·0)	1·57 (1·4–1·76)	0·25 (0·2–0·3)	0·81 (0·71–0·91)
2016	0·0 (0·0–0·0)	4·9 (4·0–6·1)	46·4 (34·9–60·8)	104·2 (92·8–117·3)	102·5 (89·0–118·5)	45·4 (34·9–58·9)	7·0 (4·6–10·1)	0·1 (0·1–0·3)	0·0 (0·0–0·0)	1·55 (1·33–1·83)	0·26 (0·2–0·33)	0·78 (0·65–0·92)
2017	0·0 (0·0–0·0)	4·9 (4·0–5·9)	46·1 (34·2–60·6)	104·1 (92·3–117·4)	100·8 (85·9–117·6)	43·8 (32·3–57·9)	6·8 (4·3–10·3)	0·1 (0·1–0·3)	0·0 (0·0–0·0)	1·53 (1·29–1·82)	0·25 (0·19–0·33)	0·76 (0·62–0·92)

Supplemental Results Table 5: Age-specific fertility rates per 1000, cumulative total fertility up to maternal age 25 (TFU25), cumulative total fertility from maternal age 30 to age 55 (TFO30), and cumulative total fertility, Saitama, 1950-2017

Year	10-14	15-19	20-24	25-29	30-34	35-39	40-44	45-49	50-54	TFR	TFU25	TFO30
1950	0·0 (0·0–0·0)	12·0 (10·6–13·4)	137·7 (125·8–150·4)	221·7 (213·6–229·8)	169·8 (159·6–180·1)	79·4 (70·5–88·1)	28·5 (24·6–32·7)	1·3 (0·8–1·8)	0·0 (0·0–0·0)	3·25 (3·14–3·37)	0·75 (0·69–0·81)	1·4 (1·32–1·47)
1951	0·0 (0·0–0·0)	9·6 (8·6–10·8)	123·4 (112·0–135·0)	206·1 (198·4–213·6)	153·8 (143·1–164·3)	68·4 (59·7–76·9)	22·1 (18·7–26·0)	0·9 (0·6–1·4)	0·0 (0·0–0·0)	2·92 (2·79–3·05)	0·67 (0·61–0·72)	1·23 (1·12–1·33)
1952	0·0 (0·0–0·0)	8·1 (7·1–9·2)	114·7 (103·4–126·8)	195·8 (187·6–204·3)	139·4 (128·5–150·4)	64·0 (54·5–75·9)	17·5 (14·4–20·9)	0·7 (0·5–1·1)	0·0 (0·0–0·0)	2·7 (2·6–2·81)	0·61 (0·56–0·67)	1·11 (1·03–1·18)
1953	0·0 (0·0–0·0)	7·1 (6·2–8·1)	108·2 (96·3–120·7)	184·5 (176·3–192·7)	124·7 (113·6–136·1)	59·0 (47·6–71·5)	13·8 (11·1–17·0)	0·6 (0·4–0·9)	0·0 (0·0–0·0)	2·49 (2·39–2·59)	0·58 (0·52–0·64)	0·99 (0·92–1·07)
1954	0·0 (0·0–0·0)	6·5 (5·6–7·5)	104·7 (92·7–116·7)	179·4 (172·0–187·3)	115·0 (105·5–126·1)	53·9 (42·3–67·6)	11·4 (9·0–14·5)	0·5 (0·3–0·7)	0·0 (0·0–0·0)	2·36 (2·26–2·45)	0·56 (0·5–0·62)	0·9 (0·84–0·98)
1955	0·0 (0·0–0·0)	5·9 (4·9–6·8)	102·0 (89·4–115·4)	178·3 (170·1–186·4)	106·0 (96·1–116·4)	48·7 (37·7–62·6)	9·5 (7·3–11·9)	0·4 (0·2–0·6)	0·0 (0·0–0·0)	2·25 (2·16–2·35)	0·54 (0·47–0·61)	0·82 (0·76–0·89)
1956	0·0 (0·0–0·0)	5·2 (4·5–6·1)	96·0 (84·5–107·5)	172·3 (164·8–180·2)	94·1 (85·1–104·0)	42·0 (31·2–55·1)	7·6 (5·9–9·6)	0·3 (0·2–0·5)	0·0 (0·0–0·0)	2·09 (2·01–2·18)	0·51 (0·45–0·56)	0·72 (0·66–0·78)
1957	0·0 (0·0–0·0)	5·0 (4·3–5·9)	96·9 (86·1–109·9)	175·6 (167·9–183·9)	89·3 (81·2–97·5)	36·1 (25·7–47·3)	6·5 (5·1–8·1)	0·3 (0·2–0·5)	0·0 (0·0–0·0)	2·05 (1·95–2·15)	0·51 (0·45–0·57)	0·66 (0·61–0·71)
1958	0·0 (0·0–0·0)	5·0 (4·3–5·8)	101·8 (89·2–115·7)	181·4 (173·1–190·0)	87·6 (79·8–96·3)	31·9 (24·1–41·2)	5·8 (4·6–7·3)	0·3 (0·2–0·5)	0·0 (0·0–0·0)	2·07 (1·98–2·16)	0·53 (0·47–0·61)	0·63 (0·58–0·69)
1959	0·0 (0·0–0·0)	5·3 (4·5–6·1)	101·9 (90·5–114·4)	180·5 (172·2–189·3)	82·4 (73·5–91·2)	27·5 (21·1–34·8)	4·9 (3·8–6·3)	0·3 (0·1–0·4)	0·0 (0·0–0·0)	2·01 (1·93–2·11)	0·54 (0·48–0·6)	0·58 (0·53–0·62)
1960	0·0 (0·0–0·0)	5·1 (4·4–6·0)	98·2 (87·5–110·2)	181·6 (173·4–189·9)	79·6 (71·9–87·2)	24·3 (19·0–30·4)	4·0 (3·0–5·2)	0·2 (0·1–0·4)	0·0 (0·0–0·0)	1·97 (1·88–2·05)	0·52 (0·46–0·58)	0·54 (0·49–0·59)
1961	0·0 (0·0–0·0)	4·8 (4·1–5·6)	95·6 (85·8–105·5)	183·9 (175·6–192·1)	78·4 (72·3–84·7)	21·8 (17·6–26·6)	3·5 (2·6–4·6)	0·2 (0·1–0·3)	0·0 (0·0–0·0)	1·94 (1·87–2·02)	0·5 (0·45–0·55)	0·52 (0·48–0·56)
1962	0·0 (0·0–0·0)	4·6 (3·9–5·4)	95·7 (85·3–106·3)	189·6 (181·8–197·5)	80·8 (74·2–87·5)	20·6 (17·2–24·8)	3·1 (2·3–4·1)	0·2 (0·1–0·3)	0·0 (0·0–0·0)	1·97 (1·9–2·05)	0·5 (0·45–0·55)	0·52 (0·49–0·57)
1963	0·0 (0·0–0·0)	4·5 (3·8–5·3)	96·5 (86·3–107·9)	191·9 (184·0–200·0)	83·2 (75·8–90·6)	19·9 (16·9–23·4)	3·1 (2·3–4·1)	0·2 (0·1–0·3)	0·0 (0·0–0·0)	2·0 (1·92–2·08)	0·51 (0·45–0·56)	0·53 (0·49–0·57)
1964	0·0 (0·0–0·0)	4·5 (3·8–5·3)	105·8 (95·1–117·7)	204·7 (196·4–213·1)	89·7 (81·6–98·2)	20·7 (17·2–24·6)	2·9 (2·2–3·9)	0·2 (0·1–0·3)	0·0 (0·0–0·0)	2·14 (2·06–2·23)	0·55 (0·5–0·61)	0·57 (0·53–0·61)
1965	0·0 (0·0–0·0)	4·4 (3·8–5·1)	98·0 (87·7–109·9)	173·3 (164·9–181·7)	75·6 (68·7–82·2)	19·1 (15·0–24·2)	2·6 (1·9–3·4)	0·1 (0·1–0·2)	0·0 (0·0–0·0)	1·87 (1·79–1·94)	0·51 (0·46–0·57)	0·49 (0·45–0·53)
1966	0·0 (0·0–0·0)	4·7 (4·0–5·4)	96·3 (86·4–107·3)	178·6 (170·5–186·6)	78·5 (71·0–87·0)	19·5 (15·4–24·0)	2·6 (1·9–3·4)	0·1 (0·1–0·2)	0·0 (0·0–0·0)	1·9 (1·82–1·98)	0·51 (0·45–0·56)	0·5 (0·46–0·54)
1967	0·0 (0·0–0·0)	5·0 (4·2–5·8)	103·3 (92·8–114·6)	218·4 (209·9–226·3)	97·1 (88·3–106·8)	22·2 (17·0–28·3)	2·6 (1·9–3·4)	0·1 (0·1–0·2)	0·0 (0·0–0·0)	2·24 (2·15–2·34)	0·54 (0·49–0·6)	0·61 (0·57–0·66)
1968	0·0 (0·0–0·0)	5·1 (4·4–5·9)	93·2 (82·7–104·5)	206·7 (198·0–215·0)	91·5 (82·9–100·2)	22·3 (16·9–29·0)	2·5 (1·9–3·4)	0·1 (0·1–0·2)	0·0 (0·0–0·0)	2·11 (2·03–2·18)	0·49 (0·44–0·55)	0·58 (0·54–0·63)
1969	0·0 (0·0–0·0)	5·5 (4·7–6·5)	92·3 (82·0–102·9)	209·5 (201·4–217·2)	92·3 (84·3–101·0)	22·0 (16·6–28·4)	2·5 (1·8–3·3)	0·1 (0·1–0·2)	0·0 (0·0–0·0)	2·12 (2·04–2·2)	0·49 (0·44–0·54)	0·58 (0·54–0·63)
1970	0·0 (0·0–0·0)	5·6 (4·6–6·7)	99·9 (88·1–112·2)	213·5 (204·8–222·1)	94·8 (86·4–104·1)	21·1 (15·9–27·4)	2·5 (1·9–3·5)	0·1 (0·1–0·2)	0·0 (0·0–0·0)	2·19 (2·1–2·27)	0·53 (0·47–0·59)	0·59 (0·55–0·64)
1971	0·0 (0·0–0·0)	5·5 (4·5–6·7)	110·1 (97·3–122·8)	211·0 (202·6–219·8)	97·3 (88·7–106·0)	19·2 (14·6–25·1)	2·8 (2·0–3·8)	0·1 (0·1–0·2)	0·0 (0·0–0·0)	2·23 (2·14–2·33)	0·58 (0·52–0·64)	0·6 (0·55–0·65)
1972	0·0 (0·0–0·0)	5·5 (4·5–6·6)	115·6 (103·3–128·9)	209·6 (201·7–218·1)	98·5 (89·3–107·5)	18·2 (13·8–23·6)	2·8 (2·0–3·9)	0·2 (0·1–0·2)	0·0 (0·0–0·0)	2·25 (2·17–2·35)	0·61 (0·55–0·67)	0·6 (0·55–0·65)
1973	0·0 (0·0–0·0)	5·4 (4·4–6·5)	115·9 (103·8–128·8)	209·3 (200·1–218·3)	94·2 (86·4–103·0)	18·1 (14·0–22·7)	2·6 (1·8–3·6)	0·1 (0·1–0·2)	0·0 (0·0–0·0)	2·23 (2·14–2·32)	0·61 (0·55–0·67)	0·58 (0·53–0·62)
1974	0·0 (0·0–0·0)	4·9 (4·0–5·9)	109·8 (98·3–121·8)	200·0 (191·1–208·6)	82·9 (75·0–90·9)	17·1 (13·6–21·3)	2·2 (1·6–2·9)	0·1 (0·0–0·2)	0·0 (0·0–0·0)	2·08 (2·0–2·17)	0·57 (0·52–0·63)	0·51 (0·47–0·55)
1975	0·0 (0·0–0·0)	4·4 (3·7–5·4)	100·3 (89·6–111·4)	190·7 (182·3–198·8)	73·5 (66·6–80·3)	16·2 (13·4–19·5)	1·8 (1·4–2·3)	0·1 (0·0–0·1)	0·0 (0·0–0·0)	1·93 (1·86–2·01)	0·52 (0·47–0·58)	0·46 (0·42–0·5)
1976	0·0 (0·0–0·0)	4·1 (3·3–5·0)	91·6 (81·8–102·4)	185·7 (177·6–193·7)	72·1 (65·8–78·8)	16·0 (12·0–20·2)	1·8 (1·3–2·4)	0·1 (0·0–0·1)	0·0 (0·0–0·0)	1·86 (1·78–1·93)	0·48 (0·43–0·53)	0·45 (0·42–0·48)
1977	0·0 (0·0–0·0)	3·9 (2·9–5·0)	83·4 (74·6–93·7)	184·3 (176·6–192·9)	75·4 (68·2–82·7)	15·6 (12·5–19·0)	1·9 (1·3–2·6)	0·1 (0·0–0·1)	0·0 (0·0–0·0)	1·82 (1·75–1·91)	0·44 (0·39–0·49)	0·46 (0·42–0·52)
1978	0·0 (0·0–0·0)	3·8 (3·1–4·7)	76·9 (68·4–86·8)	183·0 (175·7–190·4)	79·0 (72·2–86·8)	15·0 (13·1–17·0)	1·9 (1·4–2·5)	0·1 (0·0–0·1)	0·0 (0·0–0·0)	1·8 (1·73–1·87)	0·4 (0·36–0·45)	0·48 (0·44–0·52)
1979	0·0 (0·0–0·0)	3·8 (3·3–4·3)	71·6 (64·1–79·5)	180·3 (171·0–188·9)	78·5 (71·7–86·0)	14·4 (11·5–17·7)	1·9 (1·4–2·7)	0·1 (0·0–0·1)	0·0 (0·0–0·0)	1·75 (1·68–1·83)	0·38 (0·34–0·42)	0·47 (0·44–0·52)
1980	0·0 (0·0–0·0)	3·8 (3·2–4·5)	67·3 (59·6–74·9)	177·8 (170·0–186·1)	76·2 (69·3–83·5)	14·0 (11·5–16·8)	1·9 (1·4–2·6)	0·1 (0·0–0·1)	0·0 (0·0–0·0)	1·71 (1·63–1·78)	0·36 (0·32–0·39)	0·46 (0·43–0·5)
1981	0·0 (0·0–0·0)	3·9 (3·3–4·7)	64·8 (57·7–72·9)	178·8 (170·8–186·8)	76·5 (69·8–83·2)	14·5 (11·9–17·4)	1·9 (1·3–2·8)	0·1 (0·0–0·1)	0·0 (0·0–0·0)	1·7 (1·64–1·76)	0·34 (0·31–0·38)	0·47 (0·43–0·5)
1982	0·0 (0·0–0·0)	4·1 (3·3–4·9)	63·3 (56·0–70·8)	179·7 (172·1–187·7)	80·0 (73·1–87·5)	16·1 (13·7–18·9)	1·9 (1·4–2·6)	0·1 (0·0–0·1)	0·0 (0·0–0·0)	1·73 (1·66–1·79)	0·34 (0·3–0·37)	0·49 (0·45–0·53)
1983	0·0 (0·0–0·0)	4·1 (3·5–4·9)	61·3 (54·2–69·5)	178·1 (169·8–186·2)	84·2 (77·0–92·1)	17·8 (14·8–21·4)	2·0 (1·5–2·6)	0·1 (0·0–0·1)	0·0 (0·0–0·0)	1·74 (1·68–1·81)	0·33 (0·29–0·37)	0·52 (0·48–0·56)
1984	0·0 (0·0–0·0)	4·0 (3·3–4·7)	56·7 (50·6–63·8)	172·5 (165·1–180·5)	87·5 (80·3–95·3)	18·4 (15·5–22·0)	2·0 (1·4–2·7)	0·1 (0·0–0·1)	0·0 (0·0–0·0)	1·71 (1·64–1·77)	0·3 (0·27–0·34)	0·54 (0·5–0·58)
1985	0·0 (0·0–0·0)	3·8 (3·2–4·5)	53·1 (46·6–59·7)	163·6 (156·4–170·3)	89·1 (81·0–96·9)	18·3 (15·2–21·6)	2·0 (1·4–2·9)	0·1 (0·0–0·1)	0·0 (0·0–0·0)	1·65 (1·6–1·71)	0·28 (0·25–0·32)	0·55 (0·5–0·59)
1986	0·0 (0·0–0·0)	3·7 (3·1–4·3)	50·6 (44·3–57·3)	156·1 (148·9–163·8)	91·6 (84·4–98·2)	18·3 (15·2–21·8)	2·1 (1·5–2·9)	0·1 (0·0–0·1)	0·0 (0·0–0·0)	1·61 (1·56–1·67)	0·27 (0·24–0·3)	0·56 (0·52–0·6)
1987	0·0 (0·0–0·0)	3·6 (3·0–4·2)	46·9 (41·9–52·8)	149·9 (142·6–157·4)	94·4 (87·0–102·9)	19·1 (15·9–22·9)	2·3 (1·6–3·2)	0·1 (0·0–0·1)	0·0 (0·0–0·0)	1·58 (1·51–1·65)	0·25 (0·23–0·28)	0·58 (0·54–0·62)
1988	0·0 (0·0–0·0)	3·5 (2·9–4·2)	43·8 (38·4–49·9)	139·9 (133·5–147·0)	94·8 (87·1–103·2)	20·1 (16·7–24·0)	2·5 (1·7–3·4)	0·1 (0·0–0·1)	0·0 (0·0–0·0)	1·52 (1·46–1·59)	0·24 (0·21–0·27)	0·59 (0·54–0·63)
1989	0·0 (0·0–0·0)	3·5 (2·9–4·2)	40·9 (35·6–47·0)	130·2 (123·5–137·3)	93·7 (86·0–101·1)	21·2 (17·8–25·1)	2·6 (1·8–3·6)	0·1 (0·0–0·1)	0·0 (0·0–0·0)	1·46 (1·4–1·52)	0·22 (0·2–0·25)	0·59 (0·54–0·63)
1990	0·0 (0·0–0·0)	3·6 (2·9–4·3)	39·9 (35·1–44·8)	127·1 (120·1–133·9)	94·2 (86·5–101·8)	22·3 (18·8–26·6)	2·5 (1·8–3·4)	0·1 (0·1–0·1)	0·0 (0·0–0·0)	1·45 (1·39–1·51)	0·22 (0·19–0·24)	0·6 (0·55–0·64)
1991	0·0 (0·0–0·0)	3·7 (3·0–4·5)	39·9 (35·1–45·1)	123·6 (117·7–129·9)	94·1 (86·0–102·5)	23·7 (19·9–27·8)	2·5 (1·8–3·3)	0·1 (0·1–0·1)	0·0 (0·0–0·0)	1·44 (1·38–1·5)	0·22 (0·19–0·24)	0·6 (0·56–0·65)
1992	0·0 (0·0–0·0)	3·7 (3·0–4·5)	38·6 (33·2–45·0)	116·9 (110·2–123·6)	92·9 (86·0–100·4)	24·6 (20·8–28·6)	2·6 (1·9–3·5)	0·1 (0·1–0·1)	0·0 (0·0–0·0)	1·4 (1·34–1·46)	0·21 (0·18–0·24)	0·6 (0·56–0·65)
1993	0·0 (0·0–0·0)	3·7 (2·9–4·4)	37·9 (33·3–42·8)	116·4 (109·9–123·4)	97·1 (89·4–105·1)	26·8 (21·9–32·9)	2·8 (2·1–3·7)	0·1 (0·1–0·1)	0·0 (0·0–0·0)	1·42 (1·36–1·49)	0·21 (0·18–0·23)	0·63 (0·59–0·68)
1994	0·0 (0·0–0·0)	3·6 (3·0–4·4)	36·9 (32·7–41·7)	113·8 (107·5–120·0)	98·8 (90·7–107·3)	28·4 (23·9–33·4)	3·0 (2·2–4·0)	0·1 (0·1–0·2)	0·0 (0·0–0·0)	1·42 (1·35–1·49)	0·2 (0·18–0·23)	0·65 (0·6–0·7)
1995	0·0 (0·0–0·0)	3·6 (2·9–4·3)	35·3 (30·9–40·0)	106·6 (100·5–112·7)	97·5 (89·5–106·1)	29·5 (24·6–34·6)	3·2 (2·3–4·2)	0·1 (0·1–0·2)	0·0 (0·0–0·0)	1·38 (1·31–1·45)	0·19 (0·17–0·22)	0·65 (0·6–0·7)
1996	0·0 (0·0–0·0)	3·7 (3·1–4·4)	34·4 (30·1–39·0)	102·1 (96·1–108·3)	96·7 (88·7–105·4)	30·8 (25·8–37·0)	3·4 (2·4–4·4)	0·1 (0·1–0·2)	0·0 (0·0–0·0)	1·36 (1·29–1·42)	0·19 (0·17–0·21)	0·65 (0·61–0·71)
1997	0·0 (0·0–0·0)	3·9 (3·2–4·6)	33·9 (29·8–38·4)	98·1 (92·7–103·5)	94·3 (87·4–102·3)	31·3 (26·4–36·9)	3·6 (2·6–4·8)	0·1 (0·1–0·2)	0·0 (0·0–0·0)	1·33 (1·27–1·39)	0·19 (0·17–0·21)	0·65 (0·6–0·7)
1998	0·0 (0·0–0·0)	4·1 (3·4–4·8)	33·6 (29·6–38·6)	94·1 (88·5–99·7)	92·7 (85·2–101·3)	32·3 (27·1–37·6)	3·8 (2·7–5·1)	0·1 (0·1–0·2)	0·0 (0·0–0·0)	1·3 (1·24–1·37)	0·19 (0·17–0·21)	0·64 (0·6–0·69)
1999	0·0 (0·0–0·0)	4·4 (3·7–5·3)	33·7 (29·4–38·3)	91·1 (85·6–96·5)	91·7 (84·3–100·4)	32·7 (27·7–38·1)	3·9 (2·8–5·3)	0·1 (0·1–0·2)	0·0 (0·0–0·0)	1·29 (1·23–1·35)	0·19 (0·17–0·21)	0·64 (0·59–0·7)
2000	0·0 (0·0–0·0)	4·8 (4·0–5·8)	34·2 (30·4–38·4)	89·6 (84·5–95·2)	89·8 (82·6–97·2)	33·9 (28·8–39·0)	4·1 (3·0–5·4)	0·1 (0·1–0·2)	0·0 (0·0–0·0)	1·28 (1·23–1·34)	0·2 (0·17–0·22)	0·64 (0·59–0·69)
2001	0·0 (0·0–0·0)	5·1 (4·3–6·1)	34·8 (30·4–39·6)	87·5 (82·5–93·4)	86·9 (78·9–94·8)	33·5 (28·7–39·3)	4·2 (3·1–5·5)	0·1 (0·1–0·2)	0·0 (0·0–0·0)	1·26 (1·2–1·32)	0·2 (0·18–0·22)	0·62 (0·58–0·67)
2002	0·0 (0·0–0·0)	5·1 (4·3–6·1)	34·2 (29·8–38·8)	85·6 (80·3–91·1)	86·3 (79·0–94·0)	33·8 (29·0–39·3)	4·5 (3·2–5·9)	0·1 (0·1–0·2)	0·0 (0·0–0·0)	1·25 (1·19–1·31)	0·2 (0·18–0·22)	0·62 (0·58–0·67)
2003	0·0 (0·0–0·0)	4·9 (4·1–5·9)	33·1 (28·9–37·9)	83·8 (79·2–88·6)	85·8 (78·9–92·8)	35·1 (29·8–41·2)	4·8 (3·4–6·3)	0·1 (0·1–0·2)	0·0 (0·0–0·0)	1·24 (1·18–1·3)	0·19 (0·17–0·21)	0·63 (0·58–0·68)
2004	0·0 (0·0–0·0)	4·7 (4·0–5·5)	32·0 (28·1–36·8)	80·6 (75·5–86·0)	84·6 (76·7–92·5)	35·9 (30·5–41·9)	5·0 (3·7–6·6)	0·1 (0·1–0·2)	0·0 (0·0–0·0)	1·21 (1·16–1·27)	0·18 (0·16–0·21)	0·63 (0·58–0·68)
2005	0·0 (0·0–0·0)	4·6 (3·9–5·4)	31·7 (27·9–35·7)	79·6 (74·3–84·9)	85·3 (78·3–92·3)	37·1 (31·5–43·2)	5·2 (3·9–6·8)	0·1 (0·1–0·2)	0·0 (0·0–0·0)	1·22 (1·17–1·27)	0·18 (0·16–0·2)	0·64 (0·59–0·69)
2006	0·0 (0·0–0·0)	4·5 (3·8–5·3)	31·9 (27·7–36·2)	80·1 (74·6–85·2)	88·6 (81·2–96·1)	39·4 (33·9–45·9)	5·8 (4·5–7·3)	0·1 (0·1–0·2)	0·0 (0·0–0·0)	1·25 (1·2–1·31)	0·18 (0·16–0·2)	0·67 (0·63–0·72)
2007	0·0 (0·0–0·0)	4·5 (3·8–5·3)	31·6 (28·2–35·5)	79·4 (74·3–85·1)	89·3 (82·2–97·2)	41·8 (36·2–48·2)	6·3 (4·8–8·1)	0·2 (0·1–0·3)	0·0 (0·0–0·0)	1·26 (1·21–1·32)	0·18 (0·16–0·2)	0·69 (0·64–0·74)
2008	0·0 (0·0–0·0)	4·3 (3·7–5·1)	30·9 (26·7–35·6)	78·6 (73·3–83·6)	90·1 (82·9–97·4)	43·4 (37·5–50·0)	6·8 (5·3–8·6)	0·2 (0·1–0·3)	0·0 (0·0–0·0)	1·27 (1·22–1·33)	0·18 (0·16–0·2)	0·7 (0·65–0·75)
2009	0·0 (0·0–0·0)	4·1 (3·4–4·8)	29·5 (26·6–32·5)	78·1 (73·5–82·5)	90·7 (83·2–98·6)	44·7 (38·7–51·1)	7·6 (5·9–9·8)	0·2 (0·1–0·3)	0·0 (0·0–0·0)	1·27 (1·21–1·34)	0·17 (0·15–0·18)	0·72 (0·67–0·77)
2010	0·0 (0·0–0·0)	3·9 (3·3–4·6)	28·4 (23·9–33·1)	78·4 (73·4–83·7)	91·5 (84·1–100·1)	45·6 (39·1–52·3)	8·1 (6·3–10·2)	0·2 (0·1–0·3)	0·0 (0·0–0·0)	1·28 (1·21–1·35)	0·16 (0·14–0·19)	0·73 (0·67–0·78)
2011	0·0 (0·0–0·0)	3·7 (3·1–4·4)	26·8 (23·2–30·8)	77·7 (72·4–83·0)	91·4 (84·2–99·0)	46·4 (39·6–53·7)	8·5 (6·7–10·7)	0·2 (0·1–0·3)	0·0 (0·0–0·0)	1·27 (1·22–1·33)	0·15 (0·13–0·17)	0·73 (0·69–0·79)
2012	0·0 (0·0–0·0)	3·6 (2·9–4·4)	25·4 (21·9–29·4)	76·9 (72·3–81·5)	92·8 (84·7–100·8)	48·6 (42·2–55·2)	9·3 (7·3–11·6)	0·2 (0·1–0·4)	0·0 (0·0–0·0)	1·28 (1·23–1·34)	0·14 (0·13–0·16)	0·75 (0·7–0·81)
2013	0·0 (0·0–0·0)	3·5 (2·9–4·2)	24·4 (21·0–27·9)	75·3 (70·3–80·6)	93·5 (86·4–101·3)	50·2 (43·8–57·3)	9·8 (7·7–12·4)	0·2 (0·1–0·4)	0·0 (0·0–0·0)	1·28 (1·22–1·35)	0·14 (0·12–0·16)	0·77 (0·72–0·82)
2014	0·0 (0·0–0·0)	3·4 (2·9–4·1)	23·5 (19·9–27·6)	74·5 (69·4–79·8)	95·3 (87·5–103·5)	52·5 (45·8–59·9)	10·3 (8·3–12·7)	0·3 (0·2–0·4)	0·0 (0·0–0·0)	1·3 (1·23–1·38)	0·13 (0·12–0·15)	0·79 (0·73–0·86)
2015	0·0 (0·0–0·0)	3·4 (2·8–4·1)	23·9 (20·4–28·1)	75·7 (69·9–82·2)	98·6 (89·2–108·9)	51·5 (42·9–61·4)	9·6 (7·4–12·3)	0·2 (0·2–0·4)	0·0 (0·0–0·0)	1·31 (1·18–1·47)	0·14 (0·12–0·16)	0·8 (0·71–0·9)
2016	0·0 (0·0–0·0)	3·5 (2·9–4·3)	25·0 (19·6–31·6)	76·8 (69·0–86·3)	98·9 (86·2–114·6)	47·9 (37·0–61·5)	8·2 (5·9–11·0)	0·2 (0·1–0·3)	0·0 (0·0–0·0)	1·3 (1·11–1·53)	0·14 (0·11–0·18)	0·78 (0·66–0·92)
2017	0·0 (0·0–0·0)	3·5 (2·9–4·2)	25·1 (19·7–32·1)	76·5 (67·9–85·9)	98·1 (83·8–113·7)	47·0 (35·2–61·7)	8·0 (5·3–11·4)	0·2 (0·1–0·3)	0·0 (0·0–0·0)	1·29 (1·08–1·54)	0·14 (0·11–0·18)	0·77 (0·62–0·93)

Supplemental Results Table 5: Age-specific fertility rates per 1000, cumulative total fertility up to maternal age 25 (TFU25), cumulative total fertility from maternal age 30 to age 55 (TFO30), and cumulative total fertility, Shiga, 1950-2017

Year	10-14	15-19	20-24	25-29	30-34	35-39	40-44	45-49	50-54	TFR	TFU25	TFO30
1950	0·0 (0·0–0·0)	10·4 (9·2–11·6)	150·5 (137·4–164·8)	247·2 (237·7–256·3)	177·9 (167·1–188·7)	84·9 (75·4–94·2)	30·4 (26·3–34·9)	1·1 (0·8–1·7)	0·0 (0·0–0·0)	3·51 (3·39–3·64)	0·8 (0·74–0·88)	1·47 (1·4–1·55)
1951	0·0 (0·0–0·0)	8·3 (7·4–9·4)	135·0 (121·9–148·4)	230·9 (221·5–240·3)	161·1 (149·3–172·2)	68·5 (59·5–77·6)	23·6 (19·6–27·7)	0·8 (0·5–1·2)	0·0 (0·0–0·0)	3·14 (3·0–3·28)	0·72 (0·65–0·79)	1·27 (1·16–1·38)
1952	0·0 (0·0–0·0)	7·0 (6·1–8·0)	125·6 (113·2–140·7)	220·2 (210·9–229·7)	146·0 (132·0–159·6)	55·2 (47·5–63·3)	18·7 (15·2–22·5)	0·6 (0·4–1·0)	0·0 (0·0–0·0)	2·87 (2·75–2·99)	0·66 (0·6–0·74)	1·1 (1·02–1·19)
1953	0·0 (0·0–0·0)	6·1 (5·3–7·1)	118·6 (105·2–133·0)	208·0 (198·8–218·3)	130·6 (115·6–145·7)	46·5 (39·3–54·3)	14·8 (12·0–18·1)	0·5 (0·3–0·8)	0·0 (0·0–0·0)	2·63 (2·5–2·76)	0·62 (0·56–0·7)	0·96 (0·88–1·05)
1954	0·0 (0·0–0·0)	5·6 (4·8–6·5)	114·9 (100·2–131·0)	202·6 (193·8–212·3)	120·4 (105·5–135·8)	42·4 (35·5–50·5)	12·2 (9·5–15·4)	0·4 (0·3–0·7)	0·0 (0·0–0·0)	2·49 (2·36–2·64)	0·6 (0·53–0·68)	0·88 (0·8–0·96)
1955	0·0 (0·0–0·0)	5·1 (4·4–5·9)	112·0 (97·6–128·1)	201·8 (192·4–211·5)	110·9 (96·0–126·3)	38·1 (32·2–45·3)	10·1 (7·7–12·9)	0·3 (0·2–0·6)	0·0 (0·0–0·0)	2·39 (2·27–2·52)	0·59 (0·51–0·67)	0·8 (0·72–0·87)
1956	0·0 (0·0–0·0)	4·5 (3·8–5·3)	105·4 (90·8–120·4)	195·3 (186·9–204·4)	98·3 (85·4–112·7)	32·7 (27·3–38·5)	8·1 (6·1–10·3)	0·3 (0·2–0·5)	0·0 (0·0–0·0)	2·22 (2·11–2·35)	0·55 (0·48–0·63)	0·7 (0·63–0·76)
1957	0·0 (0·0–0·0)	4·3 (3·7–5·1)	106·4 (91·9–123·3)	198·8 (189·7–208·2)	93·1 (80·9–107·9)	28·0 (23·3–32·5)	6·8 (5·2–8·7)	0·3 (0·2–0·5)	0·0 (0·0–0·0)	2·19 (2·05–2·32)	0·55 (0·48–0·64)	0·64 (0·58–0·71)
1958	0·0 (0·0–0·0)	4·3 (3·7–5·1)	111·6 (96·8–127·1)	205·0 (195·1–214·8)	91·0 (78·8–104·9)	24·7 (20·4–29·3)	6·2 (4·7–7·9)	0·3 (0·1–0·5)	0·0 (0·0–0·0)	2·22 (2·1–2·34)	0·58 (0·51–0·66)	0·61 (0·55–0·68)
1959	0·0 (0·0–0·0)	4·5 (3·9–5·3)	111·5 (97·2–127·7)	204·0 (193·9–213·4)	85·3 (74·7–97·5)	21·3 (17·4–25·9)	5·1 (3·9–6·7)	0·2 (0·1–0·4)	0·0 (0·0–0·0)	2·16 (2·05–2·28)	0·58 (0·51–0·66)	0·56 (0·51–0·62)
1960	0·0 (0·0–0·0)	4·4 (3·7–5·2)	107·1 (94·4–120·5)	205·3 (195·7–215·6)	81·9 (73·0–91·5)	18·8 (15·4–22·7)	4·2 (3·1–5·4)	0·2 (0·1–0·3)	0·0 (0·0–0·0)	2·11 (2·01–2·21)	0·56 (0·5–0·63)	0·53 (0·48–0·57)
1961	0·0 (0·0–0·0)	4·1 (3·4–4·8)	103·8 (93·5–115·1)	207·7 (198·8–217·4)	80·2 (70·4–91·4)	16·9 (13·7–20·4)	3·6 (2·6–4·9)	0·2 (0·1–0·3)	0·0 (0·0–0·0)	2·08 (1·99–2·18)	0·54 (0·49–0·6)	0·5 (0·46–0·56)
1962	0·0 (0·0–0·0)	3·9 (3·2–4·7)	103·4 (92·1–115·1)	213·7 (204·5–222·9)	81·9 (74·9–88·5)	16·0 (13·1–19·1)	3·2 (2·2–4·4)	0·1 (0·1–0·3)	0·0 (0·0–0·0)	2·11 (2·03–2·19)	0·54 (0·48–0·59)	0·51 (0·47–0·54)
1963	0·0 (0·0–0·0)	3·8 (3·2–4·6)	103·7 (92·5–115·8)	215·4 (206·3–224·7)	83·7 (76·6–91·4)	15·3 (12·9–18·3)	3·1 (2·2–4·3)	0·2 (0·1–0·3)	0·0 (0·0–0·0)	2·13 (2·04–2·21)	0·54 (0·48–0·6)	0·51 (0·47–0·55)
1964	0·0 (0·0–0·0)	3·8 (3·2–4·5)	113·1 (100·3–127·3)	229·3 (219·1–239·5)	89·5 (81·1–99·0)	15·9 (13·4–18·5)	2·9 (2·1–4·1)	0·1 (0·1–0·3)	0·0 (0·0–0·0)	2·27 (2·17–2·38)	0·58 (0·52–0·66)	0·54 (0·5–0·59)
1965	0·0 (0·0–0·0)	3·7 (3·1–4·4)	104·1 (90·9–119·5)	194·2 (183·9–204·3)	74·7 (66·0–83·7)	14·6 (11·9–17·7)	2·5 (1·7–3·4)	0·1 (0·1–0·2)	0·0 (0·0–0·0)	1·97 (1·86–2·08)	0·54 (0·47–0·62)	0·46 (0·41–0·51)
1966	0·0 (0·0–0·0)	3·9 (3·3–4·6)	101·7 (85·8–118·8)	199·6 (189·4–209·2)	76·7 (67·1–86·7)	14·7 (12·4–17·5)	2·4 (1·7–3·4)	0·1 (0·1–0·2)	0·0 (0·0–0·0)	2·0 (1·88–2·13)	0·53 (0·45–0·61)	0·47 (0·42–0·52)
1967	0·0 (0·0–0·0)	4·1 (3·5–4·8)	108·3 (90·0–127·5)	243·6 (233·9–252·8)	92·8 (79·2–107·3)	16·7 (13·7–20·3)	2·3 (1·8–3·0)	0·1 (0·0–0·2)	0·0 (0·0–0·0)	2·34 (2·17–2·51)	0·56 (0·47–0·66)	0·56 (0·5–0·63)
1968	0·0 (0·0–0·0)	4·2 (3·6–4·9)	96·9 (80·3–116·3)	230·6 (220·8–240·1)	85·0 (72·5–97·6)	16·6 (13·3–20·2)	2·1 (1·6–2·8)	0·1 (0·0–0·2)	0·0 (0·0–0·0)	2·18 (2·04–2·32)	0·51 (0·42–0·6)	0·52 (0·46–0·58)
1969	0·0 (0·0–0·0)	4·6 (4·0–5·3)	95·2 (79·6–113·0)	232·9 (223·6–242·0)	85·6 (72·0–100·8)	16·1 (13·2–19·6)	2·1 (1·6–2·8)	0·1 (0·0–0·2)	0·0 (0·0–0·0)	2·18 (2·06–2·33)	0·5 (0·42–0·59)	0·52 (0·46–0·59)
1970	0·0 (0·0–0·0)	4·6 (3·9–5·4)	102·3 (86·4–120·4)	236·0 (226·7–244·8)	88·2 (75·9–102·0)	15·3 (12·2–18·9)	2·2 (1·6–2·9)	0·1 (0·0–0·2)	0·0 (0·0–0·0)	2·24 (2·11–2·38)	0·53 (0·46–0·63)	0·53 (0·47–0·59)
1971	0·0 (0·0–0·0)	4·5 (3·8–5·2)	112·0 (94·5–130·6)	232·4 (223·1–241·7)	89·7 (77·4–103·9)	13·8 (11·2–16·8)	2·4 (1·7–3·3)	0·1 (0·0–0·2)	0·0 (0·0–0·0)	2·27 (2·15–2·41)	0·58 (0·5–0·68)	0·53 (0·47–0·6)
1972	0·0 (0·0–0·0)	4·5 (3·8–5·4)	117·2 (101·4–134·8)	230·6 (221·8–239·8)	90·1 (79·2–101·3)	13·0 (10·2–15·9)	2·4 (1·7–3·4)	0·1 (0·0–0·2)	0·0 (0·0–0·0)	2·29 (2·17–2·41)	0·61 (0·53–0·7)	0·53 (0·48–0·58)
1973	0·0 (0·0–0·0)	4·4 (3·7–5·3)	117·4 (102·9–132·3)	231·2 (221·4–241·6)	85·9 (76·9–95·9)	12·7 (10·0–15·8)	2·2 (1·5–3·1)	0·1 (0·0–0·2)	0·0 (0·0–0·0)	2·27 (2·16–2·38)	0·61 (0·54–0·68)	0·5 (0·46–0·55)
1974	0·0 (0·0–0·0)	4·1 (3·3–5·0)	112·3 (100·1–125·5)	223·2 (212·9–233·4)	76·3 (68·4–84·2)	11·8 (9·1–14·9)	1·9 (1·3–2·7)	0·1 (0·0–0·1)	0·0 (0·0–0·0)	2·15 (2·07–2·24)	0·58 (0·52–0·65)	0·45 (0·41–0·49)
1975	0·0 (0·0–0·0)	3·8 (3·1–4·6)	104·8 (92·5–117·5)	216·2 (206·3–226·1)	68·0 (61·4–74·7)	11·1 (8·7–14·2)	1·6 (1·1–2·2)	0·0 (0·0–0·1)	0·0 (0·0–0·0)	2·03 (1·94–2·12)	0·54 (0·48–0·61)	0·4 (0·37–0·44)
1976	0·0 (0·0–0·0)	3·5 (2·9–4·3)	98·7 (88·0–110·7)	214·1 (203·9–225·2)	65·3 (58·8–72·3)	11·0 (8·9–13·5)	1·4 (0·9–2·3)	0·0 (0·0–0·1)	0·0 (0·0–0·0)	1·97 (1·88–2·07)	0·51 (0·46–0·57)	0·39 (0·35–0·43)
1977	0·0 (0·0–0·0)	3·4 (2·9–4·0)	92·8 (82·3–105·3)	214·9 (205·2–224·5)	67·4 (60·6–75·0)	11·1 (8·6–14·3)	1·4 (0·9–2·1)	0·0 (0·0–0·1)	0·0 (0·0–0·0)	1·96 (1·87–2·04)	0·48 (0·43–0·54)	0·4 (0·36–0·45)
1978	0·0 (0·0–0·0)	3·4 (2·7–4·2)	87·8 (79·0–97·9)	214·8 (205·3–223·9)	71·8 (65·3–78·7)	11·1 (8·2–14·7)	1·4 (0·9–2·2)	0·0 (0·0–0·1)	0·0 (0·0–0·0)	1·95 (1·88–2·03)	0·46 (0·41–0·51)	0·42 (0·39–0·46)
1979	0·0 (0·0–0·0)	3·5 (2·6–4·5)	83·1 (73·5–93·9)	212·2 (202·9–221·4)	73·5 (64·2–83·9)	11·0 (9·0–13·4)	1·5 (0·9–2·3)	0·0 (0·0–0·1)	0·0 (0·0–0·0)	1·92 (1·83–2·01)	0·43 (0·38–0·49)	0·43 (0·38–0·48)
1980	0·0 (0·0–0·0)	3·5 (2·8–4·4)	78·6 (69·3–88·3)	209·3 (199·5–219·5)	73·3 (66·8–80·0)	11·0 (8·8–13·7)	1·4 (1·0–2·1)	0·0 (0·0–0·1)	0·0 (0·0–0·0)	1·89 (1·8–1·97)	0·41 (0·36–0·46)	0·43 (0·4–0·47)
1981	0·0 (0·0–0·0)	3·7 (3·1–4·5)	76·1 (67·4–85·7)	210·7 (200·7–220·6)	74·7 (68·1–82·5)	11·6 (9·2–14·3)	1·5 (1·0–2·0)	0·0 (0·0–0·1)	0·0 (0·0–0·0)	1·89 (1·82–1·96)	0·4 (0·36–0·45)	0·44 (0·4–0·48)
1982	0·0 (0·0–0·0)	3·9 (3·2–4·7)	75·3 (65·6–85·8)	213·5 (204·6–222·2)	78·2 (71·1–85·7)	12·8 (10·3–15·9)	1·5 (1·0–2·1)	0·0 (0·0–0·1)	0·0 (0·0–0·0)	1·93 (1·85–2·02)	0·4 (0·35–0·45)	0·46 (0·43–0·51)
1983	0·0 (0·0–0·0)	4·0 (3·3–4·8)	74·2 (65·0–84·1)	214·9 (204·2–225·0)	81·9 (74·5–90·2)	14·1 (11·2–17·4)	1·5 (1·0–2·2)	0·0 (0·0–0·1)	0·0 (0·0–0·0)	1·95 (1·87–2·03)	0·39 (0·35–0·44)	0·49 (0·45–0·53)
1984	0·0 (0·0–0·0)	3·9 (3·2–4·7)	70·0 (61·7–78·6)	212·4 (203·9–222·7)	84·3 (76·6–92·3)	14·4 (11·4–18·0)	1·5 (1·1–2·1)	0·0 (0·0–0·1)	0·0 (0·0–0·0)	1·93 (1·86–2·01)	0·37 (0·33–0·41)	0·5 (0·46–0·55)
1985	0·0 (0·0–0·0)	3·7 (3·1–4·4)	66·7 (58·6–76·3)	205·6 (193·0–216·8)	85·1 (77·4–93·3)	14·3 (11·5–17·9)	1·5 (1·1–2·0)	0·0 (0·0–0·1)	0·0 (0·0–0·0)	1·88 (1·81–1·96)	0·35 (0·31–0·4)	0·5 (0·46–0·55)
1986	0·0 (0·0–0·0)	3·6 (3·0–4·3)	64·2 (56·1–72·6)	199·4 (190·6–208·7)	87·0 (79·2–94·2)	14·4 (11·2–17·9)	1·6 (1·1–2·0)	0·0 (0·0–0·1)	0·0 (0·0–0·0)	1·85 (1·78–1·93)	0·34 (0·3–0·38)	0·52 (0·47–0·56)
1987	0·0 (0·0–0·0)	3·5 (3·0–4·2)	59·5 (52·4–67·3)	194·1 (183·9–204·0)	89·7 (81·5–98·7)	15·1 (12·0–18·4)	1·7 (1·2–2·4)	0·0 (0·0–0·1)	0·0 (0·0–0·0)	1·82 (1·74–1·89)	0·31 (0·28–0·35)	0·53 (0·49–0·58)
1988	0·0 (0·0–0·0)	3·4 (2·9–4·0)	54·9 (47·4–63·3)	183·4 (173·2–194·2)	91·0 (83·7–99·2)	16·1 (12·7–19·8)	1·9 (1·3–2·7)	0·0 (0·0–0·1)	0·0 (0·0–0·0)	1·75 (1·67–1·84)	0·29 (0·25–0·33)	0·55 (0·5–0·59)
1989	0·0 (0·0–0·0)	3·4 (2·9–4·1)	50·4 (43·5–57·9)	171·6 (162·8–180·5)	91·5 (82·3–101·8)	17·1 (13·5–21·5)	2·0 (1·4–2·9)	0·0 (0·0–0·1)	0·0 (0·0–0·0)	1·68 (1·61–1·75)	0·27 (0·23–0·31)	0·55 (0·51–0·6)
1990	0·0 (0·0–0·0)	3·5 (2·9–4·1)	48·0 (42·0–54·9)	167·3 (157·5–176·6)	93·6 (85·6–101·5)	18·0 (14·5–21·8)	2·0 (1·4–2·8)	0·1 (0·0–0·1)	0·0 (0·0–0·0)	1·66 (1·59–1·74)	0·26 (0·23–0·29)	0·57 (0·52–0·61)
1991	0·0 (0·0–0·0)	3·5 (2·9–4·2)	47·2 (40·7–54·2)	162·3 (154·0–171·8)	95·0 (87·1–102·7)	19·1 (15·4–23·3)	2·1 (1·4–2·8)	0·1 (0·0–0·1)	0·0 (0·0–0·0)	1·65 (1·57–1·72)	0·25 (0·22–0·29)	0·58 (0·54–0·62)
1992	0·0 (0·0–0·0)	3·5 (2·9–4·2)	44·9 (39·6–51·4)	152·9 (143·9–161·6)	95·0 (84·8–105·6)	19·9 (16·0–24·4)	2·2 (1·5–3·0)	0·1 (0·0–0·1)	0·0 (0·0–0·0)	1·59 (1·53–1·65)	0·24 (0·22–0·28)	0·59 (0·54–0·64)
1993	0·0 (0·0–0·0)	3·5 (2·9–4·3)	44·0 (37·4–50·8)	151·9 (142·5–161·7)	100·3 (91·8–110·2)	21·7 (17·4–26·6)	2·3 (1·6–3·2)	0·1 (0·0–0·1)	0·0 (0·0–0·0)	1·62 (1·54–1·69)	0·24 (0·21–0·27)	0·62 (0·58–0·67)
1994	0·0 (0·0–0·0)	3·5 (2·8–4·4)	42·8 (37·3–49·2)	148·3 (139·7–157·2)	103·6 (94·2–113·8)	23·2 (18·5–28·5)	2·5 (1·7–3·4)	0·1 (0·0–0·1)	0·0 (0·0–0·0)	1·62 (1·55–1·7)	0·23 (0·2–0·26)	0·65 (0·6–0·7)
1995	0·0 (0·0–0·0)	3·6 (2·9–4·3)	41·3 (35·3–47·9)	139·3 (130·7–147·8)	104·2 (94·8–113·4)	24·4 (19·3–30·0)	2·6 (1·9–3·6)	0·1 (0·0–0·1)	0·0 (0·0–0·0)	1·58 (1·5–1·65)	0·22 (0·19–0·26)	0·66 (0·61–0·71)
1996	0·0 (0·0–0·0)	3·7 (2·9–4·4)	40·6 (34·8–46·8)	133·9 (125·5–142·9)	105·5 (97·0–115·1)	25·9 (20·6–31·6)	2·7 (2·0–3·7)	0·1 (0·0–0·1)	0·0 (0·0–0·0)	1·56 (1·49–1·64)	0·22 (0·19–0·25)	0·67 (0·62–0·72)
1997	0·0 (0·0–0·0)	3·8 (3·1–4·7)	40·2 (34·6–46·3)	129·2 (121·5–136·8)	105·1 (96·6–115·1)	26·8 (21·7–32·6)	2·9 (2·1–3·9)	0·1 (0·0–0·1)	0·0 (0·0–0·0)	1·54 (1·47–1·61)	0·22 (0·19–0·25)	0·67 (0·63–0·73)
1998	0·0 (0·0–0·0)	4·1 (3·2–5·0)	39·8 (34·5–46·2)	124·0 (116·2–131·6)	105·3 (95·9–115·7)	28·3 (22·7–34·3)	3·1 (2·3–4·1)	0·1 (0·0–0·1)	0·0 (0·0–0·0)	1·52 (1·45–1·6)	0·22 (0·19–0·25)	0·68 (0·63–0·74)
1999	0·0 (0·0–0·0)	4·3 (3·5–5·4)	39·5 (34·4–45·0)	119·5 (111·7–127·2)	105·5 (96·9–114·6)	29·3 (24·2–35·0)	3·3 (2·3–4·5)	0·1 (0·0–0·1)	0·0 (0·0–0·0)	1·51 (1·44–1·58)	0·22 (0·19–0·25)	0·69 (0·64–0·75)
2000	0·0 (0·0–0·0)	4·6 (3·7–5·7)	39·8 (33·1–46·7)	116·0 (108·2–123·9)	104·6 (95·5–114·2)	31·3 (25·9–37·4)	3·5 (2·5–4·8)	0·1 (0·0–0·1)	0·0 (0·0–0·0)	1·5 (1·42–1·58)	0·22 (0·19–0·26)	0·7 (0·64–0·75)
2001	0·0 (0·0–0·0)	4·8 (3·9–5·9)	39·8 (35·1–44·9)	111·7 (104·7–118·9)	102·2 (93·3–111·5)	31·9 (26·2–38·1)	3·7 (2·6–4·9)	0·1 (0·0–0·1)	0·0 (0·0–0·0)	1·47 (1·41–1·54)	0·22 (0·2–0·25)	0·69 (0·64–0·75)
2002	0·0 (0·0–0·0)	4·8 (3·9–5·9)	38·7 (33·3–44·9)	107·8 (100·7–115·0)	102·2 (93·8–111·6)	33·1 (27·6–38·9)	4·0 (2·8–5·4)	0·1 (0·0–0·2)	0·0 (0·0–0·0)	1·45 (1·38–1·52)	0·22 (0·19–0·25)	0·7 (0·64–0·75)
2003	0·0 (0·0–0·0)	4·6 (3·8–5·6)	37·1 (32·2–42·8)	104·5 (98·2–111·3)	101·6 (93·3–110·4)	35·2 (29·3–41·6)	4·3 (3·0–5·9)	0·1 (0·0–0·2)	0·0 (0·0–0·0)	1·44 (1·36–1·52)	0·21 (0·18–0·24)	0·71 (0·65–0·76)
2004	0·0 (0·0–0·0)	4·4 (3·6–5·2)	35·8 (30·8–42·0)	100·0 (93·1–107·0)	99·7 (91·3–108·6)	36·5 (30·6–43·1)	4·6 (3·3–6·2)	0·1 (0·1–0·2)	0·0 (0·0–0·0)	1·41 (1·34–1·47)	0·2 (0·18–0·23)	0·7 (0·65–0·77)
2005	0·0 (0·0–0·0)	4·3 (3·6–5·2)	35·5 (30·8–40·9)	98·6 (91·6–106·0)	99·6 (90·4–109·4)	37·9 (32·3–44·7)	4·9 (3·4–6·6)	0·1 (0·1–0·2)	0·0 (0·0–0·0)	1·4 (1·34–1·47)	0·2 (0·18–0·23)	0·71 (0·66–0·77)
2006	0·0 (0·0–0·0)	4·3 (3·6–5·1)	36·0 (31·4–41·0)	99·6 (92·6–107·0)	102·1 (93·2–111·4)	40·2 (34·2–47·1)	5·4 (3·9–7·4)	0·1 (0·1–0·2)	0·0 (0·0–0·0)	1·44 (1·37–1·51)	0·2 (0·18–0·23)	0·74 (0·68–0·8)
2007	0·0 (0·0–0·0)	4·3 (3·6–5·2)	36·1 (30·0–43·4)	99·2 (92·6–106·5)	102·1 (93·1–111·7)	42·6 (36·3–50·2)	5·9 (4·3–8·0)	0·1 (0·1–0·2)	0·0 (0·0–0·0)	1·45 (1·38–1·52)	0·2 (0·17–0·24)	0·75 (0·7–0·81)
2008	0·0 (0·0–0·0)	4·3 (3·6–5·0)	35·7 (30·9–40·7)	98·8 (91·7–105·9)	102·6 (94·2–111·3)	44·4 (37·7–51·9)	6·4 (4·7–8·9)	0·1 (0·1–0·2)	0·0 (0·0–0·0)	1·46 (1·39–1·53)	0·2 (0·18–0·23)	0·77 (0·71–0·83)
2009	0·0 (0·0–0·0)	4·1 (3·3–5·0)	34·9 (29·0–41·1)	98·6 (92·2–104·9)	103·8 (95·0–113·2)	46·3 (39·5–54·0)	7·0 (5·1–9·5)	0·1 (0·1–0·2)	0·0 (0·0–0·0)	1·47 (1·39–1·56)	0·2 (0·16–0·23)	0·79 (0·73–0·85)
2010	0·0 (0·0–0·0)	4·0 (3·0–5·2)	34·0 (29·5–38·5)	99·1 (92·2–106·3)	105·8 (96·7–115·5)	48·0 (40·9–55·4)	7·5 (5·4–9·8)	0·1 (0·1–0·3)	0·0 (0·0–0·0)	1·49 (1·42–1·56)	0·19 (0·17–0·21)	0·81 (0·75–0·87)
2011	0·0 (0·0–0·0)	3·9 (3·1–4·9)	32·5 (28·4–37·4)	98·2 (91·6–105·5)	106·7 (97·1–116·8)	49·5 (42·3–57·3)	7·8 (5·8–10·3)	0·2 (0·1–0·3)	0·0 (0·0–0·0)	1·49 (1·42–1·56)	0·18 (0·16–0·21)	0·82 (0·76–0·89)
2012	0·0 (0·0–0·0)	3·8 (3·0–4·7)	31·0 (26·7–35·7)	96·9 (90·4–103·7)	109·1 (99·7–119·1)	52·0 (44·5–60·0)	8·5 (6·3–11·0)	0·2 (0·1–0·3)	0·0 (0·0–0·0)	1·51 (1·44–1·58)	0·17 (0·15–0·2)	0·85 (0·79–0·91)
2013	0·0 (0·0–0·0)	3·8 (3·1–4·5)	29·9 (25·4–34·7)	94·7 (88·0–101·4)	109·8 (100·7–119·6)	53·3 (45·9–60·8)	8·9 (6·7–11·9)	0·2 (0·1–0·3)	0·0 (0·0–0·0)	1·5 (1·43–1·58)	0·17 (0·14–0·19)	0·86 (0·8–0·92)
2014	0·0 (0·0–0·0)	3·7 (3·1–4·4)	28·8 (24·8–33·7)	93·7 (86·8–101·3)	110·9 (101·6–121·4)	55·1 (47·2–63·8)	9·4 (6·9–12·4)	0·2 (0·1–0·3)	0·0 (0·0–0·0)	1·51 (1·43–1·6)	0·16 (0·14–0·19)	0·88 (0·81–0·95)
2015	0·0 (0·0–0·0)	3·7 (3·1–4·5)	29·5 (22·2–38·4)	95·2 (87·1–104·0)	113·2 (101·8–125·1)	52·9 (43·6–63·7)	8·7 (6·1–12·1)	0·2 (0·1–0·3)	0·0 (0·0–0·0)	1·52 (1·35–1·72)	0·17 (0·13–0·21)	0·87 (0·78–0·98)
2016	0·0 (0·0–0·0)	3·7 (3·1–4·6)	30·6 (23·6–40·1)	96·6 (86·3–108·4)	112·2 (97·5–129·5)	48·4 (36·8–63·7)	7·6 (4·2–12·3)	0·2 (0·1–0·3)	0·0 (0·0–0·0)	1·5 (1·28–1·76)	0·17 (0·13–0·22)	0·84 (0·71–1·0)
2017	0·0 (0·0–0·0)	3·7 (3·1–4·5)	30·7 (22·4–41·3)	96·6 (85·6–108·6)	110·1 (93·8–127·8)	46·8 (34·9–62·9)	7·2 (4·5–10·8)	0·2 (0·1–0·3)	0·0 (0·0–0·0)	1·48 (1·24–1·76)	0·17 (0·13–0·23)	0·82 (0·67–1·0)

Supplemental Results Table 5: Age-specific fertility rates per 1000, cumulative total fertility up to maternal age 25 (TFU25), cumulative total fertility from maternal age 30 to age 55 (TFO30), and cumulative total fertility, Shimane, 1950-2017												
**Year**	**10-14**	**15-19**	**20-24**	**25-29**	**30-34**	**35-39**	**40-44**	**45-49**	**50-54**	**TFR**	**TFU25**	**TFO30**
1950	0·0 (0·0–0·0)	8·2 (7·3–9·2)	165·9 (151·4–181·3)	235·4 (226·3–244·0)	197·1 (184·7–209·1)	130·8 (116·2–145·6)	43·2 (37·3–49·7)	1·1 (0·8–1·7)	0·0 (0·0–0·0)	3·91 (3·77–4·06)	0·87 (0·8–0·95)	1·86 (1·76–1·96)
1951	0·0 (0·0–0·0)	6·6 (5·8–7·4)	148·9 (135·3–163·8)	219·2 (209·6–228·4)	178·6 (166·8–190·6)	105·7 (92·7–118·8)	33·5 (28·2–39·5)	0·8 (0·5–1·2)	0·0 (0·0–0·0)	3·47 (3·29–3·64)	0·78 (0·71–0·85)	1·59 (1·44–1·74)
1952	0·0 (0·0–0·0)	5·6 (4·8–6·4)	138·6 (124·3–154·3)	208·8 (198·6–219·3)	161·8 (149·4–174·6)	84·7 (73·0–97·8)	26·5 (22·1–31·4)	0·6 (0·4–1·0)	0·0 (0·0–0·0)	3·13 (3·0–3·26)	0·72 (0·65–0·8)	1·37 (1·28–1·46)
1953	0·0 (0·0–0·0)	4·9 (4·1–5·8)	130·9 (116·5–147·7)	197·0 (185·5–208·5)	144·8 (131·0–157·8)	67·3 (57·4–78·6)	20·9 (17·2–25·6)	0·5 (0·3–0·8)	0·0 (0·0–0·0)	2·83 (2·69–2·98)	0·68 (0·61–0·76)	1·17 (1·07–1·26)
1954	0·0 (0·0–0·0)	4·5 (3·7–5·3)	126·9 (111·5–141·9)	191·8 (178·2–205·1)	133·4 (122·4–145·5)	55·9 (46·7–66·3)	17·2 (13·5–21·7)	0·4 (0·2–0·7)	0·0 (0·0–0·0)	2·65 (2·53–2·77)	0·66 (0·58–0·73)	1·03 (0·95–1·12)
1955	0·0 (0·0–0·0)	4·0 (3·2–4·9)	123·7 (108·5–139·4)	190·8 (177·3–205·6)	122·8 (111·5–134·5)	45·5 (38·0–54·0)	14·3 (11·1–17·9)	0·3 (0·2–0·5)	0·0 (0·0–0·0)	2·51 (2·39–2·62)	0·64 (0·56–0·72)	0·91 (0·84–0·99)
1956	0·0 (0·0–0·0)	3·6 (2·8–4·5)	116·4 (101·9–131·7)	184·5 (170·9–199·5)	108·8 (98·5–120·0)	36·7 (30·4–43·4)	11·4 (8·8–14·8)	0·3 (0·2–0·5)	0·0 (0·0–0·0)	2·31 (2·21–2·42)	0·6 (0·53–0·68)	0·79 (0·72–0·86)
1957	0·0 (0·0–0·0)	3·4 (2·7–4·3)	117·6 (103·0–134·7)	188·0 (173·4–203·2)	102·9 (93·1–113·2)	33·1 (27·5–38·7)	9·6 (7·3–12·2)	0·3 (0·2–0·5)	0·0 (0·0–0·0)	2·27 (2·15–2·42)	0·6 (0·53–0·69)	0·73 (0·67–0·79)
1958	0·0 (0·0–0·0)	3·4 (2·7–4·4)	123·4 (107·9–141·6)	194·0 (179·6–209·8)	100·4 (90·3–111·8)	30·8 (25·4–36·5)	8·6 (6·6–11·2)	0·3 (0·1–0·5)	0·0 (0·0–0·0)	2·3 (2·18–2·44)	0·63 (0·56–0·72)	0·7 (0·64–0·77)
1959	0·0 (0·0–0·0)	3·6 (2·8–4·5)	123·2 (108·5–140·4)	192·9 (180·1–205·7)	94·0 (83·9–104·6)	25·0 (20·1–30·6)	7·1 (5·4–9·4)	0·2 (0·1–0·4)	0·0 (0·0–0·0)	2·23 (2·12–2·35)	0·63 (0·56–0·72)	0·63 (0·57–0·7)
1960	0·0 (0·0–0·0)	3·5 (2·7–4·4)	118·5 (104·4–134·1)	194·0 (182·3–206·7)	90·2 (81·1–100·2)	20·9 (16·8–25·2)	5·8 (4·3–7·5)	0·2 (0·1–0·3)	0·0 (0·0–0·0)	2·17 (2·04–2·29)	0·61 (0·54–0·69)	0·59 (0·53–0·65)
1961	0·0 (0·0–0·0)	3·3 (2·5–4·1)	114·9 (102·4–129·0)	196·2 (186·6–206·4)	88·2 (79·9–96·8)	18·7 (15·0–23·3)	5·0 (3·6–6·7)	0·2 (0·1–0·3)	0·0 (0·0–0·0)	2·13 (2·04–2·24)	0·59 (0·53–0·66)	0·56 (0·51–0·62)
1962	0·0 (0·0–0·0)	3·1 (2·4–3·9)	114·6 (101·6–127·6)	201·9 (192·9–210·5)	90·0 (81·8–98·0)	17·7 (14·6–20·8)	4·4 (3·1–5·9)	0·1 (0·1–0·2)	0·0 (0·0–0·0)	2·16 (2·06–2·26)	0·59 (0·52–0·65)	0·56 (0·51–0·61)
1963	0·0 (0·0–0·0)	3·0 (2·4–3·8)	115·0 (103·4–127·7)	203·9 (195·5–212·4)	91·6 (84·0–99·9)	18·5 (14·8–23·0)	4·3 (3·0–5·9)	0·1 (0·1–0·3)	0·0 (0·0–0·0)	2·18 (2·09–2·27)	0·59 (0·53–0·65)	0·57 (0·53–0·62)
1964	0·0 (0·0–0·0)	3·0 (2·4–3·7)	125·4 (112·8–140·7)	217·2 (206·8–227·5)	97·7 (89·3–106·4)	19·0 (15·9–22·3)	4·0 (2·8–5·7)	0·1 (0·1–0·3)	0·0 (0·0–0·0)	2·33 (2·23–2·44)	0·64 (0·58–0·72)	0·6 (0·56–0·65)
1965	0·0 (0·0–0·0)	2·9 (2·4–3·6)	115·3 (99·6–132·6)	183·6 (172·0–195·4)	81·1 (72·9–89·4)	16·1 (13·6–19·0)	3·4 (2·3–4·8)	0·1 (0·1–0·2)	0·0 (0·0–0·0)	2·01 (1·88–2·16)	0·59 (0·51–0·68)	0·5 (0·46–0·55)
1966	0·0 (0·0–0·0)	3·1 (2·5–3·7)	112·4 (95·7–131·1)	188·7 (175·5–203·2)	82·8 (74·4–91·7)	16·4 (13·7–19·6)	3·3 (2·2–4·7)	0·1 (0·0–0·2)	0·0 (0·0–0·0)	2·03 (1·9–2·19)	0·58 (0·49–0·67)	0·51 (0·47–0·56)
1967	0·0 (0·0–0·0)	3·2 (2·7–3·9)	119·3 (99·9–141·1)	229·9 (212·3–247·3)	99·4 (89·8–109·7)	18·5 (15·2–22·1)	3·1 (2·2–4·4)	0·1 (0·0–0·2)	0·0 (0·0–0·0)	2·37 (2·17–2·57)	0·61 (0·51–0·72)	0·61 (0·55–0·67)
1968	0·0 (0·0–0·0)	3·3 (2·8–3·8)	106·4 (87·9–129·5)	217·1 (198·2–235·7)	90·3 (81·4–99·9)	18·4 (14·9–22·4)	2·7 (1·9–3·9)	0·1 (0·0–0·1)	0·0 (0·0–0·0)	2·19 (2·02–2·39)	0·55 (0·46–0·66)	0·56 (0·51–0·61)
1969	0·0 (0·0–0·0)	3·5 (3·0–4·1)	104·0 (86·2–123·8)	219·0 (201·3–237·8)	90·1 (81·3–100·1)	18·0 (14·4–22·3)	2·7 (1·8–3·9)	0·1 (0·0–0·1)	0·0 (0·0–0·0)	2·19 (2·02–2·37)	0·54 (0·45–0·64)	0·55 (0·5–0·62)
1970	0·0 (0·0–0·0)	3·5 (3·0–4·2)	111·3 (91·2–132·5)	222·1 (206·3–239·0)	92·2 (83·2–101·5)	17·5 (13·9–22·0)	2·8 (1·9–3·9)	0·1 (0·0–0·2)	0·0 (0·0–0·0)	2·25 (2·08–2·43)	0·57 (0·47–0·68)	0·56 (0·51–0·62)
1971	0·0 (0·0–0·0)	3·4 (2·8–4·1)	121·2 (102·3–143·3)	218·6 (203·4–233·6)	92·9 (83·8–102·5)	17·3 (13·8–21·7)	3·0 (2·1–4·1)	0·1 (0·0–0·2)	0·0 (0·0–0·0)	2·28 (2·13–2·45)	0·62 (0·53–0·73)	0·57 (0·51–0·63)
1972	0·0 (0·0–0·0)	3·5 (2·9–4·1)	126·3 (107·7–147·3)	216·7 (203·3–230·1)	92·7 (82·7–102·8)	17·3 (13·7–21·3)	2·9 (2·0–4·2)	0·1 (0·0–0·2)	0·0 (0·0–0·0)	2·3 (2·15–2·45)	0·65 (0·56–0·75)	0·56 (0·51–0·63)
1973	0·0 (0·0–0·0)	3·4 (2·7–4·1)	126·1 (109·7–143·2)	216·7 (203·5–228·9)	87·9 (78·5–97·9)	15·6 (12·3–19·5)	2·6 (1·8–3·7)	0·1 (0·0–0·1)	0·0 (0·0–0·0)	2·26 (2·14–2·39)	0·65 (0·57–0·73)	0·53 (0·48–0·59)
1974	0·0 (0·0–0·0)	3·1 (2·5–3·9)	120·5 (106·5–136·3)	208·6 (197·5–219·0)	77·9 (69·8–86·6)	13·2 (10·2–16·8)	2·2 (1·5–3·2)	0·1 (0·0–0·1)	0·0 (0·0–0·0)	2·13 (2·02–2·24)	0·62 (0·55–0·7)	0·47 (0·42–0·51)
1975	0·0 (0·0–0·0)	2·9 (2·3–3·7)	112·9 (100·6–125·4)	201·9 (192·2–211·0)	70·2 (62·7–76·9)	12·2 (9·4–15·9)	1·9 (1·3–2·7)	0·0 (0·0–0·1)	0·0 (0·0–0·0)	2·01 (1·92–2·1)	0·58 (0·52–0·64)	0·42 (0·38–0·47)
1976	0·0 (0·0–0·0)	2·8 (2·0–3·7)	107·6 (94·8–120·9)	200·4 (191·0–209·7)	68·6 (61·7–76·2)	12·0 (9·3–15·1)	1·7 (1·1–2·5)	0·0 (0·0–0·1)	0·0 (0·0–0·0)	1·97 (1·87–2·07)	0·55 (0·49–0·62)	0·41 (0·37–0·46)
1977	0·0 (0·0–0·0)	2·7 (2·1–3·3)	103·2 (91·9–117·3)	202·0 (192·9–211·6)	71·0 (63·6–79·2)	12·0 (9·1–15·7)	1·6 (1·0–2·4)	0·0 (0·0–0·1)	0·0 (0·0–0·0)	1·96 (1·87–2·06)	0·53 (0·47–0·6)	0·42 (0·38–0·47)
1978	0·0 (0·0–0·0)	2·7 (2·1–3·4)	100·0 (89·3–111·7)	203·0 (193·2–212·5)	74·5 (66·6–82·5)	11·9 (9·0–15·7)	1·4 (0·9–2·3)	0·0 (0·0–0·1)	0·0 (0·0–0·0)	1·97 (1·87–2·07)	0·51 (0·46–0·57)	0·44 (0·4–0·48)
1979	0·0 (0·0–0·0)	2·8 (2·3–3·3)	96·6 (85·1–109·1)	201·0 (191·9–210·0)	75·0 (67·2–82·9)	11·8 (9·3–14·8)	1·4 (0·8–2·6)	0·0 (0·0–0·0)	0·0 (0·0–0·0)	1·94 (1·86–2·03)	0·5 (0·44–0·56)	0·44 (0·41–0·48)
1980	0·0 (0·0–0·0)	2·8 (2·2–3·5)	92·9 (81·8–104·7)	197·9 (188·3–207·8)	74·5 (67·1–82·8)	11·9 (9·1–15·1)	1·4 (0·9–2·2)	0·0 (0·0–0·0)	0·0 (0·0–0·0)	1·91 (1·81–2·0)	0·48 (0·42–0·54)	0·44 (0·4–0·48)
1981	0·0 (0·0–0·0)	3·0 (2·4–3·8)	90·6 (80·2–102·7)	198·4 (189·0–207·8)	76·0 (68·9–83·3)	12·8 (10·1–16·0)	1·4 (1·0–2·0)	0·0 (0·0–0·0)	0·0 (0·0–0·0)	1·91 (1·83–2·0)	0·47 (0·42–0·53)	0·45 (0·41–0·49)
1982	0·0 (0·0–0·0)	3·2 (2·5–4·1)	89·6 (78·5–101·9)	200·4 (190·9–210·1)	79·6 (71·8–88·4)	14·5 (11·6–18·1)	1·5 (0·9–2·3)	0·0 (0·0–0·0)	0·0 (0·0–0·0)	1·94 (1·85–2·05)	0·46 (0·41–0·53)	0·48 (0·44–0·53)
1983	0·0 (0·0–0·0)	3·4 (2·7–4·3)	88·0 (75·6–102·0)	202·0 (191·8–211·3)	83·5 (75·5–93·3)	16·1 (12·8–19·9)	1·5 (1·0–2·2)	0·0 (0·0–0·0)	0·0 (0·0–0·0)	1·97 (1·88–2·07)	0·46 (0·4–0·53)	0·51 (0·46–0·56)
1984	0·0 (0·0–0·0)	3·4 (2·7–4·3)	82·9 (72·8–93·1)	200·7 (190·7–211·0)	86·6 (78·3–95·7)	16·7 (13·1–20·8)	1·6 (1·1–2·4)	0·0 (0·0–0·0)	0·0 (0·0–0·0)	1·96 (1·88–2·05)	0·43 (0·38–0·48)	0·52 (0·48–0·57)
1985	0·0 (0·0–0·0)	3·3 (2·6–4·1)	79·4 (69·2–91·7)	195·9 (186·1–204·7)	88·2 (79·7–97·1)	16·5 (13·1–20·4)	1·7 (1·1–2·5)	0·0 (0·0–0·0)	0·0 (0·0–0·0)	1·93 (1·84–2·01)	0·41 (0·36–0·47)	0·53 (0·49–0·58)
1986	0·0 (0·0–0·0)	3·3 (2·6–4·0)	77·3 (66·9–89·2)	191·7 (182·2–202·2)	91·2 (83·7–99·8)	16·7 (13·3–20·6)	1·7 (1·2–2·5)	0·0 (0·0–0·0)	0·0 (0·0–0·0)	1·91 (1·83–2·0)	0·4 (0·35–0·46)	0·55 (0·5–0·59)
1987	0·0 (0·0–0·0)	3·2 (2·6–4·0)	72·8 (64·2–82·9)	188·1 (177·7–198·9)	95·1 (86·4–104·6)	17·4 (13·6–21·5)	1·9 (1·3–2·9)	0·0 (0·0–0·0)	0·0 (0·0–0·0)	1·89 (1·8–1·99)	0·38 (0·34–0·43)	0·57 (0·53–0·62)
1988	0·0 (0·0–0·0)	3·2 (2·6–3·9)	69·1 (59·6–79·6)	179·0 (169·2–188·9)	97·1 (88·9–105·9)	18·3 (14·7–22·4)	2·1 (1·4–3·2)	0·0 (0·0–0·0)	0·0 (0·0–0·0)	1·84 (1·76–1·94)	0·36 (0·31–0·42)	0·59 (0·54–0·64)
1989	0·0 (0·0–0·0)	3·3 (2·7–4·1)	65·3 (55·9–76·1)	168·6 (159·4–179·1)	98·0 (86·0–110·2)	19·4 (15·5–24·1)	2·2 (1·5–3·4)	0·0 (0·0–0·1)	0·0 (0·0–0·0)	1·78 (1·69–1·88)	0·34 (0·3–0·4)	0·6 (0·54–0·66)
1990	0·0 (0·0–0·0)	3·5 (2·8–4·2)	63·9 (55·5–73·6)	165·3 (155·3–175·1)	100·3 (92·6–108·3)	20·4 (16·3–24·8)	2·2 (1·4–3·5)	0·0 (0·0–0·1)	0·0 (0·0–0·0)	1·78 (1·7–1·87)	0·34 (0·29–0·39)	0·61 (0·57–0·66)
1991	0·0 (0·0–0·0)	3·6 (3·0–4·4)	64·1 (55·2–73·8)	161·1 (152·0–171·0)	101·3 (90·7–111·7)	21·6 (16·9–27·7)	2·2 (1·4–3·3)	0·0 (0·0–0·1)	0·0 (0·0–0·0)	1·77 (1·69–1·87)	0·34 (0·29–0·39)	0·63 (0·57–0·69)
1992	0·0 (0·0–0·0)	3·7 (3·1–4·4)	62·2 (54·3–71·1)	152·6 (142·9–162·5)	100·1 (91·3–109·8)	22·4 (17·8–27·6)	2·3 (1·5–3·6)	0·0 (0·0–0·1)	0·0 (0·0–0·0)	1·72 (1·63–1·8)	0·33 (0·29–0·37)	0·62 (0·57–0·69)
1993	0·0 (0·0–0·0)	3·8 (3·1–4·6)	61·6 (52·5–71·8)	152·2 (142·2–163·0)	104·1 (94·7–113·6)	24·3 (19·2–30·2)	2·5 (1·6–3·7)	0·0 (0·0–0·1)	0·0 (0·0–0·0)	1·74 (1·63–1·85)	0·33 (0·28–0·38)	0·65 (0·59–0·72)
1994	0·0 (0·0–0·0)	3·8 (3·1–4·5)	60·4 (52·2–69·9)	149·3 (139·2–159·1)	105·5 (95·2–115·6)	25·8 (20·2–31·9)	2·6 (1·7–4·1)	0·0 (0·0–0·1)	0·0 (0·0–0·0)	1·74 (1·62–1·85)	0·32 (0·28–0·37)	0·67 (0·61–0·74)
1995	0·0 (0·0–0·0)	3·7 (3·0–4·5)	58·4 (49·8–68·0)	140·7 (131·8–150·4)	104·4 (93·9–114·6)	27·0 (21·2–33·5)	2·8 (1·8–4·2)	0·0 (0·0–0·1)	0·0 (0·0–0·0)	1·69 (1·57–1·8)	0·31 (0·27–0·36)	0·67 (0·6–0·74)
1996	0·0 (0·0–0·0)	3·8 (3·1–4·7)	57·4 (48·9–66·4)	135·8 (126·3–145·9)	104·4 (94·9–115·1)	28·6 (22·4–35·5)	3·0 (1·8–4·5)	0·0 (0·0–0·1)	0·0 (0·0–0·0)	1·67 (1·54–1·8)	0·31 (0·26–0·35)	0·68 (0·62–0·75)
1997	0·0 (0·0–0·0)	4·0 (3·3–4·8)	56·7 (47·6–66·7)	131·6 (122·5–140·3)	103·0 (92·9–113·5)	29·6 (23·7–36·6)	3·2 (2·1–4·8)	0·0 (0·0–0·1)	0·0 (0·0–0·0)	1·64 (1·54–1·75)	0·3 (0·26–0·35)	0·68 (0·62–0·74)
1998	0·0 (0·0–0·0)	4·2 (3·5–5·2)	56·0 (48·5–64·2)	127·3 (118·1–136·2)	102·4 (92·7–113·5)	31·4 (24·8–38·8)	3·4 (2·2–5·0)	0·0 (0·0–0·1)	0·0 (0·0–0·0)	1·62 (1·52–1·73)	0·3 (0·26–0·34)	0·69 (0·63–0·75)
1999	0·0 (0·0–0·0)	4·5 (3·7–5·6)	55·6 (47·5–65·1)	124·0 (115·1–133·2)	102·0 (92·8–112·5)	32·6 (26·7–40·0)	3·6 (2·3–5·2)	0·0 (0·0–0·1)	0·0 (0·0–0·0)	1·61 (1·53–1·71)	0·3 (0·26–0·35)	0·69 (0·63–0·76)
2000	0·0 (0·0–0·0)	4·9 (4·0–6·0)	55·7 (48·5–63·3)	122·0 (114·1–130·6)	100·4 (90·8–110·2)	34·7 (28·1–42·2)	3·8 (2·5–5·6)	0·1 (0·0–0·1)	0·0 (0·0–0·0)	1·61 (1·51–1·71)	0·3 (0·27–0·34)	0·69 (0·63–0·76)
2001	0·0 (0·0–0·0)	5·2 (4·2–6·3)	55·9 (48·4–64·6)	118·9 (110·7–128·0)	97·5 (88·7–107·0)	35·0 (28·7–42·5)	3·9 (2·7–5·6)	0·1 (0·0–0·1)	0·0 (0·0–0·0)	1·58 (1·5–1·67)	0·31 (0·27–0·35)	0·68 (0·62–0·75)
2002	0·0 (0·0–0·0)	5·2 (4·2–6·3)	54·5 (46·8–63·4)	115·9 (108·4–123·7)	97·3 (87·2–108·0)	35·9 (29·9–42·8)	4·2 (2·9–5·9)	0·1 (0·0–0·1)	0·0 (0·0–0·0)	1·57 (1·48–1·65)	0·3 (0·26–0·34)	0·69 (0·62–0·76)
2003	0·0 (0·0–0·0)	5·0 (4·0–6·2)	52·4 (45·5–60·6)	113·3 (103·3–124·2)	97·0 (88·2–106·8)	37·6 (30·4–45·2)	4·6 (3·1–6·2)	0·1 (0·0–0·1)	0·0 (0·0–0·0)	1·55 (1·44–1·66)	0·29 (0·25–0·33)	0·7 (0·63–0·77)
2004	0·0 (0·0–0·0)	4·8 (3·9–6·0)	50·5 (43·2–59·1)	108·8 (102·1–115·9)	95·7 (86·7–105·8)	38·5 (31·7–46·0)	4·8 (3·4–6·6)	0·1 (0·0–0·1)	0·0 (0·0–0·0)	1·52 (1·44–1·6)	0·28 (0·24–0·32)	0·7 (0·64–0·76)
2005	0·0 (0·0–0·0)	4·7 (3·8–5·7)	50·0 (43·2–58·1)	107·5 (99·8–116·1)	96·4 (87·3–106·0)	39·5 (32·3–47·7)	5·0 (3·4–7·0)	0·1 (0·0–0·1)	0·0 (0·0–0·0)	1·52 (1·44–1·6)	0·27 (0·24–0·31)	0·7 (0·64–0·77)
2006	0·0 (0·0–0·0)	4·6 (3·7–5·8)	50·5 (43·8–58·5)	108·4 (101·0–115·9)	99·4 (89·8–109·6)	41·5 (34·5–49·8)	5·5 (3·8–7·5)	0·1 (0·0–0·1)	0·0 (0·0–0·0)	1·55 (1·48–1·63)	0·28 (0·24–0·32)	0·73 (0·67–0·8)
2007	0·0 (0·0–0·0)	4·6 (3·7–5·8)	50·4 (42·1–60·8)	107·6 (100·1–115·9)	99·6 (90·8–110·8)	43·7 (36·5–51·8)	5·9 (4·0–8·0)	0·1 (0·0–0·2)	0·0 (0·0–0·0)	1·56 (1·47–1·65)	0·28 (0·23–0·33)	0·75 (0·69–0·81)
2008	0·0 (0·0–0·0)	4·6 (3·6–5·8)	49·9 (43·3–56·4)	107·1 (99·6–114·4)	100·2 (90·6–110·1)	45·3 (37·7–53·4)	6·3 (4·4–8·7)	0·1 (0·0–0·2)	0·0 (0·0–0·0)	1·57 (1·49–1·65)	0·27 (0·24–0·3)	0·76 (0·7–0·82)
2009	0·0 (0·0–0·0)	4·4 (3·5–5·7)	48·8 (39·8–58·8)	107·0 (96·8–117·7)	101·2 (91·8–111·3)	47·1 (39·4–55·9)	6·8 (4·6–9·5)	0·1 (0·0–0·2)	0·0 (0·0–0·0)	1·58 (1·46–1·7)	0·27 (0·22–0·31)	0·78 (0·71–0·85)
2010	0·0 (0·0–0·0)	4·3 (3·1–5·7)	47·7 (41·3–53·9)	107·8 (101·1–115·0)	103·0 (93·7–113·8)	48·9 (41·2–57·3)	7·1 (5·0–9·7)	0·1 (0·0–0·2)	0·0 (0·0–0·0)	1·59 (1·51–1·68)	0·26 (0·23–0·29)	0·8 (0·73–0·87)
2011	0·0 (0·0–0·0)	4·2 (3·3–5·3)	45·6 (39·4–52·9)	107·7 (99·6–116·4)	103·7 (93·8–114·3)	50·4 (42·6–59·5)	7·4 (5·0–10·2)	0·1 (0·0–0·2)	0·0 (0·0–0·0)	1·6 (1·5–1·68)	0·25 (0·22–0·28)	0·81 (0·75–0·87)
2012	0·0 (0·0–0·0)	4·1 (3·4–4·9)	43·7 (37·2–50·8)	107·7 (100·5–115·4)	105·8 (95·9–116·7)	53·0 (45·1–62·2)	7·9 (5·5–10·7)	0·1 (0·1–0·2)	0·0 (0·0–0·0)	1·61 (1·52–1·7)	0·24 (0·21–0·28)	0·83 (0·77–0·9)
2013	0·0 (0·0–0·0)	4·1 (3·3–5·0)	42·4 (35·9–50·0)	106·9 (99·2–115·6)	106·2 (97·0–116·2)	54·2 (46·0–63·0)	8·2 (5·8–11·0)	0·1 (0·1–0·2)	0·0 (0·0–0·0)	1·61 (1·51–1·72)	0·23 (0·2–0·27)	0·84 (0·78–0·91)
2014	0·0 (0·0–0·0)	4·1 (3·3–4·9)	41·2 (34·8–48·3)	107·1 (98·8–115·8)	107·1 (97·1–117·8)	55·8 (47·5–64·6)	8·6 (6·2–11·6)	0·1 (0·1–0·2)	0·0 (0·0–0·0)	1·62 (1·53–1·71)	0·23 (0·19–0·26)	0·86 (0·79–0·93)
2015	0·0 (0·0–0·0)	4·1 (3·3–5·0)	42·3 (31·0–56·0)	109·6 (98·8–121·9)	109·1 (97·6–121·6)	53·7 (43·6–64·4)	7·9 (5·4–11·0)	0·1 (0·1–0·2)	0·0 (0·0–0·0)	1·63 (1·43–1·86)	0·23 (0·17–0·3)	0·85 (0·75–0·97)
2016	0·0 (0·0–0·0)	4·1 (3·4–5·1)	43·7 (33·6–57·7)	111·2 (97·1–126·8)	108·1 (93·3–124·9)	49·1 (37·7–63·7)	6·9 (4·5–10·1)	0·1 (0·1–0·2)	0·0 (0·0–0·0)	1·62 (1·38–1·9)	0·24 (0·19–0·31)	0·82 (0·69–0·97)
2017	0·0 (0·0–0·0)	4·1 (3·4–5·0)	43·6 (31·9–58·7)	111·0 (95·9–127·6)	106·0 (90·3–123·7)	47·4 (34·9–62·6)	6·8 (4·2–10·1)	0·1 (0·1–0·2)	0·0 (0·0–0·0)	1·59 (1·32–1·9)	0·24 (0·18–0·32)	0·8 (0·66–0·97)

Supplemental Results Table 5: Age-specific fertility rates per 1000, cumulative total fertility up to maternal age 25 (TFU25), cumulative total fertility from maternal age 30 to age 55 (TFO30), and cumulative total fertility, Shizuoka, 1950-2017

Year	10-14	15-19	20-24	25-29	30-34	35-39	40-44	45-49	50-54	TFR	TFU25	TFO30
1950	0·0 (0·0–0·0)	9·6 (8·5–10·7)	151·5 (138·1–165·8)	218·5 (210·6–226·5)	156·0 (146·8–165·2)	88·9 (79·0–98·7)	29·9 (25·9–34·4)	1·5 (1·0–2·1)	0·0 (0·0–0·0)	3·28 (3·16–3·4)	0·81 (0·74–0·88)	1·38 (1·31–1·45)
1951	0·0 (0·0–0·0)	7·7 (6·9–8·7)	135·9 (123·1–149·7)	203·0 (195·5–210·5)	141·1 (131·3–150·7)	71·8 (62·4–80·8)	23·2 (19·4–27·1)	1·1 (0·7–1·6)	0·0 (0·0–0·0)	2·92 (2·79–3·05)	0·72 (0·65–0·79)	1·19 (1·08–1·3)
1952	0·0 (0·0–0·0)	6·5 (5·7–7·4)	126·5 (113·8–140·6)	192·9 (184·7–201·3)	127·7 (117·8–138·1)	57·5 (49·8–66·3)	18·4 (15·1–22·2)	0·8 (0·5–1·2)	0·0 (0·0–0·0)	2·65 (2·54–2·76)	0·67 (0·61–0·74)	1·02 (0·95–1·09)
1953	0·0 (0·0–0·0)	5·7 (4·9–6·5)	119·6 (106·3–134·2)	181·9 (173·9–190·1)	114·2 (104·6–124·1)	48·0 (40·6–56·0)	14·6 (11·7–18·3)	0·7 (0·4–1·0)	0·0 (0·0–0·0)	2·42 (2·31–2·53)	0·63 (0·56–0·7)	0·89 (0·82–0·96)
1954	0·0 (0·0–0·0)	5·2 (4·5–6·0)	115·9 (103·2–129·1)	177·0 (169·7–184·8)	105·2 (95·9–115·0)	43·4 (36·5–51·2)	12·1 (9·3–15·6)	0·6 (0·3–0·8)	0·0 (0·0–0·0)	2·3 (2·19–2·41)	0·61 (0·54–0·67)	0·81 (0·74–0·87)
1955	0·0 (0·0–0·0)	4·7 (4·0–5·5)	113·1 (99·5–127·1)	176·0 (167·3–184·3)	96·8 (87·6–106·4)	39·0 (32·7–45·8)	10·1 (7·6–13·1)	0·4 (0·3–0·7)	0·0 (0·0–0·0)	2·2 (2·09–2·31)	0·59 (0·52–0·66)	0·73 (0·67–0·8)
1956	0·0 (0·0–0·0)	4·2 (3·5–4·9)	106·6 (94·1–119·6)	170·2 (162·8–178·1)	85·8 (77·5–94·7)	33·4 (28·1–39·3)	8·1 (6·1–10·4)	0·4 (0·2–0·6)	0·0 (0·0–0·0)	2·04 (1·94–2·15)	0·55 (0·49–0·62)	0·64 (0·58–0·7)
1957	0·0 (0·0–0·0)	4·0 (3·4–4·8)	107·9 (95·5–122·4)	173·8 (166·3–181·9)	81·3 (73·6–89·6)	28·5 (23·7–33·5)	6·9 (5·3–8·8)	0·4 (0·2–0·6)	0·0 (0·0–0·0)	2·01 (1·9–2·13)	0·56 (0·5–0·63)	0·59 (0·53–0·64)
1958	0·0 (0·0–0·0)	4·1 (3·5–4·7)	113·5 (99·7–128·6)	179·8 (171·1–188·9)	79·6 (72·9–87·1)	25·1 (20·6–29·6)	6·2 (4·9–7·9)	0·4 (0·2–0·6)	0·0 (0·0–0·0)	2·04 (1·95–2·15)	0·59 (0·52–0·66)	0·56 (0·51–0·61)
1959	0·0 (0·0–0·0)	4·2 (3·6–5·0)	113·8 (101·5–127·1)	179·1 (171·0–187·7)	74·7 (66·7–82·7)	21·6 (17·7–26·1)	5·2 (4·0–6·7)	0·3 (0·2–0·5)	0·0 (0·0–0·0)	1·99 (1·9–2·09)	0·59 (0·53–0·66)	0·51 (0·46–0·56)
1960	0·0 (0·0–0·0)	4·1 (3·5–4·9)	109·7 (97·4–122·4)	180·2 (171·9–189·2)	71·9 (64·4–79·8)	19·0 (15·6–22·6)	4·3 (3·2–5·5)	0·3 (0·2–0·4)	0·0 (0·0–0·0)	1·95 (1·85–2·04)	0·57 (0·51–0·63)	0·48 (0·43–0·52)
1961	0·0 (0·0–0·0)	3·9 (3·2–4·6)	106·9 (95·4–118·0)	182·5 (174·4–190·8)	70·4 (65·4–75·8)	17·0 (14·0–20·5)	3·7 (2·7–5·0)	0·2 (0·1–0·3)	0·0 (0·0–0·0)	1·92 (1·84–2·0)	0·55 (0·5–0·61)	0·46 (0·43–0·49)
1962	0·0 (0·0–0·0)	3·7 (3·1–4·4)	107·0 (95·2–119·1)	188·2 (180·6–196·0)	72·1 (66·2–78·3)	16·0 (13·8–18·4)	3·3 (2·4–4·4)	0·2 (0·1–0·3)	0·0 (0·0–0·0)	1·95 (1·87–2·03)	0·55 (0·49–0·61)	0·46 (0·43–0·49)
1963	0·0 (0·0–0·0)	3·6 (3·1–4·3)	107·9 (96·5–120·0)	190·7 (182·6–198·8)	73·8 (67·4–80·4)	15·3 (13·0–18·0)	3·3 (2·4–4·3)	0·2 (0·1–0·3)	0·0 (0·0–0·0)	1·97 (1·9–2·05)	0·56 (0·5–0·62)	0·46 (0·43–0·5)
1964	0·0 (0·0–0·0)	3·6 (3·1–4·2)	118·4 (106·2–132·5)	203·5 (194·8–212·2)	79·0 (71·9–87·6)	15·8 (13·2–18·7)	3·1 (2·2–4·1)	0·2 (0·1–0·3)	0·0 (0·0–0·0)	2·12 (2·04–2·21)	0·61 (0·55–0·68)	0·49 (0·45–0·54)
1965	0·0 (0·0–0·0)	3·5 (2·9–4·2)	109·5 (97·7–122·6)	172·3 (163·5–180·8)	65·9 (59·1–72·5)	14·4 (12·1–17·0)	2·7 (2·0–3·5)	0·2 (0·1–0·3)	0·0 (0·0–0·0)	1·84 (1·75–1·93)	0·57 (0·51–0·63)	0·42 (0·38–0·45)
1966	0·0 (0·0–0·0)	3·8 (3·2–4·4)	107·6 (95·9–119·9)	177·8 (168·6–186·1)	67·7 (60·6–75·5)	14·6 (11·8–17·8)	2·7 (2·0–3·6)	0·2 (0·1–0·3)	0·0 (0·0–0·0)	1·87 (1·79–1·96)	0·56 (0·5–0·62)	0·43 (0·39–0·47)
1967	0·0 (0·0–0·0)	4·0 (3·4–4·6)	115·4 (103·5–127·9)	217·4 (209·1–225·8)	82·0 (73·1–92·2)	16·4 (13·4–19·9)	2·6 (2·0–3·3)	0·2 (0·1–0·2)	0·0 (0·0–0·0)	2·19 (2·08–2·31)	0·6 (0·54–0·66)	0·51 (0·46–0·56)
1968	0·0 (0·0–0·0)	4·1 (3·5–4·7)	104·0 (92·4–116·3)	206·0 (197·3–214·2)	75·0 (67·4–82·5)	16·2 (13·4–19·5)	2·3 (1·8–3·1)	0·1 (0·1–0·2)	0·0 (0·0–0·0)	2·04 (1·95–2·13)	0·54 (0·48–0·6)	0·47 (0·43–0·51)
1969	0·0 (0·0–0·0)	4·4 (3·7–5·2)	102·8 (91·4–114·9)	209·1 (200·7–217·6)	75·6 (68·8–83·5)	15·8 (13·1–18·9)	2·4 (1·8–3·2)	0·1 (0·1–0·2)	0·0 (0·0–0·0)	2·05 (1·96–2·14)	0·54 (0·48–0·6)	0·47 (0·43–0·52)
1970	0·0 (0·0–0·0)	4·5 (3·8–5·3)	111·3 (98·7–125·4)	213·5 (205·1–222·0)	78·2 (70·7–85·6)	15·5 (12·7–18·8)	2·6 (1·8–3·5)	0·2 (0·1–0·2)	0·0 (0·0–0·0)	2·13 (2·04–2·22)	0·58 (0·52–0·65)	0·48 (0·44–0·53)
1971	0·0 (0·0–0·0)	4·4 (3·6–5·3)	122·4 (108·0–136·9)	211·5 (202·5–220·4)	79·7 (71·9–87·5)	15·8 (12·8–18·9)	2·8 (1·9–3·9)	0·2 (0·1–0·3)	0·0 (0·0–0·0)	2·18 (2·09–2·27)	0·63 (0·56–0·71)	0·49 (0·45–0·54)
1972	0·0 (0·0–0·0)	4·4 (3·6–5·3)	128·3 (113·5–144·0)	210·6 (202·4–219·2)	80·3 (72·6–88·0)	16·0 (12·9–19·5)	2·8 (1·9–3·8)	0·2 (0·1–0·3)	0·0 (0·0–0·0)	2·21 (2·12–2·31)	0·66 (0·59–0·74)	0·5 (0·45–0·54)
1973	0·0 (0·0–0·0)	4·2 (3·5–5·2)	128·4 (114·9–142·6)	211·2 (202·3–220·3)	76·7 (70·1–84·0)	14·5 (11·6–17·7)	2·5 (1·7–3·6)	0·1 (0·1–0·2)	0·0 (0·0–0·0)	2·19 (2·11–2·28)	0·66 (0·59–0·73)	0·47 (0·43–0·51)
1974	0·0 (0·0–0·0)	3·9 (3·2–4·7)	121·8 (110·3–134·1)	203·1 (194·1–211·4)	68·2 (61·8–74·8)	12·1 (9·8–14·7)	2·1 (1·4–3·0)	0·1 (0·1–0·2)	0·0 (0·0–0·0)	2·06 (1·97–2·14)	0·63 (0·57–0·69)	0·41 (0·38–0·45)
1975	0·0 (0·0–0·0)	3·5 (2·9–4·3)	111·9 (98·0–125·8)	195·3 (186·7–203·7)	61·0 (55·1–66·8)	11·2 (9·1–13·6)	1·7 (1·2–2·3)	0·1 (0·0–0·1)	0·0 (0·0–0·0)	1·92 (1·84–2·01)	0·58 (0·51–0·65)	0·37 (0·34–0·4)
1976	0·0 (0·0–0·0)	3·3 (2·6–4·1)	103·2 (92·9–115·1)	192·1 (183·4–200·3)	58·7 (53·6–64·2)	11·2 (9·4–13·1)	1·4 (1·0–2·0)	0·1 (0·0–0·1)	0·0 (0·0–0·0)	1·85 (1·77–1·93)	0·53 (0·48–0·59)	0·36 (0·32–0·39)
1977	0·0 (0·0–0·0)	3·2 (2·4–4·1)	95·3 (85·4–106·9)	192·5 (184·3–201·4)	61·3 (55·5–67·3)	11·2 (9·3–13·5)	1·3 (0·9–1·9)	0·1 (0·0–0·1)	0·0 (0·0–0·0)	1·82 (1·75–1·91)	0·49 (0·44–0·55)	0·37 (0·33–0·41)
1978	0·0 (0·0–0·0)	3·2 (2·5–3·9)	89·3 (79·1–101·0)	192·9 (185·0–200·6)	66·6 (60·3–73·1)	11·1 (8·7–13·9)	1·3 (0·9–1·9)	0·1 (0·0–0·1)	0·0 (0·0–0·0)	1·82 (1·75–1·9)	0·46 (0·41–0·52)	0·4 (0·36–0·43)
1979	0·0 (0·0–0·0)	3·2 (2·8–3·7)	84·4 (75·7–93·5)	190·9 (182·0–199·7)	68·3 (62·4–75·1)	10·9 (9·1–12·9)	1·4 (0·9–2·0)	0·1 (0·0–0·1)	0·0 (0·0–0·0)	1·8 (1·72–1·87)	0·44 (0·39–0·48)	0·4 (0·37–0·44)
1980	0·0 (0·0–0·0)	3·3 (2·7–3·9)	80·2 (71·0–89·4)	188·7 (180·1–197·6)	67·9 (61·8–74·4)	10·8 (8·7–13·0)	1·4 (1·0–1·9)	0·1 (0·0–0·1)	0·0 (0·0–0·0)	1·76 (1·69–1·83)	0·42 (0·37–0·46)	0·4 (0·37–0·44)
1981	0·0 (0·0–0·0)	3·5 (2·9–4·2)	78·1 (69·7–88·2)	190·6 (182·1–198·6)	69·4 (63·2–75·7)	11·4 (9·3–13·6)	1·4 (0·9–2·1)	0·1 (0·0–0·1)	0·0 (0·0–0·0)	1·77 (1·71–1·84)	0·41 (0·37–0·46)	0·41 (0·38–0·44)
1982	0·0 (0·0–0·0)	3·7 (3·0–4·5)	77·1 (68·6–86·6)	193·5 (184·2–203·9)	73·2 (67·0–80·0)	12·7 (10·7–15·2)	1·4 (1·0–2·0)	0·0 (0·0–0·1)	0·0 (0·0–0·0)	1·81 (1·75–1·88)	0·4 (0·36–0·45)	0·44 (0·4–0·47)
1983	0·0 (0·0–0·0)	3·8 (3·1–4·7)	75·6 (67·0–85·2)	194·6 (185·6–203·2)	77·6 (71·0–86·0)	14·1 (11·6–17·1)	1·4 (1·1–1·8)	0·0 (0·0–0·1)	0·0 (0·0–0·0)	1·84 (1·77–1·91)	0·4 (0·35–0·45)	0·47 (0·43–0·51)
1984	0·0 (0·0–0·0)	3·8 (3·0–4·6)	70·5 (63·0–79·0)	191·6 (183·3–200·5)	81·1 (74·2–88·6)	14·6 (12·1–17·6)	1·5 (1·1–2·0)	0·0 (0·0–0·1)	0·0 (0·0–0·0)	1·82 (1·75–1·89)	0·37 (0·33–0·42)	0·49 (0·45–0·53)
1985	0·0 (0·0–0·0)	3·6 (3·0–4·4)	66·4 (58·1–75·2)	184·6 (176·3–192·2)	83·1 (76·2–90·1)	14·6 (12·0–17·4)	1·5 (1·0–2·1)	0·0 (0·0–0·1)	0·0 (0·0–0·0)	1·77 (1·71–1·83)	0·35 (0·31–0·39)	0·5 (0·46–0·53)
1986	0·0 (0·0–0·0)	3·5 (2·9–4·3)	63·4 (55·6–71·6)	178·1 (169·9–187·3)	86·3 (77·1–94·9)	14·8 (12·3–17·8)	1·6 (1·0–2·4)	0·1 (0·0–0·1)	0·0 (0·0–0·0)	1·74 (1·67–1·81)	0·33 (0·29–0·38)	0·51 (0·47–0·56)
1987	0·0 (0·0–0·0)	3·4 (2·8–4·2)	58·4 (51·7–65·7)	172·2 (163·8–180·7)	89·9 (82·6–98·0)	15·6 (12·9–18·6)	1·8 (1·2–2·5)	0·1 (0·0–0·1)	0·0 (0·0–0·0)	1·71 (1·63–1·79)	0·31 (0·28–0·35)	0·54 (0·5–0·58)
1988	0·0 (0·0–0·0)	3·3 (2·7–4·1)	54·0 (47·4–61·7)	161·5 (154·1–169·8)	91·7 (83·7–100·4)	16·6 (13·9–19·8)	2·0 (1·4–2·7)	0·1 (0·0–0·1)	0·0 (0·0–0·0)	1·65 (1·58–1·71)	0·29 (0·25–0·33)	0·55 (0·51–0·6)
1989	0·0 (0·0–0·0)	3·3 (2·7–4·1)	49·9 (43·3–57·5)	150·8 (142·9–159·1)	92·4 (84·8–100·1)	17·7 (14·2–21·5)	2·1 (1·5–2·9)	0·1 (0·0–0·1)	0·0 (0·0–0·0)	1·58 (1·51–1·65)	0·27 (0·23–0·3)	0·56 (0·52–0·6)
1990	0·0 (0·0–0·0)	3·4 (2·7–4·2)	48·1 (42·3–54·3)	147·8 (139·9–155·9)	95·2 (87·3–103·2)	18·6 (15·4–21·7)	2·1 (1·5–2·8)	0·1 (0·0–0·1)	0·0 (0·0–0·0)	1·58 (1·51–1·65)	0·26 (0·23–0·29)	0·58 (0·54–0·62)
1991	0·0 (0·0–0·0)	3·5 (2·9–4·4)	47·9 (41·8–54·4)	143·7 (136·9–151·0)	96·5 (88·5–105·4)	19·8 (16·4–23·6)	2·1 (1·5–2·8)	0·1 (0·0–0·1)	0·0 (0·0–0·0)	1·57 (1·51–1·63)	0·26 (0·23–0·29)	0·59 (0·55–0·64)
1992	0·0 (0·0–0·0)	3·6 (2·9–4·4)	46·2 (41·0–52·1)	132·9 (125·5–140·7)	93·7 (86·6–101·5)	20·6 (17·2–24·7)	2·3 (1·7–3·0)	0·1 (0·0–0·1)	0·0 (0·0–0·0)	1·5 (1·44–1·56)	0·25 (0·22–0·28)	0·58 (0·55–0·62)
1993	0·0 (0·0–0·0)	3·7 (3·0–4·5)	45·9 (40·5–52·0)	119·6 (112·8–126·8)	88·1 (80·9–95·2)	22·4 (18·8–26·3)	2·4 (1·8–3·2)	0·1 (0·0–0·1)	0·0 (0·0–0·0)	1·41 (1·35–1·47)	0·25 (0·22–0·28)	0·56 (0·52–0·61)
1994	0·0 (0·0–0·0)	3·8 (3·1–4·5)	45·3 (38·8–52·7)	107·1 (101·0–113·4)	82·1 (74·7–90·4)	23·9 (20·0–28·5)	2·5 (1·9–3·4)	0·1 (0·0–0·1)	0·0 (0·0–0·0)	1·32 (1·26–1·39)	0·25 (0·21–0·28)	0·54 (0·5–0·59)
1995	0·0 (0·0–0·0)	3·8 (3·2–4·6)	44·2 (39·0–49·8)	109·1 (102·4–115·9)	88·4 (81·0–96·5)	25·1 (21·1–29·4)	2·7 (2·0–3·6)	0·1 (0·0–0·1)	0·0 (0·0–0·0)	1·37 (1·31–1·43)	0·24 (0·21–0·27)	0·58 (0·54–0·62)
1996	0·0 (0·0–0·0)	4·0 (3·4–4·8)	44·0 (38·2–49·7)	117·0 (110·6–123·6)	98·2 (90·2–107·5)	26·5 (22·1–31·7)	2·8 (2·1–3·7)	0·1 (0·0–0·1)	0·0 (0·0–0·0)	1·46 (1·4–1·53)	0·24 (0·21–0·27)	0·64 (0·6–0·69)
1997	0·0 (0·0–0·0)	4·3 (3·5–5·2)	44·4 (38·9–50·5)	117·1 (108·6–126·0)	99·7 (91·9–108·5)	27·4 (22·8–32·5)	3·0 (2·2–4·0)	0·1 (0·0–0·1)	0·0 (0·0–0·0)	1·48 (1·41–1·55)	0·24 (0·22–0·27)	0·65 (0·61–0·7)
1998	0·0 (0·0–0·0)	4·6 (3·8–5·7)	44·7 (39·3–51·1)	113·2 (107·0–119·4)	99·0 (90·8–108·4)	28·6 (23·4–34·0)	3·1 (2·3–4·2)	0·1 (0·0–0·1)	0·0 (0·0–0·0)	1·47 (1·4–1·54)	0·25 (0·22–0·28)	0·65 (0·61–0·7)
1999	0·0 (0·0–0·0)	5·0 (4·1–6·1)	45·3 (39·4–51·9)	108·9 (102·3–115·4)	97·6 (89·4–106·5)	29·4 (24·7–34·6)	3·3 (2·3–4·5)	0·1 (0·0–0·1)	0·0 (0·0–0·0)	1·45 (1·38–1·52)	0·25 (0·22–0·29)	0·65 (0·6–0·7)
2000	0·0 (0·0–0·0)	5·5 (4·5–6·6)	46·2 (41·0–51·5)	105·9 (99·7–112·7)	95·3 (87·8–103·2)	31·0 (26·1–36·5)	3·5 (2·5–4·8)	0·1 (0·1–0·1)	0·0 (0·0–0·0)	1·44 (1·38–1·51)	0·26 (0·23–0·29)	0·65 (0·6–0·7)
2001	0·0 (0·0–0·0)	5·8 (4·8–6·9)	47·0 (40·6–53·5)	102·7 (96·6–109·4)	92·2 (84·1–100·7)	31·2 (26·2–36·4)	3·6 (2·6–4·9)	0·1 (0·1–0·1)	0·0 (0·0–0·0)	1·41 (1·34–1·48)	0·26 (0·23–0·3)	0·64 (0·59–0·69)
2002	0·0 (0·0–0·0)	5·8 (4·8–7·0)	46·1 (40·2–52·9)	100·2 (93·7–107·1)	91·9 (83·9–100·7)	32·1 (27·3–37·4)	3·9 (2·8–5·3)	0·1 (0·1–0·1)	0·0 (0·0–0·0)	1·4 (1·33–1·47)	0·26 (0·23–0·29)	0·64 (0·59–0·69)
2003	0·0 (0·0–0·0)	5·6 (4·6–6·8)	44·5 (38·4–51·1)	98·0 (92·7–103·6)	91·6 (84·4–99·3)	33·9 (28·5–40·0)	4·2 (3·0–5·7)	0·1 (0·1–0·1)	0·0 (0·0–0·0)	1·39 (1·33–1·45)	0·25 (0·22–0·28)	0·65 (0·6–0·7)
2004	0·0 (0·0–0·0)	5·3 (4·5–6·3)	43·1 (37·8–49·8)	94·7 (88·5–101·2)	90·4 (82·9–98·0)	35·1 (30·1–40·9)	4·4 (3·3–5·8)	0·1 (0·1–0·2)	0·0 (0·0–0·0)	1·37 (1·31–1·43)	0·24 (0·22–0·28)	0·65 (0·6–0·7)
2005	0·0 (0·0–0·0)	5·2 (4·4–6·2)	42·9 (37·8–48·4)	94·4 (88·0–100·6)	91·1 (82·8–99·1)	36·5 (30·2–43·9)	4·7 (3·5–6·2)	0·1 (0·1–0·2)	0·0 (0·0–0·0)	1·37 (1·31–1·45)	0·24 (0·21–0·27)	0·66 (0·61–0·72)
2006	0·0 (0·0–0·0)	5·2 (4·4–6·1)	43·6 (37·8–49·8)	96·1 (89·8–102·2)	94·4 (86·5–103·2)	38·8 (33·5–45·0)	5·2 (4·0–6·7)	0·1 (0·1–0·2)	0·0 (0·0–0·0)	1·42 (1·35–1·49)	0·24 (0·21–0·28)	0·69 (0·64–0·75)
2007	0·0 (0·0–0·0)	5·2 (4·4–6·1)	43·6 (39·0–49·0)	96·3 (90·4–103·4)	95·2 (86·7–103·9)	41·2 (35·5–47·5)	5·6 (4·4–7·3)	0·1 (0·1–0·2)	0·0 (0·0–0·0)	1·44 (1·37–1·5)	0·24 (0·22–0·27)	0·71 (0·66–0·76)
2008	0·0 (0·0–0·0)	5·1 (4·3–6·1)	43·2 (37·4–49·4)	96·3 (89·8–102·4)	96·3 (88·2–104·4)	42·8 (36·5–49·1)	6·1 (4·8–7·7)	0·1 (0·1–0·2)	0·0 (0·0–0·0)	1·45 (1·39–1·51)	0·24 (0·21–0·27)	0·73 (0·68–0·78)
2009	0·0 (0·0–0·0)	4·9 (4·0–5·8)	41·8 (36·3–47·4)	96·4 (90·5–101·9)	97·7 (89·2–107·1)	44·2 (38·1–50·7)	6·8 (5·4–8·5)	0·1 (0·1–0·2)	0·0 (0·0–0·0)	1·46 (1·39–1·54)	0·23 (0·21–0·26)	0·74 (0·69–0·8)
2010	0·0 (0·0–0·0)	4·6 (3·9–5·5)	40·4 (34·9–46·1)	97·2 (90·6–103·7)	99·4 (91·2–109·2)	45·3 (39·0–52·2)	7·3 (5·7–8·9)	0·1 (0·1–0·2)	0·0 (0·0–0·0)	1·47 (1·4–1·54)	0·23 (0·2–0·25)	0·76 (0·71–0·82)
2011	0·0 (0·0–0·0)	4·5 (3·6–5·4)	38·3 (33·5–43·5)	96·6 (89·9–103·7)	99·8 (91·3–108·6)	46·2 (39·9–53·5)	7·6 (6·1–9·5)	0·2 (0·1–0·2)	0·0 (0·0–0·0)	1·47 (1·4–1·53)	0·21 (0·19–0·24)	0·77 (0·72–0·83)
2012	0·0 (0·0–0·0)	4·3 (3·5–5·3)	36·2 (31·8–41·1)	95·5 (89·7–101·7)	101·3 (92·4–110·9)	48·2 (41·7–55·1)	8·3 (6·6–10·6)	0·2 (0·1–0·3)	0·0 (0·0–0·0)	1·47 (1·41–1·54)	0·2 (0·18–0·23)	0·79 (0·73–0·85)
2013	0·0 (0·0–0·0)	4·2 (3·5–5·0)	34·7 (30·5–39·4)	93·1 (86·8–100·2)	101·1 (93·2–110·0)	49·2 (42·5–56·0)	8·8 (6·8–11·3)	0·2 (0·1–0·3)	0·0 (0·0–0·0)	1·46 (1·39–1·52)	0·19 (0·17–0·22)	0·8 (0·74–0·85)
2014	0·0 (0·0–0·0)	4·1 (3·4–4·9)	33·4 (28·8–38·5)	91·4 (85·1–98·2)	101·8 (93·5–110·2)	50·7 (43·9–58·0)	9·3 (7·2–11·8)	0·2 (0·1–0·3)	0·0 (0·0–0·0)	1·45 (1·38–1·54)	0·19 (0·16–0·21)	0·81 (0·75–0·87)
2015	0·0 (0·0–0·0)	4·1 (3·4–4·9)	33·9 (28·7–39·6)	91·8 (84·4–99·6)	103·6 (93·3–114·7)	49·0 (40·9–58·1)	8·6 (6·4–11·2)	0·2 (0·1–0·3)	0·0 (0·0–0·0)	1·46 (1·31–1·62)	0·19 (0·16–0·22)	0·81 (0·72–0·9)
2016	0·0 (0·0–0·0)	4·1 (3·4–5·1)	35·2 (27·5–44·9)	91·7 (82·2–103·3)	102·2 (89·2–117·5)	45·1 (35·1–58·3)	7·4 (5·3–10·3)	0·2 (0·1–0·2)	0·0 (0·0–0·0)	1·43 (1·22–1·68)	0·2 (0·15–0·25)	0·77 (0·66–0·92)
2017	0·0 (0·0–0·0)	4·1 (3·4–5·0)	35·0 (27·4–44·9)	90·5 (80·3–101·6)	99·9 (85·3–115·8)	43·8 (32·7–57·4)	7·2 (4·9–10·7)	0·2 (0·1–0·3)	0·0 (0·0–0·0)	1·4 (1·17–1·67)	0·2 (0·15–0·25)	0·76 (0·62–0·92)

Supplemental Results Table 5: Age-specific fertility rates per 1000, cumulative total fertility up to maternal age 25 (TFU25), cumulative total fertility from maternal age 30 to age 55 (TFO30), and cumulative total fertility, Tochigi, 1950-2017												
**Year**	**10-14**	**15-19**	**20-24**	**25-29**	**30-34**	**35-39**	**40-44**	**45-49**	**50-54**	**TFR**	**TFU25**	**TFO30**
1950	0·0 (0·0–0·0)	12·4 (11·0–13·9)	155·5 (141·7–170·3)	225·7 (217·3–234·0)	185·7 (174·3–197·3)	110·1 (98·0–122·4)	41·2 (35·6–47·4)	1·6 (1·1–2·3)	0·0 (0·0–0·0)	3·66 (3·53–3·79)	0·84 (0·77–0·91)	1·69 (1·61–1·79)
1951	0·0 (0·0–0·0)	10·0 (8·9–11·2)	139·5 (126·3–153·4)	209·9 (201·8–217·8)	168·3 (156·4–179·9)	89·1 (77·5–100·7)	32·0 (26·8–37·5)	1·2 (0·8–1·7)	0·0 (0·0–0·0)	3·25 (3·09–3·41)	0·75 (0·68–0·82)	1·45 (1·32–1·59)
1952	0·0 (0·0–0·0)	8·4 (7·3–9·5)	129·9 (116·8–143·7)	199·6 (191·1–208·4)	152·6 (138·7–166·5)	71·5 (60·7–84·2)	25·3 (20·9–30·5)	0·9 (0·6–1·3)	0·0 (0·0–0·0)	2·94 (2·79–3·08)	0·69 (0·63–0·76)	1·25 (1·15–1·35)
1953	0·0 (0·0–0·0)	7·4 (6·4–8·5)	122·8 (110·1–136·8)	188·2 (179·7–197·7)	136·6 (122·0–151·7)	57·2 (46·2–69·3)	20·1 (16·4–24·3)	0·7 (0·5–1·1)	0·0 (0·0–0·0)	2·66 (2·51–2·82)	0·65 (0·59–0·72)	1·07 (0·96–1·18)
1954	0·0 (0·0–0·0)	6·8 (5·8–7·8)	119·0 (104·6–133·5)	183·2 (174·8–192·6)	126·1 (110·5–141·6)	49·7 (38·5–62·2)	16·6 (13·3–20·7)	0·6 (0·4–0·9)	0·0 (0·0–0·0)	2·51 (2·34–2·67)	0·63 (0·56–0·7)	0·96 (0·84–1·09)
1955	0·0 (0·0–0·0)	6·1 (5·2–7·1)	116·1 (102·5–131·6)	182·2 (173·4–190·4)	116·2 (100·3–131·6)	44·8 (34·8–57·5)	13·8 (10·7–17·3)	0·5 (0·3–0·7)	0·0 (0·0–0·0)	2·4 (2·24–2·56)	0·61 (0·54–0·69)	0·88 (0·75–1·01)
1956	0·0 (0·0–0·0)	5·5 (4·6–6·4)	109·4 (95·4–124·0)	176·2 (168·6–184·3)	103·0 (89·3–118·1)	38·5 (28·4–50·9)	11·0 (8·6–14·1)	0·4 (0·2–0·6)	0·0 (0·0–0·0)	2·22 (2·07–2·38)	0·57 (0·5–0·65)	0·76 (0·65–0·89)
1957	0·0 (0·0–0·0)	5·2 (4·4–6·1)	110·4 (96·4–126·2)	179·7 (171·9–188·3)	97·6 (84·3–112·5)	33·0 (23·2–43·3)	9·4 (7·3–11·8)	0·4 (0·2–0·6)	0·0 (0·0–0·0)	2·18 (2·02–2·34)	0·58 (0·51–0·66)	0·7 (0·59–0·82)
1958	0·0 (0·0–0·0)	5·2 (4·5–6·1)	115·9 (101·7–130·1)	185·6 (177·0–194·1)	95·4 (82·4–109·3)	29·1 (21·9–38·5)	8·4 (6·6–10·8)	0·4 (0·2–0·6)	0·0 (0·0–0·0)	2·2 (2·07–2·34)	0·61 (0·53–0·68)	0·67 (0·57–0·77)
1959	0·0 (0·0–0·0)	5·4 (4·6–6·4)	115·8 (102·4–130·6)	184·7 (176·3–193·5)	89·3 (78·4–101·9)	25·0 (18·9–32·1)	7·0 (5·3–9·1)	0·3 (0·2–0·5)	0·0 (0·0–0·0)	2·14 (2·02–2·26)	0·61 (0·54–0·68)	0·61 (0·53–0·7)
1960	0·0 (0·0–0·0)	5·3 (4·5–6·2)	111·3 (99·0–124·1)	185·6 (177·1–194·5)	85·9 (76·9–95·7)	22·0 (17·2–27·8)	5·7 (4·3–7·3)	0·3 (0·2–0·4)	0·0 (0·0–0·0)	2·08 (1·97–2·19)	0·58 (0·52–0·65)	0·57 (0·51–0·64)
1961	0·0 (0·0–0·0)	4·9 (4·1–5·8)	108·0 (97·4–119·3)	187·7 (179·4–196·1)	84·1 (74·0–95·3)	19·8 (15·6–24·8)	4·9 (3·7–6·6)	0·2 (0·1–0·3)	0·0 (0·0–0·0)	2·05 (1·96–2·15)	0·56 (0·51–0·62)	0·55 (0·49–0·61)
1962	0·0 (0·0–0·0)	4·7 (3·9–5·6)	107·6 (95·7–119·8)	193·2 (185·2–201·3)	85·9 (78·1–93·6)	18·7 (15·3–22·9)	4·3 (3·2–5·8)	0·2 (0·1–0·3)	0·0 (0·0–0·0)	2·07 (1·99–2·16)	0·56 (0·5–0·62)	0·55 (0·5–0·59)
1963	0·0 (0·0–0·0)	4·6 (3·9–5·6)	108·0 (96·6–120·2)	195·3 (187·0–203·8)	87·7 (80·3–95·6)	18·0 (15·2–21·3)	4·2 (3·1–5·8)	0·2 (0·1–0·3)	0·0 (0·0–0·0)	2·09 (2·01–2·17)	0·56 (0·51–0·62)	0·55 (0·51–0·59)
1964	0·0 (0·0–0·0)	4·6 (3·8–5·4)	117·8 (105·9–131·7)	208·0 (199·0–217·0)	93·7 (84·8–103·4)	18·7 (15·8–21·7)	4·0 (2·9–5·3)	0·2 (0·1–0·3)	0·0 (0·0–0·0)	2·23 (2·15–2·33)	0·61 (0·55–0·68)	0·58 (0·54–0·63)
1965	0·0 (0·0–0·0)	4·4 (3·8–5·2)	108·3 (96·6–121·2)	175·8 (166·9–185·4)	78·1 (68·8–87·7)	17·2 (14·5–20·4)	3·4 (2·5–4·5)	0·2 (0·1–0·2)	0·0 (0·0–0·0)	1·94 (1·85–2·03)	0·56 (0·51–0·63)	0·49 (0·45–0·54)
1966	0·0 (0·0–0·0)	4·7 (4·0–5·4)	105·7 (93·5–117·8)	180·9 (171·6–190·0)	80·1 (70·0–91·6)	17·5 (14·2–21·6)	3·3 (2·4–4·5)	0·2 (0·1–0·2)	0·0 (0·0–0·0)	1·96 (1·88–2·05)	0·55 (0·49–0·61)	0·51 (0·45–0·57)
1967	0·0 (0·0–0·0)	4·9 (4·2–5·7)	112·5 (101·0–124·7)	220·6 (211·2–229·7)	96·9 (83·3–111·5)	19·9 (15·7–25·1)	3·2 (2·4–4·1)	0·1 (0·1–0·2)	0·0 (0·0–0·0)	2·29 (2·17–2·41)	0·59 (0·53–0·65)	0·6 (0·52–0·69)
1968	0·0 (0·0–0·0)	5·0 (4·4–5·8)	100·6 (89·3–112·7)	208·3 (199·8–216·8)	88·7 (75·7–102·4)	19·9 (15·0–25·8)	2·8 (2·1–3·7)	0·1 (0·1–0·2)	0·0 (0·0–0·0)	2·13 (2·03–2·23)	0·53 (0·47–0·59)	0·56 (0·48–0·65)
1969	0·0 (0·0–0·0)	5·5 (4·7–6·4)	98·7 (86·7–111·7)	210·7 (202·3–219·1)	89·2 (76·1–103·9)	19·6 (15·0–25·5)	2·8 (2·1–3·7)	0·1 (0·1–0·2)	0·0 (0·0–0·0)	2·13 (2·04–2·23)	0·52 (0·46–0·59)	0·56 (0·48–0·65)
1970	0·0 (0·0–0·0)	5·5 (4·6–6·5)	105·9 (94·9–118·2)	214·3 (204·8–223·2)	92·0 (80·4–106·3)	18·6 (13·9–24·8)	3·0 (2·2–4·0)	0·1 (0·1–0·2)	0·0 (0·0–0·0)	2·2 (2·1–2·31)	0·56 (0·5–0·62)	0·57 (0·49–0·66)
1971	0·0 (0·0–0·0)	5·4 (4·5–6·4)	115·8 (101·2–130·8)	211·6 (202·3–221·0)	93·5 (81·8–107·2)	17·3 (12·9–23·0)	3·2 (2·3–4·4)	0·1 (0·1–0·2)	0·0 (0·0–0·0)	2·23 (2·13–2·34)	0·61 (0·53–0·68)	0·57 (0·5–0·66)
1972	0·0 (0·0–0·0)	5·4 (4·5–6·4)	121·0 (106·9–136·7)	210·0 (201·0–219·0)	93·9 (82·8–105·2)	16·7 (12·5–22·0)	3·2 (2·2–4·4)	0·1 (0·1–0·2)	0·0 (0·0–0·0)	2·25 (2·15–2·36)	0·63 (0·56–0·71)	0·57 (0·5–0·64)
1973	0·0 (0·0–0·0)	5·2 (4·3–6·3)	120·9 (107·9–134·8)	210·0 (200·7–220·1)	89·7 (81·0–99·1)	16·0 (11·9–20·7)	2·9 (1·9–4·2)	0·1 (0·1–0·2)	0·0 (0·0–0·0)	2·22 (2·13–2·32)	0·63 (0·57–0·7)	0·54 (0·49–0·6)
1974	0·0 (0·0–0·0)	4·9 (3·9–5·9)	115·4 (104·1–128·7)	201·8 (190·9–212·1)	79·8 (71·8–87·6)	14·6 (11·0–18·9)	2·5 (1·6–3·6)	0·1 (0·1–0·1)	0·0 (0·0–0·0)	2·09 (2·01–2·19)	0·6 (0·55–0·67)	0·48 (0·44–0·53)
1975	0·0 (0·0–0·0)	4·5 (3·8–5·4)	107·7 (96·2–119·7)	194·0 (185·1–202·8)	71·7 (64·6–78·8)	13·7 (10·7–17·4)	2·1 (1·5–2·8)	0·1 (0·0–0·1)	0·0 (0·0–0·0)	1·97 (1·89–2·06)	0·56 (0·5–0·62)	0·44 (0·39–0·48)
1976	0·0 (0·0–0·0)	4·3 (3·5–5·1)	101·6 (90·2–113·1)	190·6 (181·9–199·5)	68·8 (62·4–75·2)	13·4 (10·8–16·4)	1·8 (1·2–2·6)	0·1 (0·0–0·1)	0·0 (0·0–0·0)	1·9 (1·82–1·99)	0·53 (0·47–0·59)	0·42 (0·38–0·46)
1977	0·0 (0·0–0·0)	4·2 (3·5–4·9)	95·9 (85·7–108·6)	190·4 (182·1–198·6)	70·4 (63·5–77·7)	13·2 (10·7–16·1)	1·7 (1·2–2·4)	0·0 (0·0–0·1)	0·0 (0·0–0·0)	1·88 (1·8–1·97)	0·5 (0·45–0·56)	0·43 (0·38–0·47)
1978	0·0 (0·0–0·0)	4·2 (3·3–5·2)	91·5 (82·5–101·4)	190·4 (182·1–198·3)	74·1 (66·7–81·7)	13·0 (9·9–16·6)	1·7 (1·1–2·4)	0·0 (0·0–0·1)	0·0 (0·0–0·0)	1·87 (1·8–1·95)	0·48 (0·43–0·53)	0·44 (0·4–0·48)
1979	0·0 (0·0–0·0)	4·3 (3·2–5·6)	87·6 (77·8–98·5)	188·2 (180·3–196·3)	74·2 (67·8–81·1)	12·5 (10·5–15·0)	1·7 (1·1–2·5)	0·0 (0·0–0·1)	0·0 (0·0–0·0)	1·84 (1·76–1·92)	0·46 (0·41–0·51)	0·44 (0·41–0·48)
1980	0·0 (0·0–0·0)	4·4 (3·5–5·4)	83·8 (74·9–92·8)	185·6 (177·1–194·4)	72·5 (65·8–79·6)	12·3 (9·8–15·2)	1·7 (1·2–2·5)	0·0 (0·0–0·1)	0·0 (0·0–0·0)	1·8 (1·72–1·88)	0·44 (0·4–0·48)	0·43 (0·4–0·47)
1981	0·0 (0·0–0·0)	4·6 (3·8–5·6)	82·0 (71·6–94·3)	186·6 (178·1–194·9)	73·0 (66·0–79·7)	12·8 (10·3–15·4)	1·7 (1·1–2·5)	0·0 (0·0–0·1)	0·0 (0·0–0·0)	1·8 (1·73–1·88)	0·43 (0·38–0·5)	0·44 (0·4–0·47)
1982	0·0 (0·0–0·0)	4·9 (4·0–5·9)	81·5 (71·8–92·0)	188·7 (180·9–197·4)	76·0 (69·5–83·7)	14·1 (11·5–16·8)	1·7 (1·2–2·4)	0·0 (0·0–0·1)	0·0 (0·0–0·0)	1·83 (1·77–1·91)	0·43 (0·38–0·48)	0·46 (0·42–0·5)
1983	0·0 (0·0–0·0)	5·0 (4·2–5·9)	80·7 (72·2–90·0)	189·8 (180·3–198·8)	79·9 (72·9–88·0)	15·3 (12·3–19·0)	1·7 (1·3–2·2)	0·0 (0·0–0·1)	0·0 (0·0–0·0)	1·86 (1·79–1·94)	0·43 (0·39–0·48)	0·48 (0·45–0·53)
1984	0·0 (0·0–0·0)	4·9 (4·1–5·8)	76·7 (65·6–89·1)	187·8 (179·1–197·1)	83·2 (75·4–91·9)	15·6 (12·9–19·2)	1·8 (1·3–2·3)	0·0 (0·0–0·1)	0·0 (0·0–0·0)	1·85 (1·77–1·94)	0·41 (0·35–0·47)	0·5 (0·46–0·55)
1985	0·0 (0·0–0·0)	4·7 (3·9–5·5)	73·4 (64·8–82·7)	182·0 (173·9–189·8)	84·9 (78·2–91·8)	15·4 (12·6–18·7)	1·8 (1·3–2·3)	0·0 (0·0–0·1)	0·0 (0·0–0·0)	1·81 (1·74–1·88)	0·39 (0·35–0·44)	0·51 (0·47–0·55)
1986	0·0 (0·0–0·0)	4·5 (3·8–5·3)	71·1 (62·4–80·9)	176·8 (168·2–186·0)	87·5 (79·5–95·9)	15·5 (12·7–18·5)	1·8 (1·2–2·5)	0·0 (0·0–0·1)	0·0 (0·0–0·0)	1·79 (1·71–1·87)	0·38 (0·33–0·43)	0·52 (0·48–0·57)
1987	0·0 (0·0–0·0)	4·3 (3·6–5·1)	66·3 (58·9–74·5)	171·7 (163·8–180·0)	90·4 (83·3–98·2)	16·2 (13·4–19·2)	2·0 (1·4–2·8)	0·0 (0·0–0·1)	0·0 (0·0–0·0)	1·76 (1·69–1·82)	0·35 (0·32–0·39)	0·54 (0·5–0·59)
1988	0·0 (0·0–0·0)	4·1 (3·5–4·9)	62·0 (54·1–71·2)	161·6 (153·4–170·8)	91·5 (83·7–99·8)	17·2 (14·1–20·6)	2·1 (1·5–2·9)	0·0 (0·0–0·1)	0·0 (0·0–0·0)	1·69 (1·62–1·77)	0·33 (0·29–0·38)	0·55 (0·51–0·6)
1989	0·0 (0·0–0·0)	4·1 (3·4–5·0)	57·5 (50·2–65·7)	150·9 (143·7–158·5)	91·2 (83·6–98·7)	18·2 (15·0–22·2)	2·2 (1·6–3·1)	0·1 (0·0–0·1)	0·0 (0·0–0·0)	1·62 (1·55–1·69)	0·31 (0·27–0·35)	0·56 (0·51–0·6)
1990	0·0 (0·0–0·0)	4·2 (3·4–5·2)	55·4 (48·2–62·8)	147·1 (138·7–154·7)	92·5 (84·5–100·2)	19·2 (15·7–23·1)	2·2 (1·6–3·0)	0·1 (0·0–0·1)	0·0 (0·0–0·0)	1·6 (1·53–1·68)	0·3 (0·26–0·34)	0·57 (0·53–0·61)
1991	0·0 (0·0–0·0)	4·3 (3·5–5·1)	54·7 (47·3–62·5)	142·5 (135·3–150·7)	92·9 (84·5–101·6)	20·5 (17·1–24·6)	2·3 (1·6–3·0)	0·1 (0·0–0·1)	0·0 (0·0–0·0)	1·59 (1·51–1·66)	0·29 (0·26–0·34)	0·58 (0·53–0·62)
1992	0·0 (0·0–0·0)	4·3 (3·6–5·1)	52·3 (46·4–59·2)	134·1 (126·5–142·0)	91·7 (83·8–99·9)	21·4 (17·7–25·7)	2·4 (1·7–3·2)	0·1 (0·0–0·1)	0·0 (0·0–0·0)	1·53 (1·47–1·6)	0·28 (0·25–0·32)	0·58 (0·53–0·63)
1993	0·0 (0·0–0·0)	4·3 (3·5–5·2)	51·4 (44·3–58·8)	133·1 (124·9–141·6)	95·5 (87·5–104·6)	23·3 (19·6–27·3)	2·6 (1·8–3·4)	0·1 (0·0–0·1)	0·0 (0·0–0·0)	1·55 (1·48–1·63)	0·28 (0·24–0·32)	0·61 (0·56–0·66)
1994	0·0 (0·0–0·0)	4·3 (3·5–5·3)	50·3 (44·1–57·1)	130·0 (122·5–137·9)	97·0 (88·2–106·5)	24·7 (20·4–29·8)	2·7 (2·0–3·6)	0·1 (0·0–0·1)	0·0 (0·0–0·0)	1·55 (1·46–1·63)	0·27 (0·24–0·31)	0·62 (0·57–0·67)
1995	0·0 (0·0–0·0)	4·3 (3·5–5·2)	48·9 (42·8–55·7)	122·2 (114·4–129·8)	96·1 (87·3–104·9)	25·7 (21·6–30·4)	2·8 (2·1–3·8)	0·1 (0·0–0·1)	0·0 (0·0–0·0)	1·5 (1·42–1·59)	0·27 (0·23–0·3)	0·62 (0·57–0·67)
1996	0·0 (0·0–0·0)	4·5 (3·6–5·4)	48·5 (41·8–55·0)	117·8 (110·4–125·7)	96·0 (88·2–104·6)	27·0 (22·4–31·9)	3·0 (2·2–3·9)	0·1 (0·0–0·1)	0·0 (0·0–0·0)	1·48 (1·4–1·57)	0·26 (0·23–0·3)	0·63 (0·59–0·68)
1997	0·0 (0·0–0·0)	4·7 (3·8–5·7)	48·5 (42·6–55·1)	114·1 (107·2–121·0)	94·6 (86·6–103·4)	27·6 (23·1–32·8)	3·1 (2·3–4·3)	0·1 (0·0–0·1)	0·0 (0·0–0·0)	1·46 (1·39–1·54)	0·27 (0·24–0·3)	0·63 (0·58–0·68)
1998	0·0 (0·0–0·0)	5·0 (4·0–6·2)	48·6 (42·4–55·9)	110·3 (103·7–117·4)	93·9 (85·6–102·8)	28·9 (23·8–34·6)	3·3 (2·4–4·3)	0·1 (0·0–0·1)	0·0 (0·0–0·0)	1·45 (1·38–1·53)	0·27 (0·24–0·3)	0·63 (0·58–0·68)
1999	0·0 (0·0–0·0)	5·4 (4·3–6·7)	48·8 (43·0–55·3)	107·7 (100·6–114·6)	93·5 (85·7–102·1)	29·7 (24·9–35·1)	3·4 (2·4–4·6)	0·1 (0·0–0·1)	0·0 (0·0–0·0)	1·44 (1·37–1·51)	0·27 (0·24–0·3)	0·63 (0·58–0·68)
2000	0·0 (0·0–0·0)	5·8 (4·7–7·2)	49·5 (42·1–57·6)	106·1 (99·6–113·1)	92·0 (84·4–100·5)	31·4 (26·0–37·3)	3·6 (2·6–4·8)	0·1 (0·0–0·1)	0·0 (0·0–0·0)	1·44 (1·36–1·53)	0·28 (0·24–0·32)	0·64 (0·59–0·69)
2001	0·0 (0·0–0·0)	6·1 (4·9–7·4)	49·9 (44·3–56·4)	103·6 (96·9–111·0)	89·6 (81·4–97·7)	31·6 (26·2–37·5)	3·7 (2·7–5·1)	0·1 (0·0–0·1)	0·0 (0·0–0·0)	1·42 (1·36–1·49)	0·28 (0·25–0·31)	0·62 (0·58–0·67)
2002	0·0 (0·0–0·0)	6·0 (4·9–7·4)	48·9 (42·7–55·9)	101·3 (94·6–108·2)	89·5 (81·2–98·2)	32·5 (27·4–38·5)	4·0 (2·8–5·5)	0·1 (0·1–0·1)	0·0 (0·0–0·0)	1·41 (1·34–1·48)	0·27 (0·24–0·31)	0·63 (0·58–0·68)
2003	0·0 (0·0–0·0)	5·8 (4·7–7·0)	47·2 (40·5–54·8)	99·0 (93·2–105·3)	89·4 (81·8–97·5)	34·1 (28·3–40·4)	4·2 (2·9–5·8)	0·1 (0·1–0·1)	0·0 (0·0–0·0)	1·4 (1·33–1·47)	0·26 (0·23–0·3)	0·64 (0·59–0·69)
2004	0·0 (0·0–0·0)	5·5 (4·6–6·5)	45·5 (39·9–52·1)	95·2 (88·9–101·9)	88·3 (80·8–95·7)	35·1 (29·6–41·2)	4·5 (3·2–6·0)	0·1 (0·1–0·2)	0·0 (0·0–0·0)	1·37 (1·31–1·43)	0·25 (0·23–0·29)	0·64 (0·6–0·69)
2005	0·0 (0·0–0·0)	5·3 (4·4–6·3)	45·1 (39·5–51·2)	94·0 (87·4–101·0)	88·9 (80·8–97·2)	36·1 (30·6–42·7)	4·7 (3·3–6·4)	0·1 (0·1–0·2)	0·0 (0·0–0·0)	1·37 (1·31–1·44)	0·25 (0·22–0·28)	0·65 (0·6–0·7)
2006	0·0 (0·0–0·0)	5·2 (4·3–6·2)	45·6 (39·2–52·6)	94·7 (88·4–101·2)	91·7 (83·9–99·8)	37·9 (32·0–45·0)	5·1 (3·8–6·9)	0·1 (0·1–0·2)	0·0 (0·0–0·0)	1·4 (1·34–1·47)	0·25 (0·22–0·29)	0·67 (0·63–0·73)
2007	0·0 (0·0–0·0)	5·2 (4·3–6·2)	45·3 (40·3–51·1)	93·7 (87·8–100·6)	91·9 (83·6–100·8)	39·8 (33·9–46·4)	5·5 (4·1–7·3)	0·1 (0·1–0·2)	0·0 (0·0–0·0)	1·41 (1·34–1·48)	0·25 (0·23–0·28)	0·69 (0·64–0·74)
2008	0·0 (0·0–0·0)	5·0 (4·2–6·0)	44·6 (38·8–50·9)	92·7 (86·6–99·5)	92·1 (84·6–99·9)	41·1 (35·3–47·6)	5·9 (4·4–8·0)	0·1 (0·1–0·2)	0·0 (0·0–0·0)	1·41 (1·34–1·48)	0·25 (0·22–0·28)	0·7 (0·65–0·75)
2009	0·0 (0·0–0·0)	4·8 (3·8–5·8)	43·1 (37·1–50·2)	91·9 (84·7–99·1)	92·2 (83·8–101·3)	42·4 (36·1–49·4)	6·5 (5·0–8·4)	0·1 (0·1–0·2)	0·0 (0·0–0·0)	1·41 (1·32–1·5)	0·24 (0·21–0·28)	0·71 (0·65–0·76)
2010	0·0 (0·0–0·0)	4·6 (3·5–6·0)	41·7 (36·2–47·4)	91·9 (86·1–97·7)	92·7 (84·6–101·0)	43·6 (37·4–50·1)	6·9 (5·3–8·9)	0·1 (0·1–0·2)	0·0 (0·0–0·0)	1·41 (1·34–1·47)	0·23 (0·2–0·26)	0·72 (0·66–0·77)
2011	0·0 (0·0–0·0)	4·5 (3·6–5·7)	39·5 (34·7–44·9)	91·0 (84·6–97·7)	92·3 (84·5–101·4)	44·5 (37·9–51·5)	7·1 (5·5–9·1)	0·2 (0·1–0·3)	0·0 (0·0–0·0)	1·4 (1·33–1·46)	0·22 (0·2–0·25)	0·72 (0·67–0·77)
2012	0·0 (0·0–0·0)	4·4 (3·5–5·5)	37·6 (33·0–42·7)	90·3 (84·5–96·9)	93·4 (85·0–102·0)	46·4 (40·1–53·5)	7·7 (6·0–9·9)	0·2 (0·1–0·3)	0·0 (0·0–0·0)	1·4 (1·34–1·47)	0·21 (0·19–0·24)	0·74 (0·68–0·8)
2013	0·0 (0·0–0·0)	4·3 (3·6–5·3)	36·3 (30·1–42·8)	88·7 (82·6–94·5)	93·6 (85·4–101·9)	47·3 (40·9–54·1)	8·1 (6·2–10·4)	0·2 (0·1–0·3)	0·0 (0·0–0·0)	1·39 (1·32–1·47)	0·2 (0·17–0·24)	0·75 (0·69–0·8)
2014	0·0 (0·0–0·0)	4·2 (3·5–5·1)	34·9 (30·3–39·7)	87·8 (81·8–94·3)	94·9 (87·3–103·3)	48·7 (42·0–56·2)	8·6 (6·6–11·2)	0·2 (0·1–0·3)	0·0 (0·0–0·0)	1·4 (1·32–1·48)	0·2 (0·17–0·22)	0·76 (0·7–0·83)
2015	0·0 (0·0–0·0)	4·2 (3·5–5·2)	35·3 (29·2–42·3)	89·4 (82·0–96·9)	97·5 (88·3–108·2)	46·7 (38·8–55·7)	8·0 (5·9–10·7)	0·2 (0·1–0·3)	0·0 (0·0–0·0)	1·41 (1·26–1·57)	0·2 (0·16–0·23)	0·76 (0·68–0·86)
2016	0·0 (0·0–0·0)	4·3 (3·5–5·3)	36·7 (27·9–48·0)	90·8 (81·1–102·5)	97·4 (84·4–113·1)	42·7 (33·0–55·4)	6·9 (4·8–9·5)	0·2 (0·1–0·3)	0·0 (0·0–0·0)	1·39 (1·19–1·65)	0·2 (0·16–0·26)	0·74 (0·62–0·88)
2017	0·0 (0·0–0·0)	4·3 (3·5–5·2)	36·6 (27·2–48·1)	90·7 (80·4–102·0)	96·2 (81·9–111·6)	41·4 (30·9–54·3)	6·8 (4·3–10·1)	0·2 (0·1–0·3)	0·0 (0·0–0·0)	1·38 (1·16–1·63)	0·2 (0·15–0·27)	0·72 (0·59–0·87)

Supplemental Results Table 5: Age-specific fertility rates per 1000, cumulative total fertility up to maternal age 25 (TFU25), cumulative total fertility from maternal age 30 to age 55 (TFO30), and cumulative total fertility, Tokushima, 1950-2017

Year	10-14	15-19	20-24	25-29	30-34	35-39	40-44	45-49	50-54	TFR	TFU25	TFO30
1950	0·0 (0·0–0·0)	12·9 (11·4–14·4)	162·9 (148·7–178·0)	218·1 (210·2–226·0)	152·7 (143·7–161·7)	96·0 (85·3–106·6)	37·0 (31·9–42·4)	1·2 (0·8–1·8)	0·0 (0·0–0·0)	3·4 (3·29–3·53)	0·88 (0·81–0·95)	1·43 (1·36–1·51)
1951	0·0 (0·0–0·0)	10·3 (9·2–11·6)	146·2 (131·7–160·6)	202·5 (194·9–210·0)	137·8 (128·4–147·4)	77·4 (67·4–87·8)	28·6 (24·1–33·5)	0·9 (0·6–1·3)	0·0 (0·0–0·0)	3·02 (2·88–3·17)	0·78 (0·71–0·86)	1·22 (1·11–1·34)
1952	0·0 (0·0–0·0)	8·7 (7·6–9·9)	136·2 (121·4–151·9)	192·4 (183·9–200·7)	124·5 (114·7–134·6)	61·9 (53·2–72·0)	22·6 (18·9–26·8)	0·7 (0·4–1·1)	0·0 (0·0–0·0)	2·74 (2·62–2·86)	0·72 (0·65–0·8)	1·05 (0·98–1·12)
1953	0·0 (0·0–0·0)	7·6 (6·6–8·8)	128·7 (114·0–143·9)	181·2 (172·7–190·0)	111·2 (101·1–121·1)	49·3 (41·6–58·0)	17·9 (14·5–22·0)	0·6 (0·4–0·9)	0·0 (0·0–0·0)	2·48 (2·36–2·61)	0·68 (0·61–0·76)	0·89 (0·82–0·97)
1954	0·0 (0·0–0·0)	7·0 (5·9–8·1)	124·8 (109·0–140·5)	176·3 (168·6–184·6)	102·4 (93·4–111·4)	41·0 (33·8–49·2)	14·8 (11·5–18·8)	0·5 (0·3–0·7)	0·0 (0·0–0·0)	2·33 (2·22–2·44)	0·66 (0·58–0·74)	0·79 (0·72–0·86)
1955	0·0 (0·0–0·0)	6·3 (5·4–7·4)	121·8 (106·6–138·7)	175·1 (166·7–183·5)	94·1 (84·8–103·4)	34·2 (28·7–40·6)	12·3 (9·4–15·6)	0·4 (0·2–0·6)	0·0 (0·0–0·0)	2·22 (2·13–2·31)	0·64 (0·56–0·73)	0·7 (0·64–0·76)
1956	0·0 (0·0–0·0)	5·6 (4·8–6·6)	114·8 (98·9–131·3)	169·2 (161·3–177·5)	83·2 (75·1–92·1)	28·3 (23·3–33·9)	9·8 (7·5–12·5)	0·3 (0·2–0·5)	0·0 (0·0–0·0)	2·06 (1·96–2·15)	0·6 (0·52–0·69)	0·61 (0·55–0·67)
1957	0·0 (0·0–0·0)	5·4 (4·6–6·4)	116·1 (100·4–135·0)	172·6 (164·5–181·0)	78·8 (71·1–87·2)	25·0 (20·7–29·3)	8·3 (6·2–10·6)	0·3 (0·2–0·5)	0·0 (0·0–0·0)	2·03 (1·92–2·16)	0·61 (0·53–0·7)	0·56 (0·51–0·61)
1958	0·0 (0·0–0·0)	5·4 (4·6–6·4)	121·9 (106·7–139·2)	178·2 (169·3–187·9)	76·9 (69·2–86·2)	22·8 (18·7–27·3)	7·5 (5·6–9·7)	0·3 (0·2–0·5)	0·0 (0·0–0·0)	2·07 (1·98–2·17)	0·64 (0·56–0·72)	0·54 (0·49–0·59)
1959	0·0 (0·0–0·0)	5·7 (4·8–6·7)	121·9 (105·6–138·2)	177·2 (167·8–186·6)	71·9 (64·0–80·1)	18·9 (15·2–23·1)	6·2 (4·6–8·3)	0·3 (0·1–0·5)	0·0 (0·0–0·0)	2·01 (1·91–2·12)	0·64 (0·56–0·72)	0·49 (0·44–0·54)
1960	0·0 (0·0–0·0)	5·5 (4·7–6·5)	117·1 (103·2–132·2)	177·8 (168·3–187·3)	68·8 (61·8–76·6)	16·1 (12·8–19·7)	5·0 (3·7–6·6)	0·2 (0·1–0·4)	0·0 (0·0–0·0)	1·95 (1·86–2·05)	0·61 (0·54–0·69)	0·45 (0·41–0·49)
1961	0·0 (0·0–0·0)	5·1 (4·3–6·1)	113·6 (102·3–125·8)	179·6 (170·9–189·1)	67·1 (60·7–73·9)	14·5 (11·4–18·0)	4·3 (3·1–5·8)	0·2 (0·1–0·3)	0·0 (0·0–0·0)	1·92 (1·84–2·01)	0·59 (0·54–0·65)	0·43 (0·39–0·47)
1962	0·0 (0·0–0·0)	4·9 (4·0–5·8)	113·2 (100·5–125·9)	184·7 (175·9–193·2)	68·3 (62·3–74·3)	13·6 (11·3–16·1)	3·8 (2·7–5·2)	0·2 (0·1–0·3)	0·0 (0·0–0·0)	1·94 (1·86–2·03)	0·59 (0·53–0·65)	0·43 (0·39–0·46)
1963	0·0 (0·0–0·0)	4·8 (4·0–5·8)	113·6 (101·8–126·6)	186·7 (179·3–194·2)	69·6 (63·6–76·1)	14·0 (11·1–17·3)	3·7 (2·6–5·3)	0·2 (0·1–0·3)	0·0 (0·0–0·0)	1·96 (1·88–2·05)	0·59 (0·53–0·66)	0·44 (0·4–0·47)
1964	0·0 (0·0–0·0)	4·8 (4·0–5·6)	123·9 (109·8–140·1)	198·7 (189·7–207·8)	74·0 (67·4–81·0)	14·4 (12·0–16·8)	3·5 (2·4–5·1)	0·2 (0·1–0·3)	0·0 (0·0–0·0)	2·1 (2·0–2·2)	0·64 (0·57–0·72)	0·46 (0·43–0·5)
1965	0·0 (0·0–0·0)	4·6 (3·9–5·5)	114·0 (99·1–130·4)	167·6 (157·7–177·8)	61·2 (55·2–67·7)	12·4 (10·5–14·6)	3·0 (2·0–4·3)	0·1 (0·1–0·2)	0·0 (0·0–0·0)	1·81 (1·7–1·93)	0·59 (0·52–0·68)	0·38 (0·35–0·42)
1966	0·0 (0·0–0·0)	4·9 (4·2–5·7)	111·4 (94·1–129·9)	172·3 (160·2–184·4)	62·5 (56·2–69·4)	12·6 (10·5–15·2)	2·9 (1·9–4·3)	0·1 (0·1–0·3)	0·0 (0·0–0·0)	1·83 (1·72–1·95)	0·58 (0·5–0·68)	0·39 (0·35–0·43)
1967	0·0 (0·0–0·0)	5·2 (4·4–6·0)	118·6 (98·9–139·6)	209·6 (194·9–224·9)	74·8 (67·4–82·8)	14·2 (11·8–16·8)	2·8 (1·9–3·9)	0·1 (0·1–0·2)	0·0 (0·0–0·0)	2·13 (1·97–2·29)	0·62 (0·52–0·73)	0·46 (0·42–0·5)
1968	0·0 (0·0–0·0)	5·2 (4·5–6·1)	106·0 (87·4–126·0)	197·1 (181·4–213·4)	67·6 (60·4–75·6)	14·1 (11·5–17·3)	2·5 (1·7–3·5)	0·1 (0·0–0·2)	0·0 (0·0–0·0)	1·96 (1·84–2·1)	0·56 (0·46–0·66)	0·42 (0·38–0·46)
1969	0·0 (0·0–0·0)	5·7 (4·9–6·7)	104·0 (87·2–123·2)	198·7 (183·4–215·3)	67·4 (60·3–75·2)	13·8 (10·9–17·1)	2·4 (1·7–3·4)	0·1 (0·0–0·2)	0·0 (0·0–0·0)	1·96 (1·84–2·09)	0·55 (0·46–0·64)	0·42 (0·37–0·47)
1970	0·0 (0·0–0·0)	5·8 (5·0–6·8)	111·6 (93·3–130·9)	201·6 (187·7–216·9)	68·9 (61·9–76·7)	13·5 (10·6–17·1)	2·6 (1·8–3·6)	0·1 (0·0–0·2)	0·0 (0·0–0·0)	2·02 (1·9–2·15)	0·59 (0·5–0·69)	0·43 (0·39–0·47)
1971	0·0 (0·0–0·0)	5·6 (4·7–6·6)	121·9 (102·9–142·6)	198·5 (184·8–212·4)	69·4 (62·1–77·0)	13·5 (10·7–16·9)	2·7 (1·9–3·8)	0·1 (0·0–0·2)	0·0 (0·0–0·0)	2·06 (1·94–2·18)	0·64 (0·54–0·74)	0·43 (0·38–0·48)
1972	0·0 (0·0–0·0)	5·7 (4·7–6·7)	127·4 (109·3–146·4)	196·2 (184·3–208·3)	69·1 (61·2–77·4)	13·7 (10·7–17·0)	2·7 (1·9–3·8)	0·1 (0·0–0·2)	0·0 (0·0–0·0)	2·07 (1·96–2·19)	0·67 (0·57–0·76)	0·43 (0·38–0·48)
1973	0·0 (0·0–0·0)	5·5 (4·6–6·6)	127·5 (111·4–143·0)	195·0 (184·0–205·8)	65·1 (57·7–73·2)	12·4 (9·6–15·6)	2·5 (1·7–3·4)	0·1 (0·0–0·2)	0·0 (0·0–0·0)	2·04 (1·94–2·14)	0·67 (0·58–0·74)	0·4 (0·35–0·45)
1974	0·0 (0·0–0·0)	5·1 (4·2–6·2)	121·8 (107·5–137·9)	185·9 (176·4–195·5)	57·4 (51·0–64·5)	10·3 (7·9–13·3)	2·1 (1·4–3·0)	0·1 (0·0–0·1)	0·0 (0·0–0·0)	1·91 (1·83–2·01)	0·63 (0·56–0·72)	0·35 (0·31–0·39)
1975	0·0 (0·0–0·0)	4·7 (3·9–5·8)	113·6 (101·7–125·9)	177·6 (168·8–186·9)	51·7 (45·7–57·1)	9·4 (7·1–12·3)	1·7 (1·2–2·5)	0·0 (0·0–0·1)	0·0 (0·0–0·0)	1·79 (1·72–1·86)	0·59 (0·53–0·65)	0·31 (0·28–0·35)
1976	0·0 (0·0–0·0)	4·5 (3·7–5·4)	107·7 (95·1–121·4)	174·7 (165·9–183·4)	50·6 (45·2–56·5)	9·3 (7·1–11·7)	1·5 (1·0–2·3)	0·0 (0·0–0·1)	0·0 (0·0–0·0)	1·74 (1·65–1·84)	0·56 (0·5–0·63)	0·31 (0·27–0·34)
1977	0·0 (0·0–0·0)	4·4 (3·7–5·1)	102·5 (91·2–116·6)	175·9 (167·7–185·3)	52·6 (46·8–59·2)	9·2 (7·0–12·2)	1·4 (0·9–2·1)	0·0 (0·0–0·1)	0·0 (0·0–0·0)	1·73 (1·65–1·82)	0·53 (0·48–0·6)	0·32 (0·28–0·36)
1978	0·0 (0·0–0·0)	4·4 (3·5–5·5)	98·6 (88·0–110·9)	177·8 (169·0–186·3)	55·3 (49·4–61·9)	9·1 (6·7–12·1)	1·2 (0·8–2·0)	0·0 (0·0–0·1)	0·0 (0·0–0·0)	1·73 (1·65–1·82)	0·52 (0·46–0·58)	0·33 (0·3–0·36)
1979	0·0 (0·0–0·0)	4·5 (3·4–5·8)	95·1 (83·7–107·1)	177·9 (170·3–185·2)	56·4 (50·3–62·7)	8·9 (6·8–11·3)	1·2 (0·7–2·3)	0·0 (0·0–0·0)	0·0 (0·0–0·0)	1·72 (1·64–1·8)	0·5 (0·44–0·56)	0·33 (0·3–0·37)
1980	0·0 (0·0–0·0)	4·6 (3·6–5·7)	91·7 (80·7–102·3)	177·6 (168·7–187·0)	56·8 (50·8–63·2)	8·9 (6·8–11·5)	1·2 (0·7–1·9)	0·0 (0·0–0·1)	0·0 (0·0–0·0)	1·7 (1·62–1·79)	0·48 (0·43–0·54)	0·33 (0·3–0·37)
1981	0·0 (0·0–0·0)	4·8 (4·0–5·8)	89·8 (80·4–101·3)	180·4 (171·6–189·2)	58·3 (52·7–64·8)	9·5 (7·2–11·9)	1·2 (0·8–1·8)	0·0 (0·0–0·0)	0·0 (0·0–0·0)	1·72 (1·66–1·79)	0·47 (0·43–0·53)	0·35 (0·31–0·38)
1982	0·0 (0·0–0·0)	5·0 (4·1–6·1)	88·9 (78·3–101·0)	183·8 (175·3–192·3)	61·6 (55·5–68·3)	10·7 (8·3–13·5)	1·3 (0·8–2·0)	0·0 (0·0–0·0)	0·0 (0·0–0·0)	1·76 (1·68–1·84)	0·47 (0·42–0·53)	0·37 (0·33–0·41)
1983	0·0 (0·0–0·0)	5·2 (4·3–6·3)	86·9 (75·2–98·9)	185·7 (176·2–195·1)	65·2 (58·8–72·4)	11·9 (9·4–14·9)	1·3 (0·8–1·9)	0·0 (0·0–0·0)	0·0 (0·0–0·0)	1·78 (1·7–1·86)	0·46 (0·4–0·52)	0·39 (0·36–0·43)
1984	0·0 (0·0–0·0)	5·1 (4·2–6·1)	81·1 (71·9–90·9)	184·4 (175·1–193·8)	68·0 (61·6–75·1)	12·4 (9·7–15·9)	1·4 (0·9–2·0)	0·0 (0·0–0·0)	0·0 (0·0–0·0)	1·76 (1·68–1·84)	0·43 (0·38–0·48)	0·41 (0·37–0·45)
1985	0·0 (0·0–0·0)	4·9 (4·0–5·8)	76·7 (66·8–87·5)	179·5 (171·5–188·5)	69·6 (62·9–76·8)	12·4 (9·5–15·6)	1·4 (0·9–2·1)	0·0 (0·0–0·0)	0·0 (0·0–0·0)	1·72 (1·65–1·81)	0·41 (0·36–0·47)	0·42 (0·38–0·46)
1986	0·0 (0·0–0·0)	4·7 (4·0–5·6)	73·7 (64·4–84·5)	175·5 (165·9–185·3)	72·0 (65·5–79·2)	12·6 (9·8–15·9)	1·4 (0·9–2·1)	0·0 (0·0–0·0)	0·0 (0·0–0·0)	1·7 (1·63–1·78)	0·39 (0·35–0·45)	0·43 (0·39–0·47)
1987	0·0 (0·0–0·0)	4·5 (3·8–5·3)	68·6 (60·5–77·8)	172·0 (163·5–180·7)	75·2 (68·2–83·3)	13·2 (10·3–17·0)	1·6 (1·0–2·5)	0·0 (0·0–0·1)	0·0 (0·0–0·0)	1·68 (1·6–1·76)	0·37 (0·32–0·41)	0·45 (0·41–0·5)
1988	0·0 (0·0–0·0)	4·3 (3·6–5·0)	64·2 (55·4–74·0)	163·4 (152·8–174·8)	77·0 (69·8–85·0)	14·0 (10·9–17·5)	1·8 (1·1–2·8)	0·0 (0·0–0·1)	0·0 (0·0–0·0)	1·62 (1·54–1·71)	0·34 (0·3–0·39)	0·46 (0·42–0·51)
1989	0·0 (0·0–0·0)	4·2 (3·5–5·1)	59·6 (51·7–68·4)	153·5 (145·5–162·5)	77·8 (70·3–85·8)	14·9 (11·6–18·9)	1·9 (1·2–2·9)	0·0 (0·0–0·1)	0·0 (0·0–0·0)	1·56 (1·48–1·64)	0·32 (0·28–0·36)	0·47 (0·43–0·52)
1990	0·0 (0·0–0·0)	4·3 (3·7–5·1)	57·4 (49·4–66·7)	150·1 (141·0–159·0)	79·8 (72·2–87·6)	15·7 (12·2–19·7)	1·9 (1·2–3·0)	0·0 (0·0–0·1)	0·0 (0·0–0·0)	1·55 (1·47–1·63)	0·31 (0·27–0·36)	0·49 (0·44–0·53)
1991	0·0 (0·0–0·0)	4·4 (3·7–5·3)	56·8 (49·0–65·3)	145·9 (137·8–154·3)	80·9 (73·6–89·6)	16·8 (13·1–21·0)	2·0 (1·2–2·9)	0·0 (0·0–0·1)	0·0 (0·0–0·0)	1·53 (1·47–1·61)	0·31 (0·27–0·35)	0·5 (0·46–0·55)
1992	0·0 (0·0–0·0)	4·5 (3·7–5·4)	54·4 (47·9–62·5)	137·9 (129·3–147·3)	80·4 (73·3–88·1)	17·5 (13·6–22·6)	2·1 (1·3–3·3)	0·0 (0·0–0·1)	0·0 (0·0–0·0)	1·48 (1·42–1·56)	0·29 (0·26–0·34)	0·5 (0·46–0·54)
1993	0·0 (0·0–0·0)	4·5 (3·7–5·5)	53·5 (45·7–61·6)	137·5 (128·7–146·8)	84·1 (75·6–92·8)	19·1 (14·9–23·8)	2·2 (1·4–3·4)	0·0 (0·0–0·1)	0·0 (0·0–0·0)	1·51 (1·43–1·58)	0·29 (0·25–0·33)	0·53 (0·48–0·58)
1994	0·0 (0·0–0·0)	4·5 (3·7–5·6)	52·5 (45·6–60·2)	135·0 (126·6–143·8)	85·9 (77·3–94·6)	20·3 (15·9–25·9)	2·3 (1·5–3·6)	0·0 (0·0–0·1)	0·0 (0·0–0·0)	1·5 (1·44–1·58)	0·29 (0·25–0·32)	0·54 (0·5–0·59)
1995	0·0 (0·0–0·0)	4·6 (3·7–5·6)	51·0 (44·3–58·7)	127·7 (119·6–136·5)	85·8 (77·0–94·0)	21·2 (16·9–26·1)	2·4 (1·5–3·6)	0·0 (0·0–0·1)	0·0 (0·0–0·0)	1·46 (1·38–1·54)	0·28 (0·24–0·32)	0·55 (0·5–0·59)
1996	0·0 (0·0–0·0)	4·7 (3·8–5·7)	50·4 (43·0–57·5)	123·8 (115·2–133·1)	86·6 (77·8–96·7)	22·3 (17·6–27·6)	2·5 (1·6–3·9)	0·0 (0·0–0·1)	0·0 (0·0–0·0)	1·45 (1·37–1·54)	0·28 (0·24–0·31)	0·56 (0·51–0·61)
1997	0·0 (0·0–0·0)	4·9 (3·9–6·0)	50·1 (42·8–58·6)	120·5 (112·7–127·8)	86·3 (78·2–94·6)	23·0 (18·2–28·6)	2·7 (1·7–4·1)	0·0 (0·0–0·1)	0·0 (0·0–0·0)	1·44 (1·37–1·52)	0·28 (0·24–0·32)	0·56 (0·52–0·61)
[truncated: 3,936,401 more chars]
